# Supplementary material for: sraX: A Novel Comprehensive Resistome Analysis Tool
Source: Front Microbiol. 2020 Feb 5;11:52. doi: 10.3389/fmicb.2020.00052 (PMC7025521; doi:10.3389/fmicb.2020.00052)
Supplement: Supplementary file 4 [file Data_Sheet_3.ZIP › gyrA/gyrA_aa.html]

# gyrA

|  |
| --- |
|  |

|  |
| --- |
|  |

|  |  |
| --- | --- |
| **Definition:** | Not indicated |

|  |
| --- |
|  |

|  |
| --- |
|  |

|  |  |
| --- | --- |
| **Reference organism:** | Not indicated |

|  |
| --- |
|  |

|  |
| --- |
|  |

|  |  |
| --- | --- |
| **Summary Data** | || **Parameter** | **Value_(n)_** || Total number of genomes | 198 |
| MSA length | 823 |
| Total number of SNPs | 148 |
| SNP density (# SNPs/MSA length) | 0.180 |

|  |
| --- |
|  |

|  |
| --- |
|  |

|  |
| --- |
|  |

|  |
| --- |
|  |

|  |  |  |  |  |
| --- | --- | --- | --- | --- |
| **SNPs conferring antibiotic resistance** | | | | || **Reference gene** | **Seq. pos.** | **Ref\_base** | **Alt\_base** | **Confirmation SNP?** |||  |  |  |  |  |
| --- | --- | --- | --- | --- |
| gyrA 84 S F **Yes** | | | | |

|  |
| --- |
|  |

|  |
| --- |
|  |

|  |
| --- |
|  |

|  |  |  |  |  |  |  |  |  |  |  |  |  |  |  |  |  |  |  |  |  |  |  |  |  |  |  |  |  |  |  |  |  |  |  |  |  |  |  |  |  |  |  |  |  |  |  |  |  |  |  |  |  |  |  |  |  |  |  |  |  |  |  |  |  |  |  |  |  |  |  |  |  |  |  |  |  |  |  |  |  |  |  |  |  |  |  |  |  |  |  |  |  |  |  |  |  |  |  |  |  |  |  |  |  |  |  |  |  |  |  |  |  |  |  |  |  |  |  |  |  |  |  |  |  |  |  |  |  |  |  |  |  |  |  |  |  |  |  |  |  |  |  |  |  |  |  |  |  |  |  |  |  |  |  |  |  |  |  |  |  |  |  |  |  |  |  |  |  |  |  |  |  |  |  |  |  |  |  |  |  |  |  |  |  |  |  |  |  |  |  |  |  |  |  |  |  |  |  |  |  |  |  |  |  |  |  |  |  |  |  |  |  |  |  |  |  |  |  |  |  |  |  |  |  |  |  |  |  |  |  |  |  |  |  |  |  |  |  |  |  |  |  |  |  |  |  |  |  |  |  |  |  |  |  |  |  |  |  |  |  |  |  |  |  |  |  |  |  |  |  |  |  |  |  |  |  |  |  |  |  |  |  |  |  |  |  |  |  |  |  |  |  |  |  |  |  |  |  |  |  |  |  |  |  |  |  |  |  |  |  |  |  |  |  |  |  |  |  |  |  |  |  |  |  |  |  |  |  |  |  |  |  |  |  |  |  |  |  |  |  |  |  |  |  |  |  |  |  |  |  |  |  |  |  |  |  |  |  |  |  |  |  |  |  |  |  |  |  |  |  |  |  |  |  |  |  |  |  |  |  |  |  |  |  |  |  |  |  |  |  |  |  |  |  |  |  |  |  |  |  |  |  |  |  |  |  |  |  |  |  |  |  |  |  |  |  |  |  |  |  |  |  |  |  |  |  |  |  |  |  |  |  |  |  |  |  |  |  |  |  |  |  |  |  |  |  |  |  |  |  |  |  |  |  |  |  |  |  |  |  |  |  |  |  |  |  |  |  |  |  |  |  |  |  |  |  |  |  |  |  |  |  |  |  |  |  |  |  |  |  |  |  |  |  |  |  |  |  |  |  |  |  |  |  |  |  |  |  |  |  |  |  |  |  |  |  |  |  |  |  |  |  |  |  |  |  |  |  |  |  |  |  |  |  |  |  |  |  |  |  |  |  |  |  |  |  |  |  |  |  |  |  |  |  |  |  |  |  |  |  |  |  |  |  |  |  |  |  |  |  |  |  |  |  |  |  |  |  |  |  |  |  |  |  |  |  |  |  |  |  |  |  |  |  |  |  |  |  |  |  |  |  |  |  |  |  |  |  |  |  |  |  |  |  |  |  |  |  |  |  |  |  |  |  |  |  |  |  |  |  |  |  |  |  |  |  |  |  |  |  |  |  |  |  |  |  |  |  |  |  |  |  |  |  |  |  |  |  |  |  |  |  |  |  |  |  |  |  |  |  |  |  |  |  |  |  |  |  |  |  |  |  |  |  |  |  |  |  |  |  |  |  |  |  |  |  |  |  |  |  |  |  |  |  |  |  |  |  |  |  |  |  |  |  |  |  |  |  |  |  |  |  |  |  |  |  |  |  |  |  |  |  |  |  |  |  |  |  |  |  |  |  |  |  |  |  |  |  |  |  |  |  |  |  |  |  |  |  |  |  |  |  |  |  |  |  |  |  |  |  |  |  |  |  |  |  |  |  |  |  |  |  |  |  |  |  |  |  |  |  |  |  |  |  |  |  |  |  |  |  |  |  |  |  |  |  |  |  |  |  |  |  |  |  |  |  |  |  |  |  |  |  |  |  |
| --- | --- | --- | --- | --- | --- | --- | --- | --- | --- | --- | --- | --- | --- | --- | --- | --- | --- | --- | --- | --- | --- | --- | --- | --- | --- | --- | --- | --- | --- | --- | --- | --- | --- | --- | --- | --- | --- | --- | --- | --- | --- | --- | --- | --- | --- | --- | --- | --- | --- | --- | --- | --- | --- | --- | --- | --- | --- | --- | --- | --- | --- | --- | --- | --- | --- | --- | --- | --- | --- | --- | --- | --- | --- | --- | --- | --- | --- | --- | --- | --- | --- | --- | --- | --- | --- | --- | --- | --- | --- | --- | --- | --- | --- | --- | --- | --- | --- | --- | --- | --- | --- | --- | --- | --- | --- | --- | --- | --- | --- | --- | --- | --- | --- | --- | --- | --- | --- | --- | --- | --- | --- | --- | --- | --- | --- | --- | --- | --- | --- | --- | --- | --- | --- | --- | --- | --- | --- | --- | --- | --- | --- | --- | --- | --- | --- | --- | --- | --- | --- | --- | --- | --- | --- | --- | --- | --- | --- | --- | --- | --- | --- | --- | --- | --- | --- | --- | --- | --- | --- | --- | --- | --- | --- | --- | --- | --- | --- | --- | --- | --- | --- | --- | --- | --- | --- | --- | --- | --- | --- | --- | --- | --- | --- | --- | --- | --- | --- | --- | --- | --- | --- | --- | --- | --- | --- | --- | --- | --- | --- | --- | --- | --- | --- | --- | --- | --- | --- | --- | --- | --- | --- | --- | --- | --- | --- | --- | --- | --- | --- | --- | --- | --- | --- | --- | --- | --- | --- | --- | --- | --- | --- | --- | --- | --- | --- | --- | --- | --- | --- | --- | --- | --- | --- | --- | --- | --- | --- | --- | --- | --- | --- | --- | --- | --- | --- | --- | --- | --- | --- | --- | --- | --- | --- | --- | --- | --- | --- | --- | --- | --- | --- | --- | --- | --- | --- | --- | --- | --- | --- | --- | --- | --- | --- | --- | --- | --- | --- | --- | --- | --- | --- | --- | --- | --- | --- | --- | --- | --- | --- | --- | --- | --- | --- | --- | --- | --- | --- | --- | --- | --- | --- | --- | --- | --- | --- | --- | --- | --- | --- | --- | --- | --- | --- | --- | --- | --- | --- | --- | --- | --- | --- | --- | --- | --- | --- | --- | --- | --- | --- | --- | --- | --- | --- | --- | --- | --- | --- | --- | --- | --- | --- | --- | --- | --- | --- | --- | --- | --- | --- | --- | --- | --- | --- | --- | --- | --- | --- | --- | --- | --- | --- | --- | --- | --- | --- | --- | --- | --- | --- | --- | --- | --- | --- | --- | --- | --- | --- | --- | --- | --- | --- | --- | --- | --- | --- | --- | --- | --- | --- | --- | --- | --- | --- | --- | --- | --- | --- | --- | --- | --- | --- | --- | --- | --- | --- | --- | --- | --- | --- | --- | --- | --- | --- | --- | --- | --- | --- | --- | --- | --- | --- | --- | --- | --- | --- | --- | --- | --- | --- | --- | --- | --- | --- | --- | --- | --- | --- | --- | --- | --- | --- | --- | --- | --- | --- | --- | --- | --- | --- | --- | --- | --- | --- | --- | --- | --- | --- | --- | --- | --- | --- | --- | --- | --- | --- | --- | --- | --- | --- | --- | --- | --- | --- | --- | --- | --- | --- | --- | --- | --- | --- | --- | --- | --- | --- | --- | --- | --- | --- | --- | --- | --- | --- | --- | --- | --- | --- | --- | --- | --- | --- | --- | --- | --- | --- | --- | --- | --- | --- | --- | --- | --- | --- | --- | --- | --- | --- | --- | --- | --- | --- | --- | --- | --- | --- | --- | --- | --- | --- | --- | --- | --- | --- | --- | --- | --- | --- | --- | --- | --- | --- | --- | --- | --- | --- | --- | --- | --- | --- | --- | --- | --- | --- | --- | --- | --- | --- | --- | --- | --- | --- | --- | --- | --- | --- | --- | --- | --- | --- | --- | --- | --- | --- | --- | --- | --- | --- | --- | --- | --- | --- | --- | --- | --- | --- | --- | --- | --- | --- | --- | --- | --- | --- | --- | --- | --- | --- | --- | --- | --- | --- | --- | --- | --- | --- | --- | --- | --- | --- | --- | --- | --- | --- | --- | --- | --- | --- | --- | --- | --- | --- | --- | --- | --- | --- | --- | --- | --- | --- | --- | --- | --- | --- | --- | --- | --- | --- | --- | --- | --- | --- | --- | --- | --- | --- | --- | --- | --- | --- | --- | --- | --- | --- | --- | --- | --- | --- | --- | --- | --- | --- | --- | --- | --- | --- | --- | --- | --- | --- | --- | --- | --- | --- | --- | --- | --- | --- | --- | --- | --- | --- | --- | --- | --- | --- | --- | --- | --- | --- | --- | --- | --- | --- | --- | --- | --- | --- | --- | --- | --- | --- | --- | --- | --- | --- | --- | --- | --- | --- | --- | --- | --- | --- | --- | --- | --- | --- | --- | --- | --- | --- | --- | --- | --- | --- | --- | --- | --- | --- | --- | --- | --- | --- | --- | --- | --- | --- | --- | --- | --- | --- | --- | --- | --- | --- | --- | --- | --- | --- | --- | --- | --- | --- | --- | --- | --- | --- | --- | --- | --- | --- | --- | --- | --- | --- | --- | --- | --- | --- | --- | --- | --- | --- | --- | --- | --- | --- | --- | --- | --- | --- | --- | --- | --- | --- | --- | --- | --- | --- | --- | --- | --- | --- | --- | --- | --- | --- | --- | --- | --- | --- | --- | --- | --- |
|  || **gyrA** | **M** | **S** | **E** | **E** | **I** | **R** | **E** | **N** | **I** | **H** | **D** | **V** | **N** | **L** | **T** | **S** | **E** | **M** | **K** | **D** | **S** | **F** | **I** | **D** | **Y** | **A** | **M** | **S** | **V** | **I** | **V** | **A** | **R** | **A** | **L** | **P** | **D** | **V** | **R** | **D** | **G** | **L** | **K** | **P** | **V** | **H** | **R** | **R** | **I** | **L** | **Y** | **G** | **M** | **N** | **E** | **L** | **G** | **V** | **T** | **P** | **D** | **K** | **A** | **H** | **K** | **K** | **S** | **A** | **R** | **I** | **V** | **G** | **D** | **V** | **M** | **G** | **K** | **Y** | **H** | **P** | **H** | **G** | **D** | **S** | **A** | **I** | **Y** | **E** | **S** | **M** | **V** | **R** | **M** | **A** | **Q** | **P** | **F** | **S** | **Y** | **R** | **Y** | **M** | **L** | **V** | **D** | **G** | **H** | **G** | **N** | **F** | **G** | **S** | **V** | **D** | **G** | **D** | **G** | **A** | **A** | **A** | **M** | **R** | **Y** | **T** | **E** | **A** | **R** | **M** | **S** | **K** | **I** | **A** | **T** | **E** | **M** | **L** | **R** | **D** | **I** | **N** | **K** | **N** | **T** | **V** | **D** | **F** | **Q** | **S** | **N** | **Y** | **D** | **D** | **T** | **E** | **K** | **E** | **P** | **V** | **V** | **L** | **P** | **A** | **R** | **F** | **P** | **N** | **L** | **L** | **V** | **N** | **G** | **T** | **T** | **G** | **I** | **A** | **V** | **G** | **M** | **A** | **T** | **N** | **I** | **P** | **P** | **H** | **N** | **L** | **S** | **E** | **V** | **V** | **A** | **A** | **I** | **D** | **L** | **L** | **M** | **D** | **N** | **P** | **D** | **V** | **T** | **T** | **N** | **E** | **L** | **M** | **E** | **V** | **L** | **P** | **G** | **P** | **D** | **F** | **P** | **T** | **G** | **G** | **L** | **V** | **M** | **G** | **K** | **S** | **G** | **I** | **R** | **R** | **A** | **Y** | **E** | **T** | **G** | **K** | **G** | **S** | **I** | **T** | **V** | **R** | **A** | **K** | **V** | **E** | **I** | **T** | **E** | **M** | **P** | **N** | **G** | **K** | **E** | **R** | **I** | **I** | **V** | **T** | **E** | **L** | **P** | **Y** | **M** | **V** | **N** | **K** | **A** | **K** | **L** | **I** | **E** | **R** | **I** | **S** | **E** | **L** | **H** | **R** | **D** | **K** | **R** | **I** | **E** | **G** | **I** | **T** | **D** | **L** | **R** | **D** | **E** | **S** | **S** | **R** | **E** | **G** | **M** | **R** | **I** | **V** | **I** | **D** | **I** | **R** | **R** | **D** | **A** | **S** | **A** | **S** | **V** | **I** | **L** | **N** | **N** | **L** | **Y** | **K** | **L** | **T** | **S** | **L** | **Q** | **N** | **S** | **F** | **G** | **F** | **N** | **M** | **L** | **A** | **I** | **E** | **K** | **G** | **V** | **P** | **K** | **V** | **L** | **S** | **L** | **K** | **Q** | **I** | **L** | **E** | **N** | **Y** | **I** | **E** | **H** | **Q** | **R** | **E** | **V** | **I** | **T** | **R** | **R** | **T** | **V** | **F** | **E** | **K** | **E** | **K** | **A** | **E** | **A** | **R** | **A** | **H** | **I** | **L** | **E** | **G** | **L** | **R** | **I** | **A** | **L** | **D** | **H** | **I** | **D** | **E** | **I** | **I** | **A** | **I** | **I** | **R** | **G** | **S** | **K** | **S** | **D** | **D** | **E** | **A** | **K** | **A** | **T** | **M** | **I** | **E** | **R** | **F** | **D** | **L** | **S** | **D** | **R** | **Q** | **S** | **Q** | **A** | **I** | **L** | **D** | **M** | **R** | **L** | **R** | **R** | **L** | **T** | **G** | **L** | **E** | **R** | **E** | **K** | **I** | **E** | **N** | **E** | **Y** | **Q** | **E** | **L** | **L** | **K** | **L** | **I** | **E** | **D** | **L** | **T** | **D** | **I** | **L** | **A** | **R** | **P** | **E** | **R** | **V** | **T** | **E** | **I** | **I** | **K** | **N** | **E** | **L** | **A** | **D** | **L** | **N** | **Q** | **R** | **F** | **G** | **D** | **K** | **R** | **R** | **T** | **E** | **L** | **L** | **V** | **G** | **E** | **V** | **L** | **S** | **L** | **E** | **D** | **E** | **D** | **L** | **I** | **E** | **E** | **E** | **E** | **I** | **V** | **I** | **T** | **L** | **T** | **N** | **N** | **G** | **Y** | **I** | **K** | **R** | **L** | **A** | **N** | **N** | **E** | **F** | **R** | **A** | **Q** | **R** | **R** | **G** | **G** | **R** | **G** | **V** | **Q** | **G** | **M** | **G** | **I** | **H** | **N** | **D** | **D** | **F** | **V** | **K** | **N** | **L** | **V** | **S** | **C** | **S** | **T** | **H** | **D** | **T** | **L** | **L** | **F** | **F** | **T** | **N** | **N** | **G** | **K** | **V** | **Y** | **R** | **A** | **K** | **G** | **Y** | **E** | **I** | **P** | **E** | **Y** | **G** | **R** | **T** | **A** | **K** | **G** | **I** | **P** | **I** | **I** | **N** | **L** | **L** | **G** | **I** | **D** | **S** | **S** | **E** | **S** | **I** | **Q** | **A** | **I** | **I** | **A** | **V** | **T** | **G** | **E** | **A** | **E** | **E** | **G** | **H** | **Y** | **L** | **F** | **F** | **T** | **T** | **R** | **Q** | **G** | **T** | **V** | **K** | **R** | **T** | **S** | **V** | **T** | **A** | **F** | **A** | **N** | **I** | **R** | **S** | **N** | **G** | **L** | **I** | **A** | **I** | **G** | **L** | **K** | **E** | **D** | **D** | **E** | **L** | **V** | **N** | **V** | **L** | **L** | **T** | **D** | **G** | **Q** | **S** | **N** | **I** | **I** | **I** | **G** | **T** | **H** | **N** | **G** | **Y** | **S** | **V** | **T** | **F** | **K** | **E** | **E** | **A** | **V** | **R** | **D** | **M** | **G** | **R** | **T** | **A** | **S** | **G** | **V** | **R** | **G** | **I** | **R** | **L** | **R** | **E** | **E** | **D** | **Y** | **V** | **V** | **G** | **A** | **A** | **L** | **M** | **K** | **E** | **N** | **Q** | **E** | **V** | **L** | **V** | **I** | **T** | **E** | **K** | **G** | **Y** | **G** | **K** | **R** | **T** | **K** | **V** | **S** | **E** | **Y** | **P** | **V** | **K** | **G** | **R** | **G** | **G** | **K** | **G** | **I** | **K** | **T** | **A** | **N** | **I** | **T** | **E** | **K** | **N** | **G** | **P** | **L** | **A** | **G** | **L** | **T** | **T** | **V** | **S** | **G** | **D** | **E** | **D** | **I** | **M** | **L** | **I** | **T** | **D** | **K** | **G** | **V** | **I** | **I** | **R** | **F** | **N** | **V** | **S** | **T** | **V** | **S** | **Q** | **T** | **G** | **R** | **S** | **T** | **L** | **G** | **V** | **R** | **L** | **M** | **K** | **M** | **E** | **A** | **D** | **T** | **K** | **V** | **V** | **T** | **M** | **A** | **A** | **V** | **E** | **P** | **E** | **A** | **A** | **E** | **Q** | **T** | **V** | **E** | **E** | **Q** | **G** | **S** | **E** | **E** |
| **N55337** | **M** | **S** | **E** | **E** | **I** | **R** | **E** | **N** | **I** | **H** | **D** | **V** | **N** | **L** | **T** | **S** | **E** | **M** | **K** | **D** | **S** | **F** | **I** | **D** | **Y** | **A** | **M** | **S** | **V** | **I** | **V** | **A** | **R** | **A** | **L** | **P** | **D** | **V** | **R** | **D** | **G** | **L** | **K** | **P** | **V** | **H** | **R** | **R** | **I** | **L** | **Y** | **G** | **M** | **N** | **E** | **L** | **G** | **V** | **T** | **P** | **D** | **K** | **A** | **H** | **K** | **K** | **S** | **A** | **R** | **I** | **V** | **G** | **D** | **V** | **M** | **G** | **K** | **Y** | **H** | **P** | **H** | **G** | **D** | **S** | **A** | **I** | **Y** | **E** | **S** | **M** | **V** | **R** | **M** | **A** | **Q** | **P** | **F** | **S** | **Y** | **R** | **Y** | **M** | **L** | **V** | **D** | **G** | **H** | **G** | **N** | **F** | **G** | **S** | **V** | **D** | **G** | **D** | **G** | **A** | **A** | **A** | **M** | **R** | **Y** | **T** | **E** | **A** | **R** | **M** | **S** | **K** | **I** | **A** | **T** | **E** | **M** | **L** | **R** | **D** | **I** | **N** | **K** | **N** | **T** | **V** | **D** | **F** | **Q** | **S** | **N** | **Y** | **D** | **D** | **T** | **E** | **K** | **E** | **P** | **V** | **V** | **L** | **P** | **A** | **R** | **F** | **P** | **N** | **L** | **L** | **V** | **N** | **G** | **T** | **T** | **G** | **I** | **A** | **V** | **G** | **M** | **A** | **T** | **N** | **I** | **P** | **P** | **H** | **N** | **L** | **S** | **E** | **V** | **V** | **A** | **A** | **I** | **D** | **L** | **L** | **M** | **D** | **N** | **P** | **D** | **V** | **T** | **T** | **N** | **E** | **L** | **M** | **E** | **V** | **L** | **P** | **G** | **P** | **D** | **F** | **P** | **T** | **G** | **G** | **L** | **V** | **M** | **G** | **K** | **S** | **G** | **I** | **R** | **R** | **A** | **Y** | **E** | **T** | **G** | **K** | **G** | **S** | **I** | **T** | **V** | **R** | **A** | **K** | **V** | **E** | **I** | **T** | **E** | **M** | **P** | **N** | **G** | **K** | **E** | **R** | **I** | **I** | **V** | **T** | **E** | **L** | **P** | **Y** | **M** | **V** | **N** | **K** | **A** | **K** | **L** | **I** | **E** | **R** | **I** | **S** | **E** | **L** | **H** | **R** | **D** | **K** | **R** | **I** | **E** | **G** | **I** | **T** | **D** | **L** | **R** | **D** | **E** | **S** | **S** | **R** | **E** | **G** | **M** | **R** | **I** | **V** | **I** | **D** | **I** | **R** | **R** | **D** | **A** | **S** | **A** | **S** | **V** | **I** | **L** | **N** | **N** | **L** | **Y** | **K** | **L** | **T** | **S** | **L** | **Q** | **N** | **S** | **F** | **G** | **F** | **N** | **M** | **L** | **A** | **I** | **E** | **K** | **G** | **V** | **P** | **K** | **V** | **L** | **S** | **L** | **K** | **Q** | **I** | **L** | **E** | **N** | **Y** | **I** | **E** | **H** | **Q** | **R** | **E** | **V** | **I** | **T** | **R** | **R** | **T** | **V** | **F** | **E** | **K** | **E** | **K** | **A** | **E** | **A** | **R** | **A** | **H** | **I** | **L** | **E** | **G** | **L** | **R** | **I** | **A** | **L** | **D** | **H** | **I** | **D** | **E** | **I** | **I** | **A** | **I** | **I** | **R** | **G** | **S** | **K** | **S** | **D** | **D** | **E** | **A** | **K** | **A** | **T** | **M** | **I** | **E** | **R** | **F** | **D** | **L** | **S** | **D** | **R** | **Q** | **S** | **Q** | **A** | **I** | **L** | **D** | **M** | **R** | **L** | **R** | **R** | **L** | **T** | **G** | **L** | **E** | **R** | **E** | **K** | **I** | **E** | **N** | **E** | **Y** | **Q** | **E** | **L** | **L** | **K** | **L** | **I** | **E** | **D** | **L** | **T** | **D** | **I** | **L** | **A** | **R** | **P** | **E** | **R** | **V** | **T** | **E** | **I** | **I** | **K** | **N** | **E** | **L** | **A** | **D** | **L** | **N** | **Q** | **R** | **F** | **G** | **D** | **K** | **R** | **R** | **T** | **E** | **L** | **L** | **V** | **G** | **E** | **V** | **L** | **S** | **L** | **E** | **D** | **E** | **D** | **L** | **I** | **E** | **E** | **E** | **E** | **I** | **V** | **I** | **T** | **L** | **T** | **N** | **N** | **G** | **Y** | **I** | **K** | **R** | **L** | **A** | **N** | **N** | **E** | **F** | **R** | **A** | **Q** | **R** | **R** | **G** | **G** | **R** | **G** | **V** | **Q** | **G** | **M** | **G** | **I** | **H** | **N** | **D** | **D** | **F** | **V** | **K** | **N** | **L** | **V** | **S** | **C** | **S** | **T** | **H** | **D** | **T** | **L** | **L** | **F** | **F** | **T** | **N** | **N** | **G** | **K** | **V** | **Y** | **R** | **A** | **K** | **G** | **Y** | **E** | **I** | **P** | **E** | **Y** | **G** | **R** | **T** | **A** | **K** | **G** | **I** | **P** | **I** | **I** | **N** | **L** | **L** | **G** | **I** | **D** | **S** | **S** | **E** | **S** | **I** | **Q** | **A** | **I** | **I** | **A** | **V** | **T** | **G** | **E** | **A** | **E** | **E** | **G** | **H** | **Y** | **L** | **F** | **F** | **T** | **T** | **R** | **Q** | **G** | **T** | **V** | **K** | **R** | **T** | **S** | **V** | **T** | **A** | **F** | **A** | **N** | **I** | **R** | **S** | **N** | **G** | **L** | **I** | **A** | **I** | **G** | **L** | **K** | **E** | **D** | **D** | **E** | **L** | **V** | **N** | **V** | **L** | **L** | **T** | **D** | **G** | **Q** | **S** | **N** | **I** | **I** | **I** | **G** | **T** | **H** | **N** | **G** | **Y** | **S** | **V** | **T** | **F** | **K** | **E** | **E** | **A** | **V** | **R** | **D** | **M** | **G** | **R** | **T** | **A** | **S** | **G** | **V** | **R** | **G** | **I** | **R** | **L** | **R** | **E** | **E** | **D** | **Y** | **V** | **V** | **G** | **A** | **A** | **L** | **M** | **K** | **E** | **N** | **Q** | **E** | **V** | **L** | **V** | **I** | **T** | **E** | **K** | **G** | **Y** | **G** | **K** | **R** | **T** | **K** | **V** | **S** | **E** | **Y** | **P** | **V** | **K** | **G** | **R** | **G** | **G** | **K** | **G** | **I** | **K** | **T** | **A** | **N** | **I** | **T** | **E** | **K** | **N** | **G** | **P** | **L** | **A** | **G** | **L** | **T** | **T** | **V** | **S** | **G** | **D** | **E** | **D** | **I** | **M** | **L** | **I** | **T** | **D** | **K** | **G** | **V** | **I** | **I** | **R** | **F** | **N** | **V** | **S** | **T** | **V** | **S** | **Q** | **T** | **G** | **R** | **S** | **T** | **L** | **G** | **V** | **R** | **L** | **M** | **K** | **M** | **E** | **A** | **D** | **T** | **K** | **V** | **V** | **T** | **M** | **A** | **A** | **V** | **E** | **P** | **E** | **A** | **A** | **E** | **Q** | **T** | **V** | **E** | **E** | **Q** | **G** | **S** | **E** | **E** |
| **N59457F** | **M** | **S** | **E** | **E** | **I** | **K** | **E** | **N** | **I** | **Q** | **D** | **V** | **N** | **L** | **T** | **S** | **E** | **M** | **K** | **E** | **S** | **F** | **I** | **D** | **Y** | **A** | **M** | **S** | **V** | **I** | **V** | **A** | **R** | **A** | **L** | **P** | **D** | **V** | **R** | **D** | **G** | **L** | **K** | **P** | **V** | **H** | **R** | **R** | **I** | **L** | **Y** | **G** | **M** | **N** | **E** | **L** | **G** | **V** | **T** | **P** | **D** | **K** | **P** | **H** | **K** | **K** | **S** | **A** | **R** | **I** | **V** | **G** | **D** | **V** | **M** | **G** | **K** | **Y** | **H** | **P** | **H** | **G** | **D** | **S** | **A** | **I** | **Y** | **E** | **S** | **M** | **V** | **R** | **M** | **A** | **Q** | **P** | **F** | **S** | **Y** | **R** | **A** | **M** | **L** | **V** | **D** | **G** | **H** | **G** | **N** | **F** | **G** | **S** | **V** | **D** | **G** | **D** | **G** | **A** | **A** | **A** | **M** | **R** | **Y** | **T** | **E** | **A** | **R** | **M** | **S** | **K** | **I** | **A** | **L** | **E** | **M** | **L** | **R** | **D** | **I** | **N** | **K** | **N** | **T** | **V** | **D** | **F** | **Q** | **G** | **N** | **Y** | **D** | **D** | **S** | **E** | **Q** | **E** | **P** | **V** | **V** | **L** | **P** | **A** | **R** | **F** | **P** | **N** | **L** | **L** | **V** | **N** | **G** | **T** | **T** | **G** | **I** | **A** | **V** | **G** | **M** | **A** | **T** | **N** | **I** | **P** | **P** | **H** | **N** | **L** | **S** | **E** | **V** | **I** | **D** | **A** | **T** | **S** | **L** | **L** | **M** | **D** | **N** | **P** | **D** | **V** | **T** | **T** | **N** | **E** | **L** | **M** | **E** | **V** | **L** | **P** | **G** | **P** | **D** | **F** | **P** | **T** | **G** | **G** | **L** | **V** | **M** | **G** | **K** | **S** | **G** | **I** | **R** | **R** | **A** | **Y** | **E** | **T** | **G** | **K** | **G** | **S** | **I** | **T** | **V** | **R** | **A** | **K** | **V** | **E** | **L** | **T** | **E** | **M** | **P** | **N** | **G** | **K** | **E** | **R** | **I** | **L** | **V** | **T** | **E** | **L** | **P** | **Y** | **M** | **V** | **N** | **K** | **A** | **K** | **L** | **I** | **E** | **R** | **I** | **S** | **E** | **L** | **H** | **R** | **D** | **K** | **R** | **I** | **E** | **G** | **I** | **T** | **D** | **L** | **R** | **D** | **E** | **S** | **S** | **R** | **E** | **G** | **M** | **R** | **I** | **V** | **I** | **D** | **V** | **R** | **R** | **D** | **V** | **S** | **A** | **S** | **V** | **V** | **L** | **N** | **N** | **L** | **Y** | **K** | **M** | **T** | **A** | **L** | **Q** | **T** | **S** | **F** | **G** | **F** | **N** | **M** | **L** | **A** | **I** | **E** | **K** | **G** | **V** | **P** | **K** | **I** | **L** | **S** | **L** | **K** | **R** | **I** | **L** | **E** | **N** | **Y** | **V** | **E** | **H** | **Q** | **K** | **E** | **V** | **I** | **T** | **R** | **R** | **T** | **I** | **F** | **D** | **K** | **N** | **K** | **A** | **E** | **A** | **R** | **A** | **H** | **I** | **L** | **E** | **G** | **L** | **R** | **I** | **A** | **L** | **D** | **H** | **I** | **D** | **E** | **I** | **I** | **A** | **I** | **I** | **R** | **G** | **S** | **Q** | **S** | **D** | **D** | **E** | **A** | **K** | **A** | **T** | **L** | **I** | **E** | **R** | **F** | **E** | **F** | **S** | **D** | **R** | **Q** | **A** | **Q** | **A** | **I** | **L** | **D** | **M** | **R** | **L** | **R** | **R** | **L** | **T** | **G** | **L** | **E** | **R** | **D** | **K** | **I** | **E** | **N** | **E** | **Y** | **Q** | **E** | **L** | **L** | **K** | **F** | **I** | **A** | **D** | **L** | **E** | **D** | **I** | **L** | **A** | **R** | **P** | **E** | **R** | **V** | **I** | **E** | **I** | **I** | **K** | **T** | **E** | **L** | **N** | **D** | **V** | **R** | **T** | **K** | **F** | **G** | **D** | **A** | **R** | **R** | **T** | **E** | **L** | **L** | **V** | **G** | **E** | **V** | **L** | **S** | **L** | **E** | **D** | **E** | **D** | **L** | **I** | **E** | **E** | **E** | **E** | **V** | **V** | **I** | **T** | **L** | **T** | **N** | **N** | **G** | **Y** | **I** | **K** | **R** | **M** | **A** | **N** | **S** | **E** | **F** | **R** | **A** | **Q** | **R** | **R** | **G** | **G** | **R** | **G** | **V** | **Q** | **G** | **M** | **G** | **V** | **H** | **D** | **D** | **D** | **F** | **V** | **K** | **N** | **L** | **V** | **S** | **C** | **S** | **T** | **H** | **D** | **T** | **L** | **L** | **F** | **F** | **T** | **N** | **T** | **G** | **K** | **V** | **Y** | **R** | **A** | **K** | **G** | **Y** | **E** | **I** | **P** | **E** | **Y** | **G** | **R** | **T** | **A** | **K** | **G** | **I** | **P** | **V** | **I** | **N** | **L** | **L** | **G** | **I** | **D** | **S** | **A** | **E** | **K** | **I** | **Q** | **A** | **I** | **I** | **S** | **V** | **E** | **G** | **K** | **A** | **E** | **A** | **G** | **K** | **Y** | **L** | **F** | **F** | **T** | **T** | **L** | **K** | **G** | **T** | **V** | **K** | **R** | **T** | **A** | **V** | **T** | **A** | **F** | **S** | **N** | **I** | **R** | **S** | **N** | **G** | **L** | **I** | **A** | **I** | **S** | **L** | **K** | **E** | **D** | **D** | **E** | **L** | **V** | **N** | **V** | **V** | **T** | **T** | **N** | **G** | **N** | **Q** | **K** | **M** | **I** | **I** | **G** | **T** | **H** | **A** | **G** | **Y** | **S** | **V** | **T** | **F** | **D** | **E** | **N** | **T** | **V** | **R** | **D** | **M** | **G** | **R** | **T** | **A** | **S** | **G** | **V** | **R** | **G** | **I** | **R** | **L** | **R** | **E** | **N** | **D** | **Y** | **V** | **V** | **G** | **A** | **A** | **I** | **L** | **D** | **E** | **N** | **K** | **E** | **V** | **L** | **V** | **I** | **T** | **E** | **N** | **G** | **Y** | **G** | **K** | **R** | **T** | **K** | **A** | **S** | **E** | **Y** | **P** | **V** | **K** | **G** | **R** | **G** | **G** | **K** | **G** | **I** | **K** | **T** | **A** | **N** | **I** | **T** | **E** | **K** | **N** | **G** | **P** | **L** | **A** | **G** | **L** | **T** | **T** | **V** | **N** | **G** | **D** | **E** | **D** | **I** | **L** | **L** | **I** | **T** | **N** | **K** | **G** | **V** | **I** | **I** | **R** | **F** | **N** | **V** | **D** | **S** | **V** | **S** | **Q** | **T** | **G** | **R** | **A** | **T** | **L** | **G** | **V** | **R** | **L** | **M** | **R** | **M** | **E** | **D** | **G** | **A** | **K** | **-** | **-** | **-** | **-** | **-** | **-** | **-** | **-** | **-** | **-** | **-** | **-** | **-** | **-** | **-** | **-** | **-** | **-** | **-** | **-** | **-** | **-** | **-** |
| **N54152** | **M** | **S** | **E** | **E** | **I** | **R** | **E** | **N** | **I** | **H** | **D** | **V** | **N** | **L** | **T** | **S** | **E** | **M** | **K** | **D** | **S** | **F** | **I** | **D** | **Y** | **A** | **M** | **S** | **V** | **I** | **V** | **A** | **R** | **A** | **L** | **P** | **D** | **V** | **R** | **D** | **G** | **L** | **K** | **P** | **V** | **H** | **R** | **R** | **I** | **L** | **Y** | **G** | **M** | **N** | **E** | **L** | **G** | **V** | **T** | **P** | **D** | **K** | **A** | **H** | **K** | **K** | **S** | **A** | **R** | **I** | **V** | **G** | **D** | **V** | **M** | **G** | **K** | **Y** | **H** | **P** | **H** | **G** | **D** | **S** | **A** | **I** | **Y** | **E** | **S** | **M** | **V** | **R** | **M** | **A** | **Q** | **P** | **F** | **S** | **Y** | **R** | **Y** | **M** | **L** | **V** | **D** | **G** | **H** | **G** | **N** | **F** | **G** | **S** | **V** | **D** | **G** | **D** | **G** | **A** | **A** | **A** | **M** | **R** | **Y** | **T** | **E** | **A** | **R** | **M** | **S** | **K** | **I** | **A** | **T** | **E** | **M** | **L** | **R** | **D** | **I** | **N** | **K** | **N** | **T** | **V** | **D** | **F** | **Q** | **S** | **N** | **Y** | **D** | **D** | **T** | **E** | **K** | **E** | **P** | **V** | **V** | **L** | **P** | **A** | **R** | **F** | **P** | **N** | **L** | **L** | **V** | **N** | **G** | **T** | **T** | **G** | **I** | **A** | **V** | **G** | **M** | **A** | **T** | **N** | **I** | **P** | **P** | **H** | **N** | **L** | **S** | **E** | **V** | **V** | **A** | **A** | **I** | **D** | **L** | **L** | **M** | **D** | **N** | **P** | **D** | **V** | **T** | **T** | **N** | **E** | **L** | **M** | **E** | **V** | **L** | **P** | **G** | **P** | **D** | **F** | **P** | **T** | **G** | **G** | **L** | **V** | **M** | **G** | **K** | **S** | **G** | **I** | **R** | **R** | **A** | **Y** | **E** | **T** | **G** | **K** | **G** | **S** | **I** | **T** | **V** | **R** | **A** | **K** | **V** | **E** | **I** | **T** | **E** | **M** | **P** | **N** | **G** | **K** | **E** | **R** | **I** | **I** | **V** | **T** | **E** | **L** | **P** | **Y** | **M** | **V** | **N** | **K** | **A** | **K** | **L** | **I** | **E** | **R** | **I** | **S** | **E** | **L** | **H** | **R** | **D** | **K** | **R** | **I** | **E** | **G** | **I** | **T** | **D** | **L** | **R** | **D** | **E** | **S** | **S** | **R** | **E** | **G** | **M** | **R** | **I** | **V** | **I** | **D** | **I** | **R** | **R** | **D** | **A** | **S** | **A** | **S** | **V** | **I** | **L** | **N** | **N** | **L** | **Y** | **K** | **L** | **T** | **S** | **L** | **Q** | **N** | **S** | **F** | **G** | **F** | **N** | **M** | **L** | **A** | **I** | **E** | **K** | **G** | **V** | **P** | **K** | **V** | **L** | **S** | **L** | **K** | **Q** | **I** | **L** | **E** | **N** | **Y** | **I** | **E** | **H** | **Q** | **R** | **E** | **V** | **I** | **T** | **R** | **R** | **T** | **V** | **F** | **E** | **K** | **E** | **K** | **A** | **E** | **A** | **R** | **A** | **H** | **I** | **L** | **E** | **G** | **L** | **R** | **I** | **A** | **L** | **D** | **H** | **I** | **D** | **E** | **I** | **I** | **A** | **I** | **I** | **R** | **G** | **S** | **K** | **S** | **D** | **D** | **E** | **A** | **K** | **A** | **T** | **M** | **I** | **E** | **R** | **F** | **D** | **L** | **S** | **D** | **R** | **Q** | **S** | **Q** | **A** | **I** | **L** | **D** | **M** | **R** | **L** | **R** | **R** | **L** | **T** | **G** | **L** | **E** | **R** | **E** | **K** | **I** | **E** | **N** | **E** | **Y** | **Q** | **E** | **L** | **L** | **K** | **L** | **I** | **E** | **D** | **L** | **T** | **D** | **I** | **L** | **A** | **R** | **P** | **E** | **R** | **V** | **T** | **E** | **I** | **I** | **K** | **N** | **E** | **L** | **A** | **D** | **L** | **N** | **Q** | **R** | **F** | **G** | **D** | **K** | **R** | **R** | **T** | **E** | **L** | **L** | **V** | **G** | **E** | **V** | **L** | **S** | **L** | **E** | **D** | **E** | **D** | **L** | **I** | **E** | **E** | **E** | **E** | **I** | **V** | **I** | **T** | **L** | **T** | **N** | **N** | **G** | **Y** | **I** | **K** | **R** | **L** | **A** | **N** | **N** | **E** | **F** | **R** | **A** | **Q** | **R** | **R** | **G** | **G** | **R** | **G** | **V** | **Q** | **G** | **M** | **G** | **I** | **H** | **N** | **D** | **D** | **F** | **V** | **K** | **N** | **L** | **V** | **S** | **C** | **S** | **T** | **H** | **D** | **T** | **L** | **L** | **F** | **F** | **T** | **N** | **N** | **G** | **K** | **V** | **Y** | **R** | **A** | **K** | **G** | **Y** | **E** | **I** | **P** | **E** | **Y** | **G** | **R** | **T** | **A** | **K** | **G** | **I** | **P** | **I** | **I** | **N** | **L** | **L** | **G** | **I** | **D** | **S** | **S** | **E** | **S** | **I** | **Q** | **A** | **I** | **I** | **A** | **V** | **T** | **G** | **E** | **A** | **E** | **E** | **G** | **H** | **Y** | **L** | **F** | **F** | **T** | **T** | **R** | **Q** | **G** | **T** | **V** | **K** | **R** | **T** | **S** | **V** | **T** | **A** | **F** | **A** | **N** | **I** | **R** | **S** | **N** | **G** | **L** | **I** | **A** | **I** | **G** | **L** | **K** | **E** | **D** | **D** | **E** | **L** | **V** | **N** | **V** | **L** | **L** | **T** | **D** | **G** | **Q** | **S** | **N** | **I** | **I** | **I** | **G** | **T** | **H** | **N** | **G** | **Y** | **S** | **V** | **T** | **F** | **K** | **E** | **E** | **A** | **V** | **R** | **D** | **M** | **G** | **R** | **T** | **A** | **S** | **G** | **V** | **R** | **G** | **I** | **R** | **L** | **R** | **E** | **E** | **D** | **Y** | **V** | **V** | **G** | **A** | **A** | **L** | **M** | **K** | **E** | **N** | **Q** | **E** | **V** | **L** | **V** | **I** | **T** | **E** | **K** | **G** | **Y** | **G** | **K** | **R** | **T** | **K** | **V** | **S** | **E** | **Y** | **P** | **V** | **K** | **G** | **R** | **G** | **G** | **K** | **G** | **I** | **K** | **T** | **A** | **N** | **I** | **T** | **E** | **K** | **N** | **G** | **P** | **L** | **A** | **G** | **L** | **T** | **T** | **V** | **S** | **G** | **D** | **E** | **D** | **I** | **M** | **L** | **I** | **T** | **D** | **K** | **G** | **V** | **I** | **I** | **R** | **F** | **N** | **V** | **S** | **T** | **V** | **S** | **Q** | **T** | **G** | **R** | **S** | **T** | **L** | **G** | **V** | **R** | **L** | **M** | **K** | **M** | **E** | **A** | **D** | **T** | **K** | **V** | **V** | **T** | **M** | **A** | **A** | **V** | **E** | **P** | **E** | **A** | **A** | **E** | **Q** | **T** | **V** | **E** | **E** | **Q** | **G** | **S** | **E** | **E** |
| **N60063F** | **M** | **S** | **E** | **E** | **I** | **K** | **E** | **N** | **I** | **Q** | **D** | **V** | **N** | **L** | **T** | **S** | **E** | **M** | **K** | **E** | **S** | **F** | **I** | **D** | **Y** | **A** | **M** | **S** | **V** | **I** | **V** | **A** | **R** | **A** | **L** | **P** | **D** | **V** | **R** | **D** | **G** | **L** | **K** | **P** | **V** | **H** | **R** | **R** | **I** | **L** | **Y** | **G** | **M** | **N** | **E** | **L** | **G** | **V** | **T** | **P** | **D** | **K** | **P** | **H** | **K** | **K** | **S** | **A** | **R** | **I** | **V** | **G** | **D** | **V** | **M** | **G** | **K** | **Y** | **H** | **P** | **H** | **G** | **D** | **S** | **A** | **I** | **Y** | **E** | **S** | **M** | **V** | **R** | **M** | **A** | **Q** | **P** | **F** | **S** | **Y** | **R** | **A** | **M** | **L** | **V** | **D** | **G** | **H** | **G** | **N** | **F** | **G** | **S** | **V** | **D** | **G** | **D** | **G** | **A** | **A** | **A** | **M** | **R** | **Y** | **T** | **E** | **A** | **R** | **M** | **S** | **K** | **I** | **A** | **L** | **E** | **M** | **L** | **R** | **D** | **I** | **N** | **K** | **N** | **T** | **V** | **D** | **F** | **Q** | **G** | **N** | **Y** | **D** | **D** | **S** | **E** | **Q** | **E** | **P** | **V** | **V** | **L** | **P** | **A** | **R** | **F** | **P** | **N** | **L** | **L** | **V** | **N** | **G** | **T** | **T** | **G** | **I** | **A** | **V** | **G** | **M** | **A** | **T** | **N** | **I** | **P** | **P** | **H** | **N** | **L** | **S** | **E** | **V** | **I** | **D** | **A** | **T** | **S** | **L** | **L** | **M** | **D** | **N** | **P** | **D** | **V** | **T** | **T** | **N** | **E** | **L** | **M** | **E** | **V** | **L** | **P** | **G** | **P** | **D** | **F** | **P** | **T** | **G** | **G** | **L** | **V** | **M** | **G** | **K** | **S** | **G** | **I** | **R** | **R** | **A** | **Y** | **E** | **T** | **G** | **K** | **G** | **S** | **I** | **T** | **V** | **R** | **A** | **K** | **V** | **E** | **L** | **T** | **E** | **M** | **P** | **N** | **G** | **K** | **E** | **R** | **I** | **L** | **V** | **T** | **E** | **L** | **P** | **Y** | **M** | **V** | **N** | **K** | **A** | **K** | **L** | **I** | **E** | **R** | **I** | **S** | **E** | **L** | **H** | **R** | **D** | **K** | **R** | **I** | **E** | **G** | **I** | **T** | **D** | **L** | **R** | **D** | **E** | **S** | **S** | **R** | **E** | **G** | **M** | **R** | **I** | **V** | **I** | **D** | **V** | **R** | **R** | **D** | **V** | **S** | **A** | **S** | **V** | **V** | **L** | **N** | **N** | **L** | **Y** | **K** | **M** | **T** | **A** | **L** | **Q** | **T** | **S** | **F** | **G** | **F** | **N** | **M** | **L** | **A** | **I** | **E** | **K** | **G** | **V** | **P** | **K** | **I** | **L** | **S** | **L** | **K** | **R** | **I** | **L** | **E** | **N** | **Y** | **V** | **E** | **H** | **Q** | **K** | **E** | **V** | **I** | **T** | **R** | **R** | **T** | **I** | **F** | **D** | **K** | **N** | **K** | **A** | **E** | **A** | **R** | **A** | **H** | **I** | **L** | **E** | **G** | **L** | **R** | **I** | **A** | **L** | **D** | **H** | **I** | **D** | **E** | **I** | **I** | **A** | **I** | **I** | **R** | **G** | **S** | **Q** | **S** | **D** | **D** | **E** | **A** | **K** | **A** | **T** | **L** | **I** | **E** | **R** | **F** | **E** | **F** | **S** | **D** | **R** | **Q** | **A** | **Q** | **A** | **I** | **L** | **D** | **M** | **R** | **L** | **R** | **R** | **L** | **T** | **G** | **L** | **E** | **R** | **D** | **K** | **I** | **E** | **N** | **E** | **Y** | **Q** | **E** | **L** | **L** | **K** | **F** | **I** | **A** | **D** | **L** | **E** | **D** | **I** | **L** | **A** | **R** | **P** | **E** | **R** | **V** | **I** | **E** | **I** | **I** | **K** | **T** | **E** | **L** | **N** | **D** | **V** | **R** | **T** | **K** | **F** | **G** | **D** | **A** | **R** | **R** | **T** | **E** | **L** | **L** | **V** | **G** | **E** | **V** | **L** | **S** | **L** | **E** | **D** | **E** | **D** | **L** | **I** | **E** | **E** | **E** | **E** | **V** | **V** | **I** | **T** | **L** | **T** | **N** | **N** | **G** | **Y** | **I** | **K** | **R** | **M** | **A** | **N** | **S** | **E** | **F** | **R** | **A** | **Q** | **R** | **R** | **G** | **G** | **R** | **G** | **V** | **Q** | **G** | **M** | **G** | **V** | **H** | **D** | **D** | **D** | **F** | **V** | **K** | **N** | **L** | **V** | **S** | **C** | **S** | **T** | **H** | **D** | **T** | **L** | **L** | **F** | **F** | **T** | **N** | **T** | **G** | **K** | **V** | **Y** | **R** | **A** | **K** | **G** | **Y** | **E** | **I** | **P** | **E** | **Y** | **G** | **R** | **T** | **A** | **K** | **G** | **I** | **P** | **V** | **I** | **N** | **L** | **L** | **G** | **I** | **D** | **S** | **A** | **E** | **K** | **I** | **Q** | **A** | **I** | **I** | **S** | **V** | **E** | **G** | **K** | **A** | **E** | **A** | **G** | **K** | **Y** | **L** | **F** | **F** | **T** | **T** | **L** | **K** | **G** | **T** | **V** | **K** | **R** | **T** | **A** | **V** | **T** | **A** | **F** | **S** | **N** | **I** | **R** | **S** | **N** | **G** | **L** | **I** | **A** | **I** | **S** | **L** | **K** | **E** | **D** | **D** | **E** | **L** | **V** | **N** | **V** | **V** | **T** | **T** | **N** | **G** | **N** | **Q** | **K** | **M** | **I** | **I** | **G** | **T** | **H** | **A** | **G** | **Y** | **S** | **V** | **T** | **F** | **D** | **E** | **N** | **T** | **V** | **R** | **D** | **M** | **G** | **R** | **T** | **A** | **S** | **G** | **V** | **R** | **G** | **I** | **R** | **L** | **R** | **E** | **N** | **D** | **Y** | **V** | **V** | **G** | **A** | **A** | **I** | **L** | **D** | **E** | **N** | **K** | **E** | **V** | **L** | **V** | **I** | **T** | **E** | **N** | **G** | **Y** | **G** | **K** | **R** | **T** | **K** | **A** | **S** | **E** | **Y** | **P** | **V** | **K** | **G** | **R** | **G** | **G** | **K** | **G** | **I** | **K** | **T** | **A** | **N** | **I** | **T** | **E** | **K** | **N** | **G** | **P** | **L** | **A** | **G** | **L** | **T** | **T** | **V** | **N** | **G** | **D** | **E** | **D** | **I** | **L** | **L** | **I** | **T** | **N** | **K** | **G** | **V** | **I** | **I** | **R** | **F** | **N** | **V** | **D** | **S** | **V** | **S** | **Q** | **T** | **G** | **R** | **A** | **T** | **L** | **G** | **V** | **R** | **L** | **M** | **R** | **M** | **E** | **D** | **G** | **A** | **K** | **-** | **-** | **-** | **-** | **-** | **-** | **-** | **-** | **-** | **-** | **-** | **-** | **-** | **-** | **-** | **-** | **-** | **-** | **-** | **-** | **-** | **-** | **-** |
| **N59936F** | **M** | **S** | **E** | **E** | **I** | **K** | **E** | **N** | **I** | **Q** | **D** | **V** | **N** | **L** | **T** | **S** | **E** | **M** | **K** | **E** | **S** | **F** | **I** | **D** | **Y** | **A** | **M** | **S** | **V** | **I** | **V** | **A** | **R** | **A** | **L** | **P** | **D** | **V** | **R** | **D** | **G** | **L** | **K** | **P** | **V** | **H** | **R** | **R** | **I** | **L** | **Y** | **G** | **M** | **N** | **E** | **L** | **G** | **V** | **T** | **P** | **D** | **K** | **P** | **H** | **K** | **K** | **S** | **A** | **R** | **I** | **V** | **G** | **D** | **V** | **M** | **G** | **K** | **Y** | **H** | **P** | **H** | **G** | **D** | **S** | **A** | **I** | **Y** | **E** | **S** | **M** | **V** | **R** | **M** | **A** | **Q** | **P** | **F** | **S** | **Y** | **R** | **A** | **M** | **L** | **V** | **D** | **G** | **H** | **G** | **N** | **F** | **G** | **S** | **V** | **D** | **G** | **D** | **G** | **A** | **A** | **A** | **M** | **R** | **Y** | **T** | **E** | **A** | **R** | **M** | **S** | **K** | **I** | **A** | **L** | **E** | **M** | **L** | **R** | **D** | **I** | **N** | **K** | **N** | **T** | **V** | **D** | **F** | **Q** | **G** | **N** | **Y** | **D** | **D** | **S** | **E** | **Q** | **E** | **P** | **V** | **V** | **L** | **P** | **A** | **R** | **F** | **P** | **N** | **L** | **L** | **V** | **N** | **G** | **T** | **T** | **G** | **I** | **A** | **V** | **G** | **M** | **A** | **T** | **N** | **I** | **P** | **P** | **H** | **N** | **L** | **S** | **E** | **V** | **I** | **D** | **A** | **T** | **S** | **L** | **L** | **M** | **D** | **N** | **P** | **D** | **V** | **T** | **T** | **N** | **E** | **L** | **M** | **E** | **V** | **L** | **P** | **G** | **P** | **D** | **F** | **P** | **T** | **G** | **G** | **L** | **V** | **M** | **G** | **K** | **S** | **G** | **I** | **R** | **R** | **A** | **Y** | **E** | **T** | **G** | **K** | **G** | **S** | **I** | **T** | **V** | **R** | **A** | **K** | **V** | **E** | **L** | **T** | **E** | **M** | **P** | **N** | **G** | **K** | **E** | **R** | **I** | **L** | **V** | **T** | **E** | **L** | **P** | **Y** | **M** | **V** | **N** | **K** | **A** | **K** | **L** | **I** | **E** | **R** | **I** | **S** | **E** | **L** | **H** | **R** | **D** | **K** | **R** | **I** | **E** | **G** | **I** | **T** | **D** | **L** | **R** | **D** | **E** | **S** | **S** | **R** | **E** | **G** | **M** | **R** | **I** | **V** | **I** | **D** | **V** | **R** | **R** | **D** | **V** | **S** | **A** | **S** | **V** | **V** | **L** | **N** | **N** | **L** | **Y** | **K** | **M** | **T** | **A** | **L** | **Q** | **T** | **S** | **F** | **G** | **F** | **N** | **M** | **L** | **A** | **I** | **E** | **K** | **G** | **V** | **P** | **K** | **I** | **L** | **S** | **L** | **K** | **R** | **I** | **L** | **E** | **N** | **Y** | **V** | **E** | **H** | **Q** | **K** | **E** | **V** | **I** | **T** | **R** | **R** | **T** | **I** | **F** | **D** | **K** | **N** | **K** | **A** | **E** | **A** | **R** | **A** | **H** | **I** | **L** | **E** | **G** | **L** | **R** | **I** | **A** | **L** | **D** | **H** | **I** | **D** | **E** | **I** | **I** | **A** | **I** | **I** | **R** | **G** | **S** | **Q** | **S** | **D** | **D** | **E** | **A** | **K** | **A** | **T** | **L** | **I** | **E** | **R** | **F** | **E** | **F** | **S** | **D** | **R** | **Q** | **A** | **Q** | **A** | **I** | **L** | **D** | **M** | **R** | **L** | **R** | **R** | **L** | **T** | **G** | **L** | **E** | **R** | **D** | **K** | **I** | **E** | **N** | **E** | **Y** | **Q** | **E** | **L** | **L** | **K** | **F** | **I** | **A** | **D** | **L** | **E** | **D** | **I** | **L** | **A** | **R** | **P** | **E** | **R** | **V** | **I** | **E** | **I** | **I** | **K** | **T** | **E** | **L** | **N** | **D** | **V** | **R** | **T** | **K** | **F** | **G** | **D** | **A** | **R** | **R** | **T** | **E** | **L** | **L** | **V** | **G** | **E** | **V** | **L** | **S** | **L** | **E** | **D** | **E** | **D** | **L** | **I** | **E** | **E** | **E** | **E** | **V** | **V** | **I** | **T** | **L** | **T** | **N** | **N** | **G** | **Y** | **I** | **K** | **R** | **M** | **A** | **N** | **S** | **E** | **F** | **R** | **A** | **Q** | **R** | **R** | **G** | **G** | **R** | **G** | **V** | **Q** | **G** | **M** | **G** | **V** | **H** | **D** | **D** | **D** | **F** | **V** | **K** | **N** | **L** | **V** | **S** | **C** | **S** | **T** | **H** | **D** | **T** | **L** | **L** | **F** | **F** | **T** | **N** | **T** | **G** | **K** | **V** | **Y** | **R** | **A** | **K** | **G** | **Y** | **E** | **I** | **P** | **E** | **Y** | **G** | **R** | **T** | **A** | **K** | **G** | **I** | **P** | **V** | **I** | **N** | **L** | **L** | **G** | **I** | **D** | **S** | **A** | **E** | **K** | **I** | **Q** | **A** | **I** | **I** | **S** | **V** | **E** | **G** | **K** | **A** | **E** | **A** | **G** | **K** | **Y** | **L** | **F** | **F** | **T** | **T** | **L** | **K** | **G** | **T** | **V** | **K** | **R** | **T** | **A** | **V** | **T** | **A** | **F** | **S** | **N** | **I** | **R** | **S** | **N** | **G** | **L** | **I** | **A** | **I** | **S** | **L** | **K** | **E** | **D** | **D** | **E** | **L** | **V** | **N** | **V** | **V** | **T** | **T** | **N** | **G** | **N** | **Q** | **K** | **M** | **I** | **I** | **G** | **T** | **H** | **A** | **G** | **Y** | **S** | **V** | **T** | **F** | **D** | **E** | **N** | **T** | **V** | **R** | **D** | **M** | **G** | **R** | **T** | **A** | **S** | **G** | **V** | **R** | **G** | **I** | **R** | **L** | **R** | **E** | **N** | **D** | **Y** | **V** | **V** | **G** | **A** | **A** | **I** | **L** | **D** | **E** | **N** | **K** | **E** | **V** | **L** | **V** | **I** | **T** | **E** | **N** | **G** | **Y** | **G** | **K** | **R** | **T** | **K** | **A** | **S** | **E** | **Y** | **P** | **V** | **K** | **G** | **R** | **G** | **G** | **K** | **G** | **I** | **K** | **T** | **A** | **N** | **I** | **T** | **E** | **K** | **N** | **G** | **P** | **L** | **A** | **G** | **L** | **T** | **T** | **V** | **N** | **G** | **D** | **E** | **D** | **I** | **L** | **L** | **I** | **T** | **N** | **K** | **G** | **V** | **I** | **I** | **R** | **F** | **N** | **V** | **D** | **S** | **V** | **S** | **Q** | **T** | **G** | **R** | **A** | **T** | **L** | **G** | **V** | **R** | **L** | **M** | **R** | **M** | **E** | **D** | **G** | **A** | **K** | **-** | **-** | **-** | **-** | **-** | **-** | **-** | **-** | **-** | **-** | **-** | **-** | **-** | **-** | **-** | **-** | **-** | **-** | **-** | **-** | **-** | **-** | **-** |
| **N59409F** | **M** | **S** | **E** | **E** | **I** | **K** | **E** | **N** | **I** | **Q** | **D** | **V** | **N** | **L** | **T** | **S** | **E** | **M** | **K** | **E** | **S** | **F** | **I** | **D** | **Y** | **A** | **M** | **S** | **V** | **I** | **V** | **A** | **R** | **A** | **L** | **P** | **D** | **V** | **R** | **D** | **G** | **L** | **K** | **P** | **V** | **H** | **R** | **R** | **I** | **L** | **Y** | **G** | **M** | **N** | **E** | **L** | **G** | **V** | **T** | **P** | **D** | **K** | **P** | **H** | **K** | **K** | **S** | **A** | **R** | **I** | **V** | **G** | **D** | **V** | **M** | **G** | **K** | **Y** | **H** | **P** | **H** | **G** | **D** | **S** | **A** | **I** | **Y** | **E** | **S** | **M** | **V** | **R** | **M** | **A** | **Q** | **P** | **F** | **S** | **Y** | **R** | **A** | **M** | **L** | **V** | **D** | **G** | **H** | **G** | **N** | **F** | **G** | **S** | **V** | **D** | **G** | **D** | **G** | **A** | **A** | **A** | **M** | **R** | **Y** | **T** | **E** | **A** | **R** | **M** | **S** | **K** | **I** | **A** | **L** | **E** | **M** | **L** | **R** | **D** | **I** | **N** | **K** | **N** | **T** | **V** | **D** | **F** | **Q** | **G** | **N** | **Y** | **D** | **D** | **S** | **E** | **Q** | **E** | **P** | **V** | **V** | **L** | **P** | **A** | **R** | **F** | **P** | **N** | **L** | **L** | **V** | **N** | **G** | **T** | **T** | **G** | **I** | **A** | **V** | **G** | **M** | **A** | **T** | **N** | **I** | **P** | **P** | **H** | **N** | **L** | **S** | **E** | **V** | **I** | **D** | **A** | **T** | **S** | **L** | **L** | **M** | **D** | **N** | **P** | **D** | **V** | **T** | **T** | **N** | **E** | **L** | **M** | **E** | **V** | **L** | **P** | **G** | **P** | **D** | **F** | **P** | **T** | **G** | **G** | **L** | **V** | **M** | **G** | **K** | **S** | **G** | **I** | **R** | **R** | **A** | **Y** | **E** | **T** | **G** | **K** | **G** | **S** | **I** | **T** | **V** | **R** | **A** | **K** | **V** | **E** | **L** | **T** | **E** | **M** | **P** | **N** | **G** | **K** | **E** | **R** | **I** | **L** | **V** | **T** | **E** | **L** | **P** | **Y** | **M** | **V** | **N** | **K** | **A** | **K** | **L** | **I** | **E** | **R** | **I** | **S** | **E** | **L** | **H** | **R** | **D** | **K** | **R** | **I** | **E** | **G** | **I** | **T** | **D** | **L** | **R** | **D** | **E** | **S** | **S** | **R** | **E** | **G** | **M** | **R** | **I** | **V** | **I** | **D** | **V** | **R** | **R** | **D** | **V** | **S** | **A** | **S** | **V** | **V** | **L** | **N** | **N** | **L** | **Y** | **K** | **M** | **T** | **A** | **L** | **Q** | **T** | **S** | **F** | **G** | **F** | **N** | **M** | **L** | **A** | **I** | **E** | **K** | **G** | **V** | **P** | **K** | **I** | **L** | **S** | **L** | **K** | **R** | **I** | **L** | **E** | **N** | **Y** | **V** | **E** | **H** | **Q** | **K** | **E** | **V** | **I** | **T** | **R** | **R** | **T** | **I** | **F** | **D** | **K** | **N** | **K** | **A** | **E** | **A** | **R** | **A** | **H** | **I** | **L** | **E** | **G** | **L** | **R** | **I** | **A** | **L** | **D** | **H** | **I** | **D** | **E** | **I** | **I** | **A** | **I** | **I** | **R** | **G** | **S** | **Q** | **S** | **D** | **D** | **E** | **A** | **K** | **A** | **T** | **L** | **I** | **E** | **R** | **F** | **E** | **F** | **S** | **D** | **R** | **Q** | **A** | **Q** | **A** | **I** | **L** | **D** | **M** | **R** | **L** | **R** | **R** | **L** | **T** | **G** | **L** | **E** | **R** | **D** | **K** | **I** | **E** | **N** | **E** | **Y** | **Q** | **E** | **L** | **L** | **K** | **F** | **I** | **A** | **D** | **L** | **E** | **D** | **I** | **L** | **A** | **R** | **P** | **E** | **R** | **V** | **I** | **E** | **I** | **I** | **K** | **T** | **E** | **L** | **N** | **D** | **V** | **R** | **T** | **K** | **F** | **G** | **D** | **A** | **R** | **R** | **T** | **E** | **L** | **L** | **V** | **G** | **E** | **V** | **L** | **S** | **L** | **E** | **D** | **E** | **D** | **L** | **I** | **E** | **E** | **E** | **E** | **V** | **V** | **I** | **T** | **L** | **T** | **N** | **N** | **G** | **Y** | **I** | **K** | **R** | **M** | **A** | **N** | **S** | **E** | **F** | **R** | **A** | **Q** | **R** | **R** | **G** | **G** | **R** | **G** | **V** | **Q** | **G** | **M** | **G** | **V** | **H** | **D** | **D** | **D** | **F** | **V** | **K** | **N** | **L** | **V** | **S** | **C** | **S** | **T** | **H** | **D** | **T** | **L** | **L** | **F** | **F** | **T** | **N** | **T** | **G** | **K** | **V** | **Y** | **R** | **A** | **K** | **G** | **Y** | **E** | **I** | **P** | **E** | **Y** | **G** | **R** | **T** | **A** | **K** | **G** | **I** | **P** | **V** | **I** | **N** | **L** | **L** | **G** | **I** | **D** | **S** | **A** | **E** | **K** | **I** | **Q** | **A** | **I** | **I** | **S** | **V** | **E** | **G** | **K** | **A** | **E** | **A** | **G** | **K** | **Y** | **L** | **F** | **F** | **T** | **T** | **L** | **K** | **G** | **T** | **V** | **K** | **R** | **T** | **A** | **V** | **T** | **A** | **F** | **S** | **N** | **I** | **R** | **S** | **N** | **G** | **L** | **I** | **A** | **I** | **S** | **L** | **K** | **E** | **D** | **D** | **E** | **L** | **V** | **N** | **V** | **V** | **T** | **T** | **N** | **G** | **N** | **Q** | **K** | **M** | **I** | **I** | **G** | **T** | **H** | **A** | **G** | **Y** | **S** | **V** | **T** | **F** | **D** | **E** | **N** | **T** | **V** | **R** | **D** | **M** | **G** | **R** | **T** | **A** | **S** | **G** | **V** | **R** | **G** | **I** | **R** | **L** | **R** | **E** | **N** | **D** | **Y** | **V** | **V** | **G** | **A** | **A** | **I** | **L** | **D** | **E** | **N** | **K** | **E** | **V** | **L** | **V** | **I** | **T** | **E** | **N** | **G** | **Y** | **G** | **K** | **R** | **T** | **K** | **A** | **S** | **E** | **Y** | **P** | **V** | **K** | **G** | **R** | **G** | **G** | **K** | **G** | **I** | **K** | **T** | **A** | **N** | **I** | **T** | **E** | **K** | **N** | **G** | **P** | **L** | **A** | **G** | **L** | **T** | **T** | **V** | **N** | **G** | **D** | **E** | **D** | **I** | **L** | **L** | **I** | **T** | **N** | **K** | **G** | **V** | **I** | **I** | **R** | **F** | **N** | **V** | **D** | **S** | **V** | **S** | **Q** | **T** | **G** | **R** | **A** | **T** | **L** | **G** | **V** | **R** | **L** | **M** | **R** | **M** | **E** | **D** | **G** | **A** | **K** | **-** | **-** | **-** | **-** | **-** | **-** | **-** | **-** | **-** | **-** | **-** | **-** | **-** | **-** | **-** | **-** | **-** | **-** | **-** | **-** | **-** | **-** | **-** |
| **N59509F** | **M** | **S** | **E** | **E** | **I** | **K** | **E** | **N** | **I** | **Q** | **D** | **V** | **N** | **L** | **T** | **S** | **E** | **M** | **K** | **E** | **S** | **F** | **I** | **D** | **Y** | **A** | **M** | **S** | **V** | **I** | **V** | **A** | **R** | **A** | **L** | **P** | **D** | **V** | **R** | **D** | **G** | **L** | **K** | **P** | **V** | **H** | **R** | **R** | **I** | **L** | **Y** | **G** | **M** | **N** | **E** | **L** | **G** | **V** | **T** | **P** | **D** | **K** | **P** | **H** | **K** | **K** | **S** | **A** | **R** | **I** | **V** | **G** | **D** | **V** | **M** | **G** | **K** | **Y** | **H** | **P** | **H** | **G** | **D** | **S** | **A** | **I** | **Y** | **E** | **S** | **M** | **V** | **R** | **M** | **A** | **Q** | **P** | **F** | **S** | **Y** | **R** | **A** | **M** | **L** | **V** | **D** | **G** | **H** | **G** | **N** | **F** | **G** | **S** | **V** | **D** | **G** | **D** | **G** | **A** | **A** | **A** | **M** | **R** | **Y** | **T** | **E** | **A** | **R** | **M** | **S** | **K** | **I** | **A** | **L** | **E** | **M** | **L** | **R** | **D** | **I** | **N** | **K** | **N** | **T** | **V** | **D** | **F** | **Q** | **G** | **N** | **Y** | **D** | **D** | **S** | **E** | **Q** | **E** | **P** | **V** | **V** | **L** | **P** | **A** | **R** | **F** | **P** | **N** | **L** | **L** | **V** | **N** | **G** | **T** | **T** | **G** | **I** | **A** | **V** | **G** | **M** | **A** | **T** | **N** | **I** | **P** | **P** | **H** | **N** | **L** | **S** | **E** | **V** | **I** | **D** | **A** | **T** | **S** | **L** | **L** | **M** | **D** | **N** | **P** | **D** | **V** | **T** | **T** | **N** | **E** | **L** | **M** | **E** | **V** | **L** | **P** | **G** | **P** | **D** | **F** | **P** | **T** | **G** | **G** | **L** | **V** | **M** | **G** | **K** | **S** | **G** | **I** | **R** | **R** | **A** | **Y** | **E** | **T** | **G** | **K** | **G** | **S** | **I** | **T** | **V** | **R** | **A** | **K** | **V** | **E** | **L** | **T** | **E** | **M** | **P** | **N** | **G** | **K** | **E** | **R** | **I** | **L** | **V** | **T** | **E** | **L** | **P** | **Y** | **M** | **V** | **N** | **K** | **A** | **K** | **L** | **I** | **E** | **R** | **I** | **S** | **E** | **L** | **H** | **R** | **D** | **K** | **R** | **I** | **E** | **G** | **I** | **T** | **D** | **L** | **R** | **D** | **E** | **S** | **S** | **R** | **E** | **G** | **M** | **R** | **I** | **V** | **I** | **D** | **V** | **R** | **R** | **D** | **V** | **S** | **A** | **S** | **V** | **V** | **L** | **N** | **N** | **L** | **Y** | **K** | **M** | **T** | **A** | **L** | **Q** | **T** | **S** | **F** | **G** | **F** | **N** | **M** | **L** | **A** | **I** | **E** | **K** | **G** | **V** | **P** | **K** | **I** | **L** | **S** | **L** | **K** | **R** | **I** | **L** | **E** | **N** | **Y** | **V** | **E** | **H** | **Q** | **K** | **E** | **V** | **I** | **T** | **R** | **R** | **T** | **I** | **F** | **D** | **K** | **N** | **K** | **A** | **E** | **A** | **R** | **A** | **H** | **I** | **L** | **E** | **G** | **L** | **R** | **I** | **A** | **L** | **D** | **H** | **I** | **D** | **E** | **I** | **I** | **A** | **I** | **I** | **R** | **G** | **S** | **Q** | **S** | **D** | **D** | **E** | **A** | **K** | **A** | **T** | **L** | **I** | **E** | **R** | **F** | **E** | **F** | **S** | **D** | **R** | **Q** | **A** | **Q** | **A** | **I** | **L** | **D** | **M** | **R** | **L** | **R** | **R** | **L** | **T** | **G** | **L** | **E** | **R** | **D** | **K** | **I** | **E** | **N** | **E** | **Y** | **Q** | **E** | **L** | **L** | **K** | **F** | **I** | **A** | **D** | **L** | **E** | **D** | **I** | **L** | **A** | **R** | **P** | **E** | **R** | **V** | **I** | **E** | **I** | **I** | **K** | **T** | **E** | **L** | **N** | **D** | **V** | **R** | **T** | **K** | **F** | **G** | **D** | **A** | **R** | **R** | **T** | **E** | **L** | **L** | **V** | **G** | **E** | **V** | **L** | **S** | **L** | **E** | **D** | **E** | **D** | **L** | **I** | **E** | **E** | **E** | **E** | **V** | **V** | **I** | **T** | **L** | **T** | **N** | **N** | **G** | **Y** | **I** | **K** | **R** | **M** | **A** | **N** | **S** | **E** | **F** | **R** | **A** | **Q** | **R** | **R** | **G** | **G** | **R** | **G** | **V** | **Q** | **G** | **M** | **G** | **V** | **H** | **D** | **D** | **D** | **F** | **V** | **K** | **N** | **L** | **V** | **S** | **C** | **S** | **T** | **H** | **D** | **T** | **L** | **L** | **F** | **F** | **T** | **N** | **T** | **G** | **K** | **V** | **Y** | **R** | **A** | **K** | **G** | **Y** | **E** | **I** | **P** | **E** | **Y** | **G** | **R** | **T** | **A** | **K** | **G** | **I** | **P** | **V** | **I** | **N** | **L** | **L** | **G** | **I** | **D** | **S** | **A** | **E** | **K** | **I** | **Q** | **A** | **I** | **I** | **S** | **V** | **E** | **G** | **K** | **A** | **E** | **A** | **G** | **K** | **Y** | **L** | **F** | **F** | **T** | **T** | **L** | **K** | **G** | **T** | **V** | **K** | **R** | **T** | **A** | **V** | **T** | **A** | **F** | **S** | **N** | **I** | **R** | **S** | **N** | **G** | **L** | **I** | **A** | **I** | **S** | **L** | **K** | **E** | **D** | **D** | **E** | **L** | **V** | **N** | **V** | **V** | **T** | **T** | **N** | **G** | **N** | **Q** | **K** | **M** | **I** | **I** | **G** | **T** | **H** | **A** | **G** | **Y** | **S** | **V** | **T** | **F** | **D** | **E** | **N** | **T** | **V** | **R** | **D** | **M** | **G** | **R** | **T** | **A** | **S** | **G** | **V** | **R** | **G** | **I** | **R** | **L** | **R** | **E** | **N** | **D** | **Y** | **V** | **V** | **G** | **A** | **A** | **I** | **L** | **D** | **E** | **N** | **K** | **E** | **V** | **L** | **V** | **I** | **T** | **E** | **N** | **G** | **Y** | **G** | **K** | **R** | **T** | **K** | **A** | **S** | **E** | **Y** | **P** | **V** | **K** | **G** | **R** | **G** | **G** | **K** | **G** | **I** | **K** | **T** | **A** | **N** | **I** | **T** | **E** | **K** | **N** | **G** | **P** | **L** | **A** | **G** | **L** | **T** | **T** | **V** | **N** | **G** | **D** | **E** | **D** | **I** | **L** | **L** | **I** | **T** | **N** | **K** | **G** | **V** | **I** | **I** | **R** | **F** | **N** | **V** | **D** | **S** | **V** | **S** | **Q** | **T** | **G** | **R** | **A** | **T** | **L** | **G** | **V** | **R** | **L** | **M** | **R** | **M** | **E** | **D** | **G** | **A** | **K** | **-** | **-** | **-** | **-** | **-** | **-** | **-** | **-** | **-** | **-** | **-** | **-** | **-** | **-** | **-** | **-** | **-** | **-** | **-** | **-** | **-** | **-** | **-** |
| **N55753** | **M** | **S** | **E** | **E** | **I** | **K** | **E** | **N** | **I** | **Q** | **D** | **V** | **N** | **L** | **T** | **S** | **E** | **M** | **K** | **E** | **S** | **F** | **I** | **D** | **Y** | **A** | **M** | **S** | **V** | **I** | **V** | **A** | **R** | **A** | **L** | **P** | **D** | **V** | **R** | **D** | **G** | **L** | **K** | **P** | **V** | **H** | **R** | **R** | **I** | **L** | **Y** | **G** | **M** | **N** | **E** | **L** | **G** | **V** | **T** | **P** | **D** | **K** | **P** | **H** | **K** | **K** | **S** | **A** | **R** | **I** | **V** | **G** | **D** | **V** | **M** | **G** | **K** | **Y** | **H** | **P** | **H** | **G** | **D** | **S** | **A** | **I** | **Y** | **E** | **S** | **M** | **V** | **R** | **M** | **A** | **Q** | **P** | **F** | **S** | **Y** | **R** | **A** | **M** | **L** | **V** | **D** | **G** | **H** | **G** | **N** | **F** | **G** | **S** | **V** | **D** | **G** | **D** | **G** | **A** | **A** | **A** | **M** | **R** | **Y** | **T** | **E** | **A** | **R** | **M** | **S** | **K** | **I** | **A** | **L** | **E** | **M** | **L** | **R** | **D** | **I** | **N** | **K** | **N** | **T** | **V** | **D** | **F** | **Q** | **G** | **N** | **Y** | **D** | **D** | **S** | **E** | **Q** | **E** | **P** | **V** | **V** | **L** | **P** | **A** | **R** | **F** | **P** | **N** | **L** | **L** | **V** | **N** | **G** | **T** | **T** | **G** | **I** | **A** | **V** | **G** | **M** | **A** | **T** | **N** | **I** | **P** | **P** | **H** | **N** | **L** | **S** | **E** | **V** | **I** | **D** | **A** | **T** | **S** | **L** | **L** | **M** | **D** | **N** | **P** | **D** | **V** | **T** | **T** | **N** | **E** | **L** | **M** | **E** | **V** | **L** | **P** | **G** | **P** | **D** | **F** | **P** | **T** | **G** | **G** | **L** | **V** | **M** | **G** | **K** | **S** | **G** | **I** | **R** | **R** | **A** | **Y** | **E** | **T** | **G** | **K** | **G** | **S** | **I** | **T** | **V** | **R** | **A** | **K** | **V** | **E** | **L** | **T** | **E** | **M** | **P** | **N** | **G** | **K** | **E** | **R** | **I** | **L** | **V** | **T** | **E** | **L** | **P** | **Y** | **M** | **V** | **N** | **K** | **A** | **K** | **L** | **I** | **E** | **R** | **I** | **S** | **E** | **L** | **H** | **R** | **D** | **K** | **R** | **I** | **E** | **G** | **I** | **T** | **D** | **L** | **R** | **D** | **E** | **S** | **S** | **R** | **E** | **G** | **M** | **R** | **I** | **V** | **I** | **D** | **V** | **R** | **R** | **D** | **V** | **S** | **A** | **S** | **V** | **V** | **L** | **N** | **N** | **L** | **Y** | **K** | **M** | **T** | **A** | **L** | **Q** | **T** | **S** | **F** | **G** | **F** | **N** | **M** | **L** | **A** | **I** | **E** | **K** | **G** | **V** | **P** | **K** | **I** | **L** | **S** | **L** | **K** | **R** | **I** | **L** | **E** | **N** | **Y** | **V** | **E** | **H** | **Q** | **K** | **E** | **V** | **I** | **T** | **R** | **R** | **T** | **I** | **F** | **D** | **K** | **N** | **K** | **A** | **E** | **A** | **R** | **A** | **H** | **I** | **L** | **E** | **G** | **L** | **R** | **I** | **A** | **L** | **D** | **H** | **I** | **D** | **E** | **I** | **I** | **A** | **I** | **I** | **R** | **G** | **S** | **Q** | **S** | **D** | **D** | **E** | **A** | **K** | **A** | **T** | **L** | **I** | **E** | **R** | **F** | **E** | **F** | **S** | **D** | **R** | **Q** | **A** | **Q** | **A** | **I** | **L** | **D** | **M** | **R** | **L** | **R** | **R** | **L** | **T** | **G** | **L** | **E** | **R** | **D** | **K** | **I** | **E** | **N** | **E** | **Y** | **Q** | **E** | **L** | **L** | **K** | **F** | **I** | **A** | **D** | **L** | **E** | **D** | **I** | **L** | **A** | **R** | **P** | **E** | **R** | **V** | **I** | **E** | **I** | **I** | **K** | **T** | **E** | **L** | **N** | **D** | **V** | **R** | **T** | **K** | **F** | **G** | **D** | **A** | **R** | **R** | **T** | **E** | **L** | **L** | **V** | **G** | **E** | **V** | **L** | **S** | **L** | **E** | **D** | **E** | **D** | **L** | **I** | **E** | **E** | **E** | **E** | **V** | **V** | **I** | **T** | **L** | **T** | **N** | **N** | **G** | **Y** | **I** | **K** | **R** | **M** | **A** | **N** | **S** | **E** | **F** | **R** | **A** | **Q** | **R** | **R** | **G** | **G** | **R** | **G** | **V** | **Q** | **G** | **M** | **G** | **V** | **H** | **D** | **D** | **D** | **F** | **V** | **K** | **N** | **L** | **V** | **S** | **C** | **S** | **T** | **H** | **D** | **T** | **L** | **L** | **F** | **F** | **T** | **N** | **T** | **G** | **K** | **V** | **Y** | **R** | **A** | **K** | **G** | **Y** | **E** | **I** | **P** | **E** | **Y** | **G** | **R** | **T** | **A** | **K** | **G** | **I** | **P** | **V** | **I** | **N** | **L** | **L** | **G** | **I** | **D** | **S** | **A** | **E** | **K** | **I** | **Q** | **A** | **I** | **I** | **S** | **V** | **E** | **G** | **K** | **A** | **E** | **A** | **G** | **K** | **Y** | **L** | **F** | **F** | **T** | **T** | **L** | **K** | **G** | **T** | **V** | **K** | **R** | **T** | **A** | **V** | **T** | **A** | **F** | **S** | **N** | **I** | **R** | **S** | **N** | **G** | **L** | **I** | **A** | **I** | **S** | **L** | **K** | **E** | **D** | **D** | **E** | **L** | **V** | **N** | **V** | **V** | **T** | **T** | **N** | **G** | **N** | **Q** | **K** | **M** | **I** | **I** | **G** | **T** | **H** | **A** | **G** | **Y** | **S** | **V** | **T** | **F** | **D** | **E** | **N** | **T** | **V** | **R** | **D** | **M** | **G** | **R** | **T** | **A** | **S** | **G** | **V** | **R** | **G** | **I** | **R** | **L** | **R** | **E** | **N** | **D** | **Y** | **V** | **V** | **G** | **A** | **A** | **I** | **L** | **D** | **E** | **N** | **K** | **E** | **V** | **L** | **V** | **I** | **T** | **E** | **N** | **G** | **Y** | **G** | **K** | **R** | **T** | **K** | **A** | **S** | **E** | **Y** | **P** | **V** | **K** | **G** | **R** | **G** | **G** | **K** | **G** | **I** | **K** | **T** | **A** | **N** | **I** | **T** | **E** | **K** | **N** | **G** | **P** | **L** | **A** | **G** | **L** | **T** | **T** | **V** | **N** | **G** | **D** | **E** | **D** | **I** | **L** | **L** | **I** | **T** | **N** | **K** | **G** | **V** | **I** | **I** | **R** | **F** | **N** | **V** | **D** | **S** | **V** | **S** | **Q** | **T** | **G** | **R** | **A** | **T** | **L** | **G** | **V** | **R** | **L** | **M** | **R** | **M** | **E** | **D** | **G** | **A** | **K** | **-** | **-** | **-** | **-** | **-** | **-** | **-** | **-** | **-** | **-** | **-** | **-** | **-** | **-** | **-** | **-** | **-** | **-** | **-** | **-** | **-** | **-** | **-** |
| **N53420** | **M** | **S** | **E** | **E** | **I** | **K** | **E** | **N** | **I** | **Q** | **D** | **V** | **N** | **L** | **T** | **S** | **E** | **M** | **K** | **E** | **S** | **F** | **I** | **D** | **Y** | **A** | **M** | **S** | **V** | **I** | **V** | **A** | **R** | **A** | **L** | **P** | **D** | **V** | **R** | **D** | **G** | **L** | **K** | **P** | **V** | **H** | **R** | **R** | **I** | **L** | **Y** | **G** | **M** | **N** | **E** | **L** | **G** | **V** | **T** | **P** | **D** | **K** | **P** | **H** | **K** | **K** | **S** | **A** | **R** | **I** | **V** | **G** | **D** | **V** | **M** | **G** | **K** | **Y** | **H** | **P** | **H** | **G** | **D** | **S** | **A** | **I** | **Y** | **E** | **S** | **M** | **V** | **R** | **M** | **A** | **Q** | **P** | **F** | **S** | **Y** | **R** | **A** | **M** | **L** | **V** | **D** | **G** | **H** | **G** | **N** | **F** | **G** | **S** | **V** | **D** | **G** | **D** | **G** | **A** | **A** | **A** | **M** | **R** | **Y** | **T** | **E** | **A** | **R** | **M** | **S** | **K** | **I** | **A** | **L** | **E** | **M** | **L** | **R** | **D** | **I** | **N** | **K** | **N** | **T** | **V** | **D** | **F** | **Q** | **G** | **N** | **Y** | **D** | **D** | **S** | **E** | **Q** | **E** | **P** | **V** | **V** | **L** | **P** | **A** | **R** | **F** | **P** | **N** | **L** | **L** | **V** | **N** | **G** | **T** | **T** | **G** | **I** | **A** | **V** | **G** | **M** | **A** | **T** | **N** | **I** | **P** | **P** | **H** | **N** | **L** | **S** | **E** | **V** | **I** | **D** | **A** | **T** | **S** | **L** | **L** | **M** | **D** | **N** | **P** | **D** | **V** | **T** | **T** | **N** | **E** | **L** | **M** | **E** | **V** | **L** | **P** | **G** | **P** | **D** | **F** | **P** | **T** | **G** | **G** | **L** | **V** | **M** | **G** | **K** | **S** | **G** | **I** | **R** | **R** | **A** | **Y** | **E** | **T** | **G** | **K** | **G** | **S** | **I** | **T** | **V** | **R** | **A** | **K** | **V** | **E** | **L** | **T** | **E** | **M** | **P** | **N** | **G** | **K** | **E** | **R** | **I** | **L** | **V** | **T** | **E** | **L** | **P** | **Y** | **M** | **V** | **N** | **K** | **A** | **K** | **L** | **I** | **E** | **R** | **I** | **S** | **E** | **L** | **H** | **R** | **D** | **K** | **R** | **I** | **E** | **G** | **I** | **T** | **D** | **L** | **R** | **D** | **E** | **S** | **S** | **R** | **E** | **G** | **M** | **R** | **I** | **V** | **I** | **D** | **V** | **R** | **R** | **D** | **V** | **S** | **A** | **S** | **V** | **V** | **L** | **N** | **N** | **L** | **Y** | **K** | **M** | **T** | **A** | **L** | **Q** | **T** | **S** | **F** | **G** | **F** | **N** | **M** | **L** | **A** | **I** | **E** | **K** | **G** | **V** | **P** | **K** | **I** | **L** | **S** | **L** | **K** | **R** | **I** | **L** | **E** | **N** | **Y** | **I** | **E** | **H** | **Q** | **K** | **E** | **V** | **I** | **T** | **R** | **R** | **T** | **I** | **F** | **D** | **K** | **N** | **K** | **A** | **E** | **A** | **R** | **A** | **H** | **I** | **L** | **E** | **G** | **L** | **R** | **I** | **A** | **L** | **D** | **H** | **I** | **D** | **E** | **I** | **I** | **A** | **I** | **I** | **R** | **G** | **S** | **Q** | **S** | **D** | **D** | **E** | **A** | **K** | **A** | **T** | **L** | **I** | **E** | **R** | **F** | **E** | **F** | **S** | **D** | **R** | **Q** | **A** | **Q** | **A** | **I** | **L** | **D** | **M** | **R** | **L** | **R** | **R** | **L** | **T** | **G** | **L** | **E** | **R** | **D** | **K** | **I** | **E** | **N** | **E** | **Y** | **Q** | **E** | **L** | **L** | **K** | **F** | **I** | **A** | **D** | **L** | **E** | **D** | **I** | **L** | **A** | **R** | **P** | **E** | **R** | **V** | **I** | **E** | **I** | **I** | **K** | **T** | **E** | **L** | **N** | **D** | **V** | **R** | **T** | **K** | **F** | **G** | **D** | **A** | **R** | **R** | **T** | **E** | **L** | **L** | **V** | **G** | **E** | **V** | **L** | **S** | **L** | **E** | **D** | **E** | **D** | **L** | **I** | **E** | **E** | **E** | **E** | **V** | **V** | **I** | **T** | **L** | **T** | **N** | **N** | **G** | **Y** | **I** | **K** | **R** | **M** | **A** | **N** | **S** | **E** | **F** | **R** | **A** | **Q** | **R** | **R** | **G** | **G** | **R** | **G** | **V** | **Q** | **G** | **M** | **G** | **V** | **H** | **D** | **D** | **D** | **F** | **V** | **K** | **N** | **L** | **V** | **S** | **C** | **S** | **T** | **H** | **D** | **T** | **L** | **L** | **F** | **F** | **T** | **N** | **T** | **G** | **K** | **V** | **Y** | **R** | **A** | **K** | **G** | **Y** | **E** | **I** | **P** | **E** | **Y** | **G** | **R** | **T** | **A** | **K** | **G** | **I** | **P** | **V** | **I** | **N** | **L** | **L** | **G** | **I** | **D** | **S** | **A** | **E** | **K** | **I** | **Q** | **A** | **I** | **I** | **S** | **V** | **E** | **G** | **K** | **A** | **E** | **A** | **G** | **K** | **Y** | **L** | **F** | **F** | **T** | **T** | **L** | **K** | **G** | **T** | **V** | **K** | **R** | **T** | **A** | **V** | **T** | **A** | **F** | **S** | **N** | **I** | **R** | **S** | **N** | **G** | **L** | **I** | **A** | **I** | **S** | **L** | **K** | **E** | **D** | **D** | **E** | **L** | **V** | **N** | **V** | **V** | **T** | **T** | **N** | **G** | **N** | **Q** | **K** | **M** | **I** | **I** | **G** | **T** | **H** | **A** | **G** | **Y** | **S** | **V** | **T** | **F** | **D** | **E** | **T** | **T** | **V** | **R** | **D** | **M** | **G** | **R** | **T** | **A** | **S** | **G** | **V** | **R** | **G** | **I** | **R** | **L** | **R** | **E** | **N** | **D** | **Y** | **V** | **V** | **G** | **A** | **A** | **I** | **L** | **D** | **E** | **N** | **K** | **E** | **V** | **L** | **V** | **I** | **T** | **E** | **N** | **G** | **Y** | **G** | **K** | **R** | **T** | **K** | **A** | **S** | **E** | **Y** | **P** | **V** | **K** | **G** | **R** | **G** | **G** | **K** | **G** | **I** | **K** | **T** | **A** | **N** | **I** | **T** | **E** | **K** | **N** | **G** | **P** | **L** | **A** | **G** | **L** | **T** | **T** | **V** | **N** | **G** | **D** | **E** | **D** | **I** | **L** | **L** | **I** | **T** | **N** | **K** | **G** | **V** | **I** | **I** | **R** | **F** | **N** | **V** | **D** | **S** | **V** | **S** | **Q** | **T** | **G** | **R** | **A** | **T** | **L** | **G** | **V** | **R** | **L** | **M** | **R** | **M** | **E** | **D** | **G** | **A** | **K** | **-** | **-** | **-** | **-** | **-** | **-** | **-** | **-** | **-** | **-** | **-** | **-** | **-** | **-** | **-** | **-** | **-** | **-** | **-** | **-** | **-** | **-** | **-** |
| **N54595** | **M** | **S** | **E** | **E** | **I** | **K** | **E** | **N** | **I** | **Q** | **D** | **V** | **N** | **L** | **T** | **S** | **E** | **M** | **K** | **E** | **S** | **F** | **I** | **D** | **Y** | **A** | **M** | **S** | **V** | **I** | **V** | **A** | **R** | **A** | **L** | **P** | **D** | **V** | **R** | **D** | **G** | **L** | **K** | **P** | **V** | **H** | **R** | **R** | **I** | **L** | **Y** | **G** | **M** | **N** | **E** | **L** | **G** | **V** | **T** | **P** | **D** | **K** | **P** | **H** | **K** | **K** | **S** | **A** | **R** | **I** | **V** | **G** | **D** | **V** | **M** | **G** | **K** | **Y** | **H** | **P** | **H** | **G** | **D** | **S** | **A** | **I** | **Y** | **E** | **S** | **M** | **V** | **R** | **M** | **A** | **Q** | **P** | **F** | **S** | **Y** | **R** | **A** | **M** | **L** | **V** | **D** | **G** | **H** | **G** | **N** | **F** | **G** | **S** | **V** | **D** | **G** | **D** | **G** | **A** | **A** | **A** | **M** | **R** | **Y** | **T** | **E** | **A** | **R** | **M** | **S** | **K** | **I** | **A** | **L** | **E** | **M** | **L** | **R** | **D** | **I** | **N** | **K** | **N** | **T** | **V** | **D** | **F** | **Q** | **G** | **N** | **Y** | **D** | **D** | **S** | **E** | **Q** | **E** | **P** | **V** | **V** | **L** | **P** | **A** | **R** | **F** | **P** | **N** | **L** | **L** | **V** | **N** | **G** | **T** | **T** | **G** | **I** | **A** | **V** | **G** | **M** | **A** | **T** | **N** | **I** | **P** | **P** | **H** | **N** | **L** | **S** | **E** | **V** | **I** | **D** | **A** | **T** | **S** | **L** | **L** | **M** | **D** | **N** | **P** | **D** | **V** | **T** | **T** | **N** | **E** | **L** | **M** | **E** | **V** | **L** | **P** | **G** | **P** | **D** | **F** | **P** | **T** | **G** | **G** | **L** | **V** | **M** | **G** | **K** | **S** | **G** | **I** | **R** | **R** | **A** | **Y** | **E** | **T** | **G** | **K** | **G** | **S** | **I** | **T** | **V** | **R** | **A** | **K** | **V** | **E** | **L** | **T** | **E** | **M** | **P** | **N** | **G** | **K** | **E** | **R** | **I** | **L** | **V** | **T** | **E** | **L** | **P** | **Y** | **M** | **V** | **N** | **K** | **A** | **K** | **L** | **I** | **E** | **R** | **I** | **S** | **E** | **L** | **H** | **R** | **D** | **K** | **R** | **I** | **E** | **G** | **I** | **T** | **D** | **L** | **R** | **D** | **E** | **S** | **S** | **R** | **E** | **G** | **M** | **R** | **I** | **V** | **I** | **D** | **V** | **R** | **R** | **D** | **V** | **S** | **A** | **S** | **V** | **V** | **L** | **N** | **N** | **L** | **Y** | **K** | **M** | **T** | **A** | **L** | **Q** | **T** | **S** | **F** | **G** | **F** | **N** | **M** | **L** | **A** | **I** | **E** | **K** | **G** | **V** | **P** | **K** | **I** | **L** | **S** | **L** | **K** | **R** | **I** | **L** | **E** | **N** | **Y** | **V** | **E** | **H** | **Q** | **K** | **E** | **V** | **I** | **T** | **R** | **R** | **T** | **I** | **F** | **D** | **K** | **N** | **K** | **A** | **E** | **A** | **R** | **A** | **H** | **I** | **L** | **E** | **G** | **L** | **R** | **I** | **A** | **L** | **D** | **H** | **I** | **D** | **E** | **I** | **I** | **A** | **I** | **I** | **R** | **G** | **S** | **Q** | **S** | **D** | **D** | **E** | **A** | **K** | **A** | **T** | **L** | **I** | **E** | **R** | **F** | **E** | **F** | **S** | **D** | **R** | **Q** | **A** | **Q** | **A** | **I** | **L** | **D** | **M** | **R** | **L** | **R** | **R** | **L** | **T** | **G** | **L** | **E** | **R** | **D** | **K** | **I** | **E** | **N** | **E** | **Y** | **Q** | **E** | **L** | **L** | **K** | **F** | **I** | **A** | **D** | **L** | **E** | **D** | **I** | **L** | **A** | **R** | **P** | **E** | **R** | **V** | **I** | **E** | **I** | **I** | **K** | **T** | **E** | **L** | **N** | **D** | **V** | **R** | **T** | **K** | **F** | **G** | **D** | **A** | **R** | **R** | **T** | **E** | **L** | **L** | **V** | **G** | **E** | **V** | **L** | **S** | **L** | **E** | **D** | **E** | **D** | **L** | **I** | **E** | **E** | **E** | **E** | **V** | **V** | **I** | **T** | **L** | **T** | **N** | **N** | **G** | **Y** | **I** | **K** | **R** | **M** | **A** | **N** | **S** | **E** | **F** | **R** | **A** | **Q** | **R** | **R** | **G** | **G** | **R** | **G** | **V** | **Q** | **G** | **M** | **G** | **V** | **H** | **D** | **D** | **D** | **F** | **V** | **K** | **N** | **L** | **V** | **S** | **C** | **S** | **T** | **H** | **D** | **T** | **L** | **L** | **F** | **F** | **T** | **N** | **T** | **G** | **K** | **V** | **Y** | **R** | **A** | **K** | **G** | **Y** | **E** | **I** | **P** | **E** | **Y** | **G** | **R** | **T** | **A** | **K** | **G** | **I** | **P** | **V** | **I** | **N** | **L** | **L** | **G** | **I** | **D** | **S** | **A** | **E** | **K** | **I** | **Q** | **A** | **I** | **I** | **S** | **V** | **E** | **G** | **K** | **A** | **E** | **A** | **G** | **K** | **Y** | **L** | **F** | **F** | **T** | **T** | **L** | **K** | **G** | **T** | **V** | **K** | **R** | **T** | **A** | **V** | **T** | **A** | **F** | **S** | **N** | **I** | **R** | **S** | **N** | **G** | **L** | **I** | **A** | **I** | **S** | **L** | **K** | **E** | **D** | **D** | **E** | **L** | **V** | **N** | **V** | **V** | **T** | **T** | **N** | **G** | **N** | **Q** | **K** | **M** | **I** | **I** | **G** | **T** | **H** | **A** | **G** | **Y** | **S** | **V** | **T** | **F** | **D** | **E** | **N** | **T** | **V** | **R** | **D** | **M** | **G** | **R** | **T** | **A** | **S** | **G** | **V** | **R** | **G** | **I** | **R** | **L** | **R** | **E** | **N** | **D** | **Y** | **V** | **V** | **G** | **A** | **A** | **I** | **L** | **D** | **E** | **N** | **K** | **E** | **V** | **L** | **V** | **I** | **T** | **E** | **N** | **G** | **Y** | **G** | **K** | **R** | **T** | **K** | **A** | **S** | **E** | **Y** | **P** | **V** | **K** | **G** | **R** | **G** | **G** | **K** | **G** | **I** | **K** | **T** | **A** | **N** | **I** | **T** | **E** | **K** | **N** | **G** | **P** | **L** | **A** | **G** | **L** | **T** | **T** | **V** | **N** | **G** | **D** | **E** | **D** | **I** | **L** | **L** | **I** | **T** | **N** | **K** | **G** | **V** | **I** | **I** | **R** | **F** | **N** | **V** | **D** | **S** | **V** | **S** | **Q** | **T** | **G** | **R** | **A** | **T** | **L** | **G** | **V** | **R** | **L** | **M** | **R** | **M** | **E** | **D** | **G** | **A** | **K** | **-** | **-** | **-** | **-** | **-** | **-** | **-** | **-** | **-** | **-** | **-** | **-** | **-** | **-** | **-** | **-** | **-** | **-** | **-** | **-** | **-** | **-** | **-** |
| **N59891F** | **M** | **S** | **E** | **E** | **I** | **R** | **E** | **N** | **I** | **H** | **D** | **V** | **N** | **L** | **T** | **S** | **E** | **M** | **K** | **D** | **S** | **F** | **I** | **D** | **Y** | **A** | **M** | **S** | **V** | **I** | **V** | **A** | **R** | **A** | **L** | **P** | **D** | **V** | **R** | **D** | **G** | **L** | **K** | **P** | **V** | **H** | **R** | **R** | **I** | **L** | **Y** | **G** | **M** | **N** | **E** | **L** | **G** | **V** | **T** | **P** | **D** | **K** | **A** | **H** | **K** | **K** | **S** | **A** | **R** | **I** | **V** | **G** | **D** | **V** | **M** | **G** | **K** | **Y** | **H** | **P** | **H** | **G** | **D** | **S** | **A** | **I** | **Y** | **E** | **S** | **M** | **V** | **R** | **M** | **A** | **Q** | **P** | **F** | **S** | **Y** | **R** | **Y** | **M** | **L** | **V** | **D** | **G** | **H** | **G** | **N** | **F** | **G** | **S** | **V** | **D** | **G** | **D** | **G** | **A** | **A** | **A** | **M** | **R** | **Y** | **T** | **E** | **A** | **R** | **M** | **S** | **K** | **I** | **A** | **T** | **E** | **M** | **L** | **R** | **D** | **I** | **N** | **K** | **N** | **T** | **V** | **D** | **F** | **Q** | **S** | **N** | **Y** | **D** | **D** | **T** | **E** | **K** | **E** | **P** | **V** | **V** | **L** | **P** | **A** | **R** | **F** | **P** | **N** | **L** | **L** | **V** | **N** | **G** | **T** | **T** | **G** | **I** | **A** | **V** | **G** | **M** | **A** | **T** | **N** | **I** | **P** | **P** | **H** | **N** | **L** | **S** | **E** | **V** | **V** | **A** | **A** | **I** | **D** | **L** | **L** | **M** | **D** | **N** | **P** | **D** | **V** | **T** | **T** | **N** | **E** | **L** | **M** | **E** | **V** | **L** | **P** | **G** | **P** | **D** | **F** | **P** | **T** | **G** | **G** | **L** | **V** | **M** | **G** | **K** | **S** | **G** | **I** | **R** | **R** | **A** | **Y** | **E** | **T** | **G** | **K** | **G** | **S** | **I** | **T** | **V** | **R** | **A** | **K** | **V** | **E** | **I** | **T** | **E** | **M** | **T** | **N** | **G** | **K** | **E** | **R** | **I** | **I** | **V** | **T** | **E** | **L** | **P** | **Y** | **M** | **V** | **N** | **K** | **A** | **K** | **L** | **I** | **E** | **R** | **I** | **S** | **E** | **L** | **H** | **R** | **D** | **K** | **R** | **I** | **E** | **G** | **I** | **T** | **D** | **L** | **R** | **D** | **E** | **S** | **S** | **R** | **E** | **G** | **M** | **R** | **I** | **V** | **I** | **D** | **I** | **R** | **R** | **D** | **A** | **S** | **A** | **S** | **V** | **I** | **L** | **N** | **N** | **L** | **Y** | **K** | **L** | **T** | **S** | **L** | **Q** | **N** | **S** | **F** | **G** | **F** | **N** | **M** | **L** | **A** | **I** | **E** | **K** | **G** | **V** | **P** | **K** | **V** | **L** | **S** | **L** | **K** | **Q** | **I** | **L** | **E** | **N** | **Y** | **I** | **E** | **H** | **Q** | **R** | **E** | **V** | **I** | **T** | **R** | **R** | **T** | **V** | **F** | **E** | **K** | **E** | **K** | **A** | **E** | **A** | **R** | **A** | **H** | **I** | **L** | **E** | **G** | **L** | **R** | **I** | **A** | **L** | **D** | **H** | **I** | **D** | **E** | **I** | **I** | **A** | **I** | **I** | **R** | **G** | **S** | **K** | **S** | **D** | **D** | **E** | **A** | **K** | **A** | **T** | **M** | **I** | **E** | **R** | **F** | **D** | **L** | **S** | **D** | **R** | **Q** | **S** | **Q** | **A** | **I** | **L** | **D** | **M** | **R** | **L** | **R** | **R** | **L** | **T** | **G** | **L** | **E** | **R** | **E** | **K** | **I** | **E** | **N** | **E** | **Y** | **Q** | **E** | **L** | **L** | **K** | **L** | **I** | **E** | **D** | **L** | **T** | **D** | **I** | **L** | **A** | **R** | **P** | **E** | **R** | **V** | **T** | **E** | **I** | **I** | **K** | **N** | **E** | **L** | **A** | **D** | **L** | **N** | **Q** | **R** | **F** | **G** | **D** | **K** | **R** | **R** | **T** | **E** | **L** | **L** | **V** | **G** | **E** | **V** | **L** | **S** | **L** | **E** | **D** | **E** | **D** | **L** | **I** | **E** | **E** | **E** | **E** | **I** | **V** | **I** | **T** | **L** | **T** | **N** | **N** | **G** | **Y** | **I** | **K** | **R** | **L** | **A** | **N** | **N** | **E** | **F** | **R** | **A** | **Q** | **R** | **R** | **G** | **G** | **R** | **G** | **V** | **Q** | **G** | **M** | **G** | **I** | **H** | **N** | **D** | **D** | **F** | **V** | **K** | **N** | **L** | **V** | **S** | **C** | **S** | **T** | **H** | **D** | **T** | **L** | **L** | **F** | **F** | **T** | **N** | **N** | **G** | **K** | **V** | **Y** | **R** | **A** | **K** | **G** | **Y** | **E** | **I** | **P** | **E** | **Y** | **G** | **R** | **T** | **A** | **K** | **G** | **I** | **P** | **I** | **I** | **N** | **L** | **L** | **G** | **I** | **D** | **S** | **S** | **E** | **S** | **I** | **Q** | **A** | **I** | **I** | **A** | **V** | **T** | **G** | **E** | **A** | **E** | **E** | **G** | **H** | **Y** | **L** | **F** | **F** | **T** | **T** | **R** | **Q** | **G** | **T** | **V** | **K** | **R** | **T** | **S** | **V** | **T** | **A** | **F** | **A** | **N** | **I** | **R** | **S** | **N** | **G** | **L** | **I** | **A** | **I** | **G** | **L** | **K** | **E** | **D** | **D** | **E** | **L** | **V** | **N** | **V** | **L** | **L** | **T** | **D** | **G** | **Q** | **S** | **N** | **I** | **I** | **I** | **G** | **T** | **H** | **N** | **G** | **Y** | **S** | **V** | **T** | **F** | **K** | **E** | **E** | **A** | **V** | **R** | **D** | **M** | **G** | **R** | **T** | **A** | **S** | **G** | **V** | **R** | **G** | **I** | **R** | **L** | **R** | **E** | **E** | **D** | **Y** | **V** | **V** | **G** | **A** | **A** | **L** | **M** | **K** | **E** | **D** | **Q** | **E** | **V** | **L** | **V** | **I** | **T** | **E** | **K** | **G** | **Y** | **G** | **K** | **R** | **T** | **K** | **V** | **S** | **E** | **Y** | **P** | **V** | **K** | **G** | **R** | **G** | **G** | **K** | **G** | **I** | **K** | **T** | **A** | **N** | **I** | **T** | **E** | **K** | **N** | **G** | **P** | **L** | **A** | **G** | **L** | **T** | **T** | **V** | **S** | **G** | **D** | **E** | **D** | **I** | **M** | **L** | **I** | **T** | **D** | **K** | **G** | **V** | **I** | **I** | **R** | **F** | **N** | **V** | **S** | **T** | **V** | **S** | **Q** | **T** | **G** | **R** | **S** | **T** | **L** | **G** | **V** | **R** | **L** | **M** | **K** | **M** | **E** | **A** | **D** | **T** | **K** | **V** | **V** | **T** | **M** | **A** | **A** | **V** | **E** | **P** | **E** | **A** | **A** | **E** | **Q** | **T** | **V** | **E** | **E** | **Q** | **G** | **S** | **E** | **E** |
| **N59613F** | **M** | **S** | **E** | **E** | **I** | **R** | **E** | **N** | **I** | **H** | **D** | **V** | **N** | **L** | **T** | **S** | **E** | **M** | **K** | **D** | **S** | **F** | **I** | **D** | **Y** | **A** | **M** | **S** | **V** | **I** | **V** | **A** | **R** | **A** | **L** | **P** | **D** | **V** | **R** | **D** | **G** | **L** | **K** | **P** | **V** | **H** | **R** | **R** | **I** | **L** | **Y** | **G** | **M** | **N** | **E** | **L** | **G** | **V** | **T** | **P** | **D** | **K** | **A** | **H** | **K** | **K** | **S** | **A** | **R** | **I** | **V** | **G** | **D** | **V** | **M** | **G** | **K** | **Y** | **H** | **P** | **H** | **G** | **D** | **S** | **A** | **I** | **Y** | **E** | **S** | **M** | **V** | **R** | **M** | **A** | **Q** | **P** | **F** | **S** | **Y** | **R** | **Y** | **M** | **L** | **V** | **D** | **G** | **H** | **G** | **N** | **F** | **G** | **S** | **V** | **D** | **G** | **D** | **G** | **A** | **A** | **A** | **M** | **R** | **Y** | **T** | **E** | **A** | **R** | **M** | **S** | **K** | **I** | **A** | **T** | **E** | **M** | **L** | **R** | **D** | **I** | **N** | **K** | **N** | **T** | **V** | **D** | **F** | **Q** | **S** | **N** | **Y** | **D** | **D** | **T** | **E** | **K** | **E** | **P** | **V** | **V** | **L** | **P** | **A** | **R** | **F** | **P** | **N** | **L** | **L** | **V** | **N** | **G** | **T** | **T** | **G** | **I** | **A** | **V** | **G** | **M** | **A** | **T** | **N** | **I** | **P** | **P** | **H** | **N** | **L** | **S** | **E** | **V** | **V** | **A** | **A** | **I** | **D** | **L** | **L** | **M** | **D** | **N** | **P** | **D** | **V** | **T** | **T** | **N** | **E** | **L** | **M** | **E** | **V** | **L** | **P** | **G** | **P** | **D** | **F** | **P** | **T** | **G** | **G** | **L** | **V** | **M** | **G** | **K** | **S** | **G** | **I** | **R** | **R** | **A** | **Y** | **E** | **T** | **G** | **K** | **G** | **S** | **I** | **T** | **V** | **R** | **A** | **K** | **V** | **E** | **I** | **T** | **E** | **M** | **P** | **N** | **G** | **K** | **E** | **R** | **I** | **L** | **V** | **T** | **E** | **L** | **P** | **Y** | **M** | **V** | **N** | **K** | **A** | **K** | **L** | **I** | **E** | **R** | **I** | **S** | **E** | **L** | **H** | **R** | **D** | **K** | **R** | **I** | **E** | **G** | **I** | **T** | **D** | **L** | **R** | **D** | **E** | **S** | **S** | **R** | **E** | **G** | **M** | **R** | **I** | **V** | **I** | **D** | **I** | **R** | **R** | **D** | **A** | **S** | **A** | **S** | **V** | **I** | **L** | **N** | **N** | **L** | **Y** | **K** | **L** | **T** | **S** | **L** | **Q** | **N** | **S** | **F** | **G** | **F** | **N** | **M** | **L** | **A** | **I** | **E** | **K** | **G** | **V** | **P** | **K** | **V** | **L** | **S** | **L** | **K** | **Q** | **I** | **L** | **E** | **N** | **Y** | **I** | **E** | **H** | **Q** | **R** | **E** | **V** | **I** | **T** | **R** | **R** | **T** | **V** | **F** | **E** | **K** | **E** | **K** | **A** | **E** | **A** | **R** | **A** | **H** | **I** | **L** | **E** | **G** | **L** | **R** | **I** | **A** | **L** | **D** | **H** | **I** | **D** | **E** | **I** | **I** | **A** | **I** | **I** | **R** | **G** | **S** | **K** | **S** | **D** | **D** | **E** | **A** | **K** | **A** | **I** | **M** | **I** | **E** | **R** | **F** | **D** | **L** | **S** | **D** | **R** | **Q** | **S** | **Q** | **A** | **I** | **L** | **D** | **M** | **R** | **L** | **R** | **R** | **L** | **T** | **G** | **L** | **E** | **R** | **E** | **K** | **I** | **E** | **N** | **E** | **Y** | **Q** | **E** | **L** | **L** | **K** | **L** | **I** | **E** | **D** | **L** | **T** | **D** | **I** | **L** | **A** | **R** | **P** | **E** | **R** | **V** | **T** | **E** | **I** | **I** | **K** | **N** | **E** | **L** | **A** | **D** | **L** | **N** | **Q** | **R** | **F** | **G** | **D** | **K** | **R** | **R** | **T** | **E** | **L** | **L** | **V** | **G** | **E** | **V** | **L** | **S** | **L** | **E** | **D** | **E** | **D** | **L** | **I** | **E** | **E** | **E** | **E** | **I** | **V** | **I** | **T** | **L** | **T** | **N** | **N** | **G** | **Y** | **I** | **K** | **R** | **L** | **A** | **N** | **N** | **E** | **F** | **R** | **A** | **Q** | **R** | **R** | **G** | **G** | **R** | **G** | **V** | **Q** | **G** | **M** | **G** | **I** | **H** | **N** | **D** | **D** | **F** | **V** | **K** | **N** | **L** | **V** | **S** | **C** | **S** | **T** | **H** | **D** | **T** | **L** | **L** | **F** | **F** | **T** | **N** | **N** | **G** | **K** | **V** | **Y** | **R** | **A** | **K** | **G** | **Y** | **E** | **I** | **P** | **E** | **Y** | **G** | **R** | **T** | **A** | **K** | **G** | **I** | **P** | **I** | **I** | **N** | **L** | **L** | **G** | **I** | **D** | **S** | **S** | **E** | **S** | **I** | **Q** | **A** | **I** | **I** | **A** | **V** | **T** | **G** | **E** | **A** | **E** | **E** | **G** | **H** | **Y** | **L** | **F** | **F** | **T** | **T** | **R** | **Q** | **G** | **T** | **V** | **K** | **R** | **T** | **S** | **V** | **T** | **A** | **F** | **A** | **N** | **I** | **R** | **S** | **N** | **G** | **L** | **I** | **A** | **I** | **G** | **L** | **K** | **E** | **D** | **D** | **E** | **L** | **V** | **N** | **V** | **L** | **L** | **T** | **D** | **G** | **Q** | **S** | **N** | **I** | **I** | **I** | **G** | **T** | **H** | **N** | **G** | **Y** | **S** | **V** | **T** | **F** | **K** | **E** | **E** | **A** | **V** | **R** | **D** | **M** | **G** | **R** | **T** | **A** | **S** | **G** | **V** | **R** | **G** | **I** | **R** | **L** | **R** | **E** | **E** | **D** | **Y** | **V** | **V** | **G** | **A** | **A** | **L** | **M** | **K** | **E** | **D** | **Q** | **E** | **V** | **L** | **V** | **I** | **T** | **E** | **K** | **G** | **Y** | **G** | **K** | **R** | **T** | **K** | **V** | **S** | **E** | **Y** | **P** | **V** | **K** | **G** | **R** | **G** | **G** | **K** | **G** | **I** | **K** | **T** | **A** | **N** | **I** | **T** | **E** | **K** | **N** | **G** | **P** | **L** | **A** | **G** | **L** | **T** | **T** | **V** | **S** | **G** | **D** | **E** | **D** | **I** | **M** | **L** | **I** | **T** | **D** | **K** | **G** | **V** | **I** | **I** | **R** | **F** | **N** | **V** | **S** | **T** | **V** | **S** | **Q** | **T** | **G** | **R** | **S** | **T** | **L** | **G** | **V** | **R** | **L** | **M** | **K** | **M** | **E** | **A** | **D** | **T** | **K** | **V** | **V** | **T** | **M** | **-** | **-** | **-** | **-** | **-** | **-** | **-** | **-** | **-** | **-** | **-** | **-** | **-** | **-** | **-** | **-** | **-** | **-** | **-** |
| **N59508F** | **M** | **S** | **E** | **E** | **I** | **K** | **E** | **N** | **I** | **Q** | **D** | **V** | **N** | **L** | **T** | **S** | **E** | **M** | **K** | **E** | **S** | **F** | **I** | **D** | **Y** | **A** | **M** | **S** | **V** | **I** | **V** | **A** | **R** | **A** | **L** | **P** | **D** | **V** | **R** | **D** | **G** | **L** | **K** | **P** | **V** | **H** | **R** | **R** | **I** | **L** | **Y** | **G** | **M** | **N** | **E** | **L** | **G** | **V** | **T** | **P** | **D** | **K** | **P** | **H** | **K** | **K** | **S** | **A** | **R** | **I** | **V** | **G** | **D** | **V** | **M** | **G** | **K** | **Y** | **H** | **P** | **H** | **G** | **D** | **S** | **A** | **I** | **Y** | **E** | **S** | **M** | **V** | **R** | **M** | **A** | **Q** | **P** | **F** | **S** | **Y** | **R** | **A** | **M** | **L** | **V** | **D** | **G** | **H** | **G** | **N** | **F** | **G** | **S** | **V** | **D** | **G** | **D** | **G** | **A** | **A** | **A** | **M** | **R** | **Y** | **T** | **E** | **A** | **R** | **M** | **S** | **K** | **I** | **A** | **L** | **E** | **M** | **L** | **R** | **D** | **I** | **N** | **K** | **N** | **T** | **V** | **D** | **F** | **Q** | **G** | **N** | **Y** | **D** | **D** | **S** | **E** | **Q** | **E** | **P** | **V** | **V** | **L** | **P** | **A** | **R** | **F** | **P** | **N** | **L** | **L** | **V** | **N** | **G** | **T** | **T** | **G** | **I** | **A** | **V** | **G** | **M** | **A** | **T** | **N** | **I** | **P** | **P** | **H** | **N** | **L** | **S** | **E** | **V** | **I** | **D** | **A** | **T** | **S** | **L** | **L** | **M** | **D** | **N** | **P** | **D** | **V** | **T** | **T** | **N** | **E** | **L** | **M** | **E** | **V** | **L** | **P** | **G** | **P** | **D** | **F** | **P** | **T** | **G** | **G** | **L** | **V** | **M** | **G** | **K** | **S** | **G** | **I** | **R** | **R** | **A** | **Y** | **E** | **T** | **G** | **K** | **G** | **S** | **I** | **T** | **V** | **R** | **A** | **K** | **V** | **E** | **L** | **T** | **E** | **M** | **P** | **N** | **G** | **K** | **E** | **R** | **I** | **L** | **V** | **T** | **E** | **L** | **P** | **Y** | **M** | **V** | **N** | **K** | **A** | **K** | **L** | **I** | **E** | **R** | **I** | **S** | **E** | **L** | **H** | **R** | **D** | **K** | **R** | **I** | **E** | **G** | **I** | **T** | **D** | **L** | **R** | **D** | **E** | **S** | **S** | **R** | **E** | **G** | **M** | **R** | **I** | **V** | **I** | **D** | **V** | **R** | **R** | **D** | **V** | **S** | **A** | **S** | **V** | **V** | **L** | **N** | **N** | **L** | **Y** | **K** | **M** | **T** | **A** | **L** | **Q** | **T** | **S** | **F** | **G** | **F** | **N** | **M** | **L** | **A** | **I** | **E** | **K** | **G** | **V** | **P** | **K** | **I** | **L** | **S** | **L** | **K** | **R** | **I** | **L** | **E** | **N** | **Y** | **V** | **E** | **H** | **Q** | **K** | **E** | **V** | **I** | **T** | **R** | **R** | **T** | **I** | **F** | **D** | **K** | **N** | **K** | **A** | **E** | **A** | **R** | **A** | **H** | **I** | **L** | **E** | **G** | **L** | **R** | **I** | **A** | **L** | **D** | **H** | **I** | **D** | **E** | **I** | **I** | **A** | **I** | **I** | **R** | **G** | **S** | **Q** | **S** | **D** | **D** | **E** | **A** | **K** | **A** | **T** | **L** | **I** | **E** | **R** | **F** | **E** | **F** | **S** | **D** | **R** | **Q** | **A** | **Q** | **A** | **I** | **L** | **D** | **M** | **R** | **L** | **R** | **R** | **L** | **T** | **G** | **L** | **E** | **R** | **D** | **K** | **I** | **E** | **N** | **E** | **Y** | **Q** | **E** | **L** | **L** | **K** | **F** | **I** | **A** | **D** | **L** | **E** | **D** | **I** | **L** | **A** | **R** | **P** | **E** | **R** | **V** | **I** | **E** | **I** | **I** | **K** | **T** | **E** | **L** | **N** | **D** | **V** | **R** | **T** | **K** | **F** | **G** | **D** | **A** | **R** | **R** | **T** | **E** | **L** | **L** | **V** | **G** | **E** | **V** | **L** | **S** | **L** | **E** | **D** | **E** | **D** | **L** | **I** | **E** | **E** | **E** | **E** | **V** | **V** | **I** | **T** | **L** | **T** | **N** | **N** | **G** | **Y** | **I** | **K** | **R** | **M** | **A** | **N** | **S** | **E** | **F** | **R** | **A** | **Q** | **R** | **R** | **G** | **G** | **R** | **G** | **V** | **Q** | **G** | **M** | **G** | **V** | **H** | **D** | **D** | **D** | **F** | **V** | **K** | **N** | **L** | **V** | **S** | **C** | **S** | **T** | **H** | **D** | **T** | **L** | **L** | **F** | **F** | **T** | **N** | **T** | **G** | **K** | **V** | **Y** | **R** | **A** | **K** | **G** | **Y** | **E** | **I** | **P** | **E** | **Y** | **G** | **R** | **T** | **A** | **K** | **G** | **I** | **P** | **V** | **I** | **N** | **L** | **L** | **G** | **I** | **D** | **S** | **A** | **E** | **K** | **I** | **Q** | **A** | **I** | **I** | **S** | **V** | **E** | **G** | **K** | **A** | **E** | **A** | **G** | **K** | **Y** | **L** | **F** | **F** | **T** | **T** | **L** | **K** | **G** | **T** | **V** | **K** | **R** | **T** | **A** | **V** | **T** | **A** | **F** | **S** | **N** | **I** | **R** | **S** | **N** | **G** | **L** | **I** | **A** | **I** | **S** | **L** | **K** | **E** | **D** | **D** | **E** | **L** | **V** | **N** | **V** | **V** | **T** | **T** | **N** | **G** | **N** | **Q** | **K** | **M** | **I** | **I** | **G** | **T** | **H** | **A** | **G** | **Y** | **S** | **V** | **T** | **F** | **D** | **E** | **N** | **T** | **V** | **R** | **D** | **M** | **G** | **R** | **T** | **A** | **S** | **G** | **V** | **R** | **G** | **I** | **R** | **L** | **R** | **E** | **N** | **D** | **Y** | **V** | **V** | **G** | **A** | **A** | **I** | **L** | **D** | **E** | **N** | **K** | **E** | **V** | **L** | **V** | **I** | **T** | **E** | **N** | **G** | **Y** | **G** | **K** | **R** | **T** | **K** | **A** | **S** | **E** | **Y** | **P** | **V** | **K** | **G** | **R** | **G** | **G** | **K** | **G** | **I** | **K** | **T** | **A** | **N** | **I** | **T** | **E** | **K** | **N** | **G** | **P** | **L** | **A** | **G** | **L** | **T** | **T** | **V** | **N** | **G** | **D** | **E** | **D** | **I** | **L** | **L** | **I** | **T** | **N** | **K** | **G** | **V** | **I** | **I** | **R** | **F** | **N** | **V** | **D** | **S** | **V** | **S** | **Q** | **T** | **G** | **R** | **A** | **T** | **L** | **G** | **V** | **R** | **L** | **M** | **R** | **M** | **E** | **D** | **G** | **A** | **K** | **-** | **-** | **-** | **-** | **-** | **-** | **-** | **-** | **-** | **-** | **-** | **-** | **-** | **-** | **-** | **-** | **-** | **-** | **-** | **-** | **-** | **-** | **-** |
| **N52459** | **M** | **S** | **E** | **E** | **I** | **R** | **E** | **N** | **I** | **H** | **D** | **V** | **N** | **L** | **T** | **S** | **E** | **M** | **K** | **D** | **S** | **F** | **I** | **D** | **Y** | **A** | **M** | **S** | **V** | **I** | **V** | **A** | **R** | **A** | **L** | **P** | **D** | **V** | **R** | **D** | **G** | **L** | **K** | **P** | **V** | **H** | **R** | **R** | **I** | **L** | **Y** | **G** | **M** | **N** | **E** | **L** | **G** | **V** | **T** | **P** | **D** | **K** | **A** | **H** | **K** | **K** | **S** | **A** | **R** | **I** | **V** | **G** | **D** | **V** | **M** | **G** | **K** | **Y** | **H** | **P** | **H** | **G** | **D** | **S** | **A** | **I** | **Y** | **E** | **S** | **M** | **V** | **R** | **M** | **A** | **Q** | **P** | **F** | **S** | **Y** | **R** | **Y** | **M** | **L** | **V** | **D** | **G** | **H** | **G** | **N** | **F** | **G** | **S** | **V** | **D** | **G** | **D** | **G** | **A** | **A** | **A** | **M** | **R** | **Y** | **T** | **E** | **A** | **R** | **M** | **S** | **K** | **I** | **A** | **T** | **E** | **M** | **L** | **R** | **D** | **I** | **N** | **K** | **N** | **T** | **V** | **D** | **F** | **Q** | **S** | **N** | **Y** | **D** | **D** | **T** | **E** | **K** | **E** | **P** | **V** | **V** | **L** | **P** | **A** | **R** | **F** | **P** | **N** | **L** | **L** | **V** | **N** | **G** | **T** | **T** | **G** | **I** | **A** | **V** | **G** | **M** | **A** | **T** | **N** | **I** | **P** | **P** | **H** | **N** | **L** | **S** | **E** | **V** | **V** | **A** | **A** | **I** | **D** | **L** | **L** | **M** | **D** | **N** | **P** | **D** | **V** | **T** | **T** | **N** | **E** | **L** | **M** | **E** | **V** | **L** | **P** | **G** | **P** | **D** | **F** | **P** | **T** | **G** | **G** | **L** | **V** | **M** | **G** | **K** | **S** | **G** | **I** | **R** | **R** | **A** | **Y** | **E** | **T** | **G** | **K** | **G** | **S** | **I** | **T** | **V** | **R** | **A** | **K** | **V** | **E** | **I** | **T** | **E** | **M** | **P** | **N** | **G** | **K** | **E** | **R** | **I** | **I** | **V** | **T** | **E** | **L** | **P** | **Y** | **M** | **V** | **N** | **K** | **A** | **K** | **L** | **I** | **E** | **R** | **I** | **S** | **E** | **L** | **H** | **R** | **D** | **K** | **R** | **I** | **E** | **G** | **I** | **T** | **D** | **L** | **R** | **D** | **E** | **S** | **S** | **R** | **E** | **G** | **M** | **R** | **I** | **V** | **I** | **D** | **I** | **R** | **R** | **D** | **A** | **S** | **A** | **S** | **V** | **I** | **L** | **N** | **N** | **L** | **Y** | **K** | **L** | **T** | **S** | **L** | **Q** | **N** | **S** | **F** | **G** | **F** | **N** | **M** | **L** | **A** | **I** | **E** | **K** | **G** | **V** | **P** | **K** | **V** | **L** | **S** | **L** | **K** | **Q** | **I** | **L** | **E** | **N** | **Y** | **I** | **E** | **H** | **Q** | **R** | **K** | **V** | **I** | **T** | **R** | **R** | **T** | **V** | **F** | **E** | **K** | **E** | **K** | **A** | **E** | **A** | **R** | **A** | **H** | **I** | **L** | **E** | **G** | **L** | **R** | **I** | **A** | **L** | **D** | **H** | **I** | **D** | **E** | **I** | **I** | **A** | **I** | **I** | **R** | **G** | **S** | **K** | **S** | **D** | **D** | **E** | **A** | **K** | **A** | **T** | **M** | **I** | **E** | **R** | **F** | **D** | **L** | **S** | **D** | **R** | **Q** | **S** | **Q** | **A** | **I** | **L** | **D** | **M** | **R** | **L** | **R** | **R** | **L** | **T** | **G** | **L** | **E** | **R** | **E** | **K** | **I** | **E** | **N** | **E** | **Y** | **Q** | **E** | **L** | **L** | **K** | **L** | **I** | **E** | **D** | **L** | **T** | **D** | **I** | **L** | **A** | **R** | **P** | **E** | **R** | **V** | **T** | **E** | **I** | **I** | **K** | **N** | **E** | **L** | **A** | **D** | **L** | **N** | **Q** | **R** | **F** | **G** | **D** | **K** | **R** | **R** | **T** | **E** | **L** | **L** | **V** | **G** | **E** | **V** | **L** | **S** | **L** | **E** | **D** | **E** | **D** | **L** | **I** | **E** | **E** | **E** | **E** | **I** | **V** | **I** | **T** | **L** | **T** | **N** | **N** | **G** | **Y** | **I** | **K** | **R** | **L** | **A** | **N** | **N** | **E** | **F** | **R** | **A** | **Q** | **R** | **R** | **G** | **G** | **R** | **G** | **V** | **Q** | **G** | **M** | **G** | **I** | **H** | **N** | **D** | **D** | **F** | **V** | **K** | **N** | **L** | **V** | **S** | **C** | **S** | **T** | **H** | **D** | **T** | **L** | **L** | **F** | **F** | **T** | **N** | **N** | **G** | **K** | **V** | **Y** | **R** | **A** | **K** | **G** | **Y** | **E** | **I** | **P** | **E** | **Y** | **G** | **R** | **T** | **A** | **K** | **G** | **I** | **P** | **I** | **I** | **N** | **L** | **L** | **G** | **I** | **D** | **S** | **S** | **E** | **S** | **I** | **Q** | **A** | **I** | **I** | **A** | **V** | **T** | **G** | **E** | **A** | **E** | **E** | **G** | **H** | **Y** | **L** | **F** | **F** | **T** | **T** | **R** | **Q** | **G** | **T** | **V** | **K** | **R** | **T** | **S** | **V** | **T** | **A** | **F** | **A** | **N** | **I** | **R** | **S** | **N** | **G** | **L** | **I** | **A** | **I** | **G** | **L** | **K** | **E** | **D** | **D** | **E** | **L** | **V** | **N** | **V** | **L** | **L** | **T** | **D** | **G** | **Q** | **S** | **N** | **I** | **I** | **I** | **G** | **T** | **H** | **N** | **G** | **Y** | **S** | **V** | **T** | **F** | **K** | **E** | **E** | **A** | **V** | **R** | **D** | **M** | **G** | **R** | **T** | **A** | **S** | **G** | **V** | **R** | **G** | **I** | **R** | **L** | **R** | **E** | **E** | **D** | **Y** | **V** | **V** | **G** | **A** | **A** | **L** | **M** | **K** | **E** | **N** | **Q** | **E** | **V** | **L** | **V** | **I** | **T** | **E** | **K** | **G** | **Y** | **G** | **K** | **R** | **T** | **K** | **V** | **S** | **E** | **Y** | **P** | **V** | **K** | **G** | **R** | **G** | **G** | **K** | **G** | **I** | **K** | **T** | **A** | **N** | **I** | **T** | **E** | **K** | **N** | **G** | **P** | **L** | **A** | **G** | **L** | **T** | **T** | **V** | **S** | **G** | **D** | **E** | **D** | **I** | **M** | **L** | **I** | **T** | **D** | **K** | **G** | **V** | **I** | **I** | **R** | **F** | **N** | **V** | **S** | **T** | **V** | **S** | **Q** | **T** | **G** | **R** | **S** | **T** | **L** | **G** | **V** | **R** | **L** | **M** | **K** | **M** | **E** | **A** | **D** | **T** | **K** | **V** | **V** | **T** | **M** | **A** | **A** | **V** | **E** | **P** | **E** | **A** | **A** | **E** | **Q** | **T** | **V** | **E** | **E** | **Q** | **G** | **S** | **E** | **E** |
| **N59599F** | **M** | **S** | **E** | **E** | **I** | **K** | **E** | **N** | **I** | **Q** | **D** | **V** | **N** | **L** | **T** | **S** | **E** | **M** | **K** | **E** | **S** | **F** | **I** | **D** | **Y** | **A** | **M** | **S** | **V** | **I** | **V** | **A** | **R** | **A** | **L** | **P** | **D** | **V** | **R** | **D** | **G** | **L** | **K** | **P** | **V** | **H** | **R** | **R** | **I** | **L** | **Y** | **G** | **M** | **N** | **E** | **L** | **G** | **V** | **T** | **P** | **D** | **K** | **P** | **H** | **K** | **K** | **S** | **A** | **R** | **I** | **V** | **G** | **D** | **V** | **M** | **G** | **K** | **Y** | **H** | **P** | **H** | **G** | **D** | **S** | **A** | **I** | **Y** | **E** | **S** | **M** | **V** | **R** | **M** | **A** | **Q** | **P** | **F** | **S** | **Y** | **R** | **A** | **M** | **L** | **V** | **D** | **G** | **H** | **G** | **N** | **F** | **G** | **S** | **V** | **D** | **G** | **D** | **G** | **A** | **A** | **A** | **M** | **R** | **Y** | **T** | **E** | **A** | **R** | **M** | **S** | **K** | **I** | **A** | **L** | **E** | **M** | **L** | **R** | **D** | **I** | **N** | **K** | **N** | **T** | **V** | **D** | **F** | **Q** | **G** | **N** | **Y** | **D** | **D** | **S** | **E** | **Q** | **E** | **P** | **V** | **V** | **L** | **P** | **A** | **R** | **F** | **P** | **N** | **L** | **L** | **V** | **N** | **G** | **T** | **T** | **G** | **I** | **A** | **V** | **G** | **M** | **A** | **T** | **N** | **I** | **P** | **P** | **H** | **N** | **L** | **S** | **E** | **V** | **I** | **D** | **A** | **T** | **S** | **L** | **L** | **M** | **D** | **N** | **P** | **D** | **V** | **T** | **T** | **N** | **E** | **L** | **M** | **E** | **V** | **L** | **P** | **G** | **P** | **D** | **F** | **P** | **T** | **G** | **G** | **L** | **V** | **M** | **G** | **K** | **S** | **G** | **I** | **R** | **R** | **A** | **Y** | **E** | **T** | **G** | **K** | **G** | **S** | **I** | **T** | **V** | **R** | **A** | **K** | **V** | **E** | **L** | **T** | **E** | **M** | **P** | **N** | **G** | **K** | **E** | **R** | **I** | **L** | **V** | **T** | **E** | **L** | **P** | **Y** | **M** | **V** | **N** | **K** | **A** | **K** | **L** | **I** | **E** | **R** | **I** | **S** | **E** | **L** | **H** | **R** | **D** | **K** | **R** | **I** | **E** | **G** | **I** | **T** | **D** | **L** | **R** | **D** | **E** | **S** | **S** | **R** | **E** | **G** | **M** | **R** | **I** | **V** | **I** | **D** | **V** | **R** | **R** | **D** | **V** | **S** | **A** | **S** | **V** | **V** | **L** | **N** | **N** | **L** | **Y** | **K** | **M** | **T** | **A** | **L** | **Q** | **T** | **S** | **F** | **G** | **F** | **N** | **M** | **L** | **A** | **I** | **E** | **K** | **G** | **V** | **P** | **K** | **I** | **L** | **S** | **L** | **K** | **R** | **I** | **L** | **E** | **N** | **Y** | **V** | **E** | **H** | **Q** | **K** | **E** | **V** | **I** | **T** | **R** | **R** | **T** | **I** | **F** | **D** | **K** | **N** | **K** | **A** | **E** | **A** | **R** | **A** | **H** | **I** | **L** | **E** | **G** | **L** | **R** | **I** | **A** | **L** | **D** | **H** | **I** | **D** | **E** | **I** | **I** | **A** | **I** | **I** | **R** | **G** | **S** | **Q** | **S** | **D** | **D** | **E** | **A** | **K** | **A** | **T** | **L** | **I** | **E** | **R** | **F** | **E** | **F** | **S** | **D** | **R** | **Q** | **A** | **Q** | **A** | **I** | **L** | **D** | **M** | **R** | **L** | **R** | **R** | **L** | **T** | **G** | **L** | **E** | **R** | **D** | **K** | **I** | **E** | **N** | **E** | **Y** | **Q** | **E** | **L** | **L** | **K** | **F** | **I** | **A** | **D** | **L** | **E** | **D** | **I** | **L** | **A** | **R** | **P** | **E** | **R** | **V** | **I** | **E** | **I** | **I** | **K** | **T** | **E** | **L** | **N** | **D** | **V** | **R** | **T** | **K** | **F** | **G** | **D** | **A** | **R** | **R** | **T** | **E** | **L** | **L** | **V** | **G** | **E** | **V** | **L** | **S** | **L** | **E** | **D** | **E** | **D** | **L** | **I** | **E** | **E** | **E** | **E** | **V** | **V** | **I** | **T** | **L** | **T** | **N** | **N** | **G** | **Y** | **I** | **K** | **R** | **M** | **A** | **N** | **S** | **E** | **F** | **R** | **A** | **Q** | **R** | **R** | **G** | **G** | **R** | **G** | **V** | **Q** | **G** | **M** | **G** | **V** | **H** | **D** | **D** | **D** | **F** | **V** | **K** | **N** | **L** | **V** | **S** | **C** | **S** | **T** | **H** | **D** | **T** | **L** | **L** | **F** | **F** | **T** | **N** | **T** | **G** | **K** | **V** | **Y** | **R** | **A** | **K** | **G** | **Y** | **E** | **I** | **P** | **E** | **Y** | **G** | **R** | **T** | **A** | **K** | **G** | **I** | **P** | **V** | **I** | **N** | **L** | **L** | **G** | **I** | **D** | **S** | **A** | **E** | **K** | **I** | **Q** | **A** | **I** | **I** | **S** | **V** | **E** | **G** | **K** | **A** | **E** | **A** | **G** | **K** | **Y** | **L** | **F** | **F** | **T** | **T** | **L** | **K** | **G** | **T** | **V** | **K** | **R** | **T** | **A** | **V** | **T** | **A** | **F** | **S** | **N** | **I** | **R** | **S** | **N** | **G** | **L** | **I** | **A** | **I** | **S** | **L** | **K** | **E** | **D** | **D** | **E** | **L** | **V** | **N** | **V** | **V** | **T** | **T** | **N** | **G** | **N** | **Q** | **K** | **M** | **I** | **I** | **G** | **T** | **H** | **A** | **G** | **Y** | **S** | **V** | **T** | **F** | **D** | **E** | **N** | **T** | **V** | **R** | **D** | **M** | **G** | **R** | **T** | **A** | **S** | **G** | **V** | **R** | **G** | **I** | **R** | **L** | **R** | **E** | **N** | **D** | **Y** | **V** | **V** | **G** | **A** | **A** | **I** | **L** | **D** | **E** | **N** | **K** | **E** | **V** | **L** | **V** | **I** | **T** | **E** | **N** | **G** | **Y** | **G** | **K** | **R** | **T** | **K** | **A** | **S** | **E** | **Y** | **P** | **V** | **K** | **G** | **R** | **G** | **G** | **K** | **G** | **I** | **K** | **T** | **A** | **N** | **I** | **T** | **E** | **K** | **N** | **G** | **P** | **L** | **A** | **G** | **L** | **T** | **T** | **V** | **N** | **G** | **N** | **E** | **D** | **I** | **L** | **L** | **I** | **T** | **N** | **K** | **G** | **V** | **I** | **I** | **R** | **F** | **N** | **V** | **D** | **S** | **V** | **S** | **Q** | **T** | **G** | **R** | **A** | **T** | **L** | **G** | **V** | **R** | **L** | **M** | **R** | **M** | **E** | **D** | **G** | **A** | **K** | **-** | **-** | **-** | **-** | **-** | **-** | **-** | **-** | **-** | **-** | **-** | **-** | **-** | **-** | **-** | **-** | **-** | **-** | **-** | **-** | **-** | **-** | **-** |
| **N54121** | **M** | **S** | **E** | **E** | **I** | **K** | **E** | **N** | **I** | **Q** | **D** | **V** | **N** | **L** | **T** | **S** | **E** | **M** | **K** | **E** | **S** | **F** | **I** | **D** | **Y** | **A** | **M** | **S** | **V** | **I** | **V** | **A** | **R** | **A** | **L** | **P** | **D** | **V** | **R** | **D** | **G** | **L** | **K** | **P** | **V** | **H** | **R** | **R** | **I** | **L** | **Y** | **G** | **M** | **N** | **E** | **L** | **G** | **V** | **T** | **P** | **D** | **K** | **P** | **H** | **K** | **K** | **S** | **A** | **R** | **I** | **V** | **G** | **D** | **V** | **M** | **G** | **K** | **Y** | **H** | **P** | **H** | **G** | **D** | **S** | **A** | **I** | **Y** | **E** | **S** | **M** | **V** | **R** | **M** | **A** | **Q** | **P** | **F** | **S** | **Y** | **R** | **A** | **M** | **L** | **V** | **D** | **G** | **H** | **G** | **N** | **F** | **G** | **S** | **V** | **D** | **G** | **D** | **G** | **A** | **A** | **A** | **M** | **R** | **Y** | **T** | **E** | **A** | **R** | **M** | **S** | **K** | **I** | **A** | **L** | **E** | **M** | **L** | **R** | **D** | **I** | **N** | **K** | **N** | **T** | **V** | **D** | **F** | **Q** | **G** | **N** | **Y** | **D** | **D** | **S** | **E** | **Q** | **E** | **P** | **V** | **V** | **L** | **P** | **A** | **R** | **F** | **P** | **N** | **L** | **L** | **V** | **N** | **G** | **T** | **T** | **G** | **I** | **A** | **V** | **G** | **M** | **A** | **T** | **N** | **I** | **P** | **P** | **H** | **N** | **L** | **S** | **E** | **V** | **I** | **D** | **A** | **T** | **S** | **L** | **L** | **M** | **D** | **N** | **P** | **D** | **V** | **T** | **T** | **N** | **E** | **L** | **M** | **E** | **V** | **L** | **P** | **G** | **P** | **D** | **F** | **P** | **T** | **G** | **G** | **L** | **V** | **M** | **G** | **K** | **S** | **G** | **I** | **R** | **R** | **A** | **Y** | **E** | **T** | **G** | **K** | **G** | **S** | **I** | **T** | **V** | **R** | **A** | **K** | **V** | **E** | **L** | **T** | **E** | **M** | **P** | **N** | **G** | **K** | **E** | **R** | **I** | **L** | **V** | **T** | **E** | **L** | **P** | **Y** | **M** | **V** | **N** | **K** | **A** | **K** | **L** | **I** | **E** | **R** | **I** | **S** | **E** | **L** | **H** | **R** | **D** | **K** | **R** | **I** | **E** | **G** | **I** | **T** | **D** | **L** | **R** | **D** | **E** | **S** | **S** | **R** | **E** | **G** | **M** | **R** | **I** | **V** | **I** | **D** | **V** | **R** | **R** | **D** | **V** | **S** | **A** | **S** | **V** | **V** | **L** | **N** | **N** | **L** | **Y** | **K** | **M** | **T** | **A** | **L** | **Q** | **T** | **S** | **F** | **G** | **F** | **N** | **M** | **L** | **A** | **I** | **E** | **K** | **G** | **V** | **P** | **K** | **I** | **L** | **S** | **L** | **K** | **R** | **I** | **L** | **E** | **N** | **Y** | **V** | **E** | **H** | **Q** | **K** | **E** | **V** | **I** | **T** | **R** | **R** | **T** | **I** | **F** | **D** | **K** | **N** | **K** | **A** | **E** | **A** | **R** | **A** | **H** | **I** | **L** | **E** | **G** | **L** | **R** | **I** | **A** | **L** | **D** | **H** | **I** | **D** | **E** | **I** | **I** | **A** | **I** | **I** | **R** | **G** | **S** | **Q** | **S** | **D** | **D** | **E** | **A** | **K** | **A** | **T** | **L** | **I** | **E** | **R** | **F** | **E** | **F** | **S** | **D** | **R** | **Q** | **A** | **Q** | **A** | **I** | **L** | **D** | **M** | **R** | **L** | **R** | **R** | **L** | **T** | **G** | **L** | **E** | **R** | **D** | **K** | **I** | **E** | **N** | **E** | **Y** | **Q** | **E** | **L** | **L** | **K** | **F** | **I** | **A** | **D** | **L** | **E** | **D** | **I** | **L** | **A** | **R** | **P** | **E** | **R** | **V** | **I** | **E** | **I** | **I** | **K** | **T** | **E** | **L** | **N** | **D** | **V** | **R** | **T** | **K** | **F** | **G** | **D** | **A** | **R** | **R** | **T** | **E** | **L** | **L** | **V** | **G** | **E** | **V** | **L** | **S** | **L** | **E** | **D** | **E** | **D** | **L** | **I** | **E** | **E** | **E** | **E** | **V** | **V** | **I** | **T** | **L** | **T** | **N** | **N** | **G** | **Y** | **I** | **K** | **R** | **M** | **A** | **N** | **S** | **E** | **F** | **R** | **A** | **Q** | **R** | **R** | **G** | **G** | **R** | **G** | **V** | **Q** | **G** | **M** | **G** | **V** | **H** | **D** | **D** | **D** | **F** | **V** | **K** | **N** | **L** | **V** | **S** | **C** | **S** | **T** | **H** | **D** | **T** | **L** | **L** | **F** | **F** | **T** | **N** | **T** | **G** | **K** | **V** | **Y** | **R** | **A** | **K** | **G** | **Y** | **E** | **I** | **P** | **E** | **Y** | **G** | **R** | **T** | **A** | **K** | **G** | **I** | **P** | **V** | **I** | **N** | **L** | **L** | **G** | **I** | **D** | **S** | **A** | **E** | **K** | **I** | **Q** | **A** | **I** | **I** | **S** | **V** | **E** | **G** | **K** | **A** | **E** | **A** | **G** | **K** | **Y** | **L** | **F** | **F** | **T** | **T** | **L** | **K** | **G** | **T** | **V** | **K** | **R** | **T** | **A** | **V** | **T** | **A** | **F** | **S** | **N** | **I** | **R** | **S** | **N** | **G** | **L** | **I** | **A** | **I** | **S** | **L** | **K** | **E** | **D** | **D** | **E** | **L** | **V** | **N** | **V** | **V** | **T** | **T** | **N** | **G** | **N** | **Q** | **K** | **M** | **I** | **I** | **G** | **T** | **H** | **A** | **G** | **Y** | **S** | **V** | **T** | **F** | **D** | **E** | **N** | **T** | **V** | **R** | **D** | **M** | **G** | **R** | **T** | **A** | **S** | **G** | **V** | **R** | **G** | **I** | **R** | **L** | **R** | **E** | **N** | **D** | **Y** | **V** | **V** | **G** | **A** | **A** | **I** | **L** | **D** | **E** | **N** | **K** | **E** | **V** | **L** | **V** | **I** | **T** | **E** | **N** | **G** | **Y** | **G** | **K** | **R** | **T** | **K** | **A** | **S** | **E** | **Y** | **P** | **V** | **K** | **G** | **R** | **G** | **G** | **K** | **G** | **I** | **K** | **T** | **A** | **N** | **I** | **T** | **E** | **K** | **N** | **G** | **P** | **L** | **A** | **G** | **L** | **T** | **T** | **V** | **N** | **G** | **D** | **E** | **D** | **I** | **L** | **L** | **I** | **T** | **N** | **K** | **G** | **V** | **I** | **I** | **R** | **F** | **N** | **V** | **D** | **S** | **V** | **S** | **Q** | **T** | **G** | **R** | **A** | **T** | **L** | **G** | **V** | **R** | **L** | **M** | **R** | **M** | **E** | **D** | **G** | **A** | **K** | **-** | **-** | **-** | **-** | **-** | **-** | **-** | **-** | **-** | **-** | **-** | **-** | **-** | **-** | **-** | **-** | **-** | **-** | **-** | **-** | **-** | **-** | **-** |
| **N52702** | **M** | **S** | **E** | **E** | **I** | **R** | **E** | **N** | **I** | **H** | **D** | **V** | **N** | **L** | **T** | **S** | **E** | **M** | **K** | **D** | **S** | **F** | **I** | **D** | **Y** | **A** | **M** | **S** | **V** | **I** | **V** | **A** | **R** | **A** | **L** | **P** | **D** | **V** | **R** | **D** | **G** | **L** | **K** | **P** | **V** | **H** | **R** | **R** | **I** | **L** | **Y** | **G** | **M** | **N** | **E** | **L** | **G** | **V** | **T** | **P** | **D** | **K** | **A** | **H** | **K** | **K** | **S** | **A** | **R** | **I** | **V** | **G** | **D** | **V** | **M** | **G** | **K** | **Y** | **H** | **P** | **H** | **G** | **D** | **S** | **A** | **I** | **Y** | **E** | **S** | **M** | **V** | **R** | **M** | **A** | **Q** | **P** | **F** | **S** | **Y** | **R** | **Y** | **M** | **L** | **V** | **D** | **G** | **H** | **G** | **N** | **F** | **G** | **S** | **V** | **D** | **G** | **D** | **G** | **A** | **A** | **A** | **M** | **R** | **Y** | **T** | **E** | **A** | **R** | **M** | **S** | **K** | **I** | **A** | **T** | **E** | **M** | **L** | **R** | **D** | **I** | **N** | **K** | **N** | **T** | **V** | **D** | **F** | **Q** | **S** | **N** | **Y** | **D** | **D** | **T** | **E** | **K** | **E** | **P** | **V** | **V** | **L** | **P** | **A** | **R** | **F** | **P** | **N** | **L** | **L** | **V** | **N** | **G** | **T** | **T** | **G** | **I** | **A** | **V** | **G** | **M** | **A** | **T** | **N** | **I** | **P** | **P** | **H** | **N** | **L** | **S** | **E** | **V** | **V** | **A** | **A** | **I** | **D** | **L** | **L** | **M** | **D** | **N** | **P** | **D** | **V** | **T** | **T** | **N** | **E** | **L** | **M** | **E** | **V** | **L** | **P** | **G** | **P** | **D** | **F** | **P** | **T** | **G** | **G** | **L** | **V** | **M** | **G** | **K** | **S** | **G** | **I** | **R** | **R** | **A** | **Y** | **E** | **T** | **G** | **K** | **G** | **S** | **I** | **T** | **V** | **R** | **A** | **K** | **V** | **E** | **I** | **T** | **E** | **M** | **P** | **N** | **G** | **K** | **E** | **R** | **I** | **I** | **V** | **T** | **E** | **L** | **P** | **Y** | **M** | **V** | **N** | **K** | **A** | **K** | **L** | **I** | **E** | **R** | **I** | **S** | **E** | **L** | **H** | **R** | **D** | **K** | **R** | **I** | **E** | **G** | **I** | **T** | **D** | **L** | **R** | **D** | **E** | **S** | **S** | **R** | **E** | **G** | **M** | **R** | **I** | **V** | **I** | **D** | **I** | **R** | **R** | **D** | **A** | **S** | **A** | **S** | **V** | **I** | **L** | **N** | **N** | **L** | **Y** | **K** | **L** | **T** | **S** | **L** | **Q** | **N** | **S** | **F** | **G** | **F** | **N** | **M** | **L** | **A** | **I** | **E** | **K** | **G** | **V** | **P** | **K** | **V** | **L** | **S** | **L** | **K** | **Q** | **I** | **L** | **E** | **N** | **Y** | **I** | **E** | **H** | **Q** | **R** | **E** | **V** | **I** | **T** | **R** | **R** | **T** | **V** | **F** | **E** | **K** | **E** | **K** | **A** | **E** | **A** | **R** | **A** | **H** | **I** | **L** | **E** | **G** | **L** | **R** | **I** | **A** | **L** | **D** | **H** | **I** | **D** | **E** | **I** | **I** | **A** | **I** | **I** | **R** | **G** | **S** | **K** | **S** | **D** | **D** | **E** | **A** | **K** | **A** | **T** | **M** | **I** | **E** | **R** | **F** | **D** | **L** | **S** | **D** | **R** | **Q** | **S** | **Q** | **A** | **I** | **L** | **D** | **M** | **R** | **L** | **R** | **R** | **L** | **T** | **G** | **L** | **E** | **R** | **E** | **K** | **I** | **E** | **N** | **E** | **Y** | **Q** | **E** | **L** | **L** | **K** | **L** | **I** | **E** | **D** | **L** | **T** | **D** | **I** | **L** | **A** | **R** | **P** | **E** | **R** | **V** | **T** | **E** | **I** | **I** | **K** | **N** | **E** | **L** | **A** | **D** | **L** | **N** | **Q** | **R** | **F** | **G** | **D** | **K** | **R** | **R** | **T** | **E** | **L** | **L** | **V** | **G** | **E** | **V** | **L** | **S** | **L** | **E** | **D** | **E** | **D** | **L** | **I** | **E** | **E** | **E** | **E** | **I** | **V** | **I** | **T** | **L** | **T** | **N** | **N** | **G** | **Y** | **I** | **K** | **R** | **L** | **A** | **N** | **N** | **E** | **F** | **R** | **A** | **Q** | **R** | **R** | **G** | **G** | **R** | **G** | **V** | **Q** | **G** | **M** | **G** | **I** | **H** | **N** | **D** | **D** | **F** | **V** | **K** | **N** | **L** | **V** | **S** | **C** | **S** | **T** | **H** | **D** | **T** | **L** | **L** | **F** | **F** | **T** | **N** | **N** | **G** | **K** | **V** | **Y** | **R** | **A** | **K** | **G** | **Y** | **E** | **I** | **P** | **E** | **Y** | **G** | **R** | **T** | **A** | **K** | **G** | **I** | **P** | **I** | **I** | **N** | **L** | **L** | **G** | **I** | **D** | **S** | **S** | **E** | **S** | **I** | **Q** | **A** | **I** | **I** | **A** | **V** | **T** | **G** | **E** | **A** | **E** | **E** | **G** | **H** | **Y** | **L** | **F** | **F** | **T** | **T** | **R** | **Q** | **G** | **T** | **V** | **K** | **R** | **T** | **S** | **V** | **T** | **A** | **F** | **A** | **N** | **I** | **R** | **S** | **N** | **G** | **L** | **I** | **A** | **I** | **G** | **L** | **K** | **E** | **D** | **D** | **E** | **L** | **V** | **N** | **V** | **L** | **L** | **T** | **D** | **G** | **Q** | **S** | **N** | **I** | **I** | **I** | **G** | **T** | **H** | **N** | **G** | **Y** | **S** | **V** | **T** | **F** | **K** | **E** | **E** | **A** | **V** | **R** | **D** | **M** | **G** | **R** | **T** | **A** | **S** | **G** | **V** | **R** | **G** | **I** | **R** | **L** | **R** | **E** | **E** | **D** | **Y** | **V** | **V** | **G** | **A** | **A** | **L** | **M** | **K** | **E** | **N** | **Q** | **E** | **V** | **L** | **V** | **I** | **T** | **E** | **K** | **G** | **Y** | **G** | **K** | **R** | **T** | **K** | **V** | **S** | **E** | **Y** | **P** | **V** | **K** | **G** | **R** | **G** | **G** | **K** | **G** | **I** | **K** | **T** | **A** | **N** | **I** | **T** | **E** | **K** | **N** | **G** | **P** | **L** | **A** | **G** | **L** | **T** | **T** | **V** | **S** | **G** | **D** | **E** | **D** | **I** | **M** | **L** | **I** | **T** | **D** | **K** | **G** | **V** | **I** | **I** | **R** | **F** | **N** | **V** | **S** | **T** | **V** | **S** | **Q** | **T** | **G** | **R** | **S** | **T** | **L** | **G** | **V** | **R** | **L** | **M** | **K** | **M** | **E** | **A** | **D** | **T** | **K** | **V** | **V** | **T** | **M** | **A** | **A** | **V** | **E** | **P** | **E** | **A** | **A** | **E** | **Q** | **T** | **V** | **E** | **E** | **Q** | **G** | **S** | **E** | **E** |
| **N59469F** | **M** | **S** | **E** | **E** | **I** | **R** | **E** | **N** | **I** | **H** | **D** | **V** | **N** | **L** | **T** | **S** | **E** | **M** | **K** | **D** | **S** | **F** | **I** | **D** | **Y** | **A** | **M** | **S** | **V** | **I** | **V** | **A** | **R** | **A** | **L** | **P** | **D** | **V** | **R** | **D** | **G** | **L** | **K** | **P** | **V** | **H** | **R** | **R** | **I** | **L** | **Y** | **G** | **M** | **N** | **E** | **L** | **G** | **V** | **T** | **P** | **D** | **K** | **A** | **H** | **K** | **K** | **S** | **A** | **R** | **I** | **V** | **G** | **D** | **V** | **M** | **G** | **K** | **Y** | **H** | **P** | **H** | **G** | **D** | **S** | **A** | **I** | **Y** | **E** | **S** | **M** | **V** | **R** | **M** | **A** | **Q** | **P** | **F** | **S** | **Y** | **R** | **Y** | **M** | **L** | **V** | **D** | **G** | **H** | **G** | **N** | **F** | **G** | **S** | **V** | **D** | **G** | **D** | **G** | **A** | **A** | **A** | **M** | **R** | **Y** | **T** | **E** | **A** | **R** | **M** | **S** | **K** | **I** | **A** | **T** | **E** | **M** | **L** | **R** | **D** | **I** | **N** | **K** | **N** | **T** | **V** | **D** | **F** | **Q** | **S** | **N** | **Y** | **D** | **D** | **T** | **E** | **K** | **E** | **P** | **V** | **V** | **L** | **P** | **A** | **R** | **F** | **P** | **N** | **L** | **L** | **V** | **N** | **G** | **T** | **T** | **G** | **I** | **A** | **V** | **G** | **M** | **A** | **T** | **N** | **I** | **P** | **P** | **H** | **N** | **L** | **S** | **E** | **V** | **V** | **A** | **A** | **I** | **D** | **L** | **L** | **M** | **D** | **N** | **P** | **D** | **V** | **T** | **T** | **N** | **E** | **L** | **M** | **E** | **V** | **L** | **P** | **G** | **P** | **D** | **F** | **P** | **T** | **G** | **G** | **L** | **V** | **M** | **G** | **K** | **S** | **G** | **I** | **R** | **R** | **A** | **Y** | **E** | **T** | **G** | **K** | **G** | **S** | **I** | **T** | **V** | **R** | **A** | **K** | **V** | **E** | **I** | **T** | **E** | **M** | **P** | **N** | **G** | **K** | **E** | **R** | **I** | **I** | **V** | **T** | **E** | **L** | **P** | **Y** | **M** | **V** | **N** | **K** | **A** | **K** | **L** | **I** | **E** | **R** | **I** | **S** | **E** | **L** | **H** | **R** | **D** | **K** | **R** | **I** | **E** | **G** | **I** | **T** | **D** | **L** | **R** | **D** | **E** | **S** | **S** | **R** | **E** | **G** | **M** | **R** | **I** | **V** | **I** | **D** | **I** | **R** | **R** | **D** | **A** | **S** | **A** | **S** | **V** | **I** | **L** | **N** | **N** | **L** | **Y** | **K** | **L** | **T** | **S** | **L** | **Q** | **N** | **S** | **F** | **G** | **F** | **N** | **M** | **L** | **A** | **I** | **E** | **K** | **G** | **V** | **P** | **K** | **V** | **L** | **S** | **L** | **K** | **Q** | **I** | **L** | **E** | **N** | **Y** | **I** | **E** | **H** | **Q** | **R** | **E** | **V** | **I** | **T** | **R** | **R** | **T** | **V** | **F** | **E** | **K** | **E** | **K** | **A** | **E** | **A** | **R** | **A** | **H** | **I** | **L** | **E** | **G** | **L** | **R** | **I** | **A** | **L** | **D** | **H** | **I** | **D** | **E** | **I** | **I** | **A** | **I** | **I** | **R** | **G** | **S** | **K** | **S** | **D** | **D** | **E** | **A** | **K** | **A** | **T** | **M** | **I** | **E** | **R** | **F** | **D** | **L** | **S** | **D** | **R** | **Q** | **S** | **Q** | **A** | **I** | **L** | **D** | **M** | **R** | **L** | **R** | **R** | **L** | **T** | **G** | **L** | **E** | **R** | **E** | **K** | **I** | **E** | **N** | **E** | **Y** | **Q** | **E** | **L** | **L** | **K** | **L** | **I** | **E** | **D** | **L** | **T** | **D** | **I** | **L** | **A** | **R** | **P** | **E** | **R** | **V** | **T** | **E** | **I** | **I** | **K** | **N** | **E** | **L** | **A** | **D** | **L** | **N** | **Q** | **R** | **F** | **G** | **D** | **K** | **R** | **R** | **T** | **E** | **L** | **L** | **V** | **G** | **E** | **V** | **L** | **S** | **L** | **E** | **D** | **E** | **D** | **L** | **I** | **E** | **E** | **E** | **E** | **I** | **V** | **I** | **T** | **L** | **T** | **N** | **N** | **G** | **Y** | **I** | **K** | **R** | **L** | **A** | **N** | **N** | **E** | **F** | **R** | **A** | **Q** | **R** | **R** | **G** | **G** | **R** | **G** | **V** | **Q** | **G** | **M** | **G** | **I** | **H** | **N** | **D** | **D** | **F** | **V** | **K** | **N** | **L** | **V** | **S** | **C** | **S** | **T** | **H** | **D** | **T** | **L** | **L** | **F** | **F** | **T** | **N** | **N** | **G** | **K** | **V** | **Y** | **R** | **A** | **K** | **G** | **Y** | **E** | **I** | **P** | **E** | **Y** | **G** | **R** | **T** | **A** | **K** | **G** | **I** | **P** | **I** | **I** | **N** | **L** | **L** | **G** | **I** | **D** | **S** | **S** | **E** | **S** | **I** | **Q** | **A** | **I** | **I** | **A** | **V** | **T** | **G** | **E** | **A** | **E** | **E** | **G** | **H** | **Y** | **L** | **F** | **F** | **T** | **T** | **R** | **Q** | **G** | **T** | **V** | **K** | **R** | **T** | **S** | **V** | **T** | **A** | **F** | **A** | **N** | **I** | **R** | **S** | **N** | **G** | **L** | **I** | **A** | **I** | **G** | **L** | **K** | **E** | **D** | **D** | **E** | **L** | **V** | **N** | **V** | **L** | **L** | **T** | **D** | **G** | **Q** | **S** | **N** | **I** | **I** | **I** | **G** | **T** | **H** | **N** | **G** | **Y** | **S** | **V** | **T** | **F** | **K** | **E** | **E** | **A** | **V** | **R** | **D** | **M** | **G** | **R** | **T** | **A** | **S** | **G** | **V** | **R** | **G** | **I** | **R** | **L** | **R** | **E** | **E** | **D** | **Y** | **V** | **V** | **G** | **A** | **A** | **L** | **M** | **K** | **E** | **D** | **Q** | **E** | **V** | **L** | **V** | **I** | **T** | **E** | **K** | **G** | **Y** | **G** | **K** | **R** | **T** | **K** | **V** | **S** | **E** | **Y** | **P** | **V** | **K** | **G** | **R** | **G** | **G** | **K** | **G** | **I** | **K** | **T** | **A** | **N** | **I** | **T** | **E** | **K** | **N** | **G** | **P** | **L** | **A** | **G** | **L** | **T** | **T** | **V** | **S** | **G** | **D** | **E** | **D** | **I** | **M** | **L** | **I** | **T** | **D** | **K** | **G** | **V** | **I** | **I** | **R** | **F** | **N** | **V** | **S** | **T** | **V** | **S** | **Q** | **T** | **G** | **R** | **S** | **T** | **L** | **G** | **V** | **R** | **L** | **M** | **K** | **M** | **E** | **A** | **D** | **T** | **K** | **V** | **V** | **T** | **M** | **A** | **A** | **V** | **E** | **P** | **E** | **A** | **A** | **E** | **Q** | **T** | **V** | **E** | **E** | **Q** | **G** | **S** | **E** | **E** |
| **N55106** | **M** | **S** | **E** | **E** | **I** | **K** | **E** | **N** | **I** | **Q** | **D** | **V** | **N** | **L** | **T** | **S** | **E** | **M** | **K** | **E** | **S** | **F** | **I** | **D** | **Y** | **A** | **M** | **S** | **V** | **I** | **V** | **A** | **R** | **A** | **L** | **P** | **D** | **V** | **R** | **D** | **G** | **L** | **K** | **P** | **V** | **H** | **R** | **R** | **I** | **L** | **Y** | **G** | **M** | **N** | **E** | **L** | **G** | **V** | **T** | **P** | **D** | **K** | **P** | **H** | **K** | **K** | **S** | **A** | **R** | **I** | **V** | **G** | **D** | **V** | **M** | **G** | **K** | **Y** | **H** | **P** | **H** | **G** | **D** | **S** | **A** | **I** | **Y** | **E** | **S** | **M** | **V** | **R** | **M** | **A** | **Q** | **P** | **F** | **S** | **Y** | **R** | **A** | **M** | **L** | **V** | **D** | **G** | **H** | **G** | **N** | **F** | **G** | **S** | **V** | **D** | **G** | **D** | **G** | **A** | **A** | **A** | **M** | **R** | **Y** | **T** | **E** | **A** | **R** | **M** | **S** | **K** | **I** | **A** | **L** | **E** | **M** | **L** | **R** | **D** | **I** | **N** | **K** | **N** | **T** | **V** | **D** | **F** | **Q** | **G** | **N** | **Y** | **D** | **D** | **S** | **E** | **Q** | **E** | **P** | **V** | **V** | **L** | **P** | **A** | **R** | **F** | **P** | **N** | **L** | **L** | **V** | **N** | **G** | **T** | **T** | **G** | **I** | **A** | **V** | **G** | **M** | **A** | **T** | **N** | **I** | **P** | **P** | **H** | **N** | **L** | **S** | **E** | **V** | **I** | **D** | **A** | **T** | **S** | **L** | **L** | **M** | **D** | **N** | **P** | **D** | **V** | **T** | **T** | **N** | **E** | **L** | **M** | **E** | **V** | **L** | **P** | **G** | **P** | **D** | **F** | **P** | **T** | **G** | **G** | **L** | **V** | **M** | **G** | **K** | **S** | **G** | **I** | **R** | **R** | **A** | **Y** | **E** | **T** | **G** | **K** | **G** | **S** | **I** | **T** | **V** | **R** | **A** | **K** | **V** | **E** | **L** | **T** | **E** | **M** | **P** | **N** | **G** | **K** | **E** | **R** | **I** | **L** | **V** | **T** | **E** | **L** | **P** | **Y** | **M** | **V** | **N** | **K** | **A** | **K** | **L** | **I** | **E** | **R** | **I** | **S** | **E** | **L** | **H** | **R** | **D** | **K** | **R** | **I** | **E** | **G** | **I** | **T** | **D** | **L** | **R** | **D** | **E** | **S** | **S** | **R** | **E** | **G** | **M** | **R** | **I** | **V** | **I** | **D** | **V** | **R** | **R** | **D** | **V** | **S** | **A** | **S** | **V** | **V** | **L** | **N** | **N** | **L** | **Y** | **K** | **M** | **T** | **A** | **L** | **Q** | **T** | **S** | **F** | **G** | **F** | **N** | **M** | **L** | **A** | **I** | **E** | **K** | **G** | **V** | **P** | **K** | **I** | **L** | **S** | **L** | **K** | **R** | **I** | **L** | **E** | **N** | **Y** | **V** | **E** | **H** | **Q** | **K** | **E** | **V** | **I** | **T** | **R** | **R** | **T** | **I** | **F** | **D** | **K** | **N** | **K** | **A** | **E** | **A** | **R** | **A** | **H** | **I** | **L** | **E** | **G** | **L** | **R** | **I** | **A** | **L** | **D** | **H** | **I** | **D** | **E** | **I** | **I** | **A** | **I** | **I** | **R** | **G** | **S** | **Q** | **S** | **D** | **D** | **E** | **A** | **K** | **A** | **T** | **L** | **I** | **E** | **R** | **F** | **E** | **F** | **S** | **D** | **R** | **Q** | **A** | **Q** | **A** | **I** | **L** | **D** | **M** | **R** | **L** | **R** | **R** | **L** | **T** | **G** | **L** | **E** | **R** | **D** | **K** | **I** | **E** | **N** | **E** | **Y** | **Q** | **E** | **L** | **L** | **K** | **F** | **I** | **A** | **D** | **L** | **E** | **D** | **I** | **L** | **A** | **R** | **P** | **E** | **R** | **V** | **I** | **E** | **I** | **I** | **K** | **T** | **E** | **L** | **N** | **D** | **V** | **R** | **T** | **K** | **F** | **G** | **D** | **A** | **R** | **R** | **T** | **E** | **L** | **L** | **V** | **G** | **E** | **V** | **L** | **S** | **L** | **E** | **D** | **E** | **D** | **L** | **I** | **E** | **E** | **E** | **E** | **V** | **V** | **I** | **T** | **L** | **T** | **N** | **N** | **G** | **Y** | **I** | **K** | **R** | **M** | **A** | **N** | **S** | **E** | **F** | **R** | **A** | **Q** | **R** | **R** | **G** | **G** | **R** | **G** | **V** | **Q** | **G** | **M** | **G** | **V** | **H** | **D** | **D** | **D** | **F** | **V** | **K** | **N** | **L** | **V** | **S** | **C** | **S** | **T** | **H** | **D** | **T** | **L** | **L** | **F** | **F** | **T** | **N** | **T** | **G** | **K** | **V** | **Y** | **R** | **A** | **K** | **G** | **Y** | **E** | **I** | **P** | **E** | **Y** | **G** | **R** | **T** | **A** | **K** | **G** | **I** | **P** | **V** | **I** | **N** | **L** | **L** | **G** | **I** | **D** | **S** | **A** | **E** | **K** | **I** | **Q** | **A** | **I** | **I** | **S** | **V** | **E** | **G** | **K** | **A** | **E** | **A** | **G** | **K** | **Y** | **L** | **F** | **F** | **T** | **T** | **L** | **K** | **G** | **T** | **V** | **K** | **R** | **T** | **A** | **V** | **T** | **A** | **F** | **S** | **N** | **I** | **R** | **S** | **N** | **G** | **L** | **I** | **A** | **I** | **S** | **L** | **K** | **E** | **D** | **D** | **E** | **L** | **V** | **N** | **V** | **V** | **T** | **T** | **N** | **G** | **N** | **Q** | **K** | **M** | **I** | **I** | **G** | **T** | **H** | **A** | **G** | **Y** | **S** | **V** | **T** | **F** | **D** | **E** | **N** | **T** | **V** | **R** | **D** | **M** | **G** | **R** | **T** | **A** | **S** | **G** | **V** | **R** | **G** | **I** | **R** | **L** | **R** | **E** | **N** | **D** | **Y** | **V** | **V** | **G** | **A** | **A** | **I** | **L** | **D** | **E** | **N** | **K** | **E** | **V** | **L** | **V** | **I** | **T** | **E** | **N** | **G** | **Y** | **G** | **K** | **R** | **T** | **K** | **A** | **S** | **E** | **Y** | **P** | **V** | **K** | **G** | **R** | **G** | **G** | **K** | **G** | **I** | **K** | **T** | **A** | **N** | **I** | **T** | **E** | **K** | **N** | **G** | **P** | **L** | **A** | **G** | **L** | **T** | **T** | **V** | **N** | **G** | **D** | **E** | **D** | **I** | **L** | **L** | **I** | **T** | **N** | **K** | **G** | **V** | **I** | **I** | **R** | **F** | **N** | **V** | **D** | **S** | **V** | **S** | **Q** | **T** | **G** | **R** | **A** | **T** | **L** | **G** | **V** | **R** | **L** | **M** | **R** | **M** | **E** | **D** | **G** | **A** | **K** | **-** | **-** | **-** | **-** | **-** | **-** | **-** | **-** | **-** | **-** | **-** | **-** | **-** | **-** | **-** | **-** | **-** | **-** | **-** | **-** | **-** | **-** | **-** |
| **N54519** | **M** | **S** | **E** | **E** | **I** | **R** | **E** | **N** | **I** | **H** | **D** | **V** | **N** | **L** | **T** | **S** | **E** | **M** | **K** | **D** | **S** | **F** | **I** | **D** | **Y** | **A** | **M** | **S** | **V** | **I** | **V** | **A** | **R** | **A** | **L** | **P** | **D** | **V** | **R** | **D** | **G** | **L** | **K** | **P** | **V** | **H** | **R** | **R** | **I** | **L** | **Y** | **G** | **M** | **N** | **E** | **L** | **G** | **V** | **T** | **P** | **D** | **K** | **A** | **H** | **K** | **K** | **S** | **A** | **R** | **I** | **V** | **G** | **D** | **V** | **M** | **G** | **K** | **Y** | **H** | **P** | **H** | **G** | **D** | **S** | **A** | **I** | **Y** | **E** | **S** | **M** | **V** | **R** | **M** | **A** | **Q** | **P** | **F** | **S** | **Y** | **R** | **Y** | **M** | **L** | **V** | **D** | **G** | **H** | **G** | **N** | **F** | **G** | **S** | **V** | **D** | **G** | **D** | **G** | **A** | **A** | **A** | **M** | **R** | **Y** | **T** | **E** | **A** | **R** | **M** | **S** | **K** | **I** | **A** | **T** | **E** | **M** | **L** | **R** | **D** | **I** | **N** | **K** | **N** | **T** | **V** | **D** | **F** | **Q** | **S** | **N** | **Y** | **D** | **D** | **T** | **E** | **K** | **E** | **P** | **V** | **V** | **L** | **P** | **A** | **R** | **F** | **P** | **N** | **L** | **L** | **V** | **N** | **G** | **T** | **T** | **G** | **I** | **A** | **V** | **G** | **M** | **A** | **T** | **N** | **I** | **P** | **P** | **H** | **N** | **L** | **S** | **E** | **I** | **V** | **A** | **A** | **I** | **D** | **L** | **L** | **M** | **D** | **N** | **P** | **D** | **V** | **T** | **T** | **N** | **E** | **L** | **M** | **E** | **V** | **L** | **P** | **G** | **P** | **D** | **F** | **P** | **T** | **G** | **G** | **L** | **V** | **M** | **G** | **K** | **S** | **G** | **I** | **R** | **R** | **A** | **Y** | **E** | **T** | **G** | **K** | **G** | **S** | **I** | **T** | **V** | **R** | **A** | **K** | **V** | **E** | **I** | **T** | **E** | **M** | **P** | **N** | **G** | **K** | **E** | **R** | **I** | **L** | **V** | **T** | **E** | **L** | **P** | **Y** | **M** | **V** | **N** | **K** | **A** | **K** | **L** | **I** | **E** | **R** | **I** | **S** | **E** | **L** | **H** | **R** | **D** | **K** | **R** | **I** | **E** | **G** | **I** | **T** | **D** | **L** | **R** | **D** | **E** | **S** | **S** | **R** | **E** | **G** | **M** | **R** | **I** | **V** | **I** | **D** | **V** | **R** | **R** | **D** | **A** | **S** | **A** | **S** | **V** | **I** | **L** | **N** | **N** | **L** | **Y** | **K** | **L** | **T** | **S** | **L** | **Q** | **N** | **S** | **F** | **G** | **F** | **N** | **M** | **L** | **A** | **I** | **E** | **K** | **G** | **V** | **P** | **K** | **V** | **L** | **S** | **L** | **K** | **Q** | **I** | **L** | **E** | **N** | **Y** | **I** | **E** | **H** | **Q** | **R** | **E** | **V** | **I** | **T** | **R** | **R** | **T** | **V** | **F** | **E** | **K** | **E** | **K** | **A** | **E** | **A** | **R** | **A** | **H** | **I** | **L** | **E** | **G** | **L** | **R** | **I** | **A** | **L** | **D** | **H** | **I** | **N** | **E** | **I** | **I** | **A** | **I** | **I** | **R** | **G** | **S** | **K** | **S** | **D** | **D** | **E** | **A** | **K** | **A** | **T** | **M** | **I** | **E** | **R** | **F** | **D** | **L** | **S** | **D** | **R** | **Q** | **S** | **Q** | **A** | **I** | **L** | **D** | **M** | **R** | **L** | **R** | **R** | **L** | **T** | **G** | **L** | **E** | **R** | **E** | **K** | **I** | **E** | **N** | **E** | **Y** | **Q** | **E** | **L** | **L** | **K** | **L** | **I** | **E** | **D** | **L** | **T** | **D** | **I** | **L** | **A** | **R** | **P** | **E** | **R** | **V** | **T** | **E** | **I** | **I** | **K** | **N** | **E** | **L** | **A** | **D** | **L** | **N** | **Q** | **R** | **F** | **G** | **D** | **K** | **R** | **R** | **T** | **E** | **L** | **L** | **V** | **G** | **E** | **V** | **L** | **S** | **L** | **E** | **D** | **E** | **D** | **L** | **I** | **E** | **E** | **E** | **E** | **I** | **V** | **I** | **T** | **L** | **T** | **N** | **N** | **G** | **Y** | **I** | **K** | **R** | **L** | **A** | **N** | **N** | **E** | **F** | **R** | **A** | **Q** | **R** | **R** | **G** | **G** | **R** | **G** | **V** | **Q** | **G** | **M** | **G** | **I** | **H** | **N** | **D** | **D** | **F** | **V** | **K** | **N** | **L** | **V** | **S** | **C** | **S** | **T** | **H** | **D** | **T** | **L** | **L** | **F** | **F** | **T** | **N** | **N** | **G** | **K** | **V** | **Y** | **R** | **A** | **K** | **G** | **Y** | **E** | **I** | **P** | **E** | **Y** | **G** | **R** | **T** | **A** | **K** | **G** | **I** | **P** | **I** | **I** | **N** | **L** | **L** | **G** | **I** | **D** | **S** | **S** | **E** | **S** | **I** | **Q** | **A** | **I** | **I** | **A** | **V** | **T** | **G** | **E** | **A** | **E** | **E** | **G** | **H** | **Y** | **L** | **F** | **F** | **T** | **T** | **R** | **Q** | **G** | **T** | **V** | **K** | **R** | **T** | **S** | **V** | **T** | **A** | **F** | **A** | **N** | **I** | **R** | **S** | **N** | **G** | **L** | **I** | **A** | **I** | **G** | **L** | **K** | **E** | **D** | **D** | **E** | **L** | **V** | **N** | **V** | **L** | **L** | **T** | **D** | **G** | **Q** | **S** | **N** | **I** | **I** | **I** | **G** | **T** | **H** | **N** | **G** | **Y** | **S** | **V** | **T** | **F** | **K** | **E** | **E** | **A** | **V** | **R** | **D** | **M** | **G** | **R** | **T** | **A** | **S** | **G** | **V** | **R** | **G** | **I** | **R** | **L** | **R** | **E** | **E** | **D** | **Y** | **V** | **V** | **G** | **A** | **A** | **L** | **M** | **K** | **E** | **D** | **Q** | **E** | **V** | **L** | **V** | **I** | **T** | **E** | **K** | **G** | **Y** | **G** | **K** | **R** | **T** | **K** | **V** | **S** | **E** | **Y** | **P** | **V** | **K** | **G** | **R** | **G** | **G** | **K** | **G** | **I** | **K** | **T** | **A** | **N** | **I** | **T** | **E** | **K** | **N** | **G** | **P** | **L** | **A** | **G** | **L** | **T** | **T** | **V** | **S** | **G** | **N** | **E** | **D** | **I** | **M** | **L** | **I** | **T** | **D** | **K** | **G** | **V** | **I** | **I** | **R** | **F** | **N** | **V** | **S** | **T** | **V** | **S** | **Q** | **T** | **G** | **R** | **S** | **T** | **L** | **G** | **V** | **R** | **L** | **M** | **K** | **M** | **E** | **A** | **D** | **T** | **K** | **V** | **V** | **T** | **M** | **-** | **-** | **-** | **-** | **-** | **-** | **-** | **-** | **-** | **-** | **-** | **-** | **-** | **-** | **-** | **-** | **-** | **-** | **-** |
| **N53702** | **M** | **S** | **E** | **E** | **I** | **R** | **E** | **N** | **I** | **H** | **D** | **V** | **N** | **L** | **T** | **S** | **E** | **M** | **K** | **D** | **S** | **F** | **I** | **D** | **Y** | **A** | **M** | **S** | **V** | **I** | **V** | **A** | **R** | **A** | **L** | **P** | **D** | **V** | **R** | **D** | **G** | **L** | **K** | **P** | **V** | **H** | **R** | **R** | **I** | **L** | **Y** | **G** | **M** | **N** | **E** | **L** | **G** | **V** | **T** | **P** | **D** | **K** | **A** | **H** | **K** | **K** | **S** | **A** | **R** | **I** | **V** | **G** | **D** | **V** | **M** | **G** | **K** | **Y** | **H** | **P** | **H** | **G** | **D** | **S** | **A** | **I** | **Y** | **E** | **S** | **M** | **V** | **R** | **M** | **A** | **Q** | **P** | **F** | **S** | **Y** | **R** | **Y** | **M** | **L** | **V** | **D** | **G** | **H** | **G** | **N** | **F** | **G** | **S** | **V** | **D** | **G** | **D** | **G** | **A** | **A** | **A** | **M** | **R** | **Y** | **T** | **E** | **A** | **R** | **M** | **S** | **K** | **I** | **A** | **T** | **E** | **M** | **L** | **R** | **D** | **I** | **N** | **K** | **N** | **T** | **V** | **D** | **F** | **Q** | **S** | **N** | **Y** | **D** | **D** | **T** | **E** | **K** | **E** | **P** | **V** | **V** | **L** | **P** | **A** | **R** | **F** | **P** | **N** | **L** | **L** | **V** | **N** | **G** | **T** | **T** | **G** | **I** | **A** | **V** | **G** | **M** | **A** | **T** | **N** | **I** | **P** | **P** | **H** | **N** | **L** | **S** | **E** | **V** | **V** | **A** | **A** | **I** | **D** | **L** | **L** | **M** | **D** | **N** | **P** | **D** | **V** | **T** | **T** | **N** | **E** | **L** | **M** | **E** | **V** | **L** | **P** | **G** | **P** | **D** | **F** | **P** | **T** | **G** | **G** | **L** | **V** | **M** | **G** | **K** | **S** | **G** | **I** | **R** | **R** | **A** | **Y** | **E** | **T** | **G** | **K** | **G** | **S** | **I** | **T** | **V** | **R** | **A** | **K** | **V** | **E** | **I** | **T** | **E** | **M** | **P** | **N** | **G** | **K** | **E** | **R** | **I** | **I** | **V** | **T** | **E** | **L** | **P** | **Y** | **M** | **V** | **N** | **K** | **A** | **K** | **L** | **I** | **E** | **R** | **I** | **S** | **E** | **L** | **H** | **R** | **D** | **K** | **R** | **I** | **E** | **G** | **I** | **T** | **D** | **L** | **R** | **D** | **E** | **S** | **S** | **R** | **E** | **G** | **M** | **R** | **I** | **V** | **I** | **D** | **I** | **R** | **R** | **D** | **A** | **S** | **A** | **S** | **V** | **I** | **L** | **N** | **N** | **L** | **Y** | **K** | **L** | **T** | **S** | **L** | **Q** | **N** | **S** | **F** | **G** | **F** | **N** | **M** | **L** | **A** | **I** | **E** | **K** | **G** | **V** | **P** | **K** | **V** | **L** | **S** | **L** | **K** | **Q** | **I** | **L** | **E** | **N** | **Y** | **I** | **E** | **H** | **Q** | **R** | **E** | **V** | **I** | **T** | **R** | **R** | **T** | **V** | **F** | **E** | **K** | **E** | **K** | **A** | **E** | **A** | **R** | **A** | **H** | **I** | **L** | **E** | **G** | **L** | **R** | **I** | **A** | **L** | **D** | **H** | **I** | **D** | **E** | **I** | **I** | **A** | **I** | **I** | **R** | **G** | **S** | **K** | **S** | **D** | **D** | **E** | **A** | **K** | **A** | **T** | **M** | **I** | **E** | **R** | **F** | **D** | **L** | **S** | **D** | **R** | **Q** | **S** | **Q** | **A** | **I** | **L** | **D** | **M** | **R** | **L** | **R** | **R** | **L** | **T** | **G** | **L** | **E** | **R** | **E** | **K** | **I** | **E** | **N** | **E** | **Y** | **Q** | **E** | **L** | **L** | **K** | **L** | **I** | **E** | **D** | **L** | **T** | **D** | **I** | **L** | **A** | **R** | **P** | **E** | **R** | **V** | **T** | **E** | **I** | **I** | **K** | **N** | **E** | **L** | **A** | **D** | **L** | **N** | **Q** | **R** | **F** | **G** | **D** | **K** | **R** | **R** | **T** | **E** | **L** | **L** | **V** | **G** | **E** | **V** | **L** | **S** | **L** | **E** | **D** | **E** | **D** | **L** | **I** | **E** | **E** | **E** | **E** | **I** | **V** | **I** | **T** | **L** | **T** | **N** | **N** | **G** | **Y** | **I** | **K** | **R** | **L** | **A** | **N** | **N** | **E** | **F** | **R** | **A** | **Q** | **R** | **R** | **G** | **G** | **R** | **G** | **V** | **Q** | **G** | **M** | **G** | **I** | **H** | **N** | **D** | **D** | **F** | **V** | **K** | **N** | **L** | **V** | **S** | **C** | **S** | **T** | **H** | **D** | **T** | **L** | **L** | **F** | **F** | **T** | **N** | **N** | **G** | **K** | **V** | **Y** | **R** | **A** | **K** | **G** | **Y** | **E** | **I** | **P** | **E** | **Y** | **G** | **R** | **T** | **A** | **K** | **G** | **I** | **P** | **I** | **I** | **N** | **L** | **L** | **G** | **I** | **D** | **S** | **S** | **E** | **S** | **I** | **Q** | **A** | **I** | **I** | **A** | **V** | **T** | **G** | **E** | **A** | **E** | **E** | **G** | **H** | **Y** | **L** | **F** | **F** | **T** | **T** | **R** | **Q** | **G** | **T** | **V** | **K** | **R** | **T** | **S** | **V** | **T** | **A** | **F** | **A** | **N** | **I** | **R** | **S** | **N** | **G** | **L** | **I** | **A** | **I** | **G** | **L** | **K** | **E** | **D** | **D** | **E** | **L** | **V** | **N** | **V** | **L** | **L** | **T** | **D** | **G** | **Q** | **S** | **N** | **I** | **I** | **I** | **G** | **T** | **H** | **N** | **G** | **Y** | **S** | **V** | **T** | **F** | **K** | **E** | **E** | **A** | **V** | **R** | **D** | **M** | **G** | **R** | **T** | **A** | **S** | **G** | **V** | **R** | **G** | **I** | **R** | **L** | **R** | **E** | **E** | **D** | **Y** | **V** | **V** | **G** | **A** | **A** | **L** | **M** | **K** | **E** | **D** | **Q** | **E** | **V** | **L** | **V** | **I** | **T** | **E** | **K** | **G** | **Y** | **G** | **K** | **R** | **T** | **K** | **V** | **S** | **E** | **Y** | **P** | **V** | **K** | **G** | **R** | **G** | **G** | **K** | **G** | **I** | **K** | **T** | **A** | **N** | **I** | **T** | **E** | **K** | **N** | **G** | **P** | **L** | **A** | **G** | **L** | **T** | **T** | **V** | **S** | **G** | **D** | **E** | **D** | **I** | **M** | **L** | **I** | **T** | **D** | **K** | **G** | **V** | **I** | **I** | **R** | **F** | **N** | **V** | **S** | **T** | **V** | **S** | **Q** | **T** | **G** | **R** | **S** | **T** | **L** | **G** | **V** | **R** | **L** | **M** | **K** | **M** | **E** | **A** | **D** | **T** | **K** | **V** | **V** | **T** | **M** | **A** | **A** | **V** | **E** | **P** | **E** | **A** | **A** | **E** | **Q** | **T** | **V** | **E** | **E** | **Q** | **G** | **S** | **E** | **E** |
| **N53533** | **M** | **S** | **E** | **E** | **I** | **R** | **E** | **N** | **I** | **H** | **D** | **V** | **N** | **L** | **T** | **S** | **E** | **M** | **K** | **D** | **S** | **F** | **I** | **D** | **Y** | **A** | **M** | **S** | **V** | **I** | **V** | **A** | **R** | **A** | **L** | **P** | **D** | **V** | **R** | **D** | **G** | **L** | **K** | **P** | **V** | **H** | **R** | **R** | **I** | **L** | **Y** | **G** | **M** | **N** | **E** | **L** | **G** | **V** | **T** | **P** | **D** | **K** | **A** | **H** | **K** | **K** | **S** | **A** | **R** | **I** | **V** | **G** | **D** | **V** | **M** | **G** | **K** | **Y** | **H** | **P** | **H** | **G** | **D** | **S** | **A** | **I** | **Y** | **E** | **S** | **M** | **V** | **R** | **M** | **A** | **Q** | **P** | **F** | **S** | **Y** | **R** | **Y** | **M** | **L** | **V** | **D** | **G** | **H** | **G** | **N** | **F** | **G** | **S** | **V** | **D** | **G** | **D** | **G** | **A** | **A** | **A** | **M** | **R** | **Y** | **T** | **E** | **A** | **R** | **M** | **S** | **K** | **I** | **A** | **T** | **E** | **M** | **L** | **R** | **D** | **I** | **N** | **K** | **N** | **T** | **V** | **D** | **F** | **Q** | **S** | **N** | **Y** | **D** | **D** | **T** | **E** | **K** | **E** | **P** | **V** | **V** | **L** | **P** | **A** | **R** | **F** | **P** | **N** | **L** | **L** | **V** | **N** | **G** | **T** | **T** | **G** | **I** | **A** | **V** | **G** | **M** | **A** | **T** | **N** | **I** | **P** | **P** | **H** | **N** | **L** | **S** | **E** | **V** | **V** | **A** | **A** | **I** | **D** | **L** | **L** | **M** | **D** | **N** | **P** | **D** | **V** | **T** | **T** | **N** | **E** | **L** | **M** | **E** | **V** | **L** | **P** | **G** | **P** | **D** | **F** | **P** | **T** | **G** | **G** | **L** | **V** | **M** | **G** | **K** | **S** | **G** | **I** | **R** | **R** | **A** | **Y** | **E** | **T** | **G** | **K** | **G** | **S** | **I** | **T** | **V** | **R** | **A** | **K** | **V** | **E** | **I** | **T** | **E** | **M** | **P** | **N** | **G** | **K** | **E** | **R** | **I** | **I** | **V** | **T** | **E** | **L** | **P** | **Y** | **M** | **V** | **N** | **K** | **A** | **K** | **L** | **I** | **E** | **R** | **I** | **S** | **E** | **L** | **H** | **R** | **D** | **K** | **R** | **I** | **E** | **G** | **I** | **T** | **D** | **L** | **R** | **D** | **E** | **S** | **S** | **R** | **E** | **G** | **M** | **R** | **I** | **V** | **I** | **D** | **I** | **R** | **R** | **D** | **A** | **S** | **A** | **S** | **V** | **I** | **L** | **N** | **N** | **L** | **Y** | **K** | **L** | **T** | **S** | **L** | **Q** | **N** | **S** | **F** | **G** | **F** | **N** | **M** | **L** | **A** | **I** | **E** | **K** | **G** | **V** | **P** | **K** | **V** | **L** | **S** | **L** | **K** | **Q** | **I** | **L** | **E** | **N** | **Y** | **I** | **E** | **H** | **Q** | **R** | **E** | **V** | **I** | **T** | **R** | **R** | **T** | **V** | **F** | **E** | **K** | **E** | **K** | **A** | **E** | **A** | **R** | **A** | **H** | **I** | **L** | **E** | **G** | **L** | **R** | **I** | **A** | **L** | **D** | **H** | **I** | **D** | **E** | **I** | **I** | **A** | **I** | **I** | **R** | **G** | **S** | **K** | **S** | **D** | **D** | **E** | **A** | **K** | **A** | **T** | **M** | **I** | **E** | **R** | **F** | **D** | **L** | **S** | **D** | **R** | **Q** | **S** | **Q** | **A** | **I** | **L** | **D** | **M** | **R** | **L** | **R** | **R** | **L** | **T** | **G** | **L** | **E** | **R** | **E** | **K** | **I** | **E** | **N** | **E** | **Y** | **Q** | **E** | **L** | **L** | **K** | **L** | **I** | **E** | **D** | **L** | **T** | **D** | **I** | **L** | **A** | **R** | **P** | **E** | **R** | **V** | **T** | **E** | **I** | **I** | **K** | **N** | **E** | **L** | **A** | **D** | **L** | **N** | **Q** | **R** | **F** | **G** | **D** | **K** | **R** | **R** | **T** | **E** | **L** | **L** | **V** | **G** | **E** | **V** | **L** | **S** | **L** | **E** | **D** | **E** | **D** | **L** | **I** | **E** | **E** | **E** | **E** | **I** | **V** | **I** | **T** | **L** | **T** | **N** | **N** | **G** | **Y** | **I** | **K** | **R** | **L** | **A** | **N** | **N** | **E** | **F** | **R** | **A** | **Q** | **R** | **R** | **G** | **G** | **R** | **G** | **V** | **Q** | **G** | **M** | **G** | **I** | **H** | **N** | **D** | **D** | **F** | **V** | **K** | **N** | **L** | **V** | **S** | **C** | **S** | **T** | **H** | **D** | **T** | **L** | **L** | **F** | **F** | **T** | **N** | **N** | **G** | **K** | **V** | **Y** | **R** | **A** | **K** | **G** | **Y** | **E** | **I** | **P** | **E** | **Y** | **G** | **R** | **T** | **A** | **K** | **G** | **I** | **P** | **I** | **I** | **N** | **L** | **L** | **G** | **I** | **D** | **S** | **S** | **E** | **S** | **I** | **Q** | **A** | **I** | **I** | **A** | **V** | **T** | **G** | **E** | **A** | **E** | **E** | **G** | **H** | **Y** | **L** | **F** | **F** | **T** | **T** | **R** | **Q** | **G** | **T** | **V** | **K** | **R** | **T** | **S** | **V** | **T** | **A** | **F** | **A** | **N** | **I** | **R** | **S** | **N** | **G** | **L** | **I** | **A** | **I** | **G** | **L** | **K** | **E** | **D** | **D** | **E** | **L** | **V** | **N** | **V** | **L** | **L** | **T** | **D** | **G** | **Q** | **S** | **N** | **I** | **I** | **I** | **G** | **T** | **H** | **N** | **G** | **Y** | **S** | **V** | **T** | **F** | **K** | **E** | **E** | **A** | **V** | **R** | **D** | **M** | **G** | **R** | **T** | **A** | **S** | **G** | **V** | **R** | **G** | **I** | **R** | **L** | **R** | **E** | **E** | **D** | **Y** | **V** | **V** | **G** | **A** | **A** | **L** | **M** | **K** | **E** | **N** | **Q** | **E** | **V** | **L** | **V** | **I** | **T** | **E** | **K** | **G** | **Y** | **G** | **K** | **R** | **T** | **K** | **V** | **S** | **E** | **Y** | **P** | **V** | **K** | **G** | **R** | **G** | **G** | **K** | **G** | **I** | **K** | **T** | **A** | **N** | **I** | **T** | **E** | **K** | **N** | **G** | **P** | **L** | **A** | **G** | **L** | **T** | **T** | **V** | **S** | **G** | **D** | **E** | **D** | **I** | **M** | **L** | **I** | **T** | **D** | **K** | **G** | **V** | **I** | **I** | **R** | **F** | **N** | **V** | **S** | **T** | **V** | **S** | **Q** | **T** | **G** | **R** | **S** | **T** | **L** | **G** | **V** | **R** | **L** | **M** | **K** | **M** | **E** | **A** | **D** | **T** | **K** | **V** | **V** | **T** | **M** | **A** | **A** | **V** | **E** | **P** | **E** | **A** | **A** | **E** | **Q** | **T** | **V** | **E** | **E** | **Q** | **G** | **S** | **E** | **E** |
| **N59466F** | **M** | **S** | **E** | **E** | **I** | **K** | **E** | **N** | **I** | **Q** | **D** | **V** | **N** | **L** | **T** | **S** | **E** | **M** | **K** | **E** | **S** | **F** | **I** | **D** | **Y** | **A** | **M** | **S** | **V** | **I** | **V** | **A** | **R** | **A** | **L** | **P** | **D** | **V** | **R** | **D** | **G** | **L** | **K** | **P** | **V** | **H** | **R** | **R** | **I** | **L** | **Y** | **G** | **M** | **N** | **E** | **L** | **G** | **V** | **T** | **P** | **D** | **K** | **P** | **H** | **K** | **K** | **S** | **A** | **R** | **I** | **V** | **G** | **D** | **V** | **M** | **G** | **K** | **Y** | **H** | **P** | **H** | **G** | **D** | **S** | **A** | **I** | **Y** | **E** | **S** | **M** | **V** | **R** | **M** | **A** | **Q** | **P** | **F** | **S** | **Y** | **R** | **A** | **M** | **L** | **V** | **D** | **G** | **H** | **G** | **N** | **F** | **G** | **S** | **V** | **D** | **G** | **D** | **G** | **A** | **A** | **A** | **M** | **R** | **Y** | **T** | **E** | **A** | **R** | **M** | **S** | **K** | **I** | **A** | **L** | **E** | **M** | **L** | **R** | **D** | **I** | **N** | **K** | **N** | **T** | **V** | **D** | **F** | **Q** | **G** | **N** | **Y** | **D** | **D** | **S** | **E** | **Q** | **E** | **P** | **V** | **V** | **L** | **P** | **A** | **R** | **F** | **P** | **N** | **L** | **L** | **V** | **N** | **G** | **T** | **T** | **G** | **I** | **A** | **V** | **G** | **M** | **A** | **T** | **N** | **I** | **P** | **P** | **H** | **N** | **L** | **S** | **E** | **V** | **I** | **D** | **A** | **T** | **S** | **L** | **L** | **M** | **D** | **N** | **P** | **D** | **V** | **T** | **T** | **N** | **E** | **L** | **M** | **E** | **V** | **L** | **P** | **G** | **P** | **D** | **F** | **P** | **T** | **G** | **G** | **L** | **V** | **M** | **G** | **K** | **S** | **G** | **I** | **R** | **R** | **A** | **Y** | **E** | **T** | **G** | **K** | **G** | **S** | **I** | **T** | **V** | **R** | **A** | **K** | **V** | **E** | **L** | **T** | **E** | **M** | **P** | **N** | **G** | **K** | **E** | **R** | **I** | **L** | **V** | **T** | **E** | **L** | **P** | **Y** | **M** | **V** | **N** | **K** | **A** | **K** | **L** | **I** | **E** | **R** | **I** | **S** | **E** | **L** | **H** | **R** | **D** | **K** | **R** | **I** | **E** | **G** | **I** | **T** | **D** | **L** | **R** | **D** | **E** | **S** | **S** | **R** | **E** | **G** | **M** | **R** | **I** | **V** | **I** | **D** | **V** | **R** | **R** | **D** | **V** | **S** | **A** | **S** | **V** | **V** | **L** | **N** | **N** | **L** | **Y** | **K** | **M** | **T** | **A** | **L** | **Q** | **T** | **S** | **F** | **G** | **F** | **N** | **M** | **L** | **A** | **I** | **E** | **K** | **G** | **V** | **P** | **K** | **I** | **L** | **S** | **L** | **K** | **R** | **I** | **L** | **E** | **N** | **Y** | **V** | **E** | **H** | **Q** | **K** | **E** | **V** | **I** | **T** | **R** | **R** | **T** | **I** | **F** | **D** | **K** | **N** | **K** | **A** | **E** | **A** | **R** | **A** | **H** | **I** | **L** | **E** | **G** | **L** | **R** | **I** | **A** | **L** | **D** | **H** | **I** | **D** | **E** | **I** | **I** | **A** | **I** | **I** | **R** | **G** | **S** | **Q** | **S** | **D** | **D** | **E** | **A** | **K** | **A** | **T** | **L** | **I** | **E** | **R** | **F** | **E** | **F** | **S** | **D** | **R** | **Q** | **A** | **Q** | **A** | **I** | **L** | **D** | **M** | **R** | **L** | **R** | **R** | **L** | **T** | **G** | **L** | **E** | **R** | **D** | **K** | **I** | **E** | **N** | **E** | **Y** | **Q** | **E** | **L** | **L** | **K** | **F** | **I** | **A** | **D** | **L** | **E** | **D** | **I** | **L** | **A** | **R** | **P** | **E** | **R** | **V** | **I** | **E** | **I** | **I** | **K** | **T** | **E** | **L** | **N** | **D** | **V** | **R** | **T** | **K** | **F** | **G** | **D** | **A** | **R** | **R** | **T** | **E** | **L** | **L** | **V** | **G** | **E** | **V** | **L** | **S** | **L** | **E** | **D** | **E** | **D** | **L** | **I** | **E** | **E** | **E** | **E** | **V** | **V** | **I** | **T** | **L** | **T** | **N** | **N** | **G** | **Y** | **I** | **K** | **R** | **M** | **A** | **N** | **S** | **E** | **F** | **R** | **A** | **Q** | **R** | **R** | **G** | **G** | **R** | **G** | **V** | **Q** | **G** | **M** | **G** | **V** | **H** | **D** | **D** | **D** | **F** | **V** | **K** | **N** | **L** | **V** | **S** | **C** | **S** | **T** | **H** | **D** | **T** | **L** | **L** | **F** | **F** | **T** | **N** | **T** | **G** | **K** | **V** | **Y** | **R** | **A** | **K** | **G** | **Y** | **E** | **I** | **P** | **E** | **Y** | **G** | **R** | **T** | **A** | **K** | **G** | **I** | **P** | **V** | **I** | **N** | **L** | **L** | **G** | **I** | **D** | **S** | **A** | **E** | **K** | **I** | **Q** | **A** | **I** | **I** | **S** | **V** | **E** | **G** | **K** | **A** | **E** | **A** | **G** | **K** | **Y** | **L** | **F** | **F** | **T** | **T** | **L** | **K** | **G** | **T** | **V** | **K** | **R** | **T** | **A** | **V** | **T** | **A** | **F** | **S** | **N** | **I** | **R** | **S** | **N** | **G** | **L** | **I** | **A** | **I** | **S** | **L** | **K** | **E** | **D** | **D** | **E** | **L** | **V** | **N** | **V** | **V** | **T** | **T** | **N** | **G** | **N** | **Q** | **K** | **M** | **I** | **I** | **G** | **T** | **H** | **A** | **G** | **Y** | **S** | **V** | **T** | **F** | **D** | **E** | **N** | **T** | **V** | **R** | **D** | **M** | **G** | **R** | **T** | **A** | **S** | **G** | **V** | **R** | **G** | **I** | **R** | **L** | **R** | **E** | **N** | **D** | **Y** | **V** | **V** | **G** | **A** | **A** | **I** | **L** | **D** | **E** | **N** | **K** | **E** | **V** | **L** | **V** | **I** | **T** | **E** | **N** | **G** | **Y** | **G** | **K** | **R** | **T** | **K** | **A** | **S** | **E** | **Y** | **P** | **V** | **K** | **G** | **R** | **G** | **G** | **K** | **G** | **I** | **K** | **T** | **A** | **N** | **I** | **T** | **E** | **K** | **N** | **G** | **P** | **L** | **A** | **G** | **L** | **T** | **T** | **V** | **N** | **G** | **N** | **E** | **D** | **I** | **L** | **L** | **I** | **T** | **N** | **K** | **G** | **V** | **I** | **I** | **R** | **F** | **N** | **V** | **D** | **S** | **V** | **S** | **Q** | **T** | **G** | **R** | **A** | **T** | **L** | **G** | **V** | **R** | **L** | **M** | **R** | **M** | **E** | **D** | **G** | **A** | **K** | **-** | **-** | **-** | **-** | **-** | **-** | **-** | **-** | **-** | **-** | **-** | **-** | **-** | **-** | **-** | **-** | **-** | **-** | **-** | **-** | **-** | **-** | **-** |
| **N59439F** | **M** | **S** | **E** | **E** | **I** | **R** | **E** | **N** | **I** | **H** | **D** | **V** | **N** | **L** | **T** | **S** | **E** | **M** | **K** | **D** | **S** | **F** | **I** | **D** | **Y** | **A** | **M** | **S** | **V** | **I** | **V** | **A** | **R** | **A** | **L** | **P** | **D** | **V** | **R** | **D** | **G** | **L** | **K** | **P** | **V** | **H** | **R** | **R** | **I** | **L** | **Y** | **G** | **M** | **N** | **E** | **L** | **G** | **V** | **T** | **P** | **D** | **K** | **A** | **H** | **K** | **K** | **S** | **A** | **R** | **I** | **V** | **G** | **D** | **V** | **M** | **G** | **K** | **Y** | **H** | **P** | **H** | **G** | **D** | **S** | **A** | **I** | **Y** | **E** | **S** | **M** | **V** | **R** | **M** | **A** | **Q** | **P** | **F** | **S** | **Y** | **R** | **Y** | **M** | **L** | **V** | **D** | **G** | **H** | **G** | **N** | **F** | **G** | **S** | **V** | **D** | **G** | **D** | **G** | **A** | **A** | **A** | **M** | **R** | **Y** | **T** | **E** | **A** | **R** | **M** | **S** | **K** | **I** | **A** | **T** | **E** | **M** | **L** | **R** | **D** | **I** | **N** | **K** | **N** | **T** | **V** | **D** | **F** | **Q** | **S** | **N** | **Y** | **D** | **D** | **T** | **E** | **K** | **E** | **P** | **V** | **V** | **L** | **P** | **A** | **R** | **F** | **P** | **N** | **L** | **L** | **V** | **N** | **G** | **T** | **T** | **G** | **I** | **A** | **V** | **G** | **M** | **A** | **T** | **N** | **I** | **P** | **P** | **H** | **N** | **L** | **S** | **E** | **V** | **V** | **A** | **A** | **I** | **D** | **L** | **L** | **M** | **D** | **N** | **P** | **D** | **V** | **T** | **T** | **N** | **E** | **L** | **M** | **E** | **V** | **L** | **P** | **G** | **P** | **D** | **F** | **P** | **T** | **G** | **G** | **L** | **V** | **M** | **G** | **K** | **S** | **G** | **I** | **R** | **R** | **A** | **Y** | **E** | **T** | **G** | **K** | **G** | **S** | **I** | **T** | **V** | **R** | **A** | **K** | **V** | **E** | **I** | **T** | **E** | **M** | **P** | **N** | **G** | **K** | **E** | **R** | **I** | **I** | **V** | **T** | **E** | **L** | **P** | **Y** | **M** | **V** | **N** | **K** | **A** | **K** | **L** | **I** | **E** | **R** | **I** | **S** | **E** | **L** | **H** | **R** | **D** | **K** | **R** | **I** | **E** | **G** | **I** | **T** | **D** | **L** | **R** | **D** | **E** | **S** | **S** | **R** | **E** | **G** | **M** | **R** | **I** | **V** | **I** | **D** | **I** | **R** | **R** | **D** | **A** | **S** | **A** | **S** | **V** | **I** | **L** | **N** | **N** | **L** | **Y** | **K** | **L** | **T** | **S** | **L** | **Q** | **N** | **S** | **F** | **G** | **F** | **N** | **M** | **L** | **A** | **I** | **E** | **K** | **G** | **V** | **P** | **K** | **V** | **L** | **S** | **L** | **K** | **Q** | **I** | **L** | **E** | **N** | **Y** | **I** | **E** | **H** | **Q** | **R** | **E** | **V** | **I** | **T** | **R** | **R** | **T** | **V** | **F** | **E** | **K** | **E** | **K** | **A** | **E** | **A** | **R** | **A** | **H** | **I** | **L** | **E** | **G** | **L** | **R** | **I** | **A** | **L** | **D** | **H** | **I** | **D** | **E** | **I** | **I** | **A** | **I** | **I** | **R** | **G** | **S** | **K** | **S** | **D** | **D** | **E** | **A** | **K** | **A** | **T** | **M** | **I** | **E** | **R** | **F** | **D** | **L** | **S** | **D** | **R** | **Q** | **S** | **Q** | **A** | **I** | **L** | **D** | **M** | **R** | **L** | **R** | **R** | **L** | **T** | **G** | **L** | **E** | **R** | **E** | **K** | **I** | **E** | **N** | **E** | **Y** | **Q** | **E** | **L** | **L** | **K** | **L** | **I** | **E** | **D** | **L** | **T** | **D** | **I** | **L** | **A** | **R** | **P** | **E** | **R** | **V** | **T** | **E** | **I** | **I** | **K** | **N** | **E** | **L** | **A** | **D** | **L** | **N** | **Q** | **R** | **F** | **G** | **D** | **K** | **R** | **R** | **T** | **E** | **L** | **L** | **V** | **G** | **E** | **V** | **L** | **S** | **L** | **E** | **D** | **E** | **D** | **L** | **I** | **E** | **E** | **E** | **E** | **I** | **V** | **I** | **T** | **L** | **T** | **N** | **N** | **G** | **Y** | **I** | **K** | **R** | **L** | **A** | **N** | **N** | **E** | **F** | **R** | **A** | **Q** | **R** | **R** | **G** | **G** | **R** | **G** | **V** | **Q** | **G** | **M** | **G** | **I** | **H** | **N** | **D** | **D** | **F** | **V** | **K** | **N** | **L** | **V** | **S** | **C** | **S** | **T** | **H** | **D** | **T** | **L** | **L** | **F** | **F** | **T** | **N** | **N** | **G** | **K** | **V** | **Y** | **R** | **A** | **K** | **G** | **Y** | **E** | **I** | **P** | **E** | **Y** | **G** | **R** | **T** | **A** | **K** | **G** | **I** | **P** | **I** | **I** | **N** | **L** | **L** | **G** | **I** | **D** | **S** | **S** | **E** | **S** | **I** | **Q** | **A** | **I** | **I** | **A** | **V** | **T** | **G** | **E** | **A** | **E** | **E** | **G** | **H** | **Y** | **L** | **F** | **F** | **T** | **T** | **R** | **Q** | **G** | **T** | **V** | **K** | **R** | **T** | **S** | **V** | **T** | **A** | **F** | **A** | **N** | **I** | **R** | **S** | **N** | **G** | **L** | **I** | **A** | **I** | **G** | **L** | **K** | **E** | **D** | **D** | **E** | **L** | **V** | **N** | **V** | **L** | **L** | **T** | **D** | **G** | **Q** | **S** | **N** | **I** | **I** | **I** | **G** | **T** | **H** | **N** | **G** | **Y** | **S** | **V** | **T** | **F** | **K** | **E** | **E** | **A** | **V** | **R** | **D** | **M** | **G** | **R** | **T** | **A** | **S** | **G** | **V** | **R** | **G** | **I** | **R** | **L** | **R** | **E** | **E** | **D** | **Y** | **V** | **V** | **G** | **A** | **A** | **L** | **M** | **K** | **E** | **N** | **Q** | **E** | **V** | **L** | **V** | **I** | **T** | **E** | **K** | **G** | **Y** | **G** | **K** | **R** | **T** | **K** | **V** | **S** | **E** | **Y** | **P** | **V** | **K** | **G** | **R** | **G** | **G** | **K** | **G** | **I** | **K** | **T** | **A** | **N** | **I** | **T** | **E** | **K** | **N** | **G** | **P** | **L** | **A** | **G** | **L** | **T** | **T** | **V** | **S** | **G** | **D** | **E** | **D** | **I** | **M** | **L** | **I** | **T** | **D** | **K** | **G** | **V** | **I** | **I** | **R** | **F** | **N** | **V** | **S** | **T** | **V** | **S** | **Q** | **T** | **G** | **R** | **S** | **T** | **L** | **G** | **V** | **R** | **L** | **M** | **K** | **M** | **E** | **A** | **D** | **T** | **K** | **V** | **V** | **T** | **M** | **A** | **A** | **V** | **E** | **P** | **E** | **A** | **A** | **E** | **Q** | **T** | **V** | **E** | **E** | **Q** | **G** | **S** | **E** | **E** |
| **N54671** | **M** | **S** | **E** | **E** | **I** | **R** | **E** | **N** | **I** | **H** | **D** | **V** | **N** | **L** | **T** | **S** | **E** | **M** | **K** | **D** | **S** | **F** | **I** | **D** | **Y** | **A** | **M** | **S** | **V** | **I** | **V** | **A** | **R** | **A** | **L** | **P** | **D** | **V** | **R** | **D** | **G** | **L** | **K** | **P** | **V** | **H** | **R** | **R** | **I** | **L** | **Y** | **G** | **M** | **N** | **E** | **L** | **G** | **V** | **T** | **P** | **D** | **K** | **A** | **H** | **K** | **K** | **S** | **A** | **R** | **I** | **V** | **G** | **D** | **V** | **M** | **G** | **K** | **Y** | **H** | **P** | **H** | **G** | **D** | **S** | **A** | **I** | **Y** | **E** | **S** | **M** | **V** | **R** | **M** | **A** | **Q** | **P** | **F** | **S** | **Y** | **R** | **Y** | **M** | **L** | **V** | **D** | **G** | **H** | **G** | **N** | **F** | **G** | **S** | **V** | **D** | **G** | **D** | **G** | **A** | **A** | **A** | **M** | **R** | **Y** | **T** | **E** | **A** | **R** | **M** | **S** | **K** | **I** | **A** | **T** | **E** | **M** | **L** | **R** | **D** | **I** | **N** | **K** | **N** | **T** | **V** | **D** | **F** | **Q** | **S** | **N** | **Y** | **D** | **D** | **T** | **E** | **K** | **E** | **P** | **V** | **V** | **L** | **P** | **A** | **R** | **F** | **P** | **N** | **L** | **L** | **V** | **N** | **G** | **T** | **T** | **G** | **I** | **A** | **V** | **G** | **M** | **A** | **T** | **N** | **I** | **P** | **P** | **H** | **N** | **L** | **S** | **E** | **V** | **V** | **A** | **A** | **I** | **D** | **L** | **L** | **M** | **D** | **N** | **P** | **D** | **V** | **T** | **T** | **N** | **E** | **L** | **M** | **E** | **V** | **L** | **P** | **G** | **P** | **D** | **F** | **P** | **T** | **G** | **G** | **L** | **V** | **M** | **G** | **K** | **S** | **G** | **I** | **R** | **R** | **A** | **Y** | **E** | **T** | **G** | **K** | **G** | **S** | **I** | **T** | **V** | **R** | **A** | **K** | **V** | **E** | **I** | **T** | **E** | **M** | **P** | **N** | **G** | **K** | **E** | **R** | **I** | **I** | **V** | **T** | **E** | **L** | **P** | **Y** | **M** | **V** | **N** | **K** | **A** | **K** | **L** | **I** | **E** | **R** | **I** | **S** | **E** | **L** | **H** | **R** | **D** | **K** | **R** | **I** | **E** | **G** | **I** | **T** | **D** | **L** | **R** | **D** | **E** | **S** | **S** | **R** | **E** | **G** | **M** | **R** | **I** | **V** | **I** | **D** | **I** | **R** | **R** | **D** | **A** | **S** | **A** | **S** | **V** | **I** | **L** | **N** | **N** | **L** | **Y** | **K** | **L** | **T** | **S** | **L** | **Q** | **N** | **S** | **F** | **G** | **F** | **N** | **M** | **L** | **A** | **I** | **E** | **K** | **G** | **V** | **P** | **K** | **V** | **L** | **S** | **L** | **K** | **Q** | **I** | **L** | **E** | **N** | **Y** | **I** | **E** | **H** | **Q** | **R** | **E** | **V** | **I** | **T** | **R** | **R** | **T** | **V** | **F** | **E** | **K** | **E** | **K** | **A** | **E** | **A** | **R** | **A** | **H** | **I** | **L** | **E** | **G** | **L** | **R** | **I** | **A** | **L** | **D** | **H** | **I** | **D** | **E** | **I** | **I** | **A** | **I** | **I** | **R** | **G** | **S** | **K** | **S** | **D** | **D** | **E** | **A** | **K** | **A** | **T** | **M** | **I** | **E** | **R** | **F** | **D** | **L** | **S** | **D** | **R** | **Q** | **S** | **Q** | **A** | **I** | **L** | **D** | **M** | **R** | **L** | **R** | **R** | **L** | **T** | **G** | **L** | **E** | **R** | **E** | **K** | **I** | **E** | **N** | **E** | **Y** | **Q** | **E** | **L** | **L** | **K** | **L** | **I** | **E** | **D** | **L** | **T** | **D** | **I** | **L** | **A** | **R** | **P** | **E** | **R** | **V** | **T** | **E** | **I** | **I** | **K** | **N** | **E** | **L** | **A** | **D** | **L** | **N** | **Q** | **R** | **F** | **G** | **D** | **K** | **R** | **R** | **T** | **E** | **L** | **L** | **V** | **G** | **E** | **V** | **L** | **S** | **L** | **E** | **D** | **E** | **D** | **L** | **I** | **E** | **E** | **E** | **E** | **I** | **V** | **I** | **T** | **L** | **T** | **N** | **N** | **G** | **Y** | **I** | **K** | **R** | **L** | **A** | **N** | **N** | **E** | **F** | **R** | **A** | **Q** | **R** | **R** | **G** | **G** | **R** | **G** | **V** | **Q** | **G** | **M** | **G** | **I** | **H** | **N** | **D** | **D** | **F** | **V** | **K** | **N** | **L** | **V** | **S** | **C** | **S** | **T** | **H** | **D** | **T** | **L** | **L** | **F** | **F** | **T** | **N** | **N** | **G** | **K** | **V** | **Y** | **R** | **A** | **K** | **G** | **Y** | **E** | **I** | **P** | **E** | **Y** | **G** | **R** | **T** | **A** | **K** | **G** | **I** | **P** | **I** | **I** | **N** | **L** | **L** | **G** | **I** | **D** | **S** | **S** | **E** | **S** | **I** | **Q** | **A** | **I** | **I** | **A** | **V** | **T** | **G** | **E** | **A** | **E** | **E** | **G** | **H** | **Y** | **L** | **F** | **F** | **T** | **T** | **R** | **Q** | **G** | **T** | **V** | **K** | **R** | **T** | **S** | **V** | **T** | **A** | **F** | **A** | **N** | **I** | **R** | **S** | **N** | **G** | **L** | **I** | **A** | **I** | **G** | **L** | **K** | **E** | **D** | **D** | **E** | **L** | **V** | **N** | **V** | **L** | **L** | **T** | **D** | **G** | **Q** | **S** | **N** | **I** | **I** | **I** | **G** | **T** | **H** | **N** | **G** | **Y** | **S** | **V** | **T** | **F** | **K** | **E** | **E** | **A** | **V** | **R** | **D** | **M** | **G** | **R** | **T** | **A** | **S** | **G** | **V** | **R** | **G** | **I** | **R** | **L** | **R** | **E** | **E** | **D** | **Y** | **V** | **V** | **G** | **A** | **A** | **L** | **M** | **K** | **E** | **D** | **Q** | **E** | **V** | **L** | **V** | **I** | **T** | **E** | **K** | **G** | **Y** | **G** | **K** | **R** | **T** | **K** | **V** | **S** | **E** | **Y** | **P** | **V** | **K** | **G** | **R** | **G** | **G** | **K** | **G** | **I** | **K** | **T** | **A** | **N** | **I** | **T** | **E** | **K** | **N** | **G** | **P** | **L** | **A** | **G** | **L** | **T** | **T** | **V** | **S** | **G** | **D** | **E** | **D** | **I** | **M** | **L** | **I** | **T** | **D** | **K** | **G** | **V** | **I** | **I** | **R** | **F** | **N** | **V** | **S** | **T** | **V** | **S** | **Q** | **T** | **G** | **R** | **S** | **T** | **L** | **G** | **V** | **R** | **L** | **M** | **K** | **M** | **E** | **A** | **D** | **T** | **K** | **V** | **V** | **T** | **M** | **A** | **A** | **V** | **E** | **P** | **E** | **A** | **A** | **E** | **Q** | **T** | **V** | **E** | **E** | **Q** | **G** | **S** | **E** | **E** |
| **N52636** | **M** | **S** | **E** | **E** | **I** | **R** | **E** | **N** | **I** | **H** | **D** | **V** | **N** | **L** | **T** | **S** | **E** | **M** | **K** | **D** | **S** | **F** | **I** | **D** | **Y** | **A** | **M** | **S** | **V** | **I** | **V** | **A** | **R** | **A** | **L** | **P** | **D** | **V** | **R** | **D** | **G** | **L** | **K** | **P** | **V** | **H** | **R** | **R** | **I** | **L** | **Y** | **G** | **M** | **N** | **E** | **L** | **G** | **V** | **T** | **P** | **D** | **K** | **A** | **H** | **K** | **K** | **S** | **A** | **R** | **I** | **V** | **G** | **D** | **V** | **M** | **G** | **K** | **Y** | **H** | **P** | **H** | **G** | **D** | **S** | **A** | **I** | **Y** | **E** | **S** | **M** | **V** | **R** | **M** | **A** | **Q** | **P** | **F** | **S** | **Y** | **R** | **Y** | **M** | **L** | **V** | **D** | **G** | **H** | **G** | **N** | **F** | **G** | **S** | **V** | **D** | **G** | **D** | **G** | **A** | **A** | **A** | **M** | **R** | **Y** | **T** | **E** | **A** | **R** | **M** | **S** | **K** | **I** | **A** | **T** | **E** | **M** | **L** | **R** | **D** | **I** | **N** | **K** | **N** | **T** | **V** | **D** | **F** | **Q** | **S** | **N** | **Y** | **D** | **D** | **T** | **E** | **K** | **E** | **P** | **V** | **V** | **L** | **P** | **A** | **R** | **F** | **P** | **N** | **L** | **L** | **V** | **N** | **G** | **T** | **T** | **G** | **I** | **A** | **V** | **G** | **M** | **A** | **T** | **N** | **I** | **P** | **P** | **H** | **N** | **L** | **S** | **E** | **V** | **V** | **A** | **A** | **I** | **D** | **L** | **L** | **M** | **D** | **N** | **P** | **D** | **V** | **T** | **T** | **N** | **E** | **L** | **M** | **E** | **V** | **L** | **P** | **G** | **P** | **D** | **F** | **P** | **T** | **G** | **G** | **L** | **V** | **M** | **G** | **K** | **S** | **G** | **I** | **R** | **R** | **A** | **Y** | **E** | **T** | **G** | **K** | **G** | **S** | **I** | **T** | **V** | **R** | **A** | **K** | **V** | **E** | **I** | **T** | **E** | **M** | **P** | **N** | **G** | **K** | **E** | **R** | **I** | **I** | **V** | **T** | **E** | **L** | **P** | **Y** | **M** | **V** | **N** | **K** | **A** | **K** | **L** | **I** | **E** | **R** | **I** | **S** | **E** | **L** | **H** | **R** | **D** | **K** | **R** | **I** | **E** | **G** | **I** | **T** | **D** | **L** | **R** | **D** | **E** | **S** | **S** | **R** | **E** | **G** | **M** | **R** | **I** | **V** | **I** | **D** | **I** | **R** | **R** | **D** | **A** | **S** | **A** | **S** | **V** | **I** | **L** | **N** | **N** | **L** | **Y** | **K** | **L** | **T** | **S** | **L** | **Q** | **N** | **S** | **F** | **G** | **F** | **N** | **M** | **L** | **A** | **I** | **E** | **K** | **G** | **V** | **P** | **K** | **V** | **L** | **S** | **L** | **K** | **Q** | **I** | **L** | **E** | **N** | **Y** | **I** | **E** | **H** | **Q** | **R** | **E** | **V** | **I** | **T** | **R** | **R** | **T** | **V** | **F** | **E** | **K** | **E** | **K** | **A** | **E** | **A** | **R** | **A** | **H** | **I** | **L** | **E** | **G** | **L** | **R** | **I** | **A** | **L** | **D** | **H** | **I** | **D** | **E** | **I** | **I** | **A** | **I** | **I** | **R** | **G** | **S** | **K** | **S** | **D** | **D** | **E** | **A** | **K** | **A** | **T** | **M** | **I** | **E** | **R** | **F** | **D** | **L** | **S** | **D** | **R** | **Q** | **S** | **Q** | **A** | **I** | **L** | **D** | **M** | **R** | **L** | **R** | **R** | **L** | **T** | **G** | **L** | **E** | **R** | **E** | **K** | **I** | **E** | **N** | **E** | **Y** | **Q** | **E** | **L** | **L** | **K** | **L** | **I** | **E** | **D** | **L** | **T** | **D** | **I** | **L** | **A** | **R** | **P** | **E** | **R** | **V** | **T** | **E** | **I** | **I** | **K** | **N** | **E** | **L** | **A** | **D** | **L** | **N** | **Q** | **R** | **F** | **G** | **D** | **K** | **R** | **R** | **T** | **E** | **L** | **L** | **V** | **G** | **E** | **V** | **L** | **S** | **L** | **E** | **D** | **E** | **D** | **L** | **I** | **E** | **E** | **E** | **E** | **I** | **V** | **I** | **T** | **L** | **T** | **N** | **N** | **G** | **Y** | **I** | **K** | **R** | **L** | **A** | **N** | **N** | **E** | **F** | **R** | **A** | **Q** | **R** | **R** | **G** | **G** | **R** | **G** | **V** | **Q** | **G** | **M** | **G** | **I** | **H** | **N** | **D** | **D** | **F** | **V** | **K** | **N** | **L** | **V** | **S** | **C** | **S** | **T** | **H** | **D** | **T** | **L** | **L** | **F** | **F** | **T** | **N** | **N** | **G** | **K** | **V** | **Y** | **R** | **A** | **K** | **G** | **Y** | **E** | **I** | **P** | **E** | **Y** | **G** | **R** | **T** | **A** | **K** | **G** | **I** | **P** | **I** | **I** | **N** | **L** | **L** | **G** | **I** | **D** | **S** | **S** | **E** | **S** | **I** | **Q** | **A** | **I** | **I** | **A** | **V** | **T** | **G** | **E** | **A** | **E** | **E** | **G** | **H** | **Y** | **L** | **F** | **F** | **T** | **T** | **R** | **Q** | **G** | **T** | **V** | **K** | **R** | **T** | **S** | **V** | **T** | **A** | **F** | **A** | **N** | **I** | **R** | **S** | **N** | **G** | **L** | **I** | **A** | **I** | **G** | **L** | **K** | **E** | **D** | **D** | **E** | **L** | **V** | **N** | **V** | **L** | **L** | **T** | **D** | **G** | **Q** | **S** | **N** | **I** | **I** | **I** | **G** | **T** | **H** | **N** | **G** | **Y** | **S** | **V** | **T** | **F** | **K** | **E** | **E** | **A** | **V** | **R** | **D** | **M** | **G** | **R** | **T** | **A** | **S** | **G** | **V** | **R** | **G** | **I** | **R** | **L** | **R** | **E** | **E** | **D** | **Y** | **V** | **V** | **G** | **A** | **A** | **L** | **M** | **K** | **E** | **D** | **Q** | **E** | **V** | **L** | **V** | **I** | **T** | **E** | **K** | **G** | **Y** | **G** | **K** | **R** | **T** | **K** | **V** | **S** | **E** | **Y** | **P** | **V** | **K** | **G** | **R** | **G** | **G** | **K** | **G** | **I** | **K** | **T** | **A** | **N** | **I** | **T** | **E** | **K** | **N** | **G** | **P** | **L** | **A** | **G** | **L** | **T** | **T** | **V** | **S** | **G** | **D** | **E** | **D** | **I** | **M** | **L** | **I** | **T** | **D** | **K** | **G** | **V** | **I** | **I** | **R** | **F** | **N** | **V** | **S** | **T** | **V** | **S** | **Q** | **T** | **G** | **R** | **S** | **T** | **L** | **G** | **V** | **R** | **L** | **M** | **K** | **M** | **E** | **A** | **D** | **T** | **K** | **V** | **V** | **T** | **M** | **A** | **A** | **V** | **E** | **P** | **E** | **A** | **A** | **E** | **Q** | **T** | **V** | **E** | **E** | **Q** | **G** | **S** | **E** | **E** |
| **N59513F** | **M** | **S** | **E** | **E** | **I** | **R** | **E** | **N** | **I** | **H** | **D** | **V** | **N** | **L** | **T** | **S** | **E** | **M** | **K** | **D** | **S** | **F** | **I** | **D** | **Y** | **A** | **M** | **S** | **V** | **I** | **V** | **A** | **R** | **A** | **L** | **P** | **D** | **V** | **R** | **D** | **G** | **L** | **K** | **P** | **V** | **H** | **R** | **R** | **I** | **L** | **Y** | **G** | **M** | **N** | **E** | **L** | **G** | **V** | **T** | **P** | **D** | **K** | **A** | **H** | **K** | **K** | **S** | **A** | **R** | **I** | **V** | **G** | **D** | **V** | **M** | **G** | **K** | **Y** | **H** | **P** | **H** | **G** | **D** | **S** | **A** | **I** | **Y** | **E** | **S** | **M** | **V** | **R** | **M** | **A** | **Q** | **P** | **F** | **S** | **Y** | **R** | **Y** | **M** | **L** | **V** | **D** | **G** | **H** | **G** | **N** | **F** | **G** | **S** | **V** | **D** | **G** | **D** | **G** | **A** | **A** | **A** | **M** | **R** | **Y** | **T** | **E** | **A** | **R** | **M** | **S** | **K** | **I** | **A** | **T** | **E** | **M** | **L** | **R** | **D** | **I** | **N** | **K** | **N** | **T** | **V** | **D** | **F** | **Q** | **S** | **N** | **Y** | **D** | **D** | **T** | **E** | **K** | **E** | **P** | **V** | **V** | **L** | **P** | **A** | **R** | **F** | **P** | **N** | **L** | **L** | **V** | **N** | **G** | **T** | **T** | **G** | **I** | **A** | **V** | **G** | **M** | **A** | **T** | **N** | **I** | **P** | **P** | **H** | **N** | **L** | **S** | **E** | **V** | **V** | **A** | **A** | **I** | **D** | **L** | **L** | **M** | **D** | **N** | **P** | **D** | **V** | **T** | **T** | **N** | **E** | **L** | **M** | **E** | **V** | **L** | **P** | **G** | **P** | **D** | **F** | **P** | **T** | **G** | **G** | **L** | **V** | **M** | **G** | **K** | **S** | **G** | **I** | **R** | **R** | **A** | **Y** | **E** | **T** | **G** | **K** | **G** | **S** | **I** | **T** | **V** | **R** | **A** | **K** | **V** | **E** | **I** | **T** | **E** | **M** | **P** | **N** | **G** | **K** | **E** | **R** | **I** | **L** | **V** | **T** | **E** | **L** | **P** | **Y** | **M** | **V** | **N** | **K** | **A** | **K** | **L** | **I** | **E** | **R** | **I** | **S** | **E** | **L** | **H** | **R** | **D** | **K** | **R** | **I** | **E** | **G** | **I** | **T** | **D** | **L** | **R** | **D** | **E** | **S** | **S** | **R** | **E** | **G** | **M** | **R** | **I** | **V** | **I** | **D** | **I** | **R** | **R** | **D** | **A** | **S** | **A** | **S** | **V** | **I** | **L** | **N** | **N** | **L** | **Y** | **K** | **L** | **T** | **S** | **L** | **Q** | **N** | **S** | **F** | **G** | **F** | **N** | **M** | **L** | **A** | **I** | **E** | **K** | **G** | **V** | **P** | **K** | **V** | **L** | **S** | **L** | **K** | **Q** | **I** | **L** | **E** | **N** | **Y** | **I** | **E** | **H** | **Q** | **R** | **E** | **V** | **I** | **T** | **R** | **R** | **T** | **V** | **F** | **E** | **K** | **E** | **K** | **A** | **E** | **A** | **R** | **A** | **H** | **I** | **L** | **E** | **G** | **L** | **R** | **I** | **A** | **L** | **D** | **H** | **I** | **D** | **E** | **I** | **I** | **A** | **I** | **I** | **R** | **G** | **S** | **K** | **S** | **D** | **D** | **E** | **A** | **K** | **A** | **T** | **M** | **I** | **E** | **R** | **F** | **D** | **L** | **S** | **D** | **R** | **Q** | **S** | **Q** | **A** | **I** | **L** | **D** | **M** | **R** | **L** | **R** | **R** | **L** | **T** | **G** | **L** | **E** | **R** | **E** | **K** | **I** | **E** | **N** | **E** | **Y** | **Q** | **E** | **L** | **L** | **K** | **L** | **I** | **E** | **D** | **L** | **T** | **D** | **I** | **L** | **A** | **R** | **P** | **E** | **R** | **V** | **T** | **E** | **I** | **I** | **K** | **N** | **E** | **L** | **A** | **D** | **L** | **N** | **Q** | **R** | **F** | **G** | **D** | **K** | **R** | **R** | **T** | **E** | **L** | **L** | **V** | **G** | **E** | **V** | **L** | **S** | **L** | **E** | **D** | **E** | **D** | **L** | **I** | **E** | **E** | **E** | **E** | **I** | **V** | **I** | **T** | **L** | **T** | **N** | **N** | **G** | **Y** | **I** | **K** | **R** | **L** | **A** | **N** | **N** | **E** | **F** | **R** | **A** | **Q** | **R** | **R** | **G** | **G** | **R** | **G** | **V** | **Q** | **G** | **M** | **G** | **I** | **H** | **N** | **D** | **D** | **F** | **V** | **K** | **N** | **L** | **V** | **S** | **C** | **S** | **T** | **H** | **D** | **T** | **L** | **L** | **F** | **F** | **T** | **N** | **N** | **G** | **K** | **V** | **Y** | **R** | **A** | **K** | **G** | **Y** | **E** | **I** | **P** | **E** | **Y** | **G** | **R** | **T** | **A** | **K** | **G** | **I** | **P** | **I** | **I** | **N** | **L** | **L** | **G** | **I** | **D** | **S** | **S** | **E** | **S** | **I** | **Q** | **A** | **I** | **I** | **A** | **V** | **T** | **G** | **E** | **A** | **E** | **E** | **G** | **H** | **Y** | **L** | **F** | **F** | **T** | **T** | **R** | **Q** | **G** | **T** | **V** | **K** | **R** | **T** | **S** | **V** | **T** | **A** | **F** | **A** | **N** | **I** | **R** | **S** | **N** | **G** | **L** | **I** | **A** | **I** | **G** | **L** | **K** | **E** | **D** | **D** | **E** | **L** | **V** | **N** | **V** | **L** | **L** | **T** | **D** | **G** | **Q** | **S** | **N** | **I** | **I** | **I** | **G** | **T** | **H** | **N** | **G** | **Y** | **S** | **V** | **T** | **F** | **K** | **E** | **E** | **A** | **V** | **R** | **D** | **M** | **G** | **R** | **T** | **A** | **S** | **G** | **V** | **R** | **G** | **I** | **R** | **L** | **R** | **E** | **E** | **D** | **Y** | **V** | **V** | **G** | **A** | **A** | **L** | **M** | **K** | **E** | **D** | **Q** | **E** | **V** | **L** | **V** | **I** | **T** | **E** | **K** | **G** | **Y** | **G** | **K** | **R** | **T** | **K** | **V** | **S** | **E** | **Y** | **P** | **V** | **K** | **G** | **R** | **G** | **G** | **K** | **G** | **I** | **K** | **T** | **S** | **N** | **I** | **T** | **E** | **K** | **N** | **G** | **P** | **L** | **A** | **G** | **L** | **T** | **T** | **V** | **S** | **G** | **D** | **E** | **D** | **I** | **M** | **L** | **I** | **T** | **D** | **K** | **G** | **V** | **I** | **I** | **R** | **F** | **N** | **V** | **S** | **T** | **V** | **S** | **Q** | **T** | **G** | **R** | **S** | **T** | **L** | **G** | **V** | **R** | **L** | **M** | **K** | **M** | **E** | **A** | **D** | **T** | **K** | **V** | **V** | **T** | **M** | **-** | **-** | **-** | **-** | **-** | **-** | **-** | **-** | **-** | **-** | **-** | **-** | **-** | **-** | **-** | **-** | **-** | **-** | **-** |
| **N60417F** | **M** | **S** | **E** | **E** | **I** | **R** | **E** | **N** | **I** | **H** | **D** | **V** | **N** | **L** | **T** | **S** | **E** | **M** | **K** | **D** | **S** | **F** | **I** | **D** | **Y** | **A** | **M** | **S** | **V** | **I** | **V** | **A** | **R** | **A** | **L** | **P** | **D** | **V** | **R** | **D** | **G** | **L** | **K** | **P** | **V** | **H** | **R** | **R** | **I** | **L** | **Y** | **G** | **M** | **N** | **E** | **L** | **G** | **V** | **T** | **P** | **D** | **K** | **A** | **H** | **K** | **K** | **S** | **A** | **R** | **I** | **V** | **G** | **D** | **V** | **M** | **G** | **K** | **Y** | **H** | **P** | **H** | **G** | **D** | **S** | **A** | **I** | **Y** | **E** | **S** | **M** | **V** | **R** | **M** | **A** | **Q** | **P** | **F** | **S** | **Y** | **R** | **Y** | **M** | **L** | **V** | **D** | **G** | **H** | **G** | **N** | **F** | **G** | **S** | **V** | **D** | **G** | **D** | **G** | **A** | **A** | **A** | **M** | **R** | **Y** | **T** | **E** | **A** | **R** | **M** | **S** | **K** | **I** | **A** | **T** | **E** | **M** | **L** | **R** | **D** | **I** | **N** | **K** | **N** | **T** | **V** | **D** | **F** | **Q** | **S** | **N** | **Y** | **D** | **D** | **T** | **E** | **K** | **E** | **P** | **V** | **V** | **L** | **P** | **A** | **R** | **F** | **P** | **N** | **L** | **L** | **V** | **N** | **G** | **T** | **T** | **G** | **I** | **A** | **V** | **G** | **M** | **A** | **T** | **N** | **I** | **P** | **P** | **H** | **N** | **L** | **S** | **E** | **V** | **V** | **A** | **A** | **I** | **D** | **L** | **L** | **M** | **D** | **N** | **P** | **D** | **V** | **T** | **T** | **N** | **E** | **L** | **M** | **E** | **V** | **L** | **P** | **G** | **P** | **D** | **F** | **P** | **T** | **G** | **G** | **L** | **V** | **M** | **G** | **K** | **S** | **G** | **I** | **R** | **R** | **A** | **Y** | **E** | **T** | **G** | **K** | **G** | **S** | **I** | **T** | **V** | **R** | **A** | **K** | **V** | **E** | **I** | **T** | **E** | **M** | **P** | **N** | **G** | **K** | **E** | **R** | **I** | **L** | **V** | **T** | **E** | **L** | **P** | **Y** | **M** | **V** | **N** | **K** | **A** | **K** | **L** | **I** | **E** | **R** | **I** | **S** | **E** | **L** | **H** | **R** | **D** | **K** | **R** | **I** | **E** | **G** | **I** | **T** | **D** | **L** | **R** | **D** | **E** | **S** | **S** | **R** | **E** | **G** | **M** | **R** | **I** | **V** | **I** | **D** | **V** | **R** | **R** | **D** | **A** | **S** | **A** | **S** | **V** | **I** | **L** | **N** | **N** | **L** | **Y** | **K** | **L** | **T** | **S** | **L** | **Q** | **N** | **S** | **F** | **G** | **F** | **N** | **M** | **L** | **A** | **I** | **E** | **K** | **G** | **V** | **P** | **K** | **V** | **L** | **S** | **L** | **K** | **Q** | **I** | **L** | **E** | **N** | **Y** | **I** | **E** | **H** | **Q** | **R** | **E** | **V** | **I** | **T** | **R** | **R** | **T** | **V** | **F** | **E** | **K** | **E** | **K** | **A** | **E** | **A** | **R** | **A** | **H** | **I** | **L** | **E** | **G** | **L** | **R** | **I** | **A** | **L** | **D** | **H** | **I** | **D** | **E** | **I** | **I** | **A** | **I** | **I** | **R** | **G** | **S** | **K** | **S** | **D** | **D** | **E** | **A** | **K** | **A** | **T** | **M** | **I** | **E** | **R** | **F** | **D** | **L** | **S** | **D** | **R** | **Q** | **S** | **Q** | **A** | **I** | **L** | **D** | **M** | **R** | **L** | **R** | **R** | **L** | **T** | **G** | **L** | **E** | **R** | **E** | **K** | **I** | **E** | **N** | **E** | **Y** | **Q** | **E** | **L** | **L** | **K** | **L** | **I** | **E** | **D** | **L** | **T** | **D** | **I** | **L** | **A** | **R** | **P** | **E** | **R** | **V** | **T** | **E** | **I** | **I** | **K** | **N** | **E** | **L** | **A** | **D** | **L** | **N** | **Q** | **R** | **F** | **G** | **D** | **K** | **R** | **R** | **T** | **E** | **L** | **L** | **V** | **G** | **E** | **V** | **L** | **S** | **L** | **E** | **D** | **E** | **D** | **L** | **I** | **E** | **E** | **E** | **E** | **I** | **V** | **I** | **T** | **L** | **T** | **N** | **N** | **G** | **Y** | **I** | **K** | **R** | **L** | **A** | **N** | **N** | **E** | **F** | **R** | **A** | **Q** | **R** | **R** | **G** | **G** | **R** | **G** | **V** | **Q** | **G** | **M** | **G** | **I** | **H** | **N** | **D** | **D** | **F** | **V** | **K** | **N** | **L** | **V** | **S** | **C** | **S** | **T** | **H** | **D** | **T** | **L** | **L** | **F** | **F** | **T** | **N** | **N** | **G** | **K** | **V** | **Y** | **R** | **A** | **K** | **G** | **Y** | **E** | **I** | **P** | **E** | **Y** | **G** | **R** | **T** | **A** | **K** | **G** | **I** | **P** | **I** | **I** | **N** | **L** | **L** | **G** | **I** | **D** | **S** | **S** | **E** | **S** | **I** | **Q** | **A** | **I** | **I** | **A** | **V** | **T** | **G** | **E** | **A** | **E** | **E** | **G** | **H** | **Y** | **L** | **F** | **F** | **T** | **T** | **R** | **Q** | **G** | **T** | **V** | **K** | **R** | **T** | **S** | **V** | **T** | **A** | **F** | **A** | **N** | **I** | **R** | **S** | **N** | **G** | **L** | **I** | **A** | **I** | **G** | **L** | **K** | **E** | **D** | **D** | **E** | **L** | **V** | **N** | **V** | **L** | **L** | **T** | **D** | **G** | **Q** | **S** | **N** | **I** | **I** | **I** | **G** | **T** | **H** | **N** | **G** | **Y** | **S** | **V** | **T** | **F** | **K** | **E** | **E** | **A** | **V** | **R** | **D** | **M** | **G** | **R** | **T** | **A** | **S** | **G** | **V** | **R** | **G** | **I** | **R** | **L** | **R** | **E** | **E** | **D** | **Y** | **V** | **V** | **G** | **A** | **A** | **L** | **M** | **K** | **E** | **D** | **Q** | **E** | **V** | **L** | **V** | **I** | **T** | **E** | **K** | **G** | **Y** | **G** | **K** | **R** | **T** | **K** | **V** | **S** | **E** | **Y** | **P** | **V** | **K** | **G** | **R** | **G** | **G** | **K** | **G** | **I** | **K** | **T** | **A** | **N** | **I** | **T** | **E** | **K** | **N** | **G** | **P** | **L** | **A** | **G** | **L** | **T** | **T** | **V** | **S** | **G** | **N** | **E** | **D** | **I** | **M** | **L** | **I** | **T** | **D** | **K** | **G** | **V** | **I** | **I** | **R** | **F** | **N** | **V** | **S** | **T** | **V** | **S** | **Q** | **T** | **G** | **R** | **S** | **T** | **L** | **G** | **V** | **R** | **L** | **M** | **K** | **M** | **E** | **A** | **D** | **T** | **K** | **V** | **V** | **T** | **M** | **-** | **-** | **-** | **-** | **-** | **-** | **-** | **-** | **-** | **-** | **-** | **-** | **-** | **-** | **-** | **-** | **-** | **-** | **-** |
| **N59896F** | **M** | **S** | **E** | **E** | **I** | **K** | **E** | **N** | **I** | **Q** | **D** | **V** | **N** | **L** | **T** | **S** | **E** | **M** | **K** | **E** | **S** | **F** | **I** | **D** | **Y** | **A** | **M** | **S** | **V** | **I** | **V** | **A** | **R** | **A** | **L** | **P** | **D** | **V** | **R** | **D** | **G** | **L** | **K** | **P** | **V** | **H** | **R** | **R** | **I** | **L** | **Y** | **G** | **M** | **N** | **E** | **L** | **G** | **V** | **T** | **P** | **D** | **K** | **P** | **H** | **K** | **K** | **S** | **A** | **R** | **I** | **V** | **G** | **D** | **V** | **M** | **G** | **K** | **Y** | **H** | **P** | **H** | **G** | **D** | **S** | **A** | **I** | **Y** | **E** | **S** | **M** | **V** | **R** | **M** | **A** | **Q** | **P** | **F** | **S** | **Y** | **R** | **A** | **M** | **L** | **V** | **D** | **G** | **H** | **G** | **N** | **F** | **G** | **S** | **V** | **D** | **G** | **D** | **G** | **A** | **A** | **A** | **M** | **R** | **Y** | **T** | **E** | **A** | **R** | **M** | **S** | **K** | **I** | **A** | **L** | **E** | **M** | **L** | **R** | **D** | **I** | **N** | **K** | **N** | **T** | **V** | **D** | **F** | **Q** | **G** | **N** | **Y** | **D** | **D** | **S** | **E** | **Q** | **E** | **P** | **V** | **V** | **L** | **P** | **A** | **R** | **F** | **P** | **N** | **L** | **L** | **V** | **N** | **G** | **T** | **T** | **G** | **I** | **A** | **V** | **G** | **M** | **A** | **T** | **N** | **I** | **P** | **P** | **H** | **N** | **L** | **S** | **E** | **V** | **I** | **D** | **A** | **T** | **S** | **L** | **L** | **M** | **D** | **N** | **P** | **D** | **V** | **T** | **T** | **N** | **E** | **L** | **M** | **E** | **V** | **L** | **P** | **G** | **P** | **D** | **F** | **P** | **T** | **G** | **G** | **L** | **V** | **M** | **G** | **K** | **S** | **G** | **I** | **R** | **R** | **A** | **Y** | **E** | **T** | **G** | **K** | **G** | **S** | **I** | **T** | **V** | **R** | **A** | **K** | **V** | **E** | **L** | **T** | **E** | **M** | **P** | **N** | **G** | **K** | **E** | **R** | **I** | **L** | **V** | **T** | **E** | **L** | **P** | **Y** | **M** | **V** | **N** | **K** | **A** | **K** | **L** | **I** | **E** | **R** | **I** | **S** | **E** | **L** | **H** | **R** | **D** | **K** | **R** | **I** | **E** | **G** | **I** | **T** | **D** | **L** | **R** | **D** | **E** | **S** | **S** | **R** | **E** | **G** | **M** | **R** | **I** | **V** | **I** | **D** | **V** | **R** | **R** | **D** | **V** | **S** | **A** | **S** | **V** | **V** | **L** | **N** | **N** | **L** | **Y** | **K** | **M** | **T** | **A** | **L** | **Q** | **T** | **S** | **F** | **G** | **F** | **N** | **M** | **L** | **A** | **I** | **E** | **K** | **G** | **V** | **P** | **K** | **I** | **L** | **S** | **L** | **K** | **R** | **I** | **L** | **E** | **N** | **Y** | **V** | **E** | **H** | **Q** | **K** | **E** | **V** | **I** | **T** | **R** | **R** | **T** | **I** | **F** | **D** | **K** | **N** | **K** | **A** | **E** | **A** | **R** | **A** | **H** | **I** | **L** | **E** | **G** | **L** | **R** | **I** | **A** | **L** | **D** | **H** | **I** | **D** | **E** | **I** | **I** | **A** | **I** | **I** | **R** | **G** | **S** | **Q** | **S** | **D** | **D** | **E** | **A** | **K** | **A** | **T** | **L** | **I** | **E** | **R** | **F** | **E** | **F** | **S** | **D** | **R** | **Q** | **A** | **Q** | **A** | **I** | **L** | **D** | **M** | **R** | **L** | **R** | **R** | **L** | **T** | **G** | **L** | **E** | **R** | **D** | **K** | **I** | **E** | **N** | **E** | **Y** | **Q** | **E** | **L** | **L** | **K** | **F** | **I** | **A** | **D** | **L** | **E** | **D** | **I** | **L** | **A** | **R** | **P** | **E** | **R** | **V** | **I** | **E** | **I** | **I** | **K** | **T** | **E** | **L** | **N** | **D** | **V** | **R** | **T** | **K** | **F** | **G** | **D** | **A** | **R** | **R** | **T** | **E** | **L** | **L** | **V** | **G** | **E** | **V** | **L** | **S** | **L** | **E** | **D** | **E** | **D** | **L** | **I** | **E** | **E** | **E** | **E** | **V** | **V** | **I** | **T** | **L** | **T** | **N** | **N** | **G** | **Y** | **I** | **K** | **R** | **M** | **A** | **N** | **S** | **E** | **F** | **R** | **A** | **Q** | **R** | **R** | **G** | **G** | **R** | **G** | **V** | **Q** | **G** | **M** | **G** | **V** | **H** | **D** | **D** | **D** | **F** | **V** | **K** | **N** | **L** | **V** | **S** | **C** | **S** | **T** | **H** | **D** | **T** | **L** | **L** | **F** | **F** | **T** | **N** | **T** | **G** | **K** | **V** | **Y** | **R** | **A** | **K** | **G** | **Y** | **E** | **I** | **P** | **E** | **Y** | **G** | **R** | **T** | **A** | **K** | **G** | **I** | **P** | **V** | **I** | **N** | **L** | **L** | **G** | **I** | **D** | **S** | **A** | **E** | **K** | **I** | **Q** | **A** | **I** | **I** | **S** | **V** | **E** | **G** | **K** | **A** | **E** | **A** | **G** | **K** | **Y** | **L** | **F** | **F** | **T** | **T** | **L** | **K** | **G** | **T** | **V** | **K** | **R** | **T** | **A** | **V** | **T** | **A** | **F** | **S** | **N** | **I** | **R** | **S** | **N** | **G** | **L** | **I** | **A** | **I** | **S** | **L** | **K** | **E** | **D** | **D** | **E** | **L** | **V** | **N** | **V** | **V** | **T** | **T** | **N** | **G** | **N** | **Q** | **K** | **M** | **I** | **I** | **G** | **T** | **H** | **A** | **G** | **Y** | **S** | **V** | **T** | **F** | **D** | **E** | **N** | **T** | **V** | **R** | **D** | **M** | **G** | **R** | **T** | **A** | **S** | **G** | **V** | **R** | **G** | **I** | **R** | **L** | **R** | **E** | **N** | **D** | **Y** | **V** | **V** | **G** | **A** | **A** | **I** | **L** | **D** | **E** | **N** | **K** | **E** | **V** | **L** | **V** | **I** | **T** | **E** | **N** | **G** | **Y** | **G** | **K** | **R** | **T** | **K** | **A** | **S** | **E** | **Y** | **P** | **V** | **K** | **G** | **R** | **G** | **G** | **K** | **G** | **I** | **K** | **T** | **A** | **N** | **I** | **T** | **E** | **K** | **N** | **G** | **P** | **L** | **A** | **G** | **L** | **T** | **T** | **V** | **N** | **G** | **D** | **E** | **D** | **I** | **L** | **L** | **I** | **T** | **N** | **K** | **G** | **V** | **I** | **I** | **R** | **F** | **N** | **V** | **D** | **S** | **V** | **S** | **Q** | **T** | **G** | **R** | **A** | **T** | **L** | **G** | **V** | **R** | **L** | **M** | **R** | **M** | **E** | **D** | **G** | **A** | **K** | **-** | **-** | **-** | **-** | **-** | **-** | **-** | **-** | **-** | **-** | **-** | **-** | **-** | **-** | **-** | **-** | **-** | **-** | **-** | **-** | **-** | **-** | **-** |
| **N52751** | **M** | **S** | **E** | **E** | **I** | **R** | **E** | **N** | **I** | **H** | **D** | **V** | **N** | **L** | **T** | **S** | **E** | **M** | **K** | **D** | **S** | **F** | **I** | **D** | **Y** | **A** | **M** | **S** | **V** | **I** | **V** | **A** | **R** | **A** | **L** | **P** | **D** | **V** | **R** | **D** | **G** | **L** | **K** | **P** | **V** | **H** | **R** | **R** | **I** | **L** | **Y** | **G** | **M** | **N** | **E** | **L** | **G** | **V** | **T** | **P** | **D** | **K** | **A** | **H** | **K** | **K** | **S** | **A** | **R** | **I** | **V** | **G** | **D** | **V** | **M** | **G** | **K** | **Y** | **H** | **P** | **H** | **G** | **D** | **S** | **A** | **I** | **Y** | **E** | **S** | **M** | **V** | **R** | **M** | **A** | **Q** | **P** | **F** | **S** | **Y** | **R** | **Y** | **M** | **L** | **V** | **D** | **G** | **H** | **G** | **N** | **F** | **G** | **S** | **V** | **D** | **G** | **D** | **G** | **A** | **A** | **A** | **M** | **R** | **Y** | **T** | **E** | **A** | **R** | **M** | **S** | **K** | **I** | **A** | **T** | **E** | **M** | **L** | **R** | **D** | **I** | **N** | **K** | **N** | **T** | **V** | **D** | **F** | **Q** | **S** | **N** | **Y** | **D** | **D** | **T** | **E** | **K** | **E** | **P** | **V** | **V** | **L** | **P** | **A** | **R** | **F** | **P** | **N** | **L** | **L** | **V** | **N** | **G** | **T** | **T** | **G** | **I** | **A** | **V** | **G** | **M** | **A** | **T** | **N** | **I** | **P** | **P** | **H** | **N** | **L** | **S** | **E** | **V** | **V** | **A** | **A** | **I** | **D** | **L** | **L** | **M** | **D** | **N** | **P** | **D** | **V** | **T** | **T** | **N** | **E** | **L** | **M** | **E** | **V** | **L** | **P** | **G** | **P** | **D** | **F** | **P** | **T** | **G** | **G** | **L** | **V** | **M** | **G** | **K** | **S** | **G** | **I** | **R** | **R** | **A** | **Y** | **E** | **T** | **G** | **K** | **G** | **S** | **I** | **T** | **V** | **R** | **A** | **K** | **V** | **E** | **I** | **T** | **E** | **M** | **P** | **N** | **G** | **K** | **E** | **R** | **I** | **I** | **V** | **T** | **E** | **L** | **P** | **Y** | **M** | **V** | **N** | **K** | **A** | **K** | **L** | **I** | **E** | **R** | **I** | **S** | **E** | **L** | **H** | **R** | **D** | **K** | **R** | **I** | **E** | **G** | **I** | **T** | **D** | **L** | **R** | **D** | **E** | **S** | **S** | **R** | **E** | **G** | **M** | **R** | **I** | **V** | **I** | **D** | **I** | **R** | **R** | **D** | **A** | **S** | **A** | **S** | **V** | **I** | **L** | **N** | **N** | **L** | **Y** | **K** | **L** | **T** | **S** | **L** | **Q** | **N** | **S** | **F** | **G** | **F** | **N** | **M** | **L** | **A** | **I** | **E** | **K** | **G** | **V** | **P** | **K** | **V** | **L** | **S** | **L** | **K** | **Q** | **I** | **L** | **E** | **N** | **Y** | **I** | **E** | **H** | **Q** | **R** | **E** | **V** | **I** | **T** | **R** | **R** | **T** | **V** | **F** | **E** | **K** | **E** | **K** | **A** | **E** | **A** | **R** | **A** | **H** | **I** | **L** | **E** | **G** | **L** | **R** | **I** | **A** | **L** | **D** | **H** | **I** | **D** | **E** | **I** | **I** | **A** | **I** | **I** | **R** | **G** | **S** | **K** | **S** | **D** | **D** | **E** | **A** | **K** | **A** | **T** | **M** | **I** | **E** | **R** | **F** | **D** | **L** | **S** | **D** | **R** | **Q** | **S** | **Q** | **A** | **I** | **L** | **D** | **M** | **R** | **L** | **R** | **R** | **L** | **T** | **G** | **L** | **E** | **R** | **E** | **K** | **I** | **E** | **N** | **E** | **Y** | **Q** | **E** | **L** | **L** | **K** | **L** | **I** | **E** | **D** | **L** | **T** | **D** | **I** | **L** | **A** | **R** | **P** | **E** | **R** | **V** | **T** | **E** | **I** | **I** | **K** | **N** | **E** | **L** | **A** | **D** | **L** | **N** | **Q** | **R** | **F** | **G** | **D** | **K** | **R** | **R** | **T** | **E** | **L** | **L** | **V** | **G** | **E** | **V** | **L** | **S** | **L** | **E** | **D** | **E** | **D** | **L** | **I** | **E** | **E** | **E** | **E** | **I** | **V** | **I** | **T** | **L** | **T** | **N** | **N** | **G** | **Y** | **I** | **K** | **R** | **L** | **A** | **N** | **N** | **E** | **F** | **R** | **A** | **Q** | **R** | **R** | **G** | **G** | **R** | **G** | **V** | **Q** | **G** | **M** | **G** | **I** | **H** | **N** | **D** | **D** | **F** | **V** | **K** | **N** | **L** | **V** | **S** | **C** | **S** | **T** | **H** | **D** | **T** | **L** | **L** | **F** | **F** | **T** | **N** | **N** | **G** | **K** | **V** | **Y** | **R** | **A** | **K** | **G** | **Y** | **E** | **I** | **P** | **E** | **Y** | **G** | **R** | **T** | **A** | **K** | **G** | **I** | **P** | **I** | **I** | **N** | **L** | **L** | **G** | **I** | **D** | **S** | **S** | **E** | **S** | **I** | **Q** | **A** | **I** | **I** | **A** | **V** | **T** | **G** | **E** | **A** | **E** | **E** | **G** | **H** | **Y** | **L** | **F** | **F** | **T** | **T** | **R** | **Q** | **G** | **T** | **V** | **K** | **R** | **T** | **S** | **V** | **T** | **A** | **F** | **A** | **N** | **I** | **R** | **S** | **N** | **G** | **L** | **I** | **A** | **I** | **G** | **L** | **K** | **E** | **D** | **D** | **E** | **L** | **V** | **N** | **V** | **L** | **L** | **T** | **D** | **G** | **Q** | **S** | **N** | **I** | **I** | **I** | **G** | **T** | **H** | **N** | **G** | **Y** | **S** | **V** | **T** | **F** | **K** | **E** | **E** | **A** | **V** | **R** | **D** | **M** | **G** | **R** | **T** | **A** | **S** | **G** | **V** | **R** | **G** | **I** | **R** | **L** | **R** | **E** | **E** | **D** | **Y** | **V** | **V** | **G** | **A** | **A** | **L** | **M** | **K** | **E** | **N** | **Q** | **E** | **V** | **L** | **V** | **I** | **T** | **E** | **K** | **G** | **Y** | **G** | **K** | **R** | **T** | **K** | **V** | **S** | **E** | **Y** | **P** | **V** | **K** | **G** | **R** | **G** | **G** | **K** | **G** | **I** | **K** | **T** | **A** | **N** | **I** | **T** | **E** | **K** | **N** | **G** | **P** | **L** | **A** | **G** | **L** | **T** | **T** | **V** | **S** | **G** | **D** | **E** | **D** | **I** | **M** | **L** | **I** | **T** | **D** | **K** | **G** | **V** | **I** | **I** | **R** | **F** | **N** | **V** | **S** | **T** | **V** | **S** | **Q** | **T** | **G** | **R** | **S** | **T** | **L** | **G** | **V** | **R** | **L** | **M** | **K** | **M** | **E** | **A** | **D** | **T** | **K** | **V** | **V** | **T** | **M** | **A** | **A** | **V** | **E** | **P** | **E** | **A** | **A** | **E** | **Q** | **T** | **V** | **E** | **E** | **Q** | **G** | **S** | **E** | **E** |
| **N52729** | **M** | **S** | **E** | **E** | **I** | **K** | **E** | **N** | **I** | **Q** | **D** | **V** | **N** | **L** | **T** | **S** | **E** | **M** | **K** | **E** | **S** | **F** | **I** | **D** | **Y** | **A** | **M** | **S** | **V** | **I** | **V** | **A** | **R** | **A** | **L** | **P** | **D** | **V** | **R** | **D** | **G** | **L** | **K** | **P** | **V** | **H** | **R** | **R** | **I** | **L** | **Y** | **G** | **M** | **N** | **E** | **L** | **G** | **V** | **T** | **P** | **D** | **K** | **P** | **H** | **K** | **K** | **S** | **A** | **R** | **I** | **V** | **G** | **D** | **V** | **M** | **G** | **K** | **Y** | **H** | **P** | **H** | **G** | **D** | **S** | **A** | **I** | **Y** | **E** | **S** | **M** | **V** | **R** | **M** | **A** | **Q** | **P** | **F** | **S** | **Y** | **R** | **A** | **M** | **L** | **V** | **D** | **G** | **H** | **G** | **N** | **F** | **G** | **S** | **V** | **D** | **G** | **D** | **G** | **A** | **A** | **A** | **M** | **R** | **Y** | **T** | **E** | **A** | **R** | **M** | **S** | **K** | **I** | **A** | **L** | **E** | **M** | **L** | **R** | **D** | **I** | **N** | **K** | **N** | **T** | **V** | **D** | **F** | **Q** | **G** | **N** | **Y** | **D** | **D** | **S** | **E** | **Q** | **E** | **P** | **V** | **V** | **L** | **P** | **A** | **R** | **F** | **P** | **N** | **L** | **L** | **V** | **N** | **G** | **T** | **T** | **G** | **I** | **A** | **V** | **G** | **M** | **A** | **T** | **N** | **I** | **P** | **P** | **H** | **N** | **L** | **S** | **E** | **V** | **I** | **D** | **A** | **T** | **S** | **L** | **L** | **M** | **D** | **N** | **P** | **D** | **V** | **T** | **T** | **N** | **E** | **L** | **M** | **E** | **V** | **L** | **P** | **G** | **P** | **D** | **F** | **P** | **T** | **G** | **G** | **L** | **V** | **M** | **G** | **K** | **S** | **G** | **I** | **R** | **R** | **A** | **Y** | **E** | **T** | **G** | **K** | **G** | **S** | **I** | **T** | **V** | **R** | **A** | **K** | **V** | **E** | **L** | **T** | **E** | **M** | **P** | **N** | **G** | **K** | **E** | **R** | **I** | **L** | **V** | **T** | **E** | **L** | **P** | **Y** | **M** | **V** | **N** | **K** | **A** | **K** | **L** | **I** | **E** | **R** | **I** | **S** | **E** | **L** | **H** | **R** | **D** | **K** | **R** | **I** | **E** | **G** | **I** | **T** | **D** | **L** | **R** | **D** | **E** | **S** | **S** | **R** | **E** | **G** | **M** | **R** | **I** | **V** | **I** | **D** | **V** | **R** | **R** | **D** | **V** | **S** | **A** | **S** | **V** | **V** | **L** | **N** | **N** | **L** | **Y** | **K** | **M** | **T** | **A** | **L** | **Q** | **T** | **S** | **F** | **G** | **F** | **N** | **M** | **L** | **A** | **I** | **E** | **K** | **G** | **V** | **P** | **K** | **I** | **L** | **S** | **L** | **K** | **R** | **I** | **L** | **E** | **N** | **Y** | **V** | **E** | **H** | **Q** | **K** | **E** | **V** | **I** | **T** | **R** | **R** | **T** | **I** | **F** | **D** | **K** | **N** | **K** | **A** | **E** | **A** | **R** | **A** | **H** | **I** | **L** | **E** | **G** | **L** | **R** | **I** | **A** | **L** | **D** | **H** | **I** | **D** | **E** | **I** | **I** | **A** | **I** | **I** | **R** | **G** | **S** | **Q** | **S** | **D** | **D** | **E** | **A** | **K** | **A** | **T** | **L** | **I** | **E** | **R** | **F** | **E** | **F** | **S** | **D** | **R** | **Q** | **A** | **Q** | **A** | **I** | **L** | **D** | **M** | **R** | **L** | **R** | **R** | **L** | **T** | **G** | **L** | **E** | **R** | **D** | **K** | **I** | **E** | **N** | **E** | **Y** | **Q** | **E** | **L** | **L** | **K** | **F** | **I** | **A** | **D** | **L** | **E** | **D** | **I** | **L** | **A** | **R** | **P** | **E** | **R** | **V** | **I** | **E** | **I** | **I** | **K** | **T** | **E** | **L** | **N** | **D** | **V** | **R** | **T** | **K** | **F** | **G** | **D** | **A** | **R** | **R** | **T** | **E** | **L** | **L** | **V** | **G** | **E** | **V** | **L** | **S** | **L** | **E** | **D** | **E** | **D** | **L** | **I** | **E** | **E** | **E** | **E** | **V** | **V** | **I** | **T** | **L** | **T** | **N** | **N** | **G** | **Y** | **I** | **K** | **R** | **M** | **A** | **N** | **S** | **E** | **F** | **R** | **A** | **Q** | **R** | **R** | **G** | **G** | **R** | **G** | **V** | **Q** | **G** | **M** | **G** | **V** | **H** | **D** | **D** | **D** | **F** | **V** | **K** | **N** | **L** | **V** | **S** | **C** | **S** | **T** | **H** | **D** | **T** | **L** | **L** | **F** | **F** | **T** | **N** | **T** | **G** | **K** | **V** | **Y** | **R** | **A** | **K** | **G** | **Y** | **E** | **I** | **P** | **E** | **Y** | **G** | **R** | **T** | **A** | **K** | **G** | **I** | **P** | **V** | **I** | **N** | **L** | **L** | **G** | **I** | **D** | **S** | **A** | **E** | **K** | **I** | **Q** | **A** | **I** | **I** | **S** | **V** | **E** | **G** | **K** | **A** | **E** | **A** | **G** | **K** | **Y** | **L** | **F** | **F** | **T** | **T** | **L** | **K** | **G** | **T** | **V** | **K** | **R** | **T** | **A** | **V** | **T** | **A** | **F** | **S** | **N** | **I** | **R** | **S** | **N** | **G** | **L** | **I** | **A** | **I** | **S** | **L** | **K** | **E** | **D** | **D** | **E** | **L** | **V** | **N** | **V** | **V** | **T** | **T** | **N** | **G** | **N** | **Q** | **K** | **M** | **I** | **I** | **G** | **T** | **H** | **A** | **G** | **Y** | **S** | **V** | **T** | **F** | **D** | **E** | **N** | **T** | **V** | **R** | **D** | **M** | **G** | **R** | **T** | **A** | **S** | **G** | **V** | **R** | **G** | **I** | **R** | **L** | **R** | **E** | **N** | **D** | **Y** | **V** | **V** | **G** | **A** | **A** | **I** | **L** | **D** | **E** | **N** | **K** | **E** | **V** | **L** | **V** | **I** | **T** | **E** | **N** | **G** | **Y** | **G** | **K** | **R** | **T** | **K** | **A** | **S** | **E** | **Y** | **P** | **V** | **K** | **G** | **R** | **G** | **G** | **K** | **G** | **I** | **K** | **T** | **A** | **N** | **I** | **T** | **E** | **K** | **N** | **G** | **P** | **L** | **A** | **G** | **L** | **T** | **T** | **V** | **N** | **G** | **D** | **E** | **D** | **I** | **L** | **L** | **I** | **T** | **N** | **K** | **G** | **V** | **I** | **I** | **R** | **F** | **N** | **V** | **D** | **S** | **V** | **S** | **Q** | **T** | **G** | **R** | **A** | **T** | **L** | **G** | **V** | **R** | **L** | **M** | **R** | **M** | **E** | **D** | **G** | **A** | **K** | **-** | **-** | **-** | **-** | **-** | **-** | **-** | **-** | **-** | **-** | **-** | **-** | **-** | **-** | **-** | **-** | **-** | **-** | **-** | **-** | **-** | **-** | **-** |
| **N54150** | **M** | **S** | **E** | **E** | **I** | **R** | **E** | **N** | **I** | **H** | **D** | **V** | **N** | **L** | **T** | **S** | **E** | **M** | **K** | **D** | **S** | **F** | **I** | **D** | **Y** | **A** | **M** | **S** | **V** | **I** | **V** | **A** | **R** | **A** | **L** | **P** | **D** | **V** | **R** | **D** | **G** | **L** | **K** | **P** | **V** | **H** | **R** | **R** | **I** | **L** | **Y** | **G** | **M** | **N** | **E** | **L** | **G** | **V** | **T** | **P** | **D** | **K** | **A** | **H** | **K** | **K** | **S** | **A** | **R** | **I** | **V** | **G** | **D** | **V** | **M** | **G** | **K** | **Y** | **H** | **P** | **H** | **G** | **D** | **S** | **A** | **I** | **Y** | **E** | **S** | **M** | **V** | **R** | **M** | **A** | **Q** | **P** | **F** | **S** | **Y** | **R** | **Y** | **M** | **L** | **V** | **D** | **G** | **H** | **G** | **N** | **F** | **G** | **S** | **V** | **D** | **G** | **D** | **G** | **A** | **A** | **A** | **M** | **R** | **Y** | **T** | **E** | **A** | **R** | **M** | **S** | **K** | **I** | **A** | **T** | **E** | **M** | **L** | **R** | **D** | **I** | **N** | **K** | **N** | **T** | **V** | **D** | **F** | **Q** | **S** | **N** | **Y** | **D** | **D** | **T** | **E** | **K** | **E** | **P** | **V** | **V** | **L** | **P** | **A** | **R** | **F** | **P** | **N** | **L** | **L** | **V** | **N** | **G** | **T** | **T** | **G** | **I** | **A** | **V** | **G** | **M** | **A** | **T** | **N** | **I** | **P** | **P** | **H** | **N** | **L** | **S** | **E** | **V** | **V** | **A** | **A** | **I** | **D** | **L** | **L** | **M** | **D** | **N** | **P** | **D** | **V** | **T** | **T** | **N** | **E** | **L** | **M** | **E** | **V** | **L** | **P** | **G** | **P** | **D** | **F** | **P** | **T** | **G** | **G** | **L** | **V** | **M** | **G** | **K** | **S** | **G** | **I** | **R** | **R** | **A** | **Y** | **E** | **T** | **G** | **K** | **G** | **S** | **I** | **T** | **V** | **R** | **A** | **K** | **V** | **E** | **I** | **T** | **E** | **M** | **P** | **N** | **G** | **K** | **E** | **R** | **I** | **I** | **V** | **T** | **E** | **L** | **P** | **Y** | **M** | **V** | **N** | **K** | **A** | **K** | **L** | **I** | **E** | **R** | **I** | **S** | **E** | **L** | **H** | **R** | **D** | **K** | **R** | **I** | **E** | **G** | **I** | **T** | **D** | **L** | **R** | **D** | **E** | **S** | **S** | **R** | **E** | **G** | **M** | **R** | **I** | **V** | **I** | **D** | **I** | **R** | **R** | **D** | **A** | **S** | **A** | **S** | **V** | **I** | **L** | **N** | **N** | **L** | **Y** | **K** | **L** | **T** | **S** | **L** | **Q** | **N** | **S** | **F** | **G** | **F** | **N** | **M** | **L** | **A** | **I** | **E** | **K** | **G** | **V** | **P** | **K** | **V** | **L** | **S** | **L** | **K** | **Q** | **I** | **L** | **E** | **N** | **Y** | **I** | **E** | **H** | **Q** | **R** | **E** | **V** | **I** | **T** | **R** | **R** | **T** | **V** | **F** | **E** | **K** | **E** | **K** | **A** | **E** | **A** | **R** | **A** | **H** | **I** | **L** | **E** | **G** | **L** | **R** | **I** | **A** | **L** | **D** | **H** | **I** | **D** | **E** | **I** | **I** | **A** | **I** | **I** | **R** | **G** | **S** | **K** | **S** | **D** | **D** | **E** | **A** | **K** | **A** | **T** | **M** | **I** | **E** | **R** | **F** | **D** | **L** | **S** | **D** | **R** | **Q** | **S** | **Q** | **A** | **I** | **L** | **D** | **M** | **R** | **L** | **R** | **R** | **L** | **T** | **G** | **L** | **E** | **R** | **E** | **K** | **I** | **E** | **N** | **E** | **Y** | **Q** | **E** | **L** | **L** | **K** | **L** | **I** | **E** | **D** | **L** | **T** | **D** | **I** | **L** | **A** | **R** | **P** | **E** | **R** | **V** | **T** | **E** | **I** | **I** | **K** | **N** | **E** | **L** | **A** | **D** | **L** | **N** | **Q** | **R** | **F** | **G** | **D** | **K** | **R** | **R** | **T** | **E** | **L** | **L** | **V** | **G** | **E** | **V** | **L** | **S** | **L** | **E** | **D** | **E** | **D** | **L** | **I** | **E** | **E** | **E** | **E** | **I** | **V** | **I** | **T** | **L** | **T** | **N** | **N** | **G** | **Y** | **I** | **K** | **R** | **L** | **A** | **N** | **N** | **E** | **F** | **R** | **A** | **Q** | **R** | **R** | **G** | **G** | **R** | **G** | **V** | **Q** | **G** | **M** | **G** | **I** | **H** | **N** | **D** | **D** | **F** | **V** | **K** | **N** | **L** | **V** | **S** | **C** | **S** | **T** | **H** | **D** | **T** | **L** | **L** | **F** | **F** | **T** | **N** | **N** | **G** | **K** | **V** | **Y** | **R** | **A** | **K** | **G** | **Y** | **E** | **I** | **P** | **E** | **Y** | **G** | **R** | **T** | **A** | **K** | **G** | **I** | **P** | **I** | **I** | **N** | **L** | **L** | **G** | **I** | **D** | **S** | **S** | **E** | **S** | **I** | **Q** | **A** | **I** | **I** | **A** | **V** | **T** | **G** | **E** | **A** | **E** | **E** | **G** | **H** | **Y** | **L** | **F** | **F** | **T** | **T** | **R** | **Q** | **G** | **T** | **V** | **K** | **R** | **T** | **S** | **V** | **T** | **A** | **F** | **A** | **N** | **I** | **R** | **S** | **N** | **G** | **L** | **I** | **A** | **I** | **G** | **L** | **K** | **E** | **D** | **D** | **E** | **L** | **V** | **N** | **V** | **L** | **L** | **T** | **D** | **G** | **Q** | **S** | **N** | **I** | **I** | **I** | **G** | **T** | **H** | **N** | **G** | **Y** | **S** | **V** | **T** | **F** | **K** | **E** | **E** | **A** | **V** | **R** | **D** | **M** | **G** | **R** | **T** | **A** | **S** | **G** | **V** | **R** | **G** | **I** | **R** | **L** | **R** | **E** | **E** | **D** | **Y** | **V** | **V** | **G** | **A** | **A** | **L** | **M** | **K** | **E** | **D** | **Q** | **E** | **V** | **L** | **V** | **I** | **T** | **E** | **K** | **G** | **Y** | **G** | **K** | **R** | **T** | **K** | **V** | **S** | **E** | **Y** | **P** | **V** | **K** | **G** | **R** | **G** | **G** | **K** | **G** | **I** | **K** | **T** | **A** | **N** | **I** | **T** | **E** | **K** | **N** | **G** | **P** | **L** | **A** | **G** | **L** | **T** | **T** | **V** | **S** | **G** | **D** | **E** | **D** | **I** | **M** | **L** | **I** | **T** | **D** | **K** | **G** | **V** | **I** | **I** | **R** | **F** | **N** | **V** | **S** | **T** | **V** | **S** | **Q** | **T** | **G** | **R** | **S** | **T** | **L** | **G** | **V** | **R** | **L** | **M** | **K** | **M** | **E** | **A** | **D** | **T** | **K** | **V** | **V** | **T** | **M** | **A** | **A** | **V** | **E** | **P** | **E** | **A** | **A** | **E** | **Q** | **T** | **V** | **E** | **E** | **Q** | **G** | **S** | **E** | **E** |
| **N59504F** | **M** | **S** | **E** | **E** | **I** | **R** | **E** | **N** | **I** | **H** | **D** | **V** | **N** | **L** | **T** | **S** | **E** | **M** | **K** | **D** | **S** | **F** | **I** | **D** | **Y** | **A** | **M** | **S** | **V** | **I** | **V** | **A** | **R** | **A** | **L** | **P** | **D** | **V** | **R** | **D** | **G** | **L** | **K** | **P** | **V** | **H** | **R** | **R** | **I** | **L** | **Y** | **G** | **M** | **N** | **E** | **L** | **G** | **V** | **T** | **P** | **D** | **K** | **A** | **H** | **K** | **K** | **S** | **A** | **R** | **I** | **V** | **G** | **D** | **V** | **M** | **G** | **K** | **Y** | **H** | **P** | **H** | **G** | **D** | **S** | **A** | **I** | **Y** | **E** | **S** | **M** | **V** | **R** | **M** | **A** | **Q** | **P** | **F** | **S** | **Y** | **R** | **Y** | **M** | **L** | **V** | **D** | **G** | **H** | **G** | **N** | **F** | **G** | **S** | **V** | **D** | **G** | **D** | **G** | **A** | **A** | **A** | **M** | **R** | **Y** | **T** | **E** | **A** | **R** | **M** | **S** | **K** | **I** | **A** | **T** | **E** | **M** | **L** | **R** | **D** | **I** | **N** | **K** | **N** | **T** | **V** | **D** | **F** | **Q** | **S** | **N** | **Y** | **D** | **D** | **T** | **E** | **K** | **E** | **P** | **V** | **V** | **L** | **P** | **A** | **R** | **F** | **P** | **N** | **L** | **L** | **V** | **N** | **G** | **T** | **T** | **G** | **I** | **A** | **V** | **G** | **M** | **A** | **T** | **N** | **I** | **P** | **P** | **H** | **N** | **L** | **S** | **E** | **V** | **V** | **A** | **A** | **I** | **D** | **L** | **L** | **M** | **D** | **N** | **P** | **D** | **V** | **T** | **T** | **N** | **E** | **L** | **M** | **E** | **V** | **L** | **P** | **G** | **P** | **D** | **F** | **P** | **T** | **G** | **G** | **L** | **V** | **M** | **G** | **K** | **S** | **G** | **I** | **R** | **R** | **A** | **Y** | **E** | **T** | **G** | **K** | **G** | **S** | **I** | **T** | **V** | **R** | **A** | **K** | **V** | **E** | **I** | **T** | **E** | **M** | **P** | **N** | **G** | **K** | **E** | **R** | **I** | **I** | **V** | **T** | **E** | **L** | **P** | **Y** | **M** | **V** | **N** | **K** | **A** | **K** | **L** | **I** | **E** | **R** | **I** | **S** | **E** | **L** | **H** | **R** | **D** | **K** | **R** | **I** | **E** | **G** | **I** | **T** | **D** | **L** | **R** | **D** | **E** | **S** | **S** | **R** | **E** | **G** | **M** | **R** | **I** | **V** | **I** | **D** | **I** | **R** | **R** | **D** | **A** | **S** | **A** | **S** | **V** | **I** | **L** | **N** | **N** | **L** | **Y** | **K** | **L** | **T** | **S** | **L** | **Q** | **N** | **S** | **F** | **G** | **F** | **N** | **M** | **L** | **A** | **I** | **E** | **K** | **G** | **V** | **P** | **K** | **V** | **L** | **S** | **L** | **K** | **Q** | **I** | **L** | **E** | **N** | **Y** | **I** | **E** | **H** | **Q** | **R** | **E** | **V** | **I** | **T** | **R** | **R** | **T** | **V** | **F** | **E** | **K** | **E** | **K** | **A** | **E** | **A** | **R** | **A** | **H** | **I** | **L** | **E** | **G** | **L** | **R** | **I** | **A** | **L** | **D** | **H** | **I** | **D** | **E** | **I** | **I** | **A** | **I** | **I** | **R** | **G** | **S** | **K** | **S** | **D** | **D** | **E** | **A** | **K** | **A** | **T** | **M** | **I** | **E** | **R** | **F** | **D** | **L** | **S** | **D** | **R** | **Q** | **S** | **Q** | **A** | **I** | **L** | **D** | **M** | **R** | **L** | **R** | **R** | **L** | **T** | **G** | **L** | **E** | **R** | **E** | **K** | **I** | **E** | **N** | **E** | **Y** | **Q** | **E** | **L** | **L** | **K** | **L** | **I** | **E** | **D** | **L** | **T** | **D** | **I** | **L** | **A** | **R** | **P** | **E** | **R** | **V** | **T** | **E** | **I** | **I** | **K** | **N** | **E** | **L** | **A** | **D** | **L** | **N** | **Q** | **R** | **F** | **G** | **D** | **K** | **R** | **R** | **T** | **E** | **L** | **L** | **V** | **G** | **E** | **V** | **L** | **S** | **L** | **E** | **D** | **E** | **D** | **L** | **I** | **E** | **E** | **E** | **E** | **I** | **V** | **I** | **T** | **L** | **T** | **N** | **N** | **G** | **Y** | **I** | **K** | **R** | **L** | **A** | **N** | **N** | **E** | **F** | **R** | **A** | **Q** | **R** | **R** | **G** | **G** | **R** | **G** | **V** | **Q** | **G** | **M** | **G** | **I** | **H** | **N** | **D** | **D** | **F** | **V** | **K** | **N** | **L** | **V** | **S** | **C** | **S** | **T** | **H** | **D** | **T** | **L** | **L** | **F** | **F** | **T** | **N** | **N** | **G** | **K** | **V** | **Y** | **R** | **A** | **K** | **G** | **Y** | **E** | **I** | **P** | **E** | **Y** | **G** | **R** | **T** | **A** | **K** | **G** | **I** | **P** | **I** | **I** | **N** | **L** | **L** | **G** | **I** | **D** | **S** | **S** | **E** | **S** | **I** | **Q** | **A** | **I** | **I** | **A** | **V** | **T** | **G** | **E** | **A** | **E** | **E** | **G** | **H** | **Y** | **L** | **F** | **F** | **T** | **T** | **R** | **Q** | **G** | **T** | **V** | **K** | **R** | **T** | **S** | **V** | **T** | **A** | **F** | **A** | **N** | **I** | **R** | **S** | **N** | **G** | **L** | **I** | **A** | **I** | **G** | **L** | **K** | **E** | **D** | **D** | **E** | **L** | **V** | **N** | **V** | **L** | **L** | **T** | **D** | **G** | **Q** | **S** | **N** | **I** | **I** | **I** | **G** | **T** | **H** | **N** | **G** | **Y** | **S** | **V** | **T** | **F** | **K** | **E** | **E** | **A** | **V** | **R** | **D** | **M** | **G** | **R** | **T** | **A** | **S** | **G** | **V** | **R** | **G** | **I** | **R** | **L** | **R** | **E** | **E** | **D** | **Y** | **V** | **V** | **G** | **A** | **A** | **L** | **M** | **K** | **E** | **N** | **Q** | **E** | **V** | **L** | **V** | **I** | **T** | **E** | **K** | **G** | **Y** | **G** | **K** | **R** | **T** | **K** | **V** | **S** | **E** | **Y** | **P** | **V** | **K** | **G** | **R** | **G** | **G** | **K** | **G** | **I** | **K** | **T** | **A** | **N** | **I** | **T** | **E** | **K** | **N** | **G** | **P** | **L** | **A** | **G** | **L** | **T** | **T** | **V** | **S** | **G** | **D** | **E** | **D** | **I** | **M** | **L** | **I** | **T** | **D** | **K** | **G** | **V** | **I** | **I** | **R** | **F** | **N** | **V** | **S** | **T** | **V** | **S** | **Q** | **T** | **G** | **R** | **S** | **T** | **L** | **G** | **V** | **R** | **L** | **M** | **K** | **M** | **E** | **A** | **D** | **T** | **K** | **V** | **V** | **T** | **M** | **A** | **A** | **V** | **E** | **P** | **E** | **A** | **A** | **E** | **Q** | **T** | **V** | **E** | **E** | **Q** | **G** | **S** | **E** | **E** |
| **N59711F** | **M** | **S** | **E** | **E** | **I** | **R** | **E** | **N** | **I** | **H** | **D** | **V** | **N** | **L** | **T** | **S** | **E** | **M** | **K** | **D** | **S** | **F** | **I** | **D** | **Y** | **A** | **M** | **S** | **V** | **I** | **V** | **A** | **R** | **A** | **L** | **P** | **D** | **V** | **R** | **D** | **G** | **L** | **K** | **P** | **V** | **H** | **R** | **R** | **I** | **L** | **Y** | **G** | **M** | **N** | **E** | **L** | **G** | **V** | **T** | **P** | **D** | **K** | **A** | **H** | **K** | **K** | **S** | **A** | **R** | **I** | **V** | **G** | **D** | **V** | **M** | **G** | **K** | **Y** | **H** | **P** | **H** | **G** | **D** | **S** | **A** | **I** | **Y** | **E** | **S** | **M** | **V** | **R** | **M** | **A** | **Q** | **P** | **F** | **S** | **Y** | **R** | **Y** | **M** | **L** | **V** | **D** | **G** | **H** | **G** | **N** | **F** | **G** | **S** | **V** | **D** | **G** | **D** | **G** | **A** | **A** | **A** | **M** | **R** | **Y** | **T** | **E** | **A** | **R** | **M** | **S** | **K** | **I** | **A** | **T** | **E** | **M** | **L** | **R** | **D** | **I** | **N** | **K** | **N** | **T** | **V** | **D** | **F** | **Q** | **S** | **N** | **Y** | **D** | **D** | **T** | **E** | **K** | **E** | **P** | **V** | **V** | **L** | **P** | **A** | **R** | **F** | **P** | **N** | **L** | **L** | **V** | **N** | **G** | **T** | **T** | **G** | **I** | **A** | **V** | **G** | **M** | **A** | **T** | **N** | **I** | **P** | **P** | **H** | **N** | **L** | **S** | **E** | **V** | **V** | **A** | **A** | **I** | **D** | **L** | **L** | **M** | **D** | **N** | **P** | **D** | **V** | **T** | **T** | **N** | **E** | **L** | **M** | **E** | **V** | **L** | **P** | **G** | **P** | **D** | **F** | **P** | **T** | **G** | **G** | **L** | **V** | **M** | **G** | **K** | **S** | **G** | **I** | **R** | **R** | **A** | **Y** | **E** | **T** | **G** | **K** | **G** | **S** | **I** | **T** | **V** | **R** | **A** | **K** | **V** | **E** | **I** | **T** | **E** | **M** | **P** | **N** | **G** | **K** | **E** | **R** | **I** | **L** | **V** | **T** | **E** | **L** | **P** | **Y** | **M** | **V** | **N** | **K** | **A** | **K** | **L** | **I** | **E** | **R** | **I** | **S** | **E** | **L** | **H** | **R** | **D** | **K** | **R** | **I** | **E** | **G** | **I** | **T** | **D** | **L** | **R** | **D** | **E** | **S** | **S** | **R** | **E** | **G** | **M** | **R** | **I** | **V** | **I** | **D** | **I** | **R** | **R** | **D** | **A** | **S** | **A** | **S** | **V** | **I** | **L** | **N** | **N** | **L** | **Y** | **K** | **L** | **T** | **S** | **L** | **Q** | **N** | **S** | **F** | **G** | **F** | **N** | **M** | **L** | **A** | **I** | **E** | **K** | **G** | **V** | **P** | **K** | **V** | **L** | **S** | **L** | **K** | **Q** | **I** | **L** | **E** | **N** | **Y** | **I** | **E** | **H** | **Q** | **R** | **E** | **V** | **I** | **T** | **R** | **R** | **T** | **V** | **F** | **E** | **K** | **E** | **K** | **A** | **E** | **A** | **R** | **A** | **H** | **I** | **L** | **E** | **G** | **L** | **R** | **I** | **A** | **L** | **D** | **H** | **I** | **D** | **E** | **I** | **I** | **A** | **I** | **I** | **R** | **G** | **S** | **K** | **S** | **D** | **D** | **E** | **A** | **K** | **A** | **T** | **M** | **I** | **E** | **R** | **F** | **D** | **L** | **S** | **D** | **R** | **Q** | **S** | **Q** | **A** | **I** | **L** | **D** | **M** | **R** | **L** | **R** | **R** | **L** | **T** | **G** | **L** | **E** | **R** | **E** | **K** | **I** | **E** | **N** | **E** | **Y** | **Q** | **E** | **L** | **L** | **K** | **L** | **I** | **E** | **D** | **L** | **T** | **D** | **I** | **L** | **A** | **R** | **P** | **E** | **R** | **V** | **T** | **E** | **I** | **I** | **K** | **N** | **E** | **L** | **A** | **D** | **L** | **N** | **Q** | **R** | **F** | **G** | **D** | **K** | **R** | **R** | **T** | **E** | **L** | **L** | **V** | **G** | **E** | **V** | **L** | **S** | **L** | **E** | **D** | **E** | **D** | **L** | **I** | **E** | **E** | **E** | **E** | **I** | **V** | **I** | **T** | **L** | **T** | **N** | **N** | **G** | **Y** | **I** | **K** | **R** | **L** | **A** | **N** | **N** | **E** | **F** | **R** | **A** | **Q** | **R** | **R** | **G** | **G** | **R** | **G** | **V** | **Q** | **G** | **M** | **G** | **I** | **H** | **N** | **D** | **D** | **F** | **V** | **K** | **N** | **L** | **V** | **S** | **C** | **S** | **T** | **H** | **D** | **T** | **L** | **L** | **F** | **F** | **T** | **N** | **N** | **G** | **K** | **V** | **Y** | **R** | **A** | **K** | **G** | **Y** | **E** | **I** | **P** | **E** | **Y** | **G** | **R** | **T** | **A** | **K** | **G** | **I** | **P** | **I** | **I** | **N** | **L** | **L** | **G** | **I** | **D** | **S** | **S** | **E** | **S** | **I** | **Q** | **A** | **I** | **I** | **A** | **V** | **T** | **G** | **E** | **A** | **E** | **E** | **G** | **H** | **Y** | **L** | **F** | **F** | **T** | **T** | **R** | **Q** | **G** | **T** | **V** | **K** | **R** | **T** | **S** | **V** | **T** | **A** | **F** | **A** | **N** | **I** | **R** | **S** | **N** | **G** | **L** | **I** | **A** | **I** | **G** | **L** | **K** | **E** | **D** | **D** | **E** | **L** | **V** | **N** | **V** | **L** | **L** | **T** | **D** | **G** | **Q** | **S** | **N** | **I** | **I** | **I** | **G** | **T** | **H** | **N** | **G** | **Y** | **S** | **V** | **T** | **F** | **K** | **E** | **E** | **A** | **V** | **R** | **D** | **M** | **G** | **R** | **T** | **A** | **S** | **G** | **V** | **R** | **G** | **I** | **R** | **L** | **R** | **E** | **E** | **D** | **Y** | **V** | **V** | **G** | **A** | **A** | **L** | **M** | **K** | **E** | **D** | **Q** | **E** | **V** | **L** | **V** | **I** | **T** | **E** | **K** | **G** | **Y** | **G** | **K** | **R** | **T** | **K** | **V** | **S** | **E** | **Y** | **P** | **V** | **K** | **G** | **R** | **G** | **G** | **K** | **G** | **I** | **K** | **T** | **A** | **N** | **I** | **T** | **E** | **K** | **N** | **G** | **P** | **L** | **A** | **G** | **L** | **T** | **T** | **V** | **S** | **G** | **D** | **E** | **D** | **I** | **M** | **L** | **I** | **T** | **D** | **K** | **G** | **V** | **I** | **I** | **R** | **F** | **N** | **V** | **S** | **T** | **V** | **S** | **Q** | **T** | **G** | **R** | **S** | **T** | **L** | **G** | **V** | **R** | **L** | **M** | **K** | **M** | **E** | **A** | **D** | **T** | **K** | **V** | **V** | **T** | **M** | **-** | **-** | **-** | **-** | **-** | **-** | **-** | **-** | **-** | **-** | **-** | **-** | **-** | **-** | **-** | **-** | **-** | **-** | **-** |
| **N55724** | **M** | **S** | **E** | **E** | **I** | **K** | **E** | **N** | **I** | **Q** | **D** | **V** | **N** | **L** | **T** | **S** | **E** | **M** | **K** | **E** | **S** | **F** | **I** | **D** | **Y** | **A** | **M** | **S** | **V** | **I** | **V** | **A** | **R** | **A** | **L** | **P** | **D** | **V** | **R** | **D** | **G** | **L** | **K** | **P** | **V** | **H** | **R** | **R** | **I** | **L** | **Y** | **G** | **M** | **N** | **E** | **L** | **G** | **V** | **T** | **P** | **D** | **K** | **P** | **H** | **K** | **K** | **S** | **A** | **R** | **I** | **V** | **G** | **D** | **V** | **M** | **G** | **K** | **Y** | **H** | **P** | **H** | **G** | **D** | **S** | **A** | **I** | **Y** | **E** | **S** | **M** | **V** | **R** | **M** | **A** | **Q** | **P** | **F** | **S** | **Y** | **R** | **A** | **M** | **L** | **V** | **D** | **G** | **H** | **G** | **N** | **F** | **G** | **S** | **V** | **D** | **G** | **D** | **G** | **A** | **A** | **A** | **M** | **R** | **Y** | **T** | **E** | **A** | **R** | **M** | **S** | **K** | **I** | **A** | **L** | **E** | **M** | **L** | **R** | **D** | **I** | **N** | **K** | **N** | **T** | **V** | **D** | **F** | **Q** | **G** | **N** | **Y** | **D** | **D** | **S** | **E** | **Q** | **E** | **P** | **V** | **V** | **L** | **P** | **A** | **R** | **F** | **P** | **N** | **L** | **L** | **V** | **N** | **G** | **T** | **T** | **G** | **I** | **A** | **V** | **G** | **M** | **A** | **T** | **N** | **I** | **P** | **P** | **H** | **N** | **L** | **S** | **E** | **V** | **I** | **D** | **A** | **T** | **S** | **L** | **L** | **M** | **D** | **N** | **P** | **D** | **V** | **T** | **T** | **N** | **E** | **L** | **M** | **E** | **V** | **L** | **P** | **G** | **P** | **D** | **F** | **P** | **T** | **G** | **G** | **L** | **V** | **M** | **G** | **K** | **S** | **G** | **I** | **R** | **R** | **A** | **Y** | **E** | **T** | **G** | **K** | **G** | **S** | **I** | **T** | **V** | **R** | **A** | **K** | **V** | **E** | **L** | **T** | **E** | **M** | **P** | **N** | **G** | **K** | **E** | **R** | **I** | **L** | **V** | **T** | **E** | **L** | **P** | **Y** | **M** | **V** | **N** | **K** | **A** | **K** | **L** | **I** | **E** | **R** | **I** | **S** | **E** | **L** | **H** | **R** | **D** | **K** | **R** | **I** | **E** | **G** | **I** | **T** | **D** | **L** | **R** | **D** | **E** | **S** | **S** | **R** | **E** | **G** | **M** | **R** | **I** | **V** | **I** | **D** | **V** | **R** | **R** | **D** | **V** | **S** | **A** | **S** | **V** | **V** | **L** | **N** | **N** | **L** | **Y** | **K** | **M** | **T** | **A** | **L** | **Q** | **T** | **S** | **F** | **G** | **F** | **N** | **M** | **L** | **A** | **I** | **E** | **K** | **G** | **V** | **P** | **K** | **I** | **L** | **S** | **L** | **K** | **R** | **I** | **L** | **E** | **N** | **Y** | **V** | **E** | **H** | **Q** | **K** | **E** | **V** | **I** | **T** | **R** | **R** | **T** | **I** | **F** | **D** | **K** | **N** | **K** | **A** | **E** | **A** | **R** | **A** | **H** | **I** | **L** | **E** | **G** | **L** | **R** | **I** | **A** | **L** | **D** | **H** | **I** | **D** | **E** | **I** | **I** | **A** | **I** | **I** | **R** | **G** | **S** | **Q** | **S** | **D** | **D** | **E** | **A** | **K** | **A** | **T** | **L** | **I** | **E** | **R** | **F** | **E** | **F** | **S** | **D** | **R** | **Q** | **A** | **Q** | **A** | **I** | **L** | **D** | **M** | **R** | **L** | **R** | **R** | **L** | **T** | **G** | **L** | **E** | **R** | **D** | **K** | **I** | **E** | **N** | **E** | **Y** | **Q** | **E** | **L** | **L** | **K** | **F** | **I** | **A** | **D** | **L** | **E** | **D** | **I** | **L** | **A** | **R** | **P** | **E** | **R** | **V** | **I** | **E** | **I** | **I** | **K** | **T** | **E** | **L** | **N** | **D** | **V** | **R** | **T** | **K** | **F** | **G** | **D** | **A** | **R** | **R** | **T** | **E** | **L** | **L** | **V** | **G** | **E** | **V** | **L** | **S** | **L** | **E** | **D** | **E** | **D** | **L** | **I** | **E** | **E** | **E** | **E** | **V** | **V** | **I** | **T** | **L** | **T** | **N** | **N** | **G** | **Y** | **I** | **K** | **R** | **M** | **A** | **N** | **S** | **E** | **F** | **R** | **A** | **Q** | **R** | **R** | **G** | **G** | **R** | **G** | **V** | **Q** | **G** | **M** | **G** | **V** | **H** | **D** | **D** | **D** | **F** | **V** | **K** | **N** | **L** | **V** | **S** | **C** | **S** | **T** | **H** | **D** | **T** | **L** | **L** | **F** | **F** | **T** | **N** | **T** | **G** | **K** | **V** | **Y** | **R** | **A** | **K** | **G** | **Y** | **E** | **I** | **P** | **E** | **Y** | **G** | **R** | **T** | **A** | **K** | **G** | **I** | **P** | **V** | **I** | **N** | **L** | **L** | **G** | **I** | **D** | **S** | **A** | **E** | **K** | **I** | **Q** | **A** | **I** | **I** | **S** | **V** | **E** | **G** | **K** | **A** | **E** | **A** | **G** | **K** | **Y** | **L** | **F** | **F** | **T** | **T** | **L** | **K** | **G** | **T** | **V** | **K** | **R** | **T** | **A** | **V** | **T** | **A** | **F** | **S** | **N** | **I** | **R** | **S** | **N** | **G** | **L** | **I** | **A** | **I** | **S** | **L** | **K** | **E** | **D** | **D** | **E** | **L** | **V** | **N** | **V** | **V** | **T** | **T** | **N** | **G** | **N** | **Q** | **K** | **M** | **I** | **I** | **G** | **T** | **H** | **A** | **G** | **Y** | **S** | **V** | **T** | **F** | **D** | **E** | **N** | **T** | **V** | **R** | **D** | **M** | **G** | **R** | **T** | **A** | **S** | **G** | **V** | **R** | **G** | **I** | **R** | **L** | **R** | **E** | **N** | **D** | **Y** | **V** | **V** | **G** | **A** | **A** | **I** | **L** | **D** | **E** | **N** | **K** | **E** | **V** | **L** | **V** | **I** | **T** | **E** | **N** | **G** | **Y** | **G** | **K** | **R** | **T** | **K** | **A** | **S** | **E** | **Y** | **P** | **V** | **K** | **G** | **R** | **G** | **G** | **K** | **G** | **I** | **K** | **T** | **A** | **N** | **I** | **T** | **E** | **K** | **N** | **G** | **P** | **L** | **A** | **G** | **L** | **T** | **T** | **V** | **N** | **G** | **D** | **E** | **D** | **I** | **L** | **L** | **I** | **T** | **N** | **K** | **G** | **V** | **I** | **I** | **R** | **F** | **N** | **V** | **D** | **S** | **V** | **S** | **Q** | **T** | **G** | **R** | **A** | **T** | **L** | **G** | **V** | **R** | **L** | **M** | **R** | **M** | **E** | **D** | **G** | **A** | **K** | **-** | **-** | **-** | **-** | **-** | **-** | **-** | **-** | **-** | **-** | **-** | **-** | **-** | **-** | **-** | **-** | **-** | **-** | **-** | **-** | **-** | **-** | **-** |
| **N60280F** | **M** | **S** | **E** | **E** | **I** | **K** | **E** | **N** | **I** | **Q** | **D** | **V** | **N** | **L** | **T** | **S** | **E** | **M** | **K** | **E** | **S** | **F** | **I** | **D** | **Y** | **A** | **M** | **S** | **V** | **I** | **V** | **A** | **R** | **A** | **L** | **P** | **D** | **V** | **R** | **D** | **G** | **L** | **K** | **P** | **V** | **H** | **R** | **R** | **I** | **L** | **Y** | **G** | **M** | **N** | **E** | **L** | **G** | **V** | **T** | **P** | **D** | **K** | **P** | **H** | **K** | **K** | **S** | **A** | **R** | **I** | **V** | **G** | **D** | **V** | **M** | **G** | **K** | **Y** | **H** | **P** | **H** | **G** | **D** | **S** | **A** | **I** | **Y** | **E** | **S** | **M** | **V** | **R** | **M** | **A** | **Q** | **P** | **F** | **S** | **Y** | **R** | **A** | **M** | **L** | **V** | **D** | **G** | **H** | **G** | **N** | **F** | **G** | **S** | **V** | **D** | **G** | **D** | **G** | **A** | **A** | **A** | **M** | **R** | **Y** | **T** | **E** | **A** | **R** | **M** | **S** | **K** | **I** | **A** | **L** | **E** | **M** | **L** | **R** | **D** | **I** | **N** | **K** | **N** | **T** | **V** | **D** | **F** | **Q** | **G** | **N** | **Y** | **D** | **D** | **S** | **E** | **Q** | **E** | **P** | **V** | **V** | **L** | **P** | **A** | **R** | **F** | **P** | **N** | **L** | **L** | **V** | **N** | **G** | **T** | **T** | **G** | **I** | **A** | **V** | **G** | **M** | **A** | **T** | **N** | **I** | **P** | **P** | **H** | **N** | **L** | **S** | **E** | **V** | **I** | **D** | **A** | **T** | **S** | **L** | **L** | **M** | **D** | **N** | **P** | **D** | **V** | **T** | **T** | **N** | **E** | **L** | **M** | **E** | **V** | **L** | **P** | **G** | **P** | **D** | **F** | **P** | **T** | **G** | **G** | **L** | **V** | **M** | **G** | **K** | **S** | **G** | **I** | **R** | **R** | **A** | **Y** | **E** | **T** | **G** | **K** | **G** | **S** | **I** | **T** | **V** | **R** | **A** | **K** | **V** | **E** | **L** | **T** | **E** | **M** | **P** | **N** | **G** | **K** | **E** | **R** | **I** | **L** | **V** | **T** | **E** | **L** | **P** | **Y** | **M** | **V** | **N** | **K** | **A** | **K** | **L** | **I** | **E** | **R** | **I** | **S** | **E** | **L** | **H** | **R** | **D** | **K** | **R** | **I** | **E** | **G** | **I** | **T** | **D** | **L** | **R** | **D** | **E** | **S** | **S** | **R** | **E** | **G** | **M** | **R** | **I** | **V** | **I** | **D** | **V** | **R** | **R** | **D** | **V** | **S** | **A** | **S** | **V** | **V** | **L** | **N** | **N** | **L** | **Y** | **K** | **M** | **T** | **A** | **L** | **Q** | **T** | **S** | **F** | **G** | **F** | **N** | **M** | **L** | **A** | **I** | **E** | **K** | **G** | **V** | **P** | **K** | **I** | **L** | **S** | **L** | **K** | **R** | **I** | **L** | **E** | **N** | **Y** | **V** | **E** | **H** | **Q** | **K** | **E** | **V** | **I** | **T** | **R** | **R** | **T** | **I** | **F** | **D** | **K** | **N** | **K** | **A** | **E** | **A** | **R** | **A** | **H** | **I** | **L** | **E** | **G** | **L** | **R** | **I** | **A** | **L** | **D** | **H** | **I** | **D** | **E** | **I** | **I** | **A** | **I** | **I** | **R** | **G** | **S** | **Q** | **S** | **D** | **D** | **E** | **A** | **K** | **A** | **T** | **L** | **I** | **E** | **R** | **F** | **E** | **F** | **S** | **D** | **R** | **Q** | **A** | **Q** | **A** | **I** | **L** | **D** | **M** | **R** | **L** | **R** | **R** | **L** | **T** | **G** | **L** | **E** | **R** | **D** | **K** | **I** | **E** | **N** | **E** | **Y** | **Q** | **E** | **L** | **L** | **K** | **F** | **I** | **A** | **D** | **L** | **E** | **D** | **I** | **L** | **A** | **R** | **P** | **E** | **R** | **V** | **I** | **E** | **I** | **I** | **K** | **T** | **E** | **L** | **N** | **D** | **V** | **R** | **T** | **K** | **F** | **G** | **D** | **A** | **R** | **R** | **T** | **E** | **L** | **L** | **V** | **G** | **E** | **V** | **L** | **S** | **L** | **E** | **D** | **E** | **D** | **L** | **I** | **E** | **E** | **E** | **E** | **V** | **V** | **I** | **T** | **L** | **T** | **N** | **N** | **G** | **Y** | **I** | **K** | **R** | **M** | **A** | **N** | **S** | **E** | **F** | **R** | **A** | **Q** | **R** | **R** | **G** | **G** | **R** | **G** | **V** | **Q** | **G** | **M** | **G** | **V** | **H** | **D** | **D** | **D** | **F** | **V** | **K** | **N** | **L** | **V** | **S** | **C** | **S** | **T** | **H** | **D** | **T** | **L** | **L** | **F** | **F** | **T** | **N** | **T** | **G** | **K** | **V** | **Y** | **R** | **A** | **K** | **G** | **Y** | **E** | **I** | **P** | **E** | **Y** | **G** | **R** | **T** | **A** | **K** | **G** | **I** | **P** | **V** | **I** | **N** | **L** | **L** | **G** | **I** | **D** | **S** | **A** | **E** | **K** | **I** | **Q** | **A** | **I** | **I** | **S** | **V** | **E** | **G** | **K** | **A** | **E** | **A** | **G** | **K** | **Y** | **L** | **F** | **F** | **T** | **T** | **L** | **K** | **G** | **T** | **V** | **K** | **R** | **T** | **A** | **V** | **T** | **A** | **F** | **S** | **N** | **I** | **R** | **S** | **N** | **G** | **L** | **I** | **A** | **I** | **S** | **L** | **K** | **E** | **D** | **D** | **E** | **L** | **V** | **N** | **V** | **V** | **T** | **T** | **N** | **G** | **N** | **Q** | **K** | **M** | **I** | **I** | **G** | **T** | **H** | **A** | **G** | **Y** | **S** | **V** | **T** | **F** | **D** | **E** | **N** | **T** | **V** | **R** | **D** | **M** | **G** | **R** | **T** | **A** | **S** | **G** | **V** | **R** | **G** | **I** | **R** | **L** | **R** | **E** | **N** | **D** | **Y** | **V** | **V** | **G** | **A** | **A** | **I** | **L** | **D** | **E** | **N** | **K** | **E** | **V** | **L** | **V** | **I** | **T** | **E** | **N** | **G** | **Y** | **G** | **K** | **R** | **T** | **K** | **A** | **S** | **E** | **Y** | **P** | **V** | **K** | **G** | **R** | **G** | **G** | **K** | **G** | **I** | **K** | **T** | **A** | **N** | **I** | **T** | **E** | **K** | **N** | **G** | **P** | **L** | **A** | **G** | **L** | **T** | **T** | **V** | **N** | **G** | **D** | **E** | **D** | **I** | **L** | **L** | **I** | **T** | **N** | **K** | **G** | **V** | **I** | **I** | **R** | **F** | **N** | **V** | **D** | **S** | **V** | **S** | **Q** | **T** | **G** | **R** | **A** | **T** | **L** | **G** | **V** | **R** | **L** | **M** | **R** | **M** | **E** | **D** | **G** | **A** | **K** | **-** | **-** | **-** | **-** | **-** | **-** | **-** | **-** | **-** | **-** | **-** | **-** | **-** | **-** | **-** | **-** | **-** | **-** | **-** | **-** | **-** | **-** | **-** |
| **N54097** | **M** | **S** | **E** | **E** | **I** | **K** | **E** | **N** | **I** | **Q** | **D** | **V** | **N** | **L** | **T** | **S** | **E** | **M** | **K** | **E** | **S** | **F** | **I** | **D** | **Y** | **A** | **M** | **S** | **V** | **I** | **V** | **A** | **R** | **A** | **L** | **P** | **D** | **V** | **R** | **D** | **G** | **L** | **K** | **P** | **V** | **H** | **R** | **R** | **I** | **L** | **Y** | **G** | **M** | **N** | **E** | **L** | **G** | **V** | **T** | **P** | **D** | **K** | **P** | **H** | **K** | **K** | **S** | **A** | **R** | **I** | **V** | **G** | **D** | **V** | **M** | **G** | **K** | **Y** | **H** | **P** | **H** | **G** | **D** | **S** | **A** | **I** | **Y** | **E** | **S** | **M** | **V** | **R** | **M** | **A** | **Q** | **P** | **F** | **S** | **Y** | **R** | **A** | **M** | **L** | **V** | **D** | **G** | **H** | **G** | **N** | **F** | **G** | **S** | **V** | **D** | **G** | **D** | **G** | **A** | **A** | **A** | **M** | **R** | **Y** | **T** | **E** | **A** | **R** | **M** | **S** | **K** | **I** | **A** | **L** | **E** | **M** | **L** | **R** | **D** | **I** | **N** | **K** | **N** | **T** | **V** | **D** | **F** | **Q** | **G** | **N** | **Y** | **D** | **D** | **S** | **E** | **Q** | **E** | **P** | **V** | **V** | **L** | **P** | **A** | **R** | **F** | **P** | **N** | **L** | **L** | **V** | **N** | **G** | **T** | **T** | **G** | **I** | **A** | **V** | **G** | **M** | **A** | **T** | **N** | **I** | **P** | **P** | **H** | **N** | **L** | **S** | **E** | **V** | **I** | **D** | **A** | **T** | **S** | **L** | **L** | **M** | **D** | **N** | **P** | **D** | **V** | **T** | **T** | **N** | **E** | **L** | **M** | **E** | **V** | **L** | **P** | **G** | **P** | **D** | **F** | **P** | **T** | **G** | **G** | **L** | **V** | **M** | **G** | **K** | **S** | **G** | **I** | **R** | **R** | **A** | **Y** | **E** | **T** | **G** | **K** | **G** | **S** | **I** | **T** | **V** | **R** | **A** | **K** | **V** | **E** | **L** | **T** | **E** | **M** | **P** | **N** | **G** | **K** | **E** | **R** | **I** | **L** | **V** | **T** | **E** | **L** | **P** | **Y** | **M** | **V** | **N** | **K** | **A** | **K** | **L** | **I** | **E** | **R** | **I** | **S** | **E** | **L** | **H** | **R** | **D** | **K** | **R** | **I** | **E** | **G** | **I** | **T** | **D** | **L** | **R** | **D** | **E** | **S** | **S** | **R** | **E** | **G** | **M** | **R** | **I** | **V** | **I** | **D** | **V** | **R** | **R** | **D** | **V** | **S** | **A** | **S** | **V** | **V** | **L** | **N** | **N** | **L** | **Y** | **K** | **M** | **T** | **A** | **L** | **Q** | **T** | **S** | **F** | **G** | **F** | **N** | **M** | **L** | **A** | **I** | **E** | **K** | **G** | **V** | **P** | **K** | **I** | **L** | **S** | **L** | **K** | **R** | **I** | **L** | **E** | **N** | **Y** | **V** | **E** | **H** | **Q** | **K** | **E** | **V** | **I** | **T** | **R** | **R** | **T** | **I** | **F** | **D** | **K** | **N** | **K** | **A** | **E** | **A** | **R** | **A** | **H** | **I** | **L** | **E** | **G** | **L** | **R** | **I** | **A** | **L** | **D** | **H** | **I** | **D** | **E** | **I** | **I** | **A** | **I** | **I** | **R** | **G** | **S** | **Q** | **S** | **D** | **D** | **E** | **A** | **K** | **A** | **T** | **L** | **I** | **E** | **R** | **F** | **E** | **F** | **S** | **D** | **R** | **Q** | **A** | **Q** | **A** | **I** | **L** | **D** | **M** | **R** | **L** | **R** | **R** | **L** | **T** | **G** | **L** | **E** | **R** | **D** | **K** | **I** | **E** | **N** | **E** | **Y** | **Q** | **E** | **L** | **L** | **K** | **F** | **I** | **A** | **D** | **L** | **E** | **D** | **I** | **L** | **A** | **R** | **P** | **E** | **R** | **V** | **I** | **E** | **I** | **I** | **K** | **T** | **E** | **L** | **N** | **D** | **V** | **R** | **T** | **K** | **F** | **G** | **D** | **A** | **R** | **R** | **T** | **E** | **L** | **L** | **V** | **G** | **E** | **V** | **L** | **S** | **L** | **E** | **D** | **E** | **D** | **L** | **I** | **E** | **E** | **E** | **E** | **V** | **V** | **I** | **T** | **L** | **T** | **N** | **N** | **G** | **Y** | **I** | **K** | **R** | **M** | **A** | **N** | **S** | **E** | **F** | **R** | **A** | **Q** | **R** | **R** | **G** | **G** | **R** | **G** | **V** | **Q** | **G** | **M** | **G** | **V** | **H** | **D** | **D** | **D** | **F** | **V** | **K** | **N** | **L** | **V** | **S** | **C** | **S** | **T** | **H** | **D** | **T** | **L** | **L** | **F** | **F** | **T** | **N** | **T** | **G** | **K** | **V** | **Y** | **R** | **A** | **K** | **G** | **Y** | **E** | **I** | **P** | **E** | **Y** | **G** | **R** | **T** | **A** | **K** | **G** | **I** | **P** | **V** | **I** | **N** | **L** | **L** | **G** | **I** | **D** | **S** | **A** | **E** | **K** | **I** | **Q** | **A** | **I** | **I** | **S** | **V** | **E** | **G** | **K** | **A** | **E** | **A** | **G** | **K** | **Y** | **L** | **F** | **F** | **T** | **T** | **L** | **K** | **G** | **T** | **V** | **K** | **R** | **T** | **A** | **V** | **T** | **A** | **F** | **S** | **N** | **I** | **R** | **S** | **N** | **G** | **L** | **I** | **A** | **I** | **S** | **L** | **K** | **E** | **D** | **D** | **E** | **L** | **V** | **N** | **V** | **V** | **T** | **T** | **N** | **G** | **N** | **Q** | **K** | **M** | **I** | **I** | **G** | **T** | **H** | **A** | **G** | **Y** | **S** | **V** | **T** | **F** | **D** | **E** | **N** | **T** | **V** | **R** | **D** | **M** | **G** | **R** | **T** | **A** | **S** | **G** | **V** | **R** | **G** | **I** | **R** | **L** | **R** | **E** | **N** | **D** | **Y** | **V** | **V** | **G** | **A** | **A** | **I** | **L** | **D** | **E** | **N** | **K** | **E** | **V** | **L** | **V** | **I** | **T** | **E** | **N** | **G** | **Y** | **G** | **K** | **R** | **T** | **K** | **A** | **S** | **E** | **Y** | **P** | **V** | **K** | **G** | **R** | **G** | **G** | **K** | **G** | **I** | **K** | **T** | **A** | **N** | **I** | **T** | **E** | **K** | **N** | **G** | **P** | **L** | **A** | **G** | **L** | **T** | **T** | **V** | **N** | **G** | **D** | **E** | **D** | **I** | **L** | **L** | **I** | **T** | **N** | **K** | **G** | **V** | **I** | **I** | **R** | **F** | **N** | **V** | **D** | **S** | **V** | **S** | **Q** | **T** | **G** | **R** | **A** | **T** | **L** | **G** | **V** | **R** | **L** | **M** | **R** | **M** | **E** | **D** | **G** | **A** | **K** | **-** | **-** | **-** | **-** | **-** | **-** | **-** | **-** | **-** | **-** | **-** | **-** | **-** | **-** | **-** | **-** | **-** | **-** | **-** | **-** | **-** | **-** | **-** |
| **N55728** | **M** | **S** | **E** | **E** | **I** | **R** | **E** | **N** | **I** | **H** | **D** | **V** | **N** | **L** | **T** | **S** | **E** | **M** | **K** | **D** | **S** | **F** | **I** | **D** | **Y** | **A** | **M** | **S** | **V** | **I** | **V** | **A** | **R** | **A** | **L** | **P** | **D** | **V** | **R** | **D** | **G** | **L** | **K** | **P** | **V** | **H** | **R** | **R** | **I** | **L** | **Y** | **G** | **M** | **N** | **E** | **L** | **G** | **V** | **T** | **P** | **D** | **K** | **A** | **H** | **K** | **K** | **S** | **A** | **R** | **I** | **V** | **G** | **D** | **V** | **M** | **G** | **K** | **Y** | **H** | **P** | **H** | **G** | **D** | **S** | **A** | **I** | **Y** | **E** | **S** | **M** | **V** | **R** | **M** | **A** | **Q** | **P** | **F** | **S** | **Y** | **R** | **Y** | **M** | **L** | **V** | **D** | **G** | **H** | **G** | **N** | **F** | **G** | **S** | **V** | **D** | **G** | **D** | **G** | **A** | **A** | **A** | **M** | **R** | **Y** | **T** | **E** | **A** | **R** | **M** | **S** | **K** | **I** | **A** | **T** | **E** | **M** | **L** | **R** | **D** | **I** | **N** | **K** | **N** | **T** | **V** | **D** | **F** | **Q** | **S** | **N** | **Y** | **D** | **D** | **T** | **E** | **K** | **E** | **P** | **V** | **V** | **L** | **P** | **A** | **R** | **F** | **P** | **N** | **L** | **L** | **V** | **N** | **G** | **T** | **T** | **G** | **I** | **A** | **V** | **G** | **M** | **A** | **T** | **N** | **I** | **P** | **P** | **H** | **N** | **L** | **S** | **E** | **V** | **V** | **A** | **A** | **I** | **D** | **L** | **L** | **M** | **D** | **N** | **P** | **D** | **V** | **T** | **T** | **N** | **E** | **L** | **M** | **E** | **V** | **L** | **P** | **G** | **P** | **D** | **F** | **P** | **T** | **G** | **G** | **L** | **V** | **M** | **G** | **K** | **S** | **G** | **I** | **R** | **R** | **A** | **Y** | **E** | **T** | **G** | **K** | **G** | **S** | **I** | **T** | **V** | **R** | **A** | **K** | **V** | **E** | **I** | **T** | **E** | **M** | **P** | **N** | **G** | **K** | **E** | **R** | **I** | **I** | **V** | **T** | **E** | **L** | **P** | **Y** | **M** | **V** | **N** | **K** | **A** | **K** | **L** | **I** | **E** | **R** | **I** | **S** | **E** | **L** | **H** | **R** | **D** | **K** | **R** | **I** | **E** | **G** | **I** | **T** | **D** | **L** | **R** | **D** | **E** | **S** | **S** | **R** | **E** | **G** | **M** | **R** | **I** | **V** | **I** | **D** | **I** | **R** | **R** | **D** | **A** | **S** | **A** | **S** | **V** | **I** | **L** | **N** | **N** | **L** | **Y** | **K** | **L** | **T** | **S** | **L** | **Q** | **N** | **S** | **F** | **G** | **F** | **N** | **M** | **L** | **A** | **I** | **E** | **K** | **G** | **V** | **P** | **K** | **V** | **L** | **S** | **L** | **K** | **Q** | **I** | **L** | **E** | **N** | **Y** | **I** | **E** | **H** | **Q** | **R** | **E** | **V** | **I** | **T** | **R** | **R** | **T** | **V** | **F** | **E** | **K** | **E** | **K** | **A** | **E** | **A** | **R** | **A** | **H** | **I** | **L** | **E** | **G** | **L** | **R** | **I** | **A** | **L** | **D** | **H** | **I** | **D** | **E** | **I** | **I** | **A** | **I** | **I** | **R** | **G** | **S** | **K** | **S** | **D** | **D** | **E** | **A** | **K** | **A** | **T** | **M** | **I** | **E** | **R** | **F** | **D** | **L** | **S** | **D** | **R** | **Q** | **S** | **Q** | **A** | **I** | **L** | **D** | **M** | **R** | **L** | **R** | **R** | **L** | **T** | **G** | **L** | **E** | **R** | **E** | **K** | **I** | **E** | **N** | **E** | **Y** | **Q** | **E** | **L** | **L** | **K** | **L** | **I** | **E** | **D** | **L** | **T** | **D** | **I** | **L** | **A** | **R** | **P** | **E** | **R** | **V** | **T** | **E** | **I** | **I** | **K** | **N** | **E** | **L** | **A** | **D** | **L** | **N** | **Q** | **R** | **F** | **G** | **D** | **K** | **R** | **R** | **T** | **E** | **L** | **L** | **V** | **G** | **E** | **V** | **L** | **S** | **L** | **E** | **D** | **E** | **D** | **L** | **I** | **E** | **E** | **E** | **E** | **I** | **V** | **I** | **T** | **L** | **T** | **N** | **N** | **G** | **Y** | **I** | **K** | **R** | **L** | **A** | **N** | **N** | **E** | **F** | **R** | **A** | **Q** | **R** | **R** | **G** | **G** | **R** | **G** | **V** | **Q** | **G** | **M** | **G** | **I** | **H** | **N** | **D** | **D** | **F** | **V** | **K** | **N** | **L** | **V** | **S** | **C** | **S** | **T** | **H** | **D** | **T** | **L** | **L** | **F** | **F** | **T** | **N** | **N** | **G** | **K** | **V** | **Y** | **R** | **A** | **K** | **G** | **Y** | **E** | **I** | **P** | **E** | **Y** | **G** | **R** | **T** | **A** | **K** | **G** | **I** | **P** | **I** | **I** | **N** | **L** | **L** | **G** | **I** | **D** | **S** | **S** | **E** | **S** | **I** | **Q** | **A** | **I** | **I** | **A** | **V** | **T** | **G** | **E** | **A** | **E** | **E** | **G** | **H** | **Y** | **L** | **F** | **F** | **T** | **T** | **R** | **Q** | **G** | **T** | **V** | **K** | **R** | **T** | **S** | **V** | **T** | **A** | **F** | **A** | **N** | **I** | **R** | **S** | **N** | **G** | **L** | **I** | **A** | **I** | **G** | **L** | **K** | **E** | **D** | **D** | **E** | **L** | **V** | **N** | **V** | **L** | **L** | **T** | **D** | **G** | **Q** | **S** | **N** | **I** | **I** | **I** | **G** | **T** | **H** | **N** | **G** | **Y** | **S** | **V** | **T** | **F** | **K** | **E** | **E** | **A** | **V** | **R** | **D** | **M** | **G** | **R** | **T** | **A** | **S** | **G** | **V** | **R** | **G** | **I** | **R** | **L** | **R** | **E** | **E** | **D** | **Y** | **V** | **V** | **G** | **A** | **A** | **L** | **M** | **K** | **E** | **D** | **Q** | **E** | **V** | **L** | **V** | **I** | **T** | **E** | **K** | **G** | **Y** | **G** | **K** | **R** | **T** | **K** | **V** | **S** | **E** | **Y** | **P** | **V** | **K** | **G** | **R** | **G** | **G** | **K** | **G** | **I** | **K** | **T** | **A** | **N** | **I** | **T** | **E** | **K** | **N** | **G** | **P** | **L** | **A** | **G** | **L** | **T** | **T** | **V** | **S** | **G** | **D** | **E** | **D** | **I** | **M** | **L** | **I** | **T** | **D** | **K** | **G** | **V** | **I** | **I** | **R** | **F** | **N** | **V** | **S** | **T** | **V** | **S** | **Q** | **T** | **G** | **R** | **S** | **T** | **L** | **G** | **V** | **R** | **L** | **M** | **K** | **M** | **E** | **A** | **D** | **T** | **K** | **V** | **V** | **T** | **M** | **A** | **A** | **V** | **E** | **P** | **E** | **A** | **A** | **E** | **Q** | **T** | **V** | **E** | **E** | **Q** | **G** | **S** | **E** | **E** |
| **N59943F** | **M** | **S** | **E** | **E** | **I** | **R** | **E** | **N** | **I** | **H** | **D** | **V** | **N** | **L** | **T** | **S** | **E** | **M** | **K** | **D** | **S** | **F** | **I** | **D** | **Y** | **A** | **M** | **S** | **V** | **I** | **V** | **A** | **R** | **A** | **L** | **P** | **D** | **V** | **R** | **D** | **G** | **L** | **K** | **P** | **V** | **H** | **R** | **R** | **I** | **L** | **Y** | **G** | **M** | **N** | **E** | **L** | **G** | **V** | **T** | **P** | **D** | **K** | **A** | **H** | **K** | **K** | **S** | **A** | **R** | **I** | **V** | **G** | **D** | **V** | **M** | **G** | **K** | **Y** | **H** | **P** | **H** | **G** | **D** | **S** | **A** | **I** | **Y** | **E** | **S** | **M** | **V** | **R** | **M** | **A** | **Q** | **P** | **F** | **S** | **Y** | **R** | **Y** | **M** | **L** | **V** | **D** | **G** | **H** | **G** | **N** | **F** | **G** | **S** | **V** | **D** | **G** | **D** | **G** | **A** | **A** | **A** | **M** | **R** | **Y** | **T** | **E** | **A** | **R** | **M** | **S** | **K** | **I** | **A** | **T** | **E** | **M** | **L** | **R** | **D** | **I** | **N** | **K** | **N** | **T** | **V** | **D** | **F** | **Q** | **S** | **N** | **Y** | **D** | **D** | **T** | **E** | **K** | **E** | **P** | **V** | **V** | **L** | **P** | **A** | **R** | **F** | **P** | **N** | **L** | **L** | **V** | **N** | **G** | **T** | **T** | **G** | **I** | **A** | **V** | **G** | **M** | **A** | **T** | **N** | **I** | **P** | **P** | **H** | **N** | **L** | **S** | **E** | **V** | **V** | **A** | **A** | **I** | **D** | **L** | **L** | **M** | **D** | **N** | **P** | **D** | **V** | **T** | **T** | **N** | **E** | **L** | **M** | **E** | **V** | **L** | **P** | **G** | **P** | **D** | **F** | **P** | **T** | **G** | **G** | **L** | **V** | **M** | **G** | **K** | **S** | **G** | **I** | **R** | **R** | **A** | **Y** | **E** | **T** | **G** | **K** | **G** | **S** | **I** | **T** | **V** | **R** | **A** | **K** | **V** | **E** | **I** | **T** | **E** | **M** | **P** | **N** | **G** | **K** | **E** | **R** | **I** | **L** | **V** | **T** | **E** | **L** | **P** | **Y** | **M** | **V** | **N** | **K** | **A** | **K** | **L** | **I** | **E** | **R** | **I** | **S** | **E** | **L** | **H** | **R** | **D** | **K** | **R** | **I** | **E** | **G** | **I** | **T** | **D** | **L** | **R** | **D** | **E** | **S** | **S** | **R** | **E** | **G** | **M** | **R** | **I** | **V** | **I** | **D** | **V** | **R** | **R** | **D** | **A** | **S** | **A** | **S** | **V** | **I** | **L** | **N** | **N** | **L** | **Y** | **K** | **L** | **T** | **S** | **L** | **Q** | **N** | **S** | **F** | **G** | **F** | **N** | **M** | **L** | **A** | **I** | **E** | **K** | **G** | **V** | **P** | **K** | **V** | **L** | **S** | **L** | **K** | **Q** | **I** | **L** | **E** | **N** | **Y** | **I** | **E** | **H** | **Q** | **R** | **E** | **V** | **I** | **T** | **R** | **R** | **T** | **V** | **F** | **E** | **K** | **E** | **K** | **A** | **E** | **A** | **R** | **A** | **H** | **I** | **L** | **E** | **G** | **L** | **R** | **I** | **A** | **L** | **D** | **H** | **I** | **D** | **E** | **I** | **I** | **A** | **I** | **I** | **R** | **G** | **S** | **K** | **S** | **D** | **D** | **E** | **A** | **K** | **A** | **T** | **M** | **I** | **E** | **R** | **F** | **D** | **L** | **S** | **D** | **R** | **Q** | **S** | **Q** | **A** | **I** | **L** | **D** | **M** | **R** | **L** | **R** | **R** | **L** | **T** | **G** | **L** | **E** | **R** | **E** | **K** | **I** | **E** | **N** | **E** | **Y** | **Q** | **E** | **L** | **L** | **K** | **L** | **I** | **E** | **D** | **L** | **T** | **D** | **I** | **L** | **A** | **R** | **P** | **E** | **R** | **V** | **T** | **E** | **I** | **I** | **K** | **N** | **E** | **L** | **A** | **D** | **L** | **N** | **Q** | **R** | **F** | **G** | **D** | **K** | **R** | **R** | **T** | **E** | **L** | **L** | **V** | **G** | **E** | **V** | **L** | **S** | **L** | **E** | **D** | **E** | **D** | **L** | **I** | **E** | **E** | **E** | **E** | **I** | **V** | **I** | **T** | **L** | **T** | **N** | **N** | **G** | **Y** | **I** | **K** | **R** | **L** | **A** | **N** | **N** | **E** | **F** | **R** | **A** | **Q** | **R** | **R** | **G** | **G** | **R** | **G** | **V** | **Q** | **G** | **M** | **G** | **I** | **H** | **N** | **D** | **D** | **F** | **V** | **K** | **N** | **L** | **V** | **S** | **C** | **S** | **T** | **H** | **D** | **T** | **L** | **L** | **F** | **F** | **T** | **N** | **N** | **G** | **K** | **V** | **Y** | **R** | **A** | **K** | **G** | **Y** | **E** | **I** | **P** | **E** | **Y** | **G** | **R** | **T** | **A** | **K** | **G** | **I** | **P** | **I** | **I** | **N** | **L** | **L** | **G** | **I** | **D** | **S** | **S** | **E** | **S** | **I** | **Q** | **A** | **I** | **I** | **A** | **V** | **T** | **G** | **E** | **A** | **E** | **E** | **G** | **H** | **Y** | **L** | **F** | **F** | **T** | **T** | **R** | **Q** | **G** | **T** | **V** | **K** | **R** | **T** | **S** | **V** | **T** | **A** | **F** | **A** | **N** | **I** | **R** | **S** | **N** | **G** | **L** | **I** | **A** | **I** | **G** | **L** | **K** | **E** | **D** | **D** | **E** | **L** | **V** | **N** | **V** | **L** | **L** | **T** | **D** | **G** | **Q** | **S** | **N** | **I** | **I** | **I** | **G** | **T** | **H** | **N** | **G** | **Y** | **S** | **V** | **T** | **F** | **K** | **E** | **E** | **A** | **V** | **R** | **D** | **M** | **G** | **R** | **T** | **A** | **S** | **G** | **V** | **R** | **G** | **I** | **R** | **L** | **R** | **E** | **E** | **D** | **Y** | **V** | **V** | **G** | **A** | **A** | **L** | **M** | **K** | **E** | **D** | **Q** | **E** | **V** | **L** | **V** | **I** | **T** | **E** | **K** | **G** | **Y** | **G** | **K** | **R** | **T** | **K** | **V** | **S** | **E** | **Y** | **P** | **V** | **K** | **G** | **R** | **G** | **G** | **K** | **G** | **I** | **K** | **T** | **A** | **N** | **I** | **T** | **E** | **K** | **N** | **G** | **P** | **L** | **A** | **G** | **L** | **T** | **T** | **V** | **S** | **G** | **N** | **E** | **D** | **I** | **M** | **L** | **I** | **T** | **D** | **K** | **G** | **V** | **I** | **I** | **R** | **F** | **N** | **V** | **S** | **T** | **V** | **S** | **Q** | **T** | **G** | **R** | **S** | **T** | **L** | **G** | **V** | **R** | **L** | **M** | **K** | **M** | **E** | **A** | **D** | **T** | **K** | **V** | **V** | **T** | **M** | **-** | **-** | **-** | **-** | **-** | **-** | **-** | **-** | **-** | **-** | **-** | **-** | **-** | **-** | **-** | **-** | **-** | **-** | **-** |
| **N52643** | **M** | **S** | **E** | **E** | **I** | **R** | **E** | **N** | **I** | **H** | **D** | **V** | **N** | **L** | **T** | **S** | **E** | **M** | **K** | **D** | **S** | **F** | **I** | **D** | **Y** | **A** | **M** | **S** | **V** | **I** | **V** | **A** | **R** | **A** | **L** | **P** | **D** | **V** | **R** | **D** | **G** | **L** | **K** | **P** | **V** | **H** | **R** | **R** | **I** | **L** | **Y** | **G** | **M** | **N** | **E** | **L** | **G** | **V** | **T** | **P** | **D** | **K** | **A** | **H** | **K** | **K** | **S** | **A** | **R** | **I** | **V** | **G** | **D** | **V** | **M** | **G** | **K** | **Y** | **H** | **P** | **H** | **G** | **D** | **S** | **A** | **I** | **Y** | **E** | **S** | **M** | **V** | **R** | **M** | **A** | **Q** | **P** | **F** | **S** | **Y** | **R** | **Y** | **M** | **L** | **V** | **D** | **G** | **H** | **G** | **N** | **F** | **G** | **S** | **V** | **D** | **G** | **D** | **G** | **A** | **A** | **A** | **M** | **R** | **Y** | **T** | **E** | **A** | **R** | **M** | **S** | **K** | **I** | **A** | **T** | **E** | **M** | **L** | **R** | **D** | **I** | **N** | **K** | **N** | **T** | **V** | **D** | **F** | **Q** | **S** | **N** | **Y** | **D** | **D** | **T** | **E** | **K** | **E** | **P** | **V** | **V** | **L** | **P** | **A** | **R** | **F** | **P** | **N** | **L** | **L** | **V** | **N** | **G** | **T** | **T** | **G** | **I** | **A** | **V** | **G** | **M** | **A** | **T** | **N** | **I** | **P** | **P** | **H** | **N** | **L** | **S** | **E** | **V** | **V** | **A** | **A** | **I** | **D** | **L** | **L** | **M** | **D** | **N** | **P** | **D** | **V** | **T** | **T** | **N** | **E** | **L** | **M** | **E** | **V** | **L** | **P** | **G** | **P** | **D** | **F** | **P** | **T** | **G** | **G** | **L** | **V** | **M** | **G** | **K** | **S** | **G** | **I** | **R** | **R** | **A** | **Y** | **E** | **T** | **G** | **K** | **G** | **S** | **I** | **T** | **V** | **R** | **A** | **K** | **V** | **E** | **I** | **T** | **E** | **M** | **P** | **N** | **G** | **K** | **E** | **R** | **I** | **I** | **V** | **T** | **E** | **L** | **P** | **Y** | **M** | **V** | **N** | **K** | **A** | **K** | **L** | **I** | **E** | **R** | **I** | **S** | **E** | **L** | **H** | **R** | **D** | **K** | **R** | **I** | **E** | **G** | **I** | **T** | **D** | **L** | **R** | **D** | **E** | **S** | **S** | **R** | **E** | **G** | **M** | **R** | **I** | **V** | **I** | **D** | **I** | **R** | **R** | **D** | **A** | **S** | **A** | **S** | **V** | **I** | **L** | **N** | **N** | **L** | **Y** | **K** | **L** | **T** | **S** | **L** | **Q** | **N** | **S** | **F** | **G** | **F** | **N** | **M** | **L** | **A** | **I** | **E** | **K** | **G** | **V** | **P** | **K** | **V** | **L** | **S** | **L** | **K** | **Q** | **I** | **L** | **E** | **N** | **Y** | **I** | **E** | **H** | **Q** | **R** | **E** | **V** | **I** | **T** | **R** | **R** | **T** | **V** | **F** | **E** | **K** | **E** | **K** | **A** | **E** | **A** | **R** | **A** | **H** | **I** | **L** | **E** | **G** | **L** | **R** | **I** | **A** | **L** | **D** | **H** | **I** | **D** | **E** | **I** | **I** | **A** | **I** | **I** | **R** | **G** | **S** | **K** | **S** | **D** | **D** | **E** | **A** | **K** | **A** | **T** | **M** | **I** | **E** | **R** | **F** | **D** | **L** | **S** | **D** | **R** | **Q** | **S** | **Q** | **A** | **I** | **L** | **D** | **M** | **R** | **L** | **R** | **R** | **L** | **T** | **G** | **L** | **E** | **R** | **E** | **K** | **I** | **E** | **N** | **E** | **Y** | **Q** | **E** | **L** | **L** | **K** | **L** | **I** | **E** | **D** | **L** | **T** | **D** | **I** | **L** | **A** | **R** | **P** | **E** | **R** | **V** | **T** | **E** | **I** | **I** | **K** | **N** | **E** | **L** | **A** | **D** | **L** | **N** | **Q** | **R** | **F** | **G** | **D** | **K** | **R** | **R** | **T** | **E** | **L** | **L** | **V** | **G** | **E** | **V** | **L** | **S** | **L** | **E** | **D** | **E** | **D** | **L** | **I** | **E** | **E** | **E** | **E** | **I** | **V** | **I** | **T** | **L** | **T** | **N** | **N** | **G** | **Y** | **I** | **K** | **R** | **L** | **A** | **N** | **N** | **E** | **F** | **R** | **A** | **Q** | **R** | **R** | **G** | **G** | **R** | **G** | **V** | **Q** | **G** | **M** | **G** | **I** | **H** | **N** | **D** | **D** | **F** | **V** | **K** | **N** | **L** | **V** | **S** | **C** | **S** | **T** | **H** | **D** | **T** | **L** | **L** | **F** | **F** | **T** | **N** | **N** | **G** | **K** | **V** | **Y** | **R** | **A** | **K** | **G** | **Y** | **E** | **I** | **P** | **E** | **Y** | **G** | **R** | **T** | **A** | **K** | **G** | **I** | **P** | **I** | **I** | **N** | **L** | **L** | **G** | **I** | **D** | **S** | **S** | **E** | **S** | **I** | **Q** | **A** | **I** | **I** | **A** | **V** | **T** | **G** | **E** | **A** | **E** | **E** | **G** | **H** | **Y** | **L** | **F** | **F** | **T** | **T** | **R** | **Q** | **G** | **T** | **V** | **K** | **R** | **T** | **S** | **V** | **T** | **A** | **F** | **A** | **N** | **I** | **R** | **S** | **N** | **G** | **L** | **I** | **A** | **I** | **G** | **L** | **K** | **E** | **D** | **D** | **E** | **L** | **V** | **N** | **V** | **L** | **L** | **T** | **D** | **G** | **Q** | **S** | **N** | **I** | **I** | **I** | **G** | **T** | **H** | **N** | **G** | **Y** | **S** | **V** | **T** | **F** | **K** | **E** | **E** | **A** | **V** | **R** | **D** | **M** | **G** | **R** | **T** | **A** | **S** | **G** | **V** | **R** | **G** | **I** | **R** | **L** | **R** | **E** | **E** | **D** | **Y** | **V** | **V** | **G** | **A** | **A** | **L** | **M** | **K** | **E** | **N** | **Q** | **E** | **V** | **L** | **V** | **I** | **T** | **E** | **K** | **G** | **Y** | **G** | **K** | **R** | **T** | **K** | **V** | **S** | **E** | **Y** | **P** | **V** | **K** | **G** | **R** | **G** | **G** | **K** | **G** | **I** | **K** | **T** | **A** | **N** | **I** | **T** | **E** | **K** | **N** | **G** | **P** | **L** | **A** | **G** | **L** | **T** | **T** | **V** | **S** | **G** | **D** | **E** | **D** | **I** | **M** | **L** | **I** | **T** | **D** | **K** | **G** | **V** | **I** | **I** | **R** | **F** | **N** | **V** | **S** | **T** | **V** | **S** | **Q** | **T** | **G** | **R** | **S** | **T** | **L** | **G** | **V** | **R** | **L** | **M** | **K** | **M** | **E** | **A** | **D** | **T** | **K** | **V** | **V** | **T** | **M** | **A** | **A** | **V** | **E** | **P** | **E** | **A** | **A** | **E** | **Q** | **T** | **V** | **E** | **E** | **Q** | **G** | **S** | **E** | **E** |
| **N59609F** | **M** | **S** | **E** | **E** | **I** | **K** | **E** | **N** | **I** | **Q** | **D** | **V** | **N** | **L** | **T** | **S** | **E** | **M** | **K** | **E** | **S** | **F** | **I** | **D** | **Y** | **A** | **M** | **S** | **V** | **I** | **V** | **A** | **R** | **A** | **L** | **P** | **D** | **V** | **R** | **D** | **G** | **L** | **K** | **P** | **V** | **H** | **R** | **R** | **I** | **L** | **Y** | **G** | **M** | **N** | **E** | **L** | **G** | **V** | **T** | **P** | **D** | **K** | **P** | **H** | **K** | **K** | **S** | **A** | **R** | **I** | **V** | **G** | **D** | **V** | **M** | **G** | **K** | **Y** | **H** | **P** | **H** | **G** | **D** | **S** | **A** | **I** | **Y** | **E** | **S** | **M** | **V** | **R** | **M** | **A** | **Q** | **P** | **F** | **S** | **Y** | **R** | **A** | **M** | **L** | **V** | **D** | **G** | **H** | **G** | **N** | **F** | **G** | **S** | **V** | **D** | **G** | **D** | **G** | **A** | **A** | **A** | **M** | **R** | **Y** | **T** | **E** | **A** | **R** | **M** | **S** | **K** | **I** | **A** | **L** | **E** | **M** | **L** | **R** | **D** | **I** | **N** | **K** | **N** | **T** | **V** | **D** | **F** | **Q** | **G** | **N** | **Y** | **D** | **D** | **S** | **E** | **Q** | **E** | **P** | **V** | **V** | **L** | **P** | **A** | **R** | **F** | **P** | **N** | **L** | **L** | **V** | **N** | **G** | **T** | **T** | **G** | **I** | **A** | **V** | **G** | **M** | **A** | **T** | **N** | **I** | **P** | **P** | **H** | **N** | **L** | **S** | **E** | **V** | **I** | **D** | **A** | **T** | **S** | **L** | **L** | **M** | **D** | **N** | **P** | **D** | **V** | **T** | **T** | **N** | **E** | **L** | **M** | **E** | **V** | **L** | **P** | **G** | **P** | **D** | **F** | **P** | **T** | **G** | **G** | **L** | **V** | **M** | **G** | **K** | **S** | **G** | **I** | **R** | **R** | **A** | **Y** | **E** | **T** | **G** | **K** | **G** | **S** | **I** | **T** | **V** | **R** | **A** | **K** | **V** | **E** | **L** | **T** | **E** | **M** | **P** | **N** | **G** | **K** | **E** | **R** | **I** | **L** | **V** | **T** | **E** | **L** | **P** | **Y** | **M** | **V** | **N** | **K** | **A** | **K** | **L** | **I** | **E** | **R** | **I** | **S** | **E** | **L** | **H** | **R** | **D** | **K** | **R** | **I** | **E** | **G** | **I** | **T** | **D** | **L** | **R** | **D** | **E** | **S** | **S** | **R** | **E** | **G** | **M** | **R** | **I** | **V** | **I** | **D** | **V** | **R** | **R** | **D** | **V** | **S** | **A** | **S** | **V** | **V** | **L** | **N** | **N** | **L** | **Y** | **K** | **M** | **T** | **A** | **L** | **Q** | **T** | **S** | **F** | **G** | **F** | **N** | **M** | **L** | **A** | **I** | **E** | **K** | **G** | **V** | **P** | **K** | **I** | **L** | **S** | **L** | **K** | **R** | **I** | **L** | **E** | **N** | **Y** | **V** | **E** | **H** | **Q** | **K** | **E** | **V** | **I** | **T** | **R** | **R** | **T** | **I** | **F** | **D** | **K** | **N** | **K** | **A** | **E** | **A** | **R** | **A** | **H** | **I** | **L** | **E** | **G** | **L** | **R** | **I** | **A** | **L** | **D** | **H** | **I** | **D** | **E** | **I** | **I** | **A** | **I** | **I** | **R** | **G** | **S** | **Q** | **S** | **D** | **D** | **E** | **A** | **K** | **A** | **T** | **L** | **I** | **E** | **R** | **F** | **E** | **F** | **S** | **D** | **R** | **Q** | **A** | **Q** | **A** | **I** | **L** | **D** | **M** | **R** | **L** | **R** | **R** | **L** | **T** | **G** | **L** | **E** | **R** | **D** | **K** | **I** | **E** | **N** | **E** | **Y** | **Q** | **E** | **L** | **L** | **K** | **F** | **I** | **A** | **D** | **L** | **E** | **D** | **I** | **L** | **A** | **R** | **P** | **E** | **R** | **V** | **I** | **E** | **I** | **I** | **K** | **T** | **E** | **L** | **N** | **D** | **V** | **R** | **T** | **K** | **F** | **G** | **D** | **A** | **R** | **R** | **T** | **E** | **L** | **L** | **V** | **G** | **E** | **V** | **L** | **S** | **L** | **E** | **D** | **E** | **D** | **L** | **I** | **E** | **E** | **E** | **E** | **V** | **V** | **I** | **T** | **L** | **T** | **N** | **N** | **G** | **Y** | **I** | **K** | **R** | **M** | **A** | **N** | **S** | **E** | **F** | **R** | **A** | **Q** | **R** | **R** | **G** | **G** | **R** | **G** | **V** | **Q** | **G** | **M** | **G** | **V** | **H** | **D** | **D** | **D** | **F** | **V** | **K** | **N** | **L** | **V** | **S** | **C** | **S** | **T** | **H** | **D** | **T** | **L** | **L** | **F** | **F** | **T** | **N** | **T** | **G** | **K** | **V** | **Y** | **R** | **A** | **K** | **G** | **Y** | **E** | **I** | **P** | **E** | **Y** | **G** | **R** | **T** | **A** | **K** | **G** | **I** | **P** | **V** | **I** | **N** | **L** | **L** | **G** | **I** | **D** | **S** | **A** | **E** | **K** | **I** | **Q** | **A** | **I** | **I** | **S** | **V** | **E** | **G** | **K** | **A** | **E** | **A** | **G** | **K** | **Y** | **L** | **F** | **F** | **T** | **T** | **L** | **K** | **G** | **T** | **V** | **K** | **R** | **T** | **A** | **V** | **T** | **A** | **F** | **S** | **N** | **I** | **R** | **S** | **N** | **G** | **L** | **I** | **A** | **I** | **S** | **L** | **K** | **E** | **D** | **D** | **E** | **L** | **V** | **N** | **V** | **V** | **T** | **T** | **N** | **G** | **N** | **Q** | **K** | **M** | **I** | **I** | **G** | **T** | **H** | **A** | **G** | **Y** | **S** | **V** | **T** | **F** | **D** | **E** | **N** | **T** | **V** | **R** | **D** | **M** | **G** | **R** | **T** | **A** | **S** | **G** | **V** | **R** | **G** | **I** | **R** | **L** | **R** | **E** | **N** | **D** | **Y** | **V** | **V** | **G** | **A** | **A** | **I** | **L** | **D** | **E** | **N** | **K** | **E** | **V** | **L** | **V** | **I** | **T** | **E** | **N** | **G** | **Y** | **G** | **K** | **R** | **T** | **K** | **A** | **S** | **E** | **Y** | **P** | **V** | **K** | **G** | **R** | **G** | **G** | **K** | **G** | **I** | **K** | **T** | **A** | **N** | **I** | **T** | **E** | **K** | **N** | **G** | **P** | **L** | **A** | **G** | **L** | **T** | **T** | **V** | **N** | **G** | **N** | **E** | **D** | **I** | **L** | **L** | **I** | **T** | **N** | **K** | **G** | **V** | **I** | **I** | **R** | **F** | **N** | **V** | **D** | **S** | **V** | **S** | **Q** | **T** | **G** | **R** | **A** | **T** | **L** | **G** | **V** | **R** | **L** | **M** | **R** | **M** | **E** | **D** | **G** | **A** | **K** | **-** | **-** | **-** | **-** | **-** | **-** | **-** | **-** | **-** | **-** | **-** | **-** | **-** | **-** | **-** | **-** | **-** | **-** | **-** | **-** | **-** | **-** | **-** |
| **N53655** | **M** | **S** | **E** | **E** | **I** | **R** | **E** | **N** | **I** | **H** | **D** | **V** | **N** | **L** | **T** | **S** | **E** | **M** | **K** | **D** | **S** | **F** | **I** | **D** | **Y** | **A** | **M** | **S** | **V** | **I** | **V** | **A** | **R** | **A** | **L** | **P** | **D** | **V** | **R** | **D** | **G** | **L** | **K** | **P** | **V** | **H** | **R** | **R** | **I** | **L** | **Y** | **G** | **M** | **N** | **E** | **L** | **G** | **V** | **T** | **P** | **D** | **K** | **A** | **H** | **K** | **K** | **S** | **A** | **R** | **I** | **V** | **G** | **D** | **V** | **M** | **G** | **K** | **Y** | **H** | **P** | **H** | **G** | **D** | **S** | **A** | **I** | **Y** | **E** | **S** | **M** | **V** | **R** | **M** | **A** | **Q** | **P** | **F** | **S** | **Y** | **R** | **Y** | **M** | **L** | **V** | **D** | **G** | **H** | **G** | **N** | **F** | **G** | **S** | **V** | **D** | **G** | **D** | **G** | **A** | **A** | **A** | **M** | **R** | **Y** | **T** | **E** | **A** | **R** | **M** | **S** | **K** | **I** | **A** | **T** | **E** | **M** | **L** | **R** | **D** | **I** | **N** | **K** | **N** | **T** | **V** | **D** | **F** | **Q** | **S** | **N** | **Y** | **D** | **D** | **T** | **E** | **K** | **E** | **P** | **V** | **V** | **L** | **P** | **A** | **R** | **F** | **P** | **N** | **L** | **L** | **V** | **N** | **G** | **T** | **T** | **G** | **I** | **A** | **V** | **G** | **M** | **A** | **T** | **N** | **I** | **P** | **P** | **H** | **N** | **L** | **S** | **E** | **V** | **V** | **A** | **A** | **I** | **D** | **L** | **L** | **M** | **D** | **N** | **P** | **D** | **V** | **T** | **T** | **N** | **E** | **L** | **M** | **E** | **V** | **L** | **P** | **G** | **P** | **D** | **F** | **P** | **T** | **G** | **G** | **L** | **V** | **M** | **G** | **K** | **S** | **G** | **I** | **R** | **R** | **A** | **Y** | **E** | **T** | **G** | **K** | **G** | **S** | **I** | **T** | **V** | **R** | **A** | **K** | **V** | **E** | **I** | **T** | **E** | **M** | **P** | **N** | **G** | **K** | **E** | **R** | **I** | **I** | **V** | **T** | **E** | **L** | **P** | **Y** | **M** | **V** | **N** | **K** | **A** | **K** | **L** | **I** | **E** | **R** | **I** | **S** | **E** | **L** | **H** | **R** | **D** | **K** | **R** | **I** | **E** | **G** | **I** | **T** | **D** | **L** | **R** | **D** | **E** | **S** | **S** | **R** | **E** | **G** | **M** | **R** | **I** | **V** | **I** | **D** | **I** | **R** | **R** | **D** | **A** | **S** | **A** | **S** | **V** | **I** | **L** | **N** | **N** | **L** | **Y** | **K** | **L** | **T** | **S** | **L** | **Q** | **N** | **S** | **F** | **G** | **F** | **N** | **M** | **L** | **A** | **I** | **E** | **K** | **G** | **V** | **P** | **K** | **V** | **L** | **S** | **L** | **K** | **Q** | **I** | **L** | **E** | **N** | **Y** | **I** | **E** | **H** | **Q** | **R** | **E** | **V** | **I** | **T** | **R** | **R** | **T** | **V** | **F** | **E** | **K** | **E** | **K** | **A** | **E** | **A** | **R** | **A** | **H** | **I** | **L** | **E** | **G** | **L** | **R** | **I** | **A** | **L** | **D** | **H** | **I** | **D** | **E** | **I** | **I** | **A** | **I** | **I** | **R** | **G** | **S** | **K** | **S** | **D** | **D** | **E** | **A** | **K** | **A** | **T** | **M** | **I** | **E** | **R** | **F** | **D** | **L** | **S** | **D** | **R** | **Q** | **S** | **Q** | **A** | **I** | **L** | **D** | **M** | **R** | **L** | **R** | **R** | **L** | **T** | **G** | **L** | **E** | **R** | **E** | **K** | **I** | **E** | **N** | **E** | **Y** | **Q** | **E** | **L** | **L** | **K** | **L** | **I** | **E** | **D** | **L** | **T** | **D** | **I** | **L** | **A** | **R** | **P** | **E** | **R** | **V** | **T** | **E** | **I** | **I** | **K** | **N** | **E** | **L** | **A** | **D** | **L** | **N** | **Q** | **R** | **F** | **G** | **D** | **K** | **R** | **R** | **T** | **E** | **L** | **L** | **V** | **G** | **E** | **V** | **L** | **S** | **L** | **E** | **D** | **E** | **D** | **L** | **I** | **E** | **E** | **E** | **E** | **I** | **V** | **I** | **T** | **L** | **T** | **N** | **N** | **G** | **Y** | **I** | **K** | **R** | **L** | **A** | **N** | **N** | **E** | **F** | **R** | **A** | **Q** | **R** | **R** | **G** | **G** | **R** | **G** | **V** | **Q** | **G** | **M** | **G** | **I** | **H** | **N** | **D** | **D** | **F** | **V** | **K** | **N** | **L** | **V** | **S** | **C** | **S** | **T** | **H** | **D** | **T** | **L** | **L** | **F** | **F** | **T** | **N** | **N** | **G** | **K** | **V** | **Y** | **R** | **A** | **K** | **G** | **Y** | **E** | **I** | **P** | **E** | **Y** | **G** | **R** | **T** | **A** | **K** | **G** | **I** | **P** | **I** | **I** | **N** | **L** | **L** | **G** | **I** | **D** | **S** | **S** | **E** | **S** | **I** | **Q** | **A** | **I** | **I** | **A** | **V** | **T** | **G** | **E** | **A** | **E** | **E** | **G** | **H** | **Y** | **L** | **F** | **F** | **T** | **T** | **R** | **Q** | **G** | **T** | **V** | **K** | **R** | **T** | **S** | **V** | **T** | **A** | **F** | **A** | **N** | **I** | **R** | **S** | **N** | **G** | **L** | **I** | **A** | **I** | **G** | **L** | **K** | **E** | **D** | **D** | **E** | **L** | **V** | **N** | **V** | **L** | **L** | **T** | **D** | **G** | **Q** | **S** | **N** | **I** | **I** | **I** | **G** | **T** | **H** | **N** | **G** | **Y** | **S** | **V** | **T** | **F** | **K** | **E** | **E** | **A** | **V** | **R** | **D** | **M** | **G** | **R** | **T** | **A** | **S** | **G** | **V** | **R** | **G** | **I** | **R** | **L** | **R** | **E** | **E** | **D** | **Y** | **V** | **V** | **G** | **A** | **A** | **L** | **M** | **K** | **E** | **D** | **Q** | **E** | **V** | **L** | **V** | **I** | **T** | **E** | **K** | **G** | **Y** | **G** | **K** | **R** | **T** | **K** | **V** | **S** | **E** | **Y** | **P** | **V** | **K** | **G** | **R** | **G** | **G** | **K** | **G** | **I** | **K** | **T** | **A** | **N** | **I** | **T** | **E** | **K** | **N** | **G** | **P** | **L** | **A** | **G** | **L** | **T** | **T** | **V** | **S** | **G** | **D** | **E** | **D** | **I** | **M** | **L** | **I** | **T** | **D** | **K** | **G** | **V** | **I** | **I** | **R** | **F** | **N** | **V** | **S** | **T** | **V** | **S** | **Q** | **T** | **G** | **R** | **S** | **T** | **L** | **G** | **V** | **R** | **L** | **M** | **K** | **M** | **E** | **A** | **D** | **T** | **K** | **V** | **V** | **T** | **M** | **A** | **A** | **V** | **E** | **P** | **E** | **A** | **A** | **E** | **Q** | **T** | **V** | **E** | **E** | **Q** | **G** | **S** | **E** | **E** |
| **N52769** | **M** | **S** | **E** | **E** | **I** | **R** | **E** | **N** | **I** | **H** | **D** | **V** | **N** | **L** | **T** | **S** | **E** | **M** | **K** | **D** | **S** | **F** | **I** | **D** | **Y** | **A** | **M** | **S** | **V** | **I** | **V** | **A** | **R** | **A** | **L** | **P** | **D** | **V** | **R** | **D** | **G** | **L** | **K** | **P** | **V** | **H** | **R** | **R** | **I** | **L** | **Y** | **G** | **M** | **N** | **E** | **L** | **G** | **V** | **T** | **P** | **D** | **K** | **A** | **H** | **K** | **K** | **S** | **A** | **R** | **I** | **V** | **G** | **D** | **V** | **M** | **G** | **K** | **Y** | **H** | **P** | **H** | **G** | **D** | **S** | **A** | **I** | **Y** | **E** | **S** | **M** | **V** | **R** | **M** | **A** | **Q** | **P** | **F** | **S** | **Y** | **R** | **Y** | **M** | **L** | **V** | **D** | **G** | **H** | **G** | **N** | **F** | **G** | **S** | **V** | **D** | **G** | **D** | **G** | **A** | **A** | **A** | **M** | **R** | **Y** | **T** | **E** | **A** | **R** | **M** | **S** | **K** | **I** | **A** | **T** | **E** | **M** | **L** | **R** | **D** | **I** | **N** | **K** | **N** | **T** | **V** | **D** | **F** | **Q** | **S** | **N** | **Y** | **D** | **D** | **T** | **E** | **K** | **E** | **P** | **V** | **V** | **L** | **P** | **A** | **R** | **F** | **P** | **N** | **L** | **L** | **V** | **N** | **G** | **T** | **T** | **G** | **I** | **A** | **V** | **G** | **M** | **A** | **T** | **N** | **I** | **P** | **P** | **H** | **N** | **L** | **S** | **E** | **V** | **V** | **A** | **A** | **I** | **D** | **L** | **L** | **M** | **D** | **N** | **P** | **D** | **V** | **T** | **T** | **N** | **E** | **L** | **M** | **E** | **V** | **L** | **P** | **G** | **P** | **D** | **F** | **P** | **T** | **G** | **G** | **L** | **V** | **M** | **G** | **K** | **S** | **G** | **I** | **R** | **R** | **A** | **Y** | **E** | **T** | **G** | **K** | **G** | **S** | **I** | **T** | **V** | **R** | **A** | **K** | **V** | **E** | **I** | **T** | **E** | **M** | **P** | **N** | **G** | **K** | **E** | **R** | **I** | **L** | **V** | **T** | **E** | **L** | **P** | **Y** | **M** | **V** | **N** | **K** | **A** | **K** | **L** | **I** | **E** | **R** | **I** | **S** | **E** | **L** | **H** | **R** | **D** | **K** | **R** | **I** | **E** | **G** | **I** | **T** | **D** | **L** | **R** | **D** | **E** | **S** | **S** | **R** | **E** | **G** | **M** | **R** | **I** | **V** | **I** | **D** | **V** | **R** | **R** | **D** | **A** | **S** | **A** | **S** | **V** | **I** | **L** | **N** | **N** | **L** | **Y** | **K** | **L** | **T** | **S** | **L** | **Q** | **N** | **S** | **F** | **G** | **F** | **N** | **M** | **L** | **A** | **I** | **E** | **K** | **G** | **V** | **P** | **K** | **V** | **L** | **S** | **L** | **K** | **Q** | **I** | **L** | **E** | **N** | **Y** | **I** | **E** | **H** | **Q** | **R** | **E** | **V** | **I** | **T** | **R** | **R** | **T** | **V** | **F** | **E** | **K** | **E** | **K** | **A** | **E** | **A** | **R** | **A** | **H** | **I** | **L** | **E** | **G** | **L** | **R** | **I** | **A** | **L** | **D** | **H** | **I** | **D** | **E** | **I** | **I** | **A** | **I** | **I** | **R** | **G** | **S** | **K** | **S** | **D** | **D** | **E** | **A** | **K** | **A** | **T** | **M** | **I** | **E** | **R** | **F** | **D** | **L** | **S** | **D** | **R** | **Q** | **S** | **Q** | **A** | **I** | **L** | **D** | **M** | **R** | **L** | **R** | **R** | **L** | **T** | **G** | **L** | **E** | **R** | **E** | **K** | **I** | **E** | **N** | **E** | **Y** | **Q** | **E** | **L** | **L** | **K** | **L** | **I** | **E** | **D** | **L** | **T** | **D** | **I** | **L** | **A** | **R** | **P** | **E** | **R** | **V** | **T** | **E** | **I** | **I** | **K** | **N** | **E** | **L** | **A** | **D** | **L** | **N** | **Q** | **R** | **F** | **G** | **D** | **K** | **R** | **R** | **T** | **E** | **L** | **L** | **V** | **G** | **E** | **V** | **L** | **S** | **L** | **E** | **D** | **E** | **D** | **L** | **I** | **E** | **E** | **E** | **E** | **I** | **V** | **I** | **T** | **L** | **T** | **N** | **N** | **G** | **Y** | **I** | **K** | **R** | **L** | **A** | **N** | **N** | **E** | **F** | **R** | **A** | **Q** | **R** | **R** | **G** | **G** | **R** | **G** | **V** | **Q** | **G** | **M** | **G** | **I** | **H** | **N** | **D** | **D** | **F** | **V** | **K** | **N** | **L** | **V** | **S** | **C** | **S** | **T** | **H** | **D** | **T** | **L** | **L** | **F** | **F** | **T** | **N** | **N** | **G** | **K** | **V** | **Y** | **R** | **A** | **K** | **G** | **Y** | **E** | **I** | **P** | **E** | **Y** | **G** | **R** | **T** | **A** | **K** | **G** | **I** | **P** | **I** | **I** | **N** | **L** | **L** | **G** | **I** | **D** | **S** | **S** | **E** | **S** | **I** | **Q** | **A** | **I** | **I** | **A** | **V** | **T** | **G** | **E** | **A** | **E** | **E** | **G** | **H** | **Y** | **L** | **F** | **F** | **T** | **T** | **R** | **Q** | **G** | **T** | **V** | **K** | **R** | **T** | **S** | **V** | **T** | **A** | **F** | **A** | **N** | **I** | **R** | **S** | **N** | **G** | **L** | **I** | **A** | **I** | **G** | **L** | **K** | **E** | **D** | **D** | **E** | **L** | **I** | **N** | **V** | **L** | **L** | **T** | **D** | **G** | **Q** | **S** | **N** | **I** | **I** | **I** | **G** | **T** | **H** | **N** | **G** | **Y** | **S** | **V** | **T** | **F** | **K** | **E** | **E** | **A** | **V** | **R** | **D** | **M** | **G** | **R** | **T** | **A** | **S** | **G** | **V** | **R** | **G** | **I** | **R** | **L** | **R** | **E** | **E** | **D** | **C** | **V** | **V** | **G** | **A** | **A** | **L** | **M** | **K** | **E** | **D** | **Q** | **E** | **V** | **L** | **V** | **I** | **T** | **E** | **K** | **G** | **Y** | **G** | **K** | **R** | **T** | **K** | **V** | **S** | **E** | **Y** | **P** | **V** | **K** | **G** | **R** | **G** | **G** | **K** | **G** | **I** | **K** | **T** | **A** | **N** | **I** | **T** | **E** | **K** | **N** | **G** | **P** | **L** | **A** | **G** | **L** | **T** | **T** | **V** | **S** | **G** | **N** | **E** | **D** | **I** | **M** | **L** | **I** | **T** | **D** | **K** | **G** | **V** | **I** | **I** | **R** | **F** | **N** | **V** | **S** | **T** | **V** | **S** | **Q** | **T** | **G** | **R** | **S** | **T** | **L** | **G** | **V** | **R** | **L** | **M** | **K** | **M** | **E** | **A** | **D** | **T** | **K** | **V** | **V** | **T** | **M** | **-** | **-** | **-** | **-** | **-** | **-** | **-** | **-** | **-** | **-** | **-** | **-** | **-** | **-** | **-** | **-** | **-** | **-** | **-** |
| **N54156** | **M** | **S** | **E** | **E** | **I** | **K** | **E** | **N** | **I** | **Q** | **D** | **V** | **N** | **L** | **T** | **S** | **E** | **M** | **K** | **E** | **S** | **F** | **I** | **D** | **Y** | **A** | **M** | **S** | **V** | **I** | **V** | **A** | **R** | **A** | **L** | **P** | **D** | **V** | **R** | **D** | **G** | **L** | **K** | **P** | **V** | **H** | **R** | **R** | **I** | **L** | **Y** | **G** | **M** | **N** | **E** | **L** | **G** | **V** | **T** | **P** | **D** | **K** | **P** | **H** | **K** | **K** | **S** | **A** | **R** | **I** | **V** | **G** | **D** | **V** | **M** | **G** | **K** | **Y** | **H** | **P** | **H** | **G** | **D** | **S** | **A** | **I** | **Y** | **E** | **S** | **M** | **V** | **R** | **M** | **A** | **Q** | **P** | **F** | **S** | **Y** | **R** | **A** | **M** | **L** | **V** | **D** | **G** | **H** | **G** | **N** | **F** | **G** | **S** | **V** | **D** | **G** | **D** | **G** | **A** | **A** | **A** | **M** | **R** | **Y** | **T** | **E** | **A** | **R** | **M** | **S** | **K** | **I** | **A** | **L** | **E** | **M** | **L** | **R** | **D** | **I** | **N** | **K** | **N** | **T** | **V** | **D** | **F** | **Q** | **G** | **N** | **Y** | **D** | **D** | **S** | **E** | **Q** | **E** | **P** | **V** | **V** | **L** | **P** | **A** | **R** | **F** | **P** | **N** | **L** | **L** | **V** | **N** | **G** | **T** | **T** | **G** | **I** | **A** | **V** | **G** | **M** | **A** | **T** | **N** | **I** | **P** | **P** | **H** | **N** | **L** | **S** | **E** | **V** | **I** | **D** | **A** | **T** | **S** | **L** | **L** | **M** | **D** | **N** | **P** | **D** | **V** | **T** | **T** | **N** | **E** | **L** | **M** | **E** | **V** | **L** | **P** | **G** | **P** | **D** | **F** | **P** | **T** | **G** | **G** | **L** | **V** | **M** | **G** | **K** | **S** | **G** | **I** | **R** | **R** | **A** | **Y** | **E** | **T** | **G** | **K** | **G** | **S** | **I** | **T** | **V** | **R** | **A** | **K** | **V** | **E** | **L** | **T** | **E** | **M** | **P** | **N** | **G** | **K** | **E** | **R** | **I** | **L** | **V** | **T** | **E** | **L** | **P** | **Y** | **M** | **V** | **N** | **K** | **A** | **K** | **L** | **I** | **E** | **R** | **I** | **S** | **E** | **L** | **H** | **R** | **D** | **K** | **R** | **I** | **E** | **G** | **I** | **T** | **D** | **L** | **R** | **D** | **E** | **S** | **S** | **R** | **E** | **G** | **M** | **R** | **I** | **V** | **I** | **D** | **V** | **R** | **R** | **D** | **V** | **S** | **A** | **S** | **V** | **V** | **L** | **N** | **N** | **L** | **Y** | **K** | **M** | **T** | **A** | **L** | **Q** | **T** | **S** | **F** | **G** | **F** | **N** | **M** | **L** | **A** | **I** | **E** | **K** | **G** | **V** | **P** | **K** | **I** | **L** | **S** | **L** | **K** | **R** | **I** | **L** | **E** | **N** | **Y** | **V** | **E** | **H** | **Q** | **K** | **E** | **V** | **I** | **T** | **R** | **R** | **T** | **I** | **F** | **D** | **K** | **N** | **K** | **A** | **E** | **A** | **R** | **A** | **H** | **I** | **L** | **E** | **G** | **L** | **R** | **I** | **A** | **L** | **D** | **H** | **I** | **D** | **E** | **I** | **I** | **A** | **I** | **I** | **R** | **G** | **S** | **Q** | **S** | **D** | **D** | **E** | **A** | **K** | **A** | **T** | **L** | **I** | **E** | **R** | **F** | **E** | **F** | **S** | **D** | **R** | **Q** | **A** | **Q** | **A** | **I** | **L** | **D** | **M** | **R** | **L** | **R** | **R** | **L** | **T** | **G** | **L** | **E** | **R** | **D** | **K** | **I** | **E** | **N** | **E** | **Y** | **Q** | **E** | **L** | **L** | **K** | **F** | **I** | **A** | **D** | **L** | **E** | **D** | **I** | **L** | **A** | **R** | **P** | **E** | **R** | **V** | **I** | **E** | **I** | **I** | **K** | **T** | **E** | **L** | **N** | **D** | **V** | **R** | **T** | **K** | **F** | **G** | **D** | **A** | **R** | **R** | **T** | **E** | **L** | **L** | **V** | **G** | **E** | **V** | **L** | **S** | **L** | **E** | **D** | **E** | **D** | **L** | **I** | **E** | **E** | **E** | **E** | **V** | **V** | **I** | **T** | **L** | **T** | **N** | **N** | **G** | **Y** | **I** | **K** | **R** | **M** | **A** | **N** | **S** | **E** | **F** | **R** | **A** | **Q** | **R** | **R** | **G** | **G** | **R** | **G** | **V** | **Q** | **G** | **M** | **G** | **V** | **H** | **D** | **D** | **D** | **F** | **V** | **K** | **N** | **L** | **V** | **S** | **C** | **S** | **T** | **H** | **D** | **T** | **L** | **L** | **F** | **F** | **T** | **N** | **T** | **G** | **K** | **V** | **Y** | **R** | **A** | **K** | **G** | **Y** | **E** | **I** | **P** | **E** | **Y** | **G** | **R** | **T** | **A** | **K** | **G** | **I** | **P** | **V** | **I** | **N** | **L** | **L** | **G** | **I** | **D** | **S** | **A** | **E** | **K** | **I** | **Q** | **A** | **I** | **I** | **S** | **V** | **E** | **G** | **K** | **A** | **E** | **A** | **G** | **K** | **Y** | **L** | **F** | **F** | **T** | **T** | **L** | **K** | **G** | **T** | **V** | **K** | **R** | **T** | **A** | **V** | **T** | **A** | **F** | **S** | **N** | **I** | **R** | **S** | **N** | **G** | **L** | **I** | **A** | **I** | **S** | **L** | **K** | **E** | **D** | **D** | **E** | **L** | **V** | **N** | **V** | **V** | **T** | **T** | **N** | **G** | **N** | **Q** | **K** | **M** | **I** | **I** | **G** | **T** | **H** | **A** | **G** | **Y** | **S** | **V** | **T** | **F** | **D** | **E** | **N** | **T** | **V** | **R** | **D** | **M** | **G** | **R** | **T** | **A** | **S** | **G** | **V** | **R** | **G** | **I** | **R** | **L** | **R** | **E** | **N** | **D** | **Y** | **V** | **V** | **G** | **A** | **A** | **I** | **L** | **D** | **E** | **N** | **K** | **E** | **V** | **L** | **V** | **I** | **T** | **E** | **N** | **G** | **Y** | **G** | **K** | **R** | **T** | **K** | **A** | **S** | **E** | **Y** | **P** | **V** | **K** | **G** | **R** | **G** | **G** | **K** | **G** | **I** | **K** | **T** | **A** | **N** | **I** | **T** | **E** | **K** | **N** | **G** | **P** | **L** | **A** | **G** | **L** | **T** | **T** | **V** | **N** | **G** | **D** | **E** | **D** | **I** | **L** | **L** | **I** | **T** | **N** | **K** | **G** | **V** | **I** | **I** | **R** | **F** | **N** | **V** | **D** | **S** | **V** | **S** | **Q** | **T** | **G** | **R** | **A** | **T** | **L** | **G** | **V** | **R** | **L** | **M** | **R** | **M** | **E** | **D** | **G** | **A** | **K** | **-** | **-** | **-** | **-** | **-** | **-** | **-** | **-** | **-** | **-** | **-** | **-** | **-** | **-** | **-** | **-** | **-** | **-** | **-** | **-** | **-** | **-** | **-** |
| **N59847F** | **M** | **S** | **E** | **E** | **I** | **K** | **E** | **N** | **I** | **Q** | **D** | **V** | **N** | **L** | **T** | **S** | **E** | **M** | **K** | **E** | **S** | **F** | **I** | **D** | **Y** | **A** | **M** | **S** | **V** | **I** | **V** | **A** | **R** | **A** | **L** | **P** | **D** | **V** | **R** | **D** | **G** | **L** | **K** | **P** | **V** | **H** | **R** | **R** | **I** | **L** | **Y** | **G** | **M** | **N** | **E** | **L** | **G** | **V** | **T** | **P** | **D** | **K** | **P** | **H** | **K** | **K** | **S** | **A** | **R** | **I** | **V** | **G** | **D** | **V** | **M** | **G** | **K** | **Y** | **H** | **P** | **H** | **G** | **D** | **S** | **A** | **I** | **Y** | **E** | **S** | **M** | **V** | **R** | **M** | **A** | **Q** | **P** | **F** | **S** | **Y** | **R** | **A** | **M** | **L** | **V** | **D** | **G** | **H** | **G** | **N** | **F** | **G** | **S** | **V** | **D** | **G** | **D** | **G** | **A** | **A** | **A** | **M** | **R** | **Y** | **T** | **E** | **A** | **R** | **M** | **S** | **K** | **I** | **A** | **L** | **E** | **M** | **L** | **R** | **D** | **I** | **N** | **K** | **N** | **T** | **V** | **D** | **F** | **Q** | **G** | **N** | **Y** | **D** | **D** | **S** | **E** | **Q** | **E** | **P** | **V** | **V** | **L** | **P** | **A** | **R** | **F** | **P** | **N** | **L** | **L** | **V** | **N** | **G** | **T** | **T** | **G** | **I** | **A** | **V** | **G** | **M** | **A** | **T** | **N** | **I** | **P** | **P** | **H** | **N** | **L** | **S** | **E** | **V** | **I** | **D** | **A** | **T** | **S** | **L** | **L** | **M** | **D** | **N** | **P** | **D** | **V** | **T** | **T** | **N** | **E** | **L** | **M** | **E** | **V** | **L** | **P** | **G** | **P** | **D** | **F** | **P** | **T** | **G** | **G** | **L** | **V** | **M** | **G** | **K** | **S** | **G** | **I** | **R** | **R** | **A** | **Y** | **E** | **T** | **G** | **K** | **G** | **S** | **I** | **T** | **V** | **R** | **A** | **K** | **V** | **E** | **L** | **T** | **E** | **M** | **P** | **N** | **G** | **K** | **E** | **R** | **I** | **L** | **V** | **T** | **E** | **L** | **P** | **Y** | **M** | **V** | **N** | **K** | **A** | **K** | **L** | **I** | **E** | **R** | **I** | **S** | **E** | **L** | **H** | **R** | **D** | **K** | **R** | **I** | **E** | **G** | **I** | **T** | **D** | **L** | **R** | **D** | **E** | **S** | **S** | **R** | **E** | **G** | **M** | **R** | **I** | **V** | **I** | **D** | **V** | **R** | **R** | **D** | **V** | **S** | **A** | **S** | **V** | **V** | **L** | **N** | **N** | **L** | **Y** | **K** | **M** | **T** | **A** | **L** | **Q** | **T** | **S** | **F** | **G** | **F** | **N** | **M** | **L** | **A** | **I** | **E** | **K** | **G** | **V** | **P** | **K** | **I** | **L** | **S** | **L** | **K** | **R** | **I** | **L** | **E** | **N** | **Y** | **V** | **E** | **H** | **Q** | **K** | **E** | **V** | **I** | **T** | **R** | **R** | **T** | **I** | **F** | **D** | **K** | **N** | **K** | **A** | **E** | **A** | **R** | **A** | **H** | **I** | **L** | **E** | **G** | **L** | **R** | **I** | **A** | **L** | **D** | **H** | **I** | **D** | **E** | **I** | **I** | **A** | **I** | **I** | **R** | **G** | **S** | **Q** | **S** | **D** | **D** | **E** | **A** | **K** | **A** | **T** | **L** | **I** | **E** | **R** | **F** | **E** | **F** | **S** | **D** | **R** | **Q** | **A** | **Q** | **A** | **I** | **L** | **D** | **M** | **R** | **L** | **R** | **R** | **L** | **T** | **G** | **L** | **E** | **R** | **D** | **K** | **I** | **E** | **N** | **E** | **Y** | **Q** | **E** | **L** | **L** | **K** | **F** | **I** | **A** | **D** | **L** | **E** | **D** | **I** | **L** | **A** | **R** | **P** | **E** | **R** | **V** | **I** | **E** | **I** | **I** | **K** | **T** | **E** | **L** | **N** | **D** | **V** | **R** | **T** | **K** | **F** | **G** | **D** | **A** | **R** | **R** | **T** | **E** | **L** | **L** | **V** | **G** | **E** | **V** | **L** | **S** | **L** | **E** | **D** | **E** | **D** | **L** | **I** | **E** | **E** | **E** | **E** | **V** | **V** | **I** | **T** | **L** | **T** | **N** | **N** | **G** | **Y** | **I** | **K** | **R** | **M** | **A** | **N** | **S** | **E** | **F** | **R** | **A** | **Q** | **R** | **R** | **G** | **G** | **R** | **G** | **V** | **Q** | **G** | **M** | **G** | **V** | **H** | **D** | **D** | **D** | **F** | **V** | **K** | **N** | **L** | **V** | **S** | **C** | **S** | **T** | **H** | **D** | **T** | **L** | **L** | **F** | **F** | **T** | **N** | **T** | **G** | **K** | **V** | **Y** | **R** | **A** | **K** | **G** | **Y** | **E** | **I** | **P** | **E** | **Y** | **G** | **R** | **T** | **A** | **K** | **G** | **I** | **P** | **V** | **I** | **N** | **L** | **L** | **G** | **I** | **D** | **S** | **T** | **E** | **K** | **I** | **Q** | **A** | **I** | **I** | **S** | **V** | **E** | **G** | **K** | **A** | **E** | **A** | **G** | **K** | **Y** | **L** | **F** | **F** | **T** | **T** | **L** | **K** | **G** | **T** | **V** | **K** | **R** | **T** | **A** | **V** | **T** | **A** | **F** | **S** | **N** | **I** | **R** | **S** | **N** | **G** | **L** | **I** | **A** | **I** | **S** | **L** | **K** | **E** | **D** | **D** | **E** | **L** | **V** | **N** | **V** | **V** | **T** | **T** | **N** | **G** | **N** | **Q** | **K** | **M** | **I** | **I** | **G** | **T** | **H** | **A** | **G** | **Y** | **S** | **V** | **T** | **F** | **D** | **E** | **N** | **T** | **V** | **R** | **D** | **M** | **G** | **R** | **T** | **A** | **S** | **G** | **V** | **R** | **G** | **I** | **R** | **L** | **R** | **E** | **N** | **D** | **Y** | **V** | **V** | **G** | **A** | **A** | **I** | **L** | **D** | **E** | **N** | **K** | **E** | **V** | **L** | **V** | **I** | **T** | **E** | **N** | **G** | **Y** | **G** | **K** | **R** | **T** | **K** | **A** | **S** | **E** | **Y** | **P** | **V** | **K** | **G** | **R** | **G** | **G** | **K** | **G** | **I** | **K** | **T** | **A** | **N** | **I** | **T** | **E** | **K** | **N** | **G** | **P** | **L** | **A** | **G** | **L** | **T** | **T** | **V** | **N** | **G** | **D** | **E** | **D** | **I** | **L** | **L** | **I** | **T** | **N** | **K** | **G** | **V** | **I** | **I** | **R** | **F** | **N** | **V** | **D** | **S** | **V** | **S** | **Q** | **T** | **G** | **R** | **A** | **T** | **L** | **G** | **V** | **R** | **L** | **M** | **R** | **M** | **E** | **D** | **G** | **A** | **K** | **-** | **-** | **-** | **-** | **-** | **-** | **-** | **-** | **-** | **-** | **-** | **-** | **-** | **-** | **-** | **-** | **-** | **-** | **-** | **-** | **-** | **-** | **-** |
| **N54574** | **M** | **S** | **E** | **E** | **I** | **K** | **E** | **N** | **I** | **Q** | **D** | **V** | **N** | **L** | **T** | **S** | **E** | **M** | **K** | **E** | **S** | **F** | **I** | **D** | **Y** | **A** | **M** | **S** | **V** | **I** | **V** | **A** | **R** | **A** | **L** | **P** | **D** | **V** | **R** | **D** | **G** | **L** | **K** | **P** | **V** | **H** | **R** | **R** | **I** | **L** | **Y** | **G** | **M** | **N** | **E** | **L** | **G** | **V** | **T** | **P** | **D** | **K** | **P** | **H** | **K** | **K** | **S** | **A** | **R** | **I** | **V** | **G** | **D** | **V** | **M** | **G** | **K** | **Y** | **H** | **P** | **H** | **G** | **D** | **S** | **A** | **I** | **Y** | **E** | **S** | **M** | **V** | **R** | **M** | **A** | **Q** | **P** | **F** | **S** | **Y** | **R** | **A** | **M** | **L** | **V** | **D** | **G** | **H** | **G** | **N** | **F** | **G** | **S** | **V** | **D** | **G** | **D** | **G** | **A** | **A** | **A** | **M** | **R** | **Y** | **T** | **E** | **A** | **R** | **M** | **S** | **K** | **I** | **A** | **L** | **E** | **M** | **L** | **R** | **D** | **I** | **N** | **K** | **N** | **T** | **V** | **D** | **F** | **Q** | **G** | **N** | **Y** | **D** | **D** | **S** | **E** | **Q** | **E** | **P** | **V** | **V** | **L** | **P** | **A** | **R** | **F** | **P** | **N** | **L** | **L** | **V** | **N** | **G** | **T** | **T** | **G** | **I** | **A** | **V** | **G** | **M** | **A** | **T** | **N** | **I** | **P** | **P** | **H** | **N** | **L** | **S** | **E** | **V** | **I** | **D** | **A** | **T** | **S** | **L** | **L** | **M** | **D** | **N** | **P** | **D** | **V** | **T** | **T** | **N** | **E** | **L** | **M** | **E** | **V** | **L** | **P** | **G** | **P** | **D** | **F** | **P** | **T** | **G** | **G** | **L** | **V** | **M** | **G** | **K** | **S** | **G** | **I** | **R** | **R** | **A** | **Y** | **E** | **T** | **G** | **K** | **G** | **S** | **I** | **T** | **V** | **R** | **A** | **K** | **V** | **E** | **L** | **T** | **E** | **M** | **P** | **N** | **G** | **K** | **E** | **R** | **I** | **L** | **V** | **T** | **E** | **L** | **P** | **Y** | **M** | **V** | **N** | **K** | **A** | **K** | **L** | **I** | **E** | **R** | **I** | **S** | **E** | **L** | **H** | **R** | **D** | **K** | **R** | **I** | **E** | **G** | **I** | **T** | **D** | **L** | **R** | **D** | **E** | **S** | **S** | **R** | **E** | **G** | **M** | **R** | **I** | **V** | **I** | **D** | **V** | **R** | **R** | **D** | **V** | **S** | **A** | **S** | **V** | **V** | **L** | **N** | **N** | **L** | **Y** | **K** | **M** | **T** | **A** | **L** | **Q** | **T** | **S** | **F** | **G** | **F** | **N** | **M** | **L** | **A** | **I** | **E** | **K** | **G** | **V** | **P** | **K** | **I** | **L** | **S** | **L** | **K** | **R** | **I** | **L** | **E** | **N** | **Y** | **V** | **E** | **H** | **Q** | **K** | **E** | **V** | **I** | **T** | **R** | **R** | **T** | **I** | **F** | **D** | **K** | **N** | **K** | **A** | **E** | **A** | **R** | **A** | **H** | **I** | **L** | **E** | **G** | **L** | **R** | **I** | **A** | **L** | **D** | **H** | **I** | **D** | **E** | **I** | **I** | **A** | **I** | **I** | **R** | **G** | **S** | **Q** | **S** | **D** | **D** | **E** | **A** | **K** | **A** | **T** | **L** | **I** | **E** | **R** | **F** | **E** | **F** | **S** | **D** | **R** | **Q** | **A** | **Q** | **A** | **I** | **L** | **D** | **M** | **R** | **L** | **R** | **R** | **L** | **T** | **G** | **L** | **E** | **R** | **D** | **K** | **I** | **E** | **N** | **E** | **Y** | **Q** | **E** | **L** | **L** | **K** | **F** | **I** | **A** | **D** | **L** | **E** | **D** | **I** | **L** | **A** | **R** | **P** | **E** | **R** | **V** | **I** | **E** | **I** | **I** | **K** | **T** | **E** | **L** | **N** | **D** | **V** | **R** | **T** | **K** | **F** | **G** | **D** | **A** | **R** | **R** | **T** | **E** | **L** | **L** | **V** | **G** | **E** | **V** | **L** | **S** | **L** | **E** | **D** | **E** | **D** | **L** | **I** | **E** | **E** | **E** | **E** | **V** | **V** | **I** | **T** | **L** | **T** | **N** | **N** | **G** | **Y** | **I** | **K** | **R** | **M** | **A** | **N** | **S** | **E** | **F** | **R** | **A** | **Q** | **R** | **R** | **G** | **G** | **R** | **G** | **V** | **Q** | **G** | **M** | **G** | **V** | **H** | **D** | **D** | **D** | **F** | **V** | **K** | **N** | **L** | **I** | **S** | **C** | **S** | **T** | **H** | **D** | **T** | **L** | **L** | **F** | **F** | **T** | **N** | **T** | **G** | **K** | **V** | **Y** | **R** | **A** | **K** | **G** | **Y** | **E** | **I** | **P** | **E** | **Y** | **G** | **R** | **T** | **A** | **K** | **G** | **I** | **P** | **V** | **I** | **N** | **L** | **L** | **G** | **I** | **D** | **S** | **A** | **E** | **K** | **I** | **Q** | **A** | **I** | **I** | **S** | **V** | **E** | **G** | **K** | **A** | **E** | **A** | **G** | **K** | **Y** | **L** | **F** | **F** | **T** | **T** | **L** | **K** | **G** | **T** | **V** | **K** | **R** | **T** | **A** | **V** | **T** | **A** | **F** | **S** | **N** | **I** | **R** | **S** | **N** | **G** | **L** | **I** | **A** | **I** | **S** | **L** | **K** | **E** | **D** | **D** | **E** | **L** | **V** | **N** | **V** | **V** | **T** | **T** | **N** | **G** | **N** | **Q** | **K** | **M** | **I** | **I** | **G** | **T** | **H** | **A** | **G** | **Y** | **S** | **V** | **T** | **F** | **D** | **E** | **N** | **T** | **V** | **R** | **D** | **M** | **G** | **R** | **T** | **A** | **S** | **G** | **V** | **R** | **G** | **I** | **R** | **L** | **R** | **E** | **N** | **D** | **Y** | **V** | **V** | **G** | **A** | **A** | **I** | **L** | **D** | **E** | **N** | **K** | **E** | **V** | **L** | **V** | **I** | **T** | **E** | **N** | **G** | **Y** | **G** | **K** | **R** | **T** | **K** | **A** | **S** | **E** | **Y** | **P** | **V** | **K** | **G** | **R** | **G** | **G** | **K** | **G** | **I** | **K** | **T** | **A** | **N** | **I** | **T** | **E** | **K** | **N** | **G** | **P** | **L** | **A** | **G** | **L** | **T** | **T** | **V** | **N** | **G** | **D** | **E** | **D** | **I** | **L** | **L** | **I** | **T** | **N** | **K** | **G** | **V** | **I** | **I** | **R** | **F** | **N** | **V** | **D** | **S** | **V** | **S** | **Q** | **T** | **G** | **R** | **A** | **T** | **L** | **G** | **V** | **R** | **L** | **M** | **R** | **M** | **E** | **D** | **G** | **A** | **K** | **-** | **-** | **-** | **-** | **-** | **-** | **-** | **-** | **-** | **-** | **-** | **-** | **-** | **-** | **-** | **-** | **-** | **-** | **-** | **-** | **-** | **-** | **-** |
| **N52777** | **M** | **S** | **E** | **E** | **I** | **R** | **E** | **N** | **I** | **H** | **D** | **V** | **N** | **L** | **T** | **S** | **E** | **M** | **K** | **D** | **S** | **F** | **I** | **D** | **Y** | **A** | **M** | **S** | **V** | **I** | **V** | **A** | **R** | **A** | **L** | **P** | **D** | **V** | **R** | **D** | **G** | **L** | **K** | **P** | **V** | **H** | **R** | **R** | **I** | **L** | **Y** | **G** | **M** | **N** | **E** | **L** | **G** | **V** | **T** | **P** | **D** | **K** | **A** | **H** | **K** | **K** | **S** | **A** | **R** | **I** | **V** | **G** | **D** | **V** | **M** | **G** | **K** | **Y** | **H** | **P** | **H** | **G** | **D** | **S** | **A** | **I** | **Y** | **E** | **S** | **M** | **V** | **R** | **M** | **A** | **Q** | **P** | **F** | **S** | **Y** | **R** | **Y** | **M** | **L** | **V** | **D** | **G** | **H** | **G** | **N** | **F** | **G** | **S** | **V** | **D** | **G** | **D** | **G** | **A** | **A** | **A** | **M** | **R** | **Y** | **T** | **E** | **A** | **R** | **M** | **S** | **K** | **I** | **A** | **T** | **E** | **M** | **L** | **R** | **D** | **I** | **N** | **K** | **N** | **T** | **V** | **D** | **F** | **Q** | **S** | **N** | **Y** | **D** | **D** | **T** | **E** | **K** | **E** | **P** | **V** | **V** | **L** | **P** | **A** | **R** | **F** | **P** | **N** | **L** | **L** | **V** | **N** | **G** | **T** | **T** | **G** | **I** | **A** | **V** | **G** | **M** | **A** | **T** | **N** | **I** | **P** | **P** | **H** | **N** | **L** | **S** | **E** | **V** | **V** | **A** | **A** | **I** | **D** | **L** | **L** | **M** | **D** | **N** | **P** | **D** | **V** | **T** | **T** | **N** | **E** | **L** | **M** | **E** | **V** | **L** | **P** | **G** | **P** | **D** | **F** | **P** | **T** | **G** | **G** | **L** | **V** | **M** | **G** | **K** | **S** | **G** | **I** | **R** | **R** | **A** | **Y** | **E** | **T** | **G** | **K** | **G** | **S** | **I** | **T** | **V** | **R** | **A** | **K** | **V** | **E** | **I** | **T** | **E** | **M** | **P** | **N** | **G** | **K** | **E** | **R** | **I** | **I** | **V** | **T** | **E** | **L** | **P** | **Y** | **M** | **V** | **N** | **K** | **A** | **K** | **L** | **I** | **E** | **R** | **I** | **S** | **E** | **L** | **H** | **R** | **D** | **K** | **R** | **I** | **E** | **G** | **I** | **T** | **D** | **L** | **R** | **D** | **E** | **S** | **S** | **R** | **E** | **G** | **M** | **R** | **I** | **V** | **I** | **D** | **I** | **R** | **R** | **D** | **A** | **S** | **A** | **S** | **V** | **I** | **L** | **N** | **N** | **L** | **Y** | **K** | **L** | **T** | **S** | **L** | **Q** | **N** | **S** | **F** | **G** | **F** | **N** | **M** | **L** | **A** | **I** | **E** | **K** | **G** | **V** | **P** | **K** | **V** | **L** | **S** | **L** | **K** | **Q** | **I** | **L** | **E** | **N** | **Y** | **I** | **E** | **H** | **Q** | **R** | **E** | **V** | **I** | **T** | **R** | **R** | **T** | **V** | **F** | **E** | **K** | **E** | **K** | **A** | **E** | **A** | **R** | **A** | **H** | **I** | **L** | **E** | **G** | **L** | **R** | **I** | **A** | **L** | **D** | **H** | **I** | **D** | **E** | **I** | **I** | **A** | **I** | **I** | **R** | **G** | **S** | **K** | **S** | **D** | **D** | **E** | **A** | **K** | **A** | **T** | **M** | **I** | **E** | **R** | **F** | **D** | **L** | **S** | **D** | **R** | **Q** | **S** | **Q** | **A** | **I** | **L** | **D** | **M** | **R** | **L** | **R** | **R** | **L** | **T** | **G** | **L** | **E** | **R** | **E** | **K** | **I** | **E** | **N** | **E** | **Y** | **Q** | **E** | **L** | **L** | **K** | **L** | **I** | **E** | **D** | **L** | **T** | **D** | **I** | **L** | **A** | **R** | **P** | **E** | **R** | **V** | **T** | **E** | **I** | **I** | **K** | **N** | **E** | **L** | **A** | **D** | **L** | **N** | **Q** | **R** | **F** | **G** | **D** | **K** | **R** | **R** | **T** | **E** | **L** | **L** | **V** | **G** | **E** | **V** | **L** | **S** | **L** | **E** | **D** | **E** | **D** | **L** | **I** | **E** | **E** | **E** | **E** | **I** | **V** | **I** | **T** | **L** | **T** | **N** | **N** | **G** | **Y** | **I** | **K** | **R** | **L** | **A** | **N** | **N** | **E** | **F** | **R** | **A** | **Q** | **R** | **R** | **G** | **G** | **R** | **G** | **V** | **Q** | **G** | **M** | **G** | **I** | **H** | **N** | **D** | **D** | **F** | **V** | **K** | **N** | **L** | **V** | **S** | **C** | **S** | **T** | **H** | **D** | **T** | **L** | **L** | **F** | **F** | **T** | **N** | **N** | **G** | **K** | **V** | **Y** | **R** | **A** | **K** | **G** | **Y** | **E** | **I** | **P** | **E** | **Y** | **G** | **R** | **T** | **A** | **K** | **G** | **I** | **P** | **I** | **I** | **N** | **L** | **L** | **G** | **I** | **D** | **S** | **S** | **E** | **S** | **I** | **Q** | **A** | **I** | **I** | **A** | **V** | **T** | **G** | **E** | **A** | **E** | **E** | **G** | **H** | **Y** | **L** | **F** | **F** | **T** | **T** | **R** | **Q** | **G** | **T** | **V** | **K** | **R** | **T** | **S** | **V** | **T** | **A** | **F** | **A** | **N** | **I** | **R** | **S** | **N** | **G** | **L** | **I** | **A** | **I** | **G** | **L** | **K** | **E** | **D** | **D** | **E** | **L** | **V** | **N** | **V** | **L** | **L** | **T** | **D** | **G** | **Q** | **S** | **N** | **I** | **I** | **I** | **G** | **T** | **H** | **N** | **G** | **Y** | **S** | **V** | **T** | **F** | **K** | **E** | **E** | **A** | **V** | **R** | **D** | **M** | **G** | **R** | **T** | **A** | **S** | **G** | **V** | **R** | **G** | **I** | **R** | **L** | **R** | **E** | **E** | **D** | **Y** | **V** | **V** | **G** | **A** | **A** | **L** | **M** | **K** | **E** | **N** | **Q** | **E** | **V** | **L** | **V** | **I** | **T** | **E** | **K** | **G** | **Y** | **G** | **K** | **R** | **T** | **K** | **V** | **S** | **E** | **Y** | **P** | **V** | **K** | **G** | **R** | **G** | **G** | **K** | **G** | **I** | **K** | **T** | **A** | **N** | **I** | **T** | **E** | **K** | **N** | **G** | **P** | **L** | **A** | **G** | **L** | **T** | **T** | **V** | **S** | **G** | **D** | **E** | **D** | **I** | **M** | **L** | **I** | **T** | **D** | **K** | **G** | **V** | **I** | **I** | **R** | **F** | **N** | **V** | **S** | **T** | **V** | **S** | **Q** | **T** | **G** | **R** | **S** | **T** | **L** | **G** | **V** | **R** | **L** | **M** | **K** | **M** | **E** | **A** | **D** | **T** | **K** | **V** | **V** | **T** | **M** | **A** | **A** | **V** | **E** | **P** | **E** | **A** | **A** | **E** | **Q** | **T** | **V** | **E** | **E** | **Q** | **G** | **S** | **E** | **E** |
| **N54551** | **M** | **S** | **E** | **E** | **I** | **R** | **E** | **N** | **I** | **H** | **D** | **V** | **N** | **L** | **T** | **S** | **E** | **M** | **K** | **D** | **S** | **F** | **I** | **D** | **Y** | **A** | **M** | **S** | **V** | **I** | **V** | **A** | **R** | **A** | **L** | **P** | **D** | **V** | **R** | **D** | **G** | **L** | **K** | **P** | **V** | **H** | **R** | **R** | **I** | **L** | **Y** | **G** | **M** | **N** | **E** | **L** | **G** | **V** | **T** | **P** | **D** | **K** | **A** | **H** | **K** | **K** | **S** | **A** | **R** | **I** | **V** | **G** | **D** | **V** | **M** | **G** | **K** | **Y** | **H** | **P** | **H** | **G** | **D** | **S** | **A** | **I** | **Y** | **E** | **S** | **M** | **V** | **R** | **M** | **A** | **Q** | **P** | **F** | **S** | **Y** | **R** | **Y** | **M** | **L** | **V** | **D** | **G** | **H** | **G** | **N** | **F** | **G** | **S** | **V** | **D** | **G** | **D** | **G** | **A** | **A** | **A** | **M** | **R** | **Y** | **T** | **E** | **A** | **R** | **M** | **S** | **K** | **I** | **A** | **T** | **E** | **M** | **L** | **R** | **D** | **I** | **N** | **K** | **N** | **T** | **V** | **D** | **F** | **Q** | **S** | **N** | **Y** | **D** | **D** | **T** | **E** | **K** | **E** | **P** | **V** | **V** | **L** | **P** | **A** | **R** | **F** | **P** | **N** | **L** | **L** | **V** | **N** | **G** | **T** | **T** | **G** | **I** | **A** | **V** | **G** | **M** | **A** | **T** | **N** | **I** | **P** | **P** | **H** | **N** | **L** | **S** | **E** | **V** | **V** | **A** | **A** | **I** | **D** | **L** | **L** | **M** | **D** | **N** | **P** | **D** | **V** | **T** | **T** | **N** | **E** | **L** | **M** | **E** | **V** | **L** | **P** | **G** | **P** | **D** | **F** | **P** | **T** | **G** | **G** | **L** | **V** | **M** | **G** | **K** | **S** | **G** | **I** | **R** | **R** | **A** | **Y** | **E** | **T** | **G** | **K** | **G** | **S** | **I** | **T** | **V** | **R** | **A** | **K** | **V** | **E** | **I** | **T** | **E** | **M** | **P** | **N** | **G** | **K** | **E** | **R** | **I** | **I** | **V** | **T** | **E** | **L** | **P** | **Y** | **M** | **V** | **N** | **K** | **A** | **K** | **L** | **I** | **E** | **R** | **I** | **S** | **E** | **L** | **H** | **R** | **D** | **K** | **R** | **I** | **E** | **G** | **I** | **T** | **D** | **L** | **R** | **D** | **E** | **S** | **S** | **R** | **E** | **G** | **M** | **R** | **I** | **V** | **I** | **D** | **I** | **R** | **R** | **D** | **A** | **S** | **A** | **S** | **V** | **I** | **L** | **N** | **N** | **L** | **Y** | **K** | **L** | **T** | **S** | **L** | **Q** | **N** | **S** | **F** | **G** | **F** | **N** | **M** | **L** | **A** | **I** | **E** | **K** | **G** | **V** | **P** | **K** | **V** | **L** | **S** | **L** | **K** | **Q** | **I** | **L** | **E** | **N** | **Y** | **I** | **E** | **H** | **Q** | **R** | **E** | **V** | **I** | **T** | **R** | **R** | **T** | **V** | **F** | **E** | **K** | **E** | **K** | **A** | **E** | **A** | **R** | **A** | **H** | **I** | **L** | **E** | **G** | **L** | **R** | **I** | **A** | **L** | **D** | **H** | **I** | **D** | **E** | **I** | **I** | **A** | **I** | **I** | **R** | **G** | **S** | **K** | **S** | **D** | **D** | **E** | **A** | **K** | **A** | **T** | **M** | **I** | **E** | **R** | **F** | **D** | **L** | **S** | **D** | **R** | **Q** | **S** | **Q** | **A** | **I** | **L** | **D** | **M** | **R** | **L** | **R** | **R** | **L** | **T** | **G** | **L** | **E** | **R** | **E** | **K** | **I** | **E** | **N** | **E** | **Y** | **Q** | **E** | **L** | **L** | **K** | **L** | **I** | **E** | **D** | **L** | **T** | **D** | **I** | **L** | **A** | **R** | **P** | **E** | **R** | **V** | **T** | **E** | **I** | **I** | **K** | **N** | **E** | **L** | **A** | **D** | **L** | **N** | **Q** | **R** | **F** | **G** | **D** | **K** | **R** | **R** | **T** | **E** | **L** | **L** | **V** | **G** | **E** | **V** | **L** | **S** | **L** | **E** | **D** | **E** | **D** | **L** | **I** | **E** | **E** | **E** | **E** | **I** | **V** | **I** | **T** | **L** | **T** | **N** | **N** | **G** | **Y** | **I** | **K** | **R** | **L** | **A** | **N** | **N** | **E** | **F** | **R** | **A** | **Q** | **R** | **R** | **G** | **G** | **R** | **G** | **V** | **Q** | **G** | **M** | **G** | **I** | **H** | **N** | **D** | **D** | **F** | **V** | **K** | **N** | **L** | **V** | **S** | **C** | **S** | **T** | **H** | **D** | **T** | **L** | **L** | **F** | **F** | **T** | **N** | **N** | **G** | **K** | **V** | **Y** | **R** | **A** | **K** | **G** | **Y** | **E** | **I** | **P** | **E** | **Y** | **G** | **R** | **T** | **A** | **K** | **G** | **I** | **P** | **I** | **I** | **N** | **L** | **L** | **G** | **I** | **D** | **S** | **S** | **E** | **S** | **I** | **Q** | **A** | **I** | **I** | **A** | **V** | **T** | **G** | **E** | **A** | **E** | **E** | **G** | **H** | **Y** | **L** | **F** | **F** | **T** | **T** | **R** | **Q** | **G** | **T** | **V** | **K** | **R** | **T** | **S** | **V** | **T** | **A** | **F** | **A** | **N** | **I** | **R** | **S** | **N** | **G** | **L** | **I** | **A** | **I** | **G** | **L** | **K** | **E** | **D** | **D** | **E** | **L** | **V** | **N** | **V** | **L** | **L** | **T** | **D** | **G** | **Q** | **S** | **N** | **I** | **I** | **I** | **G** | **T** | **H** | **N** | **G** | **Y** | **S** | **V** | **T** | **F** | **K** | **E** | **E** | **A** | **V** | **R** | **D** | **M** | **G** | **R** | **T** | **A** | **S** | **G** | **V** | **R** | **G** | **I** | **R** | **L** | **R** | **E** | **E** | **D** | **Y** | **V** | **V** | **G** | **A** | **A** | **L** | **M** | **K** | **E** | **N** | **Q** | **E** | **V** | **L** | **V** | **I** | **T** | **E** | **K** | **G** | **Y** | **G** | **K** | **R** | **T** | **K** | **V** | **S** | **E** | **Y** | **P** | **V** | **K** | **G** | **R** | **G** | **G** | **K** | **G** | **I** | **K** | **T** | **A** | **N** | **I** | **T** | **E** | **K** | **N** | **G** | **P** | **L** | **A** | **G** | **L** | **T** | **T** | **V** | **S** | **G** | **D** | **E** | **D** | **I** | **M** | **L** | **I** | **T** | **D** | **K** | **G** | **V** | **I** | **I** | **R** | **F** | **N** | **V** | **S** | **T** | **V** | **S** | **Q** | **T** | **G** | **R** | **S** | **T** | **L** | **G** | **V** | **R** | **L** | **M** | **K** | **M** | **E** | **A** | **D** | **T** | **K** | **V** | **V** | **T** | **M** | **A** | **A** | **V** | **E** | **P** | **E** | **A** | **A** | **E** | **Q** | **T** | **V** | **E** | **E** | **Q** | **G** | **S** | **E** | **E** |
| **N60299F** | **M** | **S** | **E** | **E** | **I** | **R** | **E** | **N** | **I** | **H** | **D** | **V** | **N** | **L** | **T** | **S** | **E** | **M** | **K** | **D** | **S** | **F** | **I** | **D** | **Y** | **A** | **M** | **S** | **V** | **I** | **V** | **A** | **R** | **A** | **L** | **P** | **D** | **V** | **R** | **D** | **G** | **L** | **K** | **P** | **V** | **H** | **R** | **R** | **I** | **L** | **Y** | **G** | **M** | **N** | **E** | **L** | **G** | **V** | **T** | **P** | **D** | **K** | **A** | **H** | **K** | **K** | **S** | **A** | **R** | **I** | **V** | **G** | **D** | **V** | **M** | **G** | **K** | **Y** | **H** | **P** | **H** | **G** | **D** | **S** | **A** | **I** | **Y** | **E** | **S** | **M** | **V** | **R** | **M** | **A** | **Q** | **P** | **F** | **S** | **Y** | **R** | **Y** | **M** | **L** | **V** | **D** | **G** | **H** | **G** | **N** | **F** | **G** | **S** | **V** | **D** | **G** | **D** | **G** | **A** | **A** | **A** | **M** | **R** | **Y** | **T** | **E** | **A** | **R** | **M** | **S** | **K** | **I** | **A** | **T** | **E** | **M** | **L** | **R** | **D** | **I** | **N** | **K** | **N** | **T** | **V** | **D** | **F** | **Q** | **S** | **N** | **Y** | **D** | **D** | **T** | **E** | **K** | **E** | **P** | **V** | **V** | **L** | **P** | **A** | **R** | **F** | **P** | **N** | **L** | **L** | **V** | **N** | **G** | **T** | **T** | **G** | **I** | **A** | **V** | **G** | **M** | **A** | **T** | **N** | **I** | **P** | **P** | **H** | **N** | **L** | **S** | **E** | **V** | **V** | **A** | **A** | **I** | **D** | **L** | **L** | **M** | **D** | **N** | **P** | **D** | **V** | **T** | **T** | **N** | **E** | **L** | **M** | **E** | **V** | **L** | **P** | **G** | **P** | **D** | **F** | **P** | **T** | **G** | **G** | **L** | **V** | **M** | **G** | **K** | **S** | **G** | **I** | **R** | **R** | **A** | **Y** | **E** | **T** | **G** | **K** | **G** | **S** | **I** | **T** | **V** | **R** | **A** | **K** | **V** | **E** | **I** | **T** | **E** | **M** | **P** | **N** | **G** | **K** | **E** | **R** | **I** | **I** | **V** | **T** | **E** | **L** | **P** | **Y** | **M** | **V** | **N** | **K** | **A** | **K** | **L** | **I** | **E** | **R** | **I** | **S** | **E** | **L** | **H** | **R** | **D** | **K** | **R** | **I** | **E** | **G** | **I** | **T** | **D** | **L** | **R** | **D** | **E** | **S** | **S** | **R** | **E** | **G** | **M** | **R** | **I** | **V** | **I** | **D** | **I** | **R** | **R** | **D** | **A** | **S** | **A** | **S** | **V** | **I** | **L** | **N** | **N** | **L** | **Y** | **K** | **L** | **T** | **S** | **L** | **Q** | **N** | **S** | **F** | **G** | **F** | **N** | **M** | **L** | **A** | **I** | **E** | **K** | **G** | **V** | **P** | **K** | **V** | **L** | **S** | **L** | **K** | **Q** | **I** | **L** | **E** | **N** | **Y** | **I** | **E** | **H** | **Q** | **R** | **E** | **V** | **I** | **T** | **R** | **R** | **T** | **V** | **F** | **E** | **K** | **E** | **K** | **A** | **E** | **A** | **R** | **A** | **H** | **I** | **L** | **E** | **G** | **L** | **R** | **I** | **A** | **L** | **D** | **H** | **I** | **D** | **E** | **I** | **I** | **A** | **I** | **I** | **R** | **G** | **S** | **K** | **S** | **D** | **D** | **E** | **A** | **K** | **A** | **T** | **M** | **I** | **E** | **R** | **F** | **D** | **L** | **S** | **D** | **R** | **Q** | **S** | **Q** | **A** | **I** | **L** | **D** | **M** | **R** | **L** | **R** | **R** | **L** | **T** | **G** | **L** | **E** | **R** | **E** | **K** | **I** | **E** | **N** | **E** | **Y** | **Q** | **E** | **L** | **L** | **K** | **L** | **I** | **E** | **D** | **L** | **T** | **D** | **I** | **L** | **A** | **R** | **P** | **E** | **R** | **V** | **T** | **E** | **I** | **I** | **K** | **N** | **E** | **L** | **A** | **D** | **L** | **N** | **Q** | **R** | **F** | **G** | **D** | **K** | **R** | **R** | **T** | **E** | **L** | **L** | **V** | **G** | **E** | **V** | **L** | **S** | **L** | **E** | **D** | **E** | **D** | **L** | **I** | **E** | **E** | **E** | **E** | **I** | **V** | **I** | **T** | **L** | **T** | **N** | **N** | **G** | **Y** | **I** | **K** | **R** | **L** | **A** | **N** | **N** | **E** | **F** | **R** | **A** | **Q** | **R** | **R** | **G** | **G** | **R** | **G** | **V** | **Q** | **G** | **M** | **G** | **I** | **H** | **N** | **D** | **D** | **F** | **V** | **K** | **N** | **L** | **V** | **S** | **C** | **S** | **T** | **H** | **D** | **T** | **L** | **L** | **F** | **F** | **T** | **N** | **N** | **G** | **K** | **V** | **Y** | **R** | **A** | **K** | **G** | **Y** | **E** | **I** | **P** | **E** | **Y** | **G** | **R** | **T** | **A** | **K** | **G** | **I** | **P** | **I** | **I** | **N** | **L** | **L** | **G** | **I** | **D** | **S** | **S** | **E** | **S** | **I** | **Q** | **A** | **I** | **I** | **A** | **V** | **T** | **G** | **E** | **A** | **E** | **E** | **G** | **H** | **Y** | **L** | **F** | **F** | **T** | **T** | **R** | **Q** | **G** | **T** | **V** | **K** | **R** | **T** | **S** | **V** | **T** | **A** | **F** | **A** | **N** | **I** | **R** | **S** | **N** | **G** | **L** | **I** | **A** | **I** | **G** | **L** | **K** | **E** | **D** | **D** | **E** | **L** | **V** | **N** | **V** | **L** | **L** | **T** | **D** | **G** | **Q** | **S** | **N** | **I** | **I** | **I** | **G** | **T** | **H** | **N** | **G** | **Y** | **S** | **V** | **T** | **F** | **K** | **E** | **E** | **A** | **V** | **R** | **D** | **M** | **G** | **R** | **T** | **A** | **S** | **G** | **V** | **R** | **G** | **I** | **R** | **L** | **R** | **E** | **E** | **D** | **Y** | **V** | **V** | **G** | **A** | **A** | **L** | **M** | **K** | **E** | **N** | **Q** | **E** | **V** | **L** | **V** | **I** | **T** | **E** | **K** | **G** | **Y** | **G** | **K** | **R** | **T** | **K** | **V** | **S** | **E** | **Y** | **P** | **V** | **K** | **G** | **R** | **G** | **G** | **K** | **G** | **I** | **K** | **T** | **A** | **N** | **I** | **T** | **E** | **K** | **N** | **G** | **P** | **L** | **A** | **G** | **L** | **T** | **T** | **V** | **S** | **G** | **D** | **E** | **D** | **I** | **M** | **L** | **I** | **T** | **D** | **K** | **G** | **V** | **I** | **I** | **R** | **F** | **N** | **V** | **S** | **T** | **V** | **S** | **Q** | **T** | **G** | **R** | **S** | **T** | **L** | **G** | **V** | **R** | **L** | **M** | **K** | **M** | **E** | **A** | **D** | **T** | **K** | **V** | **V** | **T** | **M** | **A** | **A** | **V** | **E** | **P** | **E** | **A** | **A** | **E** | **Q** | **T** | **V** | **E** | **E** | **Q** | **G** | **S** | **E** | **E** |
| **N59600F** | **M** | **S** | **E** | **E** | **I** | **K** | **E** | **N** | **I** | **Q** | **D** | **V** | **N** | **L** | **T** | **S** | **E** | **M** | **K** | **E** | **S** | **F** | **I** | **D** | **Y** | **A** | **M** | **S** | **V** | **I** | **V** | **A** | **R** | **A** | **L** | **P** | **D** | **V** | **R** | **D** | **G** | **L** | **K** | **P** | **V** | **H** | **R** | **R** | **I** | **L** | **Y** | **G** | **M** | **N** | **E** | **L** | **G** | **V** | **T** | **P** | **D** | **K** | **P** | **H** | **K** | **K** | **S** | **A** | **R** | **I** | **V** | **G** | **D** | **V** | **M** | **G** | **K** | **Y** | **H** | **P** | **H** | **G** | **D** | **S** | **A** | **I** | **Y** | **E** | **S** | **M** | **V** | **R** | **M** | **A** | **Q** | **P** | **F** | **S** | **Y** | **R** | **A** | **M** | **L** | **V** | **D** | **G** | **H** | **G** | **N** | **F** | **G** | **S** | **V** | **D** | **G** | **D** | **G** | **A** | **A** | **A** | **M** | **R** | **Y** | **T** | **E** | **A** | **R** | **M** | **S** | **K** | **I** | **A** | **L** | **E** | **M** | **L** | **R** | **D** | **I** | **N** | **K** | **N** | **T** | **V** | **D** | **F** | **Q** | **G** | **N** | **Y** | **D** | **D** | **S** | **E** | **Q** | **E** | **P** | **V** | **V** | **L** | **P** | **A** | **R** | **F** | **P** | **N** | **L** | **L** | **V** | **N** | **G** | **T** | **T** | **G** | **I** | **A** | **V** | **G** | **M** | **A** | **T** | **N** | **I** | **P** | **P** | **H** | **N** | **L** | **S** | **E** | **V** | **I** | **D** | **A** | **T** | **S** | **L** | **L** | **M** | **D** | **N** | **P** | **D** | **V** | **T** | **T** | **N** | **E** | **L** | **M** | **E** | **V** | **L** | **P** | **G** | **P** | **D** | **F** | **P** | **T** | **G** | **G** | **L** | **V** | **M** | **G** | **K** | **S** | **G** | **I** | **R** | **R** | **A** | **Y** | **E** | **T** | **G** | **K** | **G** | **S** | **I** | **T** | **V** | **R** | **A** | **K** | **V** | **E** | **L** | **T** | **E** | **M** | **P** | **N** | **G** | **K** | **E** | **R** | **I** | **L** | **V** | **T** | **E** | **L** | **P** | **Y** | **M** | **V** | **N** | **K** | **A** | **K** | **L** | **I** | **E** | **R** | **I** | **S** | **E** | **L** | **H** | **R** | **D** | **K** | **R** | **I** | **E** | **G** | **I** | **T** | **D** | **L** | **R** | **D** | **E** | **S** | **S** | **R** | **E** | **G** | **M** | **R** | **I** | **V** | **I** | **D** | **V** | **R** | **R** | **D** | **V** | **S** | **A** | **S** | **V** | **V** | **L** | **N** | **N** | **L** | **Y** | **K** | **M** | **T** | **A** | **L** | **Q** | **T** | **S** | **F** | **G** | **F** | **N** | **M** | **L** | **A** | **I** | **E** | **K** | **G** | **V** | **P** | **K** | **I** | **L** | **S** | **L** | **K** | **R** | **I** | **L** | **E** | **N** | **Y** | **V** | **E** | **H** | **Q** | **K** | **E** | **V** | **I** | **T** | **R** | **R** | **T** | **I** | **F** | **D** | **K** | **N** | **K** | **A** | **E** | **A** | **R** | **A** | **H** | **I** | **L** | **E** | **G** | **L** | **R** | **I** | **A** | **L** | **D** | **H** | **I** | **D** | **E** | **I** | **I** | **A** | **I** | **I** | **R** | **G** | **S** | **Q** | **S** | **D** | **D** | **E** | **A** | **K** | **A** | **T** | **L** | **I** | **E** | **R** | **F** | **E** | **F** | **S** | **D** | **R** | **Q** | **A** | **Q** | **A** | **I** | **L** | **D** | **M** | **R** | **L** | **R** | **R** | **L** | **T** | **G** | **L** | **E** | **R** | **D** | **K** | **I** | **E** | **N** | **E** | **Y** | **Q** | **E** | **L** | **L** | **K** | **F** | **I** | **A** | **D** | **L** | **E** | **D** | **I** | **L** | **A** | **R** | **P** | **E** | **R** | **V** | **I** | **E** | **I** | **I** | **K** | **T** | **E** | **L** | **N** | **D** | **V** | **R** | **T** | **K** | **F** | **G** | **D** | **A** | **R** | **R** | **T** | **E** | **L** | **L** | **V** | **G** | **E** | **V** | **L** | **S** | **L** | **E** | **D** | **E** | **D** | **L** | **I** | **E** | **E** | **E** | **E** | **V** | **V** | **I** | **T** | **L** | **T** | **N** | **N** | **G** | **Y** | **I** | **K** | **R** | **M** | **A** | **N** | **S** | **E** | **F** | **R** | **A** | **Q** | **R** | **R** | **G** | **G** | **R** | **G** | **V** | **Q** | **G** | **M** | **G** | **V** | **H** | **D** | **D** | **D** | **F** | **V** | **K** | **N** | **L** | **V** | **S** | **C** | **S** | **T** | **H** | **D** | **T** | **L** | **L** | **F** | **F** | **T** | **N** | **T** | **G** | **K** | **V** | **Y** | **R** | **A** | **K** | **G** | **Y** | **E** | **I** | **P** | **E** | **Y** | **G** | **R** | **T** | **A** | **K** | **G** | **I** | **P** | **V** | **I** | **N** | **L** | **L** | **G** | **I** | **D** | **S** | **A** | **E** | **K** | **I** | **Q** | **A** | **I** | **I** | **S** | **V** | **E** | **G** | **K** | **A** | **E** | **A** | **G** | **K** | **Y** | **L** | **F** | **F** | **T** | **T** | **L** | **K** | **G** | **T** | **V** | **K** | **R** | **T** | **A** | **V** | **T** | **A** | **F** | **S** | **N** | **I** | **R** | **S** | **N** | **G** | **L** | **I** | **A** | **I** | **S** | **L** | **K** | **E** | **D** | **D** | **E** | **L** | **V** | **N** | **V** | **V** | **T** | **T** | **N** | **G** | **N** | **Q** | **K** | **M** | **I** | **I** | **G** | **T** | **H** | **A** | **G** | **Y** | **S** | **V** | **T** | **F** | **D** | **E** | **N** | **T** | **V** | **R** | **D** | **M** | **G** | **R** | **T** | **A** | **S** | **G** | **V** | **R** | **G** | **I** | **R** | **L** | **R** | **E** | **N** | **D** | **Y** | **V** | **V** | **G** | **A** | **A** | **I** | **L** | **D** | **E** | **N** | **K** | **E** | **V** | **L** | **V** | **I** | **T** | **E** | **N** | **G** | **Y** | **G** | **K** | **R** | **T** | **K** | **A** | **S** | **E** | **Y** | **P** | **V** | **K** | **G** | **R** | **G** | **G** | **K** | **G** | **I** | **K** | **T** | **A** | **N** | **I** | **T** | **E** | **K** | **N** | **G** | **P** | **L** | **A** | **G** | **L** | **T** | **T** | **V** | **N** | **G** | **D** | **E** | **D** | **I** | **L** | **L** | **I** | **T** | **N** | **K** | **G** | **V** | **I** | **I** | **R** | **F** | **N** | **V** | **D** | **S** | **V** | **S** | **Q** | **T** | **G** | **R** | **A** | **T** | **L** | **G** | **V** | **R** | **L** | **M** | **R** | **M** | **E** | **D** | **G** | **A** | **K** | **-** | **-** | **-** | **-** | **-** | **-** | **-** | **-** | **-** | **-** | **-** | **-** | **-** | **-** | **-** | **-** | **-** | **-** | **-** | **-** | **-** | **-** | **-** |
| **N54550** | **M** | **S** | **E** | **E** | **I** | **R** | **E** | **N** | **I** | **H** | **D** | **V** | **N** | **L** | **T** | **S** | **E** | **M** | **K** | **D** | **S** | **F** | **I** | **D** | **Y** | **A** | **M** | **S** | **V** | **I** | **V** | **A** | **R** | **A** | **L** | **P** | **D** | **V** | **R** | **D** | **G** | **L** | **K** | **P** | **V** | **H** | **R** | **R** | **I** | **L** | **Y** | **G** | **M** | **N** | **E** | **L** | **G** | **V** | **T** | **P** | **D** | **K** | **A** | **H** | **K** | **K** | **S** | **A** | **R** | **I** | **V** | **G** | **D** | **V** | **M** | **G** | **K** | **Y** | **H** | **P** | **H** | **G** | **D** | **S** | **A** | **I** | **Y** | **E** | **S** | **M** | **V** | **R** | **M** | **A** | **Q** | **P** | **F** | **S** | **Y** | **R** | **Y** | **M** | **L** | **V** | **D** | **G** | **H** | **G** | **N** | **F** | **G** | **S** | **V** | **D** | **G** | **D** | **G** | **A** | **A** | **A** | **M** | **R** | **Y** | **T** | **E** | **A** | **R** | **M** | **S** | **K** | **I** | **A** | **T** | **E** | **M** | **L** | **R** | **D** | **I** | **N** | **K** | **N** | **T** | **V** | **D** | **F** | **Q** | **S** | **N** | **Y** | **D** | **D** | **T** | **E** | **K** | **E** | **P** | **V** | **V** | **L** | **P** | **A** | **R** | **F** | **P** | **N** | **L** | **L** | **V** | **N** | **G** | **T** | **T** | **G** | **I** | **A** | **V** | **G** | **M** | **A** | **T** | **N** | **I** | **P** | **P** | **H** | **N** | **L** | **S** | **E** | **V** | **V** | **A** | **A** | **I** | **D** | **L** | **L** | **M** | **D** | **N** | **P** | **D** | **V** | **T** | **T** | **N** | **E** | **L** | **M** | **E** | **V** | **L** | **P** | **G** | **P** | **D** | **F** | **P** | **T** | **G** | **G** | **L** | **V** | **M** | **G** | **K** | **S** | **G** | **I** | **R** | **R** | **A** | **Y** | **E** | **T** | **G** | **K** | **G** | **S** | **I** | **T** | **V** | **R** | **A** | **K** | **V** | **E** | **I** | **T** | **E** | **M** | **P** | **N** | **G** | **K** | **E** | **R** | **I** | **I** | **V** | **T** | **E** | **L** | **P** | **Y** | **M** | **V** | **N** | **K** | **A** | **K** | **L** | **I** | **E** | **R** | **I** | **S** | **E** | **L** | **H** | **R** | **D** | **K** | **R** | **I** | **E** | **G** | **I** | **T** | **D** | **L** | **R** | **D** | **E** | **S** | **S** | **R** | **E** | **G** | **M** | **R** | **I** | **V** | **I** | **D** | **I** | **R** | **R** | **D** | **A** | **S** | **A** | **S** | **V** | **I** | **L** | **N** | **N** | **L** | **Y** | **K** | **L** | **T** | **S** | **L** | **Q** | **N** | **S** | **F** | **G** | **F** | **N** | **M** | **L** | **A** | **I** | **E** | **K** | **G** | **V** | **P** | **K** | **V** | **L** | **S** | **L** | **K** | **Q** | **I** | **L** | **E** | **N** | **Y** | **I** | **E** | **H** | **Q** | **R** | **E** | **V** | **I** | **T** | **R** | **R** | **T** | **V** | **F** | **E** | **K** | **E** | **K** | **A** | **E** | **A** | **R** | **A** | **H** | **I** | **L** | **E** | **G** | **L** | **R** | **I** | **A** | **L** | **D** | **H** | **I** | **D** | **E** | **I** | **I** | **A** | **I** | **I** | **R** | **G** | **S** | **K** | **S** | **D** | **D** | **E** | **A** | **K** | **A** | **T** | **M** | **I** | **E** | **R** | **F** | **D** | **L** | **S** | **D** | **R** | **Q** | **S** | **Q** | **A** | **I** | **L** | **D** | **M** | **R** | **L** | **R** | **R** | **L** | **T** | **G** | **L** | **E** | **R** | **E** | **K** | **I** | **E** | **N** | **E** | **Y** | **Q** | **E** | **L** | **L** | **K** | **L** | **I** | **E** | **D** | **L** | **T** | **D** | **I** | **L** | **A** | **R** | **P** | **E** | **R** | **V** | **T** | **E** | **I** | **I** | **K** | **N** | **E** | **L** | **A** | **D** | **L** | **N** | **Q** | **R** | **F** | **G** | **D** | **K** | **R** | **R** | **T** | **E** | **L** | **L** | **V** | **G** | **E** | **V** | **L** | **S** | **L** | **E** | **D** | **E** | **D** | **L** | **I** | **E** | **E** | **E** | **E** | **I** | **V** | **I** | **T** | **L** | **T** | **N** | **N** | **G** | **Y** | **I** | **K** | **R** | **L** | **A** | **N** | **N** | **E** | **F** | **R** | **A** | **Q** | **R** | **R** | **G** | **G** | **R** | **G** | **V** | **Q** | **G** | **M** | **G** | **I** | **H** | **N** | **D** | **D** | **F** | **V** | **K** | **N** | **L** | **V** | **S** | **C** | **S** | **T** | **H** | **D** | **T** | **L** | **L** | **F** | **F** | **T** | **N** | **N** | **G** | **K** | **V** | **Y** | **R** | **A** | **K** | **G** | **Y** | **E** | **I** | **P** | **E** | **Y** | **G** | **R** | **T** | **A** | **K** | **G** | **I** | **P** | **I** | **I** | **N** | **L** | **L** | **G** | **I** | **D** | **S** | **S** | **E** | **S** | **I** | **Q** | **A** | **I** | **I** | **A** | **V** | **T** | **G** | **E** | **A** | **E** | **E** | **G** | **H** | **Y** | **L** | **F** | **F** | **T** | **T** | **R** | **Q** | **G** | **T** | **V** | **K** | **R** | **T** | **S** | **V** | **T** | **A** | **F** | **A** | **N** | **I** | **R** | **S** | **N** | **G** | **L** | **I** | **A** | **I** | **G** | **L** | **K** | **E** | **D** | **D** | **E** | **L** | **V** | **N** | **V** | **L** | **L** | **T** | **D** | **G** | **Q** | **S** | **N** | **I** | **I** | **I** | **G** | **T** | **H** | **N** | **G** | **Y** | **S** | **V** | **T** | **F** | **K** | **E** | **E** | **A** | **V** | **R** | **D** | **M** | **G** | **R** | **T** | **A** | **S** | **G** | **V** | **R** | **G** | **I** | **R** | **L** | **R** | **E** | **E** | **D** | **Y** | **V** | **V** | **G** | **A** | **A** | **L** | **M** | **K** | **E** | **N** | **Q** | **E** | **V** | **L** | **V** | **I** | **T** | **E** | **K** | **G** | **Y** | **G** | **K** | **R** | **T** | **K** | **V** | **S** | **E** | **Y** | **P** | **V** | **K** | **G** | **R** | **G** | **G** | **K** | **G** | **I** | **K** | **T** | **A** | **N** | **I** | **T** | **E** | **K** | **N** | **G** | **P** | **L** | **A** | **G** | **L** | **T** | **T** | **V** | **S** | **G** | **D** | **E** | **D** | **I** | **M** | **L** | **I** | **T** | **D** | **K** | **G** | **V** | **I** | **I** | **R** | **F** | **N** | **V** | **S** | **T** | **V** | **S** | **Q** | **T** | **G** | **R** | **S** | **T** | **L** | **G** | **V** | **R** | **L** | **M** | **K** | **M** | **E** | **A** | **D** | **T** | **K** | **V** | **V** | **T** | **M** | **A** | **A** | **V** | **E** | **P** | **E** | **A** | **A** | **E** | **Q** | **T** | **V** | **E** | **E** | **Q** | **G** | **S** | **E** | **E** |
| **N59420F** | **M** | **S** | **E** | **E** | **I** | **K** | **E** | **N** | **I** | **Q** | **D** | **V** | **N** | **L** | **T** | **S** | **E** | **M** | **K** | **E** | **S** | **F** | **I** | **D** | **Y** | **A** | **M** | **S** | **V** | **I** | **V** | **A** | **R** | **A** | **L** | **P** | **D** | **V** | **R** | **D** | **G** | **L** | **K** | **P** | **V** | **H** | **R** | **R** | **I** | **L** | **Y** | **G** | **M** | **N** | **E** | **L** | **G** | **V** | **T** | **P** | **D** | **K** | **P** | **H** | **K** | **K** | **S** | **A** | **R** | **I** | **V** | **G** | **D** | **V** | **M** | **G** | **K** | **Y** | **H** | **P** | **H** | **G** | **D** | **S** | **A** | **I** | **Y** | **E** | **S** | **M** | **V** | **R** | **M** | **A** | **Q** | **P** | **F** | **S** | **Y** | **R** | **A** | **M** | **L** | **V** | **D** | **G** | **H** | **G** | **N** | **F** | **G** | **S** | **V** | **D** | **G** | **D** | **G** | **A** | **A** | **A** | **M** | **R** | **Y** | **T** | **E** | **A** | **R** | **M** | **S** | **K** | **I** | **A** | **L** | **E** | **M** | **L** | **R** | **D** | **I** | **N** | **K** | **N** | **T** | **V** | **D** | **F** | **Q** | **G** | **N** | **Y** | **D** | **D** | **S** | **E** | **Q** | **E** | **P** | **V** | **V** | **L** | **P** | **A** | **R** | **F** | **P** | **N** | **L** | **L** | **V** | **N** | **G** | **T** | **T** | **G** | **I** | **A** | **V** | **G** | **M** | **A** | **T** | **N** | **I** | **P** | **P** | **H** | **N** | **L** | **S** | **E** | **V** | **I** | **D** | **A** | **T** | **S** | **L** | **L** | **M** | **D** | **N** | **P** | **D** | **V** | **T** | **T** | **N** | **E** | **L** | **M** | **E** | **V** | **L** | **P** | **G** | **P** | **D** | **F** | **P** | **T** | **G** | **G** | **L** | **V** | **M** | **G** | **K** | **S** | **G** | **I** | **R** | **R** | **A** | **Y** | **E** | **T** | **G** | **K** | **G** | **S** | **I** | **T** | **V** | **R** | **A** | **K** | **V** | **E** | **L** | **T** | **E** | **M** | **P** | **N** | **G** | **K** | **E** | **R** | **I** | **L** | **V** | **T** | **E** | **L** | **P** | **Y** | **M** | **V** | **N** | **K** | **A** | **K** | **L** | **I** | **E** | **R** | **I** | **S** | **E** | **L** | **H** | **R** | **D** | **K** | **R** | **I** | **E** | **G** | **I** | **T** | **D** | **L** | **R** | **D** | **E** | **S** | **S** | **R** | **E** | **G** | **M** | **R** | **I** | **V** | **I** | **D** | **V** | **R** | **R** | **D** | **V** | **S** | **A** | **S** | **V** | **V** | **L** | **N** | **N** | **L** | **Y** | **K** | **M** | **T** | **A** | **L** | **Q** | **T** | **S** | **F** | **G** | **F** | **N** | **M** | **L** | **A** | **I** | **E** | **K** | **G** | **V** | **P** | **K** | **I** | **L** | **S** | **L** | **K** | **R** | **I** | **L** | **E** | **N** | **Y** | **V** | **E** | **H** | **Q** | **K** | **E** | **V** | **I** | **T** | **R** | **R** | **T** | **I** | **F** | **D** | **K** | **N** | **K** | **A** | **E** | **A** | **R** | **A** | **H** | **I** | **L** | **E** | **G** | **L** | **R** | **I** | **A** | **L** | **D** | **H** | **I** | **D** | **E** | **I** | **I** | **A** | **I** | **I** | **R** | **G** | **S** | **Q** | **S** | **D** | **D** | **E** | **A** | **K** | **A** | **T** | **L** | **I** | **E** | **R** | **F** | **E** | **F** | **S** | **D** | **R** | **Q** | **A** | **Q** | **A** | **I** | **L** | **D** | **M** | **R** | **L** | **R** | **R** | **L** | **T** | **G** | **L** | **E** | **R** | **D** | **K** | **I** | **E** | **N** | **E** | **Y** | **Q** | **E** | **L** | **L** | **K** | **F** | **I** | **A** | **D** | **L** | **E** | **D** | **I** | **L** | **A** | **R** | **P** | **E** | **R** | **V** | **I** | **E** | **I** | **I** | **K** | **T** | **E** | **L** | **N** | **D** | **V** | **R** | **T** | **K** | **F** | **G** | **D** | **A** | **R** | **R** | **T** | **E** | **L** | **L** | **V** | **G** | **E** | **V** | **L** | **S** | **L** | **E** | **D** | **E** | **D** | **L** | **I** | **E** | **E** | **E** | **E** | **V** | **V** | **I** | **T** | **L** | **T** | **N** | **N** | **G** | **Y** | **I** | **K** | **R** | **M** | **A** | **N** | **S** | **E** | **F** | **R** | **A** | **Q** | **R** | **R** | **G** | **G** | **R** | **G** | **V** | **Q** | **G** | **M** | **G** | **V** | **H** | **D** | **D** | **D** | **F** | **V** | **K** | **N** | **L** | **V** | **S** | **C** | **S** | **T** | **H** | **D** | **T** | **L** | **L** | **F** | **F** | **T** | **N** | **T** | **G** | **K** | **V** | **Y** | **R** | **A** | **K** | **G** | **Y** | **E** | **I** | **P** | **E** | **Y** | **G** | **R** | **T** | **A** | **K** | **G** | **I** | **P** | **V** | **I** | **N** | **L** | **L** | **G** | **I** | **D** | **S** | **A** | **E** | **K** | **I** | **Q** | **A** | **I** | **I** | **S** | **V** | **E** | **G** | **K** | **A** | **E** | **A** | **G** | **K** | **Y** | **L** | **F** | **F** | **T** | **T** | **L** | **K** | **G** | **T** | **V** | **K** | **R** | **T** | **S** | **V** | **T** | **A** | **F** | **S** | **N** | **I** | **R** | **S** | **N** | **G** | **L** | **I** | **A** | **I** | **S** | **L** | **K** | **E** | **D** | **D** | **E** | **L** | **V** | **N** | **V** | **V** | **T** | **T** | **N** | **G** | **N** | **Q** | **K** | **M** | **I** | **I** | **G** | **T** | **H** | **A** | **G** | **Y** | **S** | **V** | **T** | **F** | **D** | **E** | **N** | **T** | **V** | **R** | **D** | **M** | **G** | **R** | **T** | **A** | **S** | **G** | **V** | **R** | **G** | **I** | **R** | **L** | **R** | **E** | **N** | **D** | **Y** | **V** | **V** | **G** | **A** | **A** | **I** | **L** | **D** | **E** | **N** | **K** | **E** | **V** | **L** | **V** | **I** | **T** | **E** | **N** | **G** | **Y** | **G** | **K** | **R** | **T** | **K** | **A** | **S** | **E** | **Y** | **P** | **V** | **K** | **G** | **R** | **G** | **G** | **K** | **G** | **I** | **K** | **T** | **A** | **N** | **I** | **T** | **E** | **K** | **N** | **G** | **P** | **L** | **A** | **G** | **L** | **T** | **T** | **V** | **N** | **G** | **D** | **E** | **D** | **I** | **L** | **L** | **I** | **T** | **N** | **K** | **G** | **V** | **I** | **I** | **R** | **F** | **N** | **V** | **D** | **S** | **V** | **S** | **Q** | **T** | **G** | **R** | **A** | **T** | **L** | **G** | **V** | **R** | **L** | **M** | **R** | **M** | **E** | **D** | **G** | **A** | **K** | **-** | **-** | **-** | **-** | **-** | **-** | **-** | **-** | **-** | **-** | **-** | **-** | **-** | **-** | **-** | **-** | **-** | **-** | **-** | **-** | **-** | **-** | **-** |
| **N52623** | **M** | **S** | **E** | **E** | **I** | **K** | **E** | **N** | **I** | **Q** | **D** | **V** | **N** | **L** | **T** | **S** | **E** | **M** | **K** | **E** | **S** | **F** | **I** | **D** | **Y** | **A** | **M** | **S** | **V** | **I** | **V** | **A** | **R** | **A** | **L** | **P** | **D** | **V** | **R** | **D** | **G** | **L** | **K** | **P** | **V** | **H** | **R** | **R** | **I** | **L** | **Y** | **G** | **M** | **N** | **E** | **L** | **G** | **V** | **T** | **P** | **D** | **K** | **P** | **H** | **K** | **K** | **S** | **A** | **R** | **I** | **V** | **G** | **D** | **V** | **M** | **G** | **K** | **Y** | **H** | **P** | **H** | **G** | **D** | **S** | **A** | **I** | **Y** | **E** | **S** | **M** | **V** | **R** | **M** | **A** | **Q** | **P** | **F** | **S** | **Y** | **R** | **A** | **M** | **L** | **V** | **D** | **G** | **H** | **G** | **N** | **F** | **G** | **S** | **V** | **D** | **G** | **D** | **G** | **A** | **A** | **A** | **M** | **R** | **Y** | **T** | **E** | **A** | **R** | **M** | **S** | **K** | **I** | **A** | **L** | **E** | **M** | **L** | **R** | **D** | **I** | **N** | **K** | **N** | **T** | **V** | **D** | **F** | **Q** | **G** | **N** | **Y** | **D** | **D** | **S** | **E** | **Q** | **E** | **P** | **V** | **V** | **L** | **P** | **A** | **R** | **F** | **P** | **N** | **L** | **L** | **V** | **N** | **G** | **T** | **T** | **G** | **I** | **A** | **V** | **G** | **M** | **A** | **T** | **N** | **I** | **P** | **P** | **H** | **N** | **L** | **S** | **E** | **V** | **I** | **D** | **A** | **T** | **S** | **L** | **L** | **M** | **D** | **N** | **P** | **D** | **V** | **T** | **T** | **N** | **E** | **L** | **M** | **E** | **V** | **L** | **P** | **G** | **P** | **D** | **F** | **P** | **T** | **G** | **G** | **L** | **V** | **M** | **G** | **K** | **S** | **G** | **I** | **R** | **R** | **A** | **Y** | **E** | **T** | **G** | **K** | **G** | **S** | **I** | **T** | **V** | **R** | **A** | **K** | **V** | **E** | **L** | **T** | **E** | **M** | **P** | **N** | **G** | **K** | **E** | **R** | **I** | **L** | **V** | **T** | **E** | **L** | **P** | **Y** | **M** | **V** | **N** | **K** | **A** | **K** | **L** | **I** | **E** | **R** | **I** | **S** | **E** | **L** | **H** | **R** | **D** | **K** | **R** | **I** | **E** | **G** | **I** | **T** | **D** | **L** | **R** | **D** | **E** | **S** | **S** | **R** | **E** | **G** | **M** | **R** | **I** | **V** | **I** | **D** | **V** | **R** | **R** | **D** | **V** | **S** | **A** | **S** | **V** | **V** | **L** | **N** | **N** | **L** | **Y** | **K** | **M** | **T** | **A** | **L** | **Q** | **T** | **S** | **F** | **G** | **F** | **N** | **M** | **L** | **A** | **I** | **E** | **K** | **G** | **V** | **P** | **K** | **I** | **L** | **S** | **L** | **K** | **R** | **I** | **L** | **E** | **N** | **Y** | **V** | **E** | **H** | **Q** | **K** | **E** | **V** | **I** | **T** | **R** | **R** | **T** | **I** | **F** | **D** | **K** | **N** | **K** | **A** | **E** | **A** | **R** | **A** | **H** | **I** | **L** | **E** | **G** | **L** | **R** | **I** | **A** | **L** | **D** | **H** | **I** | **D** | **E** | **I** | **I** | **A** | **I** | **I** | **R** | **G** | **S** | **Q** | **S** | **D** | **D** | **E** | **A** | **K** | **A** | **T** | **L** | **I** | **E** | **R** | **F** | **E** | **F** | **S** | **D** | **R** | **Q** | **A** | **Q** | **A** | **I** | **L** | **D** | **M** | **R** | **L** | **R** | **R** | **L** | **T** | **G** | **L** | **E** | **R** | **D** | **K** | **I** | **E** | **N** | **E** | **Y** | **Q** | **E** | **L** | **L** | **K** | **F** | **I** | **A** | **D** | **L** | **E** | **D** | **I** | **L** | **A** | **R** | **P** | **E** | **R** | **V** | **I** | **E** | **I** | **I** | **K** | **T** | **E** | **L** | **N** | **D** | **V** | **R** | **T** | **K** | **F** | **G** | **D** | **A** | **R** | **R** | **T** | **E** | **L** | **L** | **V** | **G** | **E** | **V** | **L** | **S** | **L** | **E** | **D** | **E** | **D** | **L** | **I** | **E** | **E** | **E** | **E** | **V** | **V** | **I** | **T** | **L** | **T** | **N** | **N** | **G** | **Y** | **I** | **K** | **R** | **M** | **A** | **N** | **S** | **E** | **F** | **R** | **A** | **Q** | **R** | **R** | **G** | **G** | **R** | **G** | **V** | **Q** | **G** | **M** | **G** | **V** | **H** | **D** | **D** | **D** | **F** | **V** | **K** | **N** | **L** | **V** | **S** | **C** | **S** | **T** | **H** | **D** | **T** | **L** | **L** | **F** | **F** | **T** | **N** | **T** | **G** | **K** | **V** | **Y** | **R** | **A** | **K** | **G** | **Y** | **E** | **I** | **P** | **E** | **Y** | **G** | **R** | **T** | **A** | **K** | **G** | **I** | **P** | **V** | **I** | **N** | **L** | **L** | **G** | **I** | **D** | **S** | **A** | **E** | **K** | **I** | **Q** | **A** | **I** | **I** | **S** | **V** | **E** | **G** | **K** | **A** | **E** | **A** | **G** | **K** | **Y** | **L** | **F** | **F** | **T** | **T** | **L** | **K** | **G** | **T** | **V** | **K** | **R** | **T** | **A** | **V** | **T** | **A** | **F** | **S** | **N** | **I** | **R** | **S** | **N** | **G** | **L** | **I** | **A** | **I** | **S** | **L** | **K** | **E** | **D** | **D** | **E** | **L** | **V** | **N** | **V** | **V** | **T** | **T** | **N** | **G** | **N** | **Q** | **K** | **M** | **I** | **I** | **G** | **T** | **H** | **A** | **G** | **Y** | **S** | **V** | **T** | **F** | **D** | **E** | **N** | **T** | **V** | **R** | **D** | **M** | **G** | **R** | **T** | **A** | **S** | **G** | **V** | **R** | **G** | **I** | **R** | **L** | **R** | **E** | **N** | **D** | **Y** | **V** | **V** | **G** | **A** | **A** | **I** | **L** | **D** | **E** | **N** | **K** | **E** | **V** | **L** | **V** | **I** | **T** | **E** | **N** | **G** | **Y** | **G** | **K** | **R** | **T** | **K** | **A** | **S** | **E** | **Y** | **P** | **V** | **K** | **G** | **R** | **G** | **G** | **K** | **G** | **I** | **K** | **T** | **A** | **N** | **I** | **T** | **E** | **K** | **N** | **G** | **P** | **L** | **A** | **G** | **L** | **T** | **T** | **V** | **N** | **G** | **D** | **E** | **D** | **I** | **L** | **L** | **I** | **T** | **N** | **K** | **G** | **V** | **I** | **I** | **R** | **F** | **N** | **V** | **D** | **S** | **V** | **S** | **Q** | **T** | **G** | **R** | **A** | **T** | **L** | **G** | **V** | **R** | **L** | **M** | **R** | **M** | **E** | **D** | **G** | **A** | **K** | **V** | **V** | **T** | **M** | **A** | **V** | **V** | **E** | **P** | **E** | **E** | **H** | **K** | **K** | **V** | **S** | **P** | **V** | **G** | **-** | **-** | **-** | **-** |
| **N60440F** | **M** | **S** | **E** | **E** | **I** | **K** | **E** | **N** | **I** | **Q** | **D** | **V** | **N** | **L** | **T** | **S** | **E** | **M** | **K** | **E** | **S** | **F** | **I** | **D** | **Y** | **A** | **M** | **S** | **V** | **I** | **V** | **A** | **R** | **A** | **L** | **P** | **D** | **V** | **R** | **D** | **G** | **L** | **K** | **P** | **V** | **H** | **R** | **R** | **I** | **L** | **Y** | **G** | **M** | **N** | **E** | **L** | **G** | **V** | **T** | **P** | **D** | **K** | **P** | **H** | **K** | **K** | **S** | **A** | **R** | **I** | **V** | **G** | **D** | **V** | **M** | **G** | **K** | **Y** | **H** | **P** | **H** | **G** | **D** | **F** | **A** | **I** | **Y** | **E** | **S** | **M** | **V** | **R** | **M** | **A** | **Q** | **P** | **F** | **S** | **Y** | **R** | **A** | **M** | **L** | **V** | **D** | **G** | **H** | **G** | **N** | **F** | **G** | **S** | **V** | **D** | **G** | **D** | **G** | **A** | **A** | **A** | **M** | **R** | **Y** | **T** | **E** | **A** | **R** | **M** | **S** | **K** | **I** | **A** | **L** | **E** | **M** | **L** | **R** | **D** | **I** | **N** | **K** | **N** | **T** | **V** | **D** | **F** | **Q** | **G** | **N** | **Y** | **D** | **D** | **S** | **E** | **Q** | **E** | **P** | **V** | **V** | **L** | **P** | **A** | **R** | **F** | **P** | **N** | **L** | **L** | **V** | **N** | **G** | **T** | **T** | **G** | **I** | **A** | **V** | **G** | **M** | **A** | **T** | **N** | **I** | **P** | **P** | **H** | **N** | **L** | **S** | **E** | **V** | **I** | **D** | **A** | **T** | **S** | **L** | **L** | **M** | **D** | **N** | **P** | **D** | **V** | **T** | **T** | **N** | **E** | **L** | **M** | **E** | **V** | **L** | **P** | **G** | **P** | **D** | **F** | **P** | **T** | **G** | **G** | **L** | **V** | **M** | **G** | **K** | **S** | **G** | **I** | **R** | **R** | **A** | **Y** | **E** | **T** | **G** | **K** | **G** | **S** | **I** | **T** | **V** | **R** | **A** | **K** | **V** | **E** | **L** | **T** | **E** | **M** | **P** | **N** | **G** | **K** | **E** | **R** | **I** | **L** | **V** | **T** | **E** | **L** | **P** | **Y** | **M** | **V** | **N** | **K** | **A** | **K** | **L** | **I** | **E** | **R** | **I** | **S** | **E** | **L** | **H** | **R** | **D** | **K** | **R** | **I** | **E** | **G** | **I** | **T** | **D** | **L** | **R** | **D** | **E** | **S** | **S** | **R** | **E** | **G** | **M** | **R** | **I** | **V** | **I** | **D** | **V** | **R** | **R** | **D** | **V** | **S** | **A** | **S** | **V** | **V** | **L** | **N** | **N** | **L** | **Y** | **K** | **M** | **T** | **A** | **L** | **Q** | **T** | **S** | **F** | **G** | **F** | **N** | **M** | **L** | **A** | **I** | **E** | **K** | **G** | **V** | **P** | **K** | **I** | **L** | **S** | **L** | **K** | **R** | **I** | **L** | **E** | **N** | **Y** | **V** | **E** | **H** | **Q** | **K** | **E** | **V** | **I** | **T** | **R** | **R** | **T** | **I** | **F** | **D** | **K** | **N** | **K** | **A** | **E** | **A** | **R** | **A** | **H** | **I** | **L** | **E** | **G** | **L** | **R** | **I** | **A** | **L** | **D** | **H** | **I** | **D** | **E** | **I** | **I** | **A** | **I** | **I** | **R** | **G** | **S** | **Q** | **S** | **D** | **D** | **E** | **A** | **K** | **A** | **T** | **L** | **I** | **E** | **R** | **F** | **E** | **F** | **S** | **D** | **R** | **Q** | **A** | **Q** | **A** | **I** | **L** | **D** | **M** | **R** | **L** | **R** | **R** | **L** | **T** | **G** | **L** | **E** | **R** | **D** | **K** | **I** | **E** | **N** | **E** | **Y** | **Q** | **E** | **L** | **L** | **K** | **F** | **I** | **A** | **D** | **L** | **E** | **D** | **I** | **L** | **A** | **R** | **P** | **E** | **R** | **V** | **I** | **E** | **I** | **I** | **K** | **T** | **E** | **L** | **N** | **D** | **V** | **R** | **T** | **K** | **F** | **G** | **D** | **A** | **R** | **R** | **T** | **E** | **L** | **L** | **V** | **G** | **E** | **V** | **L** | **S** | **L** | **E** | **D** | **E** | **D** | **L** | **I** | **E** | **E** | **E** | **E** | **V** | **V** | **I** | **T** | **L** | **T** | **N** | **N** | **G** | **Y** | **I** | **K** | **R** | **M** | **A** | **N** | **S** | **E** | **F** | **R** | **A** | **Q** | **R** | **R** | **G** | **G** | **R** | **G** | **V** | **Q** | **G** | **M** | **G** | **V** | **H** | **D** | **D** | **D** | **F** | **V** | **K** | **N** | **L** | **V** | **S** | **C** | **S** | **T** | **H** | **D** | **T** | **L** | **L** | **F** | **F** | **T** | **N** | **T** | **G** | **K** | **V** | **Y** | **R** | **A** | **K** | **G** | **Y** | **E** | **I** | **P** | **E** | **Y** | **G** | **R** | **T** | **A** | **K** | **G** | **I** | **P** | **V** | **I** | **N** | **L** | **L** | **G** | **I** | **D** | **S** | **A** | **E** | **K** | **I** | **Q** | **A** | **I** | **I** | **S** | **V** | **E** | **G** | **K** | **A** | **E** | **A** | **G** | **K** | **Y** | **L** | **F** | **F** | **T** | **T** | **L** | **K** | **G** | **T** | **V** | **K** | **R** | **T** | **A** | **V** | **T** | **A** | **F** | **S** | **N** | **I** | **R** | **S** | **N** | **G** | **L** | **I** | **A** | **I** | **S** | **L** | **K** | **E** | **D** | **D** | **E** | **L** | **V** | **N** | **V** | **V** | **T** | **T** | **N** | **G** | **N** | **Q** | **K** | **M** | **I** | **I** | **G** | **T** | **H** | **A** | **G** | **Y** | **S** | **V** | **T** | **F** | **D** | **E** | **N** | **T** | **V** | **R** | **D** | **M** | **G** | **R** | **T** | **A** | **S** | **G** | **V** | **R** | **G** | **I** | **R** | **L** | **R** | **E** | **N** | **D** | **Y** | **V** | **V** | **G** | **A** | **A** | **I** | **L** | **D** | **E** | **N** | **K** | **E** | **V** | **L** | **V** | **I** | **T** | **E** | **N** | **G** | **Y** | **G** | **K** | **R** | **T** | **K** | **A** | **S** | **E** | **Y** | **P** | **V** | **K** | **G** | **R** | **G** | **G** | **K** | **G** | **I** | **K** | **T** | **A** | **N** | **I** | **T** | **E** | **K** | **N** | **G** | **P** | **L** | **A** | **G** | **L** | **T** | **T** | **V** | **N** | **G** | **N** | **E** | **D** | **I** | **L** | **L** | **I** | **T** | **N** | **K** | **G** | **V** | **I** | **I** | **R** | **F** | **N** | **V** | **D** | **S** | **V** | **S** | **Q** | **T** | **G** | **R** | **A** | **T** | **L** | **G** | **V** | **R** | **L** | **M** | **R** | **M** | **E** | **D** | **G** | **A** | **K** | **-** | **-** | **-** | **-** | **-** | **-** | **-** | **-** | **-** | **-** | **-** | **-** | **-** | **-** | **-** | **-** | **-** | **-** | **-** | **-** | **-** | **-** | **-** |
| **N60252F** | **M** | **S** | **E** | **E** | **I** | **R** | **E** | **N** | **I** | **H** | **D** | **V** | **N** | **L** | **T** | **S** | **E** | **M** | **K** | **D** | **S** | **F** | **I** | **D** | **Y** | **A** | **M** | **S** | **V** | **I** | **V** | **A** | **R** | **A** | **L** | **P** | **D** | **V** | **R** | **D** | **G** | **L** | **K** | **P** | **V** | **H** | **R** | **R** | **I** | **L** | **Y** | **G** | **M** | **N** | **E** | **L** | **G** | **V** | **T** | **P** | **D** | **K** | **A** | **H** | **K** | **K** | **S** | **A** | **R** | **I** | **V** | **G** | **D** | **V** | **M** | **G** | **K** | **Y** | **H** | **P** | **H** | **G** | **D** | **S** | **A** | **I** | **Y** | **E** | **S** | **M** | **V** | **R** | **M** | **A** | **Q** | **P** | **F** | **S** | **Y** | **R** | **Y** | **M** | **L** | **V** | **D** | **G** | **H** | **G** | **N** | **F** | **G** | **S** | **V** | **D** | **G** | **D** | **G** | **A** | **A** | **A** | **M** | **R** | **Y** | **T** | **E** | **A** | **R** | **M** | **S** | **K** | **I** | **A** | **T** | **E** | **M** | **L** | **R** | **D** | **I** | **N** | **K** | **N** | **T** | **V** | **D** | **F** | **Q** | **S** | **N** | **Y** | **D** | **D** | **T** | **E** | **K** | **E** | **P** | **V** | **V** | **L** | **P** | **A** | **R** | **F** | **P** | **N** | **L** | **L** | **V** | **N** | **G** | **T** | **T** | **G** | **I** | **A** | **V** | **G** | **M** | **A** | **T** | **N** | **I** | **P** | **P** | **H** | **N** | **L** | **S** | **E** | **V** | **V** | **A** | **A** | **I** | **D** | **L** | **L** | **M** | **D** | **N** | **P** | **D** | **V** | **T** | **T** | **N** | **E** | **L** | **M** | **E** | **V** | **L** | **P** | **G** | **P** | **D** | **F** | **P** | **T** | **G** | **G** | **L** | **V** | **M** | **G** | **K** | **S** | **G** | **I** | **R** | **R** | **A** | **Y** | **E** | **T** | **G** | **K** | **G** | **S** | **I** | **T** | **V** | **R** | **A** | **K** | **V** | **E** | **I** | **T** | **E** | **M** | **P** | **N** | **G** | **K** | **E** | **R** | **I** | **I** | **V** | **T** | **E** | **L** | **P** | **Y** | **M** | **V** | **N** | **K** | **A** | **K** | **L** | **I** | **E** | **R** | **I** | **S** | **E** | **L** | **H** | **R** | **D** | **K** | **R** | **I** | **E** | **G** | **I** | **T** | **D** | **L** | **R** | **D** | **E** | **S** | **S** | **R** | **E** | **G** | **M** | **R** | **I** | **V** | **I** | **D** | **I** | **R** | **R** | **D** | **A** | **S** | **A** | **S** | **V** | **I** | **L** | **N** | **N** | **L** | **Y** | **K** | **L** | **T** | **S** | **L** | **Q** | **N** | **S** | **F** | **G** | **F** | **N** | **M** | **L** | **A** | **I** | **E** | **K** | **G** | **V** | **P** | **K** | **V** | **L** | **S** | **L** | **K** | **Q** | **I** | **L** | **E** | **N** | **Y** | **I** | **E** | **H** | **Q** | **R** | **E** | **V** | **I** | **T** | **R** | **R** | **T** | **V** | **F** | **E** | **K** | **E** | **K** | **A** | **E** | **A** | **R** | **A** | **H** | **I** | **L** | **E** | **G** | **L** | **R** | **I** | **A** | **L** | **D** | **H** | **I** | **D** | **E** | **I** | **I** | **A** | **I** | **I** | **R** | **G** | **S** | **K** | **S** | **D** | **D** | **E** | **A** | **K** | **A** | **T** | **M** | **I** | **E** | **R** | **F** | **D** | **L** | **S** | **D** | **R** | **Q** | **S** | **Q** | **A** | **I** | **L** | **D** | **M** | **R** | **L** | **R** | **R** | **L** | **T** | **G** | **L** | **E** | **R** | **E** | **K** | **I** | **E** | **N** | **E** | **Y** | **Q** | **E** | **L** | **L** | **K** | **L** | **I** | **E** | **D** | **L** | **T** | **D** | **I** | **L** | **A** | **R** | **P** | **E** | **R** | **V** | **T** | **E** | **I** | **I** | **K** | **N** | **E** | **L** | **A** | **D** | **L** | **N** | **Q** | **R** | **F** | **G** | **D** | **K** | **R** | **R** | **T** | **E** | **L** | **L** | **V** | **G** | **E** | **V** | **L** | **S** | **L** | **E** | **D** | **E** | **D** | **L** | **I** | **E** | **E** | **E** | **E** | **I** | **V** | **I** | **T** | **L** | **T** | **N** | **N** | **G** | **Y** | **I** | **K** | **R** | **L** | **A** | **N** | **N** | **E** | **F** | **R** | **A** | **Q** | **R** | **R** | **G** | **G** | **R** | **G** | **V** | **Q** | **G** | **M** | **G** | **I** | **H** | **N** | **D** | **D** | **F** | **V** | **K** | **N** | **L** | **V** | **S** | **C** | **S** | **T** | **H** | **D** | **T** | **L** | **L** | **F** | **F** | **T** | **N** | **N** | **G** | **K** | **V** | **Y** | **R** | **A** | **K** | **G** | **Y** | **E** | **I** | **P** | **E** | **Y** | **G** | **R** | **T** | **A** | **K** | **G** | **I** | **P** | **I** | **I** | **N** | **L** | **L** | **G** | **I** | **D** | **S** | **S** | **E** | **S** | **I** | **Q** | **A** | **I** | **I** | **A** | **V** | **T** | **G** | **E** | **A** | **E** | **E** | **G** | **H** | **Y** | **L** | **F** | **F** | **T** | **T** | **R** | **Q** | **G** | **T** | **V** | **K** | **R** | **T** | **S** | **V** | **T** | **A** | **F** | **A** | **N** | **I** | **R** | **S** | **N** | **G** | **L** | **I** | **A** | **I** | **G** | **L** | **K** | **E** | **D** | **D** | **E** | **L** | **V** | **N** | **V** | **L** | **L** | **T** | **D** | **G** | **Q** | **S** | **N** | **I** | **I** | **I** | **G** | **T** | **H** | **N** | **G** | **Y** | **S** | **V** | **T** | **F** | **K** | **E** | **E** | **A** | **V** | **R** | **D** | **M** | **G** | **R** | **T** | **A** | **S** | **G** | **V** | **R** | **G** | **I** | **R** | **L** | **R** | **E** | **E** | **D** | **Y** | **V** | **V** | **G** | **A** | **A** | **L** | **M** | **K** | **E** | **N** | **Q** | **E** | **V** | **L** | **V** | **I** | **T** | **E** | **K** | **G** | **Y** | **G** | **K** | **R** | **T** | **K** | **V** | **S** | **E** | **Y** | **P** | **V** | **K** | **G** | **R** | **G** | **G** | **K** | **G** | **I** | **K** | **T** | **A** | **N** | **I** | **T** | **E** | **K** | **N** | **G** | **P** | **L** | **A** | **G** | **L** | **T** | **T** | **V** | **S** | **G** | **D** | **E** | **D** | **I** | **M** | **L** | **I** | **T** | **D** | **K** | **G** | **V** | **I** | **I** | **R** | **F** | **N** | **V** | **S** | **T** | **V** | **S** | **Q** | **T** | **G** | **R** | **S** | **T** | **L** | **G** | **V** | **R** | **L** | **M** | **K** | **M** | **E** | **A** | **D** | **T** | **K** | **V** | **V** | **T** | **M** | **A** | **A** | **V** | **E** | **P** | **E** | **A** | **A** | **E** | **Q** | **T** | **V** | **E** | **E** | **Q** | **G** | **S** | **E** | **E** |
| **N59959F** | **M** | **S** | **E** | **E** | **I** | **K** | **E** | **N** | **I** | **Q** | **D** | **V** | **N** | **L** | **T** | **S** | **E** | **M** | **K** | **E** | **S** | **F** | **I** | **D** | **Y** | **A** | **M** | **S** | **V** | **I** | **V** | **A** | **R** | **A** | **L** | **P** | **D** | **V** | **R** | **D** | **G** | **L** | **K** | **P** | **V** | **H** | **R** | **R** | **I** | **L** | **Y** | **G** | **M** | **N** | **E** | **L** | **G** | **V** | **T** | **P** | **D** | **K** | **P** | **H** | **K** | **K** | **S** | **A** | **R** | **I** | **V** | **G** | **D** | **V** | **M** | **G** | **K** | **Y** | **H** | **P** | **H** | **G** | **D** | **S** | **A** | **I** | **Y** | **E** | **S** | **M** | **V** | **R** | **M** | **A** | **Q** | **P** | **F** | **S** | **Y** | **R** | **A** | **M** | **L** | **V** | **D** | **G** | **H** | **G** | **N** | **F** | **G** | **S** | **V** | **D** | **G** | **D** | **G** | **A** | **A** | **A** | **M** | **R** | **Y** | **T** | **E** | **A** | **R** | **M** | **S** | **K** | **I** | **A** | **L** | **E** | **M** | **L** | **R** | **D** | **I** | **N** | **K** | **N** | **T** | **V** | **D** | **F** | **Q** | **G** | **N** | **Y** | **D** | **D** | **S** | **E** | **Q** | **E** | **P** | **V** | **V** | **L** | **P** | **A** | **R** | **F** | **P** | **N** | **L** | **L** | **V** | **N** | **G** | **T** | **T** | **G** | **I** | **A** | **V** | **G** | **M** | **A** | **T** | **N** | **I** | **P** | **P** | **H** | **N** | **L** | **S** | **E** | **V** | **I** | **D** | **A** | **T** | **S** | **L** | **L** | **M** | **D** | **N** | **P** | **D** | **V** | **T** | **T** | **N** | **E** | **L** | **M** | **E** | **V** | **L** | **P** | **G** | **P** | **D** | **F** | **P** | **T** | **G** | **G** | **L** | **V** | **M** | **G** | **K** | **S** | **G** | **I** | **R** | **R** | **A** | **Y** | **E** | **T** | **G** | **K** | **G** | **S** | **I** | **T** | **V** | **R** | **A** | **K** | **V** | **E** | **L** | **T** | **E** | **M** | **P** | **N** | **G** | **K** | **E** | **R** | **I** | **L** | **V** | **T** | **E** | **L** | **P** | **Y** | **M** | **V** | **N** | **K** | **A** | **K** | **L** | **I** | **E** | **R** | **I** | **S** | **E** | **L** | **H** | **R** | **D** | **K** | **R** | **I** | **E** | **G** | **I** | **T** | **D** | **L** | **R** | **D** | **E** | **S** | **S** | **R** | **E** | **G** | **M** | **R** | **I** | **V** | **I** | **D** | **V** | **R** | **R** | **D** | **V** | **S** | **A** | **S** | **V** | **V** | **L** | **N** | **N** | **L** | **Y** | **K** | **M** | **T** | **A** | **L** | **Q** | **T** | **S** | **F** | **G** | **F** | **N** | **M** | **L** | **A** | **I** | **E** | **K** | **G** | **V** | **P** | **K** | **I** | **L** | **S** | **L** | **K** | **R** | **I** | **L** | **E** | **N** | **Y** | **V** | **E** | **H** | **Q** | **K** | **E** | **V** | **I** | **T** | **R** | **R** | **T** | **I** | **F** | **D** | **K** | **N** | **K** | **A** | **E** | **A** | **R** | **A** | **H** | **I** | **L** | **E** | **G** | **L** | **R** | **I** | **A** | **L** | **D** | **H** | **I** | **D** | **E** | **I** | **I** | **A** | **I** | **I** | **R** | **G** | **S** | **Q** | **S** | **D** | **D** | **E** | **A** | **K** | **A** | **T** | **L** | **I** | **E** | **R** | **F** | **E** | **F** | **S** | **D** | **R** | **Q** | **A** | **Q** | **A** | **I** | **L** | **D** | **M** | **R** | **L** | **R** | **R** | **L** | **T** | **G** | **L** | **E** | **R** | **D** | **K** | **I** | **E** | **N** | **E** | **Y** | **Q** | **E** | **L** | **L** | **K** | **F** | **I** | **A** | **D** | **L** | **E** | **D** | **I** | **L** | **A** | **R** | **P** | **E** | **R** | **V** | **I** | **E** | **I** | **I** | **K** | **T** | **E** | **L** | **N** | **D** | **V** | **R** | **T** | **K** | **F** | **G** | **D** | **A** | **R** | **R** | **T** | **E** | **L** | **L** | **V** | **G** | **E** | **V** | **L** | **S** | **L** | **E** | **D** | **E** | **D** | **L** | **I** | **E** | **E** | **E** | **E** | **V** | **V** | **I** | **T** | **L** | **T** | **N** | **N** | **G** | **Y** | **I** | **K** | **R** | **M** | **A** | **N** | **S** | **E** | **F** | **R** | **A** | **Q** | **R** | **R** | **G** | **G** | **R** | **G** | **V** | **Q** | **G** | **M** | **G** | **V** | **H** | **D** | **D** | **D** | **F** | **V** | **K** | **N** | **L** | **V** | **S** | **C** | **S** | **T** | **H** | **D** | **T** | **L** | **L** | **F** | **F** | **T** | **N** | **T** | **G** | **K** | **V** | **Y** | **R** | **A** | **K** | **G** | **Y** | **E** | **I** | **P** | **E** | **Y** | **G** | **R** | **T** | **A** | **K** | **G** | **I** | **P** | **V** | **I** | **N** | **L** | **L** | **G** | **I** | **D** | **S** | **A** | **E** | **K** | **I** | **Q** | **A** | **I** | **I** | **S** | **V** | **E** | **G** | **K** | **A** | **E** | **A** | **G** | **K** | **Y** | **L** | **F** | **F** | **T** | **T** | **L** | **K** | **G** | **T** | **V** | **K** | **R** | **T** | **A** | **V** | **T** | **A** | **F** | **S** | **N** | **I** | **R** | **S** | **N** | **G** | **L** | **I** | **A** | **I** | **S** | **L** | **K** | **E** | **D** | **D** | **E** | **L** | **V** | **N** | **V** | **V** | **T** | **T** | **N** | **G** | **N** | **Q** | **K** | **M** | **I** | **I** | **G** | **T** | **H** | **A** | **G** | **Y** | **S** | **V** | **T** | **F** | **D** | **E** | **N** | **T** | **V** | **R** | **D** | **M** | **G** | **R** | **T** | **A** | **S** | **G** | **V** | **R** | **G** | **I** | **R** | **L** | **R** | **E** | **N** | **D** | **Y** | **V** | **V** | **G** | **A** | **A** | **I** | **L** | **D** | **E** | **N** | **K** | **E** | **V** | **L** | **V** | **I** | **T** | **E** | **N** | **G** | **Y** | **G** | **K** | **R** | **T** | **K** | **A** | **S** | **E** | **Y** | **P** | **V** | **K** | **G** | **R** | **G** | **G** | **K** | **G** | **I** | **K** | **T** | **A** | **N** | **I** | **T** | **E** | **K** | **N** | **G** | **P** | **L** | **A** | **G** | **L** | **T** | **T** | **V** | **N** | **G** | **D** | **E** | **D** | **I** | **L** | **L** | **I** | **T** | **N** | **K** | **G** | **V** | **I** | **I** | **R** | **F** | **N** | **V** | **D** | **S** | **V** | **S** | **Q** | **T** | **G** | **R** | **A** | **T** | **L** | **G** | **V** | **R** | **L** | **M** | **R** | **M** | **E** | **D** | **G** | **A** | **K** | **-** | **-** | **-** | **-** | **-** | **-** | **-** | **-** | **-** | **-** | **-** | **-** | **-** | **-** | **-** | **-** | **-** | **-** | **-** | **-** | **-** | **-** | **-** |
| **N59862F** | **M** | **S** | **E** | **E** | **I** | **K** | **E** | **N** | **I** | **Q** | **D** | **V** | **N** | **L** | **T** | **S** | **E** | **M** | **K** | **E** | **S** | **F** | **I** | **D** | **Y** | **A** | **M** | **S** | **V** | **I** | **V** | **A** | **R** | **A** | **L** | **P** | **D** | **V** | **R** | **D** | **G** | **L** | **K** | **P** | **V** | **H** | **R** | **R** | **I** | **L** | **Y** | **G** | **M** | **N** | **E** | **L** | **G** | **V** | **T** | **P** | **D** | **K** | **P** | **H** | **K** | **K** | **S** | **A** | **R** | **I** | **V** | **G** | **D** | **V** | **M** | **G** | **K** | **Y** | **H** | **P** | **H** | **G** | **D** | **S** | **A** | **I** | **Y** | **E** | **S** | **M** | **V** | **R** | **M** | **A** | **Q** | **P** | **F** | **S** | **Y** | **R** | **A** | **M** | **L** | **V** | **D** | **G** | **H** | **G** | **N** | **F** | **G** | **S** | **V** | **D** | **G** | **D** | **G** | **A** | **A** | **A** | **M** | **R** | **Y** | **T** | **E** | **A** | **R** | **M** | **S** | **K** | **I** | **A** | **L** | **E** | **M** | **L** | **R** | **D** | **I** | **N** | **K** | **N** | **T** | **V** | **D** | **F** | **Q** | **G** | **N** | **Y** | **D** | **D** | **S** | **E** | **Q** | **E** | **P** | **V** | **V** | **L** | **P** | **A** | **R** | **F** | **P** | **N** | **L** | **L** | **V** | **N** | **G** | **T** | **T** | **G** | **I** | **A** | **V** | **G** | **M** | **A** | **T** | **N** | **I** | **P** | **P** | **H** | **N** | **L** | **S** | **E** | **V** | **I** | **D** | **A** | **T** | **S** | **L** | **L** | **M** | **D** | **N** | **P** | **D** | **V** | **T** | **T** | **N** | **E** | **L** | **M** | **E** | **V** | **L** | **P** | **G** | **P** | **D** | **F** | **P** | **T** | **G** | **G** | **L** | **V** | **M** | **G** | **K** | **S** | **G** | **I** | **R** | **R** | **A** | **Y** | **E** | **T** | **G** | **K** | **G** | **S** | **I** | **T** | **V** | **R** | **A** | **K** | **V** | **E** | **L** | **T** | **E** | **M** | **P** | **N** | **G** | **K** | **E** | **R** | **I** | **L** | **V** | **T** | **E** | **L** | **P** | **Y** | **M** | **V** | **N** | **K** | **A** | **K** | **L** | **I** | **E** | **R** | **I** | **S** | **E** | **L** | **H** | **R** | **D** | **K** | **R** | **I** | **E** | **G** | **I** | **T** | **D** | **L** | **R** | **D** | **E** | **S** | **S** | **R** | **E** | **G** | **M** | **R** | **I** | **V** | **I** | **D** | **V** | **R** | **R** | **D** | **V** | **S** | **A** | **S** | **V** | **V** | **L** | **N** | **N** | **L** | **Y** | **K** | **M** | **T** | **A** | **L** | **Q** | **T** | **S** | **F** | **G** | **F** | **N** | **M** | **L** | **A** | **I** | **E** | **K** | **G** | **V** | **P** | **K** | **I** | **L** | **S** | **L** | **K** | **R** | **I** | **L** | **E** | **N** | **Y** | **V** | **E** | **H** | **Q** | **K** | **E** | **V** | **I** | **T** | **R** | **R** | **T** | **I** | **F** | **D** | **K** | **N** | **K** | **A** | **E** | **A** | **R** | **A** | **H** | **I** | **L** | **E** | **G** | **L** | **R** | **I** | **A** | **L** | **D** | **H** | **I** | **D** | **E** | **I** | **I** | **A** | **I** | **I** | **R** | **G** | **S** | **Q** | **S** | **D** | **D** | **E** | **A** | **K** | **A** | **T** | **L** | **I** | **E** | **R** | **F** | **E** | **F** | **S** | **D** | **R** | **Q** | **A** | **Q** | **A** | **I** | **L** | **D** | **M** | **R** | **L** | **R** | **R** | **L** | **T** | **G** | **L** | **E** | **R** | **D** | **K** | **I** | **E** | **N** | **E** | **Y** | **Q** | **E** | **L** | **L** | **K** | **F** | **I** | **A** | **D** | **L** | **E** | **D** | **I** | **L** | **A** | **R** | **P** | **E** | **R** | **V** | **I** | **E** | **I** | **I** | **K** | **T** | **E** | **L** | **N** | **D** | **V** | **R** | **T** | **K** | **F** | **G** | **D** | **A** | **R** | **R** | **T** | **E** | **L** | **L** | **V** | **G** | **E** | **V** | **L** | **S** | **L** | **E** | **D** | **E** | **D** | **L** | **I** | **E** | **E** | **E** | **E** | **V** | **V** | **I** | **T** | **L** | **T** | **N** | **N** | **G** | **Y** | **I** | **K** | **R** | **M** | **A** | **N** | **S** | **E** | **F** | **R** | **A** | **Q** | **R** | **R** | **G** | **G** | **R** | **G** | **V** | **Q** | **G** | **M** | **G** | **V** | **H** | **D** | **D** | **D** | **F** | **V** | **K** | **N** | **L** | **V** | **S** | **C** | **S** | **T** | **H** | **D** | **T** | **L** | **L** | **F** | **F** | **T** | **N** | **T** | **G** | **K** | **V** | **Y** | **R** | **A** | **K** | **G** | **Y** | **E** | **I** | **P** | **E** | **Y** | **G** | **R** | **T** | **A** | **K** | **G** | **I** | **P** | **V** | **I** | **N** | **L** | **L** | **G** | **I** | **D** | **S** | **A** | **E** | **K** | **I** | **Q** | **A** | **I** | **I** | **S** | **V** | **E** | **G** | **K** | **A** | **E** | **A** | **G** | **K** | **Y** | **L** | **F** | **F** | **T** | **T** | **L** | **K** | **G** | **T** | **V** | **K** | **R** | **T** | **A** | **V** | **T** | **A** | **F** | **S** | **N** | **I** | **R** | **S** | **N** | **G** | **L** | **I** | **A** | **I** | **S** | **L** | **K** | **E** | **D** | **D** | **E** | **L** | **V** | **N** | **V** | **V** | **T** | **T** | **N** | **G** | **N** | **Q** | **K** | **M** | **I** | **I** | **G** | **T** | **H** | **A** | **G** | **Y** | **S** | **V** | **T** | **F** | **D** | **E** | **N** | **T** | **V** | **R** | **D** | **M** | **G** | **R** | **T** | **A** | **S** | **G** | **V** | **R** | **G** | **I** | **R** | **L** | **R** | **E** | **N** | **D** | **Y** | **V** | **V** | **G** | **A** | **A** | **I** | **L** | **D** | **E** | **N** | **K** | **E** | **V** | **L** | **V** | **I** | **T** | **E** | **N** | **G** | **Y** | **G** | **K** | **R** | **T** | **K** | **A** | **S** | **E** | **Y** | **P** | **V** | **K** | **G** | **R** | **G** | **G** | **K** | **G** | **I** | **K** | **T** | **A** | **N** | **I** | **T** | **E** | **K** | **N** | **G** | **P** | **L** | **A** | **G** | **L** | **T** | **T** | **V** | **N** | **G** | **D** | **E** | **D** | **I** | **L** | **L** | **I** | **T** | **N** | **K** | **G** | **V** | **I** | **I** | **R** | **F** | **N** | **V** | **D** | **S** | **V** | **S** | **Q** | **T** | **G** | **R** | **A** | **T** | **L** | **G** | **V** | **R** | **L** | **M** | **R** | **M** | **E** | **D** | **G** | **A** | **K** | **-** | **-** | **-** | **-** | **-** | **-** | **-** | **-** | **-** | **-** | **-** | **-** | **-** | **-** | **-** | **-** | **-** | **-** | **-** | **-** | **-** | **-** | **-** |
| **N59455F** | **M** | **S** | **E** | **E** | **I** | **K** | **E** | **N** | **I** | **Q** | **D** | **V** | **N** | **L** | **T** | **S** | **E** | **M** | **K** | **E** | **S** | **F** | **I** | **D** | **Y** | **A** | **M** | **S** | **V** | **I** | **V** | **A** | **R** | **A** | **L** | **P** | **D** | **V** | **R** | **D** | **G** | **L** | **K** | **P** | **V** | **H** | **R** | **R** | **I** | **L** | **Y** | **G** | **M** | **N** | **E** | **L** | **G** | **V** | **T** | **P** | **D** | **K** | **P** | **H** | **K** | **K** | **S** | **A** | **R** | **I** | **V** | **G** | **D** | **V** | **M** | **G** | **K** | **Y** | **H** | **P** | **H** | **G** | **D** | **S** | **A** | **I** | **Y** | **E** | **S** | **M** | **V** | **R** | **M** | **A** | **Q** | **P** | **F** | **S** | **Y** | **R** | **A** | **M** | **L** | **V** | **D** | **G** | **H** | **G** | **N** | **F** | **G** | **S** | **V** | **D** | **G** | **D** | **G** | **A** | **A** | **A** | **M** | **R** | **Y** | **T** | **E** | **A** | **R** | **M** | **S** | **K** | **I** | **A** | **L** | **E** | **M** | **L** | **R** | **D** | **I** | **N** | **K** | **N** | **T** | **V** | **D** | **F** | **Q** | **G** | **N** | **Y** | **D** | **D** | **S** | **E** | **Q** | **E** | **P** | **V** | **V** | **L** | **P** | **A** | **R** | **F** | **P** | **N** | **L** | **L** | **V** | **N** | **G** | **T** | **T** | **G** | **I** | **A** | **V** | **G** | **M** | **A** | **T** | **N** | **I** | **P** | **P** | **H** | **N** | **L** | **S** | **E** | **V** | **I** | **D** | **A** | **T** | **S** | **L** | **L** | **M** | **D** | **N** | **P** | **D** | **V** | **T** | **T** | **N** | **E** | **L** | **M** | **E** | **V** | **L** | **P** | **G** | **P** | **D** | **F** | **P** | **T** | **G** | **G** | **L** | **V** | **M** | **G** | **K** | **S** | **G** | **I** | **R** | **R** | **A** | **Y** | **E** | **T** | **G** | **K** | **G** | **S** | **I** | **T** | **V** | **R** | **A** | **K** | **V** | **E** | **L** | **T** | **E** | **M** | **P** | **N** | **G** | **K** | **E** | **R** | **I** | **L** | **V** | **T** | **E** | **L** | **P** | **Y** | **M** | **V** | **N** | **K** | **A** | **K** | **L** | **I** | **E** | **R** | **I** | **S** | **E** | **L** | **H** | **R** | **D** | **K** | **R** | **I** | **E** | **G** | **I** | **T** | **D** | **L** | **R** | **D** | **E** | **S** | **S** | **R** | **E** | **G** | **M** | **R** | **I** | **V** | **I** | **D** | **V** | **R** | **R** | **D** | **V** | **S** | **A** | **S** | **V** | **V** | **L** | **N** | **N** | **L** | **Y** | **K** | **M** | **T** | **A** | **L** | **Q** | **T** | **S** | **F** | **G** | **F** | **N** | **M** | **L** | **A** | **I** | **E** | **K** | **G** | **V** | **P** | **K** | **I** | **L** | **S** | **L** | **K** | **R** | **I** | **L** | **E** | **N** | **Y** | **V** | **E** | **H** | **Q** | **K** | **E** | **V** | **I** | **T** | **R** | **R** | **T** | **I** | **F** | **D** | **K** | **N** | **K** | **A** | **E** | **A** | **R** | **A** | **H** | **I** | **L** | **E** | **G** | **L** | **R** | **I** | **A** | **L** | **D** | **H** | **I** | **D** | **E** | **I** | **I** | **A** | **I** | **I** | **R** | **G** | **S** | **Q** | **S** | **D** | **D** | **E** | **A** | **K** | **A** | **T** | **L** | **I** | **E** | **R** | **F** | **E** | **F** | **S** | **D** | **R** | **Q** | **A** | **Q** | **A** | **I** | **L** | **D** | **M** | **R** | **L** | **R** | **R** | **L** | **T** | **G** | **L** | **E** | **R** | **D** | **K** | **I** | **E** | **N** | **E** | **Y** | **Q** | **E** | **L** | **L** | **K** | **F** | **I** | **A** | **D** | **L** | **E** | **D** | **I** | **L** | **A** | **R** | **P** | **E** | **R** | **V** | **I** | **E** | **I** | **I** | **K** | **T** | **E** | **L** | **N** | **D** | **V** | **R** | **T** | **K** | **F** | **G** | **D** | **A** | **R** | **R** | **T** | **E** | **L** | **L** | **V** | **G** | **E** | **V** | **L** | **S** | **L** | **E** | **D** | **E** | **D** | **L** | **I** | **E** | **E** | **E** | **E** | **V** | **V** | **I** | **T** | **L** | **T** | **N** | **N** | **G** | **Y** | **I** | **K** | **R** | **M** | **A** | **N** | **S** | **E** | **F** | **R** | **A** | **Q** | **R** | **R** | **G** | **G** | **R** | **G** | **V** | **Q** | **G** | **M** | **G** | **V** | **H** | **D** | **D** | **D** | **F** | **V** | **K** | **K** | **L** | **V** | **S** | **C** | **S** | **T** | **H** | **D** | **T** | **L** | **L** | **F** | **F** | **T** | **N** | **T** | **G** | **K** | **V** | **Y** | **R** | **A** | **K** | **G** | **Y** | **E** | **I** | **P** | **E** | **Y** | **G** | **R** | **T** | **A** | **K** | **G** | **I** | **P** | **V** | **I** | **N** | **L** | **L** | **G** | **I** | **D** | **S** | **A** | **E** | **K** | **I** | **Q** | **A** | **I** | **I** | **S** | **V** | **E** | **G** | **K** | **A** | **E** | **A** | **G** | **K** | **Y** | **L** | **F** | **F** | **T** | **T** | **L** | **K** | **G** | **T** | **V** | **K** | **R** | **T** | **A** | **V** | **T** | **A** | **F** | **S** | **N** | **I** | **R** | **S** | **N** | **G** | **L** | **I** | **A** | **I** | **S** | **L** | **K** | **E** | **D** | **D** | **E** | **L** | **V** | **N** | **V** | **V** | **T** | **T** | **N** | **G** | **N** | **Q** | **K** | **M** | **I** | **I** | **G** | **T** | **H** | **A** | **G** | **Y** | **S** | **V** | **T** | **F** | **D** | **E** | **N** | **T** | **V** | **R** | **D** | **M** | **G** | **R** | **T** | **A** | **S** | **G** | **V** | **R** | **G** | **I** | **R** | **L** | **R** | **E** | **N** | **D** | **Y** | **V** | **V** | **G** | **A** | **A** | **I** | **L** | **D** | **E** | **N** | **K** | **E** | **V** | **L** | **V** | **I** | **T** | **E** | **N** | **G** | **Y** | **G** | **K** | **R** | **T** | **K** | **A** | **S** | **E** | **Y** | **P** | **V** | **K** | **G** | **R** | **G** | **G** | **K** | **G** | **I** | **K** | **T** | **A** | **N** | **I** | **T** | **E** | **K** | **N** | **G** | **P** | **L** | **A** | **G** | **L** | **T** | **T** | **V** | **N** | **G** | **D** | **E** | **D** | **I** | **L** | **L** | **I** | **T** | **N** | **K** | **G** | **V** | **I** | **I** | **R** | **F** | **N** | **V** | **D** | **S** | **V** | **S** | **Q** | **T** | **G** | **R** | **A** | **T** | **L** | **G** | **V** | **R** | **L** | **M** | **R** | **M** | **E** | **D** | **G** | **A** | **K** | **-** | **-** | **-** | **-** | **-** | **-** | **-** | **-** | **-** | **-** | **-** | **-** | **-** | **-** | **-** | **-** | **-** | **-** | **-** | **-** | **-** | **-** | **-** |
| **N53405** | **M** | **S** | **E** | **E** | **I** | **K** | **E** | **N** | **I** | **Q** | **D** | **V** | **N** | **L** | **T** | **S** | **E** | **M** | **K** | **E** | **S** | **F** | **I** | **D** | **Y** | **A** | **M** | **S** | **V** | **I** | **V** | **A** | **R** | **A** | **L** | **P** | **D** | **V** | **R** | **D** | **G** | **L** | **K** | **P** | **V** | **H** | **R** | **R** | **I** | **L** | **Y** | **G** | **M** | **N** | **E** | **L** | **G** | **V** | **T** | **P** | **D** | **K** | **P** | **H** | **K** | **K** | **S** | **A** | **R** | **I** | **V** | **G** | **D** | **V** | **M** | **G** | **K** | **Y** | **H** | **P** | **H** | **G** | **D** | **S** | **A** | **I** | **Y** | **E** | **S** | **M** | **V** | **R** | **M** | **A** | **Q** | **P** | **F** | **S** | **Y** | **R** | **A** | **M** | **L** | **V** | **D** | **G** | **H** | **G** | **N** | **F** | **G** | **S** | **V** | **D** | **G** | **D** | **G** | **A** | **A** | **A** | **M** | **R** | **Y** | **T** | **E** | **A** | **R** | **M** | **S** | **K** | **I** | **A** | **L** | **E** | **M** | **L** | **R** | **D** | **I** | **N** | **K** | **N** | **T** | **V** | **D** | **F** | **Q** | **G** | **N** | **Y** | **D** | **D** | **S** | **E** | **Q** | **E** | **P** | **V** | **V** | **L** | **P** | **A** | **R** | **F** | **P** | **N** | **L** | **L** | **V** | **N** | **G** | **T** | **T** | **G** | **I** | **A** | **V** | **G** | **M** | **A** | **T** | **N** | **I** | **P** | **P** | **H** | **N** | **L** | **S** | **E** | **V** | **I** | **D** | **A** | **T** | **S** | **L** | **L** | **M** | **D** | **N** | **P** | **D** | **V** | **T** | **T** | **N** | **E** | **L** | **M** | **E** | **V** | **L** | **P** | **G** | **P** | **D** | **F** | **P** | **T** | **G** | **G** | **L** | **V** | **M** | **G** | **K** | **S** | **G** | **I** | **R** | **R** | **A** | **Y** | **E** | **T** | **G** | **K** | **G** | **S** | **I** | **T** | **V** | **R** | **A** | **K** | **V** | **E** | **L** | **T** | **E** | **M** | **P** | **N** | **G** | **K** | **E** | **R** | **I** | **L** | **V** | **T** | **E** | **L** | **P** | **Y** | **M** | **V** | **N** | **K** | **A** | **K** | **L** | **I** | **E** | **R** | **I** | **S** | **E** | **L** | **H** | **R** | **D** | **K** | **R** | **I** | **E** | **G** | **I** | **T** | **D** | **L** | **R** | **D** | **E** | **S** | **S** | **R** | **E** | **G** | **M** | **R** | **I** | **V** | **I** | **D** | **V** | **R** | **R** | **D** | **V** | **S** | **A** | **S** | **V** | **V** | **L** | **N** | **N** | **L** | **Y** | **K** | **M** | **T** | **A** | **L** | **Q** | **T** | **S** | **F** | **G** | **F** | **N** | **M** | **L** | **A** | **I** | **E** | **K** | **G** | **V** | **P** | **K** | **I** | **L** | **S** | **L** | **K** | **R** | **I** | **L** | **E** | **N** | **Y** | **V** | **E** | **H** | **Q** | **K** | **E** | **V** | **I** | **T** | **R** | **R** | **T** | **I** | **F** | **D** | **K** | **N** | **K** | **A** | **E** | **A** | **R** | **A** | **H** | **I** | **L** | **E** | **G** | **L** | **R** | **I** | **A** | **L** | **D** | **H** | **I** | **D** | **E** | **I** | **I** | **A** | **I** | **I** | **R** | **G** | **S** | **Q** | **S** | **D** | **D** | **E** | **A** | **K** | **A** | **T** | **L** | **I** | **E** | **R** | **F** | **E** | **F** | **S** | **D** | **R** | **Q** | **A** | **Q** | **A** | **I** | **L** | **D** | **M** | **R** | **L** | **R** | **R** | **L** | **T** | **G** | **L** | **E** | **R** | **D** | **K** | **I** | **E** | **N** | **E** | **Y** | **Q** | **E** | **L** | **L** | **K** | **F** | **I** | **A** | **D** | **L** | **E** | **D** | **I** | **L** | **A** | **R** | **P** | **E** | **R** | **V** | **I** | **E** | **I** | **I** | **K** | **T** | **E** | **L** | **N** | **D** | **V** | **R** | **T** | **K** | **F** | **G** | **D** | **A** | **R** | **R** | **T** | **E** | **L** | **L** | **V** | **G** | **E** | **V** | **L** | **S** | **L** | **E** | **D** | **E** | **D** | **L** | **I** | **E** | **E** | **E** | **E** | **V** | **V** | **I** | **T** | **L** | **T** | **N** | **N** | **G** | **Y** | **I** | **K** | **R** | **M** | **A** | **N** | **S** | **E** | **F** | **R** | **A** | **Q** | **R** | **R** | **G** | **G** | **R** | **G** | **V** | **Q** | **G** | **M** | **G** | **V** | **H** | **D** | **D** | **D** | **F** | **V** | **K** | **N** | **L** | **V** | **S** | **C** | **S** | **T** | **H** | **D** | **T** | **L** | **L** | **F** | **F** | **T** | **N** | **T** | **G** | **K** | **V** | **Y** | **R** | **A** | **K** | **G** | **Y** | **E** | **I** | **P** | **E** | **Y** | **G** | **R** | **T** | **A** | **K** | **G** | **I** | **P** | **V** | **I** | **N** | **L** | **L** | **G** | **I** | **D** | **S** | **A** | **E** | **K** | **I** | **Q** | **A** | **I** | **I** | **S** | **V** | **E** | **G** | **K** | **A** | **E** | **A** | **G** | **K** | **Y** | **L** | **F** | **F** | **T** | **T** | **L** | **K** | **G** | **T** | **V** | **K** | **R** | **T** | **A** | **V** | **T** | **A** | **F** | **S** | **N** | **I** | **R** | **S** | **N** | **G** | **L** | **I** | **A** | **I** | **S** | **L** | **K** | **E** | **D** | **D** | **E** | **L** | **V** | **N** | **V** | **V** | **T** | **T** | **N** | **G** | **N** | **Q** | **K** | **M** | **I** | **I** | **G** | **T** | **H** | **A** | **G** | **Y** | **S** | **V** | **T** | **F** | **D** | **E** | **N** | **T** | **V** | **R** | **D** | **M** | **G** | **R** | **T** | **A** | **S** | **G** | **V** | **R** | **G** | **I** | **R** | **L** | **R** | **E** | **N** | **D** | **Y** | **V** | **V** | **G** | **A** | **A** | **I** | **L** | **D** | **E** | **N** | **K** | **E** | **V** | **L** | **V** | **I** | **T** | **E** | **N** | **G** | **Y** | **G** | **K** | **R** | **T** | **K** | **A** | **S** | **E** | **Y** | **P** | **V** | **K** | **G** | **R** | **G** | **G** | **K** | **G** | **I** | **K** | **T** | **A** | **N** | **I** | **T** | **E** | **K** | **N** | **G** | **P** | **L** | **A** | **G** | **L** | **T** | **T** | **V** | **N** | **G** | **D** | **E** | **D** | **I** | **L** | **L** | **I** | **T** | **N** | **K** | **G** | **V** | **I** | **I** | **R** | **F** | **N** | **V** | **D** | **S** | **V** | **S** | **Q** | **T** | **G** | **R** | **A** | **T** | **L** | **G** | **V** | **R** | **L** | **M** | **R** | **M** | **E** | **D** | **G** | **A** | **K** | **-** | **-** | **-** | **-** | **-** | **-** | **-** | **-** | **-** | **-** | **-** | **-** | **-** | **-** | **-** | **-** | **-** | **-** | **-** | **-** | **-** | **-** | **-** |
| **N54548** | **M** | **S** | **E** | **E** | **I** | **K** | **E** | **N** | **I** | **Q** | **D** | **V** | **N** | **L** | **T** | **S** | **E** | **M** | **K** | **E** | **S** | **F** | **I** | **D** | **Y** | **A** | **M** | **S** | **V** | **I** | **V** | **A** | **R** | **A** | **L** | **P** | **D** | **V** | **R** | **D** | **G** | **L** | **K** | **P** | **V** | **H** | **R** | **R** | **I** | **L** | **Y** | **G** | **M** | **N** | **E** | **L** | **G** | **V** | **T** | **P** | **D** | **K** | **P** | **H** | **K** | **K** | **S** | **A** | **R** | **I** | **V** | **G** | **D** | **V** | **M** | **G** | **K** | **Y** | **H** | **P** | **H** | **G** | **D** | **S** | **A** | **I** | **Y** | **E** | **S** | **M** | **V** | **R** | **M** | **A** | **Q** | **P** | **F** | **S** | **Y** | **R** | **A** | **M** | **L** | **V** | **D** | **G** | **H** | **G** | **N** | **F** | **G** | **S** | **V** | **D** | **G** | **D** | **G** | **A** | **A** | **A** | **M** | **R** | **Y** | **T** | **E** | **A** | **R** | **M** | **S** | **K** | **I** | **A** | **L** | **E** | **M** | **L** | **R** | **D** | **I** | **N** | **K** | **N** | **T** | **V** | **D** | **F** | **Q** | **G** | **N** | **Y** | **D** | **D** | **S** | **E** | **Q** | **E** | **P** | **V** | **V** | **L** | **P** | **A** | **R** | **F** | **P** | **N** | **L** | **L** | **V** | **N** | **G** | **T** | **T** | **G** | **I** | **A** | **V** | **G** | **M** | **A** | **T** | **N** | **I** | **P** | **P** | **H** | **N** | **L** | **S** | **E** | **V** | **I** | **D** | **A** | **T** | **S** | **L** | **L** | **M** | **D** | **N** | **P** | **D** | **V** | **T** | **T** | **N** | **E** | **L** | **M** | **E** | **V** | **L** | **P** | **G** | **P** | **D** | **F** | **P** | **T** | **G** | **G** | **L** | **V** | **M** | **G** | **K** | **S** | **G** | **I** | **R** | **R** | **A** | **Y** | **E** | **T** | **G** | **K** | **G** | **S** | **I** | **T** | **V** | **R** | **A** | **K** | **V** | **E** | **L** | **T** | **E** | **M** | **P** | **N** | **G** | **K** | **E** | **R** | **I** | **L** | **V** | **T** | **E** | **L** | **P** | **Y** | **M** | **V** | **N** | **K** | **A** | **K** | **L** | **I** | **E** | **R** | **I** | **S** | **E** | **L** | **H** | **R** | **D** | **K** | **R** | **I** | **E** | **G** | **I** | **T** | **D** | **L** | **R** | **D** | **E** | **S** | **S** | **R** | **E** | **G** | **M** | **R** | **I** | **V** | **I** | **D** | **V** | **R** | **R** | **D** | **V** | **S** | **A** | **S** | **V** | **V** | **L** | **N** | **N** | **L** | **Y** | **K** | **M** | **T** | **A** | **L** | **Q** | **T** | **S** | **F** | **G** | **F** | **N** | **M** | **L** | **A** | **I** | **E** | **K** | **G** | **V** | **P** | **K** | **I** | **L** | **S** | **L** | **K** | **R** | **I** | **L** | **E** | **N** | **Y** | **I** | **E** | **H** | **Q** | **K** | **E** | **V** | **I** | **T** | **R** | **R** | **T** | **I** | **F** | **D** | **K** | **N** | **K** | **A** | **E** | **A** | **R** | **A** | **H** | **I** | **L** | **E** | **G** | **L** | **R** | **I** | **A** | **L** | **D** | **H** | **I** | **D** | **E** | **I** | **I** | **A** | **I** | **I** | **R** | **G** | **S** | **Q** | **S** | **D** | **D** | **E** | **A** | **K** | **A** | **T** | **L** | **I** | **E** | **R** | **F** | **E** | **F** | **S** | **D** | **R** | **Q** | **A** | **Q** | **A** | **I** | **L** | **D** | **M** | **R** | **L** | **R** | **R** | **L** | **T** | **G** | **L** | **E** | **R** | **D** | **K** | **I** | **E** | **N** | **E** | **Y** | **Q** | **E** | **L** | **L** | **K** | **F** | **I** | **A** | **D** | **L** | **E** | **D** | **I** | **L** | **A** | **R** | **P** | **E** | **R** | **V** | **I** | **E** | **I** | **I** | **K** | **T** | **E** | **L** | **N** | **D** | **V** | **R** | **T** | **K** | **F** | **G** | **D** | **A** | **R** | **R** | **T** | **E** | **L** | **L** | **V** | **G** | **E** | **V** | **L** | **S** | **L** | **E** | **D** | **E** | **D** | **L** | **I** | **E** | **E** | **E** | **E** | **V** | **V** | **I** | **T** | **L** | **T** | **N** | **N** | **G** | **Y** | **I** | **K** | **R** | **M** | **A** | **N** | **S** | **E** | **F** | **R** | **A** | **Q** | **R** | **R** | **G** | **G** | **R** | **G** | **V** | **Q** | **G** | **M** | **G** | **V** | **H** | **D** | **D** | **D** | **F** | **V** | **K** | **N** | **L** | **V** | **S** | **C** | **S** | **T** | **H** | **D** | **T** | **L** | **L** | **F** | **F** | **T** | **N** | **T** | **G** | **K** | **V** | **Y** | **R** | **A** | **K** | **G** | **Y** | **E** | **I** | **P** | **E** | **Y** | **G** | **R** | **T** | **A** | **K** | **G** | **I** | **P** | **V** | **I** | **N** | **L** | **L** | **G** | **I** | **D** | **S** | **A** | **E** | **K** | **I** | **Q** | **A** | **I** | **I** | **S** | **V** | **E** | **G** | **K** | **A** | **E** | **A** | **G** | **K** | **Y** | **L** | **F** | **F** | **T** | **T** | **L** | **K** | **G** | **T** | **V** | **K** | **R** | **T** | **A** | **V** | **T** | **A** | **F** | **S** | **N** | **I** | **R** | **S** | **N** | **G** | **L** | **I** | **A** | **I** | **S** | **L** | **K** | **E** | **D** | **D** | **E** | **L** | **V** | **N** | **V** | **V** | **T** | **T** | **N** | **G** | **N** | **Q** | **K** | **M** | **I** | **I** | **G** | **T** | **H** | **A** | **G** | **Y** | **S** | **V** | **T** | **F** | **D** | **E** | **T** | **T** | **V** | **R** | **D** | **M** | **G** | **R** | **T** | **A** | **S** | **G** | **V** | **R** | **G** | **I** | **R** | **L** | **R** | **E** | **N** | **D** | **Y** | **V** | **V** | **G** | **A** | **A** | **I** | **L** | **D** | **E** | **N** | **K** | **E** | **V** | **L** | **V** | **I** | **T** | **E** | **N** | **G** | **Y** | **G** | **K** | **R** | **T** | **K** | **A** | **S** | **E** | **Y** | **P** | **V** | **K** | **G** | **R** | **G** | **G** | **K** | **G** | **I** | **K** | **T** | **A** | **N** | **I** | **T** | **E** | **K** | **N** | **G** | **P** | **L** | **A** | **G** | **L** | **T** | **T** | **V** | **N** | **G** | **D** | **E** | **D** | **I** | **L** | **L** | **I** | **T** | **N** | **K** | **G** | **V** | **I** | **I** | **R** | **F** | **N** | **V** | **D** | **S** | **V** | **S** | **Q** | **T** | **G** | **R** | **A** | **T** | **L** | **G** | **V** | **R** | **L** | **M** | **R** | **M** | **E** | **D** | **G** | **A** | **K** | **-** | **-** | **-** | **-** | **-** | **-** | **-** | **-** | **-** | **-** | **-** | **-** | **-** | **-** | **-** | **-** | **-** | **-** | **-** | **-** | **-** | **-** | **-** |
| **N52587** | **M** | **S** | **E** | **E** | **I** | **K** | **E** | **N** | **I** | **Q** | **D** | **V** | **N** | **L** | **T** | **S** | **E** | **M** | **K** | **E** | **S** | **F** | **I** | **D** | **Y** | **A** | **M** | **S** | **V** | **I** | **V** | **A** | **R** | **A** | **L** | **P** | **D** | **V** | **R** | **D** | **G** | **L** | **K** | **P** | **V** | **H** | **R** | **R** | **I** | **L** | **Y** | **G** | **M** | **N** | **E** | **L** | **G** | **V** | **T** | **P** | **D** | **K** | **P** | **H** | **K** | **K** | **S** | **A** | **R** | **I** | **V** | **G** | **D** | **V** | **M** | **G** | **K** | **Y** | **H** | **P** | **H** | **G** | **D** | **S** | **A** | **I** | **Y** | **E** | **S** | **M** | **V** | **R** | **M** | **A** | **Q** | **P** | **F** | **S** | **Y** | **R** | **A** | **M** | **L** | **V** | **D** | **G** | **H** | **G** | **N** | **F** | **G** | **S** | **V** | **D** | **G** | **D** | **G** | **A** | **A** | **A** | **M** | **R** | **Y** | **T** | **E** | **A** | **R** | **M** | **S** | **K** | **I** | **A** | **L** | **E** | **M** | **L** | **R** | **D** | **I** | **N** | **K** | **N** | **T** | **V** | **D** | **F** | **Q** | **G** | **N** | **Y** | **D** | **D** | **S** | **E** | **Q** | **E** | **P** | **V** | **V** | **L** | **P** | **A** | **R** | **F** | **P** | **N** | **L** | **L** | **V** | **N** | **G** | **T** | **T** | **G** | **I** | **A** | **V** | **G** | **M** | **A** | **T** | **N** | **I** | **P** | **P** | **H** | **N** | **L** | **S** | **E** | **V** | **I** | **D** | **A** | **T** | **S** | **L** | **L** | **M** | **D** | **N** | **P** | **D** | **V** | **T** | **T** | **N** | **E** | **L** | **M** | **E** | **V** | **L** | **P** | **G** | **P** | **D** | **F** | **P** | **T** | **G** | **G** | **L** | **V** | **M** | **G** | **K** | **S** | **G** | **I** | **R** | **R** | **A** | **Y** | **E** | **T** | **G** | **K** | **G** | **S** | **I** | **T** | **V** | **R** | **A** | **K** | **V** | **E** | **L** | **T** | **E** | **M** | **P** | **N** | **G** | **K** | **E** | **R** | **I** | **L** | **V** | **T** | **E** | **L** | **P** | **Y** | **M** | **V** | **N** | **K** | **A** | **K** | **L** | **I** | **E** | **R** | **I** | **S** | **E** | **L** | **H** | **R** | **D** | **K** | **R** | **I** | **E** | **G** | **I** | **T** | **D** | **L** | **R** | **D** | **E** | **S** | **S** | **R** | **E** | **G** | **M** | **R** | **I** | **V** | **I** | **D** | **V** | **R** | **R** | **D** | **V** | **S** | **A** | **S** | **V** | **V** | **L** | **N** | **N** | **L** | **Y** | **K** | **M** | **T** | **A** | **L** | **Q** | **T** | **S** | **F** | **G** | **F** | **N** | **M** | **L** | **A** | **I** | **E** | **K** | **G** | **V** | **P** | **K** | **I** | **L** | **S** | **L** | **K** | **R** | **I** | **L** | **E** | **N** | **Y** | **I** | **E** | **H** | **Q** | **K** | **E** | **V** | **I** | **T** | **R** | **R** | **T** | **I** | **F** | **D** | **K** | **N** | **K** | **A** | **E** | **A** | **R** | **A** | **H** | **I** | **L** | **E** | **G** | **L** | **R** | **I** | **A** | **L** | **D** | **H** | **I** | **D** | **E** | **I** | **I** | **A** | **I** | **I** | **R** | **G** | **S** | **Q** | **S** | **D** | **D** | **E** | **A** | **K** | **A** | **T** | **L** | **I** | **E** | **R** | **F** | **E** | **F** | **S** | **D** | **R** | **Q** | **A** | **Q** | **A** | **I** | **L** | **D** | **M** | **R** | **L** | **R** | **R** | **L** | **T** | **G** | **L** | **E** | **R** | **D** | **K** | **I** | **E** | **N** | **E** | **Y** | **Q** | **E** | **L** | **L** | **K** | **F** | **I** | **A** | **D** | **L** | **E** | **D** | **I** | **L** | **A** | **R** | **P** | **E** | **R** | **V** | **I** | **E** | **I** | **I** | **K** | **T** | **E** | **L** | **N** | **D** | **V** | **R** | **T** | **K** | **F** | **G** | **D** | **A** | **R** | **R** | **T** | **E** | **L** | **L** | **V** | **G** | **E** | **V** | **L** | **S** | **L** | **E** | **D** | **E** | **D** | **L** | **I** | **E** | **E** | **E** | **E** | **V** | **V** | **I** | **T** | **L** | **T** | **N** | **N** | **G** | **Y** | **I** | **K** | **R** | **M** | **A** | **N** | **S** | **E** | **F** | **R** | **A** | **Q** | **R** | **R** | **G** | **G** | **R** | **G** | **V** | **Q** | **G** | **M** | **G** | **V** | **H** | **D** | **D** | **D** | **F** | **V** | **K** | **N** | **L** | **V** | **S** | **C** | **S** | **T** | **H** | **D** | **T** | **L** | **L** | **F** | **F** | **T** | **N** | **T** | **G** | **K** | **V** | **Y** | **R** | **A** | **K** | **G** | **Y** | **E** | **I** | **P** | **E** | **Y** | **G** | **R** | **T** | **A** | **K** | **G** | **I** | **P** | **V** | **I** | **N** | **L** | **L** | **G** | **I** | **D** | **S** | **A** | **E** | **K** | **I** | **Q** | **A** | **I** | **I** | **S** | **V** | **E** | **G** | **K** | **A** | **E** | **A** | **G** | **K** | **Y** | **L** | **F** | **F** | **T** | **T** | **L** | **K** | **G** | **T** | **V** | **K** | **R** | **T** | **A** | **V** | **T** | **A** | **F** | **S** | **N** | **I** | **R** | **S** | **N** | **G** | **L** | **I** | **A** | **I** | **S** | **L** | **K** | **E** | **D** | **D** | **E** | **L** | **V** | **N** | **V** | **V** | **T** | **T** | **N** | **G** | **N** | **Q** | **K** | **M** | **I** | **I** | **G** | **T** | **H** | **A** | **G** | **Y** | **S** | **V** | **T** | **F** | **D** | **E** | **T** | **T** | **V** | **R** | **D** | **M** | **G** | **R** | **T** | **A** | **S** | **G** | **V** | **R** | **G** | **I** | **R** | **L** | **R** | **E** | **N** | **D** | **Y** | **V** | **V** | **G** | **A** | **A** | **I** | **L** | **D** | **E** | **N** | **K** | **E** | **V** | **L** | **V** | **I** | **T** | **E** | **N** | **G** | **Y** | **G** | **K** | **R** | **T** | **K** | **A** | **S** | **E** | **Y** | **P** | **V** | **K** | **G** | **R** | **G** | **G** | **K** | **G** | **I** | **K** | **T** | **A** | **N** | **I** | **T** | **E** | **K** | **N** | **G** | **P** | **L** | **A** | **G** | **L** | **T** | **T** | **V** | **N** | **G** | **D** | **E** | **D** | **I** | **L** | **L** | **I** | **T** | **N** | **K** | **G** | **V** | **I** | **I** | **R** | **F** | **N** | **V** | **D** | **S** | **V** | **S** | **Q** | **T** | **G** | **R** | **A** | **T** | **L** | **G** | **V** | **R** | **L** | **M** | **R** | **M** | **E** | **D** | **G** | **A** | **K** | **-** | **-** | **-** | **-** | **-** | **-** | **-** | **-** | **-** | **-** | **-** | **-** | **-** | **-** | **-** | **-** | **-** | **-** | **-** | **-** | **-** | **-** | **-** |
| **N53476** | **M** | **S** | **E** | **E** | **I** | **K** | **E** | **N** | **I** | **Q** | **D** | **V** | **N** | **L** | **T** | **S** | **E** | **M** | **K** | **E** | **S** | **F** | **I** | **D** | **Y** | **A** | **M** | **S** | **V** | **I** | **V** | **A** | **R** | **A** | **L** | **P** | **D** | **V** | **R** | **D** | **G** | **L** | **K** | **P** | **V** | **H** | **R** | **R** | **I** | **L** | **Y** | **G** | **M** | **N** | **E** | **L** | **G** | **V** | **T** | **P** | **D** | **K** | **P** | **H** | **K** | **K** | **S** | **A** | **R** | **I** | **V** | **G** | **D** | **V** | **M** | **G** | **K** | **Y** | **H** | **P** | **H** | **G** | **D** | **S** | **A** | **I** | **Y** | **E** | **S** | **M** | **V** | **R** | **M** | **A** | **Q** | **P** | **F** | **S** | **Y** | **R** | **A** | **M** | **L** | **V** | **D** | **G** | **H** | **G** | **N** | **F** | **G** | **S** | **V** | **D** | **G** | **D** | **G** | **A** | **A** | **A** | **M** | **R** | **Y** | **T** | **E** | **A** | **R** | **M** | **S** | **K** | **I** | **A** | **L** | **E** | **M** | **L** | **R** | **D** | **I** | **N** | **K** | **N** | **T** | **V** | **D** | **F** | **Q** | **G** | **N** | **Y** | **D** | **D** | **S** | **E** | **Q** | **E** | **P** | **V** | **V** | **L** | **P** | **A** | **R** | **F** | **P** | **N** | **L** | **L** | **V** | **N** | **G** | **T** | **T** | **G** | **I** | **A** | **V** | **G** | **M** | **A** | **T** | **N** | **I** | **P** | **P** | **H** | **N** | **L** | **S** | **E** | **V** | **I** | **D** | **A** | **T** | **S** | **L** | **L** | **M** | **D** | **N** | **P** | **D** | **V** | **T** | **T** | **N** | **E** | **L** | **M** | **E** | **V** | **L** | **P** | **G** | **P** | **D** | **F** | **P** | **T** | **G** | **G** | **L** | **V** | **M** | **G** | **K** | **S** | **G** | **I** | **R** | **R** | **A** | **Y** | **E** | **T** | **G** | **K** | **G** | **S** | **I** | **T** | **V** | **R** | **A** | **K** | **V** | **E** | **L** | **T** | **E** | **M** | **P** | **N** | **G** | **K** | **E** | **R** | **I** | **L** | **V** | **T** | **E** | **L** | **P** | **Y** | **M** | **V** | **N** | **K** | **A** | **K** | **L** | **I** | **E** | **R** | **I** | **S** | **E** | **L** | **H** | **R** | **D** | **K** | **R** | **I** | **E** | **G** | **I** | **T** | **D** | **L** | **R** | **D** | **E** | **S** | **S** | **R** | **E** | **G** | **M** | **R** | **I** | **V** | **I** | **D** | **V** | **R** | **R** | **D** | **V** | **S** | **A** | **S** | **V** | **V** | **L** | **N** | **N** | **L** | **Y** | **K** | **M** | **T** | **A** | **L** | **Q** | **T** | **S** | **F** | **G** | **F** | **N** | **M** | **L** | **A** | **I** | **E** | **K** | **G** | **V** | **P** | **K** | **I** | **L** | **S** | **L** | **K** | **R** | **I** | **L** | **E** | **N** | **Y** | **V** | **E** | **H** | **Q** | **K** | **E** | **V** | **I** | **T** | **R** | **R** | **T** | **I** | **F** | **D** | **K** | **N** | **K** | **A** | **E** | **A** | **R** | **A** | **H** | **I** | **L** | **E** | **G** | **L** | **R** | **I** | **A** | **L** | **D** | **H** | **I** | **D** | **E** | **I** | **I** | **A** | **I** | **I** | **R** | **G** | **S** | **Q** | **S** | **D** | **D** | **E** | **A** | **K** | **A** | **T** | **L** | **I** | **E** | **R** | **F** | **E** | **F** | **S** | **D** | **R** | **Q** | **A** | **Q** | **A** | **I** | **L** | **D** | **M** | **R** | **L** | **R** | **R** | **L** | **T** | **G** | **L** | **E** | **R** | **D** | **K** | **I** | **E** | **N** | **E** | **Y** | **Q** | **E** | **L** | **L** | **K** | **F** | **I** | **A** | **D** | **L** | **E** | **D** | **I** | **L** | **A** | **R** | **P** | **E** | **R** | **V** | **I** | **E** | **I** | **I** | **K** | **T** | **E** | **L** | **N** | **D** | **V** | **R** | **T** | **K** | **F** | **G** | **D** | **A** | **R** | **R** | **T** | **E** | **L** | **L** | **V** | **G** | **E** | **V** | **L** | **S** | **L** | **E** | **D** | **E** | **D** | **L** | **I** | **E** | **E** | **E** | **E** | **V** | **V** | **I** | **T** | **L** | **T** | **N** | **N** | **G** | **Y** | **I** | **K** | **R** | **M** | **A** | **N** | **S** | **E** | **F** | **R** | **A** | **Q** | **R** | **R** | **G** | **G** | **R** | **G** | **V** | **Q** | **G** | **M** | **G** | **V** | **H** | **D** | **D** | **D** | **F** | **V** | **K** | **N** | **L** | **V** | **S** | **C** | **S** | **T** | **H** | **D** | **T** | **L** | **L** | **F** | **F** | **T** | **N** | **T** | **G** | **K** | **V** | **Y** | **R** | **A** | **K** | **G** | **Y** | **E** | **I** | **P** | **E** | **Y** | **G** | **R** | **T** | **A** | **K** | **G** | **I** | **P** | **V** | **I** | **N** | **L** | **L** | **G** | **I** | **D** | **S** | **A** | **E** | **K** | **I** | **Q** | **A** | **I** | **I** | **S** | **V** | **E** | **G** | **K** | **A** | **E** | **A** | **G** | **K** | **Y** | **L** | **F** | **F** | **T** | **T** | **L** | **K** | **G** | **T** | **V** | **K** | **R** | **T** | **A** | **V** | **T** | **A** | **F** | **S** | **N** | **I** | **R** | **S** | **N** | **G** | **L** | **I** | **A** | **I** | **S** | **L** | **K** | **E** | **D** | **D** | **E** | **L** | **V** | **N** | **V** | **V** | **T** | **T** | **N** | **G** | **N** | **Q** | **K** | **M** | **I** | **I** | **G** | **T** | **H** | **A** | **G** | **Y** | **S** | **V** | **T** | **F** | **D** | **E** | **N** | **T** | **V** | **R** | **D** | **M** | **G** | **R** | **T** | **A** | **S** | **G** | **V** | **R** | **G** | **I** | **R** | **L** | **R** | **E** | **N** | **D** | **Y** | **V** | **V** | **G** | **A** | **A** | **I** | **L** | **D** | **E** | **N** | **K** | **E** | **V** | **L** | **V** | **I** | **T** | **E** | **N** | **G** | **Y** | **G** | **K** | **R** | **T** | **K** | **A** | **S** | **E** | **Y** | **P** | **V** | **K** | **G** | **R** | **G** | **G** | **K** | **G** | **I** | **K** | **T** | **A** | **N** | **I** | **T** | **E** | **K** | **N** | **G** | **P** | **L** | **A** | **G** | **L** | **T** | **T** | **V** | **N** | **G** | **D** | **E** | **D** | **I** | **L** | **L** | **I** | **T** | **N** | **K** | **G** | **V** | **I** | **I** | **R** | **F** | **N** | **V** | **D** | **S** | **V** | **S** | **Q** | **T** | **G** | **R** | **A** | **T** | **L** | **G** | **V** | **R** | **L** | **M** | **R** | **M** | **E** | **D** | **G** | **A** | **K** | **-** | **-** | **-** | **-** | **-** | **-** | **-** | **-** | **-** | **-** | **-** | **-** | **-** | **-** | **-** | **-** | **-** | **-** | **-** | **-** | **-** | **-** | **-** |
| **N60268F** | **M** | **S** | **E** | **E** | **I** | **K** | **E** | **N** | **I** | **Q** | **D** | **V** | **N** | **L** | **T** | **S** | **E** | **M** | **K** | **E** | **S** | **F** | **I** | **D** | **Y** | **A** | **M** | **S** | **V** | **I** | **V** | **A** | **R** | **A** | **L** | **P** | **D** | **V** | **R** | **D** | **G** | **L** | **K** | **P** | **V** | **H** | **R** | **R** | **I** | **L** | **Y** | **G** | **M** | **N** | **E** | **L** | **G** | **V** | **T** | **P** | **D** | **K** | **P** | **H** | **K** | **K** | **S** | **A** | **R** | **I** | **V** | **G** | **D** | **V** | **M** | **G** | **K** | **Y** | **H** | **P** | **H** | **G** | **D** | **S** | **A** | **I** | **Y** | **E** | **S** | **M** | **V** | **R** | **M** | **A** | **Q** | **P** | **F** | **S** | **Y** | **R** | **A** | **M** | **L** | **V** | **D** | **G** | **H** | **G** | **N** | **F** | **G** | **S** | **V** | **D** | **G** | **D** | **G** | **A** | **A** | **A** | **M** | **R** | **Y** | **T** | **E** | **A** | **R** | **M** | **S** | **K** | **I** | **A** | **L** | **E** | **M** | **L** | **R** | **D** | **I** | **N** | **K** | **N** | **T** | **V** | **D** | **F** | **Q** | **G** | **N** | **Y** | **D** | **D** | **S** | **E** | **Q** | **E** | **P** | **V** | **V** | **L** | **P** | **A** | **R** | **F** | **P** | **N** | **L** | **L** | **V** | **N** | **G** | **T** | **T** | **G** | **I** | **A** | **V** | **G** | **M** | **A** | **T** | **N** | **I** | **P** | **P** | **H** | **N** | **L** | **S** | **E** | **V** | **I** | **D** | **A** | **T** | **S** | **L** | **L** | **M** | **D** | **N** | **P** | **D** | **V** | **T** | **T** | **N** | **E** | **L** | **M** | **E** | **V** | **L** | **P** | **G** | **P** | **D** | **F** | **P** | **T** | **G** | **G** | **L** | **V** | **M** | **G** | **K** | **S** | **G** | **I** | **R** | **R** | **A** | **Y** | **E** | **T** | **G** | **K** | **G** | **S** | **I** | **T** | **V** | **R** | **A** | **K** | **V** | **E** | **L** | **T** | **E** | **M** | **P** | **N** | **G** | **K** | **E** | **R** | **I** | **L** | **V** | **T** | **E** | **L** | **P** | **Y** | **M** | **V** | **N** | **K** | **A** | **K** | **L** | **I** | **E** | **R** | **I** | **S** | **E** | **L** | **H** | **R** | **D** | **K** | **R** | **I** | **E** | **G** | **I** | **T** | **D** | **L** | **R** | **D** | **E** | **S** | **S** | **R** | **E** | **G** | **M** | **R** | **I** | **V** | **I** | **D** | **V** | **R** | **R** | **D** | **V** | **S** | **A** | **S** | **V** | **V** | **L** | **N** | **N** | **L** | **Y** | **K** | **M** | **T** | **A** | **L** | **Q** | **T** | **S** | **F** | **G** | **F** | **N** | **M** | **L** | **A** | **I** | **E** | **K** | **G** | **V** | **P** | **K** | **I** | **L** | **S** | **L** | **K** | **R** | **I** | **L** | **E** | **N** | **Y** | **V** | **E** | **H** | **Q** | **K** | **E** | **V** | **I** | **T** | **R** | **R** | **T** | **I** | **F** | **D** | **K** | **N** | **K** | **A** | **E** | **A** | **R** | **A** | **H** | **I** | **L** | **E** | **G** | **L** | **R** | **I** | **A** | **L** | **D** | **H** | **I** | **D** | **E** | **I** | **I** | **A** | **I** | **I** | **R** | **G** | **S** | **Q** | **S** | **D** | **D** | **E** | **A** | **K** | **A** | **T** | **L** | **I** | **E** | **R** | **F** | **E** | **F** | **S** | **D** | **R** | **Q** | **A** | **Q** | **A** | **I** | **L** | **D** | **M** | **R** | **L** | **R** | **R** | **L** | **T** | **G** | **L** | **E** | **R** | **D** | **K** | **I** | **E** | **N** | **E** | **Y** | **Q** | **E** | **L** | **L** | **K** | **F** | **I** | **A** | **D** | **L** | **E** | **D** | **I** | **L** | **A** | **R** | **P** | **E** | **R** | **V** | **I** | **E** | **I** | **I** | **K** | **T** | **E** | **L** | **N** | **D** | **V** | **R** | **T** | **K** | **F** | **G** | **D** | **A** | **R** | **R** | **T** | **E** | **L** | **L** | **V** | **G** | **E** | **V** | **L** | **S** | **L** | **E** | **D** | **E** | **D** | **L** | **I** | **E** | **E** | **E** | **E** | **V** | **V** | **I** | **T** | **L** | **T** | **N** | **N** | **G** | **Y** | **I** | **K** | **R** | **M** | **A** | **N** | **S** | **E** | **F** | **R** | **A** | **Q** | **R** | **R** | **G** | **G** | **R** | **G** | **V** | **Q** | **G** | **M** | **G** | **V** | **H** | **D** | **D** | **D** | **F** | **V** | **K** | **N** | **L** | **V** | **S** | **C** | **S** | **T** | **H** | **D** | **T** | **L** | **L** | **F** | **F** | **T** | **N** | **T** | **G** | **K** | **V** | **Y** | **R** | **A** | **K** | **G** | **Y** | **E** | **I** | **P** | **E** | **Y** | **G** | **R** | **T** | **A** | **K** | **G** | **I** | **P** | **V** | **I** | **N** | **L** | **L** | **G** | **I** | **D** | **S** | **A** | **E** | **K** | **I** | **Q** | **A** | **I** | **I** | **S** | **V** | **E** | **G** | **K** | **A** | **E** | **A** | **G** | **K** | **Y** | **L** | **F** | **F** | **T** | **T** | **L** | **K** | **G** | **T** | **V** | **K** | **R** | **T** | **A** | **V** | **T** | **A** | **F** | **S** | **N** | **I** | **R** | **S** | **N** | **G** | **L** | **I** | **A** | **I** | **S** | **L** | **K** | **E** | **D** | **D** | **E** | **L** | **V** | **N** | **V** | **V** | **T** | **T** | **N** | **G** | **N** | **Q** | **K** | **M** | **I** | **I** | **G** | **T** | **H** | **A** | **G** | **Y** | **S** | **V** | **T** | **F** | **D** | **E** | **N** | **T** | **V** | **R** | **D** | **M** | **G** | **R** | **T** | **A** | **S** | **G** | **V** | **R** | **G** | **I** | **R** | **L** | **R** | **E** | **N** | **D** | **Y** | **V** | **V** | **G** | **A** | **A** | **I** | **L** | **D** | **E** | **N** | **K** | **E** | **V** | **L** | **V** | **I** | **T** | **E** | **N** | **G** | **Y** | **G** | **K** | **R** | **T** | **K** | **A** | **S** | **E** | **Y** | **P** | **V** | **K** | **G** | **R** | **G** | **G** | **K** | **G** | **I** | **K** | **T** | **A** | **N** | **I** | **T** | **E** | **K** | **N** | **G** | **P** | **L** | **A** | **G** | **L** | **T** | **T** | **V** | **N** | **G** | **D** | **E** | **D** | **I** | **L** | **L** | **I** | **T** | **N** | **K** | **G** | **V** | **I** | **I** | **R** | **F** | **N** | **V** | **D** | **S** | **V** | **S** | **Q** | **T** | **G** | **R** | **A** | **T** | **L** | **G** | **V** | **R** | **L** | **M** | **R** | **M** | **E** | **D** | **G** | **A** | **K** | **-** | **-** | **-** | **-** | **-** | **-** | **-** | **-** | **-** | **-** | **-** | **-** | **-** | **-** | **-** | **-** | **-** | **-** | **-** | **-** | **-** | **-** | **-** |
| **N55725** | **M** | **S** | **E** | **E** | **I** | **K** | **E** | **N** | **I** | **Q** | **D** | **V** | **N** | **L** | **T** | **S** | **E** | **M** | **K** | **E** | **S** | **F** | **I** | **D** | **Y** | **A** | **M** | **S** | **V** | **I** | **V** | **A** | **R** | **A** | **L** | **P** | **D** | **V** | **R** | **D** | **G** | **L** | **K** | **P** | **V** | **H** | **R** | **R** | **I** | **L** | **Y** | **G** | **M** | **N** | **E** | **L** | **G** | **V** | **T** | **P** | **D** | **K** | **P** | **H** | **K** | **K** | **S** | **A** | **R** | **I** | **V** | **G** | **D** | **V** | **M** | **G** | **K** | **Y** | **H** | **P** | **H** | **G** | **D** | **S** | **A** | **I** | **Y** | **E** | **S** | **M** | **V** | **R** | **M** | **A** | **Q** | **P** | **F** | **S** | **Y** | **R** | **A** | **M** | **L** | **V** | **D** | **G** | **H** | **G** | **N** | **F** | **G** | **S** | **V** | **D** | **G** | **D** | **G** | **A** | **A** | **A** | **M** | **R** | **Y** | **T** | **E** | **A** | **R** | **M** | **S** | **K** | **I** | **A** | **L** | **E** | **M** | **L** | **R** | **D** | **I** | **N** | **K** | **N** | **T** | **V** | **D** | **F** | **Q** | **G** | **N** | **Y** | **D** | **D** | **S** | **E** | **Q** | **E** | **P** | **V** | **V** | **L** | **P** | **A** | **R** | **F** | **P** | **N** | **L** | **L** | **V** | **N** | **G** | **T** | **T** | **G** | **I** | **A** | **V** | **G** | **M** | **A** | **T** | **N** | **I** | **P** | **P** | **H** | **N** | **L** | **S** | **E** | **V** | **I** | **D** | **A** | **T** | **S** | **L** | **L** | **M** | **D** | **N** | **P** | **D** | **V** | **T** | **T** | **N** | **E** | **L** | **M** | **E** | **V** | **L** | **P** | **G** | **P** | **D** | **F** | **P** | **T** | **G** | **G** | **L** | **V** | **M** | **G** | **K** | **S** | **G** | **I** | **R** | **R** | **A** | **Y** | **E** | **T** | **G** | **K** | **G** | **S** | **I** | **T** | **V** | **R** | **A** | **K** | **V** | **E** | **L** | **T** | **E** | **M** | **P** | **N** | **G** | **K** | **E** | **R** | **I** | **L** | **V** | **T** | **E** | **L** | **P** | **Y** | **M** | **V** | **N** | **K** | **A** | **K** | **L** | **I** | **E** | **R** | **I** | **S** | **E** | **L** | **H** | **R** | **D** | **K** | **R** | **I** | **E** | **G** | **I** | **T** | **D** | **L** | **R** | **D** | **E** | **S** | **S** | **R** | **E** | **G** | **M** | **R** | **I** | **V** | **I** | **D** | **V** | **R** | **R** | **D** | **V** | **S** | **A** | **S** | **V** | **V** | **L** | **N** | **N** | **L** | **Y** | **K** | **M** | **T** | **A** | **L** | **Q** | **T** | **S** | **F** | **G** | **F** | **N** | **M** | **L** | **A** | **I** | **E** | **K** | **G** | **V** | **P** | **K** | **I** | **L** | **S** | **L** | **K** | **R** | **I** | **L** | **E** | **N** | **Y** | **V** | **E** | **H** | **Q** | **K** | **E** | **V** | **I** | **T** | **R** | **R** | **T** | **I** | **F** | **D** | **K** | **N** | **K** | **A** | **E** | **A** | **R** | **A** | **H** | **I** | **L** | **E** | **G** | **L** | **R** | **I** | **A** | **L** | **D** | **H** | **I** | **D** | **E** | **I** | **I** | **A** | **I** | **I** | **R** | **G** | **S** | **Q** | **S** | **D** | **D** | **E** | **A** | **K** | **A** | **T** | **L** | **I** | **E** | **R** | **F** | **E** | **F** | **S** | **D** | **R** | **Q** | **A** | **Q** | **A** | **I** | **L** | **D** | **M** | **R** | **L** | **R** | **R** | **L** | **T** | **G** | **L** | **E** | **R** | **D** | **K** | **I** | **E** | **N** | **E** | **Y** | **Q** | **E** | **L** | **L** | **K** | **F** | **I** | **A** | **D** | **L** | **E** | **D** | **I** | **L** | **A** | **R** | **P** | **E** | **R** | **V** | **I** | **E** | **I** | **I** | **K** | **T** | **E** | **L** | **N** | **D** | **V** | **R** | **T** | **K** | **F** | **G** | **D** | **A** | **R** | **R** | **T** | **E** | **L** | **L** | **V** | **G** | **E** | **V** | **L** | **S** | **L** | **E** | **D** | **E** | **D** | **L** | **I** | **E** | **E** | **E** | **E** | **V** | **V** | **I** | **T** | **L** | **T** | **N** | **N** | **G** | **Y** | **I** | **K** | **R** | **M** | **A** | **N** | **S** | **E** | **F** | **R** | **A** | **Q** | **R** | **R** | **G** | **G** | **R** | **G** | **V** | **Q** | **G** | **M** | **G** | **V** | **H** | **D** | **D** | **D** | **F** | **V** | **K** | **N** | **L** | **V** | **S** | **C** | **S** | **T** | **H** | **D** | **T** | **L** | **L** | **F** | **F** | **T** | **N** | **T** | **G** | **K** | **V** | **Y** | **R** | **A** | **K** | **G** | **Y** | **E** | **I** | **P** | **E** | **Y** | **G** | **R** | **T** | **A** | **K** | **G** | **I** | **P** | **V** | **I** | **N** | **L** | **L** | **G** | **I** | **D** | **S** | **A** | **E** | **K** | **I** | **Q** | **A** | **I** | **I** | **S** | **V** | **E** | **G** | **K** | **A** | **E** | **A** | **G** | **K** | **Y** | **L** | **F** | **F** | **T** | **T** | **L** | **K** | **G** | **T** | **V** | **K** | **R** | **T** | **A** | **V** | **T** | **A** | **F** | **S** | **N** | **I** | **R** | **S** | **N** | **G** | **L** | **I** | **A** | **I** | **S** | **L** | **K** | **E** | **D** | **D** | **E** | **L** | **V** | **N** | **V** | **V** | **T** | **T** | **N** | **G** | **N** | **Q** | **K** | **M** | **I** | **I** | **G** | **T** | **H** | **A** | **G** | **Y** | **S** | **V** | **T** | **F** | **D** | **E** | **N** | **T** | **V** | **R** | **D** | **M** | **G** | **R** | **T** | **A** | **S** | **G** | **V** | **R** | **G** | **I** | **R** | **L** | **R** | **E** | **N** | **D** | **Y** | **V** | **V** | **G** | **A** | **A** | **I** | **L** | **D** | **E** | **N** | **K** | **E** | **V** | **L** | **V** | **I** | **T** | **E** | **N** | **G** | **Y** | **G** | **K** | **R** | **T** | **K** | **A** | **S** | **E** | **Y** | **P** | **V** | **K** | **G** | **R** | **G** | **G** | **K** | **G** | **I** | **K** | **T** | **A** | **N** | **I** | **T** | **E** | **K** | **N** | **G** | **P** | **L** | **A** | **G** | **L** | **T** | **T** | **V** | **N** | **G** | **D** | **E** | **D** | **I** | **L** | **L** | **I** | **T** | **N** | **K** | **G** | **V** | **I** | **I** | **R** | **F** | **N** | **V** | **D** | **S** | **V** | **S** | **Q** | **T** | **G** | **R** | **A** | **T** | **L** | **G** | **V** | **R** | **L** | **M** | **R** | **M** | **E** | **D** | **G** | **A** | **K** | **-** | **-** | **-** | **-** | **-** | **-** | **-** | **-** | **-** | **-** | **-** | **-** | **-** | **-** | **-** | **-** | **-** | **-** | **-** | **-** | **-** | **-** | **-** |
| **N59845F** | **M** | **S** | **E** | **E** | **I** | **K** | **E** | **N** | **I** | **Q** | **D** | **V** | **N** | **L** | **T** | **S** | **E** | **M** | **K** | **E** | **S** | **F** | **I** | **D** | **Y** | **A** | **M** | **S** | **V** | **I** | **V** | **A** | **R** | **A** | **L** | **P** | **D** | **V** | **R** | **D** | **G** | **L** | **K** | **P** | **V** | **H** | **R** | **R** | **I** | **L** | **Y** | **G** | **M** | **N** | **E** | **L** | **G** | **V** | **T** | **P** | **D** | **K** | **P** | **H** | **K** | **K** | **S** | **A** | **R** | **I** | **V** | **G** | **D** | **V** | **M** | **G** | **K** | **Y** | **H** | **P** | **H** | **G** | **D** | **S** | **A** | **I** | **Y** | **E** | **S** | **M** | **V** | **R** | **M** | **A** | **Q** | **P** | **F** | **S** | **Y** | **R** | **A** | **M** | **L** | **V** | **D** | **G** | **H** | **G** | **N** | **F** | **G** | **S** | **V** | **D** | **G** | **D** | **G** | **A** | **A** | **A** | **M** | **R** | **Y** | **T** | **E** | **A** | **R** | **M** | **S** | **K** | **I** | **A** | **L** | **E** | **M** | **L** | **R** | **D** | **I** | **N** | **K** | **N** | **T** | **V** | **D** | **F** | **Q** | **G** | **N** | **Y** | **D** | **D** | **S** | **E** | **Q** | **E** | **P** | **V** | **V** | **L** | **P** | **A** | **R** | **F** | **P** | **N** | **L** | **L** | **V** | **N** | **G** | **T** | **T** | **G** | **I** | **A** | **V** | **G** | **M** | **A** | **T** | **N** | **I** | **P** | **P** | **H** | **N** | **L** | **S** | **E** | **V** | **I** | **D** | **A** | **T** | **S** | **L** | **L** | **M** | **D** | **N** | **P** | **D** | **V** | **T** | **T** | **N** | **E** | **L** | **M** | **E** | **V** | **L** | **P** | **G** | **P** | **D** | **F** | **P** | **T** | **G** | **G** | **L** | **V** | **M** | **G** | **K** | **S** | **G** | **I** | **R** | **R** | **A** | **Y** | **E** | **T** | **G** | **K** | **G** | **S** | **I** | **T** | **V** | **R** | **A** | **K** | **V** | **E** | **L** | **T** | **E** | **M** | **P** | **N** | **G** | **K** | **E** | **R** | **I** | **L** | **V** | **T** | **E** | **L** | **P** | **Y** | **M** | **V** | **N** | **K** | **A** | **K** | **L** | **I** | **E** | **R** | **I** | **S** | **E** | **L** | **H** | **R** | **D** | **K** | **R** | **I** | **E** | **G** | **I** | **T** | **D** | **L** | **R** | **D** | **E** | **S** | **S** | **R** | **E** | **G** | **M** | **R** | **I** | **V** | **I** | **D** | **V** | **R** | **R** | **D** | **V** | **S** | **A** | **S** | **V** | **V** | **L** | **N** | **N** | **L** | **Y** | **K** | **M** | **T** | **A** | **L** | **Q** | **T** | **S** | **F** | **G** | **F** | **N** | **M** | **L** | **A** | **I** | **E** | **K** | **G** | **V** | **P** | **K** | **I** | **L** | **S** | **L** | **K** | **R** | **I** | **L** | **E** | **N** | **Y** | **V** | **E** | **H** | **Q** | **K** | **E** | **V** | **I** | **T** | **R** | **R** | **T** | **I** | **F** | **D** | **K** | **N** | **K** | **A** | **E** | **A** | **R** | **A** | **H** | **I** | **L** | **E** | **G** | **L** | **R** | **I** | **A** | **L** | **D** | **H** | **I** | **D** | **E** | **I** | **I** | **A** | **I** | **I** | **R** | **G** | **S** | **Q** | **S** | **D** | **D** | **E** | **A** | **K** | **A** | **T** | **L** | **I** | **E** | **R** | **F** | **E** | **F** | **S** | **D** | **R** | **Q** | **A** | **Q** | **A** | **I** | **L** | **D** | **M** | **R** | **L** | **R** | **R** | **L** | **T** | **G** | **L** | **E** | **R** | **D** | **K** | **I** | **E** | **N** | **E** | **Y** | **Q** | **E** | **L** | **L** | **K** | **F** | **I** | **A** | **D** | **L** | **E** | **D** | **I** | **L** | **A** | **R** | **P** | **E** | **R** | **V** | **I** | **E** | **I** | **I** | **K** | **T** | **E** | **L** | **N** | **D** | **V** | **R** | **T** | **K** | **F** | **G** | **D** | **A** | **R** | **R** | **T** | **E** | **L** | **L** | **V** | **G** | **E** | **V** | **L** | **S** | **L** | **E** | **D** | **E** | **D** | **L** | **I** | **E** | **E** | **E** | **E** | **V** | **V** | **I** | **T** | **L** | **T** | **N** | **N** | **G** | **Y** | **I** | **K** | **R** | **M** | **A** | **N** | **S** | **E** | **F** | **R** | **A** | **Q** | **R** | **R** | **G** | **G** | **R** | **G** | **V** | **Q** | **G** | **M** | **G** | **V** | **H** | **D** | **D** | **D** | **F** | **V** | **K** | **N** | **L** | **V** | **S** | **C** | **S** | **T** | **H** | **D** | **T** | **L** | **L** | **F** | **F** | **T** | **N** | **T** | **G** | **K** | **V** | **Y** | **R** | **A** | **K** | **G** | **Y** | **E** | **I** | **P** | **E** | **Y** | **G** | **R** | **T** | **A** | **K** | **G** | **I** | **P** | **V** | **I** | **N** | **L** | **L** | **G** | **I** | **D** | **S** | **A** | **E** | **K** | **I** | **Q** | **A** | **I** | **I** | **S** | **V** | **E** | **G** | **K** | **A** | **E** | **A** | **G** | **K** | **Y** | **L** | **F** | **F** | **T** | **T** | **L** | **K** | **G** | **T** | **V** | **K** | **R** | **T** | **A** | **V** | **T** | **A** | **F** | **S** | **N** | **I** | **R** | **S** | **N** | **G** | **L** | **I** | **A** | **I** | **S** | **L** | **K** | **E** | **D** | **D** | **E** | **L** | **V** | **N** | **V** | **V** | **T** | **T** | **N** | **G** | **N** | **Q** | **K** | **M** | **I** | **I** | **G** | **T** | **H** | **A** | **G** | **Y** | **S** | **V** | **T** | **F** | **D** | **E** | **N** | **T** | **V** | **R** | **D** | **M** | **G** | **R** | **T** | **A** | **S** | **G** | **V** | **R** | **G** | **I** | **R** | **L** | **R** | **E** | **N** | **D** | **Y** | **V** | **V** | **G** | **A** | **A** | **I** | **L** | **D** | **E** | **N** | **K** | **E** | **V** | **L** | **V** | **I** | **T** | **E** | **N** | **G** | **Y** | **G** | **K** | **R** | **T** | **K** | **A** | **S** | **E** | **Y** | **P** | **V** | **K** | **G** | **R** | **G** | **G** | **K** | **G** | **I** | **K** | **T** | **A** | **N** | **I** | **T** | **E** | **K** | **N** | **G** | **P** | **L** | **A** | **G** | **L** | **T** | **T** | **V** | **N** | **G** | **D** | **E** | **D** | **I** | **L** | **L** | **I** | **T** | **N** | **K** | **G** | **V** | **I** | **I** | **R** | **F** | **N** | **V** | **D** | **S** | **V** | **S** | **Q** | **T** | **G** | **R** | **A** | **T** | **L** | **G** | **V** | **R** | **L** | **M** | **R** | **M** | **E** | **D** | **G** | **A** | **K** | **-** | **-** | **-** | **-** | **-** | **-** | **-** | **-** | **-** | **-** | **-** | **-** | **-** | **-** | **-** | **-** | **-** | **-** | **-** | **-** | **-** | **-** | **-** |
| **N59907F** | **M** | **S** | **E** | **E** | **I** | **R** | **E** | **N** | **I** | **H** | **D** | **V** | **N** | **L** | **T** | **S** | **E** | **M** | **K** | **D** | **S** | **F** | **I** | **D** | **Y** | **A** | **M** | **S** | **V** | **I** | **V** | **A** | **R** | **A** | **L** | **P** | **D** | **V** | **R** | **D** | **G** | **L** | **K** | **P** | **V** | **H** | **R** | **R** | **I** | **L** | **Y** | **G** | **M** | **N** | **E** | **L** | **G** | **V** | **T** | **P** | **D** | **K** | **A** | **H** | **K** | **K** | **S** | **A** | **R** | **I** | **V** | **G** | **D** | **V** | **M** | **G** | **K** | **Y** | **H** | **P** | **H** | **G** | **D** | **S** | **A** | **I** | **Y** | **E** | **S** | **M** | **V** | **R** | **M** | **A** | **Q** | **P** | **F** | **S** | **Y** | **R** | **Y** | **M** | **L** | **V** | **D** | **G** | **H** | **G** | **N** | **F** | **G** | **S** | **V** | **D** | **G** | **D** | **G** | **A** | **A** | **A** | **M** | **R** | **Y** | **T** | **E** | **A** | **R** | **M** | **S** | **K** | **I** | **A** | **T** | **E** | **M** | **L** | **R** | **D** | **I** | **N** | **K** | **N** | **T** | **V** | **D** | **F** | **Q** | **S** | **N** | **Y** | **D** | **D** | **T** | **E** | **K** | **E** | **P** | **V** | **V** | **L** | **P** | **A** | **R** | **F** | **P** | **N** | **L** | **L** | **V** | **N** | **G** | **T** | **T** | **G** | **I** | **A** | **V** | **G** | **M** | **A** | **T** | **N** | **I** | **P** | **P** | **H** | **N** | **L** | **S** | **E** | **V** | **V** | **A** | **A** | **I** | **D** | **L** | **L** | **M** | **D** | **N** | **P** | **D** | **V** | **T** | **T** | **N** | **E** | **L** | **M** | **E** | **V** | **L** | **P** | **G** | **P** | **D** | **F** | **P** | **T** | **G** | **G** | **L** | **V** | **M** | **G** | **K** | **S** | **G** | **I** | **R** | **R** | **A** | **Y** | **E** | **T** | **G** | **K** | **G** | **S** | **I** | **T** | **V** | **R** | **A** | **K** | **V** | **E** | **I** | **T** | **E** | **M** | **P** | **N** | **G** | **K** | **E** | **R** | **I** | **I** | **V** | **T** | **E** | **L** | **P** | **Y** | **M** | **V** | **N** | **K** | **A** | **K** | **L** | **I** | **E** | **R** | **I** | **S** | **E** | **L** | **H** | **R** | **D** | **K** | **R** | **I** | **E** | **G** | **I** | **T** | **D** | **L** | **R** | **D** | **E** | **S** | **S** | **R** | **E** | **G** | **M** | **R** | **I** | **V** | **I** | **D** | **I** | **R** | **R** | **D** | **A** | **S** | **A** | **S** | **V** | **I** | **L** | **N** | **N** | **L** | **Y** | **K** | **L** | **T** | **S** | **L** | **Q** | **N** | **S** | **F** | **G** | **F** | **N** | **M** | **L** | **A** | **I** | **E** | **K** | **G** | **V** | **P** | **K** | **V** | **L** | **S** | **L** | **K** | **Q** | **I** | **L** | **E** | **N** | **Y** | **I** | **E** | **H** | **Q** | **R** | **E** | **V** | **I** | **T** | **R** | **R** | **T** | **V** | **F** | **E** | **K** | **E** | **K** | **A** | **E** | **A** | **R** | **A** | **H** | **I** | **L** | **E** | **G** | **L** | **R** | **I** | **A** | **L** | **D** | **H** | **I** | **D** | **E** | **I** | **I** | **A** | **I** | **I** | **R** | **G** | **S** | **K** | **S** | **D** | **D** | **E** | **A** | **K** | **A** | **T** | **M** | **I** | **E** | **R** | **F** | **D** | **L** | **S** | **D** | **R** | **Q** | **S** | **Q** | **A** | **I** | **L** | **D** | **M** | **R** | **L** | **R** | **R** | **L** | **T** | **G** | **L** | **E** | **R** | **E** | **K** | **I** | **E** | **N** | **E** | **Y** | **Q** | **E** | **L** | **L** | **K** | **L** | **I** | **E** | **D** | **L** | **T** | **D** | **I** | **L** | **A** | **R** | **P** | **E** | **R** | **V** | **T** | **E** | **I** | **I** | **K** | **N** | **E** | **L** | **A** | **D** | **L** | **N** | **Q** | **R** | **F** | **G** | **D** | **K** | **R** | **R** | **T** | **E** | **L** | **L** | **V** | **G** | **E** | **V** | **L** | **S** | **L** | **E** | **D** | **E** | **D** | **L** | **I** | **E** | **E** | **E** | **E** | **I** | **V** | **I** | **T** | **L** | **T** | **N** | **N** | **G** | **Y** | **I** | **K** | **R** | **L** | **A** | **N** | **N** | **E** | **F** | **R** | **A** | **Q** | **R** | **R** | **G** | **G** | **R** | **G** | **V** | **Q** | **G** | **M** | **G** | **I** | **H** | **N** | **D** | **D** | **F** | **V** | **K** | **N** | **L** | **V** | **S** | **C** | **S** | **T** | **H** | **D** | **T** | **L** | **L** | **F** | **F** | **T** | **N** | **N** | **G** | **K** | **V** | **Y** | **R** | **A** | **K** | **G** | **Y** | **E** | **I** | **P** | **E** | **Y** | **G** | **R** | **T** | **A** | **K** | **G** | **I** | **P** | **I** | **I** | **N** | **L** | **L** | **G** | **I** | **D** | **S** | **S** | **E** | **S** | **I** | **Q** | **A** | **I** | **I** | **A** | **V** | **T** | **G** | **E** | **A** | **E** | **E** | **G** | **H** | **Y** | **L** | **F** | **F** | **T** | **T** | **R** | **Q** | **G** | **T** | **V** | **K** | **R** | **T** | **S** | **V** | **T** | **A** | **F** | **A** | **N** | **I** | **R** | **S** | **N** | **G** | **L** | **I** | **A** | **I** | **G** | **L** | **K** | **E** | **D** | **D** | **E** | **L** | **V** | **N** | **V** | **L** | **L** | **T** | **D** | **G** | **Q** | **S** | **N** | **I** | **I** | **I** | **G** | **T** | **H** | **N** | **G** | **Y** | **S** | **V** | **T** | **F** | **K** | **E** | **E** | **A** | **V** | **R** | **D** | **M** | **G** | **R** | **T** | **A** | **S** | **G** | **V** | **R** | **G** | **I** | **R** | **L** | **R** | **E** | **E** | **D** | **Y** | **V** | **V** | **G** | **A** | **A** | **L** | **M** | **K** | **E** | **D** | **Q** | **E** | **V** | **L** | **V** | **I** | **T** | **E** | **K** | **G** | **Y** | **G** | **K** | **R** | **T** | **K** | **V** | **S** | **E** | **Y** | **P** | **V** | **K** | **G** | **R** | **G** | **G** | **K** | **G** | **I** | **K** | **T** | **A** | **N** | **I** | **T** | **E** | **K** | **N** | **G** | **P** | **L** | **A** | **G** | **L** | **T** | **T** | **V** | **S** | **G** | **D** | **E** | **D** | **I** | **M** | **L** | **I** | **T** | **D** | **K** | **G** | **V** | **I** | **I** | **R** | **F** | **N** | **V** | **S** | **T** | **V** | **S** | **Q** | **T** | **G** | **R** | **S** | **T** | **L** | **G** | **V** | **R** | **L** | **M** | **K** | **M** | **E** | **A** | **D** | **T** | **K** | **V** | **V** | **T** | **M** | **A** | **A** | **V** | **E** | **P** | **E** | **A** | **A** | **E** | **Q** | **T** | **V** | **E** | **E** | **Q** | **G** | **S** | **E** | **E** |
| **N53804** | **M** | **S** | **E** | **E** | **I** | **R** | **E** | **N** | **I** | **H** | **D** | **V** | **N** | **L** | **T** | **S** | **E** | **M** | **K** | **D** | **S** | **F** | **I** | **D** | **Y** | **A** | **M** | **S** | **V** | **I** | **V** | **A** | **R** | **A** | **L** | **P** | **D** | **V** | **R** | **D** | **G** | **L** | **K** | **P** | **V** | **H** | **R** | **R** | **I** | **L** | **Y** | **G** | **M** | **N** | **E** | **L** | **G** | **V** | **T** | **P** | **D** | **K** | **A** | **H** | **K** | **K** | **S** | **A** | **R** | **I** | **V** | **G** | **D** | **V** | **M** | **G** | **K** | **Y** | **H** | **P** | **H** | **G** | **D** | **S** | **A** | **I** | **Y** | **E** | **S** | **M** | **V** | **R** | **M** | **A** | **Q** | **P** | **F** | **S** | **Y** | **R** | **Y** | **M** | **L** | **V** | **D** | **G** | **H** | **G** | **N** | **F** | **G** | **S** | **V** | **D** | **G** | **D** | **G** | **A** | **A** | **A** | **M** | **R** | **Y** | **T** | **E** | **A** | **R** | **M** | **S** | **K** | **I** | **A** | **T** | **E** | **M** | **L** | **R** | **D** | **I** | **N** | **K** | **N** | **T** | **V** | **D** | **F** | **Q** | **S** | **N** | **Y** | **D** | **D** | **T** | **E** | **K** | **E** | **P** | **V** | **V** | **L** | **P** | **A** | **R** | **F** | **P** | **N** | **L** | **L** | **V** | **N** | **G** | **T** | **T** | **G** | **I** | **A** | **V** | **G** | **M** | **A** | **T** | **N** | **I** | **P** | **P** | **H** | **N** | **L** | **S** | **E** | **V** | **V** | **A** | **A** | **I** | **D** | **L** | **L** | **M** | **D** | **N** | **P** | **D** | **V** | **T** | **T** | **N** | **E** | **L** | **M** | **E** | **V** | **L** | **P** | **G** | **P** | **D** | **F** | **P** | **T** | **G** | **G** | **L** | **V** | **M** | **G** | **K** | **S** | **G** | **I** | **R** | **R** | **A** | **Y** | **E** | **T** | **G** | **K** | **G** | **S** | **I** | **T** | **V** | **R** | **A** | **K** | **V** | **E** | **I** | **T** | **E** | **M** | **P** | **N** | **G** | **K** | **E** | **R** | **I** | **I** | **V** | **T** | **E** | **L** | **P** | **Y** | **M** | **V** | **N** | **K** | **A** | **K** | **L** | **I** | **E** | **R** | **I** | **S** | **E** | **L** | **H** | **R** | **D** | **K** | **R** | **I** | **E** | **G** | **I** | **T** | **D** | **L** | **R** | **D** | **E** | **S** | **S** | **R** | **E** | **G** | **M** | **R** | **I** | **V** | **I** | **D** | **I** | **R** | **R** | **D** | **A** | **S** | **A** | **S** | **V** | **I** | **L** | **N** | **N** | **L** | **Y** | **K** | **L** | **T** | **S** | **L** | **Q** | **N** | **S** | **F** | **G** | **F** | **N** | **M** | **L** | **A** | **I** | **E** | **K** | **G** | **V** | **P** | **K** | **V** | **L** | **S** | **L** | **K** | **Q** | **I** | **L** | **E** | **N** | **Y** | **I** | **E** | **H** | **Q** | **R** | **E** | **V** | **I** | **T** | **R** | **R** | **T** | **V** | **F** | **E** | **K** | **E** | **K** | **A** | **E** | **A** | **R** | **A** | **H** | **I** | **L** | **E** | **G** | **L** | **R** | **I** | **A** | **L** | **D** | **H** | **I** | **D** | **E** | **I** | **I** | **A** | **I** | **I** | **R** | **G** | **S** | **K** | **S** | **D** | **D** | **E** | **A** | **K** | **A** | **T** | **M** | **I** | **E** | **R** | **F** | **D** | **L** | **S** | **D** | **R** | **Q** | **S** | **Q** | **A** | **I** | **L** | **D** | **M** | **R** | **L** | **R** | **R** | **L** | **T** | **G** | **L** | **E** | **R** | **E** | **K** | **I** | **E** | **N** | **E** | **Y** | **Q** | **E** | **L** | **L** | **K** | **L** | **I** | **E** | **D** | **L** | **T** | **D** | **I** | **L** | **A** | **R** | **P** | **E** | **R** | **V** | **T** | **E** | **I** | **I** | **K** | **N** | **E** | **L** | **A** | **D** | **L** | **N** | **Q** | **R** | **F** | **G** | **D** | **K** | **R** | **R** | **T** | **E** | **L** | **L** | **V** | **G** | **E** | **V** | **L** | **S** | **L** | **E** | **D** | **E** | **D** | **L** | **I** | **E** | **E** | **E** | **E** | **I** | **V** | **I** | **T** | **L** | **T** | **N** | **N** | **G** | **Y** | **I** | **K** | **R** | **L** | **A** | **N** | **N** | **E** | **F** | **R** | **A** | **Q** | **R** | **R** | **G** | **G** | **R** | **G** | **V** | **Q** | **G** | **M** | **G** | **I** | **H** | **N** | **D** | **D** | **F** | **V** | **K** | **N** | **L** | **V** | **S** | **C** | **S** | **T** | **H** | **D** | **T** | **L** | **L** | **F** | **F** | **T** | **N** | **N** | **G** | **K** | **V** | **Y** | **R** | **A** | **K** | **G** | **Y** | **E** | **I** | **P** | **E** | **Y** | **G** | **R** | **T** | **A** | **K** | **G** | **I** | **P** | **I** | **I** | **N** | **L** | **L** | **G** | **I** | **D** | **S** | **S** | **E** | **S** | **I** | **Q** | **A** | **I** | **I** | **A** | **V** | **T** | **G** | **E** | **A** | **E** | **E** | **G** | **H** | **Y** | **L** | **F** | **F** | **T** | **T** | **R** | **Q** | **G** | **T** | **V** | **K** | **R** | **T** | **S** | **V** | **T** | **A** | **F** | **A** | **N** | **I** | **R** | **S** | **N** | **G** | **L** | **I** | **A** | **I** | **G** | **L** | **K** | **E** | **D** | **D** | **E** | **L** | **V** | **N** | **V** | **L** | **L** | **T** | **D** | **G** | **Q** | **S** | **N** | **I** | **I** | **I** | **G** | **T** | **H** | **N** | **G** | **Y** | **S** | **V** | **T** | **F** | **K** | **E** | **E** | **A** | **V** | **R** | **D** | **M** | **G** | **R** | **T** | **A** | **S** | **G** | **V** | **R** | **G** | **I** | **R** | **L** | **R** | **E** | **E** | **D** | **Y** | **V** | **V** | **G** | **A** | **A** | **L** | **M** | **K** | **E** | **N** | **Q** | **E** | **V** | **L** | **V** | **I** | **T** | **E** | **K** | **G** | **Y** | **G** | **K** | **R** | **T** | **K** | **V** | **S** | **E** | **Y** | **P** | **V** | **K** | **G** | **R** | **G** | **G** | **K** | **G** | **I** | **K** | **T** | **A** | **N** | **I** | **T** | **E** | **K** | **N** | **G** | **P** | **L** | **A** | **G** | **L** | **T** | **T** | **V** | **S** | **G** | **D** | **E** | **D** | **I** | **M** | **L** | **I** | **T** | **D** | **K** | **G** | **V** | **I** | **I** | **R** | **F** | **N** | **V** | **S** | **T** | **V** | **S** | **Q** | **T** | **G** | **R** | **S** | **T** | **L** | **G** | **V** | **R** | **L** | **M** | **K** | **M** | **E** | **A** | **D** | **T** | **K** | **V** | **V** | **T** | **M** | **A** | **A** | **V** | **E** | **P** | **E** | **A** | **A** | **E** | **Q** | **T** | **V** | **E** | **E** | **Q** | **G** | **S** | **E** | **E** |
| **N60365F** | **M** | **S** | **E** | **E** | **I** | **R** | **E** | **N** | **I** | **H** | **D** | **V** | **N** | **L** | **T** | **S** | **E** | **M** | **K** | **D** | **S** | **F** | **I** | **D** | **Y** | **A** | **M** | **S** | **V** | **I** | **V** | **A** | **R** | **A** | **L** | **P** | **D** | **V** | **R** | **D** | **G** | **L** | **K** | **P** | **V** | **H** | **R** | **R** | **I** | **L** | **Y** | **G** | **M** | **N** | **E** | **L** | **G** | **V** | **T** | **P** | **D** | **K** | **A** | **H** | **K** | **K** | **S** | **A** | **R** | **I** | **V** | **G** | **D** | **V** | **M** | **G** | **K** | **Y** | **H** | **P** | **H** | **G** | **D** | **S** | **A** | **I** | **Y** | **E** | **S** | **M** | **V** | **R** | **M** | **A** | **Q** | **P** | **F** | **S** | **Y** | **R** | **Y** | **M** | **L** | **V** | **D** | **G** | **H** | **G** | **N** | **F** | **G** | **S** | **V** | **D** | **G** | **D** | **G** | **A** | **A** | **A** | **M** | **R** | **Y** | **T** | **E** | **A** | **R** | **M** | **S** | **K** | **I** | **A** | **T** | **E** | **M** | **L** | **R** | **D** | **I** | **N** | **K** | **N** | **T** | **V** | **D** | **F** | **Q** | **S** | **N** | **Y** | **D** | **D** | **T** | **E** | **K** | **E** | **P** | **V** | **V** | **L** | **P** | **A** | **R** | **F** | **P** | **N** | **L** | **L** | **V** | **N** | **G** | **T** | **T** | **G** | **I** | **A** | **V** | **G** | **M** | **A** | **T** | **N** | **I** | **P** | **P** | **H** | **N** | **L** | **S** | **E** | **V** | **V** | **A** | **A** | **I** | **D** | **L** | **L** | **M** | **D** | **N** | **P** | **D** | **V** | **T** | **T** | **N** | **E** | **L** | **M** | **E** | **V** | **L** | **P** | **G** | **P** | **D** | **F** | **P** | **T** | **G** | **G** | **L** | **V** | **M** | **G** | **K** | **S** | **G** | **I** | **R** | **R** | **A** | **Y** | **E** | **T** | **G** | **K** | **G** | **S** | **I** | **T** | **V** | **R** | **A** | **K** | **V** | **E** | **I** | **T** | **E** | **M** | **P** | **N** | **G** | **K** | **E** | **R** | **I** | **I** | **V** | **T** | **E** | **L** | **P** | **Y** | **M** | **V** | **N** | **K** | **A** | **K** | **L** | **I** | **E** | **R** | **I** | **S** | **E** | **L** | **H** | **R** | **D** | **K** | **R** | **I** | **E** | **G** | **I** | **T** | **D** | **L** | **R** | **D** | **E** | **S** | **S** | **R** | **E** | **G** | **M** | **R** | **I** | **V** | **I** | **D** | **I** | **R** | **R** | **D** | **A** | **S** | **A** | **S** | **V** | **I** | **L** | **N** | **N** | **L** | **Y** | **K** | **L** | **T** | **S** | **L** | **Q** | **N** | **S** | **F** | **G** | **F** | **N** | **M** | **L** | **A** | **I** | **E** | **K** | **G** | **V** | **P** | **K** | **V** | **L** | **S** | **L** | **K** | **Q** | **I** | **L** | **E** | **N** | **Y** | **I** | **E** | **H** | **Q** | **R** | **E** | **V** | **I** | **T** | **R** | **R** | **T** | **V** | **F** | **E** | **K** | **E** | **K** | **A** | **E** | **A** | **R** | **A** | **H** | **I** | **L** | **E** | **G** | **L** | **R** | **I** | **A** | **L** | **D** | **H** | **I** | **D** | **E** | **I** | **I** | **A** | **I** | **I** | **R** | **G** | **S** | **K** | **S** | **D** | **D** | **E** | **A** | **K** | **A** | **T** | **M** | **I** | **E** | **R** | **F** | **D** | **L** | **S** | **D** | **R** | **Q** | **S** | **Q** | **A** | **I** | **L** | **D** | **M** | **R** | **L** | **R** | **R** | **L** | **T** | **G** | **L** | **E** | **R** | **E** | **K** | **I** | **E** | **N** | **E** | **Y** | **Q** | **E** | **L** | **L** | **K** | **L** | **I** | **E** | **D** | **L** | **T** | **D** | **I** | **L** | **A** | **R** | **P** | **E** | **R** | **V** | **T** | **E** | **I** | **I** | **K** | **N** | **E** | **L** | **A** | **D** | **L** | **N** | **Q** | **R** | **F** | **G** | **D** | **K** | **R** | **R** | **T** | **E** | **L** | **L** | **V** | **G** | **E** | **V** | **L** | **S** | **L** | **E** | **D** | **E** | **D** | **L** | **I** | **E** | **E** | **E** | **E** | **I** | **V** | **I** | **T** | **L** | **T** | **N** | **N** | **G** | **Y** | **I** | **K** | **R** | **L** | **A** | **N** | **N** | **E** | **F** | **R** | **A** | **Q** | **R** | **R** | **G** | **G** | **R** | **G** | **V** | **Q** | **G** | **M** | **G** | **I** | **H** | **N** | **D** | **D** | **F** | **V** | **K** | **N** | **L** | **V** | **S** | **C** | **S** | **T** | **H** | **D** | **T** | **L** | **L** | **F** | **F** | **T** | **N** | **N** | **G** | **K** | **V** | **Y** | **R** | **A** | **K** | **G** | **Y** | **E** | **I** | **P** | **E** | **Y** | **G** | **R** | **T** | **A** | **K** | **G** | **I** | **P** | **I** | **I** | **N** | **L** | **L** | **G** | **I** | **D** | **S** | **S** | **E** | **S** | **I** | **Q** | **A** | **I** | **I** | **A** | **V** | **T** | **G** | **E** | **A** | **E** | **E** | **G** | **H** | **Y** | **L** | **F** | **F** | **T** | **T** | **R** | **Q** | **G** | **T** | **V** | **K** | **R** | **T** | **S** | **V** | **T** | **A** | **F** | **A** | **N** | **I** | **R** | **S** | **N** | **G** | **L** | **I** | **A** | **I** | **G** | **L** | **K** | **E** | **D** | **D** | **E** | **L** | **V** | **N** | **V** | **L** | **L** | **T** | **D** | **G** | **Q** | **S** | **N** | **I** | **I** | **I** | **G** | **T** | **H** | **N** | **G** | **Y** | **S** | **V** | **T** | **F** | **K** | **E** | **E** | **A** | **V** | **R** | **D** | **M** | **G** | **R** | **T** | **A** | **S** | **G** | **V** | **R** | **G** | **I** | **R** | **L** | **R** | **E** | **E** | **D** | **Y** | **V** | **V** | **G** | **A** | **A** | **L** | **M** | **K** | **E** | **N** | **Q** | **E** | **V** | **L** | **V** | **I** | **T** | **E** | **K** | **G** | **Y** | **G** | **K** | **R** | **T** | **K** | **V** | **S** | **E** | **Y** | **P** | **V** | **K** | **G** | **R** | **G** | **G** | **K** | **G** | **I** | **K** | **T** | **A** | **N** | **I** | **T** | **E** | **K** | **N** | **G** | **P** | **L** | **A** | **G** | **L** | **T** | **T** | **V** | **S** | **G** | **D** | **E** | **D** | **I** | **M** | **L** | **I** | **T** | **D** | **K** | **G** | **V** | **I** | **I** | **R** | **F** | **N** | **V** | **S** | **T** | **V** | **S** | **Q** | **T** | **G** | **R** | **S** | **T** | **L** | **G** | **V** | **R** | **L** | **M** | **K** | **M** | **E** | **A** | **D** | **T** | **K** | **V** | **V** | **T** | **M** | **A** | **A** | **V** | **E** | **P** | **E** | **A** | **A** | **E** | **Q** | **T** | **V** | **E** | **E** | **Q** | **G** | **S** | **E** | **E** |
| **N52656** | **M** | **S** | **E** | **E** | **I** | **R** | **E** | **N** | **I** | **H** | **D** | **V** | **N** | **L** | **T** | **S** | **E** | **M** | **K** | **D** | **S** | **F** | **I** | **D** | **Y** | **A** | **M** | **S** | **V** | **I** | **V** | **A** | **R** | **A** | **L** | **P** | **D** | **V** | **R** | **D** | **G** | **L** | **K** | **P** | **V** | **H** | **R** | **R** | **I** | **L** | **Y** | **G** | **M** | **N** | **E** | **L** | **G** | **V** | **T** | **P** | **D** | **K** | **A** | **H** | **K** | **K** | **S** | **A** | **R** | **I** | **V** | **G** | **D** | **V** | **M** | **G** | **K** | **Y** | **H** | **P** | **H** | **G** | **D** | **S** | **A** | **I** | **Y** | **E** | **S** | **M** | **V** | **R** | **M** | **A** | **Q** | **P** | **F** | **S** | **Y** | **R** | **Y** | **M** | **L** | **V** | **D** | **G** | **H** | **G** | **N** | **F** | **G** | **S** | **V** | **D** | **G** | **D** | **G** | **A** | **A** | **A** | **M** | **R** | **Y** | **T** | **E** | **A** | **R** | **M** | **S** | **K** | **I** | **A** | **T** | **E** | **M** | **L** | **R** | **D** | **I** | **N** | **K** | **N** | **T** | **V** | **D** | **F** | **Q** | **S** | **N** | **Y** | **D** | **D** | **T** | **E** | **K** | **E** | **P** | **V** | **V** | **L** | **P** | **A** | **R** | **F** | **P** | **N** | **L** | **L** | **V** | **N** | **G** | **T** | **T** | **G** | **I** | **A** | **V** | **G** | **M** | **A** | **T** | **N** | **I** | **P** | **P** | **H** | **N** | **L** | **S** | **E** | **V** | **V** | **A** | **A** | **I** | **D** | **L** | **L** | **M** | **D** | **N** | **P** | **D** | **V** | **T** | **T** | **N** | **E** | **L** | **M** | **E** | **V** | **L** | **P** | **G** | **P** | **D** | **F** | **P** | **T** | **G** | **G** | **L** | **V** | **M** | **G** | **K** | **S** | **G** | **I** | **R** | **R** | **A** | **Y** | **E** | **T** | **G** | **K** | **G** | **S** | **I** | **T** | **V** | **R** | **A** | **K** | **V** | **E** | **I** | **T** | **E** | **M** | **P** | **N** | **G** | **K** | **E** | **R** | **I** | **L** | **V** | **T** | **E** | **L** | **P** | **Y** | **M** | **V** | **N** | **K** | **A** | **K** | **L** | **I** | **E** | **R** | **I** | **S** | **E** | **L** | **H** | **R** | **D** | **K** | **R** | **I** | **E** | **G** | **I** | **T** | **D** | **L** | **R** | **D** | **E** | **S** | **S** | **R** | **E** | **G** | **M** | **R** | **I** | **V** | **I** | **D** | **V** | **R** | **R** | **D** | **A** | **S** | **A** | **S** | **V** | **I** | **L** | **N** | **N** | **L** | **Y** | **K** | **L** | **T** | **S** | **L** | **Q** | **N** | **S** | **F** | **G** | **F** | **N** | **M** | **L** | **A** | **I** | **E** | **K** | **G** | **V** | **P** | **K** | **V** | **L** | **S** | **L** | **K** | **Q** | **I** | **L** | **E** | **N** | **Y** | **I** | **E** | **H** | **Q** | **R** | **E** | **V** | **I** | **T** | **R** | **R** | **T** | **V** | **F** | **E** | **K** | **E** | **K** | **A** | **E** | **A** | **R** | **A** | **H** | **I** | **L** | **E** | **G** | **L** | **R** | **I** | **A** | **L** | **D** | **H** | **I** | **D** | **E** | **I** | **I** | **A** | **I** | **I** | **R** | **G** | **S** | **K** | **S** | **D** | **D** | **E** | **A** | **K** | **A** | **T** | **M** | **I** | **E** | **R** | **F** | **D** | **L** | **S** | **D** | **R** | **Q** | **S** | **Q** | **A** | **I** | **L** | **D** | **M** | **R** | **L** | **R** | **R** | **L** | **T** | **G** | **L** | **E** | **R** | **E** | **K** | **I** | **E** | **N** | **E** | **Y** | **Q** | **E** | **L** | **L** | **K** | **L** | **I** | **E** | **D** | **L** | **T** | **D** | **I** | **L** | **A** | **R** | **P** | **E** | **R** | **V** | **T** | **E** | **I** | **I** | **K** | **N** | **E** | **L** | **A** | **D** | **L** | **N** | **Q** | **R** | **F** | **G** | **D** | **K** | **R** | **R** | **T** | **E** | **L** | **L** | **V** | **G** | **E** | **V** | **L** | **S** | **L** | **E** | **D** | **E** | **D** | **L** | **I** | **E** | **E** | **E** | **E** | **I** | **V** | **I** | **T** | **L** | **T** | **N** | **N** | **G** | **Y** | **I** | **K** | **R** | **L** | **A** | **N** | **N** | **E** | **F** | **R** | **A** | **Q** | **R** | **R** | **G** | **G** | **R** | **G** | **V** | **Q** | **G** | **M** | **G** | **I** | **H** | **N** | **D** | **D** | **F** | **V** | **K** | **N** | **L** | **V** | **S** | **C** | **S** | **T** | **H** | **D** | **T** | **L** | **L** | **F** | **F** | **T** | **N** | **N** | **G** | **K** | **V** | **Y** | **R** | **A** | **K** | **G** | **Y** | **E** | **I** | **P** | **E** | **Y** | **G** | **R** | **T** | **A** | **K** | **G** | **I** | **P** | **I** | **I** | **N** | **L** | **L** | **G** | **I** | **D** | **S** | **S** | **E** | **S** | **I** | **Q** | **A** | **I** | **I** | **A** | **V** | **T** | **G** | **E** | **A** | **E** | **E** | **G** | **H** | **Y** | **L** | **F** | **F** | **T** | **T** | **R** | **Q** | **G** | **T** | **V** | **K** | **R** | **T** | **S** | **V** | **T** | **A** | **F** | **A** | **N** | **I** | **R** | **S** | **N** | **G** | **L** | **I** | **A** | **I** | **G** | **L** | **K** | **E** | **D** | **D** | **E** | **L** | **V** | **N** | **V** | **L** | **L** | **T** | **D** | **G** | **Q** | **S** | **N** | **I** | **I** | **I** | **G** | **T** | **H** | **N** | **G** | **Y** | **S** | **V** | **T** | **F** | **K** | **E** | **E** | **A** | **V** | **R** | **D** | **M** | **G** | **R** | **T** | **A** | **S** | **G** | **V** | **R** | **G** | **I** | **R** | **L** | **R** | **E** | **E** | **D** | **Y** | **V** | **V** | **G** | **A** | **A** | **L** | **M** | **K** | **E** | **D** | **Q** | **E** | **V** | **L** | **V** | **I** | **T** | **E** | **K** | **G** | **Y** | **G** | **K** | **R** | **T** | **K** | **V** | **S** | **E** | **Y** | **P** | **V** | **K** | **G** | **R** | **G** | **G** | **K** | **G** | **I** | **K** | **T** | **A** | **N** | **I** | **T** | **E** | **K** | **N** | **G** | **P** | **L** | **A** | **G** | **L** | **T** | **T** | **V** | **S** | **G** | **N** | **E** | **D** | **I** | **M** | **L** | **I** | **T** | **D** | **K** | **G** | **V** | **I** | **I** | **R** | **F** | **N** | **V** | **S** | **T** | **V** | **S** | **Q** | **T** | **G** | **R** | **S** | **T** | **L** | **G** | **V** | **R** | **L** | **M** | **K** | **M** | **E** | **A** | **D** | **T** | **K** | **V** | **V** | **T** | **M** | **-** | **-** | **-** | **-** | **-** | **-** | **-** | **-** | **-** | **-** | **-** | **-** | **-** | **-** | **-** | **-** | **-** | **-** | **-** |
| **N52467** | **M** | **S** | **E** | **E** | **I** | **K** | **E** | **N** | **I** | **Q** | **D** | **V** | **N** | **L** | **T** | **S** | **E** | **M** | **K** | **E** | **S** | **F** | **I** | **D** | **Y** | **A** | **M** | **S** | **V** | **I** | **V** | **A** | **R** | **A** | **L** | **P** | **D** | **V** | **R** | **D** | **G** | **L** | **K** | **P** | **V** | **H** | **R** | **R** | **I** | **L** | **Y** | **G** | **M** | **N** | **E** | **L** | **G** | **V** | **T** | **P** | **D** | **K** | **P** | **H** | **K** | **K** | **S** | **A** | **R** | **I** | **V** | **G** | **D** | **V** | **M** | **G** | **K** | **Y** | **H** | **P** | **H** | **G** | **D** | **S** | **A** | **I** | **Y** | **E** | **S** | **M** | **V** | **R** | **M** | **A** | **Q** | **P** | **F** | **S** | **Y** | **R** | **A** | **M** | **L** | **V** | **D** | **G** | **H** | **G** | **N** | **F** | **G** | **S** | **V** | **D** | **G** | **D** | **G** | **A** | **A** | **A** | **M** | **R** | **Y** | **T** | **E** | **A** | **R** | **M** | **S** | **K** | **I** | **A** | **L** | **E** | **M** | **L** | **R** | **D** | **I** | **N** | **K** | **N** | **T** | **V** | **D** | **F** | **Q** | **G** | **N** | **Y** | **D** | **D** | **S** | **E** | **Q** | **E** | **P** | **V** | **V** | **L** | **P** | **A** | **R** | **F** | **P** | **N** | **L** | **L** | **V** | **N** | **G** | **T** | **T** | **G** | **I** | **A** | **V** | **G** | **M** | **A** | **T** | **N** | **I** | **P** | **P** | **H** | **N** | **L** | **S** | **E** | **V** | **I** | **D** | **A** | **T** | **S** | **L** | **L** | **M** | **D** | **N** | **P** | **D** | **V** | **T** | **T** | **N** | **E** | **L** | **M** | **E** | **V** | **L** | **P** | **G** | **P** | **D** | **F** | **P** | **T** | **G** | **G** | **L** | **V** | **M** | **G** | **K** | **S** | **G** | **I** | **R** | **R** | **A** | **Y** | **E** | **T** | **G** | **K** | **G** | **S** | **I** | **T** | **V** | **R** | **A** | **K** | **V** | **E** | **L** | **T** | **E** | **M** | **P** | **N** | **G** | **K** | **E** | **R** | **I** | **L** | **V** | **T** | **E** | **L** | **P** | **Y** | **M** | **V** | **N** | **K** | **A** | **K** | **L** | **I** | **E** | **R** | **I** | **S** | **E** | **L** | **H** | **R** | **D** | **K** | **R** | **I** | **E** | **G** | **I** | **T** | **D** | **L** | **R** | **D** | **E** | **S** | **S** | **R** | **E** | **G** | **M** | **R** | **I** | **V** | **I** | **D** | **V** | **R** | **R** | **D** | **V** | **S** | **A** | **S** | **V** | **V** | **L** | **N** | **N** | **L** | **Y** | **K** | **M** | **T** | **A** | **L** | **Q** | **T** | **S** | **F** | **G** | **F** | **N** | **M** | **L** | **A** | **I** | **E** | **K** | **G** | **V** | **P** | **K** | **I** | **L** | **S** | **L** | **K** | **R** | **I** | **L** | **E** | **N** | **Y** | **I** | **E** | **H** | **Q** | **K** | **E** | **V** | **I** | **T** | **R** | **R** | **T** | **I** | **F** | **D** | **K** | **N** | **K** | **A** | **E** | **A** | **R** | **A** | **H** | **I** | **L** | **E** | **G** | **L** | **R** | **I** | **A** | **L** | **D** | **H** | **I** | **D** | **E** | **I** | **I** | **A** | **I** | **I** | **R** | **G** | **S** | **Q** | **S** | **D** | **D** | **E** | **A** | **K** | **A** | **T** | **L** | **I** | **E** | **R** | **F** | **E** | **F** | **S** | **D** | **R** | **Q** | **A** | **Q** | **A** | **I** | **L** | **D** | **M** | **R** | **L** | **R** | **R** | **L** | **T** | **G** | **L** | **E** | **R** | **D** | **K** | **I** | **E** | **N** | **E** | **Y** | **Q** | **E** | **L** | **L** | **K** | **F** | **I** | **A** | **D** | **L** | **E** | **D** | **I** | **L** | **A** | **R** | **P** | **E** | **R** | **V** | **I** | **E** | **I** | **I** | **K** | **T** | **E** | **L** | **N** | **D** | **V** | **R** | **T** | **K** | **F** | **G** | **D** | **A** | **R** | **R** | **T** | **E** | **L** | **L** | **V** | **G** | **E** | **V** | **L** | **S** | **L** | **E** | **D** | **E** | **D** | **L** | **I** | **E** | **E** | **E** | **E** | **V** | **V** | **I** | **T** | **L** | **T** | **N** | **N** | **G** | **Y** | **I** | **K** | **R** | **M** | **A** | **N** | **S** | **E** | **F** | **R** | **A** | **Q** | **R** | **R** | **G** | **G** | **R** | **G** | **V** | **Q** | **G** | **M** | **G** | **V** | **H** | **D** | **D** | **D** | **F** | **V** | **K** | **N** | **L** | **V** | **S** | **C** | **S** | **T** | **H** | **D** | **T** | **L** | **L** | **F** | **F** | **T** | **N** | **T** | **G** | **K** | **V** | **Y** | **R** | **A** | **K** | **G** | **Y** | **E** | **I** | **P** | **E** | **Y** | **G** | **R** | **T** | **A** | **K** | **G** | **I** | **P** | **V** | **I** | **N** | **L** | **L** | **G** | **I** | **D** | **S** | **A** | **E** | **K** | **I** | **Q** | **A** | **I** | **I** | **S** | **V** | **E** | **G** | **K** | **A** | **E** | **A** | **G** | **K** | **Y** | **L** | **F** | **F** | **T** | **T** | **L** | **K** | **G** | **T** | **V** | **K** | **R** | **T** | **A** | **V** | **T** | **A** | **F** | **S** | **N** | **I** | **R** | **S** | **N** | **G** | **L** | **I** | **A** | **I** | **S** | **L** | **K** | **E** | **D** | **D** | **E** | **L** | **V** | **N** | **V** | **V** | **T** | **T** | **N** | **G** | **N** | **Q** | **K** | **M** | **I** | **I** | **G** | **T** | **H** | **A** | **G** | **Y** | **S** | **V** | **T** | **F** | **D** | **E** | **T** | **T** | **V** | **R** | **D** | **M** | **G** | **R** | **T** | **A** | **S** | **G** | **V** | **R** | **G** | **I** | **R** | **L** | **R** | **E** | **N** | **D** | **Y** | **V** | **V** | **G** | **A** | **A** | **I** | **L** | **D** | **E** | **N** | **K** | **E** | **V** | **L** | **V** | **I** | **T** | **E** | **N** | **G** | **Y** | **G** | **K** | **R** | **T** | **K** | **A** | **S** | **E** | **Y** | **P** | **V** | **K** | **G** | **R** | **G** | **G** | **K** | **G** | **I** | **K** | **T** | **A** | **N** | **I** | **T** | **E** | **K** | **N** | **G** | **P** | **L** | **A** | **G** | **L** | **T** | **T** | **V** | **N** | **G** | **D** | **E** | **D** | **I** | **L** | **L** | **I** | **T** | **N** | **K** | **G** | **V** | **I** | **I** | **R** | **F** | **N** | **V** | **D** | **S** | **V** | **S** | **Q** | **T** | **G** | **R** | **A** | **T** | **L** | **G** | **V** | **R** | **L** | **M** | **R** | **M** | **E** | **D** | **G** | **A** | **K** | **-** | **-** | **-** | **-** | **-** | **-** | **-** | **-** | **-** | **-** | **-** | **-** | **-** | **-** | **-** | **-** | **-** | **-** | **-** | **-** | **-** | **-** | **-** |
| **N55290** | **M** | **S** | **E** | **E** | **I** | **R** | **E** | **N** | **I** | **H** | **D** | **V** | **N** | **L** | **T** | **S** | **E** | **M** | **K** | **D** | **S** | **F** | **I** | **D** | **Y** | **A** | **M** | **S** | **V** | **I** | **V** | **A** | **R** | **A** | **L** | **P** | **D** | **V** | **R** | **D** | **G** | **L** | **K** | **P** | **V** | **H** | **R** | **R** | **I** | **L** | **Y** | **G** | **M** | **N** | **E** | **L** | **G** | **V** | **T** | **P** | **D** | **K** | **A** | **H** | **K** | **K** | **S** | **A** | **R** | **I** | **V** | **G** | **D** | **V** | **M** | **G** | **K** | **Y** | **H** | **P** | **H** | **G** | **D** | **S** | **A** | **I** | **Y** | **E** | **S** | **M** | **V** | **R** | **M** | **A** | **Q** | **P** | **F** | **S** | **Y** | **R** | **Y** | **M** | **L** | **V** | **D** | **G** | **H** | **G** | **N** | **F** | **G** | **S** | **V** | **D** | **G** | **D** | **G** | **A** | **A** | **A** | **M** | **R** | **Y** | **T** | **E** | **A** | **R** | **M** | **S** | **K** | **I** | **A** | **T** | **E** | **M** | **L** | **R** | **D** | **I** | **N** | **K** | **N** | **T** | **V** | **D** | **F** | **Q** | **S** | **N** | **Y** | **D** | **D** | **T** | **E** | **K** | **E** | **P** | **V** | **V** | **L** | **P** | **A** | **R** | **F** | **P** | **N** | **L** | **L** | **V** | **N** | **G** | **T** | **T** | **G** | **I** | **A** | **V** | **G** | **M** | **A** | **T** | **N** | **I** | **P** | **P** | **H** | **N** | **L** | **S** | **E** | **V** | **V** | **A** | **A** | **I** | **D** | **L** | **L** | **M** | **D** | **N** | **P** | **D** | **V** | **T** | **T** | **N** | **E** | **L** | **M** | **E** | **V** | **L** | **P** | **G** | **P** | **D** | **F** | **P** | **T** | **G** | **G** | **L** | **V** | **M** | **G** | **K** | **S** | **G** | **I** | **R** | **R** | **A** | **Y** | **E** | **T** | **G** | **K** | **G** | **S** | **I** | **T** | **V** | **R** | **A** | **K** | **V** | **E** | **I** | **T** | **E** | **M** | **P** | **N** | **G** | **K** | **E** | **R** | **I** | **L** | **V** | **T** | **E** | **L** | **P** | **Y** | **M** | **V** | **N** | **K** | **A** | **K** | **L** | **I** | **E** | **R** | **I** | **S** | **E** | **L** | **H** | **R** | **D** | **K** | **R** | **I** | **E** | **G** | **I** | **T** | **D** | **L** | **R** | **D** | **E** | **S** | **S** | **R** | **E** | **G** | **M** | **R** | **I** | **V** | **I** | **D** | **V** | **R** | **R** | **D** | **A** | **S** | **A** | **S** | **V** | **I** | **L** | **N** | **N** | **L** | **Y** | **K** | **L** | **T** | **S** | **L** | **Q** | **N** | **S** | **F** | **G** | **F** | **N** | **M** | **L** | **A** | **I** | **E** | **K** | **G** | **V** | **P** | **K** | **V** | **L** | **S** | **L** | **K** | **Q** | **I** | **L** | **E** | **N** | **Y** | **I** | **E** | **H** | **Q** | **R** | **E** | **V** | **I** | **T** | **R** | **R** | **T** | **V** | **F** | **E** | **K** | **E** | **K** | **A** | **E** | **A** | **R** | **A** | **H** | **I** | **L** | **E** | **G** | **L** | **R** | **I** | **A** | **L** | **D** | **H** | **I** | **D** | **E** | **I** | **I** | **A** | **I** | **I** | **R** | **G** | **S** | **K** | **S** | **D** | **D** | **E** | **A** | **K** | **A** | **T** | **M** | **I** | **E** | **R** | **F** | **D** | **L** | **S** | **D** | **R** | **Q** | **S** | **Q** | **A** | **I** | **L** | **D** | **M** | **R** | **L** | **R** | **R** | **L** | **T** | **G** | **L** | **E** | **R** | **E** | **K** | **I** | **E** | **N** | **E** | **Y** | **Q** | **E** | **L** | **L** | **K** | **L** | **I** | **E** | **D** | **L** | **T** | **D** | **I** | **L** | **A** | **R** | **P** | **E** | **R** | **V** | **T** | **E** | **I** | **I** | **K** | **N** | **E** | **L** | **A** | **D** | **L** | **N** | **Q** | **R** | **F** | **G** | **D** | **K** | **R** | **R** | **T** | **E** | **L** | **L** | **V** | **G** | **E** | **V** | **L** | **S** | **L** | **E** | **D** | **E** | **D** | **L** | **I** | **E** | **E** | **E** | **E** | **I** | **V** | **I** | **T** | **L** | **T** | **N** | **N** | **G** | **Y** | **I** | **K** | **R** | **L** | **A** | **N** | **N** | **E** | **F** | **R** | **A** | **Q** | **R** | **R** | **G** | **G** | **R** | **G** | **V** | **Q** | **G** | **M** | **G** | **I** | **H** | **N** | **D** | **D** | **F** | **V** | **K** | **N** | **L** | **V** | **S** | **C** | **S** | **T** | **H** | **D** | **T** | **L** | **L** | **F** | **F** | **T** | **N** | **N** | **G** | **K** | **V** | **Y** | **R** | **A** | **K** | **G** | **Y** | **E** | **I** | **P** | **E** | **Y** | **G** | **R** | **T** | **A** | **K** | **G** | **I** | **P** | **I** | **I** | **N** | **L** | **L** | **G** | **I** | **D** | **S** | **S** | **E** | **S** | **I** | **Q** | **A** | **I** | **I** | **A** | **V** | **T** | **G** | **E** | **A** | **E** | **E** | **G** | **H** | **Y** | **L** | **F** | **F** | **T** | **T** | **R** | **Q** | **G** | **T** | **V** | **K** | **R** | **T** | **S** | **V** | **T** | **A** | **F** | **A** | **N** | **I** | **R** | **S** | **N** | **G** | **L** | **I** | **A** | **I** | **G** | **L** | **K** | **E** | **D** | **D** | **E** | **L** | **I** | **N** | **V** | **L** | **L** | **T** | **D** | **G** | **Q** | **S** | **N** | **I** | **I** | **I** | **G** | **T** | **H** | **N** | **G** | **Y** | **S** | **V** | **T** | **F** | **K** | **E** | **E** | **A** | **V** | **R** | **D** | **M** | **G** | **R** | **T** | **A** | **S** | **G** | **V** | **R** | **G** | **I** | **R** | **L** | **R** | **E** | **E** | **D** | **C** | **V** | **V** | **G** | **A** | **A** | **L** | **M** | **K** | **E** | **D** | **Q** | **E** | **V** | **L** | **V** | **I** | **T** | **E** | **K** | **G** | **Y** | **G** | **K** | **R** | **T** | **K** | **V** | **S** | **E** | **Y** | **P** | **V** | **K** | **G** | **R** | **G** | **G** | **K** | **G** | **I** | **K** | **T** | **A** | **N** | **I** | **T** | **E** | **K** | **N** | **G** | **P** | **L** | **A** | **G** | **L** | **T** | **T** | **V** | **S** | **G** | **N** | **E** | **D** | **I** | **M** | **L** | **I** | **T** | **D** | **K** | **G** | **V** | **I** | **I** | **R** | **F** | **N** | **V** | **S** | **T** | **V** | **S** | **Q** | **T** | **G** | **R** | **S** | **T** | **L** | **G** | **V** | **R** | **L** | **M** | **K** | **M** | **E** | **A** | **D** | **T** | **K** | **V** | **V** | **T** | **M** | **-** | **-** | **-** | **-** | **-** | **-** | **-** | **-** | **-** | **-** | **-** | **-** | **-** | **-** | **-** | **-** | **-** | **-** | **-** |
| **N54118** | **M** | **S** | **E** | **E** | **I** | **K** | **E** | **N** | **I** | **Q** | **D** | **V** | **N** | **L** | **T** | **S** | **E** | **M** | **K** | **E** | **S** | **F** | **I** | **D** | **Y** | **A** | **M** | **S** | **V** | **I** | **V** | **A** | **R** | **A** | **L** | **P** | **D** | **V** | **R** | **D** | **G** | **L** | **K** | **P** | **V** | **H** | **R** | **R** | **I** | **L** | **Y** | **G** | **M** | **N** | **E** | **L** | **G** | **V** | **T** | **P** | **D** | **K** | **P** | **H** | **K** | **K** | **S** | **A** | **R** | **I** | **V** | **G** | **D** | **V** | **M** | **G** | **K** | **Y** | **H** | **P** | **H** | **G** | **D** | **S** | **A** | **I** | **Y** | **E** | **S** | **M** | **V** | **R** | **M** | **A** | **Q** | **P** | **F** | **S** | **Y** | **R** | **A** | **M** | **L** | **V** | **D** | **G** | **H** | **G** | **N** | **F** | **G** | **S** | **V** | **D** | **G** | **D** | **G** | **A** | **A** | **A** | **M** | **R** | **Y** | **T** | **E** | **A** | **R** | **M** | **S** | **K** | **I** | **A** | **L** | **E** | **M** | **L** | **R** | **D** | **I** | **N** | **K** | **N** | **T** | **V** | **D** | **F** | **Q** | **G** | **N** | **Y** | **D** | **D** | **S** | **E** | **Q** | **E** | **P** | **V** | **V** | **L** | **P** | **A** | **R** | **F** | **P** | **N** | **L** | **L** | **V** | **N** | **G** | **T** | **T** | **G** | **I** | **A** | **V** | **G** | **M** | **A** | **T** | **N** | **I** | **P** | **P** | **H** | **N** | **L** | **S** | **E** | **V** | **I** | **D** | **A** | **T** | **S** | **L** | **L** | **M** | **D** | **N** | **P** | **D** | **V** | **T** | **T** | **N** | **E** | **L** | **M** | **E** | **V** | **L** | **P** | **G** | **P** | **D** | **F** | **P** | **T** | **G** | **G** | **L** | **V** | **M** | **G** | **K** | **S** | **G** | **I** | **R** | **R** | **A** | **Y** | **E** | **T** | **G** | **K** | **G** | **S** | **I** | **T** | **V** | **R** | **A** | **K** | **V** | **E** | **L** | **T** | **E** | **M** | **P** | **N** | **G** | **K** | **E** | **R** | **I** | **L** | **V** | **T** | **E** | **L** | **P** | **Y** | **M** | **V** | **N** | **K** | **A** | **K** | **L** | **I** | **E** | **R** | **I** | **S** | **E** | **L** | **H** | **R** | **D** | **K** | **R** | **I** | **E** | **G** | **I** | **T** | **D** | **L** | **R** | **D** | **E** | **S** | **S** | **R** | **E** | **G** | **M** | **R** | **I** | **V** | **I** | **D** | **V** | **R** | **R** | **D** | **V** | **S** | **A** | **S** | **V** | **V** | **L** | **N** | **N** | **L** | **Y** | **K** | **M** | **T** | **A** | **L** | **Q** | **T** | **S** | **F** | **G** | **F** | **N** | **M** | **L** | **A** | **I** | **E** | **K** | **G** | **V** | **P** | **K** | **I** | **L** | **S** | **L** | **K** | **R** | **I** | **L** | **E** | **N** | **Y** | **V** | **E** | **H** | **Q** | **K** | **E** | **V** | **I** | **T** | **R** | **R** | **T** | **I** | **F** | **D** | **K** | **N** | **K** | **A** | **E** | **A** | **R** | **A** | **H** | **I** | **L** | **E** | **G** | **L** | **R** | **I** | **A** | **L** | **D** | **H** | **I** | **D** | **E** | **I** | **I** | **A** | **I** | **I** | **R** | **G** | **S** | **Q** | **S** | **D** | **D** | **E** | **A** | **K** | **A** | **T** | **L** | **I** | **E** | **R** | **F** | **E** | **F** | **S** | **D** | **R** | **Q** | **A** | **Q** | **A** | **I** | **L** | **D** | **M** | **R** | **L** | **R** | **R** | **L** | **T** | **G** | **L** | **E** | **R** | **D** | **K** | **I** | **E** | **N** | **E** | **Y** | **Q** | **E** | **L** | **L** | **K** | **F** | **I** | **A** | **D** | **L** | **E** | **D** | **I** | **L** | **A** | **R** | **P** | **E** | **R** | **V** | **I** | **E** | **I** | **I** | **K** | **T** | **E** | **L** | **N** | **D** | **V** | **R** | **T** | **K** | **F** | **G** | **D** | **A** | **R** | **R** | **T** | **E** | **L** | **L** | **V** | **G** | **E** | **V** | **L** | **S** | **L** | **E** | **D** | **E** | **D** | **L** | **I** | **E** | **E** | **E** | **E** | **V** | **V** | **I** | **T** | **L** | **T** | **N** | **N** | **G** | **Y** | **I** | **K** | **R** | **M** | **A** | **N** | **S** | **E** | **F** | **R** | **A** | **Q** | **R** | **R** | **G** | **G** | **R** | **G** | **V** | **Q** | **G** | **M** | **G** | **V** | **H** | **D** | **D** | **D** | **F** | **V** | **K** | **N** | **L** | **V** | **S** | **C** | **S** | **T** | **H** | **D** | **T** | **L** | **L** | **F** | **F** | **T** | **N** | **T** | **G** | **K** | **V** | **Y** | **R** | **A** | **K** | **G** | **Y** | **E** | **I** | **P** | **E** | **Y** | **G** | **R** | **T** | **A** | **K** | **G** | **I** | **P** | **V** | **I** | **N** | **L** | **L** | **G** | **I** | **D** | **S** | **A** | **E** | **K** | **I** | **Q** | **A** | **I** | **I** | **S** | **V** | **E** | **G** | **K** | **A** | **E** | **A** | **G** | **K** | **Y** | **L** | **F** | **F** | **T** | **T** | **L** | **K** | **G** | **T** | **V** | **K** | **R** | **T** | **A** | **V** | **T** | **A** | **F** | **S** | **N** | **I** | **R** | **S** | **N** | **G** | **L** | **I** | **A** | **I** | **S** | **L** | **K** | **E** | **D** | **D** | **E** | **L** | **V** | **N** | **V** | **V** | **T** | **T** | **N** | **G** | **N** | **Q** | **K** | **M** | **I** | **I** | **G** | **T** | **H** | **A** | **G** | **Y** | **S** | **V** | **T** | **F** | **D** | **E** | **N** | **T** | **V** | **R** | **D** | **M** | **G** | **R** | **T** | **A** | **S** | **G** | **V** | **R** | **G** | **I** | **R** | **L** | **R** | **E** | **N** | **D** | **Y** | **V** | **V** | **G** | **A** | **A** | **I** | **L** | **D** | **E** | **N** | **K** | **E** | **V** | **L** | **V** | **I** | **T** | **E** | **N** | **G** | **Y** | **G** | **K** | **R** | **T** | **K** | **A** | **S** | **E** | **Y** | **P** | **V** | **K** | **G** | **R** | **G** | **G** | **K** | **G** | **I** | **K** | **T** | **A** | **N** | **I** | **T** | **E** | **K** | **N** | **G** | **P** | **L** | **A** | **G** | **L** | **T** | **T** | **V** | **N** | **G** | **D** | **E** | **D** | **I** | **L** | **L** | **I** | **T** | **N** | **K** | **G** | **V** | **I** | **I** | **R** | **F** | **N** | **V** | **D** | **S** | **V** | **S** | **Q** | **T** | **G** | **R** | **A** | **T** | **L** | **G** | **V** | **R** | **L** | **M** | **R** | **M** | **E** | **D** | **G** | **A** | **K** | **-** | **-** | **-** | **-** | **-** | **-** | **-** | **-** | **-** | **-** | **-** | **-** | **-** | **-** | **-** | **-** | **-** | **-** | **-** | **-** | **-** | **-** | **-** |
| **N60027F** | **M** | **S** | **E** | **E** | **I** | **K** | **E** | **N** | **I** | **Q** | **D** | **V** | **N** | **L** | **T** | **S** | **E** | **M** | **K** | **E** | **S** | **F** | **I** | **D** | **Y** | **A** | **M** | **S** | **V** | **I** | **V** | **A** | **R** | **A** | **L** | **P** | **D** | **V** | **R** | **D** | **G** | **L** | **K** | **P** | **V** | **H** | **R** | **R** | **I** | **L** | **Y** | **G** | **M** | **N** | **E** | **L** | **G** | **V** | **T** | **P** | **D** | **K** | **P** | **H** | **K** | **K** | **S** | **A** | **R** | **I** | **V** | **G** | **D** | **V** | **M** | **G** | **K** | **Y** | **H** | **P** | **H** | **G** | **D** | **S** | **A** | **I** | **Y** | **E** | **S** | **M** | **V** | **R** | **M** | **A** | **Q** | **P** | **F** | **S** | **Y** | **R** | **A** | **M** | **L** | **V** | **D** | **G** | **H** | **G** | **N** | **F** | **G** | **S** | **V** | **D** | **G** | **D** | **G** | **A** | **A** | **A** | **M** | **R** | **Y** | **T** | **E** | **A** | **R** | **M** | **S** | **K** | **I** | **A** | **L** | **E** | **M** | **L** | **R** | **D** | **I** | **N** | **K** | **N** | **T** | **V** | **D** | **F** | **Q** | **G** | **N** | **Y** | **D** | **D** | **S** | **E** | **Q** | **E** | **P** | **V** | **V** | **L** | **P** | **A** | **R** | **F** | **P** | **N** | **L** | **L** | **V** | **N** | **G** | **T** | **T** | **G** | **I** | **A** | **V** | **G** | **M** | **A** | **T** | **N** | **I** | **P** | **P** | **H** | **N** | **L** | **S** | **E** | **V** | **I** | **D** | **A** | **T** | **S** | **L** | **L** | **M** | **D** | **N** | **P** | **D** | **V** | **T** | **T** | **N** | **E** | **L** | **M** | **E** | **V** | **L** | **P** | **G** | **P** | **D** | **F** | **P** | **T** | **G** | **G** | **L** | **V** | **M** | **G** | **K** | **S** | **G** | **I** | **R** | **R** | **A** | **Y** | **E** | **T** | **G** | **K** | **G** | **S** | **I** | **T** | **V** | **R** | **A** | **K** | **V** | **E** | **L** | **T** | **E** | **M** | **P** | **N** | **G** | **K** | **E** | **R** | **I** | **L** | **V** | **T** | **E** | **L** | **P** | **Y** | **M** | **V** | **N** | **K** | **A** | **K** | **L** | **I** | **E** | **R** | **I** | **S** | **E** | **L** | **H** | **R** | **D** | **K** | **R** | **I** | **E** | **G** | **I** | **T** | **D** | **L** | **R** | **D** | **E** | **S** | **S** | **R** | **E** | **G** | **M** | **R** | **I** | **V** | **I** | **D** | **V** | **R** | **R** | **D** | **V** | **S** | **A** | **S** | **V** | **V** | **L** | **N** | **N** | **L** | **Y** | **K** | **M** | **T** | **A** | **L** | **Q** | **T** | **S** | **F** | **G** | **F** | **N** | **M** | **L** | **A** | **I** | **E** | **K** | **G** | **V** | **P** | **K** | **I** | **L** | **S** | **L** | **K** | **R** | **I** | **L** | **E** | **N** | **Y** | **V** | **E** | **H** | **Q** | **K** | **E** | **V** | **I** | **T** | **R** | **R** | **T** | **I** | **F** | **D** | **K** | **N** | **K** | **A** | **E** | **A** | **R** | **A** | **H** | **I** | **L** | **E** | **G** | **L** | **R** | **I** | **A** | **L** | **D** | **H** | **I** | **D** | **E** | **I** | **I** | **A** | **I** | **I** | **R** | **G** | **S** | **Q** | **S** | **D** | **D** | **E** | **A** | **K** | **A** | **T** | **L** | **I** | **E** | **R** | **F** | **E** | **F** | **S** | **D** | **R** | **Q** | **A** | **Q** | **A** | **I** | **L** | **D** | **M** | **R** | **L** | **R** | **R** | **L** | **T** | **G** | **L** | **E** | **R** | **D** | **K** | **I** | **E** | **N** | **E** | **Y** | **Q** | **E** | **L** | **L** | **K** | **F** | **I** | **A** | **D** | **L** | **E** | **D** | **I** | **L** | **A** | **R** | **P** | **E** | **R** | **V** | **I** | **E** | **I** | **I** | **K** | **T** | **E** | **L** | **N** | **D** | **V** | **R** | **T** | **K** | **F** | **G** | **D** | **A** | **R** | **R** | **T** | **E** | **L** | **L** | **V** | **G** | **E** | **V** | **L** | **S** | **L** | **E** | **D** | **E** | **D** | **L** | **I** | **E** | **E** | **E** | **E** | **V** | **V** | **I** | **T** | **L** | **T** | **N** | **N** | **G** | **Y** | **I** | **K** | **R** | **M** | **A** | **N** | **S** | **E** | **F** | **R** | **A** | **Q** | **R** | **R** | **G** | **G** | **R** | **G** | **V** | **Q** | **G** | **M** | **G** | **V** | **H** | **D** | **D** | **D** | **F** | **V** | **K** | **N** | **L** | **V** | **S** | **C** | **S** | **T** | **H** | **D** | **T** | **L** | **L** | **F** | **F** | **T** | **N** | **T** | **G** | **K** | **V** | **Y** | **R** | **A** | **K** | **G** | **Y** | **E** | **I** | **P** | **E** | **Y** | **G** | **R** | **T** | **A** | **K** | **G** | **I** | **P** | **V** | **I** | **N** | **L** | **L** | **G** | **I** | **D** | **S** | **A** | **E** | **K** | **I** | **Q** | **A** | **I** | **I** | **S** | **V** | **E** | **G** | **K** | **A** | **E** | **A** | **G** | **K** | **Y** | **L** | **F** | **F** | **T** | **T** | **L** | **K** | **G** | **T** | **V** | **K** | **R** | **T** | **A** | **V** | **T** | **A** | **F** | **S** | **N** | **I** | **R** | **S** | **N** | **G** | **L** | **I** | **A** | **I** | **S** | **L** | **K** | **E** | **D** | **D** | **E** | **L** | **V** | **N** | **V** | **V** | **T** | **T** | **N** | **G** | **N** | **Q** | **K** | **M** | **I** | **I** | **G** | **T** | **H** | **A** | **G** | **Y** | **S** | **V** | **T** | **F** | **D** | **E** | **N** | **T** | **V** | **R** | **D** | **M** | **G** | **R** | **T** | **A** | **S** | **G** | **V** | **R** | **G** | **I** | **R** | **L** | **R** | **E** | **N** | **D** | **Y** | **V** | **V** | **G** | **A** | **A** | **I** | **L** | **D** | **E** | **N** | **K** | **E** | **V** | **L** | **V** | **I** | **T** | **E** | **N** | **G** | **Y** | **G** | **K** | **R** | **T** | **K** | **A** | **S** | **E** | **Y** | **P** | **V** | **K** | **G** | **R** | **G** | **G** | **K** | **G** | **I** | **K** | **T** | **A** | **N** | **I** | **T** | **E** | **K** | **N** | **G** | **P** | **L** | **A** | **G** | **L** | **T** | **T** | **V** | **N** | **G** | **D** | **E** | **D** | **I** | **L** | **L** | **I** | **T** | **N** | **K** | **G** | **V** | **I** | **I** | **R** | **F** | **N** | **V** | **D** | **S** | **V** | **S** | **Q** | **T** | **G** | **R** | **A** | **T** | **L** | **G** | **V** | **R** | **L** | **M** | **R** | **M** | **E** | **D** | **G** | **A** | **K** | **-** | **-** | **-** | **-** | **-** | **-** | **-** | **-** | **-** | **-** | **-** | **-** | **-** | **-** | **-** | **-** | **-** | **-** | **-** | **-** | **-** | **-** | **-** |
| **N52824** | **M** | **S** | **E** | **E** | **I** | **R** | **E** | **N** | **I** | **H** | **D** | **V** | **N** | **L** | **T** | **S** | **E** | **M** | **K** | **D** | **S** | **F** | **I** | **D** | **Y** | **A** | **M** | **S** | **V** | **I** | **V** | **A** | **R** | **A** | **L** | **P** | **D** | **V** | **R** | **D** | **G** | **L** | **K** | **P** | **V** | **H** | **R** | **R** | **I** | **L** | **Y** | **G** | **M** | **N** | **E** | **L** | **G** | **V** | **T** | **P** | **D** | **K** | **A** | **H** | **K** | **K** | **S** | **A** | **R** | **I** | **V** | **G** | **D** | **V** | **M** | **G** | **K** | **Y** | **H** | **P** | **H** | **G** | **D** | **S** | **A** | **I** | **Y** | **E** | **S** | **M** | **V** | **R** | **M** | **A** | **Q** | **P** | **F** | **S** | **Y** | **R** | **Y** | **M** | **L** | **V** | **D** | **G** | **H** | **G** | **N** | **F** | **G** | **S** | **V** | **D** | **G** | **D** | **G** | **A** | **A** | **A** | **M** | **R** | **Y** | **T** | **E** | **A** | **R** | **M** | **S** | **K** | **I** | **A** | **T** | **E** | **M** | **L** | **R** | **D** | **I** | **N** | **K** | **N** | **T** | **V** | **D** | **F** | **Q** | **S** | **N** | **Y** | **D** | **D** | **T** | **E** | **K** | **E** | **P** | **V** | **V** | **L** | **P** | **A** | **R** | **F** | **P** | **N** | **L** | **L** | **V** | **N** | **G** | **T** | **T** | **G** | **I** | **A** | **V** | **G** | **M** | **A** | **T** | **N** | **I** | **P** | **P** | **H** | **N** | **L** | **S** | **E** | **V** | **V** | **A** | **A** | **I** | **D** | **L** | **L** | **M** | **D** | **N** | **P** | **D** | **V** | **T** | **T** | **N** | **E** | **L** | **M** | **E** | **V** | **L** | **P** | **G** | **P** | **D** | **F** | **P** | **T** | **G** | **G** | **L** | **V** | **M** | **G** | **K** | **S** | **G** | **I** | **R** | **R** | **A** | **Y** | **E** | **T** | **G** | **K** | **G** | **S** | **I** | **T** | **V** | **R** | **A** | **K** | **V** | **E** | **I** | **T** | **E** | **M** | **P** | **N** | **G** | **K** | **E** | **R** | **I** | **I** | **V** | **T** | **E** | **L** | **P** | **Y** | **M** | **V** | **N** | **K** | **A** | **K** | **L** | **I** | **E** | **R** | **I** | **S** | **E** | **L** | **H** | **R** | **D** | **K** | **R** | **I** | **E** | **G** | **I** | **T** | **D** | **L** | **R** | **D** | **E** | **S** | **S** | **R** | **E** | **G** | **M** | **R** | **I** | **V** | **I** | **D** | **I** | **R** | **R** | **D** | **A** | **S** | **A** | **S** | **V** | **I** | **L** | **N** | **N** | **L** | **Y** | **K** | **L** | **T** | **S** | **L** | **Q** | **N** | **S** | **F** | **G** | **F** | **N** | **M** | **L** | **A** | **I** | **E** | **K** | **G** | **V** | **P** | **K** | **V** | **L** | **S** | **L** | **K** | **Q** | **I** | **L** | **E** | **N** | **Y** | **I** | **E** | **H** | **Q** | **R** | **E** | **V** | **I** | **T** | **R** | **R** | **T** | **V** | **F** | **E** | **K** | **E** | **K** | **A** | **E** | **A** | **R** | **A** | **H** | **I** | **L** | **E** | **G** | **L** | **R** | **I** | **A** | **L** | **D** | **H** | **I** | **D** | **E** | **I** | **I** | **A** | **I** | **I** | **R** | **G** | **S** | **K** | **S** | **D** | **D** | **E** | **A** | **K** | **A** | **T** | **M** | **I** | **E** | **R** | **F** | **D** | **L** | **S** | **D** | **R** | **Q** | **S** | **Q** | **A** | **I** | **L** | **D** | **M** | **R** | **L** | **R** | **R** | **L** | **T** | **G** | **L** | **E** | **R** | **E** | **K** | **I** | **E** | **N** | **E** | **Y** | **Q** | **E** | **L** | **L** | **K** | **L** | **I** | **E** | **D** | **L** | **T** | **D** | **I** | **L** | **A** | **R** | **P** | **E** | **R** | **V** | **T** | **E** | **I** | **I** | **K** | **N** | **E** | **L** | **A** | **D** | **L** | **N** | **Q** | **R** | **F** | **G** | **D** | **K** | **R** | **R** | **T** | **E** | **L** | **L** | **V** | **G** | **E** | **V** | **L** | **S** | **L** | **E** | **D** | **E** | **D** | **L** | **I** | **E** | **E** | **E** | **E** | **I** | **V** | **I** | **T** | **L** | **T** | **N** | **N** | **G** | **Y** | **I** | **K** | **R** | **L** | **A** | **N** | **N** | **E** | **F** | **R** | **A** | **Q** | **R** | **R** | **G** | **G** | **R** | **G** | **V** | **Q** | **G** | **M** | **G** | **I** | **H** | **N** | **D** | **D** | **F** | **V** | **K** | **N** | **L** | **V** | **S** | **C** | **S** | **T** | **H** | **D** | **T** | **L** | **L** | **F** | **F** | **T** | **N** | **N** | **G** | **K** | **V** | **Y** | **R** | **A** | **K** | **G** | **Y** | **E** | **I** | **P** | **E** | **Y** | **G** | **R** | **T** | **A** | **K** | **G** | **I** | **P** | **I** | **I** | **N** | **L** | **L** | **G** | **I** | **D** | **S** | **S** | **E** | **S** | **I** | **Q** | **A** | **I** | **I** | **A** | **V** | **T** | **G** | **E** | **A** | **E** | **E** | **G** | **H** | **Y** | **L** | **F** | **F** | **T** | **T** | **R** | **Q** | **G** | **T** | **V** | **K** | **R** | **T** | **S** | **V** | **T** | **A** | **F** | **A** | **N** | **I** | **R** | **S** | **N** | **G** | **L** | **I** | **A** | **I** | **G** | **L** | **K** | **E** | **D** | **D** | **E** | **L** | **V** | **N** | **V** | **L** | **L** | **T** | **D** | **G** | **Q** | **S** | **N** | **I** | **I** | **I** | **G** | **T** | **H** | **N** | **G** | **Y** | **S** | **V** | **T** | **F** | **K** | **E** | **E** | **A** | **V** | **R** | **D** | **M** | **G** | **R** | **T** | **A** | **S** | **G** | **V** | **R** | **G** | **I** | **R** | **L** | **R** | **E** | **E** | **D** | **Y** | **V** | **V** | **G** | **A** | **A** | **L** | **M** | **K** | **E** | **D** | **Q** | **E** | **V** | **L** | **V** | **I** | **T** | **E** | **K** | **G** | **Y** | **G** | **K** | **R** | **T** | **K** | **V** | **S** | **E** | **Y** | **P** | **V** | **K** | **G** | **R** | **G** | **G** | **K** | **G** | **I** | **K** | **T** | **A** | **N** | **I** | **T** | **E** | **K** | **N** | **G** | **P** | **L** | **A** | **G** | **L** | **T** | **T** | **V** | **S** | **G** | **D** | **E** | **D** | **I** | **M** | **L** | **I** | **T** | **D** | **K** | **G** | **V** | **I** | **I** | **R** | **F** | **N** | **V** | **S** | **T** | **V** | **S** | **Q** | **T** | **G** | **R** | **S** | **T** | **L** | **G** | **V** | **R** | **L** | **M** | **K** | **M** | **E** | **A** | **D** | **T** | **K** | **V** | **V** | **T** | **M** | **A** | **A** | **V** | **E** | **P** | **E** | **A** | **A** | **E** | **Q** | **T** | **V** | **E** | **E** | **Q** | **G** | **S** | **E** | **E** |
| **N59963F** | **M** | **S** | **E** | **E** | **I** | **K** | **E** | **N** | **I** | **Q** | **D** | **V** | **N** | **L** | **T** | **S** | **E** | **M** | **K** | **E** | **S** | **F** | **I** | **D** | **Y** | **A** | **M** | **S** | **V** | **I** | **V** | **A** | **R** | **A** | **L** | **P** | **D** | **V** | **R** | **D** | **G** | **L** | **K** | **P** | **V** | **H** | **R** | **R** | **I** | **L** | **Y** | **G** | **M** | **N** | **E** | **L** | **G** | **V** | **T** | **P** | **D** | **K** | **P** | **H** | **K** | **K** | **S** | **A** | **R** | **I** | **V** | **G** | **D** | **V** | **M** | **G** | **K** | **Y** | **H** | **P** | **H** | **G** | **D** | **S** | **A** | **I** | **Y** | **E** | **S** | **M** | **V** | **R** | **M** | **A** | **Q** | **P** | **F** | **S** | **Y** | **R** | **A** | **M** | **L** | **V** | **D** | **G** | **H** | **G** | **N** | **F** | **G** | **S** | **V** | **D** | **G** | **D** | **G** | **A** | **A** | **A** | **M** | **R** | **Y** | **T** | **E** | **A** | **R** | **M** | **S** | **K** | **I** | **A** | **L** | **E** | **M** | **L** | **R** | **D** | **I** | **N** | **K** | **N** | **T** | **V** | **D** | **F** | **Q** | **G** | **N** | **Y** | **D** | **D** | **S** | **E** | **Q** | **E** | **P** | **V** | **V** | **L** | **P** | **A** | **R** | **F** | **P** | **N** | **L** | **L** | **V** | **N** | **G** | **T** | **T** | **G** | **I** | **A** | **V** | **G** | **M** | **A** | **T** | **N** | **I** | **P** | **P** | **H** | **N** | **L** | **S** | **E** | **V** | **I** | **D** | **A** | **T** | **S** | **L** | **L** | **M** | **D** | **N** | **P** | **D** | **V** | **T** | **T** | **N** | **E** | **L** | **M** | **E** | **V** | **L** | **P** | **G** | **P** | **D** | **F** | **P** | **T** | **G** | **G** | **L** | **V** | **M** | **G** | **K** | **S** | **G** | **I** | **R** | **R** | **A** | **Y** | **E** | **T** | **G** | **K** | **G** | **S** | **I** | **T** | **V** | **R** | **A** | **K** | **V** | **E** | **L** | **T** | **E** | **M** | **P** | **N** | **G** | **K** | **E** | **R** | **I** | **L** | **V** | **T** | **E** | **L** | **P** | **Y** | **M** | **V** | **N** | **K** | **A** | **K** | **L** | **I** | **E** | **R** | **I** | **S** | **E** | **L** | **H** | **R** | **D** | **K** | **R** | **I** | **E** | **G** | **I** | **T** | **D** | **L** | **R** | **D** | **E** | **S** | **S** | **R** | **E** | **G** | **M** | **R** | **I** | **V** | **I** | **D** | **V** | **R** | **R** | **D** | **V** | **S** | **A** | **S** | **V** | **V** | **L** | **N** | **N** | **L** | **Y** | **K** | **M** | **T** | **A** | **L** | **Q** | **T** | **S** | **F** | **G** | **F** | **N** | **M** | **L** | **A** | **I** | **E** | **K** | **G** | **V** | **P** | **K** | **I** | **L** | **S** | **L** | **K** | **R** | **I** | **L** | **E** | **N** | **Y** | **V** | **E** | **H** | **Q** | **K** | **E** | **V** | **I** | **T** | **R** | **R** | **T** | **I** | **F** | **D** | **K** | **N** | **K** | **A** | **E** | **A** | **R** | **A** | **H** | **I** | **L** | **E** | **G** | **L** | **R** | **I** | **A** | **L** | **D** | **H** | **I** | **D** | **E** | **I** | **I** | **A** | **I** | **I** | **R** | **G** | **S** | **Q** | **S** | **D** | **D** | **E** | **A** | **K** | **A** | **T** | **L** | **I** | **E** | **R** | **F** | **E** | **F** | **S** | **D** | **R** | **Q** | **A** | **Q** | **A** | **I** | **L** | **D** | **M** | **R** | **L** | **R** | **R** | **L** | **T** | **G** | **L** | **E** | **R** | **D** | **K** | **I** | **E** | **N** | **E** | **Y** | **Q** | **E** | **L** | **L** | **K** | **F** | **I** | **A** | **D** | **L** | **E** | **D** | **I** | **L** | **A** | **R** | **P** | **E** | **R** | **V** | **I** | **E** | **I** | **I** | **K** | **T** | **E** | **L** | **N** | **D** | **V** | **R** | **T** | **K** | **F** | **G** | **D** | **A** | **R** | **R** | **T** | **E** | **L** | **L** | **V** | **G** | **E** | **V** | **L** | **S** | **L** | **E** | **D** | **E** | **D** | **L** | **I** | **E** | **E** | **E** | **E** | **V** | **V** | **I** | **T** | **L** | **T** | **N** | **N** | **G** | **Y** | **I** | **K** | **R** | **M** | **A** | **N** | **S** | **E** | **F** | **R** | **A** | **Q** | **R** | **R** | **G** | **G** | **R** | **G** | **V** | **Q** | **G** | **M** | **G** | **V** | **H** | **D** | **D** | **D** | **F** | **V** | **K** | **N** | **L** | **V** | **S** | **C** | **S** | **T** | **H** | **D** | **T** | **L** | **L** | **F** | **F** | **T** | **N** | **T** | **G** | **K** | **V** | **Y** | **R** | **A** | **K** | **G** | **Y** | **E** | **I** | **P** | **E** | **Y** | **G** | **R** | **T** | **A** | **K** | **G** | **I** | **P** | **V** | **I** | **N** | **L** | **L** | **G** | **I** | **D** | **S** | **A** | **E** | **K** | **I** | **Q** | **A** | **I** | **I** | **S** | **V** | **E** | **G** | **K** | **A** | **E** | **A** | **G** | **K** | **Y** | **L** | **F** | **F** | **T** | **T** | **L** | **K** | **G** | **T** | **V** | **K** | **R** | **T** | **A** | **V** | **T** | **A** | **F** | **S** | **N** | **I** | **R** | **S** | **N** | **G** | **L** | **I** | **A** | **I** | **S** | **L** | **K** | **E** | **D** | **D** | **E** | **L** | **V** | **N** | **V** | **V** | **T** | **T** | **N** | **G** | **N** | **Q** | **K** | **M** | **I** | **I** | **G** | **T** | **H** | **A** | **G** | **Y** | **S** | **V** | **T** | **F** | **D** | **E** | **N** | **T** | **V** | **R** | **D** | **M** | **G** | **R** | **T** | **A** | **S** | **G** | **V** | **R** | **G** | **I** | **R** | **L** | **R** | **E** | **N** | **D** | **Y** | **V** | **V** | **G** | **A** | **A** | **I** | **L** | **D** | **E** | **N** | **K** | **E** | **V** | **L** | **V** | **I** | **T** | **E** | **N** | **G** | **Y** | **G** | **K** | **R** | **T** | **K** | **A** | **S** | **E** | **Y** | **P** | **V** | **K** | **G** | **R** | **G** | **G** | **K** | **G** | **I** | **K** | **T** | **A** | **N** | **I** | **T** | **E** | **K** | **N** | **G** | **P** | **L** | **A** | **G** | **L** | **T** | **T** | **V** | **N** | **G** | **D** | **E** | **D** | **I** | **L** | **L** | **I** | **T** | **N** | **K** | **G** | **V** | **I** | **I** | **R** | **F** | **N** | **V** | **D** | **S** | **V** | **S** | **Q** | **T** | **G** | **R** | **A** | **T** | **L** | **G** | **V** | **R** | **L** | **M** | **R** | **M** | **E** | **D** | **G** | **A** | **K** | **-** | **-** | **-** | **-** | **-** | **-** | **-** | **-** | **-** | **-** | **-** | **-** | **-** | **-** | **-** | **-** | **-** | **-** | **-** | **-** | **-** | **-** | **-** |
| **N52829** | **M** | **S** | **E** | **E** | **I** | **R** | **E** | **N** | **I** | **H** | **D** | **V** | **N** | **L** | **T** | **S** | **E** | **M** | **K** | **D** | **S** | **F** | **I** | **D** | **Y** | **A** | **M** | **S** | **V** | **I** | **V** | **A** | **R** | **A** | **L** | **P** | **D** | **V** | **R** | **D** | **G** | **L** | **K** | **P** | **V** | **H** | **R** | **R** | **I** | **L** | **Y** | **G** | **M** | **N** | **E** | **L** | **G** | **V** | **T** | **P** | **D** | **K** | **A** | **H** | **K** | **K** | **S** | **A** | **R** | **I** | **V** | **G** | **D** | **V** | **M** | **G** | **K** | **Y** | **H** | **P** | **H** | **G** | **D** | **S** | **A** | **I** | **Y** | **E** | **S** | **M** | **V** | **R** | **M** | **A** | **Q** | **P** | **F** | **S** | **Y** | **R** | **Y** | **M** | **L** | **V** | **D** | **G** | **H** | **G** | **N** | **F** | **G** | **S** | **V** | **D** | **G** | **D** | **G** | **A** | **A** | **A** | **M** | **R** | **Y** | **T** | **E** | **A** | **R** | **M** | **S** | **K** | **I** | **A** | **T** | **E** | **M** | **L** | **R** | **D** | **I** | **N** | **K** | **N** | **T** | **V** | **D** | **F** | **Q** | **S** | **N** | **Y** | **D** | **D** | **T** | **E** | **K** | **E** | **P** | **V** | **V** | **L** | **P** | **A** | **R** | **F** | **P** | **N** | **L** | **L** | **V** | **N** | **G** | **T** | **T** | **G** | **I** | **A** | **V** | **G** | **M** | **A** | **T** | **N** | **I** | **P** | **P** | **H** | **N** | **L** | **S** | **E** | **V** | **V** | **A** | **A** | **I** | **D** | **L** | **L** | **M** | **D** | **N** | **P** | **D** | **V** | **T** | **T** | **N** | **E** | **L** | **M** | **E** | **V** | **L** | **P** | **G** | **P** | **D** | **F** | **P** | **T** | **G** | **G** | **L** | **V** | **M** | **G** | **K** | **S** | **G** | **I** | **R** | **R** | **A** | **Y** | **E** | **T** | **G** | **K** | **G** | **S** | **I** | **T** | **V** | **R** | **A** | **K** | **V** | **E** | **I** | **T** | **E** | **M** | **P** | **N** | **G** | **K** | **E** | **R** | **I** | **I** | **V** | **T** | **E** | **L** | **P** | **Y** | **M** | **V** | **N** | **K** | **A** | **K** | **L** | **I** | **E** | **R** | **I** | **S** | **E** | **L** | **H** | **R** | **D** | **K** | **R** | **I** | **E** | **G** | **I** | **T** | **D** | **L** | **R** | **D** | **E** | **S** | **S** | **R** | **E** | **G** | **M** | **R** | **I** | **V** | **I** | **D** | **I** | **R** | **R** | **D** | **A** | **S** | **A** | **S** | **V** | **I** | **L** | **N** | **N** | **L** | **Y** | **K** | **L** | **T** | **S** | **L** | **Q** | **N** | **S** | **F** | **G** | **F** | **N** | **M** | **L** | **A** | **I** | **E** | **K** | **G** | **V** | **P** | **K** | **V** | **L** | **S** | **L** | **K** | **Q** | **I** | **L** | **E** | **N** | **Y** | **I** | **E** | **H** | **Q** | **R** | **E** | **V** | **I** | **T** | **R** | **R** | **T** | **V** | **F** | **E** | **K** | **E** | **K** | **A** | **E** | **A** | **R** | **A** | **H** | **I** | **L** | **E** | **G** | **L** | **R** | **I** | **A** | **L** | **D** | **H** | **I** | **D** | **E** | **I** | **I** | **A** | **I** | **I** | **R** | **G** | **S** | **K** | **S** | **D** | **D** | **E** | **A** | **K** | **A** | **T** | **M** | **I** | **E** | **R** | **F** | **D** | **L** | **S** | **D** | **R** | **Q** | **S** | **Q** | **A** | **I** | **L** | **D** | **M** | **R** | **L** | **R** | **R** | **L** | **T** | **G** | **L** | **E** | **R** | **E** | **K** | **I** | **E** | **N** | **E** | **Y** | **Q** | **E** | **L** | **L** | **K** | **L** | **I** | **E** | **D** | **L** | **T** | **D** | **I** | **L** | **A** | **R** | **P** | **E** | **R** | **V** | **T** | **E** | **I** | **I** | **K** | **N** | **E** | **L** | **A** | **D** | **L** | **N** | **Q** | **R** | **F** | **G** | **D** | **K** | **R** | **R** | **T** | **E** | **L** | **L** | **V** | **G** | **E** | **V** | **L** | **S** | **L** | **E** | **D** | **E** | **D** | **L** | **I** | **E** | **E** | **E** | **E** | **I** | **V** | **I** | **T** | **L** | **T** | **N** | **N** | **G** | **Y** | **I** | **K** | **R** | **L** | **A** | **N** | **N** | **E** | **F** | **R** | **A** | **Q** | **R** | **R** | **G** | **G** | **R** | **G** | **V** | **Q** | **G** | **M** | **G** | **I** | **H** | **N** | **D** | **D** | **F** | **V** | **K** | **N** | **L** | **V** | **S** | **C** | **S** | **T** | **H** | **D** | **T** | **L** | **L** | **F** | **F** | **T** | **N** | **N** | **G** | **K** | **V** | **Y** | **R** | **A** | **K** | **G** | **Y** | **E** | **I** | **P** | **E** | **Y** | **G** | **R** | **T** | **A** | **K** | **G** | **I** | **P** | **I** | **I** | **N** | **L** | **L** | **G** | **I** | **D** | **S** | **S** | **E** | **S** | **I** | **Q** | **A** | **I** | **I** | **A** | **V** | **T** | **G** | **E** | **A** | **E** | **E** | **G** | **H** | **Y** | **L** | **F** | **F** | **T** | **T** | **R** | **Q** | **G** | **T** | **V** | **K** | **R** | **T** | **S** | **V** | **T** | **A** | **F** | **A** | **N** | **I** | **R** | **S** | **N** | **G** | **L** | **I** | **A** | **I** | **G** | **L** | **K** | **E** | **D** | **D** | **E** | **L** | **V** | **N** | **V** | **L** | **L** | **T** | **D** | **G** | **Q** | **S** | **N** | **I** | **I** | **I** | **G** | **T** | **H** | **N** | **G** | **Y** | **S** | **V** | **T** | **F** | **K** | **E** | **E** | **A** | **V** | **R** | **D** | **M** | **G** | **R** | **T** | **A** | **S** | **G** | **V** | **R** | **G** | **I** | **R** | **L** | **R** | **E** | **E** | **D** | **Y** | **V** | **V** | **G** | **A** | **A** | **L** | **M** | **K** | **E** | **N** | **Q** | **E** | **V** | **L** | **V** | **I** | **T** | **E** | **K** | **G** | **Y** | **G** | **K** | **R** | **T** | **K** | **V** | **S** | **E** | **Y** | **P** | **V** | **K** | **G** | **R** | **G** | **G** | **K** | **G** | **I** | **K** | **T** | **A** | **N** | **I** | **T** | **E** | **K** | **N** | **G** | **P** | **L** | **A** | **G** | **L** | **T** | **T** | **V** | **S** | **G** | **D** | **E** | **D** | **I** | **M** | **L** | **I** | **T** | **D** | **K** | **G** | **V** | **I** | **I** | **R** | **F** | **N** | **V** | **S** | **T** | **V** | **S** | **Q** | **T** | **G** | **R** | **S** | **T** | **L** | **G** | **V** | **R** | **L** | **M** | **K** | **M** | **E** | **A** | **D** | **T** | **K** | **V** | **V** | **T** | **M** | **A** | **A** | **V** | **E** | **P** | **E** | **A** | **A** | **E** | **Q** | **T** | **V** | **E** | **E** | **Q** | **G** | **S** | **E** | **E** |
| **N60260F** | **M** | **S** | **E** | **E** | **I** | **R** | **E** | **N** | **I** | **H** | **D** | **V** | **N** | **L** | **T** | **S** | **E** | **M** | **K** | **D** | **S** | **F** | **I** | **D** | **Y** | **A** | **M** | **S** | **V** | **I** | **V** | **A** | **R** | **A** | **L** | **P** | **D** | **V** | **R** | **D** | **G** | **L** | **K** | **P** | **V** | **H** | **R** | **R** | **I** | **L** | **Y** | **G** | **M** | **N** | **E** | **L** | **G** | **V** | **T** | **P** | **D** | **K** | **A** | **H** | **K** | **K** | **S** | **A** | **R** | **I** | **V** | **G** | **D** | **V** | **M** | **G** | **K** | **Y** | **H** | **P** | **H** | **G** | **D** | **S** | **A** | **I** | **Y** | **E** | **S** | **M** | **V** | **R** | **M** | **A** | **Q** | **P** | **F** | **S** | **Y** | **R** | **Y** | **M** | **L** | **V** | **D** | **G** | **H** | **G** | **N** | **F** | **G** | **S** | **V** | **D** | **G** | **D** | **G** | **A** | **A** | **A** | **M** | **R** | **Y** | **T** | **E** | **A** | **R** | **M** | **S** | **K** | **I** | **A** | **T** | **E** | **M** | **L** | **R** | **D** | **I** | **N** | **K** | **N** | **T** | **V** | **D** | **F** | **Q** | **S** | **N** | **Y** | **D** | **D** | **T** | **E** | **K** | **E** | **P** | **V** | **V** | **L** | **P** | **A** | **R** | **F** | **P** | **N** | **L** | **L** | **V** | **N** | **G** | **T** | **T** | **G** | **I** | **A** | **V** | **G** | **M** | **A** | **T** | **N** | **I** | **P** | **P** | **H** | **N** | **L** | **S** | **E** | **V** | **V** | **A** | **A** | **I** | **D** | **L** | **L** | **M** | **D** | **N** | **P** | **D** | **V** | **T** | **T** | **N** | **E** | **L** | **M** | **E** | **V** | **L** | **P** | **G** | **P** | **D** | **F** | **P** | **T** | **G** | **G** | **L** | **V** | **M** | **G** | **K** | **S** | **G** | **I** | **R** | **R** | **A** | **Y** | **E** | **T** | **G** | **K** | **G** | **S** | **I** | **T** | **V** | **R** | **A** | **K** | **V** | **E** | **I** | **T** | **E** | **M** | **P** | **N** | **G** | **K** | **E** | **R** | **I** | **I** | **V** | **T** | **E** | **L** | **P** | **Y** | **M** | **V** | **N** | **K** | **A** | **K** | **L** | **I** | **E** | **R** | **I** | **S** | **E** | **L** | **H** | **R** | **D** | **K** | **R** | **I** | **E** | **G** | **I** | **T** | **D** | **L** | **R** | **D** | **E** | **S** | **S** | **R** | **E** | **G** | **M** | **R** | **I** | **V** | **I** | **D** | **I** | **R** | **R** | **D** | **A** | **S** | **A** | **S** | **V** | **I** | **L** | **N** | **N** | **L** | **Y** | **K** | **L** | **T** | **S** | **L** | **Q** | **N** | **S** | **F** | **G** | **F** | **N** | **M** | **L** | **A** | **I** | **E** | **K** | **G** | **V** | **P** | **K** | **V** | **L** | **S** | **L** | **K** | **Q** | **I** | **L** | **E** | **N** | **Y** | **I** | **E** | **H** | **Q** | **R** | **E** | **V** | **I** | **T** | **R** | **R** | **T** | **V** | **F** | **E** | **K** | **E** | **K** | **A** | **E** | **A** | **R** | **A** | **H** | **I** | **L** | **E** | **G** | **L** | **R** | **I** | **A** | **L** | **D** | **H** | **I** | **D** | **E** | **I** | **I** | **A** | **I** | **I** | **R** | **G** | **S** | **K** | **S** | **D** | **D** | **E** | **A** | **K** | **A** | **T** | **M** | **I** | **E** | **R** | **F** | **D** | **L** | **S** | **D** | **R** | **Q** | **S** | **Q** | **A** | **I** | **L** | **D** | **M** | **R** | **L** | **R** | **R** | **L** | **T** | **G** | **L** | **E** | **R** | **E** | **K** | **I** | **E** | **N** | **E** | **Y** | **Q** | **E** | **L** | **L** | **K** | **L** | **I** | **E** | **D** | **L** | **T** | **D** | **I** | **L** | **A** | **R** | **P** | **E** | **R** | **V** | **T** | **E** | **I** | **I** | **K** | **N** | **E** | **L** | **A** | **D** | **L** | **N** | **Q** | **R** | **F** | **G** | **D** | **K** | **R** | **R** | **T** | **E** | **L** | **L** | **V** | **G** | **E** | **V** | **L** | **S** | **L** | **E** | **D** | **E** | **D** | **L** | **I** | **E** | **E** | **E** | **E** | **I** | **V** | **I** | **T** | **L** | **T** | **N** | **N** | **G** | **Y** | **I** | **K** | **R** | **L** | **A** | **N** | **N** | **E** | **F** | **R** | **A** | **Q** | **R** | **R** | **G** | **G** | **R** | **G** | **V** | **Q** | **G** | **M** | **G** | **I** | **H** | **N** | **D** | **D** | **F** | **V** | **K** | **N** | **L** | **V** | **S** | **C** | **S** | **T** | **H** | **D** | **T** | **L** | **L** | **F** | **F** | **T** | **N** | **N** | **G** | **K** | **V** | **Y** | **R** | **A** | **K** | **G** | **Y** | **E** | **I** | **P** | **E** | **Y** | **G** | **R** | **T** | **A** | **K** | **G** | **I** | **P** | **I** | **I** | **N** | **L** | **L** | **G** | **I** | **D** | **S** | **S** | **E** | **S** | **I** | **Q** | **A** | **I** | **I** | **A** | **V** | **T** | **G** | **E** | **A** | **E** | **E** | **G** | **H** | **Y** | **L** | **F** | **F** | **T** | **T** | **R** | **Q** | **G** | **T** | **V** | **K** | **R** | **T** | **S** | **V** | **T** | **A** | **F** | **A** | **N** | **I** | **R** | **S** | **N** | **G** | **L** | **I** | **A** | **I** | **G** | **L** | **K** | **E** | **D** | **D** | **E** | **L** | **V** | **N** | **V** | **L** | **L** | **T** | **D** | **G** | **Q** | **S** | **N** | **I** | **I** | **I** | **G** | **T** | **H** | **N** | **G** | **Y** | **S** | **V** | **T** | **F** | **K** | **E** | **E** | **A** | **V** | **R** | **D** | **M** | **G** | **R** | **T** | **A** | **S** | **G** | **V** | **R** | **G** | **I** | **R** | **L** | **R** | **E** | **E** | **D** | **Y** | **V** | **V** | **G** | **A** | **A** | **L** | **M** | **K** | **E** | **N** | **Q** | **E** | **V** | **L** | **V** | **I** | **T** | **E** | **K** | **G** | **Y** | **G** | **K** | **R** | **T** | **K** | **V** | **S** | **E** | **Y** | **P** | **V** | **K** | **G** | **R** | **G** | **G** | **K** | **G** | **I** | **K** | **T** | **A** | **N** | **I** | **T** | **E** | **K** | **N** | **G** | **P** | **L** | **A** | **G** | **L** | **T** | **T** | **V** | **S** | **G** | **D** | **E** | **D** | **I** | **M** | **L** | **I** | **T** | **D** | **K** | **G** | **V** | **I** | **I** | **R** | **F** | **N** | **V** | **S** | **T** | **V** | **S** | **Q** | **T** | **G** | **R** | **S** | **T** | **L** | **G** | **V** | **R** | **L** | **M** | **K** | **M** | **E** | **A** | **D** | **T** | **K** | **V** | **V** | **T** | **M** | **A** | **A** | **V** | **E** | **P** | **E** | **A** | **A** | **E** | **Q** | **T** | **V** | **E** | **E** | **Q** | **G** | **S** | **E** | **E** |
| **N52724** | **M** | **S** | **E** | **E** | **I** | **K** | **E** | **N** | **I** | **Q** | **D** | **V** | **N** | **L** | **T** | **S** | **E** | **M** | **K** | **E** | **S** | **F** | **I** | **D** | **Y** | **A** | **M** | **S** | **V** | **I** | **V** | **A** | **R** | **A** | **L** | **P** | **D** | **V** | **R** | **D** | **G** | **L** | **K** | **P** | **V** | **H** | **R** | **R** | **I** | **L** | **Y** | **G** | **M** | **N** | **E** | **L** | **G** | **V** | **T** | **P** | **D** | **K** | **P** | **H** | **K** | **K** | **S** | **A** | **R** | **I** | **V** | **G** | **D** | **V** | **M** | **G** | **K** | **Y** | **H** | **P** | **H** | **G** | **D** | **S** | **A** | **I** | **Y** | **E** | **S** | **M** | **V** | **R** | **M** | **A** | **Q** | **P** | **F** | **S** | **Y** | **R** | **A** | **M** | **L** | **V** | **D** | **G** | **H** | **G** | **N** | **F** | **G** | **S** | **V** | **D** | **G** | **D** | **G** | **A** | **A** | **A** | **M** | **R** | **Y** | **T** | **E** | **A** | **R** | **M** | **S** | **K** | **I** | **A** | **L** | **E** | **M** | **L** | **R** | **D** | **I** | **N** | **K** | **N** | **T** | **V** | **D** | **F** | **Q** | **G** | **N** | **Y** | **D** | **D** | **S** | **E** | **Q** | **E** | **P** | **V** | **V** | **L** | **P** | **A** | **R** | **F** | **P** | **N** | **L** | **L** | **V** | **N** | **G** | **T** | **T** | **G** | **I** | **A** | **V** | **G** | **M** | **A** | **T** | **N** | **I** | **P** | **P** | **H** | **N** | **L** | **S** | **E** | **V** | **I** | **D** | **A** | **T** | **S** | **L** | **L** | **M** | **D** | **N** | **P** | **D** | **V** | **T** | **T** | **N** | **E** | **L** | **M** | **E** | **V** | **L** | **P** | **G** | **P** | **D** | **F** | **P** | **T** | **G** | **G** | **L** | **V** | **M** | **G** | **K** | **S** | **G** | **I** | **R** | **R** | **A** | **Y** | **E** | **T** | **G** | **K** | **G** | **S** | **I** | **T** | **V** | **R** | **A** | **K** | **V** | **E** | **L** | **T** | **E** | **M** | **P** | **N** | **G** | **K** | **E** | **R** | **I** | **L** | **V** | **T** | **E** | **L** | **P** | **Y** | **M** | **V** | **N** | **K** | **A** | **K** | **L** | **I** | **E** | **R** | **I** | **S** | **E** | **L** | **H** | **R** | **D** | **K** | **R** | **I** | **E** | **G** | **I** | **T** | **D** | **L** | **R** | **D** | **E** | **S** | **S** | **R** | **E** | **G** | **M** | **R** | **I** | **V** | **I** | **D** | **V** | **R** | **R** | **D** | **V** | **S** | **A** | **S** | **V** | **V** | **L** | **N** | **N** | **L** | **Y** | **K** | **M** | **T** | **A** | **L** | **Q** | **T** | **S** | **F** | **G** | **F** | **N** | **M** | **L** | **A** | **I** | **E** | **K** | **G** | **V** | **P** | **K** | **I** | **L** | **S** | **L** | **K** | **R** | **I** | **L** | **E** | **N** | **Y** | **V** | **E** | **H** | **Q** | **K** | **E** | **V** | **I** | **T** | **R** | **R** | **T** | **I** | **F** | **D** | **K** | **N** | **K** | **A** | **E** | **A** | **R** | **A** | **H** | **I** | **L** | **E** | **G** | **L** | **R** | **I** | **A** | **L** | **D** | **H** | **I** | **D** | **E** | **I** | **I** | **A** | **I** | **I** | **R** | **G** | **S** | **Q** | **S** | **D** | **D** | **E** | **A** | **K** | **A** | **T** | **L** | **I** | **E** | **R** | **F** | **E** | **F** | **S** | **D** | **R** | **Q** | **A** | **Q** | **A** | **I** | **L** | **D** | **M** | **R** | **L** | **R** | **R** | **L** | **T** | **G** | **L** | **E** | **R** | **D** | **K** | **I** | **E** | **N** | **E** | **Y** | **Q** | **E** | **L** | **L** | **K** | **F** | **I** | **A** | **D** | **L** | **E** | **D** | **I** | **L** | **A** | **R** | **P** | **E** | **R** | **V** | **I** | **E** | **I** | **I** | **K** | **T** | **E** | **L** | **N** | **D** | **V** | **R** | **T** | **K** | **F** | **G** | **D** | **A** | **R** | **R** | **T** | **E** | **L** | **L** | **V** | **G** | **E** | **V** | **L** | **S** | **L** | **E** | **D** | **E** | **D** | **L** | **I** | **E** | **E** | **E** | **E** | **V** | **V** | **I** | **T** | **L** | **T** | **N** | **N** | **G** | **Y** | **I** | **K** | **R** | **M** | **A** | **N** | **S** | **E** | **F** | **R** | **A** | **Q** | **R** | **R** | **G** | **G** | **R** | **G** | **V** | **Q** | **G** | **M** | **G** | **V** | **H** | **D** | **D** | **D** | **F** | **V** | **K** | **N** | **L** | **V** | **S** | **C** | **S** | **T** | **H** | **D** | **T** | **L** | **L** | **F** | **F** | **T** | **N** | **T** | **G** | **K** | **V** | **Y** | **R** | **A** | **K** | **G** | **Y** | **E** | **I** | **P** | **E** | **Y** | **G** | **R** | **T** | **A** | **K** | **G** | **I** | **P** | **V** | **I** | **N** | **L** | **L** | **G** | **I** | **D** | **S** | **A** | **E** | **K** | **I** | **Q** | **A** | **I** | **I** | **S** | **V** | **E** | **G** | **K** | **A** | **E** | **A** | **G** | **K** | **Y** | **L** | **F** | **F** | **T** | **T** | **L** | **K** | **G** | **T** | **V** | **K** | **R** | **T** | **A** | **V** | **T** | **A** | **F** | **S** | **N** | **I** | **R** | **S** | **N** | **G** | **L** | **I** | **A** | **I** | **S** | **L** | **K** | **E** | **D** | **D** | **E** | **L** | **V** | **N** | **V** | **V** | **T** | **T** | **N** | **G** | **N** | **Q** | **K** | **M** | **I** | **I** | **G** | **T** | **H** | **A** | **G** | **Y** | **S** | **V** | **T** | **F** | **D** | **E** | **N** | **T** | **A** | **R** | **D** | **M** | **G** | **R** | **T** | **A** | **S** | **G** | **V** | **R** | **G** | **I** | **R** | **L** | **R** | **E** | **N** | **D** | **Y** | **V** | **V** | **G** | **A** | **A** | **I** | **L** | **D** | **E** | **N** | **K** | **E** | **V** | **L** | **V** | **I** | **T** | **E** | **N** | **G** | **Y** | **G** | **K** | **R** | **T** | **K** | **A** | **S** | **E** | **Y** | **P** | **V** | **K** | **G** | **R** | **G** | **G** | **K** | **G** | **I** | **K** | **T** | **A** | **N** | **I** | **T** | **E** | **K** | **N** | **G** | **P** | **L** | **A** | **G** | **L** | **T** | **T** | **V** | **N** | **G** | **D** | **E** | **D** | **I** | **L** | **L** | **I** | **T** | **N** | **K** | **G** | **V** | **I** | **I** | **R** | **F** | **N** | **V** | **D** | **S** | **V** | **S** | **Q** | **T** | **G** | **R** | **A** | **T** | **L** | **G** | **V** | **R** | **L** | **M** | **R** | **M** | **E** | **D** | **G** | **A** | **K** | **-** | **-** | **-** | **-** | **-** | **-** | **-** | **-** | **-** | **-** | **-** | **-** | **-** | **-** | **-** | **-** | **-** | **-** | **-** | **-** | **-** | **-** | **-** |
| **N52662** | **M** | **S** | **E** | **E** | **I** | **K** | **E** | **N** | **I** | **Q** | **D** | **V** | **N** | **L** | **T** | **S** | **E** | **M** | **K** | **E** | **S** | **F** | **I** | **D** | **Y** | **A** | **M** | **S** | **V** | **I** | **V** | **A** | **R** | **A** | **L** | **P** | **D** | **V** | **R** | **D** | **G** | **L** | **K** | **P** | **V** | **H** | **R** | **R** | **I** | **L** | **Y** | **G** | **M** | **N** | **E** | **L** | **G** | **V** | **T** | **P** | **D** | **K** | **P** | **H** | **K** | **K** | **S** | **A** | **R** | **I** | **V** | **G** | **D** | **V** | **M** | **G** | **K** | **Y** | **H** | **P** | **H** | **G** | **D** | **S** | **A** | **I** | **Y** | **E** | **S** | **M** | **V** | **R** | **M** | **A** | **Q** | **P** | **F** | **S** | **Y** | **R** | **A** | **M** | **L** | **V** | **D** | **G** | **H** | **G** | **N** | **F** | **G** | **S** | **V** | **D** | **G** | **D** | **G** | **A** | **A** | **A** | **M** | **R** | **Y** | **T** | **E** | **A** | **R** | **M** | **S** | **K** | **I** | **A** | **L** | **E** | **M** | **L** | **R** | **D** | **I** | **N** | **K** | **N** | **T** | **V** | **D** | **F** | **Q** | **G** | **N** | **Y** | **D** | **D** | **S** | **E** | **Q** | **E** | **P** | **V** | **V** | **L** | **P** | **A** | **R** | **F** | **P** | **N** | **L** | **L** | **V** | **N** | **G** | **T** | **T** | **G** | **I** | **A** | **V** | **G** | **M** | **A** | **T** | **N** | **I** | **P** | **P** | **H** | **N** | **L** | **S** | **E** | **V** | **I** | **D** | **A** | **T** | **S** | **L** | **L** | **M** | **D** | **N** | **P** | **D** | **V** | **T** | **T** | **N** | **E** | **L** | **M** | **E** | **V** | **L** | **P** | **G** | **P** | **D** | **F** | **P** | **T** | **G** | **G** | **L** | **V** | **M** | **G** | **K** | **S** | **G** | **I** | **R** | **R** | **A** | **Y** | **E** | **T** | **G** | **K** | **G** | **S** | **I** | **T** | **V** | **R** | **A** | **K** | **V** | **E** | **L** | **T** | **E** | **M** | **P** | **N** | **G** | **K** | **E** | **R** | **I** | **L** | **V** | **T** | **E** | **L** | **P** | **Y** | **M** | **V** | **N** | **K** | **A** | **K** | **L** | **I** | **E** | **R** | **I** | **S** | **E** | **L** | **H** | **R** | **D** | **K** | **R** | **I** | **E** | **G** | **I** | **T** | **D** | **L** | **R** | **D** | **E** | **S** | **S** | **R** | **E** | **G** | **M** | **R** | **I** | **V** | **I** | **D** | **V** | **R** | **R** | **D** | **V** | **S** | **A** | **S** | **V** | **V** | **L** | **N** | **N** | **L** | **Y** | **K** | **M** | **T** | **A** | **L** | **Q** | **T** | **S** | **F** | **G** | **F** | **N** | **M** | **L** | **A** | **I** | **E** | **K** | **G** | **V** | **P** | **K** | **I** | **L** | **S** | **L** | **K** | **R** | **I** | **L** | **E** | **N** | **Y** | **I** | **E** | **H** | **Q** | **K** | **E** | **V** | **I** | **T** | **R** | **R** | **T** | **I** | **F** | **D** | **K** | **N** | **K** | **A** | **E** | **A** | **R** | **A** | **H** | **I** | **L** | **E** | **G** | **L** | **R** | **I** | **A** | **L** | **D** | **H** | **I** | **D** | **E** | **I** | **I** | **A** | **I** | **I** | **R** | **G** | **S** | **Q** | **S** | **D** | **D** | **E** | **A** | **K** | **A** | **T** | **L** | **I** | **E** | **R** | **F** | **E** | **F** | **S** | **D** | **R** | **Q** | **A** | **Q** | **A** | **I** | **L** | **D** | **M** | **R** | **L** | **R** | **R** | **L** | **T** | **G** | **L** | **E** | **R** | **D** | **K** | **I** | **E** | **N** | **E** | **Y** | **Q** | **E** | **L** | **L** | **K** | **F** | **I** | **A** | **D** | **L** | **E** | **D** | **I** | **L** | **A** | **R** | **P** | **E** | **R** | **V** | **I** | **E** | **I** | **I** | **K** | **T** | **E** | **L** | **N** | **D** | **V** | **R** | **T** | **K** | **F** | **G** | **D** | **A** | **R** | **R** | **T** | **E** | **L** | **L** | **V** | **G** | **E** | **V** | **L** | **S** | **L** | **E** | **D** | **E** | **D** | **L** | **I** | **E** | **E** | **E** | **E** | **V** | **V** | **I** | **T** | **L** | **T** | **N** | **N** | **G** | **Y** | **I** | **K** | **R** | **M** | **A** | **N** | **S** | **E** | **F** | **R** | **A** | **Q** | **R** | **R** | **G** | **G** | **R** | **G** | **V** | **Q** | **G** | **M** | **G** | **V** | **H** | **D** | **D** | **D** | **F** | **V** | **K** | **N** | **L** | **V** | **S** | **C** | **S** | **T** | **H** | **D** | **T** | **L** | **L** | **F** | **F** | **T** | **N** | **T** | **G** | **K** | **V** | **Y** | **R** | **A** | **K** | **G** | **Y** | **E** | **I** | **P** | **E** | **Y** | **G** | **R** | **T** | **A** | **K** | **G** | **I** | **P** | **V** | **I** | **N** | **L** | **L** | **G** | **I** | **D** | **S** | **A** | **E** | **K** | **I** | **Q** | **A** | **I** | **I** | **S** | **V** | **E** | **G** | **K** | **A** | **E** | **A** | **G** | **K** | **Y** | **L** | **F** | **F** | **T** | **T** | **L** | **K** | **G** | **T** | **V** | **K** | **R** | **T** | **A** | **V** | **T** | **A** | **F** | **S** | **N** | **I** | **R** | **S** | **N** | **G** | **L** | **I** | **A** | **I** | **S** | **L** | **K** | **E** | **D** | **D** | **E** | **L** | **V** | **N** | **V** | **V** | **T** | **T** | **N** | **G** | **N** | **Q** | **K** | **M** | **I** | **I** | **G** | **T** | **H** | **A** | **G** | **Y** | **S** | **V** | **T** | **F** | **D** | **E** | **T** | **T** | **V** | **R** | **D** | **M** | **G** | **R** | **T** | **A** | **S** | **G** | **V** | **R** | **G** | **I** | **R** | **L** | **R** | **E** | **N** | **D** | **Y** | **V** | **V** | **G** | **A** | **A** | **I** | **L** | **D** | **E** | **N** | **K** | **E** | **V** | **L** | **V** | **I** | **T** | **E** | **N** | **G** | **Y** | **G** | **K** | **R** | **T** | **K** | **A** | **S** | **E** | **Y** | **P** | **V** | **K** | **G** | **R** | **G** | **G** | **K** | **G** | **I** | **K** | **T** | **A** | **N** | **I** | **T** | **E** | **K** | **N** | **G** | **P** | **L** | **A** | **G** | **L** | **T** | **T** | **V** | **N** | **G** | **D** | **E** | **D** | **I** | **L** | **L** | **I** | **T** | **N** | **K** | **G** | **V** | **I** | **I** | **R** | **F** | **N** | **V** | **D** | **S** | **V** | **S** | **Q** | **T** | **G** | **R** | **A** | **T** | **L** | **G** | **V** | **R** | **L** | **M** | **R** | **M** | **E** | **D** | **G** | **A** | **K** | **-** | **-** | **-** | **-** | **-** | **-** | **-** | **-** | **-** | **-** | **-** | **-** | **-** | **-** | **-** | **-** | **-** | **-** | **-** | **-** | **-** | **-** | **-** |
| **N55299** | **M** | **S** | **E** | **E** | **I** | **R** | **E** | **N** | **I** | **H** | **D** | **V** | **N** | **L** | **T** | **S** | **E** | **M** | **K** | **D** | **S** | **F** | **I** | **D** | **Y** | **A** | **M** | **S** | **V** | **I** | **V** | **A** | **R** | **A** | **L** | **P** | **D** | **V** | **R** | **D** | **G** | **L** | **K** | **P** | **V** | **H** | **R** | **R** | **I** | **L** | **Y** | **G** | **M** | **N** | **E** | **L** | **G** | **V** | **T** | **P** | **D** | **K** | **A** | **H** | **K** | **K** | **S** | **A** | **R** | **I** | **V** | **G** | **D** | **V** | **M** | **G** | **K** | **Y** | **H** | **P** | **H** | **G** | **D** | **S** | **A** | **I** | **Y** | **E** | **S** | **M** | **V** | **R** | **M** | **A** | **Q** | **P** | **F** | **S** | **Y** | **R** | **Y** | **M** | **L** | **V** | **D** | **G** | **H** | **G** | **N** | **F** | **G** | **S** | **V** | **D** | **G** | **D** | **G** | **A** | **A** | **A** | **M** | **R** | **Y** | **T** | **E** | **A** | **R** | **M** | **S** | **K** | **I** | **A** | **T** | **E** | **M** | **L** | **R** | **D** | **I** | **N** | **K** | **N** | **T** | **V** | **D** | **F** | **Q** | **S** | **N** | **Y** | **D** | **D** | **T** | **E** | **K** | **E** | **P** | **V** | **V** | **L** | **P** | **A** | **R** | **F** | **P** | **N** | **L** | **L** | **V** | **N** | **G** | **T** | **T** | **G** | **I** | **A** | **V** | **G** | **M** | **A** | **T** | **N** | **I** | **P** | **P** | **H** | **N** | **L** | **S** | **E** | **V** | **V** | **A** | **A** | **I** | **D** | **L** | **L** | **M** | **D** | **N** | **P** | **D** | **V** | **T** | **T** | **N** | **E** | **L** | **M** | **E** | **V** | **L** | **P** | **G** | **P** | **D** | **F** | **P** | **T** | **G** | **G** | **L** | **V** | **M** | **G** | **K** | **S** | **G** | **I** | **R** | **R** | **A** | **Y** | **E** | **T** | **G** | **K** | **G** | **S** | **I** | **T** | **V** | **R** | **A** | **K** | **V** | **E** | **I** | **T** | **E** | **M** | **P** | **N** | **G** | **K** | **E** | **R** | **I** | **I** | **V** | **T** | **E** | **L** | **P** | **Y** | **M** | **V** | **N** | **K** | **A** | **K** | **L** | **I** | **E** | **R** | **I** | **S** | **E** | **L** | **H** | **R** | **D** | **K** | **R** | **I** | **E** | **G** | **I** | **T** | **D** | **L** | **R** | **D** | **E** | **S** | **S** | **R** | **E** | **G** | **M** | **R** | **I** | **V** | **I** | **D** | **I** | **R** | **R** | **D** | **A** | **S** | **A** | **S** | **V** | **I** | **L** | **N** | **N** | **L** | **Y** | **K** | **L** | **T** | **S** | **L** | **Q** | **N** | **S** | **F** | **G** | **F** | **N** | **M** | **L** | **A** | **I** | **E** | **K** | **G** | **V** | **P** | **K** | **V** | **L** | **S** | **L** | **K** | **Q** | **I** | **L** | **E** | **N** | **Y** | **I** | **E** | **H** | **Q** | **R** | **E** | **V** | **I** | **T** | **R** | **R** | **T** | **V** | **F** | **E** | **K** | **E** | **K** | **A** | **E** | **A** | **R** | **A** | **H** | **I** | **L** | **E** | **G** | **L** | **R** | **I** | **A** | **L** | **D** | **H** | **I** | **D** | **E** | **I** | **I** | **A** | **I** | **I** | **R** | **G** | **S** | **K** | **S** | **D** | **D** | **E** | **A** | **K** | **A** | **T** | **M** | **I** | **E** | **R** | **F** | **D** | **L** | **S** | **D** | **R** | **Q** | **S** | **Q** | **A** | **I** | **L** | **D** | **M** | **R** | **L** | **R** | **R** | **L** | **T** | **G** | **L** | **E** | **R** | **E** | **K** | **I** | **E** | **N** | **E** | **Y** | **Q** | **E** | **L** | **L** | **K** | **L** | **I** | **E** | **D** | **L** | **T** | **D** | **I** | **L** | **A** | **R** | **P** | **E** | **R** | **V** | **T** | **E** | **I** | **I** | **K** | **N** | **E** | **L** | **A** | **D** | **L** | **N** | **Q** | **R** | **F** | **G** | **D** | **K** | **R** | **R** | **T** | **E** | **L** | **L** | **V** | **G** | **E** | **V** | **L** | **S** | **L** | **E** | **D** | **E** | **D** | **L** | **I** | **E** | **E** | **E** | **E** | **I** | **V** | **I** | **T** | **L** | **T** | **N** | **N** | **G** | **Y** | **I** | **K** | **R** | **L** | **A** | **N** | **N** | **E** | **F** | **R** | **A** | **Q** | **R** | **R** | **G** | **G** | **R** | **G** | **V** | **Q** | **G** | **M** | **G** | **I** | **H** | **N** | **D** | **D** | **F** | **V** | **K** | **N** | **L** | **V** | **S** | **C** | **S** | **T** | **H** | **D** | **T** | **L** | **L** | **F** | **F** | **T** | **N** | **N** | **G** | **K** | **V** | **Y** | **R** | **A** | **K** | **G** | **Y** | **E** | **I** | **P** | **E** | **Y** | **G** | **R** | **T** | **A** | **K** | **G** | **I** | **P** | **I** | **I** | **N** | **L** | **L** | **G** | **I** | **D** | **S** | **S** | **E** | **S** | **I** | **Q** | **A** | **I** | **I** | **A** | **V** | **T** | **G** | **E** | **A** | **E** | **E** | **G** | **H** | **Y** | **L** | **F** | **F** | **T** | **T** | **R** | **Q** | **G** | **T** | **V** | **K** | **R** | **T** | **S** | **V** | **T** | **A** | **F** | **A** | **N** | **I** | **R** | **S** | **N** | **G** | **L** | **I** | **A** | **I** | **G** | **L** | **K** | **E** | **D** | **D** | **E** | **L** | **V** | **N** | **V** | **L** | **L** | **T** | **D** | **G** | **Q** | **S** | **N** | **I** | **I** | **I** | **G** | **T** | **H** | **N** | **G** | **Y** | **S** | **V** | **T** | **F** | **K** | **E** | **E** | **A** | **V** | **R** | **D** | **M** | **G** | **R** | **T** | **A** | **S** | **G** | **V** | **R** | **G** | **I** | **R** | **L** | **R** | **E** | **E** | **D** | **Y** | **V** | **V** | **G** | **A** | **A** | **L** | **M** | **K** | **E** | **N** | **Q** | **E** | **V** | **L** | **V** | **I** | **T** | **E** | **K** | **G** | **Y** | **G** | **K** | **R** | **T** | **K** | **V** | **S** | **E** | **Y** | **P** | **V** | **K** | **G** | **R** | **G** | **G** | **K** | **G** | **I** | **K** | **T** | **A** | **N** | **I** | **T** | **E** | **K** | **N** | **G** | **P** | **L** | **A** | **G** | **L** | **T** | **T** | **V** | **S** | **G** | **D** | **E** | **D** | **I** | **M** | **L** | **I** | **T** | **D** | **K** | **G** | **V** | **I** | **I** | **R** | **F** | **N** | **V** | **S** | **T** | **V** | **S** | **Q** | **T** | **G** | **R** | **S** | **T** | **L** | **G** | **V** | **R** | **L** | **M** | **K** | **M** | **E** | **A** | **D** | **T** | **K** | **V** | **V** | **T** | **M** | **A** | **A** | **V** | **E** | **P** | **E** | **A** | **A** | **E** | **Q** | **T** | **V** | **E** | **E** | **Q** | **G** | **S** | **E** | **E** |
| **N52454** | **M** | **S** | **E** | **E** | **I** | **K** | **E** | **N** | **I** | **Q** | **D** | **V** | **N** | **L** | **T** | **S** | **E** | **M** | **K** | **E** | **S** | **F** | **I** | **D** | **Y** | **A** | **M** | **S** | **V** | **I** | **V** | **A** | **R** | **A** | **L** | **P** | **D** | **V** | **R** | **D** | **G** | **L** | **K** | **P** | **V** | **H** | **R** | **R** | **I** | **L** | **Y** | **G** | **M** | **N** | **E** | **L** | **G** | **V** | **T** | **P** | **D** | **K** | **P** | **H** | **K** | **K** | **S** | **A** | **R** | **I** | **V** | **G** | **D** | **V** | **M** | **G** | **K** | **Y** | **H** | **P** | **H** | **G** | **D** | **S** | **A** | **I** | **Y** | **E** | **S** | **M** | **V** | **R** | **M** | **A** | **Q** | **P** | **F** | **S** | **Y** | **R** | **A** | **M** | **L** | **V** | **D** | **G** | **H** | **G** | **N** | **F** | **G** | **S** | **V** | **D** | **G** | **D** | **G** | **A** | **A** | **A** | **M** | **R** | **Y** | **T** | **E** | **A** | **R** | **M** | **S** | **K** | **I** | **A** | **L** | **E** | **M** | **L** | **R** | **D** | **I** | **N** | **K** | **N** | **T** | **V** | **D** | **F** | **Q** | **G** | **N** | **Y** | **D** | **D** | **S** | **E** | **Q** | **E** | **P** | **V** | **V** | **L** | **P** | **A** | **R** | **F** | **P** | **N** | **L** | **L** | **V** | **N** | **G** | **T** | **T** | **G** | **I** | **A** | **V** | **G** | **M** | **A** | **T** | **N** | **I** | **P** | **P** | **H** | **N** | **L** | **S** | **E** | **V** | **I** | **D** | **A** | **T** | **S** | **L** | **L** | **M** | **D** | **N** | **P** | **D** | **V** | **T** | **T** | **N** | **E** | **L** | **M** | **E** | **V** | **L** | **P** | **G** | **P** | **D** | **F** | **P** | **T** | **G** | **G** | **L** | **V** | **M** | **G** | **K** | **S** | **G** | **I** | **R** | **R** | **A** | **Y** | **E** | **T** | **G** | **K** | **G** | **S** | **I** | **T** | **V** | **R** | **A** | **K** | **V** | **E** | **L** | **T** | **E** | **M** | **P** | **N** | **G** | **K** | **E** | **R** | **I** | **L** | **V** | **T** | **E** | **L** | **P** | **Y** | **M** | **V** | **N** | **K** | **A** | **K** | **L** | **I** | **E** | **R** | **I** | **S** | **E** | **L** | **H** | **R** | **D** | **K** | **R** | **I** | **E** | **G** | **I** | **T** | **D** | **L** | **R** | **D** | **E** | **S** | **S** | **R** | **E** | **G** | **M** | **R** | **I** | **V** | **I** | **D** | **V** | **R** | **R** | **D** | **V** | **S** | **A** | **S** | **V** | **V** | **L** | **N** | **N** | **L** | **Y** | **K** | **M** | **T** | **A** | **L** | **Q** | **T** | **S** | **F** | **G** | **F** | **N** | **M** | **L** | **A** | **I** | **E** | **K** | **G** | **V** | **P** | **K** | **I** | **L** | **S** | **L** | **K** | **R** | **I** | **L** | **E** | **N** | **Y** | **V** | **E** | **H** | **Q** | **K** | **E** | **V** | **I** | **T** | **R** | **R** | **T** | **I** | **F** | **D** | **K** | **N** | **K** | **A** | **E** | **A** | **R** | **A** | **H** | **I** | **L** | **E** | **G** | **L** | **R** | **I** | **A** | **L** | **D** | **H** | **I** | **D** | **E** | **I** | **I** | **A** | **I** | **I** | **R** | **G** | **S** | **Q** | **S** | **D** | **D** | **E** | **A** | **K** | **A** | **T** | **L** | **I** | **E** | **R** | **F** | **E** | **F** | **S** | **D** | **R** | **Q** | **A** | **Q** | **A** | **I** | **L** | **D** | **M** | **R** | **L** | **R** | **R** | **L** | **T** | **G** | **L** | **E** | **R** | **D** | **K** | **I** | **E** | **N** | **E** | **Y** | **Q** | **E** | **L** | **L** | **K** | **F** | **I** | **A** | **D** | **L** | **E** | **D** | **I** | **L** | **A** | **R** | **P** | **E** | **R** | **V** | **I** | **E** | **I** | **I** | **K** | **T** | **E** | **L** | **N** | **D** | **V** | **R** | **T** | **K** | **F** | **G** | **D** | **A** | **R** | **R** | **T** | **E** | **L** | **L** | **V** | **G** | **E** | **V** | **L** | **S** | **L** | **E** | **D** | **E** | **D** | **L** | **I** | **E** | **E** | **E** | **E** | **V** | **V** | **I** | **T** | **L** | **T** | **N** | **N** | **G** | **Y** | **I** | **K** | **R** | **M** | **A** | **N** | **S** | **E** | **F** | **R** | **A** | **Q** | **R** | **R** | **G** | **G** | **R** | **G** | **V** | **Q** | **G** | **M** | **G** | **V** | **H** | **D** | **D** | **D** | **F** | **V** | **K** | **N** | **L** | **V** | **S** | **C** | **S** | **T** | **H** | **D** | **T** | **L** | **L** | **F** | **F** | **T** | **N** | **T** | **G** | **K** | **V** | **Y** | **R** | **A** | **K** | **G** | **Y** | **E** | **I** | **P** | **E** | **Y** | **G** | **R** | **T** | **A** | **K** | **G** | **I** | **P** | **V** | **I** | **N** | **L** | **L** | **G** | **I** | **D** | **S** | **A** | **E** | **K** | **I** | **Q** | **A** | **I** | **I** | **S** | **V** | **E** | **G** | **K** | **A** | **E** | **A** | **G** | **K** | **Y** | **L** | **F** | **F** | **T** | **T** | **L** | **K** | **G** | **T** | **V** | **K** | **R** | **T** | **A** | **V** | **T** | **A** | **F** | **S** | **N** | **I** | **R** | **S** | **N** | **G** | **L** | **I** | **A** | **I** | **S** | **L** | **K** | **E** | **D** | **D** | **E** | **L** | **V** | **N** | **V** | **V** | **T** | **T** | **N** | **G** | **N** | **Q** | **K** | **M** | **I** | **I** | **G** | **T** | **H** | **A** | **G** | **Y** | **S** | **V** | **T** | **F** | **D** | **E** | **N** | **T** | **V** | **R** | **D** | **M** | **G** | **R** | **T** | **A** | **S** | **G** | **V** | **R** | **G** | **I** | **R** | **L** | **R** | **E** | **N** | **D** | **Y** | **V** | **V** | **G** | **A** | **A** | **I** | **L** | **D** | **E** | **N** | **K** | **E** | **V** | **L** | **V** | **I** | **T** | **E** | **N** | **G** | **Y** | **G** | **K** | **R** | **T** | **K** | **A** | **S** | **E** | **Y** | **P** | **V** | **K** | **G** | **R** | **G** | **G** | **K** | **G** | **I** | **K** | **T** | **A** | **N** | **I** | **T** | **E** | **K** | **N** | **G** | **P** | **L** | **A** | **G** | **L** | **T** | **T** | **V** | **N** | **G** | **D** | **E** | **D** | **I** | **L** | **L** | **I** | **T** | **N** | **K** | **G** | **V** | **I** | **I** | **R** | **F** | **N** | **V** | **D** | **S** | **V** | **S** | **Q** | **T** | **G** | **R** | **A** | **T** | **L** | **G** | **V** | **R** | **L** | **M** | **R** | **M** | **E** | **D** | **G** | **A** | **K** | **-** | **-** | **-** | **-** | **-** | **-** | **-** | **-** | **-** | **-** | **-** | **-** | **-** | **-** | **-** | **-** | **-** | **-** | **-** | **-** | **-** | **-** | **-** |
| **N60449F** | **M** | **S** | **E** | **E** | **I** | **R** | **E** | **N** | **I** | **H** | **D** | **V** | **N** | **L** | **T** | **S** | **E** | **M** | **K** | **D** | **S** | **F** | **I** | **D** | **Y** | **A** | **M** | **S** | **V** | **I** | **V** | **A** | **R** | **A** | **L** | **P** | **D** | **V** | **R** | **D** | **G** | **L** | **K** | **P** | **V** | **H** | **R** | **R** | **I** | **L** | **Y** | **G** | **M** | **N** | **E** | **L** | **G** | **V** | **T** | **P** | **D** | **K** | **A** | **H** | **K** | **K** | **S** | **A** | **R** | **I** | **V** | **G** | **D** | **V** | **M** | **G** | **K** | **Y** | **H** | **P** | **H** | **G** | **D** | **S** | **A** | **I** | **Y** | **E** | **S** | **M** | **V** | **R** | **M** | **A** | **Q** | **P** | **F** | **S** | **Y** | **R** | **Y** | **M** | **L** | **V** | **D** | **G** | **H** | **G** | **N** | **F** | **G** | **S** | **V** | **D** | **G** | **D** | **G** | **A** | **A** | **A** | **M** | **R** | **Y** | **T** | **E** | **A** | **R** | **M** | **S** | **K** | **I** | **A** | **T** | **E** | **M** | **L** | **R** | **D** | **I** | **N** | **K** | **N** | **T** | **V** | **D** | **F** | **Q** | **S** | **N** | **Y** | **D** | **D** | **T** | **E** | **K** | **E** | **P** | **V** | **V** | **L** | **P** | **A** | **R** | **F** | **P** | **N** | **L** | **L** | **V** | **N** | **G** | **T** | **T** | **G** | **I** | **A** | **V** | **G** | **M** | **A** | **T** | **N** | **I** | **P** | **P** | **H** | **N** | **L** | **S** | **E** | **V** | **V** | **A** | **A** | **I** | **D** | **L** | **L** | **M** | **D** | **N** | **P** | **D** | **V** | **T** | **T** | **N** | **E** | **L** | **M** | **E** | **V** | **L** | **P** | **G** | **P** | **D** | **F** | **P** | **T** | **G** | **G** | **L** | **V** | **M** | **G** | **K** | **S** | **G** | **I** | **R** | **R** | **A** | **Y** | **E** | **T** | **G** | **K** | **G** | **S** | **I** | **T** | **V** | **R** | **A** | **K** | **V** | **E** | **I** | **T** | **E** | **M** | **T** | **N** | **G** | **K** | **E** | **R** | **I** | **I** | **V** | **T** | **E** | **L** | **P** | **Y** | **M** | **V** | **N** | **K** | **A** | **K** | **L** | **I** | **E** | **R** | **I** | **S** | **E** | **L** | **H** | **R** | **D** | **K** | **R** | **I** | **E** | **G** | **I** | **T** | **D** | **L** | **R** | **D** | **E** | **S** | **S** | **R** | **E** | **G** | **M** | **R** | **I** | **V** | **I** | **D** | **I** | **R** | **R** | **D** | **A** | **S** | **A** | **S** | **V** | **I** | **L** | **N** | **N** | **L** | **Y** | **K** | **L** | **T** | **S** | **L** | **Q** | **N** | **S** | **F** | **G** | **F** | **N** | **M** | **L** | **A** | **I** | **E** | **K** | **G** | **V** | **P** | **K** | **V** | **L** | **S** | **L** | **K** | **Q** | **I** | **L** | **E** | **N** | **Y** | **I** | **E** | **H** | **Q** | **R** | **E** | **V** | **I** | **T** | **R** | **R** | **T** | **V** | **F** | **E** | **K** | **E** | **K** | **A** | **E** | **A** | **R** | **A** | **H** | **I** | **L** | **E** | **G** | **L** | **R** | **I** | **A** | **L** | **D** | **H** | **I** | **D** | **E** | **I** | **I** | **A** | **I** | **I** | **R** | **G** | **S** | **K** | **S** | **D** | **D** | **E** | **A** | **K** | **A** | **T** | **M** | **I** | **E** | **R** | **F** | **D** | **L** | **S** | **D** | **R** | **Q** | **S** | **Q** | **A** | **I** | **L** | **D** | **M** | **R** | **L** | **R** | **R** | **L** | **T** | **G** | **L** | **E** | **R** | **E** | **K** | **I** | **E** | **N** | **E** | **Y** | **Q** | **E** | **L** | **L** | **K** | **L** | **I** | **E** | **D** | **L** | **T** | **D** | **I** | **L** | **A** | **R** | **P** | **E** | **R** | **V** | **T** | **E** | **I** | **I** | **K** | **N** | **E** | **L** | **A** | **D** | **L** | **N** | **Q** | **R** | **F** | **G** | **D** | **K** | **R** | **R** | **T** | **E** | **L** | **L** | **V** | **G** | **E** | **V** | **L** | **S** | **L** | **E** | **D** | **E** | **D** | **L** | **I** | **E** | **E** | **E** | **E** | **I** | **V** | **I** | **T** | **L** | **T** | **N** | **N** | **G** | **Y** | **I** | **K** | **R** | **L** | **A** | **N** | **N** | **E** | **F** | **R** | **A** | **Q** | **R** | **R** | **G** | **G** | **R** | **G** | **V** | **Q** | **G** | **M** | **G** | **I** | **H** | **N** | **D** | **D** | **F** | **V** | **K** | **N** | **L** | **V** | **S** | **C** | **S** | **T** | **H** | **D** | **T** | **L** | **L** | **F** | **F** | **T** | **N** | **N** | **G** | **K** | **V** | **Y** | **R** | **A** | **K** | **G** | **Y** | **E** | **I** | **P** | **E** | **Y** | **G** | **R** | **T** | **A** | **K** | **G** | **I** | **P** | **I** | **I** | **N** | **L** | **L** | **G** | **I** | **D** | **S** | **S** | **E** | **S** | **I** | **Q** | **A** | **I** | **I** | **A** | **V** | **T** | **G** | **E** | **A** | **E** | **E** | **G** | **H** | **Y** | **L** | **F** | **F** | **T** | **T** | **R** | **Q** | **G** | **T** | **V** | **K** | **R** | **T** | **S** | **V** | **T** | **A** | **F** | **A** | **N** | **I** | **R** | **S** | **N** | **G** | **L** | **I** | **A** | **I** | **G** | **L** | **K** | **E** | **D** | **D** | **E** | **L** | **V** | **N** | **V** | **L** | **L** | **T** | **D** | **G** | **Q** | **S** | **N** | **I** | **I** | **I** | **G** | **T** | **H** | **N** | **G** | **Y** | **S** | **V** | **T** | **F** | **K** | **E** | **E** | **A** | **V** | **R** | **D** | **M** | **G** | **R** | **T** | **A** | **S** | **G** | **V** | **R** | **G** | **I** | **R** | **L** | **R** | **E** | **E** | **D** | **Y** | **V** | **V** | **G** | **A** | **A** | **L** | **M** | **K** | **E** | **D** | **Q** | **E** | **V** | **L** | **V** | **I** | **T** | **E** | **K** | **G** | **Y** | **G** | **K** | **R** | **T** | **K** | **V** | **S** | **E** | **Y** | **P** | **V** | **K** | **G** | **R** | **G** | **G** | **K** | **G** | **I** | **K** | **T** | **A** | **N** | **I** | **T** | **E** | **K** | **N** | **G** | **P** | **L** | **A** | **G** | **L** | **T** | **T** | **V** | **S** | **G** | **D** | **E** | **D** | **I** | **M** | **L** | **I** | **T** | **D** | **K** | **G** | **V** | **I** | **I** | **R** | **F** | **N** | **V** | **S** | **T** | **V** | **S** | **Q** | **T** | **G** | **R** | **S** | **T** | **L** | **G** | **V** | **R** | **L** | **M** | **K** | **M** | **E** | **A** | **D** | **T** | **K** | **V** | **V** | **T** | **M** | **A** | **A** | **V** | **E** | **P** | **E** | **A** | **A** | **E** | **Q** | **T** | **V** | **E** | **E** | **Q** | **G** | **S** | **E** | **E** |
| **N53548** | **M** | **S** | **E** | **E** | **I** | **R** | **E** | **N** | **I** | **H** | **D** | **V** | **N** | **L** | **T** | **S** | **E** | **M** | **K** | **D** | **S** | **F** | **I** | **D** | **Y** | **A** | **M** | **S** | **V** | **I** | **V** | **A** | **R** | **A** | **L** | **P** | **D** | **V** | **R** | **D** | **G** | **L** | **K** | **P** | **V** | **H** | **R** | **R** | **I** | **L** | **Y** | **G** | **M** | **N** | **E** | **L** | **G** | **V** | **T** | **P** | **D** | **K** | **A** | **H** | **K** | **K** | **S** | **A** | **R** | **I** | **V** | **G** | **D** | **V** | **M** | **G** | **K** | **Y** | **H** | **P** | **H** | **G** | **D** | **S** | **A** | **I** | **Y** | **E** | **S** | **M** | **V** | **R** | **M** | **A** | **Q** | **P** | **F** | **S** | **Y** | **R** | **Y** | **M** | **L** | **V** | **D** | **G** | **H** | **G** | **N** | **F** | **G** | **S** | **V** | **D** | **G** | **D** | **G** | **A** | **A** | **A** | **M** | **R** | **Y** | **T** | **E** | **A** | **R** | **M** | **S** | **K** | **I** | **A** | **T** | **E** | **M** | **L** | **R** | **D** | **I** | **N** | **K** | **N** | **T** | **V** | **D** | **F** | **Q** | **S** | **N** | **Y** | **D** | **D** | **T** | **E** | **K** | **E** | **P** | **V** | **V** | **L** | **P** | **A** | **R** | **F** | **P** | **N** | **L** | **L** | **V** | **N** | **G** | **T** | **T** | **G** | **I** | **A** | **V** | **G** | **M** | **A** | **T** | **N** | **I** | **P** | **P** | **H** | **N** | **L** | **S** | **E** | **V** | **V** | **A** | **A** | **I** | **D** | **L** | **L** | **M** | **D** | **N** | **P** | **D** | **V** | **T** | **T** | **N** | **E** | **L** | **M** | **E** | **V** | **L** | **P** | **G** | **P** | **D** | **F** | **P** | **T** | **G** | **G** | **L** | **V** | **M** | **G** | **K** | **S** | **G** | **I** | **R** | **R** | **A** | **Y** | **E** | **T** | **G** | **K** | **G** | **S** | **I** | **T** | **V** | **R** | **A** | **K** | **V** | **E** | **I** | **T** | **E** | **M** | **P** | **N** | **G** | **K** | **E** | **R** | **I** | **I** | **V** | **T** | **E** | **L** | **P** | **Y** | **M** | **V** | **N** | **K** | **A** | **K** | **L** | **I** | **E** | **R** | **I** | **S** | **E** | **L** | **H** | **R** | **D** | **K** | **R** | **I** | **E** | **G** | **I** | **T** | **D** | **L** | **R** | **D** | **E** | **S** | **S** | **R** | **E** | **G** | **M** | **R** | **I** | **V** | **I** | **D** | **I** | **R** | **R** | **D** | **A** | **S** | **A** | **S** | **V** | **I** | **L** | **N** | **N** | **L** | **Y** | **K** | **L** | **T** | **S** | **L** | **Q** | **N** | **S** | **F** | **G** | **F** | **N** | **M** | **L** | **A** | **I** | **E** | **K** | **G** | **V** | **P** | **K** | **V** | **L** | **S** | **L** | **K** | **Q** | **I** | **L** | **E** | **N** | **Y** | **I** | **E** | **H** | **Q** | **R** | **E** | **V** | **I** | **T** | **R** | **R** | **T** | **V** | **F** | **E** | **K** | **E** | **K** | **A** | **E** | **A** | **R** | **A** | **H** | **I** | **L** | **E** | **G** | **L** | **R** | **I** | **A** | **L** | **D** | **H** | **I** | **D** | **E** | **I** | **I** | **A** | **I** | **I** | **R** | **G** | **S** | **K** | **S** | **D** | **D** | **E** | **A** | **K** | **A** | **T** | **M** | **I** | **E** | **R** | **F** | **D** | **L** | **S** | **D** | **R** | **Q** | **S** | **Q** | **A** | **I** | **L** | **D** | **M** | **R** | **L** | **R** | **R** | **L** | **T** | **G** | **L** | **E** | **R** | **E** | **K** | **I** | **E** | **N** | **E** | **Y** | **Q** | **E** | **L** | **L** | **K** | **L** | **I** | **E** | **D** | **L** | **T** | **D** | **I** | **L** | **A** | **R** | **P** | **E** | **R** | **V** | **T** | **E** | **I** | **I** | **K** | **N** | **E** | **L** | **A** | **D** | **L** | **N** | **Q** | **R** | **F** | **G** | **D** | **K** | **R** | **R** | **T** | **E** | **L** | **L** | **V** | **G** | **E** | **V** | **L** | **S** | **L** | **E** | **D** | **E** | **D** | **L** | **I** | **E** | **E** | **E** | **E** | **I** | **V** | **I** | **T** | **L** | **T** | **N** | **N** | **G** | **Y** | **I** | **K** | **R** | **L** | **A** | **N** | **N** | **E** | **F** | **R** | **A** | **Q** | **R** | **R** | **G** | **G** | **R** | **G** | **V** | **Q** | **G** | **M** | **G** | **I** | **H** | **N** | **D** | **D** | **F** | **V** | **K** | **N** | **L** | **V** | **S** | **C** | **S** | **T** | **H** | **D** | **T** | **L** | **L** | **F** | **F** | **T** | **N** | **N** | **G** | **K** | **V** | **Y** | **R** | **A** | **K** | **G** | **Y** | **E** | **I** | **P** | **E** | **Y** | **G** | **R** | **T** | **A** | **K** | **G** | **I** | **P** | **I** | **I** | **N** | **L** | **L** | **G** | **I** | **D** | **S** | **S** | **E** | **S** | **I** | **Q** | **A** | **I** | **I** | **A** | **V** | **T** | **G** | **E** | **A** | **E** | **E** | **G** | **H** | **Y** | **L** | **F** | **F** | **T** | **T** | **R** | **Q** | **G** | **T** | **V** | **K** | **R** | **T** | **S** | **V** | **T** | **A** | **F** | **A** | **N** | **I** | **R** | **S** | **N** | **G** | **L** | **I** | **A** | **I** | **G** | **L** | **K** | **E** | **D** | **D** | **E** | **L** | **V** | **N** | **V** | **L** | **L** | **T** | **D** | **G** | **Q** | **S** | **N** | **I** | **I** | **I** | **G** | **T** | **H** | **N** | **G** | **Y** | **S** | **V** | **T** | **F** | **K** | **E** | **E** | **A** | **V** | **R** | **D** | **M** | **G** | **R** | **T** | **A** | **S** | **G** | **V** | **R** | **G** | **I** | **R** | **L** | **R** | **E** | **E** | **D** | **Y** | **V** | **V** | **G** | **A** | **A** | **L** | **M** | **K** | **E** | **D** | **Q** | **E** | **V** | **L** | **V** | **I** | **T** | **E** | **K** | **G** | **Y** | **G** | **K** | **R** | **T** | **K** | **V** | **S** | **E** | **Y** | **P** | **V** | **K** | **G** | **R** | **G** | **G** | **K** | **G** | **I** | **K** | **T** | **A** | **N** | **I** | **T** | **E** | **K** | **N** | **G** | **P** | **L** | **A** | **G** | **L** | **T** | **T** | **V** | **S** | **G** | **D** | **E** | **D** | **I** | **M** | **L** | **I** | **T** | **D** | **K** | **G** | **V** | **I** | **I** | **R** | **F** | **N** | **V** | **S** | **T** | **V** | **S** | **Q** | **T** | **G** | **R** | **S** | **T** | **L** | **G** | **V** | **R** | **L** | **M** | **K** | **M** | **E** | **A** | **D** | **T** | **K** | **V** | **V** | **T** | **M** | **A** | **A** | **V** | **E** | **P** | **E** | **A** | **A** | **E** | **Q** | **T** | **V** | **E** | **E** | **Q** | **G** | **S** | **E** | **E** |
| **N53434** | **M** | **S** | **E** | **E** | **I** | **K** | **E** | **N** | **I** | **Q** | **D** | **V** | **N** | **L** | **T** | **S** | **E** | **M** | **K** | **E** | **S** | **F** | **I** | **D** | **Y** | **A** | **M** | **S** | **V** | **I** | **V** | **A** | **R** | **A** | **L** | **P** | **D** | **V** | **R** | **D** | **G** | **L** | **K** | **P** | **V** | **H** | **R** | **R** | **I** | **L** | **Y** | **G** | **M** | **N** | **E** | **L** | **G** | **V** | **T** | **P** | **D** | **K** | **P** | **H** | **K** | **K** | **S** | **A** | **R** | **I** | **V** | **G** | **D** | **V** | **M** | **G** | **K** | **Y** | **H** | **P** | **H** | **G** | **D** | **S** | **A** | **I** | **Y** | **E** | **S** | **M** | **V** | **R** | **M** | **A** | **Q** | **P** | **F** | **S** | **Y** | **R** | **A** | **M** | **L** | **V** | **D** | **G** | **H** | **G** | **N** | **F** | **G** | **S** | **V** | **D** | **G** | **D** | **G** | **A** | **A** | **A** | **M** | **R** | **Y** | **T** | **E** | **A** | **R** | **M** | **S** | **K** | **I** | **A** | **L** | **E** | **M** | **L** | **R** | **D** | **I** | **N** | **K** | **N** | **T** | **V** | **D** | **F** | **Q** | **G** | **N** | **Y** | **D** | **D** | **S** | **E** | **Q** | **E** | **P** | **V** | **V** | **L** | **P** | **A** | **R** | **F** | **P** | **N** | **L** | **L** | **V** | **N** | **G** | **T** | **T** | **G** | **I** | **A** | **V** | **G** | **M** | **A** | **T** | **N** | **I** | **P** | **P** | **H** | **N** | **L** | **S** | **E** | **V** | **I** | **D** | **A** | **T** | **S** | **L** | **L** | **M** | **D** | **N** | **P** | **D** | **V** | **T** | **T** | **N** | **E** | **L** | **M** | **E** | **V** | **L** | **P** | **G** | **P** | **D** | **F** | **P** | **T** | **G** | **G** | **L** | **V** | **M** | **G** | **K** | **S** | **G** | **I** | **R** | **R** | **A** | **Y** | **E** | **T** | **G** | **K** | **G** | **S** | **I** | **T** | **V** | **R** | **A** | **K** | **V** | **E** | **L** | **T** | **E** | **M** | **P** | **N** | **G** | **K** | **E** | **R** | **I** | **L** | **V** | **T** | **E** | **L** | **P** | **Y** | **M** | **V** | **N** | **K** | **A** | **K** | **L** | **I** | **E** | **R** | **I** | **S** | **E** | **L** | **H** | **R** | **D** | **K** | **R** | **I** | **E** | **G** | **I** | **T** | **D** | **L** | **R** | **D** | **E** | **S** | **S** | **R** | **E** | **G** | **M** | **R** | **I** | **V** | **I** | **D** | **V** | **R** | **R** | **D** | **V** | **S** | **A** | **S** | **V** | **V** | **L** | **N** | **N** | **L** | **Y** | **K** | **M** | **T** | **A** | **L** | **Q** | **T** | **S** | **F** | **G** | **F** | **N** | **M** | **L** | **A** | **I** | **E** | **K** | **G** | **V** | **P** | **K** | **I** | **L** | **S** | **L** | **K** | **R** | **I** | **L** | **E** | **N** | **Y** | **V** | **E** | **H** | **Q** | **K** | **E** | **V** | **I** | **T** | **R** | **R** | **T** | **I** | **F** | **D** | **K** | **N** | **K** | **A** | **E** | **A** | **R** | **A** | **H** | **I** | **L** | **E** | **G** | **L** | **R** | **I** | **A** | **L** | **D** | **H** | **I** | **D** | **E** | **I** | **I** | **A** | **I** | **I** | **R** | **G** | **S** | **Q** | **S** | **D** | **D** | **E** | **A** | **K** | **A** | **T** | **L** | **I** | **E** | **R** | **F** | **E** | **F** | **S** | **D** | **R** | **Q** | **A** | **Q** | **A** | **I** | **L** | **D** | **M** | **R** | **L** | **R** | **R** | **L** | **T** | **G** | **L** | **E** | **R** | **D** | **K** | **I** | **E** | **N** | **E** | **Y** | **Q** | **E** | **L** | **L** | **K** | **F** | **I** | **A** | **D** | **L** | **E** | **D** | **I** | **L** | **A** | **R** | **P** | **E** | **R** | **V** | **I** | **E** | **I** | **I** | **K** | **T** | **E** | **L** | **N** | **D** | **V** | **R** | **T** | **K** | **F** | **G** | **D** | **A** | **R** | **R** | **T** | **E** | **L** | **L** | **V** | **G** | **E** | **V** | **L** | **S** | **L** | **E** | **D** | **E** | **D** | **L** | **I** | **E** | **E** | **E** | **E** | **V** | **V** | **I** | **T** | **L** | **T** | **N** | **N** | **G** | **Y** | **I** | **K** | **R** | **M** | **A** | **N** | **S** | **E** | **F** | **R** | **A** | **Q** | **R** | **R** | **G** | **G** | **R** | **G** | **V** | **Q** | **G** | **M** | **G** | **V** | **H** | **D** | **D** | **D** | **F** | **V** | **K** | **N** | **L** | **V** | **S** | **C** | **S** | **T** | **H** | **D** | **T** | **L** | **L** | **F** | **F** | **T** | **N** | **T** | **G** | **K** | **V** | **Y** | **R** | **A** | **K** | **G** | **Y** | **E** | **I** | **P** | **E** | **Y** | **G** | **R** | **T** | **A** | **K** | **G** | **I** | **P** | **V** | **I** | **N** | **L** | **L** | **G** | **I** | **D** | **S** | **A** | **E** | **K** | **I** | **Q** | **A** | **I** | **I** | **S** | **V** | **E** | **G** | **K** | **A** | **E** | **A** | **G** | **K** | **Y** | **L** | **F** | **F** | **T** | **T** | **L** | **K** | **G** | **T** | **V** | **K** | **R** | **T** | **A** | **V** | **T** | **A** | **F** | **S** | **N** | **I** | **R** | **S** | **N** | **G** | **L** | **I** | **A** | **I** | **S** | **L** | **K** | **E** | **D** | **D** | **E** | **L** | **V** | **N** | **V** | **V** | **T** | **T** | **N** | **G** | **N** | **Q** | **K** | **M** | **I** | **I** | **G** | **T** | **H** | **A** | **G** | **Y** | **S** | **V** | **T** | **F** | **D** | **E** | **N** | **T** | **V** | **R** | **D** | **M** | **G** | **R** | **T** | **A** | **S** | **G** | **V** | **R** | **G** | **I** | **R** | **L** | **R** | **E** | **N** | **D** | **Y** | **V** | **V** | **G** | **A** | **A** | **I** | **L** | **D** | **E** | **N** | **K** | **E** | **V** | **L** | **V** | **I** | **T** | **E** | **N** | **G** | **Y** | **G** | **K** | **R** | **T** | **K** | **A** | **S** | **E** | **Y** | **P** | **V** | **K** | **G** | **R** | **G** | **G** | **K** | **G** | **I** | **K** | **T** | **A** | **N** | **I** | **T** | **E** | **K** | **N** | **G** | **P** | **L** | **A** | **G** | **L** | **T** | **T** | **V** | **N** | **G** | **D** | **E** | **D** | **I** | **L** | **L** | **I** | **T** | **N** | **K** | **G** | **V** | **I** | **I** | **R** | **F** | **N** | **V** | **D** | **S** | **V** | **S** | **Q** | **T** | **G** | **R** | **A** | **T** | **L** | **G** | **V** | **R** | **L** | **M** | **R** | **M** | **E** | **D** | **G** | **A** | **K** | **-** | **-** | **-** | **-** | **-** | **-** | **-** | **-** | **-** | **-** | **-** | **-** | **-** | **-** | **-** | **-** | **-** | **-** | **-** | **-** | **-** | **-** | **-** |
| **N59544F** | **M** | **S** | **E** | **E** | **I** | **K** | **E** | **N** | **I** | **Q** | **D** | **V** | **N** | **L** | **T** | **S** | **E** | **M** | **K** | **E** | **S** | **F** | **I** | **D** | **Y** | **A** | **M** | **S** | **V** | **I** | **V** | **A** | **R** | **A** | **L** | **P** | **D** | **V** | **R** | **D** | **G** | **L** | **K** | **P** | **V** | **H** | **R** | **R** | **I** | **L** | **Y** | **G** | **M** | **N** | **E** | **L** | **G** | **V** | **T** | **P** | **D** | **K** | **P** | **H** | **K** | **K** | **S** | **A** | **R** | **I** | **V** | **G** | **D** | **V** | **M** | **G** | **K** | **Y** | **H** | **P** | **H** | **G** | **D** | **S** | **A** | **I** | **Y** | **E** | **S** | **M** | **V** | **R** | **M** | **A** | **Q** | **P** | **F** | **S** | **Y** | **R** | **A** | **M** | **L** | **V** | **D** | **G** | **H** | **G** | **N** | **F** | **G** | **S** | **V** | **D** | **G** | **D** | **G** | **A** | **A** | **A** | **M** | **R** | **Y** | **T** | **E** | **A** | **R** | **M** | **S** | **K** | **I** | **A** | **L** | **E** | **M** | **L** | **R** | **D** | **I** | **N** | **K** | **N** | **T** | **V** | **D** | **F** | **Q** | **G** | **N** | **Y** | **D** | **D** | **S** | **E** | **Q** | **E** | **P** | **V** | **V** | **L** | **P** | **A** | **R** | **F** | **P** | **N** | **L** | **L** | **V** | **N** | **G** | **T** | **T** | **G** | **I** | **A** | **V** | **G** | **M** | **A** | **T** | **N** | **I** | **P** | **P** | **H** | **N** | **L** | **S** | **E** | **V** | **I** | **D** | **A** | **T** | **S** | **L** | **L** | **M** | **D** | **N** | **P** | **D** | **V** | **T** | **T** | **N** | **E** | **L** | **M** | **E** | **V** | **L** | **P** | **G** | **P** | **D** | **F** | **P** | **T** | **G** | **G** | **L** | **V** | **M** | **G** | **K** | **S** | **G** | **I** | **R** | **R** | **A** | **Y** | **E** | **T** | **G** | **K** | **G** | **S** | **I** | **T** | **V** | **R** | **A** | **K** | **V** | **E** | **L** | **T** | **E** | **M** | **P** | **N** | **G** | **K** | **E** | **R** | **I** | **L** | **V** | **T** | **E** | **L** | **P** | **Y** | **M** | **V** | **N** | **K** | **A** | **K** | **L** | **I** | **E** | **R** | **I** | **S** | **E** | **L** | **H** | **R** | **D** | **K** | **R** | **I** | **E** | **G** | **I** | **T** | **D** | **L** | **R** | **D** | **E** | **S** | **S** | **R** | **E** | **G** | **M** | **R** | **I** | **V** | **I** | **D** | **V** | **R** | **R** | **D** | **V** | **S** | **A** | **S** | **V** | **V** | **L** | **N** | **N** | **L** | **Y** | **K** | **M** | **T** | **A** | **L** | **Q** | **T** | **S** | **F** | **G** | **F** | **N** | **M** | **L** | **A** | **I** | **E** | **K** | **G** | **V** | **P** | **K** | **I** | **L** | **S** | **L** | **K** | **R** | **I** | **L** | **E** | **N** | **Y** | **V** | **E** | **H** | **Q** | **K** | **E** | **V** | **I** | **T** | **R** | **R** | **T** | **I** | **F** | **D** | **K** | **N** | **K** | **A** | **E** | **A** | **R** | **A** | **H** | **I** | **L** | **E** | **G** | **L** | **R** | **I** | **A** | **L** | **D** | **H** | **I** | **D** | **E** | **I** | **I** | **A** | **I** | **I** | **R** | **G** | **S** | **Q** | **S** | **D** | **D** | **E** | **A** | **K** | **A** | **T** | **L** | **I** | **E** | **R** | **F** | **E** | **F** | **S** | **D** | **R** | **Q** | **A** | **Q** | **A** | **I** | **L** | **D** | **M** | **R** | **L** | **R** | **R** | **L** | **T** | **G** | **L** | **E** | **R** | **D** | **K** | **I** | **E** | **N** | **E** | **Y** | **Q** | **E** | **L** | **L** | **K** | **F** | **I** | **A** | **D** | **L** | **E** | **D** | **I** | **L** | **A** | **R** | **P** | **E** | **R** | **V** | **I** | **E** | **I** | **I** | **K** | **T** | **E** | **L** | **N** | **D** | **V** | **R** | **T** | **K** | **F** | **G** | **D** | **A** | **R** | **R** | **T** | **E** | **L** | **L** | **V** | **G** | **E** | **V** | **L** | **S** | **L** | **E** | **D** | **E** | **D** | **L** | **I** | **E** | **E** | **E** | **E** | **V** | **V** | **I** | **T** | **L** | **T** | **N** | **N** | **G** | **Y** | **I** | **K** | **R** | **M** | **A** | **N** | **S** | **E** | **F** | **R** | **A** | **Q** | **R** | **R** | **G** | **G** | **R** | **G** | **V** | **Q** | **G** | **M** | **G** | **V** | **H** | **D** | **D** | **D** | **F** | **V** | **K** | **N** | **L** | **V** | **S** | **C** | **S** | **T** | **H** | **D** | **T** | **L** | **L** | **F** | **F** | **T** | **N** | **T** | **G** | **K** | **V** | **Y** | **R** | **A** | **K** | **G** | **Y** | **E** | **I** | **P** | **E** | **Y** | **G** | **R** | **T** | **A** | **K** | **G** | **I** | **P** | **V** | **I** | **N** | **L** | **L** | **G** | **I** | **D** | **S** | **T** | **E** | **K** | **I** | **Q** | **A** | **I** | **I** | **S** | **V** | **E** | **G** | **K** | **A** | **E** | **A** | **G** | **K** | **Y** | **L** | **F** | **F** | **T** | **T** | **L** | **K** | **G** | **T** | **V** | **K** | **R** | **T** | **A** | **V** | **T** | **A** | **F** | **S** | **N** | **I** | **R** | **S** | **N** | **G** | **L** | **I** | **A** | **I** | **S** | **L** | **K** | **E** | **D** | **D** | **E** | **L** | **V** | **N** | **V** | **V** | **T** | **T** | **N** | **G** | **N** | **Q** | **K** | **M** | **I** | **I** | **G** | **T** | **H** | **A** | **G** | **Y** | **S** | **V** | **T** | **F** | **D** | **E** | **N** | **T** | **V** | **R** | **D** | **M** | **G** | **R** | **T** | **A** | **S** | **G** | **V** | **R** | **G** | **I** | **R** | **L** | **R** | **E** | **N** | **D** | **Y** | **V** | **V** | **G** | **A** | **A** | **I** | **L** | **D** | **E** | **N** | **K** | **E** | **V** | **L** | **V** | **I** | **T** | **E** | **N** | **G** | **Y** | **G** | **K** | **R** | **T** | **K** | **A** | **S** | **E** | **Y** | **P** | **V** | **K** | **G** | **R** | **G** | **G** | **K** | **G** | **I** | **K** | **T** | **A** | **N** | **I** | **T** | **E** | **K** | **N** | **G** | **P** | **L** | **A** | **G** | **L** | **T** | **T** | **V** | **N** | **G** | **D** | **E** | **D** | **I** | **L** | **L** | **I** | **T** | **N** | **K** | **G** | **V** | **I** | **I** | **R** | **F** | **N** | **V** | **D** | **S** | **V** | **S** | **Q** | **T** | **G** | **R** | **A** | **T** | **L** | **G** | **V** | **R** | **L** | **M** | **R** | **M** | **E** | **D** | **G** | **A** | **K** | **-** | **-** | **-** | **-** | **-** | **-** | **-** | **-** | **-** | **-** | **-** | **-** | **-** | **-** | **-** | **-** | **-** | **-** | **-** | **-** | **-** | **-** | **-** |
| **N60392F** | **M** | **S** | **E** | **E** | **I** | **K** | **E** | **N** | **I** | **Q** | **D** | **V** | **N** | **L** | **T** | **S** | **E** | **M** | **K** | **E** | **S** | **F** | **I** | **D** | **Y** | **A** | **M** | **S** | **V** | **I** | **V** | **A** | **R** | **A** | **L** | **P** | **D** | **V** | **R** | **D** | **G** | **L** | **K** | **P** | **V** | **H** | **R** | **R** | **I** | **L** | **Y** | **G** | **M** | **N** | **E** | **L** | **G** | **V** | **T** | **P** | **D** | **K** | **P** | **H** | **K** | **K** | **S** | **A** | **R** | **I** | **V** | **G** | **D** | **V** | **M** | **G** | **K** | **Y** | **H** | **P** | **H** | **G** | **D** | **S** | **A** | **I** | **Y** | **E** | **S** | **M** | **V** | **R** | **M** | **A** | **Q** | **P** | **F** | **S** | **Y** | **R** | **A** | **M** | **L** | **V** | **D** | **G** | **H** | **G** | **N** | **F** | **G** | **S** | **V** | **D** | **G** | **D** | **G** | **A** | **A** | **A** | **M** | **R** | **Y** | **T** | **E** | **A** | **R** | **M** | **S** | **K** | **I** | **A** | **L** | **E** | **M** | **L** | **R** | **D** | **I** | **N** | **K** | **N** | **T** | **V** | **D** | **F** | **Q** | **G** | **N** | **Y** | **D** | **D** | **S** | **E** | **Q** | **E** | **P** | **V** | **V** | **L** | **P** | **A** | **R** | **F** | **P** | **N** | **L** | **L** | **V** | **N** | **G** | **T** | **T** | **G** | **I** | **A** | **V** | **G** | **M** | **A** | **T** | **N** | **I** | **P** | **P** | **H** | **N** | **L** | **S** | **E** | **V** | **I** | **D** | **A** | **T** | **S** | **L** | **L** | **M** | **D** | **N** | **P** | **D** | **V** | **T** | **T** | **N** | **E** | **L** | **M** | **E** | **V** | **L** | **P** | **G** | **P** | **D** | **F** | **P** | **T** | **G** | **G** | **L** | **V** | **M** | **G** | **K** | **S** | **G** | **I** | **R** | **R** | **A** | **Y** | **E** | **T** | **G** | **K** | **G** | **S** | **I** | **T** | **V** | **R** | **A** | **K** | **V** | **E** | **L** | **T** | **E** | **M** | **P** | **N** | **G** | **K** | **E** | **R** | **I** | **L** | **V** | **T** | **E** | **L** | **P** | **Y** | **M** | **V** | **N** | **K** | **A** | **K** | **L** | **I** | **E** | **R** | **I** | **S** | **E** | **L** | **H** | **R** | **D** | **K** | **R** | **I** | **E** | **G** | **I** | **T** | **D** | **L** | **R** | **D** | **E** | **S** | **S** | **R** | **E** | **G** | **M** | **R** | **I** | **V** | **I** | **D** | **V** | **R** | **R** | **D** | **V** | **S** | **A** | **S** | **V** | **V** | **L** | **N** | **N** | **L** | **Y** | **K** | **M** | **T** | **A** | **L** | **Q** | **T** | **S** | **F** | **G** | **F** | **N** | **M** | **L** | **A** | **I** | **E** | **K** | **G** | **V** | **P** | **K** | **I** | **L** | **S** | **L** | **K** | **R** | **I** | **L** | **E** | **N** | **Y** | **V** | **E** | **H** | **Q** | **K** | **E** | **V** | **I** | **T** | **R** | **R** | **T** | **I** | **F** | **D** | **K** | **N** | **K** | **A** | **E** | **A** | **R** | **A** | **H** | **I** | **L** | **E** | **G** | **L** | **R** | **I** | **A** | **L** | **D** | **H** | **I** | **D** | **E** | **I** | **I** | **A** | **I** | **I** | **R** | **G** | **S** | **Q** | **S** | **D** | **D** | **E** | **A** | **K** | **A** | **T** | **L** | **I** | **E** | **R** | **F** | **E** | **F** | **S** | **D** | **R** | **Q** | **A** | **Q** | **A** | **I** | **L** | **D** | **M** | **R** | **L** | **R** | **R** | **L** | **T** | **G** | **L** | **E** | **R** | **D** | **K** | **I** | **E** | **N** | **E** | **Y** | **Q** | **E** | **L** | **L** | **I** | **F** | **I** | **A** | **D** | **L** | **E** | **D** | **I** | **L** | **A** | **R** | **P** | **E** | **R** | **V** | **I** | **E** | **I** | **I** | **K** | **T** | **E** | **L** | **N** | **D** | **V** | **R** | **T** | **K** | **F** | **G** | **D** | **A** | **R** | **R** | **T** | **E** | **L** | **L** | **V** | **G** | **E** | **V** | **L** | **S** | **L** | **E** | **D** | **E** | **D** | **L** | **I** | **E** | **E** | **E** | **E** | **V** | **V** | **I** | **T** | **L** | **T** | **N** | **N** | **G** | **Y** | **I** | **K** | **R** | **M** | **A** | **N** | **S** | **E** | **F** | **R** | **A** | **Q** | **R** | **R** | **G** | **G** | **R** | **G** | **V** | **Q** | **G** | **M** | **G** | **V** | **H** | **D** | **D** | **D** | **F** | **V** | **K** | **N** | **L** | **V** | **S** | **C** | **S** | **T** | **H** | **D** | **T** | **L** | **L** | **F** | **F** | **T** | **N** | **T** | **G** | **K** | **V** | **Y** | **R** | **A** | **K** | **G** | **Y** | **E** | **I** | **P** | **E** | **Y** | **G** | **R** | **T** | **A** | **K** | **G** | **I** | **P** | **V** | **I** | **N** | **L** | **L** | **G** | **I** | **D** | **S** | **A** | **E** | **K** | **I** | **Q** | **A** | **I** | **I** | **S** | **V** | **E** | **G** | **K** | **A** | **E** | **A** | **G** | **K** | **Y** | **L** | **F** | **F** | **T** | **T** | **L** | **K** | **G** | **T** | **V** | **K** | **R** | **T** | **A** | **V** | **T** | **A** | **F** | **S** | **N** | **I** | **R** | **S** | **N** | **G** | **L** | **I** | **A** | **I** | **S** | **L** | **K** | **E** | **D** | **D** | **E** | **L** | **V** | **N** | **V** | **V** | **T** | **T** | **N** | **G** | **N** | **Q** | **K** | **M** | **I** | **I** | **G** | **T** | **H** | **A** | **G** | **Y** | **S** | **V** | **T** | **F** | **D** | **E** | **N** | **T** | **V** | **R** | **D** | **M** | **G** | **R** | **T** | **A** | **S** | **G** | **V** | **R** | **G** | **I** | **R** | **L** | **R** | **E** | **N** | **D** | **Y** | **V** | **V** | **G** | **A** | **A** | **I** | **L** | **D** | **E** | **N** | **K** | **E** | **V** | **L** | **V** | **I** | **T** | **E** | **N** | **G** | **Y** | **G** | **K** | **R** | **T** | **K** | **A** | **S** | **E** | **Y** | **P** | **V** | **K** | **G** | **R** | **G** | **G** | **K** | **G** | **I** | **K** | **T** | **A** | **N** | **I** | **T** | **E** | **K** | **N** | **G** | **P** | **L** | **A** | **G** | **L** | **T** | **T** | **V** | **N** | **G** | **D** | **E** | **D** | **I** | **L** | **L** | **I** | **T** | **N** | **K** | **G** | **V** | **I** | **I** | **R** | **F** | **N** | **V** | **D** | **S** | **V** | **S** | **Q** | **T** | **G** | **R** | **A** | **T** | **L** | **G** | **V** | **R** | **L** | **M** | **R** | **M** | **E** | **D** | **G** | **A** | **K** | **-** | **-** | **-** | **-** | **-** | **-** | **-** | **-** | **-** | **-** | **-** | **-** | **-** | **-** | **-** | **-** | **-** | **-** | **-** | **-** | **-** | **-** | **-** |
| **N59653F** | **M** | **S** | **E** | **E** | **I** | **R** | **E** | **N** | **I** | **H** | **D** | **V** | **N** | **L** | **T** | **S** | **E** | **M** | **K** | **D** | **S** | **F** | **I** | **D** | **Y** | **A** | **M** | **S** | **V** | **I** | **V** | **A** | **R** | **A** | **L** | **P** | **D** | **V** | **R** | **D** | **G** | **L** | **K** | **P** | **V** | **H** | **R** | **R** | **I** | **L** | **Y** | **G** | **M** | **N** | **E** | **L** | **G** | **V** | **T** | **P** | **D** | **K** | **A** | **H** | **K** | **K** | **S** | **A** | **R** | **I** | **V** | **G** | **D** | **V** | **M** | **G** | **K** | **Y** | **H** | **P** | **H** | **G** | **D** | **S** | **A** | **I** | **Y** | **E** | **S** | **M** | **V** | **R** | **M** | **A** | **Q** | **P** | **F** | **S** | **Y** | **R** | **Y** | **M** | **L** | **V** | **D** | **G** | **H** | **G** | **N** | **F** | **G** | **S** | **V** | **D** | **G** | **D** | **G** | **A** | **A** | **A** | **M** | **R** | **Y** | **T** | **E** | **A** | **R** | **M** | **S** | **K** | **I** | **A** | **T** | **E** | **M** | **L** | **R** | **D** | **I** | **N** | **K** | **N** | **T** | **V** | **D** | **F** | **Q** | **S** | **N** | **Y** | **D** | **D** | **T** | **E** | **K** | **E** | **P** | **V** | **V** | **L** | **P** | **A** | **R** | **F** | **P** | **N** | **L** | **L** | **V** | **N** | **G** | **T** | **T** | **G** | **I** | **A** | **V** | **G** | **M** | **A** | **T** | **N** | **I** | **P** | **P** | **H** | **N** | **L** | **S** | **E** | **V** | **V** | **A** | **A** | **I** | **D** | **L** | **L** | **M** | **D** | **N** | **P** | **D** | **V** | **T** | **T** | **N** | **E** | **L** | **M** | **E** | **V** | **L** | **P** | **G** | **P** | **D** | **F** | **P** | **T** | **G** | **G** | **L** | **V** | **M** | **G** | **K** | **S** | **G** | **I** | **R** | **R** | **A** | **Y** | **E** | **T** | **G** | **K** | **G** | **S** | **I** | **T** | **V** | **R** | **A** | **K** | **V** | **E** | **I** | **T** | **E** | **M** | **P** | **N** | **G** | **K** | **E** | **R** | **I** | **L** | **V** | **T** | **E** | **L** | **P** | **Y** | **M** | **V** | **N** | **K** | **A** | **K** | **L** | **I** | **E** | **R** | **I** | **S** | **E** | **L** | **H** | **R** | **D** | **K** | **R** | **I** | **E** | **G** | **I** | **T** | **D** | **L** | **R** | **D** | **E** | **S** | **S** | **R** | **E** | **G** | **M** | **R** | **I** | **V** | **I** | **D** | **I** | **R** | **R** | **D** | **A** | **S** | **A** | **S** | **V** | **I** | **L** | **N** | **N** | **L** | **Y** | **K** | **L** | **T** | **S** | **L** | **Q** | **N** | **S** | **F** | **G** | **F** | **N** | **M** | **L** | **A** | **I** | **E** | **K** | **G** | **V** | **P** | **K** | **V** | **L** | **S** | **L** | **K** | **Q** | **I** | **L** | **E** | **N** | **Y** | **I** | **E** | **H** | **Q** | **R** | **E** | **V** | **I** | **T** | **R** | **R** | **T** | **V** | **F** | **E** | **K** | **E** | **K** | **A** | **E** | **A** | **R** | **A** | **H** | **I** | **L** | **E** | **G** | **L** | **R** | **I** | **A** | **L** | **D** | **H** | **I** | **D** | **E** | **I** | **I** | **A** | **I** | **I** | **R** | **G** | **S** | **K** | **S** | **D** | **D** | **E** | **A** | **K** | **A** | **T** | **M** | **I** | **E** | **R** | **F** | **D** | **L** | **S** | **D** | **R** | **Q** | **S** | **Q** | **A** | **I** | **L** | **D** | **M** | **R** | **L** | **R** | **R** | **L** | **T** | **G** | **L** | **E** | **R** | **E** | **K** | **I** | **E** | **N** | **E** | **Y** | **Q** | **E** | **L** | **L** | **K** | **L** | **I** | **E** | **D** | **L** | **T** | **D** | **I** | **L** | **A** | **R** | **P** | **E** | **R** | **V** | **T** | **E** | **I** | **I** | **K** | **N** | **E** | **L** | **A** | **D** | **L** | **N** | **Q** | **R** | **F** | **G** | **D** | **K** | **R** | **R** | **T** | **E** | **L** | **L** | **V** | **G** | **E** | **V** | **L** | **S** | **L** | **E** | **D** | **E** | **D** | **L** | **I** | **E** | **E** | **E** | **E** | **I** | **V** | **I** | **T** | **L** | **T** | **N** | **N** | **G** | **Y** | **I** | **K** | **R** | **L** | **A** | **N** | **N** | **E** | **F** | **R** | **A** | **Q** | **R** | **R** | **G** | **G** | **R** | **G** | **V** | **Q** | **G** | **M** | **G** | **I** | **H** | **N** | **D** | **D** | **F** | **V** | **K** | **N** | **L** | **V** | **S** | **C** | **S** | **T** | **H** | **D** | **T** | **L** | **L** | **F** | **F** | **T** | **N** | **N** | **G** | **K** | **V** | **Y** | **R** | **A** | **K** | **G** | **Y** | **E** | **I** | **P** | **E** | **Y** | **G** | **R** | **T** | **A** | **K** | **G** | **I** | **P** | **I** | **I** | **N** | **L** | **L** | **G** | **I** | **D** | **S** | **S** | **E** | **S** | **I** | **Q** | **A** | **I** | **I** | **A** | **V** | **T** | **G** | **E** | **A** | **E** | **E** | **G** | **H** | **Y** | **L** | **F** | **F** | **T** | **T** | **R** | **Q** | **G** | **T** | **V** | **K** | **R** | **T** | **S** | **V** | **T** | **A** | **F** | **A** | **N** | **I** | **R** | **S** | **N** | **G** | **L** | **I** | **A** | **I** | **G** | **L** | **K** | **E** | **D** | **D** | **E** | **L** | **V** | **N** | **V** | **L** | **L** | **T** | **D** | **G** | **Q** | **S** | **N** | **I** | **I** | **I** | **G** | **T** | **H** | **N** | **G** | **Y** | **S** | **V** | **T** | **F** | **K** | **E** | **E** | **A** | **V** | **R** | **D** | **M** | **G** | **R** | **T** | **A** | **S** | **G** | **V** | **R** | **G** | **I** | **R** | **L** | **R** | **E** | **E** | **D** | **Y** | **V** | **V** | **G** | **A** | **A** | **L** | **M** | **K** | **E** | **D** | **Q** | **E** | **V** | **L** | **V** | **I** | **T** | **E** | **K** | **G** | **Y** | **G** | **K** | **R** | **T** | **K** | **V** | **S** | **E** | **Y** | **P** | **V** | **K** | **G** | **R** | **G** | **G** | **K** | **G** | **I** | **K** | **T** | **A** | **N** | **I** | **T** | **E** | **K** | **N** | **G** | **P** | **L** | **A** | **G** | **L** | **T** | **T** | **V** | **S** | **G** | **D** | **E** | **D** | **I** | **M** | **L** | **I** | **T** | **D** | **K** | **G** | **V** | **I** | **I** | **R** | **F** | **N** | **V** | **S** | **T** | **V** | **S** | **Q** | **T** | **G** | **R** | **S** | **T** | **L** | **G** | **V** | **R** | **L** | **M** | **K** | **M** | **E** | **A** | **D** | **T** | **K** | **V** | **V** | **T** | **M** | **-** | **-** | **-** | **-** | **-** | **-** | **-** | **-** | **-** | **-** | **-** | **-** | **-** | **-** | **-** | **-** | **-** | **-** | **-** |
| **N52469** | **M** | **S** | **E** | **E** | **I** | **K** | **E** | **N** | **I** | **Q** | **D** | **V** | **N** | **L** | **T** | **S** | **E** | **M** | **K** | **E** | **S** | **F** | **I** | **D** | **Y** | **A** | **M** | **S** | **V** | **I** | **V** | **A** | **R** | **A** | **L** | **P** | **D** | **V** | **R** | **D** | **G** | **L** | **K** | **P** | **V** | **H** | **R** | **R** | **I** | **L** | **Y** | **G** | **M** | **N** | **E** | **L** | **G** | **V** | **T** | **P** | **D** | **K** | **P** | **H** | **K** | **K** | **S** | **A** | **R** | **I** | **V** | **G** | **D** | **V** | **M** | **G** | **K** | **Y** | **H** | **P** | **H** | **G** | **D** | **S** | **A** | **I** | **Y** | **E** | **S** | **M** | **V** | **R** | **M** | **A** | **Q** | **P** | **F** | **S** | **Y** | **R** | **A** | **M** | **L** | **V** | **D** | **G** | **H** | **G** | **N** | **F** | **G** | **S** | **V** | **D** | **G** | **D** | **G** | **A** | **A** | **A** | **M** | **R** | **Y** | **T** | **E** | **A** | **R** | **M** | **S** | **K** | **I** | **A** | **L** | **E** | **M** | **L** | **R** | **D** | **I** | **N** | **K** | **N** | **T** | **V** | **D** | **F** | **Q** | **G** | **N** | **Y** | **D** | **D** | **S** | **E** | **Q** | **E** | **P** | **V** | **V** | **L** | **P** | **A** | **R** | **F** | **P** | **N** | **L** | **L** | **V** | **N** | **G** | **T** | **T** | **G** | **I** | **A** | **V** | **G** | **M** | **A** | **T** | **N** | **I** | **P** | **P** | **H** | **N** | **L** | **S** | **E** | **V** | **I** | **D** | **A** | **T** | **S** | **L** | **L** | **M** | **D** | **N** | **P** | **D** | **V** | **T** | **T** | **N** | **E** | **L** | **M** | **E** | **V** | **L** | **P** | **G** | **P** | **D** | **F** | **P** | **T** | **G** | **G** | **L** | **V** | **M** | **G** | **K** | **S** | **G** | **I** | **R** | **R** | **A** | **Y** | **E** | **T** | **G** | **K** | **G** | **S** | **I** | **T** | **V** | **R** | **A** | **K** | **V** | **E** | **L** | **T** | **E** | **M** | **P** | **N** | **G** | **K** | **E** | **R** | **I** | **L** | **V** | **T** | **E** | **L** | **P** | **Y** | **M** | **V** | **N** | **K** | **A** | **K** | **L** | **I** | **E** | **R** | **I** | **S** | **E** | **L** | **H** | **R** | **D** | **K** | **R** | **I** | **E** | **G** | **I** | **T** | **D** | **L** | **R** | **D** | **E** | **S** | **S** | **R** | **E** | **G** | **M** | **R** | **I** | **V** | **I** | **D** | **V** | **R** | **R** | **D** | **V** | **S** | **A** | **S** | **V** | **V** | **L** | **N** | **N** | **L** | **Y** | **K** | **M** | **T** | **A** | **L** | **Q** | **T** | **S** | **F** | **G** | **F** | **N** | **M** | **L** | **A** | **I** | **E** | **K** | **G** | **V** | **P** | **K** | **I** | **L** | **S** | **L** | **K** | **R** | **I** | **L** | **E** | **N** | **Y** | **V** | **E** | **H** | **Q** | **K** | **E** | **V** | **I** | **T** | **R** | **R** | **T** | **I** | **F** | **D** | **K** | **N** | **K** | **A** | **E** | **A** | **R** | **A** | **H** | **I** | **L** | **E** | **G** | **L** | **R** | **I** | **A** | **L** | **D** | **H** | **I** | **D** | **E** | **I** | **I** | **A** | **I** | **I** | **R** | **G** | **S** | **Q** | **S** | **D** | **D** | **E** | **A** | **K** | **A** | **T** | **L** | **I** | **E** | **R** | **F** | **E** | **F** | **S** | **D** | **R** | **Q** | **A** | **Q** | **A** | **I** | **L** | **D** | **M** | **R** | **L** | **R** | **R** | **L** | **T** | **G** | **L** | **E** | **R** | **D** | **K** | **I** | **E** | **N** | **E** | **Y** | **Q** | **E** | **L** | **L** | **K** | **F** | **I** | **A** | **D** | **L** | **E** | **D** | **I** | **L** | **A** | **R** | **P** | **E** | **R** | **V** | **I** | **E** | **I** | **I** | **K** | **T** | **E** | **L** | **N** | **D** | **V** | **R** | **T** | **K** | **F** | **G** | **D** | **A** | **R** | **R** | **T** | **E** | **L** | **L** | **V** | **G** | **E** | **V** | **L** | **S** | **L** | **E** | **D** | **E** | **D** | **L** | **I** | **E** | **E** | **E** | **E** | **V** | **V** | **I** | **T** | **L** | **T** | **N** | **N** | **G** | **Y** | **I** | **K** | **R** | **M** | **A** | **N** | **S** | **E** | **F** | **R** | **A** | **Q** | **R** | **R** | **G** | **G** | **R** | **G** | **V** | **Q** | **G** | **M** | **G** | **V** | **H** | **D** | **D** | **D** | **F** | **V** | **K** | **N** | **L** | **V** | **S** | **C** | **S** | **T** | **H** | **D** | **T** | **L** | **L** | **F** | **F** | **T** | **N** | **T** | **G** | **K** | **V** | **Y** | **R** | **A** | **K** | **G** | **Y** | **E** | **I** | **P** | **E** | **Y** | **G** | **R** | **T** | **A** | **K** | **G** | **I** | **P** | **V** | **I** | **N** | **L** | **L** | **G** | **I** | **D** | **S** | **A** | **E** | **K** | **I** | **Q** | **A** | **I** | **I** | **S** | **V** | **E** | **G** | **K** | **A** | **E** | **A** | **G** | **K** | **Y** | **L** | **F** | **F** | **T** | **T** | **L** | **K** | **G** | **T** | **V** | **K** | **R** | **T** | **A** | **V** | **T** | **A** | **F** | **S** | **N** | **I** | **R** | **S** | **N** | **G** | **L** | **I** | **A** | **I** | **S** | **L** | **K** | **E** | **D** | **D** | **E** | **L** | **V** | **N** | **V** | **V** | **T** | **T** | **N** | **G** | **N** | **Q** | **K** | **M** | **I** | **I** | **G** | **T** | **H** | **A** | **G** | **Y** | **S** | **V** | **T** | **F** | **D** | **E** | **N** | **T** | **V** | **R** | **D** | **M** | **G** | **R** | **T** | **A** | **S** | **G** | **V** | **R** | **G** | **I** | **R** | **L** | **R** | **E** | **N** | **D** | **Y** | **V** | **V** | **G** | **A** | **A** | **I** | **L** | **D** | **E** | **N** | **K** | **E** | **V** | **L** | **V** | **I** | **T** | **E** | **N** | **G** | **Y** | **G** | **K** | **R** | **T** | **K** | **A** | **S** | **E** | **Y** | **P** | **V** | **K** | **G** | **R** | **G** | **G** | **K** | **G** | **I** | **K** | **T** | **A** | **N** | **I** | **T** | **E** | **K** | **N** | **G** | **P** | **L** | **A** | **G** | **L** | **T** | **T** | **V** | **N** | **G** | **D** | **E** | **D** | **I** | **L** | **L** | **I** | **T** | **N** | **K** | **G** | **V** | **I** | **I** | **R** | **F** | **N** | **V** | **D** | **S** | **V** | **S** | **Q** | **T** | **G** | **R** | **A** | **T** | **L** | **G** | **V** | **R** | **L** | **M** | **R** | **M** | **E** | **D** | **G** | **A** | **K** | **-** | **-** | **-** | **-** | **-** | **-** | **-** | **-** | **-** | **-** | **-** | **-** | **-** | **-** | **-** | **-** | **-** | **-** | **-** | **-** | **-** | **-** | **-** |
| **N59779F** | **M** | **S** | **E** | **E** | **I** | **R** | **E** | **N** | **I** | **H** | **D** | **V** | **N** | **L** | **T** | **S** | **E** | **M** | **K** | **D** | **S** | **F** | **I** | **D** | **Y** | **A** | **M** | **S** | **V** | **I** | **V** | **A** | **R** | **A** | **L** | **P** | **D** | **V** | **R** | **D** | **G** | **L** | **K** | **P** | **V** | **H** | **R** | **R** | **I** | **L** | **Y** | **G** | **M** | **N** | **E** | **L** | **G** | **V** | **T** | **P** | **D** | **K** | **A** | **H** | **K** | **K** | **S** | **A** | **R** | **I** | **V** | **G** | **D** | **V** | **M** | **G** | **K** | **Y** | **H** | **P** | **H** | **G** | **D** | **S** | **A** | **I** | **Y** | **E** | **S** | **M** | **V** | **R** | **M** | **A** | **Q** | **P** | **F** | **S** | **Y** | **R** | **Y** | **M** | **L** | **V** | **D** | **G** | **H** | **G** | **N** | **F** | **G** | **S** | **V** | **D** | **G** | **D** | **G** | **A** | **A** | **A** | **M** | **R** | **Y** | **T** | **E** | **A** | **R** | **M** | **S** | **K** | **I** | **A** | **T** | **E** | **M** | **L** | **R** | **D** | **I** | **N** | **K** | **N** | **T** | **V** | **D** | **F** | **Q** | **S** | **N** | **Y** | **D** | **D** | **T** | **E** | **K** | **E** | **P** | **V** | **V** | **L** | **P** | **A** | **R** | **F** | **P** | **N** | **L** | **L** | **V** | **N** | **G** | **T** | **T** | **G** | **I** | **A** | **V** | **G** | **M** | **A** | **T** | **N** | **I** | **P** | **P** | **H** | **N** | **L** | **S** | **E** | **V** | **V** | **A** | **A** | **I** | **D** | **L** | **L** | **M** | **D** | **N** | **P** | **D** | **V** | **T** | **T** | **N** | **E** | **L** | **M** | **E** | **V** | **L** | **P** | **G** | **P** | **D** | **F** | **P** | **T** | **G** | **G** | **L** | **V** | **M** | **G** | **K** | **S** | **G** | **I** | **R** | **R** | **A** | **Y** | **E** | **T** | **G** | **K** | **G** | **S** | **I** | **T** | **V** | **R** | **A** | **K** | **V** | **E** | **I** | **T** | **E** | **M** | **P** | **N** | **G** | **K** | **E** | **R** | **I** | **I** | **V** | **T** | **E** | **L** | **P** | **Y** | **M** | **V** | **N** | **K** | **A** | **K** | **L** | **I** | **E** | **R** | **I** | **S** | **E** | **L** | **H** | **R** | **D** | **K** | **R** | **I** | **E** | **G** | **I** | **T** | **D** | **L** | **R** | **D** | **E** | **S** | **S** | **R** | **E** | **G** | **M** | **R** | **I** | **V** | **I** | **D** | **I** | **R** | **R** | **D** | **A** | **S** | **A** | **S** | **V** | **I** | **L** | **N** | **N** | **L** | **Y** | **K** | **L** | **T** | **S** | **L** | **Q** | **N** | **S** | **F** | **G** | **F** | **N** | **M** | **L** | **A** | **I** | **E** | **K** | **G** | **V** | **P** | **K** | **V** | **L** | **S** | **L** | **K** | **Q** | **I** | **L** | **E** | **N** | **Y** | **I** | **E** | **H** | **Q** | **R** | **E** | **V** | **I** | **T** | **R** | **R** | **T** | **V** | **F** | **E** | **K** | **E** | **K** | **A** | **E** | **A** | **R** | **A** | **H** | **I** | **L** | **E** | **G** | **L** | **R** | **I** | **A** | **L** | **D** | **H** | **I** | **D** | **E** | **I** | **I** | **A** | **I** | **I** | **R** | **G** | **S** | **K** | **S** | **D** | **D** | **E** | **A** | **K** | **A** | **T** | **M** | **I** | **E** | **R** | **F** | **D** | **L** | **S** | **D** | **R** | **Q** | **S** | **Q** | **A** | **I** | **L** | **D** | **M** | **R** | **L** | **R** | **R** | **L** | **T** | **G** | **L** | **E** | **R** | **E** | **K** | **I** | **E** | **N** | **E** | **Y** | **Q** | **E** | **L** | **L** | **K** | **L** | **I** | **E** | **D** | **L** | **T** | **D** | **I** | **L** | **A** | **R** | **P** | **E** | **R** | **V** | **T** | **E** | **I** | **I** | **K** | **N** | **E** | **L** | **A** | **D** | **L** | **N** | **Q** | **R** | **F** | **G** | **D** | **K** | **R** | **R** | **T** | **E** | **L** | **L** | **V** | **G** | **E** | **V** | **L** | **S** | **L** | **E** | **D** | **E** | **D** | **L** | **I** | **E** | **E** | **E** | **E** | **I** | **V** | **I** | **T** | **L** | **T** | **N** | **N** | **G** | **Y** | **I** | **K** | **R** | **L** | **A** | **N** | **N** | **E** | **F** | **R** | **A** | **Q** | **R** | **R** | **G** | **G** | **R** | **G** | **V** | **Q** | **G** | **M** | **G** | **I** | **H** | **N** | **D** | **D** | **F** | **V** | **K** | **N** | **L** | **V** | **S** | **C** | **S** | **T** | **H** | **D** | **T** | **L** | **L** | **F** | **F** | **T** | **N** | **N** | **G** | **K** | **V** | **Y** | **R** | **A** | **K** | **G** | **Y** | **E** | **I** | **P** | **E** | **Y** | **G** | **R** | **T** | **A** | **K** | **G** | **I** | **P** | **I** | **I** | **N** | **L** | **L** | **G** | **I** | **D** | **S** | **S** | **E** | **S** | **I** | **Q** | **A** | **I** | **I** | **A** | **V** | **T** | **G** | **E** | **A** | **E** | **E** | **G** | **H** | **Y** | **L** | **F** | **F** | **T** | **T** | **R** | **Q** | **G** | **T** | **V** | **K** | **R** | **T** | **S** | **V** | **T** | **A** | **F** | **A** | **N** | **I** | **R** | **S** | **N** | **G** | **L** | **I** | **A** | **I** | **G** | **L** | **K** | **E** | **D** | **D** | **E** | **L** | **V** | **N** | **V** | **L** | **L** | **T** | **D** | **G** | **Q** | **S** | **N** | **I** | **I** | **I** | **G** | **T** | **H** | **N** | **G** | **Y** | **S** | **V** | **T** | **F** | **K** | **E** | **E** | **A** | **V** | **R** | **D** | **M** | **G** | **R** | **T** | **A** | **S** | **G** | **V** | **R** | **G** | **I** | **R** | **L** | **R** | **E** | **E** | **D** | **Y** | **V** | **V** | **G** | **A** | **A** | **L** | **M** | **K** | **E** | **N** | **Q** | **E** | **V** | **L** | **V** | **I** | **T** | **E** | **K** | **G** | **Y** | **G** | **K** | **R** | **T** | **K** | **V** | **S** | **E** | **Y** | **P** | **V** | **K** | **G** | **R** | **G** | **G** | **K** | **G** | **I** | **K** | **T** | **A** | **N** | **I** | **T** | **E** | **K** | **N** | **G** | **P** | **L** | **A** | **G** | **L** | **T** | **T** | **V** | **S** | **G** | **D** | **E** | **D** | **I** | **M** | **L** | **I** | **T** | **D** | **K** | **G** | **V** | **I** | **I** | **R** | **F** | **N** | **V** | **S** | **T** | **V** | **S** | **Q** | **T** | **G** | **R** | **S** | **T** | **L** | **G** | **V** | **R** | **L** | **M** | **K** | **M** | **E** | **A** | **D** | **T** | **K** | **V** | **V** | **T** | **M** | **A** | **A** | **V** | **E** | **P** | **E** | **A** | **A** | **E** | **Q** | **T** | **V** | **E** | **E** | **Q** | **G** | **S** | **E** | **E** |
| **N59505F** | **M** | **S** | **E** | **E** | **I** | **K** | **E** | **N** | **I** | **Q** | **D** | **V** | **N** | **L** | **T** | **S** | **E** | **M** | **K** | **E** | **S** | **F** | **I** | **D** | **Y** | **A** | **M** | **S** | **V** | **I** | **V** | **A** | **R** | **A** | **L** | **P** | **D** | **V** | **R** | **D** | **G** | **L** | **K** | **P** | **V** | **H** | **R** | **R** | **I** | **L** | **Y** | **G** | **M** | **N** | **E** | **L** | **G** | **V** | **T** | **P** | **D** | **K** | **P** | **H** | **K** | **K** | **S** | **A** | **R** | **I** | **V** | **G** | **D** | **V** | **M** | **G** | **K** | **Y** | **H** | **P** | **H** | **G** | **D** | **S** | **A** | **I** | **Y** | **E** | **S** | **M** | **V** | **R** | **M** | **A** | **Q** | **P** | **F** | **S** | **Y** | **R** | **A** | **M** | **L** | **V** | **D** | **G** | **H** | **G** | **N** | **F** | **G** | **S** | **V** | **D** | **G** | **D** | **G** | **A** | **A** | **A** | **M** | **R** | **Y** | **T** | **E** | **A** | **R** | **M** | **S** | **K** | **I** | **A** | **L** | **E** | **M** | **L** | **R** | **D** | **I** | **N** | **K** | **N** | **T** | **V** | **D** | **F** | **Q** | **G** | **N** | **Y** | **D** | **D** | **S** | **E** | **Q** | **E** | **P** | **V** | **V** | **L** | **P** | **A** | **R** | **F** | **P** | **N** | **L** | **L** | **V** | **N** | **G** | **T** | **T** | **G** | **I** | **A** | **V** | **G** | **M** | **A** | **T** | **N** | **I** | **P** | **P** | **H** | **N** | **L** | **S** | **E** | **V** | **I** | **D** | **A** | **T** | **S** | **L** | **L** | **M** | **D** | **N** | **P** | **D** | **V** | **T** | **T** | **N** | **E** | **L** | **M** | **E** | **V** | **L** | **P** | **G** | **P** | **D** | **F** | **P** | **T** | **G** | **G** | **L** | **V** | **M** | **G** | **K** | **S** | **G** | **I** | **R** | **R** | **A** | **Y** | **E** | **T** | **G** | **K** | **G** | **S** | **I** | **T** | **V** | **R** | **A** | **K** | **V** | **E** | **L** | **T** | **E** | **M** | **P** | **N** | **G** | **K** | **E** | **R** | **I** | **L** | **V** | **T** | **E** | **L** | **P** | **Y** | **M** | **V** | **N** | **K** | **A** | **K** | **L** | **I** | **E** | **R** | **I** | **S** | **E** | **L** | **H** | **R** | **D** | **K** | **R** | **I** | **E** | **G** | **I** | **T** | **D** | **L** | **R** | **D** | **E** | **S** | **S** | **R** | **E** | **G** | **M** | **R** | **I** | **V** | **I** | **D** | **V** | **R** | **R** | **D** | **V** | **S** | **A** | **S** | **V** | **V** | **L** | **N** | **N** | **L** | **Y** | **K** | **M** | **T** | **A** | **L** | **Q** | **T** | **S** | **F** | **G** | **F** | **N** | **M** | **L** | **A** | **I** | **E** | **K** | **G** | **V** | **P** | **K** | **I** | **L** | **S** | **L** | **K** | **R** | **I** | **L** | **E** | **N** | **Y** | **V** | **E** | **H** | **Q** | **K** | **E** | **V** | **I** | **T** | **R** | **R** | **T** | **I** | **F** | **D** | **K** | **N** | **K** | **A** | **E** | **A** | **R** | **A** | **H** | **I** | **L** | **E** | **G** | **L** | **R** | **I** | **A** | **L** | **D** | **H** | **I** | **D** | **E** | **I** | **I** | **A** | **I** | **I** | **R** | **G** | **S** | **Q** | **S** | **D** | **D** | **E** | **A** | **K** | **A** | **T** | **L** | **I** | **E** | **R** | **F** | **E** | **F** | **S** | **D** | **R** | **Q** | **A** | **Q** | **A** | **I** | **L** | **D** | **M** | **R** | **L** | **R** | **R** | **L** | **T** | **G** | **L** | **E** | **R** | **D** | **K** | **I** | **E** | **N** | **E** | **Y** | **Q** | **E** | **L** | **L** | **K** | **F** | **I** | **A** | **D** | **L** | **E** | **D** | **I** | **L** | **A** | **R** | **P** | **E** | **R** | **V** | **I** | **E** | **I** | **I** | **K** | **T** | **E** | **L** | **N** | **D** | **V** | **R** | **T** | **K** | **F** | **G** | **D** | **A** | **R** | **R** | **T** | **E** | **L** | **L** | **V** | **G** | **E** | **V** | **L** | **S** | **L** | **E** | **D** | **E** | **D** | **L** | **I** | **E** | **E** | **E** | **E** | **V** | **V** | **I** | **T** | **L** | **T** | **N** | **N** | **G** | **Y** | **I** | **K** | **R** | **M** | **A** | **N** | **S** | **E** | **F** | **R** | **A** | **Q** | **R** | **R** | **G** | **G** | **R** | **G** | **V** | **Q** | **G** | **M** | **G** | **V** | **H** | **D** | **D** | **D** | **F** | **V** | **K** | **N** | **L** | **V** | **S** | **C** | **S** | **T** | **H** | **D** | **T** | **L** | **L** | **F** | **F** | **T** | **N** | **T** | **G** | **K** | **V** | **Y** | **R** | **A** | **K** | **G** | **Y** | **E** | **I** | **P** | **E** | **Y** | **G** | **R** | **T** | **A** | **K** | **G** | **I** | **P** | **V** | **I** | **N** | **L** | **L** | **G** | **I** | **D** | **S** | **A** | **E** | **K** | **I** | **Q** | **A** | **I** | **I** | **S** | **V** | **E** | **G** | **K** | **A** | **E** | **A** | **G** | **K** | **Y** | **L** | **F** | **F** | **T** | **T** | **L** | **K** | **G** | **T** | **V** | **K** | **R** | **T** | **A** | **V** | **T** | **A** | **F** | **S** | **N** | **I** | **R** | **S** | **N** | **G** | **L** | **I** | **A** | **I** | **S** | **L** | **K** | **E** | **D** | **D** | **E** | **L** | **V** | **N** | **V** | **V** | **T** | **T** | **N** | **G** | **N** | **Q** | **K** | **M** | **I** | **I** | **G** | **T** | **H** | **A** | **G** | **Y** | **S** | **V** | **T** | **F** | **D** | **E** | **N** | **T** | **V** | **R** | **D** | **M** | **G** | **R** | **T** | **A** | **S** | **G** | **V** | **R** | **G** | **I** | **R** | **L** | **R** | **E** | **N** | **D** | **Y** | **V** | **V** | **G** | **A** | **A** | **I** | **L** | **D** | **E** | **N** | **K** | **E** | **V** | **L** | **V** | **I** | **T** | **E** | **N** | **G** | **Y** | **G** | **K** | **R** | **T** | **K** | **A** | **S** | **E** | **Y** | **P** | **V** | **K** | **G** | **R** | **G** | **G** | **K** | **G** | **I** | **K** | **T** | **A** | **N** | **I** | **T** | **E** | **K** | **N** | **G** | **P** | **L** | **A** | **G** | **L** | **T** | **T** | **V** | **N** | **G** | **D** | **E** | **D** | **I** | **L** | **L** | **I** | **T** | **N** | **K** | **G** | **V** | **I** | **I** | **R** | **F** | **N** | **V** | **D** | **S** | **V** | **S** | **Q** | **T** | **G** | **R** | **A** | **T** | **L** | **G** | **V** | **R** | **L** | **M** | **R** | **M** | **E** | **D** | **G** | **A** | **K** | **-** | **-** | **-** | **-** | **-** | **-** | **-** | **-** | **-** | **-** | **-** | **-** | **-** | **-** | **-** | **-** | **-** | **-** | **-** | **-** | **-** | **-** | **-** |
| **N55072** | **M** | **S** | **E** | **E** | **I** | **R** | **E** | **N** | **I** | **H** | **D** | **V** | **N** | **L** | **T** | **S** | **E** | **M** | **K** | **D** | **S** | **F** | **I** | **D** | **Y** | **A** | **M** | **S** | **V** | **I** | **V** | **A** | **R** | **A** | **L** | **P** | **D** | **V** | **R** | **D** | **G** | **L** | **K** | **P** | **V** | **H** | **R** | **R** | **I** | **L** | **Y** | **G** | **M** | **N** | **E** | **L** | **G** | **V** | **T** | **P** | **D** | **K** | **A** | **H** | **K** | **K** | **S** | **A** | **R** | **I** | **V** | **G** | **D** | **V** | **M** | **G** | **K** | **Y** | **H** | **P** | **H** | **G** | **D** | **S** | **A** | **I** | **Y** | **E** | **S** | **M** | **V** | **R** | **M** | **A** | **Q** | **P** | **F** | **S** | **Y** | **R** | **Y** | **M** | **L** | **V** | **D** | **G** | **H** | **G** | **N** | **F** | **G** | **S** | **V** | **D** | **G** | **D** | **G** | **A** | **A** | **A** | **M** | **R** | **Y** | **T** | **E** | **A** | **R** | **M** | **S** | **K** | **I** | **A** | **T** | **E** | **M** | **L** | **R** | **D** | **I** | **N** | **K** | **N** | **T** | **V** | **D** | **F** | **Q** | **S** | **N** | **Y** | **D** | **D** | **T** | **E** | **K** | **E** | **P** | **V** | **V** | **L** | **P** | **A** | **R** | **F** | **P** | **N** | **L** | **L** | **V** | **N** | **G** | **T** | **T** | **G** | **I** | **A** | **V** | **G** | **M** | **A** | **T** | **N** | **I** | **P** | **P** | **H** | **N** | **L** | **S** | **E** | **V** | **V** | **A** | **A** | **I** | **D** | **L** | **L** | **M** | **D** | **N** | **P** | **D** | **V** | **T** | **T** | **N** | **E** | **L** | **M** | **E** | **V** | **L** | **P** | **G** | **P** | **D** | **F** | **P** | **T** | **G** | **G** | **L** | **V** | **M** | **G** | **K** | **S** | **G** | **I** | **R** | **R** | **A** | **Y** | **E** | **T** | **G** | **K** | **G** | **S** | **I** | **T** | **V** | **R** | **A** | **K** | **V** | **E** | **I** | **T** | **E** | **M** | **P** | **N** | **G** | **K** | **E** | **R** | **I** | **I** | **V** | **T** | **E** | **L** | **P** | **Y** | **M** | **V** | **N** | **K** | **A** | **K** | **L** | **I** | **E** | **R** | **I** | **S** | **E** | **L** | **H** | **R** | **D** | **K** | **R** | **I** | **E** | **G** | **I** | **T** | **D** | **L** | **R** | **D** | **E** | **S** | **S** | **R** | **E** | **G** | **M** | **R** | **I** | **V** | **I** | **D** | **I** | **R** | **R** | **D** | **A** | **S** | **A** | **S** | **V** | **I** | **L** | **N** | **N** | **L** | **Y** | **K** | **L** | **T** | **S** | **L** | **Q** | **N** | **S** | **F** | **G** | **F** | **N** | **M** | **L** | **A** | **I** | **E** | **K** | **G** | **V** | **P** | **K** | **V** | **L** | **S** | **L** | **K** | **Q** | **I** | **L** | **E** | **N** | **Y** | **I** | **E** | **H** | **Q** | **R** | **E** | **V** | **I** | **T** | **R** | **R** | **T** | **V** | **F** | **E** | **K** | **E** | **K** | **A** | **E** | **A** | **R** | **A** | **H** | **I** | **L** | **E** | **G** | **L** | **R** | **I** | **A** | **L** | **D** | **H** | **I** | **D** | **E** | **I** | **I** | **A** | **I** | **I** | **R** | **G** | **S** | **K** | **S** | **D** | **D** | **E** | **A** | **K** | **A** | **T** | **M** | **I** | **E** | **R** | **F** | **D** | **L** | **S** | **D** | **R** | **Q** | **S** | **Q** | **A** | **I** | **L** | **D** | **M** | **R** | **L** | **R** | **R** | **L** | **T** | **G** | **L** | **E** | **R** | **E** | **K** | **I** | **E** | **N** | **E** | **Y** | **Q** | **E** | **L** | **L** | **K** | **L** | **I** | **E** | **D** | **L** | **T** | **D** | **I** | **L** | **A** | **R** | **P** | **E** | **R** | **V** | **T** | **E** | **I** | **I** | **K** | **N** | **E** | **L** | **A** | **D** | **L** | **N** | **Q** | **R** | **F** | **G** | **D** | **K** | **R** | **R** | **T** | **E** | **L** | **L** | **V** | **G** | **E** | **V** | **L** | **S** | **L** | **E** | **D** | **E** | **D** | **L** | **I** | **E** | **E** | **E** | **E** | **I** | **V** | **I** | **T** | **L** | **T** | **N** | **N** | **G** | **Y** | **I** | **K** | **R** | **L** | **A** | **N** | **N** | **E** | **F** | **R** | **A** | **Q** | **R** | **R** | **G** | **G** | **R** | **G** | **V** | **Q** | **G** | **M** | **G** | **I** | **H** | **N** | **D** | **D** | **F** | **V** | **K** | **N** | **L** | **V** | **S** | **C** | **S** | **T** | **H** | **D** | **T** | **L** | **L** | **F** | **F** | **T** | **N** | **N** | **G** | **K** | **V** | **Y** | **R** | **A** | **K** | **G** | **Y** | **E** | **I** | **P** | **E** | **Y** | **G** | **R** | **T** | **A** | **K** | **G** | **I** | **P** | **I** | **I** | **N** | **L** | **L** | **G** | **I** | **D** | **S** | **S** | **E** | **S** | **I** | **Q** | **A** | **I** | **I** | **A** | **V** | **T** | **G** | **E** | **A** | **E** | **E** | **G** | **H** | **Y** | **L** | **F** | **F** | **T** | **T** | **R** | **Q** | **G** | **T** | **V** | **K** | **R** | **T** | **S** | **V** | **T** | **A** | **F** | **A** | **N** | **I** | **R** | **S** | **N** | **G** | **L** | **I** | **A** | **I** | **G** | **L** | **K** | **E** | **D** | **D** | **E** | **L** | **V** | **N** | **V** | **L** | **L** | **T** | **D** | **G** | **Q** | **S** | **N** | **I** | **I** | **I** | **G** | **T** | **H** | **N** | **G** | **Y** | **S** | **V** | **T** | **F** | **K** | **E** | **E** | **A** | **V** | **R** | **D** | **M** | **G** | **R** | **T** | **A** | **S** | **G** | **V** | **R** | **G** | **I** | **R** | **L** | **R** | **E** | **E** | **D** | **Y** | **V** | **V** | **G** | **T** | **A** | **L** | **M** | **K** | **E** | **N** | **Q** | **E** | **V** | **L** | **V** | **I** | **T** | **E** | **K** | **G** | **Y** | **G** | **K** | **R** | **T** | **K** | **V** | **S** | **E** | **Y** | **P** | **V** | **K** | **G** | **R** | **G** | **G** | **K** | **G** | **I** | **K** | **T** | **A** | **N** | **I** | **T** | **E** | **K** | **N** | **G** | **P** | **L** | **A** | **G** | **L** | **T** | **T** | **V** | **S** | **G** | **D** | **E** | **D** | **I** | **M** | **L** | **I** | **T** | **D** | **K** | **G** | **V** | **I** | **I** | **R** | **F** | **N** | **V** | **S** | **T** | **V** | **S** | **Q** | **T** | **G** | **R** | **S** | **T** | **L** | **G** | **V** | **R** | **L** | **M** | **K** | **M** | **E** | **A** | **D** | **T** | **K** | **V** | **V** | **T** | **M** | **A** | **A** | **V** | **E** | **P** | **E** | **A** | **A** | **E** | **Q** | **T** | **V** | **E** | **E** | **Q** | **G** | **S** | **E** | **E** |
| **N59555F** | **M** | **S** | **E** | **E** | **I** | **K** | **E** | **N** | **I** | **Q** | **D** | **V** | **N** | **L** | **T** | **S** | **E** | **M** | **K** | **E** | **S** | **F** | **I** | **D** | **Y** | **A** | **M** | **S** | **V** | **I** | **V** | **A** | **R** | **A** | **L** | **P** | **D** | **V** | **R** | **D** | **G** | **L** | **K** | **P** | **V** | **H** | **R** | **R** | **I** | **L** | **Y** | **G** | **M** | **N** | **E** | **L** | **G** | **V** | **T** | **P** | **D** | **K** | **P** | **H** | **K** | **K** | **S** | **A** | **R** | **I** | **V** | **G** | **D** | **V** | **M** | **G** | **K** | **Y** | **H** | **P** | **H** | **G** | **D** | **S** | **A** | **I** | **Y** | **E** | **S** | **M** | **V** | **R** | **M** | **A** | **Q** | **P** | **F** | **S** | **Y** | **R** | **A** | **M** | **L** | **V** | **D** | **G** | **H** | **G** | **N** | **F** | **G** | **S** | **V** | **D** | **G** | **D** | **G** | **A** | **A** | **A** | **M** | **R** | **Y** | **T** | **E** | **A** | **R** | **M** | **S** | **K** | **I** | **A** | **L** | **E** | **M** | **L** | **R** | **D** | **I** | **N** | **K** | **N** | **T** | **V** | **D** | **F** | **Q** | **G** | **N** | **Y** | **D** | **D** | **S** | **E** | **Q** | **E** | **P** | **V** | **V** | **L** | **P** | **A** | **R** | **F** | **P** | **N** | **L** | **L** | **V** | **N** | **G** | **T** | **T** | **G** | **I** | **A** | **V** | **G** | **M** | **A** | **T** | **N** | **I** | **P** | **P** | **H** | **N** | **L** | **S** | **E** | **V** | **I** | **D** | **A** | **T** | **S** | **L** | **L** | **M** | **D** | **N** | **P** | **D** | **V** | **T** | **T** | **N** | **E** | **L** | **M** | **E** | **V** | **L** | **P** | **G** | **P** | **D** | **F** | **P** | **T** | **G** | **G** | **L** | **V** | **M** | **G** | **K** | **S** | **G** | **I** | **R** | **R** | **A** | **Y** | **E** | **T** | **G** | **K** | **G** | **S** | **I** | **T** | **V** | **R** | **A** | **K** | **V** | **E** | **L** | **T** | **E** | **M** | **P** | **N** | **G** | **K** | **E** | **R** | **I** | **L** | **V** | **T** | **E** | **L** | **P** | **Y** | **M** | **V** | **N** | **K** | **A** | **K** | **L** | **I** | **E** | **R** | **I** | **S** | **E** | **L** | **H** | **R** | **D** | **K** | **R** | **I** | **E** | **G** | **I** | **T** | **D** | **L** | **R** | **D** | **E** | **S** | **S** | **R** | **E** | **G** | **M** | **R** | **I** | **V** | **I** | **D** | **V** | **R** | **R** | **D** | **V** | **S** | **A** | **S** | **V** | **V** | **L** | **N** | **N** | **L** | **Y** | **K** | **M** | **T** | **A** | **L** | **Q** | **T** | **S** | **F** | **G** | **F** | **N** | **M** | **L** | **A** | **I** | **E** | **K** | **G** | **V** | **P** | **K** | **I** | **L** | **S** | **L** | **K** | **R** | **I** | **L** | **E** | **N** | **Y** | **V** | **E** | **H** | **Q** | **K** | **E** | **V** | **I** | **T** | **R** | **R** | **T** | **I** | **F** | **D** | **K** | **N** | **K** | **A** | **E** | **A** | **R** | **A** | **H** | **I** | **L** | **E** | **G** | **L** | **R** | **I** | **A** | **L** | **D** | **H** | **I** | **D** | **E** | **I** | **I** | **A** | **I** | **I** | **R** | **G** | **S** | **Q** | **S** | **D** | **D** | **E** | **A** | **K** | **A** | **T** | **L** | **I** | **E** | **R** | **F** | **E** | **F** | **S** | **D** | **R** | **Q** | **A** | **Q** | **A** | **I** | **L** | **D** | **M** | **R** | **L** | **R** | **R** | **L** | **T** | **G** | **L** | **E** | **R** | **D** | **K** | **I** | **E** | **N** | **E** | **Y** | **Q** | **E** | **L** | **L** | **K** | **F** | **I** | **A** | **D** | **L** | **E** | **D** | **I** | **L** | **A** | **R** | **P** | **E** | **R** | **V** | **I** | **E** | **I** | **I** | **K** | **T** | **E** | **L** | **N** | **D** | **V** | **R** | **T** | **K** | **F** | **G** | **D** | **A** | **R** | **R** | **T** | **E** | **L** | **L** | **V** | **G** | **E** | **V** | **L** | **S** | **L** | **E** | **D** | **E** | **D** | **L** | **I** | **E** | **E** | **E** | **E** | **V** | **V** | **I** | **T** | **L** | **T** | **N** | **N** | **G** | **Y** | **I** | **K** | **R** | **M** | **A** | **N** | **S** | **E** | **F** | **R** | **A** | **Q** | **R** | **R** | **G** | **G** | **R** | **G** | **V** | **Q** | **G** | **M** | **G** | **V** | **H** | **D** | **D** | **D** | **F** | **V** | **K** | **N** | **L** | **V** | **S** | **C** | **S** | **T** | **H** | **D** | **T** | **L** | **L** | **F** | **F** | **T** | **N** | **T** | **G** | **K** | **V** | **Y** | **R** | **A** | **K** | **G** | **Y** | **E** | **I** | **P** | **E** | **Y** | **G** | **R** | **T** | **A** | **K** | **G** | **I** | **P** | **V** | **I** | **N** | **L** | **L** | **G** | **I** | **D** | **S** | **A** | **E** | **K** | **I** | **Q** | **A** | **I** | **I** | **S** | **V** | **E** | **G** | **K** | **A** | **E** | **A** | **G** | **K** | **Y** | **L** | **F** | **F** | **T** | **T** | **L** | **K** | **G** | **T** | **V** | **K** | **R** | **T** | **A** | **V** | **T** | **A** | **F** | **S** | **N** | **I** | **R** | **S** | **N** | **G** | **L** | **I** | **A** | **I** | **S** | **L** | **K** | **E** | **D** | **D** | **E** | **L** | **V** | **N** | **V** | **V** | **T** | **T** | **N** | **G** | **N** | **Q** | **K** | **M** | **I** | **I** | **G** | **T** | **H** | **A** | **G** | **Y** | **S** | **V** | **T** | **F** | **D** | **E** | **N** | **T** | **V** | **R** | **D** | **M** | **G** | **R** | **T** | **A** | **S** | **G** | **V** | **R** | **G** | **I** | **R** | **L** | **R** | **E** | **N** | **D** | **Y** | **V** | **V** | **G** | **A** | **A** | **I** | **L** | **D** | **E** | **N** | **K** | **E** | **V** | **L** | **V** | **I** | **T** | **E** | **N** | **G** | **Y** | **G** | **K** | **R** | **T** | **K** | **A** | **S** | **E** | **Y** | **P** | **V** | **K** | **G** | **R** | **G** | **G** | **K** | **G** | **I** | **K** | **T** | **A** | **N** | **I** | **T** | **E** | **K** | **N** | **G** | **P** | **L** | **A** | **G** | **L** | **T** | **T** | **V** | **N** | **G** | **D** | **E** | **D** | **I** | **L** | **L** | **I** | **T** | **N** | **K** | **G** | **V** | **I** | **I** | **R** | **F** | **N** | **V** | **D** | **S** | **V** | **S** | **Q** | **T** | **G** | **R** | **A** | **T** | **L** | **G** | **V** | **R** | **L** | **M** | **R** | **M** | **E** | **D** | **G** | **A** | **K** | **-** | **-** | **-** | **-** | **-** | **-** | **-** | **-** | **-** | **-** | **-** | **-** | **-** | **-** | **-** | **-** | **-** | **-** | **-** | **-** | **-** | **-** | **-** |
| **N59517F** | **M** | **S** | **E** | **E** | **I** | **R** | **E** | **N** | **I** | **H** | **D** | **V** | **N** | **L** | **T** | **S** | **E** | **M** | **K** | **D** | **S** | **F** | **I** | **D** | **Y** | **A** | **M** | **S** | **V** | **I** | **V** | **A** | **R** | **A** | **L** | **P** | **D** | **V** | **R** | **D** | **G** | **L** | **K** | **P** | **V** | **H** | **R** | **R** | **I** | **L** | **Y** | **G** | **M** | **N** | **E** | **L** | **G** | **V** | **T** | **P** | **D** | **K** | **A** | **H** | **K** | **K** | **S** | **A** | **R** | **I** | **V** | **G** | **D** | **V** | **M** | **G** | **K** | **Y** | **H** | **P** | **H** | **G** | **D** | **S** | **A** | **I** | **Y** | **E** | **S** | **M** | **V** | **R** | **M** | **A** | **Q** | **P** | **F** | **S** | **Y** | **R** | **Y** | **M** | **L** | **V** | **D** | **G** | **H** | **G** | **N** | **F** | **G** | **S** | **V** | **D** | **G** | **D** | **G** | **A** | **A** | **A** | **M** | **R** | **Y** | **T** | **E** | **A** | **R** | **M** | **S** | **K** | **I** | **A** | **T** | **E** | **M** | **L** | **R** | **D** | **I** | **N** | **K** | **N** | **T** | **V** | **D** | **F** | **Q** | **S** | **N** | **Y** | **D** | **D** | **T** | **E** | **K** | **E** | **P** | **V** | **V** | **L** | **P** | **A** | **R** | **F** | **P** | **N** | **L** | **L** | **V** | **N** | **G** | **T** | **T** | **G** | **I** | **A** | **V** | **G** | **M** | **A** | **T** | **N** | **I** | **P** | **P** | **H** | **N** | **L** | **S** | **E** | **V** | **V** | **A** | **A** | **I** | **D** | **L** | **L** | **M** | **D** | **N** | **P** | **D** | **V** | **T** | **T** | **N** | **E** | **L** | **M** | **E** | **V** | **L** | **P** | **G** | **P** | **D** | **F** | **P** | **T** | **G** | **G** | **L** | **V** | **M** | **G** | **K** | **S** | **G** | **I** | **R** | **R** | **A** | **Y** | **E** | **T** | **G** | **K** | **G** | **S** | **I** | **T** | **V** | **R** | **A** | **K** | **V** | **E** | **I** | **T** | **E** | **M** | **P** | **N** | **G** | **K** | **E** | **R** | **I** | **I** | **V** | **T** | **E** | **L** | **P** | **Y** | **M** | **V** | **N** | **K** | **A** | **K** | **L** | **I** | **E** | **R** | **I** | **S** | **E** | **L** | **H** | **R** | **D** | **K** | **R** | **I** | **E** | **G** | **I** | **T** | **D** | **L** | **R** | **D** | **E** | **S** | **S** | **R** | **E** | **G** | **M** | **R** | **I** | **V** | **I** | **D** | **I** | **R** | **R** | **D** | **A** | **S** | **A** | **S** | **V** | **I** | **L** | **N** | **N** | **L** | **Y** | **K** | **L** | **T** | **S** | **L** | **Q** | **N** | **S** | **F** | **G** | **F** | **N** | **M** | **L** | **A** | **I** | **E** | **K** | **G** | **V** | **P** | **K** | **V** | **L** | **S** | **L** | **K** | **Q** | **I** | **L** | **E** | **N** | **Y** | **I** | **E** | **H** | **Q** | **R** | **E** | **V** | **I** | **T** | **R** | **R** | **T** | **V** | **F** | **E** | **K** | **E** | **K** | **A** | **E** | **A** | **R** | **A** | **H** | **I** | **L** | **E** | **G** | **L** | **R** | **I** | **A** | **L** | **D** | **H** | **I** | **D** | **E** | **I** | **I** | **A** | **I** | **I** | **R** | **G** | **S** | **K** | **S** | **D** | **D** | **E** | **A** | **K** | **A** | **T** | **M** | **I** | **E** | **R** | **F** | **D** | **L** | **S** | **D** | **R** | **Q** | **S** | **Q** | **A** | **I** | **L** | **D** | **M** | **R** | **L** | **R** | **R** | **L** | **T** | **G** | **L** | **E** | **R** | **E** | **K** | **I** | **E** | **N** | **E** | **Y** | **Q** | **E** | **L** | **L** | **K** | **L** | **I** | **E** | **D** | **L** | **T** | **D** | **I** | **L** | **A** | **R** | **P** | **E** | **R** | **V** | **T** | **E** | **I** | **I** | **K** | **N** | **E** | **L** | **A** | **D** | **L** | **N** | **Q** | **R** | **F** | **G** | **D** | **K** | **R** | **R** | **T** | **E** | **L** | **L** | **V** | **G** | **E** | **V** | **L** | **S** | **L** | **E** | **D** | **E** | **D** | **L** | **I** | **E** | **E** | **E** | **E** | **I** | **V** | **I** | **T** | **L** | **T** | **N** | **N** | **G** | **Y** | **I** | **K** | **R** | **L** | **A** | **N** | **N** | **E** | **F** | **R** | **A** | **Q** | **R** | **R** | **G** | **G** | **R** | **G** | **V** | **Q** | **G** | **M** | **G** | **I** | **H** | **N** | **D** | **D** | **F** | **V** | **K** | **N** | **L** | **V** | **S** | **C** | **S** | **T** | **H** | **D** | **T** | **L** | **L** | **F** | **F** | **T** | **N** | **N** | **G** | **K** | **V** | **Y** | **R** | **A** | **K** | **G** | **Y** | **E** | **I** | **P** | **E** | **Y** | **G** | **R** | **T** | **A** | **K** | **G** | **I** | **P** | **I** | **I** | **N** | **L** | **L** | **G** | **I** | **D** | **S** | **S** | **E** | **S** | **I** | **Q** | **A** | **I** | **I** | **A** | **V** | **T** | **G** | **E** | **A** | **E** | **E** | **G** | **H** | **Y** | **L** | **F** | **F** | **T** | **T** | **R** | **Q** | **G** | **T** | **V** | **K** | **R** | **T** | **S** | **V** | **T** | **A** | **F** | **A** | **N** | **I** | **R** | **S** | **N** | **G** | **L** | **I** | **A** | **I** | **G** | **L** | **K** | **E** | **D** | **D** | **E** | **L** | **V** | **N** | **V** | **L** | **L** | **T** | **D** | **G** | **Q** | **S** | **N** | **I** | **I** | **I** | **G** | **T** | **H** | **N** | **G** | **Y** | **S** | **V** | **T** | **F** | **K** | **E** | **E** | **A** | **V** | **R** | **D** | **M** | **G** | **R** | **T** | **A** | **S** | **G** | **V** | **R** | **G** | **I** | **R** | **L** | **R** | **E** | **E** | **D** | **Y** | **V** | **V** | **G** | **A** | **A** | **L** | **M** | **K** | **E** | **N** | **Q** | **E** | **V** | **L** | **V** | **I** | **T** | **E** | **K** | **G** | **Y** | **G** | **K** | **R** | **T** | **K** | **V** | **S** | **E** | **Y** | **P** | **V** | **K** | **G** | **R** | **G** | **G** | **K** | **G** | **I** | **K** | **T** | **A** | **N** | **I** | **T** | **E** | **K** | **N** | **G** | **P** | **L** | **A** | **G** | **L** | **T** | **T** | **V** | **S** | **G** | **D** | **E** | **D** | **I** | **M** | **L** | **I** | **T** | **D** | **K** | **G** | **V** | **I** | **I** | **R** | **F** | **N** | **V** | **S** | **T** | **V** | **S** | **Q** | **T** | **G** | **R** | **S** | **T** | **L** | **G** | **V** | **R** | **L** | **M** | **K** | **M** | **E** | **A** | **D** | **T** | **K** | **V** | **V** | **T** | **M** | **A** | **A** | **V** | **E** | **P** | **E** | **A** | **A** | **E** | **Q** | **T** | **V** | **E** | **E** | **Q** | **G** | **S** | **E** | **E** |
| **N60045F** | **M** | **S** | **E** | **E** | **I** | **R** | **E** | **N** | **I** | **H** | **D** | **V** | **N** | **L** | **T** | **S** | **E** | **M** | **K** | **D** | **S** | **F** | **I** | **D** | **Y** | **A** | **M** | **S** | **V** | **I** | **V** | **A** | **R** | **A** | **L** | **P** | **D** | **V** | **R** | **D** | **G** | **L** | **K** | **P** | **V** | **H** | **R** | **R** | **I** | **L** | **Y** | **G** | **M** | **N** | **E** | **L** | **G** | **V** | **T** | **P** | **D** | **K** | **A** | **H** | **K** | **K** | **S** | **A** | **R** | **I** | **V** | **G** | **D** | **V** | **M** | **G** | **K** | **Y** | **H** | **P** | **H** | **G** | **D** | **S** | **A** | **I** | **Y** | **E** | **S** | **M** | **V** | **R** | **M** | **A** | **Q** | **P** | **F** | **S** | **Y** | **R** | **Y** | **M** | **L** | **V** | **D** | **G** | **H** | **G** | **N** | **F** | **G** | **S** | **V** | **D** | **G** | **D** | **G** | **A** | **A** | **A** | **M** | **R** | **Y** | **T** | **E** | **A** | **R** | **M** | **S** | **K** | **I** | **A** | **T** | **E** | **M** | **L** | **R** | **D** | **I** | **N** | **K** | **N** | **T** | **V** | **D** | **F** | **Q** | **S** | **N** | **Y** | **D** | **D** | **T** | **E** | **K** | **E** | **P** | **V** | **V** | **L** | **P** | **A** | **R** | **F** | **P** | **N** | **L** | **L** | **V** | **N** | **G** | **T** | **T** | **G** | **I** | **A** | **V** | **G** | **M** | **A** | **T** | **N** | **I** | **P** | **P** | **H** | **N** | **L** | **S** | **E** | **V** | **V** | **A** | **A** | **I** | **D** | **L** | **L** | **M** | **D** | **N** | **P** | **D** | **V** | **T** | **T** | **N** | **E** | **L** | **M** | **E** | **V** | **L** | **P** | **G** | **P** | **D** | **F** | **P** | **T** | **G** | **G** | **L** | **V** | **M** | **G** | **K** | **S** | **G** | **I** | **R** | **R** | **A** | **Y** | **E** | **T** | **G** | **K** | **G** | **S** | **I** | **T** | **V** | **R** | **A** | **K** | **V** | **E** | **I** | **T** | **E** | **M** | **P** | **N** | **G** | **K** | **E** | **R** | **I** | **I** | **V** | **T** | **E** | **L** | **P** | **Y** | **M** | **V** | **N** | **K** | **A** | **K** | **L** | **I** | **E** | **R** | **I** | **S** | **E** | **L** | **H** | **R** | **D** | **K** | **R** | **I** | **E** | **G** | **I** | **T** | **D** | **L** | **R** | **D** | **E** | **S** | **S** | **R** | **E** | **G** | **M** | **R** | **I** | **V** | **I** | **D** | **I** | **R** | **R** | **D** | **A** | **S** | **A** | **S** | **V** | **I** | **L** | **N** | **N** | **L** | **Y** | **K** | **L** | **T** | **S** | **L** | **Q** | **N** | **S** | **F** | **G** | **F** | **N** | **M** | **L** | **A** | **I** | **E** | **K** | **G** | **V** | **P** | **K** | **V** | **L** | **S** | **L** | **K** | **Q** | **I** | **L** | **E** | **N** | **Y** | **I** | **E** | **H** | **Q** | **R** | **E** | **V** | **I** | **T** | **R** | **R** | **T** | **V** | **F** | **E** | **K** | **E** | **K** | **A** | **E** | **A** | **R** | **A** | **H** | **I** | **L** | **E** | **G** | **L** | **R** | **I** | **A** | **L** | **D** | **H** | **I** | **D** | **E** | **I** | **I** | **A** | **I** | **I** | **R** | **G** | **S** | **K** | **S** | **D** | **D** | **E** | **A** | **K** | **A** | **T** | **M** | **I** | **E** | **R** | **F** | **D** | **L** | **S** | **D** | **R** | **Q** | **S** | **Q** | **A** | **I** | **L** | **D** | **M** | **R** | **L** | **R** | **R** | **L** | **T** | **G** | **L** | **E** | **R** | **E** | **K** | **I** | **E** | **N** | **E** | **Y** | **Q** | **E** | **L** | **L** | **K** | **L** | **I** | **E** | **D** | **L** | **T** | **D** | **I** | **L** | **A** | **R** | **P** | **E** | **R** | **V** | **T** | **E** | **I** | **I** | **K** | **N** | **E** | **L** | **A** | **D** | **L** | **N** | **Q** | **R** | **F** | **G** | **D** | **K** | **R** | **R** | **T** | **E** | **L** | **L** | **V** | **G** | **E** | **V** | **L** | **S** | **L** | **E** | **D** | **E** | **D** | **L** | **I** | **E** | **E** | **E** | **E** | **I** | **V** | **I** | **T** | **L** | **T** | **N** | **N** | **G** | **Y** | **I** | **K** | **R** | **L** | **A** | **N** | **N** | **E** | **F** | **R** | **A** | **Q** | **R** | **R** | **G** | **G** | **R** | **G** | **V** | **Q** | **G** | **M** | **G** | **I** | **H** | **N** | **D** | **D** | **F** | **V** | **K** | **N** | **L** | **V** | **S** | **C** | **S** | **T** | **H** | **D** | **T** | **L** | **L** | **F** | **F** | **T** | **N** | **N** | **G** | **K** | **V** | **Y** | **R** | **A** | **K** | **G** | **Y** | **E** | **I** | **P** | **E** | **Y** | **G** | **R** | **T** | **A** | **K** | **G** | **I** | **P** | **I** | **I** | **N** | **L** | **L** | **G** | **I** | **D** | **S** | **S** | **E** | **S** | **I** | **Q** | **A** | **I** | **I** | **A** | **V** | **T** | **G** | **E** | **A** | **E** | **E** | **G** | **H** | **Y** | **L** | **F** | **F** | **T** | **T** | **R** | **Q** | **G** | **T** | **V** | **K** | **R** | **T** | **S** | **V** | **T** | **A** | **F** | **A** | **N** | **I** | **R** | **S** | **N** | **G** | **L** | **I** | **A** | **I** | **G** | **L** | **K** | **E** | **D** | **D** | **E** | **L** | **V** | **N** | **V** | **L** | **L** | **T** | **D** | **G** | **Q** | **S** | **N** | **I** | **I** | **I** | **G** | **T** | **H** | **N** | **G** | **Y** | **S** | **V** | **T** | **F** | **K** | **E** | **E** | **A** | **V** | **R** | **D** | **M** | **G** | **R** | **T** | **A** | **S** | **G** | **V** | **R** | **G** | **I** | **R** | **L** | **R** | **E** | **E** | **D** | **Y** | **V** | **V** | **G** | **A** | **A** | **L** | **M** | **K** | **E** | **D** | **Q** | **E** | **V** | **L** | **V** | **I** | **T** | **E** | **K** | **G** | **Y** | **G** | **K** | **R** | **T** | **K** | **V** | **S** | **E** | **Y** | **P** | **V** | **K** | **G** | **R** | **G** | **G** | **K** | **G** | **I** | **K** | **T** | **A** | **N** | **I** | **T** | **E** | **K** | **N** | **G** | **P** | **L** | **A** | **G** | **L** | **T** | **T** | **V** | **S** | **G** | **D** | **E** | **D** | **I** | **M** | **L** | **I** | **T** | **D** | **K** | **G** | **V** | **I** | **I** | **R** | **F** | **N** | **V** | **S** | **T** | **V** | **S** | **Q** | **T** | **G** | **R** | **S** | **T** | **L** | **G** | **V** | **R** | **L** | **M** | **K** | **M** | **E** | **A** | **D** | **T** | **K** | **V** | **V** | **T** | **M** | **A** | **A** | **V** | **E** | **P** | **E** | **A** | **A** | **E** | **Q** | **T** | **V** | **E** | **E** | **Q** | **G** | **S** | **E** | **E** |
| **N59841F** | **M** | **S** | **E** | **E** | **I** | **K** | **E** | **N** | **I** | **Q** | **D** | **V** | **N** | **L** | **T** | **S** | **E** | **M** | **K** | **E** | **S** | **F** | **I** | **D** | **Y** | **A** | **M** | **S** | **V** | **I** | **V** | **A** | **R** | **A** | **L** | **P** | **D** | **V** | **R** | **D** | **G** | **L** | **K** | **P** | **V** | **H** | **R** | **R** | **I** | **L** | **Y** | **G** | **M** | **N** | **E** | **L** | **G** | **V** | **T** | **P** | **D** | **K** | **P** | **H** | **K** | **K** | **S** | **A** | **R** | **I** | **V** | **G** | **D** | **V** | **M** | **G** | **K** | **Y** | **H** | **P** | **H** | **G** | **D** | **S** | **A** | **I** | **Y** | **E** | **S** | **M** | **V** | **R** | **M** | **A** | **Q** | **P** | **F** | **S** | **Y** | **R** | **A** | **M** | **L** | **V** | **D** | **G** | **H** | **G** | **N** | **F** | **G** | **S** | **V** | **D** | **G** | **D** | **G** | **A** | **A** | **A** | **M** | **R** | **Y** | **T** | **E** | **A** | **R** | **M** | **S** | **K** | **I** | **A** | **L** | **E** | **M** | **L** | **R** | **D** | **I** | **N** | **K** | **N** | **T** | **V** | **D** | **F** | **Q** | **G** | **N** | **Y** | **D** | **D** | **S** | **E** | **Q** | **E** | **P** | **V** | **V** | **L** | **P** | **A** | **R** | **F** | **P** | **N** | **L** | **L** | **V** | **N** | **G** | **T** | **T** | **G** | **I** | **A** | **V** | **G** | **M** | **A** | **T** | **N** | **I** | **P** | **P** | **H** | **N** | **L** | **S** | **E** | **V** | **I** | **D** | **A** | **T** | **S** | **L** | **L** | **M** | **D** | **N** | **P** | **D** | **V** | **T** | **T** | **N** | **E** | **L** | **M** | **E** | **V** | **L** | **P** | **G** | **P** | **D** | **F** | **P** | **T** | **G** | **G** | **L** | **V** | **M** | **G** | **K** | **S** | **G** | **I** | **R** | **R** | **A** | **Y** | **E** | **T** | **G** | **K** | **G** | **S** | **I** | **T** | **V** | **R** | **A** | **K** | **V** | **E** | **L** | **T** | **E** | **M** | **P** | **N** | **G** | **K** | **E** | **R** | **I** | **L** | **V** | **T** | **E** | **L** | **P** | **Y** | **M** | **V** | **N** | **K** | **A** | **K** | **L** | **I** | **E** | **R** | **I** | **S** | **E** | **L** | **H** | **R** | **D** | **K** | **R** | **I** | **E** | **G** | **I** | **T** | **D** | **L** | **R** | **D** | **E** | **S** | **S** | **R** | **E** | **G** | **M** | **R** | **I** | **V** | **I** | **D** | **V** | **R** | **R** | **D** | **V** | **S** | **A** | **S** | **V** | **V** | **L** | **N** | **N** | **L** | **Y** | **K** | **M** | **T** | **A** | **L** | **Q** | **T** | **S** | **F** | **G** | **F** | **N** | **M** | **L** | **A** | **I** | **E** | **K** | **G** | **V** | **P** | **K** | **I** | **L** | **S** | **L** | **K** | **R** | **I** | **L** | **E** | **N** | **Y** | **V** | **E** | **H** | **Q** | **K** | **E** | **V** | **I** | **T** | **R** | **R** | **T** | **I** | **F** | **D** | **K** | **N** | **K** | **A** | **E** | **A** | **R** | **A** | **H** | **I** | **L** | **E** | **G** | **L** | **R** | **I** | **A** | **L** | **D** | **H** | **I** | **D** | **E** | **I** | **I** | **A** | **I** | **I** | **R** | **G** | **S** | **Q** | **S** | **D** | **D** | **E** | **A** | **K** | **A** | **T** | **L** | **I** | **E** | **R** | **F** | **E** | **F** | **S** | **D** | **R** | **Q** | **A** | **Q** | **A** | **I** | **L** | **D** | **M** | **R** | **L** | **R** | **R** | **L** | **T** | **G** | **L** | **E** | **R** | **D** | **K** | **I** | **E** | **N** | **E** | **Y** | **Q** | **E** | **L** | **L** | **K** | **F** | **I** | **A** | **D** | **L** | **E** | **D** | **I** | **L** | **A** | **R** | **P** | **E** | **R** | **V** | **I** | **E** | **I** | **I** | **K** | **T** | **E** | **L** | **N** | **D** | **V** | **R** | **T** | **K** | **F** | **G** | **D** | **A** | **R** | **R** | **T** | **E** | **L** | **L** | **V** | **G** | **E** | **V** | **L** | **S** | **L** | **E** | **D** | **E** | **D** | **L** | **I** | **E** | **E** | **E** | **E** | **V** | **V** | **I** | **T** | **L** | **T** | **N** | **N** | **G** | **Y** | **I** | **K** | **R** | **M** | **A** | **N** | **S** | **E** | **F** | **R** | **A** | **Q** | **R** | **R** | **G** | **G** | **R** | **G** | **V** | **Q** | **G** | **M** | **G** | **V** | **H** | **D** | **D** | **D** | **F** | **V** | **K** | **N** | **L** | **V** | **S** | **C** | **S** | **T** | **H** | **D** | **T** | **L** | **L** | **F** | **F** | **T** | **N** | **T** | **G** | **K** | **V** | **Y** | **R** | **A** | **K** | **G** | **Y** | **E** | **I** | **P** | **E** | **Y** | **G** | **R** | **T** | **A** | **K** | **G** | **I** | **P** | **V** | **I** | **N** | **L** | **L** | **G** | **I** | **D** | **S** | **A** | **E** | **K** | **I** | **Q** | **A** | **I** | **I** | **S** | **V** | **E** | **G** | **K** | **A** | **E** | **A** | **G** | **K** | **Y** | **L** | **F** | **F** | **T** | **T** | **L** | **K** | **G** | **T** | **V** | **K** | **R** | **T** | **A** | **V** | **T** | **A** | **F** | **S** | **N** | **I** | **R** | **S** | **N** | **G** | **L** | **I** | **A** | **I** | **S** | **L** | **K** | **E** | **D** | **D** | **E** | **L** | **V** | **N** | **V** | **V** | **T** | **T** | **N** | **G** | **N** | **Q** | **K** | **M** | **I** | **I** | **G** | **T** | **H** | **A** | **G** | **Y** | **S** | **V** | **T** | **F** | **D** | **E** | **N** | **T** | **V** | **R** | **D** | **M** | **G** | **R** | **T** | **A** | **S** | **G** | **V** | **R** | **G** | **I** | **R** | **L** | **R** | **E** | **N** | **D** | **Y** | **V** | **V** | **G** | **A** | **A** | **I** | **L** | **D** | **E** | **N** | **K** | **E** | **V** | **L** | **V** | **I** | **T** | **E** | **N** | **G** | **Y** | **G** | **K** | **R** | **T** | **K** | **A** | **S** | **E** | **Y** | **P** | **V** | **K** | **G** | **R** | **G** | **G** | **K** | **G** | **I** | **K** | **T** | **A** | **N** | **I** | **T** | **E** | **K** | **N** | **G** | **P** | **L** | **A** | **G** | **L** | **T** | **T** | **V** | **N** | **G** | **D** | **E** | **D** | **I** | **L** | **L** | **I** | **T** | **N** | **K** | **G** | **V** | **I** | **I** | **R** | **F** | **N** | **V** | **D** | **S** | **V** | **S** | **Q** | **T** | **G** | **R** | **A** | **T** | **L** | **G** | **V** | **R** | **L** | **M** | **R** | **M** | **E** | **D** | **G** | **A** | **K** | **-** | **-** | **-** | **-** | **-** | **-** | **-** | **-** | **-** | **-** | **-** | **-** | **-** | **-** | **-** | **-** | **-** | **-** | **-** | **-** | **-** | **-** | **-** |
| **N54190** | **M** | **S** | **E** | **E** | **I** | **R** | **E** | **N** | **I** | **H** | **D** | **V** | **N** | **L** | **T** | **S** | **E** | **M** | **K** | **D** | **S** | **F** | **I** | **D** | **Y** | **A** | **M** | **S** | **V** | **I** | **V** | **A** | **R** | **A** | **L** | **P** | **D** | **V** | **R** | **D** | **G** | **L** | **K** | **P** | **V** | **H** | **R** | **R** | **I** | **L** | **Y** | **G** | **M** | **N** | **E** | **L** | **G** | **V** | **T** | **P** | **D** | **K** | **A** | **H** | **K** | **K** | **S** | **A** | **R** | **I** | **V** | **G** | **D** | **V** | **M** | **G** | **K** | **Y** | **H** | **P** | **H** | **G** | **D** | **S** | **A** | **I** | **Y** | **E** | **S** | **M** | **V** | **R** | **M** | **A** | **Q** | **P** | **F** | **S** | **Y** | **R** | **Y** | **M** | **L** | **V** | **D** | **G** | **H** | **G** | **N** | **F** | **G** | **S** | **V** | **D** | **G** | **D** | **G** | **A** | **A** | **A** | **M** | **R** | **Y** | **T** | **E** | **A** | **R** | **M** | **S** | **K** | **I** | **A** | **T** | **E** | **M** | **L** | **R** | **D** | **I** | **N** | **K** | **N** | **T** | **V** | **D** | **F** | **Q** | **S** | **N** | **Y** | **D** | **D** | **T** | **E** | **K** | **E** | **P** | **V** | **V** | **L** | **P** | **A** | **R** | **F** | **P** | **N** | **L** | **L** | **V** | **N** | **G** | **T** | **T** | **G** | **I** | **A** | **V** | **G** | **M** | **A** | **T** | **N** | **I** | **P** | **P** | **H** | **N** | **L** | **S** | **E** | **V** | **V** | **A** | **A** | **I** | **D** | **L** | **L** | **M** | **D** | **N** | **P** | **D** | **V** | **T** | **T** | **N** | **E** | **L** | **M** | **E** | **V** | **L** | **P** | **G** | **P** | **D** | **F** | **P** | **T** | **G** | **G** | **L** | **V** | **M** | **G** | **K** | **S** | **G** | **I** | **R** | **R** | **A** | **Y** | **E** | **T** | **G** | **K** | **G** | **S** | **I** | **T** | **V** | **R** | **A** | **K** | **V** | **E** | **I** | **T** | **E** | **M** | **P** | **N** | **G** | **K** | **E** | **R** | **I** | **I** | **V** | **T** | **E** | **L** | **P** | **Y** | **M** | **V** | **N** | **K** | **A** | **K** | **L** | **I** | **E** | **R** | **I** | **S** | **E** | **L** | **H** | **R** | **D** | **K** | **R** | **I** | **E** | **G** | **I** | **T** | **D** | **L** | **R** | **D** | **E** | **S** | **S** | **R** | **E** | **G** | **M** | **R** | **I** | **V** | **I** | **D** | **I** | **R** | **R** | **D** | **A** | **S** | **A** | **S** | **V** | **I** | **L** | **N** | **N** | **L** | **Y** | **K** | **L** | **T** | **S** | **L** | **Q** | **N** | **S** | **F** | **G** | **F** | **N** | **M** | **L** | **A** | **I** | **E** | **K** | **G** | **V** | **P** | **K** | **V** | **L** | **S** | **L** | **K** | **Q** | **I** | **L** | **E** | **N** | **Y** | **I** | **E** | **H** | **Q** | **R** | **E** | **V** | **I** | **T** | **R** | **R** | **T** | **V** | **F** | **E** | **K** | **E** | **K** | **A** | **E** | **A** | **R** | **A** | **H** | **I** | **L** | **E** | **G** | **L** | **R** | **I** | **A** | **L** | **D** | **H** | **I** | **D** | **E** | **I** | **I** | **A** | **I** | **I** | **R** | **G** | **S** | **K** | **S** | **D** | **D** | **E** | **A** | **K** | **A** | **T** | **M** | **I** | **E** | **R** | **F** | **D** | **L** | **S** | **D** | **R** | **Q** | **S** | **Q** | **A** | **I** | **L** | **D** | **M** | **R** | **L** | **R** | **R** | **L** | **T** | **G** | **L** | **E** | **R** | **E** | **K** | **I** | **E** | **N** | **E** | **Y** | **Q** | **E** | **L** | **L** | **K** | **L** | **I** | **E** | **D** | **L** | **T** | **D** | **I** | **L** | **A** | **R** | **P** | **E** | **R** | **V** | **T** | **E** | **I** | **I** | **K** | **N** | **E** | **L** | **A** | **D** | **L** | **N** | **Q** | **R** | **F** | **G** | **D** | **K** | **R** | **R** | **T** | **E** | **L** | **L** | **V** | **G** | **E** | **V** | **L** | **S** | **L** | **E** | **D** | **E** | **D** | **L** | **I** | **E** | **E** | **E** | **E** | **I** | **V** | **I** | **T** | **L** | **T** | **N** | **N** | **G** | **Y** | **I** | **K** | **R** | **L** | **A** | **N** | **N** | **E** | **F** | **R** | **A** | **Q** | **R** | **R** | **G** | **G** | **R** | **G** | **V** | **Q** | **G** | **M** | **G** | **I** | **H** | **N** | **D** | **D** | **F** | **V** | **K** | **N** | **L** | **V** | **S** | **C** | **S** | **T** | **H** | **D** | **T** | **L** | **L** | **F** | **F** | **T** | **N** | **N** | **G** | **K** | **V** | **Y** | **R** | **A** | **K** | **G** | **Y** | **E** | **I** | **P** | **E** | **Y** | **G** | **R** | **T** | **A** | **K** | **G** | **I** | **P** | **I** | **I** | **N** | **L** | **L** | **G** | **I** | **D** | **S** | **S** | **E** | **S** | **I** | **Q** | **A** | **I** | **I** | **A** | **V** | **T** | **G** | **E** | **A** | **E** | **E** | **G** | **H** | **Y** | **L** | **F** | **F** | **T** | **T** | **R** | **Q** | **G** | **T** | **V** | **K** | **R** | **T** | **S** | **V** | **T** | **A** | **F** | **A** | **N** | **I** | **R** | **S** | **N** | **G** | **L** | **I** | **A** | **I** | **G** | **L** | **K** | **E** | **D** | **D** | **E** | **L** | **V** | **N** | **V** | **L** | **L** | **T** | **D** | **G** | **Q** | **S** | **N** | **I** | **I** | **I** | **G** | **T** | **H** | **N** | **G** | **Y** | **S** | **V** | **T** | **F** | **K** | **E** | **E** | **A** | **V** | **R** | **D** | **M** | **G** | **R** | **T** | **A** | **S** | **G** | **V** | **R** | **G** | **I** | **R** | **L** | **R** | **E** | **E** | **D** | **Y** | **V** | **V** | **G** | **A** | **A** | **L** | **M** | **K** | **E** | **N** | **Q** | **E** | **V** | **L** | **V** | **I** | **T** | **E** | **K** | **G** | **Y** | **G** | **K** | **R** | **T** | **K** | **V** | **S** | **E** | **Y** | **P** | **V** | **K** | **G** | **R** | **G** | **G** | **K** | **G** | **I** | **K** | **T** | **A** | **N** | **I** | **T** | **E** | **K** | **N** | **G** | **P** | **L** | **A** | **G** | **L** | **T** | **T** | **V** | **S** | **G** | **D** | **E** | **D** | **I** | **M** | **L** | **I** | **T** | **D** | **K** | **G** | **V** | **I** | **I** | **R** | **F** | **N** | **V** | **S** | **T** | **V** | **S** | **Q** | **T** | **G** | **R** | **S** | **T** | **L** | **G** | **V** | **R** | **L** | **M** | **K** | **M** | **E** | **A** | **D** | **T** | **K** | **V** | **V** | **T** | **M** | **A** | **A** | **V** | **E** | **P** | **E** | **A** | **A** | **E** | **Q** | **T** | **V** | **E** | **E** | **Q** | **G** | **S** | **E** | **E** |
| **N55279** | **M** | **S** | **E** | **E** | **I** | **R** | **E** | **N** | **I** | **H** | **D** | **V** | **N** | **L** | **T** | **S** | **E** | **M** | **K** | **D** | **S** | **F** | **I** | **D** | **Y** | **A** | **M** | **S** | **V** | **I** | **V** | **A** | **R** | **A** | **L** | **P** | **D** | **V** | **R** | **D** | **G** | **L** | **K** | **P** | **V** | **H** | **R** | **R** | **I** | **L** | **Y** | **G** | **M** | **N** | **E** | **L** | **G** | **V** | **T** | **P** | **D** | **K** | **A** | **H** | **K** | **K** | **S** | **A** | **R** | **I** | **V** | **G** | **D** | **V** | **M** | **G** | **K** | **Y** | **H** | **P** | **H** | **G** | **D** | **S** | **A** | **I** | **Y** | **E** | **S** | **M** | **V** | **R** | **M** | **A** | **Q** | **P** | **F** | **S** | **Y** | **R** | **Y** | **M** | **L** | **V** | **D** | **G** | **H** | **G** | **N** | **F** | **G** | **S** | **V** | **D** | **G** | **D** | **G** | **A** | **A** | **A** | **M** | **R** | **Y** | **T** | **E** | **A** | **R** | **M** | **S** | **K** | **I** | **A** | **T** | **E** | **M** | **L** | **R** | **D** | **I** | **N** | **K** | **N** | **T** | **V** | **D** | **F** | **Q** | **S** | **N** | **Y** | **D** | **D** | **T** | **E** | **K** | **E** | **P** | **V** | **V** | **L** | **P** | **A** | **R** | **F** | **P** | **N** | **L** | **L** | **V** | **N** | **G** | **T** | **T** | **G** | **I** | **A** | **V** | **G** | **M** | **A** | **T** | **N** | **I** | **P** | **P** | **H** | **N** | **L** | **S** | **E** | **V** | **V** | **A** | **A** | **I** | **D** | **L** | **L** | **M** | **D** | **N** | **P** | **D** | **V** | **T** | **T** | **N** | **E** | **L** | **M** | **E** | **V** | **L** | **P** | **G** | **P** | **D** | **F** | **P** | **T** | **G** | **G** | **L** | **V** | **M** | **G** | **K** | **S** | **G** | **I** | **R** | **R** | **A** | **Y** | **E** | **T** | **G** | **K** | **G** | **S** | **I** | **T** | **V** | **R** | **A** | **K** | **V** | **E** | **I** | **T** | **E** | **M** | **P** | **N** | **G** | **K** | **E** | **R** | **I** | **L** | **V** | **T** | **E** | **L** | **P** | **Y** | **M** | **V** | **N** | **K** | **A** | **K** | **L** | **I** | **E** | **R** | **I** | **S** | **E** | **L** | **H** | **R** | **D** | **K** | **R** | **I** | **E** | **G** | **I** | **T** | **D** | **L** | **R** | **D** | **E** | **S** | **S** | **R** | **E** | **G** | **M** | **R** | **I** | **V** | **I** | **D** | **I** | **R** | **R** | **D** | **A** | **S** | **A** | **S** | **V** | **I** | **L** | **N** | **N** | **L** | **Y** | **K** | **L** | **T** | **S** | **L** | **Q** | **N** | **S** | **F** | **G** | **F** | **N** | **M** | **L** | **A** | **I** | **E** | **K** | **G** | **V** | **P** | **K** | **V** | **L** | **S** | **L** | **K** | **Q** | **I** | **L** | **E** | **N** | **Y** | **I** | **E** | **H** | **Q** | **R** | **E** | **V** | **I** | **T** | **R** | **R** | **T** | **V** | **F** | **E** | **K** | **E** | **K** | **A** | **E** | **A** | **R** | **A** | **H** | **I** | **L** | **E** | **G** | **L** | **R** | **I** | **A** | **L** | **D** | **H** | **I** | **D** | **E** | **I** | **I** | **A** | **I** | **I** | **R** | **G** | **S** | **K** | **S** | **D** | **D** | **E** | **A** | **K** | **A** | **T** | **M** | **I** | **E** | **R** | **F** | **D** | **L** | **S** | **D** | **R** | **Q** | **S** | **Q** | **A** | **I** | **L** | **D** | **M** | **R** | **L** | **R** | **R** | **L** | **T** | **G** | **L** | **E** | **R** | **E** | **K** | **I** | **E** | **N** | **E** | **Y** | **Q** | **E** | **L** | **L** | **K** | **L** | **I** | **E** | **D** | **L** | **T** | **D** | **I** | **L** | **A** | **R** | **P** | **E** | **R** | **V** | **T** | **E** | **I** | **I** | **K** | **N** | **E** | **L** | **A** | **D** | **L** | **N** | **Q** | **R** | **F** | **G** | **D** | **K** | **R** | **R** | **T** | **E** | **L** | **L** | **V** | **G** | **E** | **V** | **L** | **S** | **L** | **E** | **D** | **E** | **D** | **L** | **I** | **E** | **E** | **E** | **E** | **I** | **V** | **I** | **T** | **L** | **T** | **N** | **N** | **G** | **Y** | **I** | **K** | **R** | **L** | **A** | **N** | **N** | **E** | **F** | **R** | **A** | **Q** | **R** | **R** | **G** | **G** | **R** | **G** | **V** | **Q** | **G** | **M** | **G** | **I** | **H** | **N** | **D** | **D** | **F** | **V** | **K** | **N** | **L** | **V** | **S** | **C** | **S** | **T** | **H** | **D** | **T** | **L** | **L** | **F** | **F** | **T** | **N** | **N** | **G** | **K** | **V** | **Y** | **R** | **A** | **K** | **G** | **Y** | **E** | **I** | **P** | **E** | **Y** | **G** | **R** | **T** | **A** | **K** | **G** | **I** | **P** | **I** | **I** | **N** | **L** | **L** | **G** | **I** | **D** | **S** | **S** | **E** | **S** | **I** | **Q** | **A** | **I** | **I** | **A** | **V** | **T** | **G** | **E** | **A** | **E** | **E** | **G** | **H** | **Y** | **L** | **F** | **F** | **T** | **T** | **R** | **Q** | **G** | **T** | **V** | **K** | **R** | **T** | **S** | **V** | **T** | **A** | **F** | **A** | **N** | **I** | **R** | **S** | **N** | **G** | **L** | **I** | **A** | **I** | **G** | **L** | **K** | **E** | **D** | **D** | **E** | **L** | **V** | **N** | **V** | **L** | **L** | **T** | **D** | **G** | **Q** | **S** | **N** | **I** | **I** | **I** | **G** | **T** | **H** | **N** | **G** | **Y** | **S** | **V** | **T** | **F** | **K** | **E** | **E** | **A** | **V** | **R** | **D** | **M** | **G** | **R** | **T** | **A** | **S** | **G** | **V** | **R** | **G** | **I** | **R** | **L** | **R** | **E** | **E** | **D** | **Y** | **V** | **V** | **G** | **A** | **A** | **L** | **M** | **K** | **E** | **D** | **Q** | **E** | **V** | **L** | **V** | **I** | **T** | **E** | **K** | **G** | **Y** | **G** | **K** | **R** | **T** | **K** | **V** | **S** | **E** | **Y** | **P** | **V** | **K** | **G** | **R** | **G** | **G** | **K** | **G** | **I** | **K** | **T** | **A** | **N** | **I** | **T** | **E** | **K** | **N** | **G** | **P** | **L** | **A** | **G** | **L** | **T** | **T** | **V** | **S** | **G** | **D** | **E** | **D** | **I** | **M** | **L** | **I** | **T** | **D** | **K** | **G** | **V** | **I** | **I** | **R** | **F** | **N** | **V** | **S** | **T** | **V** | **S** | **Q** | **T** | **G** | **R** | **S** | **T** | **L** | **G** | **V** | **R** | **L** | **M** | **K** | **M** | **E** | **A** | **D** | **T** | **K** | **V** | **V** | **T** | **M** | **-** | **-** | **-** | **-** | **-** | **-** | **-** | **-** | **-** | **-** | **-** | **-** | **-** | **-** | **-** | **-** | **-** | **-** | **-** |
| **N59591F** | **M** | **S** | **E** | **E** | **I** | **K** | **E** | **N** | **I** | **Q** | **D** | **V** | **N** | **L** | **T** | **S** | **E** | **M** | **K** | **E** | **S** | **F** | **I** | **D** | **Y** | **A** | **M** | **S** | **V** | **I** | **V** | **A** | **R** | **A** | **L** | **P** | **D** | **V** | **R** | **D** | **G** | **L** | **K** | **P** | **V** | **H** | **R** | **R** | **I** | **L** | **Y** | **G** | **M** | **N** | **E** | **L** | **G** | **V** | **T** | **P** | **D** | **K** | **P** | **H** | **K** | **K** | **S** | **A** | **R** | **I** | **V** | **G** | **D** | **V** | **M** | **G** | **K** | **Y** | **H** | **P** | **H** | **G** | **D** | **S** | **A** | **I** | **Y** | **E** | **S** | **M** | **V** | **R** | **M** | **A** | **Q** | **P** | **F** | **S** | **Y** | **R** | **A** | **M** | **L** | **V** | **D** | **G** | **H** | **G** | **N** | **F** | **G** | **S** | **V** | **D** | **G** | **D** | **G** | **A** | **A** | **A** | **M** | **R** | **Y** | **T** | **E** | **A** | **R** | **M** | **S** | **K** | **I** | **A** | **L** | **E** | **M** | **L** | **R** | **D** | **I** | **N** | **K** | **N** | **T** | **V** | **D** | **F** | **Q** | **G** | **N** | **Y** | **D** | **D** | **S** | **E** | **Q** | **E** | **P** | **V** | **V** | **L** | **P** | **A** | **R** | **F** | **P** | **N** | **L** | **L** | **V** | **N** | **G** | **T** | **T** | **G** | **I** | **A** | **V** | **G** | **M** | **A** | **T** | **N** | **I** | **P** | **P** | **H** | **N** | **L** | **S** | **E** | **V** | **I** | **D** | **A** | **T** | **S** | **L** | **L** | **M** | **D** | **N** | **P** | **D** | **V** | **T** | **T** | **N** | **E** | **L** | **M** | **E** | **V** | **L** | **P** | **G** | **P** | **D** | **F** | **P** | **T** | **G** | **G** | **L** | **V** | **M** | **G** | **K** | **S** | **G** | **I** | **R** | **R** | **A** | **Y** | **E** | **T** | **G** | **K** | **G** | **S** | **I** | **T** | **V** | **R** | **A** | **K** | **V** | **E** | **L** | **T** | **E** | **M** | **P** | **N** | **G** | **K** | **E** | **R** | **I** | **L** | **V** | **T** | **E** | **L** | **P** | **Y** | **M** | **V** | **N** | **K** | **A** | **K** | **L** | **I** | **E** | **R** | **I** | **S** | **E** | **L** | **H** | **R** | **D** | **K** | **R** | **I** | **E** | **G** | **I** | **T** | **D** | **L** | **R** | **D** | **E** | **S** | **S** | **R** | **E** | **G** | **M** | **R** | **I** | **V** | **I** | **D** | **V** | **R** | **R** | **D** | **V** | **S** | **A** | **S** | **V** | **V** | **L** | **N** | **N** | **L** | **Y** | **K** | **M** | **T** | **A** | **L** | **Q** | **T** | **S** | **F** | **G** | **F** | **N** | **M** | **L** | **A** | **I** | **E** | **K** | **G** | **V** | **P** | **K** | **I** | **L** | **S** | **L** | **K** | **R** | **I** | **L** | **E** | **N** | **Y** | **V** | **E** | **H** | **Q** | **K** | **E** | **V** | **I** | **T** | **R** | **R** | **T** | **I** | **F** | **D** | **K** | **N** | **K** | **A** | **E** | **A** | **R** | **A** | **H** | **I** | **L** | **E** | **G** | **L** | **R** | **I** | **A** | **L** | **D** | **H** | **I** | **D** | **E** | **I** | **I** | **A** | **I** | **I** | **R** | **G** | **S** | **Q** | **S** | **D** | **D** | **E** | **A** | **K** | **A** | **T** | **L** | **I** | **E** | **R** | **F** | **E** | **F** | **S** | **D** | **R** | **Q** | **A** | **Q** | **A** | **I** | **L** | **D** | **M** | **R** | **L** | **R** | **R** | **L** | **T** | **G** | **L** | **E** | **R** | **D** | **K** | **I** | **E** | **N** | **E** | **Y** | **Q** | **E** | **L** | **L** | **K** | **F** | **I** | **A** | **D** | **L** | **E** | **D** | **I** | **L** | **A** | **R** | **P** | **E** | **R** | **V** | **I** | **E** | **I** | **I** | **K** | **T** | **E** | **L** | **N** | **D** | **V** | **R** | **T** | **K** | **F** | **G** | **D** | **A** | **R** | **R** | **T** | **E** | **L** | **L** | **V** | **G** | **E** | **V** | **L** | **S** | **L** | **E** | **D** | **E** | **D** | **L** | **I** | **E** | **E** | **E** | **E** | **V** | **V** | **I** | **T** | **L** | **T** | **N** | **N** | **G** | **Y** | **I** | **K** | **R** | **M** | **A** | **N** | **S** | **E** | **F** | **R** | **A** | **Q** | **R** | **R** | **G** | **G** | **R** | **G** | **V** | **Q** | **G** | **M** | **G** | **V** | **H** | **D** | **D** | **D** | **F** | **V** | **K** | **N** | **L** | **V** | **S** | **C** | **S** | **T** | **H** | **D** | **T** | **L** | **L** | **F** | **F** | **T** | **N** | **T** | **G** | **K** | **V** | **Y** | **R** | **A** | **K** | **G** | **Y** | **E** | **I** | **P** | **E** | **Y** | **G** | **R** | **T** | **A** | **K** | **G** | **I** | **P** | **V** | **I** | **N** | **L** | **L** | **G** | **I** | **D** | **S** | **A** | **E** | **K** | **I** | **Q** | **A** | **I** | **I** | **S** | **V** | **E** | **G** | **K** | **A** | **E** | **A** | **G** | **K** | **Y** | **L** | **F** | **F** | **T** | **T** | **L** | **K** | **G** | **T** | **V** | **K** | **R** | **T** | **A** | **V** | **T** | **A** | **F** | **S** | **N** | **I** | **R** | **S** | **N** | **G** | **L** | **I** | **A** | **I** | **S** | **L** | **K** | **E** | **D** | **D** | **E** | **L** | **V** | **N** | **V** | **V** | **T** | **T** | **N** | **G** | **N** | **Q** | **K** | **M** | **I** | **I** | **G** | **T** | **H** | **A** | **G** | **Y** | **S** | **V** | **T** | **F** | **D** | **E** | **N** | **T** | **V** | **R** | **D** | **M** | **G** | **R** | **T** | **A** | **S** | **G** | **V** | **R** | **G** | **I** | **R** | **L** | **R** | **E** | **N** | **D** | **Y** | **V** | **V** | **G** | **A** | **A** | **I** | **L** | **D** | **E** | **N** | **K** | **E** | **V** | **L** | **V** | **I** | **T** | **E** | **N** | **G** | **Y** | **G** | **K** | **R** | **T** | **K** | **A** | **S** | **E** | **Y** | **P** | **V** | **K** | **G** | **R** | **G** | **G** | **K** | **G** | **I** | **K** | **T** | **A** | **N** | **I** | **T** | **E** | **K** | **N** | **G** | **P** | **L** | **A** | **G** | **L** | **T** | **T** | **V** | **N** | **G** | **D** | **E** | **D** | **I** | **L** | **L** | **I** | **T** | **N** | **K** | **G** | **V** | **I** | **I** | **R** | **F** | **N** | **V** | **D** | **S** | **V** | **S** | **Q** | **T** | **G** | **R** | **A** | **T** | **L** | **G** | **V** | **R** | **L** | **M** | **R** | **M** | **E** | **D** | **G** | **A** | **K** | **-** | **-** | **-** | **-** | **-** | **-** | **-** | **-** | **-** | **-** | **-** | **-** | **-** | **-** | **-** | **-** | **-** | **-** | **-** | **-** | **-** | **-** | **-** |
| **N53453** | **M** | **S** | **E** | **E** | **I** | **K** | **E** | **N** | **I** | **Q** | **D** | **V** | **N** | **L** | **T** | **S** | **E** | **M** | **K** | **E** | **S** | **F** | **I** | **D** | **Y** | **A** | **M** | **S** | **V** | **I** | **V** | **A** | **R** | **A** | **L** | **P** | **D** | **V** | **R** | **D** | **G** | **L** | **K** | **P** | **V** | **H** | **R** | **R** | **I** | **L** | **Y** | **G** | **M** | **N** | **E** | **L** | **G** | **V** | **T** | **P** | **D** | **K** | **P** | **H** | **K** | **K** | **S** | **A** | **R** | **I** | **V** | **G** | **D** | **V** | **M** | **G** | **K** | **Y** | **H** | **P** | **H** | **G** | **D** | **S** | **A** | **I** | **Y** | **E** | **S** | **M** | **V** | **R** | **M** | **A** | **Q** | **P** | **F** | **S** | **Y** | **R** | **A** | **M** | **L** | **V** | **D** | **G** | **H** | **G** | **N** | **F** | **G** | **S** | **V** | **D** | **G** | **D** | **G** | **A** | **A** | **A** | **M** | **R** | **Y** | **T** | **E** | **A** | **R** | **M** | **S** | **K** | **I** | **A** | **L** | **E** | **M** | **L** | **R** | **D** | **I** | **N** | **K** | **N** | **T** | **V** | **D** | **F** | **Q** | **G** | **N** | **Y** | **D** | **D** | **S** | **E** | **Q** | **E** | **P** | **V** | **V** | **L** | **P** | **A** | **R** | **F** | **P** | **N** | **L** | **L** | **V** | **N** | **G** | **T** | **T** | **G** | **I** | **A** | **V** | **G** | **M** | **A** | **T** | **N** | **I** | **P** | **P** | **H** | **N** | **L** | **S** | **E** | **V** | **I** | **D** | **A** | **T** | **S** | **L** | **L** | **M** | **D** | **N** | **P** | **D** | **V** | **T** | **T** | **N** | **E** | **L** | **M** | **E** | **V** | **L** | **P** | **G** | **P** | **D** | **F** | **P** | **T** | **G** | **G** | **L** | **V** | **M** | **G** | **K** | **S** | **G** | **I** | **R** | **R** | **A** | **Y** | **E** | **T** | **G** | **K** | **G** | **S** | **I** | **T** | **V** | **R** | **A** | **K** | **V** | **E** | **L** | **T** | **E** | **M** | **P** | **N** | **G** | **K** | **E** | **R** | **I** | **L** | **V** | **T** | **E** | **L** | **P** | **Y** | **M** | **V** | **N** | **K** | **A** | **K** | **L** | **I** | **E** | **R** | **I** | **S** | **E** | **L** | **H** | **R** | **D** | **K** | **R** | **I** | **E** | **G** | **I** | **T** | **D** | **L** | **R** | **D** | **E** | **S** | **S** | **R** | **E** | **G** | **M** | **R** | **I** | **V** | **I** | **D** | **V** | **R** | **R** | **D** | **V** | **S** | **A** | **S** | **V** | **V** | **L** | **N** | **N** | **L** | **Y** | **K** | **M** | **T** | **A** | **L** | **Q** | **T** | **S** | **F** | **G** | **F** | **N** | **M** | **L** | **A** | **I** | **E** | **K** | **G** | **V** | **P** | **K** | **I** | **L** | **S** | **L** | **K** | **R** | **I** | **L** | **E** | **N** | **Y** | **V** | **E** | **H** | **Q** | **K** | **E** | **V** | **I** | **T** | **R** | **R** | **T** | **I** | **F** | **D** | **K** | **N** | **K** | **A** | **E** | **A** | **R** | **A** | **H** | **I** | **L** | **E** | **G** | **L** | **R** | **I** | **A** | **L** | **D** | **H** | **I** | **D** | **E** | **I** | **I** | **A** | **I** | **I** | **R** | **G** | **S** | **Q** | **S** | **D** | **D** | **E** | **A** | **K** | **A** | **T** | **L** | **I** | **E** | **R** | **F** | **E** | **F** | **S** | **D** | **R** | **Q** | **A** | **Q** | **A** | **I** | **L** | **D** | **M** | **R** | **L** | **R** | **R** | **L** | **T** | **G** | **L** | **E** | **R** | **D** | **K** | **I** | **E** | **N** | **E** | **Y** | **Q** | **E** | **L** | **L** | **K** | **F** | **I** | **A** | **D** | **L** | **E** | **D** | **I** | **L** | **A** | **R** | **P** | **E** | **R** | **V** | **I** | **E** | **I** | **I** | **K** | **T** | **E** | **L** | **N** | **D** | **V** | **R** | **T** | **K** | **F** | **G** | **D** | **A** | **R** | **R** | **T** | **E** | **L** | **L** | **V** | **G** | **E** | **V** | **L** | **S** | **L** | **E** | **D** | **E** | **D** | **L** | **I** | **E** | **E** | **E** | **E** | **V** | **V** | **I** | **T** | **L** | **T** | **N** | **N** | **G** | **Y** | **I** | **K** | **R** | **M** | **A** | **N** | **S** | **E** | **F** | **R** | **A** | **Q** | **R** | **R** | **G** | **G** | **R** | **G** | **V** | **Q** | **G** | **M** | **G** | **V** | **H** | **D** | **D** | **D** | **F** | **V** | **K** | **N** | **L** | **V** | **S** | **C** | **S** | **T** | **H** | **D** | **T** | **L** | **L** | **F** | **F** | **T** | **N** | **T** | **G** | **K** | **V** | **Y** | **R** | **A** | **K** | **G** | **Y** | **E** | **I** | **P** | **E** | **Y** | **G** | **R** | **T** | **A** | **K** | **G** | **I** | **P** | **V** | **I** | **N** | **L** | **L** | **G** | **I** | **D** | **S** | **A** | **E** | **K** | **I** | **Q** | **A** | **I** | **I** | **S** | **V** | **E** | **G** | **K** | **A** | **E** | **A** | **G** | **K** | **Y** | **L** | **F** | **F** | **T** | **T** | **L** | **K** | **G** | **T** | **V** | **K** | **R** | **T** | **A** | **V** | **T** | **A** | **F** | **S** | **N** | **I** | **R** | **S** | **N** | **G** | **L** | **I** | **A** | **I** | **S** | **L** | **K** | **E** | **D** | **D** | **E** | **L** | **V** | **N** | **V** | **V** | **T** | **T** | **N** | **G** | **N** | **Q** | **K** | **M** | **I** | **I** | **G** | **T** | **H** | **A** | **G** | **Y** | **S** | **V** | **T** | **F** | **D** | **E** | **N** | **T** | **V** | **R** | **D** | **M** | **G** | **R** | **T** | **A** | **S** | **G** | **V** | **R** | **G** | **I** | **R** | **L** | **R** | **E** | **N** | **D** | **Y** | **V** | **V** | **G** | **A** | **A** | **I** | **L** | **D** | **E** | **N** | **K** | **E** | **V** | **L** | **V** | **I** | **T** | **E** | **N** | **G** | **Y** | **G** | **K** | **R** | **T** | **K** | **A** | **S** | **E** | **Y** | **P** | **V** | **K** | **G** | **R** | **G** | **G** | **K** | **G** | **I** | **K** | **T** | **A** | **N** | **I** | **T** | **E** | **K** | **N** | **G** | **P** | **L** | **A** | **G** | **L** | **T** | **T** | **V** | **N** | **G** | **D** | **E** | **D** | **I** | **L** | **L** | **I** | **T** | **N** | **K** | **G** | **V** | **I** | **I** | **R** | **F** | **N** | **V** | **D** | **S** | **V** | **S** | **Q** | **T** | **G** | **R** | **A** | **T** | **L** | **G** | **V** | **R** | **L** | **M** | **R** | **M** | **E** | **D** | **G** | **A** | **K** | **-** | **-** | **-** | **-** | **-** | **-** | **-** | **-** | **-** | **-** | **-** | **-** | **-** | **-** | **-** | **-** | **-** | **-** | **-** | **-** | **-** | **-** | **-** |
| **N59788F** | **M** | **S** | **E** | **E** | **I** | **R** | **E** | **N** | **I** | **H** | **D** | **V** | **N** | **L** | **T** | **S** | **E** | **M** | **K** | **D** | **S** | **F** | **I** | **D** | **Y** | **A** | **M** | **S** | **V** | **I** | **V** | **A** | **R** | **A** | **L** | **P** | **D** | **V** | **R** | **D** | **G** | **L** | **K** | **P** | **V** | **H** | **R** | **R** | **I** | **L** | **Y** | **G** | **M** | **N** | **E** | **L** | **G** | **V** | **T** | **P** | **D** | **K** | **A** | **H** | **K** | **K** | **S** | **A** | **R** | **I** | **V** | **G** | **D** | **V** | **M** | **G** | **K** | **Y** | **H** | **P** | **H** | **G** | **D** | **S** | **A** | **I** | **Y** | **E** | **S** | **M** | **V** | **R** | **M** | **A** | **Q** | **P** | **F** | **S** | **Y** | **R** | **Y** | **M** | **L** | **V** | **D** | **G** | **H** | **G** | **N** | **F** | **G** | **S** | **V** | **D** | **G** | **D** | **G** | **A** | **A** | **A** | **M** | **R** | **Y** | **T** | **E** | **A** | **R** | **M** | **S** | **K** | **I** | **A** | **T** | **E** | **M** | **L** | **R** | **D** | **I** | **N** | **K** | **N** | **T** | **V** | **D** | **F** | **Q** | **S** | **N** | **Y** | **D** | **D** | **T** | **E** | **K** | **E** | **P** | **V** | **V** | **L** | **P** | **A** | **R** | **F** | **P** | **N** | **L** | **L** | **V** | **N** | **G** | **T** | **T** | **G** | **I** | **A** | **V** | **G** | **M** | **A** | **T** | **N** | **I** | **P** | **P** | **H** | **N** | **L** | **S** | **E** | **V** | **V** | **A** | **A** | **I** | **D** | **L** | **L** | **M** | **D** | **N** | **P** | **D** | **V** | **T** | **T** | **N** | **E** | **L** | **M** | **E** | **V** | **L** | **P** | **G** | **P** | **D** | **F** | **P** | **T** | **G** | **G** | **L** | **V** | **M** | **G** | **K** | **S** | **G** | **I** | **R** | **R** | **A** | **Y** | **E** | **T** | **G** | **K** | **G** | **S** | **I** | **T** | **V** | **R** | **A** | **K** | **V** | **E** | **I** | **T** | **E** | **M** | **P** | **N** | **G** | **K** | **E** | **R** | **I** | **I** | **V** | **T** | **E** | **L** | **P** | **Y** | **M** | **V** | **N** | **K** | **A** | **K** | **L** | **I** | **E** | **R** | **I** | **S** | **E** | **L** | **H** | **R** | **D** | **K** | **R** | **I** | **E** | **G** | **I** | **T** | **D** | **L** | **R** | **D** | **E** | **S** | **S** | **R** | **E** | **G** | **M** | **R** | **I** | **V** | **I** | **D** | **I** | **R** | **R** | **D** | **A** | **S** | **A** | **S** | **V** | **I** | **L** | **N** | **N** | **L** | **Y** | **K** | **L** | **T** | **S** | **L** | **Q** | **N** | **S** | **F** | **G** | **F** | **N** | **M** | **L** | **A** | **I** | **E** | **K** | **G** | **V** | **P** | **K** | **V** | **L** | **S** | **L** | **K** | **Q** | **I** | **L** | **E** | **N** | **Y** | **I** | **E** | **H** | **Q** | **R** | **E** | **V** | **I** | **T** | **R** | **R** | **T** | **V** | **F** | **E** | **K** | **E** | **K** | **A** | **E** | **A** | **R** | **A** | **H** | **I** | **L** | **E** | **G** | **L** | **R** | **I** | **A** | **L** | **D** | **H** | **I** | **D** | **E** | **I** | **I** | **A** | **I** | **I** | **R** | **G** | **S** | **K** | **S** | **D** | **D** | **E** | **A** | **K** | **A** | **T** | **M** | **I** | **E** | **R** | **F** | **D** | **L** | **S** | **D** | **R** | **Q** | **S** | **Q** | **A** | **I** | **L** | **D** | **M** | **R** | **L** | **R** | **R** | **L** | **T** | **G** | **L** | **E** | **R** | **E** | **K** | **I** | **E** | **N** | **E** | **Y** | **Q** | **E** | **L** | **L** | **K** | **L** | **I** | **E** | **D** | **L** | **T** | **D** | **I** | **L** | **A** | **R** | **P** | **E** | **R** | **V** | **T** | **E** | **I** | **I** | **K** | **N** | **E** | **L** | **A** | **D** | **L** | **N** | **Q** | **R** | **F** | **G** | **D** | **K** | **R** | **R** | **T** | **E** | **L** | **L** | **V** | **G** | **E** | **V** | **L** | **S** | **L** | **E** | **D** | **E** | **D** | **L** | **I** | **E** | **E** | **E** | **E** | **I** | **V** | **I** | **T** | **L** | **T** | **N** | **N** | **G** | **Y** | **I** | **K** | **R** | **L** | **A** | **N** | **N** | **E** | **F** | **R** | **A** | **Q** | **R** | **R** | **G** | **G** | **R** | **G** | **V** | **Q** | **G** | **M** | **G** | **I** | **H** | **N** | **D** | **D** | **F** | **V** | **K** | **N** | **L** | **V** | **S** | **C** | **S** | **T** | **H** | **D** | **T** | **L** | **L** | **F** | **F** | **T** | **N** | **N** | **G** | **K** | **V** | **Y** | **R** | **A** | **K** | **G** | **Y** | **E** | **I** | **P** | **E** | **Y** | **G** | **R** | **T** | **A** | **K** | **G** | **I** | **P** | **I** | **I** | **N** | **L** | **L** | **G** | **I** | **D** | **S** | **S** | **E** | **S** | **I** | **Q** | **A** | **I** | **I** | **A** | **V** | **T** | **G** | **E** | **A** | **E** | **E** | **G** | **H** | **Y** | **L** | **F** | **F** | **T** | **T** | **R** | **Q** | **G** | **T** | **V** | **K** | **R** | **T** | **S** | **V** | **T** | **A** | **F** | **A** | **N** | **I** | **R** | **S** | **N** | **G** | **L** | **I** | **A** | **I** | **G** | **L** | **K** | **E** | **D** | **D** | **E** | **L** | **V** | **N** | **V** | **L** | **L** | **T** | **D** | **G** | **Q** | **S** | **N** | **I** | **I** | **I** | **G** | **T** | **H** | **N** | **G** | **Y** | **S** | **V** | **T** | **F** | **K** | **E** | **E** | **A** | **V** | **R** | **D** | **M** | **G** | **R** | **T** | **A** | **S** | **G** | **V** | **R** | **G** | **I** | **R** | **L** | **R** | **E** | **E** | **D** | **Y** | **V** | **V** | **G** | **A** | **A** | **L** | **M** | **K** | **E** | **D** | **Q** | **E** | **V** | **L** | **V** | **I** | **T** | **E** | **K** | **G** | **Y** | **G** | **K** | **R** | **T** | **K** | **V** | **S** | **E** | **Y** | **P** | **V** | **K** | **G** | **R** | **G** | **G** | **K** | **G** | **I** | **K** | **T** | **A** | **N** | **I** | **T** | **E** | **K** | **N** | **G** | **P** | **L** | **A** | **G** | **L** | **T** | **T** | **V** | **S** | **G** | **D** | **E** | **D** | **I** | **M** | **L** | **I** | **T** | **D** | **K** | **G** | **V** | **I** | **I** | **R** | **F** | **N** | **V** | **S** | **T** | **V** | **S** | **Q** | **T** | **G** | **R** | **S** | **T** | **L** | **G** | **V** | **R** | **L** | **M** | **K** | **M** | **E** | **A** | **D** | **T** | **K** | **V** | **V** | **T** | **M** | **A** | **A** | **V** | **E** | **P** | **E** | **A** | **A** | **E** | **Q** | **T** | **V** | **E** | **E** | **Q** | **G** | **S** | **E** | **E** |
| **N53519** | **M** | **S** | **E** | **E** | **I** | **K** | **E** | **N** | **I** | **Q** | **D** | **V** | **N** | **L** | **T** | **S** | **E** | **M** | **K** | **E** | **S** | **F** | **I** | **D** | **Y** | **A** | **M** | **S** | **V** | **I** | **V** | **A** | **R** | **A** | **L** | **P** | **D** | **V** | **R** | **D** | **G** | **L** | **K** | **P** | **V** | **H** | **R** | **R** | **I** | **L** | **Y** | **G** | **M** | **N** | **E** | **L** | **G** | **V** | **T** | **P** | **D** | **K** | **P** | **H** | **K** | **K** | **S** | **A** | **R** | **I** | **V** | **G** | **D** | **V** | **M** | **G** | **K** | **Y** | **H** | **P** | **H** | **G** | **D** | **S** | **A** | **I** | **Y** | **E** | **S** | **M** | **V** | **R** | **M** | **A** | **Q** | **P** | **F** | **S** | **Y** | **R** | **A** | **M** | **L** | **V** | **D** | **G** | **H** | **G** | **N** | **F** | **G** | **S** | **V** | **D** | **G** | **D** | **G** | **A** | **A** | **A** | **M** | **R** | **Y** | **T** | **E** | **A** | **R** | **M** | **S** | **K** | **I** | **A** | **L** | **E** | **M** | **L** | **R** | **D** | **I** | **N** | **K** | **N** | **T** | **V** | **D** | **F** | **Q** | **G** | **N** | **Y** | **D** | **D** | **S** | **E** | **Q** | **E** | **P** | **V** | **V** | **L** | **P** | **A** | **R** | **F** | **P** | **N** | **L** | **L** | **V** | **N** | **G** | **T** | **T** | **G** | **I** | **A** | **V** | **G** | **M** | **A** | **T** | **N** | **I** | **P** | **P** | **H** | **N** | **L** | **S** | **E** | **V** | **I** | **D** | **A** | **T** | **S** | **L** | **L** | **M** | **D** | **N** | **P** | **D** | **V** | **T** | **T** | **N** | **E** | **L** | **M** | **E** | **V** | **L** | **P** | **G** | **P** | **D** | **F** | **P** | **T** | **G** | **G** | **L** | **V** | **M** | **G** | **K** | **S** | **G** | **I** | **R** | **R** | **A** | **Y** | **E** | **T** | **G** | **K** | **G** | **S** | **I** | **T** | **V** | **R** | **A** | **K** | **V** | **E** | **L** | **T** | **E** | **M** | **P** | **N** | **G** | **K** | **E** | **R** | **I** | **L** | **V** | **T** | **E** | **L** | **P** | **Y** | **M** | **V** | **N** | **K** | **A** | **K** | **L** | **I** | **E** | **R** | **I** | **S** | **E** | **L** | **H** | **R** | **D** | **K** | **R** | **I** | **E** | **G** | **I** | **T** | **D** | **L** | **R** | **D** | **E** | **S** | **S** | **R** | **E** | **G** | **M** | **R** | **I** | **V** | **I** | **D** | **V** | **R** | **R** | **D** | **V** | **S** | **A** | **S** | **V** | **V** | **L** | **N** | **N** | **L** | **Y** | **K** | **M** | **T** | **A** | **L** | **Q** | **T** | **S** | **F** | **G** | **F** | **N** | **M** | **L** | **A** | **I** | **E** | **K** | **G** | **V** | **P** | **K** | **I** | **L** | **S** | **L** | **K** | **R** | **I** | **L** | **E** | **N** | **Y** | **V** | **E** | **H** | **Q** | **K** | **E** | **V** | **I** | **T** | **R** | **R** | **T** | **I** | **F** | **D** | **K** | **N** | **K** | **A** | **E** | **A** | **R** | **A** | **H** | **I** | **L** | **E** | **G** | **L** | **R** | **I** | **A** | **L** | **D** | **H** | **I** | **D** | **E** | **I** | **I** | **A** | **I** | **I** | **R** | **G** | **S** | **Q** | **S** | **D** | **D** | **E** | **A** | **K** | **A** | **T** | **L** | **I** | **E** | **R** | **F** | **E** | **F** | **S** | **D** | **R** | **Q** | **A** | **Q** | **A** | **I** | **L** | **D** | **M** | **R** | **L** | **R** | **R** | **L** | **T** | **G** | **L** | **E** | **R** | **D** | **K** | **I** | **E** | **N** | **E** | **Y** | **Q** | **E** | **L** | **L** | **K** | **F** | **I** | **A** | **D** | **L** | **E** | **D** | **I** | **L** | **A** | **R** | **P** | **E** | **R** | **V** | **I** | **E** | **I** | **I** | **K** | **T** | **E** | **L** | **N** | **D** | **V** | **R** | **T** | **K** | **F** | **G** | **D** | **A** | **R** | **R** | **T** | **E** | **L** | **L** | **V** | **G** | **E** | **V** | **L** | **S** | **L** | **E** | **D** | **E** | **D** | **L** | **I** | **E** | **E** | **E** | **E** | **V** | **V** | **I** | **T** | **L** | **T** | **N** | **N** | **G** | **Y** | **I** | **K** | **R** | **M** | **A** | **N** | **S** | **E** | **F** | **R** | **A** | **Q** | **R** | **R** | **G** | **G** | **R** | **G** | **V** | **Q** | **G** | **M** | **G** | **V** | **H** | **D** | **D** | **D** | **F** | **V** | **K** | **N** | **L** | **V** | **S** | **C** | **S** | **T** | **H** | **D** | **T** | **L** | **L** | **F** | **F** | **T** | **N** | **T** | **G** | **K** | **V** | **Y** | **R** | **A** | **K** | **G** | **Y** | **E** | **I** | **P** | **E** | **Y** | **G** | **R** | **T** | **A** | **K** | **G** | **I** | **P** | **V** | **I** | **N** | **L** | **L** | **G** | **I** | **D** | **S** | **A** | **E** | **K** | **I** | **Q** | **A** | **I** | **I** | **S** | **V** | **E** | **G** | **K** | **A** | **E** | **A** | **G** | **K** | **Y** | **L** | **F** | **F** | **T** | **T** | **L** | **K** | **G** | **T** | **V** | **K** | **R** | **T** | **A** | **V** | **T** | **A** | **F** | **S** | **N** | **I** | **R** | **S** | **N** | **G** | **L** | **I** | **A** | **I** | **S** | **L** | **K** | **E** | **D** | **D** | **E** | **L** | **V** | **N** | **V** | **V** | **T** | **T** | **N** | **G** | **N** | **Q** | **K** | **M** | **I** | **I** | **G** | **T** | **H** | **A** | **G** | **Y** | **S** | **V** | **T** | **F** | **D** | **E** | **N** | **T** | **V** | **R** | **D** | **M** | **G** | **R** | **T** | **A** | **S** | **G** | **V** | **R** | **G** | **I** | **R** | **L** | **R** | **E** | **N** | **D** | **Y** | **V** | **V** | **G** | **A** | **A** | **I** | **L** | **D** | **E** | **N** | **K** | **E** | **V** | **L** | **V** | **I** | **T** | **E** | **N** | **G** | **Y** | **G** | **K** | **R** | **T** | **K** | **A** | **S** | **E** | **Y** | **P** | **V** | **K** | **G** | **R** | **G** | **G** | **K** | **G** | **I** | **K** | **T** | **A** | **N** | **I** | **T** | **E** | **K** | **N** | **G** | **P** | **L** | **A** | **G** | **L** | **T** | **T** | **V** | **N** | **G** | **D** | **E** | **D** | **I** | **L** | **L** | **I** | **T** | **N** | **K** | **G** | **V** | **I** | **I** | **R** | **F** | **N** | **V** | **D** | **S** | **V** | **S** | **Q** | **T** | **G** | **R** | **A** | **T** | **L** | **G** | **V** | **R** | **L** | **M** | **R** | **M** | **E** | **D** | **G** | **A** | **K** | **-** | **-** | **-** | **-** | **-** | **-** | **-** | **-** | **-** | **-** | **-** | **-** | **-** | **-** | **-** | **-** | **-** | **-** | **-** | **-** | **-** | **-** | **-** |
| **N60463F** | **M** | **S** | **E** | **E** | **I** | **R** | **E** | **N** | **I** | **H** | **D** | **V** | **N** | **L** | **T** | **S** | **E** | **M** | **K** | **D** | **S** | **F** | **I** | **D** | **Y** | **A** | **M** | **S** | **V** | **I** | **V** | **A** | **R** | **A** | **L** | **P** | **D** | **V** | **R** | **D** | **G** | **L** | **K** | **P** | **V** | **H** | **R** | **R** | **I** | **L** | **Y** | **G** | **M** | **N** | **E** | **L** | **G** | **V** | **T** | **P** | **D** | **K** | **A** | **H** | **K** | **K** | **S** | **A** | **R** | **I** | **V** | **G** | **D** | **V** | **M** | **G** | **K** | **Y** | **H** | **P** | **H** | **G** | **D** | **S** | **A** | **I** | **Y** | **E** | **S** | **M** | **V** | **R** | **M** | **A** | **Q** | **P** | **F** | **S** | **Y** | **R** | **Y** | **M** | **L** | **V** | **D** | **G** | **H** | **G** | **N** | **F** | **G** | **S** | **V** | **D** | **G** | **D** | **G** | **A** | **A** | **A** | **M** | **R** | **Y** | **T** | **E** | **A** | **R** | **M** | **S** | **K** | **I** | **A** | **T** | **E** | **M** | **L** | **R** | **D** | **I** | **N** | **K** | **N** | **T** | **V** | **D** | **F** | **Q** | **S** | **N** | **Y** | **D** | **D** | **T** | **E** | **K** | **E** | **P** | **V** | **V** | **L** | **P** | **A** | **R** | **F** | **P** | **N** | **L** | **L** | **V** | **N** | **G** | **T** | **T** | **G** | **I** | **A** | **V** | **G** | **M** | **A** | **T** | **N** | **I** | **P** | **P** | **H** | **N** | **L** | **S** | **E** | **V** | **V** | **A** | **A** | **I** | **D** | **L** | **L** | **M** | **D** | **N** | **P** | **D** | **V** | **T** | **T** | **N** | **E** | **L** | **M** | **E** | **V** | **L** | **P** | **G** | **P** | **D** | **F** | **P** | **T** | **G** | **G** | **L** | **V** | **M** | **G** | **K** | **S** | **G** | **I** | **R** | **R** | **A** | **Y** | **E** | **T** | **G** | **K** | **G** | **S** | **I** | **T** | **V** | **R** | **A** | **K** | **V** | **E** | **I** | **T** | **E** | **M** | **P** | **N** | **G** | **K** | **E** | **R** | **I** | **I** | **V** | **T** | **E** | **L** | **P** | **Y** | **M** | **V** | **N** | **K** | **A** | **K** | **L** | **I** | **E** | **R** | **I** | **S** | **E** | **L** | **H** | **R** | **D** | **K** | **R** | **I** | **E** | **G** | **I** | **T** | **D** | **L** | **R** | **D** | **E** | **S** | **S** | **R** | **E** | **G** | **M** | **R** | **I** | **V** | **I** | **D** | **I** | **R** | **R** | **D** | **A** | **S** | **A** | **S** | **V** | **I** | **L** | **N** | **N** | **L** | **Y** | **K** | **L** | **T** | **S** | **L** | **Q** | **N** | **S** | **F** | **G** | **F** | **N** | **M** | **L** | **A** | **I** | **E** | **K** | **G** | **V** | **P** | **K** | **V** | **L** | **S** | **L** | **K** | **Q** | **I** | **L** | **E** | **N** | **Y** | **I** | **E** | **H** | **Q** | **R** | **E** | **V** | **I** | **T** | **R** | **R** | **T** | **V** | **F** | **E** | **K** | **E** | **K** | **A** | **E** | **A** | **R** | **A** | **H** | **I** | **L** | **E** | **G** | **L** | **R** | **I** | **A** | **L** | **D** | **H** | **I** | **D** | **E** | **I** | **I** | **A** | **I** | **I** | **R** | **G** | **S** | **K** | **S** | **D** | **D** | **E** | **A** | **K** | **A** | **T** | **M** | **I** | **E** | **R** | **F** | **D** | **L** | **S** | **D** | **R** | **Q** | **S** | **Q** | **A** | **I** | **L** | **D** | **M** | **R** | **L** | **R** | **R** | **L** | **T** | **G** | **L** | **E** | **R** | **E** | **K** | **I** | **E** | **N** | **E** | **Y** | **Q** | **E** | **L** | **L** | **K** | **L** | **I** | **E** | **D** | **L** | **T** | **D** | **I** | **L** | **A** | **R** | **P** | **E** | **R** | **V** | **T** | **E** | **I** | **I** | **K** | **N** | **E** | **L** | **A** | **D** | **L** | **N** | **Q** | **R** | **F** | **G** | **D** | **K** | **R** | **R** | **T** | **E** | **L** | **L** | **V** | **G** | **E** | **V** | **L** | **S** | **L** | **E** | **D** | **E** | **D** | **L** | **I** | **E** | **E** | **E** | **E** | **I** | **V** | **I** | **T** | **L** | **T** | **N** | **N** | **G** | **Y** | **I** | **K** | **R** | **L** | **A** | **N** | **N** | **E** | **F** | **R** | **A** | **Q** | **R** | **R** | **G** | **G** | **R** | **G** | **V** | **Q** | **G** | **M** | **G** | **I** | **H** | **N** | **D** | **D** | **F** | **V** | **K** | **N** | **L** | **V** | **S** | **C** | **S** | **T** | **H** | **D** | **T** | **L** | **L** | **F** | **F** | **T** | **N** | **N** | **G** | **K** | **V** | **Y** | **R** | **A** | **K** | **G** | **Y** | **E** | **I** | **P** | **E** | **Y** | **G** | **R** | **T** | **A** | **K** | **G** | **I** | **P** | **I** | **I** | **N** | **L** | **L** | **G** | **I** | **D** | **S** | **S** | **E** | **S** | **I** | **Q** | **A** | **I** | **I** | **A** | **V** | **T** | **G** | **E** | **A** | **E** | **E** | **G** | **H** | **Y** | **L** | **F** | **F** | **T** | **T** | **R** | **Q** | **G** | **T** | **V** | **K** | **R** | **T** | **S** | **V** | **T** | **A** | **F** | **A** | **N** | **I** | **R** | **S** | **N** | **G** | **L** | **I** | **A** | **I** | **G** | **L** | **K** | **E** | **D** | **D** | **E** | **L** | **V** | **N** | **V** | **L** | **L** | **T** | **D** | **G** | **Q** | **S** | **N** | **I** | **I** | **I** | **G** | **T** | **H** | **N** | **G** | **Y** | **S** | **V** | **T** | **F** | **K** | **E** | **E** | **A** | **V** | **R** | **D** | **M** | **G** | **R** | **T** | **A** | **S** | **G** | **V** | **R** | **G** | **I** | **R** | **L** | **R** | **E** | **E** | **D** | **Y** | **V** | **V** | **G** | **A** | **A** | **L** | **M** | **K** | **E** | **N** | **Q** | **E** | **V** | **L** | **V** | **I** | **T** | **E** | **K** | **G** | **Y** | **G** | **K** | **R** | **T** | **K** | **V** | **S** | **E** | **Y** | **P** | **V** | **K** | **G** | **R** | **G** | **G** | **K** | **G** | **I** | **K** | **T** | **A** | **N** | **I** | **T** | **E** | **K** | **N** | **G** | **P** | **L** | **A** | **G** | **L** | **T** | **T** | **V** | **S** | **G** | **D** | **E** | **D** | **I** | **M** | **L** | **I** | **T** | **D** | **K** | **G** | **V** | **I** | **I** | **R** | **F** | **N** | **V** | **S** | **T** | **V** | **S** | **Q** | **T** | **G** | **R** | **S** | **T** | **L** | **G** | **V** | **R** | **L** | **M** | **K** | **M** | **E** | **A** | **D** | **T** | **K** | **V** | **V** | **T** | **M** | **A** | **A** | **V** | **E** | **P** | **E** | **A** | **A** | **E** | **Q** | **T** | **V** | **E** | **E** | **Q** | **G** | **S** | **E** | **E** |
| **N59435F** | **M** | **S** | **E** | **E** | **I** | **R** | **E** | **N** | **I** | **H** | **D** | **V** | **N** | **L** | **T** | **S** | **E** | **M** | **K** | **D** | **S** | **F** | **I** | **D** | **Y** | **A** | **M** | **S** | **V** | **I** | **V** | **A** | **R** | **A** | **L** | **P** | **D** | **V** | **R** | **D** | **G** | **L** | **K** | **P** | **V** | **H** | **R** | **R** | **I** | **L** | **Y** | **G** | **M** | **N** | **E** | **L** | **G** | **V** | **T** | **P** | **D** | **K** | **A** | **H** | **K** | **K** | **S** | **A** | **R** | **I** | **V** | **G** | **D** | **V** | **M** | **G** | **K** | **Y** | **H** | **P** | **H** | **G** | **D** | **S** | **A** | **I** | **Y** | **E** | **S** | **M** | **V** | **R** | **M** | **A** | **Q** | **P** | **F** | **S** | **Y** | **R** | **Y** | **M** | **L** | **V** | **D** | **G** | **H** | **G** | **N** | **F** | **G** | **S** | **V** | **D** | **G** | **D** | **G** | **A** | **A** | **A** | **M** | **R** | **Y** | **T** | **E** | **A** | **R** | **M** | **S** | **K** | **I** | **A** | **T** | **E** | **M** | **L** | **R** | **D** | **I** | **N** | **K** | **N** | **T** | **V** | **D** | **F** | **Q** | **S** | **N** | **Y** | **D** | **D** | **T** | **E** | **K** | **E** | **P** | **V** | **V** | **L** | **P** | **A** | **R** | **F** | **P** | **N** | **L** | **L** | **V** | **N** | **G** | **T** | **T** | **G** | **I** | **A** | **V** | **G** | **M** | **A** | **T** | **N** | **I** | **P** | **P** | **H** | **N** | **L** | **S** | **E** | **V** | **V** | **A** | **A** | **I** | **D** | **L** | **L** | **M** | **D** | **N** | **P** | **D** | **V** | **T** | **T** | **N** | **E** | **L** | **M** | **E** | **V** | **L** | **P** | **G** | **P** | **D** | **F** | **P** | **T** | **G** | **G** | **L** | **V** | **M** | **G** | **K** | **S** | **G** | **I** | **R** | **R** | **A** | **Y** | **E** | **T** | **G** | **K** | **G** | **S** | **I** | **T** | **V** | **R** | **A** | **K** | **V** | **E** | **I** | **T** | **E** | **M** | **P** | **N** | **G** | **K** | **E** | **R** | **I** | **I** | **V** | **T** | **E** | **L** | **P** | **Y** | **M** | **V** | **N** | **K** | **A** | **K** | **L** | **I** | **E** | **R** | **I** | **S** | **E** | **L** | **H** | **R** | **D** | **K** | **R** | **I** | **E** | **G** | **I** | **T** | **D** | **L** | **R** | **D** | **E** | **S** | **S** | **R** | **E** | **G** | **M** | **R** | **I** | **V** | **I** | **D** | **I** | **R** | **R** | **D** | **A** | **S** | **A** | **S** | **V** | **I** | **L** | **N** | **N** | **L** | **Y** | **K** | **L** | **T** | **S** | **L** | **Q** | **N** | **S** | **F** | **G** | **F** | **N** | **M** | **L** | **A** | **I** | **E** | **K** | **G** | **V** | **P** | **K** | **V** | **L** | **S** | **L** | **K** | **Q** | **I** | **L** | **E** | **N** | **Y** | **I** | **E** | **H** | **Q** | **R** | **E** | **V** | **I** | **T** | **R** | **R** | **T** | **V** | **F** | **E** | **K** | **E** | **K** | **A** | **E** | **A** | **R** | **A** | **H** | **I** | **L** | **E** | **G** | **L** | **R** | **I** | **A** | **L** | **D** | **H** | **I** | **D** | **E** | **I** | **I** | **A** | **I** | **I** | **R** | **G** | **S** | **K** | **S** | **D** | **D** | **E** | **A** | **K** | **A** | **T** | **M** | **I** | **E** | **R** | **F** | **D** | **L** | **S** | **D** | **R** | **Q** | **S** | **Q** | **A** | **I** | **L** | **D** | **M** | **R** | **L** | **R** | **R** | **L** | **T** | **G** | **L** | **E** | **R** | **E** | **K** | **I** | **E** | **N** | **E** | **Y** | **Q** | **E** | **L** | **L** | **K** | **L** | **I** | **E** | **D** | **L** | **T** | **D** | **I** | **L** | **A** | **R** | **P** | **E** | **R** | **V** | **T** | **E** | **I** | **I** | **K** | **N** | **E** | **L** | **A** | **D** | **L** | **N** | **Q** | **R** | **F** | **G** | **D** | **K** | **R** | **R** | **T** | **E** | **L** | **L** | **V** | **G** | **E** | **V** | **L** | **S** | **L** | **E** | **D** | **E** | **D** | **L** | **I** | **E** | **E** | **E** | **E** | **I** | **V** | **I** | **T** | **L** | **T** | **N** | **N** | **G** | **Y** | **I** | **K** | **R** | **L** | **A** | **N** | **N** | **E** | **F** | **R** | **A** | **Q** | **R** | **R** | **G** | **G** | **R** | **G** | **V** | **Q** | **G** | **M** | **G** | **I** | **H** | **N** | **D** | **D** | **F** | **V** | **K** | **N** | **L** | **V** | **S** | **C** | **S** | **T** | **H** | **D** | **T** | **L** | **L** | **F** | **F** | **T** | **N** | **N** | **G** | **K** | **V** | **Y** | **R** | **A** | **K** | **G** | **Y** | **E** | **I** | **P** | **E** | **Y** | **G** | **R** | **T** | **A** | **K** | **G** | **I** | **P** | **I** | **I** | **N** | **L** | **L** | **G** | **I** | **D** | **S** | **S** | **E** | **S** | **I** | **Q** | **A** | **I** | **I** | **A** | **V** | **T** | **G** | **E** | **A** | **E** | **E** | **G** | **H** | **Y** | **L** | **F** | **F** | **T** | **T** | **R** | **Q** | **G** | **T** | **V** | **K** | **R** | **T** | **S** | **V** | **T** | **A** | **F** | **A** | **N** | **I** | **R** | **S** | **N** | **G** | **L** | **I** | **A** | **I** | **G** | **L** | **K** | **E** | **D** | **D** | **E** | **L** | **V** | **N** | **V** | **L** | **L** | **T** | **D** | **G** | **Q** | **S** | **N** | **I** | **I** | **I** | **G** | **T** | **H** | **N** | **G** | **Y** | **S** | **V** | **T** | **F** | **K** | **E** | **E** | **A** | **V** | **R** | **D** | **M** | **G** | **R** | **T** | **A** | **S** | **G** | **V** | **R** | **G** | **I** | **R** | **L** | **R** | **E** | **E** | **D** | **Y** | **V** | **V** | **G** | **A** | **A** | **L** | **M** | **K** | **E** | **D** | **Q** | **E** | **V** | **L** | **V** | **I** | **T** | **E** | **K** | **G** | **Y** | **G** | **K** | **R** | **T** | **K** | **V** | **S** | **E** | **Y** | **P** | **V** | **K** | **G** | **R** | **G** | **G** | **K** | **G** | **I** | **K** | **T** | **A** | **N** | **I** | **T** | **E** | **K** | **N** | **G** | **P** | **L** | **A** | **G** | **L** | **T** | **T** | **V** | **S** | **G** | **D** | **E** | **D** | **I** | **M** | **L** | **I** | **T** | **D** | **K** | **G** | **V** | **I** | **I** | **R** | **F** | **N** | **V** | **S** | **T** | **V** | **S** | **Q** | **T** | **G** | **R** | **S** | **T** | **L** | **G** | **V** | **R** | **L** | **M** | **K** | **M** | **E** | **A** | **D** | **T** | **K** | **V** | **V** | **T** | **M** | **A** | **A** | **V** | **E** | **P** | **E** | **A** | **A** | **E** | **Q** | **T** | **V** | **E** | **E** | **Q** | **G** | **S** | **E** | **E** |
| **N54107** | **M** | **S** | **E** | **E** | **I** | **R** | **E** | **N** | **I** | **H** | **D** | **V** | **N** | **L** | **T** | **S** | **E** | **M** | **K** | **D** | **S** | **F** | **I** | **D** | **Y** | **A** | **M** | **S** | **V** | **I** | **V** | **A** | **R** | **A** | **L** | **P** | **D** | **V** | **R** | **D** | **G** | **L** | **K** | **P** | **V** | **H** | **R** | **R** | **I** | **L** | **Y** | **G** | **M** | **N** | **E** | **L** | **G** | **V** | **T** | **P** | **D** | **K** | **A** | **H** | **K** | **K** | **S** | **A** | **R** | **I** | **V** | **G** | **D** | **V** | **M** | **G** | **K** | **Y** | **H** | **P** | **H** | **G** | **D** | **S** | **A** | **I** | **Y** | **E** | **S** | **M** | **V** | **R** | **M** | **A** | **Q** | **P** | **F** | **S** | **Y** | **R** | **Y** | **M** | **L** | **V** | **D** | **G** | **H** | **G** | **N** | **F** | **G** | **S** | **V** | **D** | **G** | **D** | **G** | **A** | **A** | **A** | **M** | **R** | **Y** | **T** | **E** | **A** | **R** | **M** | **S** | **K** | **I** | **A** | **T** | **E** | **M** | **L** | **R** | **D** | **I** | **N** | **K** | **N** | **T** | **V** | **D** | **F** | **Q** | **S** | **N** | **Y** | **D** | **D** | **T** | **E** | **K** | **E** | **P** | **V** | **V** | **L** | **P** | **A** | **R** | **F** | **P** | **N** | **L** | **L** | **V** | **N** | **G** | **T** | **T** | **G** | **I** | **A** | **V** | **G** | **M** | **A** | **T** | **N** | **I** | **P** | **P** | **H** | **N** | **L** | **S** | **E** | **V** | **V** | **A** | **A** | **I** | **D** | **L** | **L** | **M** | **D** | **N** | **P** | **D** | **V** | **T** | **T** | **N** | **E** | **L** | **M** | **E** | **V** | **L** | **P** | **G** | **P** | **D** | **F** | **P** | **T** | **G** | **G** | **L** | **V** | **M** | **G** | **K** | **S** | **G** | **I** | **R** | **R** | **A** | **Y** | **E** | **T** | **G** | **K** | **G** | **S** | **I** | **T** | **V** | **R** | **A** | **K** | **V** | **E** | **I** | **T** | **E** | **M** | **P** | **N** | **G** | **K** | **E** | **R** | **I** | **I** | **V** | **T** | **E** | **L** | **P** | **Y** | **M** | **V** | **N** | **K** | **A** | **K** | **L** | **I** | **E** | **R** | **I** | **S** | **E** | **L** | **H** | **R** | **D** | **K** | **R** | **I** | **E** | **G** | **I** | **T** | **D** | **L** | **R** | **D** | **E** | **S** | **S** | **R** | **E** | **G** | **M** | **R** | **I** | **V** | **I** | **D** | **I** | **R** | **R** | **D** | **A** | **S** | **A** | **S** | **V** | **I** | **L** | **N** | **N** | **L** | **Y** | **K** | **L** | **T** | **S** | **L** | **Q** | **N** | **S** | **F** | **G** | **F** | **N** | **M** | **L** | **A** | **I** | **E** | **K** | **G** | **V** | **P** | **K** | **V** | **L** | **S** | **L** | **K** | **Q** | **I** | **L** | **E** | **N** | **Y** | **I** | **E** | **H** | **Q** | **R** | **E** | **V** | **I** | **T** | **R** | **R** | **T** | **V** | **F** | **E** | **K** | **E** | **K** | **A** | **E** | **A** | **R** | **A** | **H** | **I** | **L** | **E** | **G** | **L** | **R** | **I** | **A** | **L** | **D** | **H** | **I** | **D** | **E** | **I** | **I** | **A** | **I** | **I** | **R** | **G** | **S** | **K** | **S** | **D** | **D** | **E** | **A** | **K** | **A** | **T** | **M** | **I** | **E** | **R** | **F** | **D** | **L** | **S** | **D** | **R** | **Q** | **S** | **Q** | **A** | **I** | **L** | **D** | **M** | **R** | **L** | **R** | **R** | **L** | **T** | **G** | **L** | **E** | **R** | **E** | **K** | **I** | **E** | **N** | **E** | **Y** | **Q** | **E** | **L** | **L** | **K** | **L** | **I** | **E** | **D** | **L** | **T** | **D** | **I** | **L** | **A** | **R** | **P** | **E** | **R** | **V** | **T** | **E** | **I** | **I** | **K** | **N** | **E** | **L** | **A** | **D** | **L** | **N** | **Q** | **R** | **F** | **G** | **D** | **K** | **R** | **R** | **T** | **E** | **L** | **L** | **V** | **G** | **E** | **V** | **L** | **S** | **L** | **E** | **D** | **E** | **D** | **L** | **I** | **E** | **E** | **E** | **E** | **I** | **V** | **I** | **T** | **L** | **T** | **N** | **N** | **G** | **Y** | **I** | **K** | **R** | **L** | **A** | **N** | **N** | **E** | **F** | **R** | **A** | **Q** | **R** | **R** | **G** | **G** | **R** | **G** | **V** | **Q** | **G** | **M** | **G** | **I** | **H** | **N** | **D** | **D** | **F** | **V** | **K** | **N** | **L** | **V** | **S** | **C** | **S** | **T** | **H** | **D** | **T** | **L** | **L** | **F** | **F** | **T** | **N** | **N** | **G** | **K** | **V** | **Y** | **R** | **A** | **K** | **G** | **Y** | **E** | **I** | **P** | **E** | **Y** | **G** | **R** | **T** | **A** | **K** | **G** | **I** | **P** | **I** | **I** | **N** | **L** | **L** | **G** | **I** | **D** | **S** | **S** | **E** | **S** | **I** | **Q** | **A** | **I** | **I** | **A** | **V** | **T** | **G** | **E** | **A** | **E** | **E** | **G** | **H** | **Y** | **L** | **F** | **F** | **T** | **T** | **R** | **Q** | **G** | **T** | **V** | **K** | **R** | **T** | **S** | **V** | **T** | **A** | **F** | **A** | **N** | **I** | **R** | **S** | **N** | **G** | **L** | **I** | **A** | **I** | **G** | **L** | **K** | **E** | **D** | **D** | **E** | **L** | **V** | **N** | **V** | **L** | **L** | **T** | **D** | **G** | **Q** | **S** | **N** | **I** | **I** | **I** | **G** | **T** | **H** | **N** | **G** | **Y** | **S** | **V** | **T** | **F** | **K** | **E** | **E** | **A** | **V** | **R** | **D** | **M** | **G** | **R** | **T** | **A** | **S** | **G** | **V** | **R** | **G** | **I** | **R** | **L** | **R** | **E** | **E** | **D** | **Y** | **V** | **V** | **G** | **A** | **A** | **L** | **M** | **K** | **E** | **N** | **Q** | **E** | **V** | **L** | **V** | **I** | **T** | **E** | **K** | **G** | **Y** | **G** | **K** | **R** | **T** | **K** | **V** | **S** | **E** | **Y** | **P** | **V** | **K** | **G** | **R** | **G** | **G** | **K** | **G** | **I** | **K** | **T** | **A** | **N** | **I** | **T** | **E** | **K** | **N** | **G** | **P** | **L** | **A** | **G** | **L** | **T** | **T** | **V** | **S** | **G** | **D** | **E** | **D** | **I** | **M** | **L** | **I** | **T** | **D** | **K** | **G** | **V** | **I** | **I** | **R** | **F** | **N** | **V** | **S** | **T** | **V** | **S** | **Q** | **T** | **G** | **R** | **S** | **T** | **L** | **G** | **V** | **R** | **L** | **M** | **K** | **M** | **E** | **A** | **D** | **T** | **K** | **V** | **V** | **T** | **M** | **A** | **A** | **V** | **E** | **P** | **E** | **A** | **A** | **E** | **Q** | **T** | **V** | **E** | **E** | **Q** | **G** | **S** | **E** | **E** |
| **N59584F** | **M** | **S** | **E** | **E** | **I** | **K** | **E** | **N** | **I** | **Q** | **D** | **V** | **N** | **L** | **T** | **S** | **E** | **M** | **K** | **E** | **S** | **F** | **I** | **D** | **Y** | **A** | **M** | **S** | **V** | **I** | **V** | **A** | **R** | **A** | **L** | **P** | **D** | **V** | **R** | **D** | **G** | **L** | **K** | **P** | **V** | **H** | **R** | **R** | **I** | **L** | **Y** | **G** | **M** | **N** | **E** | **L** | **G** | **V** | **T** | **P** | **D** | **K** | **P** | **H** | **K** | **K** | **S** | **A** | **R** | **I** | **V** | **G** | **D** | **V** | **M** | **G** | **K** | **Y** | **H** | **P** | **H** | **G** | **D** | **S** | **A** | **I** | **Y** | **E** | **S** | **M** | **V** | **R** | **M** | **A** | **Q** | **P** | **F** | **S** | **Y** | **R** | **A** | **M** | **L** | **V** | **D** | **G** | **H** | **G** | **N** | **F** | **G** | **S** | **V** | **D** | **G** | **D** | **G** | **A** | **A** | **A** | **M** | **R** | **Y** | **T** | **E** | **A** | **R** | **M** | **S** | **K** | **I** | **A** | **L** | **E** | **M** | **L** | **R** | **D** | **I** | **N** | **K** | **N** | **T** | **V** | **D** | **F** | **Q** | **G** | **N** | **Y** | **D** | **D** | **S** | **E** | **Q** | **E** | **P** | **V** | **V** | **L** | **P** | **A** | **R** | **F** | **P** | **N** | **L** | **L** | **V** | **N** | **G** | **T** | **T** | **G** | **I** | **A** | **V** | **G** | **M** | **A** | **T** | **N** | **I** | **P** | **P** | **H** | **N** | **L** | **S** | **E** | **V** | **I** | **D** | **A** | **T** | **S** | **L** | **L** | **M** | **D** | **N** | **P** | **D** | **V** | **T** | **T** | **N** | **E** | **L** | **M** | **E** | **V** | **L** | **P** | **G** | **P** | **D** | **F** | **P** | **T** | **G** | **G** | **L** | **V** | **M** | **G** | **K** | **S** | **G** | **I** | **R** | **R** | **A** | **Y** | **E** | **T** | **G** | **K** | **G** | **S** | **I** | **T** | **V** | **R** | **A** | **K** | **V** | **E** | **L** | **T** | **E** | **M** | **P** | **N** | **G** | **K** | **E** | **R** | **I** | **L** | **V** | **T** | **E** | **L** | **P** | **Y** | **M** | **V** | **N** | **K** | **A** | **K** | **L** | **I** | **E** | **R** | **I** | **S** | **E** | **L** | **H** | **R** | **D** | **K** | **R** | **I** | **E** | **G** | **I** | **T** | **D** | **L** | **R** | **D** | **E** | **S** | **S** | **R** | **E** | **G** | **M** | **R** | **I** | **V** | **I** | **D** | **V** | **R** | **R** | **D** | **V** | **S** | **A** | **S** | **V** | **V** | **L** | **N** | **N** | **L** | **Y** | **K** | **M** | **T** | **A** | **L** | **Q** | **T** | **S** | **F** | **G** | **F** | **N** | **M** | **L** | **A** | **I** | **E** | **K** | **G** | **V** | **P** | **K** | **I** | **L** | **S** | **L** | **K** | **R** | **I** | **L** | **E** | **N** | **Y** | **V** | **E** | **H** | **Q** | **K** | **E** | **V** | **I** | **T** | **R** | **R** | **T** | **I** | **F** | **D** | **K** | **N** | **K** | **A** | **E** | **A** | **R** | **A** | **H** | **I** | **L** | **E** | **G** | **L** | **R** | **I** | **A** | **L** | **D** | **H** | **I** | **D** | **E** | **I** | **I** | **A** | **I** | **I** | **R** | **G** | **S** | **Q** | **S** | **D** | **D** | **E** | **A** | **K** | **A** | **T** | **L** | **I** | **E** | **R** | **F** | **E** | **F** | **S** | **D** | **R** | **Q** | **A** | **Q** | **A** | **I** | **L** | **D** | **M** | **R** | **L** | **R** | **R** | **L** | **T** | **G** | **L** | **E** | **R** | **D** | **K** | **I** | **E** | **N** | **E** | **Y** | **Q** | **E** | **L** | **L** | **K** | **F** | **I** | **A** | **D** | **L** | **E** | **D** | **I** | **L** | **A** | **R** | **P** | **E** | **R** | **V** | **I** | **E** | **I** | **I** | **K** | **T** | **E** | **L** | **N** | **D** | **V** | **R** | **T** | **K** | **F** | **G** | **D** | **A** | **R** | **R** | **T** | **E** | **L** | **L** | **V** | **G** | **E** | **V** | **L** | **S** | **L** | **E** | **D** | **E** | **D** | **L** | **I** | **E** | **E** | **E** | **E** | **V** | **V** | **I** | **T** | **L** | **T** | **N** | **N** | **G** | **Y** | **I** | **K** | **R** | **M** | **A** | **N** | **S** | **E** | **F** | **R** | **A** | **Q** | **R** | **R** | **G** | **G** | **R** | **G** | **V** | **Q** | **G** | **M** | **G** | **V** | **H** | **D** | **D** | **D** | **F** | **V** | **K** | **N** | **L** | **V** | **S** | **C** | **S** | **T** | **H** | **D** | **T** | **L** | **L** | **F** | **F** | **T** | **N** | **T** | **G** | **K** | **V** | **Y** | **R** | **A** | **K** | **G** | **Y** | **E** | **I** | **P** | **E** | **Y** | **G** | **R** | **T** | **A** | **K** | **G** | **I** | **P** | **V** | **I** | **N** | **L** | **L** | **G** | **I** | **D** | **S** | **A** | **E** | **K** | **I** | **Q** | **A** | **I** | **I** | **S** | **V** | **E** | **G** | **K** | **A** | **E** | **A** | **G** | **K** | **Y** | **L** | **F** | **F** | **T** | **T** | **L** | **K** | **G** | **T** | **V** | **K** | **R** | **T** | **A** | **V** | **T** | **A** | **F** | **S** | **N** | **I** | **R** | **S** | **N** | **G** | **L** | **I** | **A** | **I** | **S** | **L** | **K** | **E** | **D** | **D** | **E** | **L** | **V** | **N** | **V** | **V** | **T** | **T** | **N** | **G** | **N** | **Q** | **K** | **M** | **I** | **I** | **G** | **T** | **H** | **A** | **G** | **Y** | **S** | **V** | **T** | **F** | **D** | **E** | **N** | **T** | **V** | **R** | **D** | **M** | **G** | **R** | **T** | **A** | **S** | **G** | **V** | **R** | **G** | **I** | **R** | **L** | **R** | **E** | **N** | **D** | **Y** | **V** | **V** | **G** | **A** | **A** | **I** | **L** | **D** | **E** | **N** | **K** | **E** | **V** | **L** | **V** | **I** | **T** | **E** | **N** | **G** | **Y** | **G** | **K** | **R** | **T** | **K** | **A** | **S** | **E** | **Y** | **P** | **V** | **K** | **G** | **R** | **G** | **G** | **K** | **G** | **I** | **K** | **T** | **A** | **N** | **I** | **T** | **E** | **K** | **N** | **G** | **P** | **L** | **A** | **G** | **L** | **T** | **T** | **V** | **N** | **G** | **D** | **E** | **D** | **I** | **L** | **L** | **I** | **T** | **N** | **K** | **G** | **V** | **I** | **I** | **R** | **F** | **N** | **V** | **D** | **S** | **V** | **S** | **Q** | **T** | **G** | **R** | **A** | **T** | **L** | **G** | **V** | **R** | **L** | **M** | **R** | **M** | **E** | **D** | **G** | **A** | **K** | **-** | **-** | **-** | **-** | **-** | **-** | **-** | **-** | **-** | **-** | **-** | **-** | **-** | **-** | **-** | **-** | **-** | **-** | **-** | **-** | **-** | **-** | **-** |
| **N52755** | **M** | **S** | **E** | **E** | **I** | **K** | **E** | **N** | **I** | **Q** | **D** | **V** | **N** | **L** | **T** | **S** | **E** | **M** | **K** | **E** | **S** | **F** | **I** | **D** | **Y** | **A** | **M** | **S** | **V** | **I** | **V** | **A** | **R** | **A** | **L** | **P** | **D** | **V** | **R** | **D** | **G** | **L** | **K** | **P** | **V** | **H** | **R** | **R** | **I** | **L** | **Y** | **G** | **M** | **N** | **E** | **L** | **G** | **V** | **T** | **P** | **D** | **K** | **P** | **H** | **K** | **K** | **S** | **A** | **R** | **I** | **V** | **G** | **D** | **V** | **M** | **G** | **K** | **Y** | **H** | **P** | **H** | **G** | **D** | **S** | **A** | **I** | **Y** | **E** | **S** | **M** | **V** | **R** | **M** | **A** | **Q** | **P** | **F** | **S** | **Y** | **R** | **A** | **M** | **L** | **V** | **D** | **G** | **H** | **G** | **N** | **F** | **G** | **S** | **V** | **D** | **G** | **D** | **G** | **A** | **A** | **A** | **M** | **R** | **Y** | **T** | **E** | **A** | **R** | **M** | **S** | **K** | **I** | **A** | **L** | **E** | **M** | **L** | **R** | **D** | **I** | **N** | **K** | **N** | **T** | **V** | **D** | **F** | **Q** | **G** | **N** | **Y** | **D** | **D** | **S** | **E** | **Q** | **E** | **P** | **V** | **V** | **L** | **P** | **A** | **R** | **F** | **P** | **N** | **L** | **L** | **V** | **N** | **G** | **T** | **T** | **G** | **I** | **A** | **V** | **G** | **M** | **A** | **T** | **N** | **I** | **P** | **P** | **H** | **N** | **L** | **S** | **E** | **V** | **I** | **D** | **A** | **T** | **S** | **L** | **L** | **M** | **D** | **N** | **P** | **D** | **V** | **T** | **T** | **N** | **E** | **L** | **M** | **E** | **V** | **L** | **P** | **G** | **P** | **D** | **F** | **P** | **T** | **G** | **G** | **L** | **V** | **M** | **G** | **K** | **S** | **G** | **I** | **R** | **R** | **A** | **Y** | **E** | **T** | **G** | **K** | **G** | **S** | **I** | **T** | **V** | **R** | **A** | **K** | **V** | **E** | **L** | **T** | **E** | **M** | **P** | **N** | **G** | **K** | **E** | **R** | **I** | **L** | **V** | **T** | **E** | **L** | **P** | **Y** | **M** | **V** | **N** | **K** | **A** | **K** | **L** | **I** | **E** | **R** | **I** | **S** | **E** | **L** | **H** | **R** | **D** | **K** | **R** | **I** | **E** | **G** | **I** | **T** | **D** | **L** | **R** | **D** | **E** | **S** | **S** | **R** | **E** | **G** | **M** | **R** | **I** | **V** | **I** | **D** | **V** | **R** | **R** | **D** | **V** | **S** | **A** | **S** | **V** | **V** | **L** | **N** | **N** | **L** | **Y** | **K** | **M** | **T** | **A** | **L** | **Q** | **T** | **S** | **F** | **G** | **F** | **N** | **M** | **L** | **A** | **I** | **E** | **K** | **G** | **V** | **P** | **K** | **I** | **L** | **S** | **L** | **K** | **R** | **I** | **L** | **E** | **N** | **Y** | **V** | **E** | **H** | **Q** | **K** | **E** | **V** | **I** | **T** | **R** | **R** | **T** | **I** | **F** | **D** | **K** | **N** | **K** | **A** | **E** | **A** | **R** | **A** | **H** | **I** | **L** | **E** | **G** | **L** | **R** | **I** | **A** | **L** | **D** | **H** | **I** | **D** | **E** | **I** | **I** | **A** | **I** | **I** | **R** | **G** | **S** | **Q** | **S** | **D** | **D** | **E** | **A** | **K** | **A** | **T** | **L** | **I** | **E** | **R** | **F** | **E** | **F** | **S** | **D** | **R** | **Q** | **A** | **Q** | **A** | **I** | **L** | **D** | **M** | **R** | **L** | **R** | **R** | **L** | **T** | **G** | **L** | **E** | **R** | **D** | **K** | **I** | **E** | **N** | **E** | **Y** | **Q** | **E** | **L** | **L** | **K** | **F** | **I** | **A** | **D** | **L** | **E** | **D** | **I** | **L** | **A** | **R** | **P** | **E** | **R** | **V** | **I** | **E** | **I** | **I** | **K** | **T** | **E** | **L** | **N** | **D** | **V** | **R** | **T** | **K** | **F** | **G** | **D** | **A** | **R** | **R** | **T** | **E** | **L** | **L** | **V** | **G** | **E** | **V** | **L** | **S** | **L** | **E** | **D** | **E** | **D** | **L** | **I** | **E** | **E** | **E** | **E** | **V** | **V** | **I** | **T** | **L** | **T** | **N** | **N** | **G** | **Y** | **I** | **K** | **R** | **M** | **V** | **N** | **S** | **E** | **F** | **R** | **A** | **Q** | **R** | **R** | **G** | **G** | **R** | **G** | **V** | **Q** | **G** | **M** | **G** | **V** | **H** | **D** | **D** | **D** | **F** | **V** | **K** | **N** | **L** | **V** | **S** | **C** | **S** | **T** | **H** | **D** | **T** | **L** | **L** | **F** | **F** | **T** | **N** | **T** | **G** | **K** | **V** | **Y** | **R** | **A** | **K** | **G** | **Y** | **E** | **I** | **P** | **E** | **Y** | **G** | **R** | **T** | **A** | **K** | **G** | **I** | **P** | **V** | **I** | **N** | **L** | **L** | **G** | **I** | **D** | **S** | **A** | **E** | **K** | **I** | **Q** | **A** | **I** | **I** | **S** | **V** | **E** | **G** | **K** | **A** | **E** | **A** | **G** | **K** | **Y** | **L** | **F** | **F** | **T** | **T** | **L** | **K** | **G** | **T** | **V** | **K** | **R** | **T** | **A** | **V** | **T** | **A** | **F** | **S** | **N** | **I** | **R** | **S** | **N** | **G** | **L** | **I** | **A** | **I** | **S** | **L** | **K** | **E** | **D** | **D** | **E** | **L** | **V** | **N** | **V** | **V** | **T** | **T** | **N** | **G** | **N** | **Q** | **K** | **M** | **I** | **I** | **G** | **T** | **H** | **A** | **G** | **Y** | **S** | **V** | **T** | **F** | **D** | **E** | **N** | **T** | **V** | **R** | **D** | **M** | **G** | **R** | **T** | **A** | **S** | **G** | **V** | **R** | **G** | **I** | **R** | **L** | **R** | **E** | **N** | **D** | **Y** | **V** | **V** | **G** | **A** | **A** | **I** | **L** | **D** | **E** | **N** | **K** | **E** | **V** | **L** | **V** | **I** | **T** | **E** | **N** | **G** | **Y** | **G** | **K** | **R** | **T** | **K** | **A** | **S** | **E** | **Y** | **P** | **V** | **K** | **G** | **R** | **G** | **G** | **K** | **G** | **I** | **K** | **T** | **A** | **N** | **I** | **T** | **E** | **K** | **N** | **G** | **P** | **L** | **A** | **G** | **L** | **T** | **T** | **V** | **N** | **G** | **D** | **E** | **D** | **I** | **L** | **L** | **I** | **T** | **N** | **K** | **G** | **V** | **I** | **I** | **R** | **F** | **N** | **V** | **D** | **S** | **V** | **S** | **Q** | **T** | **G** | **R** | **A** | **T** | **L** | **G** | **V** | **R** | **L** | **M** | **R** | **M** | **E** | **D** | **G** | **A** | **K** | **-** | **-** | **-** | **-** | **-** | **-** | **-** | **-** | **-** | **-** | **-** | **-** | **-** | **-** | **-** | **-** | **-** | **-** | **-** | **-** | **-** | **-** | **-** |
| **N60100F** | **M** | **S** | **E** | **E** | **I** | **K** | **E** | **N** | **I** | **Q** | **D** | **V** | **N** | **L** | **T** | **S** | **E** | **M** | **K** | **E** | **S** | **F** | **I** | **D** | **Y** | **A** | **M** | **S** | **V** | **I** | **V** | **A** | **R** | **A** | **L** | **P** | **D** | **V** | **R** | **D** | **G** | **L** | **K** | **P** | **V** | **H** | **R** | **R** | **I** | **L** | **Y** | **G** | **M** | **N** | **E** | **L** | **G** | **V** | **T** | **P** | **D** | **K** | **P** | **H** | **K** | **K** | **S** | **A** | **R** | **I** | **V** | **G** | **D** | **V** | **M** | **G** | **K** | **Y** | **H** | **P** | **H** | **G** | **D** | **S** | **A** | **I** | **Y** | **E** | **S** | **M** | **V** | **R** | **M** | **A** | **Q** | **P** | **F** | **S** | **Y** | **R** | **A** | **M** | **L** | **V** | **D** | **G** | **H** | **G** | **N** | **F** | **G** | **S** | **V** | **D** | **G** | **D** | **G** | **A** | **A** | **A** | **M** | **R** | **Y** | **T** | **E** | **A** | **R** | **M** | **S** | **K** | **I** | **A** | **L** | **E** | **M** | **L** | **R** | **D** | **I** | **N** | **K** | **N** | **T** | **V** | **D** | **F** | **Q** | **G** | **N** | **Y** | **D** | **D** | **S** | **E** | **Q** | **E** | **P** | **V** | **V** | **L** | **P** | **A** | **R** | **F** | **P** | **N** | **L** | **L** | **V** | **N** | **G** | **T** | **T** | **G** | **I** | **A** | **V** | **G** | **M** | **A** | **T** | **N** | **I** | **P** | **P** | **H** | **N** | **L** | **S** | **E** | **V** | **I** | **D** | **A** | **T** | **S** | **L** | **L** | **M** | **D** | **N** | **P** | **D** | **V** | **T** | **T** | **N** | **E** | **L** | **M** | **E** | **V** | **L** | **P** | **G** | **P** | **D** | **F** | **P** | **T** | **G** | **G** | **L** | **V** | **M** | **G** | **K** | **S** | **G** | **I** | **R** | **R** | **A** | **Y** | **E** | **T** | **G** | **K** | **G** | **S** | **I** | **T** | **V** | **R** | **A** | **K** | **V** | **E** | **L** | **T** | **E** | **M** | **P** | **N** | **G** | **K** | **E** | **R** | **I** | **L** | **V** | **T** | **E** | **L** | **P** | **Y** | **M** | **V** | **N** | **K** | **A** | **K** | **L** | **I** | **E** | **R** | **I** | **S** | **E** | **L** | **H** | **R** | **D** | **K** | **R** | **I** | **E** | **G** | **I** | **T** | **D** | **L** | **R** | **D** | **E** | **S** | **S** | **R** | **E** | **G** | **M** | **R** | **I** | **V** | **I** | **D** | **V** | **R** | **R** | **D** | **V** | **S** | **A** | **S** | **V** | **V** | **L** | **N** | **N** | **L** | **Y** | **K** | **M** | **T** | **A** | **L** | **Q** | **T** | **S** | **F** | **G** | **F** | **N** | **M** | **L** | **A** | **I** | **E** | **K** | **G** | **V** | **P** | **K** | **I** | **L** | **S** | **L** | **K** | **R** | **I** | **L** | **E** | **N** | **Y** | **V** | **E** | **H** | **Q** | **K** | **E** | **V** | **I** | **T** | **R** | **R** | **T** | **I** | **F** | **D** | **K** | **N** | **K** | **A** | **E** | **A** | **R** | **A** | **H** | **I** | **L** | **E** | **G** | **L** | **R** | **I** | **A** | **L** | **D** | **H** | **I** | **D** | **E** | **I** | **I** | **A** | **I** | **I** | **R** | **G** | **S** | **Q** | **S** | **D** | **D** | **E** | **A** | **K** | **A** | **T** | **L** | **I** | **E** | **R** | **F** | **E** | **F** | **S** | **D** | **R** | **Q** | **A** | **Q** | **A** | **I** | **L** | **D** | **M** | **R** | **L** | **R** | **R** | **L** | **T** | **G** | **L** | **E** | **R** | **D** | **K** | **I** | **E** | **N** | **E** | **Y** | **Q** | **E** | **L** | **L** | **K** | **F** | **I** | **A** | **D** | **L** | **E** | **D** | **I** | **L** | **A** | **R** | **P** | **E** | **R** | **V** | **I** | **E** | **I** | **I** | **K** | **T** | **E** | **L** | **N** | **D** | **V** | **R** | **T** | **K** | **F** | **G** | **D** | **A** | **R** | **R** | **T** | **E** | **L** | **L** | **V** | **G** | **E** | **V** | **L** | **S** | **L** | **E** | **D** | **E** | **D** | **L** | **I** | **E** | **E** | **E** | **E** | **V** | **V** | **I** | **T** | **L** | **T** | **N** | **N** | **G** | **Y** | **I** | **K** | **R** | **M** | **A** | **N** | **S** | **E** | **F** | **R** | **A** | **Q** | **R** | **R** | **G** | **G** | **R** | **G** | **V** | **Q** | **G** | **M** | **G** | **V** | **H** | **D** | **D** | **D** | **F** | **V** | **K** | **N** | **L** | **V** | **S** | **C** | **S** | **T** | **H** | **D** | **T** | **L** | **L** | **F** | **F** | **T** | **N** | **T** | **G** | **K** | **V** | **Y** | **R** | **A** | **K** | **G** | **Y** | **E** | **I** | **P** | **E** | **Y** | **G** | **R** | **T** | **A** | **K** | **G** | **I** | **P** | **V** | **I** | **N** | **L** | **L** | **G** | **I** | **D** | **S** | **A** | **E** | **K** | **I** | **Q** | **A** | **I** | **I** | **S** | **V** | **E** | **G** | **K** | **A** | **E** | **A** | **G** | **K** | **Y** | **L** | **F** | **F** | **T** | **T** | **L** | **K** | **G** | **T** | **V** | **K** | **R** | **T** | **A** | **V** | **T** | **A** | **F** | **S** | **N** | **I** | **R** | **S** | **N** | **G** | **L** | **I** | **A** | **I** | **S** | **L** | **K** | **E** | **D** | **D** | **E** | **L** | **V** | **N** | **V** | **V** | **T** | **T** | **N** | **G** | **N** | **Q** | **K** | **M** | **I** | **I** | **G** | **T** | **H** | **A** | **G** | **Y** | **S** | **V** | **T** | **F** | **D** | **E** | **N** | **T** | **V** | **R** | **D** | **M** | **G** | **R** | **T** | **A** | **S** | **G** | **V** | **R** | **G** | **I** | **R** | **L** | **R** | **E** | **N** | **D** | **Y** | **V** | **V** | **G** | **A** | **A** | **I** | **L** | **D** | **E** | **N** | **K** | **E** | **V** | **L** | **V** | **I** | **T** | **E** | **N** | **G** | **Y** | **G** | **K** | **R** | **T** | **K** | **A** | **S** | **E** | **Y** | **P** | **V** | **K** | **G** | **R** | **G** | **G** | **K** | **G** | **I** | **K** | **T** | **A** | **N** | **I** | **T** | **E** | **K** | **N** | **G** | **P** | **L** | **A** | **G** | **L** | **T** | **T** | **V** | **N** | **G** | **D** | **E** | **D** | **I** | **L** | **L** | **I** | **T** | **N** | **K** | **G** | **V** | **I** | **I** | **R** | **F** | **N** | **V** | **D** | **S** | **V** | **S** | **Q** | **T** | **G** | **R** | **A** | **T** | **L** | **G** | **V** | **R** | **L** | **M** | **R** | **M** | **E** | **D** | **G** | **A** | **K** | **-** | **-** | **-** | **-** | **-** | **-** | **-** | **-** | **-** | **-** | **-** | **-** | **-** | **-** | **-** | **-** | **-** | **-** | **-** | **-** | **-** | **-** | **-** |
| **N60357F** | **M** | **S** | **E** | **E** | **I** | **R** | **E** | **N** | **I** | **H** | **D** | **V** | **N** | **L** | **T** | **S** | **E** | **M** | **K** | **D** | **S** | **F** | **I** | **D** | **Y** | **A** | **M** | **S** | **V** | **I** | **V** | **A** | **R** | **A** | **L** | **P** | **D** | **V** | **R** | **D** | **G** | **L** | **K** | **P** | **V** | **H** | **R** | **R** | **I** | **L** | **Y** | **G** | **M** | **N** | **E** | **L** | **G** | **V** | **T** | **P** | **D** | **K** | **A** | **H** | **K** | **K** | **S** | **A** | **R** | **I** | **V** | **G** | **D** | **V** | **M** | **G** | **K** | **Y** | **H** | **P** | **H** | **G** | **D** | **S** | **A** | **I** | **Y** | **E** | **S** | **M** | **V** | **R** | **M** | **A** | **Q** | **P** | **F** | **S** | **Y** | **R** | **Y** | **M** | **L** | **V** | **D** | **G** | **H** | **G** | **N** | **F** | **G** | **S** | **V** | **D** | **G** | **D** | **G** | **A** | **A** | **A** | **M** | **R** | **Y** | **T** | **E** | **A** | **R** | **M** | **S** | **K** | **I** | **A** | **T** | **E** | **M** | **L** | **R** | **D** | **I** | **N** | **K** | **N** | **T** | **V** | **D** | **F** | **Q** | **S** | **N** | **Y** | **D** | **D** | **T** | **E** | **K** | **E** | **P** | **V** | **V** | **L** | **P** | **A** | **R** | **F** | **P** | **N** | **L** | **L** | **V** | **N** | **G** | **T** | **T** | **G** | **I** | **A** | **V** | **G** | **M** | **A** | **T** | **N** | **I** | **P** | **P** | **H** | **N** | **L** | **S** | **E** | **V** | **V** | **A** | **A** | **I** | **D** | **L** | **L** | **M** | **D** | **N** | **P** | **D** | **V** | **T** | **T** | **N** | **E** | **L** | **M** | **E** | **V** | **L** | **P** | **G** | **P** | **D** | **F** | **P** | **T** | **G** | **G** | **L** | **V** | **M** | **G** | **K** | **S** | **G** | **I** | **R** | **R** | **A** | **Y** | **E** | **T** | **G** | **K** | **G** | **S** | **I** | **T** | **V** | **R** | **A** | **K** | **V** | **E** | **I** | **I** | **E** | **M** | **P** | **N** | **G** | **K** | **E** | **R** | **I** | **I** | **V** | **T** | **E** | **L** | **P** | **Y** | **M** | **V** | **N** | **K** | **A** | **K** | **L** | **I** | **E** | **R** | **I** | **S** | **E** | **L** | **H** | **R** | **D** | **K** | **R** | **I** | **E** | **G** | **I** | **T** | **D** | **L** | **R** | **D** | **E** | **S** | **S** | **R** | **E** | **G** | **M** | **R** | **I** | **V** | **I** | **D** | **I** | **R** | **R** | **D** | **A** | **S** | **A** | **S** | **V** | **I** | **L** | **N** | **N** | **L** | **Y** | **K** | **L** | **T** | **S** | **L** | **Q** | **N** | **S** | **F** | **G** | **F** | **N** | **M** | **L** | **A** | **I** | **E** | **K** | **G** | **V** | **P** | **K** | **V** | **L** | **S** | **L** | **K** | **Q** | **I** | **L** | **E** | **N** | **Y** | **I** | **E** | **H** | **Q** | **R** | **E** | **V** | **I** | **T** | **R** | **R** | **T** | **V** | **F** | **E** | **K** | **E** | **K** | **A** | **E** | **A** | **R** | **A** | **H** | **I** | **L** | **E** | **G** | **L** | **R** | **I** | **A** | **L** | **D** | **H** | **I** | **D** | **E** | **I** | **I** | **A** | **I** | **I** | **R** | **G** | **S** | **K** | **S** | **D** | **D** | **E** | **A** | **K** | **A** | **T** | **M** | **I** | **E** | **R** | **F** | **D** | **L** | **S** | **D** | **R** | **Q** | **S** | **Q** | **A** | **I** | **L** | **D** | **M** | **R** | **L** | **R** | **R** | **L** | **T** | **G** | **L** | **E** | **R** | **E** | **K** | **I** | **E** | **N** | **E** | **Y** | **Q** | **E** | **L** | **L** | **K** | **L** | **I** | **E** | **D** | **L** | **T** | **D** | **I** | **L** | **A** | **R** | **P** | **E** | **R** | **V** | **T** | **E** | **I** | **I** | **K** | **N** | **E** | **L** | **A** | **D** | **L** | **N** | **Q** | **R** | **F** | **G** | **D** | **K** | **R** | **R** | **T** | **E** | **L** | **L** | **V** | **G** | **E** | **V** | **L** | **S** | **L** | **E** | **D** | **E** | **D** | **L** | **I** | **E** | **E** | **E** | **E** | **I** | **V** | **I** | **T** | **L** | **T** | **N** | **N** | **G** | **Y** | **I** | **K** | **R** | **L** | **A** | **N** | **N** | **E** | **F** | **R** | **A** | **Q** | **R** | **R** | **G** | **G** | **R** | **G** | **V** | **Q** | **G** | **M** | **G** | **I** | **H** | **N** | **D** | **D** | **F** | **V** | **K** | **N** | **L** | **V** | **S** | **C** | **S** | **T** | **H** | **D** | **T** | **L** | **L** | **F** | **F** | **T** | **N** | **N** | **G** | **K** | **V** | **Y** | **R** | **A** | **K** | **G** | **Y** | **E** | **I** | **P** | **E** | **Y** | **G** | **R** | **T** | **A** | **K** | **G** | **I** | **P** | **I** | **I** | **N** | **L** | **L** | **G** | **I** | **D** | **S** | **S** | **E** | **S** | **I** | **Q** | **A** | **I** | **I** | **A** | **V** | **T** | **G** | **E** | **A** | **E** | **E** | **G** | **H** | **Y** | **L** | **F** | **F** | **T** | **T** | **R** | **Q** | **G** | **T** | **V** | **K** | **R** | **T** | **S** | **V** | **T** | **A** | **F** | **A** | **N** | **I** | **R** | **S** | **N** | **G** | **L** | **I** | **A** | **I** | **G** | **L** | **K** | **E** | **D** | **D** | **E** | **L** | **V** | **N** | **V** | **L** | **L** | **T** | **D** | **G** | **Q** | **S** | **N** | **I** | **I** | **I** | **G** | **T** | **H** | **N** | **G** | **Y** | **S** | **V** | **T** | **F** | **K** | **E** | **E** | **A** | **V** | **R** | **D** | **M** | **G** | **R** | **T** | **A** | **S** | **G** | **V** | **R** | **G** | **I** | **R** | **L** | **R** | **E** | **E** | **D** | **Y** | **V** | **V** | **G** | **A** | **A** | **L** | **M** | **K** | **E** | **N** | **Q** | **E** | **V** | **L** | **V** | **I** | **T** | **E** | **K** | **G** | **Y** | **G** | **K** | **R** | **T** | **K** | **V** | **S** | **E** | **Y** | **P** | **V** | **K** | **G** | **R** | **G** | **G** | **K** | **G** | **I** | **K** | **T** | **A** | **N** | **I** | **T** | **E** | **K** | **N** | **G** | **P** | **L** | **A** | **G** | **L** | **T** | **T** | **V** | **S** | **G** | **D** | **E** | **D** | **I** | **M** | **L** | **I** | **T** | **D** | **K** | **G** | **V** | **I** | **I** | **R** | **F** | **N** | **V** | **S** | **T** | **V** | **S** | **Q** | **T** | **G** | **R** | **S** | **T** | **L** | **G** | **V** | **R** | **L** | **M** | **K** | **M** | **E** | **A** | **D** | **T** | **K** | **V** | **V** | **T** | **M** | **A** | **A** | **V** | **E** | **P** | **E** | **A** | **A** | **E** | **Q** | **T** | **V** | **E** | **E** | **Q** | **G** | **S** | **E** | **E** |
| **N54564** | **M** | **S** | **E** | **E** | **I** | **K** | **E** | **N** | **I** | **Q** | **D** | **V** | **N** | **L** | **T** | **S** | **E** | **M** | **K** | **E** | **S** | **F** | **I** | **D** | **Y** | **A** | **M** | **S** | **V** | **I** | **V** | **A** | **R** | **A** | **L** | **P** | **D** | **V** | **R** | **D** | **G** | **L** | **K** | **P** | **V** | **H** | **R** | **R** | **I** | **L** | **Y** | **G** | **M** | **N** | **E** | **L** | **G** | **V** | **T** | **P** | **D** | **K** | **P** | **H** | **K** | **K** | **S** | **A** | **R** | **I** | **V** | **G** | **D** | **V** | **M** | **G** | **K** | **Y** | **H** | **P** | **H** | **G** | **D** | **S** | **A** | **I** | **Y** | **E** | **S** | **M** | **V** | **R** | **M** | **A** | **Q** | **P** | **F** | **S** | **Y** | **R** | **A** | **M** | **L** | **V** | **D** | **G** | **H** | **G** | **N** | **F** | **G** | **S** | **V** | **D** | **G** | **D** | **G** | **A** | **A** | **A** | **M** | **R** | **Y** | **T** | **E** | **A** | **R** | **M** | **S** | **K** | **I** | **A** | **L** | **E** | **M** | **L** | **R** | **D** | **I** | **N** | **K** | **N** | **T** | **V** | **D** | **F** | **Q** | **G** | **N** | **Y** | **D** | **D** | **S** | **E** | **Q** | **E** | **P** | **V** | **V** | **L** | **P** | **A** | **R** | **F** | **P** | **N** | **L** | **L** | **V** | **N** | **G** | **T** | **T** | **G** | **I** | **A** | **V** | **G** | **M** | **A** | **T** | **N** | **I** | **P** | **P** | **H** | **N** | **L** | **S** | **E** | **V** | **I** | **D** | **A** | **T** | **S** | **L** | **L** | **M** | **D** | **N** | **P** | **D** | **V** | **T** | **T** | **N** | **E** | **L** | **M** | **E** | **V** | **L** | **P** | **G** | **P** | **D** | **F** | **P** | **T** | **G** | **G** | **L** | **V** | **M** | **G** | **K** | **S** | **G** | **I** | **R** | **R** | **A** | **Y** | **E** | **T** | **G** | **K** | **G** | **S** | **I** | **T** | **V** | **R** | **A** | **K** | **V** | **E** | **L** | **T** | **E** | **M** | **P** | **N** | **G** | **K** | **E** | **R** | **I** | **L** | **V** | **T** | **E** | **L** | **P** | **Y** | **M** | **V** | **N** | **K** | **A** | **K** | **L** | **I** | **E** | **R** | **I** | **S** | **E** | **L** | **H** | **R** | **D** | **K** | **R** | **I** | **E** | **G** | **I** | **T** | **D** | **L** | **R** | **D** | **E** | **S** | **S** | **R** | **E** | **G** | **M** | **R** | **I** | **V** | **I** | **D** | **V** | **R** | **R** | **D** | **V** | **S** | **A** | **S** | **V** | **V** | **L** | **N** | **N** | **L** | **Y** | **K** | **M** | **T** | **A** | **L** | **Q** | **T** | **S** | **F** | **G** | **F** | **N** | **M** | **L** | **A** | **I** | **E** | **K** | **G** | **V** | **P** | **K** | **I** | **L** | **S** | **L** | **K** | **R** | **I** | **L** | **E** | **N** | **Y** | **V** | **E** | **H** | **Q** | **K** | **E** | **V** | **I** | **T** | **R** | **R** | **T** | **I** | **F** | **D** | **K** | **N** | **K** | **A** | **E** | **A** | **R** | **A** | **H** | **I** | **L** | **E** | **G** | **L** | **R** | **I** | **A** | **L** | **D** | **H** | **I** | **D** | **E** | **I** | **I** | **A** | **I** | **I** | **R** | **G** | **S** | **Q** | **S** | **D** | **D** | **E** | **A** | **K** | **A** | **T** | **L** | **I** | **E** | **R** | **F** | **E** | **F** | **S** | **D** | **R** | **Q** | **A** | **Q** | **A** | **I** | **L** | **D** | **M** | **R** | **L** | **R** | **R** | **L** | **T** | **G** | **L** | **E** | **R** | **D** | **K** | **I** | **E** | **N** | **E** | **Y** | **Q** | **E** | **L** | **L** | **I** | **F** | **I** | **A** | **D** | **L** | **E** | **D** | **I** | **L** | **A** | **R** | **P** | **E** | **R** | **V** | **I** | **E** | **I** | **I** | **K** | **T** | **E** | **L** | **N** | **D** | **V** | **R** | **T** | **K** | **F** | **G** | **D** | **A** | **R** | **R** | **T** | **E** | **L** | **L** | **V** | **G** | **E** | **V** | **L** | **S** | **L** | **E** | **D** | **E** | **D** | **L** | **I** | **E** | **E** | **E** | **E** | **V** | **V** | **I** | **T** | **L** | **T** | **N** | **N** | **G** | **Y** | **I** | **K** | **R** | **M** | **A** | **N** | **S** | **E** | **F** | **R** | **A** | **Q** | **R** | **R** | **G** | **G** | **R** | **G** | **V** | **Q** | **G** | **M** | **G** | **V** | **H** | **D** | **D** | **D** | **F** | **V** | **K** | **N** | **L** | **V** | **S** | **C** | **S** | **T** | **H** | **D** | **T** | **L** | **L** | **F** | **F** | **T** | **N** | **T** | **G** | **K** | **V** | **Y** | **R** | **A** | **K** | **G** | **Y** | **E** | **I** | **P** | **E** | **Y** | **G** | **R** | **T** | **A** | **K** | **G** | **I** | **P** | **V** | **I** | **N** | **L** | **L** | **G** | **I** | **D** | **S** | **A** | **E** | **K** | **I** | **Q** | **A** | **I** | **I** | **S** | **V** | **E** | **G** | **K** | **A** | **E** | **A** | **G** | **K** | **Y** | **L** | **F** | **F** | **T** | **T** | **L** | **K** | **G** | **T** | **V** | **K** | **R** | **T** | **A** | **V** | **T** | **A** | **F** | **S** | **N** | **I** | **R** | **S** | **N** | **G** | **L** | **I** | **A** | **I** | **S** | **L** | **K** | **E** | **D** | **D** | **E** | **L** | **V** | **N** | **V** | **V** | **T** | **T** | **N** | **G** | **N** | **Q** | **K** | **M** | **I** | **I** | **G** | **T** | **H** | **A** | **G** | **Y** | **S** | **V** | **T** | **F** | **D** | **E** | **N** | **T** | **V** | **R** | **D** | **M** | **G** | **R** | **T** | **A** | **S** | **G** | **V** | **R** | **G** | **I** | **R** | **L** | **R** | **E** | **N** | **D** | **Y** | **V** | **V** | **G** | **A** | **A** | **I** | **L** | **D** | **E** | **N** | **K** | **E** | **V** | **L** | **V** | **I** | **T** | **E** | **N** | **G** | **Y** | **G** | **K** | **R** | **T** | **K** | **A** | **S** | **E** | **Y** | **P** | **V** | **K** | **G** | **R** | **G** | **G** | **K** | **G** | **I** | **K** | **T** | **A** | **N** | **I** | **T** | **E** | **K** | **N** | **G** | **P** | **L** | **A** | **G** | **L** | **T** | **T** | **V** | **N** | **G** | **D** | **E** | **D** | **I** | **L** | **L** | **I** | **T** | **N** | **K** | **G** | **V** | **I** | **I** | **R** | **F** | **N** | **V** | **D** | **S** | **V** | **S** | **Q** | **T** | **G** | **R** | **A** | **T** | **L** | **G** | **V** | **R** | **L** | **M** | **R** | **M** | **E** | **D** | **G** | **A** | **K** | **-** | **-** | **-** | **-** | **-** | **-** | **-** | **-** | **-** | **-** | **-** | **-** | **-** | **-** | **-** | **-** | **-** | **-** | **-** | **-** | **-** | **-** | **-** |
| **N60034F** | **M** | **S** | **E** | **E** | **I** | **K** | **E** | **N** | **I** | **Q** | **D** | **V** | **N** | **L** | **T** | **S** | **E** | **M** | **K** | **E** | **S** | **F** | **I** | **D** | **Y** | **A** | **M** | **S** | **V** | **I** | **V** | **A** | **R** | **A** | **L** | **P** | **D** | **V** | **R** | **D** | **G** | **L** | **K** | **P** | **V** | **H** | **R** | **R** | **I** | **L** | **Y** | **G** | **M** | **N** | **E** | **L** | **G** | **V** | **T** | **P** | **D** | **K** | **P** | **H** | **K** | **K** | **S** | **A** | **R** | **I** | **V** | **G** | **D** | **V** | **M** | **G** | **K** | **Y** | **H** | **P** | **H** | **G** | **D** | **S** | **A** | **I** | **Y** | **E** | **S** | **M** | **V** | **R** | **M** | **A** | **Q** | **P** | **F** | **S** | **Y** | **R** | **A** | **M** | **L** | **V** | **D** | **G** | **H** | **G** | **N** | **F** | **G** | **S** | **V** | **D** | **G** | **D** | **G** | **A** | **A** | **A** | **M** | **R** | **Y** | **T** | **E** | **A** | **R** | **M** | **S** | **K** | **I** | **A** | **L** | **E** | **M** | **L** | **R** | **D** | **I** | **N** | **K** | **N** | **T** | **V** | **D** | **F** | **Q** | **G** | **N** | **Y** | **D** | **D** | **S** | **E** | **Q** | **E** | **P** | **V** | **V** | **L** | **P** | **A** | **R** | **F** | **P** | **N** | **L** | **L** | **V** | **N** | **G** | **T** | **T** | **G** | **I** | **A** | **V** | **G** | **M** | **A** | **T** | **N** | **I** | **P** | **P** | **H** | **N** | **L** | **S** | **E** | **V** | **I** | **D** | **A** | **T** | **S** | **L** | **L** | **M** | **D** | **N** | **P** | **D** | **V** | **T** | **T** | **N** | **E** | **L** | **M** | **E** | **V** | **L** | **P** | **G** | **P** | **D** | **F** | **P** | **T** | **G** | **G** | **L** | **V** | **M** | **G** | **K** | **S** | **G** | **I** | **R** | **R** | **A** | **Y** | **E** | **T** | **G** | **K** | **G** | **S** | **I** | **T** | **V** | **R** | **A** | **K** | **V** | **E** | **L** | **T** | **E** | **M** | **P** | **N** | **G** | **K** | **E** | **R** | **I** | **L** | **V** | **T** | **E** | **L** | **P** | **Y** | **M** | **V** | **N** | **K** | **A** | **K** | **L** | **I** | **E** | **R** | **I** | **S** | **E** | **L** | **H** | **R** | **D** | **K** | **R** | **I** | **E** | **G** | **I** | **T** | **D** | **L** | **R** | **D** | **E** | **S** | **S** | **R** | **E** | **G** | **M** | **R** | **I** | **V** | **I** | **D** | **V** | **R** | **R** | **D** | **V** | **S** | **A** | **S** | **V** | **V** | **L** | **N** | **N** | **L** | **Y** | **K** | **M** | **T** | **A** | **L** | **Q** | **T** | **S** | **F** | **G** | **F** | **N** | **M** | **L** | **A** | **I** | **E** | **K** | **G** | **V** | **P** | **K** | **I** | **L** | **S** | **L** | **K** | **R** | **I** | **L** | **E** | **N** | **Y** | **V** | **E** | **H** | **Q** | **K** | **E** | **V** | **I** | **T** | **R** | **R** | **T** | **I** | **F** | **D** | **K** | **N** | **K** | **A** | **E** | **A** | **R** | **A** | **H** | **I** | **L** | **E** | **G** | **L** | **R** | **I** | **A** | **L** | **D** | **H** | **I** | **D** | **E** | **I** | **I** | **A** | **I** | **I** | **R** | **G** | **S** | **Q** | **S** | **D** | **D** | **E** | **A** | **K** | **A** | **T** | **L** | **I** | **E** | **R** | **F** | **E** | **F** | **S** | **D** | **R** | **Q** | **A** | **Q** | **A** | **I** | **L** | **D** | **M** | **R** | **L** | **R** | **R** | **L** | **T** | **G** | **L** | **E** | **R** | **D** | **K** | **I** | **E** | **N** | **E** | **Y** | **Q** | **E** | **L** | **L** | **K** | **F** | **I** | **A** | **D** | **L** | **E** | **D** | **I** | **L** | **A** | **R** | **P** | **E** | **R** | **V** | **I** | **E** | **I** | **I** | **K** | **T** | **E** | **L** | **N** | **D** | **V** | **R** | **T** | **K** | **F** | **G** | **D** | **A** | **R** | **R** | **T** | **E** | **L** | **L** | **V** | **G** | **E** | **V** | **L** | **S** | **L** | **E** | **D** | **E** | **D** | **L** | **I** | **E** | **E** | **E** | **E** | **V** | **V** | **I** | **T** | **L** | **T** | **N** | **N** | **G** | **Y** | **I** | **K** | **R** | **M** | **A** | **N** | **S** | **E** | **F** | **R** | **A** | **Q** | **R** | **R** | **G** | **G** | **R** | **G** | **V** | **Q** | **G** | **M** | **G** | **V** | **H** | **D** | **D** | **D** | **F** | **V** | **K** | **N** | **L** | **V** | **S** | **C** | **S** | **T** | **H** | **D** | **T** | **L** | **L** | **F** | **F** | **T** | **N** | **T** | **G** | **K** | **V** | **Y** | **R** | **A** | **K** | **G** | **Y** | **E** | **I** | **P** | **E** | **Y** | **G** | **R** | **T** | **A** | **K** | **G** | **I** | **P** | **V** | **I** | **N** | **L** | **L** | **G** | **I** | **D** | **S** | **A** | **E** | **K** | **I** | **Q** | **A** | **I** | **I** | **S** | **V** | **E** | **G** | **K** | **A** | **E** | **A** | **G** | **K** | **Y** | **L** | **F** | **F** | **T** | **T** | **L** | **K** | **G** | **T** | **V** | **K** | **R** | **T** | **A** | **V** | **T** | **A** | **F** | **S** | **N** | **I** | **R** | **S** | **N** | **G** | **L** | **I** | **A** | **I** | **S** | **L** | **K** | **E** | **D** | **D** | **E** | **L** | **V** | **N** | **V** | **V** | **T** | **T** | **N** | **G** | **N** | **Q** | **K** | **M** | **I** | **I** | **G** | **T** | **H** | **A** | **G** | **Y** | **S** | **V** | **T** | **F** | **D** | **E** | **N** | **T** | **V** | **R** | **D** | **M** | **G** | **R** | **T** | **A** | **S** | **G** | **V** | **R** | **G** | **I** | **R** | **L** | **R** | **E** | **N** | **D** | **Y** | **V** | **V** | **G** | **A** | **A** | **I** | **L** | **D** | **E** | **N** | **K** | **E** | **V** | **L** | **V** | **I** | **T** | **E** | **N** | **G** | **Y** | **G** | **K** | **R** | **T** | **K** | **A** | **S** | **E** | **Y** | **P** | **V** | **K** | **G** | **R** | **G** | **G** | **K** | **G** | **I** | **K** | **T** | **A** | **N** | **I** | **T** | **E** | **K** | **N** | **G** | **P** | **L** | **A** | **G** | **L** | **T** | **T** | **V** | **N** | **G** | **D** | **E** | **D** | **I** | **L** | **L** | **I** | **T** | **N** | **K** | **G** | **V** | **I** | **I** | **R** | **F** | **N** | **V** | **D** | **S** | **V** | **S** | **Q** | **T** | **G** | **R** | **A** | **T** | **L** | **G** | **V** | **R** | **L** | **M** | **R** | **M** | **E** | **D** | **G** | **A** | **K** | **-** | **-** | **-** | **-** | **-** | **-** | **-** | **-** | **-** | **-** | **-** | **-** | **-** | **-** | **-** | **-** | **-** | **-** | **-** | **-** | **-** | **-** | **-** |
| **N59559F** | **M** | **S** | **E** | **E** | **I** | **R** | **E** | **N** | **I** | **H** | **D** | **V** | **N** | **L** | **T** | **S** | **E** | **M** | **K** | **D** | **S** | **F** | **I** | **D** | **Y** | **A** | **M** | **S** | **V** | **I** | **V** | **A** | **R** | **A** | **L** | **P** | **D** | **V** | **R** | **D** | **G** | **L** | **K** | **P** | **V** | **H** | **R** | **R** | **I** | **L** | **Y** | **G** | **M** | **N** | **E** | **L** | **G** | **V** | **T** | **P** | **D** | **K** | **A** | **H** | **K** | **K** | **S** | **A** | **R** | **I** | **V** | **G** | **D** | **V** | **M** | **G** | **K** | **Y** | **H** | **P** | **H** | **G** | **D** | **S** | **A** | **I** | **Y** | **E** | **S** | **M** | **V** | **R** | **M** | **A** | **Q** | **P** | **F** | **S** | **Y** | **R** | **Y** | **M** | **L** | **V** | **D** | **G** | **H** | **G** | **N** | **F** | **G** | **S** | **V** | **D** | **G** | **D** | **G** | **A** | **A** | **A** | **M** | **R** | **Y** | **T** | **E** | **A** | **R** | **M** | **S** | **K** | **I** | **A** | **T** | **E** | **M** | **L** | **R** | **D** | **I** | **N** | **K** | **N** | **T** | **V** | **D** | **F** | **Q** | **S** | **N** | **Y** | **D** | **D** | **T** | **E** | **K** | **E** | **P** | **V** | **V** | **L** | **P** | **A** | **R** | **F** | **P** | **N** | **L** | **L** | **V** | **N** | **G** | **T** | **T** | **G** | **I** | **A** | **V** | **G** | **M** | **A** | **T** | **N** | **I** | **P** | **P** | **H** | **N** | **L** | **S** | **E** | **V** | **V** | **A** | **A** | **I** | **D** | **L** | **L** | **M** | **D** | **N** | **P** | **D** | **V** | **T** | **T** | **N** | **E** | **L** | **M** | **E** | **V** | **L** | **P** | **G** | **P** | **D** | **F** | **P** | **T** | **G** | **G** | **L** | **V** | **M** | **G** | **K** | **S** | **G** | **I** | **R** | **R** | **A** | **Y** | **E** | **T** | **G** | **K** | **G** | **S** | **I** | **T** | **V** | **R** | **A** | **K** | **V** | **E** | **I** | **T** | **E** | **M** | **P** | **N** | **G** | **K** | **E** | **R** | **I** | **L** | **V** | **T** | **E** | **L** | **P** | **Y** | **M** | **V** | **N** | **K** | **A** | **K** | **L** | **I** | **E** | **R** | **I** | **S** | **E** | **L** | **H** | **R** | **D** | **K** | **R** | **I** | **E** | **G** | **I** | **T** | **D** | **L** | **R** | **D** | **E** | **S** | **S** | **R** | **E** | **G** | **M** | **R** | **I** | **V** | **I** | **D** | **V** | **R** | **R** | **D** | **A** | **S** | **A** | **S** | **V** | **I** | **L** | **N** | **N** | **L** | **Y** | **K** | **L** | **T** | **S** | **L** | **Q** | **N** | **S** | **F** | **G** | **F** | **N** | **M** | **L** | **A** | **I** | **E** | **K** | **G** | **V** | **P** | **K** | **V** | **L** | **S** | **L** | **K** | **Q** | **I** | **L** | **E** | **N** | **Y** | **I** | **E** | **H** | **Q** | **R** | **E** | **V** | **I** | **T** | **R** | **R** | **T** | **V** | **F** | **E** | **K** | **E** | **K** | **A** | **E** | **A** | **R** | **A** | **H** | **I** | **L** | **E** | **G** | **L** | **R** | **I** | **A** | **L** | **D** | **H** | **I** | **D** | **E** | **I** | **I** | **A** | **I** | **I** | **R** | **G** | **S** | **K** | **S** | **D** | **D** | **E** | **A** | **K** | **A** | **T** | **M** | **I** | **E** | **R** | **F** | **D** | **L** | **S** | **D** | **R** | **Q** | **S** | **Q** | **A** | **I** | **L** | **D** | **M** | **R** | **L** | **R** | **R** | **L** | **T** | **G** | **L** | **E** | **R** | **E** | **K** | **I** | **E** | **N** | **E** | **Y** | **Q** | **E** | **L** | **L** | **K** | **L** | **I** | **E** | **D** | **L** | **T** | **D** | **I** | **L** | **A** | **R** | **P** | **E** | **R** | **V** | **T** | **E** | **I** | **I** | **K** | **N** | **E** | **L** | **A** | **D** | **L** | **N** | **Q** | **R** | **F** | **G** | **D** | **K** | **R** | **R** | **T** | **E** | **L** | **L** | **V** | **G** | **E** | **V** | **L** | **S** | **L** | **E** | **D** | **E** | **D** | **L** | **I** | **E** | **E** | **E** | **E** | **I** | **V** | **I** | **T** | **L** | **T** | **N** | **N** | **G** | **Y** | **I** | **K** | **R** | **L** | **A** | **N** | **N** | **E** | **F** | **R** | **A** | **Q** | **R** | **R** | **G** | **G** | **R** | **G** | **V** | **Q** | **G** | **M** | **G** | **I** | **H** | **N** | **D** | **D** | **F** | **V** | **K** | **N** | **L** | **V** | **S** | **C** | **S** | **T** | **H** | **D** | **T** | **L** | **L** | **F** | **F** | **T** | **N** | **N** | **G** | **K** | **V** | **Y** | **R** | **A** | **K** | **G** | **Y** | **E** | **I** | **P** | **E** | **Y** | **G** | **R** | **T** | **A** | **K** | **G** | **I** | **P** | **I** | **I** | **N** | **L** | **L** | **G** | **I** | **D** | **S** | **S** | **E** | **S** | **I** | **Q** | **A** | **I** | **I** | **A** | **V** | **T** | **G** | **E** | **A** | **E** | **E** | **G** | **H** | **Y** | **L** | **F** | **F** | **T** | **T** | **R** | **Q** | **G** | **T** | **V** | **K** | **R** | **T** | **S** | **V** | **T** | **A** | **F** | **A** | **N** | **I** | **R** | **S** | **N** | **G** | **L** | **I** | **A** | **I** | **G** | **L** | **K** | **E** | **D** | **D** | **E** | **L** | **V** | **N** | **V** | **L** | **L** | **T** | **D** | **G** | **Q** | **S** | **N** | **I** | **I** | **I** | **G** | **T** | **H** | **N** | **G** | **Y** | **S** | **V** | **T** | **F** | **K** | **E** | **E** | **A** | **V** | **R** | **D** | **M** | **G** | **R** | **T** | **A** | **S** | **G** | **V** | **R** | **G** | **I** | **R** | **L** | **R** | **E** | **E** | **D** | **Y** | **V** | **V** | **G** | **A** | **A** | **L** | **M** | **K** | **E** | **D** | **Q** | **E** | **V** | **L** | **V** | **I** | **T** | **E** | **K** | **G** | **Y** | **G** | **K** | **R** | **T** | **K** | **V** | **S** | **E** | **Y** | **P** | **V** | **K** | **G** | **R** | **G** | **G** | **K** | **G** | **I** | **K** | **T** | **A** | **N** | **I** | **T** | **E** | **K** | **N** | **G** | **P** | **L** | **A** | **G** | **L** | **T** | **T** | **V** | **S** | **G** | **N** | **E** | **D** | **I** | **M** | **L** | **I** | **T** | **D** | **K** | **G** | **V** | **I** | **I** | **R** | **F** | **N** | **V** | **S** | **T** | **V** | **S** | **Q** | **T** | **G** | **R** | **S** | **T** | **L** | **G** | **V** | **R** | **L** | **M** | **K** | **M** | **E** | **A** | **D** | **T** | **K** | **V** | **V** | **T** | **M** | **-** | **-** | **-** | **-** | **-** | **-** | **-** | **-** | **-** | **-** | **-** | **-** | **-** | **-** | **-** | **-** | **-** | **-** | **-** |
| **N60490F** | **M** | **S** | **E** | **E** | **I** | **R** | **E** | **N** | **I** | **H** | **D** | **V** | **N** | **L** | **T** | **S** | **E** | **M** | **K** | **D** | **S** | **F** | **I** | **D** | **Y** | **A** | **M** | **S** | **V** | **I** | **V** | **A** | **R** | **A** | **L** | **P** | **D** | **V** | **R** | **D** | **G** | **L** | **K** | **P** | **V** | **H** | **R** | **R** | **I** | **L** | **Y** | **G** | **M** | **N** | **E** | **L** | **G** | **V** | **T** | **P** | **D** | **K** | **A** | **H** | **K** | **K** | **S** | **A** | **R** | **I** | **V** | **G** | **D** | **V** | **M** | **G** | **K** | **Y** | **H** | **P** | **H** | **G** | **D** | **S** | **A** | **I** | **Y** | **E** | **S** | **M** | **V** | **R** | **M** | **A** | **Q** | **P** | **F** | **S** | **Y** | **R** | **Y** | **M** | **L** | **V** | **D** | **G** | **H** | **G** | **N** | **F** | **G** | **S** | **V** | **D** | **G** | **D** | **G** | **A** | **A** | **A** | **M** | **R** | **Y** | **T** | **E** | **A** | **R** | **M** | **S** | **K** | **I** | **A** | **T** | **E** | **M** | **L** | **R** | **D** | **I** | **N** | **K** | **N** | **T** | **V** | **D** | **F** | **Q** | **S** | **N** | **Y** | **D** | **D** | **T** | **E** | **K** | **E** | **P** | **V** | **V** | **L** | **P** | **A** | **R** | **F** | **P** | **N** | **L** | **L** | **V** | **N** | **G** | **T** | **T** | **G** | **I** | **A** | **V** | **G** | **M** | **A** | **T** | **N** | **I** | **P** | **P** | **H** | **N** | **L** | **S** | **E** | **V** | **V** | **A** | **A** | **I** | **D** | **L** | **L** | **M** | **D** | **N** | **P** | **D** | **V** | **T** | **T** | **N** | **E** | **L** | **M** | **E** | **V** | **L** | **P** | **G** | **P** | **D** | **F** | **P** | **T** | **G** | **G** | **L** | **V** | **M** | **G** | **K** | **S** | **G** | **I** | **R** | **R** | **A** | **Y** | **E** | **T** | **G** | **K** | **G** | **S** | **I** | **T** | **V** | **R** | **A** | **K** | **V** | **E** | **I** | **T** | **E** | **M** | **P** | **N** | **G** | **K** | **E** | **R** | **I** | **I** | **V** | **T** | **E** | **L** | **P** | **Y** | **M** | **V** | **N** | **K** | **A** | **K** | **L** | **I** | **E** | **R** | **I** | **S** | **E** | **L** | **H** | **R** | **D** | **K** | **R** | **I** | **E** | **G** | **I** | **T** | **D** | **L** | **R** | **D** | **E** | **S** | **S** | **R** | **E** | **G** | **M** | **R** | **I** | **V** | **I** | **D** | **I** | **R** | **R** | **D** | **A** | **S** | **A** | **S** | **V** | **I** | **L** | **N** | **N** | **L** | **Y** | **K** | **L** | **T** | **S** | **L** | **Q** | **N** | **S** | **F** | **G** | **F** | **N** | **M** | **L** | **A** | **I** | **E** | **K** | **G** | **V** | **P** | **K** | **V** | **L** | **S** | **L** | **K** | **Q** | **I** | **L** | **E** | **N** | **Y** | **I** | **E** | **H** | **Q** | **R** | **E** | **V** | **I** | **T** | **R** | **R** | **T** | **V** | **F** | **E** | **K** | **E** | **K** | **A** | **E** | **A** | **R** | **A** | **H** | **I** | **L** | **E** | **G** | **L** | **R** | **I** | **A** | **L** | **D** | **H** | **I** | **D** | **E** | **I** | **I** | **A** | **I** | **I** | **R** | **G** | **S** | **K** | **S** | **D** | **D** | **E** | **A** | **K** | **A** | **T** | **M** | **I** | **E** | **R** | **F** | **D** | **L** | **S** | **D** | **R** | **Q** | **S** | **Q** | **A** | **I** | **L** | **D** | **M** | **R** | **L** | **R** | **R** | **L** | **T** | **G** | **L** | **E** | **R** | **E** | **K** | **I** | **E** | **N** | **E** | **Y** | **Q** | **E** | **L** | **L** | **K** | **L** | **I** | **E** | **D** | **L** | **T** | **D** | **I** | **L** | **A** | **R** | **P** | **E** | **R** | **V** | **T** | **E** | **I** | **I** | **K** | **N** | **E** | **L** | **A** | **D** | **L** | **N** | **Q** | **R** | **F** | **G** | **D** | **K** | **R** | **R** | **T** | **E** | **L** | **L** | **V** | **G** | **E** | **V** | **L** | **S** | **L** | **E** | **D** | **E** | **D** | **L** | **I** | **E** | **E** | **E** | **E** | **I** | **V** | **I** | **T** | **L** | **T** | **N** | **N** | **G** | **Y** | **I** | **K** | **R** | **L** | **A** | **N** | **N** | **E** | **F** | **R** | **A** | **Q** | **R** | **R** | **G** | **G** | **R** | **G** | **V** | **Q** | **G** | **M** | **G** | **I** | **H** | **N** | **D** | **D** | **F** | **V** | **K** | **N** | **L** | **V** | **S** | **C** | **S** | **T** | **H** | **D** | **T** | **L** | **L** | **F** | **F** | **T** | **N** | **N** | **G** | **K** | **V** | **Y** | **R** | **A** | **K** | **G** | **Y** | **E** | **I** | **P** | **E** | **Y** | **G** | **R** | **T** | **A** | **K** | **G** | **I** | **P** | **I** | **I** | **N** | **L** | **L** | **G** | **I** | **D** | **S** | **S** | **E** | **S** | **I** | **Q** | **A** | **I** | **I** | **A** | **V** | **T** | **G** | **E** | **A** | **E** | **E** | **G** | **H** | **Y** | **L** | **F** | **F** | **T** | **T** | **R** | **Q** | **G** | **T** | **V** | **K** | **R** | **T** | **S** | **V** | **T** | **A** | **F** | **A** | **N** | **I** | **R** | **S** | **N** | **G** | **L** | **I** | **A** | **I** | **G** | **L** | **K** | **E** | **D** | **D** | **E** | **L** | **V** | **N** | **V** | **L** | **L** | **T** | **D** | **G** | **Q** | **S** | **N** | **I** | **I** | **I** | **G** | **T** | **H** | **N** | **G** | **Y** | **S** | **V** | **T** | **F** | **K** | **E** | **E** | **A** | **V** | **R** | **D** | **M** | **G** | **R** | **T** | **A** | **S** | **G** | **V** | **R** | **G** | **I** | **R** | **L** | **R** | **E** | **E** | **D** | **Y** | **V** | **V** | **G** | **A** | **A** | **L** | **M** | **K** | **E** | **D** | **Q** | **E** | **V** | **L** | **V** | **I** | **T** | **E** | **K** | **G** | **Y** | **G** | **K** | **R** | **T** | **K** | **V** | **S** | **E** | **Y** | **P** | **V** | **K** | **G** | **R** | **G** | **G** | **K** | **G** | **I** | **K** | **T** | **A** | **N** | **I** | **T** | **E** | **K** | **N** | **G** | **P** | **L** | **A** | **G** | **L** | **T** | **T** | **V** | **S** | **G** | **D** | **E** | **D** | **I** | **M** | **L** | **I** | **T** | **D** | **K** | **G** | **V** | **I** | **I** | **R** | **F** | **N** | **V** | **S** | **T** | **V** | **S** | **Q** | **T** | **G** | **R** | **S** | **T** | **L** | **G** | **V** | **R** | **L** | **M** | **K** | **M** | **E** | **A** | **D** | **T** | **K** | **V** | **V** | **T** | **M** | **A** | **A** | **V** | **E** | **P** | **E** | **A** | **A** | **E** | **Q** | **T** | **V** | **E** | **E** | **Q** | **G** | **S** | **E** | **E** |
| **N55240** | **M** | **S** | **E** | **E** | **I** | **K** | **E** | **N** | **I** | **Q** | **D** | **V** | **N** | **L** | **T** | **S** | **E** | **M** | **K** | **E** | **S** | **F** | **I** | **D** | **Y** | **A** | **M** | **S** | **V** | **I** | **V** | **A** | **R** | **A** | **L** | **P** | **D** | **V** | **R** | **D** | **G** | **L** | **K** | **P** | **V** | **H** | **R** | **R** | **I** | **L** | **Y** | **G** | **M** | **N** | **E** | **L** | **G** | **V** | **T** | **P** | **D** | **K** | **P** | **H** | **K** | **K** | **S** | **A** | **R** | **I** | **V** | **G** | **D** | **V** | **M** | **G** | **K** | **Y** | **H** | **P** | **H** | **G** | **D** | **S** | **A** | **I** | **Y** | **E** | **S** | **M** | **V** | **R** | **M** | **A** | **Q** | **P** | **F** | **S** | **Y** | **R** | **A** | **M** | **L** | **V** | **D** | **G** | **H** | **G** | **N** | **F** | **G** | **S** | **V** | **D** | **G** | **D** | **G** | **A** | **A** | **A** | **M** | **R** | **Y** | **T** | **E** | **A** | **R** | **M** | **S** | **K** | **I** | **A** | **L** | **E** | **M** | **L** | **R** | **D** | **I** | **N** | **K** | **N** | **T** | **V** | **D** | **F** | **Q** | **G** | **N** | **Y** | **D** | **D** | **S** | **E** | **Q** | **E** | **P** | **V** | **V** | **L** | **P** | **A** | **R** | **F** | **P** | **N** | **L** | **L** | **V** | **N** | **G** | **T** | **T** | **G** | **I** | **A** | **V** | **G** | **M** | **A** | **T** | **N** | **I** | **P** | **P** | **H** | **N** | **L** | **S** | **E** | **V** | **I** | **D** | **A** | **T** | **S** | **L** | **L** | **M** | **D** | **N** | **P** | **D** | **V** | **T** | **T** | **N** | **E** | **L** | **M** | **E** | **V** | **L** | **P** | **G** | **P** | **D** | **F** | **P** | **T** | **G** | **G** | **L** | **V** | **M** | **G** | **K** | **S** | **G** | **I** | **R** | **R** | **A** | **Y** | **E** | **T** | **G** | **K** | **G** | **S** | **I** | **T** | **V** | **R** | **A** | **K** | **V** | **E** | **L** | **T** | **E** | **M** | **P** | **N** | **G** | **K** | **E** | **R** | **I** | **L** | **V** | **T** | **E** | **L** | **P** | **Y** | **M** | **V** | **N** | **K** | **A** | **K** | **L** | **I** | **E** | **R** | **I** | **S** | **E** | **L** | **H** | **R** | **D** | **K** | **R** | **I** | **E** | **G** | **I** | **T** | **D** | **L** | **R** | **D** | **E** | **S** | **S** | **R** | **E** | **G** | **M** | **R** | **I** | **V** | **I** | **D** | **V** | **R** | **R** | **D** | **V** | **S** | **A** | **S** | **V** | **V** | **L** | **N** | **N** | **L** | **Y** | **K** | **M** | **T** | **A** | **L** | **Q** | **T** | **S** | **F** | **G** | **F** | **N** | **M** | **L** | **A** | **I** | **E** | **K** | **G** | **V** | **P** | **K** | **I** | **L** | **S** | **L** | **K** | **R** | **I** | **L** | **E** | **N** | **Y** | **V** | **E** | **H** | **Q** | **K** | **E** | **V** | **I** | **T** | **R** | **R** | **T** | **I** | **F** | **D** | **K** | **N** | **K** | **A** | **E** | **A** | **R** | **A** | **H** | **I** | **L** | **E** | **G** | **L** | **R** | **I** | **A** | **L** | **D** | **H** | **I** | **D** | **E** | **I** | **I** | **A** | **I** | **I** | **R** | **G** | **S** | **Q** | **S** | **D** | **D** | **E** | **A** | **K** | **A** | **T** | **L** | **I** | **E** | **R** | **F** | **E** | **F** | **S** | **D** | **R** | **Q** | **A** | **Q** | **A** | **I** | **L** | **D** | **M** | **R** | **L** | **R** | **R** | **L** | **T** | **G** | **L** | **E** | **R** | **D** | **K** | **I** | **E** | **N** | **E** | **Y** | **Q** | **E** | **L** | **L** | **K** | **F** | **I** | **A** | **D** | **L** | **E** | **D** | **I** | **L** | **A** | **R** | **P** | **E** | **R** | **V** | **I** | **E** | **I** | **I** | **K** | **T** | **E** | **L** | **N** | **D** | **V** | **R** | **T** | **K** | **F** | **G** | **D** | **A** | **R** | **R** | **T** | **E** | **L** | **L** | **V** | **G** | **E** | **V** | **L** | **S** | **L** | **E** | **D** | **E** | **D** | **L** | **I** | **E** | **E** | **E** | **E** | **V** | **V** | **I** | **T** | **L** | **T** | **N** | **N** | **G** | **Y** | **I** | **K** | **R** | **M** | **A** | **N** | **S** | **E** | **F** | **R** | **A** | **Q** | **R** | **R** | **G** | **G** | **R** | **G** | **V** | **Q** | **G** | **M** | **G** | **V** | **H** | **D** | **D** | **D** | **F** | **V** | **K** | **N** | **L** | **V** | **S** | **C** | **S** | **T** | **H** | **D** | **T** | **L** | **L** | **F** | **F** | **T** | **N** | **T** | **G** | **K** | **V** | **Y** | **R** | **A** | **K** | **G** | **Y** | **E** | **I** | **P** | **E** | **Y** | **G** | **R** | **T** | **A** | **K** | **G** | **I** | **P** | **V** | **I** | **N** | **L** | **L** | **G** | **I** | **D** | **S** | **A** | **E** | **K** | **I** | **Q** | **A** | **I** | **I** | **S** | **V** | **E** | **G** | **K** | **A** | **E** | **A** | **G** | **K** | **Y** | **L** | **F** | **F** | **T** | **T** | **L** | **K** | **G** | **T** | **V** | **K** | **R** | **T** | **A** | **V** | **T** | **A** | **F** | **S** | **N** | **I** | **R** | **S** | **N** | **G** | **L** | **I** | **A** | **I** | **S** | **L** | **K** | **E** | **D** | **D** | **E** | **L** | **V** | **N** | **V** | **V** | **T** | **T** | **N** | **G** | **N** | **Q** | **K** | **M** | **I** | **I** | **G** | **T** | **H** | **A** | **G** | **Y** | **S** | **V** | **T** | **F** | **D** | **E** | **N** | **T** | **V** | **R** | **D** | **M** | **G** | **R** | **T** | **A** | **S** | **G** | **V** | **R** | **G** | **I** | **R** | **L** | **R** | **E** | **N** | **D** | **Y** | **V** | **V** | **G** | **A** | **A** | **I** | **L** | **D** | **E** | **N** | **K** | **E** | **V** | **L** | **V** | **I** | **T** | **E** | **N** | **G** | **Y** | **G** | **K** | **R** | **T** | **K** | **A** | **S** | **E** | **Y** | **P** | **V** | **K** | **G** | **R** | **G** | **G** | **K** | **G** | **I** | **K** | **T** | **A** | **N** | **I** | **T** | **E** | **K** | **N** | **G** | **P** | **L** | **A** | **G** | **L** | **T** | **T** | **V** | **N** | **G** | **D** | **E** | **D** | **I** | **L** | **L** | **I** | **T** | **N** | **K** | **G** | **V** | **I** | **I** | **R** | **F** | **N** | **V** | **D** | **S** | **V** | **S** | **Q** | **T** | **G** | **R** | **A** | **T** | **L** | **G** | **V** | **R** | **L** | **M** | **R** | **M** | **E** | **D** | **G** | **A** | **K** | **-** | **-** | **-** | **-** | **-** | **-** | **-** | **-** | **-** | **-** | **-** | **-** | **-** | **-** | **-** | **-** | **-** | **-** | **-** | **-** | **-** | **-** | **-** |
| **N59503F** | **M** | **S** | **E** | **E** | **I** | **R** | **E** | **N** | **I** | **H** | **D** | **V** | **N** | **L** | **T** | **S** | **E** | **M** | **K** | **D** | **S** | **F** | **I** | **D** | **Y** | **A** | **M** | **S** | **V** | **I** | **V** | **A** | **R** | **A** | **L** | **P** | **D** | **V** | **R** | **D** | **G** | **L** | **K** | **P** | **V** | **H** | **R** | **R** | **I** | **L** | **Y** | **G** | **M** | **N** | **E** | **L** | **G** | **V** | **T** | **P** | **D** | **K** | **A** | **H** | **K** | **K** | **S** | **A** | **R** | **I** | **V** | **G** | **D** | **V** | **M** | **G** | **K** | **Y** | **H** | **P** | **H** | **G** | **D** | **S** | **A** | **I** | **Y** | **E** | **S** | **M** | **V** | **R** | **M** | **A** | **Q** | **P** | **F** | **S** | **Y** | **R** | **Y** | **M** | **L** | **V** | **D** | **G** | **H** | **G** | **N** | **F** | **G** | **S** | **V** | **D** | **G** | **D** | **G** | **A** | **A** | **A** | **M** | **R** | **Y** | **T** | **E** | **A** | **R** | **M** | **S** | **K** | **I** | **A** | **T** | **E** | **M** | **L** | **R** | **D** | **I** | **N** | **K** | **N** | **T** | **V** | **D** | **F** | **Q** | **S** | **N** | **Y** | **D** | **D** | **T** | **E** | **K** | **E** | **P** | **V** | **V** | **L** | **P** | **A** | **R** | **F** | **P** | **N** | **L** | **L** | **V** | **N** | **G** | **T** | **T** | **G** | **I** | **A** | **V** | **G** | **M** | **A** | **T** | **N** | **I** | **P** | **P** | **H** | **N** | **L** | **S** | **E** | **V** | **V** | **A** | **A** | **I** | **D** | **L** | **L** | **M** | **D** | **N** | **P** | **D** | **V** | **T** | **T** | **N** | **E** | **L** | **M** | **E** | **V** | **L** | **P** | **G** | **P** | **D** | **F** | **P** | **T** | **G** | **G** | **L** | **V** | **M** | **G** | **K** | **S** | **G** | **I** | **R** | **R** | **A** | **Y** | **E** | **T** | **G** | **K** | **G** | **S** | **I** | **T** | **V** | **R** | **A** | **K** | **V** | **E** | **I** | **T** | **E** | **M** | **P** | **N** | **G** | **K** | **E** | **R** | **I** | **I** | **V** | **T** | **E** | **L** | **P** | **Y** | **M** | **V** | **N** | **K** | **A** | **K** | **L** | **I** | **E** | **R** | **I** | **S** | **E** | **L** | **H** | **R** | **D** | **K** | **R** | **I** | **E** | **G** | **I** | **T** | **D** | **L** | **R** | **D** | **E** | **S** | **S** | **R** | **E** | **G** | **M** | **R** | **I** | **V** | **I** | **D** | **I** | **R** | **R** | **D** | **A** | **S** | **A** | **S** | **V** | **I** | **L** | **N** | **N** | **L** | **Y** | **K** | **L** | **T** | **S** | **L** | **Q** | **N** | **S** | **F** | **G** | **F** | **N** | **M** | **L** | **A** | **I** | **E** | **K** | **G** | **V** | **P** | **K** | **V** | **L** | **S** | **L** | **K** | **Q** | **I** | **L** | **E** | **N** | **Y** | **I** | **E** | **H** | **Q** | **R** | **E** | **V** | **I** | **T** | **R** | **R** | **T** | **V** | **F** | **E** | **K** | **E** | **K** | **A** | **E** | **A** | **R** | **A** | **H** | **I** | **L** | **E** | **G** | **L** | **R** | **I** | **A** | **L** | **D** | **H** | **I** | **D** | **E** | **I** | **I** | **A** | **I** | **I** | **R** | **G** | **S** | **K** | **S** | **D** | **D** | **E** | **A** | **K** | **A** | **T** | **M** | **I** | **E** | **R** | **F** | **D** | **L** | **S** | **D** | **R** | **Q** | **S** | **Q** | **A** | **I** | **L** | **D** | **M** | **R** | **L** | **R** | **R** | **L** | **T** | **G** | **L** | **E** | **R** | **E** | **K** | **I** | **E** | **N** | **E** | **Y** | **Q** | **E** | **L** | **L** | **K** | **L** | **I** | **E** | **D** | **L** | **T** | **D** | **I** | **L** | **A** | **R** | **P** | **E** | **R** | **V** | **T** | **E** | **I** | **I** | **K** | **N** | **E** | **L** | **A** | **D** | **L** | **N** | **Q** | **R** | **F** | **G** | **D** | **K** | **R** | **R** | **T** | **E** | **L** | **L** | **V** | **G** | **E** | **V** | **L** | **S** | **L** | **E** | **D** | **E** | **D** | **L** | **I** | **E** | **E** | **E** | **E** | **I** | **V** | **I** | **T** | **L** | **T** | **N** | **N** | **G** | **Y** | **I** | **K** | **R** | **L** | **A** | **N** | **N** | **E** | **F** | **R** | **A** | **Q** | **R** | **R** | **G** | **G** | **R** | **G** | **V** | **Q** | **G** | **M** | **G** | **I** | **H** | **N** | **D** | **D** | **F** | **V** | **K** | **N** | **L** | **V** | **S** | **C** | **S** | **T** | **H** | **D** | **T** | **L** | **L** | **F** | **F** | **T** | **N** | **N** | **G** | **K** | **V** | **Y** | **R** | **A** | **K** | **G** | **Y** | **E** | **I** | **P** | **E** | **Y** | **G** | **R** | **T** | **A** | **K** | **G** | **I** | **P** | **I** | **I** | **N** | **L** | **L** | **G** | **I** | **D** | **S** | **S** | **E** | **S** | **I** | **Q** | **A** | **I** | **I** | **A** | **V** | **T** | **G** | **E** | **A** | **E** | **E** | **G** | **H** | **Y** | **L** | **F** | **F** | **T** | **T** | **R** | **Q** | **G** | **T** | **V** | **K** | **R** | **T** | **S** | **V** | **T** | **A** | **F** | **A** | **N** | **I** | **R** | **S** | **N** | **G** | **L** | **I** | **A** | **I** | **G** | **L** | **K** | **E** | **D** | **D** | **E** | **L** | **V** | **N** | **V** | **L** | **L** | **T** | **D** | **G** | **Q** | **S** | **N** | **I** | **I** | **I** | **G** | **T** | **H** | **N** | **G** | **Y** | **S** | **V** | **T** | **F** | **K** | **E** | **E** | **A** | **V** | **R** | **D** | **M** | **G** | **R** | **T** | **A** | **S** | **G** | **V** | **R** | **G** | **I** | **R** | **L** | **R** | **E** | **E** | **D** | **Y** | **V** | **V** | **G** | **A** | **A** | **L** | **M** | **K** | **E** | **D** | **Q** | **E** | **V** | **L** | **V** | **I** | **T** | **E** | **K** | **G** | **Y** | **G** | **K** | **R** | **T** | **K** | **V** | **S** | **E** | **Y** | **P** | **V** | **K** | **G** | **R** | **G** | **G** | **K** | **G** | **I** | **K** | **T** | **A** | **N** | **I** | **T** | **E** | **K** | **N** | **G** | **P** | **L** | **A** | **G** | **L** | **T** | **T** | **V** | **S** | **G** | **D** | **E** | **D** | **I** | **M** | **L** | **I** | **T** | **D** | **K** | **G** | **V** | **I** | **I** | **R** | **F** | **N** | **V** | **S** | **T** | **V** | **S** | **Q** | **T** | **G** | **R** | **S** | **T** | **L** | **G** | **V** | **R** | **L** | **M** | **K** | **M** | **E** | **A** | **D** | **T** | **K** | **V** | **V** | **T** | **M** | **A** | **A** | **V** | **E** | **P** | **E** | **A** | **A** | **E** | **Q** | **T** | **V** | **E** | **E** | **Q** | **G** | **S** | **E** | **E** |
| **N55229** | **M** | **S** | **E** | **E** | **I** | **R** | **E** | **N** | **I** | **H** | **D** | **V** | **N** | **L** | **T** | **S** | **E** | **M** | **K** | **D** | **S** | **F** | **I** | **D** | **Y** | **A** | **M** | **S** | **V** | **I** | **V** | **A** | **R** | **A** | **L** | **P** | **D** | **V** | **R** | **D** | **G** | **L** | **K** | **P** | **V** | **H** | **R** | **R** | **I** | **L** | **Y** | **G** | **M** | **N** | **E** | **L** | **G** | **V** | **T** | **P** | **D** | **K** | **A** | **H** | **K** | **K** | **S** | **A** | **R** | **I** | **V** | **G** | **D** | **V** | **M** | **G** | **K** | **Y** | **H** | **P** | **H** | **G** | **D** | **S** | **A** | **I** | **Y** | **E** | **S** | **M** | **V** | **R** | **M** | **A** | **Q** | **P** | **F** | **S** | **Y** | **R** | **Y** | **M** | **L** | **V** | **D** | **G** | **H** | **G** | **N** | **F** | **G** | **S** | **V** | **D** | **G** | **D** | **G** | **A** | **A** | **A** | **M** | **R** | **Y** | **T** | **E** | **A** | **R** | **M** | **S** | **K** | **I** | **A** | **T** | **E** | **M** | **L** | **R** | **D** | **I** | **N** | **K** | **N** | **T** | **V** | **D** | **F** | **Q** | **S** | **N** | **Y** | **D** | **D** | **T** | **E** | **K** | **E** | **P** | **V** | **V** | **L** | **P** | **A** | **R** | **F** | **P** | **N** | **L** | **L** | **V** | **N** | **G** | **T** | **T** | **G** | **I** | **A** | **V** | **G** | **M** | **A** | **T** | **N** | **I** | **P** | **P** | **H** | **N** | **L** | **S** | **E** | **V** | **V** | **A** | **A** | **I** | **D** | **L** | **L** | **M** | **D** | **N** | **P** | **D** | **V** | **T** | **T** | **N** | **E** | **L** | **M** | **E** | **V** | **L** | **P** | **G** | **P** | **D** | **F** | **P** | **T** | **G** | **G** | **L** | **V** | **M** | **G** | **K** | **S** | **G** | **I** | **R** | **R** | **A** | **Y** | **E** | **T** | **G** | **K** | **G** | **S** | **I** | **T** | **V** | **R** | **A** | **K** | **V** | **E** | **I** | **T** | **E** | **M** | **P** | **N** | **G** | **K** | **E** | **R** | **I** | **I** | **V** | **T** | **E** | **L** | **P** | **Y** | **M** | **V** | **N** | **K** | **A** | **K** | **L** | **I** | **E** | **R** | **I** | **S** | **E** | **L** | **H** | **R** | **D** | **K** | **R** | **I** | **E** | **G** | **I** | **T** | **D** | **L** | **R** | **D** | **E** | **S** | **S** | **R** | **E** | **G** | **M** | **R** | **I** | **V** | **I** | **D** | **I** | **R** | **R** | **D** | **A** | **S** | **A** | **S** | **V** | **I** | **L** | **N** | **N** | **L** | **Y** | **K** | **L** | **T** | **S** | **L** | **Q** | **N** | **S** | **F** | **G** | **F** | **N** | **M** | **L** | **A** | **I** | **E** | **K** | **G** | **V** | **P** | **K** | **V** | **L** | **S** | **L** | **K** | **Q** | **I** | **L** | **E** | **N** | **Y** | **I** | **E** | **H** | **Q** | **R** | **E** | **V** | **I** | **T** | **R** | **R** | **T** | **V** | **F** | **E** | **K** | **E** | **K** | **A** | **E** | **A** | **R** | **A** | **H** | **I** | **L** | **E** | **G** | **L** | **R** | **I** | **A** | **L** | **D** | **H** | **I** | **D** | **E** | **I** | **I** | **A** | **I** | **I** | **R** | **G** | **S** | **K** | **S** | **D** | **D** | **E** | **A** | **K** | **A** | **T** | **M** | **I** | **E** | **R** | **F** | **D** | **L** | **S** | **D** | **R** | **Q** | **S** | **Q** | **A** | **I** | **L** | **D** | **M** | **R** | **L** | **R** | **R** | **L** | **T** | **G** | **L** | **E** | **R** | **E** | **K** | **I** | **E** | **N** | **E** | **Y** | **Q** | **E** | **L** | **L** | **K** | **L** | **I** | **E** | **D** | **L** | **T** | **D** | **I** | **L** | **A** | **R** | **P** | **E** | **R** | **V** | **T** | **E** | **I** | **I** | **K** | **N** | **E** | **L** | **A** | **D** | **L** | **N** | **Q** | **R** | **F** | **G** | **D** | **K** | **R** | **R** | **T** | **E** | **L** | **L** | **V** | **G** | **E** | **V** | **L** | **S** | **L** | **E** | **D** | **E** | **D** | **L** | **I** | **E** | **E** | **E** | **E** | **I** | **V** | **I** | **T** | **L** | **T** | **N** | **N** | **G** | **Y** | **I** | **K** | **R** | **L** | **A** | **N** | **N** | **E** | **F** | **R** | **A** | **Q** | **R** | **R** | **G** | **G** | **R** | **G** | **V** | **Q** | **G** | **M** | **G** | **I** | **H** | **N** | **D** | **D** | **F** | **V** | **K** | **N** | **L** | **V** | **S** | **C** | **S** | **T** | **H** | **D** | **T** | **L** | **L** | **F** | **F** | **T** | **N** | **N** | **G** | **K** | **V** | **Y** | **R** | **A** | **K** | **G** | **Y** | **E** | **I** | **P** | **E** | **Y** | **G** | **R** | **T** | **A** | **K** | **G** | **I** | **P** | **I** | **I** | **N** | **L** | **L** | **G** | **I** | **D** | **S** | **S** | **E** | **S** | **I** | **Q** | **A** | **I** | **I** | **A** | **V** | **T** | **G** | **E** | **A** | **E** | **E** | **G** | **H** | **Y** | **L** | **F** | **F** | **T** | **T** | **R** | **Q** | **G** | **T** | **V** | **K** | **R** | **T** | **S** | **V** | **T** | **A** | **F** | **A** | **N** | **I** | **R** | **S** | **N** | **G** | **L** | **I** | **A** | **I** | **G** | **L** | **K** | **E** | **D** | **D** | **E** | **L** | **V** | **N** | **V** | **L** | **L** | **T** | **D** | **G** | **Q** | **S** | **N** | **I** | **I** | **I** | **G** | **T** | **H** | **N** | **G** | **Y** | **S** | **V** | **T** | **F** | **K** | **E** | **E** | **A** | **V** | **R** | **D** | **M** | **G** | **R** | **T** | **A** | **S** | **G** | **V** | **R** | **G** | **I** | **R** | **L** | **R** | **E** | **E** | **D** | **Y** | **V** | **V** | **G** | **A** | **A** | **L** | **M** | **K** | **E** | **N** | **Q** | **E** | **V** | **L** | **V** | **I** | **T** | **E** | **K** | **G** | **Y** | **G** | **K** | **R** | **T** | **K** | **V** | **S** | **E** | **Y** | **P** | **V** | **K** | **G** | **R** | **G** | **G** | **K** | **G** | **I** | **K** | **T** | **A** | **N** | **I** | **T** | **E** | **K** | **N** | **G** | **P** | **L** | **A** | **G** | **L** | **T** | **T** | **V** | **S** | **G** | **D** | **E** | **D** | **I** | **M** | **L** | **I** | **T** | **D** | **K** | **G** | **V** | **I** | **I** | **R** | **F** | **N** | **V** | **S** | **T** | **V** | **S** | **Q** | **T** | **G** | **R** | **S** | **T** | **L** | **G** | **V** | **R** | **L** | **M** | **K** | **M** | **E** | **A** | **D** | **T** | **K** | **V** | **V** | **T** | **M** | **A** | **A** | **V** | **E** | **P** | **E** | **A** | **A** | **E** | **Q** | **T** | **V** | **E** | **E** | **Q** | **G** | **S** | **E** | **E** |
| **N59624F** | **M** | **S** | **E** | **E** | **I** | **R** | **E** | **N** | **I** | **H** | **D** | **V** | **N** | **L** | **T** | **S** | **E** | **M** | **K** | **D** | **S** | **F** | **I** | **D** | **Y** | **A** | **M** | **S** | **V** | **I** | **V** | **A** | **R** | **A** | **L** | **P** | **D** | **V** | **R** | **D** | **G** | **L** | **K** | **P** | **V** | **H** | **R** | **R** | **I** | **L** | **Y** | **G** | **M** | **N** | **E** | **L** | **G** | **V** | **T** | **P** | **D** | **K** | **A** | **H** | **K** | **K** | **S** | **A** | **R** | **I** | **V** | **G** | **D** | **V** | **M** | **G** | **K** | **Y** | **H** | **P** | **H** | **G** | **D** | **S** | **A** | **I** | **Y** | **E** | **S** | **M** | **V** | **R** | **M** | **A** | **Q** | **P** | **F** | **S** | **Y** | **R** | **Y** | **M** | **L** | **V** | **D** | **G** | **H** | **G** | **N** | **F** | **G** | **S** | **V** | **D** | **G** | **D** | **G** | **A** | **A** | **A** | **M** | **R** | **Y** | **T** | **E** | **A** | **R** | **M** | **S** | **K** | **I** | **A** | **T** | **E** | **M** | **L** | **R** | **D** | **I** | **N** | **K** | **N** | **T** | **V** | **D** | **F** | **Q** | **S** | **N** | **Y** | **D** | **D** | **T** | **E** | **K** | **E** | **P** | **V** | **V** | **L** | **P** | **A** | **R** | **F** | **P** | **N** | **L** | **L** | **V** | **N** | **G** | **T** | **T** | **G** | **I** | **A** | **V** | **G** | **M** | **A** | **T** | **N** | **I** | **P** | **P** | **H** | **N** | **L** | **S** | **E** | **V** | **V** | **A** | **A** | **I** | **D** | **L** | **L** | **M** | **D** | **N** | **P** | **D** | **V** | **T** | **T** | **N** | **E** | **L** | **M** | **E** | **V** | **L** | **P** | **G** | **P** | **D** | **F** | **P** | **T** | **G** | **G** | **L** | **V** | **M** | **G** | **K** | **S** | **G** | **I** | **R** | **R** | **A** | **Y** | **E** | **T** | **G** | **K** | **G** | **S** | **I** | **T** | **V** | **R** | **A** | **K** | **V** | **E** | **I** | **T** | **E** | **M** | **P** | **N** | **G** | **K** | **E** | **R** | **I** | **L** | **V** | **T** | **E** | **L** | **P** | **Y** | **M** | **V** | **N** | **K** | **A** | **K** | **L** | **I** | **E** | **R** | **I** | **S** | **E** | **L** | **H** | **R** | **D** | **K** | **R** | **I** | **E** | **G** | **I** | **T** | **D** | **L** | **R** | **D** | **E** | **S** | **S** | **R** | **E** | **G** | **M** | **R** | **I** | **V** | **I** | **D** | **I** | **R** | **R** | **D** | **A** | **S** | **A** | **S** | **V** | **I** | **L** | **N** | **N** | **L** | **Y** | **K** | **L** | **T** | **S** | **L** | **Q** | **N** | **S** | **F** | **G** | **F** | **N** | **M** | **L** | **A** | **I** | **E** | **K** | **G** | **V** | **P** | **K** | **V** | **L** | **S** | **L** | **K** | **Q** | **I** | **L** | **E** | **N** | **Y** | **I** | **E** | **H** | **Q** | **R** | **E** | **V** | **I** | **T** | **R** | **R** | **T** | **V** | **F** | **E** | **K** | **E** | **K** | **A** | **E** | **A** | **R** | **A** | **H** | **I** | **L** | **E** | **G** | **L** | **R** | **I** | **A** | **L** | **D** | **H** | **I** | **D** | **E** | **I** | **I** | **A** | **I** | **I** | **R** | **G** | **S** | **K** | **S** | **D** | **D** | **E** | **A** | **K** | **A** | **T** | **M** | **I** | **E** | **R** | **F** | **D** | **L** | **S** | **D** | **R** | **Q** | **S** | **Q** | **A** | **I** | **L** | **D** | **M** | **R** | **L** | **R** | **R** | **L** | **T** | **G** | **L** | **E** | **R** | **E** | **K** | **I** | **E** | **N** | **E** | **Y** | **Q** | **E** | **L** | **L** | **K** | **L** | **I** | **E** | **D** | **L** | **T** | **D** | **I** | **L** | **A** | **R** | **P** | **E** | **R** | **V** | **T** | **E** | **I** | **I** | **K** | **N** | **E** | **L** | **A** | **D** | **L** | **N** | **Q** | **R** | **F** | **G** | **D** | **K** | **R** | **R** | **T** | **E** | **L** | **L** | **V** | **G** | **E** | **V** | **L** | **S** | **L** | **E** | **D** | **E** | **D** | **L** | **I** | **E** | **E** | **E** | **E** | **I** | **V** | **I** | **T** | **L** | **T** | **N** | **N** | **G** | **Y** | **I** | **K** | **R** | **L** | **A** | **N** | **N** | **E** | **F** | **R** | **A** | **Q** | **R** | **R** | **G** | **G** | **R** | **G** | **V** | **Q** | **G** | **M** | **G** | **I** | **H** | **N** | **D** | **D** | **F** | **V** | **K** | **N** | **L** | **V** | **S** | **C** | **S** | **T** | **H** | **D** | **T** | **L** | **L** | **F** | **F** | **T** | **N** | **N** | **G** | **K** | **V** | **Y** | **R** | **A** | **K** | **G** | **Y** | **E** | **I** | **P** | **E** | **Y** | **G** | **R** | **T** | **A** | **K** | **G** | **I** | **P** | **I** | **I** | **N** | **L** | **L** | **G** | **I** | **D** | **S** | **S** | **E** | **S** | **I** | **Q** | **A** | **I** | **I** | **A** | **V** | **T** | **G** | **E** | **A** | **E** | **E** | **G** | **H** | **Y** | **L** | **F** | **F** | **T** | **T** | **R** | **Q** | **G** | **T** | **V** | **K** | **R** | **T** | **S** | **V** | **T** | **A** | **F** | **A** | **N** | **I** | **R** | **S** | **N** | **G** | **L** | **I** | **A** | **I** | **G** | **L** | **K** | **E** | **D** | **D** | **E** | **L** | **V** | **N** | **V** | **L** | **L** | **T** | **D** | **G** | **Q** | **S** | **N** | **I** | **I** | **I** | **G** | **T** | **H** | **N** | **G** | **Y** | **S** | **V** | **T** | **F** | **K** | **E** | **E** | **A** | **V** | **R** | **D** | **M** | **G** | **R** | **T** | **A** | **S** | **G** | **V** | **R** | **G** | **I** | **R** | **L** | **R** | **E** | **E** | **D** | **Y** | **V** | **V** | **G** | **A** | **A** | **L** | **M** | **K** | **E** | **D** | **Q** | **E** | **V** | **L** | **V** | **I** | **T** | **E** | **K** | **G** | **Y** | **G** | **K** | **R** | **T** | **K** | **V** | **S** | **E** | **Y** | **P** | **V** | **K** | **G** | **R** | **G** | **G** | **K** | **G** | **I** | **K** | **T** | **A** | **N** | **I** | **T** | **E** | **K** | **N** | **G** | **P** | **L** | **A** | **G** | **L** | **T** | **T** | **V** | **S** | **G** | **D** | **E** | **D** | **I** | **M** | **L** | **I** | **T** | **D** | **K** | **G** | **V** | **I** | **I** | **R** | **F** | **N** | **V** | **S** | **T** | **V** | **S** | **Q** | **T** | **G** | **R** | **S** | **T** | **L** | **G** | **V** | **R** | **L** | **M** | **K** | **M** | **E** | **A** | **D** | **T** | **K** | **V** | **V** | **T** | **M** | **-** | **-** | **-** | **-** | **-** | **-** | **-** | **-** | **-** | **-** | **-** | **-** | **-** | **-** | **-** | **-** | **-** | **-** | **-** |
| **N59754F** | **M** | **S** | **E** | **E** | **I** | **R** | **E** | **N** | **I** | **H** | **D** | **V** | **N** | **L** | **T** | **S** | **E** | **M** | **K** | **D** | **S** | **F** | **I** | **D** | **Y** | **A** | **M** | **S** | **V** | **I** | **V** | **A** | **R** | **A** | **L** | **P** | **D** | **V** | **R** | **D** | **G** | **L** | **K** | **P** | **V** | **H** | **R** | **R** | **I** | **L** | **Y** | **G** | **M** | **N** | **E** | **L** | **G** | **V** | **T** | **P** | **D** | **K** | **A** | **H** | **K** | **K** | **S** | **A** | **R** | **I** | **V** | **G** | **D** | **V** | **M** | **G** | **K** | **Y** | **H** | **P** | **H** | **G** | **D** | **S** | **A** | **I** | **Y** | **E** | **S** | **M** | **V** | **R** | **M** | **A** | **Q** | **P** | **F** | **S** | **Y** | **R** | **Y** | **M** | **L** | **V** | **D** | **G** | **H** | **G** | **N** | **F** | **G** | **S** | **V** | **D** | **G** | **D** | **G** | **A** | **A** | **A** | **M** | **R** | **Y** | **T** | **E** | **A** | **R** | **M** | **S** | **K** | **I** | **A** | **T** | **E** | **M** | **L** | **R** | **D** | **I** | **N** | **K** | **N** | **T** | **V** | **D** | **F** | **Q** | **S** | **N** | **Y** | **D** | **D** | **T** | **E** | **K** | **E** | **P** | **V** | **V** | **L** | **P** | **A** | **R** | **F** | **P** | **N** | **L** | **L** | **V** | **N** | **G** | **T** | **T** | **G** | **I** | **A** | **V** | **G** | **M** | **A** | **T** | **N** | **I** | **P** | **P** | **H** | **N** | **L** | **S** | **E** | **V** | **V** | **A** | **A** | **I** | **D** | **L** | **L** | **M** | **D** | **N** | **P** | **D** | **V** | **T** | **T** | **N** | **E** | **L** | **M** | **E** | **V** | **L** | **P** | **G** | **P** | **D** | **F** | **P** | **T** | **G** | **G** | **L** | **V** | **M** | **G** | **K** | **S** | **G** | **I** | **R** | **R** | **A** | **Y** | **E** | **T** | **G** | **K** | **G** | **S** | **I** | **T** | **V** | **R** | **A** | **K** | **V** | **E** | **I** | **T** | **E** | **M** | **P** | **N** | **G** | **K** | **E** | **R** | **I** | **I** | **V** | **T** | **E** | **L** | **P** | **Y** | **M** | **V** | **N** | **K** | **A** | **K** | **L** | **I** | **E** | **R** | **I** | **S** | **E** | **L** | **H** | **R** | **D** | **K** | **R** | **I** | **E** | **G** | **I** | **T** | **D** | **L** | **R** | **D** | **E** | **S** | **S** | **R** | **E** | **G** | **M** | **R** | **I** | **V** | **I** | **D** | **I** | **R** | **R** | **D** | **A** | **S** | **A** | **S** | **V** | **I** | **L** | **N** | **N** | **L** | **Y** | **K** | **L** | **T** | **S** | **L** | **Q** | **N** | **S** | **F** | **G** | **F** | **N** | **M** | **L** | **A** | **I** | **E** | **K** | **G** | **V** | **P** | **K** | **V** | **L** | **S** | **L** | **K** | **Q** | **I** | **L** | **E** | **N** | **Y** | **I** | **E** | **H** | **Q** | **R** | **E** | **V** | **I** | **T** | **R** | **R** | **T** | **V** | **F** | **E** | **K** | **E** | **K** | **A** | **E** | **A** | **R** | **A** | **H** | **I** | **L** | **E** | **G** | **L** | **R** | **I** | **A** | **L** | **D** | **H** | **I** | **D** | **E** | **I** | **I** | **A** | **I** | **I** | **R** | **G** | **S** | **K** | **S** | **D** | **D** | **E** | **A** | **K** | **A** | **T** | **M** | **I** | **E** | **R** | **F** | **D** | **L** | **S** | **D** | **R** | **Q** | **S** | **Q** | **A** | **I** | **L** | **D** | **M** | **R** | **L** | **R** | **R** | **L** | **T** | **G** | **L** | **E** | **R** | **E** | **K** | **I** | **E** | **N** | **E** | **Y** | **Q** | **E** | **L** | **L** | **K** | **L** | **I** | **E** | **D** | **L** | **T** | **D** | **I** | **L** | **A** | **R** | **P** | **E** | **R** | **V** | **T** | **E** | **I** | **I** | **K** | **N** | **E** | **L** | **A** | **D** | **L** | **N** | **Q** | **R** | **F** | **G** | **D** | **K** | **R** | **R** | **T** | **E** | **L** | **L** | **V** | **G** | **E** | **V** | **L** | **S** | **L** | **E** | **D** | **E** | **D** | **L** | **I** | **E** | **E** | **E** | **E** | **I** | **V** | **I** | **T** | **L** | **T** | **N** | **N** | **G** | **Y** | **I** | **K** | **R** | **L** | **A** | **N** | **N** | **E** | **F** | **R** | **A** | **Q** | **R** | **R** | **G** | **G** | **R** | **G** | **V** | **Q** | **G** | **M** | **G** | **I** | **H** | **N** | **D** | **D** | **F** | **V** | **K** | **N** | **L** | **V** | **S** | **C** | **S** | **T** | **H** | **D** | **T** | **L** | **L** | **F** | **F** | **T** | **N** | **N** | **G** | **K** | **V** | **Y** | **R** | **A** | **K** | **G** | **Y** | **E** | **I** | **P** | **E** | **Y** | **G** | **R** | **T** | **A** | **K** | **G** | **I** | **P** | **I** | **I** | **N** | **L** | **L** | **G** | **I** | **D** | **S** | **S** | **E** | **S** | **I** | **Q** | **A** | **I** | **I** | **A** | **V** | **T** | **G** | **E** | **A** | **E** | **E** | **G** | **H** | **Y** | **L** | **F** | **F** | **T** | **T** | **R** | **Q** | **G** | **T** | **V** | **K** | **R** | **T** | **S** | **V** | **T** | **A** | **F** | **A** | **N** | **I** | **R** | **S** | **N** | **G** | **L** | **I** | **A** | **I** | **G** | **L** | **K** | **E** | **D** | **D** | **E** | **L** | **V** | **N** | **V** | **L** | **L** | **T** | **D** | **G** | **Q** | **S** | **N** | **I** | **I** | **I** | **G** | **T** | **H** | **N** | **G** | **Y** | **S** | **V** | **T** | **F** | **K** | **E** | **E** | **A** | **V** | **R** | **D** | **M** | **G** | **R** | **T** | **A** | **S** | **G** | **V** | **R** | **G** | **I** | **R** | **L** | **R** | **E** | **E** | **D** | **Y** | **V** | **V** | **G** | **A** | **A** | **L** | **M** | **K** | **E** | **N** | **Q** | **E** | **V** | **L** | **V** | **I** | **T** | **E** | **K** | **G** | **Y** | **G** | **K** | **R** | **T** | **K** | **V** | **S** | **E** | **Y** | **P** | **V** | **K** | **G** | **R** | **G** | **G** | **K** | **G** | **I** | **K** | **T** | **A** | **N** | **I** | **T** | **E** | **K** | **N** | **G** | **P** | **L** | **A** | **G** | **L** | **T** | **T** | **V** | **S** | **G** | **D** | **E** | **D** | **I** | **M** | **L** | **I** | **T** | **D** | **K** | **G** | **V** | **I** | **I** | **R** | **F** | **N** | **V** | **S** | **T** | **V** | **S** | **Q** | **T** | **G** | **R** | **S** | **T** | **L** | **G** | **V** | **R** | **L** | **M** | **K** | **M** | **E** | **A** | **D** | **T** | **K** | **V** | **V** | **T** | **M** | **A** | **A** | **V** | **E** | **P** | **E** | **A** | **A** | **E** | **Q** | **T** | **V** | **E** | **E** | **Q** | **G** | **S** | **E** | **E** |
| **N54599** | **M** | **S** | **E** | **E** | **I** | **R** | **E** | **N** | **I** | **H** | **D** | **V** | **N** | **L** | **T** | **S** | **E** | **M** | **K** | **D** | **S** | **F** | **I** | **D** | **Y** | **A** | **M** | **S** | **V** | **I** | **V** | **A** | **R** | **A** | **L** | **P** | **D** | **V** | **R** | **D** | **G** | **L** | **K** | **P** | **V** | **H** | **R** | **R** | **I** | **L** | **Y** | **G** | **M** | **N** | **E** | **L** | **G** | **V** | **T** | **P** | **D** | **K** | **A** | **H** | **K** | **K** | **S** | **A** | **R** | **I** | **V** | **G** | **D** | **V** | **M** | **G** | **K** | **Y** | **H** | **P** | **H** | **G** | **D** | **S** | **A** | **I** | **Y** | **E** | **S** | **M** | **V** | **R** | **M** | **A** | **Q** | **P** | **F** | **S** | **Y** | **R** | **Y** | **M** | **L** | **V** | **D** | **G** | **H** | **G** | **N** | **F** | **G** | **S** | **V** | **D** | **G** | **D** | **G** | **A** | **A** | **A** | **M** | **R** | **Y** | **T** | **E** | **A** | **R** | **M** | **S** | **K** | **I** | **A** | **T** | **E** | **M** | **L** | **R** | **D** | **I** | **N** | **K** | **N** | **T** | **V** | **D** | **F** | **Q** | **S** | **N** | **Y** | **D** | **D** | **T** | **E** | **K** | **E** | **P** | **V** | **V** | **L** | **P** | **A** | **R** | **F** | **P** | **N** | **L** | **L** | **V** | **N** | **G** | **T** | **T** | **G** | **I** | **A** | **V** | **G** | **M** | **A** | **T** | **N** | **I** | **P** | **P** | **H** | **N** | **L** | **S** | **E** | **V** | **V** | **A** | **A** | **I** | **D** | **L** | **L** | **M** | **D** | **N** | **P** | **D** | **V** | **T** | **T** | **N** | **E** | **L** | **M** | **E** | **V** | **L** | **P** | **G** | **P** | **D** | **F** | **P** | **T** | **G** | **G** | **L** | **V** | **M** | **G** | **K** | **S** | **G** | **I** | **R** | **R** | **A** | **Y** | **E** | **T** | **G** | **K** | **G** | **S** | **I** | **T** | **V** | **R** | **A** | **K** | **V** | **E** | **I** | **T** | **E** | **M** | **P** | **N** | **G** | **K** | **E** | **R** | **I** | **L** | **V** | **T** | **E** | **L** | **P** | **Y** | **M** | **V** | **N** | **K** | **A** | **K** | **L** | **I** | **E** | **R** | **I** | **S** | **E** | **L** | **H** | **R** | **D** | **K** | **R** | **I** | **E** | **G** | **I** | **T** | **D** | **L** | **R** | **D** | **E** | **S** | **S** | **R** | **E** | **G** | **M** | **R** | **I** | **V** | **I** | **D** | **V** | **R** | **R** | **D** | **A** | **S** | **A** | **S** | **V** | **I** | **L** | **N** | **N** | **L** | **Y** | **K** | **L** | **T** | **S** | **L** | **Q** | **N** | **S** | **F** | **G** | **F** | **N** | **M** | **L** | **A** | **I** | **E** | **K** | **G** | **V** | **P** | **K** | **V** | **L** | **S** | **L** | **K** | **Q** | **I** | **L** | **E** | **N** | **Y** | **I** | **E** | **H** | **Q** | **R** | **E** | **V** | **I** | **T** | **R** | **R** | **T** | **V** | **F** | **E** | **K** | **E** | **K** | **A** | **E** | **A** | **R** | **A** | **H** | **I** | **L** | **E** | **G** | **L** | **R** | **I** | **A** | **L** | **D** | **H** | **I** | **D** | **E** | **I** | **I** | **A** | **I** | **I** | **R** | **G** | **S** | **K** | **S** | **D** | **D** | **E** | **A** | **K** | **A** | **T** | **M** | **I** | **E** | **R** | **F** | **D** | **L** | **S** | **D** | **R** | **Q** | **S** | **Q** | **A** | **I** | **L** | **D** | **M** | **R** | **L** | **R** | **R** | **L** | **T** | **G** | **L** | **E** | **R** | **E** | **K** | **I** | **E** | **N** | **E** | **Y** | **Q** | **E** | **L** | **L** | **K** | **L** | **I** | **E** | **D** | **L** | **T** | **D** | **I** | **L** | **A** | **R** | **P** | **E** | **R** | **V** | **T** | **E** | **I** | **I** | **K** | **N** | **E** | **L** | **A** | **D** | **L** | **N** | **Q** | **R** | **F** | **G** | **D** | **K** | **R** | **R** | **T** | **E** | **L** | **L** | **V** | **G** | **E** | **V** | **L** | **S** | **L** | **E** | **D** | **E** | **D** | **L** | **I** | **E** | **E** | **E** | **E** | **I** | **V** | **I** | **T** | **L** | **T** | **N** | **N** | **G** | **Y** | **I** | **K** | **R** | **L** | **A** | **N** | **N** | **E** | **F** | **R** | **A** | **Q** | **R** | **R** | **G** | **G** | **R** | **G** | **V** | **Q** | **G** | **M** | **G** | **I** | **H** | **N** | **D** | **D** | **F** | **V** | **K** | **N** | **L** | **V** | **S** | **C** | **S** | **T** | **H** | **D** | **T** | **L** | **L** | **F** | **F** | **T** | **N** | **N** | **G** | **K** | **V** | **Y** | **R** | **A** | **K** | **G** | **Y** | **E** | **I** | **P** | **E** | **Y** | **G** | **R** | **T** | **A** | **K** | **G** | **I** | **P** | **I** | **I** | **N** | **L** | **L** | **G** | **I** | **D** | **S** | **S** | **E** | **S** | **I** | **Q** | **A** | **I** | **I** | **A** | **V** | **T** | **G** | **E** | **A** | **E** | **E** | **G** | **H** | **Y** | **L** | **F** | **F** | **T** | **T** | **R** | **Q** | **G** | **T** | **V** | **K** | **R** | **T** | **S** | **V** | **T** | **A** | **F** | **A** | **N** | **I** | **R** | **S** | **N** | **G** | **L** | **I** | **A** | **I** | **G** | **L** | **K** | **E** | **D** | **D** | **E** | **L** | **V** | **N** | **V** | **L** | **L** | **T** | **D** | **G** | **Q** | **S** | **N** | **I** | **I** | **I** | **G** | **T** | **H** | **N** | **G** | **Y** | **S** | **V** | **T** | **F** | **K** | **E** | **E** | **A** | **V** | **R** | **D** | **M** | **G** | **R** | **T** | **A** | **S** | **G** | **V** | **R** | **G** | **I** | **R** | **L** | **R** | **E** | **E** | **D** | **Y** | **V** | **V** | **G** | **A** | **A** | **L** | **M** | **K** | **E** | **D** | **Q** | **E** | **V** | **L** | **V** | **I** | **T** | **E** | **K** | **G** | **Y** | **G** | **K** | **R** | **T** | **K** | **V** | **S** | **E** | **Y** | **P** | **V** | **K** | **G** | **R** | **G** | **G** | **K** | **G** | **I** | **K** | **T** | **A** | **N** | **I** | **T** | **E** | **K** | **N** | **G** | **P** | **L** | **A** | **G** | **L** | **T** | **T** | **V** | **S** | **G** | **N** | **E** | **D** | **I** | **M** | **L** | **I** | **T** | **D** | **K** | **G** | **V** | **I** | **I** | **R** | **F** | **N** | **V** | **S** | **T** | **V** | **S** | **Q** | **T** | **G** | **R** | **S** | **T** | **L** | **G** | **V** | **R** | **L** | **M** | **K** | **M** | **E** | **A** | **D** | **T** | **K** | **V** | **V** | **T** | **M** | **-** | **-** | **-** | **-** | **-** | **-** | **-** | **-** | **-** | **-** | **-** | **-** | **-** | **-** | **-** | **-** | **-** | **-** | **-** |
| **N54538** | **M** | **S** | **E** | **E** | **I** | **R** | **E** | **N** | **I** | **H** | **D** | **V** | **N** | **L** | **T** | **S** | **E** | **M** | **K** | **D** | **S** | **F** | **I** | **D** | **Y** | **A** | **M** | **S** | **V** | **I** | **V** | **A** | **R** | **A** | **L** | **P** | **D** | **V** | **R** | **D** | **G** | **L** | **K** | **P** | **V** | **H** | **R** | **R** | **I** | **L** | **Y** | **G** | **M** | **N** | **E** | **L** | **G** | **V** | **T** | **P** | **D** | **K** | **A** | **H** | **K** | **K** | **S** | **A** | **R** | **I** | **V** | **G** | **D** | **V** | **M** | **G** | **K** | **Y** | **H** | **P** | **H** | **G** | **D** | **S** | **A** | **I** | **Y** | **E** | **S** | **M** | **V** | **R** | **M** | **A** | **Q** | **P** | **F** | **S** | **Y** | **R** | **Y** | **M** | **L** | **V** | **D** | **G** | **H** | **G** | **N** | **F** | **G** | **S** | **V** | **D** | **G** | **D** | **G** | **A** | **A** | **A** | **M** | **R** | **Y** | **T** | **E** | **A** | **R** | **M** | **S** | **K** | **I** | **A** | **T** | **E** | **M** | **L** | **R** | **D** | **I** | **N** | **K** | **N** | **T** | **V** | **D** | **F** | **Q** | **S** | **N** | **Y** | **D** | **D** | **T** | **E** | **K** | **E** | **P** | **V** | **V** | **L** | **P** | **A** | **R** | **F** | **P** | **N** | **L** | **L** | **V** | **N** | **G** | **T** | **T** | **G** | **I** | **A** | **V** | **G** | **M** | **A** | **T** | **N** | **I** | **P** | **P** | **H** | **N** | **L** | **S** | **E** | **V** | **V** | **A** | **A** | **I** | **D** | **L** | **L** | **M** | **D** | **N** | **P** | **D** | **V** | **T** | **T** | **N** | **E** | **L** | **M** | **E** | **V** | **L** | **P** | **G** | **P** | **D** | **F** | **P** | **T** | **G** | **G** | **L** | **V** | **M** | **G** | **K** | **S** | **G** | **I** | **R** | **R** | **A** | **Y** | **E** | **T** | **G** | **K** | **G** | **S** | **I** | **T** | **V** | **R** | **A** | **K** | **V** | **E** | **I** | **T** | **E** | **M** | **P** | **N** | **G** | **K** | **E** | **R** | **I** | **I** | **V** | **T** | **E** | **L** | **P** | **Y** | **M** | **V** | **N** | **K** | **A** | **K** | **L** | **I** | **E** | **R** | **I** | **S** | **E** | **L** | **H** | **R** | **D** | **K** | **R** | **I** | **E** | **G** | **I** | **T** | **D** | **L** | **R** | **D** | **E** | **S** | **S** | **R** | **E** | **G** | **M** | **R** | **I** | **V** | **I** | **D** | **I** | **R** | **R** | **D** | **A** | **S** | **A** | **S** | **V** | **I** | **L** | **N** | **N** | **L** | **Y** | **K** | **L** | **T** | **S** | **L** | **Q** | **N** | **S** | **F** | **G** | **F** | **N** | **M** | **L** | **A** | **I** | **E** | **K** | **G** | **V** | **P** | **K** | **V** | **L** | **S** | **L** | **K** | **Q** | **I** | **L** | **E** | **N** | **Y** | **I** | **E** | **H** | **Q** | **R** | **E** | **V** | **I** | **T** | **R** | **R** | **T** | **V** | **F** | **E** | **K** | **E** | **K** | **A** | **E** | **A** | **R** | **A** | **H** | **I** | **L** | **E** | **G** | **L** | **R** | **I** | **A** | **L** | **D** | **H** | **I** | **D** | **E** | **I** | **I** | **A** | **I** | **I** | **R** | **G** | **S** | **K** | **S** | **D** | **D** | **E** | **A** | **K** | **A** | **T** | **M** | **I** | **E** | **R** | **F** | **D** | **L** | **S** | **D** | **R** | **Q** | **S** | **Q** | **A** | **I** | **L** | **D** | **M** | **R** | **L** | **R** | **R** | **L** | **T** | **G** | **L** | **E** | **R** | **E** | **K** | **I** | **E** | **N** | **E** | **Y** | **Q** | **E** | **L** | **L** | **K** | **L** | **I** | **E** | **D** | **L** | **T** | **D** | **I** | **L** | **A** | **R** | **P** | **E** | **R** | **V** | **T** | **E** | **I** | **I** | **K** | **N** | **E** | **L** | **A** | **D** | **L** | **N** | **Q** | **R** | **F** | **G** | **D** | **K** | **R** | **R** | **T** | **E** | **L** | **L** | **V** | **G** | **E** | **V** | **L** | **S** | **L** | **E** | **D** | **E** | **D** | **L** | **I** | **E** | **E** | **E** | **E** | **I** | **V** | **I** | **T** | **L** | **T** | **N** | **N** | **G** | **Y** | **I** | **K** | **R** | **L** | **A** | **N** | **N** | **E** | **F** | **R** | **A** | **Q** | **R** | **R** | **G** | **G** | **R** | **G** | **V** | **Q** | **G** | **M** | **G** | **I** | **H** | **N** | **D** | **D** | **F** | **V** | **K** | **N** | **L** | **V** | **S** | **C** | **S** | **T** | **H** | **D** | **T** | **L** | **L** | **F** | **F** | **T** | **N** | **N** | **G** | **K** | **V** | **Y** | **R** | **A** | **K** | **G** | **Y** | **E** | **I** | **P** | **E** | **Y** | **G** | **R** | **T** | **A** | **K** | **G** | **I** | **P** | **I** | **I** | **N** | **L** | **L** | **G** | **I** | **D** | **S** | **S** | **E** | **S** | **I** | **Q** | **A** | **I** | **I** | **A** | **V** | **T** | **G** | **E** | **A** | **E** | **E** | **G** | **H** | **Y** | **L** | **F** | **F** | **T** | **T** | **R** | **Q** | **G** | **T** | **V** | **K** | **R** | **T** | **S** | **V** | **T** | **A** | **F** | **A** | **N** | **I** | **R** | **S** | **N** | **G** | **L** | **I** | **A** | **I** | **G** | **L** | **K** | **E** | **D** | **D** | **E** | **L** | **V** | **N** | **V** | **L** | **L** | **T** | **D** | **G** | **Q** | **S** | **N** | **I** | **I** | **I** | **G** | **T** | **H** | **N** | **G** | **Y** | **S** | **V** | **T** | **F** | **K** | **E** | **E** | **A** | **V** | **R** | **D** | **M** | **G** | **R** | **T** | **A** | **S** | **G** | **V** | **R** | **G** | **I** | **R** | **L** | **R** | **E** | **E** | **D** | **Y** | **V** | **V** | **G** | **A** | **A** | **L** | **M** | **K** | **E** | **N** | **Q** | **E** | **V** | **L** | **V** | **I** | **T** | **E** | **K** | **G** | **Y** | **G** | **K** | **R** | **T** | **K** | **V** | **S** | **E** | **Y** | **P** | **V** | **K** | **G** | **R** | **G** | **G** | **K** | **G** | **I** | **K** | **T** | **A** | **N** | **I** | **T** | **E** | **K** | **N** | **G** | **P** | **L** | **A** | **G** | **L** | **T** | **T** | **V** | **S** | **G** | **D** | **E** | **D** | **I** | **M** | **L** | **I** | **T** | **D** | **K** | **G** | **V** | **I** | **I** | **R** | **F** | **N** | **V** | **S** | **T** | **V** | **S** | **Q** | **T** | **G** | **R** | **S** | **T** | **L** | **G** | **V** | **R** | **L** | **M** | **K** | **M** | **E** | **A** | **D** | **T** | **K** | **V** | **V** | **T** | **M** | **A** | **A** | **V** | **E** | **P** | **E** | **A** | **A** | **E** | **Q** | **T** | **V** | **E** | **E** | **Q** | **G** | **S** | **E** | **E** |
| **N60114F** | **M** | **S** | **E** | **E** | **I** | **K** | **E** | **N** | **I** | **Q** | **D** | **V** | **N** | **L** | **T** | **S** | **E** | **M** | **K** | **E** | **S** | **F** | **I** | **D** | **Y** | **A** | **M** | **S** | **V** | **I** | **V** | **A** | **R** | **A** | **L** | **P** | **D** | **V** | **R** | **D** | **G** | **L** | **K** | **P** | **V** | **H** | **R** | **R** | **I** | **L** | **Y** | **G** | **M** | **N** | **E** | **L** | **G** | **V** | **T** | **P** | **D** | **K** | **P** | **H** | **K** | **K** | **S** | **A** | **R** | **I** | **V** | **G** | **D** | **V** | **M** | **G** | **K** | **Y** | **H** | **P** | **H** | **G** | **D** | **S** | **A** | **I** | **Y** | **E** | **S** | **M** | **V** | **R** | **M** | **A** | **Q** | **P** | **F** | **S** | **Y** | **R** | **A** | **M** | **L** | **V** | **D** | **G** | **H** | **G** | **N** | **F** | **G** | **S** | **V** | **D** | **G** | **D** | **G** | **A** | **A** | **A** | **M** | **R** | **Y** | **T** | **E** | **A** | **R** | **M** | **S** | **K** | **I** | **A** | **L** | **E** | **M** | **L** | **R** | **D** | **I** | **N** | **K** | **N** | **T** | **V** | **D** | **F** | **Q** | **G** | **N** | **Y** | **D** | **D** | **S** | **E** | **Q** | **E** | **P** | **V** | **V** | **L** | **P** | **A** | **R** | **F** | **P** | **N** | **L** | **L** | **V** | **N** | **G** | **T** | **T** | **G** | **I** | **A** | **V** | **G** | **M** | **A** | **T** | **N** | **I** | **P** | **P** | **H** | **N** | **L** | **S** | **E** | **V** | **I** | **D** | **A** | **T** | **S** | **L** | **L** | **M** | **D** | **N** | **P** | **D** | **V** | **T** | **T** | **N** | **E** | **L** | **M** | **E** | **V** | **L** | **P** | **G** | **P** | **D** | **F** | **P** | **T** | **G** | **G** | **L** | **V** | **M** | **G** | **K** | **S** | **G** | **I** | **R** | **R** | **A** | **Y** | **E** | **T** | **G** | **K** | **G** | **S** | **I** | **T** | **V** | **R** | **A** | **K** | **V** | **E** | **L** | **T** | **E** | **M** | **P** | **N** | **G** | **K** | **E** | **R** | **I** | **L** | **V** | **T** | **E** | **L** | **P** | **Y** | **M** | **V** | **N** | **K** | **A** | **K** | **L** | **I** | **E** | **R** | **I** | **S** | **E** | **L** | **H** | **R** | **D** | **K** | **R** | **I** | **E** | **G** | **I** | **T** | **D** | **L** | **R** | **D** | **E** | **S** | **S** | **R** | **E** | **G** | **M** | **R** | **I** | **V** | **I** | **D** | **V** | **R** | **R** | **D** | **V** | **S** | **A** | **S** | **V** | **V** | **L** | **N** | **N** | **L** | **Y** | **K** | **M** | **T** | **A** | **L** | **Q** | **T** | **S** | **F** | **G** | **F** | **N** | **M** | **L** | **A** | **I** | **E** | **K** | **G** | **V** | **P** | **K** | **I** | **L** | **S** | **L** | **K** | **R** | **I** | **L** | **E** | **N** | **Y** | **V** | **E** | **H** | **Q** | **K** | **E** | **V** | **I** | **T** | **R** | **R** | **T** | **I** | **F** | **D** | **K** | **N** | **K** | **A** | **E** | **A** | **R** | **A** | **H** | **I** | **L** | **E** | **G** | **L** | **R** | **I** | **A** | **L** | **D** | **H** | **I** | **D** | **E** | **I** | **I** | **A** | **I** | **I** | **R** | **G** | **S** | **Q** | **S** | **D** | **D** | **E** | **A** | **K** | **A** | **T** | **L** | **I** | **E** | **R** | **F** | **E** | **F** | **S** | **D** | **R** | **Q** | **A** | **Q** | **A** | **I** | **L** | **D** | **M** | **R** | **L** | **R** | **R** | **L** | **T** | **G** | **L** | **E** | **R** | **D** | **K** | **I** | **E** | **N** | **E** | **Y** | **Q** | **E** | **L** | **L** | **K** | **F** | **I** | **A** | **D** | **L** | **E** | **D** | **I** | **L** | **A** | **R** | **P** | **E** | **R** | **V** | **I** | **E** | **I** | **I** | **K** | **T** | **E** | **L** | **N** | **D** | **V** | **R** | **T** | **K** | **F** | **G** | **D** | **A** | **R** | **R** | **T** | **E** | **L** | **L** | **V** | **G** | **E** | **V** | **L** | **S** | **L** | **E** | **D** | **E** | **D** | **L** | **I** | **E** | **E** | **E** | **E** | **V** | **V** | **I** | **T** | **L** | **T** | **N** | **N** | **G** | **Y** | **I** | **K** | **R** | **M** | **A** | **N** | **S** | **E** | **F** | **R** | **A** | **Q** | **R** | **R** | **G** | **G** | **R** | **G** | **V** | **Q** | **G** | **M** | **G** | **V** | **H** | **D** | **D** | **D** | **F** | **V** | **K** | **N** | **L** | **V** | **S** | **C** | **S** | **T** | **H** | **D** | **T** | **L** | **L** | **F** | **F** | **T** | **N** | **T** | **G** | **K** | **V** | **Y** | **R** | **A** | **K** | **G** | **Y** | **E** | **I** | **P** | **E** | **Y** | **G** | **R** | **T** | **A** | **K** | **G** | **I** | **P** | **V** | **I** | **N** | **L** | **L** | **G** | **I** | **D** | **S** | **A** | **E** | **K** | **I** | **Q** | **A** | **I** | **I** | **S** | **V** | **E** | **G** | **K** | **A** | **E** | **A** | **G** | **K** | **Y** | **L** | **F** | **F** | **T** | **T** | **L** | **K** | **G** | **T** | **V** | **K** | **R** | **T** | **A** | **V** | **T** | **A** | **F** | **S** | **N** | **I** | **R** | **S** | **N** | **G** | **L** | **I** | **A** | **I** | **S** | **L** | **K** | **E** | **D** | **D** | **E** | **L** | **V** | **N** | **V** | **V** | **T** | **T** | **N** | **G** | **N** | **Q** | **K** | **M** | **I** | **I** | **G** | **T** | **H** | **A** | **G** | **Y** | **S** | **V** | **T** | **F** | **D** | **E** | **N** | **T** | **V** | **R** | **D** | **M** | **G** | **R** | **T** | **A** | **S** | **G** | **V** | **R** | **G** | **I** | **R** | **L** | **R** | **E** | **N** | **D** | **Y** | **V** | **V** | **G** | **A** | **A** | **I** | **L** | **D** | **E** | **N** | **K** | **E** | **V** | **L** | **V** | **I** | **T** | **E** | **N** | **G** | **Y** | **G** | **K** | **R** | **T** | **K** | **A** | **S** | **E** | **Y** | **P** | **V** | **K** | **G** | **R** | **G** | **G** | **K** | **G** | **I** | **K** | **T** | **A** | **N** | **I** | **T** | **E** | **K** | **N** | **G** | **P** | **L** | **A** | **G** | **L** | **T** | **T** | **V** | **N** | **G** | **D** | **E** | **D** | **I** | **L** | **L** | **I** | **T** | **N** | **K** | **G** | **V** | **I** | **I** | **R** | **F** | **N** | **V** | **D** | **S** | **V** | **S** | **Q** | **T** | **G** | **R** | **A** | **T** | **L** | **G** | **V** | **R** | **L** | **M** | **R** | **M** | **E** | **D** | **G** | **A** | **K** | **-** | **-** | **-** | **-** | **-** | **-** | **-** | **-** | **-** | **-** | **-** | **-** | **-** | **-** | **-** | **-** | **-** | **-** | **-** | **-** | **-** | **-** | **-** |
| **N59491F** | **M** | **S** | **E** | **E** | **I** | **K** | **E** | **N** | **I** | **Q** | **D** | **V** | **N** | **L** | **T** | **S** | **E** | **M** | **K** | **E** | **S** | **F** | **I** | **D** | **Y** | **A** | **M** | **S** | **V** | **I** | **V** | **A** | **R** | **A** | **L** | **P** | **D** | **V** | **R** | **D** | **G** | **L** | **K** | **P** | **V** | **H** | **R** | **R** | **I** | **L** | **Y** | **G** | **M** | **N** | **E** | **L** | **G** | **V** | **T** | **P** | **D** | **K** | **P** | **H** | **K** | **K** | **S** | **A** | **R** | **I** | **V** | **G** | **D** | **V** | **M** | **G** | **K** | **Y** | **H** | **P** | **H** | **G** | **D** | **S** | **A** | **I** | **Y** | **E** | **S** | **M** | **V** | **R** | **M** | **A** | **Q** | **P** | **F** | **S** | **Y** | **R** | **A** | **M** | **L** | **V** | **D** | **G** | **H** | **G** | **N** | **F** | **G** | **S** | **V** | **D** | **G** | **D** | **G** | **A** | **A** | **A** | **M** | **R** | **Y** | **T** | **E** | **A** | **R** | **M** | **S** | **K** | **I** | **A** | **L** | **E** | **M** | **L** | **R** | **D** | **I** | **N** | **K** | **N** | **T** | **V** | **D** | **F** | **Q** | **G** | **N** | **Y** | **D** | **D** | **S** | **E** | **Q** | **E** | **P** | **V** | **V** | **L** | **P** | **A** | **R** | **F** | **P** | **N** | **L** | **L** | **V** | **N** | **G** | **T** | **T** | **G** | **I** | **A** | **V** | **G** | **M** | **A** | **T** | **N** | **I** | **P** | **P** | **H** | **N** | **L** | **S** | **E** | **V** | **I** | **D** | **A** | **T** | **S** | **L** | **L** | **M** | **D** | **N** | **P** | **D** | **V** | **T** | **T** | **N** | **E** | **L** | **M** | **E** | **V** | **L** | **P** | **G** | **P** | **D** | **F** | **P** | **T** | **G** | **G** | **L** | **V** | **M** | **G** | **K** | **S** | **G** | **I** | **R** | **R** | **A** | **Y** | **E** | **T** | **G** | **K** | **G** | **S** | **I** | **T** | **V** | **R** | **A** | **K** | **V** | **E** | **L** | **T** | **E** | **M** | **P** | **N** | **G** | **K** | **E** | **R** | **I** | **L** | **V** | **T** | **E** | **L** | **P** | **Y** | **M** | **V** | **N** | **K** | **A** | **K** | **L** | **I** | **E** | **R** | **I** | **S** | **E** | **L** | **H** | **R** | **D** | **K** | **R** | **I** | **E** | **G** | **I** | **T** | **D** | **L** | **R** | **D** | **E** | **S** | **S** | **R** | **E** | **G** | **M** | **R** | **I** | **V** | **I** | **D** | **V** | **R** | **R** | **D** | **V** | **S** | **A** | **S** | **V** | **V** | **L** | **N** | **N** | **L** | **Y** | **K** | **M** | **T** | **A** | **L** | **Q** | **T** | **S** | **F** | **G** | **F** | **N** | **M** | **L** | **A** | **I** | **E** | **K** | **G** | **V** | **P** | **K** | **I** | **L** | **S** | **L** | **K** | **R** | **I** | **L** | **E** | **N** | **Y** | **V** | **E** | **H** | **Q** | **K** | **E** | **V** | **I** | **T** | **R** | **R** | **T** | **I** | **F** | **D** | **K** | **N** | **K** | **A** | **E** | **A** | **R** | **A** | **H** | **I** | **L** | **E** | **G** | **L** | **R** | **I** | **A** | **L** | **D** | **H** | **I** | **D** | **E** | **I** | **I** | **A** | **I** | **I** | **R** | **G** | **S** | **Q** | **S** | **D** | **D** | **E** | **A** | **K** | **A** | **T** | **L** | **I** | **E** | **R** | **F** | **E** | **F** | **S** | **D** | **R** | **Q** | **A** | **Q** | **A** | **I** | **L** | **D** | **M** | **R** | **L** | **R** | **R** | **L** | **T** | **G** | **L** | **E** | **R** | **D** | **K** | **I** | **E** | **N** | **E** | **Y** | **Q** | **E** | **L** | **L** | **K** | **F** | **I** | **A** | **D** | **L** | **E** | **D** | **I** | **L** | **A** | **R** | **P** | **E** | **R** | **V** | **I** | **E** | **I** | **I** | **K** | **T** | **E** | **L** | **N** | **D** | **V** | **R** | **T** | **K** | **F** | **G** | **D** | **A** | **R** | **R** | **T** | **E** | **L** | **L** | **V** | **G** | **E** | **V** | **L** | **S** | **L** | **E** | **D** | **E** | **D** | **L** | **I** | **E** | **E** | **E** | **E** | **V** | **V** | **I** | **T** | **L** | **T** | **N** | **N** | **G** | **Y** | **I** | **K** | **R** | **M** | **A** | **N** | **S** | **E** | **F** | **R** | **A** | **Q** | **R** | **R** | **G** | **G** | **R** | **G** | **V** | **Q** | **G** | **M** | **G** | **V** | **H** | **D** | **D** | **D** | **F** | **V** | **K** | **K** | **L** | **V** | **S** | **C** | **S** | **T** | **H** | **D** | **T** | **L** | **L** | **F** | **F** | **T** | **N** | **T** | **G** | **K** | **V** | **Y** | **R** | **A** | **K** | **G** | **Y** | **E** | **I** | **P** | **E** | **Y** | **G** | **R** | **T** | **A** | **K** | **G** | **I** | **P** | **V** | **I** | **N** | **L** | **L** | **G** | **I** | **D** | **S** | **A** | **E** | **K** | **I** | **Q** | **A** | **I** | **I** | **S** | **V** | **E** | **G** | **K** | **A** | **E** | **A** | **G** | **K** | **Y** | **L** | **F** | **F** | **T** | **T** | **L** | **K** | **G** | **T** | **V** | **K** | **R** | **T** | **A** | **V** | **T** | **A** | **F** | **S** | **N** | **I** | **R** | **S** | **N** | **G** | **L** | **I** | **A** | **I** | **S** | **L** | **K** | **E** | **D** | **D** | **E** | **L** | **V** | **N** | **V** | **V** | **T** | **T** | **N** | **G** | **N** | **Q** | **K** | **M** | **I** | **I** | **G** | **T** | **H** | **A** | **G** | **Y** | **S** | **V** | **T** | **F** | **D** | **E** | **N** | **T** | **V** | **R** | **D** | **M** | **G** | **R** | **T** | **A** | **S** | **G** | **V** | **R** | **G** | **I** | **R** | **L** | **R** | **E** | **N** | **D** | **Y** | **V** | **V** | **G** | **A** | **A** | **I** | **L** | **D** | **E** | **N** | **K** | **E** | **V** | **L** | **V** | **I** | **T** | **E** | **N** | **G** | **Y** | **G** | **K** | **R** | **T** | **K** | **A** | **S** | **E** | **Y** | **P** | **V** | **K** | **G** | **R** | **G** | **G** | **K** | **G** | **I** | **K** | **T** | **A** | **N** | **I** | **T** | **E** | **K** | **N** | **G** | **P** | **L** | **A** | **G** | **L** | **T** | **T** | **V** | **N** | **G** | **D** | **E** | **D** | **I** | **L** | **L** | **I** | **T** | **N** | **K** | **G** | **V** | **I** | **I** | **R** | **F** | **N** | **V** | **D** | **S** | **V** | **S** | **Q** | **T** | **G** | **R** | **A** | **T** | **L** | **G** | **V** | **R** | **L** | **M** | **R** | **M** | **E** | **D** | **G** | **A** | **K** | **-** | **-** | **-** | **-** | **-** | **-** | **-** | **-** | **-** | **-** | **-** | **-** | **-** | **-** | **-** | **-** | **-** | **-** | **-** | **-** | **-** | **-** | **-** |
| **N59947F** | **M** | **S** | **E** | **E** | **I** | **R** | **E** | **N** | **I** | **H** | **D** | **V** | **N** | **L** | **T** | **S** | **E** | **M** | **K** | **D** | **S** | **F** | **I** | **D** | **Y** | **A** | **M** | **S** | **V** | **I** | **V** | **A** | **R** | **A** | **L** | **P** | **D** | **V** | **R** | **D** | **G** | **L** | **K** | **P** | **V** | **H** | **R** | **R** | **I** | **L** | **Y** | **G** | **M** | **N** | **E** | **L** | **G** | **V** | **T** | **P** | **D** | **K** | **A** | **H** | **K** | **K** | **S** | **A** | **R** | **I** | **V** | **G** | **D** | **V** | **M** | **G** | **K** | **Y** | **H** | **P** | **H** | **G** | **D** | **S** | **A** | **I** | **Y** | **E** | **S** | **M** | **V** | **R** | **M** | **A** | **Q** | **P** | **F** | **S** | **Y** | **R** | **Y** | **M** | **L** | **V** | **D** | **G** | **H** | **G** | **N** | **F** | **G** | **S** | **V** | **D** | **G** | **D** | **G** | **A** | **A** | **A** | **M** | **R** | **Y** | **T** | **E** | **A** | **R** | **M** | **S** | **K** | **I** | **A** | **T** | **E** | **M** | **L** | **R** | **D** | **I** | **N** | **K** | **N** | **T** | **V** | **D** | **F** | **Q** | **S** | **N** | **Y** | **D** | **D** | **T** | **E** | **K** | **E** | **P** | **V** | **V** | **L** | **P** | **A** | **R** | **F** | **P** | **N** | **L** | **L** | **V** | **N** | **G** | **T** | **T** | **G** | **I** | **A** | **V** | **G** | **M** | **A** | **T** | **N** | **I** | **P** | **P** | **H** | **N** | **L** | **S** | **E** | **V** | **V** | **A** | **A** | **I** | **D** | **L** | **L** | **M** | **D** | **N** | **P** | **D** | **V** | **T** | **T** | **N** | **E** | **L** | **M** | **E** | **V** | **L** | **P** | **G** | **P** | **D** | **F** | **P** | **T** | **G** | **G** | **L** | **V** | **M** | **G** | **K** | **S** | **G** | **I** | **R** | **R** | **A** | **Y** | **E** | **T** | **G** | **K** | **G** | **S** | **I** | **T** | **V** | **R** | **A** | **K** | **V** | **E** | **I** | **T** | **E** | **M** | **P** | **N** | **G** | **K** | **E** | **R** | **I** | **L** | **V** | **T** | **E** | **L** | **P** | **Y** | **M** | **V** | **N** | **K** | **A** | **K** | **L** | **I** | **E** | **R** | **I** | **S** | **E** | **L** | **H** | **R** | **D** | **K** | **R** | **I** | **E** | **G** | **I** | **T** | **D** | **L** | **R** | **D** | **E** | **S** | **S** | **R** | **E** | **G** | **M** | **R** | **I** | **V** | **I** | **D** | **V** | **R** | **R** | **D** | **A** | **S** | **A** | **S** | **V** | **I** | **L** | **N** | **N** | **L** | **Y** | **K** | **L** | **T** | **S** | **L** | **Q** | **N** | **S** | **F** | **G** | **F** | **N** | **M** | **L** | **A** | **I** | **E** | **K** | **G** | **V** | **P** | **K** | **V** | **L** | **S** | **L** | **K** | **Q** | **I** | **L** | **E** | **N** | **Y** | **I** | **E** | **H** | **Q** | **R** | **E** | **V** | **I** | **T** | **R** | **R** | **T** | **V** | **F** | **E** | **K** | **E** | **K** | **A** | **E** | **A** | **R** | **A** | **H** | **I** | **L** | **E** | **G** | **L** | **R** | **I** | **A** | **L** | **D** | **H** | **I** | **D** | **E** | **I** | **I** | **A** | **I** | **I** | **R** | **G** | **S** | **K** | **S** | **D** | **D** | **E** | **A** | **K** | **A** | **T** | **M** | **I** | **E** | **R** | **F** | **D** | **L** | **S** | **D** | **R** | **Q** | **S** | **Q** | **A** | **I** | **L** | **D** | **M** | **R** | **L** | **R** | **R** | **L** | **T** | **G** | **L** | **E** | **R** | **E** | **K** | **I** | **E** | **N** | **E** | **Y** | **Q** | **E** | **L** | **L** | **K** | **L** | **I** | **E** | **D** | **L** | **T** | **D** | **I** | **L** | **A** | **R** | **P** | **E** | **R** | **V** | **T** | **E** | **I** | **I** | **K** | **N** | **E** | **L** | **A** | **D** | **L** | **N** | **Q** | **R** | **F** | **G** | **D** | **K** | **R** | **R** | **T** | **E** | **L** | **L** | **V** | **G** | **E** | **V** | **L** | **S** | **L** | **E** | **D** | **E** | **D** | **L** | **I** | **E** | **E** | **E** | **E** | **I** | **V** | **I** | **T** | **L** | **T** | **N** | **N** | **G** | **Y** | **I** | **K** | **R** | **L** | **A** | **N** | **N** | **E** | **F** | **R** | **A** | **Q** | **R** | **R** | **G** | **G** | **R** | **G** | **V** | **Q** | **G** | **M** | **G** | **I** | **H** | **N** | **D** | **D** | **F** | **V** | **K** | **N** | **L** | **V** | **S** | **C** | **S** | **T** | **H** | **D** | **T** | **L** | **L** | **F** | **F** | **T** | **N** | **N** | **G** | **K** | **V** | **Y** | **R** | **A** | **K** | **G** | **Y** | **E** | **I** | **P** | **E** | **Y** | **G** | **R** | **T** | **A** | **K** | **G** | **I** | **P** | **I** | **I** | **N** | **L** | **L** | **G** | **I** | **D** | **S** | **S** | **E** | **S** | **I** | **Q** | **A** | **I** | **I** | **A** | **V** | **T** | **G** | **E** | **A** | **E** | **E** | **G** | **H** | **Y** | **L** | **F** | **F** | **T** | **T** | **R** | **Q** | **G** | **T** | **V** | **K** | **R** | **T** | **S** | **V** | **T** | **A** | **F** | **A** | **N** | **I** | **R** | **S** | **N** | **G** | **L** | **I** | **A** | **I** | **G** | **L** | **K** | **E** | **D** | **D** | **E** | **L** | **V** | **N** | **V** | **L** | **L** | **T** | **D** | **G** | **Q** | **S** | **N** | **I** | **I** | **I** | **G** | **T** | **H** | **N** | **G** | **Y** | **S** | **V** | **T** | **F** | **K** | **E** | **E** | **A** | **V** | **R** | **D** | **M** | **G** | **R** | **T** | **A** | **S** | **G** | **V** | **R** | **G** | **I** | **R** | **L** | **R** | **E** | **E** | **D** | **Y** | **V** | **V** | **G** | **A** | **A** | **L** | **M** | **K** | **E** | **D** | **Q** | **E** | **V** | **L** | **V** | **I** | **T** | **E** | **K** | **G** | **Y** | **G** | **K** | **R** | **T** | **K** | **V** | **S** | **E** | **Y** | **P** | **V** | **K** | **G** | **R** | **G** | **G** | **K** | **G** | **I** | **K** | **T** | **A** | **N** | **I** | **T** | **E** | **K** | **N** | **G** | **P** | **L** | **A** | **G** | **L** | **T** | **T** | **V** | **S** | **G** | **N** | **E** | **D** | **I** | **M** | **L** | **I** | **T** | **D** | **K** | **G** | **V** | **I** | **I** | **R** | **F** | **N** | **V** | **S** | **T** | **V** | **S** | **Q** | **T** | **G** | **R** | **S** | **T** | **L** | **G** | **V** | **R** | **L** | **M** | **K** | **M** | **E** | **A** | **D** | **T** | **K** | **V** | **V** | **T** | **M** | **-** | **-** | **-** | **-** | **-** | **-** | **-** | **-** | **-** | **-** | **-** | **-** | **-** | **-** | **-** | **-** | **-** | **-** | **-** |
| **N59454F** | **M** | **S** | **E** | **E** | **I** | **K** | **E** | **N** | **I** | **Q** | **D** | **V** | **N** | **L** | **T** | **S** | **E** | **M** | **K** | **E** | **S** | **F** | **I** | **D** | **Y** | **A** | **M** | **S** | **V** | **I** | **V** | **A** | **R** | **A** | **L** | **P** | **D** | **V** | **R** | **D** | **G** | **L** | **K** | **P** | **V** | **H** | **R** | **R** | **I** | **L** | **Y** | **G** | **M** | **N** | **E** | **L** | **G** | **V** | **T** | **P** | **D** | **K** | **P** | **H** | **K** | **K** | **S** | **A** | **R** | **I** | **V** | **G** | **D** | **V** | **M** | **G** | **K** | **Y** | **H** | **P** | **H** | **G** | **D** | **S** | **A** | **I** | **Y** | **E** | **S** | **M** | **V** | **R** | **M** | **A** | **Q** | **P** | **F** | **S** | **Y** | **R** | **A** | **M** | **L** | **V** | **D** | **G** | **H** | **G** | **N** | **F** | **G** | **S** | **V** | **D** | **G** | **D** | **G** | **A** | **A** | **A** | **M** | **R** | **Y** | **T** | **E** | **A** | **R** | **M** | **S** | **K** | **I** | **A** | **L** | **E** | **M** | **L** | **R** | **D** | **I** | **N** | **K** | **N** | **T** | **V** | **D** | **F** | **Q** | **G** | **N** | **Y** | **D** | **D** | **S** | **E** | **Q** | **E** | **P** | **V** | **V** | **L** | **P** | **A** | **R** | **F** | **P** | **N** | **L** | **L** | **V** | **N** | **G** | **T** | **T** | **G** | **I** | **A** | **V** | **G** | **M** | **A** | **T** | **N** | **I** | **P** | **P** | **H** | **N** | **L** | **S** | **E** | **V** | **I** | **D** | **A** | **T** | **S** | **L** | **L** | **M** | **D** | **N** | **P** | **D** | **V** | **T** | **T** | **N** | **E** | **L** | **M** | **E** | **V** | **L** | **P** | **G** | **P** | **D** | **F** | **P** | **T** | **G** | **G** | **L** | **V** | **M** | **G** | **K** | **S** | **G** | **I** | **R** | **R** | **A** | **Y** | **E** | **T** | **G** | **K** | **G** | **S** | **I** | **T** | **V** | **R** | **A** | **K** | **V** | **E** | **L** | **T** | **E** | **M** | **P** | **N** | **G** | **K** | **E** | **R** | **I** | **L** | **V** | **T** | **E** | **L** | **P** | **Y** | **M** | **V** | **N** | **K** | **A** | **K** | **L** | **I** | **E** | **R** | **I** | **S** | **E** | **L** | **H** | **R** | **D** | **K** | **R** | **I** | **E** | **G** | **I** | **T** | **D** | **L** | **R** | **D** | **E** | **S** | **S** | **R** | **E** | **G** | **M** | **R** | **I** | **V** | **I** | **D** | **V** | **R** | **R** | **D** | **V** | **S** | **A** | **S** | **V** | **V** | **L** | **N** | **N** | **L** | **Y** | **K** | **M** | **T** | **A** | **L** | **Q** | **T** | **S** | **F** | **G** | **F** | **N** | **M** | **L** | **A** | **I** | **E** | **K** | **G** | **V** | **P** | **K** | **I** | **L** | **S** | **L** | **K** | **R** | **I** | **L** | **E** | **N** | **Y** | **V** | **E** | **H** | **Q** | **K** | **E** | **V** | **I** | **T** | **R** | **R** | **T** | **I** | **F** | **D** | **K** | **N** | **K** | **A** | **E** | **A** | **R** | **A** | **H** | **I** | **L** | **E** | **G** | **L** | **R** | **I** | **A** | **L** | **D** | **H** | **I** | **D** | **E** | **I** | **I** | **A** | **I** | **I** | **R** | **G** | **S** | **Q** | **S** | **D** | **D** | **E** | **A** | **K** | **A** | **T** | **L** | **I** | **E** | **R** | **F** | **E** | **F** | **S** | **D** | **R** | **Q** | **A** | **Q** | **A** | **I** | **L** | **D** | **M** | **R** | **L** | **R** | **R** | **L** | **T** | **G** | **L** | **E** | **R** | **D** | **K** | **I** | **E** | **N** | **E** | **Y** | **Q** | **E** | **L** | **L** | **K** | **F** | **I** | **A** | **D** | **L** | **E** | **D** | **I** | **L** | **A** | **R** | **P** | **E** | **R** | **V** | **I** | **E** | **I** | **I** | **K** | **T** | **E** | **L** | **N** | **D** | **V** | **R** | **T** | **K** | **F** | **G** | **D** | **A** | **R** | **R** | **T** | **E** | **L** | **L** | **V** | **G** | **E** | **V** | **L** | **S** | **L** | **E** | **D** | **E** | **D** | **L** | **I** | **E** | **E** | **E** | **E** | **V** | **V** | **I** | **T** | **L** | **T** | **N** | **N** | **G** | **Y** | **I** | **K** | **R** | **M** | **A** | **N** | **S** | **E** | **F** | **R** | **A** | **Q** | **R** | **R** | **G** | **G** | **R** | **G** | **V** | **Q** | **G** | **M** | **G** | **V** | **H** | **D** | **D** | **D** | **F** | **V** | **K** | **N** | **L** | **V** | **S** | **C** | **S** | **T** | **H** | **D** | **T** | **L** | **L** | **F** | **F** | **T** | **N** | **T** | **G** | **K** | **V** | **Y** | **R** | **A** | **K** | **G** | **Y** | **E** | **I** | **P** | **E** | **Y** | **G** | **R** | **T** | **A** | **K** | **G** | **I** | **P** | **V** | **I** | **N** | **L** | **L** | **G** | **I** | **D** | **S** | **A** | **E** | **K** | **I** | **Q** | **A** | **I** | **I** | **S** | **V** | **E** | **G** | **K** | **A** | **E** | **A** | **G** | **K** | **Y** | **L** | **F** | **F** | **T** | **T** | **L** | **K** | **G** | **T** | **V** | **K** | **R** | **T** | **A** | **V** | **T** | **A** | **F** | **S** | **N** | **I** | **R** | **S** | **N** | **G** | **L** | **I** | **A** | **I** | **S** | **L** | **K** | **E** | **D** | **D** | **E** | **L** | **V** | **N** | **V** | **V** | **T** | **T** | **N** | **G** | **N** | **Q** | **K** | **M** | **I** | **I** | **G** | **T** | **H** | **A** | **G** | **Y** | **S** | **V** | **T** | **F** | **D** | **E** | **N** | **T** | **V** | **R** | **D** | **M** | **G** | **R** | **T** | **A** | **S** | **G** | **V** | **R** | **G** | **I** | **R** | **L** | **R** | **E** | **N** | **D** | **Y** | **V** | **V** | **G** | **A** | **A** | **I** | **L** | **D** | **E** | **N** | **K** | **E** | **V** | **L** | **V** | **I** | **T** | **E** | **N** | **G** | **Y** | **G** | **K** | **R** | **T** | **K** | **A** | **S** | **E** | **Y** | **P** | **V** | **K** | **G** | **R** | **G** | **G** | **K** | **G** | **I** | **K** | **T** | **A** | **N** | **I** | **T** | **E** | **K** | **N** | **G** | **P** | **L** | **A** | **G** | **L** | **T** | **T** | **V** | **N** | **G** | **D** | **E** | **D** | **I** | **L** | **L** | **I** | **T** | **N** | **K** | **G** | **V** | **I** | **I** | **R** | **F** | **N** | **V** | **D** | **S** | **V** | **S** | **Q** | **T** | **G** | **R** | **A** | **T** | **L** | **G** | **V** | **R** | **L** | **M** | **R** | **M** | **E** | **D** | **G** | **A** | **K** | **-** | **-** | **-** | **-** | **-** | **-** | **-** | **-** | **-** | **-** | **-** | **-** | **-** | **-** | **-** | **-** | **-** | **-** | **-** | **-** | **-** | **-** | **-** |
| **N55113** | **M** | **S** | **E** | **E** | **I** | **K** | **E** | **N** | **I** | **Q** | **D** | **V** | **N** | **L** | **T** | **S** | **E** | **M** | **K** | **E** | **S** | **F** | **I** | **D** | **Y** | **A** | **M** | **S** | **V** | **I** | **V** | **A** | **R** | **A** | **L** | **P** | **D** | **V** | **R** | **D** | **G** | **L** | **K** | **P** | **V** | **H** | **R** | **R** | **I** | **L** | **Y** | **G** | **M** | **N** | **E** | **L** | **G** | **V** | **T** | **P** | **D** | **K** | **P** | **H** | **K** | **K** | **S** | **A** | **R** | **I** | **V** | **G** | **D** | **V** | **M** | **G** | **K** | **Y** | **H** | **P** | **H** | **G** | **D** | **S** | **A** | **I** | **Y** | **E** | **S** | **M** | **V** | **R** | **M** | **A** | **Q** | **P** | **F** | **S** | **Y** | **R** | **A** | **M** | **L** | **V** | **D** | **G** | **H** | **G** | **N** | **F** | **G** | **S** | **V** | **D** | **G** | **D** | **G** | **A** | **A** | **A** | **M** | **R** | **Y** | **T** | **E** | **A** | **R** | **M** | **S** | **K** | **I** | **A** | **L** | **E** | **M** | **L** | **R** | **D** | **I** | **N** | **K** | **N** | **T** | **V** | **D** | **F** | **Q** | **G** | **N** | **Y** | **D** | **D** | **S** | **E** | **Q** | **E** | **P** | **V** | **V** | **L** | **P** | **A** | **R** | **F** | **P** | **N** | **L** | **L** | **V** | **N** | **G** | **T** | **T** | **G** | **I** | **A** | **V** | **G** | **M** | **A** | **T** | **N** | **I** | **P** | **P** | **H** | **N** | **L** | **S** | **E** | **V** | **I** | **D** | **A** | **T** | **S** | **L** | **L** | **M** | **D** | **N** | **P** | **D** | **V** | **T** | **T** | **N** | **E** | **L** | **M** | **E** | **V** | **L** | **P** | **G** | **P** | **D** | **F** | **P** | **T** | **G** | **G** | **L** | **V** | **M** | **G** | **K** | **S** | **G** | **I** | **R** | **R** | **A** | **Y** | **E** | **T** | **G** | **K** | **G** | **S** | **I** | **T** | **V** | **R** | **A** | **K** | **V** | **E** | **L** | **T** | **E** | **M** | **P** | **N** | **G** | **K** | **E** | **R** | **I** | **L** | **V** | **T** | **E** | **L** | **P** | **Y** | **M** | **V** | **N** | **K** | **A** | **K** | **L** | **I** | **E** | **R** | **I** | **S** | **E** | **L** | **H** | **R** | **D** | **K** | **R** | **I** | **E** | **G** | **I** | **T** | **D** | **L** | **R** | **D** | **E** | **S** | **S** | **R** | **E** | **G** | **M** | **R** | **I** | **V** | **I** | **D** | **V** | **R** | **R** | **D** | **V** | **S** | **A** | **S** | **V** | **V** | **L** | **N** | **N** | **L** | **Y** | **K** | **M** | **T** | **A** | **L** | **Q** | **T** | **S** | **F** | **G** | **F** | **N** | **M** | **L** | **A** | **I** | **E** | **K** | **G** | **V** | **P** | **K** | **I** | **L** | **S** | **L** | **K** | **R** | **I** | **L** | **E** | **N** | **Y** | **V** | **E** | **H** | **Q** | **K** | **E** | **V** | **I** | **T** | **R** | **R** | **T** | **I** | **F** | **D** | **K** | **N** | **K** | **A** | **E** | **A** | **R** | **A** | **H** | **I** | **L** | **E** | **G** | **L** | **R** | **I** | **A** | **L** | **D** | **H** | **I** | **D** | **E** | **I** | **I** | **A** | **I** | **I** | **R** | **G** | **S** | **Q** | **S** | **D** | **D** | **E** | **A** | **K** | **A** | **T** | **L** | **I** | **E** | **R** | **F** | **E** | **F** | **S** | **D** | **R** | **Q** | **A** | **Q** | **A** | **I** | **L** | **D** | **M** | **R** | **L** | **R** | **R** | **L** | **T** | **G** | **L** | **E** | **R** | **D** | **K** | **I** | **E** | **N** | **E** | **Y** | **Q** | **E** | **L** | **L** | **K** | **F** | **I** | **A** | **D** | **L** | **E** | **D** | **I** | **L** | **A** | **R** | **P** | **E** | **R** | **V** | **I** | **E** | **I** | **I** | **K** | **T** | **E** | **L** | **N** | **D** | **V** | **R** | **T** | **K** | **F** | **G** | **D** | **A** | **R** | **R** | **T** | **E** | **L** | **L** | **V** | **G** | **E** | **V** | **L** | **S** | **L** | **E** | **D** | **E** | **D** | **L** | **I** | **E** | **E** | **E** | **E** | **V** | **V** | **I** | **T** | **L** | **T** | **N** | **N** | **G** | **Y** | **I** | **K** | **R** | **M** | **A** | **N** | **S** | **E** | **F** | **R** | **A** | **Q** | **R** | **R** | **G** | **G** | **R** | **G** | **V** | **Q** | **G** | **M** | **G** | **V** | **H** | **D** | **D** | **D** | **F** | **V** | **K** | **N** | **L** | **V** | **S** | **C** | **S** | **T** | **H** | **D** | **T** | **L** | **L** | **F** | **F** | **T** | **N** | **T** | **G** | **K** | **V** | **Y** | **R** | **A** | **K** | **G** | **Y** | **E** | **I** | **P** | **E** | **Y** | **G** | **R** | **T** | **A** | **K** | **G** | **I** | **P** | **V** | **I** | **N** | **L** | **L** | **G** | **I** | **D** | **S** | **A** | **E** | **K** | **I** | **Q** | **A** | **I** | **I** | **S** | **V** | **E** | **G** | **K** | **A** | **E** | **A** | **G** | **K** | **Y** | **L** | **F** | **F** | **T** | **T** | **L** | **K** | **G** | **T** | **V** | **K** | **R** | **T** | **A** | **V** | **T** | **A** | **F** | **S** | **N** | **I** | **R** | **S** | **N** | **G** | **L** | **I** | **A** | **I** | **S** | **L** | **K** | **E** | **D** | **D** | **E** | **L** | **V** | **N** | **V** | **V** | **T** | **T** | **N** | **G** | **N** | **Q** | **K** | **M** | **I** | **I** | **G** | **T** | **H** | **A** | **G** | **Y** | **S** | **V** | **T** | **F** | **D** | **E** | **N** | **T** | **V** | **R** | **D** | **M** | **G** | **R** | **T** | **A** | **S** | **G** | **V** | **R** | **G** | **I** | **R** | **L** | **R** | **E** | **N** | **D** | **Y** | **V** | **V** | **G** | **A** | **A** | **I** | **L** | **D** | **E** | **N** | **K** | **E** | **V** | **L** | **V** | **I** | **T** | **E** | **N** | **G** | **Y** | **G** | **K** | **R** | **T** | **K** | **A** | **S** | **E** | **Y** | **P** | **V** | **K** | **G** | **R** | **G** | **G** | **K** | **G** | **I** | **K** | **T** | **A** | **N** | **I** | **T** | **E** | **K** | **N** | **G** | **P** | **L** | **A** | **G** | **L** | **T** | **T** | **V** | **N** | **G** | **D** | **E** | **D** | **I** | **L** | **L** | **I** | **T** | **N** | **K** | **G** | **V** | **I** | **I** | **R** | **F** | **N** | **V** | **D** | **S** | **V** | **S** | **Q** | **T** | **G** | **R** | **A** | **T** | **L** | **G** | **V** | **R** | **L** | **M** | **R** | **M** | **E** | **D** | **G** | **A** | **K** | **-** | **-** | **-** | **-** | **-** | **-** | **-** | **-** | **-** | **-** | **-** | **-** | **-** | **-** | **-** | **-** | **-** | **-** | **-** | **-** | **-** | **-** | **-** |
| **N59592F** | **M** | **S** | **E** | **E** | **I** | **K** | **E** | **N** | **I** | **Q** | **D** | **V** | **N** | **L** | **T** | **S** | **E** | **M** | **K** | **E** | **S** | **F** | **I** | **D** | **Y** | **A** | **M** | **S** | **V** | **I** | **V** | **A** | **R** | **A** | **L** | **P** | **D** | **V** | **R** | **D** | **G** | **L** | **K** | **P** | **V** | **H** | **R** | **R** | **I** | **L** | **Y** | **G** | **M** | **N** | **E** | **L** | **G** | **V** | **T** | **P** | **D** | **K** | **P** | **H** | **K** | **K** | **S** | **A** | **R** | **I** | **V** | **G** | **D** | **V** | **M** | **G** | **K** | **Y** | **H** | **P** | **H** | **G** | **D** | **S** | **A** | **I** | **Y** | **E** | **S** | **M** | **V** | **R** | **M** | **A** | **Q** | **P** | **F** | **S** | **Y** | **R** | **A** | **M** | **L** | **V** | **D** | **G** | **H** | **G** | **N** | **F** | **G** | **S** | **V** | **D** | **G** | **D** | **G** | **A** | **A** | **A** | **M** | **R** | **Y** | **T** | **E** | **A** | **R** | **M** | **S** | **K** | **I** | **A** | **L** | **E** | **M** | **L** | **R** | **D** | **I** | **N** | **K** | **N** | **T** | **V** | **D** | **F** | **Q** | **G** | **N** | **Y** | **D** | **D** | **S** | **E** | **Q** | **E** | **P** | **V** | **V** | **L** | **P** | **A** | **R** | **F** | **P** | **N** | **L** | **L** | **V** | **N** | **G** | **T** | **T** | **G** | **I** | **A** | **V** | **G** | **M** | **A** | **T** | **N** | **I** | **P** | **P** | **H** | **N** | **L** | **S** | **E** | **V** | **I** | **D** | **A** | **T** | **S** | **L** | **L** | **M** | **D** | **N** | **P** | **D** | **V** | **T** | **T** | **N** | **E** | **L** | **M** | **E** | **V** | **L** | **P** | **G** | **P** | **D** | **F** | **P** | **T** | **G** | **G** | **L** | **V** | **M** | **G** | **K** | **S** | **G** | **I** | **R** | **R** | **A** | **Y** | **E** | **T** | **G** | **K** | **G** | **S** | **I** | **T** | **V** | **R** | **A** | **K** | **V** | **E** | **L** | **T** | **E** | **M** | **P** | **N** | **G** | **K** | **E** | **R** | **I** | **L** | **V** | **T** | **E** | **L** | **P** | **Y** | **M** | **V** | **N** | **K** | **A** | **K** | **L** | **I** | **E** | **R** | **I** | **S** | **E** | **L** | **H** | **R** | **D** | **K** | **R** | **I** | **E** | **G** | **I** | **T** | **D** | **L** | **R** | **D** | **E** | **S** | **S** | **R** | **E** | **G** | **M** | **R** | **I** | **V** | **I** | **D** | **V** | **R** | **R** | **D** | **V** | **S** | **A** | **S** | **V** | **V** | **L** | **N** | **N** | **L** | **Y** | **K** | **M** | **T** | **A** | **L** | **Q** | **T** | **S** | **F** | **G** | **F** | **N** | **M** | **L** | **A** | **I** | **E** | **K** | **G** | **V** | **P** | **K** | **I** | **L** | **S** | **L** | **K** | **R** | **I** | **L** | **E** | **N** | **Y** | **V** | **E** | **H** | **Q** | **K** | **E** | **V** | **I** | **T** | **R** | **R** | **T** | **I** | **F** | **D** | **K** | **N** | **K** | **A** | **E** | **A** | **R** | **A** | **H** | **I** | **L** | **E** | **G** | **L** | **R** | **I** | **A** | **L** | **D** | **H** | **I** | **D** | **E** | **I** | **I** | **A** | **I** | **I** | **R** | **G** | **S** | **Q** | **S** | **D** | **D** | **E** | **A** | **K** | **A** | **T** | **L** | **I** | **E** | **R** | **F** | **E** | **F** | **S** | **D** | **R** | **Q** | **A** | **Q** | **A** | **I** | **L** | **D** | **M** | **R** | **L** | **R** | **R** | **L** | **T** | **G** | **L** | **E** | **R** | **D** | **K** | **I** | **E** | **N** | **E** | **Y** | **Q** | **E** | **L** | **L** | **K** | **F** | **I** | **A** | **D** | **L** | **E** | **D** | **I** | **L** | **A** | **R** | **P** | **E** | **R** | **V** | **I** | **E** | **I** | **I** | **K** | **T** | **E** | **L** | **N** | **D** | **V** | **R** | **T** | **K** | **F** | **G** | **D** | **A** | **R** | **R** | **T** | **E** | **L** | **L** | **V** | **G** | **E** | **V** | **L** | **S** | **L** | **E** | **D** | **E** | **D** | **L** | **I** | **E** | **E** | **E** | **E** | **V** | **V** | **I** | **T** | **L** | **T** | **N** | **N** | **G** | **Y** | **I** | **K** | **R** | **M** | **A** | **N** | **S** | **E** | **F** | **R** | **A** | **Q** | **R** | **R** | **G** | **G** | **R** | **G** | **V** | **Q** | **G** | **M** | **G** | **V** | **H** | **D** | **D** | **D** | **F** | **V** | **K** | **N** | **L** | **V** | **S** | **C** | **S** | **T** | **H** | **D** | **T** | **L** | **L** | **F** | **F** | **T** | **N** | **T** | **G** | **K** | **V** | **Y** | **R** | **A** | **K** | **G** | **Y** | **E** | **I** | **P** | **E** | **Y** | **G** | **R** | **T** | **A** | **K** | **G** | **I** | **P** | **V** | **I** | **N** | **L** | **L** | **G** | **I** | **D** | **S** | **A** | **E** | **K** | **I** | **Q** | **A** | **I** | **I** | **S** | **V** | **E** | **G** | **K** | **A** | **E** | **A** | **G** | **K** | **Y** | **L** | **F** | **F** | **T** | **T** | **L** | **K** | **G** | **T** | **V** | **K** | **R** | **T** | **A** | **V** | **T** | **A** | **F** | **S** | **N** | **I** | **R** | **S** | **N** | **G** | **L** | **I** | **A** | **I** | **S** | **L** | **K** | **E** | **D** | **D** | **E** | **L** | **V** | **N** | **V** | **V** | **T** | **T** | **N** | **G** | **N** | **Q** | **K** | **M** | **I** | **I** | **G** | **T** | **H** | **A** | **G** | **Y** | **S** | **V** | **T** | **F** | **D** | **E** | **N** | **T** | **V** | **R** | **D** | **M** | **G** | **R** | **T** | **A** | **S** | **G** | **V** | **R** | **G** | **I** | **R** | **L** | **R** | **E** | **N** | **D** | **Y** | **V** | **V** | **G** | **A** | **A** | **I** | **L** | **D** | **E** | **N** | **K** | **E** | **V** | **L** | **V** | **I** | **T** | **E** | **N** | **G** | **Y** | **G** | **K** | **R** | **T** | **K** | **A** | **S** | **E** | **Y** | **P** | **V** | **K** | **G** | **R** | **G** | **G** | **K** | **G** | **I** | **K** | **T** | **A** | **N** | **I** | **T** | **E** | **K** | **N** | **G** | **P** | **L** | **A** | **G** | **L** | **T** | **T** | **V** | **N** | **G** | **D** | **E** | **D** | **I** | **L** | **L** | **I** | **T** | **N** | **K** | **G** | **V** | **I** | **I** | **R** | **F** | **N** | **V** | **D** | **S** | **V** | **S** | **Q** | **T** | **G** | **R** | **A** | **T** | **L** | **G** | **V** | **R** | **L** | **M** | **R** | **M** | **E** | **D** | **G** | **A** | **K** | **-** | **-** | **-** | **-** | **-** | **-** | **-** | **-** | **-** | **-** | **-** | **-** | **-** | **-** | **-** | **-** | **-** | **-** | **-** | **-** | **-** | **-** | **-** |
| **N52649** | **M** | **S** | **E** | **E** | **I** | **R** | **E** | **N** | **I** | **H** | **D** | **V** | **N** | **L** | **T** | **S** | **E** | **M** | **K** | **D** | **S** | **F** | **I** | **D** | **Y** | **A** | **M** | **S** | **V** | **I** | **V** | **A** | **R** | **A** | **L** | **P** | **D** | **V** | **R** | **D** | **G** | **L** | **K** | **P** | **V** | **H** | **R** | **R** | **I** | **L** | **Y** | **G** | **M** | **N** | **E** | **L** | **G** | **V** | **T** | **P** | **D** | **K** | **A** | **H** | **K** | **K** | **S** | **A** | **R** | **I** | **V** | **G** | **D** | **V** | **M** | **G** | **K** | **Y** | **H** | **P** | **H** | **G** | **D** | **S** | **A** | **I** | **Y** | **E** | **S** | **M** | **V** | **R** | **M** | **A** | **Q** | **P** | **F** | **S** | **Y** | **R** | **Y** | **M** | **L** | **V** | **D** | **G** | **H** | **G** | **N** | **F** | **G** | **S** | **V** | **D** | **G** | **D** | **G** | **A** | **A** | **A** | **M** | **R** | **Y** | **T** | **E** | **A** | **R** | **M** | **S** | **K** | **I** | **A** | **T** | **E** | **M** | **L** | **R** | **D** | **I** | **N** | **K** | **N** | **T** | **V** | **D** | **F** | **Q** | **S** | **N** | **Y** | **D** | **D** | **T** | **E** | **K** | **E** | **P** | **V** | **V** | **L** | **P** | **A** | **R** | **F** | **P** | **N** | **L** | **L** | **V** | **N** | **G** | **T** | **T** | **G** | **I** | **A** | **V** | **G** | **M** | **A** | **T** | **N** | **I** | **P** | **P** | **H** | **N** | **L** | **S** | **E** | **V** | **V** | **A** | **A** | **I** | **D** | **L** | **L** | **M** | **D** | **N** | **P** | **D** | **V** | **T** | **T** | **N** | **E** | **L** | **M** | **E** | **V** | **L** | **P** | **G** | **P** | **D** | **F** | **P** | **T** | **G** | **G** | **L** | **V** | **M** | **G** | **K** | **S** | **G** | **I** | **R** | **R** | **A** | **Y** | **E** | **T** | **G** | **K** | **G** | **S** | **I** | **T** | **V** | **R** | **A** | **K** | **V** | **E** | **I** | **T** | **E** | **M** | **P** | **N** | **G** | **K** | **E** | **R** | **I** | **I** | **V** | **T** | **E** | **L** | **P** | **Y** | **M** | **V** | **N** | **K** | **A** | **K** | **L** | **I** | **E** | **R** | **I** | **S** | **E** | **L** | **H** | **R** | **D** | **K** | **R** | **I** | **E** | **G** | **I** | **T** | **D** | **L** | **R** | **D** | **E** | **S** | **S** | **R** | **E** | **G** | **M** | **R** | **I** | **V** | **I** | **D** | **I** | **R** | **R** | **D** | **A** | **S** | **A** | **S** | **V** | **I** | **L** | **N** | **N** | **L** | **Y** | **K** | **L** | **T** | **S** | **L** | **Q** | **N** | **S** | **F** | **G** | **F** | **N** | **M** | **L** | **A** | **I** | **E** | **K** | **G** | **V** | **P** | **K** | **V** | **L** | **S** | **L** | **K** | **Q** | **I** | **L** | **E** | **N** | **Y** | **I** | **E** | **H** | **Q** | **R** | **E** | **V** | **I** | **T** | **R** | **R** | **T** | **V** | **F** | **E** | **K** | **E** | **K** | **A** | **E** | **A** | **R** | **A** | **H** | **I** | **L** | **E** | **G** | **L** | **R** | **I** | **A** | **L** | **D** | **H** | **I** | **D** | **E** | **I** | **I** | **A** | **I** | **I** | **R** | **G** | **S** | **K** | **S** | **D** | **D** | **E** | **A** | **K** | **A** | **T** | **M** | **I** | **E** | **R** | **F** | **D** | **L** | **S** | **D** | **R** | **Q** | **S** | **Q** | **A** | **I** | **L** | **D** | **M** | **R** | **L** | **R** | **R** | **L** | **T** | **G** | **L** | **E** | **R** | **E** | **K** | **I** | **E** | **N** | **E** | **Y** | **Q** | **E** | **L** | **L** | **K** | **L** | **I** | **E** | **D** | **L** | **T** | **D** | **I** | **L** | **A** | **R** | **P** | **E** | **R** | **V** | **T** | **E** | **I** | **I** | **K** | **N** | **E** | **L** | **A** | **D** | **L** | **N** | **Q** | **R** | **F** | **G** | **D** | **K** | **R** | **R** | **T** | **E** | **L** | **L** | **V** | **G** | **E** | **V** | **L** | **S** | **L** | **E** | **D** | **E** | **D** | **L** | **I** | **E** | **E** | **E** | **E** | **I** | **V** | **I** | **T** | **L** | **T** | **N** | **N** | **G** | **Y** | **I** | **K** | **R** | **L** | **A** | **N** | **N** | **E** | **F** | **R** | **A** | **Q** | **R** | **R** | **G** | **G** | **R** | **G** | **V** | **Q** | **G** | **M** | **G** | **I** | **H** | **N** | **D** | **D** | **F** | **V** | **K** | **N** | **L** | **V** | **S** | **C** | **S** | **T** | **H** | **D** | **T** | **L** | **L** | **F** | **F** | **T** | **N** | **N** | **G** | **K** | **V** | **Y** | **R** | **A** | **K** | **G** | **Y** | **E** | **I** | **P** | **E** | **Y** | **G** | **R** | **T** | **A** | **K** | **G** | **I** | **P** | **I** | **I** | **N** | **L** | **L** | **G** | **I** | **D** | **S** | **S** | **E** | **S** | **I** | **Q** | **A** | **I** | **I** | **A** | **V** | **T** | **G** | **E** | **A** | **E** | **E** | **G** | **H** | **Y** | **L** | **F** | **F** | **T** | **T** | **R** | **Q** | **G** | **T** | **V** | **K** | **R** | **T** | **S** | **V** | **T** | **A** | **F** | **A** | **N** | **I** | **R** | **S** | **N** | **G** | **L** | **I** | **A** | **I** | **G** | **L** | **K** | **E** | **D** | **D** | **E** | **L** | **V** | **N** | **V** | **L** | **L** | **T** | **D** | **G** | **Q** | **S** | **N** | **I** | **I** | **I** | **G** | **T** | **H** | **N** | **G** | **Y** | **S** | **V** | **T** | **F** | **K** | **E** | **E** | **A** | **V** | **R** | **D** | **M** | **G** | **R** | **T** | **A** | **S** | **G** | **V** | **R** | **G** | **I** | **R** | **L** | **R** | **E** | **E** | **D** | **Y** | **V** | **V** | **G** | **A** | **A** | **L** | **M** | **K** | **E** | **N** | **Q** | **E** | **V** | **L** | **V** | **I** | **T** | **E** | **K** | **G** | **Y** | **G** | **K** | **R** | **T** | **K** | **V** | **S** | **E** | **Y** | **P** | **V** | **K** | **G** | **R** | **G** | **G** | **K** | **G** | **I** | **K** | **T** | **A** | **N** | **I** | **T** | **E** | **K** | **N** | **G** | **P** | **L** | **A** | **G** | **L** | **T** | **T** | **V** | **S** | **G** | **D** | **E** | **D** | **I** | **M** | **L** | **I** | **T** | **D** | **K** | **G** | **V** | **I** | **I** | **R** | **F** | **N** | **V** | **S** | **T** | **V** | **S** | **Q** | **T** | **G** | **R** | **S** | **T** | **L** | **G** | **V** | **R** | **L** | **M** | **K** | **M** | **E** | **A** | **D** | **T** | **K** | **V** | **V** | **T** | **M** | **A** | **A** | **V** | **E** | **P** | **E** | **A** | **A** | **E** | **Q** | **T** | **V** | **E** | **E** | **Q** | **G** | **S** | **E** | **E** |
| **N55706** | **M** | **S** | **E** | **E** | **I** | **K** | **E** | **N** | **I** | **Q** | **D** | **V** | **N** | **L** | **T** | **S** | **E** | **M** | **K** | **E** | **S** | **F** | **I** | **D** | **Y** | **A** | **M** | **S** | **V** | **I** | **V** | **A** | **R** | **A** | **L** | **P** | **D** | **V** | **R** | **D** | **G** | **L** | **K** | **P** | **V** | **H** | **R** | **R** | **I** | **L** | **Y** | **G** | **M** | **N** | **E** | **L** | **G** | **V** | **T** | **P** | **D** | **K** | **P** | **H** | **K** | **K** | **S** | **A** | **R** | **I** | **V** | **G** | **D** | **V** | **M** | **G** | **K** | **Y** | **H** | **P** | **H** | **G** | **D** | **S** | **A** | **I** | **Y** | **E** | **S** | **M** | **V** | **R** | **M** | **A** | **Q** | **P** | **F** | **S** | **Y** | **R** | **A** | **M** | **L** | **V** | **D** | **G** | **H** | **G** | **N** | **F** | **G** | **S** | **V** | **D** | **G** | **D** | **G** | **A** | **A** | **A** | **M** | **R** | **Y** | **T** | **E** | **A** | **R** | **M** | **S** | **K** | **I** | **A** | **L** | **E** | **M** | **L** | **R** | **D** | **I** | **N** | **K** | **N** | **T** | **V** | **D** | **F** | **Q** | **G** | **N** | **Y** | **D** | **D** | **S** | **E** | **Q** | **E** | **P** | **V** | **V** | **L** | **P** | **A** | **R** | **F** | **P** | **N** | **L** | **L** | **V** | **N** | **G** | **T** | **T** | **G** | **I** | **A** | **V** | **G** | **M** | **A** | **T** | **N** | **I** | **P** | **P** | **H** | **N** | **L** | **S** | **E** | **V** | **I** | **D** | **A** | **T** | **S** | **L** | **L** | **M** | **D** | **N** | **P** | **D** | **V** | **T** | **T** | **N** | **E** | **L** | **M** | **E** | **V** | **L** | **P** | **G** | **P** | **D** | **F** | **P** | **T** | **G** | **G** | **L** | **V** | **M** | **G** | **K** | **S** | **G** | **I** | **R** | **R** | **A** | **Y** | **E** | **T** | **G** | **K** | **G** | **S** | **I** | **T** | **V** | **R** | **A** | **K** | **V** | **E** | **L** | **T** | **E** | **M** | **P** | **N** | **G** | **K** | **E** | **R** | **I** | **L** | **V** | **T** | **E** | **L** | **P** | **Y** | **M** | **V** | **N** | **K** | **A** | **K** | **L** | **I** | **E** | **R** | **I** | **S** | **E** | **L** | **H** | **R** | **D** | **K** | **R** | **I** | **E** | **G** | **I** | **T** | **D** | **L** | **R** | **D** | **E** | **S** | **S** | **R** | **E** | **G** | **M** | **R** | **I** | **V** | **I** | **D** | **V** | **R** | **R** | **D** | **V** | **S** | **A** | **S** | **V** | **V** | **L** | **N** | **N** | **L** | **Y** | **K** | **M** | **T** | **A** | **L** | **Q** | **T** | **S** | **F** | **G** | **F** | **N** | **M** | **L** | **A** | **I** | **E** | **K** | **G** | **V** | **P** | **K** | **I** | **L** | **S** | **L** | **K** | **R** | **I** | **L** | **E** | **N** | **Y** | **V** | **E** | **H** | **Q** | **K** | **E** | **V** | **I** | **T** | **R** | **R** | **T** | **I** | **F** | **D** | **K** | **N** | **K** | **A** | **E** | **A** | **R** | **A** | **H** | **I** | **L** | **E** | **G** | **L** | **R** | **I** | **A** | **L** | **D** | **H** | **I** | **D** | **E** | **I** | **I** | **A** | **I** | **I** | **R** | **G** | **S** | **Q** | **S** | **D** | **D** | **E** | **A** | **K** | **A** | **T** | **L** | **I** | **E** | **R** | **F** | **E** | **F** | **S** | **D** | **R** | **Q** | **A** | **Q** | **A** | **I** | **L** | **D** | **M** | **R** | **L** | **R** | **R** | **L** | **T** | **G** | **L** | **E** | **R** | **D** | **K** | **I** | **E** | **N** | **E** | **Y** | **Q** | **E** | **L** | **L** | **K** | **F** | **I** | **A** | **D** | **L** | **E** | **D** | **I** | **L** | **A** | **R** | **P** | **E** | **R** | **V** | **I** | **E** | **I** | **I** | **K** | **T** | **E** | **L** | **N** | **D** | **V** | **R** | **T** | **K** | **F** | **G** | **D** | **A** | **R** | **R** | **T** | **E** | **L** | **L** | **V** | **G** | **E** | **V** | **L** | **S** | **L** | **E** | **D** | **E** | **D** | **L** | **I** | **E** | **E** | **E** | **E** | **V** | **V** | **I** | **T** | **L** | **T** | **N** | **N** | **G** | **Y** | **I** | **K** | **R** | **M** | **A** | **N** | **S** | **E** | **F** | **R** | **A** | **Q** | **R** | **R** | **G** | **G** | **R** | **G** | **V** | **Q** | **G** | **M** | **G** | **V** | **H** | **D** | **D** | **D** | **F** | **V** | **K** | **N** | **L** | **V** | **S** | **C** | **S** | **T** | **H** | **D** | **T** | **L** | **L** | **F** | **F** | **T** | **N** | **T** | **G** | **K** | **V** | **Y** | **R** | **A** | **K** | **G** | **Y** | **E** | **I** | **P** | **E** | **Y** | **G** | **R** | **T** | **A** | **K** | **G** | **I** | **P** | **V** | **I** | **N** | **L** | **L** | **G** | **I** | **D** | **S** | **A** | **E** | **K** | **I** | **Q** | **A** | **I** | **I** | **S** | **V** | **E** | **G** | **K** | **A** | **E** | **A** | **G** | **K** | **Y** | **L** | **F** | **F** | **T** | **T** | **L** | **K** | **G** | **T** | **V** | **K** | **R** | **T** | **A** | **V** | **T** | **A** | **F** | **S** | **N** | **I** | **R** | **S** | **N** | **G** | **L** | **I** | **A** | **I** | **S** | **L** | **K** | **E** | **D** | **D** | **E** | **L** | **V** | **N** | **V** | **V** | **T** | **T** | **N** | **G** | **N** | **Q** | **K** | **M** | **I** | **I** | **G** | **T** | **H** | **A** | **G** | **Y** | **S** | **V** | **T** | **F** | **D** | **E** | **N** | **T** | **V** | **R** | **D** | **M** | **G** | **R** | **T** | **A** | **S** | **G** | **V** | **R** | **G** | **I** | **R** | **L** | **R** | **E** | **N** | **D** | **Y** | **V** | **V** | **G** | **A** | **A** | **I** | **L** | **D** | **E** | **N** | **K** | **E** | **V** | **L** | **V** | **I** | **T** | **E** | **N** | **G** | **Y** | **G** | **K** | **R** | **T** | **K** | **A** | **S** | **E** | **Y** | **P** | **V** | **K** | **G** | **R** | **G** | **G** | **K** | **G** | **I** | **K** | **T** | **A** | **N** | **I** | **T** | **E** | **K** | **N** | **G** | **P** | **L** | **A** | **G** | **L** | **T** | **T** | **V** | **N** | **G** | **D** | **E** | **D** | **I** | **L** | **L** | **I** | **T** | **N** | **K** | **G** | **V** | **I** | **I** | **R** | **F** | **N** | **V** | **D** | **S** | **V** | **S** | **Q** | **T** | **G** | **R** | **A** | **T** | **L** | **G** | **V** | **R** | **L** | **M** | **R** | **M** | **E** | **D** | **G** | **A** | **K** | **-** | **-** | **-** | **-** | **-** | **-** | **-** | **-** | **-** | **-** | **-** | **-** | **-** | **-** | **-** | **-** | **-** | **-** | **-** | **-** | **-** | **-** | **-** |
| **N60284F** | **M** | **S** | **E** | **E** | **I** | **R** | **E** | **N** | **I** | **H** | **D** | **V** | **N** | **L** | **T** | **S** | **E** | **M** | **K** | **D** | **S** | **F** | **I** | **D** | **Y** | **A** | **M** | **S** | **V** | **I** | **V** | **A** | **R** | **A** | **L** | **P** | **D** | **V** | **R** | **D** | **G** | **L** | **K** | **P** | **V** | **H** | **R** | **R** | **I** | **L** | **Y** | **G** | **M** | **N** | **E** | **L** | **G** | **V** | **T** | **P** | **D** | **K** | **A** | **H** | **K** | **K** | **S** | **A** | **R** | **I** | **V** | **G** | **D** | **V** | **M** | **G** | **K** | **Y** | **H** | **P** | **H** | **G** | **D** | **S** | **A** | **I** | **Y** | **E** | **S** | **M** | **V** | **R** | **M** | **A** | **Q** | **P** | **F** | **S** | **Y** | **R** | **Y** | **M** | **L** | **V** | **D** | **G** | **H** | **G** | **N** | **F** | **G** | **S** | **V** | **D** | **G** | **D** | **G** | **A** | **A** | **A** | **M** | **R** | **Y** | **T** | **E** | **A** | **R** | **M** | **S** | **K** | **I** | **A** | **T** | **E** | **M** | **L** | **R** | **D** | **I** | **N** | **K** | **N** | **T** | **V** | **D** | **F** | **Q** | **S** | **N** | **Y** | **D** | **D** | **T** | **E** | **K** | **E** | **P** | **V** | **V** | **L** | **P** | **A** | **R** | **F** | **P** | **N** | **L** | **L** | **V** | **N** | **G** | **T** | **T** | **G** | **I** | **A** | **V** | **G** | **M** | **A** | **T** | **N** | **I** | **P** | **P** | **H** | **N** | **L** | **S** | **E** | **V** | **V** | **A** | **A** | **I** | **D** | **L** | **L** | **M** | **D** | **N** | **P** | **D** | **V** | **T** | **T** | **N** | **E** | **L** | **M** | **E** | **V** | **L** | **P** | **G** | **P** | **D** | **F** | **P** | **T** | **G** | **G** | **L** | **V** | **M** | **G** | **K** | **S** | **G** | **I** | **R** | **R** | **A** | **Y** | **E** | **T** | **G** | **K** | **G** | **S** | **I** | **T** | **V** | **R** | **A** | **K** | **V** | **E** | **I** | **T** | **E** | **M** | **P** | **N** | **G** | **K** | **E** | **R** | **I** | **I** | **V** | **T** | **E** | **L** | **P** | **Y** | **M** | **V** | **N** | **K** | **A** | **K** | **L** | **I** | **E** | **R** | **I** | **S** | **E** | **L** | **H** | **R** | **D** | **K** | **R** | **I** | **E** | **G** | **I** | **T** | **D** | **L** | **R** | **D** | **E** | **S** | **S** | **R** | **E** | **G** | **M** | **R** | **I** | **V** | **I** | **D** | **I** | **R** | **R** | **D** | **A** | **S** | **A** | **S** | **V** | **I** | **L** | **N** | **N** | **L** | **Y** | **K** | **L** | **T** | **S** | **L** | **Q** | **N** | **S** | **F** | **G** | **F** | **N** | **M** | **L** | **A** | **I** | **E** | **K** | **G** | **V** | **P** | **K** | **V** | **L** | **S** | **L** | **K** | **Q** | **I** | **L** | **E** | **N** | **Y** | **I** | **E** | **H** | **Q** | **R** | **E** | **V** | **I** | **T** | **R** | **R** | **T** | **V** | **F** | **E** | **K** | **E** | **K** | **A** | **E** | **A** | **R** | **A** | **H** | **I** | **L** | **E** | **G** | **L** | **R** | **I** | **A** | **L** | **D** | **H** | **I** | **D** | **E** | **I** | **I** | **A** | **I** | **I** | **R** | **G** | **S** | **K** | **S** | **D** | **D** | **E** | **A** | **K** | **A** | **T** | **M** | **I** | **E** | **R** | **F** | **D** | **L** | **S** | **D** | **R** | **Q** | **S** | **Q** | **A** | **I** | **L** | **D** | **M** | **R** | **L** | **R** | **R** | **L** | **T** | **G** | **L** | **E** | **R** | **E** | **K** | **I** | **E** | **N** | **E** | **Y** | **Q** | **E** | **L** | **L** | **K** | **L** | **I** | **E** | **D** | **L** | **T** | **D** | **I** | **L** | **A** | **R** | **P** | **E** | **R** | **V** | **T** | **E** | **I** | **I** | **K** | **N** | **E** | **L** | **A** | **D** | **L** | **N** | **Q** | **R** | **F** | **G** | **D** | **K** | **R** | **R** | **T** | **E** | **L** | **L** | **V** | **G** | **E** | **V** | **L** | **S** | **L** | **E** | **D** | **E** | **D** | **L** | **I** | **E** | **E** | **E** | **E** | **I** | **V** | **I** | **T** | **L** | **T** | **N** | **N** | **G** | **Y** | **I** | **K** | **R** | **L** | **A** | **N** | **N** | **E** | **F** | **R** | **A** | **Q** | **R** | **R** | **G** | **G** | **R** | **G** | **V** | **Q** | **G** | **M** | **G** | **I** | **H** | **N** | **D** | **D** | **F** | **V** | **K** | **N** | **L** | **V** | **S** | **C** | **S** | **T** | **H** | **D** | **T** | **L** | **L** | **F** | **F** | **T** | **N** | **N** | **G** | **K** | **V** | **Y** | **R** | **A** | **K** | **G** | **Y** | **E** | **I** | **P** | **E** | **Y** | **G** | **R** | **T** | **A** | **K** | **G** | **I** | **P** | **I** | **I** | **N** | **L** | **L** | **G** | **I** | **D** | **S** | **S** | **E** | **S** | **I** | **Q** | **A** | **I** | **I** | **A** | **V** | **T** | **G** | **E** | **A** | **E** | **E** | **G** | **H** | **Y** | **L** | **F** | **F** | **T** | **T** | **R** | **Q** | **G** | **T** | **V** | **K** | **R** | **T** | **S** | **V** | **T** | **A** | **F** | **A** | **N** | **I** | **R** | **S** | **N** | **G** | **L** | **I** | **A** | **I** | **G** | **L** | **K** | **E** | **D** | **D** | **E** | **L** | **V** | **N** | **V** | **L** | **L** | **T** | **D** | **G** | **Q** | **S** | **N** | **I** | **I** | **I** | **G** | **T** | **H** | **N** | **G** | **Y** | **S** | **V** | **T** | **F** | **K** | **E** | **E** | **A** | **V** | **R** | **D** | **M** | **G** | **R** | **T** | **A** | **S** | **G** | **V** | **R** | **G** | **I** | **R** | **L** | **R** | **E** | **E** | **D** | **Y** | **V** | **V** | **G** | **A** | **A** | **L** | **M** | **K** | **E** | **N** | **Q** | **E** | **V** | **L** | **V** | **I** | **T** | **E** | **K** | **G** | **Y** | **G** | **K** | **R** | **T** | **K** | **V** | **S** | **E** | **Y** | **P** | **V** | **K** | **G** | **R** | **G** | **G** | **K** | **G** | **I** | **K** | **T** | **A** | **N** | **I** | **T** | **E** | **K** | **N** | **G** | **P** | **L** | **A** | **G** | **L** | **T** | **T** | **V** | **S** | **G** | **D** | **E** | **D** | **I** | **M** | **L** | **I** | **T** | **D** | **K** | **G** | **V** | **I** | **I** | **R** | **F** | **N** | **V** | **S** | **T** | **V** | **S** | **Q** | **T** | **G** | **R** | **S** | **T** | **L** | **G** | **V** | **R** | **L** | **M** | **K** | **M** | **E** | **A** | **D** | **T** | **K** | **V** | **V** | **T** | **M** | **A** | **A** | **V** | **E** | **P** | **E** | **A** | **A** | **E** | **Q** | **T** | **V** | **E** | **E** | **Q** | **G** | **S** | **E** | **E** |
| **N59531F** | **M** | **S** | **E** | **E** | **I** | **R** | **E** | **N** | **I** | **H** | **D** | **V** | **N** | **L** | **T** | **S** | **E** | **M** | **K** | **D** | **S** | **F** | **I** | **D** | **Y** | **A** | **M** | **S** | **V** | **I** | **V** | **A** | **R** | **A** | **L** | **P** | **D** | **V** | **R** | **D** | **G** | **L** | **K** | **P** | **V** | **H** | **R** | **R** | **I** | **L** | **Y** | **G** | **M** | **N** | **E** | **L** | **G** | **V** | **T** | **P** | **D** | **K** | **A** | **H** | **K** | **K** | **S** | **A** | **R** | **I** | **V** | **G** | **D** | **V** | **M** | **G** | **K** | **Y** | **H** | **P** | **H** | **G** | **D** | **S** | **A** | **I** | **Y** | **E** | **S** | **M** | **V** | **R** | **M** | **A** | **Q** | **P** | **F** | **S** | **Y** | **R** | **Y** | **M** | **L** | **V** | **D** | **G** | **H** | **G** | **N** | **F** | **G** | **S** | **V** | **D** | **G** | **D** | **G** | **A** | **A** | **A** | **M** | **R** | **Y** | **T** | **E** | **A** | **R** | **M** | **S** | **K** | **I** | **A** | **T** | **E** | **M** | **L** | **R** | **D** | **I** | **N** | **K** | **N** | **T** | **V** | **D** | **F** | **Q** | **S** | **N** | **Y** | **D** | **D** | **T** | **E** | **K** | **E** | **P** | **V** | **V** | **L** | **P** | **A** | **R** | **F** | **P** | **N** | **L** | **L** | **V** | **N** | **G** | **T** | **T** | **G** | **I** | **A** | **V** | **G** | **M** | **A** | **T** | **N** | **I** | **P** | **P** | **H** | **N** | **L** | **S** | **E** | **V** | **V** | **A** | **A** | **I** | **D** | **L** | **L** | **M** | **D** | **N** | **P** | **D** | **V** | **T** | **T** | **N** | **E** | **L** | **M** | **E** | **V** | **L** | **P** | **G** | **P** | **D** | **F** | **P** | **T** | **G** | **G** | **L** | **V** | **M** | **G** | **K** | **S** | **G** | **I** | **R** | **R** | **A** | **Y** | **E** | **T** | **G** | **K** | **G** | **S** | **I** | **T** | **V** | **R** | **A** | **K** | **V** | **E** | **I** | **T** | **E** | **M** | **P** | **N** | **G** | **K** | **E** | **R** | **I** | **L** | **V** | **T** | **E** | **L** | **P** | **Y** | **M** | **V** | **N** | **K** | **A** | **K** | **L** | **I** | **E** | **R** | **I** | **S** | **E** | **L** | **H** | **R** | **D** | **K** | **R** | **I** | **E** | **G** | **I** | **T** | **D** | **L** | **R** | **D** | **E** | **S** | **S** | **R** | **E** | **G** | **M** | **R** | **I** | **V** | **I** | **D** | **V** | **R** | **R** | **D** | **A** | **S** | **A** | **S** | **V** | **I** | **L** | **N** | **N** | **L** | **Y** | **K** | **L** | **T** | **S** | **L** | **Q** | **N** | **S** | **F** | **G** | **F** | **N** | **M** | **L** | **A** | **I** | **E** | **K** | **G** | **V** | **P** | **K** | **V** | **L** | **S** | **L** | **K** | **Q** | **I** | **L** | **E** | **N** | **Y** | **I** | **E** | **H** | **Q** | **R** | **E** | **V** | **I** | **T** | **R** | **R** | **T** | **V** | **F** | **E** | **K** | **E** | **K** | **A** | **E** | **A** | **R** | **A** | **H** | **I** | **L** | **E** | **G** | **L** | **R** | **I** | **A** | **L** | **D** | **H** | **I** | **D** | **E** | **I** | **I** | **A** | **I** | **I** | **R** | **G** | **S** | **K** | **S** | **D** | **D** | **E** | **A** | **K** | **A** | **T** | **M** | **I** | **E** | **R** | **F** | **D** | **L** | **S** | **D** | **R** | **Q** | **S** | **Q** | **A** | **I** | **L** | **D** | **M** | **R** | **L** | **R** | **R** | **L** | **T** | **G** | **L** | **E** | **R** | **E** | **K** | **I** | **E** | **N** | **E** | **Y** | **Q** | **E** | **L** | **L** | **K** | **L** | **I** | **E** | **D** | **L** | **T** | **D** | **I** | **L** | **A** | **R** | **P** | **E** | **R** | **V** | **T** | **E** | **I** | **I** | **K** | **N** | **E** | **L** | **A** | **D** | **L** | **N** | **Q** | **R** | **F** | **G** | **D** | **K** | **R** | **R** | **T** | **E** | **L** | **L** | **V** | **G** | **E** | **V** | **L** | **S** | **L** | **E** | **D** | **E** | **D** | **L** | **I** | **E** | **E** | **E** | **E** | **I** | **V** | **I** | **T** | **L** | **T** | **N** | **N** | **G** | **Y** | **I** | **K** | **R** | **L** | **A** | **N** | **N** | **E** | **F** | **R** | **A** | **Q** | **R** | **R** | **G** | **G** | **R** | **G** | **V** | **Q** | **G** | **M** | **G** | **I** | **H** | **N** | **D** | **D** | **F** | **V** | **K** | **N** | **L** | **V** | **S** | **C** | **S** | **T** | **H** | **D** | **T** | **L** | **L** | **F** | **F** | **T** | **N** | **N** | **G** | **K** | **V** | **Y** | **R** | **A** | **K** | **G** | **Y** | **E** | **I** | **P** | **E** | **Y** | **G** | **R** | **T** | **A** | **K** | **G** | **I** | **P** | **I** | **I** | **N** | **L** | **L** | **G** | **I** | **D** | **S** | **S** | **E** | **S** | **I** | **Q** | **A** | **I** | **I** | **A** | **V** | **T** | **G** | **E** | **A** | **E** | **E** | **G** | **H** | **Y** | **L** | **F** | **F** | **T** | **T** | **R** | **Q** | **G** | **T** | **V** | **K** | **R** | **T** | **S** | **V** | **T** | **A** | **F** | **A** | **N** | **I** | **R** | **S** | **N** | **G** | **L** | **I** | **A** | **I** | **G** | **L** | **K** | **E** | **D** | **D** | **E** | **L** | **V** | **N** | **V** | **L** | **L** | **T** | **D** | **G** | **Q** | **S** | **N** | **I** | **I** | **I** | **G** | **T** | **H** | **N** | **G** | **Y** | **S** | **V** | **T** | **F** | **K** | **E** | **E** | **A** | **V** | **R** | **D** | **M** | **G** | **R** | **T** | **A** | **S** | **G** | **V** | **R** | **G** | **I** | **R** | **L** | **R** | **E** | **E** | **D** | **Y** | **V** | **V** | **G** | **A** | **A** | **L** | **M** | **K** | **E** | **D** | **Q** | **E** | **V** | **L** | **V** | **I** | **T** | **E** | **K** | **G** | **Y** | **G** | **K** | **R** | **T** | **K** | **V** | **S** | **E** | **Y** | **P** | **V** | **K** | **G** | **R** | **G** | **G** | **K** | **G** | **I** | **K** | **T** | **A** | **N** | **I** | **T** | **E** | **K** | **N** | **G** | **P** | **L** | **A** | **G** | **L** | **T** | **T** | **V** | **S** | **G** | **N** | **E** | **D** | **I** | **M** | **L** | **I** | **T** | **D** | **K** | **G** | **V** | **I** | **I** | **R** | **F** | **N** | **V** | **S** | **T** | **V** | **S** | **Q** | **T** | **G** | **R** | **S** | **T** | **L** | **G** | **V** | **R** | **L** | **M** | **K** | **M** | **E** | **A** | **D** | **T** | **K** | **V** | **V** | **T** | **M** | **-** | **-** | **-** | **-** | **-** | **-** | **-** | **-** | **-** | **-** | **-** | **-** | **-** | **-** | **-** | **-** | **-** | **-** | **-** |
| **N60001F** | **M** | **S** | **E** | **E** | **I** | **R** | **E** | **N** | **I** | **H** | **D** | **V** | **N** | **L** | **T** | **S** | **E** | **M** | **K** | **D** | **S** | **F** | **I** | **D** | **Y** | **A** | **M** | **S** | **V** | **I** | **V** | **A** | **R** | **A** | **L** | **P** | **D** | **V** | **R** | **D** | **G** | **L** | **K** | **P** | **V** | **H** | **R** | **R** | **I** | **L** | **Y** | **G** | **M** | **N** | **E** | **L** | **G** | **V** | **T** | **P** | **D** | **K** | **A** | **H** | **K** | **K** | **S** | **A** | **R** | **I** | **V** | **G** | **D** | **V** | **M** | **G** | **K** | **Y** | **H** | **P** | **H** | **G** | **D** | **S** | **A** | **I** | **Y** | **E** | **S** | **M** | **V** | **R** | **M** | **A** | **Q** | **P** | **F** | **S** | **Y** | **R** | **Y** | **M** | **L** | **V** | **D** | **G** | **H** | **G** | **N** | **F** | **G** | **S** | **V** | **D** | **G** | **D** | **G** | **A** | **A** | **A** | **M** | **R** | **Y** | **T** | **E** | **A** | **R** | **M** | **S** | **K** | **I** | **A** | **T** | **E** | **M** | **L** | **R** | **D** | **I** | **N** | **K** | **N** | **T** | **V** | **D** | **F** | **Q** | **S** | **N** | **Y** | **D** | **D** | **T** | **E** | **K** | **E** | **P** | **V** | **V** | **L** | **P** | **A** | **R** | **F** | **P** | **N** | **L** | **L** | **V** | **N** | **G** | **T** | **T** | **G** | **I** | **A** | **V** | **G** | **M** | **A** | **T** | **N** | **I** | **P** | **P** | **H** | **N** | **L** | **S** | **E** | **V** | **V** | **A** | **A** | **I** | **D** | **L** | **L** | **M** | **D** | **N** | **P** | **D** | **V** | **T** | **T** | **N** | **E** | **L** | **M** | **E** | **V** | **L** | **P** | **G** | **P** | **D** | **F** | **P** | **T** | **G** | **G** | **L** | **V** | **M** | **G** | **K** | **S** | **G** | **I** | **R** | **R** | **A** | **Y** | **E** | **T** | **G** | **K** | **G** | **S** | **I** | **T** | **V** | **R** | **A** | **K** | **V** | **E** | **I** | **T** | **E** | **M** | **P** | **N** | **G** | **K** | **E** | **R** | **I** | **L** | **V** | **T** | **E** | **L** | **P** | **Y** | **M** | **V** | **N** | **K** | **A** | **K** | **L** | **I** | **E** | **R** | **I** | **S** | **E** | **L** | **H** | **R** | **D** | **K** | **R** | **I** | **E** | **G** | **I** | **T** | **D** | **L** | **R** | **D** | **E** | **S** | **S** | **R** | **E** | **G** | **M** | **R** | **I** | **V** | **I** | **D** | **I** | **R** | **R** | **D** | **A** | **S** | **A** | **S** | **V** | **I** | **L** | **N** | **N** | **L** | **Y** | **K** | **L** | **T** | **S** | **L** | **Q** | **N** | **S** | **F** | **G** | **F** | **N** | **M** | **L** | **A** | **I** | **E** | **K** | **G** | **V** | **P** | **K** | **V** | **L** | **S** | **L** | **K** | **Q** | **I** | **L** | **E** | **N** | **Y** | **I** | **E** | **H** | **Q** | **R** | **E** | **V** | **I** | **T** | **R** | **R** | **T** | **V** | **F** | **E** | **K** | **E** | **K** | **A** | **E** | **A** | **R** | **A** | **H** | **I** | **L** | **E** | **G** | **L** | **R** | **I** | **A** | **L** | **D** | **H** | **I** | **D** | **E** | **I** | **I** | **A** | **I** | **I** | **R** | **G** | **S** | **K** | **S** | **D** | **D** | **E** | **A** | **K** | **A** | **T** | **M** | **I** | **E** | **R** | **F** | **D** | **L** | **S** | **D** | **R** | **Q** | **S** | **Q** | **A** | **I** | **L** | **D** | **M** | **R** | **L** | **R** | **R** | **L** | **T** | **G** | **L** | **E** | **R** | **E** | **K** | **I** | **E** | **N** | **E** | **Y** | **Q** | **E** | **L** | **L** | **K** | **L** | **I** | **E** | **D** | **L** | **T** | **D** | **I** | **L** | **A** | **R** | **P** | **E** | **R** | **V** | **T** | **E** | **I** | **I** | **K** | **N** | **E** | **L** | **A** | **D** | **L** | **N** | **Q** | **R** | **F** | **G** | **D** | **K** | **R** | **R** | **T** | **E** | **L** | **L** | **V** | **G** | **E** | **V** | **L** | **S** | **L** | **E** | **D** | **E** | **D** | **L** | **I** | **E** | **E** | **E** | **E** | **I** | **V** | **I** | **T** | **L** | **T** | **N** | **N** | **G** | **Y** | **I** | **K** | **R** | **L** | **A** | **N** | **N** | **E** | **F** | **R** | **A** | **Q** | **R** | **R** | **G** | **G** | **R** | **G** | **V** | **Q** | **G** | **M** | **G** | **I** | **H** | **N** | **D** | **D** | **F** | **V** | **K** | **N** | **L** | **V** | **S** | **C** | **S** | **T** | **H** | **D** | **T** | **L** | **L** | **F** | **F** | **T** | **N** | **N** | **G** | **K** | **V** | **Y** | **R** | **A** | **K** | **G** | **Y** | **E** | **I** | **P** | **E** | **Y** | **G** | **R** | **T** | **A** | **K** | **G** | **I** | **P** | **I** | **I** | **N** | **L** | **L** | **G** | **I** | **D** | **S** | **S** | **E** | **S** | **I** | **Q** | **A** | **I** | **I** | **A** | **V** | **T** | **G** | **E** | **A** | **E** | **E** | **G** | **H** | **Y** | **L** | **F** | **F** | **T** | **T** | **R** | **Q** | **G** | **T** | **V** | **K** | **R** | **T** | **S** | **V** | **T** | **A** | **F** | **A** | **N** | **I** | **R** | **S** | **N** | **G** | **L** | **I** | **A** | **I** | **G** | **L** | **K** | **E** | **D** | **D** | **E** | **L** | **V** | **N** | **V** | **L** | **L** | **T** | **D** | **G** | **Q** | **S** | **N** | **I** | **I** | **I** | **G** | **T** | **H** | **N** | **G** | **Y** | **S** | **V** | **T** | **F** | **K** | **E** | **E** | **A** | **V** | **R** | **D** | **M** | **G** | **R** | **T** | **A** | **S** | **G** | **V** | **R** | **G** | **I** | **R** | **L** | **R** | **E** | **E** | **D** | **Y** | **V** | **V** | **G** | **A** | **A** | **L** | **M** | **K** | **E** | **D** | **Q** | **E** | **V** | **L** | **V** | **I** | **T** | **E** | **K** | **G** | **Y** | **G** | **K** | **R** | **T** | **K** | **V** | **S** | **E** | **Y** | **P** | **V** | **K** | **G** | **R** | **G** | **G** | **K** | **G** | **I** | **K** | **T** | **A** | **N** | **I** | **T** | **E** | **K** | **N** | **G** | **P** | **L** | **A** | **G** | **L** | **T** | **T** | **V** | **S** | **G** | **D** | **E** | **D** | **I** | **M** | **L** | **I** | **T** | **D** | **K** | **G** | **V** | **I** | **I** | **R** | **F** | **N** | **V** | **S** | **T** | **V** | **S** | **Q** | **T** | **G** | **R** | **S** | **T** | **L** | **G** | **V** | **R** | **L** | **M** | **K** | **M** | **E** | **A** | **D** | **T** | **K** | **V** | **V** | **T** | **M** | **-** | **-** | **-** | **-** | **-** | **-** | **-** | **-** | **-** | **-** | **-** | **-** | **-** | **-** | **-** | **-** | **-** | **-** | **-** |
| **N52690** | **M** | **S** | **E** | **E** | **I** | **R** | **E** | **N** | **I** | **H** | **D** | **V** | **N** | **L** | **T** | **S** | **E** | **M** | **K** | **D** | **S** | **F** | **I** | **D** | **Y** | **A** | **M** | **S** | **V** | **I** | **V** | **A** | **R** | **A** | **L** | **P** | **D** | **V** | **R** | **D** | **G** | **L** | **K** | **P** | **V** | **H** | **R** | **R** | **I** | **L** | **Y** | **G** | **M** | **N** | **E** | **L** | **G** | **V** | **T** | **P** | **D** | **K** | **A** | **H** | **K** | **K** | **S** | **A** | **R** | **I** | **V** | **G** | **D** | **V** | **M** | **G** | **K** | **Y** | **H** | **P** | **H** | **G** | **D** | **S** | **A** | **I** | **Y** | **E** | **S** | **M** | **V** | **R** | **M** | **A** | **Q** | **P** | **F** | **S** | **Y** | **R** | **Y** | **M** | **L** | **V** | **D** | **G** | **H** | **G** | **N** | **F** | **G** | **S** | **V** | **D** | **G** | **D** | **G** | **A** | **A** | **A** | **M** | **R** | **Y** | **T** | **E** | **A** | **R** | **M** | **S** | **K** | **I** | **A** | **T** | **E** | **M** | **L** | **R** | **D** | **I** | **N** | **K** | **N** | **T** | **V** | **D** | **F** | **Q** | **S** | **N** | **Y** | **D** | **D** | **T** | **E** | **K** | **E** | **P** | **V** | **V** | **L** | **P** | **A** | **R** | **F** | **P** | **N** | **L** | **L** | **V** | **N** | **G** | **T** | **T** | **G** | **I** | **A** | **V** | **G** | **M** | **A** | **T** | **N** | **I** | **P** | **P** | **H** | **N** | **L** | **S** | **E** | **V** | **V** | **A** | **A** | **I** | **D** | **L** | **L** | **M** | **D** | **N** | **P** | **D** | **V** | **T** | **T** | **N** | **E** | **L** | **M** | **E** | **V** | **L** | **P** | **G** | **P** | **D** | **F** | **P** | **T** | **G** | **G** | **L** | **V** | **M** | **G** | **K** | **S** | **G** | **I** | **R** | **R** | **A** | **Y** | **E** | **T** | **G** | **K** | **G** | **S** | **I** | **T** | **V** | **R** | **A** | **K** | **V** | **E** | **I** | **T** | **E** | **M** | **P** | **N** | **G** | **K** | **E** | **R** | **I** | **I** | **V** | **T** | **E** | **L** | **P** | **Y** | **M** | **V** | **N** | **K** | **A** | **K** | **L** | **I** | **E** | **R** | **I** | **S** | **E** | **L** | **H** | **R** | **D** | **K** | **R** | **I** | **E** | **G** | **I** | **T** | **D** | **L** | **R** | **D** | **E** | **S** | **S** | **R** | **E** | **G** | **M** | **R** | **I** | **V** | **I** | **D** | **I** | **R** | **R** | **D** | **A** | **S** | **A** | **S** | **V** | **I** | **L** | **N** | **N** | **L** | **Y** | **K** | **L** | **T** | **S** | **L** | **Q** | **N** | **S** | **F** | **G** | **F** | **N** | **M** | **L** | **A** | **I** | **E** | **K** | **G** | **V** | **P** | **K** | **V** | **L** | **S** | **L** | **K** | **Q** | **I** | **L** | **E** | **N** | **Y** | **I** | **E** | **H** | **Q** | **R** | **E** | **V** | **I** | **T** | **R** | **R** | **T** | **V** | **F** | **E** | **K** | **E** | **K** | **A** | **E** | **A** | **R** | **A** | **H** | **I** | **L** | **E** | **G** | **L** | **R** | **I** | **A** | **L** | **D** | **H** | **I** | **D** | **E** | **I** | **I** | **A** | **I** | **I** | **R** | **G** | **S** | **K** | **S** | **D** | **D** | **E** | **A** | **K** | **A** | **T** | **M** | **I** | **E** | **R** | **F** | **D** | **L** | **S** | **D** | **R** | **Q** | **S** | **Q** | **A** | **I** | **L** | **D** | **M** | **R** | **L** | **R** | **R** | **L** | **T** | **G** | **L** | **E** | **R** | **E** | **K** | **I** | **E** | **N** | **E** | **Y** | **Q** | **E** | **L** | **L** | **K** | **L** | **I** | **E** | **D** | **L** | **T** | **D** | **I** | **L** | **A** | **R** | **P** | **E** | **R** | **V** | **T** | **E** | **I** | **I** | **K** | **N** | **E** | **L** | **A** | **D** | **L** | **N** | **Q** | **R** | **F** | **G** | **D** | **K** | **R** | **R** | **T** | **E** | **L** | **L** | **V** | **G** | **E** | **V** | **L** | **S** | **L** | **E** | **D** | **E** | **D** | **L** | **I** | **E** | **E** | **E** | **E** | **I** | **V** | **I** | **T** | **L** | **T** | **N** | **N** | **G** | **Y** | **I** | **K** | **R** | **L** | **A** | **N** | **N** | **E** | **F** | **R** | **A** | **Q** | **R** | **R** | **G** | **G** | **R** | **G** | **V** | **Q** | **G** | **M** | **G** | **I** | **H** | **N** | **D** | **D** | **F** | **V** | **K** | **N** | **L** | **V** | **S** | **C** | **S** | **T** | **H** | **D** | **T** | **L** | **L** | **F** | **F** | **T** | **N** | **N** | **G** | **K** | **V** | **Y** | **R** | **A** | **K** | **G** | **Y** | **E** | **I** | **P** | **E** | **Y** | **G** | **R** | **T** | **A** | **K** | **G** | **I** | **P** | **I** | **I** | **N** | **L** | **L** | **G** | **I** | **D** | **S** | **S** | **E** | **S** | **I** | **Q** | **A** | **I** | **I** | **A** | **V** | **T** | **G** | **E** | **A** | **E** | **E** | **G** | **H** | **Y** | **L** | **F** | **F** | **T** | **T** | **R** | **Q** | **G** | **T** | **V** | **K** | **R** | **T** | **S** | **V** | **T** | **A** | **F** | **A** | **N** | **I** | **R** | **S** | **N** | **G** | **L** | **I** | **A** | **I** | **G** | **L** | **K** | **E** | **D** | **D** | **E** | **L** | **V** | **N** | **V** | **L** | **L** | **T** | **D** | **G** | **Q** | **S** | **N** | **I** | **I** | **I** | **G** | **T** | **H** | **N** | **G** | **Y** | **S** | **V** | **T** | **F** | **K** | **E** | **E** | **A** | **V** | **R** | **D** | **M** | **G** | **R** | **T** | **A** | **S** | **G** | **V** | **R** | **G** | **I** | **R** | **L** | **R** | **E** | **E** | **D** | **Y** | **V** | **V** | **G** | **A** | **A** | **L** | **M** | **K** | **G** | **N** | **Q** | **E** | **V** | **L** | **V** | **I** | **T** | **E** | **K** | **G** | **Y** | **G** | **K** | **R** | **T** | **K** | **V** | **S** | **E** | **Y** | **P** | **V** | **K** | **G** | **R** | **G** | **G** | **K** | **G** | **I** | **K** | **T** | **A** | **N** | **I** | **T** | **E** | **K** | **N** | **G** | **P** | **L** | **A** | **G** | **L** | **T** | **T** | **V** | **S** | **G** | **D** | **E** | **D** | **I** | **M** | **L** | **I** | **T** | **D** | **K** | **G** | **V** | **I** | **I** | **R** | **F** | **N** | **V** | **S** | **T** | **V** | **S** | **Q** | **T** | **G** | **R** | **S** | **T** | **L** | **G** | **V** | **R** | **L** | **M** | **K** | **M** | **E** | **A** | **D** | **T** | **K** | **V** | **V** | **T** | **M** | **A** | **A** | **V** | **E** | **P** | **E** | **A** | **A** | **E** | **Q** | **T** | **V** | **E** | **E** | **Q** | **G** | **S** | **E** | **E** |
| **N53457** | **M** | **S** | **E** | **E** | **I** | **K** | **E** | **N** | **I** | **Q** | **D** | **V** | **N** | **L** | **T** | **S** | **E** | **M** | **K** | **E** | **S** | **F** | **I** | **D** | **Y** | **A** | **M** | **S** | **V** | **I** | **V** | **A** | **R** | **A** | **L** | **P** | **D** | **V** | **R** | **D** | **G** | **L** | **K** | **P** | **V** | **H** | **R** | **R** | **I** | **L** | **Y** | **G** | **M** | **N** | **E** | **L** | **G** | **V** | **T** | **P** | **D** | **K** | **P** | **H** | **K** | **K** | **S** | **A** | **R** | **I** | **V** | **G** | **D** | **V** | **M** | **G** | **K** | **Y** | **H** | **P** | **H** | **G** | **D** | **S** | **A** | **I** | **Y** | **E** | **S** | **M** | **V** | **R** | **M** | **A** | **Q** | **P** | **F** | **S** | **Y** | **R** | **A** | **M** | **L** | **V** | **D** | **G** | **H** | **G** | **N** | **F** | **G** | **S** | **V** | **D** | **G** | **D** | **G** | **A** | **A** | **A** | **M** | **R** | **Y** | **T** | **E** | **A** | **R** | **M** | **S** | **K** | **I** | **A** | **L** | **E** | **M** | **L** | **R** | **D** | **I** | **N** | **K** | **N** | **T** | **V** | **D** | **F** | **Q** | **G** | **N** | **Y** | **D** | **D** | **S** | **E** | **Q** | **E** | **P** | **V** | **V** | **L** | **P** | **A** | **R** | **F** | **P** | **N** | **L** | **L** | **V** | **N** | **G** | **T** | **T** | **G** | **I** | **A** | **V** | **G** | **M** | **A** | **T** | **N** | **I** | **P** | **P** | **H** | **N** | **L** | **S** | **E** | **V** | **I** | **D** | **A** | **T** | **S** | **L** | **L** | **M** | **D** | **N** | **P** | **D** | **V** | **T** | **T** | **N** | **E** | **L** | **M** | **E** | **V** | **L** | **P** | **G** | **P** | **D** | **F** | **P** | **T** | **G** | **G** | **L** | **V** | **M** | **G** | **K** | **S** | **G** | **I** | **R** | **R** | **A** | **Y** | **E** | **T** | **G** | **K** | **G** | **S** | **I** | **T** | **V** | **R** | **A** | **K** | **V** | **E** | **L** | **T** | **E** | **M** | **P** | **N** | **G** | **K** | **E** | **R** | **I** | **L** | **V** | **T** | **E** | **L** | **P** | **Y** | **M** | **V** | **N** | **K** | **A** | **K** | **L** | **I** | **E** | **R** | **I** | **S** | **E** | **L** | **H** | **R** | **D** | **K** | **R** | **I** | **E** | **G** | **I** | **T** | **D** | **L** | **R** | **D** | **E** | **S** | **S** | **R** | **E** | **G** | **M** | **R** | **I** | **V** | **I** | **D** | **V** | **R** | **R** | **D** | **V** | **S** | **A** | **S** | **V** | **V** | **L** | **N** | **N** | **L** | **Y** | **K** | **M** | **T** | **A** | **L** | **Q** | **T** | **S** | **F** | **G** | **F** | **N** | **M** | **L** | **A** | **I** | **E** | **K** | **G** | **V** | **P** | **K** | **I** | **L** | **S** | **L** | **K** | **R** | **I** | **L** | **E** | **N** | **Y** | **V** | **E** | **H** | **Q** | **K** | **E** | **V** | **I** | **T** | **R** | **R** | **T** | **I** | **F** | **D** | **K** | **N** | **K** | **A** | **E** | **A** | **R** | **A** | **H** | **I** | **L** | **E** | **G** | **L** | **R** | **I** | **A** | **L** | **D** | **H** | **I** | **D** | **E** | **I** | **I** | **A** | **I** | **I** | **R** | **G** | **S** | **Q** | **S** | **D** | **D** | **E** | **A** | **K** | **A** | **T** | **L** | **I** | **E** | **R** | **F** | **E** | **F** | **S** | **D** | **R** | **Q** | **A** | **Q** | **A** | **I** | **L** | **D** | **M** | **R** | **L** | **R** | **R** | **L** | **T** | **G** | **L** | **E** | **R** | **D** | **K** | **I** | **E** | **N** | **E** | **Y** | **Q** | **E** | **L** | **L** | **K** | **F** | **I** | **A** | **D** | **L** | **E** | **D** | **I** | **L** | **A** | **R** | **P** | **E** | **R** | **V** | **I** | **E** | **I** | **I** | **K** | **T** | **E** | **L** | **N** | **D** | **V** | **R** | **T** | **K** | **F** | **G** | **D** | **A** | **R** | **R** | **T** | **E** | **L** | **L** | **V** | **G** | **E** | **V** | **L** | **S** | **L** | **E** | **D** | **E** | **D** | **L** | **I** | **E** | **E** | **E** | **E** | **V** | **V** | **I** | **T** | **L** | **T** | **N** | **N** | **G** | **Y** | **I** | **K** | **R** | **M** | **A** | **N** | **S** | **E** | **F** | **R** | **A** | **Q** | **R** | **R** | **G** | **G** | **R** | **G** | **V** | **Q** | **G** | **M** | **G** | **V** | **H** | **D** | **D** | **D** | **F** | **V** | **K** | **N** | **L** | **V** | **S** | **C** | **S** | **T** | **H** | **D** | **T** | **L** | **L** | **F** | **F** | **T** | **N** | **T** | **G** | **K** | **V** | **Y** | **R** | **A** | **K** | **G** | **Y** | **E** | **I** | **P** | **E** | **Y** | **G** | **R** | **T** | **A** | **K** | **G** | **I** | **P** | **V** | **I** | **N** | **L** | **L** | **G** | **I** | **D** | **S** | **A** | **E** | **K** | **I** | **Q** | **A** | **I** | **I** | **S** | **V** | **E** | **G** | **K** | **A** | **E** | **A** | **G** | **K** | **Y** | **L** | **F** | **F** | **T** | **T** | **L** | **K** | **G** | **T** | **V** | **K** | **R** | **T** | **A** | **V** | **T** | **A** | **F** | **S** | **N** | **I** | **R** | **S** | **N** | **G** | **L** | **I** | **A** | **I** | **S** | **L** | **K** | **E** | **D** | **D** | **E** | **L** | **V** | **N** | **V** | **V** | **T** | **T** | **N** | **G** | **N** | **Q** | **K** | **M** | **I** | **I** | **G** | **T** | **H** | **A** | **G** | **Y** | **S** | **V** | **T** | **F** | **D** | **E** | **N** | **T** | **V** | **R** | **D** | **M** | **G** | **R** | **T** | **A** | **S** | **G** | **V** | **R** | **G** | **I** | **R** | **L** | **R** | **E** | **N** | **D** | **Y** | **V** | **V** | **G** | **A** | **A** | **I** | **L** | **D** | **E** | **N** | **K** | **E** | **V** | **L** | **V** | **I** | **T** | **E** | **N** | **G** | **Y** | **G** | **K** | **R** | **T** | **K** | **A** | **S** | **E** | **Y** | **P** | **V** | **K** | **G** | **R** | **G** | **G** | **K** | **G** | **I** | **K** | **T** | **A** | **N** | **I** | **T** | **E** | **K** | **N** | **G** | **P** | **L** | **A** | **G** | **L** | **T** | **T** | **V** | **N** | **G** | **D** | **E** | **D** | **I** | **L** | **L** | **I** | **T** | **N** | **K** | **G** | **V** | **I** | **I** | **R** | **F** | **N** | **V** | **D** | **S** | **V** | **S** | **Q** | **T** | **G** | **R** | **A** | **T** | **L** | **G** | **V** | **R** | **L** | **M** | **R** | **M** | **E** | **D** | **G** | **A** | **K** | **-** | **-** | **-** | **-** | **-** | **-** | **-** | **-** | **-** | **-** | **-** | **-** | **-** | **-** | **-** | **-** | **-** | **-** | **-** | **-** | **-** | **-** | **-** |
| **N59910F** | **M** | **S** | **E** | **E** | **I** | **K** | **E** | **N** | **I** | **Q** | **D** | **V** | **N** | **L** | **T** | **S** | **E** | **M** | **K** | **E** | **S** | **F** | **I** | **D** | **Y** | **A** | **M** | **S** | **V** | **I** | **V** | **A** | **R** | **A** | **L** | **P** | **D** | **V** | **R** | **D** | **G** | **L** | **K** | **P** | **V** | **H** | **R** | **R** | **I** | **L** | **Y** | **G** | **M** | **N** | **E** | **L** | **G** | **V** | **T** | **P** | **D** | **K** | **P** | **H** | **K** | **K** | **S** | **A** | **R** | **I** | **V** | **G** | **D** | **V** | **M** | **G** | **K** | **Y** | **H** | **P** | **H** | **G** | **D** | **S** | **A** | **I** | **Y** | **E** | **S** | **M** | **V** | **R** | **M** | **A** | **Q** | **P** | **F** | **S** | **Y** | **R** | **A** | **M** | **L** | **V** | **D** | **G** | **H** | **G** | **N** | **F** | **G** | **S** | **V** | **D** | **G** | **D** | **G** | **A** | **A** | **A** | **M** | **R** | **Y** | **T** | **E** | **A** | **R** | **M** | **S** | **K** | **I** | **A** | **L** | **E** | **M** | **L** | **R** | **D** | **I** | **N** | **K** | **N** | **T** | **V** | **D** | **F** | **Q** | **G** | **N** | **Y** | **D** | **D** | **S** | **E** | **Q** | **E** | **P** | **V** | **V** | **L** | **P** | **A** | **R** | **F** | **P** | **N** | **L** | **L** | **V** | **N** | **G** | **T** | **T** | **G** | **I** | **A** | **V** | **G** | **M** | **A** | **T** | **N** | **I** | **P** | **P** | **H** | **N** | **L** | **S** | **E** | **V** | **I** | **D** | **A** | **T** | **S** | **L** | **L** | **M** | **D** | **N** | **P** | **D** | **V** | **T** | **T** | **N** | **E** | **L** | **M** | **E** | **V** | **L** | **P** | **G** | **P** | **D** | **F** | **P** | **T** | **G** | **G** | **L** | **V** | **M** | **G** | **K** | **S** | **G** | **I** | **R** | **R** | **A** | **Y** | **E** | **T** | **G** | **K** | **G** | **S** | **I** | **T** | **V** | **R** | **A** | **K** | **V** | **E** | **L** | **T** | **E** | **M** | **P** | **N** | **G** | **K** | **E** | **R** | **I** | **L** | **V** | **T** | **E** | **L** | **P** | **Y** | **M** | **V** | **N** | **K** | **A** | **K** | **L** | **I** | **E** | **R** | **I** | **S** | **E** | **L** | **H** | **R** | **D** | **K** | **R** | **I** | **E** | **G** | **I** | **T** | **D** | **L** | **R** | **D** | **E** | **S** | **S** | **R** | **E** | **G** | **M** | **R** | **I** | **V** | **I** | **D** | **V** | **R** | **R** | **D** | **V** | **S** | **A** | **S** | **V** | **V** | **L** | **N** | **N** | **L** | **Y** | **K** | **M** | **T** | **A** | **L** | **Q** | **T** | **S** | **F** | **G** | **F** | **N** | **M** | **L** | **A** | **I** | **E** | **K** | **G** | **V** | **P** | **K** | **I** | **L** | **S** | **L** | **K** | **R** | **I** | **L** | **E** | **N** | **Y** | **V** | **E** | **H** | **Q** | **K** | **E** | **V** | **I** | **T** | **R** | **R** | **T** | **I** | **F** | **D** | **K** | **N** | **K** | **A** | **E** | **A** | **R** | **A** | **H** | **I** | **L** | **E** | **G** | **L** | **R** | **I** | **A** | **L** | **D** | **H** | **I** | **D** | **E** | **I** | **I** | **A** | **I** | **I** | **R** | **G** | **S** | **Q** | **S** | **D** | **D** | **E** | **A** | **K** | **A** | **T** | **L** | **I** | **E** | **R** | **F** | **E** | **F** | **S** | **D** | **R** | **Q** | **A** | **Q** | **A** | **I** | **L** | **D** | **M** | **R** | **L** | **R** | **R** | **L** | **T** | **G** | **L** | **E** | **R** | **D** | **K** | **I** | **E** | **N** | **E** | **Y** | **Q** | **E** | **L** | **L** | **K** | **F** | **I** | **A** | **D** | **L** | **E** | **D** | **I** | **L** | **A** | **R** | **P** | **E** | **R** | **V** | **I** | **E** | **I** | **I** | **K** | **T** | **E** | **L** | **N** | **D** | **V** | **R** | **T** | **K** | **F** | **G** | **D** | **A** | **R** | **R** | **T** | **E** | **L** | **L** | **V** | **G** | **E** | **V** | **L** | **S** | **L** | **E** | **D** | **E** | **D** | **L** | **I** | **E** | **E** | **E** | **E** | **V** | **V** | **I** | **T** | **L** | **T** | **N** | **N** | **G** | **Y** | **I** | **K** | **R** | **M** | **A** | **N** | **S** | **E** | **F** | **R** | **A** | **Q** | **R** | **R** | **G** | **G** | **R** | **G** | **V** | **Q** | **G** | **M** | **G** | **V** | **H** | **D** | **D** | **D** | **F** | **V** | **K** | **N** | **L** | **V** | **S** | **C** | **S** | **T** | **H** | **D** | **T** | **L** | **L** | **F** | **F** | **T** | **N** | **T** | **G** | **K** | **V** | **Y** | **R** | **A** | **K** | **G** | **Y** | **E** | **I** | **P** | **E** | **Y** | **G** | **R** | **T** | **A** | **K** | **G** | **I** | **P** | **V** | **I** | **N** | **L** | **L** | **G** | **I** | **D** | **S** | **A** | **E** | **K** | **I** | **Q** | **A** | **I** | **I** | **S** | **V** | **E** | **G** | **K** | **A** | **E** | **A** | **G** | **K** | **Y** | **L** | **F** | **F** | **T** | **T** | **L** | **K** | **G** | **T** | **V** | **K** | **R** | **T** | **A** | **V** | **T** | **A** | **F** | **S** | **N** | **I** | **R** | **S** | **N** | **G** | **L** | **I** | **A** | **I** | **S** | **L** | **K** | **E** | **D** | **D** | **E** | **L** | **V** | **N** | **V** | **V** | **T** | **T** | **N** | **G** | **N** | **Q** | **K** | **M** | **I** | **I** | **G** | **T** | **H** | **A** | **G** | **Y** | **S** | **V** | **T** | **F** | **D** | **E** | **N** | **T** | **V** | **R** | **D** | **M** | **G** | **R** | **T** | **A** | **S** | **G** | **V** | **R** | **G** | **I** | **R** | **L** | **R** | **E** | **N** | **D** | **Y** | **V** | **V** | **G** | **A** | **A** | **I** | **L** | **D** | **E** | **N** | **K** | **E** | **V** | **L** | **V** | **I** | **T** | **E** | **N** | **G** | **Y** | **G** | **K** | **R** | **T** | **K** | **A** | **S** | **E** | **Y** | **P** | **V** | **K** | **G** | **R** | **G** | **G** | **K** | **G** | **I** | **K** | **T** | **A** | **N** | **I** | **T** | **E** | **K** | **N** | **G** | **P** | **L** | **A** | **G** | **L** | **T** | **T** | **V** | **N** | **G** | **D** | **E** | **D** | **I** | **L** | **L** | **I** | **T** | **N** | **K** | **G** | **V** | **I** | **I** | **R** | **F** | **N** | **V** | **D** | **S** | **V** | **S** | **Q** | **T** | **G** | **R** | **A** | **T** | **L** | **G** | **V** | **R** | **L** | **M** | **R** | **M** | **E** | **D** | **G** | **A** | **K** | **-** | **-** | **-** | **-** | **-** | **-** | **-** | **-** | **-** | **-** | **-** | **-** | **-** | **-** | **-** | **-** | **-** | **-** | **-** | **-** | **-** | **-** | **-** |
| **N60336F** | **M** | **S** | **E** | **E** | **I** | **R** | **E** | **N** | **I** | **H** | **D** | **V** | **N** | **L** | **T** | **S** | **E** | **M** | **K** | **D** | **S** | **F** | **I** | **D** | **Y** | **A** | **M** | **S** | **V** | **I** | **V** | **A** | **R** | **A** | **L** | **P** | **D** | **V** | **R** | **D** | **G** | **L** | **K** | **P** | **V** | **H** | **R** | **R** | **I** | **L** | **Y** | **G** | **M** | **N** | **E** | **L** | **G** | **V** | **T** | **P** | **D** | **K** | **A** | **H** | **K** | **K** | **S** | **A** | **R** | **I** | **V** | **G** | **D** | **V** | **M** | **G** | **K** | **Y** | **H** | **P** | **H** | **G** | **D** | **S** | **A** | **I** | **Y** | **E** | **S** | **M** | **V** | **R** | **M** | **A** | **Q** | **P** | **F** | **S** | **Y** | **R** | **Y** | **M** | **L** | **V** | **D** | **G** | **H** | **G** | **N** | **F** | **G** | **S** | **V** | **D** | **G** | **D** | **G** | **A** | **A** | **A** | **M** | **R** | **Y** | **T** | **E** | **A** | **R** | **M** | **S** | **K** | **I** | **A** | **T** | **E** | **M** | **L** | **R** | **D** | **I** | **N** | **K** | **N** | **T** | **V** | **D** | **F** | **Q** | **S** | **N** | **Y** | **D** | **D** | **T** | **E** | **K** | **E** | **P** | **V** | **V** | **L** | **P** | **A** | **R** | **F** | **P** | **N** | **L** | **L** | **V** | **N** | **G** | **T** | **T** | **G** | **I** | **A** | **V** | **G** | **M** | **A** | **T** | **N** | **I** | **P** | **P** | **H** | **N** | **L** | **S** | **E** | **V** | **V** | **A** | **A** | **I** | **D** | **L** | **L** | **M** | **D** | **N** | **P** | **D** | **V** | **T** | **T** | **N** | **E** | **L** | **M** | **E** | **V** | **L** | **P** | **G** | **P** | **D** | **F** | **P** | **T** | **G** | **G** | **L** | **V** | **M** | **G** | **K** | **S** | **G** | **I** | **R** | **R** | **A** | **Y** | **E** | **T** | **G** | **K** | **G** | **S** | **I** | **T** | **V** | **R** | **A** | **K** | **V** | **E** | **I** | **T** | **E** | **M** | **P** | **N** | **G** | **K** | **E** | **R** | **I** | **I** | **V** | **T** | **E** | **L** | **P** | **Y** | **M** | **V** | **N** | **K** | **A** | **K** | **L** | **I** | **E** | **R** | **I** | **S** | **E** | **L** | **H** | **R** | **D** | **K** | **R** | **I** | **E** | **G** | **I** | **T** | **D** | **L** | **R** | **D** | **E** | **S** | **S** | **R** | **E** | **G** | **M** | **R** | **I** | **V** | **I** | **D** | **I** | **R** | **R** | **D** | **A** | **S** | **A** | **S** | **V** | **I** | **L** | **N** | **N** | **L** | **Y** | **K** | **L** | **T** | **S** | **L** | **Q** | **N** | **S** | **F** | **G** | **F** | **N** | **M** | **L** | **A** | **I** | **E** | **K** | **G** | **V** | **P** | **K** | **V** | **L** | **S** | **L** | **K** | **Q** | **I** | **L** | **E** | **N** | **Y** | **I** | **E** | **H** | **Q** | **R** | **E** | **V** | **I** | **T** | **R** | **R** | **T** | **V** | **F** | **E** | **K** | **E** | **K** | **A** | **E** | **A** | **R** | **A** | **H** | **I** | **L** | **E** | **G** | **L** | **R** | **I** | **A** | **L** | **D** | **H** | **I** | **D** | **E** | **I** | **I** | **A** | **I** | **I** | **R** | **G** | **S** | **K** | **S** | **D** | **D** | **E** | **A** | **K** | **A** | **T** | **M** | **I** | **E** | **R** | **F** | **D** | **L** | **S** | **D** | **R** | **Q** | **S** | **Q** | **A** | **I** | **L** | **D** | **M** | **R** | **L** | **R** | **R** | **L** | **T** | **G** | **L** | **E** | **R** | **E** | **K** | **I** | **E** | **N** | **E** | **Y** | **Q** | **E** | **L** | **L** | **K** | **L** | **I** | **E** | **D** | **L** | **T** | **D** | **I** | **L** | **A** | **R** | **P** | **E** | **R** | **V** | **T** | **E** | **I** | **I** | **K** | **N** | **E** | **L** | **A** | **D** | **L** | **N** | **Q** | **R** | **F** | **G** | **D** | **K** | **R** | **R** | **T** | **E** | **L** | **L** | **V** | **G** | **E** | **V** | **L** | **S** | **L** | **E** | **D** | **E** | **D** | **L** | **I** | **E** | **E** | **E** | **E** | **I** | **V** | **I** | **T** | **L** | **T** | **N** | **N** | **G** | **Y** | **I** | **K** | **R** | **L** | **A** | **N** | **N** | **E** | **F** | **R** | **A** | **Q** | **R** | **R** | **G** | **G** | **R** | **G** | **V** | **Q** | **G** | **M** | **G** | **I** | **H** | **N** | **D** | **D** | **F** | **V** | **K** | **N** | **L** | **V** | **S** | **C** | **S** | **T** | **H** | **D** | **T** | **L** | **L** | **F** | **F** | **T** | **N** | **N** | **G** | **K** | **V** | **Y** | **R** | **A** | **K** | **G** | **Y** | **E** | **I** | **P** | **E** | **Y** | **G** | **R** | **T** | **A** | **K** | **G** | **I** | **P** | **I** | **I** | **N** | **L** | **L** | **G** | **I** | **D** | **S** | **S** | **E** | **S** | **I** | **Q** | **A** | **I** | **I** | **A** | **V** | **T** | **G** | **E** | **A** | **E** | **E** | **G** | **H** | **Y** | **L** | **F** | **F** | **T** | **T** | **R** | **Q** | **G** | **T** | **V** | **K** | **R** | **T** | **S** | **V** | **T** | **A** | **F** | **A** | **N** | **I** | **R** | **S** | **N** | **G** | **L** | **I** | **A** | **I** | **G** | **L** | **K** | **E** | **D** | **D** | **E** | **L** | **V** | **N** | **V** | **L** | **L** | **T** | **D** | **G** | **Q** | **S** | **N** | **I** | **I** | **I** | **G** | **T** | **H** | **N** | **G** | **Y** | **S** | **V** | **T** | **F** | **K** | **E** | **E** | **A** | **V** | **R** | **D** | **M** | **G** | **R** | **T** | **A** | **S** | **G** | **V** | **R** | **G** | **I** | **R** | **L** | **R** | **E** | **E** | **D** | **Y** | **V** | **V** | **G** | **A** | **A** | **L** | **M** | **K** | **G** | **N** | **Q** | **E** | **V** | **L** | **V** | **I** | **T** | **E** | **K** | **G** | **Y** | **G** | **K** | **R** | **T** | **K** | **V** | **S** | **E** | **Y** | **P** | **V** | **K** | **G** | **R** | **G** | **G** | **K** | **G** | **I** | **K** | **T** | **A** | **N** | **I** | **T** | **E** | **K** | **N** | **G** | **P** | **L** | **A** | **G** | **L** | **T** | **T** | **V** | **S** | **G** | **D** | **E** | **D** | **I** | **M** | **L** | **I** | **T** | **D** | **K** | **G** | **V** | **I** | **I** | **R** | **F** | **N** | **V** | **S** | **T** | **V** | **S** | **Q** | **T** | **G** | **R** | **S** | **T** | **L** | **G** | **V** | **R** | **L** | **M** | **K** | **M** | **E** | **A** | **D** | **T** | **K** | **V** | **V** | **T** | **M** | **A** | **A** | **V** | **E** | **P** | **E** | **A** | **A** | **E** | **Q** | **T** | **V** | **E** | **E** | **Q** | **G** | **S** | **E** | **E** |
| **N55655** | **M** | **S** | **E** | **E** | **I** | **R** | **E** | **N** | **I** | **H** | **D** | **V** | **N** | **L** | **T** | **S** | **E** | **M** | **K** | **D** | **S** | **F** | **I** | **D** | **Y** | **A** | **M** | **S** | **V** | **I** | **V** | **A** | **R** | **A** | **L** | **P** | **D** | **V** | **R** | **D** | **G** | **L** | **K** | **P** | **V** | **H** | **R** | **R** | **I** | **L** | **Y** | **G** | **M** | **N** | **E** | **L** | **G** | **V** | **T** | **P** | **D** | **K** | **A** | **H** | **K** | **K** | **S** | **A** | **R** | **I** | **V** | **G** | **D** | **V** | **M** | **G** | **K** | **Y** | **H** | **P** | **H** | **G** | **D** | **S** | **A** | **I** | **Y** | **E** | **S** | **M** | **V** | **R** | **M** | **A** | **Q** | **P** | **F** | **S** | **Y** | **R** | **Y** | **M** | **L** | **V** | **D** | **G** | **H** | **G** | **N** | **F** | **G** | **S** | **V** | **D** | **G** | **D** | **G** | **A** | **A** | **A** | **M** | **R** | **Y** | **T** | **E** | **A** | **R** | **M** | **S** | **K** | **I** | **A** | **T** | **E** | **M** | **L** | **R** | **D** | **I** | **N** | **K** | **N** | **T** | **V** | **D** | **F** | **Q** | **S** | **N** | **Y** | **D** | **D** | **T** | **E** | **K** | **E** | **P** | **V** | **V** | **L** | **P** | **A** | **R** | **F** | **P** | **N** | **L** | **L** | **V** | **N** | **G** | **T** | **T** | **G** | **I** | **A** | **V** | **G** | **M** | **A** | **T** | **N** | **I** | **P** | **P** | **H** | **N** | **L** | **S** | **E** | **V** | **V** | **A** | **A** | **I** | **D** | **L** | **L** | **M** | **D** | **N** | **P** | **D** | **V** | **T** | **T** | **N** | **E** | **L** | **M** | **E** | **V** | **L** | **P** | **G** | **P** | **D** | **F** | **P** | **T** | **G** | **G** | **L** | **V** | **M** | **G** | **K** | **S** | **G** | **I** | **R** | **R** | **A** | **Y** | **E** | **T** | **G** | **K** | **G** | **S** | **I** | **T** | **V** | **R** | **A** | **K** | **V** | **E** | **I** | **T** | **E** | **M** | **P** | **N** | **G** | **K** | **E** | **R** | **I** | **I** | **V** | **T** | **E** | **L** | **P** | **Y** | **M** | **V** | **N** | **K** | **A** | **K** | **L** | **I** | **E** | **R** | **I** | **S** | **E** | **L** | **H** | **R** | **D** | **K** | **R** | **I** | **E** | **G** | **I** | **T** | **D** | **L** | **R** | **D** | **E** | **S** | **S** | **R** | **E** | **G** | **M** | **R** | **I** | **V** | **I** | **D** | **I** | **R** | **R** | **D** | **A** | **S** | **A** | **S** | **V** | **I** | **L** | **N** | **N** | **L** | **Y** | **K** | **L** | **T** | **S** | **L** | **Q** | **N** | **S** | **F** | **G** | **F** | **N** | **M** | **L** | **A** | **I** | **E** | **K** | **G** | **V** | **P** | **K** | **V** | **L** | **S** | **L** | **K** | **Q** | **I** | **L** | **E** | **N** | **Y** | **I** | **E** | **H** | **Q** | **R** | **E** | **V** | **I** | **T** | **R** | **R** | **T** | **V** | **F** | **E** | **K** | **E** | **K** | **A** | **E** | **A** | **R** | **A** | **H** | **I** | **L** | **E** | **G** | **L** | **R** | **I** | **A** | **L** | **D** | **H** | **I** | **D** | **E** | **I** | **I** | **A** | **I** | **I** | **R** | **G** | **S** | **K** | **S** | **D** | **D** | **E** | **A** | **K** | **A** | **T** | **M** | **I** | **E** | **R** | **F** | **D** | **L** | **S** | **D** | **R** | **Q** | **S** | **Q** | **A** | **I** | **L** | **D** | **M** | **R** | **L** | **R** | **R** | **L** | **T** | **G** | **L** | **E** | **R** | **E** | **K** | **I** | **E** | **N** | **E** | **Y** | **Q** | **E** | **L** | **L** | **K** | **L** | **I** | **E** | **D** | **L** | **T** | **D** | **I** | **L** | **A** | **R** | **P** | **E** | **R** | **V** | **T** | **E** | **I** | **I** | **K** | **N** | **E** | **L** | **A** | **D** | **L** | **N** | **Q** | **R** | **F** | **G** | **D** | **K** | **R** | **R** | **T** | **E** | **L** | **L** | **V** | **G** | **E** | **V** | **L** | **S** | **L** | **E** | **D** | **E** | **D** | **L** | **I** | **E** | **E** | **E** | **E** | **I** | **V** | **I** | **T** | **L** | **T** | **N** | **N** | **G** | **Y** | **I** | **K** | **R** | **L** | **A** | **N** | **N** | **E** | **F** | **R** | **A** | **Q** | **R** | **R** | **G** | **G** | **R** | **G** | **V** | **Q** | **G** | **M** | **G** | **I** | **H** | **N** | **D** | **D** | **F** | **V** | **K** | **N** | **L** | **V** | **S** | **C** | **S** | **T** | **H** | **D** | **T** | **L** | **L** | **F** | **F** | **T** | **N** | **N** | **G** | **K** | **V** | **Y** | **R** | **A** | **K** | **G** | **Y** | **E** | **I** | **P** | **E** | **Y** | **G** | **R** | **T** | **A** | **K** | **G** | **I** | **P** | **I** | **I** | **N** | **L** | **L** | **G** | **I** | **D** | **S** | **S** | **E** | **S** | **I** | **Q** | **A** | **I** | **I** | **A** | **V** | **T** | **G** | **E** | **A** | **E** | **E** | **G** | **H** | **Y** | **L** | **F** | **F** | **T** | **T** | **R** | **Q** | **G** | **T** | **V** | **K** | **R** | **T** | **S** | **V** | **T** | **A** | **F** | **A** | **N** | **I** | **R** | **S** | **N** | **G** | **L** | **I** | **A** | **I** | **G** | **L** | **K** | **E** | **D** | **D** | **E** | **L** | **V** | **N** | **V** | **L** | **L** | **T** | **D** | **G** | **Q** | **S** | **N** | **I** | **I** | **I** | **G** | **T** | **H** | **N** | **G** | **Y** | **S** | **V** | **T** | **F** | **K** | **E** | **E** | **A** | **V** | **R** | **D** | **M** | **G** | **R** | **T** | **A** | **S** | **G** | **V** | **R** | **G** | **I** | **R** | **L** | **R** | **E** | **E** | **D** | **Y** | **V** | **V** | **G** | **A** | **A** | **L** | **M** | **K** | **E** | **D** | **Q** | **E** | **V** | **L** | **V** | **I** | **T** | **E** | **K** | **G** | **Y** | **G** | **K** | **R** | **T** | **K** | **V** | **S** | **E** | **Y** | **P** | **V** | **K** | **G** | **R** | **G** | **G** | **K** | **G** | **I** | **K** | **T** | **A** | **N** | **I** | **T** | **E** | **K** | **N** | **G** | **P** | **L** | **A** | **G** | **L** | **T** | **T** | **V** | **S** | **G** | **D** | **E** | **D** | **I** | **M** | **L** | **I** | **T** | **D** | **K** | **G** | **V** | **I** | **I** | **R** | **F** | **N** | **V** | **S** | **T** | **V** | **S** | **Q** | **T** | **G** | **R** | **S** | **T** | **L** | **G** | **V** | **R** | **L** | **M** | **K** | **M** | **E** | **A** | **D** | **T** | **K** | **V** | **V** | **T** | **M** | **A** | **A** | **V** | **E** | **P** | **E** | **A** | **A** | **E** | **Q** | **T** | **V** | **E** | **E** | **Q** | **G** | **S** | **E** | **E** |
| **N60324F** | **M** | **S** | **E** | **E** | **I** | **R** | **E** | **N** | **I** | **H** | **D** | **V** | **N** | **L** | **T** | **S** | **E** | **M** | **K** | **D** | **S** | **F** | **I** | **D** | **Y** | **A** | **M** | **S** | **V** | **I** | **V** | **A** | **R** | **A** | **L** | **P** | **D** | **V** | **R** | **D** | **G** | **L** | **K** | **P** | **V** | **H** | **R** | **R** | **I** | **L** | **Y** | **G** | **M** | **N** | **E** | **L** | **G** | **V** | **T** | **P** | **D** | **K** | **A** | **H** | **K** | **K** | **S** | **A** | **R** | **I** | **V** | **G** | **D** | **V** | **M** | **G** | **K** | **Y** | **H** | **P** | **H** | **G** | **D** | **S** | **A** | **I** | **Y** | **E** | **S** | **M** | **V** | **R** | **M** | **A** | **Q** | **P** | **F** | **S** | **Y** | **R** | **Y** | **M** | **L** | **V** | **D** | **G** | **H** | **G** | **N** | **F** | **G** | **S** | **V** | **D** | **G** | **D** | **G** | **A** | **A** | **A** | **M** | **R** | **Y** | **T** | **E** | **A** | **R** | **M** | **S** | **K** | **I** | **A** | **T** | **E** | **M** | **L** | **R** | **D** | **I** | **N** | **K** | **N** | **T** | **V** | **D** | **F** | **Q** | **S** | **N** | **Y** | **D** | **D** | **T** | **E** | **K** | **E** | **P** | **V** | **V** | **L** | **P** | **A** | **R** | **F** | **P** | **N** | **L** | **L** | **V** | **N** | **G** | **T** | **T** | **G** | **I** | **A** | **V** | **G** | **M** | **A** | **T** | **N** | **I** | **P** | **P** | **H** | **N** | **L** | **S** | **E** | **V** | **V** | **A** | **A** | **I** | **D** | **L** | **L** | **M** | **D** | **N** | **P** | **D** | **V** | **T** | **T** | **N** | **E** | **L** | **M** | **E** | **V** | **L** | **P** | **G** | **P** | **D** | **F** | **P** | **T** | **G** | **G** | **L** | **V** | **M** | **G** | **K** | **S** | **G** | **I** | **R** | **R** | **A** | **Y** | **E** | **T** | **G** | **K** | **G** | **S** | **I** | **T** | **V** | **R** | **A** | **K** | **V** | **E** | **I** | **T** | **E** | **M** | **P** | **N** | **G** | **K** | **E** | **R** | **I** | **I** | **V** | **T** | **E** | **L** | **P** | **Y** | **M** | **V** | **N** | **K** | **A** | **K** | **L** | **I** | **E** | **R** | **I** | **S** | **E** | **L** | **H** | **R** | **D** | **K** | **R** | **I** | **E** | **G** | **I** | **T** | **D** | **L** | **R** | **D** | **E** | **S** | **S** | **R** | **E** | **G** | **M** | **R** | **I** | **V** | **I** | **D** | **I** | **R** | **R** | **D** | **A** | **S** | **A** | **S** | **V** | **I** | **L** | **N** | **N** | **L** | **Y** | **K** | **L** | **T** | **S** | **L** | **Q** | **N** | **S** | **F** | **G** | **F** | **N** | **M** | **L** | **A** | **I** | **E** | **K** | **G** | **V** | **P** | **K** | **V** | **L** | **S** | **L** | **K** | **Q** | **I** | **L** | **E** | **N** | **Y** | **I** | **E** | **H** | **Q** | **R** | **E** | **V** | **I** | **T** | **R** | **R** | **T** | **V** | **F** | **E** | **K** | **E** | **K** | **A** | **E** | **A** | **R** | **A** | **H** | **I** | **L** | **E** | **G** | **L** | **R** | **I** | **A** | **L** | **D** | **H** | **I** | **D** | **E** | **I** | **I** | **A** | **I** | **I** | **R** | **G** | **S** | **K** | **S** | **D** | **D** | **E** | **A** | **K** | **A** | **T** | **M** | **I** | **E** | **R** | **F** | **D** | **L** | **S** | **D** | **R** | **Q** | **S** | **Q** | **A** | **I** | **L** | **D** | **M** | **R** | **L** | **R** | **R** | **L** | **T** | **G** | **L** | **E** | **R** | **E** | **K** | **I** | **E** | **N** | **E** | **Y** | **Q** | **E** | **L** | **L** | **K** | **L** | **I** | **E** | **D** | **L** | **T** | **D** | **I** | **L** | **A** | **R** | **P** | **E** | **R** | **V** | **T** | **E** | **I** | **I** | **K** | **N** | **E** | **L** | **A** | **D** | **L** | **N** | **Q** | **R** | **F** | **G** | **D** | **K** | **R** | **R** | **T** | **E** | **L** | **L** | **V** | **G** | **E** | **V** | **L** | **S** | **L** | **E** | **D** | **E** | **D** | **L** | **I** | **E** | **E** | **E** | **E** | **I** | **V** | **I** | **T** | **L** | **T** | **N** | **N** | **G** | **Y** | **I** | **K** | **R** | **L** | **A** | **N** | **N** | **E** | **F** | **R** | **A** | **Q** | **R** | **R** | **G** | **G** | **R** | **G** | **V** | **Q** | **G** | **M** | **G** | **I** | **H** | **N** | **D** | **D** | **F** | **V** | **K** | **N** | **L** | **V** | **S** | **C** | **S** | **T** | **H** | **D** | **T** | **L** | **L** | **F** | **F** | **T** | **N** | **N** | **G** | **K** | **V** | **Y** | **R** | **A** | **K** | **G** | **Y** | **E** | **I** | **P** | **E** | **Y** | **G** | **R** | **T** | **A** | **K** | **G** | **I** | **P** | **I** | **I** | **N** | **L** | **L** | **G** | **I** | **D** | **S** | **S** | **E** | **S** | **I** | **Q** | **A** | **I** | **I** | **A** | **V** | **T** | **G** | **E** | **A** | **E** | **E** | **G** | **H** | **Y** | **L** | **F** | **F** | **T** | **T** | **R** | **Q** | **G** | **T** | **V** | **K** | **R** | **T** | **S** | **V** | **T** | **A** | **F** | **A** | **N** | **I** | **R** | **S** | **N** | **G** | **L** | **I** | **A** | **I** | **G** | **L** | **K** | **E** | **D** | **D** | **E** | **L** | **V** | **N** | **V** | **L** | **L** | **T** | **D** | **G** | **Q** | **S** | **N** | **I** | **I** | **I** | **G** | **T** | **H** | **N** | **G** | **Y** | **S** | **V** | **T** | **F** | **K** | **E** | **E** | **A** | **V** | **R** | **D** | **M** | **G** | **R** | **T** | **A** | **S** | **G** | **V** | **R** | **G** | **I** | **R** | **L** | **R** | **E** | **E** | **D** | **Y** | **V** | **V** | **G** | **A** | **A** | **L** | **M** | **K** | **E** | **N** | **Q** | **E** | **V** | **L** | **V** | **I** | **T** | **E** | **K** | **G** | **Y** | **G** | **K** | **R** | **T** | **K** | **V** | **S** | **E** | **Y** | **P** | **V** | **K** | **G** | **R** | **G** | **G** | **K** | **G** | **I** | **K** | **T** | **A** | **N** | **I** | **T** | **E** | **K** | **N** | **G** | **P** | **L** | **A** | **G** | **L** | **T** | **T** | **V** | **S** | **G** | **D** | **E** | **D** | **I** | **M** | **L** | **I** | **T** | **D** | **K** | **G** | **V** | **I** | **I** | **R** | **F** | **N** | **V** | **S** | **T** | **V** | **S** | **Q** | **T** | **G** | **R** | **S** | **T** | **L** | **G** | **V** | **R** | **L** | **M** | **K** | **M** | **E** | **A** | **D** | **T** | **K** | **V** | **V** | **T** | **M** | **A** | **A** | **V** | **E** | **P** | **E** | **A** | **A** | **E** | **Q** | **T** | **V** | **E** | **E** | **Q** | **G** | **S** | **E** | **E** |
| **N53559** | **M** | **S** | **E** | **E** | **I** | **R** | **E** | **N** | **I** | **H** | **D** | **V** | **N** | **L** | **T** | **S** | **E** | **M** | **K** | **D** | **S** | **F** | **I** | **D** | **Y** | **A** | **M** | **S** | **V** | **I** | **V** | **A** | **R** | **A** | **L** | **P** | **D** | **V** | **R** | **D** | **G** | **L** | **K** | **P** | **V** | **H** | **R** | **R** | **I** | **L** | **Y** | **G** | **M** | **N** | **E** | **L** | **G** | **V** | **T** | **P** | **D** | **K** | **A** | **H** | **K** | **K** | **S** | **A** | **R** | **I** | **V** | **G** | **D** | **V** | **M** | **G** | **K** | **Y** | **H** | **P** | **H** | **G** | **D** | **S** | **A** | **I** | **Y** | **E** | **S** | **M** | **V** | **R** | **M** | **A** | **Q** | **P** | **F** | **S** | **Y** | **R** | **Y** | **M** | **L** | **V** | **D** | **G** | **H** | **G** | **N** | **F** | **G** | **S** | **V** | **D** | **G** | **D** | **G** | **A** | **A** | **A** | **M** | **R** | **Y** | **T** | **E** | **A** | **R** | **M** | **S** | **K** | **I** | **A** | **T** | **E** | **M** | **L** | **R** | **D** | **I** | **N** | **K** | **N** | **T** | **V** | **D** | **F** | **Q** | **S** | **N** | **Y** | **D** | **D** | **T** | **E** | **K** | **E** | **P** | **V** | **V** | **L** | **P** | **A** | **R** | **F** | **P** | **N** | **L** | **L** | **V** | **N** | **G** | **T** | **T** | **G** | **I** | **A** | **V** | **G** | **M** | **A** | **T** | **N** | **I** | **P** | **P** | **H** | **N** | **L** | **S** | **E** | **V** | **V** | **A** | **A** | **I** | **D** | **L** | **L** | **M** | **D** | **N** | **P** | **D** | **V** | **T** | **T** | **N** | **E** | **L** | **M** | **E** | **V** | **L** | **P** | **G** | **P** | **D** | **F** | **P** | **T** | **G** | **G** | **L** | **V** | **M** | **G** | **K** | **S** | **G** | **I** | **R** | **R** | **A** | **Y** | **E** | **T** | **G** | **K** | **G** | **S** | **I** | **T** | **V** | **R** | **A** | **K** | **V** | **E** | **I** | **T** | **E** | **M** | **P** | **N** | **G** | **K** | **E** | **R** | **I** | **I** | **V** | **T** | **E** | **L** | **P** | **Y** | **M** | **V** | **N** | **K** | **A** | **K** | **L** | **I** | **E** | **R** | **I** | **S** | **E** | **L** | **H** | **R** | **D** | **K** | **R** | **I** | **E** | **G** | **I** | **T** | **D** | **L** | **R** | **D** | **E** | **S** | **S** | **R** | **E** | **G** | **M** | **R** | **I** | **V** | **I** | **D** | **I** | **R** | **R** | **D** | **A** | **S** | **A** | **S** | **V** | **I** | **L** | **N** | **N** | **L** | **Y** | **K** | **L** | **T** | **S** | **L** | **Q** | **N** | **S** | **F** | **G** | **F** | **N** | **M** | **L** | **A** | **I** | **E** | **K** | **G** | **V** | **P** | **K** | **V** | **L** | **S** | **L** | **K** | **Q** | **I** | **L** | **E** | **N** | **Y** | **I** | **E** | **H** | **Q** | **R** | **E** | **V** | **I** | **T** | **R** | **R** | **T** | **V** | **F** | **E** | **K** | **E** | **K** | **A** | **E** | **A** | **R** | **A** | **H** | **I** | **L** | **E** | **G** | **L** | **R** | **I** | **A** | **L** | **D** | **H** | **I** | **D** | **E** | **I** | **I** | **A** | **I** | **I** | **R** | **G** | **S** | **K** | **S** | **D** | **D** | **E** | **A** | **K** | **A** | **T** | **M** | **I** | **E** | **R** | **F** | **D** | **L** | **S** | **D** | **R** | **Q** | **S** | **Q** | **A** | **I** | **L** | **D** | **M** | **R** | **L** | **R** | **R** | **L** | **T** | **G** | **L** | **E** | **R** | **E** | **K** | **I** | **E** | **N** | **E** | **Y** | **Q** | **E** | **L** | **L** | **K** | **L** | **I** | **E** | **D** | **L** | **T** | **D** | **I** | **L** | **A** | **R** | **P** | **E** | **R** | **V** | **T** | **E** | **I** | **I** | **K** | **N** | **E** | **L** | **A** | **D** | **L** | **N** | **Q** | **R** | **F** | **G** | **D** | **K** | **R** | **R** | **T** | **E** | **L** | **L** | **V** | **G** | **E** | **V** | **L** | **S** | **L** | **E** | **D** | **E** | **D** | **L** | **I** | **E** | **E** | **E** | **E** | **I** | **V** | **I** | **T** | **L** | **T** | **N** | **N** | **G** | **Y** | **I** | **K** | **R** | **L** | **A** | **N** | **N** | **E** | **F** | **R** | **A** | **Q** | **R** | **R** | **G** | **G** | **R** | **G** | **V** | **Q** | **G** | **M** | **G** | **I** | **H** | **N** | **D** | **D** | **F** | **V** | **K** | **N** | **L** | **V** | **S** | **C** | **S** | **T** | **H** | **D** | **T** | **L** | **L** | **F** | **F** | **T** | **N** | **N** | **G** | **K** | **V** | **Y** | **R** | **A** | **K** | **G** | **Y** | **E** | **I** | **P** | **E** | **Y** | **G** | **R** | **T** | **A** | **K** | **G** | **I** | **P** | **I** | **I** | **N** | **L** | **L** | **G** | **I** | **D** | **S** | **S** | **E** | **S** | **I** | **Q** | **A** | **I** | **I** | **A** | **V** | **T** | **G** | **E** | **A** | **E** | **E** | **G** | **H** | **Y** | **L** | **F** | **F** | **T** | **T** | **R** | **Q** | **G** | **T** | **V** | **K** | **R** | **T** | **S** | **V** | **T** | **A** | **F** | **A** | **N** | **I** | **R** | **S** | **N** | **G** | **L** | **I** | **A** | **I** | **G** | **L** | **K** | **E** | **D** | **D** | **E** | **L** | **V** | **N** | **V** | **L** | **L** | **T** | **D** | **G** | **Q** | **S** | **N** | **I** | **I** | **I** | **G** | **T** | **H** | **N** | **G** | **Y** | **S** | **V** | **T** | **F** | **K** | **E** | **E** | **A** | **V** | **R** | **D** | **M** | **G** | **R** | **T** | **A** | **S** | **G** | **V** | **R** | **G** | **I** | **R** | **L** | **R** | **E** | **E** | **D** | **Y** | **V** | **V** | **G** | **A** | **A** | **L** | **M** | **K** | **E** | **N** | **Q** | **E** | **V** | **L** | **V** | **I** | **T** | **E** | **K** | **G** | **Y** | **G** | **K** | **R** | **T** | **K** | **V** | **S** | **E** | **Y** | **P** | **V** | **K** | **G** | **R** | **G** | **G** | **K** | **G** | **I** | **K** | **T** | **A** | **N** | **I** | **T** | **E** | **K** | **N** | **G** | **P** | **L** | **A** | **G** | **L** | **T** | **T** | **V** | **S** | **G** | **D** | **E** | **D** | **I** | **M** | **L** | **I** | **T** | **D** | **K** | **G** | **V** | **I** | **I** | **R** | **F** | **N** | **V** | **S** | **T** | **V** | **S** | **Q** | **T** | **G** | **R** | **S** | **T** | **L** | **G** | **V** | **R** | **L** | **M** | **K** | **M** | **E** | **A** | **D** | **T** | **K** | **V** | **V** | **T** | **M** | **A** | **A** | **V** | **E** | **P** | **E** | **A** | **A** | **E** | **Q** | **T** | **V** | **E** | **E** | **Q** | **G** | **S** | **E** | **E** |
| **N52648** | **M** | **S** | **E** | **E** | **I** | **K** | **E** | **N** | **I** | **Q** | **D** | **V** | **N** | **L** | **T** | **S** | **E** | **M** | **K** | **E** | **S** | **F** | **I** | **D** | **Y** | **A** | **M** | **S** | **V** | **I** | **V** | **A** | **R** | **A** | **L** | **P** | **D** | **V** | **R** | **D** | **G** | **L** | **K** | **P** | **V** | **H** | **R** | **R** | **I** | **L** | **Y** | **G** | **M** | **N** | **E** | **L** | **G** | **V** | **T** | **P** | **D** | **K** | **P** | **H** | **K** | **K** | **S** | **A** | **R** | **I** | **V** | **G** | **D** | **V** | **M** | **G** | **K** | **Y** | **H** | **P** | **H** | **G** | **D** | **S** | **A** | **I** | **Y** | **E** | **S** | **M** | **V** | **R** | **M** | **A** | **Q** | **P** | **F** | **S** | **Y** | **R** | **A** | **M** | **L** | **V** | **D** | **G** | **H** | **G** | **N** | **F** | **G** | **S** | **V** | **D** | **G** | **D** | **G** | **A** | **A** | **A** | **M** | **R** | **Y** | **T** | **E** | **A** | **R** | **M** | **S** | **K** | **I** | **A** | **L** | **E** | **M** | **L** | **R** | **D** | **I** | **N** | **K** | **N** | **T** | **V** | **D** | **F** | **Q** | **G** | **N** | **Y** | **D** | **D** | **S** | **E** | **Q** | **E** | **P** | **V** | **V** | **L** | **P** | **A** | **R** | **F** | **P** | **N** | **L** | **L** | **V** | **N** | **G** | **T** | **T** | **G** | **I** | **A** | **V** | **G** | **M** | **A** | **T** | **N** | **I** | **P** | **P** | **H** | **N** | **L** | **S** | **E** | **V** | **I** | **D** | **A** | **T** | **S** | **L** | **L** | **M** | **D** | **N** | **P** | **D** | **V** | **T** | **T** | **N** | **E** | **L** | **M** | **E** | **V** | **L** | **P** | **G** | **P** | **D** | **F** | **P** | **T** | **G** | **G** | **L** | **V** | **M** | **G** | **K** | **S** | **G** | **I** | **R** | **R** | **A** | **Y** | **E** | **T** | **G** | **K** | **G** | **S** | **I** | **T** | **V** | **R** | **A** | **K** | **V** | **E** | **L** | **T** | **E** | **M** | **P** | **N** | **G** | **K** | **E** | **R** | **I** | **L** | **V** | **T** | **E** | **L** | **P** | **Y** | **M** | **V** | **N** | **K** | **A** | **K** | **L** | **I** | **E** | **R** | **I** | **S** | **E** | **L** | **H** | **R** | **D** | **K** | **R** | **I** | **E** | **G** | **I** | **T** | **D** | **L** | **R** | **D** | **E** | **S** | **S** | **R** | **E** | **G** | **M** | **R** | **I** | **V** | **I** | **D** | **V** | **R** | **R** | **D** | **V** | **S** | **A** | **S** | **V** | **V** | **L** | **N** | **N** | **L** | **Y** | **K** | **M** | **T** | **A** | **L** | **Q** | **T** | **S** | **F** | **G** | **F** | **N** | **M** | **L** | **A** | **I** | **E** | **K** | **G** | **V** | **P** | **K** | **I** | **L** | **S** | **L** | **K** | **R** | **I** | **L** | **E** | **N** | **Y** | **V** | **E** | **H** | **Q** | **K** | **E** | **V** | **I** | **T** | **R** | **R** | **T** | **I** | **F** | **D** | **K** | **N** | **K** | **A** | **E** | **A** | **R** | **A** | **H** | **I** | **L** | **E** | **G** | **L** | **R** | **I** | **A** | **L** | **D** | **H** | **I** | **D** | **E** | **I** | **I** | **A** | **I** | **I** | **R** | **G** | **S** | **Q** | **S** | **D** | **D** | **E** | **A** | **K** | **A** | **T** | **L** | **I** | **E** | **R** | **F** | **E** | **F** | **S** | **D** | **R** | **Q** | **A** | **Q** | **A** | **I** | **L** | **D** | **M** | **R** | **L** | **R** | **R** | **L** | **T** | **G** | **L** | **E** | **R** | **D** | **K** | **I** | **E** | **N** | **E** | **Y** | **Q** | **E** | **L** | **L** | **K** | **F** | **I** | **A** | **D** | **L** | **E** | **D** | **I** | **L** | **A** | **R** | **P** | **E** | **R** | **V** | **I** | **E** | **I** | **I** | **K** | **T** | **E** | **L** | **N** | **D** | **V** | **R** | **T** | **K** | **F** | **G** | **D** | **A** | **R** | **R** | **T** | **E** | **L** | **L** | **V** | **G** | **E** | **V** | **L** | **S** | **L** | **E** | **D** | **E** | **D** | **L** | **I** | **E** | **E** | **E** | **E** | **V** | **V** | **I** | **T** | **L** | **T** | **N** | **N** | **G** | **Y** | **I** | **K** | **R** | **M** | **A** | **N** | **S** | **E** | **F** | **R** | **A** | **Q** | **R** | **R** | **G** | **G** | **R** | **G** | **V** | **Q** | **G** | **M** | **G** | **V** | **H** | **D** | **D** | **D** | **F** | **V** | **K** | **N** | **L** | **V** | **S** | **C** | **S** | **T** | **H** | **D** | **T** | **L** | **L** | **F** | **F** | **T** | **N** | **T** | **G** | **K** | **V** | **Y** | **R** | **A** | **K** | **G** | **Y** | **E** | **I** | **P** | **E** | **Y** | **G** | **R** | **T** | **A** | **K** | **G** | **I** | **P** | **V** | **I** | **N** | **L** | **L** | **G** | **I** | **D** | **S** | **A** | **E** | **K** | **I** | **Q** | **A** | **I** | **I** | **S** | **V** | **E** | **G** | **K** | **A** | **E** | **A** | **G** | **K** | **Y** | **L** | **F** | **F** | **T** | **T** | **L** | **K** | **G** | **T** | **V** | **K** | **R** | **T** | **A** | **V** | **T** | **A** | **F** | **S** | **N** | **I** | **R** | **S** | **N** | **G** | **L** | **I** | **A** | **I** | **S** | **L** | **K** | **E** | **D** | **D** | **E** | **L** | **V** | **N** | **V** | **V** | **T** | **T** | **N** | **G** | **N** | **Q** | **K** | **M** | **I** | **I** | **G** | **T** | **H** | **A** | **G** | **Y** | **S** | **V** | **T** | **F** | **D** | **E** | **N** | **T** | **V** | **R** | **D** | **M** | **G** | **R** | **T** | **A** | **S** | **G** | **V** | **R** | **G** | **I** | **R** | **L** | **R** | **E** | **N** | **D** | **Y** | **V** | **V** | **G** | **A** | **A** | **I** | **L** | **D** | **E** | **N** | **K** | **E** | **V** | **L** | **V** | **I** | **T** | **E** | **N** | **G** | **Y** | **G** | **K** | **R** | **T** | **K** | **A** | **S** | **E** | **Y** | **P** | **V** | **K** | **G** | **R** | **G** | **G** | **K** | **G** | **I** | **K** | **T** | **A** | **N** | **I** | **T** | **E** | **K** | **N** | **G** | **P** | **L** | **A** | **G** | **L** | **T** | **T** | **V** | **N** | **G** | **D** | **E** | **D** | **I** | **L** | **L** | **I** | **T** | **N** | **K** | **G** | **V** | **I** | **I** | **R** | **F** | **N** | **V** | **D** | **S** | **V** | **S** | **Q** | **T** | **G** | **R** | **A** | **T** | **L** | **G** | **V** | **R** | **L** | **M** | **R** | **M** | **E** | **D** | **G** | **A** | **K** | **-** | **-** | **-** | **-** | **-** | **-** | **-** | **-** | **-** | **-** | **-** | **-** | **-** | **-** | **-** | **-** | **-** | **-** | **-** | **-** | **-** | **-** | **-** |
| **N60123F** | **M** | **S** | **E** | **E** | **I** | **R** | **E** | **N** | **I** | **H** | **D** | **V** | **N** | **L** | **T** | **S** | **E** | **M** | **K** | **D** | **S** | **F** | **I** | **D** | **Y** | **A** | **M** | **S** | **V** | **I** | **V** | **A** | **R** | **A** | **L** | **P** | **D** | **V** | **R** | **D** | **G** | **L** | **K** | **P** | **V** | **H** | **R** | **R** | **I** | **L** | **Y** | **G** | **M** | **N** | **E** | **L** | **G** | **V** | **T** | **P** | **D** | **K** | **A** | **H** | **K** | **K** | **S** | **A** | **R** | **I** | **V** | **G** | **D** | **V** | **M** | **G** | **K** | **Y** | **H** | **P** | **H** | **G** | **D** | **S** | **A** | **I** | **Y** | **E** | **S** | **M** | **V** | **R** | **M** | **A** | **Q** | **P** | **F** | **S** | **Y** | **R** | **Y** | **M** | **L** | **V** | **D** | **G** | **H** | **G** | **N** | **F** | **G** | **S** | **V** | **D** | **G** | **D** | **G** | **A** | **A** | **A** | **M** | **R** | **Y** | **T** | **E** | **A** | **R** | **M** | **S** | **K** | **I** | **A** | **T** | **E** | **M** | **L** | **R** | **D** | **I** | **N** | **K** | **N** | **T** | **V** | **D** | **F** | **Q** | **S** | **N** | **Y** | **D** | **D** | **T** | **E** | **K** | **E** | **P** | **V** | **V** | **L** | **P** | **A** | **R** | **F** | **P** | **N** | **L** | **L** | **V** | **N** | **G** | **T** | **T** | **G** | **I** | **A** | **V** | **G** | **M** | **A** | **T** | **N** | **I** | **P** | **P** | **H** | **N** | **L** | **S** | **E** | **V** | **V** | **A** | **A** | **I** | **D** | **L** | **L** | **M** | **D** | **N** | **P** | **D** | **V** | **T** | **T** | **N** | **E** | **L** | **M** | **E** | **V** | **L** | **P** | **G** | **P** | **D** | **F** | **P** | **T** | **G** | **G** | **L** | **V** | **M** | **G** | **K** | **S** | **G** | **I** | **R** | **R** | **A** | **Y** | **E** | **T** | **G** | **K** | **G** | **S** | **I** | **T** | **V** | **R** | **A** | **K** | **V** | **E** | **I** | **T** | **E** | **M** | **P** | **N** | **G** | **K** | **E** | **R** | **I** | **I** | **V** | **T** | **E** | **L** | **P** | **Y** | **M** | **V** | **N** | **K** | **A** | **K** | **L** | **I** | **E** | **R** | **I** | **S** | **E** | **L** | **H** | **R** | **D** | **K** | **R** | **I** | **E** | **G** | **I** | **T** | **D** | **L** | **R** | **D** | **E** | **S** | **S** | **R** | **E** | **G** | **M** | **R** | **I** | **V** | **I** | **D** | **I** | **R** | **R** | **D** | **A** | **S** | **A** | **S** | **V** | **I** | **L** | **N** | **N** | **L** | **Y** | **K** | **L** | **T** | **S** | **L** | **Q** | **N** | **S** | **F** | **G** | **F** | **N** | **M** | **L** | **A** | **I** | **E** | **K** | **G** | **V** | **P** | **K** | **V** | **L** | **S** | **L** | **K** | **Q** | **I** | **L** | **E** | **N** | **Y** | **I** | **E** | **H** | **Q** | **R** | **E** | **V** | **I** | **T** | **R** | **R** | **T** | **V** | **F** | **E** | **K** | **E** | **K** | **A** | **E** | **A** | **R** | **A** | **H** | **I** | **L** | **E** | **G** | **L** | **R** | **I** | **A** | **L** | **D** | **H** | **I** | **D** | **E** | **I** | **I** | **A** | **I** | **I** | **R** | **G** | **S** | **K** | **S** | **D** | **D** | **E** | **A** | **K** | **A** | **T** | **M** | **I** | **E** | **R** | **F** | **D** | **L** | **S** | **D** | **R** | **Q** | **S** | **Q** | **A** | **I** | **L** | **D** | **M** | **R** | **L** | **R** | **R** | **L** | **T** | **G** | **L** | **E** | **R** | **E** | **K** | **I** | **E** | **N** | **E** | **Y** | **Q** | **E** | **L** | **L** | **K** | **L** | **I** | **E** | **D** | **L** | **T** | **D** | **I** | **L** | **A** | **R** | **P** | **E** | **R** | **V** | **T** | **E** | **I** | **I** | **K** | **N** | **E** | **L** | **A** | **D** | **L** | **N** | **Q** | **R** | **F** | **G** | **D** | **K** | **R** | **R** | **T** | **E** | **L** | **L** | **V** | **G** | **E** | **V** | **L** | **S** | **L** | **E** | **D** | **E** | **D** | **L** | **I** | **E** | **E** | **E** | **E** | **I** | **V** | **I** | **T** | **L** | **T** | **N** | **N** | **G** | **Y** | **I** | **K** | **R** | **L** | **A** | **N** | **N** | **E** | **F** | **R** | **A** | **Q** | **R** | **R** | **G** | **G** | **R** | **G** | **V** | **Q** | **G** | **M** | **G** | **I** | **H** | **N** | **D** | **D** | **F** | **V** | **K** | **N** | **L** | **V** | **S** | **C** | **S** | **T** | **H** | **D** | **T** | **L** | **L** | **F** | **F** | **T** | **N** | **N** | **G** | **K** | **V** | **Y** | **R** | **A** | **K** | **G** | **Y** | **E** | **I** | **P** | **E** | **Y** | **G** | **R** | **T** | **A** | **K** | **G** | **I** | **P** | **I** | **I** | **N** | **L** | **L** | **G** | **I** | **D** | **S** | **S** | **E** | **S** | **I** | **Q** | **A** | **I** | **I** | **A** | **V** | **T** | **G** | **E** | **A** | **E** | **E** | **G** | **H** | **Y** | **L** | **F** | **F** | **T** | **T** | **R** | **Q** | **G** | **T** | **V** | **K** | **R** | **T** | **S** | **V** | **T** | **A** | **F** | **A** | **N** | **I** | **R** | **S** | **N** | **G** | **L** | **I** | **A** | **I** | **G** | **L** | **K** | **E** | **D** | **D** | **E** | **L** | **V** | **N** | **V** | **L** | **L** | **T** | **D** | **G** | **Q** | **S** | **N** | **I** | **I** | **I** | **G** | **T** | **H** | **N** | **G** | **Y** | **S** | **V** | **T** | **F** | **K** | **E** | **E** | **A** | **V** | **R** | **D** | **M** | **G** | **R** | **T** | **A** | **S** | **G** | **V** | **R** | **G** | **I** | **R** | **L** | **R** | **E** | **E** | **D** | **Y** | **V** | **V** | **G** | **A** | **A** | **L** | **M** | **K** | **E** | **D** | **Q** | **E** | **V** | **L** | **V** | **I** | **T** | **E** | **K** | **G** | **Y** | **G** | **K** | **R** | **T** | **K** | **V** | **S** | **E** | **Y** | **P** | **V** | **K** | **G** | **R** | **G** | **G** | **K** | **G** | **I** | **K** | **T** | **A** | **N** | **I** | **T** | **E** | **K** | **N** | **G** | **P** | **L** | **A** | **G** | **L** | **T** | **T** | **V** | **S** | **G** | **D** | **E** | **D** | **I** | **M** | **L** | **I** | **T** | **D** | **K** | **G** | **V** | **I** | **I** | **R** | **F** | **N** | **V** | **S** | **T** | **V** | **S** | **Q** | **T** | **G** | **R** | **S** | **T** | **L** | **G** | **V** | **R** | **L** | **M** | **K** | **M** | **E** | **A** | **D** | **T** | **K** | **V** | **V** | **T** | **M** | **A** | **A** | **V** | **E** | **P** | **E** | **A** | **A** | **E** | **Q** | **T** | **V** | **E** | **E** | **Q** | **G** | **S** | **E** | **E** |
| **N53439** | **M** | **S** | **E** | **E** | **I** | **R** | **E** | **N** | **I** | **H** | **D** | **V** | **N** | **L** | **T** | **S** | **E** | **M** | **K** | **D** | **S** | **F** | **I** | **D** | **Y** | **A** | **M** | **S** | **V** | **I** | **V** | **A** | **R** | **A** | **L** | **P** | **D** | **V** | **R** | **D** | **G** | **L** | **K** | **P** | **V** | **H** | **R** | **R** | **I** | **L** | **Y** | **G** | **M** | **N** | **E** | **L** | **G** | **V** | **T** | **P** | **D** | **K** | **A** | **H** | **K** | **K** | **S** | **A** | **R** | **I** | **V** | **G** | **D** | **V** | **M** | **G** | **K** | **Y** | **H** | **P** | **H** | **G** | **D** | **S** | **A** | **I** | **Y** | **E** | **S** | **M** | **V** | **R** | **M** | **A** | **Q** | **P** | **F** | **S** | **Y** | **R** | **Y** | **M** | **L** | **V** | **D** | **G** | **H** | **G** | **N** | **F** | **G** | **S** | **V** | **D** | **G** | **D** | **G** | **A** | **A** | **A** | **M** | **R** | **Y** | **T** | **E** | **A** | **R** | **M** | **S** | **K** | **I** | **A** | **T** | **E** | **M** | **L** | **R** | **D** | **I** | **N** | **K** | **N** | **T** | **V** | **D** | **F** | **Q** | **S** | **N** | **Y** | **D** | **D** | **T** | **E** | **K** | **E** | **P** | **V** | **V** | **L** | **P** | **A** | **R** | **F** | **P** | **N** | **L** | **L** | **V** | **N** | **G** | **T** | **T** | **G** | **I** | **A** | **V** | **G** | **M** | **A** | **T** | **N** | **I** | **P** | **P** | **H** | **N** | **L** | **S** | **E** | **V** | **V** | **A** | **A** | **I** | **D** | **L** | **L** | **M** | **D** | **N** | **P** | **D** | **V** | **T** | **T** | **N** | **E** | **L** | **M** | **E** | **V** | **L** | **P** | **G** | **P** | **D** | **F** | **P** | **T** | **G** | **G** | **L** | **V** | **M** | **G** | **K** | **S** | **G** | **I** | **R** | **R** | **A** | **Y** | **E** | **T** | **G** | **K** | **G** | **S** | **I** | **T** | **V** | **R** | **A** | **K** | **V** | **E** | **I** | **T** | **E** | **M** | **P** | **N** | **G** | **K** | **E** | **R** | **I** | **I** | **V** | **T** | **E** | **L** | **P** | **Y** | **M** | **V** | **N** | **K** | **A** | **K** | **L** | **I** | **E** | **R** | **I** | **S** | **E** | **L** | **H** | **R** | **D** | **K** | **R** | **I** | **E** | **G** | **I** | **T** | **D** | **L** | **R** | **D** | **E** | **S** | **S** | **R** | **E** | **G** | **M** | **R** | **I** | **V** | **I** | **D** | **I** | **R** | **R** | **D** | **A** | **S** | **A** | **S** | **V** | **I** | **L** | **N** | **N** | **L** | **Y** | **K** | **L** | **T** | **S** | **L** | **Q** | **N** | **S** | **F** | **G** | **F** | **N** | **M** | **L** | **A** | **I** | **E** | **K** | **G** | **V** | **P** | **K** | **V** | **L** | **S** | **L** | **K** | **Q** | **I** | **L** | **E** | **N** | **Y** | **I** | **E** | **H** | **Q** | **R** | **E** | **V** | **I** | **T** | **R** | **R** | **T** | **V** | **F** | **E** | **K** | **E** | **K** | **A** | **E** | **A** | **R** | **A** | **H** | **I** | **L** | **E** | **G** | **L** | **R** | **I** | **A** | **L** | **D** | **H** | **I** | **D** | **E** | **I** | **I** | **A** | **I** | **I** | **R** | **G** | **S** | **K** | **S** | **D** | **D** | **E** | **A** | **K** | **A** | **T** | **M** | **I** | **E** | **R** | **F** | **D** | **L** | **S** | **D** | **R** | **Q** | **S** | **Q** | **A** | **I** | **L** | **D** | **M** | **R** | **L** | **R** | **R** | **L** | **T** | **G** | **L** | **E** | **R** | **E** | **K** | **I** | **E** | **N** | **E** | **Y** | **Q** | **E** | **L** | **L** | **K** | **L** | **I** | **E** | **D** | **L** | **T** | **D** | **I** | **L** | **A** | **R** | **P** | **E** | **R** | **V** | **T** | **E** | **I** | **I** | **K** | **N** | **E** | **L** | **A** | **D** | **L** | **N** | **Q** | **R** | **F** | **G** | **D** | **K** | **R** | **R** | **T** | **E** | **L** | **L** | **V** | **G** | **E** | **V** | **L** | **S** | **L** | **E** | **D** | **E** | **D** | **L** | **I** | **E** | **E** | **E** | **E** | **I** | **V** | **I** | **T** | **L** | **T** | **N** | **N** | **G** | **Y** | **I** | **K** | **R** | **L** | **A** | **N** | **N** | **E** | **F** | **R** | **A** | **Q** | **R** | **R** | **G** | **G** | **R** | **G** | **V** | **Q** | **G** | **M** | **G** | **I** | **H** | **N** | **D** | **D** | **F** | **V** | **K** | **N** | **L** | **V** | **S** | **C** | **S** | **T** | **H** | **D** | **T** | **L** | **L** | **F** | **F** | **T** | **N** | **N** | **G** | **K** | **V** | **Y** | **R** | **A** | **K** | **G** | **Y** | **E** | **I** | **P** | **E** | **Y** | **G** | **R** | **T** | **A** | **K** | **G** | **I** | **P** | **I** | **I** | **N** | **L** | **L** | **G** | **I** | **D** | **S** | **S** | **E** | **S** | **I** | **Q** | **A** | **I** | **I** | **A** | **V** | **T** | **G** | **E** | **A** | **E** | **E** | **G** | **H** | **Y** | **L** | **F** | **F** | **T** | **T** | **R** | **Q** | **G** | **T** | **V** | **K** | **R** | **T** | **S** | **V** | **T** | **A** | **F** | **A** | **N** | **I** | **R** | **S** | **N** | **G** | **L** | **I** | **A** | **I** | **G** | **L** | **K** | **E** | **D** | **D** | **E** | **L** | **V** | **N** | **V** | **L** | **L** | **T** | **D** | **G** | **Q** | **S** | **N** | **I** | **I** | **I** | **G** | **T** | **H** | **N** | **G** | **Y** | **S** | **V** | **T** | **F** | **K** | **E** | **E** | **A** | **V** | **R** | **D** | **M** | **G** | **R** | **T** | **A** | **S** | **G** | **V** | **R** | **G** | **I** | **R** | **L** | **R** | **E** | **E** | **D** | **Y** | **V** | **V** | **G** | **A** | **A** | **L** | **M** | **K** | **E** | **D** | **Q** | **E** | **V** | **L** | **V** | **I** | **T** | **E** | **K** | **G** | **Y** | **G** | **K** | **R** | **T** | **K** | **V** | **S** | **E** | **Y** | **P** | **V** | **K** | **G** | **R** | **G** | **G** | **K** | **G** | **I** | **K** | **T** | **A** | **N** | **I** | **T** | **E** | **K** | **N** | **G** | **P** | **L** | **A** | **G** | **L** | **T** | **T** | **V** | **S** | **G** | **D** | **E** | **D** | **I** | **M** | **L** | **I** | **T** | **D** | **K** | **G** | **V** | **I** | **I** | **R** | **F** | **N** | **V** | **S** | **T** | **V** | **S** | **Q** | **T** | **G** | **R** | **S** | **T** | **L** | **G** | **V** | **R** | **L** | **M** | **K** | **M** | **E** | **A** | **D** | **T** | **K** | **V** | **V** | **T** | **M** | **A** | **A** | **V** | **E** | **P** | **E** | **A** | **A** | **E** | **Q** | **T** | **V** | **E** | **E** | **Q** | **G** | **S** | **E** | **E** |
| **N59611F** | **M** | **S** | **E** | **E** | **I** | **K** | **E** | **N** | **I** | **Q** | **D** | **V** | **N** | **L** | **T** | **S** | **E** | **M** | **K** | **E** | **S** | **F** | **I** | **D** | **Y** | **A** | **M** | **S** | **V** | **I** | **V** | **A** | **R** | **A** | **L** | **P** | **D** | **V** | **R** | **D** | **G** | **L** | **K** | **P** | **V** | **H** | **R** | **R** | **I** | **L** | **Y** | **G** | **M** | **N** | **E** | **L** | **G** | **V** | **T** | **P** | **D** | **K** | **P** | **H** | **K** | **K** | **S** | **A** | **R** | **I** | **V** | **G** | **D** | **V** | **M** | **G** | **K** | **Y** | **H** | **P** | **H** | **G** | **D** | **S** | **A** | **I** | **Y** | **E** | **S** | **M** | **V** | **R** | **M** | **A** | **Q** | **P** | **F** | **S** | **Y** | **R** | **A** | **M** | **L** | **V** | **D** | **G** | **H** | **G** | **N** | **F** | **G** | **S** | **V** | **D** | **G** | **D** | **G** | **A** | **A** | **A** | **M** | **R** | **Y** | **T** | **E** | **A** | **R** | **M** | **S** | **K** | **I** | **A** | **L** | **E** | **M** | **L** | **R** | **D** | **I** | **N** | **K** | **N** | **T** | **V** | **D** | **F** | **Q** | **G** | **N** | **Y** | **D** | **D** | **S** | **E** | **Q** | **E** | **P** | **V** | **V** | **L** | **P** | **A** | **R** | **F** | **P** | **N** | **L** | **L** | **V** | **N** | **G** | **T** | **T** | **G** | **I** | **A** | **V** | **G** | **M** | **A** | **T** | **N** | **I** | **P** | **P** | **H** | **N** | **L** | **S** | **E** | **V** | **I** | **D** | **A** | **T** | **S** | **L** | **L** | **M** | **D** | **N** | **P** | **D** | **V** | **T** | **T** | **N** | **E** | **L** | **M** | **E** | **V** | **L** | **P** | **G** | **P** | **D** | **F** | **P** | **T** | **G** | **G** | **L** | **V** | **M** | **G** | **K** | **S** | **G** | **I** | **R** | **R** | **A** | **Y** | **E** | **T** | **G** | **K** | **G** | **S** | **I** | **T** | **V** | **R** | **A** | **K** | **V** | **E** | **L** | **T** | **E** | **M** | **P** | **N** | **G** | **K** | **E** | **R** | **I** | **L** | **V** | **T** | **E** | **L** | **P** | **Y** | **M** | **V** | **N** | **K** | **A** | **K** | **L** | **I** | **E** | **R** | **I** | **S** | **E** | **L** | **H** | **R** | **D** | **K** | **R** | **I** | **E** | **G** | **I** | **T** | **D** | **L** | **R** | **D** | **E** | **S** | **S** | **R** | **E** | **G** | **M** | **R** | **I** | **V** | **I** | **D** | **V** | **R** | **R** | **D** | **V** | **S** | **A** | **S** | **V** | **V** | **L** | **N** | **N** | **L** | **Y** | **K** | **M** | **T** | **A** | **L** | **Q** | **T** | **S** | **F** | **G** | **F** | **N** | **M** | **L** | **A** | **I** | **E** | **K** | **G** | **V** | **P** | **K** | **I** | **L** | **S** | **L** | **K** | **R** | **I** | **L** | **E** | **N** | **Y** | **V** | **E** | **H** | **Q** | **K** | **E** | **V** | **I** | **T** | **R** | **R** | **T** | **I** | **F** | **D** | **K** | **N** | **K** | **A** | **E** | **A** | **R** | **A** | **H** | **I** | **L** | **E** | **G** | **L** | **R** | **I** | **A** | **L** | **D** | **H** | **I** | **D** | **E** | **I** | **I** | **A** | **I** | **I** | **R** | **G** | **S** | **Q** | **S** | **D** | **D** | **E** | **A** | **K** | **A** | **T** | **L** | **I** | **E** | **R** | **F** | **E** | **F** | **S** | **D** | **R** | **Q** | **A** | **Q** | **A** | **I** | **L** | **D** | **M** | **R** | **L** | **R** | **R** | **L** | **T** | **G** | **L** | **E** | **R** | **D** | **K** | **I** | **E** | **N** | **E** | **Y** | **Q** | **E** | **L** | **L** | **K** | **F** | **I** | **A** | **D** | **L** | **E** | **D** | **I** | **L** | **A** | **R** | **P** | **E** | **R** | **V** | **I** | **E** | **I** | **I** | **K** | **T** | **E** | **L** | **N** | **D** | **V** | **R** | **T** | **K** | **F** | **G** | **D** | **A** | **R** | **R** | **T** | **E** | **L** | **L** | **V** | **G** | **E** | **V** | **L** | **S** | **L** | **E** | **D** | **E** | **D** | **L** | **I** | **E** | **E** | **E** | **E** | **V** | **V** | **I** | **T** | **L** | **T** | **N** | **N** | **G** | **Y** | **I** | **K** | **R** | **M** | **A** | **N** | **S** | **E** | **F** | **R** | **A** | **Q** | **R** | **R** | **G** | **G** | **R** | **G** | **V** | **Q** | **G** | **M** | **G** | **V** | **H** | **D** | **D** | **D** | **F** | **V** | **K** | **N** | **L** | **V** | **S** | **C** | **S** | **T** | **H** | **D** | **T** | **L** | **L** | **F** | **F** | **T** | **N** | **T** | **G** | **K** | **V** | **Y** | **R** | **A** | **K** | **G** | **Y** | **E** | **I** | **P** | **E** | **Y** | **G** | **R** | **T** | **A** | **K** | **G** | **I** | **P** | **V** | **I** | **N** | **L** | **L** | **G** | **I** | **D** | **S** | **A** | **E** | **K** | **I** | **Q** | **A** | **I** | **I** | **S** | **V** | **E** | **G** | **K** | **A** | **E** | **A** | **G** | **K** | **Y** | **L** | **F** | **F** | **T** | **T** | **L** | **K** | **G** | **T** | **V** | **K** | **R** | **T** | **A** | **V** | **T** | **A** | **F** | **S** | **N** | **I** | **R** | **S** | **N** | **G** | **L** | **I** | **A** | **I** | **S** | **L** | **K** | **E** | **D** | **D** | **E** | **L** | **V** | **N** | **V** | **V** | **T** | **T** | **N** | **G** | **N** | **Q** | **K** | **M** | **I** | **I** | **G** | **T** | **H** | **A** | **G** | **Y** | **S** | **V** | **T** | **F** | **D** | **E** | **N** | **T** | **V** | **R** | **D** | **M** | **G** | **R** | **T** | **A** | **S** | **G** | **V** | **R** | **G** | **I** | **R** | **L** | **R** | **E** | **N** | **D** | **Y** | **V** | **V** | **G** | **A** | **A** | **I** | **L** | **D** | **E** | **N** | **K** | **E** | **V** | **L** | **V** | **I** | **T** | **E** | **N** | **G** | **Y** | **G** | **K** | **R** | **T** | **K** | **A** | **S** | **E** | **Y** | **P** | **V** | **K** | **G** | **R** | **G** | **G** | **K** | **G** | **I** | **K** | **T** | **A** | **N** | **I** | **T** | **E** | **K** | **N** | **G** | **P** | **L** | **A** | **G** | **L** | **T** | **T** | **V** | **N** | **G** | **D** | **E** | **D** | **I** | **L** | **L** | **I** | **T** | **N** | **K** | **G** | **V** | **I** | **I** | **R** | **F** | **N** | **V** | **D** | **S** | **V** | **S** | **Q** | **T** | **G** | **R** | **A** | **T** | **L** | **G** | **V** | **R** | **L** | **M** | **R** | **M** | **E** | **D** | **G** | **A** | **K** | **-** | **-** | **-** | **-** | **-** | **-** | **-** | **-** | **-** | **-** | **-** | **-** | **-** | **-** | **-** | **-** | **-** | **-** | **-** | **-** | **-** | **-** | **-** |
| **N52773** | **M** | **S** | **E** | **E** | **I** | **K** | **E** | **N** | **I** | **Q** | **D** | **V** | **N** | **L** | **T** | **S** | **E** | **M** | **K** | **E** | **S** | **F** | **I** | **D** | **Y** | **A** | **M** | **S** | **V** | **I** | **V** | **A** | **R** | **A** | **L** | **P** | **D** | **V** | **R** | **D** | **G** | **L** | **K** | **P** | **V** | **H** | **R** | **R** | **I** | **L** | **Y** | **G** | **M** | **N** | **E** | **L** | **G** | **V** | **T** | **P** | **D** | **K** | **P** | **H** | **K** | **K** | **S** | **A** | **R** | **I** | **V** | **G** | **D** | **V** | **M** | **G** | **K** | **Y** | **H** | **P** | **H** | **G** | **D** | **S** | **A** | **I** | **Y** | **E** | **S** | **M** | **V** | **R** | **M** | **A** | **Q** | **P** | **F** | **S** | **Y** | **R** | **A** | **M** | **L** | **V** | **D** | **G** | **H** | **G** | **N** | **F** | **G** | **S** | **V** | **D** | **G** | **D** | **G** | **A** | **A** | **A** | **M** | **R** | **Y** | **T** | **E** | **A** | **R** | **M** | **S** | **K** | **I** | **A** | **L** | **E** | **M** | **L** | **R** | **D** | **I** | **N** | **K** | **N** | **T** | **V** | **D** | **F** | **Q** | **G** | **N** | **Y** | **D** | **D** | **S** | **E** | **Q** | **E** | **P** | **V** | **V** | **L** | **P** | **A** | **R** | **F** | **P** | **N** | **L** | **L** | **V** | **N** | **G** | **T** | **T** | **G** | **I** | **A** | **V** | **G** | **M** | **A** | **T** | **N** | **I** | **P** | **P** | **H** | **N** | **L** | **S** | **E** | **V** | **I** | **D** | **A** | **T** | **S** | **L** | **L** | **M** | **D** | **N** | **P** | **D** | **V** | **T** | **T** | **N** | **E** | **L** | **M** | **E** | **V** | **L** | **P** | **G** | **P** | **D** | **F** | **P** | **T** | **G** | **G** | **L** | **V** | **M** | **G** | **K** | **S** | **G** | **I** | **R** | **R** | **A** | **Y** | **E** | **T** | **G** | **K** | **G** | **S** | **I** | **T** | **V** | **R** | **A** | **K** | **V** | **E** | **L** | **T** | **E** | **M** | **P** | **N** | **G** | **K** | **E** | **R** | **I** | **L** | **V** | **T** | **E** | **L** | **P** | **Y** | **M** | **V** | **N** | **K** | **A** | **K** | **L** | **I** | **E** | **R** | **I** | **S** | **E** | **L** | **H** | **R** | **D** | **K** | **R** | **I** | **E** | **G** | **I** | **T** | **D** | **L** | **R** | **D** | **E** | **S** | **S** | **R** | **E** | **G** | **M** | **R** | **I** | **V** | **I** | **D** | **V** | **R** | **R** | **D** | **V** | **S** | **A** | **S** | **V** | **V** | **L** | **N** | **N** | **L** | **Y** | **K** | **M** | **T** | **A** | **L** | **Q** | **T** | **S** | **F** | **G** | **F** | **N** | **M** | **L** | **A** | **I** | **E** | **K** | **G** | **V** | **P** | **K** | **I** | **L** | **S** | **L** | **K** | **R** | **I** | **L** | **E** | **N** | **Y** | **V** | **E** | **H** | **Q** | **K** | **E** | **V** | **I** | **T** | **R** | **R** | **T** | **I** | **F** | **D** | **K** | **N** | **K** | **A** | **E** | **A** | **R** | **A** | **H** | **I** | **L** | **E** | **G** | **L** | **R** | **I** | **A** | **L** | **D** | **H** | **I** | **D** | **E** | **I** | **I** | **A** | **I** | **I** | **R** | **G** | **S** | **Q** | **S** | **D** | **D** | **E** | **A** | **K** | **A** | **T** | **L** | **I** | **E** | **R** | **F** | **E** | **F** | **S** | **D** | **R** | **Q** | **A** | **Q** | **A** | **I** | **L** | **D** | **M** | **R** | **L** | **R** | **R** | **L** | **T** | **G** | **L** | **E** | **R** | **D** | **K** | **I** | **E** | **N** | **E** | **Y** | **Q** | **E** | **L** | **L** | **K** | **F** | **I** | **A** | **D** | **L** | **E** | **D** | **I** | **L** | **A** | **R** | **P** | **E** | **R** | **V** | **I** | **E** | **I** | **I** | **K** | **T** | **E** | **L** | **N** | **D** | **V** | **R** | **T** | **K** | **F** | **G** | **D** | **A** | **R** | **R** | **T** | **E** | **L** | **L** | **V** | **G** | **E** | **V** | **L** | **S** | **L** | **E** | **D** | **E** | **D** | **L** | **I** | **E** | **E** | **E** | **E** | **V** | **V** | **I** | **T** | **L** | **T** | **N** | **N** | **G** | **Y** | **I** | **K** | **R** | **M** | **A** | **N** | **S** | **E** | **F** | **R** | **A** | **Q** | **R** | **R** | **G** | **G** | **R** | **G** | **V** | **Q** | **G** | **M** | **G** | **V** | **H** | **D** | **D** | **D** | **F** | **V** | **K** | **N** | **L** | **V** | **S** | **C** | **S** | **T** | **H** | **D** | **T** | **L** | **L** | **F** | **F** | **T** | **N** | **T** | **G** | **K** | **V** | **Y** | **R** | **A** | **K** | **G** | **Y** | **E** | **I** | **P** | **E** | **Y** | **G** | **R** | **T** | **A** | **K** | **G** | **I** | **P** | **V** | **I** | **N** | **L** | **L** | **G** | **I** | **D** | **S** | **A** | **E** | **K** | **I** | **Q** | **A** | **I** | **I** | **S** | **V** | **E** | **G** | **K** | **A** | **E** | **A** | **G** | **K** | **Y** | **L** | **F** | **F** | **T** | **T** | **L** | **K** | **G** | **T** | **V** | **K** | **R** | **T** | **A** | **V** | **T** | **A** | **F** | **S** | **N** | **I** | **R** | **S** | **N** | **G** | **L** | **I** | **A** | **I** | **S** | **L** | **K** | **E** | **D** | **D** | **E** | **L** | **V** | **N** | **V** | **V** | **T** | **T** | **N** | **G** | **N** | **Q** | **K** | **M** | **I** | **I** | **G** | **T** | **H** | **A** | **G** | **Y** | **S** | **V** | **T** | **F** | **D** | **E** | **N** | **T** | **V** | **R** | **D** | **M** | **G** | **R** | **T** | **A** | **S** | **G** | **V** | **R** | **G** | **I** | **R** | **L** | **R** | **E** | **N** | **D** | **Y** | **V** | **V** | **G** | **A** | **A** | **I** | **L** | **D** | **E** | **N** | **K** | **E** | **V** | **L** | **V** | **I** | **T** | **E** | **N** | **G** | **Y** | **G** | **K** | **R** | **T** | **K** | **A** | **S** | **E** | **Y** | **P** | **V** | **K** | **G** | **R** | **G** | **G** | **K** | **G** | **I** | **K** | **T** | **A** | **N** | **I** | **T** | **E** | **K** | **N** | **G** | **P** | **L** | **A** | **G** | **L** | **T** | **T** | **V** | **N** | **G** | **D** | **E** | **D** | **I** | **L** | **L** | **I** | **T** | **N** | **K** | **G** | **V** | **I** | **I** | **R** | **F** | **N** | **V** | **D** | **S** | **V** | **S** | **Q** | **T** | **G** | **R** | **A** | **T** | **L** | **G** | **V** | **R** | **L** | **M** | **R** | **M** | **E** | **D** | **G** | **A** | **K** | **-** | **-** | **-** | **-** | **-** | **-** | **-** | **-** | **-** | **-** | **-** | **-** | **-** | **-** | **-** | **-** | **-** | **-** | **-** | **-** | **-** | **-** | **-** |
| **N55185** | **M** | **S** | **E** | **E** | **I** | **K** | **E** | **N** | **I** | **Q** | **D** | **V** | **N** | **L** | **T** | **S** | **E** | **M** | **K** | **E** | **S** | **F** | **I** | **D** | **Y** | **A** | **M** | **S** | **V** | **I** | **V** | **A** | **R** | **A** | **L** | **P** | **D** | **V** | **R** | **D** | **G** | **L** | **K** | **P** | **V** | **H** | **R** | **R** | **I** | **L** | **Y** | **G** | **M** | **N** | **E** | **L** | **G** | **V** | **T** | **P** | **D** | **K** | **P** | **H** | **K** | **K** | **S** | **A** | **R** | **I** | **V** | **G** | **D** | **V** | **M** | **G** | **K** | **Y** | **H** | **P** | **H** | **G** | **D** | **S** | **A** | **I** | **Y** | **E** | **S** | **M** | **V** | **R** | **M** | **A** | **Q** | **P** | **F** | **S** | **Y** | **R** | **A** | **M** | **L** | **V** | **D** | **G** | **H** | **G** | **N** | **F** | **G** | **S** | **V** | **D** | **G** | **D** | **G** | **A** | **A** | **A** | **M** | **R** | **Y** | **T** | **E** | **A** | **R** | **M** | **S** | **K** | **I** | **A** | **L** | **E** | **M** | **L** | **R** | **D** | **I** | **N** | **K** | **N** | **T** | **V** | **D** | **F** | **Q** | **G** | **N** | **Y** | **D** | **D** | **S** | **E** | **Q** | **E** | **P** | **V** | **V** | **L** | **P** | **A** | **R** | **F** | **P** | **N** | **L** | **L** | **V** | **N** | **G** | **T** | **T** | **G** | **I** | **A** | **V** | **G** | **M** | **A** | **T** | **N** | **I** | **P** | **P** | **H** | **N** | **L** | **S** | **E** | **V** | **I** | **D** | **A** | **T** | **S** | **L** | **L** | **M** | **D** | **N** | **P** | **D** | **V** | **T** | **T** | **N** | **E** | **L** | **M** | **E** | **V** | **L** | **P** | **G** | **P** | **D** | **F** | **P** | **T** | **G** | **G** | **L** | **V** | **M** | **G** | **K** | **S** | **G** | **I** | **R** | **R** | **A** | **Y** | **E** | **T** | **G** | **K** | **G** | **S** | **I** | **T** | **V** | **R** | **A** | **K** | **V** | **E** | **L** | **T** | **E** | **M** | **P** | **N** | **G** | **K** | **E** | **R** | **I** | **L** | **V** | **T** | **E** | **L** | **P** | **Y** | **M** | **V** | **N** | **K** | **A** | **K** | **L** | **I** | **E** | **R** | **I** | **S** | **E** | **L** | **H** | **R** | **D** | **K** | **R** | **I** | **E** | **G** | **I** | **T** | **D** | **L** | **R** | **D** | **E** | **S** | **S** | **R** | **E** | **G** | **M** | **R** | **I** | **V** | **I** | **D** | **V** | **R** | **R** | **D** | **V** | **S** | **A** | **S** | **V** | **V** | **L** | **N** | **N** | **L** | **Y** | **K** | **M** | **T** | **A** | **L** | **Q** | **T** | **S** | **F** | **G** | **F** | **N** | **M** | **L** | **A** | **I** | **E** | **K** | **G** | **V** | **P** | **K** | **I** | **L** | **S** | **L** | **K** | **R** | **I** | **L** | **E** | **N** | **Y** | **V** | **E** | **H** | **Q** | **K** | **E** | **V** | **I** | **T** | **R** | **R** | **T** | **I** | **F** | **D** | **K** | **N** | **K** | **A** | **E** | **A** | **R** | **A** | **H** | **I** | **L** | **E** | **G** | **L** | **R** | **I** | **A** | **L** | **D** | **H** | **I** | **D** | **E** | **I** | **I** | **A** | **I** | **I** | **R** | **G** | **S** | **Q** | **S** | **D** | **D** | **E** | **A** | **K** | **A** | **T** | **L** | **I** | **E** | **R** | **F** | **E** | **F** | **S** | **D** | **R** | **Q** | **A** | **Q** | **A** | **I** | **L** | **D** | **M** | **R** | **L** | **R** | **R** | **L** | **T** | **G** | **L** | **E** | **R** | **D** | **K** | **I** | **E** | **N** | **E** | **Y** | **Q** | **E** | **L** | **L** | **K** | **F** | **I** | **A** | **D** | **L** | **E** | **D** | **I** | **L** | **A** | **R** | **P** | **E** | **R** | **V** | **I** | **E** | **I** | **I** | **K** | **T** | **E** | **L** | **N** | **D** | **V** | **R** | **T** | **K** | **F** | **G** | **D** | **A** | **R** | **R** | **T** | **E** | **L** | **L** | **V** | **G** | **E** | **V** | **L** | **S** | **L** | **E** | **D** | **E** | **D** | **L** | **I** | **E** | **E** | **E** | **E** | **V** | **V** | **I** | **T** | **L** | **T** | **N** | **N** | **G** | **Y** | **I** | **K** | **R** | **M** | **A** | **N** | **S** | **E** | **F** | **R** | **A** | **Q** | **R** | **R** | **G** | **G** | **R** | **G** | **V** | **Q** | **G** | **M** | **G** | **V** | **H** | **D** | **D** | **D** | **F** | **V** | **K** | **N** | **L** | **V** | **S** | **C** | **S** | **T** | **H** | **D** | **T** | **L** | **L** | **F** | **F** | **T** | **N** | **T** | **G** | **K** | **V** | **Y** | **R** | **A** | **K** | **G** | **Y** | **E** | **I** | **P** | **E** | **Y** | **G** | **R** | **T** | **A** | **K** | **G** | **I** | **P** | **V** | **I** | **N** | **L** | **L** | **G** | **I** | **D** | **S** | **A** | **E** | **K** | **I** | **Q** | **A** | **I** | **I** | **S** | **V** | **E** | **G** | **K** | **A** | **E** | **A** | **G** | **K** | **Y** | **L** | **F** | **F** | **T** | **T** | **L** | **K** | **G** | **T** | **V** | **K** | **R** | **T** | **A** | **V** | **T** | **A** | **F** | **S** | **N** | **I** | **R** | **S** | **N** | **G** | **L** | **I** | **A** | **I** | **S** | **L** | **K** | **E** | **D** | **D** | **E** | **L** | **V** | **N** | **V** | **V** | **T** | **T** | **N** | **G** | **N** | **Q** | **K** | **M** | **I** | **I** | **G** | **T** | **H** | **A** | **G** | **Y** | **S** | **V** | **T** | **F** | **D** | **E** | **N** | **T** | **V** | **R** | **D** | **M** | **G** | **R** | **T** | **A** | **S** | **G** | **V** | **R** | **G** | **I** | **R** | **L** | **R** | **E** | **N** | **D** | **Y** | **V** | **V** | **G** | **A** | **A** | **I** | **L** | **D** | **E** | **N** | **K** | **E** | **V** | **L** | **V** | **I** | **T** | **E** | **N** | **G** | **Y** | **G** | **K** | **R** | **T** | **K** | **A** | **S** | **E** | **Y** | **P** | **V** | **K** | **G** | **R** | **G** | **G** | **K** | **G** | **I** | **K** | **T** | **A** | **N** | **I** | **T** | **E** | **K** | **N** | **G** | **P** | **L** | **A** | **G** | **L** | **T** | **T** | **V** | **N** | **G** | **D** | **E** | **D** | **I** | **L** | **L** | **I** | **T** | **N** | **K** | **G** | **V** | **I** | **I** | **R** | **F** | **N** | **V** | **D** | **S** | **V** | **S** | **Q** | **T** | **G** | **R** | **A** | **T** | **L** | **G** | **V** | **R** | **L** | **M** | **R** | **M** | **E** | **D** | **G** | **A** | **K** | **-** | **-** | **-** | **-** | **-** | **-** | **-** | **-** | **-** | **-** | **-** | **-** | **-** | **-** | **-** | **-** | **-** | **-** | **-** | **-** | **-** | **-** | **-** |
| **N60271F** | **M** | **S** | **E** | **E** | **I** | **K** | **E** | **N** | **I** | **Q** | **D** | **V** | **N** | **L** | **T** | **S** | **E** | **M** | **K** | **E** | **S** | **F** | **I** | **D** | **Y** | **A** | **M** | **S** | **V** | **I** | **V** | **A** | **R** | **A** | **L** | **P** | **D** | **V** | **R** | **D** | **G** | **L** | **K** | **P** | **V** | **H** | **R** | **R** | **I** | **L** | **Y** | **G** | **M** | **N** | **E** | **L** | **G** | **V** | **T** | **P** | **D** | **K** | **P** | **H** | **K** | **K** | **S** | **A** | **R** | **I** | **V** | **G** | **D** | **V** | **M** | **G** | **K** | **Y** | **H** | **P** | **H** | **G** | **D** | **S** | **A** | **I** | **Y** | **E** | **S** | **M** | **V** | **R** | **M** | **A** | **Q** | **P** | **F** | **S** | **Y** | **R** | **A** | **M** | **L** | **V** | **D** | **G** | **H** | **G** | **N** | **F** | **G** | **S** | **V** | **D** | **G** | **D** | **G** | **A** | **A** | **A** | **M** | **R** | **Y** | **T** | **E** | **A** | **R** | **M** | **S** | **K** | **I** | **A** | **L** | **E** | **M** | **L** | **R** | **D** | **I** | **N** | **K** | **N** | **T** | **V** | **D** | **F** | **Q** | **G** | **N** | **Y** | **D** | **D** | **S** | **E** | **Q** | **E** | **P** | **V** | **V** | **L** | **P** | **A** | **R** | **F** | **P** | **N** | **L** | **L** | **V** | **N** | **G** | **T** | **T** | **G** | **I** | **A** | **V** | **G** | **M** | **A** | **T** | **N** | **I** | **P** | **P** | **H** | **N** | **L** | **S** | **E** | **V** | **I** | **D** | **A** | **T** | **S** | **L** | **L** | **M** | **D** | **N** | **P** | **D** | **V** | **T** | **T** | **N** | **E** | **L** | **M** | **E** | **V** | **L** | **P** | **G** | **P** | **D** | **F** | **P** | **T** | **G** | **G** | **L** | **V** | **M** | **G** | **K** | **S** | **G** | **I** | **R** | **R** | **A** | **Y** | **E** | **T** | **G** | **K** | **G** | **S** | **I** | **T** | **V** | **R** | **A** | **K** | **V** | **E** | **L** | **T** | **E** | **M** | **P** | **N** | **G** | **K** | **E** | **R** | **I** | **L** | **V** | **T** | **E** | **L** | **P** | **Y** | **M** | **V** | **N** | **K** | **A** | **K** | **L** | **I** | **E** | **R** | **I** | **S** | **E** | **L** | **H** | **R** | **D** | **K** | **R** | **I** | **E** | **G** | **I** | **T** | **D** | **L** | **R** | **D** | **E** | **S** | **S** | **R** | **E** | **G** | **M** | **R** | **I** | **V** | **I** | **D** | **V** | **R** | **R** | **D** | **V** | **S** | **A** | **S** | **V** | **V** | **L** | **N** | **N** | **L** | **Y** | **K** | **M** | **T** | **A** | **L** | **Q** | **T** | **S** | **F** | **G** | **F** | **N** | **M** | **L** | **A** | **I** | **E** | **K** | **G** | **V** | **P** | **K** | **I** | **L** | **S** | **L** | **K** | **R** | **I** | **L** | **E** | **N** | **Y** | **V** | **E** | **H** | **Q** | **K** | **E** | **V** | **I** | **T** | **R** | **R** | **T** | **I** | **F** | **D** | **K** | **N** | **K** | **A** | **E** | **A** | **R** | **A** | **H** | **I** | **L** | **E** | **G** | **L** | **R** | **I** | **A** | **L** | **D** | **H** | **I** | **D** | **E** | **I** | **I** | **A** | **I** | **I** | **R** | **G** | **S** | **Q** | **S** | **D** | **D** | **E** | **A** | **K** | **A** | **T** | **L** | **I** | **E** | **R** | **F** | **E** | **F** | **S** | **D** | **R** | **Q** | **A** | **Q** | **A** | **I** | **L** | **D** | **M** | **R** | **L** | **R** | **R** | **L** | **T** | **G** | **L** | **E** | **R** | **D** | **K** | **I** | **E** | **N** | **E** | **Y** | **Q** | **E** | **L** | **L** | **K** | **F** | **I** | **A** | **D** | **L** | **E** | **D** | **I** | **L** | **A** | **R** | **P** | **E** | **R** | **V** | **I** | **E** | **I** | **I** | **K** | **T** | **E** | **L** | **N** | **D** | **V** | **R** | **T** | **K** | **F** | **G** | **D** | **A** | **R** | **R** | **T** | **E** | **L** | **L** | **V** | **G** | **E** | **V** | **L** | **S** | **L** | **E** | **D** | **E** | **D** | **L** | **I** | **E** | **E** | **E** | **E** | **V** | **V** | **I** | **T** | **L** | **T** | **N** | **N** | **G** | **Y** | **I** | **K** | **R** | **M** | **A** | **N** | **S** | **E** | **F** | **R** | **A** | **Q** | **R** | **R** | **G** | **G** | **R** | **G** | **V** | **Q** | **G** | **M** | **G** | **V** | **H** | **D** | **D** | **D** | **F** | **V** | **K** | **N** | **L** | **V** | **S** | **C** | **S** | **T** | **H** | **D** | **T** | **L** | **L** | **F** | **F** | **T** | **N** | **T** | **G** | **K** | **V** | **Y** | **R** | **A** | **K** | **G** | **Y** | **E** | **I** | **P** | **E** | **Y** | **G** | **R** | **T** | **A** | **K** | **G** | **I** | **P** | **V** | **I** | **N** | **L** | **L** | **G** | **I** | **D** | **S** | **A** | **E** | **K** | **I** | **Q** | **A** | **I** | **I** | **S** | **V** | **E** | **G** | **K** | **A** | **E** | **A** | **G** | **K** | **Y** | **L** | **F** | **F** | **T** | **T** | **L** | **K** | **G** | **T** | **V** | **K** | **R** | **T** | **A** | **V** | **T** | **A** | **F** | **S** | **N** | **I** | **R** | **S** | **N** | **G** | **L** | **I** | **A** | **I** | **S** | **L** | **K** | **E** | **D** | **D** | **E** | **L** | **V** | **N** | **V** | **V** | **T** | **T** | **N** | **G** | **N** | **Q** | **K** | **M** | **I** | **I** | **G** | **T** | **H** | **A** | **G** | **Y** | **S** | **V** | **T** | **F** | **D** | **E** | **N** | **T** | **V** | **R** | **D** | **M** | **G** | **R** | **T** | **A** | **S** | **G** | **V** | **R** | **G** | **I** | **R** | **L** | **R** | **E** | **N** | **D** | **Y** | **V** | **V** | **G** | **A** | **A** | **I** | **L** | **D** | **E** | **N** | **K** | **E** | **V** | **L** | **V** | **I** | **T** | **E** | **N** | **G** | **Y** | **G** | **K** | **R** | **T** | **K** | **A** | **S** | **E** | **Y** | **P** | **V** | **K** | **G** | **R** | **G** | **G** | **K** | **G** | **I** | **K** | **T** | **A** | **N** | **I** | **T** | **E** | **K** | **N** | **G** | **P** | **L** | **A** | **G** | **L** | **T** | **T** | **V** | **N** | **G** | **D** | **E** | **D** | **I** | **L** | **L** | **I** | **T** | **N** | **K** | **G** | **V** | **I** | **I** | **R** | **F** | **N** | **V** | **D** | **S** | **V** | **S** | **Q** | **T** | **G** | **R** | **A** | **T** | **L** | **G** | **V** | **R** | **L** | **M** | **R** | **M** | **E** | **D** | **G** | **A** | **K** | **-** | **-** | **-** | **-** | **-** | **-** | **-** | **-** | **-** | **-** | **-** | **-** | **-** | **-** | **-** | **-** | **-** | **-** | **-** | **-** | **-** | **-** | **-** |
| **N59745F** | **M** | **S** | **E** | **E** | **I** | **R** | **E** | **N** | **I** | **H** | **D** | **V** | **N** | **L** | **T** | **S** | **E** | **M** | **K** | **D** | **S** | **F** | **I** | **D** | **Y** | **A** | **M** | **S** | **V** | **I** | **V** | **A** | **R** | **A** | **L** | **P** | **D** | **V** | **R** | **D** | **G** | **L** | **K** | **P** | **V** | **H** | **R** | **R** | **I** | **L** | **Y** | **G** | **M** | **N** | **E** | **L** | **G** | **V** | **T** | **P** | **D** | **K** | **A** | **H** | **K** | **K** | **S** | **A** | **R** | **I** | **V** | **G** | **D** | **V** | **M** | **G** | **K** | **Y** | **H** | **P** | **H** | **G** | **D** | **S** | **A** | **I** | **Y** | **E** | **S** | **M** | **V** | **R** | **M** | **A** | **Q** | **P** | **F** | **S** | **Y** | **R** | **Y** | **M** | **L** | **V** | **D** | **G** | **H** | **G** | **N** | **F** | **G** | **S** | **V** | **D** | **G** | **D** | **G** | **A** | **A** | **A** | **M** | **R** | **Y** | **T** | **E** | **A** | **R** | **M** | **S** | **K** | **I** | **A** | **T** | **E** | **M** | **L** | **R** | **D** | **I** | **N** | **K** | **N** | **T** | **V** | **D** | **F** | **Q** | **S** | **N** | **Y** | **D** | **D** | **T** | **E** | **K** | **E** | **P** | **V** | **V** | **L** | **P** | **A** | **R** | **F** | **P** | **N** | **L** | **L** | **V** | **N** | **G** | **T** | **T** | **G** | **I** | **A** | **V** | **G** | **M** | **A** | **T** | **N** | **I** | **P** | **P** | **H** | **N** | **L** | **S** | **E** | **V** | **V** | **A** | **A** | **I** | **D** | **L** | **L** | **M** | **D** | **N** | **P** | **D** | **V** | **T** | **T** | **N** | **E** | **L** | **M** | **E** | **V** | **L** | **P** | **G** | **P** | **D** | **F** | **P** | **T** | **G** | **G** | **L** | **V** | **M** | **G** | **K** | **S** | **G** | **I** | **R** | **R** | **A** | **Y** | **E** | **T** | **G** | **K** | **G** | **S** | **I** | **T** | **V** | **R** | **A** | **K** | **V** | **E** | **I** | **T** | **E** | **M** | **P** | **N** | **G** | **K** | **E** | **R** | **I** | **L** | **V** | **T** | **E** | **L** | **P** | **Y** | **M** | **V** | **N** | **K** | **A** | **K** | **L** | **I** | **E** | **R** | **I** | **S** | **E** | **L** | **H** | **R** | **D** | **K** | **R** | **I** | **E** | **G** | **I** | **T** | **D** | **L** | **R** | **D** | **E** | **S** | **S** | **R** | **E** | **G** | **M** | **R** | **I** | **V** | **I** | **D** | **I** | **R** | **R** | **D** | **A** | **S** | **A** | **S** | **V** | **I** | **L** | **N** | **N** | **L** | **Y** | **K** | **L** | **T** | **S** | **L** | **Q** | **N** | **S** | **F** | **G** | **F** | **N** | **M** | **L** | **A** | **I** | **E** | **K** | **G** | **V** | **P** | **K** | **V** | **L** | **S** | **L** | **K** | **Q** | **I** | **L** | **E** | **N** | **Y** | **I** | **E** | **H** | **Q** | **R** | **E** | **V** | **I** | **T** | **R** | **R** | **T** | **V** | **F** | **E** | **K** | **E** | **K** | **A** | **E** | **A** | **R** | **A** | **H** | **I** | **L** | **E** | **G** | **L** | **R** | **I** | **A** | **L** | **D** | **H** | **I** | **D** | **E** | **I** | **I** | **A** | **I** | **I** | **R** | **G** | **S** | **K** | **S** | **D** | **D** | **E** | **A** | **K** | **A** | **T** | **M** | **I** | **E** | **R** | **F** | **D** | **L** | **S** | **D** | **R** | **Q** | **S** | **Q** | **A** | **I** | **L** | **D** | **M** | **R** | **L** | **R** | **R** | **L** | **T** | **G** | **L** | **E** | **R** | **E** | **K** | **I** | **E** | **N** | **E** | **Y** | **Q** | **E** | **L** | **L** | **K** | **L** | **I** | **E** | **D** | **L** | **T** | **D** | **I** | **L** | **A** | **R** | **P** | **E** | **R** | **V** | **T** | **E** | **I** | **I** | **K** | **N** | **E** | **L** | **A** | **D** | **L** | **N** | **Q** | **R** | **F** | **G** | **D** | **K** | **R** | **R** | **T** | **E** | **L** | **L** | **V** | **G** | **E** | **V** | **L** | **S** | **L** | **E** | **D** | **E** | **D** | **L** | **I** | **E** | **E** | **E** | **E** | **I** | **V** | **I** | **T** | **L** | **T** | **N** | **N** | **G** | **Y** | **I** | **K** | **R** | **L** | **A** | **N** | **N** | **E** | **F** | **R** | **A** | **Q** | **R** | **R** | **G** | **G** | **R** | **G** | **V** | **Q** | **G** | **M** | **G** | **I** | **H** | **N** | **D** | **D** | **F** | **V** | **K** | **N** | **L** | **V** | **S** | **C** | **S** | **T** | **H** | **D** | **T** | **L** | **L** | **F** | **F** | **T** | **N** | **N** | **G** | **K** | **V** | **Y** | **R** | **A** | **K** | **G** | **Y** | **E** | **I** | **P** | **E** | **Y** | **G** | **R** | **T** | **A** | **K** | **G** | **I** | **P** | **I** | **I** | **N** | **L** | **L** | **G** | **I** | **D** | **S** | **S** | **E** | **S** | **I** | **Q** | **A** | **I** | **I** | **A** | **V** | **T** | **G** | **E** | **A** | **E** | **E** | **G** | **H** | **Y** | **L** | **F** | **F** | **T** | **T** | **R** | **Q** | **G** | **T** | **V** | **K** | **R** | **T** | **S** | **V** | **T** | **A** | **F** | **A** | **N** | **I** | **R** | **S** | **N** | **G** | **L** | **I** | **A** | **I** | **G** | **L** | **K** | **E** | **D** | **D** | **E** | **L** | **V** | **N** | **V** | **L** | **L** | **T** | **D** | **G** | **Q** | **S** | **N** | **I** | **I** | **I** | **G** | **T** | **H** | **N** | **G** | **Y** | **S** | **V** | **T** | **F** | **K** | **E** | **E** | **A** | **V** | **R** | **D** | **M** | **G** | **R** | **T** | **A** | **S** | **G** | **V** | **R** | **G** | **I** | **R** | **L** | **R** | **E** | **E** | **D** | **Y** | **V** | **V** | **G** | **A** | **A** | **L** | **M** | **K** | **E** | **D** | **Q** | **E** | **V** | **L** | **V** | **I** | **T** | **E** | **K** | **G** | **Y** | **G** | **K** | **R** | **T** | **K** | **V** | **S** | **E** | **Y** | **P** | **V** | **K** | **G** | **R** | **G** | **G** | **K** | **G** | **I** | **K** | **T** | **A** | **N** | **I** | **T** | **E** | **K** | **N** | **G** | **P** | **L** | **A** | **G** | **L** | **T** | **T** | **V** | **S** | **G** | **D** | **E** | **D** | **I** | **M** | **L** | **I** | **T** | **D** | **K** | **G** | **V** | **I** | **I** | **R** | **F** | **N** | **V** | **S** | **T** | **V** | **S** | **Q** | **T** | **G** | **R** | **S** | **T** | **L** | **G** | **V** | **R** | **L** | **M** | **K** | **M** | **E** | **A** | **D** | **T** | **K** | **V** | **V** | **T** | **M** | **-** | **-** | **-** | **-** | **-** | **-** | **-** | **-** | **-** | **-** | **-** | **-** | **-** | **-** | **-** | **-** | **-** | **-** | **-** |
| **N54070** | **M** | **S** | **E** | **E** | **I** | **R** | **E** | **N** | **I** | **H** | **D** | **V** | **N** | **L** | **T** | **S** | **E** | **M** | **K** | **D** | **S** | **F** | **I** | **D** | **Y** | **A** | **M** | **S** | **V** | **I** | **V** | **A** | **R** | **A** | **L** | **P** | **D** | **V** | **R** | **D** | **G** | **L** | **K** | **P** | **V** | **H** | **R** | **R** | **I** | **L** | **Y** | **G** | **M** | **N** | **E** | **L** | **G** | **V** | **T** | **P** | **D** | **K** | **A** | **H** | **K** | **K** | **S** | **A** | **R** | **I** | **V** | **G** | **D** | **V** | **M** | **G** | **K** | **Y** | **H** | **P** | **H** | **G** | **D** | **S** | **A** | **I** | **Y** | **E** | **S** | **M** | **V** | **R** | **M** | **A** | **Q** | **P** | **F** | **S** | **Y** | **R** | **Y** | **M** | **L** | **V** | **D** | **G** | **H** | **G** | **N** | **F** | **G** | **S** | **V** | **D** | **G** | **D** | **G** | **A** | **A** | **A** | **M** | **R** | **Y** | **T** | **E** | **A** | **R** | **M** | **S** | **K** | **I** | **A** | **T** | **E** | **M** | **L** | **R** | **D** | **I** | **N** | **K** | **N** | **T** | **V** | **D** | **F** | **Q** | **S** | **N** | **Y** | **D** | **D** | **T** | **E** | **K** | **E** | **P** | **V** | **V** | **L** | **P** | **A** | **R** | **F** | **P** | **N** | **L** | **L** | **V** | **N** | **G** | **T** | **T** | **G** | **I** | **A** | **V** | **G** | **M** | **A** | **T** | **N** | **I** | **P** | **P** | **H** | **N** | **L** | **S** | **E** | **V** | **V** | **A** | **A** | **I** | **D** | **L** | **L** | **M** | **D** | **N** | **P** | **D** | **V** | **T** | **T** | **N** | **E** | **L** | **M** | **E** | **V** | **L** | **P** | **G** | **P** | **D** | **F** | **P** | **T** | **G** | **G** | **L** | **V** | **M** | **G** | **K** | **S** | **G** | **I** | **R** | **R** | **A** | **Y** | **E** | **T** | **G** | **K** | **G** | **S** | **I** | **T** | **V** | **R** | **A** | **K** | **V** | **E** | **I** | **T** | **E** | **M** | **P** | **N** | **G** | **K** | **E** | **R** | **I** | **I** | **V** | **T** | **E** | **L** | **P** | **Y** | **M** | **V** | **N** | **K** | **A** | **K** | **L** | **I** | **E** | **R** | **I** | **S** | **E** | **L** | **H** | **R** | **D** | **K** | **R** | **I** | **E** | **G** | **I** | **T** | **D** | **L** | **R** | **D** | **E** | **S** | **S** | **R** | **E** | **G** | **M** | **R** | **I** | **V** | **I** | **D** | **I** | **R** | **R** | **D** | **A** | **S** | **A** | **S** | **V** | **I** | **L** | **N** | **N** | **L** | **Y** | **K** | **L** | **T** | **S** | **L** | **Q** | **N** | **S** | **F** | **G** | **F** | **N** | **M** | **L** | **A** | **I** | **E** | **K** | **G** | **V** | **P** | **K** | **V** | **L** | **S** | **L** | **K** | **Q** | **I** | **L** | **E** | **N** | **Y** | **I** | **E** | **H** | **Q** | **R** | **E** | **V** | **I** | **T** | **R** | **R** | **T** | **V** | **F** | **E** | **K** | **E** | **K** | **A** | **E** | **A** | **R** | **A** | **H** | **I** | **L** | **E** | **G** | **L** | **R** | **I** | **A** | **L** | **D** | **H** | **I** | **D** | **E** | **I** | **I** | **A** | **I** | **I** | **R** | **G** | **S** | **K** | **S** | **D** | **D** | **E** | **A** | **K** | **A** | **T** | **M** | **I** | **E** | **R** | **F** | **D** | **L** | **S** | **D** | **R** | **Q** | **S** | **Q** | **A** | **I** | **L** | **D** | **M** | **R** | **L** | **R** | **R** | **L** | **T** | **G** | **L** | **E** | **R** | **E** | **K** | **I** | **E** | **N** | **E** | **Y** | **Q** | **E** | **L** | **L** | **K** | **L** | **I** | **E** | **D** | **L** | **T** | **D** | **I** | **L** | **A** | **R** | **P** | **E** | **R** | **V** | **T** | **E** | **I** | **I** | **K** | **N** | **E** | **L** | **A** | **D** | **L** | **N** | **Q** | **R** | **F** | **G** | **D** | **K** | **R** | **R** | **T** | **E** | **L** | **L** | **V** | **G** | **E** | **V** | **L** | **S** | **L** | **E** | **D** | **E** | **D** | **L** | **I** | **E** | **E** | **E** | **E** | **I** | **V** | **I** | **T** | **L** | **T** | **N** | **N** | **G** | **Y** | **I** | **K** | **R** | **L** | **A** | **N** | **N** | **E** | **F** | **R** | **A** | **Q** | **R** | **R** | **G** | **G** | **R** | **G** | **V** | **Q** | **G** | **M** | **G** | **I** | **H** | **N** | **D** | **D** | **F** | **V** | **K** | **N** | **L** | **V** | **S** | **C** | **S** | **T** | **H** | **D** | **T** | **L** | **L** | **F** | **F** | **T** | **N** | **N** | **G** | **K** | **V** | **Y** | **R** | **A** | **K** | **G** | **Y** | **E** | **I** | **P** | **E** | **Y** | **G** | **R** | **T** | **A** | **K** | **G** | **I** | **P** | **I** | **I** | **N** | **L** | **L** | **G** | **I** | **D** | **S** | **S** | **E** | **S** | **I** | **Q** | **A** | **I** | **I** | **A** | **V** | **T** | **G** | **E** | **A** | **E** | **E** | **G** | **H** | **Y** | **L** | **F** | **F** | **T** | **T** | **R** | **Q** | **G** | **T** | **V** | **K** | **R** | **T** | **S** | **V** | **T** | **A** | **F** | **A** | **N** | **I** | **R** | **S** | **N** | **G** | **L** | **I** | **A** | **I** | **G** | **L** | **K** | **E** | **D** | **D** | **E** | **L** | **V** | **N** | **V** | **L** | **L** | **T** | **D** | **G** | **Q** | **S** | **N** | **I** | **I** | **I** | **G** | **T** | **H** | **N** | **G** | **Y** | **S** | **V** | **T** | **F** | **K** | **E** | **E** | **A** | **V** | **R** | **D** | **M** | **G** | **R** | **T** | **A** | **S** | **G** | **V** | **R** | **G** | **I** | **R** | **L** | **R** | **E** | **E** | **D** | **Y** | **V** | **V** | **G** | **A** | **A** | **L** | **M** | **K** | **E** | **N** | **Q** | **E** | **V** | **L** | **V** | **I** | **T** | **E** | **K** | **G** | **Y** | **G** | **K** | **R** | **T** | **K** | **V** | **S** | **E** | **Y** | **P** | **V** | **K** | **G** | **R** | **G** | **G** | **K** | **G** | **I** | **K** | **T** | **A** | **N** | **I** | **T** | **E** | **K** | **N** | **G** | **P** | **L** | **A** | **G** | **L** | **T** | **T** | **V** | **S** | **G** | **D** | **E** | **D** | **I** | **M** | **L** | **I** | **T** | **D** | **K** | **G** | **V** | **I** | **I** | **R** | **F** | **N** | **V** | **S** | **T** | **V** | **S** | **Q** | **T** | **G** | **R** | **S** | **T** | **L** | **G** | **V** | **R** | **L** | **M** | **K** | **M** | **E** | **A** | **D** | **T** | **K** | **V** | **V** | **T** | **M** | **A** | **A** | **V** | **E** | **P** | **E** | **A** | **A** | **E** | **Q** | **T** | **V** | **E** | **E** | **Q** | **G** | **S** | **E** | **E** |
| **N52789** | **M** | **S** | **E** | **E** | **I** | **K** | **E** | **N** | **I** | **Q** | **D** | **V** | **N** | **L** | **T** | **S** | **E** | **M** | **K** | **E** | **S** | **F** | **I** | **D** | **Y** | **A** | **M** | **S** | **V** | **I** | **V** | **A** | **R** | **A** | **L** | **P** | **D** | **V** | **R** | **D** | **G** | **L** | **K** | **P** | **V** | **H** | **R** | **R** | **I** | **L** | **Y** | **G** | **M** | **N** | **E** | **L** | **G** | **V** | **T** | **P** | **D** | **K** | **P** | **H** | **K** | **K** | **S** | **A** | **R** | **I** | **V** | **G** | **D** | **V** | **M** | **G** | **K** | **Y** | **H** | **P** | **H** | **G** | **D** | **S** | **A** | **I** | **Y** | **E** | **S** | **M** | **V** | **R** | **M** | **A** | **Q** | **P** | **F** | **S** | **Y** | **R** | **A** | **M** | **L** | **V** | **D** | **G** | **H** | **G** | **N** | **F** | **G** | **S** | **V** | **D** | **G** | **D** | **G** | **A** | **A** | **A** | **M** | **R** | **Y** | **T** | **E** | **A** | **R** | **M** | **S** | **K** | **I** | **A** | **L** | **E** | **M** | **L** | **R** | **D** | **I** | **N** | **K** | **N** | **T** | **V** | **D** | **F** | **Q** | **G** | **N** | **Y** | **D** | **D** | **S** | **E** | **Q** | **E** | **P** | **V** | **V** | **L** | **P** | **A** | **R** | **F** | **P** | **N** | **L** | **L** | **V** | **N** | **G** | **T** | **T** | **G** | **I** | **A** | **V** | **G** | **M** | **A** | **T** | **N** | **I** | **P** | **P** | **H** | **N** | **L** | **S** | **E** | **V** | **I** | **D** | **A** | **T** | **S** | **L** | **L** | **M** | **D** | **N** | **P** | **D** | **V** | **T** | **T** | **N** | **E** | **L** | **M** | **E** | **V** | **L** | **P** | **G** | **P** | **D** | **F** | **P** | **T** | **G** | **G** | **L** | **V** | **M** | **G** | **K** | **S** | **G** | **I** | **R** | **R** | **A** | **Y** | **E** | **T** | **G** | **K** | **G** | **S** | **I** | **T** | **V** | **R** | **A** | **K** | **V** | **E** | **L** | **T** | **E** | **M** | **P** | **N** | **G** | **K** | **E** | **R** | **I** | **L** | **V** | **T** | **E** | **L** | **P** | **Y** | **M** | **V** | **N** | **K** | **A** | **K** | **L** | **I** | **E** | **R** | **I** | **S** | **E** | **L** | **H** | **R** | **D** | **K** | **R** | **I** | **E** | **G** | **I** | **T** | **D** | **L** | **R** | **D** | **E** | **S** | **S** | **R** | **E** | **G** | **M** | **R** | **I** | **V** | **I** | **D** | **V** | **R** | **R** | **D** | **V** | **S** | **A** | **S** | **V** | **V** | **L** | **N** | **N** | **L** | **Y** | **K** | **M** | **T** | **A** | **L** | **Q** | **T** | **S** | **F** | **G** | **F** | **N** | **M** | **L** | **A** | **I** | **E** | **K** | **G** | **V** | **P** | **K** | **I** | **L** | **S** | **L** | **K** | **R** | **I** | **L** | **E** | **N** | **Y** | **V** | **E** | **H** | **Q** | **K** | **E** | **V** | **I** | **T** | **R** | **R** | **T** | **I** | **F** | **D** | **K** | **N** | **K** | **A** | **E** | **A** | **R** | **A** | **H** | **I** | **L** | **E** | **G** | **L** | **R** | **I** | **A** | **L** | **D** | **H** | **I** | **D** | **E** | **I** | **I** | **A** | **I** | **I** | **R** | **G** | **S** | **Q** | **S** | **D** | **D** | **E** | **A** | **K** | **A** | **T** | **L** | **I** | **E** | **R** | **F** | **E** | **F** | **S** | **D** | **R** | **Q** | **A** | **Q** | **A** | **I** | **L** | **D** | **M** | **R** | **L** | **R** | **R** | **L** | **T** | **G** | **L** | **E** | **R** | **D** | **K** | **I** | **E** | **N** | **E** | **Y** | **Q** | **E** | **L** | **L** | **K** | **F** | **I** | **A** | **D** | **L** | **E** | **D** | **I** | **L** | **A** | **R** | **P** | **E** | **R** | **V** | **I** | **E** | **I** | **I** | **K** | **T** | **E** | **L** | **N** | **D** | **V** | **R** | **T** | **K** | **F** | **G** | **D** | **A** | **R** | **R** | **T** | **E** | **L** | **L** | **V** | **G** | **E** | **V** | **L** | **S** | **L** | **E** | **D** | **E** | **D** | **L** | **I** | **E** | **E** | **E** | **E** | **V** | **V** | **I** | **T** | **L** | **T** | **N** | **N** | **G** | **Y** | **I** | **K** | **R** | **M** | **A** | **N** | **S** | **E** | **F** | **R** | **A** | **Q** | **R** | **R** | **G** | **G** | **R** | **G** | **V** | **Q** | **G** | **M** | **G** | **V** | **H** | **D** | **D** | **D** | **F** | **V** | **K** | **N** | **L** | **V** | **S** | **C** | **S** | **T** | **H** | **D** | **T** | **L** | **L** | **F** | **F** | **T** | **N** | **T** | **G** | **K** | **V** | **Y** | **R** | **A** | **K** | **G** | **Y** | **E** | **I** | **P** | **E** | **Y** | **G** | **R** | **T** | **A** | **K** | **G** | **I** | **P** | **V** | **I** | **N** | **L** | **L** | **G** | **I** | **D** | **S** | **A** | **E** | **K** | **I** | **Q** | **A** | **I** | **I** | **S** | **V** | **E** | **G** | **K** | **A** | **E** | **A** | **G** | **K** | **Y** | **L** | **F** | **F** | **T** | **T** | **L** | **K** | **G** | **T** | **V** | **K** | **R** | **T** | **A** | **V** | **T** | **A** | **F** | **S** | **N** | **I** | **R** | **S** | **N** | **G** | **L** | **I** | **A** | **I** | **S** | **L** | **K** | **E** | **D** | **D** | **E** | **L** | **V** | **N** | **V** | **V** | **T** | **T** | **N** | **G** | **N** | **Q** | **K** | **M** | **I** | **I** | **G** | **T** | **H** | **A** | **G** | **Y** | **S** | **V** | **T** | **F** | **D** | **E** | **N** | **T** | **V** | **R** | **D** | **M** | **G** | **R** | **T** | **A** | **S** | **G** | **V** | **R** | **G** | **I** | **R** | **L** | **R** | **E** | **N** | **D** | **Y** | **V** | **V** | **G** | **A** | **A** | **I** | **L** | **D** | **E** | **N** | **K** | **E** | **V** | **L** | **V** | **I** | **T** | **E** | **N** | **G** | **Y** | **G** | **K** | **R** | **T** | **K** | **A** | **S** | **E** | **Y** | **P** | **V** | **K** | **G** | **R** | **G** | **G** | **K** | **G** | **I** | **K** | **T** | **A** | **N** | **I** | **T** | **E** | **K** | **N** | **G** | **P** | **L** | **A** | **G** | **L** | **T** | **T** | **V** | **N** | **G** | **D** | **E** | **D** | **I** | **L** | **L** | **I** | **T** | **N** | **K** | **G** | **V** | **I** | **I** | **R** | **F** | **N** | **V** | **D** | **S** | **V** | **S** | **Q** | **T** | **G** | **R** | **A** | **T** | **L** | **G** | **V** | **R** | **L** | **M** | **R** | **M** | **E** | **D** | **G** | **A** | **K** | **-** | **-** | **-** | **-** | **-** | **-** | **-** | **-** | **-** | **-** | **-** | **-** | **-** | **-** | **-** | **-** | **-** | **-** | **-** | **-** | **-** | **-** | **-** |
| **N54553** | **M** | **S** | **E** | **E** | **I** | **K** | **E** | **N** | **I** | **Q** | **D** | **V** | **N** | **L** | **T** | **S** | **E** | **M** | **K** | **E** | **S** | **F** | **I** | **D** | **Y** | **A** | **M** | **S** | **V** | **I** | **V** | **A** | **R** | **A** | **L** | **P** | **D** | **V** | **R** | **D** | **G** | **L** | **K** | **P** | **V** | **H** | **R** | **R** | **I** | **L** | **Y** | **G** | **M** | **N** | **E** | **L** | **G** | **V** | **T** | **P** | **D** | **K** | **P** | **H** | **K** | **K** | **S** | **A** | **R** | **I** | **V** | **G** | **D** | **V** | **M** | **G** | **K** | **Y** | **H** | **P** | **H** | **G** | **D** | **S** | **A** | **I** | **Y** | **E** | **S** | **M** | **V** | **R** | **M** | **A** | **Q** | **P** | **F** | **S** | **Y** | **R** | **A** | **M** | **L** | **V** | **D** | **G** | **H** | **G** | **N** | **F** | **G** | **S** | **V** | **D** | **G** | **D** | **G** | **A** | **A** | **A** | **M** | **R** | **Y** | **T** | **E** | **A** | **R** | **M** | **S** | **K** | **I** | **A** | **L** | **E** | **M** | **L** | **R** | **D** | **I** | **N** | **K** | **N** | **T** | **V** | **D** | **F** | **Q** | **G** | **N** | **Y** | **D** | **D** | **S** | **E** | **Q** | **E** | **P** | **V** | **V** | **L** | **P** | **A** | **R** | **F** | **P** | **N** | **L** | **L** | **V** | **N** | **G** | **T** | **T** | **G** | **I** | **A** | **V** | **G** | **M** | **A** | **T** | **N** | **I** | **P** | **P** | **H** | **N** | **L** | **S** | **E** | **V** | **I** | **D** | **A** | **T** | **S** | **L** | **L** | **M** | **D** | **N** | **P** | **D** | **V** | **T** | **T** | **N** | **E** | **L** | **M** | **E** | **V** | **L** | **P** | **G** | **P** | **D** | **F** | **P** | **T** | **G** | **G** | **L** | **V** | **M** | **G** | **K** | **S** | **G** | **I** | **R** | **R** | **A** | **Y** | **E** | **T** | **G** | **K** | **G** | **S** | **I** | **T** | **V** | **R** | **A** | **K** | **V** | **E** | **L** | **T** | **E** | **M** | **P** | **N** | **G** | **K** | **E** | **R** | **I** | **L** | **V** | **T** | **E** | **L** | **P** | **Y** | **M** | **V** | **N** | **K** | **A** | **K** | **L** | **I** | **E** | **R** | **I** | **S** | **E** | **L** | **H** | **R** | **D** | **K** | **R** | **I** | **E** | **G** | **I** | **T** | **D** | **L** | **R** | **D** | **E** | **S** | **S** | **R** | **E** | **G** | **M** | **R** | **I** | **V** | **I** | **D** | **V** | **R** | **R** | **D** | **V** | **S** | **A** | **S** | **V** | **V** | **L** | **N** | **N** | **L** | **Y** | **K** | **M** | **T** | **A** | **L** | **Q** | **T** | **S** | **F** | **G** | **F** | **N** | **M** | **L** | **A** | **I** | **E** | **K** | **G** | **V** | **P** | **K** | **I** | **L** | **S** | **L** | **K** | **R** | **I** | **L** | **E** | **N** | **Y** | **V** | **E** | **H** | **Q** | **K** | **E** | **V** | **I** | **T** | **R** | **R** | **T** | **I** | **F** | **D** | **K** | **N** | **K** | **A** | **E** | **A** | **R** | **A** | **H** | **I** | **L** | **E** | **G** | **L** | **R** | **I** | **A** | **L** | **D** | **H** | **I** | **D** | **E** | **I** | **I** | **A** | **I** | **I** | **R** | **G** | **S** | **Q** | **S** | **D** | **D** | **E** | **A** | **K** | **A** | **T** | **L** | **I** | **E** | **R** | **F** | **E** | **F** | **S** | **D** | **R** | **Q** | **A** | **Q** | **A** | **I** | **L** | **D** | **M** | **R** | **L** | **R** | **R** | **L** | **T** | **G** | **L** | **E** | **R** | **D** | **K** | **I** | **E** | **N** | **E** | **Y** | **Q** | **E** | **L** | **L** | **K** | **F** | **I** | **A** | **D** | **L** | **E** | **D** | **I** | **L** | **A** | **R** | **P** | **E** | **R** | **V** | **I** | **E** | **I** | **I** | **K** | **T** | **E** | **L** | **N** | **D** | **V** | **R** | **T** | **K** | **F** | **G** | **D** | **A** | **R** | **R** | **T** | **E** | **L** | **L** | **V** | **G** | **E** | **V** | **L** | **S** | **L** | **E** | **D** | **E** | **D** | **L** | **I** | **E** | **E** | **E** | **E** | **V** | **V** | **I** | **T** | **L** | **T** | **N** | **N** | **G** | **Y** | **I** | **K** | **R** | **M** | **A** | **N** | **S** | **E** | **F** | **R** | **A** | **Q** | **R** | **R** | **G** | **G** | **R** | **G** | **V** | **Q** | **G** | **M** | **G** | **V** | **H** | **D** | **D** | **D** | **F** | **V** | **K** | **N** | **L** | **V** | **S** | **C** | **S** | **T** | **H** | **D** | **T** | **L** | **L** | **F** | **F** | **T** | **N** | **T** | **G** | **K** | **V** | **Y** | **R** | **A** | **K** | **G** | **Y** | **E** | **I** | **P** | **E** | **Y** | **G** | **R** | **T** | **A** | **K** | **G** | **I** | **P** | **V** | **I** | **N** | **L** | **L** | **G** | **I** | **D** | **S** | **A** | **E** | **K** | **I** | **Q** | **A** | **I** | **I** | **S** | **V** | **E** | **G** | **K** | **A** | **E** | **A** | **G** | **K** | **Y** | **L** | **F** | **F** | **T** | **T** | **L** | **K** | **G** | **T** | **V** | **K** | **R** | **T** | **A** | **V** | **T** | **A** | **F** | **S** | **N** | **I** | **R** | **S** | **N** | **G** | **L** | **I** | **A** | **I** | **S** | **L** | **K** | **E** | **D** | **D** | **E** | **L** | **V** | **N** | **V** | **V** | **T** | **T** | **N** | **G** | **N** | **Q** | **K** | **M** | **I** | **I** | **G** | **T** | **H** | **A** | **G** | **Y** | **S** | **V** | **T** | **F** | **D** | **E** | **N** | **T** | **V** | **R** | **D** | **M** | **G** | **R** | **T** | **A** | **S** | **G** | **V** | **R** | **G** | **I** | **R** | **L** | **R** | **E** | **N** | **D** | **Y** | **V** | **V** | **G** | **A** | **A** | **I** | **L** | **D** | **E** | **N** | **K** | **E** | **V** | **L** | **V** | **I** | **T** | **E** | **N** | **G** | **Y** | **G** | **K** | **R** | **T** | **K** | **A** | **S** | **E** | **Y** | **P** | **V** | **K** | **G** | **R** | **G** | **G** | **K** | **G** | **I** | **K** | **T** | **A** | **N** | **I** | **T** | **E** | **K** | **N** | **G** | **P** | **L** | **A** | **G** | **L** | **T** | **T** | **V** | **N** | **G** | **D** | **E** | **D** | **I** | **L** | **L** | **I** | **T** | **N** | **K** | **G** | **V** | **I** | **I** | **R** | **F** | **N** | **V** | **D** | **S** | **V** | **S** | **Q** | **T** | **G** | **R** | **A** | **T** | **L** | **G** | **V** | **R** | **L** | **M** | **R** | **M** | **E** | **D** | **G** | **A** | **K** | **-** | **-** | **-** | **-** | **-** | **-** | **-** | **-** | **-** | **-** | **-** | **-** | **-** | **-** | **-** | **-** | **-** | **-** | **-** | **-** | **-** | **-** | **-** |
| **N54546** | **M** | **S** | **E** | **E** | **I** | **R** | **E** | **N** | **I** | **H** | **D** | **V** | **N** | **L** | **T** | **S** | **E** | **M** | **K** | **D** | **S** | **F** | **I** | **D** | **Y** | **A** | **M** | **S** | **V** | **I** | **V** | **A** | **R** | **A** | **L** | **P** | **D** | **V** | **R** | **D** | **G** | **L** | **K** | **P** | **V** | **H** | **R** | **R** | **I** | **L** | **Y** | **G** | **M** | **N** | **E** | **L** | **G** | **V** | **T** | **P** | **D** | **K** | **A** | **H** | **K** | **K** | **S** | **A** | **R** | **I** | **V** | **G** | **D** | **V** | **M** | **G** | **K** | **Y** | **H** | **P** | **H** | **G** | **D** | **S** | **A** | **I** | **Y** | **E** | **S** | **M** | **V** | **R** | **M** | **A** | **Q** | **P** | **F** | **S** | **Y** | **R** | **Y** | **M** | **L** | **V** | **D** | **G** | **H** | **G** | **N** | **F** | **G** | **S** | **V** | **D** | **G** | **D** | **G** | **A** | **A** | **A** | **M** | **R** | **Y** | **T** | **E** | **A** | **R** | **M** | **S** | **K** | **I** | **A** | **T** | **E** | **M** | **L** | **R** | **D** | **I** | **N** | **K** | **N** | **T** | **V** | **D** | **F** | **Q** | **S** | **N** | **Y** | **D** | **D** | **T** | **E** | **K** | **E** | **P** | **V** | **V** | **L** | **P** | **A** | **R** | **F** | **P** | **N** | **L** | **L** | **V** | **N** | **G** | **T** | **T** | **G** | **I** | **A** | **V** | **G** | **M** | **A** | **T** | **N** | **I** | **P** | **P** | **H** | **N** | **L** | **S** | **E** | **V** | **V** | **A** | **A** | **I** | **D** | **L** | **L** | **M** | **D** | **N** | **P** | **D** | **V** | **T** | **T** | **N** | **E** | **L** | **M** | **E** | **V** | **L** | **P** | **G** | **P** | **D** | **F** | **P** | **T** | **G** | **G** | **L** | **V** | **M** | **G** | **K** | **S** | **G** | **I** | **R** | **R** | **A** | **Y** | **E** | **T** | **G** | **K** | **G** | **S** | **I** | **T** | **V** | **R** | **A** | **K** | **V** | **E** | **I** | **T** | **E** | **M** | **P** | **N** | **G** | **K** | **E** | **R** | **I** | **I** | **V** | **T** | **E** | **L** | **P** | **Y** | **M** | **V** | **N** | **K** | **A** | **K** | **L** | **I** | **E** | **R** | **I** | **S** | **E** | **L** | **H** | **R** | **D** | **K** | **R** | **I** | **E** | **G** | **I** | **T** | **D** | **L** | **R** | **D** | **E** | **S** | **S** | **R** | **E** | **G** | **M** | **R** | **I** | **V** | **I** | **D** | **I** | **R** | **R** | **D** | **A** | **S** | **A** | **S** | **V** | **I** | **L** | **N** | **N** | **L** | **Y** | **K** | **L** | **T** | **S** | **L** | **Q** | **N** | **S** | **F** | **G** | **F** | **N** | **M** | **L** | **A** | **I** | **E** | **K** | **G** | **V** | **P** | **K** | **V** | **L** | **S** | **L** | **K** | **Q** | **I** | **L** | **E** | **N** | **Y** | **I** | **E** | **H** | **Q** | **R** | **E** | **V** | **I** | **T** | **R** | **R** | **T** | **V** | **F** | **E** | **K** | **E** | **K** | **A** | **E** | **A** | **R** | **A** | **H** | **I** | **L** | **E** | **G** | **L** | **R** | **I** | **A** | **L** | **D** | **H** | **I** | **D** | **E** | **I** | **I** | **A** | **I** | **I** | **R** | **G** | **S** | **K** | **S** | **D** | **D** | **E** | **A** | **K** | **A** | **T** | **M** | **I** | **E** | **R** | **F** | **D** | **L** | **S** | **D** | **R** | **Q** | **S** | **Q** | **A** | **I** | **L** | **D** | **M** | **R** | **L** | **R** | **R** | **L** | **T** | **G** | **L** | **E** | **R** | **E** | **K** | **I** | **E** | **N** | **E** | **Y** | **Q** | **E** | **L** | **L** | **K** | **L** | **I** | **E** | **D** | **L** | **T** | **D** | **I** | **L** | **A** | **R** | **P** | **E** | **R** | **V** | **T** | **E** | **I** | **I** | **K** | **N** | **E** | **L** | **A** | **D** | **L** | **N** | **Q** | **R** | **F** | **G** | **D** | **K** | **R** | **R** | **T** | **E** | **L** | **L** | **V** | **G** | **E** | **V** | **L** | **S** | **L** | **E** | **D** | **E** | **D** | **L** | **I** | **E** | **E** | **E** | **E** | **I** | **V** | **I** | **T** | **L** | **T** | **N** | **N** | **G** | **Y** | **I** | **K** | **R** | **L** | **A** | **N** | **N** | **E** | **F** | **R** | **A** | **Q** | **R** | **R** | **G** | **G** | **R** | **G** | **V** | **Q** | **G** | **M** | **G** | **I** | **H** | **N** | **D** | **D** | **F** | **V** | **K** | **N** | **L** | **V** | **S** | **C** | **S** | **T** | **H** | **D** | **T** | **L** | **L** | **F** | **F** | **T** | **N** | **N** | **G** | **K** | **V** | **Y** | **R** | **A** | **K** | **G** | **Y** | **E** | **I** | **P** | **E** | **Y** | **G** | **R** | **T** | **A** | **K** | **G** | **I** | **P** | **I** | **I** | **N** | **L** | **L** | **G** | **I** | **D** | **S** | **S** | **E** | **S** | **I** | **Q** | **A** | **I** | **I** | **A** | **V** | **T** | **G** | **E** | **A** | **E** | **E** | **G** | **H** | **Y** | **L** | **F** | **F** | **T** | **T** | **R** | **Q** | **G** | **T** | **V** | **K** | **R** | **T** | **S** | **V** | **T** | **A** | **F** | **A** | **N** | **I** | **R** | **S** | **N** | **G** | **L** | **I** | **A** | **I** | **G** | **L** | **K** | **E** | **D** | **D** | **E** | **L** | **V** | **N** | **V** | **L** | **L** | **T** | **D** | **G** | **Q** | **S** | **N** | **I** | **I** | **I** | **G** | **T** | **H** | **N** | **G** | **Y** | **S** | **V** | **T** | **F** | **K** | **E** | **E** | **A** | **V** | **R** | **D** | **M** | **G** | **R** | **T** | **A** | **S** | **G** | **V** | **R** | **G** | **I** | **R** | **L** | **R** | **E** | **E** | **D** | **Y** | **V** | **V** | **G** | **A** | **A** | **L** | **M** | **K** | **E** | **N** | **Q** | **E** | **V** | **L** | **V** | **I** | **T** | **E** | **K** | **G** | **Y** | **G** | **K** | **R** | **T** | **K** | **V** | **S** | **E** | **Y** | **P** | **V** | **K** | **G** | **R** | **G** | **G** | **K** | **G** | **I** | **K** | **T** | **A** | **N** | **I** | **T** | **E** | **K** | **N** | **G** | **P** | **L** | **A** | **G** | **L** | **T** | **T** | **V** | **S** | **G** | **D** | **E** | **D** | **I** | **M** | **L** | **I** | **T** | **D** | **K** | **G** | **V** | **I** | **I** | **R** | **F** | **N** | **V** | **S** | **T** | **V** | **S** | **Q** | **T** | **G** | **R** | **S** | **T** | **L** | **G** | **V** | **R** | **L** | **M** | **K** | **M** | **E** | **A** | **D** | **T** | **K** | **V** | **V** | **T** | **M** | **A** | **A** | **V** | **E** | **P** | **E** | **A** | **A** | **E** | **Q** | **T** | **V** | **E** | **E** | **Q** | **G** | **S** | **E** | **E** |
| **N60363F** | **M** | **S** | **E** | **E** | **I** | **R** | **E** | **N** | **I** | **H** | **D** | **V** | **N** | **L** | **T** | **S** | **E** | **M** | **K** | **D** | **S** | **F** | **I** | **D** | **Y** | **A** | **M** | **S** | **V** | **I** | **V** | **A** | **R** | **A** | **L** | **P** | **D** | **V** | **R** | **D** | **G** | **L** | **K** | **P** | **V** | **H** | **R** | **R** | **I** | **L** | **Y** | **G** | **M** | **N** | **E** | **L** | **G** | **V** | **T** | **P** | **D** | **K** | **A** | **H** | **K** | **K** | **S** | **A** | **R** | **I** | **V** | **G** | **D** | **V** | **M** | **G** | **K** | **Y** | **H** | **P** | **H** | **G** | **D** | **S** | **A** | **I** | **Y** | **E** | **S** | **M** | **V** | **R** | **M** | **A** | **Q** | **P** | **F** | **S** | **Y** | **R** | **Y** | **M** | **L** | **V** | **D** | **G** | **H** | **G** | **N** | **F** | **G** | **S** | **V** | **D** | **G** | **D** | **G** | **A** | **A** | **A** | **M** | **R** | **Y** | **T** | **E** | **A** | **R** | **M** | **S** | **K** | **I** | **A** | **T** | **E** | **M** | **L** | **R** | **D** | **I** | **N** | **K** | **N** | **T** | **V** | **D** | **F** | **Q** | **S** | **N** | **Y** | **D** | **D** | **T** | **E** | **K** | **E** | **P** | **V** | **V** | **L** | **P** | **A** | **R** | **F** | **P** | **N** | **L** | **L** | **V** | **N** | **G** | **T** | **T** | **G** | **I** | **A** | **V** | **G** | **M** | **A** | **T** | **N** | **I** | **P** | **P** | **H** | **N** | **L** | **S** | **E** | **V** | **V** | **A** | **A** | **I** | **D** | **L** | **L** | **M** | **D** | **N** | **P** | **D** | **V** | **T** | **T** | **N** | **E** | **L** | **M** | **E** | **V** | **L** | **P** | **G** | **P** | **D** | **F** | **P** | **T** | **G** | **G** | **L** | **V** | **M** | **G** | **K** | **S** | **G** | **I** | **R** | **R** | **A** | **Y** | **E** | **T** | **G** | **K** | **G** | **S** | **I** | **T** | **V** | **R** | **A** | **K** | **V** | **E** | **I** | **T** | **E** | **M** | **P** | **N** | **G** | **K** | **E** | **R** | **I** | **I** | **V** | **T** | **E** | **L** | **P** | **Y** | **M** | **V** | **N** | **K** | **A** | **K** | **L** | **I** | **E** | **R** | **I** | **S** | **E** | **L** | **H** | **R** | **D** | **K** | **R** | **I** | **E** | **G** | **I** | **T** | **D** | **L** | **R** | **D** | **E** | **S** | **S** | **R** | **E** | **G** | **M** | **R** | **I** | **V** | **I** | **D** | **I** | **R** | **R** | **D** | **A** | **S** | **A** | **S** | **V** | **I** | **L** | **N** | **N** | **L** | **Y** | **K** | **L** | **T** | **S** | **L** | **Q** | **N** | **S** | **F** | **G** | **F** | **N** | **M** | **L** | **A** | **I** | **E** | **K** | **G** | **V** | **P** | **K** | **V** | **L** | **S** | **L** | **K** | **Q** | **I** | **L** | **E** | **N** | **Y** | **I** | **E** | **H** | **Q** | **R** | **E** | **V** | **I** | **T** | **R** | **R** | **T** | **V** | **F** | **E** | **K** | **E** | **K** | **A** | **E** | **A** | **R** | **A** | **H** | **I** | **L** | **E** | **G** | **L** | **R** | **I** | **A** | **L** | **D** | **H** | **I** | **D** | **E** | **I** | **I** | **A** | **I** | **I** | **R** | **G** | **S** | **K** | **S** | **D** | **D** | **E** | **A** | **K** | **A** | **T** | **M** | **I** | **E** | **R** | **F** | **D** | **L** | **S** | **D** | **R** | **Q** | **S** | **Q** | **A** | **I** | **L** | **D** | **M** | **R** | **L** | **R** | **R** | **L** | **T** | **G** | **L** | **E** | **R** | **E** | **K** | **I** | **E** | **N** | **E** | **Y** | **Q** | **E** | **L** | **L** | **K** | **L** | **I** | **E** | **D** | **L** | **T** | **D** | **I** | **L** | **A** | **R** | **P** | **E** | **R** | **V** | **T** | **E** | **I** | **I** | **K** | **N** | **E** | **L** | **A** | **D** | **L** | **N** | **Q** | **R** | **F** | **G** | **D** | **K** | **R** | **R** | **T** | **E** | **L** | **L** | **V** | **G** | **E** | **V** | **L** | **S** | **L** | **E** | **D** | **E** | **D** | **L** | **I** | **E** | **E** | **E** | **E** | **I** | **V** | **I** | **T** | **L** | **T** | **N** | **N** | **G** | **Y** | **I** | **K** | **R** | **L** | **A** | **N** | **N** | **E** | **F** | **R** | **A** | **Q** | **R** | **R** | **G** | **G** | **R** | **G** | **V** | **Q** | **G** | **M** | **G** | **I** | **H** | **N** | **D** | **D** | **F** | **V** | **K** | **N** | **L** | **V** | **S** | **C** | **S** | **T** | **H** | **D** | **T** | **L** | **L** | **F** | **F** | **T** | **N** | **N** | **G** | **K** | **V** | **Y** | **R** | **A** | **K** | **G** | **Y** | **E** | **I** | **P** | **E** | **Y** | **G** | **R** | **T** | **A** | **K** | **G** | **I** | **P** | **I** | **I** | **N** | **L** | **L** | **G** | **I** | **D** | **S** | **S** | **E** | **S** | **I** | **Q** | **A** | **I** | **I** | **A** | **V** | **T** | **G** | **E** | **A** | **E** | **E** | **G** | **H** | **Y** | **L** | **F** | **F** | **T** | **T** | **R** | **Q** | **G** | **T** | **V** | **K** | **R** | **T** | **S** | **V** | **T** | **A** | **F** | **A** | **N** | **I** | **R** | **S** | **N** | **G** | **L** | **I** | **A** | **I** | **G** | **L** | **K** | **E** | **D** | **D** | **E** | **L** | **V** | **N** | **V** | **L** | **L** | **T** | **D** | **G** | **Q** | **S** | **N** | **I** | **I** | **I** | **G** | **T** | **H** | **N** | **G** | **Y** | **S** | **V** | **T** | **F** | **K** | **E** | **E** | **A** | **V** | **R** | **D** | **M** | **G** | **R** | **T** | **A** | **S** | **G** | **V** | **R** | **G** | **I** | **R** | **L** | **R** | **E** | **E** | **D** | **Y** | **V** | **V** | **G** | **A** | **A** | **L** | **M** | **K** | **E** | **D** | **Q** | **E** | **V** | **L** | **V** | **I** | **T** | **E** | **K** | **G** | **Y** | **G** | **K** | **R** | **T** | **K** | **V** | **S** | **E** | **Y** | **P** | **V** | **K** | **G** | **R** | **G** | **G** | **K** | **G** | **I** | **K** | **T** | **A** | **N** | **I** | **T** | **E** | **K** | **N** | **G** | **P** | **L** | **A** | **G** | **L** | **T** | **T** | **V** | **S** | **G** | **D** | **E** | **D** | **I** | **M** | **L** | **I** | **T** | **D** | **K** | **G** | **V** | **I** | **I** | **R** | **F** | **N** | **V** | **S** | **T** | **V** | **S** | **Q** | **T** | **G** | **R** | **S** | **T** | **L** | **G** | **V** | **R** | **L** | **M** | **K** | **M** | **E** | **A** | **D** | **T** | **K** | **V** | **V** | **T** | **M** | **A** | **A** | **V** | **E** | **P** | **E** | **A** | **A** | **E** | **Q** | **T** | **V** | **E** | **E** | **Q** | **G** | **S** | **E** | **E** |
| **N59458F** | **M** | **S** | **E** | **E** | **I** | **K** | **E** | **N** | **I** | **Q** | **D** | **V** | **N** | **L** | **T** | **S** | **E** | **M** | **K** | **E** | **S** | **F** | **I** | **D** | **Y** | **A** | **M** | **S** | **V** | **I** | **V** | **A** | **R** | **A** | **L** | **P** | **D** | **V** | **R** | **D** | **G** | **L** | **K** | **P** | **V** | **H** | **R** | **R** | **I** | **L** | **Y** | **G** | **M** | **N** | **E** | **L** | **G** | **V** | **T** | **P** | **D** | **K** | **P** | **H** | **K** | **K** | **S** | **A** | **R** | **I** | **V** | **G** | **D** | **V** | **M** | **G** | **K** | **Y** | **H** | **P** | **H** | **G** | **D** | **S** | **A** | **I** | **Y** | **E** | **S** | **M** | **V** | **R** | **M** | **A** | **Q** | **P** | **F** | **S** | **Y** | **R** | **A** | **M** | **L** | **V** | **D** | **G** | **H** | **G** | **N** | **F** | **G** | **S** | **V** | **D** | **G** | **D** | **G** | **A** | **A** | **A** | **M** | **R** | **Y** | **T** | **E** | **A** | **R** | **M** | **S** | **K** | **I** | **A** | **L** | **E** | **M** | **L** | **R** | **D** | **I** | **N** | **K** | **N** | **T** | **V** | **D** | **F** | **Q** | **G** | **N** | **Y** | **D** | **D** | **S** | **E** | **Q** | **E** | **P** | **V** | **V** | **L** | **P** | **A** | **R** | **F** | **P** | **N** | **L** | **L** | **V** | **N** | **G** | **T** | **T** | **G** | **I** | **A** | **V** | **G** | **M** | **A** | **T** | **N** | **I** | **P** | **P** | **H** | **N** | **L** | **S** | **E** | **V** | **I** | **D** | **A** | **T** | **S** | **L** | **L** | **M** | **D** | **N** | **P** | **D** | **V** | **T** | **T** | **N** | **E** | **L** | **M** | **E** | **V** | **L** | **P** | **G** | **P** | **D** | **F** | **P** | **T** | **G** | **G** | **L** | **V** | **M** | **G** | **K** | **S** | **G** | **I** | **R** | **R** | **A** | **Y** | **E** | **T** | **G** | **K** | **G** | **S** | **I** | **T** | **V** | **R** | **A** | **K** | **V** | **E** | **L** | **T** | **E** | **M** | **P** | **N** | **G** | **K** | **E** | **R** | **I** | **L** | **V** | **T** | **E** | **L** | **P** | **Y** | **M** | **V** | **N** | **K** | **A** | **K** | **L** | **I** | **E** | **R** | **I** | **S** | **E** | **L** | **H** | **R** | **D** | **K** | **R** | **I** | **E** | **G** | **I** | **T** | **D** | **L** | **R** | **D** | **E** | **S** | **S** | **R** | **E** | **G** | **M** | **R** | **I** | **V** | **I** | **D** | **V** | **R** | **R** | **D** | **V** | **S** | **A** | **S** | **V** | **V** | **L** | **N** | **N** | **L** | **Y** | **K** | **M** | **T** | **A** | **L** | **Q** | **T** | **S** | **F** | **G** | **F** | **N** | **M** | **L** | **A** | **I** | **E** | **K** | **G** | **V** | **P** | **K** | **I** | **L** | **S** | **L** | **K** | **R** | **I** | **L** | **E** | **N** | **Y** | **V** | **E** | **H** | **Q** | **K** | **E** | **V** | **I** | **T** | **R** | **R** | **T** | **I** | **F** | **D** | **K** | **N** | **K** | **A** | **E** | **A** | **R** | **A** | **H** | **I** | **L** | **E** | **G** | **L** | **R** | **I** | **A** | **L** | **D** | **H** | **I** | **D** | **E** | **I** | **I** | **A** | **I** | **I** | **R** | **G** | **S** | **Q** | **S** | **D** | **D** | **E** | **A** | **K** | **A** | **T** | **L** | **I** | **E** | **R** | **F** | **E** | **F** | **S** | **D** | **R** | **Q** | **A** | **Q** | **A** | **I** | **L** | **D** | **M** | **R** | **L** | **R** | **R** | **L** | **T** | **G** | **L** | **E** | **R** | **D** | **K** | **I** | **E** | **N** | **E** | **Y** | **Q** | **E** | **L** | **L** | **K** | **F** | **I** | **A** | **D** | **L** | **E** | **D** | **I** | **L** | **A** | **R** | **P** | **E** | **R** | **V** | **I** | **E** | **I** | **I** | **K** | **T** | **E** | **L** | **N** | **D** | **V** | **R** | **T** | **K** | **F** | **G** | **D** | **A** | **R** | **R** | **T** | **E** | **L** | **L** | **V** | **G** | **E** | **V** | **L** | **S** | **L** | **E** | **D** | **E** | **D** | **L** | **I** | **E** | **E** | **E** | **E** | **V** | **V** | **I** | **T** | **L** | **T** | **N** | **N** | **G** | **Y** | **I** | **K** | **R** | **M** | **A** | **N** | **S** | **E** | **F** | **R** | **A** | **Q** | **R** | **R** | **G** | **G** | **R** | **G** | **V** | **Q** | **G** | **M** | **G** | **V** | **H** | **D** | **D** | **D** | **F** | **V** | **K** | **N** | **L** | **V** | **S** | **C** | **S** | **T** | **H** | **D** | **T** | **L** | **L** | **F** | **F** | **T** | **N** | **T** | **G** | **K** | **V** | **Y** | **R** | **A** | **K** | **G** | **Y** | **E** | **I** | **P** | **E** | **Y** | **G** | **R** | **T** | **A** | **K** | **G** | **I** | **P** | **V** | **I** | **N** | **L** | **L** | **G** | **I** | **D** | **S** | **A** | **E** | **K** | **I** | **Q** | **A** | **I** | **I** | **S** | **V** | **E** | **G** | **K** | **A** | **E** | **A** | **G** | **K** | **Y** | **L** | **F** | **F** | **T** | **T** | **L** | **K** | **G** | **T** | **V** | **K** | **R** | **T** | **A** | **V** | **T** | **A** | **F** | **S** | **N** | **I** | **R** | **S** | **N** | **G** | **L** | **I** | **A** | **I** | **S** | **L** | **K** | **E** | **D** | **D** | **E** | **L** | **V** | **N** | **V** | **V** | **T** | **T** | **N** | **G** | **N** | **Q** | **K** | **M** | **I** | **I** | **G** | **T** | **H** | **A** | **G** | **Y** | **S** | **V** | **T** | **F** | **D** | **E** | **N** | **T** | **V** | **R** | **D** | **M** | **G** | **R** | **T** | **A** | **S** | **G** | **V** | **R** | **G** | **I** | **R** | **L** | **R** | **E** | **N** | **D** | **Y** | **V** | **V** | **G** | **A** | **A** | **I** | **L** | **D** | **E** | **N** | **K** | **E** | **V** | **L** | **V** | **I** | **T** | **E** | **N** | **G** | **Y** | **G** | **K** | **R** | **T** | **K** | **A** | **S** | **E** | **Y** | **P** | **V** | **K** | **G** | **R** | **G** | **G** | **K** | **G** | **I** | **K** | **T** | **A** | **N** | **I** | **T** | **E** | **K** | **N** | **G** | **P** | **L** | **A** | **G** | **L** | **T** | **T** | **V** | **N** | **G** | **D** | **E** | **D** | **I** | **L** | **L** | **I** | **T** | **N** | **K** | **G** | **V** | **I** | **I** | **R** | **F** | **N** | **V** | **D** | **S** | **V** | **S** | **Q** | **T** | **G** | **R** | **A** | **T** | **L** | **G** | **V** | **R** | **L** | **M** | **R** | **M** | **E** | **D** | **G** | **A** | **K** | **-** | **-** | **-** | **-** | **-** | **-** | **-** | **-** | **-** | **-** | **-** | **-** | **-** | **-** | **-** | **-** | **-** | **-** | **-** | **-** | **-** | **-** | **-** |
| **N53761** | **M** | **S** | **E** | **E** | **I** | **K** | **E** | **N** | **I** | **Q** | **D** | **V** | **N** | **L** | **T** | **S** | **E** | **M** | **K** | **E** | **S** | **F** | **I** | **D** | **Y** | **A** | **M** | **S** | **V** | **I** | **V** | **A** | **R** | **A** | **L** | **P** | **D** | **V** | **R** | **D** | **G** | **L** | **K** | **P** | **V** | **H** | **R** | **R** | **I** | **L** | **Y** | **G** | **M** | **N** | **E** | **L** | **G** | **V** | **T** | **P** | **D** | **K** | **P** | **H** | **K** | **K** | **S** | **A** | **R** | **I** | **V** | **G** | **D** | **V** | **M** | **G** | **K** | **Y** | **H** | **P** | **H** | **G** | **D** | **S** | **A** | **I** | **Y** | **E** | **S** | **M** | **V** | **R** | **M** | **A** | **Q** | **P** | **F** | **S** | **Y** | **R** | **A** | **M** | **L** | **V** | **D** | **G** | **H** | **G** | **N** | **F** | **G** | **S** | **V** | **D** | **G** | **D** | **G** | **A** | **A** | **A** | **M** | **R** | **Y** | **T** | **E** | **A** | **R** | **M** | **S** | **K** | **I** | **A** | **L** | **E** | **M** | **L** | **R** | **D** | **I** | **N** | **K** | **N** | **T** | **V** | **D** | **F** | **Q** | **G** | **N** | **Y** | **D** | **D** | **S** | **E** | **Q** | **E** | **P** | **V** | **V** | **L** | **P** | **A** | **R** | **F** | **P** | **N** | **L** | **L** | **V** | **N** | **G** | **T** | **T** | **G** | **I** | **A** | **V** | **G** | **M** | **A** | **T** | **N** | **I** | **P** | **P** | **H** | **N** | **L** | **S** | **E** | **V** | **I** | **D** | **A** | **T** | **S** | **L** | **L** | **M** | **D** | **N** | **P** | **D** | **V** | **T** | **T** | **N** | **E** | **L** | **M** | **E** | **V** | **L** | **P** | **G** | **P** | **D** | **F** | **P** | **T** | **G** | **G** | **L** | **V** | **M** | **G** | **K** | **S** | **G** | **I** | **R** | **R** | **A** | **Y** | **E** | **T** | **G** | **K** | **G** | **S** | **I** | **T** | **V** | **R** | **A** | **K** | **V** | **E** | **L** | **T** | **E** | **M** | **P** | **N** | **G** | **K** | **E** | **R** | **I** | **L** | **V** | **T** | **E** | **L** | **P** | **Y** | **M** | **V** | **N** | **K** | **A** | **K** | **L** | **I** | **E** | **R** | **I** | **S** | **E** | **L** | **H** | **R** | **D** | **K** | **R** | **I** | **E** | **G** | **I** | **T** | **D** | **L** | **R** | **D** | **E** | **S** | **S** | **R** | **E** | **G** | **M** | **R** | **I** | **V** | **I** | **D** | **V** | **R** | **R** | **D** | **V** | **S** | **A** | **S** | **V** | **V** | **L** | **N** | **N** | **L** | **Y** | **K** | **M** | **T** | **A** | **L** | **Q** | **T** | **S** | **F** | **G** | **F** | **N** | **M** | **L** | **A** | **I** | **E** | **K** | **G** | **V** | **P** | **K** | **I** | **L** | **S** | **L** | **K** | **R** | **I** | **L** | **E** | **N** | **Y** | **V** | **E** | **H** | **Q** | **K** | **E** | **V** | **I** | **T** | **R** | **R** | **T** | **I** | **F** | **D** | **K** | **N** | **K** | **A** | **E** | **A** | **R** | **A** | **H** | **I** | **L** | **E** | **G** | **L** | **R** | **I** | **A** | **L** | **D** | **H** | **I** | **D** | **E** | **I** | **I** | **A** | **I** | **I** | **R** | **G** | **S** | **Q** | **S** | **D** | **D** | **E** | **A** | **K** | **A** | **T** | **L** | **I** | **E** | **R** | **F** | **E** | **F** | **S** | **D** | **R** | **Q** | **A** | **Q** | **A** | **I** | **L** | **D** | **M** | **R** | **L** | **R** | **R** | **L** | **T** | **G** | **L** | **E** | **R** | **D** | **K** | **I** | **E** | **N** | **E** | **Y** | **Q** | **E** | **L** | **L** | **K** | **F** | **I** | **A** | **D** | **L** | **E** | **D** | **I** | **L** | **A** | **R** | **P** | **E** | **R** | **V** | **I** | **E** | **I** | **I** | **K** | **T** | **E** | **L** | **N** | **D** | **V** | **R** | **T** | **K** | **F** | **G** | **D** | **A** | **R** | **R** | **T** | **E** | **L** | **L** | **V** | **G** | **E** | **V** | **L** | **S** | **L** | **E** | **D** | **E** | **D** | **L** | **I** | **E** | **E** | **E** | **E** | **V** | **V** | **I** | **T** | **L** | **T** | **N** | **N** | **G** | **Y** | **I** | **K** | **R** | **M** | **A** | **N** | **S** | **E** | **F** | **R** | **A** | **Q** | **R** | **R** | **G** | **G** | **R** | **G** | **V** | **Q** | **G** | **M** | **G** | **V** | **H** | **D** | **D** | **D** | **F** | **V** | **K** | **N** | **L** | **V** | **S** | **C** | **S** | **T** | **H** | **D** | **T** | **L** | **L** | **F** | **F** | **T** | **N** | **T** | **G** | **K** | **V** | **Y** | **R** | **A** | **K** | **G** | **Y** | **E** | **I** | **P** | **E** | **Y** | **G** | **R** | **T** | **A** | **K** | **G** | **I** | **P** | **V** | **I** | **N** | **L** | **L** | **G** | **I** | **D** | **S** | **A** | **E** | **K** | **I** | **Q** | **A** | **I** | **I** | **S** | **V** | **E** | **G** | **K** | **A** | **E** | **A** | **G** | **K** | **Y** | **L** | **F** | **F** | **T** | **T** | **L** | **K** | **G** | **T** | **V** | **K** | **R** | **T** | **A** | **V** | **T** | **A** | **F** | **S** | **N** | **I** | **R** | **S** | **N** | **G** | **L** | **I** | **A** | **I** | **S** | **L** | **K** | **E** | **D** | **D** | **E** | **L** | **V** | **N** | **V** | **V** | **T** | **T** | **N** | **G** | **N** | **Q** | **K** | **M** | **I** | **I** | **G** | **T** | **H** | **A** | **G** | **Y** | **S** | **V** | **T** | **F** | **D** | **E** | **N** | **T** | **V** | **R** | **D** | **M** | **G** | **R** | **T** | **A** | **S** | **G** | **V** | **R** | **G** | **I** | **R** | **L** | **R** | **E** | **N** | **D** | **Y** | **V** | **V** | **G** | **A** | **A** | **I** | **L** | **D** | **E** | **N** | **K** | **E** | **V** | **L** | **V** | **I** | **T** | **E** | **N** | **G** | **Y** | **G** | **K** | **R** | **T** | **K** | **A** | **S** | **E** | **Y** | **P** | **V** | **K** | **G** | **R** | **G** | **G** | **K** | **G** | **I** | **K** | **T** | **A** | **N** | **I** | **T** | **E** | **K** | **N** | **G** | **P** | **L** | **A** | **G** | **L** | **T** | **T** | **V** | **N** | **G** | **D** | **E** | **D** | **I** | **L** | **L** | **I** | **T** | **N** | **K** | **G** | **V** | **I** | **I** | **R** | **F** | **N** | **V** | **D** | **S** | **V** | **S** | **Q** | **T** | **G** | **R** | **A** | **T** | **L** | **G** | **V** | **R** | **L** | **M** | **R** | **M** | **E** | **D** | **G** | **A** | **K** | **-** | **-** | **-** | **-** | **-** | **-** | **-** | **-** | **-** | **-** | **-** | **-** | **-** | **-** | **-** | **-** | **-** | **-** | **-** | **-** | **-** | **-** | **-** |
| **N52734** | **M** | **S** | **E** | **E** | **I** | **K** | **E** | **N** | **I** | **Q** | **D** | **V** | **N** | **L** | **T** | **S** | **E** | **M** | **K** | **E** | **S** | **F** | **I** | **D** | **Y** | **A** | **M** | **S** | **V** | **I** | **V** | **A** | **R** | **A** | **L** | **P** | **D** | **V** | **R** | **D** | **G** | **L** | **K** | **P** | **V** | **H** | **R** | **R** | **I** | **L** | **Y** | **G** | **M** | **N** | **E** | **L** | **G** | **V** | **T** | **P** | **D** | **K** | **P** | **H** | **K** | **K** | **S** | **A** | **R** | **I** | **V** | **G** | **D** | **V** | **M** | **G** | **K** | **Y** | **H** | **P** | **H** | **G** | **D** | **S** | **A** | **I** | **Y** | **E** | **S** | **M** | **V** | **R** | **M** | **A** | **Q** | **P** | **F** | **S** | **Y** | **R** | **A** | **M** | **L** | **V** | **D** | **G** | **H** | **G** | **N** | **F** | **G** | **S** | **V** | **D** | **G** | **D** | **G** | **A** | **A** | **A** | **M** | **R** | **Y** | **T** | **E** | **A** | **R** | **M** | **S** | **K** | **I** | **A** | **L** | **E** | **M** | **L** | **R** | **D** | **I** | **N** | **K** | **N** | **T** | **V** | **D** | **F** | **Q** | **G** | **N** | **Y** | **D** | **D** | **S** | **E** | **Q** | **E** | **P** | **V** | **V** | **L** | **P** | **A** | **R** | **F** | **P** | **N** | **L** | **L** | **V** | **N** | **G** | **T** | **T** | **G** | **I** | **A** | **V** | **G** | **M** | **A** | **T** | **N** | **I** | **P** | **P** | **H** | **N** | **L** | **S** | **E** | **V** | **I** | **D** | **A** | **T** | **S** | **L** | **L** | **M** | **D** | **N** | **P** | **D** | **V** | **T** | **T** | **N** | **E** | **L** | **M** | **E** | **V** | **L** | **P** | **G** | **P** | **D** | **F** | **P** | **T** | **G** | **G** | **L** | **V** | **M** | **G** | **K** | **S** | **G** | **I** | **R** | **R** | **A** | **Y** | **E** | **T** | **G** | **K** | **G** | **S** | **I** | **T** | **V** | **R** | **A** | **K** | **V** | **E** | **L** | **T** | **E** | **M** | **P** | **N** | **G** | **K** | **E** | **R** | **I** | **L** | **V** | **T** | **E** | **L** | **P** | **Y** | **M** | **V** | **N** | **K** | **A** | **K** | **L** | **I** | **E** | **R** | **I** | **S** | **E** | **L** | **H** | **R** | **D** | **K** | **R** | **I** | **E** | **G** | **I** | **T** | **D** | **L** | **R** | **D** | **E** | **S** | **S** | **R** | **E** | **G** | **M** | **R** | **I** | **V** | **I** | **D** | **V** | **R** | **R** | **D** | **V** | **S** | **A** | **S** | **V** | **V** | **L** | **N** | **N** | **L** | **Y** | **K** | **M** | **T** | **A** | **L** | **Q** | **T** | **S** | **F** | **G** | **F** | **N** | **M** | **L** | **A** | **I** | **E** | **K** | **G** | **V** | **P** | **K** | **I** | **L** | **S** | **L** | **K** | **R** | **I** | **L** | **E** | **N** | **Y** | **V** | **E** | **H** | **Q** | **K** | **E** | **V** | **I** | **T** | **R** | **R** | **T** | **I** | **F** | **D** | **K** | **N** | **K** | **A** | **E** | **A** | **R** | **A** | **H** | **I** | **L** | **E** | **G** | **L** | **R** | **I** | **A** | **L** | **D** | **H** | **I** | **D** | **E** | **I** | **I** | **A** | **I** | **I** | **R** | **G** | **S** | **Q** | **S** | **D** | **D** | **E** | **A** | **K** | **A** | **T** | **L** | **I** | **E** | **R** | **F** | **E** | **F** | **S** | **D** | **R** | **Q** | **A** | **Q** | **A** | **I** | **L** | **D** | **M** | **R** | **L** | **R** | **R** | **L** | **T** | **G** | **L** | **E** | **R** | **D** | **K** | **I** | **E** | **N** | **E** | **Y** | **Q** | **E** | **L** | **L** | **K** | **F** | **I** | **A** | **D** | **L** | **E** | **D** | **I** | **L** | **A** | **R** | **P** | **E** | **R** | **V** | **I** | **E** | **I** | **I** | **K** | **T** | **E** | **L** | **N** | **D** | **V** | **R** | **T** | **K** | **F** | **G** | **D** | **A** | **R** | **R** | **T** | **E** | **L** | **L** | **V** | **G** | **E** | **V** | **L** | **S** | **L** | **E** | **D** | **E** | **D** | **L** | **I** | **E** | **E** | **E** | **E** | **V** | **V** | **I** | **T** | **L** | **T** | **N** | **N** | **G** | **Y** | **I** | **K** | **R** | **M** | **A** | **N** | **S** | **E** | **F** | **R** | **A** | **Q** | **R** | **R** | **G** | **G** | **R** | **G** | **V** | **Q** | **G** | **M** | **G** | **V** | **H** | **D** | **D** | **D** | **F** | **V** | **K** | **N** | **L** | **V** | **S** | **C** | **S** | **T** | **H** | **D** | **T** | **L** | **L** | **F** | **F** | **T** | **N** | **T** | **G** | **K** | **V** | **Y** | **R** | **A** | **K** | **G** | **Y** | **E** | **I** | **P** | **E** | **Y** | **G** | **R** | **T** | **A** | **K** | **G** | **I** | **P** | **V** | **I** | **N** | **L** | **L** | **G** | **I** | **D** | **S** | **A** | **E** | **K** | **I** | **Q** | **A** | **I** | **I** | **S** | **V** | **E** | **G** | **K** | **A** | **E** | **A** | **G** | **K** | **Y** | **L** | **F** | **F** | **T** | **T** | **L** | **K** | **G** | **T** | **V** | **K** | **R** | **T** | **A** | **V** | **T** | **A** | **F** | **S** | **N** | **I** | **R** | **S** | **N** | **G** | **L** | **I** | **A** | **I** | **S** | **L** | **K** | **E** | **D** | **D** | **E** | **L** | **V** | **N** | **V** | **V** | **T** | **T** | **N** | **G** | **N** | **Q** | **K** | **M** | **I** | **I** | **G** | **T** | **H** | **A** | **G** | **Y** | **S** | **V** | **T** | **F** | **D** | **E** | **N** | **T** | **V** | **R** | **D** | **M** | **G** | **R** | **T** | **A** | **S** | **G** | **V** | **R** | **G** | **I** | **R** | **L** | **R** | **E** | **N** | **D** | **Y** | **V** | **V** | **G** | **A** | **A** | **I** | **L** | **D** | **E** | **N** | **K** | **E** | **V** | **L** | **V** | **I** | **T** | **E** | **N** | **G** | **Y** | **G** | **K** | **R** | **T** | **K** | **A** | **S** | **E** | **Y** | **P** | **V** | **K** | **G** | **R** | **G** | **G** | **K** | **G** | **I** | **K** | **T** | **A** | **N** | **I** | **T** | **E** | **K** | **N** | **G** | **P** | **L** | **A** | **G** | **L** | **T** | **T** | **V** | **N** | **G** | **D** | **E** | **D** | **I** | **L** | **L** | **I** | **T** | **N** | **K** | **G** | **V** | **I** | **I** | **R** | **F** | **N** | **V** | **D** | **S** | **V** | **S** | **Q** | **T** | **G** | **R** | **A** | **T** | **L** | **G** | **V** | **R** | **L** | **M** | **R** | **M** | **E** | **D** | **G** | **A** | **K** | **-** | **-** | **-** | **-** | **-** | **-** | **-** | **-** | **-** | **-** | **-** | **-** | **-** | **-** | **-** | **-** | **-** | **-** | **-** | **-** | **-** | **-** | **-** |
| **N59646F** | **M** | **S** | **E** | **E** | **I** | **K** | **E** | **N** | **I** | **Q** | **D** | **V** | **N** | **L** | **T** | **S** | **E** | **M** | **K** | **E** | **S** | **F** | **I** | **D** | **Y** | **A** | **M** | **S** | **V** | **I** | **V** | **A** | **R** | **A** | **L** | **P** | **D** | **V** | **R** | **D** | **G** | **L** | **K** | **P** | **V** | **H** | **R** | **R** | **I** | **L** | **Y** | **G** | **M** | **N** | **E** | **L** | **G** | **V** | **T** | **P** | **D** | **K** | **P** | **H** | **K** | **K** | **S** | **A** | **R** | **I** | **V** | **G** | **D** | **V** | **M** | **G** | **K** | **Y** | **H** | **P** | **H** | **G** | **D** | **S** | **A** | **I** | **Y** | **E** | **S** | **M** | **V** | **R** | **M** | **A** | **Q** | **P** | **F** | **S** | **Y** | **R** | **A** | **M** | **L** | **V** | **D** | **G** | **H** | **G** | **N** | **F** | **G** | **S** | **V** | **D** | **G** | **D** | **G** | **A** | **A** | **A** | **M** | **R** | **Y** | **T** | **E** | **A** | **R** | **M** | **S** | **K** | **I** | **A** | **L** | **E** | **M** | **L** | **R** | **D** | **I** | **N** | **K** | **N** | **T** | **V** | **D** | **F** | **Q** | **G** | **N** | **Y** | **D** | **D** | **S** | **E** | **Q** | **E** | **P** | **V** | **V** | **L** | **P** | **A** | **R** | **F** | **P** | **N** | **L** | **L** | **V** | **N** | **G** | **T** | **T** | **G** | **I** | **A** | **V** | **G** | **M** | **A** | **T** | **N** | **I** | **P** | **P** | **H** | **N** | **L** | **S** | **E** | **V** | **I** | **D** | **A** | **T** | **S** | **L** | **L** | **M** | **D** | **N** | **P** | **D** | **V** | **T** | **T** | **N** | **E** | **L** | **M** | **E** | **V** | **L** | **P** | **G** | **P** | **D** | **F** | **P** | **T** | **G** | **G** | **L** | **V** | **M** | **G** | **K** | **S** | **G** | **I** | **R** | **R** | **A** | **Y** | **E** | **T** | **G** | **K** | **G** | **S** | **I** | **T** | **V** | **R** | **A** | **K** | **V** | **E** | **L** | **T** | **E** | **M** | **P** | **N** | **G** | **K** | **E** | **R** | **I** | **L** | **V** | **T** | **E** | **L** | **P** | **Y** | **M** | **V** | **N** | **K** | **A** | **K** | **L** | **I** | **E** | **R** | **I** | **S** | **E** | **L** | **H** | **R** | **D** | **K** | **R** | **I** | **E** | **G** | **I** | **T** | **D** | **L** | **R** | **D** | **E** | **S** | **S** | **R** | **E** | **G** | **M** | **R** | **I** | **V** | **I** | **D** | **V** | **R** | **R** | **D** | **V** | **S** | **A** | **S** | **V** | **V** | **L** | **N** | **N** | **L** | **Y** | **K** | **M** | **T** | **A** | **L** | **Q** | **T** | **S** | **F** | **G** | **F** | **N** | **M** | **L** | **A** | **I** | **E** | **K** | **G** | **V** | **P** | **K** | **I** | **L** | **S** | **L** | **K** | **R** | **I** | **L** | **E** | **N** | **Y** | **V** | **E** | **H** | **Q** | **K** | **E** | **V** | **I** | **T** | **R** | **R** | **T** | **I** | **F** | **D** | **K** | **N** | **K** | **A** | **E** | **A** | **R** | **A** | **H** | **I** | **L** | **E** | **G** | **L** | **R** | **I** | **A** | **L** | **D** | **H** | **I** | **D** | **E** | **I** | **I** | **A** | **I** | **I** | **R** | **G** | **S** | **Q** | **S** | **D** | **D** | **E** | **A** | **K** | **A** | **T** | **L** | **I** | **E** | **R** | **F** | **E** | **F** | **S** | **D** | **R** | **Q** | **A** | **Q** | **A** | **I** | **L** | **D** | **M** | **R** | **L** | **R** | **R** | **L** | **T** | **G** | **L** | **E** | **R** | **D** | **K** | **I** | **E** | **N** | **E** | **Y** | **Q** | **E** | **L** | **L** | **K** | **F** | **I** | **A** | **D** | **L** | **E** | **D** | **I** | **L** | **A** | **R** | **P** | **E** | **R** | **V** | **I** | **E** | **I** | **I** | **K** | **T** | **E** | **L** | **N** | **D** | **V** | **R** | **T** | **K** | **F** | **G** | **D** | **A** | **R** | **R** | **T** | **E** | **L** | **L** | **V** | **G** | **E** | **V** | **L** | **S** | **L** | **E** | **D** | **E** | **D** | **L** | **I** | **E** | **E** | **E** | **E** | **V** | **V** | **I** | **T** | **L** | **T** | **N** | **N** | **G** | **Y** | **I** | **K** | **R** | **M** | **A** | **N** | **S** | **E** | **F** | **R** | **A** | **Q** | **R** | **R** | **G** | **G** | **R** | **G** | **V** | **Q** | **G** | **M** | **G** | **V** | **H** | **D** | **D** | **D** | **F** | **V** | **K** | **N** | **L** | **V** | **S** | **C** | **S** | **T** | **H** | **D** | **T** | **L** | **L** | **F** | **F** | **T** | **N** | **T** | **G** | **K** | **V** | **Y** | **R** | **A** | **K** | **G** | **Y** | **E** | **I** | **P** | **E** | **Y** | **G** | **R** | **T** | **A** | **K** | **G** | **I** | **P** | **V** | **I** | **N** | **L** | **L** | **G** | **I** | **D** | **S** | **A** | **E** | **K** | **I** | **Q** | **A** | **I** | **I** | **S** | **V** | **E** | **G** | **K** | **A** | **E** | **A** | **G** | **K** | **Y** | **L** | **F** | **F** | **T** | **T** | **L** | **K** | **G** | **T** | **V** | **K** | **R** | **T** | **A** | **V** | **T** | **A** | **F** | **S** | **N** | **I** | **R** | **S** | **N** | **G** | **L** | **I** | **A** | **I** | **S** | **L** | **K** | **E** | **D** | **D** | **E** | **L** | **V** | **N** | **V** | **V** | **T** | **T** | **N** | **G** | **N** | **Q** | **K** | **M** | **I** | **I** | **G** | **T** | **H** | **A** | **G** | **Y** | **S** | **V** | **T** | **F** | **D** | **E** | **N** | **T** | **V** | **R** | **D** | **M** | **G** | **R** | **T** | **A** | **S** | **G** | **V** | **R** | **G** | **I** | **R** | **L** | **R** | **E** | **N** | **D** | **Y** | **V** | **V** | **G** | **A** | **A** | **I** | **L** | **D** | **E** | **N** | **K** | **E** | **V** | **L** | **V** | **I** | **T** | **E** | **N** | **G** | **Y** | **G** | **K** | **R** | **T** | **K** | **A** | **S** | **E** | **Y** | **P** | **V** | **K** | **G** | **R** | **G** | **G** | **K** | **G** | **I** | **K** | **T** | **A** | **N** | **I** | **T** | **E** | **K** | **N** | **G** | **P** | **L** | **A** | **G** | **L** | **T** | **T** | **V** | **N** | **G** | **D** | **E** | **D** | **I** | **L** | **L** | **I** | **T** | **N** | **K** | **G** | **V** | **I** | **I** | **R** | **F** | **N** | **V** | **D** | **S** | **V** | **S** | **Q** | **T** | **G** | **R** | **A** | **T** | **L** | **G** | **V** | **R** | **L** | **M** | **R** | **M** | **E** | **D** | **G** | **A** | **K** | **-** | **-** | **-** | **-** | **-** | **-** | **-** | **-** | **-** | **-** | **-** | **-** | **-** | **-** | **-** | **-** | **-** | **-** | **-** | **-** | **-** | **-** | **-** |
| **N54588** | **M** | **S** | **E** | **E** | **I** | **K** | **E** | **N** | **I** | **Q** | **D** | **V** | **N** | **L** | **T** | **S** | **E** | **M** | **K** | **E** | **S** | **F** | **I** | **D** | **Y** | **A** | **M** | **S** | **V** | **I** | **V** | **A** | **R** | **A** | **L** | **P** | **D** | **V** | **R** | **D** | **G** | **L** | **K** | **P** | **V** | **H** | **R** | **R** | **I** | **L** | **Y** | **G** | **M** | **N** | **E** | **L** | **G** | **V** | **T** | **P** | **D** | **K** | **P** | **H** | **K** | **K** | **S** | **A** | **R** | **I** | **V** | **G** | **D** | **V** | **M** | **G** | **K** | **Y** | **H** | **P** | **H** | **G** | **D** | **S** | **A** | **I** | **Y** | **E** | **S** | **M** | **V** | **R** | **M** | **A** | **Q** | **P** | **F** | **S** | **Y** | **R** | **A** | **M** | **L** | **V** | **D** | **G** | **H** | **G** | **N** | **F** | **G** | **S** | **V** | **D** | **G** | **D** | **G** | **A** | **A** | **A** | **M** | **R** | **Y** | **T** | **E** | **A** | **R** | **M** | **S** | **K** | **I** | **A** | **L** | **E** | **M** | **L** | **R** | **D** | **I** | **N** | **K** | **N** | **T** | **V** | **D** | **F** | **Q** | **G** | **N** | **Y** | **D** | **D** | **S** | **E** | **Q** | **E** | **P** | **V** | **V** | **L** | **P** | **A** | **R** | **F** | **P** | **N** | **L** | **L** | **V** | **N** | **G** | **T** | **T** | **G** | **I** | **A** | **V** | **G** | **M** | **A** | **T** | **N** | **I** | **P** | **P** | **H** | **N** | **L** | **S** | **E** | **V** | **I** | **D** | **A** | **T** | **S** | **L** | **L** | **M** | **D** | **N** | **P** | **D** | **V** | **T** | **T** | **N** | **E** | **L** | **M** | **E** | **V** | **L** | **P** | **G** | **P** | **D** | **F** | **P** | **T** | **G** | **G** | **L** | **V** | **M** | **G** | **K** | **S** | **G** | **I** | **R** | **R** | **A** | **Y** | **E** | **T** | **G** | **K** | **G** | **S** | **I** | **T** | **V** | **R** | **A** | **K** | **V** | **E** | **L** | **T** | **E** | **M** | **P** | **N** | **G** | **K** | **E** | **R** | **I** | **L** | **V** | **T** | **E** | **L** | **P** | **Y** | **M** | **V** | **N** | **K** | **A** | **K** | **L** | **I** | **E** | **R** | **I** | **S** | **E** | **L** | **H** | **R** | **D** | **K** | **R** | **I** | **E** | **G** | **I** | **T** | **D** | **L** | **R** | **D** | **E** | **S** | **S** | **R** | **E** | **G** | **M** | **R** | **I** | **V** | **I** | **D** | **V** | **R** | **R** | **D** | **V** | **S** | **A** | **S** | **V** | **V** | **L** | **N** | **N** | **L** | **Y** | **K** | **M** | **T** | **A** | **L** | **Q** | **T** | **S** | **F** | **G** | **F** | **N** | **M** | **L** | **A** | **I** | **E** | **K** | **G** | **V** | **P** | **K** | **I** | **L** | **S** | **L** | **K** | **R** | **I** | **L** | **E** | **N** | **Y** | **V** | **E** | **H** | **Q** | **K** | **E** | **V** | **I** | **T** | **R** | **R** | **T** | **I** | **F** | **D** | **K** | **N** | **K** | **A** | **E** | **A** | **R** | **A** | **H** | **I** | **L** | **E** | **G** | **L** | **R** | **I** | **A** | **L** | **D** | **H** | **I** | **D** | **E** | **I** | **I** | **A** | **I** | **I** | **R** | **G** | **S** | **Q** | **S** | **D** | **D** | **E** | **A** | **K** | **A** | **T** | **L** | **I** | **E** | **R** | **F** | **E** | **F** | **S** | **D** | **R** | **Q** | **A** | **Q** | **A** | **I** | **L** | **D** | **M** | **R** | **L** | **R** | **R** | **L** | **T** | **G** | **L** | **E** | **R** | **D** | **K** | **I** | **E** | **N** | **E** | **Y** | **Q** | **E** | **L** | **L** | **K** | **F** | **I** | **A** | **D** | **L** | **E** | **D** | **I** | **L** | **A** | **R** | **P** | **E** | **R** | **V** | **I** | **E** | **I** | **I** | **K** | **T** | **E** | **L** | **N** | **D** | **V** | **R** | **T** | **K** | **F** | **G** | **D** | **A** | **R** | **R** | **T** | **E** | **L** | **L** | **V** | **G** | **E** | **V** | **L** | **S** | **L** | **E** | **D** | **E** | **D** | **L** | **I** | **E** | **E** | **E** | **E** | **V** | **V** | **I** | **T** | **L** | **T** | **N** | **N** | **G** | **Y** | **I** | **K** | **R** | **M** | **A** | **N** | **S** | **E** | **F** | **R** | **A** | **Q** | **R** | **R** | **G** | **G** | **R** | **G** | **V** | **Q** | **G** | **M** | **G** | **V** | **H** | **D** | **D** | **D** | **F** | **V** | **K** | **N** | **L** | **V** | **S** | **C** | **S** | **T** | **H** | **D** | **T** | **L** | **L** | **F** | **F** | **T** | **N** | **T** | **G** | **K** | **V** | **Y** | **R** | **A** | **K** | **G** | **Y** | **E** | **I** | **P** | **E** | **Y** | **G** | **R** | **T** | **A** | **K** | **G** | **I** | **P** | **V** | **I** | **N** | **L** | **L** | **G** | **I** | **D** | **S** | **A** | **E** | **K** | **I** | **Q** | **A** | **I** | **I** | **S** | **V** | **E** | **G** | **K** | **A** | **E** | **A** | **G** | **K** | **Y** | **L** | **F** | **F** | **T** | **T** | **L** | **K** | **G** | **T** | **V** | **K** | **R** | **T** | **A** | **V** | **T** | **A** | **F** | **S** | **N** | **I** | **R** | **S** | **N** | **G** | **L** | **I** | **A** | **I** | **S** | **L** | **K** | **E** | **D** | **D** | **E** | **L** | **V** | **N** | **V** | **V** | **T** | **T** | **N** | **G** | **N** | **Q** | **K** | **M** | **I** | **I** | **G** | **T** | **H** | **A** | **G** | **Y** | **S** | **V** | **T** | **F** | **D** | **E** | **N** | **T** | **V** | **R** | **D** | **M** | **G** | **R** | **T** | **A** | **S** | **G** | **V** | **R** | **G** | **I** | **R** | **L** | **R** | **E** | **N** | **D** | **Y** | **V** | **V** | **G** | **A** | **A** | **I** | **L** | **D** | **E** | **N** | **K** | **E** | **V** | **L** | **V** | **I** | **T** | **E** | **N** | **G** | **Y** | **G** | **K** | **R** | **T** | **K** | **A** | **S** | **E** | **Y** | **P** | **V** | **K** | **G** | **R** | **G** | **G** | **K** | **G** | **I** | **K** | **T** | **A** | **N** | **I** | **T** | **E** | **K** | **N** | **G** | **P** | **L** | **A** | **G** | **L** | **T** | **T** | **V** | **N** | **G** | **D** | **E** | **D** | **I** | **L** | **L** | **I** | **T** | **N** | **K** | **G** | **V** | **I** | **I** | **R** | **F** | **N** | **V** | **D** | **S** | **V** | **S** | **Q** | **T** | **G** | **R** | **A** | **T** | **L** | **G** | **V** | **R** | **L** | **M** | **R** | **M** | **E** | **D** | **G** | **A** | **K** | **-** | **-** | **-** | **-** | **-** | **-** | **-** | **-** | **-** | **-** | **-** | **-** | **-** | **-** | **-** | **-** | **-** | **-** | **-** | **-** | **-** | **-** | **-** |
| **N53766** | **M** | **S** | **E** | **E** | **I** | **K** | **E** | **N** | **I** | **Q** | **D** | **V** | **N** | **L** | **T** | **S** | **E** | **M** | **K** | **E** | **S** | **F** | **I** | **D** | **Y** | **A** | **M** | **S** | **V** | **I** | **V** | **A** | **R** | **A** | **L** | **P** | **D** | **V** | **R** | **D** | **G** | **L** | **K** | **P** | **V** | **H** | **R** | **R** | **I** | **L** | **Y** | **G** | **M** | **N** | **E** | **L** | **G** | **V** | **T** | **P** | **D** | **K** | **P** | **H** | **K** | **K** | **S** | **A** | **R** | **I** | **V** | **G** | **D** | **V** | **M** | **G** | **K** | **Y** | **H** | **P** | **H** | **G** | **D** | **S** | **A** | **I** | **Y** | **E** | **S** | **M** | **V** | **R** | **M** | **A** | **Q** | **P** | **F** | **S** | **Y** | **R** | **A** | **M** | **L** | **V** | **D** | **G** | **H** | **G** | **N** | **F** | **G** | **S** | **V** | **D** | **G** | **D** | **G** | **A** | **A** | **A** | **M** | **R** | **Y** | **T** | **E** | **A** | **R** | **M** | **S** | **K** | **I** | **A** | **L** | **E** | **M** | **L** | **R** | **D** | **I** | **N** | **K** | **N** | **T** | **V** | **D** | **F** | **Q** | **G** | **N** | **Y** | **D** | **D** | **S** | **E** | **Q** | **E** | **P** | **V** | **V** | **L** | **P** | **A** | **R** | **F** | **P** | **N** | **L** | **L** | **V** | **N** | **G** | **T** | **T** | **G** | **I** | **A** | **V** | **G** | **M** | **A** | **T** | **N** | **I** | **P** | **P** | **H** | **N** | **L** | **S** | **E** | **V** | **I** | **D** | **A** | **T** | **S** | **L** | **L** | **M** | **D** | **N** | **P** | **D** | **V** | **T** | **T** | **N** | **E** | **L** | **M** | **E** | **V** | **L** | **P** | **G** | **P** | **D** | **F** | **P** | **T** | **G** | **G** | **L** | **V** | **M** | **G** | **K** | **S** | **G** | **I** | **R** | **R** | **A** | **Y** | **E** | **T** | **G** | **K** | **G** | **S** | **I** | **T** | **V** | **R** | **A** | **K** | **V** | **E** | **L** | **T** | **E** | **M** | **P** | **N** | **G** | **K** | **E** | **R** | **I** | **L** | **V** | **T** | **E** | **L** | **P** | **Y** | **M** | **V** | **N** | **K** | **A** | **K** | **L** | **I** | **E** | **R** | **I** | **S** | **E** | **L** | **H** | **R** | **D** | **K** | **R** | **I** | **E** | **G** | **I** | **T** | **D** | **L** | **R** | **D** | **E** | **S** | **S** | **R** | **E** | **G** | **M** | **R** | **I** | **V** | **I** | **D** | **V** | **R** | **R** | **D** | **V** | **S** | **A** | **S** | **V** | **V** | **L** | **N** | **N** | **L** | **Y** | **K** | **M** | **T** | **A** | **L** | **Q** | **T** | **S** | **F** | **G** | **F** | **N** | **M** | **L** | **A** | **I** | **E** | **K** | **G** | **V** | **P** | **K** | **I** | **L** | **S** | **L** | **K** | **R** | **I** | **L** | **E** | **N** | **Y** | **V** | **E** | **H** | **Q** | **K** | **E** | **V** | **I** | **T** | **R** | **R** | **T** | **I** | **F** | **D** | **K** | **N** | **K** | **A** | **E** | **A** | **R** | **A** | **H** | **I** | **L** | **E** | **G** | **L** | **R** | **I** | **A** | **L** | **D** | **H** | **I** | **D** | **E** | **I** | **I** | **A** | **I** | **I** | **R** | **G** | **S** | **Q** | **S** | **D** | **D** | **E** | **A** | **K** | **A** | **T** | **L** | **I** | **E** | **R** | **F** | **E** | **F** | **S** | **D** | **R** | **Q** | **A** | **Q** | **A** | **I** | **L** | **D** | **M** | **R** | **L** | **R** | **R** | **L** | **T** | **G** | **L** | **E** | **R** | **D** | **K** | **I** | **E** | **N** | **E** | **Y** | **Q** | **E** | **L** | **L** | **K** | **F** | **I** | **A** | **D** | **L** | **E** | **D** | **I** | **L** | **A** | **R** | **P** | **E** | **R** | **V** | **I** | **E** | **I** | **I** | **K** | **T** | **E** | **L** | **N** | **D** | **V** | **R** | **T** | **K** | **F** | **G** | **D** | **A** | **R** | **R** | **T** | **E** | **L** | **L** | **V** | **G** | **E** | **V** | **L** | **S** | **L** | **E** | **D** | **E** | **D** | **L** | **I** | **E** | **E** | **E** | **E** | **V** | **V** | **I** | **T** | **L** | **T** | **N** | **N** | **G** | **Y** | **I** | **K** | **R** | **M** | **A** | **N** | **S** | **E** | **F** | **R** | **A** | **Q** | **R** | **R** | **G** | **G** | **R** | **G** | **V** | **Q** | **G** | **M** | **G** | **V** | **H** | **D** | **D** | **D** | **F** | **V** | **K** | **N** | **L** | **V** | **S** | **C** | **S** | **T** | **H** | **D** | **T** | **L** | **L** | **F** | **F** | **T** | **N** | **T** | **G** | **K** | **V** | **Y** | **R** | **A** | **K** | **G** | **Y** | **E** | **I** | **P** | **E** | **Y** | **G** | **R** | **T** | **A** | **K** | **G** | **I** | **P** | **V** | **I** | **N** | **L** | **L** | **G** | **I** | **D** | **S** | **A** | **E** | **K** | **I** | **Q** | **A** | **I** | **I** | **S** | **V** | **E** | **G** | **K** | **A** | **E** | **A** | **G** | **K** | **Y** | **L** | **F** | **F** | **T** | **T** | **L** | **K** | **G** | **T** | **V** | **K** | **R** | **T** | **A** | **V** | **T** | **A** | **F** | **S** | **N** | **I** | **R** | **S** | **N** | **G** | **L** | **I** | **A** | **I** | **S** | **L** | **K** | **E** | **D** | **D** | **E** | **L** | **V** | **N** | **V** | **V** | **T** | **T** | **N** | **G** | **N** | **Q** | **K** | **M** | **I** | **I** | **G** | **T** | **H** | **A** | **G** | **Y** | **S** | **V** | **T** | **F** | **D** | **E** | **N** | **T** | **V** | **R** | **D** | **M** | **G** | **R** | **T** | **A** | **S** | **G** | **V** | **R** | **G** | **I** | **R** | **L** | **R** | **E** | **N** | **D** | **Y** | **V** | **V** | **G** | **A** | **A** | **I** | **L** | **D** | **E** | **N** | **K** | **E** | **V** | **L** | **V** | **I** | **T** | **E** | **N** | **G** | **Y** | **G** | **K** | **R** | **T** | **K** | **A** | **S** | **E** | **Y** | **P** | **V** | **K** | **G** | **R** | **G** | **G** | **K** | **G** | **I** | **K** | **T** | **A** | **N** | **I** | **T** | **E** | **K** | **N** | **G** | **P** | **L** | **A** | **G** | **L** | **T** | **T** | **V** | **N** | **G** | **D** | **E** | **D** | **I** | **L** | **L** | **I** | **T** | **N** | **K** | **G** | **V** | **I** | **I** | **R** | **F** | **N** | **V** | **D** | **S** | **V** | **S** | **Q** | **T** | **G** | **R** | **A** | **T** | **L** | **G** | **V** | **R** | **L** | **M** | **R** | **M** | **E** | **D** | **G** | **A** | **K** | **-** | **-** | **-** | **-** | **-** | **-** | **-** | **-** | **-** | **-** | **-** | **-** | **-** | **-** | **-** | **-** | **-** | **-** | **-** | **-** | **-** | **-** | **-** |
| **N60190F** | **M** | **S** | **E** | **E** | **I** | **R** | **E** | **N** | **I** | **H** | **D** | **V** | **N** | **L** | **T** | **S** | **E** | **M** | **K** | **D** | **S** | **F** | **I** | **D** | **Y** | **A** | **M** | **S** | **V** | **I** | **V** | **A** | **R** | **A** | **L** | **P** | **D** | **V** | **R** | **D** | **G** | **L** | **K** | **P** | **V** | **H** | **R** | **R** | **I** | **L** | **Y** | **G** | **M** | **N** | **E** | **L** | **G** | **V** | **T** | **P** | **D** | **K** | **A** | **H** | **K** | **K** | **S** | **A** | **R** | **I** | **V** | **G** | **D** | **V** | **M** | **G** | **K** | **Y** | **H** | **P** | **H** | **G** | **D** | **S** | **A** | **I** | **Y** | **E** | **S** | **M** | **V** | **R** | **M** | **A** | **Q** | **P** | **F** | **S** | **Y** | **R** | **Y** | **M** | **L** | **V** | **D** | **G** | **H** | **G** | **N** | **F** | **G** | **S** | **V** | **D** | **G** | **D** | **G** | **A** | **A** | **A** | **M** | **R** | **Y** | **T** | **E** | **A** | **R** | **M** | **S** | **K** | **I** | **A** | **T** | **E** | **M** | **L** | **R** | **D** | **I** | **N** | **K** | **N** | **T** | **V** | **D** | **F** | **Q** | **S** | **N** | **Y** | **D** | **D** | **T** | **E** | **K** | **E** | **P** | **V** | **V** | **L** | **P** | **A** | **R** | **F** | **P** | **N** | **L** | **L** | **V** | **N** | **G** | **T** | **T** | **G** | **I** | **A** | **V** | **G** | **M** | **A** | **T** | **N** | **I** | **P** | **P** | **H** | **N** | **L** | **S** | **E** | **V** | **V** | **A** | **A** | **I** | **D** | **L** | **L** | **M** | **D** | **N** | **P** | **D** | **V** | **T** | **T** | **N** | **E** | **L** | **M** | **E** | **V** | **L** | **P** | **G** | **P** | **D** | **F** | **P** | **T** | **G** | **G** | **L** | **V** | **M** | **G** | **K** | **S** | **G** | **I** | **R** | **R** | **A** | **Y** | **E** | **T** | **G** | **K** | **G** | **S** | **I** | **T** | **V** | **R** | **A** | **K** | **V** | **E** | **I** | **T** | **E** | **M** | **P** | **N** | **G** | **K** | **E** | **R** | **I** | **L** | **V** | **T** | **E** | **L** | **P** | **Y** | **M** | **V** | **N** | **K** | **A** | **K** | **L** | **I** | **E** | **R** | **I** | **S** | **E** | **L** | **H** | **R** | **D** | **K** | **R** | **I** | **E** | **G** | **I** | **T** | **D** | **L** | **R** | **D** | **E** | **S** | **S** | **R** | **E** | **G** | **M** | **R** | **I** | **V** | **I** | **D** | **V** | **R** | **R** | **D** | **A** | **S** | **A** | **S** | **V** | **I** | **L** | **N** | **N** | **L** | **Y** | **K** | **L** | **T** | **S** | **L** | **Q** | **N** | **S** | **F** | **G** | **F** | **N** | **M** | **L** | **A** | **I** | **E** | **K** | **G** | **V** | **P** | **K** | **V** | **L** | **S** | **L** | **K** | **Q** | **I** | **L** | **E** | **N** | **Y** | **I** | **E** | **H** | **Q** | **R** | **E** | **V** | **I** | **T** | **R** | **R** | **T** | **V** | **F** | **E** | **K** | **E** | **K** | **A** | **E** | **A** | **R** | **A** | **H** | **I** | **L** | **E** | **G** | **L** | **R** | **I** | **A** | **L** | **D** | **H** | **I** | **D** | **E** | **I** | **I** | **A** | **I** | **I** | **R** | **G** | **S** | **K** | **S** | **D** | **D** | **E** | **A** | **K** | **A** | **T** | **M** | **I** | **E** | **R** | **F** | **D** | **L** | **S** | **D** | **R** | **Q** | **S** | **Q** | **A** | **I** | **L** | **D** | **M** | **R** | **L** | **R** | **R** | **L** | **T** | **G** | **L** | **E** | **R** | **E** | **K** | **I** | **E** | **N** | **E** | **Y** | **Q** | **E** | **L** | **L** | **K** | **L** | **I** | **E** | **D** | **L** | **T** | **D** | **I** | **L** | **A** | **R** | **P** | **E** | **R** | **V** | **T** | **E** | **I** | **I** | **K** | **N** | **E** | **L** | **A** | **D** | **L** | **N** | **Q** | **R** | **F** | **G** | **D** | **K** | **R** | **R** | **T** | **E** | **L** | **L** | **V** | **G** | **E** | **V** | **L** | **S** | **L** | **E** | **D** | **E** | **D** | **L** | **I** | **E** | **E** | **E** | **E** | **I** | **V** | **I** | **T** | **L** | **T** | **N** | **N** | **G** | **Y** | **I** | **K** | **R** | **L** | **A** | **N** | **N** | **E** | **F** | **R** | **A** | **Q** | **R** | **R** | **G** | **G** | **R** | **G** | **V** | **Q** | **G** | **M** | **G** | **I** | **H** | **N** | **D** | **D** | **F** | **V** | **K** | **N** | **L** | **V** | **S** | **C** | **S** | **T** | **H** | **D** | **T** | **L** | **L** | **F** | **F** | **T** | **N** | **N** | **G** | **K** | **V** | **Y** | **R** | **A** | **K** | **G** | **Y** | **E** | **I** | **P** | **E** | **Y** | **G** | **R** | **T** | **A** | **K** | **G** | **I** | **P** | **I** | **I** | **N** | **L** | **L** | **G** | **I** | **D** | **S** | **S** | **E** | **S** | **I** | **Q** | **A** | **I** | **I** | **A** | **V** | **T** | **G** | **E** | **A** | **E** | **E** | **G** | **H** | **Y** | **L** | **F** | **F** | **T** | **T** | **R** | **Q** | **G** | **T** | **V** | **K** | **R** | **T** | **S** | **V** | **T** | **A** | **F** | **A** | **N** | **I** | **R** | **S** | **N** | **G** | **L** | **I** | **A** | **I** | **G** | **L** | **K** | **E** | **D** | **D** | **E** | **L** | **V** | **N** | **V** | **L** | **L** | **T** | **D** | **G** | **Q** | **S** | **N** | **I** | **I** | **I** | **G** | **T** | **H** | **N** | **G** | **Y** | **S** | **V** | **T** | **F** | **K** | **E** | **E** | **A** | **V** | **R** | **D** | **M** | **G** | **R** | **T** | **A** | **S** | **G** | **V** | **R** | **G** | **I** | **R** | **L** | **R** | **E** | **E** | **D** | **Y** | **V** | **V** | **G** | **A** | **A** | **L** | **M** | **K** | **E** | **D** | **Q** | **E** | **V** | **L** | **V** | **I** | **T** | **E** | **K** | **G** | **Y** | **G** | **K** | **R** | **T** | **K** | **V** | **S** | **E** | **Y** | **P** | **V** | **K** | **G** | **R** | **G** | **G** | **K** | **G** | **I** | **K** | **T** | **A** | **N** | **I** | **T** | **E** | **K** | **N** | **G** | **P** | **L** | **A** | **G** | **L** | **T** | **T** | **V** | **S** | **G** | **N** | **E** | **D** | **I** | **M** | **L** | **I** | **T** | **D** | **K** | **G** | **V** | **I** | **I** | **R** | **F** | **N** | **V** | **S** | **T** | **V** | **S** | **Q** | **T** | **G** | **R** | **S** | **T** | **L** | **G** | **V** | **R** | **L** | **M** | **K** | **M** | **E** | **A** | **D** | **T** | **K** | **V** | **V** | **T** | **M** | **-** | **-** | **-** | **-** | **-** | **-** | **-** | **-** | **-** | **-** | **-** | **-** | **-** | **-** | **-** | **-** | **-** | **-** | **-** |
| **N55317** | **M** | **S** | **E** | **E** | **I** | **R** | **E** | **N** | **I** | **H** | **D** | **V** | **N** | **L** | **T** | **S** | **E** | **M** | **K** | **D** | **S** | **F** | **I** | **D** | **Y** | **A** | **M** | **S** | **V** | **I** | **V** | **A** | **R** | **A** | **L** | **P** | **D** | **V** | **R** | **D** | **G** | **L** | **K** | **P** | **V** | **H** | **R** | **R** | **I** | **L** | **Y** | **G** | **M** | **N** | **E** | **L** | **G** | **V** | **T** | **P** | **D** | **K** | **A** | **H** | **K** | **K** | **S** | **A** | **R** | **I** | **V** | **G** | **D** | **V** | **M** | **G** | **K** | **Y** | **H** | **P** | **H** | **G** | **D** | **S** | **A** | **I** | **Y** | **E** | **S** | **M** | **V** | **R** | **M** | **A** | **Q** | **P** | **F** | **S** | **Y** | **R** | **Y** | **M** | **L** | **V** | **D** | **G** | **H** | **G** | **N** | **F** | **G** | **S** | **V** | **D** | **G** | **D** | **G** | **A** | **A** | **A** | **M** | **R** | **Y** | **T** | **E** | **A** | **R** | **M** | **S** | **K** | **I** | **A** | **T** | **E** | **M** | **L** | **R** | **D** | **I** | **N** | **K** | **N** | **T** | **V** | **D** | **F** | **Q** | **S** | **N** | **Y** | **D** | **D** | **T** | **E** | **K** | **E** | **P** | **V** | **V** | **L** | **P** | **A** | **R** | **F** | **P** | **N** | **L** | **L** | **V** | **N** | **G** | **T** | **T** | **G** | **I** | **A** | **V** | **G** | **M** | **A** | **T** | **N** | **I** | **P** | **P** | **H** | **N** | **L** | **S** | **E** | **V** | **V** | **A** | **A** | **I** | **D** | **L** | **L** | **M** | **D** | **N** | **P** | **D** | **V** | **T** | **T** | **N** | **E** | **L** | **M** | **E** | **V** | **L** | **P** | **G** | **P** | **D** | **F** | **P** | **T** | **G** | **G** | **L** | **V** | **M** | **G** | **K** | **S** | **G** | **I** | **R** | **R** | **A** | **Y** | **E** | **T** | **G** | **K** | **G** | **S** | **I** | **T** | **V** | **R** | **A** | **K** | **V** | **E** | **I** | **T** | **E** | **M** | **P** | **N** | **G** | **K** | **E** | **R** | **I** | **L** | **V** | **T** | **E** | **L** | **P** | **Y** | **M** | **V** | **N** | **K** | **A** | **K** | **L** | **I** | **E** | **R** | **I** | **S** | **E** | **L** | **H** | **R** | **D** | **K** | **R** | **I** | **E** | **G** | **I** | **T** | **D** | **L** | **R** | **D** | **E** | **S** | **S** | **R** | **E** | **G** | **M** | **R** | **I** | **V** | **I** | **D** | **V** | **R** | **R** | **D** | **A** | **S** | **A** | **S** | **V** | **I** | **L** | **N** | **N** | **L** | **Y** | **K** | **L** | **T** | **S** | **L** | **Q** | **N** | **S** | **F** | **G** | **F** | **N** | **M** | **L** | **A** | **I** | **E** | **K** | **G** | **V** | **P** | **K** | **V** | **L** | **S** | **L** | **K** | **Q** | **I** | **L** | **E** | **N** | **Y** | **I** | **E** | **H** | **Q** | **R** | **E** | **V** | **I** | **T** | **R** | **R** | **T** | **V** | **F** | **E** | **K** | **E** | **K** | **A** | **E** | **A** | **R** | **A** | **H** | **I** | **L** | **E** | **G** | **L** | **R** | **I** | **A** | **L** | **D** | **H** | **I** | **D** | **E** | **I** | **I** | **A** | **I** | **I** | **R** | **G** | **S** | **K** | **S** | **D** | **D** | **E** | **A** | **K** | **A** | **T** | **M** | **I** | **E** | **R** | **F** | **D** | **L** | **S** | **D** | **R** | **Q** | **S** | **Q** | **A** | **I** | **L** | **D** | **M** | **R** | **L** | **R** | **R** | **L** | **T** | **G** | **L** | **E** | **R** | **E** | **K** | **I** | **E** | **N** | **E** | **Y** | **Q** | **E** | **L** | **L** | **K** | **L** | **I** | **E** | **D** | **L** | **T** | **D** | **I** | **L** | **A** | **R** | **P** | **E** | **R** | **V** | **T** | **E** | **I** | **I** | **K** | **N** | **E** | **L** | **A** | **D** | **L** | **N** | **Q** | **R** | **F** | **G** | **D** | **K** | **R** | **R** | **T** | **E** | **L** | **L** | **V** | **G** | **E** | **V** | **L** | **S** | **L** | **E** | **D** | **E** | **D** | **L** | **I** | **E** | **E** | **E** | **E** | **I** | **V** | **I** | **T** | **L** | **T** | **N** | **N** | **G** | **Y** | **I** | **K** | **R** | **L** | **A** | **N** | **N** | **E** | **F** | **R** | **A** | **Q** | **R** | **R** | **G** | **G** | **R** | **G** | **V** | **Q** | **G** | **M** | **G** | **I** | **H** | **N** | **D** | **D** | **F** | **V** | **K** | **N** | **L** | **V** | **S** | **C** | **S** | **T** | **H** | **D** | **T** | **L** | **L** | **F** | **F** | **T** | **N** | **N** | **G** | **K** | **V** | **Y** | **R** | **A** | **K** | **G** | **Y** | **E** | **I** | **P** | **E** | **Y** | **G** | **R** | **T** | **A** | **K** | **G** | **I** | **P** | **I** | **I** | **N** | **L** | **L** | **G** | **I** | **D** | **S** | **S** | **E** | **S** | **I** | **Q** | **A** | **I** | **I** | **A** | **V** | **T** | **G** | **E** | **A** | **E** | **E** | **G** | **H** | **Y** | **L** | **F** | **F** | **T** | **T** | **R** | **Q** | **G** | **T** | **V** | **K** | **R** | **T** | **S** | **V** | **T** | **A** | **F** | **A** | **N** | **I** | **R** | **S** | **N** | **G** | **L** | **I** | **A** | **I** | **G** | **L** | **K** | **E** | **D** | **D** | **E** | **L** | **I** | **N** | **V** | **L** | **L** | **T** | **D** | **G** | **Q** | **S** | **N** | **I** | **I** | **I** | **G** | **T** | **H** | **N** | **G** | **Y** | **S** | **V** | **T** | **F** | **K** | **E** | **E** | **A** | **V** | **R** | **D** | **M** | **G** | **R** | **T** | **A** | **S** | **G** | **V** | **R** | **G** | **I** | **R** | **L** | **R** | **E** | **E** | **D** | **C** | **V** | **V** | **G** | **A** | **A** | **L** | **M** | **K** | **E** | **D** | **Q** | **E** | **V** | **L** | **V** | **I** | **T** | **E** | **K** | **G** | **Y** | **G** | **K** | **R** | **T** | **K** | **V** | **S** | **E** | **Y** | **P** | **V** | **K** | **G** | **R** | **G** | **G** | **K** | **G** | **I** | **K** | **T** | **A** | **N** | **I** | **T** | **E** | **K** | **N** | **G** | **P** | **L** | **A** | **G** | **L** | **T** | **T** | **V** | **S** | **G** | **N** | **E** | **D** | **I** | **M** | **L** | **I** | **T** | **D** | **K** | **G** | **V** | **I** | **I** | **R** | **F** | **N** | **V** | **S** | **T** | **V** | **S** | **Q** | **T** | **G** | **R** | **S** | **T** | **L** | **G** | **V** | **R** | **L** | **M** | **K** | **M** | **E** | **A** | **D** | **T** | **K** | **V** | **V** | **T** | **M** | **-** | **-** | **-** | **-** | **-** | **-** | **-** | **-** | **-** | **-** | **-** | **-** | **-** | **-** | **-** | **-** | **-** | **-** | **-** |
| **N55265** | **M** | **S** | **E** | **E** | **I** | **K** | **E** | **N** | **I** | **Q** | **D** | **V** | **N** | **L** | **T** | **S** | **E** | **M** | **K** | **E** | **S** | **F** | **I** | **D** | **Y** | **A** | **M** | **S** | **V** | **I** | **V** | **A** | **R** | **A** | **L** | **P** | **D** | **V** | **R** | **D** | **G** | **L** | **K** | **P** | **V** | **H** | **R** | **R** | **I** | **L** | **Y** | **G** | **M** | **N** | **E** | **L** | **G** | **V** | **T** | **P** | **D** | **K** | **P** | **H** | **K** | **K** | **S** | **A** | **R** | **I** | **V** | **G** | **D** | **V** | **M** | **G** | **K** | **Y** | **H** | **P** | **H** | **G** | **D** | **S** | **A** | **I** | **Y** | **E** | **S** | **M** | **V** | **R** | **M** | **A** | **Q** | **P** | **F** | **S** | **Y** | **R** | **A** | **M** | **L** | **V** | **D** | **G** | **H** | **G** | **N** | **F** | **G** | **S** | **V** | **D** | **G** | **D** | **G** | **A** | **A** | **A** | **M** | **R** | **Y** | **T** | **E** | **A** | **R** | **M** | **S** | **K** | **I** | **A** | **L** | **E** | **M** | **L** | **R** | **D** | **I** | **N** | **K** | **N** | **T** | **V** | **D** | **F** | **Q** | **G** | **N** | **Y** | **D** | **D** | **S** | **E** | **Q** | **E** | **P** | **V** | **V** | **L** | **P** | **A** | **R** | **F** | **P** | **N** | **L** | **L** | **V** | **N** | **G** | **T** | **T** | **G** | **I** | **A** | **V** | **G** | **M** | **A** | **T** | **N** | **I** | **P** | **P** | **H** | **N** | **L** | **S** | **E** | **V** | **I** | **D** | **A** | **T** | **S** | **L** | **L** | **M** | **D** | **N** | **P** | **D** | **V** | **T** | **T** | **N** | **E** | **L** | **M** | **E** | **V** | **L** | **P** | **G** | **P** | **D** | **F** | **P** | **T** | **G** | **G** | **L** | **V** | **M** | **G** | **K** | **S** | **G** | **I** | **R** | **R** | **A** | **Y** | **E** | **T** | **G** | **K** | **G** | **S** | **I** | **T** | **V** | **R** | **A** | **K** | **V** | **E** | **L** | **T** | **E** | **M** | **P** | **N** | **G** | **K** | **E** | **R** | **I** | **L** | **V** | **T** | **E** | **L** | **P** | **Y** | **M** | **V** | **N** | **K** | **A** | **K** | **L** | **I** | **E** | **R** | **I** | **S** | **E** | **L** | **H** | **R** | **D** | **K** | **R** | **I** | **E** | **G** | **I** | **T** | **D** | **L** | **R** | **D** | **E** | **S** | **S** | **R** | **E** | **G** | **M** | **R** | **I** | **V** | **I** | **D** | **V** | **R** | **R** | **D** | **V** | **S** | **A** | **S** | **V** | **V** | **L** | **N** | **N** | **L** | **Y** | **K** | **M** | **T** | **A** | **L** | **Q** | **T** | **S** | **F** | **G** | **F** | **N** | **M** | **L** | **A** | **I** | **E** | **K** | **G** | **V** | **P** | **K** | **I** | **L** | **S** | **L** | **K** | **R** | **I** | **L** | **E** | **N** | **Y** | **I** | **E** | **H** | **Q** | **K** | **E** | **V** | **I** | **T** | **R** | **R** | **T** | **I** | **F** | **D** | **K** | **N** | **K** | **A** | **E** | **A** | **R** | **A** | **H** | **I** | **L** | **E** | **G** | **L** | **R** | **I** | **A** | **L** | **D** | **H** | **I** | **D** | **E** | **I** | **I** | **A** | **I** | **I** | **R** | **G** | **S** | **Q** | **S** | **D** | **D** | **E** | **A** | **K** | **A** | **T** | **L** | **I** | **E** | **R** | **F** | **E** | **F** | **S** | **D** | **R** | **Q** | **A** | **Q** | **A** | **I** | **L** | **D** | **M** | **R** | **L** | **R** | **R** | **L** | **T** | **G** | **L** | **E** | **R** | **D** | **K** | **I** | **E** | **N** | **E** | **Y** | **Q** | **E** | **L** | **L** | **K** | **F** | **I** | **A** | **D** | **L** | **E** | **D** | **I** | **L** | **A** | **R** | **P** | **E** | **R** | **V** | **I** | **E** | **I** | **I** | **K** | **T** | **E** | **L** | **N** | **D** | **V** | **R** | **T** | **K** | **F** | **G** | **D** | **A** | **R** | **R** | **T** | **E** | **L** | **L** | **V** | **G** | **E** | **V** | **L** | **S** | **L** | **E** | **D** | **E** | **D** | **L** | **I** | **E** | **E** | **E** | **E** | **V** | **V** | **I** | **T** | **L** | **T** | **N** | **N** | **G** | **Y** | **I** | **K** | **R** | **M** | **A** | **N** | **S** | **E** | **F** | **R** | **A** | **Q** | **R** | **R** | **G** | **G** | **R** | **G** | **V** | **Q** | **G** | **M** | **G** | **V** | **H** | **D** | **D** | **D** | **F** | **V** | **K** | **N** | **L** | **V** | **S** | **C** | **S** | **T** | **H** | **D** | **T** | **L** | **L** | **F** | **F** | **T** | **N** | **T** | **G** | **K** | **V** | **Y** | **R** | **A** | **K** | **G** | **Y** | **E** | **I** | **P** | **E** | **Y** | **G** | **R** | **T** | **A** | **K** | **G** | **I** | **P** | **V** | **I** | **N** | **L** | **L** | **G** | **I** | **D** | **S** | **A** | **E** | **K** | **I** | **Q** | **A** | **I** | **I** | **S** | **V** | **E** | **G** | **K** | **A** | **E** | **A** | **G** | **K** | **Y** | **L** | **F** | **F** | **T** | **T** | **L** | **K** | **G** | **T** | **V** | **K** | **R** | **T** | **A** | **V** | **T** | **A** | **F** | **S** | **N** | **I** | **R** | **S** | **N** | **G** | **L** | **I** | **A** | **I** | **S** | **L** | **K** | **E** | **D** | **D** | **E** | **L** | **V** | **N** | **V** | **V** | **T** | **T** | **N** | **G** | **N** | **Q** | **K** | **M** | **I** | **I** | **G** | **T** | **H** | **A** | **G** | **Y** | **S** | **V** | **T** | **F** | **D** | **E** | **T** | **T** | **V** | **R** | **D** | **M** | **G** | **R** | **T** | **A** | **S** | **G** | **V** | **R** | **G** | **I** | **R** | **L** | **R** | **E** | **N** | **D** | **Y** | **V** | **V** | **G** | **A** | **A** | **I** | **L** | **D** | **E** | **N** | **K** | **E** | **V** | **L** | **V** | **I** | **T** | **E** | **N** | **G** | **Y** | **G** | **K** | **R** | **T** | **K** | **A** | **S** | **E** | **Y** | **P** | **V** | **K** | **G** | **R** | **G** | **G** | **K** | **G** | **I** | **K** | **T** | **A** | **N** | **I** | **T** | **E** | **K** | **N** | **G** | **P** | **L** | **A** | **G** | **L** | **T** | **T** | **V** | **N** | **G** | **D** | **E** | **D** | **I** | **L** | **L** | **I** | **T** | **N** | **K** | **G** | **V** | **I** | **I** | **R** | **F** | **N** | **V** | **D** | **S** | **V** | **S** | **Q** | **T** | **G** | **R** | **A** | **T** | **L** | **G** | **V** | **R** | **L** | **M** | **R** | **M** | **E** | **D** | **G** | **A** | **K** | **-** | **-** | **-** | **-** | **-** | **-** | **-** | **-** | **-** | **-** | **-** | **-** | **-** | **-** | **-** | **-** | **-** | **-** | **-** | **-** | **-** | **-** | **-** |
| **N59860F** | **M** | **S** | **E** | **E** | **I** | **K** | **E** | **N** | **I** | **Q** | **D** | **V** | **N** | **L** | **T** | **S** | **E** | **M** | **K** | **E** | **S** | **F** | **I** | **D** | **Y** | **A** | **M** | **S** | **V** | **I** | **V** | **A** | **R** | **A** | **L** | **P** | **D** | **V** | **R** | **D** | **G** | **L** | **K** | **P** | **V** | **H** | **R** | **R** | **I** | **L** | **Y** | **G** | **M** | **N** | **E** | **L** | **G** | **V** | **T** | **P** | **D** | **K** | **P** | **H** | **K** | **K** | **S** | **A** | **R** | **I** | **V** | **G** | **D** | **V** | **M** | **G** | **K** | **Y** | **H** | **P** | **H** | **G** | **D** | **S** | **A** | **I** | **Y** | **E** | **S** | **M** | **V** | **R** | **M** | **A** | **Q** | **P** | **F** | **S** | **Y** | **R** | **A** | **M** | **L** | **V** | **D** | **G** | **H** | **G** | **N** | **F** | **G** | **S** | **V** | **D** | **G** | **D** | **G** | **A** | **A** | **A** | **M** | **R** | **Y** | **T** | **E** | **A** | **R** | **M** | **S** | **K** | **I** | **A** | **L** | **E** | **M** | **L** | **R** | **D** | **I** | **N** | **K** | **N** | **T** | **V** | **D** | **F** | **Q** | **G** | **N** | **Y** | **D** | **D** | **S** | **E** | **Q** | **E** | **P** | **V** | **V** | **L** | **P** | **A** | **R** | **F** | **P** | **N** | **L** | **L** | **V** | **N** | **G** | **T** | **T** | **G** | **I** | **A** | **V** | **G** | **M** | **A** | **T** | **N** | **I** | **P** | **P** | **H** | **N** | **L** | **S** | **E** | **V** | **I** | **D** | **A** | **T** | **S** | **L** | **L** | **M** | **D** | **N** | **P** | **D** | **V** | **T** | **T** | **N** | **E** | **L** | **M** | **E** | **V** | **L** | **P** | **G** | **P** | **D** | **F** | **P** | **T** | **G** | **G** | **L** | **V** | **M** | **G** | **K** | **S** | **G** | **I** | **R** | **R** | **A** | **Y** | **E** | **T** | **G** | **K** | **G** | **S** | **I** | **T** | **V** | **R** | **A** | **K** | **V** | **E** | **L** | **T** | **E** | **M** | **P** | **N** | **G** | **K** | **E** | **R** | **I** | **L** | **V** | **T** | **E** | **L** | **P** | **Y** | **M** | **V** | **N** | **K** | **A** | **K** | **L** | **I** | **E** | **R** | **I** | **S** | **E** | **L** | **H** | **R** | **D** | **K** | **R** | **I** | **E** | **G** | **I** | **T** | **D** | **L** | **R** | **D** | **E** | **S** | **S** | **R** | **E** | **G** | **M** | **R** | **I** | **V** | **I** | **D** | **V** | **R** | **R** | **D** | **V** | **S** | **A** | **S** | **V** | **V** | **L** | **N** | **N** | **L** | **Y** | **K** | **M** | **T** | **A** | **L** | **Q** | **T** | **S** | **F** | **G** | **F** | **N** | **M** | **L** | **A** | **I** | **E** | **K** | **G** | **V** | **P** | **K** | **I** | **L** | **S** | **L** | **K** | **R** | **I** | **L** | **E** | **N** | **Y** | **V** | **E** | **H** | **Q** | **K** | **E** | **V** | **I** | **T** | **R** | **R** | **T** | **I** | **F** | **D** | **K** | **N** | **K** | **A** | **E** | **A** | **R** | **A** | **H** | **I** | **L** | **E** | **G** | **L** | **R** | **I** | **A** | **L** | **D** | **H** | **I** | **D** | **E** | **I** | **I** | **A** | **I** | **I** | **R** | **G** | **S** | **Q** | **S** | **D** | **D** | **E** | **A** | **K** | **A** | **T** | **L** | **I** | **E** | **R** | **F** | **E** | **F** | **S** | **D** | **R** | **Q** | **A** | **Q** | **A** | **I** | **L** | **D** | **M** | **R** | **L** | **R** | **R** | **L** | **T** | **G** | **L** | **E** | **R** | **D** | **K** | **I** | **E** | **N** | **E** | **Y** | **Q** | **E** | **L** | **L** | **K** | **F** | **I** | **A** | **D** | **L** | **E** | **D** | **I** | **L** | **A** | **R** | **P** | **E** | **R** | **V** | **I** | **E** | **I** | **I** | **K** | **T** | **E** | **L** | **N** | **D** | **V** | **R** | **T** | **K** | **F** | **G** | **D** | **A** | **R** | **R** | **T** | **E** | **L** | **L** | **V** | **G** | **E** | **V** | **L** | **S** | **L** | **E** | **D** | **E** | **D** | **L** | **I** | **E** | **E** | **E** | **E** | **V** | **V** | **I** | **T** | **L** | **T** | **N** | **N** | **G** | **Y** | **I** | **K** | **R** | **M** | **A** | **N** | **S** | **E** | **F** | **R** | **A** | **Q** | **R** | **R** | **G** | **G** | **R** | **G** | **V** | **Q** | **G** | **M** | **G** | **V** | **H** | **D** | **D** | **D** | **F** | **V** | **K** | **N** | **L** | **V** | **S** | **C** | **S** | **T** | **H** | **D** | **T** | **L** | **L** | **F** | **F** | **T** | **N** | **T** | **G** | **K** | **V** | **Y** | **R** | **A** | **K** | **G** | **Y** | **E** | **I** | **P** | **E** | **Y** | **G** | **R** | **T** | **A** | **K** | **G** | **I** | **P** | **V** | **I** | **N** | **L** | **L** | **G** | **I** | **D** | **S** | **A** | **E** | **K** | **I** | **Q** | **A** | **I** | **I** | **S** | **V** | **E** | **G** | **K** | **A** | **E** | **A** | **G** | **K** | **Y** | **L** | **F** | **F** | **T** | **T** | **L** | **K** | **G** | **T** | **V** | **K** | **R** | **T** | **A** | **V** | **T** | **A** | **F** | **S** | **N** | **I** | **R** | **S** | **N** | **G** | **L** | **I** | **A** | **I** | **S** | **L** | **K** | **E** | **D** | **D** | **E** | **L** | **V** | **N** | **V** | **V** | **T** | **T** | **N** | **G** | **N** | **Q** | **K** | **M** | **I** | **I** | **G** | **T** | **H** | **A** | **G** | **Y** | **S** | **V** | **T** | **F** | **D** | **E** | **N** | **T** | **V** | **R** | **D** | **M** | **G** | **R** | **T** | **A** | **S** | **G** | **V** | **R** | **G** | **I** | **R** | **L** | **R** | **E** | **N** | **D** | **Y** | **V** | **V** | **G** | **A** | **A** | **I** | **L** | **D** | **E** | **N** | **K** | **E** | **V** | **L** | **V** | **I** | **T** | **E** | **N** | **G** | **Y** | **G** | **K** | **R** | **T** | **K** | **A** | **S** | **E** | **Y** | **P** | **V** | **K** | **G** | **R** | **G** | **G** | **K** | **G** | **I** | **K** | **T** | **A** | **N** | **I** | **T** | **E** | **K** | **N** | **G** | **P** | **L** | **A** | **G** | **L** | **T** | **T** | **V** | **N** | **G** | **D** | **E** | **D** | **I** | **L** | **L** | **I** | **T** | **N** | **K** | **G** | **V** | **I** | **I** | **R** | **F** | **N** | **V** | **D** | **S** | **V** | **S** | **Q** | **T** | **G** | **R** | **A** | **T** | **L** | **G** | **V** | **R** | **L** | **M** | **R** | **M** | **E** | **D** | **G** | **A** | **K** | **-** | **-** | **-** | **-** | **-** | **-** | **-** | **-** | **-** | **-** | **-** | **-** | **-** | **-** | **-** | **-** | **-** | **-** | **-** | **-** | **-** | **-** | **-** |
| **N53418** | **M** | **S** | **E** | **E** | **I** | **K** | **E** | **N** | **I** | **Q** | **D** | **V** | **N** | **L** | **T** | **S** | **E** | **M** | **K** | **E** | **S** | **F** | **I** | **D** | **Y** | **A** | **M** | **S** | **V** | **I** | **V** | **A** | **R** | **A** | **L** | **P** | **D** | **V** | **R** | **D** | **G** | **L** | **K** | **P** | **V** | **H** | **R** | **R** | **I** | **L** | **Y** | **G** | **M** | **N** | **E** | **L** | **G** | **V** | **T** | **P** | **D** | **K** | **P** | **H** | **K** | **K** | **S** | **A** | **R** | **I** | **V** | **G** | **D** | **V** | **M** | **G** | **K** | **Y** | **H** | **P** | **H** | **G** | **D** | **S** | **A** | **I** | **Y** | **E** | **S** | **M** | **V** | **R** | **M** | **A** | **Q** | **P** | **F** | **S** | **Y** | **R** | **A** | **M** | **L** | **V** | **D** | **G** | **H** | **G** | **N** | **F** | **G** | **S** | **V** | **D** | **G** | **D** | **G** | **A** | **A** | **A** | **M** | **R** | **Y** | **T** | **E** | **A** | **R** | **M** | **S** | **K** | **I** | **A** | **L** | **E** | **M** | **L** | **R** | **D** | **I** | **N** | **K** | **N** | **T** | **V** | **D** | **F** | **Q** | **G** | **N** | **Y** | **D** | **D** | **S** | **E** | **Q** | **E** | **P** | **V** | **V** | **L** | **P** | **A** | **R** | **F** | **P** | **N** | **L** | **L** | **V** | **N** | **G** | **T** | **T** | **G** | **I** | **A** | **V** | **G** | **M** | **A** | **T** | **N** | **I** | **P** | **P** | **H** | **N** | **L** | **S** | **E** | **V** | **I** | **D** | **A** | **T** | **S** | **L** | **L** | **M** | **D** | **N** | **P** | **D** | **V** | **T** | **T** | **N** | **E** | **L** | **M** | **E** | **V** | **L** | **P** | **G** | **P** | **D** | **F** | **P** | **T** | **G** | **G** | **L** | **V** | **M** | **G** | **K** | **S** | **G** | **I** | **R** | **R** | **A** | **Y** | **E** | **T** | **G** | **K** | **G** | **S** | **I** | **T** | **V** | **R** | **A** | **K** | **V** | **E** | **L** | **T** | **E** | **M** | **P** | **N** | **G** | **K** | **E** | **R** | **I** | **L** | **V** | **T** | **E** | **L** | **P** | **Y** | **M** | **V** | **N** | **K** | **A** | **K** | **L** | **I** | **E** | **R** | **I** | **S** | **E** | **L** | **H** | **R** | **D** | **K** | **R** | **I** | **E** | **G** | **I** | **T** | **D** | **L** | **R** | **D** | **E** | **S** | **S** | **R** | **E** | **G** | **M** | **R** | **I** | **V** | **I** | **D** | **V** | **R** | **R** | **D** | **V** | **S** | **A** | **S** | **V** | **V** | **L** | **N** | **N** | **L** | **Y** | **K** | **M** | **T** | **A** | **L** | **Q** | **T** | **S** | **F** | **G** | **F** | **N** | **M** | **L** | **A** | **I** | **E** | **K** | **G** | **V** | **P** | **K** | **I** | **L** | **S** | **L** | **K** | **R** | **I** | **L** | **E** | **N** | **Y** | **V** | **E** | **H** | **Q** | **K** | **E** | **V** | **I** | **T** | **R** | **R** | **T** | **I** | **F** | **D** | **K** | **N** | **K** | **A** | **E** | **A** | **R** | **A** | **H** | **I** | **L** | **E** | **G** | **L** | **R** | **I** | **A** | **L** | **D** | **H** | **I** | **D** | **E** | **I** | **I** | **A** | **I** | **I** | **R** | **G** | **S** | **Q** | **S** | **D** | **D** | **E** | **A** | **K** | **A** | **T** | **L** | **I** | **E** | **R** | **F** | **E** | **F** | **S** | **D** | **R** | **Q** | **A** | **Q** | **A** | **I** | **L** | **D** | **M** | **R** | **L** | **R** | **R** | **L** | **T** | **G** | **L** | **E** | **R** | **D** | **K** | **I** | **E** | **N** | **E** | **Y** | **Q** | **E** | **L** | **L** | **K** | **F** | **I** | **A** | **D** | **L** | **E** | **D** | **I** | **L** | **A** | **R** | **P** | **E** | **R** | **V** | **I** | **E** | **I** | **I** | **K** | **T** | **E** | **L** | **N** | **D** | **V** | **R** | **T** | **K** | **F** | **G** | **D** | **A** | **R** | **R** | **T** | **E** | **L** | **L** | **V** | **G** | **E** | **V** | **L** | **S** | **L** | **E** | **D** | **E** | **D** | **L** | **I** | **E** | **E** | **E** | **E** | **V** | **V** | **I** | **T** | **L** | **T** | **N** | **N** | **G** | **Y** | **I** | **K** | **R** | **M** | **A** | **N** | **S** | **E** | **F** | **R** | **A** | **Q** | **R** | **R** | **G** | **G** | **R** | **G** | **V** | **Q** | **G** | **M** | **G** | **V** | **H** | **D** | **D** | **D** | **F** | **V** | **K** | **N** | **L** | **V** | **S** | **C** | **S** | **T** | **H** | **D** | **T** | **L** | **L** | **F** | **F** | **T** | **N** | **T** | **G** | **K** | **V** | **Y** | **R** | **A** | **K** | **G** | **Y** | **E** | **I** | **P** | **E** | **Y** | **G** | **R** | **T** | **A** | **K** | **G** | **I** | **P** | **V** | **I** | **N** | **L** | **L** | **G** | **I** | **D** | **S** | **A** | **E** | **K** | **I** | **Q** | **A** | **I** | **I** | **S** | **V** | **E** | **G** | **K** | **A** | **E** | **A** | **G** | **K** | **Y** | **L** | **F** | **F** | **T** | **T** | **L** | **K** | **G** | **T** | **V** | **K** | **R** | **T** | **A** | **V** | **T** | **A** | **F** | **S** | **N** | **I** | **R** | **S** | **N** | **G** | **L** | **I** | **A** | **I** | **S** | **L** | **K** | **E** | **D** | **D** | **E** | **L** | **V** | **N** | **V** | **V** | **T** | **T** | **N** | **G** | **N** | **Q** | **K** | **M** | **I** | **I** | **G** | **T** | **H** | **A** | **G** | **Y** | **S** | **V** | **T** | **F** | **D** | **E** | **N** | **T** | **V** | **R** | **D** | **M** | **G** | **R** | **T** | **A** | **S** | **G** | **V** | **R** | **G** | **I** | **R** | **L** | **R** | **E** | **N** | **D** | **Y** | **V** | **V** | **G** | **A** | **A** | **I** | **L** | **D** | **E** | **N** | **K** | **E** | **V** | **L** | **V** | **I** | **T** | **E** | **N** | **G** | **Y** | **G** | **K** | **R** | **T** | **K** | **A** | **S** | **E** | **Y** | **P** | **V** | **K** | **G** | **R** | **G** | **G** | **K** | **G** | **I** | **K** | **T** | **A** | **N** | **I** | **T** | **E** | **K** | **N** | **G** | **P** | **L** | **A** | **G** | **L** | **T** | **T** | **V** | **N** | **G** | **D** | **E** | **D** | **I** | **L** | **L** | **I** | **T** | **N** | **K** | **G** | **V** | **I** | **I** | **R** | **F** | **N** | **V** | **D** | **S** | **V** | **S** | **Q** | **T** | **G** | **R** | **A** | **T** | **L** | **G** | **V** | **R** | **L** | **M** | **R** | **M** | **E** | **D** | **G** | **A** | **K** | **-** | **-** | **-** | **-** | **-** | **-** | **-** | **-** | **-** | **-** | **-** | **-** | **-** | **-** | **-** | **-** | **-** | **-** | **-** | **-** | **-** | **-** | **-** |
| **N60519F** | **M** | **S** | **E** | **E** | **I** | **R** | **E** | **N** | **I** | **H** | **D** | **V** | **N** | **L** | **T** | **S** | **E** | **M** | **K** | **D** | **S** | **F** | **I** | **D** | **Y** | **A** | **M** | **S** | **V** | **I** | **V** | **A** | **R** | **A** | **L** | **P** | **D** | **V** | **R** | **D** | **G** | **L** | **K** | **P** | **V** | **H** | **R** | **R** | **I** | **L** | **Y** | **G** | **M** | **N** | **E** | **L** | **G** | **V** | **T** | **P** | **D** | **K** | **A** | **H** | **K** | **K** | **S** | **A** | **R** | **I** | **V** | **G** | **D** | **V** | **M** | **G** | **K** | **Y** | **H** | **P** | **H** | **G** | **D** | **S** | **A** | **I** | **Y** | **E** | **S** | **M** | **V** | **R** | **M** | **A** | **Q** | **P** | **F** | **S** | **Y** | **R** | **Y** | **M** | **L** | **V** | **D** | **G** | **H** | **G** | **N** | **F** | **G** | **S** | **V** | **D** | **G** | **D** | **G** | **A** | **A** | **A** | **M** | **R** | **Y** | **T** | **E** | **A** | **R** | **M** | **S** | **K** | **I** | **A** | **T** | **E** | **M** | **L** | **R** | **D** | **I** | **N** | **K** | **N** | **T** | **V** | **D** | **F** | **Q** | **S** | **N** | **Y** | **D** | **D** | **T** | **E** | **K** | **E** | **P** | **V** | **V** | **L** | **P** | **A** | **R** | **F** | **P** | **N** | **L** | **L** | **V** | **N** | **G** | **T** | **T** | **G** | **I** | **A** | **V** | **G** | **M** | **A** | **T** | **N** | **I** | **P** | **P** | **H** | **N** | **L** | **S** | **E** | **V** | **V** | **A** | **A** | **I** | **D** | **L** | **L** | **M** | **D** | **N** | **P** | **D** | **V** | **T** | **T** | **N** | **E** | **L** | **M** | **E** | **V** | **L** | **P** | **G** | **P** | **D** | **F** | **P** | **T** | **G** | **G** | **L** | **V** | **M** | **G** | **K** | **S** | **G** | **I** | **R** | **R** | **A** | **Y** | **E** | **T** | **G** | **K** | **G** | **S** | **I** | **T** | **V** | **R** | **A** | **K** | **V** | **E** | **I** | **T** | **E** | **M** | **P** | **N** | **G** | **K** | **E** | **R** | **I** | **I** | **V** | **T** | **E** | **L** | **P** | **Y** | **M** | **V** | **N** | **K** | **A** | **K** | **L** | **I** | **E** | **R** | **I** | **S** | **E** | **L** | **H** | **R** | **D** | **K** | **R** | **I** | **E** | **G** | **I** | **T** | **D** | **L** | **R** | **D** | **E** | **S** | **S** | **R** | **E** | **G** | **M** | **R** | **I** | **V** | **I** | **D** | **I** | **R** | **R** | **D** | **A** | **S** | **A** | **S** | **V** | **I** | **L** | **N** | **N** | **L** | **Y** | **K** | **L** | **T** | **S** | **L** | **Q** | **N** | **S** | **F** | **G** | **F** | **N** | **M** | **L** | **A** | **I** | **E** | **K** | **G** | **V** | **P** | **K** | **V** | **L** | **S** | **L** | **K** | **Q** | **I** | **L** | **E** | **N** | **Y** | **I** | **E** | **H** | **Q** | **R** | **E** | **V** | **I** | **T** | **R** | **R** | **T** | **V** | **F** | **E** | **K** | **E** | **K** | **A** | **E** | **A** | **R** | **A** | **H** | **I** | **L** | **E** | **G** | **L** | **R** | **I** | **A** | **L** | **D** | **H** | **I** | **D** | **E** | **I** | **I** | **A** | **I** | **I** | **R** | **G** | **S** | **K** | **S** | **D** | **D** | **E** | **A** | **K** | **A** | **T** | **M** | **I** | **E** | **R** | **F** | **D** | **L** | **S** | **D** | **R** | **Q** | **S** | **Q** | **A** | **I** | **L** | **D** | **M** | **R** | **L** | **R** | **R** | **L** | **T** | **G** | **L** | **E** | **R** | **E** | **K** | **I** | **E** | **N** | **E** | **Y** | **Q** | **E** | **L** | **L** | **K** | **L** | **I** | **E** | **D** | **L** | **T** | **D** | **I** | **L** | **A** | **R** | **P** | **E** | **R** | **V** | **T** | **E** | **I** | **I** | **K** | **N** | **E** | **L** | **A** | **D** | **L** | **N** | **Q** | **R** | **F** | **G** | **D** | **K** | **R** | **R** | **T** | **E** | **L** | **L** | **V** | **G** | **E** | **V** | **L** | **S** | **L** | **E** | **D** | **E** | **D** | **L** | **I** | **E** | **E** | **E** | **E** | **I** | **V** | **I** | **T** | **L** | **T** | **N** | **N** | **G** | **Y** | **I** | **K** | **R** | **L** | **A** | **N** | **N** | **E** | **F** | **R** | **A** | **Q** | **R** | **R** | **G** | **G** | **R** | **G** | **V** | **Q** | **G** | **M** | **G** | **I** | **H** | **N** | **D** | **D** | **F** | **V** | **K** | **N** | **L** | **V** | **S** | **C** | **S** | **T** | **H** | **D** | **T** | **L** | **L** | **F** | **F** | **T** | **N** | **N** | **G** | **K** | **V** | **Y** | **R** | **A** | **K** | **G** | **Y** | **E** | **I** | **P** | **E** | **Y** | **G** | **R** | **T** | **A** | **K** | **G** | **I** | **P** | **I** | **I** | **N** | **L** | **L** | **G** | **I** | **D** | **S** | **S** | **E** | **S** | **I** | **Q** | **A** | **I** | **I** | **A** | **V** | **T** | **G** | **E** | **A** | **E** | **E** | **G** | **H** | **Y** | **L** | **F** | **F** | **T** | **T** | **R** | **Q** | **G** | **T** | **V** | **K** | **R** | **T** | **S** | **V** | **T** | **A** | **F** | **A** | **N** | **I** | **R** | **S** | **N** | **G** | **L** | **I** | **A** | **I** | **G** | **L** | **K** | **E** | **D** | **D** | **E** | **L** | **V** | **N** | **V** | **L** | **L** | **T** | **D** | **G** | **Q** | **S** | **N** | **I** | **I** | **I** | **G** | **T** | **H** | **N** | **G** | **Y** | **S** | **V** | **T** | **F** | **K** | **E** | **E** | **A** | **V** | **R** | **D** | **M** | **G** | **R** | **T** | **A** | **S** | **G** | **V** | **R** | **G** | **I** | **R** | **L** | **R** | **E** | **E** | **D** | **Y** | **V** | **V** | **G** | **A** | **A** | **L** | **M** | **K** | **E** | **D** | **Q** | **E** | **V** | **L** | **V** | **I** | **T** | **E** | **K** | **G** | **Y** | **G** | **K** | **R** | **T** | **K** | **V** | **S** | **E** | **Y** | **P** | **V** | **K** | **G** | **R** | **G** | **G** | **K** | **G** | **I** | **K** | **T** | **A** | **N** | **I** | **T** | **E** | **K** | **N** | **G** | **P** | **L** | **A** | **G** | **L** | **T** | **T** | **V** | **S** | **G** | **D** | **E** | **D** | **I** | **M** | **L** | **I** | **T** | **D** | **K** | **G** | **V** | **I** | **I** | **R** | **F** | **N** | **V** | **S** | **T** | **V** | **S** | **Q** | **T** | **G** | **R** | **S** | **T** | **L** | **G** | **V** | **R** | **L** | **M** | **K** | **M** | **E** | **A** | **D** | **T** | **K** | **V** | **V** | **T** | **M** | **A** | **A** | **V** | **E** | **P** | **E** | **A** | **A** | **E** | **Q** | **T** | **V** | **E** | **E** | **Q** | **G** | **S** | **E** | **E** |
| **N55338** | **M** | **S** | **E** | **E** | **I** | **K** | **E** | **N** | **I** | **Q** | **D** | **V** | **N** | **L** | **T** | **S** | **E** | **M** | **K** | **E** | **S** | **F** | **I** | **D** | **Y** | **A** | **M** | **S** | **V** | **I** | **V** | **A** | **R** | **A** | **L** | **P** | **D** | **V** | **R** | **D** | **G** | **L** | **K** | **P** | **V** | **H** | **R** | **R** | **I** | **L** | **Y** | **G** | **M** | **N** | **E** | **L** | **G** | **V** | **T** | **P** | **D** | **K** | **P** | **H** | **K** | **K** | **S** | **A** | **R** | **I** | **V** | **G** | **D** | **V** | **M** | **G** | **K** | **Y** | **H** | **P** | **H** | **G** | **D** | **S** | **A** | **I** | **Y** | **E** | **S** | **M** | **V** | **R** | **M** | **A** | **Q** | **P** | **F** | **S** | **Y** | **R** | **A** | **M** | **L** | **V** | **D** | **G** | **H** | **G** | **N** | **F** | **G** | **S** | **V** | **D** | **G** | **D** | **G** | **A** | **A** | **A** | **M** | **R** | **Y** | **T** | **E** | **A** | **R** | **M** | **S** | **K** | **I** | **A** | **L** | **E** | **M** | **L** | **R** | **D** | **I** | **N** | **K** | **N** | **T** | **V** | **D** | **F** | **Q** | **G** | **N** | **Y** | **D** | **D** | **S** | **E** | **Q** | **E** | **P** | **V** | **V** | **L** | **P** | **A** | **R** | **F** | **P** | **N** | **L** | **L** | **V** | **N** | **G** | **T** | **T** | **G** | **I** | **A** | **V** | **G** | **M** | **A** | **T** | **N** | **I** | **P** | **P** | **H** | **N** | **L** | **S** | **E** | **V** | **I** | **D** | **A** | **T** | **S** | **L** | **L** | **M** | **D** | **N** | **P** | **D** | **V** | **T** | **T** | **N** | **E** | **L** | **M** | **E** | **V** | **L** | **P** | **G** | **P** | **D** | **F** | **P** | **T** | **G** | **G** | **L** | **V** | **M** | **G** | **K** | **S** | **G** | **I** | **R** | **R** | **A** | **Y** | **E** | **T** | **G** | **K** | **G** | **S** | **I** | **T** | **V** | **R** | **A** | **K** | **V** | **E** | **L** | **T** | **E** | **M** | **P** | **N** | **G** | **K** | **E** | **R** | **I** | **L** | **V** | **T** | **E** | **L** | **P** | **Y** | **M** | **V** | **N** | **K** | **A** | **K** | **L** | **I** | **E** | **R** | **I** | **S** | **E** | **L** | **H** | **R** | **D** | **K** | **R** | **I** | **E** | **G** | **I** | **T** | **D** | **L** | **R** | **D** | **E** | **S** | **S** | **R** | **E** | **G** | **M** | **R** | **I** | **V** | **I** | **D** | **V** | **R** | **R** | **D** | **V** | **S** | **A** | **S** | **V** | **V** | **L** | **N** | **N** | **L** | **Y** | **K** | **M** | **T** | **A** | **L** | **Q** | **T** | **S** | **F** | **G** | **F** | **N** | **M** | **L** | **A** | **I** | **E** | **K** | **G** | **V** | **P** | **K** | **I** | **L** | **S** | **L** | **K** | **R** | **I** | **L** | **E** | **N** | **Y** | **V** | **E** | **H** | **Q** | **K** | **E** | **V** | **I** | **T** | **R** | **R** | **T** | **I** | **F** | **D** | **K** | **N** | **K** | **A** | **E** | **A** | **R** | **A** | **H** | **I** | **L** | **E** | **G** | **L** | **R** | **I** | **A** | **L** | **D** | **H** | **I** | **D** | **E** | **I** | **I** | **A** | **I** | **I** | **R** | **G** | **S** | **Q** | **S** | **D** | **D** | **E** | **A** | **K** | **A** | **T** | **L** | **I** | **E** | **R** | **F** | **E** | **F** | **S** | **D** | **R** | **Q** | **A** | **Q** | **A** | **I** | **L** | **D** | **M** | **R** | **L** | **R** | **R** | **L** | **T** | **G** | **L** | **E** | **R** | **D** | **K** | **I** | **E** | **N** | **E** | **Y** | **Q** | **E** | **L** | **L** | **K** | **F** | **I** | **A** | **D** | **L** | **E** | **D** | **I** | **L** | **A** | **R** | **P** | **E** | **R** | **V** | **I** | **E** | **I** | **I** | **K** | **T** | **E** | **L** | **N** | **D** | **V** | **R** | **T** | **K** | **F** | **G** | **D** | **A** | **R** | **R** | **T** | **E** | **L** | **L** | **V** | **G** | **E** | **V** | **L** | **S** | **L** | **E** | **D** | **E** | **D** | **L** | **I** | **E** | **E** | **E** | **E** | **V** | **V** | **I** | **T** | **L** | **T** | **N** | **N** | **G** | **Y** | **I** | **K** | **R** | **M** | **A** | **N** | **S** | **E** | **F** | **R** | **A** | **Q** | **R** | **R** | **G** | **G** | **R** | **G** | **V** | **Q** | **G** | **M** | **G** | **V** | **H** | **D** | **D** | **D** | **F** | **V** | **K** | **N** | **L** | **V** | **S** | **C** | **S** | **T** | **H** | **D** | **T** | **L** | **L** | **F** | **F** | **T** | **N** | **T** | **G** | **K** | **V** | **Y** | **R** | **A** | **K** | **G** | **Y** | **E** | **I** | **P** | **E** | **Y** | **G** | **R** | **T** | **A** | **K** | **G** | **I** | **P** | **V** | **I** | **N** | **L** | **L** | **G** | **I** | **D** | **S** | **A** | **E** | **K** | **I** | **Q** | **A** | **I** | **I** | **S** | **V** | **E** | **G** | **K** | **A** | **E** | **A** | **G** | **K** | **Y** | **L** | **F** | **F** | **T** | **T** | **L** | **K** | **G** | **T** | **V** | **K** | **R** | **T** | **A** | **V** | **T** | **A** | **F** | **S** | **N** | **I** | **R** | **S** | **N** | **G** | **L** | **I** | **A** | **I** | **S** | **L** | **K** | **E** | **D** | **D** | **E** | **L** | **V** | **N** | **V** | **V** | **T** | **T** | **N** | **G** | **N** | **Q** | **K** | **M** | **I** | **I** | **G** | **T** | **H** | **A** | **G** | **Y** | **S** | **V** | **T** | **F** | **D** | **E** | **N** | **T** | **V** | **R** | **D** | **M** | **G** | **R** | **T** | **A** | **S** | **G** | **V** | **R** | **G** | **I** | **R** | **L** | **R** | **E** | **N** | **D** | **Y** | **V** | **V** | **G** | **A** | **A** | **I** | **L** | **D** | **E** | **N** | **K** | **E** | **V** | **L** | **V** | **I** | **T** | **E** | **N** | **G** | **Y** | **G** | **K** | **R** | **T** | **K** | **A** | **S** | **E** | **Y** | **P** | **V** | **K** | **G** | **R** | **G** | **G** | **K** | **G** | **I** | **K** | **T** | **A** | **N** | **I** | **T** | **E** | **K** | **N** | **G** | **P** | **L** | **A** | **G** | **L** | **T** | **T** | **V** | **N** | **G** | **D** | **E** | **D** | **I** | **L** | **L** | **I** | **T** | **N** | **K** | **G** | **V** | **I** | **I** | **R** | **F** | **N** | **V** | **D** | **S** | **V** | **S** | **Q** | **T** | **G** | **R** | **A** | **T** | **L** | **G** | **V** | **R** | **L** | **M** | **R** | **M** | **E** | **D** | **G** | **A** | **K** | **-** | **-** | **-** | **-** | **-** | **-** | **-** | **-** | **-** | **-** | **-** | **-** | **-** | **-** | **-** | **-** | **-** | **-** | **-** | **-** | **-** | **-** | **-** |
| **N59567F** | **M** | **S** | **E** | **E** | **I** | **R** | **E** | **N** | **I** | **H** | **D** | **V** | **N** | **L** | **T** | **S** | **E** | **M** | **K** | **D** | **S** | **F** | **I** | **D** | **Y** | **A** | **M** | **S** | **V** | **I** | **V** | **A** | **R** | **A** | **L** | **P** | **D** | **V** | **R** | **D** | **G** | **L** | **K** | **P** | **V** | **H** | **R** | **R** | **I** | **L** | **Y** | **G** | **M** | **N** | **E** | **L** | **G** | **V** | **T** | **P** | **D** | **K** | **A** | **H** | **K** | **K** | **S** | **A** | **R** | **I** | **V** | **G** | **D** | **V** | **M** | **G** | **K** | **Y** | **H** | **P** | **H** | **G** | **D** | **S** | **A** | **I** | **Y** | **E** | **S** | **M** | **V** | **R** | **M** | **A** | **Q** | **P** | **F** | **S** | **Y** | **R** | **Y** | **M** | **L** | **V** | **D** | **G** | **H** | **G** | **N** | **F** | **G** | **S** | **V** | **D** | **G** | **D** | **G** | **A** | **A** | **A** | **M** | **R** | **Y** | **T** | **E** | **A** | **R** | **M** | **S** | **K** | **I** | **A** | **T** | **E** | **M** | **L** | **R** | **D** | **I** | **N** | **K** | **N** | **T** | **V** | **D** | **F** | **Q** | **S** | **N** | **Y** | **D** | **D** | **T** | **E** | **K** | **E** | **P** | **V** | **V** | **L** | **P** | **A** | **R** | **F** | **P** | **N** | **L** | **L** | **V** | **N** | **G** | **T** | **T** | **G** | **I** | **A** | **V** | **G** | **M** | **A** | **T** | **N** | **I** | **P** | **P** | **H** | **N** | **L** | **S** | **E** | **V** | **V** | **A** | **A** | **I** | **D** | **L** | **L** | **M** | **D** | **N** | **P** | **D** | **V** | **T** | **T** | **N** | **E** | **L** | **M** | **E** | **V** | **L** | **P** | **G** | **P** | **D** | **F** | **P** | **T** | **G** | **G** | **L** | **V** | **M** | **G** | **K** | **S** | **G** | **I** | **R** | **R** | **A** | **Y** | **E** | **T** | **G** | **K** | **G** | **S** | **I** | **T** | **V** | **R** | **A** | **K** | **V** | **E** | **I** | **T** | **E** | **M** | **P** | **N** | **G** | **K** | **E** | **R** | **I** | **I** | **V** | **T** | **E** | **L** | **P** | **Y** | **M** | **V** | **N** | **K** | **A** | **K** | **L** | **I** | **E** | **R** | **I** | **S** | **E** | **L** | **H** | **R** | **D** | **K** | **R** | **I** | **E** | **G** | **I** | **T** | **D** | **L** | **R** | **D** | **E** | **S** | **S** | **R** | **E** | **G** | **M** | **R** | **I** | **V** | **I** | **D** | **I** | **R** | **R** | **D** | **A** | **S** | **A** | **S** | **V** | **I** | **L** | **N** | **N** | **L** | **Y** | **K** | **L** | **T** | **S** | **L** | **Q** | **N** | **S** | **F** | **G** | **F** | **N** | **M** | **L** | **A** | **I** | **E** | **K** | **G** | **V** | **P** | **K** | **V** | **L** | **S** | **L** | **K** | **Q** | **I** | **L** | **E** | **N** | **Y** | **I** | **E** | **H** | **Q** | **R** | **E** | **V** | **I** | **T** | **R** | **R** | **T** | **V** | **F** | **E** | **K** | **E** | **K** | **A** | **E** | **A** | **R** | **A** | **H** | **I** | **L** | **E** | **G** | **L** | **R** | **I** | **A** | **L** | **D** | **H** | **I** | **D** | **E** | **I** | **I** | **A** | **I** | **I** | **R** | **G** | **S** | **K** | **S** | **D** | **D** | **E** | **A** | **K** | **A** | **T** | **M** | **I** | **E** | **R** | **F** | **D** | **L** | **S** | **D** | **R** | **Q** | **S** | **Q** | **A** | **I** | **L** | **D** | **M** | **R** | **L** | **R** | **R** | **L** | **T** | **G** | **L** | **E** | **R** | **E** | **K** | **I** | **E** | **N** | **E** | **Y** | **Q** | **E** | **L** | **L** | **K** | **L** | **I** | **E** | **D** | **L** | **T** | **D** | **I** | **L** | **A** | **R** | **P** | **E** | **R** | **V** | **T** | **E** | **I** | **I** | **K** | **N** | **E** | **L** | **A** | **D** | **L** | **N** | **Q** | **R** | **F** | **G** | **D** | **K** | **R** | **R** | **T** | **E** | **L** | **L** | **V** | **G** | **E** | **V** | **L** | **S** | **L** | **E** | **D** | **E** | **D** | **L** | **I** | **E** | **E** | **E** | **E** | **I** | **V** | **I** | **T** | **L** | **T** | **N** | **N** | **G** | **Y** | **I** | **K** | **R** | **L** | **A** | **N** | **N** | **E** | **F** | **R** | **A** | **Q** | **R** | **R** | **G** | **G** | **R** | **G** | **V** | **Q** | **G** | **M** | **G** | **I** | **H** | **N** | **D** | **D** | **F** | **V** | **K** | **N** | **L** | **V** | **S** | **C** | **S** | **T** | **H** | **D** | **T** | **L** | **L** | **F** | **F** | **T** | **N** | **N** | **G** | **K** | **V** | **Y** | **R** | **A** | **K** | **G** | **Y** | **E** | **I** | **P** | **E** | **Y** | **G** | **R** | **T** | **A** | **K** | **G** | **I** | **P** | **I** | **I** | **N** | **L** | **L** | **G** | **I** | **D** | **S** | **S** | **E** | **S** | **I** | **Q** | **A** | **I** | **I** | **A** | **V** | **T** | **G** | **E** | **A** | **E** | **E** | **G** | **H** | **Y** | **L** | **F** | **F** | **T** | **T** | **R** | **Q** | **G** | **T** | **V** | **K** | **R** | **T** | **S** | **V** | **T** | **A** | **F** | **A** | **N** | **I** | **R** | **S** | **N** | **G** | **L** | **I** | **A** | **I** | **G** | **L** | **K** | **E** | **D** | **D** | **E** | **L** | **V** | **N** | **V** | **L** | **L** | **T** | **D** | **G** | **Q** | **S** | **N** | **I** | **I** | **I** | **G** | **T** | **H** | **N** | **G** | **Y** | **S** | **V** | **T** | **F** | **K** | **E** | **E** | **A** | **V** | **R** | **D** | **M** | **G** | **R** | **T** | **A** | **S** | **G** | **V** | **R** | **G** | **I** | **R** | **L** | **R** | **E** | **E** | **D** | **Y** | **V** | **V** | **G** | **A** | **A** | **L** | **M** | **K** | **E** | **D** | **Q** | **E** | **V** | **L** | **V** | **I** | **T** | **E** | **K** | **G** | **Y** | **G** | **K** | **R** | **T** | **K** | **V** | **S** | **E** | **Y** | **P** | **V** | **K** | **G** | **R** | **G** | **G** | **K** | **G** | **I** | **K** | **T** | **A** | **N** | **I** | **T** | **E** | **K** | **N** | **G** | **P** | **L** | **A** | **G** | **L** | **T** | **T** | **V** | **S** | **G** | **D** | **E** | **D** | **I** | **M** | **L** | **I** | **T** | **D** | **K** | **G** | **V** | **I** | **I** | **R** | **F** | **N** | **V** | **S** | **T** | **V** | **S** | **Q** | **T** | **G** | **R** | **S** | **T** | **L** | **G** | **V** | **R** | **L** | **M** | **K** | **M** | **E** | **A** | **D** | **T** | **K** | **V** | **V** | **T** | **M** | **A** | **A** | **V** | **E** | **P** | **E** | **A** | **A** | **E** | **Q** | **T** | **V** | **E** | **E** | **Q** | **G** | **S** | **E** | **E** |
| **N59492F** | **M** | **S** | **E** | **E** | **I** | **R** | **E** | **N** | **I** | **H** | **D** | **V** | **N** | **L** | **T** | **S** | **E** | **M** | **K** | **D** | **S** | **F** | **I** | **D** | **Y** | **A** | **M** | **S** | **V** | **I** | **V** | **A** | **R** | **A** | **L** | **P** | **D** | **V** | **R** | **D** | **G** | **L** | **K** | **P** | **V** | **H** | **R** | **R** | **I** | **L** | **Y** | **G** | **M** | **N** | **E** | **L** | **G** | **V** | **T** | **P** | **D** | **K** | **A** | **H** | **K** | **K** | **S** | **A** | **R** | **I** | **V** | **G** | **D** | **V** | **M** | **G** | **K** | **Y** | **H** | **P** | **H** | **G** | **D** | **S** | **A** | **I** | **Y** | **E** | **S** | **M** | **V** | **R** | **M** | **A** | **Q** | **P** | **F** | **S** | **Y** | **R** | **Y** | **M** | **L** | **V** | **D** | **G** | **H** | **G** | **N** | **F** | **G** | **S** | **V** | **D** | **G** | **D** | **G** | **A** | **A** | **A** | **M** | **R** | **Y** | **T** | **E** | **A** | **R** | **M** | **S** | **K** | **I** | **A** | **T** | **E** | **M** | **L** | **R** | **D** | **I** | **N** | **K** | **N** | **T** | **V** | **D** | **F** | **Q** | **S** | **N** | **Y** | **D** | **D** | **T** | **E** | **K** | **E** | **P** | **V** | **V** | **L** | **P** | **A** | **R** | **F** | **P** | **N** | **L** | **L** | **V** | **N** | **G** | **T** | **T** | **G** | **I** | **A** | **V** | **G** | **M** | **A** | **T** | **N** | **I** | **P** | **P** | **H** | **N** | **L** | **S** | **E** | **V** | **V** | **A** | **A** | **I** | **D** | **L** | **L** | **M** | **D** | **N** | **P** | **D** | **V** | **T** | **T** | **N** | **E** | **L** | **M** | **E** | **V** | **L** | **P** | **G** | **P** | **D** | **F** | **P** | **T** | **G** | **G** | **L** | **V** | **M** | **G** | **K** | **S** | **G** | **I** | **R** | **R** | **A** | **Y** | **E** | **T** | **G** | **K** | **G** | **S** | **I** | **T** | **V** | **R** | **A** | **K** | **V** | **E** | **I** | **T** | **E** | **M** | **P** | **N** | **G** | **K** | **E** | **R** | **I** | **I** | **V** | **T** | **E** | **L** | **P** | **Y** | **M** | **V** | **N** | **K** | **A** | **K** | **L** | **I** | **E** | **R** | **I** | **S** | **E** | **L** | **H** | **R** | **D** | **K** | **R** | **I** | **E** | **G** | **I** | **T** | **D** | **L** | **R** | **D** | **E** | **S** | **S** | **R** | **E** | **G** | **M** | **R** | **I** | **V** | **I** | **D** | **I** | **R** | **R** | **D** | **A** | **S** | **A** | **S** | **V** | **I** | **L** | **N** | **N** | **L** | **Y** | **K** | **L** | **T** | **S** | **L** | **Q** | **N** | **S** | **F** | **G** | **F** | **N** | **M** | **L** | **A** | **I** | **E** | **K** | **G** | **V** | **P** | **K** | **V** | **L** | **S** | **L** | **K** | **Q** | **I** | **L** | **E** | **N** | **Y** | **I** | **E** | **H** | **Q** | **R** | **E** | **V** | **I** | **T** | **R** | **R** | **T** | **V** | **F** | **E** | **K** | **E** | **K** | **A** | **E** | **A** | **R** | **A** | **H** | **I** | **L** | **E** | **G** | **L** | **R** | **I** | **A** | **L** | **D** | **H** | **I** | **D** | **E** | **I** | **I** | **A** | **I** | **I** | **R** | **G** | **S** | **K** | **S** | **D** | **D** | **E** | **A** | **K** | **A** | **T** | **M** | **I** | **E** | **R** | **F** | **D** | **L** | **S** | **D** | **R** | **Q** | **S** | **Q** | **A** | **I** | **L** | **D** | **M** | **R** | **L** | **R** | **R** | **L** | **T** | **G** | **L** | **E** | **R** | **E** | **K** | **I** | **E** | **N** | **E** | **Y** | **Q** | **E** | **L** | **L** | **K** | **L** | **I** | **E** | **D** | **L** | **T** | **D** | **I** | **L** | **A** | **R** | **P** | **E** | **R** | **V** | **T** | **E** | **I** | **I** | **K** | **N** | **E** | **L** | **A** | **D** | **L** | **N** | **Q** | **R** | **F** | **G** | **D** | **K** | **R** | **R** | **T** | **E** | **L** | **L** | **V** | **G** | **E** | **V** | **L** | **S** | **L** | **E** | **D** | **E** | **D** | **L** | **I** | **E** | **E** | **E** | **E** | **I** | **V** | **I** | **T** | **L** | **T** | **N** | **N** | **G** | **Y** | **I** | **K** | **R** | **L** | **A** | **N** | **N** | **E** | **F** | **R** | **A** | **Q** | **R** | **R** | **G** | **G** | **R** | **G** | **V** | **Q** | **G** | **M** | **G** | **I** | **H** | **N** | **D** | **D** | **F** | **V** | **K** | **N** | **L** | **V** | **S** | **C** | **S** | **T** | **H** | **D** | **T** | **L** | **L** | **F** | **F** | **T** | **N** | **N** | **G** | **K** | **V** | **Y** | **R** | **A** | **K** | **G** | **Y** | **E** | **I** | **P** | **E** | **Y** | **G** | **R** | **T** | **A** | **K** | **G** | **I** | **P** | **I** | **I** | **N** | **L** | **L** | **G** | **I** | **D** | **S** | **S** | **E** | **S** | **I** | **Q** | **A** | **I** | **I** | **A** | **V** | **T** | **G** | **E** | **A** | **E** | **E** | **G** | **H** | **Y** | **L** | **F** | **F** | **T** | **T** | **R** | **Q** | **G** | **T** | **V** | **K** | **R** | **T** | **S** | **V** | **T** | **A** | **F** | **A** | **N** | **I** | **R** | **S** | **N** | **G** | **L** | **I** | **A** | **I** | **G** | **L** | **K** | **E** | **D** | **D** | **E** | **L** | **V** | **N** | **V** | **L** | **L** | **T** | **D** | **G** | **Q** | **S** | **N** | **I** | **I** | **I** | **G** | **T** | **H** | **N** | **G** | **Y** | **S** | **V** | **T** | **F** | **K** | **E** | **E** | **A** | **V** | **R** | **D** | **M** | **G** | **R** | **T** | **A** | **S** | **G** | **V** | **R** | **G** | **I** | **R** | **L** | **R** | **E** | **E** | **D** | **Y** | **V** | **V** | **G** | **A** | **A** | **L** | **M** | **K** | **E** | **D** | **Q** | **E** | **V** | **L** | **V** | **I** | **T** | **E** | **K** | **G** | **Y** | **G** | **K** | **R** | **T** | **K** | **V** | **S** | **E** | **Y** | **P** | **V** | **K** | **G** | **R** | **G** | **G** | **K** | **G** | **I** | **K** | **T** | **A** | **N** | **I** | **T** | **E** | **K** | **N** | **G** | **P** | **L** | **A** | **G** | **L** | **T** | **T** | **V** | **S** | **G** | **D** | **E** | **D** | **I** | **M** | **L** | **I** | **T** | **D** | **K** | **G** | **V** | **I** | **I** | **R** | **F** | **N** | **V** | **S** | **T** | **V** | **S** | **Q** | **T** | **G** | **R** | **S** | **T** | **L** | **G** | **V** | **R** | **L** | **M** | **K** | **M** | **E** | **A** | **D** | **T** | **K** | **V** | **V** | **T** | **M** | **A** | **A** | **V** | **E** | **P** | **E** | **A** | **A** | **E** | **Q** | **T** | **V** | **E** | **E** | **Q** | **G** | **S** | **E** | **E** |
| **N59542F** | **-** | **-** | **-** | **-** | **-** | **-** | **-** | **-** | **-** | **-** | **-** | **-** | **-** | **-** | **-** | **-** | **-** | **-** | **-** | **-** | **-** | **-** | **-** | **-** | **-** | **-** | **-** | **-** | **-** | **-** | **-** | **-** | **-** | **-** | **-** | **-** | **-** | **-** | **-** | **-** | **-** | **-** | **-** | **-** | **-** | **-** | **-** | **-** | **-** | **-** | **-** | **-** | **-** | **-** | **-** | **-** | **-** | **-** | **-** | **-** | **-** | **-** | **-** | **-** | **-** | **-** | **-** | **-** | **-** | **-** | **-** | **-** | **-** | **-** | **-** | **-** | **-** | **-** | **-** | **-** | **-** | **-** | **-** | **-** | **-** | **-** | **-** | **-** | **-** | **-** | **-** | **-** | **-** | **-** | **-** | **-** | **-** | **-** | **-** | **-** | **-** | **-** | **-** | **-** | **-** | **-** | **-** | **-** | **-** | **-** | **-** | **-** | **-** | **-** | **-** | **-** | **-** | **-** | **-** | **-** | **-** | **-** | **-** | **-** | **-** | **-** | **-** | **-** | **-** | **-** | **-** | **-** | **-** | **-** | **-** | **-** | **-** | **-** | **-** | **-** | **-** | **-** | **-** | **-** | **-** | **-** | **-** | **-** | **-** | **-** | **-** | **-** | **-** | **-** | **-** | **-** | **-** | **-** | **-** | **-** | **-** | **-** | **-** | **-** | **-** | **-** | **-** | **-** | **-** | **-** | **-** | **-** | **-** | **-** | **-** | **-** | **-** | **-** | **-** | **-** | **-** | **-** | **-** | **-** | **-** | **-** | **-** | **-** | **-** | **-** | **-** | **-** | **-** | **-** | **-** | **-** | **-** | **-** | **-** | **-** | **-** | **-** | **-** | **-** | **-** | **-** | **-** | **-** | **-** | **-** | **-** | **-** | **-** | **-** | **-** | **-** | **-** | **-** | **-** | **-** | **-** | **-** | **-** | **-** | **-** | **-** | **K** | **S** | **G** | **I** | **R** | **R** | **A** | **Y** | **E** | **T** | **G** | **K** | **G** | **S** | **I** | **T** | **V** | **R** | **A** | **K** | **V** | **E** | **I** | **T** | **E** | **M** | **P** | **N** | **G** | **K** | **E** | **R** | **I** | **I** | **V** | **T** | **E** | **L** | **P** | **Y** | **M** | **V** | **N** | **K** | **A** | **K** | **L** | **I** | **E** | **R** | **I** | **S** | **E** | **L** | **H** | **R** | **D** | **K** | **R** | **I** | **E** | **G** | **I** | **T** | **D** | **L** | **R** | **D** | **E** | **S** | **S** | **R** | **E** | **G** | **M** | **R** | **I** | **V** | **I** | **D** | **I** | **R** | **R** | **D** | **A** | **S** | **A** | **S** | **V** | **I** | **L** | **N** | **N** | **L** | **Y** | **K** | **L** | **T** | **S** | **L** | **Q** | **N** | **S** | **F** | **G** | **F** | **N** | **M** | **L** | **A** | **I** | **E** | **K** | **G** | **V** | **P** | **K** | **V** | **L** | **S** | **L** | **K** | **Q** | **I** | **L** | **E** | **N** | **Y** | **I** | **E** | **H** | **Q** | **R** | **E** | **V** | **I** | **T** | **R** | **R** | **T** | **V** | **F** | **E** | **K** | **E** | **K** | **A** | **E** | **A** | **R** | **A** | **H** | **I** | **L** | **E** | **G** | **L** | **R** | **I** | **A** | **L** | **D** | **H** | **I** | **D** | **E** | **I** | **I** | **A** | **I** | **I** | **R** | **G** | **S** | **K** | **S** | **D** | **D** | **E** | **A** | **K** | **A** | **T** | **M** | **I** | **E** | **R** | **F** | **D** | **L** | **S** | **D** | **R** | **Q** | **S** | **Q** | **A** | **I** | **L** | **D** | **M** | **R** | **L** | **R** | **R** | **L** | **T** | **G** | **L** | **E** | **R** | **E** | **K** | **I** | **E** | **N** | **E** | **Y** | **Q** | **E** | **L** | **L** | **K** | **L** | **I** | **E** | **D** | **L** | **T** | **D** | **I** | **L** | **A** | **R** | **P** | **E** | **R** | **V** | **T** | **E** | **I** | **I** | **K** | **N** | **E** | **L** | **A** | **D** | **L** | **N** | **Q** | **R** | **F** | **G** | **D** | **K** | **R** | **R** | **T** | **E** | **L** | **L** | **V** | **G** | **E** | **V** | **L** | **S** | **L** | **E** | **D** | **E** | **D** | **L** | **I** | **E** | **E** | **E** | **E** | **I** | **V** | **I** | **T** | **L** | **T** | **N** | **N** | **G** | **Y** | **I** | **K** | **R** | **L** | **A** | **N** | **N** | **E** | **F** | **R** | **A** | **Q** | **R** | **R** | **G** | **G** | **R** | **G** | **V** | **Q** | **G** | **M** | **G** | **I** | **H** | **N** | **D** | **D** | **F** | **V** | **K** | **N** | **L** | **V** | **S** | **C** | **S** | **T** | **H** | **D** | **T** | **L** | **L** | **F** | **F** | **T** | **N** | **N** | **G** | **K** | **V** | **Y** | **R** | **A** | **K** | **G** | **Y** | **E** | **I** | **P** | **E** | **Y** | **G** | **R** | **T** | **A** | **K** | **G** | **I** | **P** | **I** | **I** | **N** | **L** | **L** | **G** | **I** | **D** | **S** | **S** | **E** | **S** | **I** | **Q** | **A** | **I** | **I** | **A** | **V** | **T** | **G** | **E** | **A** | **E** | **E** | **G** | **H** | **Y** | **L** | **F** | **F** | **T** | **T** | **R** | **Q** | **G** | **T** | **V** | **K** | **R** | **T** | **S** | **V** | **T** | **A** | **F** | **A** | **N** | **I** | **R** | **S** | **N** | **G** | **L** | **I** | **A** | **I** | **G** | **L** | **K** | **E** | **D** | **D** | **E** | **L** | **V** | **N** | **V** | **L** | **L** | **T** | **D** | **G** | **Q** | **S** | **N** | **I** | **I** | **I** | **G** | **T** | **H** | **N** | **G** | **Y** | **S** | **V** | **T** | **F** | **K** | **E** | **E** | **A** | **V** | **R** | **D** | **M** | **G** | **R** | **T** | **A** | **S** | **G** | **V** | **R** | **G** | **I** | **R** | **L** | **R** | **E** | **E** | **D** | **Y** | **V** | **V** | **G** | **A** | **A** | **L** | **M** | **K** | **E** | **N** | **Q** | **E** | **V** | **L** | **V** | **I** | **T** | **E** | **K** | **G** | **Y** | **G** | **K** | **R** | **T** | **K** | **V** | **S** | **E** | **Y** | **P** | **V** | **K** | **G** | **R** | **G** | **G** | **K** | **G** | **I** | **K** | **T** | **A** | **N** | **I** | **T** | **E** | **K** | **N** | **G** | **P** | **L** | **A** | **G** | **L** | **T** | **T** | **V** | **S** | **G** | **D** | **E** | **D** | **I** | **M** | **L** | **I** | **T** | **D** | **K** | **G** | **V** | **I** | **I** | **R** | **F** | **N** | **V** | **S** | **T** | **V** | **S** | **Q** | **T** | **G** | **R** | **S** | **T** | **L** | **G** | **V** | **R** | **L** | **M** | **K** | **M** | **E** | **A** | **D** | **T** | **K** | **V** | **V** | **T** | **M** | **A** | **A** | **V** | **E** | **P** | **E** | **A** | **A** | **E** | **Q** | **T** | **V** | **E** | **E** | **Q** | **G** | **S** | **E** | **E** |
| **N53479** | **M** | **S** | **E** | **E** | **I** | **R** | **E** | **N** | **I** | **H** | **D** | **V** | **N** | **L** | **T** | **S** | **E** | **M** | **K** | **D** | **S** | **F** | **I** | **D** | **Y** | **A** | **M** | **S** | **V** | **I** | **V** | **A** | **R** | **A** | **L** | **P** | **D** | **V** | **R** | **D** | **G** | **L** | **K** | **P** | **V** | **H** | **R** | **R** | **I** | **L** | **Y** | **G** | **M** | **N** | **E** | **L** | **G** | **V** | **T** | **P** | **D** | **K** | **A** | **H** | **K** | **K** | **S** | **A** | **R** | **I** | **V** | **G** | **D** | **V** | **M** | **G** | **K** | **Y** | **H** | **P** | **H** | **G** | **D** | **S** | **A** | **I** | **Y** | **E** | **S** | **M** | **V** | **R** | **M** | **A** | **Q** | **P** | **F** | **S** | **Y** | **R** | **Y** | **M** | **L** | **V** | **D** | **G** | **H** | **G** | **N** | **F** | **G** | **S** | **V** | **D** | **G** | **D** | **G** | **A** | **A** | **A** | **M** | **R** | **Y** | **T** | **E** | **A** | **R** | **M** | **S** | **K** | **I** | **A** | **T** | **E** | **M** | **L** | **R** | **D** | **I** | **N** | **K** | **N** | **T** | **V** | **D** | **F** | **Q** | **S** | **N** | **Y** | **D** | **D** | **T** | **E** | **K** | **E** | **P** | **V** | **V** | **L** | **P** | **A** | **R** | **F** | **P** | **N** | **L** | **L** | **V** | **N** | **G** | **T** | **T** | **G** | **I** | **A** | **V** | **G** | **M** | **A** | **T** | **N** | **I** | **P** | **P** | **H** | **N** | **L** | **S** | **E** | **V** | **V** | **A** | **A** | **I** | **D** | **L** | **L** | **M** | **D** | **N** | **P** | **D** | **V** | **T** | **T** | **N** | **E** | **L** | **M** | **E** | **V** | **L** | **P** | **G** | **P** | **D** | **F** | **P** | **T** | **G** | **G** | **L** | **V** | **M** | **G** | **K** | **S** | **G** | **I** | **R** | **R** | **A** | **Y** | **E** | **T** | **G** | **K** | **G** | **S** | **I** | **T** | **V** | **R** | **A** | **K** | **V** | **E** | **I** | **T** | **E** | **M** | **P** | **N** | **G** | **K** | **E** | **R** | **I** | **I** | **V** | **T** | **E** | **L** | **P** | **Y** | **M** | **V** | **N** | **K** | **A** | **K** | **L** | **I** | **E** | **R** | **I** | **S** | **E** | **L** | **H** | **R** | **D** | **K** | **R** | **I** | **E** | **G** | **I** | **T** | **D** | **L** | **R** | **D** | **E** | **S** | **S** | **R** | **E** | **G** | **M** | **R** | **I** | **V** | **I** | **D** | **I** | **R** | **R** | **D** | **A** | **S** | **A** | **S** | **V** | **I** | **L** | **N** | **N** | **L** | **Y** | **K** | **L** | **T** | **S** | **L** | **Q** | **N** | **S** | **F** | **G** | **F** | **N** | **M** | **L** | **A** | **I** | **E** | **K** | **G** | **V** | **P** | **K** | **V** | **L** | **S** | **L** | **K** | **Q** | **I** | **L** | **E** | **N** | **Y** | **I** | **E** | **H** | **Q** | **R** | **E** | **V** | **I** | **T** | **R** | **R** | **T** | **V** | **F** | **E** | **K** | **E** | **K** | **A** | **E** | **A** | **R** | **A** | **H** | **I** | **L** | **E** | **G** | **L** | **R** | **I** | **A** | **L** | **D** | **H** | **I** | **D** | **E** | **I** | **I** | **A** | **I** | **I** | **R** | **G** | **S** | **K** | **S** | **D** | **D** | **E** | **A** | **K** | **A** | **T** | **M** | **I** | **E** | **R** | **F** | **D** | **L** | **S** | **D** | **R** | **Q** | **S** | **Q** | **A** | **I** | **L** | **D** | **M** | **R** | **L** | **R** | **R** | **L** | **T** | **G** | **L** | **E** | **R** | **E** | **K** | **I** | **E** | **N** | **E** | **Y** | **Q** | **E** | **L** | **L** | **K** | **L** | **I** | **E** | **D** | **L** | **T** | **D** | **I** | **L** | **A** | **R** | **P** | **E** | **R** | **V** | **T** | **E** | **I** | **I** | **K** | **N** | **E** | **L** | **A** | **D** | **L** | **N** | **Q** | **R** | **F** | **G** | **D** | **K** | **R** | **R** | **T** | **E** | **L** | **L** | **V** | **G** | **E** | **V** | **L** | **S** | **L** | **E** | **D** | **E** | **D** | **L** | **I** | **E** | **E** | **E** | **E** | **I** | **V** | **I** | **T** | **L** | **T** | **N** | **N** | **G** | **Y** | **I** | **K** | **R** | **L** | **A** | **N** | **N** | **E** | **F** | **R** | **A** | **Q** | **R** | **R** | **G** | **G** | **R** | **G** | **V** | **Q** | **G** | **M** | **G** | **I** | **H** | **N** | **D** | **D** | **F** | **V** | **K** | **N** | **L** | **V** | **S** | **C** | **S** | **T** | **H** | **D** | **T** | **L** | **L** | **F** | **F** | **T** | **N** | **N** | **G** | **K** | **V** | **Y** | **R** | **A** | **K** | **G** | **Y** | **E** | **I** | **P** | **E** | **Y** | **G** | **R** | **T** | **A** | **K** | **G** | **I** | **P** | **I** | **I** | **N** | **L** | **L** | **G** | **I** | **D** | **S** | **S** | **E** | **S** | **I** | **Q** | **A** | **I** | **I** | **A** | **V** | **T** | **G** | **E** | **A** | **E** | **E** | **G** | **H** | **Y** | **L** | **F** | **F** | **T** | **T** | **R** | **Q** | **G** | **T** | **V** | **K** | **R** | **T** | **S** | **V** | **T** | **A** | **F** | **A** | **N** | **I** | **R** | **S** | **N** | **G** | **L** | **I** | **A** | **I** | **G** | **L** | **K** | **E** | **D** | **D** | **E** | **L** | **V** | **N** | **V** | **L** | **L** | **T** | **D** | **G** | **Q** | **S** | **N** | **I** | **I** | **I** | **G** | **T** | **H** | **N** | **G** | **Y** | **S** | **V** | **T** | **F** | **K** | **E** | **E** | **A** | **V** | **R** | **D** | **M** | **G** | **R** | **T** | **A** | **S** | **G** | **V** | **R** | **G** | **I** | **R** | **L** | **R** | **E** | **E** | **D** | **Y** | **V** | **V** | **G** | **A** | **A** | **L** | **M** | **K** | **E** | **N** | **Q** | **E** | **V** | **L** | **V** | **I** | **T** | **E** | **K** | **G** | **Y** | **G** | **K** | **R** | **T** | **K** | **V** | **S** | **E** | **Y** | **P** | **V** | **K** | **G** | **R** | **G** | **G** | **K** | **G** | **I** | **K** | **T** | **A** | **N** | **I** | **T** | **E** | **K** | **N** | **G** | **P** | **L** | **A** | **G** | **L** | **T** | **T** | **V** | **S** | **G** | **D** | **E** | **D** | **I** | **M** | **L** | **I** | **T** | **D** | **K** | **G** | **V** | **I** | **I** | **R** | **F** | **N** | **V** | **S** | **T** | **V** | **S** | **Q** | **T** | **G** | **R** | **S** | **T** | **L** | **G** | **V** | **R** | **L** | **M** | **K** | **M** | **E** | **A** | **D** | **T** | **K** | **V** | **V** | **T** | **M** | **A** | **A** | **V** | **E** | **P** | **E** | **A** | **A** | **E** | **Q** | **T** | **V** | **E** | **E** | **Q** | **G** | **S** | **E** | **E** |
| **N59554F** | **M** | **S** | **E** | **E** | **I** | **K** | **E** | **N** | **I** | **Q** | **D** | **V** | **N** | **L** | **T** | **S** | **E** | **M** | **K** | **E** | **S** | **F** | **I** | **D** | **Y** | **A** | **M** | **S** | **V** | **I** | **V** | **A** | **R** | **A** | **L** | **P** | **D** | **V** | **R** | **D** | **G** | **L** | **K** | **P** | **V** | **H** | **R** | **R** | **I** | **L** | **Y** | **G** | **M** | **N** | **E** | **L** | **G** | **V** | **T** | **P** | **D** | **K** | **P** | **H** | **K** | **K** | **S** | **A** | **R** | **I** | **V** | **G** | **D** | **V** | **M** | **G** | **K** | **Y** | **H** | **P** | **H** | **G** | **D** | **S** | **A** | **I** | **Y** | **E** | **S** | **M** | **V** | **R** | **M** | **A** | **Q** | **P** | **F** | **S** | **Y** | **R** | **A** | **M** | **L** | **V** | **D** | **G** | **H** | **G** | **N** | **F** | **G** | **S** | **V** | **D** | **G** | **D** | **G** | **A** | **A** | **A** | **M** | **R** | **Y** | **T** | **E** | **A** | **R** | **M** | **S** | **K** | **I** | **A** | **L** | **E** | **M** | **L** | **R** | **D** | **I** | **N** | **K** | **N** | **T** | **V** | **D** | **F** | **Q** | **G** | **N** | **Y** | **D** | **D** | **S** | **E** | **Q** | **E** | **P** | **V** | **V** | **L** | **P** | **A** | **R** | **F** | **P** | **N** | **L** | **L** | **V** | **N** | **G** | **T** | **T** | **G** | **I** | **A** | **V** | **G** | **M** | **A** | **T** | **N** | **I** | **P** | **P** | **H** | **N** | **L** | **S** | **E** | **V** | **I** | **D** | **A** | **T** | **S** | **L** | **L** | **M** | **D** | **N** | **P** | **D** | **V** | **T** | **T** | **N** | **E** | **L** | **M** | **E** | **V** | **L** | **P** | **G** | **P** | **D** | **F** | **P** | **T** | **G** | **G** | **L** | **V** | **M** | **G** | **K** | **S** | **G** | **I** | **R** | **R** | **A** | **Y** | **E** | **T** | **G** | **K** | **G** | **S** | **I** | **T** | **V** | **R** | **A** | **K** | **V** | **E** | **L** | **T** | **E** | **M** | **P** | **N** | **G** | **K** | **E** | **R** | **I** | **L** | **V** | **T** | **E** | **L** | **P** | **Y** | **M** | **V** | **N** | **K** | **A** | **K** | **L** | **I** | **E** | **R** | **I** | **S** | **E** | **L** | **H** | **R** | **D** | **K** | **R** | **I** | **E** | **G** | **I** | **T** | **D** | **L** | **R** | **D** | **E** | **S** | **S** | **R** | **E** | **G** | **M** | **R** | **I** | **V** | **I** | **D** | **V** | **R** | **R** | **D** | **V** | **S** | **A** | **S** | **V** | **V** | **L** | **N** | **N** | **L** | **Y** | **K** | **M** | **T** | **A** | **L** | **Q** | **T** | **S** | **F** | **G** | **F** | **N** | **M** | **L** | **A** | **I** | **E** | **K** | **G** | **V** | **P** | **K** | **I** | **L** | **S** | **L** | **K** | **R** | **I** | **L** | **E** | **N** | **Y** | **V** | **E** | **H** | **Q** | **K** | **E** | **V** | **I** | **T** | **R** | **R** | **T** | **I** | **F** | **D** | **K** | **N** | **K** | **A** | **E** | **A** | **R** | **A** | **H** | **I** | **L** | **E** | **G** | **L** | **R** | **I** | **A** | **L** | **D** | **H** | **I** | **D** | **E** | **I** | **I** | **A** | **I** | **I** | **R** | **G** | **S** | **Q** | **S** | **D** | **D** | **E** | **A** | **K** | **A** | **T** | **L** | **I** | **E** | **R** | **F** | **E** | **F** | **S** | **D** | **R** | **Q** | **A** | **Q** | **A** | **I** | **L** | **D** | **M** | **R** | **L** | **R** | **R** | **L** | **T** | **G** | **L** | **E** | **R** | **D** | **K** | **I** | **E** | **N** | **E** | **Y** | **Q** | **E** | **L** | **L** | **K** | **F** | **I** | **A** | **D** | **L** | **E** | **D** | **I** | **L** | **A** | **R** | **P** | **E** | **R** | **V** | **I** | **E** | **I** | **I** | **K** | **T** | **E** | **L** | **N** | **D** | **V** | **R** | **T** | **K** | **F** | **G** | **D** | **A** | **R** | **R** | **T** | **E** | **L** | **L** | **V** | **G** | **E** | **V** | **L** | **S** | **L** | **E** | **D** | **E** | **D** | **L** | **I** | **E** | **E** | **E** | **E** | **V** | **V** | **I** | **T** | **L** | **T** | **N** | **N** | **G** | **Y** | **I** | **K** | **R** | **M** | **A** | **N** | **S** | **E** | **F** | **R** | **A** | **Q** | **R** | **R** | **G** | **G** | **R** | **G** | **V** | **Q** | **G** | **M** | **G** | **V** | **H** | **D** | **D** | **D** | **F** | **V** | **K** | **N** | **L** | **V** | **S** | **C** | **S** | **T** | **H** | **D** | **T** | **L** | **L** | **F** | **F** | **T** | **N** | **T** | **G** | **K** | **V** | **Y** | **R** | **A** | **K** | **G** | **Y** | **E** | **I** | **P** | **E** | **Y** | **G** | **R** | **T** | **A** | **K** | **G** | **I** | **P** | **V** | **I** | **N** | **L** | **L** | **G** | **I** | **D** | **S** | **A** | **E** | **K** | **I** | **Q** | **A** | **I** | **I** | **S** | **V** | **E** | **G** | **K** | **A** | **E** | **A** | **G** | **K** | **Y** | **L** | **F** | **F** | **T** | **T** | **L** | **K** | **G** | **T** | **V** | **K** | **R** | **T** | **A** | **V** | **T** | **A** | **F** | **S** | **N** | **I** | **R** | **S** | **N** | **G** | **L** | **I** | **A** | **I** | **S** | **L** | **K** | **E** | **D** | **D** | **E** | **L** | **V** | **N** | **V** | **V** | **T** | **T** | **N** | **G** | **N** | **Q** | **K** | **M** | **I** | **I** | **G** | **T** | **H** | **A** | **G** | **Y** | **S** | **V** | **T** | **F** | **D** | **E** | **N** | **T** | **V** | **R** | **D** | **M** | **G** | **R** | **T** | **A** | **S** | **G** | **V** | **R** | **G** | **I** | **R** | **L** | **R** | **E** | **N** | **D** | **Y** | **V** | **V** | **G** | **A** | **A** | **I** | **L** | **D** | **E** | **N** | **K** | **E** | **V** | **L** | **V** | **I** | **T** | **E** | **N** | **G** | **Y** | **G** | **K** | **R** | **T** | **K** | **A** | **S** | **E** | **Y** | **P** | **V** | **K** | **G** | **R** | **G** | **G** | **K** | **G** | **I** | **K** | **T** | **A** | **N** | **I** | **T** | **E** | **K** | **N** | **G** | **P** | **L** | **A** | **G** | **L** | **T** | **T** | **V** | **N** | **G** | **D** | **E** | **D** | **I** | **L** | **L** | **I** | **T** | **N** | **K** | **G** | **V** | **I** | **I** | **R** | **F** | **N** | **V** | **D** | **S** | **V** | **S** | **Q** | **T** | **G** | **R** | **A** | **T** | **L** | **G** | **V** | **R** | **L** | **M** | **R** | **M** | **E** | **D** | **G** | **A** | **K** | **-** | **-** | **-** | **-** | **-** | **-** | **-** | **-** | **-** | **-** | **-** | **-** | **-** | **-** | **-** | **-** | **-** | **-** | **-** | **-** | **-** | **-** | **-** |
| **N54155** | **M** | **S** | **E** | **E** | **I** | **R** | **E** | **N** | **I** | **H** | **D** | **V** | **N** | **L** | **T** | **S** | **E** | **M** | **K** | **D** | **S** | **F** | **I** | **D** | **Y** | **A** | **M** | **S** | **V** | **I** | **V** | **A** | **R** | **A** | **L** | **P** | **D** | **V** | **R** | **D** | **G** | **L** | **K** | **P** | **V** | **H** | **R** | **R** | **I** | **L** | **Y** | **G** | **M** | **N** | **E** | **L** | **G** | **V** | **T** | **P** | **D** | **K** | **A** | **H** | **K** | **K** | **S** | **A** | **R** | **I** | **V** | **G** | **D** | **V** | **M** | **G** | **K** | **Y** | **H** | **P** | **H** | **G** | **D** | **S** | **A** | **I** | **Y** | **E** | **S** | **M** | **V** | **R** | **M** | **A** | **Q** | **P** | **F** | **S** | **Y** | **R** | **Y** | **M** | **L** | **V** | **D** | **G** | **H** | **G** | **N** | **F** | **G** | **S** | **V** | **D** | **G** | **D** | **G** | **A** | **A** | **A** | **M** | **R** | **Y** | **T** | **E** | **A** | **R** | **M** | **S** | **K** | **I** | **A** | **T** | **E** | **M** | **L** | **R** | **D** | **I** | **N** | **K** | **N** | **T** | **V** | **D** | **F** | **Q** | **S** | **N** | **Y** | **D** | **D** | **T** | **E** | **K** | **E** | **P** | **V** | **V** | **L** | **P** | **A** | **R** | **F** | **P** | **N** | **L** | **L** | **V** | **N** | **G** | **T** | **T** | **G** | **I** | **A** | **V** | **G** | **M** | **A** | **T** | **N** | **I** | **P** | **P** | **H** | **N** | **L** | **S** | **E** | **V** | **V** | **A** | **A** | **I** | **D** | **L** | **L** | **M** | **D** | **N** | **P** | **D** | **V** | **T** | **T** | **N** | **E** | **L** | **M** | **E** | **V** | **L** | **P** | **G** | **P** | **D** | **F** | **P** | **T** | **G** | **G** | **L** | **V** | **M** | **G** | **K** | **S** | **G** | **I** | **R** | **R** | **A** | **Y** | **E** | **T** | **G** | **K** | **G** | **S** | **I** | **T** | **V** | **R** | **A** | **K** | **V** | **E** | **I** | **T** | **E** | **M** | **P** | **N** | **G** | **K** | **E** | **R** | **I** | **I** | **V** | **T** | **E** | **L** | **P** | **Y** | **M** | **V** | **N** | **K** | **A** | **K** | **L** | **I** | **E** | **R** | **I** | **S** | **E** | **L** | **H** | **R** | **D** | **K** | **R** | **I** | **E** | **G** | **I** | **T** | **D** | **L** | **R** | **D** | **E** | **S** | **S** | **R** | **E** | **G** | **M** | **R** | **I** | **V** | **I** | **D** | **I** | **R** | **R** | **D** | **A** | **S** | **A** | **S** | **V** | **I** | **L** | **N** | **N** | **L** | **Y** | **K** | **L** | **T** | **S** | **L** | **Q** | **N** | **S** | **F** | **G** | **F** | **N** | **M** | **L** | **A** | **I** | **E** | **K** | **G** | **V** | **P** | **K** | **V** | **L** | **S** | **L** | **K** | **Q** | **I** | **L** | **E** | **N** | **Y** | **I** | **E** | **H** | **Q** | **R** | **E** | **V** | **I** | **T** | **R** | **R** | **T** | **V** | **F** | **E** | **K** | **E** | **K** | **A** | **E** | **A** | **R** | **A** | **H** | **I** | **L** | **E** | **G** | **L** | **R** | **I** | **A** | **L** | **D** | **H** | **I** | **D** | **E** | **I** | **I** | **A** | **I** | **I** | **R** | **G** | **S** | **K** | **S** | **D** | **D** | **E** | **A** | **K** | **A** | **T** | **M** | **I** | **E** | **R** | **F** | **D** | **L** | **S** | **D** | **R** | **Q** | **S** | **Q** | **A** | **I** | **L** | **D** | **M** | **R** | **L** | **R** | **R** | **L** | **T** | **G** | **L** | **E** | **R** | **E** | **K** | **I** | **E** | **N** | **E** | **Y** | **Q** | **E** | **L** | **L** | **K** | **L** | **I** | **E** | **D** | **L** | **T** | **D** | **I** | **L** | **A** | **R** | **P** | **E** | **R** | **V** | **T** | **E** | **I** | **I** | **K** | **N** | **E** | **L** | **A** | **D** | **L** | **N** | **Q** | **R** | **F** | **G** | **D** | **K** | **R** | **R** | **T** | **E** | **L** | **L** | **V** | **G** | **E** | **V** | **L** | **S** | **L** | **E** | **D** | **E** | **D** | **L** | **I** | **E** | **E** | **E** | **E** | **I** | **V** | **I** | **T** | **L** | **T** | **N** | **N** | **G** | **Y** | **I** | **K** | **R** | **L** | **A** | **N** | **N** | **E** | **F** | **R** | **A** | **Q** | **R** | **R** | **G** | **G** | **R** | **G** | **V** | **Q** | **G** | **M** | **G** | **I** | **H** | **N** | **D** | **D** | **F** | **V** | **K** | **N** | **L** | **V** | **S** | **C** | **S** | **T** | **H** | **D** | **T** | **L** | **L** | **F** | **F** | **T** | **N** | **N** | **G** | **K** | **V** | **Y** | **R** | **A** | **K** | **G** | **Y** | **E** | **I** | **P** | **E** | **Y** | **G** | **R** | **T** | **A** | **K** | **G** | **I** | **P** | **I** | **I** | **N** | **L** | **L** | **G** | **I** | **D** | **S** | **S** | **E** | **S** | **I** | **Q** | **A** | **I** | **I** | **A** | **V** | **T** | **G** | **E** | **A** | **E** | **E** | **G** | **H** | **Y** | **L** | **F** | **F** | **T** | **T** | **R** | **Q** | **G** | **T** | **V** | **K** | **R** | **T** | **S** | **V** | **T** | **A** | **F** | **A** | **N** | **I** | **R** | **S** | **N** | **G** | **L** | **I** | **A** | **I** | **G** | **L** | **K** | **E** | **D** | **D** | **E** | **L** | **V** | **N** | **V** | **L** | **L** | **T** | **D** | **G** | **Q** | **S** | **N** | **I** | **I** | **I** | **G** | **T** | **H** | **N** | **G** | **Y** | **S** | **V** | **T** | **F** | **K** | **E** | **E** | **A** | **V** | **R** | **D** | **M** | **G** | **R** | **T** | **A** | **S** | **G** | **V** | **R** | **G** | **I** | **R** | **L** | **R** | **E** | **E** | **D** | **Y** | **V** | **V** | **G** | **A** | **A** | **L** | **M** | **K** | **E** | **D** | **Q** | **E** | **V** | **L** | **V** | **I** | **T** | **E** | **K** | **G** | **Y** | **G** | **K** | **R** | **T** | **K** | **V** | **S** | **E** | **Y** | **P** | **V** | **K** | **G** | **R** | **G** | **G** | **K** | **G** | **I** | **K** | **T** | **A** | **N** | **I** | **T** | **E** | **K** | **N** | **G** | **P** | **L** | **A** | **G** | **L** | **T** | **T** | **V** | **S** | **G** | **D** | **E** | **D** | **I** | **M** | **L** | **I** | **T** | **D** | **K** | **G** | **V** | **I** | **I** | **R** | **F** | **N** | **V** | **S** | **T** | **V** | **S** | **Q** | **T** | **G** | **R** | **S** | **T** | **L** | **G** | **V** | **R** | **L** | **M** | **K** | **M** | **E** | **A** | **D** | **T** | **K** | **V** | **V** | **T** | **M** | **A** | **A** | **V** | **E** | **P** | **E** | **A** | **A** | **E** | **Q** | **T** | **V** | **E** | **E** | **Q** | **G** | **S** | **E** | **E** |
| **N60200F** | **M** | **S** | **E** | **E** | **I** | **R** | **E** | **N** | **I** | **H** | **D** | **V** | **N** | **L** | **T** | **S** | **E** | **M** | **K** | **D** | **S** | **F** | **I** | **D** | **Y** | **A** | **M** | **S** | **V** | **I** | **V** | **A** | **R** | **A** | **L** | **P** | **D** | **V** | **R** | **D** | **G** | **L** | **K** | **P** | **V** | **H** | **R** | **R** | **I** | **L** | **Y** | **G** | **M** | **N** | **E** | **L** | **G** | **V** | **T** | **P** | **D** | **K** | **A** | **H** | **K** | **K** | **S** | **A** | **R** | **I** | **V** | **G** | **D** | **V** | **M** | **G** | **K** | **Y** | **H** | **P** | **H** | **G** | **D** | **S** | **A** | **I** | **Y** | **E** | **S** | **M** | **V** | **R** | **M** | **A** | **Q** | **P** | **F** | **S** | **Y** | **R** | **Y** | **M** | **L** | **V** | **D** | **G** | **H** | **G** | **N** | **F** | **G** | **S** | **V** | **D** | **G** | **D** | **G** | **A** | **A** | **A** | **M** | **R** | **Y** | **T** | **E** | **A** | **R** | **M** | **S** | **K** | **I** | **A** | **T** | **E** | **M** | **L** | **R** | **D** | **I** | **N** | **K** | **N** | **T** | **V** | **D** | **F** | **Q** | **S** | **N** | **Y** | **D** | **D** | **T** | **E** | **K** | **E** | **P** | **V** | **V** | **L** | **P** | **A** | **R** | **F** | **P** | **N** | **L** | **L** | **V** | **N** | **G** | **T** | **T** | **G** | **I** | **A** | **V** | **G** | **M** | **A** | **T** | **N** | **I** | **P** | **P** | **H** | **N** | **L** | **S** | **E** | **V** | **V** | **A** | **A** | **I** | **D** | **L** | **L** | **M** | **D** | **N** | **P** | **D** | **V** | **T** | **T** | **N** | **E** | **L** | **M** | **E** | **V** | **L** | **P** | **G** | **P** | **D** | **F** | **P** | **T** | **G** | **G** | **L** | **V** | **M** | **G** | **K** | **S** | **G** | **I** | **R** | **R** | **A** | **Y** | **E** | **T** | **G** | **K** | **G** | **S** | **I** | **T** | **V** | **R** | **A** | **K** | **V** | **E** | **I** | **T** | **E** | **M** | **P** | **N** | **G** | **K** | **E** | **R** | **I** | **I** | **V** | **T** | **E** | **L** | **P** | **Y** | **M** | **V** | **N** | **K** | **A** | **K** | **L** | **I** | **E** | **R** | **I** | **S** | **E** | **L** | **H** | **R** | **D** | **K** | **R** | **I** | **E** | **G** | **I** | **T** | **D** | **L** | **R** | **D** | **E** | **S** | **S** | **R** | **E** | **G** | **M** | **R** | **I** | **V** | **I** | **D** | **I** | **R** | **R** | **D** | **A** | **S** | **A** | **S** | **V** | **I** | **L** | **N** | **N** | **L** | **Y** | **K** | **L** | **T** | **S** | **L** | **Q** | **N** | **S** | **F** | **G** | **F** | **N** | **M** | **L** | **A** | **I** | **E** | **K** | **G** | **V** | **P** | **K** | **V** | **L** | **S** | **L** | **K** | **Q** | **I** | **L** | **E** | **N** | **Y** | **I** | **E** | **H** | **Q** | **R** | **E** | **V** | **I** | **T** | **R** | **R** | **T** | **V** | **F** | **E** | **K** | **E** | **K** | **A** | **E** | **A** | **R** | **A** | **H** | **I** | **L** | **E** | **G** | **L** | **R** | **I** | **A** | **L** | **D** | **H** | **I** | **D** | **E** | **I** | **I** | **A** | **I** | **I** | **R** | **G** | **S** | **K** | **S** | **D** | **D** | **E** | **A** | **K** | **A** | **T** | **M** | **I** | **E** | **R** | **F** | **D** | **L** | **S** | **D** | **R** | **Q** | **S** | **Q** | **A** | **I** | **L** | **D** | **M** | **R** | **L** | **R** | **R** | **L** | **T** | **G** | **L** | **E** | **R** | **E** | **K** | **I** | **E** | **N** | **E** | **Y** | **Q** | **E** | **L** | **L** | **K** | **L** | **I** | **E** | **D** | **L** | **T** | **D** | **I** | **L** | **A** | **R** | **P** | **E** | **R** | **V** | **T** | **E** | **I** | **I** | **K** | **N** | **E** | **L** | **A** | **D** | **L** | **N** | **Q** | **R** | **F** | **G** | **D** | **K** | **R** | **R** | **T** | **E** | **L** | **L** | **V** | **G** | **E** | **V** | **L** | **S** | **L** | **E** | **D** | **E** | **D** | **L** | **I** | **E** | **E** | **E** | **E** | **I** | **V** | **I** | **T** | **L** | **T** | **N** | **N** | **G** | **Y** | **I** | **K** | **R** | **L** | **A** | **N** | **N** | **E** | **F** | **R** | **A** | **Q** | **R** | **R** | **G** | **G** | **R** | **G** | **V** | **Q** | **G** | **M** | **G** | **I** | **H** | **N** | **D** | **D** | **F** | **V** | **K** | **N** | **L** | **V** | **S** | **C** | **S** | **T** | **H** | **D** | **T** | **L** | **L** | **F** | **F** | **T** | **N** | **N** | **G** | **K** | **V** | **Y** | **R** | **A** | **K** | **G** | **Y** | **E** | **I** | **P** | **E** | **Y** | **G** | **R** | **T** | **A** | **K** | **G** | **I** | **P** | **I** | **I** | **N** | **L** | **L** | **G** | **I** | **D** | **S** | **S** | **E** | **S** | **I** | **Q** | **A** | **I** | **I** | **A** | **V** | **T** | **G** | **E** | **A** | **E** | **E** | **G** | **H** | **Y** | **L** | **F** | **F** | **T** | **T** | **R** | **Q** | **G** | **T** | **V** | **K** | **R** | **T** | **S** | **V** | **T** | **A** | **F** | **A** | **N** | **I** | **R** | **S** | **N** | **G** | **L** | **I** | **A** | **I** | **G** | **L** | **K** | **E** | **D** | **D** | **E** | **L** | **V** | **N** | **V** | **L** | **L** | **T** | **D** | **G** | **Q** | **S** | **N** | **I** | **I** | **I** | **G** | **T** | **H** | **N** | **G** | **Y** | **S** | **V** | **T** | **F** | **K** | **E** | **E** | **A** | **V** | **R** | **D** | **M** | **G** | **R** | **T** | **A** | **S** | **G** | **V** | **R** | **G** | **I** | **R** | **L** | **R** | **E** | **E** | **D** | **Y** | **V** | **V** | **G** | **A** | **A** | **L** | **M** | **K** | **E** | **N** | **Q** | **E** | **V** | **L** | **V** | **I** | **T** | **E** | **K** | **G** | **Y** | **G** | **K** | **R** | **T** | **K** | **V** | **S** | **E** | **Y** | **P** | **V** | **K** | **G** | **R** | **G** | **G** | **K** | **G** | **I** | **K** | **T** | **A** | **N** | **I** | **T** | **E** | **K** | **N** | **G** | **P** | **L** | **A** | **G** | **L** | **T** | **T** | **V** | **S** | **G** | **D** | **E** | **D** | **I** | **M** | **L** | **I** | **T** | **D** | **K** | **G** | **V** | **I** | **I** | **R** | **F** | **N** | **V** | **S** | **T** | **V** | **S** | **Q** | **T** | **G** | **R** | **S** | **T** | **L** | **G** | **V** | **R** | **L** | **M** | **K** | **M** | **E** | **A** | **D** | **T** | **K** | **V** | **V** | **T** | **M** | **A** | **A** | **V** | **E** | **P** | **E** | **A** | **A** | **E** | **Q** | **T** | **V** | **E** | **E** | **Q** | **G** | **S** | **E** | **E** |
| **N55077** | **M** | **S** | **E** | **E** | **I** | **R** | **E** | **N** | **I** | **H** | **D** | **V** | **N** | **L** | **T** | **S** | **E** | **M** | **K** | **D** | **S** | **F** | **I** | **D** | **Y** | **A** | **M** | **S** | **V** | **I** | **V** | **A** | **R** | **A** | **L** | **P** | **D** | **V** | **R** | **D** | **G** | **L** | **K** | **P** | **V** | **H** | **R** | **R** | **I** | **L** | **Y** | **G** | **M** | **N** | **E** | **L** | **G** | **V** | **T** | **P** | **D** | **K** | **A** | **H** | **K** | **K** | **S** | **A** | **R** | **I** | **V** | **G** | **D** | **V** | **M** | **G** | **K** | **Y** | **H** | **P** | **H** | **G** | **D** | **S** | **A** | **I** | **Y** | **E** | **S** | **M** | **V** | **R** | **M** | **A** | **Q** | **P** | **F** | **S** | **Y** | **R** | **Y** | **M** | **L** | **V** | **D** | **G** | **H** | **G** | **N** | **F** | **G** | **S** | **V** | **D** | **G** | **D** | **G** | **A** | **A** | **A** | **M** | **R** | **Y** | **T** | **E** | **A** | **R** | **M** | **S** | **K** | **I** | **A** | **T** | **E** | **M** | **L** | **R** | **D** | **I** | **N** | **K** | **N** | **T** | **V** | **D** | **F** | **Q** | **S** | **N** | **Y** | **D** | **D** | **T** | **E** | **K** | **E** | **P** | **V** | **V** | **L** | **P** | **A** | **R** | **F** | **P** | **N** | **L** | **L** | **V** | **N** | **G** | **T** | **T** | **G** | **I** | **A** | **V** | **G** | **M** | **A** | **T** | **N** | **I** | **P** | **P** | **H** | **N** | **L** | **S** | **E** | **V** | **V** | **A** | **A** | **I** | **D** | **L** | **L** | **M** | **D** | **N** | **P** | **D** | **V** | **T** | **T** | **N** | **E** | **L** | **M** | **E** | **V** | **L** | **P** | **G** | **P** | **D** | **F** | **P** | **T** | **G** | **G** | **L** | **V** | **M** | **G** | **K** | **S** | **G** | **I** | **R** | **R** | **A** | **Y** | **E** | **T** | **G** | **K** | **G** | **S** | **I** | **T** | **V** | **R** | **A** | **K** | **V** | **E** | **I** | **T** | **E** | **M** | **P** | **N** | **G** | **K** | **E** | **R** | **I** | **I** | **V** | **T** | **E** | **L** | **P** | **Y** | **M** | **V** | **N** | **K** | **A** | **K** | **L** | **I** | **E** | **R** | **I** | **S** | **E** | **L** | **H** | **R** | **D** | **K** | **R** | **I** | **E** | **G** | **I** | **T** | **D** | **L** | **R** | **D** | **E** | **S** | **S** | **R** | **E** | **G** | **M** | **R** | **I** | **V** | **I** | **D** | **I** | **R** | **R** | **D** | **A** | **S** | **A** | **S** | **V** | **I** | **L** | **N** | **N** | **L** | **Y** | **K** | **L** | **T** | **S** | **L** | **Q** | **N** | **S** | **F** | **G** | **F** | **N** | **M** | **L** | **A** | **I** | **E** | **K** | **G** | **V** | **P** | **K** | **V** | **L** | **S** | **L** | **K** | **Q** | **I** | **L** | **E** | **N** | **Y** | **I** | **E** | **H** | **Q** | **R** | **E** | **V** | **I** | **T** | **R** | **R** | **T** | **V** | **F** | **E** | **K** | **E** | **K** | **A** | **E** | **A** | **R** | **A** | **H** | **I** | **L** | **E** | **G** | **L** | **R** | **I** | **A** | **L** | **D** | **H** | **I** | **D** | **E** | **I** | **I** | **A** | **I** | **I** | **R** | **G** | **S** | **K** | **S** | **D** | **D** | **E** | **A** | **K** | **A** | **T** | **M** | **I** | **E** | **R** | **F** | **D** | **L** | **S** | **D** | **R** | **Q** | **S** | **Q** | **A** | **I** | **L** | **D** | **M** | **R** | **L** | **R** | **R** | **L** | **T** | **G** | **L** | **E** | **R** | **E** | **K** | **I** | **E** | **N** | **E** | **Y** | **Q** | **E** | **L** | **L** | **K** | **L** | **I** | **E** | **D** | **L** | **T** | **D** | **I** | **L** | **A** | **R** | **P** | **E** | **R** | **V** | **T** | **E** | **I** | **I** | **K** | **N** | **E** | **L** | **A** | **D** | **L** | **N** | **Q** | **R** | **F** | **G** | **D** | **K** | **R** | **R** | **T** | **E** | **L** | **L** | **V** | **G** | **E** | **V** | **L** | **S** | **L** | **E** | **D** | **E** | **D** | **L** | **I** | **E** | **E** | **E** | **E** | **I** | **V** | **I** | **T** | **L** | **T** | **N** | **N** | **G** | **Y** | **I** | **K** | **R** | **L** | **A** | **N** | **N** | **E** | **F** | **R** | **A** | **Q** | **R** | **R** | **G** | **G** | **R** | **G** | **V** | **Q** | **G** | **M** | **G** | **I** | **H** | **N** | **D** | **D** | **F** | **V** | **K** | **N** | **L** | **V** | **S** | **C** | **S** | **T** | **H** | **D** | **T** | **L** | **L** | **F** | **F** | **T** | **N** | **N** | **G** | **K** | **V** | **Y** | **R** | **A** | **K** | **G** | **Y** | **E** | **I** | **P** | **E** | **Y** | **G** | **R** | **T** | **A** | **K** | **G** | **I** | **P** | **I** | **I** | **N** | **L** | **L** | **G** | **I** | **D** | **S** | **S** | **E** | **S** | **I** | **Q** | **A** | **I** | **I** | **A** | **V** | **T** | **G** | **E** | **A** | **E** | **E** | **G** | **H** | **Y** | **L** | **F** | **F** | **T** | **T** | **R** | **Q** | **G** | **T** | **V** | **K** | **R** | **T** | **S** | **V** | **T** | **A** | **F** | **A** | **N** | **I** | **R** | **S** | **N** | **G** | **L** | **I** | **A** | **I** | **G** | **L** | **K** | **E** | **D** | **D** | **E** | **L** | **V** | **N** | **V** | **L** | **L** | **T** | **D** | **G** | **Q** | **S** | **N** | **I** | **I** | **I** | **G** | **T** | **H** | **N** | **G** | **Y** | **S** | **V** | **T** | **F** | **K** | **E** | **E** | **A** | **V** | **R** | **D** | **M** | **G** | **R** | **T** | **A** | **S** | **G** | **V** | **R** | **G** | **I** | **R** | **L** | **R** | **E** | **E** | **D** | **Y** | **V** | **V** | **G** | **A** | **A** | **L** | **M** | **K** | **E** | **D** | **Q** | **E** | **V** | **L** | **V** | **I** | **T** | **E** | **K** | **G** | **Y** | **G** | **K** | **R** | **T** | **K** | **V** | **S** | **E** | **Y** | **P** | **V** | **K** | **G** | **R** | **G** | **G** | **K** | **G** | **I** | **K** | **T** | **A** | **N** | **I** | **T** | **E** | **K** | **N** | **G** | **P** | **L** | **A** | **G** | **L** | **T** | **T** | **V** | **S** | **G** | **D** | **E** | **D** | **I** | **M** | **L** | **I** | **T** | **D** | **K** | **G** | **V** | **I** | **I** | **R** | **F** | **N** | **V** | **S** | **T** | **V** | **S** | **Q** | **T** | **G** | **R** | **S** | **T** | **L** | **G** | **V** | **R** | **L** | **M** | **K** | **M** | **E** | **A** | **D** | **T** | **K** | **V** | **V** | **T** | **M** | **A** | **A** | **V** | **E** | **P** | **E** | **A** | **A** | **E** | **Q** | **T** | **V** | **E** | **E** | **Q** | **G** | **S** | **E** | **E** |
| **N54676** | **M** | **S** | **E** | **E** | **I** | **K** | **E** | **N** | **I** | **Q** | **D** | **V** | **N** | **L** | **T** | **S** | **E** | **M** | **K** | **E** | **S** | **F** | **I** | **D** | **Y** | **A** | **M** | **S** | **V** | **I** | **V** | **A** | **R** | **A** | **L** | **P** | **D** | **V** | **R** | **D** | **G** | **L** | **K** | **P** | **V** | **H** | **R** | **R** | **I** | **L** | **Y** | **G** | **M** | **N** | **E** | **L** | **G** | **V** | **T** | **P** | **D** | **K** | **P** | **H** | **K** | **K** | **S** | **A** | **R** | **I** | **V** | **G** | **D** | **V** | **M** | **G** | **K** | **Y** | **H** | **P** | **H** | **G** | **D** | **S** | **A** | **I** | **Y** | **E** | **S** | **M** | **V** | **R** | **M** | **A** | **Q** | **P** | **F** | **S** | **Y** | **R** | **A** | **M** | **L** | **V** | **D** | **G** | **H** | **G** | **N** | **F** | **G** | **S** | **V** | **D** | **G** | **D** | **G** | **A** | **A** | **A** | **M** | **R** | **Y** | **T** | **E** | **A** | **R** | **M** | **S** | **K** | **I** | **A** | **L** | **E** | **M** | **L** | **R** | **D** | **I** | **N** | **K** | **N** | **T** | **V** | **D** | **F** | **Q** | **G** | **N** | **Y** | **D** | **D** | **S** | **E** | **Q** | **E** | **P** | **V** | **V** | **L** | **P** | **A** | **R** | **F** | **P** | **N** | **L** | **L** | **V** | **N** | **G** | **T** | **T** | **G** | **I** | **A** | **V** | **G** | **M** | **A** | **T** | **N** | **I** | **P** | **P** | **H** | **N** | **L** | **S** | **E** | **V** | **I** | **D** | **A** | **T** | **S** | **L** | **L** | **M** | **D** | **N** | **P** | **D** | **V** | **T** | **T** | **N** | **E** | **L** | **M** | **E** | **V** | **L** | **P** | **G** | **P** | **D** | **F** | **P** | **T** | **G** | **G** | **L** | **V** | **M** | **G** | **K** | **S** | **G** | **I** | **R** | **R** | **A** | **Y** | **E** | **T** | **G** | **K** | **G** | **S** | **I** | **T** | **V** | **R** | **A** | **K** | **V** | **E** | **L** | **T** | **E** | **M** | **P** | **N** | **G** | **K** | **E** | **R** | **I** | **L** | **V** | **T** | **E** | **L** | **P** | **Y** | **M** | **V** | **N** | **K** | **A** | **K** | **L** | **I** | **E** | **R** | **I** | **S** | **E** | **L** | **H** | **R** | **D** | **K** | **R** | **I** | **E** | **G** | **I** | **T** | **D** | **L** | **R** | **D** | **E** | **S** | **S** | **R** | **E** | **G** | **M** | **R** | **I** | **V** | **I** | **D** | **V** | **R** | **R** | **D** | **V** | **S** | **A** | **S** | **V** | **V** | **L** | **N** | **N** | **L** | **Y** | **K** | **M** | **T** | **A** | **L** | **Q** | **T** | **S** | **F** | **G** | **F** | **N** | **M** | **L** | **A** | **I** | **E** | **K** | **G** | **V** | **P** | **K** | **I** | **L** | **S** | **L** | **K** | **R** | **I** | **L** | **E** | **N** | **Y** | **V** | **E** | **H** | **Q** | **K** | **E** | **V** | **I** | **T** | **R** | **R** | **T** | **I** | **F** | **D** | **K** | **N** | **K** | **A** | **E** | **A** | **R** | **A** | **H** | **I** | **L** | **E** | **G** | **L** | **R** | **I** | **A** | **L** | **D** | **H** | **I** | **D** | **E** | **I** | **I** | **A** | **I** | **I** | **R** | **G** | **S** | **Q** | **S** | **D** | **D** | **E** | **A** | **K** | **A** | **T** | **L** | **I** | **E** | **R** | **F** | **E** | **F** | **S** | **D** | **R** | **Q** | **A** | **Q** | **A** | **I** | **L** | **D** | **M** | **R** | **L** | **R** | **R** | **L** | **T** | **G** | **L** | **E** | **R** | **D** | **K** | **I** | **E** | **N** | **E** | **Y** | **Q** | **E** | **L** | **L** | **K** | **F** | **I** | **A** | **D** | **L** | **E** | **D** | **I** | **L** | **A** | **R** | **P** | **E** | **R** | **V** | **I** | **E** | **I** | **I** | **K** | **T** | **E** | **L** | **N** | **D** | **V** | **R** | **T** | **K** | **F** | **G** | **D** | **A** | **R** | **R** | **T** | **E** | **L** | **L** | **V** | **G** | **E** | **V** | **L** | **S** | **L** | **E** | **D** | **E** | **D** | **L** | **I** | **E** | **E** | **E** | **E** | **V** | **V** | **I** | **T** | **L** | **T** | **N** | **N** | **G** | **Y** | **I** | **K** | **R** | **M** | **A** | **N** | **S** | **E** | **F** | **R** | **A** | **Q** | **R** | **R** | **G** | **G** | **R** | **G** | **V** | **Q** | **G** | **M** | **G** | **V** | **H** | **D** | **D** | **D** | **F** | **V** | **K** | **N** | **L** | **V** | **S** | **C** | **S** | **T** | **H** | **D** | **T** | **L** | **L** | **F** | **F** | **T** | **N** | **T** | **G** | **K** | **V** | **Y** | **R** | **A** | **K** | **G** | **Y** | **E** | **I** | **P** | **E** | **Y** | **G** | **R** | **T** | **A** | **K** | **G** | **I** | **P** | **V** | **I** | **N** | **L** | **L** | **G** | **I** | **D** | **S** | **A** | **E** | **K** | **I** | **Q** | **A** | **I** | **I** | **S** | **V** | **E** | **G** | **K** | **A** | **E** | **A** | **G** | **K** | **Y** | **L** | **F** | **F** | **T** | **T** | **L** | **K** | **G** | **T** | **V** | **K** | **R** | **T** | **A** | **V** | **T** | **A** | **F** | **S** | **N** | **I** | **R** | **S** | **N** | **G** | **L** | **I** | **A** | **I** | **S** | **L** | **K** | **E** | **D** | **D** | **E** | **L** | **V** | **N** | **V** | **V** | **T** | **T** | **N** | **G** | **N** | **Q** | **K** | **M** | **I** | **I** | **G** | **T** | **H** | **A** | **G** | **Y** | **S** | **V** | **T** | **F** | **D** | **E** | **N** | **T** | **V** | **R** | **D** | **M** | **G** | **R** | **T** | **A** | **S** | **G** | **V** | **R** | **G** | **I** | **R** | **L** | **R** | **E** | **N** | **D** | **Y** | **V** | **V** | **G** | **A** | **A** | **I** | **L** | **D** | **E** | **N** | **K** | **E** | **V** | **L** | **V** | **I** | **T** | **E** | **N** | **G** | **Y** | **G** | **K** | **R** | **T** | **K** | **A** | **S** | **E** | **Y** | **P** | **V** | **K** | **G** | **R** | **G** | **G** | **K** | **G** | **I** | **K** | **T** | **A** | **N** | **I** | **T** | **E** | **K** | **N** | **G** | **P** | **L** | **A** | **G** | **L** | **T** | **T** | **V** | **N** | **G** | **D** | **E** | **D** | **I** | **L** | **L** | **I** | **T** | **N** | **K** | **G** | **V** | **I** | **I** | **R** | **F** | **N** | **V** | **D** | **S** | **V** | **S** | **Q** | **T** | **G** | **R** | **A** | **T** | **L** | **G** | **V** | **R** | **L** | **M** | **R** | **M** | **E** | **D** | **G** | **A** | **K** | **-** | **-** | **-** | **-** | **-** | **-** | **-** | **-** | **-** | **-** | **-** | **-** | **-** | **-** | **-** | **-** | **-** | **-** | **-** | **-** | **-** | **-** | **-** |
| **N54567** | **M** | **S** | **E** | **E** | **I** | **R** | **E** | **N** | **I** | **H** | **D** | **V** | **N** | **L** | **T** | **S** | **E** | **M** | **K** | **D** | **S** | **F** | **I** | **D** | **Y** | **A** | **M** | **S** | **V** | **I** | **V** | **A** | **R** | **A** | **L** | **P** | **D** | **V** | **R** | **D** | **G** | **L** | **K** | **P** | **V** | **H** | **R** | **R** | **I** | **L** | **Y** | **G** | **M** | **N** | **E** | **L** | **G** | **V** | **T** | **P** | **D** | **K** | **A** | **H** | **K** | **K** | **S** | **A** | **R** | **I** | **V** | **G** | **D** | **V** | **M** | **G** | **K** | **Y** | **H** | **P** | **H** | **G** | **D** | **S** | **A** | **I** | **Y** | **E** | **S** | **M** | **V** | **R** | **M** | **A** | **Q** | **P** | **F** | **S** | **Y** | **R** | **Y** | **M** | **L** | **V** | **D** | **G** | **H** | **G** | **N** | **F** | **G** | **S** | **V** | **D** | **G** | **D** | **G** | **A** | **A** | **A** | **M** | **R** | **Y** | **T** | **E** | **A** | **R** | **M** | **S** | **K** | **I** | **A** | **T** | **E** | **M** | **L** | **R** | **D** | **I** | **N** | **K** | **N** | **T** | **V** | **D** | **F** | **Q** | **S** | **N** | **Y** | **D** | **D** | **T** | **E** | **K** | **E** | **P** | **V** | **V** | **L** | **P** | **A** | **R** | **F** | **P** | **N** | **L** | **L** | **V** | **N** | **G** | **T** | **T** | **G** | **I** | **A** | **V** | **G** | **M** | **A** | **T** | **N** | **I** | **P** | **P** | **H** | **N** | **L** | **S** | **E** | **V** | **V** | **A** | **A** | **I** | **D** | **L** | **L** | **M** | **D** | **N** | **P** | **D** | **V** | **T** | **T** | **N** | **E** | **L** | **M** | **E** | **V** | **L** | **P** | **G** | **P** | **D** | **F** | **P** | **T** | **G** | **G** | **L** | **V** | **M** | **G** | **K** | **S** | **G** | **I** | **R** | **R** | **A** | **Y** | **E** | **T** | **G** | **K** | **G** | **S** | **I** | **T** | **V** | **R** | **A** | **K** | **V** | **E** | **I** | **T** | **E** | **M** | **P** | **N** | **G** | **K** | **E** | **R** | **I** | **I** | **V** | **T** | **E** | **L** | **P** | **Y** | **M** | **V** | **N** | **K** | **A** | **K** | **L** | **I** | **E** | **R** | **I** | **S** | **E** | **L** | **H** | **R** | **D** | **K** | **R** | **I** | **E** | **G** | **I** | **T** | **D** | **L** | **R** | **D** | **E** | **S** | **S** | **R** | **E** | **G** | **M** | **R** | **I** | **V** | **I** | **D** | **I** | **R** | **R** | **D** | **A** | **S** | **A** | **S** | **V** | **I** | **L** | **N** | **N** | **L** | **Y** | **K** | **L** | **T** | **S** | **L** | **Q** | **N** | **S** | **F** | **G** | **F** | **N** | **M** | **L** | **A** | **I** | **E** | **K** | **G** | **V** | **P** | **K** | **V** | **L** | **S** | **L** | **K** | **Q** | **I** | **L** | **E** | **N** | **Y** | **I** | **E** | **H** | **Q** | **R** | **E** | **V** | **I** | **T** | **R** | **R** | **T** | **V** | **F** | **E** | **K** | **E** | **K** | **A** | **E** | **A** | **R** | **A** | **H** | **I** | **L** | **E** | **G** | **L** | **R** | **I** | **A** | **L** | **D** | **H** | **I** | **D** | **E** | **I** | **I** | **A** | **I** | **I** | **R** | **G** | **S** | **K** | **S** | **D** | **D** | **E** | **A** | **K** | **A** | **T** | **M** | **I** | **E** | **R** | **F** | **D** | **L** | **S** | **D** | **R** | **Q** | **S** | **Q** | **A** | **I** | **L** | **D** | **M** | **R** | **L** | **R** | **R** | **L** | **T** | **G** | **L** | **E** | **R** | **E** | **K** | **I** | **E** | **N** | **E** | **Y** | **Q** | **E** | **L** | **L** | **K** | **L** | **I** | **E** | **D** | **L** | **T** | **D** | **I** | **L** | **A** | **R** | **P** | **E** | **R** | **V** | **T** | **E** | **I** | **I** | **K** | **N** | **E** | **L** | **A** | **D** | **L** | **N** | **Q** | **R** | **F** | **G** | **D** | **K** | **R** | **R** | **T** | **E** | **L** | **L** | **V** | **G** | **E** | **V** | **L** | **S** | **L** | **E** | **D** | **E** | **D** | **L** | **I** | **E** | **E** | **E** | **E** | **I** | **V** | **I** | **T** | **L** | **T** | **N** | **N** | **G** | **Y** | **I** | **K** | **R** | **L** | **A** | **N** | **N** | **E** | **F** | **R** | **A** | **Q** | **R** | **R** | **G** | **G** | **R** | **G** | **V** | **Q** | **G** | **M** | **G** | **I** | **H** | **N** | **D** | **D** | **F** | **V** | **K** | **N** | **L** | **V** | **S** | **C** | **S** | **T** | **H** | **D** | **T** | **L** | **L** | **F** | **F** | **T** | **N** | **N** | **G** | **K** | **V** | **Y** | **R** | **A** | **K** | **G** | **Y** | **E** | **I** | **P** | **E** | **Y** | **G** | **R** | **T** | **A** | **K** | **G** | **I** | **P** | **I** | **I** | **N** | **L** | **L** | **G** | **I** | **D** | **S** | **S** | **E** | **S** | **I** | **Q** | **A** | **I** | **I** | **A** | **V** | **T** | **G** | **E** | **A** | **E** | **E** | **G** | **H** | **Y** | **L** | **F** | **F** | **T** | **T** | **R** | **Q** | **G** | **T** | **V** | **K** | **R** | **T** | **S** | **V** | **T** | **A** | **F** | **A** | **N** | **I** | **R** | **S** | **N** | **G** | **L** | **I** | **A** | **I** | **G** | **L** | **K** | **E** | **D** | **D** | **E** | **L** | **V** | **N** | **V** | **L** | **L** | **T** | **D** | **G** | **Q** | **S** | **N** | **I** | **I** | **I** | **G** | **T** | **H** | **N** | **G** | **Y** | **S** | **V** | **T** | **F** | **K** | **E** | **E** | **A** | **V** | **R** | **D** | **M** | **G** | **R** | **T** | **A** | **S** | **G** | **V** | **R** | **G** | **I** | **R** | **L** | **R** | **E** | **E** | **D** | **Y** | **V** | **V** | **G** | **A** | **A** | **L** | **M** | **K** | **E** | **D** | **Q** | **E** | **V** | **L** | **V** | **I** | **T** | **E** | **K** | **G** | **Y** | **G** | **K** | **R** | **T** | **K** | **V** | **S** | **E** | **Y** | **P** | **V** | **K** | **G** | **R** | **G** | **G** | **K** | **G** | **I** | **K** | **T** | **A** | **N** | **I** | **T** | **E** | **K** | **N** | **G** | **P** | **L** | **A** | **G** | **L** | **T** | **T** | **V** | **S** | **G** | **D** | **E** | **D** | **I** | **M** | **L** | **I** | **T** | **D** | **K** | **G** | **V** | **I** | **I** | **R** | **F** | **N** | **V** | **S** | **T** | **V** | **S** | **Q** | **T** | **G** | **R** | **S** | **T** | **L** | **G** | **V** | **R** | **L** | **M** | **K** | **M** | **E** | **A** | **D** | **T** | **K** | **V** | **V** | **T** | **M** | **A** | **A** | **V** | **E** | **P** | **E** | **A** | **A** | **E** | **Q** | **T** | **V** | **E** | **E** | **Q** | **G** | **S** | **E** | **E** |
| **N59550F** | **M** | **S** | **E** | **E** | **I** | **K** | **E** | **N** | **I** | **Q** | **D** | **V** | **N** | **L** | **T** | **S** | **E** | **M** | **K** | **E** | **S** | **F** | **I** | **D** | **Y** | **A** | **M** | **S** | **V** | **I** | **V** | **A** | **R** | **A** | **L** | **P** | **D** | **V** | **R** | **D** | **G** | **L** | **K** | **P** | **V** | **H** | **R** | **R** | **I** | **L** | **Y** | **G** | **M** | **N** | **E** | **L** | **G** | **V** | **T** | **P** | **D** | **K** | **P** | **H** | **K** | **K** | **S** | **A** | **R** | **I** | **V** | **G** | **D** | **V** | **M** | **G** | **K** | **Y** | **H** | **P** | **H** | **G** | **D** | **S** | **A** | **I** | **Y** | **E** | **S** | **M** | **V** | **R** | **M** | **A** | **Q** | **P** | **F** | **S** | **Y** | **R** | **A** | **M** | **L** | **V** | **D** | **G** | **H** | **G** | **N** | **F** | **G** | **S** | **V** | **D** | **G** | **D** | **G** | **A** | **A** | **A** | **M** | **R** | **Y** | **T** | **E** | **A** | **R** | **M** | **S** | **K** | **I** | **A** | **L** | **E** | **M** | **L** | **R** | **D** | **I** | **N** | **K** | **N** | **T** | **V** | **D** | **F** | **Q** | **G** | **N** | **Y** | **D** | **D** | **S** | **E** | **Q** | **E** | **P** | **V** | **V** | **L** | **P** | **A** | **R** | **F** | **P** | **N** | **L** | **L** | **V** | **N** | **G** | **T** | **T** | **G** | **I** | **A** | **V** | **G** | **M** | **A** | **T** | **N** | **I** | **P** | **P** | **H** | **N** | **L** | **S** | **E** | **V** | **I** | **D** | **A** | **T** | **S** | **L** | **L** | **M** | **D** | **N** | **P** | **D** | **V** | **T** | **T** | **N** | **E** | **L** | **M** | **E** | **V** | **L** | **P** | **G** | **P** | **D** | **F** | **P** | **T** | **G** | **G** | **L** | **V** | **M** | **G** | **K** | **S** | **G** | **I** | **R** | **R** | **A** | **Y** | **E** | **T** | **G** | **K** | **G** | **S** | **I** | **T** | **V** | **R** | **A** | **K** | **V** | **E** | **L** | **T** | **E** | **M** | **P** | **N** | **G** | **K** | **E** | **R** | **I** | **L** | **V** | **T** | **E** | **L** | **P** | **Y** | **M** | **V** | **N** | **K** | **A** | **K** | **L** | **I** | **E** | **R** | **I** | **S** | **E** | **L** | **H** | **R** | **D** | **K** | **R** | **I** | **E** | **G** | **I** | **T** | **D** | **L** | **R** | **D** | **E** | **S** | **S** | **R** | **E** | **G** | **M** | **R** | **I** | **V** | **I** | **D** | **V** | **R** | **R** | **D** | **V** | **S** | **A** | **S** | **V** | **V** | **L** | **N** | **N** | **L** | **Y** | **K** | **M** | **T** | **A** | **L** | **Q** | **T** | **S** | **F** | **G** | **F** | **N** | **M** | **L** | **A** | **I** | **E** | **K** | **G** | **V** | **P** | **K** | **I** | **L** | **S** | **L** | **K** | **R** | **I** | **L** | **E** | **N** | **Y** | **V** | **E** | **H** | **Q** | **K** | **E** | **V** | **I** | **T** | **R** | **R** | **T** | **I** | **F** | **D** | **K** | **N** | **K** | **A** | **E** | **A** | **R** | **A** | **H** | **I** | **L** | **E** | **G** | **L** | **R** | **I** | **A** | **L** | **D** | **H** | **I** | **D** | **E** | **I** | **I** | **A** | **I** | **I** | **R** | **G** | **S** | **Q** | **S** | **D** | **D** | **E** | **A** | **K** | **A** | **T** | **L** | **I** | **E** | **R** | **F** | **E** | **F** | **S** | **D** | **R** | **Q** | **A** | **Q** | **A** | **I** | **L** | **D** | **M** | **R** | **L** | **R** | **R** | **L** | **T** | **G** | **L** | **E** | **R** | **D** | **K** | **I** | **E** | **N** | **E** | **Y** | **Q** | **E** | **L** | **L** | **K** | **F** | **I** | **A** | **D** | **L** | **E** | **D** | **I** | **L** | **A** | **R** | **P** | **E** | **R** | **V** | **I** | **E** | **I** | **I** | **K** | **T** | **E** | **L** | **N** | **D** | **V** | **R** | **T** | **K** | **F** | **G** | **D** | **A** | **R** | **R** | **T** | **E** | **L** | **L** | **V** | **G** | **E** | **V** | **L** | **S** | **L** | **E** | **D** | **E** | **D** | **L** | **I** | **E** | **E** | **E** | **E** | **V** | **V** | **I** | **T** | **L** | **T** | **N** | **N** | **G** | **Y** | **I** | **K** | **R** | **M** | **A** | **N** | **S** | **E** | **F** | **R** | **A** | **Q** | **R** | **R** | **G** | **G** | **R** | **G** | **V** | **Q** | **G** | **M** | **G** | **V** | **H** | **D** | **D** | **D** | **F** | **V** | **K** | **N** | **L** | **V** | **S** | **C** | **S** | **T** | **H** | **D** | **T** | **L** | **L** | **F** | **F** | **T** | **N** | **T** | **G** | **K** | **V** | **Y** | **R** | **A** | **K** | **G** | **Y** | **E** | **I** | **P** | **E** | **Y** | **G** | **R** | **T** | **A** | **K** | **G** | **I** | **P** | **V** | **I** | **N** | **L** | **L** | **G** | **I** | **D** | **S** | **A** | **E** | **K** | **I** | **Q** | **A** | **I** | **I** | **S** | **V** | **E** | **G** | **K** | **A** | **E** | **A** | **G** | **K** | **Y** | **L** | **F** | **F** | **T** | **T** | **L** | **K** | **G** | **T** | **V** | **K** | **R** | **T** | **A** | **V** | **T** | **A** | **F** | **S** | **N** | **I** | **R** | **S** | **N** | **G** | **L** | **I** | **A** | **I** | **S** | **L** | **K** | **E** | **D** | **D** | **E** | **L** | **V** | **N** | **V** | **V** | **T** | **T** | **N** | **G** | **N** | **Q** | **K** | **M** | **I** | **I** | **G** | **T** | **H** | **A** | **G** | **Y** | **S** | **V** | **T** | **F** | **D** | **E** | **N** | **T** | **V** | **R** | **D** | **M** | **G** | **R** | **T** | **A** | **S** | **G** | **V** | **R** | **G** | **I** | **R** | **L** | **R** | **E** | **N** | **D** | **Y** | **V** | **V** | **G** | **A** | **A** | **I** | **L** | **D** | **E** | **N** | **K** | **E** | **V** | **L** | **V** | **I** | **T** | **E** | **N** | **G** | **Y** | **G** | **K** | **R** | **T** | **K** | **A** | **S** | **E** | **Y** | **P** | **V** | **K** | **G** | **R** | **G** | **G** | **K** | **G** | **I** | **K** | **T** | **A** | **N** | **I** | **T** | **E** | **K** | **N** | **G** | **P** | **L** | **A** | **G** | **L** | **T** | **T** | **V** | **N** | **G** | **D** | **E** | **D** | **I** | **L** | **L** | **I** | **T** | **N** | **K** | **G** | **V** | **I** | **I** | **R** | **F** | **N** | **V** | **D** | **S** | **V** | **S** | **Q** | **T** | **G** | **R** | **A** | **T** | **L** | **G** | **V** | **R** | **L** | **M** | **R** | **M** | **E** | **D** | **G** | **A** | **K** | **-** | **-** | **-** | **-** | **-** | **-** | **-** | **-** | **-** | **-** | **-** | **-** | **-** | **-** | **-** | **-** | **-** | **-** | **-** | **-** | **-** | **-** | **-** |
| **N53661** | **M** | **S** | **E** | **E** | **I** | **K** | **E** | **N** | **I** | **Q** | **D** | **V** | **N** | **L** | **T** | **S** | **E** | **M** | **K** | **E** | **S** | **F** | **I** | **D** | **Y** | **A** | **M** | **S** | **V** | **I** | **V** | **A** | **R** | **A** | **L** | **P** | **D** | **V** | **R** | **D** | **G** | **L** | **K** | **P** | **V** | **H** | **R** | **R** | **I** | **L** | **Y** | **G** | **M** | **N** | **E** | **L** | **G** | **V** | **T** | **P** | **D** | **K** | **P** | **H** | **K** | **K** | **S** | **A** | **R** | **I** | **V** | **G** | **D** | **V** | **M** | **G** | **K** | **Y** | **H** | **P** | **H** | **G** | **D** | **S** | **A** | **I** | **Y** | **E** | **S** | **M** | **V** | **R** | **M** | **A** | **Q** | **P** | **F** | **S** | **Y** | **R** | **A** | **M** | **L** | **V** | **D** | **G** | **H** | **G** | **N** | **F** | **G** | **S** | **V** | **D** | **G** | **D** | **G** | **A** | **A** | **A** | **M** | **R** | **Y** | **T** | **E** | **A** | **R** | **M** | **S** | **K** | **I** | **A** | **L** | **E** | **M** | **L** | **R** | **D** | **I** | **N** | **K** | **N** | **T** | **V** | **D** | **F** | **Q** | **G** | **N** | **Y** | **D** | **D** | **S** | **E** | **Q** | **E** | **P** | **V** | **V** | **L** | **P** | **A** | **R** | **F** | **P** | **N** | **L** | **L** | **V** | **N** | **G** | **T** | **T** | **G** | **I** | **A** | **V** | **G** | **M** | **A** | **T** | **N** | **I** | **P** | **P** | **H** | **N** | **L** | **S** | **E** | **V** | **I** | **D** | **A** | **T** | **S** | **L** | **L** | **M** | **D** | **N** | **P** | **D** | **V** | **T** | **T** | **N** | **E** | **L** | **M** | **E** | **V** | **L** | **P** | **G** | **P** | **D** | **F** | **P** | **T** | **G** | **G** | **L** | **V** | **M** | **G** | **K** | **S** | **G** | **I** | **R** | **R** | **A** | **Y** | **E** | **T** | **G** | **K** | **G** | **S** | **I** | **T** | **V** | **R** | **A** | **K** | **V** | **E** | **L** | **T** | **E** | **M** | **P** | **N** | **G** | **K** | **E** | **R** | **I** | **L** | **V** | **T** | **E** | **L** | **P** | **Y** | **M** | **V** | **N** | **K** | **A** | **K** | **L** | **I** | **E** | **R** | **I** | **S** | **E** | **L** | **H** | **R** | **D** | **K** | **R** | **I** | **E** | **G** | **I** | **T** | **D** | **L** | **R** | **D** | **E** | **S** | **S** | **R** | **E** | **G** | **M** | **R** | **I** | **V** | **I** | **D** | **V** | **R** | **R** | **D** | **V** | **S** | **A** | **S** | **V** | **V** | **L** | **N** | **N** | **L** | **Y** | **K** | **M** | **T** | **A** | **L** | **Q** | **T** | **S** | **F** | **G** | **F** | **N** | **M** | **L** | **A** | **I** | **E** | **K** | **G** | **V** | **P** | **K** | **I** | **L** | **S** | **L** | **K** | **R** | **I** | **L** | **E** | **N** | **Y** | **V** | **E** | **H** | **Q** | **K** | **E** | **V** | **I** | **T** | **R** | **R** | **T** | **I** | **F** | **D** | **K** | **N** | **K** | **A** | **E** | **A** | **R** | **A** | **H** | **I** | **L** | **E** | **G** | **L** | **R** | **I** | **A** | **L** | **D** | **H** | **I** | **D** | **E** | **I** | **I** | **A** | **I** | **I** | **R** | **G** | **S** | **Q** | **S** | **D** | **D** | **E** | **A** | **K** | **A** | **T** | **L** | **I** | **E** | **R** | **F** | **E** | **F** | **S** | **D** | **R** | **Q** | **A** | **Q** | **A** | **I** | **L** | **D** | **M** | **R** | **L** | **R** | **R** | **L** | **T** | **G** | **L** | **E** | **R** | **D** | **K** | **I** | **E** | **N** | **E** | **Y** | **Q** | **E** | **L** | **L** | **K** | **F** | **I** | **A** | **D** | **L** | **E** | **D** | **I** | **L** | **A** | **R** | **P** | **E** | **R** | **V** | **I** | **E** | **I** | **I** | **K** | **T** | **E** | **L** | **N** | **D** | **V** | **R** | **T** | **K** | **F** | **G** | **D** | **A** | **R** | **R** | **T** | **E** | **L** | **L** | **V** | **G** | **E** | **V** | **L** | **S** | **L** | **E** | **D** | **E** | **D** | **L** | **I** | **E** | **E** | **E** | **E** | **V** | **V** | **I** | **T** | **L** | **T** | **N** | **N** | **G** | **Y** | **I** | **K** | **R** | **M** | **A** | **N** | **S** | **E** | **F** | **R** | **A** | **Q** | **R** | **R** | **G** | **G** | **R** | **G** | **V** | **Q** | **G** | **M** | **G** | **V** | **H** | **D** | **D** | **D** | **F** | **V** | **K** | **N** | **L** | **V** | **S** | **C** | **S** | **T** | **H** | **D** | **T** | **L** | **L** | **F** | **F** | **T** | **N** | **T** | **G** | **K** | **V** | **Y** | **R** | **A** | **K** | **G** | **Y** | **E** | **I** | **P** | **E** | **Y** | **G** | **R** | **T** | **A** | **K** | **G** | **I** | **P** | **V** | **I** | **N** | **L** | **L** | **G** | **I** | **D** | **S** | **A** | **E** | **K** | **I** | **Q** | **A** | **I** | **I** | **S** | **V** | **E** | **G** | **K** | **A** | **E** | **A** | **G** | **K** | **Y** | **L** | **F** | **F** | **T** | **T** | **L** | **K** | **G** | **T** | **V** | **K** | **R** | **T** | **A** | **V** | **T** | **A** | **F** | **S** | **N** | **I** | **R** | **S** | **N** | **G** | **L** | **I** | **A** | **I** | **S** | **L** | **K** | **E** | **D** | **D** | **E** | **L** | **V** | **N** | **V** | **V** | **T** | **T** | **N** | **G** | **N** | **Q** | **K** | **M** | **I** | **I** | **G** | **T** | **H** | **A** | **G** | **Y** | **S** | **V** | **T** | **F** | **D** | **E** | **N** | **T** | **V** | **R** | **D** | **M** | **G** | **R** | **T** | **A** | **S** | **G** | **V** | **R** | **G** | **I** | **R** | **L** | **R** | **E** | **N** | **D** | **Y** | **V** | **V** | **G** | **A** | **A** | **I** | **L** | **D** | **E** | **N** | **K** | **E** | **V** | **L** | **V** | **I** | **T** | **E** | **N** | **G** | **Y** | **G** | **K** | **R** | **T** | **K** | **A** | **S** | **E** | **Y** | **P** | **V** | **K** | **G** | **R** | **G** | **G** | **K** | **G** | **I** | **K** | **T** | **A** | **N** | **I** | **T** | **E** | **K** | **N** | **G** | **P** | **L** | **A** | **G** | **L** | **T** | **T** | **V** | **N** | **G** | **D** | **E** | **D** | **I** | **L** | **L** | **I** | **T** | **N** | **K** | **G** | **V** | **I** | **I** | **R** | **F** | **N** | **V** | **D** | **S** | **V** | **S** | **Q** | **T** | **G** | **R** | **A** | **T** | **L** | **G** | **V** | **R** | **L** | **M** | **R** | **M** | **E** | **D** | **G** | **A** | **K** | **-** | **-** | **-** | **-** | **-** | **-** | **-** | **-** | **-** | **-** | **-** | **-** | **-** | **-** | **-** | **-** | **-** | **-** | **-** | **-** | **-** | **-** | **-** |
| **N54035** | **M** | **S** | **E** | **E** | **I** | **R** | **E** | **N** | **I** | **H** | **D** | **V** | **N** | **L** | **T** | **S** | **E** | **M** | **K** | **D** | **S** | **F** | **I** | **D** | **Y** | **A** | **M** | **S** | **V** | **I** | **V** | **A** | **R** | **A** | **L** | **P** | **D** | **V** | **R** | **D** | **G** | **L** | **K** | **P** | **V** | **H** | **R** | **R** | **I** | **L** | **Y** | **G** | **M** | **N** | **E** | **L** | **G** | **V** | **T** | **P** | **D** | **K** | **A** | **H** | **K** | **K** | **S** | **A** | **R** | **I** | **V** | **G** | **D** | **V** | **M** | **G** | **K** | **Y** | **H** | **P** | **H** | **G** | **D** | **S** | **A** | **I** | **Y** | **E** | **S** | **M** | **V** | **R** | **M** | **A** | **Q** | **P** | **F** | **S** | **Y** | **R** | **Y** | **M** | **L** | **V** | **D** | **G** | **H** | **G** | **N** | **F** | **G** | **S** | **V** | **D** | **G** | **D** | **G** | **A** | **A** | **A** | **M** | **R** | **Y** | **T** | **E** | **A** | **R** | **M** | **S** | **K** | **I** | **A** | **T** | **E** | **M** | **L** | **R** | **D** | **I** | **N** | **K** | **N** | **T** | **V** | **D** | **F** | **Q** | **S** | **N** | **Y** | **D** | **D** | **T** | **E** | **K** | **E** | **P** | **V** | **V** | **L** | **P** | **A** | **R** | **F** | **P** | **N** | **L** | **L** | **V** | **N** | **G** | **T** | **T** | **G** | **I** | **A** | **V** | **G** | **M** | **A** | **T** | **N** | **I** | **P** | **P** | **H** | **N** | **L** | **S** | **E** | **V** | **V** | **A** | **A** | **I** | **D** | **L** | **L** | **M** | **D** | **N** | **P** | **D** | **V** | **T** | **T** | **N** | **E** | **L** | **M** | **E** | **V** | **L** | **P** | **G** | **P** | **D** | **F** | **P** | **T** | **G** | **G** | **L** | **V** | **M** | **G** | **K** | **S** | **G** | **I** | **R** | **R** | **A** | **Y** | **E** | **T** | **G** | **K** | **G** | **S** | **I** | **T** | **V** | **R** | **A** | **K** | **V** | **E** | **I** | **T** | **E** | **M** | **P** | **N** | **G** | **K** | **E** | **R** | **I** | **I** | **V** | **T** | **E** | **L** | **P** | **Y** | **M** | **V** | **N** | **K** | **A** | **K** | **L** | **I** | **E** | **R** | **I** | **S** | **E** | **L** | **H** | **R** | **D** | **K** | **R** | **I** | **E** | **G** | **I** | **T** | **D** | **L** | **R** | **D** | **E** | **S** | **S** | **R** | **E** | **G** | **M** | **R** | **I** | **V** | **I** | **D** | **I** | **R** | **R** | **D** | **A** | **S** | **A** | **S** | **V** | **I** | **L** | **N** | **N** | **L** | **Y** | **K** | **L** | **T** | **S** | **L** | **Q** | **N** | **S** | **F** | **G** | **F** | **N** | **M** | **L** | **A** | **I** | **E** | **K** | **G** | **V** | **P** | **K** | **V** | **L** | **S** | **L** | **K** | **Q** | **I** | **L** | **E** | **N** | **Y** | **I** | **E** | **H** | **Q** | **R** | **E** | **V** | **I** | **T** | **R** | **R** | **T** | **V** | **F** | **E** | **K** | **E** | **K** | **A** | **E** | **A** | **R** | **A** | **H** | **I** | **L** | **E** | **G** | **L** | **R** | **I** | **A** | **L** | **D** | **H** | **I** | **D** | **E** | **I** | **I** | **A** | **I** | **I** | **R** | **G** | **S** | **K** | **S** | **D** | **D** | **E** | **A** | **K** | **A** | **T** | **M** | **I** | **E** | **R** | **F** | **D** | **L** | **S** | **D** | **R** | **Q** | **S** | **Q** | **A** | **I** | **L** | **D** | **M** | **R** | **L** | **R** | **R** | **L** | **T** | **G** | **L** | **E** | **R** | **E** | **K** | **I** | **E** | **N** | **E** | **Y** | **Q** | **E** | **L** | **L** | **K** | **L** | **I** | **E** | **D** | **L** | **T** | **D** | **I** | **L** | **A** | **R** | **P** | **E** | **R** | **V** | **T** | **E** | **I** | **I** | **K** | **N** | **E** | **L** | **A** | **D** | **L** | **N** | **Q** | **R** | **F** | **G** | **D** | **K** | **R** | **R** | **T** | **E** | **L** | **L** | **V** | **G** | **E** | **V** | **L** | **S** | **L** | **E** | **D** | **E** | **D** | **L** | **I** | **E** | **E** | **E** | **E** | **I** | **V** | **I** | **T** | **L** | **T** | **N** | **N** | **G** | **Y** | **I** | **K** | **R** | **L** | **A** | **N** | **N** | **E** | **F** | **R** | **A** | **Q** | **R** | **R** | **G** | **G** | **R** | **G** | **V** | **Q** | **G** | **M** | **G** | **I** | **H** | **N** | **D** | **D** | **F** | **V** | **K** | **N** | **L** | **V** | **S** | **C** | **S** | **T** | **H** | **D** | **T** | **L** | **L** | **F** | **F** | **T** | **N** | **N** | **G** | **K** | **V** | **Y** | **R** | **A** | **K** | **G** | **Y** | **E** | **I** | **P** | **E** | **Y** | **G** | **R** | **T** | **A** | **K** | **G** | **I** | **P** | **I** | **I** | **N** | **L** | **L** | **G** | **I** | **D** | **S** | **S** | **E** | **S** | **I** | **Q** | **A** | **I** | **I** | **A** | **V** | **T** | **G** | **E** | **A** | **E** | **E** | **G** | **H** | **Y** | **L** | **F** | **F** | **T** | **T** | **R** | **Q** | **G** | **T** | **V** | **K** | **R** | **T** | **S** | **V** | **T** | **A** | **F** | **A** | **N** | **I** | **R** | **S** | **N** | **G** | **L** | **I** | **A** | **I** | **G** | **L** | **K** | **E** | **D** | **D** | **E** | **L** | **V** | **N** | **V** | **L** | **L** | **T** | **D** | **G** | **Q** | **S** | **N** | **I** | **I** | **I** | **G** | **T** | **H** | **N** | **G** | **Y** | **S** | **V** | **T** | **F** | **K** | **E** | **E** | **A** | **V** | **R** | **D** | **M** | **G** | **R** | **T** | **A** | **S** | **G** | **V** | **R** | **G** | **I** | **R** | **L** | **R** | **E** | **E** | **D** | **Y** | **V** | **V** | **G** | **A** | **A** | **L** | **M** | **K** | **E** | **D** | **Q** | **E** | **V** | **L** | **V** | **I** | **T** | **E** | **K** | **G** | **Y** | **G** | **K** | **R** | **T** | **K** | **V** | **S** | **E** | **Y** | **P** | **V** | **K** | **G** | **R** | **G** | **G** | **K** | **G** | **I** | **K** | **T** | **A** | **N** | **I** | **T** | **E** | **K** | **N** | **G** | **P** | **L** | **A** | **G** | **L** | **T** | **T** | **V** | **S** | **G** | **D** | **E** | **D** | **I** | **M** | **L** | **I** | **T** | **D** | **K** | **G** | **V** | **I** | **I** | **R** | **F** | **N** | **V** | **S** | **T** | **V** | **S** | **Q** | **T** | **G** | **R** | **S** | **T** | **L** | **G** | **V** | **R** | **L** | **M** | **K** | **M** | **E** | **A** | **D** | **T** | **K** | **V** | **V** | **T** | **M** | **A** | **A** | **V** | **E** | **P** | **E** | **A** | **A** | **E** | **Q** | **T** | **V** | **E** | **E** | **Q** | **G** | **S** | **E** | **E** |
| **N60061F** | **M** | **S** | **E** | **E** | **I** | **R** | **E** | **N** | **I** | **H** | **D** | **V** | **N** | **L** | **T** | **S** | **E** | **M** | **K** | **D** | **S** | **F** | **I** | **D** | **Y** | **A** | **M** | **S** | **V** | **I** | **V** | **A** | **R** | **A** | **L** | **P** | **D** | **V** | **R** | **D** | **G** | **L** | **K** | **P** | **V** | **H** | **R** | **R** | **I** | **L** | **Y** | **G** | **M** | **N** | **E** | **L** | **G** | **V** | **T** | **P** | **D** | **K** | **A** | **H** | **K** | **K** | **S** | **A** | **R** | **I** | **V** | **G** | **D** | **V** | **M** | **G** | **K** | **Y** | **H** | **P** | **H** | **G** | **D** | **S** | **A** | **I** | **Y** | **E** | **S** | **M** | **V** | **R** | **M** | **A** | **Q** | **P** | **F** | **S** | **Y** | **R** | **Y** | **M** | **L** | **V** | **D** | **G** | **H** | **G** | **N** | **F** | **G** | **S** | **V** | **D** | **G** | **D** | **G** | **A** | **A** | **A** | **M** | **R** | **Y** | **T** | **E** | **A** | **R** | **M** | **S** | **K** | **I** | **A** | **T** | **E** | **M** | **L** | **R** | **D** | **I** | **N** | **K** | **N** | **T** | **V** | **D** | **F** | **Q** | **S** | **N** | **Y** | **D** | **D** | **T** | **E** | **K** | **E** | **P** | **V** | **V** | **L** | **P** | **A** | **R** | **F** | **P** | **N** | **L** | **L** | **V** | **N** | **G** | **T** | **T** | **G** | **I** | **A** | **V** | **G** | **M** | **A** | **T** | **N** | **I** | **P** | **P** | **H** | **N** | **L** | **S** | **E** | **V** | **V** | **A** | **A** | **I** | **D** | **L** | **L** | **M** | **D** | **N** | **P** | **D** | **V** | **T** | **T** | **N** | **E** | **L** | **M** | **E** | **V** | **L** | **P** | **G** | **P** | **D** | **F** | **P** | **T** | **G** | **G** | **L** | **V** | **M** | **G** | **K** | **S** | **G** | **I** | **R** | **R** | **A** | **Y** | **E** | **T** | **G** | **K** | **G** | **S** | **I** | **T** | **V** | **R** | **A** | **K** | **V** | **E** | **I** | **T** | **E** | **M** | **P** | **N** | **G** | **K** | **E** | **R** | **I** | **I** | **V** | **T** | **E** | **L** | **P** | **Y** | **M** | **V** | **N** | **K** | **A** | **K** | **L** | **I** | **E** | **R** | **I** | **S** | **E** | **L** | **H** | **R** | **D** | **K** | **R** | **I** | **E** | **G** | **I** | **T** | **D** | **L** | **R** | **D** | **E** | **S** | **S** | **R** | **E** | **G** | **M** | **R** | **I** | **V** | **I** | **D** | **I** | **R** | **R** | **D** | **A** | **S** | **A** | **S** | **V** | **I** | **L** | **N** | **N** | **L** | **Y** | **K** | **L** | **T** | **S** | **L** | **Q** | **N** | **S** | **F** | **G** | **F** | **N** | **M** | **L** | **A** | **I** | **E** | **K** | **G** | **V** | **P** | **K** | **V** | **L** | **S** | **L** | **K** | **Q** | **I** | **L** | **E** | **N** | **Y** | **I** | **E** | **H** | **Q** | **R** | **E** | **V** | **I** | **T** | **R** | **R** | **T** | **V** | **F** | **E** | **K** | **E** | **K** | **A** | **E** | **A** | **R** | **A** | **H** | **I** | **L** | **E** | **G** | **L** | **R** | **I** | **A** | **L** | **D** | **H** | **I** | **D** | **E** | **I** | **I** | **A** | **I** | **I** | **R** | **G** | **S** | **K** | **S** | **D** | **D** | **E** | **A** | **K** | **A** | **T** | **M** | **I** | **E** | **R** | **F** | **D** | **L** | **S** | **D** | **R** | **Q** | **S** | **Q** | **A** | **I** | **L** | **D** | **M** | **R** | **L** | **R** | **R** | **L** | **T** | **G** | **L** | **E** | **R** | **E** | **K** | **I** | **E** | **N** | **E** | **Y** | **Q** | **E** | **L** | **L** | **K** | **L** | **I** | **E** | **D** | **L** | **T** | **D** | **I** | **L** | **A** | **R** | **P** | **E** | **R** | **V** | **T** | **E** | **I** | **I** | **K** | **N** | **E** | **L** | **A** | **D** | **L** | **N** | **Q** | **R** | **F** | **G** | **D** | **K** | **R** | **R** | **T** | **E** | **L** | **L** | **V** | **G** | **E** | **V** | **L** | **S** | **L** | **E** | **D** | **E** | **D** | **L** | **I** | **E** | **E** | **E** | **E** | **I** | **V** | **I** | **T** | **L** | **T** | **N** | **N** | **G** | **Y** | **I** | **K** | **R** | **L** | **A** | **N** | **N** | **E** | **F** | **R** | **A** | **Q** | **R** | **R** | **G** | **G** | **R** | **G** | **V** | **Q** | **G** | **M** | **G** | **I** | **H** | **N** | **D** | **D** | **F** | **V** | **K** | **N** | **L** | **V** | **S** | **C** | **S** | **T** | **H** | **D** | **T** | **L** | **L** | **F** | **F** | **T** | **N** | **N** | **G** | **K** | **V** | **Y** | **R** | **A** | **K** | **G** | **Y** | **E** | **I** | **P** | **E** | **Y** | **G** | **R** | **T** | **A** | **K** | **G** | **I** | **P** | **I** | **I** | **N** | **L** | **L** | **G** | **I** | **D** | **S** | **S** | **E** | **S** | **I** | **Q** | **A** | **I** | **I** | **A** | **V** | **T** | **G** | **E** | **A** | **E** | **E** | **G** | **H** | **Y** | **L** | **F** | **F** | **T** | **T** | **R** | **Q** | **G** | **T** | **V** | **K** | **R** | **T** | **S** | **V** | **T** | **A** | **F** | **A** | **N** | **I** | **R** | **S** | **N** | **G** | **L** | **I** | **A** | **I** | **G** | **L** | **K** | **E** | **D** | **D** | **E** | **L** | **V** | **N** | **V** | **L** | **L** | **T** | **D** | **G** | **Q** | **S** | **N** | **I** | **I** | **I** | **G** | **T** | **H** | **N** | **G** | **Y** | **S** | **V** | **T** | **F** | **K** | **E** | **E** | **A** | **V** | **R** | **D** | **M** | **G** | **R** | **T** | **A** | **S** | **G** | **V** | **R** | **G** | **I** | **R** | **L** | **R** | **E** | **E** | **D** | **Y** | **V** | **V** | **G** | **A** | **A** | **L** | **M** | **K** | **E** | **D** | **Q** | **E** | **V** | **L** | **V** | **I** | **T** | **E** | **K** | **G** | **Y** | **G** | **K** | **R** | **T** | **K** | **V** | **S** | **E** | **Y** | **P** | **V** | **K** | **G** | **R** | **G** | **G** | **K** | **G** | **I** | **K** | **T** | **A** | **N** | **I** | **T** | **E** | **K** | **N** | **G** | **P** | **L** | **A** | **G** | **L** | **T** | **T** | **V** | **S** | **G** | **D** | **E** | **D** | **I** | **M** | **L** | **I** | **T** | **D** | **K** | **G** | **V** | **I** | **I** | **R** | **F** | **N** | **V** | **S** | **T** | **V** | **S** | **Q** | **T** | **G** | **R** | **S** | **T** | **L** | **G** | **V** | **R** | **L** | **M** | **K** | **M** | **E** | **A** | **D** | **T** | **K** | **V** | **V** | **T** | **M** | **A** | **A** | **V** | **E** | **P** | **E** | **A** | **A** | **E** | **Q** | **T** | **V** | **E** | **E** | **Q** | **G** | **S** | **E** | **E** |
| **N53805** | **M** | **S** | **E** | **E** | **I** | **R** | **E** | **N** | **I** | **H** | **D** | **V** | **N** | **L** | **T** | **S** | **E** | **M** | **K** | **D** | **S** | **F** | **I** | **D** | **Y** | **A** | **M** | **S** | **V** | **I** | **V** | **A** | **R** | **A** | **L** | **P** | **D** | **V** | **R** | **D** | **G** | **L** | **K** | **P** | **V** | **H** | **R** | **R** | **I** | **L** | **Y** | **G** | **M** | **N** | **E** | **L** | **G** | **V** | **T** | **P** | **D** | **K** | **A** | **H** | **K** | **K** | **S** | **A** | **R** | **I** | **V** | **G** | **D** | **V** | **M** | **G** | **K** | **Y** | **H** | **P** | **H** | **G** | **D** | **S** | **A** | **I** | **Y** | **E** | **S** | **M** | **V** | **R** | **M** | **A** | **Q** | **P** | **F** | **S** | **Y** | **R** | **Y** | **M** | **L** | **V** | **D** | **G** | **H** | **G** | **N** | **F** | **G** | **S** | **V** | **D** | **G** | **D** | **G** | **A** | **A** | **A** | **M** | **R** | **Y** | **T** | **E** | **A** | **R** | **M** | **S** | **K** | **I** | **A** | **T** | **E** | **M** | **L** | **R** | **D** | **I** | **N** | **K** | **N** | **T** | **V** | **D** | **F** | **Q** | **S** | **N** | **Y** | **D** | **D** | **T** | **E** | **K** | **E** | **P** | **V** | **V** | **L** | **P** | **A** | **R** | **F** | **P** | **N** | **L** | **L** | **V** | **N** | **G** | **T** | **T** | **G** | **I** | **A** | **V** | **G** | **M** | **A** | **T** | **N** | **I** | **P** | **P** | **H** | **N** | **L** | **S** | **E** | **V** | **V** | **A** | **A** | **I** | **D** | **L** | **L** | **M** | **D** | **N** | **P** | **D** | **V** | **T** | **T** | **N** | **E** | **L** | **M** | **E** | **V** | **L** | **P** | **G** | **P** | **D** | **F** | **P** | **T** | **G** | **G** | **L** | **V** | **M** | **G** | **K** | **S** | **G** | **I** | **R** | **R** | **A** | **Y** | **E** | **T** | **G** | **K** | **G** | **S** | **I** | **T** | **V** | **R** | **A** | **K** | **V** | **E** | **I** | **T** | **E** | **M** | **P** | **N** | **G** | **K** | **E** | **R** | **I** | **I** | **V** | **T** | **E** | **L** | **P** | **Y** | **M** | **V** | **N** | **K** | **A** | **K** | **L** | **I** | **E** | **R** | **I** | **S** | **E** | **L** | **H** | **R** | **D** | **K** | **R** | **I** | **E** | **G** | **I** | **T** | **D** | **L** | **R** | **D** | **E** | **S** | **S** | **R** | **E** | **G** | **M** | **R** | **I** | **V** | **I** | **D** | **I** | **R** | **R** | **D** | **A** | **S** | **A** | **S** | **V** | **I** | **L** | **N** | **N** | **L** | **Y** | **K** | **L** | **T** | **S** | **L** | **Q** | **N** | **S** | **F** | **G** | **F** | **N** | **M** | **L** | **A** | **I** | **E** | **K** | **G** | **V** | **P** | **K** | **V** | **L** | **S** | **L** | **K** | **Q** | **I** | **L** | **E** | **N** | **Y** | **I** | **E** | **H** | **Q** | **R** | **E** | **V** | **I** | **T** | **R** | **R** | **T** | **V** | **F** | **E** | **K** | **E** | **K** | **A** | **E** | **A** | **R** | **A** | **H** | **I** | **L** | **E** | **G** | **L** | **R** | **I** | **A** | **L** | **D** | **H** | **I** | **D** | **E** | **I** | **I** | **A** | **I** | **I** | **R** | **G** | **S** | **K** | **S** | **D** | **D** | **E** | **A** | **K** | **A** | **T** | **M** | **I** | **E** | **R** | **F** | **D** | **L** | **S** | **D** | **R** | **Q** | **S** | **Q** | **A** | **I** | **L** | **D** | **M** | **R** | **L** | **R** | **R** | **L** | **T** | **G** | **L** | **E** | **R** | **E** | **K** | **I** | **E** | **N** | **E** | **Y** | **Q** | **E** | **L** | **L** | **K** | **L** | **I** | **E** | **D** | **L** | **T** | **D** | **I** | **L** | **A** | **R** | **P** | **E** | **R** | **V** | **T** | **E** | **I** | **I** | **K** | **N** | **E** | **L** | **A** | **D** | **L** | **N** | **Q** | **R** | **F** | **G** | **D** | **K** | **R** | **R** | **T** | **E** | **L** | **L** | **V** | **G** | **E** | **V** | **L** | **S** | **L** | **E** | **D** | **E** | **D** | **L** | **I** | **E** | **E** | **E** | **E** | **I** | **V** | **I** | **T** | **L** | **T** | **N** | **N** | **G** | **Y** | **I** | **K** | **R** | **L** | **A** | **N** | **N** | **E** | **F** | **R** | **A** | **Q** | **R** | **R** | **G** | **G** | **R** | **G** | **V** | **Q** | **G** | **M** | **G** | **I** | **H** | **N** | **D** | **D** | **F** | **V** | **K** | **N** | **L** | **V** | **S** | **C** | **S** | **T** | **H** | **D** | **T** | **L** | **L** | **F** | **F** | **T** | **N** | **N** | **G** | **K** | **V** | **Y** | **R** | **A** | **K** | **G** | **Y** | **E** | **I** | **P** | **E** | **Y** | **G** | **R** | **T** | **A** | **K** | **G** | **I** | **P** | **I** | **I** | **N** | **L** | **L** | **G** | **I** | **D** | **S** | **S** | **E** | **S** | **I** | **Q** | **A** | **I** | **I** | **A** | **V** | **T** | **G** | **E** | **A** | **E** | **E** | **G** | **H** | **Y** | **L** | **F** | **F** | **T** | **T** | **R** | **Q** | **G** | **T** | **V** | **K** | **R** | **T** | **S** | **V** | **T** | **A** | **F** | **A** | **N** | **I** | **R** | **S** | **N** | **G** | **L** | **I** | **A** | **I** | **G** | **L** | **K** | **E** | **D** | **D** | **E** | **L** | **V** | **N** | **V** | **L** | **L** | **T** | **D** | **G** | **Q** | **S** | **N** | **I** | **I** | **I** | **G** | **T** | **H** | **N** | **G** | **Y** | **S** | **V** | **T** | **F** | **K** | **E** | **E** | **A** | **V** | **R** | **D** | **M** | **G** | **R** | **T** | **A** | **S** | **G** | **V** | **R** | **G** | **I** | **R** | **L** | **R** | **E** | **E** | **D** | **Y** | **V** | **V** | **G** | **A** | **A** | **L** | **M** | **K** | **E** | **N** | **Q** | **E** | **V** | **L** | **V** | **I** | **T** | **E** | **K** | **G** | **Y** | **G** | **K** | **R** | **T** | **K** | **V** | **S** | **E** | **Y** | **P** | **V** | **K** | **G** | **R** | **G** | **G** | **K** | **G** | **I** | **K** | **T** | **A** | **N** | **I** | **T** | **E** | **K** | **N** | **G** | **P** | **L** | **A** | **G** | **L** | **T** | **T** | **V** | **S** | **G** | **D** | **E** | **D** | **I** | **M** | **L** | **I** | **T** | **D** | **K** | **G** | **V** | **I** | **I** | **R** | **F** | **N** | **V** | **S** | **T** | **V** | **S** | **Q** | **T** | **G** | **R** | **S** | **T** | **L** | **G** | **V** | **R** | **L** | **M** | **K** | **M** | **E** | **A** | **D** | **T** | **K** | **V** | **V** | **T** | **M** | **A** | **A** | **V** | **E** | **P** | **E** | **A** | **A** | **E** | **Q** | **T** | **V** | **E** | **E** | **Q** | **G** | **S** | **E** | **E** |
| **N53595** | **M** | **S** | **E** | **E** | **I** | **R** | **E** | **N** | **I** | **H** | **D** | **V** | **N** | **L** | **T** | **S** | **E** | **M** | **K** | **D** | **S** | **F** | **I** | **D** | **Y** | **A** | **M** | **S** | **V** | **I** | **V** | **A** | **R** | **A** | **L** | **P** | **D** | **V** | **R** | **D** | **G** | **L** | **K** | **P** | **V** | **H** | **R** | **R** | **I** | **L** | **Y** | **G** | **M** | **N** | **E** | **L** | **G** | **V** | **T** | **P** | **D** | **K** | **A** | **H** | **K** | **K** | **S** | **A** | **R** | **I** | **V** | **G** | **D** | **V** | **M** | **G** | **K** | **Y** | **H** | **P** | **H** | **G** | **D** | **S** | **A** | **I** | **Y** | **E** | **S** | **M** | **V** | **R** | **M** | **A** | **Q** | **P** | **F** | **S** | **Y** | **R** | **Y** | **M** | **L** | **V** | **D** | **G** | **H** | **G** | **N** | **F** | **G** | **S** | **V** | **D** | **G** | **D** | **G** | **A** | **A** | **A** | **M** | **R** | **Y** | **T** | **E** | **A** | **R** | **M** | **S** | **K** | **I** | **A** | **T** | **E** | **M** | **L** | **R** | **D** | **I** | **N** | **K** | **N** | **T** | **V** | **D** | **F** | **Q** | **S** | **N** | **Y** | **D** | **D** | **T** | **E** | **K** | **E** | **P** | **V** | **V** | **L** | **P** | **A** | **R** | **F** | **P** | **N** | **L** | **L** | **V** | **N** | **G** | **T** | **T** | **G** | **I** | **A** | **V** | **G** | **M** | **A** | **T** | **N** | **I** | **P** | **P** | **H** | **N** | **L** | **S** | **E** | **V** | **V** | **A** | **A** | **I** | **D** | **L** | **L** | **M** | **D** | **N** | **P** | **D** | **V** | **T** | **T** | **N** | **E** | **L** | **M** | **E** | **V** | **L** | **P** | **G** | **P** | **D** | **F** | **P** | **T** | **G** | **G** | **L** | **V** | **M** | **G** | **K** | **S** | **G** | **I** | **R** | **R** | **A** | **Y** | **E** | **T** | **G** | **K** | **G** | **S** | **I** | **T** | **V** | **R** | **A** | **K** | **V** | **E** | **I** | **T** | **E** | **M** | **P** | **N** | **G** | **K** | **E** | **R** | **I** | **I** | **V** | **T** | **E** | **L** | **P** | **Y** | **M** | **V** | **N** | **K** | **A** | **K** | **L** | **I** | **E** | **R** | **I** | **S** | **E** | **L** | **H** | **R** | **D** | **K** | **R** | **I** | **E** | **G** | **I** | **T** | **D** | **L** | **R** | **D** | **E** | **S** | **S** | **R** | **E** | **G** | **M** | **R** | **I** | **V** | **I** | **D** | **I** | **R** | **R** | **D** | **A** | **S** | **A** | **S** | **V** | **I** | **L** | **N** | **N** | **L** | **Y** | **K** | **L** | **T** | **S** | **L** | **Q** | **N** | **S** | **F** | **G** | **F** | **N** | **M** | **L** | **A** | **I** | **E** | **K** | **G** | **V** | **P** | **K** | **V** | **L** | **S** | **L** | **K** | **Q** | **I** | **L** | **E** | **N** | **Y** | **I** | **E** | **H** | **Q** | **R** | **E** | **V** | **I** | **T** | **R** | **R** | **T** | **V** | **F** | **E** | **K** | **E** | **K** | **A** | **E** | **A** | **R** | **A** | **H** | **I** | **L** | **E** | **G** | **L** | **R** | **I** | **A** | **L** | **D** | **H** | **I** | **D** | **E** | **I** | **I** | **A** | **I** | **I** | **R** | **G** | **S** | **K** | **S** | **D** | **D** | **E** | **A** | **K** | **A** | **T** | **M** | **I** | **E** | **R** | **F** | **D** | **L** | **S** | **D** | **R** | **Q** | **S** | **Q** | **A** | **I** | **L** | **D** | **M** | **R** | **L** | **R** | **R** | **L** | **T** | **G** | **L** | **E** | **R** | **E** | **K** | **I** | **E** | **N** | **E** | **Y** | **Q** | **E** | **L** | **L** | **K** | **L** | **I** | **E** | **D** | **L** | **T** | **D** | **I** | **L** | **A** | **R** | **P** | **E** | **R** | **V** | **T** | **E** | **I** | **I** | **K** | **N** | **E** | **L** | **A** | **D** | **L** | **N** | **Q** | **R** | **F** | **G** | **D** | **K** | **R** | **R** | **T** | **E** | **L** | **L** | **V** | **G** | **E** | **V** | **L** | **S** | **L** | **E** | **D** | **E** | **D** | **L** | **I** | **E** | **E** | **E** | **E** | **I** | **V** | **I** | **T** | **L** | **T** | **N** | **N** | **G** | **Y** | **I** | **K** | **R** | **L** | **A** | **N** | **N** | **E** | **F** | **R** | **A** | **Q** | **R** | **R** | **G** | **G** | **R** | **G** | **V** | **Q** | **G** | **M** | **G** | **I** | **H** | **N** | **D** | **D** | **F** | **V** | **K** | **N** | **L** | **V** | **S** | **C** | **S** | **T** | **H** | **D** | **T** | **L** | **L** | **F** | **F** | **T** | **N** | **N** | **G** | **K** | **V** | **Y** | **R** | **A** | **K** | **G** | **Y** | **E** | **I** | **P** | **E** | **Y** | **G** | **R** | **T** | **A** | **K** | **G** | **I** | **P** | **I** | **I** | **N** | **L** | **L** | **G** | **I** | **D** | **S** | **S** | **E** | **S** | **I** | **Q** | **A** | **I** | **I** | **A** | **V** | **T** | **G** | **E** | **A** | **E** | **E** | **G** | **H** | **Y** | **L** | **F** | **F** | **T** | **T** | **R** | **Q** | **G** | **T** | **V** | **K** | **R** | **T** | **S** | **V** | **T** | **A** | **F** | **A** | **N** | **I** | **R** | **S** | **N** | **G** | **L** | **I** | **A** | **I** | **G** | **L** | **K** | **E** | **D** | **D** | **E** | **L** | **V** | **N** | **V** | **L** | **L** | **T** | **D** | **G** | **Q** | **S** | **N** | **I** | **I** | **I** | **G** | **T** | **H** | **N** | **G** | **Y** | **S** | **V** | **T** | **F** | **K** | **E** | **E** | **A** | **V** | **R** | **D** | **M** | **G** | **R** | **T** | **A** | **S** | **G** | **V** | **R** | **G** | **I** | **R** | **L** | **R** | **E** | **E** | **D** | **Y** | **V** | **V** | **G** | **A** | **A** | **L** | **M** | **K** | **E** | **D** | **Q** | **E** | **V** | **L** | **V** | **I** | **T** | **E** | **K** | **G** | **Y** | **G** | **K** | **R** | **T** | **K** | **V** | **S** | **E** | **Y** | **P** | **V** | **K** | **G** | **R** | **G** | **G** | **K** | **G** | **I** | **K** | **T** | **A** | **N** | **I** | **T** | **E** | **K** | **N** | **G** | **P** | **L** | **A** | **G** | **L** | **T** | **T** | **V** | **S** | **G** | **D** | **E** | **D** | **I** | **M** | **L** | **I** | **T** | **D** | **K** | **G** | **V** | **I** | **I** | **R** | **F** | **N** | **V** | **S** | **T** | **V** | **S** | **Q** | **T** | **G** | **R** | **S** | **T** | **L** | **G** | **V** | **R** | **L** | **M** | **K** | **M** | **E** | **A** | **D** | **T** | **K** | **V** | **V** | **T** | **M** | **A** | **A** | **V** | **E** | **P** | **E** | **A** | **A** | **E** | **Q** | **T** | **V** | **E** | **E** | **Q** | **G** | **S** | **E** | **E** |
| **N60425F** | **M** | **S** | **E** | **E** | **I** | **R** | **E** | **N** | **I** | **H** | **D** | **V** | **N** | **L** | **T** | **S** | **E** | **M** | **K** | **D** | **S** | **F** | **I** | **D** | **Y** | **A** | **M** | **S** | **V** | **I** | **V** | **A** | **R** | **A** | **L** | **P** | **D** | **V** | **R** | **D** | **G** | **L** | **K** | **P** | **V** | **H** | **R** | **R** | **I** | **L** | **Y** | **G** | **M** | **N** | **E** | **L** | **G** | **V** | **T** | **P** | **D** | **K** | **A** | **H** | **K** | **K** | **S** | **A** | **R** | **I** | **V** | **G** | **D** | **V** | **M** | **G** | **K** | **Y** | **H** | **P** | **H** | **G** | **D** | **S** | **A** | **I** | **Y** | **E** | **S** | **M** | **V** | **R** | **M** | **A** | **Q** | **P** | **F** | **S** | **Y** | **R** | **Y** | **M** | **L** | **V** | **D** | **G** | **H** | **G** | **N** | **F** | **G** | **S** | **V** | **D** | **G** | **D** | **G** | **A** | **A** | **A** | **M** | **R** | **Y** | **T** | **E** | **A** | **R** | **M** | **S** | **K** | **I** | **A** | **T** | **E** | **M** | **L** | **R** | **D** | **I** | **N** | **K** | **N** | **T** | **V** | **D** | **F** | **Q** | **S** | **N** | **Y** | **D** | **D** | **T** | **E** | **K** | **E** | **P** | **V** | **V** | **L** | **P** | **A** | **R** | **F** | **P** | **N** | **L** | **L** | **V** | **N** | **G** | **T** | **T** | **G** | **I** | **A** | **V** | **G** | **M** | **A** | **T** | **N** | **I** | **P** | **P** | **H** | **N** | **L** | **S** | **E** | **V** | **V** | **A** | **A** | **I** | **D** | **L** | **L** | **M** | **D** | **N** | **P** | **D** | **V** | **T** | **T** | **N** | **E** | **L** | **M** | **E** | **V** | **L** | **P** | **G** | **P** | **D** | **F** | **P** | **T** | **G** | **G** | **L** | **V** | **M** | **G** | **K** | **S** | **G** | **I** | **R** | **R** | **A** | **Y** | **E** | **T** | **G** | **K** | **G** | **S** | **I** | **T** | **V** | **R** | **A** | **K** | **V** | **E** | **I** | **T** | **E** | **M** | **P** | **N** | **G** | **K** | **E** | **R** | **I** | **I** | **V** | **T** | **E** | **L** | **P** | **Y** | **M** | **V** | **N** | **K** | **A** | **K** | **L** | **I** | **E** | **R** | **I** | **S** | **E** | **L** | **H** | **R** | **D** | **K** | **R** | **I** | **E** | **G** | **I** | **T** | **D** | **L** | **R** | **D** | **E** | **S** | **S** | **R** | **E** | **G** | **M** | **R** | **I** | **V** | **I** | **D** | **I** | **R** | **R** | **D** | **A** | **S** | **A** | **S** | **V** | **I** | **L** | **N** | **N** | **L** | **Y** | **K** | **L** | **T** | **S** | **L** | **Q** | **N** | **S** | **F** | **G** | **F** | **N** | **M** | **L** | **A** | **I** | **E** | **K** | **G** | **V** | **P** | **K** | **V** | **L** | **S** | **L** | **K** | **Q** | **I** | **L** | **E** | **N** | **Y** | **I** | **E** | **H** | **Q** | **R** | **E** | **V** | **I** | **T** | **R** | **R** | **T** | **V** | **F** | **E** | **K** | **E** | **K** | **A** | **E** | **A** | **R** | **A** | **H** | **I** | **L** | **E** | **G** | **L** | **R** | **I** | **A** | **L** | **D** | **H** | **I** | **D** | **E** | **I** | **I** | **A** | **I** | **I** | **R** | **G** | **S** | **K** | **S** | **D** | **D** | **E** | **A** | **K** | **A** | **T** | **M** | **I** | **E** | **R** | **F** | **D** | **L** | **S** | **D** | **R** | **Q** | **S** | **Q** | **A** | **I** | **L** | **D** | **M** | **R** | **L** | **R** | **R** | **L** | **T** | **G** | **L** | **E** | **R** | **E** | **K** | **I** | **E** | **N** | **E** | **Y** | **Q** | **E** | **L** | **L** | **K** | **L** | **I** | **E** | **D** | **L** | **T** | **D** | **I** | **L** | **A** | **R** | **P** | **E** | **R** | **V** | **T** | **E** | **I** | **I** | **K** | **N** | **E** | **L** | **A** | **D** | **L** | **N** | **Q** | **R** | **F** | **G** | **D** | **K** | **R** | **R** | **T** | **E** | **L** | **L** | **V** | **G** | **E** | **V** | **L** | **S** | **L** | **E** | **D** | **E** | **D** | **L** | **I** | **E** | **E** | **E** | **E** | **I** | **V** | **I** | **T** | **L** | **T** | **N** | **N** | **G** | **Y** | **I** | **K** | **R** | **L** | **A** | **N** | **N** | **E** | **F** | **R** | **A** | **Q** | **R** | **R** | **G** | **G** | **R** | **G** | **V** | **Q** | **G** | **M** | **G** | **I** | **H** | **N** | **D** | **D** | **F** | **V** | **K** | **N** | **L** | **V** | **S** | **C** | **S** | **T** | **H** | **D** | **T** | **L** | **L** | **F** | **F** | **T** | **N** | **N** | **G** | **K** | **V** | **Y** | **R** | **A** | **K** | **G** | **Y** | **E** | **I** | **P** | **E** | **Y** | **G** | **R** | **T** | **A** | **K** | **G** | **I** | **P** | **I** | **I** | **N** | **L** | **L** | **G** | **I** | **D** | **S** | **S** | **E** | **S** | **I** | **Q** | **A** | **I** | **I** | **A** | **V** | **T** | **G** | **E** | **A** | **E** | **E** | **G** | **H** | **Y** | **L** | **F** | **F** | **T** | **T** | **R** | **Q** | **G** | **T** | **V** | **K** | **R** | **T** | **S** | **V** | **T** | **A** | **F** | **A** | **N** | **I** | **R** | **S** | **N** | **G** | **L** | **I** | **A** | **I** | **G** | **L** | **K** | **E** | **D** | **D** | **E** | **L** | **V** | **N** | **V** | **L** | **L** | **T** | **D** | **G** | **Q** | **S** | **N** | **I** | **I** | **I** | **G** | **T** | **H** | **N** | **G** | **Y** | **S** | **V** | **T** | **F** | **K** | **E** | **E** | **A** | **V** | **R** | **D** | **M** | **G** | **R** | **T** | **A** | **S** | **G** | **V** | **R** | **G** | **I** | **R** | **L** | **R** | **E** | **E** | **D** | **Y** | **V** | **V** | **G** | **A** | **A** | **L** | **M** | **K** | **E** | **D** | **Q** | **E** | **V** | **L** | **V** | **I** | **T** | **E** | **K** | **G** | **Y** | **G** | **K** | **R** | **T** | **K** | **V** | **S** | **E** | **Y** | **P** | **V** | **K** | **G** | **R** | **G** | **G** | **K** | **G** | **I** | **K** | **T** | **A** | **N** | **I** | **T** | **E** | **K** | **N** | **G** | **P** | **L** | **A** | **G** | **L** | **T** | **T** | **V** | **S** | **G** | **D** | **E** | **D** | **I** | **M** | **L** | **I** | **T** | **D** | **K** | **G** | **V** | **I** | **I** | **R** | **F** | **N** | **V** | **S** | **T** | **V** | **S** | **Q** | **T** | **G** | **R** | **S** | **T** | **L** | **G** | **V** | **R** | **L** | **M** | **K** | **M** | **E** | **A** | **D** | **T** | **K** | **V** | **V** | **T** | **M** | **A** | **A** | **V** | **E** | **P** | **E** | **A** | **A** | **E** | **Q** | **T** | **V** | **E** | **E** | **Q** | **G** | **S** | **E** | **E** |
| **N60048F** | **M** | **S** | **E** | **E** | **I** | **R** | **E** | **N** | **I** | **H** | **D** | **V** | **N** | **L** | **T** | **S** | **E** | **M** | **K** | **D** | **S** | **F** | **I** | **D** | **Y** | **A** | **M** | **S** | **V** | **I** | **V** | **A** | **R** | **A** | **L** | **P** | **D** | **V** | **R** | **D** | **G** | **L** | **K** | **P** | **V** | **H** | **R** | **R** | **I** | **L** | **Y** | **G** | **M** | **N** | **E** | **L** | **G** | **V** | **T** | **P** | **D** | **K** | **A** | **H** | **K** | **K** | **S** | **A** | **R** | **I** | **V** | **G** | **D** | **V** | **M** | **G** | **K** | **Y** | **H** | **P** | **H** | **G** | **D** | **S** | **A** | **I** | **Y** | **E** | **S** | **M** | **V** | **R** | **M** | **A** | **Q** | **P** | **F** | **S** | **Y** | **R** | **Y** | **M** | **L** | **V** | **D** | **G** | **H** | **G** | **N** | **F** | **G** | **S** | **V** | **D** | **G** | **D** | **G** | **A** | **A** | **A** | **M** | **R** | **Y** | **T** | **E** | **A** | **R** | **M** | **S** | **K** | **I** | **A** | **T** | **E** | **M** | **L** | **R** | **D** | **I** | **N** | **K** | **N** | **T** | **V** | **D** | **F** | **Q** | **S** | **N** | **Y** | **D** | **D** | **T** | **E** | **K** | **E** | **P** | **V** | **V** | **L** | **P** | **A** | **R** | **F** | **P** | **N** | **L** | **L** | **V** | **N** | **G** | **T** | **T** | **G** | **I** | **A** | **V** | **G** | **M** | **A** | **T** | **N** | **I** | **P** | **P** | **H** | **N** | **L** | **S** | **E** | **V** | **V** | **A** | **A** | **I** | **D** | **L** | **L** | **M** | **D** | **N** | **P** | **D** | **V** | **T** | **T** | **N** | **E** | **L** | **M** | **E** | **V** | **L** | **P** | **G** | **P** | **D** | **F** | **P** | **T** | **G** | **G** | **L** | **V** | **M** | **G** | **K** | **S** | **G** | **I** | **R** | **R** | **A** | **Y** | **E** | **T** | **G** | **K** | **G** | **S** | **I** | **T** | **V** | **R** | **A** | **K** | **V** | **E** | **I** | **T** | **E** | **M** | **P** | **N** | **G** | **K** | **E** | **R** | **I** | **I** | **V** | **T** | **E** | **L** | **P** | **Y** | **M** | **V** | **N** | **K** | **A** | **K** | **L** | **I** | **E** | **R** | **I** | **S** | **E** | **L** | **H** | **R** | **D** | **K** | **R** | **I** | **E** | **G** | **I** | **T** | **D** | **L** | **R** | **D** | **E** | **S** | **S** | **R** | **E** | **G** | **M** | **R** | **I** | **V** | **I** | **D** | **I** | **R** | **R** | **D** | **A** | **S** | **A** | **S** | **V** | **I** | **L** | **N** | **N** | **L** | **Y** | **K** | **L** | **T** | **S** | **L** | **Q** | **N** | **S** | **F** | **G** | **F** | **N** | **M** | **L** | **A** | **I** | **E** | **K** | **G** | **V** | **P** | **K** | **V** | **L** | **S** | **L** | **K** | **Q** | **I** | **L** | **E** | **N** | **Y** | **I** | **E** | **H** | **Q** | **R** | **E** | **V** | **I** | **T** | **R** | **R** | **T** | **V** | **F** | **E** | **K** | **E** | **K** | **A** | **E** | **A** | **R** | **A** | **H** | **I** | **L** | **E** | **G** | **L** | **R** | **I** | **A** | **L** | **D** | **H** | **I** | **D** | **E** | **I** | **I** | **A** | **I** | **I** | **R** | **G** | **S** | **K** | **S** | **D** | **D** | **E** | **A** | **K** | **A** | **T** | **M** | **I** | **E** | **R** | **F** | **D** | **L** | **S** | **D** | **R** | **Q** | **S** | **Q** | **A** | **I** | **L** | **D** | **M** | **R** | **L** | **R** | **R** | **L** | **T** | **G** | **L** | **E** | **R** | **E** | **K** | **I** | **E** | **N** | **E** | **Y** | **Q** | **E** | **L** | **L** | **K** | **L** | **I** | **E** | **D** | **L** | **T** | **D** | **I** | **L** | **A** | **R** | **P** | **E** | **R** | **V** | **T** | **E** | **I** | **I** | **K** | **N** | **E** | **L** | **A** | **D** | **L** | **N** | **Q** | **R** | **F** | **G** | **D** | **K** | **R** | **R** | **T** | **E** | **L** | **L** | **V** | **G** | **E** | **V** | **L** | **S** | **L** | **E** | **D** | **E** | **D** | **L** | **I** | **E** | **E** | **E** | **E** | **I** | **V** | **I** | **T** | **L** | **T** | **N** | **N** | **G** | **Y** | **I** | **K** | **R** | **L** | **A** | **N** | **N** | **E** | **F** | **R** | **A** | **Q** | **R** | **R** | **G** | **G** | **R** | **G** | **V** | **Q** | **G** | **M** | **G** | **I** | **H** | **N** | **D** | **D** | **F** | **V** | **K** | **N** | **L** | **V** | **S** | **C** | **S** | **T** | **H** | **D** | **T** | **L** | **L** | **F** | **F** | **T** | **N** | **N** | **G** | **K** | **V** | **Y** | **R** | **A** | **K** | **G** | **Y** | **E** | **I** | **P** | **E** | **Y** | **G** | **R** | **T** | **A** | **K** | **G** | **I** | **P** | **I** | **I** | **N** | **L** | **L** | **G** | **I** | **D** | **S** | **S** | **E** | **S** | **I** | **Q** | **A** | **I** | **I** | **A** | **V** | **T** | **G** | **E** | **A** | **E** | **E** | **G** | **H** | **Y** | **L** | **F** | **F** | **T** | **T** | **R** | **Q** | **G** | **T** | **V** | **K** | **R** | **T** | **S** | **V** | **T** | **A** | **F** | **A** | **N** | **I** | **R** | **S** | **N** | **G** | **L** | **I** | **A** | **I** | **G** | **L** | **K** | **E** | **D** | **D** | **E** | **L** | **V** | **N** | **V** | **L** | **L** | **T** | **D** | **G** | **Q** | **S** | **N** | **I** | **I** | **I** | **G** | **T** | **H** | **N** | **G** | **Y** | **S** | **V** | **T** | **F** | **K** | **E** | **E** | **A** | **V** | **R** | **D** | **M** | **G** | **R** | **T** | **A** | **S** | **G** | **V** | **R** | **G** | **I** | **R** | **L** | **R** | **E** | **E** | **D** | **Y** | **V** | **V** | **G** | **A** | **A** | **L** | **M** | **K** | **E** | **D** | **Q** | **E** | **V** | **L** | **V** | **I** | **T** | **E** | **K** | **G** | **Y** | **G** | **K** | **R** | **T** | **K** | **V** | **S** | **E** | **Y** | **P** | **V** | **K** | **G** | **R** | **G** | **G** | **K** | **G** | **I** | **K** | **T** | **A** | **N** | **I** | **T** | **E** | **K** | **N** | **G** | **P** | **L** | **A** | **G** | **L** | **T** | **T** | **V** | **S** | **G** | **D** | **E** | **D** | **I** | **M** | **L** | **I** | **T** | **D** | **K** | **G** | **V** | **I** | **I** | **R** | **F** | **N** | **V** | **S** | **T** | **V** | **S** | **Q** | **T** | **G** | **R** | **S** | **T** | **L** | **G** | **V** | **R** | **L** | **M** | **K** | **M** | **E** | **A** | **D** | **T** | **K** | **V** | **V** | **T** | **M** | **A** | **A** | **V** | **E** | **P** | **E** | **A** | **A** | **E** | **Q** | **T** | **V** | **E** | **E** | **Q** | **G** | **S** | **E** | **E** |
| **N52564** | **M** | **S** | **E** | **E** | **I** | **K** | **E** | **N** | **I** | **Q** | **D** | **V** | **N** | **L** | **T** | **S** | **E** | **M** | **K** | **E** | **S** | **F** | **I** | **D** | **Y** | **A** | **M** | **S** | **V** | **I** | **V** | **A** | **R** | **A** | **L** | **P** | **D** | **V** | **R** | **D** | **G** | **L** | **K** | **P** | **V** | **H** | **R** | **R** | **I** | **L** | **Y** | **G** | **M** | **N** | **E** | **L** | **G** | **V** | **T** | **P** | **D** | **K** | **P** | **H** | **K** | **K** | **S** | **A** | **R** | **I** | **V** | **G** | **D** | **V** | **M** | **G** | **K** | **Y** | **H** | **P** | **H** | **G** | **D** | **S** | **A** | **I** | **Y** | **E** | **S** | **M** | **V** | **R** | **M** | **A** | **Q** | **P** | **F** | **S** | **Y** | **R** | **A** | **M** | **L** | **V** | **D** | **G** | **H** | **G** | **N** | **F** | **G** | **S** | **V** | **D** | **G** | **D** | **G** | **A** | **A** | **A** | **M** | **R** | **Y** | **T** | **E** | **A** | **R** | **M** | **S** | **K** | **I** | **A** | **L** | **E** | **M** | **L** | **R** | **D** | **I** | **N** | **K** | **N** | **T** | **V** | **D** | **F** | **Q** | **G** | **N** | **Y** | **D** | **D** | **S** | **E** | **Q** | **E** | **P** | **V** | **V** | **L** | **P** | **A** | **R** | **F** | **P** | **N** | **L** | **L** | **V** | **N** | **G** | **T** | **T** | **G** | **I** | **A** | **V** | **G** | **M** | **A** | **T** | **N** | **I** | **P** | **P** | **H** | **N** | **L** | **S** | **E** | **V** | **I** | **D** | **A** | **T** | **S** | **L** | **L** | **M** | **D** | **N** | **P** | **D** | **V** | **T** | **T** | **N** | **E** | **L** | **M** | **E** | **V** | **L** | **P** | **G** | **P** | **D** | **F** | **P** | **T** | **G** | **G** | **L** | **V** | **M** | **G** | **K** | **S** | **G** | **I** | **R** | **R** | **A** | **Y** | **E** | **T** | **G** | **K** | **G** | **S** | **I** | **T** | **V** | **R** | **A** | **K** | **V** | **E** | **L** | **T** | **E** | **M** | **P** | **N** | **G** | **K** | **E** | **R** | **I** | **L** | **V** | **T** | **E** | **L** | **P** | **Y** | **M** | **V** | **N** | **K** | **A** | **K** | **L** | **I** | **E** | **R** | **I** | **S** | **E** | **L** | **H** | **R** | **D** | **K** | **R** | **I** | **E** | **G** | **I** | **T** | **D** | **L** | **R** | **D** | **E** | **S** | **S** | **R** | **E** | **G** | **M** | **R** | **I** | **V** | **I** | **D** | **V** | **R** | **R** | **D** | **V** | **S** | **A** | **S** | **V** | **V** | **L** | **N** | **N** | **L** | **Y** | **K** | **M** | **T** | **A** | **L** | **Q** | **T** | **S** | **F** | **G** | **F** | **N** | **M** | **L** | **A** | **I** | **E** | **K** | **G** | **V** | **P** | **K** | **I** | **L** | **S** | **L** | **K** | **R** | **I** | **L** | **E** | **N** | **Y** | **V** | **E** | **H** | **Q** | **K** | **E** | **V** | **I** | **T** | **R** | **R** | **T** | **I** | **F** | **D** | **K** | **N** | **K** | **A** | **E** | **A** | **R** | **A** | **H** | **I** | **L** | **E** | **G** | **L** | **R** | **I** | **A** | **L** | **D** | **H** | **I** | **D** | **E** | **I** | **I** | **A** | **I** | **I** | **R** | **G** | **S** | **Q** | **S** | **D** | **D** | **E** | **A** | **K** | **A** | **T** | **L** | **I** | **E** | **R** | **F** | **E** | **F** | **S** | **D** | **R** | **Q** | **A** | **Q** | **A** | **I** | **L** | **D** | **M** | **R** | **L** | **R** | **R** | **L** | **T** | **G** | **L** | **E** | **R** | **D** | **K** | **I** | **E** | **N** | **E** | **Y** | **Q** | **E** | **L** | **L** | **K** | **F** | **I** | **A** | **D** | **L** | **E** | **D** | **I** | **L** | **A** | **R** | **P** | **E** | **R** | **V** | **I** | **E** | **I** | **I** | **K** | **T** | **E** | **L** | **N** | **D** | **V** | **R** | **T** | **K** | **F** | **G** | **D** | **A** | **R** | **R** | **T** | **E** | **L** | **L** | **V** | **G** | **E** | **V** | **L** | **S** | **L** | **E** | **D** | **E** | **D** | **L** | **I** | **E** | **E** | **E** | **E** | **V** | **V** | **I** | **T** | **L** | **T** | **N** | **N** | **G** | **Y** | **I** | **K** | **R** | **M** | **A** | **N** | **S** | **E** | **F** | **R** | **A** | **Q** | **R** | **R** | **G** | **G** | **R** | **G** | **V** | **Q** | **G** | **M** | **G** | **V** | **H** | **D** | **D** | **D** | **F** | **V** | **K** | **N** | **L** | **V** | **S** | **C** | **S** | **T** | **H** | **D** | **T** | **L** | **L** | **F** | **F** | **T** | **N** | **T** | **G** | **K** | **V** | **Y** | **R** | **A** | **K** | **G** | **Y** | **E** | **I** | **P** | **E** | **Y** | **G** | **R** | **T** | **A** | **K** | **G** | **I** | **P** | **V** | **I** | **N** | **L** | **L** | **G** | **I** | **D** | **S** | **A** | **E** | **K** | **I** | **Q** | **A** | **I** | **I** | **S** | **V** | **E** | **G** | **K** | **A** | **E** | **A** | **G** | **K** | **Y** | **L** | **F** | **F** | **T** | **T** | **L** | **K** | **G** | **T** | **V** | **K** | **R** | **T** | **A** | **V** | **T** | **A** | **F** | **S** | **N** | **I** | **R** | **S** | **N** | **G** | **L** | **I** | **A** | **I** | **S** | **L** | **K** | **E** | **D** | **D** | **E** | **L** | **V** | **N** | **V** | **V** | **T** | **T** | **N** | **G** | **N** | **Q** | **K** | **M** | **I** | **I** | **G** | **T** | **H** | **A** | **G** | **Y** | **S** | **V** | **T** | **F** | **D** | **E** | **N** | **T** | **V** | **R** | **D** | **M** | **G** | **R** | **T** | **A** | **S** | **G** | **V** | **R** | **G** | **I** | **R** | **L** | **R** | **E** | **N** | **D** | **Y** | **V** | **V** | **G** | **A** | **A** | **I** | **L** | **D** | **E** | **N** | **K** | **E** | **V** | **L** | **V** | **I** | **T** | **E** | **N** | **G** | **Y** | **G** | **K** | **R** | **T** | **K** | **A** | **S** | **E** | **Y** | **P** | **V** | **K** | **G** | **R** | **G** | **G** | **K** | **G** | **I** | **K** | **T** | **A** | **N** | **I** | **T** | **E** | **K** | **N** | **G** | **P** | **L** | **A** | **G** | **L** | **T** | **T** | **V** | **N** | **G** | **D** | **E** | **D** | **I** | **L** | **L** | **I** | **T** | **N** | **K** | **G** | **V** | **I** | **I** | **R** | **F** | **N** | **V** | **D** | **S** | **V** | **S** | **Q** | **T** | **G** | **R** | **A** | **T** | **L** | **G** | **V** | **R** | **L** | **M** | **R** | **M** | **E** | **D** | **G** | **A** | **K** | **-** | **-** | **-** | **-** | **-** | **-** | **-** | **-** | **-** | **-** | **-** | **-** | **-** | **-** | **-** | **-** | **-** | **-** | **-** | **-** | **-** | **-** | **-** |
| **N59836F** | **M** | **S** | **E** | **E** | **I** | **K** | **E** | **N** | **I** | **Q** | **D** | **V** | **N** | **L** | **T** | **S** | **E** | **M** | **K** | **E** | **S** | **F** | **I** | **D** | **Y** | **A** | **M** | **S** | **V** | **I** | **V** | **A** | **R** | **A** | **L** | **P** | **D** | **V** | **R** | **D** | **G** | **L** | **K** | **P** | **V** | **H** | **R** | **R** | **I** | **L** | **Y** | **G** | **M** | **N** | **E** | **L** | **G** | **V** | **T** | **P** | **D** | **K** | **P** | **H** | **K** | **K** | **S** | **A** | **R** | **I** | **V** | **G** | **D** | **V** | **M** | **G** | **K** | **Y** | **H** | **P** | **H** | **G** | **D** | **S** | **A** | **I** | **Y** | **E** | **S** | **M** | **V** | **R** | **M** | **A** | **Q** | **P** | **F** | **S** | **Y** | **R** | **A** | **M** | **L** | **V** | **D** | **G** | **H** | **G** | **N** | **F** | **G** | **S** | **V** | **D** | **G** | **D** | **G** | **A** | **A** | **A** | **M** | **R** | **Y** | **T** | **E** | **A** | **R** | **M** | **S** | **K** | **I** | **A** | **L** | **E** | **M** | **L** | **R** | **D** | **I** | **N** | **K** | **N** | **T** | **V** | **D** | **F** | **Q** | **G** | **N** | **Y** | **D** | **D** | **S** | **E** | **Q** | **E** | **P** | **V** | **V** | **L** | **P** | **A** | **R** | **F** | **P** | **N** | **L** | **L** | **V** | **N** | **G** | **T** | **T** | **G** | **I** | **A** | **V** | **G** | **M** | **A** | **T** | **N** | **I** | **P** | **P** | **H** | **N** | **L** | **S** | **E** | **V** | **I** | **D** | **A** | **T** | **S** | **L** | **L** | **M** | **D** | **N** | **P** | **D** | **V** | **T** | **T** | **N** | **E** | **L** | **M** | **E** | **V** | **L** | **P** | **G** | **P** | **D** | **F** | **P** | **T** | **G** | **G** | **L** | **V** | **M** | **G** | **K** | **S** | **G** | **I** | **R** | **R** | **A** | **Y** | **E** | **T** | **G** | **K** | **G** | **S** | **I** | **T** | **V** | **R** | **A** | **K** | **V** | **E** | **L** | **T** | **E** | **M** | **P** | **N** | **G** | **K** | **E** | **R** | **I** | **L** | **V** | **T** | **E** | **L** | **P** | **Y** | **M** | **V** | **N** | **K** | **A** | **K** | **L** | **I** | **E** | **R** | **I** | **S** | **E** | **L** | **H** | **R** | **D** | **K** | **R** | **I** | **E** | **G** | **I** | **T** | **D** | **L** | **R** | **D** | **E** | **S** | **S** | **R** | **E** | **G** | **M** | **R** | **I** | **V** | **I** | **D** | **V** | **R** | **R** | **D** | **V** | **S** | **A** | **S** | **V** | **V** | **L** | **N** | **N** | **L** | **Y** | **K** | **M** | **T** | **A** | **L** | **Q** | **T** | **S** | **F** | **G** | **F** | **N** | **M** | **L** | **A** | **I** | **E** | **K** | **G** | **V** | **P** | **K** | **I** | **L** | **S** | **L** | **K** | **R** | **I** | **L** | **E** | **N** | **Y** | **V** | **E** | **H** | **Q** | **K** | **E** | **V** | **I** | **T** | **R** | **R** | **T** | **I** | **F** | **D** | **K** | **N** | **K** | **A** | **E** | **A** | **R** | **A** | **H** | **I** | **L** | **E** | **G** | **L** | **R** | **I** | **A** | **L** | **D** | **H** | **I** | **D** | **E** | **I** | **I** | **A** | **I** | **I** | **R** | **G** | **S** | **Q** | **S** | **D** | **D** | **E** | **A** | **K** | **A** | **T** | **L** | **I** | **E** | **R** | **F** | **E** | **F** | **S** | **D** | **R** | **Q** | **A** | **Q** | **A** | **I** | **L** | **D** | **M** | **R** | **L** | **R** | **R** | **L** | **T** | **G** | **L** | **E** | **R** | **D** | **K** | **I** | **E** | **N** | **E** | **Y** | **Q** | **E** | **L** | **L** | **K** | **F** | **I** | **A** | **D** | **L** | **E** | **D** | **I** | **L** | **A** | **R** | **P** | **E** | **R** | **V** | **I** | **E** | **I** | **I** | **K** | **T** | **E** | **L** | **N** | **D** | **V** | **R** | **T** | **K** | **F** | **G** | **D** | **A** | **R** | **R** | **T** | **E** | **L** | **L** | **V** | **G** | **E** | **V** | **L** | **S** | **L** | **E** | **D** | **E** | **D** | **L** | **I** | **E** | **E** | **E** | **E** | **V** | **V** | **I** | **T** | **L** | **T** | **N** | **N** | **G** | **Y** | **I** | **K** | **R** | **M** | **A** | **N** | **S** | **E** | **F** | **R** | **A** | **Q** | **R** | **R** | **G** | **G** | **R** | **G** | **V** | **Q** | **G** | **M** | **G** | **V** | **H** | **D** | **D** | **D** | **F** | **V** | **K** | **N** | **L** | **V** | **S** | **C** | **S** | **T** | **H** | **D** | **T** | **L** | **L** | **F** | **F** | **T** | **N** | **T** | **G** | **K** | **V** | **Y** | **R** | **A** | **K** | **G** | **Y** | **E** | **I** | **P** | **E** | **Y** | **G** | **R** | **T** | **A** | **K** | **G** | **I** | **P** | **V** | **I** | **N** | **L** | **L** | **G** | **I** | **D** | **S** | **A** | **E** | **K** | **I** | **Q** | **A** | **I** | **I** | **S** | **V** | **E** | **G** | **K** | **A** | **E** | **A** | **G** | **K** | **Y** | **L** | **F** | **F** | **T** | **T** | **L** | **K** | **G** | **T** | **V** | **K** | **R** | **T** | **A** | **V** | **T** | **A** | **F** | **S** | **N** | **I** | **R** | **S** | **N** | **G** | **L** | **I** | **A** | **I** | **S** | **L** | **K** | **E** | **D** | **D** | **E** | **L** | **V** | **N** | **V** | **V** | **T** | **T** | **N** | **G** | **N** | **Q** | **K** | **M** | **I** | **I** | **G** | **T** | **H** | **A** | **G** | **Y** | **S** | **V** | **T** | **F** | **D** | **E** | **N** | **T** | **V** | **R** | **D** | **M** | **G** | **R** | **T** | **A** | **S** | **G** | **V** | **R** | **G** | **I** | **R** | **L** | **R** | **E** | **N** | **D** | **Y** | **V** | **V** | **G** | **A** | **A** | **I** | **L** | **D** | **E** | **N** | **K** | **E** | **V** | **L** | **V** | **I** | **T** | **E** | **N** | **G** | **Y** | **G** | **K** | **R** | **T** | **K** | **A** | **S** | **E** | **Y** | **P** | **V** | **K** | **G** | **R** | **G** | **G** | **K** | **G** | **I** | **K** | **T** | **A** | **N** | **I** | **T** | **E** | **K** | **N** | **G** | **P** | **L** | **A** | **G** | **L** | **T** | **T** | **V** | **N** | **G** | **D** | **E** | **D** | **I** | **L** | **L** | **I** | **T** | **N** | **K** | **G** | **V** | **I** | **I** | **R** | **F** | **N** | **V** | **D** | **S** | **V** | **S** | **Q** | **T** | **G** | **R** | **A** | **T** | **L** | **G** | **V** | **R** | **L** | **M** | **R** | **M** | **E** | **D** | **G** | **A** | **K** | **-** | **-** | **-** | **-** | **-** | **-** | **-** | **-** | **-** | **-** | **-** | **-** | **-** | **-** | **-** | **-** | **-** | **-** | **-** | **-** | **-** | **-** | **-** |
| **N59484F** | **M** | **S** | **E** | **E** | **I** | **R** | **E** | **N** | **I** | **H** | **D** | **V** | **N** | **L** | **T** | **S** | **E** | **M** | **K** | **D** | **S** | **F** | **I** | **D** | **Y** | **A** | **M** | **S** | **V** | **I** | **V** | **A** | **R** | **A** | **L** | **P** | **D** | **V** | **R** | **D** | **G** | **L** | **K** | **P** | **V** | **H** | **R** | **R** | **I** | **L** | **Y** | **G** | **M** | **N** | **E** | **L** | **G** | **V** | **T** | **P** | **D** | **K** | **A** | **H** | **K** | **K** | **S** | **A** | **R** | **I** | **V** | **G** | **D** | **V** | **M** | **G** | **K** | **Y** | **H** | **P** | **H** | **G** | **D** | **S** | **A** | **I** | **Y** | **E** | **S** | **M** | **V** | **R** | **M** | **A** | **Q** | **P** | **F** | **S** | **Y** | **R** | **Y** | **M** | **L** | **V** | **D** | **G** | **H** | **G** | **N** | **F** | **G** | **S** | **V** | **D** | **G** | **D** | **G** | **A** | **A** | **A** | **M** | **R** | **Y** | **T** | **E** | **A** | **R** | **M** | **S** | **K** | **I** | **A** | **T** | **E** | **M** | **L** | **R** | **D** | **I** | **N** | **K** | **N** | **T** | **V** | **D** | **F** | **Q** | **S** | **N** | **Y** | **D** | **D** | **T** | **E** | **K** | **E** | **P** | **V** | **V** | **L** | **P** | **A** | **R** | **F** | **P** | **N** | **L** | **L** | **V** | **N** | **G** | **T** | **T** | **G** | **I** | **A** | **V** | **G** | **M** | **A** | **T** | **N** | **I** | **P** | **P** | **H** | **N** | **L** | **S** | **E** | **V** | **V** | **A** | **A** | **I** | **D** | **L** | **L** | **M** | **D** | **N** | **P** | **D** | **V** | **T** | **T** | **N** | **E** | **L** | **M** | **E** | **V** | **L** | **P** | **G** | **P** | **D** | **F** | **P** | **T** | **G** | **G** | **L** | **V** | **M** | **G** | **K** | **S** | **G** | **I** | **R** | **R** | **A** | **Y** | **E** | **T** | **G** | **K** | **G** | **S** | **I** | **T** | **V** | **R** | **A** | **K** | **V** | **E** | **I** | **T** | **E** | **M** | **P** | **N** | **G** | **K** | **E** | **R** | **I** | **I** | **V** | **T** | **E** | **L** | **P** | **Y** | **M** | **V** | **N** | **K** | **A** | **K** | **L** | **I** | **E** | **R** | **I** | **S** | **E** | **L** | **H** | **R** | **D** | **K** | **R** | **I** | **E** | **G** | **I** | **T** | **D** | **L** | **R** | **D** | **E** | **S** | **S** | **R** | **E** | **G** | **M** | **R** | **I** | **V** | **I** | **D** | **I** | **R** | **R** | **D** | **A** | **S** | **A** | **S** | **V** | **I** | **L** | **N** | **N** | **L** | **Y** | **K** | **L** | **T** | **S** | **L** | **Q** | **N** | **S** | **F** | **G** | **F** | **N** | **M** | **L** | **A** | **I** | **E** | **K** | **G** | **V** | **P** | **K** | **V** | **L** | **S** | **L** | **K** | **Q** | **I** | **L** | **E** | **N** | **Y** | **I** | **E** | **H** | **Q** | **R** | **E** | **V** | **I** | **T** | **R** | **R** | **T** | **V** | **F** | **E** | **K** | **E** | **K** | **A** | **E** | **A** | **R** | **A** | **H** | **I** | **L** | **E** | **G** | **L** | **R** | **I** | **A** | **L** | **D** | **H** | **I** | **D** | **E** | **I** | **I** | **A** | **I** | **I** | **R** | **G** | **S** | **K** | **S** | **D** | **D** | **E** | **A** | **K** | **A** | **T** | **M** | **I** | **E** | **R** | **F** | **D** | **L** | **S** | **D** | **R** | **Q** | **S** | **Q** | **A** | **I** | **L** | **D** | **M** | **R** | **L** | **R** | **R** | **L** | **T** | **G** | **L** | **E** | **R** | **E** | **K** | **I** | **E** | **N** | **E** | **Y** | **Q** | **E** | **L** | **L** | **K** | **L** | **I** | **E** | **D** | **L** | **T** | **D** | **I** | **L** | **A** | **R** | **P** | **E** | **R** | **V** | **T** | **E** | **I** | **I** | **K** | **N** | **E** | **L** | **A** | **D** | **L** | **N** | **Q** | **R** | **F** | **G** | **D** | **K** | **R** | **R** | **T** | **E** | **L** | **L** | **V** | **G** | **E** | **V** | **L** | **S** | **L** | **E** | **D** | **E** | **D** | **L** | **I** | **E** | **E** | **E** | **E** | **I** | **V** | **I** | **T** | **L** | **T** | **N** | **N** | **G** | **Y** | **I** | **K** | **R** | **L** | **A** | **N** | **N** | **E** | **F** | **R** | **A** | **Q** | **R** | **R** | **G** | **G** | **R** | **G** | **V** | **Q** | **G** | **M** | **G** | **I** | **H** | **N** | **D** | **D** | **F** | **V** | **K** | **N** | **L** | **V** | **S** | **C** | **S** | **T** | **H** | **D** | **T** | **L** | **L** | **F** | **F** | **T** | **N** | **N** | **G** | **K** | **V** | **Y** | **R** | **A** | **K** | **G** | **Y** | **E** | **I** | **P** | **E** | **Y** | **G** | **R** | **T** | **A** | **K** | **G** | **I** | **P** | **I** | **I** | **N** | **L** | **L** | **G** | **I** | **D** | **S** | **S** | **E** | **S** | **I** | **Q** | **A** | **I** | **I** | **A** | **V** | **T** | **G** | **E** | **A** | **E** | **E** | **G** | **H** | **Y** | **L** | **F** | **F** | **T** | **T** | **R** | **Q** | **G** | **T** | **V** | **K** | **R** | **T** | **S** | **V** | **T** | **A** | **F** | **A** | **N** | **I** | **R** | **S** | **N** | **G** | **L** | **I** | **A** | **I** | **G** | **L** | **K** | **E** | **D** | **D** | **E** | **L** | **V** | **N** | **V** | **L** | **L** | **T** | **D** | **G** | **Q** | **S** | **N** | **I** | **I** | **I** | **G** | **T** | **H** | **N** | **G** | **Y** | **S** | **V** | **T** | **F** | **K** | **E** | **E** | **A** | **V** | **R** | **D** | **M** | **G** | **R** | **T** | **A** | **S** | **G** | **V** | **R** | **G** | **I** | **R** | **L** | **R** | **E** | **E** | **D** | **Y** | **V** | **V** | **G** | **A** | **A** | **L** | **M** | **K** | **E** | **D** | **Q** | **E** | **V** | **L** | **V** | **I** | **T** | **E** | **K** | **G** | **Y** | **G** | **K** | **R** | **T** | **K** | **V** | **S** | **E** | **Y** | **P** | **V** | **K** | **G** | **R** | **G** | **G** | **K** | **G** | **I** | **K** | **T** | **A** | **N** | **I** | **T** | **E** | **K** | **N** | **G** | **P** | **L** | **A** | **G** | **L** | **T** | **T** | **V** | **S** | **G** | **D** | **E** | **D** | **I** | **M** | **L** | **I** | **T** | **D** | **K** | **G** | **V** | **I** | **I** | **R** | **F** | **N** | **V** | **S** | **T** | **V** | **S** | **Q** | **T** | **G** | **R** | **S** | **T** | **L** | **G** | **V** | **R** | **L** | **M** | **K** | **M** | **E** | **A** | **D** | **T** | **K** | **V** | **V** | **T** | **M** | **A** | **A** | **V** | **E** | **P** | **E** | **A** | **A** | **E** | **Q** | **T** | **V** | **E** | **E** | **Q** | **G** | **S** | **E** | **E** |
| **N54560** | **M** | **S** | **E** | **E** | **I** | **R** | **E** | **N** | **I** | **H** | **D** | **V** | **N** | **L** | **T** | **S** | **E** | **M** | **K** | **D** | **S** | **F** | **I** | **D** | **Y** | **A** | **M** | **S** | **V** | **I** | **V** | **A** | **R** | **A** | **L** | **P** | **D** | **V** | **R** | **D** | **G** | **L** | **K** | **P** | **V** | **H** | **R** | **R** | **I** | **L** | **Y** | **G** | **M** | **N** | **E** | **L** | **G** | **V** | **T** | **P** | **D** | **K** | **A** | **H** | **K** | **K** | **S** | **A** | **R** | **I** | **V** | **G** | **D** | **V** | **M** | **G** | **K** | **Y** | **H** | **P** | **H** | **G** | **D** | **S** | **A** | **I** | **Y** | **E** | **S** | **M** | **V** | **R** | **M** | **A** | **Q** | **P** | **F** | **S** | **Y** | **R** | **Y** | **M** | **L** | **V** | **D** | **G** | **H** | **G** | **N** | **F** | **G** | **S** | **V** | **D** | **G** | **D** | **G** | **A** | **A** | **A** | **M** | **R** | **Y** | **T** | **E** | **A** | **R** | **M** | **S** | **K** | **I** | **A** | **T** | **E** | **M** | **L** | **R** | **D** | **I** | **N** | **K** | **N** | **T** | **V** | **D** | **F** | **Q** | **S** | **N** | **Y** | **D** | **D** | **T** | **E** | **K** | **E** | **P** | **V** | **V** | **L** | **P** | **A** | **R** | **F** | **P** | **N** | **L** | **L** | **V** | **N** | **G** | **T** | **T** | **G** | **I** | **A** | **V** | **G** | **M** | **A** | **T** | **N** | **I** | **P** | **P** | **H** | **N** | **L** | **S** | **E** | **V** | **V** | **A** | **A** | **I** | **D** | **L** | **L** | **M** | **D** | **N** | **P** | **D** | **V** | **T** | **T** | **N** | **E** | **L** | **M** | **E** | **V** | **L** | **P** | **G** | **P** | **D** | **F** | **P** | **T** | **G** | **G** | **L** | **V** | **M** | **G** | **K** | **S** | **G** | **I** | **R** | **R** | **A** | **Y** | **E** | **T** | **G** | **K** | **G** | **S** | **I** | **T** | **V** | **R** | **A** | **K** | **V** | **E** | **I** | **T** | **E** | **M** | **P** | **N** | **G** | **K** | **E** | **R** | **I** | **I** | **V** | **T** | **E** | **L** | **P** | **Y** | **M** | **V** | **N** | **K** | **A** | **K** | **L** | **I** | **E** | **R** | **I** | **S** | **E** | **L** | **H** | **R** | **D** | **K** | **R** | **I** | **E** | **G** | **I** | **T** | **D** | **L** | **R** | **D** | **E** | **S** | **S** | **R** | **E** | **G** | **M** | **R** | **I** | **V** | **I** | **D** | **I** | **R** | **R** | **D** | **A** | **S** | **A** | **S** | **V** | **I** | **L** | **N** | **N** | **L** | **Y** | **K** | **L** | **T** | **S** | **L** | **Q** | **N** | **S** | **F** | **G** | **F** | **N** | **M** | **L** | **A** | **I** | **E** | **K** | **G** | **V** | **P** | **K** | **V** | **L** | **S** | **L** | **K** | **Q** | **I** | **L** | **E** | **N** | **Y** | **I** | **E** | **H** | **Q** | **R** | **E** | **V** | **I** | **T** | **R** | **R** | **T** | **V** | **F** | **E** | **K** | **E** | **K** | **A** | **E** | **A** | **R** | **A** | **H** | **I** | **L** | **E** | **G** | **L** | **R** | **I** | **A** | **L** | **D** | **H** | **I** | **D** | **E** | **I** | **I** | **A** | **I** | **I** | **R** | **G** | **S** | **K** | **S** | **D** | **D** | **E** | **A** | **K** | **A** | **T** | **M** | **I** | **E** | **R** | **F** | **D** | **L** | **S** | **D** | **R** | **Q** | **S** | **Q** | **A** | **I** | **L** | **D** | **M** | **R** | **L** | **R** | **R** | **L** | **T** | **G** | **L** | **E** | **R** | **E** | **K** | **I** | **E** | **N** | **E** | **Y** | **Q** | **E** | **L** | **L** | **K** | **L** | **I** | **E** | **D** | **L** | **T** | **D** | **I** | **L** | **A** | **R** | **P** | **E** | **R** | **V** | **T** | **E** | **I** | **I** | **K** | **N** | **E** | **L** | **A** | **D** | **L** | **N** | **Q** | **R** | **F** | **G** | **D** | **K** | **R** | **R** | **T** | **E** | **L** | **L** | **V** | **G** | **E** | **V** | **L** | **S** | **L** | **E** | **D** | **E** | **D** | **L** | **I** | **E** | **E** | **E** | **E** | **I** | **V** | **I** | **T** | **L** | **T** | **N** | **N** | **G** | **Y** | **I** | **K** | **R** | **L** | **A** | **N** | **N** | **E** | **F** | **R** | **A** | **Q** | **R** | **R** | **G** | **G** | **R** | **G** | **V** | **Q** | **G** | **M** | **G** | **I** | **H** | **N** | **D** | **D** | **F** | **V** | **K** | **N** | **L** | **V** | **S** | **C** | **S** | **T** | **H** | **D** | **T** | **L** | **L** | **F** | **F** | **T** | **N** | **N** | **G** | **K** | **V** | **Y** | **R** | **A** | **K** | **G** | **Y** | **E** | **I** | **P** | **E** | **Y** | **G** | **R** | **T** | **A** | **K** | **G** | **I** | **P** | **I** | **I** | **N** | **L** | **L** | **G** | **I** | **D** | **S** | **S** | **E** | **S** | **I** | **Q** | **A** | **I** | **I** | **A** | **V** | **T** | **G** | **E** | **A** | **E** | **E** | **G** | **H** | **Y** | **L** | **F** | **F** | **T** | **T** | **R** | **Q** | **G** | **T** | **V** | **K** | **R** | **T** | **S** | **V** | **T** | **A** | **F** | **A** | **N** | **I** | **R** | **S** | **N** | **G** | **L** | **I** | **A** | **I** | **G** | **L** | **K** | **E** | **D** | **D** | **E** | **L** | **V** | **N** | **V** | **L** | **L** | **T** | **D** | **G** | **Q** | **S** | **N** | **I** | **I** | **I** | **G** | **T** | **H** | **N** | **G** | **Y** | **S** | **V** | **T** | **F** | **K** | **E** | **E** | **A** | **V** | **R** | **D** | **M** | **G** | **R** | **T** | **A** | **S** | **G** | **V** | **R** | **G** | **I** | **R** | **L** | **R** | **E** | **E** | **D** | **Y** | **V** | **V** | **G** | **A** | **A** | **L** | **M** | **K** | **E** | **D** | **Q** | **E** | **V** | **L** | **V** | **I** | **T** | **E** | **K** | **G** | **Y** | **G** | **K** | **R** | **T** | **K** | **V** | **S** | **E** | **Y** | **P** | **V** | **K** | **G** | **R** | **G** | **G** | **K** | **G** | **I** | **K** | **T** | **A** | **N** | **I** | **T** | **E** | **K** | **N** | **G** | **P** | **L** | **A** | **G** | **L** | **T** | **T** | **V** | **S** | **G** | **D** | **E** | **D** | **I** | **M** | **L** | **I** | **T** | **D** | **K** | **G** | **V** | **I** | **I** | **R** | **F** | **N** | **V** | **S** | **T** | **V** | **S** | **Q** | **T** | **G** | **R** | **S** | **T** | **L** | **G** | **V** | **R** | **L** | **M** | **K** | **M** | **E** | **A** | **D** | **T** | **K** | **V** | **V** | **T** | **M** | **A** | **A** | **V** | **E** | **P** | **E** | **A** | **A** | **E** | **Q** | **T** | **V** | **E** | **E** | **Q** | **G** | **S** | **E** | **E** |
| **N55119** | **M** | **S** | **E** | **E** | **I** | **K** | **E** | **N** | **I** | **Q** | **D** | **V** | **N** | **L** | **T** | **S** | **E** | **M** | **K** | **E** | **S** | **F** | **I** | **D** | **Y** | **A** | **M** | **S** | **V** | **I** | **V** | **A** | **R** | **A** | **L** | **P** | **D** | **V** | **R** | **D** | **G** | **L** | **K** | **P** | **V** | **H** | **R** | **R** | **I** | **L** | **Y** | **G** | **M** | **N** | **E** | **L** | **G** | **V** | **T** | **P** | **D** | **K** | **P** | **H** | **K** | **K** | **S** | **A** | **R** | **I** | **V** | **G** | **D** | **V** | **M** | **G** | **K** | **Y** | **H** | **P** | **H** | **G** | **D** | **S** | **A** | **I** | **Y** | **E** | **S** | **M** | **V** | **R** | **M** | **A** | **Q** | **P** | **F** | **S** | **Y** | **R** | **A** | **M** | **L** | **V** | **D** | **G** | **H** | **G** | **N** | **F** | **G** | **S** | **V** | **D** | **G** | **D** | **G** | **A** | **A** | **A** | **M** | **R** | **Y** | **T** | **E** | **A** | **R** | **M** | **S** | **K** | **I** | **A** | **L** | **E** | **M** | **L** | **R** | **D** | **I** | **N** | **K** | **N** | **T** | **V** | **D** | **F** | **Q** | **G** | **N** | **Y** | **D** | **D** | **S** | **E** | **Q** | **E** | **P** | **V** | **V** | **L** | **P** | **A** | **R** | **F** | **P** | **N** | **L** | **L** | **V** | **N** | **G** | **T** | **T** | **G** | **I** | **A** | **V** | **G** | **M** | **A** | **T** | **N** | **I** | **P** | **P** | **H** | **N** | **L** | **S** | **E** | **V** | **I** | **D** | **A** | **T** | **S** | **L** | **L** | **M** | **D** | **N** | **P** | **D** | **V** | **T** | **T** | **N** | **E** | **L** | **M** | **E** | **V** | **L** | **P** | **G** | **P** | **D** | **F** | **P** | **T** | **G** | **G** | **L** | **V** | **M** | **G** | **K** | **S** | **G** | **I** | **R** | **R** | **A** | **Y** | **E** | **T** | **G** | **K** | **G** | **S** | **I** | **T** | **V** | **R** | **A** | **K** | **V** | **E** | **L** | **T** | **E** | **M** | **P** | **N** | **G** | **K** | **E** | **R** | **I** | **L** | **V** | **T** | **E** | **L** | **P** | **Y** | **M** | **V** | **N** | **K** | **A** | **K** | **L** | **I** | **E** | **R** | **I** | **S** | **E** | **L** | **H** | **R** | **D** | **K** | **R** | **I** | **E** | **G** | **I** | **T** | **D** | **L** | **R** | **D** | **E** | **S** | **S** | **R** | **E** | **G** | **M** | **R** | **I** | **V** | **I** | **D** | **V** | **R** | **R** | **D** | **V** | **S** | **A** | **S** | **V** | **V** | **L** | **N** | **N** | **L** | **Y** | **K** | **M** | **T** | **A** | **L** | **Q** | **T** | **S** | **F** | **G** | **F** | **N** | **M** | **L** | **A** | **I** | **E** | **K** | **G** | **V** | **P** | **K** | **I** | **L** | **S** | **L** | **K** | **R** | **I** | **L** | **E** | **N** | **Y** | **V** | **E** | **H** | **Q** | **K** | **E** | **V** | **I** | **T** | **R** | **R** | **T** | **I** | **F** | **D** | **K** | **N** | **K** | **A** | **E** | **A** | **R** | **A** | **H** | **I** | **L** | **E** | **G** | **L** | **R** | **I** | **A** | **L** | **D** | **H** | **I** | **D** | **E** | **I** | **I** | **A** | **I** | **I** | **R** | **G** | **S** | **Q** | **S** | **D** | **D** | **E** | **A** | **K** | **A** | **T** | **L** | **I** | **E** | **R** | **F** | **E** | **F** | **S** | **D** | **R** | **Q** | **A** | **Q** | **A** | **I** | **L** | **D** | **M** | **R** | **L** | **R** | **R** | **L** | **T** | **G** | **L** | **E** | **R** | **D** | **K** | **I** | **E** | **N** | **E** | **Y** | **Q** | **E** | **L** | **L** | **K** | **F** | **I** | **A** | **D** | **L** | **E** | **D** | **I** | **L** | **A** | **R** | **P** | **E** | **R** | **V** | **I** | **E** | **I** | **I** | **K** | **T** | **E** | **L** | **N** | **D** | **V** | **R** | **T** | **K** | **F** | **G** | **D** | **A** | **R** | **R** | **T** | **E** | **L** | **L** | **V** | **G** | **E** | **V** | **L** | **S** | **L** | **E** | **D** | **E** | **D** | **L** | **I** | **E** | **E** | **E** | **E** | **V** | **V** | **I** | **T** | **L** | **T** | **N** | **N** | **G** | **Y** | **I** | **K** | **R** | **M** | **A** | **N** | **S** | **E** | **F** | **R** | **A** | **Q** | **R** | **R** | **G** | **G** | **R** | **G** | **V** | **Q** | **G** | **M** | **G** | **V** | **H** | **D** | **D** | **D** | **F** | **V** | **K** | **N** | **L** | **V** | **S** | **C** | **S** | **T** | **H** | **D** | **T** | **L** | **L** | **F** | **F** | **T** | **N** | **T** | **G** | **K** | **V** | **Y** | **R** | **A** | **K** | **G** | **Y** | **E** | **I** | **P** | **E** | **Y** | **G** | **R** | **T** | **A** | **K** | **G** | **I** | **P** | **V** | **I** | **N** | **L** | **L** | **G** | **I** | **D** | **S** | **A** | **E** | **K** | **I** | **Q** | **A** | **I** | **I** | **S** | **V** | **E** | **G** | **K** | **A** | **E** | **A** | **G** | **K** | **Y** | **L** | **F** | **F** | **T** | **T** | **L** | **K** | **G** | **T** | **V** | **K** | **R** | **T** | **A** | **V** | **T** | **A** | **F** | **S** | **N** | **I** | **R** | **S** | **N** | **G** | **L** | **I** | **A** | **I** | **S** | **L** | **K** | **E** | **D** | **D** | **E** | **L** | **V** | **N** | **V** | **V** | **T** | **T** | **N** | **G** | **N** | **Q** | **K** | **M** | **I** | **I** | **G** | **T** | **H** | **A** | **G** | **Y** | **S** | **V** | **T** | **F** | **D** | **E** | **N** | **T** | **V** | **R** | **D** | **M** | **G** | **R** | **T** | **A** | **S** | **G** | **V** | **R** | **G** | **I** | **R** | **L** | **R** | **E** | **N** | **D** | **Y** | **V** | **V** | **G** | **A** | **A** | **I** | **L** | **D** | **E** | **N** | **K** | **E** | **V** | **L** | **V** | **I** | **T** | **E** | **N** | **G** | **Y** | **G** | **K** | **R** | **T** | **K** | **A** | **S** | **E** | **Y** | **P** | **V** | **K** | **G** | **R** | **G** | **G** | **K** | **G** | **I** | **K** | **T** | **A** | **N** | **I** | **T** | **E** | **K** | **N** | **G** | **P** | **L** | **A** | **G** | **L** | **T** | **T** | **V** | **N** | **G** | **D** | **E** | **D** | **I** | **L** | **L** | **I** | **T** | **N** | **K** | **G** | **V** | **I** | **I** | **R** | **F** | **N** | **V** | **D** | **S** | **V** | **S** | **Q** | **T** | **G** | **R** | **A** | **T** | **L** | **G** | **V** | **R** | **L** | **M** | **R** | **M** | **E** | **D** | **G** | **A** | **K** | **-** | **-** | **-** | **-** | **-** | **-** | **-** | **-** | **-** | **-** | **-** | **-** | **-** | **-** | **-** | **-** | **-** | **-** | **-** | **-** | **-** | **-** | **-** |
| **N59689F** | **M** | **S** | **E** | **E** | **I** | **K** | **E** | **N** | **I** | **Q** | **D** | **V** | **N** | **L** | **T** | **S** | **E** | **M** | **K** | **E** | **S** | **F** | **I** | **D** | **Y** | **A** | **M** | **S** | **V** | **I** | **V** | **A** | **R** | **A** | **L** | **P** | **D** | **V** | **R** | **D** | **G** | **L** | **K** | **P** | **V** | **H** | **R** | **R** | **I** | **L** | **Y** | **G** | **M** | **N** | **E** | **L** | **G** | **V** | **T** | **P** | **D** | **K** | **P** | **H** | **K** | **K** | **S** | **A** | **R** | **I** | **V** | **G** | **D** | **V** | **M** | **G** | **K** | **Y** | **H** | **P** | **H** | **G** | **D** | **S** | **A** | **I** | **Y** | **E** | **S** | **M** | **V** | **R** | **M** | **A** | **Q** | **P** | **F** | **S** | **Y** | **R** | **A** | **M** | **L** | **V** | **D** | **G** | **H** | **G** | **N** | **F** | **G** | **S** | **V** | **D** | **G** | **D** | **G** | **A** | **A** | **A** | **M** | **R** | **Y** | **T** | **E** | **A** | **R** | **M** | **S** | **K** | **I** | **A** | **L** | **E** | **M** | **L** | **R** | **D** | **I** | **N** | **K** | **N** | **T** | **V** | **D** | **F** | **Q** | **G** | **N** | **Y** | **D** | **D** | **S** | **E** | **Q** | **E** | **P** | **V** | **V** | **L** | **P** | **A** | **R** | **F** | **P** | **N** | **L** | **L** | **V** | **N** | **G** | **T** | **T** | **G** | **I** | **A** | **V** | **G** | **M** | **A** | **T** | **N** | **I** | **P** | **P** | **H** | **N** | **L** | **S** | **E** | **V** | **I** | **D** | **A** | **T** | **S** | **L** | **L** | **M** | **D** | **N** | **P** | **D** | **V** | **T** | **T** | **N** | **E** | **L** | **M** | **E** | **V** | **L** | **P** | **G** | **P** | **D** | **F** | **P** | **T** | **G** | **G** | **L** | **V** | **M** | **G** | **K** | **S** | **G** | **I** | **R** | **R** | **A** | **Y** | **E** | **T** | **G** | **K** | **G** | **S** | **I** | **T** | **V** | **R** | **A** | **K** | **V** | **E** | **L** | **T** | **E** | **M** | **P** | **N** | **G** | **K** | **E** | **R** | **I** | **L** | **V** | **T** | **E** | **L** | **P** | **Y** | **M** | **V** | **N** | **K** | **A** | **K** | **L** | **I** | **E** | **R** | **I** | **S** | **E** | **L** | **H** | **R** | **D** | **K** | **R** | **I** | **E** | **G** | **I** | **T** | **D** | **L** | **R** | **D** | **E** | **S** | **S** | **R** | **E** | **G** | **M** | **R** | **I** | **V** | **I** | **D** | **V** | **R** | **R** | **D** | **V** | **S** | **A** | **S** | **V** | **V** | **L** | **N** | **N** | **L** | **Y** | **K** | **M** | **T** | **A** | **L** | **Q** | **T** | **S** | **F** | **G** | **F** | **N** | **M** | **L** | **A** | **I** | **E** | **K** | **G** | **V** | **P** | **K** | **I** | **L** | **S** | **L** | **K** | **R** | **I** | **L** | **E** | **N** | **Y** | **V** | **E** | **H** | **Q** | **K** | **E** | **V** | **I** | **T** | **R** | **R** | **T** | **I** | **F** | **D** | **K** | **N** | **K** | **A** | **E** | **A** | **R** | **A** | **H** | **I** | **L** | **E** | **G** | **L** | **R** | **I** | **A** | **L** | **D** | **H** | **I** | **D** | **E** | **I** | **I** | **A** | **I** | **I** | **R** | **G** | **S** | **Q** | **S** | **D** | **D** | **E** | **A** | **K** | **A** | **T** | **L** | **I** | **E** | **R** | **F** | **E** | **F** | **S** | **D** | **R** | **Q** | **A** | **Q** | **A** | **I** | **L** | **D** | **M** | **R** | **L** | **R** | **R** | **L** | **T** | **G** | **L** | **E** | **R** | **D** | **K** | **I** | **E** | **N** | **E** | **Y** | **Q** | **E** | **L** | **L** | **K** | **F** | **I** | **A** | **D** | **L** | **E** | **D** | **I** | **L** | **A** | **R** | **P** | **E** | **R** | **V** | **I** | **E** | **I** | **I** | **K** | **T** | **E** | **L** | **N** | **D** | **V** | **R** | **T** | **K** | **F** | **G** | **D** | **A** | **R** | **R** | **T** | **E** | **L** | **L** | **V** | **G** | **E** | **V** | **L** | **S** | **L** | **E** | **D** | **E** | **D** | **L** | **I** | **E** | **E** | **E** | **E** | **V** | **V** | **I** | **T** | **L** | **T** | **N** | **N** | **G** | **Y** | **I** | **K** | **R** | **M** | **A** | **N** | **S** | **E** | **F** | **R** | **A** | **Q** | **R** | **R** | **G** | **G** | **R** | **G** | **V** | **Q** | **G** | **M** | **G** | **V** | **H** | **D** | **D** | **D** | **F** | **V** | **K** | **N** | **L** | **V** | **S** | **C** | **S** | **T** | **H** | **D** | **T** | **L** | **L** | **F** | **F** | **T** | **N** | **T** | **G** | **K** | **V** | **Y** | **R** | **A** | **K** | **G** | **Y** | **E** | **I** | **P** | **E** | **Y** | **G** | **R** | **T** | **A** | **K** | **G** | **I** | **P** | **V** | **I** | **N** | **L** | **L** | **G** | **I** | **D** | **S** | **A** | **E** | **K** | **I** | **Q** | **A** | **I** | **I** | **S** | **V** | **E** | **G** | **K** | **A** | **E** | **A** | **G** | **K** | **Y** | **L** | **F** | **F** | **T** | **T** | **L** | **K** | **G** | **T** | **V** | **K** | **R** | **T** | **A** | **V** | **T** | **A** | **F** | **S** | **N** | **I** | **R** | **S** | **N** | **G** | **L** | **I** | **A** | **I** | **S** | **L** | **K** | **E** | **D** | **D** | **E** | **L** | **V** | **N** | **V** | **V** | **T** | **T** | **N** | **G** | **N** | **Q** | **K** | **M** | **I** | **I** | **G** | **T** | **H** | **A** | **G** | **Y** | **S** | **V** | **T** | **F** | **D** | **E** | **N** | **T** | **V** | **R** | **D** | **M** | **G** | **R** | **T** | **A** | **S** | **G** | **V** | **R** | **G** | **I** | **R** | **L** | **R** | **E** | **N** | **D** | **Y** | **V** | **V** | **G** | **A** | **A** | **I** | **L** | **D** | **E** | **N** | **K** | **E** | **V** | **L** | **V** | **I** | **T** | **E** | **N** | **G** | **Y** | **G** | **K** | **R** | **T** | **K** | **A** | **S** | **E** | **Y** | **P** | **V** | **K** | **G** | **R** | **G** | **G** | **K** | **G** | **I** | **K** | **T** | **A** | **N** | **I** | **T** | **E** | **K** | **N** | **G** | **P** | **L** | **A** | **G** | **L** | **T** | **T** | **V** | **N** | **G** | **D** | **E** | **D** | **I** | **L** | **L** | **I** | **T** | **N** | **K** | **G** | **V** | **I** | **I** | **R** | **F** | **N** | **V** | **D** | **S** | **V** | **S** | **Q** | **T** | **G** | **R** | **A** | **T** | **L** | **G** | **V** | **R** | **L** | **M** | **R** | **M** | **E** | **D** | **G** | **A** | **K** | **-** | **-** | **-** | **-** | **-** | **-** | **-** | **-** | **-** | **-** | **-** | **-** | **-** | **-** | **-** | **-** | **-** | **-** | **-** | **-** | **-** | **-** | **-** |
| **N52629** | **M** | **S** | **E** | **E** | **I** | **R** | **E** | **N** | **I** | **H** | **D** | **V** | **N** | **L** | **T** | **S** | **E** | **M** | **K** | **D** | **S** | **F** | **I** | **D** | **Y** | **A** | **M** | **S** | **V** | **I** | **V** | **A** | **R** | **A** | **L** | **P** | **D** | **V** | **R** | **D** | **G** | **L** | **K** | **P** | **V** | **H** | **R** | **R** | **I** | **L** | **Y** | **G** | **M** | **N** | **E** | **L** | **G** | **V** | **T** | **P** | **D** | **K** | **A** | **H** | **K** | **K** | **S** | **A** | **R** | **I** | **V** | **G** | **D** | **V** | **M** | **G** | **K** | **Y** | **H** | **P** | **H** | **G** | **D** | **S** | **A** | **I** | **Y** | **E** | **S** | **M** | **V** | **R** | **M** | **A** | **Q** | **P** | **F** | **S** | **Y** | **R** | **Y** | **M** | **L** | **V** | **D** | **G** | **H** | **G** | **N** | **F** | **G** | **S** | **V** | **D** | **G** | **D** | **G** | **A** | **A** | **A** | **M** | **R** | **Y** | **T** | **E** | **A** | **R** | **M** | **S** | **K** | **I** | **A** | **T** | **E** | **M** | **L** | **R** | **D** | **I** | **N** | **K** | **N** | **T** | **V** | **D** | **F** | **Q** | **S** | **N** | **Y** | **D** | **D** | **T** | **E** | **K** | **E** | **P** | **V** | **V** | **L** | **P** | **A** | **R** | **F** | **P** | **N** | **L** | **L** | **V** | **N** | **G** | **T** | **T** | **G** | **I** | **A** | **V** | **G** | **M** | **A** | **T** | **N** | **I** | **P** | **P** | **H** | **N** | **L** | **S** | **E** | **V** | **V** | **A** | **A** | **I** | **D** | **L** | **L** | **M** | **D** | **N** | **P** | **D** | **V** | **T** | **T** | **N** | **E** | **L** | **M** | **E** | **V** | **L** | **P** | **G** | **P** | **D** | **F** | **P** | **T** | **G** | **G** | **L** | **V** | **M** | **G** | **K** | **S** | **G** | **I** | **R** | **R** | **A** | **Y** | **E** | **T** | **G** | **K** | **G** | **S** | **I** | **T** | **V** | **R** | **A** | **K** | **V** | **E** | **I** | **T** | **E** | **M** | **P** | **N** | **G** | **K** | **E** | **R** | **I** | **L** | **V** | **T** | **E** | **L** | **P** | **Y** | **M** | **V** | **N** | **K** | **A** | **K** | **L** | **I** | **E** | **R** | **I** | **S** | **E** | **L** | **H** | **R** | **D** | **K** | **R** | **I** | **E** | **G** | **I** | **T** | **D** | **L** | **R** | **D** | **E** | **S** | **S** | **R** | **E** | **G** | **M** | **R** | **I** | **V** | **I** | **D** | **V** | **R** | **R** | **D** | **A** | **S** | **A** | **S** | **V** | **I** | **L** | **N** | **N** | **L** | **Y** | **K** | **L** | **T** | **S** | **L** | **Q** | **N** | **S** | **F** | **G** | **F** | **N** | **M** | **L** | **A** | **I** | **E** | **K** | **G** | **V** | **P** | **K** | **V** | **L** | **S** | **L** | **K** | **Q** | **I** | **L** | **E** | **N** | **Y** | **I** | **E** | **H** | **Q** | **R** | **E** | **V** | **I** | **T** | **R** | **R** | **T** | **V** | **F** | **E** | **K** | **E** | **K** | **A** | **E** | **A** | **R** | **A** | **H** | **I** | **L** | **E** | **G** | **L** | **R** | **I** | **A** | **L** | **D** | **H** | **I** | **D** | **E** | **I** | **I** | **A** | **I** | **I** | **R** | **G** | **S** | **K** | **S** | **D** | **D** | **E** | **A** | **K** | **A** | **T** | **M** | **I** | **E** | **R** | **F** | **D** | **L** | **S** | **D** | **R** | **Q** | **S** | **Q** | **A** | **I** | **L** | **D** | **M** | **R** | **L** | **R** | **R** | **L** | **T** | **G** | **L** | **E** | **R** | **E** | **K** | **I** | **E** | **N** | **E** | **Y** | **Q** | **E** | **L** | **L** | **K** | **L** | **I** | **E** | **D** | **L** | **T** | **D** | **I** | **L** | **A** | **R** | **P** | **E** | **R** | **V** | **T** | **E** | **I** | **I** | **K** | **N** | **E** | **L** | **A** | **D** | **L** | **N** | **Q** | **R** | **F** | **G** | **D** | **K** | **R** | **R** | **T** | **E** | **L** | **L** | **V** | **G** | **E** | **V** | **L** | **S** | **L** | **E** | **D** | **E** | **D** | **L** | **I** | **E** | **E** | **E** | **E** | **I** | **V** | **I** | **T** | **L** | **T** | **N** | **N** | **G** | **Y** | **I** | **K** | **R** | **L** | **A** | **N** | **N** | **E** | **F** | **R** | **A** | **Q** | **R** | **R** | **G** | **G** | **R** | **G** | **V** | **Q** | **G** | **M** | **G** | **I** | **H** | **N** | **D** | **D** | **F** | **V** | **K** | **N** | **L** | **V** | **S** | **C** | **S** | **T** | **H** | **D** | **T** | **L** | **L** | **F** | **F** | **T** | **N** | **N** | **G** | **K** | **V** | **Y** | **R** | **A** | **K** | **G** | **Y** | **E** | **I** | **P** | **E** | **Y** | **G** | **R** | **T** | **A** | **K** | **G** | **I** | **P** | **I** | **I** | **N** | **L** | **L** | **G** | **I** | **D** | **S** | **S** | **E** | **S** | **I** | **Q** | **A** | **I** | **I** | **A** | **V** | **T** | **G** | **E** | **A** | **E** | **E** | **G** | **H** | **Y** | **L** | **F** | **F** | **T** | **T** | **R** | **Q** | **G** | **T** | **V** | **K** | **R** | **T** | **S** | **V** | **T** | **A** | **F** | **A** | **N** | **I** | **R** | **S** | **N** | **G** | **L** | **I** | **A** | **I** | **G** | **L** | **K** | **E** | **D** | **D** | **E** | **L** | **V** | **N** | **V** | **L** | **L** | **T** | **D** | **G** | **Q** | **S** | **N** | **I** | **I** | **I** | **G** | **T** | **H** | **N** | **G** | **Y** | **S** | **V** | **T** | **F** | **K** | **E** | **E** | **A** | **V** | **R** | **D** | **M** | **G** | **R** | **T** | **A** | **S** | **G** | **V** | **R** | **G** | **I** | **R** | **L** | **R** | **E** | **E** | **D** | **Y** | **V** | **V** | **G** | **A** | **A** | **L** | **M** | **K** | **E** | **D** | **Q** | **E** | **V** | **L** | **V** | **I** | **T** | **E** | **K** | **G** | **Y** | **G** | **K** | **R** | **T** | **K** | **V** | **S** | **E** | **Y** | **P** | **V** | **K** | **G** | **R** | **G** | **G** | **K** | **G** | **I** | **K** | **T** | **A** | **N** | **I** | **T** | **E** | **K** | **N** | **G** | **P** | **L** | **A** | **G** | **L** | **T** | **T** | **V** | **S** | **G** | **N** | **E** | **D** | **I** | **M** | **L** | **I** | **T** | **D** | **K** | **G** | **V** | **I** | **I** | **R** | **F** | **N** | **V** | **S** | **T** | **V** | **S** | **Q** | **T** | **G** | **R** | **S** | **T** | **L** | **G** | **V** | **R** | **L** | **M** | **K** | **M** | **E** | **A** | **D** | **T** | **K** | **V** | **V** | **T** | **M** | **-** | **-** | **-** | **-** | **-** | **-** | **-** | **-** | **-** | **-** | **-** | **-** | **-** | **-** | **-** | **-** | **-** | **-** | **-** |
| **N54576** | **M** | **S** | **E** | **E** | **I** | **K** | **E** | **N** | **I** | **Q** | **D** | **V** | **N** | **L** | **T** | **S** | **E** | **M** | **K** | **E** | **S** | **F** | **I** | **D** | **Y** | **A** | **M** | **S** | **V** | **I** | **V** | **A** | **R** | **A** | **L** | **P** | **D** | **V** | **R** | **D** | **G** | **L** | **K** | **P** | **V** | **H** | **R** | **R** | **I** | **L** | **Y** | **G** | **M** | **N** | **E** | **L** | **G** | **V** | **T** | **P** | **D** | **K** | **P** | **H** | **K** | **K** | **S** | **A** | **R** | **I** | **V** | **G** | **D** | **V** | **M** | **G** | **K** | **Y** | **H** | **P** | **H** | **G** | **D** | **S** | **A** | **I** | **Y** | **E** | **S** | **M** | **V** | **R** | **M** | **A** | **Q** | **P** | **F** | **S** | **Y** | **R** | **A** | **M** | **L** | **V** | **D** | **G** | **H** | **G** | **N** | **F** | **G** | **S** | **V** | **D** | **G** | **D** | **G** | **A** | **A** | **A** | **M** | **R** | **Y** | **T** | **E** | **A** | **R** | **M** | **S** | **K** | **I** | **A** | **L** | **E** | **M** | **L** | **R** | **D** | **I** | **N** | **K** | **N** | **T** | **V** | **D** | **F** | **Q** | **G** | **N** | **Y** | **D** | **D** | **S** | **E** | **Q** | **E** | **P** | **V** | **V** | **L** | **P** | **A** | **R** | **F** | **P** | **N** | **L** | **L** | **V** | **N** | **G** | **T** | **T** | **G** | **I** | **A** | **V** | **G** | **M** | **A** | **T** | **N** | **I** | **P** | **P** | **H** | **N** | **L** | **S** | **E** | **V** | **I** | **D** | **A** | **T** | **S** | **L** | **L** | **M** | **D** | **N** | **P** | **D** | **V** | **T** | **T** | **N** | **E** | **L** | **M** | **E** | **V** | **L** | **P** | **G** | **P** | **D** | **F** | **P** | **T** | **G** | **G** | **L** | **V** | **M** | **G** | **K** | **S** | **G** | **I** | **R** | **R** | **A** | **Y** | **E** | **T** | **G** | **K** | **G** | **S** | **I** | **T** | **V** | **R** | **A** | **K** | **V** | **E** | **L** | **T** | **E** | **M** | **P** | **N** | **G** | **K** | **E** | **R** | **I** | **L** | **V** | **T** | **E** | **L** | **P** | **Y** | **M** | **V** | **N** | **K** | **A** | **K** | **L** | **I** | **E** | **R** | **I** | **S** | **E** | **L** | **H** | **R** | **D** | **K** | **R** | **I** | **E** | **G** | **I** | **T** | **D** | **L** | **R** | **D** | **E** | **S** | **S** | **R** | **E** | **G** | **M** | **R** | **I** | **V** | **I** | **D** | **V** | **R** | **R** | **D** | **V** | **S** | **A** | **S** | **V** | **V** | **L** | **N** | **N** | **L** | **Y** | **K** | **M** | **T** | **A** | **L** | **Q** | **T** | **S** | **F** | **G** | **F** | **N** | **M** | **L** | **A** | **I** | **E** | **K** | **G** | **V** | **P** | **K** | **I** | **L** | **S** | **L** | **K** | **R** | **I** | **L** | **E** | **N** | **Y** | **V** | **E** | **H** | **Q** | **K** | **E** | **V** | **I** | **T** | **R** | **R** | **T** | **I** | **F** | **D** | **K** | **N** | **K** | **A** | **E** | **A** | **R** | **A** | **H** | **I** | **L** | **E** | **G** | **L** | **R** | **I** | **A** | **L** | **D** | **H** | **I** | **D** | **E** | **I** | **I** | **A** | **I** | **I** | **R** | **G** | **S** | **Q** | **S** | **D** | **D** | **E** | **A** | **K** | **A** | **T** | **L** | **I** | **E** | **R** | **F** | **E** | **F** | **S** | **D** | **R** | **Q** | **A** | **Q** | **A** | **I** | **L** | **D** | **M** | **R** | **L** | **R** | **R** | **L** | **T** | **G** | **L** | **E** | **R** | **D** | **K** | **I** | **E** | **N** | **E** | **Y** | **Q** | **E** | **L** | **L** | **K** | **F** | **I** | **A** | **D** | **L** | **E** | **D** | **I** | **L** | **A** | **R** | **P** | **E** | **R** | **V** | **I** | **E** | **I** | **I** | **K** | **T** | **E** | **L** | **N** | **D** | **V** | **R** | **T** | **K** | **F** | **G** | **D** | **A** | **R** | **R** | **T** | **E** | **L** | **L** | **V** | **G** | **E** | **V** | **L** | **S** | **L** | **E** | **D** | **E** | **D** | **L** | **I** | **E** | **E** | **E** | **E** | **V** | **V** | **I** | **T** | **L** | **T** | **N** | **N** | **G** | **Y** | **I** | **K** | **R** | **M** | **A** | **N** | **S** | **E** | **F** | **R** | **A** | **Q** | **R** | **R** | **G** | **G** | **R** | **G** | **V** | **Q** | **G** | **M** | **G** | **V** | **H** | **D** | **D** | **D** | **F** | **V** | **K** | **N** | **L** | **V** | **S** | **C** | **S** | **T** | **H** | **D** | **T** | **L** | **L** | **F** | **F** | **T** | **N** | **T** | **G** | **K** | **V** | **Y** | **R** | **A** | **K** | **G** | **Y** | **E** | **I** | **P** | **E** | **Y** | **G** | **R** | **T** | **A** | **K** | **G** | **I** | **P** | **V** | **I** | **N** | **L** | **L** | **G** | **I** | **D** | **S** | **A** | **E** | **K** | **I** | **Q** | **A** | **I** | **I** | **S** | **V** | **E** | **G** | **K** | **A** | **E** | **A** | **G** | **K** | **Y** | **L** | **F** | **F** | **T** | **T** | **L** | **K** | **G** | **T** | **V** | **K** | **R** | **T** | **A** | **V** | **T** | **A** | **F** | **S** | **N** | **I** | **R** | **S** | **N** | **G** | **L** | **I** | **A** | **I** | **S** | **L** | **K** | **E** | **D** | **D** | **E** | **L** | **V** | **N** | **V** | **V** | **T** | **T** | **N** | **G** | **N** | **Q** | **K** | **M** | **I** | **I** | **G** | **T** | **H** | **A** | **G** | **Y** | **S** | **V** | **T** | **F** | **D** | **E** | **N** | **T** | **V** | **R** | **D** | **M** | **G** | **R** | **T** | **A** | **S** | **G** | **V** | **R** | **G** | **I** | **R** | **L** | **R** | **E** | **N** | **D** | **Y** | **V** | **V** | **G** | **A** | **A** | **I** | **L** | **D** | **E** | **N** | **K** | **E** | **V** | **L** | **V** | **I** | **T** | **E** | **N** | **G** | **Y** | **G** | **K** | **R** | **T** | **K** | **A** | **S** | **E** | **Y** | **P** | **V** | **K** | **G** | **R** | **G** | **G** | **K** | **G** | **I** | **K** | **T** | **A** | **N** | **I** | **T** | **E** | **K** | **N** | **G** | **P** | **L** | **A** | **G** | **L** | **T** | **T** | **V** | **N** | **G** | **D** | **E** | **D** | **I** | **L** | **L** | **I** | **T** | **N** | **K** | **G** | **V** | **I** | **I** | **R** | **F** | **N** | **V** | **D** | **S** | **V** | **S** | **Q** | **T** | **G** | **R** | **A** | **T** | **L** | **G** | **V** | **R** | **L** | **M** | **R** | **M** | **E** | **D** | **G** | **A** | **K** | **-** | **-** | **-** | **-** | **-** | **-** | **-** | **-** | **-** | **-** | **-** | **-** | **-** | **-** | **-** | **-** | **-** | **-** | **-** | **-** | **-** | **-** | **-** |
| **N59606F** | **M** | **S** | **E** | **E** | **I** | **K** | **E** | **N** | **I** | **Q** | **D** | **V** | **N** | **L** | **T** | **S** | **E** | **M** | **K** | **E** | **S** | **F** | **I** | **D** | **Y** | **A** | **M** | **S** | **V** | **I** | **V** | **A** | **R** | **A** | **L** | **P** | **D** | **V** | **R** | **D** | **G** | **L** | **K** | **P** | **V** | **H** | **R** | **R** | **I** | **L** | **Y** | **G** | **M** | **N** | **E** | **L** | **G** | **V** | **T** | **P** | **D** | **K** | **P** | **H** | **K** | **K** | **S** | **A** | **R** | **I** | **V** | **G** | **D** | **V** | **M** | **G** | **K** | **Y** | **H** | **P** | **H** | **G** | **D** | **S** | **A** | **I** | **Y** | **E** | **S** | **M** | **V** | **R** | **M** | **A** | **Q** | **P** | **F** | **S** | **Y** | **R** | **A** | **M** | **L** | **V** | **D** | **G** | **H** | **G** | **N** | **F** | **G** | **S** | **V** | **D** | **G** | **D** | **G** | **A** | **A** | **A** | **M** | **R** | **Y** | **T** | **E** | **A** | **R** | **M** | **S** | **K** | **I** | **A** | **L** | **E** | **M** | **L** | **R** | **D** | **I** | **N** | **K** | **N** | **T** | **V** | **D** | **F** | **Q** | **G** | **N** | **Y** | **D** | **D** | **S** | **E** | **Q** | **E** | **P** | **V** | **V** | **L** | **P** | **A** | **R** | **F** | **P** | **N** | **L** | **L** | **V** | **N** | **G** | **T** | **T** | **G** | **I** | **A** | **V** | **G** | **M** | **A** | **T** | **N** | **I** | **P** | **P** | **H** | **N** | **L** | **S** | **E** | **V** | **I** | **D** | **A** | **T** | **S** | **L** | **L** | **M** | **D** | **N** | **P** | **D** | **V** | **T** | **T** | **N** | **E** | **L** | **M** | **E** | **V** | **L** | **P** | **G** | **P** | **D** | **F** | **P** | **T** | **G** | **G** | **L** | **V** | **M** | **G** | **K** | **S** | **G** | **I** | **R** | **R** | **A** | **Y** | **E** | **T** | **G** | **K** | **G** | **S** | **I** | **T** | **V** | **R** | **A** | **K** | **V** | **E** | **L** | **T** | **E** | **M** | **P** | **N** | **G** | **K** | **E** | **R** | **I** | **L** | **V** | **T** | **E** | **L** | **P** | **Y** | **M** | **V** | **N** | **K** | **A** | **K** | **L** | **I** | **E** | **R** | **I** | **S** | **E** | **L** | **H** | **R** | **D** | **K** | **R** | **I** | **E** | **G** | **I** | **T** | **D** | **L** | **R** | **D** | **E** | **S** | **S** | **R** | **E** | **G** | **M** | **R** | **I** | **V** | **I** | **D** | **V** | **R** | **R** | **D** | **V** | **S** | **A** | **S** | **V** | **V** | **L** | **N** | **N** | **L** | **Y** | **K** | **M** | **T** | **A** | **L** | **Q** | **T** | **S** | **F** | **G** | **F** | **N** | **M** | **L** | **A** | **I** | **E** | **K** | **G** | **V** | **P** | **K** | **I** | **L** | **S** | **L** | **K** | **R** | **I** | **L** | **E** | **N** | **Y** | **V** | **E** | **H** | **Q** | **K** | **E** | **V** | **I** | **T** | **R** | **R** | **T** | **I** | **F** | **D** | **K** | **N** | **K** | **A** | **E** | **A** | **R** | **A** | **H** | **I** | **L** | **E** | **G** | **L** | **R** | **I** | **A** | **L** | **D** | **H** | **I** | **D** | **E** | **I** | **I** | **A** | **I** | **I** | **R** | **G** | **S** | **Q** | **S** | **D** | **D** | **E** | **A** | **K** | **A** | **T** | **L** | **I** | **E** | **R** | **F** | **E** | **F** | **S** | **D** | **R** | **Q** | **A** | **Q** | **A** | **I** | **L** | **D** | **M** | **R** | **L** | **R** | **R** | **L** | **T** | **G** | **L** | **E** | **R** | **D** | **K** | **I** | **E** | **N** | **E** | **Y** | **Q** | **E** | **L** | **L** | **K** | **F** | **I** | **A** | **D** | **L** | **E** | **D** | **I** | **L** | **A** | **R** | **P** | **E** | **R** | **V** | **I** | **E** | **I** | **I** | **K** | **T** | **E** | **L** | **N** | **D** | **V** | **R** | **T** | **K** | **F** | **G** | **D** | **A** | **R** | **R** | **T** | **E** | **L** | **L** | **V** | **G** | **E** | **V** | **L** | **S** | **L** | **E** | **D** | **E** | **D** | **L** | **I** | **E** | **E** | **E** | **E** | **V** | **V** | **I** | **T** | **L** | **T** | **N** | **N** | **G** | **Y** | **I** | **K** | **R** | **M** | **A** | **N** | **S** | **E** | **F** | **R** | **A** | **Q** | **R** | **R** | **G** | **G** | **R** | **G** | **V** | **Q** | **G** | **M** | **G** | **V** | **H** | **D** | **D** | **D** | **F** | **V** | **K** | **N** | **L** | **V** | **S** | **C** | **S** | **T** | **H** | **D** | **T** | **L** | **L** | **F** | **F** | **T** | **N** | **T** | **G** | **K** | **V** | **Y** | **R** | **A** | **K** | **G** | **Y** | **E** | **I** | **P** | **E** | **Y** | **G** | **R** | **T** | **A** | **K** | **G** | **I** | **P** | **V** | **I** | **N** | **L** | **L** | **G** | **I** | **D** | **S** | **A** | **E** | **K** | **I** | **Q** | **A** | **I** | **I** | **S** | **V** | **E** | **G** | **K** | **A** | **E** | **A** | **G** | **K** | **Y** | **L** | **F** | **F** | **T** | **T** | **L** | **K** | **G** | **T** | **V** | **K** | **R** | **T** | **A** | **V** | **T** | **A** | **F** | **S** | **N** | **I** | **R** | **S** | **N** | **G** | **L** | **I** | **A** | **I** | **S** | **L** | **K** | **E** | **D** | **D** | **E** | **L** | **V** | **N** | **V** | **V** | **T** | **T** | **N** | **G** | **N** | **Q** | **K** | **M** | **I** | **I** | **G** | **T** | **H** | **A** | **G** | **Y** | **S** | **V** | **T** | **F** | **D** | **E** | **N** | **T** | **V** | **R** | **D** | **M** | **G** | **R** | **T** | **A** | **S** | **G** | **V** | **R** | **G** | **I** | **R** | **L** | **R** | **E** | **N** | **D** | **Y** | **V** | **V** | **G** | **A** | **A** | **I** | **L** | **D** | **E** | **N** | **K** | **E** | **V** | **L** | **V** | **I** | **T** | **E** | **N** | **G** | **Y** | **G** | **K** | **R** | **T** | **K** | **A** | **S** | **E** | **Y** | **P** | **V** | **K** | **G** | **R** | **G** | **G** | **K** | **G** | **I** | **K** | **T** | **A** | **N** | **I** | **T** | **E** | **K** | **N** | **G** | **P** | **L** | **A** | **G** | **L** | **T** | **T** | **V** | **N** | **G** | **D** | **E** | **D** | **I** | **L** | **L** | **I** | **T** | **N** | **K** | **G** | **V** | **I** | **I** | **R** | **F** | **N** | **V** | **D** | **S** | **V** | **S** | **Q** | **T** | **G** | **R** | **A** | **T** | **L** | **G** | **V** | **R** | **L** | **M** | **R** | **M** | **E** | **D** | **G** | **A** | **K** | **-** | **-** | **-** | **-** | **-** | **-** | **-** | **-** | **-** | **-** | **-** | **-** | **-** | **-** | **-** | **-** | **-** | **-** | **-** | **-** | **-** | **-** | **-** |
| **N55268** | **M** | **S** | **E** | **E** | **I** | **K** | **E** | **N** | **I** | **Q** | **D** | **V** | **N** | **L** | **T** | **S** | **E** | **M** | **K** | **E** | **S** | **F** | **I** | **D** | **Y** | **A** | **M** | **S** | **V** | **I** | **V** | **A** | **R** | **A** | **L** | **P** | **D** | **V** | **R** | **D** | **G** | **L** | **K** | **P** | **V** | **H** | **R** | **R** | **I** | **L** | **Y** | **G** | **M** | **N** | **E** | **L** | **G** | **V** | **T** | **P** | **D** | **K** | **P** | **H** | **K** | **K** | **S** | **A** | **R** | **I** | **V** | **G** | **D** | **V** | **M** | **G** | **K** | **Y** | **H** | **P** | **H** | **G** | **D** | **S** | **A** | **I** | **Y** | **E** | **S** | **M** | **V** | **R** | **M** | **A** | **Q** | **P** | **F** | **S** | **Y** | **R** | **A** | **M** | **L** | **V** | **D** | **G** | **H** | **G** | **N** | **F** | **G** | **S** | **V** | **D** | **G** | **D** | **G** | **A** | **A** | **A** | **M** | **R** | **Y** | **T** | **E** | **A** | **R** | **M** | **S** | **K** | **I** | **A** | **L** | **E** | **M** | **L** | **R** | **D** | **I** | **N** | **K** | **N** | **T** | **V** | **D** | **F** | **Q** | **G** | **N** | **Y** | **D** | **D** | **S** | **E** | **Q** | **E** | **P** | **V** | **V** | **L** | **P** | **A** | **R** | **F** | **P** | **N** | **L** | **L** | **V** | **N** | **G** | **T** | **T** | **G** | **I** | **A** | **V** | **G** | **M** | **A** | **T** | **N** | **I** | **P** | **P** | **H** | **N** | **L** | **S** | **E** | **V** | **I** | **D** | **A** | **T** | **S** | **L** | **L** | **M** | **D** | **N** | **P** | **D** | **V** | **T** | **T** | **N** | **E** | **L** | **M** | **E** | **V** | **L** | **P** | **G** | **P** | **D** | **F** | **P** | **T** | **G** | **G** | **L** | **V** | **M** | **G** | **K** | **S** | **G** | **I** | **R** | **R** | **A** | **Y** | **E** | **T** | **G** | **K** | **G** | **S** | **I** | **T** | **V** | **R** | **A** | **K** | **V** | **E** | **L** | **T** | **E** | **M** | **P** | **N** | **G** | **K** | **E** | **R** | **I** | **L** | **V** | **T** | **E** | **L** | **P** | **Y** | **M** | **V** | **N** | **K** | **A** | **K** | **L** | **I** | **E** | **R** | **I** | **S** | **E** | **L** | **H** | **R** | **D** | **K** | **R** | **I** | **E** | **G** | **I** | **T** | **D** | **L** | **R** | **D** | **E** | **S** | **S** | **R** | **E** | **G** | **M** | **R** | **I** | **V** | **I** | **D** | **V** | **R** | **R** | **D** | **V** | **S** | **A** | **S** | **V** | **V** | **L** | **N** | **N** | **L** | **Y** | **K** | **M** | **T** | **A** | **L** | **Q** | **T** | **S** | **F** | **G** | **F** | **N** | **M** | **L** | **A** | **I** | **E** | **K** | **G** | **V** | **P** | **K** | **I** | **L** | **S** | **L** | **K** | **R** | **I** | **L** | **E** | **N** | **Y** | **V** | **E** | **H** | **Q** | **K** | **E** | **V** | **I** | **T** | **R** | **R** | **T** | **I** | **F** | **D** | **K** | **N** | **K** | **A** | **E** | **A** | **R** | **A** | **H** | **I** | **L** | **E** | **G** | **L** | **R** | **I** | **A** | **L** | **D** | **H** | **I** | **D** | **E** | **I** | **I** | **A** | **I** | **I** | **R** | **G** | **S** | **Q** | **S** | **D** | **D** | **E** | **A** | **K** | **A** | **T** | **L** | **I** | **E** | **R** | **F** | **E** | **F** | **S** | **D** | **R** | **Q** | **A** | **Q** | **A** | **I** | **L** | **D** | **M** | **R** | **L** | **R** | **R** | **L** | **T** | **G** | **L** | **E** | **R** | **D** | **K** | **I** | **E** | **N** | **E** | **Y** | **Q** | **E** | **L** | **L** | **K** | **F** | **I** | **A** | **D** | **L** | **E** | **D** | **I** | **L** | **A** | **R** | **P** | **E** | **R** | **V** | **I** | **E** | **I** | **I** | **K** | **T** | **E** | **L** | **N** | **D** | **V** | **R** | **T** | **K** | **F** | **G** | **D** | **A** | **R** | **R** | **T** | **E** | **L** | **L** | **V** | **G** | **E** | **V** | **L** | **S** | **L** | **E** | **D** | **E** | **D** | **L** | **I** | **E** | **E** | **E** | **E** | **V** | **V** | **I** | **T** | **L** | **T** | **N** | **N** | **G** | **Y** | **I** | **K** | **R** | **M** | **A** | **N** | **S** | **E** | **F** | **R** | **A** | **Q** | **R** | **R** | **G** | **G** | **R** | **G** | **V** | **Q** | **G** | **M** | **G** | **V** | **H** | **D** | **D** | **D** | **F** | **V** | **K** | **N** | **L** | **V** | **S** | **C** | **S** | **T** | **H** | **D** | **T** | **L** | **L** | **F** | **F** | **T** | **N** | **T** | **G** | **K** | **V** | **Y** | **R** | **A** | **K** | **G** | **Y** | **E** | **I** | **P** | **E** | **Y** | **G** | **R** | **T** | **A** | **K** | **G** | **I** | **P** | **V** | **I** | **N** | **L** | **L** | **G** | **I** | **D** | **S** | **A** | **E** | **K** | **I** | **Q** | **A** | **I** | **I** | **S** | **V** | **E** | **G** | **K** | **A** | **E** | **A** | **G** | **K** | **Y** | **L** | **F** | **F** | **T** | **T** | **L** | **K** | **G** | **T** | **V** | **K** | **R** | **T** | **A** | **V** | **T** | **A** | **F** | **S** | **N** | **I** | **R** | **S** | **N** | **G** | **L** | **I** | **A** | **I** | **S** | **L** | **K** | **E** | **D** | **D** | **E** | **L** | **V** | **N** | **V** | **V** | **T** | **T** | **N** | **G** | **N** | **Q** | **K** | **M** | **I** | **I** | **G** | **T** | **H** | **A** | **G** | **Y** | **S** | **V** | **T** | **F** | **D** | **E** | **N** | **T** | **V** | **R** | **D** | **M** | **G** | **R** | **T** | **A** | **S** | **G** | **V** | **R** | **G** | **I** | **R** | **L** | **R** | **E** | **N** | **D** | **Y** | **V** | **V** | **G** | **A** | **A** | **I** | **L** | **D** | **E** | **N** | **K** | **E** | **V** | **L** | **V** | **I** | **T** | **E** | **N** | **G** | **Y** | **G** | **K** | **R** | **T** | **K** | **A** | **S** | **E** | **Y** | **P** | **V** | **K** | **G** | **R** | **G** | **G** | **K** | **G** | **I** | **K** | **T** | **A** | **N** | **I** | **T** | **E** | **K** | **N** | **G** | **P** | **L** | **A** | **G** | **L** | **T** | **T** | **V** | **N** | **G** | **D** | **E** | **D** | **I** | **L** | **L** | **I** | **T** | **N** | **K** | **G** | **V** | **I** | **I** | **R** | **F** | **N** | **V** | **D** | **S** | **V** | **S** | **Q** | **T** | **G** | **R** | **A** | **T** | **L** | **G** | **V** | **R** | **L** | **M** | **R** | **M** | **E** | **D** | **G** | **A** | **K** | **-** | **-** | **-** | **-** | **-** | **-** | **-** | **-** | **-** | **-** | **-** | **-** | **-** | **-** | **-** | **-** | **-** | **-** | **-** | **-** | **-** | **-** | **-** |
| **N52732** | **M** | **S** | **E** | **E** | **I** | **R** | **E** | **N** | **I** | **H** | **D** | **V** | **N** | **L** | **T** | **S** | **E** | **M** | **K** | **D** | **S** | **F** | **I** | **D** | **Y** | **A** | **M** | **S** | **V** | **I** | **V** | **A** | **R** | **A** | **L** | **P** | **D** | **V** | **R** | **D** | **G** | **L** | **K** | **P** | **V** | **H** | **R** | **R** | **I** | **L** | **Y** | **G** | **M** | **N** | **E** | **L** | **G** | **V** | **T** | **P** | **D** | **K** | **A** | **H** | **K** | **K** | **S** | **A** | **R** | **I** | **V** | **G** | **D** | **V** | **M** | **G** | **K** | **Y** | **H** | **P** | **H** | **G** | **D** | **S** | **A** | **I** | **Y** | **E** | **S** | **M** | **V** | **R** | **M** | **A** | **Q** | **P** | **F** | **S** | **Y** | **R** | **Y** | **M** | **L** | **V** | **D** | **G** | **H** | **G** | **N** | **F** | **G** | **S** | **V** | **D** | **G** | **D** | **G** | **A** | **A** | **A** | **M** | **R** | **Y** | **T** | **E** | **A** | **R** | **M** | **S** | **K** | **I** | **A** | **T** | **E** | **M** | **L** | **R** | **D** | **I** | **N** | **K** | **N** | **T** | **V** | **D** | **F** | **Q** | **S** | **N** | **Y** | **D** | **D** | **T** | **E** | **K** | **E** | **P** | **V** | **V** | **L** | **P** | **A** | **R** | **F** | **P** | **N** | **L** | **L** | **V** | **N** | **G** | **T** | **T** | **G** | **I** | **A** | **V** | **G** | **M** | **A** | **T** | **N** | **I** | **P** | **P** | **H** | **N** | **L** | **S** | **E** | **I** | **V** | **A** | **A** | **I** | **D** | **L** | **L** | **M** | **D** | **N** | **P** | **D** | **V** | **T** | **T** | **N** | **E** | **L** | **M** | **E** | **V** | **L** | **P** | **G** | **P** | **D** | **F** | **P** | **T** | **G** | **G** | **L** | **V** | **M** | **G** | **K** | **S** | **G** | **I** | **R** | **R** | **A** | **Y** | **E** | **T** | **G** | **K** | **G** | **S** | **I** | **T** | **V** | **R** | **A** | **K** | **V** | **E** | **I** | **T** | **E** | **M** | **P** | **N** | **G** | **K** | **E** | **R** | **I** | **L** | **V** | **T** | **E** | **L** | **P** | **Y** | **M** | **V** | **N** | **K** | **A** | **K** | **L** | **I** | **E** | **R** | **I** | **S** | **E** | **L** | **H** | **R** | **D** | **K** | **R** | **I** | **E** | **G** | **I** | **T** | **D** | **L** | **R** | **D** | **E** | **S** | **S** | **R** | **E** | **G** | **M** | **R** | **I** | **V** | **I** | **D** | **V** | **R** | **R** | **D** | **A** | **S** | **A** | **S** | **V** | **I** | **L** | **N** | **N** | **L** | **Y** | **K** | **L** | **T** | **S** | **L** | **Q** | **N** | **S** | **F** | **G** | **F** | **N** | **M** | **L** | **A** | **I** | **E** | **K** | **G** | **V** | **P** | **K** | **V** | **L** | **S** | **L** | **K** | **Q** | **I** | **L** | **E** | **N** | **Y** | **I** | **E** | **H** | **Q** | **R** | **E** | **V** | **I** | **T** | **R** | **R** | **T** | **V** | **F** | **E** | **K** | **E** | **K** | **A** | **E** | **A** | **R** | **A** | **H** | **I** | **L** | **E** | **G** | **L** | **R** | **I** | **A** | **L** | **D** | **H** | **I** | **N** | **E** | **I** | **I** | **A** | **I** | **I** | **R** | **G** | **S** | **K** | **S** | **D** | **D** | **E** | **A** | **K** | **A** | **T** | **M** | **I** | **E** | **R** | **F** | **D** | **L** | **S** | **D** | **R** | **Q** | **S** | **Q** | **A** | **I** | **L** | **D** | **M** | **R** | **L** | **R** | **R** | **L** | **T** | **G** | **L** | **E** | **R** | **E** | **K** | **I** | **E** | **N** | **E** | **Y** | **Q** | **E** | **L** | **L** | **K** | **L** | **I** | **E** | **D** | **L** | **T** | **D** | **I** | **L** | **A** | **R** | **P** | **E** | **R** | **V** | **T** | **E** | **I** | **I** | **K** | **N** | **E** | **L** | **A** | **D** | **L** | **N** | **Q** | **R** | **F** | **G** | **D** | **K** | **R** | **R** | **T** | **E** | **L** | **L** | **V** | **G** | **E** | **V** | **L** | **S** | **L** | **E** | **D** | **E** | **D** | **L** | **I** | **E** | **E** | **E** | **E** | **I** | **V** | **I** | **T** | **L** | **T** | **N** | **N** | **G** | **Y** | **I** | **K** | **R** | **L** | **A** | **N** | **N** | **E** | **F** | **R** | **A** | **Q** | **R** | **R** | **G** | **G** | **R** | **G** | **V** | **Q** | **G** | **M** | **G** | **I** | **H** | **N** | **D** | **D** | **F** | **V** | **K** | **N** | **L** | **V** | **S** | **C** | **S** | **T** | **H** | **D** | **T** | **L** | **L** | **F** | **F** | **T** | **N** | **N** | **G** | **K** | **V** | **Y** | **R** | **A** | **K** | **G** | **Y** | **E** | **I** | **P** | **E** | **Y** | **G** | **R** | **T** | **A** | **K** | **G** | **I** | **P** | **I** | **I** | **N** | **L** | **L** | **G** | **I** | **D** | **S** | **S** | **E** | **S** | **I** | **Q** | **A** | **I** | **I** | **A** | **V** | **T** | **G** | **E** | **A** | **E** | **E** | **G** | **H** | **Y** | **L** | **F** | **F** | **T** | **T** | **R** | **Q** | **G** | **T** | **V** | **K** | **R** | **T** | **S** | **V** | **T** | **A** | **F** | **A** | **N** | **I** | **R** | **S** | **N** | **G** | **L** | **I** | **A** | **I** | **G** | **L** | **K** | **E** | **D** | **D** | **E** | **L** | **V** | **N** | **V** | **L** | **L** | **T** | **D** | **G** | **Q** | **S** | **N** | **I** | **I** | **I** | **G** | **T** | **H** | **N** | **G** | **Y** | **S** | **V** | **T** | **F** | **K** | **E** | **E** | **A** | **V** | **R** | **D** | **M** | **G** | **R** | **T** | **A** | **S** | **G** | **V** | **R** | **G** | **I** | **R** | **L** | **R** | **E** | **E** | **D** | **Y** | **V** | **V** | **G** | **A** | **A** | **L** | **M** | **K** | **E** | **D** | **Q** | **E** | **V** | **L** | **V** | **I** | **T** | **E** | **K** | **G** | **Y** | **G** | **K** | **R** | **T** | **K** | **V** | **S** | **E** | **Y** | **P** | **V** | **K** | **G** | **R** | **G** | **G** | **K** | **G** | **I** | **K** | **T** | **A** | **N** | **I** | **T** | **E** | **K** | **N** | **G** | **P** | **L** | **A** | **G** | **L** | **T** | **T** | **V** | **S** | **G** | **N** | **E** | **D** | **I** | **M** | **L** | **I** | **T** | **D** | **K** | **G** | **V** | **I** | **I** | **R** | **F** | **N** | **V** | **S** | **T** | **V** | **S** | **Q** | **T** | **G** | **R** | **S** | **T** | **L** | **G** | **V** | **R** | **L** | **M** | **K** | **M** | **E** | **A** | **D** | **T** | **K** | **V** | **V** | **T** | **M** | **-** | **-** | **-** | **-** | **-** | **-** | **-** | **-** | **-** | **-** | **-** | **-** | **-** | **-** | **-** | **-** | **-** | **-** | **-** |
| **N59998F** | **M** | **S** | **E** | **E** | **I** | **R** | **E** | **N** | **I** | **H** | **D** | **V** | **N** | **L** | **T** | **S** | **E** | **M** | **K** | **D** | **S** | **F** | **I** | **D** | **Y** | **A** | **M** | **S** | **V** | **I** | **V** | **A** | **R** | **A** | **L** | **P** | **D** | **V** | **R** | **D** | **G** | **L** | **K** | **P** | **V** | **H** | **R** | **R** | **I** | **L** | **Y** | **G** | **M** | **N** | **E** | **L** | **G** | **V** | **T** | **P** | **D** | **K** | **A** | **H** | **K** | **K** | **S** | **A** | **R** | **I** | **V** | **G** | **D** | **V** | **M** | **G** | **K** | **Y** | **H** | **P** | **H** | **G** | **D** | **S** | **A** | **I** | **Y** | **E** | **S** | **M** | **V** | **R** | **M** | **A** | **Q** | **P** | **F** | **S** | **Y** | **R** | **Y** | **M** | **L** | **V** | **D** | **G** | **H** | **G** | **N** | **F** | **G** | **S** | **V** | **D** | **G** | **D** | **G** | **A** | **A** | **A** | **M** | **R** | **Y** | **T** | **E** | **A** | **R** | **M** | **S** | **K** | **I** | **A** | **T** | **E** | **M** | **L** | **R** | **D** | **I** | **N** | **K** | **N** | **T** | **V** | **D** | **F** | **Q** | **S** | **N** | **Y** | **D** | **D** | **T** | **E** | **K** | **E** | **P** | **V** | **V** | **L** | **P** | **A** | **R** | **F** | **P** | **N** | **L** | **L** | **V** | **N** | **G** | **T** | **T** | **G** | **I** | **A** | **V** | **G** | **M** | **A** | **T** | **N** | **I** | **P** | **P** | **H** | **N** | **L** | **S** | **E** | **V** | **V** | **A** | **A** | **I** | **D** | **L** | **L** | **M** | **D** | **N** | **P** | **D** | **V** | **T** | **T** | **N** | **E** | **L** | **M** | **E** | **V** | **L** | **P** | **G** | **P** | **D** | **F** | **P** | **T** | **G** | **G** | **L** | **V** | **M** | **G** | **K** | **S** | **G** | **I** | **R** | **R** | **A** | **Y** | **E** | **T** | **G** | **K** | **G** | **S** | **I** | **T** | **V** | **R** | **A** | **K** | **V** | **E** | **I** | **T** | **E** | **M** | **P** | **N** | **G** | **K** | **E** | **R** | **I** | **I** | **V** | **T** | **E** | **L** | **P** | **Y** | **M** | **V** | **N** | **K** | **A** | **K** | **L** | **I** | **E** | **R** | **I** | **S** | **E** | **L** | **H** | **R** | **D** | **K** | **R** | **I** | **E** | **G** | **I** | **T** | **D** | **L** | **R** | **D** | **E** | **S** | **S** | **R** | **E** | **G** | **M** | **R** | **I** | **V** | **I** | **D** | **I** | **R** | **R** | **D** | **A** | **S** | **A** | **S** | **V** | **I** | **L** | **N** | **N** | **L** | **Y** | **K** | **L** | **T** | **S** | **L** | **Q** | **N** | **S** | **F** | **G** | **F** | **N** | **M** | **L** | **A** | **I** | **E** | **K** | **G** | **V** | **P** | **K** | **V** | **L** | **S** | **L** | **K** | **Q** | **I** | **L** | **E** | **N** | **Y** | **I** | **E** | **H** | **Q** | **R** | **E** | **V** | **I** | **T** | **R** | **R** | **T** | **V** | **F** | **E** | **K** | **E** | **K** | **A** | **E** | **A** | **R** | **A** | **H** | **I** | **L** | **E** | **G** | **L** | **R** | **I** | **A** | **L** | **D** | **H** | **I** | **D** | **E** | **I** | **I** | **A** | **I** | **I** | **R** | **G** | **S** | **K** | **S** | **D** | **D** | **E** | **A** | **K** | **A** | **T** | **M** | **I** | **E** | **R** | **F** | **D** | **L** | **S** | **D** | **R** | **Q** | **S** | **Q** | **A** | **I** | **L** | **D** | **M** | **R** | **L** | **R** | **R** | **L** | **T** | **G** | **L** | **E** | **R** | **E** | **K** | **I** | **E** | **N** | **E** | **Y** | **Q** | **E** | **L** | **L** | **K** | **L** | **I** | **E** | **D** | **L** | **T** | **D** | **I** | **L** | **A** | **R** | **P** | **E** | **R** | **V** | **T** | **E** | **I** | **I** | **K** | **N** | **E** | **L** | **A** | **D** | **L** | **N** | **Q** | **R** | **F** | **G** | **D** | **K** | **R** | **R** | **T** | **E** | **L** | **L** | **V** | **G** | **E** | **V** | **L** | **S** | **L** | **E** | **D** | **E** | **D** | **L** | **I** | **E** | **E** | **E** | **E** | **I** | **V** | **I** | **T** | **L** | **T** | **N** | **N** | **G** | **Y** | **I** | **K** | **R** | **L** | **A** | **N** | **N** | **E** | **F** | **R** | **A** | **Q** | **R** | **R** | **G** | **G** | **R** | **G** | **V** | **Q** | **G** | **M** | **G** | **I** | **H** | **N** | **D** | **D** | **F** | **V** | **K** | **N** | **L** | **V** | **S** | **C** | **S** | **T** | **H** | **D** | **T** | **L** | **L** | **F** | **F** | **T** | **N** | **N** | **G** | **K** | **V** | **Y** | **R** | **A** | **K** | **G** | **Y** | **E** | **I** | **P** | **E** | **Y** | **G** | **R** | **T** | **A** | **K** | **G** | **I** | **P** | **I** | **I** | **N** | **L** | **L** | **G** | **I** | **D** | **S** | **S** | **E** | **S** | **I** | **Q** | **A** | **I** | **I** | **A** | **V** | **T** | **G** | **E** | **A** | **E** | **E** | **G** | **H** | **Y** | **L** | **F** | **F** | **T** | **T** | **R** | **Q** | **G** | **T** | **V** | **K** | **R** | **T** | **S** | **V** | **T** | **A** | **F** | **A** | **N** | **I** | **R** | **S** | **N** | **G** | **L** | **I** | **A** | **I** | **G** | **L** | **K** | **E** | **D** | **D** | **E** | **L** | **V** | **N** | **V** | **L** | **L** | **T** | **D** | **G** | **Q** | **S** | **N** | **I** | **I** | **I** | **G** | **T** | **H** | **N** | **G** | **Y** | **S** | **V** | **T** | **F** | **K** | **E** | **E** | **A** | **V** | **R** | **D** | **M** | **G** | **R** | **T** | **A** | **S** | **G** | **V** | **R** | **G** | **I** | **R** | **L** | **R** | **E** | **E** | **D** | **Y** | **V** | **V** | **G** | **A** | **A** | **L** | **M** | **K** | **E** | **D** | **Q** | **E** | **V** | **L** | **V** | **I** | **T** | **E** | **K** | **G** | **Y** | **G** | **K** | **R** | **T** | **K** | **V** | **S** | **E** | **Y** | **P** | **V** | **K** | **G** | **R** | **G** | **G** | **K** | **G** | **I** | **K** | **T** | **A** | **N** | **I** | **T** | **E** | **K** | **N** | **G** | **P** | **L** | **A** | **G** | **L** | **T** | **T** | **V** | **S** | **G** | **D** | **E** | **D** | **I** | **M** | **L** | **I** | **T** | **D** | **K** | **G** | **V** | **I** | **I** | **R** | **F** | **N** | **V** | **S** | **T** | **V** | **S** | **Q** | **T** | **G** | **R** | **S** | **T** | **L** | **G** | **V** | **R** | **L** | **M** | **K** | **M** | **E** | **A** | **D** | **T** | **K** | **V** | **V** | **T** | **M** | **A** | **A** | **V** | **E** | **P** | **E** | **A** | **A** | **E** | **Q** | **T** | **V** | **E** | **E** | **Q** | **G** | **S** | **E** | **E** |
| **N59462F** | **M** | **S** | **E** | **E** | **I** | **K** | **E** | **N** | **I** | **Q** | **D** | **V** | **N** | **L** | **T** | **S** | **E** | **M** | **K** | **E** | **S** | **F** | **I** | **D** | **Y** | **A** | **M** | **S** | **V** | **I** | **V** | **A** | **R** | **A** | **L** | **P** | **D** | **V** | **R** | **D** | **G** | **L** | **K** | **P** | **V** | **H** | **R** | **R** | **I** | **L** | **Y** | **G** | **M** | **N** | **E** | **L** | **G** | **V** | **T** | **P** | **D** | **K** | **P** | **H** | **K** | **K** | **S** | **A** | **R** | **I** | **V** | **G** | **D** | **V** | **M** | **G** | **K** | **Y** | **H** | **P** | **H** | **G** | **D** | **S** | **A** | **I** | **Y** | **E** | **S** | **M** | **V** | **R** | **M** | **A** | **Q** | **P** | **F** | **S** | **Y** | **R** | **A** | **M** | **L** | **V** | **D** | **G** | **H** | **G** | **N** | **F** | **G** | **S** | **V** | **D** | **G** | **D** | **G** | **A** | **A** | **A** | **M** | **R** | **Y** | **T** | **E** | **A** | **R** | **M** | **S** | **K** | **I** | **A** | **L** | **E** | **M** | **L** | **R** | **D** | **I** | **N** | **K** | **N** | **T** | **V** | **D** | **F** | **Q** | **G** | **N** | **Y** | **D** | **D** | **S** | **E** | **Q** | **E** | **P** | **V** | **V** | **L** | **P** | **A** | **R** | **F** | **P** | **N** | **L** | **L** | **V** | **N** | **G** | **T** | **T** | **G** | **I** | **A** | **V** | **G** | **M** | **A** | **T** | **N** | **I** | **P** | **P** | **H** | **N** | **L** | **S** | **E** | **V** | **I** | **D** | **A** | **T** | **S** | **L** | **L** | **M** | **D** | **N** | **P** | **D** | **V** | **T** | **T** | **N** | **E** | **L** | **M** | **E** | **V** | **L** | **P** | **G** | **P** | **D** | **F** | **P** | **T** | **G** | **G** | **L** | **V** | **M** | **G** | **K** | **S** | **G** | **I** | **R** | **R** | **A** | **Y** | **E** | **T** | **G** | **K** | **G** | **S** | **I** | **T** | **V** | **R** | **A** | **K** | **V** | **E** | **L** | **T** | **E** | **M** | **P** | **N** | **G** | **K** | **E** | **R** | **I** | **L** | **V** | **T** | **E** | **L** | **P** | **Y** | **M** | **V** | **N** | **K** | **A** | **K** | **L** | **I** | **E** | **R** | **I** | **S** | **E** | **L** | **H** | **R** | **D** | **K** | **R** | **I** | **E** | **G** | **I** | **T** | **D** | **L** | **R** | **D** | **E** | **S** | **S** | **R** | **E** | **G** | **M** | **R** | **I** | **V** | **I** | **D** | **V** | **R** | **R** | **D** | **V** | **S** | **A** | **S** | **V** | **V** | **L** | **N** | **N** | **L** | **Y** | **K** | **M** | **T** | **A** | **L** | **Q** | **T** | **S** | **F** | **G** | **F** | **N** | **M** | **L** | **A** | **I** | **E** | **K** | **G** | **V** | **P** | **K** | **I** | **L** | **S** | **L** | **K** | **R** | **I** | **L** | **E** | **N** | **Y** | **V** | **E** | **H** | **Q** | **K** | **E** | **V** | **I** | **T** | **R** | **R** | **T** | **I** | **F** | **D** | **K** | **N** | **K** | **A** | **E** | **A** | **R** | **A** | **H** | **I** | **L** | **E** | **G** | **L** | **R** | **I** | **A** | **L** | **D** | **H** | **I** | **D** | **E** | **I** | **I** | **A** | **I** | **I** | **R** | **G** | **S** | **Q** | **S** | **D** | **D** | **E** | **A** | **K** | **A** | **T** | **L** | **I** | **E** | **R** | **F** | **E** | **F** | **S** | **D** | **R** | **Q** | **A** | **Q** | **A** | **I** | **L** | **D** | **M** | **R** | **L** | **R** | **R** | **L** | **T** | **G** | **L** | **E** | **R** | **D** | **K** | **I** | **E** | **N** | **E** | **Y** | **Q** | **E** | **L** | **L** | **K** | **F** | **I** | **A** | **D** | **L** | **E** | **D** | **I** | **L** | **A** | **R** | **P** | **E** | **R** | **V** | **I** | **E** | **I** | **I** | **K** | **T** | **E** | **L** | **N** | **D** | **V** | **R** | **T** | **K** | **F** | **G** | **D** | **A** | **R** | **R** | **T** | **E** | **L** | **L** | **V** | **G** | **E** | **V** | **L** | **S** | **L** | **E** | **D** | **E** | **D** | **L** | **I** | **E** | **E** | **E** | **E** | **V** | **V** | **I** | **T** | **L** | **T** | **N** | **N** | **G** | **Y** | **I** | **K** | **R** | **M** | **A** | **N** | **S** | **E** | **F** | **R** | **A** | **Q** | **R** | **R** | **G** | **G** | **R** | **G** | **V** | **Q** | **G** | **M** | **G** | **V** | **H** | **D** | **D** | **D** | **F** | **V** | **K** | **N** | **L** | **V** | **S** | **C** | **S** | **T** | **H** | **D** | **T** | **L** | **L** | **F** | **F** | **T** | **N** | **T** | **G** | **K** | **V** | **Y** | **R** | **A** | **K** | **G** | **Y** | **E** | **I** | **P** | **E** | **Y** | **G** | **R** | **T** | **A** | **K** | **G** | **I** | **P** | **V** | **I** | **N** | **L** | **L** | **G** | **I** | **D** | **S** | **A** | **E** | **K** | **I** | **Q** | **A** | **I** | **I** | **S** | **V** | **E** | **G** | **K** | **A** | **E** | **A** | **G** | **K** | **Y** | **L** | **F** | **F** | **T** | **T** | **L** | **K** | **G** | **T** | **V** | **K** | **R** | **T** | **A** | **V** | **T** | **A** | **F** | **S** | **N** | **I** | **R** | **S** | **N** | **G** | **L** | **I** | **A** | **I** | **S** | **L** | **K** | **E** | **D** | **D** | **E** | **L** | **V** | **N** | **V** | **V** | **T** | **T** | **N** | **G** | **N** | **Q** | **K** | **M** | **I** | **I** | **G** | **T** | **H** | **A** | **G** | **Y** | **S** | **V** | **T** | **F** | **D** | **E** | **N** | **T** | **V** | **R** | **D** | **M** | **G** | **R** | **T** | **A** | **S** | **G** | **V** | **R** | **G** | **I** | **R** | **L** | **R** | **E** | **N** | **D** | **Y** | **V** | **V** | **G** | **A** | **A** | **I** | **L** | **D** | **E** | **N** | **K** | **E** | **V** | **L** | **V** | **I** | **T** | **E** | **N** | **G** | **Y** | **G** | **K** | **R** | **T** | **K** | **A** | **S** | **E** | **Y** | **P** | **V** | **K** | **G** | **R** | **G** | **G** | **K** | **G** | **I** | **K** | **T** | **A** | **N** | **I** | **T** | **E** | **K** | **N** | **G** | **P** | **L** | **A** | **G** | **L** | **T** | **T** | **V** | **N** | **G** | **D** | **E** | **D** | **I** | **L** | **L** | **I** | **T** | **N** | **K** | **G** | **V** | **I** | **I** | **R** | **F** | **N** | **V** | **D** | **S** | **V** | **S** | **Q** | **T** | **G** | **R** | **A** | **T** | **L** | **G** | **V** | **R** | **L** | **M** | **R** | **M** | **E** | **D** | **G** | **A** | **K** | **-** | **-** | **-** | **-** | **-** | **-** | **-** | **-** | **-** | **-** | **-** | **-** | **-** | **-** | **-** | **-** | **-** | **-** | **-** | **-** | **-** | **-** | **-** |
| **N55181** | **M** | **S** | **E** | **E** | **I** | **K** | **E** | **N** | **I** | **Q** | **D** | **V** | **N** | **L** | **T** | **S** | **E** | **M** | **K** | **E** | **S** | **F** | **I** | **D** | **Y** | **A** | **M** | **S** | **V** | **I** | **V** | **A** | **R** | **A** | **L** | **P** | **D** | **V** | **R** | **D** | **G** | **L** | **K** | **P** | **V** | **H** | **R** | **R** | **I** | **L** | **Y** | **G** | **M** | **N** | **E** | **L** | **G** | **V** | **T** | **P** | **D** | **K** | **P** | **H** | **K** | **K** | **S** | **A** | **R** | **I** | **V** | **G** | **D** | **V** | **M** | **G** | **K** | **Y** | **H** | **P** | **H** | **G** | **D** | **S** | **A** | **I** | **Y** | **E** | **S** | **M** | **V** | **R** | **M** | **A** | **Q** | **P** | **F** | **S** | **Y** | **R** | **A** | **M** | **L** | **V** | **D** | **G** | **H** | **G** | **N** | **F** | **G** | **S** | **V** | **D** | **G** | **D** | **G** | **A** | **A** | **A** | **M** | **R** | **Y** | **T** | **E** | **A** | **R** | **M** | **S** | **K** | **I** | **A** | **L** | **E** | **M** | **L** | **R** | **D** | **I** | **N** | **K** | **N** | **T** | **V** | **D** | **F** | **Q** | **G** | **N** | **Y** | **D** | **D** | **S** | **E** | **Q** | **E** | **P** | **V** | **V** | **L** | **P** | **A** | **R** | **F** | **P** | **N** | **L** | **L** | **V** | **N** | **G** | **T** | **T** | **G** | **I** | **A** | **V** | **G** | **M** | **A** | **T** | **N** | **I** | **P** | **P** | **H** | **N** | **L** | **S** | **E** | **V** | **I** | **D** | **A** | **T** | **S** | **L** | **L** | **M** | **D** | **N** | **P** | **D** | **V** | **T** | **T** | **N** | **E** | **L** | **M** | **E** | **V** | **L** | **P** | **G** | **P** | **D** | **F** | **P** | **T** | **G** | **G** | **L** | **V** | **M** | **G** | **K** | **S** | **G** | **I** | **R** | **R** | **A** | **Y** | **E** | **T** | **G** | **K** | **G** | **S** | **I** | **T** | **V** | **R** | **A** | **K** | **V** | **E** | **L** | **T** | **E** | **M** | **P** | **N** | **G** | **K** | **E** | **R** | **I** | **L** | **V** | **T** | **E** | **L** | **P** | **Y** | **M** | **V** | **N** | **K** | **A** | **K** | **L** | **I** | **E** | **R** | **I** | **S** | **E** | **L** | **H** | **R** | **D** | **K** | **R** | **I** | **E** | **G** | **I** | **T** | **D** | **L** | **R** | **D** | **E** | **S** | **S** | **R** | **E** | **G** | **M** | **R** | **I** | **V** | **I** | **D** | **V** | **R** | **R** | **D** | **V** | **S** | **A** | **S** | **V** | **V** | **L** | **N** | **N** | **L** | **Y** | **K** | **M** | **T** | **A** | **L** | **Q** | **T** | **S** | **F** | **G** | **F** | **N** | **M** | **L** | **A** | **I** | **E** | **K** | **G** | **V** | **P** | **K** | **I** | **L** | **S** | **L** | **K** | **R** | **I** | **L** | **E** | **N** | **Y** | **V** | **E** | **H** | **Q** | **K** | **E** | **V** | **I** | **T** | **R** | **R** | **T** | **I** | **F** | **D** | **K** | **N** | **K** | **A** | **E** | **A** | **R** | **A** | **H** | **I** | **L** | **E** | **G** | **L** | **R** | **I** | **A** | **L** | **D** | **H** | **I** | **D** | **E** | **I** | **I** | **A** | **I** | **I** | **R** | **G** | **S** | **Q** | **S** | **D** | **D** | **E** | **A** | **K** | **A** | **T** | **L** | **I** | **E** | **R** | **F** | **E** | **F** | **S** | **D** | **R** | **Q** | **A** | **Q** | **A** | **I** | **L** | **D** | **M** | **R** | **L** | **R** | **R** | **L** | **T** | **G** | **L** | **E** | **R** | **D** | **K** | **I** | **E** | **N** | **E** | **Y** | **Q** | **E** | **L** | **L** | **K** | **F** | **I** | **A** | **D** | **L** | **E** | **D** | **I** | **L** | **A** | **R** | **P** | **E** | **R** | **V** | **I** | **E** | **I** | **I** | **K** | **T** | **E** | **L** | **N** | **D** | **V** | **R** | **T** | **K** | **F** | **G** | **D** | **A** | **R** | **R** | **T** | **E** | **L** | **L** | **V** | **G** | **E** | **V** | **L** | **S** | **L** | **E** | **D** | **E** | **D** | **L** | **I** | **E** | **E** | **E** | **E** | **V** | **V** | **I** | **T** | **L** | **T** | **N** | **N** | **G** | **Y** | **I** | **K** | **R** | **M** | **A** | **N** | **S** | **E** | **F** | **R** | **A** | **Q** | **R** | **R** | **G** | **G** | **R** | **G** | **V** | **Q** | **G** | **M** | **G** | **V** | **H** | **D** | **D** | **D** | **F** | **V** | **K** | **N** | **L** | **V** | **S** | **C** | **S** | **T** | **H** | **D** | **T** | **L** | **L** | **F** | **F** | **T** | **N** | **T** | **G** | **K** | **V** | **Y** | **R** | **A** | **K** | **G** | **Y** | **E** | **I** | **P** | **E** | **Y** | **G** | **R** | **T** | **A** | **K** | **G** | **I** | **P** | **V** | **I** | **N** | **L** | **L** | **G** | **I** | **D** | **S** | **A** | **E** | **K** | **I** | **Q** | **A** | **I** | **I** | **S** | **V** | **E** | **G** | **K** | **A** | **E** | **A** | **G** | **K** | **Y** | **L** | **F** | **F** | **T** | **T** | **L** | **K** | **G** | **T** | **V** | **K** | **R** | **T** | **A** | **V** | **T** | **A** | **F** | **S** | **N** | **I** | **R** | **S** | **N** | **G** | **L** | **I** | **A** | **I** | **S** | **L** | **K** | **E** | **D** | **D** | **E** | **L** | **V** | **N** | **V** | **V** | **T** | **T** | **N** | **G** | **N** | **Q** | **K** | **M** | **I** | **I** | **G** | **T** | **H** | **A** | **G** | **Y** | **S** | **V** | **T** | **F** | **D** | **E** | **N** | **T** | **V** | **R** | **D** | **M** | **G** | **R** | **T** | **A** | **S** | **G** | **V** | **R** | **G** | **I** | **R** | **L** | **R** | **E** | **N** | **D** | **Y** | **V** | **V** | **G** | **A** | **A** | **I** | **L** | **D** | **E** | **N** | **K** | **E** | **V** | **L** | **V** | **I** | **T** | **E** | **N** | **G** | **Y** | **G** | **K** | **R** | **T** | **K** | **A** | **S** | **E** | **Y** | **P** | **V** | **K** | **G** | **R** | **G** | **G** | **K** | **G** | **I** | **K** | **T** | **A** | **N** | **I** | **T** | **E** | **K** | **N** | **G** | **P** | **L** | **A** | **G** | **L** | **T** | **T** | **V** | **N** | **G** | **D** | **E** | **D** | **I** | **L** | **L** | **I** | **T** | **N** | **K** | **G** | **V** | **I** | **I** | **R** | **F** | **N** | **V** | **D** | **S** | **V** | **S** | **Q** | **T** | **G** | **R** | **A** | **T** | **L** | **G** | **V** | **R** | **L** | **M** | **R** | **M** | **E** | **D** | **G** | **A** | **K** | **-** | **-** | **-** | **-** | **-** | **-** | **-** | **-** | **-** | **-** | **-** | **-** | **-** | **-** | **-** | **-** | **-** | **-** | **-** | **-** | **-** | **-** | **-** |
| **N59589F** | **M** | **S** | **E** | **E** | **I** | **R** | **E** | **N** | **I** | **H** | **D** | **V** | **N** | **L** | **T** | **S** | **E** | **M** | **K** | **D** | **S** | **F** | **I** | **D** | **Y** | **A** | **M** | **S** | **V** | **I** | **V** | **A** | **R** | **A** | **L** | **P** | **D** | **V** | **R** | **D** | **G** | **L** | **K** | **P** | **V** | **H** | **R** | **R** | **I** | **L** | **Y** | **G** | **M** | **N** | **E** | **L** | **G** | **V** | **T** | **P** | **D** | **K** | **A** | **H** | **K** | **K** | **S** | **A** | **R** | **I** | **V** | **G** | **D** | **V** | **M** | **G** | **K** | **Y** | **H** | **P** | **H** | **G** | **D** | **S** | **A** | **I** | **Y** | **E** | **S** | **M** | **V** | **R** | **M** | **A** | **Q** | **P** | **F** | **S** | **Y** | **R** | **Y** | **M** | **L** | **V** | **D** | **G** | **H** | **G** | **N** | **F** | **G** | **S** | **V** | **D** | **G** | **D** | **G** | **A** | **A** | **A** | **M** | **R** | **Y** | **T** | **E** | **A** | **R** | **M** | **S** | **K** | **I** | **A** | **T** | **E** | **M** | **L** | **R** | **D** | **I** | **N** | **K** | **N** | **T** | **V** | **D** | **F** | **Q** | **S** | **N** | **Y** | **D** | **D** | **T** | **E** | **K** | **E** | **P** | **V** | **V** | **L** | **P** | **A** | **R** | **F** | **P** | **N** | **L** | **L** | **V** | **N** | **G** | **T** | **T** | **G** | **I** | **A** | **V** | **G** | **M** | **A** | **T** | **N** | **I** | **P** | **P** | **H** | **N** | **L** | **S** | **E** | **V** | **V** | **A** | **A** | **I** | **D** | **L** | **L** | **M** | **D** | **N** | **P** | **D** | **V** | **T** | **T** | **N** | **E** | **L** | **M** | **E** | **V** | **L** | **P** | **G** | **P** | **D** | **F** | **P** | **T** | **G** | **G** | **L** | **V** | **M** | **G** | **K** | **S** | **G** | **I** | **R** | **R** | **A** | **Y** | **E** | **T** | **G** | **K** | **G** | **S** | **I** | **T** | **V** | **R** | **A** | **K** | **V** | **E** | **I** | **T** | **E** | **M** | **P** | **N** | **G** | **K** | **E** | **R** | **I** | **L** | **V** | **T** | **E** | **L** | **P** | **Y** | **M** | **V** | **N** | **K** | **A** | **K** | **L** | **I** | **E** | **R** | **I** | **S** | **E** | **L** | **H** | **R** | **D** | **K** | **R** | **I** | **E** | **G** | **I** | **T** | **D** | **L** | **R** | **D** | **E** | **S** | **S** | **R** | **E** | **G** | **M** | **R** | **I** | **V** | **I** | **D** | **I** | **R** | **R** | **D** | **A** | **S** | **A** | **S** | **V** | **I** | **L** | **N** | **N** | **L** | **Y** | **K** | **L** | **T** | **S** | **L** | **Q** | **N** | **S** | **F** | **G** | **F** | **N** | **M** | **L** | **A** | **I** | **E** | **K** | **G** | **V** | **P** | **K** | **V** | **L** | **S** | **L** | **K** | **Q** | **I** | **L** | **E** | **N** | **Y** | **I** | **E** | **H** | **Q** | **R** | **E** | **V** | **I** | **T** | **R** | **R** | **T** | **V** | **F** | **E** | **K** | **E** | **K** | **A** | **E** | **A** | **R** | **A** | **H** | **I** | **L** | **E** | **G** | **L** | **R** | **I** | **A** | **L** | **D** | **H** | **I** | **D** | **E** | **I** | **I** | **A** | **I** | **I** | **R** | **G** | **S** | **K** | **S** | **D** | **D** | **E** | **A** | **K** | **A** | **T** | **M** | **I** | **E** | **R** | **F** | **D** | **L** | **S** | **D** | **R** | **Q** | **S** | **Q** | **A** | **I** | **L** | **D** | **M** | **R** | **L** | **R** | **R** | **L** | **T** | **G** | **L** | **E** | **R** | **E** | **K** | **I** | **E** | **N** | **E** | **Y** | **Q** | **E** | **L** | **L** | **K** | **L** | **I** | **E** | **D** | **L** | **T** | **D** | **I** | **L** | **A** | **R** | **P** | **E** | **R** | **V** | **T** | **E** | **I** | **I** | **K** | **N** | **E** | **L** | **A** | **D** | **L** | **N** | **Q** | **R** | **F** | **G** | **D** | **K** | **R** | **R** | **T** | **E** | **L** | **L** | **V** | **G** | **E** | **V** | **L** | **S** | **L** | **E** | **D** | **E** | **D** | **L** | **I** | **E** | **E** | **E** | **E** | **I** | **V** | **I** | **T** | **L** | **T** | **N** | **N** | **G** | **Y** | **I** | **K** | **R** | **L** | **A** | **N** | **N** | **E** | **F** | **R** | **A** | **Q** | **R** | **R** | **G** | **G** | **R** | **G** | **V** | **Q** | **G** | **M** | **G** | **I** | **H** | **N** | **D** | **D** | **F** | **V** | **K** | **N** | **L** | **V** | **S** | **C** | **S** | **T** | **H** | **D** | **T** | **L** | **L** | **F** | **F** | **T** | **N** | **N** | **G** | **K** | **V** | **Y** | **R** | **A** | **K** | **G** | **Y** | **E** | **I** | **P** | **E** | **Y** | **G** | **R** | **T** | **A** | **K** | **G** | **I** | **P** | **I** | **I** | **N** | **L** | **L** | **G** | **I** | **D** | **S** | **S** | **E** | **S** | **I** | **Q** | **A** | **I** | **I** | **A** | **V** | **T** | **G** | **E** | **A** | **E** | **E** | **G** | **H** | **Y** | **L** | **F** | **F** | **T** | **T** | **R** | **Q** | **G** | **T** | **V** | **K** | **R** | **T** | **S** | **V** | **T** | **A** | **F** | **A** | **N** | **I** | **R** | **S** | **N** | **G** | **L** | **I** | **A** | **I** | **G** | **L** | **K** | **E** | **D** | **D** | **E** | **L** | **V** | **N** | **V** | **L** | **L** | **T** | **D** | **G** | **Q** | **S** | **N** | **I** | **I** | **I** | **G** | **T** | **H** | **N** | **G** | **Y** | **S** | **V** | **T** | **F** | **K** | **E** | **E** | **A** | **V** | **R** | **D** | **M** | **G** | **R** | **T** | **A** | **S** | **G** | **V** | **R** | **G** | **I** | **R** | **L** | **R** | **E** | **E** | **D** | **Y** | **V** | **V** | **G** | **A** | **A** | **L** | **M** | **K** | **E** | **D** | **Q** | **E** | **V** | **L** | **V** | **I** | **T** | **E** | **K** | **G** | **Y** | **G** | **K** | **R** | **T** | **K** | **V** | **S** | **E** | **Y** | **P** | **V** | **K** | **G** | **R** | **G** | **G** | **K** | **G** | **I** | **K** | **T** | **A** | **N** | **I** | **T** | **E** | **K** | **N** | **G** | **P** | **L** | **A** | **G** | **L** | **T** | **T** | **V** | **S** | **G** | **D** | **E** | **D** | **I** | **M** | **L** | **I** | **T** | **D** | **K** | **G** | **V** | **I** | **I** | **R** | **F** | **N** | **V** | **S** | **T** | **V** | **S** | **Q** | **T** | **G** | **R** | **S** | **T** | **L** | **G** | **V** | **R** | **L** | **M** | **K** | **M** | **E** | **A** | **D** | **T** | **K** | **V** | **V** | **T** | **M** | **-** | **-** | **-** | **-** | **-** | **-** | **-** | **-** | **-** | **-** | **-** | **-** | **-** | **-** | **-** | **-** | **-** | **-** | **-** |
| **N59951F** | **M** | **S** | **E** | **E** | **I** | **R** | **E** | **N** | **I** | **H** | **D** | **V** | **N** | **L** | **T** | **S** | **E** | **M** | **K** | **D** | **S** | **F** | **I** | **D** | **Y** | **A** | **M** | **S** | **V** | **I** | **V** | **A** | **R** | **A** | **L** | **P** | **D** | **V** | **R** | **D** | **G** | **L** | **K** | **P** | **V** | **H** | **R** | **R** | **I** | **L** | **Y** | **G** | **M** | **N** | **E** | **L** | **G** | **V** | **T** | **P** | **D** | **K** | **A** | **H** | **K** | **K** | **S** | **A** | **R** | **I** | **V** | **G** | **D** | **V** | **M** | **G** | **K** | **Y** | **H** | **P** | **H** | **G** | **D** | **S** | **A** | **I** | **Y** | **E** | **S** | **M** | **V** | **R** | **M** | **A** | **Q** | **P** | **F** | **S** | **Y** | **R** | **Y** | **M** | **L** | **V** | **D** | **G** | **H** | **G** | **N** | **F** | **G** | **S** | **V** | **D** | **G** | **D** | **G** | **A** | **A** | **A** | **M** | **R** | **Y** | **T** | **E** | **A** | **R** | **M** | **S** | **K** | **I** | **A** | **T** | **E** | **M** | **L** | **R** | **D** | **I** | **N** | **K** | **N** | **T** | **V** | **D** | **F** | **Q** | **S** | **N** | **Y** | **D** | **D** | **T** | **E** | **K** | **E** | **P** | **V** | **V** | **L** | **P** | **A** | **R** | **F** | **P** | **N** | **L** | **L** | **V** | **N** | **G** | **T** | **T** | **G** | **I** | **A** | **V** | **G** | **M** | **A** | **T** | **N** | **I** | **P** | **P** | **H** | **N** | **L** | **S** | **E** | **V** | **V** | **A** | **A** | **I** | **D** | **L** | **L** | **M** | **D** | **N** | **P** | **D** | **V** | **T** | **T** | **N** | **E** | **L** | **M** | **E** | **V** | **L** | **P** | **G** | **P** | **D** | **F** | **P** | **T** | **G** | **G** | **L** | **V** | **M** | **G** | **K** | **S** | **G** | **I** | **R** | **R** | **A** | **Y** | **E** | **T** | **G** | **K** | **G** | **S** | **I** | **T** | **V** | **R** | **A** | **K** | **V** | **E** | **I** | **T** | **E** | **M** | **P** | **N** | **G** | **K** | **E** | **R** | **I** | **I** | **V** | **T** | **E** | **L** | **P** | **Y** | **M** | **V** | **N** | **K** | **A** | **K** | **L** | **I** | **E** | **R** | **I** | **S** | **E** | **L** | **H** | **R** | **D** | **K** | **R** | **I** | **E** | **G** | **I** | **T** | **D** | **L** | **R** | **D** | **E** | **S** | **S** | **R** | **E** | **G** | **M** | **R** | **I** | **V** | **I** | **D** | **I** | **R** | **R** | **D** | **A** | **S** | **A** | **S** | **V** | **I** | **L** | **N** | **N** | **L** | **Y** | **K** | **L** | **T** | **S** | **L** | **Q** | **N** | **S** | **F** | **G** | **F** | **N** | **M** | **L** | **A** | **I** | **E** | **K** | **G** | **V** | **P** | **K** | **V** | **L** | **S** | **L** | **K** | **Q** | **I** | **L** | **E** | **N** | **Y** | **I** | **E** | **H** | **Q** | **R** | **E** | **V** | **I** | **T** | **R** | **R** | **T** | **V** | **F** | **E** | **K** | **E** | **K** | **A** | **E** | **A** | **R** | **A** | **H** | **I** | **L** | **E** | **G** | **L** | **R** | **I** | **A** | **L** | **D** | **H** | **I** | **D** | **E** | **I** | **I** | **A** | **I** | **I** | **R** | **G** | **S** | **K** | **S** | **D** | **D** | **E** | **A** | **K** | **A** | **T** | **M** | **I** | **E** | **R** | **F** | **D** | **L** | **S** | **D** | **R** | **Q** | **S** | **Q** | **A** | **I** | **L** | **D** | **M** | **R** | **L** | **R** | **R** | **L** | **T** | **G** | **L** | **E** | **R** | **E** | **K** | **I** | **E** | **N** | **E** | **Y** | **Q** | **E** | **L** | **L** | **K** | **L** | **I** | **E** | **D** | **L** | **T** | **D** | **I** | **L** | **A** | **R** | **P** | **E** | **R** | **V** | **T** | **E** | **I** | **I** | **K** | **N** | **E** | **L** | **A** | **D** | **L** | **N** | **Q** | **R** | **F** | **G** | **D** | **K** | **R** | **R** | **T** | **E** | **L** | **L** | **V** | **G** | **E** | **V** | **L** | **S** | **L** | **E** | **D** | **E** | **D** | **L** | **I** | **E** | **E** | **E** | **E** | **I** | **V** | **I** | **T** | **L** | **T** | **N** | **N** | **G** | **Y** | **I** | **K** | **R** | **L** | **A** | **N** | **N** | **E** | **F** | **R** | **A** | **Q** | **R** | **R** | **G** | **G** | **R** | **G** | **V** | **Q** | **G** | **M** | **G** | **I** | **H** | **N** | **D** | **D** | **F** | **V** | **K** | **N** | **L** | **V** | **S** | **C** | **S** | **T** | **H** | **D** | **T** | **L** | **L** | **F** | **F** | **T** | **N** | **N** | **G** | **K** | **V** | **Y** | **R** | **A** | **K** | **G** | **Y** | **E** | **I** | **P** | **E** | **Y** | **G** | **R** | **T** | **A** | **K** | **G** | **I** | **P** | **I** | **I** | **N** | **L** | **L** | **G** | **I** | **D** | **S** | **S** | **E** | **S** | **I** | **Q** | **A** | **I** | **I** | **A** | **V** | **T** | **G** | **E** | **A** | **E** | **E** | **G** | **H** | **Y** | **L** | **F** | **F** | **T** | **T** | **R** | **Q** | **G** | **T** | **V** | **K** | **R** | **T** | **S** | **V** | **T** | **A** | **F** | **A** | **N** | **I** | **R** | **S** | **N** | **G** | **L** | **I** | **A** | **I** | **G** | **L** | **K** | **E** | **D** | **D** | **E** | **L** | **V** | **N** | **V** | **L** | **L** | **T** | **D** | **G** | **Q** | **S** | **N** | **I** | **I** | **I** | **G** | **T** | **H** | **N** | **G** | **Y** | **S** | **V** | **T** | **F** | **K** | **E** | **E** | **A** | **V** | **R** | **D** | **M** | **G** | **R** | **T** | **A** | **S** | **G** | **V** | **R** | **G** | **I** | **R** | **L** | **R** | **E** | **E** | **D** | **Y** | **V** | **V** | **G** | **A** | **A** | **L** | **M** | **K** | **E** | **D** | **Q** | **E** | **V** | **L** | **V** | **I** | **T** | **E** | **K** | **G** | **Y** | **G** | **K** | **R** | **T** | **K** | **V** | **S** | **E** | **Y** | **P** | **V** | **K** | **G** | **R** | **G** | **G** | **K** | **G** | **I** | **K** | **T** | **A** | **N** | **I** | **T** | **E** | **K** | **N** | **G** | **P** | **L** | **A** | **G** | **L** | **T** | **T** | **V** | **S** | **G** | **D** | **E** | **D** | **I** | **M** | **L** | **I** | **T** | **D** | **K** | **G** | **V** | **I** | **I** | **R** | **F** | **N** | **V** | **S** | **T** | **V** | **S** | **Q** | **T** | **G** | **R** | **S** | **T** | **L** | **G** | **V** | **R** | **L** | **M** | **K** | **M** | **E** | **A** | **D** | **T** | **K** | **V** | **V** | **T** | **M** | **A** | **A** | **V** | **E** | **P** | **E** | **A** | **A** | **E** | **Q** | **T** | **V** | **E** | **E** | **Q** | **G** | **S** | **E** | **E** |
 **SNP position** | . | . | . | . | . | . | . | . | . | . | . | . | . | . | . | . | . | . | . | . | . | . | . | . | . | . | . | . | . | . | . | . | . | . | . | . | . | . | . | . | . | . | . | . | . | . | . | . | . | . | . | . | . | . | . | . | . | . | . | . | . | . | . | . | . | . | . | . | . | . | . | . | . | . | . | . | . | . | . | . | . | . | . | 84 | . | . | . | . | . | . | . | . | . | . | . | . | . | . | . | . | . | . | . | . | . | . | . | . | . | . | . | . | . | . | . | . | . | . | . | . | . | . | . | . | . | . | . | . | . | . | . | . | . | . | . | . | . | . | . | . | . | . | . | . | . | . | . | . | . | . | . | . | . | . | . | . | . | . | . | . | . | . | . | . | . | . | . | . | . | . | . | . | . | . | . | . | . | . | . | . | . | . | . | . | . | . | . | . | . | . | . | . | . | . | . | . | . | . | . | . | . | . | . | . | . | . | . | . | . | . | . | . | . | . | . | . | . | . | . | . | . | . | . | . | . | . | . | . | . | . | . | . | . | . | . | . | . | . | . | . | . | . | . | . | . | . | . | . | 249 | 250 | . | . | 253 | . | . | . | . | . | . | 260 | . | . | . | . | . | . | . | . | . | . | . | . | . | . | . | . | . | . | . | . | . | . | . | . | . | . | . | . | . | . | . | . | . | . | . | . | . | . | . | . | . | . | . | . | . | . | 307 | . | . | . | 311 | . | . | . | . | 316 | . | . | . | . | . | . | 323 | . | 325 | . | . | 328 | . | . | . | . | . | . | . | . | . | . | . | . | . | . | . | 344 | . | . | . | . | 349 | . | . | . | . | . | 355 | . | . | . | 359 | 360 | . | . | . | . | . | . | 367 | . | 369 | . | 371 | . | . | . | . | . | . | . | . | . | . | . | . | . | . | . | . | . | . | . | 391 | . | . | . | . | . | . | . | . | . | 401 | . | . | . | . | . | . | . | 409 | 410 | . | . | . | . | 415 | 416 | . | . | . | . | 421 | . | . | . | . | . | . | . | . | . | . | . | . | . | . | . | . | 438 | . | . | . | . | . | . | . | . | . | . | 449 | 450 | . | 452 | . | . | 455 | . | . | . | . | . | . | . | . | . | 465 | . | . | . | . | 470 | . | . | 473 | . | 475 | 476 | 477 | 478 | . | . | . | 482 | . | . | . | . | . | . | . | . | . | . | . | . | . | . | . | . | . | . | . | . | . | . | . | 506 | . | . | . | . | . | . | . | . | . | . | . | . | 519 | 520 | . | 522 | . | . | . | . | . | . | . | . | . | . | . | . | . | . | . | . | 539 | . | 541 | . | . | . | . | . | 547 | . | 549 | . | . | . | . | . | . | . | . | . | . | . | . | . | 563 | . | . | . | . | . | . | . | . | . | . | . | . | . | . | . | . | . | . | . | . | . | . | 586 | . | . | . | . | . | . | . | . | 595 | . | 597 | . | . | . | . | . | 603 | . | 605 | . | 607 | . | . | 610 | . | 612 | . | . | . | . | . | . | 619 | 620 | . | . | . | . | . | . | 627 | . | . | . | . | 632 | . | . | . | . | . | . | . | . | . | . | 643 | . | . | . | . | . | . | . | 651 | . | . | 654 | 655 | . | 657 | . | 659 | 660 | 661 | 662 | . | . | . | . | . | 668 | . | . | . | . | . | . | 675 | . | 677 | 678 | 679 | . | . | . | . | . | . | . | . | . | . | . | . | . | . | . | . | . | 697 | . | 699 | . | . | . | 703 | . | 705 | 706 | 707 | 708 | 709 | 710 | . | . | . | . | . | . | . | 718 | . | . | . | . | . | . | . | 726 | . | . | . | . | . | . | . | . | . | . | . | . | . | . | . | 742 | . | . | . | . | . | . | . | . | . | . | . | . | . | . | . | 758 | . | 760 | . | . | . | 764 | . | . | . | 768 | . | . | . | . | . | . | . | . | . | 778 | 779 | . | . | . | . | . | . | 786 | . | . | . | . | . | . | . | 794 | . | . | 797 | 798 | 799 | . | . | . | . | . | . | . | . | . | . | . | . | . | . | . | . | . | . | . | . | . | . | . | . |

|  |  |
| --- | --- |
|  |

|  |  |
| --- | --- |
|  |

|  |  |
| --- | --- |
| **Legend** | |
  | **Reference Sequence** |

  |
|  | **Validated SNPs** |
|  | **Non-validated SNPs** |
|  | **New SNPs** |
|  | **GAPs** |
|  |  |

  
  |
|  |  |
