# Supplementary material for: sraX: A Novel Comprehensive Resistome Analysis Tool
Source: Front Microbiol. 2020 Feb 5;11:52. doi: 10.3389/fmicb.2020.00052 (PMC7025521; doi:10.3389/fmicb.2020.00052)
Supplement: Supplementary file 4 [file Data_Sheet_3.ZIP › gyrA/gyrA_dna.html]

 
 
 
 

   gyrA   

    
    
    Definition:  Not indicated  
    
    
    Reference organism:  Not indicated     
    
     Summary Data     Parameter    Value (n)     Total number of genomes  198    MSA length  2472    Total number of SNPs  931    SNP density (# SNPs/MSA length)  0.377      
    
    
    
 
    Enterococci (sraxID_hPul2ydnYp48)    
   A   
   T   
   G   
   A   
   G   
   T   
   G   
   A   
   A   
   G   
   A   
   A   
   A   
   T   
   C   
   A   
   G   
   A   
   G   
   A   
   A   
   A   
   A   
   C   
   A   
   T   
   C   
   C   
   A   
   T   
   G   
   A   
   T   
   G   
   T   
   C   
   A   
   A   
   T   
   C   
   T   
   A   
   A   
   C   
   C   
   A   
   G   
   C   
   G   
   A   
   A   
   A   
   T   
   G   
   A   
   A   
   A   
   G   
   A   
   C   
   T   
   C   
   C   
   T   
   T   
   C   
   A   
   T   
   C   
   G   
   A   
   T   
   T   
   A   
   T   
   G   
   C   
   G   
   A   
   T   
   G   
   A   
   G   
   T   
   G   
   T   
   T   
   A   
   T   
   T   
   G   
   T   
   C   
   G   
   C   
   A   
   C   
   G   
   T   
   G   
   C   
   A   
   C   
   T   
   T   
   C   
   C   
   T   
   G   
   A   
   T   
   G   
   T   
   C   
   A   
   G   
   A   
   G   
   A   
   T   
   G   
   G   
   T   
   C   
   T   
   G   
   A   
   A   
   A   
   C   
   C   
   C   
   G   
   T   
   T   
   C   
   A   
   T   
   C   
   G   
   C   
   C   
   G   
   T   
   A   
   T   
   T   
   T   
   T   
   A   
   T   
   A   
   C   
   G   
   G   
   C   
   A   
   T   
   G   
   A   
   A   
   C   
   G   
   A   
   A   
   T   
   T   
   A   
   G   
   G   
   T   
   G   
   T   
   A   
   A   
   C   
   A   
   C   
   C   
   G   
   G   
   A   
   T   
   A   
   A   
   A   
   G   
   C   
   C   
   C   
   A   
   T   
   A   
   A   
   A   
   A   
   A   
   A   
   T   
   C   
   T   
   G   
   C   
   C   
   A   
   G   
   A   
   A   
   T   
   C   
   G   
   T   
   C   
   G   
   G   
   A   
   G   
   A   
   T   
   G   
   T   
   C   
   A   
   T   
   G   
   G   
   G   
   T   
   A   
   A   
   A   
   T   
   A   
   C   
   C   
   A   
   T   
   C   
   C   
   T   
   C   
   A   
   T   
   G   
   G   
   A   
   G   
   A   
   C   
   A   
   G   
   T   
   G   
   C   
   G   
   A   
   T   
   C   
   T   
   A   
   T   
   G   
   A   
   G   
   T   
   C   
   A   
   A   
   T   
   G   
   G   
   T   
   A   
   C   
   G   
   G   
   A   
   T   
   G   
   G   
   C   
   A   
   C   
   A   
   G   
   C   
   C   
   A   
   T   
   T   
   C   
   A   
   G   
   C   
   T   
   A   
   C   
   C   
   G   
   T   
   T   
   A   
   T   
   A   
   T   
   G   
   C   
   T   
   A   
   G   
   T   
   G   
   G   
   A   
   T   
   G   
   G   
   T   
   C   
   A   
   T   
   G   
   G   
   A   
   A   
   A   
   C   
   T   
   T   
   T   
   G   
   G   
   T   
   T   
   C   
   T   
   G   
   T   
   T   
   G   
   A   
   C   
   G   
   G   
   T   
   G   
   A   
   C   
   G   
   G   
   T   
   G   
   C   
   C   
   G   
   C   
   C   
   G   
   C   
   T   
   A   
   T   
   G   
   C   
   G   
   T   
   T   
   A   
   T   
   A   
   C   
   C   
   G   
   A   
   A   
   G   
   C   
   A   
   C   
   G   
   G   
   A   
   T   
   G   
   A   
   G   
   C   
   A   
   A   
   A   
   A   
   T   
   C   
   G   
   C   
   T   
   A   
   C   
   A   
   G   
   A   
   A   
   A   
   T   
   G   
   C   
   T   
   G   
   C   
   G   
   T   
   G   
   A   
   T   
   A   
   T   
   C   
   A   
   A   
   T   
   A   
   A   
   G   
   A   
   A   
   C   
   A   
   C   
   T   
   G   
   T   
   T   
   G   
   A   
   T   
   T   
   T   
   C   
   C   
   A   
   A   
   A   
   G   
   C   
   A   
   A   
   C   
   T   
   A   
   T   
   G   
   A   
   T   
   G   
   A   
   T   
   A   
   C   
   A   
   G   
   A   
   A   
   A   
   A   
   A   
   G   
   A   
   A   
   C   
   C   
   A   
   G   
   T   
   T   
   G   
   T   
   A   
   C   
   T   
   A   
   C   
   C   
   T   
   G   
   C   
   T   
   C   
   G   
   C   
   T   
   T   
   C   
   C   
   C   
   G   
   A   
   A   
   T   
   C   
   T   
   T   
   T   
   T   
   A   
   G   
   T   
   G   
   A   
   A   
   C   
   G   
   G   
   A   
   A   
   C   
   A   
   A   
   C   
   G   
   G   
   G   
   G   
   A   
   T   
   C   
   G   
   C   
   C   
   G   
   T   
   A   
   G   
   G   
   T   
   A   
   T   
   G   
   G   
   C   
   C   
   A   
   C   
   G   
   A   
   A   
   T   
   A   
   T   
   C   
   C   
   C   
   A   
   C   
   C   
   T   
   C   
   A   
   T   
   A   
   A   
   C   
   T   
   T   
   G   
   T   
   C   
   T   
   G   
   A   
   G   
   G   
   T   
   A   
   G   
   T   
   A   
   G   
   C   
   G   
   G   
   C   
   T   
   A   
   T   
   C   
   G   
   A   
   T   
   T   
   T   
   G   
   C   
   T   
   G   
   A   
   T   
   G   
   G   
   A   
   T   
   A   
   A   
   T   
   C   
   C   
   T   
   G   
   A   
   T   
   G   
   T   
   G   
   A   
   C   
   G   
   A   
   C   
   C   
   A   
   A   
   T   
   G   
   A   
   A   
   T   
   T   
   G   
   A   
   T   
   G   
   G   
   A   
   A   
   G   
   T   
   A   
   T   
   T   
   A   
   C   
   C   
   T   
   G   
   G   
   A   
   C   
   C   
   A   
   G   
   A   
   T   
   T   
   T   
   T   
   C   
   C   
   A   
   A   
   C   
   A   
   G   
   G   
   T   
   G   
   G   
   T   
   T   
   T   
   G   
   G   
   T   
   T   
   A   
   T   
   G   
   G   
   G   
   G   
   A   
   A   
   A   
   T   
   C   
   A   
   G   
   G   
    A    
   A   
   T   
    C    
   C   
   G   
    A    
    A    
   G   
   A   
   G   
   C   
    G    
   T   
   A   
   T   
   G   
   A   
   A   
   A   
   C   
    A    
   G   
   G   
    T    
   A   
   A   
    G    
   G   
   G   
   T   
   T   
   C   
    T    
   A   
   T   
   T   
   A   
   C   
    C    
   G   
   T   
    A    
   C   
   G   
   T   
   G   
   C   
   A   
   A   
   A   
   A   
   G   
   T   
   T   
   G   
   A   
    G    
    A    
   T   
    T    
   A   
    C    
   T   
   G   
   A   
    G    
   A   
   T   
   G   
    C    
   C   
    A    
   A   
   A   
   T   
   G   
   G   
    T    
   A   
   A   
   A   
   G   
   A   
   A   
   C   
   G   
    G    
   A   
   T   
    C    
    A    
   T   
    C    
   G   
   T   
    G    
   A   
   C   
    A    
   G   
   A   
   A   
   T   
   T   
    G    
   C   
   C   
   T   
   T   
   A   
   T   
   A   
   T   
   G   
   G   
   T   
    C    
   A   
   A   
   T   
   A   
   A   
   A   
   G   
   C   
    A    
   A   
   A   
   A   
   T   
   T   
    G    
   A   
   T   
    C    
   G   
   A   
   A   
   C   
   G   
    G    
   A   
   T   
   T   
   T   
   C   
   T   
   G   
   A   
    A    
    C    
   T   
    G    
   C   
   A   
   C   
   A   
   G   
   A   
   G   
   A   
    C    
   A   
   A   
    G    
   C   
   G   
    T    
   A   
   T   
    C    
   G   
   A   
   A   
   G   
   G   
    G    
   A   
   T   
    C    
   A   
   C   
    A    
   G   
   A   
   T   
   T   
   T   
   G   
   C   
   G   
   G   
   G   
   A   
    C    
   G   
   A   
   A   
   T   
   C   
   T   
   T   
   C   
    C    
   C   
   G   
   T   
   G   
   A   
    A    
   G   
   G   
    T    
   A   
   T   
   G   
   C   
   G   
    G    
   A   
   T   
    C    
   G   
   T   
   C   
   A   
   T   
    T    
   G   
   A   
    C    
    A    
   T   
    C    
   C   
   G   
   T   
   C   
   G   
    T    
   G   
   A   
    C    
   G   
    C    
    C    
   A   
   G   
    C    
   G   
   C   
    A    
   T   
   C   
    C    
   G   
   T   
   T   
    A    
   T   
    C    
   T   
   T   
    G    
   A   
   A   
    T    
   A   
   A   
   C   
   T   
   T   
    A    
   T   
   A   
    T    
   A   
   A   
   A   
    T    
   T   
   G   
   A   
   C   
    A    
    T    
   C   
    C    
   T   
   T   
    G    
   C   
   A   
    A    
   A   
    A    
    T    
   T   
   C   
    A    
   T   
   T   
    T    
   G   
   G   
    A    
   T   
   T   
    C    
   A   
   A   
    T    
   A   
   T   
   G   
   T   
   T   
    G    
   G   
   C   
    G    
   A   
   T   
    C    
   G   
   A   
   A   
   A   
   A   
   A   
   G   
   G   
    T    
   G   
   T   
    G    
   C   
   C   
    G    
   A   
   A   
   A   
    G    
   T   
   T   
   T   
   T   
    G    
   A   
   G   
    C    
   T   
   T   
   G   
   A   
   A   
   A   
   C   
    A    
    A    
   A   
   T   
    C    
    C    
   T   
    T    
   G   
   A   
   A   
   A   
   A   
    C    
   T   
   A   
    T    
    A    
   T   
    C    
   G   
   A   
    A    
   C   
   A   
    T    
   C   
   A   
    A    
    C    
    G    
    C    
    G    
   A   
   A   
   G   
   T   
   C   
   A   
   T   
    C    
   A   
   C   
    A    
    C    
   G   
    A    
   C   
   G   
   G   
   A   
   C   
    G    
    G    
   T   
    C    
   T   
   T   
   T   
   G   
   A   
    A    
   A   
   A   
   A   
    G    
   A   
    A    
   A   
   A   
    A    
   G   
   C   
   A   
   G   
   A   
   A   
   G   
   C   
   A   
    A    
   G   
    A    
   G   
   C   
    A    
   C   
   A   
    C    
   A   
   T   
    C    
   T   
   T   
   A   
   G   
   A   
   A   
   G   
   G   
   T   
    T    
   T   
    G    
   C   
   G   
    T    
   A   
   T   
   T   
   G   
   C   
    T    
   T   
   T   
    G    
   G   
   A   
   T   
   C   
   A   
   T   
   A   
   T   
   C   
    G    
   A   
   T   
   G   
   A   
   A   
   A   
   T   
    C    
   A   
   T   
   T   
   G   
   C   
    G    
   A   
   T   
   T   
   A   
   T   
    C    
   C   
   G   
    C    
   G   
   G   
    G    
   T   
   C   
    T    
    A    
   A   
   A   
   T   
   C   
   A   
   G   
   A   
   T   
   G   
   A   
    C    
   G   
   A   
    G    
   G   
   C   
   A   
   A   
   A   
   A   
   G   
   C   
    A    
   A   
    C    
    A    
    A    
   T   
    G    
   A   
   T   
    C    
   G   
   A   
   A   
   C   
   G   
    G    
   T   
   T   
    C    
   G   
   A   
    T    
   T   
   T   
    A    
   T   
   C   
    C    
   G   
   A   
   T   
   C   
   G   
    C    
   C   
   A   
    G    
    T    
   C   
    T    
   C   
   A   
   A   
   G   
   C   
    T    
   A   
   T   
    T    
   T   
   T   
   A   
   G   
   A   
   T   
   A   
   T   
   G   
   C   
   G   
   T   
   T   
   T   
    A    
   C   
   G   
    C    
   C   
   G   
    A    
   T   
   T   
    G    
   A   
   C   
    C    
   G   
   G   
    T    
   T   
   T   
    G    
   G   
   A   
   A   
   C   
   G   
    G    
   G   
   A   
    A    
   A   
   A   
   A   
   A   
   T   
    C    
   G   
   A   
    A    
   A   
   A   
   T   
   G   
   A   
   A   
   T   
   A   
    C    
   C   
   A   
   A   
   G   
   A   
    A    
   C   
   T   
    C    
   T   
   T   
    A    
   A   
    A    
   A   
    C    
   T   
    G    
   A   
   T   
   T   
   G   
    A    
    A    
   G   
   A   
    T    
   T   
   T   
    A    
    A    
    C    
    T    
   G   
   A   
   C   
   A   
   T   
   T   
    C    
   T   
    G    
   G   
   C   
    A    
    A    
   G   
    A    
   C   
   C   
    A    
   G   
   A   
    A    
   C   
   G   
    T    
   G   
   T   
    T    
   A   
    C    
    A    
   G   
   A   
   A   
   A   
   T   
    C    
   A   
   T   
    C    
   A   
   A   
    G    
   A   
    A    
    T    
   G   
   A   
    A    
   T   
   T   
   A   
    G    
    C    
    A    
   G   
   A   
   C   
    T    
   T   
    G    
    A    
    A    
    T    
    C    
    A    
   A   
    C    
    G    
   A   
   T   
   T   
   T   
   G   
   G   
   T   
   G   
   A   
   T   
    A    
    A    
   A   
   C   
   G   
   A   
   C   
   G   
    C    
   A   
   C   
   A   
   G   
   A   
   A   
    T    
   T   
   A   
    C    
   T   
   A   
   G   
   T   
    C    
   G   
   G   
   T   
   G   
   A   
    G    
   G   
   T   
   C   
    T    
   T   
    A    
   A   
   G   
   T   
   C   
   T   
    G    
   G   
   A   
   A   
   G   
   A   
   T   
   G   
   A   
   A   
   G   
   A   
   T   
    T    
   T   
    A    
   A   
   T   
    C    
   G   
   A   
   A   
   G   
   A   
   A   
   G   
   A   
   A   
   G   
   A   
   A   
    A    
   T   
   C   
   G   
   T   
    T    
   A   
   T   
   T   
   A   
   C   
    A    
    T    
   T   
    G    
   A   
   C   
    A    
   A   
   A   
    C    
   A   
   A   
    T    
   G   
   G   
    T    
   T   
   A   
    C    
   A   
   T   
    C    
   A   
   A   
    A    
   C   
   G   
    T    
    T    
   T   
   G   
   G   
    C    
   A   
   A   
   A   
    T    
    A    
    A    
    T    
   G   
   A   
   A   
   T   
   T   
   C   
    A    
   G   
    A    
   G   
   C   
    T    
   C   
   A   
   A   
   C   
   G   
    A    
    A    
   G   
    G    
   G   
   G   
    T    
   G   
   G   
    A    
   C   
   G   
   T   
   G   
   G   
    T    
   G   
   T   
    C    
   C   
   A   
   A   
   G   
   G   
    A    
   A   
   T   
   G   
   G   
   G   
    T    
    A    
   T   
    C    
   C   
   A   
    C    
    A    
   A   
   T   
   G   
   A   
    C    
   G   
   A   
    C    
   T   
   T   
   C   
   G   
   T   
    C    
   A   
   A   
    A    
   A   
   A   
    T    
   C   
   T   
    G    
    G    
   T   
   T   
   T   
   C   
   T   
   T   
   G   
    T    
   T   
   C   
    T    
   A   
   C   
    C    
   C   
   A   
    T    
   G   
   A   
    T    
   A   
   C   
    A    
   T   
   T   
    G    
   T   
   T   
   A   
   T   
   T   
    C    
   T   
   T   
   T   
   A   
   C   
   G   
   A   
   A   
    T    
   A   
    A    
    C    
   G   
   G   
    A    
   A   
   A   
   A   
   G   
   T   
    G    
   T   
   A   
    T    
   C   
   G   
    G    
   G   
   C   
   A   
   A   
   A   
   A   
   G   
   G   
    A    
   T   
   A   
    C    
   G   
   A   
   A   
   A   
   T   
    T    
   C   
   C   
    A    
   G   
   A   
    A    
   T   
   A   
   C   
   G   
   G   
    G    
    C    
   G   
    T    
   A   
   C   
   A   
   G   
   C   
    C    
   A   
   A   
   A   
   G   
   G   
    A    
   A   
   T   
    C    
   C   
   C   
   A   
    A    
   T   
    C    
   A   
   T   
    C    
   A   
   A   
    T    
   T   
   T   
    A    
   C   
   T   
    A    
   G   
   G   
    T    
   A   
   T   
    C    
   G   
   A   
    T    
   T   
   C   
    G    
    A    
    G    
    T    
   G   
   A   
   A   
   A   
    G    
    C    
   A   
   T   
    C    
   C   
   A   
    G    
   G   
   C   
    A    
   A   
   T   
    C    
   A   
   T   
   T   
    G    
   C   
    G    
   G   
   T   
    G    
    A    
    C    
   G   
   G   
   G   
    A    
    G    
   A   
   A   
   G   
   C   
    A    
   G   
   A   
   A   
   G   
    A    
    A    
   G   
   G   
    C    
    C    
   A   
    C    
   T   
   A   
    T    
    C    
   T   
    C    
   T   
   T   
   C   
   T   
   T   
    T    
   A   
   C   
    G    
   A   
   C   
    G    
    C    
    G    
    T    
    C    
   A   
   A   
   G   
   G   
    T    
   A   
   C   
    A    
   G   
   T   
    A    
   A   
   A   
   A   
   C   
   G   
    T    
   A   
   C   
    T    
    T    
   C   
    T    
   G   
   T   
    C    
   A   
   C   
    T    
   G   
   C   
    T    
   T   
   T   
    T    
    G    
   C   
   T   
   A   
   A   
    C    
   A   
   T   
    C    
   C   
   G   
    A    
   A   
   G   
    T    
   A   
   A   
   T   
   G   
   G   
    T    
    C    
   T   
    G    
   A   
   T   
    C    
   G   
   C   
    A    
   A   
   T   
   T   
    G    
   G   
    T    
   T   
   T   
    G    
   A   
   A   
   A   
   G   
   A   
   A   
   G   
   A   
    C    
   G   
   A   
    T    
   G   
   A   
    G    
    C    
   T   
    C    
    G    
   T   
    C    
   A   
   A   
    T    
   G   
   T   
    G    
    C    
   T   
   A   
    C    
    T    
   G   
   A   
   C   
    A    
    G    
   A   
    T    
   G   
   G   
    T    
    C    
   A   
    A    
    T    
    C    
    T    
   A   
   A   
    T    
   A   
   T   
    C    
   A   
   T   
    T    
   A   
   T   
    T    
   G   
   G   
    A    
   A   
   C   
   A   
   C   
   A   
   T   
    A    
    A    
    C    
   G   
   G   
    C    
   T   
   A   
   C   
   T   
   C   
   T   
   G   
   T   
    C    
   A   
   C   
    T    
   T   
   T   
    C    
    A    
   A   
    A    
   G   
   A   
    A    
    G    
    A    
    A    
    G    
   C   
    A    
   G   
    T    
    C    
   C   
   G   
    C    
   G   
   A   
   T   
   A   
   T   
   G   
   G   
   G   
    A    
   C   
   G   
    T    
   A   
   C   
   A   
   G   
   C   
    T    
   T   
   C   
    C    
   G   
   G   
    G    
   G   
   T   
    C    
    A    
   G   
    A    
   G   
   G   
    G    
   A   
   T   
    A    
   C   
   G   
    C    
    C    
   T   
    G    
   C   
   G   
    T    
   G   
   A   
    G    
    G    
   A   
    A    
   G   
   A   
    T    
   T   
    A    
    T    
   G   
   T   
    C    
   G   
   T   
    T    
   G   
   G   
    G    
    G    
   C   
   A   
   G   
   C   
    C    
    T    
   T   
    A    
    A    
   T   
   G   
    A    
   A   
    G    
   G   
    A    
   A   
    A    
   A   
    T    
    C    
   A   
   A   
   G   
   A   
   A   
   G   
   T   
    A    
    T    
   T   
    A    
   G   
   T   
    G    
   A   
   T   
    C    
   A   
   C   
    A    
   G   
   A   
   A   
   A   
   A   
    A    
   G   
   G   
   T   
   T   
   A   
    C    
   G   
   G   
    A    
   A   
   A   
    A    
   C   
   G   
    G    
   A   
   C   
    A    
   A   
   A   
   A   
   G   
    T    
    A    
   T   
   C   
    G    
   G   
   A   
    A    
   T   
   A   
   T   
   C   
   C   
   A   
   G   
   T   
    G    
   A   
   A   
    A    
   G   
   G   
    C    
   C   
   G   
    C    
   G   
   G   
    A    
   G   
   G   
   T   
   A   
   A   
   A   
   G   
   G   
    G    
   A   
   T   
    C    
   A   
   A   
    A    
   A   
   C   
    G    
    G    
   C   
    G    
   A   
   A   
    C    
   A   
   T   
   C   
   A   
   C   
   T   
   G   
   A   
    A    
   A   
   A   
   A   
   A   
   A   
   T   
   G   
   G   
   T   
   C   
   C   
   A   
    C    
   T   
   A   
   G   
   C   
    T    
   G   
   G   
    A    
   T   
   T   
    G    
   A   
   C   
   C   
   A   
   C   
    T    
   G   
   T   
    T    
    T    
    C    
   T   
   G   
   G   
    A    
    G    
   A   
   T   
   G   
   A   
   A   
   G   
   A   
    C    
   A   
   T   
   C   
    A    
   T   
    G    
    C    
   T   
    G    
   A   
   T   
    T    
   A   
   C   
    A    
    G    
   A   
    T    
   A   
   A   
   A   
   G   
   G   
    T    
   G   
   T   
    C    
   A   
   T   
   T   
   A   
   T   
   C   
   C   
   G   
    T    
   T   
   T   
    C    
   A   
   A   
   C   
   G   
   T   
    A    
    T    
    C    
    G    
    A    
   C   
   T   
   G   
   T   
   T   
   T   
   C   
   T   
   C   
   A   
   A   
   A   
   C   
    T    
   G   
   G   
   A   
   C   
   G   
    T    
    T    
   C   
   A   
   A   
   C   
   A   
   T   
   T   
    A    
   G   
   G   
    C    
   G   
   T   
    A    
   C   
   G   
   T   
   T   
   T   
    G    
   A   
   T   
   G   
   A   
    A    
    G    
   A   
   T   
   G   
   G   
   A   
   A   
   G   
    C    
    T    
   G   
    A    
    T    
    A    
   C   
    G    
   A   
   A   
   A   
   G   
   T   
   T   
   G   
   T   
   C   
   A   
   C   
   G   
   A   
   T   
   G   
   G   
   C   
   A   
   G   
   C   
   A   
   G   
   T   
   C   
   G   
   A   
   A   
   C   
   C   
   A   
   G   
   A   
   A   
   G   
   C   
   T   
   G   
   C   
   A   
   G   
   A   
   A   
   C   
   A   
   A   
   A   
   C   
   A   
   G   
   T   
   T   
   G   
   A   
   A   
   G   
   A   
   A   
   C   
   A   
   A   
   G   
   G   
   A   
   T   
   C   
   A   
   G   
   A   
   A   
   G   
   A   
   A   
   T   
   A   
   A   
 

 
   N53702   
   A   
   T   
   G   
   A   
   G   
   T   
   G   
   A   
   A   
   G   
   A   
   A   
   A   
   T   
   C   
   A   
   G   
   A   
   G   
   A   
   A   
   A   
   A   
   C   
   A   
   T   
   C   
   C   
   A   
   T   
   G   
   A   
   T   
   G   
   T   
   C   
   A   
   A   
   T   
   C   
   T   
   A   
   A   
   C   
   C   
   A   
   G   
   C   
   G   
   A   
   A   
   A   
   T   
   G   
   A   
   A   
   A   
   G   
   A   
   C   
   T   
   C   
   C   
   T   
   T   
   C   
   A   
   T   
   C   
   G   
   A   
   T   
   T   
   A   
   T   
   G   
   C   
   G   
   A   
   T   
   G   
   A   
   G   
   T   
   G   
   T   
   T   
   A   
   T   
   T   
   G   
   T   
   C   
   G   
   C   
   A   
   C   
   G   
   T   
   G   
   C   
   A   
   C   
   T   
   T   
   C   
   C   
   T   
   G   
   A   
   T   
   G   
   T   
   C   
   A   
   G   
   A   
   G   
   A   
   T   
   G   
   G   
   T   
   C   
   T   
   G   
   A   
   A   
   A   
   C   
   C   
   C   
   G   
   T   
   T   
   C   
   A   
   T   
   C   
   G   
   C   
   C   
   G   
   T   
   A   
   T   
   T   
   T   
   T   
   A   
   T   
   A   
   C   
   G   
   G   
   C   
   A   
   T   
   G   
   A   
   A   
   C   
   G   
   A   
   A   
   T   
   T   
   A   
   G   
   G   
   T   
   G   
   T   
   A   
   A   
   C   
   A   
   C   
   C   
   G   
   G   
   A   
   T   
   A   
   A   
   A   
   G   
   C   
   C   
   C   
   A   
   T   
   A   
   A   
   A   
   A   
   A   
   A   
   T   
   C   
   T   
   G   
   C   
   C   
   A   
   G   
   A   
   A   
   T   
   C   
   G   
   T   
   C   
   G   
   G   
   A   
   G   
   A   
   T   
   G   
   T   
   C   
   A   
   T   
   G   
   G   
   G   
   T   
   A   
   A   
   A   
   T   
   A   
   C   
   C   
   A   
   T   
   C   
   C   
   T   
   C   
   A   
   T   
   G   
   G   
   A   
   G   
   A   
   C   
   A   
   G   
   T   
   G   
   C   
   G   
   A   
   T   
   C   
   T   
   A   
   T   
   G   
   A   
   G   
   T   
   C   
   A   
   A   
   T   
   G   
   G   
   T   
   A   
   C   
   G   
   G   
   A   
   T   
   G   
   G   
   C   
   A   
   C   
   A   
   G   
   C   
   C   
   A   
   T   
   T   
   C   
   A   
   G   
   C   
   T   
   A   
   C   
   C   
   G   
   T   
   T   
   A   
   T   
   A   
   T   
   G   
   C   
   T   
   A   
   G   
   T   
   G   
   G   
   A   
   T   
   G   
   G   
   T   
   C   
   A   
   T   
   G   
   G   
   A   
   A   
   A   
   C   
   T   
   T   
   T   
   G   
   G   
   T   
   T   
   C   
   T   
   G   
   T   
   T   
   G   
   A   
   C   
   G   
   G   
   T   
   G   
   A   
   C   
   G   
   G   
   T   
   G   
   C   
   C   
   G   
   C   
   C   
   G   
   C   
   T   
   A   
   T   
   G   
   C   
   G   
   T   
   T   
   A   
   T   
   A   
   C   
   C   
   G   
   A   
   A   
   G   
   C   
   A   
   C   
   G   
   G   
   A   
   T   
   G   
   A   
   G   
   C   
   A   
   A   
   A   
   A   
   T   
   C   
   G   
   C   
   T   
   A   
   C   
   A   
   G   
   A   
   A   
   A   
   T   
   G   
   C   
   T   
   G   
   C   
   G   
   T   
   G   
   A   
   T   
   A   
   T   
   C   
   A   
   A   
   T   
   A   
   A   
   G   
   A   
   A   
   C   
   A   
   C   
   T   
   G   
   T   
   T   
   G   
   A   
   T   
   T   
   T   
   C   
   C   
   A   
   A   
   A   
   G   
   C   
   A   
   A   
   C   
   T   
   A   
   T   
   G   
   A   
   T   
   G   
   A   
   T   
   A   
   C   
   A   
   G   
   A   
   A   
   A   
   A   
   A   
   G   
   A   
   A   
   C   
   C   
   A   
   G   
   T   
   T   
   G   
   T   
   A   
   C   
   T   
   A   
   C   
   C   
   T   
   G   
   C   
   T   
   C   
   G   
   C   
   T   
   T   
   C   
   C   
   C   
   G   
   A   
   A   
   T   
   C   
   T   
   T   
   T   
   T   
   A   
   G   
   T   
   G   
   A   
   A   
   C   
   G   
   G   
   A   
   A   
   C   
   A   
   A   
   C   
   G   
   G   
   G   
   G   
   A   
   T   
   C   
   G   
   C   
   C   
   G   
   T   
   A   
   G   
   G   
   T   
   A   
   T   
   G   
   G   
   C   
   C   
   A   
   C   
   G   
   A   
   A   
   T   
   A   
   T   
   C   
   C   
   C   
   A   
   C   
   C   
   T   
   C   
   A   
   T   
   A   
   A   
   C   
   T   
   T   
   G   
   T   
   C   
   T   
   G   
   A   
   G   
   G   
   T   
   A   
   G   
   T   
   A   
   G   
   C   
   G   
   G   
   C   
   T   
   A   
   T   
   C   
   G   
   A   
   T   
   T   
   T   
   G   
   C   
   T   
   G   
   A   
   T   
   G   
   G   
   A   
   T   
   A   
   A   
   T   
   C   
   C   
   T   
   G   
   A   
   T   
   G   
   T   
   G   
   A   
   C   
   G   
   A   
   C   
   C   
   A   
   A   
   T   
   G   
   A   
   A   
   T   
   T   
   G   
   A   
   T   
   G   
   G   
   A   
   A   
   G   
   T   
   A   
   T   
   T   
   A   
   C   
   C   
   T   
   G   
   G   
   A   
   C   
   C   
   A   
   G   
   A   
   T   
   T   
   T   
   T   
   C   
   C   
   A   
   A   
   C   
   A   
   G   
   G   
   T   
   G   
   G   
   T   
   T   
   T   
   G   
   G   
   T   
   T   
   A   
   T   
   G   
   G   
   G   
   A   
   A   
   A   
   A   
   T   
   C   
   A   
   G   
   G   
    A    
   A   
   T   
    C    
   C   
   G   
    A    
    A    
   G   
   A   
   G   
   C   
    G    
   T   
   A   
   T   
   G   
   A   
   A   
   A   
   C   
    A    
   G   
   G   
    T    
   A   
   A   
    G    
   G   
   G   
   T   
   T   
   C   
    T    
   A   
   T   
   T   
   A   
   C   
    C    
   G   
   T   
    A    
   C   
   G   
   T   
   G   
   C   
   A   
   A   
   A   
   A   
   G   
   T   
   T   
   G   
   A   
    G    
    A    
   T   
    T    
   A   
    C    
   T   
   G   
   A   
    G    
   A   
   T   
   G   
    C    
   C   
    A    
   A   
   A   
   T   
   G   
   G   
    T    
   A   
   A   
   A   
   G   
   A   
   A   
   C   
   G   
    G    
   A   
   T   
    C    
    A    
   T   
    C    
   G   
   T   
    G    
   A   
   C   
    A    
   G   
   A   
   A   
   T   
   T   
    G    
   C   
   C   
   T   
   T   
   A   
   T   
   A   
   T   
   G   
   G   
   T   
    C    
   A   
   A   
   T   
   A   
   A   
   A   
   G   
   C   
    A    
   A   
   A   
   A   
   T   
   T   
    G    
   A   
   T   
    C    
   G   
   A   
   A   
   C   
   G   
    G    
   A   
   T   
   T   
   T   
   C   
   T   
   G   
   A   
    A    
    C    
   T   
    G    
   C   
   A   
   C   
   A   
   G   
   A   
   G   
   A   
    C    
   A   
   A   
    G    
   C   
   G   
    T    
   A   
   T   
    C    
   G   
   A   
   A   
   G   
   G   
    G    
   A   
   T   
    C    
   A   
   C   
    A    
   G   
   A   
   T   
   T   
   T   
   G   
   C   
   G   
   G   
   G   
   A   
    C    
   G   
   A   
   A   
   T   
   C   
   T   
   T   
   C   
    C    
   C   
   G   
   T   
   G   
   A   
    A    
   G   
   G   
    T    
   A   
   T   
   G   
   C   
   G   
    G    
   A   
   T   
    C    
   G   
   T   
   C   
   A   
   T   
    T    
   G   
   A   
    C    
    A    
   T   
    C    
   C   
   G   
   T   
   C   
   G   
    T    
   G   
   A   
    C    
   G   
    C    
    C    
   A   
   G   
    C    
   G   
   C   
    A    
   T   
   C   
    T    
   G   
   T   
   T   
    A    
   T   
    C    
   T   
   T   
    G    
   A   
   A   
    T    
   A   
   A   
   C   
   T   
   T   
    A    
   T   
   A   
    T    
   A   
   A   
   A   
    T    
   T   
   G   
   A   
   C   
    A    
    T    
   C   
    C    
   T   
   T   
    G    
   C   
   A   
    A    
   A   
    A    
    T    
   T   
   C   
    A    
   T   
   T   
    T    
   G   
   G   
    A    
   T   
   T   
    C    
   A   
   A   
    T    
   A   
   T   
   G   
   T   
   T   
    G    
   G   
   C   
    G    
   A   
   T   
    C    
   G   
   A   
   A   
   A   
   A   
   A   
   G   
   G   
    T    
   G   
   T   
    G    
   C   
   C   
    G    
   A   
   A   
   A   
    G    
   T   
   T   
   T   
   T   
    G    
   A   
   G   
    C    
   T   
   T   
   G   
   A   
   A   
   A   
   C   
    A    
    A    
   A   
   T   
    T    
    C    
   T   
    T    
   G   
   A   
   A   
   A   
   A   
    C    
   T   
   A   
    T    
    A    
   T   
    C    
   G   
   A   
    A    
   C   
   A   
    T    
   C   
   A   
    G    
    C    
    G    
    C    
    G    
   A   
   A   
   G   
   T   
   C   
   A   
   T   
    C    
   A   
   C   
    A    
    A    
   G   
    A    
   C   
   G   
   G   
   A   
   C   
    A    
    G    
   T   
    C    
   T   
   T   
   T   
   G   
   A   
    A    
   A   
   A   
   A   
    G    
   A   
    A    
   A   
   A   
    A    
   G   
   C   
   A   
   G   
   A   
   A   
   G   
   C   
   A   
    A    
   G   
    G    
   G   
   C   
    A    
   C   
   A   
    C    
   A   
   T   
    T    
   T   
   T   
   A   
   G   
   A   
   A   
   G   
   G   
   T   
    T    
   T   
    G    
   C   
   G   
    T    
   A   
   T   
   T   
   G   
   C   
    T    
   T   
   T   
    G    
   G   
   A   
   T   
   C   
   A   
   T   
   A   
   T   
   C   
    G    
   A   
   T   
   G   
   A   
   A   
   A   
   T   
    C    
   A   
   T   
   T   
   G   
   C   
    G    
   A   
   T   
   T   
   A   
   T   
    C    
   C   
   G   
    C    
   G   
   G   
    G    
   T   
   C   
    T    
    A    
   A   
   A   
   T   
   C   
   A   
   G   
   A   
   T   
   G   
   A   
    C    
   G   
   A   
    A    
   G   
   C   
   A   
   A   
   A   
   A   
   G   
   C   
    G    
   A   
    C    
    G    
    A    
   T   
    G    
   A   
   T   
    C    
   G   
   A   
   A   
   C   
   G   
    G    
   T   
   T   
    C    
   G   
   A   
    T    
   T   
   T   
    A    
   T   
   C   
    C    
   G   
   A   
   T   
   C   
   G   
    C    
   C   
   A   
    G    
    T    
   C   
    T    
   C   
   A   
   A   
   G   
   C   
    T    
   A   
   T   
    T    
   T   
   T   
   A   
   G   
   A   
   T   
   A   
   T   
   G   
   C   
   G   
   T   
   T   
   T   
    A    
   C   
   G   
    C    
   C   
   G   
    A    
   T   
   T   
    G    
   A   
   C   
    C    
   G   
   G   
    T    
   T   
   T   
    G    
   G   
   A   
   A   
   C   
   G   
    G    
   G   
   A   
    A    
   A   
   A   
   A   
   A   
   T   
    C    
   G   
   A   
    A    
   A   
   A   
   T   
   G   
   A   
   A   
   T   
   A   
    C    
   C   
   A   
   A   
   G   
   A   
    A    
   C   
   T   
    C    
   T   
   T   
    A    
   A   
    A    
   A   
    C    
   T   
    G    
   A   
   T   
   T   
   G   
    A    
    A    
   G   
   A   
    T    
   T   
   T   
    A    
    A    
    C    
    T    
   G   
   A   
   C   
   A   
   T   
   T   
    C    
   T   
    G    
   G   
   C   
    A    
    A    
   G   
    A    
   C   
   C   
    A    
   G   
   A   
    A    
   C   
   G   
    T    
   G   
   T   
    T    
   A   
    C    
    A    
   G   
   A   
   A   
   A   
   T   
    C    
   A   
   T   
    C    
   A   
   A   
    G    
   A   
    A    
    T    
   G   
   A   
    A    
   T   
   T   
   A   
    G    
    C    
    A    
   G   
   A   
   C   
    T    
   T   
    G    
    A    
    A    
    T    
    C    
    A    
   A   
    C    
    G    
   A   
   T   
   T   
   T   
   G   
   G   
   T   
   G   
   A   
   T   
    A    
    A    
   A   
   C   
   G   
   A   
   C   
   G   
    C    
   A   
   C   
   A   
   G   
   A   
   A   
    T    
   T   
   A   
    C    
   T   
   A   
   G   
   T   
    C    
   G   
   G   
   T   
   G   
   A   
    G    
   G   
   T   
   C   
    T    
   T   
    A    
   A   
   G   
   T   
   C   
   T   
    G    
   G   
   A   
   A   
   G   
   A   
   T   
   G   
   A   
   A   
   G   
   A   
   T   
    T    
   T   
    A    
   A   
   T   
    C    
   G   
   A   
   A   
   G   
   A   
   A   
   G   
   A   
   A   
   G   
   A   
   A   
    A    
   T   
   C   
   G   
   T   
    T    
   A   
   T   
   T   
   A   
   C   
    A    
    T    
   T   
    G    
   A   
   C   
    A    
   A   
   A   
    C    
   A   
   A   
    T    
   G   
   G   
    T    
   T   
   A   
    C    
   A   
   T   
    C    
   A   
   A   
    A    
   C   
   G   
    T    
    T    
   T   
   G   
   G   
    C    
   A   
   A   
   A   
    T    
    A    
    A    
    T    
   G   
   A   
   A   
   T   
   T   
   C   
    A    
   G   
    A    
   G   
   C   
    T    
   C   
   A   
   A   
   C   
   G   
    A    
    A    
   G   
    G    
   G   
   G   
    T    
   G   
   G   
    A    
   C   
   G   
   T   
   G   
   G   
    T    
   G   
   T   
    C    
   C   
   A   
   A   
   G   
   G   
    A    
   A   
   T   
   G   
   G   
   G   
    T    
    A    
   T   
    C    
   C   
   A   
    C    
    A    
   A   
   T   
   G   
   A   
    C    
   G   
   A   
    C    
   T   
   T   
   C   
   G   
   T   
    C    
   A   
   A   
    A    
   A   
   A   
    T    
   C   
   T   
    G    
    G    
   T   
   T   
   T   
   C   
   T   
   T   
   G   
    T    
   T   
   C   
    T    
   A   
   C   
    C    
   C   
   A   
    T    
   G   
   A   
    T    
   A   
   C   
    A    
   T   
   T   
    G    
   T   
   T   
   A   
   T   
   T   
    C    
   T   
   T   
   T   
   A   
   C   
   G   
   A   
   A   
    T    
   A   
    A    
    C    
   G   
   G   
    A    
   A   
   A   
   A   
   G   
   T   
    G    
   T   
   A   
    T    
   C   
   G   
    G    
   G   
   C   
   A   
   A   
   A   
   A   
   G   
   G   
    A    
   T   
   A   
    C    
   G   
   A   
   A   
   A   
   T   
    T    
   C   
   C   
    A    
   G   
   A   
    A    
   T   
   A   
   C   
   G   
   G   
    G    
    C    
   G   
    C    
   A   
   C   
   A   
   G   
   C   
    C    
   A   
   A   
   A   
   G   
   G   
    A    
   A   
   T   
    A    
   C   
   C   
   A   
    A    
   T   
    T    
   A   
   T   
    C    
   A   
   A   
    T    
   T   
   T   
    A    
   C   
   T   
    A    
   G   
   G   
    T    
   A   
   T   
    C    
   G   
   A   
    T    
   T   
   C   
    G    
    A    
    G    
    T    
   G   
   A   
   A   
   A   
    G    
    C    
   A   
   T   
    C    
   C   
   A   
    G    
   G   
   C   
    A    
   A   
   T   
    C    
   A   
   T   
   T   
    G    
   C   
    G    
   G   
   T   
    G    
    A    
    C    
   G   
   G   
   G   
    A    
    G    
   A   
   A   
   G   
   C   
    A    
   G   
   A   
   A   
   G   
    A    
    A    
   G   
   G   
    C    
    C    
   A   
    C    
   T   
   A   
    T    
    C    
   T   
    C    
   T   
   T   
   C   
   T   
   T   
    T    
   A   
   C   
    G    
   A   
   C   
    G    
    C    
    G    
    T    
    C    
   A   
   A   
   G   
   G   
    T    
   A   
   C   
    A    
   G   
   T   
    A    
   A   
   A   
   A   
   C   
   G   
    T    
   A   
   C   
    T    
    T    
   C   
    T    
   G   
   T   
    C    
   A   
   C   
    T    
   G   
   C   
    T    
   T   
   T   
    T    
    G    
   C   
   T   
   A   
   A   
    C    
   A   
   T   
    C    
   C   
   G   
    A    
   A   
   G   
    T    
   A   
   A   
   T   
   G   
   G   
    T    
    C    
   T   
    G    
   A   
   T   
    C    
   G   
   C   
    A    
   A   
   T   
   T   
    G    
   G   
    T    
   T   
   T   
    G    
   A   
   A   
   A   
   G   
   A   
   A   
   G   
   A   
    C    
   G   
   A   
    T    
   G   
   A   
    G    
    C    
   T   
    C    
    G    
   T   
    C    
   A   
   A   
    T    
   G   
   T   
    G    
    C    
   T   
   A   
    C    
    T    
   G   
   A   
   C   
    A    
    G    
   A   
    T    
   G   
   G   
    T    
    C    
   A   
    A    
    T    
    C    
    T    
   A   
   A   
    T    
   A   
   T   
    C    
   A   
   T   
    T    
   A   
   T   
    T    
   G   
   G   
    A    
   A   
   C   
   A   
   C   
   A   
   T   
    A    
    A    
    C    
   G   
   G   
    C    
   T   
   A   
   C   
   T   
   C   
   T   
   G   
   T   
    C    
   A   
   C   
    T    
   T   
   T   
    C    
    A    
   A   
    A    
   G   
   A   
    A    
    G    
    A    
    A    
    G    
   C   
    A    
   G   
    T    
    C    
   C   
   G   
    C    
   G   
   A   
   T   
   A   
   T   
   G   
   G   
   G   
    A    
   C   
   G   
    T    
   A   
   C   
   A   
   G   
   C   
    T    
   T   
   C   
    C    
   G   
   G   
    G    
   G   
   T   
    T    
    A    
   G   
    A    
   G   
   G   
    A    
   A   
   T   
    A    
   C   
   G   
    C    
    C    
   T   
    G    
   C   
   G   
    T    
   G   
   A   
    G    
    G    
   A   
    A    
   G   
   A   
    T    
   T   
    A    
    T    
   G   
   T   
    C    
   G   
   T   
    T    
   G   
   G   
    G    
    G    
   C   
   A   
   G   
   C   
    C    
    T    
   T   
    A    
    A    
   T   
   G   
    A    
   A   
    G    
   G   
    A    
   A   
    G    
   A   
    T    
    C    
   A   
   A   
   G   
   A   
   A   
   G   
   T   
    A    
    T    
   T   
    A    
   G   
   T   
    G    
   A   
   T   
    C    
   A   
   C   
    A    
   G   
   A   
   A   
   A   
   A   
    A    
   G   
   G   
   T   
   T   
   A   
    C    
   G   
   G   
    A    
   A   
   A   
    A    
   C   
   G   
    G    
   A   
   C   
    A    
   A   
   A   
   A   
   G   
    T    
    A    
   T   
   C   
    G    
   G   
   A   
    A    
   T   
   A   
   T   
   C   
   C   
   A   
   G   
   T   
    G    
   A   
   A   
    A    
   G   
   G   
    C    
   C   
   G   
    C    
   G   
   G   
    A    
   G   
   G   
   T   
   A   
   A   
   A   
   G   
   G   
    G    
   A   
   T   
    C    
   A   
   A   
    A    
   A   
   C   
    G    
    G    
   C   
    G    
   A   
   A   
    C    
   A   
   T   
   C   
   A   
   C   
   T   
   G   
   A   
    A    
   A   
   A   
   A   
   A   
   A   
   T   
   G   
   G   
   T   
   C   
   C   
   A   
    C    
   T   
   A   
   G   
   C   
    T    
   G   
   G   
    A    
   T   
   T   
    G    
   A   
   C   
   C   
   A   
   C   
    T    
   G   
   T   
    T    
    T    
    C    
   T   
   G   
   G   
    A    
    G    
   A   
   T   
   G   
   A   
   A   
   G   
   A   
    C    
   A   
   T   
   C   
    A    
   T   
    G    
    C    
   T   
    G    
   A   
   T   
    T    
   A   
   C   
    A    
    G    
   A   
    T    
   A   
   A   
   A   
   G   
   G   
    T    
   G   
   T   
    C    
   A   
   T   
   T   
   A   
   T   
   C   
   C   
   G   
    T    
   T   
   T   
    C    
   A   
   A   
   C   
   G   
   T   
    A    
    T    
    C    
    G    
    A    
   C   
   T   
   G   
   T   
   T   
   T   
   C   
   T   
   C   
   A   
   A   
   A   
   C   
    T    
   G   
   G   
   A   
   C   
   G   
    T    
    T    
   C   
   A   
   A   
   C   
   A   
   T   
   T   
    A    
   G   
   G   
    C    
   G   
   T   
    A    
   C   
   G   
   T   
   T   
   T   
    G    
   A   
   T   
   G   
   A   
    A    
    G    
   A   
   T   
   G   
   G   
   A   
   A   
   G   
    C    
    T    
   G   
    A    
    T    
    A    
   C   
    G    
   A   
   A   
   A   
   G   
   T   
   T   
   G   
   T   
   C   
   A   
   C   
   G   
   A   
   T   
   G   
   G   
   C   
   A   
   G   
   C   
   A   
   G   
   T   
   C   
   G   
   A   
   A   
   C   
   C   
   A   
   G   
   A   
   A   
   G   
   C   
   T   
   G   
   C   
   A   
   G   
   A   
   A   
   C   
   A   
   A   
   A   
   C   
   A   
   G   
   T   
   T   
   G   
   A   
   A   
   G   
   A   
   A   
   C   
   A   
   A   
   G   
   G   
   A   
   T   
   C   
   A   
   G   
   A   
   A   
   G   
   A   
   A   
   -   
   -   
   -   
 

 
   N60061F   
   A   
   T   
   G   
   A   
   G   
   T   
   G   
   A   
   A   
   G   
   A   
   A   
   A   
   T   
   C   
   A   
   G   
   A   
   G   
   A   
   A   
   A   
   A   
   C   
   A   
   T   
   C   
   C   
   A   
   T   
   G   
   A   
   T   
   G   
   T   
   C   
   A   
   A   
   T   
   C   
   T   
   A   
   A   
   C   
   C   
   A   
   G   
   C   
   G   
   A   
   A   
   A   
   T   
   G   
   A   
   A   
   A   
   G   
   A   
   C   
   T   
   C   
   C   
   T   
   T   
   C   
   A   
   T   
   C   
   G   
   A   
   T   
   T   
   A   
   T   
   G   
   C   
   G   
   A   
   T   
   G   
   A   
   G   
   T   
   G   
   T   
   T   
   A   
   T   
   T   
   G   
   T   
   C   
   G   
   C   
   A   
   C   
   G   
   T   
   G   
   C   
   A   
   C   
   T   
   T   
   C   
   C   
   T   
   G   
   A   
   T   
   G   
   T   
   C   
   A   
   G   
   A   
   G   
   A   
   T   
   G   
   G   
   T   
   C   
   T   
   G   
   A   
   A   
   A   
   C   
   C   
   C   
   G   
   T   
   T   
   C   
   A   
   T   
   C   
   G   
   C   
   C   
   G   
   T   
   A   
   T   
   T   
   T   
   T   
   A   
   T   
   A   
   C   
   G   
   G   
   C   
   A   
   T   
   G   
   A   
   A   
   C   
   G   
   A   
   A   
   T   
   T   
   A   
   G   
   G   
   T   
   G   
   T   
   A   
   A   
   C   
   A   
   C   
   C   
   G   
   G   
   A   
   T   
   A   
   A   
   A   
   G   
   C   
   C   
   C   
   A   
   T   
   A   
   A   
   A   
   A   
   A   
   A   
   T   
   C   
   T   
   G   
   C   
   C   
   A   
   G   
   A   
   A   
   T   
   C   
   G   
   T   
   C   
   G   
   G   
   A   
   G   
   A   
   T   
   G   
   T   
   C   
   A   
   T   
   G   
   G   
   G   
   T   
   A   
   A   
   A   
   T   
   A   
   C   
   C   
   A   
   T   
   C   
   C   
   T   
   C   
   A   
   T   
   G   
   G   
   A   
   G   
   A   
   C   
   A   
   G   
   T   
   G   
   C   
   G   
   A   
   T   
   C   
   T   
   A   
   T   
   G   
   A   
   G   
   T   
   C   
   A   
   A   
   T   
   G   
   G   
   T   
   A   
   C   
   G   
   G   
   A   
   T   
   G   
   G   
   C   
   A   
   C   
   A   
   G   
   C   
   C   
   A   
   T   
   T   
   C   
   A   
   G   
   C   
   T   
   A   
   C   
   C   
   G   
   T   
   T   
   A   
   T   
   A   
   T   
   G   
   C   
   T   
   A   
   G   
   T   
   G   
   G   
   A   
   T   
   G   
   G   
   C   
   C   
   A   
   T   
   G   
   G   
   A   
   A   
   A   
   C   
   T   
   T   
   T   
   G   
   G   
   T   
   T   
   C   
   T   
   G   
   T   
   T   
   G   
   A   
   C   
   G   
   G   
   T   
   G   
   A   
   C   
   G   
   G   
   T   
   G   
   C   
   C   
   G   
   C   
   T   
   G   
   C   
   T   
   A   
   T   
   G   
   C   
   G   
   T   
   T   
   A   
   T   
   A   
   C   
   C   
   G   
   A   
   A   
   G   
   C   
   A   
   C   
   G   
   G   
   A   
   T   
   G   
   A   
   G   
   T   
   A   
   A   
   A   
   A   
   T   
   C   
   G   
   C   
   T   
   A   
   C   
   A   
   G   
   A   
   A   
   A   
   T   
   G   
   C   
   T   
   G   
   C   
   G   
   T   
   G   
   A   
   T   
   A   
   T   
   C   
   A   
   A   
   T   
   A   
   A   
   G   
   A   
   A   
   C   
   A   
   C   
   T   
   G   
   T   
   T   
   G   
   A   
   T   
   T   
   T   
   C   
   C   
   A   
   A   
   A   
   G   
   C   
   A   
   A   
   C   
   T   
   A   
   T   
   G   
   A   
   T   
   G   
   A   
   T   
   A   
   C   
   A   
   G   
   A   
   A   
   A   
   A   
   A   
   G   
   A   
   A   
   C   
   C   
   A   
   G   
   T   
   T   
   G   
   T   
   A   
   C   
   T   
   A   
   C   
   C   
   T   
   G   
   C   
   T   
   C   
   G   
   C   
   T   
   T   
   C   
   C   
   C   
   G   
   A   
   A   
   T   
   C   
   T   
   T   
   T   
   T   
   A   
   G   
   T   
   G   
   A   
   A   
   C   
   G   
   G   
   A   
   A   
   C   
   A   
   A   
   C   
   G   
   G   
   G   
   G   
   A   
   T   
   C   
   G   
   C   
   C   
   G   
   T   
   A   
   G   
   G   
   T   
   A   
   T   
   G   
   G   
   C   
   C   
   A   
   C   
   G   
   A   
   A   
   T   
   A   
   T   
   C   
   C   
   C   
   A   
   C   
   C   
   T   
   C   
   A   
   T   
   A   
   A   
   C   
   T   
   T   
   G   
   T   
   C   
   T   
   G   
   A   
   G   
   G   
   T   
   A   
   G   
   T   
   A   
   G   
   C   
   G   
   G   
   C   
   T   
   A   
   T   
   C   
   G   
   A   
   T   
   T   
   T   
   G   
   C   
   T   
   G   
   A   
   T   
   G   
   G   
   A   
   T   
   A   
   A   
   T   
   C   
   C   
   T   
   G   
   A   
   T   
   G   
   T   
   G   
   A   
   C   
   G   
   A   
   C   
   C   
   A   
   A   
   T   
   G   
   A   
   A   
   T   
   T   
   G   
   A   
   T   
   G   
   G   
   A   
   A   
   G   
   T   
   A   
   T   
   T   
   A   
   C   
   C   
   T   
   G   
   G   
   A   
   C   
   C   
   A   
   G   
   A   
   T   
   T   
   T   
   T   
   C   
   C   
   A   
   A   
   C   
   A   
   G   
   G   
   T   
   G   
   G   
   T   
   T   
   T   
   G   
   G   
   T   
   T   
   A   
   T   
   G   
   G   
   G   
   A   
   A   
   A   
   A   
   T   
   C   
   A   
   G   
   G   
    A    
   A   
   T   
    C    
   C   
   G   
    A    
    A    
   G   
   A   
   G   
   C   
    G    
   T   
   A   
   T   
   G   
   A   
   A   
   A   
   C   
    A    
   G   
   G   
    T    
   A   
   A   
    G    
   G   
   G   
   T   
   T   
   C   
    T    
   A   
   T   
   T   
   A   
   C   
    C    
   G   
   T   
    A    
   C   
   G   
   T   
   G   
   C   
   A   
   A   
   A   
   A   
   G   
   T   
   T   
   G   
   A   
    G    
    A    
   T   
    T    
   A   
    C    
   T   
   G   
   A   
    G    
   A   
   T   
   G   
    C    
   C   
    A    
   A   
   A   
   T   
   G   
   G   
    T    
   A   
   A   
   A   
   G   
   A   
   A   
   C   
   G   
    G    
   A   
   T   
    C    
    A    
   T   
    C    
   G   
   T   
    G    
   A   
   C   
    A    
   G   
   A   
   A   
   T   
   T   
    G    
   C   
   C   
   T   
   T   
   A   
   T   
   A   
   T   
   G   
   G   
   T   
    C    
   A   
   A   
   T   
   A   
   A   
   A   
   G   
   C   
    A    
   A   
   A   
   A   
   T   
   T   
    G    
   A   
   T   
    C    
   G   
   A   
   A   
   C   
   G   
    G    
   A   
   T   
   T   
   T   
   C   
   T   
   G   
   A   
    A    
    C    
   T   
    G    
   C   
   A   
   C   
   A   
   G   
   A   
   G   
   A   
    C    
   A   
   A   
    G    
   C   
   G   
    T    
   A   
   T   
    C    
   G   
   A   
   A   
   G   
   G   
    G    
   A   
   T   
    C    
   A   
   C   
    A    
   G   
   A   
   T   
   T   
   T   
   G   
   C   
   G   
   G   
   G   
   A   
    C    
   G   
   A   
   A   
   T   
   C   
   T   
   T   
   C   
    C    
   C   
   G   
   T   
   G   
   A   
    A    
   G   
   G   
    T    
   A   
   T   
   G   
   C   
   G   
    G    
   A   
   T   
    C    
   G   
   T   
   C   
   A   
   T   
    T    
   G   
   A   
    C    
    A    
   T   
    C    
   C   
   G   
   T   
   C   
   G   
    T    
   G   
   A   
    C    
   G   
    C    
    C    
   A   
   G   
    C    
   G   
   C   
    A    
   T   
   C   
    C    
   G   
   T   
   T   
    A    
   T   
    C    
   T   
   T   
    G    
   A   
   A   
    T    
   A   
   A   
   C   
   T   
   T   
    A    
   T   
   A   
    T    
   A   
   A   
   A   
    T    
   T   
   G   
   A   
   C   
    A    
    T    
   C   
    C    
   T   
   T   
    G    
   C   
   A   
    A    
   A   
    A    
    T    
   T   
   C   
    A    
   T   
   T   
    T    
   G   
   G   
    A    
   T   
   T   
    C    
   A   
   A   
    T    
   A   
   T   
   G   
   T   
   T   
    G    
   G   
   C   
    G    
   A   
   T   
    C    
   G   
   A   
   A   
   A   
   A   
   A   
   G   
   G   
    T    
   G   
   T   
    G    
   C   
   C   
    G    
   A   
   A   
   A   
    G    
   T   
   T   
   T   
   T   
    G    
   A   
   G   
    C    
   T   
   T   
   G   
   A   
   A   
   A   
   C   
    A    
    A    
   A   
   T   
    T    
    C    
   T   
    T    
   G   
   A   
   A   
   A   
   A   
    C    
   T   
   A   
    T    
    A    
   T   
    C    
   G   
   A   
    A    
   C   
   A   
    T    
   C   
   A   
    G    
    C    
    G    
    C    
    G    
   A   
   A   
   G   
   T   
   C   
   A   
   T   
    C    
   A   
   C   
    A    
    A    
   G   
    A    
   C   
   G   
   G   
   A   
   C   
    A    
    G    
   T   
    C    
   T   
   T   
   T   
   G   
   A   
    A    
   A   
   A   
   A   
    G    
   A   
    A    
   A   
   A   
    A    
   G   
   C   
   A   
   G   
   A   
   A   
   G   
   C   
   A   
    A    
   G   
    G    
   G   
   C   
    A    
   C   
   A   
    C    
   A   
   T   
    T    
   T   
   T   
   A   
   G   
   A   
   A   
   G   
   G   
   T   
    T    
   T   
    G    
   C   
   G   
    T    
   A   
   T   
   T   
   G   
   C   
    T    
   T   
   T   
    G    
   G   
   A   
   T   
   C   
   A   
   T   
   A   
   T   
   C   
    G    
   A   
   T   
   G   
   A   
   A   
   A   
   T   
    A    
   A   
   T   
   T   
   G   
   C   
    G    
   A   
   T   
   T   
   A   
   T   
    C    
   C   
   G   
    C    
   G   
   G   
    G    
   T   
   C   
    T    
    A    
   A   
   A   
   T   
   C   
   A   
   G   
   A   
   T   
   G   
   A   
    C    
   G   
   A   
    A    
   G   
   C   
   A   
   A   
   A   
   A   
   G   
   C   
    G    
   A   
    C    
    G    
    A    
   T   
    G    
   A   
   T   
    C    
   G   
   A   
   A   
   C   
   G   
    G    
   T   
   T   
    C    
   G   
   A   
    T    
   T   
   T   
    A    
   T   
   C   
    C    
   G   
   A   
   T   
   C   
   G   
    C    
   C   
   A   
    G    
    T    
   C   
    T    
   C   
   A   
   A   
   G   
   C   
    T    
   A   
   T   
    T    
   T   
   T   
   A   
   G   
   A   
   T   
   A   
   T   
   G   
   C   
   G   
   T   
   T   
   T   
    A    
   C   
   G   
    C    
   C   
   G   
    A    
   T   
   T   
    G    
   A   
   C   
    C    
   G   
   G   
    T    
   T   
   T   
    G    
   G   
   A   
   A   
   C   
   G   
    G    
   G   
   A   
    A    
   A   
   A   
   A   
   A   
   T   
    C    
   G   
   A   
    A    
   A   
   A   
   T   
   G   
   A   
   A   
   T   
   A   
    C    
   C   
   A   
   A   
   G   
   A   
    A    
   C   
   T   
    C    
   T   
   T   
    A    
   A   
    A    
   A   
    C    
   T   
    G    
   A   
   T   
   T   
   G   
    A    
    A    
   G   
   A   
    T    
   T   
   T   
    A    
    A    
    C    
    T    
   G   
   A   
   C   
   A   
   T   
   T   
    C    
   T   
    G    
   G   
   C   
    A    
    A    
   G   
    A    
   C   
   C   
    A    
   G   
   A   
    A    
   C   
   G   
    T    
   G   
   T   
    T    
   A   
    C    
    A    
   G   
   A   
   A   
   A   
   T   
    C    
   A   
   T   
    C    
   A   
   A   
    G    
   A   
    A    
    T    
   G   
   A   
    A    
   T   
   T   
   A   
    G    
    C    
    A    
   G   
   A   
   C   
    T    
   T   
    A    
    A    
    A    
    T    
    C    
    A    
   A   
    C    
    G    
   A   
   T   
   T   
   T   
   G   
   G   
   T   
   G   
   A   
   T   
    A    
    A    
   A   
   C   
   G   
   A   
   C   
   G   
    C    
   A   
   C   
   A   
   G   
   A   
   A   
    T    
   T   
   A   
    C    
   T   
   A   
   G   
   T   
    C    
   G   
   G   
   T   
   G   
   A   
    G    
   G   
   T   
   C   
    T    
   T   
    A    
   A   
   G   
   T   
   C   
   T   
    G    
   G   
   A   
   A   
   G   
   A   
   T   
   G   
   A   
   A   
   G   
   A   
   T   
    T    
   T   
    A    
   A   
   T   
    C    
   G   
   A   
   A   
   G   
   A   
   A   
   G   
   A   
   A   
   G   
   A   
   A   
    A    
   T   
   C   
   G   
   T   
    T    
   A   
   T   
   T   
   A   
   C   
    A    
    T    
   T   
    G    
   A   
   C   
    A    
   A   
   A   
    C    
   A   
   A   
    T    
   G   
   G   
    T    
   T   
   A   
    C    
   A   
   T   
    C    
   A   
   A   
    A    
   C   
   G   
    T    
    T    
   T   
   G   
   G   
    C    
   A   
   A   
   A   
    T    
    A    
    A    
    T    
   G   
   A   
   A   
   T   
   T   
   C   
    A    
   G   
    A    
   G   
   C   
    T    
   C   
   A   
   A   
   C   
   G   
    A    
    A    
   G   
    G    
   G   
   G   
    T    
   G   
   G   
    A    
   C   
   G   
   T   
   G   
   G   
    T    
   G   
   T   
    C    
   C   
   A   
   A   
   G   
   G   
    A    
   A   
   T   
   G   
   G   
   G   
    T    
    A    
   T   
    C    
   C   
   A   
    C    
    A    
   A   
   T   
   G   
   A   
    C    
   G   
   A   
    C    
   T   
   T   
   C   
   G   
   T   
    C    
   A   
   A   
    A    
   A   
   A   
    T    
   C   
   T   
    G    
    G    
   T   
   T   
   T   
   C   
   T   
   T   
   G   
    C    
   T   
   C   
    T    
   A   
   C   
    C    
   C   
   A   
    T    
   G   
   A   
    T    
   A   
   C   
    A    
   T   
   T   
    G    
   T   
   T   
   A   
   T   
   T   
    C    
   T   
   T   
   T   
   A   
   C   
   G   
   A   
   A   
    T    
   A   
    A    
    C    
   G   
   G   
    A    
   A   
   A   
   A   
   G   
   T   
    G    
   T   
   A   
    T    
   C   
   G   
    G    
   G   
   C   
   A   
   A   
   A   
   A   
   G   
   G   
    A    
   T   
   A   
    C    
   G   
   A   
   A   
   A   
   T   
    T    
   C   
   C   
    A    
   G   
   A   
    A    
   T   
   A   
   C   
   G   
   G   
    G    
    C    
   G   
    C    
   A   
   C   
   A   
   G   
   C   
    C    
   A   
   A   
   A   
   G   
   G   
    A    
   A   
   T   
    A    
   C   
   C   
   A   
    A    
   T   
    T    
   A   
   T   
    C    
   A   
   A   
    T    
   T   
   T   
    A    
   C   
   T   
    A    
   G   
   G   
    T    
   A   
   T   
    C    
   G   
   A   
    T    
   T   
   C   
    G    
    A    
    G    
    T    
   G   
   A   
   A   
   A   
    G    
    C    
   A   
   T   
    C    
   C   
   A   
    G    
   G   
   C   
    A    
   A   
   T   
    C    
   A   
   T   
   T   
    G    
   C   
    G    
   G   
   T   
    G    
    A    
    C    
   G   
   G   
   G   
    A    
    G    
   A   
   A   
   G   
   C   
    A    
   G   
   A   
   A   
   G   
    A    
    A    
   G   
   G   
    C    
    C    
   A   
    C    
   T   
   A   
    T    
    C    
   T   
    C    
   T   
   T   
   C   
   T   
   T   
    T    
   A   
   C   
    G    
   A   
   C   
    G    
    C    
    G    
    T    
    C    
   A   
   A   
   G   
   G   
    T    
   A   
   C   
    A    
   G   
   T   
    A    
   A   
   A   
   A   
   C   
   G   
    T    
   A   
   C   
    T    
    T    
   C   
    T    
   G   
   T   
    C    
   A   
   C   
    T    
   G   
   C   
    T    
   T   
   T   
    T    
    G    
   C   
   T   
   A   
   A   
    C    
   A   
   T   
    C    
   C   
   G   
    A    
   A   
   G   
    T    
   A   
   A   
   T   
   G   
   G   
    T    
    C    
   T   
    G    
   A   
   T   
    C    
   G   
   C   
    A    
   A   
   T   
   T   
    G    
   G   
    T    
   T   
   T   
    G    
   A   
   A   
   A   
   G   
   A   
   A   
   G   
   A   
    C    
   G   
   A   
    T    
   G   
   A   
    G    
    C    
   T   
    C    
    G    
   T   
    C    
   A   
   A   
    T    
   G   
   T   
    G    
    C    
   T   
   A   
    C    
    T    
   G   
   A   
   C   
    A    
    G    
   A   
    T    
   G   
   G   
    T    
    C    
   A   
    A    
    T    
    C    
    T    
   A   
   A   
    T    
   A   
   T   
    C    
   A   
   T   
    T    
   A   
   T   
    T    
   G   
   G   
    A    
   A   
   C   
   A   
   C   
   A   
   T   
    A    
    A    
    C    
   G   
   G   
    C    
   T   
   A   
   C   
   T   
   C   
   T   
   G   
   T   
    C    
   A   
   C   
    T    
   T   
   T   
    C    
    A    
   A   
    A    
   G   
   A   
    A    
    G    
    A    
    A    
    G    
   C   
    A    
   G   
    T    
    C    
   C   
   G   
    C    
   G   
   A   
   T   
   A   
   T   
   G   
   G   
   G   
    A    
   C   
   G   
    T    
   A   
   C   
   A   
   G   
   C   
    T    
   T   
   C   
    C    
   G   
   G   
    G    
   G   
   T   
    C    
    A    
   G   
    A    
   G   
   G   
    A    
   A   
   T   
    A    
   C   
   G   
    C    
    C    
   T   
    G    
   C   
   G   
    T    
   G   
   A   
    G    
    G    
   A   
    A    
   G   
   A   
    T    
   T   
    A    
    T    
   G   
   T   
    C    
   G   
   T   
    T    
   G   
   G   
    G    
    G    
   C   
   A   
   G   
   C   
    C    
    T    
   T   
    A    
    A    
   T   
   G   
    A    
   A   
    G    
   G   
    A    
   A   
    G    
   A   
    T    
    C    
   A   
   A   
   G   
   A   
   A   
   G   
   T   
    A    
    T    
   T   
    A    
   G   
   T   
    G    
   A   
   T   
    C    
   A   
   C   
    A    
   G   
   A   
   A   
   A   
   A   
    A    
   G   
   G   
   T   
   T   
   A   
    C    
   G   
   G   
    A    
   A   
   A   
    A    
   C   
   G   
    G    
   A   
   C   
    A    
   A   
   A   
   A   
   G   
    T    
    A    
   T   
   C   
    G    
   G   
   A   
    A    
   T   
   A   
   T   
   C   
   C   
   A   
   G   
   T   
    G    
   A   
   A   
    G    
   G   
   G   
    C    
   C   
   G   
    C    
   G   
   G   
    A    
   G   
   G   
   T   
   A   
   A   
   A   
   G   
   G   
    G    
   A   
   T   
    C    
   A   
   A   
    A    
   A   
   C   
    G    
    G    
   C   
    G    
   A   
   A   
    C    
   A   
   T   
   C   
   A   
   C   
   T   
   G   
   A   
    A    
   A   
   A   
   A   
   A   
   A   
   T   
   G   
   G   
   T   
   C   
   C   
   A   
    C    
   T   
   A   
   G   
   C   
    T    
   G   
   G   
    A    
   T   
   T   
    G    
   A   
   C   
   C   
   A   
   C   
    T    
   G   
   T   
    T    
    T    
    C    
   T   
   G   
   G   
    A    
    G    
   A   
   T   
   G   
   A   
   A   
   G   
   A   
    C    
   A   
   T   
   C   
    A    
   T   
    G    
    C    
   T   
    G    
   A   
   T   
    T    
   A   
   C   
    A    
    G    
   A   
    T    
   A   
   A   
   A   
   G   
   G   
    T    
   G   
   T   
    C    
   A   
   T   
   T   
   A   
   T   
   C   
   C   
   G   
    T    
   T   
   T   
    C    
   A   
   A   
   C   
   G   
   T   
    A    
    T    
    C    
    G    
    A    
   C   
   T   
   G   
   T   
   T   
   T   
   C   
   T   
   C   
   A   
   A   
   A   
   C   
    T    
   G   
   G   
   A   
   C   
   G   
    T    
    T    
   C   
   A   
   A   
   C   
   A   
   T   
   T   
    A    
   G   
   G   
    C    
   G   
   T   
    A    
   C   
   G   
   T   
   T   
   T   
    G    
   A   
   T   
   G   
   A   
    A    
    G    
   A   
   T   
   G   
   G   
   A   
   A   
   G   
    C    
    T    
   G   
    A    
    T    
    A    
   C   
    G    
   A   
   A   
   A   
   G   
   T   
   T   
   G   
   T   
   C   
   A   
   C   
   G   
   A   
   T   
   G   
   G   
   C   
   A   
   G   
   C   
   A   
   G   
   T   
   C   
   G   
   A   
   A   
   C   
   C   
   A   
   G   
   A   
   A   
   G   
   C   
   T   
   G   
   C   
   A   
   G   
   A   
   A   
   C   
   A   
   A   
   A   
   C   
   A   
   G   
   T   
   T   
   G   
   A   
   A   
   G   
   A   
   A   
   C   
   A   
   A   
   G   
   G   
   A   
   T   
   C   
   A   
   G   
   A   
   A   
   G   
   A   
   A   
   -   
   -   
   -   
 

 
   N59653F   
   A   
   T   
   G   
   A   
   G   
   T   
   G   
   A   
   A   
   G   
   A   
   A   
   A   
   T   
   C   
   A   
   G   
   A   
   G   
   A   
   A   
   A   
   A   
   C   
   A   
   T   
   C   
   C   
   A   
   T   
   G   
   A   
   T   
   G   
   T   
   C   
   A   
   A   
   T   
   C   
   T   
   A   
   A   
   C   
   C   
   A   
   G   
   C   
   G   
   A   
   A   
   A   
   T   
   G   
   A   
   A   
   A   
   G   
   A   
   C   
   T   
   C   
   C   
   T   
   T   
   C   
   A   
   T   
   C   
   G   
   A   
   T   
   T   
   A   
   T   
   G   
   C   
   G   
   A   
   T   
   G   
   A   
   G   
   T   
   G   
   T   
   T   
   A   
   T   
   T   
   G   
   T   
   C   
   G   
   C   
   A   
   C   
   G   
   T   
   G   
   C   
   A   
   C   
   T   
   G   
   C   
   C   
   T   
   G   
   A   
   T   
   G   
   T   
   C   
   A   
   G   
   A   
   G   
   A   
   T   
   G   
   G   
   T   
   C   
   T   
   G   
   A   
   A   
   A   
   C   
   C   
   C   
   G   
   T   
   T   
   C   
   A   
   T   
   C   
   G   
   C   
   C   
   G   
   T   
   A   
   T   
   T   
   T   
   T   
   A   
   T   
   A   
   C   
   G   
   G   
   C   
   A   
   T   
   G   
   A   
   A   
   C   
   G   
   A   
   A   
   T   
   T   
   A   
   G   
   G   
   T   
   G   
   T   
   G   
   A   
   C   
   A   
   C   
   C   
   G   
   G   
   A   
   T   
   A   
   A   
   A   
   G   
   C   
   C   
   C   
   A   
   T   
   A   
   A   
   A   
   A   
   A   
   A   
   T   
   C   
   T   
   G   
   C   
   C   
   A   
   G   
   A   
   A   
   T   
   C   
   G   
   T   
   C   
   G   
   G   
   A   
   G   
   A   
   T   
   G   
   T   
   C   
   A   
   T   
   G   
   G   
   G   
   T   
   A   
   A   
   A   
   T   
   A   
   C   
   C   
   A   
   T   
   C   
   C   
   T   
   C   
   A   
   T   
   G   
   G   
   G   
   G   
   A   
   T   
   A   
   G   
   T   
   G   
   C   
   G   
   A   
   T   
   C   
   T   
   A   
   T   
   G   
   A   
   G   
   T   
   C   
   A   
   A   
   T   
   G   
   G   
   T   
   A   
   C   
   G   
   G   
   A   
   T   
   G   
   G   
   C   
   G   
   C   
   A   
   G   
   C   
   C   
   A   
   T   
   T   
   T   
   A   
   G   
   C   
   T   
   A   
   C   
   C   
   G   
   T   
   T   
   A   
   T   
   A   
   T   
   G   
   C   
   T   
   A   
   G   
   T   
   G   
   G   
   A   
   T   
   G   
   G   
   C   
   C   
   A   
   T   
   G   
   G   
   A   
   A   
   A   
   C   
   T   
   T   
   T   
   G   
   G   
   T   
   T   
   C   
   T   
   G   
   T   
   T   
   G   
   A   
   C   
   G   
   G   
   T   
   G   
   A   
   C   
   G   
   G   
   T   
   G   
   C   
   C   
   G   
   C   
   T   
   G   
   C   
   T   
   A   
   T   
   G   
   C   
   G   
   T   
   T   
   A   
   T   
   A   
   C   
   C   
   G   
   A   
   A   
   G   
   C   
   A   
   C   
   G   
   G   
   A   
   T   
   G   
   A   
   G   
   T   
   A   
   A   
   A   
   A   
   T   
   C   
   G   
   C   
   T   
   A   
   C   
   A   
   G   
   A   
   A   
   A   
   T   
   G   
   C   
   T   
   G   
   C   
   G   
   T   
   G   
   A   
   T   
   A   
   T   
   C   
   A   
   A   
   T   
   A   
   A   
   G   
   A   
   A   
   C   
   A   
   C   
   T   
   G   
   T   
   T   
   G   
   A   
   T   
   T   
   T   
   C   
   C   
   A   
   A   
   A   
   G   
   C   
   A   
   A   
   C   
   T   
   A   
   T   
   G   
   A   
   T   
   G   
   A   
   C   
   A   
   C   
   A   
   G   
   A   
   A   
   A   
   A   
   A   
   G   
   A   
   A   
   C   
   C   
   C   
   G   
   T   
   T   
   G   
   T   
   A   
   T   
   T   
   A   
   C   
   C   
   T   
   G   
   C   
   T   
   C   
   G   
   C   
   T   
   T   
   C   
   C   
   C   
   C   
   A   
   A   
   T   
   C   
   T   
   T   
   T   
   T   
   G   
   G   
   T   
   G   
   A   
   A   
   C   
   G   
   G   
   A   
   A   
   C   
   G   
   A   
   C   
   G   
   G   
   G   
   G   
   A   
   T   
   A   
   G   
   C   
   C   
   G   
   T   
   G   
   G   
   G   
   T   
   A   
   T   
   G   
   G   
   C   
   A   
   A   
   C   
   G   
   A   
   A   
   T   
   A   
   T   
   C   
   C   
   C   
   A   
   C   
   C   
   T   
   C   
   A   
   T   
   A   
   A   
   T   
   T   
   T   
   A   
   T   
   C   
   T   
   G   
   A   
   G   
   G   
   T   
   A   
   G   
   T   
   A   
   G   
   C   
   G   
   G   
   C   
   T   
   A   
   T   
   C   
   G   
   A   
   T   
   T   
   T   
   G   
   C   
   T   
   G   
   A   
   T   
   G   
   G   
   A   
   T   
   A   
   A   
   T   
   C   
   C   
   T   
   G   
   A   
   T   
   G   
   T   
   G   
   A   
   C   
   G   
   A   
   C   
   C   
   A   
   A   
   T   
   G   
   A   
   A   
   T   
   T   
   G   
   A   
   T   
   G   
   G   
   A   
   A   
   G   
   T   
   A   
   T   
   T   
   A   
   C   
   C   
   T   
   G   
   G   
   A   
   C   
   C   
   A   
   G   
   A   
   T   
   T   
   T   
   T   
   C   
   C   
   A   
   A   
   C   
   A   
   G   
   G   
   T   
   G   
   G   
   T   
   T   
   T   
   A   
   G   
   T   
   C   
   A   
   T   
   G   
   G   
   G   
   A   
   A   
   A   
   A   
   T   
   C   
   A   
   G   
   G   
    A    
   A   
   T   
    C    
   C   
   G   
    A    
    A    
   G   
   A   
   G   
   C   
    G    
   T   
   A   
   T   
   G   
   A   
   A   
   A   
   C   
    A    
   G   
   G   
    T    
   A   
   A   
    A    
   G   
   G   
   T   
   T   
   C   
    T    
   A   
   T   
   T   
   A   
   C   
    C    
   G   
   T   
    A    
   C   
   G   
   T   
   G   
   C   
   A   
   A   
   A   
   A   
   G   
   T   
   T   
   G   
   A   
    G    
    A    
   T   
    C    
   A   
    C    
   T   
   G   
   A   
    G    
   A   
   T   
   G   
    C    
   C   
    A    
   A   
   A   
   T   
   G   
   G   
    C    
   A   
   A   
   A   
   G   
   A   
   A   
   C   
   G   
    G    
   A   
   T   
    C    
    C    
   T   
    C    
   G   
   T   
    G    
   A   
   C   
    G    
   G   
   A   
   A   
   T   
   T   
    A    
   C   
   C   
   T   
   T   
   A   
   T   
   A   
   T   
   G   
   G   
   T   
    C    
   A   
   A   
   T   
   A   
   A   
   A   
   G   
   C   
    C    
   A   
   A   
   A   
   T   
   T   
    G    
   A   
   T   
    T    
   G   
   A   
   A   
   C   
   G   
    G    
   A   
   T   
   T   
   T   
   C   
   T   
   G   
   A   
    G    
    T    
   T   
    G    
   C   
   A   
   C   
   A   
   G   
   A   
   G   
   A   
    C    
   A   
   A   
    G    
   C   
   G   
    T    
   A   
   T   
    C    
   G   
   A   
   A   
   G   
   G   
    A    
   A   
   T   
    C    
   A   
   C   
    G    
   G   
   A   
   T   
   T   
   T   
   G   
   C   
   G   
   G   
   G   
   A   
    T    
   G   
   A   
   A   
   T   
   C   
   T   
   T   
   C   
    C    
   C   
   G   
   T   
   G   
   A   
    A    
   G   
   G   
    C    
   A   
   T   
   G   
   C   
   G   
    A    
   A   
   T   
    T    
   G   
   T   
   C   
   A   
   T   
    T    
   G   
   A   
    C    
    A    
   T   
    T    
   C   
   G   
   T   
   C   
   G   
    T    
   G   
   A   
    C    
   G   
    C    
    C    
   A   
   G   
    C    
   G   
   C   
    A    
   T   
   C   
    A    
   G   
   T   
   T   
    A    
   T   
    T    
   T   
   T   
    G    
   A   
   A   
    T    
   A   
   A   
   C   
   T   
   T   
    A    
   T   
   A   
    C    
   A   
   A   
   A   
    T    
   T   
   G   
   A   
   C   
    A    
    T    
   C   
    C    
   T   
   T   
    A    
   C   
   A   
    G    
   A   
    A    
    T    
   T   
   C   
    A    
   T   
   T   
    T    
   G   
   G   
    A    
   T   
   T   
    C    
   A   
   A   
    T    
   A   
   T   
   G   
   T   
   T   
    G    
   G   
   C   
    C    
   A   
   T   
    C    
   G   
   A   
   A   
   A   
   A   
   A   
   G   
   G   
    C    
   G   
   T   
    G    
   C   
   C   
    G    
   A   
   A   
   A   
    G    
   T   
   T   
   T   
   T   
    A    
   A   
   G   
    T    
   T   
   T   
   G   
   A   
   A   
   A   
   C   
    A    
    A    
   A   
   T   
    C    
    C    
   T   
    T    
   G   
   A   
   A   
   A   
   A   
    C    
   T   
   A   
    T    
    A    
   T   
    C    
   G   
   A   
    A    
   C   
   A   
    T    
   C   
   A   
    A    
    C    
    G    
    C    
    G    
   A   
   A   
   G   
   T   
   C   
   A   
   T   
    C    
   A   
   C   
    A    
    C    
   G   
    A    
   C   
   G   
   G   
   A   
   C   
    G    
    G    
   T   
    C    
   T   
   T   
   T   
   G   
   A   
    A    
   A   
   A   
   A   
    G    
   A   
    A    
   A   
   A   
    A    
   G   
   C   
   A   
   G   
   A   
   A   
   G   
   C   
   A   
    A    
   G   
    A    
   G   
   C   
    A    
   C   
   A   
    C    
   A   
   T   
    C    
   T   
   T   
   A   
   G   
   A   
   A   
   G   
   G   
   T   
    T    
   T   
    G    
   C   
   G   
    T    
   A   
   T   
   T   
   G   
   C   
    T    
   T   
   T   
    G    
   G   
   A   
   T   
   C   
   A   
   T   
   A   
   T   
   C   
    G    
   A   
   T   
   G   
   A   
   A   
   A   
   T   
    C    
   A   
   T   
   T   
   G   
   C   
    G    
   A   
   T   
   T   
   A   
   T   
    C    
   C   
   G   
    C    
   G   
   G   
    G    
   T   
   C   
    T    
    A    
   A   
   A   
   T   
   C   
   A   
   G   
   A   
   T   
   G   
   A   
    T    
   G   
   A   
    A    
   G   
   C   
   A   
   A   
   A   
   A   
   G   
   C   
    A    
   A   
    C    
    A    
    A    
   T   
    G    
   A   
   T   
    C    
   G   
   A   
   A   
   C   
   G   
    G    
   T   
   T   
    C    
   G   
   A   
    T    
   T   
   T   
    A    
   T   
   C   
    C    
   G   
   A   
   T   
   C   
   G   
    C    
   C   
   A   
    A    
    T    
   C   
    A    
   C   
   A   
   A   
   G   
   C   
    T    
   A   
   T   
    C    
   T   
   T   
   A   
   G   
   A   
   T   
   A   
   T   
   G   
   C   
   G   
   T   
   T   
   T   
    G    
   C   
   G   
    T    
   C   
   G   
    A    
   T   
   T   
    G    
   A   
   C   
    C    
   G   
   G   
    T    
   T   
   T   
    G    
   G   
   A   
   A   
   C   
   G   
    G    
   G   
   A   
    A    
   A   
   A   
   A   
   A   
   T   
    C    
   G   
   A   
    A    
   A   
   A   
   T   
   G   
   A   
   A   
   T   
   A   
    T    
   C   
   A   
   A   
   G   
   A   
    A    
   C   
   T   
    A    
   T   
   T   
    G    
   A   
    A    
   A   
    C    
   T   
    G    
   A   
   T   
   T   
   G   
    A    
    A    
   G   
   A   
    T    
   T   
   T   
    G    
    A    
    C    
    T    
   G   
   A   
   C   
   A   
   T   
   T   
    C    
   T   
    G    
   G   
   C   
    A    
    A    
   G   
    A    
   C   
   C   
    C    
   G   
   A   
    A    
   C   
   G   
    T    
   G   
   T   
    C    
   A   
    C    
    G    
   G   
   A   
   A   
   A   
   T   
    C    
   A   
   T   
    C    
   A   
   A   
    G    
   A   
    A    
    T    
   G   
   A   
    G    
   T   
   T   
   A   
    G    
    C    
    A    
   G   
   A   
   C   
    T    
   T   
    G    
    A    
    A    
    T    
    C    
    A    
   A   
    C    
    G    
   A   
   T   
   T   
   T   
   G   
   G   
   T   
   G   
   A   
   T   
    A    
    A    
   A   
   C   
   G   
   A   
   C   
   G   
    C    
   A   
   C   
   A   
   G   
   A   
   A   
    T    
   T   
   A   
    C    
   T   
   A   
   G   
   T   
    C    
   G   
   G   
   T   
   G   
   A   
    G    
   G   
   T   
   C   
    T    
   T   
    A    
   A   
   G   
   T   
   C   
   T   
    A    
   G   
   A   
   A   
   G   
   A   
   T   
   G   
   A   
   A   
   G   
   A   
   T   
    T    
   T   
    G    
   A   
   T   
    C    
   G   
   A   
   A   
   G   
   A   
   A   
   G   
   A   
   A   
   G   
   A   
   A   
    A    
   T   
   C   
   G   
   T   
    T    
   A   
   T   
   T   
   A   
   C   
    A    
    C    
   T   
    G    
   A   
   C   
    A    
   A   
   A   
    C    
   A   
   A   
    T    
   G   
   G   
    T    
   T   
   A   
    T    
   A   
   T   
    C    
   A   
   A   
    A    
   C   
   G   
    T    
    T    
   T   
   G   
   G   
    C    
   A   
   A   
   A   
    T    
    A    
    A    
    T    
   G   
   A   
   A   
   T   
   T   
   C   
    A    
   G   
    A    
   G   
   C   
    T    
   C   
   A   
   A   
   C   
   G   
    A    
    A    
   G   
    A    
   G   
   G   
    C    
   G   
   G   
    G    
   C   
   G   
   T   
   G   
   G   
    C    
   G   
   T   
    C    
   C   
   A   
   A   
   G   
   G   
    A    
   A   
   T   
   G   
   G   
   G   
    T    
    A    
   T   
    C    
   C   
   A   
    C    
    A    
   A   
   T   
   G   
   A   
    C    
   G   
   A   
    C    
   T   
   T   
   C   
   G   
   T   
    C    
   A   
   A   
    G    
   A   
   A   
    T    
   C   
   T   
    A    
    G    
   T   
   T   
   T   
   C   
   T   
   T   
   G   
    T    
   T   
   C   
    T    
   A   
   C   
    C    
   C   
   A   
    T    
   G   
   A   
    T    
   A   
   C   
    A    
   T   
   T   
    G    
   T   
   T   
   A   
   T   
   T   
    C    
   T   
   T   
   T   
   A   
   C   
   G   
   A   
   A   
    T    
   A   
    A    
    C    
   G   
   G   
    A    
   A   
   A   
   A   
   G   
   T   
    G    
   T   
   A   
    T    
   C   
   G   
    G    
   G   
   C   
   A   
   A   
   A   
   A   
   G   
   G   
    A    
   T   
   A   
    C    
   G   
   A   
   A   
   A   
   T   
    T    
   C   
   C   
    A    
   G   
   A   
    A    
   T   
   A   
   C   
   G   
   G   
    A    
    C    
   G   
    T    
   A   
   C   
   A   
   G   
   C   
    C    
   A   
   A   
   A   
   G   
   G   
    T    
   A   
   T   
    C    
   C   
   C   
   A   
    A    
   T   
    C    
   A   
   T   
    C    
   A   
   A   
    T    
   T   
   T   
    G    
   C   
   T   
    G    
   G   
   G   
    T    
   A   
   T   
    C    
   G   
   A   
    C    
   T   
   C   
    G    
    A    
    G    
    T    
   G   
   A   
   A   
   A   
    G    
    C    
   A   
   T   
    C    
   C   
   A   
    A    
   G   
   C   
    A    
   A   
   T   
    T    
   A   
   T   
   T   
    G    
   C   
    G    
   G   
   T   
    G    
    A    
    C    
   G   
   G   
   G   
    A    
    G    
   A   
   A   
   G   
   C   
    A    
   G   
   A   
   A   
   G   
    A    
    A    
   G   
   G   
    C    
    C    
   A   
    C    
   T   
   A   
    C    
    C    
   T   
    C    
   T   
   T   
   C   
   T   
   T   
    T    
   A   
   C   
    G    
   A   
   C   
    G    
    C    
    G    
    T    
    C    
   A   
   A   
   G   
   G   
    T    
   A   
   C   
    A    
   G   
   T   
    T    
   A   
   A   
   A   
   C   
   G   
    T    
   A   
   C   
    G    
    T    
   C   
    T    
   G   
   T   
    C    
   A   
   C   
    T    
   G   
   C   
    T    
   T   
   T   
    C    
    G    
   C   
   T   
   A   
   A   
    C    
   A   
   T   
    C    
   C   
   G   
    A    
   A   
   G   
    C    
   A   
   A   
   T   
   G   
   G   
    T    
    C    
   T   
    G    
   A   
   T   
    T    
   G   
   C   
    A    
   A   
   T   
   T   
    G    
   G   
    T    
   T   
   T   
    G    
   A   
   A   
   A   
   G   
   A   
   A   
   G   
   A   
    T    
   G   
   A   
    C    
   G   
   A   
    A    
    T    
   T   
    A    
    G    
   T   
    C    
   A   
   A   
    T    
   G   
   T   
    T    
    C    
   T   
   A   
    T    
    T    
   G   
   A   
   C   
    A    
    G    
   A   
    T    
   G   
   G   
    A    
    C    
   A   
    G    
    T    
    C    
    A    
   A   
   A   
    T    
   A   
   T   
    C    
   A   
   T   
    C    
   A   
   T   
    T    
   G   
   G   
    C    
   A   
   C   
   A   
   C   
   A   
   T   
    A    
    A    
    C    
   G   
   G   
    C    
   T   
   A   
   C   
   T   
   C   
   T   
   G   
   T   
    G    
   A   
   C   
    C    
   T   
   T   
    C    
    A    
   A   
    A    
   G   
   A   
    G    
    G    
    A    
    A    
    G    
   C   
    A    
   G   
    T    
    C    
   C   
   G   
    C    
   G   
   A   
   T   
   A   
   T   
   G   
   G   
   G   
    A    
   C   
   G   
    T    
   A   
   C   
   A   
   G   
   C   
    T    
   T   
   C   
    C    
   G   
   G   
    C    
   G   
   T   
    C    
    A    
   G   
    A    
   G   
   G   
    G    
   A   
   T   
    A    
   C   
   G   
    T    
    T    
   T   
    G    
   C   
   G   
    T    
   G   
   A   
    G    
    G    
   A   
    A    
   G   
   A   
    C    
   T   
    A    
    C    
   G   
   T   
    T    
   G   
   T   
    T    
   G   
   G   
    G    
    G    
   C   
   A   
   G   
   C   
    C    
    C    
   T   
    G    
    A    
   T   
   G   
    A    
   A   
    G    
   G   
    A    
   A   
    G    
   A   
    C    
    C    
   A   
   A   
   G   
   A   
   A   
   G   
   T   
    A    
    C    
   T   
    A    
   G   
   T   
    G    
   A   
   T   
    T    
   A   
   C   
    A    
   G   
   A   
   A   
   A   
   A   
    A    
   G   
   G   
   T   
   T   
   A   
    C    
   G   
   G   
    A    
   A   
   A   
    A    
   C   
   G   
    G    
   A   
   C   
    C    
   A   
   A   
   A   
   G   
    T    
    A    
   T   
   C   
    G    
   G   
   A   
    A    
   T   
   A   
   T   
   C   
   C   
   A   
   G   
   T   
    G    
   A   
   A   
    A    
   G   
   G   
    C    
   C   
   G   
    C    
   G   
   G   
    A    
   G   
   G   
   T   
   A   
   A   
   A   
   G   
   G   
    A    
   A   
   T   
    C    
   A   
   A   
    A    
   A   
   C   
    G    
    G    
   C   
    G    
   A   
   A   
    T    
   A   
   T   
   C   
   A   
   C   
   T   
   G   
   A   
    A    
   A   
   A   
   A   
   A   
   A   
   T   
   G   
   G   
   T   
   C   
   C   
   A   
    C    
   T   
   A   
   G   
   C   
    C    
   G   
   G   
    A    
   T   
   T   
    G    
   A   
   C   
   C   
   A   
   C   
    T    
   G   
   T   
    T    
    T    
    C    
   T   
   G   
   G   
    A    
    G    
   A   
   T   
   G   
   A   
   A   
   G   
   A   
    C    
   A   
   T   
   C   
    A    
   T   
    G    
    C    
   T   
    G    
   A   
   T   
    T    
   A   
   C   
    A    
    G    
   A   
    T    
   A   
   A   
   A   
   G   
   G   
    T    
   G   
   T   
    G    
   A   
   T   
   T   
   A   
   T   
   C   
   C   
   G   
    T    
   T   
   T   
    C    
   A   
   A   
   C   
   G   
   T   
    A    
    T    
    C    
    G    
    A    
   C   
   T   
   G   
   T   
   T   
   T   
   C   
   T   
   C   
   A   
   A   
   A   
   C   
    T    
   G   
   G   
   A   
   C   
   G   
    T    
    T    
   C   
   A   
   A   
   C   
   A   
   T   
   T   
    G    
   G   
   G   
    T    
   G   
   T   
    A    
   C   
   G   
   T   
   T   
   T   
    G    
   A   
   T   
   G   
   A   
    A    
    G    
   A   
   T   
   G   
   G   
   A   
   A   
   G   
    C    
    T    
   G   
    A    
    T    
    A    
   C   
    G    
   A   
   A   
   A   
   G   
   T   
   C   
   G   
   T   
   C   
   A   
   C   
   A   
   A   
   T   
   G   
   -   
   -   
   -   
   -   
   -   
   -   
   -   
   -   
   -   
   -   
   -   
   -   
   -   
   -   
   -   
   -   
   -   
   -   
   -   
   -   
   -   
   -   
   -   
   -   
   -   
   -   
   -   
   -   
   -   
   -   
   -   
   -   
   -   
   -   
   -   
   -   
   -   
   -   
   -   
   -   
   -   
   -   
   -   
   -   
   -   
   -   
   -   
   -   
   -   
   -   
   -   
   -   
   -   
   -   
   -   
   -   
   -   
   -   
   -   
   -   
 

 
   N59484F   
   A   
   T   
   G   
   A   
   G   
   T   
   G   
   A   
   A   
   G   
   A   
   A   
   A   
   T   
   C   
   A   
   G   
   A   
   G   
   A   
   A   
   A   
   A   
   C   
   A   
   T   
   C   
   C   
   A   
   T   
   G   
   A   
   T   
   G   
   T   
   C   
   A   
   A   
   T   
   C   
   T   
   A   
   A   
   C   
   C   
   A   
   G   
   C   
   G   
   A   
   A   
   A   
   T   
   G   
   A   
   A   
   A   
   G   
   A   
   C   
   T   
   C   
   C   
   T   
   T   
   C   
   A   
   T   
   C   
   G   
   A   
   T   
   T   
   A   
   T   
   G   
   C   
   G   
   A   
   T   
   G   
   A   
   G   
   T   
   G   
   T   
   T   
   A   
   T   
   T   
   G   
   T   
   C   
   G   
   C   
   A   
   C   
   G   
   T   
   G   
   C   
   A   
   C   
   T   
   T   
   C   
   C   
   T   
   G   
   A   
   T   
   G   
   T   
   C   
   A   
   G   
   A   
   G   
   A   
   T   
   G   
   G   
   T   
   C   
   T   
   G   
   A   
   A   
   A   
   C   
   C   
   C   
   G   
   T   
   T   
   C   
   A   
   T   
   C   
   G   
   C   
   C   
   G   
   T   
   A   
   T   
   T   
   T   
   T   
   A   
   T   
   A   
   C   
   G   
   G   
   C   
   A   
   T   
   G   
   A   
   A   
   C   
   G   
   A   
   A   
   T   
   T   
   A   
   G   
   G   
   T   
   G   
   T   
   A   
   A   
   C   
   A   
   C   
   C   
   G   
   G   
   A   
   T   
   A   
   A   
   A   
   G   
   C   
   C   
   C   
   A   
   T   
   A   
   A   
   A   
   A   
   A   
   A   
   T   
   C   
   T   
   G   
   C   
   C   
   A   
   G   
   A   
   A   
   T   
   C   
   G   
   T   
   C   
   G   
   G   
   A   
   G   
   A   
   T   
   G   
   T   
   C   
   A   
   T   
   G   
   G   
   G   
   T   
   A   
   A   
   A   
   T   
   A   
   C   
   C   
   A   
   T   
   C   
   C   
   T   
   C   
   A   
   T   
   G   
   G   
   A   
   G   
   A   
   C   
   A   
   G   
   T   
   G   
   C   
   G   
   A   
   T   
   C   
   T   
   A   
   T   
   G   
   A   
   G   
   T   
   C   
   A   
   A   
   T   
   G   
   G   
   T   
   A   
   C   
   G   
   G   
   A   
   T   
   G   
   G   
   C   
   A   
   C   
   A   
   G   
   C   
   C   
   A   
   T   
   T   
   C   
   A   
   G   
   C   
   T   
   A   
   C   
   C   
   G   
   T   
   T   
   A   
   T   
   A   
   T   
   G   
   C   
   T   
   A   
   G   
   T   
   G   
   G   
   A   
   T   
   G   
   G   
   T   
   C   
   A   
   T   
   G   
   G   
   A   
   A   
   A   
   C   
   T   
   T   
   T   
   G   
   G   
   T   
   T   
   C   
   T   
   G   
   T   
   T   
   G   
   A   
   C   
   G   
   G   
   T   
   G   
   A   
   C   
   G   
   G   
   T   
   G   
   C   
   C   
   G   
   C   
   C   
   G   
   C   
   T   
   A   
   T   
   G   
   C   
   G   
   T   
   T   
   A   
   T   
   A   
   C   
   C   
   G   
   A   
   A   
   G   
   C   
   A   
   C   
   G   
   G   
   A   
   T   
   G   
   A   
   G   
   C   
   A   
   A   
   A   
   A   
   T   
   C   
   G   
   C   
   T   
   A   
   C   
   A   
   G   
   A   
   A   
   A   
   T   
   G   
   C   
   T   
   G   
   C   
   G   
   T   
   G   
   A   
   T   
   A   
   T   
   C   
   A   
   A   
   T   
   A   
   A   
   G   
   A   
   A   
   C   
   A   
   C   
   T   
   G   
   T   
   T   
   G   
   A   
   T   
   T   
   T   
   C   
   C   
   A   
   A   
   A   
   G   
   C   
   A   
   A   
   C   
   T   
   A   
   T   
   G   
   A   
   T   
   G   
   A   
   T   
   A   
   C   
   A   
   G   
   A   
   A   
   A   
   A   
   A   
   G   
   A   
   A   
   C   
   C   
   A   
   G   
   T   
   T   
   G   
   T   
   A   
   C   
   T   
   A   
   C   
   C   
   T   
   G   
   C   
   T   
   C   
   G   
   C   
   T   
   T   
   C   
   C   
   C   
   G   
   A   
   A   
   T   
   C   
   T   
   T   
   T   
   T   
   A   
   G   
   T   
   G   
   A   
   A   
   C   
   G   
   G   
   A   
   A   
   C   
   A   
   A   
   C   
   G   
   G   
   G   
   G   
   A   
   T   
   C   
   G   
   C   
   C   
   G   
   T   
   A   
   G   
   G   
   T   
   A   
   T   
   G   
   G   
   C   
   C   
   A   
   C   
   G   
   A   
   A   
   T   
   A   
   T   
   C   
   C   
   C   
   A   
   C   
   C   
   T   
   C   
   A   
   T   
   A   
   A   
   C   
   T   
   T   
   G   
   T   
   C   
   T   
   G   
   A   
   G   
   G   
   T   
   A   
   G   
   T   
   A   
   G   
   C   
   G   
   G   
   C   
   T   
   A   
   T   
   C   
   G   
   A   
   T   
   T   
   T   
   G   
   C   
   T   
   G   
   A   
   T   
   G   
   G   
   A   
   T   
   A   
   A   
   T   
   C   
   C   
   T   
   G   
   A   
   T   
   G   
   T   
   G   
   A   
   C   
   G   
   A   
   C   
   C   
   A   
   A   
   T   
   G   
   A   
   A   
   T   
   T   
   G   
   A   
   T   
   G   
   G   
   A   
   A   
   G   
   T   
   A   
   T   
   T   
   A   
   C   
   C   
   T   
   G   
   G   
   A   
   C   
   C   
   A   
   G   
   A   
   T   
   T   
   T   
   T   
   C   
   C   
   A   
   A   
   C   
   A   
   G   
   G   
   T   
   G   
   G   
   T   
   T   
   T   
   G   
   G   
   T   
   T   
   A   
   T   
   G   
   G   
   G   
   A   
   A   
   A   
   A   
   T   
   C   
   A   
   G   
   G   
    A    
   A   
   T   
    C    
   C   
   G   
    A    
    A    
   G   
   A   
   G   
   C   
    G    
   T   
   A   
   T   
   G   
   A   
   A   
   A   
   C   
    A    
   G   
   G   
    T    
   A   
   A   
    G    
   G   
   G   
   T   
   T   
   C   
    T    
   A   
   T   
   T   
   A   
   C   
    C    
   G   
   T   
    A    
   C   
   G   
   T   
   G   
   C   
   A   
   A   
   A   
   A   
   G   
   T   
   T   
   G   
   A   
    G    
    A    
   T   
    T    
   A   
    C    
   T   
   G   
   A   
    G    
   A   
   T   
   G   
    C    
   C   
    A    
   A   
   A   
   T   
   G   
   G   
    T    
   A   
   A   
   A   
   G   
   A   
   A   
   C   
   G   
    G    
   A   
   T   
    C    
    A    
   T   
    C    
   G   
   T   
    G    
   A   
   C   
    A    
   G   
   A   
   A   
   T   
   T   
    G    
   C   
   C   
   T   
   T   
   A   
   T   
   A   
   T   
   G   
   G   
   T   
    C    
   A   
   A   
   T   
   A   
   A   
   A   
   G   
   C   
    A    
   A   
   A   
   A   
   T   
   T   
    G    
   A   
   T   
    C    
   G   
   A   
   A   
   C   
   G   
    G    
   A   
   T   
   T   
   T   
   C   
   T   
   G   
   A   
    A    
    C    
   T   
    G    
   C   
   A   
   C   
   A   
   G   
   A   
   G   
   A   
    C    
   A   
   A   
    G    
   C   
   G   
    T    
   A   
   T   
    C    
   G   
   A   
   A   
   G   
   G   
    G    
   A   
   T   
    C    
   A   
   C   
    A    
   G   
   A   
   T   
   T   
   T   
   G   
   C   
   G   
   G   
   G   
   A   
    C    
   G   
   A   
   A   
   T   
   C   
   T   
   T   
   C   
    C    
   C   
   G   
   T   
   G   
   A   
    A    
   G   
   G   
    T    
   A   
   T   
   G   
   C   
   G   
    G    
   A   
   T   
    C    
   G   
   T   
   C   
   A   
   T   
    T    
   G   
   A   
    C    
    A    
   T   
    C    
   C   
   G   
   T   
   C   
   G   
    T    
   G   
   A   
    C    
   G   
    C    
    C    
   A   
   G   
    C    
   G   
   C   
    A    
   T   
   C   
    T    
   G   
   T   
   T   
    A    
   T   
    C    
   T   
   T   
    G    
   A   
   A   
    T    
   A   
   A   
   C   
   T   
   T   
    A    
   T   
   A   
    T    
   A   
   A   
   A   
    T    
   T   
   G   
   A   
   C   
    A    
    T    
   C   
    C    
   T   
   T   
    G    
   C   
   A   
    A    
   A   
    A    
    T    
   T   
   C   
    A    
   T   
   T   
    T    
   G   
   G   
    A    
   T   
   T   
    C    
   A   
   A   
    T    
   A   
   T   
   G   
   T   
   T   
    G    
   G   
   C   
    G    
   A   
   T   
    C    
   G   
   A   
   A   
   A   
   A   
   A   
   G   
   G   
    T    
   G   
   T   
    G    
   C   
   C   
    G    
   A   
   A   
   A   
    G    
   T   
   T   
   T   
   T   
    G    
   A   
   G   
    C    
   T   
   T   
   G   
   A   
   A   
   A   
   C   
    A    
    A    
   A   
   T   
    T    
    C    
   T   
    T    
   G   
   A   
   A   
   A   
   A   
    C    
   T   
   A   
    T    
    A    
   T   
    C    
   G   
   A   
    A    
   C   
   A   
    T    
   C   
   A   
    G    
    C    
    G    
    C    
    G    
   A   
   A   
   G   
   T   
   C   
   A   
   T   
    C    
   A   
   C   
    A    
    A    
   G   
    A    
   C   
   G   
   G   
   A   
   C   
    A    
    G    
   T   
    C    
   T   
   T   
   T   
   G   
   A   
    A    
   A   
   A   
   A   
    G    
   A   
    A    
   A   
   A   
    A    
   G   
   C   
   A   
   G   
   A   
   A   
   G   
   C   
   A   
    A    
   G   
    G    
   G   
   C   
    A    
   C   
   A   
    C    
   A   
   T   
    T    
   T   
   T   
   A   
   G   
   A   
   A   
   G   
   G   
   T   
    T    
   T   
    G    
   C   
   G   
    T    
   A   
   T   
   T   
   G   
   C   
    T    
   T   
   T   
    G    
   G   
   A   
   T   
   C   
   A   
   T   
   A   
   T   
   C   
    G    
   A   
   T   
   G   
   A   
   A   
   A   
   T   
    C    
   A   
   T   
   T   
   G   
   C   
    G    
   A   
   T   
   T   
   A   
   T   
    C    
   C   
   G   
    C    
   G   
   G   
    G    
   T   
   C   
    T    
    A    
   A   
   A   
   T   
   C   
   A   
   G   
   A   
   T   
   G   
   A   
    C    
   G   
   A   
    A    
   G   
   C   
   A   
   A   
   A   
   A   
   G   
   C   
    G    
   A   
    C    
    G    
    A    
   T   
    G    
   A   
   T   
    C    
   G   
   A   
   A   
   C   
   G   
    G    
   T   
   T   
    C    
   G   
   A   
    T    
   T   
   T   
    A    
   T   
   C   
    C    
   G   
   A   
   T   
   C   
   G   
    C    
   C   
   A   
    G    
    T    
   C   
    T    
   C   
   A   
   A   
   G   
   C   
    T    
   A   
   T   
    T    
   T   
   T   
   A   
   G   
   A   
   T   
   A   
   T   
   G   
   C   
   G   
   T   
   T   
   T   
    A    
   C   
   G   
    C    
   C   
   G   
    A    
   T   
   T   
    G    
   A   
   C   
    C    
   G   
   G   
    T    
   T   
   T   
    G    
   G   
   A   
   A   
   C   
   G   
    G    
   G   
   A   
    A    
   A   
   A   
   A   
   A   
   T   
    C    
   G   
   A   
    A    
   A   
   A   
   T   
   G   
   A   
   A   
   T   
   A   
    C    
   C   
   A   
   A   
   G   
   A   
    A    
   C   
   T   
    C    
   T   
   T   
    A    
   A   
    A    
   A   
    C    
   T   
    G    
   A   
   T   
   T   
   G   
    A    
    A    
   G   
   A   
    T    
   T   
   T   
    A    
    A    
    C    
    T    
   G   
   A   
   C   
   A   
   T   
   T   
    C    
   T   
    G    
   G   
   C   
    A    
    A    
   G   
    A    
   C   
   C   
    A    
   G   
   A   
    A    
   C   
   G   
    T    
   G   
   T   
    T    
   A   
    C    
    A    
   G   
   A   
   A   
   A   
   T   
    C    
   A   
   T   
    C    
   A   
   A   
    G    
   A   
    A    
    T    
   G   
   A   
    A    
   T   
   T   
   A   
    G    
    C    
    A    
   G   
   A   
   C   
    T    
   T   
    G    
    A    
    A    
    T    
    C    
    A    
   A   
    C    
    G    
   A   
   T   
   T   
   T   
   G   
   G   
   T   
   G   
   A   
   T   
    A    
    A    
   A   
   C   
   G   
   A   
   C   
   G   
    C    
   A   
   C   
   A   
   G   
   A   
   A   
    T    
   T   
   A   
    C    
   T   
   A   
   G   
   T   
    C    
   G   
   G   
   T   
   G   
   A   
    G    
   G   
   T   
   C   
    T    
   T   
    A    
   A   
   G   
   T   
   C   
   T   
    G    
   G   
   A   
   A   
   G   
   A   
   T   
   G   
   A   
   A   
   G   
   A   
   T   
    T    
   T   
    A    
   A   
   T   
    C    
   G   
   A   
   A   
   G   
   A   
   A   
   G   
   A   
   A   
   G   
   A   
   A   
    A    
   T   
   C   
   G   
   T   
    T    
   A   
   T   
   T   
   A   
   C   
    A    
    T    
   T   
    G    
   A   
   C   
    A    
   A   
   A   
    C    
   A   
   A   
    T    
   G   
   G   
    T    
   T   
   A   
    C    
   A   
   T   
    C    
   A   
   A   
    A    
   C   
   G   
    T    
    T    
   T   
   G   
   G   
    C    
   A   
   A   
   A   
    T    
    A    
    A    
    T    
   G   
   A   
   A   
   T   
   T   
   C   
    A    
   G   
    A    
   G   
   C   
    T    
   C   
   A   
   A   
   C   
   G   
    A    
    A    
   G   
    G    
   G   
   G   
    T    
   G   
   G   
    A    
   C   
   G   
   T   
   G   
   G   
    T    
   G   
   T   
    C    
   C   
   A   
   A   
   G   
   G   
    A    
   A   
   T   
   G   
   G   
   G   
    T    
    A    
   T   
    C    
   C   
   A   
    C    
    A    
   A   
   T   
   G   
   A   
    C    
   G   
   A   
    C    
   T   
   T   
   C   
   G   
   T   
    C    
   A   
   A   
    A    
   A   
   A   
    T    
   C   
   T   
    G    
    G    
   T   
   T   
   T   
   C   
   T   
   T   
   G   
    T    
   T   
   C   
    T    
   A   
   C   
    C    
   C   
   A   
    T    
   G   
   A   
    T    
   A   
   C   
    A    
   T   
   T   
    G    
   T   
   T   
   A   
   T   
   T   
    C    
   T   
   T   
   T   
   A   
   C   
   G   
   A   
   A   
    T    
   A   
    A    
    C    
   G   
   G   
    A    
   A   
   A   
   A   
   G   
   T   
    G    
   T   
   A   
    T    
   C   
   G   
    G    
   G   
   C   
   A   
   A   
   A   
   A   
   G   
   G   
    A    
   T   
   A   
    C    
   G   
   A   
   A   
   A   
   T   
    T    
   C   
   C   
    A    
   G   
   A   
    A    
   T   
   A   
   C   
   G   
   G   
    G    
    C    
   G   
    C    
   A   
   C   
   A   
   G   
   C   
    C    
   A   
   A   
   A   
   G   
   G   
    A    
   A   
   T   
    A    
   C   
   C   
   A   
    A    
   T   
    T    
   A   
   T   
    C    
   A   
   A   
    T    
   T   
   T   
    A    
   C   
   T   
    A    
   G   
   G   
    T    
   A   
   T   
    C    
   G   
   A   
    T    
   T   
   C   
    G    
    A    
    G    
    T    
   G   
   A   
   A   
   A   
    G    
    C    
   A   
   T   
    C    
   C   
   A   
    G    
   G   
   C   
    A    
   A   
   T   
    C    
   A   
   T   
   T   
    G    
   C   
    G    
   G   
   T   
    G    
    A    
    C    
   G   
   G   
   G   
    A    
    G    
   A   
   A   
   G   
   C   
    A    
   G   
   A   
   A   
   G   
    A    
    A    
   G   
   G   
    C    
    C    
   A   
    C    
   T   
   A   
    T    
    C    
   T   
    C    
   T   
   T   
   C   
   T   
   T   
    T    
   A   
   C   
    G    
   A   
   C   
    G    
    C    
    G    
    T    
    C    
   A   
   A   
   G   
   G   
    T    
   A   
   C   
    A    
   G   
   T   
    A    
   A   
   A   
   A   
   C   
   G   
    T    
   A   
   C   
    T    
    T    
   C   
    T    
   G   
   T   
    C    
   A   
   C   
    T    
   G   
   C   
    T    
   T   
   T   
    T    
    G    
   C   
   T   
   A   
   A   
    C    
   A   
   T   
    C    
   C   
   G   
    A    
   A   
   G   
    T    
   A   
   A   
   T   
   G   
   G   
    T    
    C    
   T   
    G    
   A   
   T   
    C    
   G   
   C   
    A    
   A   
   T   
   T   
    G    
   G   
    T    
   T   
   T   
    G    
   A   
   A   
   A   
   G   
   A   
   A   
   G   
   A   
    C    
   G   
   A   
    T    
   G   
   A   
    G    
    C    
   T   
    C    
    G    
   T   
    C    
   A   
   A   
    T    
   G   
   T   
    G    
    C    
   T   
   A   
    C    
    T    
   G   
   A   
   C   
    A    
    G    
   A   
    T    
   G   
   G   
    T    
    C    
   A   
    A    
    T    
    C    
    T    
   A   
   A   
    T    
   A   
   T   
    C    
   A   
   T   
    T    
   A   
   T   
    T    
   G   
   G   
    A    
   A   
   C   
   A   
   C   
   A   
   T   
    A    
    A    
    C    
   G   
   G   
    C    
   T   
   A   
   C   
   T   
   C   
   T   
   G   
   T   
    C    
   A   
   C   
    T    
   T   
   T   
    C    
    A    
   A   
    A    
   G   
   A   
    A    
    G    
    A    
    A    
    G    
   C   
    A    
   G   
    T    
    C    
   C   
   G   
    C    
   G   
   A   
   T   
   A   
   T   
   G   
   G   
   G   
    A    
   C   
   G   
    T    
   A   
   C   
   A   
   G   
   C   
    T    
   T   
   C   
    C    
   G   
   G   
    G    
   G   
   T   
    T    
    A    
   G   
    A    
   G   
   G   
    A    
   A   
   T   
    A    
   C   
   G   
    C    
    C    
   T   
    G    
   C   
   G   
    T    
   G   
   A   
    G    
    G    
   A   
    A    
   G   
   A   
    T    
   T   
    A    
    T    
   G   
   T   
    C    
   G   
   T   
    T    
   G   
   G   
    G    
    G    
   C   
   A   
   G   
   C   
    C    
    T    
   T   
    A    
    A    
   T   
   G   
    A    
   A   
    G    
   G   
    A    
   A   
    G    
   A   
    T    
    C    
   A   
   A   
   G   
   A   
   A   
   G   
   T   
    A    
    T    
   T   
    A    
   G   
   T   
    G    
   A   
   T   
    C    
   A   
   C   
    A    
   G   
   A   
   A   
   A   
   A   
    A    
   G   
   G   
   T   
   T   
   A   
    C    
   G   
   G   
    A    
   A   
   A   
    A    
   C   
   G   
    G    
   A   
   C   
    A    
   A   
   A   
   A   
   G   
    T    
    A    
   T   
   C   
    G    
   G   
   A   
    A    
   T   
   A   
   T   
   C   
   C   
   A   
   G   
   T   
    G    
   A   
   A   
    A    
   G   
   G   
    C    
   C   
   G   
    C    
   G   
   G   
    A    
   G   
   G   
   T   
   A   
   A   
   A   
   G   
   G   
    G    
   A   
   T   
    C    
   A   
   A   
    A    
   A   
   C   
    G    
    G    
   C   
    G    
   A   
   A   
    C    
   A   
   T   
   C   
   A   
   C   
   T   
   G   
   A   
    A    
   A   
   A   
   A   
   A   
   A   
   T   
   G   
   G   
   T   
   C   
   C   
   A   
    C    
   T   
   A   
   G   
   C   
    T    
   G   
   G   
    A    
   T   
   T   
    G    
   A   
   C   
   C   
   A   
   C   
    T    
   G   
   T   
    T    
    T    
    C    
   T   
   G   
   G   
    A    
    G    
   A   
   T   
   G   
   A   
   A   
   G   
   A   
    C    
   A   
   T   
   C   
    A    
   T   
    G    
    C    
   T   
    G    
   A   
   T   
    T    
   A   
   C   
    A    
    G    
   A   
    T    
   A   
   A   
   A   
   G   
   G   
    T    
   G   
   T   
    C    
   A   
   T   
   T   
   A   
   T   
   C   
   C   
   G   
    T    
   T   
   T   
    C    
   A   
   A   
   C   
   G   
   T   
    A    
    T    
    C    
    G    
    A    
   C   
   T   
   G   
   T   
   T   
   T   
   C   
   T   
   C   
   A   
   A   
   A   
   C   
    T    
   G   
   G   
   A   
   C   
   G   
    T    
    T    
   C   
   A   
   A   
   C   
   A   
   T   
   T   
    A    
   G   
   G   
    C    
   G   
   T   
    A    
   C   
   G   
   T   
   T   
   T   
    G    
   A   
   T   
   G   
   A   
    A    
    G    
   A   
   T   
   G   
   G   
   A   
   A   
   G   
    C    
    T    
   G   
    A    
    T    
    A    
   C   
    G    
   A   
   A   
   A   
   G   
   T   
   T   
   G   
   T   
   C   
   A   
   C   
   G   
   A   
   T   
   G   
   G   
   C   
   A   
   G   
   C   
   A   
   G   
   T   
   C   
   G   
   A   
   A   
   C   
   C   
   A   
   G   
   A   
   A   
   G   
   C   
   T   
   G   
   C   
   A   
   G   
   A   
   A   
   C   
   A   
   A   
   A   
   C   
   A   
   G   
   T   
   T   
   G   
   A   
   A   
   G   
   A   
   A   
   C   
   A   
   A   
   G   
   G   
   A   
   T   
   C   
   A   
   G   
   A   
   A   
   G   
   A   
   A   
   -   
   -   
   -   
 

 
   N59860F   
   A   
   T   
   G   
   A   
   G   
   T   
   G   
   A   
   A   
   G   
   A   
   A   
   A   
   T   
   T   
   A   
   A   
   A   
   G   
   A   
   A   
   A   
   A   
   C   
   A   
   T   
   T   
   C   
   A   
   A   
   G   
   A   
   C   
   G   
   T   
   C   
   A   
   A   
   T   
   C   
   T   
   G   
   A   
   C   
   C   
   A   
   G   
   C   
   G   
   A   
   A   
   A   
   T   
   G   
   A   
   A   
   A   
   G   
   A   
   A   
   T   
   C   
   T   
   T   
   T   
   C   
   A   
   T   
   T   
   G   
   A   
   C   
   T   
   A   
   C   
   G   
   C   
   A   
   A   
   T   
   G   
   A   
   G   
   T   
   G   
   T   
   T   
   A   
   T   
   C   
   G   
   T   
   A   
   G   
   C   
   C   
   C   
   G   
   C   
   G   
   C   
   G   
   T   
   T   
   A   
   C   
   C   
   T   
   G   
   A   
   C   
   G   
   T   
   T   
   C   
   G   
   T   
   G   
   A   
   C   
   G   
   G   
   T   
   T   
   T   
   A   
   A   
   A   
   A   
   C   
   C   
   T   
   G   
   T   
   T   
   C   
   A   
   T   
   C   
   G   
   C   
   C   
   G   
   A   
   A   
   T   
   C   
   T   
   T   
   A   
   T   
   A   
   T   
   G   
   G   
   A   
   A   
   T   
   G   
   A   
   A   
   C   
   G   
   A   
   A   
   T   
   T   
   A   
   G   
   G   
   G   
   G   
   T   
   A   
   A   
   C   
   C   
   C   
   C   
   T   
   G   
   A   
   T   
   A   
   A   
   A   
   C   
   C   
   A   
   C   
   A   
   C   
   A   
   A   
   A   
   A   
   A   
   A   
   T   
   C   
   A   
   G   
   C   
   C   
   C   
   G   
   G   
   A   
   T   
   T   
   G   
   T   
   T   
   G   
   G   
   G   
   G   
   A   
   T   
   G   
   T   
   T   
   A   
   T   
   G   
   G   
   G   
   T   
   A   
   A   
   A   
   T   
   A   
   T   
   C   
   A   
   C   
   C   
   C   
   C   
   C   
   A   
   T   
   G   
   G   
   G   
   G   
   A   
   C   
   A   
   G   
   T   
   G   
   C   
   G   
   A   
   T   
   T   
   T   
   A   
   C   
   G   
   A   
   A   
   T   
   C   
   A   
   A   
   T   
   G   
   G   
   T   
   G   
   C   
   G   
   G   
   A   
   T   
   G   
   G   
   C   
   A   
   C   
   A   
   A   
   C   
   C   
   T   
   T   
   T   
   T   
   A   
   G   
   T   
   T   
   A   
   T   
   C   
   G   
   G   
   G   
   C   
   T   
   A   
   T   
   G   
   C   
   T   
   A   
   G   
   T   
   T   
   G   
   A   
   C   
   G   
   G   
   C   
   C   
   A   
   C   
   G   
   G   
   A   
   A   
   A   
   C   
   T   
   T   
   C   
   G   
   G   
   T   
   T   
   C   
   T   
   G   
   T   
   C   
   G   
   A   
   T   
   G   
   G   
   T   
   G   
   A   
   C   
   G   
   G   
   C   
   G   
   C   
   T   
   G   
   C   
   C   
   G   
   C   
   T   
   A   
   T   
   G   
   C   
   G   
   T   
   T   
   A   
   T   
   A   
   C   
   C   
   G   
   A   
   A   
   G   
   C   
   A   
   C   
   G   
   T   
   A   
   T   
   G   
   A   
   G   
   T   
   A   
   A   
   A   
   A   
   T   
   T   
   G   
   C   
   T   
   T   
   T   
   A   
   G   
   A   
   A   
   A   
   T   
   G   
   C   
   T   
   A   
   C   
   G   
   A   
   G   
   A   
   T   
   A   
   T   
   T   
   A   
   A   
   C   
   A   
   A   
   A   
   A   
   A   
   T   
   A   
   C   
   A   
   G   
   T   
   C   
   G   
   A   
   T   
   T   
   T   
   C   
   C   
   A   
   A   
   G   
   G   
   A   
   A   
   A   
   C   
   T   
   A   
   T   
   G   
   A   
   T   
   G   
   A   
   T   
   T   
   C   
   A   
   G   
   A   
   A   
   C   
   A   
   A   
   G   
   A   
   A   
   C   
   C   
   A   
   G   
   T   
   A   
   G   
   T   
   T   
   T   
   T   
   A   
   C   
   C   
   T   
   G   
   C   
   T   
   C   
   G   
   T   
   T   
   T   
   T   
   C   
   C   
   A   
   A   
   A   
   C   
   T   
   T   
   A   
   C   
   T   
   A   
   G   
   T   
   T   
   A   
   A   
   C   
   G   
   G   
   A   
   A   
   C   
   A   
   A   
   C   
   G   
   G   
   G   
   G   
   A   
   T   
   T   
   G   
   C   
   G   
   G   
   T   
   G   
   G   
   G   
   G   
   A   
   T   
   G   
   G   
   C   
   A   
   A   
   C   
   A   
   A   
   A   
   T   
   A   
   T   
   T   
   C   
   C   
   A   
   C   
   C   
   A   
   C   
   A   
   C   
   A   
   A   
   T   
   T   
   T   
   A   
   A   
   G   
   T   
   G   
   A   
   A   
   G   
   T   
   G   
   A   
   T   
   T   
   G   
   A   
   T   
   G   
   C   
   A   
   A   
   C   
   A   
   A   
   G   
   T   
   T   
   T   
   A   
   T   
   T   
   G   
   A   
   T   
   G   
   G   
   A   
   C   
   A   
   A   
   T   
   C   
   C   
   T   
   G   
   A   
   T   
   G   
   T   
   A   
   A   
   C   
   G   
   A   
   C   
   G   
   A   
   A   
   T   
   G   
   A   
   A   
   T   
   T   
   G   
   A   
   T   
   G   
   G   
   A   
   A   
   G   
   T   
   G   
   T   
   T   
   A   
   C   
   C   
   T   
   G   
   G   
   A   
   C   
   C   
   A   
   G   
   A   
   T   
   T   
   T   
   T   
   C   
   C   
   A   
   A   
   C   
   A   
   G   
   G   
   C   
   G   
   G   
   T   
   T   
   T   
   A   
   G   
   T   
   G   
   A   
   T   
   G   
   G   
   G   
   G   
   A   
   A   
   A   
   T   
   C   
   A   
   G   
   G   
    G    
   A   
   T   
    T    
   C   
   G   
    C    
    C    
   G   
   A   
   G   
   C   
    A    
   T   
   A   
   T   
   G   
   A   
   A   
   A   
   C   
    A    
   G   
   G   
    G    
   A   
   A   
    A    
   G   
   G   
   T   
   T   
   C   
    G    
   A   
   T   
   T   
   A   
   C   
    T    
   G   
   T   
    T    
   C   
   G   
   T   
   G   
   C   
   A   
   A   
   A   
   A   
   G   
   T   
   T   
   G   
   A   
    A    
    T    
   T   
    G    
   A   
    C    
   T   
   G   
   A   
    A    
   A   
   T   
   G   
    C    
   C   
    G    
   A   
   A   
   T   
   G   
   G   
    A    
   A   
   A   
   A   
   G   
   A   
   A   
   C   
   G   
    T    
   A   
   T   
    T    
    T    
   T   
    A    
   G   
   T   
    A    
   A   
   C   
    T    
   G   
   A   
   A   
   T   
   T   
    G    
   C   
   C   
   T   
   T   
   A   
   T   
   A   
   T   
   G   
   G   
   T   
    G    
   A   
   A   
   T   
   A   
   A   
   A   
   G   
   C   
    C    
   A   
   A   
   A   
   T   
   T   
    A    
   A   
   T   
    C    
   G   
   A   
   A   
   C   
   G   
    A    
   A   
   T   
   T   
   T   
   C   
   T   
   G   
   A   
    A    
    T    
   T   
    A    
   C   
   A   
   C   
   A   
   G   
   A   
   G   
   A   
    T    
   A   
   A   
    A    
   C   
   G   
    A    
   A   
   T   
    T    
   G   
   A   
   A   
   G   
   G   
    A    
   A   
   T   
    T    
   A   
   C   
    T    
   G   
   A   
   T   
   T   
   T   
   G   
   C   
   G   
   G   
   G   
   A   
    T    
   G   
   A   
   A   
   T   
   C   
   T   
   T   
   C   
    T    
   C   
   G   
   T   
   G   
   A   
    A    
   G   
   G   
    C    
   A   
   T   
   G   
   C   
   G   
    G    
   A   
   T   
    T    
   G   
   T   
   C   
   A   
   T   
    C    
   G   
   A   
    T    
    G    
   T   
    T    
   C   
   G   
   T   
   C   
   G   
    A    
   G   
   A   
    T    
   G   
    T    
    G    
   A   
   G   
    T    
   G   
   C   
    C    
   T   
   C   
    T    
   G   
   T   
   T   
    G    
   T   
    G    
   T   
   T   
    A    
   A   
   A   
    C    
   A   
   A   
   C   
   T   
   T   
    G    
   T   
   A   
    C    
   A   
   A   
   A   
    A    
   T   
   G   
   A   
   C   
    T    
    G    
   C   
    C    
   T   
   T   
    A    
   C   
   A   
    A    
   A   
    C    
    A    
   T   
   C   
    T    
   T   
   T   
    T    
   G   
   G   
    T    
   T   
   T   
    T    
   A   
   A   
    C    
   A   
   T   
   G   
   T   
   T   
    A    
   G   
   C   
    C    
   A   
   T   
    C    
   G   
   A   
   A   
   A   
   A   
   A   
   G   
   G   
    C    
   G   
   T   
    A    
   C   
   C   
    G    
   A   
   A   
   A   
    A    
   T   
   T   
   T   
   T   
    A    
   A   
   G   
    C    
   T   
   T   
   G   
   A   
   A   
   A   
   C   
    G    
    T    
   A   
   T   
    T    
    T    
   T   
    A    
   G   
   A   
   A   
   A   
   A   
    C    
   T   
   A   
    C    
    G    
   T   
    C    
   G   
   A   
    G    
   C   
   A   
    C    
   C   
   A   
    A    
    A    
    A    
    A    
    G    
   A   
   A   
   G   
   T   
   C   
   A   
   T   
    T    
   A   
   C   
    T    
    C    
   G   
    C    
   C   
   G   
   G   
   A   
   C   
    G    
    A    
   T   
    T    
   T   
   T   
   T   
   G   
   A   
    C    
   A   
   A   
   A   
    A    
   A   
    C    
   A   
   A   
    G    
   G   
   C   
   A   
   G   
   A   
   A   
   G   
   C   
   A   
    C    
   G   
    G    
   G   
   C   
    G    
   C   
   A   
    T    
   A   
   T   
    T    
   T   
   T   
   A   
   G   
   A   
   A   
   G   
   G   
   T   
    C    
   T   
    A    
   C   
   G   
    A    
   A   
   T   
   T   
   G   
   C   
    C    
   T   
   T   
    A    
   G   
   A   
   T   
   C   
   A   
   T   
   A   
   T   
   C   
    G    
   A   
   T   
   G   
   A   
   A   
   A   
   T   
    C    
   A   
   T   
   T   
   G   
   C   
    C    
   A   
   T   
   T   
   A   
   T   
    C    
   C   
   G   
    T    
   G   
   G   
    G    
   T   
   C   
    A    
    C    
   A   
   A   
   T   
   C   
   A   
   G   
   A   
   T   
   G   
   A   
    C    
   G   
   A   
    A    
   G   
   C   
   A   
   A   
   A   
   A   
   G   
   C   
    A    
   A   
    C    
    G    
    T    
   T   
    A    
   A   
   T   
    T    
   G   
   A   
   A   
   C   
   G   
    C    
   T   
   T   
    T    
   G   
   A   
    A    
   T   
   T   
    T    
   T   
   C   
    A    
   G   
   A   
   T   
   C   
   G   
    T    
   C   
   A   
    A    
    G    
   C   
    G    
   C   
   A   
   A   
   G   
   C   
    A    
   A   
   T   
    T    
   T   
   T   
   A   
   G   
   A   
   T   
   A   
   T   
   G   
   C   
   G   
   T   
   T   
   T   
    A    
   C   
   G   
    C    
   C   
   G   
    T    
   T   
   T   
    A    
   A   
   C   
    A    
   G   
   G   
    C    
   T   
   T   
    A    
   G   
   A   
   A   
   C   
   G   
    C    
   G   
   A   
    C    
   A   
   A   
   A   
   A   
   T   
    T    
   G   
   A   
    A    
   A   
   A   
   T   
   G   
   A   
   A   
   T   
   A   
    T    
   C   
   A   
   A   
   G   
   A   
    G    
   C   
   T   
    A    
   T   
   T   
    G    
   A   
    A    
   A   
    T    
   T   
    C    
   A   
   T   
   T   
   G   
    C    
    G    
   G   
   A   
    C    
   T   
   T   
    A    
    G    
    A    
    G    
   G   
   A   
   C   
   A   
   T   
   T   
    T    
   T   
    A    
   G   
   C   
    C    
    C    
   G   
    C    
   C   
   C   
    A    
   G   
   A   
    A    
   C   
   G   
    C    
   G   
   T   
    C    
   A   
    T    
    T    
   G   
   A   
   A   
   A   
   T   
    C    
   A   
   T   
    T    
   A   
   A   
    A    
   A   
    C    
    A    
   G   
   A   
    G    
   T   
   T   
   A   
    A    
    A    
    T    
   G   
   A   
   C   
    G    
   T   
    T    
    C    
    G    
    C    
    A    
    C    
   A   
    A    
    A    
   A   
   T   
   T   
   T   
   G   
   G   
   T   
   G   
   A   
   T   
    G    
    C    
   A   
   C   
   G   
   A   
   C   
   G   
    C    
   A   
   C   
   A   
   G   
   A   
   A   
    T    
   T   
   A   
    T    
   T   
   A   
   G   
   T   
    A    
   G   
   G   
   T   
   G   
   A   
    A    
   G   
   T   
   C   
    T    
   T   
    A    
   A   
   G   
   T   
   C   
   T   
    T    
   G   
   A   
   A   
   G   
   A   
   T   
   G   
   A   
   A   
   G   
   A   
   T   
    T    
   T   
    A    
   A   
   T   
    C    
   G   
   A   
   A   
   G   
   A   
   A   
   G   
   A   
   A   
   G   
   A   
   A   
    G    
   T   
   C   
   G   
   T   
    G    
   A   
   T   
   T   
   A   
   C   
    G    
    T    
   T   
    A    
   A   
   C   
    C    
   A   
   A   
    T    
   A   
   A   
    C    
   G   
   G   
    C    
   T   
   A   
    C    
   A   
   T   
    T    
   A   
   A   
    G    
   C   
   G   
    G    
    A    
   T   
   G   
   G   
    C    
   A   
   A   
   A   
    C    
    T    
    C    
    C    
   G   
   A   
   A   
   T   
   T   
   C   
    C    
   G   
    G    
   G   
   C   
    G    
   C   
   A   
   A   
   C   
   G   
    C    
    C    
   G   
    C    
   G   
   G   
    T    
   G   
   G   
    A    
   C   
   G   
   T   
   G   
   G   
    T    
   G   
   T   
    C    
   C   
   A   
   A   
   G   
   G   
    G    
   A   
   T   
   G   
   G   
   G   
    C    
    G    
   T   
    T    
   C   
   A   
    T    
    G    
   A   
   T   
   G   
   A   
    T    
   G   
   A   
    T    
   T   
   T   
   C   
   G   
   T   
    G    
   A   
   A   
    A    
   A   
   A   
    C    
   C   
   T   
    A    
    G    
   T   
   T   
   T   
   C   
   T   
   T   
   G   
    T    
   T   
   C   
    A    
   A   
   C   
    A    
   C   
   A   
    C    
   G   
   A   
    C    
   A   
   C   
    G    
   T   
   T   
    A    
   T   
   T   
   A   
   T   
   T   
    C    
   T   
   T   
   T   
   A   
   C   
   G   
   A   
   A   
    C    
   A   
    C    
    T    
   G   
   G   
    G    
   A   
   A   
   A   
   G   
   T   
    T    
   T   
   A   
    C    
   C   
   G   
    A    
   G   
   C   
   A   
   A   
   A   
   A   
   G   
   G   
    T    
   T   
   A   
    C    
   G   
   A   
   A   
   A   
   T   
    C    
   C   
   C   
    T    
   G   
   A   
    A    
   T   
   A   
   C   
   G   
   G   
    T    
    A    
   G   
    A    
   A   
   C   
   A   
   G   
   C   
    A    
   A   
   A   
   A   
   G   
   G   
    A    
   A   
   T   
    T    
   C   
   C   
   A   
    G    
   T   
    G    
   A   
   T   
    T    
   A   
   A   
    C    
   T   
   T   
    A    
   C   
   T   
    G    
   G   
   G   
    A    
   A   
   T   
    T    
   G   
   A   
    T    
   T   
   C   
    T    
    G    
    C    
    A    
   G   
   A   
   A   
   A   
    A    
    A    
   A   
   T   
    T    
   C   
   A   
    A    
   G   
   C   
    G    
   A   
   T   
    T    
   A   
   T   
   T   
    T    
   C   
    T    
   G   
   T   
    T    
    G    
    A    
   G   
   G   
   G   
    C    
    A    
   A   
   A   
   G   
   C   
    G    
   G   
   A   
   A   
   G   
    C    
    A    
   G   
   G   
    T    
    A    
   A   
    A    
   T   
   A   
    C    
    T    
   T   
    G    
   T   
   T   
   C   
   T   
   T   
    C    
   A   
   C   
    A    
   A   
   C   
    C    
    T    
    T    
    A    
    A    
   A   
   A   
   G   
   G   
    A    
   A   
   C   
    C    
   G   
   T   
    C    
   A   
   A   
   A   
   C   
   G   
    G    
   A   
   C   
    A    
    G    
   C   
    C    
   G   
   T   
    A    
   A   
   C   
    A    
   G   
   C   
    C    
   T   
   T   
    T    
    T    
   C   
   T   
   A   
   A   
    T    
   A   
   T   
    C    
   C   
   G   
    T    
   A   
   G   
    T    
   A   
   A   
   T   
   G   
   G   
    A    
    T    
   T   
    A    
   A   
   T   
    C    
   G   
   C   
    C    
   A   
   T   
   T   
    A    
   G   
    C    
   T   
   T   
    A    
   A   
   A   
   A   
   G   
   A   
   A   
   G   
   A   
    T    
   G   
   A   
    T    
   G   
   A   
    G    
    T    
   T   
    A    
    G    
   T   
    T    
   A   
   A   
    C    
   G   
   T   
    G    
    G    
   T   
   A   
    A    
    C    
   G   
   A   
   C   
    T    
    A    
   A   
    T    
   G   
   G   
    C    
    A    
   A   
    T    
    C    
    A    
    G    
   A   
   A   
    G    
   A   
   T   
    G    
   A   
   T   
    T    
   A   
   T   
    C    
   G   
   G   
    A    
   A   
   C   
   A   
   C   
   A   
   T   
    G    
    C    
    A    
   G   
   G   
    A    
   T   
   A   
   C   
   T   
   C   
   T   
   G   
   T   
    C    
   A   
   C   
    A    
   T   
   T   
    T    
    G    
   A   
    T    
   G   
   A   
    A    
    A    
    A    
    T    
    A    
   C   
    T    
   G   
    T    
    A    
   C   
   G   
    T    
   G   
   A   
   T   
   A   
   T   
   G   
   G   
   G   
    C    
   C   
   G   
    G    
   A   
   C   
   A   
   G   
   C   
    A    
   T   
   C   
    A    
   G   
   G   
    T    
   G   
   T   
    T    
    C    
   G   
    G    
   G   
   G   
    A    
   A   
   T   
    C    
   C   
   G   
    T    
    C    
   T   
    C    
   C   
   G   
    C    
   G   
   A   
    A    
    A    
   A   
    T    
   G   
   A   
    T    
   T   
    A    
    T    
   G   
   T   
    G    
   G   
   T   
    C    
   G   
   G   
    T    
    G    
   C   
   A   
   G   
   C   
    G    
    A    
   T   
    T    
    C    
   T   
   G   
    G    
   A   
    T    
   G   
    A    
   A   
    A    
   A   
    C    
    A    
   A   
   A   
   G   
   A   
   A   
   G   
   T   
    C    
    C    
   T   
    A    
   G   
   T   
    C    
   A   
   T   
    T    
   A   
   C   
    T    
   G   
   A   
   A   
   A   
   A   
    T    
   G   
   G   
   T   
   T   
   A   
    T    
   G   
   G   
    T    
   A   
   A   
    G    
   C   
   G   
    T    
   A   
   C   
    A    
   A   
   A   
   A   
   G   
    C    
    T    
   T   
   C   
    T    
   G   
   A   
    A    
   T   
   A   
   T   
   C   
   C   
   A   
   G   
   T   
    T    
   A   
   A   
    A    
   G   
   G   
    A    
   C   
   G   
    T    
   G   
   G   
    C    
   G   
   G   
   T   
   A   
   A   
   A   
   G   
   G   
    G    
   A   
   T   
    T    
   A   
   A   
    G    
   A   
   C   
    A    
    G    
   C   
    A    
   A   
   A   
    T    
   A   
   T   
   C   
   A   
   C   
   T   
   G   
   A   
    G    
   A   
   A   
   A   
   A   
   A   
   T   
   G   
   G   
   T   
   C   
   C   
   A   
    T    
   T   
   A   
   G   
   C   
    T    
   G   
   G   
    T    
   T   
   T   
    A    
   A   
   C   
   C   
   A   
   C   
    G    
   G   
   T   
    C    
    A    
    A    
   T   
   G   
   G   
    G    
    G    
   A   
   T   
   G   
   A   
   A   
   G   
   A   
    T    
   A   
   T   
   C   
    T    
   T   
    A    
    T    
   T   
    G    
   A   
   T   
    T    
   A   
   C   
    G    
    A    
   A   
    C    
   A   
   A   
   A   
   G   
   G   
    C    
   G   
   T   
    C    
   A   
   T   
   T   
   A   
   T   
   C   
   C   
   G   
    C    
   T   
   T   
    T    
   A   
   A   
   C   
   G   
   T   
    T    
    G    
    A    
    T    
    T    
   C   
   T   
   G   
   T   
   T   
   T   
   C   
   T   
   C   
   A   
   A   
   A   
   C   
    A    
   G   
   G   
   A   
   C   
   G   
    C    
    G    
   C   
   A   
   A   
   C   
   A   
   T   
   T   
    A    
   G   
   G   
    G    
   G   
   T   
    T    
   C   
   G   
   T   
   T   
   T   
    A    
   A   
   T   
   G   
   A   
    G    
    A    
   A   
   T   
   G   
   G   
   A   
   A   
   G   
    A    
    T    
   G   
    G    
    T    
    G    
   C   
    C    
   A   
   A   
   A   
   -   
   -   
   -   
   -   
   -   
   -   
   -   
   -   
   -   
   -   
   -   
   -   
   -   
   -   
   -   
   -   
   -   
   -   
   -   
   -   
   -   
   -   
   -   
   -   
   -   
   -   
   -   
   -   
   -   
   -   
   -   
   -   
   -   
   -   
   -   
   -   
   -   
   -   
   -   
   -   
   -   
   -   
   -   
   -   
   -   
   -   
   -   
   -   
   -   
   -   
   -   
   -   
   -   
   -   
   -   
   -   
   -   
   -   
   -   
   -   
   -   
   -   
   -   
   -   
   -   
   -   
   -   
   -   
   -   
   -   
   -   
   -   
 

 
   N52829   
   A   
   T   
   G   
   A   
   G   
   T   
   G   
   A   
   A   
   G   
   A   
   A   
   A   
   T   
   C   
   A   
   G   
   A   
   G   
   A   
   A   
   A   
   A   
   C   
   A   
   T   
   C   
   C   
   A   
   T   
   G   
   A   
   T   
   G   
   T   
   C   
   A   
   A   
   T   
   C   
   T   
   A   
   A   
   C   
   C   
   A   
   G   
   C   
   G   
   A   
   A   
   A   
   T   
   G   
   A   
   A   
   A   
   G   
   A   
   C   
   T   
   C   
   C   
   T   
   T   
   C   
   A   
   T   
   C   
   G   
   A   
   T   
   T   
   A   
   T   
   G   
   C   
   G   
   A   
   T   
   G   
   A   
   G   
   T   
   G   
   T   
   T   
   A   
   T   
   T   
   G   
   T   
   C   
   G   
   C   
   A   
   C   
   G   
   T   
   G   
   C   
   A   
   C   
   T   
   T   
   C   
   C   
   T   
   G   
   A   
   T   
   G   
   T   
   C   
   A   
   G   
   A   
   G   
   A   
   T   
   G   
   G   
   T   
   C   
   T   
   G   
   A   
   A   
   A   
   C   
   C   
   C   
   G   
   T   
   T   
   C   
   A   
   T   
   C   
   G   
   C   
   C   
   G   
   T   
   A   
   T   
   T   
   T   
   T   
   A   
   T   
   A   
   C   
   G   
   G   
   C   
   A   
   T   
   G   
   A   
   A   
   C   
   G   
   A   
   A   
   T   
   T   
   A   
   G   
   G   
   T   
   G   
   T   
   A   
   A   
   C   
   A   
   C   
   C   
   G   
   G   
   A   
   T   
   A   
   A   
   A   
   G   
   C   
   C   
   C   
   A   
   T   
   A   
   A   
   A   
   A   
   A   
   A   
   T   
   C   
   T   
   G   
   C   
   C   
   A   
   G   
   A   
   A   
   T   
   C   
   G   
   T   
   C   
   G   
   G   
   A   
   G   
   A   
   T   
   G   
   T   
   C   
   A   
   T   
   G   
   G   
   G   
   T   
   A   
   A   
   A   
   T   
   A   
   C   
   C   
   A   
   T   
   C   
   C   
   T   
   C   
   A   
   T   
   G   
   G   
   A   
   G   
   A   
   C   
   A   
   G   
   T   
   G   
   C   
   G   
   A   
   T   
   C   
   T   
   A   
   T   
   G   
   A   
   G   
   T   
   C   
   A   
   A   
   T   
   G   
   G   
   T   
   A   
   C   
   G   
   G   
   A   
   T   
   G   
   G   
   C   
   A   
   C   
   A   
   G   
   C   
   C   
   A   
   T   
   T   
   C   
   A   
   G   
   C   
   T   
   A   
   C   
   C   
   G   
   T   
   T   
   A   
   T   
   A   
   T   
   G   
   C   
   T   
   A   
   G   
   T   
   G   
   G   
   A   
   T   
   G   
   G   
   T   
   C   
   A   
   T   
   G   
   G   
   A   
   A   
   A   
   C   
   T   
   T   
   T   
   G   
   G   
   T   
   T   
   C   
   T   
   G   
   T   
   T   
   G   
   A   
   C   
   G   
   G   
   T   
   G   
   A   
   C   
   G   
   G   
   T   
   G   
   C   
   C   
   G   
   C   
   C   
   G   
   C   
   T   
   A   
   T   
   G   
   C   
   G   
   T   
   T   
   A   
   T   
   A   
   C   
   C   
   G   
   A   
   A   
   G   
   C   
   A   
   C   
   G   
   G   
   A   
   T   
   G   
   A   
   G   
   C   
   A   
   A   
   A   
   A   
   T   
   C   
   G   
   C   
   T   
   A   
   C   
   A   
   G   
   A   
   A   
   A   
   T   
   G   
   C   
   T   
   G   
   C   
   G   
   T   
   G   
   A   
   T   
   A   
   T   
   C   
   A   
   A   
   T   
   A   
   A   
   G   
   A   
   A   
   C   
   A   
   C   
   T   
   G   
   T   
   T   
   G   
   A   
   T   
   T   
   T   
   C   
   C   
   A   
   A   
   A   
   G   
   C   
   A   
   A   
   C   
   T   
   A   
   T   
   G   
   A   
   T   
   G   
   A   
   T   
   A   
   C   
   A   
   G   
   A   
   A   
   A   
   A   
   A   
   G   
   A   
   A   
   C   
   C   
   A   
   G   
   T   
   T   
   G   
   T   
   A   
   C   
   T   
   A   
   C   
   C   
   T   
   G   
   C   
   T   
   C   
   G   
   C   
   T   
   T   
   C   
   C   
   C   
   G   
   A   
   A   
   T   
   C   
   T   
   T   
   T   
   T   
   A   
   G   
   T   
   G   
   A   
   A   
   C   
   G   
   G   
   A   
   A   
   C   
   A   
   A   
   C   
   G   
   G   
   G   
   G   
   A   
   T   
   C   
   G   
   C   
   C   
   G   
   T   
   A   
   G   
   G   
   T   
   A   
   T   
   G   
   G   
   C   
   C   
   A   
   C   
   G   
   A   
   A   
   T   
   A   
   T   
   C   
   C   
   C   
   A   
   C   
   C   
   T   
   C   
   A   
   T   
   A   
   A   
   C   
   T   
   T   
   G   
   T   
   C   
   T   
   G   
   A   
   G   
   G   
   T   
   A   
   G   
   T   
   A   
   G   
   C   
   G   
   G   
   C   
   T   
   A   
   T   
   C   
   G   
   A   
   T   
   T   
   T   
   G   
   C   
   T   
   G   
   A   
   T   
   G   
   G   
   A   
   T   
   A   
   A   
   T   
   C   
   C   
   T   
   G   
   A   
   T   
   G   
   T   
   G   
   A   
   C   
   G   
   A   
   C   
   C   
   A   
   A   
   T   
   G   
   A   
   A   
   T   
   T   
   G   
   A   
   T   
   G   
   G   
   A   
   A   
   G   
   T   
   A   
   T   
   T   
   A   
   C   
   C   
   T   
   G   
   G   
   A   
   C   
   C   
   A   
   G   
   A   
   T   
   T   
   T   
   T   
   C   
   C   
   A   
   A   
   C   
   A   
   G   
   G   
   T   
   G   
   G   
   T   
   T   
   T   
   G   
   G   
   T   
   T   
   A   
   T   
   G   
   G   
   G   
   G   
   A   
   A   
   A   
   T   
   C   
   A   
   G   
   G   
    A    
   A   
   T   
    C    
   C   
   G   
    A    
    A    
   G   
   A   
   G   
   C   
    G    
   T   
   A   
   T   
   G   
   A   
   A   
   A   
   C   
    A    
   G   
   G   
    T    
   A   
   A   
    G    
   G   
   G   
   T   
   T   
   C   
    T    
   A   
   T   
   T   
   A   
   C   
    C    
   G   
   T   
    A    
   C   
   G   
   T   
   G   
   C   
   A   
   A   
   A   
   A   
   G   
   T   
   T   
   G   
   A   
    G    
    A    
   T   
    T    
   A   
    C    
   T   
   G   
   A   
    G    
   A   
   T   
   G   
    C    
   C   
    A    
   A   
   A   
   T   
   G   
   G   
    T    
   A   
   A   
   A   
   G   
   A   
   A   
   C   
   G   
    G    
   A   
   T   
    C    
    A    
   T   
    C    
   G   
   T   
    G    
   A   
   C   
    A    
   G   
   A   
   A   
   T   
   T   
    G    
   C   
   C   
   T   
   T   
   A   
   T   
   A   
   T   
   G   
   G   
   T   
    C    
   A   
   A   
   T   
   A   
   A   
   A   
   G   
   C   
    A    
   A   
   A   
   A   
   T   
   T   
    G    
   A   
   T   
    C    
   G   
   A   
   A   
   C   
   G   
    G    
   A   
   T   
   T   
   T   
   C   
   T   
   G   
   A   
    A    
    C    
   T   
    G    
   C   
   A   
   C   
   A   
   G   
   A   
   G   
   A   
    C    
   A   
   A   
    G    
   C   
   G   
    T    
   A   
   T   
    C    
   G   
   A   
   A   
   G   
   G   
    G    
   A   
   T   
    C    
   A   
   C   
    A    
   G   
   A   
   T   
   T   
   T   
   G   
   C   
   G   
   G   
   G   
   A   
    C    
   G   
   A   
   A   
   T   
   C   
   T   
   T   
   C   
    C    
   C   
   G   
   T   
   G   
   A   
    A    
   G   
   G   
    T    
   A   
   T   
   G   
   C   
   G   
    G    
   A   
   T   
    C    
   G   
   T   
   C   
   A   
   T   
    T    
   G   
   A   
    C    
    A    
   T   
    C    
   C   
   G   
   T   
   C   
   G   
    T    
   G   
   A   
    C    
   G   
    C    
    C    
   A   
   G   
    C    
   G   
   C   
    A    
   T   
   C   
    C    
   G   
   T   
   T   
    A    
   T   
    C    
   T   
   T   
    G    
   A   
   A   
    T    
   A   
   A   
   C   
   T   
   T   
    A    
   T   
   A   
    T    
   A   
   A   
   A   
    T    
   T   
   G   
   A   
   C   
    A    
    T    
   C   
    C    
   T   
   T   
    G    
   C   
   A   
    A    
   A   
    A    
    T    
   T   
   C   
    A    
   T   
   T   
    T    
   G   
   G   
    A    
   T   
   T   
    C    
   A   
   A   
    T    
   A   
   T   
   G   
   T   
   T   
    G    
   G   
   C   
    G    
   A   
   T   
    C    
   G   
   A   
   A   
   A   
   A   
   A   
   G   
   G   
    T    
   G   
   T   
    G    
   C   
   C   
    G    
   A   
   A   
   A   
    G    
   T   
   T   
   T   
   T   
    G    
   A   
   G   
    C    
   T   
   T   
   G   
   A   
   A   
   A   
   C   
    A    
    A    
   A   
   T   
    C    
    C    
   T   
    T    
   G   
   A   
   A   
   A   
   A   
    C    
   T   
   A   
    T    
    A    
   T   
    C    
   G   
   A   
    A    
   C   
   A   
    T    
   C   
   A   
    A    
    C    
    G    
    C    
    G    
   A   
   A   
   G   
   T   
   C   
   A   
   T   
    C    
   A   
   C   
    A    
    C    
   G   
    A    
   C   
   G   
   G   
   A   
   C   
    G    
    G    
   T   
    C    
   T   
   T   
   T   
   G   
   A   
    A    
   A   
   A   
   A   
    G    
   A   
    A    
   A   
   A   
    A    
   G   
   C   
   A   
   G   
   A   
   A   
   G   
   C   
   A   
    A    
   G   
    A    
   G   
   C   
    A    
   C   
   A   
    C    
   A   
   T   
    C    
   T   
   T   
   A   
   G   
   A   
   A   
   G   
   G   
   T   
    T    
   T   
    G    
   C   
   G   
    T    
   A   
   T   
   T   
   G   
   C   
    T    
   T   
   T   
    G    
   G   
   A   
   T   
   C   
   A   
   T   
   A   
   T   
   C   
    G    
   A   
   T   
   G   
   A   
   A   
   A   
   T   
    C    
   A   
   T   
   T   
   G   
   C   
    G    
   A   
   T   
   T   
   A   
   T   
    C    
   C   
   G   
    C    
   G   
   G   
    G    
   T   
   C   
    T    
    A    
   A   
   A   
   T   
   C   
   A   
   G   
   A   
   T   
   G   
   A   
    C    
   G   
   A   
    G    
   G   
   C   
   A   
   A   
   A   
   A   
   G   
   C   
    A    
   A   
    C    
    A    
    A    
   T   
    G    
   A   
   T   
    C    
   G   
   A   
   A   
   C   
   G   
    G    
   T   
   T   
    C    
   G   
   A   
    T    
   T   
   T   
    A    
   T   
   C   
    C    
   G   
   A   
   T   
   C   
   G   
    C    
   C   
   A   
    G    
    T    
   C   
    T    
   C   
   A   
   A   
   G   
   C   
    T    
   A   
   T   
    T    
   T   
   T   
   A   
   G   
   A   
   T   
   A   
   T   
   G   
   C   
   G   
   T   
   T   
   T   
    A    
   C   
   G   
    C    
   C   
   G   
    A    
   T   
   T   
    G    
   A   
   C   
    C    
   G   
   G   
    T    
   T   
   T   
    G    
   G   
   A   
   A   
   C   
   G   
    G    
   G   
   A   
    A    
   A   
   A   
   A   
   A   
   T   
    C    
   G   
   A   
    A    
   A   
   A   
   T   
   G   
   A   
   A   
   T   
   A   
    C    
   C   
   A   
   A   
   G   
   A   
    A    
   C   
   T   
    C    
   T   
   T   
    A    
   A   
    A    
   A   
    C    
   T   
    G    
   A   
   T   
   T   
   G   
    A    
    A    
   G   
   A   
    T    
   T   
   T   
    A    
    A    
    C    
    T    
   G   
   A   
   C   
   A   
   T   
   T   
    C    
   T   
    G    
   G   
   C   
    A    
    A    
   G   
    A    
   C   
   C   
    A    
   G   
   A   
    A    
   C   
   G   
    T    
   G   
   T   
    T    
   A   
    C    
    A    
   G   
   A   
   A   
   A   
   T   
    C    
   A   
   T   
    C    
   A   
   A   
    G    
   A   
    A    
    T    
   G   
   A   
    A    
   T   
   T   
   A   
    G    
    C    
    A    
   G   
   A   
   C   
    T    
   T   
    G    
    A    
    A    
    T    
    C    
    A    
   A   
    C    
    G    
   A   
   T   
   T   
   T   
   G   
   G   
   T   
   G   
   A   
   T   
    A    
    A    
   A   
   C   
   G   
   A   
   C   
   G   
    C    
   A   
   C   
   A   
   G   
   A   
   A   
    T    
   T   
   A   
    C    
   T   
   A   
   G   
   T   
    C    
   G   
   G   
   T   
   G   
   A   
    G    
   G   
   T   
   C   
    T    
   T   
    A    
   A   
   G   
   T   
   C   
   T   
    G    
   G   
   A   
   A   
   G   
   A   
   T   
   G   
   A   
   A   
   G   
   A   
   T   
    T    
   T   
    A    
   A   
   T   
    C    
   G   
   A   
   A   
   G   
   A   
   A   
   G   
   A   
   A   
   G   
   A   
   A   
    A    
   T   
   C   
   G   
   T   
    T    
   A   
   T   
   T   
   A   
   C   
    A    
    T    
   T   
    G    
   A   
   C   
    A    
   A   
   A   
    C    
   A   
   A   
    T    
   G   
   G   
    T    
   T   
   A   
    C    
   A   
   T   
    C    
   A   
   A   
    A    
   C   
   G   
    T    
    T    
   T   
   G   
   G   
    C    
   A   
   A   
   A   
    T    
    A    
    A    
    T    
   G   
   A   
   A   
   T   
   T   
   C   
    A    
   G   
    A    
   G   
   C   
    T    
   C   
   A   
   A   
   C   
   G   
    A    
    A    
   G   
    G    
   G   
   G   
    T    
   G   
   G   
    A    
   C   
   G   
   T   
   G   
   G   
    T    
   G   
   T   
    C    
   C   
   A   
   A   
   G   
   G   
    A    
   A   
   T   
   G   
   G   
   G   
    T    
    A    
   T   
    C    
   C   
   A   
    C    
    A    
   A   
   T   
   G   
   A   
    C    
   G   
   A   
    C    
   T   
   T   
   C   
   G   
   T   
    C    
   A   
   A   
    A    
   A   
   A   
    T    
   C   
   T   
    G    
    G    
   T   
   T   
   T   
   C   
   T   
   T   
   G   
    T    
   T   
   C   
    T    
   A   
   C   
    C    
   C   
   A   
    T    
   G   
   A   
    T    
   A   
   C   
    A    
   T   
   T   
    G    
   T   
   T   
   A   
   T   
   T   
    C    
   T   
   T   
   T   
   A   
   C   
   G   
   A   
   A   
    T    
   A   
    A    
    C    
   G   
   G   
    A    
   A   
   A   
   A   
   G   
   T   
    G    
   T   
   A   
    T    
   C   
   G   
    G    
   G   
   C   
   A   
   A   
   A   
   A   
   G   
   G   
    A    
   T   
   A   
    C    
   G   
   A   
   A   
   A   
   T   
    T    
   C   
   C   
    A    
   G   
   A   
    A    
   T   
   A   
   C   
   G   
   G   
    G    
    C    
   G   
    T    
   A   
   C   
   A   
   G   
   C   
    C    
   A   
   A   
   A   
   G   
   G   
    A    
   A   
   T   
    C    
   C   
   C   
   A   
    A    
   T   
    C    
   A   
   T   
    C    
   A   
   A   
    T    
   T   
   T   
    A    
   C   
   T   
    A    
   G   
   G   
    T    
   A   
   T   
    C    
   G   
   A   
    T    
   T   
   C   
    G    
    A    
    G    
    T    
   G   
   A   
   A   
   A   
    G    
    C    
   A   
   T   
    C    
   C   
   A   
    G    
   G   
   C   
    A    
   A   
   T   
    C    
   A   
   T   
   T   
    G    
   C   
    G    
   G   
   T   
    G    
    A    
    C    
   G   
   G   
   G   
    A    
    G    
   A   
   A   
   G   
   C   
    A    
   G   
   A   
   A   
   G   
    A    
    A    
   G   
   G   
    C    
    C    
   A   
    C    
   T   
   A   
    T    
    C    
   T   
    C    
   T   
   T   
   C   
   T   
   T   
    T    
   A   
   C   
    G    
   A   
   C   
    G    
    C    
    G    
    T    
    C    
   A   
   A   
   G   
   G   
    T    
   A   
   C   
    A    
   G   
   T   
    A    
   A   
   A   
   A   
   C   
   G   
    T    
   A   
   C   
    T    
    T    
   C   
    T    
   G   
   T   
    C    
   A   
   C   
    T    
   G   
   C   
    T    
   T   
   T   
    T    
    G    
   C   
   T   
   A   
   A   
    C    
   A   
   T   
    C    
   C   
   G   
    A    
   A   
   G   
    T    
   A   
   A   
   T   
   G   
   G   
    T    
    C    
   T   
    G    
   A   
   T   
    C    
   G   
   C   
    A    
   A   
   T   
   T   
    G    
   G   
    T    
   T   
   T   
    G    
   A   
   A   
   A   
   G   
   A   
   A   
   G   
   A   
    C    
   G   
   A   
    T    
   G   
   A   
    G    
    C    
   T   
    C    
    G    
   T   
    C    
   A   
   A   
    T    
   G   
   T   
    G    
    C    
   T   
   A   
    C    
    T    
   G   
   A   
   C   
    A    
    G    
   A   
    T    
   G   
   G   
    T    
    C    
   A   
    A    
    T    
    C    
    T    
   A   
   A   
    T    
   A   
   T   
    C    
   A   
   T   
    T    
   A   
   T   
    T    
   G   
   G   
    A    
   A   
   C   
   A   
   C   
   A   
   T   
    A    
    A    
    C    
   G   
   G   
    C    
   T   
   A   
   C   
   T   
   C   
   T   
   G   
   T   
    C    
   A   
   C   
    T    
   T   
   T   
    C    
    A    
   A   
    A    
   G   
   A   
    A    
    G    
    A    
    A    
    G    
   C   
    A    
   G   
    T    
    C    
   C   
   G   
    C    
   G   
   A   
   T   
   A   
   T   
   G   
   G   
   G   
    A    
   C   
   G   
    T    
   A   
   C   
   A   
   G   
   C   
    T    
   T   
   C   
    C    
   G   
   G   
    G    
   G   
   T   
    C    
    A    
   G   
    A    
   G   
   G   
    G    
   A   
   T   
    A    
   C   
   G   
    C    
    C    
   T   
    G    
   C   
   G   
    T    
   G   
   A   
    G    
    G    
   A   
    A    
   G   
   A   
    T    
   T   
    A    
    T    
   G   
   T   
    C    
   G   
   T   
    T    
   G   
   G   
    G    
    G    
   C   
   A   
   G   
   C   
    C    
    T    
   T   
    A    
    A    
   T   
   G   
    A    
   A   
    G    
   G   
    A    
   A   
    A    
   A   
    T    
    C    
   A   
   A   
   G   
   A   
   A   
   G   
   T   
    A    
    T    
   T   
    A    
   G   
   T   
    G    
   A   
   T   
    C    
   A   
   C   
    A    
   G   
   A   
   A   
   A   
   A   
    A    
   G   
   G   
   T   
   T   
   A   
    C    
   G   
   G   
    A    
   A   
   A   
    A    
   C   
   G   
    G    
   A   
   C   
    A    
   A   
   A   
   A   
   G   
    T    
    A    
   T   
   C   
    G    
   G   
   A   
    A    
   T   
   A   
   T   
   C   
   C   
   A   
   G   
   T   
    G    
   A   
   A   
    A    
   G   
   G   
    C    
   C   
   G   
    C    
   G   
   G   
    A    
   G   
   G   
   T   
   A   
   A   
   A   
   G   
   G   
    G    
   A   
   T   
    C    
   A   
   A   
    A    
   A   
   C   
    G    
    G    
   C   
    G    
   A   
   A   
    C    
   A   
   T   
   C   
   A   
   C   
   T   
   G   
   A   
    A    
   A   
   A   
   A   
   A   
   A   
   T   
   G   
   G   
   T   
   C   
   C   
   A   
    C    
   T   
   A   
   G   
   C   
    T    
   G   
   G   
    A    
   T   
   T   
    G    
   A   
   C   
   C   
   A   
   C   
    T    
   G   
   T   
    T    
    T    
    C    
   T   
   G   
   G   
    A    
    G    
   A   
   T   
   G   
   A   
   A   
   G   
   A   
    C    
   A   
   T   
   C   
    A    
   T   
    G    
    C    
   T   
    G    
   A   
   T   
    T    
   A   
   C   
    A    
    G    
   A   
    T    
   A   
   A   
   A   
   G   
   G   
    T    
   G   
   T   
    C    
   A   
   T   
   T   
   A   
   T   
   C   
   C   
   G   
    T    
   T   
   T   
    C    
   A   
   A   
   C   
   G   
   T   
    A    
    T    
    C    
    G    
    A    
   C   
   T   
   G   
   T   
   T   
   T   
   C   
   T   
   C   
   A   
   A   
   A   
   C   
    T    
   G   
   G   
   A   
   C   
   G   
    T    
    T    
   C   
   A   
   A   
   C   
   A   
   T   
   T   
    A    
   G   
   G   
    C    
   G   
   T   
    A    
   C   
   G   
   T   
   T   
   T   
    G    
   A   
   T   
   G   
   A   
    A    
    G    
   A   
   T   
   G   
   G   
   A   
   A   
   G   
    C    
    T    
   G   
    A    
    T    
    A    
   C   
    G    
   A   
   A   
   A   
   G   
   T   
   T   
   G   
   T   
   C   
   A   
   C   
   G   
   A   
   T   
   G   
   G   
   C   
   A   
   G   
   C   
   A   
   G   
   T   
   C   
   G   
   A   
   A   
   C   
   C   
   A   
   G   
   A   
   A   
   G   
   C   
   T   
   G   
   C   
   A   
   G   
   A   
   A   
   C   
   A   
   A   
   A   
   C   
   A   
   G   
   T   
   T   
   G   
   A   
   A   
   G   
   A   
   A   
   C   
   A   
   A   
   G   
   G   
   A   
   T   
   C   
   A   
   G   
   A   
   A   
   G   
   A   
   A   
   -   
   -   
   -   
 

 
   N59609F   
   A   
   T   
   G   
   A   
   G   
   T   
   G   
   A   
   A   
   G   
   A   
   A   
   A   
   T   
   T   
   A   
   A   
   A   
   G   
   A   
   A   
   A   
   A   
   C   
   A   
   T   
   T   
   C   
   A   
   A   
   G   
   A   
   C   
   G   
   T   
   C   
   A   
   A   
   T   
   C   
   T   
   G   
   A   
   C   
   C   
   A   
   G   
   C   
   G   
   A   
   A   
   A   
   T   
   G   
   A   
   A   
   A   
   G   
   A   
   A   
   T   
   C   
   T   
   T   
   T   
   C   
   A   
   T   
   T   
   G   
   A   
   C   
   T   
   A   
   C   
   G   
   C   
   A   
   A   
   T   
   G   
   A   
   G   
   T   
   G   
   T   
   T   
   A   
   T   
   C   
   G   
   T   
   A   
   G   
   C   
   C   
   C   
   G   
   C   
   G   
   C   
   G   
   T   
   T   
   A   
   C   
   C   
   T   
   G   
   A   
   C   
   G   
   T   
   T   
   C   
   G   
   T   
   G   
   A   
   C   
   G   
   G   
   T   
   T   
   T   
   A   
   A   
   A   
   A   
   C   
   C   
   T   
   G   
   T   
   T   
   C   
   A   
   T   
   C   
   G   
   C   
   C   
   G   
   A   
   A   
   T   
   C   
   T   
   T   
   A   
   T   
   A   
   T   
   G   
   G   
   A   
   A   
   T   
   G   
   A   
   A   
   C   
   G   
   A   
   A   
   T   
   T   
   A   
   G   
   G   
   G   
   G   
   T   
   A   
   A   
   C   
   C   
   C   
   C   
   T   
   G   
   A   
   T   
   A   
   A   
   A   
   C   
   C   
   A   
   C   
   A   
   C   
   A   
   A   
   A   
   A   
   A   
   A   
   T   
   C   
   A   
   G   
   C   
   C   
   C   
   G   
   G   
   A   
   T   
   T   
   G   
   T   
   T   
   G   
   G   
   G   
   G   
   A   
   T   
   G   
   T   
   T   
   A   
   T   
   G   
   G   
   G   
   T   
   A   
   A   
   A   
   T   
   A   
   T   
   C   
   A   
   C   
   C   
   C   
   C   
   C   
   A   
   T   
   G   
   G   
   G   
   G   
   A   
   C   
   A   
   G   
   T   
   G   
   C   
   G   
   A   
   T   
   T   
   T   
   A   
   C   
   G   
   A   
   A   
   T   
   C   
   A   
   A   
   T   
   G   
   G   
   T   
   G   
   C   
   G   
   G   
   A   
   T   
   G   
   G   
   C   
   A   
   C   
   A   
   A   
   C   
   C   
   T   
   T   
   T   
   T   
   A   
   G   
   T   
   T   
   A   
   T   
   C   
   G   
   G   
   G   
   C   
   T   
   A   
   T   
   G   
   T   
   T   
   A   
   G   
   T   
   T   
   G   
   A   
   C   
   G   
   G   
   C   
   C   
   A   
   C   
   G   
   G   
   A   
   A   
   A   
   C   
   T   
   T   
   C   
   G   
   G   
   T   
   T   
   C   
   T   
   G   
   T   
   C   
   G   
   A   
   T   
   G   
   G   
   T   
   G   
   A   
   C   
   G   
   G   
   C   
   G   
   C   
   T   
   G   
   C   
   C   
   G   
   C   
   T   
   A   
   T   
   G   
   C   
   G   
   T   
   T   
   A   
   T   
   A   
   C   
   C   
   G   
   A   
   A   
   G   
   C   
   A   
   C   
   G   
   T   
   A   
   T   
   G   
   A   
   G   
   T   
   A   
   A   
   A   
   A   
   T   
   T   
   G   
   C   
   T   
   T   
   T   
   A   
   G   
   A   
   A   
   A   
   T   
   G   
   C   
   T   
   A   
   C   
   G   
   A   
   G   
   A   
   T   
   A   
   T   
   T   
   A   
   A   
   C   
   A   
   A   
   A   
   A   
   A   
   T   
   A   
   C   
   A   
   G   
   T   
   C   
   G   
   A   
   T   
   T   
   T   
   C   
   C   
   A   
   A   
   G   
   G   
   A   
   A   
   A   
   C   
   T   
   A   
   T   
   G   
   A   
   T   
   G   
   A   
   T   
   T   
   C   
   A   
   G   
   A   
   A   
   C   
   A   
   A   
   G   
   A   
   A   
   C   
   C   
   A   
   G   
   T   
   A   
   G   
   T   
   T   
   T   
   T   
   A   
   C   
   C   
   T   
   G   
   C   
   T   
   C   
   G   
   T   
   T   
   T   
   T   
   C   
   C   
   A   
   A   
   A   
   C   
   T   
   T   
   A   
   C   
   T   
   A   
   G   
   T   
   T   
   A   
   A   
   C   
   G   
   G   
   A   
   A   
   C   
   A   
   A   
   C   
   G   
   G   
   G   
   G   
   A   
   T   
   T   
   G   
   C   
   G   
   G   
   T   
   G   
   G   
   G   
   G   
   A   
   T   
   G   
   G   
   C   
   A   
   A   
   C   
   A   
   A   
   A   
   T   
   A   
   T   
   T   
   C   
   C   
   A   
   C   
   C   
   A   
   C   
   A   
   C   
   A   
   A   
   T   
   T   
   T   
   A   
   A   
   G   
   T   
   G   
   A   
   A   
   G   
   T   
   G   
   A   
   T   
   T   
   G   
   A   
   T   
   G   
   C   
   A   
   A   
   C   
   A   
   A   
   G   
   T   
   T   
   T   
   A   
   T   
   T   
   G   
   A   
   T   
   G   
   G   
   A   
   C   
   A   
   A   
   T   
   C   
   C   
   T   
   G   
   A   
   T   
   G   
   T   
   A   
   A   
   C   
   G   
   A   
   C   
   G   
   A   
   A   
   T   
   G   
   A   
   A   
   T   
   T   
   G   
   A   
   T   
   G   
   G   
   A   
   A   
   G   
   T   
   G   
   T   
   T   
   A   
   C   
   C   
   T   
   G   
   G   
   A   
   C   
   C   
   A   
   G   
   A   
   T   
   T   
   T   
   T   
   C   
   C   
   A   
   A   
   C   
   A   
   G   
   G   
   C   
   G   
   G   
   T   
   T   
   T   
   A   
   G   
   T   
   G   
   A   
   T   
   G   
   G   
   G   
   G   
   A   
   A   
   A   
   T   
   C   
   A   
   G   
   G   
    G    
   A   
   T   
    T    
   C   
   G   
    C    
    C    
   G   
   A   
   G   
   C   
    A    
   T   
   A   
   T   
   G   
   A   
   A   
   A   
   C   
    A    
   G   
   G   
    G    
   A   
   A   
    A    
   G   
   G   
   T   
   T   
   C   
    G    
   A   
   T   
   T   
   A   
   C   
    T    
   G   
   T   
    T    
   C   
   G   
   T   
   G   
   C   
   A   
   A   
   A   
   A   
   G   
   T   
   T   
   G   
   A   
    A    
    T    
   T   
    G    
   A   
    C    
   T   
   G   
   A   
    A    
   A   
   T   
   G   
    C    
   C   
    G    
   A   
   A   
   T   
   G   
   G   
    A    
   A   
   A   
   A   
   G   
   A   
   A   
   C   
   G   
    T    
   A   
   T   
    T    
    T    
   T   
    A    
   G   
   T   
    A    
   A   
   C   
    T    
   G   
   A   
   A   
   T   
   T   
    G    
   C   
   C   
   T   
   T   
   A   
   T   
   A   
   T   
   G   
   G   
   T   
    G    
   A   
   A   
   T   
   A   
   A   
   A   
   G   
   C   
    C    
   A   
   A   
   A   
   T   
   T   
    A    
   A   
   T   
    C    
   G   
   A   
   A   
   C   
   G   
    A    
   A   
   T   
   T   
   T   
   C   
   T   
   G   
   A   
    A    
    T    
   T   
    A    
   C   
   A   
   C   
   A   
   G   
   A   
   G   
   A   
    T    
   A   
   A   
    A    
   C   
   G   
    A    
   A   
   T   
    T    
   G   
   A   
   A   
   G   
   G   
    A    
   A   
   T   
    T    
   A   
   C   
    T    
   G   
   A   
   T   
   T   
   T   
   G   
   C   
   G   
   G   
   G   
   A   
    T    
   G   
   A   
   A   
   T   
   C   
   T   
   T   
   C   
    T    
   C   
   G   
   T   
   G   
   A   
    A    
   G   
   G   
    C    
   A   
   T   
   G   
   C   
   G   
    G    
   A   
   T   
    C    
   G   
   T   
   C   
   A   
   T   
    C    
   G   
   A   
    T    
    G    
   T   
    T    
   C   
   G   
   T   
   C   
   G   
    A    
   G   
   A   
    T    
   G   
    T    
    G    
   A   
   G   
    T    
   G   
   C   
    C    
   T   
   C   
    T    
   G   
   T   
   T   
    G    
   T   
    G    
   T   
   T   
    A    
   A   
   A   
    C    
   A   
   A   
   C   
   T   
   T   
    G    
   T   
   A   
    C    
   A   
   A   
   A   
    A    
   T   
   G   
   A   
   C   
    T    
    G    
   C   
    C    
   T   
   T   
    A    
   C   
   A   
    A    
   A   
    C    
    A    
   T   
   C   
    T    
   T   
   T   
    T    
   G   
   G   
    T    
   T   
   T   
    T    
   A   
   A   
    C    
   A   
   T   
   G   
   T   
   T   
    A    
   G   
   C   
    C    
   A   
   T   
    T    
   G   
   A   
   A   
   A   
   A   
   A   
   G   
   G   
    C    
   G   
   T   
    A    
   C   
   C   
    G    
   A   
   A   
   A   
    A    
   T   
   T   
   T   
   T   
    A    
   A   
   G   
    C    
   T   
   T   
   G   
   A   
   A   
   A   
   C   
    G    
    T    
   A   
   T   
    T    
    T    
   T   
    A    
   G   
   A   
   A   
   A   
   A   
    T    
   T   
   A   
    C    
    G    
   T   
    C    
   G   
   A   
    G    
   C   
   A   
    C    
   C   
   A   
    A    
    A    
    A    
    A    
    G    
   A   
   A   
   G   
   T   
   C   
   A   
   T   
    T    
   A   
   C   
    T    
    C    
   G   
    C    
   C   
   G   
   G   
   A   
   C   
    G    
    A    
   T   
    T    
   T   
   T   
   T   
   G   
   A   
    C    
   A   
   A   
   A   
    A    
   A   
    C    
   A   
   A   
    A    
   G   
   C   
   A   
   G   
   A   
   A   
   G   
   C   
   A   
    C    
   G   
    G    
   G   
   C   
    G    
   C   
   A   
    T    
   A   
   T   
    T    
   T   
   T   
   A   
   G   
   A   
   A   
   G   
   G   
   T   
    C    
   T   
    A    
   C   
   G   
    A    
   A   
   T   
   T   
   G   
   C   
    C    
   T   
   T   
    A    
   G   
   A   
   T   
   C   
   A   
   T   
   A   
   T   
   C   
    G    
   A   
   T   
   G   
   A   
   A   
   A   
   T   
    C    
   A   
   T   
   T   
   G   
   C   
    C    
   A   
   T   
   T   
   A   
   T   
    C    
   C   
   G   
    T    
   G   
   G   
    A    
   T   
   C   
    A    
    C    
   A   
   A   
   T   
   C   
   A   
   G   
   A   
   T   
   G   
   A   
    C    
   G   
   A   
    A    
   G   
   C   
   A   
   A   
   A   
   A   
   G   
   C   
    A    
   A   
    C    
    G    
    T    
   T   
    A    
   A   
   T   
    T    
   G   
   A   
   A   
   C   
   G   
    C    
   T   
   T   
    T    
   G   
   A   
    A    
   T   
   T   
    T    
   T   
   C   
    A    
   G   
   A   
   T   
   C   
   G   
    T    
   C   
   A   
    A    
    G    
   C   
    G    
   C   
   A   
   A   
   G   
   C   
    G    
   A   
   T   
    T    
   T   
   T   
   A   
   G   
   A   
   T   
   A   
   T   
   G   
   C   
   G   
   T   
   T   
   T   
    A    
   C   
   G   
    C    
   C   
   G   
    T    
   T   
   T   
    A    
   A   
   C   
    A    
   G   
   G   
    C    
   T   
   T   
    A    
   G   
   A   
   A   
   C   
   G   
    C    
   G   
   A   
    C    
   A   
   A   
   A   
   A   
   T   
    T    
   G   
   A   
    A    
   A   
   A   
   T   
   G   
   A   
   A   
   T   
   A   
    T    
   C   
   A   
   A   
   G   
   A   
    G    
   C   
   T   
    A    
   T   
   T   
    G    
   A   
    A    
   A   
    T    
   T   
    C    
   A   
   T   
   T   
   G   
    C    
    G    
   G   
   A   
    C    
   T   
   T   
    A    
    G    
    A    
    G    
   G   
   A   
   C   
   A   
   T   
   T   
    T    
   T   
    A    
   G   
   C   
    C    
    C    
   G   
    C    
   C   
   C   
    A    
   G   
   A   
    A    
   C   
   G   
    C    
   G   
   T   
    C    
   A   
    T    
    T    
   G   
   A   
   A   
   A   
   T   
    C    
   A   
   T   
    T    
   A   
   A   
    A    
   A   
    C    
    A    
   G   
   A   
    G    
   T   
   T   
   A   
    A    
    A    
    T    
   G   
   A   
   C   
    G    
   T   
    T    
    C    
    G    
    C    
    A    
    C    
   A   
    A    
    A    
   A   
   T   
   T   
   T   
   G   
   G   
   T   
   G   
   A   
   T   
    G    
    C    
   A   
   C   
   G   
   A   
   C   
   G   
    C    
   A   
   C   
   A   
   G   
   A   
   A   
    T    
   T   
   A   
    T    
   T   
   A   
   G   
   T   
    A    
   G   
   G   
   T   
   G   
   A   
    A    
   G   
   T   
   C   
    C    
   T   
    A    
   A   
   G   
   T   
   C   
   T   
    T    
   G   
   A   
   A   
   G   
   A   
   T   
   G   
   A   
   A   
   G   
   A   
   T   
    T    
   T   
    A    
   A   
   T   
    C    
   G   
   A   
   A   
   G   
   A   
   A   
   G   
   A   
   A   
   G   
   A   
   A   
    G    
   T   
   C   
   G   
   T   
    G    
   A   
   T   
   T   
   A   
   C   
    G    
    T    
   T   
    A    
   A   
   C   
    C    
   A   
   A   
    T    
   A   
   A   
    C    
   G   
   G   
    C    
   T   
   A   
    C    
   A   
   T   
    T    
   A   
   A   
    G    
   C   
   G   
    G    
    A    
   T   
   G   
   G   
    C    
   A   
   A   
   A   
    C    
    T    
    C    
    C    
   G   
   A   
   A   
   T   
   T   
   C   
    C    
   G   
    G    
   G   
   C   
    G    
   C   
   A   
   A   
   C   
   G   
    C    
    C    
   G   
    C    
   G   
   G   
    C    
   G   
   G   
    A    
   C   
   G   
   T   
   G   
   G   
    T    
   G   
   T   
    C    
   C   
   A   
   A   
   G   
   G   
    G    
   A   
   T   
   G   
   G   
   G   
    C    
    G    
   T   
    T    
   C   
   A   
    C    
    G    
   A   
   T   
   G   
   A   
    T    
   G   
   A   
    T    
   T   
   T   
   C   
   G   
   T   
    G    
   A   
   A   
    A    
   A   
   A   
    C    
   C   
   T   
    A    
    G    
   T   
   T   
   T   
   C   
   T   
   T   
   G   
    T    
   T   
   C   
    A    
   A   
   C   
    A    
   C   
   A   
    C    
   G   
   A   
    C    
   A   
   C   
    G    
   T   
   T   
    A    
   T   
   T   
   A   
   T   
   T   
    C    
   T   
   T   
   T   
   A   
   C   
   G   
   A   
   A   
    C    
   A   
    C    
    T    
   G   
   G   
    G    
   A   
   A   
   A   
   G   
   T   
    T    
   T   
   A   
    C    
   C   
   G   
    A    
   G   
   C   
   A   
   A   
   A   
   A   
   G   
   G   
    T    
   T   
   A   
    C    
   G   
   A   
   A   
   A   
   T   
    C    
   C   
   C   
    T    
   G   
   A   
    A    
   T   
   A   
   C   
   G   
   G   
    T    
    A    
   G   
    A    
   A   
   C   
   A   
   G   
   C   
    A    
   A   
   A   
   A   
   G   
   G   
    A    
   A   
   T   
    T    
   C   
   C   
   A   
    G    
   T   
    A    
   A   
   T   
    T    
   A   
   A   
    C    
   T   
   T   
    A    
   C   
   T   
    G    
   G   
   G   
    A    
   A   
   T   
    T    
   G   
   A   
    T    
   T   
   C   
    T    
    G    
    C    
    A    
   G   
   A   
   A   
   A   
    A    
    A    
   A   
   T   
    T    
   C   
   A   
    A    
   G   
   C   
    G    
   A   
   T   
    T    
   A   
   T   
   T   
    T    
   C   
    T    
   G   
   T   
    T    
    G    
    A    
   G   
   G   
   G   
    C    
    A    
   A   
   A   
   G   
   C   
    G    
   G   
   A   
   A   
   G   
    C    
    A    
   G   
   G   
    T    
    A    
   A   
    A    
   T   
   A   
    C    
    T    
   T   
    G    
   T   
   T   
   C   
   T   
   T   
    C    
   A   
   C   
    A    
   A   
   C   
    C    
    T    
    T    
    A    
    A    
   A   
   A   
   G   
   G   
    A    
   A   
   C   
    T    
   G   
   T   
    C    
   A   
   A   
   A   
   C   
   G   
    G    
   A   
   C   
    A    
    G    
   C   
    C    
   G   
   T   
    A    
   A   
   C   
    A    
   G   
   C   
    C    
   T   
   T   
    T    
    T    
   C   
   T   
   A   
   A   
    T    
   A   
   T   
    C    
   C   
   G   
    T    
   A   
   G   
    T    
   A   
   A   
   T   
   G   
   G   
    A    
    T    
   T   
    A    
   A   
   T   
    C    
   G   
   C   
    C    
   A   
   T   
   T   
    A    
   G   
    C    
   T   
   T   
    A    
   A   
   A   
   A   
   G   
   A   
   A   
   G   
   A   
    T    
   G   
   A   
    T    
   G   
   A   
    G    
    T    
   T   
    A    
    G    
   T   
    T    
   A   
   A   
    C    
   G   
   T   
    A    
    G    
   T   
   A   
    A    
    C    
   G   
   A   
   C   
    T    
    A    
   A   
    T    
   G   
   G   
    C    
    A    
   A   
    T    
    C    
    A    
    G    
   A   
   A   
    G    
   A   
   T   
    G    
   A   
   T   
    T    
   A   
   T   
    C    
   G   
   G   
    A    
   A   
   C   
   A   
   C   
   A   
   T   
    G    
    C    
    A    
   G   
   G   
    A    
   T   
   A   
   C   
   T   
   C   
   T   
   G   
   T   
    C    
   A   
   C   
    A    
   T   
   T   
    T    
    G    
   A   
    T    
   G   
   A   
    A    
    A    
    A    
    T    
    A    
   C   
    T    
   G   
    T    
    A    
   C   
   G   
    T    
   G   
   A   
   T   
   A   
   T   
   G   
   G   
   G   
    C    
   C   
   G   
    G    
   A   
   C   
   A   
   G   
   C   
    A    
   T   
   C   
    A    
   G   
   G   
    C    
   G   
   T   
    T    
    C    
   G   
    G    
   G   
   G   
    A    
   A   
   T   
    C    
   C   
   G   
    T    
    C    
   T   
    C    
   C   
   G   
    C    
   G   
   A   
    A    
    A    
   A   
    T    
   G   
   A   
    T    
   T   
    A    
    T    
   G   
   T   
    G    
   G   
   T   
    C    
   G   
   G   
    C    
    G    
   C   
   A   
   G   
   C   
    G    
    A    
   T   
    T    
    C    
   T   
   G   
    G    
   A   
    T    
   G   
    A    
   A   
    A    
   A   
    T    
    A    
   A   
   A   
   G   
   A   
   A   
   G   
   T   
    C    
    C    
   T   
    A    
   G   
   T   
    C    
   A   
   T   
    T    
   A   
   C   
    T    
   G   
   A   
   A   
   A   
   A   
    T    
   G   
   G   
   T   
   T   
   A   
    T    
   G   
   G   
    T    
   A   
   A   
    G    
   C   
   G   
    T    
   A   
   C   
    A    
   A   
   A   
   A   
   G   
    C    
    T    
   T   
   C   
    T    
   G   
   A   
    A    
   T   
   A   
   T   
   C   
   C   
   A   
   G   
   T   
    T    
   A   
   A   
    A    
   G   
   G   
    A    
   C   
   G   
    T    
   G   
   G   
    C    
   G   
   G   
   T   
   A   
   A   
   A   
   G   
   G   
    G    
   A   
   T   
    T    
   A   
   A   
    G    
   A   
   C   
    A    
    G    
   C   
    A    
   A   
   A   
    T    
   A   
   T   
   C   
   A   
   C   
   T   
   G   
   A   
    G    
   A   
   A   
   A   
   A   
   A   
   T   
   G   
   G   
   T   
   C   
   C   
   A   
    T    
   T   
   A   
   G   
   C   
    T    
   G   
   G   
    T    
   T   
   T   
    A    
   A   
   C   
   C   
   A   
   C   
    G    
   G   
   T   
    C    
    A    
    A    
   T   
   G   
   G   
    G    
    A    
   A   
   T   
   G   
   A   
   A   
   G   
   A   
    T    
   A   
   T   
   C   
    T    
   T   
    A    
    T    
   T   
    G    
   A   
   T   
    T    
   A   
   C   
    G    
    A    
   A   
    C    
   A   
   A   
   A   
   G   
   G   
    C    
   G   
   T   
    C    
   A   
   T   
   T   
   A   
   T   
   C   
   C   
   G   
    C    
   T   
   T   
    T    
   A   
   A   
   C   
   G   
   T   
    T    
    G    
    A    
    T    
    T    
   C   
   T   
   G   
   T   
   T   
   T   
   C   
   T   
   C   
   A   
   A   
   A   
   C   
    A    
   G   
   G   
   A   
   C   
   G   
    C    
    G    
   C   
   A   
   A   
   C   
   A   
   T   
   T   
    A    
   G   
   G   
    G    
   G   
   T   
    T    
   C   
   G   
   T   
   T   
   T   
    A    
   A   
   T   
   G   
   A   
    G    
    A    
   A   
   T   
   G   
   G   
   A   
   A   
   G   
    A    
    T    
   G   
    G    
    T    
    G    
   C   
    C    
   A   
   A   
   A   
   -   
   -   
   -   
   -   
   -   
   -   
   -   
   -   
   -   
   -   
   -   
   -   
   -   
   -   
   -   
   -   
   -   
   -   
   -   
   -   
   -   
   -   
   -   
   -   
   -   
   -   
   -   
   -   
   -   
   -   
   -   
   -   
   -   
   -   
   -   
   -   
   -   
   -   
   -   
   -   
   -   
   -   
   -   
   -   
   -   
   -   
   -   
   -   
   -   
   -   
   -   
   -   
   -   
   -   
   -   
   -   
   -   
   -   
   -   
   -   
   -   
   -   
   -   
   -   
   -   
   -   
   -   
   -   
   -   
   -   
   -   
   -   
 

 
   N52467   
   A   
   T   
   G   
   A   
   G   
   T   
   G   
   A   
   A   
   G   
   A   
   A   
   A   
   T   
   T   
   A   
   A   
   A   
   G   
   A   
   A   
   A   
   A   
   C   
   A   
   T   
   T   
   C   
   A   
   A   
   G   
   A   
   C   
   G   
   T   
   C   
   A   
   A   
   T   
   C   
   T   
   G   
   A   
   C   
   C   
   A   
   G   
   C   
   G   
   A   
   A   
   A   
   T   
   G   
   A   
   A   
   A   
   G   
   A   
   A   
   T   
   C   
   T   
   T   
   T   
   C   
   A   
   T   
   T   
   G   
   A   
   C   
   T   
   A   
   C   
   G   
   C   
   A   
   A   
   T   
   G   
   A   
   G   
   T   
   G   
   T   
   T   
   A   
   T   
   C   
   G   
   T   
   A   
   G   
   C   
   C   
   C   
   G   
   C   
   G   
   C   
   G   
   T   
   T   
   A   
   C   
   C   
   T   
   G   
   A   
   C   
   G   
   T   
   T   
   C   
   G   
   T   
   G   
   A   
   C   
   G   
   G   
   T   
   T   
   T   
   A   
   A   
   A   
   A   
   C   
   C   
   T   
   G   
   T   
   T   
   C   
   A   
   T   
   C   
   G   
   C   
   C   
   G   
   A   
   A   
   T   
   C   
   T   
   T   
   A   
   T   
   A   
   T   
   G   
   G   
   A   
   A   
   T   
   G   
   A   
   A   
   C   
   G   
   A   
   A   
   T   
   T   
   A   
   G   
   G   
   G   
   G   
   T   
   A   
   A   
   C   
   C   
   C   
   C   
   T   
   G   
   A   
   T   
   A   
   A   
   A   
   C   
   C   
   A   
   C   
   A   
   C   
   A   
   A   
   A   
   A   
   A   
   A   
   T   
   C   
   A   
   G   
   C   
   C   
   C   
   G   
   G   
   A   
   T   
   T   
   G   
   T   
   T   
   G   
   G   
   G   
   G   
   A   
   T   
   G   
   T   
   T   
   A   
   T   
   G   
   G   
   G   
   T   
   A   
   A   
   A   
   T   
   A   
   T   
   C   
   A   
   C   
   C   
   C   
   C   
   C   
   A   
   T   
   G   
   G   
   G   
   G   
   A   
   C   
   A   
   G   
   T   
   G   
   C   
   A   
   A   
   T   
   T   
   T   
   A   
   C   
   G   
   A   
   A   
   T   
   C   
   A   
   A   
   T   
   G   
   G   
   T   
   G   
   C   
   G   
   G   
   A   
   T   
   G   
   G   
   C   
   A   
   C   
   A   
   A   
   C   
   C   
   T   
   T   
   T   
   T   
   A   
   G   
   T   
   T   
   A   
   T   
   C   
   G   
   G   
   G   
   C   
   G   
   A   
   T   
   G   
   T   
   T   
   A   
   G   
   T   
   T   
   G   
   A   
   C   
   G   
   G   
   C   
   C   
   A   
   C   
   G   
   G   
   A   
   A   
   A   
   C   
   T   
   T   
   C   
   G   
   G   
   T   
   T   
   C   
   T   
   G   
   T   
   C   
   G   
   A   
   T   
   G   
   G   
   T   
   G   
   A   
   C   
   G   
   G   
   C   
   G   
   C   
   T   
   G   
   C   
   C   
   G   
   C   
   T   
   A   
   T   
   G   
   C   
   G   
   T   
   T   
   A   
   T   
   A   
   C   
   C   
   G   
   A   
   A   
   G   
   C   
   A   
   C   
   G   
   T   
   A   
   T   
   G   
   A   
   G   
   T   
   A   
   A   
   A   
   A   
   T   
   T   
   G   
   C   
   T   
   T   
   T   
   A   
   G   
   A   
   A   
   A   
   T   
   G   
   C   
   T   
   A   
   C   
   G   
   A   
   G   
   A   
   T   
   A   
   T   
   T   
   A   
   A   
   C   
   A   
   A   
   A   
   A   
   A   
   T   
   A   
   C   
   A   
   G   
   T   
   C   
   G   
   A   
   T   
   T   
   T   
   C   
   C   
   A   
   A   
   G   
   G   
   A   
   A   
   A   
   C   
   T   
   A   
   T   
   G   
   A   
   T   
   G   
   A   
   T   
   T   
   C   
   A   
   G   
   A   
   A   
   C   
   A   
   A   
   G   
   A   
   A   
   C   
   C   
   A   
   G   
   T   
   A   
   G   
   T   
   T   
   T   
   T   
   A   
   C   
   C   
   T   
   G   
   C   
   T   
   C   
   G   
   T   
   T   
   T   
   T   
   C   
   C   
   A   
   A   
   A   
   C   
   T   
   T   
   A   
   C   
   T   
   A   
   G   
   T   
   T   
   A   
   A   
   C   
   G   
   G   
   A   
   A   
   C   
   A   
   A   
   C   
   G   
   G   
   G   
   G   
   A   
   T   
   T   
   G   
   C   
   G   
   G   
   T   
   G   
   G   
   G   
   G   
   A   
   T   
   G   
   G   
   C   
   A   
   A   
   C   
   A   
   A   
   A   
   T   
   A   
   T   
   C   
   C   
   C   
   G   
   C   
   C   
   A   
   C   
   A   
   C   
   A   
   A   
   T   
   T   
   T   
   A   
   A   
   G   
   T   
   G   
   A   
   A   
   G   
   T   
   G   
   A   
   T   
   T   
   G   
   A   
   T   
   G   
   C   
   A   
   A   
   C   
   A   
   A   
   G   
   T   
   T   
   T   
   A   
   T   
   T   
   G   
   A   
   T   
   G   
   G   
   A   
   C   
   A   
   A   
   T   
   C   
   C   
   T   
   G   
   A   
   T   
   G   
   T   
   A   
   A   
   C   
   G   
   A   
   C   
   G   
   A   
   A   
   T   
   G   
   A   
   A   
   T   
   T   
   G   
   A   
   T   
   G   
   G   
   A   
   A   
   G   
   T   
   G   
   T   
   T   
   A   
   C   
   C   
   T   
   G   
   G   
   A   
   C   
   C   
   A   
   G   
   A   
   C   
   T   
   T   
   T   
   C   
   C   
   A   
   A   
   C   
   A   
   G   
   G   
   C   
   G   
   G   
   T   
   T   
   T   
   A   
   G   
   T   
   G   
   A   
   T   
   G   
   G   
   G   
   G   
   A   
   A   
   A   
   T   
   C   
   A   
   G   
   G   
    G    
   A   
   T   
    T    
   C   
   G   
    C    
    C    
   G   
   A   
   G   
   C   
    A    
   T   
   A   
   T   
   G   
   A   
   A   
   A   
   C   
    A    
   G   
   G   
    G    
   A   
   A   
    A    
   G   
   G   
   T   
   T   
   C   
    G    
   A   
   T   
   T   
   A   
   C   
    T    
   G   
   T   
    T    
   C   
   G   
   T   
   G   
   C   
   A   
   A   
   A   
   A   
   G   
   T   
   T   
   G   
   A   
    A    
    T    
   T   
    G    
   A   
    C    
   T   
   G   
   A   
    A    
   A   
   T   
   G   
    C    
   C   
    G    
   A   
   A   
   T   
   G   
   G   
    A    
   A   
   A   
   A   
   G   
   A   
   A   
   C   
   G   
    T    
   A   
   T   
    T    
    T    
   T   
    A    
   G   
   T   
    A    
   A   
   C   
    T    
   G   
   A   
   A   
   T   
   T   
    G    
   C   
   C   
   T   
   T   
   A   
   T   
   A   
   T   
   G   
   G   
   T   
    G    
   A   
   A   
   T   
   A   
   A   
   A   
   G   
   C   
    C    
   A   
   A   
   A   
   T   
   T   
    A    
   A   
   T   
    C    
   G   
   A   
   A   
   C   
   G   
    A    
   A   
   T   
   T   
   T   
   C   
   T   
   G   
   A   
    A    
    T    
   T   
    A    
   C   
   A   
   C   
   A   
   G   
   A   
   G   
   A   
    T    
   A   
   A   
    A    
   C   
   G   
    A    
   A   
   T   
    T    
   G   
   A   
   A   
   G   
   G   
    A    
   A   
   T   
    T    
   A   
   C   
    T    
   G   
   A   
   T   
   T   
   T   
   G   
   C   
   G   
   G   
   G   
   A   
    T    
   G   
   A   
   A   
   T   
   C   
   T   
   T   
   C   
    T    
   C   
   G   
   T   
   G   
   A   
    A    
   G   
   G   
    C    
   A   
   T   
   G   
   C   
   G   
    G    
   A   
   T   
    C    
   G   
   T   
   C   
   A   
   T   
    C    
   G   
   A   
    T    
    G    
   T   
    T    
   C   
   G   
   T   
   C   
   G   
    A    
   G   
   A   
    T    
   G   
    T    
    G    
   A   
   G   
    T    
   G   
   C   
    C    
   T   
   C   
    T    
   G   
   T   
   T   
    G    
   T   
    G    
   T   
   T   
    A    
   A   
   A   
    C    
   A   
   A   
   C   
   T   
   T   
    A    
   T   
   A   
    C    
   A   
   A   
   A   
    A    
   T   
   G   
   A   
   C   
    T    
    G    
   C   
    C    
   T   
   T   
    A    
   C   
   A   
    A    
   A   
    C    
    A    
   T   
   C   
    T    
   T   
   T   
    T    
   G   
   G   
    T    
   T   
   T   
    T    
   A   
   A   
    C    
   A   
   T   
   G   
   T   
   T   
    A    
   G   
   C   
    C    
   A   
   T   
    C    
   G   
   A   
   A   
   A   
   A   
   A   
   G   
   G   
    C    
   G   
   T   
    A    
   C   
   C   
    G    
   A   
   A   
   A   
    A    
   T   
   T   
   T   
   T   
    A    
   A   
   G   
    T    
   T   
   T   
   G   
   A   
   A   
   A   
   C   
    G    
    T    
   A   
   T   
    T    
    T    
   T   
    A    
   G   
   A   
   A   
   A   
   A   
    T    
   T   
   A   
    C    
    A    
   T   
    C    
   G   
   A   
    G    
   C   
   A   
    C    
   C   
   A   
    A    
    A    
    A    
    A    
    G    
   A   
   A   
   G   
   T   
   C   
   A   
   T   
    T    
   A   
   C   
    T    
    C    
   G   
    C    
   C   
   G   
   G   
   A   
   C   
    G    
    A    
   T   
    T    
   T   
   T   
   T   
   G   
   A   
    C    
   A   
   A   
   A   
    A    
   A   
    C    
   A   
   A   
    A    
   G   
   C   
   A   
   G   
   A   
   A   
   G   
   C   
   A   
    C    
   G   
    G    
   G   
   C   
    G    
   C   
   A   
    T    
   A   
   T   
    T    
   T   
   T   
   A   
   G   
   A   
   A   
   G   
   G   
   T   
    C    
   T   
    A    
   C   
   G   
    A    
   A   
   T   
   T   
   G   
   C   
    C    
   T   
   T   
    A    
   G   
   A   
   T   
   C   
   A   
   T   
   A   
   T   
   C   
    G    
   A   
   T   
   G   
   A   
   A   
   A   
   T   
    C    
   A   
   T   
   T   
   G   
   C   
    C    
   A   
   T   
   T   
   A   
   T   
    C    
   C   
   G   
    T    
   G   
   G   
    G    
   T   
   C   
    A    
    C    
   A   
   A   
   T   
   C   
   A   
   G   
   A   
   T   
   G   
   A   
    C    
   G   
   A   
    A    
   G   
   C   
   A   
   A   
   A   
   A   
   G   
   C   
    A    
   A   
    C    
    G    
    T    
   T   
    A    
   A   
   T   
    T    
   G   
   A   
   A   
   C   
   G   
    C    
   T   
   T   
    T    
   G   
   A   
    A    
   T   
   T   
    T    
   T   
   C   
    A    
   G   
   A   
   T   
   C   
   G   
    T    
   C   
   A   
    A    
    G    
   C   
    G    
   C   
   A   
   A   
   G   
   C   
    G    
   A   
   T   
    T    
   T   
   T   
   A   
   G   
   A   
   T   
   A   
   T   
   G   
   C   
   G   
   T   
   T   
   T   
    A    
   C   
   G   
    C    
   C   
   G   
    T    
   T   
   T   
    A    
   A   
   C   
    A    
   G   
   G   
    T    
   T   
   T   
    A    
   G   
   A   
   A   
   C   
   G   
    C    
   G   
   A   
    C    
   A   
   A   
   A   
   A   
   T   
    T    
   G   
   A   
    G    
   A   
   A   
   T   
   G   
   A   
   A   
   T   
   A   
    T    
   C   
   A   
   A   
   G   
   A   
    G    
   C   
   T   
    A    
   T   
   T   
    G    
   A   
    A    
   A   
    T    
   T   
    C    
   A   
   T   
   T   
   G   
    C    
    G    
   G   
   A   
    C    
   T   
   T   
    A    
    G    
    A    
    G    
   G   
   A   
   C   
   A   
   T   
   T   
    T    
   T   
    A    
   G   
   C   
    C    
    C    
   G   
    C    
   C   
   C   
    A    
   G   
   A   
    A    
   C   
   G   
    C    
   G   
   T   
    C    
   A   
    T    
    T    
   G   
   A   
   A   
   A   
   T   
    C    
   A   
   T   
    T    
   A   
   A   
    A    
   A   
    C    
    A    
   G   
   A   
    G    
   T   
   T   
   A   
    A    
    A    
    T    
   G   
   A   
   C   
    G    
   T   
    T    
    C    
    G    
    C    
    A    
    C    
   A   
    A    
    A    
   A   
   T   
   T   
   T   
   G   
   G   
   T   
   G   
   A   
   T   
    G    
    C    
   A   
   C   
   G   
   A   
   C   
   G   
    C    
   A   
   C   
   A   
   G   
   A   
   A   
    T    
   T   
   A   
    T    
   T   
   A   
   G   
   T   
    A    
   G   
   G   
   T   
   G   
   A   
    A    
   G   
   T   
   C   
    C    
   T   
    A    
   A   
   G   
   T   
   C   
   T   
    T    
   G   
   A   
   A   
   G   
   A   
   T   
   G   
   A   
   A   
   G   
   A   
   T   
    C    
   T   
    A    
   A   
   T   
    C    
   G   
   A   
   A   
   G   
   A   
   A   
   G   
   A   
   A   
   G   
   A   
   A   
    G    
   T   
   C   
   G   
   T   
    G    
   A   
   T   
   T   
   A   
   C   
    G    
    T    
   T   
    A    
   A   
   C   
    C    
   A   
   A   
    T    
   A   
   A   
    C    
   G   
   G   
    C    
   T   
   A   
    C    
   A   
   T   
    T    
   A   
   A   
    G    
   C   
   G   
    G    
    A    
   T   
   G   
   G   
    C    
   A   
   A   
   A   
    C    
    T    
    C    
    C    
   G   
   A   
   A   
   T   
   T   
   C   
    C    
   G   
    G    
   G   
   C   
    G    
   C   
   A   
   A   
   C   
   G   
    C    
    C    
   G   
    C    
   G   
   G   
    T    
   G   
   G   
    A    
   C   
   G   
   T   
   G   
   G   
    T    
   G   
   T   
    T    
   C   
   A   
   A   
   G   
   G   
    C    
   A   
   T   
   G   
   G   
   G   
    C    
    G    
   T   
    T    
   C   
   A   
    T    
    G    
   A   
   T   
   G   
   A   
    T    
   G   
   A   
    C    
   T   
   T   
   C   
   G   
   T   
    G    
   A   
   A   
    A    
   A   
   A   
    C    
   C   
   T   
    A    
    G    
   T   
   T   
   T   
   C   
   T   
   T   
   G   
    T    
   T   
   C   
    A    
   A   
   C   
    A    
   C   
   A   
    C    
   G   
   A   
    C    
   A   
   C   
    G    
   T   
   T   
    A    
   T   
   T   
   A   
   T   
   T   
    C    
   T   
   T   
   T   
   A   
   C   
   G   
   A   
   A   
    C    
   A   
    C    
    T    
   G   
   G   
    G    
   A   
   A   
   A   
   G   
   T   
    T    
   T   
   A   
    C    
   C   
   G   
    A    
   G   
   C   
   A   
   A   
   A   
   A   
   G   
   G   
    T    
   T   
   A   
    C    
   G   
   A   
   A   
   A   
   T   
    C    
   C   
   C   
    T    
   G   
   A   
    A    
   T   
   A   
   C   
   G   
   G   
    T    
    A    
   G   
    A    
   A   
   C   
   A   
   G   
   C   
    A    
   A   
   A   
   A   
   G   
   G   
    A    
   A   
   T   
    T    
   C   
   C   
   A   
    G    
   T   
    G    
   A   
   T   
    T    
   A   
   A   
    C    
   T   
   T   
    A    
   C   
   T   
    G    
   G   
   G   
    A    
   A   
   T   
    T    
   G   
   A   
    T    
   T   
   C   
    T    
    G    
    C    
    A    
   G   
   A   
   A   
   A   
    A    
    A    
   A   
   T   
    T    
   C   
   A   
    A    
   G   
   C   
    G    
   A   
   T   
    T    
   A   
   T   
   T   
    T    
   C   
    T    
   G   
   T   
    T    
    G    
    A    
   G   
   G   
   G   
    C    
    A    
   A   
   A   
   G   
   C   
    G    
   G   
   A   
   A   
   G   
    C    
    A    
   G   
   G   
    T    
    A    
   A   
    A    
   T   
   A   
    C    
    T    
   T   
    A    
   T   
   T   
   C   
   T   
   T   
    C    
   A   
   C   
    A    
   A   
   C   
    C    
    T    
    T    
    A    
    A    
   A   
   A   
   G   
   G   
    A    
   A   
   C   
    C    
   G   
   T   
    C    
   A   
   A   
   A   
   C   
   G   
    G    
   A   
   C   
    A    
    G    
   C   
    C    
   G   
   T   
    A    
   A   
   C   
    A    
   G   
   C   
    C    
   T   
   T   
    T    
    T    
   C   
   T   
   A   
   A   
    T    
   A   
   T   
    C    
   C   
   G   
    T    
   A   
   G   
    T    
   A   
   A   
   T   
   G   
   G   
    A    
    T    
   T   
    A    
   A   
   T   
    C    
   G   
   C   
    C    
   A   
   T   
   T   
    A    
   G   
    C    
   T   
   T   
    A    
   A   
   A   
   A   
   G   
   A   
   A   
   G   
   A   
    T    
   G   
   A   
    T    
   G   
   A   
    G    
    T    
   T   
    A    
    G    
   T   
    T    
   A   
   A   
    C    
   G   
   T   
    G    
    G    
   T   
   A   
    A    
    C    
   G   
   A   
   C   
    T    
    A    
   A   
    T    
   G   
   G   
    C    
    A    
   A   
    T    
    C    
    A    
    G    
   A   
   A   
    G    
   A   
   T   
    G    
   A   
   T   
    T    
   A   
   T   
    C    
   G   
   G   
    A    
   A   
   C   
   A   
   C   
   A   
   T   
    G    
    C    
    A    
   G   
   G   
    A    
   T   
   A   
   C   
   T   
   C   
   T   
   G   
   T   
    C    
   A   
   C   
    A    
   T   
   T   
    T    
    G    
   A   
    T    
   G   
   A   
    A    
    A    
    C    
    T    
    A    
   C   
    T    
   G   
    T    
    A    
   C   
   G   
    T    
   G   
   A   
   T   
   A   
   T   
   G   
   G   
   G   
    C    
   C   
   G   
    G    
   A   
   C   
   A   
   G   
   C   
    A    
   T   
   C   
    A    
   G   
   G   
    T    
   G   
   T   
    T    
    C    
   G   
    T    
   G   
   G   
    A    
   A   
   T   
    C    
   C   
   G   
    T    
    C    
   T   
    C    
   C   
   G   
    C    
   G   
   A   
    A    
    A    
   A   
    T    
   G   
   A   
    T    
   T   
    A    
    T    
   G   
   T   
    G    
   G   
   T   
    C    
   G   
   G   
    C    
    G    
   C   
   A   
   G   
   C   
    G    
    A    
   T   
    T    
    C    
   T   
   G   
    G    
   A   
    T    
   G   
    A    
   A   
    A    
   A   
    T    
    A    
   A   
   A   
   G   
   A   
   A   
   G   
   T   
    C    
    C    
   T   
    A    
   G   
   T   
    C    
   A   
   T   
    T    
   A   
   C   
    T    
   G   
   A   
   A   
   A   
   A   
    T    
   G   
   G   
   T   
   T   
   A   
    T    
   G   
   G   
    T    
   A   
   A   
    G    
   C   
   G   
    T    
   A   
   C   
    A    
   A   
   A   
   A   
   G   
    C    
    T    
   T   
   C   
    T    
   G   
   A   
    A    
   T   
   A   
   T   
   C   
   C   
   A   
   G   
   T   
    T    
   A   
   A   
    A    
   G   
   G   
    A    
   C   
   G   
    T    
   G   
   G   
    C    
   G   
   G   
   T   
   A   
   A   
   A   
   G   
   G   
    G    
   A   
   T   
    T    
   A   
   A   
    G    
   A   
   C   
    A    
    G    
   C   
    A    
   A   
   A   
    T    
   A   
   T   
   C   
   A   
   C   
   T   
   G   
   A   
    G    
   A   
   A   
   A   
   A   
   A   
   T   
   G   
   G   
   T   
   C   
   C   
   A   
    T    
   T   
   A   
   G   
   C   
    T    
   G   
   G   
    T    
   T   
   T   
    A    
   A   
   C   
   C   
   A   
   C   
    G    
   G   
   T   
    C    
    A    
    A    
   T   
   G   
   G   
    G    
    G    
   A   
   T   
   G   
   A   
   A   
   G   
   A   
    T    
   A   
   T   
   C   
    T    
   T   
    A    
    T    
   T   
    A    
   A   
   T   
    C    
   A   
   C   
    G    
    A    
   A   
    C    
   A   
   A   
   A   
   G   
   G   
    T    
   G   
   T   
    C    
   A   
   T   
   T   
   A   
   T   
   C   
   C   
   G   
    C    
   T   
   T   
    T    
   A   
   A   
   C   
   G   
   T   
    T    
    G    
    A    
    T    
    T    
   C   
   T   
   G   
   T   
   T   
   T   
   C   
   T   
   C   
   A   
   A   
   A   
   C   
    A    
   G   
   G   
   A   
   C   
   G   
    C    
    G    
   C   
   A   
   A   
   C   
   A   
   T   
   T   
    A    
   G   
   G   
    G    
   G   
   T   
    T    
   C   
   G   
   T   
   T   
   T   
    A    
   A   
   T   
   G   
   A   
    G    
    A    
   A   
   T   
   G   
   G   
   A   
   A   
   G   
    A    
    T    
   G   
    G    
    C    
    G    
   C   
    C    
   A   
   A   
   A   
   -   
   -   
   -   
   -   
   -   
   -   
   -   
   -   
   -   
   -   
   -   
   -   
   -   
   -   
   -   
   -   
   -   
   -   
   -   
   -   
   -   
   -   
   -   
   -   
   -   
   -   
   -   
   -   
   -   
   -   
   -   
   -   
   -   
   -   
   -   
   -   
   -   
   -   
   -   
   -   
   -   
   -   
   -   
   -   
   -   
   -   
   -   
   -   
   -   
   -   
   -   
   -   
   -   
   -   
   -   
   -   
   -   
   -   
   -   
   -   
   -   
   -   
   -   
   -   
   -   
   -   
   -   
   -   
   -   
   -   
   -   
   -   
 

 
   N60200F   
   A   
   T   
   G   
   A   
   G   
   T   
   G   
   A   
   A   
   G   
   A   
   A   
   A   
   T   
   C   
   A   
   G   
   A   
   G   
   A   
   A   
   A   
   A   
   C   
   A   
   T   
   C   
   C   
   A   
   T   
   G   
   A   
   T   
   G   
   T   
   C   
   A   
   A   
   T   
   C   
   T   
   A   
   A   
   C   
   C   
   A   
   G   
   C   
   G   
   A   
   A   
   A   
   T   
   G   
   A   
   A   
   A   
   G   
   A   
   C   
   T   
   C   
   C   
   T   
   T   
   C   
   A   
   T   
   C   
   G   
   A   
   T   
   T   
   A   
   T   
   G   
   C   
   G   
   A   
   T   
   G   
   A   
   G   
   T   
   G   
   T   
   T   
   A   
   T   
   T   
   G   
   T   
   C   
   G   
   C   
   A   
   C   
   G   
   T   
   G   
   C   
   A   
   C   
   T   
   T   
   C   
   C   
   T   
   G   
   A   
   T   
   G   
   T   
   C   
   A   
   G   
   A   
   G   
   A   
   T   
   G   
   G   
   T   
   C   
   T   
   G   
   A   
   A   
   A   
   C   
   C   
   C   
   G   
   T   
   T   
   C   
   A   
   C   
   C   
   G   
   C   
   C   
   G   
   T   
   A   
   T   
   T   
   T   
   T   
   A   
   T   
   A   
   C   
   G   
   G   
   C   
   A   
   T   
   G   
   A   
   A   
   C   
   G   
   A   
   A   
   T   
   T   
   A   
   G   
   G   
   T   
   G   
   T   
   A   
   A   
   C   
   A   
   C   
   C   
   G   
   G   
   A   
   T   
   A   
   A   
   A   
   G   
   C   
   C   
   C   
   A   
   T   
   A   
   A   
   A   
   A   
   A   
   A   
   T   
   C   
   T   
   G   
   C   
   C   
   A   
   G   
   A   
   A   
   T   
   C   
   G   
   T   
   C   
   G   
   G   
   A   
   G   
   A   
   T   
   G   
   T   
   C   
   A   
   T   
   G   
   G   
   G   
   T   
   A   
   A   
   A   
   T   
   A   
   C   
   C   
   A   
   T   
   C   
   C   
   T   
   C   
   A   
   T   
   G   
   G   
   A   
   G   
   A   
   C   
   A   
   G   
   T   
   G   
   C   
   G   
   A   
   T   
   C   
   T   
   A   
   T   
   G   
   A   
   G   
   T   
   C   
   A   
   A   
   T   
   G   
   G   
   T   
   A   
   C   
   G   
   G   
   A   
   T   
   G   
   G   
   C   
   G   
   C   
   A   
   G   
   C   
   C   
   A   
   T   
   T   
   C   
   A   
   G   
   C   
   T   
   A   
   C   
   C   
   G   
   T   
   T   
   A   
   T   
   A   
   T   
   G   
   C   
   T   
   A   
   G   
   T   
   G   
   G   
   A   
   T   
   G   
   G   
   T   
   C   
   A   
   T   
   G   
   G   
   A   
   A   
   A   
   C   
   T   
   T   
   T   
   G   
   G   
   T   
   T   
   C   
   T   
   G   
   T   
   T   
   G   
   A   
   C   
   G   
   G   
   T   
   G   
   A   
   C   
   G   
   G   
   T   
   G   
   C   
   C   
   G   
   C   
   C   
   G   
   C   
   T   
   A   
   T   
   G   
   C   
   G   
   T   
   T   
   A   
   T   
   A   
   C   
   C   
   G   
   A   
   A   
   G   
   C   
   A   
   C   
   G   
   G   
   A   
   T   
   G   
   A   
   G   
   C   
   A   
   A   
   A   
   A   
   T   
   C   
   G   
   C   
   T   
   A   
   C   
   A   
   G   
   A   
   A   
   A   
   T   
   G   
   C   
   T   
   G   
   C   
   G   
   T   
   G   
   A   
   T   
   A   
   T   
   C   
   A   
   A   
   T   
   A   
   A   
   G   
   A   
   A   
   C   
   A   
   C   
   T   
   G   
   T   
   T   
   G   
   A   
   T   
   T   
   T   
   C   
   C   
   A   
   A   
   A   
   G   
   C   
   A   
   A   
   C   
   T   
   A   
   T   
   G   
   A   
   T   
   G   
   A   
   T   
   A   
   C   
   A   
   G   
   A   
   A   
   A   
   A   
   A   
   G   
   A   
   A   
   C   
   C   
   A   
   G   
   T   
   T   
   G   
   T   
   A   
   C   
   T   
   A   
   C   
   C   
   T   
   G   
   C   
   T   
   C   
   G   
   C   
   T   
   T   
   C   
   C   
   C   
   G   
   A   
   A   
   T   
   C   
   T   
   T   
   T   
   T   
   A   
   G   
   T   
   G   
   A   
   A   
   C   
   G   
   G   
   A   
   A   
   C   
   A   
   A   
   C   
   G   
   G   
   G   
   G   
   A   
   T   
   C   
   G   
   C   
   C   
   G   
   T   
   A   
   G   
   G   
   T   
   A   
   T   
   G   
   G   
   C   
   C   
   A   
   C   
   G   
   A   
   A   
   T   
   A   
   T   
   C   
   C   
   C   
   A   
   C   
   C   
   T   
   C   
   A   
   T   
   A   
   A   
   C   
   T   
   T   
   G   
   T   
   C   
   T   
   G   
   A   
   G   
   G   
   T   
   A   
   G   
   T   
   A   
   G   
   C   
   G   
   G   
   C   
   T   
   A   
   T   
   C   
   G   
   A   
   T   
   T   
   T   
   G   
   C   
   T   
   G   
   A   
   T   
   G   
   G   
   A   
   T   
   A   
   A   
   T   
   C   
   C   
   T   
   G   
   A   
   T   
   G   
   T   
   G   
   A   
   C   
   G   
   A   
   C   
   C   
   A   
   A   
   T   
   G   
   A   
   A   
   T   
   T   
   G   
   A   
   T   
   G   
   G   
   A   
   A   
   G   
   T   
   A   
   T   
   T   
   A   
   C   
   C   
   T   
   G   
   G   
   A   
   C   
   C   
   A   
   G   
   A   
   T   
   T   
   T   
   T   
   C   
   C   
   A   
   A   
   C   
   A   
   G   
   G   
   T   
   G   
   G   
   T   
   T   
   T   
   G   
   G   
   T   
   T   
   A   
   T   
   G   
   G   
   G   
   A   
   A   
   A   
   A   
   T   
   C   
   A   
   G   
   G   
    A    
   A   
   T   
    C    
   C   
   G   
    A    
    A    
   G   
   A   
   G   
   C   
    G    
   T   
   A   
   T   
   G   
   A   
   A   
   A   
   C   
    A    
   G   
   G   
    T    
   A   
   A   
    G    
   G   
   G   
   T   
   T   
   C   
    T    
   A   
   T   
   T   
   A   
   C   
    C    
   G   
   T   
    A    
   C   
   G   
   T   
   G   
   C   
   A   
   A   
   A   
   A   
   G   
   T   
   T   
   G   
   A   
    G    
    A    
   T   
    T    
   A   
    C    
   T   
   G   
   A   
    G    
   A   
   T   
   G   
    C    
   C   
    A    
   A   
   A   
   T   
   G   
   G   
    T    
   A   
   A   
   A   
   G   
   A   
   A   
   C   
   G   
    G    
   A   
   T   
    C    
    A    
   T   
    C    
   G   
   T   
    G    
   A   
   C   
    A    
   G   
   A   
   A   
   T   
   T   
    G    
   C   
   C   
   T   
   T   
   A   
   T   
   A   
   T   
   G   
   G   
   T   
    C    
   A   
   A   
   T   
   A   
   A   
   A   
   G   
   C   
    A    
   A   
   A   
   A   
   T   
   T   
    G    
   A   
   T   
    C    
   G   
   A   
   A   
   C   
   G   
    G    
   A   
   T   
   T   
   T   
   C   
   T   
   G   
   A   
    A    
    C    
   T   
    G    
   C   
   A   
   C   
   A   
   G   
   A   
   G   
   A   
    C    
   A   
   A   
    G    
   C   
   G   
    T    
   A   
   T   
    C    
   G   
   A   
   A   
   G   
   G   
    G    
   A   
   T   
    C    
   A   
   C   
    A    
   G   
   A   
   T   
   T   
   T   
   G   
   C   
   G   
   G   
   G   
   A   
    C    
   G   
   A   
   A   
   T   
   C   
   T   
   T   
   C   
    C    
   C   
   G   
   T   
   G   
   A   
    A    
   G   
   G   
    T    
   A   
   T   
   G   
   C   
   G   
    G    
   A   
   T   
    C    
   G   
   T   
   C   
   A   
   T   
    T    
   G   
   A   
    C    
    A    
   T   
    C    
   C   
   G   
   T   
   C   
   G   
    T    
   G   
   A   
    C    
   G   
    C    
    C    
   A   
   G   
    C    
   G   
   C   
    A    
   T   
   C   
    C    
   G   
   T   
   T   
    A    
   T   
    C    
   T   
   T   
    G    
   A   
   A   
    T    
   A   
   A   
   C   
   T   
   T   
    A    
   T   
   A   
    T    
   A   
   A   
   A   
    T    
   T   
   G   
   A   
   C   
    A    
    T    
   C   
    C    
   T   
   T   
    G    
   C   
   A   
    A    
   A   
    A    
    T    
   T   
   C   
    A    
   T   
   T   
    T    
   G   
   G   
    A    
   T   
   T   
    C    
   A   
   A   
    T    
   A   
   T   
   G   
   T   
   T   
    G    
   G   
   C   
    G    
   A   
   T   
    C    
   G   
   A   
   A   
   A   
   A   
   A   
   G   
   G   
    T    
   G   
   T   
    G    
   C   
   C   
    G    
   A   
   A   
   A   
    G    
   T   
   T   
   T   
   T   
    G    
   A   
   G   
    C    
   T   
   T   
   G   
   A   
   A   
   A   
   C   
    A    
    A    
   A   
   T   
    T    
    C    
   T   
    T    
   G   
   A   
   A   
   A   
   A   
    C    
   T   
   A   
    T    
    A    
   T   
    C    
   G   
   A   
    A    
   C   
   A   
    T    
   C   
   A   
    G    
    C    
    G    
    C    
    G    
   A   
   A   
   G   
   T   
   C   
   A   
   T   
    C    
   A   
   C   
    A    
    A    
   G   
    A    
   C   
   G   
   G   
   A   
   C   
    A    
    G    
   T   
    C    
   T   
   T   
   T   
   G   
   A   
    A    
   A   
   A   
   A   
    G    
   A   
    A    
   A   
   A   
    A    
   G   
   C   
   A   
   G   
   A   
   A   
   G   
   C   
   A   
    A    
   G   
    G    
   G   
   C   
    A    
   C   
   A   
    C    
   A   
   T   
    T    
   T   
   T   
   A   
   G   
   A   
   A   
   G   
   G   
   T   
    T    
   T   
    G    
   C   
   G   
    T    
   A   
   T   
   T   
   G   
   C   
    T    
   T   
   T   
    G    
   G   
   A   
   T   
   C   
   A   
   T   
   A   
   T   
   C   
    G    
   A   
   T   
   G   
   A   
   A   
   A   
   T   
    A    
   A   
   T   
   T   
   G   
   C   
    G    
   A   
   T   
   T   
   A   
   T   
    C    
   C   
   G   
    C    
   G   
   G   
    G    
   T   
   C   
    T    
    A    
   A   
   A   
   T   
   C   
   A   
   G   
   A   
   T   
   G   
   A   
    C    
   G   
   A   
    A    
   G   
   C   
   A   
   A   
   A   
   A   
   G   
   C   
    G    
   A   
    C    
    G    
    A    
   T   
    G    
   A   
   T   
    C    
   G   
   A   
   A   
   C   
   G   
    G    
   T   
   T   
    C    
   G   
   A   
    T    
   T   
   T   
    A    
   T   
   C   
    C    
   G   
   A   
   T   
   C   
   G   
    C    
   C   
   A   
    G    
    T    
   C   
    T    
   C   
   A   
   A   
   G   
   C   
    T    
   A   
   T   
    T    
   T   
   T   
   A   
   G   
   A   
   T   
   A   
   T   
   G   
   C   
   G   
   T   
   T   
   T   
    A    
   C   
   G   
    C    
   C   
   G   
    A    
   T   
   T   
    G    
   A   
   C   
    C    
   G   
   G   
    T    
   T   
   T   
    G    
   G   
   A   
   A   
   C   
   G   
    G    
   G   
   A   
    A    
   A   
   A   
   A   
   A   
   T   
    C    
   G   
   A   
    A    
   A   
   A   
   T   
   G   
   A   
   A   
   T   
   A   
    C    
   C   
   A   
   A   
   G   
   A   
    A    
   C   
   T   
    C    
   T   
   T   
    A    
   A   
    A    
   A   
    C    
   T   
    G    
   A   
   T   
   T   
   G   
    A    
    A    
   G   
   A   
    T    
   T   
   T   
    A    
    A    
    C    
    T    
   G   
   A   
   C   
   A   
   T   
   T   
    C    
   T   
    G    
   G   
   C   
    A    
    A    
   G   
    A    
   C   
   C   
    A    
   G   
   A   
    A    
   C   
   G   
    T    
   G   
   T   
    T    
   A   
    C    
    A    
   G   
   A   
   A   
   A   
   T   
    C    
   A   
   T   
    C    
   A   
   A   
    G    
   A   
    A    
    T    
   G   
   A   
    A    
   T   
   T   
   A   
    G    
    C    
    A    
   G   
   A   
   C   
    T    
   T   
    G    
    A    
    A    
    T    
    C    
    A    
   A   
    C    
    G    
   A   
   T   
   T   
   T   
   G   
   G   
   T   
   G   
   A   
   T   
    A    
    A    
   A   
   C   
   G   
   A   
   C   
   G   
    C    
   A   
   C   
   A   
   G   
   A   
   A   
    T    
   T   
   A   
    C    
   T   
   A   
   G   
   T   
    C    
   G   
   G   
   T   
   G   
   A   
    G    
   G   
   T   
   C   
    T    
   T   
    A    
   A   
   G   
   T   
   C   
   T   
    G    
   G   
   A   
   A   
   G   
   A   
   T   
   G   
   A   
   A   
   G   
   A   
   T   
    T    
   T   
    A    
   A   
   T   
    C    
   G   
   A   
   A   
   G   
   A   
   A   
   G   
   A   
   A   
   G   
   A   
   A   
    A    
   T   
   C   
   G   
   T   
    T    
   A   
   T   
   T   
   A   
   C   
    A    
    T    
   T   
    G    
   A   
   C   
    A    
   A   
   A   
    C    
   A   
   A   
    T    
   G   
   G   
    T    
   T   
   A   
    C    
   A   
   T   
    C    
   A   
   A   
    A    
   C   
   G   
    T    
    T    
   T   
   G   
   G   
    C    
   A   
   A   
   A   
    T    
    A    
    A    
    T    
   G   
   A   
   A   
   T   
   T   
   C   
    A    
   G   
    A    
   G   
   C   
    T    
   C   
   A   
   A   
   C   
   G   
    A    
    A    
   G   
    G    
   G   
   G   
    T    
   G   
   G   
    A    
   C   
   G   
   T   
   G   
   G   
    T    
   G   
   T   
    C    
   C   
   A   
   A   
   G   
   G   
    A    
   A   
   T   
   G   
   G   
   G   
    T    
    A    
   T   
    C    
   C   
   A   
    C    
    A    
   A   
   T   
   G   
   A   
    C    
   G   
   A   
    C    
   T   
   T   
   C   
   G   
   T   
    C    
   A   
   A   
    A    
   A   
   A   
    T    
   C   
   T   
    G    
    G    
   T   
   T   
   T   
   C   
   T   
   T   
   G   
    T    
   T   
   C   
    T    
   A   
   C   
    C    
   C   
   A   
    T    
   G   
   A   
    T    
   A   
   C   
    A    
   T   
   T   
    G    
   T   
   T   
   A   
   T   
   T   
    C    
   T   
   T   
   T   
   A   
   C   
   G   
   A   
   A   
    T    
   A   
    A    
    C    
   G   
   G   
    A    
   A   
   A   
   A   
   G   
   T   
    G    
   T   
   A   
    T    
   C   
   G   
    G    
   G   
   C   
   A   
   A   
   A   
   A   
   G   
   G   
    A    
   T   
   A   
    C    
   G   
   A   
   A   
   A   
   T   
    T    
   C   
   C   
    A    
   G   
   A   
    A    
   T   
   A   
   C   
   G   
   G   
    G    
    C    
   G   
    T    
   A   
   C   
   A   
   G   
   C   
    C    
   A   
   A   
   A   
   G   
   G   
    A    
   A   
   T   
    A    
   C   
   C   
   A   
    A    
   T   
    T    
   A   
   T   
    C    
   A   
   A   
    T    
   T   
   T   
    A    
   C   
   T   
    A    
   G   
   G   
    T    
   A   
   T   
    C    
   G   
   A   
    T    
   T   
   C   
    G    
    A    
    G    
    T    
   G   
   A   
   A   
   A   
    G    
    C    
   A   
   T   
    C    
   C   
   A   
    G    
   G   
   C   
    A    
   A   
   T   
    C    
   A   
   T   
   T   
    G    
   C   
    G    
   G   
   T   
    G    
    A    
    C    
   G   
   G   
   G   
    A    
    G    
   A   
   A   
   G   
   C   
    A    
   G   
   A   
   A   
   G   
    A    
    A    
   G   
   G   
    C    
    C    
   A   
    C    
   T   
   A   
    T    
    C    
   T   
    C    
   T   
   T   
   C   
   T   
   T   
    T    
   A   
   C   
    G    
   A   
   C   
    G    
    C    
    G    
    T    
    C    
   A   
   A   
   G   
   G   
    T    
   A   
   C   
    A    
   G   
   T   
    A    
   A   
   A   
   A   
   C   
   G   
    T    
   A   
   C   
    T    
    T    
   C   
    T    
   G   
   T   
    C    
   A   
   C   
    T    
   G   
   C   
    T    
   T   
   T   
    T    
    G    
   C   
   T   
   A   
   A   
    C    
   A   
   T   
    C    
   C   
   G   
    A    
   A   
   G   
    T    
   A   
   A   
   T   
   G   
   G   
    T    
    C    
   T   
    G    
   A   
   T   
    C    
   G   
   C   
    A    
   A   
   T   
   T   
    G    
   G   
    T    
   T   
   T   
    G    
   A   
   A   
   A   
   G   
   A   
   A   
   G   
   A   
    C    
   G   
   A   
    T    
   G   
   A   
    G    
    C    
   T   
    C    
    G    
   T   
    C    
   A   
   A   
    T    
   G   
   T   
    G    
    C    
   T   
   A   
    C    
    T    
   G   
   A   
   C   
    A    
    G    
   A   
    T    
   G   
   G   
    T    
    C    
   A   
    A    
    T    
    C    
    T    
   A   
   A   
    T    
   A   
   T   
    C    
   A   
   T   
    T    
   A   
   T   
    T    
   G   
   G   
    A    
   A   
   C   
   A   
   C   
   A   
   T   
    A    
    A    
    C    
   G   
   G   
    C    
   T   
   A   
   C   
   T   
   C   
   T   
   G   
   T   
    C    
   A   
   C   
    T    
   T   
   T   
    C    
    A    
   A   
    A    
   G   
   A   
    A    
    G    
    A    
    A    
    G    
   C   
    A    
   G   
    T    
    C    
   C   
   G   
    C    
   G   
   A   
   T   
   A   
   T   
   G   
   G   
   G   
    A    
   C   
   G   
    T    
   A   
   C   
   A   
   G   
   C   
    T    
   T   
   C   
    C    
   G   
   G   
    G    
   G   
   T   
    C    
    A    
   G   
    A    
   G   
   G   
    A    
   A   
   T   
    A    
   C   
   G   
    C    
    C    
   T   
    G    
   C   
   G   
    T    
   G   
   A   
    G    
    G    
   A   
    A    
   G   
   A   
    T    
   T   
    A    
    T    
   G   
   T   
    C    
   G   
   T   
    T    
   G   
   G   
    G    
    G    
   C   
   A   
   G   
   C   
    C    
    T    
   T   
    A    
    A    
   T   
   G   
    A    
   A   
    G    
   G   
    A    
   A   
    A    
   A   
    T    
    C    
   A   
   A   
   G   
   A   
   A   
   G   
   T   
    A    
    T    
   T   
    A    
   G   
   T   
    G    
   A   
   T   
    C    
   A   
   C   
    A    
   G   
   A   
   A   
   A   
   A   
    A    
   G   
   G   
   T   
   T   
   A   
    C    
   G   
   G   
    A    
   A   
   A   
    A    
   C   
   G   
    G    
   A   
   C   
    A    
   A   
   A   
   A   
   G   
    T    
    A    
   T   
   C   
    G    
   G   
   A   
    A    
   T   
   A   
   T   
   C   
   C   
   A   
   G   
   T   
    G    
   A   
   A   
    A    
   G   
   G   
    C    
   C   
   G   
    C    
   G   
   G   
    A    
   G   
   G   
   T   
   A   
   A   
   A   
   G   
   G   
    G    
   A   
   T   
    C    
   A   
   A   
    A    
   A   
   C   
    G    
    G    
   C   
    G    
   A   
   A   
    C    
   A   
   T   
   C   
   A   
   C   
   T   
   G   
   A   
    A    
   A   
   A   
   A   
   A   
   A   
   T   
   G   
   G   
   T   
   C   
   C   
   A   
    C    
   T   
   A   
   G   
   C   
    T    
   G   
   G   
    A    
   T   
   T   
    G    
   A   
   C   
   C   
   A   
   C   
    T    
   G   
   T   
    T    
    T    
    C    
   T   
   G   
   G   
    A    
    G    
   A   
   T   
   G   
   A   
   A   
   G   
   A   
    C    
   A   
   T   
   C   
    A    
   T   
    G    
    C    
   T   
    G    
   A   
   T   
    T    
   A   
   C   
    A    
    G    
   A   
    T    
   A   
   A   
   A   
   G   
   G   
    T    
   G   
   T   
    C    
   A   
   T   
   T   
   A   
   T   
   C   
   C   
   G   
    T    
   T   
   T   
    C    
   A   
   A   
   C   
   G   
   T   
    A    
    T    
    C    
    G    
    A    
   C   
   T   
   G   
   T   
   T   
   T   
   C   
   T   
   C   
   A   
   A   
   A   
   C   
    T    
   G   
   G   
   A   
   C   
   G   
    T    
    T    
   C   
   A   
   A   
   C   
   A   
   T   
   T   
    A    
   G   
   G   
    C    
   G   
   T   
    A    
   C   
   G   
   T   
   T   
   T   
    G    
   A   
   T   
   G   
   A   
    A    
    G    
   A   
   T   
   G   
   G   
   A   
   A   
   G   
    C    
    T    
   G   
    A    
    T    
    A    
   C   
    G    
   A   
   A   
   A   
   G   
   T   
   T   
   G   
   T   
   C   
   A   
   C   
   G   
   A   
   T   
   G   
   G   
   C   
   A   
   G   
   C   
   A   
   G   
   T   
   C   
   G   
   A   
   A   
   C   
   C   
   A   
   G   
   A   
   A   
   G   
   C   
   T   
   G   
   C   
   A   
   G   
   A   
   A   
   C   
   A   
   A   
   A   
   C   
   A   
   G   
   T   
   T   
   G   
   A   
   A   
   G   
   A   
   A   
   C   
   A   
   A   
   G   
   G   
   A   
   T   
   C   
   A   
   G   
   A   
   A   
   G   
   A   
   A   
   -   
   -   
   -   
 

 
   N55181   
   A   
   T   
   G   
   A   
   G   
   T   
   G   
   A   
   A   
   G   
   A   
   A   
   A   
   T   
   T   
   A   
   A   
   A   
   G   
   A   
   A   
   A   
   A   
   C   
   A   
   T   
   T   
   C   
   A   
   A   
   G   
   A   
   C   
   G   
   T   
   C   
   A   
   A   
   T   
   C   
   T   
   G   
   A   
   C   
   C   
   A   
   G   
   C   
   G   
   A   
   A   
   A   
   T   
   G   
   A   
   A   
   A   
   G   
   A   
   A   
   T   
   C   
   T   
   T   
   T   
   C   
   A   
   T   
   T   
   G   
   A   
   C   
   T   
   A   
   C   
   G   
   C   
   A   
   A   
   T   
   G   
   A   
   G   
   T   
   G   
   T   
   T   
   A   
   T   
   C   
   G   
   T   
   A   
   G   
   C   
   C   
   C   
   G   
   C   
   G   
   C   
   G   
   T   
   T   
   A   
   C   
   C   
   T   
   G   
   A   
   C   
   G   
   T   
   T   
   C   
   G   
   T   
   G   
   A   
   C   
   G   
   G   
   T   
   T   
   T   
   A   
   A   
   A   
   A   
   C   
   C   
   T   
   G   
   T   
   T   
   C   
   A   
   T   
   C   
   G   
   C   
   C   
   G   
   A   
   A   
   T   
   C   
   T   
   T   
   A   
   T   
   A   
   T   
   G   
   G   
   A   
   A   
   T   
   G   
   A   
   A   
   C   
   G   
   A   
   A   
   T   
   T   
   A   
   G   
   G   
   G   
   G   
   T   
   A   
   A   
   C   
   C   
   C   
   C   
   T   
   G   
   A   
   T   
   A   
   A   
   A   
   C   
   C   
   A   
   C   
   A   
   C   
   A   
   A   
   A   
   A   
   A   
   A   
   T   
   C   
   A   
   G   
   C   
   C   
   C   
   G   
   G   
   A   
   T   
   T   
   G   
   T   
   T   
   G   
   G   
   G   
   G   
   A   
   T   
   G   
   T   
   T   
   A   
   T   
   G   
   G   
   G   
   T   
   A   
   A   
   A   
   T   
   A   
   T   
   C   
   A   
   C   
   C   
   C   
   C   
   C   
   A   
   T   
   G   
   G   
   G   
   G   
   A   
   C   
   A   
   G   
   T   
   G   
   C   
   G   
   A   
   T   
   T   
   T   
   A   
   C   
   G   
   A   
   A   
   T   
   C   
   A   
   A   
   T   
   G   
   G   
   T   
   G   
   C   
   G   
   G   
   A   
   T   
   G   
   G   
   C   
   A   
   C   
   A   
   A   
   C   
   C   
   T   
   T   
   T   
   T   
   A   
   G   
   T   
   T   
   A   
   C   
   C   
   G   
   G   
   G   
   C   
   T   
   A   
   T   
   G   
   T   
   T   
   A   
   G   
   T   
   T   
   G   
   A   
   C   
   G   
   G   
   C   
   C   
   A   
   C   
   G   
   G   
   A   
   A   
   A   
   C   
   T   
   T   
   C   
   G   
   G   
   T   
   T   
   C   
   T   
   G   
   T   
   C   
   G   
   A   
   T   
   G   
   G   
   T   
   G   
   A   
   C   
   G   
   G   
   C   
   G   
   C   
   T   
   G   
   C   
   C   
   G   
   C   
   T   
   A   
   T   
   G   
   C   
   G   
   T   
   T   
   A   
   T   
   A   
   C   
   C   
   G   
   A   
   A   
   G   
   C   
   A   
   C   
   G   
   T   
   A   
   T   
   G   
   A   
   G   
   T   
   A   
   A   
   A   
   A   
   T   
   T   
   G   
   C   
   T   
   T   
   T   
   A   
   G   
   A   
   A   
   A   
   T   
   G   
   C   
   T   
   A   
   C   
   G   
   A   
   G   
   A   
   T   
   A   
   T   
   T   
   A   
   A   
   C   
   A   
   A   
   A   
   A   
   A   
   T   
   A   
   C   
   A   
   G   
   T   
   T   
   G   
   A   
   T   
   T   
   T   
   C   
   C   
   A   
   A   
   G   
   G   
   A   
   A   
   A   
   C   
   T   
   A   
   T   
   G   
   A   
   T   
   G   
   A   
   T   
   T   
   C   
   A   
   G   
   A   
   A   
   C   
   A   
   A   
   G   
   A   
   A   
   C   
   C   
   A   
   G   
   T   
   A   
   G   
   T   
   T   
   T   
   T   
   A   
   C   
   C   
   T   
   G   
   C   
   T   
   C   
   G   
   T   
   T   
   T   
   T   
   C   
   C   
   A   
   A   
   A   
   C   
   T   
   T   
   A   
   C   
   T   
   A   
   G   
   T   
   T   
   A   
   A   
   C   
   G   
   G   
   A   
   A   
   C   
   A   
   A   
   C   
   G   
   G   
   G   
   G   
   A   
   T   
   T   
   G   
   C   
   G   
   G   
   T   
   G   
   G   
   G   
   G   
   A   
   T   
   G   
   G   
   C   
   A   
   A   
   C   
   A   
   A   
   A   
   T   
   A   
   T   
   T   
   C   
   C   
   A   
   C   
   C   
   A   
   C   
   A   
   C   
   A   
   A   
   T   
   T   
   T   
   A   
   A   
   G   
   T   
   G   
   A   
   A   
   G   
   T   
   G   
   A   
   T   
   T   
   G   
   A   
   T   
   G   
   C   
   A   
   A   
   C   
   A   
   A   
   G   
   T   
   T   
   T   
   A   
   T   
   T   
   G   
   A   
   T   
   G   
   G   
   A   
   C   
   A   
   A   
   T   
   C   
   C   
   T   
   G   
   A   
   T   
   G   
   T   
   A   
   A   
   C   
   G   
   A   
   C   
   G   
   A   
   A   
   T   
   G   
   A   
   A   
   T   
   T   
   G   
   A   
   T   
   G   
   G   
   A   
   A   
   G   
   T   
   G   
   T   
   T   
   A   
   C   
   C   
   T   
   G   
   G   
   A   
   C   
   C   
   A   
   G   
   A   
   C   
   T   
   T   
   T   
   C   
   C   
   A   
   A   
   C   
   A   
   G   
   G   
   C   
   G   
   G   
   T   
   T   
   T   
   A   
   G   
   T   
   G   
   A   
   T   
   G   
   G   
   G   
   G   
   A   
   A   
   A   
   T   
   C   
   A   
   G   
   G   
    G    
   A   
   T   
    T    
   C   
   G   
    C    
    C    
   G   
   A   
   G   
   C   
    A    
   T   
   A   
   T   
   G   
   A   
   A   
   A   
   C   
    A    
   G   
   G   
    G    
   A   
   A   
    A    
   G   
   G   
   T   
   T   
   C   
    G    
   A   
   T   
   T   
   A   
   C   
    T    
   G   
   T   
    T    
   C   
   G   
   T   
   G   
   C   
   A   
   A   
   A   
   A   
   G   
   T   
   T   
   G   
   A   
    A    
    T    
   T   
    G    
   A   
    C    
   T   
   G   
   A   
    A    
   A   
   T   
   G   
    C    
   C   
    G    
   A   
   A   
   T   
   G   
   G   
    A    
   A   
   A   
   A   
   G   
   A   
   A   
   C   
   G   
    T    
   A   
   T   
    T    
    T    
   T   
    A    
   G   
   T   
    A    
   A   
   C   
    T    
   G   
   A   
   A   
   T   
   T   
    G    
   C   
   C   
   T   
   T   
   A   
   T   
   A   
   T   
   G   
   G   
   T   
    G    
   A   
   A   
   T   
   A   
   A   
   A   
   G   
   C   
    C    
   A   
   A   
   A   
   T   
   T   
    A    
   A   
   T   
    C    
   G   
   A   
   A   
   C   
   G   
    A    
   A   
   T   
   T   
   T   
   C   
   T   
   G   
   A   
    A    
    T    
   T   
    A    
   C   
   A   
   C   
   A   
   G   
   A   
   G   
   A   
    T    
   A   
   A   
    A    
   C   
   G   
    A    
   A   
   T   
    T    
   G   
   A   
   A   
   G   
   G   
    A    
   A   
   T   
    T    
   A   
   C   
    T    
   G   
   A   
   T   
   T   
   T   
   G   
   C   
   G   
   G   
   G   
   A   
    T    
   G   
   A   
   A   
   T   
   C   
   T   
   T   
   C   
    T    
   C   
   G   
   T   
   G   
   A   
    A    
   G   
   G   
    C    
   A   
   T   
   G   
   C   
   G   
    G    
   A   
   T   
    T    
   G   
   T   
   C   
   A   
   T   
    C    
   G   
   A   
    T    
    G    
   T   
    T    
   C   
   G   
   T   
   C   
   G   
    A    
   G   
   A   
    T    
   G   
    T    
    G    
   A   
   G   
    T    
   G   
   C   
    C    
   T   
   C   
    T    
   G   
   T   
   T   
    G    
   T   
    G    
   T   
   T   
    A    
   A   
   A   
    C    
   A   
   A   
   C   
   T   
   T   
    G    
   T   
   A   
    C    
   A   
   A   
   A   
    A    
   T   
   G   
   A   
   C   
    T    
    G    
   C   
    C    
   T   
   T   
    A    
   C   
   A   
    A    
   A   
    C    
    A    
   T   
   C   
    T    
   T   
   T   
    T    
   G   
   G   
    T    
   T   
   T   
    T    
   A   
   A   
    C    
   A   
   T   
   G   
   T   
   T   
    A    
   G   
   C   
    C    
   A   
   T   
    C    
   G   
   A   
   A   
   A   
   A   
   A   
   G   
   G   
    C    
   G   
   T   
    A    
   C   
   C   
    G    
   A   
   A   
   A   
    A    
   T   
   T   
   T   
   T   
    A    
   A   
   G   
    C    
   T   
   T   
   G   
   A   
   A   
   A   
   C   
    G    
    T    
   A   
   T   
    T    
    T    
   T   
    A    
   G   
   A   
   A   
   A   
   A   
    C    
   T   
   A   
    C    
    G    
   T   
    C    
   G   
   A   
    G    
   C   
   A   
    T    
   C   
   A   
    A    
    A    
    A    
    A    
    G    
   A   
   A   
   G   
   T   
   C   
   A   
   T   
    T    
   A   
   C   
    T    
    C    
   G   
    C    
   C   
   G   
   G   
   A   
   C   
    G    
    A    
   T   
    T    
   T   
   T   
   T   
   G   
   A   
    C    
   A   
   A   
   A   
    A    
   A   
    C    
   A   
   A   
    A    
   G   
   C   
   A   
   G   
   A   
   A   
   G   
   C   
   A   
    C    
   G   
    G    
   G   
   C   
    G    
   C   
   A   
    T    
   A   
   T   
    T    
   T   
   T   
   A   
   G   
   A   
   A   
   G   
   G   
   T   
    C    
   T   
    A    
   C   
   G   
    A    
   A   
   T   
   T   
   G   
   C   
    C    
   T   
   T   
    A    
   G   
   A   
   T   
   C   
   A   
   T   
   A   
   T   
   C   
    G    
   A   
   T   
   G   
   A   
   A   
   A   
   T   
    C    
   A   
   T   
   T   
   G   
   C   
    C    
   A   
   T   
   T   
   A   
   T   
    C    
   C   
   G   
    T    
   G   
   G   
    A    
   T   
   C   
    A    
    C    
   A   
   A   
   T   
   C   
   A   
   G   
   A   
   T   
   G   
   A   
    C    
   G   
   A   
    A    
   G   
   C   
   A   
   A   
   A   
   A   
   G   
   C   
    A    
   A   
    C    
    G    
    T    
   T   
    A    
   A   
   T   
    T    
   G   
   A   
   A   
   C   
   G   
    C    
   T   
   T   
    T    
   G   
   A   
    A    
   T   
   T   
    T    
   T   
   C   
    A    
   G   
   A   
   T   
   C   
   G   
    T    
   C   
   A   
    A    
    G    
   C   
    G    
   C   
   A   
   A   
   G   
   C   
    A    
   A   
   T   
    T    
   T   
   T   
   A   
   G   
   A   
   T   
   A   
   T   
   G   
   C   
   G   
   T   
   T   
   T   
    A    
   C   
   G   
    C    
   C   
   G   
    T    
   T   
   T   
    A    
   A   
   C   
    A    
   G   
   G   
    C    
   T   
   T   
    A    
   G   
   A   
   A   
   C   
   G   
    C    
   G   
   A   
    C    
   A   
   A   
   A   
   A   
   T   
    T    
   G   
   A   
    A    
   A   
   A   
   T   
   G   
   A   
   A   
   T   
   A   
    T    
   C   
   A   
   A   
   G   
   A   
    G    
   C   
   T   
    A    
   T   
   T   
    G    
   A   
    A    
   A   
    T    
   T   
    C    
   A   
   T   
   T   
   G   
    C    
    G    
   G   
   A   
    C    
   T   
   T   
    A    
    G    
    A    
    G    
   G   
   A   
   C   
   A   
   T   
   T   
    T    
   T   
    A    
   G   
   C   
    C    
    C    
   G   
    C    
   C   
   C   
    A    
   G   
   A   
    A    
   C   
   G   
    C    
   G   
   T   
    C    
   A   
    T    
    T    
   G   
   A   
   A   
   A   
   T   
    C    
   A   
   T   
    T    
   A   
   A   
    A    
   A   
    C    
    A    
   G   
   A   
    G    
   T   
   T   
   A   
    A    
    A    
    T    
   G   
   A   
   C   
    G    
   T   
    C    
    C    
    G    
    T    
    A    
    C    
   A   
    A    
    A    
   A   
   T   
   T   
   T   
   G   
   G   
   T   
   G   
   A   
   T   
    G    
    C    
   A   
   C   
   G   
   A   
   C   
   G   
    C    
   A   
   C   
   A   
   G   
   A   
   A   
    T    
   T   
   A   
    T    
   T   
   A   
   G   
   T   
    A    
   G   
   G   
   T   
   G   
   A   
    A    
   G   
   T   
   C   
    T    
   T   
    A    
   A   
   G   
   T   
   C   
   T   
    T    
   G   
   A   
   A   
   G   
   A   
   T   
   G   
   A   
   A   
   G   
   A   
   T   
    T    
   T   
    A    
   A   
   T   
    C    
   G   
   A   
   A   
   G   
   A   
   A   
   G   
   A   
   A   
   G   
   A   
   A   
    G    
   T   
   C   
   G   
   T   
    G    
   A   
   T   
   T   
   A   
   C   
    G    
    T    
   T   
    A    
   A   
   C   
    C    
   A   
   A   
    T    
   A   
   A   
    C    
   G   
   G   
    C    
   T   
   A   
    C    
   A   
   T   
    T    
   A   
   A   
    G    
   C   
   G   
    G    
    A    
   T   
   G   
   G   
    C    
   A   
   A   
   A   
    C    
    T    
    C    
    T    
   G   
   A   
   A   
   T   
   T   
   C   
    C    
   G   
    G    
   G   
   C   
    G    
   C   
   A   
   A   
   C   
   G   
    C    
    C    
   G   
    T    
   G   
   G   
    C    
   G   
   G   
    A    
   C   
   G   
   T   
   G   
   G   
    T    
   G   
   T   
    C    
   C   
   A   
   A   
   G   
   G   
    G    
   A   
   T   
   G   
   G   
   G   
    C    
    G    
   T   
    T    
   C   
   A   
    T    
    G    
   A   
   T   
   G   
   A   
    T    
   G   
   A   
    T    
   T   
   T   
   C   
   G   
   T   
    G    
   A   
   A   
    A    
   A   
   A   
    C    
   C   
   T   
    A    
    G    
   T   
   T   
   T   
   C   
   T   
   T   
   G   
    T    
   T   
   C   
    A    
   A   
   C   
    A    
   C   
   A   
    C    
   G   
   A   
    C    
   A   
   C   
    G    
   T   
   T   
    A    
   T   
   T   
   A   
   T   
   T   
    C    
   T   
   T   
   T   
   A   
   C   
   G   
   A   
   A   
    C    
   A   
    C    
    T    
   G   
   G   
    G    
   A   
   A   
   A   
   G   
   T   
    T    
   T   
   A   
    C    
   C   
   G   
    A    
   G   
   C   
   A   
   A   
   A   
   A   
   G   
   G   
    T    
   T   
   A   
    C    
   G   
   A   
   A   
   A   
   T   
    C    
   C   
   C   
    T    
   G   
   A   
    G    
   T   
   A   
   C   
   G   
   G   
    T    
    A    
   G   
    A    
   A   
   C   
   A   
   G   
   C   
    A    
   A   
   A   
   A   
   G   
   G   
    A    
   A   
   T   
    T    
   C   
   C   
   A   
    G    
   T   
    G    
   A   
   T   
    C    
   A   
   A   
    C    
   T   
   T   
    A    
   C   
   T   
    G    
   G   
   G   
    A    
   A   
   T   
    T    
   G   
   A   
    T    
   T   
   C   
    T    
    G    
    C    
    A    
   G   
   A   
   A   
   A   
    A    
    A    
   A   
   T   
    T    
   C   
   A   
    A    
   G   
   C   
    G    
   A   
   T   
    T    
   A   
   T   
   T   
    T    
   C   
    T    
   G   
   T   
    T    
    G    
    A    
   G   
   G   
   G   
    C    
    A    
   A   
   A   
   G   
   C   
    G    
   G   
   A   
   A   
   G   
    C    
    A    
   G   
   G   
    T    
    A    
   A   
    A    
   T   
   A   
    C    
    T    
   T   
    G    
   T   
   T   
   C   
   T   
   T   
    C    
   A   
   C   
    A    
   A   
   C   
    C    
    T    
    T    
    A    
    A    
   A   
   A   
   G   
   G   
    A    
   A   
   C   
    C    
   G   
   T   
    C    
   A   
   A   
   A   
   C   
   G   
    G    
   A   
   C   
    A    
    G    
   C   
    C    
   G   
   T   
    A    
   A   
   C   
    A    
   G   
   C   
    C    
   T   
   T   
    T    
    T    
   C   
   T   
   A   
   A   
    T    
   A   
   T   
    C    
   C   
   G   
    T    
   A   
   G   
    T    
   A   
   A   
   T   
   G   
   G   
    A    
    T    
   T   
    A    
   A   
   T   
    C    
   G   
   C   
    C    
   A   
   T   
   T   
    A    
   G   
    C    
   T   
   T   
    A    
   A   
   A   
   A   
   G   
   A   
   A   
   G   
   A   
    T    
   G   
   A   
    T    
   G   
   A   
    G    
    T    
   T   
    A    
    G    
   T   
    T    
   A   
   A   
    C    
   G   
   T   
    G    
    G    
   T   
   A   
    A    
    C    
   G   
   A   
   C   
    T    
    A    
   A   
    T    
   G   
   G   
    C    
    A    
   A   
    T    
    C    
    A    
    G    
   A   
   A   
    G    
   A   
   T   
    G    
   A   
   T   
    T    
   A   
   T   
    C    
   G   
   G   
    A    
   A   
   C   
   A   
   C   
   A   
   T   
    G    
    C    
    A    
   G   
   G   
    A    
   T   
   A   
   C   
   T   
   C   
   T   
   G   
   T   
    C    
   A   
   C   
    A    
   T   
   T   
    T    
    G    
   A   
    T    
   G   
   A   
    A    
    A    
    A    
    T    
    A    
   C   
    T    
   G   
    T    
    A    
   C   
   G   
    T    
   G   
   A   
   T   
   A   
   T   
   G   
   G   
   G   
    C    
   C   
   G   
    G    
   A   
   C   
   A   
   G   
   C   
    A    
   T   
   C   
    A    
   G   
   G   
    T    
   G   
   T   
    T    
    C    
   G   
    T    
   G   
   G   
    A    
   A   
   T   
    C    
   C   
   G   
    T    
    C    
   T   
    C    
   C   
   G   
    C    
   G   
   A   
    A    
    A    
   A   
    T    
   G   
   A   
    T    
   T   
    A    
    T    
   G   
   T   
    G    
   G   
   T   
    C    
   G   
   G   
    C    
    G    
   C   
   A   
   G   
   C   
    G    
    A    
   T   
    T    
    C    
   T   
   G   
    G    
   A   
    T    
   G   
    A    
   A   
    A    
   A   
    T    
    A    
   A   
   A   
   G   
   A   
   A   
   G   
   T   
    C    
    C    
   T   
    A    
   G   
   T   
    C    
   A   
   T   
    T    
   A   
   C   
    T    
   G   
   A   
   A   
   A   
   A   
    T    
   G   
   G   
   T   
   T   
   A   
    T    
   G   
   G   
    T    
   A   
   A   
    G    
   C   
   G   
    T    
   A   
   C   
    A    
   A   
   A   
   A   
   G   
    C    
    T    
   T   
   C   
    T    
   G   
   A   
    A    
   T   
   A   
   T   
   C   
   C   
   A   
   G   
   T   
    T    
   A   
   A   
    A    
   G   
   G   
    A    
   C   
   G   
    T    
   G   
   G   
    C    
   G   
   G   
   T   
   A   
   A   
   A   
   G   
   G   
    G    
   A   
   T   
    T    
   A   
   A   
    G    
   A   
   C   
    A    
    G    
   C   
    A    
   A   
   A   
    T    
   A   
   T   
   C   
   A   
   C   
   T   
   G   
   A   
    G    
   A   
   A   
   A   
   A   
   A   
   T   
   G   
   G   
   T   
   C   
   C   
   A   
    T    
   T   
   A   
   G   
   C   
    T    
   G   
   G   
    T    
   T   
   T   
    A    
   A   
   C   
   C   
   A   
   C   
    G    
   G   
   T   
    C    
    A    
    A    
   T   
   G   
   G   
    G    
    G    
   A   
   T   
   G   
   A   
   A   
   G   
   A   
    T    
   A   
   T   
   C   
    T    
   T   
    A    
    T    
   T   
    G    
   A   
   T   
    T    
   A   
   C   
    G    
    A    
   A   
    C    
   A   
   A   
   A   
   G   
   G   
    C    
   G   
   T   
    C    
   A   
   T   
   T   
   A   
   T   
   C   
   C   
   G   
    C    
   T   
   T   
    T    
   A   
   A   
   C   
   G   
   T   
    T    
    G    
    A    
    T    
    T    
   C   
   T   
   G   
   T   
   T   
   T   
   C   
   T   
   C   
   A   
   A   
   A   
   C   
    A    
   G   
   G   
   A   
   C   
   G   
    C    
    G    
   C   
   A   
   A   
   C   
   A   
   T   
   T   
    A    
   G   
   G   
    G    
   G   
   T   
    T    
   C   
   G   
   T   
   T   
   T   
    A    
   A   
   T   
   G   
   A   
    G    
    A    
   A   
   T   
   G   
   G   
   A   
   A   
   G   
    A    
    T    
   G   
    G    
    T    
    G    
   C   
    C    
   A   
   A   
   A   
   -   
   -   
   -   
   -   
   -   
   -   
   -   
   -   
   -   
   -   
   -   
   -   
   -   
   -   
   -   
   -   
   -   
   -   
   -   
   -   
   -   
   -   
   -   
   -   
   -   
   -   
   -   
   -   
   -   
   -   
   -   
   -   
   -   
   -   
   -   
   -   
   -   
   -   
   -   
   -   
   -   
   -   
   -   
   -   
   -   
   -   
   -   
   -   
   -   
   -   
   -   
   -   
   -   
   -   
   -   
   -   
   -   
   -   
   -   
   -   
   -   
   -   
   -   
   -   
   -   
   -   
   -   
   -   
   -   
   -   
   -   
   -   
 

 
   N54097   
   A   
   T   
   G   
   A   
   G   
   T   
   G   
   A   
   A   
   G   
   A   
   A   
   A   
   T   
   T   
   A   
   A   
   A   
   G   
   A   
   A   
   A   
   A   
   C   
   A   
   T   
   T   
   C   
   A   
   A   
   G   
   A   
   C   
   G   
   T   
   C   
   A   
   A   
   T   
   C   
   T   
   G   
   A   
   C   
   C   
   A   
   G   
   C   
   G   
   A   
   A   
   A   
   T   
   G   
   A   
   A   
   A   
   G   
   A   
   A   
   T   
   C   
   T   
   T   
   T   
   C   
   A   
   T   
   T   
   G   
   A   
   C   
   T   
   A   
   C   
   G   
   C   
   A   
   A   
   T   
   G   
   A   
   G   
   T   
   G   
   T   
   T   
   A   
   T   
   C   
   G   
   T   
   A   
   G   
   C   
   C   
   C   
   G   
   C   
   G   
   C   
   G   
   T   
   T   
   A   
   C   
   C   
   T   
   G   
   A   
   C   
   G   
   T   
   T   
   C   
   G   
   T   
   G   
   A   
   C   
   G   
   G   
   T   
   T   
   T   
   A   
   A   
   A   
   A   
   C   
   C   
   T   
   G   
   T   
   T   
   C   
   A   
   T   
   C   
   G   
   C   
   C   
   G   
   A   
   A   
   T   
   C   
   T   
   T   
   A   
   T   
   A   
   T   
   G   
   G   
   A   
   A   
   T   
   G   
   A   
   A   
   C   
   G   
   A   
   A   
   T   
   T   
   A   
   G   
   G   
   G   
   G   
   T   
   A   
   A   
   C   
   C   
   C   
   C   
   T   
   G   
   A   
   T   
   A   
   A   
   A   
   C   
   C   
   A   
   C   
   A   
   C   
   A   
   A   
   A   
   A   
   A   
   A   
   T   
   C   
   A   
   G   
   C   
   C   
   C   
   G   
   G   
   A   
   T   
   T   
   G   
   T   
   T   
   G   
   G   
   G   
   G   
   A   
   T   
   G   
   T   
   T   
   A   
   T   
   G   
   G   
   G   
   T   
   A   
   A   
   A   
   T   
   A   
   T   
   C   
   A   
   C   
   C   
   C   
   C   
   C   
   A   
   T   
   G   
   G   
   G   
   G   
   A   
   C   
   A   
   G   
   T   
   G   
   C   
   G   
   A   
   T   
   T   
   T   
   A   
   C   
   G   
   A   
   A   
   T   
   C   
   A   
   A   
   T   
   G   
   G   
   T   
   G   
   C   
   G   
   G   
   A   
   T   
   G   
   G   
   C   
   A   
   C   
   A   
   A   
   C   
   C   
   T   
   T   
   T   
   T   
   A   
   G   
   T   
   T   
   A   
   T   
   C   
   G   
   G   
   G   
   C   
   T   
   A   
   T   
   G   
   T   
   T   
   A   
   G   
   T   
   T   
   G   
   A   
   C   
   G   
   G   
   C   
   C   
   A   
   C   
   G   
   G   
   A   
   A   
   A   
   C   
   T   
   T   
   C   
   G   
   G   
   T   
   T   
   C   
   T   
   G   
   T   
   C   
   G   
   A   
   T   
   G   
   G   
   T   
   G   
   A   
   C   
   G   
   G   
   C   
   G   
   C   
   T   
   G   
   C   
   C   
   G   
   C   
   T   
   A   
   T   
   G   
   C   
   G   
   T   
   T   
   A   
   T   
   A   
   C   
   C   
   G   
   A   
   A   
   G   
   C   
   A   
   C   
   G   
   T   
   A   
   T   
   G   
   A   
   G   
   T   
   A   
   A   
   A   
   A   
   T   
   T   
   G   
   C   
   T   
   T   
   T   
   A   
   G   
   A   
   A   
   A   
   T   
   G   
   C   
   T   
   A   
   C   
   G   
   A   
   G   
   A   
   T   
   A   
   T   
   T   
   A   
   A   
   C   
   A   
   A   
   A   
   A   
   A   
   T   
   A   
   C   
   A   
   G   
   T   
   C   
   G   
   A   
   T   
   T   
   T   
   C   
   C   
   A   
   A   
   G   
   G   
   A   
   A   
   A   
   C   
   T   
   A   
   T   
   G   
   A   
   T   
   G   
   A   
   T   
   T   
   C   
   A   
   G   
   A   
   A   
   C   
   A   
   A   
   G   
   A   
   A   
   C   
   C   
   A   
   G   
   T   
   A   
   G   
   T   
   T   
   T   
   T   
   A   
   C   
   C   
   T   
   G   
   C   
   T   
   C   
   G   
   T   
   T   
   T   
   T   
   C   
   C   
   A   
   A   
   A   
   C   
   T   
   T   
   A   
   C   
   T   
   A   
   G   
   T   
   T   
   A   
   A   
   C   
   G   
   G   
   A   
   A   
   C   
   A   
   A   
   C   
   G   
   G   
   G   
   G   
   A   
   T   
   T   
   G   
   C   
   G   
   G   
   T   
   G   
   G   
   G   
   G   
   A   
   T   
   G   
   G   
   C   
   A   
   A   
   C   
   A   
   A   
   A   
   T   
   A   
   T   
   T   
   C   
   C   
   A   
   C   
   C   
   A   
   C   
   A   
   C   
   A   
   A   
   T   
   T   
   T   
   A   
   A   
   G   
   T   
   G   
   A   
   A   
   G   
   T   
   G   
   A   
   T   
   T   
   G   
   A   
   T   
   G   
   C   
   A   
   A   
   C   
   A   
   A   
   G   
   T   
   T   
   T   
   A   
   T   
   T   
   G   
   A   
   T   
   G   
   G   
   A   
   C   
   A   
   A   
   T   
   C   
   C   
   T   
   G   
   A   
   T   
   G   
   T   
   A   
   A   
   C   
   G   
   A   
   C   
   G   
   A   
   A   
   T   
   G   
   A   
   A   
   T   
   T   
   G   
   A   
   T   
   G   
   G   
   A   
   A   
   G   
   T   
   G   
   T   
   T   
   A   
   C   
   C   
   T   
   G   
   G   
   A   
   C   
   C   
   A   
   G   
   A   
   T   
   T   
   T   
   T   
   C   
   C   
   A   
   A   
   C   
   A   
   G   
   G   
   C   
   G   
   G   
   T   
   T   
   T   
   A   
   G   
   T   
   G   
   A   
   T   
   G   
   G   
   G   
   G   
   A   
   A   
   A   
   T   
   C   
   A   
   G   
   G   
    G    
   A   
   T   
    T    
   C   
   G   
    C    
    C    
   G   
   A   
   G   
   C   
    A    
   T   
   A   
   T   
   G   
   A   
   A   
   A   
   C   
    A    
   G   
   G   
    G    
   A   
   A   
    A    
   G   
   G   
   T   
   T   
   C   
    G    
   A   
   T   
   T   
   A   
   C   
    T    
   G   
   T   
    T    
   C   
   G   
   T   
   G   
   C   
   A   
   A   
   A   
   A   
   G   
   T   
   T   
   G   
   A   
    A    
    T    
   T   
    G    
   A   
    C    
   T   
   G   
   A   
    A    
   A   
   T   
   G   
    C    
   C   
    G    
   A   
   A   
   T   
   G   
   G   
    A    
   A   
   A   
   A   
   G   
   A   
   A   
   C   
   G   
    T    
   A   
   T   
    T    
    T    
   T   
    A    
   G   
   T   
    A    
   A   
   C   
    T    
   G   
   A   
   A   
   T   
   T   
    G    
   C   
   C   
   T   
   T   
   A   
   T   
   A   
   T   
   G   
   G   
   T   
    G    
   A   
   A   
   T   
   A   
   A   
   A   
   G   
   C   
    C    
   A   
   A   
   A   
   T   
   T   
    A    
   A   
   T   
    C    
   G   
   A   
   A   
   C   
   G   
    A    
   A   
   T   
   T   
   T   
   C   
   T   
   G   
   A   
    A    
    T    
   T   
    A    
   C   
   A   
   C   
   A   
   G   
   A   
   G   
   A   
    T    
   A   
   A   
    A    
   C   
   G   
    A    
   A   
   T   
    T    
   G   
   A   
   A   
   G   
   G   
    A    
   A   
   T   
    T    
   A   
   C   
    T    
   G   
   A   
   T   
   T   
   T   
   G   
   C   
   G   
   G   
   G   
   A   
    T    
   G   
   A   
   A   
   T   
   C   
   T   
   T   
   C   
    T    
   C   
   G   
   T   
   G   
   A   
    A    
   G   
   G   
    C    
   A   
   T   
   G   
   C   
   G   
    G    
   A   
   T   
    C    
   G   
   T   
   C   
   A   
   T   
    C    
   G   
   A   
    T    
    G    
   T   
    T    
   C   
   G   
   T   
   C   
   G   
    A    
   G   
   A   
    T    
   G   
    T    
    G    
   A   
   G   
    T    
   G   
   C   
    C    
   T   
   C   
    T    
   G   
   T   
   T   
    G    
   T   
    G    
   T   
   T   
    A    
   A   
   A   
    C    
   A   
   A   
   C   
   T   
   T   
    G    
   T   
   A   
    C    
   A   
   A   
   A   
    A    
   T   
   G   
   A   
   C   
    T    
    G    
   C   
    C    
   T   
   T   
    A    
   C   
   A   
    A    
   A   
    C    
    A    
   T   
   C   
    T    
   T   
   T   
    T    
   G   
   G   
    T    
   T   
   T   
    T    
   A   
   A   
    C    
   A   
   T   
   G   
   T   
   T   
    A    
   G   
   C   
    C    
   A   
   T   
    T    
   G   
   A   
   A   
   A   
   A   
   A   
   G   
   G   
    C    
   G   
   T   
    A    
   C   
   C   
    G    
   A   
   A   
   A   
    A    
   T   
   T   
   T   
   T   
    A    
   A   
   G   
    C    
   T   
   T   
   G   
   A   
   A   
   A   
   C   
    G    
    T    
   A   
   T   
    T    
    T    
   T   
    A    
   G   
   A   
   A   
   A   
   A   
    T    
   T   
   A   
    C    
    G    
   T   
    C    
   G   
   A   
    G    
   C   
   A   
    C    
   C   
   A   
    A    
    A    
    A    
    A    
    G    
   A   
   A   
   G   
   T   
   C   
   A   
   T   
    T    
   A   
   C   
    T    
    C    
   G   
    C    
   C   
   G   
   G   
   A   
   C   
    G    
    A    
   T   
    T    
   T   
   T   
   T   
   G   
   A   
    C    
   A   
   A   
   A   
    A    
   A   
    C    
   A   
   A   
    A    
   G   
   C   
   A   
   G   
   A   
   A   
   G   
   C   
   A   
    C    
   G   
    G    
   G   
   C   
    G    
   C   
   A   
    T    
   A   
   T   
    T    
   T   
   T   
   A   
   G   
   A   
   A   
   G   
   G   
   T   
    C    
   T   
    A    
   C   
   G   
    A    
   A   
   T   
   T   
   G   
   C   
    C    
   T   
   T   
    A    
   G   
   A   
   T   
   C   
   A   
   T   
   A   
   T   
   C   
    G    
   A   
   T   
   G   
   A   
   A   
   A   
   T   
    C    
   A   
   T   
   T   
   G   
   C   
    C    
   A   
   T   
   T   
   A   
   T   
    C    
   C   
   G   
    T    
   G   
   G   
    A    
   T   
   C   
    A    
    C    
   A   
   A   
   T   
   C   
   A   
   G   
   A   
   T   
   G   
   A   
    C    
   G   
   A   
    A    
   G   
   C   
   A   
   A   
   A   
   A   
   G   
   C   
    A    
   A   
    C    
    G    
    T    
   T   
    A    
   A   
   T   
    T    
   G   
   A   
   A   
   C   
   G   
    C    
   T   
   T   
    T    
   G   
   A   
    A    
   T   
   T   
    T    
   T   
   C   
    A    
   G   
   A   
   T   
   C   
   G   
    T    
   C   
   A   
    A    
    G    
   C   
    G    
   C   
   A   
   A   
   G   
   C   
    G    
   A   
   T   
    T    
   T   
   T   
   A   
   G   
   A   
   T   
   A   
   T   
   G   
   C   
   G   
   T   
   T   
   T   
    A    
   C   
   G   
    C    
   C   
   G   
    T    
   T   
   T   
    A    
   A   
   C   
    A    
   G   
   G   
    C    
   T   
   T   
    A    
   G   
   A   
   A   
   C   
   G   
    C    
   G   
   A   
    C    
   A   
   A   
   A   
   A   
   T   
    T    
   G   
   A   
    A    
   A   
   A   
   T   
   G   
   A   
   A   
   T   
   A   
    T    
   C   
   A   
   A   
   G   
   A   
    G    
   C   
   T   
    A    
   T   
   T   
    G    
   A   
    A    
   A   
    T    
   T   
    C    
   A   
   T   
   T   
   G   
    C    
    G    
   G   
   A   
    C    
   T   
   T   
    A    
    G    
    A    
    G    
   G   
   A   
   C   
   A   
   T   
   T   
    T    
   T   
    A    
   G   
   C   
    C    
    C    
   G   
    C    
   C   
   C   
    A    
   G   
   A   
    A    
   C   
   G   
    C    
   G   
   T   
    C    
   A   
    T    
    T    
   G   
   A   
   A   
   A   
   T   
    C    
   A   
   T   
    T    
   A   
   A   
    A    
   A   
    C    
    A    
   G   
   A   
    G    
   T   
   T   
   A   
    A    
    A    
    T    
   G   
   A   
   C   
    G    
   T   
    T    
    C    
    G    
    C    
    A    
    C    
   A   
    A    
    A    
   A   
   T   
   T   
   T   
   G   
   G   
   T   
   G   
   A   
   T   
    G    
    C    
   A   
   C   
   G   
   A   
   C   
   G   
    C    
   A   
   C   
   A   
   G   
   A   
   A   
    T    
   T   
   A   
    T    
   T   
   A   
   G   
   T   
    A    
   G   
   G   
   T   
   G   
   A   
    A    
   G   
   T   
   C   
    C    
   T   
    A    
   A   
   G   
   T   
   C   
   T   
    T    
   G   
   A   
   A   
   G   
   A   
   T   
   G   
   A   
   A   
   G   
   A   
   T   
    T    
   T   
    A    
   A   
   T   
    C    
   G   
   A   
   A   
   G   
   A   
   A   
   G   
   A   
   A   
   G   
   A   
   A   
    G    
   T   
   C   
   G   
   T   
    G    
   A   
   T   
   T   
   A   
   C   
    G    
    T    
   T   
    A    
   A   
   C   
    C    
   A   
   A   
    T    
   A   
   A   
    C    
   G   
   G   
    C    
   T   
   A   
    C    
   A   
   T   
    T    
   A   
   A   
    G    
   C   
   G   
    G    
    A    
   T   
   G   
   G   
    C    
   A   
   A   
   A   
    C    
    T    
    C    
    C    
   G   
   A   
   A   
   T   
   T   
   C   
    C    
   G   
    G    
   G   
   C   
    G    
   C   
   A   
   A   
   C   
   G   
    C    
    C    
   G   
    T    
   G   
   G   
    C    
   G   
   G   
    A    
   C   
   G   
   T   
   G   
   G   
    T    
   G   
   T   
    C    
   C   
   A   
   A   
   G   
   G   
    C    
   A   
   T   
   G   
   G   
   G   
    C    
    G    
   T   
    T    
   C   
   A   
    T    
    G    
   A   
   T   
   G   
   A   
    T    
   G   
   A   
    T    
   T   
   T   
   C   
   G   
   T   
    G    
   A   
   A   
    A    
   A   
   A   
    C    
   C   
   T   
    A    
    G    
   T   
   T   
   T   
   C   
   T   
   T   
   G   
    T    
   T   
   C   
    A    
   A   
   C   
    A    
   C   
   A   
    C    
   G   
   A   
    C    
   A   
   C   
    G    
   T   
   T   
    A    
   T   
   T   
   A   
   T   
   T   
    C    
   T   
   T   
   T   
   A   
   C   
   G   
   A   
   A   
    C    
   A   
    C    
    T    
   G   
   G   
    G    
   A   
   A   
   A   
   G   
   T   
    T    
   T   
   A   
    C    
   C   
   G   
    A    
   G   
   C   
   A   
   A   
   A   
   A   
   G   
   G   
    T    
   T   
   A   
    C    
   G   
   A   
   A   
   A   
   T   
    C    
   C   
   C   
    T    
   G   
   A   
    A    
   T   
   A   
   C   
   G   
   G   
    T    
    A    
   G   
    A    
   A   
   C   
   A   
   G   
   C   
    A    
   A   
   A   
   A   
   G   
   G   
    A    
   A   
   T   
    T    
   C   
   C   
   A   
    G    
   T   
    A    
   A   
   T   
    T    
   A   
   A   
    C    
   T   
   T   
    A    
   C   
   T   
    G    
   G   
   G   
    A    
   A   
   T   
    T    
   G   
   A   
    T    
   T   
   C   
    T    
    G    
    C    
    A    
   G   
   A   
   A   
   A   
    A    
    A    
   A   
   T   
    T    
   C   
   A   
    A    
   G   
   C   
    G    
   A   
   T   
    T    
   A   
   T   
   T   
    T    
   C   
    T    
   G   
   T   
    T    
    G    
    A    
   G   
   G   
   G   
    C    
    A    
   A   
   A   
   G   
   C   
    G    
   G   
   A   
   A   
   G   
    C    
    A    
   G   
   G   
    T    
    A    
   A   
    A    
   T   
   A   
    C    
    T    
   T   
    G    
   T   
   T   
   C   
   T   
   T   
    C    
   A   
   C   
    A    
   A   
   C   
    C    
    T    
    T    
    A    
    A    
   A   
   A   
   G   
   G   
    A    
   A   
   C   
    C    
   G   
   T   
    C    
   A   
   A   
   A   
   C   
   G   
    G    
   A   
   C   
    A    
    G    
   C   
    C    
   G   
   T   
    A    
   A   
   C   
    A    
   G   
   C   
    C    
   T   
   T   
    T    
    T    
   C   
   T   
   A   
   A   
    T    
   A   
   T   
    C    
   C   
   G   
    T    
   A   
   G   
    T    
   A   
   A   
   T   
   G   
   G   
    A    
    T    
   T   
    A    
   A   
   T   
    C    
   G   
   C   
    C    
   A   
   T   
   T   
    A    
   G   
    C    
   T   
   T   
    A    
   A   
   A   
   A   
   G   
   A   
   A   
   G   
   A   
    T    
   G   
   A   
    T    
   G   
   A   
    G    
    T    
   T   
    A    
    G    
   T   
    T    
   A   
   A   
    C    
   G   
   T   
    G    
    G    
   T   
   A   
    A    
    C    
   G   
   A   
   C   
    T    
    A    
   A   
    T    
   G   
   G   
    C    
    A    
   A   
    T    
    C    
    A    
    G    
   A   
   A   
    G    
   A   
   T   
    G    
   A   
   T   
    T    
   A   
   T   
    C    
   G   
   G   
    A    
   A   
   C   
   A   
   C   
   A   
   T   
    G    
    C    
    A    
   G   
   G   
    A    
   T   
   A   
   C   
   T   
   C   
   T   
   G   
   T   
    C    
   A   
   C   
    A    
   T   
   T   
    T    
    G    
   A   
    T    
   G   
   A   
    A    
    A    
    A    
    T    
    A    
   C   
    T    
   G   
    T    
    A    
   C   
   G   
    T    
   G   
   A   
   T   
   A   
   T   
   G   
   G   
   G   
    C    
   C   
   G   
    G    
   A   
   C   
   A   
   G   
   C   
    A    
   T   
   C   
    A    
   G   
   G   
    T    
   G   
   T   
    T    
    C    
   G   
    G    
   G   
   G   
    A    
   A   
   T   
    C    
   C   
   G   
    T    
    C    
   T   
    C    
   C   
   G   
    C    
   G   
   A   
    A    
    A    
   A   
    T    
   G   
   A   
    T    
   T   
    A    
    T    
   G   
   T   
    G    
   G   
   T   
    C    
   G   
   G   
    T    
    G    
   C   
   A   
   G   
   C   
    G    
    A    
   T   
    T    
    C    
   T   
   G   
    G    
   A   
    T    
   G   
    A    
   A   
    A    
   A   
    C    
    A    
   A   
   A   
   G   
   A   
   A   
   G   
   T   
    C    
    C    
   T   
    A    
   G   
   T   
    C    
   A   
   T   
    T    
   A   
   C   
    T    
   G   
   A   
   A   
   A   
   A   
    T    
   G   
   G   
   T   
   T   
   A   
    T    
   G   
   G   
    T    
   A   
   A   
    G    
   C   
   G   
    T    
   A   
   C   
    A    
   A   
   A   
   A   
   G   
    C    
    T    
   T   
   C   
    T    
   G   
   A   
    A    
   T   
   A   
   T   
   C   
   C   
   A   
   G   
   T   
    T    
   A   
   A   
    A    
   G   
   G   
    A    
   C   
   G   
    T    
   G   
   G   
    C    
   G   
   G   
   T   
   A   
   A   
   A   
   G   
   G   
    G    
   A   
   T   
    T    
   A   
   A   
    G    
   A   
   C   
    A    
    G    
   C   
    A    
   A   
   A   
    T    
   A   
   T   
   C   
   A   
   C   
   T   
   G   
   A   
    G    
   A   
   A   
   A   
   A   
   A   
   T   
   G   
   G   
   T   
   C   
   C   
   A   
    T    
   T   
   A   
   G   
   C   
    T    
   G   
   G   
    T    
   T   
   T   
    A    
   A   
   C   
   C   
   A   
   C   
    G    
   G   
   T   
    C    
    A    
    A    
   T   
   G   
   G   
    G    
    G    
   A   
   T   
   G   
   A   
   A   
   G   
   A   
    T    
   A   
   T   
   C   
    T    
   T   
    A    
    T    
   T   
    G    
   A   
   T   
    T    
   A   
   C   
    G    
    A    
   A   
    C    
   A   
   A   
   A   
   G   
   G   
    C    
   G   
   T   
    C    
   A   
   T   
   T   
   A   
   T   
   C   
   C   
   G   
    C    
   T   
   T   
    T    
   A   
   A   
   C   
   G   
   T   
    T    
    G    
    A    
    T    
    T    
   C   
   T   
   G   
   T   
   T   
   T   
   C   
   T   
   C   
   A   
   A   
   A   
   C   
    A    
   G   
   G   
   A   
   C   
   G   
    C    
    G    
   C   
   A   
   A   
   C   
   A   
   T   
   T   
    A    
   G   
   G   
    G    
   G   
   T   
    T    
   C   
   G   
   T   
   T   
   T   
    A    
   A   
   T   
   G   
   A   
    G    
    A    
   A   
   T   
   G   
   G   
   A   
   A   
   G   
    A    
    T    
   G   
    G    
    T    
    G    
   C   
    C    
   A   
   A   
   A   
   -   
   -   
   -   
   -   
   -   
   -   
   -   
   -   
   -   
   -   
   -   
   -   
   -   
   -   
   -   
   -   
   -   
   -   
   -   
   -   
   -   
   -   
   -   
   -   
   -   
   -   
   -   
   -   
   -   
   -   
   -   
   -   
   -   
   -   
   -   
   -   
   -   
   -   
   -   
   -   
   -   
   -   
   -   
   -   
   -   
   -   
   -   
   -   
   -   
   -   
   -   
   -   
   -   
   -   
   -   
   -   
   -   
   -   
   -   
   -   
   -   
   -   
   -   
   -   
   -   
   -   
   -   
   -   
   -   
   -   
   -   
   -   
 

 
   N60001F   
   A   
   T   
   G   
   A   
   G   
   T   
   G   
   A   
   A   
   G   
   A   
   A   
   A   
   T   
   C   
   A   
   G   
   A   
   G   
   A   
   A   
   A   
   A   
   C   
   A   
   T   
   C   
   C   
   A   
   T   
   G   
   A   
   T   
   G   
   T   
   C   
   A   
   A   
   T   
   C   
   T   
   A   
   A   
   C   
   C   
   A   
   G   
   C   
   G   
   A   
   A   
   A   
   T   
   G   
   A   
   A   
   A   
   G   
   A   
   C   
   T   
   C   
   C   
   T   
   T   
   C   
   A   
   T   
   C   
   G   
   A   
   T   
   T   
   A   
   T   
   G   
   C   
   G   
   A   
   T   
   G   
   A   
   G   
   T   
   G   
   T   
   T   
   A   
   T   
   T   
   G   
   T   
   C   
   G   
   C   
   A   
   C   
   G   
   T   
   G   
   C   
   A   
   C   
   T   
   G   
   C   
   C   
   T   
   G   
   A   
   T   
   G   
   T   
   C   
   A   
   G   
   A   
   G   
   A   
   T   
   G   
   G   
   T   
   C   
   T   
   G   
   A   
   A   
   A   
   C   
   C   
   C   
   G   
   T   
   T   
   C   
   A   
   T   
   C   
   G   
   C   
   C   
   G   
   T   
   A   
   T   
   T   
   T   
   T   
   A   
   T   
   A   
   C   
   G   
   G   
   C   
   A   
   T   
   G   
   A   
   A   
   C   
   G   
   A   
   A   
   T   
   T   
   A   
   G   
   G   
   T   
   G   
   T   
   G   
   A   
   C   
   A   
   C   
   C   
   G   
   G   
   A   
   T   
   A   
   A   
   A   
   G   
   C   
   C   
   C   
   A   
   T   
   A   
   A   
   A   
   A   
   A   
   A   
   T   
   C   
   T   
   G   
   C   
   C   
   A   
   G   
   A   
   A   
   T   
   C   
   G   
   T   
   C   
   G   
   G   
   A   
   G   
   A   
   T   
   G   
   T   
   C   
   A   
   T   
   G   
   G   
   G   
   T   
   A   
   A   
   A   
   T   
   A   
   C   
   C   
   A   
   T   
   C   
   C   
   T   
   C   
   A   
   T   
   G   
   G   
   G   
   G   
   A   
   T   
   A   
   G   
   T   
   G   
   C   
   G   
   A   
   T   
   C   
   T   
   A   
   T   
   G   
   A   
   G   
   T   
   C   
   A   
   A   
   T   
   G   
   G   
   T   
   A   
   C   
   G   
   G   
   A   
   T   
   G   
   G   
   C   
   G   
   C   
   A   
   G   
   C   
   C   
   A   
   T   
   T   
   T   
   A   
   G   
   C   
   T   
   A   
   C   
   C   
   G   
   T   
   T   
   A   
   T   
   A   
   T   
   G   
   C   
   T   
   A   
   G   
   T   
   G   
   G   
   A   
   T   
   G   
   G   
   C   
   C   
   A   
   T   
   G   
   G   
   A   
   A   
   A   
   C   
   T   
   T   
   T   
   G   
   G   
   T   
   T   
   C   
   T   
   G   
   T   
   T   
   G   
   A   
   C   
   G   
   G   
   T   
   G   
   A   
   C   
   G   
   G   
   T   
   G   
   C   
   C   
   G   
   C   
   T   
   G   
   C   
   T   
   A   
   T   
   G   
   C   
   G   
   T   
   T   
   A   
   T   
   A   
   C   
   C   
   G   
   A   
   A   
   G   
   C   
   A   
   C   
   G   
   G   
   A   
   T   
   G   
   A   
   G   
   T   
   A   
   A   
   A   
   A   
   T   
   C   
   G   
   C   
   T   
   A   
   C   
   A   
   G   
   A   
   A   
   A   
   T   
   G   
   C   
   T   
   G   
   C   
   G   
   T   
   G   
   A   
   T   
   A   
   T   
   C   
   A   
   A   
   T   
   A   
   A   
   G   
   A   
   A   
   C   
   A   
   C   
   T   
   G   
   T   
   T   
   G   
   A   
   T   
   T   
   T   
   C   
   C   
   A   
   A   
   A   
   G   
   C   
   A   
   A   
   C   
   T   
   A   
   T   
   G   
   A   
   T   
   G   
   A   
   C   
   A   
   C   
   A   
   G   
   A   
   A   
   A   
   A   
   A   
   G   
   A   
   A   
   C   
   C   
   C   
   G   
   T   
   T   
   G   
   T   
   A   
   T   
   T   
   A   
   C   
   C   
   T   
   G   
   C   
   T   
   C   
   G   
   C   
   T   
   T   
   C   
   C   
   C   
   C   
   A   
   A   
   T   
   C   
   T   
   T   
   T   
   T   
   G   
   G   
   T   
   G   
   A   
   A   
   C   
   G   
   G   
   A   
   A   
   C   
   G   
   A   
   C   
   G   
   G   
   G   
   G   
   A   
   T   
   A   
   G   
   C   
   C   
   G   
   T   
   G   
   G   
   G   
   T   
   A   
   T   
   G   
   G   
   C   
   A   
   A   
   C   
   G   
   A   
   A   
   T   
   A   
   T   
   C   
   C   
   C   
   A   
   C   
   C   
   T   
   C   
   A   
   T   
   A   
   A   
   T   
   T   
   T   
   A   
   T   
   C   
   T   
   G   
   A   
   G   
   G   
   T   
   A   
   G   
   T   
   A   
   G   
   C   
   G   
   G   
   C   
   T   
   A   
   T   
   C   
   G   
   A   
   T   
   T   
   T   
   G   
   C   
   T   
   G   
   A   
   T   
   G   
   G   
   A   
   T   
   A   
   A   
   T   
   C   
   C   
   T   
   G   
   A   
   T   
   G   
   T   
   G   
   A   
   C   
   G   
   A   
   C   
   C   
   A   
   A   
   T   
   G   
   A   
   A   
   T   
   T   
   G   
   A   
   T   
   G   
   G   
   A   
   A   
   G   
   T   
   A   
   T   
   T   
   A   
   C   
   C   
   T   
   G   
   G   
   A   
   C   
   C   
   A   
   G   
   A   
   T   
   T   
   T   
   T   
   C   
   C   
   A   
   A   
   C   
   A   
   G   
   G   
   T   
   G   
   G   
   T   
   T   
   T   
   A   
   G   
   T   
   C   
   A   
   T   
   G   
   G   
   G   
   A   
   A   
   A   
   A   
   T   
   C   
   A   
   G   
   G   
    A    
   A   
   T   
    C    
   C   
   G   
    A    
    A    
   G   
   A   
   G   
   C   
    G    
   T   
   A   
   T   
   G   
   A   
   A   
   A   
   C   
    A    
   G   
   G   
    T    
   A   
   A   
    A    
   G   
   G   
   T   
   T   
   C   
    T    
   A   
   T   
   T   
   A   
   C   
    C    
   G   
   T   
    A    
   C   
   G   
   T   
   G   
   C   
   A   
   A   
   A   
   A   
   G   
   T   
   T   
   G   
   A   
    G    
    A    
   T   
    C    
   A   
    C    
   T   
   G   
   A   
    G    
   A   
   T   
   G   
    C    
   C   
    A    
   A   
   A   
   T   
   G   
   G   
    C    
   A   
   A   
   A   
   G   
   A   
   A   
   C   
   G   
    G    
   A   
   T   
    C    
    C    
   T   
    C    
   G   
   T   
    G    
   A   
   C   
    G    
   G   
   A   
   A   
   T   
   T   
    A    
   C   
   C   
   T   
   T   
   A   
   T   
   A   
   T   
   G   
   G   
   T   
    C    
   A   
   A   
   T   
   A   
   A   
   A   
   G   
   C   
    C    
   A   
   A   
   A   
   T   
   T   
    G    
   A   
   T   
    T    
   G   
   A   
   A   
   C   
   G   
    G    
   A   
   T   
   T   
   T   
   C   
   T   
   G   
   A   
    G    
    T    
   T   
    G    
   C   
   A   
   C   
   A   
   G   
   A   
   G   
   A   
    C    
   A   
   A   
    G    
   C   
   G   
    T    
   A   
   T   
    C    
   G   
   A   
   A   
   G   
   G   
    A    
   A   
   T   
    C    
   A   
   C   
    G    
   G   
   A   
   T   
   T   
   T   
   G   
   C   
   G   
   G   
   G   
   A   
    T    
   G   
   A   
   A   
   T   
   C   
   T   
   T   
   C   
    C    
   C   
   G   
   T   
   G   
   A   
    A    
   G   
   G   
    C    
   A   
   T   
   G   
   C   
   G   
    A    
   A   
   T   
    T    
   G   
   T   
   C   
   A   
   T   
    T    
   G   
   A   
    C    
    A    
   T   
    T    
   C   
   G   
   T   
   C   
   G   
    T    
   G   
   A   
    C    
   G   
    C    
    C    
   A   
   G   
    C    
   G   
   C   
    A    
   T   
   C   
    A    
   G   
   T   
   T   
    A    
   T   
    T    
   T   
   T   
    G    
   A   
   A   
    T    
   A   
   A   
   C   
   T   
   T   
    A    
   T   
   A   
    C    
   A   
   A   
   A   
    T    
   T   
   G   
   A   
   C   
    A    
    T    
   C   
    C    
   T   
   T   
    A    
   C   
   A   
    G    
   A   
    A    
    T    
   T   
   C   
    A    
   T   
   T   
    T    
   G   
   G   
    A    
   T   
   T   
    C    
   A   
   A   
    T    
   A   
   T   
   G   
   T   
   T   
    G    
   G   
   C   
    C    
   A   
   T   
    C    
   G   
   A   
   A   
   A   
   A   
   A   
   G   
   G   
    C    
   G   
   T   
    G    
   C   
   C   
    G    
   A   
   A   
   A   
    G    
   T   
   T   
   T   
   T   
    A    
   A   
   G   
    T    
   T   
   T   
   G   
   A   
   A   
   A   
   C   
    A    
    A    
   A   
   T   
    C    
    C    
   T   
    T    
   G   
   A   
   A   
   A   
   A   
    C    
   T   
   A   
    T    
    A    
   T   
    C    
   G   
   A   
    A    
   C   
   A   
    T    
   C   
   A   
    A    
    C    
    G    
    C    
    G    
   A   
   A   
   G   
   T   
   C   
   A   
   T   
    C    
   A   
   C   
    A    
    C    
   G   
    A    
   C   
   G   
   G   
   A   
   C   
    G    
    G    
   T   
    C    
   T   
   T   
   T   
   G   
   A   
    A    
   A   
   A   
   A   
    G    
   A   
    A    
   A   
   A   
    A    
   G   
   C   
   A   
   G   
   A   
   A   
   G   
   C   
   A   
    A    
   G   
    A    
   G   
   C   
    A    
   C   
   A   
    C    
   A   
   T   
    C    
   T   
   T   
   A   
   G   
   A   
   A   
   G   
   G   
   T   
    T    
   T   
    G    
   C   
   G   
    T    
   A   
   T   
   T   
   G   
   C   
    T    
   T   
   T   
    G    
   G   
   A   
   T   
   C   
   A   
   T   
   A   
   T   
   C   
    G    
   A   
   T   
   G   
   A   
   A   
   A   
   T   
    C    
   A   
   T   
   T   
   G   
   C   
    G    
   A   
   T   
   T   
   A   
   T   
    C    
   C   
   G   
    C    
   G   
   G   
    G    
   T   
   C   
    T    
    A    
   A   
   A   
   T   
   C   
   A   
   G   
   A   
   T   
   G   
   A   
    T    
   G   
   A   
    A    
   G   
   C   
   A   
   A   
   A   
   A   
   G   
   C   
    A    
   A   
    C    
    A    
    A    
   T   
    G    
   A   
   T   
    C    
   G   
   A   
   A   
   C   
   G   
    G    
   T   
   T   
    C    
   G   
   A   
    T    
   T   
   T   
    A    
   T   
   C   
    C    
   G   
   A   
   T   
   C   
   G   
    C    
   C   
   A   
    A    
    T    
   C   
    A    
   C   
   A   
   A   
   G   
   C   
    T    
   A   
   T   
    C    
   T   
   T   
   A   
   G   
   A   
   T   
   A   
   T   
   G   
   C   
   G   
   T   
   T   
   T   
    G    
   C   
   G   
    T    
   C   
   G   
    A    
   T   
   T   
    G    
   A   
   C   
    C    
   G   
   G   
    T    
   T   
   T   
    G    
   G   
   A   
   A   
   C   
   G   
    G    
   G   
   A   
    A    
   A   
   A   
   A   
   A   
   T   
    C    
   G   
   A   
    A    
   A   
   A   
   T   
   G   
   A   
   A   
   T   
   A   
    T    
   C   
   A   
   A   
   G   
   A   
    A    
   C   
   T   
    A    
   T   
   T   
    G    
   A   
    A    
   A   
    C    
   T   
    G    
   A   
   T   
   T   
   G   
    A    
    A    
   G   
   A   
    T    
   T   
   T   
    G    
    A    
    C    
    T    
   G   
   A   
   C   
   A   
   T   
   T   
    C    
   T   
    G    
   G   
   C   
    A    
    A    
   G   
    A    
   C   
   C   
    C    
   G   
   A   
    A    
   C   
   G   
    T    
   G   
   T   
    C    
   A   
    C    
    G    
   G   
   A   
   A   
   A   
   T   
    C    
   A   
   T   
    C    
   A   
   A   
    G    
   A   
    A    
    T    
   G   
   A   
    G    
   T   
   T   
   A   
    G    
    C    
    A    
   G   
   A   
   C   
    T    
   T   
    G    
    A    
    A    
    T    
    C    
    A    
   A   
    C    
    G    
   A   
   T   
   T   
   T   
   G   
   G   
   T   
   G   
   A   
   T   
    A    
    A    
   A   
   C   
   G   
   A   
   C   
   G   
    C    
   A   
   C   
   A   
   G   
   A   
   A   
    T    
   T   
   A   
    C    
   T   
   A   
   G   
   T   
    C    
   G   
   G   
   T   
   G   
   A   
    G    
   G   
   T   
   C   
    T    
   T   
    A    
   A   
   G   
   T   
   C   
   T   
    A    
   G   
   A   
   A   
   G   
   A   
   T   
   G   
   A   
   A   
   G   
   A   
   T   
    T    
   T   
    G    
   A   
   T   
    C    
   G   
   A   
   A   
   G   
   A   
   A   
   G   
   A   
   A   
   G   
   A   
   A   
    A    
   T   
   C   
   G   
   T   
    T    
   A   
   T   
   T   
   A   
   C   
    A    
    C    
   T   
    G    
   A   
   C   
    A    
   A   
   A   
    C    
   A   
   A   
    T    
   G   
   G   
    T    
   T   
   A   
    T    
   A   
   T   
    C    
   A   
   A   
    A    
   C   
   G   
    T    
    T    
   T   
   G   
   G   
    C    
   A   
   A   
   A   
    T    
    A    
    A    
    T    
   G   
   A   
   A   
   T   
   T   
   C   
    A    
   G   
    A    
   G   
   C   
    T    
   C   
   A   
   A   
   C   
   G   
    A    
    A    
   G   
    A    
   G   
   G   
    C    
   G   
   G   
    G    
   C   
   G   
   T   
   G   
   G   
    T    
   G   
   T   
    C    
   C   
   A   
   A   
   G   
   G   
    A    
   A   
   T   
   G   
   G   
   G   
    T    
    A    
   T   
    C    
   C   
   A   
    C    
    A    
   A   
   T   
   G   
   A   
    C    
   G   
   A   
    C    
   T   
   T   
   C   
   G   
   T   
    C    
   A   
   A   
    G    
   A   
   A   
    T    
   C   
   T   
    A    
    G    
   T   
   T   
   T   
   C   
   T   
   T   
   G   
    T    
   T   
   C   
    T    
   A   
   C   
    C    
   C   
   A   
    T    
   G   
   A   
    T    
   A   
   C   
    A    
   T   
   T   
    G    
   T   
   T   
   A   
   T   
   T   
    C    
   T   
   T   
   T   
   A   
   C   
   G   
   A   
   A   
    T    
   A   
    A    
    C    
   G   
   G   
    A    
   A   
   A   
   A   
   G   
   T   
    G    
   T   
   A   
    T    
   C   
   G   
    G    
   G   
   C   
   A   
   A   
   A   
   A   
   G   
   G   
    A    
   T   
   A   
    C    
   G   
   A   
   A   
   A   
   T   
    T    
   C   
   C   
    A    
   G   
   A   
    A    
   T   
   A   
   C   
   G   
   G   
    A    
    C    
   G   
    T    
   A   
   C   
   A   
   G   
   C   
    C    
   A   
   A   
   A   
   G   
   G   
    T    
   A   
   T   
    C    
   C   
   C   
   A   
    A    
   T   
    C    
   A   
   T   
    C    
   A   
   A   
    T    
   T   
   T   
    G    
   C   
   T   
    G    
   G   
   G   
    T    
   A   
   T   
    C    
   G   
   A   
    C    
   T   
   C   
    G    
    A    
    G    
    T    
   G   
   A   
   A   
   A   
    G    
    C    
   A   
   T   
    C    
   C   
   A   
    A    
   G   
   C   
    A    
   A   
   T   
    T    
   A   
   T   
   T   
    G    
   C   
    G    
   G   
   T   
    G    
    A    
    C    
   G   
   G   
   G   
    A    
    G    
   A   
   A   
   G   
   C   
    A    
   G   
   A   
   A   
   G   
    A    
    A    
   G   
   G   
    C    
    C    
   A   
    C    
   T   
   A   
    T    
    C    
   T   
    C    
   T   
   T   
   C   
   T   
   T   
    T    
   A   
   C   
    G    
   A   
   C   
    G    
    C    
    G    
    T    
    C    
   A   
   A   
   G   
   G   
    T    
   A   
   C   
    A    
   G   
   T   
    T    
   A   
   A   
   A   
   C   
   G   
    T    
   A   
   C   
    G    
    T    
   C   
    T    
   G   
   T   
    C    
   A   
   C   
    T    
   G   
   C   
    T    
   T   
   T   
    C    
    G    
   C   
   T   
   A   
   A   
    C    
   A   
   T   
    C    
   C   
   G   
    A    
   A   
   G   
    C    
   A   
   A   
   T   
   G   
   G   
    T    
    C    
   T   
    G    
   A   
   T   
    T    
   G   
   C   
    A    
   A   
   T   
   T   
    G    
   G   
    T    
   T   
   T   
    G    
   A   
   A   
   A   
   G   
   A   
   A   
   G   
   A   
    T    
   G   
   A   
    C    
   G   
   A   
    A    
    T    
   T   
    A    
    G    
   T   
    C    
   A   
   A   
    T    
   G   
   T   
    T    
    C    
   T   
   A   
    T    
    T    
   G   
   A   
   C   
    A    
    G    
   A   
    T    
   G   
   G   
    A    
    C    
   A   
    G    
    T    
    C    
    A    
   A   
   A   
    T    
   A   
   T   
    C    
   A   
   T   
    C    
   A   
   T   
    T    
   G   
   G   
    C    
   A   
   C   
   A   
   C   
   A   
   T   
    A    
    A    
    C    
   G   
   G   
    C    
   T   
   A   
   C   
   T   
   C   
   T   
   G   
   T   
    G    
   A   
   C   
    C    
   T   
   T   
    C    
    A    
   A   
    A    
   G   
   A   
    G    
    G    
    A    
    A    
    G    
   C   
    A    
   G   
    T    
    C    
   C   
   G   
    C    
   G   
   A   
   T   
   A   
   T   
   G   
   G   
   G   
    A    
   C   
   G   
    T    
   A   
   C   
   A   
   G   
   C   
    T    
   T   
   C   
    C    
   G   
   G   
    C    
   G   
   T   
    C    
    A    
   G   
    A    
   G   
   G   
    G    
   A   
   T   
    A    
   C   
   G   
    T    
    T    
   T   
    G    
   C   
   G   
    T    
   G   
   A   
    G    
    G    
   A   
    A    
   G   
   A   
    C    
   T   
    A    
    C    
   G   
   T   
    T    
   G   
   T   
    T    
   G   
   G   
    G    
    G    
   C   
   A   
   G   
   C   
    C    
    C    
   T   
    G    
    A    
   T   
   G   
    A    
   A   
    G    
   G   
    A    
   A   
    G    
   A   
    C    
    C    
   A   
   A   
   G   
   A   
   A   
   G   
   T   
    A    
    C    
   T   
    A    
   G   
   T   
    G    
   A   
   T   
    T    
   A   
   C   
    A    
   G   
   A   
   A   
   A   
   A   
    A    
   G   
   G   
   T   
   T   
   A   
    C    
   G   
   G   
    A    
   A   
   A   
    A    
   C   
   G   
    G    
   A   
   C   
    C    
   A   
   A   
   A   
   G   
    T    
    A    
   T   
   C   
    G    
   G   
   A   
    A    
   T   
   A   
   T   
   C   
   C   
   A   
   G   
   T   
    G    
   A   
   A   
    A    
   G   
   G   
    C    
   C   
   G   
    C    
   G   
   G   
    T    
   G   
   G   
   T   
   A   
   A   
   A   
   G   
   G   
    A    
   A   
   T   
    C    
   A   
   A   
    A    
   A   
   C   
    G    
    G    
   C   
    G    
   A   
   A   
    T    
   A   
   T   
   C   
   A   
   C   
   T   
   G   
   A   
    A    
   A   
   A   
   A   
   A   
   A   
   T   
   G   
   G   
   T   
   C   
   C   
   A   
    C    
   T   
   A   
   G   
   C   
    C    
   G   
   G   
    A    
   T   
   T   
    G    
   A   
   C   
   C   
   A   
   C   
    T    
   G   
   T   
    T    
    T    
    C    
   T   
   G   
   G   
    A    
    G    
   A   
   T   
   G   
   A   
   A   
   G   
   A   
    C    
   A   
   T   
   C   
    A    
   T   
    G    
    C    
   T   
    G    
   A   
   T   
    T    
   A   
   C   
    A    
    G    
   A   
    T    
   A   
   A   
   A   
   G   
   G   
    T    
   G   
   T   
    G    
   A   
   T   
   T   
   A   
   T   
   C   
   C   
   G   
    T    
   T   
   T   
    C    
   A   
   A   
   C   
   G   
   T   
    A    
    T    
    C    
    G    
    A    
   C   
   T   
   G   
   T   
   T   
   T   
   C   
   T   
   C   
   A   
   A   
   A   
   C   
    T    
   G   
   G   
   A   
   C   
   G   
    T    
    T    
   C   
   A   
   A   
   C   
   A   
   T   
   T   
    G    
   G   
   G   
    T    
   G   
   T   
    A    
   C   
   G   
   T   
   T   
   T   
    G    
   A   
   T   
   G   
   A   
    A    
    G    
   A   
   T   
   G   
   G   
   A   
   A   
   G   
    C    
    T    
   G   
    A    
    T    
    A    
   C   
    G    
   A   
   A   
   A   
   G   
   T   
   C   
   G   
   T   
   C   
   A   
   C   
   A   
   A   
   T   
   G   
   -   
   -   
   -   
   -   
   -   
   -   
   -   
   -   
   -   
   -   
   -   
   -   
   -   
   -   
   -   
   -   
   -   
   -   
   -   
   -   
   -   
   -   
   -   
   -   
   -   
   -   
   -   
   -   
   -   
   -   
   -   
   -   
   -   
   -   
   -   
   -   
   -   
   -   
   -   
   -   
   -   
   -   
   -   
   -   
   -   
   -   
   -   
   -   
   -   
   -   
   -   
   -   
   -   
   -   
   -   
   -   
   -   
   -   
   -   
   -   
 

 
   N54035   
   A   
   T   
   G   
   A   
   G   
   T   
   G   
   A   
   A   
   G   
   A   
   A   
   A   
   T   
   C   
   A   
   G   
   A   
   G   
   A   
   A   
   A   
   A   
   C   
   A   
   T   
   C   
   C   
   A   
   T   
   G   
   A   
   T   
   G   
   T   
   C   
   A   
   A   
   T   
   C   
   T   
   A   
   A   
   C   
   C   
   A   
   G   
   C   
   G   
   A   
   A   
   A   
   T   
   G   
   A   
   A   
   A   
   G   
   A   
   C   
   T   
   C   
   C   
   T   
   T   
   C   
   A   
   T   
   C   
   G   
   A   
   T   
   T   
   A   
   T   
   G   
   C   
   G   
   A   
   T   
   G   
   A   
   G   
   T   
   G   
   T   
   T   
   A   
   T   
   T   
   G   
   T   
   C   
   G   
   C   
   A   
   C   
   G   
   T   
   G   
   C   
   A   
   C   
   T   
   T   
   C   
   C   
   T   
   G   
   A   
   T   
   G   
   T   
   C   
   A   
   G   
   A   
   G   
   A   
   T   
   G   
   G   
   T   
   C   
   T   
   G   
   A   
   A   
   A   
   C   
   C   
   C   
   G   
   T   
   T   
   C   
   A   
   T   
   C   
   G   
   C   
   C   
   G   
   T   
   A   
   T   
   T   
   T   
   T   
   A   
   T   
   A   
   C   
   G   
   G   
   C   
   A   
   T   
   G   
   A   
   A   
   C   
   G   
   A   
   A   
   T   
   T   
   A   
   G   
   G   
   T   
   G   
   T   
   A   
   A   
   C   
   A   
   C   
   C   
   G   
   G   
   A   
   T   
   A   
   A   
   A   
   G   
   C   
   C   
   C   
   A   
   T   
   A   
   A   
   A   
   A   
   A   
   A   
   T   
   C   
   T   
   G   
   C   
   C   
   A   
   G   
   A   
   A   
   T   
   C   
   G   
   T   
   C   
   G   
   G   
   A   
   G   
   A   
   T   
   G   
   T   
   C   
   A   
   T   
   G   
   G   
   G   
   T   
   A   
   A   
   A   
   T   
   A   
   C   
   C   
   A   
   T   
   C   
   C   
   T   
   C   
   A   
   T   
   G   
   G   
   A   
   G   
   A   
   C   
   A   
   G   
   T   
   G   
   C   
   G   
   A   
   T   
   C   
   T   
   A   
   T   
   G   
   A   
   G   
   T   
   C   
   A   
   A   
   T   
   G   
   G   
   T   
   A   
   C   
   G   
   G   
   A   
   T   
   G   
   G   
   C   
   A   
   C   
   A   
   G   
   C   
   C   
   A   
   T   
   T   
   C   
   A   
   G   
   C   
   T   
   A   
   C   
   C   
   G   
   T   
   T   
   A   
   T   
   A   
   T   
   G   
   C   
   T   
   A   
   G   
   T   
   G   
   G   
   A   
   T   
   G   
   G   
   C   
   C   
   A   
   T   
   G   
   G   
   A   
   A   
   A   
   C   
   T   
   T   
   T   
   G   
   G   
   T   
   T   
   C   
   T   
   G   
   T   
   T   
   G   
   A   
   C   
   G   
   G   
   T   
   G   
   A   
   C   
   G   
   G   
   T   
   G   
   C   
   C   
   G   
   C   
   T   
   G   
   C   
   T   
   A   
   T   
   G   
   C   
   G   
   T   
   T   
   A   
   T   
   A   
   C   
   C   
   G   
   A   
   A   
   G   
   C   
   A   
   C   
   G   
   G   
   A   
   T   
   G   
   A   
   G   
   T   
   A   
   A   
   A   
   A   
   T   
   C   
   G   
   C   
   T   
   A   
   C   
   A   
   G   
   A   
   A   
   A   
   T   
   G   
   C   
   T   
   G   
   C   
   G   
   T   
   G   
   A   
   T   
   A   
   T   
   C   
   A   
   A   
   T   
   A   
   A   
   G   
   A   
   A   
   C   
   A   
   C   
   T   
   G   
   T   
   T   
   G   
   A   
   T   
   T   
   T   
   C   
   C   
   A   
   A   
   A   
   G   
   C   
   A   
   A   
   C   
   T   
   A   
   T   
   G   
   A   
   T   
   G   
   A   
   T   
   A   
   C   
   A   
   G   
   A   
   A   
   A   
   A   
   A   
   G   
   A   
   A   
   C   
   C   
   A   
   G   
   T   
   T   
   G   
   T   
   A   
   C   
   T   
   A   
   C   
   C   
   T   
   G   
   C   
   T   
   C   
   G   
   C   
   T   
   T   
   C   
   C   
   C   
   G   
   A   
   A   
   T   
   C   
   T   
   T   
   T   
   T   
   A   
   G   
   T   
   G   
   A   
   A   
   C   
   G   
   G   
   A   
   A   
   C   
   A   
   A   
   C   
   G   
   G   
   G   
   G   
   A   
   T   
   C   
   G   
   C   
   C   
   G   
   T   
   A   
   G   
   G   
   T   
   A   
   T   
   G   
   G   
   C   
   C   
   A   
   C   
   G   
   A   
   A   
   T   
   A   
   T   
   C   
   C   
   C   
   A   
   C   
   C   
   T   
   C   
   A   
   T   
   A   
   A   
   C   
   T   
   T   
   G   
   T   
   C   
   T   
   G   
   A   
   G   
   G   
   T   
   A   
   G   
   T   
   A   
   G   
   C   
   G   
   G   
   C   
   T   
   A   
   T   
   C   
   G   
   A   
   T   
   T   
   T   
   G   
   C   
   T   
   G   
   A   
   T   
   G   
   G   
   A   
   T   
   A   
   A   
   T   
   C   
   C   
   T   
   G   
   A   
   T   
   G   
   T   
   G   
   A   
   C   
   G   
   A   
   C   
   C   
   A   
   A   
   T   
   G   
   A   
   A   
   T   
   T   
   G   
   A   
   T   
   G   
   G   
   A   
   A   
   G   
   T   
   A   
   T   
   T   
   A   
   C   
   C   
   T   
   G   
   G   
   A   
   C   
   C   
   A   
   G   
   A   
   T   
   T   
   T   
   T   
   C   
   C   
   A   
   A   
   C   
   A   
   G   
   G   
   T   
   G   
   G   
   T   
   T   
   T   
   G   
   G   
   T   
   T   
   A   
   T   
   G   
   G   
   G   
   A   
   A   
   A   
   A   
   T   
   C   
   A   
   G   
   G   
    A    
   A   
   T   
    C    
   C   
   G   
    A    
    A    
   G   
   A   
   G   
   C   
    G    
   T   
   A   
   T   
   G   
   A   
   A   
   A   
   C   
    A    
   G   
   G   
    T    
   A   
   A   
    G    
   G   
   G   
   T   
   T   
   C   
    T    
   A   
   T   
   T   
   A   
   C   
    C    
   G   
   T   
    A    
   C   
   G   
   T   
   G   
   C   
   A   
   A   
   A   
   A   
   G   
   T   
   T   
   G   
   A   
    G    
    A    
   T   
    T    
   A   
    C    
   T   
   G   
   A   
    G    
   A   
   T   
   G   
    C    
   C   
    A    
   A   
   A   
   T   
   G   
   G   
    T    
   A   
   A   
   A   
   G   
   A   
   A   
   C   
   G   
    G    
   A   
   T   
    C    
    A    
   T   
    C    
   G   
   T   
    G    
   A   
   C   
    A    
   G   
   A   
   A   
   T   
   T   
    G    
   C   
   C   
   T   
   T   
   A   
   T   
   A   
   T   
   G   
   G   
   T   
    C    
   A   
   A   
   T   
   A   
   A   
   A   
   G   
   C   
    A    
   A   
   A   
   A   
   T   
   T   
    G    
   A   
   T   
    C    
   G   
   A   
   A   
   C   
   G   
    G    
   A   
   T   
   T   
   T   
   C   
   T   
   G   
   A   
    A    
    C    
   T   
    G    
   C   
   A   
   C   
   A   
   G   
   A   
   G   
   A   
    C    
   A   
   A   
    G    
   C   
   G   
    T    
   A   
   T   
    C    
   G   
   A   
   A   
   G   
   G   
    G    
   A   
   T   
    C    
   A   
   C   
    A    
   G   
   A   
   T   
   T   
   T   
   G   
   C   
   G   
   G   
   G   
   A   
    C    
   G   
   A   
   A   
   T   
   C   
   T   
   T   
   C   
    C    
   C   
   G   
   T   
   G   
   A   
    A    
   G   
   G   
    T    
   A   
   T   
   G   
   C   
   G   
    G    
   A   
   T   
    C    
   G   
   T   
   C   
   A   
   T   
    T    
   G   
   A   
    C    
    A    
   T   
    C    
   C   
   G   
   T   
   C   
   G   
    T    
   G   
   A   
    C    
   G   
    C    
    C    
   A   
   G   
    C    
   G   
   C   
    A    
   T   
   C   
    C    
   G   
   T   
   T   
    A    
   T   
    C    
   T   
   T   
    G    
   A   
   A   
    T    
   A   
   A   
   C   
   T   
   T   
    A    
   T   
   A   
    T    
   A   
   A   
   A   
    T    
   T   
   G   
   A   
   C   
    A    
    T    
   C   
    C    
   T   
   T   
    G    
   C   
   A   
    A    
   A   
    A    
    T    
   T   
   C   
    A    
   T   
   T   
    T    
   G   
   G   
    A    
   T   
   T   
    C    
   A   
   A   
    T    
   A   
   T   
   G   
   T   
   T   
    G    
   G   
   C   
    G    
   A   
   T   
    C    
   G   
   A   
   A   
   A   
   A   
   A   
   G   
   G   
    T    
   G   
   T   
    G    
   C   
   C   
    G    
   A   
   A   
   A   
    G    
   T   
   T   
   T   
   T   
    G    
   A   
   G   
    C    
   T   
   T   
   G   
   A   
   A   
   A   
   C   
    A    
    A    
   A   
   T   
    T    
    C    
   T   
    T    
   G   
   A   
   A   
   A   
   A   
    C    
   T   
   A   
    T    
    A    
   T   
    C    
   G   
   A   
    A    
   C   
   A   
    T    
   C   
   A   
    G    
    C    
    G    
    C    
    G    
   A   
   A   
   G   
   T   
   C   
   A   
   T   
    C    
   A   
   C   
    A    
    A    
   G   
    A    
   C   
   G   
   G   
   A   
   C   
    A    
    G    
   T   
    C    
   T   
   T   
   T   
   G   
   A   
    A    
   A   
   A   
   A   
    G    
   A   
    A    
   A   
   A   
    A    
   G   
   C   
   A   
   G   
   A   
   A   
   G   
   C   
   A   
    A    
   G   
    G    
   G   
   C   
    A    
   C   
   A   
    C    
   A   
   T   
    T    
   T   
   T   
   A   
   G   
   A   
   A   
   G   
   G   
   T   
    T    
   T   
    G    
   C   
   G   
    T    
   A   
   T   
   T   
   G   
   C   
    T    
   T   
   T   
    G    
   G   
   A   
   T   
   C   
   A   
   T   
   A   
   T   
   C   
    G    
   A   
   T   
   G   
   A   
   A   
   A   
   T   
    A    
   A   
   T   
   T   
   G   
   C   
    G    
   A   
   T   
   T   
   A   
   T   
    C    
   C   
   G   
    C    
   G   
   G   
    G    
   T   
   C   
    T    
    A    
   A   
   A   
   T   
   C   
   A   
   G   
   A   
   T   
   G   
   A   
    C    
   G   
   A   
    A    
   G   
   C   
   A   
   A   
   A   
   A   
   G   
   C   
    G    
   A   
    C    
    G    
    A    
   T   
    G    
   A   
   T   
    C    
   G   
   A   
   A   
   C   
   G   
    G    
   T   
   T   
    C    
   G   
   A   
    T    
   T   
   T   
    A    
   T   
   C   
    C    
   G   
   A   
   T   
   C   
   G   
    C    
   C   
   A   
    G    
    T    
   C   
    T    
   C   
   A   
   A   
   G   
   C   
    T    
   A   
   T   
    T    
   T   
   T   
   A   
   G   
   A   
   T   
   A   
   T   
   G   
   C   
   G   
   T   
   T   
   T   
    A    
   C   
   G   
    C    
   C   
   G   
    A    
   T   
   T   
    G    
   A   
   C   
    C    
   G   
   G   
    T    
   T   
   T   
    G    
   G   
   A   
   A   
   C   
   G   
    G    
   G   
   A   
    A    
   A   
   A   
   A   
   A   
   T   
    C    
   G   
   A   
    A    
   A   
   A   
   T   
   G   
   A   
   A   
   T   
   A   
    C    
   C   
   A   
   A   
   G   
   A   
    A    
   C   
   T   
    C    
   T   
   T   
    A    
   A   
    A    
   A   
    C    
   T   
    G    
   A   
   T   
   T   
   G   
    A    
    A    
   G   
   A   
    T    
   T   
   T   
    A    
    A    
    C    
    T    
   G   
   A   
   C   
   A   
   T   
   T   
    C    
   T   
    G    
   G   
   C   
    A    
    A    
   G   
    A    
   C   
   C   
    A    
   G   
   A   
    A    
   C   
   G   
    T    
   G   
   T   
    T    
   A   
    C    
    A    
   G   
   A   
   A   
   A   
   T   
    C    
   A   
   T   
    C    
   A   
   A   
    G    
   A   
    A    
    T    
   G   
   A   
    A    
   T   
   T   
   A   
    G    
    C    
    A    
   G   
   A   
   C   
    T    
   T   
    A    
    A    
    A    
    T    
    C    
    A    
   A   
    C    
    G    
   A   
   T   
   T   
   T   
   G   
   G   
   T   
   G   
   A   
   T   
    A    
    A    
   A   
   C   
   G   
   A   
   C   
   G   
    C    
   A   
   C   
   A   
   G   
   A   
   A   
    T    
   T   
   A   
    C    
   T   
   A   
   G   
   T   
    C    
   G   
   G   
   T   
   G   
   A   
    G    
   G   
   T   
   C   
    T    
   T   
    A    
   A   
   G   
   T   
   C   
   T   
    G    
   G   
   A   
   A   
   G   
   A   
   T   
   G   
   A   
   A   
   G   
   A   
   T   
    T    
   T   
    A    
   A   
   T   
    C    
   G   
   A   
   A   
   G   
   A   
   A   
   G   
   A   
   A   
   G   
   A   
   A   
    A    
   T   
   C   
   G   
   T   
    T    
   A   
   T   
   T   
   A   
   C   
    A    
    T    
   T   
    G    
   A   
   C   
    A    
   A   
   A   
    C    
   A   
   A   
    T    
   G   
   G   
    T    
   T   
   A   
    C    
   A   
   T   
    C    
   A   
   A   
    A    
   C   
   G   
    T    
    T    
   T   
   G   
   G   
    C    
   A   
   A   
   A   
    T    
    A    
    A    
    T    
   G   
   A   
   A   
   T   
   T   
   C   
    A    
   G   
    A    
   G   
   C   
    T    
   C   
   A   
   A   
   C   
   G   
    A    
    A    
   G   
    G    
   G   
   G   
    T    
   G   
   G   
    A    
   C   
   G   
   T   
   G   
   G   
    T    
   G   
   T   
    C    
   C   
   A   
   A   
   G   
   G   
    A    
   A   
   T   
   G   
   G   
   G   
    T    
    A    
   T   
    C    
   C   
   A   
    C    
    A    
   A   
   T   
   G   
   A   
    C    
   G   
   A   
    C    
   T   
   T   
   C   
   G   
   T   
    C    
   A   
   A   
    A    
   A   
   A   
    T    
   C   
   T   
    G    
    G    
   T   
   T   
   T   
   C   
   T   
   T   
   G   
    C    
   T   
   C   
    T    
   A   
   C   
    C    
   C   
   A   
    T    
   G   
   A   
    T    
   A   
   C   
    A    
   T   
   T   
    G    
   T   
   T   
   A   
   T   
   T   
    C    
   T   
   T   
   T   
   A   
   C   
   G   
   A   
   A   
    T    
   A   
    A    
    C    
   G   
   G   
    A    
   A   
   A   
   A   
   G   
   T   
    G    
   T   
   A   
    T    
   C   
   G   
    G    
   G   
   C   
   A   
   A   
   A   
   A   
   G   
   G   
    A    
   T   
   A   
    C    
   G   
   A   
   A   
   A   
   T   
    T    
   C   
   C   
    A    
   G   
   A   
    A    
   T   
   A   
   C   
   G   
   G   
    G    
    C    
   G   
    C    
   A   
   C   
   A   
   G   
   C   
    C    
   A   
   A   
   A   
   G   
   G   
    A    
   A   
   T   
    A    
   C   
   C   
   A   
    A    
   T   
    T    
   A   
   T   
    C    
   A   
   A   
    T    
   T   
   T   
    A    
   C   
   T   
    A    
   G   
   G   
    T    
   A   
   T   
    C    
   G   
   A   
    T    
   T   
   C   
    G    
    A    
    G    
    T    
   G   
   A   
   A   
   A   
    G    
    C    
   A   
   T   
    C    
   C   
   A   
    G    
   G   
   C   
    A    
   A   
   T   
    C    
   A   
   T   
   T   
    G    
   C   
    G    
   G   
   T   
    G    
    A    
    C    
   G   
   G   
   G   
    A    
    G    
   A   
   A   
   G   
   C   
    A    
   G   
   A   
   A   
   G   
    A    
    A    
   G   
   G   
    C    
    C    
   A   
    C    
   T   
   A   
    T    
    C    
   T   
    C    
   T   
   T   
   C   
   T   
   T   
    T    
   A   
   C   
    G    
   A   
   C   
    G    
    C    
    G    
    T    
    C    
   A   
   A   
   G   
   G   
    T    
   A   
   C   
    A    
   G   
   T   
    A    
   A   
   A   
   A   
   C   
   G   
    T    
   A   
   C   
    T    
    T    
   C   
    T    
   G   
   T   
    C    
   A   
   C   
    T    
   G   
   C   
    T    
   T   
   T   
    T    
    G    
   C   
   T   
   A   
   A   
    C    
   A   
   T   
    C    
   C   
   G   
    A    
   A   
   G   
    T    
   A   
   A   
   T   
   G   
   G   
    T    
    C    
   T   
    G    
   A   
   T   
    C    
   G   
   C   
    A    
   A   
   T   
   T   
    G    
   G   
    T    
   T   
   T   
    G    
   A   
   A   
   A   
   G   
   A   
   A   
   G   
   A   
    C    
   G   
   A   
    T    
   G   
   A   
    G    
    C    
   T   
    C    
    G    
   T   
    C    
   A   
   A   
    T    
   G   
   T   
    G    
    C    
   T   
   A   
    C    
    T    
   G   
   A   
   C   
    A    
    G    
   A   
    T    
   G   
   G   
    T    
    C    
   A   
    A    
    T    
    C    
    T    
   A   
   A   
    T    
   A   
   T   
    C    
   A   
   T   
    T    
   A   
   T   
    T    
   G   
   G   
    A    
   A   
   C   
   A   
   C   
   A   
   T   
    A    
    A    
    C    
   G   
   G   
    C    
   T   
   A   
   C   
   T   
   C   
   T   
   G   
   T   
    C    
   A   
   C   
    T    
   T   
   T   
    C    
    A    
   A   
    A    
   G   
   A   
    A    
    G    
    A    
    A    
    G    
   C   
    A    
   G   
    T    
    C    
   C   
   G   
    C    
   G   
   A   
   T   
   A   
   T   
   G   
   G   
   G   
    A    
   C   
   G   
    T    
   A   
   C   
   A   
   G   
   C   
    T    
   T   
   C   
    C    
   G   
   G   
    G    
   G   
   T   
    C    
    A    
   G   
    A    
   G   
   G   
    A    
   A   
   T   
    A    
   C   
   G   
    C    
    C    
   T   
    G    
   C   
   G   
    T    
   G   
   A   
    G    
    G    
   A   
    A    
   G   
   A   
    T    
   T   
    A    
    T    
   G   
   T   
    C    
   G   
   T   
    T    
   G   
   G   
    G    
    G    
   C   
   A   
   G   
   C   
    C    
    T    
   T   
    A    
    A    
   T   
   G   
    A    
   A   
    G    
   G   
    A    
   A   
    G    
   A   
    T    
    C    
   A   
   A   
   G   
   A   
   A   
   G   
   T   
    A    
    T    
   T   
    A    
   G   
   T   
    G    
   A   
   T   
    C    
   A   
   C   
    A    
   G   
   A   
   A   
   A   
   A   
    A    
   G   
   G   
   T   
   T   
   A   
    C    
   G   
   G   
    A    
   A   
   A   
    A    
   C   
   G   
    G    
   A   
   C   
    A    
   A   
   A   
   A   
   G   
    T    
    A    
   T   
   C   
    G    
   G   
   A   
    A    
   T   
   A   
   T   
   C   
   C   
   A   
   G   
   T   
    G    
   A   
   A   
    G    
   G   
   G   
    C    
   C   
   G   
    C    
   G   
   G   
    A    
   G   
   G   
   T   
   A   
   A   
   A   
   G   
   G   
    G    
   A   
   T   
    C    
   A   
   A   
    A    
   A   
   C   
    G    
    G    
   C   
    G    
   A   
   A   
    C    
   A   
   T   
   C   
   A   
   C   
   T   
   G   
   A   
    A    
   A   
   A   
   A   
   A   
   A   
   T   
   G   
   G   
   T   
   C   
   C   
   A   
    C    
   T   
   A   
   G   
   C   
    T    
   G   
   G   
    A    
   T   
   T   
    G    
   A   
   C   
   C   
   A   
   C   
    T    
   G   
   T   
    T    
    T    
    C    
   T   
   G   
   G   
    A    
    G    
   A   
   T   
   G   
   A   
   A   
   G   
   A   
    C    
   A   
   T   
   C   
    A    
   T   
    G    
    C    
   T   
    G    
   A   
   T   
    T    
   A   
   C   
    A    
    G    
   A   
    T    
   A   
   A   
   A   
   G   
   G   
    T    
   G   
   T   
    C    
   A   
   T   
   T   
   A   
   T   
   C   
   C   
   G   
    T    
   T   
   T   
    C    
   A   
   A   
   C   
   G   
   T   
    A    
    T    
    C    
    G    
    A    
   C   
   T   
   G   
   T   
   T   
   T   
   C   
   T   
   C   
   A   
   A   
   A   
   C   
    T    
   G   
   G   
   A   
   C   
   G   
    T    
    T    
   C   
   A   
   A   
   C   
   A   
   T   
   T   
    A    
   G   
   G   
    C    
   G   
   T   
    A    
   C   
   G   
   T   
   T   
   T   
    G    
   A   
   T   
   G   
   A   
    A    
    G    
   A   
   T   
   G   
   G   
   A   
   A   
   G   
    C    
    T    
   G   
    A    
    T    
    A    
   C   
    G    
   A   
   A   
   A   
   G   
   T   
   T   
   G   
   T   
   C   
   A   
   C   
   G   
   A   
   T   
   G   
   G   
   C   
   A   
   G   
   C   
   A   
   G   
   T   
   C   
   G   
   A   
   A   
   C   
   C   
   A   
   G   
   A   
   A   
   G   
   C   
   T   
   G   
   C   
   A   
   G   
   A   
   A   
   C   
   A   
   A   
   A   
   C   
   A   
   G   
   T   
   T   
   G   
   A   
   A   
   G   
   A   
   A   
   C   
   A   
   A   
   G   
   G   
   A   
   T   
   C   
   A   
   G   
   A   
   A   
   G   
   A   
   A   
   -   
   -   
   -   
 

 
   N52564   
   A   
   T   
   G   
   A   
   G   
   T   
   G   
   A   
   A   
   G   
   A   
   A   
   A   
   T   
   T   
   A   
   A   
   A   
   G   
   A   
   A   
   A   
   A   
   C   
   A   
   T   
   T   
   C   
   A   
   A   
   G   
   A   
   C   
   G   
   T   
   C   
   A   
   A   
   T   
   C   
   T   
   G   
   A   
   C   
   C   
   A   
   G   
   C   
   G   
   A   
   A   
   A   
   T   
   G   
   A   
   A   
   A   
   G   
   A   
   A   
   T   
   C   
   T   
   T   
   T   
   C   
   A   
   T   
   T   
   G   
   A   
   C   
   T   
   A   
   C   
   G   
   C   
   A   
   A   
   T   
   G   
   A   
   G   
   T   
   G   
   T   
   T   
   A   
   T   
   C   
   G   
   T   
   A   
   G   
   C   
   C   
   C   
   G   
   C   
   G   
   C   
   G   
   T   
   T   
   A   
   C   
   C   
   T   
   G   
   A   
   C   
   G   
   T   
   T   
   C   
   G   
   T   
   G   
   A   
   C   
   G   
   G   
   T   
   T   
   T   
   A   
   A   
   A   
   A   
   C   
   C   
   T   
   G   
   T   
   T   
   C   
   A   
   T   
   C   
   G   
   C   
   C   
   G   
   A   
   A   
   T   
   C   
   T   
   T   
   A   
   T   
   A   
   T   
   G   
   G   
   A   
   A   
   T   
   G   
   A   
   A   
   C   
   G   
   A   
   A   
   T   
   T   
   A   
   G   
   G   
   G   
   G   
   T   
   A   
   A   
   C   
   C   
   C   
   C   
   T   
   G   
   A   
   T   
   A   
   A   
   A   
   C   
   C   
   A   
   C   
   A   
   C   
   A   
   A   
   A   
   A   
   A   
   A   
   T   
   C   
   A   
   G   
   C   
   C   
   C   
   G   
   G   
   A   
   T   
   T   
   G   
   T   
   T   
   G   
   G   
   G   
   G   
   A   
   T   
   G   
   T   
   T   
   A   
   T   
   G   
   G   
   G   
   T   
   A   
   A   
   A   
   T   
   A   
   T   
   C   
   A   
   C   
   C   
   C   
   C   
   C   
   A   
   T   
   G   
   G   
   G   
   G   
   A   
   C   
   A   
   G   
   T   
   G   
   C   
   G   
   A   
   T   
   T   
   T   
   A   
   C   
   G   
   A   
   A   
   T   
   C   
   A   
   A   
   T   
   G   
   G   
   T   
   G   
   C   
   G   
   G   
   A   
   T   
   G   
   G   
   C   
   A   
   C   
   A   
   A   
   C   
   C   
   T   
   T   
   T   
   T   
   A   
   G   
   T   
   T   
   A   
   T   
   C   
   G   
   G   
   G   
   C   
   T   
   A   
   T   
   G   
   C   
   T   
   A   
   G   
   T   
   T   
   G   
   A   
   C   
   G   
   G   
   C   
   C   
   A   
   C   
   G   
   G   
   A   
   A   
   A   
   C   
   T   
   T   
   C   
   G   
   G   
   T   
   T   
   C   
   T   
   G   
   T   
   C   
   G   
   A   
   T   
   G   
   G   
   T   
   G   
   A   
   C   
   G   
   G   
   C   
   G   
   C   
   T   
   G   
   C   
   C   
   G   
   C   
   T   
   A   
   T   
   G   
   C   
   G   
   T   
   T   
   A   
   T   
   A   
   C   
   C   
   G   
   A   
   A   
   G   
   C   
   A   
   C   
   G   
   T   
   A   
   T   
   G   
   A   
   G   
   T   
   A   
   A   
   A   
   A   
   T   
   T   
   G   
   C   
   T   
   T   
   T   
   A   
   G   
   A   
   A   
   A   
   T   
   G   
   C   
   T   
   A   
   C   
   G   
   A   
   G   
   A   
   T   
   A   
   T   
   T   
   A   
   A   
   C   
   A   
   A   
   A   
   A   
   A   
   T   
   A   
   C   
   A   
   G   
   T   
   C   
   G   
   A   
   T   
   T   
   T   
   C   
   C   
   A   
   A   
   G   
   G   
   A   
   A   
   A   
   C   
   T   
   A   
   T   
   G   
   A   
   T   
   G   
   A   
   T   
   T   
   C   
   A   
   G   
   A   
   A   
   C   
   A   
   A   
   G   
   A   
   A   
   C   
   C   
   A   
   G   
   T   
   A   
   G   
   T   
   T   
   T   
   T   
   A   
   C   
   C   
   T   
   G   
   C   
   T   
   C   
   G   
   T   
   T   
   T   
   T   
   C   
   C   
   A   
   A   
   A   
   C   
   T   
   T   
   A   
   C   
   T   
   A   
   G   
   T   
   T   
   A   
   A   
   C   
   G   
   G   
   A   
   A   
   C   
   A   
   A   
   C   
   G   
   G   
   G   
   G   
   A   
   T   
   T   
   G   
   C   
   G   
   G   
   T   
   G   
   G   
   G   
   G   
   A   
   T   
   G   
   G   
   C   
   A   
   A   
   C   
   A   
   A   
   A   
   T   
   A   
   T   
   T   
   C   
   C   
   A   
   C   
   C   
   A   
   C   
   A   
   C   
   A   
   A   
   T   
   T   
   T   
   A   
   A   
   G   
   T   
   G   
   A   
   A   
   G   
   T   
   G   
   A   
   T   
   T   
   G   
   A   
   T   
   G   
   C   
   A   
   A   
   C   
   A   
   A   
   G   
   T   
   T   
   T   
   A   
   T   
   T   
   G   
   A   
   T   
   G   
   G   
   A   
   C   
   A   
   A   
   T   
   C   
   C   
   T   
   G   
   A   
   T   
   G   
   T   
   A   
   A   
   C   
   G   
   A   
   C   
   G   
   A   
   A   
   T   
   G   
   A   
   A   
   T   
   T   
   G   
   A   
   T   
   G   
   G   
   A   
   A   
   G   
   T   
   G   
   T   
   T   
   A   
   C   
   C   
   T   
   G   
   G   
   A   
   C   
   C   
   A   
   G   
   A   
   T   
   T   
   T   
   T   
   C   
   C   
   A   
   A   
   C   
   A   
   G   
   G   
   C   
   G   
   G   
   T   
   T   
   T   
   A   
   G   
   T   
   G   
   A   
   T   
   G   
   G   
   G   
   G   
   A   
   A   
   A   
   T   
   C   
   A   
   G   
   G   
    G    
   A   
   T   
    T    
   C   
   G   
    C    
    C    
   G   
   A   
   G   
   C   
    A    
   T   
   A   
   T   
   G   
   A   
   A   
   A   
   C   
    A    
   G   
   G   
    G    
   A   
   A   
    A    
   G   
   G   
   T   
   T   
   C   
    G    
   A   
   T   
   T   
   A   
   C   
    T    
   G   
   T   
    T    
   C   
   G   
   T   
   G   
   C   
   A   
   A   
   A   
   A   
   G   
   T   
   T   
   G   
   A   
    A    
    T    
   T   
    G    
   A   
    C    
   T   
   G   
   A   
    A    
   A   
   T   
   G   
    C    
   C   
    G    
   A   
   A   
   T   
   G   
   G   
    A    
   A   
   A   
   A   
   G   
   A   
   A   
   C   
   G   
    T    
   A   
   T   
    T    
    T    
   T   
    A    
   G   
   T   
    A    
   A   
   C   
    T    
   G   
   A   
   A   
   T   
   T   
    G    
   C   
   C   
   T   
   T   
   A   
   T   
   A   
   T   
   G   
   G   
   T   
    G    
   A   
   A   
   T   
   A   
   A   
   A   
   G   
   C   
    C    
   A   
   A   
   A   
   T   
   T   
    A    
   A   
   T   
    C    
   G   
   A   
   A   
   C   
   G   
    A    
   A   
   T   
   T   
   T   
   C   
   T   
   G   
   A   
    A    
    T    
   T   
    A    
   C   
   A   
   C   
   A   
   G   
   A   
   G   
   A   
    T    
   A   
   A   
    A    
   C   
   G   
    A    
   A   
   T   
    T    
   G   
   A   
   A   
   G   
   G   
    A    
   A   
   T   
    T    
   A   
   C   
    T    
   G   
   A   
   T   
   T   
   T   
   G   
   C   
   G   
   G   
   G   
   A   
    T    
   G   
   A   
   A   
   T   
   C   
   T   
   T   
   C   
    T    
   C   
   G   
   T   
   G   
   A   
    A    
   G   
   G   
    C    
   A   
   T   
   G   
   C   
   G   
    G    
   A   
   T   
    T    
   G   
   T   
   C   
   A   
   T   
    C    
   G   
   A   
    T    
    G    
   T   
    T    
   C   
   G   
   T   
   C   
   G   
    A    
   G   
   A   
    T    
   G   
    T    
    G    
   A   
   G   
    T    
   G   
   C   
    C    
   T   
   C   
    T    
   G   
   T   
   T   
    G    
   T   
    G    
   T   
   T   
    A    
   A   
   A   
    C    
   A   
   A   
   C   
   T   
   T   
    G    
   T   
   A   
    C    
   A   
   A   
   A   
    A    
   T   
   G   
   A   
   C   
    T    
    G    
   C   
    C    
   T   
   T   
    A    
   C   
   A   
    A    
   A   
    C    
    A    
   T   
   C   
    T    
   T   
   T   
    T    
   G   
   G   
    T    
   T   
   T   
    T    
   A   
   A   
    C    
   A   
   T   
   G   
   T   
   T   
    A    
   G   
   C   
    C    
   A   
   T   
    C    
   G   
   A   
   A   
   A   
   A   
   A   
   G   
   G   
    C    
   G   
   T   
    A    
   C   
   C   
    G    
   A   
   A   
   A   
    A    
   T   
   T   
   T   
   T   
    A    
   A   
   G   
    C    
   T   
   T   
   G   
   A   
   A   
   A   
   C   
    G    
    T    
   A   
   T   
    T    
    T    
   T   
    A    
   G   
   A   
   A   
   A   
   A   
    C    
   T   
   A   
    C    
    G    
   T   
    C    
   G   
   A   
    G    
   C   
   A   
    C    
   C   
   A   
    A    
    A    
    A    
    A    
    G    
   A   
   A   
   G   
   T   
   C   
   A   
   T   
    T    
   A   
   C   
    T    
    C    
   G   
    C    
   C   
   G   
   G   
   A   
   C   
    G    
    A    
   T   
    T    
   T   
   T   
   T   
   G   
   A   
    C    
   A   
   A   
   A   
    A    
   A   
    C    
   A   
   A   
    G    
   G   
   C   
   A   
   G   
   A   
   A   
   G   
   C   
   A   
    C    
   G   
    G    
   G   
   C   
    G    
   C   
   A   
    T    
   A   
   T   
    T    
   T   
   T   
   A   
   G   
   A   
   A   
   G   
   G   
   T   
    C    
   T   
    A    
   C   
   G   
    A    
   A   
   T   
   T   
   G   
   C   
    C    
   T   
   T   
    A    
   G   
   A   
   T   
   C   
   A   
   T   
   A   
   T   
   C   
    G    
   A   
   T   
   G   
   A   
   A   
   A   
   T   
    C    
   A   
   T   
   T   
   G   
   C   
    C    
   A   
   T   
   T   
   A   
   T   
    C    
   C   
   G   
    T    
   G   
   G   
    G    
   T   
   C   
    A    
    C    
   A   
   A   
   T   
   C   
   A   
   G   
   A   
   T   
   G   
   A   
    C    
   G   
   A   
    A    
   G   
   C   
   A   
   A   
   A   
   A   
   G   
   C   
    A    
   A   
    C    
    G    
    T    
   T   
    A    
   A   
   T   
    T    
   G   
   A   
   A   
   C   
   G   
    C    
   T   
   T   
    T    
   G   
   A   
    A    
   T   
   T   
    T    
   T   
   C   
    A    
   G   
   A   
   T   
   C   
   G   
    T    
   C   
   A   
    A    
    G    
   C   
    G    
   C   
   A   
   A   
   G   
   C   
    A    
   A   
   T   
    T    
   T   
   T   
   A   
   G   
   A   
   T   
   A   
   T   
   G   
   C   
   G   
   T   
   T   
   T   
    A    
   C   
   G   
    C    
   C   
   G   
    T    
   T   
   T   
    A    
   A   
   C   
    A    
   G   
   G   
    C    
   T   
   T   
    A    
   G   
   A   
   A   
   C   
   G   
    C    
   G   
   A   
    C    
   A   
   A   
   A   
   A   
   T   
    T    
   G   
   A   
    A    
   A   
   A   
   T   
   G   
   A   
   A   
   T   
   A   
    T    
   C   
   A   
   A   
   G   
   A   
    G    
   C   
   T   
    A    
   T   
   T   
    G    
   A   
    A    
   A   
    T    
   T   
    C    
   A   
   T   
   T   
   G   
    C    
    G    
   G   
   A   
    C    
   T   
   T   
    A    
    G    
    A    
    G    
   G   
   A   
   C   
   A   
   T   
   T   
    T    
   T   
    A    
   G   
   C   
    C    
    C    
   G   
    C    
   C   
   C   
    A    
   G   
   A   
    A    
   C   
   G   
    C    
   G   
   T   
    C    
   A   
    T    
    T    
   G   
   A   
   A   
   A   
   T   
    C    
   A   
   T   
    T    
   A   
   A   
    A    
   A   
    C    
    A    
   G   
   A   
    G    
   T   
   T   
   A   
    A    
    A    
    T    
   G   
   A   
   C   
    G    
   T   
    T    
    C    
    G    
    C    
    A    
    C    
   A   
    A    
    A    
   A   
   T   
   T   
   T   
   G   
   G   
   T   
   G   
   A   
   T   
    G    
    C    
   A   
   C   
   G   
   A   
   C   
   G   
    C    
   A   
   C   
   A   
   G   
   A   
   A   
    T    
   T   
   A   
    T    
   T   
   A   
   G   
   T   
    A    
   G   
   G   
   T   
   G   
   A   
    A    
   G   
   T   
   C   
    T    
   T   
    A    
   A   
   G   
   T   
   C   
   T   
    T    
   G   
   A   
   A   
   G   
   A   
   T   
   G   
   A   
   A   
   G   
   A   
   T   
    T    
   T   
    A    
   A   
   T   
    C    
   G   
   A   
   A   
   G   
   A   
   A   
   G   
   A   
   A   
   G   
   A   
   A   
    G    
   T   
   C   
   G   
   T   
    G    
   A   
   T   
   T   
   A   
   C   
    G    
    T    
   T   
    A    
   A   
   C   
    C    
   A   
   A   
    T    
   A   
   A   
    C    
   G   
   G   
    C    
   T   
   A   
    C    
   A   
   T   
    T    
   A   
   A   
    G    
   C   
   G   
    G    
    A    
   T   
   G   
   G   
    C    
   A   
   A   
   A   
    C    
    T    
    C    
    C    
   G   
   A   
   A   
   T   
   T   
   C   
    C    
   G   
    G    
   G   
   C   
    G    
   C   
   A   
   A   
   C   
   G   
    C    
    C    
   G   
    C    
   G   
   G   
    T    
   G   
   G   
    A    
   C   
   G   
   T   
   G   
   G   
    T    
   G   
   T   
    C    
   C   
   A   
   A   
   G   
   G   
    G    
   A   
   T   
   G   
   G   
   G   
    C    
    G    
   T   
    T    
   C   
   A   
    T    
    G    
   A   
   T   
   G   
   A   
    T    
   G   
   A   
    T    
   T   
   T   
   C   
   G   
   T   
    G    
   A   
   A   
    A    
   A   
   A   
    C    
   C   
   T   
    A    
    G    
   T   
   T   
   T   
   C   
   T   
   T   
   G   
    T    
   T   
   C   
    A    
   A   
   C   
    A    
   C   
   A   
    C    
   G   
   A   
    C    
   A   
   C   
    G    
   T   
   T   
    A    
   T   
   T   
   A   
   T   
   T   
    C    
   T   
   T   
   T   
   A   
   C   
   G   
   A   
   A   
    C    
   A   
    C    
    T    
   G   
   G   
    G    
   A   
   A   
   A   
   G   
   T   
    T    
   T   
   A   
    C    
   C   
   G   
    A    
   G   
   C   
   A   
   A   
   A   
   A   
   G   
   G   
    T    
   T   
   A   
    C    
   G   
   A   
   A   
   A   
   T   
    C    
   C   
   C   
    T    
   G   
   A   
    A    
   T   
   A   
   C   
   G   
   G   
    T    
    A    
   G   
    A    
   A   
   C   
   A   
   G   
   C   
    A    
   A   
   A   
   A   
   G   
   G   
    A    
   A   
   T   
    T    
   C   
   C   
   A   
    G    
   T   
    G    
   A   
   T   
    T    
   A   
   A   
    C    
   T   
   T   
    A    
   C   
   T   
    G    
   G   
   G   
    A    
   A   
   T   
    T    
   G   
   A   
    T    
   T   
   C   
    T    
    G    
    C    
    A    
   G   
   A   
   A   
   A   
    A    
    A    
   A   
   T   
    T    
   C   
   A   
    A    
   G   
   C   
    G    
   A   
   T   
    T    
   A   
   T   
   T   
    T    
   C   
    T    
   G   
   T   
    T    
    G    
    A    
   G   
   G   
   G   
    C    
    A    
   A   
   A   
   G   
   C   
    G    
   G   
   A   
   A   
   G   
    C    
    A    
   G   
   G   
    T    
    A    
   A   
    A    
   T   
   A   
    C    
    T    
   T   
    G    
   T   
   T   
   C   
   T   
   T   
    C    
   A   
   C   
    A    
   A   
   C   
    C    
    T    
    T    
    A    
    A    
   A   
   A   
   G   
   G   
    A    
   A   
   C   
    C    
   G   
   T   
    C    
   A   
   A   
   A   
   C   
   G   
    G    
   A   
   C   
    A    
    G    
   C   
    C    
   G   
   T   
    A    
   A   
   C   
    A    
   G   
   C   
    C    
   T   
   T   
    T    
    T    
   C   
   T   
   A   
   A   
    T    
   A   
   T   
    C    
   C   
   G   
    T    
   A   
   G   
    T    
   A   
   A   
   T   
   G   
   G   
    A    
    T    
   T   
    A    
   A   
   T   
    C    
   G   
   C   
    C    
   A   
   T   
   T   
    A    
   G   
    C    
   T   
   T   
    A    
   A   
   A   
   A   
   G   
   A   
   A   
   G   
   A   
    T    
   G   
   A   
    T    
   G   
   A   
    G    
    T    
   T   
    A    
    G    
   T   
    T    
   A   
   A   
    C    
   G   
   T   
    G    
    G    
   T   
   A   
    A    
    C    
   G   
   A   
   C   
    T    
    A    
   A   
    T    
   G   
   G   
    C    
    A    
   A   
    T    
    C    
    A    
    G    
   A   
   A   
    G    
   A   
   T   
    G    
   A   
   T   
    T    
   A   
   T   
    C    
   G   
   G   
    A    
   A   
   C   
   A   
   C   
   A   
   T   
    G    
    C    
    A    
   G   
   G   
    A    
   T   
   A   
   C   
   T   
   C   
   T   
   G   
   T   
    C    
   A   
   C   
    A    
   T   
   T   
    T    
    G    
   A   
    T    
   G   
   A   
    A    
    A    
    A    
    T    
    A    
   C   
    T    
   G   
    T    
    A    
   C   
   G   
    T    
   G   
   A   
   T   
   A   
   T   
   G   
   G   
   G   
    C    
   C   
   G   
    G    
   A   
   C   
   A   
   G   
   C   
    A    
   T   
   C   
    A    
   G   
   G   
    T    
   G   
   T   
    T    
    C    
   G   
    G    
   G   
   G   
    A    
   A   
   T   
    C    
   C   
   G   
    T    
    C    
   T   
    C    
   C   
   G   
    C    
   G   
   A   
    A    
    A    
   A   
    T    
   G   
   A   
    T    
   T   
    A    
    T    
   G   
   T   
    G    
   G   
   T   
    C    
   G   
   G   
    T    
    G    
   C   
   A   
   G   
   C   
    G    
    A    
   T   
    T    
    C    
   T   
   G   
    G    
   A   
    T    
   G   
    A    
   A   
    A    
   A   
    C    
    A    
   A   
   A   
   G   
   A   
   A   
   G   
   T   
    C    
    C    
   T   
    A    
   G   
   T   
    C    
   A   
   T   
    T    
   A   
   C   
    T    
   G   
   A   
   A   
   A   
   A   
    T    
   G   
   G   
   T   
   T   
   A   
    T    
   G   
   G   
    T    
   A   
   A   
    G    
   C   
   G   
    T    
   A   
   C   
    A    
   A   
   A   
   A   
   G   
    C    
    T    
   T   
   C   
    T    
   G   
   A   
    A    
   T   
   A   
   T   
   C   
   C   
   A   
   G   
   T   
    T    
   A   
   A   
    A    
   G   
   G   
    A    
   C   
   G   
    T    
   G   
   G   
    C    
   G   
   G   
   T   
   A   
   A   
   A   
   G   
   G   
    G    
   A   
   T   
    T    
   A   
   A   
    G    
   A   
   C   
    A    
    G    
   C   
    A    
   A   
   A   
    T    
   A   
   T   
   C   
   A   
   C   
   T   
   G   
   A   
    G    
   A   
   A   
   A   
   A   
   A   
   T   
   G   
   G   
   T   
   C   
   C   
   A   
    T    
   T   
   A   
   G   
   C   
    T    
   G   
   G   
    T    
   T   
   T   
    A    
   A   
   C   
   C   
   A   
   C   
    G    
   G   
   T   
    C    
    A    
    A    
   T   
   G   
   G   
    G    
    G    
   A   
   T   
   G   
   A   
   A   
   G   
   A   
    T    
   A   
   T   
   C   
    T    
   T   
    A    
    T    
   T   
    G    
   A   
   T   
    T    
   A   
   C   
    G    
    A    
   A   
    C    
   A   
   A   
   A   
   G   
   G   
    C    
   G   
   T   
    C    
   A   
   T   
   T   
   A   
   T   
   C   
   C   
   G   
    C    
   T   
   T   
    T    
   A   
   A   
   C   
   G   
   T   
    T    
    G    
    A    
    T    
    T    
   C   
   T   
   G   
   T   
   T   
   T   
   C   
   T   
   C   
   A   
   A   
   A   
   C   
    A    
   G   
   G   
   A   
   C   
   G   
    C    
    G    
   C   
   A   
   A   
   C   
   A   
   T   
   T   
    A    
   G   
   G   
    G    
   G   
   T   
    T    
   C   
   G   
   T   
   T   
   T   
    A    
   A   
   T   
   G   
   A   
    G    
    A    
   A   
   T   
   G   
   G   
   A   
   A   
   G   
    A    
    T    
   G   
    G    
    T    
    G    
   C   
    C    
   A   
   A   
   A   
   -   
   -   
   -   
   -   
   -   
   -   
   -   
   -   
   -   
   -   
   -   
   -   
   -   
   -   
   -   
   -   
   -   
   -   
   -   
   -   
   -   
   -   
   -   
   -   
   -   
   -   
   -   
   -   
   -   
   -   
   -   
   -   
   -   
   -   
   -   
   -   
   -   
   -   
   -   
   -   
   -   
   -   
   -   
   -   
   -   
   -   
   -   
   -   
   -   
   -   
   -   
   -   
   -   
   -   
   -   
   -   
   -   
   -   
   -   
   -   
   -   
   -   
   -   
   -   
   -   
   -   
   -   
   -   
   -   
   -   
   -   
   -   
 

 
   N59592F   
   A   
   T   
   G   
   A   
   G   
   T   
   G   
   A   
   A   
   G   
   A   
   A   
   A   
   T   
   T   
   A   
   A   
   A   
   G   
   A   
   A   
   A   
   A   
   C   
   A   
   T   
   T   
   C   
   A   
   A   
   G   
   A   
   C   
   G   
   T   
   C   
   A   
   A   
   T   
   C   
   T   
   G   
   A   
   C   
   C   
   A   
   G   
   C   
   G   
   A   
   A   
   A   
   T   
   G   
   A   
   A   
   A   
   G   
   A   
   A   
   T   
   C   
   T   
   T   
   T   
   C   
   A   
   T   
   T   
   G   
   A   
   C   
   T   
   A   
   C   
   G   
   C   
   A   
   A   
   T   
   G   
   A   
   G   
   T   
   G   
   T   
   T   
   A   
   T   
   C   
   G   
   T   
   A   
   G   
   C   
   C   
   C   
   G   
   C   
   G   
   C   
   G   
   T   
   T   
   A   
   C   
   C   
   T   
   G   
   A   
   C   
   G   
   T   
   T   
   C   
   G   
   T   
   G   
   A   
   C   
   G   
   G   
   T   
   T   
   T   
   A   
   A   
   A   
   A   
   C   
   C   
   T   
   G   
   T   
   T   
   C   
   A   
   T   
   C   
   G   
   C   
   C   
   G   
   A   
   A   
   T   
   C   
   T   
   T   
   A   
   T   
   A   
   T   
   G   
   G   
   A   
   A   
   T   
   G   
   A   
   A   
   C   
   G   
   A   
   A   
   T   
   T   
   A   
   G   
   G   
   G   
   G   
   T   
   A   
   A   
   C   
   C   
   C   
   C   
   T   
   G   
   A   
   T   
   A   
   A   
   A   
   C   
   C   
   A   
   C   
   A   
   C   
   A   
   A   
   A   
   A   
   A   
   A   
   T   
   C   
   A   
   G   
   C   
   C   
   C   
   G   
   G   
   A   
   T   
   T   
   G   
   T   
   T   
   G   
   G   
   G   
   G   
   A   
   T   
   G   
   T   
   T   
   A   
   T   
   G   
   G   
   G   
   T   
   A   
   A   
   A   
   T   
   A   
   T   
   C   
   A   
   C   
   C   
   C   
   C   
   C   
   A   
   T   
   G   
   G   
   G   
   G   
   A   
   C   
   A   
   G   
   T   
   G   
   C   
   G   
   A   
   T   
   T   
   T   
   A   
   C   
   G   
   A   
   A   
   T   
   C   
   A   
   A   
   T   
   G   
   G   
   T   
   G   
   C   
   G   
   G   
   A   
   T   
   G   
   G   
   C   
   A   
   C   
   A   
   A   
   C   
   C   
   T   
   T   
   T   
   T   
   A   
   G   
   T   
   T   
   A   
   T   
   C   
   G   
   G   
   G   
   C   
   T   
   A   
   T   
   G   
   T   
   T   
   A   
   G   
   T   
   T   
   G   
   A   
   C   
   G   
   G   
   C   
   C   
   A   
   C   
   G   
   G   
   A   
   A   
   A   
   C   
   T   
   T   
   C   
   G   
   G   
   T   
   T   
   C   
   T   
   G   
   T   
   C   
   G   
   A   
   C   
   G   
   G   
   T   
   G   
   A   
   C   
   G   
   G   
   C   
   G   
   C   
   T   
   G   
   C   
   C   
   G   
   C   
   T   
   A   
   T   
   G   
   C   
   G   
   T   
   T   
   A   
   T   
   A   
   C   
   C   
   G   
   A   
   A   
   G   
   C   
   A   
   C   
   G   
   T   
   A   
   T   
   G   
   A   
   G   
   T   
   A   
   A   
   A   
   A   
   T   
   T   
   G   
   C   
   T   
   T   
   T   
   A   
   G   
   A   
   A   
   A   
   T   
   G   
   C   
   T   
   A   
   C   
   G   
   A   
   G   
   A   
   T   
   A   
   T   
   T   
   A   
   A   
   C   
   A   
   A   
   A   
   A   
   A   
   T   
   A   
   C   
   A   
   G   
   T   
   C   
   G   
   A   
   T   
   T   
   T   
   C   
   C   
   A   
   A   
   G   
   G   
   A   
   A   
   A   
   C   
   T   
   A   
   T   
   G   
   A   
   T   
   G   
   A   
   T   
   T   
   C   
   A   
   G   
   A   
   A   
   C   
   A   
   A   
   G   
   A   
   A   
   C   
   C   
   A   
   G   
   T   
   A   
   G   
   T   
   T   
   T   
   T   
   A   
   C   
   C   
   T   
   G   
   C   
   T   
   C   
   G   
   T   
   T   
   T   
   T   
   C   
   C   
   A   
   A   
   A   
   C   
   T   
   T   
   A   
   C   
   T   
   A   
   G   
   T   
   T   
   A   
   A   
   C   
   G   
   G   
   A   
   A   
   C   
   A   
   A   
   C   
   G   
   G   
   G   
   G   
   A   
   T   
   T   
   G   
   C   
   G   
   G   
   T   
   G   
   G   
   G   
   G   
   A   
   T   
   G   
   G   
   C   
   A   
   A   
   C   
   A   
   A   
   A   
   T   
   A   
   T   
   T   
   C   
   C   
   A   
   C   
   C   
   A   
   C   
   A   
   C   
   A   
   A   
   T   
   T   
   T   
   A   
   A   
   G   
   T   
   G   
   A   
   A   
   G   
   T   
   G   
   A   
   T   
   T   
   G   
   A   
   T   
   G   
   C   
   A   
   A   
   C   
   A   
   A   
   G   
   T   
   T   
   T   
   A   
   T   
   T   
   G   
   A   
   T   
   G   
   G   
   A   
   C   
   A   
   A   
   T   
   C   
   C   
   T   
   G   
   A   
   T   
   G   
   T   
   A   
   A   
   C   
   G   
   A   
   C   
   G   
   A   
   A   
   T   
   G   
   A   
   A   
   T   
   T   
   G   
   A   
   T   
   G   
   G   
   A   
   A   
   G   
   T   
   G   
   T   
   T   
   A   
   C   
   C   
   T   
   G   
   G   
   A   
   C   
   C   
   A   
   G   
   A   
   C   
   T   
   T   
   T   
   C   
   C   
   A   
   A   
   C   
   A   
   G   
   G   
   C   
   G   
   G   
   T   
   T   
   T   
   A   
   G   
   T   
   G   
   A   
   T   
   G   
   G   
   G   
   G   
   A   
   A   
   A   
   T   
   C   
   A   
   G   
   G   
    G    
   A   
   T   
    T    
   C   
   G   
    T    
    C    
   G   
   A   
   G   
   C   
    A    
   T   
   A   
   T   
   G   
   A   
   A   
   A   
   C   
    A    
   G   
   G   
    G    
   A   
   A   
    A    
   G   
   G   
   T   
   T   
   C   
    G    
   A   
   T   
   T   
   A   
   C   
    T    
   G   
   T   
    T    
   C   
   G   
   T   
   G   
   C   
   A   
   A   
   A   
   A   
   G   
   T   
   T   
   G   
   A   
    A    
    T    
   T   
    G    
   A   
    C    
   T   
   G   
   A   
    A    
   A   
   T   
   G   
    C    
   C   
    G    
   A   
   A   
   T   
   G   
   G   
    A    
   A   
   A   
   A   
   G   
   A   
   A   
   C   
   G   
    T    
   A   
   T   
    T    
    T    
   T   
    A    
   G   
   T   
    A    
   A   
   C   
    T    
   G   
   A   
   A   
   T   
   T   
    G    
   C   
   C   
   T   
   T   
   A   
   T   
   A   
   T   
   G   
   G   
   T   
    G    
   A   
   A   
   T   
   A   
   A   
   A   
   G   
   C   
    C    
   A   
   A   
   A   
   T   
   T   
    A    
   A   
   T   
    C    
   G   
   A   
   A   
   C   
   G   
    A    
   A   
   T   
   T   
   T   
   C   
   T   
   G   
   A   
    A    
    T    
   T   
    A    
   C   
   A   
   C   
   A   
   G   
   A   
   G   
   A   
    T    
   A   
   A   
    A    
   C   
   G   
    A    
   A   
   T   
    T    
   G   
   A   
   A   
   G   
   G   
    A    
   A   
   T   
    T    
   A   
   C   
    T    
   G   
   A   
   T   
   T   
   T   
   G   
   C   
   G   
   G   
   G   
   A   
    T    
   G   
   A   
   A   
   T   
   C   
   T   
   T   
   C   
    T    
   C   
   G   
   T   
   G   
   A   
    A    
   G   
   G   
    C    
   A   
   T   
   G   
   C   
   G   
    G    
   A   
   T   
    C    
   G   
   T   
   C   
   A   
   T   
    C    
   G   
   A   
    T    
    G    
   T   
    T    
   C   
   G   
   T   
   C   
   G   
    A    
   G   
   A   
    T    
   G   
    T    
    G    
   A   
   G   
    T    
   G   
   C   
    C    
   T   
   C   
    T    
   G   
   T   
   T   
    G    
   T   
    G    
   T   
   T   
    A    
   A   
   A   
    C    
   A   
   A   
   C   
   T   
   T   
    G    
   T   
   A   
    C    
   A   
   A   
   A   
    A    
   T   
   G   
   A   
   C   
    T    
    G    
   C   
    C    
   T   
   T   
    A    
   C   
   A   
    A    
   A   
    C    
    A    
   T   
   C   
    T    
   T   
   T   
    T    
   G   
   G   
    T    
   T   
   T   
    T    
   A   
   A   
    C    
   A   
   T   
   G   
   T   
   T   
    A    
   G   
   C   
    C    
   A   
   T   
    T    
   G   
   A   
   A   
   A   
   A   
   A   
   G   
   G   
    T    
   G   
   T   
    A    
   C   
   C   
    G    
   A   
   A   
   A   
    A    
   T   
   T   
   T   
   T   
    A    
   A   
   G   
    C    
   T   
   T   
   G   
   A   
   A   
   A   
   C   
    G    
    T    
   A   
   T   
    T    
    T    
   T   
    A    
   G   
   A   
   A   
   A   
   A   
    C    
   T   
   A   
    C    
    G    
   T   
    C    
   G   
   A   
    G    
   C   
   A   
    T    
   C   
   A   
    A    
    A    
    A    
    A    
    G    
   A   
   A   
   G   
   T   
   C   
   A   
   T   
    T    
   A   
   C   
    T    
    C    
   G   
    C    
   C   
   G   
   G   
   A   
   C   
    G    
    A    
   T   
    T    
   T   
   T   
   T   
   G   
   A   
    C    
   A   
   A   
   A   
    A    
   A   
    C    
   A   
   A   
    A    
   G   
   C   
   A   
   G   
   A   
   A   
   G   
   C   
   A   
    C    
   G   
    G    
   G   
   C   
    A    
   C   
   A   
    T    
   A   
   T   
    T    
   T   
   T   
   A   
   G   
   A   
   A   
   G   
   G   
   T   
    C    
   T   
    A    
   C   
   G   
    A    
   A   
   T   
   T   
   G   
   C   
    C    
   T   
   T   
    A    
   G   
   A   
   T   
   C   
   A   
   T   
   A   
   T   
   C   
    G    
   A   
   T   
   G   
   A   
   A   
   A   
   T   
    C    
   A   
   T   
   T   
   G   
   C   
    C    
   A   
   T   
   T   
   A   
   T   
    C    
   C   
   G   
    T    
   G   
   G   
    A    
   T   
   C   
    A    
    C    
   A   
   A   
   T   
   C   
   A   
   G   
   A   
   T   
   G   
   A   
    C    
   G   
   A   
    A    
   G   
   C   
   A   
   A   
   A   
   A   
   G   
   C   
    A    
   A   
    C    
    G    
    T    
   T   
    A    
   A   
   T   
    T    
   G   
   A   
   A   
   C   
   G   
    C    
   T   
   T   
    T    
   G   
   A   
    A    
   T   
   T   
    T    
   T   
   C   
    A    
   G   
   A   
   T   
   C   
   G   
    T    
   C   
   A   
    A    
    G    
   C   
    G    
   C   
   A   
   A   
   G   
   C   
    G    
   A   
   T   
    T    
   T   
   T   
   A   
   G   
   A   
   T   
   A   
   T   
   G   
   C   
   G   
   T   
   T   
   T   
    A    
   C   
   G   
    C    
   C   
   G   
    T    
   T   
   T   
    A    
   A   
   C   
    A    
   G   
   G   
    C    
   T   
   T   
    A    
   G   
   A   
   A   
   C   
   G   
    C    
   G   
   A   
    C    
   A   
   A   
   A   
   A   
   T   
    T    
   G   
   A   
    A    
   A   
   A   
   T   
   G   
   A   
   A   
   T   
   A   
    T    
   C   
   A   
   A   
   G   
   A   
    G    
   C   
   T   
    A    
   T   
   T   
    G    
   A   
    A    
   A   
    T    
   T   
    C    
   A   
   T   
   T   
   G   
    C    
    G    
   G   
   A   
    C    
   T   
   T   
    A    
    G    
    A    
    G    
   G   
   A   
   C   
   A   
   T   
   T   
    T    
   T   
    A    
   G   
   C   
    C    
    C    
   G   
    C    
   C   
   C   
    A    
   G   
   A   
    A    
   C   
   G   
    C    
   G   
   T   
    C    
   A   
    T    
    T    
   G   
   A   
   A   
   A   
   T   
    C    
   A   
   T   
    T    
   A   
   A   
    A    
   A   
    C    
    A    
   G   
   A   
    G    
   T   
   T   
   A   
    A    
    A    
    T    
   G   
   A   
   C   
    G    
   T   
    T    
    C    
    G    
    C    
    A    
    C    
   A   
    A    
    A    
   A   
   T   
   T   
   T   
   G   
   G   
   T   
   G   
   A   
   T   
    G    
    C    
   A   
   C   
   G   
   A   
   C   
   G   
    C    
   A   
   C   
   A   
   G   
   A   
   A   
    T    
   T   
   A   
    T    
   T   
   A   
   G   
   T   
    A    
   G   
   G   
   T   
   G   
   A   
    A    
   G   
   T   
   C   
    C    
   T   
    A    
   A   
   G   
   T   
   C   
   T   
    T    
   G   
   A   
   A   
   G   
   A   
   T   
   G   
   A   
   A   
   G   
   A   
   T   
    T    
   T   
    A    
   A   
   T   
    C    
   G   
   A   
   A   
   G   
   A   
   A   
   G   
   A   
   A   
   G   
   A   
   A   
    G    
   T   
   C   
   G   
   T   
    G    
   A   
   T   
   T   
   A   
   C   
    A    
    T    
   T   
    A    
   A   
   C   
    C    
   A   
   A   
    T    
   A   
   A   
    C    
   G   
   G   
    C    
   T   
   A   
    C    
   A   
   T   
    T    
   A   
   A   
    G    
   C   
   G   
    G    
    A    
   T   
   G   
   G   
    C    
   A   
   A   
   A   
    C    
    T    
    C    
    C    
   G   
   A   
   A   
   T   
   T   
   C   
    C    
   G   
    G    
   G   
   C   
    G    
   C   
   A   
   A   
   C   
   G   
    C    
    C    
   G   
    C    
   G   
   G   
    C    
   G   
   G   
    A    
   C   
   G   
   T   
   G   
   G   
    T    
   G   
   T   
    C    
   C   
   A   
   A   
   G   
   G   
    G    
   A   
   T   
   G   
   G   
   G   
    C    
    G    
   T   
    T    
   C   
   A   
    T    
    G    
   A   
   T   
   G   
   A   
    T    
   G   
   A   
    T    
   T   
   T   
   C   
   G   
   T   
    G    
   A   
   A   
    A    
   A   
   A   
    C    
   C   
   T   
    A    
    G    
   T   
   T   
   T   
   C   
   T   
   T   
   G   
    T    
   T   
   C   
    A    
   A   
   C   
    A    
   C   
   A   
    C    
   G   
   A   
    C    
   A   
   C   
    G    
   T   
   T   
    A    
   T   
   T   
   A   
   T   
   T   
    C    
   T   
   T   
   T   
   A   
   C   
   G   
   A   
   A   
    C    
   A   
    C    
    T    
   G   
   G   
    G    
   A   
   A   
   A   
   G   
   T   
    T    
   T   
   A   
    C    
   C   
   G   
    A    
   G   
   C   
   A   
   A   
   A   
   A   
   G   
   G   
    T    
   T   
   A   
    C    
   G   
   A   
   A   
   A   
   T   
    C    
   C   
   C   
    T    
   G   
   A   
    A    
   T   
   A   
   C   
   G   
   G   
    T    
    A    
   G   
    A    
   A   
   C   
   A   
   G   
   C   
    A    
   A   
   A   
   A   
   G   
   G   
    A    
   A   
   T   
    T    
   C   
   C   
   A   
    G    
   T   
    A    
   A   
   T   
    T    
   A   
   A   
    C    
   T   
   T   
    A    
   C   
   T   
    G    
   G   
   G   
    A    
   A   
   T   
    T    
   G   
   A   
    T    
   T   
   C   
    T    
    G    
    C    
    A    
   G   
   A   
   A   
   A   
    A    
    A    
   A   
   T   
    T    
   C   
   A   
    A    
   G   
   C   
    G    
   A   
   T   
    T    
   A   
   T   
   T   
    T    
   C   
    T    
   G   
   T   
    T    
    G    
    A    
   G   
   G   
   G   
    C    
    A    
   A   
   A   
   G   
   C   
    G    
   G   
   A   
   A   
   G   
    C    
    A    
   G   
   G   
    T    
    A    
   A   
    A    
   T   
   A   
    C    
    T    
   T   
    G    
   T   
   T   
   C   
   T   
   T   
    C    
   A   
   C   
    A    
   A   
   C   
    C    
    T    
    T    
    A    
    A    
   A   
   A   
   G   
   G   
    A    
   A   
   C   
    C    
   G   
   T   
    C    
   A   
   A   
   A   
   C   
   G   
    G    
   A   
   C   
    A    
    G    
   C   
    C    
   G   
   T   
    A    
   A   
   C   
    A    
   G   
   C   
    C    
   T   
   T   
    T    
    T    
   C   
   T   
   A   
   A   
    T    
   A   
   T   
    C    
   C   
   G   
    T    
   A   
   G   
    T    
   A   
   A   
   T   
   G   
   G   
    A    
    T    
   T   
    A    
   A   
   T   
    C    
   G   
   C   
    C    
   A   
   T   
   T   
    A    
   G   
    C    
   T   
   T   
    A    
   A   
   A   
   A   
   G   
   A   
   A   
   G   
   A   
    T    
   G   
   A   
    T    
   G   
   A   
    G    
    T    
   T   
    A    
    G    
   T   
    T    
   A   
   A   
    C    
   G   
   T   
    G    
    G    
   T   
   A   
    A    
    C    
   G   
   A   
   C   
    T    
    A    
   A   
    T    
   G   
   G   
    C    
    A    
   A   
    T    
    C    
    A    
    G    
   A   
   A   
    G    
   A   
   T   
    G    
   A   
   T   
    T    
   A   
   T   
    C    
   G   
   G   
    A    
   A   
   C   
   A   
   C   
   A   
   T   
    G    
    C    
    A    
   G   
   G   
    A    
   T   
   A   
   C   
   T   
   C   
   T   
   G   
   T   
    C    
   A   
   C   
    A    
   T   
   T   
    T    
    G    
   A   
    T    
   G   
   A   
    A    
    A    
    A    
    T    
    A    
   C   
    T    
   G   
    T    
    A    
   C   
   G   
    T    
   G   
   A   
   T   
   A   
   T   
   G   
   G   
   G   
    C    
   C   
   G   
    G    
   A   
   C   
   A   
   G   
   C   
    A    
   T   
   C   
    A    
   G   
   G   
    T    
   G   
   T   
    T    
    C    
   G   
    G    
   G   
   G   
    A    
   A   
   T   
    C    
   C   
   G   
    T    
    C    
   T   
    C    
   C   
   G   
    C    
   G   
   A   
    A    
    A    
   A   
    T    
   G   
   A   
    T    
   T   
    A    
    T    
   G   
   T   
    G    
   G   
   T   
    C    
   G   
   G   
    C    
    G    
   C   
   A   
   G   
   C   
    G    
    A    
   T   
    T    
    C    
   T   
   G   
    G    
   A   
    T    
   G   
    A    
   A   
    A    
   A   
    T    
    A    
   A   
   A   
   G   
   A   
   A   
   G   
   T   
    C    
    C    
   T   
    A    
   G   
   T   
    C    
   A   
   T   
    T    
   A   
   C   
    T    
   G   
   A   
   A   
   A   
   A   
    T    
   G   
   G   
   T   
   T   
   A   
    T    
   G   
   G   
    T    
   A   
   A   
    G    
   C   
   G   
    T    
   A   
   C   
    A    
   A   
   A   
   A   
   G   
    C    
    T    
   T   
   C   
    T    
   G   
   A   
    A    
   T   
   A   
   T   
   C   
   C   
   A   
   G   
   T   
    T    
   A   
   A   
    A    
   G   
   G   
    A    
   C   
   G   
    T    
   G   
   G   
    C    
   G   
   G   
   T   
   A   
   A   
   A   
   G   
   G   
    G    
   A   
   T   
    T    
   A   
   A   
    G    
   A   
   C   
    A    
    G    
   C   
    A    
   A   
   A   
    T    
   A   
   T   
   C   
   A   
   C   
   T   
   G   
   A   
    G    
   A   
   A   
   A   
   A   
   A   
   T   
   G   
   G   
   T   
   C   
   C   
   A   
    T    
   T   
   A   
   G   
   C   
    T    
   G   
   G   
    T    
   T   
   T   
    A    
   A   
   C   
   C   
   A   
   C   
    G    
   G   
   T   
    C    
    A    
    A    
   T   
   G   
   G   
    G    
    G    
   A   
   T   
   G   
   A   
   A   
   G   
   A   
    T    
   A   
   T   
   C   
    T    
   T   
    A    
    T    
   T   
    G    
   A   
   T   
    T    
   A   
   C   
    G    
    A    
   A   
    C    
   A   
   A   
   A   
   G   
   G   
    C    
   G   
   T   
    C    
   A   
   T   
   T   
   A   
   T   
   C   
   C   
   G   
    C    
   T   
   T   
    T    
   A   
   A   
   C   
   G   
   T   
    T    
    G    
    A    
    T    
    T    
   C   
   T   
   G   
   T   
   T   
   T   
   C   
   T   
   C   
   A   
   A   
   A   
   C   
    A    
   G   
   G   
   A   
   C   
   G   
    C    
    G    
   C   
   A   
   A   
   C   
   A   
   T   
   T   
    A    
   G   
   G   
    G    
   G   
   T   
    T    
   C   
   G   
   T   
   T   
   T   
    A    
   A   
   T   
   G   
   A   
    G    
    A    
   A   
   T   
   G   
   G   
   A   
   A   
   G   
    A    
    T    
   G   
    G    
    T    
    G    
   C   
    C    
   A   
   A   
   A   
   -   
   -   
   -   
   -   
   -   
   -   
   -   
   -   
   -   
   -   
   -   
   -   
   -   
   -   
   -   
   -   
   -   
   -   
   -   
   -   
   -   
   -   
   -   
   -   
   -   
   -   
   -   
   -   
   -   
   -   
   -   
   -   
   -   
   -   
   -   
   -   
   -   
   -   
   -   
   -   
   -   
   -   
   -   
   -   
   -   
   -   
   -   
   -   
   -   
   -   
   -   
   -   
   -   
   -   
   -   
   -   
   -   
   -   
   -   
   -   
   -   
   -   
   -   
   -   
   -   
   -   
   -   
   -   
   -   
   -   
   -   
   -   
 

 
   N60280F   
   A   
   T   
   G   
   A   
   G   
   T   
   G   
   A   
   A   
   G   
   A   
   A   
   A   
   T   
   T   
   A   
   A   
   A   
   G   
   A   
   A   
   A   
   A   
   C   
   A   
   T   
   T   
   C   
   A   
   A   
   G   
   A   
   C   
   G   
   T   
   C   
   A   
   A   
   T   
   C   
   T   
   G   
   A   
   C   
   C   
   A   
   G   
   C   
   G   
   A   
   A   
   A   
   T   
   G   
   A   
   A   
   A   
   G   
   A   
   A   
   T   
   C   
   T   
   T   
   T   
   C   
   A   
   T   
   T   
   G   
   A   
   C   
   T   
   A   
   C   
   G   
   C   
   A   
   A   
   T   
   G   
   A   
   G   
   T   
   G   
   T   
   T   
   A   
   T   
   C   
   G   
   T   
   A   
   G   
   C   
   C   
   C   
   G   
   C   
   G   
   C   
   G   
   T   
   T   
   A   
   C   
   C   
   T   
   G   
   A   
   C   
   G   
   T   
   T   
   C   
   G   
   T   
   G   
   A   
   C   
   G   
   G   
   T   
   T   
   T   
   A   
   A   
   A   
   A   
   C   
   C   
   T   
   G   
   T   
   T   
   C   
   A   
   T   
   C   
   G   
   C   
   C   
   G   
   A   
   A   
   T   
   C   
   T   
   T   
   A   
   T   
   A   
   T   
   G   
   G   
   A   
   A   
   T   
   G   
   A   
   A   
   C   
   G   
   A   
   A   
   T   
   T   
   A   
   G   
   G   
   G   
   G   
   T   
   A   
   A   
   C   
   C   
   C   
   C   
   T   
   G   
   A   
   T   
   A   
   A   
   A   
   C   
   C   
   A   
   C   
   A   
   C   
   A   
   A   
   A   
   A   
   A   
   A   
   T   
   C   
   A   
   G   
   C   
   C   
   C   
   G   
   G   
   A   
   T   
   T   
   G   
   T   
   T   
   G   
   G   
   G   
   G   
   A   
   T   
   G   
   T   
   T   
   A   
   T   
   G   
   G   
   G   
   T   
   A   
   A   
   A   
   T   
   A   
   T   
   C   
   A   
   C   
   C   
   C   
   C   
   C   
   A   
   T   
   G   
   G   
   G   
   G   
   A   
   C   
   A   
   G   
   T   
   G   
   C   
   G   
   A   
   T   
   T   
   T   
   A   
   C   
   G   
   A   
   A   
   T   
   C   
   A   
   A   
   T   
   G   
   G   
   T   
   G   
   C   
   G   
   G   
   A   
   T   
   G   
   G   
   C   
   A   
   C   
   A   
   A   
   C   
   C   
   T   
   T   
   T   
   T   
   A   
   G   
   T   
   T   
   A   
   T   
   C   
   G   
   G   
   G   
   C   
   T   
   A   
   T   
   G   
   T   
   T   
   A   
   G   
   T   
   T   
   G   
   A   
   C   
   G   
   G   
   C   
   C   
   A   
   C   
   G   
   G   
   A   
   A   
   A   
   C   
   T   
   T   
   C   
   G   
   G   
   T   
   T   
   C   
   T   
   G   
   T   
   C   
   G   
   A   
   C   
   G   
   G   
   T   
   G   
   A   
   C   
   G   
   G   
   C   
   G   
   C   
   T   
   G   
   C   
   C   
   G   
   C   
   T   
   A   
   T   
   G   
   C   
   G   
   T   
   T   
   A   
   T   
   A   
   C   
   C   
   G   
   A   
   A   
   G   
   C   
   A   
   C   
   G   
   T   
   A   
   T   
   G   
   A   
   G   
   T   
   A   
   A   
   A   
   A   
   T   
   T   
   G   
   C   
   T   
   T   
   T   
   A   
   G   
   A   
   A   
   A   
   T   
   G   
   C   
   T   
   A   
   C   
   G   
   A   
   G   
   A   
   T   
   A   
   T   
   T   
   A   
   A   
   C   
   A   
   A   
   A   
   A   
   A   
   T   
   A   
   C   
   A   
   G   
   T   
   C   
   G   
   A   
   T   
   T   
   T   
   C   
   C   
   A   
   A   
   G   
   G   
   A   
   A   
   A   
   C   
   T   
   A   
   T   
   G   
   A   
   T   
   G   
   A   
   T   
   T   
   C   
   A   
   G   
   A   
   A   
   C   
   A   
   A   
   G   
   A   
   A   
   C   
   C   
   A   
   G   
   T   
   A   
   G   
   T   
   T   
   T   
   T   
   A   
   C   
   C   
   T   
   G   
   C   
   T   
   C   
   G   
   T   
   T   
   T   
   T   
   C   
   C   
   A   
   A   
   A   
   C   
   T   
   T   
   A   
   C   
   T   
   A   
   G   
   T   
   T   
   A   
   A   
   C   
   G   
   G   
   A   
   A   
   C   
   A   
   A   
   C   
   G   
   G   
   G   
   G   
   A   
   T   
   T   
   G   
   C   
   G   
   G   
   T   
   G   
   G   
   G   
   G   
   A   
   T   
   G   
   G   
   C   
   A   
   A   
   C   
   A   
   A   
   A   
   T   
   A   
   T   
   T   
   C   
   C   
   A   
   C   
   C   
   A   
   C   
   A   
   C   
   A   
   A   
   T   
   T   
   T   
   A   
   A   
   G   
   T   
   G   
   A   
   A   
   G   
   T   
   G   
   A   
   T   
   T   
   G   
   A   
   T   
   G   
   C   
   A   
   A   
   C   
   A   
   A   
   G   
   T   
   T   
   T   
   A   
   T   
   T   
   G   
   A   
   T   
   G   
   G   
   A   
   C   
   A   
   A   
   T   
   C   
   C   
   T   
   G   
   A   
   T   
   G   
   T   
   A   
   A   
   C   
   G   
   A   
   C   
   G   
   A   
   A   
   T   
   G   
   A   
   A   
   T   
   T   
   G   
   A   
   T   
   G   
   G   
   A   
   A   
   G   
   T   
   G   
   T   
   T   
   A   
   C   
   C   
   T   
   G   
   G   
   A   
   C   
   C   
   A   
   G   
   A   
   C   
   T   
   T   
   T   
   C   
   C   
   A   
   A   
   C   
   A   
   G   
   G   
   C   
   G   
   G   
   T   
   T   
   T   
   A   
   G   
   T   
   G   
   A   
   T   
   G   
   G   
   G   
   G   
   A   
   A   
   A   
   T   
   C   
   A   
   G   
   G   
    G    
   A   
   T   
    T    
   C   
   G   
    T    
    C    
   G   
   A   
   G   
   C   
    A    
   T   
   A   
   T   
   G   
   A   
   A   
   A   
   C   
    A    
   G   
   G   
    G    
   A   
   A   
    A    
   G   
   G   
   T   
   T   
   C   
    G    
   A   
   T   
   T   
   A   
   C   
    T    
   G   
   T   
    T    
   C   
   G   
   T   
   G   
   C   
   A   
   A   
   A   
   A   
   G   
   T   
   T   
   G   
   A   
    A    
    T    
   T   
    G    
   A   
    C    
   T   
   G   
   A   
    A    
   A   
   T   
   G   
    C    
   C   
    G    
   A   
   A   
   T   
   G   
   G   
    A    
   A   
   A   
   A   
   G   
   A   
   A   
   C   
   G   
    T    
   A   
   T   
    T    
    T    
   T   
    A    
   G   
   T   
    A    
   A   
   C   
    T    
   G   
   A   
   A   
   T   
   T   
    G    
   C   
   C   
   T   
   T   
   A   
   T   
   A   
   T   
   G   
   G   
   T   
    G    
   A   
   A   
   T   
   A   
   A   
   A   
   G   
   C   
    C    
   A   
   A   
   A   
   T   
   T   
    A    
   A   
   T   
    C    
   G   
   A   
   A   
   C   
   G   
    A    
   A   
   T   
   T   
   T   
   C   
   T   
   G   
   A   
    A    
    T    
   T   
    A    
   C   
   A   
   C   
   A   
   G   
   A   
   G   
   A   
    T    
   A   
   A   
    A    
   C   
   G   
    A    
   A   
   T   
    T    
   G   
   A   
   A   
   G   
   G   
    A    
   A   
   T   
    T    
   A   
   C   
    T    
   G   
   A   
   T   
   T   
   T   
   G   
   C   
   G   
   G   
   G   
   A   
    T    
   G   
   A   
   A   
   T   
   C   
   T   
   T   
   C   
    T    
   C   
   G   
   T   
   G   
   A   
    A    
   G   
   G   
    C    
   A   
   T   
   G   
   C   
   G   
    G    
   A   
   T   
    C    
   G   
   T   
   C   
   A   
   T   
    C    
   G   
   A   
    T    
    G    
   T   
    T    
   C   
   G   
   T   
   C   
   G   
    A    
   G   
   A   
    T    
   G   
    T    
    G    
   A   
   G   
    T    
   G   
   C   
    C    
   T   
   C   
    T    
   G   
   T   
   T   
    G    
   T   
    G    
   T   
   T   
    A    
   A   
   A   
    C    
   A   
   A   
   C   
   T   
   T   
    G    
   T   
   A   
    C    
   A   
   A   
   A   
    A    
   T   
   G   
   A   
   C   
    T    
    G    
   C   
    C    
   T   
   T   
    A    
   C   
   A   
    A    
   A   
    C    
    A    
   T   
   C   
    T    
   T   
   T   
    T    
   G   
   G   
    T    
   T   
   T   
    T    
   A   
   A   
    C    
   A   
   T   
   G   
   T   
   T   
    A    
   G   
   C   
    C    
   A   
   T   
    T    
   G   
   A   
   A   
   A   
   A   
   A   
   G   
   G   
    T    
   G   
   T   
    A    
   C   
   C   
    G    
   A   
   A   
   A   
    A    
   T   
   T   
   T   
   T   
    A    
   A   
   G   
    C    
   T   
   T   
   G   
   A   
   A   
   A   
   C   
    G    
    T    
   A   
   T   
    T    
    T    
   T   
    A    
   G   
   A   
   A   
   A   
   A   
    C    
   T   
   A   
    C    
    G    
   T   
    C    
   G   
   A   
    G    
   C   
   A   
    T    
   C   
   A   
    A    
    A    
    A    
    A    
    G    
   A   
   A   
   G   
   T   
   C   
   A   
   T   
    T    
   A   
   C   
    T    
    C    
   G   
    C    
   C   
   G   
   G   
   A   
   C   
    G    
    A    
   T   
    T    
   T   
   T   
   T   
   G   
   A   
    C    
   A   
   A   
   A   
    A    
   A   
    C    
   A   
   A   
    A    
   G   
   C   
   A   
   G   
   A   
   A   
   G   
   C   
   A   
    C    
   G   
    G    
   G   
   C   
    A    
   C   
   A   
    T    
   A   
   T   
    T    
   T   
   T   
   A   
   G   
   A   
   A   
   G   
   G   
   T   
    C    
   T   
    A    
   C   
   G   
    A    
   A   
   T   
   T   
   G   
   C   
    C    
   T   
   T   
    A    
   G   
   A   
   T   
   C   
   A   
   T   
   A   
   T   
   C   
    G    
   A   
   T   
   G   
   A   
   A   
   A   
   T   
    C    
   A   
   T   
   T   
   G   
   C   
    C    
   A   
   T   
   T   
   A   
   T   
    C    
   C   
   G   
    T    
   G   
   G   
    A    
   T   
   C   
    A    
    C    
   A   
   A   
   T   
   C   
   A   
   G   
   A   
   T   
   G   
   A   
    C    
   G   
   A   
    A    
   G   
   C   
   A   
   A   
   A   
   A   
   G   
   C   
    A    
   A   
    C    
    G    
    T    
   T   
    A    
   A   
   T   
    T    
   G   
   A   
   A   
   C   
   G   
    C    
   T   
   T   
    T    
   G   
   A   
    A    
   T   
   T   
    T    
   T   
   C   
    A    
   G   
   A   
   T   
   C   
   G   
    T    
   C   
   A   
    A    
    G    
   C   
    G    
   C   
   A   
   A   
   G   
   C   
    G    
   A   
   T   
    T    
   T   
   T   
   A   
   G   
   A   
   T   
   A   
   T   
   G   
   C   
   G   
   T   
   T   
   T   
    A    
   C   
   G   
    C    
   C   
   G   
    T    
   T   
   T   
    A    
   A   
   C   
    A    
   G   
   G   
    C    
   T   
   T   
    A    
   G   
   A   
   A   
   C   
   G   
    C    
   G   
   A   
    C    
   A   
   A   
   A   
   A   
   T   
    T    
   G   
   A   
    A    
   A   
   A   
   T   
   G   
   A   
   A   
   T   
   A   
    T    
   C   
   A   
   A   
   G   
   A   
    G    
   C   
   T   
    A    
   T   
   T   
    G    
   A   
    A    
   A   
    T    
   T   
    C    
   A   
   T   
   T   
   G   
    C    
    G    
   G   
   A   
    C    
   T   
   T   
    A    
    G    
    A    
    G    
   G   
   A   
   C   
   A   
   T   
   T   
    T    
   T   
    A    
   G   
   C   
    C    
    C    
   G   
    C    
   C   
   C   
    A    
   G   
   A   
    A    
   C   
   G   
    C    
   G   
   T   
    C    
   A   
    T    
    T    
   G   
   A   
   A   
   A   
   T   
    C    
   A   
   T   
    T    
   A   
   A   
    A    
   A   
    C    
    A    
   G   
   A   
    G    
   T   
   T   
   A   
    A    
    A    
    T    
   G   
   A   
   C   
    G    
   T   
    T    
    C    
    G    
    C    
    A    
    C    
   A   
    A    
    A    
   A   
   T   
   T   
   T   
   G   
   G   
   T   
   G   
   A   
   T   
    G    
    C    
   A   
   C   
   G   
   A   
   C   
   G   
    C    
   A   
   C   
   A   
   G   
   A   
   A   
    T    
   T   
   A   
    T    
   T   
   A   
   G   
   T   
    A    
   G   
   G   
   T   
   G   
   A   
    A    
   G   
   T   
   C   
    C    
   T   
    A    
   A   
   G   
   T   
   C   
   T   
    T    
   G   
   A   
   A   
   G   
   A   
   T   
   G   
   A   
   A   
   G   
   A   
   T   
    T    
   T   
    A    
   A   
   T   
    C    
   G   
   A   
   A   
   G   
   A   
   A   
   G   
   A   
   A   
   G   
   A   
   A   
    G    
   T   
   C   
   G   
   T   
    G    
   A   
   T   
   T   
   A   
   C   
    A    
    T    
   T   
    A    
   A   
   C   
    C    
   A   
   A   
    T    
   A   
   A   
    C    
   G   
   G   
    C    
   T   
   A   
    C    
   A   
   T   
    T    
   A   
   A   
    G    
   C   
   G   
    G    
    A    
   T   
   G   
   G   
    C    
   A   
   A   
   A   
    C    
    T    
    C    
    C    
   G   
   A   
   A   
   T   
   T   
   C   
    C    
   G   
    G    
   G   
   C   
    G    
   C   
   A   
   A   
   C   
   G   
    C    
    C    
   G   
    C    
   G   
   G   
    C    
   G   
   G   
    A    
   C   
   G   
   T   
   G   
   G   
    T    
   G   
   T   
    C    
   C   
   A   
   A   
   G   
   G   
    G    
   A   
   T   
   G   
   G   
   G   
    C    
    G    
   T   
    T    
   C   
   A   
    T    
    G    
   A   
   T   
   G   
   A   
    T    
   G   
   A   
    T    
   T   
   T   
   C   
   G   
   T   
    G    
   A   
   A   
    A    
   A   
   A   
    C    
   C   
   T   
    A    
    G    
   T   
   T   
   T   
   C   
   T   
   T   
   G   
    T    
   T   
   C   
    A    
   A   
   C   
    A    
   C   
   A   
    C    
   G   
   A   
    C    
   A   
   C   
    G    
   T   
   T   
    A    
   T   
   T   
   A   
   T   
   T   
    C    
   T   
   T   
   T   
   A   
   C   
   G   
   A   
   A   
    C    
   A   
    C    
    T    
   G   
   G   
    G    
   A   
   A   
   A   
   G   
   T   
    T    
   T   
   A   
    C    
   C   
   G   
    A    
   G   
   C   
   A   
   A   
   A   
   A   
   G   
   G   
    T    
   T   
   A   
    C    
   G   
   A   
   A   
   A   
   T   
    C    
   C   
   C   
    T    
   G   
   A   
    A    
   T   
   A   
   C   
   G   
   G   
    T    
    A    
   G   
    A    
   A   
   C   
   A   
   G   
   C   
    A    
   A   
   A   
   A   
   G   
   G   
    A    
   A   
   T   
    T    
   C   
   C   
   A   
    G    
   T   
    A    
   A   
   T   
    T    
   A   
   A   
    C    
   T   
   T   
    A    
   C   
   T   
    G    
   G   
   G   
    A    
   A   
   T   
    T    
   G   
   A   
    T    
   T   
   C   
    T    
    G    
    C    
    A    
   G   
   A   
   A   
   A   
    A    
    A    
   A   
   T   
    T    
   C   
   A   
    A    
   G   
   C   
    G    
   A   
   T   
    T    
   A   
   T   
   T   
    T    
   C   
    T    
   G   
   T   
    T    
    G    
    A    
   G   
   G   
   G   
    C    
    A    
   A   
   A   
   G   
   C   
    G    
   G   
   A   
   A   
   G   
    C    
    A    
   G   
   G   
    T    
    A    
   A   
    A    
   T   
   A   
    C    
    T    
   T   
    G    
   T   
   T   
   C   
   T   
   T   
    C    
   A   
   C   
    A    
   A   
   C   
    C    
    T    
    T    
    A    
    A    
   A   
   A   
   G   
   G   
    A    
   A   
   C   
    C    
   G   
   T   
    C    
   A   
   A   
   A   
   C   
   G   
    G    
   A   
   C   
    A    
    G    
   C   
    C    
   G   
   T   
    A    
   A   
   C   
    A    
   G   
   C   
    C    
   T   
   T   
    T    
    T    
   C   
   T   
   A   
   A   
    T    
   A   
   T   
    C    
   C   
   G   
    T    
   A   
   G   
    T    
   A   
   A   
   T   
   G   
   G   
    A    
    T    
   T   
    A    
   A   
   T   
    C    
   G   
   C   
    C    
   A   
   T   
   T   
    A    
   G   
    C    
   T   
   T   
    A    
   A   
   A   
   A   
   G   
   A   
   A   
   G   
   A   
    T    
   G   
   A   
    T    
   G   
   A   
    G    
    T    
   T   
    A    
    G    
   T   
    T    
   A   
   A   
    C    
   G   
   T   
    G    
    G    
   T   
   A   
    A    
    C    
   G   
   A   
   C   
    T    
    A    
   A   
    T    
   G   
   G   
    C    
    A    
   A   
    T    
    C    
    A    
    G    
   A   
   A   
    G    
   A   
   T   
    G    
   A   
   T   
    T    
   A   
   T   
    C    
   G   
   G   
    A    
   A   
   C   
   A   
   C   
   A   
   T   
    G    
    C    
    A    
   G   
   G   
    A    
   T   
   A   
   C   
   T   
   C   
   T   
   G   
   T   
    C    
   A   
   C   
    A    
   T   
   T   
    T    
    G    
   A   
    T    
   G   
   A   
    A    
    A    
    A    
    T    
    A    
   C   
    T    
   G   
    T    
    A    
   C   
   G   
    T    
   G   
   A   
   T   
   A   
   T   
   G   
   G   
   G   
    C    
   C   
   G   
    G    
   A   
   C   
   A   
   G   
   C   
    A    
   T   
   C   
    A    
   G   
   G   
    T    
   G   
   T   
    T    
    C    
   G   
    G    
   G   
   G   
    A    
   A   
   T   
    C    
   C   
   G   
    T    
    C    
   T   
    C    
   C   
   G   
    C    
   G   
   A   
    A    
    A    
   A   
    T    
   G   
   A   
    T    
   T   
    A    
    T    
   G   
   T   
    G    
   G   
   T   
    C    
   G   
   G   
    C    
    G    
   C   
   A   
   G   
   C   
    G    
    A    
   T   
    T    
    C    
   T   
   G   
    G    
   A   
    T    
   G   
    A    
   A   
    A    
   A   
    T    
    A    
   A   
   A   
   G   
   A   
   A   
   G   
   T   
    C    
    C    
   T   
    A    
   G   
   T   
    C    
   A   
   T   
    T    
   A   
   C   
    T    
   G   
   A   
   A   
   A   
   A   
    T    
   G   
   G   
   T   
   T   
   A   
    T    
   G   
   G   
    T    
   A   
   A   
    G    
   C   
   G   
    T    
   A   
   C   
    A    
   A   
   A   
   A   
   G   
    C    
    T    
   T   
   C   
    T    
   G   
   A   
    A    
   T   
   A   
   T   
   C   
   C   
   A   
   G   
   T   
    T    
   A   
   A   
    A    
   G   
   G   
    A    
   C   
   G   
    T    
   G   
   G   
    C    
   G   
   G   
   T   
   A   
   A   
   A   
   G   
   G   
    G    
   A   
   T   
    T    
   A   
   A   
    G    
   A   
   C   
    A    
    G    
   C   
    A    
   A   
   A   
    T    
   A   
   T   
   C   
   A   
   C   
   T   
   G   
   A   
    G    
   A   
   A   
   A   
   A   
   A   
   T   
   G   
   G   
   T   
   C   
   C   
   A   
    T    
   T   
   A   
   G   
   C   
    T    
   G   
   G   
    T    
   T   
   T   
    A    
   A   
   C   
   C   
   A   
   C   
    G    
   G   
   T   
    C    
    A    
    A    
   T   
   G   
   G   
    G    
    G    
   A   
   T   
   G   
   A   
   A   
   G   
   A   
    T    
   A   
   T   
   C   
    T    
   T   
    A    
    T    
   T   
    G    
   A   
   T   
    T    
   A   
   C   
    G    
    A    
   A   
    C    
   A   
   A   
   A   
   G   
   G   
    C    
   G   
   T   
    C    
   A   
   T   
   T   
   A   
   T   
   C   
   C   
   G   
    C    
   T   
   T   
    T    
   A   
   A   
   C   
   G   
   T   
    T    
    G    
    A    
    T    
    T    
   C   
   T   
   G   
   T   
   T   
   T   
   C   
   T   
   C   
   A   
   A   
   A   
   C   
    A    
   G   
   G   
   A   
   C   
   G   
    C    
    G    
   C   
   A   
   A   
   C   
   A   
   T   
   T   
    A    
   G   
   G   
    G    
   G   
   T   
    T    
   C   
   G   
   T   
   T   
   T   
    A    
   A   
   T   
   G   
   A   
    G    
    A    
   A   
   T   
   G   
   G   
   A   
   A   
   G   
    A    
    T    
   G   
    G    
    T    
    G    
   C   
    C    
   A   
   A   
   A   
   -   
   -   
   -   
   -   
   -   
   -   
   -   
   -   
   -   
   -   
   -   
   -   
   -   
   -   
   -   
   -   
   -   
   -   
   -   
   -   
   -   
   -   
   -   
   -   
   -   
   -   
   -   
   -   
   -   
   -   
   -   
   -   
   -   
   -   
   -   
   -   
   -   
   -   
   -   
   -   
   -   
   -   
   -   
   -   
   -   
   -   
   -   
   -   
   -   
   -   
   -   
   -   
   -   
   -   
   -   
   -   
   -   
   -   
   -   
   -   
   -   
   -   
   -   
   -   
   -   
   -   
   -   
   -   
   -   
   -   
   -   
   -   
 

 
   N52702   
   A   
   T   
   G   
   A   
   G   
   T   
   G   
   A   
   A   
   G   
   A   
   A   
   A   
   T   
   C   
   A   
   G   
   A   
   G   
   A   
   A   
   A   
   A   
   C   
   A   
   T   
   C   
   C   
   A   
   T   
   G   
   A   
   T   
   G   
   T   
   C   
   A   
   A   
   T   
   C   
   T   
   A   
   A   
   C   
   C   
   A   
   G   
   C   
   G   
   A   
   A   
   A   
   T   
   G   
   A   
   A   
   A   
   G   
   A   
   C   
   T   
   C   
   C   
   T   
   T   
   C   
   A   
   T   
   C   
   G   
   A   
   T   
   T   
   A   
   T   
   G   
   C   
   G   
   A   
   T   
   G   
   A   
   G   
   T   
   G   
   T   
   T   
   A   
   T   
   T   
   G   
   T   
   C   
   G   
   C   
   A   
   C   
   G   
   T   
   G   
   C   
   A   
   C   
   T   
   T   
   C   
   C   
   T   
   G   
   A   
   T   
   G   
   T   
   C   
   A   
   G   
   A   
   G   
   A   
   T   
   G   
   G   
   T   
   C   
   T   
   G   
   A   
   A   
   A   
   C   
   C   
   C   
   G   
   T   
   T   
   C   
   A   
   C   
   C   
   G   
   C   
   C   
   G   
   T   
   A   
   T   
   T   
   T   
   T   
   A   
   T   
   A   
   C   
   G   
   G   
   C   
   A   
   T   
   G   
   A   
   A   
   C   
   G   
   A   
   A   
   T   
   T   
   A   
   G   
   G   
   T   
   G   
   T   
   A   
   A   
   C   
   A   
   C   
   C   
   G   
   G   
   A   
   T   
   A   
   A   
   A   
   G   
   C   
   C   
   C   
   A   
   T   
   A   
   A   
   A   
   A   
   A   
   A   
   T   
   C   
   T   
   G   
   C   
   C   
   A   
   G   
   A   
   A   
   T   
   C   
   G   
   T   
   C   
   G   
   G   
   A   
   G   
   A   
   T   
   G   
   T   
   C   
   A   
   T   
   G   
   G   
   G   
   T   
   A   
   A   
   A   
   T   
   A   
   C   
   C   
   A   
   T   
   C   
   C   
   T   
   C   
   A   
   T   
   G   
   G   
   A   
   G   
   A   
   C   
   A   
   G   
   T   
   G   
   C   
   G   
   A   
   T   
   C   
   T   
   A   
   T   
   G   
   A   
   G   
   T   
   C   
   A   
   A   
   T   
   G   
   G   
   T   
   A   
   C   
   G   
   G   
   A   
   T   
   G   
   G   
   C   
   G   
   C   
   A   
   G   
   C   
   C   
   A   
   T   
   T   
   C   
   A   
   G   
   C   
   T   
   A   
   C   
   C   
   G   
   T   
   T   
   A   
   T   
   A   
   T   
   G   
   C   
   T   
   A   
   G   
   T   
   G   
   G   
   A   
   T   
   G   
   G   
   T   
   C   
   A   
   T   
   G   
   G   
   A   
   A   
   A   
   C   
   T   
   T   
   T   
   G   
   G   
   T   
   T   
   C   
   T   
   G   
   T   
   T   
   G   
   A   
   C   
   G   
   G   
   T   
   G   
   A   
   C   
   G   
   G   
   T   
   G   
   C   
   C   
   G   
   C   
   C   
   G   
   C   
   T   
   A   
   T   
   G   
   C   
   G   
   T   
   T   
   A   
   T   
   A   
   C   
   C   
   G   
   A   
   A   
   G   
   C   
   A   
   C   
   G   
   G   
   A   
   T   
   G   
   A   
   G   
   C   
   A   
   A   
   A   
   A   
   T   
   C   
   G   
   C   
   T   
   A   
   C   
   A   
   G   
   A   
   A   
   A   
   T   
   G   
   C   
   T   
   G   
   C   
   G   
   T   
   G   
   A   
   T   
   A   
   T   
   C   
   A   
   A   
   T   
   A   
   A   
   G   
   A   
   A   
   C   
   A   
   C   
   T   
   G   
   T   
   T   
   G   
   A   
   T   
   T   
   T   
   C   
   C   
   A   
   A   
   A   
   G   
   C   
   A   
   A   
   C   
   T   
   A   
   T   
   G   
   A   
   T   
   G   
   A   
   T   
   A   
   C   
   A   
   G   
   A   
   A   
   A   
   A   
   A   
   G   
   A   
   A   
   C   
   C   
   A   
   G   
   T   
   T   
   G   
   T   
   A   
   C   
   T   
   A   
   C   
   C   
   T   
   G   
   C   
   T   
   C   
   G   
   C   
   T   
   T   
   C   
   C   
   C   
   G   
   A   
   A   
   T   
   C   
   T   
   T   
   T   
   T   
   A   
   G   
   T   
   G   
   A   
   A   
   C   
   G   
   G   
   A   
   A   
   C   
   A   
   A   
   C   
   G   
   G   
   G   
   G   
   A   
   T   
   C   
   G   
   C   
   C   
   G   
   T   
   A   
   G   
   G   
   T   
   A   
   T   
   G   
   G   
   C   
   C   
   A   
   C   
   G   
   A   
   A   
   T   
   A   
   T   
   C   
   C   
   C   
   A   
   C   
   C   
   T   
   C   
   A   
   T   
   A   
   A   
   C   
   T   
   T   
   G   
   T   
   C   
   T   
   G   
   A   
   G   
   G   
   T   
   A   
   G   
   T   
   A   
   G   
   C   
   G   
   G   
   C   
   T   
   A   
   T   
   C   
   G   
   A   
   T   
   T   
   T   
   G   
   C   
   T   
   G   
   A   
   T   
   G   
   G   
   A   
   T   
   A   
   A   
   T   
   C   
   C   
   T   
   G   
   A   
   T   
   G   
   T   
   G   
   A   
   C   
   G   
   A   
   C   
   C   
   A   
   A   
   T   
   G   
   A   
   A   
   T   
   T   
   G   
   A   
   T   
   G   
   G   
   A   
   A   
   G   
   T   
   A   
   T   
   T   
   A   
   C   
   C   
   T   
   G   
   G   
   A   
   C   
   C   
   A   
   G   
   A   
   T   
   T   
   T   
   T   
   C   
   C   
   A   
   A   
   C   
   A   
   G   
   G   
   T   
   G   
   G   
   T   
   T   
   T   
   G   
   G   
   T   
   T   
   A   
   T   
   G   
   G   
   G   
   A   
   A   
   A   
   A   
   T   
   C   
   A   
   G   
   G   
    A    
   A   
   T   
    C    
   C   
   G   
    A    
    A    
   G   
   A   
   G   
   C   
    G    
   T   
   A   
   T   
   G   
   A   
   A   
   A   
   C   
    A    
   G   
   G   
    T    
   A   
   A   
    G    
   G   
   G   
   T   
   T   
   C   
    T    
   A   
   T   
   T   
   A   
   C   
    C    
   G   
   T   
    A    
   C   
   G   
   T   
   G   
   C   
   A   
   A   
   A   
   A   
   G   
   T   
   T   
   G   
   A   
    G    
    A    
   T   
    T    
   A   
    C    
   T   
   G   
   A   
    G    
   A   
   T   
   G   
    C    
   C   
    A    
   A   
   A   
   T   
   G   
   G   
    T    
   A   
   A   
   A   
   G   
   A   
   A   
   C   
   G   
    G    
   A   
   T   
    C    
    A    
   T   
    C    
   G   
   T   
    G    
   A   
   C   
    A    
   G   
   A   
   A   
   T   
   T   
    G    
   C   
   C   
   T   
   T   
   A   
   T   
   A   
   T   
   G   
   G   
   T   
    C    
   A   
   A   
   T   
   A   
   A   
   A   
   G   
   C   
    A    
   A   
   A   
   A   
   T   
   T   
    G    
   A   
   T   
    C    
   G   
   A   
   A   
   C   
   G   
    G    
   A   
   T   
   T   
   T   
   C   
   T   
   G   
   A   
    A    
    C    
   T   
    G    
   C   
   A   
   C   
   A   
   G   
   A   
   G   
   A   
    C    
   A   
   A   
    G    
   C   
   G   
    T    
   A   
   T   
    C    
   G   
   A   
   A   
   G   
   G   
    G    
   A   
   T   
    C    
   A   
   C   
    A    
   G   
   A   
   T   
   T   
   T   
   G   
   C   
   G   
   G   
   G   
   A   
    C    
   G   
   A   
   A   
   T   
   C   
   T   
   T   
   C   
    C    
   C   
   G   
   T   
   G   
   A   
    A    
   G   
   G   
    T    
   A   
   T   
   G   
   C   
   G   
    G    
   A   
   T   
    C    
   G   
   T   
   C   
   A   
   T   
    T    
   G   
   A   
    C    
    A    
   T   
    C    
   C   
   G   
   T   
   C   
   G   
    T    
   G   
   A   
    C    
   G   
    C    
    C    
   A   
   G   
    C    
   G   
   C   
    A    
   T   
   C   
    C    
   G   
   T   
   T   
    A    
   T   
    C    
   T   
   T   
    G    
   A   
   A   
    T    
   A   
   A   
   C   
   T   
   T   
    A    
   T   
   A   
    T    
   A   
   A   
   A   
    T    
   T   
   G   
   A   
   C   
    A    
    T    
   C   
    C    
   T   
   T   
    G    
   C   
   A   
    A    
   A   
    A    
    T    
   T   
   C   
    A    
   T   
   T   
    T    
   G   
   G   
    A    
   T   
   T   
    C    
   A   
   A   
    T    
   A   
   T   
   G   
   T   
   T   
    G    
   G   
   C   
    G    
   A   
   T   
    C    
   G   
   A   
   A   
   A   
   A   
   A   
   G   
   G   
    T    
   G   
   T   
    G    
   C   
   C   
    G    
   A   
   A   
   A   
    G    
   T   
   T   
   T   
   T   
    G    
   A   
   G   
    C    
   T   
   T   
   G   
   A   
   A   
   A   
   C   
    A    
    A    
   A   
   T   
    T    
    C    
   T   
    T    
   G   
   A   
   A   
   A   
   A   
    C    
   T   
   A   
    T    
    A    
   T   
    C    
   G   
   A   
    A    
   C   
   A   
    T    
   C   
   A   
    G    
    C    
    G    
    C    
    G    
   A   
   A   
   G   
   T   
   C   
   A   
   T   
    C    
   A   
   C   
    A    
    A    
   G   
    A    
   C   
   G   
   G   
   A   
   C   
    A    
    G    
   T   
    C    
   T   
   T   
   T   
   G   
   A   
    A    
   A   
   A   
   A   
    G    
   A   
    A    
   A   
   A   
    A    
   G   
   C   
   A   
   G   
   A   
   A   
   G   
   C   
   A   
    A    
   G   
    G    
   G   
   C   
    A    
   C   
   A   
    C    
   A   
   T   
    T    
   T   
   T   
   A   
   G   
   A   
   A   
   G   
   G   
   T   
    T    
   T   
    G    
   C   
   G   
    T    
   A   
   T   
   T   
   G   
   C   
    T    
   T   
   T   
    G    
   G   
   A   
   T   
   C   
   A   
   T   
   A   
   T   
   C   
    G    
   A   
   T   
   G   
   A   
   A   
   A   
   T   
    A    
   A   
   T   
   T   
   G   
   C   
    G    
   A   
   T   
   T   
   A   
   T   
    C    
   C   
   G   
    C    
   G   
   G   
    G    
   T   
   C   
    T    
    A    
   A   
   A   
   T   
   C   
   A   
   G   
   A   
   T   
   G   
   A   
    C    
   G   
   A   
    A    
   G   
   C   
   A   
   A   
   A   
   A   
   G   
   C   
    G    
   A   
    C    
    G    
    A    
   T   
    G    
   A   
   T   
    C    
   G   
   A   
   A   
   C   
   G   
    G    
   T   
   T   
    C    
   G   
   A   
    T    
   T   
   T   
    A    
   T   
   C   
    C    
   G   
   A   
   T   
   C   
   G   
    C    
   C   
   A   
    G    
    T    
   C   
    T    
   C   
   A   
   A   
   G   
   C   
    T    
   A   
   T   
    T    
   T   
   T   
   A   
   G   
   A   
   T   
   A   
   T   
   G   
   C   
   G   
   T   
   T   
   T   
    A    
   C   
   G   
    C    
   C   
   G   
    A    
   T   
   T   
    G    
   A   
   C   
    C    
   G   
   G   
    T    
   T   
   T   
    G    
   G   
   A   
   A   
   C   
   G   
    G    
   G   
   A   
    A    
   A   
   A   
   A   
   A   
   T   
    C    
   G   
   A   
    A    
   A   
   A   
   T   
   G   
   A   
   A   
   T   
   A   
    C    
   C   
   A   
   A   
   G   
   A   
    A    
   C   
   T   
    C    
   T   
   T   
    A    
   A   
    A    
   A   
    C    
   T   
    G    
   A   
   T   
   T   
   G   
    A    
    A    
   G   
   A   
    T    
   T   
   T   
    A    
    A    
    C    
    T    
   G   
   A   
   C   
   A   
   T   
   T   
    C    
   T   
    G    
   G   
   C   
    A    
    A    
   G   
    A    
   C   
   C   
    A    
   G   
   A   
    A    
   C   
   G   
    T    
   G   
   T   
    T    
   A   
    C    
    A    
   G   
   A   
   A   
   A   
   T   
    C    
   A   
   T   
    C    
   A   
   A   
    G    
   A   
    A    
    T    
   G   
   A   
    A    
   T   
   T   
   A   
    G    
    C    
    A    
   G   
   A   
   C   
    T    
   T   
    G    
    A    
    A    
    T    
    C    
    A    
   A   
    C    
    G    
   A   
   T   
   T   
   T   
   G   
   G   
   T   
   G   
   A   
   T   
    A    
    A    
   A   
   C   
   G   
   A   
   C   
   G   
    C    
   A   
   C   
   A   
   G   
   A   
   A   
    T    
   T   
   A   
    C    
   T   
   A   
   G   
   T   
    C    
   G   
   G   
   T   
   G   
   A   
    G    
   G   
   T   
   C   
    T    
   T   
    A    
   A   
   G   
   T   
   C   
   T   
    G    
   G   
   A   
   A   
   G   
   A   
   T   
   G   
   A   
   A   
   G   
   A   
   T   
    T    
   T   
    A    
   A   
   T   
    C    
   G   
   A   
   A   
   G   
   A   
   A   
   G   
   A   
   A   
   G   
   A   
   A   
    A    
   T   
   C   
   G   
   T   
    T    
   A   
   T   
   T   
   A   
   C   
    A    
    T    
   T   
    G    
   A   
   C   
    A    
   A   
   A   
    C    
   A   
   A   
    T    
   G   
   G   
    T    
   T   
   A   
    C    
   A   
   T   
    C    
   A   
   A   
    A    
   C   
   G   
    T    
    T    
   T   
   G   
   G   
    C    
   A   
   A   
   A   
    T    
    A    
    A    
    T    
   G   
   A   
   A   
   T   
   T   
   C   
    A    
   G   
    A    
   G   
   C   
    T    
   C   
   A   
   A   
   C   
   G   
    A    
    A    
   G   
    G    
   G   
   G   
    T    
   G   
   G   
    A    
   C   
   G   
   T   
   G   
   G   
    T    
   G   
   T   
    C    
   C   
   A   
   A   
   G   
   G   
    A    
   A   
   T   
   G   
   G   
   G   
    T    
    A    
   T   
    C    
   C   
   A   
    C    
    A    
   A   
   T   
   G   
   A   
    C    
   G   
   A   
    C    
   T   
   T   
   C   
   G   
   T   
    C    
   A   
   A   
    A    
   A   
   A   
    T    
   C   
   T   
    G    
    G    
   T   
   T   
   T   
   C   
   T   
   T   
   G   
    T    
   T   
   C   
    T    
   A   
   C   
    C    
   C   
   A   
    T    
   G   
   A   
    T    
   A   
   C   
    A    
   T   
   T   
    G    
   T   
   T   
   A   
   T   
   T   
    C    
   T   
   T   
   T   
   A   
   C   
   G   
   A   
   A   
    T    
   A   
    A    
    C    
   G   
   G   
    A    
   A   
   A   
   A   
   G   
   T   
    G    
   T   
   A   
    T    
   C   
   G   
    G    
   G   
   C   
   A   
   A   
   A   
   A   
   G   
   G   
    A    
   T   
   A   
    C    
   G   
   A   
   A   
   A   
   T   
    T    
   C   
   C   
    A    
   G   
   A   
    A    
   T   
   A   
   C   
   G   
   G   
    G    
    C    
   G   
    T    
   A   
   C   
   A   
   G   
   C   
    C    
   A   
   A   
   A   
   G   
   G   
    A    
   A   
   T   
    A    
   C   
   C   
   A   
    A    
   T   
    T    
   A   
   T   
    C    
   A   
   A   
    T    
   T   
   T   
    A    
   C   
   T   
    A    
   G   
   G   
    T    
   A   
   T   
    C    
   G   
   A   
    T    
   T   
   C   
    G    
    A    
    G    
    T    
   G   
   A   
   A   
   A   
    G    
    C    
   A   
   T   
    C    
   C   
   A   
    G    
   G   
   C   
    A    
   A   
   T   
    C    
   A   
   T   
   T   
    G    
   C   
    G    
   G   
   T   
    G    
    A    
    C    
   G   
   G   
   G   
    A    
    G    
   A   
   A   
   G   
   C   
    A    
   G   
   A   
   A   
   G   
    A    
    A    
   G   
   G   
    C    
    C    
   A   
    C    
   T   
   A   
    T    
    C    
   T   
    C    
   T   
   T   
   C   
   T   
   T   
    T    
   A   
   C   
    G    
   A   
   C   
    G    
    C    
    G    
    T    
    C    
   A   
   A   
   G   
   G   
    T    
   A   
   C   
    A    
   G   
   T   
    A    
   A   
   A   
   A   
   C   
   G   
    T    
   A   
   C   
    T    
    T    
   C   
    T    
   G   
   T   
    C    
   A   
   C   
    T    
   G   
   C   
    T    
   T   
   T   
    T    
    G    
   C   
   T   
   A   
   A   
    C    
   A   
   T   
    C    
   C   
   G   
    A    
   A   
   G   
    T    
   A   
   A   
   T   
   G   
   G   
    T    
    C    
   T   
    G    
   A   
   T   
    C    
   G   
   C   
    A    
   A   
   T   
   T   
    G    
   G   
    T    
   T   
   T   
    G    
   A   
   A   
   A   
   G   
   A   
   A   
   G   
   A   
    C    
   G   
   A   
    T    
   G   
   A   
    G    
    C    
   T   
    C    
    G    
   T   
    C    
   A   
   A   
    T    
   G   
   T   
    G    
    C    
   T   
   A   
    C    
    T    
   G   
   A   
   C   
    A    
    G    
   A   
    T    
   G   
   G   
    T    
    C    
   A   
    A    
    T    
    C    
    T    
   A   
   A   
    T    
   A   
   T   
    C    
   A   
   T   
    T    
   A   
   T   
    T    
   G   
   G   
    A    
   A   
   C   
   A   
   C   
   A   
   T   
    A    
    A    
    C    
   G   
   G   
    C    
   T   
   A   
   C   
   T   
   C   
   T   
   G   
   T   
    C    
   A   
   C   
    T    
   T   
   T   
    C    
    A    
   A   
    A    
   G   
   A   
    A    
    G    
    A    
    A    
    G    
   C   
    A    
   G   
    T    
    C    
   C   
   G   
    C    
   G   
   A   
   T   
   A   
   T   
   G   
   G   
   G   
    A    
   C   
   G   
    T    
   A   
   C   
   A   
   G   
   C   
    T    
   T   
   C   
    C    
   G   
   G   
    G    
   G   
   T   
    C    
    A    
   G   
    A    
   G   
   G   
    G    
   A   
   T   
    A    
   C   
   G   
    C    
    C    
   T   
    G    
   C   
   G   
    T    
   G   
   A   
    G    
    G    
   A   
    A    
   G   
   A   
    T    
   T   
    A    
    T    
   G   
   T   
    C    
   G   
   T   
    T    
   G   
   G   
    G    
    G    
   C   
   A   
   G   
   C   
    C    
    T    
   T   
    A    
    A    
   T   
   G   
    A    
   A   
    G    
   G   
    A    
   A   
    A    
   A   
    T    
    C    
   A   
   A   
   G   
   A   
   A   
   G   
   T   
    A    
    T    
   T   
    A    
   G   
   T   
    G    
   A   
   T   
    C    
   A   
   C   
    A    
   G   
   A   
   A   
   A   
   A   
    A    
   G   
   G   
   T   
   T   
   A   
    C    
   G   
   G   
    A    
   A   
   A   
    A    
   C   
   G   
    G    
   A   
   C   
    A    
   A   
   A   
   A   
   G   
    T    
    A    
   T   
   C   
    G    
   G   
   A   
    A    
   T   
   A   
   T   
   C   
   C   
   A   
   G   
   T   
    G    
   A   
   A   
    A    
   G   
   G   
    C    
   C   
   G   
    C    
   G   
   G   
    A    
   G   
   G   
   T   
   A   
   A   
   A   
   G   
   G   
    G    
   A   
   T   
    C    
   A   
   A   
    A    
   A   
   C   
    G    
    G    
   C   
    G    
   A   
   A   
    C    
   A   
   T   
   C   
   A   
   C   
   T   
   G   
   A   
    A    
   A   
   A   
   A   
   A   
   A   
   T   
   G   
   G   
   T   
   C   
   C   
   A   
    C    
   T   
   A   
   G   
   C   
    T    
   G   
   G   
    A    
   T   
   T   
    G    
   A   
   C   
   C   
   A   
   C   
    T    
   G   
   T   
    T    
    T    
    C    
   T   
   G   
   G   
    A    
    G    
   A   
   T   
   G   
   A   
   A   
   G   
   A   
    C    
   A   
   T   
   C   
    A    
   T   
    G    
    C    
   T   
    G    
   A   
   T   
    T    
   A   
   C   
    A    
    G    
   A   
    T    
   A   
   A   
   A   
   G   
   G   
    T    
   G   
   T   
    C    
   A   
   T   
   T   
   A   
   T   
   C   
   C   
   G   
    T    
   T   
   T   
    C    
   A   
   A   
   C   
   G   
   T   
    A    
    T    
    C    
    G    
    A    
   C   
   T   
   G   
   T   
   T   
   T   
   C   
   T   
   C   
   A   
   A   
   A   
   C   
    T    
   G   
   G   
   A   
   C   
   G   
    T    
    T    
   C   
   A   
   A   
   C   
   A   
   T   
   T   
    A    
   G   
   G   
    C    
   G   
   T   
    A    
   C   
   G   
   T   
   T   
   T   
    G    
   A   
   T   
   G   
   A   
    A    
    G    
   A   
   T   
   G   
   G   
   A   
   A   
   G   
    C    
    T    
   G   
    A    
    T    
    A    
   C   
    G    
   A   
   A   
   A   
   G   
   T   
   T   
   G   
   T   
   C   
   A   
   C   
   G   
   A   
   T   
   G   
   G   
   C   
   A   
   G   
   C   
   A   
   G   
   T   
   C   
   G   
   A   
   A   
   C   
   C   
   A   
   G   
   A   
   A   
   G   
   C   
   T   
   G   
   C   
   A   
   G   
   A   
   A   
   C   
   A   
   A   
   A   
   C   
   A   
   G   
   T   
   T   
   G   
   A   
   A   
   G   
   A   
   A   
   C   
   A   
   A   
   G   
   G   
   A   
   T   
   C   
   A   
   G   
   A   
   A   
   G   
   A   
   A   
   -   
   -   
   -   
 

 
   N53655   
   A   
   T   
   G   
   A   
   G   
   T   
   G   
   A   
   A   
   G   
   A   
   A   
   A   
   T   
   C   
   A   
   G   
   A   
   G   
   A   
   A   
   A   
   A   
   C   
   A   
   T   
   C   
   C   
   A   
   T   
   G   
   A   
   T   
   G   
   T   
   C   
   A   
   A   
   T   
   C   
   T   
   A   
   A   
   C   
   C   
   A   
   G   
   C   
   G   
   A   
   A   
   A   
   T   
   G   
   A   
   A   
   A   
   G   
   A   
   C   
   T   
   C   
   C   
   T   
   T   
   C   
   A   
   T   
   C   
   G   
   A   
   T   
   T   
   A   
   T   
   G   
   C   
   G   
   A   
   T   
   G   
   A   
   G   
   T   
   G   
   T   
   T   
   A   
   T   
   T   
   G   
   T   
   C   
   G   
   C   
   A   
   C   
   G   
   T   
   G   
   C   
   A   
   C   
   T   
   T   
   C   
   C   
   T   
   G   
   A   
   T   
   G   
   T   
   C   
   A   
   G   
   A   
   G   
   A   
   T   
   G   
   G   
   T   
   C   
   T   
   G   
   A   
   A   
   A   
   C   
   C   
   C   
   G   
   T   
   T   
   C   
   A   
   C   
   C   
   G   
   C   
   C   
   G   
   T   
   A   
   T   
   T   
   T   
   T   
   A   
   T   
   A   
   C   
   G   
   G   
   C   
   A   
   T   
   G   
   A   
   A   
   C   
   G   
   A   
   A   
   T   
   T   
   A   
   G   
   G   
   T   
   G   
   T   
   A   
   A   
   C   
   A   
   C   
   C   
   G   
   G   
   A   
   T   
   A   
   A   
   A   
   G   
   C   
   C   
   C   
   A   
   T   
   A   
   A   
   A   
   A   
   A   
   A   
   T   
   C   
   T   
   G   
   C   
   C   
   A   
   G   
   A   
   A   
   T   
   C   
   G   
   T   
   C   
   G   
   G   
   A   
   G   
   A   
   T   
   G   
   T   
   C   
   A   
   T   
   G   
   G   
   G   
   T   
   A   
   A   
   A   
   T   
   A   
   C   
   C   
   A   
   T   
   C   
   C   
   T   
   C   
   A   
   T   
   G   
   G   
   A   
   G   
   A   
   C   
   A   
   G   
   T   
   G   
   C   
   G   
   A   
   T   
   C   
   T   
   A   
   T   
   G   
   A   
   G   
   T   
   C   
   A   
   A   
   T   
   G   
   G   
   T   
   A   
   C   
   G   
   G   
   A   
   T   
   G   
   G   
   C   
   G   
   C   
   A   
   G   
   C   
   C   
   A   
   T   
   T   
   C   
   A   
   G   
   C   
   T   
   A   
   C   
   C   
   G   
   T   
   T   
   A   
   T   
   A   
   T   
   G   
   C   
   T   
   A   
   G   
   T   
   G   
   G   
   A   
   T   
   G   
   G   
   T   
   C   
   A   
   T   
   G   
   G   
   A   
   A   
   A   
   C   
   T   
   T   
   T   
   G   
   G   
   T   
   T   
   C   
   T   
   G   
   T   
   T   
   G   
   A   
   C   
   G   
   G   
   T   
   G   
   A   
   C   
   G   
   G   
   T   
   G   
   C   
   C   
   G   
   C   
   C   
   G   
   C   
   T   
   A   
   T   
   G   
   C   
   G   
   T   
   T   
   A   
   T   
   A   
   C   
   C   
   G   
   A   
   A   
   G   
   C   
   A   
   C   
   G   
   G   
   A   
   T   
   G   
   A   
   G   
   C   
   A   
   A   
   A   
   A   
   T   
   C   
   G   
   C   
   T   
   A   
   C   
   A   
   G   
   A   
   A   
   A   
   T   
   G   
   C   
   T   
   G   
   C   
   G   
   T   
   G   
   A   
   T   
   A   
   T   
   C   
   A   
   A   
   T   
   A   
   A   
   G   
   A   
   A   
   C   
   A   
   C   
   T   
   G   
   T   
   T   
   G   
   A   
   T   
   T   
   T   
   C   
   C   
   A   
   A   
   A   
   G   
   C   
   A   
   A   
   C   
   T   
   A   
   T   
   G   
   A   
   T   
   G   
   A   
   T   
   A   
   C   
   A   
   G   
   A   
   A   
   A   
   A   
   A   
   G   
   A   
   A   
   C   
   C   
   A   
   G   
   T   
   T   
   G   
   T   
   A   
   C   
   T   
   A   
   C   
   C   
   T   
   G   
   C   
   T   
   C   
   G   
   C   
   T   
   T   
   C   
   C   
   C   
   G   
   A   
   A   
   T   
   C   
   T   
   T   
   T   
   T   
   A   
   G   
   T   
   G   
   A   
   A   
   C   
   G   
   G   
   A   
   A   
   C   
   A   
   A   
   C   
   G   
   G   
   G   
   G   
   A   
   T   
   C   
   G   
   C   
   C   
   G   
   T   
   A   
   G   
   G   
   T   
   A   
   T   
   G   
   G   
   C   
   C   
   A   
   C   
   G   
   A   
   A   
   T   
   A   
   T   
   C   
   C   
   C   
   A   
   C   
   C   
   T   
   C   
   A   
   T   
   A   
   A   
   C   
   T   
   T   
   G   
   T   
   C   
   T   
   G   
   A   
   G   
   G   
   T   
   A   
   G   
   T   
   A   
   G   
   C   
   G   
   G   
   C   
   T   
   A   
   T   
   C   
   G   
   A   
   T   
   T   
   T   
   G   
   C   
   T   
   G   
   A   
   T   
   G   
   G   
   A   
   T   
   A   
   A   
   T   
   C   
   C   
   T   
   G   
   A   
   T   
   G   
   T   
   G   
   A   
   C   
   G   
   A   
   C   
   C   
   A   
   A   
   T   
   G   
   A   
   A   
   T   
   T   
   G   
   A   
   T   
   G   
   G   
   A   
   A   
   G   
   T   
   A   
   T   
   T   
   A   
   C   
   C   
   T   
   G   
   G   
   A   
   C   
   C   
   A   
   G   
   A   
   T   
   T   
   T   
   T   
   C   
   C   
   A   
   A   
   C   
   A   
   G   
   G   
   T   
   G   
   G   
   T   
   T   
   T   
   G   
   G   
   T   
   T   
   A   
   T   
   G   
   G   
   G   
   G   
   A   
   A   
   A   
   T   
   C   
   A   
   G   
   G   
    A    
   A   
   T   
    C    
   C   
   G   
    A    
    A    
   G   
   A   
   G   
   C   
    G    
   T   
   A   
   T   
   G   
   A   
   A   
   A   
   C   
    A    
   G   
   G   
    T    
   A   
   A   
    G    
   G   
   G   
   T   
   T   
   C   
    T    
   A   
   T   
   T   
   A   
   C   
    C    
   G   
   T   
    A    
   C   
   G   
   T   
   G   
   C   
   A   
   A   
   A   
   A   
   G   
   T   
   T   
   G   
   A   
    G    
    A    
   T   
    T    
   A   
    C    
   T   
   G   
   A   
    G    
   A   
   T   
   G   
    C    
   C   
    A    
   A   
   A   
   T   
   G   
   G   
    T    
   A   
   A   
   A   
   G   
   A   
   A   
   C   
   G   
    G    
   A   
   T   
    C    
    A    
   T   
    C    
   G   
   T   
    G    
   A   
   C   
    A    
   G   
   A   
   A   
   T   
   T   
    G    
   C   
   C   
   T   
   T   
   A   
   T   
   A   
   T   
   G   
   G   
   T   
    C    
   A   
   A   
   T   
   A   
   A   
   A   
   G   
   C   
    A    
   A   
   A   
   A   
   T   
   T   
    G    
   A   
   T   
    C    
   G   
   A   
   A   
   C   
   G   
    G    
   A   
   T   
   T   
   T   
   C   
   T   
   G   
   A   
    A    
    C    
   T   
    G    
   C   
   A   
   C   
   A   
   G   
   A   
   G   
   A   
    C    
   A   
   A   
    G    
   C   
   G   
    T    
   A   
   T   
    C    
   G   
   A   
   A   
   G   
   G   
    G    
   A   
   T   
    C    
   A   
   C   
    A    
   G   
   A   
   T   
   T   
   T   
   G   
   C   
   G   
   G   
   G   
   A   
    C    
   G   
   A   
   A   
   T   
   C   
   T   
   T   
   C   
    C    
   C   
   G   
   T   
   G   
   A   
    G    
   G   
   G   
    T    
   A   
   T   
   G   
   C   
   G   
    G    
   A   
   T   
    C    
   G   
   T   
   C   
   A   
   T   
    T    
   G   
   A   
    C    
    A    
   T   
    C    
   C   
   G   
   T   
   C   
   G   
    T    
   G   
   A   
    C    
   G   
    C    
    C    
   A   
   G   
    C    
   G   
   C   
    A    
   T   
   C   
    C    
   G   
   T   
   T   
    A    
   T   
    C    
   T   
   T   
    G    
   A   
   A   
    T    
   A   
   A   
   C   
   T   
   T   
    A    
   T   
   A   
    T    
   A   
   A   
   A   
    T    
   T   
   G   
   A   
   C   
    A    
    T    
   C   
    C    
   T   
   T   
    G    
   C   
   A   
    A    
   A   
    A    
    T    
   T   
   C   
    A    
   T   
   T   
    T    
   G   
   G   
    A    
   T   
   T   
    C    
   A   
   A   
    T    
   A   
   T   
   G   
   T   
   T   
    G    
   G   
   C   
    G    
   A   
   T   
    C    
   G   
   A   
   A   
   A   
   A   
   A   
   G   
   G   
    T    
   G   
   T   
    G    
   C   
   C   
    G    
   A   
   A   
   A   
    G    
   T   
   T   
   T   
   T   
    G    
   A   
   G   
    C    
   T   
   T   
   G   
   A   
   A   
   A   
   C   
    A    
    A    
   A   
   T   
    T    
    C    
   T   
    T    
   G   
   A   
   A   
   A   
   A   
    C    
   T   
   A   
    T    
    A    
   T   
    C    
   G   
   A   
    A    
   C   
   A   
    T    
   C   
   A   
    G    
    C    
    G    
    C    
    G    
   A   
   A   
   G   
   T   
   C   
   A   
   T   
    C    
   A   
   C   
    A    
    A    
   G   
    A    
   C   
   G   
   G   
   A   
   C   
    G    
    G    
   T   
    C    
   T   
   T   
   T   
   G   
   A   
    A    
   A   
   A   
   A   
    G    
   A   
    A    
   A   
   A   
    A    
   G   
   C   
   A   
   G   
   A   
   A   
   G   
   C   
   A   
    A    
   G   
    G    
   G   
   C   
    A    
   C   
   A   
    C    
   A   
   T   
    T    
   T   
   T   
   A   
   G   
   A   
   A   
   G   
   G   
   T   
    T    
   T   
    G    
   C   
   G   
    T    
   A   
   T   
   T   
   G   
   C   
    T    
   T   
   T   
    G    
   G   
   A   
   T   
   C   
   A   
   T   
   A   
   T   
   C   
    G    
   A   
   T   
   G   
   A   
   A   
   A   
   T   
    C    
   A   
   T   
   T   
   G   
   C   
    G    
   A   
   T   
   T   
   A   
   T   
    C    
   C   
   G   
    C    
   G   
   G   
    G    
   T   
   C   
    T    
    A    
   A   
   A   
   T   
   C   
   A   
   G   
   A   
   T   
   G   
   A   
    C    
   G   
   A   
    A    
   G   
   C   
   A   
   A   
   A   
   A   
   G   
   C   
    G    
   A   
    C    
    G    
    A    
   T   
    G    
   A   
   T   
    C    
   G   
   A   
   A   
   C   
   G   
    G    
   T   
   T   
    C    
   G   
   A   
    T    
   T   
   T   
    A    
   T   
   C   
    C    
   G   
   A   
   T   
   C   
   G   
    C    
   C   
   A   
    G    
    T    
   C   
    T    
   C   
   A   
   A   
   G   
   C   
    T    
   A   
   T   
    T    
   T   
   T   
   A   
   G   
   A   
   T   
   A   
   T   
   G   
   C   
   G   
   T   
   T   
   T   
    A    
   C   
   G   
    C    
   C   
   G   
    A    
   T   
   T   
    G    
   A   
   C   
    C    
   G   
   G   
    T    
   T   
   T   
    G    
   G   
   A   
   A   
   C   
   G   
    G    
   G   
   A   
    A    
   A   
   A   
   A   
   A   
   T   
    C    
   G   
   A   
    A    
   A   
   A   
   T   
   G   
   A   
   A   
   T   
   A   
    C    
   C   
   A   
   A   
   G   
   A   
    A    
   C   
   T   
    C    
   T   
   T   
    A    
   A   
    A    
   A   
    C    
   T   
    G    
   A   
   T   
   T   
   G   
    A    
    A    
   G   
   A   
    T    
   T   
   T   
    A    
    A    
    C    
    T    
   G   
   A   
   C   
   A   
   T   
   T   
    C    
   T   
    G    
   G   
   C   
    A    
    A    
   G   
    A    
   C   
   C   
    A    
   G   
   A   
    A    
   C   
   G   
    T    
   G   
   T   
    T    
   A   
    C    
    A    
   G   
   A   
   A   
   A   
   T   
    C    
   A   
   T   
    C    
   A   
   A   
    G    
   A   
    A    
    T    
   G   
   A   
    A    
   T   
   T   
   A   
    G    
    C    
    A    
   G   
   A   
   C   
    T    
   T   
    G    
    A    
    A    
    T    
    C    
    A    
   A   
    C    
    G    
   A   
   T   
   T   
   T   
   G   
   G   
   T   
   G   
   A   
   T   
    A    
    A    
   A   
   C   
   G   
   A   
   C   
   G   
    C    
   A   
   C   
   A   
   G   
   A   
   A   
    T    
   T   
   A   
    C    
   T   
   A   
   G   
   T   
    C    
   G   
   G   
   T   
   G   
   A   
    G    
   G   
   T   
   C   
    T    
   T   
    A    
   A   
   G   
   T   
   C   
   T   
    G    
   G   
   A   
   A   
   G   
   A   
   T   
   G   
   A   
   A   
   G   
   A   
   T   
    T    
   T   
    A    
   A   
   T   
    C    
   G   
   A   
   A   
   G   
   A   
   A   
   G   
   A   
   A   
   G   
   A   
   A   
    A    
   T   
   C   
   G   
   T   
    T    
   A   
   T   
   T   
   A   
   C   
    A    
    T    
   T   
    G    
   A   
   C   
    A    
   A   
   A   
    C    
   A   
   A   
    T    
   G   
   G   
    T    
   T   
   A   
    C    
   A   
   T   
    C    
   A   
   A   
    A    
   C   
   G   
    T    
    T    
   T   
   G   
   G   
    C    
   A   
   A   
   A   
    T    
    A    
    A    
    T    
   G   
   A   
   A   
   T   
   T   
   C   
    A    
   G   
    A    
   G   
   C   
    T    
   C   
   A   
   A   
   C   
   G   
    A    
    A    
   G   
    G    
   G   
   G   
    T    
   G   
   G   
    A    
   C   
   G   
   T   
   G   
   G   
    T    
   G   
   T   
    C    
   C   
   A   
   A   
   G   
   G   
    A    
   A   
   T   
   G   
   G   
   G   
    T    
    A    
   T   
    C    
   C   
   A   
    C    
    A    
   A   
   T   
   G   
   A   
    C    
   G   
   A   
    C    
   T   
   T   
   C   
   G   
   T   
    C    
   A   
   A   
    A    
   A   
   A   
    T    
   C   
   T   
    G    
    G    
   T   
   T   
   T   
   C   
   T   
   T   
   G   
    T    
   T   
   C   
    T    
   A   
   C   
    C    
   C   
   A   
    T    
   G   
   A   
    T    
   A   
   C   
    A    
   T   
   T   
    G    
   T   
   T   
   A   
   T   
   T   
    C    
   T   
   T   
   T   
   A   
   C   
   G   
   A   
   A   
    T    
   A   
    A    
    C    
   G   
   G   
    A    
   A   
   A   
   A   
   G   
   T   
    G    
   T   
   A   
    T    
   C   
   G   
    G    
   G   
   C   
   A   
   A   
   A   
   A   
   G   
   G   
    A    
   T   
   A   
    C    
   G   
   A   
   A   
   A   
   T   
    T    
   C   
   C   
    A    
   G   
   A   
    A    
   T   
   A   
   C   
   G   
   G   
    G    
    C    
   G   
    C    
   A   
   C   
   A   
   G   
   C   
    C    
   A   
   A   
   A   
   G   
   G   
    A    
   A   
   T   
    A    
   C   
   C   
   A   
    A    
   T   
    T    
   A   
   T   
    C    
   A   
   A   
    T    
   T   
   T   
    A    
   C   
   T   
    A    
   G   
   G   
    T    
   A   
   T   
    C    
   G   
   A   
    T    
   T   
   C   
    G    
    A    
    G    
    T    
   G   
   A   
   A   
   A   
    G    
    C    
   A   
   T   
    C    
   C   
   A   
    G    
   G   
   C   
    A    
   A   
   T   
    C    
   A   
   T   
   T   
    G    
   C   
    G    
   G   
   T   
    G    
    A    
    C    
   G   
   G   
   G   
    A    
    G    
   A   
   A   
   G   
   C   
    A    
   G   
   A   
   A   
   G   
    A    
    A    
   G   
   G   
    C    
    C    
   A   
    C    
   T   
   A   
    T    
    C    
   T   
    C    
   T   
   T   
   C   
   T   
   T   
    T    
   A   
   C   
    G    
   A   
   C   
    G    
    C    
    G    
    T    
    C    
   A   
   A   
   G   
   G   
    T    
   A   
   C   
    A    
   G   
   T   
    A    
   A   
   A   
   A   
   C   
   G   
    T    
   A   
   C   
    T    
    T    
   C   
    T    
   G   
   T   
    C    
   A   
   C   
    T    
   G   
   C   
    T    
   T   
   T   
    T    
    G    
   C   
   T   
   A   
   A   
    C    
   A   
   T   
    C    
   C   
   G   
    A    
   A   
   G   
    T    
   A   
   A   
   T   
   G   
   G   
    T    
    C    
   T   
    G    
   A   
   T   
    C    
   G   
   C   
    A    
   A   
   T   
   T   
    G    
   G   
    T    
   T   
   T   
    G    
   A   
   A   
   A   
   G   
   A   
   A   
   G   
   A   
    C    
   G   
   A   
    T    
   G   
   A   
    G    
    C    
   T   
    C    
    G    
   T   
    C    
   A   
   A   
    T    
   G   
   T   
    G    
    C    
   T   
   A   
    C    
    T    
   G   
   A   
   C   
    A    
    G    
   A   
    T    
   G   
   G   
    T    
    C    
   A   
    A    
    T    
    C    
    T    
   A   
   A   
    T    
   A   
   T   
    C    
   A   
   T   
    T    
   A   
   T   
    T    
   G   
   G   
    A    
   A   
   C   
   A   
   C   
   A   
   T   
    A    
    A    
    C    
   G   
   G   
    C    
   T   
   A   
   C   
   T   
   C   
   T   
   G   
   T   
    C    
   A   
   C   
    T    
   T   
   T   
    C    
    A    
   A   
    A    
   G   
   A   
    A    
    G    
    A    
    A    
    G    
   C   
    A    
   G   
    T    
    C    
   C   
   G   
    C    
   G   
   A   
   T   
   A   
   T   
   G   
   G   
   G   
    A    
   C   
   G   
    T    
   A   
   C   
   A   
   G   
   C   
    T    
   T   
   C   
    C    
   G   
   G   
    G    
   G   
   T   
    C    
    A    
   G   
    A    
   G   
   G   
    A    
   A   
   T   
    A    
   C   
   G   
    C    
    C    
   T   
    G    
   C   
   G   
    T    
   G   
   A   
    G    
    G    
   A   
    A    
   G   
   A   
    T    
   T   
    A    
    T    
   G   
   T   
    C    
   G   
   T   
    T    
   G   
   G   
    G    
    G    
   C   
   A   
   G   
   C   
    C    
    T    
   T   
    A    
    A    
   T   
   G   
    A    
   A   
    G    
   G   
    A    
   A   
    G    
   A   
    T    
    C    
   A   
   A   
   G   
   A   
   A   
   G   
   T   
    A    
    T    
   T   
    A    
   G   
   T   
    G    
   A   
   T   
    C    
   A   
   C   
    A    
   G   
   A   
   A   
   A   
   A   
    A    
   G   
   G   
   T   
   T   
   A   
    C    
   G   
   G   
    A    
   A   
   A   
    A    
   C   
   G   
    G    
   A   
   C   
    A    
   A   
   A   
   A   
   G   
    T    
    A    
   T   
   C   
    G    
   G   
   A   
    A    
   T   
   A   
   T   
   C   
   C   
   A   
   G   
   T   
    G    
   A   
   A   
    A    
   G   
   G   
    C    
   C   
   G   
    C    
   G   
   G   
    A    
   G   
   G   
   T   
   A   
   A   
   A   
   G   
   G   
    G    
   A   
   T   
    C    
   A   
   A   
    A    
   A   
   C   
    G    
    G    
   C   
    G    
   A   
   A   
    C    
   A   
   T   
   C   
   A   
   C   
   T   
   G   
   A   
    A    
   A   
   A   
   A   
   A   
   A   
   T   
   G   
   G   
   T   
   C   
   C   
   A   
    C    
   T   
   A   
   G   
   C   
    T    
   G   
   G   
    A    
   T   
   T   
    G    
   A   
   C   
   C   
   A   
   C   
    T    
   G   
   T   
    T    
    T    
    C    
   T   
   G   
   G   
    A    
    G    
   A   
   T   
   G   
   A   
   A   
   G   
   A   
    C    
   A   
   T   
   C   
    A    
   T   
    G    
    C    
   T   
    G    
   A   
   T   
    T    
   A   
   C   
    A    
    G    
   A   
    T    
   A   
   A   
   A   
   G   
   G   
    T    
   G   
   T   
    C    
   A   
   T   
   T   
   A   
   T   
   C   
   C   
   G   
    T    
   T   
   T   
    C    
   A   
   A   
   C   
   G   
   T   
    A    
    T    
    C    
    G    
    A    
   C   
   T   
   G   
   T   
   T   
   T   
   C   
   T   
   C   
   A   
   A   
   A   
   C   
    T    
   G   
   G   
   A   
   C   
   G   
    T    
    T    
   C   
   A   
   A   
   C   
   A   
   T   
   T   
    A    
   G   
   G   
    C    
   G   
   T   
    A    
   C   
   G   
   T   
   T   
   T   
    G    
   A   
   T   
   G   
   A   
    A    
    G    
   A   
   T   
   G   
   G   
   A   
   A   
   G   
    C    
    T    
   G   
    A    
    T    
    A    
   C   
    G    
   A   
   A   
   A   
   G   
   T   
   T   
   G   
   T   
   C   
   A   
   C   
   G   
   A   
   T   
   G   
   G   
   C   
   A   
   G   
   C   
   A   
   G   
   T   
   C   
   G   
   A   
   A   
   C   
   C   
   A   
   G   
   A   
   A   
   G   
   C   
   T   
   G   
   C   
   A   
   G   
   A   
   A   
   C   
   A   
   A   
   A   
   C   
   A   
   G   
   T   
   T   
   G   
   A   
   A   
   G   
   A   
   A   
   C   
   A   
   A   
   G   
   G   
   A   
   T   
   C   
   A   
   G   
   A   
   A   
   G   
   A   
   A   
   -   
   -   
   -   
 

 
   N59555F   
   A   
   T   
   G   
   A   
   G   
   T   
   G   
   A   
   A   
   G   
   A   
   A   
   A   
   T   
   T   
   A   
   A   
   A   
   G   
   A   
   A   
   A   
   A   
   C   
   A   
   T   
   T   
   C   
   A   
   A   
   G   
   A   
   C   
   G   
   T   
   C   
   A   
   A   
   T   
   C   
   T   
   G   
   A   
   C   
   C   
   A   
   G   
   C   
   G   
   A   
   A   
   A   
   T   
   G   
   A   
   A   
   A   
   G   
   A   
   A   
   T   
   C   
   T   
   T   
   T   
   C   
   A   
   T   
   T   
   G   
   A   
   C   
   T   
   A   
   C   
   G   
   C   
   A   
   A   
   T   
   G   
   A   
   G   
   T   
   G   
   T   
   T   
   A   
   T   
   C   
   G   
   T   
   A   
   G   
   C   
   C   
   C   
   G   
   C   
   G   
   C   
   G   
   T   
   T   
   A   
   C   
   C   
   T   
   G   
   A   
   C   
   G   
   T   
   T   
   C   
   G   
   T   
   G   
   A   
   C   
   G   
   G   
   T   
   T   
   T   
   A   
   A   
   A   
   A   
   C   
   C   
   T   
   G   
   T   
   T   
   C   
   A   
   T   
   C   
   G   
   C   
   C   
   G   
   A   
   A   
   T   
   C   
   T   
   T   
   A   
   T   
   A   
   T   
   G   
   G   
   A   
   A   
   T   
   G   
   A   
   A   
   C   
   G   
   A   
   A   
   T   
   T   
   A   
   G   
   G   
   G   
   G   
   T   
   A   
   A   
   C   
   C   
   C   
   C   
   T   
   G   
   A   
   T   
   A   
   A   
   A   
   C   
   C   
   A   
   C   
   A   
   C   
   A   
   A   
   A   
   A   
   A   
   A   
   T   
   C   
   A   
   G   
   C   
   C   
   C   
   G   
   G   
   A   
   T   
   T   
   G   
   T   
   T   
   G   
   G   
   G   
   G   
   A   
   T   
   G   
   T   
   T   
   A   
   T   
   G   
   G   
   G   
   T   
   A   
   A   
   A   
   T   
   A   
   T   
   C   
   A   
   C   
   C   
   C   
   C   
   C   
   A   
   T   
   G   
   G   
   G   
   G   
   A   
   C   
   A   
   G   
   T   
   G   
   C   
   G   
   A   
   T   
   T   
   T   
   A   
   C   
   G   
   A   
   A   
   T   
   C   
   A   
   A   
   T   
   G   
   G   
   T   
   G   
   C   
   G   
   G   
   A   
   T   
   G   
   G   
   C   
   A   
   C   
   A   
   A   
   C   
   C   
   T   
   T   
   T   
   T   
   A   
   G   
   T   
   T   
   A   
   T   
   C   
   G   
   G   
   G   
   C   
   T   
   A   
   T   
   G   
   T   
   T   
   A   
   G   
   T   
   T   
   G   
   A   
   C   
   G   
   G   
   C   
   C   
   A   
   C   
   G   
   G   
   A   
   A   
   A   
   C   
   T   
   T   
   C   
   G   
   G   
   T   
   T   
   C   
   T   
   G   
   T   
   C   
   G   
   A   
   T   
   G   
   G   
   T   
   G   
   A   
   C   
   G   
   G   
   C   
   G   
   C   
   T   
   G   
   C   
   C   
   G   
   C   
   T   
   A   
   T   
   G   
   C   
   G   
   T   
   T   
   A   
   T   
   A   
   C   
   C   
   G   
   A   
   A   
   G   
   C   
   A   
   C   
   G   
   T   
   A   
   T   
   G   
   A   
   G   
   T   
   A   
   A   
   A   
   A   
   T   
   T   
   G   
   C   
   T   
   T   
   T   
   A   
   G   
   A   
   A   
   A   
   T   
   G   
   C   
   T   
   A   
   C   
   G   
   A   
   G   
   A   
   T   
   A   
   T   
   T   
   A   
   A   
   C   
   A   
   A   
   A   
   A   
   A   
   T   
   A   
   C   
   A   
   G   
   T   
   C   
   G   
   A   
   T   
   T   
   T   
   C   
   C   
   A   
   A   
   G   
   G   
   A   
   A   
   A   
   C   
   T   
   A   
   T   
   G   
   A   
   T   
   G   
   A   
   T   
   T   
   C   
   A   
   G   
   A   
   A   
   C   
   A   
   A   
   G   
   A   
   A   
   C   
   C   
   A   
   G   
   T   
   A   
   G   
   T   
   T   
   T   
   T   
   A   
   C   
   C   
   T   
   G   
   C   
   T   
   C   
   G   
   T   
   T   
   T   
   T   
   C   
   C   
   A   
   A   
   A   
   C   
   T   
   T   
   A   
   C   
   T   
   A   
   G   
   T   
   T   
   A   
   A   
   C   
   G   
   G   
   A   
   A   
   C   
   A   
   A   
   C   
   G   
   G   
   G   
   G   
   A   
   T   
   T   
   G   
   C   
   G   
   G   
   T   
   G   
   G   
   G   
   G   
   A   
   T   
   G   
   G   
   C   
   A   
   A   
   C   
   A   
   A   
   A   
   T   
   A   
   T   
   T   
   C   
   C   
   A   
   C   
   C   
   A   
   C   
   A   
   C   
   A   
   A   
   T   
   T   
   T   
   A   
   A   
   G   
   T   
   G   
   A   
   A   
   G   
   T   
   G   
   A   
   T   
   T   
   G   
   A   
   T   
   G   
   C   
   A   
   A   
   C   
   A   
   A   
   G   
   T   
   T   
   T   
   A   
   T   
   T   
   G   
   A   
   T   
   G   
   G   
   A   
   C   
   A   
   A   
   T   
   C   
   C   
   T   
   G   
   A   
   T   
   G   
   T   
   A   
   A   
   C   
   G   
   A   
   C   
   G   
   A   
   A   
   T   
   G   
   A   
   A   
   T   
   T   
   G   
   A   
   T   
   G   
   G   
   A   
   A   
   G   
   T   
   G   
   T   
   T   
   A   
   C   
   C   
   T   
   G   
   G   
   A   
   C   
   C   
   A   
   G   
   A   
   T   
   T   
   T   
   T   
   C   
   C   
   A   
   A   
   C   
   A   
   G   
   G   
   C   
   G   
   G   
   T   
   T   
   T   
   A   
   G   
   T   
   G   
   A   
   T   
   G   
   G   
   G   
   G   
   A   
   A   
   A   
   T   
   C   
   A   
   G   
   G   
    G    
   A   
   T   
    T    
   C   
   G   
    C    
    C    
   G   
   A   
   G   
   C   
    A    
   T   
   A   
   T   
   G   
   A   
   A   
   A   
   C   
    G    
   G   
   G   
    G    
   A   
   A   
    A    
   G   
   G   
   T   
   T   
   C   
    G    
   A   
   T   
   T   
   A   
   C   
    A    
   G   
   T   
    T    
   C   
   G   
   T   
   G   
   C   
   A   
   A   
   A   
   A   
   G   
   T   
   T   
   G   
   A   
    A    
    T    
   T   
    G    
   A   
    C    
   T   
   G   
   A   
    A    
   A   
   T   
   G   
    C    
   C   
    G    
   A   
   A   
   T   
   G   
   G   
    A    
   A   
   A   
   A   
   G   
   A   
   A   
   C   
   G   
    T    
   A   
   T   
    T    
    T    
   T   
    A    
   G   
   T   
    A    
   A   
   C   
    T    
   G   
   A   
   A   
   T   
   T   
    G    
   C   
   C   
   T   
   T   
   A   
   T   
   A   
   T   
   G   
   G   
   T   
    G    
   A   
   A   
   T   
   A   
   A   
   A   
   G   
   C   
    C    
   A   
   A   
   A   
   T   
   T   
    A    
   A   
   T   
    C    
   G   
   A   
   A   
   C   
   G   
    A    
   A   
   T   
   T   
   T   
   C   
   T   
   G   
   A   
    A    
    T    
   T   
    A    
   C   
   A   
   C   
   A   
   G   
   A   
   G   
   A   
    T    
   A   
   A   
    A    
   C   
   G   
    A    
   A   
   T   
    T    
   G   
   A   
   A   
   G   
   G   
    A    
   A   
   T   
    T    
   A   
   C   
    T    
   G   
   A   
   T   
   T   
   T   
   G   
   C   
   G   
   G   
   G   
   A   
    T    
   G   
   A   
   A   
   T   
   C   
   T   
   T   
   C   
    T    
   C   
   G   
   T   
   G   
   A   
    A    
   G   
   G   
    C    
   A   
   T   
   G   
   C   
   G   
    G    
   A   
   T   
    T    
   G   
   T   
   C   
   A   
   T   
    C    
   G   
   A   
    T    
    G    
   T   
    T    
   C   
   G   
   T   
   C   
   G   
    A    
   G   
   A   
    T    
   G   
    T    
    G    
   A   
   G   
    T    
   G   
   C   
    C    
   T   
   C   
    T    
   G   
   T   
   T   
    G    
   T   
    G    
   T   
   T   
    A    
   A   
   A   
    C    
   A   
   A   
   C   
   T   
   T   
    G    
   T   
   A   
    C    
   A   
   A   
   A   
    A    
   T   
   G   
   A   
   C   
    T    
    G    
   C   
    C    
   T   
   T   
    A    
   C   
   A   
    A    
   A   
    C    
    A    
   T   
   C   
    T    
   T   
   T   
    T    
   G   
   G   
    T    
   T   
   T   
    T    
   A   
   A   
    C    
   A   
   T   
   G   
   T   
   T   
    A    
   G   
   C   
    C    
   A   
   T   
    C    
   G   
   A   
   A   
   A   
   A   
   A   
   G   
   G   
    C    
   G   
   T   
    A    
   C   
   C   
    G    
   A   
   A   
   A   
    A    
   T   
   T   
   T   
   T   
    A    
   A   
   G   
    C    
   T   
   T   
   G   
   A   
   A   
   A   
   C   
    G    
    T    
   A   
   T   
    T    
    T    
   T   
    A    
   G   
   A   
   A   
   A   
   A   
    T    
   T   
   A   
    C    
    G    
   T   
    C    
   G   
   A   
    G    
   C   
   A   
    C    
   C   
   A   
    A    
    A    
    A    
    A    
    G    
   A   
   A   
   G   
   T   
   C   
   A   
   T   
    T    
   A   
   C   
    T    
    C    
   G   
    C    
   C   
   G   
   G   
   A   
   C   
    G    
    A    
   T   
    T    
   T   
   T   
   T   
   G   
   A   
    C    
   A   
   A   
   A   
    A    
   A   
    C    
   A   
   A   
    A    
   G   
   C   
   A   
   G   
   A   
   A   
   G   
   C   
   A   
    C    
   G   
    G    
   G   
   C   
    G    
   C   
   A   
    T    
   A   
   T   
    T    
   T   
   T   
   A   
   G   
   A   
   A   
   G   
   G   
   T   
    C    
   T   
    C    
   C   
   G   
    A    
   A   
   T   
   T   
   G   
   C   
    C    
   T   
   T   
    A    
   G   
   A   
   T   
   C   
   A   
   T   
   A   
   T   
   C   
    G    
   A   
   T   
   G   
   A   
   A   
   A   
   T   
    C    
   A   
   T   
   T   
   G   
   C   
    C    
   A   
   T   
   T   
   A   
   T   
    C    
   C   
   G   
    T    
   G   
   G   
    A    
   T   
   C   
    A    
    C    
   A   
   A   
   T   
   C   
   A   
   G   
   A   
   T   
   G   
   A   
    C    
   G   
   A   
    A    
   G   
   C   
   A   
   A   
   A   
   A   
   G   
   C   
    A    
   A   
    C    
    G    
    T    
   T   
    A    
   A   
   T   
    T    
   G   
   A   
   A   
   C   
   G   
    C    
   T   
   T   
    T    
   G   
   A   
    A    
   T   
   T   
    T    
   T   
   C   
    A    
   G   
   A   
   T   
   C   
   G   
    T    
   C   
   A   
    A    
    G    
   C   
    G    
   C   
   A   
   A   
   G   
   C   
    G    
   A   
   T   
    T    
   T   
   T   
   A   
   G   
   A   
   T   
   A   
   T   
   G   
   C   
   G   
   T   
   T   
   T   
    A    
   C   
   G   
    C    
   C   
   G   
    T    
   T   
   T   
    A    
   A   
   C   
    A    
   G   
   G   
    C    
   T   
   T   
    A    
   G   
   A   
   A   
   C   
   G   
    C    
   G   
   A   
    C    
   A   
   A   
   A   
   A   
   T   
    T    
   G   
   A   
    A    
   A   
   A   
   T   
   G   
   A   
   A   
   T   
   A   
    T    
   C   
   A   
   A   
   G   
   A   
    G    
   C   
   T   
    A    
   T   
   T   
    G    
   A   
    A    
   A   
    T    
   T   
    C    
   A   
   T   
   T   
   G   
    C    
    G    
   G   
   A   
    C    
   T   
   T   
    A    
    G    
    A    
    G    
   G   
   A   
   C   
   A   
   T   
   T   
    T    
   T   
    A    
   G   
   C   
    C    
    C    
   G   
    C    
   C   
   C   
    A    
   G   
   A   
    A    
   C   
   G   
    C    
   G   
   T   
    C    
   A   
    T    
    T    
   G   
   A   
   A   
   A   
   T   
    C    
   A   
   T   
    T    
   A   
   A   
    A    
   A   
    C    
    A    
   G   
   A   
    G    
   T   
   T   
   A   
    A    
    A    
    T    
   G   
   A   
   C   
    G    
   T   
    T    
    C    
    G    
    C    
    A    
    C    
   A   
    A    
    A    
   A   
   T   
   T   
   T   
   G   
   G   
   T   
   G   
   A   
   T   
    G    
    C    
   A   
   C   
   G   
   A   
   C   
   G   
    C    
   A   
   C   
   A   
   G   
   A   
   A   
    T    
   T   
   A   
    T    
   T   
   A   
   G   
   T   
    A    
   G   
   G   
   T   
   G   
   A   
    A    
   G   
   T   
   C   
    T    
   T   
    A    
   A   
   G   
   T   
   C   
   T   
    T    
   G   
   A   
   A   
   G   
   A   
   T   
   G   
   A   
   A   
   G   
   A   
   T   
    T    
   T   
    A    
   A   
   T   
    C    
   G   
   A   
   A   
   G   
   A   
   A   
   G   
   A   
   A   
   G   
   A   
   A   
    G    
   T   
   C   
   G   
   T   
    G    
   A   
   T   
   T   
   A   
   C   
    A    
    T    
   T   
    A    
   A   
   C   
    C    
   A   
   A   
    T    
   A   
   A   
    C    
   G   
   G   
    C    
   T   
   A   
    C    
   A   
   T   
    T    
   A   
   A   
    G    
   C   
   G   
    G    
    A    
   T   
   G   
   G   
    C    
   A   
   A   
   A   
    C    
    T    
    C    
    T    
   G   
   A   
   A   
   T   
   T   
   C   
    C    
   G   
    G    
   G   
   C   
    G    
   C   
   A   
   A   
   C   
   G   
    C    
    C    
   G   
    T    
   G   
   G   
    C    
   G   
   G   
    A    
   C   
   G   
   T   
   G   
   G   
    T    
   G   
   T   
    C    
   C   
   A   
   A   
   G   
   G   
    C    
   A   
   T   
   G   
   G   
   G   
    C    
    G    
   T   
    T    
   C   
   A   
    T    
    G    
   A   
   T   
   G   
   A   
    T    
   G   
   A   
    T    
   T   
   T   
   C   
   G   
   T   
    G    
   A   
   A   
    A    
   A   
   A   
    C    
   C   
   T   
    A    
    G    
   T   
   T   
   T   
   C   
   T   
   T   
   G   
    T    
   T   
   C   
    A    
   A   
   C   
    A    
   C   
   A   
    C    
   G   
   A   
    C    
   A   
   C   
    G    
   T   
   T   
    A    
   T   
   T   
   A   
   T   
   T   
    C    
   T   
   T   
   T   
   A   
   C   
   G   
   A   
   A   
    C    
   A   
    C    
    T    
   G   
   G   
    G    
   A   
   A   
   A   
   G   
   T   
    T    
   T   
   A   
    C    
   C   
   G   
    A    
   G   
   C   
   A   
   A   
   A   
   A   
   G   
   G   
    T    
   T   
   A   
    C    
   G   
   A   
   A   
   A   
   T   
    C    
   C   
   C   
    T    
   G   
   A   
    A    
   T   
   A   
   C   
   G   
   G   
    T    
    A    
   G   
    A    
   A   
   C   
   A   
   G   
   C   
    A    
   A   
   A   
   A   
   G   
   G   
    A    
   A   
   T   
    T    
   C   
   C   
   A   
    G    
   T   
    G    
   A   
   T   
    T    
   A   
   A   
    C    
   T   
   T   
    A    
   C   
   T   
    G    
   G   
   G   
    A    
   A   
   T   
    T    
   G   
   A   
    T    
   T   
   C   
    T    
    G    
    C    
    A    
   G   
   A   
   A   
   A   
    A    
    A    
   A   
   T   
    T    
   C   
   A   
    A    
   G   
   C   
    G    
   A   
   T   
    T    
   A   
   T   
   T   
    T    
   C   
    T    
   G   
   T   
    T    
    G    
    A    
   G   
   G   
   G   
    C    
    A    
   A   
   A   
   G   
   C   
    G    
   G   
   A   
   A   
   G   
    C    
    A    
   G   
   G   
    T    
    A    
   A   
    A    
   T   
   A   
    C    
    T    
   T   
    G    
   T   
   T   
   C   
   T   
   T   
    C    
   A   
   C   
    A    
   A   
   C   
    C    
    T    
    T    
    A    
    A    
   A   
   A   
   G   
   G   
    A    
   A   
   C   
    C    
   G   
   T   
    C    
   A   
   A   
   A   
   C   
   G   
    G    
   A   
   C   
    A    
    G    
   C   
    C    
   G   
   T   
    A    
   A   
   C   
    A    
   G   
   C   
    C    
   T   
   T   
    T    
    T    
   C   
   T   
   A   
   A   
    T    
   A   
   T   
    C    
   C   
   G   
    T    
   A   
   G   
    T    
   A   
   A   
   T   
   G   
   G   
    T    
    T    
   T   
    A    
   A   
   T   
    C    
   G   
   C   
    C    
   A   
   T   
   T   
    A    
   G   
    C    
   T   
   T   
    A    
   A   
   A   
   A   
   G   
   A   
   A   
   G   
   A   
    T    
   G   
   A   
    T    
   G   
   A   
    G    
    T    
   T   
    A    
    G    
   T   
    T    
   A   
   A   
    C    
   G   
   T   
    G    
    G    
   T   
   A   
    A    
    C    
   G   
   A   
   C   
    T    
    A    
   A   
    T    
   G   
   G   
    C    
    A    
   A   
    T    
    C    
    A    
    G    
   A   
   A   
    G    
   A   
   T   
    G    
   A   
   T   
    T    
   A   
   T   
    C    
   G   
   G   
    A    
   A   
   C   
   A   
   C   
   A   
   T   
    G    
    C    
    A    
   G   
   G   
    A    
   T   
   A   
   C   
   T   
   C   
   T   
   G   
   T   
    C    
   A   
   C   
    A    
   T   
   T   
    T    
    G    
   A   
    T    
   G   
   A   
    A    
    A    
    A    
    T    
    A    
   C   
    T    
   G   
    T    
    A    
   C   
   G   
    T    
   G   
   A   
   T   
   A   
   T   
   G   
   G   
   G   
    C    
   C   
   G   
    G    
   A   
   C   
   A   
   G   
   C   
    A    
   T   
   C   
    C    
   G   
   G   
    T    
   G   
   T   
    T    
    C    
   G   
    G    
   G   
   G   
    A    
   A   
   T   
    C    
   C   
   G   
    T    
    C    
   T   
    C    
   C   
   G   
    C    
   G   
   A   
    A    
    A    
   A   
    T    
   G   
   A   
    T    
   T   
    A    
    T    
   G   
   T   
    G    
   G   
   T   
    C    
   G   
   G   
    C    
    G    
   C   
   A   
   G   
   C   
    G    
    A    
   T   
    T    
    C    
   T   
   G   
    G    
   A   
    T    
   G   
    A    
   A   
    A    
   A   
    T    
    A    
   A   
   A   
   G   
   A   
   A   
   G   
   T   
    C    
    C    
   T   
    A    
   G   
   T   
    C    
   A   
   T   
    T    
   A   
   C   
    T    
   G   
   A   
   A   
   A   
   A   
    T    
   G   
   G   
   T   
   T   
   A   
    T    
   G   
   G   
    T    
   A   
   A   
    G    
   C   
   G   
    T    
   A   
   C   
    A    
   A   
   A   
   A   
   G   
    C    
    T    
   T   
   C   
    T    
   G   
   A   
    A    
   T   
   A   
   T   
   C   
   C   
   A   
   G   
   T   
    T    
   A   
   A   
    A    
   G   
   G   
    A    
   C   
   G   
    T    
   G   
   G   
    C    
   G   
   G   
   T   
   A   
   A   
   A   
   G   
   G   
    G    
   A   
   T   
    T    
   A   
   A   
    G    
   A   
   C   
    A    
    G    
   C   
    A    
   A   
   A   
    T    
   A   
   T   
   C   
   A   
   C   
   T   
   G   
   A   
    G    
   A   
   A   
   A   
   A   
   A   
   T   
   G   
   G   
   T   
   C   
   C   
   A   
    T    
   T   
   A   
   G   
   C   
    T    
   G   
   G   
    T    
   T   
   T   
    A    
   A   
   C   
   C   
   A   
   C   
    G    
   G   
   T   
    C    
    A    
    A    
   T   
   G   
   G   
    G    
    G    
   A   
   T   
   G   
   A   
   A   
   G   
   A   
    T    
   A   
   T   
   C   
    T    
   T   
    A    
    T    
   T   
    G    
   A   
   T   
    T    
   A   
   C   
    G    
    A    
   A   
    C    
   A   
   A   
   A   
   G   
   G   
    C    
   G   
   T   
    C    
   A   
   T   
   T   
   A   
   T   
   C   
   C   
   G   
    C    
   T   
   T   
    T    
   A   
   A   
   C   
   G   
   T   
    T    
    G    
    A    
    T    
    T    
   C   
   T   
   G   
   T   
   T   
   T   
   C   
   T   
   C   
   A   
   A   
   A   
   C   
    A    
   G   
   G   
   A   
   C   
   G   
    C    
    G    
   C   
   A   
   A   
   C   
   A   
   T   
   T   
    A    
   G   
   G   
    G    
   G   
   T   
    T    
   C   
   G   
   T   
   T   
   T   
    A    
   A   
   T   
   G   
   A   
    G    
    A    
   A   
   T   
   G   
   G   
   A   
   A   
   G   
    A    
    T    
   G   
    G    
    T    
    G    
   C   
    C    
   A   
   A   
   A   
   -   
   -   
   -   
   -   
   -   
   -   
   -   
   -   
   -   
   -   
   -   
   -   
   -   
   -   
   -   
   -   
   -   
   -   
   -   
   -   
   -   
   -   
   -   
   -   
   -   
   -   
   -   
   -   
   -   
   -   
   -   
   -   
   -   
   -   
   -   
   -   
   -   
   -   
   -   
   -   
   -   
   -   
   -   
   -   
   -   
   -   
   -   
   -   
   -   
   -   
   -   
   -   
   -   
   -   
   -   
   -   
   -   
   -   
   -   
   -   
   -   
   -   
   -   
   -   
   -   
   -   
   -   
   -   
   -   
   -   
   -   
   -   
 

 
   N59567F   
   A   
   T   
   G   
   A   
   G   
   T   
   G   
   A   
   A   
   G   
   A   
   A   
   A   
   T   
   C   
   A   
   G   
   A   
   G   
   A   
   A   
   A   
   A   
   C   
   A   
   T   
   C   
   C   
   A   
   T   
   G   
   A   
   T   
   G   
   T   
   C   
   A   
   A   
   T   
   C   
   T   
   A   
   A   
   C   
   C   
   A   
   G   
   C   
   G   
   A   
   A   
   A   
   T   
   G   
   A   
   A   
   A   
   G   
   A   
   C   
   T   
   C   
   C   
   T   
   T   
   C   
   A   
   T   
   C   
   G   
   A   
   T   
   T   
   A   
   T   
   G   
   C   
   G   
   A   
   T   
   G   
   A   
   G   
   T   
   G   
   T   
   T   
   A   
   T   
   T   
   G   
   T   
   C   
   G   
   C   
   A   
   C   
   G   
   T   
   G   
   C   
   A   
   C   
   T   
   T   
   C   
   C   
   T   
   G   
   A   
   T   
   G   
   T   
   C   
   A   
   G   
   A   
   G   
   A   
   T   
   G   
   G   
   T   
   C   
   T   
   G   
   A   
   A   
   A   
   C   
   C   
   C   
   G   
   T   
   T   
   C   
   A   
   T   
   C   
   G   
   C   
   C   
   G   
   T   
   A   
   T   
   T   
   T   
   T   
   A   
   T   
   A   
   C   
   G   
   G   
   C   
   A   
   T   
   G   
   A   
   A   
   C   
   G   
   A   
   A   
   T   
   T   
   A   
   G   
   G   
   T   
   G   
   T   
   A   
   A   
   C   
   A   
   C   
   C   
   G   
   G   
   A   
   T   
   A   
   A   
   A   
   G   
   C   
   C   
   C   
   A   
   T   
   A   
   A   
   A   
   A   
   A   
   A   
   T   
   C   
   T   
   G   
   C   
   C   
   A   
   G   
   A   
   A   
   T   
   C   
   G   
   T   
   C   
   G   
   G   
   A   
   G   
   A   
   T   
   G   
   T   
   C   
   A   
   T   
   G   
   G   
   G   
   T   
   A   
   A   
   A   
   T   
   A   
   C   
   C   
   A   
   T   
   C   
   C   
   T   
   C   
   A   
   T   
   G   
   G   
   A   
   G   
   A   
   C   
   A   
   G   
   T   
   G   
   C   
   G   
   A   
   T   
   C   
   T   
   A   
   T   
   G   
   A   
   G   
   T   
   C   
   A   
   A   
   T   
   G   
   G   
   T   
   A   
   C   
   G   
   G   
   A   
   T   
   G   
   G   
   C   
   A   
   C   
   A   
   G   
   C   
   C   
   A   
   T   
   T   
   C   
   A   
   G   
   C   
   T   
   A   
   C   
   C   
   G   
   T   
   T   
   A   
   T   
   A   
   T   
   G   
   C   
   T   
   A   
   G   
   T   
   G   
   G   
   A   
   T   
   G   
   G   
   T   
   C   
   A   
   T   
   G   
   G   
   A   
   A   
   A   
   C   
   T   
   T   
   T   
   G   
   G   
   T   
   T   
   C   
   T   
   G   
   T   
   T   
   G   
   A   
   C   
   G   
   G   
   T   
   G   
   A   
   C   
   G   
   G   
   T   
   G   
   C   
   C   
   G   
   C   
   C   
   G   
   C   
   T   
   A   
   T   
   G   
   C   
   G   
   T   
   T   
   A   
   T   
   A   
   C   
   C   
   G   
   A   
   A   
   G   
   C   
   A   
   C   
   G   
   G   
   A   
   T   
   G   
   A   
   G   
   C   
   A   
   A   
   A   
   A   
   T   
   C   
   G   
   C   
   T   
   A   
   C   
   A   
   G   
   A   
   A   
   A   
   T   
   G   
   C   
   T   
   G   
   C   
   G   
   T   
   G   
   A   
   T   
   A   
   T   
   C   
   A   
   A   
   T   
   A   
   A   
   G   
   A   
   A   
   C   
   A   
   C   
   T   
   G   
   T   
   T   
   G   
   A   
   T   
   T   
   T   
   C   
   C   
   A   
   A   
   A   
   G   
   C   
   A   
   A   
   C   
   T   
   A   
   T   
   G   
   A   
   T   
   G   
   A   
   T   
   A   
   C   
   A   
   G   
   A   
   A   
   A   
   A   
   A   
   G   
   A   
   A   
   C   
   C   
   A   
   G   
   T   
   T   
   G   
   T   
   A   
   C   
   T   
   A   
   C   
   C   
   T   
   G   
   C   
   T   
   C   
   G   
   C   
   T   
   T   
   C   
   C   
   C   
   G   
   A   
   A   
   T   
   C   
   T   
   T   
   T   
   T   
   A   
   G   
   T   
   G   
   A   
   A   
   C   
   G   
   G   
   A   
   A   
   C   
   A   
   A   
   C   
   G   
   G   
   G   
   G   
   A   
   T   
   C   
   G   
   C   
   C   
   G   
   T   
   A   
   G   
   G   
   T   
   A   
   T   
   G   
   G   
   C   
   C   
   A   
   C   
   G   
   A   
   A   
   T   
   A   
   T   
   C   
   C   
   C   
   A   
   C   
   C   
   T   
   C   
   A   
   T   
   A   
   A   
   C   
   T   
   T   
   G   
   T   
   C   
   T   
   G   
   A   
   G   
   G   
   T   
   A   
   G   
   T   
   A   
   G   
   C   
   G   
   G   
   C   
   T   
   A   
   T   
   C   
   G   
   A   
   T   
   T   
   T   
   G   
   C   
   T   
   G   
   A   
   T   
   G   
   G   
   A   
   T   
   A   
   A   
   T   
   C   
   C   
   T   
   G   
   A   
   T   
   G   
   T   
   G   
   A   
   C   
   G   
   A   
   C   
   C   
   A   
   A   
   T   
   G   
   A   
   A   
   T   
   T   
   G   
   A   
   T   
   G   
   G   
   A   
   A   
   G   
   T   
   A   
   T   
   T   
   A   
   C   
   C   
   T   
   G   
   G   
   A   
   C   
   C   
   A   
   G   
   A   
   T   
   T   
   T   
   T   
   C   
   C   
   A   
   A   
   C   
   A   
   G   
   G   
   T   
   G   
   G   
   T   
   T   
   T   
   G   
   G   
   T   
   T   
   A   
   T   
   G   
   G   
   G   
   A   
   A   
   A   
   A   
   T   
   C   
   A   
   G   
   G   
    A    
   A   
   T   
    C    
   C   
   G   
    A    
    A    
   G   
   A   
   G   
   C   
    G    
   T   
   A   
   T   
   G   
   A   
   A   
   A   
   C   
    A    
   G   
   G   
    T    
   A   
   A   
    G    
   G   
   G   
   T   
   T   
   C   
    T    
   A   
   T   
   T   
   A   
   C   
    C    
   G   
   T   
    A    
   C   
   G   
   T   
   G   
   C   
   A   
   A   
   A   
   A   
   G   
   T   
   T   
   G   
   A   
    G    
    A    
   T   
    T    
   A   
    C    
   T   
   G   
   A   
    G    
   A   
   T   
   G   
    C    
   C   
    A    
   A   
   A   
   T   
   G   
   G   
    T    
   A   
   A   
   A   
   G   
   A   
   A   
   C   
   G   
    G    
   A   
   T   
    C    
    A    
   T   
    C    
   G   
   T   
    G    
   A   
   C   
    A    
   G   
   A   
   A   
   T   
   T   
    G    
   C   
   C   
   T   
   T   
   A   
   T   
   A   
   T   
   G   
   G   
   T   
    C    
   A   
   A   
   T   
   A   
   A   
   A   
   G   
   C   
    A    
   A   
   A   
   A   
   T   
   T   
    G    
   A   
   T   
    C    
   G   
   A   
   A   
   C   
   G   
    G    
   A   
   T   
   T   
   T   
   C   
   T   
   G   
   A   
    A    
    C    
   T   
    G    
   C   
   A   
   C   
   A   
   G   
   A   
   G   
   A   
    C    
   A   
   A   
    G    
   C   
   G   
    T    
   A   
   T   
    C    
   G   
   A   
   A   
   G   
   G   
    G    
   A   
   T   
    C    
   A   
   C   
    A    
   G   
   A   
   T   
   T   
   T   
   G   
   C   
   G   
   G   
   G   
   A   
    C    
   G   
   A   
   A   
   T   
   C   
   T   
   T   
   C   
    C    
   C   
   G   
   T   
   G   
   A   
    A    
   G   
   G   
    T    
   A   
   T   
   G   
   C   
   G   
    G    
   A   
   T   
    C    
   G   
   T   
   C   
   A   
   T   
    T    
   G   
   A   
    C    
    A    
   T   
    C    
   C   
   G   
   T   
   C   
   G   
    T    
   G   
   A   
    C    
   G   
    C    
    C    
   A   
   G   
    C    
   G   
   C   
    A    
   T   
   C   
    T    
   G   
   T   
   T   
    A    
   T   
    C    
   T   
   T   
    G    
   A   
   A   
    T    
   A   
   A   
   C   
   T   
   T   
    A    
   T   
   A   
    T    
   A   
   A   
   A   
    T    
   T   
   G   
   A   
   C   
    A    
    T    
   C   
    C    
   T   
   T   
    G    
   C   
   A   
    A    
   A   
    A    
    T    
   T   
   C   
    A    
   T   
   T   
    T    
   G   
   G   
    A    
   T   
   T   
    C    
   A   
   A   
    T    
   A   
   T   
   G   
   T   
   T   
    G    
   G   
   C   
    G    
   A   
   T   
    C    
   G   
   A   
   A   
   A   
   A   
   A   
   G   
   G   
    T    
   G   
   T   
    G    
   C   
   C   
    G    
   A   
   A   
   A   
    G    
   T   
   T   
   T   
   T   
    G    
   A   
   G   
    C    
   T   
   T   
   G   
   A   
   A   
   A   
   C   
    A    
    A    
   A   
   T   
    T    
    C    
   T   
    T    
   G   
   A   
   A   
   A   
   A   
    C    
   T   
   A   
    T    
    A    
   T   
    C    
   G   
   A   
    A    
   C   
   A   
    T    
   C   
   A   
    G    
    C    
    G    
    C    
    G    
   A   
   A   
   G   
   T   
   C   
   A   
   T   
    C    
   A   
   C   
    A    
    A    
   G   
    A    
   C   
   G   
   G   
   A   
   C   
    A    
    G    
   T   
    C    
   T   
   T   
   T   
   G   
   A   
    A    
   A   
   A   
   A   
    G    
   A   
    A    
   A   
   A   
    A    
   G   
   C   
   A   
   G   
   A   
   A   
   G   
   C   
   A   
    A    
   G   
    G    
   G   
   C   
    A    
   C   
   A   
    C    
   A   
   T   
    T    
   T   
   T   
   A   
   G   
   A   
   A   
   G   
   G   
   T   
    T    
   T   
    G    
   C   
   G   
    T    
   A   
   T   
   T   
   G   
   C   
    T    
   T   
   T   
    G    
   G   
   A   
   T   
   C   
   A   
   T   
   A   
   T   
   C   
    G    
   A   
   T   
   G   
   A   
   A   
   A   
   T   
    C    
   A   
   T   
   T   
   G   
   C   
    G    
   A   
   T   
   T   
   A   
   T   
    C    
   C   
   G   
    C    
   G   
   G   
    G    
   T   
   C   
    T    
    A    
   A   
   A   
   T   
   C   
   A   
   G   
   A   
   T   
   G   
   A   
    C    
   G   
   A   
    A    
   G   
   C   
   A   
   A   
   A   
   A   
   G   
   C   
    G    
   A   
    C    
    G    
    A    
   T   
    G    
   A   
   T   
    C    
   G   
   A   
   A   
   C   
   G   
    G    
   T   
   T   
    C    
   G   
   A   
    T    
   T   
   T   
    A    
   T   
   C   
    C    
   G   
   A   
   T   
   C   
   G   
    C    
   C   
   A   
    G    
    T    
   C   
    T    
   C   
   A   
   A   
   G   
   C   
    T    
   A   
   T   
    T    
   T   
   T   
   A   
   G   
   A   
   T   
   A   
   T   
   G   
   C   
   G   
   T   
   T   
   T   
    A    
   C   
   G   
    C    
   C   
   G   
    A    
   T   
   T   
    G    
   A   
   C   
    C    
   G   
   G   
    T    
   T   
   T   
    G    
   G   
   A   
   A   
   C   
   G   
    G    
   G   
   A   
    A    
   A   
   A   
   A   
   A   
   T   
    C    
   G   
   A   
    A    
   A   
   A   
   T   
   G   
   A   
   A   
   T   
   A   
    C    
   C   
   A   
   A   
   G   
   A   
    A    
   C   
   T   
    C    
   T   
   T   
    A    
   A   
    A    
   A   
    C    
   T   
    G    
   A   
   T   
   T   
   G   
    A    
    A    
   G   
   A   
    T    
   T   
   T   
    A    
    A    
    C    
    T    
   G   
   A   
   C   
   A   
   T   
   T   
    C    
   T   
    G    
   G   
   C   
    A    
    A    
   G   
    A    
   C   
   C   
    A    
   G   
   A   
    A    
   C   
   G   
    T    
   G   
   T   
    T    
   A   
    C    
    A    
   G   
   A   
   A   
   A   
   T   
    C    
   A   
   T   
    C    
   A   
   A   
    G    
   A   
    A    
    T    
   G   
   A   
    A    
   T   
   T   
   A   
    G    
    C    
    A    
   G   
   A   
   C   
    T    
   T   
    G    
    A    
    A    
    T    
    C    
    A    
   A   
    C    
    G    
   A   
   T   
   T   
   T   
   G   
   G   
   T   
   G   
   A   
   T   
    A    
    A    
   A   
   C   
   G   
   A   
   C   
   G   
    C    
   A   
   C   
   A   
   G   
   A   
   A   
    T    
   T   
   A   
    C    
   T   
   A   
   G   
   T   
    C    
   G   
   G   
   T   
   G   
   A   
    G    
   G   
   T   
   C   
    T    
   T   
    A    
   A   
   G   
   T   
   C   
   T   
    G    
   G   
   A   
   A   
   G   
   A   
   T   
   G   
   A   
   A   
   G   
   A   
   T   
    T    
   T   
    A    
   A   
   T   
    C    
   G   
   A   
   A   
   G   
   A   
   A   
   G   
   A   
   A   
   G   
   A   
   A   
    A    
   T   
   C   
   G   
   T   
    T    
   A   
   T   
   T   
   A   
   C   
    A    
    T    
   T   
    G    
   A   
   C   
    A    
   A   
   A   
    C    
   A   
   A   
    T    
   G   
   G   
    T    
   T   
   A   
    C    
   A   
   T   
    C    
   A   
   A   
    A    
   C   
   G   
    T    
    T    
   T   
   G   
   G   
    C    
   A   
   A   
   A   
    T    
    A    
    A    
    T    
   G   
   A   
   A   
   T   
   T   
   C   
    A    
   G   
    A    
   G   
   C   
    T    
   C   
   A   
   A   
   C   
   G   
    A    
    A    
   G   
    G    
   G   
   G   
    T    
   G   
   G   
    A    
   C   
   G   
   T   
   G   
   G   
    T    
   G   
   T   
    C    
   C   
   A   
   A   
   G   
   G   
    A    
   A   
   T   
   G   
   G   
   G   
    T    
    A    
   T   
    C    
   C   
   A   
    C    
    A    
   A   
   T   
   G   
   A   
    C    
   G   
   A   
    C    
   T   
   T   
   C   
   G   
   T   
    C    
   A   
   A   
    A    
   A   
   A   
    T    
   C   
   T   
    G    
    G    
   T   
   T   
   T   
   C   
   T   
   T   
   G   
    T    
   T   
   C   
    T    
   A   
   C   
    C    
   C   
   A   
    T    
   G   
   A   
    T    
   A   
   C   
    A    
   T   
   T   
    G    
   T   
   T   
   A   
   T   
   T   
    C    
   T   
   T   
   T   
   A   
   C   
   G   
   A   
   A   
    T    
   A   
    A    
    C    
   G   
   G   
    A    
   A   
   A   
   A   
   G   
   T   
    G    
   T   
   A   
    T    
   C   
   G   
    G    
   G   
   C   
   A   
   A   
   A   
   A   
   G   
   G   
    A    
   T   
   A   
    C    
   G   
   A   
   A   
   A   
   T   
    T    
   C   
   C   
    A    
   G   
   A   
    A    
   T   
   A   
   C   
   G   
   G   
    G    
    C    
   G   
    C    
   A   
   C   
   A   
   G   
   C   
    C    
   A   
   A   
   A   
   G   
   G   
    A    
   A   
   T   
    A    
   C   
   C   
   A   
    A    
   T   
    T    
   A   
   T   
    C    
   A   
   A   
    T    
   T   
   T   
    A    
   C   
   T   
    A    
   G   
   G   
    T    
   A   
   T   
    C    
   G   
   A   
    T    
   T   
   C   
    G    
    A    
    G    
    T    
   G   
   A   
   A   
   A   
    G    
    C    
   A   
   T   
    C    
   C   
   A   
    G    
   G   
   C   
    A    
   A   
   T   
    C    
   A   
   T   
   T   
    G    
   C   
    G    
   G   
   T   
    G    
    A    
    C    
   G   
   G   
   G   
    A    
    G    
   A   
   A   
   G   
   C   
    A    
   G   
   A   
   A   
   G   
    A    
    A    
   G   
   G   
    C    
    C    
   A   
    C    
   T   
   A   
    T    
    C    
   T   
    C    
   T   
   T   
   C   
   T   
   T   
    T    
   A   
   C   
    G    
   A   
   C   
    G    
    C    
    G    
    T    
    C    
   A   
   A   
   G   
   G   
    T    
   A   
   C   
    A    
   G   
   T   
    A    
   A   
   A   
   A   
   C   
   G   
    T    
   A   
   C   
    T    
    T    
   C   
    T    
   G   
   T   
    C    
   A   
   C   
    T    
   G   
   C   
    T    
   T   
   T   
    T    
    G    
   C   
   T   
   A   
   A   
    C    
   A   
   T   
    C    
   C   
   G   
    A    
   A   
   G   
    T    
   A   
   A   
   T   
   G   
   G   
    T    
    C    
   T   
    G    
   A   
   T   
    C    
   G   
   C   
    A    
   A   
   T   
   T   
    G    
   G   
    T    
   T   
   T   
    G    
   A   
   A   
   A   
   G   
   A   
   A   
   G   
   A   
    C    
   G   
   A   
    T    
   G   
   A   
    G    
    C    
   T   
    C    
    G    
   T   
    C    
   A   
   A   
    T    
   G   
   T   
    G    
    C    
   T   
   A   
    C    
    T    
   G   
   A   
   C   
    A    
    G    
   A   
    T    
   G   
   G   
    T    
    C    
   A   
    A    
    T    
    C    
    T    
   A   
   A   
    T    
   A   
   T   
    C    
   A   
   T   
    T    
   A   
   T   
    T    
   G   
   G   
    A    
   A   
   C   
   A   
   C   
   A   
   T   
    A    
    A    
    C    
   G   
   G   
    C    
   T   
   A   
   C   
   T   
   C   
   T   
   G   
   T   
    C    
   A   
   C   
    T    
   T   
   T   
    C    
    A    
   A   
    A    
   G   
   A   
    A    
    G    
    A    
    A    
    G    
   C   
    A    
   G   
    T    
    C    
   C   
   G   
    C    
   G   
   A   
   T   
   A   
   T   
   G   
   G   
   G   
    A    
   C   
   G   
    T    
   A   
   C   
   A   
   G   
   C   
    T    
   T   
   C   
    C    
   G   
   G   
    G    
   G   
   T   
    T    
    A    
   G   
    A    
   G   
   G   
    A    
   A   
   T   
    A    
   C   
   G   
    C    
    C    
   T   
    G    
   C   
   G   
    T    
   G   
   A   
    G    
    G    
   A   
    A    
   G   
   A   
    T    
   T   
    A    
    T    
   G   
   T   
    C    
   G   
   T   
    T    
   G   
   G   
    G    
    G    
   C   
   A   
   G   
   C   
    C    
    T    
   T   
    A    
    A    
   T   
   G   
    A    
   A   
    G    
   G   
    A    
   A   
    G    
   A   
    T    
    C    
   A   
   A   
   G   
   A   
   A   
   G   
   T   
    A    
    T    
   T   
    A    
   G   
   T   
    G    
   A   
   T   
    C    
   A   
   C   
    A    
   G   
   A   
   A   
   A   
   A   
    A    
   G   
   G   
   T   
   T   
   A   
    C    
   G   
   G   
    A    
   A   
   A   
    A    
   C   
   G   
    G    
   A   
   C   
    A    
   A   
   A   
   A   
   G   
    T    
    A    
   T   
   C   
    G    
   G   
   A   
    A    
   T   
   A   
   T   
   C   
   C   
   A   
   G   
   T   
    G    
   A   
   A   
    A    
   G   
   G   
    C    
   C   
   G   
    C    
   G   
   G   
    A    
   G   
   G   
   T   
   A   
   A   
   A   
   G   
   G   
    G    
   A   
   T   
    C    
   A   
   A   
    A    
   A   
   C   
    G    
    G    
   C   
    G    
   A   
   A   
    C    
   A   
   T   
   C   
   A   
   C   
   T   
   G   
   A   
    A    
   A   
   A   
   A   
   A   
   A   
   T   
   G   
   G   
   T   
   C   
   C   
   A   
    C    
   T   
   A   
   G   
   C   
    T    
   G   
   G   
    A    
   T   
   T   
    G    
   A   
   C   
   C   
   A   
   C   
    T    
   G   
   T   
    T    
    T    
    C    
   T   
   G   
   G   
    A    
    G    
   A   
   T   
   G   
   A   
   A   
   G   
   A   
    C    
   A   
   T   
   C   
    A    
   T   
    G    
    C    
   T   
    G    
   A   
   T   
    T    
   A   
   C   
    A    
    G    
   A   
    T    
   A   
   A   
   A   
   G   
   G   
    T    
   G   
   T   
    C    
   A   
   T   
   T   
   A   
   T   
   C   
   C   
   G   
    T    
   T   
   T   
    C    
   A   
   A   
   C   
   G   
   T   
    A    
    T    
    C    
    G    
    A    
   C   
   T   
   G   
   T   
   T   
   T   
   C   
   T   
   C   
   A   
   A   
   A   
   C   
    T    
   G   
   G   
   A   
   C   
   G   
    T    
    T    
   C   
   A   
   A   
   C   
   A   
   T   
   T   
    A    
   G   
   G   
    C    
   G   
   T   
    A    
   C   
   G   
   T   
   T   
   T   
    G    
   A   
   T   
   G   
   A   
    A    
    G    
   A   
   T   
   G   
   G   
   A   
   A   
   G   
    C    
    T    
   G   
    A    
    T    
    A    
   C   
    G    
   A   
   A   
   A   
   G   
   T   
   T   
   G   
   T   
   C   
   A   
   C   
   G   
   A   
   T   
   G   
   G   
   C   
   A   
   G   
   C   
   A   
   G   
   T   
   C   
   G   
   A   
   A   
   C   
   C   
   A   
   G   
   A   
   A   
   G   
   C   
   T   
   G   
   C   
   A   
   G   
   A   
   A   
   C   
   A   
   A   
   A   
   C   
   A   
   G   
   T   
   T   
   G   
   A   
   A   
   G   
   A   
   A   
   C   
   A   
   A   
   G   
   G   
   A   
   T   
   C   
   A   
   G   
   A   
   A   
   G   
   A   
   A   
   -   
   -   
   -   
 

 
   N59910F   
   A   
   T   
   G   
   A   
   G   
   T   
   G   
   A   
   A   
   G   
   A   
   A   
   A   
   T   
   T   
   A   
   A   
   A   
   G   
   A   
   A   
   A   
   A   
   C   
   A   
   T   
   T   
   C   
   A   
   A   
   G   
   A   
   C   
   G   
   T   
   C   
   A   
   A   
   T   
   C   
   T   
   G   
   A   
   C   
   C   
   A   
   G   
   C   
   G   
   A   
   A   
   A   
   T   
   G   
   A   
   A   
   A   
   G   
   A   
   A   
   T   
   C   
   T   
   T   
   T   
   C   
   A   
   T   
   T   
   G   
   A   
   C   
   T   
   A   
   C   
   G   
   C   
   A   
   A   
   T   
   G   
   A   
   G   
   T   
   G   
   T   
   T   
   A   
   T   
   C   
   G   
   T   
   A   
   G   
   C   
   C   
   C   
   G   
   C   
   G   
   C   
   G   
   T   
   T   
   A   
   C   
   C   
   T   
   G   
   A   
   C   
   G   
   T   
   T   
   C   
   G   
   T   
   G   
   A   
   C   
   G   
   G   
   T   
   T   
   T   
   A   
   A   
   A   
   A   
   C   
   C   
   T   
   G   
   T   
   T   
   C   
   A   
   T   
   C   
   G   
   C   
   C   
   G   
   A   
   A   
   T   
   C   
   T   
   T   
   A   
   T   
   A   
   T   
   G   
   G   
   A   
   A   
   T   
   G   
   A   
   A   
   C   
   G   
   A   
   A   
   T   
   T   
   A   
   G   
   G   
   G   
   G   
   T   
   A   
   A   
   C   
   C   
   C   
   C   
   T   
   G   
   A   
   T   
   A   
   A   
   A   
   C   
   C   
   A   
   C   
   A   
   C   
   A   
   A   
   A   
   A   
   A   
   A   
   T   
   C   
   A   
   G   
   C   
   C   
   C   
   G   
   G   
   A   
   T   
   T   
   G   
   T   
   T   
   G   
   G   
   G   
   G   
   A   
   T   
   G   
   T   
   T   
   A   
   T   
   G   
   G   
   G   
   T   
   A   
   A   
   A   
   T   
   A   
   T   
   C   
   A   
   T   
   C   
   C   
   C   
   C   
   A   
   T   
   G   
   G   
   G   
   G   
   A   
   C   
   A   
   G   
   T   
   G   
   C   
   G   
   A   
   T   
   T   
   T   
   A   
   C   
   G   
   A   
   A   
   T   
   C   
   A   
   A   
   T   
   G   
   G   
   T   
   G   
   C   
   G   
   G   
   A   
   T   
   G   
   G   
   C   
   A   
   C   
   A   
   A   
   C   
   C   
   T   
   T   
   T   
   T   
   A   
   G   
   T   
   T   
   A   
   T   
   C   
   G   
   G   
   G   
   C   
   T   
   A   
   T   
   G   
   T   
   T   
   A   
   G   
   T   
   T   
   G   
   A   
   C   
   G   
   G   
   C   
   C   
   A   
   C   
   G   
   G   
   A   
   A   
   A   
   C   
   T   
   T   
   C   
   G   
   G   
   T   
   T   
   C   
   T   
   G   
   T   
   C   
   G   
   A   
   T   
   G   
   G   
   T   
   G   
   A   
   C   
   G   
   G   
   C   
   G   
   C   
   T   
   G   
   C   
   C   
   G   
   C   
   T   
   A   
   T   
   G   
   C   
   G   
   T   
   T   
   A   
   T   
   A   
   C   
   C   
   G   
   A   
   A   
   G   
   C   
   A   
   C   
   G   
   T   
   A   
   T   
   G   
   A   
   G   
   T   
   A   
   A   
   A   
   A   
   T   
   T   
   G   
   C   
   T   
   T   
   T   
   A   
   G   
   A   
   A   
   A   
   T   
   G   
   C   
   T   
   A   
   C   
   G   
   A   
   G   
   A   
   T   
   A   
   T   
   T   
   A   
   A   
   C   
   A   
   A   
   A   
   A   
   A   
   T   
   A   
   C   
   A   
   G   
   T   
   C   
   G   
   A   
   T   
   T   
   T   
   C   
   C   
   A   
   A   
   G   
   G   
   A   
   A   
   A   
   C   
   T   
   A   
   T   
   G   
   A   
   T   
   G   
   A   
   T   
   T   
   C   
   A   
   G   
   A   
   A   
   C   
   A   
   A   
   G   
   A   
   A   
   C   
   C   
   A   
   G   
   T   
   A   
   G   
   T   
   T   
   T   
   T   
   A   
   C   
   C   
   T   
   G   
   C   
   T   
   C   
   G   
   T   
   T   
   T   
   T   
   C   
   C   
   A   
   A   
   A   
   C   
   T   
   T   
   A   
   C   
   T   
   A   
   G   
   T   
   T   
   A   
   A   
   C   
   G   
   G   
   A   
   A   
   C   
   A   
   A   
   C   
   G   
   G   
   G   
   G   
   A   
   T   
   T   
   G   
   C   
   G   
   G   
   T   
   G   
   G   
   G   
   G   
   A   
   T   
   G   
   G   
   C   
   A   
   A   
   C   
   A   
   A   
   A   
   T   
   A   
   T   
   T   
   C   
   C   
   A   
   C   
   C   
   A   
   C   
   A   
   T   
   A   
   A   
   T   
   T   
   T   
   A   
   A   
   G   
   T   
   G   
   A   
   A   
   G   
   T   
   G   
   A   
   T   
   T   
   G   
   A   
   T   
   G   
   C   
   A   
   A   
   C   
   A   
   A   
   G   
   T   
   T   
   T   
   A   
   T   
   T   
   G   
   A   
   T   
   G   
   G   
   A   
   C   
   A   
   A   
   T   
   C   
   C   
   T   
   G   
   A   
   T   
   G   
   T   
   A   
   A   
   C   
   G   
   A   
   C   
   G   
   A   
   A   
   T   
   G   
   A   
   A   
   T   
   T   
   G   
   A   
   T   
   G   
   G   
   A   
   A   
   G   
   T   
   G   
   T   
   T   
   A   
   C   
   C   
   T   
   G   
   G   
   A   
   C   
   C   
   A   
   G   
   A   
   T   
   T   
   T   
   T   
   C   
   C   
   A   
   A   
   C   
   A   
   G   
   G   
   C   
   G   
   G   
   T   
   T   
   T   
   A   
   G   
   T   
   G   
   A   
   T   
   G   
   G   
   G   
   G   
   A   
   A   
   A   
   T   
   C   
   A   
   G   
   G   
    G    
   A   
   T   
    T    
   C   
   G   
    C    
    C    
   G   
   A   
   G   
   C   
    A    
   T   
   A   
   T   
   G   
   A   
   A   
   A   
   C   
    A    
   G   
   G   
    G    
   A   
   A   
    A    
   G   
   G   
   T   
   T   
   C   
    G    
   A   
   T   
   T   
   A   
   C   
    T    
   G   
   T   
    T    
   C   
   G   
   T   
   G   
   C   
   A   
   A   
   A   
   A   
   G   
   T   
   T   
   G   
   A   
    A    
    T    
   T   
    G    
   A   
    C    
   T   
   G   
   A   
    A    
   A   
   T   
   G   
    C    
   C   
    G    
   A   
   A   
   T   
   G   
   G   
    A    
   A   
   A   
   A   
   G   
   A   
   A   
   C   
   G   
    T    
   A   
   T   
    T    
    T    
   T   
    A    
   G   
   T   
    A    
   A   
   C   
    T    
   G   
   A   
   A   
   T   
   T   
    G    
   C   
   C   
   T   
   T   
   A   
   T   
   A   
   T   
   G   
   G   
   T   
    G    
   A   
   A   
   T   
   A   
   A   
   A   
   G   
   C   
    C    
   A   
   A   
   A   
   T   
   T   
    A    
   A   
   T   
    C    
   G   
   A   
   A   
   C   
   G   
    A    
   A   
   T   
   T   
   T   
   C   
   T   
   G   
   A   
    A    
    T    
   T   
    A    
   C   
   A   
   C   
   A   
   G   
   A   
   G   
   A   
    T    
   A   
   A   
    A    
   C   
   G   
    A    
   A   
   T   
    T    
   G   
   A   
   A   
   G   
   G   
    A    
   A   
   T   
    T    
   A   
   C   
    T    
   G   
   A   
   T   
   T   
   T   
   G   
   C   
   G   
   G   
   G   
   A   
    T    
   G   
   A   
   A   
   T   
   C   
   T   
   T   
   C   
    T    
   C   
   G   
   T   
   G   
   A   
    A    
   G   
   G   
    C    
   A   
   T   
   G   
   C   
   G   
    G    
   A   
   T   
    C    
   G   
   T   
   C   
   A   
   T   
    C    
   G   
   A   
    T    
    G    
   T   
    T    
   C   
   G   
   T   
   C   
   G   
    A    
   G   
   A   
    T    
   G   
    T    
    G    
   A   
   G   
    T    
   G   
   C   
    C    
   T   
   C   
    T    
   G   
   T   
   T   
    G    
   T   
    G    
   T   
   T   
    A    
   A   
   A   
    C    
   A   
   A   
   C   
   T   
   T   
    G    
   T   
   A   
    C    
   A   
   A   
   A   
    A    
   T   
   G   
   A   
   C   
    T    
    G    
   C   
    C    
   T   
   T   
    A    
   C   
   A   
    A    
   A   
    C    
    A    
   T   
   C   
    T    
   T   
   T   
    T    
   G   
   G   
    T    
   T   
   T   
    T    
   A   
   A   
    C    
   A   
   T   
   G   
   T   
   T   
    A    
   G   
   C   
    C    
   A   
   T   
    T    
   G   
   A   
   A   
   A   
   A   
   A   
   G   
   G   
    C    
   G   
   T   
    A    
   C   
   C   
    G    
   A   
   A   
   A   
    A    
   T   
   T   
   T   
   T   
    A    
   A   
   G   
    C    
   T   
   T   
   G   
   A   
   A   
   A   
   C   
    G    
    T    
   A   
   T   
    T    
    T    
   T   
    A    
   G   
   A   
   A   
   A   
   A   
    T    
   T   
   A   
    C    
    G    
   T   
    C    
   G   
   A   
    G    
   C   
   A   
    C    
   C   
   A   
    A    
    A    
    A    
    A    
    G    
   A   
   A   
   G   
   T   
   C   
   A   
   T   
    T    
   A   
   C   
    T    
    C    
   G   
    C    
   C   
   G   
   G   
   A   
   C   
    G    
    A    
   T   
    T    
   T   
   T   
   T   
   G   
   A   
    C    
   A   
   A   
   A   
    A    
   A   
    C    
   A   
   A   
    A    
   G   
   C   
   A   
   G   
   A   
   A   
   G   
   C   
   A   
    C    
   G   
    G    
   G   
   C   
    G    
   C   
   A   
    T    
   A   
   T   
    T    
   T   
   T   
   A   
   G   
   A   
   A   
   G   
   G   
   T   
    C    
   T   
    A    
   C   
   G   
    A    
   A   
   T   
   T   
   G   
   C   
    C    
   T   
   T   
    A    
   G   
   A   
   T   
   C   
   A   
   T   
   A   
   T   
   C   
    G    
   A   
   T   
   G   
   A   
   A   
   A   
   T   
    C    
   A   
   T   
   T   
   G   
   C   
    C    
   A   
   T   
   T   
   A   
   T   
    C    
   C   
   G   
    T    
   G   
   G   
    A    
   T   
   C   
    A    
    C    
   A   
   A   
   T   
   C   
   A   
   G   
   A   
   T   
   G   
   A   
    C    
   G   
   A   
    A    
   G   
   C   
   A   
   A   
   A   
   A   
   G   
   C   
    A    
   A   
    C    
    G    
    T    
   T   
    A    
   A   
   T   
    T    
   G   
   A   
   A   
   C   
   G   
    C    
   T   
   T   
    T    
   G   
   A   
    A    
   T   
   T   
    T    
   T   
   C   
    A    
   G   
   A   
   T   
   C   
   G   
    T    
   C   
   A   
    A    
    G    
   C   
    G    
   C   
   A   
   A   
   G   
   C   
    G    
   A   
   T   
    T    
   T   
   T   
   A   
   G   
   A   
   T   
   A   
   T   
   G   
   C   
   G   
   T   
   T   
   T   
    A    
   C   
   G   
    C    
   C   
   G   
    T    
   T   
   T   
    A    
   A   
   C   
    A    
   G   
   G   
    C    
   T   
   T   
    A    
   G   
   A   
   A   
   C   
   G   
    C    
   G   
   A   
    C    
   A   
   A   
   A   
   A   
   T   
    T    
   G   
   A   
    A    
   A   
   A   
   T   
   G   
   A   
   A   
   T   
   A   
    T    
   C   
   A   
   A   
   G   
   A   
    G    
   C   
   T   
    A    
   T   
   T   
    G    
   A   
    A    
   A   
    T    
   T   
    C    
   A   
   T   
   T   
   G   
    C    
    G    
   G   
   A   
    C    
   T   
   T   
    A    
    G    
    A    
    G    
   G   
   A   
   C   
   A   
   T   
   T   
    T    
   T   
    A    
   G   
   C   
    C    
    C    
   G   
    C    
   C   
   C   
    A    
   G   
   A   
    A    
   C   
   G   
    C    
   G   
   T   
    C    
   A   
    T    
    T    
   G   
   A   
   A   
   A   
   T   
    C    
   A   
   T   
    T    
   A   
   A   
    A    
   A   
    C    
    A    
   G   
   A   
    G    
   T   
   T   
   A   
    A    
    A    
    T    
   G   
   A   
   C   
    G    
   T   
    T    
    C    
    G    
    C    
    A    
    C    
   A   
    A    
    A    
   A   
   T   
   T   
   T   
   G   
   G   
   T   
   G   
   A   
   T   
    G    
    C    
   A   
   C   
   G   
   A   
   C   
   G   
    C    
   A   
   C   
   A   
   G   
   A   
   A   
    T    
   T   
   A   
    T    
   T   
   A   
   G   
   T   
    A    
   G   
   G   
   T   
   G   
   A   
    A    
   G   
   T   
   C   
    T    
   T   
    A    
   A   
   G   
   T   
   C   
   T   
    T    
   G   
   A   
   A   
   G   
   A   
   T   
   G   
   A   
   A   
   G   
   A   
   T   
    T    
   T   
    A    
   A   
   T   
    C    
   G   
   A   
   A   
   G   
   A   
   A   
   G   
   A   
   A   
   G   
   A   
   A   
    G    
   T   
   C   
   G   
   T   
    G    
   A   
   T   
   T   
   A   
   C   
    G    
    T    
   T   
    A    
   A   
   C   
    C    
   A   
   A   
    T    
   A   
   A   
    C    
   G   
   G   
    C    
   T   
   A   
    C    
   A   
   T   
    T    
   A   
   A   
    G    
   C   
   G   
    G    
    A    
   T   
   G   
   G   
    C    
   A   
   A   
   A   
    C    
    T    
    C    
    C    
   G   
   A   
   A   
   T   
   T   
   C   
    C    
   G   
    G    
   G   
   C   
    G    
   C   
   A   
   A   
   C   
   G   
    C    
    C    
   G   
    C    
   G   
   G   
    T    
   G   
   G   
    A    
   C   
   G   
   T   
   G   
   G   
    T    
   G   
   T   
    C    
   C   
   A   
   A   
   G   
   G   
    G    
   A   
   T   
   G   
   G   
   G   
    C    
    G    
   T   
    T    
   C   
   A   
    T    
    G    
   A   
   T   
   G   
   A   
    T    
   G   
   A   
    T    
   T   
   T   
   C   
   G   
   T   
    G    
   A   
   A   
    A    
   A   
   A   
    C    
   C   
   T   
    A    
    G    
   T   
   T   
   T   
   C   
   T   
   T   
   G   
    T    
   T   
   C   
    A    
   A   
   C   
    A    
   C   
   A   
    C    
   G   
   A   
    C    
   A   
   C   
    G    
   T   
   T   
    A    
   T   
   T   
   A   
   T   
   T   
    C    
   T   
   T   
   T   
   A   
   C   
   G   
   A   
   A   
    C    
   A   
    C    
    T    
   G   
   G   
    G    
   A   
   A   
   A   
   G   
   T   
    T    
   T   
   A   
    C    
   C   
   G   
    A    
   G   
   C   
   A   
   A   
   A   
   A   
   G   
   G   
    T    
   T   
   A   
    C    
   G   
   A   
   A   
   A   
   T   
    C    
   C   
   C   
    T    
   G   
   A   
    A    
   T   
   A   
   C   
   G   
   G   
    T    
    A    
   G   
    A    
   A   
   C   
   A   
   G   
   C   
    A    
   A   
   A   
   A   
   G   
   G   
    A    
   A   
   T   
    T    
   C   
   C   
   A   
    G    
   T   
    G    
   A   
   T   
    T    
   A   
   A   
    C    
   T   
   T   
    A    
   C   
   T   
    G    
   G   
   G   
    A    
   A   
   T   
    T    
   G   
   A   
    T    
   T   
   C   
    T    
    G    
    C    
    A    
   G   
   A   
   A   
   A   
    A    
    A    
   A   
   T   
    T    
   C   
   A   
    A    
   G   
   C   
    G    
   A   
   T   
    T    
   A   
   T   
   T   
    T    
   C   
    T    
   G   
   T   
    T    
    G    
    A    
   G   
   G   
   G   
    C    
    A    
   A   
   A   
   G   
   C   
    G    
   G   
   A   
   A   
   G   
    C    
    A    
   G   
   G   
    T    
    A    
   A   
    A    
   T   
   A   
    C    
    T    
   T   
    G    
   T   
   T   
   C   
   T   
   T   
    C    
   A   
   C   
    A    
   A   
   C   
    C    
    T    
    T    
    A    
    A    
   A   
   A   
   G   
   G   
    A    
   A   
   C   
    C    
   G   
   T   
    C    
   A   
   A   
   A   
   C   
   G   
    G    
   A   
   C   
    A    
    G    
   C   
    C    
   G   
   T   
    A    
   A   
   C   
    A    
   G   
   C   
    C    
   T   
   T   
    T    
    T    
   C   
   T   
   A   
   A   
    T    
   A   
   T   
    C    
   C   
   G   
    T    
   A   
   G   
    T    
   A   
   A   
   T   
   G   
   G   
    A    
    T    
   T   
    A    
   A   
   T   
    C    
   G   
   C   
    C    
   A   
   T   
   T   
    A    
   G   
    C    
   T   
   T   
    A    
   A   
   A   
   A   
   G   
   A   
   A   
   G   
   A   
    T    
   G   
   A   
    T    
   G   
   A   
    G    
    T    
   T   
    A    
    G    
   T   
    T    
   A   
   A   
    C    
   G   
   T   
    G    
    G    
   T   
   A   
    A    
    C    
   G   
   A   
   C   
    T    
    A    
   A   
    T    
   G   
   G   
    C    
    A    
   A   
    T    
    C    
    A    
    G    
   A   
   A   
    G    
   A   
   T   
    G    
   A   
   T   
    T    
   A   
   T   
    C    
   G   
   G   
    A    
   A   
   C   
   A   
   C   
   A   
   T   
    G    
    C    
    A    
   G   
   G   
    A    
   T   
   A   
   C   
   T   
   C   
   T   
   G   
   T   
    C    
   A   
   C   
    G    
   T   
   T   
    T    
    G    
   A   
    T    
   G   
   A   
    A    
    A    
    A    
    T    
    A    
   C   
    T    
   G   
    T    
    A    
   C   
   G   
    T    
   G   
   A   
   T   
   A   
   T   
   G   
   G   
   G   
    C    
   C   
   G   
    G    
   A   
   C   
   A   
   G   
   C   
    A    
   T   
   C   
    A    
   G   
   G   
    T    
   G   
   T   
    T    
    C    
   G   
    G    
   G   
   G   
    A    
   A   
   T   
    C    
   C   
   G   
    T    
    C    
   T   
    C    
   C   
   G   
    C    
   G   
   A   
    A    
    A    
   A   
    T    
   G   
   A   
    T    
   T   
    A    
    T    
   G   
   T   
    G    
   G   
   T   
    C    
   G   
   G   
    T    
    G    
   C   
   A   
   G   
   C   
    G    
    A    
   T   
    T    
    C    
   T   
   G   
    G    
   A   
    T    
   G   
    A    
   A   
    A    
   A   
    C    
    A    
   A   
   A   
   G   
   A   
   A   
   G   
   T   
    C    
    C    
   T   
    A    
   G   
   T   
    C    
   A   
   T   
    T    
   A   
   C   
    T    
   G   
   A   
   A   
   A   
   A   
    T    
   G   
   G   
   T   
   T   
   A   
    T    
   G   
   G   
    T    
   A   
   A   
    G    
   C   
   G   
    T    
   A   
   C   
    A    
   A   
   A   
   A   
   G   
    C    
    T    
   T   
   C   
    T    
   G   
   A   
    A    
   T   
   A   
   T   
   C   
   C   
   A   
   G   
   T   
    T    
   A   
   A   
    A    
   G   
   G   
    A    
   C   
   G   
    T    
   G   
   G   
    C    
   G   
   G   
   T   
   A   
   A   
   A   
   G   
   G   
    G    
   A   
   T   
    T    
   A   
   A   
    G    
   A   
   C   
    A    
    G    
   C   
    A    
   A   
   A   
    T    
   A   
   T   
   C   
   A   
   C   
   T   
   G   
   A   
    G    
   A   
   A   
   A   
   A   
   A   
   T   
   G   
   G   
   T   
   C   
   C   
   A   
    T    
   T   
   A   
   G   
   C   
    T    
   G   
   G   
    T    
   T   
   T   
    A    
   A   
   C   
   C   
   A   
   C   
    G    
   G   
   T   
    C    
    A    
    A    
   T   
   G   
   G   
    G    
    G    
   A   
   T   
   G   
   A   
   A   
   G   
   A   
    T    
   A   
   T   
   C   
    T    
   T   
    A    
    T    
   T   
    G    
   A   
   T   
    T    
   A   
   C   
    G    
    A    
   A   
    C    
   A   
   A   
   A   
   G   
   G   
    C    
   G   
   T   
    C    
   A   
   T   
   T   
   A   
   T   
   C   
   C   
   G   
    C    
   T   
   T   
    T    
   A   
   A   
   C   
   G   
   T   
    T    
    G    
    A    
    T    
    T    
   C   
   T   
   G   
   T   
   T   
   T   
   C   
   T   
   C   
   A   
   A   
   A   
   C   
    A    
   G   
   G   
   A   
   C   
   G   
    C    
    G    
   C   
   A   
   A   
   C   
   A   
   T   
   T   
    A    
   G   
   G   
    G    
   G   
   T   
    T    
   C   
   G   
   T   
   T   
   T   
    A    
   A   
   T   
   G   
   A   
    G    
    A    
   A   
   T   
   G   
   G   
   A   
   A   
   G   
    A    
    T    
   G   
    G    
    T    
    G    
   C   
    C    
   A   
   A   
   A   
   -   
   -   
   -   
   -   
   -   
   -   
   -   
   -   
   -   
   -   
   -   
   -   
   -   
   -   
   -   
   -   
   -   
   -   
   -   
   -   
   -   
   -   
   -   
   -   
   -   
   -   
   -   
   -   
   -   
   -   
   -   
   -   
   -   
   -   
   -   
   -   
   -   
   -   
   -   
   -   
   -   
   -   
   -   
   -   
   -   
   -   
   -   
   -   
   -   
   -   
   -   
   -   
   -   
   -   
   -   
   -   
   -   
   -   
   -   
   -   
   -   
   -   
   -   
   -   
   -   
   -   
   -   
   -   
   -   
   -   
   -   
   -   
 

 
   N59559F   
   A   
   T   
   G   
   A   
   G   
   T   
   G   
   A   
   A   
   G   
   A   
   A   
   A   
   T   
   C   
   A   
   G   
   A   
   G   
   A   
   A   
   A   
   A   
   C   
   A   
   T   
   C   
   C   
   A   
   T   
   G   
   A   
   T   
   G   
   T   
   C   
   A   
   A   
   T   
   C   
   T   
   A   
   A   
   C   
   C   
   A   
   G   
   C   
   G   
   A   
   A   
   A   
   T   
   G   
   A   
   A   
   A   
   G   
   A   
   C   
   T   
   C   
   C   
   T   
   T   
   C   
   A   
   T   
   C   
   G   
   A   
   T   
   T   
   A   
   T   
   G   
   C   
   G   
   A   
   T   
   G   
   A   
   G   
   T   
   G   
   T   
   T   
   A   
   T   
   T   
   G   
   T   
   C   
   G   
   C   
   A   
   C   
   G   
   T   
   G   
   C   
   A   
   C   
   T   
   G   
   C   
   C   
   T   
   G   
   A   
   T   
   G   
   T   
   C   
   A   
   G   
   A   
   G   
   A   
   T   
   G   
   G   
   T   
   C   
   T   
   G   
   A   
   A   
   A   
   C   
   C   
   C   
   G   
   T   
   T   
   C   
   A   
   T   
   C   
   G   
   C   
   C   
   G   
   T   
   A   
   T   
   T   
   T   
   T   
   A   
   T   
   A   
   C   
   G   
   G   
   C   
   A   
   T   
   G   
   A   
   A   
   C   
   G   
   A   
   A   
   T   
   T   
   A   
   G   
   G   
   T   
   G   
   T   
   G   
   A   
   C   
   A   
   C   
   C   
   G   
   G   
   A   
   T   
   A   
   A   
   A   
   G   
   C   
   C   
   C   
   A   
   T   
   A   
   A   
   A   
   A   
   A   
   A   
   T   
   C   
   T   
   G   
   C   
   C   
   A   
   G   
   A   
   A   
   T   
   C   
   G   
   T   
   C   
   G   
   G   
   A   
   G   
   A   
   T   
   G   
   T   
   C   
   A   
   T   
   G   
   G   
   G   
   T   
   A   
   A   
   A   
   T   
   A   
   C   
   C   
   A   
   T   
   C   
   C   
   T   
   C   
   A   
   T   
   G   
   G   
   G   
   G   
   A   
   T   
   A   
   G   
   T   
   G   
   C   
   A   
   A   
   T   
   C   
   T   
   A   
   T   
   G   
   A   
   G   
   T   
   C   
   A   
   A   
   T   
   G   
   G   
   T   
   A   
   C   
   G   
   G   
   A   
   T   
   G   
   G   
   C   
   G   
   C   
   A   
   G   
   C   
   C   
   A   
   T   
   T   
   T   
   A   
   G   
   C   
   T   
   A   
   C   
   C   
   G   
   T   
   T   
   A   
   T   
   A   
   T   
   G   
   C   
   T   
   A   
   G   
   T   
   G   
   G   
   A   
   T   
   G   
   G   
   T   
   C   
   A   
   T   
   G   
   G   
   A   
   A   
   A   
   C   
   T   
   T   
   T   
   G   
   G   
   T   
   T   
   C   
   T   
   G   
   T   
   T   
   G   
   A   
   C   
   G   
   G   
   T   
   G   
   A   
   C   
   G   
   G   
   T   
   G   
   C   
   C   
   G   
   C   
   T   
   G   
   C   
   T   
   A   
   T   
   G   
   C   
   G   
   T   
   T   
   A   
   T   
   A   
   C   
   C   
   G   
   A   
   A   
   G   
   C   
   A   
   C   
   G   
   G   
   A   
   T   
   G   
   A   
   G   
   C   
   A   
   A   
   A   
   A   
   T   
   C   
   G   
   C   
   T   
   A   
   C   
   A   
   G   
   A   
   A   
   A   
   T   
   G   
   C   
   T   
   G   
   C   
   G   
   T   
   G   
   A   
   T   
   A   
   T   
   C   
   A   
   A   
   T   
   A   
   A   
   G   
   A   
   A   
   T   
   A   
   C   
   T   
   G   
   T   
   G   
   G   
   A   
   T   
   T   
   T   
   C   
   C   
   A   
   A   
   A   
   G   
   C   
   A   
   A   
   C   
   T   
   A   
   T   
   G   
   A   
   T   
   G   
   A   
   C   
   A   
   C   
   A   
   G   
   A   
   A   
   A   
   A   
   A   
   G   
   A   
   A   
   C   
   C   
   C   
   G   
   T   
   T   
   G   
   T   
   A   
   T   
   T   
   A   
   C   
   C   
   T   
   G   
   C   
   T   
   C   
   G   
   C   
   T   
   T   
   C   
   C   
   C   
   T   
   A   
   A   
   T   
   C   
   T   
   T   
   T   
   T   
   G   
   G   
   T   
   G   
   A   
   A   
   C   
   G   
   G   
   A   
   A   
   C   
   G   
   A   
   C   
   G   
   G   
   G   
   G   
   A   
   T   
   C   
   G   
   C   
   C   
   G   
   T   
   G   
   G   
   G   
   T   
   A   
   T   
   G   
   G   
   C   
   A   
   A   
   C   
   G   
   A   
   A   
   T   
   A   
   T   
   C   
   C   
   C   
   A   
   C   
   C   
   T   
   C   
   A   
   T   
   A   
   A   
   T   
   T   
   T   
   G   
   T   
   C   
   T   
   G   
   A   
   G   
   G   
   T   
   A   
   G   
   T   
   A   
   G   
   C   
   G   
   G   
   C   
   T   
   A   
   T   
   C   
   G   
   A   
   T   
   T   
   T   
   G   
   C   
   T   
   G   
   A   
   T   
   G   
   G   
   A   
   T   
   A   
   A   
   T   
   C   
   C   
   T   
   G   
   A   
   T   
   G   
   T   
   G   
   A   
   C   
   G   
   A   
   C   
   C   
   A   
   A   
   T   
   G   
   A   
   A   
   T   
   T   
   G   
   A   
   T   
   G   
   G   
   A   
   A   
   G   
   T   
   A   
   T   
   T   
   A   
   C   
   C   
   T   
   G   
   G   
   A   
   C   
   C   
   A   
   G   
   A   
   T   
   T   
   T   
   T   
   C   
   C   
   A   
   A   
   C   
   A   
   G   
   G   
   T   
   G   
   G   
   T   
   T   
   T   
   G   
   G   
   T   
   C   
   A   
   T   
   G   
   G   
   G   
   A   
   A   
   A   
   A   
   T   
   C   
   A   
   G   
   G   
    A    
   A   
   T   
    C    
   C   
   G   
    A    
    A    
   G   
   A   
   G   
   C   
    G    
   T   
   A   
   T   
   G   
   A   
   A   
   A   
   C   
    A    
   G   
   G   
    T    
   A   
   A   
    A    
   G   
   G   
   T   
   T   
   C   
    T    
   A   
   T   
   T   
   A   
   C   
    C    
   G   
   T   
    A    
   C   
   G   
   T   
   G   
   C   
   A   
   A   
   A   
   A   
   G   
   T   
   T   
   G   
   A   
    G    
    A    
   T   
    C    
   A   
    C    
   T   
   G   
   A   
    G    
   A   
   T   
   G   
    C    
   C   
    A    
   A   
   A   
   T   
   G   
   G   
    C    
   A   
   A   
   A   
   G   
   A   
   A   
   C   
   G   
    G    
   A   
   T   
    C    
    C    
   T   
    C    
   G   
   T   
    G    
   A   
   C   
    G    
   G   
   A   
   A   
   T   
   T   
    A    
   C   
   C   
   T   
   T   
   A   
   T   
   A   
   T   
   G   
   G   
   T   
    C    
   A   
   A   
   T   
   A   
   A   
   A   
   G   
   C   
    A    
   A   
   A   
   A   
   T   
   T   
    G    
   A   
   T   
    T    
   G   
   A   
   A   
   C   
   G   
    G    
   A   
   T   
   T   
   T   
   C   
   T   
   G   
   A   
    G    
    T    
   T   
    G    
   C   
   A   
   C   
   A   
   G   
   A   
   G   
   A   
    C    
   A   
   A   
    G    
   C   
   G   
    T    
   A   
   T   
    C    
   G   
   A   
   A   
   G   
   G   
    A    
   A   
   T   
    T    
   A   
   C   
    G    
   G   
   A   
   T   
   T   
   T   
   G   
   C   
   G   
   G   
   G   
   A   
    T    
   G   
   A   
   A   
   T   
   C   
   T   
   T   
   C   
    C    
   C   
   G   
   T   
   G   
   A   
    A    
   G   
   G   
    C    
   A   
   T   
   G   
   C   
   G   
    A    
   A   
   T   
    T    
   G   
   T   
   C   
   A   
   T   
    T    
   G   
   A   
    C    
    G    
   T   
    T    
   C   
   G   
   T   
   C   
   G   
    T    
   G   
   A   
    C    
   G   
    C    
    C    
   A   
   G   
    C    
   G   
   C   
    A    
   T   
   C   
    A    
   G   
   T   
   T   
    A    
   T   
    T    
   T   
   T   
    G    
   A   
   A   
    T    
   A   
   A   
   C   
   T   
   T   
    A    
   T   
   A   
    C    
   A   
   A   
   A   
    T    
   T   
   G   
   A   
   C   
    A    
    T    
   C   
    C    
   T   
   T   
    G    
   C   
   A   
    G    
   A   
    A    
    T    
   T   
   C   
    A    
   T   
   T   
    C    
   G   
   G   
    A    
   T   
   T   
    C    
   A   
   A   
    T    
   A   
   T   
   G   
   T   
   T   
    G    
   G   
   C   
    A    
   A   
   T   
    C    
   G   
   A   
   A   
   A   
   A   
   A   
   G   
   G   
    C    
   G   
   T   
    G    
   C   
   C   
    G    
   A   
   A   
   A   
    G    
   T   
   T   
   T   
   T   
    A    
   A   
   G   
    T    
   T   
   T   
   G   
   A   
   A   
   A   
   C   
    A    
    A    
   A   
   T   
    C    
    C    
   T   
    T    
   G   
   A   
   A   
   A   
   A   
    T    
   T   
   A   
    T    
    A    
   T   
    C    
   G   
   A   
    A    
   C   
   A   
    T    
   C   
   A   
    A    
    C    
    G    
    C    
    G    
   A   
   A   
   G   
   T   
   C   
   A   
   T   
    C    
   A   
   C   
    A    
    C    
   G   
    A    
   C   
   G   
   G   
   A   
   C   
    G    
    G    
   T   
    C    
   T   
   T   
   T   
   G   
   A   
    A    
   A   
   A   
   A   
    G    
   A   
    A    
   A   
   A   
    A    
   G   
   C   
   A   
   G   
   A   
   A   
   G   
   C   
   A   
    A    
   G   
    A    
   G   
   C   
    A    
   C   
   A   
    C    
   A   
   T   
    C    
   T   
   T   
   A   
   G   
   A   
   A   
   G   
   G   
   T   
    T    
   T   
    G    
   C   
   G   
    T    
   A   
   T   
   T   
   G   
   C   
    T    
   T   
   T   
    G    
   G   
   A   
   T   
   C   
   A   
   T   
   A   
   T   
   C   
    G    
   A   
   T   
   G   
   A   
   A   
   A   
   T   
    C    
   A   
   T   
   T   
   G   
   C   
    G    
   A   
   T   
   T   
   A   
   T   
    C    
   C   
   G   
    C    
   G   
   G   
    G    
   T   
   C   
    T    
    A    
   A   
   A   
   T   
   C   
   A   
   G   
   A   
   T   
   G   
   A   
    C    
   G   
   A   
    A    
   G   
   C   
   A   
   A   
   A   
   A   
   G   
   C   
    A    
   A   
    C    
    A    
    A    
   T   
    G    
   A   
   T   
    C    
   G   
   A   
   A   
   C   
   G   
    G    
   T   
   T   
    C    
   G   
   A   
    T    
   T   
   T   
    A    
   T   
   C   
    C    
   G   
   A   
   T   
   C   
   G   
    C    
   C   
   A   
    A    
    T    
   C   
    A    
   C   
   A   
   A   
   G   
   C   
    T    
   A   
   T   
    C    
   T   
   T   
   A   
   G   
   A   
   T   
   A   
   T   
   G   
   C   
   G   
   T   
   T   
   T   
    G    
   C   
   G   
    T    
   C   
   G   
    A    
   T   
   T   
    G    
   A   
   C   
    C    
   G   
   G   
    T    
   T   
   T   
    G    
   G   
   A   
   A   
   C   
   G   
    G    
   G   
   A   
    A    
   A   
   A   
   A   
   A   
   T   
    C    
   G   
   A   
    A    
   A   
   A   
   T   
   G   
   A   
   A   
   T   
   A   
    T    
   C   
   A   
   A   
   G   
   A   
    A    
   C   
   T   
    A    
   T   
   T   
    G    
   A   
    A    
   A   
    C    
   T   
    G    
   A   
   T   
   T   
   G   
    A    
    A    
   G   
   A   
    T    
   T   
   T   
    G    
    A    
    C    
    T    
   G   
   A   
   C   
   A   
   T   
   T   
    C    
   T   
    G    
   G   
   C   
    A    
    A    
   G   
    A    
   C   
   C   
    C    
   G   
   A   
    A    
   C   
   G   
    T    
   G   
   T   
    C    
   A   
    C    
    A    
   G   
   A   
   A   
   A   
   T   
    C    
   A   
   T   
    C    
   A   
   A   
    G    
   A   
    A    
    T    
   G   
   A   
    A    
   T   
   T   
   A   
    G    
    C    
    A    
   G   
   A   
   C   
    T    
   T   
    G    
    A    
    A    
    T    
    C    
    A    
   A   
    C    
    G    
   A   
   T   
   T   
   T   
   G   
   G   
   T   
   G   
   A   
   T   
    A    
    A    
   A   
   C   
   G   
   A   
   C   
   G   
    C    
   A   
   C   
   A   
   G   
   A   
   A   
    T    
   T   
   A   
    C    
   T   
   A   
   G   
   T   
    C    
   G   
   G   
   T   
   G   
   A   
    G    
   G   
   T   
   C   
    T    
   T   
    A    
   A   
   G   
   T   
   C   
   T   
    A    
   G   
   A   
   A   
   G   
   A   
   T   
   G   
   A   
   A   
   G   
   A   
   T   
    T    
   T   
    G    
   A   
   T   
    T    
   G   
   A   
   A   
   G   
   A   
   A   
   G   
   A   
   A   
   G   
   A   
   A   
    A    
   T   
   C   
   G   
   T   
    T    
   A   
   T   
   T   
   A   
   C   
    A    
    T    
   T   
    G    
   A   
   C   
    A    
   A   
   A   
    C    
   A   
   A   
    T    
   G   
   G   
    T    
   T   
   A   
    T    
   A   
   T   
    C    
   A   
   A   
    A    
   C   
   G   
    T    
    T    
   T   
   G   
   G   
    C    
   A   
   A   
   A   
    T    
    A    
    A    
    T    
   G   
   A   
   A   
   T   
   T   
   C   
    A    
   G   
    A    
   G   
   C   
    T    
   C   
   A   
   A   
   C   
   G   
    A    
    A    
   G   
    A    
   G   
   G   
    C    
   G   
   G   
    G    
   C   
   G   
   T   
   G   
   G   
    T    
   G   
   T   
    C    
   C   
   A   
   A   
   G   
   G   
    A    
   A   
   T   
   G   
   G   
   G   
    T    
    A    
   T   
    C    
   C   
   A   
    C    
    A    
   A   
   T   
   G   
   A   
    C    
   G   
   A   
    C    
   T   
   T   
   C   
   G   
   T   
    C    
   A   
   A   
    G    
   A   
   A   
    T    
   C   
   T   
    A    
    G    
   T   
   T   
   T   
   C   
   T   
   T   
   G   
    T    
   T   
   C   
    T    
   A   
   C   
    C    
   C   
   A   
    T    
   G   
   A   
    T    
   A   
   C   
    A    
   T   
   T   
    G    
   T   
   T   
   A   
   T   
   T   
    C    
   T   
   T   
   T   
   A   
   C   
   G   
   A   
   A   
    T    
   A   
    A    
    C    
   G   
   G   
    A    
   A   
   A   
   A   
   G   
   T   
    G    
   T   
   A   
    T    
   C   
   G   
    G    
   G   
   C   
   A   
   A   
   A   
   A   
   G   
   G   
    A    
   T   
   A   
    C    
   G   
   A   
   A   
   A   
   T   
    T    
   C   
   C   
    A    
   G   
   A   
    A    
   T   
   A   
   C   
   G   
   G   
    G    
    C    
   G   
    T    
   A   
   C   
   A   
   G   
   C   
    C    
   A   
   A   
   A   
   G   
   G   
    A    
   A   
   T   
    C    
   C   
   C   
   A   
    A    
   T   
    C    
   A   
   T   
    C    
   A   
   A   
    T    
   T   
   T   
    G    
   C   
   T   
    G    
   G   
   G   
    T    
   A   
   T   
    C    
   G   
   A   
    C    
   T   
   C   
    G    
    A    
    G    
    T    
   G   
   A   
   A   
   A   
    G    
    C    
   A   
   T   
    C    
   C   
   A   
    A    
   G   
   C   
    A    
   A   
   T   
    T    
   A   
   T   
   T   
    G    
   C   
    G    
   G   
   T   
    G    
    A    
    C    
   G   
   G   
   G   
    A    
    G    
   A   
   A   
   G   
   C   
    A    
   G   
   A   
   A   
   G   
    A    
    A    
   G   
   G   
    C    
    C    
   A   
    C    
   T   
   A   
    T    
    C    
   T   
    T    
   T   
   T   
   C   
   T   
   T   
    T    
   A   
   C   
    G    
   A   
   C   
    G    
    C    
    G    
    T    
    C    
   A   
   A   
   G   
   G   
    T    
   A   
   C   
    A    
   G   
   T   
    T    
   A   
   A   
   A   
   C   
   G   
    T    
   A   
   C   
    G    
    T    
   C   
    T    
   G   
   T   
    C    
   A   
   C   
    T    
   G   
   C   
    T    
   T   
   T   
    C    
    G    
   C   
   T   
   A   
   A   
    C    
   A   
   T   
    T    
   C   
   G   
    A    
   A   
   G   
    C    
   A   
   A   
   T   
   G   
   G   
    C    
    C    
   T   
    G    
   A   
   T   
    T    
   G   
   C   
    A    
   A   
   T   
   T   
    G    
   G   
    T    
   T   
   T   
    G    
   A   
   A   
   A   
   G   
   A   
   A   
   G   
   A   
    T    
   G   
   A   
    C    
   G   
   A   
    A    
    T    
   T   
    A    
    G    
   T   
    C    
   A   
   A   
    T    
   G   
   T   
    T    
    C    
   T   
   A   
    T    
    T    
   G   
   A   
   C   
    A    
    G    
   A   
    C    
   G   
   G   
    A    
    C    
   A   
    G    
    T    
    C    
    A    
   A   
   A   
    T    
   A   
   T   
    C    
   A   
   T   
    C    
   A   
   T   
    T    
   G   
   G   
    C    
   A   
   C   
   A   
   C   
   A   
   T   
    A    
    A    
    C    
   G   
   G   
    C    
   T   
   A   
   C   
   T   
   C   
   T   
   G   
   T   
    G    
   A   
   C   
    C    
   T   
   T   
    C    
    A    
   A   
    A    
   G   
   A   
    A    
    G    
    A    
    A    
    G    
   C   
    A    
   G   
    T    
    C    
   C   
   G   
    C    
   G   
   A   
   T   
   A   
   T   
   G   
   G   
   G   
    A    
   C   
   G   
    T    
   A   
   C   
   A   
   G   
   C   
    T    
   T   
   C   
    C    
   G   
   G   
    C    
   G   
   T   
    C    
    A    
   G   
    A    
   G   
   G   
    G    
   A   
   T   
    A    
   C   
   G   
    T    
    C    
   T   
    G    
   C   
   G   
    T    
   G   
   A   
    G    
    G    
   A   
    A    
   G   
   A   
    T    
   T   
    A    
    C    
   G   
   T   
    C    
   G   
   T   
    T    
   G   
   G   
    G    
    G    
   C   
   A   
   G   
   C   
    C    
    T    
   T   
    G    
    A    
   T   
   G   
    A    
   A   
    G    
   G   
    A    
   A   
    G    
   A   
    C    
    C    
   A   
   A   
   G   
   A   
   A   
   G   
   T   
    A    
    C    
   T   
    G    
   G   
   T   
    G    
   A   
   T   
    T    
   A   
   C   
    A    
   G   
   A   
   A   
   A   
   A   
    A    
   G   
   G   
   T   
   T   
   A   
    C    
   G   
   G   
    A    
   A   
   A   
    A    
   C   
   G   
    G    
   A   
   C   
    C    
   A   
   A   
   A   
   G   
    T    
    A    
   T   
   C   
    G    
   G   
   A   
    A    
   T   
   A   
   T   
   C   
   C   
   A   
   G   
   T   
    G    
   A   
   A   
    A    
   G   
   G   
    C    
   C   
   G   
    C    
   G   
   G   
    A    
   G   
   G   
   T   
   A   
   A   
   A   
   G   
   G   
    G    
   A   
   T   
    C    
   A   
   A   
    A    
   A   
   C   
    G    
    G    
   C   
    G    
   A   
   A   
    T    
   A   
   T   
   C   
   A   
   C   
   T   
   G   
   A   
    A    
   A   
   A   
   A   
   A   
   A   
   T   
   G   
   G   
   T   
   C   
   C   
   A   
    C    
   T   
   A   
   G   
   C   
    C    
   G   
   G   
    A    
   T   
   T   
    G    
   A   
   C   
   C   
   A   
   C   
    T    
   G   
   T   
    T    
    T    
    C    
   T   
   G   
   G   
    A    
    A    
   A   
   T   
   G   
   A   
   A   
   G   
   A   
    C    
   A   
   T   
   C   
    A    
   T   
    G    
    C    
   T   
    G    
   A   
   T   
    T    
   A   
   C   
    A    
    G    
   A   
    T    
   A   
   A   
   A   
   G   
   G   
    T    
   G   
   T   
    G    
   A   
   T   
   T   
   A   
   T   
   C   
   C   
   G   
    T    
   T   
   T   
    C    
   A   
   A   
   C   
   G   
   T   
    A    
    T    
    C    
    G    
    A    
   C   
   T   
   G   
   T   
   T   
   T   
   C   
   T   
   C   
   A   
   A   
   A   
   C   
    T    
   G   
   G   
   A   
   C   
   G   
    T    
    T    
   C   
   A   
   A   
   C   
   A   
   T   
   T   
    G    
   G   
   G   
    T    
   G   
   T   
    A    
   C   
   G   
   T   
   T   
   T   
    G    
   A   
   T   
   G   
   A   
    A    
    G    
   A   
   T   
   G   
   G   
   A   
   A   
   G   
    C    
    T    
   G   
    A    
    T    
    A    
   C   
    G    
   A   
   A   
   A   
   G   
   T   
   C   
   G   
   T   
   C   
   A   
   C   
   A   
   A   
   T   
   G   
   -   
   -   
   -   
   -   
   -   
   -   
   -   
   -   
   -   
   -   
   -   
   -   
   -   
   -   
   -   
   -   
   -   
   -   
   -   
   -   
   -   
   -   
   -   
   -   
   -   
   -   
   -   
   -   
   -   
   -   
   -   
   -   
   -   
   -   
   -   
   -   
   -   
   -   
   -   
   -   
   -   
   -   
   -   
   -   
   -   
   -   
   -   
   -   
   -   
   -   
   -   
   -   
   -   
   -   
   -   
   -   
   -   
   -   
   -   
   -   
 

 
   N59947F   
   A   
   T   
   G   
   A   
   G   
   T   
   G   
   A   
   A   
   G   
   A   
   A   
   A   
   T   
   C   
   A   
   G   
   A   
   G   
   A   
   A   
   A   
   A   
   C   
   A   
   T   
   C   
   C   
   A   
   T   
   G   
   A   
   T   
   G   
   T   
   C   
   A   
   A   
   T   
   C   
   T   
   A   
   A   
   C   
   C   
   A   
   G   
   C   
   G   
   A   
   A   
   A   
   T   
   G   
   A   
   A   
   A   
   G   
   A   
   C   
   T   
   C   
   C   
   T   
   T   
   C   
   A   
   T   
   C   
   G   
   A   
   T   
   T   
   A   
   T   
   G   
   C   
   G   
   A   
   T   
   G   
   A   
   G   
   T   
   G   
   T   
   T   
   A   
   T   
   T   
   G   
   T   
   C   
   G   
   C   
   A   
   C   
   G   
   T   
   G   
   C   
   A   
   C   
   T   
   G   
   C   
   C   
   T   
   G   
   A   
   T   
   G   
   T   
   C   
   A   
   G   
   A   
   G   
   A   
   T   
   G   
   G   
   T   
   C   
   T   
   G   
   A   
   A   
   A   
   C   
   C   
   C   
   G   
   T   
   T   
   C   
   A   
   T   
   C   
   G   
   C   
   C   
   G   
   T   
   A   
   T   
   T   
   T   
   T   
   A   
   T   
   A   
   C   
   G   
   G   
   C   
   A   
   T   
   G   
   A   
   A   
   C   
   G   
   A   
   A   
   T   
   T   
   A   
   G   
   G   
   T   
   G   
   T   
   G   
   A   
   C   
   A   
   C   
   C   
   G   
   G   
   A   
   T   
   A   
   A   
   A   
   G   
   C   
   C   
   C   
   A   
   T   
   A   
   A   
   A   
   A   
   A   
   A   
   T   
   C   
   T   
   G   
   C   
   C   
   A   
   G   
   A   
   A   
   T   
   C   
   G   
   T   
   C   
   G   
   G   
   A   
   G   
   A   
   T   
   G   
   T   
   C   
   A   
   T   
   G   
   G   
   G   
   T   
   A   
   A   
   A   
   T   
   A   
   C   
   C   
   A   
   T   
   C   
   C   
   T   
   C   
   A   
   T   
   G   
   G   
   G   
   G   
   A   
   T   
   A   
   G   
   T   
   G   
   C   
   A   
   A   
   T   
   C   
   T   
   A   
   T   
   G   
   A   
   G   
   T   
   C   
   A   
   A   
   T   
   G   
   G   
   T   
   A   
   C   
   G   
   G   
   A   
   T   
   G   
   G   
   C   
   G   
   C   
   A   
   G   
   C   
   C   
   A   
   T   
   T   
   T   
   A   
   G   
   C   
   T   
   A   
   C   
   C   
   G   
   T   
   T   
   A   
   T   
   A   
   T   
   G   
   C   
   T   
   A   
   G   
   T   
   G   
   G   
   A   
   T   
   G   
   G   
   T   
   C   
   A   
   T   
   G   
   G   
   A   
   A   
   A   
   C   
   T   
   T   
   T   
   G   
   G   
   T   
   T   
   C   
   T   
   G   
   T   
   T   
   G   
   A   
   C   
   G   
   G   
   T   
   G   
   A   
   C   
   G   
   G   
   T   
   G   
   C   
   C   
   G   
   C   
   T   
   G   
   C   
   T   
   A   
   T   
   G   
   C   
   G   
   T   
   T   
   A   
   T   
   A   
   C   
   C   
   G   
   A   
   A   
   G   
   C   
   A   
   C   
   G   
   G   
   A   
   T   
   G   
   A   
   G   
   C   
   A   
   A   
   A   
   A   
   T   
   C   
   G   
   C   
   T   
   A   
   C   
   A   
   G   
   A   
   A   
   A   
   T   
   G   
   C   
   T   
   G   
   C   
   G   
   T   
   G   
   A   
   T   
   A   
   T   
   C   
   A   
   A   
   T   
   A   
   A   
   G   
   A   
   A   
   T   
   A   
   C   
   T   
   G   
   T   
   G   
   G   
   A   
   T   
   T   
   T   
   C   
   C   
   A   
   A   
   A   
   G   
   C   
   A   
   A   
   C   
   T   
   A   
   T   
   G   
   A   
   T   
   G   
   A   
   C   
   A   
   C   
   A   
   G   
   A   
   A   
   A   
   A   
   A   
   G   
   A   
   A   
   C   
   C   
   C   
   G   
   T   
   T   
   G   
   T   
   A   
   T   
   T   
   A   
   C   
   C   
   T   
   G   
   C   
   T   
   C   
   G   
   C   
   T   
   T   
   C   
   C   
   C   
   T   
   A   
   A   
   T   
   C   
   T   
   T   
   T   
   T   
   G   
   G   
   T   
   G   
   A   
   A   
   C   
   G   
   G   
   A   
   A   
   C   
   G   
   A   
   C   
   G   
   G   
   G   
   G   
   A   
   T   
   C   
   G   
   C   
   C   
   G   
   T   
   G   
   G   
   G   
   T   
   A   
   T   
   G   
   G   
   C   
   A   
   A   
   C   
   G   
   A   
   A   
   T   
   A   
   T   
   C   
   C   
   C   
   A   
   C   
   C   
   T   
   C   
   A   
   T   
   A   
   A   
   T   
   T   
   T   
   G   
   T   
   C   
   T   
   G   
   A   
   G   
   G   
   T   
   A   
   G   
   T   
   A   
   G   
   C   
   G   
   G   
   C   
   T   
   A   
   T   
   C   
   G   
   A   
   T   
   T   
   T   
   G   
   C   
   T   
   G   
   A   
   T   
   G   
   G   
   A   
   T   
   A   
   A   
   T   
   C   
   C   
   T   
   G   
   A   
   T   
   G   
   T   
   G   
   A   
   C   
   G   
   A   
   C   
   C   
   A   
   A   
   T   
   G   
   A   
   A   
   T   
   T   
   G   
   A   
   T   
   G   
   G   
   A   
   A   
   G   
   T   
   A   
   T   
   T   
   A   
   C   
   C   
   T   
   G   
   G   
   A   
   C   
   C   
   A   
   G   
   A   
   T   
   T   
   T   
   T   
   C   
   C   
   A   
   A   
   C   
   A   
   G   
   G   
   T   
   G   
   G   
   T   
   T   
   T   
   G   
   G   
   T   
   C   
   A   
   T   
   G   
   G   
   G   
   A   
   A   
   A   
   A   
   T   
   C   
   A   
   G   
   G   
    A    
   A   
   T   
    C    
   C   
   G   
    A    
    A    
   G   
   A   
   G   
   C   
    G    
   T   
   A   
   T   
   G   
   A   
   A   
   A   
   C   
    A    
   G   
   G   
    T    
   A   
   A   
    A    
   G   
   G   
   T   
   T   
   C   
    T    
   A   
   T   
   T   
   A   
   C   
    C    
   G   
   T   
    A    
   C   
   G   
   T   
   G   
   C   
   A   
   A   
   A   
   A   
   G   
   T   
   T   
   G   
   A   
    G    
    A    
   T   
    C    
   A   
    C    
   T   
   G   
   A   
    G    
   A   
   T   
   G   
    C    
   C   
    A    
   A   
   A   
   T   
   G   
   G   
    C    
   A   
   A   
   A   
   G   
   A   
   A   
   C   
   G   
    G    
   A   
   T   
    C    
    C    
   T   
    C    
   G   
   T   
    G    
   A   
   C   
    G    
   G   
   A   
   A   
   T   
   T   
    A    
   C   
   C   
   T   
   T   
   A   
   T   
   A   
   T   
   G   
   G   
   T   
    C    
   A   
   A   
   T   
   A   
   A   
   A   
   G   
   C   
    A    
   A   
   A   
   A   
   T   
   T   
    G    
   A   
   T   
    T    
   G   
   A   
   A   
   C   
   G   
    G    
   A   
   T   
   T   
   T   
   C   
   T   
   G   
   A   
    G    
    T    
   T   
    G    
   C   
   A   
   C   
   A   
   G   
   A   
   G   
   A   
    C    
   A   
   A   
    G    
   C   
   G   
    T    
   A   
   T   
    C    
   G   
   A   
   A   
   G   
   G   
    A    
   A   
   T   
    T    
   A   
   C   
    G    
   G   
   A   
   T   
   T   
   T   
   G   
   C   
   G   
   G   
   G   
   A   
    T    
   G   
   A   
   A   
   T   
   C   
   T   
   T   
   C   
    C    
   C   
   G   
   T   
   G   
   A   
    A    
   G   
   G   
    C    
   A   
   T   
   G   
   C   
   G   
    A    
   A   
   T   
    T    
   G   
   T   
   C   
   A   
   T   
    T    
   G   
   A   
    C    
    G    
   T   
    T    
   C   
   G   
   T   
   C   
   G   
    T    
   G   
   A   
    C    
   G   
    C    
    C    
   A   
   G   
    C    
   G   
   C   
    A    
   T   
   C   
    A    
   G   
   T   
   T   
    A    
   T   
    T    
   T   
   T   
    G    
   A   
   A   
    T    
   A   
   A   
   C   
   T   
   T   
    A    
   T   
   A   
    C    
   A   
   A   
   A   
    T    
   T   
   G   
   A   
   C   
    A    
    T    
   C   
    C    
   T   
   T   
    G    
   C   
   A   
    G    
   A   
    A    
    T    
   T   
   C   
    A    
   T   
   T   
    C    
   G   
   G   
    A    
   T   
   T   
    C    
   A   
   A   
    T    
   A   
   T   
   G   
   T   
   T   
    G    
   G   
   C   
    A    
   A   
   T   
    C    
   G   
   A   
   A   
   A   
   A   
   A   
   G   
   G   
    C    
   G   
   T   
    G    
   C   
   C   
    G    
   A   
   A   
   A   
    G    
   T   
   T   
   T   
   T   
    A    
   A   
   G   
    T    
   T   
   T   
   G   
   A   
   A   
   A   
   C   
    A    
    A    
   A   
   T   
    C    
    C    
   T   
    T    
   G   
   A   
   A   
   A   
   A   
    T    
   T   
   A   
    T    
    A    
   T   
    C    
   G   
   A   
    A    
   C   
   A   
    T    
   C   
   A   
    A    
    C    
    G    
    C    
    G    
   A   
   A   
   G   
   T   
   C   
   A   
   T   
    C    
   A   
   C   
    A    
    C    
   G   
    A    
   C   
   G   
   G   
   A   
   C   
    G    
    G    
   T   
    C    
   T   
   T   
   T   
   G   
   A   
    A    
   A   
   A   
   A   
    G    
   A   
    A    
   A   
   A   
    A    
   G   
   C   
   A   
   G   
   A   
   A   
   G   
   C   
   A   
    A    
   G   
    A    
   G   
   C   
    A    
   C   
   A   
    C    
   A   
   T   
    C    
   T   
   T   
   A   
   G   
   A   
   A   
   G   
   G   
   T   
    T    
   T   
    G    
   C   
   G   
    T    
   A   
   T   
   T   
   G   
   C   
    T    
   T   
   T   
    G    
   G   
   A   
   T   
   C   
   A   
   T   
   A   
   T   
   C   
    G    
   A   
   T   
   G   
   A   
   A   
   A   
   T   
    C    
   A   
   T   
   T   
   G   
   C   
    G    
   A   
   T   
   T   
   A   
   T   
    C    
   C   
   G   
    C    
   G   
   G   
    G    
   T   
   C   
    T    
    A    
   A   
   A   
   T   
   C   
   A   
   G   
   A   
   T   
   G   
   A   
    C    
   G   
   A   
    A    
   G   
   C   
   A   
   A   
   A   
   A   
   G   
   C   
    A    
   A   
    C    
    A    
    A    
   T   
    G    
   A   
   T   
    C    
   G   
   A   
   A   
   C   
   G   
    G    
   T   
   T   
    C    
   G   
   A   
    T    
   T   
   T   
    A    
   T   
   C   
    C    
   G   
   A   
   T   
   C   
   G   
    C    
   C   
   A   
    A    
    T    
   C   
    A    
   C   
   A   
   A   
   G   
   C   
    T    
   A   
   T   
    C    
   T   
   T   
   A   
   G   
   A   
   T   
   A   
   T   
   G   
   C   
   G   
   T   
   T   
   T   
    G    
   C   
   G   
    T    
   C   
   G   
    A    
   T   
   T   
    G    
   A   
   C   
    C    
   G   
   G   
    T    
   T   
   T   
    G    
   G   
   A   
   A   
   C   
   G   
    G    
   G   
   A   
    A    
   A   
   A   
   A   
   A   
   T   
    C    
   G   
   A   
    A    
   A   
   A   
   T   
   G   
   A   
   A   
   T   
   A   
    T    
   C   
   A   
   A   
   G   
   A   
    A    
   C   
   T   
    A    
   T   
   T   
    G    
   A   
    A    
   A   
    C    
   T   
    G    
   A   
   T   
   T   
   G   
    A    
    A    
   G   
   A   
    T    
   T   
   T   
    G    
    A    
    C    
    T    
   G   
   A   
   C   
   A   
   T   
   T   
    C    
   T   
    G    
   G   
   C   
    A    
    A    
   G   
    A    
   C   
   C   
    C    
   G   
   A   
    A    
   C   
   G   
    T    
   G   
   T   
    C    
   A   
    C    
    A    
   G   
   A   
   A   
   A   
   T   
    C    
   A   
   T   
    C    
   A   
   A   
    G    
   A   
    A    
    T    
   G   
   A   
    A    
   T   
   T   
   A   
    G    
    C    
    A    
   G   
   A   
   C   
    T    
   T   
    G    
    A    
    A    
    T    
    C    
    A    
   A   
    C    
    G    
   A   
   T   
   T   
   T   
   G   
   G   
   T   
   G   
   A   
   T   
    A    
    A    
   A   
   C   
   G   
   A   
   C   
   G   
    C    
   A   
   C   
   A   
   G   
   A   
   A   
    T    
   T   
   A   
    C    
   T   
   A   
   G   
   T   
    C    
   G   
   G   
   T   
   G   
   A   
    G    
   G   
   T   
   C   
    T    
   T   
    A    
   A   
   G   
   T   
   C   
   T   
    A    
   G   
   A   
   A   
   G   
   A   
   T   
   G   
   A   
   A   
   G   
   A   
   T   
    T    
   T   
    G    
   A   
   T   
    T    
   G   
   A   
   A   
   G   
   A   
   A   
   G   
   A   
   A   
   G   
   A   
   A   
    A    
   T   
   C   
   G   
   T   
    T    
   A   
   T   
   T   
   A   
   C   
    A    
    T    
   T   
    G    
   A   
   C   
    A    
   A   
   A   
    C    
   A   
   A   
    T    
   G   
   G   
    T    
   T   
   A   
    T    
   A   
   T   
    C    
   A   
   A   
    A    
   C   
   G   
    T    
    T    
   T   
   G   
   G   
    C    
   A   
   A   
   A   
    T    
    A    
    A    
    T    
   G   
   A   
   A   
   T   
   T   
   C   
    A    
   G   
    A    
   G   
   C   
    T    
   C   
   A   
   A   
   C   
   G   
    A    
    A    
   G   
    A    
   G   
   G   
    C    
   G   
   G   
    G    
   C   
   G   
   T   
   G   
   G   
    T    
   G   
   T   
    C    
   C   
   A   
   A   
   G   
   G   
    A    
   A   
   T   
   G   
   G   
   G   
    T    
    A    
   T   
    C    
   C   
   A   
    C    
    A    
   A   
   T   
   G   
   A   
    C    
   G   
   A   
    C    
   T   
   T   
   C   
   G   
   T   
    C    
   A   
   A   
    G    
   A   
   A   
    T    
   C   
   T   
    A    
    G    
   T   
   T   
   T   
   C   
   T   
   T   
   G   
    T    
   T   
   C   
    T    
   A   
   C   
    C    
   C   
   A   
    T    
   G   
   A   
    T    
   A   
   C   
    A    
   T   
   T   
    G    
   T   
   T   
   A   
   T   
   T   
    C    
   T   
   T   
   T   
   A   
   C   
   G   
   A   
   A   
    T    
   A   
    A    
    C    
   G   
   G   
    A    
   A   
   A   
   A   
   G   
   T   
    G    
   T   
   A   
    T    
   C   
   G   
    G    
   G   
   C   
   A   
   A   
   A   
   A   
   G   
   G   
    A    
   T   
   A   
    C    
   G   
   A   
   A   
   A   
   T   
    T    
   C   
   C   
    A    
   G   
   A   
    A    
   T   
   A   
   C   
   G   
   G   
    G    
    C    
   G   
    T    
   A   
   C   
   A   
   G   
   C   
    C    
   A   
   A   
   A   
   G   
   G   
    A    
   A   
   T   
    C    
   C   
   C   
   A   
    A    
   T   
    C    
   A   
   T   
    C    
   A   
   A   
    T    
   T   
   T   
    G    
   C   
   T   
    G    
   G   
   G   
    T    
   A   
   T   
    C    
   G   
   A   
    C    
   T   
   C   
    G    
    A    
    G    
    T    
   G   
   A   
   A   
   A   
    G    
    C    
   A   
   T   
    C    
   C   
   A   
    A    
   G   
   C   
    A    
   A   
   T   
    T    
   A   
   T   
   T   
    G    
   C   
    G    
   G   
   T   
    G    
    A    
    C    
   G   
   G   
   G   
    A    
    G    
   A   
   A   
   G   
   C   
    A    
   G   
   A   
   A   
   G   
    A    
    A    
   G   
   G   
    C    
    C    
   A   
    C    
   T   
   A   
    T    
    C    
   T   
    T    
   T   
   T   
   C   
   T   
   T   
    T    
   A   
   C   
    G    
   A   
   C   
    G    
    C    
    G    
    T    
    C    
   A   
   A   
   G   
   G   
    T    
   A   
   C   
    A    
   G   
   T   
    T    
   A   
   A   
   A   
   C   
   G   
    T    
   A   
   C   
    G    
    T    
   C   
    T    
   G   
   T   
    C    
   A   
   C   
    T    
   G   
   C   
    T    
   T   
   T   
    C    
    G    
   C   
   T   
   A   
   A   
    C    
   A   
   T   
    T    
   C   
   G   
    A    
   A   
   G   
    C    
   A   
   A   
   T   
   G   
   G   
    C    
    C    
   T   
    G    
   A   
   T   
    T    
   G   
   C   
    A    
   A   
   T   
   T   
    G    
   G   
    T    
   T   
   T   
    G    
   A   
   A   
   A   
   G   
   A   
   A   
   G   
   A   
    T    
   G   
   A   
    C    
   G   
   A   
    A    
    T    
   T   
    A    
    G    
   T   
    C    
   A   
   A   
    T    
   G   
   T   
    T    
    C    
   T   
   A   
    T    
    T    
   G   
   A   
   C   
    A    
    G    
   A   
    C    
   G   
   G   
    A    
    C    
   A   
    G    
    T    
    C    
    A    
   A   
   A   
    T    
   A   
   T   
    C    
   A   
   T   
    C    
   A   
   T   
    T    
   G   
   G   
    C    
   A   
   C   
   A   
   C   
   A   
   T   
    A    
    A    
    C    
   G   
   G   
    C    
   T   
   A   
   C   
   T   
   C   
   T   
   G   
   T   
    G    
   A   
   C   
    C    
   T   
   T   
    C    
    A    
   A   
    A    
   G   
   A   
    A    
    G    
    A    
    A    
    G    
   C   
    A    
   G   
    T    
    C    
   C   
   G   
    C    
   G   
   A   
   T   
   A   
   T   
   G   
   G   
   G   
    A    
   C   
   G   
    T    
   A   
   C   
   A   
   G   
   C   
    T    
   T   
   C   
    C    
   G   
   G   
    C    
   G   
   T   
    C    
    A    
   G   
    A    
   G   
   G   
    G    
   A   
   T   
    A    
   C   
   G   
    T    
    C    
   T   
    G    
   C   
   G   
    T    
   G   
   A   
    G    
    G    
   A   
    A    
   G   
   A   
    T    
   T   
    A    
    C    
   G   
   T   
    C    
   G   
   T   
    T    
   G   
   G   
    G    
    G    
   C   
   A   
   G   
   C   
    C    
    T    
   T   
    G    
    A    
   T   
   G   
    A    
   A   
    G    
   G   
    A    
   A   
    G    
   A   
    C    
    C    
   A   
   A   
   G   
   A   
   A   
   G   
   T   
    A    
    C    
   T   
    G    
   G   
   T   
    G    
   A   
   T   
    T    
   A   
   C   
    A    
   G   
   A   
   A   
   A   
   A   
    A    
   G   
   G   
   T   
   T   
   A   
    C    
   G   
   G   
    A    
   A   
   A   
    A    
   C   
   G   
    G    
   A   
   C   
    C    
   A   
   A   
   A   
   G   
    T    
    A    
   T   
   C   
    G    
   G   
   A   
    A    
   T   
   A   
   T   
   C   
   C   
   A   
   G   
   T   
    G    
   A   
   A   
    A    
   G   
   G   
    C    
   C   
   G   
    C    
   G   
   G   
    A    
   G   
   G   
   T   
   A   
   A   
   A   
   G   
   G   
    G    
   A   
   T   
    C    
   A   
   A   
    A    
   A   
   C   
    G    
    G    
   C   
    G    
   A   
   A   
    T    
   A   
   T   
   C   
   A   
   C   
   T   
   G   
   A   
    A    
   A   
   A   
   A   
   A   
   A   
   T   
   G   
   G   
   T   
   C   
   C   
   A   
    C    
   T   
   A   
   G   
   C   
    C    
   G   
   G   
    A    
   T   
   T   
    G    
   A   
   C   
   C   
   A   
   C   
    T    
   G   
   T   
    T    
    T    
    C    
   T   
   G   
   G   
    A    
    A    
   A   
   T   
   G   
   A   
   A   
   G   
   A   
    C    
   A   
   T   
   C   
    A    
   T   
    G    
    C    
   T   
    G    
   A   
   T   
    T    
   A   
   C   
    A    
    G    
   A   
    T    
   A   
   A   
   A   
   G   
   G   
    T    
   G   
   T   
    G    
   A   
   T   
   T   
   A   
   T   
   C   
   C   
   G   
    T    
   T   
   T   
    C    
   A   
   A   
   C   
   G   
   T   
    A    
    T    
    C    
    G    
    A    
   C   
   T   
   G   
   T   
   T   
   T   
   C   
   T   
   C   
   A   
   A   
   A   
   C   
    T    
   G   
   G   
   A   
   C   
   G   
    T    
    T    
   C   
   A   
   A   
   C   
   A   
   T   
   T   
    G    
   G   
   G   
    T    
   G   
   T   
    A    
   C   
   G   
   T   
   T   
   T   
    G    
   A   
   T   
   G   
   A   
    A    
    G    
   A   
   T   
   G   
   G   
   A   
   A   
   G   
    C    
    T    
   G   
    A    
    T    
    A    
   C   
    G    
   A   
   A   
   A   
   G   
   T   
   C   
   G   
   T   
   C   
   A   
   C   
   A   
   A   
   T   
   G   
   -   
   -   
   -   
   -   
   -   
   -   
   -   
   -   
   -   
   -   
   -   
   -   
   -   
   -   
   -   
   -   
   -   
   -   
   -   
   -   
   -   
   -   
   -   
   -   
   -   
   -   
   -   
   -   
   -   
   -   
   -   
   -   
   -   
   -   
   -   
   -   
   -   
   -   
   -   
   -   
   -   
   -   
   -   
   -   
   -   
   -   
   -   
   -   
   -   
   -   
   -   
   -   
   -   
   -   
   -   
   -   
   -   
   -   
   -   
   -   
 

 
   N55317   
   A   
   T   
   G   
   A   
   G   
   T   
   G   
   A   
   A   
   G   
   A   
   A   
   A   
   T   
   C   
   A   
   G   
   A   
   G   
   A   
   A   
   A   
   A   
   C   
   A   
   T   
   C   
   C   
   A   
   T   
   G   
   A   
   T   
   G   
   T   
   C   
   A   
   A   
   T   
   C   
   T   
   A   
   A   
   C   
   C   
   A   
   G   
   C   
   G   
   A   
   A   
   A   
   T   
   G   
   A   
   A   
   A   
   G   
   A   
   C   
   T   
   C   
   C   
   T   
   T   
   C   
   A   
   T   
   C   
   G   
   A   
   T   
   T   
   A   
   T   
   G   
   C   
   G   
   A   
   T   
   G   
   A   
   G   
   T   
   G   
   T   
   T   
   A   
   T   
   T   
   G   
   T   
   C   
   G   
   C   
   A   
   C   
   G   
   T   
   G   
   C   
   A   
   C   
   T   
   G   
   C   
   C   
   T   
   G   
   A   
   T   
   G   
   T   
   C   
   A   
   G   
   A   
   G   
   A   
   T   
   G   
   G   
   T   
   C   
   T   
   G   
   A   
   A   
   A   
   C   
   C   
   C   
   G   
   T   
   T   
   C   
   A   
   T   
   C   
   G   
   C   
   C   
   G   
   T   
   A   
   T   
   T   
   T   
   T   
   A   
   T   
   A   
   C   
   G   
   G   
   C   
   A   
   T   
   G   
   A   
   A   
   C   
   G   
   A   
   A   
   T   
   T   
   A   
   G   
   G   
   T   
   G   
   T   
   G   
   A   
   C   
   A   
   C   
   C   
   G   
   G   
   A   
   T   
   A   
   A   
   A   
   G   
   C   
   C   
   C   
   A   
   T   
   A   
   A   
   A   
   A   
   A   
   A   
   T   
   C   
   T   
   G   
   C   
   C   
   A   
   G   
   A   
   A   
   T   
   C   
   G   
   T   
   C   
   G   
   G   
   A   
   G   
   A   
   T   
   G   
   T   
   C   
   A   
   T   
   G   
   G   
   G   
   T   
   A   
   A   
   A   
   T   
   A   
   C   
   C   
   A   
   T   
   C   
   C   
   T   
   C   
   A   
   T   
   G   
   G   
   G   
   G   
   A   
   T   
   A   
   G   
   T   
   G   
   C   
   A   
   A   
   T   
   C   
   T   
   A   
   T   
   G   
   A   
   G   
   T   
   C   
   A   
   A   
   T   
   G   
   G   
   T   
   A   
   C   
   G   
   G   
   A   
   T   
   G   
   G   
   C   
   G   
   C   
   A   
   G   
   C   
   C   
   A   
   T   
   T   
   T   
   A   
   G   
   C   
   T   
   A   
   C   
   C   
   G   
   T   
   T   
   A   
   T   
   A   
   T   
   G   
   C   
   T   
   A   
   G   
   T   
   G   
   G   
   A   
   T   
   G   
   G   
   T   
   C   
   A   
   T   
   G   
   G   
   A   
   A   
   A   
   C   
   T   
   T   
   T   
   G   
   G   
   T   
   T   
   C   
   T   
   G   
   T   
   T   
   G   
   A   
   C   
   G   
   G   
   T   
   G   
   A   
   C   
   G   
   G   
   T   
   G   
   C   
   C   
   G   
   C   
   T   
   G   
   C   
   T   
   A   
   T   
   G   
   C   
   G   
   T   
   T   
   A   
   T   
   A   
   C   
   C   
   G   
   A   
   A   
   G   
   C   
   A   
   C   
   G   
   G   
   A   
   T   
   G   
   A   
   G   
   C   
   A   
   A   
   A   
   A   
   T   
   C   
   G   
   C   
   T   
   A   
   C   
   A   
   G   
   A   
   A   
   A   
   T   
   G   
   C   
   T   
   G   
   C   
   G   
   T   
   G   
   A   
   T   
   A   
   T   
   C   
   A   
   A   
   T   
   A   
   A   
   G   
   A   
   A   
   T   
   A   
   C   
   T   
   G   
   T   
   T   
   G   
   A   
   T   
   T   
   T   
   C   
   C   
   A   
   A   
   A   
   G   
   C   
   A   
   A   
   C   
   T   
   A   
   T   
   G   
   A   
   T   
   G   
   A   
   C   
   A   
   C   
   A   
   G   
   A   
   A   
   A   
   A   
   A   
   G   
   A   
   A   
   C   
   C   
   C   
   G   
   T   
   T   
   G   
   T   
   A   
   T   
   T   
   A   
   C   
   C   
   T   
   G   
   C   
   T   
   C   
   G   
   C   
   T   
   T   
   C   
   C   
   C   
   T   
   A   
   A   
   T   
   C   
   T   
   T   
   T   
   T   
   G   
   G   
   T   
   G   
   A   
   A   
   C   
   G   
   G   
   A   
   A   
   C   
   G   
   A   
   C   
   G   
   G   
   G   
   G   
   A   
   T   
   C   
   G   
   C   
   C   
   G   
   T   
   G   
   G   
   G   
   T   
   A   
   T   
   G   
   G   
   C   
   A   
   A   
   C   
   G   
   A   
   A   
   T   
   A   
   T   
   C   
   C   
   C   
   G   
   C   
   C   
   T   
   C   
   A   
   T   
   A   
   A   
   T   
   T   
   T   
   G   
   T   
   C   
   T   
   G   
   A   
   G   
   G   
   T   
   A   
   G   
   T   
   A   
   G   
   C   
   G   
   G   
   C   
   T   
   A   
   T   
   C   
   G   
   A   
   T   
   T   
   T   
   G   
   C   
   T   
   G   
   A   
   T   
   G   
   G   
   A   
   T   
   A   
   A   
   T   
   C   
   C   
   T   
   G   
   A   
   T   
   G   
   T   
   G   
   A   
   C   
   G   
   A   
   C   
   C   
   A   
   A   
   T   
   G   
   A   
   A   
   T   
   T   
   G   
   A   
   T   
   G   
   G   
   A   
   A   
   G   
   T   
   A   
   T   
   T   
   A   
   C   
   C   
   T   
   G   
   G   
   G   
   C   
   C   
   A   
   G   
   A   
   T   
   T   
   T   
   T   
   C   
   C   
   A   
   A   
   C   
   A   
   G   
   G   
   T   
   G   
   G   
   T   
   T   
   T   
   A   
   G   
   T   
   C   
   A   
   T   
   G   
   G   
   G   
   A   
   A   
   A   
   A   
   T   
   C   
   A   
   G   
   G   
    A    
   A   
   T   
    C    
   C   
   G   
    A    
    A    
   G   
   A   
   G   
   C   
    G    
   T   
   A   
   T   
   G   
   A   
   A   
   A   
   C   
    A    
   G   
   G   
    T    
   A   
   A   
    A    
   G   
   G   
   T   
   T   
   C   
    T    
   A   
   T   
   T   
   A   
   C   
    C    
   G   
   T   
    A    
   C   
   G   
   T   
   G   
   C   
   A   
   A   
   A   
   A   
   G   
   T   
   T   
   G   
   A   
    G    
    A    
   T   
    C    
   A   
    C    
   T   
   G   
   A   
    G    
   A   
   T   
   G   
    C    
   C   
    A    
   A   
   A   
   T   
   G   
   G   
    C    
   A   
   A   
   A   
   G   
   A   
   A   
   C   
   G   
    G    
   A   
   T   
    C    
    C    
   T   
    C    
   G   
   T   
    G    
   A   
   C   
    G    
   G   
   A   
   A   
   T   
   T   
    A    
   C   
   C   
   T   
   T   
   A   
   T   
   A   
   T   
   G   
   G   
   T   
    C    
   A   
   A   
   T   
   A   
   A   
   A   
   G   
   C   
    A    
   A   
   A   
   A   
   T   
   T   
    G    
   A   
   T   
    T    
   G   
   A   
   A   
   C   
   G   
    G    
   A   
   T   
   T   
   T   
   C   
   T   
   G   
   A   
    G    
    T    
   T   
    G    
   C   
   A   
   C   
   A   
   G   
   A   
   G   
   A   
    C    
   A   
   A   
    G    
   C   
   G   
    T    
   A   
   T   
    T    
   G   
   A   
   A   
   G   
   G   
    A    
   A   
   T   
    T    
   A   
   C   
    G    
   G   
   A   
   T   
   T   
   T   
   G   
   C   
   G   
   G   
   G   
   A   
    T    
   G   
   A   
   A   
   T   
   C   
   T   
   T   
   C   
    C    
   C   
   G   
   T   
   G   
   A   
    A    
   G   
   G   
    C    
   A   
   T   
   G   
   C   
   G   
    A    
   A   
   T   
    T    
   G   
   T   
   C   
   A   
   T   
    T    
   G   
   A   
    C    
    G    
   T   
    T    
   C   
   G   
   T   
   C   
   G   
    T    
   G   
   A   
    C    
   G   
    C    
    C    
   A   
   G   
    C    
   G   
   C   
    A    
   T   
   C   
    A    
   G   
   T   
   T   
    A    
   T   
    T    
   T   
   T   
    G    
   A   
   A   
    T    
   A   
   A   
   C   
   T   
   T   
    A    
   T   
   A   
    C    
   A   
   A   
   A   
    T    
   T   
   G   
   A   
   C   
    A    
    T    
   C   
    C    
   T   
   T   
    G    
   C   
   A   
    G    
   A   
    A    
    T    
   T   
   C   
    A    
   T   
   T   
    T    
   G   
   G   
    A    
   T   
   T   
    C    
   A   
   A   
    T    
   A   
   T   
   G   
   T   
   T   
    G    
   G   
   C   
    A    
   A   
   T   
    C    
   G   
   A   
   A   
   A   
   A   
   A   
   G   
   G   
    C    
   G   
   T   
    G    
   C   
   C   
    G    
   A   
   A   
   A   
    G    
   T   
   T   
   T   
   T   
    A    
   A   
   G   
    T    
   T   
   T   
   G   
   A   
   A   
   A   
   C   
    A    
    A    
   A   
   T   
    C    
    C    
   T   
    T    
   G   
   A   
   A   
   A   
   A   
    C    
   T   
   A   
    T    
    A    
   T   
    C    
   G   
   A   
    A    
   C   
   A   
    T    
   C   
   A   
    A    
    C    
    G    
    C    
    G    
   A   
   A   
   G   
   T   
   C   
   A   
   T   
    C    
   A   
   C   
    A    
    C    
   G   
    A    
   C   
   G   
   G   
   A   
   C   
    G    
    G    
   T   
    C    
   T   
   T   
   T   
   G   
   A   
    A    
   A   
   A   
   A   
    G    
   A   
    A    
   A   
   A   
    A    
   G   
   C   
   A   
   G   
   A   
   A   
   G   
   C   
   A   
    A    
   G   
    A    
   G   
   C   
    A    
   C   
   A   
    C    
   A   
   T   
    C    
   T   
   T   
   A   
   G   
   A   
   A   
   G   
   G   
   T   
    T    
   T   
    G    
   C   
   G   
    T    
   A   
   T   
   T   
   G   
   C   
    T    
   T   
   T   
    G    
   G   
   A   
   T   
   C   
   A   
   T   
   A   
   T   
   C   
    G    
   A   
   T   
   G   
   A   
   A   
   A   
   T   
    C    
   A   
   T   
   T   
   G   
   C   
    G    
   A   
   T   
   T   
   A   
   T   
    C    
   C   
   G   
    C    
   G   
   G   
    G    
   T   
   C   
    T    
    A    
   A   
   A   
   T   
   C   
   A   
   G   
   A   
   T   
   G   
   A   
    T    
   G   
   A   
    A    
   G   
   C   
   A   
   A   
   A   
   A   
   G   
   C   
    A    
   A   
    C    
    A    
    A    
   T   
    G    
   A   
   T   
    C    
   G   
   A   
   A   
   C   
   G   
    G    
   T   
   T   
    C    
   G   
   A   
    T    
   T   
   T   
    A    
   T   
   C   
    C    
   G   
   A   
   T   
   C   
   G   
    C    
   C   
   A   
    A    
    T    
   C   
    A    
   C   
   A   
   A   
   G   
   C   
    T    
   A   
   T   
    C    
   T   
   T   
   A   
   G   
   A   
   T   
   A   
   T   
   G   
   C   
   G   
   T   
   T   
   T   
    G    
   C   
   G   
    T    
   C   
   G   
    A    
   T   
   T   
    G    
   A   
   C   
    C    
   G   
   G   
    T    
   T   
   T   
    G    
   G   
   A   
   A   
   C   
   G   
    G    
   G   
   A   
    A    
   A   
   A   
   A   
   A   
   T   
    C    
   G   
   A   
    A    
   A   
   A   
   T   
   G   
   A   
   A   
   T   
   A   
    T    
   C   
   A   
   A   
   G   
   A   
    A    
   C   
   T   
    A    
   T   
   T   
    G    
   A   
    A    
   A   
    C    
   T   
    G    
   A   
   T   
   T   
   G   
    A    
    A    
   G   
   A   
    T    
   T   
   T   
    G    
    A    
    C    
    T    
   G   
   A   
   C   
   A   
   T   
   T   
    C    
   T   
    G    
   G   
   C   
    A    
    A    
   G   
    A    
   C   
   C   
    C    
   G   
   A   
    A    
   C   
   G   
    T    
   G   
   T   
    C    
   A   
    C    
    A    
   G   
   A   
   A   
   A   
   T   
    C    
   A   
   T   
    C    
   A   
   A   
    G    
   A   
    A    
    T    
   G   
   A   
    A    
   T   
   T   
   A   
    G    
    C    
    A    
   G   
   A   
   C   
    T    
   T   
    G    
    A    
    A    
    T    
    C    
    A    
   A   
    C    
    G    
   A   
   T   
   T   
   T   
   G   
   G   
   T   
   G   
   A   
   T   
    A    
    A    
   A   
   C   
   G   
   A   
   C   
   G   
    C    
   A   
   C   
   A   
   G   
   A   
   A   
    T    
   T   
   A   
    C    
   T   
   A   
   G   
   T   
    C    
   G   
   G   
   T   
   G   
   A   
    G    
   G   
   T   
   C   
    T    
   T   
    A    
   A   
   G   
   T   
   C   
   T   
    A    
   G   
   A   
   A   
   G   
   A   
   T   
   G   
   A   
   A   
   G   
   A   
   T   
    T    
   T   
    G    
   A   
   T   
    T    
   G   
   A   
   A   
   G   
   A   
   A   
   G   
   A   
   A   
   G   
   A   
   A   
    A    
   T   
   C   
   G   
   T   
    T    
   A   
   T   
   T   
   A   
   C   
    A    
    T    
   T   
    G    
   A   
   C   
    A    
   A   
   A   
    C    
   A   
   A   
    T    
   G   
   G   
    T    
   T   
   A   
    T    
   A   
   T   
    C    
   A   
   A   
    A    
   C   
   G   
    T    
    T    
   T   
   G   
   G   
    C    
   A   
   A   
   A   
    T    
    A    
    A    
    T    
   G   
   A   
   A   
   T   
   T   
   C   
    A    
   G   
    A    
   G   
   C   
    T    
   C   
   A   
   A   
   C   
   G   
    A    
    A    
   G   
    A    
   G   
   G   
    C    
   G   
   G   
    G    
   C   
   G   
   T   
   G   
   G   
    T    
   G   
   T   
    C    
   C   
   A   
   A   
   G   
   G   
    A    
   A   
   T   
   G   
   G   
   G   
    T    
    A    
   T   
    C    
   C   
   A   
    C    
    A    
   A   
   T   
   G   
   A   
    C    
   G   
   A   
    C    
   T   
   T   
   C   
   G   
   T   
    C    
   A   
   A   
    G    
   A   
   A   
    T    
   C   
   T   
    A    
    G    
   T   
   T   
   T   
   C   
   T   
   T   
   G   
    T    
   T   
   C   
    T    
   A   
   C   
    C    
   C   
   A   
    T    
   G   
   A   
    T    
   A   
   C   
    A    
   T   
   T   
    G    
   T   
   T   
   A   
   T   
   T   
    C    
   T   
   T   
   T   
   A   
   C   
   G   
   A   
   A   
    T    
   A   
    A    
    C    
   G   
   G   
    A    
   A   
   A   
   A   
   G   
   T   
    G    
   T   
   A   
    T    
   C   
   G   
    G    
   G   
   C   
   A   
   A   
   A   
   A   
   G   
   G   
    A    
   T   
   A   
    C    
   G   
   A   
   A   
   A   
   T   
    T    
   C   
   C   
    A    
   G   
   A   
    A    
   T   
   A   
   C   
   G   
   G   
    G    
    C    
   G   
    T    
   A   
   C   
   A   
   G   
   C   
    C    
   A   
   A   
   A   
   G   
   G   
    A    
   A   
   T   
    C    
   C   
   C   
   A   
    A    
   T   
    C    
   A   
   T   
    C    
   A   
   A   
    T    
   T   
   T   
    G    
   C   
   T   
    G    
   G   
   G   
    T    
   A   
   T   
    C    
   G   
   A   
    C    
   T   
   C   
    G    
    A    
    G    
    T    
   G   
   A   
   A   
   A   
    G    
    C    
   A   
   T   
    C    
   C   
   A   
    A    
   G   
   C   
    A    
   A   
   T   
    T    
   A   
   T   
   T   
    G    
   C   
    G    
   G   
   T   
    G    
    A    
    C    
   G   
   G   
   G   
    A    
    G    
   A   
   A   
   G   
   C   
    A    
   G   
   A   
   A   
   G   
    A    
    A    
   G   
   G   
    C    
    C    
   A   
    C    
   T   
   A   
    T    
    C    
   T   
    C    
   T   
   T   
   C   
   T   
   T   
    T    
   A   
   C   
    G    
   A   
   C   
    G    
    C    
    G    
    T    
    C    
   A   
   A   
   G   
   G   
    T    
   A   
   C   
    A    
   G   
   T   
    T    
   A   
   A   
   A   
   C   
   G   
    T    
   A   
   C   
    G    
    T    
   C   
    T    
   G   
   T   
    C    
   A   
   C   
    T    
   G   
   C   
    T    
   T   
   T   
    C    
    G    
   C   
   T   
   A   
   A   
    C    
   A   
   T   
    C    
   C   
   G   
    A    
   A   
   G   
    C    
   A   
   A   
   T   
   G   
   G   
    C    
    C    
   T   
    G    
   A   
   T   
    T    
   G   
   C   
    A    
   A   
   T   
   T   
    G    
   G   
    T    
   T   
   T   
    G    
   A   
   A   
   A   
   G   
   A   
   A   
   G   
   A   
    T    
   G   
   A   
    C    
   G   
   A   
    A    
    T    
   T   
    A    
    A    
   T   
    C    
   A   
   A   
    T    
   G   
   T   
    T    
    C    
   T   
   A   
    T    
    T    
   G   
   A   
   C   
    A    
    G    
   A   
    C    
   G   
   G   
    A    
    C    
   A   
    G    
    T    
    C    
    A    
   A   
   A   
    T    
   A   
   T   
    C    
   A   
   T   
    C    
   A   
   T   
    T    
   G   
   G   
    C    
   A   
   C   
   A   
   C   
   A   
   T   
    A    
    A    
    C    
   G   
   G   
    C    
   T   
   A   
   C   
   T   
   C   
   T   
   G   
   T   
    G    
   A   
   C   
    C    
   T   
   T   
    C    
    A    
   A   
    A    
   G   
   A   
    A    
    G    
    A    
    A    
    G    
   C   
    A    
   G   
    T    
    C    
   C   
   G   
    C    
   G   
   A   
   T   
   A   
   T   
   G   
   G   
   G   
    A    
   C   
   G   
    T    
   A   
   C   
   A   
   G   
   C   
    T    
   T   
   C   
    C    
   G   
   G   
    C    
   G   
   T   
    C    
    A    
   G   
    A    
   G   
   G   
    G    
   A   
   T   
    A    
   C   
   G   
    T    
    C    
   T   
    G    
   C   
   G   
    T    
   G   
   A   
    G    
    G    
   A   
    A    
   G   
   A   
    T    
   T   
    G    
    C    
   G   
   T   
    C    
   G   
   T   
    T    
   G   
   G   
    A    
    G    
   C   
   A   
   G   
   C   
    C    
    C    
   T   
    G    
    A    
   T   
   G   
    A    
   A   
    G    
   G   
    A    
   A   
    G    
   A   
    C    
    C    
   A   
   A   
   G   
   A   
   A   
   G   
   T   
    A    
    C    
   T   
    A    
   G   
   T   
    G    
   A   
   T   
    T    
   A   
   C   
    A    
   G   
   A   
   A   
   A   
   A   
    A    
   G   
   G   
   T   
   T   
   A   
    C    
   G   
   G   
    A    
   A   
   A   
    A    
   C   
   G   
    G    
   A   
   C   
    C    
   A   
   A   
   A   
   G   
    T    
    A    
   T   
   C   
    G    
   G   
   A   
    G    
   T   
   A   
   T   
   C   
   C   
   A   
   G   
   T   
    G    
   A   
   A   
    A    
   G   
   G   
    C    
   C   
   G   
    C    
   G   
   G   
    A    
   G   
   G   
   T   
   A   
   A   
   A   
   G   
   G   
    A    
   A   
   T   
    C    
   A   
   A   
    A    
   A   
   C   
    G    
    G    
   C   
    G    
   A   
   A   
    T    
   A   
   T   
   C   
   A   
   C   
   T   
   G   
   A   
    A    
   A   
   A   
   A   
   A   
   A   
   T   
   G   
   G   
   T   
   C   
   C   
   A   
    C    
   T   
   A   
   G   
   C   
    C    
   G   
   G   
    A    
   T   
   T   
    G    
   A   
   C   
   C   
   A   
   C   
    T    
   G   
   T   
    T    
    T    
    C    
   T   
   G   
   G   
    A    
    A    
   A   
   T   
   G   
   A   
   A   
   G   
   A   
    C    
   A   
   T   
   C   
    A    
   T   
    G    
    C    
   T   
    G    
   A   
   T   
    T    
   A   
   C   
    A    
    G    
   A   
    T    
   A   
   A   
   A   
   G   
   G   
    T    
   G   
   T   
    G    
   A   
   T   
   T   
   A   
   T   
   C   
   C   
   G   
    T    
   T   
   T   
    C    
   A   
   A   
   C   
   G   
   T   
    A    
    T    
    C    
    G    
    A    
   C   
   T   
   G   
   T   
   T   
   T   
   C   
   T   
   C   
   A   
   A   
   A   
   C   
    T    
   G   
   G   
   A   
   C   
   G   
    T    
    T    
   C   
   A   
   A   
   C   
   A   
   T   
   T   
    G    
   G   
   G   
    T    
   G   
   T   
    A    
   C   
   G   
   T   
   T   
   T   
    G    
   A   
   T   
   G   
   A   
    A    
    G    
   A   
   T   
   G   
   G   
   A   
   A   
   G   
    C    
    T    
   G   
    A    
    T    
    A    
   C   
    G    
   A   
   A   
   A   
   G   
   T   
   C   
   G   
   T   
   C   
   A   
   C   
   A   
   A   
   T   
   G   
   -   
   -   
   -   
   -   
   -   
   -   
   -   
   -   
   -   
   -   
   -   
   -   
   -   
   -   
   -   
   -   
   -   
   -   
   -   
   -   
   -   
   -   
   -   
   -   
   -   
   -   
   -   
   -   
   -   
   -   
   -   
   -   
   -   
   -   
   -   
   -   
   -   
   -   
   -   
   -   
   -   
   -   
   -   
   -   
   -   
   -   
   -   
   -   
   -   
   -   
   -   
   -   
   -   
   -   
   -   
   -   
   -   
   -   
   -   
   -   
 

 
   N52643   
   A   
   T   
   G   
   A   
   G   
   T   
   G   
   A   
   A   
   G   
   A   
   A   
   A   
   T   
   C   
   A   
   G   
   A   
   G   
   A   
   A   
   A   
   A   
   C   
   A   
   T   
   C   
   C   
   A   
   T   
   G   
   A   
   T   
   G   
   T   
   C   
   A   
   A   
   T   
   C   
   T   
   A   
   A   
   C   
   C   
   A   
   G   
   C   
   G   
   A   
   A   
   A   
   T   
   G   
   A   
   A   
   A   
   G   
   A   
   C   
   T   
   C   
   C   
   T   
   T   
   C   
   A   
   T   
   C   
   G   
   A   
   T   
   T   
   A   
   T   
   G   
   C   
   G   
   A   
   T   
   G   
   A   
   G   
   T   
   G   
   T   
   T   
   A   
   T   
   T   
   G   
   T   
   C   
   G   
   C   
   A   
   C   
   G   
   T   
   G   
   C   
   A   
   C   
   T   
   T   
   C   
   C   
   T   
   G   
   A   
   T   
   G   
   T   
   C   
   A   
   G   
   A   
   G   
   A   
   T   
   G   
   G   
   T   
   C   
   T   
   G   
   A   
   A   
   A   
   C   
   C   
   C   
   G   
   T   
   T   
   C   
   A   
   C   
   C   
   G   
   C   
   C   
   G   
   T   
   A   
   T   
   T   
   T   
   T   
   A   
   T   
   A   
   C   
   G   
   G   
   C   
   A   
   T   
   G   
   A   
   A   
   C   
   G   
   A   
   A   
   T   
   T   
   A   
   G   
   G   
   T   
   G   
   T   
   A   
   A   
   C   
   A   
   C   
   C   
   G   
   G   
   A   
   T   
   A   
   A   
   A   
   G   
   C   
   C   
   C   
   A   
   T   
   A   
   A   
   A   
   A   
   A   
   A   
   T   
   C   
   T   
   G   
   C   
   C   
   A   
   G   
   A   
   A   
   T   
   C   
   G   
   T   
   C   
   G   
   G   
   A   
   G   
   A   
   T   
   G   
   T   
   C   
   A   
   T   
   G   
   G   
   G   
   T   
   A   
   A   
   A   
   T   
   A   
   C   
   C   
   A   
   T   
   C   
   C   
   T   
   C   
   A   
   T   
   G   
   G   
   A   
   G   
   A   
   C   
   A   
   G   
   T   
   G   
   C   
   G   
   A   
   T   
   C   
   T   
   A   
   T   
   G   
   A   
   G   
   T   
   C   
   A   
   A   
   T   
   G   
   G   
   T   
   A   
   C   
   G   
   G   
   A   
   T   
   G   
   G   
   C   
   G   
   C   
   A   
   G   
   C   
   C   
   A   
   T   
   T   
   C   
   A   
   G   
   C   
   T   
   A   
   C   
   C   
   G   
   T   
   T   
   A   
   T   
   A   
   T   
   G   
   C   
   T   
   A   
   G   
   T   
   G   
   G   
   A   
   T   
   G   
   G   
   T   
   C   
   A   
   T   
   G   
   G   
   A   
   A   
   A   
   C   
   T   
   T   
   T   
   G   
   G   
   T   
   T   
   C   
   T   
   G   
   T   
   T   
   G   
   A   
   C   
   G   
   G   
   T   
   G   
   A   
   C   
   G   
   G   
   T   
   G   
   C   
   C   
   G   
   C   
   C   
   G   
   C   
   T   
   A   
   T   
   G   
   C   
   G   
   T   
   T   
   A   
   T   
   A   
   C   
   C   
   G   
   A   
   A   
   G   
   C   
   A   
   C   
   G   
   G   
   A   
   T   
   G   
   A   
   G   
   C   
   A   
   A   
   A   
   A   
   T   
   C   
   G   
   C   
   T   
   A   
   C   
   A   
   G   
   A   
   A   
   A   
   T   
   G   
   C   
   T   
   G   
   C   
   G   
   T   
   G   
   A   
   T   
   A   
   T   
   C   
   A   
   A   
   T   
   A   
   A   
   G   
   A   
   A   
   C   
   A   
   C   
   T   
   G   
   T   
   T   
   G   
   A   
   T   
   T   
   T   
   C   
   C   
   A   
   A   
   A   
   G   
   C   
   A   
   A   
   C   
   T   
   A   
   T   
   G   
   A   
   T   
   G   
   A   
   T   
   A   
   C   
   A   
   G   
   A   
   A   
   A   
   A   
   A   
   G   
   A   
   A   
   C   
   C   
   A   
   G   
   T   
   T   
   G   
   T   
   A   
   C   
   T   
   A   
   C   
   C   
   T   
   G   
   C   
   T   
   C   
   G   
   C   
   T   
   T   
   C   
   C   
   C   
   G   
   A   
   A   
   T   
   C   
   T   
   T   
   T   
   T   
   A   
   G   
   T   
   G   
   A   
   A   
   C   
   G   
   G   
   A   
   A   
   C   
   A   
   A   
   C   
   G   
   G   
   G   
   G   
   A   
   T   
   C   
   G   
   C   
   C   
   G   
   T   
   A   
   G   
   G   
   T   
   A   
   T   
   G   
   G   
   C   
   C   
   A   
   C   
   G   
   A   
   A   
   T   
   A   
   T   
   C   
   C   
   C   
   A   
   C   
   C   
   T   
   C   
   A   
   T   
   A   
   A   
   C   
   T   
   T   
   G   
   T   
   C   
   T   
   G   
   A   
   G   
   G   
   T   
   A   
   G   
   T   
   A   
   G   
   C   
   G   
   G   
   C   
   T   
   A   
   T   
   C   
   G   
   A   
   T   
   T   
   T   
   G   
   C   
   T   
   G   
   A   
   T   
   G   
   G   
   A   
   T   
   A   
   A   
   T   
   C   
   C   
   T   
   G   
   A   
   T   
   G   
   T   
   G   
   A   
   C   
   G   
   A   
   C   
   C   
   A   
   A   
   T   
   G   
   A   
   A   
   T   
   T   
   G   
   A   
   T   
   G   
   G   
   A   
   A   
   G   
   T   
   A   
   T   
   T   
   A   
   C   
   C   
   T   
   G   
   G   
   A   
   C   
   C   
   A   
   G   
   A   
   T   
   T   
   T   
   T   
   C   
   C   
   A   
   A   
   C   
   A   
   G   
   G   
   T   
   G   
   G   
   T   
   T   
   T   
   G   
   G   
   T   
   T   
   A   
   T   
   G   
   G   
   G   
   A   
   A   
   A   
   A   
   T   
   C   
   A   
   G   
   G   
    A    
   A   
   T   
    C    
   C   
   G   
    A    
    A    
   G   
   A   
   G   
   C   
    G    
   T   
   A   
   T   
   G   
   A   
   A   
   A   
   C   
    A    
   G   
   G   
    T    
   A   
   A   
    G    
   G   
   G   
   T   
   T   
   C   
    T    
   A   
   T   
   T   
   A   
   C   
    C    
   G   
   T   
    A    
   C   
   G   
   T   
   G   
   C   
   A   
   A   
   A   
   A   
   G   
   T   
   T   
   G   
   A   
    G    
    A    
   T   
    T    
   A   
    C    
   T   
   G   
   A   
    G    
   A   
   T   
   G   
    C    
   C   
    A    
   A   
   A   
   T   
   G   
   G   
    T    
   A   
   A   
   A   
   G   
   A   
   A   
   C   
   G   
    G    
   A   
   T   
    C    
    A    
   T   
    C    
   G   
   T   
    G    
   A   
   C   
    A    
   G   
   A   
   A   
   T   
   T   
    G    
   C   
   C   
   T   
   T   
   A   
   T   
   A   
   T   
   G   
   G   
   T   
    C    
   A   
   A   
   T   
   A   
   A   
   A   
   G   
   C   
    A    
   A   
   A   
   A   
   T   
   T   
    G    
   A   
   T   
    C    
   G   
   A   
   A   
   C   
   G   
    G    
   A   
   T   
   T   
   T   
   C   
   T   
   G   
   A   
    A    
    C    
   T   
    G    
   C   
   A   
   C   
   A   
   G   
   A   
   G   
   A   
    C    
   A   
   A   
    G    
   C   
   G   
    T    
   A   
   T   
    C    
   G   
   A   
   A   
   G   
   G   
    G    
   A   
   T   
    C    
   A   
   C   
    A    
   G   
   A   
   T   
   T   
   T   
   G   
   C   
   G   
   G   
   G   
   A   
    C    
   G   
   A   
   A   
   T   
   C   
   T   
   T   
   C   
    C    
   C   
   G   
   T   
   G   
   A   
    A    
   G   
   G   
    T    
   A   
   T   
   G   
   C   
   G   
    G    
   A   
   T   
    C    
   G   
   T   
   C   
   A   
   T   
    T    
   G   
   A   
    C    
    A    
   T   
    C    
   C   
   G   
   T   
   C   
   G   
    T    
   G   
   A   
    C    
   G   
    C    
    C    
   A   
   G   
    C    
   G   
   C   
    A    
   T   
   C   
    C    
   G   
   T   
   T   
    A    
   T   
    C    
   T   
   T   
    G    
   A   
   A   
    T    
   A   
   A   
   C   
   T   
   T   
    A    
   T   
   A   
    T    
   A   
   A   
   A   
    T    
   T   
   G   
   A   
   C   
    A    
    T    
   C   
    C    
   T   
   T   
    G    
   C   
   A   
    A    
   A   
    A    
    T    
   T   
   C   
    A    
   T   
   T   
    T    
   G   
   G   
    A    
   T   
   T   
    C    
   A   
   A   
    T    
   A   
   T   
   G   
   T   
   T   
    G    
   G   
   C   
    G    
   A   
   T   
    C    
   G   
   A   
   A   
   A   
   A   
   A   
   G   
   G   
    T    
   G   
   T   
    G    
   C   
   C   
    G    
   A   
   A   
   A   
    G    
   T   
   T   
   T   
   T   
    G    
   A   
   G   
    C    
   T   
   T   
   G   
   A   
   A   
   A   
   C   
    A    
    A    
   A   
   T   
    T    
    C    
   T   
    T    
   G   
   A   
   A   
   A   
   A   
    C    
   T   
   A   
    T    
    A    
   T   
    C    
   G   
   A   
    A    
   C   
   A   
    T    
   C   
   A   
    G    
    C    
    G    
    C    
    G    
   A   
   A   
   G   
   T   
   C   
   A   
   T   
    C    
   A   
   C   
    A    
    A    
   G   
    A    
   C   
   G   
   G   
   A   
   C   
    A    
    G    
   T   
    C    
   T   
   T   
   T   
   G   
   A   
    A    
   A   
   A   
   A   
    G    
   A   
    A    
   A   
   A   
    A    
   G   
   C   
   A   
   G   
   A   
   A   
   G   
   C   
   A   
    A    
   G   
    G    
   G   
   C   
    A    
   C   
   A   
    C    
   A   
   T   
    T    
   T   
   T   
   A   
   G   
   A   
   A   
   G   
   G   
   T   
    T    
   T   
    G    
   C   
   G   
    T    
   A   
   T   
   T   
   G   
   C   
    T    
   T   
   T   
    G    
   G   
   A   
   T   
   C   
   A   
   T   
   A   
   T   
   C   
    G    
   A   
   T   
   G   
   A   
   A   
   A   
   T   
    A    
   A   
   T   
   T   
   G   
   C   
    G    
   A   
   T   
   T   
   A   
   T   
    C    
   C   
   G   
    C    
   G   
   G   
    G    
   T   
   C   
    T    
    A    
   A   
   A   
   T   
   C   
   A   
   G   
   A   
   T   
   G   
   A   
    C    
   G   
   A   
    A    
   G   
   C   
   A   
   A   
   A   
   A   
   G   
   C   
    G    
   A   
    C    
    G    
    A    
   T   
    G    
   A   
   T   
    C    
   G   
   A   
   A   
   C   
   G   
    G    
   T   
   T   
    C    
   G   
   A   
    T    
   T   
   T   
    A    
   T   
   C   
    C    
   G   
   A   
   T   
   C   
   G   
    C    
   C   
   A   
    G    
    T    
   C   
    T    
   C   
   A   
   A   
   G   
   C   
    T    
   A   
   T   
    T    
   T   
   T   
   A   
   G   
   A   
   T   
   A   
   T   
   G   
   C   
   G   
   T   
   T   
   T   
    A    
   C   
   G   
    C    
   C   
   G   
    A    
   T   
   T   
    G    
   A   
   C   
    C    
   G   
   G   
    T    
   T   
   T   
    G    
   G   
   A   
   A   
   C   
   G   
    G    
   G   
   A   
    A    
   A   
   A   
   A   
   A   
   T   
    C    
   G   
   A   
    A    
   A   
   A   
   T   
   G   
   A   
   A   
   T   
   A   
    C    
   C   
   A   
   A   
   G   
   A   
    A    
   C   
   T   
    C    
   T   
   T   
    A    
   A   
    A    
   A   
    C    
   T   
    G    
   A   
   T   
   T   
   G   
    A    
    A    
   G   
   A   
    T    
   T   
   T   
    A    
    A    
    C    
    T    
   G   
   A   
   C   
   A   
   T   
   T   
    C    
   T   
    G    
   G   
   C   
    A    
    A    
   G   
    A    
   C   
   C   
    A    
   G   
   A   
    A    
   C   
   G   
    T    
   G   
   T   
    T    
   A   
    C    
    A    
   G   
   A   
   A   
   A   
   T   
    C    
   A   
   T   
    C    
   A   
   A   
    G    
   A   
    A    
    T    
   G   
   A   
    A    
   T   
   T   
   A   
    G    
    C    
    A    
   G   
   A   
   C   
    T    
   T   
    G    
    A    
    A    
    T    
    C    
    A    
   A   
    C    
    G    
   A   
   T   
   T   
   T   
   G   
   G   
   T   
   G   
   A   
   T   
    A    
    A    
   A   
   C   
   G   
   A   
   C   
   G   
    C    
   A   
   C   
   A   
   G   
   A   
   A   
    T    
   T   
   A   
    C    
   T   
   A   
   G   
   T   
    C    
   G   
   G   
   T   
   G   
   A   
    G    
   G   
   T   
   C   
    T    
   T   
    A    
   A   
   G   
   T   
   C   
   T   
    G    
   G   
   A   
   A   
   G   
   A   
   T   
   G   
   A   
   A   
   G   
   A   
   T   
    T    
   T   
    A    
   A   
   T   
    C    
   G   
   A   
   A   
   G   
   A   
   A   
   G   
   A   
   A   
   G   
   A   
   A   
    A    
   T   
   C   
   G   
   T   
    T    
   A   
   T   
   T   
   A   
   C   
    A    
    T    
   T   
    G    
   A   
   C   
    A    
   A   
   A   
    C    
   A   
   A   
    T    
   G   
   G   
    T    
   T   
   A   
    C    
   A   
   T   
    C    
   A   
   A   
    A    
   C   
   G   
    T    
    T    
   T   
   G   
   G   
    C    
   A   
   A   
   A   
    T    
    A    
    A    
    T    
   G   
   A   
   A   
   T   
   T   
   C   
    A    
   G   
    A    
   G   
   C   
    T    
   C   
   A   
   A   
   C   
   G   
    A    
    A    
   G   
    G    
   G   
   G   
    T    
   G   
   G   
    A    
   C   
   G   
   T   
   G   
   G   
    T    
   G   
   T   
    C    
   C   
   A   
   A   
   G   
   G   
    A    
   A   
   T   
   G   
   G   
   G   
    T    
    A    
   T   
    C    
   C   
   A   
    C    
    A    
   A   
   T   
   G   
   A   
    C    
   G   
   A   
    C    
   T   
   T   
   C   
   G   
   T   
    C    
   A   
   A   
    A    
   A   
   A   
    T    
   C   
   T   
    G    
    G    
   T   
   T   
   T   
   C   
   T   
   T   
   G   
    T    
   T   
   C   
    T    
   A   
   C   
    C    
   C   
   A   
    T    
   G   
   A   
    T    
   A   
   C   
    A    
   T   
   T   
    G    
   T   
   T   
   A   
   T   
   T   
    C    
   T   
   T   
   T   
   A   
   C   
   G   
   A   
   A   
    T    
   A   
    A    
    C    
   G   
   G   
    A    
   A   
   A   
   A   
   G   
   T   
    G    
   T   
   A   
    T    
   C   
   G   
    G    
   G   
   C   
   A   
   A   
   A   
   A   
   G   
   G   
    A    
   T   
   A   
    C    
   G   
   A   
   A   
   A   
   T   
    T    
   C   
   C   
    A    
   G   
   A   
    A    
   T   
   A   
   C   
   G   
   G   
    G    
    C    
   G   
    T    
   A   
   C   
   A   
   G   
   C   
    C    
   A   
   A   
   A   
   G   
   G   
    A    
   A   
   T   
    A    
   C   
   C   
   A   
    A    
   T   
    T    
   A   
   T   
    C    
   A   
   A   
    T    
   T   
   T   
    A    
   C   
   T   
    A    
   G   
   G   
    T    
   A   
   T   
    C    
   G   
   A   
    T    
   T   
   C   
    G    
    A    
    G    
    T    
   G   
   A   
   A   
   A   
    G    
    C    
   A   
   T   
    C    
   C   
   A   
    G    
   G   
   C   
    A    
   A   
   T   
    C    
   A   
   T   
   T   
    G    
   C   
    G    
   G   
   T   
    G    
    A    
    C    
   G   
   G   
   G   
    A    
    G    
   A   
   A   
   G   
   C   
    A    
   G   
   A   
   A   
   G   
    A    
    A    
   G   
   G   
    C    
    C    
   A   
    C    
   T   
   A   
    T    
    C    
   T   
    C    
   T   
   T   
   C   
   T   
   T   
    T    
   A   
   C   
    G    
   A   
   C   
    G    
    C    
    G    
    T    
    C    
   A   
   A   
   G   
   G   
    T    
   A   
   C   
    A    
   G   
   T   
    A    
   A   
   A   
   A   
   C   
   G   
    T    
   A   
   C   
    T    
    T    
   C   
    T    
   G   
   T   
    C    
   A   
   C   
    T    
   G   
   C   
    T    
   T   
   T   
    T    
    G    
   C   
   T   
   A   
   A   
    C    
   A   
   T   
    C    
   C   
   G   
    A    
   A   
   G   
    T    
   A   
   A   
   T   
   G   
   G   
    T    
    C    
   T   
    G    
   A   
   T   
    C    
   G   
   C   
    A    
   A   
   T   
   T   
    G    
   G   
    T    
   T   
   T   
    G    
   A   
   A   
   A   
   G   
   A   
   A   
   G   
   A   
    C    
   G   
   A   
    T    
   G   
   A   
    G    
    C    
   T   
    C    
    G    
   T   
    C    
   A   
   A   
    T    
   G   
   T   
    G    
    C    
   T   
   A   
    C    
    T    
   G   
   A   
   C   
    A    
    G    
   A   
    T    
   G   
   G   
    T    
    C    
   A   
    A    
    T    
    C    
    T    
   A   
   A   
    T    
   A   
   T   
    C    
   A   
   T   
    T    
   A   
   T   
    T    
   G   
   G   
    A    
   A   
   C   
   A   
   C   
   A   
   T   
    A    
    A    
    C    
   G   
   G   
    C    
   T   
   A   
   C   
   T   
   C   
   T   
   G   
   T   
    C    
   A   
   C   
    T    
   T   
   T   
    C    
    A    
   A   
    A    
   G   
   A   
    A    
    G    
    A    
    A    
    G    
   C   
    A    
   G   
    T    
    C    
   C   
   G   
    C    
   G   
   A   
   T   
   A   
   T   
   G   
   G   
   G   
    A    
   C   
   G   
    T    
   A   
   C   
   A   
   G   
   C   
    T    
   T   
   C   
    C    
   G   
   G   
    G    
   G   
   T   
    C    
    A    
   G   
    A    
   G   
   G   
    G    
   A   
   T   
    A    
   C   
   G   
    C    
    C    
   T   
    G    
   C   
   G   
    T    
   G   
   A   
    G    
    G    
   A   
    A    
   G   
   A   
    T    
   T   
    A    
    T    
   G   
   T   
    C    
   G   
   T   
    T    
   G   
   G   
    G    
    G    
   C   
   A   
   G   
   C   
    C    
    T    
   T   
    A    
    A    
   T   
   G   
    A    
   A   
    G    
   G   
    A    
   A   
    A    
   A   
    T    
    C    
   A   
   A   
   G   
   A   
   A   
   G   
   T   
    A    
    T    
   T   
    A    
   G   
   T   
    G    
   A   
   T   
    C    
   A   
   C   
    A    
   G   
   A   
   A   
   A   
   A   
    A    
   G   
   G   
   T   
   T   
   A   
    C    
   G   
   G   
    A    
   A   
   A   
    A    
   C   
   G   
    G    
   A   
   C   
    A    
   A   
   A   
   A   
   G   
    T    
    A    
   T   
   C   
    G    
   G   
   A   
    A    
   T   
   A   
   T   
   C   
   C   
   A   
   G   
   T   
    G    
   A   
   A   
    A    
   G   
   G   
    C    
   C   
   G   
    C    
   G   
   G   
    A    
   G   
   G   
   T   
   A   
   A   
   A   
   G   
   G   
    G    
   A   
   T   
    C    
   A   
   A   
    A    
   A   
   C   
    G    
    G    
   C   
    G    
   A   
   A   
    C    
   A   
   T   
   C   
   A   
   C   
   T   
   G   
   A   
    A    
   A   
   A   
   A   
   A   
   A   
   T   
   G   
   G   
   T   
   C   
   C   
   A   
    C    
   T   
   A   
   G   
   C   
    T    
   G   
   G   
    A    
   T   
   T   
    G    
   A   
   C   
   C   
   A   
   C   
    T    
   G   
   T   
    T    
    T    
    C    
   T   
   G   
   G   
    A    
    G    
   A   
   T   
   G   
   A   
   A   
   G   
   A   
    C    
   A   
   T   
   C   
    A    
   T   
    G    
    C    
   T   
    G    
   A   
   T   
    T    
   A   
   C   
    A    
    G    
   A   
    T    
   A   
   A   
   A   
   G   
   G   
    T    
   G   
   T   
    C    
   A   
   T   
   T   
   A   
   T   
   C   
   C   
   G   
    T    
   T   
   T   
    C    
   A   
   A   
   C   
   G   
   T   
    A    
    T    
    C    
    G    
    A    
   C   
   T   
   G   
   T   
   T   
   T   
   C   
   T   
   C   
   A   
   A   
   A   
   C   
    T    
   G   
   G   
   A   
   C   
   G   
    T    
    T    
   C   
   A   
   A   
   C   
   A   
   T   
   T   
    A    
   G   
   G   
    C    
   G   
   T   
    A    
   C   
   G   
   T   
   T   
   T   
    G    
   A   
   T   
   G   
   A   
    A    
    G    
   A   
   T   
   G   
   G   
   A   
   A   
   G   
    C    
    T    
   G   
    A    
    T    
    A    
   C   
    G    
   A   
   A   
   A   
   G   
   T   
   T   
   G   
   T   
   C   
   A   
   C   
   G   
   A   
   T   
   G   
   G   
   C   
   A   
   G   
   C   
   A   
   G   
   T   
   C   
   G   
   A   
   A   
   C   
   C   
   A   
   G   
   A   
   A   
   G   
   C   
   T   
   G   
   C   
   A   
   G   
   A   
   A   
   C   
   A   
   A   
   A   
   C   
   A   
   G   
   T   
   T   
   G   
   A   
   A   
   G   
   A   
   A   
   C   
   A   
   A   
   G   
   G   
   A   
   T   
   C   
   A   
   G   
   A   
   A   
   G   
   A   
   A   
   -   
   -   
   -   
 

 
   N53439   
   A   
   T   
   G   
   A   
   G   
   T   
   G   
   A   
   A   
   G   
   A   
   A   
   A   
   T   
   C   
   A   
   G   
   A   
   G   
   A   
   A   
   A   
   A   
   C   
   A   
   T   
   C   
   C   
   A   
   T   
   G   
   A   
   T   
   G   
   T   
   C   
   A   
   A   
   T   
   C   
   T   
   A   
   A   
   C   
   C   
   A   
   G   
   C   
   G   
   A   
   A   
   A   
   T   
   G   
   A   
   A   
   A   
   G   
   A   
   C   
   T   
   C   
   C   
   T   
   T   
   C   
   A   
   T   
   C   
   G   
   A   
   T   
   T   
   A   
   T   
   G   
   C   
   G   
   A   
   T   
   G   
   A   
   G   
   T   
   G   
   T   
   T   
   A   
   T   
   T   
   G   
   T   
   C   
   G   
   C   
   A   
   C   
   G   
   T   
   G   
   C   
   A   
   C   
   T   
   T   
   C   
   C   
   T   
   G   
   A   
   T   
   G   
   T   
   C   
   A   
   G   
   A   
   G   
   A   
   T   
   G   
   G   
   T   
   C   
   T   
   G   
   A   
   A   
   A   
   C   
   C   
   C   
   G   
   T   
   T   
   C   
   A   
   C   
   C   
   G   
   C   
   C   
   G   
   T   
   A   
   T   
   T   
   T   
   T   
   A   
   T   
   A   
   C   
   G   
   G   
   C   
   A   
   T   
   G   
   A   
   A   
   C   
   G   
   A   
   A   
   T   
   T   
   A   
   G   
   G   
   T   
   G   
   T   
   A   
   A   
   C   
   A   
   C   
   C   
   G   
   G   
   A   
   T   
   A   
   A   
   A   
   G   
   C   
   C   
   C   
   A   
   T   
   A   
   A   
   A   
   A   
   A   
   A   
   T   
   C   
   T   
   G   
   C   
   C   
   A   
   G   
   A   
   A   
   T   
   C   
   G   
   T   
   C   
   G   
   G   
   A   
   G   
   A   
   T   
   G   
   T   
   C   
   A   
   T   
   G   
   G   
   G   
   T   
   A   
   A   
   A   
   T   
   A   
   C   
   C   
   A   
   T   
   C   
   C   
   T   
   C   
   A   
   T   
   G   
   G   
   A   
   G   
   A   
   C   
   A   
   G   
   T   
   G   
   C   
   G   
   A   
   T   
   C   
   T   
   A   
   T   
   G   
   A   
   G   
   T   
   C   
   A   
   A   
   T   
   G   
   G   
   T   
   A   
   C   
   G   
   G   
   A   
   T   
   G   
   G   
   C   
   G   
   C   
   A   
   G   
   C   
   C   
   A   
   T   
   T   
   C   
   A   
   G   
   C   
   T   
   A   
   C   
   C   
   G   
   T   
   T   
   A   
   T   
   A   
   T   
   G   
   C   
   T   
   A   
   G   
   T   
   G   
   G   
   A   
   T   
   G   
   G   
   T   
   C   
   A   
   T   
   G   
   G   
   A   
   A   
   A   
   C   
   T   
   T   
   T   
   G   
   G   
   T   
   T   
   C   
   T   
   G   
   T   
   T   
   G   
   A   
   C   
   G   
   G   
   T   
   G   
   A   
   C   
   G   
   G   
   T   
   G   
   C   
   C   
   G   
   C   
   C   
   G   
   C   
   T   
   A   
   T   
   G   
   C   
   G   
   T   
   T   
   A   
   T   
   A   
   C   
   C   
   G   
   A   
   A   
   G   
   C   
   A   
   C   
   G   
   G   
   A   
   T   
   G   
   A   
   G   
   C   
   A   
   A   
   A   
   A   
   T   
   C   
   G   
   C   
   T   
   A   
   C   
   A   
   G   
   A   
   A   
   A   
   T   
   G   
   C   
   T   
   G   
   C   
   G   
   T   
   G   
   A   
   T   
   A   
   T   
   C   
   A   
   A   
   T   
   A   
   A   
   G   
   A   
   A   
   C   
   A   
   C   
   T   
   G   
   T   
   T   
   G   
   A   
   T   
   T   
   T   
   C   
   C   
   A   
   A   
   A   
   G   
   C   
   A   
   A   
   C   
   T   
   A   
   T   
   G   
   A   
   T   
   G   
   A   
   T   
   A   
   C   
   A   
   G   
   A   
   A   
   A   
   A   
   A   
   G   
   A   
   A   
   C   
   C   
   A   
   G   
   T   
   T   
   G   
   T   
   A   
   C   
   T   
   A   
   C   
   C   
   T   
   G   
   C   
   T   
   C   
   G   
   C   
   T   
   T   
   C   
   C   
   C   
   G   
   A   
   A   
   T   
   C   
   T   
   T   
   T   
   T   
   A   
   G   
   T   
   G   
   A   
   A   
   C   
   G   
   G   
   A   
   A   
   C   
   A   
   A   
   C   
   G   
   G   
   G   
   G   
   A   
   T   
   C   
   G   
   C   
   C   
   G   
   T   
   A   
   G   
   G   
   T   
   A   
   T   
   G   
   G   
   C   
   C   
   A   
   C   
   G   
   A   
   A   
   T   
   A   
   T   
   C   
   C   
   C   
   A   
   C   
   C   
   T   
   C   
   A   
   T   
   A   
   A   
   C   
   T   
   T   
   G   
   T   
   C   
   T   
   G   
   A   
   G   
   G   
   T   
   A   
   G   
   T   
   A   
   G   
   C   
   G   
   G   
   C   
   T   
   A   
   T   
   C   
   G   
   A   
   T   
   T   
   T   
   G   
   C   
   T   
   G   
   A   
   T   
   G   
   G   
   A   
   T   
   A   
   A   
   T   
   C   
   C   
   T   
   G   
   A   
   T   
   G   
   T   
   G   
   A   
   C   
   G   
   A   
   C   
   C   
   A   
   A   
   T   
   G   
   A   
   A   
   T   
   T   
   G   
   A   
   T   
   G   
   G   
   A   
   A   
   G   
   T   
   A   
   T   
   T   
   A   
   C   
   C   
   T   
   G   
   G   
   A   
   C   
   C   
   A   
   G   
   A   
   T   
   T   
   T   
   T   
   C   
   C   
   A   
   A   
   C   
   A   
   G   
   G   
   T   
   G   
   G   
   T   
   T   
   T   
   G   
   G   
   T   
   T   
   A   
   T   
   G   
   G   
   G   
   G   
   A   
   A   
   A   
   T   
   C   
   A   
   G   
   G   
    A    
   A   
   T   
    C    
   C   
   G   
    A    
    A    
   G   
   A   
   G   
   C   
    G    
   T   
   A   
   T   
   G   
   A   
   A   
   A   
   C   
    A    
   G   
   G   
    T    
   A   
   A   
    G    
   G   
   G   
   T   
   T   
   C   
    T    
   A   
   T   
   T   
   A   
   C   
    C    
   G   
   T   
    A    
   C   
   G   
   T   
   G   
   C   
   A   
   A   
   A   
   A   
   G   
   T   
   T   
   G   
   A   
    G    
    A    
   T   
    T    
   A   
    C    
   T   
   G   
   A   
    G    
   A   
   T   
   G   
    C    
   C   
    A    
   A   
   A   
   T   
   G   
   G   
    T    
   A   
   A   
   A   
   G   
   A   
   A   
   C   
   G   
    G    
   A   
   T   
    C    
    A    
   T   
    C    
   G   
   T   
    G    
   A   
   C   
    A    
   G   
   A   
   A   
   T   
   T   
    G    
   C   
   C   
   T   
   T   
   A   
   T   
   A   
   T   
   G   
   G   
   T   
    C    
   A   
   A   
   T   
   A   
   A   
   A   
   G   
   C   
    A    
   A   
   A   
   A   
   T   
   T   
    G    
   A   
   T   
    C    
   G   
   A   
   A   
   C   
   G   
    G    
   A   
   T   
   T   
   T   
   C   
   T   
   G   
   A   
    A    
    C    
   T   
    G    
   C   
   A   
   C   
   A   
   G   
   A   
   G   
   A   
    C    
   A   
   A   
    G    
   C   
   G   
    T    
   A   
   T   
    C    
   G   
   A   
   A   
   G   
   G   
    G    
   A   
   T   
    C    
   A   
   C   
    A    
   G   
   A   
   T   
   T   
   T   
   G   
   C   
   G   
   G   
   G   
   A   
    C    
   G   
   A   
   A   
   T   
   C   
   T   
   T   
   C   
    C    
   C   
   G   
   T   
   G   
   A   
    G    
   G   
   G   
    T    
   A   
   T   
   G   
   C   
   G   
    G    
   A   
   T   
    C    
   G   
   T   
   C   
   A   
   T   
    T    
   G   
   A   
    C    
    A    
   T   
    C    
   C   
   G   
   T   
   C   
   G   
    T    
   G   
   A   
    C    
   G   
    C    
    C    
   A   
   G   
    C    
   G   
   C   
    A    
   T   
   C   
    C    
   G   
   T   
   T   
    A    
   T   
    C    
   T   
   T   
    G    
   A   
   A   
    T    
   A   
   A   
   C   
   T   
   T   
    A    
   T   
   A   
    T    
   A   
   A   
   A   
    T    
   T   
   G   
   A   
   C   
    A    
    T    
   C   
    C    
   T   
   T   
    G    
   C   
   A   
    A    
   A   
    A    
    T    
   T   
   C   
    A    
   T   
   T   
    T    
   G   
   G   
    A    
   T   
   T   
    C    
   A   
   A   
    T    
   A   
   T   
   G   
   T   
   T   
    G    
   G   
   C   
    G    
   A   
   T   
    C    
   G   
   A   
   A   
   A   
   A   
   A   
   G   
   G   
    T    
   G   
   T   
    G    
   C   
   C   
    G    
   A   
   A   
   A   
    G    
   T   
   T   
   T   
   T   
    G    
   A   
   G   
    C    
   T   
   T   
   G   
   A   
   A   
   A   
   C   
    A    
    A    
   A   
   T   
    T    
    C    
   T   
    T    
   G   
   A   
   A   
   A   
   A   
    C    
   T   
   A   
    T    
    A    
   T   
    C    
   G   
   A   
    A    
   C   
   A   
    T    
   C   
   A   
    G    
    C    
    G    
    C    
    G    
   A   
   A   
   G   
   T   
   C   
   A   
   T   
    C    
   A   
   C   
    A    
    A    
   G   
    A    
   C   
   G   
   G   
   A   
   C   
    G    
    G    
   T   
    C    
   T   
   T   
   T   
   G   
   A   
    A    
   A   
   A   
   A   
    G    
   A   
    A    
   A   
   A   
    A    
   G   
   C   
   A   
   G   
   A   
   A   
   G   
   C   
   A   
    A    
   G   
    G    
   G   
   C   
    A    
   C   
   A   
    C    
   A   
   T   
    T    
   T   
   T   
   A   
   G   
   A   
   A   
   G   
   G   
   T   
    T    
   T   
    G    
   C   
   G   
    T    
   A   
   T   
   T   
   G   
   C   
    T    
   T   
   T   
    G    
   G   
   A   
   T   
   C   
   A   
   T   
   A   
   T   
   C   
    G    
   A   
   T   
   G   
   A   
   A   
   A   
   T   
    C    
   A   
   T   
   T   
   G   
   C   
    G    
   A   
   T   
   T   
   A   
   T   
    C    
   C   
   G   
    C    
   G   
   G   
    G    
   T   
   C   
    T    
    A    
   A   
   A   
   T   
   C   
   A   
   G   
   A   
   T   
   G   
   A   
    C    
   G   
   A   
    A    
   G   
   C   
   A   
   A   
   A   
   A   
   G   
   C   
    G    
   A   
    C    
    G    
    A    
   T   
    G    
   A   
   T   
    C    
   G   
   A   
   A   
   C   
   G   
    G    
   T   
   T   
    C    
   G   
   A   
    T    
   T   
   T   
    A    
   T   
   C   
    C    
   G   
   A   
   T   
   C   
   G   
    C    
   C   
   A   
    G    
    T    
   C   
    T    
   C   
   A   
   A   
   G   
   C   
    T    
   A   
   T   
    T    
   T   
   T   
   A   
   G   
   A   
   T   
   A   
   T   
   G   
   C   
   G   
   T   
   T   
   T   
    A    
   C   
   G   
    C    
   C   
   G   
    A    
   T   
   T   
    G    
   A   
   C   
    C    
   G   
   G   
    T    
   T   
   T   
    G    
   G   
   A   
   A   
   C   
   G   
    G    
   G   
   A   
    A    
   A   
   A   
   A   
   A   
   T   
    C    
   G   
   A   
    A    
   A   
   A   
   T   
   G   
   A   
   A   
   T   
   A   
    C    
   C   
   A   
   A   
   G   
   A   
    A    
   C   
   T   
    C    
   T   
   T   
    A    
   A   
    A    
   A   
    C    
   T   
    G    
   A   
   T   
   T   
   G   
    A    
    A    
   G   
   A   
    T    
   T   
   T   
    A    
    A    
    C    
    T    
   G   
   A   
   C   
   A   
   T   
   T   
    C    
   T   
    G    
   G   
   C   
    A    
    A    
   G   
    A    
   C   
   C   
    A    
   G   
   A   
    A    
   C   
   G   
    T    
   G   
   T   
    T    
   A   
    C    
    A    
   G   
   A   
   A   
   A   
   T   
    C    
   A   
   T   
    C    
   A   
   A   
    G    
   A   
    A    
    T    
   G   
   A   
    A    
   T   
   T   
   A   
    G    
    C    
    A    
   G   
   A   
   C   
    T    
   T   
    G    
    A    
    A    
    T    
    C    
    A    
   A   
    C    
    G    
   A   
   T   
   T   
   T   
   G   
   G   
   T   
   G   
   A   
   T   
    A    
    A    
   A   
   C   
   G   
   A   
   C   
   G   
    C    
   A   
   C   
   A   
   G   
   A   
   A   
    T    
   T   
   A   
    C    
   T   
   A   
   G   
   T   
    C    
   G   
   G   
   T   
   G   
   A   
    G    
   G   
   T   
   C   
    T    
   T   
    A    
   A   
   G   
   T   
   C   
   T   
    G    
   G   
   A   
   A   
   G   
   A   
   T   
   G   
   A   
   A   
   G   
   A   
   T   
    T    
   T   
    A    
   A   
   T   
    C    
   G   
   A   
   A   
   G   
   A   
   A   
   G   
   A   
   A   
   G   
   A   
   A   
    A    
   T   
   C   
   G   
   T   
    T    
   A   
   T   
   T   
   A   
   C   
    A    
    T    
   T   
    G    
   A   
   C   
    A    
   A   
   A   
    C    
   A   
   A   
    T    
   G   
   G   
    T    
   T   
   A   
    C    
   A   
   T   
    C    
   A   
   A   
    A    
   C   
   G   
    T    
    T    
   T   
   G   
   G   
    C    
   A   
   A   
   A   
    T    
    A    
    A    
    T    
   G   
   A   
   A   
   T   
   T   
   C   
    A    
   G   
    A    
   G   
   C   
    T    
   C   
   A   
   A   
   C   
   G   
    A    
    A    
   G   
    G    
   G   
   G   
    T    
   G   
   G   
    A    
   C   
   G   
   T   
   G   
   G   
    T    
   G   
   T   
    C    
   C   
   A   
   A   
   G   
   G   
    A    
   A   
   T   
   G   
   G   
   G   
    T    
    A    
   T   
    C    
   C   
   A   
    C    
    A    
   A   
   T   
   G   
   A   
    C    
   G   
   A   
    C    
   T   
   T   
   C   
   G   
   T   
    C    
   A   
   A   
    A    
   A   
   A   
    T    
   C   
   T   
    G    
    G    
   T   
   T   
   T   
   C   
   T   
   T   
   G   
    T    
   T   
   C   
    T    
   A   
   C   
    C    
   C   
   A   
    T    
   G   
   A   
    T    
   A   
   C   
    A    
   T   
   T   
    G    
   T   
   T   
   A   
   T   
   T   
    C    
   T   
   T   
   T   
   A   
   C   
   G   
   A   
   A   
    T    
   A   
    A    
    C    
   G   
   G   
    A    
   A   
   A   
   A   
   G   
   T   
    G    
   T   
   A   
    T    
   C   
   G   
    G    
   G   
   C   
   A   
   A   
   A   
   A   
   G   
   G   
    A    
   T   
   A   
    C    
   G   
   A   
   A   
   A   
   T   
    T    
   C   
   C   
    A    
   G   
   A   
    A    
   T   
   A   
   C   
   G   
   G   
    G    
    C    
   G   
    C    
   A   
   C   
   A   
   G   
   C   
    C    
   A   
   A   
   A   
   G   
   G   
    A    
   A   
   T   
    A    
   C   
   C   
   A   
    A    
   T   
    T    
   A   
   T   
    C    
   A   
   A   
    T    
   T   
   T   
    A    
   C   
   T   
    A    
   G   
   G   
    T    
   A   
   T   
    C    
   G   
   A   
    T    
   T   
   C   
    G    
    A    
    G    
    T    
   G   
   A   
   A   
   A   
    G    
    C    
   A   
   T   
    C    
   C   
   A   
    G    
   G   
   C   
    A    
   A   
   T   
    C    
   A   
   T   
   T   
    G    
   C   
    G    
   G   
   T   
    G    
    A    
    C    
   G   
   G   
   G   
    A    
    G    
   A   
   A   
   G   
   C   
    A    
   G   
   A   
   A   
   G   
    A    
    A    
   G   
   G   
    C    
    C    
   A   
    C    
   T   
   A   
    T    
    C    
   T   
    C    
   T   
   T   
   C   
   T   
   T   
    T    
   A   
   C   
    G    
   A   
   C   
    G    
    C    
    G    
    T    
    C    
   A   
   A   
   G   
   G   
    T    
   A   
   C   
    A    
   G   
   T   
    A    
   A   
   A   
   A   
   C   
   G   
    T    
   A   
   C   
    T    
    T    
   C   
    T    
   G   
   T   
    C    
   A   
   C   
    T    
   G   
   C   
    T    
   T   
   T   
    T    
    G    
   C   
   T   
   A   
   A   
    C    
   A   
   T   
    C    
   C   
   G   
    A    
   A   
   G   
    T    
   A   
   A   
   T   
   G   
   G   
    T    
    C    
   T   
    G    
   A   
   T   
    C    
   G   
   C   
    A    
   A   
   T   
   T   
    G    
   G   
    T    
   T   
   T   
    G    
   A   
   A   
   A   
   G   
   A   
   A   
   G   
   A   
    C    
   G   
   A   
    T    
   G   
   A   
    G    
    C    
   T   
    C    
    G    
   T   
    C    
   A   
   A   
    T    
   G   
   T   
    G    
    C    
   T   
   A   
    C    
    T    
   G   
   A   
   C   
    A    
    G    
   A   
    T    
   G   
   G   
    T    
    C    
   A   
    A    
    T    
    C    
    T    
   A   
   A   
    T    
   A   
   T   
    C    
   A   
   T   
    T    
   A   
   T   
    T    
   G   
   G   
    A    
   A   
   C   
   A   
   C   
   A   
   T   
    A    
    A    
    C    
   G   
   G   
    C    
   T   
   A   
   C   
   T   
   C   
   T   
   G   
   T   
    C    
   A   
   C   
    T    
   T   
   T   
    C    
    A    
   A   
    A    
   G   
   A   
    A    
    G    
    A    
    A    
    G    
   C   
    A    
   G   
    T    
    C    
   C   
   G   
    C    
   G   
   A   
   T   
   A   
   T   
   G   
   G   
   G   
    A    
   C   
   G   
    T    
   A   
   C   
   A   
   G   
   C   
    T    
   T   
   C   
    C    
   G   
   G   
    G    
   G   
   T   
    C    
    A    
   G   
    A    
   G   
   G   
    A    
   A   
   T   
    A    
   C   
   G   
    C    
    C    
   T   
    G    
   C   
   G   
    T    
   G   
   A   
    G    
    G    
   A   
    A    
   G   
   A   
    T    
   T   
    A    
    T    
   G   
   T   
    C    
   G   
   T   
    T    
   G   
   G   
    G    
    G    
   C   
   A   
   G   
   C   
    C    
    T    
   T   
    A    
    A    
   T   
   G   
    A    
   A   
    G    
   G   
    A    
   A   
    G    
   A   
    T    
    C    
   A   
   A   
   G   
   A   
   A   
   G   
   T   
    A    
    T    
   T   
    A    
   G   
   T   
    G    
   A   
   T   
    C    
   A   
   C   
    A    
   G   
   A   
   A   
   A   
   A   
    A    
   G   
   G   
   T   
   T   
   A   
    C    
   G   
   G   
    A    
   A   
   A   
    A    
   C   
   G   
    G    
   A   
   C   
    A    
   A   
   A   
   A   
   G   
    T    
    A    
   T   
   C   
    G    
   G   
   A   
    A    
   T   
   A   
   T   
   C   
   C   
   A   
   G   
   T   
    G    
   A   
   A   
    A    
   G   
   G   
    C    
   C   
   G   
    C    
   G   
   G   
    A    
   G   
   G   
   T   
   A   
   A   
   A   
   G   
   G   
    G    
   A   
   T   
    C    
   A   
   A   
    A    
   A   
   C   
    G    
    G    
   C   
    G    
   A   
   A   
    C    
   A   
   T   
   C   
   A   
   C   
   T   
   G   
   A   
    A    
   A   
   A   
   A   
   A   
   A   
   T   
   G   
   G   
   T   
   C   
   C   
   A   
    C    
   T   
   A   
   G   
   C   
    T    
   G   
   G   
    A    
   T   
   T   
    G    
   A   
   C   
   C   
   A   
   C   
    T    
   G   
   T   
    T    
    T    
    C    
   T   
   G   
   G   
    A    
    G    
   A   
   T   
   G   
   A   
   A   
   G   
   A   
    C    
   A   
   T   
   C   
    A    
   T   
    G    
    C    
   T   
    G    
   A   
   T   
    T    
   A   
   C   
    A    
    G    
   A   
    T    
   A   
   A   
   A   
   G   
   G   
    T    
   G   
   T   
    C    
   A   
   T   
   T   
   A   
   T   
   C   
   C   
   G   
    T    
   T   
   T   
    C    
   A   
   A   
   C   
   G   
   T   
    A    
    T    
    C    
    G    
    A    
   C   
   T   
   G   
   T   
   T   
   T   
   C   
   T   
   C   
   A   
   A   
   A   
   C   
    T    
   G   
   G   
   A   
   C   
   G   
    T    
    T    
   C   
   A   
   A   
   C   
   A   
   T   
   T   
    A    
   G   
   G   
    C    
   G   
   T   
    A    
   C   
   G   
   T   
   T   
   T   
    G    
   A   
   T   
   G   
   A   
    A    
    G    
   A   
   T   
   G   
   G   
   A   
   A   
   G   
    C    
    T    
   G   
    A    
    T    
    A    
   C   
    G    
   A   
   A   
   A   
   G   
   T   
   T   
   G   
   T   
   C   
   A   
   C   
   G   
   A   
   T   
   G   
   G   
   C   
   A   
   G   
   C   
   A   
   G   
   T   
   C   
   G   
   A   
   A   
   C   
   C   
   A   
   G   
   A   
   A   
   G   
   C   
   T   
   G   
   C   
   A   
   G   
   A   
   A   
   C   
   A   
   A   
   A   
   C   
   A   
   G   
   T   
   T   
   G   
   A   
   A   
   G   
   A   
   A   
   C   
   A   
   A   
   G   
   G   
   A   
   T   
   C   
   A   
   G   
   A   
   A   
   G   
   A   
   A   
   -   
   -   
   -   
 

 
   N59998F   
   A   
   T   
   G   
   A   
   G   
   T   
   G   
   A   
   A   
   G   
   A   
   A   
   A   
   T   
   C   
   A   
   G   
   A   
   G   
   A   
   A   
   A   
   A   
   C   
   A   
   T   
   C   
   C   
   A   
   T   
   G   
   A   
   T   
   G   
   T   
   C   
   A   
   A   
   T   
   C   
   T   
   A   
   A   
   C   
   C   
   A   
   G   
   C   
   G   
   A   
   A   
   A   
   T   
   G   
   A   
   A   
   A   
   G   
   A   
   C   
   T   
   C   
   C   
   T   
   T   
   C   
   A   
   T   
   C   
   G   
   A   
   T   
   T   
   A   
   T   
   G   
   C   
   G   
   A   
   T   
   G   
   A   
   G   
   T   
   G   
   T   
   T   
   A   
   T   
   T   
   G   
   T   
   C   
   G   
   C   
   A   
   C   
   G   
   T   
   G   
   C   
   A   
   C   
   T   
   T   
   C   
   C   
   T   
   G   
   A   
   T   
   G   
   T   
   C   
   A   
   G   
   A   
   G   
   A   
   T   
   G   
   G   
   T   
   C   
   T   
   G   
   A   
   A   
   A   
   C   
   C   
   C   
   G   
   T   
   T   
   C   
   A   
   T   
   C   
   G   
   C   
   C   
   G   
   T   
   A   
   T   
   T   
   T   
   T   
   A   
   T   
   A   
   C   
   G   
   G   
   C   
   A   
   T   
   G   
   A   
   A   
   C   
   G   
   A   
   A   
   T   
   T   
   A   
   G   
   G   
   T   
   G   
   T   
   A   
   A   
   C   
   A   
   C   
   C   
   G   
   G   
   A   
   T   
   A   
   A   
   A   
   G   
   C   
   C   
   C   
   A   
   T   
   A   
   A   
   A   
   A   
   A   
   A   
   T   
   C   
   T   
   G   
   C   
   C   
   A   
   G   
   A   
   A   
   T   
   C   
   G   
   T   
   C   
   G   
   G   
   A   
   G   
   A   
   T   
   G   
   T   
   C   
   A   
   T   
   G   
   G   
   G   
   T   
   A   
   A   
   A   
   T   
   A   
   C   
   C   
   A   
   T   
   C   
   C   
   T   
   C   
   A   
   T   
   G   
   G   
   A   
   G   
   A   
   C   
   A   
   G   
   T   
   G   
   C   
   G   
   A   
   T   
   C   
   T   
   A   
   T   
   G   
   A   
   G   
   T   
   C   
   A   
   A   
   T   
   G   
   G   
   T   
   A   
   C   
   G   
   G   
   A   
   T   
   G   
   G   
   C   
   A   
   C   
   A   
   G   
   C   
   C   
   A   
   T   
   T   
   C   
   A   
   G   
   C   
   T   
   A   
   C   
   C   
   G   
   T   
   T   
   A   
   T   
   A   
   T   
   G   
   C   
   T   
   A   
   G   
   T   
   G   
   G   
   A   
   T   
   G   
   G   
   T   
   C   
   A   
   T   
   G   
   G   
   A   
   A   
   A   
   C   
   T   
   T   
   T   
   G   
   G   
   T   
   T   
   C   
   T   
   G   
   T   
   T   
   G   
   A   
   C   
   G   
   G   
   T   
   G   
   A   
   C   
   G   
   G   
   T   
   G   
   C   
   C   
   G   
   C   
   C   
   G   
   C   
   T   
   A   
   T   
   G   
   C   
   G   
   T   
   T   
   A   
   T   
   A   
   C   
   C   
   G   
   A   
   A   
   G   
   C   
   A   
   C   
   G   
   G   
   A   
   T   
   G   
   A   
   G   
   C   
   A   
   A   
   A   
   A   
   T   
   C   
   G   
   C   
   T   
   A   
   C   
   A   
   G   
   A   
   A   
   A   
   T   
   G   
   C   
   T   
   G   
   C   
   G   
   T   
   G   
   A   
   T   
   A   
   T   
   C   
   A   
   A   
   T   
   A   
   A   
   G   
   A   
   A   
   C   
   A   
   C   
   T   
   G   
   T   
   T   
   G   
   A   
   T   
   T   
   T   
   C   
   C   
   A   
   A   
   A   
   G   
   C   
   A   
   A   
   C   
   T   
   A   
   T   
   G   
   A   
   T   
   G   
   A   
   T   
   A   
   C   
   A   
   G   
   A   
   A   
   A   
   A   
   A   
   G   
   A   
   A   
   C   
   C   
   A   
   G   
   T   
   T   
   G   
   T   
   A   
   C   
   T   
   A   
   C   
   C   
   T   
   G   
   C   
   T   
   C   
   G   
   C   
   T   
   T   
   C   
   C   
   C   
   G   
   A   
   A   
   T   
   C   
   T   
   T   
   T   
   T   
   A   
   G   
   T   
   G   
   A   
   A   
   C   
   G   
   G   
   A   
   A   
   C   
   A   
   A   
   C   
   G   
   G   
   G   
   G   
   A   
   T   
   C   
   G   
   C   
   C   
   G   
   T   
   A   
   G   
   G   
   T   
   A   
   T   
   G   
   G   
   C   
   C   
   A   
   C   
   G   
   A   
   A   
   T   
   A   
   T   
   C   
   C   
   C   
   A   
   C   
   C   
   T   
   C   
   A   
   T   
   A   
   A   
   C   
   T   
   T   
   G   
   T   
   C   
   T   
   G   
   A   
   G   
   G   
   T   
   A   
   G   
   T   
   A   
   G   
   C   
   G   
   G   
   C   
   T   
   A   
   T   
   C   
   G   
   A   
   T   
   T   
   T   
   G   
   C   
   T   
   G   
   A   
   T   
   G   
   G   
   A   
   T   
   A   
   A   
   T   
   C   
   C   
   T   
   G   
   A   
   T   
   G   
   T   
   G   
   A   
   C   
   G   
   A   
   C   
   C   
   A   
   A   
   T   
   G   
   A   
   A   
   T   
   T   
   G   
   A   
   T   
   G   
   G   
   A   
   A   
   G   
   T   
   A   
   T   
   T   
   A   
   C   
   C   
   T   
   G   
   G   
   A   
   C   
   C   
   A   
   G   
   A   
   T   
   T   
   T   
   T   
   C   
   C   
   A   
   A   
   C   
   A   
   G   
   G   
   T   
   G   
   G   
   T   
   T   
   T   
   G   
   G   
   T   
   T   
   A   
   T   
   G   
   G   
   G   
   A   
   A   
   A   
   A   
   T   
   C   
   A   
   G   
   G   
    A    
   A   
   T   
    C    
   C   
   G   
    A    
    A    
   G   
   A   
   G   
   C   
    G    
   T   
   A   
   T   
   G   
   A   
   A   
   A   
   C   
    A    
   G   
   G   
    T    
   A   
   A   
    G    
   G   
   G   
   T   
   T   
   C   
    T    
   A   
   T   
   T   
   A   
   C   
    C    
   G   
   T   
    A    
   C   
   G   
   T   
   G   
   C   
   A   
   A   
   A   
   A   
   G   
   T   
   T   
   G   
   A   
    G    
    A    
   T   
    T    
   A   
    C    
   T   
   G   
   A   
    G    
   A   
   T   
   G   
    C    
   C   
    A    
   A   
   A   
   T   
   G   
   G   
    T    
   A   
   A   
   A   
   G   
   A   
   A   
   C   
   G   
    G    
   A   
   T   
    C    
    A    
   T   
    C    
   G   
   T   
    G    
   A   
   C   
    A    
   G   
   A   
   A   
   T   
   T   
    G    
   C   
   C   
   T   
   T   
   A   
   T   
   A   
   T   
   G   
   G   
   T   
    C    
   A   
   A   
   T   
   A   
   A   
   A   
   G   
   C   
    A    
   A   
   A   
   A   
   T   
   T   
    G    
   A   
   T   
    C    
   G   
   A   
   A   
   C   
   G   
    G    
   A   
   T   
   T   
   T   
   C   
   T   
   G   
   A   
    A    
    C    
   T   
    G    
   C   
   A   
   C   
   A   
   G   
   A   
   G   
   A   
    C    
   A   
   A   
    G    
   C   
   G   
    T    
   A   
   T   
    C    
   G   
   A   
   A   
   G   
   G   
    G    
   A   
   T   
    C    
   A   
   C   
    A    
   G   
   A   
   T   
   T   
   T   
   G   
   C   
   G   
   G   
   G   
   A   
    C    
   G   
   A   
   A   
   T   
   C   
   T   
   T   
   C   
    C    
   C   
   G   
   T   
   G   
   A   
    A    
   G   
   G   
    T    
   A   
   T   
   G   
   C   
   G   
    G    
   A   
   T   
    C    
   G   
   T   
   C   
   A   
   T   
    T    
   G   
   A   
    C    
    A    
   T   
    C    
   C   
   G   
   T   
   C   
   G   
    T    
   G   
   A   
    C    
   G   
    C    
    C    
   A   
   G   
    C    
   G   
   C   
    A    
   T   
   C   
    T    
   G   
   T   
   T   
    A    
   T   
    C    
   T   
   T   
    G    
   A   
   A   
    T    
   A   
   A   
   C   
   T   
   T   
    A    
   T   
   A   
    T    
   A   
   A   
   A   
    T    
   T   
   G   
   A   
   C   
    A    
    T    
   C   
    C    
   T   
   T   
    G    
   C   
   A   
    A    
   A   
    A    
    T    
   T   
   C   
    A    
   T   
   T   
    T    
   G   
   G   
    A    
   T   
   T   
    C    
   A   
   A   
    T    
   A   
   T   
   G   
   T   
   T   
    G    
   G   
   C   
    G    
   A   
   T   
    C    
   G   
   A   
   A   
   A   
   A   
   A   
   G   
   G   
    T    
   G   
   T   
    G    
   C   
   C   
    G    
   A   
   A   
   A   
    G    
   T   
   T   
   T   
   T   
    G    
   A   
   G   
    C    
   T   
   T   
   G   
   A   
   A   
   A   
   C   
    A    
    A    
   A   
   T   
    T    
    C    
   T   
    T    
   G   
   A   
   A   
   A   
   A   
    C    
   T   
   A   
    T    
    A    
   T   
    C    
   G   
   A   
    A    
   C   
   A   
    T    
   C   
   A   
    G    
    C    
    G    
    C    
    G    
   A   
   A   
   G   
   T   
   C   
   A   
   T   
    C    
   A   
   C   
    A    
    A    
   G   
    A    
   C   
   G   
   G   
   A   
   C   
    A    
    G    
   T   
    C    
   T   
   T   
   T   
   G   
   A   
    A    
   A   
   A   
   A   
    G    
   A   
    A    
   A   
   A   
    A    
   G   
   C   
   A   
   G   
   A   
   A   
   G   
   C   
   A   
    A    
   G   
    G    
   G   
   C   
    A    
   C   
   A   
    C    
   A   
   T   
    T    
   T   
   T   
   A   
   G   
   A   
   A   
   G   
   G   
   T   
    T    
   T   
    A    
   C   
   G   
    T    
   A   
   T   
   T   
   G   
   C   
    T    
   T   
   T   
    G    
   G   
   A   
   T   
   C   
   A   
   T   
   A   
   T   
   C   
    G    
   A   
   T   
   G   
   A   
   A   
   A   
   T   
    C    
   A   
   T   
   T   
   G   
   C   
    G    
   A   
   T   
   T   
   A   
   T   
    C    
   C   
   G   
    C    
   G   
   G   
    G    
   T   
   C   
    T    
    A    
   A   
   A   
   T   
   C   
   A   
   G   
   A   
   T   
   G   
   A   
    C    
   G   
   A   
    A    
   G   
   C   
   A   
   A   
   A   
   A   
   G   
   C   
    G    
   A   
    C    
    G    
    A    
   T   
    G    
   A   
   T   
    C    
   G   
   A   
   A   
   C   
   G   
    G    
   T   
   T   
    C    
   G   
   A   
    T    
   T   
   T   
    A    
   T   
   C   
    C    
   G   
   A   
   T   
   C   
   G   
    C    
   C   
   A   
    G    
    T    
   C   
    T    
   C   
   A   
   A   
   G   
   C   
    T    
   A   
   T   
    T    
   T   
   T   
   A   
   G   
   A   
   T   
   A   
   T   
   G   
   C   
   G   
   T   
   T   
   T   
    A    
   C   
   G   
    C    
   C   
   G   
    A    
   T   
   T   
    G    
   A   
   C   
    C    
   G   
   G   
    T    
   T   
   T   
    G    
   G   
   A   
   A   
   C   
   G   
    G    
   G   
   A   
    A    
   A   
   A   
   A   
   A   
   T   
    C    
   G   
   A   
    A    
   A   
   A   
   T   
   G   
   A   
   A   
   T   
   A   
    C    
   C   
   A   
   A   
   G   
   A   
    A    
   C   
   T   
    C    
   T   
   T   
    A    
   A   
    A    
   A   
    C    
   T   
    G    
   A   
   T   
   T   
   G   
    A    
    A    
   G   
   A   
    T    
   T   
   T   
    A    
    A    
    C    
    T    
   G   
   A   
   C   
   A   
   T   
   T   
    C    
   T   
    G    
   G   
   C   
    A    
    A    
   G   
    A    
   C   
   C   
    A    
   G   
   A   
    A    
   C   
   G   
    T    
   G   
   T   
    T    
   A   
    C    
    A    
   G   
   A   
   A   
   A   
   T   
    C    
   A   
   T   
    C    
   A   
   A   
    G    
   A   
    A    
    T    
   G   
   A   
    A    
   T   
   T   
   A   
    G    
    C    
    A    
   G   
   A   
   C   
    T    
   T   
    G    
    A    
    A    
    T    
    C    
    A    
   A   
    C    
    G    
   A   
   T   
   T   
   T   
   G   
   G   
   T   
   G   
   A   
   T   
    A    
    A    
   A   
   C   
   G   
   A   
   C   
   G   
    C    
   A   
   C   
   A   
   G   
   A   
   A   
    T    
   T   
   A   
    C    
   T   
   A   
   G   
   T   
    C    
   G   
   G   
   T   
   G   
   A   
    G    
   G   
   T   
   C   
    T    
   T   
    A    
   A   
   G   
   T   
   C   
   T   
    G    
   G   
   A   
   A   
   G   
   A   
   T   
   G   
   A   
   A   
   G   
   A   
   T   
    T    
   T   
    A    
   A   
   T   
    C    
   G   
   A   
   A   
   G   
   A   
   A   
   G   
   A   
   A   
   G   
   A   
   A   
    A    
   T   
   C   
   G   
   T   
    T    
   A   
   T   
   T   
   A   
   C   
    A    
    T    
   T   
    G    
   A   
   C   
    A    
   A   
   A   
    C    
   A   
   A   
    T    
   G   
   G   
    T    
   T   
   A   
    C    
   A   
   T   
    C    
   A   
   A   
    A    
   C   
   G   
    T    
    T    
   T   
   G   
   G   
    C    
   A   
   A   
   A   
    T    
    A    
    A    
    T    
   G   
   A   
   A   
   T   
   T   
   C   
    A    
   G   
    A    
   G   
   C   
    T    
   C   
   A   
   A   
   C   
   G   
    A    
    A    
   G   
    G    
   G   
   G   
    T    
   G   
   G   
    A    
   C   
   G   
   T   
   G   
   G   
    T    
   G   
   T   
    C    
   C   
   A   
   A   
   G   
   G   
    A    
   A   
   T   
   G   
   G   
   G   
    T    
    A    
   T   
    C    
   C   
   A   
    C    
    A    
   A   
   T   
   G   
   A   
    C    
   G   
   A   
    C    
   T   
   T   
   C   
   G   
   T   
    C    
   A   
   A   
    A    
   A   
   A   
    T    
   C   
   T   
    G    
    G    
   T   
   T   
   T   
   C   
   T   
   T   
   G   
    T    
   T   
   C   
    T    
   A   
   C   
    C    
   C   
   A   
    T    
   G   
   A   
    T    
   A   
   C   
    A    
   T   
   T   
    G    
   T   
   T   
   A   
   T   
   T   
    C    
   T   
   T   
   T   
   A   
   C   
   G   
   A   
   A   
    T    
   A   
    A    
    C    
   G   
   G   
    A    
   A   
   A   
   A   
   G   
   T   
    G    
   T   
   A   
    T    
   C   
   G   
    G    
   G   
   C   
   A   
   A   
   A   
   A   
   G   
   G   
    A    
   T   
   A   
    C    
   G   
   A   
   A   
   A   
   T   
    T    
   C   
   C   
    A    
   G   
   A   
    A    
   T   
   A   
   C   
   G   
   G   
    G    
    C    
   G   
    C    
   A   
   C   
   A   
   G   
   C   
    C    
   A   
   A   
   A   
   G   
   G   
    A    
   A   
   T   
    A    
   C   
   C   
   A   
    A    
   T   
    T    
   A   
   T   
    C    
   A   
   A   
    T    
   T   
   T   
    A    
   C   
   T   
    A    
   G   
   G   
    T    
   A   
   T   
    C    
   G   
   A   
    T    
   T   
   C   
    G    
    A    
    G    
    T    
   G   
   A   
   A   
   A   
    G    
    C    
   A   
   T   
    C    
   C   
   A   
    G    
   G   
   C   
    A    
   A   
   T   
    C    
   A   
   T   
   T   
    G    
   C   
    G    
   G   
   T   
    G    
    A    
    C    
   G   
   G   
   G   
    A    
    G    
   A   
   A   
   G   
   C   
    A    
   G   
   A   
   A   
   G   
    A    
    A    
   G   
   G   
    C    
    C    
   A   
    C    
   T   
   A   
    T    
    C    
   T   
    C    
   T   
   T   
   C   
   T   
   T   
    T    
   A   
   C   
    G    
   A   
   C   
    G    
    C    
    G    
    T    
    C    
   A   
   A   
   G   
   G   
    T    
   A   
   C   
    A    
   G   
   T   
    A    
   A   
   A   
   A   
   C   
   G   
    T    
   A   
   C   
    T    
    T    
   C   
    T    
   G   
   T   
    C    
   A   
   C   
    T    
   G   
   C   
    T    
   T   
   T   
    T    
    G    
   C   
   T   
   A   
   A   
    C    
   A   
   T   
    C    
   C   
   G   
    A    
   A   
   G   
    T    
   A   
   A   
   T   
   G   
   G   
    T    
    C    
   T   
    G    
   A   
   T   
    C    
   G   
   C   
    A    
   A   
   T   
   T   
    G    
   G   
    T    
   T   
   T   
    G    
   A   
   A   
   A   
   G   
   A   
   A   
   G   
   A   
    C    
   G   
   A   
    T    
   G   
   A   
    G    
    C    
   T   
    C    
    G    
   T   
    C    
   A   
   A   
    T    
   G   
   T   
    G    
    C    
   T   
   A   
    C    
    T    
   G   
   A   
   C   
    A    
    G    
   A   
    T    
   G   
   G   
    T    
    C    
   A   
    A    
    T    
    C    
    T    
   A   
   A   
    T    
   A   
   T   
    C    
   A   
   T   
    T    
   A   
   T   
    T    
   G   
   G   
    A    
   A   
   C   
   A   
   C   
   A   
   T   
    A    
    A    
    C    
   G   
   G   
    C    
   T   
   A   
   C   
   T   
   C   
   T   
   G   
   T   
    C    
   A   
   C   
    T    
   T   
   T   
    C    
    A    
   A   
    A    
   G   
   A   
    A    
    G    
    A    
    A    
    G    
   C   
    A    
   G   
    T    
    C    
   C   
   G   
    C    
   G   
   A   
   T   
   A   
   T   
   G   
   G   
   G   
    A    
   C   
   G   
    T    
   A   
   C   
   A   
   G   
   C   
    T    
   T   
   C   
    C    
   G   
   G   
    G    
   G   
   T   
    T    
    A    
   G   
    A    
   G   
   G   
    A    
   A   
   T   
    A    
   C   
   G   
    C    
    C    
   T   
    G    
   C   
   G   
    T    
   G   
   A   
    G    
    G    
   A   
    A    
   G   
   A   
    T    
   T   
    A    
    T    
   G   
   T   
    C    
   G   
   T   
    T    
   G   
   G   
    G    
    G    
   C   
   A   
   G   
   C   
    C    
    T    
   T   
    A    
    A    
   T   
   G   
    A    
   A   
    G    
   G   
    A    
   A   
    G    
   A   
    T    
    C    
   A   
   A   
   G   
   A   
   A   
   G   
   T   
    A    
    T    
   T   
    A    
   G   
   T   
    G    
   A   
   T   
    C    
   A   
   C   
    A    
   G   
   A   
   A   
   A   
   A   
    A    
   G   
   G   
   T   
   T   
   A   
    C    
   G   
   G   
    A    
   A   
   A   
    A    
   C   
   G   
    G    
   A   
   C   
    A    
   A   
   A   
   A   
   G   
    T    
    A    
   T   
   C   
    G    
   G   
   A   
    A    
   T   
   A   
   T   
   C   
   C   
   A   
   G   
   T   
    G    
   A   
   A   
    A    
   G   
   G   
    C    
   C   
   G   
    C    
   G   
   G   
    A    
   G   
   G   
   T   
   A   
   A   
   A   
   G   
   G   
    G    
   A   
   T   
    C    
   A   
   A   
    A    
   A   
   C   
    G    
    G    
   C   
    G    
   A   
   A   
    C    
   A   
   T   
   C   
   A   
   C   
   T   
   G   
   A   
    A    
   A   
   A   
   A   
   A   
   A   
   T   
   G   
   G   
   T   
   C   
   C   
   A   
    C    
   T   
   A   
   G   
   C   
    T    
   G   
   G   
    A    
   T   
   T   
    G    
   A   
   C   
   C   
   A   
   C   
    T    
   G   
   T   
    T    
    T    
    C    
   T   
   G   
   G   
    A    
    G    
   A   
   T   
   G   
   A   
   A   
   G   
   A   
    C    
   A   
   T   
   C   
    A    
   T   
    G    
    C    
   T   
    G    
   A   
   T   
    T    
   A   
   C   
    A    
    G    
   A   
    T    
   A   
   A   
   A   
   G   
   G   
    T    
   G   
   T   
    C    
   A   
   T   
   T   
   A   
   T   
   C   
   C   
   G   
    T    
   T   
   T   
    C    
   A   
   A   
   C   
   G   
   T   
    A    
    T    
    C    
    G    
    A    
   C   
   T   
   G   
   T   
   T   
   T   
   C   
   T   
   C   
   A   
   A   
   A   
   C   
    T    
   G   
   G   
   A   
   C   
   G   
    T    
    T    
   C   
   A   
   A   
   C   
   A   
   T   
   T   
    A    
   G   
   G   
    C    
   G   
   T   
    A    
   C   
   G   
   T   
   T   
   T   
    G    
   A   
   T   
   G   
   A   
    A    
    G    
   A   
   T   
   G   
   G   
   A   
   A   
   G   
    C    
    T    
   G   
    A    
    T    
    A    
   C   
    G    
   A   
   A   
   A   
   G   
   T   
   T   
   G   
   T   
   C   
   A   
   C   
   G   
   A   
   T   
   G   
   G   
   C   
   A   
   G   
   C   
   A   
   G   
   T   
   C   
   G   
   A   
   A   
   C   
   C   
   A   
   G   
   A   
   A   
   G   
   C   
   T   
   G   
   C   
   A   
   G   
   A   
   A   
   C   
   A   
   A   
   A   
   C   
   A   
   G   
   T   
   T   
   G   
   A   
   A   
   G   
   A   
   A   
   C   
   A   
   A   
   G   
   G   
   A   
   T   
   C   
   A   
   G   
   A   
   A   
   G   
   A   
   A   
   -   
   -   
   -   
 

 
   N55185   
   A   
   T   
   G   
   A   
   G   
   T   
   G   
   A   
   A   
   G   
   A   
   A   
   A   
   T   
   T   
   A   
   A   
   A   
   G   
   A   
   A   
   A   
   A   
   C   
   A   
   T   
   T   
   C   
   A   
   A   
   G   
   A   
   C   
   G   
   T   
   C   
   A   
   A   
   T   
   C   
   T   
   G   
   A   
   C   
   C   
   A   
   G   
   C   
   G   
   A   
   A   
   A   
   T   
   G   
   A   
   A   
   A   
   G   
   A   
   A   
   T   
   C   
   T   
   T   
   T   
   C   
   A   
   T   
   T   
   G   
   A   
   C   
   T   
   A   
   C   
   G   
   C   
   A   
   A   
   T   
   G   
   A   
   G   
   T   
   G   
   T   
   T   
   A   
   T   
   C   
   G   
   T   
   A   
   G   
   C   
   C   
   C   
   G   
   C   
   G   
   C   
   G   
   T   
   T   
   A   
   C   
   C   
   T   
   G   
   A   
   C   
   G   
   T   
   T   
   C   
   G   
   T   
   G   
   A   
   C   
   G   
   G   
   T   
   T   
   T   
   A   
   A   
   A   
   A   
   C   
   C   
   T   
   G   
   T   
   T   
   C   
   A   
   T   
   C   
   G   
   C   
   C   
   G   
   A   
   A   
   T   
   C   
   T   
   T   
   A   
   T   
   A   
   T   
   G   
   G   
   A   
   A   
   T   
   G   
   A   
   A   
   C   
   G   
   A   
   A   
   T   
   T   
   A   
   G   
   G   
   G   
   G   
   T   
   A   
   A   
   C   
   C   
   C   
   C   
   T   
   G   
   A   
   T   
   A   
   A   
   A   
   C   
   C   
   A   
   C   
   A   
   C   
   A   
   A   
   A   
   A   
   A   
   A   
   T   
   C   
   A   
   G   
   C   
   C   
   C   
   G   
   G   
   A   
   T   
   T   
   G   
   T   
   T   
   G   
   G   
   G   
   G   
   A   
   T   
   G   
   T   
   T   
   A   
   T   
   G   
   G   
   G   
   T   
   A   
   A   
   A   
   T   
   A   
   T   
   C   
   A   
   C   
   C   
   C   
   C   
   C   
   A   
   T   
   G   
   G   
   G   
   G   
   A   
   C   
   A   
   G   
   T   
   G   
   C   
   G   
   A   
   T   
   T   
   T   
   A   
   C   
   G   
   A   
   A   
   T   
   C   
   A   
   A   
   T   
   G   
   G   
   T   
   G   
   C   
   G   
   G   
   A   
   T   
   G   
   G   
   C   
   A   
   C   
   A   
   A   
   C   
   C   
   T   
   T   
   T   
   T   
   A   
   G   
   T   
   T   
   A   
   T   
   C   
   G   
   G   
   G   
   C   
   T   
   A   
   T   
   G   
   T   
   T   
   A   
   G   
   T   
   T   
   G   
   A   
   C   
   G   
   G   
   C   
   C   
   A   
   C   
   G   
   G   
   A   
   A   
   A   
   C   
   T   
   T   
   C   
   G   
   G   
   T   
   T   
   C   
   T   
   G   
   T   
   C   
   G   
   A   
   T   
   G   
   G   
   T   
   G   
   A   
   C   
   G   
   G   
   C   
   G   
   C   
   T   
   G   
   C   
   C   
   G   
   C   
   T   
   A   
   T   
   G   
   C   
   G   
   T   
   T   
   A   
   T   
   A   
   C   
   C   
   G   
   A   
   A   
   G   
   C   
   A   
   C   
   G   
   T   
   A   
   T   
   G   
   A   
   G   
   T   
   A   
   A   
   A   
   A   
   T   
   T   
   G   
   C   
   T   
   T   
   T   
   A   
   G   
   A   
   A   
   A   
   T   
   G   
   C   
   T   
   A   
   C   
   G   
   A   
   G   
   A   
   T   
   A   
   T   
   T   
   A   
   A   
   C   
   A   
   A   
   A   
   A   
   A   
   T   
   A   
   C   
   A   
   G   
   T   
   C   
   G   
   A   
   T   
   T   
   T   
   C   
   C   
   A   
   A   
   G   
   G   
   A   
   A   
   A   
   C   
   T   
   A   
   T   
   G   
   A   
   T   
   G   
   A   
   T   
   T   
   C   
   A   
   G   
   A   
   A   
   C   
   A   
   A   
   G   
   A   
   A   
   C   
   C   
   A   
   G   
   T   
   A   
   G   
   T   
   T   
   T   
   T   
   A   
   C   
   C   
   T   
   G   
   C   
   T   
   C   
   G   
   T   
   T   
   T   
   T   
   C   
   C   
   A   
   A   
   A   
   C   
   T   
   T   
   A   
   C   
   T   
   A   
   G   
   T   
   T   
   A   
   A   
   C   
   G   
   G   
   A   
   A   
   C   
   A   
   A   
   C   
   G   
   G   
   G   
   G   
   A   
   T   
   T   
   G   
   C   
   G   
   G   
   T   
   G   
   G   
   G   
   G   
   A   
   T   
   G   
   G   
   C   
   A   
   A   
   C   
   A   
   A   
   A   
   T   
   A   
   T   
   T   
   C   
   C   
   A   
   C   
   C   
   A   
   C   
   A   
   C   
   A   
   A   
   T   
   T   
   T   
   A   
   A   
   G   
   T   
   G   
   A   
   A   
   G   
   T   
   G   
   A   
   T   
   T   
   G   
   A   
   T   
   G   
   C   
   A   
   A   
   C   
   A   
   A   
   G   
   T   
   T   
   T   
   A   
   T   
   T   
   G   
   A   
   T   
   G   
   G   
   A   
   C   
   A   
   A   
   T   
   C   
   C   
   T   
   G   
   A   
   T   
   G   
   T   
   A   
   A   
   C   
   G   
   A   
   C   
   G   
   A   
   A   
   T   
   G   
   A   
   A   
   T   
   T   
   G   
   A   
   T   
   G   
   G   
   A   
   A   
   G   
   T   
   G   
   T   
   T   
   A   
   C   
   C   
   T   
   G   
   G   
   A   
   C   
   C   
   A   
   G   
   A   
   T   
   T   
   T   
   T   
   C   
   C   
   A   
   A   
   C   
   A   
   G   
   G   
   C   
   G   
   G   
   T   
   T   
   T   
   A   
   G   
   T   
   G   
   A   
   T   
   G   
   G   
   G   
   G   
   A   
   A   
   A   
   T   
   C   
   A   
   G   
   G   
    G    
   A   
   T   
    T    
   C   
   G   
    C    
    C    
   G   
   A   
   G   
   C   
    A    
   T   
   A   
   T   
   G   
   A   
   A   
   A   
   C   
    A    
   G   
   G   
    G    
   A   
   A   
    A    
   G   
   G   
   T   
   T   
   C   
    G    
   A   
   T   
   T   
   A   
   C   
    T    
   G   
   T   
    T    
   C   
   G   
   T   
   G   
   C   
   A   
   A   
   A   
   A   
   G   
   T   
   T   
   G   
   A   
    A    
    T    
   T   
    G    
   A   
    C    
   T   
   G   
   A   
    A    
   A   
   T   
   G   
    C    
   C   
    G    
   A   
   A   
   T   
   G   
   G   
    A    
   A   
   A   
   A   
   G   
   A   
   A   
   C   
   G   
    T    
   A   
   T   
    T    
    T    
   T   
    A    
   G   
   T   
    A    
   A   
   C   
    T    
   G   
   A   
   A   
   T   
   T   
    G    
   C   
   C   
   T   
   T   
   A   
   T   
   A   
   T   
   G   
   G   
   T   
    G    
   A   
   A   
   T   
   A   
   A   
   A   
   G   
   C   
    C    
   A   
   A   
   A   
   T   
   T   
    A    
   A   
   T   
    C    
   G   
   A   
   A   
   C   
   G   
    A    
   A   
   T   
   T   
   T   
   C   
   T   
   G   
   A   
    A    
    T    
   T   
    A    
   C   
   A   
   C   
   A   
   G   
   A   
   G   
   A   
    T    
   A   
   A   
    A    
   C   
   G   
    A    
   A   
   T   
    T    
   G   
   A   
   A   
   G   
   G   
    A    
   A   
   T   
    T    
   A   
   C   
    T    
   G   
   A   
   T   
   T   
   T   
   G   
   C   
   G   
   G   
   G   
   A   
    T    
   G   
   A   
   A   
   T   
   C   
   T   
   T   
   C   
    T    
   C   
   G   
   T   
   G   
   A   
    A    
   G   
   G   
    C    
   A   
   T   
   G   
   C   
   G   
    G    
   A   
   T   
    C    
   G   
   T   
   C   
   A   
   T   
    C    
   G   
   A   
    T    
    G    
   T   
    T    
   C   
   G   
   T   
   C   
   G   
    A    
   G   
   A   
    T    
   G   
    T    
    G    
   A   
   G   
    T    
   G   
   C   
    C    
   T   
   C   
    T    
   G   
   T   
   T   
    G    
   T   
    G    
   T   
   T   
    A    
   A   
   A   
    C    
   A   
   A   
   C   
   T   
   T   
    G    
   T   
   A   
    C    
   A   
   A   
   A   
    A    
   T   
   G   
   A   
   C   
    T    
    G    
   C   
    C    
   T   
   T   
    A    
   C   
   A   
    A    
   A   
    C    
    A    
   T   
   C   
    T    
   T   
   T   
    T    
   G   
   G   
    T    
   T   
   T   
    T    
   A   
   A   
    C    
   A   
   T   
   G   
   T   
   T   
    A    
   G   
   C   
    C    
   A   
   T   
    T    
   G   
   A   
   A   
   A   
   A   
   A   
   G   
   G   
    C    
   G   
   T   
    A    
   C   
   C   
    G    
   A   
   A   
   A   
    A    
   T   
   T   
   T   
   T   
    A    
   A   
   G   
    C    
   T   
   T   
   G   
   A   
   A   
   A   
   C   
    G    
    T    
   A   
   T   
    T    
    T    
   T   
    A    
   G   
   A   
   A   
   A   
   A   
    T    
   T   
   A   
    C    
    G    
   T   
    C    
   G   
   A   
    G    
   C   
   A   
    C    
   C   
   A   
    A    
    A    
    A    
    A    
    G    
   A   
   A   
   G   
   T   
   C   
   A   
   T   
    T    
   A   
   C   
    T    
    C    
   G   
    C    
   C   
   G   
   G   
   A   
   C   
    G    
    A    
   T   
    T    
   T   
   T   
   T   
   G   
   A   
    C    
   A   
   A   
   A   
    A    
   A   
    C    
   A   
   A   
    A    
   G   
   C   
   A   
   G   
   A   
   A   
   G   
   C   
   A   
    C    
   G   
    G    
   G   
   C   
    G    
   C   
   A   
    T    
   A   
   T   
    T    
   T   
   T   
   A   
   G   
   A   
   A   
   G   
   G   
   T   
    C    
   T   
    A    
   C   
   G   
    A    
   A   
   T   
   T   
   G   
   C   
    C    
   T   
   T   
    A    
   G   
   A   
   T   
   C   
   A   
   T   
   A   
   T   
   C   
    G    
   A   
   T   
   G   
   A   
   A   
   A   
   T   
    C    
   A   
   T   
   T   
   G   
   C   
    C    
   A   
   T   
   T   
   A   
   T   
    C    
   C   
   G   
    T    
   G   
   G   
    A    
   T   
   C   
    A    
    C    
   A   
   A   
   T   
   C   
   A   
   G   
   A   
   T   
   G   
   A   
    C    
   G   
   A   
    A    
   G   
   C   
   A   
   A   
   A   
   A   
   G   
   C   
    A    
   A   
    C    
    G    
    T    
   T   
    A    
   A   
   T   
    T    
   G   
   A   
   A   
   C   
   G   
    C    
   T   
   T   
    T    
   G   
   A   
    A    
   T   
   T   
    T    
   T   
   C   
    A    
   G   
   A   
   T   
   C   
   G   
    T    
   C   
   A   
    A    
    G    
   C   
    G    
   C   
   A   
   A   
   G   
   C   
    G    
   A   
   T   
    T    
   T   
   T   
   A   
   G   
   A   
   T   
   A   
   T   
   G   
   C   
   G   
   T   
   T   
   T   
    A    
   C   
   G   
    C    
   C   
   G   
    T    
   T   
   T   
    A    
   A   
   C   
    A    
   G   
   G   
    C    
   T   
   T   
    A    
   G   
   A   
   A   
   C   
   G   
    C    
   G   
   A   
    C    
   A   
   A   
   A   
   A   
   T   
    T    
   G   
   A   
    A    
   A   
   A   
   T   
   G   
   A   
   A   
   T   
   A   
    T    
   C   
   A   
   A   
   G   
   A   
    G    
   C   
   T   
    A    
   T   
   T   
    G    
   A   
    A    
   A   
    T    
   T   
    C    
   A   
   T   
   T   
   G   
    C    
    G    
   G   
   A   
    C    
   T   
   T   
    A    
    G    
    A    
    G    
   G   
   A   
   C   
   A   
   T   
   T   
    T    
   T   
    A    
   G   
   C   
    C    
    C    
   G   
    C    
   C   
   C   
    A    
   G   
   A   
    A    
   C   
   G   
    C    
   G   
   T   
    C    
   A   
    T    
    T    
   G   
   A   
   A   
   A   
   T   
    C    
   A   
   T   
    T    
   A   
   A   
    A    
   A   
    C    
    A    
   G   
   A   
    G    
   T   
   T   
   A   
    A    
    A    
    T    
   G   
   A   
   C   
    G    
   T   
    T    
    C    
    G    
    C    
    A    
    C    
   A   
    A    
    A    
   A   
   T   
   T   
   T   
   G   
   G   
   T   
   G   
   A   
   T   
    G    
    C    
   A   
   C   
   G   
   A   
   C   
   G   
    C    
   A   
   C   
   A   
   G   
   A   
   A   
    C    
   T   
   A   
    T    
   T   
   A   
   G   
   T   
    A    
   G   
   G   
   T   
   G   
   A   
    A    
   G   
   T   
   C   
    C    
   T   
    A    
   A   
   G   
   T   
   C   
   T   
    T    
   G   
   A   
   A   
   G   
   A   
   T   
   G   
   A   
   A   
   G   
   A   
   T   
    T    
   T   
    A    
   A   
   T   
    C    
   G   
   A   
   A   
   G   
   A   
   A   
   G   
   A   
   A   
   G   
   A   
   A   
    G    
   T   
   C   
   G   
   T   
    G    
   A   
   T   
   T   
   A   
   C   
    A    
    T    
   T   
    A    
   A   
   C   
    C    
   A   
   A   
    T    
   A   
   A   
    C    
   G   
   G   
    C    
   T   
   A   
    C    
   A   
   T   
    T    
   A   
   A   
    G    
   C   
   G   
    G    
    A    
   T   
   G   
   G   
    C    
   A   
   A   
   A   
    C    
    T    
    C    
    C    
   G   
   A   
   A   
   T   
   T   
   C   
    C    
   G   
    G    
   G   
   C   
    G    
   C   
   A   
   A   
   C   
   G   
    C    
    C    
   G   
    C    
   G   
   G   
    C    
   G   
   G   
    A    
   C   
   G   
   T   
   G   
   G   
    T    
   G   
   T   
    C    
   C   
   A   
   A   
   G   
   G   
    C    
   A   
   T   
   G   
   G   
   G   
    C    
    G    
   T   
    T    
   C   
   A   
    T    
    G    
   A   
   T   
   G   
   A   
    T    
   G   
   A   
    T    
   T   
   T   
   C   
   G   
   T   
    G    
   A   
   A   
    A    
   A   
   A   
    C    
   C   
   T   
    A    
    G    
   T   
   T   
   T   
   C   
   T   
   T   
   G   
    T    
   T   
   C   
    A    
   A   
   C   
    A    
   C   
   A   
    C    
   G   
   A   
    C    
   A   
   C   
    A    
   T   
   T   
    A    
   T   
   T   
   A   
   T   
   T   
    C    
   T   
   T   
   T   
   A   
   C   
   G   
   A   
   A   
    C    
   A   
    C    
    T    
   G   
   G   
    G    
   A   
   A   
   A   
   G   
   T   
    T    
   T   
   A   
    C    
   C   
   G   
    A    
   G   
   C   
   A   
   A   
   A   
   A   
   G   
   G   
    T    
   T   
   A   
    C    
   G   
   A   
   A   
   A   
   T   
    C    
   C   
   C   
    T    
   G   
   A   
    A    
   T   
   A   
   C   
   G   
   G   
    T    
    A    
   G   
    A    
   A   
   C   
   A   
   G   
   C   
    A    
   A   
   A   
   A   
   G   
   G   
    A    
   A   
   T   
    T    
   C   
   C   
   A   
    G    
   T   
    G    
   A   
   T   
    T    
   A   
   A   
    C    
   T   
   T   
    A    
   C   
   T   
    G    
   G   
   G   
    A    
   A   
   T   
    T    
   G   
   A   
    T    
   T   
   C   
    T    
    G    
    C    
    A    
   G   
   A   
   A   
   A   
    A    
    A    
   A   
   T   
    T    
   C   
   A   
    A    
   G   
   C   
    G    
   A   
   T   
    T    
   A   
   T   
   T   
    T    
   C   
    T    
   G   
   T   
    T    
    G    
    A    
   G   
   G   
   G   
    C    
    A    
   A   
   A   
   G   
   C   
    G    
   G   
   A   
   A   
   G   
    C    
    A    
   G   
   G   
    T    
    A    
   A   
    A    
   T   
   A   
    C    
    T    
   T   
    G    
   T   
   T   
   C   
   T   
   T   
    C    
   A   
   C   
    A    
   A   
   C   
    C    
    T    
    T    
    A    
    A    
   A   
   A   
   G   
   G   
    A    
   A   
   C   
    T    
   G   
   T   
    C    
   A   
   A   
   A   
   C   
   G   
    G    
   A   
   C   
    A    
    G    
   C   
    C    
   G   
   T   
    A    
   A   
   C   
    A    
   G   
   C   
    C    
   T   
   T   
    T    
    T    
   C   
   T   
   A   
   A   
    T    
   A   
   T   
    C    
   C   
   G   
    T    
   A   
   G   
    T    
   A   
   A   
   T   
   G   
   G   
    A    
    T    
   T   
    A    
   A   
   T   
    C    
   G   
   C   
    C    
   A   
   T   
   T   
    A    
   G   
    C    
   T   
   T   
    A    
   A   
   A   
   A   
   G   
   A   
   A   
   G   
   A   
    T    
   G   
   A   
    T    
   G   
   A   
    G    
    T    
   T   
    A    
    G    
   T   
    T    
   A   
   A   
    C    
   G   
   T   
    A    
    G    
   T   
   A   
    A    
    C    
   G   
   A   
   C   
    T    
    A    
   A   
    T    
   G   
   G   
    C    
    A    
   A   
    T    
    C    
    A    
    G    
   A   
   A   
    G    
   A   
   T   
    G    
   A   
   T   
    T    
   A   
   T   
    C    
   G   
   G   
    A    
   A   
   C   
   A   
   C   
   A   
   T   
    G    
    C    
    G    
   G   
   G   
    A    
   T   
   A   
   C   
   T   
   C   
   T   
   G   
   T   
    C    
   A   
   C   
    A    
   T   
   T   
    T    
    G    
   A   
    T    
   G   
   A   
    A    
    A    
    A    
    T    
    A    
   C   
    T    
   G   
    T    
    A    
   C   
   G   
    T    
   G   
   A   
   T   
   A   
   T   
   G   
   G   
   G   
    C    
   C   
   G   
    G    
   A   
   C   
   A   
   G   
   C   
    A    
   T   
   C   
    A    
   G   
   G   
    C    
   G   
   T   
    T    
    C    
   G   
    G    
   G   
   G   
    A    
   A   
   T   
    C    
   C   
   G   
    T    
    C    
   T   
    C    
   C   
   G   
    C    
   G   
   A   
    A    
    A    
   A   
    T    
   G   
   A   
    T    
   T   
    A    
    T    
   G   
   T   
    G    
   G   
   T   
    C    
   G   
   G   
    C    
    G    
   C   
   A   
   G   
   C   
    G    
    A    
   T   
    T    
    C    
   T   
   G   
    G    
   A   
    T    
   G   
    A    
   A   
    A    
   A   
    T    
    A    
   A   
   A   
   G   
   A   
   A   
   G   
   T   
    C    
    C    
   T   
    A    
   G   
   T   
    C    
   A   
   T   
    T    
   A   
   C   
    T    
   G   
   A   
   A   
   A   
   A   
    T    
   G   
   G   
   T   
   T   
   A   
    T    
   G   
   G   
    T    
   A   
   A   
    G    
   C   
   G   
    T    
   A   
   C   
    A    
   A   
   A   
   A   
   G   
    C    
    T    
   T   
   C   
    T    
   G   
   A   
    A    
   T   
   A   
   T   
   C   
   C   
   A   
   G   
   T   
    T    
   A   
   A   
    A    
   G   
   G   
    A    
   C   
   G   
    T    
   G   
   G   
    C    
   G   
   G   
   T   
   A   
   A   
   A   
   G   
   G   
    G    
   A   
   T   
    T    
   A   
   A   
    G    
   A   
   C   
    A    
    G    
   C   
    A    
   A   
   A   
    T    
   A   
   T   
   C   
   A   
   C   
   T   
   G   
   A   
    G    
   A   
   A   
   A   
   A   
   A   
   T   
   G   
   G   
   T   
   C   
   C   
   A   
    T    
   T   
   A   
   G   
   C   
    T    
   G   
   G   
    T    
   T   
   T   
    A    
   A   
   C   
   C   
   A   
   C   
    A    
   G   
   T   
    C    
    A    
    A    
   T   
   G   
   G   
    G    
    G    
   A   
   T   
   G   
   A   
   A   
   G   
   A   
    T    
   A   
   T   
   C   
    T    
   T   
    A    
    T    
   T   
    G    
   A   
   T   
    T    
   A   
   C   
    G    
    A    
   A   
    C    
   A   
   A   
   A   
   G   
   G   
    C    
   G   
   T   
    C    
   A   
   T   
   T   
   A   
   T   
   C   
   C   
   G   
    C    
   T   
   T   
    T    
   A   
   A   
   C   
   G   
   T   
    T    
    G    
    A    
    T    
    T    
   C   
   T   
   G   
   T   
   T   
   T   
   C   
   T   
   C   
   A   
   A   
   A   
   C   
    A    
   G   
   G   
   A   
   C   
   G   
    C    
    G    
   C   
   A   
   A   
   C   
   A   
   T   
   T   
    A    
   G   
   G   
    G    
   G   
   T   
    T    
   C   
   G   
   T   
   T   
   T   
    A    
   A   
   T   
   G   
   A   
    G    
    A    
   A   
   T   
   G   
   G   
   A   
   A   
   G   
    A    
    T    
   G   
    G    
    T    
    G    
   C   
    C    
   A   
   A   
   A   
   -   
   -   
   -   
   -   
   -   
   -   
   -   
   -   
   -   
   -   
   -   
   -   
   -   
   -   
   -   
   -   
   -   
   -   
   -   
   -   
   -   
   -   
   -   
   -   
   -   
   -   
   -   
   -   
   -   
   -   
   -   
   -   
   -   
   -   
   -   
   -   
   -   
   -   
   -   
   -   
   -   
   -   
   -   
   -   
   -   
   -   
   -   
   -   
   -   
   -   
   -   
   -   
   -   
   -   
   -   
   -   
   -   
   -   
   -   
   -   
   -   
   -   
   -   
   -   
   -   
   -   
   -   
   -   
   -   
   -   
   -   
   -   
 

 
   N54546   
   A   
   T   
   G   
   A   
   G   
   T   
   G   
   A   
   A   
   G   
   A   
   A   
   A   
   T   
   C   
   A   
   G   
   A   
   G   
   A   
   A   
   A   
   A   
   C   
   A   
   T   
   C   
   C   
   A   
   T   
   G   
   A   
   T   
   G   
   T   
   C   
   A   
   A   
   T   
   C   
   T   
   A   
   A   
   C   
   C   
   A   
   G   
   C   
   G   
   A   
   A   
   A   
   T   
   G   
   A   
   A   
   A   
   G   
   A   
   C   
   T   
   C   
   C   
   T   
   T   
   C   
   A   
   T   
   C   
   G   
   A   
   T   
   T   
   A   
   T   
   G   
   C   
   G   
   A   
   T   
   G   
   A   
   G   
   T   
   G   
   T   
   T   
   A   
   T   
   T   
   G   
   T   
   C   
   G   
   C   
   A   
   C   
   G   
   T   
   G   
   C   
   A   
   C   
   T   
   T   
   C   
   C   
   T   
   G   
   A   
   T   
   G   
   T   
   C   
   A   
   G   
   A   
   G   
   A   
   T   
   G   
   G   
   T   
   C   
   T   
   G   
   A   
   A   
   A   
   C   
   C   
   C   
   G   
   T   
   T   
   C   
   A   
   C   
   C   
   G   
   C   
   C   
   G   
   T   
   A   
   T   
   T   
   T   
   T   
   A   
   T   
   A   
   C   
   G   
   G   
   C   
   A   
   T   
   G   
   A   
   A   
   C   
   G   
   A   
   A   
   T   
   T   
   A   
   G   
   G   
   T   
   G   
   T   
   A   
   A   
   C   
   A   
   C   
   C   
   G   
   G   
   A   
   T   
   A   
   A   
   A   
   G   
   C   
   C   
   C   
   A   
   T   
   A   
   A   
   A   
   A   
   A   
   A   
   T   
   C   
   T   
   G   
   C   
   C   
   A   
   G   
   A   
   A   
   T   
   C   
   G   
   T   
   C   
   G   
   G   
   A   
   G   
   A   
   T   
   G   
   T   
   C   
   A   
   T   
   G   
   G   
   G   
   T   
   A   
   A   
   A   
   T   
   A   
   C   
   C   
   A   
   T   
   C   
   C   
   T   
   C   
   A   
   T   
   G   
   G   
   A   
   G   
   A   
   C   
   A   
   G   
   T   
   G   
   C   
   G   
   A   
   T   
   C   
   T   
   A   
   T   
   G   
   A   
   G   
   T   
   C   
   A   
   A   
   T   
   G   
   G   
   T   
   A   
   C   
   G   
   G   
   A   
   T   
   G   
   G   
   C   
   G   
   C   
   A   
   G   
   C   
   C   
   A   
   T   
   T   
   C   
   A   
   G   
   C   
   T   
   A   
   C   
   C   
   G   
   T   
   T   
   A   
   T   
   A   
   T   
   G   
   C   
   T   
   A   
   G   
   T   
   G   
   G   
   A   
   T   
   G   
   G   
   T   
   C   
   A   
   T   
   G   
   G   
   A   
   A   
   A   
   C   
   T   
   T   
   T   
   G   
   G   
   T   
   T   
   C   
   T   
   G   
   T   
   T   
   G   
   A   
   C   
   G   
   G   
   T   
   G   
   A   
   C   
   G   
   G   
   T   
   G   
   C   
   C   
   G   
   C   
   C   
   G   
   C   
   T   
   A   
   T   
   G   
   C   
   G   
   T   
   T   
   A   
   T   
   A   
   C   
   C   
   G   
   A   
   A   
   G   
   C   
   A   
   C   
   G   
   G   
   A   
   T   
   G   
   A   
   G   
   C   
   A   
   A   
   A   
   A   
   T   
   C   
   G   
   C   
   T   
   A   
   C   
   A   
   G   
   A   
   A   
   A   
   T   
   G   
   C   
   T   
   G   
   C   
   G   
   T   
   G   
   A   
   T   
   A   
   T   
   C   
   A   
   A   
   T   
   A   
   A   
   G   
   A   
   A   
   C   
   A   
   C   
   T   
   G   
   T   
   T   
   G   
   A   
   T   
   T   
   T   
   C   
   C   
   A   
   A   
   A   
   G   
   C   
   A   
   A   
   C   
   T   
   A   
   T   
   G   
   A   
   T   
   G   
   A   
   T   
   A   
   C   
   A   
   G   
   A   
   A   
   A   
   A   
   A   
   G   
   A   
   A   
   C   
   C   
   A   
   G   
   T   
   T   
   G   
   T   
   A   
   C   
   T   
   A   
   C   
   C   
   T   
   G   
   C   
   T   
   C   
   G   
   C   
   T   
   T   
   C   
   C   
   C   
   G   
   A   
   A   
   T   
   C   
   T   
   T   
   T   
   T   
   A   
   G   
   T   
   G   
   A   
   A   
   C   
   G   
   G   
   A   
   A   
   C   
   A   
   A   
   C   
   G   
   G   
   G   
   G   
   A   
   T   
   C   
   G   
   C   
   C   
   G   
   T   
   A   
   G   
   G   
   T   
   A   
   T   
   G   
   G   
   C   
   C   
   A   
   C   
   G   
   A   
   A   
   T   
   A   
   T   
   C   
   C   
   C   
   A   
   C   
   C   
   T   
   C   
   A   
   T   
   A   
   A   
   C   
   T   
   T   
   G   
   T   
   C   
   T   
   G   
   A   
   G   
   G   
   T   
   A   
   G   
   T   
   A   
   G   
   C   
   G   
   G   
   C   
   T   
   A   
   T   
   C   
   G   
   A   
   T   
   T   
   T   
   G   
   C   
   T   
   G   
   A   
   T   
   G   
   G   
   A   
   T   
   A   
   A   
   T   
   C   
   C   
   T   
   G   
   A   
   T   
   G   
   T   
   G   
   A   
   C   
   G   
   A   
   C   
   C   
   A   
   A   
   T   
   G   
   A   
   A   
   T   
   T   
   G   
   A   
   T   
   G   
   G   
   A   
   A   
   G   
   T   
   A   
   T   
   T   
   A   
   C   
   C   
   T   
   G   
   G   
   A   
   C   
   C   
   A   
   G   
   A   
   T   
   T   
   T   
   T   
   C   
   C   
   A   
   A   
   C   
   A   
   G   
   G   
   T   
   G   
   G   
   T   
   T   
   T   
   G   
   G   
   T   
   T   
   A   
   T   
   G   
   G   
   G   
   A   
   A   
   A   
   A   
   T   
   C   
   A   
   G   
   G   
    A    
   A   
   T   
    C    
   C   
   G   
    A    
    A    
   G   
   A   
   G   
   C   
    G    
   T   
   A   
   T   
   G   
   A   
   A   
   A   
   C   
    A    
   G   
   G   
    T    
   A   
   A   
    G    
   G   
   G   
   T   
   T   
   C   
    T    
   A   
   T   
   T   
   A   
   C   
    C    
   G   
   T   
    A    
   C   
   G   
   T   
   G   
   C   
   A   
   A   
   A   
   A   
   G   
   T   
   T   
   G   
   A   
    G    
    A    
   T   
    T    
   A   
    C    
   T   
   G   
   A   
    G    
   A   
   T   
   G   
    C    
   C   
    A    
   A   
   A   
   T   
   G   
   G   
    T    
   A   
   A   
   A   
   G   
   A   
   A   
   C   
   G   
    G    
   A   
   T   
    C    
    A    
   T   
    C    
   G   
   T   
    G    
   A   
   C   
    A    
   G   
   A   
   A   
   T   
   T   
    G    
   C   
   C   
   T   
   T   
   A   
   T   
   A   
   T   
   G   
   G   
   T   
    C    
   A   
   A   
   T   
   A   
   A   
   A   
   G   
   C   
    A    
   A   
   A   
   A   
   T   
   T   
    G    
   A   
   T   
    C    
   G   
   A   
   A   
   C   
   G   
    G    
   A   
   T   
   T   
   T   
   C   
   T   
   G   
   A   
    A    
    C    
   T   
    G    
   C   
   A   
   C   
   A   
   G   
   A   
   G   
   A   
    C    
   A   
   A   
    G    
   C   
   G   
    T    
   A   
   T   
    C    
   G   
   A   
   A   
   G   
   G   
    G    
   A   
   T   
    C    
   A   
   C   
    A    
   G   
   A   
   T   
   T   
   T   
   G   
   C   
   G   
   G   
   G   
   A   
    C    
   G   
   A   
   A   
   T   
   C   
   T   
   T   
   C   
    C    
   C   
   G   
   T   
   G   
   A   
    A    
   G   
   G   
    T    
   A   
   T   
   G   
   C   
   G   
    G    
   A   
   T   
    C    
   G   
   T   
   C   
   A   
   T   
    T    
   G   
   A   
    C    
    A    
   T   
    C    
   C   
   G   
   T   
   C   
   G   
    T    
   G   
   A   
    C    
   G   
    C    
    C    
   A   
   G   
    C    
   G   
   C   
    A    
   T   
   C   
    C    
   G   
   T   
   T   
    A    
   T   
    C    
   T   
   T   
    G    
   A   
   A   
    T    
   A   
   A   
   C   
   T   
   T   
    A    
   T   
   A   
    T    
   A   
   A   
   A   
    T    
   T   
   G   
   A   
   C   
    A    
    T    
   C   
    C    
   T   
   T   
    G    
   C   
   A   
    A    
   A   
    A    
    T    
   T   
   C   
    A    
   T   
   T   
    T    
   G   
   G   
    A    
   T   
   T   
    C    
   A   
   A   
    T    
   A   
   T   
   G   
   T   
   T   
    G    
   G   
   C   
    G    
   A   
   T   
    C    
   G   
   A   
   A   
   A   
   A   
   A   
   G   
   G   
    T    
   G   
   T   
    G    
   C   
   C   
    G    
   A   
   A   
   A   
    G    
   T   
   T   
   T   
   T   
    G    
   A   
   G   
    C    
   T   
   T   
   G   
   A   
   A   
   A   
   C   
    A    
    A    
   A   
   T   
    T    
    C    
   T   
    T    
   G   
   A   
   A   
   A   
   A   
    C    
   T   
   A   
    T    
    A    
   T   
    C    
   G   
   A   
    A    
   C   
   A   
    T    
   C   
   A   
    G    
    C    
    G    
    C    
    G    
   A   
   A   
   G   
   T   
   C   
   A   
   T   
    C    
   A   
   C   
    A    
    A    
   G   
    A    
   C   
   G   
   G   
   A   
   C   
    A    
    G    
   T   
    C    
   T   
   T   
   T   
   G   
   A   
    A    
   A   
   A   
   A   
    G    
   A   
    A    
   A   
   A   
    A    
   G   
   C   
   A   
   G   
   A   
   A   
   G   
   C   
   A   
    A    
   G   
    G    
   G   
   C   
    A    
   C   
   A   
    C    
   A   
   T   
    T    
   T   
   T   
   A   
   G   
   A   
   A   
   G   
   G   
   T   
    T    
   T   
    G    
   C   
   G   
    T    
   A   
   T   
   T   
   G   
   C   
    T    
   T   
   T   
    G    
   G   
   A   
   T   
   C   
   A   
   T   
   A   
   T   
   C   
    G    
   A   
   T   
   G   
   A   
   A   
   A   
   T   
    A    
   A   
   T   
   T   
   G   
   C   
    G    
   A   
   T   
   T   
   A   
   T   
    C    
   C   
   G   
    C    
   G   
   G   
    G    
   T   
   C   
    T    
    A    
   A   
   A   
   T   
   C   
   A   
   G   
   A   
   T   
   G   
   A   
    C    
   G   
   A   
    A    
   G   
   C   
   A   
   A   
   A   
   A   
   G   
   C   
    G    
   A   
    C    
    G    
    A    
   T   
    G    
   A   
   T   
    C    
   G   
   A   
   A   
   C   
   G   
    G    
   T   
   T   
    C    
   G   
   A   
    T    
   T   
   T   
    A    
   T   
   C   
    C    
   G   
   A   
   T   
   C   
   G   
    C    
   C   
   A   
    G    
    T    
   C   
    T    
   C   
   A   
   A   
   G   
   C   
    T    
   A   
   T   
    T    
   T   
   T   
   A   
   G   
   A   
   T   
   A   
   T   
   G   
   C   
   G   
   T   
   T   
   T   
    A    
   C   
   G   
    C    
   C   
   G   
    A    
   T   
   T   
    G    
   A   
   C   
    C    
   G   
   G   
    T    
   T   
   T   
    G    
   G   
   A   
   A   
   C   
   G   
    G    
   G   
   A   
    A    
   A   
   A   
   A   
   A   
   T   
    C    
   G   
   A   
    A    
   A   
   A   
   T   
   G   
   A   
   A   
   T   
   A   
    C    
   C   
   A   
   A   
   G   
   A   
    A    
   C   
   T   
    C    
   T   
   T   
    A    
   A   
    A    
   A   
    C    
   T   
    G    
   A   
   T   
   T   
   G   
    A    
    A    
   G   
   A   
    T    
   T   
   T   
    A    
    A    
    C    
    T    
   G   
   A   
   C   
   A   
   T   
   T   
    C    
   T   
    G    
   G   
   C   
    A    
    A    
   G   
    A    
   C   
   C   
    A    
   G   
   A   
    A    
   C   
   G   
    T    
   G   
   T   
    T    
   A   
    C    
    A    
   G   
   A   
   A   
   A   
   T   
    C    
   A   
   T   
    C    
   A   
   A   
    G    
   A   
    A    
    T    
   G   
   A   
    A    
   T   
   T   
   A   
    G    
    C    
    A    
   G   
   A   
   C   
    T    
   T   
    G    
    A    
    A    
    T    
    C    
    A    
   A   
    C    
    G    
   A   
   T   
   T   
   T   
   G   
   G   
   T   
   G   
   A   
   T   
    A    
    A    
   A   
   C   
   G   
   A   
   C   
   G   
    C    
   A   
   C   
   A   
   G   
   A   
   A   
    T    
   T   
   A   
    C    
   T   
   A   
   G   
   T   
    C    
   G   
   G   
   T   
   G   
   A   
    G    
   G   
   T   
   C   
    T    
   T   
    A    
   A   
   G   
   T   
   C   
   T   
    G    
   G   
   A   
   A   
   G   
   A   
   T   
   G   
   A   
   A   
   G   
   A   
   T   
    T    
   T   
    A    
   A   
   T   
    C    
   G   
   A   
   A   
   G   
   A   
   A   
   G   
   A   
   A   
   G   
   A   
   A   
    A    
   T   
   C   
   G   
   T   
    T    
   A   
   T   
   T   
   A   
   C   
    A    
    T    
   T   
    G    
   A   
   C   
    A    
   A   
   A   
    C    
   A   
   A   
    T    
   G   
   G   
    T    
   T   
   A   
    C    
   A   
   T   
    C    
   A   
   A   
    A    
   C   
   G   
    T    
    T    
   T   
   G   
   G   
    C    
   A   
   A   
   A   
    T    
    A    
    A    
    T    
   G   
   A   
   A   
   T   
   T   
   C   
    A    
   G   
    A    
   G   
   C   
    T    
   C   
   A   
   A   
   C   
   G   
    A    
    A    
   G   
    G    
   G   
   G   
    T    
   G   
   G   
    A    
   C   
   G   
   T   
   G   
   G   
    T    
   G   
   T   
    C    
   C   
   A   
   A   
   G   
   G   
    A    
   A   
   T   
   G   
   G   
   G   
    T    
    A    
   T   
    C    
   C   
   A   
    C    
    A    
   A   
   T   
   G   
   A   
    C    
   G   
   A   
    C    
   T   
   T   
   C   
   G   
   T   
    C    
   A   
   A   
    A    
   A   
   A   
    T    
   C   
   T   
    G    
    G    
   T   
   T   
   T   
   C   
   T   
   T   
   G   
    T    
   T   
   C   
    T    
   A   
   C   
    C    
   C   
   A   
    T    
   G   
   A   
    T    
   A   
   C   
    A    
   T   
   T   
    G    
   T   
   T   
   A   
   T   
   T   
    C    
   T   
   T   
   T   
   A   
   C   
   G   
   A   
   A   
    T    
   A   
    A    
    C    
   G   
   G   
    A    
   A   
   A   
   A   
   G   
   T   
    G    
   T   
   A   
    T    
   C   
   G   
    G    
   G   
   C   
   A   
   A   
   A   
   A   
   G   
   G   
    A    
   T   
   A   
    C    
   G   
   A   
   A   
   A   
   T   
    T    
   C   
   C   
    A    
   G   
   A   
    A    
   T   
   A   
   C   
   G   
   G   
    G    
    C    
   G   
    T    
   A   
   C   
   A   
   G   
   C   
    C    
   A   
   A   
   A   
   G   
   G   
    A    
   A   
   T   
    A    
   C   
   C   
   A   
    A    
   T   
    T    
   A   
   T   
    C    
   A   
   A   
    T    
   T   
   T   
    A    
   C   
   T   
    A    
   G   
   G   
    T    
   A   
   T   
    C    
   G   
   A   
    T    
   T   
   C   
    G    
    A    
    G    
    T    
   G   
   A   
   A   
   A   
    G    
    C    
   A   
   T   
    C    
   C   
   A   
    G    
   G   
   C   
    A    
   A   
   T   
    C    
   A   
   T   
   T   
    G    
   C   
    G    
   G   
   T   
    G    
    A    
    C    
   G   
   G   
   G   
    A    
    G    
   A   
   A   
   G   
   C   
    A    
   G   
   A   
   A   
   G   
    A    
    A    
   G   
   G   
    C    
    C    
   A   
    C    
   T   
   A   
    T    
    C    
   T   
    C    
   T   
   T   
   C   
   T   
   T   
    T    
   A   
   C   
    G    
   A   
   C   
    G    
    C    
    G    
    T    
    C    
   A   
   A   
   G   
   G   
    T    
   A   
   C   
    A    
   G   
   T   
    A    
   A   
   A   
   A   
   C   
   G   
    T    
   A   
   C   
    T    
    T    
   C   
    T    
   G   
   T   
    C    
   A   
   C   
    T    
   G   
   C   
    T    
   T   
   T   
    T    
    G    
   C   
   T   
   A   
   A   
    C    
   A   
   T   
    C    
   C   
   G   
    A    
   A   
   G   
    T    
   A   
   A   
   T   
   G   
   G   
    T    
    C    
   T   
    G    
   A   
   T   
    C    
   G   
   C   
    A    
   A   
   T   
   T   
    G    
   G   
    T    
   T   
   T   
    G    
   A   
   A   
   A   
   G   
   A   
   A   
   G   
   A   
    C    
   G   
   A   
    T    
   G   
   A   
    G    
    C    
   T   
    C    
    G    
   T   
    C    
   A   
   A   
    T    
   G   
   T   
    G    
    C    
   T   
   A   
    C    
    T    
   G   
   A   
   C   
    A    
    G    
   A   
    T    
   G   
   G   
    T    
    C    
   A   
    A    
    T    
    C    
    T    
   A   
   A   
    T    
   A   
   T   
    C    
   A   
   T   
    T    
   A   
   T   
    T    
   G   
   G   
    A    
   A   
   C   
   A   
   C   
   A   
   T   
    A    
    A    
    C    
   G   
   G   
    C    
   T   
   A   
   C   
   T   
   C   
   T   
   G   
   T   
    C    
   A   
   C   
    T    
   T   
   T   
    C    
    A    
   A   
    A    
   G   
   A   
    A    
    G    
    A    
    A    
    G    
   C   
    A    
   G   
    T    
    C    
   C   
   G   
    C    
   G   
   A   
   T   
   A   
   T   
   G   
   G   
   G   
    A    
   C   
   G   
    T    
   A   
   C   
   A   
   G   
   C   
    T    
   T   
   C   
    C    
   G   
   G   
    G    
   G   
   T   
    C    
    A    
   G   
    A    
   G   
   G   
    G    
   A   
   T   
    A    
   C   
   G   
    C    
    C    
   T   
    G    
   C   
   G   
    T    
   G   
   A   
    G    
    G    
   A   
    A    
   G   
   A   
    T    
   T   
    A    
    T    
   G   
   T   
    C    
   G   
   T   
    T    
   G   
   G   
    G    
    G    
   C   
   A   
   G   
   C   
    C    
    T    
   T   
    A    
    A    
   T   
   G   
    A    
   A   
    G    
   G   
    A    
   A   
    A    
   A   
    T    
    C    
   A   
   A   
   G   
   A   
   A   
   G   
   T   
    A    
    T    
   T   
    A    
   G   
   T   
    G    
   A   
   T   
    C    
   A   
   C   
    A    
   G   
   A   
   A   
   A   
   A   
    A    
   G   
   G   
   T   
   T   
   A   
    C    
   G   
   G   
    A    
   A   
   A   
    A    
   C   
   G   
    G    
   A   
   C   
    A    
   A   
   A   
   A   
   G   
    T    
    A    
   T   
   C   
    G    
   G   
   A   
    A    
   T   
   A   
   T   
   C   
   C   
   A   
   G   
   T   
    G    
   A   
   A   
    A    
   G   
   G   
    C    
   C   
   G   
    C    
   G   
   G   
    A    
   G   
   G   
   T   
   A   
   A   
   A   
   G   
   G   
    G    
   A   
   T   
    C    
   A   
   A   
    A    
   A   
   C   
    G    
    G    
   C   
    G    
   A   
   A   
    C    
   A   
   T   
   C   
   A   
   C   
   T   
   G   
   A   
    A    
   A   
   A   
   A   
   A   
   A   
   T   
   G   
   G   
   T   
   C   
   C   
   A   
    C    
   T   
   A   
   G   
   C   
    T    
   G   
   G   
    A    
   T   
   T   
    G    
   A   
   C   
   C   
   A   
   C   
    T    
   G   
   T   
    T    
    T    
    C    
   T   
   G   
   G   
    A    
    G    
   A   
   T   
   G   
   A   
   A   
   G   
   A   
    C    
   A   
   T   
   C   
    A    
   T   
    G    
    C    
   T   
    G    
   A   
   T   
    T    
   A   
   C   
    A    
    G    
   A   
    T    
   A   
   A   
   A   
   G   
   G   
    T    
   G   
   T   
    C    
   A   
   T   
   T   
   A   
   T   
   C   
   C   
   G   
    T    
   T   
   T   
    C    
   A   
   A   
   C   
   G   
   T   
    A    
    T    
    C    
    G    
    A    
   C   
   T   
   G   
   T   
   T   
   T   
   C   
   T   
   C   
   A   
   A   
   A   
   C   
    T    
   G   
   G   
   A   
   C   
   G   
    T    
    T    
   C   
   A   
   A   
   C   
   A   
   T   
   T   
    A    
   G   
   G   
    C    
   G   
   T   
    A    
   C   
   G   
   T   
   T   
   T   
    G    
   A   
   T   
   G   
   A   
    A    
    G    
   A   
   T   
   G   
   G   
   A   
   A   
   G   
    C    
    T    
   G   
    A    
    T    
    A    
   C   
    G    
   A   
   A   
   A   
   G   
   T   
   T   
   G   
   T   
   C   
   A   
   C   
   G   
   A   
   T   
   G   
   G   
   C   
   A   
   G   
   C   
   A   
   G   
   T   
   C   
   G   
   A   
   A   
   C   
   C   
   A   
   G   
   A   
   A   
   G   
   C   
   T   
   G   
   C   
   A   
   G   
   A   
   A   
   C   
   A   
   A   
   A   
   C   
   A   
   G   
   T   
   T   
   G   
   A   
   A   
   G   
   A   
   A   
   C   
   A   
   A   
   G   
   G   
   A   
   T   
   C   
   A   
   G   
   A   
   A   
   G   
   A   
   A   
   -   
   -   
   -   
 

 
   N59509F   
   A   
   T   
   G   
   A   
   G   
   T   
   G   
   A   
   A   
   G   
   A   
   A   
   A   
   T   
   T   
   A   
   A   
   A   
   G   
   A   
   A   
   A   
   A   
   C   
   A   
   T   
   T   
   C   
   A   
   A   
   G   
   A   
   C   
   G   
   T   
   C   
   A   
   A   
   T   
   C   
   T   
   G   
   A   
   C   
   C   
   A   
   G   
   C   
   G   
   A   
   A   
   A   
   T   
   G   
   A   
   A   
   A   
   G   
   A   
   A   
   T   
   C   
   T   
   T   
   T   
   C   
   A   
   T   
   T   
   G   
   A   
   C   
   T   
   A   
   C   
   G   
   C   
   A   
   A   
   T   
   G   
   A   
   G   
   T   
   G   
   T   
   T   
   A   
   T   
   C   
   G   
   T   
   A   
   G   
   C   
   C   
   C   
   G   
   C   
   G   
   C   
   G   
   T   
   T   
   A   
   C   
   C   
   T   
   G   
   A   
   C   
   G   
   T   
   T   
   C   
   G   
   T   
   G   
   A   
   C   
   G   
   G   
   T   
   T   
   T   
   A   
   A   
   A   
   A   
   C   
   C   
   T   
   G   
   T   
   T   
   C   
   A   
   T   
   C   
   G   
   C   
   C   
   G   
   A   
   A   
   T   
   C   
   T   
   T   
   A   
   T   
   A   
   T   
   G   
   G   
   A   
   A   
   T   
   G   
   A   
   A   
   C   
   G   
   A   
   A   
   T   
   T   
   A   
   G   
   G   
   G   
   G   
   T   
   A   
   A   
   C   
   C   
   C   
   C   
   T   
   G   
   A   
   T   
   A   
   A   
   A   
   C   
   C   
   A   
   C   
   A   
   C   
   A   
   A   
   A   
   A   
   A   
   A   
   T   
   C   
   A   
   G   
   C   
   C   
   C   
   G   
   G   
   A   
   T   
   T   
   G   
   T   
   T   
   G   
   G   
   G   
   G   
   A   
   T   
   G   
   T   
   T   
   A   
   T   
   G   
   G   
   G   
   T   
   A   
   A   
   A   
   T   
   A   
   T   
   C   
   A   
   T   
   C   
   C   
   C   
   C   
   A   
   T   
   G   
   G   
   G   
   G   
   A   
   C   
   A   
   G   
   T   
   G   
   C   
   G   
   A   
   T   
   T   
   T   
   A   
   C   
   G   
   A   
   A   
   T   
   C   
   A   
   A   
   T   
   G   
   G   
   T   
   G   
   C   
   G   
   G   
   A   
   T   
   G   
   G   
   C   
   A   
   C   
   A   
   A   
   C   
   C   
   T   
   T   
   T   
   T   
   A   
   G   
   T   
   T   
   A   
   T   
   C   
   G   
   G   
   G   
   C   
   T   
   A   
   T   
   G   
   T   
   T   
   A   
   G   
   T   
   T   
   G   
   A   
   C   
   G   
   G   
   C   
   C   
   A   
   C   
   G   
   G   
   A   
   A   
   A   
   C   
   T   
   T   
   C   
   G   
   G   
   T   
   T   
   C   
   T   
   G   
   T   
   C   
   G   
   A   
   T   
   G   
   G   
   T   
   G   
   A   
   C   
   G   
   G   
   C   
   G   
   C   
   T   
   G   
   C   
   C   
   G   
   C   
   T   
   A   
   T   
   G   
   C   
   G   
   T   
   T   
   A   
   T   
   A   
   C   
   C   
   G   
   A   
   A   
   G   
   C   
   A   
   C   
   G   
   T   
   A   
   T   
   G   
   A   
   G   
   T   
   A   
   A   
   A   
   A   
   T   
   T   
   G   
   C   
   T   
   T   
   T   
   A   
   G   
   A   
   A   
   A   
   T   
   G   
   C   
   T   
   A   
   C   
   G   
   A   
   G   
   A   
   T   
   A   
   T   
   T   
   A   
   A   
   C   
   A   
   A   
   A   
   A   
   A   
   T   
   A   
   C   
   A   
   G   
   T   
   C   
   G   
   A   
   T   
   T   
   T   
   C   
   C   
   A   
   A   
   G   
   G   
   A   
   A   
   A   
   C   
   T   
   A   
   T   
   G   
   A   
   T   
   G   
   A   
   T   
   T   
   C   
   A   
   G   
   A   
   A   
   C   
   A   
   A   
   G   
   A   
   A   
   C   
   C   
   A   
   G   
   T   
   A   
   G   
   T   
   T   
   T   
   T   
   A   
   C   
   C   
   T   
   G   
   C   
   T   
   C   
   G   
   T   
   T   
   T   
   T   
   C   
   C   
   A   
   A   
   A   
   C   
   T   
   T   
   A   
   C   
   T   
   A   
   G   
   T   
   T   
   A   
   A   
   C   
   G   
   G   
   A   
   A   
   C   
   A   
   A   
   C   
   G   
   G   
   G   
   G   
   A   
   T   
   T   
   G   
   C   
   G   
   G   
   T   
   G   
   G   
   G   
   G   
   A   
   T   
   G   
   G   
   C   
   A   
   A   
   C   
   A   
   A   
   A   
   T   
   A   
   T   
   T   
   C   
   C   
   A   
   C   
   C   
   A   
   C   
   A   
   T   
   A   
   A   
   T   
   T   
   T   
   A   
   A   
   G   
   T   
   G   
   A   
   A   
   G   
   T   
   G   
   A   
   T   
   T   
   G   
   A   
   T   
   G   
   C   
   A   
   A   
   C   
   A   
   A   
   G   
   T   
   T   
   T   
   A   
   T   
   T   
   G   
   A   
   T   
   G   
   G   
   A   
   C   
   A   
   A   
   T   
   C   
   C   
   T   
   G   
   A   
   T   
   G   
   T   
   A   
   A   
   C   
   G   
   A   
   C   
   G   
   A   
   A   
   T   
   G   
   A   
   A   
   T   
   T   
   G   
   A   
   T   
   G   
   G   
   A   
   A   
   G   
   T   
   G   
   T   
   T   
   A   
   C   
   C   
   T   
   G   
   G   
   A   
   C   
   C   
   A   
   G   
   A   
   T   
   T   
   T   
   T   
   C   
   C   
   A   
   A   
   C   
   A   
   G   
   G   
   C   
   G   
   G   
   T   
   T   
   T   
   A   
   G   
   T   
   G   
   A   
   T   
   G   
   G   
   G   
   G   
   A   
   A   
   A   
   T   
   C   
   A   
   G   
   G   
    G    
   A   
   T   
    T    
   C   
   G   
    C    
    C    
   G   
   A   
   G   
   C   
    A    
   T   
   A   
   T   
   G   
   A   
   A   
   A   
   C   
    A    
   G   
   G   
    G    
   A   
   A   
    A    
   G   
   G   
   T   
   T   
   C   
    G    
   A   
   T   
   T   
   A   
   C   
    T    
   G   
   T   
    T    
   C   
   G   
   T   
   G   
   C   
   A   
   A   
   A   
   A   
   G   
   T   
   T   
   G   
   A   
    A    
    T    
   T   
    G    
   A   
    C    
   T   
   G   
   A   
    A    
   A   
   T   
   G   
    C    
   C   
    G    
   A   
   A   
   T   
   G   
   G   
    A    
   A   
   A   
   A   
   G   
   A   
   A   
   C   
   G   
    T    
   A   
   T   
    T    
    T    
   T   
    A    
   G   
   T   
    A    
   A   
   C   
    T    
   G   
   A   
   A   
   T   
   T   
    G    
   C   
   C   
   T   
   T   
   A   
   T   
   A   
   T   
   G   
   G   
   T   
    G    
   A   
   A   
   T   
   A   
   A   
   A   
   G   
   C   
    C    
   A   
   A   
   A   
   T   
   T   
    A    
   A   
   T   
    C    
   G   
   A   
   A   
   C   
   G   
    A    
   A   
   T   
   T   
   T   
   C   
   T   
   G   
   A   
    A    
    T    
   T   
    A    
   C   
   A   
   C   
   A   
   G   
   A   
   G   
   A   
    T    
   A   
   A   
    A    
   C   
   G   
    A    
   A   
   T   
    T    
   G   
   A   
   A   
   G   
   G   
    A    
   A   
   T   
    T    
   A   
   C   
    T    
   G   
   A   
   T   
   T   
   T   
   G   
   C   
   G   
   G   
   G   
   A   
    T    
   G   
   A   
   A   
   T   
   C   
   T   
   T   
   C   
    T    
   C   
   G   
   T   
   G   
   A   
    A    
   G   
   G   
    C    
   A   
   T   
   G   
   C   
   G   
    G    
   A   
   T   
    C    
   G   
   T   
   C   
   A   
   T   
    C    
   G   
   A   
    T    
    G    
   T   
    T    
   C   
   G   
   T   
   C   
   G   
    A    
   G   
   A   
    T    
   G   
    T    
    G    
   A   
   G   
    T    
   G   
   C   
    C    
   T   
   C   
    T    
   G   
   T   
   T   
    G    
   T   
    G    
   T   
   T   
    A    
   A   
   A   
    C    
   A   
   A   
   C   
   T   
   T   
    G    
   T   
   A   
    C    
   A   
   A   
   A   
    A    
   T   
   G   
   A   
   C   
    T    
    G    
   C   
    C    
   T   
   T   
    A    
   C   
   A   
    A    
   A   
    C    
    A    
   T   
   C   
    T    
   T   
   T   
    T    
   G   
   G   
    T    
   T   
   T   
    T    
   A   
   A   
    C    
   A   
   T   
   G   
   T   
   T   
    A    
   G   
   C   
    C    
   A   
   T   
    T    
   G   
   A   
   A   
   A   
   A   
   A   
   G   
   G   
    C    
   G   
   T   
    A    
   C   
   C   
    G    
   A   
   A   
   A   
    A    
   T   
   T   
   T   
   T   
    A    
   A   
   G   
    C    
   T   
   T   
   G   
   A   
   A   
   A   
   C   
    G    
    T    
   A   
   T   
    T    
    T    
   T   
    A    
   G   
   A   
   A   
   A   
   A   
    T    
   T   
   A   
    C    
    G    
   T   
    C    
   G   
   A   
    G    
   C   
   A   
    C    
   C   
   A   
    A    
    A    
    A    
    A    
    G    
   A   
   A   
   G   
   T   
   C   
   A   
   T   
    T    
   A   
   C   
    T    
    C    
   G   
    C    
   C   
   G   
   G   
   A   
   C   
    G    
    A    
   T   
    T    
   T   
   T   
   T   
   G   
   A   
    C    
   A   
   A   
   A   
    A    
   A   
    C    
   A   
   A   
    A    
   G   
   C   
   A   
   G   
   A   
   A   
   G   
   C   
   A   
    C    
   G   
    G    
   G   
   C   
    G    
   C   
   A   
    T    
   A   
   T   
    T    
   T   
   T   
   A   
   G   
   A   
   A   
   G   
   G   
   T   
    C    
   T   
    A    
   C   
   G   
    A    
   A   
   T   
   T   
   G   
   C   
    C    
   T   
   T   
    A    
   G   
   A   
   T   
   C   
   A   
   T   
   A   
   T   
   C   
    G    
   A   
   T   
   G   
   A   
   A   
   A   
   T   
    C    
   A   
   T   
   T   
   G   
   C   
    C    
   A   
   T   
   T   
   A   
   T   
    C    
   C   
   G   
    T    
   G   
   G   
    A    
   T   
   C   
    A    
    C    
   A   
   A   
   T   
   C   
   A   
   G   
   A   
   T   
   G   
   A   
    C    
   G   
   A   
    A    
   G   
   C   
   A   
   A   
   A   
   A   
   G   
   C   
    A    
   A   
    C    
    G    
    T    
   T   
    A    
   A   
   T   
    T    
   G   
   A   
   A   
   C   
   G   
    C    
   T   
   T   
    T    
   G   
   A   
    A    
   T   
   T   
    T    
   T   
   C   
    A    
   G   
   A   
   T   
   C   
   G   
    T    
   C   
   A   
    A    
    G    
   C   
    G    
   C   
   A   
   A   
   G   
   C   
    G    
   A   
   T   
    T    
   T   
   T   
   A   
   G   
   A   
   T   
   A   
   T   
   G   
   C   
   G   
   T   
   T   
   T   
    A    
   C   
   G   
    C    
   C   
   G   
    T    
   T   
   T   
    A    
   A   
   C   
    A    
   G   
   G   
    C    
   T   
   T   
    A    
   G   
   A   
   A   
   C   
   G   
    C    
   G   
   A   
    C    
   A   
   A   
   A   
   A   
   T   
    T    
   G   
   A   
    A    
   A   
   A   
   T   
   G   
   A   
   A   
   T   
   A   
    T    
   C   
   A   
   A   
   G   
   A   
    G    
   C   
   T   
    A    
   T   
   T   
    G    
   A   
    A    
   A   
    T    
   T   
    C    
   A   
   T   
   T   
   G   
    C    
    G    
   G   
   A   
    C    
   T   
   T   
    A    
    G    
    A    
    G    
   G   
   A   
   C   
   A   
   T   
   T   
    T    
   T   
    A    
   G   
   C   
    C    
    C    
   G   
    C    
   C   
   C   
    A    
   G   
   A   
    A    
   C   
   G   
    C    
   G   
   T   
    C    
   A   
    T    
    T    
   G   
   A   
   A   
   A   
   T   
    C    
   A   
   T   
    T    
   A   
   A   
    A    
   A   
    C    
    A    
   G   
   A   
    G    
   T   
   T   
   A   
    A    
    A    
    T    
   G   
   A   
   C   
    G    
   T   
    T    
    C    
    G    
    C    
    A    
    C    
   A   
    A    
    A    
   A   
   T   
   T   
   T   
   G   
   G   
   T   
   G   
   A   
   T   
    G    
    C    
   A   
   C   
   G   
   A   
   C   
   G   
    C    
   A   
   C   
   A   
   G   
   A   
   A   
    T    
   T   
   A   
    T    
   T   
   A   
   G   
   T   
    A    
   G   
   G   
   T   
   G   
   A   
    A    
   G   
   T   
   C   
    T    
   T   
    A    
   A   
   G   
   T   
   C   
   T   
    T    
   G   
   A   
   A   
   G   
   A   
   T   
   G   
   A   
   A   
   G   
   A   
   T   
    T    
   T   
    A    
   A   
   T   
    C    
   G   
   A   
   A   
   G   
   A   
   A   
   G   
   A   
   A   
   G   
   A   
   A   
    G    
   T   
   C   
   G   
   T   
    G    
   A   
   T   
   T   
   A   
   C   
    G    
    T    
   T   
    A    
   A   
   C   
    C    
   A   
   A   
    T    
   A   
   A   
    C    
   G   
   G   
    C    
   T   
   A   
    C    
   A   
   T   
    T    
   A   
   A   
    G    
   C   
   G   
    G    
    A    
   T   
   G   
   G   
    C    
   A   
   A   
   A   
    C    
    T    
    C    
    C    
   G   
   A   
   A   
   T   
   T   
   C   
    C    
   G   
    G    
   G   
   C   
    G    
   C   
   A   
   A   
   C   
   G   
    C    
    C    
   G   
    C    
   G   
   G   
    T    
   G   
   G   
    A    
   C   
   G   
   T   
   G   
   G   
    T    
   G   
   T   
    C    
   C   
   A   
   A   
   G   
   G   
    G    
   A   
   T   
   G   
   G   
   G   
    C    
    G    
   T   
    T    
   C   
   A   
    T    
    G    
   A   
   T   
   G   
   A   
    T    
   G   
   A   
    T    
   T   
   T   
   C   
   G   
   T   
    G    
   A   
   A   
    A    
   A   
   A   
    C    
   C   
   T   
    A    
    G    
   T   
   T   
   T   
   C   
   T   
   T   
   G   
    T    
   T   
   C   
    A    
   A   
   C   
    A    
   C   
   A   
    C    
   G   
   A   
    C    
   A   
   C   
    G    
   T   
   T   
    A    
   T   
   T   
   A   
   T   
   T   
    C    
   T   
   T   
   T   
   A   
   C   
   G   
   A   
   A   
    C    
   A   
    C    
    T    
   G   
   G   
    G    
   A   
   A   
   A   
   G   
   T   
    T    
   T   
   A   
    C    
   C   
   G   
    A    
   G   
   C   
   A   
   A   
   A   
   A   
   G   
   G   
    T    
   T   
   A   
    C    
   G   
   A   
   A   
   A   
   T   
    C    
   C   
   C   
    T    
   G   
   A   
    A    
   T   
   A   
   C   
   G   
   G   
    T    
    A    
   G   
    A    
   A   
   C   
   A   
   G   
   C   
    A    
   A   
   A   
   A   
   G   
   G   
    A    
   A   
   T   
    T    
   C   
   C   
   A   
    G    
   T   
    G    
   A   
   T   
    T    
   A   
   A   
    C    
   T   
   T   
    A    
   C   
   T   
    G    
   G   
   G   
    A    
   A   
   T   
    T    
   G   
   A   
    T    
   T   
   C   
    T    
    G    
    C    
    A    
   G   
   A   
   A   
   A   
    A    
    A    
   A   
   T   
    T    
   C   
   A   
    A    
   G   
   C   
    G    
   A   
   T   
    T    
   A   
   T   
   T   
    T    
   C   
    T    
   G   
   T   
    T    
    G    
    A    
   G   
   G   
   G   
    C    
    A    
   A   
   A   
   G   
   C   
    G    
   G   
   A   
   A   
   G   
    C    
    A    
   G   
   G   
    T    
    A    
   A   
    A    
   T   
   A   
    C    
    T    
   T   
    G    
   T   
   T   
   C   
   T   
   T   
    C    
   A   
   C   
    A    
   A   
   C   
    C    
    T    
    T    
    A    
    A    
   A   
   A   
   G   
   G   
    A    
   A   
   C   
    C    
   G   
   T   
    C    
   A   
   A   
   A   
   C   
   G   
    G    
   A   
   C   
    A    
    G    
   C   
    C    
   G   
   T   
    A    
   A   
   C   
    A    
   G   
   C   
    C    
   T   
   T   
    T    
    T    
   C   
   T   
   A   
   A   
    T    
   A   
   T   
    C    
   C   
   G   
    T    
   A   
   G   
    T    
   A   
   A   
   T   
   G   
   G   
    A    
    T    
   T   
    A    
   A   
   T   
    C    
   G   
   C   
    C    
   A   
   T   
   T   
    A    
   G   
    C    
   T   
   T   
    A    
   A   
   A   
   A   
   G   
   A   
   A   
   G   
   A   
    T    
   G   
   A   
    T    
   G   
   A   
    G    
    T    
   T   
    A    
    G    
   T   
    T    
   A   
   A   
    C    
   G   
   T   
    G    
    G    
   T   
   A   
    A    
    C    
   G   
   A   
   C   
    T    
    A    
   A   
    T    
   G   
   G   
    C    
    A    
   A   
    T    
    C    
    A    
    G    
   A   
   A   
    G    
   A   
   T   
    G    
   A   
   T   
    T    
   A   
   T   
    C    
   G   
   G   
    A    
   A   
   C   
   A   
   C   
   A   
   T   
    G    
    C    
    A    
   G   
   G   
    A    
   T   
   A   
   C   
   T   
   C   
   T   
   G   
   T   
    C    
   A   
   C   
    G    
   T   
   T   
    T    
    G    
   A   
    T    
   G   
   A   
    A    
    A    
    A    
    T    
    A    
   C   
    T    
   G   
    T    
    A    
   C   
   G   
    T    
   G   
   A   
   T   
   A   
   T   
   G   
   G   
   G   
    C    
   C   
   G   
    G    
   A   
   C   
   A   
   G   
   C   
    A    
   T   
   C   
    A    
   G   
   G   
    T    
   G   
   T   
    T    
    C    
   G   
    G    
   G   
   G   
    A    
   A   
   T   
    C    
   C   
   G   
    T    
    C    
   T   
    C    
   C   
   G   
    C    
   G   
   A   
    A    
    A    
   A   
    T    
   G   
   A   
    T    
   T   
    A    
    T    
   G   
   T   
    G    
   G   
   T   
    C    
   G   
   G   
    T    
    G    
   C   
   A   
   G   
   C   
    G    
    A    
   T   
    T    
    C    
   T   
   G   
    G    
   A   
    T    
   G   
    A    
   A   
    A    
   A   
    C    
    A    
   A   
   A   
   G   
   A   
   A   
   G   
   T   
    C    
    C    
   T   
    A    
   G   
   T   
    C    
   A   
   T   
    T    
   A   
   C   
    T    
   G   
   A   
   A   
   A   
   A   
    T    
   G   
   G   
   T   
   T   
   A   
    T    
   G   
   G   
    T    
   A   
   A   
    G    
   C   
   G   
    T    
   A   
   C   
    A    
   A   
   A   
   A   
   G   
    C    
    T    
   T   
   C   
    T    
   G   
   A   
    A    
   T   
   A   
   T   
   C   
   C   
   A   
   G   
   T   
    T    
   A   
   A   
    A    
   G   
   G   
    A    
   C   
   G   
    T    
   G   
   G   
    C    
   G   
   G   
   T   
   A   
   A   
   A   
   G   
   G   
    G    
   A   
   T   
    T    
   A   
   A   
    G    
   A   
   C   
    A    
    G    
   C   
    A    
   A   
   A   
    T    
   A   
   T   
   C   
   A   
   C   
   T   
   G   
   A   
    G    
   A   
   A   
   A   
   A   
   A   
   T   
   G   
   G   
   T   
   C   
   C   
   A   
    T    
   T   
   A   
   G   
   C   
    T    
   G   
   G   
    T    
   T   
   T   
    A    
   A   
   C   
   C   
   A   
   C   
    G    
   G   
   T   
    C    
    A    
    A    
   T   
   G   
   G   
    G    
    G    
   A   
   T   
   G   
   A   
   A   
   G   
   A   
    T    
   A   
   T   
   C   
    T    
   T   
    A    
    T    
   T   
    G    
   A   
   T   
    T    
   A   
   C   
    G    
    A    
   A   
    C    
   A   
   A   
   A   
   G   
   G   
    C    
   G   
   T   
    C    
   A   
   T   
   T   
   A   
   T   
   C   
   C   
   G   
    C    
   T   
   T   
    T    
   A   
   A   
   C   
   G   
   T   
    T    
    G    
    A    
    T    
    T    
   C   
   T   
   G   
   T   
   T   
   T   
   C   
   T   
   C   
   A   
   A   
   A   
   C   
    A    
   G   
   G   
   A   
   C   
   G   
    C    
    G    
   C   
   A   
   A   
   C   
   A   
   T   
   T   
    A    
   G   
   G   
    G    
   G   
   T   
    T    
   C   
   G   
   T   
   T   
   T   
    A    
   A   
   T   
   G   
   A   
    G    
    A    
   A   
   T   
   G   
   G   
   A   
   A   
   G   
    A    
    T    
   G   
    G    
    T    
    G    
   C   
    C    
   A   
   A   
   A   
   -   
   -   
   -   
   -   
   -   
   -   
   -   
   -   
   -   
   -   
   -   
   -   
   -   
   -   
   -   
   -   
   -   
   -   
   -   
   -   
   -   
   -   
   -   
   -   
   -   
   -   
   -   
   -   
   -   
   -   
   -   
   -   
   -   
   -   
   -   
   -   
   -   
   -   
   -   
   -   
   -   
   -   
   -   
   -   
   -   
   -   
   -   
   -   
   -   
   -   
   -   
   -   
   -   
   -   
   -   
   -   
   -   
   -   
   -   
   -   
   -   
   -   
   -   
   -   
   -   
   -   
   -   
   -   
   -   
   -   
   -   
   -   
 

 
   N55265   
   A   
   T   
   G   
   A   
   G   
   T   
   G   
   A   
   A   
   G   
   A   
   A   
   A   
   T   
   T   
   A   
   A   
   A   
   G   
   A   
   A   
   A   
   A   
   C   
   A   
   T   
   T   
   C   
   A   
   A   
   G   
   A   
   C   
   G   
   T   
   C   
   A   
   A   
   T   
   C   
   T   
   G   
   A   
   C   
   C   
   A   
   G   
   C   
   G   
   A   
   A   
   A   
   T   
   G   
   A   
   A   
   A   
   G   
   A   
   A   
   T   
   C   
   T   
   T   
   T   
   C   
   A   
   T   
   T   
   G   
   A   
   C   
   T   
   A   
   C   
   G   
   C   
   A   
   A   
   T   
   G   
   A   
   G   
   T   
   G   
   T   
   T   
   A   
   T   
   C   
   G   
   T   
   A   
   G   
   C   
   C   
   C   
   G   
   C   
   G   
   C   
   G   
   T   
   T   
   A   
   C   
   C   
   T   
   G   
   A   
   C   
   G   
   T   
   T   
   C   
   G   
   T   
   G   
   A   
   C   
   G   
   G   
   T   
   T   
   T   
   A   
   A   
   A   
   A   
   C   
   C   
   T   
   G   
   T   
   T   
   C   
   A   
   T   
   C   
   G   
   C   
   C   
   G   
   A   
   A   
   T   
   C   
   T   
   T   
   A   
   T   
   A   
   T   
   G   
   G   
   A   
   A   
   T   
   G   
   A   
   A   
   C   
   G   
   A   
   A   
   T   
   T   
   A   
   G   
   G   
   G   
   G   
   T   
   A   
   A   
   C   
   C   
   C   
   C   
   T   
   G   
   A   
   T   
   A   
   A   
   A   
   C   
   C   
   A   
   C   
   A   
   C   
   A   
   A   
   A   
   A   
   A   
   A   
   T   
   C   
   A   
   G   
   C   
   C   
   C   
   G   
   G   
   A   
   T   
   T   
   G   
   T   
   T   
   G   
   G   
   G   
   G   
   A   
   T   
   G   
   T   
   T   
   A   
   T   
   G   
   G   
   G   
   T   
   A   
   A   
   A   
   T   
   A   
   T   
   C   
   A   
   C   
   C   
   C   
   C   
   C   
   A   
   T   
   G   
   G   
   G   
   G   
   A   
   C   
   A   
   G   
   T   
   G   
   C   
   A   
   A   
   T   
   T   
   T   
   A   
   C   
   G   
   A   
   A   
   T   
   C   
   A   
   A   
   T   
   G   
   G   
   T   
   G   
   C   
   G   
   G   
   A   
   T   
   G   
   G   
   C   
   A   
   C   
   A   
   A   
   C   
   C   
   T   
   T   
   T   
   T   
   A   
   G   
   T   
   T   
   A   
   T   
   C   
   G   
   G   
   G   
   C   
   G   
   A   
   T   
   G   
   T   
   T   
   A   
   G   
   T   
   T   
   G   
   A   
   C   
   G   
   G   
   C   
   C   
   A   
   C   
   G   
   G   
   A   
   A   
   A   
   C   
   T   
   T   
   C   
   G   
   G   
   T   
   T   
   C   
   T   
   G   
   T   
   C   
   G   
   A   
   T   
   G   
   G   
   T   
   G   
   A   
   C   
   G   
   G   
   C   
   G   
   C   
   T   
   G   
   C   
   C   
   G   
   C   
   T   
   A   
   T   
   G   
   C   
   G   
   T   
   T   
   A   
   T   
   A   
   C   
   C   
   G   
   A   
   A   
   G   
   C   
   A   
   C   
   G   
   T   
   A   
   T   
   G   
   A   
   G   
   T   
   A   
   A   
   A   
   A   
   T   
   T   
   G   
   C   
   T   
   T   
   T   
   A   
   G   
   A   
   A   
   A   
   T   
   G   
   C   
   T   
   A   
   C   
   G   
   A   
   G   
   A   
   T   
   A   
   T   
   T   
   A   
   A   
   C   
   A   
   A   
   A   
   A   
   A   
   T   
   A   
   C   
   A   
   G   
   T   
   C   
   G   
   A   
   T   
   T   
   T   
   C   
   C   
   A   
   A   
   G   
   G   
   A   
   A   
   A   
   C   
   T   
   A   
   T   
   G   
   A   
   T   
   G   
   A   
   T   
   T   
   C   
   A   
   G   
   A   
   A   
   C   
   A   
   A   
   G   
   A   
   A   
   C   
   C   
   A   
   G   
   T   
   A   
   G   
   T   
   T   
   T   
   T   
   A   
   C   
   C   
   T   
   G   
   C   
   T   
   C   
   G   
   T   
   T   
   T   
   T   
   C   
   C   
   A   
   A   
   A   
   C   
   T   
   T   
   A   
   C   
   T   
   A   
   G   
   T   
   T   
   A   
   A   
   C   
   G   
   G   
   A   
   A   
   C   
   A   
   A   
   C   
   G   
   G   
   G   
   G   
   A   
   T   
   T   
   G   
   C   
   G   
   G   
   T   
   G   
   G   
   G   
   G   
   A   
   T   
   G   
   G   
   C   
   A   
   A   
   C   
   A   
   A   
   A   
   T   
   A   
   T   
   C   
   C   
   C   
   G   
   C   
   C   
   A   
   C   
   A   
   C   
   A   
   A   
   T   
   T   
   T   
   A   
   A   
   G   
   T   
   G   
   A   
   A   
   G   
   T   
   G   
   A   
   T   
   T   
   G   
   A   
   T   
   G   
   C   
   A   
   A   
   C   
   A   
   A   
   G   
   T   
   T   
   T   
   A   
   T   
   T   
   G   
   A   
   T   
   G   
   G   
   A   
   C   
   A   
   A   
   T   
   C   
   C   
   T   
   G   
   A   
   T   
   G   
   T   
   A   
   A   
   C   
   G   
   A   
   C   
   G   
   A   
   A   
   T   
   G   
   A   
   A   
   T   
   T   
   G   
   A   
   T   
   G   
   G   
   A   
   A   
   G   
   T   
   G   
   T   
   T   
   A   
   C   
   C   
   T   
   G   
   G   
   A   
   C   
   C   
   A   
   G   
   A   
   C   
   T   
   T   
   T   
   C   
   C   
   A   
   A   
   C   
   A   
   G   
   G   
   C   
   G   
   G   
   T   
   T   
   T   
   A   
   G   
   T   
   G   
   A   
   T   
   G   
   G   
   G   
   G   
   A   
   A   
   A   
   T   
   C   
   A   
   G   
   G   
    G    
   A   
   T   
    T    
   C   
   G   
    C    
    C    
   G   
   A   
   G   
   C   
    A    
   T   
   A   
   T   
   G   
   A   
   A   
   A   
   C   
    A    
   G   
   G   
    G    
   A   
   A   
    A    
   G   
   G   
   T   
   T   
   C   
    G    
   A   
   T   
   T   
   A   
   C   
    T    
   G   
   T   
    T    
   C   
   G   
   T   
   G   
   C   
   A   
   A   
   A   
   A   
   G   
   T   
   T   
   G   
   A   
    A    
    T    
   T   
    G    
   A   
    C    
   T   
   G   
   A   
    A    
   A   
   T   
   G   
    C    
   C   
    G    
   A   
   A   
   T   
   G   
   G   
    A    
   A   
   A   
   A   
   G   
   A   
   A   
   C   
   G   
    T    
   A   
   T   
    T    
    T    
   T   
    A    
   G   
   T   
    A    
   A   
   C   
    T    
   G   
   A   
   A   
   T   
   T   
    G    
   C   
   C   
   T   
   T   
   A   
   T   
   A   
   T   
   G   
   G   
   T   
    G    
   A   
   A   
   T   
   A   
   A   
   A   
   G   
   C   
    C    
   A   
   A   
   A   
   T   
   T   
    A    
   A   
   T   
    C    
   G   
   A   
   A   
   C   
   G   
    A    
   A   
   T   
   T   
   T   
   C   
   T   
   G   
   A   
    A    
    T    
   T   
    A    
   C   
   A   
   C   
   A   
   G   
   A   
   G   
   A   
    T    
   A   
   A   
    A    
   C   
   G   
    A    
   A   
   T   
    T    
   G   
   A   
   A   
   G   
   G   
    A    
   A   
   T   
    T    
   A   
   C   
    T    
   G   
   A   
   T   
   T   
   T   
   G   
   C   
   G   
   G   
   G   
   A   
    T    
   G   
   A   
   A   
   T   
   C   
   T   
   T   
   C   
    T    
   C   
   G   
   T   
   G   
   A   
    A    
   G   
   G   
    C    
   A   
   T   
   G   
   C   
   G   
    G    
   A   
   T   
    C    
   G   
   T   
   C   
   A   
   T   
    C    
   G   
   A   
    T    
    G    
   T   
    T    
   C   
   G   
   T   
   C   
   G   
    A    
   G   
   A   
    T    
   G   
    T    
    G    
   A   
   G   
    T    
   G   
   C   
    C    
   T   
   C   
    T    
   G   
   T   
   T   
    G    
   T   
    G    
   T   
   T   
    A    
   A   
   A   
    C    
   A   
   A   
   C   
   T   
   T   
    A    
   T   
   A   
    C    
   A   
   A   
   A   
    A    
   T   
   G   
   A   
   C   
    T    
    G    
   C   
    C    
   T   
   T   
    A    
   C   
   A   
    A    
   A   
    C    
    A    
   T   
   C   
    T    
   T   
   T   
    T    
   G   
   G   
    T    
   T   
   T   
    T    
   A   
   A   
    C    
   A   
   T   
   G   
   T   
   T   
    A    
   G   
   C   
    C    
   A   
   T   
    C    
   G   
   A   
   A   
   A   
   A   
   A   
   G   
   G   
    C    
   G   
   T   
    A    
   C   
   C   
    G    
   A   
   A   
   A   
    A    
   T   
   T   
   T   
   T   
    A    
   A   
   G   
    T    
   T   
   T   
   G   
   A   
   A   
   A   
   C   
    G    
    T    
   A   
   T   
    T    
    T    
   T   
    A    
   G   
   A   
   A   
   A   
   A   
    T    
   T   
   A   
    C    
    A    
   T   
    C    
   G   
   A   
    G    
   C   
   A   
    C    
   C   
   A   
    A    
    A    
    A    
    A    
    G    
   A   
   A   
   G   
   T   
   C   
   A   
   T   
    T    
   A   
   C   
    T    
    C    
   G   
    C    
   C   
   G   
   G   
   A   
   C   
    G    
    A    
   T   
    T    
   T   
   T   
   T   
   G   
   A   
    C    
   A   
   A   
   A   
    A    
   A   
    C    
   A   
   A   
    A    
   G   
   C   
   A   
   G   
   A   
   A   
   G   
   C   
   A   
    C    
   G   
    G    
   G   
   C   
    G    
   C   
   A   
    T    
   A   
   T   
    T    
   T   
   T   
   A   
   G   
   A   
   A   
   G   
   G   
   T   
    C    
   T   
    A    
   C   
   G   
    A    
   A   
   T   
   T   
   G   
   C   
    C    
   T   
   T   
    A    
   G   
   A   
   T   
   C   
   A   
   T   
   A   
   T   
   C   
    G    
   A   
   T   
   G   
   A   
   A   
   A   
   T   
    C    
   A   
   T   
   T   
   G   
   C   
    C    
   A   
   T   
   T   
   A   
   T   
    C    
   C   
   G   
    T    
   G   
   G   
    G    
   T   
   C   
    A    
    C    
   A   
   A   
   T   
   C   
   A   
   G   
   A   
   T   
   G   
   A   
    C    
   G   
   A   
    A    
   G   
   C   
   A   
   A   
   A   
   A   
   G   
   C   
    A    
   A   
    C    
    G    
    T    
   T   
    A    
   A   
   T   
    T    
   G   
   A   
   A   
   C   
   G   
    C    
   T   
   T   
    T    
   G   
   A   
    A    
   T   
   T   
    T    
   T   
   C   
    A    
   G   
   A   
   T   
   C   
   G   
    T    
   C   
   A   
    A    
    G    
   C   
    G    
   C   
   A   
   A   
   G   
   C   
    G    
   A   
   T   
    T    
   T   
   T   
   A   
   G   
   A   
   T   
   A   
   T   
   G   
   C   
   G   
   T   
   T   
   T   
    A    
   C   
   G   
    C    
   C   
   G   
    T    
   T   
   T   
    A    
   A   
   C   
    A    
   G   
   G   
    T    
   T   
   T   
    A    
   G   
   A   
   A   
   C   
   G   
    C    
   G   
   A   
    C    
   A   
   A   
   A   
   A   
   T   
    T    
   G   
   A   
    G    
   A   
   A   
   T   
   G   
   A   
   A   
   T   
   A   
    T    
   C   
   A   
   A   
   G   
   A   
    G    
   C   
   T   
    A    
   T   
   T   
    G    
   A   
    A    
   A   
    T    
   T   
    C    
   A   
   T   
   T   
   G   
    C    
    G    
   G   
   A   
    C    
   T   
   T   
    A    
    G    
    A    
    G    
   G   
   A   
   C   
   A   
   T   
   T   
    T    
   T   
    A    
   G   
   C   
    C    
    C    
   G   
    C    
   C   
   C   
    A    
   G   
   A   
    A    
   C   
   G   
    C    
   G   
   T   
    C    
   A   
    T    
    T    
   G   
   A   
   A   
   A   
   T   
    C    
   A   
   T   
    T    
   A   
   A   
    A    
   A   
    C    
    A    
   G   
   A   
    G    
   T   
   T   
   A   
    A    
    A    
    T    
   G   
   A   
   C   
    G    
   T   
    T    
    C    
    G    
    C    
    A    
    C    
   A   
    A    
    A    
   A   
   T   
   T   
   T   
   G   
   G   
   T   
   G   
   A   
   T   
    G    
    C    
   A   
   C   
   G   
   A   
   C   
   G   
    C    
   A   
   C   
   A   
   G   
   A   
   A   
    T    
   T   
   A   
    T    
   T   
   A   
   G   
   T   
    A    
   G   
   G   
   T   
   G   
   A   
    A    
   G   
   T   
   C   
    C    
   T   
    A    
   A   
   G   
   T   
   C   
   T   
    T    
   G   
   A   
   A   
   G   
   A   
   T   
   G   
   A   
   A   
   G   
   A   
   T   
    C    
   T   
    A    
   A   
   T   
    C    
   G   
   A   
   A   
   G   
   A   
   A   
   G   
   A   
   A   
   G   
   A   
   A   
    G    
   T   
   C   
   G   
   T   
    G    
   A   
   T   
   T   
   A   
   C   
    G    
    T    
   T   
    A    
   A   
   C   
    C    
   A   
   A   
    T    
   A   
   A   
    C    
   G   
   G   
    C    
   T   
   A   
    C    
   A   
   T   
    T    
   A   
   A   
    G    
   C   
   G   
    G    
    A    
   T   
   G   
   G   
    C    
   A   
   A   
   A   
    C    
    T    
    C    
    C    
   G   
   A   
   A   
   T   
   T   
   C   
    C    
   G   
    G    
   G   
   C   
    G    
   C   
   A   
   A   
   C   
   G   
    C    
    C    
   G   
    C    
   G   
   G   
    T    
   G   
   G   
    A    
   C   
   G   
   T   
   G   
   G   
    T    
   G   
   T   
    T    
   C   
   A   
   A   
   G   
   G   
    C    
   A   
   T   
   G   
   G   
   G   
    C    
    G    
   T   
    T    
   C   
   A   
    T    
    G    
   A   
   T   
   G   
   A   
    T    
   G   
   A   
    C    
   T   
   T   
   C   
   G   
   T   
    G    
   A   
   A   
    A    
   A   
   A   
    C    
   C   
   T   
    A    
    G    
   T   
   T   
   T   
   C   
   T   
   T   
   G   
    T    
   T   
   C   
    A    
   A   
   C   
    A    
   C   
   A   
    C    
   G   
   A   
    C    
   A   
   C   
    G    
   T   
   T   
    A    
   T   
   T   
   A   
   T   
   T   
    C    
   T   
   T   
   T   
   A   
   C   
   G   
   A   
   A   
    C    
   A   
    C    
    T    
   G   
   G   
    G    
   A   
   A   
   A   
   G   
   T   
    T    
   T   
   A   
    C    
   C   
   G   
    A    
   G   
   C   
   A   
   A   
   A   
   A   
   G   
   G   
    T    
   T   
   A   
    C    
   G   
   A   
   A   
   A   
   T   
    C    
   C   
   C   
    T    
   G   
   A   
    A    
   T   
   A   
   C   
   G   
   G   
    T    
    A    
   G   
    A    
   A   
   C   
   A   
   G   
   C   
    A    
   A   
   A   
   A   
   G   
   G   
    A    
   A   
   T   
    T    
   C   
   C   
   A   
    G    
   T   
    G    
   A   
   T   
    T    
   A   
   A   
    C    
   T   
   T   
    A    
   C   
   T   
    G    
   G   
   G   
    A    
   A   
   T   
    T    
   G   
   A   
    T    
   T   
   C   
    T    
    G    
    C    
    A    
   G   
   A   
   A   
   A   
    A    
    A    
   A   
   T   
    T    
   C   
   A   
    A    
   G   
   C   
    G    
   A   
   T   
    T    
   A   
   T   
   T   
    T    
   C   
    T    
   G   
   T   
    T    
    G    
    A    
   G   
   G   
   G   
    C    
    A    
   A   
   A   
   G   
   C   
    G    
   G   
   A   
   A   
   G   
    C    
    A    
   G   
   G   
    T    
    A    
   A   
    A    
   T   
   A   
    C    
    T    
   T   
    A    
   T   
   T   
   C   
   T   
   T   
    C    
   A   
   C   
    A    
   A   
   C   
    C    
    T    
    T    
    A    
    A    
   A   
   A   
   G   
   G   
    A    
   A   
   C   
    C    
   G   
   T   
    C    
   A   
   A   
   A   
   C   
   G   
    G    
   A   
   C   
    A    
    G    
   C   
    C    
   G   
   T   
    A    
   A   
   C   
    A    
   G   
   C   
    C    
   T   
   T   
    T    
    T    
   C   
   T   
   A   
   A   
    T    
   A   
   T   
    C    
   C   
   G   
    T    
   A   
   G   
    T    
   A   
   A   
   T   
   G   
   G   
    A    
    T    
   T   
    A    
   A   
   T   
    C    
   G   
   C   
    C    
   A   
   T   
   T   
    A    
   G   
    C    
   T   
   T   
    A    
   A   
   A   
   A   
   G   
   A   
   A   
   G   
   A   
    T    
   G   
   A   
    T    
   G   
   A   
    G    
    T    
   T   
    A    
    G    
   T   
    T    
   A   
   A   
    C    
   G   
   T   
    G    
    G    
   T   
   A   
    A    
    C    
   G   
   A   
   C   
    T    
    A    
   A   
    T    
   G   
   G   
    C    
    A    
   A   
    T    
    C    
    A    
    G    
   A   
   A   
    G    
   A   
   T   
    G    
   A   
   T   
    T    
   A   
   T   
    C    
   G   
   G   
    A    
   A   
   C   
   A   
   C   
   A   
   T   
    G    
    C    
    A    
   G   
   G   
    A    
   T   
   A   
   C   
   T   
   C   
   T   
   G   
   T   
    C    
   A   
   C   
    A    
   T   
   T   
    T    
    G    
   A   
    T    
   G   
   A   
    A    
    A    
    C    
    T    
    A    
   C   
    T    
   G   
    T    
    A    
   C   
   G   
    T    
   G   
   A   
   T   
   A   
   T   
   G   
   G   
   G   
    C    
   C   
   G   
    G    
   A   
   C   
   A   
   G   
   C   
    A    
   T   
   C   
    A    
   G   
   G   
    T    
   G   
   T   
    T    
    C    
   G   
    T    
   G   
   G   
    A    
   A   
   T   
    C    
   C   
   G   
    T    
    C    
   T   
    C    
   C   
   G   
    C    
   G   
   A   
    A    
    A    
   A   
    T    
   G   
   A   
    T    
   T   
    A    
    T    
   G   
   T   
    G    
   G   
   T   
    C    
   G   
   G   
    C    
    G    
   C   
   A   
   G   
   C   
    G    
    A    
   T   
    T    
    C    
   T   
   G   
    G    
   A   
    T    
   G   
    A    
   A   
    A    
   A   
    T    
    A    
   A   
   A   
   G   
   A   
   A   
   G   
   T   
    C    
    C    
   T   
    A    
   G   
   T   
    C    
   A   
   T   
    T    
   A   
   C   
    T    
   G   
   A   
   A   
   A   
   A   
    T    
   G   
   G   
   T   
   T   
   A   
    T    
   G   
   G   
    T    
   A   
   A   
    G    
   C   
   G   
    T    
   A   
   C   
    A    
   A   
   A   
   A   
   G   
    C    
    T    
   T   
   C   
    T    
   G   
   A   
    A    
   T   
   A   
   T   
   C   
   C   
   A   
   G   
   T   
    T    
   A   
   A   
    A    
   G   
   G   
    A    
   C   
   G   
    T    
   G   
   G   
    C    
   G   
   G   
   T   
   A   
   A   
   A   
   G   
   G   
    G    
   A   
   T   
    T    
   A   
   A   
    G    
   A   
   C   
    A    
    G    
   C   
    A    
   A   
   A   
    T    
   A   
   T   
   C   
   A   
   C   
   T   
   G   
   A   
    G    
   A   
   A   
   A   
   A   
   A   
   T   
   G   
   G   
   T   
   C   
   C   
   A   
    T    
   T   
   A   
   G   
   C   
    T    
   G   
   G   
    T    
   T   
   T   
    A    
   A   
   C   
   C   
   A   
   C   
    G    
   G   
   T   
    C    
    A    
    A    
   T   
   G   
   G   
    G    
    G    
   A   
   T   
   G   
   A   
   A   
   G   
   A   
    T    
   A   
   T   
   C   
    T    
   T   
    A    
    T    
   T   
    A    
   A   
   T   
    C    
   A   
   C   
    G    
    A    
   A   
    C    
   A   
   A   
   A   
   G   
   G   
    T    
   G   
   T   
    C    
   A   
   T   
   T   
   A   
   T   
   C   
   C   
   G   
    C    
   T   
   T   
    T    
   A   
   A   
   C   
   G   
   T   
    T    
    G    
    A    
    T    
    T    
   C   
   T   
   G   
   T   
   T   
   T   
   C   
   T   
   C   
   A   
   A   
   A   
   C   
    A    
   G   
   G   
   A   
   C   
   G   
    C    
    G    
   C   
   A   
   A   
   C   
   A   
   T   
   T   
    A    
   G   
   G   
    G    
   G   
   T   
    T    
   C   
   G   
   T   
   T   
   T   
    A    
   A   
   T   
   G   
   A   
    G    
    A    
   A   
   T   
   G   
   G   
   A   
   A   
   G   
    A    
    T    
   G   
    G    
    C    
    G    
   C   
    C    
   A   
   A   
   A   
   -   
   -   
   -   
   -   
   -   
   -   
   -   
   -   
   -   
   -   
   -   
   -   
   -   
   -   
   -   
   -   
   -   
   -   
   -   
   -   
   -   
   -   
   -   
   -   
   -   
   -   
   -   
   -   
   -   
   -   
   -   
   -   
   -   
   -   
   -   
   -   
   -   
   -   
   -   
   -   
   -   
   -   
   -   
   -   
   -   
   -   
   -   
   -   
   -   
   -   
   -   
   -   
   -   
   -   
   -   
   -   
   -   
   -   
   -   
   -   
   -   
   -   
   -   
   -   
   -   
   -   
   -   
   -   
   -   
   -   
   -   
   -   
 

 
   N59959F   
   A   
   T   
   G   
   A   
   G   
   T   
   G   
   A   
   A   
   G   
   A   
   A   
   A   
   T   
   T   
   A   
   A   
   A   
   G   
   A   
   A   
   A   
   A   
   C   
   A   
   T   
   T   
   C   
   A   
   A   
   G   
   A   
   C   
   G   
   T   
   C   
   A   
   A   
   T   
   C   
   T   
   G   
   A   
   C   
   C   
   A   
   G   
   C   
   G   
   A   
   A   
   A   
   T   
   G   
   A   
   A   
   A   
   G   
   A   
   A   
   T   
   C   
   T   
   T   
   T   
   C   
   A   
   T   
   T   
   G   
   A   
   C   
   T   
   A   
   C   
   G   
   C   
   A   
   A   
   T   
   G   
   A   
   G   
   T   
   G   
   T   
   T   
   A   
   T   
   C   
   G   
   T   
   A   
   G   
   C   
   C   
   C   
   G   
   C   
   G   
   C   
   G   
   T   
   T   
   A   
   C   
   C   
   T   
   G   
   A   
   C   
   G   
   T   
   T   
   C   
   G   
   T   
   G   
   A   
   C   
   G   
   G   
   T   
   T   
   T   
   A   
   A   
   A   
   A   
   C   
   C   
   T   
   G   
   T   
   T   
   C   
   A   
   T   
   C   
   G   
   C   
   C   
   G   
   A   
   A   
   T   
   C   
   T   
   T   
   A   
   T   
   A   
   T   
   G   
   G   
   A   
   A   
   T   
   G   
   A   
   A   
   C   
   G   
   A   
   A   
   T   
   T   
   A   
   G   
   G   
   G   
   G   
   T   
   A   
   A   
   C   
   C   
   C   
   C   
   T   
   G   
   A   
   T   
   A   
   A   
   A   
   C   
   C   
   A   
   C   
   A   
   C   
   A   
   A   
   A   
   A   
   A   
   A   
   T   
   C   
   A   
   G   
   C   
   C   
   C   
   G   
   G   
   A   
   T   
   T   
   G   
   T   
   T   
   G   
   G   
   G   
   G   
   A   
   T   
   G   
   T   
   T   
   A   
   T   
   G   
   G   
   G   
   T   
   A   
   A   
   A   
   T   
   A   
   T   
   C   
   A   
   C   
   C   
   C   
   C   
   C   
   A   
   T   
   G   
   G   
   G   
   G   
   A   
   C   
   A   
   G   
   T   
   G   
   C   
   G   
   A   
   T   
   T   
   T   
   A   
   C   
   G   
   A   
   A   
   T   
   C   
   A   
   A   
   T   
   G   
   G   
   T   
   G   
   C   
   G   
   G   
   A   
   T   
   G   
   G   
   C   
   A   
   C   
   A   
   A   
   C   
   C   
   T   
   T   
   T   
   T   
   A   
   G   
   T   
   T   
   A   
   T   
   C   
   G   
   G   
   G   
   C   
   T   
   A   
   T   
   G   
   T   
   T   
   A   
   G   
   T   
   T   
   G   
   A   
   C   
   G   
   G   
   C   
   C   
   A   
   C   
   G   
   G   
   A   
   A   
   A   
   C   
   T   
   T   
   C   
   G   
   G   
   T   
   T   
   C   
   T   
   G   
   T   
   C   
   G   
   A   
   T   
   G   
   G   
   T   
   G   
   A   
   C   
   G   
   G   
   C   
   G   
   C   
   T   
   G   
   C   
   C   
   G   
   C   
   T   
   A   
   T   
   G   
   C   
   G   
   T   
   T   
   A   
   T   
   A   
   C   
   C   
   G   
   A   
   A   
   G   
   C   
   A   
   C   
   G   
   T   
   A   
   T   
   G   
   A   
   G   
   T   
   A   
   A   
   A   
   A   
   T   
   T   
   G   
   C   
   T   
   T   
   T   
   A   
   G   
   A   
   A   
   A   
   T   
   G   
   C   
   T   
   A   
   C   
   G   
   A   
   G   
   A   
   T   
   A   
   T   
   T   
   A   
   A   
   C   
   A   
   A   
   A   
   A   
   A   
   T   
   A   
   C   
   A   
   G   
   T   
   C   
   G   
   A   
   T   
   T   
   T   
   C   
   C   
   A   
   A   
   G   
   G   
   A   
   A   
   A   
   C   
   T   
   A   
   T   
   G   
   A   
   T   
   G   
   A   
   T   
   T   
   C   
   A   
   G   
   A   
   A   
   C   
   A   
   A   
   G   
   A   
   A   
   C   
   C   
   A   
   G   
   T   
   A   
   G   
   T   
   T   
   T   
   T   
   A   
   C   
   C   
   T   
   G   
   C   
   T   
   C   
   G   
   T   
   T   
   T   
   T   
   C   
   C   
   A   
   A   
   A   
   C   
   T   
   T   
   A   
   C   
   T   
   A   
   G   
   T   
   T   
   A   
   A   
   C   
   G   
   G   
   A   
   A   
   C   
   A   
   A   
   C   
   G   
   G   
   G   
   G   
   A   
   T   
   T   
   G   
   C   
   G   
   G   
   T   
   G   
   G   
   G   
   G   
   A   
   T   
   G   
   G   
   C   
   A   
   A   
   C   
   A   
   A   
   A   
   T   
   A   
   T   
   T   
   C   
   C   
   A   
   C   
   C   
   A   
   C   
   A   
   C   
   A   
   A   
   T   
   T   
   T   
   A   
   A   
   G   
   T   
   G   
   A   
   A   
   G   
   T   
   G   
   A   
   T   
   T   
   G   
   A   
   T   
   G   
   C   
   A   
   A   
   C   
   A   
   A   
   G   
   T   
   T   
   T   
   A   
   T   
   T   
   G   
   A   
   T   
   G   
   G   
   A   
   C   
   A   
   A   
   T   
   C   
   C   
   T   
   G   
   A   
   T   
   G   
   T   
   A   
   A   
   C   
   G   
   A   
   C   
   G   
   A   
   A   
   T   
   G   
   A   
   A   
   T   
   T   
   G   
   A   
   T   
   G   
   G   
   A   
   A   
   G   
   T   
   G   
   T   
   T   
   A   
   C   
   C   
   T   
   G   
   G   
   A   
   C   
   C   
   A   
   G   
   A   
   C   
   T   
   T   
   T   
   C   
   C   
   A   
   A   
   C   
   A   
   G   
   G   
   C   
   G   
   G   
   T   
   T   
   T   
   A   
   G   
   T   
   G   
   A   
   T   
   G   
   G   
   G   
   G   
   A   
   A   
   A   
   T   
   C   
   A   
   G   
   G   
    G    
   A   
   T   
    T    
   C   
   G   
    T    
    C    
   G   
   A   
   G   
   C   
    A    
   T   
   A   
   T   
   G   
   A   
   A   
   A   
   C   
    A    
   G   
   G   
    G    
   A   
   A   
    A    
   G   
   G   
   T   
   T   
   C   
    G    
   A   
   T   
   T   
   A   
   C   
    T    
   G   
   T   
    T    
   C   
   G   
   T   
   G   
   C   
   A   
   A   
   A   
   A   
   G   
   T   
   T   
   G   
   A   
    A    
    T    
   T   
    G    
   A   
    C    
   T   
   G   
   A   
    A    
   A   
   T   
   G   
    C    
   C   
    G    
   A   
   A   
   T   
   G   
   G   
    A    
   A   
   A   
   A   
   G   
   A   
   A   
   C   
   G   
    T    
   A   
   T   
    T    
    T    
   T   
    A    
   G   
   T   
    A    
   A   
   C   
    T    
   G   
   A   
   A   
   T   
   T   
    G    
   C   
   C   
   T   
   T   
   A   
   T   
   A   
   T   
   G   
   G   
   T   
    G    
   A   
   A   
   T   
   A   
   A   
   A   
   G   
   C   
    C    
   A   
   A   
   A   
   T   
   T   
    A    
   A   
   T   
    C    
   G   
   A   
   A   
   C   
   G   
    A    
   A   
   T   
   T   
   T   
   C   
   T   
   G   
   A   
    A    
    T    
   T   
    A    
   C   
   A   
   C   
   A   
   G   
   A   
   G   
   A   
    T    
   A   
   A   
    A    
   C   
   G   
    A    
   A   
   T   
    T    
   G   
   A   
   A   
   G   
   G   
    A    
   A   
   T   
    T    
   A   
   C   
    T    
   G   
   A   
   T   
   T   
   T   
   G   
   C   
   G   
   G   
   G   
   A   
    T    
   G   
   A   
   A   
   T   
   C   
   T   
   T   
   C   
    T    
   C   
   G   
   T   
   G   
   A   
    A    
   G   
   G   
    C    
   A   
   T   
   G   
   C   
   G   
    G    
   A   
   T   
    C    
   G   
   T   
   C   
   A   
   T   
    C    
   G   
   A   
    T    
    G    
   T   
    T    
   C   
   G   
   T   
   C   
   G   
    A    
   G   
   A   
    T    
   G   
    T    
    G    
   A   
   G   
    T    
   G   
   C   
    C    
   T   
   C   
    T    
   G   
   T   
   T   
    G    
   T   
    G    
   T   
   T   
    A    
   A   
   A   
    C    
   A   
   A   
   C   
   T   
   T   
    G    
   T   
   A   
    C    
   A   
   A   
   A   
    A    
   T   
   G   
   A   
   C   
    T    
    G    
   C   
    C    
   T   
   T   
    A    
   C   
   A   
    A    
   A   
    C    
    A    
   T   
   C   
    T    
   T   
   T   
    T    
   G   
   G   
    T    
   T   
   T   
    T    
   A   
   A   
    C    
   A   
   T   
   G   
   T   
   T   
    A    
   G   
   C   
    C    
   A   
   T   
    T    
   G   
   A   
   A   
   A   
   A   
   A   
   G   
   G   
    T    
   G   
   T   
    A    
   C   
   C   
    G    
   A   
   A   
   A   
    A    
   T   
   T   
   T   
   T   
    A    
   A   
   G   
    C    
   T   
   T   
   G   
   A   
   A   
   A   
   C   
    G    
    T    
   A   
   T   
    T    
    T    
   T   
    A    
   G   
   A   
   A   
   A   
   A   
    T    
   T   
   A   
    C    
    G    
   T   
    C    
   G   
   A   
    G    
   C   
   A   
    C    
   C   
   A   
    A    
    A    
    A    
    A    
    G    
   A   
   A   
   G   
   T   
   C   
   A   
   T   
    T    
   A   
   C   
    T    
    C    
   G   
    C    
   C   
   G   
   G   
   A   
   C   
    G    
    A    
   T   
    T    
   T   
   T   
   T   
   G   
   A   
    C    
   A   
   A   
   A   
    A    
   A   
    C    
   A   
   A   
    A    
   G   
   C   
   A   
   G   
   A   
   A   
   G   
   C   
   A   
    C    
   G   
    G    
   G   
   C   
    G    
   C   
   A   
    T    
   A   
   T   
    T    
   T   
   T   
   A   
   G   
   A   
   A   
   G   
   G   
   T   
    C    
   T   
    A    
   C   
   G   
    A    
   A   
   T   
   T   
   G   
   C   
    C    
   T   
   T   
    A    
   G   
   A   
   T   
   C   
   A   
   T   
   A   
   T   
   C   
    G    
   A   
   T   
   G   
   A   
   A   
   A   
   T   
    C    
   A   
   T   
   T   
   G   
   C   
    C    
   A   
   T   
   T   
   A   
   T   
    C    
   C   
   G   
    T    
   G   
   G   
    G    
   T   
   C   
    A    
    C    
   A   
   A   
   T   
   C   
   A   
   G   
   A   
   T   
   G   
   A   
    T    
   G   
   A   
    A    
   G   
   C   
   A   
   A   
   A   
   A   
   G   
   C   
    A    
   A   
    C    
    G    
    T    
   T   
    A    
   A   
   T   
    T    
   G   
   A   
   A   
   C   
   G   
    C    
   T   
   T   
    T    
   G   
   A   
    A    
   T   
   T   
    T    
   T   
   C   
    A    
   G   
   A   
   T   
   C   
   G   
    T    
   C   
   A   
    A    
    G    
   C   
    G    
   C   
   A   
   A   
   G   
   C   
    G    
   A   
   T   
    T    
   T   
   T   
   A   
   G   
   A   
   T   
   A   
   T   
   G   
   C   
   G   
   T   
   T   
   T   
    A    
   C   
   G   
    C    
   C   
   G   
    T    
   T   
   T   
    A    
   A   
   C   
    A    
   G   
   G   
    C    
   T   
   T   
    A    
   G   
   A   
   A   
   C   
   G   
    C    
   G   
   A   
    C    
   A   
   A   
   A   
   A   
   T   
    T    
   G   
   A   
    A    
   A   
   A   
   T   
   G   
   A   
   A   
   T   
   A   
    T    
   C   
   A   
   A   
   G   
   A   
    G    
   C   
   T   
    A    
   T   
   T   
    G    
   A   
    A    
   A   
    T    
   T   
    C    
   A   
   T   
   T   
   G   
    C    
    G    
   G   
   A   
    C    
   T   
   T   
    A    
    G    
    A    
    G    
   G   
   A   
   C   
   A   
   T   
   T   
    T    
   T   
    A    
   G   
   C   
    C    
    C    
   G   
    C    
   C   
   C   
    A    
   G   
   A   
    A    
   C   
   G   
    C    
   G   
   T   
    C    
   A   
    T    
    T    
   G   
   A   
   A   
   A   
   T   
    C    
   A   
   T   
    T    
   A   
   A   
    A    
   A   
    C    
    A    
   G   
   A   
    G    
   T   
   T   
   A   
    A    
    A    
    T    
   G   
   A   
   C   
    G    
   T   
    T    
    C    
    G    
    C    
    A    
    C    
   A   
    A    
    A    
   A   
   T   
   T   
   T   
   G   
   G   
   T   
   G   
   A   
   T   
    G    
    C    
   A   
   C   
   G   
   A   
   C   
   G   
    T    
   A   
   C   
   A   
   G   
   A   
   A   
    C    
   T   
   A   
    T    
   T   
   A   
   G   
   T   
    A    
   G   
   G   
   T   
   G   
   A   
    A    
   G   
   T   
   C   
    C    
   T   
    A    
   A   
   G   
   T   
   C   
   T   
    T    
   G   
   A   
   A   
   G   
   A   
   T   
   G   
   A   
   A   
   G   
   A   
   T   
    T    
   T   
    A    
   A   
   T   
    C    
   G   
   A   
   A   
   G   
   A   
   A   
   G   
   A   
   A   
   G   
   A   
   A   
    G    
   T   
   C   
   G   
   T   
    G    
   A   
   T   
   T   
   A   
   C   
    A    
    T    
   T   
    A    
   A   
   C   
    C    
   A   
   A   
    T    
   A   
   A   
    C    
   G   
   G   
    C    
   T   
   A   
    C    
   A   
   T   
    T    
   A   
   A   
    G    
   C   
   G   
    G    
    A    
   T   
   G   
   G   
    C    
   A   
   A   
   A   
    C    
    T    
    C    
    C    
   G   
   A   
   A   
   T   
   T   
   C   
    C    
   G   
    G    
   G   
   C   
    G    
   C   
   A   
   A   
   C   
   G   
    C    
    C    
   G   
    C    
   G   
   G   
    C    
   G   
   G   
    A    
   C   
   G   
   T   
   G   
   G   
    T    
   G   
   T   
    C    
   C   
   A   
   A   
   G   
   G   
    G    
   A   
   T   
   G   
   G   
   G   
    C    
    G    
   T   
    T    
   C   
   A   
    T    
    G    
   A   
   T   
   G   
   A   
    T    
   G   
   A   
    T    
   T   
   T   
   C   
   G   
   T   
    G    
   A   
   A   
    A    
   A   
   A   
    C    
   C   
   T   
    A    
    G    
   T   
   T   
   T   
   C   
   T   
   T   
   G   
    T    
   T   
   C   
    A    
   A   
   C   
    A    
   C   
   A   
    C    
   G   
   A   
    C    
   A   
   C   
    G    
   T   
   T   
    A    
   T   
   T   
   A   
   T   
   T   
    C    
   T   
   T   
   T   
   A   
   C   
   G   
   A   
   A   
    C    
   A   
    C    
    T    
   G   
   G   
    G    
   A   
   A   
   A   
   G   
   T   
    T    
   T   
   A   
    C    
   C   
   G   
    A    
   G   
   C   
   A   
   A   
   A   
   A   
   G   
   G   
    T    
   T   
   A   
    C    
   G   
   A   
   A   
   A   
   T   
    C    
   C   
   C   
    T    
   G   
   A   
    A    
   T   
   A   
   C   
   G   
   G   
    T    
    A    
   G   
    A    
   A   
   C   
   A   
   G   
   C   
    A    
   A   
   A   
   A   
   G   
   G   
    A    
   A   
   T   
    T    
   C   
   C   
   A   
    G    
   T   
    A    
   A   
   T   
    T    
   A   
   A   
    C    
   T   
   T   
    A    
   C   
   T   
    G    
   G   
   G   
    A    
   A   
   T   
    T    
   G   
   A   
    T    
   T   
   C   
    T    
    G    
    C    
    A    
   G   
   A   
   A   
   A   
    A    
    A    
   A   
   T   
    T    
   C   
   A   
    A    
   G   
   C   
    G    
   A   
   T   
    T    
   A   
   T   
   T   
    T    
   C   
    T    
   G   
   T   
    T    
    G    
    A    
   G   
   G   
   G   
    C    
    A    
   A   
   A   
   G   
   C   
    G    
   G   
   A   
   A   
   G   
    C    
    A    
   G   
   G   
    T    
    A    
   A   
    A    
   T   
   A   
    C    
    T    
   T   
    G    
   T   
   T   
   C   
   T   
   T   
    C    
   A   
   C   
    A    
   A   
   C   
    C    
    T    
    T    
    A    
    A    
   A   
   A   
   G   
   G   
    A    
   A   
   C   
    T    
   G   
   T   
    C    
   A   
   A   
   A   
   C   
   G   
    G    
   A   
   C   
    A    
    G    
   C   
    C    
   G   
   T   
    A    
   A   
   C   
    A    
   G   
   C   
    C    
   T   
   T   
    T    
    T    
   C   
   T   
   A   
   A   
    T    
   A   
   T   
    C    
   C   
   G   
    T    
   A   
   G   
    T    
   A   
   A   
   T   
   G   
   G   
    A    
    T    
   T   
    A    
   A   
   T   
    C    
   G   
   C   
    C    
   A   
   T   
   T   
    A    
   G   
    C    
   T   
   T   
    A    
   A   
   A   
   A   
   G   
   A   
   A   
   G   
   A   
    T    
   G   
   A   
    T    
   G   
   A   
    G    
    T    
   T   
    A    
    G    
   T   
    T    
   A   
   A   
    C    
   G   
   T   
    A    
    G    
   T   
   A   
    A    
    C    
   G   
   A   
   C   
    T    
    A    
   A   
    T    
   G   
   G   
    C    
    A    
   A   
    T    
    C    
    A    
    G    
   A   
   A   
    G    
   A   
   T   
    G    
   A   
   T   
    T    
   A   
   T   
    C    
   G   
   G   
    A    
   A   
   C   
   A   
   C   
   A   
   T   
    G    
    C    
    A    
   G   
   G   
    A    
   T   
   A   
   C   
   T   
   C   
   T   
   G   
   T   
    C    
   A   
   C   
    A    
   T   
   T   
    T    
    G    
   A   
    T    
   G   
   A   
    A    
    A    
    A    
    T    
    A    
   C   
    T    
   G   
    T    
    A    
   C   
   G   
    T    
   G   
   A   
   T   
   A   
   T   
   G   
   G   
   G   
    C    
   C   
   G   
    G    
   A   
   C   
   A   
   G   
   C   
    A    
   T   
   C   
    A    
   G   
   G   
    C    
   G   
   T   
    T    
    C    
   G   
    T    
   G   
   G   
    A    
   A   
   T   
    C    
   C   
   G   
    T    
    C    
   T   
    C    
   C   
   G   
    C    
   G   
   A   
    A    
    A    
   A   
    T    
   G   
   A   
    T    
   T   
    A    
    T    
   G   
   T   
    G    
   G   
   T   
    C    
   G   
   G   
    C    
    G    
   C   
   A   
   G   
   C   
    G    
    A    
   T   
    T    
    C    
   T   
   G   
    G    
   A   
    T    
   G   
    A    
   A   
    A    
   A   
    T    
    A    
   A   
   A   
   G   
   A   
   A   
   G   
   T   
    C    
    C    
   T   
    A    
   G   
   T   
    C    
   A   
   T   
    T    
   A   
   C   
    T    
   G   
   A   
   A   
   A   
   A   
    T    
   G   
   G   
   T   
   T   
   A   
    T    
   G   
   G   
    T    
   A   
   A   
    G    
   C   
   G   
    T    
   A   
   C   
    A    
   A   
   A   
   A   
   G   
    C    
    T    
   T   
   C   
    T    
   G   
   A   
    A    
   T   
   A   
   T   
   C   
   C   
   A   
   G   
   T   
    T    
   A   
   A   
    A    
   G   
   G   
    A    
   C   
   G   
    T    
   G   
   G   
    C    
   G   
   G   
   T   
   A   
   A   
   A   
   G   
   G   
    G    
   A   
   T   
    T    
   A   
   A   
    G    
   A   
   C   
    A    
    G    
   C   
    A    
   A   
   A   
    T    
   A   
   T   
   C   
   A   
   C   
   T   
   G   
   A   
    G    
   A   
   A   
   A   
   A   
   A   
   T   
   G   
   G   
   T   
   C   
   C   
   A   
    T    
   T   
   A   
   G   
   C   
    T    
   G   
   G   
    T    
   T   
   T   
    A    
   A   
   C   
   C   
   A   
   C   
    G    
   G   
   T   
    C    
    A    
    A    
   T   
   G   
   G   
    G    
    G    
   A   
   T   
   G   
   A   
   A   
   G   
   A   
    T    
   A   
   T   
   C   
    T    
   T   
    A    
    T    
   T   
    G    
   A   
   T   
    T    
   A   
   C   
    G    
    A    
   A   
    C    
   A   
   A   
   A   
   G   
   G   
    C    
   G   
   T   
    C    
   A   
   T   
   T   
   A   
   T   
   C   
   C   
   G   
    C    
   T   
   T   
    T    
   A   
   A   
   C   
   G   
   T   
    T    
    G    
    A    
    T    
    T    
   C   
   T   
   G   
   T   
   T   
   T   
   C   
   T   
   C   
   A   
   A   
   A   
   C   
    A    
   G   
   G   
   A   
   C   
   G   
    C    
    G    
   C   
   A   
   A   
   C   
   A   
   T   
   T   
    A    
   G   
   G   
    G    
   G   
   T   
    T    
   C   
   G   
   T   
   T   
   T   
    A    
   A   
   T   
   G   
   A   
    G    
    A    
   A   
   T   
   G   
   G   
   A   
   A   
   G   
    A    
    T    
   G   
    G    
    T    
    G    
   C   
    C    
   A   
   A   
   A   
   -   
   -   
   -   
   -   
   -   
   -   
   -   
   -   
   -   
   -   
   -   
   -   
   -   
   -   
   -   
   -   
   -   
   -   
   -   
   -   
   -   
   -   
   -   
   -   
   -   
   -   
   -   
   -   
   -   
   -   
   -   
   -   
   -   
   -   
   -   
   -   
   -   
   -   
   -   
   -   
   -   
   -   
   -   
   -   
   -   
   -   
   -   
   -   
   -   
   -   
   -   
   -   
   -   
   -   
   -   
   -   
   -   
   -   
   -   
   -   
   -   
   -   
   -   
   -   
   -   
   -   
   -   
   -   
   -   
   -   
   -   
   -   
 

 
   N59435F   
   A   
   T   
   G   
   A   
   G   
   T   
   G   
   A   
   A   
   G   
   A   
   A   
   A   
   T   
   C   
   A   
   G   
   A   
   G   
   A   
   A   
   A   
   A   
   C   
   A   
   T   
   C   
   C   
   A   
   T   
   G   
   A   
   T   
   G   
   T   
   C   
   A   
   A   
   T   
   C   
   T   
   A   
   A   
   C   
   C   
   A   
   G   
   C   
   G   
   A   
   A   
   A   
   T   
   G   
   A   
   A   
   A   
   G   
   A   
   C   
   T   
   C   
   C   
   T   
   T   
   C   
   A   
   T   
   C   
   G   
   A   
   T   
   T   
   A   
   T   
   G   
   C   
   G   
   A   
   T   
   G   
   A   
   G   
   T   
   G   
   T   
   T   
   A   
   T   
   T   
   G   
   T   
   C   
   G   
   C   
   A   
   C   
   G   
   T   
   G   
   C   
   A   
   C   
   T   
   T   
   C   
   C   
   T   
   G   
   A   
   T   
   G   
   T   
   C   
   A   
   G   
   A   
   G   
   A   
   T   
   G   
   G   
   T   
   C   
   T   
   G   
   A   
   A   
   A   
   C   
   C   
   C   
   G   
   T   
   T   
   C   
   A   
   T   
   C   
   G   
   C   
   C   
   G   
   T   
   A   
   T   
   T   
   T   
   T   
   A   
   T   
   A   
   C   
   G   
   G   
   C   
   A   
   T   
   G   
   A   
   A   
   C   
   G   
   A   
   A   
   T   
   T   
   A   
   G   
   G   
   T   
   G   
   T   
   A   
   A   
   C   
   A   
   C   
   C   
   G   
   G   
   A   
   T   
   A   
   A   
   A   
   G   
   C   
   C   
   C   
   A   
   T   
   A   
   A   
   A   
   A   
   A   
   A   
   T   
   C   
   T   
   G   
   C   
   C   
   A   
   G   
   A   
   A   
   T   
   C   
   G   
   T   
   C   
   G   
   G   
   A   
   G   
   A   
   T   
   G   
   T   
   C   
   A   
   T   
   G   
   G   
   G   
   T   
   A   
   A   
   A   
   T   
   A   
   C   
   C   
   A   
   T   
   C   
   C   
   T   
   C   
   A   
   T   
   G   
   G   
   A   
   G   
   A   
   C   
   A   
   G   
   T   
   G   
   C   
   G   
   A   
   T   
   C   
   T   
   A   
   T   
   G   
   A   
   G   
   T   
   C   
   A   
   A   
   T   
   G   
   G   
   T   
   A   
   C   
   G   
   G   
   A   
   T   
   G   
   G   
   C   
   A   
   C   
   A   
   G   
   C   
   C   
   A   
   T   
   T   
   C   
   A   
   G   
   C   
   T   
   A   
   C   
   C   
   G   
   T   
   T   
   A   
   T   
   A   
   T   
   G   
   C   
   T   
   A   
   G   
   T   
   G   
   G   
   A   
   T   
   G   
   G   
   T   
   C   
   A   
   T   
   G   
   G   
   A   
   A   
   A   
   C   
   T   
   T   
   T   
   G   
   G   
   T   
   T   
   C   
   T   
   G   
   T   
   T   
   G   
   A   
   C   
   G   
   G   
   T   
   G   
   A   
   C   
   G   
   G   
   T   
   G   
   C   
   C   
   G   
   C   
   C   
   G   
   C   
   T   
   A   
   T   
   G   
   C   
   G   
   T   
   T   
   A   
   T   
   A   
   C   
   C   
   G   
   A   
   A   
   G   
   C   
   A   
   C   
   G   
   G   
   A   
   T   
   G   
   A   
   G   
   C   
   A   
   A   
   A   
   A   
   T   
   C   
   G   
   C   
   T   
   A   
   C   
   A   
   G   
   A   
   A   
   A   
   T   
   G   
   C   
   T   
   G   
   C   
   G   
   T   
   G   
   A   
   T   
   A   
   T   
   C   
   A   
   A   
   T   
   A   
   A   
   G   
   A   
   A   
   C   
   A   
   C   
   T   
   G   
   T   
   T   
   G   
   A   
   T   
   T   
   T   
   C   
   C   
   A   
   A   
   A   
   G   
   C   
   A   
   A   
   C   
   T   
   A   
   T   
   G   
   A   
   T   
   G   
   A   
   T   
   A   
   C   
   A   
   G   
   A   
   A   
   A   
   A   
   A   
   G   
   A   
   A   
   C   
   C   
   A   
   G   
   T   
   T   
   G   
   T   
   A   
   C   
   T   
   A   
   C   
   C   
   T   
   G   
   C   
   T   
   C   
   G   
   C   
   T   
   T   
   C   
   C   
   C   
   G   
   A   
   A   
   T   
   C   
   T   
   T   
   T   
   T   
   A   
   G   
   T   
   G   
   A   
   A   
   C   
   G   
   G   
   A   
   A   
   C   
   A   
   A   
   C   
   G   
   G   
   G   
   G   
   A   
   T   
   C   
   G   
   C   
   C   
   G   
   T   
   A   
   G   
   G   
   T   
   A   
   T   
   G   
   G   
   C   
   C   
   A   
   C   
   G   
   A   
   A   
   T   
   A   
   T   
   C   
   C   
   C   
   A   
   C   
   C   
   T   
   C   
   A   
   T   
   A   
   A   
   C   
   T   
   T   
   G   
   T   
   C   
   T   
   G   
   A   
   G   
   G   
   T   
   A   
   G   
   T   
   A   
   G   
   C   
   G   
   G   
   C   
   T   
   A   
   T   
   C   
   G   
   A   
   T   
   T   
   T   
   G   
   C   
   T   
   G   
   A   
   T   
   G   
   G   
   A   
   T   
   A   
   A   
   T   
   C   
   C   
   T   
   G   
   A   
   T   
   G   
   T   
   G   
   A   
   C   
   G   
   A   
   C   
   C   
   A   
   A   
   T   
   G   
   A   
   A   
   T   
   T   
   G   
   A   
   T   
   G   
   G   
   A   
   A   
   G   
   T   
   A   
   T   
   T   
   A   
   C   
   C   
   T   
   G   
   G   
   A   
   C   
   C   
   A   
   G   
   A   
   T   
   T   
   T   
   T   
   C   
   C   
   A   
   A   
   C   
   A   
   G   
   G   
   T   
   G   
   G   
   T   
   T   
   T   
   G   
   G   
   T   
   T   
   A   
   T   
   G   
   G   
   G   
   A   
   A   
   A   
   A   
   T   
   C   
   A   
   G   
   G   
    A    
   A   
   T   
    C    
   C   
   G   
    A    
    A    
   G   
   A   
   G   
   C   
    G    
   T   
   A   
   T   
   G   
   A   
   A   
   A   
   C   
    A    
   G   
   G   
    T    
   A   
   A   
    G    
   G   
   G   
   T   
   T   
   C   
    T    
   A   
   T   
   T   
   A   
   C   
    C    
   G   
   T   
    A    
   C   
   G   
   T   
   G   
   C   
   A   
   A   
   A   
   A   
   G   
   T   
   T   
   G   
   A   
    G    
    A    
   T   
    T    
   A   
    C    
   T   
   G   
   A   
    G    
   A   
   T   
   G   
    C    
   C   
    A    
   A   
   A   
   T   
   G   
   G   
    T    
   A   
   A   
   A   
   G   
   A   
   A   
   C   
   G   
    G    
   A   
   T   
    C    
    A    
   T   
    C    
   G   
   T   
    G    
   A   
   C   
    A    
   G   
   A   
   A   
   T   
   T   
    G    
   C   
   C   
   T   
   T   
   A   
   T   
   A   
   T   
   G   
   G   
   T   
    C    
   A   
   A   
   T   
   A   
   A   
   A   
   G   
   C   
    A    
   A   
   A   
   A   
   T   
   T   
    G    
   A   
   T   
    C    
   G   
   A   
   A   
   C   
   G   
    G    
   A   
   T   
   T   
   T   
   C   
   T   
   G   
   A   
    A    
    C    
   T   
    G    
   C   
   A   
   C   
   A   
   G   
   A   
   G   
   A   
    C    
   A   
   A   
    G    
   C   
   G   
    T    
   A   
   T   
    C    
   G   
   A   
   A   
   G   
   G   
    G    
   A   
   T   
    C    
   A   
   C   
    A    
   G   
   A   
   T   
   T   
   T   
   G   
   C   
   G   
   G   
   G   
   A   
    C    
   G   
   A   
   A   
   T   
   C   
   T   
   T   
   C   
    C    
   C   
   G   
   T   
   G   
   A   
    A    
   G   
   G   
    T    
   A   
   T   
   G   
   C   
   G   
    G    
   A   
   T   
    C    
   G   
   T   
   C   
   A   
   T   
    T    
   G   
   A   
    C    
    A    
   T   
    C    
   C   
   G   
   T   
   C   
   G   
    T    
   G   
   A   
    C    
   G   
    C    
    C    
   A   
   G   
    C    
   G   
   C   
    A    
   T   
   C   
    T    
   G   
   T   
   T   
    A    
   T   
    C    
   T   
   T   
    G    
   A   
   A   
    T    
   A   
   A   
   C   
   T   
   T   
    A    
   T   
   A   
    T    
   A   
   A   
   A   
    T    
   T   
   G   
   A   
   C   
    A    
    T    
   C   
    C    
   T   
   T   
    G    
   C   
   A   
    A    
   A   
    A    
    T    
   T   
   C   
    A    
   T   
   T   
    T    
   G   
   G   
    A    
   T   
   T   
    C    
   A   
   A   
    T    
   A   
   T   
   G   
   T   
   T   
    G    
   G   
   C   
    G    
   A   
   T   
    C    
   G   
   A   
   A   
   A   
   A   
   A   
   G   
   G   
    T    
   G   
   T   
    G    
   C   
   C   
    G    
   A   
   A   
   A   
    G    
   T   
   T   
   T   
   T   
    G    
   A   
   G   
    C    
   T   
   T   
   G   
   A   
   A   
   A   
   C   
    A    
    A    
   A   
   T   
    T    
    C    
   T   
    T    
   G   
   A   
   A   
   A   
   A   
    C    
   T   
   A   
    T    
    A    
   T   
    C    
   G   
   A   
    A    
   C   
   A   
    T    
   C   
   A   
    G    
    C    
    G    
    C    
    G    
   A   
   A   
   G   
   T   
   C   
   A   
   T   
    C    
   A   
   C   
    A    
    A    
   G   
    A    
   C   
   G   
   G   
   A   
   C   
    A    
    G    
   T   
    C    
   T   
   T   
   T   
   G   
   A   
    A    
   A   
   A   
   A   
    G    
   A   
    A    
   A   
   A   
    A    
   G   
   C   
   A   
   G   
   A   
   A   
   G   
   C   
   A   
    A    
   G   
    G    
   G   
   C   
    A    
   C   
   A   
    C    
   A   
   T   
    T    
   T   
   T   
   A   
   G   
   A   
   A   
   G   
   G   
   T   
    T    
   T   
    G    
   C   
   G   
    T    
   A   
   T   
   T   
   G   
   C   
    T    
   T   
   T   
    G    
   G   
   A   
   T   
   C   
   A   
   T   
   A   
   T   
   C   
    G    
   A   
   T   
   G   
   A   
   A   
   A   
   T   
    C    
   A   
   T   
   T   
   G   
   C   
    G    
   A   
   T   
   T   
   A   
   T   
    C    
   C   
   G   
    C    
   G   
   G   
    G    
   T   
   C   
    T    
    A    
   A   
   A   
   T   
   C   
   A   
   G   
   A   
   T   
   G   
   A   
    C    
   G   
   A   
    A    
   G   
   C   
   A   
   A   
   A   
   A   
   G   
   C   
    G    
   A   
    C    
    G    
    A    
   T   
    G    
   A   
   T   
    C    
   G   
   A   
   A   
   C   
   G   
    G    
   T   
   T   
    C    
   G   
   A   
    T    
   T   
   T   
    A    
   T   
   C   
    C    
   G   
   A   
   T   
   C   
   G   
    C    
   C   
   A   
    G    
    T    
   C   
    T    
   C   
   A   
   A   
   G   
   C   
    T    
   A   
   T   
    T    
   T   
   T   
   A   
   G   
   A   
   T   
   A   
   T   
   G   
   C   
   G   
   T   
   T   
   T   
    A    
   C   
   G   
    C    
   C   
   G   
    A    
   T   
   T   
    G    
   A   
   C   
    C    
   G   
   G   
    T    
   T   
   T   
    G    
   G   
   A   
   A   
   C   
   G   
    G    
   G   
   A   
    A    
   A   
   A   
   A   
   A   
   T   
    C    
   G   
   A   
    A    
   A   
   A   
   T   
   G   
   A   
   A   
   T   
   A   
    C    
   C   
   A   
   A   
   G   
   A   
    A    
   C   
   T   
    C    
   T   
   T   
    A    
   A   
    A    
   A   
    C    
   T   
    G    
   A   
   T   
   T   
   G   
    A    
    A    
   G   
   A   
    T    
   T   
   T   
    A    
    A    
    C    
    T    
   G   
   A   
   C   
   A   
   T   
   T   
    C    
   T   
    G    
   G   
   C   
    A    
    A    
   G   
    A    
   C   
   C   
    A    
   G   
   A   
    A    
   C   
   G   
    T    
   G   
   T   
    T    
   A   
    C    
    A    
   G   
   A   
   A   
   A   
   T   
    C    
   A   
   T   
    C    
   A   
   A   
    G    
   A   
    A    
    T    
   G   
   A   
    A    
   T   
   T   
   A   
    G    
    C    
    A    
   G   
   A   
   C   
    T    
   T   
    G    
    A    
    A    
    T    
    C    
    A    
   A   
    C    
    G    
   A   
   T   
   T   
   T   
   G   
   G   
   T   
   G   
   A   
   T   
    A    
    A    
   A   
   C   
   G   
   A   
   C   
   G   
    C    
   A   
   C   
   A   
   G   
   A   
   A   
    T    
   T   
   A   
    C    
   T   
   A   
   G   
   T   
    C    
   G   
   G   
   T   
   G   
   A   
    G    
   G   
   T   
   C   
    T    
   T   
    A    
   A   
   G   
   T   
   C   
   T   
    G    
   G   
   A   
   A   
   G   
   A   
   T   
   G   
   A   
   A   
   G   
   A   
   T   
    T    
   T   
    A    
   A   
   T   
    C    
   G   
   A   
   A   
   G   
   A   
   A   
   G   
   A   
   A   
   G   
   A   
   A   
    A    
   T   
   C   
   G   
   T   
    T    
   A   
   T   
   T   
   A   
   C   
    A    
    T    
   T   
    G    
   A   
   C   
    A    
   A   
   A   
    C    
   A   
   A   
    T    
   G   
   G   
    T    
   T   
   A   
    C    
   A   
   T   
    C    
   A   
   A   
    A    
   C   
   G   
    T    
    T    
   T   
   G   
   G   
    C    
   A   
   A   
   A   
    T    
    A    
    A    
    T    
   G   
   A   
   A   
   T   
   T   
   C   
    A    
   G   
    A    
   G   
   C   
    T    
   C   
   A   
   A   
   C   
   G   
    A    
    A    
   G   
    G    
   G   
   G   
    T    
   G   
   G   
    A    
   C   
   G   
   T   
   G   
   G   
    T    
   G   
   T   
    C    
   C   
   A   
   A   
   G   
   G   
    A    
   A   
   T   
   G   
   G   
   G   
    T    
    A    
   T   
    C    
   C   
   A   
    C    
    A    
   A   
   T   
   G   
   A   
    C    
   G   
   A   
    C    
   T   
   T   
   C   
   G   
   T   
    C    
   A   
   A   
    A    
   A   
   A   
    T    
   C   
   T   
    G    
    G    
   T   
   T   
   T   
   C   
   T   
   T   
   G   
    T    
   T   
   C   
    T    
   A   
   C   
    C    
   C   
   A   
    T    
   G   
   A   
    T    
   A   
   C   
    A    
   T   
   T   
    G    
   T   
   T   
   A   
   T   
   T   
    C    
   T   
   T   
   T   
   A   
   C   
   G   
   A   
   A   
    T    
   A   
    A    
    C    
   G   
   G   
    A    
   A   
   A   
   A   
   G   
   T   
    G    
   T   
   A   
    T    
   C   
   G   
    G    
   G   
   C   
   A   
   A   
   A   
   A   
   G   
   G   
    A    
   T   
   A   
    C    
   G   
   A   
   A   
   A   
   T   
    T    
   C   
   C   
    A    
   G   
   A   
    A    
   T   
   A   
   C   
   G   
   G   
    G    
    C    
   G   
    C    
   A   
   C   
   A   
   G   
   C   
    C    
   A   
   A   
   A   
   G   
   G   
    A    
   A   
   T   
    A    
   C   
   C   
   A   
    A    
   T   
    T    
   A   
   T   
    C    
   A   
   A   
    T    
   T   
   T   
    A    
   C   
   T   
    A    
   G   
   G   
    T    
   A   
   T   
    C    
   G   
   A   
    T    
   T   
   C   
    G    
    A    
    G    
    T    
   G   
   A   
   A   
   A   
    G    
    C    
   A   
   T   
    C    
   C   
   A   
    G    
   G   
   C   
    A    
   A   
   T   
    C    
   A   
   T   
   T   
    G    
   C   
    G    
   G   
   T   
    G    
    A    
    C    
   G   
   G   
   G   
    A    
    G    
   A   
   A   
   G   
   C   
    A    
   G   
   A   
   A   
   G   
    A    
    A    
   G   
   G   
    C    
    C    
   A   
    C    
   T   
   A   
    T    
    C    
   T   
    C    
   T   
   T   
   C   
   T   
   T   
    T    
   A   
   C   
    G    
   A   
   C   
    G    
    C    
    G    
    T    
    C    
   A   
   A   
   G   
   G   
    T    
   A   
   C   
    A    
   G   
   T   
    A    
   A   
   A   
   A   
   C   
   G   
    T    
   A   
   C   
    T    
    T    
   C   
    T    
   G   
   T   
    C    
   A   
   C   
    T    
   G   
   C   
    T    
   T   
   T   
    T    
    G    
   C   
   T   
   A   
   A   
    C    
   A   
   T   
    C    
   C   
   G   
    A    
   A   
   G   
    T    
   A   
   A   
   T   
   G   
   G   
    T    
    C    
   T   
    G    
   A   
   T   
    C    
   G   
   C   
    A    
   A   
   T   
   T   
    G    
   G   
    T    
   T   
   T   
    G    
   A   
   A   
   A   
   G   
   A   
   A   
   G   
   A   
    C    
   G   
   A   
    T    
   G   
   A   
    G    
    C    
   T   
    C    
    G    
   T   
    C    
   A   
   A   
    T    
   G   
   T   
    G    
    C    
   T   
   A   
    C    
    T    
   G   
   A   
   C   
    A    
    G    
   A   
    T    
   G   
   G   
    T    
    C    
   A   
    A    
    T    
    C    
    T    
   A   
   A   
    T    
   A   
   T   
    C    
   A   
   T   
    T    
   A   
   T   
    T    
   G   
   G   
    A    
   A   
   C   
   A   
   C   
   A   
   T   
    A    
    A    
    C    
   G   
   G   
    C    
   T   
   A   
   C   
   T   
   C   
   T   
   G   
   T   
    C    
   A   
   C   
    T    
   T   
   T   
    C    
    A    
   A   
    A    
   G   
   A   
    A    
    G    
    A    
    A    
    G    
   C   
    A    
   G   
    T    
    C    
   C   
   G   
    C    
   G   
   A   
   T   
   A   
   T   
   G   
   G   
   G   
    A    
   C   
   G   
    T    
   A   
   C   
   A   
   G   
   C   
    T    
   T   
   C   
    C    
   G   
   G   
    G    
   G   
   T   
    T    
    A    
   G   
    A    
   G   
   G   
    A    
   A   
   T   
    A    
   C   
   G   
    C    
    C    
   T   
    G    
   C   
   G   
    T    
   G   
   A   
    G    
    G    
   A   
    A    
   G   
   A   
    T    
   T   
    A    
    T    
   G   
   T   
    C    
   G   
   T   
    T    
   G   
   G   
    G    
    G    
   C   
   A   
   G   
   C   
    C    
    T    
   T   
    A    
    A    
   T   
   G   
    A    
   A   
    G    
   G   
    A    
   A   
    G    
   A   
    T    
    C    
   A   
   A   
   G   
   A   
   A   
   G   
   T   
    A    
    T    
   T   
    A    
   G   
   T   
    G    
   A   
   T   
    C    
   A   
   C   
    A    
   G   
   A   
   A   
   A   
   A   
    A    
   G   
   G   
   T   
   T   
   A   
    C    
   G   
   G   
    A    
   A   
   A   
    A    
   C   
   G   
    G    
   A   
   C   
    A    
   A   
   A   
   A   
   G   
    T    
    A    
   T   
   C   
    G    
   G   
   A   
    A    
   T   
   A   
   T   
   C   
   C   
   A   
   G   
   T   
    G    
   A   
   A   
    A    
   G   
   G   
    C    
   C   
   G   
    C    
   G   
   G   
    A    
   G   
   G   
   T   
   A   
   A   
   A   
   G   
   G   
    G    
   A   
   T   
    C    
   A   
   A   
    A    
   A   
   C   
    G    
    G    
   C   
    G    
   A   
   A   
    C    
   A   
   T   
   C   
   A   
   C   
   T   
   G   
   A   
    A    
   A   
   A   
   A   
   A   
   A   
   T   
   G   
   G   
   T   
   C   
   C   
   A   
    C    
   T   
   A   
   G   
   C   
    T    
   G   
   G   
    A    
   T   
   T   
    G    
   A   
   C   
   C   
   A   
   C   
    T    
   G   
   T   
    T    
    T    
    C    
   T   
   G   
   G   
    A    
    G    
   A   
   T   
   G   
   A   
   A   
   G   
   A   
    C    
   A   
   T   
   C   
    A    
   T   
    G    
    C    
   T   
    G    
   A   
   T   
    T    
   A   
   C   
    A    
    G    
   A   
    T    
   A   
   A   
   A   
   G   
   G   
    T    
   G   
   T   
    C    
   A   
   T   
   T   
   A   
   T   
   C   
   C   
   G   
    T    
   T   
   T   
    C    
   A   
   A   
   C   
   G   
   T   
    A    
    T    
    C    
    G    
    A    
   C   
   T   
   G   
   T   
   T   
   T   
   C   
   T   
   C   
   A   
   A   
   A   
   C   
    T    
   G   
   G   
   A   
   C   
   G   
    T    
    T    
   C   
   A   
   A   
   C   
   A   
   T   
   T   
    A    
   G   
   G   
    C    
   G   
   T   
    A    
   C   
   G   
   T   
   T   
   T   
    G    
   A   
   T   
   G   
   A   
    A    
    G    
   A   
   T   
   G   
   G   
   A   
   A   
   G   
    C    
    T    
   G   
    A    
    T    
    A    
   C   
    G    
   A   
   A   
   A   
   G   
   T   
   T   
   G   
   T   
   C   
   A   
   C   
   G   
   A   
   T   
   G   
   G   
   C   
   A   
   G   
   C   
   A   
   G   
   T   
   C   
   G   
   A   
   A   
   C   
   C   
   A   
   G   
   A   
   A   
   G   
   C   
   T   
   G   
   C   
   A   
   G   
   A   
   A   
   C   
   A   
   A   
   A   
   C   
   A   
   G   
   T   
   T   
   G   
   A   
   A   
   G   
   A   
   A   
   C   
   A   
   A   
   G   
   G   
   A   
   T   
   C   
   A   
   G   
   A   
   A   
   G   
   A   
   A   
   -   
   -   
   -   
 

 
   N55229   
   A   
   T   
   G   
   A   
   G   
   T   
   G   
   A   
   A   
   G   
   A   
   A   
   A   
   T   
   C   
   A   
   G   
   A   
   G   
   A   
   A   
   A   
   A   
   C   
   A   
   T   
   C   
   C   
   A   
   T   
   G   
   A   
   T   
   G   
   T   
   C   
   A   
   A   
   T   
   C   
   T   
   A   
   A   
   C   
   C   
   A   
   G   
   C   
   G   
   A   
   A   
   A   
   T   
   G   
   A   
   A   
   A   
   G   
   A   
   C   
   T   
   C   
   C   
   T   
   T   
   C   
   A   
   T   
   C   
   G   
   A   
   T   
   T   
   A   
   T   
   G   
   C   
   G   
   A   
   T   
   G   
   A   
   G   
   T   
   G   
   T   
   T   
   A   
   T   
   T   
   G   
   T   
   C   
   G   
   C   
   A   
   C   
   G   
   T   
   G   
   C   
   A   
   C   
   T   
   T   
   C   
   C   
   T   
   G   
   A   
   T   
   G   
   T   
   C   
   A   
   G   
   A   
   G   
   A   
   T   
   G   
   G   
   T   
   C   
   T   
   G   
   A   
   A   
   A   
   C   
   C   
   C   
   G   
   T   
   T   
   C   
   A   
   T   
   C   
   G   
   C   
   C   
   G   
   T   
   A   
   T   
   T   
   T   
   T   
   A   
   T   
   A   
   C   
   G   
   G   
   C   
   A   
   T   
   G   
   A   
   A   
   C   
   G   
   A   
   A   
   T   
   T   
   A   
   G   
   G   
   T   
   G   
   T   
   A   
   A   
   C   
   A   
   C   
   C   
   G   
   G   
   A   
   T   
   A   
   A   
   A   
   G   
   C   
   C   
   C   
   A   
   T   
   A   
   A   
   A   
   A   
   A   
   A   
   T   
   C   
   T   
   G   
   C   
   C   
   A   
   G   
   A   
   A   
   T   
   C   
   G   
   T   
   C   
   G   
   G   
   A   
   G   
   A   
   T   
   G   
   T   
   C   
   A   
   T   
   G   
   G   
   G   
   T   
   A   
   A   
   A   
   T   
   A   
   C   
   C   
   A   
   T   
   C   
   C   
   T   
   C   
   A   
   T   
   G   
   G   
   A   
   G   
   A   
   C   
   A   
   G   
   T   
   G   
   C   
   G   
   A   
   T   
   C   
   T   
   A   
   T   
   G   
   A   
   G   
   T   
   C   
   A   
   A   
   T   
   G   
   G   
   T   
   A   
   C   
   G   
   G   
   A   
   T   
   G   
   G   
   C   
   A   
   C   
   A   
   G   
   C   
   C   
   A   
   T   
   T   
   C   
   A   
   G   
   C   
   T   
   A   
   C   
   C   
   G   
   T   
   T   
   A   
   T   
   A   
   T   
   G   
   C   
   T   
   A   
   G   
   T   
   G   
   G   
   A   
   T   
   G   
   G   
   T   
   C   
   A   
   T   
   G   
   G   
   A   
   A   
   A   
   C   
   T   
   T   
   T   
   G   
   G   
   T   
   T   
   C   
   T   
   G   
   T   
   T   
   G   
   A   
   C   
   G   
   G   
   T   
   G   
   A   
   C   
   G   
   G   
   T   
   G   
   C   
   C   
   G   
   C   
   C   
   G   
   C   
   T   
   A   
   T   
   G   
   C   
   G   
   T   
   T   
   A   
   T   
   A   
   C   
   T   
   G   
   A   
   A   
   G   
   C   
   A   
   C   
   G   
   G   
   A   
   T   
   G   
   A   
   G   
   C   
   A   
   A   
   A   
   A   
   T   
   C   
   G   
   C   
   T   
   A   
   C   
   A   
   G   
   A   
   A   
   A   
   T   
   G   
   C   
   T   
   G   
   C   
   G   
   T   
   G   
   A   
   T   
   A   
   T   
   C   
   A   
   A   
   T   
   A   
   A   
   G   
   A   
   A   
   C   
   A   
   C   
   T   
   G   
   T   
   T   
   G   
   A   
   T   
   T   
   T   
   C   
   C   
   A   
   A   
   A   
   G   
   C   
   A   
   A   
   C   
   T   
   A   
   T   
   G   
   A   
   T   
   G   
   A   
   T   
   A   
   C   
   A   
   G   
   A   
   A   
   A   
   A   
   A   
   G   
   A   
   A   
   C   
   C   
   A   
   G   
   T   
   T   
   G   
   T   
   A   
   C   
   T   
   A   
   C   
   C   
   T   
   G   
   C   
   T   
   C   
   G   
   C   
   T   
   T   
   C   
   C   
   C   
   G   
   A   
   A   
   T   
   C   
   T   
   T   
   T   
   T   
   A   
   G   
   T   
   G   
   A   
   A   
   C   
   G   
   G   
   A   
   A   
   C   
   A   
   A   
   C   
   G   
   G   
   G   
   G   
   A   
   T   
   C   
   G   
   C   
   C   
   G   
   T   
   A   
   G   
   G   
   T   
   A   
   T   
   G   
   G   
   C   
   C   
   A   
   C   
   G   
   A   
   A   
   T   
   A   
   T   
   C   
   C   
   C   
   A   
   C   
   C   
   T   
   C   
   A   
   T   
   A   
   A   
   C   
   T   
   T   
   G   
   T   
   C   
   T   
   G   
   A   
   G   
   G   
   T   
   A   
   G   
   T   
   A   
   G   
   C   
   G   
   G   
   C   
   T   
   A   
   T   
   C   
   G   
   A   
   T   
   T   
   T   
   G   
   C   
   T   
   G   
   A   
   T   
   G   
   G   
   A   
   T   
   A   
   A   
   T   
   C   
   C   
   T   
   G   
   A   
   T   
   G   
   T   
   G   
   A   
   C   
   G   
   A   
   C   
   C   
   A   
   A   
   T   
   G   
   A   
   A   
   T   
   T   
   G   
   A   
   T   
   G   
   G   
   A   
   A   
   G   
   T   
   A   
   T   
   T   
   A   
   C   
   C   
   T   
   G   
   G   
   A   
   C   
   C   
   A   
   G   
   A   
   T   
   T   
   T   
   T   
   C   
   C   
   A   
   A   
   C   
   A   
   G   
   G   
   T   
   G   
   G   
   T   
   T   
   T   
   G   
   G   
   T   
   T   
   A   
   T   
   G   
   G   
   G   
   G   
   A   
   A   
   A   
   T   
   C   
   A   
   G   
   G   
    A    
   A   
   T   
    C    
   C   
   G   
    A    
    A    
   G   
   A   
   G   
   C   
    G    
   T   
   A   
   T   
   G   
   A   
   A   
   A   
   C   
    A    
   G   
   G   
    T    
   A   
   A   
    G    
   G   
   G   
   T   
   T   
   C   
    T    
   A   
   T   
   T   
   A   
   C   
    C    
   G   
   T   
    A    
   C   
   G   
   T   
   G   
   C   
   A   
   A   
   A   
   A   
   G   
   T   
   T   
   G   
   A   
    G    
    A    
   T   
    T    
   A   
    C    
   T   
   G   
   A   
    G    
   A   
   T   
   G   
    C    
   C   
    A    
   A   
   A   
   T   
   G   
   G   
    T    
   A   
   A   
   A   
   G   
   A   
   A   
   C   
   G   
    G    
   A   
   T   
    C    
    A    
   T   
    C    
   G   
   T   
    G    
   A   
   C   
    A    
   G   
   A   
   A   
   T   
   T   
    G    
   C   
   C   
   T   
   T   
   A   
   T   
   A   
   T   
   G   
   G   
   T   
    C    
   A   
   A   
   T   
   A   
   A   
   A   
   G   
   C   
    A    
   A   
   A   
   A   
   T   
   T   
    G    
   A   
   T   
    C    
   G   
   A   
   A   
   C   
   G   
    G    
   A   
   T   
   T   
   T   
   C   
   T   
   G   
   A   
    A    
    C    
   T   
    G    
   C   
   A   
   C   
   A   
   G   
   A   
   G   
   A   
    C    
   A   
   A   
    G    
   C   
   G   
    T    
   A   
   T   
    C    
   G   
   A   
   A   
   G   
   G   
    G    
   A   
   T   
    C    
   A   
   C   
    A    
   G   
   A   
   T   
   T   
   T   
   G   
   C   
   G   
   G   
   G   
   A   
    C    
   G   
   A   
   A   
   T   
   C   
   T   
   T   
   C   
    C    
   C   
   G   
   T   
   G   
   A   
    A    
   G   
   G   
    T    
   A   
   T   
   G   
   C   
   G   
    G    
   A   
   T   
    C    
   G   
   T   
   C   
   A   
   T   
    T    
   G   
   A   
    C    
    A    
   T   
    C    
   C   
   G   
   T   
   C   
   G   
    T    
   G   
   A   
    C    
   G   
    C    
    C    
   A   
   G   
    C    
   G   
   C   
    A    
   T   
   C   
    C    
   G   
   T   
   T   
    A    
   T   
    C    
   T   
   T   
    G    
   A   
   A   
    T    
   A   
   A   
   C   
   T   
   T   
    A    
   T   
   A   
    T    
   A   
   A   
   A   
    T    
   T   
   G   
   A   
   C   
    A    
    T    
   C   
    C    
   T   
   T   
    G    
   C   
   A   
    A    
   A   
    A    
    T    
   T   
   C   
    A    
   T   
   T   
    T    
   G   
   G   
    A    
   T   
   T   
    C    
   A   
   A   
    T    
   A   
   T   
   G   
   T   
   T   
    G    
   G   
   C   
    G    
   A   
   T   
    C    
   G   
   A   
   A   
   A   
   A   
   A   
   G   
   G   
    T    
   G   
   T   
    G    
   C   
   C   
    G    
   A   
   A   
   A   
    G    
   T   
   T   
   T   
   T   
    G    
   A   
   G   
    C    
   T   
   T   
   G   
   A   
   A   
   A   
   C   
    A    
    A    
   A   
   T   
    C    
    C    
   T   
    T    
   G   
   A   
   A   
   A   
   A   
    C    
   T   
   A   
    T    
    A    
   T   
    C    
   G   
   A   
    A    
   C   
   A   
    T    
   C   
   A   
    A    
    C    
    G    
    C    
    G    
   A   
   A   
   G   
   T   
   C   
   A   
   T   
    C    
   A   
   C   
    A    
    C    
   G   
    A    
   C   
   G   
   G   
   A   
   C   
    G    
    G    
   T   
    C    
   T   
   T   
   T   
   G   
   A   
    A    
   A   
   A   
   A   
    G    
   A   
    A    
   A   
   A   
    A    
   G   
   C   
   A   
   G   
   A   
   A   
   G   
   C   
   A   
    A    
   G   
    A    
   G   
   C   
    A    
   C   
   A   
    C    
   A   
   T   
    C    
   T   
   T   
   A   
   G   
   A   
   A   
   G   
   G   
   T   
    T    
   T   
    G    
   C   
   G   
    T    
   A   
   T   
   T   
   G   
   C   
    T    
   T   
   T   
    G    
   G   
   A   
   T   
   C   
   A   
   T   
   A   
   T   
   C   
    G    
   A   
   T   
   G   
   A   
   A   
   A   
   T   
    C    
   A   
   T   
   T   
   G   
   C   
    G    
   A   
   T   
   T   
   A   
   T   
    C    
   C   
   G   
    C    
   G   
   G   
    G    
   T   
   C   
    T    
    A    
   A   
   A   
   T   
   C   
   A   
   G   
   A   
   T   
   G   
   A   
    C    
   G   
   A   
    G    
   G   
   C   
   A   
   A   
   A   
   A   
   G   
   C   
    A    
   A   
    C    
    A    
    A    
   T   
    G    
   A   
   T   
    C    
   G   
   A   
   A   
   C   
   G   
    G    
   T   
   T   
    C    
   G   
   A   
    T    
   T   
   T   
    A    
   T   
   C   
    C    
   G   
   A   
   T   
   C   
   G   
    C    
   C   
   A   
    G    
    T    
   C   
    T    
   C   
   A   
   A   
   G   
   C   
    T    
   A   
   T   
    T    
   T   
   T   
   A   
   G   
   A   
   T   
   A   
   T   
   G   
   C   
   G   
   T   
   T   
   T   
    A    
   C   
   G   
    C    
   C   
   G   
    A    
   T   
   T   
    G    
   A   
   C   
    C    
   G   
   G   
    T    
   T   
   T   
    G    
   G   
   A   
   A   
   C   
   G   
    G    
   G   
   A   
    A    
   A   
   A   
   A   
   A   
   T   
    C    
   G   
   A   
    A    
   A   
   A   
   T   
   G   
   A   
   A   
   T   
   A   
    C    
   C   
   A   
   A   
   G   
   A   
    A    
   C   
   T   
    C    
   T   
   T   
    A    
   A   
    A    
   A   
    C    
   T   
    G    
   A   
   T   
   T   
   G   
    A    
    A    
   G   
   A   
    T    
   T   
   T   
    A    
    A    
    C    
    T    
   G   
   A   
   C   
   A   
   T   
   T   
    C    
   T   
    G    
   G   
   C   
    A    
    A    
   G   
    A    
   C   
   C   
    A    
   G   
   A   
    A    
   C   
   G   
    T    
   G   
   T   
    T    
   A   
    C    
    A    
   G   
   A   
   A   
   A   
   T   
    C    
   A   
   T   
    C    
   A   
   A   
    G    
   A   
    A    
    T    
   G   
   A   
    A    
   T   
   T   
   A   
    G    
    C    
    A    
   G   
   A   
   C   
    T    
   T   
    G    
    A    
    A    
    T    
    C    
    A    
   A   
    C    
    G    
   A   
   T   
   T   
   T   
   G   
   G   
   T   
   G   
   A   
   T   
    A    
    A    
   A   
   C   
   G   
   A   
   C   
   G   
    C    
   A   
   C   
   A   
   G   
   A   
   A   
    T    
   T   
   A   
    C    
   T   
   A   
   G   
   T   
    C    
   G   
   G   
   T   
   G   
   A   
    G    
   G   
   T   
   C   
    T    
   T   
    A    
   A   
   G   
   T   
   C   
   T   
    G    
   G   
   A   
   A   
   G   
   A   
   T   
   G   
   A   
   A   
   G   
   A   
   T   
    T    
   T   
    A    
   A   
   T   
    C    
   G   
   A   
   A   
   G   
   A   
   A   
   G   
   A   
   A   
   G   
   A   
   A   
    A    
   T   
   C   
   G   
   T   
    T    
   A   
   T   
   T   
   A   
   C   
    A    
    T    
   T   
    G    
   A   
   C   
    A    
   A   
   A   
    C    
   A   
   A   
    T    
   G   
   G   
    T    
   T   
   A   
    C    
   A   
   T   
    C    
   A   
   A   
    A    
   C   
   G   
    T    
    T    
   T   
   G   
   G   
    C    
   A   
   A   
   A   
    T    
    A    
    A    
    T    
   G   
   A   
   A   
   T   
   T   
   C   
    A    
   G   
    A    
   G   
   C   
    T    
   C   
   A   
   A   
   C   
   G   
    A    
    A    
   G   
    G    
   G   
   G   
    T    
   G   
   G   
    A    
   C   
   G   
   T   
   G   
   G   
    T    
   G   
   T   
    C    
   C   
   A   
   A   
   G   
   G   
    A    
   A   
   T   
   G   
   G   
   G   
    T    
    A    
   T   
    C    
   C   
   A   
    C    
    A    
   A   
   T   
   G   
   A   
    C    
   G   
   A   
    C    
   T   
   T   
   C   
   G   
   T   
    C    
   A   
   A   
    A    
   A   
   A   
    T    
   C   
   T   
    G    
    G    
   T   
   T   
   T   
   C   
   T   
   T   
   G   
    T    
   T   
   C   
    T    
   A   
   C   
    C    
   C   
   A   
    T    
   G   
   A   
    T    
   A   
   C   
    A    
   T   
   T   
    G    
   T   
   T   
   A   
   T   
   T   
    C    
   T   
   T   
   T   
   A   
   C   
   G   
   A   
   A   
    T    
   A   
    A    
    C    
   G   
   G   
    A    
   A   
   A   
   A   
   G   
   T   
    G    
   T   
   A   
    T    
   C   
   G   
    G    
   G   
   C   
   A   
   A   
   A   
   A   
   G   
   G   
    A    
   T   
   A   
    C    
   G   
   A   
   A   
   A   
   T   
    T    
   C   
   C   
    A    
   G   
   A   
    A    
   T   
   A   
   C   
   G   
   G   
    G    
    C    
   G   
    T    
   A   
   C   
   A   
   G   
   C   
    C    
   A   
   A   
   A   
   G   
   G   
    A    
   A   
   T   
    C    
   C   
   C   
   A   
    A    
   T   
    C    
   A   
   T   
    C    
   A   
   A   
    T    
   T   
   T   
    A    
   C   
   T   
    A    
   G   
   G   
    T    
   A   
   T   
    C    
   G   
   A   
    T    
   T   
   C   
    G    
    A    
    G    
    T    
   G   
   A   
   A   
   A   
    G    
    C    
   A   
   T   
    C    
   C   
   A   
    G    
   G   
   C   
    A    
   A   
   T   
    C    
   A   
   T   
   T   
    G    
   C   
    G    
   G   
   T   
    G    
    A    
    C    
   G   
   G   
   G   
    A    
    G    
   A   
   A   
   G   
   C   
    A    
   G   
   A   
   A   
   G   
    A    
    A    
   G   
   G   
    C    
    C    
   A   
    C    
   T   
   A   
    T    
    C    
   T   
    C    
   T   
   T   
   C   
   T   
   T   
    T    
   A   
   C   
    G    
   A   
   C   
    G    
    C    
    G    
    T    
    C    
   A   
   A   
   G   
   G   
    T    
   A   
   C   
    A    
   G   
   T   
    A    
   A   
   A   
   A   
   C   
   G   
    T    
   A   
   C   
    T    
    T    
   C   
    T    
   G   
   T   
    C    
   A   
   C   
    T    
   G   
   C   
    T    
   T   
   T   
    T    
    G    
   C   
   T   
   A   
   A   
    C    
   A   
   T   
    C    
   C   
   G   
    A    
   A   
   G   
    T    
   A   
   A   
   T   
   G   
   G   
    T    
    C    
   T   
    G    
   A   
   T   
    C    
   G   
   C   
    A    
   A   
   T   
   T   
    G    
   G   
    T    
   T   
   T   
    G    
   A   
   A   
   A   
   G   
   A   
   A   
   G   
   A   
    C    
   G   
   A   
    T    
   G   
   A   
    G    
    C    
   T   
    C    
    G    
   T   
    C    
   A   
   A   
    T    
   G   
   T   
    G    
    C    
   T   
   A   
    C    
    T    
   G   
   A   
   C   
    A    
    G    
   A   
    T    
   G   
   G   
    T    
    C    
   A   
    A    
    T    
    C    
    T    
   A   
   A   
    T    
   A   
   T   
    C    
   A   
   T   
    T    
   A   
   T   
    T    
   G   
   G   
    A    
   A   
   C   
   A   
   C   
   A   
   T   
    A    
    A    
    C    
   G   
   G   
    C    
   T   
   A   
   C   
   T   
   C   
   T   
   G   
   T   
    C    
   A   
   C   
    T    
   T   
   T   
    C    
    A    
   A   
    A    
   G   
   A   
    A    
    G    
    A    
    A    
    G    
   C   
    A    
   G   
    T    
    C    
   C   
   G   
    C    
   G   
   A   
   T   
   A   
   T   
   G   
   G   
   G   
    A    
   C   
   G   
    T    
   A   
   C   
   A   
   G   
   C   
    T    
   T   
   C   
    C    
   G   
   G   
    G    
   G   
   T   
    C    
    A    
   G   
    A    
   G   
   G   
    G    
   A   
   T   
    A    
   C   
   G   
    C    
    C    
   T   
    G    
   C   
   G   
    T    
   G   
   A   
    G    
    G    
   A   
    A    
   G   
   A   
    T    
   T   
    A    
    T    
   G   
   T   
    C    
   G   
   T   
    T    
   G   
   G   
    G    
    G    
   C   
   A   
   G   
   C   
    C    
    T    
   T   
    A    
    A    
   T   
   G   
    A    
   A   
    G    
   G   
    A    
   A   
    A    
   A   
    T    
    C    
   A   
   A   
   G   
   A   
   A   
   G   
   T   
    A    
    T    
   T   
    A    
   G   
   T   
    G    
   A   
   T   
    C    
   A   
   C   
    A    
   G   
   A   
   A   
   A   
   A   
    A    
   G   
   G   
   T   
   T   
   A   
    C    
   G   
   G   
    A    
   A   
   A   
    A    
   C   
   G   
    G    
   A   
   C   
    A    
   A   
   A   
   A   
   G   
    T    
    A    
   T   
   C   
    G    
   G   
   A   
    A    
   T   
   A   
   T   
   C   
   C   
   A   
   G   
   T   
    G    
   A   
   A   
    A    
   G   
   G   
    C    
   C   
   G   
    C    
   G   
   G   
    A    
   G   
   G   
   T   
   A   
   A   
   A   
   G   
   G   
    G    
   A   
   T   
    C    
   A   
   A   
    A    
   A   
   C   
    G    
    G    
   C   
    G    
   A   
   A   
    C    
   A   
   T   
   C   
   A   
   C   
   T   
   G   
   A   
    A    
   A   
   A   
   A   
   A   
   A   
   T   
   G   
   G   
   T   
   C   
   C   
   A   
    C    
   T   
   A   
   G   
   C   
    T    
   G   
   G   
    A    
   T   
   T   
    G    
   A   
   C   
   C   
   A   
   C   
    T    
   G   
   T   
    T    
    T    
    C    
   T   
   G   
   G   
    A    
    G    
   A   
   T   
   G   
   A   
   A   
   G   
   A   
    C    
   A   
   T   
   C   
    A    
   T   
    G    
    C    
   T   
    G    
   A   
   T   
    T    
   A   
   C   
    A    
    G    
   A   
    T    
   A   
   A   
   A   
   G   
   G   
    T    
   G   
   T   
    C    
   A   
   T   
   T   
   A   
   T   
   C   
   C   
   G   
    T    
   T   
   T   
    C    
   A   
   A   
   C   
   G   
   T   
    A    
    T    
    C    
    G    
    A    
   C   
   T   
   G   
   T   
   T   
   T   
   C   
   T   
   C   
   A   
   A   
   A   
   C   
    T    
   G   
   G   
   A   
   C   
   G   
    T    
    T    
   C   
   A   
   A   
   C   
   A   
   T   
   T   
    A    
   G   
   G   
    C    
   G   
   T   
    A    
   C   
   G   
   T   
   T   
   T   
    G    
   A   
   T   
   G   
   A   
    A    
    G    
   A   
   T   
   G   
   G   
   A   
   A   
   G   
    C    
    C    
   G   
    A    
    T    
    A    
   C   
    G    
   A   
   A   
   A   
   G   
   T   
   T   
   G   
   T   
   C   
   A   
   C   
   G   
   A   
   T   
   G   
   G   
   C   
   A   
   G   
   C   
   A   
   G   
   T   
   C   
   G   
   A   
   A   
   C   
   C   
   A   
   G   
   A   
   A   
   G   
   C   
   T   
   G   
   C   
   A   
   G   
   A   
   A   
   C   
   A   
   A   
   A   
   C   
   A   
   G   
   T   
   T   
   G   
   A   
   A   
   G   
   A   
   A   
   C   
   A   
   A   
   G   
   G   
   A   
   T   
   C   
   A   
   G   
   A   
   A   
   G   
   A   
   A   
   -   
   -   
   -   
 

 
   N60440F   
   A   
   T   
   G   
   A   
   G   
   T   
   G   
   A   
   A   
   G   
   A   
   A   
   A   
   T   
   T   
   A   
   A   
   A   
   G   
   A   
   A   
   A   
   A   
   C   
   A   
   T   
   T   
   C   
   A   
   A   
   G   
   A   
   C   
   G   
   T   
   C   
   A   
   A   
   T   
   C   
   T   
   G   
   A   
   C   
   C   
   A   
   G   
   C   
   G   
   A   
   A   
   A   
   T   
   G   
   A   
   A   
   A   
   G   
   A   
   A   
   T   
   C   
   T   
   T   
   T   
   C   
   A   
   T   
   T   
   G   
   A   
   C   
   T   
   A   
   C   
   G   
   C   
   A   
   A   
   T   
   G   
   A   
   G   
   T   
   G   
   T   
   T   
   A   
   T   
   C   
   G   
   T   
   A   
   G   
   C   
   C   
   C   
   G   
   C   
   G   
   C   
   G   
   T   
   T   
   A   
   C   
   C   
   T   
   G   
   A   
   C   
   G   
   T   
   T   
   C   
   G   
   T   
   G   
   A   
   C   
   G   
   G   
   T   
   T   
   T   
   A   
   A   
   A   
   A   
   C   
   C   
   T   
   G   
   T   
   T   
   C   
   A   
   T   
   C   
   G   
   C   
   C   
   G   
   A   
   A   
   T   
   C   
   T   
   T   
   A   
   T   
   A   
   T   
   G   
   G   
   A   
   A   
   T   
   G   
   A   
   A   
   C   
   G   
   A   
   A   
   T   
   T   
   A   
   G   
   G   
   G   
   G   
   T   
   A   
   A   
   C   
   C   
   C   
   C   
   T   
   G   
   A   
   T   
   A   
   A   
   A   
   C   
   C   
   A   
   C   
   A   
   C   
   A   
   A   
   A   
   A   
   A   
   A   
   T   
   C   
   A   
   G   
   C   
   C   
   C   
   G   
   G   
   A   
   T   
   T   
   G   
   T   
   T   
   G   
   G   
   G   
   G   
   A   
   T   
   G   
   T   
   T   
   A   
   T   
   G   
   G   
   G   
   T   
   A   
   A   
   A   
   T   
   A   
   T   
   C   
   A   
   C   
   C   
   C   
   C   
   C   
   A   
   T   
   G   
   G   
   G   
   G   
   A   
   C   
   T   
   T   
   T   
   G   
   C   
   G   
   A   
   T   
   T   
   T   
   A   
   C   
   G   
   A   
   A   
   T   
   C   
   A   
   A   
   T   
   G   
   G   
   T   
   G   
   C   
   G   
   G   
   A   
   T   
   G   
   G   
   C   
   A   
   C   
   A   
   A   
   C   
   C   
   T   
   T   
   T   
   T   
   A   
   G   
   T   
   T   
   A   
   T   
   C   
   G   
   G   
   G   
   C   
   T   
   A   
   T   
   G   
   T   
   T   
   A   
   G   
   T   
   T   
   G   
   A   
   C   
   G   
   G   
   C   
   C   
   A   
   C   
   G   
   G   
   A   
   A   
   A   
   C   
   T   
   T   
   C   
   G   
   G   
   T   
   T   
   C   
   T   
   G   
   T   
   C   
   G   
   A   
   T   
   G   
   G   
   T   
   G   
   A   
   C   
   G   
   G   
   C   
   G   
   C   
   T   
   G   
   C   
   C   
   G   
   C   
   T   
   A   
   T   
   G   
   C   
   G   
   T   
   T   
   A   
   T   
   A   
   C   
   C   
   G   
   A   
   A   
   G   
   C   
   A   
   C   
   G   
   T   
   A   
   T   
   G   
   A   
   G   
   T   
   A   
   A   
   A   
   A   
   T   
   T   
   G   
   C   
   T   
   T   
   T   
   A   
   G   
   A   
   A   
   A   
   T   
   G   
   C   
   T   
   A   
   C   
   G   
   A   
   G   
   A   
   T   
   A   
   T   
   T   
   A   
   A   
   C   
   A   
   A   
   A   
   A   
   A   
   T   
   A   
   C   
   A   
   G   
   T   
   C   
   G   
   A   
   T   
   T   
   T   
   C   
   C   
   A   
   A   
   G   
   G   
   A   
   A   
   A   
   C   
   T   
   A   
   T   
   G   
   A   
   T   
   G   
   A   
   T   
   T   
   C   
   A   
   G   
   A   
   A   
   C   
   A   
   A   
   G   
   A   
   A   
   C   
   C   
   A   
   G   
   T   
   A   
   G   
   T   
   T   
   T   
   T   
   A   
   C   
   C   
   T   
   G   
   C   
   T   
   C   
   G   
   T   
   T   
   T   
   T   
   C   
   C   
   A   
   A   
   A   
   C   
   T   
   T   
   A   
   C   
   T   
   A   
   G   
   T   
   T   
   A   
   A   
   C   
   G   
   G   
   A   
   A   
   C   
   A   
   A   
   C   
   G   
   G   
   G   
   G   
   A   
   T   
   T   
   G   
   C   
   G   
   G   
   T   
   G   
   G   
   G   
   G   
   A   
   T   
   G   
   G   
   C   
   A   
   A   
   C   
   A   
   A   
   A   
   T   
   A   
   T   
   T   
   C   
   C   
   A   
   C   
   C   
   A   
   C   
   A   
   C   
   A   
   A   
   T   
   T   
   T   
   A   
   A   
   G   
   T   
   G   
   A   
   A   
   G   
   T   
   G   
   A   
   T   
   T   
   G   
   A   
   T   
   G   
   C   
   A   
   A   
   C   
   A   
   A   
   G   
   T   
   T   
   T   
   A   
   T   
   T   
   G   
   A   
   T   
   G   
   G   
   A   
   C   
   A   
   A   
   T   
   C   
   C   
   T   
   G   
   A   
   T   
   G   
   T   
   A   
   A   
   C   
   G   
   A   
   C   
   G   
   A   
   A   
   T   
   G   
   A   
   A   
   T   
   T   
   G   
   A   
   T   
   G   
   G   
   A   
   A   
   G   
   T   
   G   
   T   
   T   
   A   
   C   
   C   
   T   
   G   
   G   
   A   
   C   
   C   
   A   
   G   
   A   
   T   
   T   
   T   
   T   
   C   
   C   
   A   
   A   
   C   
   A   
   G   
   G   
   C   
   G   
   G   
   T   
   T   
   T   
   A   
   G   
   T   
   G   
   A   
   T   
   G   
   G   
   G   
   G   
   A   
   A   
   A   
   T   
   C   
   A   
   G   
   G   
    G    
   A   
   T   
    T    
   C   
   G   
    C    
    C    
   G   
   A   
   G   
   C   
    A    
   T   
   A   
   T   
   G   
   A   
   A   
   A   
   C   
    A    
   G   
   G   
    G    
   A   
   A   
    A    
   G   
   G   
   T   
   T   
   C   
    G    
   A   
   T   
   T   
   A   
   C   
    T    
   G   
   T   
    T    
   C   
   G   
   T   
   G   
   C   
   A   
   A   
   A   
   A   
   G   
   T   
   T   
   G   
   A   
    A    
    T    
   T   
    G    
   A   
    C    
   T   
   G   
   A   
    A    
   A   
   T   
   G   
    C    
   C   
    G    
   A   
   A   
   T   
   G   
   G   
    A    
   A   
   A   
   A   
   G   
   A   
   A   
   C   
   G   
    T    
   A   
   T   
    T    
    T    
   T   
    A    
   G   
   T   
    A    
   A   
   C   
    T    
   G   
   A   
   A   
   T   
   T   
    G    
   C   
   C   
   T   
   T   
   A   
   T   
   A   
   T   
   G   
   G   
   T   
    G    
   A   
   A   
   T   
   A   
   A   
   A   
   G   
   C   
    C    
   A   
   A   
   A   
   T   
   T   
    A    
   A   
   T   
    C    
   G   
   A   
   A   
   C   
   G   
    A    
   A   
   T   
   T   
   T   
   C   
   T   
   G   
   A   
    A    
    T    
   T   
    A    
   C   
   A   
   C   
   A   
   G   
   A   
   G   
   A   
    T    
   A   
   A   
    A    
   C   
   G   
    A    
   A   
   T   
    T    
   G   
   A   
   A   
   G   
   G   
    A    
   A   
   T   
    T    
   A   
   C   
    T    
   G   
   A   
   T   
   T   
   T   
   G   
   C   
   G   
   G   
   G   
   A   
    T    
   G   
   A   
   A   
   T   
   C   
   T   
   T   
   C   
    T    
   C   
   G   
   T   
   G   
   A   
    A    
   G   
   G   
    C    
   A   
   T   
   G   
   C   
   G   
    G    
   A   
   T   
    C    
   G   
   T   
   C   
   A   
   T   
    C    
   G   
   A   
    T    
    G    
   T   
    T    
   C   
   G   
   T   
   C   
   G   
    A    
   G   
   A   
    T    
   G   
    T    
    G    
   A   
   G   
    T    
   G   
   C   
    C    
   T   
   C   
    T    
   G   
   T   
   T   
    G    
   T   
    G    
   T   
   T   
    A    
   A   
   A   
    C    
   A   
   A   
   C   
   T   
   T   
    G    
   T   
   A   
    C    
   A   
   A   
   A   
    A    
   T   
   G   
   A   
   C   
    T    
    G    
   C   
    C    
   T   
   T   
    A    
   C   
   A   
    A    
   A   
    C    
    A    
   T   
   C   
    T    
   T   
   T   
    T    
   G   
   G   
    T    
   T   
   T   
    T    
   A   
   A   
    C    
   A   
   T   
   G   
   T   
   T   
    A    
   G   
   C   
    C    
   A   
   T   
    T    
   G   
   A   
   A   
   A   
   A   
   A   
   G   
   G   
    C    
   G   
   T   
    A    
   C   
   C   
    G    
   A   
   A   
   A   
    A    
   T   
   T   
   T   
   T   
    A    
   A   
   G   
    C    
   T   
   T   
   G   
   A   
   A   
   A   
   C   
    G    
    T    
   A   
   T   
    T    
    T    
   T   
    A    
   G   
   A   
   A   
   A   
   A   
    T    
   T   
   A   
    C    
    G    
   T   
    C    
   G   
   A   
    G    
   C   
   A   
    C    
   C   
   A   
    A    
    A    
    A    
    A    
    G    
   A   
   A   
   G   
   T   
   C   
   A   
   T   
    T    
   A   
   C   
    T    
    C    
   G   
    C    
   C   
   G   
   G   
   A   
   C   
    G    
    A    
   T   
    T    
   T   
   T   
   T   
   G   
   A   
    C    
   A   
   A   
   A   
    A    
   A   
    C    
   A   
   A   
    A    
   G   
   C   
   A   
   G   
   A   
   A   
   G   
   C   
   A   
    C    
   G   
    G    
   G   
   C   
    G    
   C   
   A   
    T    
   A   
   T   
    T    
   T   
   T   
   A   
   G   
   A   
   A   
   G   
   G   
   T   
    C    
   T   
    A    
   C   
   G   
    A    
   A   
   T   
   T   
   G   
   C   
    C    
   T   
   T   
    A    
   G   
   A   
   T   
   C   
   A   
   T   
   A   
   T   
   C   
    G    
   A   
   T   
   G   
   A   
   A   
   A   
   T   
    C    
   A   
   T   
   T   
   G   
   C   
    C    
   A   
   T   
   T   
   A   
   T   
    C    
   C   
   G   
    T    
   G   
   G   
    A    
   T   
   C   
    A    
    C    
   A   
   A   
   T   
   C   
   A   
   G   
   A   
   T   
   G   
   A   
    C    
   G   
   A   
    A    
   G   
   C   
   A   
   A   
   A   
   A   
   G   
   C   
    A    
   A   
    C    
    G    
    T    
   T   
    A    
   A   
   T   
    T    
   G   
   A   
   A   
   C   
   G   
    C    
   T   
   T   
    T    
   G   
   A   
    A    
   T   
   T   
    T    
   T   
   C   
    A    
   G   
   A   
   T   
   C   
   G   
    T    
   C   
   A   
    A    
    G    
   C   
    G    
   C   
   A   
   A   
   G   
   C   
    G    
   A   
   T   
    T    
   T   
   T   
   A   
   G   
   A   
   T   
   A   
   T   
   G   
   C   
   G   
   T   
   T   
   T   
    A    
   C   
   G   
    C    
   C   
   G   
    T    
   T   
   T   
    A    
   A   
   C   
    A    
   G   
   G   
    C    
   T   
   T   
    A    
   G   
   A   
   A   
   C   
   G   
    C    
   G   
   A   
    C    
   A   
   A   
   A   
   A   
   T   
    T    
   G   
   A   
    A    
   A   
   A   
   T   
   G   
   A   
   A   
   T   
   A   
    T    
   C   
   A   
   A   
   G   
   A   
    G    
   C   
   T   
    A    
   T   
   T   
    G    
   A   
    A    
   A   
    T    
   T   
    C    
   A   
   T   
   T   
   G   
    C    
    G    
   G   
   A   
    C    
   T   
   T   
    A    
    G    
    A    
    G    
   G   
   A   
   C   
   A   
   T   
   T   
    T    
   T   
    A    
   G   
   C   
    C    
    C    
   G   
    C    
   C   
   C   
    A    
   G   
   A   
    A    
   C   
   G   
    C    
   G   
   T   
    C    
   A   
    T    
    T    
   G   
   A   
   A   
   A   
   T   
    C    
   A   
   T   
    T    
   A   
   A   
    A    
   A   
    C    
    A    
   G   
   A   
    G    
   T   
   T   
   A   
    A    
    A    
    T    
   G   
   A   
   C   
    G    
   T   
    T    
    C    
    G    
    C    
    A    
    C    
   A   
    A    
    A    
   A   
   T   
   T   
   T   
   G   
   G   
   T   
   G   
   A   
   T   
    G    
    C    
   A   
   C   
   G   
   A   
   C   
   G   
    C    
   A   
   C   
   A   
   G   
   A   
   A   
    T    
   T   
   A   
    T    
   T   
   A   
   G   
   T   
    A    
   G   
   G   
   T   
   G   
   A   
    A    
   G   
   T   
   C   
    C    
   T   
    A    
   A   
   G   
   T   
   C   
   T   
    T    
   G   
   A   
   A   
   G   
   A   
   T   
   G   
   A   
   A   
   G   
   A   
   T   
    T    
   T   
    A    
   A   
   T   
    C    
   G   
   A   
   A   
   G   
   A   
   A   
   G   
   A   
   A   
   G   
   A   
   A   
    G    
   T   
   C   
   G   
   T   
    G    
   A   
   T   
   T   
   A   
   C   
    G    
    T    
   T   
    A    
   A   
   C   
    C    
   A   
   A   
    T    
   A   
   A   
    C    
   G   
   G   
    C    
   T   
   A   
    C    
   A   
   T   
    T    
   A   
   A   
    G    
   C   
   G   
    G    
    A    
   T   
   G   
   G   
    C    
   A   
   A   
   A   
    C    
    T    
    C    
    C    
   G   
   A   
   A   
   T   
   T   
   C   
    C    
   G   
    G    
   G   
   C   
    G    
   C   
   A   
   A   
   C   
   G   
    C    
    C    
   G   
    C    
   G   
   G   
    C    
   G   
   G   
    A    
   C   
   G   
   T   
   G   
   G   
    T    
   G   
   T   
    C    
   C   
   A   
   A   
   G   
   G   
    G    
   A   
   T   
   G   
   G   
   G   
    C    
    G    
   T   
    T    
   C   
   A   
    C    
    G    
   A   
   T   
   G   
   A   
    T    
   G   
   A   
    T    
   T   
   T   
   C   
   G   
   T   
    G    
   A   
   A   
    A    
   A   
   A   
    C    
   C   
   T   
    A    
    G    
   T   
   T   
   T   
   C   
   T   
   T   
   G   
    T    
   T   
   C   
    A    
   A   
   C   
    A    
   C   
   A   
    C    
   G   
   A   
    C    
   A   
   C   
    G    
   T   
   T   
    A    
   T   
   T   
   A   
   T   
   T   
    C    
   T   
   T   
   T   
   A   
   C   
   G   
   A   
   A   
    C    
   A   
    C    
    T    
   G   
   G   
    G    
   A   
   A   
   A   
   G   
   T   
    T    
   T   
   A   
    C    
   C   
   G   
    A    
   G   
   C   
   A   
   A   
   A   
   A   
   G   
   G   
    T    
   T   
   A   
    C    
   G   
   A   
   A   
   A   
   T   
    C    
   C   
   C   
    T    
   G   
   A   
    A    
   T   
   A   
   C   
   G   
   G   
    T    
    A    
   G   
    A    
   A   
   C   
   A   
   G   
   C   
    A    
   A   
   A   
   A   
   G   
   G   
    A    
   A   
   T   
    T    
   C   
   C   
   A   
    G    
   T   
    A    
   A   
   T   
    T    
   A   
   A   
    C    
   T   
   T   
    A    
   C   
   T   
    G    
   G   
   G   
    A    
   A   
   T   
    T    
   G   
   A   
    T    
   T   
   C   
    T    
    G    
    C    
    A    
   G   
   A   
   A   
   A   
    A    
    A    
   A   
   T   
    T    
   C   
   A   
    A    
   G   
   C   
    G    
   A   
   T   
    T    
   A   
   T   
   T   
    T    
   C   
    T    
   G   
   T   
    T    
    G    
    A    
   G   
   G   
   G   
    C    
    A    
   A   
   A   
   G   
   C   
    G    
   G   
   A   
   A   
   G   
    C    
    A    
   G   
   G   
    T    
    A    
   A   
    A    
   T   
   A   
    C    
    T    
   T   
    G    
   T   
   T   
   C   
   T   
   T   
    C    
   A   
   C   
    A    
   A   
   C   
    C    
    T    
    T    
    A    
    A    
   A   
   A   
   G   
   G   
    A    
   A   
   C   
    T    
   G   
   T   
    C    
   A   
   A   
   A   
   C   
   G   
    G    
   A   
   C   
    A    
    G    
   C   
    C    
   G   
   T   
    A    
   A   
   C   
    A    
   G   
   C   
    C    
   T   
   T   
    T    
    T    
   C   
   T   
   A   
   A   
    T    
   A   
   T   
    C    
   C   
   G   
    T    
   A   
   G   
    T    
   A   
   A   
   T   
   G   
   G   
    A    
    T    
   T   
    A    
   A   
   T   
    C    
   G   
   C   
    C    
   A   
   T   
   T   
    A    
   G   
    C    
   T   
   T   
    A    
   A   
   A   
   A   
   G   
   A   
   A   
   G   
   A   
    T    
   G   
   A   
    T    
   G   
   A   
    G    
    T    
   T   
    A    
    G    
   T   
    T    
   A   
   A   
    C    
   G   
   T   
    A    
    G    
   T   
   A   
    A    
    C    
   G   
   A   
   C   
    T    
    A    
   A   
    T    
   G   
   G   
    C    
    A    
   A   
    T    
    C    
    A    
    G    
   A   
   A   
    G    
   A   
   T   
    G    
   A   
   T   
    T    
   A   
   T   
    C    
   G   
   G   
    A    
   A   
   C   
   A   
   C   
   A   
   T   
    G    
    C    
    A    
   G   
   G   
    A    
   T   
   A   
   C   
   T   
   C   
   T   
   G   
   T   
    C    
   A   
   C   
    A    
   T   
   T   
    T    
    G    
   A   
    T    
   G   
   A   
    A    
    A    
    A    
    T    
    A    
   C   
    T    
   G   
    T    
    A    
   C   
   G   
    T    
   G   
   A   
   T   
   A   
   T   
   G   
   G   
   G   
    C    
   C   
   G   
    G    
   A   
   C   
   A   
   G   
   C   
    A    
   T   
   C   
    A    
   G   
   G   
    C    
   G   
   T   
    T    
    C    
   G   
    G    
   G   
   G   
    A    
   A   
   T   
    C    
   C   
   G   
    T    
    C    
   T   
    C    
   C   
   G   
    C    
   G   
   A   
    A    
    A    
   A   
    T    
   G   
   A   
    T    
   T   
    A    
    T    
   G   
   T   
    G    
   G   
   T   
    C    
   G   
   G   
    C    
    G    
   C   
   A   
   G   
   C   
    G    
    A    
   T   
    T    
    C    
   T   
   G   
    G    
   A   
    T    
   G   
    A    
   A   
    A    
   A   
    T    
    A    
   A   
   A   
   G   
   A   
   A   
   G   
   T   
    C    
    C    
   T   
    A    
   G   
   T   
    C    
   A   
   T   
    T    
   A   
   C   
    T    
   G   
   A   
   A   
   A   
   A   
    T    
   G   
   G   
   T   
   T   
   A   
    T    
   G   
   G   
    T    
   A   
   A   
    G    
   C   
   G   
    T    
   A   
   C   
    A    
   A   
   A   
   A   
   G   
    C    
    T    
   T   
   C   
    T    
   G   
   A   
    A    
   T   
   A   
   T   
   C   
   C   
   A   
   G   
   T   
    T    
   A   
   A   
    A    
   G   
   G   
    A    
   C   
   G   
    T    
   G   
   G   
    C    
   G   
   G   
   T   
   A   
   A   
   A   
   G   
   G   
    G    
   A   
   T   
    T    
   A   
   A   
    G    
   A   
   C   
    A    
    G    
   C   
    A    
   A   
   A   
    T    
   A   
   T   
   C   
   A   
   C   
   T   
   G   
   A   
    G    
   A   
   A   
   A   
   A   
   A   
   T   
   G   
   G   
   T   
   C   
   C   
   A   
    T    
   T   
   A   
   G   
   C   
    T    
   G   
   G   
    T    
   T   
   T   
    A    
   A   
   C   
   C   
   A   
   C   
    G    
   G   
   T   
    C    
    A    
    A    
   T   
   G   
   G   
    G    
    A    
   A   
   T   
   G   
   A   
   A   
   G   
   A   
    T    
   A   
   T   
   C   
    T    
   T   
    A    
    T    
   T   
    G    
   A   
   T   
    T    
   A   
   C   
    G    
    A    
   A   
    C    
   A   
   A   
   A   
   G   
   G   
    C    
   G   
   T   
    C    
   A   
   T   
   T   
   A   
   T   
   C   
   C   
   G   
    C    
   T   
   T   
    T    
   A   
   A   
   C   
   G   
   T   
    T    
    G    
    A    
    T    
    T    
   C   
   T   
   G   
   T   
   T   
   T   
   C   
   T   
   C   
   A   
   A   
   A   
   C   
    A    
   G   
   G   
   A   
   C   
   G   
    C    
    G    
   C   
   A   
   A   
   C   
   A   
   T   
   T   
    A    
   G   
   G   
    G    
   G   
   T   
    T    
   C   
   G   
   T   
   T   
   T   
    A    
   A   
   T   
   G   
   A   
    G    
    A    
   A   
   T   
   G   
   G   
   A   
   A   
   G   
    A    
    T    
   G   
    G    
    T    
    G    
   C   
    C    
   A   
   A   
   A   
   -   
   -   
   -   
   -   
   -   
   -   
   -   
   -   
   -   
   -   
   -   
   -   
   -   
   -   
   -   
   -   
   -   
   -   
   -   
   -   
   -   
   -   
   -   
   -   
   -   
   -   
   -   
   -   
   -   
   -   
   -   
   -   
   -   
   -   
   -   
   -   
   -   
   -   
   -   
   -   
   -   
   -   
   -   
   -   
   -   
   -   
   -   
   -   
   -   
   -   
   -   
   -   
   -   
   -   
   -   
   -   
   -   
   -   
   -   
   -   
   -   
   -   
   -   
   -   
   -   
   -   
   -   
   -   
   -   
   -   
   -   
   -   
 

 
   N54155   
   A   
   T   
   G   
   A   
   G   
   T   
   G   
   A   
   A   
   G   
   A   
   A   
   A   
   T   
   C   
   A   
   G   
   A   
   G   
   A   
   A   
   A   
   A   
   C   
   A   
   T   
   C   
   C   
   A   
   T   
   G   
   A   
   T   
   G   
   T   
   C   
   A   
   A   
   T   
   C   
   T   
   A   
   A   
   C   
   C   
   A   
   G   
   C   
   G   
   A   
   A   
   A   
   T   
   G   
   A   
   A   
   A   
   G   
   A   
   C   
   T   
   C   
   C   
   T   
   T   
   C   
   A   
   T   
   C   
   G   
   A   
   T   
   T   
   A   
   T   
   G   
   C   
   G   
   A   
   T   
   G   
   A   
   G   
   T   
   G   
   T   
   T   
   A   
   T   
   T   
   G   
   T   
   C   
   G   
   C   
   A   
   C   
   G   
   T   
   G   
   C   
   A   
   C   
   T   
   T   
   C   
   C   
   T   
   G   
   A   
   T   
   G   
   T   
   C   
   A   
   G   
   A   
   G   
   A   
   T   
   G   
   G   
   T   
   C   
   T   
   G   
   A   
   A   
   A   
   C   
   C   
   C   
   G   
   T   
   T   
   C   
   A   
   C   
   C   
   G   
   C   
   C   
   G   
   T   
   A   
   T   
   T   
   T   
   T   
   A   
   T   
   A   
   C   
   G   
   G   
   C   
   A   
   T   
   G   
   A   
   A   
   C   
   G   
   A   
   A   
   T   
   T   
   A   
   G   
   G   
   T   
   G   
   T   
   A   
   A   
   C   
   A   
   C   
   C   
   G   
   G   
   A   
   T   
   A   
   A   
   A   
   G   
   C   
   C   
   C   
   A   
   T   
   A   
   A   
   A   
   A   
   A   
   A   
   T   
   C   
   T   
   G   
   C   
   C   
   A   
   G   
   A   
   A   
   T   
   C   
   G   
   T   
   C   
   G   
   G   
   A   
   G   
   A   
   T   
   G   
   T   
   C   
   A   
   T   
   G   
   G   
   G   
   T   
   A   
   A   
   A   
   T   
   A   
   C   
   C   
   A   
   T   
   C   
   C   
   T   
   C   
   A   
   T   
   G   
   G   
   A   
   G   
   A   
   C   
   A   
   G   
   T   
   G   
   C   
   G   
   A   
   T   
   C   
   T   
   A   
   T   
   G   
   A   
   G   
   T   
   C   
   A   
   A   
   T   
   G   
   G   
   T   
   A   
   C   
   G   
   G   
   A   
   T   
   G   
   G   
   C   
   G   
   C   
   A   
   G   
   C   
   C   
   A   
   T   
   T   
   C   
   A   
   G   
   C   
   T   
   A   
   C   
   C   
   G   
   T   
   T   
   A   
   T   
   A   
   T   
   G   
   C   
   T   
   A   
   G   
   T   
   G   
   G   
   A   
   T   
   G   
   G   
   T   
   C   
   A   
   T   
   G   
   G   
   A   
   A   
   A   
   C   
   T   
   T   
   T   
   G   
   G   
   T   
   T   
   C   
   T   
   G   
   T   
   T   
   G   
   A   
   C   
   G   
   G   
   T   
   G   
   A   
   C   
   G   
   G   
   T   
   G   
   C   
   C   
   G   
   C   
   C   
   G   
   C   
   T   
   A   
   T   
   G   
   C   
   G   
   T   
   T   
   A   
   T   
   A   
   C   
   C   
   G   
   A   
   A   
   G   
   C   
   A   
   C   
   G   
   G   
   A   
   T   
   G   
   A   
   G   
   C   
   A   
   A   
   A   
   A   
   T   
   C   
   G   
   C   
   T   
   A   
   C   
   A   
   G   
   A   
   A   
   A   
   T   
   G   
   C   
   T   
   G   
   C   
   G   
   T   
   G   
   A   
   T   
   A   
   T   
   C   
   A   
   A   
   T   
   A   
   A   
   G   
   A   
   A   
   C   
   A   
   C   
   T   
   G   
   T   
   T   
   G   
   A   
   T   
   T   
   T   
   C   
   C   
   A   
   A   
   A   
   G   
   C   
   A   
   A   
   C   
   T   
   A   
   T   
   G   
   A   
   T   
   G   
   A   
   T   
   A   
   C   
   A   
   G   
   A   
   A   
   A   
   A   
   A   
   G   
   A   
   A   
   C   
   C   
   A   
   G   
   T   
   T   
   G   
   T   
   A   
   C   
   T   
   A   
   C   
   C   
   T   
   G   
   C   
   T   
   C   
   G   
   C   
   T   
   T   
   C   
   C   
   C   
   G   
   A   
   A   
   T   
   C   
   T   
   T   
   T   
   T   
   A   
   G   
   T   
   G   
   A   
   A   
   C   
   G   
   G   
   A   
   A   
   C   
   A   
   A   
   C   
   G   
   G   
   G   
   G   
   A   
   T   
   C   
   G   
   C   
   C   
   G   
   T   
   A   
   G   
   G   
   T   
   A   
   T   
   G   
   G   
   C   
   C   
   A   
   C   
   G   
   A   
   A   
   T   
   A   
   T   
   C   
   C   
   C   
   A   
   C   
   C   
   T   
   C   
   A   
   T   
   A   
   A   
   C   
   T   
   T   
   G   
   T   
   C   
   T   
   G   
   A   
   G   
   G   
   T   
   A   
   G   
   T   
   A   
   G   
   C   
   G   
   G   
   C   
   T   
   A   
   T   
   C   
   G   
   A   
   T   
   T   
   T   
   G   
   C   
   T   
   G   
   A   
   T   
   G   
   G   
   A   
   T   
   A   
   A   
   T   
   C   
   C   
   T   
   G   
   A   
   T   
   G   
   T   
   G   
   A   
   C   
   G   
   A   
   C   
   C   
   A   
   A   
   T   
   G   
   A   
   A   
   T   
   T   
   G   
   A   
   T   
   G   
   G   
   A   
   A   
   G   
   T   
   A   
   T   
   T   
   A   
   C   
   C   
   T   
   G   
   G   
   A   
   C   
   C   
   A   
   G   
   A   
   T   
   T   
   T   
   T   
   C   
   C   
   A   
   A   
   C   
   A   
   G   
   G   
   T   
   G   
   G   
   T   
   T   
   T   
   G   
   G   
   T   
   T   
   A   
   T   
   G   
   G   
   G   
   G   
   A   
   A   
   A   
   T   
   C   
   A   
   G   
   G   
    A    
   A   
   T   
    C    
   C   
   G   
    A    
    A    
   G   
   A   
   G   
   C   
    G    
   T   
   A   
   T   
   G   
   A   
   A   
   A   
   C   
    A    
   G   
   G   
    T    
   A   
   A   
    G    
   G   
   G   
   T   
   T   
   C   
    T    
   A   
   T   
   T   
   A   
   C   
    C    
   G   
   T   
    A    
   C   
   G   
   T   
   G   
   C   
   A   
   A   
   A   
   A   
   G   
   T   
   T   
   G   
   A   
    G    
    A    
   T   
    T    
   A   
    C    
   T   
   G   
   A   
    G    
   A   
   T   
   G   
    C    
   C   
    A    
   A   
   A   
   T   
   G   
   G   
    T    
   A   
   A   
   A   
   G   
   A   
   A   
   C   
   G   
    G    
   A   
   T   
    C    
    A    
   T   
    C    
   G   
   T   
    G    
   A   
   C   
    A    
   G   
   A   
   A   
   T   
   T   
    G    
   C   
   C   
   T   
   T   
   A   
   T   
   A   
   T   
   G   
   G   
   T   
    C    
   A   
   A   
   T   
   A   
   A   
   A   
   G   
   C   
    A    
   A   
   A   
   A   
   T   
   T   
    G    
   A   
   T   
    C    
   G   
   A   
   A   
   C   
   G   
    G    
   A   
   T   
   T   
   T   
   C   
   T   
   G   
   A   
    A    
    C    
   T   
    G    
   C   
   A   
   C   
   A   
   G   
   A   
   G   
   A   
    C    
   A   
   A   
    G    
   C   
   G   
    T    
   A   
   T   
    C    
   G   
   A   
   A   
   G   
   G   
    G    
   A   
   T   
    C    
   A   
   C   
    A    
   G   
   A   
   T   
   T   
   T   
   G   
   C   
   G   
   G   
   G   
   A   
    C    
   G   
   A   
   A   
   T   
   C   
   T   
   T   
   C   
    C    
   C   
   G   
   T   
   G   
   A   
    G    
   G   
   G   
    T    
   A   
   T   
   G   
   C   
   G   
    G    
   A   
   T   
    C    
   G   
   T   
   C   
   A   
   T   
    T    
   G   
   A   
    C    
    A    
   T   
    C    
   C   
   G   
   T   
   C   
   G   
    T    
   G   
   A   
    C    
   G   
    C    
    C    
   A   
   G   
    C    
   G   
   C   
    A    
   T   
   C   
    C    
   G   
   T   
   T   
    A    
   T   
    C    
   T   
   T   
    G    
   A   
   A   
    T    
   A   
   A   
   C   
   T   
   T   
    A    
   T   
   A   
    T    
   A   
   A   
   A   
    T    
   T   
   G   
   A   
   C   
    A    
    T    
   C   
    C    
   T   
   T   
    G    
   C   
   A   
    A    
   A   
    A    
    T    
   T   
   C   
    A    
   T   
   T   
    T    
   G   
   G   
    A    
   T   
   T   
    C    
   A   
   A   
    T    
   A   
   T   
   G   
   T   
   T   
    G    
   G   
   C   
    G    
   A   
   T   
    C    
   G   
   A   
   A   
   A   
   A   
   A   
   G   
   G   
    T    
   G   
   T   
    G    
   C   
   C   
    G    
   A   
   A   
   A   
    G    
   T   
   T   
   T   
   T   
    G    
   A   
   G   
    C    
   T   
   T   
   G   
   A   
   A   
   A   
   C   
    A    
    A    
   A   
   T   
    T    
    C    
   T   
    T    
   G   
   A   
   A   
   A   
   A   
    C    
   T   
   A   
    T    
    A    
   T   
    C    
   G   
   A   
    A    
   C   
   A   
    T    
   C   
   A   
    G    
    C    
    G    
    C    
    G    
   A   
   A   
   G   
   T   
   C   
   A   
   T   
    C    
   A   
   C   
    A    
    A    
   G   
    A    
   C   
   G   
   G   
   A   
   C   
    G    
    G    
   T   
    C    
   T   
   T   
   T   
   G   
   A   
    A    
   A   
   A   
   A   
    G    
   A   
    A    
   A   
   A   
    A    
   G   
   C   
   A   
   G   
   A   
   A   
   G   
   C   
   A   
    A    
   G   
    G    
   G   
   C   
    A    
   C   
   A   
    C    
   A   
   T   
    T    
   T   
   T   
   A   
   G   
   A   
   A   
   G   
   G   
   T   
    T    
   T   
    G    
   C   
   G   
    T    
   A   
   T   
   T   
   G   
   C   
    T    
   T   
   T   
    G    
   G   
   A   
   T   
   C   
   A   
   T   
   A   
   T   
   C   
    G    
   A   
   T   
   G   
   A   
   A   
   A   
   T   
    C    
   A   
   T   
   T   
   G   
   C   
    G    
   A   
   T   
   T   
   A   
   T   
    C    
   C   
   G   
    C    
   G   
   G   
    G    
   T   
   C   
    T    
    A    
   A   
   A   
   T   
   C   
   A   
   G   
   A   
   T   
   G   
   A   
    C    
   G   
   A   
    A    
   G   
   C   
   A   
   A   
   A   
   A   
   G   
   C   
    G    
   A   
    C    
    G    
    A    
   T   
    G    
   A   
   T   
    C    
   G   
   A   
   A   
   C   
   G   
    G    
   T   
   T   
    C    
   G   
   A   
    T    
   T   
   T   
    A    
   T   
   C   
    C    
   G   
   A   
   T   
   C   
   G   
    C    
   C   
   A   
    G    
    T    
   C   
    T    
   C   
   A   
   A   
   G   
   C   
    T    
   A   
   T   
    T    
   T   
   T   
   A   
   G   
   A   
   T   
   A   
   T   
   G   
   C   
   G   
   T   
   T   
   T   
    A    
   C   
   G   
    C    
   C   
   G   
    A    
   T   
   T   
    G    
   A   
   C   
    C    
   G   
   G   
    T    
   T   
   T   
    G    
   G   
   A   
   A   
   C   
   G   
    G    
   G   
   A   
    A    
   A   
   A   
   A   
   A   
   T   
    C    
   G   
   A   
    A    
   A   
   A   
   T   
   G   
   A   
   A   
   T   
   A   
    C    
   C   
   A   
   A   
   G   
   A   
    A    
   C   
   T   
    C    
   T   
   T   
    A    
   A   
    A    
   A   
    C    
   T   
    G    
   A   
   T   
   T   
   G   
    A    
    A    
   G   
   A   
    T    
   T   
   T   
    A    
    A    
    C    
    T    
   G   
   A   
   C   
   A   
   T   
   T   
    C    
   T   
    G    
   G   
   C   
    A    
    A    
   G   
    A    
   C   
   C   
    A    
   G   
   A   
    A    
   C   
   G   
    T    
   G   
   T   
    T    
   A   
    C    
    A    
   G   
   A   
   A   
   A   
   T   
    C    
   A   
   T   
    C    
   A   
   A   
    G    
   A   
    A    
    T    
   G   
   A   
    A    
   T   
   T   
   A   
    G    
    C    
    A    
   G   
   A   
   C   
    T    
   T   
    G    
    A    
    A    
    T    
    C    
    A    
   A   
    C    
    G    
   A   
   T   
   T   
   T   
   G   
   G   
   T   
   G   
   A   
   T   
    A    
    A    
   A   
   C   
   G   
   A   
   C   
   G   
    C    
   A   
   C   
   A   
   G   
   A   
   A   
    T    
   T   
   A   
    C    
   T   
   A   
   G   
   T   
    C    
   G   
   G   
   T   
   G   
   A   
    G    
   G   
   T   
   C   
    T    
   T   
    A    
   A   
   G   
   T   
   C   
   T   
    G    
   G   
   A   
   A   
   G   
   A   
   T   
   G   
   A   
   A   
   G   
   A   
   T   
    T    
   T   
    A    
   A   
   T   
    C    
   G   
   A   
   A   
   G   
   A   
   A   
   G   
   A   
   A   
   G   
   A   
   A   
    A    
   T   
   C   
   G   
   T   
    T    
   A   
   T   
   T   
   A   
   C   
    A    
    T    
   T   
    G    
   A   
   C   
    A    
   A   
   A   
    C    
   A   
   A   
    T    
   G   
   G   
    T    
   T   
   A   
    C    
   A   
   T   
    C    
   A   
   A   
    A    
   C   
   G   
    T    
    T    
   T   
   G   
   G   
    C    
   A   
   A   
   A   
    T    
    A    
    A    
    T    
   G   
   A   
   A   
   T   
   T   
   C   
    A    
   G   
    A    
   G   
   C   
    T    
   C   
   A   
   A   
   C   
   G   
    A    
    A    
   G   
    G    
   G   
   G   
    T    
   G   
   G   
    A    
   C   
   G   
   T   
   G   
   G   
    T    
   G   
   T   
    C    
   C   
   A   
   A   
   G   
   G   
    A    
   A   
   T   
   G   
   G   
   G   
    T    
    A    
   T   
    C    
   C   
   A   
    C    
    A    
   A   
   T   
   G   
   A   
    C    
   G   
   A   
    C    
   T   
   T   
   C   
   G   
   T   
    C    
   A   
   A   
    A    
   A   
   A   
    T    
   C   
   T   
    G    
    G    
   T   
   T   
   T   
   C   
   T   
   T   
   G   
    T    
   T   
   C   
    T    
   A   
   C   
    C    
   C   
   A   
    T    
   G   
   A   
    T    
   A   
   C   
    A    
   T   
   T   
    G    
   T   
   T   
   A   
   T   
   T   
    C    
   T   
   T   
   T   
   A   
   C   
   G   
   A   
   A   
    T    
   A   
    A    
    C    
   G   
   G   
    A    
   A   
   A   
   A   
   G   
   T   
    G    
   T   
   A   
    T    
   C   
   G   
    G    
   G   
   C   
   A   
   A   
   A   
   A   
   G   
   G   
    A    
   T   
   A   
    C    
   G   
   A   
   A   
   A   
   T   
    T    
   C   
   C   
    A    
   G   
   A   
    A    
   T   
   A   
   C   
   G   
   G   
    G    
    C    
   G   
    C    
   A   
   C   
   A   
   G   
   C   
    C    
   A   
   A   
   A   
   G   
   G   
    A    
   A   
   T   
    A    
   C   
   C   
   A   
    A    
   T   
    T    
   A   
   T   
    C    
   A   
   A   
    T    
   T   
   T   
    A    
   C   
   T   
    A    
   G   
   G   
    T    
   A   
   T   
    C    
   G   
   A   
    T    
   T   
   C   
    G    
    A    
    G    
    T    
   G   
   A   
   A   
   A   
    G    
    C    
   A   
   T   
    C    
   C   
   A   
    G    
   G   
   C   
    A    
   A   
   T   
    C    
   A   
   T   
   T   
    G    
   C   
    G    
   G   
   T   
    G    
    A    
    C    
   G   
   G   
   G   
    A    
    G    
   A   
   A   
   G   
   C   
    A    
   G   
   A   
   A   
   G   
    A    
    A    
   G   
   G   
    C    
    C    
   A   
    C    
   T   
   A   
    T    
    C    
   T   
    C    
   T   
   T   
   C   
   T   
   T   
    T    
   A   
   C   
    G    
   A   
   C   
    G    
    C    
    G    
    T    
    C    
   A   
   A   
   G   
   G   
    T    
   A   
   C   
    A    
   G   
   T   
    A    
   A   
   A   
   A   
   C   
   G   
    T    
   A   
   C   
    T    
    T    
   C   
    T    
   G   
   T   
    C    
   A   
   C   
    T    
   G   
   C   
    T    
   T   
   T   
    T    
    G    
   C   
   T   
   A   
   A   
    C    
   A   
   T   
    C    
   C   
   G   
    A    
   A   
   G   
    T    
   A   
   A   
   T   
   G   
   G   
    T    
    C    
   T   
    G    
   A   
   T   
    C    
   G   
   C   
    A    
   A   
   T   
   T   
    G    
   G   
    T    
   T   
   T   
    G    
   A   
   A   
   A   
   G   
   A   
   A   
   G   
   A   
    C    
   G   
   A   
    T    
   G   
   A   
    G    
    C    
   T   
    C    
    G    
   T   
    C    
   A   
   A   
    T    
   G   
   T   
    G    
    C    
   T   
   A   
    C    
    T    
   G   
   A   
   C   
    A    
    G    
   A   
    T    
   G   
   G   
    T    
    C    
   A   
    A    
    T    
    C    
    T    
   A   
   A   
    T    
   A   
   T   
    C    
   A   
   T   
    T    
   A   
   T   
    T    
   G   
   G   
    A    
   A   
   C   
   A   
   C   
   A   
   T   
    A    
    A    
    C    
   G   
   G   
    C    
   T   
   A   
   C   
   T   
   C   
   T   
   G   
   T   
    C    
   A   
   C   
    T    
   T   
   T   
    C    
    A    
   A   
    A    
   G   
   A   
    A    
    G    
    A    
    A    
    G    
   C   
    A    
   G   
    T    
    C    
   C   
   G   
    C    
   G   
   A   
   T   
   A   
   T   
   G   
   G   
   G   
    A    
   C   
   G   
    T    
   A   
   C   
   A   
   G   
   C   
    T    
   T   
   C   
    C    
   G   
   G   
    G    
   G   
   T   
    C    
    A    
   G   
    A    
   G   
   G   
    A    
   A   
   T   
    A    
   C   
   G   
    C    
    C    
   T   
    G    
   C   
   G   
    T    
   G   
   A   
    G    
    G    
   A   
    A    
   G   
   A   
    T    
   T   
    A    
    T    
   G   
   T   
    C    
   G   
   T   
    T    
   G   
   G   
    G    
    G    
   C   
   A   
   G   
   C   
    C    
    T    
   T   
    A    
    A    
   T   
   G   
    A    
   A   
    G    
   G   
    A    
   A   
    G    
   A   
    T    
    C    
   A   
   A   
   G   
   A   
   A   
   G   
   T   
    A    
    T    
   T   
    A    
   G   
   T   
    G    
   A   
   T   
    C    
   A   
   C   
    A    
   G   
   A   
   A   
   A   
   A   
    A    
   G   
   G   
   T   
   T   
   A   
    C    
   G   
   G   
    A    
   A   
   A   
    A    
   C   
   G   
    G    
   A   
   C   
    A    
   A   
   A   
   A   
   G   
    T    
    A    
   T   
   C   
    G    
   G   
   A   
    A    
   T   
   A   
   T   
   C   
   C   
   A   
   G   
   T   
    G    
   A   
   A   
    A    
   G   
   G   
    C    
   C   
   G   
    C    
   G   
   G   
    A    
   G   
   G   
   T   
   A   
   A   
   A   
   G   
   G   
    G    
   A   
   T   
    C    
   A   
   A   
    A    
   A   
   C   
    G    
    G    
   C   
    G    
   A   
   A   
    C    
   A   
   T   
   C   
   A   
   C   
   T   
   G   
   A   
    A    
   A   
   A   
   A   
   A   
   A   
   T   
   G   
   G   
   T   
   C   
   C   
   A   
    C    
   T   
   A   
   G   
   C   
    T    
   G   
   G   
    A    
   T   
   T   
    G    
   A   
   C   
   C   
   A   
   C   
    T    
   G   
   T   
    T    
    T    
    C    
   T   
   G   
   G   
    A    
    G    
   A   
   T   
   G   
   A   
   A   
   G   
   A   
    C    
   A   
   T   
   C   
    A    
   T   
    G    
    C    
   T   
    G    
   A   
   T   
    T    
   A   
   C   
    A    
    G    
   A   
    T    
   A   
   A   
   A   
   G   
   G   
    T    
   G   
   T   
    C    
   A   
   T   
   T   
   A   
   T   
   C   
   C   
   G   
    T    
   T   
   T   
    C    
   A   
   A   
   C   
   G   
   T   
    A    
    T    
    C    
    G    
    A    
   C   
   T   
   G   
   T   
   T   
   T   
   C   
   T   
   C   
   A   
   A   
   A   
   C   
    T    
   G   
   G   
   A   
   C   
   G   
    T    
    T    
   C   
   A   
   A   
   C   
   A   
   T   
   T   
    A    
   G   
   G   
    C    
   G   
   T   
    A    
   C   
   G   
   T   
   T   
   T   
    G    
   A   
   T   
   G   
   A   
    A    
    G    
   A   
   T   
   G   
   G   
   A   
   A   
   G   
    C    
    T    
   G   
    A    
    T    
    A    
   C   
    G    
   A   
   A   
   A   
   G   
   T   
   T   
   G   
   T   
   C   
   A   
   C   
   G   
   A   
   T   
   G   
   G   
   C   
   A   
   G   
   C   
   A   
   G   
   T   
   C   
   G   
   A   
   A   
   C   
   C   
   A   
   G   
   A   
   A   
   G   
   C   
   T   
   G   
   C   
   A   
   G   
   A   
   A   
   C   
   A   
   A   
   A   
   C   
   A   
   G   
   T   
   T   
   G   
   A   
   A   
   G   
   A   
   A   
   C   
   A   
   A   
   G   
   G   
   A   
   T   
   C   
   A   
   G   
   A   
   A   
   G   
   A   
   A   
   -   
   -   
   -   
 

 
   N52773   
   A   
   T   
   G   
   A   
   G   
   T   
   G   
   A   
   A   
   G   
   A   
   A   
   A   
   T   
   T   
   A   
   A   
   A   
   G   
   A   
   A   
   A   
   A   
   C   
   A   
   T   
   T   
   C   
   A   
   A   
   G   
   A   
   C   
   G   
   T   
   C   
   A   
   A   
   T   
   C   
   T   
   G   
   A   
   C   
   C   
   A   
   G   
   C   
   G   
   A   
   A   
   A   
   T   
   G   
   A   
   A   
   A   
   G   
   A   
   A   
   T   
   C   
   T   
   T   
   T   
   C   
   A   
   T   
   T   
   G   
   A   
   C   
   T   
   A   
   C   
   G   
   C   
   A   
   A   
   T   
   G   
   A   
   G   
   T   
   G   
   T   
   T   
   A   
   T   
   C   
   G   
   T   
   A   
   G   
   C   
   C   
   C   
   G   
   C   
   G   
   C   
   G   
   T   
   T   
   A   
   C   
   C   
   T   
   G   
   A   
   C   
   G   
   T   
   T   
   C   
   G   
   T   
   G   
   A   
   C   
   G   
   G   
   T   
   T   
   T   
   A   
   A   
   A   
   A   
   C   
   C   
   T   
   G   
   T   
   T   
   C   
   A   
   T   
   C   
   G   
   C   
   C   
   G   
   A   
   A   
   T   
   C   
   T   
   T   
   A   
   T   
   A   
   T   
   G   
   G   
   A   
   A   
   T   
   G   
   A   
   A   
   C   
   G   
   A   
   A   
   T   
   T   
   A   
   G   
   G   
   G   
   G   
   T   
   A   
   A   
   C   
   C   
   C   
   C   
   T   
   G   
   A   
   T   
   A   
   A   
   A   
   C   
   C   
   A   
   C   
   A   
   C   
   A   
   A   
   A   
   A   
   A   
   A   
   T   
   C   
   A   
   G   
   C   
   C   
   C   
   G   
   G   
   A   
   T   
   T   
   G   
   T   
   T   
   G   
   G   
   G   
   G   
   A   
   T   
   G   
   T   
   T   
   A   
   T   
   G   
   G   
   G   
   T   
   A   
   A   
   A   
   T   
   A   
   T   
   C   
   A   
   C   
   C   
   C   
   C   
   C   
   A   
   T   
   G   
   G   
   G   
   G   
   A   
   C   
   A   
   G   
   T   
   G   
   C   
   G   
   A   
   T   
   T   
   T   
   A   
   C   
   G   
   A   
   A   
   T   
   C   
   A   
   A   
   T   
   G   
   G   
   T   
   G   
   C   
   G   
   G   
   A   
   T   
   G   
   G   
   C   
   A   
   C   
   A   
   A   
   C   
   C   
   T   
   T   
   T   
   T   
   A   
   G   
   T   
   T   
   A   
   T   
   C   
   G   
   G   
   G   
   C   
   T   
   A   
   T   
   G   
   T   
   T   
   A   
   G   
   T   
   T   
   G   
   A   
   C   
   G   
   G   
   C   
   C   
   A   
   C   
   G   
   G   
   A   
   A   
   A   
   C   
   T   
   T   
   C   
   G   
   G   
   T   
   T   
   C   
   T   
   G   
   T   
   C   
   G   
   A   
   T   
   G   
   G   
   T   
   G   
   A   
   C   
   G   
   G   
   C   
   G   
   C   
   T   
   G   
   C   
   C   
   G   
   C   
   T   
   A   
   T   
   G   
   C   
   G   
   T   
   T   
   A   
   T   
   A   
   C   
   C   
   G   
   A   
   A   
   G   
   C   
   A   
   C   
   G   
   T   
   A   
   T   
   G   
   A   
   G   
   T   
   A   
   A   
   A   
   A   
   T   
   T   
   G   
   C   
   T   
   T   
   T   
   A   
   G   
   A   
   A   
   A   
   T   
   G   
   C   
   T   
   A   
   C   
   G   
   A   
   G   
   A   
   T   
   A   
   T   
   T   
   A   
   A   
   C   
   A   
   A   
   A   
   A   
   A   
   T   
   A   
   C   
   A   
   G   
   T   
   C   
   G   
   A   
   T   
   T   
   T   
   C   
   C   
   A   
   A   
   G   
   G   
   A   
   A   
   A   
   C   
   T   
   A   
   T   
   G   
   A   
   T   
   G   
   A   
   T   
   T   
   C   
   A   
   G   
   A   
   A   
   C   
   A   
   A   
   G   
   A   
   A   
   C   
   C   
   A   
   G   
   T   
   A   
   G   
   T   
   T   
   T   
   T   
   A   
   C   
   C   
   T   
   G   
   C   
   T   
   C   
   G   
   T   
   T   
   T   
   T   
   C   
   C   
   A   
   A   
   A   
   C   
   T   
   T   
   A   
   C   
   T   
   A   
   G   
   T   
   T   
   A   
   A   
   C   
   G   
   G   
   A   
   A   
   C   
   A   
   A   
   C   
   G   
   G   
   G   
   G   
   A   
   T   
   T   
   G   
   C   
   G   
   G   
   T   
   G   
   G   
   G   
   G   
   A   
   T   
   G   
   G   
   C   
   A   
   A   
   C   
   A   
   A   
   A   
   T   
   A   
   T   
   T   
   C   
   C   
   A   
   C   
   C   
   A   
   C   
   A   
   C   
   A   
   A   
   T   
   T   
   T   
   A   
   A   
   G   
   T   
   G   
   A   
   A   
   G   
   T   
   G   
   A   
   T   
   T   
   G   
   A   
   T   
   G   
   C   
   A   
   A   
   C   
   A   
   A   
   G   
   T   
   T   
   T   
   A   
   T   
   T   
   G   
   A   
   T   
   G   
   G   
   A   
   C   
   A   
   A   
   T   
   C   
   C   
   T   
   G   
   A   
   T   
   G   
   T   
   A   
   A   
   C   
   G   
   A   
   C   
   G   
   A   
   A   
   T   
   G   
   A   
   A   
   T   
   T   
   G   
   A   
   T   
   G   
   G   
   A   
   A   
   G   
   T   
   G   
   T   
   T   
   A   
   C   
   C   
   T   
   G   
   G   
   A   
   C   
   C   
   A   
   G   
   A   
   T   
   T   
   T   
   T   
   C   
   C   
   A   
   A   
   C   
   A   
   G   
   G   
   C   
   G   
   G   
   T   
   T   
   T   
   A   
   G   
   T   
   G   
   A   
   T   
   G   
   G   
   G   
   G   
   A   
   A   
   A   
   T   
   C   
   A   
   G   
   G   
    G    
   A   
   T   
    T    
   C   
   G   
    C    
    C    
   G   
   A   
   G   
   C   
    A    
   T   
   A   
   T   
   G   
   A   
   A   
   A   
   C   
    A    
   G   
   G   
    G    
   A   
   A   
    A    
   G   
   G   
   T   
   T   
   C   
    G    
   A   
   T   
   T   
   A   
   C   
    T    
   G   
   T   
    T    
   C   
   G   
   T   
   G   
   C   
   A   
   A   
   A   
   A   
   G   
   T   
   T   
   G   
   A   
    A    
    T    
   T   
    G    
   A   
    C    
   T   
   G   
   A   
    A    
   A   
   T   
   G   
    C    
   C   
    G    
   A   
   A   
   T   
   G   
   G   
    A    
   A   
   A   
   A   
   G   
   A   
   A   
   C   
   G   
    T    
   A   
   T   
    T    
    T    
   T   
    A    
   G   
   T   
    A    
   A   
   C   
    T    
   G   
   A   
   A   
   T   
   T   
    G    
   C   
   C   
   T   
   T   
   A   
   T   
   A   
   T   
   G   
   G   
   T   
    G    
   A   
   A   
   T   
   A   
   A   
   A   
   G   
   C   
    C    
   A   
   A   
   A   
   T   
   T   
    A    
   A   
   T   
    C    
   G   
   A   
   A   
   C   
   G   
    A    
   A   
   T   
   T   
   T   
   C   
   T   
   G   
   A   
    A    
    T    
   T   
    A    
   C   
   A   
   C   
   A   
   G   
   A   
   G   
   A   
    T    
   A   
   A   
    A    
   C   
   G   
    A    
   A   
   T   
    T    
   G   
   A   
   A   
   G   
   G   
    A    
   A   
   T   
    T    
   A   
   C   
    T    
   G   
   A   
   T   
   T   
   T   
   G   
   C   
   G   
   G   
   G   
   A   
    T    
   G   
   A   
   A   
   T   
   C   
   T   
   T   
   C   
    T    
   C   
   G   
   T   
   G   
   A   
    A    
   G   
   G   
    C    
   A   
   T   
   G   
   C   
   G   
    G    
   A   
   T   
    C    
   G   
   T   
   C   
   A   
   T   
    C    
   G   
   A   
    T    
    G    
   T   
    T    
   C   
   G   
   T   
   C   
   G   
    A    
   G   
   A   
    T    
   G   
    T    
    G    
   A   
   G   
    T    
   G   
   C   
    C    
   T   
   C   
    T    
   G   
   T   
   T   
    G    
   T   
    G    
   T   
   T   
    A    
   A   
   A   
    C    
   A   
   A   
   C   
   T   
   T   
    G    
   T   
   A   
    C    
   A   
   A   
   A   
    A    
   T   
   G   
   A   
   C   
    T    
    G    
   C   
    C    
   T   
   T   
    A    
   C   
   A   
    A    
   A   
    C    
    A    
   T   
   C   
    T    
   T   
   T   
    T    
   G   
   G   
    T    
   T   
   T   
    T    
   A   
   A   
    C    
   A   
   T   
   G   
   T   
   T   
    A    
   G   
   C   
    C    
   A   
   T   
    C    
   G   
   A   
   A   
   A   
   A   
   A   
   G   
   G   
    C    
   G   
   T   
    A    
   C   
   C   
    G    
   A   
   A   
   A   
    A    
   T   
   T   
   T   
   T   
    A    
   A   
   G   
    C    
   T   
   T   
   G   
   A   
   A   
   A   
   C   
    G    
    T    
   A   
   T   
    T    
    T    
   T   
    A    
   G   
   A   
   A   
   A   
   A   
    T    
   T   
   A   
    C    
    G    
   T   
    C    
   G   
   A   
    G    
   C   
   A   
    C    
   C   
   A   
    A    
    A    
    A    
    A    
    G    
   A   
   A   
   G   
   T   
   C   
   A   
   T   
    T    
   A   
   C   
    T    
    C    
   G   
    C    
   C   
   G   
   G   
   A   
   C   
    G    
    A    
   T   
    T    
   T   
   T   
   T   
   G   
   A   
    C    
   A   
   A   
   A   
    A    
   A   
    C    
   A   
   A   
    A    
   G   
   C   
   A   
   G   
   A   
   A   
   G   
   C   
   A   
    C    
   G   
    G    
   G   
   C   
    G    
   C   
   A   
    T    
   A   
   T   
    T    
   T   
   T   
   A   
   G   
   A   
   A   
   G   
   G   
   T   
    C    
   T   
    A    
   C   
   G   
    A    
   A   
   T   
   T   
   G   
   C   
    C    
   T   
   T   
    A    
   G   
   A   
   T   
   C   
   A   
   T   
   A   
   T   
   C   
    G    
   A   
   T   
   G   
   A   
   A   
   A   
   T   
    C    
   A   
   T   
   T   
   G   
   C   
    C    
   A   
   T   
   T   
   A   
   T   
    C    
   C   
   G   
    T    
   G   
   G   
    A    
   T   
   C   
    A    
    C    
   A   
   A   
   T   
   C   
   A   
   G   
   A   
   T   
   G   
   A   
    C    
   G   
   A   
    A    
   G   
   C   
   A   
   A   
   A   
   A   
   G   
   C   
    A    
   A   
    C    
    G    
    T    
   T   
    A    
   A   
   T   
    T    
   G   
   A   
   A   
   C   
   G   
    C    
   T   
   T   
    T    
   G   
   A   
    A    
   T   
   T   
    T    
   T   
   C   
    A    
   G   
   A   
   T   
   C   
   G   
    T    
   C   
   A   
    A    
    G    
   C   
    G    
   C   
   A   
   A   
   G   
   C   
    G    
   A   
   T   
    T    
   T   
   T   
   A   
   G   
   A   
   T   
   A   
   T   
   G   
   C   
   G   
   T   
   T   
   T   
    A    
   C   
   G   
    C    
   C   
   G   
    T    
   T   
   T   
    A    
   A   
   C   
    A    
   G   
   G   
    C    
   T   
   T   
    A    
   G   
   A   
   A   
   C   
   G   
    C    
   G   
   A   
    C    
   A   
   A   
   A   
   A   
   T   
    T    
   G   
   A   
    A    
   A   
   A   
   T   
   G   
   A   
   A   
   T   
   A   
    T    
   C   
   A   
   A   
   G   
   A   
    G    
   C   
   T   
    A    
   T   
   T   
    G    
   A   
    A    
   A   
    T    
   T   
    C    
   A   
   T   
   T   
   G   
    C    
    G    
   G   
   A   
    C    
   T   
   T   
    A    
    G    
    A    
    G    
   G   
   A   
   C   
   A   
   T   
   T   
    T    
   T   
    A    
   G   
   C   
    C    
    C    
   G   
    C    
   C   
   C   
    A    
   G   
   A   
    A    
   C   
   G   
    C    
   G   
   T   
    C    
   A   
    T    
    T    
   G   
   A   
   A   
   A   
   T   
    C    
   A   
   T   
    T    
   A   
   A   
    A    
   A   
    C    
    A    
   G   
   A   
    G    
   T   
   T   
   A   
    A    
    A    
    T    
   G   
   A   
   C   
    G    
   T   
    T    
    C    
    G    
    C    
    A    
    C    
   A   
    A    
    A    
   A   
   T   
   T   
   T   
   G   
   G   
   T   
   G   
   A   
   T   
    G    
    C    
   A   
   C   
   G   
   A   
   C   
   G   
    C    
   A   
   C   
   A   
   G   
   A   
   A   
    T    
   T   
   A   
    T    
   T   
   A   
   G   
   T   
    A    
   G   
   G   
   T   
   G   
   A   
    A    
   G   
   T   
   C   
    C    
   T   
    A    
   A   
   G   
   T   
   C   
   T   
    T    
   G   
   A   
   A   
   G   
   A   
   T   
   G   
   A   
   A   
   G   
   A   
   T   
    T    
   T   
    A    
   A   
   T   
    C    
   G   
   A   
   A   
   G   
   A   
   A   
   G   
   A   
   A   
   G   
   A   
   A   
    G    
   T   
   C   
   G   
   T   
    G    
   A   
   T   
   T   
   A   
   C   
    G    
    T    
   T   
    A    
   A   
   C   
    C    
   A   
   A   
    T    
   A   
   A   
    C    
   G   
   G   
    C    
   T   
   A   
    C    
   A   
   T   
    T    
   A   
   A   
    G    
   C   
   G   
    G    
    A    
   T   
   G   
   G   
    C    
   A   
   A   
   A   
    C    
    T    
    C    
    C    
   G   
   A   
   A   
   T   
   T   
   C   
    C    
   G   
    G    
   G   
   C   
    G    
   C   
   A   
   A   
   C   
   G   
    C    
    C    
   G   
    C    
   G   
   G   
    C    
   G   
   G   
    A    
   C   
   G   
   T   
   G   
   G   
    T    
   G   
   T   
    C    
   C   
   A   
   A   
   G   
   G   
    G    
   A   
   T   
   G   
   G   
   G   
    C    
    G    
   T   
    T    
   C   
   A   
    C    
    G    
   A   
   T   
   G   
   A   
    T    
   G   
   A   
    T    
   T   
   T   
   C   
   G   
   T   
    G    
   A   
   A   
    A    
   A   
   A   
    C    
   C   
   T   
    A    
    G    
   T   
   T   
   T   
   C   
   T   
   T   
   G   
    T    
   T   
   C   
    A    
   A   
   C   
    A    
   C   
   A   
    C    
   G   
   A   
    C    
   A   
   C   
    G    
   T   
   T   
    A    
   T   
   T   
   A   
   T   
   T   
    C    
   T   
   T   
   T   
   A   
   C   
   G   
   A   
   A   
    C    
   A   
    C    
    T    
   G   
   G   
    G    
   A   
   A   
   A   
   G   
   T   
    T    
   T   
   A   
    C    
   C   
   G   
    A    
   G   
   C   
   A   
   A   
   A   
   A   
   G   
   G   
    T    
   T   
   A   
    C    
   G   
   A   
   A   
   A   
   T   
    C    
   C   
   C   
    T    
   G   
   A   
    A    
   T   
   A   
   C   
   G   
   G   
    T    
    A    
   G   
    A    
   A   
   C   
   A   
   G   
   C   
    A    
   A   
   A   
   A   
   G   
   G   
    A    
   A   
   T   
    T    
   C   
   C   
   A   
    G    
   T   
    G    
   A   
   T   
    T    
   A   
   A   
    C    
   T   
   T   
    A    
   C   
   T   
    G    
   G   
   G   
    A    
   A   
   T   
    T    
   G   
   A   
    T    
   T   
   C   
    T    
    G    
    C    
    A    
   G   
   A   
   A   
   A   
    A    
    A    
   A   
   T   
    T    
   C   
   A   
    A    
   G   
   C   
    G    
   A   
   T   
    T    
   A   
   T   
   T   
    T    
   C   
    T    
   G   
   T   
    T    
    G    
    A    
   G   
   G   
   G   
    C    
    A    
   A   
   A   
   G   
   C   
    G    
   G   
   A   
   A   
   G   
    C    
    A    
   G   
   G   
    T    
    A    
   A   
    A    
   T   
   A   
    C    
    T    
   T   
    G    
   T   
   T   
   C   
   T   
   T   
    C    
   A   
   C   
    A    
   A   
   C   
    C    
    T    
    T    
    A    
    A    
   A   
   A   
   G   
   G   
    A    
   A   
   C   
    T    
   G   
   T   
    C    
   A   
   A   
   A   
   C   
   G   
    G    
   A   
   C   
    A    
    G    
   C   
    C    
   G   
   T   
    A    
   A   
   C   
    A    
   G   
   C   
    C    
   T   
   T   
    T    
    T    
   C   
   T   
   A   
   A   
    T    
   A   
   T   
    C    
   C   
   G   
    T    
   A   
   G   
    T    
   A   
   A   
   T   
   G   
   G   
    A    
    T    
   T   
    A    
   A   
   T   
    C    
   G   
   C   
    C    
   A   
   T   
   T   
    A    
   G   
    C    
   T   
   T   
    A    
   A   
   A   
   A   
   G   
   A   
   A   
   G   
   A   
    T    
   G   
   A   
    T    
   G   
   A   
    G    
    T    
   T   
    A    
    G    
   T   
    T    
   A   
   A   
    C    
   G   
   T   
    A    
    G    
   T   
   A   
    A    
    C    
   G   
   A   
   C   
    T    
    A    
   A   
    T    
   G   
   G   
    C    
    A    
   A   
    T    
    C    
    A    
    G    
   A   
   A   
    G    
   A   
   T   
    G    
   A   
   T   
    T    
   A   
   T   
    C    
   G   
   G   
    A    
   A   
   C   
   A   
   C   
   A   
   T   
    G    
    C    
    A    
   G   
   G   
    A    
   T   
   A   
   C   
   T   
   C   
   T   
   G   
   T   
    C    
   A   
   C   
    A    
   T   
   T   
    T    
    G    
   A   
    T    
   G   
   A   
    A    
    A    
    A    
    T    
    A    
   C   
    T    
   G   
    T    
    A    
   C   
   G   
    T    
   G   
   A   
   T   
   A   
   T   
   G   
   G   
   G   
    C    
   C   
   G   
    G    
   A   
   C   
   A   
   G   
   C   
    A    
   T   
   C   
    A    
   G   
   G   
    T    
   G   
   T   
    T    
    C    
   G   
    T    
   G   
   G   
    A    
   A   
   T   
    C    
   C   
   G   
    T    
    C    
   T   
    C    
   C   
   G   
    C    
   G   
   A   
    A    
    A    
   A   
    T    
   G   
   A   
    T    
   T   
    A    
    T    
   G   
   T   
    G    
   G   
   T   
    C    
   G   
   G   
    C    
    G    
   C   
   A   
   G   
   C   
    G    
    A    
   T   
    T    
    C    
   T   
   G   
    G    
   A   
    T    
   G   
    A    
   A   
    A    
   A   
    T    
    A    
   A   
   A   
   G   
   A   
   A   
   G   
   T   
    C    
    C    
   T   
    A    
   G   
   T   
    C    
   A   
   T   
    T    
   A   
   C   
    T    
   G   
   A   
   A   
   A   
   A   
    T    
   G   
   G   
   T   
   T   
   A   
    T    
   G   
   G   
    T    
   A   
   A   
    G    
   C   
   G   
    T    
   A   
   C   
    A    
   A   
   A   
   A   
   G   
    C    
    T    
   T   
   C   
    T    
   G   
   A   
    A    
   T   
   A   
   T   
   C   
   C   
   A   
   G   
   T   
    T    
   A   
   A   
    A    
   G   
   G   
    A    
   C   
   G   
    T    
   G   
   G   
    C    
   G   
   G   
   T   
   A   
   A   
   A   
   G   
   G   
    G    
   A   
   T   
    T    
   A   
   A   
    G    
   A   
   C   
    A    
    G    
   C   
    A    
   A   
   A   
    T    
   A   
   T   
   C   
   A   
   C   
   T   
   G   
   A   
    G    
   A   
   A   
   A   
   A   
   A   
   T   
   G   
   G   
   T   
   C   
   C   
   A   
    T    
   T   
   A   
   G   
   C   
    T    
   G   
   G   
    T    
   T   
   T   
    A    
   A   
   C   
   C   
   A   
   C   
    G    
   G   
   T   
    C    
    A    
    A    
   T   
   G   
   G   
    G    
    G    
   A   
   T   
   G   
   A   
   A   
   G   
   A   
    T    
   A   
   T   
   C   
    T    
   T   
    A    
    T    
   T   
    G    
   A   
   T   
    T    
   A   
   C   
    G    
    A    
   A   
    C    
   A   
   A   
   A   
   G   
   G   
    C    
   G   
   T   
    C    
   A   
   T   
   T   
   A   
   T   
   C   
   C   
   G   
    C    
   T   
   T   
    T    
   A   
   A   
   C   
   G   
   T   
    T    
    G    
    A    
    T    
    T    
   C   
   T   
   G   
   T   
   T   
   T   
   C   
   T   
   C   
   A   
   A   
   A   
   C   
    A    
   G   
   G   
   A   
   C   
   G   
    C    
    G    
   C   
   A   
   A   
   C   
   A   
   T   
   T   
    A    
   G   
   G   
    G    
   G   
   T   
    T    
   C   
   G   
   T   
   T   
   T   
    A    
   A   
   T   
   G   
   A   
    G    
    A    
   A   
   T   
   G   
   G   
   A   
   A   
   G   
    A    
    T    
   G   
    G    
    T    
    G    
   C   
    C    
   A   
   A   
   A   
   -   
   -   
   -   
   -   
   -   
   -   
   -   
   -   
   -   
   -   
   -   
   -   
   -   
   -   
   -   
   -   
   -   
   -   
   -   
   -   
   -   
   -   
   -   
   -   
   -   
   -   
   -   
   -   
   -   
   -   
   -   
   -   
   -   
   -   
   -   
   -   
   -   
   -   
   -   
   -   
   -   
   -   
   -   
   -   
   -   
   -   
   -   
   -   
   -   
   -   
   -   
   -   
   -   
   -   
   -   
   -   
   -   
   -   
   -   
   -   
   -   
   -   
   -   
   -   
   -   
   -   
   -   
   -   
   -   
   -   
   -   
   -   
 

 
   N59508F   
   A   
   T   
   G   
   A   
   G   
   T   
   G   
   A   
   A   
   G   
   A   
   A   
   A   
   T   
   T   
   A   
   A   
   A   
   G   
   A   
   A   
   A   
   A   
   C   
   A   
   T   
   T   
   C   
   A   
   A   
   G   
   A   
   C   
   G   
   T   
   C   
   A   
   A   
   T   
   C   
   T   
   G   
   A   
   C   
   C   
   A   
   G   
   C   
   G   
   A   
   A   
   A   
   T   
   G   
   A   
   A   
   A   
   G   
   A   
   A   
   T   
   C   
   T   
   T   
   T   
   C   
   A   
   T   
   T   
   G   
   A   
   C   
   T   
   A   
   C   
   G   
   C   
   A   
   A   
   T   
   G   
   A   
   G   
   T   
   G   
   T   
   T   
   A   
   T   
   C   
   G   
   T   
   A   
   G   
   C   
   C   
   C   
   G   
   C   
   G   
   C   
   G   
   T   
   T   
   A   
   C   
   C   
   T   
   G   
   A   
   C   
   G   
   T   
   T   
   C   
   G   
   T   
   G   
   A   
   C   
   G   
   G   
   T   
   T   
   T   
   A   
   A   
   A   
   A   
   C   
   C   
   T   
   G   
   T   
   T   
   C   
   A   
   T   
   C   
   G   
   C   
   C   
   G   
   A   
   A   
   T   
   C   
   T   
   T   
   A   
   T   
   A   
   T   
   G   
   G   
   A   
   A   
   T   
   G   
   A   
   A   
   C   
   G   
   A   
   A   
   T   
   T   
   A   
   G   
   G   
   G   
   G   
   T   
   A   
   A   
   C   
   C   
   C   
   C   
   T   
   G   
   A   
   T   
   A   
   A   
   A   
   C   
   C   
   A   
   C   
   A   
   C   
   A   
   A   
   A   
   A   
   A   
   A   
   T   
   C   
   A   
   G   
   C   
   C   
   C   
   G   
   G   
   A   
   T   
   T   
   G   
   T   
   T   
   G   
   G   
   G   
   G   
   A   
   T   
   G   
   T   
   T   
   A   
   T   
   G   
   G   
   G   
   T   
   A   
   A   
   A   
   T   
   A   
   T   
   C   
   A   
   C   
   C   
   C   
   C   
   C   
   A   
   T   
   G   
   G   
   G   
   G   
   A   
   C   
   A   
   G   
   T   
   G   
   C   
   G   
   A   
   T   
   T   
   T   
   A   
   C   
   G   
   A   
   A   
   T   
   C   
   A   
   A   
   T   
   G   
   G   
   T   
   G   
   C   
   G   
   G   
   A   
   T   
   G   
   G   
   C   
   A   
   C   
   A   
   A   
   C   
   C   
   T   
   T   
   T   
   T   
   A   
   G   
   T   
   T   
   A   
   T   
   C   
   G   
   G   
   G   
   C   
   T   
   A   
   T   
   G   
   T   
   T   
   A   
   G   
   T   
   T   
   G   
   A   
   C   
   G   
   G   
   C   
   C   
   A   
   C   
   G   
   G   
   A   
   A   
   A   
   C   
   T   
   T   
   C   
   G   
   G   
   T   
   T   
   C   
   C   
   G   
   T   
   C   
   G   
   A   
   T   
   G   
   G   
   T   
   G   
   A   
   C   
   G   
   G   
   C   
   G   
   C   
   T   
   G   
   C   
   C   
   G   
   C   
   T   
   A   
   T   
   G   
   C   
   G   
   T   
   T   
   A   
   T   
   A   
   C   
   C   
   G   
   A   
   A   
   G   
   C   
   A   
   C   
   G   
   T   
   A   
   T   
   G   
   A   
   G   
   T   
   A   
   A   
   A   
   A   
   T   
   T   
   G   
   C   
   T   
   T   
   T   
   A   
   G   
   A   
   A   
   A   
   T   
   G   
   C   
   T   
   A   
   C   
   G   
   A   
   G   
   A   
   T   
   A   
   T   
   T   
   A   
   A   
   C   
   A   
   A   
   A   
   A   
   A   
   T   
   A   
   C   
   A   
   G   
   T   
   C   
   G   
   A   
   T   
   T   
   T   
   C   
   C   
   A   
   A   
   G   
   G   
   A   
   A   
   A   
   C   
   T   
   A   
   T   
   G   
   A   
   T   
   G   
   A   
   T   
   T   
   C   
   A   
   G   
   A   
   A   
   C   
   A   
   A   
   G   
   A   
   A   
   C   
   C   
   A   
   G   
   T   
   A   
   G   
   T   
   T   
   T   
   T   
   A   
   C   
   C   
   T   
   G   
   C   
   T   
   C   
   G   
   T   
   T   
   T   
   T   
   C   
   C   
   A   
   A   
   A   
   C   
   T   
   T   
   A   
   C   
   T   
   A   
   G   
   T   
   T   
   A   
   A   
   C   
   G   
   G   
   A   
   A   
   C   
   A   
   A   
   C   
   G   
   G   
   G   
   G   
   A   
   T   
   T   
   G   
   C   
   G   
   G   
   T   
   G   
   G   
   G   
   G   
   A   
   T   
   G   
   G   
   C   
   A   
   A   
   C   
   A   
   A   
   A   
   T   
   A   
   T   
   T   
   C   
   C   
   A   
   C   
   C   
   A   
   C   
   A   
   C   
   A   
   A   
   T   
   T   
   T   
   A   
   A   
   G   
   T   
   G   
   A   
   A   
   G   
   T   
   G   
   A   
   T   
   T   
   G   
   A   
   T   
   G   
   C   
   A   
   A   
   C   
   A   
   A   
   G   
   T   
   T   
   T   
   A   
   T   
   T   
   G   
   A   
   T   
   G   
   G   
   A   
   C   
   A   
   A   
   T   
   C   
   C   
   T   
   G   
   A   
   T   
   G   
   T   
   A   
   A   
   C   
   G   
   A   
   C   
   G   
   A   
   A   
   T   
   G   
   A   
   A   
   T   
   T   
   G   
   A   
   T   
   G   
   G   
   A   
   A   
   G   
   T   
   G   
   T   
   T   
   A   
   C   
   C   
   T   
   G   
   G   
   A   
   C   
   C   
   A   
   G   
   A   
   T   
   T   
   T   
   T   
   C   
   C   
   A   
   A   
   C   
   A   
   G   
   G   
   C   
   G   
   G   
   T   
   T   
   T   
   A   
   G   
   T   
   G   
   A   
   T   
   G   
   G   
   G   
   G   
   A   
   A   
   A   
   T   
   C   
   A   
   G   
   G   
    G    
   A   
   T   
    T    
   C   
   G   
    C    
    C    
   G   
   A   
   G   
   C   
    A    
   T   
   A   
   T   
   G   
   A   
   A   
   A   
   C   
    G    
   G   
   G   
    G    
   A   
   A   
    A    
   G   
   G   
   T   
   T   
   C   
    G    
   A   
   T   
   T   
   A   
   C   
    A    
   G   
   T   
    T    
   C   
   G   
   T   
   G   
   C   
   A   
   A   
   A   
   A   
   G   
   T   
   T   
   G   
   A   
    A    
    T    
   T   
    G    
   A   
    C    
   T   
   G   
   A   
    A    
   A   
   T   
   G   
    C    
   C   
    G    
   A   
   A   
   T   
   G   
   G   
    A    
   A   
   A   
   A   
   G   
   A   
   A   
   C   
   G   
    T    
   A   
   T   
    T    
    T    
   T   
    A    
   G   
   T   
    A    
   A   
   C   
    T    
   G   
   A   
   A   
   T   
   T   
    G    
   C   
   C   
   T   
   T   
   A   
   T   
   A   
   T   
   G   
   G   
   T   
    G    
   A   
   A   
   T   
   A   
   A   
   A   
   G   
   C   
    C    
   A   
   A   
   A   
   T   
   T   
    A    
   A   
   T   
    C    
   G   
   A   
   A   
   C   
   G   
    A    
   A   
   T   
   T   
   T   
   C   
   T   
   G   
   A   
    A    
    T    
   T   
    A    
   C   
   A   
   C   
   A   
   G   
   A   
   G   
   A   
    T    
   A   
   A   
    A    
   C   
   G   
    A    
   A   
   T   
    T    
   G   
   A   
   A   
   G   
   G   
    A    
   A   
   T   
    T    
   A   
   C   
    T    
   G   
   A   
   T   
   T   
   T   
   G   
   C   
   G   
   G   
   G   
   A   
    T    
   G   
   A   
   A   
   T   
   C   
   T   
   T   
   C   
    T    
   C   
   G   
   T   
   G   
   A   
    A    
   G   
   G   
    C    
   A   
   T   
   G   
   C   
   G   
    G    
   A   
   T   
    C    
   G   
   T   
   C   
   A   
   T   
    C    
   G   
   A   
    T    
    G    
   T   
    T    
   C   
   G   
   T   
   C   
   G   
    A    
   G   
   A   
    T    
   G   
    T    
    G    
   A   
   G   
    T    
   G   
   C   
    C    
   T   
   C   
    T    
   G   
   T   
   T   
    G    
   T   
    A    
   T   
   T   
    A    
   A   
   A   
    C    
   A   
   A   
   C   
   T   
   T   
    A    
   T   
   A   
    C    
   A   
   A   
   A   
    A    
   T   
   G   
   A   
   C   
    T    
    G    
   C   
    C    
   T   
   T   
    A    
   C   
   A   
    A    
   A   
    C    
    A    
   T   
   C   
    T    
   T   
   T   
    T    
   G   
   G   
    T    
   T   
   T   
    T    
   A   
   A   
    C    
   A   
   T   
   G   
   T   
   T   
    A    
   G   
   C   
    C    
   A   
   T   
    C    
   G   
   A   
   A   
   A   
   A   
   A   
   G   
   G   
    C    
   G   
   T   
    A    
   C   
   C   
    G    
   A   
   A   
   A   
    A    
   T   
   T   
   T   
   T   
    A    
   A   
   G   
    C    
   T   
   T   
   G   
   A   
   A   
   A   
   C   
    G    
    T    
   A   
   T   
    T    
    T    
   T   
    A    
   G   
   A   
   A   
   A   
   A   
    T    
   T   
   A   
    C    
    G    
   T   
    C    
   G   
   A   
    G    
   C   
   A   
    T    
   C   
   A   
    A    
    A    
    A    
    A    
    G    
   A   
   A   
   G   
   T   
   C   
   A   
   T   
    T    
   A   
   C   
    T    
    C    
   G   
    C    
   C   
   G   
   G   
   A   
   C   
    G    
    A    
   T   
    T    
   T   
   T   
   T   
   G   
   A   
    C    
   A   
   A   
   A   
    A    
   A   
    C    
   A   
   A   
    A    
   G   
   C   
   A   
   G   
   A   
   A   
   G   
   C   
   A   
    C    
   G   
    G    
   G   
   C   
    G    
   C   
   A   
    T    
   A   
   T   
    T    
   T   
   T   
   A   
   G   
   A   
   A   
   G   
   G   
   T   
    C    
   T   
    A    
   C   
   G   
    A    
   A   
   T   
   T   
   G   
   C   
    C    
   T   
   T   
    A    
   G   
   A   
   T   
   C   
   A   
   T   
   A   
   T   
   C   
    G    
   A   
   T   
   G   
   A   
   A   
   A   
   T   
    C    
   A   
   T   
   T   
   G   
   C   
    C    
   A   
   T   
   T   
   A   
   T   
    C    
   C   
   G   
    T    
   G   
   G   
    A    
   T   
   C   
    A    
    C    
   A   
   A   
   T   
   C   
   A   
   G   
   A   
   T   
   G   
   A   
    C    
   G   
   A   
    A    
   G   
   C   
   A   
   A   
   A   
   A   
   G   
   C   
    A    
   A   
    C    
    G    
    T    
   T   
    A    
   A   
   T   
    T    
   G   
   A   
   A   
   C   
   G   
    C    
   T   
   T   
    T    
   G   
   A   
    A    
   T   
   T   
    T    
   T   
   C   
    A    
   G   
   A   
   T   
   C   
   G   
    T    
   C   
   A   
    A    
    G    
   C   
    G    
   C   
   A   
   A   
   G   
   C   
    G    
   A   
   T   
    T    
   T   
   T   
   A   
   G   
   A   
   T   
   A   
   T   
   G   
   C   
   G   
   T   
   T   
   T   
    A    
   C   
   G   
    C    
   C   
   G   
    T    
   T   
   T   
    A    
   A   
   C   
    A    
   G   
   G   
    C    
   T   
   T   
    A    
   G   
   A   
   A   
   C   
   G   
    C    
   G   
   A   
    C    
   A   
   A   
   A   
   A   
   T   
    T    
   G   
   A   
    A    
   A   
   A   
   T   
   G   
   A   
   A   
   T   
   A   
    T    
   C   
   A   
   A   
   G   
   A   
    G    
   C   
   T   
    A    
   T   
   T   
    G    
   A   
    A    
   A   
    T    
   T   
    C    
   A   
   T   
   T   
   G   
    C    
    G    
   G   
   A   
    C    
   T   
   T   
    A    
    G    
    A    
    G    
   G   
   A   
   C   
   A   
   T   
   T   
    T    
   T   
    A    
   G   
   C   
    C    
    C    
   G   
    C    
   C   
   C   
    A    
   G   
   A   
    A    
   C   
   G   
    C    
   G   
   T   
    C    
   A   
    T    
    T    
   G   
   A   
   A   
   A   
   T   
    C    
   A   
   T   
    T    
   A   
   A   
    A    
   A   
    C    
    A    
   G   
   A   
    G    
   T   
   T   
   A   
    A    
    A    
    T    
   G   
   A   
   C   
    G    
   T   
    T    
    C    
    G    
    C    
    A    
    C    
   A   
    A    
    A    
   A   
   T   
   T   
   T   
   G   
   G   
   T   
   G   
   A   
   T   
    G    
    C    
   A   
   C   
   G   
   A   
   C   
   G   
    C    
   A   
   C   
   A   
   G   
   A   
   A   
    T    
   T   
   A   
    T    
   T   
   A   
   G   
   T   
    A    
   G   
   G   
   T   
   G   
   A   
    A    
   G   
   T   
   C   
    T    
   T   
    A    
   A   
   G   
   T   
   C   
   T   
    T    
   G   
   A   
   A   
   G   
   A   
   T   
   G   
   A   
   A   
   G   
   A   
   T   
    T    
   T   
    A    
   A   
   T   
    C    
   G   
   A   
   A   
   G   
   A   
   A   
   G   
   A   
   A   
   G   
   A   
   A   
    G    
   T   
   C   
   G   
   T   
    G    
   A   
   T   
   T   
   A   
   C   
    A    
    T    
   T   
    A    
   A   
   C   
    C    
   A   
   A   
    T    
   A   
   A   
    C    
   G   
   G   
    C    
   T   
   A   
    C    
   A   
   T   
    T    
   A   
   A   
    G    
   C   
   G   
    G    
    A    
   T   
   G   
   G   
    C    
   A   
   A   
   A   
    C    
    T    
    C    
    T    
   G   
   A   
   A   
   T   
   T   
   C   
    C    
   G   
    G    
   G   
   C   
    G    
   C   
   A   
   A   
   C   
   G   
    C    
    C    
   G   
    T    
   G   
   G   
    C    
   G   
   G   
    A    
   C   
   G   
   T   
   G   
   G   
    T    
   G   
   T   
    C    
   C   
   A   
   A   
   G   
   G   
    C    
   A   
   T   
   G   
   G   
   G   
    C    
    G    
   T   
    T    
   C   
   A   
    T    
    G    
   A   
   T   
   G   
   A   
    T    
   G   
   A   
    T    
   T   
   T   
   C   
   G   
   T   
    G    
   A   
   A   
    A    
   A   
   A   
    C    
   C   
   T   
    A    
    G    
   T   
   T   
   T   
   C   
   T   
   T   
   G   
    T    
   T   
   C   
    A    
   A   
   C   
    A    
   C   
   A   
    C    
   G   
   A   
    C    
   A   
   C   
    A    
   T   
   T   
    A    
   T   
   T   
   A   
   T   
   T   
    C    
   T   
   T   
   T   
   A   
   C   
   G   
   A   
   A   
    C    
   A   
    C    
    T    
   G   
   G   
    G    
   A   
   A   
   A   
   G   
   T   
    T    
   T   
   A   
    C    
   C   
   G   
    A    
   G   
   C   
   A   
   A   
   A   
   A   
   G   
   G   
    T    
   T   
   A   
    C    
   G   
   A   
   A   
   A   
   T   
    C    
   C   
   C   
    T    
   G   
   A   
    A    
   T   
   A   
   C   
   G   
   G   
    T    
    A    
   G   
    A    
   A   
   C   
   A   
   G   
   C   
    A    
   A   
   A   
   A   
   G   
   G   
    A    
   A   
   T   
    T    
   C   
   C   
   A   
    G    
   T   
    G    
   A   
   T   
    T    
   A   
   A   
    C    
   T   
   T   
    A    
   C   
   T   
    G    
   G   
   G   
    A    
   A   
   T   
    T    
   G   
   A   
    T    
   T   
   C   
    T    
    G    
    C    
    A    
   G   
   A   
   A   
   A   
    A    
    A    
   A   
   T   
    T    
   C   
   A   
    A    
   G   
   C   
    G    
   A   
   T   
    T    
   A   
   T   
   T   
    T    
   C   
    T    
   G   
   T   
    T    
    G    
    A    
   G   
   G   
   G   
    C    
    A    
   A   
   A   
   G   
   C   
    G    
   G   
   A   
   A   
   G   
    C    
    A    
   G   
   G   
    T    
    A    
   A   
    A    
   T   
   A   
    C    
    T    
   T   
    G    
   T   
   T   
   C   
   T   
   T   
    C    
   A   
   C   
    A    
   A   
   C   
    C    
    T    
    T    
    A    
    A    
   A   
   A   
   G   
   G   
    A    
   A   
   C   
    C    
   G   
   T   
    C    
   A   
   A   
   A   
   C   
   G   
    G    
   A   
   C   
    A    
    G    
   C   
    T    
   G   
   T   
    A    
   A   
   C   
    A    
   G   
   C   
    C    
   T   
   T   
    T    
    T    
   C   
   T   
   A   
   A   
    T    
   A   
   T   
    C    
   C   
   G   
    T    
   A   
   G   
    T    
   A   
   A   
   T   
   G   
   G   
    A    
    T    
   T   
    A    
   A   
   T   
    C    
   G   
   C   
    C    
   A   
   T   
   T   
    A    
   G   
    C    
   T   
   T   
    A    
   A   
   A   
   A   
   G   
   A   
   A   
   G   
   A   
    T    
   G   
   A   
    T    
   G   
   A   
    G    
    T    
   T   
    A    
    G    
   T   
    T    
   A   
   A   
    C    
   G   
   T   
    A    
    G    
   T   
   A   
    A    
    C    
   G   
   A   
   C   
    T    
    A    
   A   
    T    
   G   
   G   
    C    
    A    
   A   
    T    
    C    
    A    
    G    
   A   
   A   
    G    
   A   
   T   
    G    
   A   
   T   
    T    
   A   
   T   
    C    
   G   
   G   
    A    
   A   
   C   
   A   
   C   
   A   
   T   
    G    
    C    
    A    
   G   
   G   
    A    
   T   
   A   
   C   
   T   
   C   
   T   
   G   
   T   
    C    
   A   
   C   
    A    
   T   
   T   
    T    
    G    
   A   
    T    
   G   
   A   
    A    
    A    
    A    
    T    
    A    
   C   
    T    
   G   
    T    
    A    
   C   
   G   
    T    
   G   
   A   
   T   
   A   
   T   
   G   
   G   
   G   
    C    
   C   
   G   
    G    
   A   
   C   
   A   
   G   
   C   
    A    
   T   
   C   
    A    
   G   
   G   
    A    
   G   
   T   
    T    
    C    
   G   
    G    
   G   
   G   
    A    
   A   
   T   
    C    
   C   
   G   
    T    
    C    
   T   
    C    
   C   
   G   
    C    
   G   
   A   
    A    
    A    
   A   
    T    
   G   
   A   
    T    
   T   
    A    
    T    
   G   
   T   
    G    
   G   
   T   
    C    
   G   
   G   
    T    
    G    
   C   
   A   
   G   
   C   
    G    
    A    
   T   
    T    
    C    
   T   
   G   
    G    
   A   
    T    
   G   
    A    
   A   
    A    
   A   
    C    
    A    
   A   
   A   
   G   
   A   
   A   
   G   
   T   
    C    
    C    
   T   
    A    
   G   
   T   
    C    
   A   
   T   
    T    
   A   
   C   
    T    
   G   
   A   
   A   
   A   
   A   
    T    
   G   
   G   
   T   
   T   
   A   
    T    
   G   
   G   
    T    
   A   
   A   
    G    
   C   
   G   
    T    
   A   
   C   
    A    
   A   
   A   
   A   
   G   
    C    
    T    
   T   
   C   
    T    
   G   
   A   
    A    
   T   
   A   
   T   
   C   
   C   
   A   
   G   
   T   
    T    
   A   
   A   
    A    
   G   
   G   
    A    
   C   
   G   
    T    
   G   
   G   
    C    
   G   
   G   
   T   
   A   
   A   
   A   
   G   
   G   
    G    
   A   
   T   
    T    
   A   
   A   
    G    
   A   
   C   
    A    
    G    
   C   
    A    
   A   
   A   
    T    
   A   
   T   
   C   
   A   
   C   
   T   
   G   
   A   
    G    
   A   
   A   
   A   
   A   
   A   
   T   
   G   
   G   
   T   
   C   
   C   
   A   
    T    
   T   
   A   
   G   
   C   
    T    
   G   
   G   
    T    
   T   
   T   
    A    
   A   
   C   
   C   
   A   
   C   
    G    
   G   
   T   
    C    
    A    
    A    
   T   
   G   
   G   
    G    
    G    
   A   
   T   
   G   
   A   
   A   
   G   
   A   
    T    
   A   
   T   
   C   
    T    
   T   
    A    
    T    
   T   
    G    
   A   
   T   
    T    
   A   
   C   
    G    
    A    
   A   
    C    
   A   
   A   
   A   
   G   
   G   
    C    
   G   
   T   
    C    
   A   
   T   
   T   
   A   
   T   
   C   
   C   
   G   
    C    
   T   
   T   
    T    
   A   
   A   
   C   
   G   
   T   
    T    
    G    
    A    
    T    
    T    
   C   
   T   
   G   
   T   
   T   
   T   
   C   
   T   
   C   
   A   
   A   
   A   
   C   
    A    
   G   
   G   
   A   
   C   
   G   
    C    
    G    
   C   
   A   
   A   
   C   
   A   
   T   
   T   
    A    
   G   
   G   
    G    
   G   
   T   
    T    
   C   
   G   
   T   
   T   
   T   
    A    
   A   
   T   
   G   
   A   
    G    
    A    
   A   
   T   
   G   
   G   
   A   
   A   
   G   
    A    
    T    
   G   
    G    
    T    
    G    
   C   
    C    
   A   
   A   
   A   
   -   
   -   
   -   
   -   
   -   
   -   
   -   
   -   
   -   
   -   
   -   
   -   
   -   
   -   
   -   
   -   
   -   
   -   
   -   
   -   
   -   
   -   
   -   
   -   
   -   
   -   
   -   
   -   
   -   
   -   
   -   
   -   
   -   
   -   
   -   
   -   
   -   
   -   
   -   
   -   
   -   
   -   
   -   
   -   
   -   
   -   
   -   
   -   
   -   
   -   
   -   
   -   
   -   
   -   
   -   
   -   
   -   
   -   
   -   
   -   
   -   
   -   
   -   
   -   
   -   
   -   
   -   
   -   
   -   
   -   
   -   
   -   
 

 
   N54190   
   A   
   T   
   G   
   A   
   G   
   T   
   G   
   A   
   A   
   G   
   A   
   A   
   A   
   T   
   C   
   A   
   G   
   A   
   G   
   A   
   A   
   A   
   A   
   C   
   A   
   T   
   C   
   C   
   A   
   T   
   G   
   A   
   T   
   G   
   T   
   C   
   A   
   A   
   T   
   C   
   T   
   A   
   A   
   C   
   C   
   A   
   G   
   C   
   G   
   A   
   A   
   A   
   T   
   G   
   A   
   A   
   A   
   G   
   A   
   C   
   T   
   C   
   C   
   T   
   T   
   C   
   A   
   T   
   C   
   G   
   A   
   T   
   T   
   A   
   T   
   G   
   C   
   G   
   A   
   T   
   G   
   A   
   G   
   T   
   G   
   T   
   T   
   A   
   T   
   T   
   G   
   T   
   C   
   G   
   C   
   A   
   C   
   G   
   T   
   G   
   C   
   A   
   C   
   T   
   T   
   C   
   C   
   T   
   G   
   A   
   T   
   G   
   T   
   C   
   A   
   G   
   A   
   G   
   A   
   T   
   G   
   G   
   T   
   C   
   T   
   G   
   A   
   A   
   A   
   C   
   C   
   C   
   G   
   T   
   T   
   C   
   A   
   C   
   C   
   G   
   C   
   C   
   G   
   T   
   A   
   T   
   T   
   T   
   T   
   A   
   T   
   A   
   C   
   G   
   G   
   C   
   A   
   T   
   G   
   A   
   A   
   C   
   G   
   A   
   A   
   T   
   T   
   A   
   G   
   G   
   T   
   G   
   T   
   A   
   A   
   C   
   A   
   C   
   C   
   G   
   G   
   A   
   T   
   A   
   A   
   A   
   G   
   C   
   C   
   C   
   A   
   T   
   A   
   A   
   A   
   A   
   A   
   A   
   T   
   C   
   T   
   G   
   C   
   C   
   A   
   G   
   A   
   A   
   T   
   C   
   G   
   T   
   C   
   G   
   G   
   A   
   G   
   A   
   T   
   G   
   T   
   C   
   A   
   T   
   G   
   G   
   G   
   T   
   A   
   A   
   A   
   T   
   A   
   C   
   C   
   A   
   T   
   C   
   C   
   T   
   C   
   A   
   T   
   G   
   G   
   A   
   G   
   A   
   C   
   A   
   G   
   T   
   G   
   C   
   G   
   A   
   T   
   C   
   T   
   A   
   T   
   G   
   A   
   G   
   T   
   C   
   A   
   A   
   T   
   G   
   G   
   T   
   A   
   C   
   G   
   G   
   A   
   T   
   G   
   G   
   C   
   G   
   C   
   A   
   G   
   C   
   C   
   A   
   T   
   T   
   C   
   A   
   G   
   C   
   T   
   A   
   C   
   C   
   G   
   T   
   T   
   A   
   T   
   A   
   T   
   G   
   C   
   T   
   A   
   G   
   T   
   G   
   G   
   A   
   T   
   G   
   G   
   T   
   C   
   A   
   T   
   G   
   G   
   A   
   A   
   A   
   C   
   T   
   T   
   T   
   G   
   G   
   T   
   T   
   C   
   T   
   G   
   T   
   T   
   G   
   A   
   C   
   G   
   G   
   T   
   G   
   A   
   C   
   G   
   G   
   T   
   G   
   C   
   C   
   G   
   C   
   C   
   G   
   C   
   T   
   A   
   T   
   G   
   C   
   G   
   T   
   T   
   A   
   T   
   A   
   C   
   C   
   G   
   A   
   A   
   G   
   C   
   A   
   C   
   G   
   G   
   A   
   T   
   G   
   A   
   G   
   C   
   A   
   A   
   A   
   A   
   T   
   C   
   G   
   C   
   T   
   A   
   C   
   A   
   G   
   A   
   A   
   A   
   T   
   G   
   C   
   T   
   G   
   C   
   G   
   T   
   G   
   A   
   T   
   A   
   T   
   C   
   A   
   A   
   T   
   A   
   A   
   G   
   A   
   A   
   C   
   A   
   C   
   T   
   G   
   T   
   T   
   G   
   A   
   T   
   T   
   T   
   C   
   C   
   A   
   A   
   A   
   G   
   C   
   A   
   A   
   C   
   T   
   A   
   T   
   G   
   A   
   T   
   G   
   A   
   T   
   A   
   C   
   A   
   G   
   A   
   A   
   A   
   A   
   A   
   G   
   A   
   A   
   C   
   C   
   A   
   G   
   T   
   T   
   G   
   T   
   A   
   C   
   T   
   A   
   C   
   C   
   T   
   G   
   C   
   T   
   C   
   G   
   C   
   T   
   T   
   C   
   C   
   C   
   G   
   A   
   A   
   T   
   C   
   T   
   T   
   T   
   T   
   A   
   G   
   T   
   G   
   A   
   A   
   C   
   G   
   G   
   A   
   A   
   C   
   A   
   A   
   C   
   G   
   G   
   G   
   G   
   A   
   T   
   C   
   G   
   C   
   C   
   G   
   T   
   A   
   G   
   G   
   T   
   A   
   T   
   G   
   G   
   C   
   C   
   A   
   C   
   G   
   A   
   A   
   T   
   A   
   T   
   C   
   C   
   C   
   A   
   C   
   C   
   T   
   C   
   A   
   T   
   A   
   A   
   C   
   T   
   T   
   G   
   T   
   C   
   T   
   G   
   A   
   G   
   G   
   T   
   A   
   G   
   T   
   A   
   G   
   C   
   G   
   G   
   C   
   T   
   A   
   T   
   C   
   G   
   A   
   T   
   T   
   T   
   G   
   C   
   T   
   G   
   A   
   T   
   G   
   G   
   A   
   T   
   A   
   A   
   T   
   C   
   C   
   T   
   G   
   A   
   T   
   G   
   T   
   G   
   A   
   C   
   G   
   A   
   C   
   C   
   A   
   A   
   T   
   G   
   A   
   A   
   T   
   T   
   G   
   A   
   T   
   G   
   G   
   A   
   A   
   G   
   T   
   A   
   T   
   T   
   A   
   C   
   C   
   T   
   G   
   G   
   A   
   C   
   C   
   A   
   G   
   A   
   T   
   T   
   T   
   T   
   C   
   C   
   A   
   A   
   C   
   A   
   G   
   G   
   T   
   G   
   G   
   T   
   T   
   T   
   G   
   G   
   T   
   T   
   A   
   T   
   G   
   G   
   G   
   A   
   A   
   A   
   A   
   T   
   C   
   A   
   G   
   G   
    A    
   A   
   T   
    C    
   C   
   G   
    A    
    A    
   G   
   A   
   G   
   C   
    G    
   T   
   A   
   T   
   G   
   A   
   A   
   A   
   C   
    A    
   G   
   G   
    T    
   A   
   A   
    G    
   G   
   G   
   T   
   T   
   C   
    T    
   A   
   T   
   T   
   A   
   C   
    C    
   G   
   T   
    A    
   C   
   G   
   T   
   G   
   C   
   A   
   A   
   A   
   A   
   G   
   T   
   T   
   G   
   A   
    G    
    A    
   T   
    T    
   A   
    C    
   T   
   G   
   A   
    G    
   A   
   T   
   G   
    C    
   C   
    A    
   A   
   A   
   T   
   G   
   G   
    T    
   A   
   A   
   A   
   G   
   A   
   A   
   C   
   G   
    G    
   A   
   T   
    C    
    A    
   T   
    C    
   G   
   T   
    G    
   A   
   C   
    A    
   G   
   A   
   A   
   T   
   T   
    G    
   C   
   C   
   T   
   T   
   A   
   T   
   A   
   T   
   G   
   G   
   T   
    C    
   A   
   A   
   T   
   A   
   A   
   A   
   G   
   C   
    A    
   A   
   A   
   A   
   T   
   T   
    G    
   A   
   T   
    C    
   G   
   A   
   A   
   C   
   G   
    G    
   A   
   T   
   T   
   T   
   C   
   T   
   G   
   A   
    A    
    C    
   T   
    G    
   C   
   A   
   C   
   A   
   G   
   A   
   G   
   A   
    C    
   A   
   A   
    G    
   C   
   G   
    T    
   A   
   T   
    C    
   G   
   A   
   A   
   G   
   G   
    G    
   A   
   T   
    C    
   A   
   C   
    A    
   G   
   A   
   T   
   T   
   T   
   G   
   C   
   G   
   G   
   G   
   A   
    C    
   G   
   A   
   A   
   T   
   C   
   T   
   T   
   C   
    C    
   C   
   G   
   T   
   G   
   A   
    A    
   G   
   G   
    T    
   A   
   T   
   G   
   C   
   G   
    G    
   A   
   T   
    C    
   G   
   T   
   C   
   A   
   T   
    T    
   G   
   A   
    C    
    A    
   T   
    C    
   C   
   G   
   T   
   C   
   G   
    T    
   G   
   A   
    C    
   G   
    C    
    C    
   A   
   G   
    C    
   G   
   C   
    A    
   T   
   C   
    C    
   G   
   T   
   T   
    A    
   T   
    C    
   T   
   T   
    G    
   A   
   A   
    T    
   A   
   A   
   C   
   T   
   T   
    A    
   T   
   A   
    T    
   A   
   A   
   A   
    T    
   T   
   G   
   A   
   C   
    A    
    T    
   C   
    C    
   T   
   T   
    G    
   C   
   A   
    A    
   A   
    A    
    T    
   T   
   C   
    A    
   T   
   T   
    T    
   G   
   G   
    A    
   T   
   T   
    C    
   A   
   A   
    T    
   A   
   T   
   G   
   T   
   T   
    G    
   G   
   C   
    G    
   A   
   T   
    C    
   G   
   A   
   A   
   A   
   A   
   A   
   G   
   G   
    T    
   G   
   T   
    G    
   C   
   C   
    G    
   A   
   A   
   A   
    G    
   T   
   T   
   T   
   T   
    G    
   A   
   G   
    C    
   T   
   T   
   G   
   A   
   A   
   A   
   C   
    A    
    A    
   A   
   T   
    T    
    C    
   T   
    T    
   G   
   A   
   A   
   A   
   A   
    C    
   T   
   A   
    T    
    A    
   T   
    C    
   G   
   A   
    A    
   C   
   A   
    T    
   C   
   A   
    G    
    C    
    G    
    C    
    G    
   A   
   A   
   G   
   T   
   C   
   A   
   T   
    C    
   A   
   C   
    A    
    A    
   G   
    A    
   C   
   G   
   G   
   A   
   C   
    A    
    G    
   T   
    C    
   T   
   T   
   T   
   G   
   A   
    A    
   A   
   A   
   A   
    G    
   A   
    A    
   A   
   A   
    A    
   G   
   C   
   A   
   G   
   A   
   A   
   G   
   C   
   A   
    A    
   G   
    G    
   G   
   C   
    A    
   C   
   A   
    C    
   A   
   T   
    T    
   T   
   T   
   A   
   G   
   A   
   A   
   G   
   G   
   T   
    T    
   T   
    G    
   C   
   G   
    T    
   A   
   T   
   T   
   G   
   C   
    T    
   T   
   T   
    G    
   G   
   A   
   T   
   C   
   A   
   T   
   A   
   T   
   C   
    G    
   A   
   T   
   G   
   A   
   A   
   A   
   T   
    A    
   A   
   T   
   T   
   G   
   C   
    G    
   A   
   T   
   T   
   A   
   T   
    C    
   C   
   G   
    C    
   G   
   G   
    G    
   T   
   C   
    T    
    A    
   A   
   A   
   T   
   C   
   A   
   G   
   A   
   T   
   G   
   A   
    C    
   G   
   A   
    A    
   G   
   C   
   A   
   A   
   A   
   A   
   G   
   C   
    G    
   A   
    C    
    G    
    A    
   T   
    G    
   A   
   T   
    C    
   G   
   A   
   A   
   C   
   G   
    G    
   T   
   T   
    C    
   G   
   A   
    T    
   T   
   T   
    A    
   T   
   C   
    C    
   G   
   A   
   T   
   C   
   G   
    C    
   C   
   A   
    G    
    T    
   C   
    T    
   C   
   A   
   A   
   G   
   C   
    T    
   A   
   T   
    T    
   T   
   T   
   A   
   G   
   A   
   T   
   A   
   T   
   G   
   C   
   G   
   T   
   T   
   T   
    A    
   C   
   G   
    C    
   C   
   G   
    A    
   T   
   T   
    G    
   A   
   C   
    C    
   G   
   G   
    T    
   T   
   T   
    G    
   G   
   A   
   A   
   C   
   G   
    G    
   G   
   A   
    A    
   A   
   A   
   A   
   A   
   T   
    C    
   G   
   A   
    A    
   A   
   A   
   T   
   G   
   A   
   A   
   T   
   A   
    C    
   C   
   A   
   A   
   G   
   A   
    A    
   C   
   T   
    C    
   T   
   T   
    A    
   A   
    A    
   A   
    C    
   T   
    G    
   A   
   T   
   T   
   G   
    A    
    A    
   G   
   A   
    T    
   T   
   T   
    A    
    A    
    C    
    T    
   G   
   A   
   C   
   A   
   T   
   T   
    C    
   T   
    G    
   G   
   C   
    A    
    A    
   G   
    A    
   C   
   C   
    A    
   G   
   A   
    A    
   C   
   G   
    T    
   G   
   T   
    T    
   A   
    C    
    A    
   G   
   A   
   A   
   A   
   T   
    C    
   A   
   T   
    C    
   A   
   A   
    G    
   A   
    A    
    T    
   G   
   A   
    A    
   T   
   T   
   A   
    G    
    C    
    A    
   G   
   A   
   C   
    T    
   T   
    G    
    A    
    A    
    T    
    C    
    A    
   A   
    C    
    G    
   A   
   T   
   T   
   T   
   G   
   G   
   T   
   G   
   A   
   T   
    A    
    A    
   A   
   C   
   G   
   A   
   C   
   G   
    C    
   A   
   C   
   A   
   G   
   A   
   A   
    T    
   T   
   A   
    C    
   T   
   A   
   G   
   T   
    C    
   G   
   G   
   T   
   G   
   A   
    G    
   G   
   T   
   C   
    T    
   T   
    A    
   A   
   G   
   T   
   C   
   T   
    G    
   G   
   A   
   A   
   G   
   A   
   T   
   G   
   A   
   A   
   G   
   A   
   T   
    T    
   T   
    A    
   A   
   T   
    C    
   G   
   A   
   A   
   G   
   A   
   A   
   G   
   A   
   A   
   G   
   A   
   A   
    A    
   T   
   C   
   G   
   T   
    T    
   A   
   T   
   T   
   A   
   C   
    A    
    T    
   T   
    G    
   A   
   C   
    A    
   A   
   A   
    C    
   A   
   A   
    T    
   G   
   G   
    T    
   T   
   A   
    C    
   A   
   T   
    C    
   A   
   A   
    A    
   C   
   G   
    T    
    T    
   T   
   G   
   G   
    C    
   A   
   A   
   A   
    T    
    A    
    A    
    T    
   G   
   A   
   A   
   T   
   T   
   C   
    A    
   G   
    A    
   G   
   C   
    T    
   C   
   A   
   A   
   C   
   G   
    A    
    A    
   G   
    G    
   G   
   G   
    T    
   G   
   G   
    A    
   C   
   G   
   T   
   G   
   G   
    T    
   G   
   T   
    C    
   C   
   A   
   A   
   G   
   G   
    A    
   A   
   T   
   G   
   G   
   G   
    T    
    A    
   T   
    C    
   C   
   A   
    C    
    A    
   A   
   T   
   G   
   A   
    C    
   G   
   A   
    C    
   T   
   T   
   C   
   G   
   T   
    C    
   A   
   A   
    A    
   A   
   A   
    T    
   C   
   T   
    G    
    G    
   T   
   T   
   T   
   C   
   T   
   T   
   G   
    T    
   T   
   C   
    T    
   A   
   C   
    C    
   C   
   A   
    T    
   G   
   A   
    T    
   A   
   C   
    A    
   T   
   T   
    G    
   T   
   T   
   A   
   T   
   T   
    C    
   T   
   T   
   T   
   A   
   C   
   G   
   A   
   A   
    T    
   A   
    A    
    C    
   G   
   G   
    A    
   A   
   A   
   A   
   G   
   T   
    G    
   T   
   A   
    T    
   C   
   G   
    G    
   G   
   C   
   A   
   A   
   A   
   A   
   G   
   G   
    A    
   T   
   A   
    C    
   G   
   A   
   A   
   A   
   T   
    T    
   C   
   C   
    A    
   G   
   A   
    A    
   T   
   A   
   C   
   G   
   G   
    G    
    C    
   G   
    T    
   A   
   C   
   A   
   G   
   C   
    C    
   A   
   A   
   A   
   G   
   G   
    A    
   A   
   T   
    A    
   C   
   C   
   A   
    A    
   T   
    T    
   A   
   T   
    C    
   A   
   A   
    T    
   T   
   T   
    A    
   C   
   T   
    A    
   G   
   G   
    T    
   A   
   T   
    C    
   G   
   A   
    T    
   T   
   C   
    G    
    A    
    G    
    T    
   G   
   A   
   A   
   A   
    G    
    C    
   A   
   T   
    C    
   C   
   A   
    G    
   G   
   C   
    A    
   A   
   T   
    C    
   A   
   T   
   T   
    G    
   C   
    G    
   G   
   T   
    G    
    A    
    C    
   G   
   G   
   G   
    A    
    G    
   A   
   A   
   G   
   C   
    A    
   G   
   A   
   A   
   G   
    A    
    A    
   G   
   G   
    C    
    C    
   A   
    C    
   T   
   A   
    T    
    C    
   T   
    C    
   T   
   T   
   C   
   T   
   T   
    T    
   A   
   C   
    G    
   A   
   C   
    G    
    C    
    G    
    T    
    C    
   A   
   A   
   G   
   G   
    T    
   A   
   C   
    A    
   G   
   T   
    A    
   A   
   A   
   A   
   C   
   G   
    T    
   A   
   C   
    T    
    T    
   C   
    T    
   G   
   T   
    C    
   A   
   C   
    T    
   G   
   C   
    T    
   T   
   T   
    T    
    G    
   C   
   T   
   A   
   A   
    C    
   A   
   T   
    C    
   C   
   G   
    A    
   A   
   G   
    T    
   A   
   A   
   T   
   G   
   G   
    T    
    C    
   T   
    G    
   A   
   T   
    C    
   G   
   C   
    A    
   A   
   T   
   T   
    G    
   G   
    T    
   T   
   T   
    G    
   A   
   A   
   A   
   G   
   A   
   A   
   G   
   A   
    C    
   G   
   A   
    T    
   G   
   A   
    G    
    C    
   T   
    C    
    G    
   T   
    C    
   A   
   A   
    T    
   G   
   T   
    G    
    C    
   T   
   A   
    C    
    T    
   G   
   A   
   C   
    A    
    G    
   A   
    T    
   G   
   G   
    T    
    C    
   A   
    A    
    T    
    C    
    T    
   A   
   A   
    T    
   A   
   T   
    C    
   A   
   T   
    T    
   A   
   T   
    T    
   G   
   G   
    A    
   A   
   C   
   A   
   C   
   A   
   T   
    A    
    A    
    C    
   G   
   G   
    C    
   T   
   A   
   C   
   T   
   C   
   T   
   G   
   T   
    C    
   A   
   C   
    T    
   T   
   T   
    C    
    A    
   A   
    A    
   G   
   A   
    A    
    G    
    A    
    A    
    G    
   C   
    A    
   G   
    T    
    C    
   C   
   G   
    C    
   G   
   A   
   T   
   A   
   T   
   G   
   G   
   G   
    A    
   C   
   G   
    T    
   A   
   C   
   A   
   G   
   C   
    T    
   T   
   C   
    C    
   G   
   G   
    G    
   G   
   T   
    C    
    A    
   G   
    A    
   G   
   G   
    A    
   A   
   T   
    A    
   C   
   G   
    C    
    C    
   T   
    G    
   C   
   G   
    T    
   G   
   A   
    G    
    G    
   A   
    A    
   G   
   A   
    T    
   T   
    A    
    T    
   G   
   T   
    C    
   G   
   T   
    T    
   G   
   G   
    G    
    G    
   C   
   A   
   G   
   C   
    C    
    T    
   T   
    A    
    A    
   T   
   G   
    A    
   A   
    G    
   G   
    A    
   A   
    A    
   A   
    T    
    C    
   A   
   A   
   G   
   A   
   A   
   G   
   T   
    A    
    T    
   T   
    A    
   G   
   T   
    G    
   A   
   T   
    C    
   A   
   C   
    A    
   G   
   A   
   A   
   A   
   A   
    A    
   G   
   G   
   T   
   T   
   A   
    C    
   G   
   G   
    A    
   A   
   A   
    A    
   C   
   G   
    G    
   A   
   C   
    A    
   A   
   A   
   A   
   G   
    T    
    A    
   T   
   C   
    G    
   G   
   A   
    A    
   T   
   A   
   T   
   C   
   C   
   A   
   G   
   T   
    G    
   A   
   A   
    A    
   G   
   G   
    C    
   C   
   G   
    C    
   G   
   G   
    A    
   G   
   G   
   T   
   A   
   A   
   A   
   G   
   G   
    G    
   A   
   T   
    C    
   A   
   A   
    A    
   A   
   C   
    G    
    G    
   C   
    G    
   A   
   A   
    C    
   A   
   T   
   C   
   A   
   C   
   T   
   G   
   A   
    A    
   A   
   A   
   A   
   A   
   A   
   T   
   G   
   G   
   T   
   C   
   C   
   A   
    C    
   T   
   A   
   G   
   C   
    T    
   G   
   G   
    A    
   T   
   T   
    G    
   A   
   C   
   C   
   A   
   C   
    T    
   G   
   T   
    T    
    T    
    C    
   T   
   G   
   G   
    A    
    G    
   A   
   T   
   G   
   A   
   A   
   G   
   A   
    C    
   A   
   T   
   C   
    A    
   T   
    G    
    C    
   T   
    G    
   A   
   T   
    T    
   A   
   C   
    A    
    G    
   A   
    T    
   A   
   A   
   A   
   G   
   G   
    T    
   G   
   T   
    C    
   A   
   T   
   T   
   A   
   T   
   C   
   C   
   G   
    T    
   T   
   T   
    C    
   A   
   A   
   C   
   G   
   T   
    A    
    T    
    C    
    G    
    A    
   C   
   T   
   G   
   T   
   T   
   T   
   C   
   T   
   C   
   A   
   A   
   A   
   C   
    T    
   G   
   G   
   A   
   C   
   G   
    T    
    T    
   C   
   A   
   A   
   C   
   A   
   T   
   T   
    A    
   G   
   G   
    C    
   G   
   T   
    A    
   C   
   G   
   T   
   T   
   T   
    G    
   A   
   T   
   G   
   A   
    A    
    G    
   A   
   T   
   G   
   G   
   A   
   A   
   G   
    C    
    T    
   G   
    A    
    T    
    A    
   C   
    G    
   A   
   A   
   A   
   G   
   T   
   T   
   G   
   T   
   C   
   A   
   C   
   G   
   A   
   T   
   G   
   G   
   C   
   A   
   G   
   C   
   A   
   G   
   T   
   C   
   G   
   A   
   A   
   C   
   C   
   A   
   G   
   A   
   A   
   G   
   C   
   T   
   G   
   C   
   A   
   G   
   A   
   A   
   C   
   A   
   A   
   A   
   C   
   A   
   G   
   T   
   T   
   G   
   A   
   A   
   G   
   A   
   A   
   C   
   A   
   A   
   G   
   G   
   A   
   T   
   C   
   A   
   G   
   A   
   A   
   G   
   A   
   A   
   -   
   -   
   -   
 

 
   N60449F   
   A   
   T   
   G   
   A   
   G   
   T   
   G   
   A   
   A   
   G   
   A   
   A   
   A   
   T   
   C   
   A   
   G   
   A   
   G   
   A   
   A   
   A   
   A   
   C   
   A   
   T   
   C   
   C   
   A   
   T   
   G   
   A   
   T   
   G   
   T   
   C   
   A   
   A   
   T   
   C   
   T   
   A   
   A   
   C   
   C   
   A   
   G   
   C   
   G   
   A   
   A   
   A   
   T   
   G   
   A   
   A   
   A   
   G   
   A   
   C   
   T   
   C   
   C   
   T   
   T   
   C   
   A   
   T   
   C   
   G   
   A   
   T   
   T   
   A   
   T   
   G   
   C   
   G   
   A   
   T   
   G   
   A   
   G   
   T   
   G   
   T   
   T   
   A   
   T   
   T   
   G   
   T   
   C   
   G   
   C   
   A   
   C   
   G   
   T   
   G   
   C   
   A   
   C   
   T   
   T   
   C   
   C   
   T   
   G   
   A   
   T   
   G   
   T   
   C   
   A   
   G   
   A   
   G   
   A   
   T   
   G   
   G   
   T   
   C   
   T   
   G   
   A   
   A   
   A   
   C   
   C   
   C   
   G   
   T   
   T   
   C   
   A   
   T   
   C   
   G   
   C   
   C   
   G   
   T   
   A   
   T   
   T   
   T   
   T   
   A   
   T   
   A   
   C   
   G   
   G   
   C   
   A   
   T   
   G   
   A   
   A   
   C   
   G   
   A   
   A   
   T   
   T   
   A   
   G   
   G   
   T   
   G   
   T   
   G   
   A   
   C   
   A   
   C   
   C   
   G   
   G   
   A   
   T   
   A   
   A   
   A   
   G   
   C   
   C   
   C   
   A   
   T   
   A   
   A   
   A   
   A   
   A   
   A   
   T   
   C   
   T   
   G   
   C   
   C   
   A   
   G   
   A   
   A   
   T   
   C   
   G   
   T   
   C   
   G   
   G   
   A   
   G   
   A   
   T   
   G   
   T   
   C   
   A   
   T   
   G   
   G   
   G   
   T   
   A   
   A   
   A   
   T   
   A   
   C   
   C   
   A   
   T   
   C   
   C   
   T   
   C   
   A   
   T   
   G   
   G   
   A   
   G   
   A   
   C   
   A   
   G   
   T   
   G   
   C   
   G   
   A   
   T   
   C   
   T   
   A   
   T   
   G   
   A   
   G   
   T   
   C   
   A   
   A   
   T   
   G   
   G   
   T   
   A   
   C   
   G   
   G   
   A   
   T   
   G   
   G   
   C   
   G   
   C   
   A   
   G   
   C   
   C   
   A   
   T   
   T   
   C   
   A   
   G   
   C   
   T   
   A   
   C   
   C   
   G   
   T   
   T   
   A   
   T   
   A   
   T   
   G   
   C   
   T   
   A   
   G   
   T   
   G   
   G   
   A   
   T   
   G   
   G   
   T   
   C   
   A   
   T   
   G   
   G   
   A   
   A   
   A   
   C   
   T   
   T   
   T   
   G   
   G   
   T   
   T   
   C   
   T   
   G   
   T   
   T   
   G   
   A   
   C   
   G   
   G   
   T   
   G   
   A   
   C   
   G   
   G   
   T   
   G   
   C   
   C   
   G   
   C   
   C   
   G   
   C   
   T   
   A   
   T   
   G   
   C   
   G   
   T   
   T   
   A   
   T   
   A   
   C   
   C   
   G   
   A   
   A   
   G   
   C   
   A   
   C   
   G   
   G   
   A   
   T   
   G   
   A   
   G   
   C   
   A   
   A   
   A   
   A   
   T   
   C   
   G   
   C   
   T   
   A   
   C   
   A   
   G   
   A   
   A   
   A   
   T   
   G   
   C   
   T   
   G   
   C   
   G   
   T   
   G   
   A   
   T   
   A   
   T   
   C   
   A   
   A   
   T   
   A   
   A   
   G   
   A   
   A   
   C   
   A   
   C   
   T   
   G   
   T   
   T   
   G   
   A   
   T   
   T   
   T   
   C   
   C   
   A   
   A   
   A   
   G   
   C   
   A   
   A   
   C   
   T   
   A   
   T   
   G   
   A   
   T   
   G   
   A   
   T   
   A   
   C   
   A   
   G   
   A   
   A   
   A   
   A   
   A   
   G   
   A   
   A   
   C   
   C   
   A   
   G   
   T   
   T   
   G   
   T   
   A   
   C   
   T   
   A   
   C   
   C   
   T   
   G   
   C   
   T   
   C   
   G   
   C   
   T   
   T   
   C   
   C   
   C   
   G   
   A   
   A   
   T   
   C   
   T   
   T   
   T   
   T   
   A   
   G   
   T   
   G   
   A   
   A   
   C   
   G   
   G   
   A   
   A   
   C   
   A   
   A   
   C   
   G   
   G   
   G   
   G   
   A   
   T   
   C   
   G   
   C   
   C   
   G   
   T   
   A   
   G   
   G   
   T   
   A   
   T   
   G   
   G   
   C   
   C   
   A   
   C   
   G   
   A   
   A   
   T   
   A   
   T   
   C   
   C   
   C   
   A   
   C   
   C   
   T   
   C   
   A   
   T   
   A   
   A   
   C   
   T   
   T   
   G   
   T   
   C   
   T   
   G   
   A   
   G   
   G   
   T   
   A   
   G   
   T   
   A   
   G   
   C   
   G   
   G   
   C   
   T   
   A   
   T   
   C   
   G   
   A   
   T   
   T   
   T   
   G   
   C   
   T   
   G   
   A   
   T   
   G   
   G   
   A   
   T   
   A   
   A   
   T   
   C   
   C   
   T   
   G   
   A   
   T   
   G   
   T   
   G   
   A   
   C   
   G   
   A   
   C   
   C   
   A   
   A   
   T   
   G   
   A   
   A   
   T   
   T   
   G   
   A   
   T   
   G   
   G   
   A   
   A   
   G   
   T   
   A   
   T   
   T   
   A   
   C   
   C   
   T   
   G   
   G   
   A   
   C   
   C   
   A   
   G   
   A   
   T   
   T   
   T   
   T   
   C   
   C   
   A   
   A   
   C   
   A   
   G   
   G   
   T   
   G   
   G   
   T   
   T   
   T   
   G   
   G   
   T   
   T   
   A   
   T   
   G   
   G   
   G   
   A   
   A   
   A   
   A   
   T   
   C   
   A   
   G   
   G   
    A    
   A   
   T   
    C    
   C   
   G   
    A    
    A    
   G   
   A   
   G   
   C   
    G    
   T   
   A   
   T   
   G   
   A   
   A   
   A   
   C   
    A    
   G   
   G   
    T    
   A   
   A   
    G    
   G   
   G   
   T   
   T   
   C   
    T    
   A   
   T   
   T   
   A   
   C   
    C    
   G   
   T   
    A    
   C   
   G   
   T   
   G   
   C   
   A   
   A   
   A   
   A   
   G   
   T   
   T   
   G   
   A   
    G    
    A    
   T   
    T    
   A   
    C    
   T   
   G   
   A   
    G    
   A   
   T   
   G   
    A    
   C   
    A    
   A   
   A   
   T   
   G   
   G   
    T    
   A   
   A   
   A   
   G   
   A   
   A   
   C   
   G   
    G    
   A   
   T   
    C    
    A    
   T   
    C    
   G   
   T   
    G    
   A   
   C   
    A    
   G   
   A   
   A   
   T   
   T   
    G    
   C   
   C   
   T   
   T   
   A   
   T   
   A   
   T   
   G   
   G   
   T   
    C    
   A   
   A   
   T   
   A   
   A   
   A   
   G   
   C   
    A    
   A   
   A   
   A   
   T   
   T   
    G    
   A   
   T   
    C    
   G   
   A   
   A   
   C   
   G   
    G    
   A   
   T   
   T   
   T   
   C   
   T   
   G   
   A   
    A    
    C    
   T   
    G    
   C   
   A   
   C   
   A   
   G   
   A   
   G   
   A   
    C    
   A   
   A   
    G    
   C   
   G   
    T    
   A   
   T   
    C    
   G   
   A   
   A   
   G   
   G   
    G    
   A   
   T   
    C    
   A   
   C   
    A    
   G   
   A   
   T   
   T   
   T   
   G   
   C   
   G   
   G   
   G   
   A   
    C    
   G   
   A   
   A   
   T   
   C   
   T   
   T   
   C   
    C    
   C   
   G   
   T   
   G   
   A   
    A    
   G   
   G   
    T    
   A   
   T   
   G   
   C   
   G   
    G    
   A   
   T   
    C    
   G   
   T   
   C   
   A   
   T   
    T    
   G   
   A   
    C    
    A    
   T   
    C    
   C   
   G   
   T   
   C   
   G   
    T    
   G   
   A   
    C    
   G   
    C    
    C    
   A   
   G   
    C    
   G   
   C   
    A    
   T   
   C   
    T    
   G   
   T   
   T   
    A    
   T   
    C    
   T   
   T   
    G    
   A   
   A   
    T    
   A   
   A   
   C   
   T   
   T   
    A    
   T   
   A   
    T    
   A   
   A   
   A   
    T    
   T   
   G   
   A   
   C   
    A    
    T    
   C   
    C    
   T   
   T   
    G    
   C   
   A   
    A    
   A   
    A    
    T    
   T   
   C   
    A    
   T   
   T   
    T    
   G   
   G   
    A    
   T   
   T   
    C    
   A   
   A   
    T    
   A   
   T   
   G   
   T   
   T   
    G    
   G   
   C   
    G    
   A   
   T   
    C    
   G   
   A   
   A   
   A   
   A   
   A   
   G   
   G   
    T    
   G   
   T   
    G    
   C   
   C   
    G    
   A   
   A   
   A   
    G    
   T   
   T   
   T   
   T   
    G    
   A   
   G   
    C    
   T   
   T   
   G   
   A   
   A   
   A   
   C   
    A    
    A    
   A   
   T   
    T    
    C    
   T   
    T    
   G   
   A   
   A   
   A   
   A   
    C    
   T   
   A   
    T    
    A    
   T   
    C    
   G   
   A   
    A    
   C   
   A   
    T    
   C   
   A   
    G    
    C    
    G    
    C    
    G    
   A   
   A   
   G   
   T   
   C   
   A   
   T   
    C    
   A   
   C   
    A    
    A    
   G   
    A    
   C   
   G   
   G   
   A   
   C   
    A    
    G    
   T   
    C    
   T   
   T   
   T   
   G   
   A   
    A    
   A   
   A   
   A   
    G    
   A   
    A    
   A   
   A   
    A    
   G   
   C   
   A   
   G   
   A   
   A   
   G   
   C   
   A   
    A    
   G   
    G    
   G   
   C   
    A    
   C   
   A   
    C    
   A   
   T   
    T    
   T   
   T   
   A   
   G   
   A   
   A   
   G   
   G   
   T   
    T    
   T   
    G    
   C   
   G   
    T    
   A   
   T   
   T   
   G   
   C   
    T    
   T   
   T   
    G    
   G   
   A   
   T   
   C   
   A   
   T   
   A   
   T   
   C   
    G    
   A   
   T   
   G   
   A   
   A   
   A   
   T   
    A    
   A   
   T   
   T   
   G   
   C   
    G    
   A   
   T   
   T   
   A   
   T   
    T    
   C   
   G   
    C    
   G   
   G   
    G    
   T   
   C   
    T    
    A    
   A   
   A   
   T   
   C   
   A   
   G   
   A   
   T   
   G   
   A   
    C    
   G   
   A   
    A    
   G   
   C   
   A   
   A   
   A   
   A   
   G   
   C   
    G    
   A   
    C    
    G    
    A    
   T   
    G    
   A   
   T   
    C    
   G   
   A   
   A   
   C   
   G   
    G    
   T   
   T   
    C    
   G   
   A   
    T    
   T   
   T   
    A    
   T   
   C   
    C    
   G   
   A   
   T   
   C   
   G   
    C    
   C   
   A   
    G    
    T    
   C   
    T    
   C   
   A   
   A   
   G   
   C   
    T    
   A   
   T   
    T    
   T   
   T   
   A   
   G   
   A   
   T   
   A   
   T   
   G   
   C   
   G   
   T   
   T   
   T   
    A    
   C   
   G   
    C    
   C   
   G   
    A    
   T   
   T   
    G    
   A   
   C   
    C    
   G   
   G   
    T    
   T   
   T   
    G    
   G   
   A   
   A   
   C   
   G   
    G    
   G   
   A   
    A    
   A   
   A   
   A   
   A   
   T   
    C    
   G   
   A   
    A    
   A   
   A   
   T   
   G   
   A   
   A   
   T   
   A   
    C    
   C   
   A   
   A   
   G   
   A   
    A    
   C   
   T   
    C    
   T   
   T   
    A    
   A   
    A    
   A   
    C    
   T   
    G    
   A   
   T   
   T   
   G   
    A    
    A    
   G   
   A   
    T    
   T   
   T   
    A    
    A    
    C    
    T    
   G   
   A   
   C   
   A   
   T   
   T   
    C    
   T   
    G    
   G   
   C   
    A    
    A    
   G   
    A    
   C   
   C   
    A    
   G   
   A   
    A    
   C   
   G   
    T    
   G   
   T   
    T    
   A   
    C    
    A    
   G   
   A   
   A   
   A   
   T   
    C    
   A   
   T   
    C    
   A   
   A   
    G    
   A   
    A    
    T    
   G   
   A   
    A    
   T   
   T   
   A   
    G    
    C    
    A    
   G   
   A   
   C   
    T    
   T   
    G    
    A    
    A    
    T    
    C    
    A    
   A   
    C    
    G    
   A   
   T   
   T   
   T   
   G   
   G   
   T   
   G   
   A   
   T   
    A    
    A    
   A   
   C   
   G   
   A   
   C   
   G   
    C    
   A   
   C   
   A   
   G   
   A   
   A   
    T    
   T   
   A   
    C    
   T   
   A   
   G   
   T   
    C    
   G   
   G   
   T   
   G   
   A   
    G    
   G   
   T   
   C   
    T    
   T   
    A    
   A   
   G   
   T   
   C   
   T   
    G    
   G   
   A   
   A   
   G   
   A   
   T   
   G   
   A   
   A   
   G   
   A   
   T   
    T    
   T   
    A    
   A   
   T   
    C    
   G   
   A   
   A   
   G   
   A   
   A   
   G   
   A   
   A   
   G   
   A   
   A   
    A    
   T   
   C   
   G   
   T   
    T    
   A   
   T   
   T   
   A   
   C   
    A    
    T    
   T   
    G    
   A   
   C   
    A    
   A   
   A   
    C    
   A   
   A   
    T    
   G   
   G   
    T    
   T   
   A   
    C    
   A   
   T   
    C    
   A   
   A   
    A    
   C   
   G   
    T    
    T    
   T   
   G   
   G   
    C    
   A   
   A   
   A   
    T    
    A    
    A    
    T    
   G   
   A   
   A   
   T   
   T   
   C   
    A    
   G   
    A    
   G   
   C   
    T    
   C   
   A   
   A   
   C   
   G   
    A    
    A    
   G   
    G    
   G   
   G   
    T    
   G   
   G   
    A    
   C   
   G   
   T   
   G   
   G   
    T    
   G   
   T   
    C    
   C   
   A   
   A   
   G   
   G   
    A    
   A   
   T   
   G   
   G   
   G   
    T    
    A    
   T   
    C    
   C   
   A   
    C    
    A    
   A   
   T   
   G   
   A   
    C    
   G   
   A   
    C    
   T   
   T   
   C   
   G   
   T   
    C    
   A   
   A   
    A    
   A   
   A   
    T    
   C   
   T   
    G    
    G    
   T   
   T   
   T   
   C   
   T   
   T   
   G   
    T    
   T   
   C   
    T    
   A   
   C   
    C    
   C   
   A   
    T    
   G   
   A   
    T    
   A   
   C   
    A    
   T   
   T   
    G    
   T   
   T   
   A   
   T   
   T   
    C    
   T   
   T   
   T   
   A   
   C   
   G   
   A   
   A   
    T    
   A   
    A    
    C    
   G   
   G   
    A    
   A   
   A   
   A   
   G   
   T   
    G    
   T   
   A   
    T    
   C   
   G   
    G    
   G   
   C   
   A   
   A   
   A   
   A   
   G   
   G   
    A    
   T   
   A   
    C    
   G   
   A   
   A   
   A   
   T   
    T    
   C   
   C   
    A    
   G   
   A   
    A    
   T   
   A   
   C   
   G   
   G   
    G    
    C    
   G   
    C    
   A   
   C   
   A   
   G   
   C   
    C    
   A   
   A   
   A   
   G   
   G   
    A    
   A   
   T   
    A    
   C   
   C   
   A   
    A    
   T   
    T    
   A   
   T   
    C    
   A   
   A   
    T    
   T   
   T   
    A    
   C   
   T   
    A    
   G   
   G   
    T    
   A   
   T   
    C    
   G   
   A   
    T    
   T   
   C   
    G    
    A    
    G    
    T    
   G   
   A   
   A   
   A   
    G    
    C    
   A   
   T   
    C    
   C   
   A   
    G    
   G   
   C   
    A    
   A   
   T   
    C    
   A   
   T   
   T   
    G    
   C   
    G    
   G   
   T   
    G    
    A    
    C    
   G   
   G   
   G   
    A    
    G    
   A   
   A   
   G   
   C   
    A    
   G   
   A   
   A   
   G   
    A    
    A    
   G   
   G   
    C    
    C    
   A   
    C    
   T   
   A   
    T    
    C    
   T   
    C    
   T   
   T   
   C   
   T   
   T   
    T    
   A   
   C   
    G    
   A   
   C   
    G    
    C    
    G    
    T    
    C    
   A   
   A   
   G   
   G   
    T    
   A   
   C   
    A    
   G   
   T   
    A    
   A   
   A   
   A   
   C   
   G   
    T    
   A   
   C   
    T    
    T    
   C   
    T    
   G   
   T   
    C    
   A   
   C   
    T    
   G   
   C   
    T    
   T   
   T   
    T    
    G    
   C   
   T   
   A   
   A   
    C    
   A   
   T   
    C    
   C   
   G   
    A    
   A   
   G   
    T    
   A   
   A   
   T   
   G   
   G   
    T    
    C    
   T   
    G    
   A   
   T   
    C    
   G   
   C   
    A    
   A   
   T   
   T   
    G    
   G   
    T    
   T   
   T   
    G    
   A   
   A   
   A   
   G   
   A   
   A   
   G   
   A   
    C    
   G   
   A   
    T    
   G   
   A   
    G    
    C    
   T   
    C    
    G    
   T   
    C    
   A   
   A   
    T    
   G   
   T   
    G    
    C    
   T   
   A   
    C    
    T    
   G   
   A   
   C   
    A    
    G    
   A   
    T    
   G   
   G   
    T    
    C    
   A   
    A    
    T    
    C    
    T    
   A   
   A   
    T    
   A   
   T   
    C    
   A   
   T   
    T    
   A   
   T   
    T    
   G   
   G   
    A    
   A   
   C   
   A   
   C   
   A   
   T   
    A    
    A    
    C    
   G   
   G   
    C    
   T   
   A   
   C   
   T   
   C   
   T   
   G   
   T   
    C    
   A   
   C   
    T    
   T   
   T   
    C    
    A    
   A   
    A    
   G   
   A   
    A    
    G    
    A    
    A    
    G    
   C   
    A    
   G   
    T    
    C    
   C   
   G   
    C    
   G   
   A   
   T   
   A   
   T   
   G   
   G   
   G   
    A    
   C   
   G   
    T    
   A   
   C   
   A   
   G   
   C   
    T    
   T   
   C   
    C    
   G   
   G   
    G    
   G   
   T   
    C    
    A    
   G   
    A    
   G   
   G   
    A    
   A   
   T   
    A    
   C   
   G   
    C    
    C    
   T   
    G    
   C   
   G   
    T    
   G   
   A   
    G    
    G    
   A   
    A    
   G   
   A   
    T    
   T   
    A    
    T    
   G   
   T   
    C    
   G   
   T   
    T    
   G   
   G   
    G    
    G    
   C   
   A   
   G   
   C   
    C    
    T    
   T   
    A    
    A    
   T   
   G   
    A    
   A   
    G    
   G   
    A    
   A   
    G    
   A   
    T    
    C    
   A   
   A   
   G   
   A   
   A   
   G   
   T   
    A    
    T    
   T   
    A    
   G   
   T   
    G    
   A   
   T   
    C    
   A   
   C   
    A    
   G   
   A   
   A   
   A   
   A   
    A    
   G   
   G   
   T   
   T   
   A   
    C    
   G   
   G   
    A    
   A   
   A   
    A    
   C   
   G   
    G    
   A   
   C   
    A    
   A   
   A   
   A   
   G   
    T    
    A    
   T   
   C   
    G    
   G   
   A   
    A    
   T   
   A   
   T   
   C   
   C   
   A   
   G   
   T   
    G    
   A   
   A   
    A    
   G   
   G   
    C    
   C   
   G   
    C    
   G   
   G   
    A    
   G   
   G   
   T   
   A   
   A   
   A   
   G   
   G   
    G    
   A   
   T   
    C    
   A   
   A   
    A    
   A   
   C   
    G    
    G    
   C   
    G    
   A   
   A   
    C    
   A   
   T   
   C   
   A   
   C   
   T   
   G   
   A   
    A    
   A   
   A   
   A   
   A   
   A   
   T   
   G   
   G   
   T   
   C   
   C   
   A   
    C    
   T   
   A   
   G   
   C   
    T    
   G   
   G   
    A    
   T   
   T   
    G    
   A   
   C   
   C   
   A   
   C   
    T    
   G   
   T   
    T    
    T    
    C    
   T   
   G   
   G   
    A    
    G    
   A   
   T   
   G   
   A   
   A   
   G   
   A   
    C    
   A   
   T   
   C   
    A    
   T   
    G    
    C    
   T   
    G    
   A   
   T   
    T    
   A   
   C   
    A    
    G    
   A   
    T    
   A   
   A   
   A   
   G   
   G   
    T    
   G   
   T   
    C    
   A   
   T   
   T   
   A   
   T   
   C   
   C   
   G   
    T    
   T   
   T   
    C    
   A   
   A   
   C   
   G   
   T   
    A    
    T    
    C    
    G    
    A    
   C   
   T   
   G   
   T   
   T   
   T   
   C   
   T   
   C   
   A   
   A   
   A   
   C   
    T    
   G   
   G   
   A   
   C   
   G   
    T    
    T    
   C   
   A   
   A   
   C   
   A   
   T   
   T   
    A    
   G   
   G   
    C    
   G   
   T   
    A    
   C   
   G   
   T   
   T   
   T   
    G    
   A   
   T   
   G   
   A   
    A    
    G    
   A   
   T   
   G   
   G   
   A   
   A   
   G   
    C    
    T    
   G   
    A    
    T    
    A    
   C   
    G    
   A   
   A   
   A   
   G   
   T   
   T   
   G   
   T   
   C   
   A   
   C   
   G   
   A   
   T   
   G   
   G   
   C   
   A   
   G   
   C   
   A   
   G   
   T   
   C   
   G   
   A   
   A   
   C   
   C   
   A   
   G   
   A   
   A   
   G   
   C   
   T   
   G   
   C   
   A   
   G   
   A   
   A   
   C   
   A   
   A   
   A   
   C   
   A   
   G   
   T   
   T   
   G   
   A   
   A   
   G   
   A   
   A   
   C   
   A   
   A   
   G   
   G   
   A   
   T   
   C   
   A   
   G   
   A   
   A   
   G   
   A   
   A   
   -   
   -   
   -   
 

 
   N60268F   
   A   
   T   
   G   
   A   
   G   
   T   
   G   
   A   
   A   
   G   
   A   
   A   
   A   
   T   
   T   
   A   
   A   
   A   
   G   
   A   
   A   
   A   
   A   
   C   
   A   
   T   
   T   
   C   
   A   
   A   
   G   
   A   
   C   
   G   
   T   
   C   
   A   
   A   
   T   
   C   
   T   
   G   
   A   
   C   
   C   
   A   
   G   
   C   
   G   
   A   
   A   
   A   
   T   
   G   
   A   
   A   
   A   
   G   
   A   
   A   
   T   
   C   
   T   
   T   
   T   
   C   
   A   
   T   
   T   
   G   
   A   
   C   
   T   
   A   
   C   
   G   
   C   
   A   
   A   
   T   
   G   
   A   
   G   
   T   
   G   
   T   
   T   
   A   
   T   
   C   
   G   
   T   
   A   
   G   
   C   
   C   
   C   
   G   
   C   
   G   
   C   
   G   
   T   
   T   
   A   
   C   
   C   
   T   
   G   
   A   
   C   
   G   
   T   
   T   
   C   
   G   
   T   
   G   
   A   
   C   
   G   
   G   
   T   
   T   
   T   
   A   
   A   
   A   
   A   
   C   
   C   
   T   
   G   
   T   
   T   
   C   
   A   
   T   
   C   
   G   
   C   
   C   
   G   
   A   
   A   
   T   
   C   
   T   
   T   
   A   
   T   
   A   
   T   
   G   
   G   
   A   
   A   
   T   
   G   
   A   
   A   
   C   
   G   
   A   
   A   
   T   
   T   
   A   
   G   
   G   
   G   
   G   
   T   
   A   
   A   
   C   
   C   
   C   
   C   
   T   
   G   
   A   
   T   
   A   
   A   
   A   
   C   
   C   
   A   
   C   
   A   
   C   
   A   
   A   
   A   
   A   
   A   
   A   
   T   
   C   
   A   
   G   
   C   
   C   
   C   
   G   
   G   
   A   
   T   
   T   
   G   
   T   
   T   
   G   
   G   
   G   
   G   
   A   
   T   
   G   
   T   
   T   
   A   
   T   
   G   
   G   
   G   
   T   
   A   
   A   
   A   
   T   
   A   
   T   
   C   
   A   
   T   
   C   
   C   
   C   
   C   
   A   
   T   
   G   
   G   
   G   
   G   
   A   
   C   
   A   
   G   
   T   
   G   
   C   
   G   
   A   
   T   
   T   
   T   
   A   
   C   
   G   
   A   
   A   
   T   
   C   
   A   
   A   
   T   
   G   
   G   
   T   
   G   
   C   
   G   
   G   
   A   
   T   
   G   
   G   
   C   
   A   
   C   
   A   
   A   
   C   
   C   
   T   
   T   
   T   
   T   
   A   
   G   
   T   
   T   
   A   
   T   
   C   
   G   
   G   
   G   
   C   
   T   
   A   
   T   
   G   
   T   
   T   
   A   
   G   
   T   
   T   
   G   
   A   
   C   
   G   
   G   
   C   
   C   
   A   
   C   
   G   
   G   
   A   
   A   
   A   
   C   
   T   
   T   
   C   
   G   
   G   
   T   
   T   
   C   
   T   
   G   
   T   
   C   
   G   
   A   
   T   
   G   
   G   
   T   
   G   
   A   
   C   
   G   
   G   
   C   
   G   
   C   
   T   
   G   
   C   
   C   
   G   
   C   
   T   
   A   
   T   
   G   
   C   
   G   
   T   
   T   
   A   
   T   
   A   
   C   
   C   
   G   
   A   
   A   
   G   
   C   
   A   
   C   
   G   
   T   
   A   
   T   
   G   
   A   
   G   
   T   
   A   
   A   
   A   
   A   
   T   
   T   
   G   
   C   
   T   
   T   
   T   
   A   
   G   
   A   
   A   
   A   
   T   
   G   
   C   
   T   
   A   
   C   
   G   
   A   
   G   
   A   
   T   
   A   
   T   
   T   
   A   
   A   
   C   
   A   
   A   
   A   
   A   
   A   
   T   
   A   
   C   
   A   
   G   
   T   
   C   
   G   
   A   
   T   
   T   
   T   
   C   
   C   
   A   
   A   
   G   
   G   
   A   
   A   
   A   
   C   
   T   
   A   
   T   
   G   
   A   
   T   
   G   
   A   
   T   
   T   
   C   
   A   
   G   
   A   
   A   
   C   
   A   
   A   
   G   
   A   
   A   
   C   
   C   
   A   
   G   
   T   
   A   
   G   
   T   
   T   
   T   
   T   
   A   
   C   
   C   
   T   
   G   
   C   
   T   
   C   
   G   
   T   
   T   
   T   
   T   
   C   
   C   
   A   
   A   
   A   
   C   
   T   
   T   
   A   
   C   
   T   
   A   
   G   
   T   
   T   
   A   
   A   
   C   
   G   
   G   
   A   
   A   
   C   
   A   
   A   
   C   
   G   
   G   
   G   
   G   
   A   
   T   
   T   
   G   
   C   
   G   
   G   
   T   
   G   
   G   
   G   
   G   
   A   
   T   
   G   
   G   
   C   
   A   
   A   
   C   
   A   
   A   
   A   
   T   
   A   
   T   
   T   
   C   
   C   
   A   
   C   
   C   
   A   
   C   
   A   
   T   
   A   
   A   
   T   
   T   
   T   
   A   
   A   
   G   
   T   
   G   
   A   
   A   
   G   
   T   
   G   
   A   
   T   
   T   
   G   
   A   
   T   
   G   
   C   
   A   
   A   
   C   
   A   
   A   
   G   
   T   
   T   
   T   
   A   
   T   
   T   
   G   
   A   
   T   
   G   
   G   
   A   
   C   
   A   
   A   
   T   
   C   
   C   
   T   
   G   
   A   
   T   
   G   
   T   
   A   
   A   
   C   
   G   
   A   
   C   
   G   
   A   
   A   
   T   
   G   
   A   
   A   
   T   
   T   
   G   
   A   
   T   
   G   
   G   
   A   
   A   
   G   
   T   
   G   
   T   
   T   
   A   
   C   
   C   
   T   
   G   
   G   
   A   
   C   
   C   
   A   
   G   
   A   
   T   
   T   
   T   
   T   
   C   
   C   
   A   
   A   
   C   
   A   
   G   
   G   
   C   
   G   
   G   
   T   
   T   
   T   
   A   
   G   
   T   
   G   
   A   
   T   
   G   
   G   
   G   
   G   
   A   
   A   
   A   
   T   
   C   
   A   
   G   
   G   
    G    
   A   
   T   
    T    
   C   
   G   
    C    
    C    
   G   
   A   
   G   
   C   
    A    
   T   
   A   
   T   
   G   
   A   
   A   
   A   
   C   
    A    
   G   
   G   
    G    
   A   
   A   
    A    
   G   
   G   
   T   
   T   
   C   
    G    
   A   
   T   
   T   
   A   
   C   
    T    
   G   
   T   
    T    
   C   
   G   
   T   
   G   
   C   
   A   
   A   
   A   
   A   
   G   
   T   
   T   
   G   
   A   
    A    
    T    
   T   
    G    
   A   
    C    
   T   
   G   
   A   
    A    
   A   
   T   
   G   
    C    
   C   
    G    
   A   
   A   
   T   
   G   
   G   
    A    
   A   
   A   
   A   
   G   
   A   
   A   
   C   
   G   
    T    
   A   
   T   
    T    
    T    
   T   
    A    
   G   
   T   
    A    
   A   
   C   
    T    
   G   
   A   
   A   
   T   
   T   
    G    
   C   
   C   
   T   
   T   
   A   
   T   
   A   
   T   
   G   
   G   
   T   
    G    
   A   
   A   
   T   
   A   
   A   
   A   
   G   
   C   
    C    
   A   
   A   
   A   
   T   
   T   
    A    
   A   
   T   
    C    
   G   
   A   
   A   
   C   
   G   
    A    
   A   
   T   
   T   
   T   
   C   
   T   
   G   
   A   
    A    
    T    
   T   
    A    
   C   
   A   
   C   
   A   
   G   
   A   
   G   
   A   
    T    
   A   
   A   
    A    
   C   
   G   
    A    
   A   
   T   
    T    
   G   
   A   
   A   
   G   
   G   
    A    
   A   
   T   
    T    
   A   
   C   
    T    
   G   
   A   
   T   
   T   
   T   
   G   
   C   
   G   
   G   
   G   
   A   
    T    
   G   
   A   
   A   
   T   
   C   
   T   
   T   
   C   
    T    
   C   
   G   
   T   
   G   
   A   
    A    
   G   
   G   
    C    
   A   
   T   
   G   
   C   
   G   
    G    
   A   
   T   
    C    
   G   
   T   
   C   
   A   
   T   
    C    
   G   
   A   
    T    
    G    
   T   
    T    
   C   
   G   
   T   
   C   
   G   
    A    
   G   
   A   
    T    
   G   
    T    
    G    
   A   
   G   
    T    
   G   
   C   
    C    
   T   
   C   
    T    
   G   
   T   
   T   
    G    
   T   
    G    
   T   
   T   
    A    
   A   
   A   
    C    
   A   
   A   
   C   
   T   
   T   
    G    
   T   
   A   
    C    
   A   
   A   
   A   
    A    
   T   
   G   
   A   
   C   
    T    
    G    
   C   
    C    
   T   
   T   
    A    
   C   
   A   
    A    
   A   
    C    
    A    
   T   
   C   
    T    
   T   
   T   
    T    
   G   
   G   
    T    
   T   
   T   
    T    
   A   
   A   
    C    
   A   
   T   
   G   
   T   
   T   
    A    
   G   
   C   
    C    
   A   
   T   
    T    
   G   
   A   
   A   
   A   
   A   
   A   
   G   
   G   
    C    
   G   
   T   
    A    
   C   
   C   
    G    
   A   
   A   
   A   
    A    
   T   
   T   
   T   
   T   
    A    
   A   
   G   
    C    
   T   
   T   
   G   
   A   
   A   
   A   
   C   
    G    
    T    
   A   
   T   
    T    
    T    
   T   
    A    
   G   
   A   
   A   
   A   
   A   
    T    
   T   
   A   
    C    
    G    
   T   
    C    
   G   
   A   
    G    
   C   
   A   
    C    
   C   
   A   
    A    
    A    
    A    
    A    
    G    
   A   
   A   
   G   
   T   
   C   
   A   
   T   
    T    
   A   
   C   
    T    
    C    
   G   
    C    
   C   
   G   
   G   
   A   
   C   
    G    
    A    
   T   
    T    
   T   
   T   
   T   
   G   
   A   
    C    
   A   
   A   
   A   
    A    
   A   
    C    
   A   
   A   
    A    
   G   
   C   
   A   
   G   
   A   
   A   
   G   
   C   
   A   
    C    
   G   
    G    
   G   
   C   
    G    
   C   
   A   
    T    
   A   
   T   
    T    
   T   
   T   
   A   
   G   
   A   
   A   
   G   
   G   
   T   
    C    
   T   
    A    
   C   
   G   
    A    
   A   
   T   
   T   
   G   
   C   
    C    
   T   
   T   
    A    
   G   
   A   
   T   
   C   
   A   
   T   
   A   
   T   
   C   
    G    
   A   
   T   
   G   
   A   
   A   
   A   
   T   
    C    
   A   
   T   
   T   
   G   
   C   
    C    
   A   
   T   
   T   
   A   
   T   
    C    
   C   
   G   
    T    
   G   
   G   
    A    
   T   
   C   
    A    
    C    
   A   
   A   
   T   
   C   
   A   
   G   
   A   
   T   
   G   
   A   
    C    
   G   
   A   
    A    
   G   
   C   
   A   
   A   
   A   
   A   
   G   
   C   
    A    
   A   
    C    
    G    
    T    
   T   
    A    
   A   
   T   
    T    
   G   
   A   
   A   
   C   
   G   
    C    
   T   
   T   
    T    
   G   
   A   
    A    
   T   
   T   
    T    
   T   
   C   
    A    
   G   
   A   
   T   
   C   
   G   
    T    
   C   
   A   
    A    
    G    
   C   
    G    
   C   
   A   
   A   
   G   
   C   
    G    
   A   
   T   
    T    
   T   
   T   
   A   
   G   
   A   
   T   
   A   
   T   
   G   
   C   
   G   
   T   
   T   
   T   
    A    
   C   
   G   
    C    
   C   
   G   
    T    
   T   
   T   
    A    
   A   
   C   
    A    
   G   
   G   
    C    
   T   
   T   
    A    
   G   
   A   
   A   
   C   
   G   
    C    
   G   
   A   
    C    
   A   
   A   
   A   
   A   
   T   
    T    
   G   
   A   
    A    
   A   
   A   
   T   
   G   
   A   
   A   
   T   
   A   
    T    
   C   
   A   
   A   
   G   
   A   
    G    
   C   
   T   
    A    
   T   
   T   
    G    
   A   
    A    
   A   
    T    
   T   
    C    
   A   
   T   
   T   
   G   
    C    
    G    
   G   
   A   
    C    
   T   
   T   
    A    
    G    
    A    
    G    
   G   
   A   
   C   
   A   
   T   
   T   
    T    
   T   
    A    
   G   
   C   
    C    
    C    
   G   
    C    
   C   
   C   
    A    
   G   
   A   
    A    
   C   
   G   
    C    
   G   
   T   
    C    
   A   
    T    
    T    
   G   
   A   
   A   
   A   
   T   
    C    
   A   
   T   
    T    
   A   
   A   
    A    
   A   
    C    
    A    
   G   
   A   
    G    
   T   
   T   
   A   
    A    
    A    
    T    
   G   
   A   
   C   
    G    
   T   
    T    
    C    
    G    
    C    
    A    
    C    
   A   
    A    
    A    
   A   
   T   
   T   
   T   
   G   
   G   
   T   
   G   
   A   
   T   
    G    
    C    
   A   
   C   
   G   
   A   
   C   
   G   
    C    
   A   
   C   
   A   
   G   
   A   
   A   
    T    
   T   
   A   
    T    
   T   
   A   
   G   
   T   
    A    
   G   
   G   
   T   
   G   
   A   
    A    
   G   
   T   
   C   
    T    
   T   
    A    
   A   
   G   
   T   
   C   
   T   
    T    
   G   
   A   
   A   
   G   
   A   
   T   
   G   
   A   
   A   
   G   
   A   
   T   
    T    
   T   
    A    
   A   
   T   
    C    
   G   
   A   
   A   
   G   
   A   
   A   
   G   
   A   
   A   
   G   
   A   
   A   
    G    
   T   
   C   
   G   
   T   
    G    
   A   
   T   
   T   
   A   
   C   
    G    
    T    
   T   
    A    
   A   
   C   
    C    
   A   
   A   
    T    
   A   
   A   
    C    
   G   
   G   
    C    
   T   
   A   
    C    
   A   
   T   
    T    
   A   
   A   
    G    
   C   
   G   
    G    
    A    
   T   
   G   
   G   
    C    
   A   
   A   
   A   
    C    
    T    
    C    
    C    
   G   
   A   
   A   
   T   
   T   
   C   
    C    
   G   
    G    
   G   
   C   
    G    
   C   
   A   
   A   
   C   
   G   
    C    
    C    
   G   
    C    
   G   
   G   
    T    
   G   
   G   
    A    
   C   
   G   
   T   
   G   
   G   
    T    
   G   
   T   
    C    
   C   
   A   
   A   
   G   
   G   
    G    
   A   
   T   
   G   
   G   
   G   
    C    
    G    
   T   
    T    
   C   
   A   
    T    
    G    
   A   
   T   
   G   
   A   
    T    
   G   
   A   
    T    
   T   
   T   
   C   
   G   
   T   
    G    
   A   
   A   
    A    
   A   
   A   
    C    
   C   
   T   
    A    
    G    
   T   
   T   
   T   
   C   
   T   
   T   
   G   
    T    
   T   
   C   
    A    
   A   
   C   
    A    
   C   
   A   
    C    
   G   
   A   
    C    
   A   
   C   
    G    
   T   
   T   
    A    
   T   
   T   
   A   
   T   
   T   
    C    
   T   
   T   
   T   
   A   
   C   
   G   
   A   
   A   
    C    
   A   
    C    
    T    
   G   
   G   
    G    
   A   
   A   
   A   
   G   
   T   
    T    
   T   
   A   
    C    
   C   
   G   
    A    
   G   
   C   
   A   
   A   
   A   
   A   
   G   
   G   
    T    
   T   
   A   
    C    
   G   
   A   
   A   
   A   
   T   
    C    
   C   
   C   
    T    
   G   
   A   
    A    
   T   
   A   
   C   
   G   
   G   
    T    
    A    
   G   
    A    
   A   
   C   
   A   
   G   
   C   
    A    
   A   
   A   
   A   
   G   
   G   
    A    
   A   
   T   
    T    
   C   
   C   
   A   
    G    
   T   
    G    
   A   
   T   
    T    
   A   
   A   
    C    
   T   
   T   
    A    
   C   
   T   
    G    
   G   
   G   
    A    
   A   
   T   
    T    
   G   
   A   
    T    
   T   
   C   
    T    
    G    
    C    
    A    
   G   
   A   
   A   
   A   
    A    
    A    
   A   
   T   
    T    
   C   
   A   
    A    
   G   
   C   
    G    
   A   
   T   
    T    
   A   
   T   
   T   
    T    
   C   
    T    
   G   
   T   
    T    
    G    
    A    
   G   
   G   
   G   
    C    
    A    
   A   
   A   
   G   
   C   
    G    
   G   
   A   
   A   
   G   
    C    
    A    
   G   
   G   
    T    
    A    
   A   
    A    
   T   
   A   
    C    
    T    
   T   
    G    
   T   
   T   
   C   
   T   
   T   
    C    
   A   
   C   
    A    
   A   
   C   
    C    
    T    
    T    
    A    
    A    
   A   
   A   
   G   
   G   
    A    
   A   
   C   
    C    
   G   
   T   
    C    
   A   
   A   
   A   
   C   
   G   
    G    
   A   
   C   
    A    
    G    
   C   
    C    
   G   
   T   
    A    
   A   
   C   
    A    
   G   
   C   
    C    
   T   
   T   
    T    
    T    
   C   
   T   
   A   
   A   
    T    
   A   
   T   
    C    
   C   
   G   
    T    
   A   
   G   
    T    
   A   
   A   
   T   
   G   
   G   
    A    
    T    
   T   
    A    
   A   
   T   
    C    
   G   
   C   
    C    
   A   
   T   
   T   
    A    
   G   
    C    
   T   
   T   
    A    
   A   
   A   
   A   
   G   
   A   
   A   
   G   
   A   
    T    
   G   
   A   
    T    
   G   
   A   
    G    
    T    
   T   
    A    
    G    
   T   
    T    
   A   
   A   
    C    
   G   
   T   
    G    
    G    
   T   
   A   
    A    
    C    
   G   
   A   
   C   
    T    
    A    
   A   
    T    
   G   
   G   
    C    
    A    
   A   
    T    
    C    
    A    
    G    
   A   
   A   
    G    
   A   
   T   
    G    
   A   
   T   
    T    
   A   
   T   
    C    
   G   
   G   
    A    
   A   
   C   
   A   
   C   
   A   
   T   
    G    
    C    
    A    
   G   
   G   
    A    
   T   
   A   
   C   
   T   
   C   
   T   
   G   
   T   
    C    
   A   
   C   
    G    
   T   
   T   
    T    
    G    
   A   
    T    
   G   
   A   
    A    
    A    
    A    
    T    
    A    
   C   
    T    
   G   
    T    
    A    
   C   
   G   
    T    
   G   
   A   
   T   
   A   
   T   
   G   
   G   
   G   
    C    
   C   
   G   
    G    
   A   
   C   
   A   
   G   
   C   
    A    
   T   
   C   
    A    
   G   
   G   
    T    
   G   
   T   
    T    
    C    
   G   
    G    
   G   
   G   
    A    
   A   
   T   
    C    
   C   
   G   
    T    
    C    
   T   
    C    
   C   
   G   
    C    
   G   
   A   
    A    
    A    
   A   
    T    
   G   
   A   
    T    
   T   
    A    
    T    
   G   
   T   
    G    
   G   
   T   
    C    
   G   
   G   
    T    
    G    
   C   
   A   
   G   
   C   
    G    
    A    
   T   
    T    
    C    
   T   
   G   
    G    
   A   
    T    
   G   
    A    
   A   
    A    
   A   
    C    
    A    
   A   
   A   
   G   
   A   
   A   
   G   
   T   
    C    
    C    
   T   
    A    
   G   
   T   
    C    
   A   
   T   
    T    
   A   
   C   
    T    
   G   
   A   
   A   
   A   
   A   
    T    
   G   
   G   
   T   
   T   
   A   
    T    
   G   
   G   
    T    
   A   
   A   
    G    
   C   
   G   
    T    
   A   
   C   
    A    
   A   
   A   
   A   
   G   
    C    
    T    
   T   
   C   
    T    
   G   
   A   
    A    
   T   
   A   
   T   
   C   
   C   
   A   
   G   
   T   
    T    
   A   
   A   
    A    
   G   
   G   
    A    
   C   
   G   
    T    
   G   
   G   
    C    
   G   
   G   
   T   
   A   
   A   
   A   
   G   
   G   
    G    
   A   
   T   
    T    
   A   
   A   
    G    
   A   
   C   
    A    
    G    
   C   
    A    
   A   
   A   
    T    
   A   
   T   
   C   
   A   
   C   
   T   
   G   
   A   
    G    
   A   
   A   
   A   
   A   
   A   
   T   
   G   
   G   
   T   
   C   
   C   
   A   
    T    
   T   
   A   
   G   
   C   
    T    
   G   
   G   
    T    
   T   
   T   
    A    
   A   
   C   
   C   
   A   
   C   
    G    
   G   
   T   
    C    
    A    
    A    
   T   
   G   
   G   
    G    
    G    
   A   
   T   
   G   
   A   
   A   
   G   
   A   
    T    
   A   
   T   
   C   
    T    
   T   
    A    
    T    
   T   
    G    
   A   
   T   
    T    
   A   
   C   
    G    
    A    
   A   
    C    
   A   
   A   
   A   
   G   
   G   
    C    
   G   
   T   
    C    
   A   
   T   
   T   
   A   
   T   
   C   
   C   
   G   
    C    
   T   
   T   
    T    
   A   
   A   
   C   
   G   
   T   
    T    
    G    
    A    
    T    
    T    
   C   
   T   
   G   
   T   
   T   
   T   
   C   
   T   
   C   
   A   
   A   
   A   
   C   
    A    
   G   
   G   
   A   
   C   
   G   
    C    
    G    
   C   
   A   
   A   
   C   
   A   
   T   
   T   
    A    
   G   
   G   
    G    
   G   
   T   
    T    
   C   
   G   
   T   
   T   
   T   
    A    
   A   
   T   
   G   
   A   
    G    
    A    
   A   
   T   
   G   
   G   
   A   
   A   
   G   
    A    
    T    
   G   
    G    
    T    
    G    
   C   
    C    
   A   
   A   
   A   
   -   
   -   
   -   
   -   
   -   
   -   
   -   
   -   
   -   
   -   
   -   
   -   
   -   
   -   
   -   
   -   
   -   
   -   
   -   
   -   
   -   
   -   
   -   
   -   
   -   
   -   
   -   
   -   
   -   
   -   
   -   
   -   
   -   
   -   
   -   
   -   
   -   
   -   
   -   
   -   
   -   
   -   
   -   
   -   
   -   
   -   
   -   
   -   
   -   
   -   
   -   
   -   
   -   
   -   
   -   
   -   
   -   
   -   
   -   
   -   
   -   
   -   
   -   
   -   
   -   
   -   
   -   
   -   
   -   
   -   
   -   
   -   
 

 
   N52656   
   A   
   T   
   G   
   A   
   G   
   T   
   G   
   A   
   A   
   G   
   A   
   A   
   A   
   T   
   C   
   A   
   G   
   A   
   G   
   A   
   A   
   A   
   A   
   C   
   A   
   T   
   C   
   C   
   A   
   T   
   G   
   A   
   T   
   G   
   T   
   C   
   A   
   A   
   T   
   C   
   T   
   A   
   A   
   C   
   C   
   A   
   G   
   C   
   G   
   A   
   A   
   A   
   T   
   G   
   A   
   A   
   A   
   G   
   A   
   C   
   T   
   C   
   C   
   T   
   T   
   C   
   A   
   T   
   C   
   G   
   A   
   T   
   T   
   A   
   T   
   G   
   C   
   G   
   A   
   T   
   G   
   A   
   G   
   T   
   G   
   T   
   T   
   A   
   T   
   T   
   G   
   T   
   C   
   G   
   C   
   A   
   C   
   G   
   T   
   G   
   C   
   A   
   C   
   T   
   G   
   C   
   C   
   T   
   G   
   A   
   T   
   G   
   T   
   C   
   A   
   G   
   A   
   G   
   A   
   T   
   G   
   G   
   T   
   C   
   T   
   G   
   A   
   A   
   A   
   C   
   C   
   C   
   G   
   T   
   T   
   C   
   A   
   T   
   C   
   G   
   C   
   C   
   G   
   T   
   A   
   T   
   T   
   T   
   T   
   A   
   T   
   A   
   C   
   G   
   G   
   C   
   A   
   T   
   G   
   A   
   A   
   C   
   G   
   A   
   A   
   T   
   T   
   A   
   G   
   G   
   T   
   G   
   T   
   G   
   A   
   C   
   A   
   C   
   C   
   G   
   G   
   A   
   T   
   A   
   A   
   A   
   G   
   C   
   C   
   C   
   A   
   T   
   A   
   A   
   A   
   A   
   A   
   A   
   T   
   C   
   T   
   G   
   C   
   C   
   A   
   G   
   A   
   A   
   T   
   C   
   G   
   T   
   C   
   G   
   G   
   A   
   G   
   A   
   T   
   G   
   T   
   C   
   A   
   T   
   G   
   G   
   G   
   T   
   A   
   A   
   A   
   T   
   A   
   C   
   C   
   A   
   T   
   C   
   C   
   T   
   C   
   A   
   T   
   G   
   G   
   G   
   G   
   A   
   T   
   A   
   G   
   T   
   G   
   C   
   A   
   A   
   T   
   C   
   T   
   A   
   T   
   G   
   A   
   G   
   T   
   C   
   A   
   A   
   T   
   G   
   G   
   T   
   A   
   C   
   G   
   G   
   A   
   T   
   G   
   G   
   C   
   G   
   C   
   A   
   G   
   C   
   C   
   A   
   T   
   T   
   T   
   A   
   G   
   C   
   T   
   A   
   C   
   C   
   G   
   T   
   T   
   A   
   T   
   A   
   T   
   G   
   C   
   T   
   A   
   G   
   T   
   G   
   G   
   A   
   T   
   G   
   G   
   T   
   C   
   A   
   T   
   G   
   G   
   A   
   A   
   A   
   C   
   T   
   T   
   T   
   G   
   G   
   T   
   T   
   C   
   T   
   G   
   T   
   T   
   G   
   A   
   C   
   G   
   G   
   T   
   G   
   A   
   C   
   G   
   G   
   T   
   G   
   C   
   C   
   G   
   C   
   T   
   G   
   C   
   T   
   A   
   T   
   G   
   C   
   G   
   T   
   T   
   A   
   T   
   A   
   C   
   C   
   G   
   A   
   A   
   G   
   C   
   A   
   C   
   G   
   G   
   A   
   T   
   G   
   A   
   G   
   C   
   A   
   A   
   A   
   A   
   T   
   C   
   G   
   C   
   T   
   A   
   C   
   A   
   G   
   A   
   A   
   A   
   T   
   G   
   C   
   T   
   G   
   C   
   G   
   T   
   G   
   A   
   T   
   A   
   T   
   C   
   A   
   A   
   T   
   A   
   A   
   G   
   A   
   A   
   T   
   A   
   C   
   T   
   G   
   T   
   G   
   G   
   A   
   T   
   T   
   T   
   C   
   C   
   A   
   A   
   A   
   G   
   C   
   A   
   A   
   C   
   T   
   A   
   T   
   G   
   A   
   T   
   G   
   A   
   C   
   A   
   C   
   A   
   G   
   A   
   A   
   A   
   A   
   A   
   G   
   A   
   A   
   C   
   C   
   C   
   G   
   T   
   T   
   G   
   T   
   A   
   T   
   T   
   A   
   C   
   C   
   T   
   G   
   C   
   T   
   C   
   G   
   C   
   T   
   T   
   C   
   C   
   C   
   T   
   A   
   A   
   T   
   C   
   T   
   T   
   T   
   T   
   G   
   G   
   T   
   G   
   A   
   A   
   C   
   G   
   G   
   A   
   A   
   C   
   G   
   A   
   C   
   G   
   G   
   G   
   G   
   A   
   T   
   C   
   G   
   C   
   C   
   G   
   T   
   G   
   G   
   G   
   T   
   A   
   T   
   G   
   G   
   C   
   A   
   A   
   C   
   G   
   A   
   A   
   T   
   A   
   T   
   C   
   C   
   C   
   A   
   C   
   C   
   T   
   C   
   A   
   T   
   A   
   A   
   T   
   T   
   T   
   G   
   T   
   C   
   T   
   G   
   A   
   G   
   G   
   T   
   A   
   G   
   T   
   A   
   G   
   C   
   G   
   G   
   C   
   T   
   A   
   T   
   C   
   G   
   A   
   T   
   T   
   T   
   G   
   C   
   T   
   G   
   A   
   T   
   G   
   G   
   A   
   T   
   A   
   A   
   T   
   C   
   C   
   T   
   G   
   A   
   T   
   G   
   T   
   G   
   A   
   C   
   G   
   A   
   C   
   C   
   A   
   A   
   T   
   G   
   A   
   A   
   T   
   T   
   G   
   A   
   T   
   G   
   G   
   A   
   A   
   G   
   T   
   A   
   T   
   T   
   A   
   C   
   C   
   T   
   G   
   G   
   A   
   C   
   C   
   A   
   G   
   A   
   T   
   T   
   T   
   T   
   C   
   C   
   A   
   A   
   C   
   A   
   G   
   G   
   T   
   G   
   G   
   T   
   T   
   T   
   G   
   G   
   T   
   C   
   A   
   T   
   G   
   G   
   G   
   A   
   A   
   A   
   A   
   T   
   C   
   A   
   G   
   G   
    A    
   A   
   T   
    C    
   C   
   G   
    A    
    A    
   G   
   A   
   G   
   C   
    G    
   T   
   A   
   T   
   G   
   A   
   A   
   A   
   C   
    A    
   G   
   G   
    T    
   A   
   A   
    A    
   G   
   G   
   T   
   T   
   C   
    T    
   A   
   T   
   T   
   A   
   C   
    C    
   G   
   T   
    A    
   C   
   G   
   T   
   G   
   C   
   A   
   A   
   A   
   A   
   G   
   T   
   T   
   G   
   A   
    G    
    A    
   T   
    C    
   A   
    C    
   T   
   G   
   A   
    G    
   A   
   T   
   G   
    C    
   C   
    A    
   A   
   A   
   T   
   G   
   G   
    C    
   A   
   A   
   A   
   G   
   A   
   A   
   C   
   G   
    G    
   A   
   T   
    C    
    C    
   T   
    C    
   G   
   T   
    G    
   A   
   C   
    G    
   G   
   A   
   A   
   T   
   T   
    A    
   C   
   C   
   T   
   T   
   A   
   T   
   A   
   T   
   G   
   G   
   T   
    C    
   A   
   A   
   T   
   A   
   A   
   A   
   G   
   C   
    A    
   A   
   A   
   A   
   T   
   T   
    G    
   A   
   T   
    T    
   G   
   A   
   A   
   C   
   G   
    G    
   A   
   T   
   T   
   T   
   C   
   T   
   G   
   A   
    G    
    T    
   T   
    G    
   C   
   A   
   C   
   A   
   G   
   A   
   G   
   A   
    C    
   A   
   A   
    G    
   C   
   G   
    T    
   A   
   T   
    C    
   G   
   A   
   A   
   G   
   G   
    A    
   A   
   T   
    T    
   A   
   C   
    G    
   G   
   A   
   T   
   T   
   T   
   G   
   C   
   G   
   G   
   G   
   A   
    T    
   G   
   A   
   A   
   T   
   C   
   T   
   T   
   C   
    C    
   C   
   G   
   T   
   G   
   A   
    A    
   G   
   G   
    C    
   A   
   T   
   G   
   C   
   G   
    A    
   A   
   T   
    T    
   G   
   T   
   C   
   A   
   T   
    T    
   G   
   A   
    C    
    G    
   T   
    T    
   C   
   G   
   T   
   C   
   G   
    T    
   G   
   A   
    C    
   G   
    C    
    C    
   A   
   G   
    C    
   G   
   C   
    A    
   T   
   C   
    A    
   G   
   T   
   T   
    A    
   T   
    T    
   T   
   T   
    G    
   A   
   A   
    T    
   A   
   A   
   C   
   T   
   T   
    A    
   T   
   A   
    C    
   A   
   A   
   A   
    T    
   T   
   G   
   A   
   C   
    A    
    T    
   C   
    C    
   T   
   T   
    G    
   C   
   A   
    G    
   A   
    A    
    T    
   T   
   C   
    A    
   T   
   T   
    C    
   G   
   G   
    A    
   T   
   T   
    C    
   A   
   A   
    T    
   A   
   T   
   G   
   T   
   T   
    G    
   G   
   C   
    A    
   A   
   T   
    C    
   G   
   A   
   A   
   A   
   A   
   A   
   G   
   G   
    C    
   G   
   T   
    G    
   C   
   C   
    G    
   A   
   A   
   A   
    G    
   T   
   T   
   T   
   T   
    A    
   A   
   G   
    T    
   T   
   T   
   G   
   A   
   A   
   A   
   C   
    A    
    A    
   A   
   T   
    C    
    C    
   T   
    T    
   G   
   A   
   A   
   A   
   A   
    T    
   T   
   A   
    T    
    A    
   T   
    C    
   G   
   A   
    A    
   C   
   A   
    T    
   C   
   A   
    A    
    C    
    G    
    C    
    G    
   A   
   A   
   G   
   T   
   C   
   A   
   T   
    C    
   A   
   C   
    A    
    C    
   G   
    A    
   C   
   G   
   G   
   A   
   C   
    G    
    G    
   T   
    C    
   T   
   T   
   T   
   G   
   A   
    A    
   A   
   A   
   A   
    G    
   A   
    A    
   A   
   A   
    A    
   G   
   C   
   A   
   G   
   A   
   A   
   G   
   C   
   A   
    A    
   G   
    A    
   G   
   C   
    A    
   C   
   A   
    C    
   A   
   T   
    C    
   T   
   T   
   A   
   G   
   A   
   A   
   G   
   G   
   T   
    T    
   T   
    G    
   C   
   G   
    T    
   A   
   T   
   T   
   G   
   C   
    T    
   T   
   T   
    G    
   G   
   A   
   T   
   C   
   A   
   T   
   A   
   T   
   C   
    G    
   A   
   T   
   G   
   A   
   A   
   A   
   T   
    C    
   A   
   T   
   T   
   G   
   C   
    G    
   A   
   T   
   T   
   A   
   T   
    C    
   C   
   G   
    C    
   G   
   G   
    G    
   T   
   C   
    T    
    A    
   A   
   A   
   T   
   C   
   A   
   G   
   A   
   T   
   G   
   A   
    C    
   G   
   A   
    A    
   G   
   C   
   A   
   A   
   A   
   A   
   G   
   C   
    A    
   A   
    C    
    A    
    A    
   T   
    G    
   A   
   T   
    C    
   G   
   A   
   A   
   C   
   G   
    G    
   T   
   T   
    C    
   G   
   A   
    T    
   T   
   T   
    A    
   T   
   C   
    C    
   G   
   A   
   T   
   C   
   G   
    C    
   C   
   A   
    A    
    T    
   C   
    A    
   C   
   A   
   A   
   G   
   C   
    T    
   A   
   T   
    C    
   T   
   T   
   A   
   G   
   A   
   T   
   A   
   T   
   G   
   C   
   G   
   T   
   T   
   T   
    G    
   C   
   G   
    T    
   C   
   G   
    A    
   T   
   T   
    G    
   A   
   C   
    C    
   G   
   G   
    T    
   T   
   T   
    G    
   G   
   A   
   A   
   C   
   G   
    G    
   G   
   A   
    A    
   A   
   A   
   A   
   A   
   T   
    C    
   G   
   A   
    A    
   A   
   A   
   T   
   G   
   A   
   A   
   T   
   A   
    T    
   C   
   A   
   A   
   G   
   A   
    A    
   C   
   T   
    A    
   T   
   T   
    G    
   A   
    A    
   A   
    C    
   T   
    G    
   A   
   T   
   T   
   G   
    A    
    A    
   G   
   A   
    T    
   T   
   T   
    G    
    A    
    C    
    T    
   G   
   A   
   C   
   A   
   T   
   T   
    C    
   T   
    G    
   G   
   C   
    A    
    A    
   G   
    A    
   C   
   C   
    C    
   G   
   A   
    A    
   C   
   G   
    T    
   G   
   T   
    C    
   A   
    C    
    A    
   G   
   A   
   A   
   A   
   T   
    C    
   A   
   T   
    C    
   A   
   A   
    G    
   A   
    A    
    T    
   G   
   A   
    A    
   T   
   T   
   A   
    G    
    C    
    A    
   G   
   A   
   C   
    T    
   T   
    G    
    A    
    A    
    T    
    C    
    A    
   A   
    C    
    G    
   A   
   T   
   T   
   T   
   G   
   G   
   T   
   G   
   A   
   T   
    A    
    A    
   A   
   C   
   G   
   A   
   C   
   G   
    C    
   A   
   C   
   A   
   G   
   A   
   A   
    T    
   T   
   A   
    C    
   T   
   A   
   G   
   T   
    C    
   G   
   G   
   T   
   G   
   A   
    G    
   G   
   T   
   C   
    T    
   T   
    A    
   A   
   G   
   T   
   C   
   T   
    A    
   G   
   A   
   A   
   G   
   A   
   T   
   G   
   A   
   A   
   G   
   A   
   T   
    T    
   T   
    G    
   A   
   T   
    T    
   G   
   A   
   A   
   G   
   A   
   A   
   G   
   A   
   A   
   G   
   A   
   A   
    A    
   T   
   C   
   G   
   T   
    T    
   A   
   T   
   T   
   A   
   C   
    A    
    T    
   T   
    G    
   A   
   C   
    A    
   A   
   A   
    C    
   A   
   A   
    T    
   G   
   G   
    T    
   T   
   A   
    T    
   A   
   T   
    C    
   A   
   A   
    A    
   C   
   G   
    T    
    T    
   T   
   G   
   G   
    C    
   A   
   A   
   A   
    T    
    A    
    A    
    T    
   G   
   A   
   A   
   T   
   T   
   C   
    A    
   G   
    A    
   G   
   C   
    T    
   C   
   A   
   A   
   C   
   G   
    A    
    A    
   G   
    A    
   G   
   G   
    C    
   G   
   G   
    G    
   C   
   G   
   T   
   G   
   G   
    T    
   G   
   T   
    C    
   C   
   A   
   A   
   G   
   G   
    A    
   A   
   T   
   G   
   G   
   G   
    T    
    A    
   T   
    C    
   C   
   A   
    C    
    A    
   A   
   T   
   G   
   A   
    C    
   G   
   A   
    C    
   T   
   T   
   C   
   G   
   T   
    C    
   A   
   A   
    G    
   A   
   A   
    T    
   C   
   T   
    A    
    G    
   T   
   T   
   T   
   C   
   T   
   T   
   G   
    T    
   T   
   C   
    T    
   A   
   C   
    C    
   C   
   A   
    T    
   G   
   A   
    T    
   A   
   C   
    A    
   T   
   T   
    G    
   T   
   T   
   A   
   T   
   T   
    C    
   T   
   T   
   T   
   A   
   C   
   G   
   A   
   A   
    T    
   A   
    A    
    C    
   G   
   G   
    A    
   A   
   A   
   A   
   G   
   T   
    G    
   T   
   A   
    T    
   C   
   G   
    G    
   G   
   C   
   A   
   A   
   A   
   A   
   G   
   G   
    A    
   T   
   A   
    C    
   G   
   A   
   A   
   A   
   T   
    T    
   C   
   C   
    A    
   G   
   A   
    A    
   T   
   A   
   C   
   G   
   G   
    G    
    C    
   G   
    T    
   A   
   C   
   A   
   G   
   C   
    C    
   A   
   A   
   A   
   G   
   G   
    A    
   A   
   T   
    C    
   C   
   C   
   A   
    A    
   T   
    C    
   A   
   T   
    C    
   A   
   A   
    T    
   T   
   T   
    G    
   C   
   T   
    G    
   G   
   G   
    T    
   A   
   T   
    C    
   G   
   A   
    C    
   T   
   C   
    G    
    A    
    G    
    T    
   G   
   A   
   A   
   A   
    G    
    C    
   A   
   T   
    C    
   C   
   A   
    A    
   G   
   C   
    A    
   A   
   T   
    T    
   A   
   T   
   T   
    G    
   C   
    G    
   G   
   T   
    G    
    A    
    C    
   G   
   G   
   G   
    A    
    G    
   A   
   A   
   G   
   C   
    A    
   G   
   A   
   A   
   G   
    A    
    A    
   G   
   G   
    C    
    C    
   A   
    C    
   T   
   A   
    T    
    C    
   T   
    T    
   T   
   T   
   C   
   T   
   T   
    T    
   A   
   C   
    G    
   A   
   C   
    G    
    C    
    G    
    T    
    C    
   A   
   A   
   G   
   G   
    T    
   A   
   C   
    A    
   G   
   T   
    T    
   A   
   A   
   A   
   C   
   G   
    T    
   A   
   C   
    G    
    T    
   C   
    T    
   G   
   T   
    C    
   A   
   C   
    T    
   G   
   C   
    T    
   T   
   T   
    C    
    G    
   C   
   T   
   A   
   A   
    C    
   A   
   T   
    T    
   C   
   G   
    A    
   A   
   G   
    C    
   A   
   A   
   T   
   G   
   G   
    C    
    C    
   T   
    G    
   A   
   T   
    T    
   G   
   C   
    A    
   A   
   T   
   T   
    G    
   G   
    T    
   T   
   T   
    G    
   A   
   A   
   A   
   G   
   A   
   A   
   G   
   A   
    T    
   G   
   A   
    C    
   G   
   A   
    A    
    T    
   T   
    A    
    G    
   T   
    C    
   A   
   A   
    T    
   G   
   T   
    T    
    C    
   T   
   A   
    T    
    T    
   G   
   A   
   C   
    A    
    G    
   A   
    C    
   G   
   G   
    A    
    C    
   A   
    G    
    T    
    C    
    A    
   A   
   A   
    T    
   A   
   T   
    C    
   A   
   T   
    C    
   A   
   T   
    T    
   G   
   G   
    C    
   A   
   C   
   A   
   C   
   A   
   T   
    A    
    A    
    C    
   G   
   G   
    C    
   T   
   A   
   C   
   T   
   C   
   T   
   G   
   T   
    G    
   A   
   C   
    C    
   T   
   T   
    C    
    A    
   A   
    A    
   G   
   A   
    A    
    G    
    A    
    A    
    G    
   C   
    A    
   G   
    T    
    C    
   C   
   G   
    C    
   G   
   A   
   T   
   A   
   T   
   G   
   G   
   G   
    A    
   C   
   G   
    T    
   A   
   C   
   A   
   G   
   C   
    T    
   T   
   C   
    C    
   G   
   G   
    C    
   G   
   T   
    C    
    A    
   G   
    A    
   G   
   G   
    G    
   A   
   T   
    A    
   C   
   G   
    T    
    C    
   T   
    G    
   C   
   G   
    T    
   G   
   A   
    G    
    G    
   A   
    A    
   G   
   A   
    T    
   T   
    A    
    C    
   G   
   T   
    C    
   G   
   T   
    T    
   G   
   G   
    G    
    G    
   C   
   A   
   G   
   C   
    C    
    T    
   T   
    G    
    A    
   T   
   G   
    A    
   A   
    G    
   G   
    A    
   A   
    G    
   A   
    C    
    C    
   A   
   A   
   G   
   A   
   A   
   G   
   T   
    A    
    C    
   T   
    G    
   G   
   T   
    G    
   A   
   T   
    T    
   A   
   C   
    A    
   G   
   A   
   A   
   A   
   A   
    A    
   G   
   G   
   T   
   T   
   A   
    C    
   G   
   G   
    A    
   A   
   A   
    A    
   C   
   G   
    G    
   A   
   C   
    C    
   A   
   A   
   A   
   G   
    T    
    A    
   T   
   C   
    G    
   G   
   A   
    A    
   T   
   A   
   T   
   C   
   C   
   A   
   G   
   T   
    G    
   A   
   A   
    A    
   G   
   G   
    C    
   C   
   G   
    C    
   G   
   G   
    A    
   G   
   G   
   T   
   A   
   A   
   A   
   G   
   G   
    G    
   A   
   T   
    C    
   A   
   A   
    A    
   A   
   C   
    G    
    G    
   C   
    G    
   A   
   A   
    T    
   A   
   T   
   C   
   A   
   C   
   T   
   G   
   A   
    A    
   A   
   A   
   A   
   A   
   A   
   T   
   G   
   G   
   T   
   C   
   C   
   A   
    C    
   T   
   A   
   G   
   C   
    C    
   G   
   G   
    A    
   T   
   T   
    G    
   A   
   C   
   C   
   A   
   C   
    T    
   G   
   T   
    T    
    T    
    C    
   T   
   G   
   G   
    A    
    A    
   A   
   T   
   G   
   A   
   A   
   G   
   A   
    C    
   A   
   T   
   C   
    A    
   T   
    G    
    C    
   T   
    G    
   A   
   T   
    T    
   A   
   C   
    A    
    G    
   A   
    T    
   A   
   A   
   A   
   G   
   G   
    T    
   G   
   T   
    G    
   A   
   T   
   T   
   A   
   T   
   C   
   C   
   G   
    T    
   T   
   T   
    C    
   A   
   A   
   C   
   G   
   T   
    A    
    T    
    C    
    G    
    A    
   C   
   T   
   G   
   T   
   T   
   T   
   C   
   T   
   C   
   A   
   A   
   A   
   C   
    T    
   G   
   G   
   A   
   C   
   G   
    T    
    T    
   C   
   A   
   A   
   C   
   A   
   T   
   T   
    G    
   G   
   G   
    T    
   G   
   T   
    A    
   C   
   G   
   T   
   T   
   T   
    G    
   A   
   T   
   G   
   A   
    A    
    G    
   A   
   T   
   G   
   G   
   A   
   A   
   G   
    C    
    T    
   G   
    A    
    T    
    A    
   C   
    G    
   A   
   A   
   A   
   G   
   T   
   C   
   G   
   T   
   C   
   A   
   C   
   A   
   A   
   T   
   G   
   -   
   -   
   -   
   -   
   -   
   -   
   -   
   -   
   -   
   -   
   -   
   -   
   -   
   -   
   -   
   -   
   -   
   -   
   -   
   -   
   -   
   -   
   -   
   -   
   -   
   -   
   -   
   -   
   -   
   -   
   -   
   -   
   -   
   -   
   -   
   -   
   -   
   -   
   -   
   -   
   -   
   -   
   -   
   -   
   -   
   -   
   -   
   -   
   -   
   -   
   -   
   -   
   -   
   -   
   -   
   -   
   -   
   -   
   -   
   -   
 

 
   N54121   
   A   
   T   
   G   
   A   
   G   
   T   
   G   
   A   
   A   
   G   
   A   
   A   
   A   
   T   
   T   
   A   
   A   
   A   
   G   
   A   
   A   
   A   
   A   
   C   
   A   
   T   
   T   
   C   
   A   
   A   
   G   
   A   
   C   
   G   
   T   
   C   
   A   
   A   
   T   
   C   
   T   
   G   
   A   
   C   
   C   
   A   
   G   
   C   
   G   
   A   
   A   
   A   
   T   
   G   
   A   
   A   
   A   
   G   
   A   
   A   
   T   
   C   
   T   
   T   
   T   
   C   
   A   
   T   
   T   
   G   
   A   
   C   
   T   
   A   
   C   
   G   
   C   
   A   
   A   
   T   
   G   
   A   
   G   
   T   
   G   
   T   
   T   
   A   
   T   
   C   
   G   
   T   
   A   
   G   
   C   
   C   
   C   
   G   
   C   
   G   
   C   
   G   
   T   
   T   
   A   
   C   
   C   
   T   
   G   
   A   
   C   
   G   
   T   
   T   
   C   
   G   
   T   
   G   
   A   
   C   
   G   
   G   
   T   
   T   
   T   
   A   
   A   
   A   
   A   
   C   
   C   
   T   
   G   
   T   
   T   
   C   
   A   
   T   
   C   
   G   
   C   
   C   
   G   
   A   
   A   
   T   
   C   
   T   
   T   
   A   
   T   
   A   
   T   
   G   
   G   
   A   
   A   
   T   
   G   
   A   
   A   
   C   
   G   
   A   
   A   
   T   
   T   
   A   
   G   
   G   
   G   
   G   
   T   
   A   
   A   
   C   
   C   
   C   
   C   
   T   
   G   
   A   
   T   
   A   
   A   
   A   
   C   
   C   
   A   
   C   
   A   
   C   
   A   
   A   
   A   
   A   
   A   
   A   
   T   
   C   
   A   
   G   
   C   
   C   
   C   
   G   
   G   
   A   
   T   
   T   
   G   
   T   
   T   
   G   
   G   
   G   
   G   
   A   
   T   
   G   
   T   
   T   
   A   
   T   
   G   
   G   
   G   
   T   
   A   
   A   
   A   
   T   
   A   
   T   
   C   
   A   
   C   
   C   
   C   
   C   
   C   
   A   
   T   
   G   
   G   
   G   
   G   
   A   
   C   
   A   
   G   
   T   
   G   
   C   
   G   
   A   
   T   
   T   
   T   
   A   
   C   
   G   
   A   
   A   
   T   
   C   
   A   
   A   
   T   
   G   
   G   
   T   
   G   
   C   
   G   
   G   
   A   
   T   
   G   
   G   
   C   
   A   
   C   
   A   
   A   
   C   
   C   
   T   
   T   
   T   
   T   
   A   
   G   
   T   
   T   
   A   
   T   
   C   
   G   
   G   
   G   
   C   
   T   
   A   
   T   
   G   
   T   
   T   
   A   
   G   
   T   
   T   
   G   
   A   
   C   
   G   
   G   
   C   
   C   
   A   
   C   
   G   
   G   
   A   
   A   
   A   
   C   
   T   
   T   
   C   
   G   
   G   
   T   
   T   
   C   
   C   
   G   
   T   
   C   
   G   
   A   
   T   
   G   
   G   
   T   
   G   
   A   
   C   
   G   
   G   
   C   
   G   
   C   
   T   
   G   
   C   
   C   
   G   
   C   
   T   
   A   
   T   
   G   
   C   
   G   
   T   
   T   
   A   
   T   
   A   
   C   
   C   
   G   
   A   
   A   
   G   
   C   
   A   
   C   
   G   
   T   
   A   
   T   
   G   
   A   
   G   
   T   
   A   
   A   
   A   
   A   
   T   
   T   
   G   
   C   
   T   
   T   
   T   
   A   
   G   
   A   
   A   
   A   
   T   
   G   
   C   
   T   
   A   
   C   
   G   
   A   
   G   
   A   
   T   
   A   
   T   
   T   
   A   
   A   
   C   
   A   
   A   
   A   
   A   
   A   
   T   
   A   
   C   
   A   
   G   
   T   
   C   
   G   
   A   
   T   
   T   
   T   
   C   
   C   
   A   
   A   
   G   
   G   
   A   
   A   
   A   
   C   
   T   
   A   
   T   
   G   
   A   
   T   
   G   
   A   
   T   
   T   
   C   
   A   
   G   
   A   
   A   
   C   
   A   
   A   
   G   
   A   
   A   
   C   
   C   
   A   
   G   
   T   
   A   
   G   
   T   
   T   
   T   
   T   
   A   
   C   
   C   
   T   
   G   
   C   
   T   
   C   
   G   
   T   
   T   
   T   
   T   
   C   
   C   
   A   
   A   
   A   
   C   
   T   
   T   
   A   
   C   
   T   
   A   
   G   
   T   
   T   
   A   
   A   
   C   
   G   
   G   
   A   
   A   
   C   
   A   
   A   
   C   
   G   
   G   
   G   
   G   
   A   
   T   
   T   
   G   
   C   
   G   
   G   
   T   
   G   
   G   
   G   
   G   
   A   
   T   
   G   
   G   
   C   
   A   
   A   
   C   
   A   
   A   
   A   
   T   
   A   
   T   
   T   
   C   
   C   
   A   
   C   
   C   
   A   
   C   
   A   
   C   
   A   
   A   
   T   
   T   
   T   
   A   
   A   
   G   
   T   
   G   
   A   
   A   
   G   
   T   
   G   
   A   
   T   
   T   
   G   
   A   
   T   
   G   
   C   
   A   
   A   
   C   
   A   
   A   
   G   
   T   
   T   
   T   
   A   
   T   
   T   
   G   
   A   
   T   
   G   
   G   
   A   
   C   
   A   
   A   
   T   
   C   
   C   
   T   
   G   
   A   
   T   
   G   
   T   
   A   
   A   
   C   
   G   
   A   
   C   
   G   
   A   
   A   
   T   
   G   
   A   
   A   
   T   
   T   
   G   
   A   
   T   
   G   
   G   
   A   
   A   
   G   
   T   
   G   
   T   
   T   
   A   
   C   
   C   
   T   
   G   
   G   
   A   
   C   
   C   
   A   
   G   
   A   
   T   
   T   
   T   
   T   
   C   
   C   
   A   
   A   
   C   
   A   
   G   
   G   
   C   
   G   
   G   
   T   
   T   
   T   
   A   
   G   
   T   
   G   
   A   
   T   
   G   
   G   
   G   
   G   
   A   
   A   
   A   
   T   
   C   
   A   
   G   
   G   
    G    
   A   
   T   
    T    
   C   
   G   
    C    
    C    
   G   
   A   
   G   
   C   
    A    
   T   
   A   
   T   
   G   
   A   
   A   
   A   
   C   
    G    
   G   
   G   
    G    
   A   
   A   
    A    
   G   
   G   
   T   
   T   
   C   
    G    
   A   
   T   
   T   
   A   
   C   
    A    
   G   
   T   
    T    
   C   
   G   
   T   
   G   
   C   
   A   
   A   
   A   
   A   
   G   
   T   
   T   
   G   
   A   
    A    
    T    
   T   
    G    
   A   
    C    
   T   
   G   
   A   
    A    
   A   
   T   
   G   
    C    
   C   
    G    
   A   
   A   
   T   
   G   
   G   
    A    
   A   
   A   
   A   
   G   
   A   
   A   
   C   
   G   
    T    
   A   
   T   
    T    
    T    
   T   
    A    
   G   
   T   
    A    
   A   
   C   
    T    
   G   
   A   
   A   
   T   
   T   
    G    
   C   
   C   
   T   
   T   
   A   
   T   
   A   
   T   
   G   
   G   
   T   
    G    
   A   
   A   
   T   
   A   
   A   
   A   
   G   
   C   
    C    
   A   
   A   
   A   
   T   
   T   
    A    
   A   
   T   
    C    
   G   
   A   
   A   
   C   
   G   
    A    
   A   
   T   
   T   
   T   
   C   
   T   
   G   
   A   
    A    
    T    
   T   
    A    
   C   
   A   
   C   
   A   
   G   
   A   
   G   
   A   
    T    
   A   
   A   
    A    
   C   
   G   
    A    
   A   
   T   
    T    
   G   
   A   
   A   
   G   
   G   
    A    
   A   
   T   
    T    
   A   
   C   
    T    
   G   
   A   
   T   
   T   
   T   
   G   
   C   
   G   
   G   
   G   
   A   
    T    
   G   
   A   
   A   
   T   
   C   
   T   
   T   
   C   
    T    
   C   
   G   
   T   
   G   
   A   
    A    
   G   
   G   
    C    
   A   
   T   
   G   
   C   
   G   
    G    
   A   
   T   
    C    
   G   
   T   
   C   
   A   
   T   
    C    
   G   
   A   
    T    
    G    
   T   
    T    
   C   
   G   
   T   
   C   
   G   
    A    
   G   
   A   
    T    
   G   
    T    
    G    
   A   
   G   
    T    
   G   
   C   
    C    
   T   
   C   
    T    
   G   
   T   
   T   
    G    
   T   
    A    
   T   
   T   
    A    
   A   
   A   
    C    
   A   
   A   
   C   
   T   
   T   
    A    
   T   
   A   
    C    
   A   
   A   
   A   
    A    
   T   
   G   
   A   
   C   
    T    
    G    
   C   
    C    
   T   
   T   
    A    
   C   
   A   
    A    
   A   
    C    
    A    
   T   
   C   
    T    
   T   
   T   
    T    
   G   
   G   
    T    
   T   
   T   
    T    
   A   
   A   
    C    
   A   
   T   
   G   
   T   
   T   
    A    
   G   
   C   
    C    
   A   
   T   
    C    
   G   
   A   
   A   
   A   
   A   
   A   
   G   
   G   
    C    
   G   
   T   
    A    
   C   
   C   
    G    
   A   
   A   
   A   
    A    
   T   
   T   
   T   
   T   
    A    
   A   
   G   
    C    
   T   
   T   
   G   
   A   
   A   
   A   
   C   
    G    
    T    
   A   
   T   
    T    
    T    
   T   
    A    
   G   
   A   
   A   
   A   
   A   
    T    
   T   
   A   
    C    
    G    
   T   
    C    
   G   
   A   
    G    
   C   
   A   
    T    
   C   
   A   
    A    
    A    
    A    
    A    
    G    
   A   
   A   
   G   
   T   
   C   
   A   
   T   
    T    
   A   
   C   
    T    
    C    
   G   
    C    
   C   
   G   
   G   
   A   
   C   
    G    
    A    
   T   
    T    
   T   
   T   
   T   
   G   
   A   
    C    
   A   
   A   
   A   
    A    
   A   
    C    
   A   
   A   
    A    
   G   
   C   
   A   
   G   
   A   
   A   
   G   
   C   
   A   
    C    
   G   
    G    
   G   
   C   
    G    
   C   
   A   
    T    
   A   
   T   
    T    
   T   
   T   
   A   
   G   
   A   
   A   
   G   
   G   
   T   
    C    
   T   
    A    
   C   
   G   
    A    
   A   
   T   
   T   
   G   
   C   
    C    
   T   
   T   
    A    
   G   
   A   
   T   
   C   
   A   
   T   
   A   
   T   
   C   
    G    
   A   
   T   
   G   
   A   
   A   
   A   
   T   
    C    
   A   
   T   
   T   
   G   
   C   
    C    
   A   
   T   
   T   
   A   
   T   
    C    
   C   
   G   
    T    
   G   
   G   
    A    
   T   
   C   
    A    
    C    
   A   
   A   
   T   
   C   
   A   
   G   
   A   
   T   
   G   
   A   
    C    
   G   
   A   
    A    
   G   
   C   
   A   
   A   
   A   
   A   
   G   
   C   
    A    
   A   
    C    
    G    
    T    
   T   
    A    
   A   
   T   
    T    
   G   
   A   
   A   
   C   
   G   
    C    
   T   
   T   
    T    
   G   
   A   
    A    
   T   
   T   
    T    
   T   
   C   
    A    
   G   
   A   
   T   
   C   
   G   
    T    
   C   
   A   
    A    
    G    
   C   
    G    
   C   
   A   
   A   
   G   
   C   
    G    
   A   
   T   
    T    
   T   
   T   
   A   
   G   
   A   
   T   
   A   
   T   
   G   
   C   
   G   
   T   
   T   
   T   
    A    
   C   
   G   
    C    
   C   
   G   
    T    
   T   
   T   
    A    
   A   
   C   
    A    
   G   
   G   
    C    
   T   
   T   
    A    
   G   
   A   
   A   
   C   
   G   
    C    
   G   
   A   
    C    
   A   
   A   
   A   
   A   
   T   
    T    
   G   
   A   
    A    
   A   
   A   
   T   
   G   
   A   
   A   
   T   
   A   
    T    
   C   
   A   
   A   
   G   
   A   
    G    
   C   
   T   
    A    
   T   
   T   
    G    
   A   
    A    
   A   
    T    
   T   
    C    
   A   
   T   
   T   
   G   
    C    
    G    
   G   
   A   
    C    
   T   
   T   
    A    
    G    
    A    
    G    
   G   
   A   
   C   
   A   
   T   
   T   
    T    
   T   
    A    
   G   
   C   
    C    
    C    
   G   
    C    
   C   
   C   
    A    
   G   
   A   
    A    
   C   
   G   
    C    
   G   
   T   
    C    
   A   
    T    
    T    
   G   
   A   
   A   
   A   
   T   
    C    
   A   
   T   
    T    
   A   
   A   
    A    
   A   
    C    
    A    
   G   
   A   
    G    
   T   
   T   
   A   
    A    
    A    
    T    
   G   
   A   
   C   
    G    
   T   
    T    
    C    
    G    
    C    
    A    
    C    
   A   
    A    
    A    
   A   
   T   
   T   
   T   
   G   
   G   
   T   
   G   
   A   
   T   
    G    
    C    
   A   
   C   
   G   
   A   
   C   
   G   
    C    
   A   
   C   
   A   
   G   
   A   
   A   
    T    
   T   
   A   
    T    
   T   
   A   
   G   
   T   
    A    
   G   
   G   
   T   
   G   
   A   
    A    
   G   
   T   
   C   
    T    
   T   
    A    
   A   
   G   
   T   
   C   
   T   
    T    
   G   
   A   
   A   
   G   
   A   
   T   
   G   
   A   
   A   
   G   
   A   
   T   
    T    
   T   
    A    
   A   
   T   
    C    
   G   
   A   
   A   
   G   
   A   
   A   
   G   
   A   
   A   
   G   
   A   
   A   
    G    
   T   
   C   
   G   
   T   
    G    
   A   
   T   
   T   
   A   
   C   
    A    
    T    
   T   
    A    
   A   
   C   
    C    
   A   
   A   
    T    
   A   
   A   
    C    
   G   
   G   
    C    
   T   
   A   
    C    
   A   
   T   
    T    
   A   
   A   
    G    
   C   
   G   
    G    
    A    
   T   
   G   
   G   
    C    
   A   
   A   
   A   
    C    
    T    
    C    
    T    
   G   
   A   
   A   
   T   
   T   
   C   
    C    
   G   
    G    
   G   
   C   
    G    
   C   
   A   
   A   
   C   
   G   
    C    
    C    
   G   
    T    
   G   
   G   
    C    
   G   
   G   
    A    
   C   
   G   
   T   
   G   
   G   
    T    
   G   
   T   
    C    
   C   
   A   
   A   
   G   
   G   
    C    
   A   
   T   
   G   
   G   
   G   
    C    
    G    
   T   
    T    
   C   
   A   
    T    
    G    
   A   
   T   
   G   
   A   
    T    
   G   
   A   
    T    
   T   
   T   
   C   
   G   
   T   
    G    
   A   
   A   
    A    
   A   
   A   
    C    
   C   
   T   
    A    
    G    
   T   
   T   
   T   
   C   
   T   
   T   
   G   
    T    
   T   
   C   
    A    
   A   
   C   
    A    
   C   
   A   
    C    
   G   
   A   
    C    
   A   
   C   
    A    
   T   
   T   
    A    
   T   
   T   
   A   
   T   
   T   
    C    
   T   
   T   
   T   
   A   
   C   
   G   
   A   
   A   
    C    
   A   
    C    
    T    
   G   
   G   
    G    
   A   
   A   
   A   
   G   
   T   
    T    
   T   
   A   
    C    
   C   
   G   
    A    
   G   
   C   
   A   
   A   
   A   
   A   
   G   
   G   
    T    
   T   
   A   
    C    
   G   
   A   
   A   
   A   
   T   
    C    
   C   
   C   
    T    
   G   
   A   
    A    
   T   
   A   
   C   
   G   
   G   
    T    
    A    
   G   
    A    
   A   
   C   
   A   
   G   
   C   
    A    
   A   
   A   
   A   
   G   
   G   
    A    
   A   
   T   
    T    
   C   
   C   
   A   
    G    
   T   
    G    
   A   
   T   
    T    
   A   
   A   
    C    
   T   
   T   
    A    
   C   
   T   
    G    
   G   
   G   
    A    
   A   
   T   
    T    
   G   
   A   
    T    
   T   
   C   
    T    
    G    
    C    
    A    
   G   
   A   
   A   
   A   
    A    
    A    
   A   
   T   
    T    
   C   
   A   
    A    
   G   
   C   
    G    
   A   
   T   
    T    
   A   
   T   
   T   
    T    
   C   
    T    
   G   
   T   
    T    
    G    
    A    
   G   
   G   
   G   
    C    
    A    
   A   
   A   
   G   
   C   
    G    
   G   
   A   
   A   
   G   
    C    
    C    
   G   
   G   
    T    
    A    
   A   
    A    
   T   
   A   
    C    
    T    
   T   
    G    
   T   
   T   
   C   
   T   
   T   
    C    
   A   
   C   
    A    
   A   
   C   
    C    
    T    
    T    
    A    
    A    
   A   
   A   
   G   
   G   
    A    
   A   
   C   
    C    
   G   
   T   
    C    
   A   
   A   
   A   
   C   
   G   
    G    
   A   
   C   
    A    
    G    
   C   
    C    
   G   
   T   
    A    
   A   
   C   
    A    
   G   
   C   
    C    
   T   
   T   
    T    
    T    
   C   
   T   
   A   
   A   
    T    
   A   
   T   
    C    
   C   
   G   
    T    
   A   
   G   
    T    
   A   
   A   
   T   
   G   
   G   
    A    
    T    
   T   
    A    
   A   
   T   
    C    
   G   
   C   
    C    
   A   
   T   
   T   
    A    
   G   
    C    
   T   
   T   
    A    
   A   
   A   
   A   
   G   
   A   
   A   
   G   
   A   
    T    
   G   
   A   
    T    
   G   
   A   
    G    
    T    
   T   
    A    
    G    
   T   
    T    
   A   
   A   
    C    
   G   
   T   
    A    
    G    
   T   
   A   
    A    
    C    
   G   
   A   
   C   
    T    
    A    
   A   
    T    
   G   
   G   
    C    
    A    
   A   
    T    
    C    
    A    
    G    
   A   
   A   
    G    
   A   
   T   
    G    
   A   
   T   
    T    
   A   
   T   
    C    
   G   
   G   
    A    
   A   
   C   
   A   
   C   
   A   
   T   
    G    
    C    
    A    
   G   
   G   
    A    
   T   
   A   
   C   
   T   
   C   
   T   
   G   
   T   
    C    
   A   
   C   
    A    
   T   
   T   
    T    
    G    
   A   
    T    
   G   
   A   
    A    
    A    
    A    
    T    
    A    
   C   
    T    
   G   
    T    
    A    
   C   
   G   
    T    
   G   
   A   
   T   
   A   
   T   
   G   
   G   
   G   
    C    
   C   
   G   
    G    
   A   
   C   
   A   
   G   
   C   
    A    
   T   
   C   
    A    
   G   
   G   
    T    
   G   
   T   
    T    
    C    
   G   
    T    
   G   
   G   
    A    
   A   
   T   
    C    
   C   
   G   
    T    
    C    
   T   
    C    
   C   
   G   
    C    
   G   
   A   
    A    
    A    
   A   
    T    
   G   
   A   
    T    
   T   
    A    
    T    
   G   
   T   
    G    
   G   
   T   
    C    
   G   
   G   
    C    
    G    
   C   
   A   
   G   
   C   
    G    
    A    
   T   
    T    
    C    
   T   
   G   
    G    
   A   
    T    
   G   
    A    
   A   
    A    
   A   
    T    
    A    
   A   
   A   
   G   
   A   
   A   
   G   
   T   
    C    
    C    
   T   
    A    
   G   
   T   
    C    
   A   
   T   
    T    
   A   
   C   
    T    
   G   
   A   
   A   
   A   
   A   
    T    
   G   
   G   
   T   
   T   
   A   
    T    
   G   
   G   
    T    
   A   
   A   
    G    
   C   
   G   
    T    
   A   
   C   
    A    
   A   
   A   
   A   
   G   
    C    
    T    
   T   
   C   
    T    
   G   
   A   
    A    
   T   
   A   
   T   
   C   
   C   
   A   
   G   
   T   
    T    
   A   
   A   
    A    
   G   
   G   
    A    
   C   
   G   
    T    
   G   
   G   
    C    
   G   
   G   
   T   
   A   
   A   
   A   
   G   
   G   
    G    
   A   
   T   
    T    
   A   
   A   
    G    
   A   
   C   
    A    
    G    
   C   
    A    
   A   
   A   
    T    
   A   
   T   
   C   
   A   
   C   
   T   
   G   
   A   
    G    
   A   
   A   
   A   
   A   
   A   
   T   
   G   
   G   
   T   
   C   
   C   
   A   
    T    
   T   
   A   
   G   
   C   
    T    
   G   
   G   
    T    
   T   
   T   
    A    
   A   
   C   
   C   
   A   
   C   
    G    
   G   
   T   
    C    
    A    
    A    
   T   
   G   
   G   
    G    
    G    
   A   
   T   
   G   
   A   
   A   
   G   
   A   
    T    
   A   
   T   
   C   
    T    
   T   
    A    
    T    
   T   
    G    
   A   
   T   
    T    
   A   
   C   
    G    
    A    
   A   
    C    
   A   
   A   
   A   
   G   
   G   
    C    
   G   
   T   
    C    
   A   
   T   
   T   
   A   
   T   
   C   
   C   
   G   
    C    
   T   
   T   
    T    
   A   
   A   
   C   
   G   
   T   
    T    
    G    
    A    
    T    
    T    
   C   
   T   
   G   
   T   
   T   
   T   
   C   
   T   
   C   
   A   
   A   
   A   
   C   
    A    
   G   
   G   
   A   
   C   
   G   
    C    
    G    
   C   
   A   
   A   
   C   
   A   
   T   
   T   
    A    
   G   
   G   
    G    
   G   
   T   
    T    
   C   
   G   
   T   
   T   
   T   
    A    
   A   
   T   
   G   
   A   
    G    
    A    
   A   
   T   
   G   
   G   
   A   
   A   
   G   
    A    
    T    
   G   
    G    
    T    
    G    
   C   
    C    
   A   
   A   
   A   
   -   
   -   
   -   
   -   
   -   
   -   
   -   
   -   
   -   
   -   
   -   
   -   
   -   
   -   
   -   
   -   
   -   
   -   
   -   
   -   
   -   
   -   
   -   
   -   
   -   
   -   
   -   
   -   
   -   
   -   
   -   
   -   
   -   
   -   
   -   
   -   
   -   
   -   
   -   
   -   
   -   
   -   
   -   
   -   
   -   
   -   
   -   
   -   
   -   
   -   
   -   
   -   
   -   
   -   
   -   
   -   
   -   
   -   
   -   
   -   
   -   
   -   
   -   
   -   
   -   
   -   
   -   
   -   
   -   
   -   
   -   
   -   
 

 
   N53661   
   A   
   T   
   G   
   A   
   G   
   T   
   G   
   A   
   A   
   G   
   A   
   A   
   A   
   T   
   T   
   A   
   A   
   A   
   G   
   A   
   A   
   A   
   A   
   C   
   A   
   T   
   T   
   C   
   A   
   A   
   G   
   A   
   C   
   G   
   T   
   C   
   A   
   A   
   T   
   C   
   T   
   G   
   A   
   C   
   C   
   A   
   G   
   C   
   G   
   A   
   A   
   A   
   T   
   G   
   A   
   A   
   A   
   G   
   A   
   A   
   T   
   C   
   T   
   T   
   T   
   C   
   A   
   T   
   T   
   G   
   A   
   C   
   T   
   A   
   C   
   G   
   C   
   A   
   A   
   T   
   G   
   A   
   G   
   T   
   G   
   T   
   T   
   A   
   T   
   C   
   G   
   T   
   A   
   G   
   C   
   C   
   C   
   G   
   C   
   G   
   C   
   G   
   T   
   T   
   A   
   C   
   C   
   T   
   G   
   A   
   C   
   G   
   T   
   T   
   C   
   G   
   T   
   G   
   A   
   C   
   G   
   G   
   T   
   T   
   T   
   A   
   A   
   A   
   A   
   C   
   C   
   T   
   G   
   T   
   T   
   C   
   A   
   T   
   C   
   G   
   C   
   C   
   G   
   A   
   A   
   T   
   C   
   T   
   T   
   A   
   T   
   A   
   T   
   G   
   G   
   A   
   A   
   T   
   G   
   A   
   A   
   C   
   G   
   A   
   A   
   T   
   T   
   A   
   G   
   G   
   G   
   G   
   T   
   A   
   A   
   C   
   C   
   C   
   C   
   T   
   G   
   A   
   T   
   A   
   A   
   A   
   C   
   C   
   A   
   C   
   A   
   C   
   A   
   A   
   A   
   A   
   A   
   A   
   T   
   C   
   A   
   G   
   C   
   C   
   C   
   G   
   G   
   A   
   T   
   T   
   G   
   T   
   T   
   G   
   G   
   G   
   G   
   A   
   T   
   G   
   T   
   T   
   A   
   T   
   G   
   G   
   G   
   T   
   A   
   A   
   A   
   T   
   A   
   T   
   C   
   A   
   C   
   C   
   C   
   C   
   C   
   A   
   T   
   G   
   G   
   G   
   G   
   A   
   C   
   A   
   G   
   T   
   G   
   C   
   G   
   A   
   T   
   T   
   T   
   A   
   C   
   G   
   A   
   A   
   T   
   C   
   A   
   A   
   T   
   G   
   G   
   T   
   G   
   C   
   G   
   G   
   A   
   T   
   G   
   G   
   C   
   A   
   C   
   A   
   A   
   C   
   C   
   T   
   T   
   T   
   T   
   A   
   G   
   T   
   T   
   A   
   T   
   C   
   G   
   G   
   G   
   C   
   T   
   A   
   T   
   G   
   T   
   T   
   A   
   G   
   T   
   T   
   G   
   A   
   C   
   G   
   G   
   C   
   C   
   A   
   C   
   G   
   G   
   A   
   A   
   A   
   C   
   T   
   T   
   C   
   G   
   G   
   T   
   T   
   C   
   C   
   G   
   T   
   C   
   G   
   A   
   T   
   G   
   G   
   T   
   G   
   A   
   C   
   G   
   G   
   C   
   G   
   C   
   T   
   G   
   C   
   C   
   G   
   C   
   T   
   A   
   T   
   G   
   C   
   G   
   T   
   T   
   A   
   T   
   A   
   C   
   C   
   G   
   A   
   A   
   G   
   C   
   A   
   C   
   G   
   T   
   A   
   T   
   G   
   A   
   G   
   T   
   A   
   A   
   A   
   A   
   T   
   T   
   G   
   C   
   T   
   T   
   T   
   A   
   G   
   A   
   A   
   A   
   T   
   G   
   C   
   T   
   A   
   C   
   G   
   A   
   G   
   A   
   T   
   A   
   T   
   T   
   A   
   A   
   C   
   A   
   A   
   A   
   A   
   A   
   T   
   A   
   C   
   A   
   G   
   T   
   C   
   G   
   A   
   T   
   T   
   T   
   C   
   C   
   A   
   A   
   G   
   G   
   A   
   A   
   A   
   C   
   T   
   A   
   T   
   G   
   A   
   T   
   G   
   A   
   T   
   T   
   C   
   A   
   G   
   A   
   A   
   C   
   A   
   A   
   G   
   A   
   A   
   C   
   C   
   A   
   G   
   T   
   A   
   G   
   T   
   T   
   T   
   T   
   A   
   C   
   C   
   T   
   G   
   C   
   T   
   C   
   G   
   T   
   T   
   T   
   T   
   C   
   C   
   A   
   A   
   A   
   C   
   T   
   T   
   A   
   C   
   T   
   A   
   G   
   T   
   T   
   A   
   A   
   C   
   G   
   G   
   A   
   A   
   C   
   A   
   A   
   C   
   G   
   G   
   G   
   G   
   A   
   T   
   T   
   G   
   C   
   G   
   G   
   T   
   G   
   G   
   G   
   G   
   A   
   T   
   G   
   G   
   C   
   A   
   A   
   C   
   A   
   A   
   A   
   T   
   A   
   T   
   T   
   C   
   C   
   A   
   C   
   C   
   A   
   C   
   A   
   C   
   A   
   A   
   T   
   T   
   T   
   A   
   A   
   G   
   T   
   G   
   A   
   A   
   G   
   T   
   G   
   A   
   T   
   T   
   G   
   A   
   T   
   G   
   C   
   A   
   A   
   C   
   A   
   A   
   G   
   T   
   T   
   T   
   A   
   T   
   T   
   G   
   A   
   T   
   G   
   G   
   A   
   C   
   A   
   A   
   T   
   C   
   C   
   T   
   G   
   A   
   T   
   G   
   T   
   A   
   A   
   C   
   G   
   A   
   C   
   G   
   A   
   A   
   T   
   G   
   A   
   A   
   T   
   T   
   G   
   A   
   T   
   G   
   G   
   A   
   A   
   G   
   T   
   G   
   T   
   T   
   A   
   C   
   C   
   T   
   G   
   G   
   A   
   C   
   C   
   A   
   G   
   A   
   T   
   T   
   T   
   T   
   C   
   C   
   A   
   A   
   C   
   A   
   G   
   G   
   C   
   G   
   G   
   T   
   T   
   T   
   A   
   G   
   T   
   G   
   A   
   T   
   G   
   G   
   G   
   G   
   A   
   A   
   A   
   T   
   C   
   A   
   G   
   G   
    G    
   A   
   T   
    T    
   C   
   G   
    C    
    C    
   G   
   A   
   G   
   C   
    A    
   T   
   A   
   T   
   G   
   A   
   A   
   A   
   C   
    A    
   G   
   G   
    G    
   A   
   A   
    A    
   G   
   G   
   T   
   T   
   C   
    G    
   A   
   T   
   T   
   A   
   C   
    T    
   G   
   T   
    T    
   C   
   G   
   T   
   G   
   C   
   A   
   A   
   A   
   A   
   G   
   T   
   T   
   G   
   A   
    A    
    T    
   T   
    G    
   A   
    C    
   T   
   G   
   A   
    A    
   A   
   T   
   G   
    C    
   C   
    G    
   A   
   A   
   T   
   G   
   G   
    A    
   A   
   A   
   A   
   G   
   A   
   A   
   C   
   G   
    T    
   A   
   T   
    T    
    T    
   T   
    A    
   G   
   T   
    A    
   A   
   C   
    T    
   G   
   A   
   A   
   T   
   T   
    G    
   C   
   C   
   T   
   T   
   A   
   T   
   A   
   T   
   G   
   G   
   T   
    G    
   A   
   A   
   T   
   A   
   A   
   A   
   G   
   C   
    C    
   A   
   A   
   A   
   T   
   T   
    A    
   A   
   T   
    C    
   G   
   A   
   A   
   C   
   G   
    A    
   A   
   T   
   T   
   T   
   C   
   T   
   G   
   A   
    A    
    T    
   T   
    A    
   C   
   A   
   C   
   A   
   G   
   A   
   G   
   A   
    T    
   A   
   A   
    A    
   C   
   G   
    A    
   A   
   T   
    T    
   G   
   A   
   A   
   G   
   G   
    A    
   A   
   T   
    T    
   A   
   C   
    T    
   G   
   A   
   T   
   T   
   T   
   G   
   C   
   G   
   G   
   G   
   A   
    T    
   G   
   A   
   A   
   T   
   C   
   T   
   T   
   C   
    T    
   C   
   G   
   T   
   G   
   A   
    A    
   G   
   G   
    C    
   A   
   T   
   G   
   C   
   G   
    G    
   A   
   T   
    T    
   G   
   T   
   C   
   A   
   T   
    C    
   G   
   A   
    T    
    G    
   T   
    T    
   C   
   G   
   T   
   C   
   G   
    A    
   G   
   A   
    T    
   G   
    T    
    G    
   A   
   G   
    T    
   G   
   C   
    C    
   T   
   C   
    T    
   G   
   T   
   T   
    G    
   T   
    G    
   T   
   T   
    A    
   A   
   A   
    C    
   A   
   A   
   C   
   T   
   T   
    G    
   T   
   A   
    C    
   A   
   A   
   A   
    A    
   T   
   G   
   A   
   C   
    T    
    G    
   C   
    C    
   T   
   T   
    A    
   C   
   A   
    A    
   A   
    C    
    A    
   T   
   C   
    T    
   T   
   T   
    T    
   G   
   G   
    T    
   T   
   T   
    T    
   A   
   A   
    C    
   A   
   T   
   G   
   T   
   T   
    A    
   G   
   C   
    C    
   A   
   T   
    C    
   G   
   A   
   A   
   A   
   A   
   A   
   G   
   G   
    C    
   G   
   T   
    A    
   C   
   C   
    G    
   A   
   A   
   A   
    A    
   T   
   T   
   T   
   T   
    A    
   A   
   G   
    C    
   T   
   T   
   G   
   A   
   A   
   A   
   C   
    G    
    T    
   A   
   T   
    T    
    T    
   T   
    A    
   G   
   A   
   A   
   A   
   A   
    C    
   T   
   A   
    C    
    G    
   T   
    C    
   G   
   A   
    G    
   C   
   A   
    C    
   C   
   A   
    A    
    A    
    A    
    A    
    G    
   A   
   A   
   G   
   T   
   C   
   A   
   T   
    T    
   A   
   C   
    T    
    C    
   G   
    C    
   C   
   G   
   G   
   A   
   C   
    G    
    A    
   T   
    T    
   T   
   T   
   T   
   G   
   A   
    C    
   A   
   A   
   A   
    A    
   A   
    C    
   A   
   A   
    A    
   G   
   C   
   A   
   G   
   A   
   A   
   G   
   C   
   A   
    C    
   G   
    G    
   G   
   C   
    G    
   C   
   A   
    T    
   A   
   T   
    T    
   T   
   T   
   A   
   G   
   A   
   A   
   G   
   G   
   T   
    C    
   T   
    A    
   C   
   G   
    A    
   A   
   T   
   T   
   G   
   C   
    C    
   T   
   T   
    A    
   G   
   A   
   T   
   C   
   A   
   T   
   A   
   T   
   C   
    G    
   A   
   T   
   G   
   A   
   A   
   A   
   T   
    C    
   A   
   T   
   T   
   G   
   C   
    C    
   A   
   T   
   T   
   A   
   T   
    C    
   C   
   G   
    T    
   G   
   G   
    G    
   T   
   C   
    A    
    C    
   A   
   A   
   T   
   C   
   A   
   G   
   A   
   T   
   G   
   A   
    C    
   G   
   A   
    A    
   G   
   C   
   A   
   A   
   A   
   A   
   G   
   C   
    A    
   A   
    C    
    G    
    T    
   T   
    A    
   A   
   T   
    T    
   G   
   A   
   A   
   C   
   G   
    C    
   T   
   T   
    T    
   G   
   A   
    A    
   T   
   T   
    T    
   T   
   C   
    A    
   G   
   A   
   T   
   C   
   G   
    T    
   C   
   A   
    A    
    G    
   C   
    G    
   C   
   A   
   A   
   G   
   C   
    G    
   A   
   T   
    T    
   T   
   T   
   A   
   G   
   A   
   T   
   A   
   T   
   G   
   C   
   G   
   T   
   T   
   T   
    A    
   C   
   G   
    C    
   C   
   G   
    T    
   T   
   T   
    A    
   A   
   C   
    A    
   G   
   G   
    C    
   T   
   T   
    A    
   G   
   A   
   A   
   C   
   G   
    C    
   G   
   A   
    C    
   A   
   A   
   A   
   A   
   T   
    T    
   G   
   A   
    A    
   A   
   A   
   T   
   G   
   A   
   A   
   T   
   A   
    T    
   C   
   A   
   A   
   G   
   A   
    G    
   C   
   T   
    A    
   T   
   T   
    G    
   A   
    A    
   A   
    T    
   T   
    C    
   A   
   T   
   T   
   G   
    C    
    G    
   G   
   A   
    C    
   T   
   T   
    A    
    G    
    A    
    G    
   G   
   A   
   C   
   A   
   T   
   T   
    T    
   T   
    A    
   G   
   C   
    C    
    C    
   G   
    C    
   C   
   C   
    A    
   G   
   A   
    A    
   C   
   G   
    C    
   G   
   T   
    C    
   A   
    T    
    T    
   G   
   A   
   A   
   A   
   T   
    C    
   A   
   T   
    T    
   A   
   A   
    A    
   A   
    C    
    A    
   G   
   A   
    G    
   T   
   T   
   A   
    A    
    A    
    T    
   G   
   A   
   C   
    G    
   T   
    T    
    C    
    G    
    C    
    A    
    C    
   A   
    A    
    A    
   A   
   T   
   T   
   T   
   G   
   G   
   T   
   G   
   A   
   T   
    G    
    C    
   A   
   C   
   G   
   A   
   C   
   G   
    C    
   A   
   C   
   A   
   G   
   A   
   A   
    T    
   T   
   A   
    T    
   T   
   A   
   G   
   T   
    A    
   G   
   G   
   T   
   G   
   A   
    A    
   G   
   T   
   C   
    C    
   T   
    A    
   A   
   G   
   T   
   C   
   T   
    T    
   G   
   A   
   A   
   G   
   A   
   T   
   G   
   A   
   A   
   G   
   A   
   T   
    T    
   T   
    A    
   A   
   T   
    C    
   G   
   A   
   A   
   G   
   A   
   A   
   G   
   A   
   A   
   G   
   A   
   A   
    G    
   T   
   C   
   G   
   T   
    G    
   A   
   T   
   T   
   A   
   C   
    A    
    T    
   T   
    A    
   A   
   C   
    C    
   A   
   A   
    T    
   A   
   A   
    C    
   G   
   G   
    C    
   T   
   A   
    C    
   A   
   T   
    T    
   A   
   A   
    G    
   C   
   G   
    G    
    A    
   T   
   G   
   G   
    C    
   A   
   A   
   A   
    C    
    T    
    C    
    C    
   G   
   A   
   A   
   T   
   T   
   C   
    C    
   G   
    G    
   G   
   C   
    G    
   C   
   A   
   A   
   C   
   G   
    C    
    C    
   G   
    C    
   G   
   G   
    C    
   G   
   G   
    A    
   C   
   G   
   T   
   G   
   G   
    T    
   G   
   T   
    C    
   C   
   A   
   A   
   G   
   G   
    C    
   A   
   T   
   G   
   G   
   G   
    C    
    G    
   T   
    T    
   C   
   A   
    T    
    G    
   A   
   T   
   G   
   A   
    T    
   G   
   A   
    T    
   T   
   T   
   C   
   G   
   T   
    G    
   A   
   A   
    A    
   A   
   A   
    C    
   C   
   T   
    A    
    G    
   T   
   T   
   T   
   C   
   T   
   T   
   G   
    T    
   T   
   C   
    A    
   A   
   C   
    A    
   C   
   A   
    T    
   G   
   A   
    C    
   A   
   C   
    G    
   T   
   T   
    A    
   T   
   T   
   A   
   T   
   T   
    C    
   T   
   T   
   T   
   A   
   C   
   G   
   A   
   A   
    C    
   A   
    C    
    T    
   G   
   G   
    G    
   A   
   A   
   A   
   G   
   T   
    T    
   T   
   A   
    C    
   C   
   G   
    A    
   G   
   C   
   A   
   A   
   A   
   A   
   G   
   G   
    T    
   T   
   A   
    C    
   G   
   A   
   A   
   A   
   T   
    C    
   C   
   C   
    T    
   G   
   A   
    A    
   T   
   A   
   C   
   G   
   G   
    T    
    A    
   G   
    A    
   A   
   C   
   A   
   G   
   C   
    A    
   A   
   A   
   A   
   G   
   G   
    A    
   A   
   T   
    T    
   C   
   C   
   A   
    G    
   T   
    G    
   A   
   T   
    T    
   A   
   A   
    C    
   T   
   T   
    A    
   C   
   T   
    G    
   G   
   G   
    A    
   A   
   T   
    T    
   G   
   A   
    T    
   T   
   C   
    T    
    G    
    C    
    A    
   G   
   A   
   A   
   A   
    A    
    A    
   A   
   T   
    T    
   C   
   A   
    A    
   G   
   C   
    G    
   A   
   T   
    T    
   A   
   T   
   T   
    T    
   C   
    T    
   G   
   T   
    T    
    G    
    A    
   G   
   G   
   G   
    C    
    A    
   A   
   A   
   G   
   C   
    G    
   G   
   A   
   A   
   G   
    C    
    C    
   G   
   G   
    T    
    A    
   A   
    A    
   T   
   A   
    C    
    T    
   T   
    G    
   T   
   T   
   C   
   T   
   T   
    C    
   A   
   C   
    A    
   A   
   C   
    C    
    T    
    T    
    A    
    A    
   A   
   A   
   G   
   G   
    A    
   A   
   C   
    C    
   G   
   T   
    C    
   A   
   A   
   A   
   C   
   G   
    G    
   A   
   C   
    A    
    G    
   C   
    C    
   G   
   T   
    A    
   A   
   C   
    A    
   G   
   C   
    C    
   T   
   T   
    T    
    T    
   C   
   T   
   A   
   A   
    T    
   A   
   T   
    C    
   C   
   G   
    T    
   A   
   G   
    T    
   A   
   A   
   T   
   G   
   G   
    A    
    T    
   T   
    A    
   A   
   T   
    C    
   G   
   C   
    C    
   A   
   T   
   T   
    A    
   G   
    C    
   T   
   T   
    A    
   A   
   A   
   A   
   G   
   A   
   A   
   G   
   A   
    T    
   G   
   A   
    T    
   G   
   A   
    G    
    T    
   T   
    A    
    G    
   T   
    T    
   A   
   A   
    C    
   G   
   T   
    A    
    G    
   T   
   A   
    A    
    C    
   G   
   A   
   C   
    T    
    A    
   A   
    T    
   G   
   G   
    C    
    A    
   A   
    T    
    C    
    A    
    G    
   A   
   A   
    G    
   A   
   T   
    G    
   A   
   T   
    T    
   A   
   T   
    C    
   G   
   G   
    A    
   A   
   C   
   A   
   C   
   A   
   T   
    G    
    C    
    A    
   G   
   G   
    A    
   T   
   A   
   C   
   T   
   C   
   T   
   G   
   T   
    C    
   A   
   C   
    A    
   T   
   T   
    T    
    G    
   A   
    T    
   G   
   A   
    A    
    A    
    A    
    T    
    A    
   C   
    T    
   G   
    T    
    A    
   C   
   G   
    T    
   G   
   A   
   T   
   A   
   T   
   G   
   G   
   G   
    C    
   C   
   G   
    G    
   A   
   C   
   A   
   G   
   C   
    A    
   T   
   C   
    A    
   G   
   G   
    T    
   G   
   T   
    T    
    C    
   G   
    G    
   G   
   G   
    A    
   A   
   T   
    C    
   C   
   G   
    T    
    C    
   T   
    C    
   C   
   G   
    C    
   G   
   A   
    A    
    A    
   A   
    T    
   G   
   A   
    T    
   T   
    A    
    T    
   G   
   T   
    G    
   G   
   T   
    C    
   G   
   G   
    T    
    G    
   C   
   A   
   G   
   C   
    G    
    A    
   T   
    T    
    C    
   T   
   G   
    G    
   A   
    T    
   G   
    A    
   A   
    A    
   A   
    T    
    A    
   A   
   A   
   G   
   A   
   A   
   G   
   T   
    C    
    C    
   T   
    A    
   G   
   T   
    C    
   A   
   T   
    T    
   A   
   C   
    T    
   G   
   A   
   A   
   A   
   A   
    T    
   G   
   G   
   T   
   T   
   A   
    T    
   G   
   G   
    T    
   A   
   A   
    G    
   C   
   G   
    T    
   A   
   C   
    A    
   A   
   A   
   A   
   G   
    C    
    T    
   T   
   C   
    T    
   G   
   A   
    A    
   T   
   A   
   T   
   C   
   C   
   A   
   G   
   T   
    T    
   A   
   A   
    A    
   G   
   G   
    A    
   C   
   G   
    T    
   G   
   G   
    C    
   G   
   G   
   T   
   A   
   A   
   A   
   G   
   G   
    G    
   A   
   T   
    T    
   A   
   A   
    G    
   A   
   C   
    A    
    G    
   C   
    A    
   A   
   A   
    T    
   A   
   T   
   C   
   A   
   C   
   T   
   G   
   A   
    G    
   A   
   A   
   A   
   A   
   A   
   T   
   G   
   G   
   T   
   C   
   C   
   A   
    T    
   T   
   A   
   G   
   C   
    T    
   G   
   G   
    T    
   T   
   T   
    A    
   A   
   C   
   C   
   A   
   C   
    G    
   G   
   T   
    C    
    A    
    A    
   T   
   G   
   G   
    G    
    G    
   A   
   T   
   G   
   A   
   A   
   G   
   A   
    T    
   A   
   T   
   C   
    T    
   T   
    G    
    T    
   T   
    G    
   A   
   T   
    T    
   A   
   C   
    G    
    A    
   A   
    C    
   A   
   A   
   A   
   G   
   G   
    C    
   G   
   T   
    C    
   A   
   T   
   T   
   A   
   T   
   C   
   C   
   G   
    C    
   T   
   T   
    T    
   A   
   A   
   C   
   G   
   T   
    A    
    G    
    A    
    T    
    T    
   C   
   T   
   G   
   T   
   T   
   T   
   C   
   T   
   C   
   A   
   A   
   A   
   C   
    A    
   G   
   G   
   A   
   C   
   G   
    C    
    G    
   C   
   A   
   A   
   C   
   A   
   T   
   T   
    A    
   G   
   G   
    G    
   G   
   T   
    T    
   C   
   G   
   T   
   T   
   T   
    A    
   A   
   T   
   G   
   A   
    G    
    A    
   A   
   T   
   G   
   G   
   A   
   A   
   G   
    A    
    T    
   G   
    G    
    T    
    G    
   C   
    C    
   A   
   A   
   A   
   -   
   -   
   -   
   -   
   -   
   -   
   -   
   -   
   -   
   -   
   -   
   -   
   -   
   -   
   -   
   -   
   -   
   -   
   -   
   -   
   -   
   -   
   -   
   -   
   -   
   -   
   -   
   -   
   -   
   -   
   -   
   -   
   -   
   -   
   -   
   -   
   -   
   -   
   -   
   -   
   -   
   -   
   -   
   -   
   -   
   -   
   -   
   -   
   -   
   -   
   -   
   -   
   -   
   -   
   -   
   -   
   -   
   -   
   -   
   -   
   -   
   -   
   -   
   -   
   -   
   -   
   -   
   -   
   -   
   -   
   -   
   -   
 

 
   N52789   
   A   
   T   
   G   
   A   
   G   
   T   
   G   
   A   
   A   
   G   
   A   
   A   
   A   
   T   
   T   
   A   
   A   
   A   
   G   
   A   
   A   
   A   
   A   
   C   
   A   
   T   
   T   
   C   
   A   
   A   
   G   
   A   
   C   
   G   
   T   
   C   
   A   
   A   
   T   
   C   
   T   
   G   
   A   
   C   
   C   
   A   
   G   
   C   
   G   
   A   
   A   
   A   
   T   
   G   
   A   
   A   
   A   
   G   
   A   
   A   
   T   
   C   
   T   
   T   
   T   
   C   
   A   
   T   
   T   
   G   
   A   
   C   
   T   
   A   
   C   
   G   
   C   
   A   
   A   
   T   
   G   
   A   
   G   
   T   
   G   
   T   
   T   
   A   
   T   
   C   
   G   
   T   
   A   
   G   
   C   
   C   
   C   
   G   
   C   
   G   
   C   
   G   
   T   
   T   
   A   
   C   
   C   
   T   
   G   
   A   
   C   
   G   
   T   
   T   
   C   
   G   
   T   
   G   
   A   
   C   
   G   
   G   
   T   
   T   
   T   
   A   
   A   
   A   
   A   
   C   
   C   
   T   
   G   
   T   
   T   
   C   
   A   
   T   
   C   
   G   
   C   
   C   
   G   
   A   
   A   
   T   
   C   
   T   
   T   
   A   
   T   
   A   
   T   
   G   
   G   
   A   
   A   
   T   
   G   
   A   
   A   
   C   
   G   
   A   
   A   
   T   
   T   
   A   
   G   
   G   
   G   
   G   
   T   
   A   
   A   
   C   
   C   
   C   
   C   
   T   
   G   
   A   
   T   
   A   
   A   
   A   
   C   
   C   
   A   
   C   
   A   
   C   
   A   
   A   
   A   
   A   
   A   
   A   
   T   
   C   
   A   
   G   
   C   
   C   
   C   
   G   
   G   
   A   
   T   
   T   
   G   
   T   
   T   
   G   
   G   
   G   
   G   
   A   
   T   
   G   
   T   
   T   
   A   
   T   
   G   
   G   
   G   
   T   
   A   
   A   
   A   
   T   
   A   
   T   
   C   
   A   
   C   
   C   
   C   
   C   
   C   
   A   
   T   
   G   
   G   
   G   
   G   
   A   
   C   
   A   
   G   
   T   
   G   
   C   
   G   
   A   
   T   
   T   
   T   
   A   
   C   
   G   
   A   
   A   
   T   
   C   
   A   
   A   
   T   
   G   
   G   
   T   
   G   
   C   
   G   
   G   
   A   
   T   
   G   
   G   
   C   
   A   
   C   
   A   
   A   
   C   
   C   
   T   
   T   
   T   
   T   
   A   
   G   
   T   
   T   
   A   
   C   
   C   
   G   
   G   
   G   
   C   
   T   
   A   
   T   
   G   
   T   
   T   
   A   
   G   
   T   
   T   
   G   
   A   
   C   
   G   
   G   
   C   
   C   
   A   
   C   
   G   
   G   
   A   
   A   
   A   
   C   
   T   
   T   
   C   
   G   
   G   
   T   
   T   
   C   
   T   
   G   
   T   
   C   
   G   
   A   
   T   
   G   
   G   
   T   
   G   
   A   
   C   
   G   
   G   
   C   
   G   
   C   
   T   
   G   
   C   
   C   
   G   
   C   
   T   
   A   
   T   
   G   
   C   
   G   
   T   
   T   
   A   
   T   
   A   
   C   
   C   
   G   
   A   
   A   
   G   
   C   
   A   
   C   
   G   
   T   
   A   
   T   
   G   
   A   
   G   
   T   
   A   
   A   
   A   
   A   
   T   
   T   
   G   
   C   
   T   
   T   
   T   
   A   
   G   
   A   
   A   
   A   
   T   
   G   
   C   
   T   
   A   
   C   
   G   
   A   
   G   
   A   
   T   
   A   
   T   
   T   
   A   
   A   
   C   
   A   
   A   
   A   
   A   
   A   
   T   
   A   
   C   
   A   
   G   
   T   
   T   
   G   
   A   
   T   
   T   
   T   
   C   
   C   
   A   
   A   
   G   
   G   
   A   
   A   
   A   
   C   
   T   
   A   
   T   
   G   
   A   
   T   
   G   
   A   
   T   
   T   
   C   
   A   
   G   
   A   
   A   
   C   
   A   
   A   
   G   
   A   
   A   
   C   
   C   
   A   
   G   
   T   
   A   
   G   
   T   
   T   
   T   
   T   
   A   
   C   
   C   
   T   
   G   
   C   
   T   
   C   
   G   
   T   
   T   
   T   
   T   
   C   
   C   
   A   
   A   
   A   
   C   
   T   
   T   
   A   
   C   
   T   
   A   
   G   
   T   
   T   
   A   
   A   
   C   
   G   
   G   
   A   
   A   
   C   
   A   
   A   
   C   
   G   
   G   
   G   
   G   
   A   
   T   
   T   
   G   
   C   
   G   
   G   
   T   
   G   
   G   
   G   
   G   
   A   
   T   
   G   
   G   
   C   
   A   
   A   
   C   
   A   
   A   
   A   
   T   
   A   
   T   
   T   
   C   
   C   
   A   
   C   
   C   
   A   
   C   
   A   
   C   
   A   
   A   
   T   
   T   
   T   
   A   
   A   
   G   
   T   
   G   
   A   
   A   
   G   
   T   
   G   
   A   
   T   
   T   
   G   
   A   
   T   
   G   
   C   
   A   
   A   
   C   
   A   
   A   
   G   
   T   
   T   
   T   
   A   
   T   
   T   
   G   
   A   
   T   
   G   
   G   
   A   
   C   
   A   
   A   
   T   
   C   
   C   
   T   
   G   
   A   
   T   
   G   
   T   
   A   
   A   
   C   
   G   
   A   
   C   
   G   
   A   
   A   
   T   
   G   
   A   
   A   
   T   
   T   
   G   
   A   
   T   
   G   
   G   
   A   
   A   
   G   
   T   
   G   
   T   
   T   
   A   
   C   
   C   
   T   
   G   
   G   
   A   
   C   
   C   
   A   
   G   
   A   
   C   
   T   
   T   
   T   
   C   
   C   
   A   
   A   
   C   
   A   
   G   
   G   
   C   
   G   
   G   
   T   
   T   
   T   
   A   
   G   
   T   
   G   
   A   
   T   
   G   
   G   
   G   
   G   
   A   
   A   
   A   
   T   
   C   
   A   
   G   
   G   
    G    
   A   
   T   
    T    
   C   
   G   
    C    
    C    
   G   
   A   
   G   
   C   
    A    
   T   
   A   
   T   
   G   
   A   
   A   
   A   
   C   
    A    
   G   
   G   
    G    
   A   
   A   
    A    
   G   
   G   
   T   
   T   
   C   
    G    
   A   
   T   
   T   
   A   
   C   
    T    
   G   
   T   
    T    
   C   
   G   
   T   
   G   
   C   
   A   
   A   
   A   
   A   
   G   
   T   
   T   
   G   
   A   
    A    
    T    
   T   
    G    
   A   
    C    
   T   
   G   
   A   
    A    
   A   
   T   
   G   
    C    
   C   
    G    
   A   
   A   
   T   
   G   
   G   
    A    
   A   
   A   
   A   
   G   
   A   
   A   
   C   
   G   
    T    
   A   
   T   
    T    
    T    
   T   
    A    
   G   
   T   
    A    
   A   
   C   
    T    
   G   
   A   
   A   
   T   
   T   
    G    
   C   
   C   
   T   
   T   
   A   
   T   
   A   
   T   
   G   
   G   
   T   
    G    
   A   
   A   
   T   
   A   
   A   
   A   
   G   
   C   
    C    
   A   
   A   
   A   
   T   
   T   
    A    
   A   
   T   
    C    
   G   
   A   
   A   
   C   
   G   
    A    
   A   
   T   
   T   
   T   
   C   
   T   
   G   
   A   
    A    
    T    
   T   
    A    
   C   
   A   
   C   
   A   
   G   
   A   
   G   
   A   
    T    
   A   
   A   
    A    
   C   
   G   
    A    
   A   
   T   
    T    
   G   
   A   
   A   
   G   
   G   
    A    
   A   
   T   
    T    
   A   
   C   
    T    
   G   
   A   
   T   
   T   
   T   
   G   
   C   
   G   
   G   
   G   
   A   
    T    
   G   
   A   
   A   
   T   
   C   
   T   
   T   
   C   
    T    
   C   
   G   
   T   
   G   
   A   
    A    
   G   
   G   
    C    
   A   
   T   
   G   
   C   
   G   
    G    
   A   
   T   
    T    
   G   
   T   
   C   
   A   
   T   
    C    
   G   
   A   
    T    
    G    
   T   
    T    
   C   
   G   
   T   
   C   
   G   
    A    
   G   
   A   
    T    
   G   
    T    
    G    
   A   
   G   
    T    
   G   
   C   
    C    
   T   
   C   
    T    
   G   
   T   
   T   
    G    
   T   
    G    
   T   
   T   
    A    
   A   
   A   
    C    
   A   
   A   
   C   
   T   
   T   
    G    
   T   
   A   
    C    
   A   
   A   
   A   
    A    
   T   
   G   
   A   
   C   
    T    
    G    
   C   
    C    
   T   
   T   
    A    
   C   
   A   
    A    
   A   
    C    
    A    
   T   
   C   
    T    
   T   
   T   
    T    
   G   
   G   
    T    
   T   
   T   
    T    
   A   
   A   
    C    
   A   
   T   
   G   
   T   
   T   
    A    
   G   
   C   
    C    
   A   
   T   
    C    
   G   
   A   
   A   
   A   
   A   
   A   
   G   
   G   
    C    
   G   
   T   
    A    
   C   
   C   
    G    
   A   
   A   
   A   
    A    
   T   
   T   
   T   
   T   
    A    
   A   
   G   
    C    
   T   
   T   
   G   
   A   
   A   
   A   
   C   
    G    
    T    
   A   
   T   
    T    
    T    
   T   
    A    
   G   
   A   
   A   
   A   
   A   
    C    
   T   
   A   
    C    
    G    
   T   
    C    
   G   
   A   
    G    
   C   
   A   
    T    
   C   
   A   
    A    
    A    
    A    
    A    
    G    
   A   
   A   
   G   
   T   
   C   
   A   
   T   
    T    
   A   
   C   
    T    
    C    
   G   
    C    
   C   
   G   
   G   
   A   
   C   
    G    
    A    
   T   
    T    
   T   
   T   
   T   
   G   
   A   
    C    
   A   
   A   
   A   
    A    
   A   
    C    
   A   
   A   
    A    
   G   
   C   
   A   
   G   
   A   
   A   
   G   
   C   
   A   
    C    
   G   
    G    
   G   
   C   
    G    
   C   
   A   
    T    
   A   
   T   
    T    
   T   
   T   
   A   
   G   
   A   
   A   
   G   
   G   
   T   
    C    
   T   
    A    
   C   
   G   
    A    
   A   
   T   
   T   
   G   
   C   
    C    
   T   
   T   
    A    
   G   
   A   
   T   
   C   
   A   
   T   
   A   
   T   
   C   
    G    
   A   
   T   
   G   
   A   
   A   
   A   
   T   
    C    
   A   
   T   
   T   
   G   
   C   
    C    
   A   
   T   
   T   
   A   
   T   
    C    
   C   
   G   
    T    
   G   
   G   
    A    
   T   
   C   
    A    
    C    
   A   
   A   
   T   
   C   
   A   
   G   
   A   
   T   
   G   
   A   
    C    
   G   
   A   
    A    
   G   
   C   
   A   
   A   
   A   
   A   
   G   
   C   
    A    
   A   
    C    
    G    
    T    
   T   
    A    
   A   
   T   
    T    
   G   
   A   
   A   
   C   
   G   
    C    
   T   
   T   
    T    
   G   
   A   
    A    
   T   
   T   
    T    
   T   
   C   
    A    
   G   
   A   
   T   
   C   
   G   
    T    
   C   
   A   
    A    
    G    
   C   
    G    
   C   
   A   
   A   
   G   
   C   
    A    
   A   
   T   
    T    
   T   
   T   
   A   
   G   
   A   
   T   
   A   
   T   
   G   
   C   
   G   
   T   
   T   
   T   
    A    
   C   
   G   
    C    
   C   
   G   
    T    
   T   
   T   
    A    
   A   
   C   
    A    
   G   
   G   
    C    
   T   
   T   
    A    
   G   
   A   
   A   
   C   
   G   
    C    
   G   
   A   
    C    
   A   
   A   
   A   
   A   
   T   
    T    
   G   
   A   
    A    
   A   
   A   
   T   
   G   
   A   
   A   
   T   
   A   
    T    
   C   
   A   
   A   
   G   
   A   
    G    
   C   
   T   
    A    
   T   
   T   
    G    
   A   
    A    
   A   
    T    
   T   
    C    
   A   
   T   
   T   
   G   
    C    
    G    
   G   
   A   
    C    
   T   
   T   
    A    
    G    
    A    
    G    
   G   
   A   
   C   
   A   
   T   
   T   
    T    
   T   
    A    
   G   
   C   
    C    
    C    
   G   
    C    
   C   
   C   
    A    
   G   
   A   
    A    
   C   
   G   
    C    
   G   
   T   
    C    
   A   
    T    
    T    
   G   
   A   
   A   
   A   
   T   
    C    
   A   
   T   
    T    
   A   
   A   
    A    
   A   
    C    
    A    
   G   
   A   
    G    
   T   
   T   
   A   
    A    
    A    
    T    
   G   
   A   
   C   
    G    
   T   
    C    
    C    
    G    
    T    
    A    
    C    
   A   
    A    
    A    
   A   
   T   
   T   
   T   
   G   
   G   
   T   
   G   
   A   
   T   
    G    
    C    
   A   
   C   
   G   
   A   
   C   
   G   
    C    
   A   
   C   
   A   
   G   
   A   
   A   
    T    
   T   
   A   
    T    
   T   
   A   
   G   
   T   
    A    
   G   
   G   
   T   
   G   
   A   
    A    
   G   
   T   
   C   
    T    
   T   
    A    
   A   
   G   
   T   
   C   
   T   
    T    
   G   
   A   
   A   
   G   
   A   
   T   
   G   
   A   
   A   
   G   
   A   
   T   
    T    
   T   
    A    
   A   
   T   
    C    
   G   
   A   
   A   
   G   
   A   
   A   
   G   
   A   
   A   
   G   
   A   
   A   
    G    
   T   
   C   
   G   
   T   
    G    
   A   
   T   
   T   
   A   
   C   
    G    
    T    
   T   
    A    
   A   
   C   
    C    
   A   
   A   
    T    
   A   
   A   
    C    
   G   
   G   
    C    
   T   
   A   
    C    
   A   
   T   
    T    
   A   
   A   
    G    
   C   
   G   
    G    
    A    
   T   
   G   
   G   
    C    
   A   
   A   
   A   
    C    
    T    
    C    
    T    
   G   
   A   
   A   
   T   
   T   
   C   
    C    
   G   
    G    
   G   
   C   
    G    
   C   
   A   
   A   
   C   
   G   
    C    
    C    
   G   
    T    
   G   
   G   
    C    
   G   
   G   
    A    
   C   
   G   
   T   
   G   
   G   
    T    
   G   
   T   
    C    
   C   
   A   
   A   
   G   
   G   
    G    
   A   
   T   
   G   
   G   
   G   
    C    
    G    
   T   
    T    
   C   
   A   
    T    
    G    
   A   
   T   
   G   
   A   
    T    
   G   
   A   
    T    
   T   
   T   
   C   
   G   
   T   
    G    
   A   
   A   
    A    
   A   
   A   
    C    
   C   
   T   
    A    
    G    
   T   
   T   
   T   
   C   
   T   
   T   
   G   
    T    
   T   
   C   
    A    
   A   
   C   
    A    
   C   
   A   
    C    
   G   
   A   
    C    
   A   
   C   
    G    
   T   
   T   
    A    
   T   
   T   
   A   
   T   
   T   
    C    
   T   
   T   
   T   
   A   
   C   
   G   
   A   
   A   
    C    
   A   
    C    
    T    
   G   
   G   
    G    
   A   
   A   
   A   
   G   
   T   
    T    
   T   
   A   
    C    
   C   
   G   
    A    
   G   
   C   
   A   
   A   
   A   
   A   
   G   
   G   
    T    
   T   
   A   
    C    
   G   
   A   
   A   
   A   
   T   
    C    
   C   
   C   
    T    
   G   
   A   
    G    
   T   
   A   
   C   
   G   
   G   
    T    
    A    
   G   
    A    
   A   
   C   
   A   
   G   
   C   
    A    
   A   
   A   
   A   
   G   
   G   
    A    
   A   
   T   
    T    
   C   
   C   
   A   
    G    
   T   
    G    
   A   
   T   
    C    
   A   
   A   
    C    
   T   
   T   
    A    
   C   
   T   
    G    
   G   
   G   
    A    
   A   
   T   
    T    
   G   
   A   
    T    
   T   
   C   
    T    
    G    
    C    
    A    
   G   
   A   
   A   
   A   
    A    
    A    
   A   
   T   
    T    
   C   
   A   
    A    
   G   
   C   
    G    
   A   
   T   
    T    
   A   
   T   
   T   
    T    
   C   
    T    
   G   
   T   
    T    
    G    
    A    
   G   
   G   
   G   
    C    
    A    
   A   
   A   
   G   
   C   
    G    
   G   
   A   
   A   
   G   
    C    
    A    
   G   
   G   
    T    
    A    
   A   
    A    
   T   
   A   
    C    
    T    
   T   
    G    
   T   
   T   
   C   
   T   
   T   
    C    
   A   
   C   
    A    
   A   
   C   
    C    
    T    
    T    
    A    
    A    
   A   
   A   
   G   
   G   
    A    
   A   
   C   
    C    
   G   
   T   
    C    
   A   
   A   
   A   
   C   
   G   
    G    
   A   
   C   
    A    
    G    
   C   
    C    
   G   
   T   
    A    
   A   
   C   
    A    
   G   
   C   
    C    
   T   
   T   
    T    
    T    
   C   
   T   
   A   
   A   
    T    
   A   
   T   
    C    
   C   
   G   
    T    
   A   
   G   
    T    
   A   
   A   
   T   
   G   
   G   
    A    
    T    
   T   
    A    
   A   
   T   
    C    
   G   
   C   
    C    
   A   
   T   
   T   
    A    
   G   
    C    
   T   
   T   
    A    
   A   
   A   
   A   
   G   
   A   
   A   
   G   
   A   
    T    
   G   
   A   
    T    
   G   
   A   
    G    
    T    
   T   
    A    
    G    
   T   
    T    
   A   
   A   
    C    
   G   
   T   
    G    
    G    
   T   
   A   
    A    
    C    
   G   
   A   
   C   
    T    
    A    
   A   
    T    
   G   
   G   
    C    
    A    
   A   
    T    
    C    
    A    
    G    
   A   
   A   
    G    
   A   
   T   
    G    
   A   
   T   
    T    
   A   
   T   
    C    
   G   
   G   
    A    
   A   
   C   
   A   
   C   
   A   
   T   
    G    
    C    
    A    
   G   
   G   
    A    
   T   
   A   
   C   
   T   
   C   
   T   
   G   
   T   
    C    
   A   
   C   
    A    
   T   
   T   
    T    
    G    
   A   
    T    
   G   
   A   
    A    
    A    
    A    
    T    
    A    
   C   
    T    
   G   
    T    
    A    
   C   
   G   
    T    
   G   
   A   
   T   
   A   
   T   
   G   
   G   
   G   
    C    
   C   
   G   
    G    
   A   
   C   
   A   
   G   
   C   
    A    
   T   
   C   
    A    
   G   
   G   
    T    
   G   
   T   
    T    
    C    
   G   
    T    
   G   
   G   
    A    
   A   
   T   
    C    
   C   
   G   
    T    
    C    
   T   
    C    
   C   
   G   
    C    
   G   
   A   
    A    
    A    
   A   
    T    
   G   
   A   
    T    
   T   
    A    
    T    
   G   
   T   
    G    
   G   
   T   
    C    
   G   
   G   
    C    
    G    
   C   
   A   
   G   
   C   
    G    
    A    
   T   
    T    
    C    
   T   
   G   
    G    
   A   
    T    
   G   
    A    
   A   
    A    
   A   
    T    
    A    
   A   
   A   
   G   
   A   
   A   
   G   
   T   
    C    
    C    
   T   
    A    
   G   
   T   
    C    
   A   
   T   
    T    
   A   
   C   
    T    
   G   
   A   
   A   
   A   
   A   
    T    
   G   
   G   
   T   
   T   
   A   
    T    
   G   
   G   
    T    
   A   
   A   
    G    
   C   
   G   
    T    
   A   
   C   
    A    
   A   
   A   
   A   
   G   
    C    
    T    
   T   
   C   
    T    
   G   
   A   
    A    
   T   
   A   
   T   
   C   
   C   
   A   
   G   
   T   
    T    
   A   
   A   
    A    
   G   
   G   
    A    
   C   
   G   
    T    
   G   
   G   
    C    
   G   
   G   
   T   
   A   
   A   
   A   
   G   
   G   
    G    
   A   
   T   
    T    
   A   
   A   
    G    
   A   
   C   
    A    
    G    
   C   
    A    
   A   
   A   
    T    
   A   
   T   
   C   
   A   
   C   
   T   
   G   
   A   
    G    
   A   
   A   
   A   
   A   
   A   
   T   
   G   
   G   
   T   
   C   
   C   
   A   
    T    
   T   
   A   
   G   
   C   
    T    
   G   
   G   
    T    
   T   
   T   
    A    
   A   
   C   
   C   
   A   
   C   
    G    
   G   
   T   
    C    
    A    
    A    
   T   
   G   
   G   
    G    
    G    
   A   
   T   
   G   
   A   
   A   
   G   
   A   
    T    
   A   
   T   
   C   
    T    
   T   
    A    
    T    
   T   
    G    
   A   
   T   
    T    
   A   
   C   
    G    
    A    
   A   
    C    
   A   
   A   
   A   
   G   
   G   
    C    
   G   
   T   
    C    
   A   
   T   
   T   
   A   
   T   
   C   
   C   
   G   
    C    
   T   
   T   
    T    
   A   
   A   
   C   
   G   
   T   
    T    
    G    
    A    
    T    
    T    
   C   
   T   
   G   
   T   
   T   
   T   
   C   
   T   
   C   
   A   
   A   
   A   
   C   
    A    
   G   
   G   
   A   
   C   
   G   
    C    
    G    
   C   
   A   
   A   
   C   
   A   
   T   
   T   
    A    
   G   
   G   
    G    
   G   
   T   
    T    
   C   
   G   
   T   
   T   
   T   
    A    
   A   
   T   
   G   
   A   
    G    
    A    
   A   
   T   
   G   
   G   
   A   
   A   
   G   
    A    
    T    
   G   
    G    
    T    
    G    
   C   
    C    
   A   
   A   
   A   
   -   
   -   
   -   
   -   
   -   
   -   
   -   
   -   
   -   
   -   
   -   
   -   
   -   
   -   
   -   
   -   
   -   
   -   
   -   
   -   
   -   
   -   
   -   
   -   
   -   
   -   
   -   
   -   
   -   
   -   
   -   
   -   
   -   
   -   
   -   
   -   
   -   
   -   
   -   
   -   
   -   
   -   
   -   
   -   
   -   
   -   
   -   
   -   
   -   
   -   
   -   
   -   
   -   
   -   
   -   
   -   
   -   
   -   
   -   
   -   
   -   
   -   
   -   
   -   
   -   
   -   
   -   
   -   
   -   
   -   
   -   
   -   
 

 
   N59599F   
   A   
   T   
   G   
   A   
   G   
   T   
   G   
   A   
   A   
   G   
   A   
   A   
   A   
   T   
   T   
   A   
   A   
   A   
   G   
   A   
   A   
   A   
   A   
   C   
   A   
   T   
   T   
   C   
   A   
   A   
   G   
   A   
   C   
   G   
   T   
   C   
   A   
   A   
   T   
   C   
   T   
   G   
   A   
   C   
   C   
   A   
   G   
   C   
   G   
   A   
   A   
   A   
   T   
   G   
   A   
   A   
   A   
   G   
   A   
   A   
   T   
   C   
   T   
   T   
   T   
   C   
   A   
   T   
   T   
   G   
   A   
   C   
   T   
   A   
   C   
   G   
   C   
   A   
   A   
   T   
   G   
   A   
   G   
   T   
   G   
   T   
   T   
   A   
   T   
   C   
   G   
   T   
   A   
   G   
   C   
   C   
   C   
   G   
   C   
   G   
   C   
   G   
   T   
   T   
   A   
   C   
   C   
   T   
   G   
   A   
   C   
   G   
   T   
   T   
   C   
   G   
   T   
   G   
   A   
   C   
   G   
   G   
   T   
   T   
   T   
   A   
   A   
   A   
   A   
   C   
   C   
   T   
   G   
   T   
   T   
   C   
   A   
   T   
   C   
   G   
   C   
   C   
   G   
   A   
   A   
   T   
   C   
   T   
   T   
   A   
   T   
   A   
   T   
   G   
   G   
   A   
   A   
   T   
   G   
   A   
   A   
   C   
   G   
   A   
   A   
   T   
   T   
   A   
   G   
   G   
   G   
   G   
   T   
   A   
   A   
   C   
   C   
   C   
   C   
   T   
   G   
   A   
   T   
   A   
   A   
   A   
   C   
   C   
   A   
   C   
   A   
   C   
   A   
   A   
   A   
   A   
   A   
   A   
   T   
   C   
   A   
   G   
   C   
   C   
   C   
   G   
   G   
   A   
   T   
   T   
   G   
   T   
   T   
   G   
   G   
   G   
   G   
   A   
   T   
   G   
   T   
   T   
   A   
   T   
   G   
   G   
   G   
   T   
   A   
   A   
   A   
   T   
   A   
   T   
   C   
   A   
   C   
   C   
   C   
   C   
   C   
   A   
   T   
   G   
   G   
   G   
   G   
   A   
   C   
   A   
   G   
   T   
   G   
   C   
   G   
   A   
   T   
   T   
   T   
   A   
   C   
   G   
   A   
   A   
   T   
   C   
   A   
   A   
   T   
   G   
   G   
   T   
   G   
   C   
   G   
   G   
   A   
   T   
   G   
   G   
   C   
   A   
   C   
   A   
   A   
   C   
   C   
   T   
   T   
   T   
   T   
   A   
   G   
   T   
   T   
   A   
   T   
   C   
   G   
   G   
   G   
   C   
   T   
   A   
   T   
   G   
   T   
   T   
   A   
   G   
   T   
   T   
   G   
   A   
   C   
   G   
   G   
   C   
   C   
   A   
   C   
   G   
   G   
   A   
   A   
   A   
   C   
   T   
   T   
   C   
   G   
   G   
   T   
   T   
   C   
   T   
   G   
   T   
   C   
   G   
   A   
   T   
   G   
   G   
   T   
   G   
   A   
   C   
   G   
   G   
   C   
   G   
   C   
   T   
   G   
   C   
   C   
   G   
   C   
   T   
   A   
   T   
   G   
   C   
   G   
   T   
   T   
   A   
   T   
   A   
   C   
   C   
   G   
   A   
   A   
   G   
   C   
   A   
   C   
   G   
   T   
   A   
   T   
   G   
   A   
   G   
   T   
   A   
   A   
   A   
   A   
   T   
   T   
   G   
   C   
   T   
   T   
   T   
   A   
   G   
   A   
   A   
   A   
   T   
   G   
   C   
   T   
   A   
   C   
   G   
   A   
   G   
   A   
   T   
   A   
   T   
   T   
   A   
   A   
   C   
   A   
   A   
   A   
   A   
   A   
   T   
   A   
   C   
   A   
   G   
   T   
   C   
   G   
   A   
   T   
   T   
   T   
   C   
   C   
   A   
   A   
   G   
   G   
   A   
   A   
   A   
   C   
   T   
   A   
   T   
   G   
   A   
   T   
   G   
   A   
   T   
   T   
   C   
   A   
   G   
   A   
   A   
   C   
   A   
   A   
   G   
   A   
   A   
   C   
   C   
   A   
   G   
   T   
   A   
   G   
   T   
   T   
   T   
   T   
   A   
   C   
   C   
   T   
   G   
   C   
   T   
   C   
   G   
   T   
   T   
   T   
   T   
   C   
   C   
   A   
   A   
   A   
   C   
   T   
   T   
   A   
   C   
   T   
   A   
   G   
   T   
   T   
   A   
   A   
   C   
   G   
   G   
   A   
   A   
   C   
   A   
   A   
   C   
   G   
   G   
   G   
   G   
   A   
   T   
   T   
   G   
   C   
   G   
   G   
   T   
   G   
   G   
   G   
   G   
   A   
   T   
   G   
   G   
   C   
   A   
   A   
   C   
   A   
   A   
   A   
   T   
   A   
   T   
   T   
   C   
   C   
   A   
   C   
   C   
   A   
   C   
   A   
   C   
   A   
   A   
   T   
   T   
   T   
   A   
   A   
   G   
   T   
   G   
   A   
   A   
   G   
   T   
   G   
   A   
   T   
   T   
   G   
   A   
   T   
   G   
   C   
   A   
   A   
   C   
   A   
   A   
   G   
   T   
   T   
   T   
   A   
   T   
   T   
   G   
   A   
   T   
   G   
   G   
   A   
   C   
   A   
   A   
   T   
   C   
   C   
   T   
   G   
   A   
   T   
   G   
   T   
   A   
   A   
   C   
   G   
   A   
   C   
   G   
   A   
   A   
   T   
   G   
   A   
   A   
   T   
   T   
   G   
   A   
   T   
   G   
   G   
   A   
   A   
   G   
   T   
   G   
   T   
   T   
   A   
   C   
   C   
   T   
   G   
   G   
   A   
   C   
   C   
   A   
   G   
   A   
   T   
   T   
   T   
   T   
   C   
   C   
   A   
   A   
   C   
   A   
   G   
   G   
   C   
   G   
   G   
   T   
   T   
   T   
   A   
   G   
   T   
   G   
   A   
   T   
   G   
   G   
   G   
   G   
   A   
   A   
   A   
   T   
   C   
   A   
   G   
   G   
    G    
   A   
   T   
    T    
   C   
   G   
    C    
    C    
   G   
   A   
   G   
   C   
    A    
   T   
   A   
   T   
   G   
   A   
   A   
   A   
   C   
    A    
   G   
   G   
    G    
   A   
   A   
    A    
   G   
   G   
   T   
   T   
   C   
    G    
   A   
   T   
   T   
   A   
   C   
    T    
   G   
   T   
    T    
   C   
   G   
   T   
   G   
   C   
   A   
   A   
   A   
   A   
   G   
   T   
   T   
   G   
   A   
    A    
    T    
   T   
    G    
   A   
    C    
   T   
   G   
   A   
    A    
   A   
   T   
   G   
    C    
   C   
    G    
   A   
   A   
   T   
   G   
   G   
    A    
   A   
   A   
   A   
   G   
   A   
   A   
   C   
   G   
    T    
   A   
   T   
    T    
    T    
   T   
    A    
   G   
   T   
    A    
   A   
   C   
    T    
   G   
   A   
   A   
   T   
   T   
    G    
   C   
   C   
   T   
   T   
   A   
   T   
   A   
   T   
   G   
   G   
   T   
    G    
   A   
   A   
   T   
   A   
   A   
   A   
   G   
   C   
    C    
   A   
   A   
   A   
   T   
   T   
    A    
   A   
   T   
    C    
   G   
   A   
   A   
   C   
   G   
    A    
   A   
   T   
   T   
   T   
   C   
   T   
   G   
   A   
    A    
    T    
   T   
    A    
   C   
   A   
   C   
   A   
   G   
   A   
   G   
   A   
    T    
   A   
   A   
    A    
   C   
   G   
    A    
   A   
   T   
    T    
   G   
   A   
   A   
   G   
   G   
    A    
   A   
   T   
    T    
   A   
   C   
    T    
   G   
   A   
   T   
   T   
   T   
   G   
   C   
   G   
   G   
   G   
   A   
    T    
   G   
   A   
   A   
   T   
   C   
   T   
   T   
   C   
    T    
   C   
   G   
   T   
   G   
   A   
    A    
   G   
   G   
    C    
   A   
   T   
   G   
   C   
   G   
    G    
   A   
   T   
    C    
   G   
   T   
   C   
   A   
   T   
    C    
   G   
   A   
    T    
    G    
   T   
    T    
   C   
   G   
   T   
   C   
   G   
    A    
   G   
   A   
    T    
   G   
    T    
    G    
   A   
   G   
    T    
   G   
   C   
    C    
   T   
   C   
    T    
   G   
   T   
   T   
    G    
   T   
    G    
   T   
   T   
    A    
   A   
   A   
    C    
   A   
   A   
   C   
   T   
   T   
    G    
   T   
   A   
    C    
   A   
   A   
   A   
    A    
   T   
   G   
   A   
   C   
    T    
    G    
   C   
    C    
   T   
   T   
    A    
   C   
   A   
    A    
   A   
    C    
    A    
   T   
   C   
    T    
   T   
   T   
    T    
   G   
   G   
    T    
   T   
   T   
    T    
   A   
   A   
    C    
   A   
   T   
   G   
   T   
   T   
    A    
   G   
   C   
    C    
   A   
   T   
    T    
   G   
   A   
   A   
   A   
   A   
   A   
   G   
   G   
    C    
   G   
   T   
    A    
   C   
   C   
    G    
   A   
   A   
   A   
    A    
   T   
   T   
   T   
   T   
    A    
   A   
   G   
    C    
   T   
   T   
   G   
   A   
   A   
   A   
   C   
    G    
    T    
   A   
   T   
    T    
    T    
   T   
    A    
   G   
   A   
   A   
   A   
   A   
    T    
   T   
   A   
    C    
    G    
   T   
    C    
   G   
   A   
    G    
   C   
   A   
    C    
   C   
   A   
    A    
    A    
    A    
    A    
    G    
   A   
   A   
   G   
   T   
   C   
   A   
   T   
    T    
   A   
   C   
    T    
    C    
   G   
    C    
   C   
   G   
   G   
   A   
   C   
    G    
    A    
   T   
    T    
   T   
   T   
   T   
   G   
   A   
    C    
   A   
   A   
   A   
    A    
   A   
    C    
   A   
   A   
    A    
   G   
   C   
   A   
   G   
   A   
   A   
   G   
   C   
   A   
    C    
   G   
    G    
   G   
   C   
    G    
   C   
   A   
    T    
   A   
   T   
    T    
   T   
   T   
   A   
   G   
   A   
   A   
   G   
   G   
   T   
    C    
   T   
    A    
   C   
   G   
    A    
   A   
   T   
   T   
   G   
   C   
    C    
   T   
   T   
    A    
   G   
   A   
   T   
   C   
   A   
   T   
   A   
   T   
   C   
    G    
   A   
   T   
   G   
   A   
   A   
   A   
   T   
    C    
   A   
   T   
   T   
   G   
   C   
    C    
   A   
   T   
   T   
   A   
   T   
    C    
   C   
   G   
    T    
   G   
   G   
    A    
   T   
   C   
    A    
    C    
   A   
   A   
   T   
   C   
   A   
   G   
   A   
   T   
   G   
   A   
    C    
   G   
   A   
    A    
   G   
   C   
   A   
   A   
   A   
   A   
   G   
   C   
    A    
   A   
    C    
    G    
    T    
   T   
    A    
   A   
   T   
    T    
   G   
   A   
   A   
   C   
   G   
    C    
   T   
   T   
    T    
   G   
   A   
    A    
   T   
   T   
    T    
   T   
   C   
    A    
   G   
   A   
   T   
   C   
   G   
    T    
   C   
   A   
    A    
    G    
   C   
    G    
   C   
   A   
   A   
   G   
   C   
    G    
   A   
   T   
    T    
   T   
   T   
   A   
   G   
   A   
   T   
   A   
   T   
   G   
   C   
   G   
   T   
   T   
   T   
    A    
   C   
   G   
    C    
   C   
   G   
    T    
   T   
   T   
    A    
   A   
   C   
    A    
   G   
   G   
    C    
   T   
   T   
    A    
   G   
   A   
   A   
   C   
   G   
    C    
   G   
   A   
    C    
   A   
   A   
   A   
   A   
   T   
    T    
   G   
   A   
    A    
   A   
   A   
   T   
   G   
   A   
   A   
   T   
   A   
    T    
   C   
   A   
   A   
   G   
   A   
    G    
   C   
   T   
    A    
   T   
   T   
    G    
   A   
    A    
   A   
    T    
   T   
    C    
   A   
   T   
   T   
   G   
    C    
    G    
   G   
   A   
    C    
   T   
   T   
    A    
    G    
    A    
    G    
   G   
   A   
   C   
   A   
   T   
   T   
    T    
   T   
    A    
   G   
   C   
    C    
    C    
   G   
    C    
   C   
   C   
    A    
   G   
   A   
    A    
   C   
   G   
    C    
   G   
   T   
    C    
   A   
    T    
    T    
   G   
   A   
   A   
   A   
   T   
    C    
   A   
   T   
    T    
   A   
   A   
    A    
   A   
    C    
    A    
   G   
   A   
    G    
   T   
   T   
   A   
    A    
    A    
    T    
   G   
   A   
   C   
    G    
   T   
    T    
    C    
    G    
    C    
    A    
    C    
   A   
    A    
    A    
   A   
   T   
   T   
   T   
   G   
   G   
   T   
   G   
   A   
   T   
    G    
    C    
   A   
   C   
   G   
   A   
   C   
   G   
    C    
   A   
   C   
   A   
   G   
   A   
   A   
    T    
   T   
   A   
    T    
   T   
   A   
   G   
   T   
    A    
   G   
   G   
   T   
   G   
   A   
    A    
   G   
   T   
   C   
    C    
   T   
    A    
   A   
   G   
   T   
   C   
   T   
    T    
   G   
   A   
   A   
   G   
   A   
   T   
   G   
   A   
   A   
   G   
   A   
   T   
    T    
   T   
    A    
   A   
   T   
    C    
   G   
   A   
   A   
   G   
   A   
   A   
   G   
   A   
   A   
   G   
   A   
   A   
    G    
   T   
   C   
   G   
   T   
    G    
   A   
   T   
   T   
   A   
   C   
    G    
    T    
   T   
    A    
   A   
   C   
    C    
   A   
   A   
    T    
   A   
   A   
    C    
   G   
   G   
    C    
   T   
   A   
    C    
   A   
   T   
    T    
   A   
   A   
    G    
   C   
   G   
    G    
    A    
   T   
   G   
   G   
    C    
   A   
   A   
   A   
    C    
    T    
    C    
    C    
   G   
   A   
   A   
   T   
   T   
   C   
    C    
   G   
    G    
   G   
   C   
    G    
   C   
   A   
   A   
   C   
   G   
    C    
    C    
   G   
    C    
   G   
   G   
    C    
   G   
   G   
    A    
   C   
   G   
   T   
   G   
   G   
    T    
   G   
   T   
    C    
   C   
   A   
   A   
   G   
   G   
    G    
   A   
   T   
   G   
   G   
   G   
    C    
    G    
   T   
    T    
   C   
   A   
    C    
    G    
   A   
   T   
   G   
   A   
    T    
   G   
   A   
    T    
   T   
   T   
   C   
   G   
   T   
    G    
   A   
   A   
    A    
   A   
   A   
    C    
   C   
   T   
    A    
    G    
   T   
   T   
   T   
   C   
   T   
   T   
   G   
    T    
   T   
   C   
    A    
   A   
   C   
    A    
   C   
   A   
    C    
   G   
   A   
    C    
   A   
   C   
    G    
   T   
   T   
    A    
   T   
   T   
   A   
   T   
   T   
    C    
   T   
   T   
   T   
   A   
   C   
   G   
   A   
   A   
    C    
   A   
    C    
    T    
   G   
   G   
    G    
   A   
   A   
   A   
   G   
   T   
    T    
   T   
   A   
    C    
   C   
   G   
    A    
   G   
   C   
   A   
   A   
   A   
   A   
   G   
   G   
    T    
   T   
   A   
    C    
   G   
   A   
   A   
   A   
   T   
    C    
   C   
   C   
    T    
   G   
   A   
    A    
   T   
   A   
   C   
   G   
   G   
    T    
    A    
   G   
    A    
   A   
   C   
   A   
   G   
   C   
    A    
   A   
   A   
   A   
   G   
   G   
    A    
   A   
   T   
    T    
   C   
   C   
   A   
    G    
   T   
    A    
   A   
   T   
    T    
   A   
   A   
    C    
   T   
   T   
    A    
   C   
   T   
    G    
   G   
   G   
    A    
   A   
   T   
    T    
   G   
   A   
    T    
   T   
   C   
    T    
    G    
    C    
    A    
   G   
   A   
   A   
   A   
    A    
    A    
   A   
   T   
    T    
   C   
   A   
    A    
   G   
   C   
    G    
   A   
   T   
    T    
   A   
   T   
   T   
    T    
   C   
    T    
   G   
   T   
    T    
    G    
    A    
   G   
   G   
   G   
    C    
    A    
   A   
   A   
   G   
   C   
    G    
   G   
   A   
   A   
   G   
    C    
    A    
   G   
   G   
    T    
    A    
   A   
    A    
   T   
   A   
    C    
    T    
   T   
    G    
   T   
   T   
   C   
   T   
   T   
    C    
   A   
   C   
    A    
   A   
   C   
    C    
    T    
    T    
    A    
    A    
   A   
   A   
   G   
   G   
    A    
   A   
   C   
    T    
   G   
   T   
    C    
   A   
   A   
   A   
   C   
   G   
    G    
   A   
   C   
    A    
    G    
   C   
    C    
   G   
   T   
    A    
   A   
   C   
    A    
   G   
   C   
    C    
   T   
   T   
    T    
    T    
   C   
   T   
   A   
   A   
    T    
   A   
   T   
    C    
   C   
   G   
    T    
   A   
   G   
    T    
   A   
   A   
   T   
   G   
   G   
    A    
    T    
   T   
    A    
   A   
   T   
    C    
   G   
   C   
    C    
   A   
   T   
   T   
    A    
   G   
    C    
   T   
   T   
    A    
   A   
   A   
   A   
   G   
   A   
   A   
   G   
   A   
    T    
   G   
   A   
    T    
   G   
   A   
    G    
    T    
   T   
    A    
    G    
   T   
    T    
   A   
   A   
    C    
   G   
   T   
    A    
    G    
   T   
   A   
    A    
    C    
   G   
   A   
   C   
    T    
    A    
   A   
    T    
   G   
   G   
    C    
    A    
   A   
    T    
    C    
    A    
    G    
   A   
   A   
    G    
   A   
   T   
    G    
   A   
   T   
    T    
   A   
   T   
    C    
   G   
   G   
    A    
   A   
   C   
   A   
   C   
   A   
   T   
    G    
    C    
    A    
   G   
   G   
    A    
   T   
   A   
   C   
   T   
   C   
   T   
   G   
   T   
    C    
   A   
   C   
    A    
   T   
   T   
    T    
    G    
   A   
    T    
   G   
   A   
    A    
    A    
    A    
    T    
    A    
   C   
    T    
   G   
    T    
    A    
   C   
   G   
    T    
   G   
   A   
   T   
   A   
   T   
   G   
   G   
   G   
    C    
   C   
   G   
    G    
   A   
   C   
   A   
   G   
   C   
    A    
   T   
   C   
    A    
   G   
   G   
    C    
   G   
   T   
    T    
    C    
   G   
    G    
   G   
   G   
    A    
   A   
   T   
    C    
   C   
   G   
    T    
    C    
   T   
    C    
   C   
   G   
    C    
   G   
   A   
    A    
    A    
   A   
    T    
   G   
   A   
    T    
   T   
    A    
    T    
   G   
   T   
    G    
   G   
   T   
    C    
   G   
   G   
    C    
    G    
   C   
   A   
   G   
   C   
    G    
    A    
   T   
    T    
    C    
   T   
   G   
    G    
   A   
    T    
   G   
    A    
   A   
    A    
   A   
    T    
    A    
   A   
   A   
   G   
   A   
   A   
   G   
   T   
    C    
    C    
   T   
    A    
   G   
   T   
    C    
   A   
   T   
    T    
   A   
   C   
    T    
   G   
   A   
   A   
   A   
   A   
    T    
   G   
   G   
   T   
   T   
   A   
    T    
   G   
   G   
    T    
   A   
   A   
    G    
   C   
   G   
    T    
   A   
   C   
    A    
   A   
   A   
   A   
   G   
    C    
    T    
   T   
   C   
    T    
   G   
   A   
    A    
   T   
   A   
   T   
   C   
   C   
   A   
   G   
   T   
    T    
   A   
   A   
    A    
   G   
   G   
    A    
   C   
   G   
    T    
   G   
   G   
    C    
   G   
   G   
   T   
   A   
   A   
   A   
   G   
   G   
    G    
   A   
   T   
    T    
   A   
   A   
    G    
   A   
   C   
    A    
    G    
   C   
    A    
   A   
   A   
    T    
   A   
   T   
   C   
   A   
   C   
   T   
   G   
   A   
    G    
   A   
   A   
   A   
   A   
   A   
   T   
   G   
   G   
   T   
   C   
   C   
   A   
    T    
   T   
   A   
   G   
   C   
    T    
   G   
   G   
    T    
   T   
   T   
    A    
   A   
   C   
   C   
   A   
   C   
    G    
   G   
   T   
    C    
    A    
    A    
   T   
   G   
   G   
    G    
    A    
   A   
   T   
   G   
   A   
   A   
   G   
   A   
    T    
   A   
   T   
   C   
    T    
   T   
    A    
    T    
   T   
    G    
   A   
   T   
    T    
   A   
   C   
    G    
    A    
   A   
    C    
   A   
   A   
   A   
   G   
   G   
    C    
   G   
   T   
    C    
   A   
   T   
   T   
   A   
   T   
   C   
   C   
   G   
    C    
   T   
   T   
    T    
   A   
   A   
   C   
   G   
   T   
    T    
    G    
    A    
    T    
    T    
   C   
   T   
   G   
   T   
   T   
   T   
   C   
   T   
   C   
   A   
   A   
   A   
   C   
    A    
   G   
   G   
   A   
   C   
   G   
    C    
    G    
   C   
   A   
   A   
   C   
   A   
   T   
   T   
    A    
   G   
   G   
    G    
   G   
   T   
    T    
   C   
   G   
   T   
   T   
   T   
    A    
   A   
   T   
   G   
   A   
    G    
    A    
   A   
   T   
   G   
   G   
   A   
   A   
   G   
    A    
    T    
   G   
    G    
    T    
    G    
   C   
    C    
   A   
   A   
   A   
   -   
   -   
   -   
   -   
   -   
   -   
   -   
   -   
   -   
   -   
   -   
   -   
   -   
   -   
   -   
   -   
   -   
   -   
   -   
   -   
   -   
   -   
   -   
   -   
   -   
   -   
   -   
   -   
   -   
   -   
   -   
   -   
   -   
   -   
   -   
   -   
   -   
   -   
   -   
   -   
   -   
   -   
   -   
   -   
   -   
   -   
   -   
   -   
   -   
   -   
   -   
   -   
   -   
   -   
   -   
   -   
   -   
   -   
   -   
   -   
   -   
   -   
   -   
   -   
   -   
   -   
   -   
   -   
   -   
   -   
   -   
   -   
 

 
   N54553   
   A   
   T   
   G   
   A   
   G   
   T   
   G   
   A   
   A   
   G   
   A   
   A   
   A   
   T   
   T   
   A   
   A   
   A   
   G   
   A   
   A   
   A   
   A   
   C   
   A   
   T   
   T   
   C   
   A   
   A   
   G   
   A   
   C   
   G   
   T   
   C   
   A   
   A   
   T   
   C   
   T   
   G   
   A   
   C   
   C   
   A   
   G   
   C   
   G   
   A   
   A   
   A   
   T   
   G   
   A   
   A   
   A   
   G   
   A   
   A   
   T   
   C   
   T   
   T   
   T   
   C   
   A   
   T   
   T   
   G   
   A   
   C   
   T   
   A   
   C   
   G   
   C   
   A   
   A   
   T   
   G   
   A   
   G   
   T   
   G   
   T   
   T   
   A   
   T   
   C   
   G   
   T   
   A   
   G   
   C   
   C   
   C   
   G   
   C   
   G   
   C   
   G   
   T   
   T   
   A   
   C   
   C   
   T   
   G   
   A   
   C   
   G   
   T   
   T   
   C   
   G   
   T   
   G   
   A   
   C   
   G   
   G   
   T   
   T   
   T   
   A   
   A   
   A   
   A   
   C   
   C   
   T   
   G   
   T   
   T   
   C   
   A   
   T   
   C   
   G   
   C   
   C   
   G   
   A   
   A   
   T   
   C   
   T   
   T   
   A   
   T   
   A   
   T   
   G   
   G   
   A   
   A   
   T   
   G   
   A   
   A   
   C   
   G   
   A   
   A   
   T   
   T   
   A   
   G   
   G   
   G   
   G   
   T   
   A   
   A   
   C   
   C   
   C   
   C   
   T   
   G   
   A   
   T   
   A   
   A   
   A   
   C   
   C   
   A   
   C   
   A   
   C   
   A   
   A   
   A   
   A   
   A   
   A   
   T   
   C   
   A   
   G   
   C   
   C   
   C   
   G   
   G   
   A   
   T   
   T   
   G   
   T   
   T   
   G   
   G   
   G   
   G   
   A   
   T   
   G   
   T   
   T   
   A   
   T   
   G   
   G   
   G   
   T   
   A   
   A   
   A   
   T   
   A   
   T   
   C   
   A   
   C   
   C   
   C   
   C   
   C   
   A   
   T   
   G   
   G   
   G   
   G   
   A   
   C   
   A   
   G   
   T   
   G   
   C   
   G   
   A   
   T   
   T   
   T   
   A   
   C   
   G   
   A   
   A   
   T   
   C   
   A   
   A   
   T   
   G   
   G   
   T   
   G   
   C   
   G   
   G   
   A   
   T   
   G   
   G   
   C   
   A   
   C   
   A   
   A   
   C   
   C   
   T   
   T   
   T   
   T   
   A   
   G   
   T   
   T   
   A   
   T   
   C   
   G   
   G   
   G   
   C   
   T   
   A   
   T   
   G   
   T   
   T   
   A   
   G   
   T   
   T   
   G   
   A   
   C   
   G   
   G   
   C   
   C   
   A   
   C   
   G   
   G   
   A   
   A   
   A   
   C   
   T   
   T   
   C   
   G   
   G   
   T   
   T   
   C   
   C   
   G   
   T   
   C   
   G   
   A   
   T   
   G   
   G   
   T   
   G   
   A   
   C   
   G   
   G   
   C   
   G   
   C   
   T   
   G   
   C   
   C   
   G   
   C   
   T   
   A   
   T   
   G   
   C   
   G   
   T   
   T   
   A   
   T   
   A   
   C   
   C   
   G   
   A   
   A   
   G   
   C   
   A   
   C   
   G   
   T   
   A   
   T   
   G   
   A   
   G   
   T   
   A   
   A   
   A   
   A   
   T   
   T   
   G   
   C   
   T   
   T   
   T   
   A   
   G   
   A   
   A   
   A   
   T   
   G   
   C   
   T   
   A   
   C   
   G   
   A   
   G   
   A   
   T   
   A   
   T   
   T   
   A   
   A   
   C   
   A   
   A   
   A   
   A   
   A   
   T   
   A   
   C   
   A   
   G   
   T   
   C   
   G   
   A   
   T   
   T   
   T   
   C   
   C   
   A   
   A   
   G   
   G   
   A   
   A   
   A   
   C   
   T   
   A   
   T   
   G   
   A   
   T   
   G   
   A   
   T   
   T   
   C   
   A   
   G   
   A   
   A   
   C   
   A   
   A   
   G   
   A   
   A   
   C   
   C   
   A   
   G   
   T   
   A   
   G   
   T   
   T   
   T   
   T   
   A   
   C   
   C   
   T   
   G   
   C   
   T   
   C   
   G   
   T   
   T   
   T   
   T   
   C   
   C   
   A   
   A   
   A   
   C   
   T   
   T   
   A   
   C   
   T   
   A   
   G   
   T   
   T   
   A   
   A   
   C   
   G   
   G   
   A   
   A   
   C   
   A   
   A   
   C   
   G   
   G   
   G   
   G   
   A   
   T   
   T   
   G   
   C   
   G   
   G   
   T   
   G   
   G   
   G   
   G   
   A   
   T   
   G   
   G   
   C   
   A   
   A   
   C   
   A   
   A   
   A   
   T   
   A   
   T   
   T   
   C   
   C   
   A   
   C   
   C   
   A   
   C   
   A   
   C   
   A   
   A   
   T   
   T   
   T   
   A   
   A   
   G   
   T   
   G   
   A   
   A   
   G   
   T   
   G   
   A   
   T   
   T   
   G   
   A   
   T   
   G   
   C   
   A   
   A   
   C   
   A   
   A   
   G   
   T   
   T   
   T   
   A   
   T   
   T   
   G   
   A   
   T   
   G   
   G   
   A   
   C   
   A   
   A   
   T   
   C   
   C   
   T   
   G   
   A   
   T   
   G   
   T   
   A   
   A   
   C   
   G   
   A   
   C   
   G   
   A   
   A   
   T   
   G   
   A   
   A   
   T   
   T   
   G   
   A   
   T   
   G   
   G   
   A   
   A   
   G   
   T   
   G   
   T   
   T   
   A   
   C   
   C   
   T   
   G   
   G   
   A   
   C   
   C   
   A   
   G   
   A   
   T   
   T   
   T   
   T   
   C   
   C   
   A   
   A   
   C   
   A   
   G   
   G   
   C   
   G   
   G   
   T   
   T   
   T   
   A   
   G   
   T   
   G   
   A   
   T   
   G   
   G   
   G   
   G   
   A   
   A   
   A   
   T   
   C   
   A   
   G   
   G   
    G    
   A   
   T   
    T    
   C   
   G   
    C    
    C    
   G   
   A   
   G   
   C   
    A    
   T   
   A   
   T   
   G   
   A   
   A   
   A   
   C   
    A    
   G   
   G   
    G    
   A   
   A   
    A    
   G   
   G   
   T   
   T   
   C   
    G    
   A   
   T   
   T   
   A   
   C   
    T    
   G   
   T   
    T    
   C   
   G   
   T   
   G   
   C   
   A   
   A   
   A   
   A   
   G   
   T   
   T   
   G   
   A   
    A    
    T    
   T   
    G    
   A   
    C    
   T   
   G   
   A   
    A    
   A   
   T   
   G   
    C    
   C   
    G    
   A   
   A   
   T   
   G   
   G   
    A    
   A   
   A   
   A   
   G   
   A   
   A   
   C   
   G   
    T    
   A   
   T   
    T    
    T    
   T   
    A    
   G   
   T   
    A    
   A   
   C   
    T    
   G   
   A   
   A   
   T   
   T   
    G    
   C   
   C   
   T   
   T   
   A   
   T   
   A   
   T   
   G   
   G   
   T   
    G    
   A   
   A   
   T   
   A   
   A   
   A   
   G   
   C   
    C    
   A   
   A   
   A   
   T   
   T   
    A    
   A   
   T   
    C    
   G   
   A   
   A   
   C   
   G   
    A    
   A   
   T   
   T   
   T   
   C   
   T   
   G   
   A   
    A    
    T    
   T   
    A    
   C   
   A   
   C   
   A   
   G   
   A   
   G   
   A   
    T    
   A   
   A   
    A    
   C   
   G   
    A    
   A   
   T   
    T    
   G   
   A   
   A   
   G   
   G   
    A    
   A   
   T   
    T    
   A   
   C   
    T    
   G   
   A   
   T   
   T   
   T   
   G   
   C   
   G   
   G   
   G   
   A   
    T    
   G   
   A   
   A   
   T   
   C   
   T   
   T   
   C   
    T    
   C   
   G   
   T   
   G   
   A   
    A    
   G   
   G   
    C    
   A   
   T   
   G   
   C   
   G   
    G    
   A   
   T   
    T    
   G   
   T   
   C   
   A   
   T   
    C    
   G   
   A   
    T    
    G    
   T   
    T    
   C   
   G   
   T   
   C   
   G   
    A    
   G   
   A   
    T    
   G   
    T    
    G    
   A   
   G   
    T    
   G   
   C   
    C    
   T   
   C   
    T    
   G   
   T   
   T   
    G    
   T   
    G    
   T   
   T   
    A    
   A   
   A   
    C    
   A   
   A   
   C   
   T   
   T   
    G    
   T   
   A   
    C    
   A   
   A   
   A   
    A    
   T   
   G   
   A   
   C   
    T    
    G    
   C   
    C    
   T   
   T   
    A    
   C   
   A   
    A    
   A   
    C    
    A    
   T   
   C   
    T    
   T   
   T   
    T    
   G   
   G   
    T    
   T   
   T   
    T    
   A   
   A   
    C    
   A   
   T   
   G   
   T   
   T   
    A    
   G   
   C   
    C    
   A   
   T   
    C    
   G   
   A   
   A   
   A   
   A   
   A   
   G   
   G   
    C    
   G   
   T   
    A    
   C   
   C   
    G    
   A   
   A   
   A   
    A    
   T   
   T   
   T   
   T   
    A    
   A   
   G   
    C    
   T   
   T   
   G   
   A   
   A   
   A   
   C   
    G    
    T    
   A   
   T   
    T    
    T    
   T   
    A    
   G   
   A   
   A   
   A   
   A   
    C    
   T   
   A   
    C    
    G    
   T   
    C    
   G   
   A   
    G    
   C   
   A   
    C    
   C   
   A   
    A    
    A    
    A    
    A    
    G    
   A   
   A   
   G   
   T   
   C   
   A   
   T   
    T    
   A   
   C   
    T    
    C    
   G   
    C    
   C   
   G   
   G   
   A   
   C   
    G    
    A    
   T   
    T    
   T   
   T   
   T   
   G   
   A   
    C    
   A   
   A   
   A   
    A    
   A   
    C    
   A   
   A   
    A    
   G   
   C   
   A   
   G   
   A   
   A   
   G   
   C   
   A   
    C    
   G   
    G    
   G   
   C   
    G    
   C   
   A   
    T    
   A   
   T   
    T    
   T   
   T   
   A   
   G   
   A   
   A   
   G   
   G   
   T   
    C    
   T   
    A    
   C   
   G   
    A    
   A   
   T   
   T   
   G   
   C   
    C    
   T   
   T   
    A    
   G   
   A   
   T   
   C   
   A   
   T   
   A   
   T   
   C   
    G    
   A   
   T   
   G   
   A   
   A   
   A   
   T   
    C    
   A   
   T   
   T   
   G   
   C   
    C    
   A   
   T   
   T   
   A   
   T   
    C    
   C   
   G   
    T    
   G   
   G   
    G    
   T   
   C   
    A    
    C    
   A   
   A   
   T   
   C   
   A   
   G   
   A   
   T   
   G   
   A   
    C    
   G   
   A   
    A    
   G   
   C   
   A   
   A   
   A   
   A   
   G   
   C   
    A    
   A   
    C    
    G    
    T    
   T   
    A    
   A   
   T   
    T    
   G   
   A   
   A   
   C   
   G   
    C    
   T   
   T   
    T    
   G   
   A   
    A    
   T   
   T   
    T    
   T   
   C   
    A    
   G   
   A   
   T   
   C   
   G   
    T    
   C   
   A   
    A    
    G    
   C   
    G    
   C   
   A   
   A   
   G   
   C   
    G    
   A   
   T   
    T    
   T   
   T   
   A   
   G   
   A   
   T   
   A   
   T   
   G   
   C   
   G   
   T   
   T   
   T   
    A    
   C   
   G   
    C    
   C   
   G   
    T    
   T   
   T   
    A    
   A   
   C   
    A    
   G   
   G   
    C    
   T   
   T   
    A    
   G   
   A   
   A   
   C   
   G   
    C    
   G   
   A   
    C    
   A   
   A   
   A   
   A   
   T   
    T    
   G   
   A   
    A    
   A   
   A   
   T   
   G   
   A   
   A   
   T   
   A   
    T    
   C   
   A   
   A   
   G   
   A   
    G    
   C   
   T   
    A    
   T   
   T   
    G    
   A   
    A    
   A   
    T    
   T   
    C    
   A   
   T   
   T   
   G   
    C    
    G    
   G   
   A   
    C    
   T   
   T   
    A    
    G    
    A    
    G    
   G   
   A   
   C   
   A   
   T   
   T   
    T    
   T   
    A    
   G   
   C   
    C    
    C    
   G   
    C    
   C   
   C   
    A    
   G   
   A   
    G    
   C   
   G   
    C    
   G   
   T   
    C    
   A   
    T    
    T    
   G   
   A   
   A   
   A   
   T   
    C    
   A   
   T   
    T    
   A   
   A   
    A    
   A   
    C    
    A    
   G   
   A   
    G    
   T   
   T   
   A   
    A    
    A    
    T    
   G   
   A   
   C   
    G    
   T   
    T    
    C    
    G    
    C    
    A    
    C    
   A   
    A    
    A    
   A   
   T   
   T   
   T   
   G   
   G   
   T   
   G   
   A   
   T   
    G    
    C    
   A   
   C   
   G   
   A   
   C   
   G   
    C    
   A   
   C   
   A   
   G   
   A   
   A   
    T    
   T   
   A   
    T    
   T   
   A   
   G   
   T   
    A    
   G   
   G   
   T   
   G   
   A   
    A    
   G   
   T   
   C   
    C    
   T   
    A    
   A   
   G   
   T   
   C   
   T   
    T    
   G   
   A   
   A   
   G   
   A   
   T   
   G   
   A   
   A   
   G   
   A   
   T   
    T    
   T   
    A    
   A   
   T   
    C    
   G   
   A   
   A   
   G   
   A   
   A   
   G   
   A   
   A   
   G   
   A   
   A   
    G    
   T   
   C   
   G   
   T   
    G    
   A   
   T   
   T   
   A   
   C   
    G    
    T    
   T   
    A    
   A   
   C   
    C    
   A   
   A   
    T    
   A   
   A   
    C    
   G   
   G   
    C    
   T   
   A   
    C    
   A   
   T   
    T    
   A   
   A   
    G    
   C   
   G   
    G    
    A    
   T   
   G   
   G   
    C    
   A   
   A   
   A   
    C    
    T    
    C    
    C    
   G   
   A   
   A   
   T   
   T   
   C   
    C    
   G   
    G    
   G   
   C   
    G    
   C   
   A   
   A   
   C   
   G   
    C    
    C    
   G   
    C    
   G   
   G   
    C    
   G   
   G   
    A    
   C   
   G   
   T   
   G   
   G   
    T    
   G   
   T   
    C    
   C   
   A   
   A   
   G   
   G   
    G    
   A   
   T   
   G   
   G   
   G   
    C    
    G    
   T   
    T    
   C   
   A   
    C    
    G    
   A   
   T   
   G   
   A   
    T    
   G   
   A   
    T    
   T   
   T   
   C   
   G   
   T   
    G    
   A   
   A   
    A    
   A   
   A   
    C    
   C   
   T   
    A    
    G    
   T   
   T   
   T   
   C   
   T   
   T   
   G   
    T    
   T   
   C   
    A    
   A   
   C   
    A    
   C   
   A   
    C    
   G   
   A   
    C    
   A   
   C   
    G    
   T   
   T   
    A    
   T   
   T   
   A   
   T   
   T   
    C    
   T   
   T   
   T   
   A   
   C   
   G   
   A   
   A   
    C    
   A   
    C    
    T    
   G   
   G   
    G    
   A   
   A   
   A   
   G   
   T   
    T    
   T   
   A   
    C    
   C   
   G   
    A    
   G   
   C   
   A   
   A   
   A   
   A   
   G   
   G   
    T    
   T   
   A   
    C    
   G   
   A   
   A   
   A   
   T   
    C    
   C   
   C   
    T    
   G   
   A   
    A    
   T   
   A   
   C   
   G   
   G   
    T    
    A    
   G   
    A    
   A   
   C   
   A   
   G   
   C   
    A    
   A   
   A   
   A   
   G   
   G   
    A    
   A   
   T   
    T    
   C   
   C   
   A   
    G    
   T   
    G    
   A   
   T   
    T    
   A   
   A   
    C    
   T   
   T   
    A    
   C   
   T   
    G    
   G   
   G   
    A    
   A   
   T   
    T    
   G   
   A   
    T    
   T   
   C   
    T    
    G    
    C    
    A    
   G   
   A   
   A   
   A   
    A    
    A    
   A   
   T   
    T    
   C   
   A   
    A    
   G   
   C   
    G    
   A   
   T   
    T    
   A   
   T   
   T   
    T    
   C   
    T    
   G   
   T   
    T    
    G    
    A    
   G   
   G   
   G   
    C    
    A    
   A   
   A   
   G   
   C   
    G    
   G   
   A   
   A   
   G   
    C    
    A    
   G   
   G   
    T    
    A    
   A   
    A    
   T   
   A   
    C    
    T    
   T   
    G    
   T   
   T   
   C   
   T   
   T   
    C    
   A   
   C   
    A    
   A   
   C   
    C    
    T    
    T    
    A    
    A    
   A   
   A   
   G   
   G   
    A    
   A   
   C   
    C    
   G   
   T   
    C    
   A   
   A   
   A   
   C   
   G   
    G    
   A   
   C   
    A    
    G    
   C   
    C    
   G   
   T   
    A    
   A   
   C   
    A    
   G   
   C   
    C    
   T   
   T   
    T    
    T    
   C   
   T   
   A   
   A   
    T    
   A   
   T   
    C    
   C   
   G   
    T    
   A   
   G   
    T    
   A   
   A   
   T   
   G   
   G   
    A    
    T    
   T   
    A    
   A   
   T   
    C    
   G   
   C   
    C    
   A   
   T   
   T   
    A    
   G   
    C    
   T   
   T   
    A    
   A   
   A   
   A   
   G   
   A   
   A   
   G   
   A   
    T    
   G   
   A   
    T    
   G   
   A   
    G    
    T    
   T   
    A    
    G    
   T   
    T    
   A   
   A   
    C    
   G   
   T   
    G    
    G    
   T   
   A   
    A    
    C    
   G   
   A   
   C   
    T    
    A    
   A   
    T    
   G   
   G   
    C    
    A    
   A   
    T    
    C    
    A    
    G    
   A   
   A   
    G    
   A   
   T   
    G    
   A   
   T   
    T    
   A   
   T   
    C    
   G   
   G   
    A    
   A   
   C   
   A   
   C   
   A   
   T   
    G    
    C    
    A    
   G   
   G   
    A    
   T   
   A   
   C   
   T   
   C   
   T   
   G   
   T   
    C    
   A   
   C   
    A    
   T   
   T   
    T    
    G    
   A   
    T    
   G   
   A   
    A    
    A    
    A    
    T    
    A    
   C   
    T    
   G   
    T    
    A    
   C   
   G   
    T    
   G   
   A   
   T   
   A   
   T   
   G   
   G   
   G   
    C    
   C   
   G   
    G    
   A   
   C   
   A   
   G   
   C   
    A    
   T   
   C   
    A    
   G   
   G   
    C    
   G   
   T   
    T    
    C    
   G   
    G    
   G   
   G   
    A    
   A   
   T   
    C    
   C   
   G   
    T    
    C    
   T   
    C    
   C   
   G   
    C    
   G   
   A   
    A    
    A    
   A   
    T    
   G   
   A   
    T    
   T   
    A    
    T    
   G   
   T   
    G    
   G   
   T   
    C    
   G   
   G   
    T    
    G    
   C   
   A   
   G   
   C   
    G    
    A    
   T   
    T    
    C    
   T   
   G   
    G    
   A   
    T    
   G   
    A    
   A   
    A    
   A   
    C    
    A    
   A   
   A   
   G   
   A   
   A   
   G   
   T   
    C    
    C    
   T   
    A    
   G   
   T   
    C    
   A   
   T   
    T    
   A   
   C   
    T    
   G   
   A   
   A   
   A   
   A   
    T    
   G   
   G   
   T   
   T   
   A   
    T    
   G   
   G   
    T    
   A   
   A   
    G    
   C   
   G   
    T    
   A   
   C   
    A    
   A   
   A   
   A   
   G   
    C    
    T    
   T   
   C   
    T    
   G   
   A   
    A    
   T   
   A   
   T   
   C   
   C   
   A   
   G   
   T   
    T    
   A   
   A   
    A    
   G   
   G   
    A    
   C   
   G   
    T    
   G   
   G   
    C    
   G   
   G   
   T   
   A   
   A   
   A   
   G   
   G   
    G    
   A   
   T   
    T    
   A   
   A   
    G    
   A   
   C   
    A    
    G    
   C   
    A    
   A   
   A   
    T    
   A   
   T   
   C   
   A   
   C   
   T   
   G   
   A   
    G    
   A   
   A   
   A   
   A   
   A   
   T   
   G   
   G   
   T   
   C   
   C   
   A   
    T    
   T   
   A   
   G   
   C   
    T    
   G   
   G   
    T    
   T   
   T   
    A    
   A   
   C   
   C   
   A   
   C   
    G    
   G   
   T   
    C    
    A    
    A    
   T   
   G   
   G   
    G    
    G    
   A   
   T   
   G   
   A   
   A   
   G   
   A   
    T    
   A   
   T   
   C   
    T    
   T   
    A    
    T    
   T   
    G    
   A   
   T   
    T    
   A   
   C   
    G    
    A    
   A   
    C    
   A   
   A   
   A   
   G   
   G   
    C    
   G   
   T   
    C    
   A   
   T   
   T   
   A   
   T   
   C   
   C   
   G   
    C    
   T   
   T   
    T    
   A   
   A   
   C   
   G   
   T   
    T    
    G    
    A    
    T    
    T    
   C   
   T   
   G   
   T   
   T   
   T   
   C   
   T   
   C   
   A   
   A   
   A   
   C   
    A    
   G   
   G   
   A   
   C   
   G   
    C    
    G    
   C   
   A   
   A   
   C   
   A   
   T   
   T   
    A    
   G   
   G   
    G    
   G   
   T   
    T    
   C   
   G   
   T   
   T   
   T   
    A    
   A   
   T   
   G   
   A   
    G    
    A    
   A   
   T   
   G   
   G   
   A   
   A   
   G   
    A    
    T    
   G   
    G    
    T    
    G    
   C   
    C    
   A   
   A   
   A   
   -   
   -   
   -   
   -   
   -   
   -   
   -   
   -   
   -   
   -   
   -   
   -   
   -   
   -   
   -   
   -   
   -   
   -   
   -   
   -   
   -   
   -   
   -   
   -   
   -   
   -   
   -   
   -   
   -   
   -   
   -   
   -   
   -   
   -   
   -   
   -   
   -   
   -   
   -   
   -   
   -   
   -   
   -   
   -   
   -   
   -   
   -   
   -   
   -   
   -   
   -   
   -   
   -   
   -   
   -   
   -   
   -   
   -   
   -   
   -   
   -   
   -   
   -   
   -   
   -   
   -   
   -   
   -   
   -   
   -   
   -   
   -   
 

 
   N59896F   
   A   
   T   
   G   
   A   
   G   
   T   
   G   
   A   
   A   
   G   
   A   
   A   
   A   
   T   
   T   
   A   
   A   
   A   
   G   
   A   
   A   
   A   
   A   
   C   
   A   
   T   
   T   
   C   
   A   
   A   
   G   
   A   
   C   
   G   
   T   
   C   
   A   
   A   
   T   
   C   
   T   
   G   
   A   
   C   
   C   
   A   
   G   
   C   
   G   
   A   
   A   
   A   
   T   
   G   
   A   
   A   
   A   
   G   
   A   
   A   
   T   
   C   
   T   
   T   
   T   
   C   
   A   
   T   
   T   
   G   
   A   
   C   
   T   
   A   
   C   
   G   
   C   
   A   
   A   
   T   
   G   
   A   
   G   
   T   
   G   
   T   
   T   
   A   
   T   
   C   
   G   
   T   
   A   
   G   
   C   
   C   
   C   
   G   
   C   
   G   
   C   
   G   
   T   
   T   
   A   
   C   
   C   
   T   
   G   
   A   
   C   
   G   
   T   
   T   
   C   
   G   
   T   
   G   
   A   
   C   
   G   
   G   
   T   
   T   
   T   
   A   
   A   
   A   
   A   
   C   
   C   
   T   
   G   
   T   
   T   
   C   
   A   
   T   
   C   
   G   
   C   
   C   
   G   
   A   
   A   
   T   
   C   
   T   
   T   
   A   
   T   
   A   
   T   
   G   
   G   
   A   
   A   
   T   
   G   
   A   
   A   
   C   
   G   
   A   
   A   
   T   
   T   
   A   
   G   
   G   
   G   
   G   
   T   
   A   
   A   
   C   
   C   
   C   
   C   
   T   
   G   
   A   
   T   
   A   
   A   
   A   
   C   
   C   
   A   
   C   
   A   
   C   
   A   
   A   
   A   
   A   
   A   
   A   
   T   
   C   
   A   
   G   
   C   
   C   
   C   
   G   
   G   
   A   
   T   
   T   
   G   
   T   
   T   
   G   
   G   
   G   
   G   
   A   
   T   
   G   
   T   
   T   
   A   
   T   
   G   
   G   
   G   
   T   
   A   
   A   
   A   
   T   
   A   
   T   
   C   
   A   
   C   
   C   
   C   
   C   
   C   
   A   
   T   
   G   
   G   
   G   
   G   
   A   
   C   
   A   
   G   
   T   
   G   
   C   
   G   
   A   
   T   
   T   
   T   
   A   
   C   
   G   
   A   
   A   
   T   
   C   
   A   
   A   
   T   
   G   
   G   
   T   
   G   
   C   
   G   
   G   
   A   
   T   
   G   
   G   
   C   
   A   
   C   
   A   
   A   
   C   
   C   
   T   
   T   
   T   
   T   
   A   
   G   
   T   
   T   
   A   
   T   
   C   
   G   
   G   
   G   
   C   
   T   
   A   
   T   
   G   
   C   
   T   
   A   
   G   
   T   
   T   
   G   
   A   
   C   
   G   
   G   
   C   
   C   
   A   
   C   
   G   
   G   
   A   
   A   
   A   
   C   
   T   
   T   
   C   
   G   
   G   
   T   
   T   
   C   
   T   
   G   
   T   
   C   
   G   
   A   
   T   
   G   
   G   
   T   
   G   
   A   
   C   
   G   
   G   
   C   
   G   
   C   
   T   
   G   
   C   
   C   
   G   
   C   
   T   
   A   
   T   
   G   
   C   
   G   
   T   
   T   
   A   
   T   
   A   
   C   
   C   
   G   
   A   
   A   
   G   
   C   
   A   
   C   
   G   
   T   
   A   
   T   
   G   
   A   
   G   
   T   
   A   
   A   
   A   
   A   
   T   
   T   
   G   
   C   
   T   
   T   
   T   
   A   
   G   
   A   
   A   
   A   
   T   
   G   
   C   
   T   
   A   
   C   
   G   
   A   
   G   
   A   
   T   
   A   
   T   
   T   
   A   
   A   
   C   
   A   
   A   
   A   
   A   
   A   
   T   
   A   
   C   
   A   
   G   
   T   
   C   
   G   
   A   
   T   
   T   
   T   
   C   
   C   
   A   
   A   
   G   
   G   
   A   
   A   
   A   
   C   
   T   
   A   
   T   
   G   
   A   
   T   
   G   
   A   
   T   
   T   
   C   
   A   
   G   
   A   
   A   
   C   
   A   
   A   
   G   
   A   
   A   
   C   
   C   
   A   
   G   
   T   
   A   
   G   
   T   
   T   
   T   
   T   
   A   
   C   
   C   
   T   
   G   
   C   
   T   
   C   
   G   
   T   
   T   
   T   
   T   
   C   
   C   
   A   
   A   
   A   
   C   
   T   
   T   
   A   
   C   
   T   
   A   
   G   
   T   
   T   
   A   
   A   
   C   
   G   
   G   
   A   
   A   
   C   
   A   
   A   
   C   
   G   
   G   
   G   
   G   
   A   
   T   
   T   
   G   
   C   
   G   
   G   
   T   
   G   
   G   
   G   
   G   
   A   
   T   
   G   
   G   
   C   
   A   
   A   
   C   
   A   
   A   
   A   
   T   
   A   
   T   
   T   
   C   
   C   
   A   
   C   
   C   
   A   
   C   
   A   
   C   
   A   
   A   
   T   
   T   
   T   
   A   
   A   
   G   
   T   
   G   
   A   
   A   
   G   
   T   
   G   
   A   
   T   
   T   
   G   
   A   
   T   
   G   
   C   
   A   
   A   
   C   
   A   
   A   
   G   
   T   
   T   
   T   
   A   
   T   
   T   
   G   
   A   
   T   
   G   
   G   
   A   
   C   
   A   
   A   
   T   
   C   
   C   
   T   
   G   
   A   
   T   
   G   
   T   
   A   
   A   
   C   
   G   
   A   
   C   
   G   
   A   
   A   
   T   
   G   
   A   
   A   
   T   
   T   
   G   
   A   
   T   
   G   
   G   
   A   
   A   
   G   
   T   
   G   
   T   
   T   
   A   
   C   
   C   
   T   
   G   
   G   
   A   
   C   
   C   
   A   
   G   
   A   
   T   
   T   
   T   
   T   
   C   
   C   
   A   
   A   
   C   
   A   
   G   
   G   
   C   
   G   
   G   
   T   
   T   
   T   
   A   
   G   
   T   
   G   
   A   
   T   
   G   
   G   
   G   
   G   
   A   
   A   
   A   
   T   
   C   
   A   
   G   
   G   
    G    
   A   
   T   
    T    
   C   
   G   
    C    
    C    
   G   
   A   
   G   
   C   
    A    
   T   
   A   
   T   
   G   
   A   
   A   
   A   
   C   
    A    
   G   
   G   
    G    
   A   
   A   
    A    
   G   
   G   
   T   
   T   
   C   
    G    
   A   
   T   
   T   
   A   
   C   
    T    
   G   
   T   
    T    
   C   
   G   
   T   
   G   
   C   
   A   
   A   
   A   
   A   
   G   
   T   
   T   
   G   
   A   
    A    
    T    
   T   
    G    
   A   
    C    
   T   
   G   
   A   
    A    
   A   
   T   
   G   
    C    
   C   
    G    
   A   
   A   
   T   
   G   
   G   
    A    
   A   
   A   
   A   
   G   
   A   
   A   
   C   
   G   
    T    
   A   
   T   
    T    
    T    
   T   
    A    
   G   
   T   
    A    
   A   
   C   
    T    
   G   
   A   
   A   
   T   
   T   
    G    
   C   
   C   
   T   
   T   
   A   
   T   
   A   
   T   
   G   
   G   
   T   
    G    
   A   
   A   
   T   
   A   
   A   
   A   
   G   
   C   
    C    
   A   
   A   
   A   
   T   
   T   
    A    
   A   
   T   
    C    
   G   
   A   
   A   
   C   
   G   
    A    
   A   
   T   
   T   
   T   
   C   
   T   
   G   
   A   
    A    
    T    
   T   
    A    
   C   
   A   
   C   
   A   
   G   
   A   
   G   
   A   
    T    
   A   
   A   
    A    
   C   
   G   
    A    
   A   
   T   
    T    
   G   
   A   
   A   
   G   
   G   
    A    
   A   
   T   
    T    
   A   
   C   
    T    
   G   
   A   
   T   
   T   
   T   
   G   
   C   
   G   
   G   
   G   
   A   
    T    
   G   
   A   
   A   
   T   
   C   
   T   
   T   
   C   
    T    
   C   
   G   
   T   
   G   
   A   
    A    
   G   
   G   
    C    
   A   
   T   
   G   
   C   
   G   
    G    
   A   
   T   
    T    
   G   
   T   
   C   
   A   
   T   
    C    
   G   
   A   
    T    
    G    
   T   
    T    
   C   
   G   
   T   
   C   
   G   
    A    
   G   
   A   
    T    
   G   
    T    
    G    
   A   
   G   
    T    
   G   
   C   
    C    
   T   
   C   
    T    
   G   
   T   
   T   
    G    
   T   
    G    
   T   
   T   
    A    
   A   
   A   
    C    
   A   
   A   
   C   
   T   
   T   
    G    
   T   
   A   
    C    
   A   
   A   
   A   
    A    
   T   
   G   
   A   
   C   
    T    
    G    
   C   
    C    
   T   
   T   
    A    
   C   
   A   
    A    
   A   
    C    
    A    
   T   
   C   
    T    
   T   
   T   
    T    
   G   
   G   
    T    
   T   
   T   
    T    
   A   
   A   
    C    
   A   
   T   
   G   
   T   
   T   
    A    
   G   
   C   
    C    
   A   
   T   
    C    
   G   
   A   
   A   
   A   
   A   
   A   
   G   
   G   
    C    
   G   
   T   
    A    
   C   
   C   
    G    
   A   
   A   
   A   
    A    
   T   
   T   
   T   
   T   
    A    
   A   
   G   
    C    
   T   
   T   
   G   
   A   
   A   
   A   
   C   
    G    
    T    
   A   
   T   
    T    
    T    
   T   
    A    
   G   
   A   
   A   
   A   
   A   
    C    
   T   
   A   
    C    
    G    
   T   
    C    
   G   
   A   
    G    
   C   
   A   
    C    
   C   
   A   
    A    
    A    
    A    
    A    
    G    
   A   
   A   
   G   
   T   
   C   
   A   
   T   
    T    
   A   
   C   
    T    
    C    
   G   
    C    
   C   
   G   
   G   
   A   
   C   
    G    
    A    
   T   
    T    
   T   
   T   
   T   
   G   
   A   
    C    
   A   
   A   
   A   
    A    
   A   
    C    
   A   
   A   
    G    
   G   
   C   
   A   
   G   
   A   
   A   
   G   
   C   
   A   
    C    
   G   
    G    
   G   
   C   
    G    
   C   
   A   
    T    
   A   
   T   
    T    
   T   
   T   
   A   
   G   
   A   
   A   
   G   
   G   
   T   
    C    
   T   
    A    
   C   
   G   
    A    
   A   
   T   
   T   
   G   
   C   
    C    
   T   
   T   
    A    
   G   
   A   
   T   
   C   
   A   
   T   
   A   
   T   
   C   
    G    
   A   
   T   
   G   
   A   
   A   
   A   
   T   
    C    
   A   
   T   
   T   
   G   
   C   
    C    
   A   
   T   
   T   
   A   
   T   
    C    
   C   
   G   
    T    
   G   
   G   
    G    
   T   
   C   
    A    
    C    
   A   
   A   
   T   
   C   
   A   
   G   
   A   
   T   
   G   
   A   
    C    
   G   
   A   
    A    
   G   
   C   
   A   
   A   
   A   
   A   
   G   
   C   
    A    
   A   
    C    
    G    
    T    
   T   
    A    
   A   
   T   
    T    
   G   
   A   
   A   
   C   
   G   
    C    
   T   
   T   
    T    
   G   
   A   
    A    
   T   
   T   
    T    
   T   
   C   
    A    
   G   
   A   
   T   
   C   
   G   
    T    
   C   
   A   
    A    
    G    
   C   
    G    
   C   
   A   
   A   
   G   
   C   
    A    
   A   
   T   
    T    
   T   
   T   
   A   
   G   
   A   
   T   
   A   
   T   
   G   
   C   
   G   
   T   
   T   
   T   
    A    
   C   
   G   
    C    
   C   
   G   
    T    
   T   
   T   
    A    
   A   
   C   
    A    
   G   
   G   
    C    
   T   
   T   
    A    
   G   
   A   
   A   
   C   
   G   
    C    
   G   
   A   
    C    
   A   
   A   
   A   
   A   
   T   
    T    
   G   
   A   
    A    
   A   
   A   
   T   
   G   
   A   
   A   
   T   
   A   
    T    
   C   
   A   
   A   
   G   
   A   
    G    
   C   
   T   
    A    
   T   
   T   
    G    
   A   
    A    
   A   
    T    
   T   
    C    
   A   
   T   
   T   
   G   
    C    
    G    
   G   
   A   
    C    
   T   
   T   
    A    
    G    
    A    
    G    
   G   
   A   
   C   
   A   
   T   
   T   
    T    
   T   
    A    
   G   
   C   
    C    
    C    
   G   
    C    
   C   
   C   
    A    
   G   
   A   
    A    
   C   
   G   
    C    
   G   
   T   
    C    
   A   
    T    
    T    
   G   
   A   
   A   
   A   
   T   
    C    
   A   
   T   
    T    
   A   
   A   
    A    
   A   
    C    
    A    
   G   
   A   
    G    
   T   
   T   
   A   
    A    
    A    
    T    
   G   
   A   
   C   
    G    
   T   
    T    
    C    
    G    
    C    
    A    
    C    
   A   
    A    
    A    
   A   
   T   
   T   
   T   
   G   
   G   
   T   
   G   
   A   
   T   
    G    
    C    
   A   
   C   
   G   
   A   
   C   
   G   
    C    
   A   
   C   
   A   
   G   
   A   
   A   
    T    
   T   
   A   
    T    
   T   
   A   
   G   
   T   
    A    
   G   
   G   
   T   
   G   
   A   
    A    
   G   
   T   
   C   
    T    
   T   
    A    
   A   
   G   
   T   
   C   
   T   
    T    
   G   
   A   
   A   
   G   
   A   
   T   
   G   
   A   
   A   
   G   
   A   
   T   
    T    
   T   
    A    
   A   
   T   
    C    
   G   
   A   
   A   
   G   
   A   
   A   
   G   
   A   
   A   
   G   
   A   
   A   
    G    
   T   
   C   
   G   
   T   
    G    
   A   
   T   
   T   
   A   
   C   
    G    
    T    
   T   
    A    
   A   
   C   
    C    
   A   
   A   
    T    
   A   
   A   
    C    
   G   
   G   
    C    
   T   
   A   
    C    
   A   
   T   
    T    
   A   
   A   
    G    
   C   
   G   
    G    
    A    
   T   
   G   
   G   
    C    
   A   
   A   
   A   
    C    
    T    
    C    
    C    
   G   
   A   
   A   
   T   
   T   
   C   
    C    
   G   
    G    
   G   
   C   
    G    
   C   
   A   
   A   
   C   
   G   
    C    
    C    
   G   
    C    
   G   
   G   
    T    
   G   
   G   
    A    
   C   
   G   
   T   
   G   
   G   
    T    
   G   
   T   
    C    
   C   
   A   
   A   
   G   
   G   
    G    
   A   
   T   
   G   
   G   
   G   
    C    
    G    
   T   
    T    
   C   
   A   
    T    
    G    
   A   
   T   
   G   
   A   
    T    
   G   
   A   
    T    
   T   
   T   
   C   
   G   
   T   
    G    
   A   
   A   
    A    
   A   
   A   
    C    
   C   
   T   
    A    
    G    
   T   
   T   
   T   
   C   
   T   
   T   
   G   
    T    
   T   
   C   
    A    
   A   
   C   
    A    
   C   
   A   
    C    
   G   
   A   
    C    
   A   
   C   
    G    
   T   
   T   
    A    
   T   
   T   
   A   
   T   
   T   
    C    
   T   
   T   
   T   
   A   
   C   
   G   
   A   
   A   
    C    
   A   
    C    
    T    
   G   
   G   
    G    
   A   
   A   
   A   
   G   
   T   
    T    
   T   
   A   
    C    
   C   
   G   
    A    
   G   
   C   
   A   
   A   
   A   
   A   
   G   
   G   
    T    
   T   
   A   
    C    
   G   
   A   
   A   
   A   
   T   
    C    
   C   
   C   
    T    
   G   
   A   
    A    
   T   
   A   
   C   
   G   
   G   
    T    
    A    
   G   
    A    
   A   
   C   
   A   
   G   
   C   
    A    
   A   
   A   
   A   
   G   
   G   
    A    
   A   
   T   
    T    
   C   
   C   
   A   
    G    
   T   
    G    
   A   
   T   
    T    
   A   
   A   
    C    
   T   
   T   
    A    
   C   
   T   
    G    
   G   
   G   
    A    
   A   
   T   
    T    
   G   
   A   
    T    
   T   
   C   
    T    
    G    
    C    
    A    
   G   
   A   
   A   
   A   
    A    
    A    
   A   
   T   
    T    
   C   
   A   
    A    
   G   
   C   
    G    
   A   
   T   
    T    
   A   
   T   
   T   
    T    
   C   
    T    
   G   
   T   
    T    
    G    
    A    
   G   
   G   
   G   
    C    
    A    
   A   
   A   
   G   
   C   
    G    
   G   
   A   
   A   
   G   
    C    
    A    
   G   
   G   
    T    
    A    
   A   
    A    
   T   
   A   
    C    
    T    
   T   
    G    
   T   
   T   
   C   
   T   
   T   
    C    
   A   
   C   
    A    
   A   
   C   
    C    
    T    
    T    
    A    
    A    
   A   
   A   
   G   
   G   
    A    
   A   
   C   
    C    
   G   
   T   
    C    
   A   
   A   
   A   
   C   
   G   
    G    
   A   
   C   
    A    
    G    
   C   
    C    
   G   
   T   
    A    
   A   
   C   
    A    
   G   
   C   
    C    
   T   
   T   
    T    
    T    
   C   
   T   
   A   
   A   
    T    
   A   
   T   
    C    
   C   
   G   
    T    
   A   
   G   
    T    
   A   
   A   
   T   
   G   
   G   
    A    
    T    
   T   
    A    
   A   
   T   
    C    
   G   
   C   
    C    
   A   
   T   
   T   
    A    
   G   
    C    
   T   
   T   
    A    
   A   
   A   
   A   
   G   
   A   
   A   
   G   
   A   
    T    
   G   
   A   
    T    
   G   
   A   
    G    
    T    
   T   
    A    
    G    
   T   
    T    
   A   
   A   
    C    
   G   
   T   
    G    
    G    
   T   
   A   
    A    
    C    
   G   
   A   
   C   
    T    
    A    
   A   
    T    
   G   
   G   
    C    
    A    
   A   
    T    
    C    
    A    
    G    
   A   
   A   
    G    
   A   
   T   
    G    
   A   
   T   
    T    
   A   
   T   
    C    
   G   
   G   
    A    
   A   
   C   
   A   
   C   
   A   
   T   
    G    
    C    
    A    
   G   
   G   
    A    
   T   
   A   
   C   
   T   
   C   
   T   
   G   
   T   
    C    
   A   
   C   
    A    
   T   
   T   
    T    
    G    
   A   
    T    
   G   
   A   
    A    
    A    
    A    
    T    
    A    
   C   
    T    
   G   
    T    
    A    
   C   
   G   
    T    
   G   
   A   
   T   
   A   
   T   
   G   
   G   
   G   
    C    
   C   
   G   
    G    
   A   
   C   
   A   
   G   
   C   
    A    
   T   
   C   
    A    
   G   
   G   
    T    
   G   
   T   
    T    
    C    
   G   
    G    
   G   
   G   
    A    
   A   
   T   
    C    
   C   
   G   
    T    
    C    
   T   
    C    
   C   
   G   
    C    
   G   
   A   
    A    
    A    
   A   
    T    
   G   
   A   
    T    
   T   
    A    
    T    
   G   
   T   
    G    
   G   
   T   
    C    
   G   
   G   
    T    
    G    
   C   
   A   
   G   
   C   
    G    
    A    
   T   
    T    
    C    
   T   
   G   
    G    
   A   
    T    
   G   
    A    
   A   
    A    
   A   
    C    
    A    
   A   
   A   
   G   
   A   
   A   
   G   
   T   
    C    
    C    
   T   
    A    
   G   
   T   
    C    
   A   
   T   
    T    
   A   
   C   
    T    
   G   
   A   
   A   
   A   
   A   
    T    
   G   
   G   
   T   
   T   
   A   
    T    
   G   
   G   
    T    
   A   
   A   
    G    
   C   
   G   
    T    
   A   
   C   
    A    
   A   
   A   
   A   
   G   
    C    
    T    
   T   
   C   
    T    
   G   
   A   
    A    
   T   
   A   
   T   
   C   
   C   
   A   
   G   
   T   
    T    
   A   
   A   
    A    
   G   
   G   
    A    
   C   
   G   
    T    
   G   
   G   
    C    
   G   
   G   
   T   
   A   
   A   
   A   
   G   
   G   
    G    
   A   
   T   
    T    
   A   
   A   
    G    
   A   
   C   
    A    
    G    
   C   
    A    
   A   
   A   
    T    
   A   
   T   
   C   
   A   
   C   
   T   
   G   
   A   
    G    
   A   
   A   
   A   
   A   
   A   
   T   
   G   
   G   
   T   
   C   
   C   
   A   
    T    
   T   
   A   
   G   
   C   
    T    
   G   
   G   
    T    
   T   
   T   
    A    
   A   
   C   
   C   
   A   
   C   
    G    
   G   
   T   
    C    
    A    
    A    
   T   
   G   
   G   
    G    
    G    
   A   
   T   
   G   
   A   
   A   
   G   
   A   
    T    
   A   
   T   
   C   
    T    
   T   
    A    
    T    
   T   
    G    
   A   
   T   
    T    
   A   
   C   
    G    
    A    
   A   
    C    
   A   
   A   
   A   
   G   
   G   
    C    
   G   
   T   
    C    
   A   
   T   
   T   
   A   
   T   
   C   
   C   
   G   
    C    
   T   
   T   
    T    
   A   
   A   
   C   
   G   
   T   
    T    
    G    
    A    
    T    
    T    
   C   
   T   
   G   
   T   
   T   
   T   
   C   
   T   
   C   
   A   
   A   
   A   
   C   
    A    
   G   
   G   
   A   
   C   
   G   
    C    
    G    
   C   
   A   
   A   
   C   
   A   
   T   
   T   
    A    
   G   
   G   
    G    
   G   
   T   
    T    
   C   
   G   
   T   
   T   
   T   
    A    
   A   
   T   
   G   
   A   
    G    
    A    
   A   
   T   
   G   
   G   
   A   
   A   
   G   
    A    
    T    
   G   
    G    
    T    
    G    
   C   
    C    
   A   
   A   
   A   
   -   
   -   
   -   
   -   
   -   
   -   
   -   
   -   
   -   
   -   
   -   
   -   
   -   
   -   
   -   
   -   
   -   
   -   
   -   
   -   
   -   
   -   
   -   
   -   
   -   
   -   
   -   
   -   
   -   
   -   
   -   
   -   
   -   
   -   
   -   
   -   
   -   
   -   
   -   
   -   
   -   
   -   
   -   
   -   
   -   
   -   
   -   
   -   
   -   
   -   
   -   
   -   
   -   
   -   
   -   
   -   
   -   
   -   
   -   
   -   
   -   
   -   
   -   
   -   
   -   
   -   
   -   
   -   
   -   
   -   
   -   
   -   
 

 
   N59454F   
   A   
   T   
   G   
   A   
   G   
   T   
   G   
   A   
   A   
   G   
   A   
   A   
   A   
   T   
   T   
   A   
   A   
   A   
   G   
   A   
   A   
   A   
   A   
   C   
   A   
   T   
   T   
   C   
   A   
   A   
   G   
   A   
   C   
   G   
   T   
   C   
   A   
   A   
   T   
   C   
   T   
   G   
   A   
   C   
   C   
   A   
   G   
   C   
   G   
   A   
   A   
   A   
   T   
   G   
   A   
   A   
   A   
   G   
   A   
   A   
   T   
   C   
   T   
   T   
   T   
   C   
   A   
   T   
   T   
   G   
   A   
   C   
   T   
   A   
   C   
   G   
   C   
   A   
   A   
   T   
   G   
   A   
   G   
   T   
   G   
   T   
   T   
   A   
   T   
   C   
   G   
   T   
   A   
   G   
   C   
   C   
   C   
   G   
   C   
   G   
   C   
   G   
   T   
   T   
   A   
   C   
   C   
   T   
   G   
   A   
   C   
   G   
   T   
   T   
   C   
   G   
   T   
   G   
   A   
   C   
   G   
   G   
   T   
   T   
   T   
   A   
   A   
   A   
   A   
   C   
   C   
   T   
   G   
   T   
   T   
   C   
   A   
   T   
   C   
   G   
   C   
   C   
   G   
   A   
   A   
   T   
   C   
   T   
   T   
   A   
   T   
   A   
   T   
   G   
   G   
   A   
   A   
   T   
   G   
   A   
   A   
   C   
   G   
   A   
   A   
   T   
   T   
   A   
   G   
   G   
   G   
   G   
   T   
   A   
   A   
   C   
   C   
   C   
   C   
   T   
   G   
   A   
   T   
   A   
   A   
   A   
   C   
   C   
   A   
   C   
   A   
   C   
   A   
   A   
   A   
   A   
   A   
   A   
   T   
   C   
   A   
   G   
   C   
   C   
   C   
   G   
   G   
   A   
   T   
   T   
   G   
   T   
   T   
   G   
   G   
   G   
   G   
   A   
   T   
   G   
   T   
   T   
   A   
   T   
   G   
   G   
   G   
   T   
   A   
   A   
   A   
   T   
   A   
   T   
   C   
   A   
   T   
   C   
   C   
   C   
   C   
   A   
   T   
   G   
   G   
   G   
   G   
   A   
   C   
   A   
   G   
   T   
   G   
   C   
   G   
   A   
   T   
   T   
   T   
   A   
   C   
   G   
   A   
   A   
   T   
   C   
   A   
   A   
   T   
   G   
   G   
   T   
   G   
   C   
   G   
   G   
   A   
   T   
   G   
   G   
   C   
   A   
   C   
   A   
   A   
   C   
   C   
   T   
   T   
   T   
   T   
   A   
   G   
   T   
   T   
   A   
   T   
   C   
   G   
   G   
   G   
   C   
   T   
   A   
   T   
   G   
   T   
   T   
   A   
   G   
   T   
   T   
   G   
   A   
   C   
   G   
   G   
   C   
   C   
   A   
   C   
   G   
   G   
   A   
   A   
   A   
   C   
   T   
   T   
   C   
   G   
   G   
   T   
   T   
   C   
   T   
   G   
   T   
   C   
   G   
   A   
   T   
   G   
   G   
   T   
   G   
   A   
   C   
   G   
   G   
   C   
   G   
   C   
   T   
   G   
   C   
   C   
   G   
   C   
   T   
   A   
   T   
   G   
   C   
   G   
   T   
   T   
   A   
   T   
   A   
   C   
   C   
   G   
   A   
   A   
   G   
   C   
   A   
   C   
   G   
   T   
   A   
   T   
   G   
   A   
   G   
   T   
   A   
   A   
   A   
   A   
   T   
   T   
   G   
   C   
   T   
   T   
   T   
   A   
   G   
   A   
   A   
   A   
   T   
   G   
   C   
   T   
   A   
   C   
   G   
   A   
   G   
   A   
   T   
   A   
   T   
   T   
   A   
   A   
   C   
   A   
   A   
   A   
   A   
   A   
   T   
   A   
   C   
   A   
   G   
   T   
   C   
   G   
   A   
   T   
   T   
   T   
   C   
   C   
   A   
   A   
   G   
   G   
   A   
   A   
   A   
   C   
   T   
   A   
   T   
   G   
   A   
   T   
   G   
   A   
   T   
   T   
   C   
   A   
   G   
   A   
   A   
   C   
   A   
   A   
   G   
   A   
   A   
   C   
   C   
   A   
   G   
   T   
   A   
   G   
   T   
   T   
   T   
   T   
   A   
   C   
   C   
   T   
   G   
   C   
   T   
   C   
   G   
   T   
   T   
   T   
   T   
   C   
   C   
   A   
   A   
   A   
   C   
   T   
   T   
   A   
   C   
   T   
   A   
   G   
   T   
   T   
   A   
   A   
   C   
   G   
   G   
   A   
   A   
   C   
   A   
   A   
   C   
   G   
   G   
   G   
   G   
   A   
   T   
   T   
   G   
   C   
   G   
   G   
   T   
   G   
   G   
   G   
   G   
   A   
   T   
   G   
   G   
   C   
   A   
   A   
   C   
   A   
   A   
   A   
   T   
   A   
   T   
   T   
   C   
   C   
   A   
   C   
   C   
   A   
   C   
   A   
   T   
   A   
   A   
   T   
   T   
   T   
   A   
   A   
   G   
   T   
   G   
   A   
   A   
   G   
   T   
   G   
   A   
   T   
   T   
   G   
   A   
   T   
   G   
   C   
   A   
   A   
   C   
   A   
   A   
   G   
   T   
   T   
   T   
   A   
   T   
   T   
   G   
   A   
   T   
   G   
   G   
   A   
   C   
   A   
   A   
   T   
   C   
   C   
   T   
   G   
   A   
   T   
   G   
   T   
   A   
   A   
   C   
   G   
   A   
   C   
   G   
   A   
   A   
   T   
   G   
   A   
   A   
   T   
   T   
   G   
   A   
   T   
   G   
   G   
   A   
   A   
   G   
   T   
   G   
   T   
   T   
   A   
   C   
   C   
   T   
   G   
   G   
   A   
   C   
   C   
   A   
   G   
   A   
   T   
   T   
   T   
   T   
   C   
   C   
   A   
   A   
   C   
   A   
   G   
   G   
   C   
   G   
   G   
   T   
   T   
   T   
   A   
   G   
   T   
   G   
   A   
   T   
   G   
   G   
   G   
   G   
   A   
   A   
   A   
   T   
   C   
   A   
   G   
   G   
    G    
   A   
   T   
    T    
   C   
   G   
    C    
    C    
   G   
   A   
   G   
   C   
    A    
   T   
   A   
   T   
   G   
   A   
   A   
   A   
   C   
    A    
   G   
   G   
    G    
   A   
   A   
    A    
   G   
   G   
   T   
   T   
   C   
    G    
   A   
   T   
   T   
   A   
   C   
    T    
   G   
   T   
    T    
   C   
   G   
   T   
   G   
   C   
   A   
   A   
   A   
   A   
   G   
   T   
   T   
   G   
   A   
    A    
    T    
   T   
    G    
   A   
    C    
   T   
   G   
   A   
    A    
   A   
   T   
   G   
    C    
   C   
    G    
   A   
   A   
   T   
   G   
   G   
    A    
   A   
   A   
   A   
   G   
   A   
   A   
   C   
   G   
    G    
   A   
   T   
    T    
    T    
   T   
    A    
   G   
   T   
    A    
   A   
   C   
    T    
   G   
   A   
   A   
   T   
   T   
    G    
   C   
   C   
   T   
   T   
   A   
   T   
   A   
   T   
   G   
   G   
   T   
    G    
   A   
   A   
   T   
   A   
   A   
   A   
   G   
   C   
    C    
   A   
   A   
   A   
   T   
   T   
    A    
   A   
   T   
    C    
   G   
   A   
   A   
   C   
   G   
    A    
   A   
   T   
   T   
   T   
   C   
   T   
   G   
   A   
    A    
    T    
   T   
    A    
   C   
   A   
   C   
   A   
   G   
   A   
   G   
   A   
    T    
   A   
   A   
    A    
   C   
   G   
    A    
   A   
   T   
    T    
   G   
   A   
   A   
   G   
   G   
    A    
   A   
   T   
    T    
   A   
   C   
    T    
   G   
   A   
   T   
   T   
   T   
   G   
   C   
   G   
   G   
   G   
   A   
    T    
   G   
   A   
   A   
   T   
   C   
   T   
   T   
   C   
    T    
   C   
   G   
   T   
   G   
   A   
    A    
   G   
   G   
    C    
   A   
   T   
   G   
   C   
   G   
    G    
   A   
   T   
    C    
   G   
   T   
   C   
   A   
   T   
    C    
   G   
   A   
    T    
    G    
   T   
    T    
   C   
   G   
   T   
   C   
   G   
    A    
   G   
   A   
    T    
   G   
    T    
    G    
   A   
   G   
    T    
   G   
   C   
    C    
   T   
   C   
    T    
   G   
   T   
   T   
    G    
   T   
    G    
   T   
   T   
    A    
   A   
   A   
    C    
   A   
   A   
   C   
   T   
   T   
    G    
   T   
   A   
    C    
   A   
   A   
   A   
    A    
   T   
   G   
   A   
   C   
    T    
    G    
   C   
    C    
   T   
   T   
    A    
   C   
   A   
    A    
   A   
    C    
    A    
   T   
   C   
    T    
   T   
   T   
    T    
   G   
   G   
    T    
   T   
   T   
    T    
   A   
   A   
    C    
   A   
   T   
   G   
   T   
   T   
    A    
   G   
   C   
    C    
   A   
   T   
    T    
   G   
   A   
   A   
   A   
   A   
   A   
   G   
   G   
    C    
   G   
   T   
    A    
   C   
   C   
    G    
   A   
   A   
   A   
    A    
   T   
   T   
   T   
   T   
    A    
   A   
   G   
    C    
   T   
   T   
   G   
   A   
   A   
   A   
   C   
    G    
    T    
   A   
   T   
    T    
    T    
   T   
    A    
   G   
   A   
   A   
   A   
   A   
    T    
   T   
   A   
    C    
    G    
   T   
    C    
   G   
   A   
    G    
   C   
   A   
    C    
   C   
   A   
    A    
    A    
    A    
    A    
    G    
   A   
   A   
   G   
   T   
   C   
   A   
   T   
    T    
   A   
   C   
    T    
    C    
   G   
    C    
   C   
   G   
   G   
   A   
   C   
    G    
    A    
   T   
    T    
   T   
   T   
   T   
   G   
   A   
    C    
   A   
   A   
   A   
    A    
   A   
    C    
   A   
   A   
    A    
   G   
   C   
   A   
   G   
   A   
   A   
   G   
   C   
   A   
    C    
   G   
    G    
   G   
   C   
    G    
   C   
   A   
    T    
   A   
   T   
    T    
   T   
   T   
   A   
   G   
   A   
   A   
   G   
   G   
   T   
    C    
   T   
    A    
   C   
   G   
    A    
   A   
   T   
   T   
   G   
   C   
    C    
   T   
   T   
    A    
   G   
   A   
   T   
   C   
   A   
   T   
   A   
   T   
   C   
    G    
   A   
   T   
   G   
   A   
   A   
   A   
   T   
    C    
   A   
   T   
   T   
   G   
   C   
    C    
   A   
   T   
   T   
   A   
   T   
    C    
   C   
   G   
    T    
   G   
   G   
    A    
   T   
   C   
    A    
    C    
   A   
   A   
   T   
   C   
   A   
   G   
   A   
   T   
   G   
   A   
    C    
   G   
   A   
    A    
   G   
   C   
   A   
   A   
   A   
   A   
   G   
   C   
    A    
   A   
    C    
    G    
    T    
   T   
    A    
   A   
   T   
    T    
   G   
   A   
   A   
   C   
   G   
    C    
   T   
   T   
    T    
   G   
   A   
    A    
   T   
   T   
    T    
   T   
   C   
    A    
   G   
   A   
   T   
   C   
   G   
    T    
   C   
   A   
    A    
    G    
   C   
    G    
   C   
   A   
   A   
   G   
   C   
    G    
   A   
   T   
    T    
   T   
   T   
   A   
   G   
   A   
   T   
   A   
   T   
   G   
   C   
   G   
   T   
   T   
   T   
    A    
   C   
   G   
    C    
   C   
   G   
    T    
   T   
   T   
    A    
   A   
   C   
    A    
   G   
   G   
    C    
   T   
   T   
    A    
   G   
   A   
   A   
   C   
   G   
    C    
   G   
   A   
    C    
   A   
   A   
   A   
   A   
   T   
    T    
   G   
   A   
    A    
   A   
   A   
   T   
   G   
   A   
   A   
   T   
   A   
    T    
   C   
   A   
   A   
   G   
   A   
    G    
   C   
   T   
    A    
   T   
   T   
    G    
   A   
    A    
   A   
    T    
   T   
    C    
   A   
   T   
   T   
   G   
    C    
    G    
   G   
   A   
    C    
   T   
   T   
    A    
    G    
    A    
    G    
   G   
   A   
   C   
   A   
   T   
   T   
    T    
   T   
    A    
   G   
   C   
    C    
    C    
   G   
    C    
   C   
   C   
    A    
   G   
   A   
    A    
   C   
   G   
    C    
   G   
   T   
    C    
   A   
    T    
    T    
   G   
   A   
   A   
   A   
   T   
    C    
   A   
   T   
    T    
   A   
   A   
    A    
   A   
    C    
    A    
   G   
   A   
    G    
   T   
   T   
   A   
    A    
    A    
    T    
   G   
   A   
   C   
    G    
   T   
    T    
    C    
    G    
    C    
    A    
    C    
   A   
    A    
    A    
   A   
   T   
   T   
   T   
   G   
   G   
   T   
   G   
   A   
   T   
    G    
    C    
   A   
   C   
   G   
   A   
   C   
   G   
    C    
   A   
   C   
   A   
   G   
   A   
   A   
    T    
   T   
   A   
    T    
   T   
   A   
   G   
   T   
    A    
   G   
   G   
   T   
   G   
   A   
    A    
   G   
   T   
   C   
    C    
   T   
    A    
   A   
   G   
   T   
   C   
   T   
    T    
   G   
   A   
   A   
   G   
   A   
   T   
   G   
   A   
   A   
   G   
   A   
   T   
    T    
   T   
    A    
   A   
   T   
    C    
   G   
   A   
   A   
   G   
   A   
   A   
   G   
   A   
   A   
   G   
   A   
   A   
    G    
   T   
   C   
   G   
   T   
    G    
   A   
   T   
   T   
   A   
   C   
    G    
    T    
   T   
    A    
   A   
   C   
    C    
   A   
   A   
    T    
   A   
   A   
    C    
   G   
   G   
    C    
   T   
   A   
    C    
   A   
   T   
    T    
   A   
   A   
    G    
   C   
   G   
    G    
    A    
   T   
   G   
   G   
    C    
   A   
   A   
   A   
    C    
    T    
    C    
    C    
   G   
   A   
   A   
   T   
   T   
   C   
    C    
   G   
    G    
   G   
   C   
    G    
   C   
   A   
   A   
   C   
   G   
    C    
    C    
   G   
    T    
   G   
   G   
    C    
   G   
   G   
    A    
   C   
   G   
   T   
   G   
   G   
    T    
   G   
   T   
    C    
   C   
   A   
   A   
   G   
   G   
    C    
   A   
   T   
   G   
   G   
   G   
    C    
    G    
   T   
    T    
   C   
   A   
    T    
    G    
   A   
   T   
   G   
   A   
    T    
   G   
   A   
    T    
   T   
   T   
   C   
   G   
   T   
    G    
   A   
   A   
    A    
   A   
   A   
    C    
   C   
   T   
    A    
    G    
   T   
   T   
   T   
   C   
   T   
   T   
   G   
    T    
   T   
   C   
    A    
   A   
   C   
    A    
   C   
   A   
    C    
   G   
   A   
    C    
   A   
   C   
    G    
   T   
   T   
    A    
   T   
   T   
   A   
   T   
   T   
    C    
   T   
   T   
   T   
   A   
   C   
   G   
   A   
   A   
    C    
   A   
    C    
    T    
   G   
   G   
    G    
   A   
   A   
   A   
   G   
   T   
    T    
   T   
   A   
    C    
   C   
   G   
    A    
   G   
   C   
   A   
   A   
   A   
   A   
   G   
   G   
    T    
   T   
   A   
    C    
   G   
   A   
   A   
   A   
   T   
    C    
   C   
   C   
    T    
   G   
   A   
    A    
   T   
   A   
   C   
   G   
   G   
    T    
    A    
   G   
    A    
   A   
   C   
   A   
   G   
   C   
    A    
   A   
   A   
   A   
   G   
   G   
    A    
   A   
   T   
    T    
   C   
   C   
   A   
    G    
   T   
    A    
   A   
   T   
    T    
   A   
   A   
    C    
   T   
   T   
    A    
   C   
   T   
    G    
   G   
   G   
    A    
   A   
   T   
    T    
   G   
   A   
    T    
   T   
   C   
    T    
    G    
    C    
    A    
   G   
   A   
   A   
   A   
    A    
    A    
   A   
   T   
    T    
   C   
   A   
    A    
   G   
   C   
    G    
   A   
   T   
    T    
   A   
   T   
   T   
    T    
   C   
    T    
   G   
   T   
    T    
    G    
    A    
   G   
   G   
   G   
    C    
    A    
   A   
   A   
   G   
   C   
    G    
   G   
   A   
   A   
   G   
    C    
    A    
   G   
   G   
    T    
    A    
   A   
    A    
   T   
   A   
    C    
    T    
   T   
    G    
   T   
   T   
   C   
   T   
   T   
    C    
   A   
   C   
    A    
   A   
   C   
    C    
    T    
    T    
    A    
    A    
   A   
   A   
   G   
   G   
    A    
   A   
   C   
    C    
   G   
   T   
    C    
   A   
   A   
   A   
   C   
   G   
    G    
   A   
   C   
    A    
    G    
   C   
    C    
   G   
   T   
    A    
   A   
   C   
    A    
   G   
   C   
    C    
   T   
   T   
    T    
    T    
   C   
   T   
   A   
   A   
    T    
   A   
   T   
    C    
   C   
   G   
    T    
   A   
   G   
    T    
   A   
   A   
   T   
   G   
   G   
    A    
    T    
   T   
    A    
   A   
   T   
    C    
   G   
   C   
    C    
   A   
   T   
   T   
    A    
   G   
    C    
   T   
   T   
    A    
   A   
   A   
   A   
   G   
   A   
   A   
   G   
   A   
    T    
   G   
   A   
    T    
   G   
   A   
    G    
    T    
   T   
    A    
    G    
   T   
    T    
   A   
   A   
    C    
   G   
   T   
    G    
    G    
   T   
   A   
    A    
    C    
   G   
   A   
   C   
    T    
    A    
   A   
    T    
   G   
   G   
    C    
    A    
   A   
    T    
    C    
    A    
    G    
   A   
   A   
    G    
   A   
   T   
    G    
   A   
   T   
    T    
   A   
   T   
    C    
   G   
   G   
    A    
   A   
   C   
   A   
   C   
   A   
   T   
    G    
    C    
    A    
   G   
   G   
    A    
   T   
   A   
   C   
   T   
   C   
   T   
   G   
   T   
    C    
   A   
   C   
    A    
   T   
   T   
    T    
    G    
   A   
    T    
   G   
   A   
    A    
    A    
    A    
    T    
    A    
   C   
    T    
   G   
    T    
    A    
   C   
   G   
    T    
   G   
   A   
   T   
   A   
   T   
   G   
   G   
   G   
    C    
   C   
   G   
    G    
   A   
   C   
   A   
   G   
   C   
    A    
   T   
   C   
    A    
   G   
   G   
    T    
   G   
   T   
    T    
    C    
   G   
    G    
   G   
   G   
    A    
   A   
   T   
    C    
   C   
   G   
    T    
    C    
   T   
    C    
   C   
   G   
    C    
   G   
   A   
    A    
    A    
   A   
    T    
   G   
   A   
    T    
   T   
    A    
    T    
   G   
   T   
    G    
   G   
   T   
    C    
   G   
   G   
    T    
    G    
   C   
   A   
   G   
   C   
    G    
    A    
   T   
    T    
    C    
   T   
   G   
    G    
   A   
    T    
   G   
    A    
   A   
    A    
   A   
    C    
    A    
   A   
   A   
   G   
   A   
   A   
   G   
   T   
    C    
    C    
   T   
    A    
   G   
   T   
    C    
   A   
   T   
    T    
   A   
   C   
    T    
   G   
   A   
   A   
   A   
   A   
    T    
   G   
   G   
   T   
   T   
   A   
    T    
   G   
   G   
    T    
   A   
   A   
    G    
   C   
   G   
    T    
   A   
   C   
    A    
   A   
   A   
   A   
   G   
    C    
    T    
   T   
   C   
    T    
   G   
   A   
    A    
   T   
   A   
   T   
   C   
   C   
   A   
   G   
   T   
    T    
   A   
   A   
    A    
   G   
   G   
    A    
   C   
   G   
    T    
   G   
   G   
    C    
   G   
   G   
   T   
   A   
   A   
   A   
   G   
   G   
    G    
   A   
   T   
    T    
   A   
   A   
    G    
   A   
   C   
    A    
    G    
   C   
    A    
   A   
   A   
    T    
   A   
   T   
   C   
   A   
   C   
   T   
   G   
   A   
    G    
   A   
   A   
   A   
   A   
   A   
   T   
   G   
   G   
   T   
   C   
   C   
   A   
    T    
   T   
   A   
   G   
   C   
    T    
   G   
   G   
    T    
   T   
   T   
    A    
   A   
   C   
   C   
   A   
   C   
    G    
   G   
   T   
    C    
    A    
    A    
   T   
   G   
   G   
    G    
    G    
   A   
   T   
   G   
   A   
   A   
   G   
   A   
    T    
   A   
   T   
   C   
    T    
   T   
    A    
    T    
   T   
    G    
   A   
   T   
    T    
   A   
   C   
    G    
    A    
   A   
    C    
   A   
   A   
   A   
   G   
   G   
    C    
   G   
   T   
    C    
   A   
   T   
   T   
   A   
   T   
   C   
   C   
   G   
    C    
   T   
   T   
    T    
   A   
   A   
   C   
   G   
   T   
    T    
    G    
    A    
    T    
    T    
   C   
   T   
   G   
   T   
   T   
   T   
   C   
   T   
   C   
   A   
   A   
   A   
   C   
    A    
   G   
   G   
   A   
   C   
   G   
    C    
    G    
   C   
   A   
   A   
   C   
   A   
   T   
   T   
    A    
   G   
   G   
    G    
   G   
   T   
    T    
   C   
   G   
   T   
   T   
   T   
    A    
   A   
   T   
   G   
   A   
    G    
    A    
   A   
   T   
   G   
   G   
   A   
   A   
   G   
    A    
    T    
   G   
    G    
    T    
    G    
   C   
    C    
   A   
   A   
   A   
   -   
   -   
   -   
   -   
   -   
   -   
   -   
   -   
   -   
   -   
   -   
   -   
   -   
   -   
   -   
   -   
   -   
   -   
   -   
   -   
   -   
   -   
   -   
   -   
   -   
   -   
   -   
   -   
   -   
   -   
   -   
   -   
   -   
   -   
   -   
   -   
   -   
   -   
   -   
   -   
   -   
   -   
   -   
   -   
   -   
   -   
   -   
   -   
   -   
   -   
   -   
   -   
   -   
   -   
   -   
   -   
   -   
   -   
   -   
   -   
   -   
   -   
   -   
   -   
   -   
   -   
   -   
   -   
   -   
   -   
   -   
   -   
 

 
   N55113   
   A   
   T   
   G   
   A   
   G   
   T   
   G   
   A   
   A   
   G   
   A   
   A   
   A   
   T   
   T   
   A   
   A   
   A   
   G   
   A   
   A   
   A   
   A   
   C   
   A   
   T   
   T   
   C   
   A   
   A   
   G   
   A   
   C   
   G   
   T   
   C   
   A   
   A   
   T   
   C   
   T   
   G   
   A   
   C   
   C   
   A   
   G   
   C   
   G   
   A   
   A   
   A   
   T   
   G   
   A   
   A   
   A   
   G   
   A   
   A   
   T   
   C   
   T   
   T   
   T   
   C   
   A   
   T   
   T   
   G   
   A   
   C   
   T   
   A   
   C   
   G   
   C   
   A   
   A   
   T   
   G   
   A   
   G   
   T   
   G   
   T   
   T   
   A   
   T   
   C   
   G   
   T   
   A   
   G   
   C   
   C   
   C   
   G   
   C   
   G   
   C   
   G   
   T   
   T   
   A   
   C   
   C   
   T   
   G   
   A   
   C   
   G   
   T   
   T   
   C   
   G   
   T   
   G   
   A   
   C   
   G   
   G   
   T   
   T   
   T   
   A   
   A   
   A   
   A   
   C   
   C   
   T   
   G   
   T   
   T   
   C   
   A   
   T   
   C   
   G   
   C   
   C   
   G   
   A   
   A   
   T   
   C   
   T   
   T   
   A   
   T   
   A   
   T   
   G   
   G   
   A   
   A   
   T   
   G   
   A   
   A   
   C   
   G   
   A   
   A   
   T   
   T   
   A   
   G   
   G   
   G   
   G   
   T   
   A   
   A   
   C   
   C   
   C   
   C   
   T   
   G   
   A   
   T   
   A   
   A   
   A   
   C   
   C   
   A   
   C   
   A   
   C   
   A   
   A   
   A   
   A   
   A   
   A   
   T   
   C   
   A   
   G   
   C   
   C   
   C   
   G   
   G   
   A   
   T   
   T   
   G   
   T   
   T   
   G   
   G   
   G   
   G   
   A   
   T   
   G   
   T   
   T   
   A   
   T   
   G   
   G   
   G   
   T   
   A   
   A   
   A   
   T   
   A   
   T   
   C   
   A   
   C   
   C   
   C   
   C   
   C   
   A   
   T   
   G   
   G   
   G   
   G   
   A   
   C   
   A   
   G   
   T   
   G   
   C   
   G   
   A   
   T   
   T   
   T   
   A   
   C   
   G   
   A   
   A   
   T   
   C   
   A   
   A   
   T   
   G   
   G   
   T   
   G   
   C   
   G   
   G   
   A   
   T   
   G   
   G   
   C   
   A   
   C   
   A   
   A   
   C   
   C   
   T   
   T   
   T   
   T   
   A   
   G   
   T   
   T   
   A   
   T   
   C   
   G   
   G   
   G   
   C   
   T   
   A   
   T   
   G   
   T   
   T   
   A   
   G   
   T   
   T   
   G   
   A   
   C   
   G   
   G   
   C   
   C   
   A   
   C   
   G   
   G   
   A   
   A   
   A   
   C   
   T   
   T   
   C   
   G   
   G   
   T   
   T   
   C   
   T   
   G   
   T   
   C   
   G   
   A   
   T   
   G   
   G   
   T   
   G   
   A   
   C   
   G   
   G   
   C   
   G   
   C   
   T   
   G   
   C   
   C   
   G   
   C   
   T   
   A   
   T   
   G   
   C   
   G   
   T   
   T   
   A   
   T   
   A   
   C   
   C   
   G   
   A   
   A   
   G   
   C   
   A   
   C   
   G   
   T   
   A   
   T   
   G   
   A   
   G   
   T   
   A   
   A   
   A   
   A   
   T   
   T   
   G   
   C   
   T   
   T   
   T   
   A   
   G   
   A   
   A   
   A   
   T   
   G   
   C   
   T   
   A   
   C   
   G   
   A   
   G   
   A   
   T   
   A   
   T   
   T   
   A   
   A   
   C   
   A   
   A   
   A   
   A   
   A   
   T   
   A   
   C   
   A   
   G   
   T   
   C   
   G   
   A   
   T   
   T   
   T   
   C   
   C   
   A   
   A   
   G   
   G   
   A   
   A   
   A   
   C   
   T   
   A   
   T   
   G   
   A   
   T   
   G   
   A   
   T   
   T   
   C   
   A   
   G   
   A   
   A   
   C   
   A   
   A   
   G   
   A   
   A   
   C   
   C   
   A   
   G   
   T   
   A   
   G   
   T   
   T   
   T   
   T   
   A   
   C   
   C   
   T   
   G   
   C   
   T   
   C   
   G   
   T   
   T   
   T   
   T   
   C   
   C   
   A   
   A   
   A   
   C   
   T   
   T   
   A   
   C   
   T   
   A   
   G   
   T   
   T   
   A   
   A   
   C   
   G   
   G   
   A   
   A   
   C   
   A   
   A   
   C   
   G   
   G   
   G   
   G   
   A   
   T   
   T   
   G   
   C   
   G   
   G   
   T   
   G   
   G   
   G   
   G   
   A   
   T   
   G   
   G   
   C   
   A   
   A   
   C   
   A   
   A   
   A   
   T   
   A   
   T   
   T   
   C   
   C   
   A   
   C   
   C   
   A   
   C   
   A   
   T   
   A   
   A   
   T   
   T   
   T   
   A   
   A   
   G   
   T   
   G   
   A   
   A   
   G   
   T   
   G   
   A   
   T   
   T   
   G   
   A   
   T   
   G   
   C   
   A   
   A   
   C   
   A   
   A   
   G   
   T   
   T   
   T   
   A   
   T   
   T   
   G   
   A   
   T   
   G   
   G   
   A   
   C   
   A   
   A   
   T   
   C   
   C   
   T   
   G   
   A   
   T   
   G   
   T   
   A   
   A   
   C   
   G   
   A   
   C   
   G   
   A   
   A   
   T   
   G   
   A   
   A   
   T   
   T   
   G   
   A   
   T   
   G   
   G   
   A   
   A   
   G   
   T   
   G   
   T   
   T   
   A   
   C   
   C   
   T   
   G   
   G   
   A   
   C   
   C   
   A   
   G   
   A   
   T   
   T   
   T   
   T   
   C   
   C   
   A   
   A   
   C   
   A   
   G   
   G   
   C   
   G   
   G   
   T   
   T   
   T   
   A   
   G   
   T   
   G   
   A   
   T   
   G   
   G   
   G   
   G   
   A   
   A   
   A   
   T   
   C   
   A   
   G   
   G   
    G    
   A   
   T   
    T    
   C   
   G   
    C    
    C    
   G   
   A   
   G   
   C   
    A    
   T   
   A   
   T   
   G   
   A   
   A   
   A   
   C   
    A    
   G   
   G   
    G    
   A   
   A   
    A    
   G   
   G   
   T   
   T   
   C   
    G    
   A   
   T   
   T   
   A   
   C   
    T    
   G   
   T   
    T    
   C   
   G   
   T   
   G   
   C   
   A   
   A   
   A   
   A   
   G   
   T   
   T   
   G   
   A   
    A    
    T    
   T   
    G    
   A   
    C    
   T   
   G   
   A   
    A    
   A   
   T   
   G   
    C    
   C   
    G    
   A   
   A   
   T   
   G   
   G   
    A    
   A   
   A   
   A   
   G   
   A   
   A   
   C   
   G   
    T    
   A   
   T   
    T    
    T    
   T   
    A    
   G   
   T   
    A    
   A   
   C   
    T    
   G   
   A   
   A   
   T   
   T   
    G    
   C   
   C   
   T   
   T   
   A   
   T   
   A   
   T   
   G   
   G   
   T   
    G    
   A   
   A   
   T   
   A   
   A   
   A   
   G   
   C   
    C    
   A   
   A   
   A   
   T   
   T   
    A    
   A   
   T   
    C    
   G   
   A   
   A   
   C   
   G   
    A    
   A   
   T   
   T   
   T   
   C   
   T   
   G   
   A   
    A    
    T    
   T   
    A    
   C   
   A   
   C   
   A   
   G   
   A   
   G   
   A   
    T    
   A   
   A   
    A    
   C   
   G   
    A    
   A   
   T   
    T    
   G   
   A   
   A   
   G   
   G   
    A    
   A   
   T   
    T    
   A   
   C   
    T    
   G   
   A   
   T   
   T   
   T   
   G   
   C   
   G   
   G   
   G   
   A   
    T    
   G   
   A   
   A   
   T   
   C   
   T   
   T   
   C   
    T    
   C   
   G   
   T   
   G   
   A   
    A    
   G   
   G   
    C    
   A   
   T   
   G   
   C   
   G   
    G    
   A   
   T   
    C    
   G   
   T   
   C   
   A   
   T   
    C    
   G   
   A   
    T    
    G    
   T   
    T    
   C   
   G   
   T   
   C   
   G   
    A    
   G   
   A   
    T    
   G   
    T    
    G    
   A   
   G   
    T    
   G   
   C   
    C    
   T   
   C   
    T    
   G   
   T   
   T   
    G    
   T   
    G    
   T   
   T   
    A    
   A   
   A   
    C    
   A   
   A   
   C   
   T   
   T   
    G    
   T   
   A   
    C    
   A   
   A   
   A   
    A    
   T   
   G   
   A   
   C   
    T    
    G    
   C   
    C    
   T   
   T   
    A    
   C   
   A   
    A    
   A   
    C    
    A    
   T   
   C   
    T    
   T   
   T   
    T    
   G   
   G   
    T    
   T   
   T   
    T    
   A   
   A   
    C    
   A   
   T   
   G   
   T   
   T   
    A    
   G   
   C   
    C    
   A   
   T   
    C    
   G   
   A   
   A   
   A   
   A   
   A   
   G   
   G   
    C    
   G   
   T   
    A    
   C   
   C   
    A    
   A   
   A   
   A   
    A    
   T   
   T   
   T   
   T   
    A    
   A   
   G   
    C    
   T   
   T   
   G   
   A   
   A   
   A   
   C   
    G    
    T    
   A   
   T   
    T    
    T    
   T   
    A    
   G   
   A   
   A   
   A   
   A   
    C    
   T   
   A   
    C    
    G    
   T   
    C    
   G   
   A   
    G    
   C   
   A   
    C    
   C   
   A   
    A    
    A    
    A    
    A    
    G    
   A   
   A   
   G   
   T   
   C   
   A   
   T   
    T    
   A   
   C   
    T    
    C    
   G   
    C    
   C   
   G   
   G   
   A   
   C   
    G    
    A    
   T   
    T    
   T   
   T   
   T   
   G   
   A   
    C    
   A   
   A   
   A   
    A    
   A   
    C    
   A   
   A   
    A    
   G   
   C   
   A   
   G   
   A   
   A   
   G   
   C   
   A   
    C    
   G   
    G    
   G   
   C   
    G    
   C   
   A   
    T    
   A   
   T   
    T    
   T   
   T   
   A   
   G   
   A   
   A   
   G   
   G   
   T   
    C    
   T   
    A    
   C   
   G   
    A    
   A   
   T   
   T   
   G   
   C   
    C    
   T   
   T   
    A    
   G   
   A   
   T   
   C   
   A   
   T   
   A   
   T   
   C   
    G    
   A   
   T   
   G   
   A   
   A   
   A   
   T   
    C    
   A   
   T   
   T   
   G   
   C   
    C    
   A   
   T   
   T   
   A   
   T   
    C    
   C   
   G   
    T    
   G   
   G   
    A    
   T   
   C   
    A    
    C    
   A   
   A   
   T   
   C   
   A   
   G   
   A   
   T   
   G   
   A   
    C    
   G   
   A   
    A    
   G   
   C   
   A   
   A   
   A   
   A   
   G   
   C   
    A    
   A   
    C    
    G    
    T    
   T   
    A    
   A   
   T   
    T    
   G   
   A   
   A   
   C   
   G   
    C    
   T   
   T   
    T    
   G   
   A   
    A    
   T   
   T   
    T    
   T   
   C   
    A    
   G   
   A   
   T   
   C   
   G   
    T    
   C   
   A   
    A    
    G    
   C   
    G    
   C   
   A   
   A   
   G   
   C   
    G    
   A   
   T   
    T    
   T   
   T   
   A   
   G   
   A   
   T   
   A   
   T   
   G   
   C   
   G   
   T   
   T   
   T   
    A    
   C   
   G   
    C    
   C   
   G   
    T    
   T   
   T   
    A    
   A   
   C   
    A    
   G   
   G   
    C    
   T   
   T   
    A    
   G   
   A   
   A   
   C   
   G   
    C    
   G   
   A   
    C    
   A   
   A   
   A   
   A   
   T   
    T    
   G   
   A   
    A    
   A   
   A   
   T   
   G   
   A   
   A   
   T   
   A   
    T    
   C   
   A   
   A   
   G   
   A   
    G    
   C   
   T   
    A    
   T   
   T   
    G    
   A   
    A    
   A   
    T    
   T   
    C    
   A   
   T   
   T   
   G   
    C    
    G    
   G   
   A   
    C    
   T   
   T   
    A    
    G    
    A    
    G    
   G   
   A   
   C   
   A   
   T   
   T   
    T    
   T   
    A    
   G   
   C   
    C    
    C    
   G   
    C    
   C   
   C   
    A    
   G   
   A   
    A    
   C   
   G   
    C    
   G   
   T   
    C    
   A   
    T    
    T    
   G   
   A   
   A   
   A   
   T   
    C    
   A   
   T   
    T    
   A   
   A   
    A    
   A   
    C    
    A    
   G   
   A   
    G    
   T   
   T   
   A   
    A    
    A    
    T    
   G   
   A   
   C   
    G    
   T   
    T    
    C    
    G    
    C    
    A    
    C    
   A   
    A    
    A    
   A   
   T   
   T   
   T   
   G   
   G   
   T   
   G   
   A   
   T   
    G    
    C    
   A   
   C   
   G   
   A   
   C   
   G   
    C    
   A   
   C   
   A   
   G   
   A   
   A   
    T    
   T   
   A   
    T    
   T   
   A   
   G   
   T   
    A    
   G   
   G   
   T   
   G   
   A   
    A    
   G   
   T   
   C   
    T    
   T   
    A    
   A   
   G   
   T   
   C   
   T   
    T    
   G   
   A   
   A   
   G   
   A   
   T   
   G   
   A   
   A   
   G   
   A   
   T   
    T    
   T   
    A    
   A   
   T   
    C    
   G   
   A   
   A   
   G   
   A   
   A   
   G   
   A   
   A   
   G   
   A   
   A   
    G    
   T   
   C   
   G   
   T   
    G    
   A   
   T   
   T   
   A   
   C   
    A    
    T    
   T   
    A    
   A   
   C   
    C    
   A   
   A   
    T    
   A   
   A   
    C    
   G   
   G   
    C    
   T   
   A   
    C    
   A   
   T   
    T    
   A   
   A   
    G    
   C   
   G   
    G    
    A    
   T   
   G   
   G   
    C    
   A   
   A   
   A   
    C    
    T    
    C    
    T    
   G   
   A   
   A   
   T   
   T   
   C   
    C    
   G   
    G    
   G   
   C   
    G    
   C   
   A   
   A   
   C   
   G   
    C    
    C    
   G   
    T    
   G   
   G   
    C    
   G   
   G   
    A    
   C   
   G   
   T   
   G   
   G   
    T    
   G   
   T   
    C    
   C   
   A   
   A   
   G   
   G   
    C    
   A   
   T   
   G   
   G   
   G   
    C    
    G    
   T   
    T    
   C   
   A   
    T    
    G    
   A   
   T   
   G   
   A   
    T    
   G   
   A   
    T    
   T   
   T   
   C   
   G   
   T   
    G    
   A   
   A   
    A    
   A   
   A   
    C    
   C   
   T   
    A    
    G    
   T   
   T   
   T   
   C   
   T   
   T   
   G   
    T    
   T   
   C   
    A    
   A   
   C   
    A    
   C   
   A   
    C    
   G   
   A   
    C    
   A   
   C   
    G    
   T   
   T   
    A    
   T   
   T   
   A   
   T   
   T   
    C    
   T   
   T   
   T   
   A   
   C   
   G   
   A   
   A   
    C    
   A   
    C    
    T    
   G   
   G   
    G    
   A   
   A   
   A   
   G   
   T   
    T    
   T   
   A   
    C    
   C   
   G   
    A    
   G   
   C   
   A   
   A   
   A   
   A   
   G   
   G   
    T    
   T   
   A   
    C    
   G   
   A   
   A   
   A   
   T   
    C    
   C   
   C   
    T    
   G   
   A   
    A    
   T   
   A   
   C   
   G   
   G   
    T    
    A    
   G   
    A    
   A   
   C   
   A   
   G   
   C   
    A    
   A   
   A   
   A   
   G   
   G   
    A    
   A   
   T   
    T    
   C   
   C   
   A   
    G    
   T   
    G    
   A   
   T   
    T    
   A   
   A   
    C    
   T   
   T   
    A    
   C   
   T   
    G    
   G   
   G   
    A    
   A   
   T   
    T    
   G   
   A   
    T    
   T   
   C   
    T    
    G    
    C    
    A    
   G   
   A   
   A   
   A   
    A    
    A    
   A   
   T   
    T    
   C   
   A   
    A    
   G   
   C   
    A    
   A   
   T   
    T    
   A   
   T   
   T   
    T    
   C   
    T    
   G   
   T   
    T    
    G    
    A    
   G   
   G   
   G   
    C    
    A    
   A   
   A   
   G   
   C   
    G    
   G   
   A   
   A   
   G   
    C    
    A    
   G   
   G   
    T    
    A    
   A   
    A    
   T   
   A   
    C    
    T    
   T   
    G    
   T   
   T   
   C   
   T   
   T   
    C    
   A   
   C   
    A    
   A   
   C   
    C    
    T    
    T    
    A    
    A    
   A   
   A   
   G   
   G   
    A    
   A   
   C   
    C    
   G   
   T   
    C    
   A   
   A   
   A   
   C   
   G   
    G    
   A   
   C   
    A    
    G    
   C   
    C    
   G   
   T   
    A    
   A   
   C   
    A    
   G   
   C   
    C    
   T   
   T   
    T    
    T    
   C   
   T   
   A   
   A   
    T    
   A   
   T   
    C    
   C   
   G   
    T    
   A   
   G   
    T    
   A   
   A   
   T   
   G   
   G   
    A    
    T    
   T   
    A    
   A   
   T   
    C    
   G   
   C   
    C    
   A   
   T   
   T   
    A    
   G   
    C    
   T   
   T   
    A    
   A   
   A   
   A   
   G   
   A   
   A   
   G   
   A   
    T    
   G   
   A   
    T    
   G   
   A   
    G    
    T    
   T   
    A    
    G    
   T   
    T    
   A   
   A   
    C    
   G   
   T   
    G    
    G    
   T   
   A   
    A    
    C    
   G   
   A   
   C   
    T    
    A    
   A   
    T    
   G   
   G   
    C    
    A    
   A   
    T    
    C    
    A    
    G    
   A   
   A   
    G    
   A   
   T   
    G    
   A   
   T   
    T    
   A   
   T   
    C    
   G   
   G   
    A    
   A   
   C   
   A   
   C   
   A   
   T   
    G    
    C    
    A    
   G   
   G   
    A    
   T   
   A   
   C   
   T   
   C   
   T   
   G   
   T   
    C    
   A   
   C   
    A    
   T   
   T   
    T    
    G    
   A   
    T    
   G   
   A   
    A    
    A    
    A    
    T    
    A    
   C   
    T    
   G   
    T    
    A    
   C   
   G   
    T    
   G   
   A   
   T   
   A   
   T   
   G   
   G   
   G   
    C    
   C   
   G   
    G    
   A   
   C   
   A   
   G   
   C   
    A    
   T   
   C   
    A    
   G   
   G   
    T    
   G   
   T   
    T    
    C    
   G   
    G    
   G   
   G   
    A    
   A   
   T   
    C    
   C   
   G   
    T    
    C    
   T   
    C    
   C   
   G   
    C    
   G   
   A   
    A    
    A    
   A   
    T    
   G   
   A   
    T    
   T   
    A    
    T    
   G   
   T   
    G    
   G   
   T   
    C    
   G   
   G   
    C    
    G    
   C   
   A   
   G   
   C   
    G    
    A    
   T   
    T    
    C    
   T   
   G   
    G    
   A   
    T    
   G   
    A    
   A   
    A    
   A   
    T    
    A    
   A   
   A   
   G   
   A   
   A   
   G   
   T   
    C    
    C    
   T   
    A    
   G   
   T   
    C    
   A   
   T   
    T    
   A   
   C   
    T    
   G   
   A   
   A   
   A   
   A   
    T    
   G   
   G   
   T   
   T   
   A   
    T    
   G   
   G   
    T    
   A   
   A   
    G    
   C   
   G   
    T    
   A   
   C   
    A    
   A   
   A   
   A   
   G   
    C    
    T    
   T   
   C   
    T    
   G   
   A   
    A    
   T   
   A   
   T   
   C   
   C   
   A   
   G   
   T   
    T    
   A   
   A   
    A    
   G   
   G   
    A    
   C   
   G   
    T    
   G   
   G   
    C    
   G   
   G   
   T   
   A   
   A   
   A   
   G   
   G   
    G    
   A   
   T   
    T    
   A   
   A   
    G    
   A   
   C   
    A    
    G    
   C   
    A    
   A   
   A   
    T    
   A   
   T   
   C   
   A   
   C   
   T   
   G   
   A   
    G    
   A   
   A   
   A   
   A   
   A   
   T   
   G   
   G   
   T   
   C   
   C   
   A   
    T    
   T   
   A   
   G   
   C   
    T    
   G   
   G   
    T    
   T   
   T   
    A    
   A   
   C   
   C   
   A   
   C   
    G    
   G   
   T   
    C    
    A    
    A    
   T   
   G   
   G   
    G    
    G    
   A   
   T   
   G   
   A   
   A   
   G   
   A   
    T    
   A   
   T   
   C   
    T    
   T   
    A    
    T    
   T   
    G    
   A   
   T   
    T    
   A   
   C   
    G    
    A    
   A   
    C    
   A   
   A   
   A   
   G   
   G   
    C    
   G   
   T   
    C    
   A   
   T   
   T   
   A   
   T   
   C   
   C   
   G   
    C    
   T   
   T   
    T    
   A   
   A   
   C   
   G   
   T   
    A    
    G    
    A    
    T    
    T    
   C   
   T   
   G   
   T   
   T   
   T   
   C   
   T   
   C   
   A   
   A   
   A   
   C   
    A    
   G   
   G   
   A   
   C   
   G   
    C    
    G    
   C   
   A   
   A   
   C   
   A   
   T   
   T   
    A    
   G   
   G   
    A    
   G   
   T   
    T    
   C   
   G   
   T   
   T   
   T   
    A    
   A   
   T   
   G   
   A   
    G    
    A    
   A   
   T   
   G   
   G   
   A   
   A   
   G   
    A    
    T    
   G   
    G    
    T    
    G    
   C   
    C    
   A   
   A   
   A   
   -   
   -   
   -   
   -   
   -   
   -   
   -   
   -   
   -   
   -   
   -   
   -   
   -   
   -   
   -   
   -   
   -   
   -   
   -   
   -   
   -   
   -   
   -   
   -   
   -   
   -   
   -   
   -   
   -   
   -   
   -   
   -   
   -   
   -   
   -   
   -   
   -   
   -   
   -   
   -   
   -   
   -   
   -   
   -   
   -   
   -   
   -   
   -   
   -   
   -   
   -   
   -   
   -   
   -   
   -   
   -   
   -   
   -   
   -   
   -   
   -   
   -   
   -   
   -   
   -   
   -   
   -   
   -   
   -   
   -   
   -   
   -   
 

 
   N59891F   
   A   
   T   
   G   
   A   
   G   
   T   
   G   
   A   
   A   
   G   
   A   
   A   
   A   
   T   
   C   
   A   
   G   
   A   
   G   
   A   
   A   
   A   
   A   
   C   
   A   
   T   
   C   
   C   
   A   
   T   
   G   
   A   
   T   
   G   
   T   
   C   
   A   
   A   
   T   
   C   
   T   
   A   
   A   
   C   
   C   
   A   
   G   
   C   
   G   
   A   
   A   
   A   
   T   
   G   
   A   
   A   
   A   
   G   
   A   
   C   
   T   
   C   
   C   
   T   
   T   
   C   
   A   
   T   
   C   
   G   
   A   
   T   
   T   
   A   
   T   
   G   
   C   
   G   
   A   
   T   
   G   
   A   
   G   
   T   
   G   
   T   
   T   
   A   
   T   
   T   
   G   
   T   
   C   
   G   
   C   
   A   
   C   
   G   
   T   
   G   
   C   
   A   
   C   
   T   
   T   
   C   
   C   
   T   
   G   
   A   
   T   
   G   
   T   
   C   
   A   
   G   
   A   
   G   
   A   
   T   
   G   
   G   
   T   
   C   
   T   
   G   
   A   
   A   
   A   
   C   
   C   
   C   
   G   
   T   
   T   
   C   
   A   
   T   
   C   
   G   
   C   
   C   
   G   
   T   
   A   
   T   
   T   
   T   
   T   
   A   
   T   
   A   
   C   
   G   
   G   
   C   
   A   
   T   
   G   
   A   
   A   
   C   
   G   
   A   
   A   
   T   
   T   
   A   
   G   
   G   
   T   
   G   
   T   
   G   
   A   
   C   
   A   
   C   
   C   
   G   
   G   
   A   
   T   
   A   
   A   
   A   
   G   
   C   
   C   
   C   
   A   
   T   
   A   
   A   
   A   
   A   
   A   
   A   
   T   
   C   
   T   
   G   
   C   
   C   
   A   
   G   
   A   
   A   
   T   
   C   
   G   
   T   
   C   
   G   
   G   
   A   
   G   
   A   
   T   
   G   
   T   
   C   
   A   
   T   
   G   
   G   
   G   
   T   
   A   
   A   
   A   
   T   
   A   
   C   
   C   
   A   
   T   
   C   
   C   
   T   
   C   
   A   
   T   
   G   
   G   
   A   
   G   
   A   
   C   
   A   
   G   
   T   
   G   
   C   
   G   
   A   
   T   
   C   
   T   
   A   
   T   
   G   
   A   
   G   
   T   
   C   
   A   
   A   
   T   
   G   
   G   
   T   
   A   
   C   
   G   
   G   
   A   
   T   
   G   
   G   
   C   
   G   
   C   
   A   
   G   
   C   
   C   
   A   
   T   
   T   
   C   
   A   
   G   
   C   
   T   
   A   
   C   
   C   
   G   
   T   
   T   
   A   
   T   
   A   
   T   
   G   
   C   
   T   
   A   
   G   
   T   
   G   
   G   
   A   
   T   
   G   
   G   
   T   
   C   
   A   
   T   
   G   
   G   
   A   
   A   
   A   
   C   
   T   
   T   
   T   
   G   
   G   
   T   
   T   
   C   
   T   
   G   
   T   
   T   
   G   
   A   
   C   
   G   
   G   
   T   
   G   
   A   
   C   
   G   
   G   
   T   
   G   
   C   
   C   
   G   
   C   
   C   
   G   
   C   
   T   
   A   
   T   
   G   
   C   
   G   
   T   
   T   
   A   
   T   
   A   
   C   
   C   
   G   
   A   
   A   
   G   
   C   
   A   
   C   
   G   
   G   
   A   
   T   
   G   
   A   
   G   
   C   
   A   
   A   
   A   
   A   
   T   
   C   
   G   
   C   
   T   
   A   
   C   
   A   
   G   
   A   
   A   
   A   
   T   
   G   
   C   
   T   
   G   
   C   
   G   
   T   
   G   
   A   
   T   
   A   
   T   
   C   
   A   
   A   
   T   
   A   
   A   
   G   
   A   
   A   
   C   
   A   
   C   
   T   
   G   
   T   
   T   
   G   
   A   
   T   
   T   
   T   
   C   
   C   
   A   
   A   
   A   
   G   
   C   
   A   
   A   
   C   
   T   
   A   
   T   
   G   
   A   
   T   
   G   
   A   
   T   
   A   
   C   
   A   
   G   
   A   
   A   
   A   
   A   
   A   
   G   
   A   
   A   
   C   
   C   
   A   
   G   
   T   
   T   
   G   
   T   
   A   
   C   
   T   
   A   
   C   
   C   
   T   
   G   
   C   
   T   
   C   
   G   
   C   
   T   
   T   
   C   
   C   
   C   
   G   
   A   
   A   
   T   
   C   
   T   
   T   
   T   
   T   
   A   
   G   
   T   
   G   
   A   
   A   
   C   
   G   
   G   
   A   
   A   
   C   
   A   
   A   
   C   
   G   
   G   
   G   
   G   
   A   
   T   
   C   
   G   
   C   
   C   
   G   
   T   
   A   
   G   
   G   
   T   
   A   
   T   
   G   
   G   
   C   
   C   
   A   
   C   
   G   
   A   
   A   
   T   
   A   
   T   
   C   
   C   
   C   
   A   
   C   
   C   
   T   
   C   
   A   
   T   
   A   
   A   
   C   
   T   
   T   
   G   
   T   
   C   
   T   
   G   
   A   
   G   
   G   
   T   
   A   
   G   
   T   
   A   
   G   
   C   
   G   
   G   
   C   
   T   
   A   
   T   
   C   
   G   
   A   
   T   
   T   
   T   
   G   
   C   
   T   
   G   
   A   
   T   
   G   
   G   
   A   
   T   
   A   
   A   
   T   
   C   
   C   
   T   
   G   
   A   
   T   
   G   
   T   
   G   
   A   
   C   
   G   
   A   
   C   
   C   
   A   
   A   
   T   
   G   
   A   
   A   
   T   
   T   
   G   
   A   
   T   
   G   
   G   
   A   
   A   
   G   
   T   
   A   
   T   
   T   
   A   
   C   
   C   
   T   
   G   
   G   
   A   
   C   
   C   
   A   
   G   
   A   
   T   
   T   
   T   
   T   
   C   
   C   
   A   
   A   
   C   
   A   
   G   
   G   
   T   
   G   
   G   
   T   
   T   
   T   
   G   
   G   
   T   
   T   
   A   
   T   
   G   
   G   
   G   
   A   
   A   
   A   
   A   
   T   
   C   
   A   
   G   
   G   
    A    
   A   
   T   
    C    
   C   
   G   
    A    
    A    
   G   
   A   
   G   
   C   
    G    
   T   
   A   
   T   
   G   
   A   
   A   
   A   
   C   
    A    
   G   
   G   
    T    
   A   
   A   
    G    
   G   
   G   
   T   
   T   
   C   
    T    
   A   
   T   
   T   
   A   
   C   
    C    
   G   
   T   
    A    
   C   
   G   
   T   
   G   
   C   
   A   
   A   
   A   
   A   
   G   
   T   
   T   
   G   
   A   
    G    
    A    
   T   
    T    
   A   
    C    
   T   
   G   
   A   
    G    
   A   
   T   
   G   
    A    
   C   
    A    
   A   
   A   
   T   
   G   
   G   
    T    
   A   
   A   
   A   
   G   
   A   
   A   
   C   
   G   
    G    
   A   
   T   
    C    
    A    
   T   
    C    
   G   
   T   
    G    
   A   
   C   
    A    
   G   
   A   
   A   
   T   
   T   
    G    
   C   
   C   
   T   
   T   
   A   
   T   
   A   
   T   
   G   
   G   
   T   
    C    
   A   
   A   
   T   
   A   
   A   
   A   
   G   
   C   
    A    
   A   
   A   
   A   
   T   
   T   
    G    
   A   
   T   
    C    
   G   
   A   
   A   
   C   
   G   
    G    
   A   
   T   
   T   
   T   
   C   
   T   
   G   
   A   
    A    
    C    
   T   
    G    
   C   
   A   
   C   
   A   
   G   
   A   
   G   
   A   
    C    
   A   
   A   
    G    
   C   
   G   
    T    
   A   
   T   
    C    
   G   
   A   
   A   
   G   
   G   
    G    
   A   
   T   
    C    
   A   
   C   
    A    
   G   
   A   
   T   
   T   
   T   
   G   
   C   
   G   
   G   
   G   
   A   
    C    
   G   
   A   
   A   
   T   
   C   
   T   
   T   
   C   
    C    
   C   
   G   
   T   
   G   
   A   
    A    
   G   
   G   
    T    
   A   
   T   
   G   
   C   
   G   
    G    
   A   
   T   
    C    
   G   
   T   
   C   
   A   
   T   
    T    
   G   
   A   
    C    
    A    
   T   
    C    
   C   
   G   
   T   
   C   
   G   
    T    
   G   
   A   
    C    
   G   
    C    
    C    
   A   
   G   
    C    
   G   
   C   
    A    
   T   
   C   
    T    
   G   
   T   
   T   
    A    
   T   
    C    
   T   
   T   
    G    
   A   
   A   
    T    
   A   
   A   
   C   
   T   
   T   
    A    
   T   
   A   
    T    
   A   
   A   
   A   
    T    
   T   
   G   
   A   
   C   
    A    
    T    
   C   
    C    
   T   
   T   
    G    
   C   
   A   
    A    
   A   
    A    
    T    
   T   
   C   
    A    
   T   
   T   
    T    
   G   
   G   
    A    
   T   
   T   
    C    
   A   
   A   
    T    
   A   
   T   
   G   
   T   
   T   
    G    
   G   
   C   
    G    
   A   
   T   
    C    
   G   
   A   
   A   
   A   
   A   
   A   
   G   
   G   
    T    
   G   
   T   
    G    
   C   
   C   
    G    
   A   
   A   
   A   
    G    
   T   
   T   
   T   
   T   
    G    
   A   
   G   
    C    
   T   
   T   
   G   
   A   
   A   
   A   
   C   
    A    
    A    
   A   
   T   
    T    
    C    
   T   
    T    
   G   
   A   
   A   
   A   
   A   
    C    
   T   
   A   
    T    
    A    
   T   
    C    
   G   
   A   
    A    
   C   
   A   
    T    
   C   
   A   
    G    
    C    
    G    
    C    
    G    
   A   
   A   
   G   
   T   
   C   
   A   
   T   
    C    
   A   
   C   
    A    
    A    
   G   
    A    
   C   
   G   
   G   
   A   
   C   
    A    
    G    
   T   
    C    
   T   
   T   
   T   
   G   
   A   
    A    
   A   
   A   
   A   
    G    
   A   
    A    
   A   
   A   
    A    
   G   
   C   
   A   
   G   
   A   
   A   
   G   
   C   
   A   
    A    
   G   
    G    
   G   
   C   
    A    
   C   
   A   
    C    
   A   
   T   
    T    
   T   
   T   
   A   
   G   
   A   
   A   
   G   
   G   
   T   
    T    
   T   
    G    
   C   
   G   
    T    
   A   
   T   
   T   
   G   
   C   
    T    
   T   
   T   
    G    
   G   
   A   
   T   
   C   
   A   
   T   
   A   
   T   
   C   
    G    
   A   
   T   
   G   
   A   
   A   
   A   
   T   
    A    
   A   
   T   
   T   
   G   
   C   
    G    
   A   
   T   
   T   
   A   
   T   
    T    
   C   
   G   
    C    
   G   
   G   
    G    
   T   
   C   
    T    
    A    
   A   
   A   
   T   
   C   
   A   
   G   
   A   
   T   
   G   
   A   
    C    
   G   
   A   
    A    
   G   
   C   
   A   
   A   
   A   
   A   
   G   
   C   
    G    
   A   
    C    
    G    
    A    
   T   
    G    
   A   
   T   
    C    
   G   
   A   
   A   
   C   
   G   
    G    
   T   
   T   
    C    
   G   
   A   
    T    
   T   
   T   
    A    
   T   
   C   
    C    
   G   
   A   
   T   
   C   
   G   
    C    
   C   
   A   
    G    
    T    
   C   
    T    
   C   
   A   
   A   
   G   
   C   
    T    
   A   
   T   
    T    
   T   
   T   
   A   
   G   
   A   
   T   
   A   
   T   
   G   
   C   
   G   
   T   
   T   
   T   
    A    
   C   
   G   
    C    
   C   
   G   
    A    
   T   
   T   
    G    
   A   
   C   
    C    
   G   
   G   
    T    
   T   
   T   
    G    
   G   
   A   
   A   
   C   
   G   
    G    
   G   
   A   
    A    
   A   
   A   
   A   
   A   
   T   
    C    
   G   
   A   
    A    
   A   
   A   
   T   
   G   
   A   
   A   
   T   
   A   
    C    
   C   
   A   
   A   
   G   
   A   
    A    
   C   
   T   
    C    
   T   
   T   
    A    
   A   
    A    
   A   
    C    
   T   
    G    
   A   
   T   
   T   
   G   
    A    
    A    
   G   
   A   
    T    
   T   
   T   
    A    
    A    
    C    
    T    
   G   
   A   
   C   
   A   
   T   
   T   
    C    
   T   
    G    
   G   
   C   
    A    
    A    
   G   
    A    
   C   
   C   
    A    
   G   
   A   
    A    
   C   
   G   
    T    
   G   
   T   
    T    
   A   
    C    
    A    
   G   
   A   
   A   
   A   
   T   
    C    
   A   
   T   
    C    
   A   
   A   
    G    
   A   
    A    
    T    
   G   
   A   
    A    
   T   
   T   
   A   
    G    
    C    
    A    
   G   
   A   
   C   
    T    
   T   
    G    
    A    
    A    
    T    
    C    
    A    
   A   
    C    
    G    
   A   
   T   
   T   
   T   
   G   
   G   
   T   
   G   
   A   
   T   
    A    
    A    
   A   
   C   
   G   
   A   
   C   
   G   
    C    
   A   
   C   
   A   
   G   
   A   
   A   
    T    
   T   
   A   
    C    
   T   
   A   
   G   
   T   
    C    
   G   
   G   
   T   
   G   
   A   
    G    
   G   
   T   
   C   
    T    
   T   
    A    
   A   
   G   
   T   
   C   
   T   
    G    
   G   
   A   
   A   
   G   
   A   
   T   
   G   
   A   
   A   
   G   
   A   
   T   
    T    
   T   
    A    
   A   
   T   
    C    
   G   
   A   
   A   
   G   
   A   
   A   
   G   
   A   
   A   
   G   
   A   
   A   
    A    
   T   
   C   
   G   
   T   
    T    
   A   
   T   
   T   
   A   
   C   
    A    
    T    
   T   
    G    
   A   
   C   
    A    
   A   
   A   
    C    
   A   
   A   
    T    
   G   
   G   
    T    
   T   
   A   
    C    
   A   
   T   
    C    
   A   
   A   
    A    
   C   
   G   
    T    
    T    
   T   
   G   
   G   
    C    
   A   
   A   
   A   
    T    
    A    
    A    
    T    
   G   
   A   
   A   
   T   
   T   
   C   
    A    
   G   
    A    
   G   
   C   
    T    
   C   
   A   
   A   
   C   
   G   
    A    
    A    
   G   
    G    
   G   
   G   
    T    
   G   
   G   
    A    
   C   
   G   
   T   
   G   
   G   
    T    
   G   
   T   
    C    
   C   
   A   
   A   
   G   
   G   
    A    
   A   
   T   
   G   
   G   
   G   
    T    
    A    
   T   
    C    
   C   
   A   
    C    
    A    
   A   
   T   
   G   
   A   
    C    
   G   
   A   
    C    
   T   
   T   
   C   
   G   
   T   
    C    
   A   
   A   
    A    
   A   
   A   
    T    
   C   
   T   
    G    
    G    
   T   
   T   
   T   
   C   
   T   
   T   
   G   
    T    
   T   
   C   
    T    
   A   
   C   
    C    
   C   
   A   
    T    
   G   
   A   
    T    
   A   
   C   
    A    
   T   
   T   
    G    
   T   
   T   
   A   
   T   
   T   
    C    
   T   
   T   
   T   
   A   
   C   
   G   
   A   
   A   
    T    
   A   
    A    
    C    
   G   
   G   
    A    
   A   
   A   
   A   
   G   
   T   
    G    
   T   
   A   
    T    
   C   
   G   
    G    
   G   
   C   
   A   
   A   
   A   
   A   
   G   
   G   
    A    
   T   
   A   
    C    
   G   
   A   
   A   
   A   
   T   
    T    
   C   
   C   
    A    
   G   
   A   
    A    
   T   
   A   
   C   
   G   
   G   
    G    
    C    
   G   
    C    
   A   
   C   
   A   
   G   
   C   
    C    
   A   
   A   
   A   
   G   
   G   
    A    
   A   
   T   
    A    
   C   
   C   
   A   
    A    
   T   
    T    
   A   
   T   
    C    
   A   
   A   
    T    
   T   
   T   
    A    
   C   
   T   
    A    
   G   
   G   
    T    
   A   
   T   
    C    
   G   
   A   
    T    
   T   
   C   
    G    
    A    
    G    
    T    
   G   
   A   
   A   
   A   
    G    
    C    
   A   
   T   
    C    
   C   
   A   
    G    
   G   
   C   
    A    
   A   
   T   
    C    
   A   
   T   
   T   
    G    
   C   
    G    
   G   
   T   
    G    
    A    
    C    
   G   
   G   
   G   
    A    
    G    
   A   
   A   
   G   
   C   
    A    
   G   
   A   
   A   
   G   
    A    
    A    
   G   
   G   
    C    
    C    
   A   
    C    
   T   
   A   
    T    
    C    
   T   
    C    
   T   
   T   
   C   
   T   
   T   
    T    
   A   
   C   
    G    
   A   
   C   
    G    
    C    
    G    
    T    
    C    
   A   
   A   
   G   
   G   
    T    
   A   
   C   
    A    
   G   
   T   
    A    
   A   
   A   
   A   
   C   
   G   
    T    
   A   
   C   
    T    
    T    
   C   
    T    
   G   
   T   
    C    
   A   
   C   
    T    
   G   
   C   
    T    
   T   
   T   
    T    
    G    
   C   
   T   
   A   
   A   
    C    
   A   
   T   
    C    
   C   
   G   
    A    
   A   
   G   
    T    
   A   
   A   
   T   
   G   
   G   
    T    
    C    
   T   
    G    
   A   
   T   
    C    
   G   
   C   
    A    
   A   
   T   
   T   
    G    
   G   
    T    
   T   
   T   
    G    
   A   
   A   
   A   
   G   
   A   
   A   
   G   
   A   
    C    
   G   
   A   
    T    
   G   
   A   
    G    
    C    
   T   
    C    
    G    
   T   
    C    
   A   
   A   
    T    
   G   
   T   
    G    
    C    
   T   
   A   
    C    
    T    
   G   
   A   
   C   
    A    
    G    
   A   
    T    
   G   
   G   
    T    
    C    
   A   
    A    
    T    
    C    
    T    
   A   
   A   
    T    
   A   
   T   
    C    
   A   
   T   
    T    
   A   
   T   
    T    
   G   
   G   
    A    
   A   
   C   
   A   
   C   
   A   
   T   
    A    
    A    
    C    
   G   
   G   
    C    
   T   
   A   
   C   
   T   
   C   
   T   
   G   
   T   
    C    
   A   
   C   
    T    
   T   
   T   
    C    
    A    
   A   
    A    
   G   
   A   
    A    
    G    
    A    
    A    
    G    
   C   
    A    
   G   
    T    
    C    
   C   
   G   
    C    
   G   
   A   
   T   
   A   
   T   
   G   
   G   
   G   
    A    
   C   
   G   
    T    
   A   
   C   
   A   
   G   
   C   
    T    
   T   
   C   
    C    
   G   
   G   
    G    
   G   
   T   
    C    
    A    
   G   
    A    
   G   
   G   
    A    
   A   
   T   
    A    
   C   
   G   
    C    
    C    
   T   
    G    
   C   
   G   
    T    
   G   
   A   
    G    
    G    
   A   
    A    
   G   
   A   
    T    
   T   
    A    
    T    
   G   
   T   
    C    
   G   
   T   
    T    
   G   
   G   
    G    
    G    
   C   
   A   
   G   
   C   
    C    
    T    
   T   
    A    
    A    
   T   
   G   
    A    
   A   
    G    
   G   
    A    
   A   
    G    
   A   
    T    
    C    
   A   
   A   
   G   
   A   
   A   
   G   
   T   
    A    
    T    
   T   
    A    
   G   
   T   
    G    
   A   
   T   
    C    
   A   
   C   
    A    
   G   
   A   
   A   
   A   
   A   
    A    
   G   
   G   
   T   
   T   
   A   
    C    
   G   
   G   
    A    
   A   
   A   
    A    
   C   
   G   
    G    
   A   
   C   
    A    
   A   
   A   
   A   
   G   
    T    
    A    
   T   
   C   
    G    
   G   
   A   
    A    
   T   
   A   
   T   
   C   
   C   
   A   
   G   
   T   
    G    
   A   
   A   
    A    
   G   
   G   
    C    
   C   
   G   
    C    
   G   
   G   
    A    
   G   
   G   
   T   
   A   
   A   
   A   
   G   
   G   
    G    
   A   
   T   
    C    
   A   
   A   
    A    
   A   
   C   
    G    
    G    
   C   
    G    
   A   
   A   
    C    
   A   
   T   
   C   
   A   
   C   
   T   
   G   
   A   
    A    
   A   
   A   
   A   
   A   
   A   
   T   
   G   
   G   
   T   
   C   
   C   
   A   
    C    
   T   
   A   
   G   
   C   
    T    
   G   
   G   
    A    
   T   
   T   
    G    
   A   
   C   
   C   
   A   
   C   
    T    
   G   
   T   
    T    
    T    
    C    
   T   
   G   
   G   
    A    
    G    
   A   
   T   
   G   
   A   
   A   
   G   
   A   
    C    
   A   
   T   
   C   
    A    
   T   
    G    
    C    
   T   
    G    
   A   
   T   
    T    
   A   
   C   
    A    
    G    
   A   
    T    
   A   
   A   
   A   
   G   
   G   
    T    
   G   
   T   
    C    
   A   
   T   
   T   
   A   
   T   
   C   
   C   
   G   
    T    
   T   
   T   
    C    
   A   
   A   
   C   
   G   
   T   
    A    
    T    
    C    
    G    
    A    
   C   
   T   
   G   
   T   
   T   
   T   
   C   
   T   
   C   
   A   
   A   
   A   
   C   
    T    
   G   
   G   
   A   
   C   
   G   
    T    
    T    
   C   
   A   
   A   
   C   
   A   
   T   
   T   
    A    
   G   
   G   
    C    
   G   
   T   
    A    
   C   
   G   
   T   
   T   
   T   
    G    
   A   
   T   
   G   
   A   
    A    
    G    
   A   
   T   
   G   
   G   
   A   
   A   
   G   
    C    
    T    
   G   
    A    
    T    
    A    
   C   
    G    
   A   
   A   
   A   
   G   
   T   
   T   
   G   
   T   
   C   
   A   
   C   
   G   
   A   
   T   
   G   
   G   
   C   
   A   
   G   
   C   
   A   
   G   
   T   
   C   
   G   
   A   
   A   
   C   
   C   
   A   
   G   
   A   
   A   
   G   
   C   
   T   
   G   
   C   
   A   
   G   
   A   
   A   
   C   
   A   
   A   
   A   
   C   
   A   
   G   
   T   
   T   
   G   
   A   
   A   
   G   
   A   
   A   
   C   
   A   
   A   
   G   
   G   
   A   
   T   
   C   
   A   
   G   
   A   
   A   
   G   
   A   
   A   
   -   
   -   
   -   
 

 
   N55337   
   A   
   T   
   G   
   A   
   G   
   T   
   G   
   A   
   A   
   G   
   A   
   A   
   A   
   T   
   C   
   A   
   G   
   A   
   G   
   A   
   A   
   A   
   A   
   C   
   A   
   T   
   C   
   C   
   A   
   T   
   G   
   A   
   T   
   G   
   T   
   C   
   A   
   A   
   T   
   C   
   T   
   A   
   A   
   C   
   C   
   A   
   G   
   C   
   G   
   A   
   A   
   A   
   T   
   G   
   A   
   A   
   A   
   G   
   A   
   C   
   T   
   C   
   C   
   T   
   T   
   C   
   A   
   T   
   C   
   G   
   A   
   T   
   T   
   A   
   T   
   G   
   C   
   G   
   A   
   T   
   G   
   A   
   G   
   T   
   G   
   T   
   T   
   A   
   T   
   T   
   G   
   T   
   C   
   G   
   C   
   A   
   C   
   G   
   T   
   G   
   C   
   A   
   C   
   T   
   T   
   C   
   C   
   T   
   G   
   A   
   T   
   G   
   T   
   C   
   A   
   G   
   A   
   G   
   A   
   T   
   G   
   G   
   T   
   C   
   T   
   G   
   A   
   A   
   A   
   C   
   C   
   C   
   G   
   T   
   T   
   C   
   A   
   C   
   C   
   G   
   C   
   C   
   G   
   T   
   A   
   T   
   T   
   T   
   T   
   A   
   T   
   A   
   C   
   G   
   G   
   C   
   A   
   T   
   G   
   A   
   A   
   C   
   G   
   A   
   A   
   T   
   T   
   A   
   G   
   G   
   T   
   G   
   T   
   A   
   A   
   C   
   A   
   C   
   C   
   G   
   G   
   A   
   T   
   A   
   A   
   A   
   G   
   C   
   C   
   C   
   A   
   T   
   A   
   A   
   A   
   A   
   A   
   A   
   T   
   C   
   T   
   G   
   C   
   C   
   A   
   G   
   A   
   A   
   T   
   C   
   G   
   T   
   C   
   G   
   G   
   A   
   G   
   A   
   T   
   G   
   T   
   C   
   A   
   T   
   G   
   G   
   G   
   T   
   A   
   A   
   A   
   T   
   A   
   C   
   C   
   A   
   T   
   C   
   C   
   T   
   C   
   A   
   T   
   G   
   G   
   A   
   G   
   A   
   C   
   A   
   G   
   T   
   G   
   C   
   G   
   A   
   T   
   C   
   T   
   A   
   T   
   G   
   A   
   G   
   T   
   C   
   A   
   A   
   T   
   G   
   G   
   T   
   A   
   C   
   G   
   G   
   A   
   T   
   G   
   G   
   C   
   G   
   C   
   A   
   G   
   C   
   C   
   A   
   T   
   T   
   C   
   A   
   G   
   C   
   T   
   A   
   C   
   C   
   G   
   T   
   T   
   A   
   T   
   A   
   T   
   G   
   C   
   T   
   A   
   G   
   T   
   G   
   G   
   A   
   T   
   G   
   G   
   T   
   C   
   A   
   T   
   G   
   G   
   A   
   A   
   A   
   C   
   T   
   T   
   T   
   G   
   G   
   T   
   T   
   C   
   T   
   G   
   T   
   T   
   G   
   A   
   C   
   G   
   G   
   T   
   G   
   A   
   C   
   G   
   G   
   T   
   G   
   C   
   C   
   G   
   C   
   C   
   G   
   C   
   T   
   A   
   T   
   G   
   C   
   G   
   T   
   T   
   A   
   T   
   A   
   C   
   C   
   G   
   A   
   A   
   G   
   C   
   A   
   C   
   G   
   G   
   A   
   T   
   G   
   A   
   G   
   C   
   A   
   A   
   A   
   A   
   T   
   C   
   G   
   C   
   T   
   A   
   C   
   A   
   G   
   A   
   A   
   A   
   T   
   G   
   C   
   T   
   G   
   C   
   G   
   T   
   G   
   A   
   T   
   A   
   T   
   C   
   A   
   A   
   T   
   A   
   A   
   G   
   A   
   A   
   C   
   A   
   C   
   T   
   G   
   T   
   T   
   G   
   A   
   T   
   T   
   T   
   C   
   C   
   A   
   A   
   A   
   G   
   C   
   A   
   A   
   C   
   T   
   A   
   T   
   G   
   A   
   T   
   G   
   A   
   T   
   A   
   C   
   A   
   G   
   A   
   A   
   A   
   A   
   A   
   G   
   A   
   A   
   C   
   C   
   A   
   G   
   T   
   T   
   G   
   T   
   A   
   C   
   T   
   A   
   C   
   C   
   T   
   G   
   C   
   T   
   C   
   G   
   C   
   T   
   T   
   C   
   C   
   C   
   G   
   A   
   A   
   T   
   C   
   T   
   T   
   T   
   T   
   A   
   G   
   T   
   G   
   A   
   A   
   C   
   G   
   G   
   A   
   A   
   C   
   A   
   A   
   C   
   G   
   G   
   G   
   G   
   A   
   T   
   C   
   G   
   C   
   C   
   G   
   T   
   A   
   G   
   G   
   T   
   A   
   T   
   G   
   G   
   C   
   C   
   A   
   C   
   G   
   A   
   A   
   T   
   A   
   T   
   C   
   C   
   C   
   A   
   C   
   C   
   T   
   C   
   A   
   T   
   A   
   A   
   C   
   T   
   T   
   G   
   T   
   C   
   T   
   G   
   A   
   G   
   G   
   T   
   A   
   G   
   T   
   A   
   G   
   C   
   G   
   G   
   C   
   T   
   A   
   T   
   C   
   G   
   A   
   T   
   T   
   T   
   G   
   C   
   T   
   G   
   A   
   T   
   G   
   G   
   A   
   T   
   A   
   A   
   T   
   C   
   C   
   T   
   G   
   A   
   T   
   G   
   T   
   G   
   A   
   C   
   G   
   A   
   C   
   C   
   A   
   A   
   T   
   G   
   A   
   A   
   T   
   T   
   G   
   A   
   T   
   G   
   G   
   A   
   A   
   G   
   T   
   A   
   T   
   T   
   A   
   C   
   C   
   T   
   G   
   G   
   A   
   C   
   C   
   A   
   G   
   A   
   T   
   T   
   T   
   T   
   C   
   C   
   A   
   A   
   C   
   A   
   G   
   G   
   T   
   G   
   G   
   T   
   T   
   T   
   G   
   G   
   T   
   T   
   A   
   T   
   G   
   G   
   G   
   A   
   A   
   A   
   A   
   T   
   C   
   A   
   G   
   G   
    A    
   A   
   T   
    C    
   C   
   G   
    A    
    A    
   G   
   A   
   G   
   C   
    G    
   T   
   A   
   T   
   G   
   A   
   A   
   A   
   C   
    A    
   G   
   G   
    T    
   A   
   A   
    G    
   G   
   G   
   T   
   T   
   C   
    T    
   A   
   T   
   T   
   A   
   C   
    C    
   G   
   T   
    A    
   C   
   G   
   T   
   G   
   C   
   A   
   A   
   A   
   A   
   G   
   T   
   T   
   G   
   A   
    G    
    A    
   T   
    T    
   A   
    C    
   T   
   G   
   A   
    G    
   A   
   T   
   G   
    C    
   C   
    A    
   A   
   A   
   T   
   G   
   G   
    T    
   A   
   A   
   A   
   G   
   A   
   A   
   C   
   G   
    G    
   A   
   T   
    C    
    A    
   T   
    C    
   G   
   T   
    G    
   A   
   C   
    A    
   G   
   A   
   A   
   T   
   T   
    G    
   C   
   C   
   T   
   T   
   A   
   T   
   A   
   T   
   G   
   G   
   T   
    C    
   A   
   A   
   T   
   A   
   A   
   A   
   G   
   C   
    A    
   A   
   A   
   A   
   T   
   T   
    G    
   A   
   T   
    C    
   G   
   A   
   A   
   C   
   G   
    G    
   A   
   T   
   T   
   T   
   C   
   T   
   G   
   A   
    A    
    C    
   T   
    G    
   C   
   A   
   C   
   A   
   G   
   A   
   G   
   A   
    C    
   A   
   A   
    G    
   C   
   G   
    T    
   A   
   T   
    C    
   G   
   A   
   A   
   G   
   G   
    G    
   A   
   T   
    C    
   A   
   C   
    A    
   G   
   A   
   T   
   T   
   T   
   G   
   C   
   G   
   G   
   G   
   A   
    C    
   G   
   A   
   A   
   T   
   C   
   T   
   T   
   C   
    C    
   C   
   G   
   T   
   G   
   A   
    A    
   G   
   G   
    T    
   A   
   T   
   G   
   C   
   G   
    G    
   A   
   T   
    C    
   G   
   T   
   C   
   A   
   T   
    T    
   G   
   A   
    C    
    A    
   T   
    C    
   C   
   G   
   T   
   C   
   G   
    T    
   G   
   A   
    C    
   G   
    C    
    C    
   A   
   G   
    C    
   G   
   C   
    A    
   T   
   C   
    C    
   G   
   T   
   T   
    A    
   T   
    C    
   T   
   T   
    G    
   A   
   A   
    T    
   A   
   A   
   C   
   T   
   T   
    A    
   T   
   A   
    T    
   A   
   A   
   A   
    T    
   T   
   G   
   A   
   C   
    A    
    T    
   C   
    C    
   T   
   T   
    G    
   C   
   A   
    A    
   A   
    A    
    T    
   T   
   C   
    A    
   T   
   T   
    T    
   G   
   G   
    A    
   T   
   T   
    C    
   A   
   A   
    T    
   A   
   T   
   G   
   T   
   T   
    G    
   G   
   C   
    G    
   A   
   T   
    C    
   G   
   A   
   A   
   A   
   A   
   A   
   G   
   G   
    T    
   G   
   T   
    G    
   C   
   C   
    G    
   A   
   A   
   A   
    G    
   T   
   T   
   T   
   T   
    G    
   A   
   G   
    C    
   T   
   T   
   G   
   A   
   A   
   A   
   C   
    A    
    A    
   A   
   T   
    T    
    C    
   T   
    T    
   G   
   A   
   A   
   A   
   A   
    C    
   T   
   A   
    T    
    A    
   T   
    C    
   G   
   A   
    A    
   C   
   A   
    T    
   C   
   A   
    G    
    C    
    G    
    C    
    G    
   A   
   A   
   G   
   T   
   C   
   A   
   T   
    C    
   A   
   C   
    A    
    A    
   G   
    A    
   C   
   G   
   G   
   A   
   C   
    A    
    G    
   T   
    C    
   T   
   T   
   T   
   G   
   A   
    A    
   A   
   A   
   A   
    G    
   A   
    A    
   A   
   A   
    A    
   G   
   C   
   A   
   G   
   A   
   A   
   G   
   C   
   A   
    A    
   G   
    G    
   G   
   C   
    A    
   C   
   A   
    C    
   A   
   T   
    T    
   T   
   T   
   A   
   G   
   A   
   A   
   G   
   G   
   T   
    T    
   T   
    G    
   C   
   G   
    T    
   A   
   T   
   T   
   G   
   C   
    T    
   T   
   T   
    G    
   G   
   A   
   T   
   C   
   A   
   T   
   A   
   T   
   C   
    G    
   A   
   T   
   G   
   A   
   A   
   A   
   T   
    A    
   A   
   T   
   T   
   G   
   C   
    G    
   A   
   T   
   T   
   A   
   T   
    C    
   C   
   G   
    C    
   G   
   G   
    G    
   T   
   C   
    T    
    A    
   A   
   A   
   T   
   C   
   A   
   G   
   A   
   T   
   G   
   A   
    C    
   G   
   A   
    A    
   G   
   C   
   A   
   A   
   A   
   A   
   G   
   C   
    G    
   A   
    C    
    G    
    A    
   T   
    G    
   A   
   T   
    C    
   G   
   A   
   A   
   C   
   G   
    G    
   T   
   T   
    C    
   G   
   A   
    T    
   T   
   T   
    A    
   T   
   C   
    C    
   G   
   A   
   T   
   C   
   G   
    C    
   C   
   A   
    G    
    T    
   C   
    T    
   C   
   A   
   A   
   G   
   C   
    T    
   A   
   T   
    T    
   T   
   T   
   A   
   G   
   A   
   T   
   A   
   T   
   G   
   C   
   G   
   T   
   T   
   T   
    A    
   C   
   G   
    C    
   C   
   G   
    A    
   T   
   T   
    G    
   A   
   C   
    C    
   G   
   G   
    T    
   T   
   T   
    G    
   G   
   A   
   A   
   C   
   G   
    G    
   G   
   A   
    A    
   A   
   A   
   A   
   A   
   T   
    C    
   G   
   A   
    A    
   A   
   A   
   T   
   G   
   A   
   A   
   T   
   A   
    C    
   C   
   A   
   A   
   G   
   A   
    A    
   C   
   T   
    C    
   T   
   T   
    A    
   A   
    A    
   A   
    C    
   T   
    G    
   A   
   T   
   T   
   G   
    A    
    A    
   G   
   A   
    T    
   T   
   T   
    A    
    A    
    C    
    T    
   G   
   A   
   C   
   A   
   T   
   T   
    C    
   T   
    G    
   G   
   C   
    A    
    A    
   G   
    A    
   C   
   C   
    A    
   G   
   A   
    A    
   C   
   G   
    T    
   G   
   T   
    T    
   A   
    C    
    A    
   G   
   A   
   A   
   A   
   T   
    C    
   A   
   T   
    C    
   A   
   A   
    G    
   A   
    A    
    T    
   G   
   A   
    A    
   T   
   T   
   A   
    G    
    C    
    A    
   G   
   A   
   C   
    T    
   T   
    G    
    A    
    A    
    T    
    C    
    A    
   A   
    C    
    G    
   A   
   T   
   T   
   T   
   G   
   G   
   T   
   G   
   A   
   T   
    A    
    A    
   A   
   C   
   G   
   A   
   C   
   G   
    C    
   A   
   C   
   A   
   G   
   A   
   A   
    T    
   T   
   A   
    C    
   T   
   A   
   G   
   T   
    C    
   G   
   G   
   T   
   G   
   A   
    G    
   G   
   T   
   C   
    T    
   T   
    A    
   A   
   G   
   T   
   C   
   T   
    G    
   G   
   A   
   A   
   G   
   A   
   T   
   G   
   A   
   A   
   G   
   A   
   T   
    T    
   T   
    A    
   A   
   T   
    C    
   G   
   A   
   A   
   G   
   A   
   A   
   G   
   A   
   A   
   G   
   A   
   A   
    A    
   T   
   C   
   G   
   T   
    T    
   A   
   T   
   T   
   A   
   C   
    A    
    T    
   T   
    G    
   A   
   C   
    A    
   A   
   A   
    C    
   A   
   A   
    T    
   G   
   G   
    T    
   T   
   A   
    C    
   A   
   T   
    C    
   A   
   A   
    A    
   C   
   G   
    T    
    T    
   T   
   G   
   G   
    C    
   A   
   A   
   A   
    T    
    A    
    A    
    T    
   G   
   A   
   A   
   T   
   T   
   C   
    A    
   G   
    A    
   G   
   C   
    T    
   C   
   A   
   A   
   C   
   G   
    A    
    A    
   G   
    G    
   G   
   G   
    T    
   G   
   G   
    A    
   C   
   G   
   T   
   G   
   G   
    T    
   G   
   T   
    C    
   C   
   A   
   A   
   G   
   G   
    A    
   A   
   T   
   G   
   G   
   G   
    T    
    A    
   T   
    C    
   C   
   A   
    C    
    A    
   A   
   T   
   G   
   A   
    C    
   G   
   A   
    C    
   T   
   T   
   C   
   G   
   T   
    C    
   A   
   A   
    A    
   A   
   A   
    T    
   C   
   T   
    G    
    G    
   T   
   T   
   T   
   C   
   T   
   T   
   G   
    T    
   T   
   C   
    T    
   A   
   C   
    C    
   C   
   A   
    T    
   G   
   A   
    T    
   A   
   C   
    A    
   T   
   T   
    G    
   T   
   T   
   A   
   T   
   T   
    C    
   T   
   T   
   T   
   A   
   C   
   G   
   A   
   A   
    T    
   A   
    A    
    C    
   G   
   G   
    A    
   A   
   A   
   A   
   G   
   T   
    G    
   T   
   A   
    T    
   C   
   G   
    G    
   G   
   C   
   A   
   A   
   A   
   A   
   G   
   G   
    A    
   T   
   A   
    C    
   G   
   A   
   A   
   A   
   T   
    T    
   C   
   C   
    A    
   G   
   A   
    A    
   T   
   A   
   C   
   G   
   G   
    G    
    C    
   G   
    T    
   A   
   C   
   A   
   G   
   C   
    C    
   A   
   A   
   A   
   G   
   G   
    A    
   A   
   T   
    A    
   C   
   C   
   A   
    A    
   T   
    T    
   A   
   T   
    C    
   A   
   A   
    T    
   T   
   T   
    A    
   C   
   T   
    A    
   G   
   G   
    T    
   A   
   T   
    C    
   G   
   A   
    T    
   T   
   C   
    G    
    A    
    G    
    T    
   G   
   A   
   A   
   A   
    G    
    C    
   A   
   T   
    C    
   C   
   A   
    G    
   G   
   C   
    A    
   A   
   T   
    C    
   A   
   T   
   T   
    G    
   C   
    G    
   G   
   T   
    G    
    A    
    C    
   G   
   G   
   G   
    A    
    G    
   A   
   A   
   G   
   C   
    A    
   G   
   A   
   A   
   G   
    A    
    A    
   G   
   G   
    C    
    C    
   A   
    C    
   T   
   A   
    T    
    C    
   T   
    C    
   T   
   T   
   C   
   T   
   T   
    T    
   A   
   C   
    G    
   A   
   C   
    G    
    C    
    G    
    T    
    C    
   A   
   A   
   G   
   G   
    T    
   A   
   C   
    A    
   G   
   T   
    A    
   A   
   A   
   A   
   C   
   G   
    T    
   A   
   C   
    T    
    T    
   C   
    T    
   G   
   T   
    C    
   A   
   C   
    T    
   G   
   C   
    T    
   T   
   T   
    T    
    G    
   C   
   T   
   A   
   A   
    C    
   A   
   T   
    C    
   C   
   G   
    A    
   A   
   G   
    T    
   A   
   A   
   T   
   G   
   G   
    T    
    C    
   T   
    G    
   A   
   T   
    C    
   G   
   C   
    A    
   A   
   T   
   T   
    G    
   G   
    T    
   T   
   T   
    G    
   A   
   A   
   A   
   G   
   A   
   A   
   G   
   A   
    C    
   G   
   A   
    T    
   G   
   A   
    G    
    C    
   T   
    C    
    G    
   T   
    C    
   A   
   A   
    T    
   G   
   T   
    G    
    C    
   T   
   A   
    C    
    T    
   G   
   A   
   C   
    A    
    G    
   A   
    T    
   G   
   G   
    T    
    C    
   A   
    A    
    T    
    C    
    T    
   A   
   A   
    T    
   A   
   T   
    C    
   A   
   T   
    T    
   A   
   T   
    T    
   G   
   G   
    A    
   A   
   C   
   A   
   C   
   A   
   T   
    A    
    A    
    C    
   G   
   G   
    C    
   T   
   A   
   C   
   T   
   C   
   T   
   G   
   T   
    C    
   A   
   C   
    T    
   T   
   T   
    C    
    A    
   A   
    A    
   G   
   A   
    A    
    G    
    A    
    A    
    G    
   C   
    A    
   G   
    T    
    C    
   C   
   G   
    C    
   G   
   A   
   T   
   A   
   T   
   G   
   G   
   G   
    A    
   C   
   G   
    T    
   A   
   C   
   A   
   G   
   C   
    T    
   T   
   C   
    C    
   G   
   G   
    G    
   G   
   T   
    C    
    A    
   G   
    A    
   G   
   G   
    G    
   A   
   T   
    A    
   C   
   G   
    C    
    C    
   T   
    G    
   C   
   G   
    T    
   G   
   A   
    G    
    G    
   A   
    A    
   G   
   A   
    T    
   T   
    A    
    T    
   G   
   T   
    C    
   G   
   T   
    T    
   G   
   G   
    G    
    G    
   C   
   A   
   G   
   C   
    C    
    T    
   T   
    A    
    A    
   T   
   G   
    A    
   A   
    G    
   G   
    A    
   A   
    A    
   A   
    T    
    C    
   A   
   A   
   G   
   A   
   A   
   G   
   T   
    A    
    T    
   T   
    A    
   G   
   T   
    G    
   A   
   T   
    C    
   A   
   C   
    A    
   G   
   A   
   A   
   A   
   A   
    A    
   G   
   G   
   T   
   T   
   A   
    C    
   G   
   G   
    A    
   A   
   A   
    A    
   C   
   G   
    G    
   A   
   C   
    A    
   A   
   A   
   A   
   G   
    T    
    A    
   T   
   C   
    G    
   G   
   A   
    A    
   T   
   A   
   T   
   C   
   C   
   A   
   G   
   T   
    G    
   A   
   A   
    A    
   G   
   G   
    C    
   C   
   G   
    C    
   G   
   G   
    A    
   G   
   G   
   T   
   A   
   A   
   A   
   G   
   G   
    G    
   A   
   T   
    C    
   A   
   A   
    A    
   A   
   C   
    G    
    G    
   C   
    G    
   A   
   A   
    C    
   A   
   T   
   C   
   A   
   C   
   T   
   G   
   A   
    A    
   A   
   A   
   A   
   A   
   A   
   T   
   G   
   G   
   T   
   C   
   C   
   A   
    C    
   T   
   A   
   G   
   C   
    T    
   G   
   G   
    A    
   T   
   T   
    G    
   A   
   C   
   C   
   A   
   C   
    T    
   G   
   T   
    T    
    T    
    C    
   T   
   G   
   G   
    A    
    G    
   A   
   T   
   G   
   A   
   A   
   G   
   A   
    C    
   A   
   T   
   C   
    A    
   T   
    G    
    C    
   T   
    G    
   A   
   T   
    T    
   A   
   C   
    A    
    G    
   A   
    T    
   A   
   A   
   A   
   G   
   G   
    T    
   G   
   T   
    C    
   A   
   T   
   T   
   A   
   T   
   C   
   C   
   G   
    T    
   T   
   T   
    C    
   A   
   A   
   C   
   G   
   T   
    A    
    T    
    C    
    G    
    A    
   C   
   T   
   G   
   T   
   T   
   T   
   C   
   T   
   C   
   A   
   A   
   A   
   C   
    T    
   G   
   G   
   A   
   C   
   G   
    T    
    T    
   C   
   A   
   A   
   C   
   A   
   T   
   T   
    A    
   G   
   G   
    C    
   G   
   T   
    A    
   C   
   G   
   T   
   T   
   T   
    G    
   A   
   T   
   G   
   A   
    A    
    G    
   A   
   T   
   G   
   G   
   A   
   A   
   G   
    C    
    T    
   G   
    A    
    T    
    A    
   C   
    G    
   A   
   A   
   A   
   G   
   T   
   T   
   G   
   T   
   C   
   A   
   C   
   G   
   A   
   T   
   G   
   G   
   C   
   A   
   G   
   C   
   A   
   G   
   T   
   C   
   G   
   A   
   A   
   C   
   C   
   A   
   G   
   A   
   A   
   G   
   C   
   T   
   G   
   C   
   A   
   G   
   A   
   A   
   C   
   A   
   A   
   A   
   C   
   A   
   G   
   T   
   T   
   G   
   A   
   A   
   G   
   A   
   A   
   C   
   A   
   A   
   G   
   G   
   A   
   T   
   C   
   A   
   G   
   A   
   A   
   G   
   A   
   A   
   -   
   -   
   -   
 

 
   N54676   
   A   
   T   
   G   
   A   
   G   
   T   
   G   
   A   
   A   
   G   
   A   
   A   
   A   
   T   
   T   
   A   
   A   
   A   
   G   
   A   
   A   
   A   
   A   
   C   
   A   
   T   
   T   
   C   
   A   
   A   
   G   
   A   
   C   
   G   
   T   
   C   
   A   
   A   
   T   
   C   
   T   
   G   
   A   
   C   
   C   
   A   
   G   
   C   
   G   
   A   
   A   
   A   
   T   
   G   
   A   
   A   
   A   
   G   
   A   
   A   
   T   
   C   
   T   
   T   
   T   
   C   
   A   
   T   
   T   
   G   
   A   
   C   
   T   
   A   
   C   
   G   
   C   
   A   
   A   
   T   
   G   
   A   
   G   
   T   
   G   
   T   
   T   
   A   
   T   
   C   
   G   
   T   
   A   
   G   
   C   
   C   
   C   
   G   
   C   
   G   
   C   
   G   
   T   
   T   
   A   
   C   
   C   
   T   
   G   
   A   
   C   
   G   
   T   
   T   
   C   
   G   
   T   
   G   
   A   
   C   
   G   
   G   
   T   
   T   
   T   
   A   
   A   
   A   
   A   
   C   
   C   
   T   
   G   
   T   
   T   
   C   
   A   
   T   
   C   
   G   
   C   
   C   
   G   
   A   
   A   
   T   
   C   
   T   
   T   
   A   
   T   
   A   
   T   
   G   
   G   
   A   
   A   
   T   
   G   
   A   
   A   
   C   
   G   
   A   
   A   
   T   
   T   
   A   
   G   
   G   
   G   
   G   
   T   
   A   
   A   
   C   
   C   
   C   
   C   
   T   
   G   
   A   
   T   
   A   
   A   
   A   
   C   
   C   
   A   
   C   
   A   
   C   
   A   
   A   
   A   
   A   
   A   
   A   
   T   
   C   
   A   
   G   
   C   
   C   
   C   
   G   
   G   
   A   
   T   
   T   
   G   
   T   
   T   
   G   
   G   
   G   
   G   
   A   
   T   
   G   
   T   
   T   
   A   
   T   
   G   
   G   
   G   
   T   
   A   
   A   
   A   
   T   
   A   
   T   
   C   
   A   
   C   
   C   
   C   
   C   
   C   
   A   
   T   
   G   
   G   
   G   
   G   
   A   
   C   
   A   
   G   
   T   
   G   
   C   
   G   
   A   
   T   
   T   
   T   
   A   
   C   
   G   
   A   
   A   
   T   
   C   
   A   
   A   
   T   
   G   
   G   
   T   
   G   
   C   
   G   
   G   
   A   
   T   
   G   
   G   
   C   
   A   
   C   
   A   
   A   
   C   
   C   
   T   
   T   
   T   
   T   
   A   
   G   
   T   
   T   
   A   
   C   
   C   
   G   
   G   
   G   
   C   
   T   
   A   
   T   
   G   
   T   
   T   
   A   
   G   
   T   
   T   
   G   
   A   
   C   
   G   
   G   
   C   
   C   
   A   
   C   
   G   
   G   
   A   
   A   
   A   
   C   
   T   
   T   
   C   
   G   
   G   
   T   
   T   
   C   
   T   
   G   
   T   
   C   
   G   
   A   
   T   
   G   
   G   
   T   
   G   
   A   
   C   
   G   
   G   
   C   
   G   
   C   
   T   
   G   
   C   
   C   
   G   
   C   
   T   
   A   
   T   
   G   
   C   
   G   
   T   
   T   
   A   
   T   
   A   
   C   
   C   
   G   
   A   
   A   
   G   
   C   
   A   
   C   
   G   
   T   
   A   
   T   
   G   
   A   
   G   
   T   
   A   
   A   
   A   
   A   
   T   
   T   
   G   
   C   
   T   
   T   
   T   
   A   
   G   
   A   
   A   
   A   
   T   
   G   
   C   
   T   
   A   
   C   
   G   
   A   
   G   
   A   
   T   
   A   
   T   
   T   
   A   
   A   
   C   
   A   
   A   
   A   
   A   
   A   
   T   
   A   
   C   
   A   
   G   
   T   
   T   
   G   
   A   
   T   
   T   
   T   
   C   
   C   
   A   
   A   
   G   
   G   
   A   
   A   
   A   
   C   
   T   
   A   
   T   
   G   
   A   
   T   
   G   
   A   
   T   
   T   
   C   
   A   
   G   
   A   
   A   
   C   
   A   
   A   
   G   
   A   
   A   
   C   
   C   
   A   
   G   
   T   
   A   
   G   
   T   
   T   
   T   
   T   
   A   
   C   
   C   
   T   
   G   
   C   
   T   
   C   
   G   
   T   
   T   
   T   
   T   
   C   
   C   
   A   
   A   
   A   
   C   
   T   
   T   
   A   
   C   
   T   
   A   
   G   
   T   
   T   
   A   
   A   
   C   
   G   
   G   
   A   
   A   
   C   
   A   
   A   
   C   
   G   
   G   
   G   
   G   
   A   
   T   
   T   
   G   
   C   
   G   
   G   
   T   
   G   
   G   
   G   
   G   
   A   
   T   
   G   
   G   
   C   
   A   
   A   
   C   
   A   
   A   
   A   
   T   
   A   
   T   
   T   
   C   
   C   
   A   
   C   
   C   
   A   
   C   
   A   
   C   
   A   
   A   
   T   
   T   
   T   
   A   
   A   
   G   
   T   
   G   
   A   
   A   
   G   
   T   
   G   
   A   
   T   
   T   
   G   
   A   
   T   
   G   
   C   
   A   
   A   
   C   
   A   
   A   
   G   
   T   
   T   
   T   
   A   
   T   
   T   
   G   
   A   
   T   
   G   
   G   
   A   
   C   
   A   
   A   
   T   
   C   
   C   
   T   
   G   
   A   
   T   
   G   
   T   
   A   
   A   
   C   
   G   
   A   
   C   
   G   
   A   
   A   
   T   
   G   
   A   
   A   
   T   
   T   
   G   
   A   
   T   
   G   
   G   
   A   
   A   
   G   
   T   
   G   
   T   
   T   
   A   
   C   
   C   
   T   
   G   
   G   
   A   
   C   
   C   
   A   
   G   
   A   
   C   
   T   
   T   
   T   
   C   
   C   
   A   
   A   
   C   
   A   
   G   
   G   
   C   
   G   
   G   
   T   
   T   
   T   
   A   
   G   
   T   
   G   
   A   
   T   
   G   
   G   
   G   
   G   
   A   
   A   
   A   
   T   
   C   
   A   
   G   
   G   
    G    
   A   
   T   
    T    
   C   
   G   
    C    
    C    
   G   
   A   
   G   
   C   
    A    
   T   
   A   
   T   
   G   
   A   
   A   
   A   
   C   
    A    
   G   
   G   
    G    
   A   
   A   
    A    
   G   
   G   
   T   
   T   
   C   
    G    
   A   
   T   
   T   
   A   
   C   
    T    
   G   
   T   
    T    
   C   
   G   
   T   
   G   
   C   
   A   
   A   
   A   
   A   
   G   
   T   
   T   
   G   
   A   
    A    
    T    
   T   
    G    
   A   
    C    
   T   
   G   
   A   
    A    
   A   
   T   
   G   
    C    
   C   
    G    
   A   
   A   
   T   
   G   
   G   
    A    
   A   
   A   
   A   
   G   
   A   
   A   
   C   
   G   
    T    
   A   
   T   
    T    
    T    
   T   
    A    
   G   
   T   
    A    
   A   
   C   
    T    
   G   
   A   
   A   
   T   
   T   
    G    
   C   
   C   
   T   
   T   
   A   
   T   
   A   
   T   
   G   
   G   
   T   
    G    
   A   
   A   
   T   
   A   
   A   
   A   
   G   
   C   
    C    
   A   
   A   
   A   
   T   
   T   
    A    
   A   
   T   
    C    
   G   
   A   
   A   
   C   
   G   
    A    
   A   
   T   
   T   
   T   
   C   
   T   
   G   
   A   
    A    
    T    
   T   
    A    
   C   
   A   
   C   
   A   
   G   
   A   
   G   
   A   
    T    
   A   
   A   
    A    
   C   
   G   
    A    
   A   
   T   
    T    
   G   
   A   
   A   
   G   
   G   
    A    
   A   
   T   
    T    
   A   
   C   
    T    
   G   
   A   
   T   
   T   
   T   
   G   
   C   
   G   
   G   
   G   
   A   
    T    
   G   
   A   
   A   
   T   
   C   
   T   
   T   
   C   
    T    
   C   
   G   
   T   
   G   
   A   
    A    
   G   
   G   
    C    
   A   
   T   
   G   
   C   
   G   
    G    
   A   
   T   
    T    
   G   
   T   
   C   
   A   
   T   
    C    
   G   
   A   
    T    
    G    
   T   
    T    
   C   
   G   
   T   
   C   
   G   
    A    
   G   
   A   
    T    
   G   
    T    
    G    
   A   
   G   
    T    
   G   
   C   
    C    
   T   
   C   
    T    
   G   
   T   
   T   
    G    
   T   
    G    
   T   
   T   
    A    
   A   
   A   
    C    
   A   
   A   
   C   
   T   
   T   
    G    
   T   
   A   
    C    
   A   
   A   
   A   
    A    
   T   
   G   
   A   
   C   
    T    
    G    
   C   
    C    
   T   
   T   
    A    
   C   
   A   
    A    
   A   
    C    
    A    
   T   
   C   
    T    
   T   
   T   
    T    
   G   
   G   
    T    
   T   
   T   
    T    
   A   
   A   
    C    
   A   
   T   
   G   
   T   
   T   
    A    
   G   
   C   
    C    
   A   
   T   
    C    
   G   
   A   
   A   
   A   
   A   
   A   
   G   
   G   
    C    
   G   
   T   
    A    
   C   
   C   
    G    
   A   
   A   
   A   
    A    
   T   
   T   
   T   
   T   
    A    
   A   
   G   
    C    
   T   
   T   
   G   
   A   
   A   
   A   
   C   
    G    
    T    
   A   
   T   
    T    
    T    
   T   
    A    
   G   
   A   
   A   
   A   
   A   
    C    
   T   
   A   
    C    
    G    
   T   
    C    
   G   
   A   
    G    
   C   
   A   
    T    
   C   
   A   
    A    
    A    
    A    
    A    
    G    
   A   
   A   
   G   
   T   
   C   
   A   
   T   
    T    
   A   
   C   
    T    
    C    
   G   
    C    
   C   
   G   
   G   
   A   
   C   
    G    
    A    
   T   
    T    
   T   
   T   
   T   
   G   
   A   
    C    
   A   
   A   
   A   
    A    
   A   
    C    
   A   
   A   
    A    
   G   
   C   
   A   
   G   
   A   
   A   
   G   
   C   
   A   
    C    
   G   
    G    
   G   
   C   
    G    
   C   
   A   
    T    
   A   
   T   
    T    
   T   
   T   
   A   
   G   
   A   
   A   
   G   
   G   
   T   
    C    
   T   
    A    
   C   
   G   
    A    
   A   
   T   
   T   
   G   
   C   
    C    
   T   
   T   
    A    
   G   
   A   
   T   
   C   
   A   
   T   
   A   
   T   
   C   
    G    
   A   
   T   
   G   
   A   
   A   
   A   
   T   
    C    
   A   
   T   
   T   
   G   
   C   
    C    
   A   
   T   
   T   
   A   
   T   
    C    
   C   
   G   
    T    
   G   
   G   
    A    
   T   
   C   
    A    
    C    
   A   
   A   
   T   
   C   
   A   
   G   
   A   
   T   
   G   
   A   
    C    
   G   
   A   
    A    
   G   
   C   
   A   
   A   
   A   
   A   
   G   
   C   
    A    
   A   
    C    
    G    
    T    
   T   
    A    
   A   
   T   
    T    
   G   
   A   
   A   
   C   
   G   
    C    
   T   
   T   
    T    
   G   
   A   
    A    
   T   
   T   
    T    
   T   
   C   
    A    
   G   
   A   
   T   
   C   
   G   
    T    
   C   
   A   
    A    
    G    
   C   
    G    
   C   
   A   
   A   
   G   
   C   
    A    
   A   
   T   
    T    
   T   
   T   
   A   
   G   
   A   
   T   
   A   
   T   
   G   
   C   
   G   
   T   
   T   
   T   
    A    
   C   
   G   
    C    
   C   
   G   
    T    
   T   
   T   
    A    
   A   
   C   
    A    
   G   
   G   
    C    
   T   
   T   
    A    
   G   
   A   
   A   
   C   
   G   
    C    
   G   
   A   
    C    
   A   
   A   
   A   
   A   
   T   
    T    
   G   
   A   
    A    
   A   
   A   
   T   
   G   
   A   
   A   
   T   
   A   
    T    
   C   
   A   
   A   
   G   
   A   
    G    
   C   
   T   
    A    
   T   
   T   
    G    
   A   
    A    
   A   
    T    
   T   
    C    
   A   
   T   
   T   
   G   
    C    
    G    
   G   
   A   
    C    
   T   
   T   
    A    
    G    
    A    
    G    
   G   
   A   
   C   
   A   
   T   
   T   
    T    
   T   
    A    
   G   
   C   
    C    
    C    
   G   
    C    
   C   
   C   
    A    
   G   
   A   
    A    
   C   
   G   
    C    
   G   
   T   
    C    
   A   
    T    
    T    
   G   
   A   
   A   
   A   
   T   
    C    
   A   
   T   
    T    
   A   
   A   
    A    
   A   
    C    
    A    
   G   
   A   
    G    
   T   
   T   
   A   
    A    
    A    
    T    
   G   
   A   
   C   
    G    
   T   
    C    
    C    
    G    
    T    
    A    
    C    
   A   
    A    
    A    
   A   
   T   
   T   
   T   
   G   
   G   
   T   
   G   
   A   
   T   
    G    
    C    
   A   
   C   
   G   
   A   
   C   
   G   
    C    
   A   
   C   
   A   
   G   
   A   
   A   
    T    
   T   
   A   
    T    
   T   
   A   
   G   
   T   
    A    
   G   
   G   
   T   
   G   
   A   
    A    
   G   
   T   
   C   
    T    
   T   
    A    
   A   
   G   
   T   
   C   
   T   
    T    
   G   
   A   
   A   
   G   
   A   
   T   
   G   
   A   
   A   
   G   
   A   
   T   
    T    
   T   
    A    
   A   
   T   
    C    
   G   
   A   
   A   
   G   
   A   
   A   
   G   
   A   
   A   
   G   
   A   
   A   
    G    
   T   
   C   
   G   
   T   
    G    
   A   
   T   
   T   
   A   
   C   
    G    
    T    
   T   
    A    
   A   
   C   
    C    
   A   
   A   
    T    
   A   
   A   
    C    
   G   
   G   
    C    
   T   
   A   
    C    
   A   
   T   
    T    
   A   
   A   
    G    
   C   
   G   
    G    
    A    
   T   
   G   
   G   
    C    
   A   
   A   
   A   
    C    
    T    
    C    
    T    
   G   
   A   
   A   
   T   
   T   
   C   
    C    
   G   
    G    
   G   
   C   
    G    
   C   
   A   
   A   
   C   
   G   
    C    
    C    
   G   
    T    
   G   
   G   
    C    
   G   
   G   
    A    
   C   
   G   
   T   
   G   
   G   
    T    
   G   
   T   
    C    
   C   
   A   
   A   
   G   
   G   
    G    
   A   
   T   
   G   
   G   
   G   
    C    
    G    
   T   
    T    
   C   
   A   
    T    
    G    
   A   
   T   
   G   
   A   
    T    
   G   
   A   
    T    
   T   
   T   
   C   
   G   
   T   
    G    
   A   
   A   
    A    
   A   
   A   
    C    
   C   
   T   
    A    
    G    
   T   
   T   
   T   
   C   
   T   
   T   
   G   
    T    
   T   
   C   
    A    
   A   
   C   
    A    
   C   
   A   
    C    
   G   
   A   
    C    
   A   
   C   
    G    
   T   
   T   
    A    
   T   
   T   
   A   
   T   
   T   
    C    
   T   
   T   
   T   
   A   
   C   
   G   
   A   
   A   
    C    
   A   
    C    
    T    
   G   
   G   
    G    
   A   
   A   
   A   
   G   
   T   
    T    
   T   
   A   
    C    
   C   
   G   
    A    
   G   
   C   
   A   
   A   
   A   
   A   
   G   
   G   
    T    
   T   
   A   
    C    
   G   
   A   
   A   
   A   
   T   
    C    
   C   
   C   
    T    
   G   
   A   
    G    
   T   
   A   
   C   
   G   
   G   
    T    
    A    
   G   
    A    
   A   
   C   
   A   
   G   
   C   
    A    
   A   
   A   
   A   
   G   
   G   
    A    
   A   
   T   
    T    
   C   
   C   
   A   
    G    
   T   
    G    
   A   
   T   
    C    
   A   
   A   
    C    
   T   
   T   
    A    
   C   
   T   
    G    
   G   
   G   
    A    
   A   
   T   
    T    
   G   
   A   
    T    
   T   
   C   
    T    
    G    
    C    
    A    
   G   
   A   
   A   
   A   
    A    
    A    
   A   
   T   
    T    
   C   
   A   
    A    
   G   
   C   
    G    
   A   
   T   
    T    
   A   
   T   
   T   
    T    
   C   
    T    
   G   
   T   
    T    
    G    
    A    
   G   
   G   
   G   
    C    
    A    
   A   
   A   
   G   
   C   
    G    
   G   
   A   
   A   
   G   
    C    
    A    
   G   
   G   
    T    
    A    
   A   
    A    
   T   
   A   
    C    
    T    
   T   
    G    
   T   
   T   
   C   
   T   
   T   
    C    
   A   
   C   
    A    
   A   
   C   
    C    
    T    
    T    
    A    
    A    
   A   
   A   
   G   
   G   
    A    
   A   
   C   
    C    
   G   
   T   
    C    
   A   
   A   
   A   
   C   
   G   
    G    
   A   
   C   
    A    
    G    
   C   
    C    
   G   
   T   
    A    
   A   
   C   
    A    
   G   
   C   
    C    
   T   
   T   
    T    
    T    
   C   
   T   
   A   
   A   
    T    
   A   
   T   
    C    
   C   
   G   
    T    
   A   
   G   
    T    
   A   
   A   
   T   
   G   
   G   
    A    
    T    
   T   
    A    
   A   
   T   
    C    
   G   
   C   
    C    
   A   
   T   
   T   
    A    
   G   
    C    
   T   
   T   
    A    
   A   
   A   
   A   
   G   
   A   
   A   
   G   
   A   
    T    
   G   
   A   
    T    
   G   
   A   
    G    
    T    
   T   
    A    
    G    
   T   
    T    
   A   
   A   
    C    
   G   
   T   
    G    
    G    
   T   
   A   
    A    
    C    
   G   
   A   
   C   
    T    
    A    
   A   
    T    
   G   
   G   
    C    
    A    
   A   
    T    
    C    
    A    
    G    
   A   
   A   
    G    
   A   
   T   
    G    
   A   
   T   
    T    
   A   
   T   
    C    
   G   
   G   
    A    
   A   
   C   
   A   
   C   
   A   
   T   
    G    
    C    
    A    
   G   
   G   
    A    
   T   
   A   
   C   
   T   
   C   
   T   
   G   
   T   
    C    
   A   
   C   
    A    
   T   
   T   
    T    
    G    
   A   
    T    
   G   
   A   
    A    
    A    
    A    
    T    
    A    
   C   
    T    
   G   
    T    
    A    
   C   
   G   
    T    
   G   
   A   
   T   
   A   
   T   
   G   
   G   
   G   
    C    
   C   
   G   
    G    
   A   
   C   
   A   
   G   
   C   
    A    
   T   
   C   
    A    
   G   
   G   
    T    
   G   
   T   
    T    
    C    
   G   
    T    
   G   
   G   
    A    
   A   
   T   
    C    
   C   
   G   
    T    
    C    
   T   
    C    
   C   
   G   
    C    
   G   
   A   
    A    
    A    
   A   
    T    
   G   
   A   
    T    
   T   
    A    
    T    
   G   
   T   
    G    
   G   
   T   
    C    
   G   
   G   
    C    
    G    
   C   
   A   
   G   
   C   
    G    
    A    
   T   
    T    
    C    
   T   
   G   
    G    
   A   
    T    
   G   
    A    
   A   
    A    
   A   
    T    
    A    
   A   
   A   
   G   
   A   
   A   
   G   
   T   
    C    
    C    
   T   
    A    
   G   
   T   
    C    
   A   
   T   
    T    
   A   
   C   
    T    
   G   
   A   
   A   
   A   
   A   
    T    
   G   
   G   
   T   
   T   
   A   
    T    
   G   
   G   
    T    
   A   
   A   
    G    
   C   
   G   
    T    
   A   
   C   
    A    
   A   
   A   
   A   
   G   
    C    
    T    
   T   
   C   
    T    
   G   
   A   
    A    
   T   
   A   
   T   
   C   
   C   
   A   
   G   
   T   
    T    
   A   
   A   
    A    
   G   
   G   
    A    
   C   
   G   
    T    
   G   
   G   
    C    
   G   
   G   
   T   
   A   
   A   
   A   
   G   
   G   
    G    
   A   
   T   
    T    
   A   
   A   
    G    
   A   
   C   
    A    
    G    
   C   
    A    
   A   
   A   
    T    
   A   
   T   
   C   
   A   
   C   
   T   
   G   
   A   
    G    
   A   
   A   
   A   
   A   
   A   
   T   
   G   
   G   
   T   
   C   
   C   
   A   
    T    
   T   
   A   
   G   
   C   
    T    
   G   
   G   
    T    
   T   
   T   
    A    
   A   
   C   
   C   
   A   
   C   
    G    
   G   
   T   
    C    
    A    
    A    
   T   
   G   
   G   
    G    
    G    
   A   
   T   
   G   
   A   
   A   
   G   
   A   
    T    
   A   
   T   
   C   
    T    
   T   
    A    
    T    
   T   
    G    
   A   
   T   
    T    
   A   
   C   
    G    
    A    
   A   
    C    
   A   
   A   
   A   
   G   
   G   
    C    
   G   
   T   
    C    
   A   
   T   
   T   
   A   
   T   
   C   
   C   
   G   
    C    
   T   
   T   
    T    
   A   
   A   
   C   
   G   
   T   
    T    
    G    
    A    
    T    
    T    
   C   
   T   
   G   
   T   
   T   
   T   
   C   
   T   
   C   
   A   
   A   
   A   
   C   
    A    
   G   
   G   
   A   
   C   
   G   
    C    
    G    
   C   
   A   
   A   
   C   
   A   
   T   
   T   
    A    
   G   
   G   
    G    
   G   
   T   
    T    
   C   
   G   
   T   
   T   
   T   
    A    
   A   
   T   
   G   
   A   
    G    
    A    
   A   
   T   
   G   
   G   
   A   
   A   
   G   
    A    
    T    
   G   
    G    
    T    
    G    
   C   
    C    
   A   
   A   
   A   
   -   
   -   
   -   
   -   
   -   
   -   
   -   
   -   
   -   
   -   
   -   
   -   
   -   
   -   
   -   
   -   
   -   
   -   
   -   
   -   
   -   
   -   
   -   
   -   
   -   
   -   
   -   
   -   
   -   
   -   
   -   
   -   
   -   
   -   
   -   
   -   
   -   
   -   
   -   
   -   
   -   
   -   
   -   
   -   
   -   
   -   
   -   
   -   
   -   
   -   
   -   
   -   
   -   
   -   
   -   
   -   
   -   
   -   
   -   
   -   
   -   
   -   
   -   
   -   
   -   
   -   
   -   
   -   
   -   
   -   
   -   
   -   
 

 
   N55728   
   A   
   T   
   G   
   A   
   G   
   T   
   G   
   A   
   A   
   G   
   A   
   A   
   A   
   T   
   C   
   A   
   G   
   A   
   G   
   A   
   A   
   A   
   A   
   C   
   A   
   T   
   C   
   C   
   A   
   T   
   G   
   A   
   T   
   G   
   T   
   C   
   A   
   A   
   T   
   C   
   T   
   A   
   A   
   C   
   C   
   A   
   G   
   C   
   G   
   A   
   A   
   A   
   T   
   G   
   A   
   A   
   A   
   G   
   A   
   C   
   T   
   C   
   C   
   T   
   T   
   C   
   A   
   T   
   C   
   G   
   A   
   T   
   T   
   A   
   T   
   G   
   C   
   G   
   A   
   T   
   G   
   A   
   G   
   T   
   G   
   T   
   T   
   A   
   T   
   T   
   G   
   T   
   C   
   G   
   C   
   A   
   C   
   G   
   T   
   G   
   C   
   A   
   C   
   T   
   T   
   C   
   C   
   T   
   G   
   A   
   T   
   G   
   T   
   C   
   A   
   G   
   A   
   G   
   A   
   T   
   G   
   G   
   T   
   C   
   T   
   G   
   A   
   A   
   A   
   C   
   C   
   C   
   G   
   T   
   T   
   C   
   A   
   C   
   C   
   G   
   C   
   C   
   G   
   T   
   A   
   T   
   T   
   T   
   T   
   A   
   T   
   A   
   C   
   G   
   G   
   C   
   A   
   T   
   G   
   A   
   A   
   C   
   G   
   A   
   A   
   T   
   T   
   A   
   G   
   G   
   T   
   G   
   T   
   A   
   A   
   C   
   A   
   C   
   C   
   G   
   G   
   A   
   T   
   A   
   A   
   A   
   G   
   C   
   C   
   C   
   A   
   T   
   A   
   A   
   A   
   A   
   A   
   A   
   T   
   C   
   T   
   G   
   C   
   C   
   A   
   G   
   A   
   A   
   T   
   C   
   G   
   T   
   C   
   G   
   G   
   A   
   G   
   A   
   T   
   G   
   T   
   C   
   A   
   T   
   G   
   G   
   G   
   T   
   A   
   A   
   A   
   T   
   A   
   C   
   C   
   A   
   T   
   C   
   C   
   T   
   C   
   A   
   T   
   G   
   G   
   A   
   G   
   A   
   C   
   A   
   G   
   T   
   G   
   C   
   G   
   A   
   T   
   C   
   T   
   A   
   T   
   G   
   A   
   G   
   T   
   C   
   A   
   A   
   T   
   G   
   G   
   T   
   A   
   C   
   G   
   G   
   A   
   T   
   G   
   G   
   C   
   G   
   C   
   A   
   G   
   C   
   C   
   A   
   T   
   T   
   C   
   A   
   G   
   C   
   T   
   A   
   C   
   C   
   G   
   T   
   T   
   A   
   T   
   A   
   T   
   G   
   C   
   T   
   A   
   G   
   T   
   G   
   G   
   A   
   T   
   G   
   G   
   T   
   C   
   A   
   T   
   G   
   G   
   A   
   A   
   A   
   C   
   T   
   T   
   T   
   G   
   G   
   T   
   T   
   C   
   T   
   G   
   T   
   T   
   G   
   A   
   C   
   G   
   G   
   T   
   G   
   A   
   C   
   G   
   G   
   T   
   G   
   C   
   C   
   G   
   C   
   C   
   G   
   C   
   T   
   A   
   T   
   G   
   C   
   G   
   T   
   T   
   A   
   T   
   A   
   C   
   C   
   G   
   A   
   A   
   G   
   C   
   A   
   C   
   G   
   G   
   A   
   T   
   G   
   A   
   G   
   C   
   A   
   A   
   A   
   A   
   T   
   C   
   G   
   C   
   T   
   A   
   C   
   A   
   G   
   A   
   A   
   A   
   T   
   G   
   C   
   T   
   G   
   C   
   G   
   T   
   G   
   A   
   T   
   A   
   T   
   C   
   A   
   A   
   T   
   A   
   A   
   G   
   A   
   A   
   C   
   A   
   C   
   T   
   G   
   T   
   T   
   G   
   A   
   T   
   T   
   T   
   C   
   C   
   A   
   A   
   A   
   G   
   C   
   A   
   A   
   C   
   T   
   A   
   T   
   G   
   A   
   T   
   G   
   A   
   T   
   A   
   C   
   A   
   G   
   A   
   A   
   A   
   A   
   A   
   G   
   A   
   A   
   C   
   C   
   A   
   G   
   T   
   T   
   G   
   T   
   A   
   C   
   T   
   A   
   C   
   C   
   T   
   G   
   C   
   T   
   C   
   G   
   C   
   T   
   T   
   C   
   C   
   C   
   G   
   A   
   A   
   T   
   C   
   T   
   T   
   T   
   T   
   A   
   G   
   T   
   G   
   A   
   A   
   C   
   G   
   G   
   A   
   A   
   C   
   A   
   A   
   C   
   G   
   G   
   G   
   G   
   A   
   T   
   C   
   G   
   C   
   C   
   G   
   T   
   A   
   G   
   G   
   T   
   A   
   T   
   G   
   G   
   C   
   C   
   A   
   C   
   G   
   A   
   A   
   T   
   A   
   T   
   C   
   C   
   C   
   A   
   C   
   C   
   T   
   C   
   A   
   T   
   A   
   A   
   C   
   T   
   T   
   G   
   T   
   C   
   T   
   G   
   A   
   G   
   G   
   T   
   A   
   G   
   T   
   A   
   G   
   C   
   G   
   G   
   C   
   T   
   A   
   T   
   C   
   G   
   A   
   T   
   T   
   T   
   G   
   C   
   T   
   G   
   A   
   T   
   G   
   G   
   A   
   T   
   A   
   A   
   T   
   C   
   C   
   T   
   G   
   A   
   T   
   G   
   T   
   G   
   A   
   C   
   G   
   A   
   C   
   C   
   A   
   A   
   T   
   G   
   A   
   A   
   T   
   T   
   G   
   A   
   T   
   G   
   G   
   A   
   A   
   G   
   T   
   A   
   T   
   T   
   A   
   C   
   C   
   T   
   G   
   G   
   A   
   C   
   C   
   A   
   G   
   A   
   T   
   T   
   T   
   T   
   C   
   C   
   A   
   A   
   C   
   A   
   G   
   G   
   T   
   G   
   G   
   T   
   T   
   T   
   G   
   G   
   T   
   T   
   A   
   T   
   G   
   G   
   G   
   G   
   A   
   A   
   A   
   T   
   C   
   A   
   G   
   G   
    A    
   A   
   T   
    C    
   C   
   G   
    A    
    A    
   G   
   A   
   G   
   C   
    G    
   T   
   A   
   T   
   G   
   A   
   A   
   A   
   C   
    A    
   G   
   G   
    T    
   A   
   A   
    G    
   G   
   G   
   T   
   T   
   C   
    T    
   A   
   T   
   T   
   A   
   C   
    C    
   G   
   T   
    A    
   C   
   G   
   T   
   G   
   C   
   A   
   A   
   A   
   A   
   G   
   T   
   T   
   G   
   A   
    G    
    A    
   T   
    T    
   A   
    C    
   T   
   G   
   A   
    G    
   A   
   T   
   G   
    C    
   C   
    A    
   A   
   A   
   T   
   G   
   G   
    T    
   A   
   A   
   A   
   G   
   A   
   A   
   C   
   G   
    G    
   A   
   T   
    C    
    A    
   T   
    C    
   G   
   T   
    G    
   A   
   C   
    A    
   G   
   A   
   A   
   T   
   T   
    G    
   C   
   C   
   T   
   T   
   A   
   T   
   A   
   T   
   G   
   G   
   T   
    C    
   A   
   A   
   T   
   A   
   A   
   A   
   G   
   C   
    A    
   A   
   A   
   A   
   T   
   T   
    G    
   A   
   T   
    C    
   G   
   A   
   A   
   C   
   G   
    G    
   A   
   T   
   T   
   T   
   C   
   T   
   G   
   A   
    A    
    C    
   T   
    G    
   C   
   A   
   C   
   A   
   G   
   A   
   G   
   A   
    C    
   A   
   A   
    G    
   C   
   G   
    T    
   A   
   T   
    C    
   G   
   A   
   A   
   G   
   G   
    G    
   A   
   T   
    C    
   A   
   C   
    A    
   G   
   A   
   T   
   T   
   T   
   G   
   C   
   G   
   G   
   G   
   A   
    C    
   G   
   A   
   A   
   T   
   C   
   T   
   T   
   C   
    C    
   C   
   G   
   T   
   G   
   A   
    G    
   G   
   G   
    T    
   A   
   T   
   G   
   C   
   G   
    G    
   A   
   T   
    C    
   G   
   T   
   C   
   A   
   T   
    T    
   G   
   A   
    C    
    A    
   T   
    C    
   C   
   G   
   T   
   C   
   G   
    T    
   G   
   A   
    C    
   G   
    C    
    C    
   A   
   G   
    C    
   G   
   C   
    A    
   T   
   C   
    C    
   G   
   T   
   T   
    A    
   T   
    C    
   T   
   T   
    G    
   A   
   A   
    T    
   A   
   A   
   C   
   T   
   T   
    A    
   T   
   A   
    T    
   A   
   A   
   A   
    T    
   T   
   G   
   A   
   C   
    A    
    T    
   C   
    C    
   T   
   T   
    G    
   C   
   A   
    A    
   A   
    A    
    T    
   T   
   C   
    A    
   T   
   T   
    T    
   G   
   G   
    A    
   T   
   T   
    C    
   A   
   A   
    T    
   A   
   T   
   G   
   T   
   T   
    G    
   G   
   C   
    G    
   A   
   T   
    C    
   G   
   A   
   A   
   A   
   A   
   A   
   G   
   G   
    T    
   G   
   T   
    G    
   C   
   C   
    G    
   A   
   A   
   A   
    G    
   T   
   T   
   T   
   T   
    G    
   A   
   G   
    C    
   T   
   T   
   G   
   A   
   A   
   A   
   C   
    A    
    A    
   A   
   T   
    T    
    C    
   T   
    T    
   G   
   A   
   A   
   A   
   A   
    C    
   T   
   A   
    T    
    A    
   T   
    C    
   G   
   A   
    A    
   C   
   A   
    T    
   C   
   A   
    G    
    C    
    G    
    C    
    G    
   A   
   A   
   G   
   T   
   C   
   A   
   T   
    C    
   A   
   C   
    A    
    A    
   G   
    A    
   C   
   G   
   G   
   A   
   C   
    G    
    G    
   T   
    C    
   T   
   T   
   T   
   G   
   A   
    A    
   A   
   A   
   A   
    G    
   A   
    A    
   A   
   A   
    A    
   G   
   C   
   A   
   G   
   A   
   A   
   G   
   C   
   A   
    A    
   G   
    G    
   G   
   C   
    A    
   C   
   A   
    C    
   A   
   T   
    T    
   T   
   T   
   A   
   G   
   A   
   A   
   G   
   G   
   T   
    T    
   T   
    G    
   C   
   G   
    T    
   A   
   T   
   T   
   G   
   C   
    T    
   T   
   T   
    G    
   G   
   A   
   T   
   C   
   A   
   T   
   A   
   T   
   C   
    G    
   A   
   T   
   G   
   A   
   A   
   A   
   T   
    C    
   A   
   T   
   T   
   G   
   C   
    G    
   A   
   T   
   T   
   A   
   T   
    C    
   C   
   G   
    C    
   G   
   G   
    G    
   T   
   C   
    T    
    A    
   A   
   A   
   T   
   C   
   A   
   G   
   A   
   T   
   G   
   A   
    C    
   G   
   A   
    A    
   G   
   C   
   A   
   A   
   A   
   A   
   G   
   C   
    G    
   A   
    C    
    G    
    A    
   T   
    G    
   A   
   T   
    C    
   G   
   A   
   A   
   C   
   G   
    G    
   T   
   T   
    C    
   G   
   A   
    T    
   T   
   T   
    A    
   T   
   C   
    C    
   G   
   A   
   T   
   C   
   G   
    C    
   C   
   A   
    G    
    T    
   C   
    T    
   C   
   A   
   A   
   G   
   C   
    T    
   A   
   T   
    T    
   T   
   T   
   A   
   G   
   A   
   T   
   A   
   T   
   G   
   C   
   G   
   T   
   T   
   T   
    A    
   C   
   G   
    C    
   C   
   G   
    A    
   T   
   T   
    G    
   A   
   C   
    C    
   G   
   G   
    T    
   T   
   T   
    G    
   G   
   A   
   A   
   C   
   G   
    G    
   G   
   A   
    A    
   A   
   A   
   A   
   A   
   T   
    C    
   G   
   A   
    A    
   A   
   A   
   T   
   G   
   A   
   A   
   T   
   A   
    C    
   C   
   A   
   A   
   G   
   A   
    A    
   C   
   T   
    C    
   T   
   T   
    A    
   A   
    A    
   A   
    C    
   T   
    G    
   A   
   T   
   T   
   G   
    A    
    A    
   G   
   A   
    T    
   T   
   T   
    A    
    A    
    C    
    T    
   G   
   A   
   C   
   A   
   T   
   T   
    C    
   T   
    G    
   G   
   C   
    A    
    A    
   G   
    A    
   C   
   C   
    A    
   G   
   A   
    A    
   C   
   G   
    T    
   G   
   T   
    T    
   A   
    C    
    A    
   G   
   A   
   A   
   A   
   T   
    C    
   A   
   T   
    C    
   A   
   A   
    G    
   A   
    A    
    T    
   G   
   A   
    A    
   T   
   T   
   A   
    G    
    C    
    A    
   G   
   A   
   C   
    T    
   T   
    G    
    A    
    A    
    T    
    C    
    A    
   A   
    C    
    G    
   A   
   T   
   T   
   T   
   G   
   G   
   T   
   G   
   A   
   T   
    A    
    A    
   A   
   C   
   G   
   A   
   C   
   G   
    C    
   A   
   C   
   A   
   G   
   A   
   A   
    T    
   T   
   A   
    C    
   T   
   A   
   G   
   T   
    C    
   G   
   G   
   T   
   G   
   A   
    G    
   G   
   T   
   C   
    T    
   T   
    A    
   A   
   G   
   T   
   C   
   T   
    G    
   G   
   A   
   A   
   G   
   A   
   T   
   G   
   A   
   A   
   G   
   A   
   T   
    T    
   T   
    A    
   A   
   T   
    C    
   G   
   A   
   A   
   G   
   A   
   A   
   G   
   A   
   A   
   G   
   A   
   A   
    A    
   T   
   C   
   G   
   T   
    T    
   A   
   T   
   T   
   A   
   C   
    A    
    T    
   T   
    G    
   A   
   C   
    A    
   A   
   A   
    C    
   A   
   A   
    T    
   G   
   G   
    T    
   T   
   A   
    C    
   A   
   T   
    C    
   A   
   A   
    A    
   C   
   G   
    T    
    T    
   T   
   G   
   G   
    C    
   A   
   A   
   A   
    T    
    A    
    A    
    T    
   G   
   A   
   A   
   T   
   T   
   C   
    A    
   G   
    A    
   G   
   C   
    T    
   C   
   A   
   A   
   C   
   G   
    A    
    A    
   G   
    G    
   G   
   G   
    T    
   G   
   G   
    A    
   C   
   G   
   T   
   G   
   G   
    T    
   G   
   T   
    C    
   C   
   A   
   A   
   G   
   G   
    A    
   A   
   T   
   G   
   G   
   G   
    T    
    A    
   T   
    C    
   C   
   A   
    C    
    A    
   A   
   T   
   G   
   A   
    C    
   G   
   A   
    C    
   T   
   T   
   C   
   G   
   T   
    C    
   A   
   A   
    A    
   A   
   A   
    T    
   C   
   T   
    G    
    G    
   T   
   T   
   T   
   C   
   T   
   T   
   G   
    T    
   T   
   C   
    T    
   A   
   C   
    C    
   C   
   A   
    T    
   G   
   A   
    T    
   A   
   C   
    A    
   T   
   T   
    G    
   T   
   T   
   A   
   T   
   T   
    C    
   T   
   T   
   T   
   A   
   C   
   G   
   A   
   A   
    T    
   A   
    A    
    C    
   G   
   G   
    A    
   A   
   A   
   A   
   G   
   T   
    G    
   T   
   A   
    T    
   C   
   G   
    G    
   G   
   C   
   A   
   A   
   A   
   A   
   G   
   G   
    A    
   T   
   A   
    C    
   G   
   A   
   A   
   A   
   T   
    T    
   C   
   C   
    A    
   G   
   A   
    A    
   T   
   A   
   C   
   G   
   G   
    G    
    C    
   G   
    C    
   A   
   C   
   A   
   G   
   C   
    C    
   A   
   A   
   A   
   G   
   G   
    A    
   A   
   T   
    A    
   C   
   C   
   A   
    A    
   T   
    T    
   A   
   T   
    C    
   A   
   A   
    T    
   T   
   T   
    A    
   C   
   T   
    A    
   G   
   G   
    T    
   A   
   T   
    C    
   G   
   A   
    T    
   T   
   C   
    G    
    A    
    G    
    T    
   G   
   A   
   A   
   A   
    G    
    C    
   A   
   T   
    C    
   C   
   A   
    G    
   G   
   C   
    A    
   A   
   T   
    C    
   A   
   T   
   T   
    G    
   C   
    G    
   G   
   T   
    G    
    A    
    C    
   G   
   G   
   G   
    A    
    G    
   A   
   A   
   G   
   C   
    A    
   G   
   A   
   A   
   G   
    A    
    A    
   G   
   G   
    C    
    C    
   A   
    C    
   T   
   A   
    T    
    C    
   T   
    C    
   T   
   T   
   C   
   T   
   T   
    T    
   A   
   C   
    G    
   A   
   C   
    G    
    C    
    G    
    T    
    C    
   A   
   A   
   G   
   G   
    T    
   A   
   C   
    A    
   G   
   T   
    A    
   A   
   A   
   A   
   C   
   G   
    T    
   A   
   C   
    T    
    T    
   C   
    T    
   G   
   T   
    C    
   A   
   C   
    T    
   G   
   C   
    T    
   T   
   T   
    T    
    G    
   C   
   T   
   A   
   A   
    C    
   A   
   T   
    C    
   C   
   G   
    A    
   A   
   G   
    T    
   A   
   A   
   T   
   G   
   G   
    T    
    C    
   T   
    G    
   A   
   T   
    C    
   G   
   C   
    A    
   A   
   T   
   T   
    G    
   G   
    T    
   T   
   T   
    G    
   A   
   A   
   A   
   G   
   A   
   A   
   G   
   A   
    C    
   G   
   A   
    T    
   G   
   A   
    G    
    C    
   T   
    C    
    G    
   T   
    C    
   A   
   A   
    T    
   G   
   T   
    G    
    C    
   T   
   A   
    C    
    T    
   G   
   A   
   C   
    A    
    G    
   A   
    T    
   G   
   G   
    T    
    C    
   A   
    A    
    T    
    C    
    T    
   A   
   A   
    T    
   A   
   T   
    C    
   A   
   T   
    T    
   A   
   T   
    T    
   G   
   G   
    A    
   A   
   C   
   A   
   C   
   A   
   T   
    A    
    A    
    C    
   G   
   G   
    C    
   T   
   A   
   C   
   T   
   C   
   T   
   G   
   T   
    C    
   A   
   C   
    T    
   T   
   T   
    C    
    A    
   A   
    A    
   G   
   A   
    A    
    G    
    A    
    A    
    G    
   C   
    A    
   G   
    T    
    C    
   C   
   G   
    C    
   G   
   A   
   T   
   A   
   T   
   G   
   G   
   G   
    A    
   C   
   G   
    T    
   A   
   C   
   A   
   G   
   C   
    T    
   T   
   C   
    C    
   G   
   G   
    G    
   G   
   T   
    C    
    A    
   G   
    A    
   G   
   G   
    A    
   A   
   T   
    A    
   C   
   G   
    C    
    C    
   T   
    G    
   C   
   G   
    T    
   G   
   A   
    G    
    G    
   A   
    A    
   G   
   A   
    T    
   T   
    A    
    T    
   G   
   T   
    C    
   G   
   T   
    T    
   G   
   G   
    G    
    G    
   C   
   A   
   G   
   C   
    C    
    T    
   T   
    A    
    A    
   T   
   G   
    A    
   A   
    G    
   G   
    A    
   A   
    G    
   A   
    T    
    C    
   A   
   A   
   G   
   A   
   A   
   G   
   T   
    A    
    T    
   T   
    A    
   G   
   T   
    G    
   A   
   T   
    C    
   A   
   C   
    A    
   G   
   A   
   A   
   A   
   A   
    A    
   G   
   G   
   T   
   T   
   A   
    C    
   G   
   G   
    A    
   A   
   A   
    A    
   C   
   G   
    G    
   A   
   C   
    A    
   A   
   A   
   A   
   G   
    T    
    A    
   T   
   C   
    G    
   G   
   A   
    A    
   T   
   A   
   T   
   C   
   C   
   A   
   G   
   T   
    G    
   A   
   A   
    A    
   G   
   G   
    C    
   C   
   G   
    C    
   G   
   G   
    A    
   G   
   G   
   T   
   A   
   A   
   A   
   G   
   G   
    G    
   A   
   T   
    C    
   A   
   A   
    A    
   A   
   C   
    G    
    G    
   C   
    G    
   A   
   A   
    C    
   A   
   T   
   C   
   A   
   C   
   T   
   G   
   A   
    A    
   A   
   A   
   A   
   A   
   A   
   T   
   G   
   G   
   T   
   C   
   C   
   A   
    C    
   T   
   A   
   G   
   C   
    T    
   G   
   G   
    A    
   T   
   T   
    G    
   A   
   C   
   C   
   A   
   C   
    T    
   G   
   T   
    T    
    T    
    C    
   T   
   G   
   G   
    A    
    G    
   A   
   T   
   G   
   A   
   A   
   G   
   A   
    C    
   A   
   T   
   C   
    A    
   T   
    G    
    C    
   T   
    G    
   A   
   T   
    T    
   A   
   C   
    A    
    G    
   A   
    T    
   A   
   A   
   A   
   G   
   G   
    T    
   G   
   T   
    C    
   A   
   T   
   T   
   A   
   T   
   C   
   C   
   G   
    T    
   T   
   T   
    C    
   A   
   A   
   C   
   G   
   T   
    A    
    T    
    C    
    G    
    A    
   C   
   T   
   G   
   T   
   T   
   T   
   C   
   T   
   C   
   A   
   A   
   A   
   C   
    T    
   G   
   G   
   A   
   C   
   G   
    T    
    T    
   C   
   A   
   A   
   C   
   A   
   T   
   T   
    A    
   G   
   G   
    C    
   G   
   T   
    A    
   C   
   G   
   T   
   T   
   T   
    G    
   A   
   T   
   G   
   A   
    A    
    G    
   A   
   T   
   G   
   G   
   A   
   A   
   G   
    C    
    T    
   G   
    A    
    T    
    A    
   C   
    G    
   A   
   A   
   A   
   G   
   T   
   T   
   G   
   T   
   C   
   A   
   C   
   G   
   A   
   T   
   G   
   G   
   C   
   A   
   G   
   C   
   A   
   G   
   T   
   C   
   G   
   A   
   A   
   C   
   C   
   A   
   G   
   A   
   A   
   G   
   C   
   T   
   G   
   C   
   A   
   G   
   A   
   A   
   C   
   A   
   A   
   A   
   C   
   A   
   G   
   T   
   T   
   G   
   A   
   A   
   G   
   A   
   A   
   C   
   A   
   A   
   G   
   G   
   A   
   T   
   C   
   A   
   G   
   A   
   A   
   G   
   A   
   A   
   -   
   -   
   -   
 

 
   N59589F   
   A   
   T   
   G   
   A   
   G   
   T   
   G   
   A   
   A   
   G   
   A   
   A   
   A   
   T   
   C   
   A   
   G   
   A   
   G   
   A   
   A   
   A   
   A   
   C   
   A   
   T   
   C   
   C   
   A   
   T   
   G   
   A   
   T   
   G   
   T   
   C   
   A   
   A   
   T   
   C   
   T   
   A   
   A   
   C   
   C   
   A   
   G   
   C   
   G   
   A   
   A   
   A   
   T   
   G   
   A   
   A   
   A   
   G   
   A   
   C   
   T   
   C   
   C   
   T   
   T   
   C   
   A   
   T   
   C   
   G   
   A   
   T   
   T   
   A   
   T   
   G   
   C   
   G   
   A   
   T   
   G   
   A   
   G   
   T   
   G   
   T   
   T   
   A   
   T   
   T   
   G   
   T   
   C   
   G   
   C   
   A   
   C   
   G   
   T   
   G   
   C   
   A   
   C   
   T   
   G   
   C   
   C   
   T   
   G   
   A   
   T   
   G   
   T   
   C   
   A   
   G   
   A   
   G   
   A   
   T   
   G   
   G   
   T   
   C   
   T   
   G   
   A   
   A   
   A   
   C   
   C   
   C   
   G   
   T   
   T   
   C   
   A   
   T   
   C   
   G   
   C   
   C   
   G   
   T   
   A   
   T   
   T   
   T   
   T   
   A   
   T   
   A   
   C   
   G   
   G   
   C   
   A   
   T   
   G   
   A   
   A   
   C   
   G   
   A   
   A   
   T   
   T   
   A   
   G   
   G   
   T   
   G   
   T   
   G   
   A   
   C   
   A   
   C   
   C   
   G   
   G   
   A   
   T   
   A   
   A   
   A   
   G   
   C   
   C   
   C   
   A   
   T   
   A   
   A   
   A   
   A   
   A   
   A   
   T   
   C   
   T   
   G   
   C   
   C   
   A   
   G   
   A   
   A   
   T   
   C   
   G   
   T   
   C   
   G   
   G   
   A   
   G   
   A   
   T   
   G   
   T   
   C   
   A   
   T   
   G   
   G   
   G   
   T   
   A   
   A   
   A   
   T   
   A   
   C   
   C   
   A   
   T   
   C   
   C   
   T   
   C   
   A   
   T   
   G   
   G   
   G   
   G   
   A   
   T   
   A   
   G   
   T   
   G   
   C   
   G   
   A   
   T   
   C   
   T   
   A   
   T   
   G   
   A   
   G   
   T   
   C   
   A   
   A   
   T   
   G   
   G   
   T   
   A   
   C   
   G   
   G   
   A   
   T   
   G   
   G   
   C   
   G   
   C   
   A   
   G   
   C   
   C   
   A   
   T   
   T   
   T   
   A   
   G   
   C   
   T   
   A   
   C   
   C   
   G   
   T   
   T   
   A   
   T   
   A   
   T   
   G   
   C   
   T   
   A   
   G   
   T   
   G   
   G   
   A   
   T   
   G   
   G   
   C   
   C   
   A   
   T   
   G   
   G   
   A   
   A   
   A   
   C   
   T   
   T   
   T   
   G   
   G   
   T   
   T   
   C   
   T   
   G   
   T   
   T   
   G   
   A   
   C   
   G   
   G   
   T   
   G   
   A   
   C   
   G   
   G   
   T   
   G   
   C   
   C   
   G   
   C   
   T   
   G   
   C   
   T   
   A   
   T   
   G   
   C   
   G   
   T   
   T   
   A   
   T   
   A   
   C   
   C   
   G   
   A   
   A   
   G   
   C   
   A   
   C   
   G   
   G   
   A   
   T   
   G   
   A   
   G   
   T   
   A   
   A   
   A   
   A   
   T   
   C   
   G   
   C   
   T   
   A   
   C   
   A   
   G   
   A   
   A   
   A   
   T   
   G   
   C   
   T   
   G   
   C   
   G   
   T   
   G   
   A   
   T   
   A   
   T   
   C   
   A   
   A   
   T   
   A   
   A   
   G   
   A   
   A   
   C   
   A   
   C   
   T   
   G   
   T   
   T   
   G   
   A   
   T   
   T   
   T   
   C   
   C   
   A   
   A   
   A   
   G   
   C   
   A   
   A   
   C   
   T   
   A   
   T   
   G   
   A   
   T   
   G   
   A   
   C   
   A   
   C   
   A   
   G   
   A   
   A   
   A   
   A   
   A   
   G   
   A   
   A   
   C   
   C   
   C   
   G   
   T   
   T   
   G   
   T   
   A   
   T   
   T   
   A   
   C   
   C   
   T   
   G   
   C   
   T   
   C   
   G   
   C   
   T   
   T   
   C   
   C   
   C   
   C   
   A   
   A   
   T   
   C   
   T   
   T   
   T   
   T   
   G   
   G   
   T   
   G   
   A   
   A   
   C   
   G   
   G   
   A   
   A   
   C   
   G   
   A   
   C   
   G   
   G   
   G   
   G   
   A   
   T   
   A   
   G   
   C   
   C   
   G   
   T   
   G   
   G   
   G   
   T   
   A   
   T   
   G   
   G   
   C   
   A   
   A   
   C   
   G   
   A   
   A   
   T   
   A   
   T   
   C   
   C   
   C   
   A   
   C   
   C   
   T   
   C   
   A   
   T   
   A   
   A   
   T   
   T   
   T   
   A   
   T   
   C   
   T   
   G   
   A   
   G   
   G   
   T   
   A   
   G   
   T   
   A   
   G   
   C   
   G   
   G   
   C   
   T   
   A   
   T   
   C   
   G   
   A   
   T   
   T   
   T   
   G   
   C   
   T   
   G   
   A   
   T   
   G   
   G   
   A   
   T   
   A   
   A   
   T   
   C   
   C   
   T   
   G   
   A   
   T   
   G   
   T   
   G   
   A   
   C   
   G   
   A   
   C   
   C   
   A   
   A   
   T   
   G   
   A   
   A   
   T   
   T   
   G   
   A   
   T   
   G   
   G   
   A   
   A   
   G   
   T   
   A   
   T   
   T   
   A   
   C   
   C   
   T   
   G   
   G   
   A   
   C   
   C   
   A   
   G   
   A   
   T   
   T   
   T   
   T   
   C   
   C   
   A   
   A   
   C   
   A   
   G   
   G   
   T   
   G   
   G   
   T   
   T   
   T   
   A   
   G   
   T   
   C   
   A   
   T   
   G   
   G   
   G   
   A   
   A   
   A   
   A   
   T   
   C   
   A   
   G   
   G   
    A    
   A   
   T   
    C    
   C   
   G   
    A    
    A    
   G   
   A   
   G   
   C   
    G    
   T   
   A   
   T   
   G   
   A   
   A   
   A   
   C   
    A    
   G   
   G   
    T    
   A   
   A   
    A    
   G   
   G   
   T   
   T   
   C   
    T    
   A   
   T   
   T   
   A   
   C   
    C    
   G   
   T   
    A    
   C   
   G   
   T   
   G   
   C   
   A   
   A   
   A   
   A   
   G   
   T   
   T   
   G   
   A   
    G    
    A    
   T   
    C    
   A   
    C    
   T   
   G   
   A   
    G    
   A   
   T   
   G   
    C    
   C   
    A    
   A   
   A   
   T   
   G   
   G   
    C    
   A   
   A   
   A   
   G   
   A   
   A   
   C   
   G   
    G    
   A   
   T   
    C    
    C    
   T   
    C    
   G   
   T   
    G    
   A   
   C   
    G    
   G   
   A   
   A   
   T   
   T   
    A    
   C   
   C   
   T   
   T   
   A   
   T   
   A   
   T   
   G   
   G   
   T   
    C    
   A   
   A   
   T   
   A   
   A   
   A   
   G   
   C   
    C    
   A   
   A   
   A   
   T   
   T   
    G    
   A   
   T   
    T    
   G   
   A   
   A   
   C   
   G   
    G    
   A   
   T   
   T   
   T   
   C   
   T   
   G   
   A   
    G    
    T    
   T   
    G    
   C   
   A   
   C   
   A   
   G   
   A   
   G   
   A   
    C    
   A   
   A   
    G    
   C   
   G   
    T    
   A   
   T   
    C    
   G   
   A   
   A   
   G   
   G   
    A    
   A   
   T   
    C    
   A   
   C   
    G    
   G   
   A   
   T   
   T   
   T   
   G   
   C   
   G   
   G   
   G   
   A   
    T    
   G   
   A   
   A   
   T   
   C   
   T   
   T   
   C   
    C    
   C   
   G   
   T   
   G   
   A   
    A    
   G   
   G   
    C    
   A   
   T   
   G   
   C   
   G   
    A    
   A   
   T   
    T    
   G   
   T   
   C   
   A   
   T   
    T    
   G   
   A   
    C    
    A    
   T   
    T    
   C   
   G   
   T   
   C   
   G   
    T    
   G   
   A   
    C    
   G   
    C    
    C    
   A   
   G   
    C    
   G   
   C   
    A    
   T   
   C   
    A    
   G   
   T   
   T   
    A    
   T   
    T    
   T   
   T   
    G    
   A   
   A   
    T    
   A   
   A   
   C   
   T   
   T   
    A    
   T   
   A   
    C    
   A   
   A   
   A   
    T    
   T   
   G   
   A   
   C   
    A    
    T    
   C   
    C    
   T   
   T   
    A    
   C   
   A   
    G    
   A   
    A    
    T    
   T   
   C   
    A    
   T   
   T   
    T    
   G   
   G   
    A    
   T   
   T   
    C    
   A   
   A   
    T    
   A   
   T   
   G   
   T   
   T   
    G    
   G   
   C   
    C    
   A   
   T   
    C    
   G   
   A   
   A   
   A   
   A   
   A   
   G   
   G   
    C    
   G   
   T   
    G    
   C   
   C   
    G    
   A   
   A   
   A   
    G    
   T   
   T   
   T   
   T   
    A    
   A   
   G   
    T    
   T   
   T   
   G   
   A   
   A   
   A   
   C   
    A    
    A    
   A   
   T   
    C    
    C    
   T   
    T    
   G   
   A   
   A   
   A   
   A   
    C    
   T   
   A   
    T    
    A    
   T   
    C    
   G   
   A   
    A    
   C   
   A   
    T    
   C   
   A   
    A    
    C    
    G    
    C    
    G    
   A   
   A   
   G   
   T   
   C   
   A   
   T   
    C    
   A   
   C   
    A    
    C    
   G   
    A    
   C   
   G   
   G   
   A   
   C   
    G    
    G    
   T   
    C    
   T   
   T   
   T   
   G   
   A   
    A    
   A   
   A   
   A   
    G    
   A   
    A    
   A   
   A   
    A    
   G   
   C   
   A   
   G   
   A   
   A   
   G   
   C   
   A   
    A    
   G   
    A    
   G   
   C   
    A    
   C   
   A   
    C    
   A   
   T   
    C    
   T   
   T   
   A   
   G   
   A   
   A   
   G   
   G   
   T   
    T    
   T   
    G    
   C   
   G   
    T    
   A   
   T   
   T   
   G   
   C   
    T    
   T   
   T   
    G    
   G   
   A   
   T   
   C   
   A   
   T   
   A   
   T   
   C   
    G    
   A   
   T   
   G   
   A   
   A   
   A   
   T   
    C    
   A   
   T   
   T   
   G   
   C   
    G    
   A   
   T   
   T   
   A   
   T   
    C    
   C   
   G   
    C    
   G   
   G   
    G    
   T   
   C   
    T    
    A    
   A   
   A   
   T   
   C   
   A   
   G   
   A   
   T   
   G   
   A   
    T    
   G   
   A   
    A    
   G   
   C   
   A   
   A   
   A   
   A   
   G   
   C   
    A    
   A   
    C    
    A    
    A    
   T   
    G    
   A   
   T   
    C    
   G   
   A   
   A   
   C   
   G   
    G    
   T   
   T   
    C    
   G   
   A   
    T    
   T   
   T   
    A    
   T   
   C   
    C    
   G   
   A   
   T   
   C   
   G   
    C    
   C   
   A   
    A    
    T    
   C   
    A    
   C   
   A   
   A   
   G   
   C   
    T    
   A   
   T   
    C    
   T   
   T   
   A   
   G   
   A   
   T   
   A   
   T   
   G   
   C   
   G   
   T   
   T   
   T   
    G    
   C   
   G   
    T    
   C   
   G   
    A    
   T   
   T   
    G    
   A   
   C   
    C    
   G   
   G   
    T    
   T   
   T   
    G    
   G   
   A   
   A   
   C   
   G   
    G    
   G   
   A   
    A    
   A   
   A   
   A   
   A   
   T   
    C    
   G   
   A   
    A    
   A   
   A   
   T   
   G   
   A   
   A   
   T   
   A   
    T    
   C   
   A   
   A   
   G   
   A   
    A    
   C   
   T   
    A    
   T   
   T   
    G    
   A   
    A    
   A   
    C    
   T   
    G    
   A   
   T   
   T   
   G   
    A    
    A    
   G   
   A   
    T    
   T   
   T   
    G    
    A    
    C    
    T    
   G   
   A   
   C   
   A   
   T   
   T   
    C    
   T   
    G    
   G   
   C   
    A    
    A    
   G   
    A    
   C   
   C   
    C    
   G   
   A   
    A    
   C   
   G   
    T    
   G   
   T   
    C    
   A   
    C    
    G    
   G   
   A   
   A   
   A   
   T   
    C    
   A   
   T   
    C    
   A   
   A   
    G    
   A   
    A    
    T    
   G   
   A   
    G    
   T   
   T   
   A   
    G    
    C    
    A    
   G   
   A   
   C   
    T    
   T   
    G    
    A    
    A    
    T    
    C    
    A    
   A   
    C    
    G    
   A   
   T   
   T   
   T   
   G   
   G   
   T   
   G   
   A   
   T   
    A    
    A    
   A   
   C   
   G   
   A   
   C   
   G   
    C    
   A   
   C   
   A   
   G   
   A   
   A   
    T    
   T   
   A   
    C    
   T   
   A   
   G   
   T   
    C    
   G   
   G   
   T   
   G   
   A   
    G    
   G   
   T   
   C   
    T    
   T   
    A    
   A   
   G   
   T   
   C   
   T   
    A    
   G   
   A   
   A   
   G   
   A   
   T   
   G   
   A   
   A   
   G   
   A   
   T   
    T    
   T   
    G    
   A   
   T   
    C    
   G   
   A   
   A   
   G   
   A   
   A   
   G   
   A   
   A   
   G   
   A   
   A   
    A    
   T   
   C   
   G   
   T   
    T    
   A   
   T   
   T   
   A   
   C   
    A    
    C    
   T   
    G    
   A   
   C   
    A    
   A   
   A   
    C    
   A   
   A   
    T    
   G   
   G   
    T    
   T   
   A   
    T    
   A   
   T   
    C    
   A   
   A   
    A    
   C   
   G   
    T    
    T    
   T   
   G   
   G   
    C    
   A   
   A   
   A   
    T    
    A    
    A    
    T    
   G   
   A   
   A   
   T   
   T   
   C   
    A    
   G   
    A    
   G   
   C   
    T    
   C   
   A   
   A   
   C   
   G   
    A    
    A    
   G   
    A    
   G   
   G   
    C    
   G   
   G   
    G    
   C   
   G   
   T   
   G   
   G   
    T    
   G   
   T   
    C    
   C   
   A   
   A   
   G   
   G   
    A    
   A   
   T   
   G   
   G   
   G   
    T    
    A    
   T   
    C    
   C   
   A   
    C    
    A    
   A   
   T   
   G   
   A   
    C    
   G   
   A   
    C    
   T   
   T   
   C   
   G   
   T   
    C    
   A   
   A   
    G    
   A   
   A   
    T    
   C   
   T   
    A    
    G    
   T   
   T   
   T   
   C   
   T   
   T   
   G   
    T    
   T   
   C   
    T    
   A   
   C   
    C    
   C   
   A   
    T    
   G   
   A   
    T    
   A   
   C   
    A    
   T   
   T   
    G    
   T   
   T   
   A   
   T   
   T   
    C    
   T   
   T   
   T   
   A   
   C   
   G   
   A   
   A   
    T    
   A   
    A    
    C    
   G   
   G   
    A    
   A   
   A   
   A   
   G   
   T   
    G    
   T   
   A   
    T    
   C   
   G   
    G    
   G   
   C   
   A   
   A   
   A   
   A   
   G   
   G   
    A    
   T   
   A   
    C    
   G   
   A   
   A   
   A   
   T   
    T    
   C   
   C   
    A    
   G   
   A   
    A    
   T   
   A   
   C   
   G   
   G   
    A    
    C    
   G   
    T    
   A   
   C   
   A   
   G   
   C   
    C    
   A   
   A   
   A   
   G   
   G   
    T    
   A   
   T   
    C    
   C   
   C   
   A   
    A    
   T   
    C    
   A   
   T   
    C    
   A   
   A   
    T    
   T   
   T   
    G    
   C   
   T   
    G    
   G   
   G   
    T    
   A   
   T   
    C    
   G   
   A   
    C    
   T   
   C   
    G    
    A    
    G    
    T    
   G   
   A   
   A   
   A   
    G    
    C    
   A   
   T   
    C    
   C   
   A   
    A    
   G   
   C   
    A    
   A   
   T   
    T    
   A   
   T   
   T   
    G    
   C   
    G    
   G   
   T   
    G    
    A    
    C    
   G   
   G   
   G   
    A    
    G    
   A   
   A   
   G   
   C   
    A    
   G   
   A   
   A   
   G   
    A    
    A    
   G   
   G   
    C    
    C    
   A   
    C    
   T   
   A   
    C    
    C    
   T   
    C    
   T   
   T   
   C   
   T   
   T   
    T    
   A   
   C   
    G    
   A   
   C   
    G    
    C    
    G    
    T    
    C    
   A   
   A   
   G   
   G   
    T    
   A   
   C   
    A    
   G   
   T   
    T    
   A   
   A   
   A   
   C   
   G   
    T    
   A   
   C   
    G    
    T    
   C   
    T    
   G   
   T   
    C    
   A   
   C   
    T    
   G   
   C   
    T    
   T   
   T   
    C    
    G    
   C   
   T   
   A   
   A   
    C    
   A   
   T   
    C    
   C   
   G   
    A    
   A   
   G   
    C    
   A   
   A   
   T   
   G   
   G   
    T    
    C    
   T   
    G    
   A   
   T   
    T    
   G   
   C   
    A    
   A   
   T   
   T   
    G    
   G   
    T    
   T   
   T   
    G    
   A   
   A   
   A   
   G   
   A   
   A   
   G   
   A   
    T    
   G   
   A   
    C    
   G   
   A   
    A    
    T    
   T   
    A    
    G    
   T   
    C    
   A   
   A   
    T    
   G   
   T   
    T    
    C    
   T   
   A   
    T    
    T    
   G   
   A   
   C   
    A    
    G    
   A   
    T    
   G   
   G   
    A    
    C    
   A   
    G    
    T    
    C    
    A    
   A   
   A   
    T    
   A   
   T   
    C    
   A   
   T   
    C    
   A   
   T   
    T    
   G   
   G   
    C    
   A   
   C   
   A   
   C   
   A   
   T   
    A    
    A    
    C    
   G   
   G   
    C    
   T   
   A   
   C   
   T   
   C   
   T   
   G   
   T   
    G    
   A   
   C   
    C    
   T   
   T   
    C    
    A    
   A   
    A    
   G   
   A   
    G    
    G    
    A    
    A    
    G    
   C   
    A    
   G   
    T    
    C    
   C   
   G   
    C    
   G   
   A   
   T   
   A   
   T   
   G   
   G   
   G   
    A    
   C   
   G   
    T    
   A   
   C   
   A   
   G   
   C   
    T    
   T   
   C   
    C    
   G   
   G   
    C    
   G   
   T   
    C    
    A    
   G   
    A    
   G   
   G   
    G    
   A   
   T   
    A    
   C   
   G   
    T    
    T    
   T   
    G    
   C   
   G   
    T    
   G   
   A   
    G    
    G    
   A   
    A    
   G   
   A   
    C    
   T   
    A    
    C    
   G   
   T   
    T    
   G   
   T   
    T    
   G   
   G   
    G    
    G    
   C   
   A   
   G   
   C   
    C    
    C    
   T   
    G    
    A    
   T   
   G   
    A    
   A   
    G    
   G   
    A    
   A   
    G    
   A   
    C    
    C    
   A   
   A   
   G   
   A   
   A   
   G   
   T   
    A    
    C    
   T   
    A    
   G   
   T   
    G    
   A   
   T   
    T    
   A   
   C   
    A    
   G   
   A   
   A   
   A   
   A   
    A    
   G   
   G   
   T   
   T   
   A   
    C    
   G   
   G   
    A    
   A   
   A   
    A    
   C   
   G   
    G    
   A   
   C   
    C    
   A   
   A   
   A   
   G   
    T    
    A    
   T   
   C   
    G    
   G   
   A   
    A    
   T   
   A   
   T   
   C   
   C   
   A   
   G   
   T   
    G    
   A   
   A   
    A    
   G   
   G   
    C    
   C   
   G   
    C    
   G   
   G   
    T    
   G   
   G   
   T   
   A   
   A   
   A   
   G   
   G   
    A    
   A   
   T   
    C    
   A   
   A   
    A    
   A   
   C   
    G    
    G    
   C   
    G    
   A   
   A   
    T    
   A   
   T   
   C   
   A   
   C   
   T   
   G   
   A   
    A    
   A   
   A   
   A   
   A   
   A   
   T   
   G   
   G   
   T   
   C   
   C   
   A   
    C    
   T   
   A   
   G   
   C   
    C    
   G   
   G   
    A    
   T   
   T   
    G    
   A   
   C   
   C   
   A   
   C   
    T    
   G   
   T   
    T    
    T    
    C    
   T   
   G   
   G   
    A    
    G    
   A   
   T   
   G   
   A   
   A   
   G   
   A   
    C    
   A   
   T   
   C   
    A    
   T   
    G    
    C    
   T   
    G    
   A   
   T   
    T    
   A   
   C   
    A    
    G    
   A   
    T    
   A   
   A   
   A   
   G   
   G   
    T    
   G   
   T   
    G    
   A   
   T   
   T   
   A   
   T   
   C   
   C   
   G   
    T    
   T   
   T   
    C    
   A   
   A   
   C   
   G   
   T   
    A    
    T    
    C    
    G    
    A    
   C   
   T   
   G   
   T   
   T   
   T   
   C   
   T   
   C   
   A   
   A   
   A   
   C   
    T    
   G   
   G   
   A   
   C   
   G   
    T    
    T    
   C   
   A   
   A   
   C   
   A   
   T   
   T   
    G    
   G   
   G   
    T    
   G   
   T   
    A    
   C   
   G   
   T   
   T   
   T   
    G    
   A   
   T   
   G   
   A   
    A    
    G    
   A   
   T   
   G   
   G   
   A   
   A   
   G   
    C    
    T    
   G   
    A    
    T    
    A    
   C   
    G    
   A   
   A   
   A   
   G   
   T   
   C   
   G   
   T   
   C   
   A   
   C   
   A   
   A   
   T   
   G   
   -   
   -   
   -   
   -   
   -   
   -   
   -   
   -   
   -   
   -   
   -   
   -   
   -   
   -   
   -   
   -   
   -   
   -   
   -   
   -   
   -   
   -   
   -   
   -   
   -   
   -   
   -   
   -   
   -   
   -   
   -   
   -   
   -   
   -   
   -   
   -   
   -   
   -   
   -   
   -   
   -   
   -   
   -   
   -   
   -   
   -   
   -   
   -   
   -   
   -   
   -   
   -   
   -   
   -   
   -   
   -   
   -   
   -   
   -   
   -   
 

 
   N52690   
   A   
   T   
   G   
   A   
   G   
   T   
   G   
   A   
   A   
   G   
   A   
   A   
   A   
   T   
   C   
   A   
   G   
   A   
   G   
   A   
   A   
   A   
   A   
   C   
   A   
   T   
   C   
   C   
   A   
   T   
   G   
   A   
   T   
   G   
   T   
   C   
   A   
   A   
   T   
   C   
   T   
   A   
   A   
   C   
   C   
   A   
   G   
   C   
   G   
   A   
   A   
   A   
   T   
   G   
   A   
   A   
   A   
   G   
   A   
   C   
   T   
   C   
   C   
   T   
   T   
   C   
   A   
   T   
   C   
   G   
   A   
   T   
   T   
   A   
   T   
   G   
   C   
   G   
   A   
   T   
   G   
   A   
   G   
   T   
   G   
   T   
   T   
   A   
   T   
   T   
   G   
   T   
   C   
   G   
   C   
   A   
   C   
   G   
   T   
   G   
   C   
   A   
   C   
   T   
   T   
   C   
   C   
   T   
   G   
   A   
   T   
   G   
   T   
   C   
   A   
   G   
   A   
   G   
   A   
   T   
   G   
   G   
   T   
   C   
   T   
   G   
   A   
   A   
   A   
   C   
   C   
   C   
   G   
   T   
   T   
   C   
   A   
   C   
   C   
   G   
   C   
   C   
   G   
   T   
   A   
   T   
   T   
   T   
   T   
   A   
   T   
   A   
   C   
   G   
   G   
   C   
   A   
   T   
   G   
   A   
   A   
   C   
   G   
   A   
   A   
   T   
   T   
   A   
   G   
   G   
   T   
   G   
   T   
   A   
   A   
   C   
   A   
   C   
   C   
   G   
   G   
   A   
   T   
   A   
   A   
   A   
   G   
   C   
   C   
   C   
   A   
   T   
   A   
   A   
   A   
   A   
   A   
   A   
   T   
   C   
   T   
   G   
   C   
   C   
   A   
   G   
   A   
   A   
   T   
   C   
   G   
   T   
   C   
   G   
   G   
   A   
   G   
   A   
   T   
   G   
   T   
   C   
   A   
   T   
   G   
   G   
   G   
   T   
   A   
   A   
   A   
   T   
   A   
   C   
   C   
   A   
   T   
   C   
   C   
   T   
   C   
   A   
   T   
   G   
   G   
   A   
   G   
   A   
   C   
   A   
   G   
   T   
   G   
   C   
   G   
   A   
   T   
   C   
   T   
   A   
   T   
   G   
   A   
   G   
   T   
   C   
   A   
   A   
   T   
   G   
   G   
   T   
   A   
   C   
   G   
   G   
   A   
   T   
   G   
   G   
   C   
   G   
   C   
   A   
   G   
   C   
   C   
   A   
   T   
   T   
   C   
   A   
   G   
   C   
   T   
   A   
   C   
   C   
   G   
   T   
   T   
   A   
   T   
   A   
   T   
   G   
   C   
   T   
   A   
   G   
   T   
   G   
   G   
   A   
   T   
   G   
   G   
   T   
   C   
   A   
   T   
   G   
   G   
   A   
   A   
   A   
   C   
   T   
   T   
   T   
   G   
   G   
   T   
   T   
   C   
   T   
   G   
   T   
   T   
   G   
   A   
   C   
   G   
   G   
   T   
   G   
   A   
   C   
   G   
   G   
   T   
   G   
   C   
   C   
   G   
   C   
   C   
   G   
   C   
   T   
   A   
   T   
   G   
   C   
   G   
   T   
   T   
   A   
   T   
   A   
   C   
   C   
   G   
   A   
   A   
   G   
   C   
   A   
   C   
   G   
   G   
   A   
   T   
   G   
   A   
   G   
   C   
   A   
   A   
   A   
   A   
   T   
   C   
   G   
   C   
   T   
   A   
   C   
   A   
   G   
   A   
   A   
   A   
   T   
   G   
   C   
   T   
   G   
   C   
   G   
   T   
   G   
   A   
   T   
   A   
   T   
   C   
   A   
   A   
   T   
   A   
   A   
   G   
   A   
   A   
   C   
   A   
   C   
   T   
   G   
   T   
   T   
   G   
   A   
   T   
   T   
   T   
   C   
   C   
   A   
   A   
   A   
   G   
   C   
   A   
   A   
   C   
   T   
   A   
   T   
   G   
   A   
   T   
   G   
   A   
   T   
   A   
   C   
   A   
   G   
   A   
   A   
   A   
   A   
   A   
   G   
   A   
   A   
   C   
   C   
   A   
   G   
   T   
   T   
   G   
   T   
   A   
   C   
   T   
   A   
   C   
   C   
   T   
   G   
   C   
   T   
   C   
   G   
   C   
   T   
   T   
   C   
   C   
   C   
   G   
   A   
   A   
   T   
   C   
   T   
   T   
   T   
   T   
   A   
   G   
   T   
   G   
   A   
   A   
   C   
   G   
   G   
   A   
   A   
   C   
   A   
   A   
   C   
   G   
   G   
   G   
   G   
   A   
   T   
   C   
   G   
   C   
   C   
   G   
   T   
   A   
   G   
   G   
   T   
   A   
   T   
   G   
   G   
   C   
   C   
   A   
   C   
   G   
   A   
   A   
   T   
   A   
   T   
   C   
   C   
   C   
   A   
   C   
   C   
   T   
   C   
   A   
   T   
   A   
   A   
   C   
   T   
   T   
   G   
   T   
   C   
   T   
   G   
   A   
   G   
   G   
   T   
   A   
   G   
   T   
   A   
   G   
   C   
   G   
   G   
   C   
   T   
   A   
   T   
   C   
   G   
   A   
   T   
   T   
   T   
   G   
   C   
   T   
   G   
   A   
   T   
   G   
   G   
   A   
   T   
   A   
   A   
   T   
   C   
   C   
   T   
   G   
   A   
   T   
   G   
   T   
   G   
   A   
   C   
   G   
   A   
   C   
   C   
   A   
   A   
   T   
   G   
   A   
   A   
   T   
   T   
   G   
   A   
   T   
   G   
   G   
   A   
   A   
   G   
   T   
   A   
   T   
   T   
   A   
   C   
   C   
   T   
   G   
   G   
   A   
   C   
   C   
   A   
   G   
   A   
   T   
   T   
   T   
   T   
   C   
   C   
   A   
   A   
   C   
   A   
   G   
   G   
   T   
   G   
   G   
   T   
   T   
   T   
   G   
   G   
   T   
   T   
   A   
   T   
   G   
   G   
   G   
   G   
   A   
   A   
   A   
   T   
   C   
   A   
   G   
   G   
    A    
   A   
   T   
    C    
   C   
   G   
    A    
    A    
   G   
   A   
   G   
   C   
    G    
   T   
   A   
   T   
   G   
   A   
   A   
   A   
   C   
    A    
   G   
   G   
    T    
   A   
   A   
    G    
   G   
   G   
   T   
   T   
   C   
    T    
   A   
   T   
   T   
   A   
   C   
    C    
   G   
   T   
    A    
   C   
   G   
   T   
   G   
   C   
   A   
   A   
   A   
   A   
   G   
   T   
   T   
   G   
   A   
    G    
    A    
   T   
    T    
   A   
    C    
   T   
   G   
   A   
    G    
   A   
   T   
   G   
    C    
   C   
    A    
   A   
   A   
   T   
   G   
   G   
    T    
   A   
   A   
   A   
   G   
   A   
   A   
   C   
   G   
    G    
   A   
   T   
    C    
    A    
   T   
    C    
   G   
   T   
    G    
   A   
   C   
    A    
   G   
   A   
   A   
   T   
   T   
    G    
   C   
   C   
   T   
   T   
   A   
   T   
   A   
   T   
   G   
   G   
   T   
    C    
   A   
   A   
   T   
   A   
   A   
   A   
   G   
   C   
    A    
   A   
   A   
   A   
   T   
   T   
    G    
   A   
   T   
    C    
   G   
   A   
   A   
   C   
   G   
    G    
   A   
   T   
   T   
   T   
   C   
   T   
   G   
   A   
    A    
    C    
   T   
    G    
   C   
   A   
   C   
   A   
   G   
   A   
   G   
   A   
    C    
   A   
   A   
    G    
   C   
   G   
    T    
   A   
   T   
    C    
   G   
   A   
   A   
   G   
   G   
    G    
   A   
   T   
    C    
   A   
   C   
    A    
   G   
   A   
   T   
   T   
   T   
   G   
   C   
   G   
   G   
   G   
   A   
    C    
   G   
   A   
   A   
   T   
   C   
   T   
   T   
   C   
    C    
   C   
   G   
   T   
   G   
   A   
    A    
   G   
   G   
    T    
   A   
   T   
   G   
   C   
   G   
    G    
   A   
   T   
    C    
   G   
   T   
   C   
   A   
   T   
    T    
   G   
   A   
    C    
    A    
   T   
    C    
   C   
   G   
   T   
   C   
   G   
    T    
   G   
   A   
    C    
   G   
    C    
    C    
   A   
   G   
    C    
   G   
   C   
    A    
   T   
   C   
    C    
   G   
   T   
   T   
    A    
   T   
    C    
   T   
   T   
    G    
   A   
   A   
    T    
   A   
   A   
   C   
   T   
   T   
    A    
   T   
   A   
    T    
   A   
   A   
   A   
    T    
   T   
   G   
   A   
   C   
    A    
    T    
   C   
    C    
   T   
   T   
    G    
   C   
   A   
    A    
   A   
    A    
    T    
   T   
   C   
    A    
   T   
   T   
    T    
   G   
   G   
    A    
   T   
   T   
    C    
   A   
   A   
    T    
   A   
   T   
   G   
   T   
   T   
    G    
   G   
   C   
    G    
   A   
   T   
    C    
   G   
   A   
   A   
   A   
   A   
   A   
   G   
   G   
    T    
   G   
   T   
    G    
   C   
   C   
    G    
   A   
   A   
   A   
    G    
   T   
   T   
   T   
   T   
    G    
   A   
   G   
    C    
   T   
   T   
   G   
   A   
   A   
   A   
   C   
    A    
    A    
   A   
   T   
    T    
    C    
   T   
    T    
   G   
   A   
   A   
   A   
   A   
    C    
   T   
   A   
    T    
    A    
   T   
    C    
   G   
   A   
    A    
   C   
   A   
    T    
   C   
   A   
    G    
    C    
    G    
    C    
    G    
   A   
   A   
   G   
   T   
   C   
   A   
   T   
    C    
   A   
   C   
    A    
    A    
   G   
    A    
   C   
   G   
   G   
   A   
   C   
    G    
    G    
   T   
    C    
   T   
   T   
   T   
   G   
   A   
    A    
   A   
   A   
   A   
    G    
   A   
    A    
   A   
   A   
    A    
   G   
   C   
   A   
   G   
   A   
   A   
   G   
   C   
   A   
    A    
   G   
    G    
   G   
   C   
    A    
   C   
   A   
    C    
   A   
   T   
    T    
   T   
   T   
   A   
   G   
   A   
   A   
   G   
   G   
   T   
    T    
   T   
    G    
   C   
   G   
    T    
   A   
   T   
   T   
   G   
   C   
    T    
   T   
   T   
    G    
   G   
   A   
   T   
   C   
   A   
   T   
   A   
   T   
   C   
    G    
   A   
   T   
   G   
   A   
   A   
   A   
   T   
    T    
   A   
   T   
   T   
   G   
   C   
    G    
   A   
   T   
   T   
   A   
   T   
    C    
   C   
   G   
    T    
   G   
   G   
    G    
   T   
   C   
    T    
    A    
   A   
   A   
   T   
   C   
   A   
   G   
   A   
   T   
   G   
   A   
    C    
   G   
   A   
    A    
   G   
   C   
   A   
   A   
   A   
   A   
   G   
   C   
    G    
   A   
    C    
    G    
    A    
   T   
    G    
   A   
   T   
    C    
   G   
   A   
   A   
   C   
   G   
    G    
   T   
   T   
    C    
   G   
   A   
    T    
   T   
   T   
    A    
   T   
   C   
    C    
   G   
   A   
   T   
   C   
   G   
    C    
   C   
   A   
    G    
    T    
   C   
    C    
   C   
   A   
   A   
   G   
   C   
    T    
   A   
   T   
    T    
   T   
   T   
   A   
   G   
   A   
   T   
   A   
   T   
   G   
   C   
   G   
   T   
   T   
   T   
    A    
   C   
   G   
    C    
   C   
   G   
    A    
   T   
   T   
    G    
   A   
   C   
    C    
   G   
   G   
    T    
   T   
   T   
    G    
   G   
   A   
   A   
   C   
   G   
    G    
   G   
   A   
    A    
   A   
   A   
   A   
   A   
   T   
    C    
   G   
   A   
    A    
   A   
   A   
   T   
   G   
   A   
   A   
   T   
   A   
    C    
   C   
   A   
   A   
   G   
   A   
    A    
   C   
   T   
    C    
   T   
   T   
    A    
   A   
    A    
   A   
    C    
   T   
    G    
   A   
   T   
   T   
   G   
    A    
    A    
   G   
   A   
    T    
   T   
   T   
    A    
    A    
    C    
    T    
   G   
   A   
   C   
   A   
   T   
   T   
    C    
   T   
    G    
   G   
   C   
    A    
    A    
   G   
    A    
   C   
   C   
    A    
   G   
   A   
    A    
   C   
   G   
    T    
   G   
   T   
    T    
   A   
    C    
    A    
   G   
   A   
   A   
   A   
   T   
    T    
   A   
   T   
    C    
   A   
   A   
    G    
   A   
    A    
    T    
   G   
   A   
    A    
   T   
   T   
   A   
    G    
    C    
    A    
   G   
   A   
   C   
    T    
   T   
    G    
    A    
    A    
    T    
    C    
    A    
   A   
    C    
    G    
   A   
   T   
   T   
   T   
   G   
   G   
   T   
   G   
   A   
   T   
    A    
    A    
   A   
   C   
   G   
   A   
   C   
   G   
    C    
   A   
   C   
   A   
   G   
   A   
   A   
    T    
   T   
   A   
    C    
   T   
   A   
   G   
   T   
    C    
   G   
   G   
   T   
   G   
   A   
    G    
   G   
   T   
   C   
    T    
   T   
    A    
   A   
   G   
   T   
   C   
   T   
    G    
   G   
   A   
   A   
   G   
   A   
   T   
   G   
   A   
   A   
   G   
   A   
   T   
    T    
   T   
    A    
   A   
   T   
    C    
   G   
   A   
   A   
   G   
   A   
   A   
   G   
   A   
   A   
   G   
   A   
   A   
    A    
   T   
   C   
   G   
   T   
    T    
   A   
   T   
   T   
   A   
   C   
    A    
    T    
   T   
    G    
   A   
   C   
    A    
   A   
   A   
    C    
   A   
   A   
    T    
   G   
   G   
    T    
   T   
   A   
    C    
   A   
   T   
    C    
   A   
   A   
    A    
   C   
   G   
    T    
    T    
   T   
   G   
   G   
    C    
   A   
   A   
   A   
    T    
    A    
    A    
    T    
   G   
   A   
   A   
   T   
   T   
   C   
    A    
   G   
    A    
   G   
   C   
    T    
   C   
   A   
   A   
   C   
   G   
    A    
    A    
   G   
    G    
   G   
   G   
    T    
   G   
   G   
    A    
   C   
   G   
   T   
   G   
   G   
    T    
   G   
   T   
    C    
   C   
   A   
   A   
   G   
   G   
    A    
   A   
   T   
   G   
   G   
   G   
    T    
    A    
   T   
    C    
   C   
   A   
    C    
    A    
   A   
   T   
   G   
   A   
    C    
   G   
   A   
    C    
   T   
   T   
   C   
   G   
   T   
    C    
   A   
   A   
    A    
   A   
   A   
    T    
   C   
   T   
    G    
    G    
   T   
   T   
   T   
   C   
   T   
   T   
   G   
    T    
   T   
   C   
    T    
   A   
   C   
    C    
   C   
   A   
    T    
   G   
   A   
    T    
   A   
   C   
    A    
   T   
   T   
    G    
   T   
   T   
   A   
   T   
   T   
    C    
   T   
   T   
   T   
   A   
   C   
   G   
   A   
   A   
    T    
   A   
    A    
    C    
   G   
   G   
    A    
   A   
   A   
   A   
   G   
   T   
    G    
   T   
   A   
    T    
   C   
   G   
    G    
   G   
   C   
   A   
   A   
   A   
   A   
   G   
   G   
    A    
   T   
   A   
    T    
   G   
   A   
   A   
   A   
   T   
    T    
   C   
   C   
    A    
   G   
   A   
    A    
   T   
   A   
   C   
   G   
   G   
    G    
    C    
   G   
    T    
   A   
   C   
   A   
   G   
   C   
    C    
   A   
   A   
   A   
   G   
   G   
    A    
   A   
   T   
    C    
   C   
   C   
   A   
    A    
   T   
    C    
   A   
   T   
    C    
   A   
   A   
    T    
   T   
   T   
    A    
   C   
   T   
    A    
   G   
   G   
    T    
   A   
   T   
    C    
   G   
   A   
    T    
   T   
   C   
    G    
    A    
    G    
    T    
   G   
   A   
   A   
   A   
    G    
    C    
   A   
   T   
    C    
   C   
   A   
    G    
   G   
   C   
    A    
   A   
   T   
    C    
   A   
   T   
   T   
    G    
   C   
    G    
   G   
   T   
    G    
    A    
    C    
   G   
   G   
   G   
    A    
    G    
   A   
   A   
   G   
   C   
    A    
   G   
   A   
   A   
   G   
    A    
    A    
   G   
   G   
    C    
    C    
   A   
    C    
   T   
   A   
    T    
    C    
   T   
    C    
   T   
   T   
   C   
   T   
   T   
    T    
   A   
   C   
    G    
   A   
   C   
    G    
    C    
    G    
    T    
    C    
   A   
   A   
   G   
   G   
    T    
   A   
   C   
    A    
   G   
   T   
    A    
   A   
   A   
   A   
   C   
   G   
    T    
   A   
   C   
    T    
    T    
   C   
    T    
   G   
   T   
    C    
   A   
   C   
    T    
   G   
   C   
    T    
   T   
   T   
    T    
    G    
   C   
   T   
   A   
   A   
    C    
   A   
   T   
    C    
   C   
   G   
    A    
   A   
   G   
    T    
   A   
   A   
   T   
   G   
   G   
    T    
    C    
   T   
    G    
   A   
   T   
    C    
   G   
   C   
    A    
   A   
   T   
   T   
    G    
   G   
    T    
   T   
   T   
    G    
   A   
   A   
   A   
   G   
   A   
   A   
   G   
   A   
    C    
   G   
   A   
    T    
   G   
   A   
    G    
    C    
   T   
    C    
    G    
   T   
    C    
   A   
   A   
    T    
   G   
   T   
    G    
    C    
   T   
   A   
    C    
    T    
   G   
   A   
   C   
    A    
    G    
   A   
    T    
   G   
   G   
    T    
    C    
   A   
    A    
    T    
    C    
    T    
   A   
   A   
    T    
   A   
   T   
    C    
   A   
   T   
    T    
   A   
   T   
    C    
   G   
   G   
    A    
   A   
   C   
   A   
   C   
   A   
   T   
    A    
    A    
    C    
   G   
   G   
    C    
   T   
   A   
   C   
   T   
   C   
   T   
   G   
   T   
    C    
   A   
   C   
    T    
   T   
   T   
    C    
    A    
   A   
    A    
   G   
   A   
    A    
    G    
    A    
    A    
    G    
   C   
    A    
   G   
    T    
    C    
   C   
   G   
    C    
   G   
   A   
   T   
   A   
   T   
   G   
   G   
   G   
    A    
   C   
   G   
    T    
   A   
   C   
   A   
   G   
   C   
    T    
   T   
   C   
    C    
   G   
   G   
    G    
   G   
   T   
    C    
    A    
   G   
    A    
   G   
   G   
    G    
   A   
   T   
    A    
   C   
   G   
    C    
    C    
   T   
    G    
   C   
   G   
    T    
   G   
   A   
    G    
    G    
   A   
    A    
   G   
   A   
    T    
   T   
    A    
    T    
   G   
   T   
    C    
   G   
   T   
    T    
   G   
   G   
    G    
    G    
   C   
   A   
   G   
   C   
    C    
    T    
   T   
    A    
    A    
   T   
   G   
    A    
   A   
    G    
   G   
    G    
   A   
    A    
   A   
    T    
    C    
   A   
   A   
   G   
   A   
   A   
   G   
   T   
    A    
    T    
   T   
    A    
   G   
   T   
    G    
   A   
   T   
    C    
   A   
   C   
    A    
   G   
   A   
   A   
   A   
   A   
    A    
   G   
   G   
   T   
   T   
   A   
    C    
   G   
   G   
    A    
   A   
   A   
    A    
   C   
   G   
    G    
   A   
   C   
    A    
   A   
   A   
   A   
   G   
    T    
    A    
   T   
   C   
    G    
   G   
   A   
    A    
   T   
   A   
   T   
   C   
   C   
   A   
   G   
   T   
    G    
   A   
   A   
    A    
   G   
   G   
    C    
   C   
   G   
    C    
   G   
   G   
    A    
   G   
   G   
   T   
   A   
   A   
   A   
   G   
   G   
    G    
   A   
   T   
    C    
   A   
   A   
    A    
   A   
   C   
    G    
    G    
   C   
    G    
   A   
   A   
    C    
   A   
   T   
   C   
   A   
   C   
   T   
   G   
   A   
    A    
   A   
   A   
   A   
   A   
   A   
   T   
   G   
   G   
   T   
   C   
   C   
   A   
    C    
   T   
   A   
   G   
   C   
    T    
   G   
   G   
    G    
   T   
   T   
    G    
   A   
   C   
   C   
   A   
   C   
    T    
   G   
   T   
    T    
    T    
    C    
   T   
   G   
   G   
    A    
    G    
   A   
   T   
   G   
   A   
   A   
   G   
   A   
    C    
   A   
   T   
   C   
    A    
   T   
    G    
    C    
   T   
    G    
   A   
   T   
    T    
   A   
   C   
    A    
    G    
   A   
    T    
   A   
   A   
   A   
   G   
   G   
    T    
   G   
   T   
    C    
   A   
   T   
   T   
   A   
   T   
   C   
   C   
   G   
    T    
   T   
   T   
    C    
   A   
   A   
   C   
   G   
   T   
    A    
    T    
    C    
    G    
    A    
   C   
   T   
   G   
   T   
   T   
   T   
   C   
   T   
   C   
   A   
   A   
   A   
   C   
    T    
   G   
   G   
   A   
   C   
   G   
    T    
    T    
   C   
   A   
   A   
   C   
   A   
   T   
   T   
    A    
   G   
   G   
    C    
   G   
   T   
    A    
   C   
   G   
   T   
   T   
   T   
    G    
   A   
   T   
   G   
   A   
    A    
    G    
   A   
   T   
   G   
   G   
   A   
   A   
   G   
    C    
    T    
   G   
    A    
    T    
    A    
   C   
    G    
   A   
   A   
   A   
   G   
   T   
   T   
   G   
   T   
   C   
   A   
   C   
   G   
   A   
   T   
   G   
   G   
   C   
   A   
   G   
   C   
   A   
   G   
   T   
   C   
   G   
   A   
   A   
   C   
   C   
   A   
   G   
   A   
   A   
   G   
   C   
   T   
   G   
   C   
   A   
   G   
   A   
   A   
   C   
   A   
   A   
   A   
   C   
   A   
   G   
   T   
   T   
   G   
   A   
   A   
   G   
   A   
   A   
   C   
   A   
   A   
   G   
   G   
   A   
   T   
   C   
   A   
   G   
   A   
   A   
   G   
   A   
   A   
   -   
   -   
   -   
 

 
   N59513F   
   A   
   T   
   G   
   A   
   G   
   T   
   G   
   A   
   A   
   G   
   A   
   A   
   A   
   T   
   C   
   A   
   G   
   A   
   G   
   A   
   A   
   A   
   A   
   C   
   A   
   T   
   C   
   C   
   A   
   T   
   G   
   A   
   T   
   G   
   T   
   C   
   A   
   A   
   T   
   C   
   T   
   A   
   A   
   C   
   C   
   A   
   G   
   C   
   G   
   A   
   A   
   A   
   T   
   G   
   A   
   A   
   A   
   G   
   A   
   C   
   T   
   C   
   C   
   T   
   T   
   C   
   A   
   T   
   C   
   G   
   A   
   T   
   T   
   A   
   T   
   G   
   C   
   G   
   A   
   T   
   G   
   A   
   G   
   T   
   G   
   T   
   T   
   A   
   T   
   T   
   G   
   T   
   C   
   G   
   C   
   A   
   C   
   G   
   T   
   G   
   C   
   A   
   C   
   T   
   G   
   C   
   C   
   T   
   G   
   A   
   T   
   G   
   T   
   C   
   A   
   G   
   A   
   G   
   A   
   T   
   G   
   G   
   T   
   C   
   T   
   G   
   A   
   A   
   A   
   C   
   C   
   C   
   G   
   T   
   T   
   C   
   A   
   T   
   C   
   G   
   C   
   C   
   G   
   T   
   A   
   T   
   T   
   T   
   T   
   A   
   T   
   A   
   C   
   G   
   G   
   C   
   A   
   T   
   G   
   A   
   A   
   C   
   G   
   A   
   A   
   T   
   T   
   A   
   G   
   G   
   T   
   G   
   T   
   G   
   A   
   C   
   A   
   C   
   C   
   G   
   G   
   A   
   T   
   A   
   A   
   A   
   G   
   C   
   C   
   C   
   A   
   T   
   A   
   A   
   A   
   A   
   A   
   A   
   T   
   C   
   T   
   G   
   C   
   C   
   A   
   G   
   A   
   A   
   T   
   C   
   G   
   T   
   C   
   G   
   G   
   A   
   G   
   A   
   T   
   G   
   T   
   C   
   A   
   T   
   G   
   G   
   G   
   T   
   A   
   A   
   A   
   T   
   A   
   C   
   C   
   A   
   T   
   C   
   C   
   T   
   C   
   A   
   T   
   G   
   G   
   G   
   G   
   A   
   T   
   A   
   G   
   T   
   G   
   C   
   G   
   A   
   T   
   C   
   T   
   A   
   T   
   G   
   A   
   G   
   T   
   C   
   A   
   A   
   T   
   G   
   G   
   T   
   A   
   C   
   G   
   G   
   A   
   T   
   G   
   G   
   C   
   G   
   C   
   A   
   G   
   C   
   C   
   A   
   T   
   T   
   T   
   A   
   G   
   C   
   T   
   A   
   C   
   C   
   G   
   T   
   T   
   A   
   T   
   A   
   T   
   G   
   C   
   T   
   A   
   G   
   T   
   G   
   G   
   A   
   T   
   G   
   G   
   C   
   C   
   A   
   T   
   G   
   G   
   A   
   A   
   A   
   C   
   T   
   T   
   T   
   G   
   G   
   T   
   T   
   C   
   T   
   G   
   T   
   T   
   G   
   A   
   C   
   G   
   G   
   T   
   G   
   A   
   C   
   G   
   G   
   T   
   G   
   C   
   C   
   G   
   C   
   T   
   G   
   C   
   T   
   A   
   T   
   G   
   C   
   G   
   T   
   T   
   A   
   T   
   A   
   C   
   C   
   G   
   A   
   A   
   G   
   C   
   A   
   C   
   G   
   G   
   A   
   T   
   G   
   A   
   G   
   T   
   A   
   A   
   A   
   A   
   T   
   C   
   G   
   C   
   T   
   A   
   C   
   A   
   G   
   A   
   A   
   A   
   T   
   G   
   C   
   T   
   G   
   C   
   G   
   T   
   G   
   A   
   T   
   A   
   T   
   C   
   A   
   A   
   T   
   A   
   A   
   G   
   A   
   A   
   C   
   A   
   C   
   T   
   G   
   T   
   T   
   G   
   A   
   T   
   T   
   T   
   C   
   C   
   A   
   A   
   A   
   G   
   C   
   A   
   A   
   C   
   T   
   A   
   T   
   G   
   A   
   T   
   G   
   A   
   C   
   A   
   C   
   A   
   G   
   A   
   A   
   A   
   A   
   A   
   G   
   A   
   A   
   C   
   C   
   C   
   G   
   T   
   T   
   G   
   T   
   A   
   T   
   T   
   A   
   C   
   C   
   T   
   G   
   C   
   T   
   C   
   G   
   C   
   T   
   T   
   C   
   C   
   C   
   C   
   A   
   A   
   T   
   C   
   T   
   T   
   T   
   T   
   G   
   G   
   T   
   G   
   A   
   A   
   C   
   G   
   G   
   A   
   A   
   C   
   G   
   A   
   C   
   G   
   G   
   G   
   G   
   A   
   T   
   A   
   G   
   C   
   C   
   G   
   T   
   G   
   G   
   G   
   T   
   A   
   T   
   G   
   G   
   C   
   A   
   A   
   C   
   G   
   A   
   A   
   T   
   A   
   T   
   C   
   C   
   C   
   A   
   C   
   C   
   T   
   C   
   A   
   T   
   A   
   A   
   T   
   T   
   T   
   A   
   T   
   C   
   T   
   G   
   A   
   G   
   G   
   T   
   A   
   G   
   T   
   A   
   G   
   C   
   G   
   G   
   C   
   T   
   A   
   T   
   C   
   G   
   A   
   T   
   T   
   T   
   G   
   C   
   T   
   G   
   A   
   T   
   G   
   G   
   A   
   T   
   A   
   A   
   T   
   C   
   C   
   T   
   G   
   A   
   T   
   G   
   T   
   G   
   A   
   C   
   G   
   A   
   C   
   C   
   A   
   A   
   T   
   G   
   A   
   A   
   T   
   T   
   G   
   A   
   T   
   G   
   G   
   A   
   A   
   G   
   T   
   A   
   T   
   T   
   A   
   C   
   C   
   T   
   G   
   G   
   A   
   C   
   C   
   A   
   G   
   A   
   T   
   T   
   T   
   T   
   C   
   C   
   A   
   A   
   C   
   A   
   G   
   G   
   T   
   G   
   G   
   T   
   T   
   T   
   A   
   G   
   T   
   C   
   A   
   T   
   G   
   G   
   G   
   A   
   A   
   A   
   A   
   T   
   C   
   A   
   G   
   G   
    A    
   A   
   T   
    C    
   C   
   G   
    A    
    A    
   G   
   A   
   G   
   C   
    G    
   T   
   A   
   T   
   G   
   A   
   A   
   A   
   C   
    A    
   G   
   G   
    T    
   A   
   A   
    A    
   G   
   G   
   T   
   T   
   C   
    T    
   A   
   T   
   T   
   A   
   C   
    C    
   G   
   T   
    A    
   C   
   G   
   T   
   G   
   C   
   A   
   A   
   A   
   A   
   G   
   T   
   T   
   G   
   A   
    G    
    A    
   T   
    C    
   A   
    C    
   T   
   G   
   A   
    G    
   A   
   T   
   G   
    C    
   C   
    A    
   A   
   A   
   T   
   G   
   G   
    C    
   A   
   A   
   A   
   G   
   A   
   A   
   C   
   G   
    G    
   A   
   T   
    C    
    C    
   T   
    C    
   G   
   T   
    G    
   A   
   C   
    G    
   G   
   A   
   A   
   T   
   T   
    A    
   C   
   C   
   T   
   T   
   A   
   T   
   A   
   T   
   G   
   G   
   T   
    C    
   A   
   A   
   T   
   A   
   A   
   A   
   G   
   C   
    C    
   A   
   A   
   A   
   T   
   T   
    G    
   A   
   T   
    T    
   G   
   A   
   A   
   C   
   G   
    G    
   A   
   T   
   T   
   T   
   C   
   T   
   G   
   A   
    G    
    T    
   T   
    G    
   C   
   A   
   C   
   A   
   G   
   A   
   G   
   A   
    C    
   A   
   A   
    G    
   C   
   G   
    T    
   A   
   T   
    C    
   G   
   A   
   A   
   G   
   G   
    A    
   A   
   T   
    C    
   A   
   C   
    G    
   G   
   A   
   T   
   T   
   T   
   G   
   C   
   G   
   G   
   G   
   A   
    T    
   G   
   A   
   A   
   T   
   C   
   T   
   T   
   C   
    C    
   C   
   G   
   T   
   G   
   A   
    A    
   G   
   G   
    C    
   A   
   T   
   G   
   C   
   G   
    A    
   A   
   T   
    T    
   G   
   T   
   C   
   A   
   T   
    T    
   G   
   A   
    C    
    A    
   T   
    T    
   C   
   G   
   T   
   C   
   G   
    T    
   G   
   A   
    C    
   G   
    C    
    C    
   A   
   G   
    C    
   G   
   C   
    A    
   T   
   C   
    A    
   G   
   T   
   T   
    A    
   T   
    T    
   T   
   T   
    G    
   A   
   A   
    T    
   A   
   A   
   C   
   T   
   T   
    A    
   T   
   A   
    C    
   A   
   A   
   A   
    T    
   T   
   G   
   A   
   C   
    A    
    T    
   C   
    C    
   T   
   T   
    A    
   C   
   A   
    G    
   A   
    A    
    T    
   T   
   C   
    A    
   T   
   T   
    T    
   G   
   G   
    A    
   T   
   T   
    C    
   A   
   A   
    T    
   A   
   T   
   G   
   T   
   T   
    G    
   G   
   C   
    C    
   A   
   T   
    C    
   G   
   A   
   A   
   A   
   A   
   A   
   G   
   G   
    C    
   G   
   T   
    G    
   C   
   C   
    G    
   A   
   A   
   A   
    G    
   T   
   T   
   T   
   T   
    A    
   A   
   G   
    T    
   T   
   T   
   G   
   A   
   A   
   A   
   C   
    A    
    A    
   A   
   T   
    C    
    C    
   T   
    T    
   G   
   A   
   A   
   A   
   A   
    C    
   T   
   A   
    T    
    A    
   T   
    C    
   G   
   A   
    A    
   C   
   A   
    T    
   C   
   A   
    A    
    C    
    G    
    C    
    G    
   A   
   A   
   G   
   T   
   C   
   A   
   T   
    C    
   A   
   C   
    A    
    C    
   G   
    A    
   C   
   G   
   G   
   A   
   C   
    G    
    G    
   T   
    C    
   T   
   T   
   T   
   G   
   A   
    A    
   A   
   A   
   A   
    G    
   A   
    A    
   A   
   A   
    A    
   G   
   C   
   A   
   G   
   A   
   A   
   G   
   C   
   A   
    A    
   G   
    A    
   G   
   C   
    A    
   C   
   A   
    C    
   A   
   T   
    C    
   T   
   T   
   A   
   G   
   A   
   A   
   G   
   G   
   T   
    T    
   T   
    G    
   C   
   G   
    T    
   A   
   T   
   T   
   G   
   C   
    T    
   T   
   T   
    G    
   G   
   A   
   T   
   C   
   A   
   T   
   A   
   T   
   C   
    G    
   A   
   T   
   G   
   A   
   A   
   A   
   T   
    C    
   A   
   T   
   T   
   G   
   C   
    G    
   A   
   T   
   T   
   A   
   T   
    C    
   C   
   G   
    C    
   G   
   G   
    G    
   T   
   C   
    T    
    A    
   A   
   A   
   T   
   C   
   A   
   G   
   A   
   T   
   G   
   A   
    T    
   G   
   A   
    A    
   G   
   C   
   A   
   A   
   A   
   A   
   G   
   C   
    A    
   A   
    C    
    A    
    A    
   T   
    G    
   A   
   T   
    C    
   G   
   A   
   A   
   C   
   G   
    G    
   T   
   T   
    C    
   G   
   A   
    T    
   T   
   T   
    A    
   T   
   C   
    C    
   G   
   A   
   T   
   C   
   G   
    C    
   C   
   A   
    A    
    T    
   C   
    A    
   C   
   A   
   A   
   G   
   C   
    T    
   A   
   T   
    C    
   T   
   T   
   A   
   G   
   A   
   T   
   A   
   T   
   G   
   C   
   G   
   T   
   T   
   T   
    G    
   C   
   G   
    T    
   C   
   G   
    A    
   T   
   T   
    G    
   A   
   C   
    C    
   G   
   G   
    T    
   T   
   T   
    G    
   G   
   A   
   A   
   C   
   G   
    G    
   G   
   A   
    A    
   A   
   A   
   A   
   A   
   T   
    C    
   G   
   A   
    A    
   A   
   A   
   T   
   G   
   A   
   A   
   T   
   A   
    T    
   C   
   A   
   A   
   G   
   A   
    A    
   C   
   T   
    A    
   T   
   T   
    G    
   A   
    A    
   A   
    C    
   T   
    G    
   A   
   T   
   T   
   G   
    A    
    A    
   G   
   A   
    T    
   T   
   T   
    G    
    A    
    C    
    T    
   G   
   A   
   C   
   A   
   T   
   T   
    C    
   T   
    G    
   G   
   C   
    A    
    A    
   G   
    A    
   C   
   C   
    C    
   G   
   A   
    A    
   C   
   G   
    T    
   G   
   T   
    C    
   A   
    C    
    G    
   G   
   A   
   A   
   A   
   T   
    C    
   A   
   T   
    C    
   A   
   A   
    G    
   A   
    A    
    T    
   G   
   A   
    G    
   T   
   T   
   A   
    G    
    C    
    A    
   G   
   A   
   C   
    T    
   T   
    G    
    A    
    A    
    T    
    C    
    A    
   A   
    C    
    G    
   A   
   T   
   T   
   T   
   G   
   G   
   T   
   G   
   A   
   T   
    A    
    A    
   A   
   C   
   G   
   A   
   C   
   G   
    C    
   A   
   C   
   A   
   G   
   A   
   A   
    T    
   T   
   A   
    C    
   T   
   A   
   G   
   T   
    C    
   G   
   G   
   T   
   G   
   A   
    G    
   G   
   T   
   C   
    T    
   T   
    A    
   A   
   G   
   T   
   C   
   T   
    A    
   G   
   A   
   A   
   G   
   A   
   T   
   G   
   A   
   A   
   G   
   A   
   T   
    T    
   T   
    G    
   A   
   T   
    C    
   G   
   A   
   A   
   G   
   A   
   A   
   G   
   A   
   A   
   G   
   A   
   A   
    A    
   T   
   C   
   G   
   T   
    T    
   A   
   T   
   T   
   A   
   C   
    A    
    C    
   T   
    G    
   A   
   C   
    A    
   A   
   A   
    C    
   A   
   A   
    T    
   G   
   G   
    T    
   T   
   A   
    T    
   A   
   T   
    C    
   A   
   A   
    A    
   C   
   G   
    T    
    T    
   T   
   G   
   G   
    C    
   A   
   A   
   A   
    T    
    A    
    A    
    T    
   G   
   A   
   A   
   T   
   T   
   C   
    A    
   G   
    A    
   G   
   C   
    T    
   C   
   A   
   A   
   C   
   G   
    A    
    A    
   G   
    A    
   G   
   G   
    C    
   G   
   G   
    G    
   C   
   G   
   T   
   G   
   G   
    C    
   G   
   T   
    C    
   C   
   A   
   A   
   G   
   G   
    A    
   A   
   T   
   G   
   G   
   G   
    C    
    A    
   T   
    C    
   C   
   A   
    C    
    A    
   A   
   T   
   G   
   A   
    C    
   G   
   A   
    C    
   T   
   T   
   C   
   G   
   T   
    C    
   A   
   A   
    G    
   A   
   A   
    T    
   C   
   T   
    A    
    G    
   T   
   T   
   T   
   C   
   T   
   T   
   G   
    T    
   T   
   C   
    T    
   A   
   C   
    C    
   C   
   A   
    T    
   G   
   A   
    T    
   A   
   C   
    A    
   T   
   T   
    G    
   T   
   T   
   A   
   T   
   T   
    C    
   T   
   T   
   T   
   A   
   C   
   G   
   A   
   A   
    T    
   A   
    A    
    C    
   G   
   G   
    A    
   A   
   A   
   A   
   G   
   T   
    G    
   T   
   A   
    T    
   C   
   G   
    G    
   G   
   C   
   A   
   A   
   A   
   A   
   G   
   G   
    A    
   T   
   A   
    C    
   G   
   A   
   A   
   A   
   T   
    T    
   C   
   C   
    A    
   G   
   A   
    A    
   T   
   A   
   C   
   G   
   G   
    A    
    C    
   G   
    T    
   A   
   C   
   A   
   G   
   C   
    C    
   A   
   A   
   A   
   G   
   G   
    T    
   A   
   T   
    C    
   C   
   C   
   A   
    A    
   T   
    C    
   A   
   T   
    C    
   A   
   A   
    T    
   T   
   T   
    G    
   C   
   T   
    G    
   G   
   G   
    T    
   A   
   T   
    C    
   G   
   A   
    C    
   T   
   C   
    G    
    A    
    G    
    T    
   G   
   A   
   A   
   A   
    G    
    C    
   A   
   T   
    C    
   C   
   A   
    A    
   G   
   C   
    A    
   A   
   T   
    T    
   A   
   T   
   T   
    G    
   C   
    G    
   G   
   T   
    G    
    A    
    C    
   G   
   G   
   G   
    A    
    G    
   A   
   A   
   G   
   C   
    A    
   G   
   A   
   A   
   G   
    A    
    A    
   G   
   G   
    C    
    C    
   A   
    C    
   T   
   A   
    C    
    C    
   T   
    C    
   T   
   T   
   C   
   T   
   T   
    T    
   A   
   C   
    G    
   A   
   C   
    G    
    C    
    G    
    T    
    C    
   A   
   A   
   G   
   G   
    T    
   A   
   C   
    A    
   G   
   T   
    T    
   A   
   A   
   A   
   C   
   G   
    T    
   A   
   C   
    G    
    T    
   C   
    T    
   G   
   T   
    C    
   A   
   C   
    T    
   G   
   C   
    T    
   T   
   T   
    C    
    G    
   C   
   T   
   A   
   A   
    C    
   A   
   T   
    C    
   C   
   G   
    A    
   A   
   G   
    C    
   A   
   A   
   T   
   G   
   G   
    T    
    C    
   T   
    G    
   A   
   T   
    T    
   G   
   C   
    A    
   A   
   T   
   T   
    G    
   G   
    T    
   T   
   T   
    G    
   A   
   A   
   A   
   G   
   A   
   A   
   G   
   A   
    T    
   G   
   A   
    C    
   G   
   A   
    A    
    T    
   T   
    A    
    G    
   T   
    C    
   A   
   A   
    T    
   G   
   T   
    T    
    C    
   T   
   A   
    T    
    T    
   G   
   A   
   C   
    A    
    G    
   A   
    T    
   G   
   G   
    A    
    C    
   A   
    G    
    T    
    C    
    A    
   A   
   A   
    T    
   A   
   T   
    C    
   A   
   T   
    C    
   A   
   T   
    T    
   G   
   G   
    C    
   A   
   C   
   A   
   C   
   A   
   T   
    A    
    A    
    C    
   G   
   G   
    C    
   T   
   A   
   C   
   T   
   C   
   T   
   G   
   T   
    G    
   A   
   C   
    C    
   T   
   T   
    C    
    A    
   A   
    A    
   G   
   A   
    G    
    G    
    A    
    A    
    G    
   C   
    A    
   G   
    T    
    C    
   C   
   G   
    C    
   G   
   A   
   T   
   A   
   T   
   G   
   G   
   G   
    A    
   C   
   G   
    T    
   A   
   C   
   A   
   G   
   C   
    T    
   T   
   C   
    C    
   G   
   G   
    C    
   G   
   T   
    C    
    A    
   G   
    A    
   G   
   G   
    G    
   A   
   T   
    A    
   C   
   G   
    T    
    T    
   T   
    G    
   C   
   G   
    T    
   G   
   A   
    G    
    G    
   A   
    A    
   G   
   A   
    C    
   T   
    A    
    C    
   G   
   T   
    T    
   G   
   T   
    T    
   G   
   G   
    G    
    G    
   C   
   A   
   G   
   C   
    C    
    C    
   T   
    G    
    A    
   T   
   G   
    A    
   A   
    G    
   G   
    A    
   A   
    G    
   A   
    C    
    C    
   A   
   A   
   G   
   A   
   A   
   G   
   T   
    A    
    C    
   T   
    A    
   G   
   T   
    G    
   A   
   T   
    T    
   A   
   C   
    A    
   G   
   A   
   A   
   A   
   A   
    A    
   G   
   G   
   T   
   T   
   A   
    C    
   G   
   G   
    A    
   A   
   A   
    A    
   C   
   G   
    G    
   A   
   C   
    C    
   A   
   A   
   A   
   G   
    T    
    A    
   T   
   C   
    G    
   G   
   A   
    A    
   T   
   A   
   T   
   C   
   C   
   A   
   G   
   T   
    G    
   A   
   A   
    A    
   G   
   G   
    C    
   C   
   G   
    C    
   G   
   G   
    A    
   G   
   G   
   T   
   A   
   A   
   A   
   G   
   G   
    A    
   A   
   T   
    C    
   A   
   A   
    A    
   A   
   C   
    G    
    T    
   C   
    G    
   A   
   A   
    T    
   A   
   T   
   C   
   A   
   C   
   T   
   G   
   A   
    A    
   A   
   A   
   A   
   A   
   A   
   T   
   G   
   G   
   T   
   C   
   C   
   A   
    C    
   T   
   A   
   G   
   C   
    C    
   G   
   G   
    A    
   T   
   T   
    G    
   A   
   C   
   C   
   A   
   C   
    T    
   G   
   T   
    T    
    T    
    C    
   T   
   G   
   G   
    A    
    G    
   A   
   T   
   G   
   A   
   A   
   G   
   A   
    C    
   A   
   T   
   C   
    A    
   T   
    G    
    C    
   T   
    G    
   A   
   T   
    T    
   A   
   C   
    A    
    G    
   A   
    T    
   A   
   A   
   A   
   G   
   G   
    T    
   G   
   T   
    G    
   A   
   T   
   T   
   A   
   T   
   C   
   C   
   G   
    T    
   T   
   T   
    C    
   A   
   A   
   C   
   G   
   T   
    A    
    T    
    C    
    G    
    A    
   C   
   T   
   G   
   T   
   T   
   T   
   C   
   T   
   C   
   A   
   A   
   A   
   C   
    T    
   G   
   G   
   A   
   C   
   G   
    T    
    T    
   C   
   A   
   A   
   C   
   A   
   T   
   T   
    G    
   G   
   G   
    T    
   G   
   T   
    A    
   C   
   G   
   T   
   T   
   T   
    G    
   A   
   T   
   G   
   A   
    A    
    G    
   A   
   T   
   G   
   G   
   A   
   A   
   G   
    C    
    T    
   G   
    A    
    T    
    A    
   C   
    G    
   A   
   A   
   A   
   G   
   T   
   C   
   G   
   T   
   C   
   A   
   C   
   A   
   A   
   T   
   G   
   -   
   -   
   -   
   -   
   -   
   -   
   -   
   -   
   -   
   -   
   -   
   -   
   -   
   -   
   -   
   -   
   -   
   -   
   -   
   -   
   -   
   -   
   -   
   -   
   -   
   -   
   -   
   -   
   -   
   -   
   -   
   -   
   -   
   -   
   -   
   -   
   -   
   -   
   -   
   -   
   -   
   -   
   -   
   -   
   -   
   -   
   -   
   -   
   -   
   -   
   -   
   -   
   -   
   -   
   -   
   -   
   -   
   -   
   -   
   -   
 

 
   N52636   
   A   
   T   
   G   
   A   
   G   
   T   
   G   
   A   
   A   
   G   
   A   
   A   
   A   
   T   
   C   
   A   
   G   
   A   
   G   
   A   
   A   
   A   
   A   
   C   
   A   
   T   
   C   
   C   
   A   
   T   
   G   
   A   
   T   
   G   
   T   
   C   
   A   
   A   
   T   
   C   
   T   
   A   
   A   
   C   
   C   
   A   
   G   
   C   
   G   
   A   
   A   
   A   
   T   
   G   
   A   
   A   
   A   
   G   
   A   
   C   
   T   
   C   
   C   
   T   
   T   
   C   
   A   
   T   
   C   
   G   
   A   
   T   
   T   
   A   
   T   
   G   
   C   
   G   
   A   
   T   
   G   
   A   
   G   
   T   
   G   
   T   
   T   
   A   
   T   
   T   
   G   
   T   
   C   
   G   
   C   
   A   
   C   
   G   
   T   
   G   
   C   
   A   
   C   
   T   
   T   
   C   
   C   
   T   
   G   
   A   
   T   
   G   
   T   
   C   
   A   
   G   
   A   
   G   
   A   
   T   
   G   
   G   
   T   
   C   
   T   
   G   
   A   
   A   
   A   
   C   
   C   
   C   
   G   
   T   
   T   
   C   
   A   
   T   
   C   
   G   
   C   
   C   
   G   
   T   
   A   
   T   
   T   
   T   
   T   
   A   
   T   
   A   
   C   
   G   
   G   
   C   
   A   
   T   
   G   
   A   
   A   
   C   
   G   
   A   
   A   
   T   
   T   
   A   
   G   
   G   
   T   
   G   
   T   
   A   
   A   
   C   
   A   
   C   
   C   
   G   
   G   
   A   
   T   
   A   
   A   
   A   
   G   
   C   
   C   
   C   
   A   
   T   
   A   
   A   
   A   
   A   
   A   
   A   
   T   
   C   
   T   
   G   
   C   
   C   
   A   
   G   
   A   
   A   
   T   
   C   
   G   
   T   
   C   
   G   
   G   
   A   
   G   
   A   
   T   
   G   
   T   
   C   
   A   
   T   
   G   
   G   
   G   
   T   
   A   
   A   
   A   
   T   
   A   
   C   
   C   
   A   
   T   
   C   
   C   
   T   
   C   
   A   
   T   
   G   
   G   
   A   
   G   
   A   
   C   
   A   
   G   
   T   
   G   
   C   
   G   
   A   
   T   
   C   
   T   
   A   
   T   
   G   
   A   
   G   
   T   
   C   
   A   
   A   
   T   
   G   
   G   
   T   
   A   
   C   
   G   
   G   
   A   
   T   
   G   
   G   
   C   
   A   
   C   
   A   
   G   
   C   
   C   
   A   
   T   
   T   
   C   
   A   
   G   
   C   
   T   
   A   
   C   
   C   
   G   
   T   
   T   
   A   
   T   
   A   
   T   
   G   
   C   
   T   
   A   
   G   
   T   
   G   
   G   
   A   
   T   
   G   
   G   
   T   
   C   
   A   
   T   
   G   
   G   
   A   
   A   
   A   
   C   
   T   
   T   
   T   
   G   
   G   
   T   
   T   
   C   
   T   
   G   
   T   
   T   
   G   
   A   
   C   
   G   
   G   
   T   
   G   
   A   
   C   
   G   
   G   
   T   
   G   
   C   
   C   
   G   
   C   
   C   
   G   
   C   
   T   
   A   
   T   
   G   
   C   
   G   
   T   
   T   
   A   
   T   
   A   
   C   
   C   
   G   
   A   
   A   
   G   
   C   
   A   
   C   
   G   
   G   
   A   
   T   
   G   
   A   
   G   
   C   
   A   
   A   
   A   
   A   
   T   
   C   
   G   
   C   
   T   
   A   
   C   
   A   
   G   
   A   
   A   
   A   
   T   
   G   
   C   
   T   
   G   
   C   
   G   
   T   
   G   
   A   
   T   
   A   
   T   
   C   
   A   
   A   
   T   
   A   
   A   
   G   
   A   
   A   
   C   
   A   
   C   
   T   
   G   
   T   
   T   
   G   
   A   
   T   
   T   
   T   
   C   
   C   
   A   
   A   
   A   
   G   
   C   
   A   
   A   
   C   
   T   
   A   
   T   
   G   
   A   
   T   
   G   
   A   
   T   
   A   
   C   
   A   
   G   
   A   
   A   
   A   
   A   
   A   
   G   
   A   
   A   
   C   
   C   
   A   
   G   
   T   
   T   
   G   
   T   
   A   
   C   
   T   
   A   
   C   
   C   
   T   
   G   
   C   
   T   
   C   
   G   
   C   
   T   
   T   
   C   
   C   
   C   
   G   
   A   
   A   
   T   
   C   
   T   
   T   
   T   
   T   
   A   
   G   
   T   
   G   
   A   
   A   
   C   
   G   
   G   
   A   
   A   
   C   
   A   
   A   
   C   
   G   
   G   
   G   
   G   
   A   
   T   
   C   
   G   
   C   
   C   
   G   
   T   
   A   
   G   
   G   
   T   
   A   
   T   
   G   
   G   
   C   
   C   
   A   
   C   
   G   
   A   
   A   
   T   
   A   
   T   
   C   
   C   
   C   
   A   
   C   
   C   
   T   
   C   
   A   
   T   
   A   
   A   
   C   
   T   
   T   
   G   
   T   
   C   
   T   
   G   
   A   
   G   
   G   
   T   
   A   
   G   
   T   
   A   
   G   
   C   
   G   
   G   
   C   
   T   
   A   
   T   
   C   
   G   
   A   
   T   
   T   
   T   
   G   
   C   
   T   
   G   
   A   
   T   
   G   
   G   
   A   
   T   
   A   
   A   
   T   
   C   
   C   
   T   
   G   
   A   
   T   
   G   
   T   
   G   
   A   
   C   
   G   
   A   
   C   
   C   
   A   
   A   
   T   
   G   
   A   
   A   
   T   
   T   
   G   
   A   
   T   
   G   
   G   
   A   
   A   
   G   
   T   
   A   
   T   
   T   
   A   
   C   
   C   
   T   
   G   
   G   
   A   
   C   
   C   
   A   
   G   
   A   
   T   
   T   
   T   
   T   
   C   
   C   
   A   
   A   
   C   
   A   
   G   
   G   
   T   
   G   
   G   
   T   
   T   
   T   
   G   
   G   
   T   
   T   
   A   
   T   
   G   
   G   
   G   
   A   
   A   
   A   
   A   
   T   
   C   
   A   
   G   
   G   
    A    
   A   
   T   
    C    
   C   
   G   
    A    
    A    
   G   
   A   
   G   
   C   
    G    
   T   
   A   
   T   
   G   
   A   
   A   
   A   
   C   
    A    
   G   
   G   
    T    
   A   
   A   
    G    
   G   
   G   
   T   
   T   
   C   
    T    
   A   
   T   
   T   
   A   
   C   
    C    
   G   
   T   
    A    
   C   
   G   
   T   
   G   
   C   
   A   
   A   
   A   
   A   
   G   
   T   
   T   
   G   
   A   
    G    
    A    
   T   
    T    
   A   
    C    
   T   
   G   
   A   
    G    
   A   
   T   
   G   
    C    
   C   
    A    
   A   
   A   
   T   
   G   
   G   
    T    
   A   
   A   
   A   
   G   
   A   
   A   
   C   
   G   
    G    
   A   
   T   
    C    
    A    
   T   
    C    
   G   
   T   
    G    
   A   
   C   
    A    
   G   
   A   
   A   
   T   
   T   
    G    
   C   
   C   
   T   
   T   
   A   
   T   
   A   
   T   
   G   
   G   
   T   
    C    
   A   
   A   
   T   
   A   
   A   
   A   
   G   
   C   
    A    
   A   
   A   
   A   
   T   
   T   
    G    
   A   
   T   
    C    
   G   
   A   
   A   
   C   
   G   
    G    
   A   
   T   
   T   
   T   
   C   
   T   
   G   
   A   
    A    
    C    
   T   
    G    
   C   
   A   
   C   
   A   
   G   
   A   
   G   
   A   
    C    
   A   
   A   
    G    
   C   
   G   
    T    
   A   
   T   
    C    
   G   
   A   
   A   
   G   
   G   
    G    
   A   
   T   
    C    
   A   
   C   
    A    
   G   
   A   
   T   
   T   
   T   
   G   
   C   
   G   
   G   
   G   
   A   
    C    
   G   
   A   
   A   
   T   
   C   
   T   
   T   
   C   
    C    
   C   
   G   
   T   
   G   
   A   
    A    
   G   
   G   
    T    
   A   
   T   
   G   
   C   
   G   
    G    
   A   
   T   
    C    
   G   
   T   
   C   
   A   
   T   
    T    
   G   
   A   
    C    
    A    
   T   
    C    
   C   
   G   
   T   
   C   
   G   
    T    
   G   
   A   
    C    
   G   
    C    
    C    
   A   
   G   
    C    
   G   
   C   
    A    
   T   
   C   
    C    
   G   
   T   
   T   
    A    
   T   
    C    
   T   
   T   
    G    
   A   
   A   
    T    
   A   
   A   
   C   
   T   
   T   
    A    
   T   
   A   
    T    
   A   
   A   
   A   
    T    
   T   
   G   
   A   
   C   
    A    
    T    
   C   
    C    
   T   
   T   
    G    
   C   
   A   
    A    
   A   
    A    
    T    
   T   
   C   
    A    
   T   
   T   
    T    
   G   
   G   
    A    
   T   
   T   
    C    
   A   
   A   
    T    
   A   
   T   
   G   
   T   
   T   
    G    
   G   
   C   
    G    
   A   
   T   
    C    
   G   
   A   
   A   
   A   
   A   
   A   
   G   
   G   
    T    
   G   
   T   
    G    
   C   
   C   
    G    
   A   
   A   
   A   
    G    
   T   
   T   
   T   
   T   
    G    
   A   
   G   
    C    
   T   
   T   
   G   
   A   
   A   
   A   
   C   
    A    
    A    
   A   
   T   
    T    
    C    
   T   
    T    
   G   
   A   
   A   
   A   
   A   
    C    
   T   
   A   
    T    
    A    
   T   
    C    
   G   
   A   
    A    
   C   
   A   
    T    
   C   
   A   
    G    
    C    
    G    
    C    
    G    
   A   
   A   
   G   
   T   
   C   
   A   
   T   
    C    
   A   
   C   
    A    
    A    
   G   
    A    
   C   
   G   
   G   
   A   
   C   
    A    
    G    
   T   
    C    
   T   
   T   
   T   
   G   
   A   
    A    
   A   
   A   
   A   
    G    
   A   
    A    
   A   
   A   
    A    
   G   
   C   
   A   
   G   
   A   
   A   
   G   
   C   
   A   
    A    
   G   
    G    
   G   
   C   
    A    
   C   
   A   
    C    
   A   
   T   
    T    
   T   
   T   
   A   
   G   
   A   
   A   
   G   
   G   
   T   
    T    
   T   
    G    
   C   
   G   
    T    
   A   
   T   
   T   
   G   
   C   
    T    
   T   
   T   
    G    
   G   
   A   
   T   
   C   
   A   
   T   
   A   
   T   
   C   
    G    
   A   
   T   
   G   
   A   
   A   
   A   
   T   
    C    
   A   
   T   
   T   
   G   
   C   
    G    
   A   
   T   
   T   
   A   
   T   
    C    
   C   
   G   
    C    
   G   
   G   
    G    
   T   
   C   
    T    
    A    
   A   
   A   
   T   
   C   
   A   
   G   
   A   
   T   
   G   
   A   
    C    
   G   
   A   
    A    
   G   
   C   
   A   
   A   
   A   
   A   
   G   
   C   
    G    
   A   
    C    
    G    
    A    
   T   
    G    
   A   
   T   
    C    
   G   
   A   
   A   
   C   
   G   
    G    
   T   
   T   
    C    
   G   
   A   
    T    
   T   
   T   
    A    
   T   
   C   
    C    
   G   
   A   
   T   
   C   
   G   
    C    
   C   
   A   
    G    
    T    
   C   
    T    
   C   
   A   
   A   
   G   
   C   
    T    
   A   
   T   
    T    
   T   
   T   
   A   
   G   
   A   
   T   
   A   
   T   
   G   
   C   
   G   
   T   
   T   
   T   
    A    
   C   
   G   
    C    
   C   
   G   
    A    
   T   
   T   
    G    
   A   
   C   
    C    
   G   
   G   
    T    
   T   
   T   
    G    
   G   
   A   
   A   
   C   
   G   
    G    
   G   
   A   
    A    
   A   
   A   
   A   
   A   
   T   
    C    
   G   
   A   
    A    
   A   
   A   
   T   
   G   
   A   
   A   
   T   
   A   
    C    
   C   
   A   
   A   
   G   
   A   
    A    
   C   
   T   
    C    
   T   
   T   
    A    
   A   
    A    
   A   
    C    
   T   
    G    
   A   
   T   
   T   
   G   
    A    
    A    
   G   
   A   
    T    
   T   
   T   
    A    
    A    
    C    
    T    
   G   
   A   
   C   
   A   
   T   
   T   
    C    
   T   
    G    
   G   
   C   
    A    
    A    
   G   
    A    
   C   
   C   
    A    
   G   
   A   
    A    
   C   
   G   
    T    
   G   
   T   
    T    
   A   
    C    
    A    
   G   
   A   
   A   
   A   
   T   
    C    
   A   
   T   
    C    
   A   
   A   
    G    
   A   
    A    
    T    
   G   
   A   
    A    
   T   
   T   
   A   
    G    
    C    
    A    
   G   
   A   
   C   
    T    
   T   
    G    
    A    
    A    
    T    
    C    
    A    
   A   
    C    
    G    
   A   
   T   
   T   
   T   
   G   
   G   
   T   
   G   
   A   
   T   
    A    
    A    
   A   
   C   
   G   
   A   
   C   
   G   
    C    
   A   
   C   
   A   
   G   
   A   
   A   
    T    
   T   
   A   
    C    
   T   
   A   
   G   
   T   
    C    
   G   
   G   
   T   
   G   
   A   
    G    
   G   
   T   
   C   
    T    
   T   
    A    
   A   
   G   
   T   
   C   
   T   
    G    
   G   
   A   
   A   
   G   
   A   
   T   
   G   
   A   
   A   
   G   
   A   
   T   
    T    
   T   
    A    
   A   
   T   
    C    
   G   
   A   
   A   
   G   
   A   
   A   
   G   
   A   
   A   
   G   
   A   
   A   
    A    
   T   
   C   
   G   
   T   
    T    
   A   
   T   
   T   
   A   
   C   
    A    
    T    
   T   
    G    
   A   
   C   
    A    
   A   
   A   
    C    
   A   
   A   
    T    
   G   
   G   
    T    
   T   
   A   
    C    
   A   
   T   
    C    
   A   
   A   
    A    
   C   
   G   
    T    
    T    
   T   
   G   
   G   
    C    
   A   
   A   
   A   
    T    
    A    
    A    
    T    
   G   
   A   
   A   
   T   
   T   
   C   
    A    
   G   
    A    
   G   
   C   
    T    
   C   
   A   
   A   
   C   
   G   
    A    
    A    
   G   
    G    
   G   
   G   
    T    
   G   
   G   
    A    
   C   
   G   
   T   
   G   
   G   
    T    
   G   
   T   
    C    
   C   
   A   
   A   
   G   
   G   
    A    
   A   
   T   
   G   
   G   
   G   
    T    
    A    
   T   
    C    
   C   
   A   
    C    
    A    
   A   
   T   
   G   
   A   
    C    
   G   
   A   
    C    
   T   
   T   
   C   
   G   
   T   
    C    
   A   
   A   
    A    
   A   
   A   
    T    
   C   
   T   
    G    
    G    
   T   
   T   
   T   
   C   
   T   
   T   
   G   
    T    
   T   
   C   
    T    
   A   
   C   
    C    
   C   
   A   
    T    
   G   
   A   
    T    
   A   
   C   
    A    
   T   
   T   
    G    
   T   
   T   
   A   
   T   
   T   
    C    
   T   
   T   
   T   
   A   
   C   
   G   
   A   
   A   
    T    
   A   
    A    
    C    
   G   
   G   
    A    
   A   
   A   
   A   
   G   
   T   
    G    
   T   
   A   
    T    
   C   
   G   
    G    
   G   
   C   
   A   
   A   
   A   
   A   
   G   
   G   
    A    
   T   
   A   
    C    
   G   
   A   
   A   
   A   
   T   
    T    
   C   
   C   
    A    
   G   
   A   
    A    
   T   
   A   
   C   
   G   
   G   
    G    
    C    
   G   
    C    
   A   
   C   
   A   
   G   
   C   
    C    
   A   
   A   
   A   
   G   
   G   
    A    
   A   
   T   
    A    
   C   
   C   
   A   
    A    
   T   
    T    
   A   
   T   
    C    
   A   
   A   
    T    
   T   
   T   
    A    
   C   
   T   
    A    
   G   
   G   
    T    
   A   
   T   
    C    
   G   
   A   
    T    
   T   
   C   
    G    
    A    
    G    
    T    
   G   
   A   
   A   
   A   
    G    
    C    
   A   
   T   
    C    
   C   
   A   
    G    
   G   
   C   
    A    
   A   
   T   
    C    
   A   
   T   
   T   
    G    
   C   
    G    
   G   
   T   
    G    
    A    
    C    
   G   
   G   
   G   
    A    
    G    
   A   
   A   
   G   
   C   
    A    
   G   
   A   
   A   
   G   
    A    
    A    
   G   
   G   
    C    
    C    
   A   
    C    
   T   
   A   
    T    
    C    
   T   
    C    
   T   
   T   
   C   
   T   
   T   
    T    
   A   
   C   
    G    
   A   
   C   
    G    
    C    
    G    
    T    
    C    
   A   
   A   
   G   
   G   
    T    
   A   
   C   
    A    
   G   
   T   
    A    
   A   
   A   
   A   
   C   
   G   
    T    
   A   
   C   
    T    
    T    
   C   
    T    
   G   
   T   
    C    
   A   
   C   
    T    
   G   
   C   
    T    
   T   
   T   
    T    
    G    
   C   
   T   
   A   
   A   
    C    
   A   
   T   
    C    
   C   
   G   
    A    
   A   
   G   
    T    
   A   
   A   
   T   
   G   
   G   
    T    
    C    
   T   
    G    
   A   
   T   
    C    
   G   
   C   
    A    
   A   
   T   
   T   
    G    
   G   
    T    
   T   
   T   
    G    
   A   
   A   
   A   
   G   
   A   
   A   
   G   
   A   
    C    
   G   
   A   
    T    
   G   
   A   
    G    
    C    
   T   
    C    
    G    
   T   
    C    
   A   
   A   
    T    
   G   
   T   
    G    
    C    
   T   
   A   
    C    
    T    
   G   
   A   
   C   
    A    
    G    
   A   
    T    
   G   
   G   
    T    
    C    
   A   
    A    
    T    
    C    
    T    
   A   
   A   
    T    
   A   
   T   
    C    
   A   
   T   
    T    
   A   
   T   
    T    
   G   
   G   
    A    
   A   
   C   
   A   
   C   
   A   
   T   
    A    
    A    
    C    
   G   
   G   
    C    
   T   
   A   
   C   
   T   
   C   
   T   
   G   
   T   
    C    
   A   
   C   
    T    
   T   
   T   
    C    
    A    
   A   
    A    
   G   
   A   
    A    
    G    
    A    
    A    
    G    
   C   
    A    
   G   
    T    
    C    
   C   
   G   
    C    
   G   
   A   
   T   
   A   
   T   
   G   
   G   
   G   
    A    
   C   
   G   
    T    
   A   
   C   
   A   
   G   
   C   
    T    
   T   
   C   
    C    
   G   
   G   
    G    
   G   
   T   
    T    
    A    
   G   
    A    
   G   
   G   
    A    
   A   
   T   
    A    
   C   
   G   
    C    
    C    
   T   
    G    
   C   
   G   
    T    
   G   
   A   
    G    
    G    
   A   
    A    
   G   
   A   
    T    
   T   
    A    
    T    
   G   
   T   
    C    
   G   
   T   
    T    
   G   
   G   
    G    
    G    
   C   
   A   
   G   
   C   
    C    
    T    
   T   
    A    
    A    
   T   
   G   
    A    
   A   
    G    
   G   
    A    
   A   
    G    
   A   
    T    
    C    
   A   
   A   
   G   
   A   
   A   
   G   
   T   
    A    
    T    
   T   
    A    
   G   
   T   
    G    
   A   
   T   
    C    
   A   
   C   
    A    
   G   
   A   
   A   
   A   
   A   
    A    
   G   
   G   
   T   
   T   
   A   
    C    
   G   
   G   
    A    
   A   
   A   
    A    
   C   
   G   
    G    
   A   
   C   
    A    
   A   
   A   
   A   
   G   
    T    
    A    
   T   
   C   
    G    
   G   
   A   
    A    
   T   
   A   
   T   
   C   
   C   
   A   
   G   
   T   
    G    
   A   
   A   
    A    
   G   
   G   
    C    
   C   
   G   
    C    
   G   
   G   
    A    
   G   
   G   
   T   
   A   
   A   
   A   
   G   
   G   
    G    
   A   
   T   
    C    
   A   
   A   
    A    
   A   
   C   
    G    
    G    
   C   
    G    
   A   
   A   
    C    
   A   
   T   
   C   
   A   
   C   
   T   
   G   
   A   
    A    
   A   
   A   
   A   
   A   
   A   
   T   
   G   
   G   
   T   
   C   
   C   
   A   
    C    
   T   
   A   
   G   
   C   
    T    
   G   
   G   
    A    
   T   
   T   
    G    
   A   
   C   
   C   
   A   
   C   
    T    
   G   
   T   
    T    
    T    
    C    
   T   
   G   
   G   
    A    
    G    
   A   
   T   
   G   
   A   
   A   
   G   
   A   
    C    
   A   
   T   
   C   
    A    
   T   
    G    
    C    
   T   
    G    
   A   
   T   
    T    
   A   
   C   
    A    
    G    
   A   
    T    
   A   
   A   
   A   
   G   
   G   
    T    
   G   
   T   
    C    
   A   
   T   
   T   
   A   
   T   
   C   
   C   
   G   
    T    
   T   
   T   
    C    
   A   
   A   
   C   
   G   
   T   
    A    
    T    
    C    
    G    
    A    
   C   
   T   
   G   
   T   
   T   
   T   
   C   
   T   
   C   
   A   
   A   
   A   
   C   
    T    
   G   
   G   
   A   
   C   
   G   
    T    
    T    
   C   
   A   
   A   
   C   
   A   
   T   
   T   
    A    
   G   
   G   
    C    
   G   
   T   
    A    
   C   
   G   
   T   
   T   
   T   
    G    
   A   
   T   
   G   
   A   
    A    
    G    
   A   
   T   
   G   
   G   
   A   
   A   
   G   
    C    
    T    
   G   
    A    
    T    
    A    
   C   
    G    
   A   
   A   
   A   
   G   
   T   
   T   
   G   
   T   
   C   
   A   
   C   
   G   
   A   
   T   
   G   
   G   
   C   
   A   
   G   
   C   
   A   
   G   
   T   
   C   
   G   
   A   
   A   
   C   
   C   
   A   
   G   
   A   
   A   
   G   
   C   
   T   
   G   
   C   
   A   
   G   
   A   
   A   
   C   
   A   
   A   
   A   
   C   
   A   
   G   
   T   
   T   
   G   
   A   
   A   
   G   
   A   
   A   
   C   
   A   
   A   
   G   
   G   
   A   
   T   
   C   
   A   
   G   
   A   
   A   
   G   
   A   
   A   
   -   
   -   
   -   
 

 
   N60357F   
   A   
   T   
   G   
   A   
   G   
   T   
   G   
   A   
   A   
   G   
   A   
   A   
   A   
   T   
   C   
   A   
   G   
   A   
   G   
   A   
   A   
   A   
   A   
   C   
   A   
   T   
   C   
   C   
   A   
   T   
   G   
   A   
   T   
   G   
   T   
   C   
   A   
   A   
   T   
   C   
   T   
   A   
   A   
   C   
   C   
   A   
   G   
   C   
   G   
   A   
   A   
   A   
   T   
   G   
   A   
   A   
   A   
   G   
   A   
   C   
   T   
   C   
   C   
   T   
   T   
   C   
   A   
   T   
   C   
   G   
   A   
   T   
   T   
   A   
   T   
   G   
   C   
   G   
   A   
   T   
   G   
   A   
   G   
   T   
   G   
   T   
   T   
   A   
   T   
   T   
   G   
   T   
   C   
   G   
   C   
   A   
   C   
   G   
   T   
   G   
   C   
   A   
   C   
   T   
   T   
   C   
   C   
   T   
   G   
   A   
   T   
   G   
   T   
   C   
   A   
   G   
   A   
   G   
   A   
   T   
   G   
   G   
   T   
   C   
   T   
   G   
   A   
   A   
   A   
   C   
   C   
   C   
   G   
   T   
   T   
   C   
   A   
   C   
   C   
   G   
   C   
   C   
   G   
   T   
   A   
   T   
   T   
   T   
   T   
   A   
   T   
   A   
   C   
   G   
   G   
   C   
   A   
   T   
   G   
   A   
   A   
   C   
   G   
   A   
   A   
   T   
   T   
   A   
   G   
   G   
   T   
   G   
   T   
   A   
   A   
   C   
   A   
   C   
   C   
   G   
   G   
   A   
   T   
   A   
   A   
   A   
   G   
   C   
   C   
   C   
   A   
   T   
   A   
   A   
   A   
   A   
   A   
   A   
   T   
   C   
   T   
   G   
   C   
   C   
   A   
   G   
   A   
   A   
   T   
   C   
   G   
   T   
   C   
   G   
   G   
   A   
   G   
   A   
   T   
   G   
   T   
   C   
   A   
   T   
   G   
   G   
   G   
   T   
   A   
   A   
   A   
   T   
   A   
   C   
   C   
   A   
   T   
   C   
   C   
   T   
   C   
   A   
   T   
   G   
   G   
   A   
   G   
   A   
   C   
   A   
   G   
   T   
   G   
   C   
   G   
   A   
   T   
   C   
   T   
   A   
   T   
   G   
   A   
   G   
   T   
   C   
   A   
   A   
   T   
   G   
   G   
   T   
   A   
   C   
   G   
   G   
   A   
   T   
   G   
   G   
   C   
   G   
   C   
   A   
   G   
   C   
   C   
   A   
   T   
   T   
   C   
   A   
   G   
   C   
   T   
   A   
   C   
   C   
   G   
   T   
   T   
   A   
   T   
   A   
   T   
   G   
   C   
   T   
   A   
   G   
   T   
   G   
   G   
   A   
   T   
   G   
   G   
   T   
   C   
   A   
   T   
   G   
   G   
   A   
   A   
   A   
   C   
   T   
   T   
   T   
   G   
   G   
   T   
   T   
   C   
   T   
   G   
   T   
   T   
   G   
   A   
   C   
   G   
   G   
   T   
   G   
   A   
   C   
   G   
   G   
   T   
   G   
   C   
   C   
   G   
   C   
   C   
   G   
   C   
   T   
   A   
   T   
   G   
   C   
   G   
   T   
   T   
   A   
   T   
   A   
   C   
   C   
   G   
   A   
   A   
   G   
   C   
   A   
   C   
   G   
   G   
   A   
   T   
   G   
   A   
   G   
   C   
   A   
   A   
   A   
   A   
   T   
   C   
   G   
   C   
   T   
   A   
   C   
   A   
   G   
   A   
   A   
   A   
   T   
   G   
   C   
   T   
   G   
   C   
   G   
   T   
   G   
   A   
   T   
   A   
   T   
   C   
   A   
   A   
   T   
   A   
   A   
   G   
   A   
   A   
   C   
   A   
   C   
   T   
   G   
   T   
   T   
   G   
   A   
   T   
   T   
   T   
   C   
   C   
   A   
   A   
   A   
   G   
   C   
   A   
   A   
   C   
   T   
   A   
   T   
   G   
   A   
   T   
   G   
   A   
   T   
   A   
   C   
   A   
   G   
   A   
   A   
   A   
   A   
   A   
   G   
   A   
   A   
   C   
   C   
   A   
   G   
   T   
   T   
   G   
   T   
   A   
   C   
   T   
   A   
   C   
   C   
   T   
   G   
   C   
   T   
   C   
   G   
   C   
   T   
   T   
   C   
   C   
   C   
   G   
   A   
   A   
   T   
   C   
   T   
   T   
   T   
   T   
   A   
   G   
   T   
   G   
   A   
   A   
   C   
   G   
   G   
   A   
   A   
   C   
   A   
   A   
   C   
   G   
   G   
   G   
   G   
   A   
   T   
   C   
   G   
   C   
   C   
   G   
   T   
   A   
   G   
   G   
   T   
   A   
   T   
   G   
   G   
   C   
   C   
   A   
   C   
   G   
   A   
   A   
   T   
   A   
   T   
   C   
   C   
   C   
   A   
   C   
   C   
   T   
   C   
   A   
   T   
   A   
   A   
   C   
   T   
   T   
   G   
   T   
   C   
   T   
   G   
   A   
   G   
   G   
   T   
   A   
   G   
   T   
   A   
   G   
   C   
   G   
   G   
   C   
   T   
   A   
   T   
   C   
   G   
   A   
   T   
   T   
   T   
   G   
   C   
   T   
   G   
   A   
   T   
   G   
   G   
   A   
   T   
   A   
   A   
   T   
   C   
   C   
   T   
   G   
   A   
   T   
   G   
   T   
   G   
   A   
   C   
   G   
   A   
   C   
   C   
   A   
   A   
   T   
   G   
   A   
   A   
   T   
   T   
   G   
   A   
   T   
   G   
   G   
   A   
   A   
   G   
   T   
   A   
   T   
   T   
   A   
   C   
   C   
   T   
   G   
   G   
   A   
   C   
   C   
   A   
   G   
   A   
   T   
   T   
   T   
   T   
   C   
   C   
   A   
   A   
   C   
   A   
   G   
   G   
   T   
   G   
   G   
   T   
   T   
   T   
   G   
   G   
   T   
   T   
   A   
   T   
   G   
   G   
   G   
   A   
   A   
   A   
   A   
   T   
   C   
   A   
   G   
   G   
    A    
   A   
   T   
    C    
   C   
   G   
    A    
    A    
   G   
   A   
   G   
   C   
    G    
   T   
   A   
   T   
   G   
   A   
   A   
   A   
   C   
    A    
   G   
   G   
    T    
   A   
   A   
    G    
   G   
   G   
   T   
   T   
   C   
    T    
   A   
   T   
   T   
   A   
   C   
    C    
   G   
   T   
    A    
   C   
   G   
   T   
   G   
   C   
   A   
   A   
   A   
   A   
   G   
   T   
   T   
   G   
   A   
    G    
    A    
   T   
    T    
   A   
    T    
   T   
   G   
   A   
    G    
   A   
   T   
   G   
    C    
   C   
    A    
   A   
   A   
   T   
   G   
   G   
    T    
   A   
   A   
   A   
   G   
   A   
   A   
   C   
   G   
    G    
   A   
   T   
    C    
    A    
   T   
    C    
   G   
   T   
    G    
   A   
   C   
    A    
   G   
   A   
   A   
   T   
   T   
    G    
   C   
   C   
   T   
   T   
   A   
   T   
   A   
   T   
   G   
   G   
   T   
    C    
   A   
   A   
   T   
   A   
   A   
   A   
   G   
   C   
    A    
   A   
   A   
   A   
   T   
   T   
    G    
   A   
   T   
    C    
   G   
   A   
   A   
   C   
   G   
    G    
   A   
   T   
   T   
   T   
   C   
   T   
   G   
   A   
    A    
    C    
   T   
    G    
   C   
   A   
   C   
   A   
   G   
   A   
   G   
   A   
    C    
   A   
   A   
    G    
   C   
   G   
    T    
   A   
   T   
    C    
   G   
   A   
   A   
   G   
   G   
    G    
   A   
   T   
    C    
   A   
   C   
    A    
   G   
   A   
   T   
   T   
   T   
   G   
   C   
   G   
   G   
   G   
   A   
    C    
   G   
   A   
   A   
   T   
   C   
   T   
   T   
   C   
    C    
   C   
   G   
   T   
   G   
   A   
    A    
   G   
   G   
    T    
   A   
   T   
   G   
   C   
   G   
    G    
   A   
   T   
    C    
   G   
   T   
   C   
   A   
   T   
    T    
   G   
   A   
    C    
    A    
   T   
    C    
   C   
   G   
   T   
   C   
   G   
    T    
   G   
   A   
    C    
   G   
    C    
    C    
   A   
   G   
    C    
   G   
   C   
    A    
   T   
   C   
    C    
   G   
   T   
   T   
    A    
   T   
    C    
   T   
   T   
    G    
   A   
   A   
    T    
   A   
   A   
   C   
   T   
   T   
    A    
   T   
   A   
    T    
   A   
   A   
   A   
    T    
   T   
   G   
   A   
   C   
    A    
    T    
   C   
    C    
   T   
   T   
    G    
   C   
   A   
    A    
   A   
    A    
    T    
   T   
   C   
    A    
   T   
   T   
    T    
   G   
   G   
    A    
   T   
   T   
    C    
   A   
   A   
    T    
   A   
   T   
   G   
   T   
   T   
    G    
   G   
   C   
    G    
   A   
   T   
    C    
   G   
   A   
   A   
   A   
   A   
   A   
   G   
   G   
    T    
   G   
   T   
    G    
   C   
   C   
    G    
   A   
   A   
   A   
    G    
   T   
   T   
   T   
   T   
    G    
   A   
   G   
    C    
   T   
   T   
   G   
   A   
   A   
   A   
   C   
    A    
    A    
   A   
   T   
    T    
    C    
   T   
    T    
   G   
   A   
   A   
   A   
   A   
    C    
   T   
   A   
    T    
    A    
   T   
    C    
   G   
   A   
    A    
   C   
   A   
    T    
   C   
   A   
    G    
    C    
    G    
    C    
    G    
   A   
   A   
   G   
   T   
   C   
   A   
   T   
    C    
   A   
   C   
    A    
    A    
   G   
    A    
   C   
   G   
   G   
   A   
   C   
    A    
    G    
   T   
    C    
   T   
   T   
   T   
   G   
   A   
    A    
   A   
   A   
   A   
    G    
   A   
    A    
   A   
   A   
    A    
   G   
   C   
   A   
   G   
   A   
   A   
   G   
   C   
   A   
    A    
   G   
    G    
   G   
   C   
    A    
   C   
   A   
    C    
   A   
   T   
    T    
   T   
   T   
   A   
   G   
   A   
   A   
   G   
   G   
   T   
    T    
   T   
    G    
   C   
   G   
    T    
   A   
   T   
   T   
   G   
   C   
    T    
   T   
   T   
    G    
   G   
   A   
   T   
   C   
   A   
   T   
   A   
   T   
   C   
    G    
   A   
   T   
   G   
   A   
   A   
   A   
   T   
    A    
   A   
   T   
   T   
   G   
   C   
    G    
   A   
   T   
   T   
   A   
   T   
    C    
   C   
   G   
    C    
   G   
   G   
    G    
   T   
   C   
    T    
    A    
   A   
   A   
   T   
   C   
   A   
   G   
   A   
   T   
   G   
   A   
    C    
   G   
   A   
    A    
   G   
   C   
   A   
   A   
   A   
   A   
   G   
   C   
    G    
   A   
    C    
    G    
    A    
   T   
    G    
   A   
   T   
    C    
   G   
   A   
   A   
   C   
   G   
    G    
   T   
   T   
    C    
   G   
   A   
    T    
   T   
   T   
    A    
   T   
   C   
    C    
   G   
   A   
   T   
   C   
   G   
    C    
   C   
   A   
    G    
    T    
   C   
    T    
   C   
   A   
   A   
   G   
   C   
    T    
   A   
   T   
    T    
   T   
   T   
   A   
   G   
   A   
   T   
   A   
   T   
   G   
   C   
   G   
   T   
   T   
   T   
    A    
   C   
   G   
    C    
   C   
   G   
    A    
   T   
   T   
    G    
   A   
   C   
    C    
   G   
   G   
    T    
   T   
   T   
    G    
   G   
   A   
   A   
   C   
   G   
    G    
   G   
   A   
    A    
   A   
   A   
   A   
   A   
   T   
    C    
   G   
   A   
    A    
   A   
   A   
   T   
   G   
   A   
   A   
   T   
   A   
    C    
   C   
   A   
   A   
   G   
   A   
    A    
   C   
   T   
    C    
   T   
   T   
    A    
   A   
    A    
   A   
    C    
   T   
    G    
   A   
   T   
   T   
   G   
    A    
    A    
   G   
   A   
    T    
   T   
   T   
    A    
    A    
    C    
    T    
   G   
   A   
   C   
   A   
   T   
   T   
    C    
   T   
    G    
   G   
   C   
    A    
    A    
   G   
    A    
   C   
   C   
    A    
   G   
   A   
    A    
   C   
   G   
    T    
   G   
   T   
    T    
   A   
    C    
    A    
   G   
   A   
   A   
   A   
   T   
    C    
   A   
   T   
    C    
   A   
   A   
    G    
   A   
    A    
    T    
   G   
   A   
    A    
   T   
   T   
   A   
    G    
    C    
    A    
   G   
   A   
   C   
    T    
   T   
    G    
    A    
    A    
    T    
    C    
    A    
   A   
    C    
    G    
   A   
   T   
   T   
   T   
   G   
   G   
   T   
   G   
   A   
   T   
    A    
    A    
   A   
   C   
   G   
   A   
   C   
   G   
    C    
   A   
   C   
   A   
   G   
   A   
   A   
    T    
   T   
   A   
    C    
   T   
   A   
   G   
   T   
    C    
   G   
   G   
   T   
   G   
   A   
    G    
   G   
   T   
   C   
    T    
   T   
    A    
   A   
   G   
   T   
   C   
   T   
    G    
   G   
   A   
   A   
   G   
   A   
   T   
   G   
   A   
   A   
   G   
   A   
   T   
    T    
   T   
    A    
   A   
   T   
    C    
   G   
   A   
   A   
   G   
   A   
   A   
   G   
   A   
   A   
   G   
   A   
   A   
    A    
   T   
   C   
   G   
   T   
    T    
   A   
   T   
   T   
   A   
   C   
    A    
    T    
   T   
    G    
   A   
   C   
    A    
   A   
   A   
    C    
   A   
   A   
    T    
   G   
   G   
    T    
   T   
   A   
    C    
   A   
   T   
    C    
   A   
   A   
    A    
   C   
   G   
    T    
    T    
   T   
   G   
   G   
    C    
   A   
   A   
   A   
    T    
    A    
    A    
    T    
   G   
   A   
   A   
   T   
   T   
   C   
    A    
   G   
    A    
   G   
   C   
    T    
   C   
   A   
   A   
   C   
   G   
    A    
    A    
   G   
    G    
   G   
   G   
    T    
   G   
   G   
    A    
   C   
   G   
   T   
   G   
   G   
    T    
   G   
   T   
    C    
   C   
   A   
   A   
   G   
   G   
    A    
   A   
   T   
   G   
   G   
   G   
    T    
    A    
   T   
    C    
   C   
   A   
    C    
    A    
   A   
   T   
   G   
   A   
    C    
   G   
   A   
    C    
   T   
   T   
   C   
   G   
   T   
    C    
   A   
   A   
    A    
   A   
   A   
    T    
   C   
   T   
    G    
    G    
   T   
   T   
   T   
   C   
   T   
   T   
   G   
    T    
   T   
   C   
    T    
   A   
   C   
    C    
   C   
   A   
    T    
   G   
   A   
    T    
   A   
   C   
    A    
   T   
   T   
    G    
   T   
   T   
   A   
   T   
   T   
    C    
   T   
   T   
   T   
   A   
   C   
   G   
   A   
   A   
    T    
   A   
    A    
    C    
   G   
   G   
    A    
   A   
   A   
   A   
   G   
   T   
    G    
   T   
   A   
    T    
   C   
   G   
    G    
   G   
   C   
   A   
   A   
   A   
   A   
   G   
   G   
    A    
   T   
   A   
    C    
   G   
   A   
   A   
   A   
   T   
    T    
   C   
   C   
    A    
   G   
   A   
    A    
   T   
   A   
   C   
   G   
   G   
    G    
    C    
   G   
    T    
   A   
   C   
   A   
   G   
   C   
    C    
   A   
   A   
   A   
   G   
   G   
    A    
   A   
   T   
    A    
   C   
   C   
   A   
    A    
   T   
    T    
   A   
   T   
    C    
   A   
   A   
    T    
   T   
   T   
    A    
   C   
   T   
    A    
   G   
   G   
    T    
   A   
   T   
    C    
   G   
   A   
    T    
   T   
   C   
    G    
    A    
    G    
    T    
   G   
   A   
   A   
   A   
    G    
    C    
   A   
   T   
    C    
   C   
   A   
    G    
   G   
   C   
    A    
   A   
   T   
    C    
   A   
   T   
   T   
    G    
   C   
    G    
   G   
   T   
    G    
    A    
    C    
   G   
   G   
   G   
    A    
    G    
   A   
   A   
   G   
   C   
    A    
   G   
   A   
   A   
   G   
    A    
    A    
   G   
   G   
    C    
    C    
   A   
    C    
   T   
   A   
    T    
    C    
   T   
    C    
   T   
   T   
   C   
   T   
   T   
    T    
   A   
   C   
    G    
   A   
   C   
    G    
    C    
    G    
    T    
    C    
   A   
   A   
   G   
   G   
    T    
   A   
   C   
    A    
   G   
   T   
    A    
   A   
   A   
   A   
   C   
   G   
    T    
   A   
   C   
    T    
    T    
   C   
    T    
   G   
   T   
    C    
   A   
   C   
    T    
   G   
   C   
    T    
   T   
   T   
    T    
    G    
   C   
   T   
   A   
   A   
    C    
   A   
   T   
    C    
   C   
   G   
    A    
   A   
   G   
    T    
   A   
   A   
   T   
   G   
   G   
    T    
    C    
   T   
    G    
   A   
   T   
    C    
   G   
   C   
    A    
   A   
   T   
   T   
    G    
   G   
    T    
   T   
   T   
    G    
   A   
   A   
   A   
   G   
   A   
   A   
   G   
   A   
    C    
   G   
   A   
    T    
   G   
   A   
    G    
    C    
   T   
    C    
    G    
   T   
    C    
   A   
   A   
    T    
   G   
   T   
    G    
    C    
   T   
   A   
    C    
    T    
   G   
   A   
   C   
    A    
    G    
   A   
    T    
   G   
   G   
    T    
    C    
   A   
    A    
    T    
    C    
    T    
   A   
   A   
    T    
   A   
   T   
    C    
   A   
   T   
    T    
   A   
   T   
    T    
   G   
   G   
    A    
   A   
   C   
   A   
   C   
   A   
   T   
    A    
    A    
    C    
   G   
   G   
    C    
   T   
   A   
   C   
   T   
   C   
   T   
   G   
   T   
    C    
   A   
   C   
    T    
   T   
   T   
    C    
    A    
   A   
    A    
   G   
   A   
    A    
    G    
    A    
    A    
    G    
   C   
    A    
   G   
    T    
    C    
   C   
   G   
    C    
   G   
   A   
   T   
   A   
   T   
   G   
   G   
   G   
    A    
   C   
   G   
    T    
   A   
   C   
   A   
   G   
   C   
    T    
   T   
   C   
    C    
   G   
   G   
    G    
   G   
   T   
    C    
    A    
   G   
    A    
   G   
   G   
    G    
   A   
   T   
    A    
   C   
   G   
    C    
    C    
   T   
    G    
   C   
   G   
    T    
   G   
   A   
    G    
    G    
   A   
    A    
   G   
   A   
    T    
   T   
    A    
    T    
   G   
   T   
    C    
   G   
   T   
    T    
   G   
   G   
    G    
    G    
   C   
   A   
   G   
   C   
    C    
    T    
   T   
    A    
    A    
   T   
   G   
    A    
   A   
    G    
   G   
    A    
   A   
    A    
   A   
    T    
    C    
   A   
   A   
   G   
   A   
   A   
   G   
   T   
    A    
    T    
   T   
    A    
   G   
   T   
    G    
   A   
   T   
    C    
   A   
   C   
    A    
   G   
   A   
   A   
   A   
   A   
    A    
   G   
   G   
   T   
   T   
   A   
    C    
   G   
   G   
    A    
   A   
   A   
    A    
   C   
   G   
    G    
   A   
   C   
    A    
   A   
   A   
   A   
   G   
    T    
    A    
   T   
   C   
    G    
   G   
   A   
    A    
   T   
   A   
   T   
   C   
   C   
   A   
   G   
   T   
    G    
   A   
   A   
    A    
   G   
   G   
    C    
   C   
   G   
    C    
   G   
   G   
    A    
   G   
   G   
   T   
   A   
   A   
   A   
   G   
   G   
    G    
   A   
   T   
    C    
   A   
   A   
    A    
   A   
   C   
    G    
    G    
   C   
    G    
   A   
   A   
    C    
   A   
   T   
   C   
   A   
   C   
   T   
   G   
   A   
    A    
   A   
   A   
   A   
   A   
   A   
   T   
   G   
   G   
   T   
   C   
   C   
   A   
    C    
   T   
   A   
   G   
   C   
    T    
   G   
   G   
    A    
   T   
   T   
    G    
   A   
   C   
   C   
   A   
   C   
    T    
   G   
   T   
    T    
    T    
    C    
   T   
   G   
   G   
    A    
    G    
   A   
   T   
   G   
   A   
   A   
   G   
   A   
    C    
   A   
   T   
   C   
    A    
   T   
    G    
    C    
   T   
    G    
   A   
   T   
    T    
   A   
   C   
    A    
    G    
   A   
    T    
   A   
   A   
   A   
   G   
   G   
    T    
   G   
   T   
    C    
   A   
   T   
   T   
   A   
   T   
   C   
   C   
   G   
    T    
   T   
   T   
    C    
   A   
   A   
   C   
   G   
   T   
    A    
    T    
    C    
    G    
    A    
   C   
   T   
   G   
   T   
   T   
   T   
   C   
   T   
   C   
   A   
   A   
   A   
   C   
    T    
   G   
   G   
   A   
   C   
   G   
    T    
    T    
   C   
   A   
   A   
   C   
   A   
   T   
   T   
    A    
   G   
   G   
    C    
   G   
   T   
    A    
   C   
   G   
   T   
   T   
   T   
    G    
   A   
   T   
   G   
   A   
    A    
    G    
   A   
   T   
   G   
   G   
   A   
   A   
   G   
    C    
    T    
   G   
    A    
    T    
    A    
   C   
    G    
   A   
   A   
   A   
   G   
   T   
   T   
   G   
   T   
   C   
   A   
   C   
   G   
   A   
   T   
   G   
   G   
   C   
   A   
   G   
   C   
   A   
   G   
   T   
   C   
   G   
   A   
   A   
   C   
   C   
   A   
   G   
   A   
   A   
   G   
   C   
   T   
   G   
   C   
   A   
   G   
   A   
   A   
   C   
   A   
   A   
   A   
   C   
   A   
   G   
   T   
   T   
   G   
   A   
   A   
   G   
   A   
   A   
   C   
   A   
   A   
   G   
   G   
   A   
   T   
   C   
   A   
   G   
   A   
   A   
   G   
   A   
   A   
   -   
   -   
   -   
 

 
   N52629   
   A   
   T   
   G   
   A   
   G   
   T   
   G   
   A   
   A   
   G   
   A   
   A   
   A   
   T   
   C   
   A   
   G   
   A   
   G   
   A   
   A   
   A   
   A   
   C   
   A   
   T   
   C   
   C   
   A   
   T   
   G   
   A   
   T   
   G   
   T   
   C   
   A   
   A   
   T   
   C   
   T   
   A   
   A   
   C   
   C   
   A   
   G   
   C   
   G   
   A   
   A   
   A   
   T   
   G   
   A   
   A   
   A   
   G   
   A   
   C   
   T   
   C   
   C   
   T   
   T   
   C   
   A   
   T   
   C   
   G   
   A   
   T   
   T   
   A   
   T   
   G   
   C   
   G   
   A   
   T   
   G   
   A   
   G   
   T   
   G   
   T   
   T   
   A   
   T   
   T   
   G   
   T   
   C   
   G   
   C   
   A   
   C   
   G   
   T   
   G   
   C   
   A   
   C   
   T   
   T   
   C   
   C   
   T   
   G   
   A   
   T   
   G   
   T   
   C   
   A   
   G   
   A   
   G   
   A   
   T   
   G   
   G   
   T   
   C   
   T   
   G   
   A   
   A   
   A   
   C   
   C   
   C   
   G   
   T   
   T   
   C   
   A   
   T   
   C   
   G   
   C   
   C   
   G   
   T   
   A   
   T   
   T   
   T   
   T   
   A   
   T   
   A   
   C   
   G   
   G   
   C   
   A   
   T   
   G   
   A   
   A   
   C   
   G   
   A   
   A   
   T   
   T   
   A   
   G   
   G   
   T   
   G   
   T   
   A   
   A   
   C   
   A   
   C   
   C   
   G   
   G   
   A   
   T   
   A   
   A   
   A   
   G   
   C   
   C   
   C   
   A   
   T   
   A   
   A   
   A   
   A   
   A   
   A   
   T   
   C   
   T   
   G   
   C   
   C   
   A   
   G   
   A   
   A   
   T   
   C   
   G   
   T   
   C   
   G   
   G   
   A   
   G   
   A   
   T   
   G   
   T   
   C   
   A   
   T   
   G   
   G   
   G   
   T   
   A   
   A   
   A   
   T   
   A   
   C   
   C   
   A   
   T   
   C   
   C   
   T   
   C   
   A   
   T   
   G   
   G   
   A   
   G   
   A   
   C   
   A   
   G   
   T   
   G   
   C   
   G   
   A   
   T   
   C   
   T   
   A   
   T   
   G   
   A   
   G   
   T   
   C   
   A   
   A   
   T   
   G   
   G   
   T   
   A   
   C   
   G   
   G   
   A   
   T   
   G   
   G   
   C   
   G   
   C   
   A   
   G   
   C   
   C   
   A   
   T   
   T   
   C   
   A   
   G   
   C   
   T   
   A   
   C   
   C   
   G   
   T   
   T   
   A   
   T   
   A   
   T   
   G   
   C   
   T   
   A   
   G   
   T   
   G   
   G   
   A   
   T   
   G   
   G   
   T   
   C   
   A   
   T   
   G   
   G   
   A   
   A   
   A   
   C   
   T   
   T   
   T   
   G   
   G   
   T   
   T   
   C   
   T   
   G   
   T   
   T   
   G   
   A   
   C   
   G   
   G   
   T   
   G   
   A   
   C   
   G   
   G   
   T   
   G   
   C   
   C   
   G   
   C   
   T   
   G   
   C   
   T   
   A   
   T   
   G   
   C   
   G   
   T   
   T   
   A   
   T   
   A   
   C   
   C   
   G   
   A   
   A   
   G   
   C   
   A   
   C   
   G   
   G   
   A   
   T   
   G   
   A   
   G   
   C   
   A   
   A   
   A   
   A   
   T   
   C   
   G   
   C   
   T   
   A   
   C   
   A   
   G   
   A   
   A   
   A   
   T   
   G   
   C   
   T   
   G   
   C   
   G   
   T   
   G   
   A   
   T   
   A   
   T   
   C   
   A   
   A   
   T   
   A   
   A   
   G   
   A   
   A   
   C   
   A   
   C   
   T   
   G   
   T   
   T   
   G   
   A   
   T   
   T   
   T   
   C   
   C   
   A   
   A   
   A   
   G   
   C   
   A   
   A   
   C   
   T   
   A   
   T   
   G   
   A   
   T   
   G   
   A   
   C   
   A   
   C   
   A   
   G   
   A   
   A   
   A   
   A   
   A   
   G   
   A   
   A   
   C   
   C   
   C   
   G   
   T   
   T   
   G   
   T   
   A   
   T   
   T   
   A   
   C   
   C   
   T   
   G   
   C   
   T   
   C   
   G   
   C   
   T   
   T   
   C   
   C   
   C   
   T   
   A   
   A   
   T   
   C   
   T   
   T   
   T   
   T   
   G   
   G   
   T   
   G   
   A   
   A   
   C   
   G   
   G   
   A   
   A   
   C   
   G   
   A   
   C   
   G   
   G   
   G   
   G   
   A   
   T   
   C   
   G   
   C   
   C   
   G   
   T   
   G   
   G   
   G   
   T   
   A   
   T   
   G   
   G   
   C   
   A   
   A   
   C   
   G   
   A   
   A   
   T   
   A   
   T   
   C   
   C   
   C   
   A   
   C   
   C   
   T   
   C   
   A   
   T   
   A   
   A   
   T   
   T   
   T   
   G   
   T   
   C   
   T   
   G   
   A   
   G   
   G   
   T   
   A   
   G   
   T   
   A   
   G   
   C   
   G   
   G   
   C   
   T   
   A   
   T   
   C   
   G   
   A   
   T   
   T   
   T   
   G   
   C   
   T   
   G   
   A   
   T   
   G   
   G   
   A   
   T   
   A   
   A   
   T   
   C   
   C   
   T   
   G   
   A   
   T   
   G   
   T   
   G   
   A   
   C   
   G   
   A   
   C   
   C   
   A   
   A   
   T   
   G   
   A   
   A   
   T   
   T   
   G   
   A   
   T   
   G   
   G   
   A   
   A   
   G   
   T   
   A   
   T   
   T   
   A   
   C   
   C   
   T   
   G   
   G   
   A   
   C   
   C   
   A   
   G   
   A   
   T   
   T   
   T   
   T   
   C   
   C   
   A   
   A   
   C   
   A   
   G   
   G   
   T   
   G   
   G   
   T   
   T   
   T   
   G   
   G   
   T   
   C   
   A   
   T   
   G   
   G   
   G   
   A   
   A   
   A   
   A   
   T   
   C   
   A   
   G   
   G   
    A    
   A   
   T   
    C    
   C   
   G   
    A    
    A    
   G   
   A   
   G   
   C   
    G    
   T   
   A   
   T   
   G   
   A   
   A   
   A   
   C   
    A    
   G   
   G   
    T    
   A   
   A   
    A    
   G   
   G   
   T   
   T   
   C   
    T    
   A   
   T   
   T   
   A   
   C   
    C    
   G   
   T   
    A    
   C   
   G   
   T   
   G   
   C   
   A   
   A   
   A   
   A   
   G   
   T   
   T   
   G   
   A   
    G    
    A    
   T   
    C    
   A   
    C    
   T   
   G   
   A   
    G    
   A   
   T   
   G   
    C    
   C   
    A    
   A   
   A   
   T   
   G   
   G   
    C    
   A   
   A   
   A   
   G   
   A   
   A   
   C   
   G   
    G    
   A   
   T   
    C    
    C    
   T   
    C    
   G   
   T   
    G    
   A   
   C   
    G    
   G   
   A   
   A   
   T   
   T   
    A    
   C   
   C   
   T   
   T   
   A   
   T   
   A   
   T   
   G   
   G   
   T   
    C    
   A   
   A   
   T   
   A   
   A   
   A   
   G   
   C   
    A    
   A   
   A   
   A   
   T   
   T   
    G    
   A   
   T   
    T    
   G   
   A   
   A   
   C   
   G   
    G    
   A   
   T   
   T   
   T   
   C   
   T   
   G   
   A   
    G    
    T    
   T   
    G    
   C   
   A   
   C   
   A   
   G   
   A   
   G   
   A   
    C    
   A   
   A   
    G    
   C   
   G   
    T    
   A   
   T   
    C    
   G   
   A   
   A   
   G   
   G   
    A    
   A   
   T   
    T    
   A   
   C   
    G    
   G   
   A   
   T   
   T   
   T   
   G   
   C   
   G   
   G   
   G   
   A   
    T    
   G   
   A   
   A   
   T   
   C   
   T   
   T   
   C   
    C    
   C   
   G   
   T   
   G   
   A   
    A    
   G   
   G   
    C    
   A   
   T   
   G   
   C   
   G   
    A    
   A   
   T   
    T    
   G   
   T   
   C   
   A   
   T   
    T    
   G   
   A   
    C    
    G    
   T   
    T    
   C   
   G   
   T   
   C   
   G   
    T    
   G   
   A   
    C    
   G   
    C    
    C    
   A   
   G   
    C    
   G   
   C   
    A    
   T   
   C   
    A    
   G   
   T   
   T   
    A    
   T   
    T    
   T   
   T   
    G    
   A   
   A   
    T    
   A   
   A   
   C   
   T   
   T   
    A    
   T   
   A   
    C    
   A   
   A   
   A   
    T    
   T   
   G   
   A   
   C   
    A    
    T    
   C   
    C    
   T   
   T   
    G    
   C   
   A   
    G    
   A   
    A    
    T    
   T   
   C   
    A    
   T   
   T   
    T    
   G   
   G   
    A    
   T   
   T   
    C    
   A   
   A   
    T    
   A   
   T   
   G   
   T   
   T   
    G    
   G   
   C   
    A    
   A   
   T   
    C    
   G   
   A   
   A   
   A   
   A   
   A   
   G   
   G   
    C    
   G   
   T   
    G    
   C   
   C   
    G    
   A   
   A   
   A   
    G    
   T   
   T   
   T   
   T   
    A    
   A   
   G   
    T    
   T   
   T   
   G   
   A   
   A   
   A   
   C   
    A    
    A    
   A   
   T   
    C    
    C    
   T   
    T    
   G   
   A   
   A   
   A   
   A   
    T    
   T   
   A   
    T    
    A    
   T   
    C    
   G   
   A   
    A    
   C   
   A   
    T    
   C   
   A   
    A    
    C    
    G    
    C    
    G    
   A   
   A   
   G   
   T   
   C   
   A   
   T   
    C    
   A   
   C   
    A    
    C    
   G   
    A    
   C   
   G   
   G   
   A   
   C   
    G    
    G    
   T   
    C    
   T   
   T   
   T   
   G   
   A   
    A    
   A   
   A   
   A   
    G    
   A   
    A    
   A   
   A   
    A    
   G   
   C   
   A   
   G   
   A   
   A   
   G   
   C   
   A   
    A    
   G   
    A    
   G   
   C   
    A    
   C   
   A   
    C    
   A   
   T   
    C    
   T   
   T   
   A   
   G   
   A   
   A   
   G   
   G   
   T   
    T    
   T   
    G    
   C   
   G   
    T    
   A   
   T   
   T   
   G   
   C   
    T    
   T   
   T   
    G    
   G   
   A   
   T   
   C   
   A   
   T   
   A   
   T   
   C   
    G    
   A   
   T   
   G   
   A   
   A   
   A   
   T   
    C    
   A   
   T   
   T   
   G   
   C   
    G    
   A   
   T   
   T   
   A   
   T   
    C    
   C   
   G   
    C    
   G   
   G   
    G    
   T   
   C   
    T    
    A    
   A   
   A   
   T   
   C   
   A   
   G   
   A   
   T   
   G   
   A   
    C    
   G   
   A   
    A    
   G   
   C   
   A   
   A   
   A   
   A   
   G   
   C   
    A    
   A   
    C    
    A    
    A    
   T   
    G    
   A   
   T   
    C    
   G   
   A   
   A   
   C   
   G   
    G    
   T   
   T   
    C    
   G   
   A   
    T    
   T   
   T   
    A    
   T   
   C   
    C    
   G   
   A   
   T   
   C   
   G   
    C    
   C   
   A   
    A    
    T    
   C   
    A    
   C   
   A   
   A   
   G   
   C   
    T    
   A   
   T   
    C    
   T   
   T   
   A   
   G   
   A   
   T   
   A   
   T   
   G   
   C   
   G   
   T   
   T   
   T   
    G    
   C   
   G   
    T    
   C   
   G   
    A    
   T   
   T   
    G    
   A   
   C   
    C    
   G   
   G   
    T    
   T   
   T   
    G    
   G   
   A   
   A   
   C   
   G   
    G    
   G   
   A   
    A    
   A   
   A   
   A   
   A   
   T   
    C    
   G   
   A   
    A    
   A   
   A   
   T   
   G   
   A   
   A   
   T   
   A   
    T    
   C   
   A   
   A   
   G   
   A   
    A    
   C   
   T   
    A    
   T   
   T   
    G    
   A   
    A    
   A   
    C    
   T   
    G    
   A   
   T   
   T   
   G   
    A    
    A    
   G   
   A   
    T    
   T   
   T   
    G    
    A    
    C    
    T    
   G   
   A   
   C   
   A   
   T   
   T   
    C    
   T   
    G    
   G   
   C   
    A    
    A    
   G   
    A    
   C   
   C   
    C    
   G   
   A   
    A    
   C   
   G   
    T    
   G   
   T   
    C    
   A   
    C    
    A    
   G   
   A   
   A   
   A   
   T   
    C    
   A   
   T   
    C    
   A   
   A   
    G    
   A   
    A    
    T    
   G   
   A   
    A    
   T   
   T   
   A   
    G    
    C    
    A    
   G   
   A   
   C   
    T    
   T   
    G    
    A    
    A    
    T    
    C    
    A    
   A   
    C    
    G    
   A   
   T   
   T   
   T   
   G   
   G   
   T   
   G   
   A   
   T   
    A    
    A    
   A   
   C   
   G   
   A   
   C   
   G   
    C    
   A   
   C   
   A   
   G   
   A   
   A   
    T    
   T   
   A   
    C    
   T   
   A   
   G   
   T   
    C    
   G   
   G   
   T   
   G   
   A   
    G    
   G   
   T   
   C   
    T    
   T   
    A    
   A   
   G   
   T   
   C   
   T   
    A    
   G   
   A   
   A   
   G   
   A   
   T   
   G   
   A   
   A   
   G   
   A   
   T   
    T    
   T   
    G    
   A   
   T   
    T    
   G   
   A   
   A   
   G   
   A   
   A   
   G   
   A   
   A   
   G   
   A   
   A   
    A    
   T   
   C   
   G   
   T   
    T    
   A   
   T   
   T   
   A   
   C   
    A    
    T    
   T   
    G    
   A   
   C   
    A    
   A   
   A   
    C    
   A   
   A   
    T    
   G   
   G   
    T    
   T   
   A   
    T    
   A   
   T   
    C    
   A   
   A   
    A    
   C   
   G   
    T    
    T    
   T   
   G   
   G   
    C    
   A   
   A   
   A   
    T    
    A    
    A    
    T    
   G   
   A   
   A   
   T   
   T   
   C   
    A    
   G   
    A    
   G   
   C   
    T    
   C   
   A   
   A   
   C   
   G   
    A    
    A    
   G   
    A    
   G   
   G   
    C    
   G   
   G   
    G    
   C   
   G   
   T   
   G   
   G   
    T    
   G   
   T   
    C    
   C   
   A   
   A   
   G   
   G   
    A    
   A   
   T   
   G   
   G   
   G   
    T    
    A    
   T   
    C    
   C   
   A   
    C    
    A    
   A   
   T   
   G   
   A   
    C    
   G   
   A   
    C    
   T   
   T   
   C   
   G   
   T   
    C    
   A   
   A   
    G    
   A   
   A   
    T    
   C   
   T   
    A    
    G    
   T   
   T   
   T   
   C   
   T   
   T   
   G   
    T    
   T   
   C   
    T    
   A   
   C   
    C    
   C   
   A   
    T    
   G   
   A   
    T    
   A   
   C   
    A    
   T   
   T   
    G    
   T   
   T   
   A   
   T   
   T   
    C    
   T   
   T   
   T   
   A   
   C   
   G   
   A   
   A   
    T    
   A   
    A    
    C    
   G   
   G   
    A    
   A   
   A   
   A   
   G   
   T   
    G    
   T   
   A   
    T    
   C   
   G   
    G    
   G   
   C   
   A   
   A   
   A   
   A   
   G   
   G   
    A    
   T   
   A   
    C    
   G   
   A   
   A   
   A   
   T   
    T    
   C   
   C   
    A    
   G   
   A   
    A    
   T   
   A   
   C   
   G   
   G   
    G    
    C    
   G   
    T    
   A   
   C   
   A   
   G   
   C   
    C    
   A   
   A   
   A   
   G   
   G   
    A    
   A   
   T   
    C    
   C   
   C   
   A   
    A    
   T   
    C    
   A   
   T   
    C    
   A   
   A   
    T    
   T   
   T   
    G    
   C   
   T   
    G    
   G   
   G   
    T    
   A   
   T   
    C    
   G   
   A   
    C    
   T   
   C   
    G    
    A    
    G    
    T    
   G   
   A   
   A   
   A   
    G    
    C    
   A   
   T   
    C    
   C   
   A   
    A    
   G   
   C   
    A    
   A   
   T   
    T    
   A   
   T   
   T   
    G    
   C   
    G    
   G   
   T   
    G    
    A    
    C    
   G   
   G   
   G   
    A    
    G    
   A   
   A   
   G   
   C   
    A    
   G   
   A   
   A   
   G   
    A    
    A    
   G   
   G   
    C    
    C    
   A   
    C    
   T   
   A   
    T    
    C    
   T   
    C    
   T   
   T   
   C   
   T   
   T   
    T    
   A   
   C   
    G    
   A   
   C   
    G    
    C    
    G    
    T    
    C    
   A   
   A   
   G   
   G   
    T    
   A   
   C   
    A    
   G   
   T   
    T    
   A   
   A   
   A   
   C   
   G   
    T    
   A   
   C   
    G    
    T    
   C   
    T    
   G   
   T   
    C    
   A   
   C   
    T    
   G   
   C   
    T    
   T   
   T   
    C    
    G    
   C   
   T   
   A   
   A   
    C    
   A   
   T   
    C    
   C   
   G   
    A    
   A   
   G   
    C    
   A   
   A   
   T   
   G   
   G   
    C    
    C    
   T   
    G    
   A   
   T   
    T    
   G   
   C   
    A    
   A   
   T   
   T   
    G    
   G   
    T    
   T   
   T   
    G    
   A   
   A   
   A   
   G   
   A   
   A   
   G   
   A   
    T    
   G   
   A   
    C    
   G   
   A   
    A    
    T    
   T   
    A    
    G    
   T   
    C    
   A   
   A   
    T    
   G   
   T   
    T    
    C    
   T   
   A   
    T    
    T    
   G   
   A   
   C   
    A    
    G    
   A   
    C    
   G   
   G   
    A    
    C    
   A   
    G    
    T    
    C    
    A    
   A   
   A   
    T    
   A   
   T   
    C    
   A   
   T   
    C    
   A   
   T   
    T    
   G   
   G   
    C    
   A   
   C   
   A   
   C   
   A   
   T   
    A    
    A    
    C    
   G   
   G   
    T    
   T   
   A   
   C   
   T   
   C   
   T   
   G   
   T   
    G    
   A   
   C   
    C    
   T   
   T   
    C    
    A    
   A   
    A    
   G   
   A   
    A    
    G    
    A    
    A    
    G    
   C   
    A    
   G   
    T    
    C    
   C   
   G   
    C    
   G   
   A   
   T   
   A   
   T   
   G   
   G   
   G   
    A    
   C   
   G   
    T    
   A   
   C   
   A   
   G   
   C   
    T    
   T   
   C   
    C    
   G   
   G   
    C    
   G   
   T   
    C    
    A    
   G   
    A    
   G   
   G   
    G    
   A   
   T   
    A    
   C   
   G   
    T    
    C    
   T   
    G    
   C   
   G   
    T    
   G   
   A   
    G    
    G    
   A   
    A    
   G   
   A   
    T    
   T   
    A    
    C    
   G   
   T   
    C    
   G   
   T   
    T    
   G   
   G   
    G    
    G    
   C   
   A   
   G   
   C   
    C    
    C    
   T   
    G    
    A    
   T   
   G   
    A    
   A   
    G    
   G   
    A    
   A   
    G    
   A   
    C    
    C    
   A   
   A   
   G   
   A   
   A   
   G   
   T   
    A    
    C    
   T   
    A    
   G   
   T   
    G    
   A   
   T   
    T    
   A   
   C   
    A    
   G   
   A   
   A   
   A   
   A   
    A    
   G   
   G   
   T   
   T   
   A   
    C    
   G   
   G   
    A    
   A   
   A   
    A    
   C   
   G   
    G    
   A   
   C   
    C    
   A   
   A   
   A   
   G   
    T    
    A    
   T   
   C   
    G    
   G   
   A   
    A    
   T   
   A   
   T   
   C   
   C   
   A   
   G   
   T   
    G    
   A   
   A   
    A    
   G   
   G   
    C    
   C   
   G   
    C    
   G   
   G   
    A    
   G   
   G   
   T   
   A   
   A   
   A   
   G   
   G   
    A    
   A   
   T   
    C    
   A   
   A   
    A    
   A   
   C   
    G    
    G    
   C   
    G    
   A   
   A   
    T    
   A   
   T   
   C   
   A   
   C   
   T   
   G   
   A   
    A    
   A   
   A   
   A   
   A   
   A   
   T   
   G   
   G   
   T   
   C   
   C   
   A   
    C    
   T   
   A   
   G   
   C   
    C    
   G   
   G   
    A    
   T   
   T   
    G    
   A   
   C   
   C   
   A   
   C   
    T    
   G   
   T   
    T    
    T    
    C    
   T   
   G   
   G   
    A    
    A    
   A   
   T   
   G   
   A   
   A   
   G   
   A   
    C    
   A   
   T   
   C   
    A    
   T   
    G    
    C    
   T   
    G    
   A   
   T   
    T    
   A   
   C   
    A    
    G    
   A   
    T    
   A   
   A   
   A   
   G   
   G   
    T    
   G   
   T   
    G    
   A   
   T   
   T   
   A   
   T   
   C   
   C   
   G   
    T    
   T   
   T   
    C    
   A   
   A   
   C   
   G   
   T   
    A    
    T    
    C    
    G    
    A    
   C   
   T   
   G   
   T   
   T   
   T   
   C   
   T   
   C   
   A   
   A   
   A   
   C   
    T    
   G   
   G   
   A   
   C   
   G   
    T    
    T    
   C   
   A   
   A   
   C   
   A   
   T   
   T   
    G    
   G   
   G   
    T    
   G   
   T   
    A    
   C   
   G   
   T   
   T   
   T   
    G    
   A   
   T   
   G   
   A   
    A    
    G    
   A   
   T   
   G   
   G   
   A   
   A   
   G   
    C    
    T    
   G   
    A    
    T    
    A    
   C   
    G    
   A   
   A   
   A   
   G   
   T   
   C   
   G   
   T   
   C   
   A   
   C   
   A   
   A   
   T   
   G   
   -   
   -   
   -   
   -   
   -   
   -   
   -   
   -   
   -   
   -   
   -   
   -   
   -   
   -   
   -   
   -   
   -   
   -   
   -   
   -   
   -   
   -   
   -   
   -   
   -   
   -   
   -   
   -   
   -   
   -   
   -   
   -   
   -   
   -   
   -   
   -   
   -   
   -   
   -   
   -   
   -   
   -   
   -   
   -   
   -   
   -   
   -   
   -   
   -   
   -   
   -   
   -   
   -   
   -   
   -   
   -   
   -   
   -   
   -   
   -   
 

 
   N52587   
   A   
   T   
   G   
   A   
   G   
   T   
   G   
   A   
   A   
   G   
   A   
   A   
   A   
   T   
   T   
   A   
   A   
   A   
   G   
   A   
   A   
   A   
   A   
   C   
   A   
   T   
   T   
   C   
   A   
   A   
   G   
   A   
   C   
   G   
   T   
   C   
   A   
   A   
   T   
   C   
   T   
   G   
   A   
   C   
   C   
   A   
   G   
   C   
   G   
   A   
   A   
   A   
   T   
   G   
   A   
   A   
   A   
   G   
   A   
   A   
   T   
   C   
   T   
   T   
   T   
   C   
   A   
   T   
   T   
   G   
   A   
   C   
   T   
   A   
   C   
   G   
   C   
   A   
   A   
   T   
   G   
   A   
   G   
   T   
   G   
   T   
   T   
   A   
   T   
   C   
   G   
   T   
   A   
   G   
   C   
   C   
   C   
   G   
   C   
   G   
   C   
   G   
   T   
   T   
   A   
   C   
   C   
   T   
   G   
   A   
   C   
   G   
   T   
   T   
   C   
   G   
   T   
   G   
   A   
   C   
   G   
   G   
   T   
   T   
   T   
   A   
   A   
   A   
   A   
   C   
   C   
   T   
   G   
   T   
   T   
   C   
   A   
   T   
   C   
   G   
   C   
   C   
   G   
   A   
   A   
   T   
   C   
   T   
   T   
   A   
   T   
   A   
   T   
   G   
   G   
   A   
   A   
   T   
   G   
   A   
   A   
   C   
   G   
   A   
   A   
   T   
   T   
   A   
   G   
   G   
   G   
   G   
   T   
   A   
   A   
   C   
   C   
   C   
   C   
   T   
   G   
   A   
   T   
   A   
   A   
   A   
   C   
   C   
   A   
   C   
   A   
   C   
   A   
   A   
   A   
   A   
   A   
   A   
   T   
   C   
   A   
   G   
   C   
   C   
   C   
   G   
   G   
   A   
   T   
   T   
   G   
   T   
   T   
   G   
   G   
   G   
   G   
   A   
   T   
   G   
   T   
   T   
   A   
   T   
   G   
   G   
   G   
   T   
   A   
   A   
   A   
   T   
   A   
   T   
   C   
   A   
   C   
   C   
   C   
   C   
   C   
   A   
   T   
   G   
   G   
   G   
   G   
   A   
   C   
   A   
   G   
   T   
   G   
   C   
   A   
   A   
   T   
   T   
   T   
   A   
   C   
   G   
   A   
   A   
   T   
   C   
   A   
   A   
   T   
   G   
   G   
   T   
   G   
   C   
   G   
   G   
   A   
   T   
   G   
   G   
   C   
   A   
   C   
   A   
   A   
   C   
   C   
   T   
   T   
   T   
   T   
   A   
   G   
   T   
   T   
   A   
   T   
   C   
   G   
   G   
   G   
   C   
   G   
   A   
   T   
   G   
   T   
   T   
   A   
   G   
   T   
   T   
   G   
   A   
   C   
   G   
   G   
   C   
   C   
   A   
   C   
   G   
   G   
   A   
   A   
   A   
   C   
   T   
   T   
   C   
   G   
   G   
   T   
   T   
   C   
   T   
   G   
   T   
   C   
   G   
   A   
   T   
   G   
   G   
   T   
   G   
   A   
   C   
   G   
   G   
   C   
   G   
   C   
   T   
   G   
   C   
   C   
   G   
   C   
   T   
   A   
   T   
   G   
   C   
   G   
   T   
   T   
   A   
   T   
   A   
   C   
   C   
   G   
   A   
   A   
   G   
   C   
   A   
   C   
   G   
   T   
   A   
   T   
   G   
   A   
   G   
   T   
   A   
   A   
   A   
   A   
   T   
   T   
   G   
   C   
   T   
   T   
   T   
   A   
   G   
   A   
   A   
   A   
   T   
   G   
   C   
   T   
   A   
   C   
   G   
   A   
   G   
   A   
   T   
   A   
   T   
   T   
   A   
   A   
   C   
   A   
   A   
   A   
   A   
   A   
   T   
   A   
   C   
   A   
   G   
   T   
   C   
   G   
   A   
   T   
   T   
   T   
   C   
   C   
   A   
   A   
   G   
   G   
   A   
   A   
   A   
   C   
   T   
   A   
   T   
   G   
   A   
   T   
   G   
   A   
   T   
   T   
   C   
   A   
   G   
   A   
   A   
   C   
   A   
   A   
   G   
   A   
   A   
   C   
   C   
   A   
   G   
   T   
   A   
   G   
   T   
   T   
   T   
   T   
   A   
   C   
   C   
   T   
   G   
   C   
   T   
   C   
   G   
   T   
   T   
   T   
   T   
   C   
   C   
   A   
   A   
   A   
   C   
   T   
   T   
   A   
   C   
   T   
   A   
   G   
   T   
   T   
   A   
   A   
   C   
   G   
   G   
   A   
   A   
   C   
   A   
   A   
   C   
   G   
   G   
   G   
   G   
   A   
   T   
   T   
   G   
   C   
   G   
   G   
   T   
   G   
   G   
   G   
   G   
   A   
   T   
   G   
   G   
   C   
   A   
   A   
   C   
   A   
   A   
   A   
   T   
   A   
   T   
   C   
   C   
   C   
   G   
   C   
   C   
   A   
   C   
   A   
   C   
   A   
   A   
   T   
   T   
   T   
   A   
   A   
   G   
   T   
   G   
   A   
   A   
   G   
   T   
   G   
   A   
   T   
   T   
   G   
   A   
   T   
   G   
   C   
   A   
   A   
   C   
   A   
   A   
   G   
   T   
   T   
   T   
   A   
   T   
   T   
   G   
   A   
   T   
   G   
   G   
   A   
   C   
   A   
   A   
   T   
   C   
   C   
   T   
   G   
   A   
   T   
   G   
   T   
   A   
   A   
   C   
   G   
   A   
   C   
   G   
   A   
   A   
   T   
   G   
   A   
   A   
   T   
   T   
   G   
   A   
   T   
   G   
   G   
   A   
   A   
   G   
   T   
   G   
   T   
   T   
   A   
   C   
   C   
   T   
   G   
   G   
   A   
   C   
   C   
   A   
   G   
   A   
   C   
   T   
   T   
   T   
   C   
   C   
   A   
   A   
   C   
   A   
   G   
   G   
   C   
   G   
   G   
   T   
   T   
   T   
   A   
   G   
   T   
   G   
   A   
   T   
   G   
   G   
   G   
   G   
   A   
   A   
   A   
   T   
   C   
   A   
   G   
   G   
    G    
   A   
   T   
    T    
   C   
   G   
    C    
    C    
   G   
   A   
   G   
   C   
    A    
   T   
   A   
   T   
   G   
   A   
   A   
   A   
   C   
    A    
   G   
   G   
    G    
   A   
   A   
    A    
   G   
   G   
   T   
   T   
   C   
    G    
   A   
   T   
   T   
   A   
   C   
    T    
   G   
   T   
    T    
   C   
   G   
   T   
   G   
   C   
   A   
   A   
   A   
   A   
   G   
   T   
   T   
   G   
   A   
    A    
    T    
   T   
    G    
   A   
    C    
   T   
   G   
   A   
    A    
   A   
   T   
   G   
    C    
   C   
    G    
   A   
   A   
   T   
   G   
   G   
    A    
   A   
   A   
   A   
   G   
   A   
   A   
   C   
   G   
    T    
   A   
   T   
    T    
    T    
   T   
    A    
   G   
   T   
    A    
   A   
   C   
    T    
   G   
   A   
   A   
   T   
   T   
    G    
   C   
   C   
   T   
   T   
   A   
   T   
   A   
   T   
   G   
   G   
   T   
    G    
   A   
   A   
   T   
   A   
   A   
   A   
   G   
   C   
    C    
   A   
   A   
   A   
   T   
   T   
    A    
   A   
   T   
    C    
   G   
   A   
   A   
   C   
   G   
    A    
   A   
   T   
   T   
   T   
   C   
   T   
   G   
   A   
    A    
    T    
   T   
    A    
   C   
   A   
   C   
   A   
   G   
   A   
   G   
   A   
    T    
   A   
   A   
    A    
   C   
   G   
    A    
   A   
   T   
    T    
   G   
   A   
   A   
   G   
   G   
    A    
   A   
   T   
    T    
   A   
   C   
    T    
   G   
   A   
   T   
   T   
   T   
   G   
   C   
   G   
   G   
   G   
   A   
    T    
   G   
   A   
   A   
   T   
   C   
   T   
   T   
   C   
    T    
   C   
   G   
   T   
   G   
   A   
    A    
   G   
   G   
    C    
   A   
   T   
   G   
   C   
   G   
    G    
   A   
   T   
    C    
   G   
   T   
   C   
   A   
   T   
    C    
   G   
   A   
    T    
    G    
   T   
    T    
   C   
   G   
   T   
   C   
   G   
    A    
   G   
   A   
    T    
   G   
    T    
    G    
   A   
   G   
    T    
   G   
   C   
    C    
   T   
   C   
    T    
   G   
   T   
   T   
    G    
   T   
    G    
   T   
   T   
    A    
   A   
   A   
    C    
   A   
   A   
   C   
   T   
   T   
    A    
   T   
   A   
    C    
   A   
   A   
   A   
    A    
   T   
   G   
   A   
   C   
    T    
    G    
   C   
    C    
   T   
   T   
    A    
   C   
   A   
    A    
   A   
    C    
    A    
   T   
   C   
    T    
   T   
   T   
    T    
   G   
   G   
    T    
   T   
   T   
    T    
   A   
   A   
    C    
   A   
   T   
   G   
   T   
   T   
    A    
   G   
   C   
    C    
   A   
   T   
    C    
   G   
   A   
   A   
   A   
   A   
   A   
   G   
   G   
    C    
   G   
   T   
    A    
   C   
   C   
    G    
   A   
   A   
   A   
    A    
   T   
   T   
   T   
   T   
    A    
   A   
   G   
    T    
   T   
   T   
   G   
   A   
   A   
   A   
   C   
    G    
    T    
   A   
   T   
    T    
    T    
   T   
    A    
   G   
   A   
   A   
   A   
   A   
    T    
   T   
   A   
    C    
    A    
   T   
    C    
   G   
   A   
    G    
   C   
   A   
    C    
   C   
   A   
    A    
    A    
    A    
    A    
    G    
   A   
   A   
   G   
   T   
   C   
   A   
   T   
    T    
   A   
   C   
    T    
    C    
   G   
    C    
   C   
   G   
   G   
   A   
   C   
    G    
    A    
   T   
    T    
   T   
   T   
   T   
   G   
   A   
    C    
   A   
   A   
   A   
    A    
   A   
    C    
   A   
   A   
    A    
   G   
   C   
   A   
   G   
   A   
   A   
   G   
   C   
   A   
    C    
   G   
    G    
   G   
   C   
    G    
   C   
   A   
    T    
   A   
   T   
    T    
   T   
   T   
   A   
   G   
   A   
   A   
   G   
   G   
   T   
    C    
   T   
    A    
   C   
   G   
    A    
   A   
   T   
   T   
   G   
   C   
    C    
   T   
   T   
    A    
   G   
   A   
   T   
   C   
   A   
   T   
   A   
   T   
   C   
    G    
   A   
   T   
   G   
   A   
   A   
   A   
   T   
    C    
   A   
   T   
   T   
   G   
   C   
    C    
   A   
   T   
   T   
   A   
   T   
    C    
   C   
   G   
    T    
   G   
   G   
    G    
   T   
   C   
    A    
    C    
   A   
   A   
   T   
   C   
   A   
   G   
   A   
   T   
   G   
   A   
    C    
   G   
   A   
    A    
   G   
   C   
   A   
   A   
   A   
   A   
   G   
   C   
    A    
   A   
    C    
    G    
    T    
   T   
    A    
   A   
   T   
    T    
   G   
   A   
   A   
   C   
   G   
    C    
   T   
   T   
    T    
   G   
   A   
    A    
   T   
   T   
    T    
   T   
   C   
    A    
   G   
   A   
   T   
   C   
   G   
    T    
   C   
   A   
    A    
    G    
   C   
    G    
   C   
   A   
   A   
   G   
   C   
    G    
   A   
   T   
    T    
   T   
   T   
   A   
   G   
   A   
   T   
   A   
   T   
   G   
   C   
   G   
   T   
   T   
   T   
    A    
   C   
   G   
    C    
   C   
   G   
    T    
   T   
   T   
    A    
   A   
   C   
    A    
   G   
   G   
    T    
   T   
   T   
    A    
   G   
   A   
   A   
   C   
   G   
    C    
   G   
   A   
    C    
   A   
   A   
   A   
   A   
   T   
    T    
   G   
   A   
    G    
   A   
   A   
   T   
   G   
   A   
   A   
   T   
   A   
    T    
   C   
   A   
   A   
   G   
   A   
    G    
   C   
   T   
    A    
   T   
   T   
    G    
   A   
    A    
   A   
    T    
   T   
    C    
   A   
   T   
   T   
   G   
    C    
    G    
   G   
   A   
    C    
   T   
   T   
    A    
    G    
    A    
    G    
   G   
   A   
   C   
   A   
   T   
   T   
    T    
   T   
    A    
   G   
   C   
    C    
    C    
   G   
    C    
   C   
   C   
    A    
   G   
   A   
    A    
   C   
   G   
    C    
   G   
   T   
    C    
   A   
    T    
    T    
   G   
   A   
   A   
   A   
   T   
    C    
   A   
   T   
    T    
   A   
   A   
    A    
   A   
    C    
    A    
   G   
   A   
    G    
   T   
   T   
   A   
    A    
    A    
    T    
   G   
   A   
   C   
    G    
   T   
    T    
    C    
    G    
    C    
    A    
    C    
   A   
    A    
    A    
   A   
   T   
   T   
   T   
   G   
   G   
   T   
   G   
   A   
   T   
    G    
    C    
   A   
   C   
   G   
   A   
   C   
   G   
    C    
   A   
   C   
   A   
   G   
   A   
   A   
    T    
   T   
   A   
    T    
   T   
   A   
   G   
   T   
    A    
   G   
   G   
   T   
   G   
   A   
    A    
   G   
   T   
   C   
    C    
   T   
    A    
   A   
   G   
   T   
   C   
   T   
    T    
   G   
   A   
   A   
   G   
   A   
   T   
   G   
   A   
   A   
   G   
   A   
   T   
    C    
   T   
    A    
   A   
   T   
    C    
   G   
   A   
   A   
   G   
   A   
   A   
   G   
   A   
   A   
   G   
   A   
   A   
    G    
   T   
   C   
   G   
   T   
    G    
   A   
   T   
   T   
   A   
   C   
    G    
    T    
   T   
    A    
   A   
   C   
    C    
   A   
   A   
    T    
   A   
   A   
    C    
   G   
   G   
    C    
   T   
   A   
    C    
   A   
   T   
    T    
   A   
   A   
    G    
   C   
   G   
    G    
    A    
   T   
   G   
   G   
    C    
   A   
   A   
   A   
    C    
    T    
    C    
    C    
   G   
   A   
   A   
   T   
   T   
   C   
    C    
   G   
    G    
   G   
   C   
    G    
   C   
   A   
   A   
   C   
   G   
    C    
    C    
   G   
    C    
   G   
   G   
    T    
   G   
   G   
    A    
   C   
   G   
   T   
   G   
   G   
    T    
   G   
   T   
    T    
   C   
   A   
   A   
   G   
   G   
    C    
   A   
   T   
   G   
   G   
   G   
    C    
    G    
   T   
    T    
   C   
   A   
    T    
    G    
   A   
   T   
   G   
   A   
    T    
   G   
   A   
    C    
   T   
   T   
   C   
   G   
   T   
    G    
   A   
   A   
    A    
   A   
   A   
    C    
   C   
   T   
    A    
    G    
   T   
   T   
   T   
   C   
   T   
   T   
   G   
    T    
   T   
   C   
    A    
   A   
   C   
    A    
   C   
   A   
    C    
   G   
   A   
    C    
   A   
   C   
    G    
   T   
   T   
    A    
   T   
   T   
   A   
   T   
   T   
    C    
   T   
   T   
   T   
   A   
   C   
   G   
   A   
   A   
    C    
   A   
    C    
    T    
   G   
   G   
    G    
   A   
   A   
   A   
   G   
   T   
    T    
   T   
   A   
    C    
   C   
   G   
    A    
   G   
   C   
   A   
   A   
   A   
   A   
   G   
   G   
    T    
   T   
   A   
    C    
   G   
   A   
   A   
   A   
   T   
    C    
   C   
   C   
    T    
   G   
   A   
    A    
   T   
   A   
   C   
   G   
   G   
    T    
    A    
   G   
    A    
   A   
   C   
   A   
   G   
   C   
    A    
   A   
   A   
   A   
   G   
   G   
    A    
   A   
   T   
    T    
   C   
   C   
   A   
    G    
   T   
    G    
   A   
   T   
    T    
   A   
   A   
    C    
   T   
   T   
    A    
   C   
   T   
    G    
   G   
   G   
    A    
   A   
   T   
    T    
   G   
   A   
    T    
   T   
   C   
    T    
    G    
    C    
    A    
   G   
   A   
   A   
   A   
    A    
    A    
   A   
   T   
    T    
   C   
   A   
    A    
   G   
   C   
    G    
   A   
   T   
    T    
   A   
   T   
   T   
    T    
   C   
    T    
   G   
   T   
    T    
    G    
    A    
   G   
   G   
   G   
    C    
    A    
   A   
   A   
   G   
   C   
    G    
   G   
   A   
   A   
   G   
    C    
    A    
   G   
   G   
    T    
    A    
   A   
    A    
   T   
   A   
    C    
    T    
   T   
    A    
   T   
   T   
   C   
   T   
   T   
    C    
   A   
   C   
    A    
   A   
   C   
    C    
    T    
    T    
    A    
    A    
   A   
   A   
   G   
   G   
    A    
   A   
   C   
    C    
   G   
   T   
    C    
   A   
   A   
   A   
   C   
   G   
    G    
   A   
   C   
    A    
    G    
   C   
    C    
   G   
   T   
    A    
   A   
   C   
    A    
   G   
   C   
    C    
   T   
   T   
    T    
    T    
   C   
   T   
   A   
   A   
    T    
   A   
   T   
    C    
   C   
   G   
    T    
   A   
   G   
    T    
   A   
   A   
   T   
   G   
   G   
    A    
    T    
   T   
    A    
   A   
   T   
    C    
   G   
   C   
    C    
   A   
   T   
   T   
    A    
   G   
    C    
   T   
   T   
    A    
   A   
   A   
   A   
   G   
   A   
   A   
   G   
   A   
    T    
   G   
   A   
    T    
   G   
   A   
    G    
    T    
   T   
    A    
    G    
   T   
    T    
   A   
   A   
    C    
   G   
   T   
    G    
    G    
   T   
   A   
    A    
    C    
   G   
   A   
   C   
    T    
    A    
   A   
    T    
   G   
   G   
    C    
    A    
   A   
    T    
    C    
    A    
    G    
   A   
   A   
    G    
   A   
   T   
    G    
   A   
   T   
    T    
   A   
   T   
    C    
   G   
   G   
    A    
   A   
   C   
   A   
   C   
   A   
   T   
    G    
    C    
    A    
   G   
   G   
    A    
   T   
   A   
   C   
   T   
   C   
   T   
   G   
   T   
    C    
   A   
   C   
    A    
   T   
   T   
    T    
    G    
   A   
    T    
   G   
   A   
    A    
    A    
    C    
    T    
    A    
   C   
    T    
   G   
    T    
    A    
   C   
   G   
    T    
   G   
   A   
   T   
   A   
   T   
   G   
   G   
   G   
    C    
   C   
   G   
    G    
   A   
   C   
   A   
   G   
   C   
    A    
   T   
   C   
    A    
   G   
   G   
    T    
   G   
   T   
    T    
    C    
   G   
    T    
   G   
   G   
    A    
   A   
   T   
    C    
   C   
   G   
    T    
    C    
   T   
    C    
   C   
   G   
    C    
   G   
   A   
    A    
    A    
   A   
    T    
   G   
   A   
    T    
   T   
    A    
    T    
   G   
   T   
    G    
   G   
   T   
    C    
   G   
   G   
    C    
    G    
   C   
   A   
   G   
   C   
    G    
    A    
   T   
    T    
    C    
   T   
   G   
    G    
   A   
    T    
   G   
    A    
   A   
    A    
   A   
    T    
    A    
   A   
   A   
   G   
   A   
   A   
   G   
   T   
    C    
    C    
   T   
    A    
   G   
   T   
    C    
   A   
   T   
    T    
   A   
   C   
    T    
   G   
   A   
   A   
   A   
   A   
    T    
   G   
   G   
   T   
   T   
   A   
    T    
   G   
   G   
    T    
   A   
   A   
    G    
   C   
   G   
    T    
   A   
   C   
    A    
   A   
   A   
   A   
   G   
    C    
    T    
   T   
   C   
    T    
   G   
   A   
    A    
   T   
   A   
   T   
   C   
   C   
   A   
   G   
   T   
    T    
   A   
   A   
    A    
   G   
   G   
    A    
   C   
   G   
    T    
   G   
   G   
    C    
   G   
   G   
   T   
   A   
   A   
   A   
   G   
   G   
    G    
   A   
   T   
    T    
   A   
   A   
    G    
   A   
   C   
    A    
    G    
   C   
    A    
   A   
   A   
    T    
   A   
   T   
   C   
   A   
   C   
   T   
   G   
   A   
    G    
   A   
   A   
   A   
   A   
   A   
   T   
   G   
   G   
   T   
   C   
   C   
   A   
    T    
   T   
   A   
   G   
   C   
    T    
   G   
   G   
    T    
   T   
   T   
    A    
   A   
   C   
   C   
   A   
   C   
    G    
   G   
   T   
    C    
    A    
    A    
   T   
   G   
   G   
    G    
    G    
   A   
   T   
   G   
   A   
   A   
   G   
   A   
    T    
   A   
   T   
   C   
    T    
   T   
    A    
    T    
   T   
    A    
   A   
   T   
    C    
   A   
   C   
    G    
    A    
   A   
    C    
   A   
   A   
   A   
   G   
   G   
    T    
   G   
   T   
    C    
   A   
   T   
   T   
   A   
   T   
   C   
   C   
   G   
    C    
   T   
   T   
    T    
   A   
   A   
   C   
   G   
   T   
    T    
    G    
    A    
    T    
    T    
   C   
   T   
   G   
   T   
   T   
   T   
   C   
   T   
   C   
   A   
   A   
   A   
   C   
    A    
   G   
   G   
   A   
   C   
   G   
    C    
    G    
   C   
   A   
   A   
   C   
   A   
   T   
   T   
    A    
   G   
   G   
    G    
   G   
   T   
    T    
   C   
   G   
   T   
   T   
   T   
    A    
   A   
   T   
   G   
   A   
    G    
    A    
   A   
   T   
   G   
   G   
   A   
   A   
   G   
    A    
    T    
   G   
    G    
    C    
    G    
   C   
    C    
   A   
   A   
   A   
   -   
   -   
   -   
   -   
   -   
   -   
   -   
   -   
   -   
   -   
   -   
   -   
   -   
   -   
   -   
   -   
   -   
   -   
   -   
   -   
   -   
   -   
   -   
   -   
   -   
   -   
   -   
   -   
   -   
   -   
   -   
   -   
   -   
   -   
   -   
   -   
   -   
   -   
   -   
   -   
   -   
   -   
   -   
   -   
   -   
   -   
   -   
   -   
   -   
   -   
   -   
   -   
   -   
   -   
   -   
   -   
   -   
   -   
   -   
   -   
   -   
   -   
   -   
   -   
   -   
   -   
   -   
   -   
   -   
   -   
   -   
   -   
 

 
   N52454   
   A   
   T   
   G   
   A   
   G   
   T   
   G   
   A   
   A   
   G   
   A   
   A   
   A   
   T   
   T   
   A   
   A   
   A   
   G   
   A   
   A   
   A   
   A   
   C   
   A   
   T   
   T   
   C   
   A   
   A   
   G   
   A   
   C   
   G   
   T   
   C   
   A   
   A   
   T   
   C   
   T   
   G   
   A   
   C   
   C   
   A   
   G   
   C   
   G   
   A   
   A   
   A   
   T   
   G   
   A   
   A   
   A   
   G   
   A   
   A   
   T   
   C   
   T   
   T   
   T   
   C   
   A   
   T   
   T   
   G   
   A   
   C   
   T   
   A   
   C   
   G   
   C   
   A   
   A   
   T   
   G   
   A   
   G   
   T   
   G   
   T   
   T   
   A   
   T   
   C   
   G   
   T   
   A   
   G   
   C   
   C   
   C   
   G   
   C   
   G   
   C   
   G   
   T   
   T   
   A   
   C   
   C   
   T   
   G   
   A   
   C   
   G   
   T   
   T   
   C   
   G   
   T   
   G   
   A   
   C   
   G   
   G   
   T   
   T   
   T   
   A   
   A   
   A   
   A   
   C   
   C   
   T   
   G   
   T   
   T   
   C   
   A   
   T   
   C   
   G   
   C   
   C   
   G   
   A   
   A   
   T   
   C   
   T   
   T   
   A   
   T   
   A   
   T   
   G   
   G   
   A   
   A   
   T   
   G   
   A   
   A   
   C   
   G   
   A   
   A   
   T   
   T   
   A   
   G   
   G   
   G   
   G   
   T   
   A   
   A   
   C   
   C   
   C   
   C   
   T   
   G   
   A   
   T   
   A   
   A   
   A   
   C   
   C   
   A   
   C   
   A   
   C   
   A   
   A   
   A   
   A   
   A   
   A   
   T   
   C   
   A   
   G   
   C   
   C   
   C   
   G   
   G   
   A   
   T   
   T   
   G   
   T   
   T   
   G   
   G   
   G   
   G   
   A   
   T   
   G   
   T   
   T   
   A   
   T   
   G   
   G   
   G   
   T   
   A   
   A   
   A   
   T   
   A   
   T   
   C   
   A   
   C   
   C   
   C   
   C   
   C   
   A   
   T   
   G   
   G   
   G   
   G   
   A   
   C   
   A   
   G   
   T   
   G   
   C   
   G   
   A   
   T   
   T   
   T   
   A   
   C   
   G   
   A   
   A   
   T   
   C   
   A   
   A   
   T   
   G   
   G   
   T   
   G   
   C   
   G   
   G   
   A   
   T   
   G   
   G   
   C   
   A   
   C   
   A   
   A   
   C   
   C   
   T   
   T   
   T   
   T   
   A   
   G   
   T   
   T   
   A   
   T   
   C   
   G   
   G   
   G   
   C   
   T   
   A   
   T   
   G   
   C   
   T   
   A   
   G   
   T   
   T   
   G   
   A   
   C   
   G   
   G   
   C   
   C   
   A   
   C   
   G   
   G   
   A   
   A   
   A   
   C   
   T   
   T   
   C   
   G   
   G   
   T   
   T   
   C   
   T   
   G   
   T   
   C   
   G   
   A   
   T   
   G   
   G   
   T   
   G   
   A   
   C   
   G   
   G   
   C   
   G   
   C   
   T   
   G   
   C   
   C   
   G   
   C   
   T   
   A   
   T   
   G   
   C   
   G   
   T   
   T   
   A   
   T   
   A   
   C   
   C   
   G   
   A   
   A   
   G   
   C   
   A   
   C   
   G   
   T   
   A   
   T   
   G   
   A   
   G   
   T   
   A   
   A   
   A   
   A   
   T   
   T   
   G   
   C   
   T   
   T   
   T   
   A   
   G   
   A   
   A   
   A   
   T   
   G   
   C   
   T   
   A   
   C   
   G   
   A   
   G   
   A   
   T   
   A   
   T   
   T   
   A   
   A   
   C   
   A   
   A   
   A   
   A   
   A   
   T   
   A   
   C   
   A   
   G   
   T   
   C   
   G   
   A   
   T   
   T   
   T   
   C   
   C   
   A   
   A   
   G   
   G   
   A   
   A   
   A   
   C   
   T   
   A   
   T   
   G   
   A   
   T   
   G   
   A   
   T   
   T   
   C   
   A   
   G   
   A   
   A   
   C   
   A   
   A   
   G   
   A   
   A   
   C   
   C   
   A   
   G   
   T   
   A   
   G   
   T   
   T   
   T   
   T   
   A   
   C   
   C   
   T   
   G   
   C   
   T   
   C   
   G   
   T   
   T   
   T   
   T   
   C   
   C   
   A   
   A   
   A   
   C   
   T   
   T   
   A   
   C   
   T   
   A   
   G   
   T   
   T   
   A   
   A   
   C   
   G   
   G   
   A   
   A   
   C   
   A   
   A   
   C   
   G   
   G   
   G   
   G   
   A   
   T   
   T   
   G   
   C   
   G   
   G   
   T   
   G   
   G   
   G   
   G   
   A   
   T   
   G   
   G   
   C   
   A   
   A   
   C   
   A   
   A   
   A   
   T   
   A   
   T   
   T   
   C   
   C   
   A   
   C   
   C   
   A   
   C   
   A   
   C   
   A   
   A   
   T   
   T   
   T   
   A   
   A   
   G   
   T   
   G   
   A   
   A   
   G   
   T   
   G   
   A   
   T   
   T   
   G   
   A   
   T   
   G   
   C   
   A   
   A   
   C   
   A   
   A   
   G   
   T   
   T   
   T   
   A   
   T   
   T   
   G   
   A   
   T   
   G   
   G   
   A   
   C   
   A   
   A   
   T   
   C   
   C   
   T   
   G   
   A   
   T   
   G   
   T   
   A   
   A   
   C   
   G   
   A   
   C   
   G   
   A   
   A   
   T   
   G   
   A   
   A   
   T   
   T   
   G   
   A   
   T   
   G   
   G   
   A   
   A   
   G   
   T   
   G   
   T   
   T   
   A   
   C   
   C   
   T   
   G   
   G   
   A   
   C   
   C   
   A   
   G   
   A   
   T   
   T   
   T   
   T   
   C   
   C   
   A   
   A   
   C   
   A   
   G   
   G   
   C   
   G   
   G   
   T   
   T   
   T   
   A   
   G   
   T   
   G   
   A   
   T   
   G   
   G   
   G   
   G   
   A   
   A   
   A   
   T   
   C   
   A   
   G   
   G   
    G    
   A   
   T   
    T    
   C   
   G   
    C    
    C    
   G   
   A   
   G   
   C   
    A    
   T   
   A   
   T   
   G   
   A   
   A   
   A   
   C   
    A    
   G   
   G   
    G    
   A   
   A   
    A    
   G   
   G   
   T   
   T   
   C   
    G    
   A   
   T   
   T   
   A   
   C   
    T    
   G   
   T   
    T    
   C   
   G   
   T   
   G   
   C   
   A   
   A   
   A   
   A   
   G   
   T   
   T   
   G   
   A   
    A    
    T    
   T   
    G    
   A   
    C    
   T   
   G   
   A   
    A    
   A   
   T   
   G   
    C    
   C   
    G    
   A   
   A   
   T   
   G   
   G   
    A    
   A   
   A   
   A   
   G   
   A   
   A   
   C   
   G   
    T    
   A   
   T   
    T    
    T    
   T   
    A    
   G   
   T   
    A    
   A   
   C   
    T    
   G   
   A   
   A   
   T   
   T   
    G    
   C   
   C   
   T   
   T   
   A   
   T   
   A   
   T   
   G   
   G   
   T   
    G    
   A   
   A   
   T   
   A   
   A   
   A   
   G   
   C   
    C    
   A   
   A   
   A   
   T   
   T   
    A    
   A   
   T   
    C    
   G   
   A   
   A   
   C   
   G   
    A    
   A   
   T   
   T   
   T   
   C   
   T   
   G   
   A   
    A    
    T    
   T   
    A    
   C   
   A   
   C   
   A   
   G   
   A   
   G   
   A   
    T    
   A   
   A   
    A    
   C   
   G   
    A    
   A   
   T   
    T    
   G   
   A   
   A   
   G   
   G   
    A    
   A   
   T   
    T    
   A   
   C   
    T    
   G   
   A   
   T   
   T   
   T   
   G   
   C   
   G   
   G   
   G   
   A   
    T    
   G   
   A   
   A   
   T   
   C   
   T   
   T   
   C   
    T    
   C   
   G   
   T   
   G   
   A   
    A    
   G   
   G   
    C    
   A   
   T   
   G   
   C   
   G   
    G    
   A   
   T   
    T    
   G   
   T   
   C   
   A   
   T   
    C    
   G   
   A   
    T    
    G    
   T   
    T    
   C   
   G   
   T   
   C   
   G   
    A    
   G   
   A   
    T    
   G   
    T    
    G    
   A   
   G   
    T    
   G   
   C   
    C    
   T   
   C   
    T    
   G   
   T   
   T   
    G    
   T   
    G    
   T   
   T   
    A    
   A   
   A   
    C    
   A   
   A   
   C   
   T   
   T   
    G    
   T   
   A   
    C    
   A   
   A   
   A   
    A    
   T   
   G   
   A   
   C   
    T    
    G    
   C   
    C    
   T   
   T   
    A    
   C   
   A   
    A    
   A   
    C    
    A    
   T   
   C   
    T    
   T   
   T   
    T    
   G   
   G   
    T    
   T   
   T   
    T    
   A   
   A   
    C    
   A   
   T   
   G   
   T   
   T   
    A    
   G   
   C   
    C    
   A   
   T   
    C    
   G   
   A   
   A   
   A   
   A   
   A   
   G   
   G   
    C    
   G   
   T   
    A    
   C   
   C   
    G    
   A   
   A   
   A   
    A    
   T   
   T   
   T   
   T   
    A    
   A   
   G   
    C    
   T   
   T   
   G   
   A   
   A   
   A   
   C   
    G    
    T    
   A   
   T   
    T    
    T    
   T   
    A    
   G   
   A   
   A   
   A   
   A   
    C    
   T   
   A   
    C    
    G    
   T   
    C    
   G   
   A   
    G    
   C   
   A   
    C    
   C   
   A   
    A    
    A    
    A    
    A    
    G    
   A   
   A   
   G   
   T   
   C   
   A   
   T   
    T    
   A   
   C   
    T    
    C    
   G   
    C    
   C   
   G   
   G   
   A   
   C   
    G    
    A    
   T   
    T    
   T   
   T   
   T   
   G   
   A   
    C    
   A   
   A   
   A   
    A    
   A   
    C    
   A   
   A   
    G    
   G   
   C   
   A   
   G   
   A   
   A   
   G   
   C   
   A   
    C    
   G   
    G    
   G   
   C   
    G    
   C   
   A   
    T    
   A   
   T   
    T    
   T   
   T   
   A   
   G   
   A   
   A   
   G   
   G   
   T   
    C    
   T   
    A    
   C   
   G   
    A    
   A   
   T   
   T   
   G   
   C   
    C    
   T   
   T   
    A    
   G   
   A   
   T   
   C   
   A   
   T   
   A   
   T   
   C   
    G    
   A   
   T   
   G   
   A   
   A   
   A   
   T   
    C    
   A   
   T   
   T   
   G   
   C   
    C    
   A   
   T   
   T   
   A   
   T   
    C    
   C   
   G   
    T    
   G   
   G   
    G    
   T   
   C   
    A    
    C    
   A   
   A   
   T   
   C   
   A   
   G   
   A   
   T   
   G   
   A   
    C    
   G   
   A   
    A    
   G   
   C   
   A   
   A   
   A   
   A   
   G   
   C   
    A    
   A   
    C    
    G    
    T    
   T   
    A    
   A   
   T   
    T    
   G   
   A   
   A   
   C   
   G   
    C    
   T   
   T   
    T    
   G   
   A   
    A    
   T   
   T   
    T    
   T   
   C   
    A    
   G   
   A   
   T   
   C   
   G   
    T    
   C   
   A   
    A    
    G    
   C   
    G    
   C   
   A   
   A   
   G   
   C   
    A    
   A   
   T   
    T    
   T   
   T   
   A   
   G   
   A   
   T   
   A   
   T   
   G   
   C   
   G   
   T   
   T   
   T   
    A    
   C   
   G   
    C    
   C   
   G   
    T    
   T   
   T   
    A    
   A   
   C   
    A    
   G   
   G   
    C    
   T   
   T   
    A    
   G   
   A   
   A   
   C   
   G   
    C    
   G   
   A   
    C    
   A   
   A   
   A   
   A   
   T   
    T    
   G   
   A   
    A    
   A   
   A   
   T   
   G   
   A   
   A   
   T   
   A   
    T    
   C   
   A   
   A   
   G   
   A   
    G    
   C   
   T   
    A    
   T   
   T   
    G    
   A   
    A    
   A   
    T    
   T   
    C    
   A   
   T   
   T   
   G   
    C    
    G    
   G   
   A   
    C    
   T   
   T   
    A    
    G    
    A    
    G    
   G   
   A   
   C   
   A   
   T   
   T   
    T    
   T   
    A    
   G   
   C   
    C    
    C    
   G   
    C    
   C   
   C   
    A    
   G   
   A   
    A    
   C   
   G   
    C    
   G   
   T   
    C    
   A   
    T    
    T    
   G   
   A   
   A   
   A   
   T   
    C    
   A   
   T   
    T    
   A   
   A   
    A    
   A   
    C    
    A    
   G   
   A   
    G    
   T   
   T   
   A   
    A    
    A    
    T    
   G   
   A   
   C   
    G    
   T   
    T    
    C    
    G    
    C    
    A    
    C    
   A   
    A    
    A    
   A   
   T   
   T   
   T   
   G   
   G   
   T   
   G   
   A   
   T   
    G    
    C    
   A   
   C   
   G   
   A   
   C   
   G   
    C    
   A   
   C   
   A   
   G   
   A   
   A   
    T    
   T   
   A   
    T    
   T   
   A   
   G   
   T   
    A    
   G   
   G   
   T   
   G   
   A   
    A    
   G   
   T   
   C   
    T    
   T   
    A    
   A   
   G   
   T   
   C   
   T   
    T    
   G   
   A   
   A   
   G   
   A   
   T   
   G   
   A   
   A   
   G   
   A   
   T   
    T    
   T   
    A    
   A   
   T   
    C    
   G   
   A   
   A   
   G   
   A   
   A   
   G   
   A   
   A   
   G   
   A   
   A   
    G    
   T   
   C   
   G   
   T   
    G    
   A   
   T   
   T   
   A   
   C   
    G    
    T    
   T   
    A    
   A   
   C   
    C    
   A   
   A   
    T    
   A   
   A   
    C    
   G   
   G   
    C    
   T   
   A   
    C    
   A   
   T   
    T    
   A   
   A   
    G    
   C   
   G   
    G    
    A    
   T   
   G   
   G   
    C    
   A   
   A   
   A   
    C    
    T    
    C    
    C    
   G   
   A   
   A   
   T   
   T   
   C   
    C    
   G   
    G    
   G   
   C   
    G    
   C   
   A   
   A   
   C   
   G   
    C    
    C    
   G   
    C    
   G   
   G   
    T    
   G   
   G   
    A    
   C   
   G   
   T   
   G   
   G   
    T    
   G   
   T   
    C    
   C   
   A   
   A   
   G   
   G   
    G    
   A   
   T   
   G   
   G   
   G   
    C    
    G    
   T   
    T    
   C   
   A   
    T    
    G    
   A   
   T   
   G   
   A   
    T    
   G   
   A   
    T    
   T   
   T   
   C   
   G   
   T   
    G    
   A   
   A   
    A    
   A   
   A   
    C    
   C   
   T   
    A    
    G    
   T   
   T   
   T   
   C   
   T   
   T   
   G   
    T    
   T   
   C   
    A    
   A   
   C   
    A    
   C   
   A   
    C    
   G   
   A   
    C    
   A   
   C   
    G    
   T   
   T   
    A    
   T   
   T   
   A   
   T   
   T   
    C    
   T   
   T   
   T   
   A   
   C   
   G   
   A   
   A   
    C    
   A   
    C    
    T    
   G   
   G   
    G    
   A   
   A   
   A   
   G   
   T   
    T    
   T   
   A   
    C    
   C   
   G   
    A    
   G   
   C   
   A   
   A   
   A   
   A   
   G   
   G   
    T    
   T   
   A   
    C    
   G   
   A   
   A   
   A   
   T   
    C    
   C   
   C   
    T    
   G   
   A   
    A    
   T   
   A   
   C   
   G   
   G   
    T    
    A    
   G   
    A    
   A   
   C   
   A   
   G   
   C   
    A    
   A   
   A   
   A   
   G   
   G   
    A    
   A   
   T   
    T    
   C   
   C   
   A   
    G    
   T   
    G    
   A   
   T   
    T    
   A   
   A   
    C    
   T   
   T   
    A    
   C   
   T   
    G    
   G   
   G   
    A    
   A   
   T   
    T    
   G   
   A   
    T    
   T   
   C   
    T    
    G    
    C    
    A    
   G   
   A   
   A   
   A   
    A    
    A    
   A   
   T   
    T    
   C   
   A   
    A    
   G   
   C   
    G    
   A   
   T   
    T    
   A   
   T   
   T   
    T    
   C   
    T    
   G   
   T   
    T    
    G    
    A    
   G   
   G   
   G   
    C    
    A    
   A   
   A   
   G   
   C   
    G    
   G   
   A   
   A   
   G   
    C    
    A    
   G   
   G   
    T    
    A    
   A   
    A    
   T   
   A   
    C    
    T    
   T   
    G    
   T   
   T   
   C   
   T   
   T   
    C    
   A   
   C   
    A    
   A   
   C   
    C    
    T    
    T    
    A    
    A    
   A   
   A   
   G   
   G   
    A    
   A   
   C   
    C    
   G   
   T   
    C    
   A   
   A   
   A   
   C   
   G   
    G    
   A   
   C   
    A    
    G    
   C   
    C    
   G   
   T   
    A    
   A   
   C   
    A    
   G   
   C   
    C    
   T   
   T   
    T    
    T    
   C   
   T   
   A   
   A   
    T    
   A   
   T   
    C    
   C   
   G   
    T    
   A   
   G   
    T    
   A   
   A   
   T   
   G   
   G   
    A    
    T    
   T   
    A    
   A   
   T   
    C    
   G   
   C   
    C    
   A   
   T   
   T   
    A    
   G   
    C    
   T   
   T   
    A    
   A   
   A   
   A   
   G   
   A   
   A   
   G   
   A   
    T    
   G   
   A   
    T    
   G   
   A   
    G    
    T    
   T   
    A    
    G    
   T   
    T    
   A   
   A   
    C    
   G   
   T   
    G    
    G    
   T   
   A   
    A    
    C    
   G   
   A   
   C   
    T    
    A    
   A   
    T    
   G   
   G   
    C    
    A    
   A   
    T    
    C    
    A    
    G    
   A   
   A   
    G    
   A   
   T   
    G    
   A   
   T   
    T    
   A   
   T   
    C    
   G   
   G   
    A    
   A   
   C   
   A   
   C   
   A   
   T   
    G    
    C    
    A    
   G   
   G   
    A    
   T   
   A   
   C   
   T   
   C   
   T   
   G   
   T   
    C    
   A   
   C   
    A    
   T   
   T   
    T    
    G    
   A   
    T    
   G   
   A   
    A    
    A    
    A    
    T    
    A    
   C   
    T    
   G   
    T    
    A    
   C   
   G   
    T    
   G   
   A   
   T   
   A   
   T   
   G   
   G   
   G   
    C    
   C   
   G   
    G    
   A   
   C   
   A   
   G   
   C   
    A    
   T   
   C   
    A    
   G   
   G   
    T    
   G   
   T   
    T    
    C    
   G   
    G    
   G   
   G   
    A    
   A   
   T   
    C    
   C   
   G   
    T    
    C    
   T   
    C    
   C   
   G   
    C    
   G   
   A   
    A    
    A    
   A   
    T    
   G   
   A   
    T    
   T   
    A    
    T    
   G   
   T   
    G    
   G   
   T   
    C    
   G   
   G   
    T    
    G    
   C   
   A   
   G   
   C   
    G    
    A    
   T   
    T    
    C    
   T   
   G   
    G    
   A   
    T    
   G   
    A    
   A   
    A    
   A   
    C    
    A    
   A   
   A   
   G   
   A   
   A   
   G   
   T   
    C    
    C    
   T   
    A    
   G   
   T   
    C    
   A   
   T   
    T    
   A   
   C   
    T    
   G   
   A   
   A   
   A   
   A   
    T    
   G   
   G   
   T   
   T   
   A   
    T    
   G   
   G   
    T    
   A   
   A   
    G    
   C   
   G   
    T    
   A   
   C   
    A    
   A   
   A   
   A   
   G   
    C    
    T    
   T   
   C   
    T    
   G   
   A   
    A    
   T   
   A   
   T   
   C   
   C   
   A   
   G   
   T   
    T    
   A   
   A   
    A    
   G   
   G   
    A    
   C   
   G   
    T    
   G   
   G   
    C    
   G   
   G   
   T   
   A   
   A   
   A   
   G   
   G   
    G    
   A   
   T   
    T    
   A   
   A   
    G    
   A   
   C   
    A    
    G    
   C   
    A    
   A   
   A   
    T    
   A   
   T   
   C   
   A   
   C   
   T   
   G   
   A   
    G    
   A   
   A   
   A   
   A   
   A   
   T   
   G   
   G   
   T   
   C   
   C   
   A   
    T    
   T   
   A   
   G   
   C   
    T    
   G   
   G   
    T    
   T   
   T   
    A    
   A   
   C   
   C   
   A   
   C   
    G    
   G   
   T   
    C    
    A    
    A    
   T   
   G   
   G   
    G    
    G    
   A   
   T   
   G   
   A   
   A   
   G   
   A   
    T    
   A   
   T   
   C   
    T    
   T   
    A    
    T    
   T   
    G    
   A   
   T   
    T    
   A   
   C   
    G    
    A    
   A   
    C    
   A   
   A   
   A   
   G   
   G   
    C    
   G   
   T   
    C    
   A   
   T   
   T   
   A   
   T   
   C   
   C   
   G   
    C    
   T   
   T   
    T    
   A   
   A   
   C   
   G   
   T   
    T    
    G    
    A    
    T    
    T    
   C   
   T   
   G   
   T   
   T   
   T   
   C   
   T   
   C   
   A   
   A   
   A   
   C   
    A    
   G   
   G   
   A   
   C   
   G   
    C    
    G    
   C   
   A   
   A   
   C   
   A   
   T   
   T   
    A    
   G   
   G   
    G    
   G   
   T   
    T    
   C   
   G   
   T   
   T   
   T   
    A    
   A   
   T   
   G   
   A   
    G    
    A    
   A   
   T   
   G   
   G   
   A   
   A   
   G   
    A    
    T    
   G   
    G    
    T    
    G    
   C   
    C    
   A   
   A   
   A   
   -   
   -   
   -   
   -   
   -   
   -   
   -   
   -   
   -   
   -   
   -   
   -   
   -   
   -   
   -   
   -   
   -   
   -   
   -   
   -   
   -   
   -   
   -   
   -   
   -   
   -   
   -   
   -   
   -   
   -   
   -   
   -   
   -   
   -   
   -   
   -   
   -   
   -   
   -   
   -   
   -   
   -   
   -   
   -   
   -   
   -   
   -   
   -   
   -   
   -   
   -   
   -   
   -   
   -   
   -   
   -   
   -   
   -   
   -   
   -   
   -   
   -   
   -   
   -   
   -   
   -   
   -   
   -   
   -   
   -   
   -   
   -   
 

 
   N59466F   
   A   
   T   
   G   
   A   
   G   
   T   
   G   
   A   
   A   
   G   
   A   
   A   
   A   
   T   
   T   
   A   
   A   
   A   
   G   
   A   
   A   
   A   
   A   
   C   
   A   
   T   
   T   
   C   
   A   
   A   
   G   
   A   
   C   
   G   
   T   
   C   
   A   
   A   
   T   
   C   
   T   
   G   
   A   
   C   
   C   
   A   
   G   
   C   
   G   
   A   
   A   
   A   
   T   
   G   
   A   
   A   
   A   
   G   
   A   
   A   
   T   
   C   
   T   
   T   
   T   
   C   
   A   
   T   
   T   
   G   
   A   
   C   
   T   
   A   
   C   
   G   
   C   
   A   
   A   
   T   
   G   
   A   
   G   
   T   
   G   
   T   
   T   
   A   
   T   
   C   
   G   
   T   
   A   
   G   
   C   
   C   
   C   
   G   
   C   
   G   
   C   
   G   
   T   
   T   
   A   
   C   
   C   
   T   
   G   
   A   
   C   
   G   
   T   
   T   
   C   
   G   
   T   
   G   
   A   
   C   
   G   
   G   
   T   
   T   
   T   
   A   
   A   
   A   
   A   
   C   
   C   
   T   
   G   
   T   
   T   
   C   
   A   
   T   
   C   
   G   
   C   
   C   
   G   
   A   
   A   
   T   
   C   
   T   
   T   
   A   
   T   
   A   
   T   
   G   
   G   
   A   
   A   
   T   
   G   
   A   
   A   
   C   
   G   
   A   
   A   
   T   
   T   
   A   
   G   
   G   
   G   
   G   
   T   
   A   
   A   
   C   
   C   
   C   
   C   
   T   
   G   
   A   
   T   
   A   
   A   
   A   
   C   
   C   
   A   
   C   
   A   
   C   
   A   
   A   
   A   
   A   
   A   
   A   
   T   
   C   
   A   
   G   
   C   
   C   
   C   
   G   
   G   
   A   
   T   
   T   
   G   
   T   
   T   
   G   
   G   
   G   
   G   
   A   
   T   
   G   
   T   
   T   
   A   
   T   
   G   
   G   
   G   
   T   
   A   
   A   
   A   
   T   
   A   
   T   
   C   
   A   
   C   
   C   
   C   
   C   
   C   
   A   
   T   
   G   
   G   
   G   
   G   
   A   
   C   
   A   
   G   
   T   
   G   
   C   
   G   
   A   
   T   
   T   
   T   
   A   
   C   
   G   
   A   
   A   
   T   
   C   
   A   
   A   
   T   
   G   
   G   
   T   
   G   
   C   
   G   
   G   
   A   
   T   
   G   
   G   
   C   
   A   
   C   
   A   
   A   
   C   
   C   
   T   
   T   
   T   
   T   
   A   
   G   
   T   
   T   
   A   
   T   
   C   
   G   
   G   
   G   
   C   
   T   
   A   
   T   
   G   
   T   
   T   
   A   
   G   
   T   
   T   
   G   
   A   
   C   
   G   
   G   
   C   
   C   
   A   
   C   
   G   
   G   
   A   
   A   
   A   
   C   
   T   
   T   
   C   
   G   
   G   
   T   
   T   
   C   
   T   
   G   
   T   
   C   
   G   
   A   
   T   
   G   
   G   
   T   
   G   
   A   
   C   
   G   
   G   
   C   
   G   
   C   
   T   
   G   
   C   
   C   
   G   
   C   
   T   
   A   
   T   
   G   
   C   
   G   
   T   
   T   
   A   
   T   
   A   
   C   
   C   
   G   
   A   
   A   
   G   
   C   
   A   
   C   
   G   
   T   
   A   
   T   
   G   
   A   
   G   
   T   
   A   
   A   
   A   
   A   
   T   
   T   
   G   
   C   
   T   
   T   
   T   
   A   
   G   
   A   
   A   
   A   
   T   
   G   
   C   
   T   
   A   
   C   
   G   
   A   
   G   
   A   
   T   
   A   
   T   
   T   
   A   
   A   
   C   
   A   
   A   
   A   
   A   
   A   
   T   
   A   
   C   
   A   
   G   
   T   
   C   
   G   
   A   
   T   
   T   
   T   
   C   
   C   
   A   
   A   
   G   
   G   
   A   
   A   
   A   
   C   
   T   
   A   
   T   
   G   
   A   
   T   
   G   
   A   
   T   
   T   
   C   
   A   
   G   
   A   
   A   
   C   
   A   
   A   
   G   
   A   
   A   
   C   
   C   
   A   
   G   
   T   
   A   
   G   
   T   
   T   
   T   
   T   
   A   
   C   
   C   
   T   
   G   
   C   
   T   
   C   
   G   
   T   
   T   
   T   
   T   
   C   
   C   
   A   
   A   
   A   
   C   
   T   
   T   
   A   
   C   
   T   
   A   
   G   
   T   
   T   
   A   
   A   
   C   
   G   
   G   
   A   
   A   
   C   
   A   
   A   
   C   
   G   
   G   
   G   
   G   
   A   
   T   
   T   
   G   
   C   
   G   
   G   
   T   
   G   
   G   
   G   
   G   
   A   
   T   
   G   
   G   
   C   
   A   
   A   
   C   
   A   
   A   
   A   
   T   
   A   
   T   
   T   
   C   
   C   
   A   
   C   
   C   
   A   
   C   
   A   
   T   
   A   
   A   
   T   
   T   
   T   
   A   
   A   
   G   
   T   
   G   
   A   
   A   
   G   
   T   
   G   
   A   
   T   
   T   
   G   
   A   
   T   
   G   
   C   
   A   
   A   
   C   
   A   
   A   
   G   
   T   
   T   
   T   
   A   
   T   
   T   
   G   
   A   
   T   
   G   
   G   
   A   
   C   
   A   
   A   
   T   
   C   
   C   
   T   
   G   
   A   
   T   
   G   
   T   
   A   
   A   
   C   
   G   
   A   
   C   
   G   
   A   
   A   
   T   
   G   
   A   
   A   
   T   
   T   
   G   
   A   
   T   
   G   
   G   
   A   
   A   
   G   
   T   
   G   
   T   
   T   
   A   
   C   
   C   
   T   
   G   
   G   
   A   
   C   
   C   
   A   
   G   
   A   
   T   
   T   
   T   
   T   
   C   
   C   
   A   
   A   
   C   
   A   
   G   
   G   
   C   
   G   
   G   
   T   
   T   
   T   
   A   
   G   
   T   
   G   
   A   
   T   
   G   
   G   
   G   
   G   
   A   
   A   
   A   
   T   
   C   
   A   
   G   
   G   
    G    
   A   
   T   
    T    
   C   
   G   
    C    
    C    
   G   
   A   
   G   
   C   
    A    
   T   
   A   
   T   
   G   
   A   
   A   
   A   
   C   
    A    
   G   
   G   
    G    
   A   
   A   
    A    
   G   
   G   
   T   
   T   
   C   
    G    
   A   
   T   
   T   
   A   
   C   
    T    
   G   
   T   
    T    
   C   
   G   
   T   
   G   
   C   
   A   
   A   
   A   
   A   
   G   
   T   
   T   
   G   
   A   
    A    
    T    
   T   
    G    
   A   
    C    
   T   
   G   
   A   
    A    
   A   
   T   
   G   
    C    
   C   
    G    
   A   
   A   
   T   
   G   
   G   
    A    
   A   
   A   
   A   
   G   
   A   
   A   
   C   
   G   
    T    
   A   
   T   
    T    
    T    
   T   
    A    
   G   
   T   
    A    
   A   
   C   
    T    
   G   
   A   
   A   
   T   
   T   
    G    
   C   
   C   
   T   
   T   
   A   
   T   
   A   
   T   
   G   
   G   
   T   
    G    
   A   
   A   
   T   
   A   
   A   
   A   
   G   
   C   
    C    
   A   
   A   
   A   
   T   
   T   
    A    
   A   
   T   
    C    
   G   
   A   
   A   
   C   
   G   
    A    
   A   
   T   
   T   
   T   
   C   
   T   
   G   
   A   
    A    
    T    
   T   
    A    
   C   
   A   
   C   
   A   
   G   
   A   
   G   
   A   
    T    
   A   
   A   
    A    
   C   
   G   
    A    
   A   
   T   
    T    
   G   
   A   
   A   
   G   
   G   
    A    
   A   
   T   
    T    
   A   
   C   
    T    
   G   
   A   
   T   
   T   
   T   
   G   
   C   
   G   
   G   
   G   
   A   
    T    
   G   
   A   
   A   
   T   
   C   
   T   
   T   
   C   
    T    
   C   
   G   
   T   
   G   
   A   
    A    
   G   
   G   
    C    
   A   
   T   
   G   
   C   
   G   
    G    
   A   
   T   
    C    
   G   
   T   
   C   
   A   
   T   
    C    
   G   
   A   
    T    
    G    
   T   
    T    
   C   
   G   
   T   
   C   
   G   
    A    
   G   
   A   
    T    
   G   
    T    
    G    
   A   
   G   
    T    
   G   
   C   
    C    
   T   
   C   
    T    
   G   
   T   
   T   
    G    
   T   
    G    
   T   
   T   
    A    
   A   
   A   
    C    
   A   
   A   
   C   
   T   
   T   
    G    
   T   
   A   
    C    
   A   
   A   
   A   
    A    
   T   
   G   
   A   
   C   
    T    
    G    
   C   
    C    
   T   
   T   
    A    
   C   
   A   
    A    
   A   
    C    
    A    
   T   
   C   
    T    
   T   
   T   
    T    
   G   
   G   
    T    
   T   
   T   
    T    
   A   
   A   
    C    
   A   
   T   
   G   
   T   
   T   
    A    
   G   
   C   
    C    
   A   
   T   
    C    
   G   
   A   
   A   
   A   
   A   
   A   
   G   
   G   
    C    
   G   
   T   
    A    
   C   
   C   
    A    
   A   
   A   
   A   
    A    
   T   
   T   
   T   
   T   
    A    
   A   
   G   
    C    
   T   
   T   
   G   
   A   
   A   
   A   
   C   
    G    
    T    
   A   
   T   
    T    
    T    
   T   
    A    
   G   
   A   
   A   
   A   
   A   
    C    
   T   
   A   
    C    
    G    
   T   
    C    
   G   
   A   
    G    
   C   
   A   
    C    
   C   
   A   
    A    
    A    
    A    
    A    
    G    
   A   
   A   
   G   
   T   
   C   
   A   
   T   
    T    
   A   
   C   
    T    
    C    
   G   
    C    
   C   
   G   
   G   
   A   
   C   
    G    
    A    
   T   
    T    
   T   
   T   
   T   
   G   
   A   
    C    
   A   
   A   
   A   
    A    
   A   
    C    
   A   
   A   
    A    
   G   
   C   
   A   
   G   
   A   
   A   
   G   
   C   
   A   
    C    
   G   
    G    
   G   
   C   
    G    
   C   
   A   
    T    
   A   
   T   
    T    
   T   
   T   
   A   
   G   
   A   
   A   
   G   
   G   
   T   
    C    
   T   
    A    
   C   
   G   
    A    
   A   
   T   
   T   
   G   
   C   
    C    
   T   
   T   
    A    
   G   
   A   
   T   
   C   
   A   
   T   
   A   
   T   
   C   
    G    
   A   
   T   
   G   
   A   
   A   
   A   
   T   
    C    
   A   
   T   
   T   
   G   
   C   
    C    
   A   
   T   
   T   
   A   
   T   
    C    
   C   
   G   
    T    
   G   
   G   
    A    
   T   
   C   
    A    
    C    
   A   
   A   
   T   
   C   
   A   
   G   
   A   
   T   
   G   
   A   
    C    
   G   
   A   
    A    
   G   
   C   
   A   
   A   
   A   
   A   
   G   
   C   
    A    
   A   
    C    
    G    
    T    
   T   
    A    
   A   
   T   
    T    
   G   
   A   
   A   
   C   
   G   
    C    
   T   
   T   
    T    
   G   
   A   
    A    
   T   
   T   
    T    
   T   
   C   
    A    
   G   
   A   
   T   
   C   
   G   
    T    
   C   
   A   
    A    
    G    
   C   
    G    
   C   
   A   
   A   
   G   
   C   
    G    
   A   
   T   
    T    
   T   
   T   
   A   
   G   
   A   
   T   
   A   
   T   
   G   
   C   
   G   
   T   
   T   
   T   
    A    
   C   
   G   
    C    
   C   
   G   
    T    
   T   
   T   
    A    
   A   
   C   
    A    
   G   
   G   
    C    
   T   
   T   
    A    
   G   
   A   
   A   
   C   
   G   
    C    
   G   
   A   
    C    
   A   
   A   
   A   
   A   
   T   
    T    
   G   
   A   
    A    
   A   
   A   
   T   
   G   
   A   
   A   
   T   
   A   
    T    
   C   
   A   
   A   
   G   
   A   
    G    
   C   
   T   
    A    
   T   
   T   
    G    
   A   
    A    
   A   
    T    
   T   
    C    
   A   
   T   
   T   
   G   
    C    
    G    
   G   
   A   
    C    
   T   
   T   
    A    
    G    
    A    
    G    
   G   
   A   
   C   
   A   
   T   
   T   
    T    
   T   
    A    
   G   
   C   
    C    
    C    
   G   
    C    
   C   
   C   
    A    
   G   
   A   
    A    
   C   
   G   
    C    
   G   
   T   
    C    
   A   
    T    
    T    
   G   
   A   
   A   
   A   
   T   
    C    
   A   
   T   
    T    
   A   
   A   
    A    
   A   
    C    
    A    
   G   
   A   
    G    
   T   
   T   
   A   
    A    
    A    
    T    
   G   
   A   
   C   
    G    
   T   
    T    
    C    
    G    
    C    
    A    
    C    
   A   
    A    
    A    
   A   
   T   
   T   
   T   
   G   
   G   
   T   
   G   
   A   
   T   
    G    
    C    
   A   
   C   
   G   
   A   
   C   
   G   
    C    
   A   
   C   
   A   
   G   
   A   
   A   
    T    
   T   
   A   
    T    
   T   
   A   
   G   
   T   
    A    
   G   
   G   
   T   
   G   
   A   
    A    
   G   
   T   
   C   
    C    
   T   
    A    
   A   
   G   
   T   
   C   
   T   
    T    
   G   
   A   
   A   
   G   
   A   
   T   
   G   
   A   
   A   
   G   
   A   
   T   
    T    
   T   
    A    
   A   
   T   
    C    
   G   
   A   
   A   
   G   
   A   
   A   
   G   
   A   
   A   
   G   
   A   
   A   
    G    
   T   
   C   
   G   
   T   
    G    
   A   
   T   
   T   
   A   
   C   
    G    
    T    
   T   
    A    
   A   
   C   
    C    
   A   
   A   
    T    
   A   
   A   
    C    
   G   
   G   
    C    
   T   
   A   
    C    
   A   
   T   
    T    
   A   
   A   
    G    
   C   
   G   
    G    
    A    
   T   
   G   
   G   
    C    
   A   
   A   
   A   
    C    
    T    
    C    
    C    
   G   
   A   
   A   
   T   
   T   
   C   
    C    
   G   
    G    
   G   
   C   
    G    
   C   
   A   
   A   
   C   
   G   
    C    
    C    
   G   
    C    
   G   
   G   
    C    
   G   
   G   
    A    
   C   
   G   
   T   
   G   
   G   
    T    
   G   
   T   
    C    
   C   
   A   
   A   
   G   
   G   
    G    
   A   
   T   
   G   
   G   
   G   
    C    
    G    
   T   
    T    
   C   
   A   
    C    
    G    
   A   
   T   
   G   
   A   
    T    
   G   
   A   
    T    
   T   
   T   
   C   
   G   
   T   
    G    
   A   
   A   
    A    
   A   
   A   
    C    
   C   
   T   
    A    
    G    
   T   
   T   
   T   
   C   
   T   
   T   
   G   
    T    
   T   
   C   
    A    
   A   
   C   
    A    
   C   
   A   
    C    
   G   
   A   
    C    
   A   
   C   
    G    
   T   
   T   
    A    
   T   
   T   
   A   
   T   
   T   
    C    
   T   
   T   
   T   
   A   
   C   
   G   
   A   
   A   
    C    
   A   
    C    
    T    
   G   
   G   
    G    
   A   
   A   
   A   
   G   
   T   
    T    
   T   
   A   
    C    
   C   
   G   
    A    
   G   
   C   
   A   
   A   
   A   
   A   
   G   
   G   
    T    
   T   
   A   
    C    
   G   
   A   
   A   
   A   
   T   
    C    
   C   
   C   
    T    
   G   
   A   
    A    
   T   
   A   
   C   
   G   
   G   
    T    
    A    
   G   
    A    
   A   
   C   
   A   
   G   
   C   
    A    
   A   
   A   
   A   
   G   
   G   
    A    
   A   
   T   
    T    
   C   
   C   
   A   
    G    
   T   
    A    
   A   
   T   
    T    
   A   
   A   
    C    
   T   
   T   
    A    
   C   
   T   
    G    
   G   
   G   
    A    
   A   
   T   
    T    
   G   
   A   
    T    
   T   
   C   
    T    
    G    
    C    
    A    
   G   
   A   
   A   
   A   
    A    
    A    
   A   
   T   
    T    
   C   
   A   
    A    
   G   
   C   
    G    
   A   
   T   
    T    
   A   
   T   
   T   
    T    
   C   
    T    
   G   
   T   
    T    
    G    
    A    
   G   
   G   
   G   
    C    
    A    
   A   
   A   
   G   
   C   
    G    
   G   
   A   
   A   
   G   
    C    
    A    
   G   
   G   
    T    
    A    
   A   
    A    
   T   
   A   
    C    
    T    
   T   
    G    
   T   
   T   
   C   
   T   
   T   
    C    
   A   
   C   
    A    
   A   
   C   
    C    
    T    
    T    
    A    
    A    
   A   
   A   
   G   
   G   
    A    
   A   
   C   
    T    
   G   
   T   
    C    
   A   
   A   
   A   
   C   
   G   
    G    
   A   
   C   
    A    
    G    
   C   
    C    
   G   
   T   
    A    
   A   
   C   
    A    
   G   
   C   
    C    
   T   
   T   
    T    
    T    
   C   
   T   
   A   
   A   
    T    
   A   
   T   
    C    
   C   
   G   
    T    
   A   
   G   
    T    
   A   
   A   
   T   
   G   
   G   
    A    
    T    
   T   
    A    
   A   
   T   
    C    
   G   
   C   
    C    
   A   
   T   
   T   
    A    
   G   
    C    
   T   
   T   
    A    
   A   
   A   
   A   
   G   
   A   
   A   
   G   
   A   
    T    
   G   
   A   
    T    
   G   
   A   
    G    
    T    
   T   
    A    
    G    
   T   
    T    
   A   
   A   
    C    
   G   
   T   
    A    
    G    
   T   
   A   
    A    
    C    
   G   
   A   
   C   
    T    
    A    
   A   
    T    
   G   
   G   
    C    
    A    
   A   
    T    
    C    
    A    
    G    
   A   
   A   
    G    
   A   
   T   
    G    
   A   
   T   
    T    
   A   
   T   
    C    
   G   
   G   
    A    
   A   
   C   
   A   
   C   
   A   
   T   
    G    
    C    
    A    
   G   
   G   
    A    
   T   
   A   
   C   
   T   
   C   
   T   
   G   
   T   
    C    
   A   
   C   
    A    
   T   
   T   
    T    
    G    
   A   
    T    
   G   
   A   
    A    
    A    
    A    
    T    
    A    
   C   
    T    
   G   
    T    
    A    
   C   
   G   
    T    
   G   
   A   
   T   
   A   
   T   
   G   
   G   
   G   
    C    
   C   
   G   
    G    
   A   
   C   
   A   
   G   
   C   
    A    
   T   
   C   
    A    
   G   
   G   
    C    
   G   
   T   
    T    
    C    
   G   
    G    
   G   
   G   
    A    
   A   
   T   
    C    
   C   
   G   
    T    
    C    
   T   
    C    
   C   
   G   
    C    
   G   
   A   
    A    
    A    
   A   
    T    
   G   
   A   
    T    
   T   
    A    
    T    
   G   
   T   
    G    
   G   
   T   
    C    
   G   
   G   
    C    
    G    
   C   
   A   
   G   
   C   
    G    
    A    
   T   
    T    
    C    
   T   
   G   
    G    
   A   
    T    
   G   
    A    
   A   
    A    
   A   
    T    
    A    
   A   
   A   
   G   
   A   
   A   
   G   
   T   
    C    
    C    
   T   
    A    
   G   
   T   
    C    
   A   
   T   
    T    
   A   
   C   
    T    
   G   
   A   
   A   
   A   
   A   
    T    
   G   
   G   
   T   
   T   
   A   
    T    
   G   
   G   
    T    
   A   
   A   
    G    
   C   
   G   
    T    
   A   
   C   
    A    
   A   
   A   
   A   
   G   
    C    
    T    
   T   
   C   
    T    
   G   
   A   
    A    
   T   
   A   
   T   
   C   
   C   
   A   
   G   
   T   
    T    
   A   
   A   
    A    
   G   
   G   
    A    
   C   
   G   
    T    
   G   
   G   
    C    
   G   
   G   
   T   
   A   
   A   
   A   
   G   
   G   
    G    
   A   
   T   
    T    
   A   
   A   
    G    
   A   
   C   
    A    
    G    
   C   
    A    
   A   
   A   
    T    
   A   
   T   
   C   
   A   
   C   
   T   
   G   
   A   
    G    
   A   
   A   
   A   
   A   
   A   
   T   
   G   
   G   
   T   
   C   
   C   
   A   
    T    
   T   
   A   
   G   
   C   
    T    
   G   
   G   
    T    
   T   
   T   
    A    
   A   
   C   
   C   
   A   
   C   
    G    
   G   
   T   
    C    
    A    
    A    
   T   
   G   
   G   
    G    
    A    
   A   
   T   
   G   
   A   
   A   
   G   
   A   
    T    
   A   
   T   
   C   
    T    
   T   
    A    
    T    
   T   
    G    
   A   
   T   
    T    
   A   
   C   
    G    
    A    
   A   
    C    
   A   
   A   
   A   
   G   
   G   
    C    
   G   
   T   
    C    
   A   
   T   
   T   
   A   
   T   
   C   
   C   
   G   
    C    
   T   
   T   
    T    
   A   
   A   
   C   
   G   
   T   
    T    
    G    
    A    
    T    
    T    
   C   
   T   
   G   
   T   
   T   
   T   
   C   
   T   
   C   
   A   
   A   
   A   
   C   
    A    
   G   
   G   
   A   
   C   
   G   
    C    
    G    
   C   
   A   
   A   
   C   
   A   
   T   
   T   
    A    
   G   
   G   
    G    
   G   
   T   
    T    
   C   
   G   
   T   
   T   
   T   
    A    
   A   
   T   
   G   
   A   
    G    
    A    
   A   
   T   
   G   
   G   
   A   
   A   
   G   
    A    
    T    
   G   
    G    
    T    
    G    
   C   
    C    
   A   
   A   
   A   
   -   
   -   
   -   
   -   
   -   
   -   
   -   
   -   
   -   
   -   
   -   
   -   
   -   
   -   
   -   
   -   
   -   
   -   
   -   
   -   
   -   
   -   
   -   
   -   
   -   
   -   
   -   
   -   
   -   
   -   
   -   
   -   
   -   
   -   
   -   
   -   
   -   
   -   
   -   
   -   
   -   
   -   
   -   
   -   
   -   
   -   
   -   
   -   
   -   
   -   
   -   
   -   
   -   
   -   
   -   
   -   
   -   
   -   
   -   
   -   
   -   
   -   
   -   
   -   
   -   
   -   
   -   
   -   
   -   
   -   
   -   
   -   
 

 
   N53457   
   A   
   T   
   G   
   A   
   G   
   T   
   G   
   A   
   A   
   G   
   A   
   A   
   A   
   T   
   T   
   A   
   A   
   A   
   G   
   A   
   A   
   A   
   A   
   C   
   A   
   T   
   T   
   C   
   A   
   A   
   G   
   A   
   C   
   G   
   T   
   C   
   A   
   A   
   T   
   C   
   T   
   G   
   A   
   C   
   C   
   A   
   G   
   C   
   G   
   A   
   A   
   A   
   T   
   G   
   A   
   A   
   A   
   G   
   A   
   A   
   T   
   C   
   T   
   T   
   T   
   C   
   A   
   T   
   T   
   G   
   A   
   C   
   T   
   A   
   C   
   G   
   C   
   A   
   A   
   T   
   G   
   A   
   G   
   T   
   G   
   T   
   T   
   A   
   T   
   C   
   G   
   T   
   A   
   G   
   C   
   C   
   C   
   G   
   C   
   G   
   C   
   G   
   T   
   T   
   A   
   C   
   C   
   T   
   G   
   A   
   C   
   G   
   T   
   T   
   C   
   G   
   T   
   G   
   A   
   C   
   G   
   G   
   T   
   T   
   T   
   A   
   A   
   A   
   A   
   C   
   C   
   T   
   G   
   T   
   T   
   C   
   A   
   T   
   C   
   G   
   C   
   C   
   G   
   A   
   A   
   T   
   C   
   T   
   T   
   A   
   T   
   A   
   T   
   G   
   G   
   A   
   A   
   T   
   G   
   A   
   A   
   C   
   G   
   A   
   A   
   T   
   T   
   A   
   G   
   G   
   G   
   G   
   T   
   A   
   A   
   C   
   C   
   C   
   C   
   T   
   G   
   A   
   T   
   A   
   A   
   A   
   C   
   C   
   A   
   C   
   A   
   C   
   A   
   A   
   A   
   A   
   A   
   A   
   T   
   C   
   A   
   G   
   C   
   C   
   C   
   G   
   G   
   A   
   T   
   T   
   G   
   T   
   T   
   G   
   G   
   G   
   G   
   A   
   T   
   G   
   T   
   T   
   A   
   T   
   G   
   G   
   G   
   T   
   A   
   A   
   A   
   T   
   A   
   T   
   C   
   A   
   C   
   C   
   C   
   C   
   C   
   A   
   T   
   G   
   G   
   G   
   G   
   A   
   C   
   A   
   G   
   T   
   G   
   C   
   G   
   A   
   T   
   T   
   T   
   A   
   C   
   G   
   A   
   A   
   T   
   C   
   A   
   A   
   T   
   G   
   G   
   T   
   G   
   C   
   G   
   G   
   A   
   T   
   G   
   G   
   C   
   A   
   C   
   A   
   A   
   C   
   C   
   T   
   T   
   T   
   T   
   A   
   G   
   T   
   T   
   A   
   T   
   C   
   G   
   G   
   G   
   C   
   T   
   A   
   T   
   G   
   T   
   T   
   A   
   G   
   T   
   T   
   G   
   A   
   C   
   G   
   G   
   C   
   C   
   A   
   C   
   G   
   G   
   A   
   A   
   A   
   C   
   T   
   T   
   C   
   G   
   G   
   T   
   T   
   C   
   T   
   G   
   T   
   C   
   G   
   A   
   T   
   G   
   G   
   T   
   G   
   A   
   C   
   G   
   G   
   C   
   G   
   C   
   T   
   G   
   C   
   C   
   G   
   C   
   T   
   A   
   T   
   G   
   C   
   G   
   T   
   T   
   A   
   T   
   A   
   C   
   C   
   G   
   A   
   A   
   G   
   C   
   A   
   C   
   G   
   T   
   A   
   T   
   G   
   A   
   G   
   T   
   A   
   A   
   A   
   A   
   T   
   T   
   G   
   C   
   T   
   T   
   T   
   A   
   G   
   A   
   A   
   A   
   T   
   G   
   C   
   T   
   A   
   C   
   G   
   A   
   G   
   A   
   T   
   A   
   T   
   T   
   A   
   A   
   C   
   A   
   A   
   A   
   A   
   A   
   T   
   A   
   C   
   A   
   G   
   T   
   C   
   G   
   A   
   T   
   T   
   T   
   C   
   C   
   A   
   A   
   G   
   G   
   A   
   A   
   A   
   C   
   T   
   A   
   T   
   G   
   A   
   T   
   G   
   A   
   T   
   T   
   C   
   A   
   G   
   A   
   A   
   C   
   A   
   A   
   G   
   A   
   A   
   C   
   C   
   A   
   G   
   T   
   A   
   G   
   T   
   T   
   T   
   T   
   A   
   C   
   C   
   T   
   G   
   C   
   T   
   C   
   G   
   T   
   T   
   T   
   T   
   C   
   C   
   A   
   A   
   A   
   C   
   T   
   T   
   A   
   C   
   T   
   A   
   G   
   T   
   T   
   A   
   A   
   C   
   G   
   G   
   A   
   A   
   C   
   A   
   A   
   C   
   G   
   G   
   G   
   G   
   A   
   T   
   T   
   G   
   C   
   G   
   G   
   T   
   G   
   G   
   G   
   G   
   A   
   T   
   G   
   G   
   C   
   A   
   A   
   C   
   A   
   A   
   A   
   T   
   A   
   T   
   T   
   C   
   C   
   A   
   C   
   C   
   A   
   C   
   A   
   C   
   A   
   A   
   T   
   T   
   T   
   A   
   A   
   G   
   T   
   G   
   A   
   A   
   G   
   T   
   G   
   A   
   T   
   T   
   G   
   A   
   T   
   G   
   C   
   A   
   A   
   C   
   A   
   A   
   G   
   T   
   T   
   T   
   A   
   T   
   T   
   G   
   A   
   T   
   G   
   G   
   A   
   C   
   A   
   A   
   T   
   C   
   C   
   T   
   G   
   A   
   T   
   G   
   T   
   A   
   A   
   C   
   G   
   A   
   C   
   G   
   A   
   A   
   T   
   G   
   A   
   A   
   T   
   T   
   G   
   A   
   T   
   G   
   G   
   A   
   A   
   G   
   T   
   G   
   T   
   T   
   A   
   C   
   C   
   T   
   G   
   G   
   A   
   C   
   C   
   A   
   G   
   A   
   C   
   T   
   T   
   T   
   C   
   C   
   A   
   A   
   C   
   A   
   G   
   G   
   C   
   G   
   G   
   T   
   T   
   T   
   A   
   G   
   T   
   G   
   A   
   T   
   G   
   G   
   G   
   G   
   A   
   A   
   A   
   T   
   C   
   A   
   G   
   G   
    G    
   A   
   T   
    T    
   C   
   G   
    T    
    C    
   G   
   A   
   G   
   C   
    A    
   T   
   A   
   T   
   G   
   A   
   A   
   A   
   C   
    A    
   G   
   G   
    G    
   A   
   A   
    A    
   G   
   G   
   T   
   T   
   C   
    G    
   A   
   T   
   T   
   A   
   C   
    T    
   G   
   T   
    T    
   C   
   G   
   T   
   G   
   C   
   A   
   A   
   A   
   A   
   G   
   T   
   T   
   G   
   A   
    A    
    T    
   T   
    G    
   A   
    C    
   T   
   G   
   A   
    A    
   A   
   T   
   G   
    C    
   C   
    G    
   A   
   A   
   T   
   G   
   G   
    A    
   A   
   A   
   A   
   G   
   A   
   A   
   C   
   G   
    T    
   A   
   T   
    T    
    T    
   T   
    A    
   G   
   T   
    A    
   A   
   C   
    T    
   G   
   A   
   A   
   T   
   T   
    G    
   C   
   C   
   T   
   T   
   A   
   T   
   A   
   T   
   G   
   G   
   T   
    G    
   A   
   A   
   T   
   A   
   A   
   A   
   G   
   C   
    C    
   A   
   A   
   A   
   T   
   T   
    A    
   A   
   T   
    C    
   G   
   A   
   A   
   C   
   G   
    A    
   A   
   T   
   T   
   T   
   C   
   T   
   G   
   A   
    A    
    T    
   T   
    A    
   C   
   A   
   C   
   A   
   G   
   A   
   G   
   A   
    T    
   A   
   A   
    A    
   C   
   G   
    A    
   A   
   T   
    T    
   G   
   A   
   A   
   G   
   G   
    A    
   A   
   T   
    T    
   A   
   C   
    T    
   G   
   A   
   T   
   T   
   T   
   G   
   C   
   G   
   G   
   G   
   A   
    T    
   G   
   A   
   A   
   T   
   C   
   T   
   T   
   C   
    T    
   C   
   G   
   T   
   G   
   A   
    A    
   G   
   G   
    C    
   A   
   T   
   G   
   C   
   G   
    G    
   A   
   T   
    C    
   G   
   T   
   C   
   A   
   T   
    C    
   G   
   A   
    T    
    G    
   T   
    T    
   C   
   G   
   T   
   C   
   G   
    A    
   G   
   A   
    T    
   G   
    T    
    G    
   A   
   G   
    T    
   G   
   C   
    C    
   T   
   C   
    T    
   G   
   T   
   T   
    G    
   T   
    G    
   T   
   T   
    A    
   A   
   A   
    C    
   A   
   A   
   C   
   T   
   T   
    G    
   T   
   A   
    C    
   A   
   A   
   A   
    A    
   T   
   G   
   A   
   C   
    T    
    G    
   C   
    C    
   T   
   T   
    A    
   C   
   A   
    A    
   A   
    C    
    A    
   T   
   C   
    T    
   T   
   T   
    T    
   G   
   G   
    T    
   T   
   T   
    T    
   A   
   A   
    C    
   A   
   T   
   G   
   T   
   T   
    A    
   G   
   C   
    C    
   A   
   T   
    T    
   G   
   A   
   A   
   A   
   A   
   A   
   G   
   G   
    T    
   G   
   T   
    A    
   C   
   C   
    G    
   A   
   A   
   A   
    A    
   T   
   T   
   T   
   T   
    A    
   A   
   G   
    C    
   T   
   T   
   G   
   A   
   A   
   A   
   C   
    G    
    T    
   A   
   T   
    T    
    T    
   T   
    A    
   G   
   A   
   A   
   A   
   A   
    T    
   T   
   A   
    C    
    G    
   T   
    C    
   G   
   A   
    G    
   C   
   A   
    C    
   C   
   A   
    A    
    A    
    A    
    A    
    G    
   A   
   A   
   G   
   T   
   C   
   A   
   T   
    T    
   A   
   C   
    T    
    C    
   G   
    C    
   C   
   G   
   G   
   A   
   C   
    G    
    A    
   T   
    T    
   T   
   T   
   T   
   G   
   A   
    C    
   A   
   A   
   A   
    A    
   A   
    C    
   A   
   A   
    A    
   G   
   C   
   A   
   G   
   A   
   A   
   G   
   C   
   A   
    C    
   G   
    G    
   G   
   C   
    G    
   C   
   A   
    T    
   A   
   T   
    T    
   T   
   T   
   A   
   G   
   A   
   A   
   G   
   G   
   T   
    C    
   T   
    A    
   C   
   G   
    A    
   A   
   T   
   T   
   G   
   C   
    C    
   T   
   T   
    A    
   G   
   A   
   T   
   C   
   A   
   T   
   A   
   T   
   C   
    G    
   A   
   T   
   G   
   A   
   A   
   A   
   T   
    C    
   A   
   T   
   T   
   G   
   C   
    C    
   A   
   T   
   T   
   A   
   T   
    C    
   C   
   G   
    T    
   G   
   G   
    G    
   T   
   C   
    A    
    C    
   A   
   A   
   T   
   C   
   A   
   G   
   A   
   T   
   G   
   A   
    T    
   G   
   A   
    A    
   G   
   C   
   A   
   A   
   A   
   A   
   G   
   C   
    A    
   A   
    C    
    G    
    T    
   T   
    A    
   A   
   T   
    T    
   G   
   A   
   A   
   C   
   G   
    C    
   T   
   T   
    T    
   G   
   A   
    A    
   T   
   T   
    T    
   T   
   C   
    A    
   G   
   A   
   T   
   C   
   G   
    T    
   C   
   A   
    A    
    G    
   C   
    G    
   C   
   A   
   A   
   G   
   C   
    G    
   A   
   T   
    T    
   T   
   T   
   A   
   G   
   A   
   T   
   A   
   T   
   G   
   C   
   G   
   T   
   T   
   T   
    A    
   C   
   G   
    C    
   C   
   G   
    T    
   T   
   T   
    A    
   A   
   C   
    A    
   G   
   G   
    C    
   T   
   T   
    A    
   G   
   A   
   A   
   C   
   G   
    C    
   G   
   A   
    C    
   A   
   A   
   A   
   A   
   T   
    T    
   G   
   A   
    A    
   A   
   A   
   T   
   G   
   A   
   A   
   T   
   A   
    T    
   C   
   A   
   A   
   G   
   A   
    G    
   C   
   T   
    A    
   T   
   T   
    G    
   A   
    A    
   A   
    T    
   T   
    C    
   A   
   T   
   T   
   G   
    C    
    G    
   G   
   A   
    C    
   T   
   T   
    A    
    G    
    A    
    G    
   G   
   A   
   C   
   A   
   T   
   T   
    T    
   T   
    A    
   G   
   C   
    C    
    C    
   G   
    C    
   C   
   C   
    A    
   G   
   A   
    A    
   C   
   G   
    C    
   G   
   T   
    C    
   A   
    T    
    T    
   G   
   A   
   A   
   A   
   T   
    C    
   A   
   T   
    T    
   A   
   A   
    A    
   A   
    C    
    A    
   G   
   A   
    G    
   T   
   T   
   A   
    A    
    A    
    T    
   G   
   A   
   C   
    G    
   T   
    T    
    C    
    G    
    C    
    A    
    C    
   A   
    A    
    A    
   A   
   T   
   T   
   T   
   G   
   G   
   T   
   G   
   A   
   T   
    G    
    C    
   A   
   C   
   G   
   A   
   C   
   G   
    T    
   A   
   C   
   A   
   G   
   A   
   A   
    C    
   T   
   A   
    T    
   T   
   A   
   G   
   T   
    A    
   G   
   G   
   T   
   G   
   A   
    A    
   G   
   T   
   C   
    C    
   T   
    A    
   A   
   G   
   T   
   C   
   T   
    T    
   G   
   A   
   A   
   G   
   A   
   T   
   G   
   A   
   A   
   G   
   A   
   T   
    T    
   T   
    A    
   A   
   T   
    C    
   G   
   A   
   A   
   G   
   A   
   A   
   G   
   A   
   A   
   G   
   A   
   A   
    G    
   T   
   C   
   G   
   T   
    G    
   A   
   T   
   T   
   A   
   C   
    A    
    T    
   T   
    A    
   A   
   C   
    C    
   A   
   A   
    T    
   A   
   A   
    C    
   G   
   G   
    C    
   T   
   A   
    C    
   A   
   T   
    T    
   A   
   A   
    G    
   C   
   G   
    G    
    A    
   T   
   G   
   G   
    C    
   A   
   A   
   A   
    C    
    T    
    C    
    C    
   G   
   A   
   A   
   T   
   T   
   C   
    C    
   G   
    G    
   G   
   C   
    G    
   C   
   A   
   A   
   C   
   G   
    C    
    C    
   G   
    C    
   G   
   G   
    C    
   G   
   G   
    A    
   C   
   G   
   T   
   G   
   G   
    T    
   G   
   T   
    C    
   C   
   A   
   A   
   G   
   G   
    G    
   A   
   T   
   G   
   G   
   G   
    C    
    G    
   T   
    T    
   C   
   A   
    T    
    G    
   A   
   T   
   G   
   A   
    T    
   G   
   A   
    T    
   T   
   T   
   C   
   G   
   T   
    G    
   A   
   A   
    A    
   A   
   A   
    C    
   C   
   T   
    A    
    G    
   T   
   T   
   T   
   C   
   T   
   T   
   G   
    T    
   T   
   C   
    A    
   A   
   C   
    A    
   C   
   A   
    C    
   G   
   A   
    C    
   A   
   C   
    G    
   T   
   T   
    A    
   T   
   T   
   A   
   T   
   T   
    C    
   T   
   T   
   T   
   A   
   C   
   G   
   A   
   A   
    C    
   A   
    C    
    T    
   G   
   G   
    G    
   A   
   A   
   A   
   G   
   T   
    T    
   T   
   A   
    C    
   C   
   G   
    A    
   G   
   C   
   A   
   A   
   A   
   A   
   G   
   G   
    T    
   T   
   A   
    C    
   G   
   A   
   A   
   A   
   T   
    C    
   C   
   C   
    T    
   G   
   A   
    A    
   T   
   A   
   C   
   G   
   G   
    T    
    A    
   G   
    A    
   A   
   C   
   A   
   G   
   C   
    A    
   A   
   A   
   A   
   G   
   G   
    A    
   A   
   T   
    T    
   C   
   C   
   A   
    G    
   T   
    A    
   A   
   T   
    T    
   A   
   A   
    C    
   T   
   T   
    A    
   C   
   T   
    G    
   G   
   G   
    A    
   A   
   T   
    T    
   G   
   A   
    T    
   T   
   C   
    T    
    G    
    C    
    A    
   G   
   A   
   A   
   A   
    A    
    A    
   A   
   T   
    T    
   C   
   A   
    A    
   G   
   C   
    G    
   A   
   T   
    T    
   A   
   T   
   T   
    T    
   C   
    T    
   G   
   T   
    T    
    G    
    A    
   G   
   G   
   G   
    C    
    A    
   A   
   A   
   G   
   C   
    G    
   G   
   A   
   A   
   G   
    C    
    A    
   G   
   G   
    T    
    A    
   A   
    A    
   T   
   A   
    C    
    T    
   T   
    G    
   T   
   T   
   C   
   T   
   T   
    C    
   A   
   C   
    A    
   A   
   C   
    C    
    T    
    T    
    A    
    A    
   A   
   A   
   G   
   G   
    A    
   A   
   C   
    T    
   G   
   T   
    C    
   A   
   A   
   A   
   C   
   G   
    G    
   A   
   C   
    A    
    G    
   C   
    C    
   G   
   T   
    A    
   A   
   C   
    A    
   G   
   C   
    C    
   T   
   T   
    T    
    T    
   C   
   T   
   A   
   A   
    T    
   A   
   T   
    C    
   C   
   G   
    T    
   A   
   G   
    T    
   A   
   A   
   T   
   G   
   G   
    A    
    T    
   T   
    A    
   A   
   T   
    C    
   G   
   C   
    C    
   A   
   T   
   T   
    A    
   G   
    C    
   T   
   T   
    A    
   A   
   A   
   A   
   G   
   A   
   A   
   G   
   A   
    T    
   G   
   A   
    T    
   G   
   A   
    G    
    T    
   T   
    A    
    G    
   T   
    T    
   A   
   A   
    C    
   G   
   T   
    A    
    G    
   T   
   A   
    A    
    C    
   G   
   A   
   C   
    T    
    A    
   A   
    T    
   G   
   G   
    C    
    A    
   A   
    T    
    C    
    A    
    G    
   A   
   A   
    G    
   A   
   T   
    G    
   A   
   T   
    T    
   A   
   T   
    C    
   G   
   G   
    A    
   A   
   C   
   A   
   C   
   A   
   T   
    G    
    C    
    A    
   G   
   G   
    A    
   T   
   A   
   C   
   T   
   C   
   T   
   G   
   T   
    C    
   A   
   C   
    A    
   T   
   T   
    T    
    G    
   A   
    T    
   G   
   A   
    A    
    A    
    A    
    T    
    A    
   C   
    T    
   G   
    T    
    A    
   C   
   G   
    T    
   G   
   A   
   T   
   A   
   T   
   G   
   G   
   G   
    C    
   C   
   G   
    G    
   A   
   C   
   A   
   G   
   C   
    A    
   T   
   C   
    A    
   G   
   G   
    C    
   G   
   T   
    T    
    C    
   G   
    T    
   G   
   G   
    A    
   A   
   T   
    C    
   C   
   G   
    T    
    C    
   T   
    C    
   C   
   G   
    C    
   G   
   A   
    A    
    A    
   A   
    T    
   G   
   A   
    T    
   T   
    A    
    T    
   G   
   T   
    G    
   G   
   T   
    C    
   G   
   G   
    C    
    G    
   C   
   A   
   G   
   C   
    G    
    A    
   T   
    T    
    C    
   T   
   G   
    G    
   A   
    T    
   G   
    A    
   A   
    A    
   A   
    T    
    A    
   A   
   A   
   G   
   A   
   A   
   G   
   T   
    C    
    C    
   T   
    A    
   G   
   T   
    C    
   A   
   T   
    T    
   A   
   C   
    T    
   G   
   A   
   A   
   A   
   A   
    T    
   G   
   G   
   T   
   T   
   A   
    T    
   G   
   G   
    T    
   A   
   A   
    G    
   C   
   G   
    T    
   A   
   C   
    A    
   A   
   A   
   A   
   G   
    C    
    T    
   T   
   C   
    T    
   G   
   A   
    A    
   T   
   A   
   T   
   C   
   C   
   A   
   G   
   T   
    T    
   A   
   A   
    A    
   G   
   G   
    A    
   C   
   G   
    T    
   G   
   G   
    C    
   G   
   G   
   T   
   A   
   A   
   A   
   G   
   G   
    G    
   A   
   T   
    T    
   A   
   A   
    G    
   A   
   C   
    A    
    G    
   C   
    A    
   A   
   A   
    T    
   A   
   T   
   C   
   A   
   C   
   T   
   G   
   A   
    G    
   A   
   A   
   A   
   A   
   A   
   T   
   G   
   G   
   T   
   C   
   C   
   A   
    T    
   T   
   A   
   G   
   C   
    T    
   G   
   G   
    T    
   T   
   T   
    A    
   A   
   C   
   C   
   A   
   C   
    G    
   G   
   T   
    C    
    A    
    A    
   T   
   G   
   G   
    G    
    G    
   A   
   T   
   G   
   A   
   A   
   G   
   A   
    T    
   A   
   T   
   C   
    T    
   T   
    A    
    T    
   T   
    G    
   A   
   T   
    T    
   A   
   C   
    G    
    A    
   A   
    C    
   A   
   A   
   A   
   G   
   G   
    C    
   G   
   T   
    C    
   A   
   T   
   T   
   A   
   T   
   C   
   C   
   G   
    C    
   T   
   T   
    T    
   A   
   A   
   C   
   G   
   T   
    T    
    G    
    A    
    T    
    T    
   C   
   T   
   G   
   T   
   T   
   T   
   C   
   T   
   C   
   A   
   A   
   A   
   C   
    A    
   G   
   G   
   A   
   C   
   G   
    C    
    G    
   C   
   A   
   A   
   C   
   A   
   T   
   T   
    A    
   G   
   G   
    G    
   G   
   T   
    T    
   C   
   G   
   T   
   T   
   T   
    A    
   A   
   T   
   G   
   A   
    G    
    A    
   A   
   T   
   G   
   G   
   A   
   A   
   G   
    A    
    T    
   G   
    G    
    T    
    G    
   C   
    C    
   A   
   A   
   A   
   -   
   -   
   -   
   -   
   -   
   -   
   -   
   -   
   -   
   -   
   -   
   -   
   -   
   -   
   -   
   -   
   -   
   -   
   -   
   -   
   -   
   -   
   -   
   -   
   -   
   -   
   -   
   -   
   -   
   -   
   -   
   -   
   -   
   -   
   -   
   -   
   -   
   -   
   -   
   -   
   -   
   -   
   -   
   -   
   -   
   -   
   -   
   -   
   -   
   -   
   -   
   -   
   -   
   -   
   -   
   -   
   -   
   -   
   -   
   -   
   -   
   -   
   -   
   -   
   -   
   -   
   -   
   -   
   -   
   -   
   -   
   -   
 

 
   N54595   
   A   
   T   
   G   
   A   
   G   
   T   
   G   
   A   
   A   
   G   
   A   
   A   
   A   
   T   
   T   
   A   
   A   
   A   
   G   
   A   
   A   
   A   
   A   
   C   
   A   
   T   
   T   
   C   
   A   
   A   
   G   
   A   
   C   
   G   
   T   
   C   
   A   
   A   
   T   
   C   
   T   
   G   
   A   
   C   
   C   
   A   
   G   
   C   
   G   
   A   
   A   
   A   
   T   
   G   
   A   
   A   
   A   
   G   
   A   
   A   
   T   
   C   
   T   
   T   
   T   
   C   
   A   
   T   
   T   
   G   
   A   
   C   
   T   
   A   
   C   
   G   
   C   
   A   
   A   
   T   
   G   
   A   
   G   
   T   
   G   
   T   
   T   
   A   
   T   
   C   
   G   
   T   
   A   
   G   
   C   
   C   
   C   
   G   
   C   
   G   
   C   
   G   
   T   
   T   
   A   
   C   
   C   
   T   
   G   
   A   
   C   
   G   
   T   
   T   
   C   
   G   
   T   
   G   
   A   
   C   
   G   
   G   
   T   
   T   
   T   
   A   
   A   
   A   
   A   
   C   
   C   
   T   
   G   
   T   
   T   
   C   
   A   
   T   
   C   
   G   
   C   
   C   
   G   
   A   
   A   
   T   
   C   
   T   
   T   
   A   
   T   
   A   
   T   
   G   
   G   
   A   
   A   
   T   
   G   
   A   
   A   
   C   
   G   
   A   
   A   
   T   
   T   
   A   
   G   
   G   
   G   
   G   
   T   
   A   
   A   
   C   
   C   
   C   
   C   
   T   
   G   
   A   
   T   
   A   
   A   
   A   
   C   
   C   
   A   
   C   
   A   
   C   
   A   
   A   
   A   
   A   
   A   
   A   
   T   
   C   
   A   
   G   
   C   
   C   
   C   
   G   
   G   
   A   
   T   
   T   
   G   
   T   
   T   
   G   
   G   
   G   
   G   
   A   
   T   
   G   
   T   
   T   
   A   
   T   
   G   
   G   
   G   
   T   
   A   
   A   
   A   
   T   
   A   
   T   
   C   
   A   
   C   
   C   
   C   
   C   
   C   
   A   
   T   
   G   
   G   
   G   
   G   
   A   
   C   
   A   
   G   
   T   
   G   
   C   
   G   
   A   
   T   
   T   
   T   
   A   
   C   
   G   
   A   
   A   
   T   
   C   
   A   
   A   
   T   
   G   
   G   
   T   
   G   
   C   
   G   
   G   
   A   
   T   
   G   
   G   
   C   
   A   
   C   
   A   
   A   
   C   
   C   
   T   
   T   
   T   
   T   
   A   
   G   
   T   
   T   
   A   
   T   
   C   
   G   
   G   
   G   
   C   
   T   
   A   
   T   
   G   
   T   
   T   
   A   
   G   
   T   
   T   
   G   
   A   
   C   
   G   
   G   
   C   
   C   
   A   
   C   
   G   
   G   
   A   
   A   
   A   
   C   
   T   
   T   
   C   
   G   
   G   
   T   
   T   
   C   
   C   
   G   
   T   
   C   
   G   
   A   
   T   
   G   
   G   
   T   
   G   
   A   
   C   
   G   
   G   
   C   
   G   
   C   
   T   
   G   
   C   
   C   
   G   
   C   
   T   
   A   
   T   
   G   
   C   
   G   
   T   
   T   
   A   
   T   
   A   
   C   
   C   
   G   
   A   
   A   
   G   
   C   
   A   
   C   
   G   
   T   
   A   
   T   
   G   
   A   
   G   
   T   
   A   
   A   
   A   
   A   
   T   
   T   
   G   
   C   
   T   
   T   
   T   
   A   
   G   
   A   
   A   
   A   
   T   
   G   
   C   
   T   
   A   
   C   
   G   
   A   
   G   
   A   
   T   
   A   
   T   
   T   
   A   
   A   
   C   
   A   
   A   
   A   
   A   
   A   
   T   
   A   
   C   
   A   
   G   
   T   
   C   
   G   
   A   
   T   
   T   
   T   
   C   
   C   
   A   
   A   
   G   
   G   
   A   
   A   
   A   
   C   
   T   
   A   
   T   
   G   
   A   
   T   
   G   
   A   
   T   
   T   
   C   
   A   
   G   
   A   
   A   
   C   
   A   
   A   
   G   
   A   
   A   
   C   
   C   
   A   
   G   
   T   
   A   
   G   
   T   
   T   
   T   
   T   
   A   
   C   
   C   
   T   
   G   
   C   
   T   
   C   
   G   
   T   
   T   
   T   
   T   
   C   
   C   
   A   
   A   
   A   
   C   
   T   
   T   
   A   
   C   
   T   
   A   
   G   
   T   
   T   
   A   
   A   
   C   
   G   
   G   
   A   
   A   
   C   
   A   
   A   
   C   
   G   
   G   
   G   
   G   
   A   
   T   
   T   
   G   
   C   
   G   
   G   
   T   
   G   
   G   
   G   
   G   
   A   
   T   
   G   
   G   
   C   
   A   
   A   
   C   
   A   
   A   
   A   
   T   
   A   
   T   
   T   
   C   
   C   
   A   
   C   
   C   
   A   
   C   
   A   
   C   
   A   
   A   
   T   
   T   
   T   
   A   
   A   
   G   
   T   
   G   
   A   
   A   
   G   
   T   
   G   
   A   
   T   
   T   
   G   
   A   
   T   
   G   
   C   
   A   
   A   
   C   
   A   
   A   
   G   
   T   
   T   
   T   
   A   
   T   
   T   
   G   
   A   
   T   
   G   
   G   
   A   
   C   
   A   
   A   
   T   
   C   
   C   
   T   
   G   
   A   
   T   
   G   
   T   
   A   
   A   
   C   
   G   
   A   
   C   
   G   
   A   
   A   
   T   
   G   
   A   
   A   
   T   
   T   
   G   
   A   
   T   
   G   
   G   
   A   
   A   
   G   
   T   
   G   
   T   
   T   
   A   
   C   
   C   
   T   
   G   
   G   
   A   
   C   
   C   
   A   
   G   
   A   
   T   
   T   
   T   
   T   
   C   
   C   
   A   
   A   
   C   
   A   
   G   
   G   
   C   
   G   
   G   
   T   
   T   
   T   
   A   
   G   
   T   
   G   
   A   
   T   
   G   
   G   
   G   
   G   
   A   
   A   
   A   
   T   
   C   
   A   
   G   
   G   
    G    
   A   
   T   
    T    
   C   
   G   
    C    
    C    
   G   
   A   
   G   
   C   
    A    
   T   
   A   
   T   
   G   
   A   
   A   
   A   
   C   
    A    
   G   
   G   
    G    
   A   
   A   
    A    
   G   
   G   
   T   
   T   
   C   
    G    
   A   
   T   
   T   
   A   
   C   
    T    
   G   
   T   
    T    
   C   
   G   
   T   
   G   
   C   
   A   
   A   
   A   
   A   
   G   
   T   
   T   
   G   
   A   
    A    
    T    
   T   
    G    
   A   
    C    
   T   
   G   
   A   
    A    
   A   
   T   
   G   
    C    
   C   
    G    
   A   
   A   
   T   
   G   
   G   
    A    
   A   
   A   
   A   
   G   
   A   
   A   
   C   
   G   
    T    
   A   
   T   
    T    
    T    
   T   
    A    
   G   
   T   
    A    
   A   
   C   
    T    
   G   
   A   
   A   
   T   
   T   
    G    
   C   
   C   
   T   
   T   
   A   
   T   
   A   
   T   
   G   
   G   
   T   
    G    
   A   
   A   
   T   
   A   
   A   
   A   
   G   
   C   
    C    
   A   
   A   
   A   
   T   
   T   
    A    
   A   
   T   
    C    
   G   
   A   
   A   
   C   
   G   
    A    
   A   
   T   
   T   
   T   
   C   
   T   
   G   
   A   
    A    
    T    
   T   
    A    
   C   
   A   
   C   
   A   
   G   
   A   
   G   
   A   
    T    
   A   
   A   
    A    
   C   
   G   
    A    
   A   
   T   
    T    
   G   
   A   
   A   
   G   
   G   
    A    
   A   
   T   
    T    
   A   
   C   
    T    
   G   
   A   
   T   
   T   
   T   
   G   
   C   
   G   
   G   
   G   
   A   
    T    
   G   
   A   
   A   
   T   
   C   
   T   
   T   
   C   
    T    
   C   
   G   
   T   
   G   
   A   
    A    
   G   
   G   
    C    
   A   
   T   
   G   
   C   
   G   
    G    
   A   
   T   
    T    
   G   
   T   
   C   
   A   
   T   
    C    
   G   
   A   
    T    
    G    
   T   
    T    
   C   
   G   
   T   
   C   
   G   
    A    
   G   
   A   
    T    
   G   
    T    
    G    
   A   
   G   
    T    
   G   
   C   
    C    
   T   
   C   
    T    
   G   
   T   
   T   
    G    
   T   
    G    
   T   
   T   
    A    
   A   
   A   
    C    
   A   
   A   
   C   
   T   
   T   
    G    
   T   
   A   
    C    
   A   
   A   
   A   
    A    
   T   
   G   
   A   
   C   
    T    
    G    
   C   
    C    
   T   
   T   
    A    
   C   
   A   
    A    
   A   
    C    
    A    
   T   
   C   
    T    
   T   
   T   
    T    
   G   
   G   
    T    
   T   
   T   
    T    
   A   
   A   
    C    
   A   
   T   
   G   
   T   
   T   
    A    
   G   
   C   
    C    
   A   
   T   
    C    
   G   
   A   
   A   
   A   
   A   
   A   
   G   
   G   
    C    
   G   
   T   
    A    
   C   
   C   
    G    
   A   
   A   
   A   
    A    
   T   
   T   
   T   
   T   
    A    
   A   
   G   
    C    
   T   
   T   
   G   
   A   
   A   
   A   
   C   
    G    
    T    
   A   
   T   
    T    
    T    
   T   
    A    
   G   
   A   
   A   
   A   
   A   
    T    
   T   
   A   
    C    
    G    
   T   
    C    
   G   
   A   
    G    
   C   
   A   
    C    
   C   
   A   
    A    
    A    
    A    
    A    
    G    
   A   
   A   
   G   
   T   
   C   
   A   
   T   
    T    
   A   
   C   
    T    
    C    
   G   
    C    
   C   
   G   
   G   
   A   
   C   
    G    
    A    
   T   
    T    
   T   
   T   
   T   
   G   
   A   
    C    
   A   
   A   
   A   
    A    
   A   
    C    
   A   
   A   
    A    
   G   
   C   
   A   
   G   
   A   
   A   
   G   
   C   
   A   
    C    
   G   
    G    
   G   
   C   
    G    
   C   
   A   
    T    
   A   
   T   
    T    
   T   
   T   
   A   
   G   
   A   
   A   
   G   
   G   
   T   
    C    
   T   
    A    
   C   
   G   
    A    
   A   
   T   
   T   
   G   
   C   
    C    
   T   
   T   
    A    
   G   
   A   
   T   
   C   
   A   
   T   
   A   
   T   
   C   
    G    
   A   
   T   
   G   
   A   
   A   
   A   
   T   
    C    
   A   
   T   
   T   
   G   
   C   
    C    
   A   
   T   
   T   
   A   
   T   
    C    
   C   
   G   
    T    
   G   
   G   
    A    
   T   
   C   
    A    
    C    
   A   
   A   
   T   
   C   
   A   
   G   
   A   
   T   
   G   
   A   
    C    
   G   
   A   
    A    
   G   
   C   
   A   
   A   
   A   
   A   
   G   
   C   
    A    
   A   
    C    
    G    
    T    
   T   
    A    
   A   
   T   
    T    
   G   
   A   
   A   
   C   
   G   
    C    
   T   
   T   
    T    
   G   
   A   
    A    
   T   
   T   
    T    
   T   
   C   
    A    
   G   
   A   
   T   
   C   
   G   
    T    
   C   
   A   
    A    
    G    
   C   
    G    
   C   
   A   
   A   
   G   
   C   
    A    
   A   
   T   
    T    
   T   
   T   
   A   
   G   
   A   
   T   
   A   
   T   
   G   
   C   
   G   
   T   
   T   
   T   
    A    
   C   
   G   
    C    
   C   
   G   
    T    
   T   
   T   
    A    
   A   
   C   
    A    
   G   
   G   
    C    
   T   
   T   
    A    
   G   
   A   
   A   
   C   
   G   
    C    
   G   
   A   
    C    
   A   
   A   
   A   
   A   
   T   
    T    
   G   
   A   
    A    
   A   
   A   
   T   
   G   
   A   
   A   
   T   
   A   
    T    
   C   
   A   
   A   
   G   
   A   
    G    
   C   
   T   
    A    
   T   
   T   
    G    
   A   
    A    
   A   
    T    
   T   
    C    
   A   
   T   
   T   
   G   
    C    
    G    
   G   
   A   
    C    
   T   
   T   
    A    
    G    
    A    
    G    
   G   
   A   
   C   
   A   
   T   
   T   
    T    
   T   
    A    
   G   
   C   
    C    
    C    
   G   
    C    
   C   
   C   
    A    
   G   
   A   
    A    
   C   
   G   
    C    
   G   
   T   
    C    
   A   
    T    
    T    
   G   
   A   
   A   
   A   
   T   
    C    
   A   
   T   
    T    
   A   
   A   
    A    
   A   
    C    
    A    
   G   
   A   
    G    
   T   
   T   
   A   
    A    
    A    
    T    
   G   
   A   
   C   
    G    
   T   
    T    
    C    
    G    
    C    
    A    
    C    
   A   
    A    
    A    
   A   
   T   
   T   
   T   
   G   
   G   
   T   
   G   
   A   
   T   
    G    
    C    
   A   
   C   
   G   
   A   
   C   
   G   
    C    
   A   
   C   
   A   
   G   
   A   
   A   
    C    
   T   
   A   
    T    
   T   
   A   
   G   
   T   
    A    
   G   
   G   
   T   
   G   
   A   
    A    
   G   
   T   
   C   
    C    
   T   
    C    
   A   
   G   
   T   
   C   
   T   
    T    
   G   
   A   
   A   
   G   
   A   
   T   
   G   
   A   
   A   
   G   
   A   
   T   
    T    
   T   
    A    
   A   
   T   
    C    
   G   
   A   
   A   
   G   
   A   
   A   
   G   
   A   
   A   
   G   
   A   
   A   
    G    
   T   
   C   
   G   
   T   
    G    
   A   
   T   
   T   
   A   
   C   
    A    
    T    
   T   
    A    
   A   
   C   
    C    
   A   
   A   
    T    
   A   
   A   
    C    
   G   
   G   
    C    
   T   
   A   
    C    
   A   
   T   
    T    
   A   
   A   
    G    
   C   
   G   
    G    
    A    
   T   
   G   
   G   
    C    
   A   
   A   
   A   
    C    
    T    
    C    
    C    
   G   
   A   
   A   
   T   
   T   
   C   
    C    
   G   
    G    
   G   
   C   
    G    
   C   
   A   
   A   
   C   
   G   
    C    
    C    
   G   
    C    
   G   
   G   
    C    
   G   
   G   
    A    
   C   
   G   
   T   
   G   
   G   
    T    
   G   
   T   
    C    
   C   
   A   
   A   
   G   
   G   
    G    
   A   
   T   
   G   
   G   
   G   
    C    
    G    
   T   
    T    
   C   
   A   
    C    
    G    
   A   
   T   
   G   
   A   
    T    
   G   
   A   
    T    
   T   
   T   
   C   
   G   
   T   
    G    
   A   
   A   
    A    
   A   
   A   
    C    
   C   
   T   
    A    
    G    
   T   
   T   
   T   
   C   
   T   
   T   
   G   
    T    
   T   
   C   
    A    
   A   
   C   
    A    
   C   
   A   
    T    
   G   
   A   
    C    
   A   
   C   
    G    
   T   
   T   
    A    
   T   
   T   
   A   
   T   
   T   
    C    
   T   
   T   
   T   
   A   
   C   
   G   
   A   
   A   
    C    
   A   
    C    
    T    
   G   
   G   
    G    
   A   
   A   
   A   
   G   
   T   
    T    
   T   
   A   
    C    
   C   
   G   
    A    
   G   
   C   
   A   
   A   
   A   
   A   
   G   
   G   
    T    
   T   
   A   
    C    
   G   
   A   
   A   
   A   
   T   
    C    
   C   
   C   
    T    
   G   
   A   
    A    
   T   
   A   
   C   
   G   
   G   
    T    
    A    
   G   
    A    
   A   
   C   
   A   
   G   
   C   
    A    
   A   
   A   
   A   
   G   
   G   
    A    
   A   
   T   
    T    
   C   
   C   
   A   
    G    
   T   
    G    
   A   
   T   
    T    
   A   
   A   
    C    
   T   
   T   
    A    
   C   
   T   
    G    
   G   
   G   
    A    
   A   
   T   
    T    
   G   
   A   
    T    
   T   
   C   
    T    
    G    
    C    
    A    
   G   
   A   
   A   
   A   
    A    
    A    
   A   
   T   
    T    
   C   
   A   
    A    
   G   
   C   
    G    
   A   
   T   
    T    
   A   
   T   
   T   
    T    
   C   
    T    
   G   
   T   
    T    
    G    
    A    
   G   
   G   
   G   
    C    
    A    
   A   
   A   
   G   
   C   
    G    
   G   
   A   
   A   
   G   
    C    
    A    
   G   
   G   
    T    
    A    
   A   
    A    
   T   
   A   
    C    
    T    
   T   
    G    
   T   
   T   
   C   
   T   
   T   
    C    
   A   
   C   
    A    
   A   
   C   
    C    
    T    
    T    
    A    
    A    
   A   
   A   
   G   
   G   
    A    
   A   
   C   
    C    
   G   
   T   
    C    
   A   
   A   
   A   
   C   
   G   
    G    
   A   
   C   
    A    
    G    
   C   
    C    
   G   
   T   
    A    
   A   
   C   
    A    
   G   
   C   
    C    
   T   
   T   
    T    
    T    
   C   
   T   
   A   
   A   
    T    
   A   
   T   
    C    
   C   
   G   
    T    
   A   
   G   
    T    
   A   
   A   
   T   
   G   
   G   
    A    
    T    
   T   
    A    
   A   
   T   
    C    
   G   
   C   
    C    
   A   
   T   
   T   
    A    
   G   
    C    
   T   
   T   
    A    
   A   
   A   
   A   
   G   
   A   
   A   
   G   
   A   
    T    
   G   
   A   
    T    
   G   
   A   
    G    
    T    
   T   
    A    
    G    
   T   
    T    
   A   
   A   
    C    
   G   
   T   
    G    
    G    
   T   
   A   
    A    
    C    
   G   
   A   
   C   
    T    
    A    
   A   
    T    
   G   
   G   
    C    
    A    
   A   
    T    
    C    
    A    
    G    
   A   
   A   
    G    
   A   
   T   
    G    
   A   
   T   
    T    
   A   
   T   
    C    
   G   
   G   
    A    
   A   
   C   
   A   
   C   
   A   
   T   
    G    
    C    
    A    
   G   
   G   
    A    
   T   
   A   
   C   
   T   
   C   
   T   
   G   
   T   
    C    
   A   
   C   
    A    
   T   
   T   
    T    
    G    
   A   
    T    
   G   
   A   
    A    
    A    
    A    
    T    
    A    
   C   
    T    
   G   
    T    
    A    
   C   
   G   
    T    
   G   
   A   
   T   
   A   
   T   
   G   
   G   
   G   
    C    
   C   
   G   
    G    
   A   
   C   
   A   
   G   
   C   
    A    
   T   
   C   
    A    
   G   
   G   
    C    
   G   
   T   
    T    
    C    
   G   
    G    
   G   
   G   
    A    
   A   
   T   
    C    
   C   
   G   
    T    
    C    
   T   
    C    
   C   
   G   
    C    
   G   
   A   
    A    
    A    
   A   
    T    
   G   
   A   
    T    
   T   
    A    
    T    
   G   
   T   
    G    
   G   
   T   
    C    
   G   
   G   
    T    
    G    
   C   
   A   
   G   
   C   
    G    
    A    
   T   
    T    
    C    
   T   
   G   
    G    
   A   
    T    
   G   
    A    
   A   
    A    
   A   
    T    
    A    
   A   
   A   
   G   
   A   
   A   
   G   
   T   
    C    
    C    
   T   
    A    
   G   
   T   
    C    
   A   
   T   
    T    
   A   
   C   
    T    
   G   
   A   
   A   
   A   
   A   
    T    
   G   
   G   
   T   
   T   
   A   
    T    
   G   
   G   
    T    
   A   
   A   
    G    
   C   
   G   
    T    
   A   
   C   
    A    
   A   
   A   
   A   
   G   
    C    
    T    
   T   
   C   
    T    
   G   
   A   
    A    
   T   
   A   
   T   
   C   
   C   
   A   
   G   
   T   
    T    
   A   
   A   
    A    
   G   
   G   
    A    
   C   
   G   
    T    
   G   
   G   
    C    
   G   
   G   
   T   
   A   
   A   
   A   
   G   
   G   
    G    
   A   
   T   
    T    
   A   
   A   
    G    
   A   
   C   
    A    
    G    
   C   
    A    
   A   
   A   
    T    
   A   
   T   
   C   
   A   
   C   
   T   
   G   
   A   
    G    
   A   
   A   
   A   
   A   
   A   
   T   
   G   
   G   
   T   
   C   
   C   
   A   
    T    
   T   
   A   
   G   
   C   
    T    
   G   
   G   
    T    
   T   
   T   
    A    
   A   
   C   
   C   
   A   
   C   
    G    
   G   
   T   
    C    
    A    
    A    
   T   
   G   
   G   
    G    
    G    
   A   
   T   
   G   
   A   
   A   
   G   
   A   
    T    
   A   
   T   
   C   
    T    
   T   
    A    
    T    
   T   
    G    
   A   
   T   
    T    
   A   
   C   
    G    
    A    
   A   
    C    
   A   
   A   
   A   
   G   
   G   
    C    
   G   
   T   
    C    
   A   
   T   
   T   
   A   
   T   
   C   
   C   
   G   
    C    
   T   
   T   
    T    
   A   
   A   
   C   
   G   
   T   
    T    
    G    
    A    
    T    
    T    
   C   
   T   
   G   
   T   
   T   
   T   
   C   
   T   
   C   
   A   
   A   
   A   
   C   
    A    
   G   
   G   
   A   
   C   
   G   
    C    
    G    
   C   
   A   
   A   
   C   
   A   
   T   
   T   
    A    
   G   
   G   
    A    
   G   
   T   
    T    
   C   
   G   
   T   
   T   
   T   
    A    
   A   
   T   
   G   
   A   
    G    
    A    
   A   
   T   
   G   
   G   
   A   
   A   
   G   
    A    
    T    
   G   
    G    
    T    
    G    
   C   
    C    
   A   
   A   
   A   
   -   
   -   
   -   
   -   
   -   
   -   
   -   
   -   
   -   
   -   
   -   
   -   
   -   
   -   
   -   
   -   
   -   
   -   
   -   
   -   
   -   
   -   
   -   
   -   
   -   
   -   
   -   
   -   
   -   
   -   
   -   
   -   
   -   
   -   
   -   
   -   
   -   
   -   
   -   
   -   
   -   
   -   
   -   
   -   
   -   
   -   
   -   
   -   
   -   
   -   
   -   
   -   
   -   
   -   
   -   
   -   
   -   
   -   
   -   
   -   
   -   
   -   
   -   
   -   
   -   
   -   
   -   
   -   
   -   
   -   
   -   
   -   
 

 
   N59624F   
   A   
   T   
   G   
   A   
   G   
   T   
   G   
   A   
   A   
   G   
   A   
   A   
   A   
   T   
   C   
   A   
   G   
   A   
   G   
   A   
   A   
   A   
   A   
   C   
   A   
   T   
   C   
   C   
   A   
   T   
   G   
   A   
   T   
   G   
   T   
   C   
   A   
   A   
   T   
   C   
   T   
   A   
   A   
   C   
   C   
   A   
   G   
   C   
   G   
   A   
   A   
   A   
   T   
   G   
   A   
   A   
   A   
   G   
   A   
   C   
   T   
   C   
   C   
   T   
   T   
   C   
   A   
   T   
   C   
   G   
   A   
   T   
   T   
   A   
   T   
   G   
   C   
   G   
   A   
   T   
   G   
   A   
   G   
   T   
   G   
   T   
   T   
   A   
   T   
   T   
   G   
   T   
   C   
   G   
   C   
   A   
   C   
   G   
   T   
   G   
   C   
   A   
   C   
   T   
   G   
   C   
   C   
   T   
   G   
   A   
   T   
   G   
   T   
   C   
   A   
   G   
   A   
   G   
   A   
   T   
   G   
   G   
   T   
   C   
   T   
   G   
   A   
   A   
   A   
   C   
   C   
   C   
   G   
   T   
   T   
   C   
   A   
   T   
   C   
   G   
   C   
   C   
   G   
   T   
   A   
   T   
   T   
   T   
   T   
   A   
   T   
   A   
   C   
   G   
   G   
   C   
   A   
   T   
   G   
   A   
   A   
   C   
   G   
   A   
   A   
   T   
   T   
   A   
   G   
   G   
   T   
   G   
   T   
   G   
   A   
   C   
   A   
   C   
   C   
   G   
   G   
   A   
   T   
   A   
   A   
   A   
   G   
   C   
   C   
   C   
   A   
   T   
   A   
   A   
   A   
   A   
   A   
   A   
   T   
   C   
   T   
   G   
   C   
   C   
   A   
   G   
   A   
   A   
   T   
   C   
   G   
   T   
   C   
   G   
   G   
   A   
   G   
   A   
   T   
   G   
   T   
   C   
   A   
   T   
   G   
   G   
   G   
   T   
   A   
   A   
   A   
   T   
   A   
   C   
   C   
   A   
   T   
   C   
   C   
   T   
   C   
   A   
   T   
   G   
   G   
   G   
   G   
   A   
   T   
   A   
   G   
   T   
   G   
   C   
   G   
   A   
   T   
   C   
   T   
   A   
   T   
   G   
   A   
   G   
   T   
   C   
   A   
   A   
   T   
   G   
   G   
   T   
   A   
   C   
   G   
   G   
   A   
   T   
   G   
   G   
   C   
   G   
   C   
   A   
   G   
   C   
   C   
   A   
   T   
   T   
   T   
   A   
   G   
   C   
   T   
   A   
   C   
   C   
   G   
   T   
   T   
   A   
   T   
   A   
   T   
   G   
   C   
   T   
   A   
   G   
   T   
   G   
   G   
   A   
   T   
   G   
   G   
   C   
   C   
   A   
   T   
   G   
   G   
   A   
   A   
   A   
   C   
   T   
   T   
   T   
   G   
   G   
   T   
   T   
   C   
   T   
   G   
   T   
   T   
   G   
   A   
   C   
   G   
   G   
   T   
   G   
   A   
   C   
   G   
   G   
   T   
   G   
   C   
   C   
   G   
   C   
   T   
   G   
   C   
   T   
   A   
   T   
   G   
   C   
   G   
   T   
   T   
   A   
   T   
   A   
   C   
   C   
   G   
   A   
   A   
   G   
   C   
   A   
   C   
   G   
   G   
   A   
   T   
   G   
   A   
   G   
   T   
   A   
   A   
   A   
   A   
   T   
   C   
   G   
   C   
   T   
   A   
   C   
   A   
   G   
   A   
   A   
   A   
   T   
   G   
   C   
   T   
   G   
   C   
   G   
   T   
   G   
   A   
   T   
   A   
   T   
   C   
   A   
   A   
   T   
   A   
   A   
   G   
   A   
   A   
   C   
   A   
   C   
   T   
   G   
   T   
   T   
   G   
   A   
   T   
   T   
   T   
   C   
   C   
   A   
   A   
   A   
   G   
   C   
   A   
   A   
   C   
   T   
   A   
   T   
   G   
   A   
   T   
   G   
   A   
   C   
   A   
   C   
   A   
   G   
   A   
   A   
   A   
   A   
   A   
   G   
   A   
   A   
   C   
   C   
   C   
   G   
   T   
   T   
   G   
   T   
   A   
   T   
   T   
   A   
   C   
   C   
   T   
   G   
   C   
   T   
   C   
   G   
   C   
   T   
   T   
   C   
   C   
   C   
   C   
   A   
   A   
   T   
   C   
   T   
   T   
   T   
   T   
   G   
   G   
   T   
   G   
   A   
   A   
   C   
   G   
   G   
   A   
   A   
   C   
   G   
   A   
   C   
   G   
   G   
   G   
   G   
   A   
   T   
   A   
   G   
   C   
   C   
   G   
   T   
   G   
   G   
   G   
   T   
   A   
   T   
   G   
   G   
   C   
   A   
   A   
   C   
   G   
   A   
   A   
   T   
   A   
   T   
   C   
   C   
   C   
   A   
   C   
   C   
   T   
   C   
   A   
   T   
   A   
   A   
   T   
   T   
   T   
   A   
   T   
   C   
   T   
   G   
   A   
   G   
   G   
   T   
   A   
   G   
   T   
   A   
   G   
   C   
   G   
   G   
   C   
   T   
   A   
   T   
   C   
   G   
   A   
   T   
   T   
   T   
   G   
   C   
   T   
   G   
   A   
   T   
   G   
   G   
   A   
   T   
   A   
   A   
   T   
   C   
   C   
   T   
   G   
   A   
   T   
   G   
   T   
   G   
   A   
   C   
   G   
   A   
   C   
   C   
   A   
   A   
   T   
   G   
   A   
   A   
   T   
   T   
   G   
   A   
   T   
   G   
   G   
   A   
   A   
   G   
   T   
   A   
   T   
   T   
   A   
   C   
   C   
   T   
   G   
   G   
   A   
   C   
   C   
   A   
   G   
   A   
   T   
   T   
   T   
   T   
   C   
   C   
   A   
   A   
   C   
   A   
   G   
   G   
   T   
   G   
   G   
   T   
   T   
   T   
   A   
   G   
   T   
   C   
   A   
   T   
   G   
   G   
   G   
   A   
   A   
   A   
   A   
   T   
   C   
   A   
   G   
   G   
    A    
   A   
   T   
    C    
   C   
   G   
    A    
    A    
   G   
   A   
   G   
   C   
    G    
   T   
   A   
   T   
   G   
   A   
   A   
   A   
   C   
    A    
   G   
   G   
    T    
   A   
   A   
    A    
   G   
   G   
   T   
   T   
   C   
    T    
   A   
   T   
   T   
   A   
   C   
    C    
   G   
   T   
    A    
   C   
   G   
   T   
   G   
   C   
   A   
   A   
   A   
   A   
   G   
   T   
   T   
   G   
   A   
    G    
    A    
   T   
    C    
   A   
    C    
   T   
   G   
   A   
    G    
   A   
   T   
   G   
    C    
   C   
    A    
   A   
   A   
   T   
   G   
   G   
    C    
   A   
   A   
   A   
   G   
   A   
   A   
   C   
   G   
    G    
   A   
   T   
    C    
    C    
   T   
    C    
   G   
   T   
    G    
   A   
   C   
    G    
   G   
   A   
   A   
   T   
   T   
    A    
   C   
   C   
   T   
   T   
   A   
   T   
   A   
   T   
   G   
   G   
   T   
    C    
   A   
   A   
   T   
   A   
   A   
   A   
   G   
   C   
    C    
   A   
   A   
   A   
   T   
   T   
    G    
   A   
   T   
    T    
   G   
   A   
   A   
   C   
   G   
    G    
   A   
   T   
   T   
   T   
   C   
   T   
   G   
   A   
    G    
    T    
   T   
    G    
   C   
   A   
   C   
   A   
   G   
   A   
   G   
   A   
    C    
   A   
   A   
    G    
   C   
   G   
    T    
   A   
   T   
    C    
   G   
   A   
   A   
   G   
   G   
    A    
   A   
   T   
    C    
   A   
   C   
    G    
   G   
   A   
   T   
   T   
   T   
   G   
   C   
   G   
   G   
   G   
   A   
    T    
   G   
   A   
   A   
   T   
   C   
   T   
   T   
   C   
    C    
   C   
   G   
   T   
   G   
   A   
    A    
   G   
   G   
    C    
   A   
   T   
   G   
   C   
   G   
    A    
   A   
   T   
    T    
   G   
   T   
   C   
   A   
   T   
    T    
   G   
   A   
    C    
    A    
   T   
    T    
   C   
   G   
   T   
   C   
   G   
    T    
   G   
   A   
    C    
   G   
    C    
    C    
   A   
   G   
    C    
   G   
   C   
    A    
   T   
   C   
    A    
   G   
   T   
   T   
    A    
   T   
    T    
   T   
   T   
    G    
   A   
   A   
    T    
   A   
   A   
   C   
   T   
   T   
    A    
   T   
   A   
    C    
   A   
   A   
   A   
    T    
   T   
   G   
   A   
   C   
    A    
    T    
   C   
    C    
   T   
   T   
    A    
   C   
   A   
    G    
   A   
    A    
    T    
   T   
   C   
    A    
   T   
   T   
    T    
   G   
   G   
    A    
   T   
   T   
    C    
   A   
   A   
    T    
   A   
   T   
   G   
   T   
   T   
    G    
   G   
   C   
    C    
   A   
   T   
    C    
   G   
   A   
   A   
   A   
   A   
   A   
   G   
   G   
    C    
   G   
   T   
    G    
   C   
   C   
    G    
   A   
   A   
   A   
    G    
   T   
   T   
   T   
   T   
    A    
   A   
   G   
    T    
   T   
   T   
   G   
   A   
   A   
   A   
   C   
    A    
    A    
   A   
   T   
    C    
    C    
   T   
    T    
   G   
   A   
   A   
   A   
   A   
    C    
   T   
   A   
    T    
    A    
   T   
    C    
   G   
   A   
    A    
   C   
   A   
    T    
   C   
   A   
    A    
    C    
    G    
    C    
    G    
   A   
   A   
   G   
   T   
   C   
   A   
   T   
    C    
   A   
   C   
    A    
    C    
   G   
    A    
   C   
   G   
   G   
   A   
   C   
    G    
    G    
   T   
    C    
   T   
   T   
   T   
   G   
   A   
    A    
   A   
   A   
   A   
    G    
   A   
    A    
   A   
   A   
    A    
   G   
   C   
   A   
   G   
   A   
   A   
   G   
   C   
   A   
    A    
   G   
    A    
   G   
   C   
    A    
   C   
   A   
    C    
   A   
   T   
    C    
   T   
   T   
   A   
   G   
   A   
   A   
   G   
   G   
   T   
    T    
   T   
    G    
   C   
   G   
    T    
   A   
   T   
   T   
   G   
   C   
    T    
   T   
   T   
    G    
   G   
   A   
   T   
   C   
   A   
   T   
   A   
   T   
   C   
    G    
   A   
   T   
   G   
   A   
   A   
   A   
   T   
    C    
   A   
   T   
   T   
   G   
   C   
    G    
   A   
   T   
   T   
   A   
   T   
    C    
   C   
   G   
    C    
   G   
   G   
    G    
   T   
   C   
    T    
    A    
   A   
   A   
   T   
   C   
   A   
   G   
   A   
   T   
   G   
   A   
    T    
   G   
   A   
    A    
   G   
   C   
   A   
   A   
   A   
   A   
   G   
   C   
    A    
   A   
    C    
    A    
    A    
   T   
    G    
   A   
   T   
    C    
   G   
   A   
   A   
   C   
   G   
    G    
   T   
   T   
    C    
   G   
   A   
    T    
   T   
   T   
    A    
   T   
   C   
    C    
   G   
   A   
   T   
   C   
   G   
    C    
   C   
   A   
    A    
    T    
   C   
    A    
   C   
   A   
   A   
   G   
   C   
    T    
   A   
   T   
    C    
   T   
   T   
   A   
   G   
   A   
   T   
   A   
   T   
   G   
   C   
   G   
   T   
   T   
   T   
    G    
   C   
   G   
    T    
   C   
   G   
    A    
   T   
   T   
    G    
   A   
   C   
    C    
   G   
   G   
    T    
   T   
   T   
    G    
   G   
   A   
   A   
   C   
   G   
    G    
   G   
   A   
    A    
   A   
   A   
   A   
   A   
   T   
    C    
   G   
   A   
    A    
   A   
   A   
   T   
   G   
   A   
   A   
   T   
   A   
    T    
   C   
   A   
   A   
   G   
   A   
    A    
   C   
   T   
    A    
   T   
   T   
    G    
   A   
    A    
   A   
    C    
   T   
    G    
   A   
   T   
   T   
   G   
    A    
    A    
   G   
   A   
    T    
   T   
   T   
    G    
    A    
    C    
    T    
   G   
   A   
   C   
   A   
   T   
   T   
    C    
   T   
    G    
   G   
   C   
    A    
    A    
   G   
    A    
   C   
   C   
    C    
   G   
   A   
    A    
   C   
   G   
    T    
   G   
   T   
    C    
   A   
    C    
    G    
   G   
   A   
   A   
   A   
   T   
    C    
   A   
   T   
    C    
   A   
   A   
    G    
   A   
    A    
    T    
   G   
   A   
    G    
   T   
   T   
   A   
    G    
    C    
    A    
   G   
   A   
   C   
    T    
   T   
    G    
    A    
    A    
    T    
    C    
    A    
   A   
    C    
    G    
   A   
   T   
   T   
   T   
   G   
   G   
   T   
   G   
   A   
   T   
    A    
    A    
   A   
   C   
   G   
   A   
   C   
   G   
    C    
   A   
   C   
   A   
   G   
   A   
   A   
    T    
   T   
   A   
    C    
   T   
   A   
   G   
   T   
    C    
   G   
   G   
   T   
   G   
   A   
    G    
   G   
   T   
   C   
    T    
   T   
    A    
   A   
   G   
   T   
   C   
   T   
    A    
   G   
   A   
   A   
   G   
   A   
   T   
   G   
   A   
   A   
   G   
   A   
   T   
    T    
   T   
    G    
   A   
   T   
    C    
   G   
   A   
   A   
   G   
   A   
   A   
   G   
   A   
   A   
   G   
   A   
   A   
    A    
   T   
   C   
   G   
   T   
    T    
   A   
   T   
   T   
   A   
   C   
    A    
    C    
   T   
    G    
   A   
   C   
    A    
   A   
   A   
    C    
   A   
   A   
    T    
   G   
   G   
    T    
   T   
   A   
    T    
   A   
   T   
    C    
   A   
   A   
    A    
   C   
   G   
    T    
    T    
   T   
   G   
   G   
    C    
   A   
   A   
   A   
    T    
    A    
    A    
    T    
   G   
   A   
   A   
   T   
   T   
   C   
    A    
   G   
    A    
   G   
   C   
    T    
   C   
   A   
   A   
   C   
   G   
    A    
    A    
   G   
    A    
   G   
   G   
    C    
   G   
   G   
    G    
   C   
   G   
   T   
   G   
   G   
    T    
   G   
   T   
    C    
   C   
   A   
   A   
   G   
   G   
    A    
   A   
   T   
   G   
   G   
   G   
    T    
    A    
   T   
    C    
   C   
   A   
    C    
    A    
   A   
   T   
   G   
   A   
    C    
   G   
   A   
    C    
   T   
   T   
   C   
   G   
   T   
    C    
   A   
   A   
    G    
   A   
   A   
    T    
   C   
   T   
    A    
    G    
   T   
   T   
   T   
   C   
   T   
   T   
   G   
    T    
   T   
   C   
    T    
   A   
   C   
    C    
   C   
   A   
    T    
   G   
   A   
    T    
   A   
   C   
    A    
   T   
   T   
    G    
   T   
   T   
   A   
   T   
   T   
    C    
   T   
   T   
   T   
   A   
   C   
   G   
   A   
   A   
    T    
   A   
    A    
    C    
   G   
   G   
    A    
   A   
   A   
   A   
   G   
   T   
    G    
   T   
   A   
    T    
   C   
   G   
    G    
   G   
   C   
   A   
   A   
   A   
   A   
   G   
   G   
    A    
   T   
   A   
    C    
   G   
   A   
   A   
   A   
   T   
    T    
   C   
   C   
    A    
   G   
   A   
    A    
   T   
   A   
   C   
   G   
   G   
    A    
    C    
   G   
    T    
   A   
   C   
   A   
   G   
   C   
    C    
   A   
   A   
   A   
   G   
   G   
    T    
   A   
   T   
    C    
   C   
   C   
   A   
    A    
   T   
    C    
   A   
   T   
    C    
   A   
   A   
    T    
   T   
   T   
    G    
   C   
   T   
    G    
   G   
   G   
    T    
   A   
   T   
    C    
   G   
   A   
    C    
   T   
   C   
    G    
    A    
    G    
    T    
   G   
   A   
   A   
   A   
    G    
    C    
   A   
   T   
    C    
   C   
   A   
    A    
   G   
   C   
    A    
   A   
   T   
    T    
   A   
   T   
   T   
    G    
   C   
    G    
   G   
   T   
    G    
    A    
    C    
   G   
   G   
   G   
    A    
    G    
   A   
   A   
   G   
   C   
    A    
   G   
   A   
   A   
   G   
    A    
    A    
   G   
   G   
    C    
    C    
   A   
    C    
   T   
   A   
    C    
    C    
   T   
    C    
   T   
   T   
   C   
   T   
   T   
    T    
   A   
   C   
    G    
   A   
   C   
    G    
    C    
    G    
    T    
    C    
   A   
   A   
   G   
   G   
    T    
   A   
   C   
    A    
   G   
   T   
    T    
   A   
   A   
   A   
   C   
   G   
    T    
   A   
   C   
    G    
    T    
   C   
    T    
   G   
   T   
    C    
   A   
   C   
    T    
   G   
   C   
    T    
   T   
   T   
    C    
    G    
   C   
   T   
   A   
   A   
    C    
   A   
   T   
    C    
   C   
   G   
    A    
   A   
   G   
    C    
   A   
   A   
   T   
   G   
   G   
    T    
    C    
   T   
    G    
   A   
   T   
    T    
   G   
   C   
    A    
   A   
   T   
   T   
    G    
   G   
    T    
   T   
   T   
    G    
   A   
   A   
   A   
   G   
   A   
   A   
   G   
   A   
    T    
   G   
   A   
    C    
   G   
   A   
    A    
    T    
   T   
    A    
    G    
   T   
    C    
   A   
   A   
    T    
   G   
   T   
    T    
    C    
   T   
   A   
    T    
    T    
   G   
   A   
   C   
    A    
    G    
   A   
    T    
   G   
   G   
    A    
    C    
   A   
    G    
    T    
    C    
    A    
   A   
   A   
    T    
   A   
   T   
    C    
   A   
   T   
    C    
   A   
   T   
    T    
   G   
   G   
    C    
   A   
   C   
   A   
   C   
   A   
   T   
    A    
    A    
    C    
   G   
   G   
    C    
   T   
   A   
   C   
   T   
   C   
   T   
   G   
   T   
    G    
   A   
   C   
    C    
   T   
   T   
    C    
    A    
   A   
    A    
   G   
   A   
    G    
    G    
    A    
    A    
    G    
   C   
    A    
   G   
    T    
    C    
   C   
   G   
    C    
   G   
   A   
   T   
   A   
   T   
   G   
   G   
   G   
    A    
   C   
   G   
    T    
   A   
   C   
   A   
   G   
   C   
    T    
   T   
   C   
    C    
   G   
   G   
    C    
   G   
   T   
    C    
    A    
   G   
    A    
   G   
   G   
    G    
   A   
   T   
    A    
   C   
   G   
    T    
    T    
   T   
    G    
   C   
   G   
    T    
   G   
   A   
    G    
    G    
   A   
    A    
   G   
   A   
    C    
   T   
    A    
    C    
   G   
   T   
    T    
   G   
   T   
    T    
   G   
   G   
    G    
    G    
   C   
   A   
   G   
   C   
    C    
    C    
   T   
    G    
    A    
   T   
   G   
    A    
   A   
    G    
   G   
    A    
   A   
    G    
   A   
    C    
    C    
   A   
   A   
   G   
   A   
   A   
   G   
   T   
    A    
    C    
   T   
    A    
   G   
   T   
    G    
   A   
   T   
    T    
   A   
   C   
    A    
   G   
   A   
   A   
   A   
   A   
    A    
   G   
   G   
   T   
   T   
   A   
    C    
   G   
   G   
    A    
   A   
   A   
    A    
   C   
   G   
    G    
   A   
   C   
    C    
   A   
   A   
   A   
   G   
    T    
    A    
   T   
   C   
    G    
   G   
   A   
    A    
   T   
   A   
   T   
   C   
   C   
   A   
   G   
   T   
    G    
   A   
   A   
    A    
   G   
   G   
    C    
   C   
   G   
    C    
   G   
   G   
    T    
   G   
   G   
   T   
   A   
   A   
   A   
   G   
   G   
    A    
   A   
   T   
    C    
   A   
   A   
    A    
   A   
   C   
    G    
    G    
   C   
    G    
   A   
   A   
    T    
   A   
   T   
   C   
   A   
   C   
   T   
   G   
   A   
    A    
   A   
   A   
   A   
   A   
   A   
   T   
   G   
   G   
   T   
   C   
   C   
   A   
    C    
   T   
   A   
   G   
   C   
    C    
   G   
   G   
    A    
   T   
   T   
    G    
   A   
   C   
   C   
   A   
   C   
    T    
   G   
   T   
    T    
    T    
    C    
   T   
   G   
   G   
    A    
    G    
   A   
   T   
   G   
   A   
   A   
   G   
   A   
    C    
   A   
   T   
   C   
    A    
   T   
    G    
    C    
   T   
    G    
   A   
   T   
    T    
   A   
   C   
    A    
    G    
   A   
    T    
   A   
   A   
   A   
   G   
   G   
    T    
   G   
   T   
    G    
   A   
   T   
   T   
   A   
   T   
   C   
   C   
   G   
    T    
   T   
   T   
    C    
   A   
   A   
   C   
   G   
   T   
    A    
    T    
    C    
    G    
    A    
   C   
   T   
   G   
   T   
   T   
   T   
   C   
   T   
   C   
   A   
   A   
   A   
   C   
    T    
   G   
   G   
   A   
   C   
   G   
    T    
    T    
   C   
   A   
   A   
   C   
   A   
   T   
   T   
    G    
   G   
   G   
    T    
   G   
   T   
    A    
   C   
   G   
   T   
   T   
   T   
    G    
   A   
   T   
   G   
   A   
    A    
    G    
   A   
   T   
   G   
   G   
   A   
   A   
   G   
    C    
    T    
   G   
    A    
    T    
    A    
   C   
    G    
   A   
   A   
   A   
   G   
   T   
   C   
   G   
   T   
   C   
   A   
   C   
   A   
   A   
   T   
   G   
   -   
   -   
   -   
   -   
   -   
   -   
   -   
   -   
   -   
   -   
   -   
   -   
   -   
   -   
   -   
   -   
   -   
   -   
   -   
   -   
   -   
   -   
   -   
   -   
   -   
   -   
   -   
   -   
   -   
   -   
   -   
   -   
   -   
   -   
   -   
   -   
   -   
   -   
   -   
   -   
   -   
   -   
   -   
   -   
   -   
   -   
   -   
   -   
   -   
   -   
   -   
   -   
   -   
   -   
   -   
   -   
   -   
   -   
   -   
   -   
 

 
   N55753   
   A   
   T   
   G   
   A   
   G   
   T   
   G   
   A   
   A   
   G   
   A   
   A   
   A   
   T   
   T   
   A   
   A   
   A   
   G   
   A   
   A   
   A   
   A   
   C   
   A   
   T   
   T   
   C   
   A   
   A   
   G   
   A   
   C   
   G   
   T   
   C   
   A   
   A   
   T   
   C   
   T   
   G   
   A   
   C   
   C   
   A   
   G   
   C   
   G   
   A   
   A   
   A   
   T   
   G   
   A   
   A   
   A   
   G   
   A   
   A   
   T   
   C   
   T   
   T   
   T   
   C   
   A   
   T   
   T   
   G   
   A   
   C   
   T   
   A   
   C   
   G   
   C   
   A   
   A   
   T   
   G   
   A   
   G   
   T   
   G   
   T   
   T   
   A   
   T   
   C   
   G   
   T   
   A   
   G   
   C   
   C   
   C   
   G   
   C   
   G   
   C   
   G   
   T   
   T   
   A   
   C   
   C   
   T   
   G   
   A   
   C   
   G   
   T   
   T   
   C   
   G   
   T   
   G   
   A   
   C   
   G   
   G   
   T   
   T   
   T   
   A   
   A   
   A   
   A   
   C   
   C   
   T   
   G   
   T   
   T   
   C   
   A   
   T   
   C   
   G   
   C   
   C   
   G   
   A   
   A   
   T   
   C   
   T   
   T   
   A   
   T   
   A   
   T   
   G   
   G   
   A   
   A   
   T   
   G   
   A   
   A   
   C   
   G   
   A   
   A   
   T   
   T   
   A   
   G   
   G   
   G   
   G   
   T   
   A   
   A   
   C   
   C   
   C   
   C   
   T   
   G   
   A   
   T   
   A   
   A   
   A   
   C   
   C   
   A   
   C   
   A   
   C   
   A   
   A   
   A   
   A   
   A   
   A   
   T   
   C   
   A   
   G   
   C   
   C   
   C   
   G   
   G   
   A   
   T   
   T   
   G   
   T   
   T   
   G   
   G   
   G   
   G   
   A   
   T   
   G   
   T   
   T   
   A   
   T   
   G   
   G   
   G   
   T   
   A   
   A   
   A   
   T   
   A   
   T   
   C   
   A   
   C   
   C   
   C   
   C   
   C   
   A   
   T   
   G   
   G   
   G   
   G   
   A   
   C   
   A   
   G   
   T   
   G   
   C   
   G   
   A   
   T   
   T   
   T   
   A   
   C   
   G   
   A   
   A   
   T   
   C   
   A   
   A   
   T   
   G   
   G   
   T   
   G   
   C   
   G   
   G   
   A   
   T   
   G   
   G   
   C   
   A   
   C   
   A   
   A   
   C   
   C   
   T   
   T   
   T   
   T   
   A   
   G   
   T   
   T   
   A   
   T   
   C   
   G   
   G   
   G   
   C   
   T   
   A   
   T   
   G   
   T   
   T   
   A   
   G   
   T   
   T   
   G   
   A   
   C   
   G   
   G   
   C   
   C   
   A   
   C   
   G   
   G   
   A   
   A   
   A   
   C   
   T   
   T   
   C   
   G   
   G   
   T   
   T   
   C   
   T   
   G   
   T   
   C   
   G   
   A   
   T   
   G   
   G   
   T   
   G   
   A   
   C   
   G   
   G   
   C   
   G   
   C   
   T   
   G   
   C   
   C   
   G   
   C   
   T   
   A   
   T   
   G   
   C   
   G   
   T   
   T   
   A   
   T   
   A   
   C   
   C   
   G   
   A   
   A   
   G   
   C   
   A   
   C   
   G   
   T   
   A   
   T   
   G   
   A   
   G   
   T   
   A   
   A   
   A   
   A   
   T   
   T   
   G   
   C   
   T   
   T   
   T   
   A   
   G   
   A   
   A   
   A   
   T   
   G   
   T   
   T   
   A   
   C   
   G   
   A   
   G   
   A   
   T   
   A   
   T   
   T   
   A   
   A   
   C   
   A   
   A   
   A   
   A   
   A   
   T   
   A   
   C   
   A   
   G   
   T   
   C   
   G   
   A   
   T   
   T   
   T   
   C   
   C   
   A   
   A   
   G   
   G   
   A   
   A   
   A   
   C   
   T   
   A   
   T   
   G   
   A   
   T   
   G   
   A   
   T   
   T   
   C   
   A   
   G   
   A   
   A   
   C   
   A   
   A   
   G   
   A   
   A   
   C   
   C   
   A   
   G   
   T   
   A   
   G   
   T   
   T   
   T   
   T   
   A   
   C   
   C   
   T   
   G   
   C   
   T   
   C   
   G   
   T   
   T   
   T   
   T   
   C   
   C   
   A   
   A   
   A   
   C   
   T   
   T   
   A   
   C   
   T   
   A   
   G   
   T   
   T   
   A   
   A   
   C   
   G   
   G   
   A   
   A   
   C   
   A   
   A   
   C   
   G   
   G   
   G   
   G   
   A   
   T   
   T   
   G   
   C   
   A   
   G   
   T   
   G   
   G   
   G   
   G   
   A   
   T   
   G   
   G   
   C   
   A   
   A   
   C   
   A   
   A   
   A   
   T   
   A   
   T   
   T   
   C   
   C   
   A   
   C   
   C   
   A   
   C   
   A   
   C   
   A   
   A   
   T   
   T   
   T   
   A   
   A   
   G   
   T   
   G   
   A   
   A   
   G   
   T   
   G   
   A   
   T   
   T   
   G   
   A   
   T   
   G   
   C   
   A   
   A   
   C   
   A   
   A   
   G   
   T   
   T   
   T   
   A   
   T   
   T   
   G   
   A   
   T   
   G   
   G   
   A   
   C   
   A   
   A   
   T   
   C   
   C   
   T   
   G   
   A   
   T   
   G   
   T   
   A   
   A   
   C   
   G   
   A   
   C   
   G   
   A   
   A   
   T   
   G   
   A   
   A   
   T   
   T   
   G   
   A   
   T   
   G   
   G   
   A   
   A   
   G   
   T   
   G   
   T   
   T   
   A   
   C   
   C   
   T   
   G   
   G   
   A   
   C   
   C   
   A   
   G   
   A   
   T   
   T   
   T   
   T   
   C   
   C   
   A   
   A   
   C   
   A   
   G   
   G   
   T   
   G   
   G   
   T   
   T   
   T   
   A   
   G   
   T   
   G   
   A   
   T   
   G   
   G   
   G   
   G   
   A   
   A   
   A   
   T   
   C   
   A   
   G   
   G   
    G    
   A   
   T   
    T    
   C   
   G   
    T    
    C    
   G   
   A   
   G   
   C   
    A    
   T   
   A   
   T   
   G   
   A   
   A   
   A   
   C   
    A    
   G   
   G   
    G    
   A   
   A   
    A    
   G   
   G   
   T   
   T   
   C   
    G    
   A   
   T   
   T   
   A   
   C   
    T    
   G   
   T   
    T    
   C   
   G   
   T   
   G   
   C   
   A   
   A   
   A   
   A   
   G   
   T   
   T   
   G   
   A   
    A    
    T    
   T   
    G    
   A   
    C    
   T   
   G   
   A   
    A    
   A   
   T   
   G   
    C    
   C   
    G    
   A   
   A   
   T   
   G   
   G   
    A    
   A   
   A   
   A   
   G   
   A   
   A   
   C   
   G   
    T    
   A   
   T   
    T    
    T    
   T   
    A    
   G   
   T   
    A    
   A   
   C   
    T    
   G   
   A   
   A   
   T   
   T   
    G    
   C   
   C   
   T   
   T   
   A   
   T   
   A   
   T   
   G   
   G   
   T   
    G    
   A   
   A   
   T   
   A   
   A   
   A   
   G   
   C   
    C    
   A   
   A   
   A   
   T   
   T   
    A    
   A   
   T   
    C    
   G   
   A   
   A   
   C   
   G   
    A    
   A   
   T   
   T   
   T   
   C   
   T   
   G   
   A   
    A    
    T    
   T   
    A    
   C   
   A   
   C   
   A   
   G   
   A   
   G   
   A   
    T    
   A   
   A   
    A    
   C   
   G   
    A    
   A   
   T   
    T    
   G   
   A   
   A   
   G   
   G   
    A    
   A   
   T   
    T    
   A   
   C   
    T    
   G   
   A   
   T   
   T   
   T   
   G   
   C   
   G   
   G   
   G   
   A   
    T    
   G   
   A   
   A   
   T   
   C   
   T   
   T   
   C   
    T    
   C   
   G   
   T   
   G   
   A   
    A    
   G   
   G   
    C    
   A   
   T   
   G   
   C   
   G   
    G    
   A   
   T   
    C    
   G   
   T   
   C   
   A   
   T   
    C    
   G   
   A   
    T    
    G    
   T   
    T    
   C   
   G   
   T   
   C   
   G   
    A    
   G   
   A   
    T    
   G   
    T    
    G    
   A   
   G   
    T    
   G   
   C   
    C    
   T   
   C   
    T    
   G   
   T   
   T   
    G    
   T   
    G    
   T   
   T   
    A    
   A   
   A   
    C    
   A   
   A   
   C   
   T   
   T   
    G    
   T   
   A   
    C    
   A   
   A   
   A   
    A    
   T   
   G   
   A   
   C   
    T    
    G    
   C   
    C    
   T   
   T   
    A    
   C   
   A   
    A    
   A   
    C    
    A    
   T   
   C   
    T    
   T   
   T   
    T    
   G   
   G   
    T    
   T   
   T   
    T    
   A   
   A   
    C    
   A   
   T   
   G   
   T   
   T   
    A    
   G   
   C   
    C    
   A   
   T   
    T    
   G   
   A   
   A   
   A   
   A   
   A   
   G   
   G   
    T    
   G   
   T   
    A    
   C   
   C   
    G    
   A   
   A   
   A   
    A    
   T   
   T   
   T   
   T   
    A    
   A   
   G   
    C    
   T   
   T   
   G   
   A   
   A   
   A   
   C   
    G    
    T    
   A   
   T   
    T    
    T    
   T   
    A    
   G   
   A   
   A   
   A   
   A   
    C    
   T   
   A   
    C    
    G    
   T   
    T    
   G   
   A   
    G    
   C   
   A   
    C    
   C   
   A   
    A    
    A    
    A    
    A    
    G    
   A   
   A   
   G   
   T   
   C   
   A   
   T   
    T    
   A   
   C   
    T    
    C    
   G   
    C    
   C   
   G   
   G   
   A   
   C   
    G    
    A    
   T   
    T    
   T   
   T   
   T   
   G   
   A   
    C    
   A   
   A   
   A   
    A    
   A   
    C    
   A   
   A   
    A    
   G   
   C   
   A   
   G   
   A   
   A   
   G   
   C   
   A   
    C    
   G   
    G    
   G   
   C   
    G    
   C   
   A   
    T    
   A   
   T   
    T    
   T   
   T   
   A   
   G   
   A   
   A   
   G   
   G   
   T   
    C    
   T   
    A    
   C   
   G   
    A    
   A   
   T   
   T   
   G   
   C   
    C    
   T   
   T   
    A    
   G   
   A   
   T   
   C   
   A   
   T   
   A   
   T   
   C   
    G    
   A   
   T   
   G   
   A   
   A   
   A   
   T   
    C    
   A   
   T   
   T   
   G   
   C   
    C    
   A   
   T   
   T   
   A   
   T   
    C    
   C   
   G   
    T    
   G   
   G   
    A    
   T   
   C   
    A    
    C    
   A   
   A   
   T   
   C   
   A   
   G   
   A   
   T   
   G   
   A   
    C    
   G   
   A   
    A    
   G   
   C   
   A   
   A   
   A   
   A   
   G   
   C   
    A    
   A   
    C    
    G    
    T    
   T   
    A    
   A   
   T   
    T    
   G   
   A   
   A   
   C   
   G   
    C    
   T   
   T   
    T    
   G   
   A   
    A    
   T   
   T   
    T    
   T   
   C   
    A    
   G   
   A   
   T   
   C   
   G   
    T    
   C   
   A   
    A    
    G    
   C   
    G    
   C   
   A   
   A   
   G   
   C   
    G    
   A   
   T   
    T    
   T   
   T   
   A   
   G   
   A   
   T   
   A   
   T   
   G   
   C   
   G   
   T   
   T   
   T   
    A    
   C   
   G   
    C    
   C   
   G   
    T    
   T   
   T   
    A    
   A   
   C   
    A    
   G   
   G   
    C    
   T   
   T   
    A    
   G   
   A   
   A   
   C   
   G   
    C    
   G   
   A   
    C    
   A   
   A   
   A   
   A   
   T   
    T    
   G   
   A   
    A    
   A   
   A   
   T   
   G   
   A   
   A   
   T   
   A   
    T    
   C   
   A   
   A   
   G   
   A   
    G    
   C   
   T   
    A    
   T   
   T   
    G    
   A   
    A    
   A   
    T    
   T   
    C    
   A   
   T   
   T   
   G   
    C    
    G    
   G   
   A   
    C    
   T   
   T   
    A    
    G    
    A    
    G    
   G   
   A   
   C   
   A   
   T   
   T   
    T    
   T   
    A    
   G   
   C   
    C    
    C    
   G   
    C    
   C   
   C   
    A    
   G   
   A   
    A    
   C   
   G   
    C    
   G   
   T   
    C    
   A   
    T    
    T    
   G   
   A   
   A   
   A   
   T   
    C    
   A   
   T   
    T    
   A   
   A   
    A    
   A   
    C    
    A    
   G   
   A   
    G    
   T   
   T   
   A   
    A    
    A    
    T    
   G   
   A   
   C   
    G    
   T   
    T    
    C    
    G    
    C    
    A    
    C    
   A   
    A    
    A    
   A   
   T   
   T   
   T   
   G   
   G   
   T   
   G   
   A   
   T   
    G    
    C    
   A   
   C   
   G   
   A   
   C   
   G   
    C    
   A   
   C   
   A   
   G   
   A   
   A   
    T    
   T   
   A   
    T    
   T   
   A   
   G   
   T   
    A    
   G   
   G   
   T   
   G   
   A   
    A    
   G   
   T   
   C   
    C    
   T   
    A    
   A   
   G   
   T   
   C   
   T   
    T    
   G   
   A   
   A   
   G   
   A   
   T   
   G   
   A   
   A   
   G   
   A   
   T   
    T    
   T   
    A    
   A   
   T   
    C    
   G   
   A   
   A   
   G   
   A   
   A   
   G   
   A   
   A   
   G   
   A   
   A   
    G    
   T   
   C   
   G   
   T   
    G    
   A   
   T   
   T   
   A   
   C   
    A    
    T    
   T   
    A    
   A   
   C   
    C    
   A   
   A   
    T    
   A   
   A   
    C    
   G   
   G   
    C    
   T   
   A   
    C    
   A   
   T   
    T    
   A   
   A   
    G    
   C   
   G   
    G    
    A    
   T   
   G   
   G   
    C    
   A   
   A   
   A   
    C    
    T    
    C    
    C    
   G   
   A   
   A   
   T   
   T   
   C   
    C    
   G   
    G    
   G   
   C   
    G    
   C   
   A   
   A   
   C   
   G   
    C    
    C    
   G   
    C    
   G   
   G   
    C    
   G   
   G   
    A    
   C   
   G   
   T   
   G   
   G   
    T    
   G   
   T   
    C    
   C   
   A   
   A   
   G   
   G   
    C    
   A   
   T   
   G   
   G   
   G   
    T    
    G    
   T   
    A    
   C   
   A   
    T    
    G    
   A   
   T   
   G   
   A   
    T    
   G   
   A   
    T    
   T   
   T   
   C   
   G   
   T   
    G    
   A   
   A   
    A    
   A   
   A   
    C    
   C   
   T   
    A    
    G    
   T   
   T   
   T   
   C   
   T   
   T   
   G   
    T    
   T   
   C   
    A    
   A   
   C   
    A    
   C   
   A   
    C    
   G   
   A   
    C    
   A   
   C   
    G    
   T   
   T   
    A    
   T   
   T   
   A   
   T   
   T   
    C    
   T   
   T   
   T   
   A   
   C   
   G   
   A   
   A   
    C    
   A   
    C    
    T    
   G   
   G   
    G    
   A   
   A   
   A   
   G   
   T   
    T    
   T   
   A   
    C    
   C   
   G   
    A    
   G   
   C   
   A   
   A   
   A   
   A   
   G   
   G   
    T    
   T   
   A   
    C    
   G   
   A   
   A   
   A   
   T   
    C    
   C   
   C   
    T    
   G   
   A   
    A    
   T   
   A   
   C   
   G   
   G   
    T    
    A    
   G   
    A    
   A   
   C   
   A   
   G   
   C   
    A    
   A   
   A   
   A   
   G   
   G   
    A    
   A   
   T   
    T    
   C   
   C   
   A   
    G    
   T   
    G    
   A   
   T   
    T    
   A   
   A   
    C    
   T   
   T   
    A    
   C   
   T   
    G    
   G   
   G   
    A    
   A   
   T   
    T    
   G   
   A   
    T    
   T   
   C   
    T    
    G    
    C    
    A    
   G   
   A   
   A   
   A   
    A    
    A    
   A   
   T   
    T    
   C   
   A   
    A    
   G   
   C   
    G    
   A   
   T   
    T    
   A   
   T   
   T   
    T    
   C   
    T    
   G   
   T   
    T    
    G    
    A    
   G   
   G   
   G   
    C    
    A    
   A   
   A   
   G   
   C   
    G    
   G   
   A   
   A   
   G   
    C    
    A    
   G   
   G   
    T    
    A    
   A   
    A    
   T   
   A   
    C    
    T    
   T   
    G    
   T   
   T   
   C   
   T   
   T   
    C    
   A   
   C   
    A    
   A   
   C   
    C    
    T    
    T    
    A    
    A    
   A   
   A   
   G   
   G   
    A    
   A   
   C   
    T    
   G   
   T   
    C    
   A   
   A   
   A   
   C   
   G   
    G    
   A   
   C   
    A    
    G    
   C   
    C    
   G   
   T   
    A    
   A   
   C   
    A    
   G   
   C   
    C    
   T   
   T   
    T    
    T    
   C   
   T   
   A   
   A   
    T    
   A   
   T   
    C    
   C   
   G   
    T    
   A   
   G   
    T    
   A   
   A   
   T   
   G   
   G   
    A    
    T    
   T   
    A    
   A   
   T   
    C    
   G   
   C   
    C    
   A   
   T   
   T   
    A    
   G   
    C    
   T   
   T   
    A    
   A   
   A   
   A   
   G   
   A   
   A   
   G   
   A   
    T    
   G   
   A   
    T    
   G   
   A   
    G    
    T    
   T   
    A    
    G    
   T   
    T    
   A   
   A   
    C    
   G   
   T   
    A    
    G    
   T   
   A   
    A    
    C    
   G   
   A   
   C   
    T    
    A    
   A   
    T    
   G   
   G   
    C    
    A    
   A   
    T    
    C    
    A    
    G    
   A   
   A   
    G    
   A   
   T   
    G    
   A   
   T   
    T    
   A   
   T   
    C    
   G   
   G   
    A    
   A   
   C   
   A   
   C   
   A   
   T   
    G    
    C    
    G    
   G   
   G   
    A    
   T   
   A   
   C   
   T   
   C   
   T   
   G   
   T   
    C    
   A   
   C   
    A    
   T   
   T   
    T    
    G    
   A   
    T    
   G   
   A   
    A    
    A    
    A    
    T    
    A    
   C   
    T    
   G   
    T    
    A    
   C   
   G   
    T    
   G   
   A   
   T   
   A   
   T   
   G   
   G   
   G   
    C    
   C   
   G   
    G    
   A   
   C   
   A   
   G   
   C   
    A    
   T   
   C   
    A    
   G   
   G   
    T    
   G   
   T   
    T    
    C    
   G   
    G    
   G   
   G   
    A    
   A   
   T   
    C    
   C   
   G   
    T    
    C    
   T   
    C    
   C   
   G   
    C    
   G   
   A   
    A    
    A    
   A   
    T    
   G   
   A   
    T    
   T   
    A    
    T    
   G   
   T   
    G    
   G   
   T   
    C    
   G   
   G   
    C    
    G    
   C   
   A   
   G   
   C   
    G    
    A    
   T   
    T    
    C    
   T   
   G   
    G    
   A   
    T    
   G   
    A    
   A   
    A    
   A   
    T    
    A    
   A   
   A   
   G   
   A   
   A   
   G   
   T   
    C    
    C    
   T   
    A    
   G   
   T   
    C    
   A   
   T   
    T    
   A   
   C   
    T    
   G   
   A   
   A   
   A   
   A   
    T    
   G   
   G   
   T   
   T   
   A   
    T    
   G   
   G   
    T    
   A   
   A   
    G    
   C   
   G   
    T    
   A   
   C   
    A    
   A   
   A   
   A   
   G   
    C    
    T    
   T   
   C   
    T    
   G   
   A   
    A    
   T   
   A   
   T   
   C   
   C   
   A   
   G   
   T   
    T    
   A   
   A   
    A    
   G   
   G   
    A    
   C   
   G   
    T    
   G   
   G   
    C    
   G   
   G   
   T   
   A   
   A   
   A   
   G   
   G   
    G    
   A   
   T   
    T    
   A   
   A   
    G    
   A   
   C   
    A    
    G    
   C   
    A    
   A   
   A   
    T    
   A   
   T   
   C   
   A   
   C   
   T   
   G   
   A   
    G    
   A   
   A   
   A   
   A   
   A   
   T   
   G   
   G   
   T   
   C   
   C   
   A   
    T    
   T   
   A   
   G   
   C   
    T    
   G   
   G   
    T    
   T   
   T   
    A    
   A   
   C   
   C   
   A   
   C   
    G    
   G   
   T   
    C    
    A    
    A    
   T   
   G   
   G   
    G    
    G    
   A   
   T   
   G   
   A   
   A   
   G   
   A   
    T    
   A   
   T   
   C   
    T    
   T   
    A    
    T    
   T   
    G    
   A   
   T   
    T    
   A   
   C   
    G    
    A    
   A   
    C    
   A   
   A   
   A   
   G   
   G   
    C    
   G   
   T   
    C    
   A   
   T   
   T   
   A   
   T   
   C   
   C   
   G   
    C    
   T   
   T   
    T    
   A   
   A   
   C   
   G   
   T   
    T    
    G    
    A    
    T    
    T    
   C   
   T   
   G   
   T   
   T   
   T   
   C   
   T   
   C   
   A   
   A   
   A   
   C   
    A    
   G   
   G   
   A   
   C   
   G   
    C    
    G    
   C   
   A   
   A   
   C   
   A   
   T   
   T   
    A    
   G   
   G   
    A    
   G   
   T   
    T    
   C   
   G   
   T   
   T   
   T   
    A    
   A   
   T   
   G   
   A   
    G    
    A    
   A   
   T   
   G   
   G   
   A   
   A   
   G   
    A    
    T    
   G   
    G    
    T    
    G    
   C   
    C    
   A   
   A   
   A   
   -   
   -   
   -   
   -   
   -   
   -   
   -   
   -   
   -   
   -   
   -   
   -   
   -   
   -   
   -   
   -   
   -   
   -   
   -   
   -   
   -   
   -   
   -   
   -   
   -   
   -   
   -   
   -   
   -   
   -   
   -   
   -   
   -   
   -   
   -   
   -   
   -   
   -   
   -   
   -   
   -   
   -   
   -   
   -   
   -   
   -   
   -   
   -   
   -   
   -   
   -   
   -   
   -   
   -   
   -   
   -   
   -   
   -   
   -   
   -   
   -   
   -   
   -   
   -   
   -   
   -   
   -   
   -   
   -   
   -   
   -   
   -   
 

 
   N60365F   
   A   
   T   
   G   
   A   
   G   
   T   
   G   
   A   
   A   
   G   
   A   
   A   
   A   
   T   
   C   
   A   
   G   
   A   
   G   
   A   
   A   
   A   
   A   
   C   
   A   
   T   
   C   
   C   
   A   
   T   
   G   
   A   
   T   
   G   
   T   
   C   
   A   
   A   
   T   
   C   
   T   
   A   
   A   
   C   
   C   
   A   
   G   
   C   
   G   
   A   
   A   
   A   
   T   
   G   
   A   
   A   
   A   
   G   
   A   
   C   
   T   
   C   
   C   
   T   
   T   
   C   
   A   
   T   
   C   
   G   
   A   
   T   
   T   
   A   
   T   
   G   
   C   
   G   
   A   
   T   
   G   
   A   
   G   
   T   
   G   
   T   
   T   
   A   
   T   
   T   
   G   
   T   
   C   
   G   
   C   
   A   
   C   
   G   
   T   
   G   
   C   
   A   
   C   
   T   
   T   
   C   
   C   
   T   
   G   
   A   
   T   
   G   
   T   
   C   
   A   
   G   
   A   
   G   
   A   
   T   
   G   
   G   
   T   
   C   
   T   
   G   
   A   
   A   
   A   
   C   
   C   
   C   
   G   
   T   
   T   
   C   
   A   
   T   
   C   
   G   
   C   
   C   
   G   
   T   
   A   
   T   
   T   
   T   
   T   
   A   
   T   
   A   
   C   
   G   
   G   
   C   
   A   
   T   
   G   
   A   
   A   
   C   
   G   
   A   
   A   
   T   
   T   
   A   
   G   
   G   
   T   
   G   
   T   
   A   
   A   
   C   
   A   
   C   
   C   
   G   
   G   
   A   
   T   
   A   
   A   
   A   
   G   
   C   
   C   
   C   
   A   
   T   
   A   
   A   
   A   
   A   
   A   
   A   
   T   
   C   
   T   
   G   
   C   
   C   
   A   
   G   
   A   
   A   
   T   
   C   
   G   
   T   
   C   
   G   
   G   
   A   
   G   
   A   
   T   
   G   
   T   
   C   
   A   
   T   
   G   
   G   
   G   
   T   
   A   
   A   
   A   
   T   
   A   
   C   
   C   
   A   
   T   
   C   
   C   
   T   
   C   
   A   
   T   
   G   
   G   
   A   
   G   
   A   
   C   
   A   
   G   
   T   
   G   
   C   
   G   
   A   
   T   
   C   
   T   
   A   
   T   
   G   
   A   
   G   
   T   
   C   
   A   
   A   
   T   
   G   
   G   
   T   
   A   
   C   
   G   
   G   
   A   
   T   
   G   
   G   
   C   
   A   
   C   
   A   
   G   
   C   
   C   
   A   
   T   
   T   
   C   
   A   
   G   
   C   
   T   
   A   
   C   
   C   
   G   
   T   
   T   
   A   
   T   
   A   
   T   
   G   
   C   
   T   
   A   
   G   
   T   
   G   
   G   
   A   
   T   
   G   
   G   
   T   
   C   
   A   
   T   
   G   
   G   
   A   
   A   
   A   
   C   
   T   
   T   
   T   
   G   
   G   
   T   
   T   
   C   
   T   
   G   
   T   
   T   
   G   
   A   
   C   
   G   
   G   
   T   
   G   
   A   
   C   
   G   
   G   
   T   
   G   
   C   
   C   
   G   
   C   
   C   
   G   
   C   
   T   
   A   
   T   
   G   
   C   
   G   
   T   
   T   
   A   
   T   
   A   
   C   
   C   
   G   
   A   
   A   
   G   
   C   
   A   
   C   
   G   
   G   
   A   
   T   
   G   
   A   
   G   
   C   
   A   
   A   
   A   
   A   
   T   
   C   
   G   
   C   
   T   
   A   
   C   
   A   
   G   
   A   
   A   
   A   
   T   
   G   
   C   
   T   
   G   
   C   
   G   
   T   
   G   
   A   
   T   
   A   
   T   
   C   
   A   
   A   
   T   
   A   
   A   
   G   
   A   
   A   
   C   
   A   
   C   
   T   
   G   
   T   
   T   
   G   
   A   
   T   
   T   
   T   
   C   
   C   
   A   
   A   
   A   
   G   
   C   
   A   
   A   
   C   
   T   
   A   
   T   
   G   
   A   
   T   
   G   
   A   
   T   
   A   
   C   
   A   
   G   
   A   
   A   
   A   
   A   
   A   
   G   
   A   
   A   
   C   
   C   
   A   
   G   
   T   
   T   
   G   
   T   
   A   
   C   
   T   
   A   
   C   
   C   
   T   
   G   
   C   
   T   
   C   
   G   
   C   
   T   
   T   
   C   
   C   
   C   
   G   
   A   
   A   
   T   
   C   
   T   
   T   
   T   
   T   
   A   
   G   
   T   
   G   
   A   
   A   
   C   
   G   
   G   
   A   
   A   
   C   
   A   
   A   
   C   
   G   
   G   
   G   
   G   
   A   
   T   
   C   
   G   
   C   
   C   
   G   
   T   
   A   
   G   
   G   
   T   
   A   
   T   
   G   
   G   
   C   
   C   
   A   
   C   
   G   
   A   
   A   
   T   
   A   
   T   
   C   
   C   
   C   
   A   
   C   
   C   
   T   
   C   
   A   
   T   
   A   
   A   
   C   
   T   
   T   
   G   
   T   
   C   
   T   
   G   
   A   
   G   
   G   
   T   
   A   
   G   
   T   
   A   
   G   
   C   
   G   
   G   
   C   
   T   
   A   
   T   
   C   
   G   
   A   
   T   
   T   
   T   
   A   
   C   
   T   
   G   
   A   
   T   
   G   
   G   
   A   
   T   
   A   
   A   
   T   
   C   
   C   
   T   
   G   
   A   
   T   
   G   
   T   
   G   
   A   
   C   
   G   
   A   
   C   
   C   
   A   
   A   
   T   
   G   
   A   
   A   
   T   
   T   
   G   
   A   
   T   
   G   
   G   
   A   
   A   
   G   
   T   
   A   
   T   
   T   
   A   
   C   
   C   
   T   
   G   
   G   
   A   
   C   
   C   
   A   
   G   
   A   
   T   
   T   
   T   
   T   
   C   
   C   
   A   
   A   
   C   
   A   
   G   
   G   
   T   
   G   
   G   
   T   
   T   
   T   
   G   
   G   
   T   
   T   
   A   
   T   
   G   
   G   
   G   
   G   
   A   
   A   
   A   
   T   
   C   
   A   
   G   
   G   
    A    
   A   
   T   
    C    
   C   
   G   
    A    
    A    
   G   
   A   
   G   
   C   
    G    
   T   
   A   
   T   
   G   
   A   
   A   
   A   
   C   
    A    
   G   
   G   
    T    
   A   
   A   
    G    
   G   
   G   
   T   
   T   
   C   
    T    
   A   
   T   
   T   
   A   
   C   
    C    
   G   
   T   
    A    
   C   
   G   
   T   
   G   
   C   
   A   
   A   
   A   
   A   
   G   
   T   
   T   
   G   
   A   
    G    
    A    
   T   
    T    
   A   
    C    
   T   
   G   
   A   
    G    
   A   
   T   
   G   
    C    
   C   
    A    
   A   
   A   
   T   
   G   
   G   
    T    
   A   
   A   
   A   
   G   
   A   
   A   
   C   
   G   
    G    
   A   
   T   
    C    
    A    
   T   
    C    
   G   
   T   
    G    
   A   
   C   
    A    
   G   
   A   
   A   
   T   
   T   
    G    
   C   
   C   
   T   
   T   
   A   
   T   
   A   
   T   
   G   
   G   
   T   
    C    
   A   
   A   
   T   
   A   
   A   
   A   
   G   
   C   
    A    
   A   
   A   
   A   
   T   
   T   
    G    
   A   
   T   
    C    
   G   
   A   
   A   
   C   
   G   
    G    
   A   
   T   
   T   
   T   
   C   
   T   
   G   
   A   
    A    
    C    
   T   
    G    
   C   
   A   
   C   
   A   
   G   
   A   
   G   
   A   
    C    
   A   
   A   
    G    
   C   
   G   
    T    
   A   
   T   
    C    
   G   
   A   
   A   
   G   
   G   
    G    
   A   
   T   
    C    
   A   
   C   
    A    
   G   
   A   
   T   
   T   
   T   
   G   
   C   
   G   
   G   
   G   
   A   
    C    
   G   
   A   
   A   
   T   
   C   
   T   
   T   
   C   
    C    
   C   
   G   
   T   
   G   
   A   
    A    
   G   
   G   
    T    
   A   
   T   
   G   
   C   
   G   
    G    
   A   
   T   
    C    
   G   
   T   
   C   
   A   
   T   
    T    
   G   
   A   
    C    
    A    
   T   
    C    
   C   
   G   
   T   
   C   
   G   
    T    
   G   
   A   
    C    
   G   
    C    
    C    
   A   
   G   
    C    
   G   
   C   
    A    
   T   
   C   
    C    
   G   
   T   
   T   
    A    
   T   
    C    
   T   
   T   
    G    
   A   
   A   
    T    
   A   
   A   
   C   
   T   
   T   
    A    
   T   
   A   
    T    
   A   
   A   
   A   
    T    
   T   
   G   
   A   
   C   
    A    
    T    
   C   
    C    
   T   
   T   
    G    
   C   
   A   
    A    
   A   
    A    
    T    
   T   
   C   
    A    
   T   
   T   
    T    
   G   
   G   
    A    
   T   
   T   
    C    
   A   
   A   
    T    
   A   
   T   
   G   
   T   
   T   
    G    
   G   
   C   
    G    
   A   
   T   
    C    
   G   
   A   
   A   
   A   
   A   
   A   
   G   
   G   
    T    
   G   
   T   
    G    
   C   
   C   
    G    
   A   
   A   
   A   
    G    
   T   
   T   
   T   
   T   
    G    
   A   
   G   
    C    
   T   
   T   
   G   
   A   
   A   
   A   
   C   
    A    
    A    
   A   
   T   
    C    
    C    
   T   
    T    
   G   
   A   
   A   
   A   
   A   
    C    
   T   
   A   
    T    
    A    
   T   
    C    
   G   
   A   
    A    
   C   
   A   
    T    
   C   
   A   
    A    
    C    
    G    
    C    
    G    
   A   
   A   
   G   
   T   
   C   
   A   
   T   
    C    
   A   
   C   
    A    
    C    
   G   
    A    
   C   
   G   
   G   
   A   
   C   
    G    
    G    
   T   
    C    
   T   
   T   
   T   
   G   
   A   
    A    
   A   
   A   
   A   
    G    
   A   
    A    
   A   
   A   
    A    
   G   
   C   
   A   
   G   
   A   
   A   
   G   
   C   
   A   
    A    
   G   
    A    
   G   
   C   
    A    
   C   
   A   
    C    
   A   
   T   
    C    
   T   
   T   
   A   
   G   
   A   
   A   
   G   
   G   
   T   
    T    
   T   
    G    
   C   
   G   
    T    
   A   
   T   
   T   
   G   
   C   
    T    
   T   
   T   
    G    
   G   
   A   
   T   
   C   
   A   
   T   
   A   
   T   
   C   
    G    
   A   
   T   
   G   
   A   
   A   
   A   
   T   
    C    
   A   
   T   
   T   
   G   
   C   
    G    
   A   
   T   
   T   
   A   
   T   
    C    
   C   
   G   
    C    
   G   
   G   
    G    
   T   
   C   
    T    
    A    
   A   
   A   
   T   
   C   
   A   
   G   
   A   
   T   
   G   
   A   
    C    
   G   
   A   
    G    
   G   
   C   
   A   
   A   
   A   
   A   
   G   
   C   
    A    
   A   
    C    
    A    
    A    
   T   
    G    
   A   
   T   
    C    
   G   
   A   
   A   
   C   
   G   
    G    
   T   
   T   
    C    
   G   
   A   
    T    
   T   
   T   
    A    
   T   
   C   
    C    
   G   
   A   
   T   
   C   
   G   
    C    
   C   
   A   
    G    
    T    
   C   
    T    
   C   
   A   
   A   
   G   
   C   
    T    
   A   
   T   
    T    
   T   
   T   
   A   
   G   
   A   
   T   
   A   
   T   
   G   
   C   
   G   
   T   
   T   
   T   
    A    
   C   
   G   
    C    
   C   
   G   
    A    
   T   
   T   
    G    
   A   
   C   
    C    
   G   
   G   
    T    
   T   
   T   
    G    
   G   
   A   
   A   
   C   
   G   
    G    
   G   
   A   
    A    
   A   
   A   
   A   
   A   
   T   
    C    
   G   
   A   
    A    
   A   
   A   
   T   
   G   
   A   
   A   
   T   
   A   
    C    
   C   
   A   
   A   
   G   
   A   
    A    
   C   
   T   
    C    
   T   
   T   
    A    
   A   
    A    
   A   
    C    
   T   
    G    
   A   
   T   
   T   
   G   
    A    
    A    
   G   
   A   
    T    
   T   
   T   
    A    
    A    
    C    
    T    
   G   
   A   
   C   
   A   
   T   
   T   
    C    
   T   
    G    
   G   
   C   
    A    
    A    
   G   
    A    
   C   
   C   
    A    
   G   
   A   
    A    
   C   
   G   
    T    
   G   
   T   
    T    
   A   
    C    
    A    
   G   
   A   
   A   
   A   
   T   
    C    
   A   
   T   
    C    
   A   
   A   
    G    
   A   
    A    
    T    
   G   
   A   
    A    
   T   
   T   
   A   
    G    
    C    
    A    
   G   
   A   
   C   
    T    
   T   
    G    
    A    
    A    
    T    
    C    
    A    
   A   
    C    
    G    
   A   
   T   
   T   
   T   
   G   
   G   
   T   
   G   
   A   
   T   
    A    
    A    
   A   
   C   
   G   
   A   
   C   
   G   
    C    
   A   
   C   
   A   
   G   
   A   
   A   
    T    
   T   
   A   
    C    
   T   
   A   
   G   
   T   
    C    
   G   
   G   
   T   
   G   
   A   
    G    
   G   
   T   
   C   
    T    
   T   
    A    
   A   
   G   
   T   
   C   
   T   
    G    
   G   
   A   
   A   
   G   
   A   
   T   
   G   
   A   
   A   
   G   
   A   
   T   
    T    
   T   
    A    
   A   
   T   
    C    
   G   
   A   
   A   
   G   
   A   
   A   
   G   
   A   
   A   
   G   
   A   
   A   
    A    
   T   
   C   
   G   
   T   
    T    
   A   
   T   
   T   
   A   
   C   
    A    
    T    
   T   
    G    
   A   
   C   
    A    
   A   
   A   
    C    
   A   
   A   
    T    
   G   
   G   
    T    
   T   
   A   
    C    
   A   
   T   
    C    
   A   
   A   
    A    
   C   
   G   
    T    
    T    
   T   
   G   
   G   
    C    
   A   
   A   
   A   
    T    
    A    
    A    
    T    
   G   
   A   
   A   
   T   
   T   
   C   
    A    
   G   
    A    
   G   
   C   
    T    
   C   
   A   
   A   
   C   
   G   
    A    
    A    
   G   
    G    
   G   
   G   
    T    
   G   
   G   
    A    
   C   
   G   
   T   
   G   
   G   
    T    
   G   
   T   
    C    
   C   
   A   
   A   
   G   
   G   
    A    
   A   
   T   
   G   
   G   
   G   
    T    
    A    
   T   
    C    
   C   
   A   
    C    
    A    
   A   
   T   
   G   
   A   
    C    
   G   
   A   
    C    
   T   
   T   
   C   
   G   
   T   
    C    
   A   
   A   
    A    
   A   
   A   
    T    
   C   
   T   
    G    
    G    
   T   
   T   
   T   
   C   
   T   
   T   
   G   
    T    
   T   
   C   
    T    
   A   
   C   
    C    
   C   
   A   
    T    
   G   
   A   
    T    
   A   
   C   
    A    
   T   
   T   
    G    
   T   
   T   
   A   
   T   
   T   
    C    
   T   
   T   
   T   
   A   
   C   
   G   
   A   
   A   
    T    
   A   
    A    
    C    
   G   
   G   
    A    
   A   
   A   
   A   
   G   
   T   
    G    
   T   
   A   
    T    
   C   
   G   
    G    
   G   
   C   
   A   
   A   
   A   
   A   
   G   
   G   
    A    
   T   
   A   
    C    
   G   
   A   
   A   
   A   
   T   
    T    
   C   
   C   
    A    
   G   
   A   
    A    
   T   
   A   
   C   
   G   
   G   
    G    
    C    
   G   
    T    
   A   
   C   
   A   
   G   
   C   
    C    
   A   
   A   
   A   
   G   
   G   
    A    
   A   
   T   
    C    
   C   
   C   
   A   
    A    
   T   
    C    
   A   
   T   
    C    
   A   
   A   
    T    
   T   
   T   
    A    
   C   
   T   
    A    
   G   
   G   
    T    
   A   
   T   
    C    
   G   
   A   
    T    
   T   
   C   
    G    
    A    
    G    
    T    
   G   
   A   
   A   
   A   
    G    
    C    
   A   
   T   
    C    
   C   
   A   
    G    
   G   
   C   
    A    
   A   
   T   
    C    
   A   
   T   
   T   
    G    
   C   
    G    
   G   
   T   
    G    
    A    
    C    
   G   
   G   
   G   
    A    
    G    
   A   
   A   
   G   
   C   
    A    
   G   
   A   
   A   
   G   
    A    
    A    
   G   
   G   
    C    
    C    
   A   
    C    
   T   
   A   
    T    
    C    
   T   
    C    
   T   
   T   
   C   
   T   
   T   
    T    
   A   
   C   
    G    
   A   
   C   
    G    
    C    
    G    
    T    
    C    
   A   
   A   
   G   
   G   
    T    
   A   
   C   
    A    
   G   
   T   
    A    
   A   
   A   
   A   
   C   
   G   
    T    
   A   
   C   
    T    
    T    
   C   
    T    
   G   
   T   
    C    
   A   
   C   
    T    
   G   
   C   
    T    
   T   
   T   
    T    
    G    
   C   
   T   
   A   
   A   
    C    
   A   
   T   
    C    
   C   
   G   
    A    
   A   
   G   
    T    
   A   
   A   
   T   
   G   
   G   
    T    
    C    
   T   
    G    
   A   
   T   
    C    
   G   
   C   
    A    
   A   
   T   
   T   
    G    
   G   
    T    
   T   
   T   
    G    
   A   
   A   
   A   
   G   
   A   
   A   
   G   
   A   
    C    
   G   
   A   
    T    
   G   
   A   
    G    
    C    
   T   
    C    
    G    
   T   
    C    
   A   
   A   
    T    
   G   
   T   
    G    
    C    
   T   
   A   
    C    
    T    
   G   
   A   
   C   
    A    
    G    
   A   
    T    
   G   
   G   
    T    
    C    
   A   
    A    
    T    
    C    
    T    
   A   
   A   
    T    
   A   
   T   
    C    
   A   
   T   
    T    
   A   
   T   
    T    
   G   
   G   
    A    
   A   
   C   
   A   
   C   
   A   
   T   
    A    
    A    
    C    
   G   
   G   
    C    
   T   
   A   
   C   
   T   
   C   
   T   
   G   
   T   
    C    
   A   
   C   
    T    
   T   
   T   
    C    
    A    
   A   
    A    
   G   
   A   
    A    
    G    
    A    
    A    
    G    
   C   
    A    
   G   
    T    
    C    
   C   
   G   
    C    
   G   
   A   
   T   
   A   
   T   
   G   
   G   
   G   
    A    
   C   
   G   
    T    
   A   
   C   
   A   
   G   
   C   
    T    
   T   
   C   
    C    
   G   
   G   
    G    
   G   
   T   
    C    
    A    
   G   
    A    
   G   
   G   
    G    
   A   
   T   
    A    
   C   
   G   
    C    
    C    
   T   
    G    
   C   
   G   
    T    
   G   
   A   
    G    
    G    
   A   
    A    
   G   
   A   
    T    
   T   
    A    
    T    
   G   
   T   
    C    
   G   
   T   
    T    
   G   
   G   
    G    
    G    
   C   
   A   
   G   
   C   
    C    
    T    
   T   
    A    
    A    
   T   
   G   
    A    
   A   
    G    
   G   
    A    
   A   
    A    
   A   
    T    
    C    
   A   
   A   
   G   
   A   
   A   
   G   
   T   
    A    
    T    
   T   
    A    
   G   
   T   
    G    
   A   
   T   
    C    
   A   
   C   
    A    
   G   
   A   
   A   
   A   
   A   
    A    
   G   
   G   
   T   
   T   
   A   
    C    
   G   
   G   
    A    
   A   
   A   
    A    
   C   
   G   
    G    
   A   
   C   
    A    
   A   
   A   
   A   
   G   
    T    
    A    
   T   
   C   
    G    
   G   
   A   
    A    
   T   
   A   
   T   
   C   
   C   
   A   
   G   
   T   
    G    
   A   
   A   
    A    
   G   
   G   
    C    
   C   
   G   
    C    
   G   
   G   
    A    
   G   
   G   
   T   
   A   
   A   
   A   
   G   
   G   
    G    
   A   
   T   
    C    
   A   
   A   
    A    
   A   
   C   
    G    
    G    
   C   
    G    
   A   
   A   
    C    
   A   
   T   
   C   
   A   
   C   
   T   
   G   
   A   
    A    
   A   
   A   
   A   
   A   
   A   
   T   
   G   
   G   
   T   
   C   
   C   
   A   
    C    
   T   
   A   
   G   
   C   
    T    
   G   
   G   
    A    
   T   
   T   
    G    
   A   
   C   
   C   
   A   
   C   
    T    
   G   
   T   
    T    
    T    
    C    
   T   
   G   
   G   
    A    
    G    
   A   
   T   
   G   
   A   
   A   
   G   
   A   
    C    
   A   
   T   
   C   
    A    
   T   
    G    
    C    
   T   
    G    
   A   
   T   
    T    
   A   
   C   
    A    
    G    
   A   
    T    
   A   
   A   
   A   
   G   
   G   
    T    
   G   
   T   
    C    
   A   
   T   
   T   
   A   
   T   
   C   
   C   
   G   
    T    
   T   
   T   
    C    
   A   
   A   
   C   
   G   
   T   
    A    
    T    
    C    
    G    
    A    
   C   
   T   
   G   
   T   
   T   
   T   
   C   
   T   
   C   
   A   
   A   
   A   
   C   
    T    
   G   
   G   
   A   
   C   
   G   
    T    
    T    
   C   
   A   
   A   
   C   
   A   
   T   
   T   
    A    
   G   
   G   
    C    
   G   
   T   
    A    
   C   
   G   
   T   
   T   
   T   
    G    
   A   
   T   
   G   
   A   
    A    
    G    
   A   
   T   
   G   
   G   
   A   
   A   
   G   
    C    
    T    
   G   
    A    
    T    
    A    
   C   
    G    
   A   
   A   
   A   
   G   
   T   
   T   
   G   
   T   
   C   
   A   
   C   
   G   
   A   
   T   
   G   
   G   
   C   
   A   
   G   
   C   
   A   
   G   
   T   
   C   
   G   
   A   
   A   
   C   
   C   
   A   
   G   
   A   
   A   
   G   
   C   
   T   
   G   
   C   
   A   
   G   
   A   
   A   
   C   
   A   
   A   
   A   
   C   
   A   
   G   
   T   
   T   
   G   
   A   
   A   
   G   
   A   
   A   
   C   
   A   
   A   
   G   
   G   
   A   
   T   
   C   
   A   
   G   
   A   
   A   
   G   
   A   
   A   
   -   
   -   
   -   
 

 
   N59611F   
   A   
   T   
   G   
   A   
   G   
   T   
   G   
   A   
   A   
   G   
   A   
   A   
   A   
   T   
   T   
   A   
   A   
   A   
   G   
   A   
   A   
   A   
   A   
   C   
   A   
   T   
   T   
   C   
   A   
   A   
   G   
   A   
   C   
   G   
   T   
   C   
   A   
   A   
   T   
   C   
   T   
   G   
   A   
   C   
   C   
   A   
   G   
   C   
   G   
   A   
   A   
   A   
   T   
   G   
   A   
   A   
   A   
   G   
   A   
   A   
   T   
   C   
   T   
   T   
   T   
   C   
   A   
   T   
   T   
   G   
   A   
   C   
   T   
   A   
   C   
   G   
   C   
   A   
   A   
   T   
   G   
   A   
   G   
   T   
   G   
   T   
   T   
   A   
   T   
   C   
   G   
   T   
   A   
   G   
   C   
   C   
   C   
   G   
   C   
   G   
   C   
   G   
   T   
   T   
   A   
   C   
   C   
   T   
   G   
   A   
   C   
   G   
   T   
   T   
   C   
   G   
   T   
   G   
   A   
   C   
   G   
   G   
   T   
   T   
   T   
   A   
   A   
   A   
   A   
   C   
   C   
   T   
   G   
   T   
   T   
   C   
   A   
   T   
   C   
   G   
   C   
   C   
   G   
   A   
   A   
   T   
   C   
   T   
   T   
   A   
   T   
   A   
   T   
   G   
   G   
   A   
   A   
   T   
   G   
   A   
   A   
   C   
   G   
   A   
   A   
   T   
   T   
   A   
   G   
   G   
   G   
   G   
   T   
   A   
   A   
   C   
   C   
   C   
   C   
   T   
   G   
   A   
   T   
   A   
   A   
   A   
   C   
   C   
   A   
   C   
   A   
   C   
   A   
   A   
   A   
   A   
   A   
   A   
   T   
   C   
   A   
   G   
   C   
   C   
   C   
   G   
   G   
   A   
   T   
   T   
   G   
   T   
   T   
   G   
   G   
   G   
   G   
   A   
   T   
   G   
   T   
   T   
   A   
   T   
   G   
   G   
   G   
   T   
   A   
   A   
   A   
   T   
   A   
   T   
   C   
   A   
   T   
   C   
   C   
   C   
   C   
   A   
   T   
   G   
   G   
   G   
   G   
   A   
   C   
   A   
   G   
   T   
   G   
   C   
   G   
   A   
   T   
   T   
   T   
   A   
   C   
   G   
   A   
   A   
   T   
   C   
   A   
   A   
   T   
   G   
   G   
   T   
   G   
   C   
   G   
   G   
   A   
   T   
   G   
   G   
   C   
   A   
   C   
   A   
   A   
   C   
   C   
   T   
   T   
   T   
   T   
   A   
   G   
   T   
   T   
   A   
   T   
   C   
   G   
   G   
   G   
   C   
   T   
   A   
   T   
   G   
   T   
   T   
   A   
   G   
   T   
   T   
   G   
   A   
   C   
   G   
   G   
   C   
   C   
   A   
   C   
   G   
   G   
   A   
   A   
   A   
   C   
   T   
   T   
   C   
   G   
   G   
   T   
   T   
   C   
   T   
   G   
   T   
   C   
   G   
   A   
   T   
   G   
   G   
   T   
   G   
   A   
   C   
   G   
   G   
   C   
   G   
   C   
   T   
   G   
   C   
   C   
   G   
   C   
   T   
   A   
   T   
   G   
   C   
   G   
   T   
   T   
   A   
   T   
   A   
   C   
   C   
   G   
   A   
   A   
   G   
   C   
   A   
   C   
   G   
   T   
   A   
   T   
   G   
   A   
   G   
   T   
   A   
   A   
   A   
   A   
   T   
   T   
   G   
   C   
   T   
   T   
   T   
   A   
   G   
   A   
   A   
   A   
   T   
   G   
   C   
   T   
   A   
   C   
   G   
   A   
   G   
   A   
   T   
   A   
   T   
   T   
   A   
   A   
   C   
   A   
   A   
   A   
   A   
   A   
   T   
   A   
   C   
   A   
   G   
   T   
   C   
   G   
   A   
   T   
   T   
   T   
   C   
   C   
   A   
   A   
   G   
   G   
   A   
   A   
   A   
   C   
   T   
   A   
   T   
   G   
   A   
   T   
   G   
   A   
   T   
   T   
   C   
   A   
   G   
   A   
   A   
   C   
   A   
   A   
   G   
   A   
   A   
   C   
   C   
   A   
   G   
   T   
   A   
   G   
   T   
   T   
   T   
   T   
   A   
   C   
   C   
   T   
   G   
   C   
   T   
   C   
   G   
   T   
   T   
   T   
   T   
   C   
   C   
   A   
   A   
   A   
   C   
   T   
   T   
   A   
   C   
   T   
   A   
   G   
   T   
   T   
   A   
   A   
   C   
   G   
   G   
   A   
   A   
   C   
   A   
   A   
   C   
   G   
   G   
   G   
   G   
   A   
   T   
   T   
   G   
   C   
   G   
   G   
   T   
   G   
   G   
   G   
   G   
   A   
   T   
   G   
   G   
   C   
   A   
   A   
   C   
   A   
   A   
   A   
   T   
   A   
   T   
   T   
   C   
   C   
   A   
   C   
   C   
   A   
   C   
   A   
   T   
   A   
   A   
   T   
   T   
   T   
   A   
   A   
   G   
   T   
   G   
   A   
   A   
   G   
   T   
   G   
   A   
   T   
   T   
   G   
   A   
   T   
   G   
   C   
   A   
   A   
   C   
   A   
   A   
   G   
   T   
   T   
   T   
   A   
   T   
   T   
   G   
   A   
   T   
   G   
   G   
   A   
   C   
   A   
   A   
   T   
   C   
   C   
   T   
   G   
   A   
   T   
   G   
   T   
   A   
   A   
   C   
   G   
   A   
   C   
   G   
   A   
   A   
   T   
   G   
   A   
   A   
   T   
   T   
   G   
   A   
   T   
   G   
   G   
   A   
   A   
   G   
   T   
   G   
   T   
   T   
   A   
   C   
   C   
   T   
   G   
   G   
   A   
   C   
   C   
   A   
   G   
   A   
   T   
   T   
   T   
   T   
   C   
   C   
   A   
   A   
   C   
   A   
   G   
   G   
   C   
   G   
   G   
   T   
   T   
   T   
   A   
   G   
   T   
   G   
   A   
   T   
   G   
   G   
   G   
   G   
   A   
   A   
   A   
   T   
   C   
   A   
   G   
   G   
    G    
   A   
   T   
    T    
   C   
   G   
    C    
    C    
   G   
   A   
   G   
   C   
    A    
   T   
   A   
   T   
   G   
   A   
   A   
   A   
   C   
    A    
   G   
   G   
    G    
   A   
   A   
    A    
   G   
   G   
   T   
   T   
   C   
    G    
   A   
   T   
   T   
   A   
   C   
    T    
   G   
   T   
    T    
   C   
   G   
   T   
   G   
   C   
   A   
   A   
   A   
   A   
   G   
   T   
   T   
   G   
   A   
    A    
    T    
   T   
    G    
   A   
    C    
   T   
   G   
   A   
    A    
   A   
   T   
   G   
    C    
   C   
    G    
   A   
   A   
   T   
   G   
   G   
    A    
   A   
   A   
   A   
   G   
   A   
   A   
   C   
   G   
    G    
   A   
   T   
    T    
    T    
   T   
    A    
   G   
   T   
    A    
   A   
   C   
    T    
   G   
   A   
   A   
   T   
   T   
    G    
   C   
   C   
   T   
   T   
   A   
   T   
   A   
   T   
   G   
   G   
   T   
    G    
   A   
   A   
   T   
   A   
   A   
   A   
   G   
   C   
    C    
   A   
   A   
   A   
   T   
   T   
    A    
   A   
   T   
    C    
   G   
   A   
   A   
   C   
   G   
    A    
   A   
   T   
   T   
   T   
   C   
   T   
   G   
   A   
    A    
    T    
   T   
    A    
   C   
   A   
   C   
   A   
   G   
   A   
   G   
   A   
    T    
   A   
   A   
    A    
   C   
   G   
    A    
   A   
   T   
    T    
   G   
   A   
   A   
   G   
   G   
    A    
   A   
   T   
    T    
   A   
   C   
    T    
   G   
   A   
   T   
   T   
   T   
   G   
   C   
   G   
   G   
   G   
   A   
    T    
   G   
   A   
   A   
   T   
   C   
   T   
   T   
   C   
    T    
   C   
   G   
   T   
   G   
   A   
    A    
   G   
   G   
    C    
   A   
   T   
   G   
   C   
   G   
    G    
   A   
   T   
    C    
   G   
   T   
   C   
   A   
   T   
    C    
   G   
   A   
    T    
    G    
   T   
    T    
   C   
   G   
   T   
   C   
   G   
    A    
   G   
   A   
    T    
   G   
    T    
    G    
   A   
   G   
    T    
   G   
   C   
    C    
   T   
   C   
    T    
   G   
   T   
   T   
    G    
   T   
    G    
   T   
   T   
    A    
   A   
   A   
    C    
   A   
   A   
   C   
   T   
   T   
    G    
   T   
   A   
    C    
   A   
   A   
   A   
    A    
   T   
   G   
   A   
   C   
    T    
    G    
   C   
    C    
   T   
   T   
    A    
   C   
   A   
    A    
   A   
    C    
    A    
   T   
   C   
    T    
   T   
   T   
    T    
   G   
   G   
    T    
   T   
   T   
    T    
   A   
   A   
    C    
   A   
   T   
   G   
   T   
   T   
    A    
   G   
   C   
    C    
   A   
   T   
    T    
   G   
   A   
   A   
   A   
   A   
   A   
   G   
   G   
    C    
   G   
   T   
    A    
   C   
   C   
    G    
   A   
   A   
   A   
    A    
   T   
   T   
   T   
   T   
    A    
   A   
   G   
    C    
   T   
   T   
   G   
   A   
   A   
   A   
   C   
    G    
    T    
   A   
   T   
    T    
    T    
   T   
    A    
   G   
   A   
   A   
   A   
   A   
    T    
   T   
   A   
    C    
    G    
   T   
    C    
   G   
   A   
    G    
   C   
   A   
    C    
   C   
   A   
    A    
    A    
    A    
    A    
    G    
   A   
   A   
   G   
   T   
   C   
   A   
   T   
    T    
   A   
   C   
    T    
    C    
   G   
    C    
   C   
   G   
   G   
   A   
   C   
    G    
    A    
   T   
    T    
   T   
   T   
   T   
   G   
   A   
    C    
   A   
   A   
   A   
    A    
   A   
    C    
   A   
   A   
    A    
   G   
   C   
   A   
   G   
   A   
   A   
   G   
   C   
   A   
    C    
   G   
    G    
   G   
   C   
    G    
   C   
   A   
    T    
   A   
   T   
    T    
   T   
   T   
   A   
   G   
   A   
   A   
   G   
   G   
   T   
    C    
   T   
    A    
   C   
   G   
    A    
   A   
   T   
   T   
   G   
   C   
    C    
   T   
   T   
    A    
   G   
   A   
   T   
   C   
   A   
   T   
   A   
   T   
   C   
    G    
   A   
   T   
   G   
   A   
   A   
   A   
   T   
    C    
   A   
   T   
   T   
   G   
   C   
    C    
   A   
   T   
   T   
   A   
   T   
    C    
   C   
   G   
    T    
   G   
   G   
    A    
   T   
   C   
    A    
    C    
   A   
   A   
   T   
   C   
   A   
   G   
   A   
   T   
   G   
   A   
    C    
   G   
   A   
    A    
   G   
   C   
   A   
   A   
   A   
   A   
   G   
   C   
    A    
   A   
    C    
    G    
    T    
   T   
    A    
   A   
   T   
    T    
   G   
   A   
   A   
   C   
   G   
    C    
   T   
   T   
    T    
   G   
   A   
    A    
   T   
   T   
    T    
   T   
   C   
    A    
   G   
   A   
   T   
   C   
   G   
    T    
   C   
   A   
    A    
    G    
   C   
    G    
   C   
   A   
   A   
   G   
   C   
    G    
   A   
   T   
    T    
   T   
   T   
   A   
   G   
   A   
   T   
   A   
   T   
   G   
   C   
   G   
   T   
   T   
   T   
    A    
   C   
   G   
    C    
   C   
   G   
    T    
   T   
   T   
    A    
   A   
   C   
    A    
   G   
   G   
    C    
   T   
   T   
    A    
   G   
   A   
   A   
   C   
   G   
    C    
   G   
   A   
    C    
   A   
   A   
   A   
   A   
   T   
    T    
   G   
   A   
    A    
   A   
   A   
   T   
   G   
   A   
   A   
   T   
   A   
    T    
   C   
   A   
   A   
   G   
   A   
    G    
   C   
   T   
    A    
   T   
   T   
    G    
   A   
    A    
   A   
    T    
   T   
    C    
   A   
   T   
   T   
   G   
    C    
    G    
   G   
   A   
    C    
   T   
   T   
    A    
    G    
    A    
    G    
   G   
   A   
   C   
   A   
   T   
   T   
    T    
   T   
    A    
   G   
   C   
    C    
    C    
   G   
    C    
   C   
   C   
    A    
   G   
   A   
    A    
   C   
   G   
    C    
   G   
   T   
    C    
   A   
    T    
    T    
   G   
   A   
   A   
   A   
   T   
    C    
   A   
   T   
    T    
   A   
   A   
    A    
   A   
    C    
    A    
   G   
   A   
    G    
   T   
   T   
   A   
    A    
    A    
    T    
   G   
   A   
   C   
    G    
   T   
    T    
    C    
    G    
    C    
    A    
    C    
   A   
    A    
    A    
   A   
   T   
   T   
   T   
   G   
   G   
   T   
   G   
   A   
   T   
    G    
    C    
   A   
   C   
   G   
   A   
   C   
   G   
    C    
   A   
   C   
   A   
   G   
   A   
   A   
    T    
   T   
   A   
    T    
   T   
   A   
   G   
   T   
    A    
   G   
   G   
   T   
   G   
   A   
    A    
   G   
   T   
   C   
    C    
   T   
    A    
   A   
   G   
   T   
   C   
   T   
    T    
   G   
   A   
   A   
   G   
   A   
   T   
   G   
   A   
   A   
   G   
   A   
   T   
    T    
   T   
    A    
   A   
   T   
    C    
   G   
   A   
   A   
   G   
   A   
   A   
   G   
   A   
   A   
   G   
   A   
   A   
    G    
   T   
   C   
   G   
   T   
    G    
   A   
   T   
   T   
   A   
   C   
    G    
    T    
   T   
    A    
   A   
   C   
    C    
   A   
   A   
    T    
   A   
   A   
    C    
   G   
   G   
    C    
   T   
   A   
    C    
   A   
   T   
    T    
   A   
   A   
    G    
   C   
   G   
    G    
    A    
   T   
   G   
   G   
    C    
   A   
   A   
   A   
    C    
    T    
    C    
    C    
   G   
   A   
   A   
   T   
   T   
   C   
    C    
   G   
    G    
   G   
   C   
    G    
   C   
   A   
   A   
   C   
   G   
    C    
    C    
   G   
    T    
   G   
   G   
    C    
   G   
   G   
    A    
   C   
   G   
   T   
   G   
   G   
    T    
   G   
   T   
    C    
   C   
   A   
   A   
   G   
   G   
    C    
   A   
   T   
   G   
   G   
   G   
    C    
    G    
   T   
    T    
   C   
   A   
    T    
    G    
   A   
   T   
   G   
   A   
    T    
   G   
   A   
    T    
   T   
   T   
   C   
   G   
   T   
    G    
   A   
   A   
    A    
   A   
   A   
    C    
   C   
   T   
    A    
    G    
   T   
   T   
   T   
   C   
   T   
   T   
   G   
    T    
   T   
   C   
    A    
   A   
   C   
    A    
   C   
   A   
    C    
   G   
   A   
    C    
   A   
   C   
    G    
   T   
   T   
    A    
   T   
   T   
   A   
   T   
   T   
    C    
   T   
   T   
   T   
   A   
   C   
   G   
   A   
   A   
    C    
   A   
    C    
    T    
   G   
   G   
    G    
   A   
   A   
   A   
   G   
   T   
    T    
   T   
   A   
    C    
   C   
   G   
    A    
   G   
   C   
   A   
   A   
   A   
   A   
   G   
   G   
    T    
   T   
   A   
    C    
   G   
   A   
   A   
   A   
   T   
    C    
   C   
   C   
    T    
   G   
   A   
    A    
   T   
   A   
   C   
   G   
   G   
    T    
    A    
   G   
    A    
   A   
   C   
   A   
   G   
   C   
    A    
   A   
   A   
   A   
   G   
   G   
    A    
   A   
   T   
    T    
   C   
   C   
   A   
    G    
   T   
    A    
   A   
   T   
    T    
   A   
   A   
    C    
   T   
   T   
    A    
   C   
   T   
    G    
   G   
   G   
    A    
   A   
   T   
    T    
   G   
   A   
    T    
   T   
   C   
    T    
    G    
    C    
    A    
   G   
   A   
   A   
   A   
    A    
    A    
   A   
   T   
    T    
   C   
   A   
    A    
   G   
   C   
    G    
   A   
   T   
    T    
   A   
   T   
   T   
    T    
   C   
    T    
   G   
   T   
    T    
    G    
    A    
   G   
   G   
   G   
    C    
    A    
   A   
   A   
   G   
   C   
    G    
   G   
   A   
   A   
   G   
    C    
    A    
   G   
   G   
    T    
    A    
   A   
    A    
   T   
   A   
    C    
    T    
   T   
    G    
   T   
   T   
   C   
   T   
   T   
    C    
   A   
   C   
    A    
   A   
   C   
    C    
    T    
    T    
    A    
    A    
   A   
   A   
   G   
   G   
    A    
   A   
   C   
    C    
   G   
   T   
    C    
   A   
   A   
   A   
   C   
   G   
    G    
   A   
   C   
    A    
    G    
   C   
    C    
   G   
   T   
    A    
   A   
   C   
    A    
   G   
   C   
    C    
   T   
   T   
    T    
    T    
   C   
   T   
   A   
   A   
    T    
   A   
   T   
    C    
   C   
   G   
    T    
   A   
   G   
    T    
   A   
   A   
   T   
   G   
   G   
    A    
    T    
   T   
    A    
   A   
   T   
    C    
   G   
   C   
    C    
   A   
   T   
   T   
    A    
   G   
    C    
   T   
   T   
    A    
   A   
   A   
   A   
   G   
   A   
   A   
   G   
   A   
    T    
   G   
   A   
    T    
   G   
   A   
    G    
    T    
   T   
    A    
    G    
   T   
    T    
   A   
   A   
    C    
   G   
   T   
    G    
    G    
   T   
   A   
    A    
    C    
   G   
   A   
   C   
    T    
    A    
   A   
    T    
   G   
   G   
    C    
    A    
   A   
    T    
    C    
    A    
    G    
   A   
   A   
    G    
   A   
   T   
    G    
   A   
   T   
    T    
   A   
   T   
    C    
   G   
   G   
    A    
   A   
   C   
   A   
   C   
   A   
   T   
    G    
    C    
    A    
   G   
   G   
    A    
   T   
   A   
   C   
   T   
   C   
   T   
   G   
   T   
    C    
   A   
   C   
    A    
   T   
   T   
    T    
    G    
   A   
    T    
   G   
   A   
    A    
    A    
    A    
    T    
    A    
   C   
    T    
   G   
    T    
    A    
   C   
   G   
    T    
   G   
   A   
   T   
   A   
   T   
   G   
   G   
   G   
    C    
   C   
   G   
    G    
   A   
   C   
   A   
   G   
   C   
    A    
   T   
   C   
    A    
   G   
   G   
    T    
   G   
   T   
    T    
    C    
   G   
    G    
   G   
   G   
    A    
   A   
   T   
    C    
   C   
   G   
    T    
    C    
   T   
    C    
   C   
   G   
    C    
   G   
   A   
    A    
    A    
   A   
    T    
   G   
   A   
    T    
   T   
    A    
    T    
   G   
   T   
    G    
   G   
   T   
    C    
   G   
   G   
    T    
    G    
   C   
   A   
   G   
   C   
    G    
    A    
   T   
    T    
    C    
   T   
   G   
    G    
   A   
    T    
   G   
    A    
   A   
    A    
   A   
    C    
    A    
   A   
   A   
   G   
   A   
   A   
   G   
   T   
    C    
    C    
   T   
    A    
   G   
   T   
    C    
   A   
   T   
    T    
   A   
   C   
    T    
   G   
   A   
   A   
   A   
   A   
    T    
   G   
   G   
   T   
   T   
   A   
    T    
   G   
   G   
    T    
   A   
   A   
    G    
   C   
   G   
    T    
   A   
   C   
    A    
   A   
   A   
   A   
   G   
    C    
    T    
   T   
   C   
    T    
   G   
   A   
    A    
   T   
   A   
   T   
   C   
   C   
   A   
   G   
   T   
    T    
   A   
   A   
    A    
   G   
   G   
    A    
   C   
   G   
    T    
   G   
   G   
    C    
   G   
   G   
   T   
   A   
   A   
   A   
   G   
   G   
    G    
   A   
   T   
    T    
   A   
   A   
    G    
   A   
   C   
    A    
    G    
   C   
    A    
   A   
   A   
    T    
   A   
   T   
   C   
   A   
   C   
   T   
   G   
   A   
    G    
   A   
   A   
   A   
   A   
   A   
   T   
   G   
   G   
   T   
   C   
   C   
   A   
    T    
   T   
   A   
   G   
   C   
    T    
   G   
   G   
    T    
   T   
   T   
    A    
   A   
   C   
   C   
   A   
   C   
    G    
   G   
   T   
    C    
    A    
    A    
   T   
   G   
   G   
    G    
    G    
   A   
   T   
   G   
   A   
   A   
   G   
   A   
    T    
   A   
   T   
   C   
    T    
   T   
    A    
    T    
   T   
    G    
   A   
   T   
    T    
   A   
   C   
    G    
    A    
   A   
    C    
   A   
   A   
   A   
   G   
   G   
    C    
   G   
   T   
    C    
   A   
   T   
   T   
   A   
   T   
   C   
   C   
   G   
    C    
   T   
   T   
    T    
   A   
   A   
   C   
   G   
   T   
    T    
    G    
    A    
    T    
    T    
   C   
   T   
   G   
   T   
   T   
   T   
   C   
   T   
   C   
   A   
   A   
   A   
   C   
    A    
   G   
   G   
   A   
   C   
   G   
    C    
    G    
   C   
   A   
   A   
   C   
   A   
   T   
   T   
    A    
   G   
   G   
    G    
   G   
   T   
    T    
   C   
   G   
   T   
   T   
   T   
    A    
   A   
   T   
   G   
   A   
    G    
    A    
   A   
   T   
   G   
   G   
   A   
   A   
   G   
    A    
    T    
   G   
    G    
    T    
    G    
   C   
    C    
   A   
   A   
   A   
   -   
   -   
   -   
   -   
   -   
   -   
   -   
   -   
   -   
   -   
   -   
   -   
   -   
   -   
   -   
   -   
   -   
   -   
   -   
   -   
   -   
   -   
   -   
   -   
   -   
   -   
   -   
   -   
   -   
   -   
   -   
   -   
   -   
   -   
   -   
   -   
   -   
   -   
   -   
   -   
   -   
   -   
   -   
   -   
   -   
   -   
   -   
   -   
   -   
   -   
   -   
   -   
   -   
   -   
   -   
   -   
   -   
   -   
   -   
   -   
   -   
   -   
   -   
   -   
   -   
   -   
   -   
   -   
   -   
   -   
   -   
   -   
 

 
   N55240   
   A   
   T   
   G   
   A   
   G   
   T   
   G   
   A   
   A   
   G   
   A   
   A   
   A   
   T   
   T   
   A   
   A   
   A   
   G   
   A   
   A   
   A   
   A   
   C   
   A   
   T   
   T   
   C   
   A   
   A   
   G   
   A   
   C   
   G   
   T   
   C   
   A   
   A   
   T   
   C   
   T   
   G   
   A   
   C   
   C   
   A   
   G   
   C   
   G   
   A   
   A   
   A   
   T   
   G   
   A   
   A   
   A   
   G   
   A   
   A   
   T   
   C   
   T   
   T   
   T   
   C   
   A   
   T   
   T   
   G   
   A   
   C   
   T   
   A   
   C   
   G   
   C   
   A   
   A   
   T   
   G   
   A   
   G   
   T   
   G   
   T   
   T   
   A   
   T   
   C   
   G   
   T   
   A   
   G   
   C   
   C   
   C   
   G   
   C   
   G   
   C   
   G   
   T   
   T   
   A   
   C   
   C   
   T   
   G   
   A   
   C   
   G   
   T   
   T   
   C   
   G   
   T   
   G   
   A   
   C   
   G   
   G   
   T   
   T   
   T   
   A   
   A   
   A   
   A   
   C   
   C   
   T   
   G   
   T   
   T   
   C   
   A   
   T   
   C   
   G   
   C   
   C   
   G   
   A   
   A   
   T   
   C   
   T   
   T   
   A   
   T   
   A   
   T   
   G   
   G   
   A   
   A   
   T   
   G   
   A   
   A   
   C   
   G   
   A   
   A   
   T   
   T   
   A   
   G   
   G   
   G   
   G   
   T   
   A   
   A   
   C   
   C   
   C   
   C   
   T   
   G   
   A   
   T   
   A   
   A   
   A   
   C   
   C   
   A   
   C   
   A   
   C   
   A   
   A   
   A   
   A   
   A   
   A   
   T   
   C   
   A   
   G   
   C   
   C   
   C   
   G   
   G   
   A   
   T   
   T   
   G   
   T   
   T   
   G   
   G   
   G   
   G   
   A   
   T   
   G   
   T   
   T   
   A   
   T   
   G   
   G   
   G   
   T   
   A   
   A   
   A   
   T   
   A   
   T   
   C   
   A   
   C   
   C   
   C   
   C   
   C   
   A   
   T   
   G   
   G   
   G   
   G   
   A   
   C   
   A   
   G   
   T   
   G   
   C   
   G   
   A   
   T   
   T   
   T   
   A   
   C   
   G   
   A   
   A   
   T   
   C   
   A   
   A   
   T   
   G   
   G   
   T   
   G   
   C   
   G   
   G   
   A   
   T   
   G   
   G   
   C   
   A   
   C   
   A   
   A   
   C   
   C   
   T   
   T   
   T   
   T   
   A   
   G   
   T   
   T   
   A   
   T   
   C   
   G   
   G   
   G   
   C   
   T   
   A   
   T   
   G   
   C   
   T   
   A   
   G   
   T   
   T   
   G   
   A   
   C   
   G   
   G   
   C   
   C   
   A   
   C   
   G   
   G   
   A   
   A   
   A   
   C   
   T   
   T   
   C   
   G   
   G   
   T   
   T   
   C   
   T   
   G   
   T   
   C   
   G   
   A   
   T   
   G   
   G   
   T   
   G   
   A   
   C   
   G   
   G   
   C   
   G   
   C   
   T   
   G   
   C   
   C   
   G   
   C   
   T   
   A   
   T   
   G   
   C   
   G   
   T   
   T   
   A   
   T   
   A   
   C   
   C   
   G   
   A   
   A   
   G   
   C   
   A   
   C   
   G   
   T   
   A   
   T   
   G   
   A   
   G   
   T   
   A   
   A   
   A   
   A   
   T   
   T   
   G   
   C   
   T   
   T   
   T   
   A   
   G   
   A   
   A   
   A   
   T   
   G   
   C   
   T   
   A   
   C   
   G   
   A   
   G   
   A   
   T   
   A   
   T   
   T   
   A   
   A   
   C   
   A   
   A   
   A   
   A   
   A   
   T   
   A   
   C   
   A   
   G   
   T   
   C   
   G   
   A   
   T   
   T   
   T   
   C   
   C   
   A   
   A   
   G   
   G   
   A   
   A   
   A   
   C   
   T   
   A   
   T   
   G   
   A   
   T   
   G   
   A   
   T   
   T   
   C   
   A   
   G   
   A   
   A   
   C   
   A   
   A   
   G   
   A   
   A   
   C   
   C   
   A   
   G   
   T   
   A   
   G   
   T   
   T   
   T   
   T   
   A   
   C   
   C   
   T   
   G   
   C   
   T   
   C   
   G   
   T   
   T   
   T   
   T   
   C   
   C   
   A   
   A   
   A   
   C   
   T   
   T   
   A   
   C   
   T   
   A   
   G   
   T   
   T   
   A   
   A   
   C   
   G   
   G   
   A   
   A   
   C   
   A   
   A   
   C   
   G   
   G   
   G   
   G   
   A   
   T   
   T   
   G   
   C   
   G   
   G   
   T   
   G   
   G   
   G   
   G   
   A   
   T   
   G   
   G   
   C   
   A   
   A   
   C   
   A   
   A   
   A   
   T   
   A   
   T   
   T   
   C   
   C   
   A   
   C   
   C   
   A   
   C   
   A   
   C   
   A   
   A   
   T   
   T   
   T   
   A   
   A   
   G   
   T   
   G   
   A   
   A   
   G   
   T   
   G   
   A   
   T   
   T   
   G   
   A   
   T   
   G   
   C   
   A   
   A   
   C   
   A   
   A   
   G   
   T   
   T   
   T   
   A   
   T   
   T   
   G   
   A   
   T   
   G   
   G   
   A   
   C   
   A   
   A   
   T   
   C   
   C   
   T   
   G   
   A   
   T   
   G   
   T   
   A   
   A   
   C   
   G   
   A   
   C   
   G   
   A   
   A   
   T   
   G   
   A   
   A   
   T   
   T   
   G   
   A   
   T   
   G   
   G   
   A   
   A   
   G   
   T   
   G   
   T   
   T   
   A   
   C   
   C   
   T   
   G   
   G   
   A   
   C   
   C   
   A   
   G   
   A   
   T   
   T   
   T   
   T   
   C   
   C   
   A   
   A   
   C   
   A   
   G   
   G   
   C   
   G   
   G   
   T   
   T   
   T   
   A   
   G   
   T   
   G   
   A   
   T   
   G   
   G   
   G   
   G   
   A   
   A   
   A   
   T   
   C   
   A   
   G   
   G   
    G    
   A   
   T   
    T    
   C   
   G   
    C    
    C    
   G   
   A   
   G   
   C   
    A    
   T   
   A   
   T   
   G   
   A   
   A   
   A   
   C   
    A    
   G   
   G   
    G    
   A   
   A   
    A    
   G   
   G   
   T   
   T   
   C   
    G    
   A   
   T   
   T   
   A   
   C   
    T    
   G   
   T   
    T    
   C   
   G   
   T   
   G   
   C   
   A   
   A   
   A   
   A   
   G   
   T   
   T   
   G   
   A   
    A    
    T    
   T   
    G    
   A   
    C    
   T   
   G   
   A   
    A    
   A   
   T   
   G   
    C    
   C   
    G    
   A   
   A   
   T   
   G   
   G   
    A    
   A   
   A   
   A   
   G   
   A   
   A   
   C   
   G   
    T    
   A   
   T   
    T    
    T    
   T   
    A    
   G   
   T   
    A    
   A   
   C   
    T    
   G   
   A   
   A   
   T   
   T   
    G    
   C   
   C   
   T   
   T   
   A   
   T   
   A   
   T   
   G   
   G   
   T   
    G    
   A   
   A   
   T   
   A   
   A   
   A   
   G   
   C   
    C    
   A   
   A   
   A   
   T   
   T   
    A    
   A   
   T   
    C    
   G   
   A   
   A   
   C   
   G   
    A    
   A   
   T   
   T   
   T   
   C   
   T   
   G   
   A   
    A    
    T    
   T   
    A    
   C   
   A   
   C   
   A   
   G   
   A   
   G   
   A   
    T    
   A   
   A   
    A    
   C   
   G   
    A    
   A   
   T   
    T    
   G   
   A   
   A   
   G   
   G   
    A    
   A   
   T   
    T    
   A   
   C   
    T    
   G   
   A   
   T   
   T   
   T   
   G   
   C   
   G   
   G   
   G   
   A   
    T    
   G   
   A   
   A   
   T   
   C   
   T   
   T   
   C   
    T    
   C   
   G   
   T   
   G   
   A   
    A    
   G   
   G   
    C    
   A   
   T   
   G   
   C   
   G   
    G    
   A   
   T   
    T    
   G   
   T   
   C   
   A   
   T   
    C    
   G   
   A   
    T    
    G    
   T   
    T    
   C   
   G   
   T   
   C   
   G   
    A    
   G   
   A   
    T    
   G   
    T    
    G    
   A   
   G   
    T    
   G   
   C   
    C    
   T   
   C   
    T    
   G   
   T   
   T   
    G    
   T   
    G    
   T   
   T   
    A    
   A   
   A   
    C    
   A   
   A   
   C   
   T   
   T   
    G    
   T   
   A   
    C    
   A   
   A   
   A   
    A    
   T   
   G   
   A   
   C   
    T    
    G    
   C   
    C    
   T   
   T   
    A    
   C   
   A   
    A    
   A   
    C    
    A    
   T   
   C   
    T    
   T   
   T   
    T    
   G   
   G   
    T    
   T   
   T   
    T    
   A   
   A   
    C    
   A   
   T   
   G   
   T   
   T   
    A    
   G   
   C   
    C    
   A   
   T   
    C    
   G   
   A   
   A   
   A   
   A   
   A   
   G   
   G   
    C    
   G   
   T   
    A    
   C   
   C   
    G    
   A   
   A   
   A   
    A    
   T   
   T   
   T   
   T   
    A    
   A   
   G   
    C    
   T   
   T   
   G   
   A   
   A   
   A   
   C   
    G    
    T    
   A   
   T   
    T    
    T    
   T   
    A    
   G   
   A   
   A   
   A   
   A   
    C    
   T   
   A   
    C    
    G    
   T   
    C    
   G   
   A   
    G    
   C   
   A   
    C    
   C   
   A   
    A    
    A    
    A    
    A    
    G    
   A   
   A   
   G   
   T   
   C   
   A   
   T   
    T    
   A   
   C   
    T    
    C    
   G   
    C    
   C   
   G   
   G   
   A   
   C   
    G    
    A    
   T   
    T    
   T   
   T   
   T   
   G   
   A   
    C    
   A   
   A   
   A   
    A    
   A   
    C    
   A   
   A   
    G    
   G   
   C   
   A   
   G   
   A   
   A   
   G   
   C   
   A   
    C    
   G   
    G    
   G   
   C   
    G    
   C   
   A   
    T    
   A   
   T   
    T    
   T   
   T   
   A   
   G   
   A   
   A   
   G   
   G   
   T   
    C    
   T   
    A    
   C   
   G   
    A    
   A   
   T   
   T   
   G   
   C   
    C    
   T   
   T   
    A    
   G   
   A   
   T   
   C   
   A   
   T   
   A   
   T   
   C   
    G    
   A   
   T   
   G   
   A   
   A   
   A   
   T   
    C    
   A   
   T   
   T   
   G   
   C   
    C    
   A   
   T   
   T   
   A   
   T   
    C    
   C   
   G   
    T    
   G   
   G   
    G    
   T   
   C   
    A    
    C    
   A   
   A   
   T   
   C   
   A   
   G   
   A   
   T   
   G   
   A   
    C    
   G   
   A   
    A    
   G   
   C   
   A   
   A   
   A   
   A   
   G   
   C   
    A    
   A   
    C    
    G    
    T    
   T   
    A    
   A   
   T   
    T    
   G   
   A   
   A   
   C   
   G   
    C    
   T   
   T   
    T    
   G   
   A   
    A    
   T   
   T   
    T    
   T   
   C   
    A    
   G   
   A   
   T   
   C   
   G   
    T    
   C   
   A   
    A    
    G    
   C   
    G    
   C   
   A   
   A   
   G   
   C   
    A    
   A   
   T   
    T    
   T   
   T   
   A   
   G   
   A   
   T   
   A   
   T   
   G   
   C   
   G   
   T   
   T   
   T   
    A    
   C   
   G   
    C    
   C   
   G   
    T    
   T   
   T   
    A    
   A   
   C   
    A    
   G   
   G   
    C    
   T   
   T   
    A    
   G   
   A   
   A   
   C   
   G   
    C    
   G   
   A   
    C    
   A   
   A   
   A   
   A   
   T   
    T    
   G   
   A   
    A    
   A   
   A   
   T   
   G   
   A   
   A   
   T   
   A   
    T    
   C   
   A   
   A   
   G   
   A   
    G    
   C   
   T   
    A    
   T   
   T   
    G    
   A   
    A    
   A   
    T    
   T   
    C    
   A   
   T   
   T   
   G   
    C    
    G    
   G   
   A   
    C    
   T   
   T   
    A    
    G    
    A    
    G    
   G   
   A   
   C   
   A   
   T   
   T   
    T    
   T   
    A    
   G   
   C   
    C    
    C    
   G   
    C    
   C   
   C   
    A    
   G   
   A   
    A    
   C   
   G   
    C    
   G   
   T   
    C    
   A   
    T    
    T    
   G   
   A   
   A   
   A   
   T   
    C    
   A   
   T   
    T    
   A   
   A   
    A    
   A   
    C    
    A    
   G   
   A   
    G    
   T   
   T   
   A   
    A    
    A    
    T    
   G   
   A   
   C   
    G    
   T   
    T    
    C    
    G    
    C    
    A    
    C    
   A   
    A    
    A    
   A   
   T   
   T   
   T   
   G   
   G   
   T   
   G   
   A   
   T   
    G    
    C    
   A   
   C   
   G   
   A   
   C   
   G   
    C    
   A   
   C   
   A   
   G   
   A   
   A   
    T    
   T   
   A   
    T    
   T   
   A   
   G   
   T   
    A    
   G   
   G   
   T   
   G   
   A   
    A    
   G   
   T   
   C   
    T    
   T   
    A    
   A   
   G   
   T   
   C   
   T   
    T    
   G   
   A   
   A   
   G   
   A   
   T   
   G   
   A   
   A   
   G   
   A   
   T   
    T    
   T   
    A    
   A   
   T   
    C    
   G   
   A   
   A   
   G   
   A   
   A   
   G   
   A   
   A   
   G   
   A   
   A   
    G    
   T   
   C   
   G   
   T   
    G    
   A   
   T   
   T   
   A   
   C   
    G    
    T    
   T   
    A    
   A   
   C   
    C    
   A   
   A   
    T    
   A   
   A   
    C    
   G   
   G   
    C    
   T   
   A   
    C    
   A   
   T   
    T    
   A   
   A   
    G    
   C   
   G   
    G    
    A    
   T   
   G   
   G   
    C    
   A   
   A   
   A   
    C    
    T    
    C    
    C    
   G   
   A   
   A   
   T   
   T   
   C   
    C    
   G   
    G    
   G   
   C   
    G    
   C   
   A   
   A   
   C   
   G   
    C    
    C    
   G   
    C    
   G   
   G   
    T    
   G   
   G   
    A    
   C   
   G   
   T   
   G   
   G   
    T    
   G   
   T   
    C    
   C   
   A   
   A   
   G   
   G   
    G    
   A   
   T   
   G   
   G   
   G   
    C    
    G    
   T   
    T    
   C   
   A   
    T    
    G    
   A   
   T   
   G   
   A   
    T    
   G   
   A   
    T    
   T   
   T   
   C   
   G   
   T   
    G    
   A   
   A   
    A    
   A   
   A   
    C    
   C   
   T   
    A    
    G    
   T   
   T   
   T   
   C   
   T   
   T   
   G   
    T    
   T   
   C   
    A    
   A   
   C   
    A    
   C   
   A   
    C    
   G   
   A   
    C    
   A   
   C   
    G    
   T   
   T   
    A    
   T   
   T   
   A   
   T   
   T   
    C    
   T   
   T   
   T   
   A   
   C   
   G   
   A   
   A   
    C    
   A   
    C    
    T    
   G   
   G   
    G    
   A   
   A   
   A   
   G   
   T   
    T    
   T   
   A   
    C    
   C   
   G   
    A    
   G   
   C   
   A   
   A   
   A   
   A   
   G   
   G   
    T    
   T   
   A   
    C    
   G   
   A   
   A   
   A   
   T   
    C    
   C   
   C   
    T    
   G   
   A   
    A    
   T   
   A   
   C   
   G   
   G   
    T    
    A    
   G   
    A    
   A   
   C   
   A   
   G   
   C   
    A    
   A   
   A   
   A   
   G   
   G   
    A    
   A   
   T   
    T    
   C   
   C   
   A   
    G    
   T   
    G    
   A   
   T   
    T    
   A   
   A   
    C    
   T   
   T   
    A    
   C   
   T   
    G    
   G   
   G   
    A    
   A   
   T   
    T    
   G   
   A   
    T    
   T   
   C   
    T    
    G    
    C    
    A    
   G   
   A   
   A   
   A   
    A    
    A    
   A   
   T   
    T    
   C   
   A   
    A    
   G   
   C   
    G    
   A   
   T   
    T    
   A   
   T   
   T   
    T    
   C   
    T    
   G   
   T   
    T    
    G    
    A    
   G   
   G   
   G   
    C    
    A    
   A   
   A   
   G   
   C   
    G    
   G   
   A   
   A   
   G   
    C    
    A    
   G   
   G   
    T    
    A    
   A   
    A    
   T   
   A   
    C    
    T    
   T   
    G    
   T   
   T   
   C   
   T   
   T   
    C    
   A   
   C   
    A    
   A   
   C   
    C    
    T    
    T    
    A    
    A    
   A   
   A   
   G   
   G   
    A    
   A   
   C   
    C    
   G   
   T   
    C    
   A   
   A   
   A   
   C   
   G   
    G    
   A   
   C   
    A    
    G    
   C   
    C    
   G   
   T   
    A    
   A   
   C   
    A    
   G   
   C   
    C    
   T   
   T   
    T    
    T    
   C   
   T   
   A   
   A   
    T    
   A   
   T   
    C    
   C   
   G   
    T    
   A   
   G   
    T    
   A   
   A   
   T   
   G   
   G   
    A    
    T    
   T   
    A    
   A   
   T   
    C    
   G   
   C   
    C    
   A   
   T   
   T   
    A    
   G   
    C    
   T   
   T   
    A    
   A   
   A   
   A   
   G   
   A   
   A   
   G   
   A   
    T    
   G   
   A   
    T    
   G   
   A   
    G    
    T    
   T   
    A    
    G    
   T   
    T    
   A   
   A   
    C    
   G   
   T   
    G    
    G    
   T   
   A   
    A    
    C    
   G   
   A   
   C   
    T    
    A    
   A   
    T    
   G   
   G   
    C    
    A    
   A   
    T    
    C    
    A    
    G    
   A   
   A   
    G    
   A   
   T   
    G    
   A   
   T   
    T    
   A   
   T   
    C    
   G   
   G   
    A    
   A   
   C   
   A   
   C   
   A   
   T   
    G    
    C    
    A    
   G   
   G   
    A    
   T   
   A   
   C   
   T   
   C   
   T   
   G   
   T   
    C    
   A   
   C   
    A    
   T   
   T   
    T    
    G    
   A   
    T    
   G   
   A   
    A    
    A    
    A    
    T    
    A    
   C   
    T    
   G   
    T    
    A    
   C   
   G   
    T    
   G   
   A   
   T   
   A   
   T   
   G   
   G   
   G   
    C    
   C   
   G   
    G    
   A   
   C   
   A   
   G   
   C   
    A    
   T   
   C   
    A    
   G   
   G   
    T    
   G   
   T   
    T    
    C    
   G   
    G    
   G   
   G   
    A    
   A   
   T   
    C    
   C   
   G   
    T    
    C    
   T   
    C    
   C   
   G   
    C    
   G   
   A   
    A    
    A    
   A   
    T    
   G   
   A   
    T    
   T   
    A    
    T    
   G   
   T   
    G    
   G   
   T   
    C    
   G   
   G   
    T    
    G    
   C   
   A   
   G   
   C   
    G    
    A    
   T   
    T    
    C    
   T   
   G   
    G    
   A   
    T    
   G   
    A    
   A   
    A    
   A   
    C    
    A    
   A   
   A   
   G   
   A   
   A   
   G   
   T   
    C    
    C    
   T   
    A    
   G   
   T   
    C    
   A   
   T   
    T    
   A   
   C   
    T    
   G   
   A   
   A   
   A   
   A   
    T    
   G   
   G   
   T   
   T   
   A   
    T    
   G   
   G   
    T    
   A   
   A   
    G    
   C   
   G   
    T    
   A   
   C   
    A    
   A   
   A   
   A   
   G   
    C    
    T    
   T   
   C   
    T    
   G   
   A   
    A    
   T   
   A   
   T   
   C   
   C   
   A   
   G   
   T   
    T    
   A   
   A   
    A    
   G   
   G   
    A    
   C   
   G   
    T    
   G   
   G   
    C    
   G   
   G   
   T   
   A   
   A   
   A   
   G   
   G   
    G    
   A   
   T   
    T    
   A   
   A   
    G    
   A   
   C   
    A    
    G    
   C   
    A    
   A   
   A   
    T    
   A   
   T   
   C   
   A   
   C   
   T   
   G   
   A   
    G    
   A   
   A   
   A   
   A   
   A   
   T   
   G   
   G   
   T   
   C   
   C   
   A   
    T    
   T   
   A   
   G   
   C   
    T    
   G   
   G   
    T    
   T   
   T   
    A    
   A   
   C   
   C   
   A   
   C   
    G    
   G   
   T   
    C    
    A    
    A    
   T   
   G   
   G   
    G    
    G    
   A   
   T   
   G   
   A   
   A   
   G   
   A   
    T    
   A   
   T   
   C   
    T    
   T   
    A    
    T    
   T   
    G    
   A   
   T   
    T    
   A   
   C   
    G    
    A    
   A   
    C    
   A   
   A   
   A   
   G   
   G   
    C    
   G   
   T   
    C    
   A   
   T   
   T   
   A   
   T   
   C   
   C   
   G   
    C    
   T   
   T   
    T    
   A   
   A   
   C   
   G   
   T   
    T    
    G    
    A    
    T    
    T    
   C   
   T   
   G   
   T   
   T   
   T   
   C   
   T   
   C   
   A   
   A   
   A   
   C   
    A    
   G   
   G   
   A   
   C   
   G   
    C    
    G    
   C   
   A   
   A   
   C   
   A   
   T   
   T   
    A    
   G   
   G   
    G    
   G   
   T   
    T    
   C   
   G   
   T   
   T   
   T   
    A    
   A   
   T   
   G   
   A   
    G    
    A    
   A   
   T   
   G   
   G   
   A   
   A   
   G   
    A    
    T    
   G   
    G    
    T    
    G    
   C   
    C    
   A   
   A   
   A   
   -   
   -   
   -   
   -   
   -   
   -   
   -   
   -   
   -   
   -   
   -   
   -   
   -   
   -   
   -   
   -   
   -   
   -   
   -   
   -   
   -   
   -   
   -   
   -   
   -   
   -   
   -   
   -   
   -   
   -   
   -   
   -   
   -   
   -   
   -   
   -   
   -   
   -   
   -   
   -   
   -   
   -   
   -   
   -   
   -   
   -   
   -   
   -   
   -   
   -   
   -   
   -   
   -   
   -   
   -   
   -   
   -   
   -   
   -   
   -   
   -   
   -   
   -   
   -   
   -   
   -   
   -   
   -   
   -   
   -   
   -   
   -   
 

 
   N53453   
   A   
   T   
   G   
   A   
   G   
   T   
   G   
   A   
   A   
   G   
   A   
   A   
   A   
   T   
   T   
   A   
   A   
   A   
   G   
   A   
   A   
   A   
   A   
   C   
   A   
   T   
   T   
   C   
   A   
   A   
   G   
   A   
   C   
   G   
   T   
   C   
   A   
   A   
   T   
   C   
   T   
   G   
   A   
   C   
   C   
   A   
   G   
   C   
   G   
   A   
   A   
   A   
   T   
   G   
   A   
   A   
   A   
   G   
   A   
   A   
   T   
   C   
   T   
   T   
   T   
   C   
   A   
   T   
   T   
   G   
   A   
   C   
   T   
   A   
   C   
   G   
   C   
   A   
   A   
   T   
   G   
   A   
   G   
   T   
   G   
   T   
   T   
   A   
   T   
   C   
   G   
   T   
   A   
   G   
   C   
   C   
   C   
   G   
   C   
   G   
   C   
   G   
   T   
   T   
   A   
   C   
   C   
   T   
   G   
   A   
   C   
   G   
   T   
   T   
   C   
   G   
   T   
   G   
   A   
   C   
   G   
   G   
   T   
   T   
   T   
   A   
   A   
   A   
   A   
   C   
   C   
   T   
   G   
   T   
   T   
   C   
   A   
   T   
   C   
   G   
   C   
   C   
   G   
   A   
   A   
   T   
   C   
   T   
   T   
   A   
   T   
   A   
   T   
   G   
   G   
   A   
   A   
   T   
   G   
   A   
   A   
   C   
   G   
   A   
   A   
   T   
   T   
   A   
   G   
   G   
   G   
   G   
   T   
   A   
   A   
   C   
   C   
   C   
   C   
   T   
   G   
   A   
   T   
   A   
   A   
   A   
   C   
   C   
   A   
   C   
   A   
   C   
   A   
   A   
   A   
   A   
   A   
   A   
   T   
   C   
   A   
   G   
   C   
   C   
   C   
   G   
   G   
   A   
   T   
   T   
   G   
   T   
   T   
   G   
   G   
   G   
   G   
   A   
   T   
   G   
   T   
   T   
   A   
   T   
   G   
   G   
   G   
   T   
   A   
   A   
   A   
   T   
   A   
   T   
   C   
   A   
   C   
   C   
   C   
   C   
   C   
   A   
   T   
   G   
   G   
   G   
   G   
   A   
   C   
   A   
   G   
   T   
   G   
   C   
   G   
   A   
   T   
   T   
   T   
   A   
   C   
   G   
   A   
   A   
   T   
   C   
   A   
   A   
   T   
   G   
   G   
   T   
   G   
   C   
   G   
   G   
   A   
   T   
   G   
   G   
   C   
   A   
   C   
   A   
   A   
   C   
   C   
   T   
   T   
   T   
   T   
   A   
   G   
   T   
   T   
   A   
   T   
   C   
   G   
   G   
   G   
   C   
   T   
   A   
   T   
   G   
   T   
   T   
   A   
   G   
   T   
   T   
   G   
   A   
   C   
   G   
   G   
   C   
   C   
   A   
   C   
   G   
   G   
   A   
   A   
   A   
   C   
   T   
   T   
   C   
   G   
   G   
   T   
   T   
   C   
   C   
   G   
   T   
   C   
   G   
   A   
   T   
   G   
   G   
   T   
   G   
   A   
   C   
   G   
   G   
   C   
   G   
   C   
   T   
   G   
   C   
   C   
   G   
   C   
   T   
   A   
   T   
   G   
   C   
   G   
   T   
   T   
   A   
   T   
   A   
   C   
   C   
   G   
   A   
   A   
   G   
   C   
   A   
   C   
   G   
   T   
   A   
   T   
   G   
   A   
   G   
   T   
   A   
   A   
   A   
   A   
   T   
   T   
   G   
   C   
   T   
   T   
   T   
   A   
   G   
   A   
   A   
   A   
   T   
   G   
   C   
   T   
   A   
   C   
   G   
   A   
   G   
   A   
   T   
   A   
   T   
   T   
   A   
   A   
   C   
   A   
   A   
   A   
   A   
   A   
   T   
   A   
   C   
   A   
   G   
   T   
   C   
   G   
   A   
   T   
   T   
   T   
   C   
   C   
   A   
   A   
   G   
   G   
   A   
   A   
   A   
   C   
   T   
   A   
   T   
   G   
   A   
   T   
   G   
   A   
   T   
   T   
   C   
   A   
   G   
   A   
   A   
   C   
   A   
   A   
   G   
   A   
   A   
   C   
   C   
   A   
   G   
   T   
   A   
   G   
   T   
   T   
   T   
   T   
   A   
   C   
   C   
   T   
   G   
   C   
   T   
   C   
   G   
   T   
   T   
   T   
   T   
   C   
   C   
   A   
   A   
   A   
   C   
   T   
   T   
   A   
   C   
   T   
   A   
   G   
   T   
   T   
   A   
   A   
   C   
   G   
   G   
   A   
   A   
   C   
   A   
   A   
   C   
   G   
   G   
   G   
   G   
   A   
   T   
   T   
   G   
   C   
   G   
   G   
   T   
   G   
   G   
   G   
   G   
   A   
   T   
   G   
   G   
   C   
   A   
   A   
   C   
   A   
   A   
   A   
   T   
   A   
   T   
   T   
   C   
   C   
   A   
   C   
   C   
   A   
   C   
   A   
   C   
   A   
   A   
   T   
   T   
   T   
   A   
   A   
   G   
   T   
   G   
   A   
   A   
   G   
   T   
   G   
   A   
   T   
   T   
   G   
   A   
   T   
   G   
   C   
   A   
   A   
   C   
   A   
   A   
   G   
   T   
   T   
   T   
   A   
   T   
   T   
   G   
   A   
   T   
   G   
   G   
   A   
   C   
   A   
   A   
   T   
   C   
   C   
   T   
   G   
   A   
   T   
   G   
   T   
   A   
   A   
   C   
   G   
   A   
   C   
   G   
   A   
   A   
   T   
   G   
   A   
   A   
   T   
   T   
   G   
   A   
   T   
   G   
   G   
   A   
   A   
   G   
   T   
   G   
   T   
   T   
   A   
   C   
   C   
   T   
   G   
   G   
   A   
   C   
   C   
   A   
   G   
   A   
   T   
   T   
   T   
   T   
   C   
   C   
   A   
   A   
   C   
   A   
   G   
   G   
   C   
   G   
   G   
   T   
   T   
   T   
   A   
   G   
   T   
   G   
   A   
   T   
   G   
   G   
   G   
   G   
   A   
   A   
   A   
   T   
   C   
   A   
   G   
   G   
    G    
   A   
   T   
    T    
   C   
   G   
    C    
    C    
   G   
   A   
   G   
   C   
    A    
   T   
   A   
   T   
   G   
   A   
   A   
   A   
   C   
    A    
   G   
   G   
    G    
   A   
   A   
    A    
   G   
   G   
   T   
   T   
   C   
    G    
   A   
   T   
   T   
   A   
   C   
    T    
   G   
   T   
    T    
   C   
   G   
   T   
   G   
   C   
   A   
   A   
   A   
   A   
   G   
   T   
   T   
   G   
   A   
    A    
    T    
   T   
    G    
   A   
    C    
   T   
   G   
   A   
    A    
   A   
   T   
   G   
    C    
   C   
    G    
   A   
   A   
   T   
   G   
   G   
    A    
   A   
   A   
   A   
   G   
   A   
   A   
   C   
   G   
    T    
   A   
   T   
    T    
    T    
   T   
    A    
   G   
   T   
    A    
   A   
   C   
    T    
   G   
   A   
   A   
   T   
   T   
    G    
   C   
   C   
   T   
   T   
   A   
   T   
   A   
   T   
   G   
   G   
   T   
    G    
   A   
   A   
   T   
   A   
   A   
   A   
   G   
   C   
    C    
   A   
   A   
   A   
   T   
   T   
    A    
   A   
   T   
    C    
   G   
   A   
   A   
   C   
   G   
    A    
   A   
   T   
   T   
   T   
   C   
   T   
   G   
   A   
    A    
    T    
   T   
    A    
   C   
   A   
   C   
   A   
   G   
   A   
   G   
   A   
    T    
   A   
   A   
    A    
   C   
   G   
    A    
   A   
   T   
    T    
   G   
   A   
   A   
   G   
   G   
    A    
   A   
   T   
    T    
   A   
   C   
    T    
   G   
   A   
   T   
   T   
   T   
   G   
   C   
   G   
   G   
   G   
   A   
    T    
   G   
   A   
   A   
   T   
   C   
   T   
   T   
   C   
    T    
   C   
   G   
   T   
   G   
   A   
    A    
   G   
   G   
    C    
   A   
   T   
   G   
   C   
   G   
    G    
   A   
   T   
    T    
   G   
   T   
   C   
   A   
   T   
    C    
   G   
   A   
    T    
    G    
   T   
    T    
   C   
   G   
   T   
   C   
   G   
    A    
   G   
   A   
    T    
   G   
    T    
    G    
   A   
   G   
    T    
   G   
   C   
    C    
   T   
   C   
    T    
   G   
   T   
   T   
    G    
   T   
    G    
   T   
   T   
    A    
   A   
   A   
    C    
   A   
   A   
   C   
   T   
   T   
    G    
   T   
   A   
    C    
   A   
   A   
   A   
    A    
   T   
   G   
   A   
   C   
    T    
    G    
   C   
    C    
   T   
   T   
    A    
   C   
   A   
    A    
   A   
    C    
    A    
   T   
   C   
    T    
   T   
   T   
    T    
   G   
   G   
    T    
   T   
   T   
    T    
   A   
   A   
    C    
   A   
   T   
   G   
   T   
   T   
    A    
   G   
   C   
    C    
   A   
   T   
    C    
   G   
   A   
   A   
   A   
   A   
   A   
   G   
   G   
    C    
   G   
   T   
    A    
   C   
   C   
    G    
   A   
   A   
   A   
    A    
   T   
   T   
   T   
   T   
    A    
   A   
   G   
    C    
   T   
   T   
   G   
   A   
   A   
   A   
   C   
    G    
    T    
   A   
   T   
    T    
    T    
   T   
    A    
   G   
   A   
   A   
   A   
   A   
    T    
   T   
   A   
    C    
    G    
   T   
    C    
   G   
   A   
    G    
   C   
   A   
    C    
   C   
   A   
    A    
    A    
    A    
    A    
    G    
   A   
   A   
   G   
   T   
   C   
   A   
   T   
    T    
   A   
   C   
    T    
    C    
   G   
    C    
   C   
   G   
   G   
   A   
   C   
    G    
    A    
   T   
    T    
   T   
   T   
   T   
   G   
   A   
    C    
   A   
   A   
   A   
    A    
   A   
    C    
   A   
   A   
    A    
   G   
   C   
   A   
   G   
   A   
   A   
   G   
   C   
   A   
    C    
   G   
    G    
   G   
   C   
    G    
   C   
   A   
    T    
   A   
   T   
    T    
   T   
   T   
   A   
   G   
   A   
   A   
   G   
   G   
   T   
    C    
   T   
    A    
   C   
   G   
    A    
   A   
   T   
   T   
   G   
   C   
    C    
   T   
   T   
    A    
   G   
   A   
   T   
   C   
   A   
   T   
   A   
   T   
   C   
    G    
   A   
   T   
   G   
   A   
   A   
   A   
   T   
    C    
   A   
   T   
   T   
   G   
   C   
    C    
   A   
   T   
   T   
   A   
   T   
    C    
   C   
   G   
    T    
   G   
   G   
    A    
   T   
   C   
    A    
    C    
   A   
   A   
   T   
   C   
   A   
   G   
   A   
   T   
   G   
   A   
    C    
   G   
   A   
    A    
   G   
   C   
   A   
   A   
   A   
   A   
   G   
   C   
    A    
   A   
    C    
    G    
    T    
   T   
    A    
   A   
   T   
    T    
   G   
   A   
   A   
   C   
   G   
    C    
   T   
   T   
    T    
   G   
   A   
    A    
   T   
   T   
    T    
   T   
   C   
    A    
   G   
   A   
   T   
   C   
   G   
    T    
   C   
   A   
    A    
    G    
   C   
    G    
   C   
   A   
   A   
   G   
   C   
    A    
   A   
   T   
    T    
   T   
   T   
   A   
   G   
   A   
   T   
   A   
   T   
   G   
   C   
   G   
   T   
   T   
   T   
    A    
   C   
   G   
    C    
   C   
   G   
    T    
   T   
   T   
    A    
   A   
   C   
    A    
   G   
   G   
    C    
   T   
   T   
    A    
   G   
   A   
   A   
   C   
   G   
    C    
   G   
   A   
    C    
   A   
   A   
   A   
   A   
   T   
    T    
   G   
   A   
    A    
   A   
   A   
   T   
   G   
   A   
   A   
   T   
   A   
    T    
   C   
   A   
   A   
   G   
   A   
    G    
   C   
   T   
    A    
   T   
   T   
    G    
   A   
    A    
   A   
    T    
   T   
    C    
   A   
   T   
   T   
   G   
    C    
    G    
   G   
   A   
    C    
   T   
   T   
    A    
    G    
    A    
    G    
   G   
   A   
   C   
   A   
   T   
   T   
    T    
   T   
    A    
   G   
   C   
    C    
    C    
   G   
    C    
   C   
   C   
    A    
   G   
   A   
    A    
   C   
   G   
    C    
   G   
   T   
    C    
   A   
    T    
    T    
   G   
   A   
   A   
   A   
   T   
    C    
   A   
   T   
    T    
   A   
   A   
    A    
   A   
    C    
    A    
   G   
   A   
    G    
   T   
   T   
   A   
    A    
    A    
    T    
   G   
   A   
   C   
    G    
   T   
    T    
    C    
    G    
    C    
    A    
    C    
   A   
    A    
    A    
   A   
   T   
   T   
   T   
   G   
   G   
   T   
   G   
   A   
   T   
    G    
    C    
   A   
   C   
   G   
   A   
   C   
   G   
    C    
   A   
   C   
   A   
   G   
   A   
   A   
    C    
   T   
   A   
    T    
   T   
   A   
   G   
   T   
    A    
   G   
   G   
   T   
   G   
   A   
    A    
   G   
   T   
   C   
    C    
   T   
    C    
   A   
   G   
   T   
   C   
   T   
    T    
   G   
   A   
   A   
   G   
   A   
   T   
   G   
   A   
   A   
   G   
   A   
   T   
    T    
   T   
    A    
   A   
   T   
    C    
   G   
   A   
   A   
   G   
   A   
   A   
   G   
   A   
   A   
   G   
   A   
   A   
    G    
   T   
   C   
   G   
   T   
    G    
   A   
   T   
   T   
   A   
   C   
    A    
    T    
   T   
    A    
   A   
   C   
    C    
   A   
   A   
    T    
   A   
   A   
    C    
   G   
   G   
    C    
   T   
   A   
    C    
   A   
   T   
    T    
   A   
   A   
    G    
   C   
   G   
    G    
    A    
   T   
   G   
   G   
    C    
   A   
   A   
   A   
    C    
    T    
    C    
    C    
   G   
   A   
   A   
   T   
   T   
   C   
    C    
   G   
    G    
   G   
   C   
    G    
   C   
   A   
   A   
   C   
   G   
    C    
    C    
   G   
    C    
   G   
   G   
    C    
   G   
   G   
    A    
   C   
   G   
   T   
   G   
   G   
    T    
   G   
   T   
    C    
   C   
   A   
   A   
   G   
   G   
    G    
   A   
   T   
   G   
   G   
   G   
    C    
    G    
   T   
    T    
   C   
   A   
    C    
    G    
   A   
   T   
   G   
   A   
    T    
   G   
   A   
    T    
   T   
   T   
   C   
   G   
   T   
    G    
   A   
   A   
    A    
   A   
   A   
    C    
   C   
   T   
    A    
    G    
   T   
   T   
   T   
   C   
   T   
   T   
   G   
    T    
   T   
   C   
    A    
   A   
   C   
    A    
   C   
   A   
    T    
   G   
   A   
    C    
   A   
   C   
    G    
   T   
   T   
    A    
   T   
   T   
   A   
   T   
   T   
    C    
   T   
   T   
   T   
   A   
   C   
   G   
   A   
   A   
    C    
   A   
    C    
    T    
   G   
   G   
    G    
   A   
   A   
   A   
   G   
   T   
    T    
   T   
   A   
    C    
   C   
   G   
    A    
   G   
   C   
   A   
   A   
   A   
   A   
   G   
   G   
    T    
   T   
   A   
    C    
   G   
   A   
   A   
   A   
   T   
    C    
   C   
   C   
    T    
   G   
   A   
    A    
   T   
   A   
   C   
   G   
   G   
    T    
    A    
   G   
    A    
   A   
   C   
   A   
   G   
   C   
    A    
   A   
   A   
   A   
   G   
   G   
    A    
   A   
   T   
    T    
   C   
   C   
   A   
    G    
   T   
    G    
   A   
   T   
    T    
   A   
   A   
    C    
   T   
   T   
    A    
   C   
   T   
    G    
   G   
   G   
    A    
   A   
   T   
    T    
   G   
   A   
    T    
   T   
   C   
    T    
    G    
    C    
    A    
   G   
   A   
   A   
   A   
    A    
    A    
   A   
   T   
    T    
   C   
   A   
    A    
   G   
   C   
    G    
   A   
   T   
    T    
   A   
   T   
   T   
    T    
   C   
    T    
   G   
   T   
    T    
    G    
    A    
   G   
   G   
   G   
    C    
    A    
   A   
   A   
   G   
   C   
    G    
   G   
   A   
   A   
   G   
    C    
    A    
   G   
   G   
    T    
    A    
   A   
    A    
   T   
   A   
    C    
    T    
   T   
    G    
   T   
   T   
   C   
   T   
   T   
    C    
   A   
   C   
    A    
   A   
   C   
    C    
    T    
    T    
    A    
    A    
   A   
   A   
   G   
   G   
    A    
   A   
   C   
    C    
   G   
   T   
    C    
   A   
   A   
   A   
   C   
   G   
    G    
   A   
   C   
    A    
    G    
   C   
    C    
   G   
   T   
    A    
   A   
   C   
    A    
   G   
   C   
    C    
   T   
   T   
    T    
    T    
   C   
   T   
   A   
   A   
    T    
   A   
   T   
    C    
   C   
   G   
    T    
   A   
   G   
    T    
   A   
   A   
   T   
   G   
   G   
    A    
    T    
   T   
    A    
   A   
   T   
    C    
   G   
   C   
    C    
   A   
   T   
   T   
    A    
   G   
    C    
   T   
   T   
    A    
   A   
   A   
   A   
   G   
   A   
   A   
   G   
   A   
    T    
   G   
   A   
    T    
   G   
   A   
    G    
    T    
   T   
    A    
    G    
   T   
    T    
   A   
   A   
    C    
   G   
   T   
    G    
    G    
   T   
   A   
    A    
    C    
   G   
   A   
   C   
    T    
    A    
   A   
    T    
   G   
   G   
    C    
    A    
   A   
    T    
    C    
    A    
    G    
   A   
   A   
    G    
   A   
   T   
    G    
   A   
   T   
    T    
   A   
   T   
    C    
   G   
   G   
    A    
   A   
   C   
   A   
   C   
   A   
   T   
    G    
    C    
    A    
   G   
   G   
    A    
   T   
   A   
   C   
   T   
   C   
   T   
   G   
   T   
    C    
   A   
   C   
    A    
   T   
   T   
    T    
    G    
   A   
    T    
   G   
   A   
    A    
    A    
    A    
    T    
    A    
   C   
    T    
   G   
    T    
    A    
   C   
   G   
    T    
   G   
   A   
   T   
   A   
   T   
   G   
   G   
   G   
    C    
   C   
   G   
    G    
   A   
   C   
   A   
   G   
   C   
    A    
   T   
   C   
    A    
   G   
   G   
    C    
   G   
   T   
    T    
    C    
   G   
    G    
   G   
   G   
    A    
   A   
   T   
    C    
   C   
   G   
    T    
    C    
   T   
    C    
   C   
   G   
    C    
   G   
   A   
    A    
    A    
   A   
    T    
   G   
   A   
    T    
   T   
    A    
    T    
   G   
   T   
    G    
   G   
   T   
    C    
   G   
   G   
    T    
    G    
   C   
   A   
   G   
   C   
    G    
    A    
   T   
    T    
    C    
   T   
   G   
    G    
   A   
    T    
   G   
    A    
   A   
    A    
   A   
    T    
    A    
   A   
   A   
   G   
   A   
   A   
   G   
   T   
    C    
    C    
   T   
    A    
   G   
   T   
    C    
   A   
   T   
    T    
   A   
   C   
    T    
   G   
   A   
   A   
   A   
   A   
    T    
   G   
   G   
   T   
   T   
   A   
    T    
   G   
   G   
    T    
   A   
   A   
    G    
   C   
   G   
    T    
   A   
   C   
    A    
   A   
   A   
   A   
   G   
    C    
    T    
   T   
   C   
    T    
   G   
   A   
    A    
   T   
   A   
   T   
   C   
   C   
   A   
   G   
   T   
    T    
   A   
   A   
    A    
   G   
   G   
    A    
   C   
   G   
    T    
   G   
   G   
    C    
   G   
   G   
   T   
   A   
   A   
   A   
   G   
   G   
    G    
   A   
   T   
    T    
   A   
   A   
    G    
   A   
   C   
    A    
    G    
   C   
    A    
   A   
   A   
    T    
   A   
   T   
   C   
   A   
   C   
   T   
   G   
   A   
    G    
   A   
   A   
   A   
   A   
   A   
   T   
   G   
   G   
   T   
   C   
   C   
   A   
    T    
   T   
   A   
   G   
   C   
    T    
   G   
   G   
    T    
   T   
   T   
    A    
   A   
   C   
   C   
   A   
   C   
    G    
   G   
   T   
    C    
    A    
    A    
   T   
   G   
   G   
    G    
    G    
   A   
   T   
   G   
   A   
   A   
   G   
   A   
    T    
   A   
   T   
   C   
    T    
   T   
    A    
    T    
   T   
    G    
   A   
   T   
    T    
   A   
   C   
    G    
    A    
   A   
    C    
   A   
   A   
   A   
   G   
   G   
    C    
   G   
   T   
    C    
   A   
   T   
   T   
   A   
   T   
   C   
   C   
   G   
    C    
   T   
   T   
    T    
   A   
   A   
   C   
   G   
   T   
    T    
    G    
    A    
    T    
    T    
   C   
   T   
   G   
   T   
   T   
   T   
   C   
   T   
   C   
   A   
   A   
   A   
   C   
    A    
   G   
   G   
   A   
   C   
   G   
    C    
    G    
   C   
   A   
   A   
   C   
   A   
   T   
   T   
    A    
   G   
   G   
    A    
   G   
   T   
    T    
   C   
   G   
   T   
   T   
   T   
    A    
   A   
   T   
   G   
   A   
    G    
    A    
   A   
   T   
   G   
   G   
   A   
   A   
   G   
    A    
    T    
   G   
    G    
    T    
    G    
   C   
    C    
   A   
   A   
   A   
   -   
   -   
   -   
   -   
   -   
   -   
   -   
   -   
   -   
   -   
   -   
   -   
   -   
   -   
   -   
   -   
   -   
   -   
   -   
   -   
   -   
   -   
   -   
   -   
   -   
   -   
   -   
   -   
   -   
   -   
   -   
   -   
   -   
   -   
   -   
   -   
   -   
   -   
   -   
   -   
   -   
   -   
   -   
   -   
   -   
   -   
   -   
   -   
   -   
   -   
   -   
   -   
   -   
   -   
   -   
   -   
   -   
   -   
   -   
   -   
   -   
   -   
   -   
   -   
   -   
   -   
   -   
   -   
   -   
   -   
   -   
   -   
 

 
   N60190F   
   A   
   T   
   G   
   A   
   G   
   T   
   G   
   A   
   A   
   G   
   A   
   A   
   A   
   T   
   C   
   A   
   G   
   A   
   G   
   A   
   A   
   A   
   A   
   C   
   A   
   T   
   C   
   C   
   A   
   T   
   G   
   A   
   T   
   G   
   T   
   C   
   A   
   A   
   T   
   C   
   T   
   A   
   A   
   C   
   C   
   A   
   G   
   C   
   G   
   A   
   A   
   A   
   T   
   G   
   A   
   A   
   A   
   G   
   A   
   C   
   T   
   C   
   C   
   T   
   T   
   C   
   A   
   T   
   C   
   G   
   A   
   T   
   T   
   A   
   T   
   G   
   C   
   G   
   A   
   T   
   G   
   A   
   G   
   T   
   G   
   T   
   T   
   A   
   T   
   T   
   G   
   T   
   C   
   G   
   C   
   A   
   C   
   G   
   T   
   G   
   C   
   A   
   C   
   T   
   G   
   C   
   C   
   T   
   G   
   A   
   T   
   G   
   T   
   C   
   A   
   G   
   A   
   G   
   A   
   T   
   G   
   G   
   T   
   C   
   T   
   G   
   A   
   A   
   A   
   C   
   C   
   C   
   G   
   T   
   T   
   C   
   A   
   T   
   C   
   G   
   C   
   C   
   G   
   T   
   A   
   T   
   T   
   T   
   T   
   A   
   T   
   A   
   C   
   G   
   G   
   C   
   A   
   T   
   G   
   A   
   A   
   C   
   G   
   A   
   A   
   T   
   T   
   A   
   G   
   G   
   T   
   G   
   T   
   G   
   A   
   C   
   A   
   C   
   C   
   G   
   G   
   A   
   T   
   A   
   A   
   A   
   G   
   C   
   C   
   C   
   A   
   T   
   A   
   A   
   A   
   A   
   A   
   A   
   T   
   C   
   T   
   G   
   C   
   C   
   A   
   G   
   A   
   A   
   T   
   C   
   G   
   T   
   C   
   G   
   G   
   A   
   G   
   A   
   T   
   G   
   T   
   C   
   A   
   T   
   G   
   G   
   G   
   T   
   A   
   A   
   A   
   T   
   A   
   C   
   C   
   A   
   T   
   C   
   C   
   T   
   C   
   A   
   T   
   G   
   G   
   G   
   G   
   A   
   T   
   A   
   G   
   T   
   G   
   C   
   A   
   A   
   T   
   C   
   T   
   A   
   T   
   G   
   A   
   G   
   T   
   C   
   A   
   A   
   T   
   G   
   G   
   T   
   A   
   C   
   G   
   G   
   A   
   T   
   G   
   G   
   C   
   G   
   C   
   A   
   G   
   C   
   C   
   A   
   T   
   T   
   T   
   A   
   G   
   C   
   T   
   A   
   C   
   C   
   G   
   T   
   T   
   A   
   T   
   A   
   T   
   G   
   C   
   T   
   A   
   G   
   T   
   G   
   G   
   A   
   T   
   G   
   G   
   T   
   C   
   A   
   T   
   G   
   G   
   A   
   A   
   A   
   C   
   T   
   T   
   T   
   G   
   G   
   T   
   T   
   C   
   T   
   G   
   T   
   T   
   G   
   A   
   C   
   G   
   G   
   T   
   G   
   A   
   C   
   G   
   G   
   T   
   G   
   C   
   C   
   G   
   C   
   T   
   G   
   C   
   T   
   A   
   T   
   G   
   C   
   G   
   T   
   T   
   A   
   T   
   A   
   C   
   C   
   G   
   A   
   A   
   G   
   C   
   A   
   C   
   G   
   G   
   A   
   T   
   G   
   A   
   G   
   C   
   A   
   A   
   A   
   A   
   T   
   C   
   G   
   C   
   T   
   A   
   C   
   A   
   G   
   A   
   A   
   A   
   T   
   G   
   C   
   T   
   G   
   C   
   G   
   T   
   G   
   A   
   T   
   A   
   T   
   C   
   A   
   A   
   T   
   A   
   A   
   G   
   A   
   A   
   T   
   A   
   C   
   T   
   G   
   T   
   G   
   G   
   A   
   T   
   T   
   T   
   C   
   C   
   A   
   A   
   A   
   G   
   C   
   A   
   A   
   C   
   T   
   A   
   T   
   G   
   A   
   T   
   G   
   A   
   C   
   A   
   C   
   A   
   G   
   A   
   A   
   A   
   A   
   A   
   G   
   A   
   A   
   C   
   C   
   C   
   G   
   T   
   T   
   G   
   T   
   A   
   T   
   T   
   A   
   C   
   C   
   T   
   G   
   C   
   T   
   C   
   G   
   C   
   T   
   T   
   C   
   C   
   C   
   T   
   A   
   A   
   T   
   C   
   T   
   T   
   T   
   T   
   G   
   G   
   T   
   G   
   A   
   A   
   C   
   G   
   G   
   A   
   A   
   C   
   G   
   A   
   C   
   G   
   G   
   G   
   G   
   A   
   T   
   C   
   G   
   C   
   C   
   G   
   T   
   G   
   G   
   G   
   T   
   A   
   T   
   G   
   G   
   C   
   A   
   A   
   C   
   G   
   A   
   A   
   T   
   A   
   T   
   C   
   C   
   C   
   A   
   C   
   C   
   T   
   C   
   A   
   T   
   A   
   A   
   T   
   T   
   T   
   G   
   T   
   C   
   T   
   G   
   A   
   G   
   G   
   T   
   A   
   G   
   T   
   A   
   G   
   C   
   G   
   G   
   C   
   T   
   A   
   T   
   C   
   G   
   A   
   T   
   T   
   T   
   G   
   C   
   T   
   G   
   A   
   T   
   G   
   G   
   A   
   T   
   A   
   A   
   T   
   C   
   C   
   T   
   G   
   A   
   T   
   G   
   T   
   G   
   A   
   C   
   G   
   A   
   C   
   C   
   A   
   A   
   T   
   G   
   A   
   A   
   T   
   T   
   G   
   A   
   T   
   G   
   G   
   A   
   A   
   G   
   T   
   A   
   T   
   T   
   A   
   C   
   C   
   T   
   G   
   G   
   A   
   C   
   C   
   A   
   G   
   A   
   T   
   T   
   T   
   T   
   C   
   C   
   A   
   A   
   C   
   A   
   G   
   G   
   T   
   G   
   G   
   T   
   T   
   T   
   G   
   G   
   T   
   C   
   A   
   T   
   G   
   G   
   G   
   A   
   A   
   A   
   A   
   T   
   C   
   A   
   G   
   G   
    A    
   A   
   T   
    C    
   C   
   G   
    A    
    A    
   G   
   A   
   G   
   C   
    G    
   T   
   A   
   T   
   G   
   A   
   A   
   A   
   C   
    A    
   G   
   G   
    T    
   A   
   A   
    A    
   G   
   G   
   T   
   T   
   C   
    T    
   A   
   T   
   T   
   A   
   C   
    C    
   G   
   T   
    A    
   C   
   G   
   T   
   G   
   C   
   A   
   A   
   A   
   A   
   G   
   T   
   T   
   G   
   A   
    G    
    A    
   T   
    C    
   A   
    C    
   T   
   G   
   A   
    G    
   A   
   T   
   G   
    C    
   C   
    A    
   A   
   A   
   T   
   G   
   G   
    C    
   A   
   A   
   A   
   G   
   A   
   A   
   C   
   G   
    G    
   A   
   T   
    C    
    C    
   T   
    C    
   G   
   T   
    G    
   A   
   C   
    G    
   G   
   A   
   A   
   T   
   T   
    A    
   C   
   C   
   T   
   T   
   A   
   T   
   A   
   T   
   G   
   G   
   T   
    C    
   A   
   A   
   T   
   A   
   A   
   A   
   G   
   C   
    A    
   A   
   A   
   A   
   T   
   T   
    G    
   A   
   T   
    T    
   G   
   A   
   A   
   C   
   G   
    G    
   A   
   T   
   T   
   T   
   C   
   T   
   G   
   A   
    G    
    T    
   T   
    G    
   C   
   A   
   C   
   A   
   G   
   A   
   G   
   A   
    C    
   A   
   A   
    G    
   C   
   G   
    T    
   A   
   T   
    C    
   G   
   A   
   A   
   G   
   G   
    A    
   A   
   T   
    T    
   A   
   C   
    G    
   G   
   A   
   T   
   T   
   T   
   G   
   C   
   G   
   G   
   G   
   A   
    T    
   G   
   A   
   A   
   T   
   C   
   T   
   T   
   C   
    C    
   C   
   G   
   T   
   G   
   A   
    A    
   G   
   G   
    C    
   A   
   T   
   G   
   C   
   G   
    A    
   A   
   T   
    T    
   G   
   T   
   C   
   A   
   T   
    T    
   G   
   A   
    C    
    G    
   T   
    T    
   C   
   G   
   T   
   C   
   G   
    T    
   G   
   A   
    C    
   G   
    C    
    C    
   A   
   G   
    C    
   G   
   C   
    A    
   T   
   C   
    A    
   G   
   T   
   T   
    A    
   T   
    T    
   T   
   T   
    G    
   A   
   A   
    T    
   A   
   A   
   C   
   T   
   T   
    A    
   T   
   A   
    C    
   A   
   A   
   A   
    T    
   T   
   G   
   A   
   C   
    A    
    T    
   C   
    C    
   T   
   T   
    G    
   C   
   A   
    G    
   A   
    A    
    T    
   T   
   C   
    A    
   T   
   T   
    C    
   G   
   G   
    A    
   T   
   T   
    C    
   A   
   A   
    T    
   A   
   T   
   G   
   T   
   T   
    G    
   G   
   C   
    A    
   A   
   T   
    C    
   G   
   A   
   A   
   A   
   A   
   A   
   G   
   G   
    C    
   G   
   T   
    G    
   C   
   C   
    G    
   A   
   A   
   A   
    G    
   T   
   T   
   T   
   T   
    A    
   A   
   G   
    T    
   T   
   T   
   G   
   A   
   A   
   A   
   C   
    A    
    A    
   A   
   T   
    C    
    C    
   T   
    T    
   G   
   A   
   A   
   A   
   A   
    T    
   T   
   A   
    T    
    A    
   T   
    C    
   G   
   A   
    A    
   C   
   A   
    T    
   C   
   A   
    A    
    C    
    G    
    C    
    G    
   A   
   A   
   G   
   T   
   C   
   A   
   T   
    C    
   A   
   C   
    A    
    C    
   G   
    A    
   C   
   G   
   G   
   A   
   C   
    G    
    G    
   T   
    C    
   T   
   T   
   T   
   G   
   A   
    A    
   A   
   A   
   A   
    G    
   A   
    A    
   A   
   A   
    A    
   G   
   C   
   A   
   G   
   A   
   A   
   G   
   C   
   A   
    A    
   G   
    A    
   G   
   C   
    A    
   C   
   A   
    C    
   A   
   T   
    C    
   T   
   T   
   A   
   G   
   A   
   A   
   G   
   G   
   T   
    T    
   T   
    G    
   C   
   G   
    T    
   A   
   T   
   T   
   G   
   C   
    T    
   T   
   T   
    G    
   G   
   A   
   T   
   C   
   A   
   T   
   A   
   T   
   C   
    G    
   A   
   T   
   G   
   A   
   A   
   A   
   T   
    C    
   A   
   T   
   T   
   G   
   C   
    G    
   A   
   T   
   T   
   A   
   T   
    C    
   C   
   G   
    C    
   G   
   G   
    G    
   T   
   C   
    T    
    A    
   A   
   A   
   T   
   C   
   A   
   G   
   A   
   T   
   G   
   A   
    C    
   G   
   A   
    A    
   G   
   C   
   A   
   A   
   A   
   A   
   G   
   C   
    A    
   A   
    C    
    A    
    A    
   T   
    G    
   A   
   T   
    C    
   G   
   A   
   A   
   C   
   G   
    G    
   T   
   T   
    C    
   G   
   A   
    T    
   T   
   T   
    A    
   T   
   C   
    C    
   G   
   A   
   T   
   C   
   G   
    C    
   C   
   A   
    A    
    T    
   C   
    A    
   C   
   A   
   A   
   G   
   C   
    T    
   A   
   T   
    C    
   T   
   T   
   A   
   G   
   A   
   T   
   A   
   T   
   G   
   C   
   G   
   T   
   T   
   T   
    G    
   C   
   G   
    T    
   C   
   G   
    A    
   T   
   T   
    G    
   A   
   C   
    C    
   G   
   G   
    T    
   T   
   T   
    G    
   G   
   A   
   A   
   C   
   G   
    G    
   G   
   A   
    A    
   A   
   A   
   A   
   A   
   T   
    C    
   G   
   A   
    A    
   A   
   A   
   T   
   G   
   A   
   A   
   T   
   A   
    T    
   C   
   A   
   A   
   G   
   A   
    A    
   C   
   T   
    A    
   T   
   T   
    G    
   A   
    A    
   A   
    C    
   T   
    G    
   A   
   T   
   T   
   G   
    A    
    A    
   G   
   A   
    T    
   T   
   T   
    G    
    A    
    C    
    T    
   G   
   A   
   C   
   A   
   T   
   T   
    C    
   T   
    G    
   G   
   C   
    A    
    A    
   G   
    A    
   C   
   C   
    C    
   G   
   A   
    A    
   C   
   G   
    T    
   G   
   T   
    C    
   A   
    C    
    A    
   G   
   A   
   A   
   A   
   T   
    C    
   A   
   T   
    C    
   A   
   A   
    G    
   A   
    A    
    T    
   G   
   A   
    A    
   T   
   T   
   A   
    G    
    C    
    A    
   G   
   A   
   C   
    T    
   T   
    G    
    A    
    A    
    T    
    C    
    A    
   A   
    C    
    G    
   A   
   T   
   T   
   T   
   G   
   G   
   T   
   G   
   A   
   T   
    A    
    A    
   A   
   C   
   G   
   A   
   C   
   G   
    C    
   A   
   C   
   A   
   G   
   A   
   A   
    T    
   T   
   A   
    C    
   T   
   A   
   G   
   T   
    C    
   G   
   G   
   T   
   G   
   A   
    G    
   G   
   T   
   C   
    T    
   T   
    A    
   A   
   G   
   T   
   C   
   T   
    A    
   G   
   A   
   A   
   G   
   A   
   T   
   G   
   A   
   A   
   G   
   A   
   T   
    T    
   T   
    G    
   A   
   T   
    T    
   G   
   A   
   A   
   G   
   A   
   A   
   G   
   A   
   A   
   G   
   A   
   A   
    A    
   T   
   C   
   G   
   T   
    T    
   A   
   T   
   T   
   A   
   C   
    A    
    T    
   T   
    G    
   A   
   C   
    A    
   A   
   A   
    C    
   A   
   A   
    T    
   G   
   G   
    T    
   T   
   A   
    T    
   A   
   T   
    C    
   A   
   A   
    A    
   C   
   G   
    T    
    T    
   T   
   G   
   G   
    C    
   A   
   A   
   A   
    T    
    A    
    A    
    T    
   G   
   A   
   A   
   T   
   T   
   C   
    A    
   G   
    A    
   G   
   C   
    T    
   C   
   A   
   A   
   C   
   G   
    A    
    A    
   G   
    A    
   G   
   G   
    C    
   G   
   G   
    G    
   C   
   G   
   T   
   G   
   G   
    T    
   G   
   T   
    C    
   C   
   A   
   A   
   G   
   G   
    A    
   A   
   T   
   G   
   G   
   G   
    T    
    A    
   T   
    C    
   C   
   A   
    C    
    A    
   A   
   T   
   G   
   A   
    C    
   G   
   A   
    C    
   T   
   T   
   C   
   G   
   T   
    C    
   A   
   A   
    G    
   A   
   A   
    T    
   C   
   T   
    A    
    G    
   T   
   T   
   T   
   C   
   T   
   T   
   G   
    T    
   T   
   C   
    T    
   A   
   C   
    C    
   C   
   A   
    T    
   G   
   A   
    T    
   A   
   C   
    A    
   T   
   T   
    G    
   T   
   T   
   A   
   T   
   T   
    C    
   T   
   T   
   T   
   A   
   C   
   G   
   A   
   A   
    T    
   A   
    A    
    C    
   G   
   G   
    A    
   A   
   A   
   A   
   G   
   T   
    G    
   T   
   A   
    T    
   C   
   G   
    G    
   G   
   C   
   A   
   A   
   A   
   A   
   G   
   G   
    A    
   T   
   A   
    C    
   G   
   A   
   A   
   A   
   T   
    T    
   C   
   C   
    A    
   G   
   A   
    A    
   T   
   A   
   C   
   G   
   G   
    G    
    C    
   G   
    T    
   A   
   C   
   A   
   G   
   C   
    C    
   A   
   A   
   A   
   G   
   G   
    A    
   A   
   T   
    C    
   C   
   C   
   A   
    A    
   T   
    C    
   A   
   T   
    C    
   A   
   A   
    T    
   T   
   T   
    G    
   C   
   T   
    G    
   G   
   G   
    T    
   A   
   T   
    C    
   G   
   A   
    C    
   T   
   C   
    G    
    A    
    G    
    T    
   G   
   A   
   A   
   A   
    G    
    C    
   A   
   T   
    C    
   C   
   A   
    A    
   G   
   C   
    A    
   A   
   T   
    T    
   A   
   T   
   T   
    G    
   C   
    G    
   G   
   T   
    G    
    A    
    C    
   G   
   G   
   G   
    A    
    G    
   A   
   A   
   G   
   C   
    A    
   G   
   A   
   A   
   G   
    A    
    A    
   G   
   G   
    C    
    C    
   A   
    C    
   T   
   A   
    T    
    C    
   T   
    T    
   T   
   T   
   C   
   T   
   T   
    T    
   A   
   C   
    G    
   A   
   C   
    G    
    C    
    G    
    T    
    C    
   A   
   A   
   G   
   G   
    T    
   A   
   C   
    A    
   G   
   T   
    T    
   A   
   A   
   A   
   C   
   G   
    T    
   A   
   C   
    G    
    T    
   C   
    T    
   G   
   T   
    C    
   A   
   C   
    T    
   G   
   C   
    T    
   T   
   T   
    C    
    G    
   C   
   T   
   A   
   A   
    C    
   A   
   T   
    T    
   C   
   G   
    A    
   A   
   G   
    C    
   A   
   A   
   T   
   G   
   G   
    C    
    C    
   T   
    G    
   A   
   T   
    T    
   G   
   C   
    A    
   A   
   T   
   T   
    G    
   G   
    T    
   T   
   T   
    G    
   A   
   A   
   A   
   G   
   A   
   A   
   G   
   A   
    T    
   G   
   A   
    C    
   G   
   A   
    A    
    T    
   T   
    A    
    G    
   T   
    C    
   A   
   A   
    T    
   G   
   T   
    T    
    C    
   T   
   A   
    T    
    T    
   G   
   A   
   C   
    A    
    G    
   A   
    C    
   G   
   G   
    A    
    C    
   A   
    G    
    T    
    C    
    A    
   A   
   A   
    T    
   A   
   T   
    C    
   A   
   T   
    C    
   A   
   T   
    T    
   G   
   G   
    C    
   A   
   C   
   A   
   C   
   A   
   T   
    A    
    A    
    C    
   G   
   G   
    C    
   T   
   A   
   C   
   T   
   C   
   T   
   G   
   T   
    G    
   A   
   C   
    C    
   T   
   T   
    C    
    A    
   A   
    A    
   G   
   A   
    A    
    G    
    A    
    A    
    G    
   C   
    A    
   G   
    T    
    C    
   C   
   G   
    C    
   G   
   A   
   T   
   A   
   T   
   G   
   G   
   G   
    A    
   C   
   G   
    T    
   A   
   C   
   A   
   G   
   C   
    T    
   T   
   C   
    C    
   G   
   G   
    C    
   G   
   T   
    C    
    A    
   G   
    A    
   G   
   G   
    G    
   A   
   T   
    A    
   C   
   G   
    T    
    C    
   T   
    G    
   C   
   G   
    T    
   G   
   A   
    G    
    G    
   A   
    A    
   G   
   A   
    T    
   T   
    A    
    C    
   G   
   T   
    C    
   G   
   T   
    T    
   G   
   G   
    G    
    G    
   C   
   A   
   G   
   C   
    C    
    T    
   T   
    G    
    A    
   T   
   G   
    A    
   A   
    G    
   G   
    A    
   A   
    G    
   A   
    C    
    C    
   A   
   A   
   G   
   A   
   A   
   G   
   T   
    A    
    C    
   T   
    G    
   G   
   T   
    G    
   A   
   T   
    T    
   A   
   C   
    A    
   G   
   A   
   A   
   A   
   A   
    A    
   G   
   G   
   T   
   T   
   A   
    C    
   G   
   G   
    A    
   A   
   A   
    A    
   C   
   G   
    G    
   A   
   C   
    C    
   A   
   A   
   A   
   G   
    T    
    A    
   T   
   C   
    G    
   G   
   A   
    A    
   T   
   A   
   T   
   C   
   C   
   A   
   G   
   T   
    G    
   A   
   A   
    A    
   G   
   G   
    C    
   C   
   G   
    C    
   G   
   G   
    A    
   G   
   G   
   T   
   A   
   A   
   A   
   G   
   G   
    G    
   A   
   T   
    C    
   A   
   A   
    A    
   A   
   C   
    G    
    G    
   C   
    G    
   A   
   A   
    T    
   A   
   T   
   C   
   A   
   C   
   T   
   G   
   A   
    A    
   A   
   A   
   A   
   A   
   A   
   T   
   G   
   G   
   T   
   C   
   C   
   A   
    C    
   T   
   A   
   G   
   C   
    C    
   G   
   G   
    A    
   T   
   T   
    G    
   A   
   C   
   C   
   A   
   C   
    T    
   G   
   T   
    T    
    T    
    C    
   T   
   G   
   G   
    A    
    A    
   A   
   T   
   G   
   A   
   A   
   G   
   A   
    C    
   A   
   T   
   C   
    A    
   T   
    G    
    C    
   T   
    G    
   A   
   T   
    T    
   A   
   C   
    A    
    G    
   A   
    T    
   A   
   A   
   A   
   G   
   G   
    T    
   G   
   T   
    G    
   A   
   T   
   T   
   A   
   T   
   C   
   C   
   G   
    T    
   T   
   T   
    C    
   A   
   A   
   C   
   G   
   T   
    A    
    T    
    C    
    G    
    A    
   C   
   T   
   G   
   T   
   T   
   T   
   C   
   T   
   C   
   A   
   A   
   A   
   C   
    T    
   G   
   G   
   A   
   C   
   G   
    T    
    T    
   C   
   A   
   A   
   C   
   A   
   T   
   T   
    G    
   G   
   G   
    T    
   G   
   T   
    A    
   C   
   G   
   T   
   T   
   T   
    G    
   A   
   T   
   G   
   A   
    A    
    G    
   A   
   T   
   G   
   G   
   A   
   A   
   G   
    C    
    T    
   G   
    A    
    T    
    A    
   C   
    G    
   A   
   A   
   A   
   G   
   T   
   C   
   G   
   T   
   C   
   A   
   C   
   A   
   A   
   T   
   G   
   -   
   -   
   -   
   -   
   -   
   -   
   -   
   -   
   -   
   -   
   -   
   -   
   -   
   -   
   -   
   -   
   -   
   -   
   -   
   -   
   -   
   -   
   -   
   -   
   -   
   -   
   -   
   -   
   -   
   -   
   -   
   -   
   -   
   -   
   -   
   -   
   -   
   -   
   -   
   -   
   -   
   -   
   -   
   -   
   -   
   -   
   -   
   -   
   -   
   -   
   -   
   -   
   -   
   -   
   -   
   -   
   -   
   -   
   -   
   -   
 

 
   N54070   
   A   
   T   
   G   
   A   
   G   
   T   
   G   
   A   
   A   
   G   
   A   
   A   
   A   
   T   
   C   
   A   
   G   
   A   
   G   
   A   
   A   
   A   
   A   
   C   
   A   
   T   
   C   
   C   
   A   
   T   
   G   
   A   
   T   
   G   
   T   
   C   
   A   
   A   
   T   
   C   
   T   
   A   
   A   
   C   
   C   
   A   
   G   
   C   
   G   
   A   
   A   
   A   
   T   
   G   
   A   
   A   
   A   
   G   
   A   
   C   
   T   
   C   
   C   
   T   
   T   
   C   
   A   
   T   
   C   
   G   
   A   
   T   
   T   
   A   
   T   
   G   
   C   
   G   
   A   
   T   
   G   
   A   
   G   
   T   
   G   
   T   
   T   
   A   
   T   
   T   
   G   
   T   
   C   
   G   
   C   
   A   
   C   
   G   
   T   
   G   
   C   
   A   
   C   
   T   
   T   
   C   
   C   
   T   
   G   
   A   
   T   
   G   
   T   
   C   
   A   
   G   
   A   
   G   
   A   
   T   
   G   
   G   
   T   
   C   
   T   
   G   
   A   
   A   
   A   
   C   
   C   
   C   
   G   
   T   
   T   
   C   
   A   
   C   
   C   
   G   
   C   
   C   
   G   
   T   
   A   
   T   
   T   
   T   
   T   
   A   
   T   
   A   
   C   
   G   
   G   
   C   
   A   
   T   
   G   
   A   
   A   
   C   
   G   
   A   
   A   
   T   
   T   
   A   
   G   
   G   
   T   
   G   
   T   
   A   
   A   
   C   
   A   
   C   
   C   
   G   
   G   
   A   
   T   
   A   
   A   
   A   
   G   
   C   
   C   
   C   
   A   
   T   
   A   
   A   
   A   
   A   
   A   
   A   
   T   
   C   
   T   
   G   
   C   
   C   
   A   
   G   
   A   
   A   
   T   
   C   
   G   
   T   
   C   
   G   
   G   
   A   
   G   
   A   
   T   
   G   
   T   
   C   
   A   
   T   
   G   
   G   
   G   
   T   
   A   
   A   
   A   
   T   
   A   
   C   
   C   
   A   
   T   
   C   
   C   
   T   
   C   
   A   
   T   
   G   
   G   
   A   
   G   
   A   
   C   
   A   
   G   
   T   
   G   
   C   
   G   
   A   
   T   
   C   
   T   
   A   
   T   
   G   
   A   
   G   
   T   
   C   
   A   
   A   
   T   
   G   
   G   
   T   
   A   
   C   
   G   
   G   
   A   
   T   
   G   
   G   
   C   
   G   
   C   
   A   
   G   
   C   
   C   
   A   
   T   
   T   
   C   
   A   
   G   
   C   
   T   
   A   
   C   
   C   
   G   
   T   
   T   
   A   
   T   
   A   
   T   
   G   
   C   
   T   
   A   
   G   
   T   
   G   
   G   
   A   
   T   
   G   
   G   
   T   
   C   
   A   
   T   
   G   
   G   
   A   
   A   
   A   
   C   
   T   
   T   
   T   
   G   
   G   
   T   
   T   
   C   
   T   
   G   
   T   
   T   
   G   
   A   
   C   
   G   
   G   
   T   
   G   
   A   
   C   
   G   
   G   
   T   
   G   
   C   
   C   
   G   
   C   
   C   
   G   
   C   
   T   
   A   
   T   
   G   
   C   
   G   
   T   
   T   
   A   
   T   
   A   
   C   
   C   
   G   
   A   
   A   
   G   
   C   
   A   
   C   
   G   
   G   
   A   
   T   
   G   
   A   
   G   
   C   
   A   
   A   
   A   
   A   
   T   
   C   
   G   
   C   
   T   
   A   
   C   
   A   
   G   
   A   
   A   
   A   
   T   
   G   
   C   
   T   
   G   
   C   
   G   
   T   
   G   
   A   
   T   
   A   
   T   
   C   
   A   
   A   
   T   
   A   
   A   
   G   
   A   
   A   
   C   
   A   
   C   
   T   
   G   
   T   
   T   
   G   
   A   
   T   
   T   
   T   
   C   
   C   
   A   
   A   
   A   
   G   
   C   
   A   
   A   
   C   
   T   
   A   
   T   
   G   
   A   
   T   
   G   
   A   
   T   
   A   
   C   
   A   
   G   
   A   
   A   
   A   
   A   
   A   
   G   
   A   
   A   
   C   
   C   
   A   
   G   
   T   
   T   
   G   
   T   
   A   
   C   
   T   
   A   
   C   
   C   
   T   
   G   
   C   
   T   
   C   
   G   
   C   
   T   
   T   
   C   
   C   
   C   
   G   
   A   
   A   
   T   
   C   
   T   
   T   
   T   
   T   
   A   
   G   
   T   
   G   
   A   
   A   
   C   
   G   
   G   
   A   
   A   
   C   
   A   
   A   
   C   
   G   
   G   
   G   
   G   
   A   
   T   
   C   
   G   
   C   
   C   
   G   
   T   
   A   
   G   
   G   
   T   
   A   
   T   
   G   
   G   
   C   
   C   
   A   
   C   
   G   
   A   
   A   
   T   
   A   
   T   
   C   
   C   
   C   
   A   
   C   
   C   
   T   
   C   
   A   
   T   
   A   
   A   
   C   
   T   
   T   
   G   
   T   
   C   
   T   
   G   
   A   
   G   
   G   
   T   
   A   
   G   
   T   
   A   
   G   
   C   
   G   
   G   
   C   
   T   
   A   
   T   
   C   
   G   
   A   
   T   
   T   
   T   
   G   
   C   
   T   
   G   
   A   
   T   
   G   
   G   
   A   
   T   
   A   
   A   
   T   
   C   
   C   
   T   
   G   
   A   
   T   
   G   
   T   
   G   
   A   
   C   
   G   
   A   
   C   
   C   
   A   
   A   
   T   
   G   
   A   
   A   
   T   
   T   
   G   
   A   
   T   
   G   
   G   
   A   
   A   
   G   
   T   
   A   
   T   
   T   
   A   
   C   
   C   
   T   
   G   
   G   
   A   
   C   
   C   
   A   
   G   
   A   
   T   
   T   
   T   
   T   
   C   
   C   
   A   
   A   
   C   
   A   
   G   
   G   
   T   
   G   
   G   
   T   
   T   
   T   
   G   
   G   
   T   
   T   
   A   
   T   
   G   
   G   
   G   
   A   
   A   
   A   
   A   
   T   
   C   
   A   
   G   
   G   
    A    
   A   
   T   
    C    
   C   
   G   
    A    
    A    
   G   
   A   
   G   
   C   
    G    
   T   
   A   
   T   
   G   
   A   
   A   
   A   
   C   
    A    
   G   
   G   
    T    
   A   
   A   
    G    
   G   
   G   
   T   
   T   
   C   
    T    
   A   
   T   
   T   
   A   
   C   
    C    
   G   
   T   
    A    
   C   
   G   
   T   
   G   
   C   
   A   
   A   
   A   
   A   
   G   
   T   
   T   
   G   
   A   
    G    
    A    
   T   
    T    
   A   
    C    
   T   
   G   
   A   
    G    
   A   
   T   
   G   
    C    
   C   
    A    
   A   
   A   
   T   
   G   
   G   
    T    
   A   
   A   
   A   
   G   
   A   
   A   
   C   
   G   
    G    
   A   
   T   
    C    
    A    
   T   
    C    
   G   
   T   
    G    
   A   
   C   
    A    
   G   
   A   
   A   
   T   
   T   
    G    
   C   
   C   
   T   
   T   
   A   
   T   
   A   
   T   
   G   
   G   
   T   
    C    
   A   
   A   
   T   
   A   
   A   
   A   
   G   
   C   
    A    
   A   
   A   
   A   
   T   
   T   
    G    
   A   
   T   
    C    
   G   
   A   
   A   
   C   
   G   
    G    
   A   
   T   
   T   
   T   
   C   
   T   
   G   
   A   
    A    
    C    
   T   
    G    
   C   
   A   
   C   
   A   
   G   
   A   
   G   
   A   
    C    
   A   
   A   
    G    
   C   
   G   
    T    
   A   
   T   
    C    
   G   
   A   
   A   
   G   
   G   
    G    
   A   
   T   
    C    
   A   
   C   
    A    
   G   
   A   
   T   
   T   
   T   
   G   
   C   
   G   
   G   
   G   
   A   
    C    
   G   
   A   
   A   
   T   
   C   
   T   
   T   
   C   
    C    
   C   
   G   
   T   
   G   
   A   
    A    
   G   
   G   
    T    
   A   
   T   
   G   
   C   
   G   
    G    
   A   
   T   
    C    
   G   
   T   
   C   
   A   
   T   
    T    
   G   
   A   
    C    
    A    
   T   
    C    
   C   
   G   
   T   
   C   
   G   
    T    
   G   
   A   
    C    
   G   
    C    
    C    
   A   
   G   
    C    
   G   
   C   
    A    
   T   
   C   
    C    
   G   
   T   
   T   
    A    
   T   
    C    
   T   
   T   
    G    
   A   
   A   
    T    
   A   
   A   
   C   
   T   
   T   
    A    
   T   
   A   
    T    
   A   
   A   
   A   
    T    
   T   
   G   
   A   
   C   
    A    
    T    
   C   
    C    
   T   
   T   
    G    
   C   
   A   
    A    
   A   
    A    
    T    
   T   
   C   
    A    
   T   
   T   
    T    
   G   
   G   
    A    
   T   
   T   
    C    
   A   
   A   
    T    
   A   
   T   
   G   
   T   
   T   
    G    
   G   
   C   
    G    
   A   
   T   
    C    
   G   
   A   
   A   
   A   
   A   
   A   
   G   
   G   
    T    
   G   
   T   
    G    
   C   
   C   
    G    
   A   
   A   
   A   
    G    
   T   
   T   
   T   
   T   
    G    
   A   
   G   
    C    
   T   
   T   
   G   
   A   
   A   
   A   
   C   
    A    
    A    
   A   
   T   
    T    
    C    
   T   
    T    
   G   
   A   
   A   
   A   
   A   
    C    
   T   
   A   
    T    
    A    
   T   
    C    
   G   
   A   
    A    
   C   
   A   
    T    
   C   
   A   
    G    
    C    
    G    
    C    
    G    
   A   
   A   
   G   
   T   
   C   
   A   
   T   
    C    
   A   
   C   
    A    
    A    
   G   
    A    
   C   
   G   
   G   
   A   
   C   
    A    
    G    
   T   
    C    
   T   
   T   
   T   
   G   
   A   
    A    
   A   
   A   
   A   
    G    
   A   
    A    
   A   
   A   
    A    
   G   
   C   
   A   
   G   
   A   
   A   
   G   
   C   
   A   
    A    
   G   
    G    
   G   
   C   
    A    
   C   
   A   
    C    
   A   
   T   
    T    
   T   
   T   
   A   
   G   
   A   
   A   
   G   
   G   
   T   
    T    
   T   
    G    
   C   
   G   
    T    
   A   
   T   
   T   
   G   
   C   
    T    
   T   
   T   
    G    
   G   
   A   
   T   
   C   
   A   
   T   
   A   
   T   
   C   
    G    
   A   
   T   
   G   
   A   
   A   
   A   
   T   
    A    
   A   
   T   
   T   
   G   
   C   
    G    
   A   
   T   
   T   
   A   
   T   
    C    
   C   
   G   
    C    
   G   
   G   
    G    
   T   
   C   
    T    
    A    
   A   
   A   
   T   
   C   
   A   
   G   
   A   
   T   
   G   
   A   
    C    
   G   
   A   
    A    
   G   
   C   
   A   
   A   
   A   
   A   
   G   
   C   
    G    
   A   
    C    
    G    
    A    
   T   
    G    
   A   
   T   
    C    
   G   
   A   
   A   
   C   
   G   
    G    
   T   
   T   
    C    
   G   
   A   
    T    
   T   
   T   
    A    
   T   
   C   
    C    
   G   
   A   
   T   
   C   
   G   
    C    
   C   
   A   
    G    
    T    
   C   
    T    
   C   
   A   
   A   
   G   
   C   
    T    
   A   
   T   
    T    
   T   
   T   
   A   
   G   
   A   
   T   
   A   
   T   
   G   
   C   
   G   
   T   
   T   
   T   
    A    
   C   
   G   
    C    
   C   
   G   
    A    
   T   
   T   
    G    
   A   
   C   
    C    
   G   
   G   
    T    
   T   
   T   
    G    
   G   
   A   
   A   
   C   
   G   
    G    
   G   
   A   
    A    
   A   
   A   
   A   
   A   
   T   
    C    
   G   
   A   
    A    
   A   
   A   
   T   
   G   
   A   
   A   
   T   
   A   
    C    
   C   
   A   
   A   
   G   
   A   
    A    
   C   
   T   
    C    
   T   
   T   
    A    
   A   
    A    
   A   
    C    
   T   
    G    
   A   
   T   
   T   
   G   
    A    
    A    
   G   
   A   
    T    
   T   
   T   
    A    
    A    
    C    
    T    
   G   
   A   
   C   
   A   
   T   
   T   
    C    
   T   
    G    
   G   
   C   
    A    
    A    
   G   
    A    
   C   
   C   
    A    
   G   
   A   
    A    
   C   
   G   
    T    
   G   
   T   
    T    
   A   
    C    
    A    
   G   
   A   
   A   
   A   
   T   
    C    
   A   
   T   
    C    
   A   
   A   
    G    
   A   
    A    
    T    
   G   
   A   
    A    
   T   
   T   
   A   
    G    
    C    
    A    
   G   
   A   
   C   
    T    
   T   
    G    
    A    
    A    
    T    
    C    
    A    
   A   
    C    
    G    
   A   
   T   
   T   
   T   
   G   
   G   
   T   
   G   
   A   
   T   
    A    
    A    
   A   
   C   
   G   
   A   
   C   
   G   
    C    
   A   
   C   
   A   
   G   
   A   
   A   
    T    
   T   
   A   
    C    
   T   
   A   
   G   
   T   
    C    
   G   
   G   
   T   
   G   
   A   
    G    
   G   
   T   
   C   
    T    
   T   
    A    
   A   
   G   
   T   
   C   
   T   
    G    
   G   
   A   
   A   
   G   
   A   
   T   
   G   
   A   
   A   
   G   
   A   
   T   
    T    
   T   
    A    
   A   
   T   
    C    
   G   
   A   
   A   
   G   
   A   
   A   
   G   
   A   
   A   
   G   
   A   
   A   
    A    
   T   
   C   
   G   
   T   
    T    
   A   
   T   
   T   
   A   
   C   
    A    
    T    
   T   
    G    
   A   
   C   
    A    
   A   
   A   
    C    
   A   
   A   
    T    
   G   
   G   
    T    
   T   
   A   
    C    
   A   
   T   
    C    
   A   
   A   
    A    
   C   
   G   
    T    
    T    
   T   
   G   
   G   
    C    
   A   
   A   
   A   
    T    
    A    
    A    
    T    
   G   
   A   
   A   
   T   
   T   
   C   
    A    
   G   
    A    
   G   
   C   
    T    
   C   
   A   
   A   
   C   
   G   
    A    
    A    
   G   
    G    
   G   
   G   
    T    
   G   
   G   
    A    
   C   
   G   
   T   
   G   
   G   
    T    
   G   
   T   
    C    
   C   
   A   
   A   
   G   
   G   
    A    
   A   
   T   
   G   
   G   
   G   
    T    
    A    
   T   
    C    
   C   
   A   
    C    
    A    
   A   
   T   
   G   
   A   
    C    
   G   
   A   
    C    
   T   
   T   
   C   
   G   
   T   
    C    
   A   
   A   
    A    
   A   
   A   
    T    
   C   
   T   
    G    
    G    
   T   
   T   
   T   
   C   
   T   
   T   
   G   
    T    
   T   
   C   
    T    
   A   
   C   
    C    
   C   
   A   
    T    
   G   
   A   
    T    
   A   
   C   
    A    
   T   
   T   
    G    
   T   
   T   
   A   
   T   
   T   
    C    
   T   
   T   
   T   
   A   
   C   
   G   
   A   
   A   
    T    
   A   
    A    
    C    
   G   
   G   
    A    
   A   
   A   
   A   
   G   
   T   
    G    
   T   
   A   
    T    
   C   
   G   
    G    
   G   
   C   
   A   
   A   
   A   
   A   
   G   
   G   
    A    
   T   
   A   
    C    
   G   
   A   
   A   
   A   
   T   
    T    
   C   
   C   
    A    
   G   
   A   
    A    
   T   
   A   
   C   
   G   
   G   
    G    
    C    
   G   
    T    
   A   
   C   
   A   
   G   
   C   
    C    
   A   
   A   
   A   
   G   
   G   
    A    
   A   
   T   
    A    
   C   
   C   
   A   
    A    
   T   
    T    
   A   
   T   
    C    
   A   
   A   
    T    
   T   
   T   
    A    
   C   
   T   
    A    
   G   
   G   
    T    
   A   
   T   
    C    
   G   
   A   
    T    
   T   
   C   
    G    
    A    
    G    
    T    
   G   
   A   
   A   
   A   
    G    
    C    
   A   
   T   
    C    
   C   
   A   
    G    
   G   
   C   
    A    
   A   
   T   
    C    
   A   
   T   
   T   
    G    
   C   
    G    
   G   
   T   
    G    
    A    
    C    
   G   
   G   
   G   
    A    
    G    
   A   
   A   
   G   
   C   
    A    
   G   
   A   
   A   
   G   
    A    
    A    
   G   
   G   
    C    
    C    
   A   
    C    
   T   
   A   
    T    
    C    
   T   
    C    
   T   
   T   
   C   
   T   
   T   
    T    
   A   
   C   
    G    
   A   
   C   
    G    
    C    
    G    
    T    
    C    
   A   
   A   
   G   
   G   
    T    
   A   
   C   
    A    
   G   
   T   
    A    
   A   
   A   
   A   
   C   
   G   
    T    
   A   
   C   
    T    
    T    
   C   
    T    
   G   
   T   
    C    
   A   
   C   
    T    
   G   
   C   
    T    
   T   
   T   
    T    
    G    
   C   
   T   
   A   
   A   
    C    
   A   
   T   
    C    
   C   
   G   
    A    
   A   
   G   
    T    
   A   
   A   
   T   
   G   
   G   
    T    
    C    
   T   
    G    
   A   
   T   
    C    
   G   
   C   
    A    
   A   
   T   
   T   
    G    
   G   
    T    
   T   
   T   
    G    
   A   
   A   
   A   
   G   
   A   
   A   
   G   
   A   
    C    
   G   
   A   
    T    
   G   
   A   
    G    
    C    
   T   
    C    
    G    
   T   
    C    
   A   
   A   
    T    
   G   
   T   
    G    
    C    
   T   
   A   
    C    
    T    
   G   
   A   
   C   
    A    
    G    
   A   
    T    
   G   
   G   
    T    
    C    
   A   
    A    
    T    
    C    
    T    
   A   
   A   
    T    
   A   
   T   
    C    
   A   
   T   
    T    
   A   
   T   
    T    
   G   
   G   
    A    
   A   
   C   
   A   
   C   
   A   
   T   
    A    
    A    
    C    
   G   
   G   
    C    
   T   
   A   
   C   
   T   
   C   
   T   
   G   
   T   
    C    
   A   
   C   
    T    
   T   
   T   
    C    
    A    
   A   
    A    
   G   
   A   
    A    
    G    
    A    
    A    
    G    
   C   
    A    
   G   
    T    
    C    
   C   
   G   
    C    
   G   
   A   
   T   
   A   
   T   
   G   
   G   
   G   
    A    
   C   
   G   
    T    
   A   
   C   
   A   
   G   
   C   
    T    
   T   
   C   
    C    
   G   
   G   
    G    
   G   
   T   
    C    
    A    
   G   
    A    
   G   
   G   
    A    
   A   
   T   
    A    
   C   
   G   
    C    
    C    
   T   
    G    
   C   
   G   
    T    
   G   
   A   
    G    
    G    
   A   
    A    
   G   
   A   
    T    
   T   
    A    
    T    
   G   
   T   
    C    
   G   
   T   
    T    
   G   
   G   
    G    
    G    
   C   
   A   
   G   
   C   
    C    
    T    
   T   
    A    
    A    
   T   
   G   
    A    
   A   
    G    
   G   
    A    
   A   
    A    
   A   
    T    
    C    
   A   
   A   
   G   
   A   
   A   
   G   
   T   
    A    
    T    
   T   
    A    
   G   
   T   
    G    
   A   
   T   
    C    
   A   
   C   
    A    
   G   
   A   
   A   
   A   
   A   
    A    
   G   
   G   
   T   
   T   
   A   
    C    
   G   
   G   
    A    
   A   
   A   
    A    
   C   
   G   
    G    
   A   
   C   
    A    
   A   
   A   
   A   
   G   
    T    
    A    
   T   
   C   
    G    
   G   
   A   
    A    
   T   
   A   
   T   
   C   
   C   
   A   
   G   
   T   
    G    
   A   
   A   
    A    
   G   
   G   
    C    
   C   
   G   
    C    
   G   
   G   
    A    
   G   
   G   
   T   
   A   
   A   
   A   
   G   
   G   
    G    
   A   
   T   
    C    
   A   
   A   
    A    
   A   
   C   
    G    
    G    
   C   
    G    
   A   
   A   
    C    
   A   
   T   
   C   
   A   
   C   
   T   
   G   
   A   
    A    
   A   
   A   
   A   
   A   
   A   
   T   
   G   
   G   
   T   
   C   
   C   
   A   
    C    
   T   
   A   
   G   
   C   
    T    
   G   
   G   
    A    
   T   
   T   
    G    
   A   
   C   
   C   
   A   
   C   
    T    
   G   
   T   
    T    
    T    
    C    
   T   
   G   
   G   
    A    
    G    
   A   
   T   
   G   
   A   
   A   
   G   
   A   
    C    
   A   
   T   
   C   
    A    
   T   
    G    
    C    
   T   
    G    
   A   
   T   
    T    
   A   
   C   
    A    
    G    
   A   
    T    
   A   
   A   
   A   
   G   
   G   
    T    
   G   
   T   
    C    
   A   
   T   
   T   
   A   
   T   
   C   
   C   
   G   
    T    
   T   
   T   
    C    
   A   
   A   
   C   
   G   
   T   
    A    
    T    
    C    
    G    
    A    
   C   
   T   
   G   
   T   
   T   
   T   
   C   
   T   
   C   
   A   
   A   
   A   
   C   
    T    
   G   
   G   
   A   
   C   
   G   
    T    
    T    
   C   
   A   
   A   
   C   
   A   
   T   
   T   
    A    
   G   
   G   
    C    
   G   
   T   
    A    
   C   
   G   
   T   
   T   
   T   
    G    
   A   
   T   
   G   
   A   
    A    
    G    
   A   
   T   
   G   
   G   
   A   
   A   
   G   
    C    
    T    
   G   
    A    
    T    
    A    
   C   
    G    
   A   
   A   
   A   
   G   
   T   
   T   
   G   
   T   
   C   
   A   
   C   
   G   
   A   
   T   
   G   
   G   
   C   
   A   
   G   
   C   
   A   
   G   
   T   
   C   
   G   
   A   
   A   
   C   
   C   
   A   
   G   
   A   
   A   
   G   
   C   
   T   
   G   
   C   
   A   
   G   
   A   
   A   
   C   
   A   
   A   
   A   
   C   
   A   
   G   
   T   
   T   
   G   
   A   
   A   
   G   
   A   
   A   
   C   
   A   
   A   
   G   
   G   
   A   
   T   
   C   
   A   
   G   
   A   
   A   
   G   
   A   
   A   
   -   
   -   
   -   
 

 
   N59420F   
   A   
   T   
   G   
   A   
   G   
   T   
   G   
   A   
   A   
   G   
   A   
   A   
   A   
   T   
   T   
   A   
   A   
   A   
   G   
   A   
   A   
   A   
   A   
   C   
   A   
   T   
   T   
   C   
   A   
   A   
   G   
   A   
   C   
   G   
   T   
   C   
   A   
   A   
   T   
   C   
   T   
   G   
   A   
   C   
   C   
   A   
   G   
   C   
   G   
   A   
   A   
   A   
   T   
   G   
   A   
   A   
   A   
   G   
   A   
   A   
   T   
   C   
   T   
   T   
   T   
   C   
   A   
   T   
   T   
   G   
   A   
   C   
   T   
   A   
   C   
   G   
   C   
   A   
   A   
   T   
   G   
   A   
   G   
   T   
   G   
   T   
   T   
   A   
   T   
   C   
   G   
   T   
   A   
   G   
   C   
   C   
   C   
   G   
   C   
   G   
   C   
   G   
   T   
   T   
   A   
   C   
   C   
   T   
   G   
   A   
   C   
   G   
   T   
   T   
   C   
   G   
   T   
   G   
   A   
   C   
   G   
   G   
   T   
   T   
   T   
   A   
   A   
   A   
   A   
   C   
   C   
   T   
   G   
   T   
   T   
   C   
   A   
   T   
   C   
   G   
   C   
   C   
   G   
   A   
   A   
   T   
   C   
   T   
   T   
   A   
   T   
   A   
   T   
   G   
   G   
   A   
   A   
   T   
   G   
   A   
   A   
   C   
   G   
   A   
   A   
   T   
   T   
   A   
   G   
   G   
   G   
   G   
   T   
   A   
   A   
   C   
   C   
   C   
   C   
   T   
   G   
   A   
   T   
   A   
   A   
   A   
   C   
   C   
   A   
   C   
   A   
   C   
   A   
   A   
   A   
   A   
   A   
   A   
   T   
   C   
   A   
   G   
   C   
   C   
   C   
   G   
   G   
   A   
   T   
   T   
   G   
   T   
   T   
   G   
   G   
   G   
   G   
   A   
   T   
   G   
   T   
   T   
   A   
   T   
   G   
   G   
   G   
   T   
   A   
   A   
   A   
   T   
   A   
   T   
   C   
   A   
   T   
   C   
   C   
   C   
   C   
   A   
   T   
   G   
   G   
   G   
   G   
   A   
   C   
   A   
   G   
   T   
   G   
   C   
   G   
   A   
   T   
   T   
   T   
   A   
   C   
   G   
   A   
   A   
   T   
   C   
   A   
   A   
   T   
   G   
   G   
   T   
   G   
   C   
   G   
   G   
   A   
   T   
   G   
   G   
   C   
   A   
   C   
   A   
   A   
   C   
   C   
   T   
   T   
   T   
   T   
   A   
   G   
   T   
   T   
   A   
   T   
   C   
   G   
   G   
   G   
   C   
   T   
   A   
   T   
   G   
   T   
   T   
   A   
   G   
   T   
   T   
   G   
   A   
   C   
   G   
   G   
   C   
   C   
   A   
   C   
   G   
   G   
   A   
   A   
   A   
   C   
   T   
   T   
   C   
   G   
   G   
   T   
   T   
   C   
   T   
   G   
   T   
   C   
   G   
   A   
   T   
   G   
   G   
   T   
   G   
   A   
   C   
   G   
   G   
   C   
   G   
   C   
   T   
   G   
   C   
   C   
   G   
   C   
   T   
   A   
   T   
   G   
   C   
   G   
   T   
   T   
   A   
   T   
   A   
   C   
   C   
   G   
   A   
   A   
   G   
   C   
   A   
   C   
   G   
   T   
   A   
   T   
   G   
   A   
   G   
   T   
   A   
   A   
   A   
   A   
   T   
   T   
   G   
   C   
   T   
   T   
   T   
   A   
   G   
   A   
   A   
   A   
   T   
   G   
   C   
   T   
   A   
   C   
   G   
   A   
   G   
   A   
   T   
   A   
   T   
   T   
   A   
   A   
   C   
   A   
   A   
   A   
   A   
   A   
   T   
   A   
   C   
   A   
   G   
   T   
   C   
   G   
   A   
   T   
   T   
   T   
   C   
   C   
   A   
   A   
   G   
   G   
   A   
   A   
   A   
   C   
   T   
   A   
   T   
   G   
   A   
   T   
   G   
   A   
   T   
   T   
   C   
   A   
   G   
   A   
   A   
   C   
   A   
   A   
   G   
   A   
   A   
   C   
   C   
   A   
   G   
   T   
   A   
   G   
   T   
   T   
   T   
   T   
   A   
   C   
   C   
   T   
   G   
   C   
   T   
   C   
   G   
   T   
   T   
   T   
   T   
   C   
   C   
   A   
   A   
   A   
   C   
   T   
   T   
   A   
   C   
   T   
   A   
   G   
   T   
   T   
   A   
   A   
   C   
   G   
   G   
   A   
   A   
   C   
   A   
   A   
   C   
   G   
   G   
   G   
   G   
   A   
   T   
   T   
   G   
   C   
   G   
   G   
   T   
   G   
   G   
   G   
   G   
   A   
   T   
   G   
   G   
   C   
   A   
   A   
   C   
   A   
   A   
   A   
   T   
   A   
   T   
   T   
   C   
   C   
   A   
   C   
   C   
   A   
   C   
   A   
   T   
   A   
   A   
   T   
   T   
   T   
   A   
   A   
   G   
   T   
   G   
   A   
   A   
   G   
   T   
   G   
   A   
   T   
   T   
   G   
   A   
   T   
   G   
   C   
   A   
   A   
   C   
   A   
   A   
   G   
   T   
   T   
   T   
   A   
   T   
   T   
   G   
   A   
   T   
   G   
   G   
   A   
   C   
   A   
   A   
   T   
   C   
   C   
   T   
   G   
   A   
   T   
   G   
   T   
   A   
   A   
   C   
   G   
   A   
   C   
   G   
   A   
   A   
   T   
   G   
   A   
   A   
   T   
   T   
   G   
   A   
   T   
   G   
   G   
   A   
   A   
   G   
   T   
   G   
   T   
   T   
   A   
   C   
   C   
   T   
   G   
   G   
   A   
   C   
   C   
   A   
   G   
   A   
   T   
   T   
   T   
   T   
   C   
   C   
   A   
   A   
   C   
   A   
   G   
   G   
   C   
   G   
   G   
   T   
   T   
   T   
   A   
   G   
   T   
   G   
   A   
   T   
   G   
   G   
   G   
   G   
   A   
   A   
   A   
   T   
   C   
   A   
   G   
   G   
    G    
   A   
   T   
    T    
   C   
   G   
    C    
    C    
   G   
   A   
   G   
   C   
    A    
   T   
   A   
   T   
   G   
   A   
   A   
   A   
   C   
    A    
   G   
   G   
    G    
   A   
   A   
    A    
   G   
   G   
   T   
   T   
   C   
    G    
   A   
   T   
   T   
   A   
   C   
    T    
   G   
   T   
    T    
   C   
   G   
   T   
   G   
   C   
   A   
   A   
   A   
   A   
   G   
   T   
   T   
   G   
   A   
    A    
    T    
   T   
    G    
   A   
    C    
   T   
   G   
   A   
    A    
   A   
   T   
   G   
    C    
   C   
    G    
   A   
   A   
   T   
   G   
   G   
    A    
   A   
   A   
   A   
   G   
   A   
   A   
   C   
   G   
    T    
   A   
   T   
    T    
    T    
   T   
    A    
   G   
   T   
    A    
   A   
   C   
    T    
   G   
   A   
   A   
   T   
   T   
    G    
   C   
   C   
   T   
   T   
   A   
   T   
   A   
   T   
   G   
   G   
   T   
    G    
   A   
   A   
   T   
   A   
   A   
   A   
   G   
   C   
    C    
   A   
   A   
   A   
   T   
   T   
    A    
   A   
   T   
    C    
   G   
   A   
   A   
   C   
   G   
    A    
   A   
   T   
   T   
   T   
   C   
   T   
   G   
   A   
    A    
    T    
   T   
    A    
   C   
   A   
   C   
   A   
   G   
   A   
   G   
   A   
    T    
   A   
   A   
    A    
   C   
   G   
    A    
   A   
   T   
    T    
   G   
   A   
   A   
   G   
   G   
    A    
   A   
   T   
    T    
   A   
   C   
    T    
   G   
   A   
   T   
   T   
   T   
   G   
   C   
   G   
   G   
   G   
   A   
    T    
   G   
   A   
   A   
   T   
   C   
   T   
   T   
   C   
    T    
   C   
   G   
   T   
   G   
   A   
    A    
   G   
   G   
    C    
   A   
   T   
   G   
   C   
   G   
    G    
   A   
   T   
    C    
   G   
   T   
   C   
   A   
   T   
    C    
   G   
   A   
    T    
    G    
   T   
    T    
   C   
   G   
   T   
   C   
   G   
    A    
   G   
   A   
    T    
   G   
    T    
    G    
   A   
   G   
    T    
   G   
   C   
    C    
   T   
   C   
    T    
   G   
   T   
   T   
    G    
   T   
    G    
   T   
   T   
    A    
   A   
   A   
    C    
   A   
   A   
   C   
   T   
   T   
    G    
   T   
   A   
    C    
   A   
   A   
   A   
    A    
   T   
   G   
   A   
   C   
    T    
    G    
   C   
    C    
   T   
   T   
    A    
   C   
   A   
    A    
   A   
    C    
    A    
   T   
   C   
    T    
   T   
   T   
    T    
   G   
   G   
    T    
   T   
   T   
    T    
   A   
   A   
    C    
   A   
   T   
   G   
   T   
   T   
    A    
   G   
   C   
    C    
   A   
   T   
    T    
   G   
   A   
   A   
   A   
   A   
   A   
   G   
   G   
    C    
   G   
   T   
    A    
   C   
   C   
    G    
   A   
   A   
   A   
    A    
   T   
   T   
   T   
   T   
    A    
   A   
   G   
    C    
   T   
   T   
   G   
   A   
   A   
   A   
   C   
    G    
    T    
   A   
   T   
    T    
    T    
   T   
    A    
   G   
   A   
   A   
   A   
   A   
    C    
   T   
   A   
    C    
    G    
   T   
    C    
   G   
   A   
    G    
   C   
   A   
    T    
   C   
   A   
    A    
    A    
    A    
    A    
    G    
   A   
   A   
   G   
   T   
   C   
   A   
   T   
    T    
   A   
   C   
    T    
    C    
   G   
    C    
   C   
   G   
   G   
   A   
   C   
    G    
    A    
   T   
    T    
   T   
   T   
   T   
   G   
   A   
    C    
   A   
   A   
   A   
    A    
   A   
    C    
   A   
   A   
    A    
   G   
   C   
   A   
   G   
   A   
   A   
   G   
   C   
   A   
    C    
   G   
    G    
   G   
   C   
    G    
   C   
   A   
    T    
   A   
   T   
    T    
   T   
   T   
   A   
   G   
   A   
   A   
   G   
   G   
   T   
    C    
   T   
    A    
   C   
   G   
    A    
   A   
   T   
   T   
   G   
   C   
    C    
   T   
   T   
    A    
   G   
   A   
   T   
   C   
   A   
   T   
   A   
   T   
   C   
    G    
   A   
   T   
   G   
   A   
   A   
   A   
   T   
    C    
   A   
   T   
   T   
   G   
   C   
    C    
   A   
   T   
   T   
   A   
   T   
    C    
   C   
   G   
    T    
   G   
   G   
    G    
   T   
   C   
    A    
    C    
   A   
   A   
   T   
   C   
   A   
   G   
   A   
   T   
   G   
   A   
    C    
   G   
   A   
    A    
   G   
   C   
   A   
   A   
   A   
   A   
   G   
   C   
    A    
   A   
    C    
    G    
    T    
   T   
    A    
   A   
   T   
    T    
   G   
   A   
   A   
   C   
   G   
    C    
   T   
   T   
    T    
   G   
   A   
    A    
   T   
   T   
    T    
   T   
   C   
    A    
   G   
   A   
   T   
   C   
   G   
    T    
   C   
   A   
    A    
    G    
   C   
    G    
   C   
   A   
   A   
   G   
   C   
    G    
   A   
   T   
    T    
   T   
   T   
   A   
   G   
   A   
   T   
   A   
   T   
   G   
   C   
   G   
   T   
   T   
   T   
    A    
   C   
   G   
    C    
   C   
   G   
    T    
   T   
   T   
    A    
   A   
   C   
    A    
   G   
   G   
    C    
   T   
   T   
    A    
   G   
   A   
   A   
   C   
   G   
    C    
   G   
   A   
    C    
   A   
   A   
   A   
   A   
   T   
    T    
   G   
   A   
    A    
   A   
   A   
   T   
   G   
   A   
   A   
   T   
   A   
    T    
   C   
   A   
   A   
   G   
   A   
    G    
   C   
   T   
    A    
   T   
   T   
    G    
   A   
    A    
   A   
    T    
   T   
    C    
   A   
   T   
   T   
   G   
    C    
    G    
   G   
   A   
    C    
   T   
   T   
    A    
    G    
    A    
    G    
   G   
   A   
   C   
   A   
   T   
   T   
    T    
   T   
    A    
   G   
   C   
    C    
    C    
   G   
    C    
   C   
   C   
    A    
   G   
   A   
    G    
   C   
   G   
    C    
   G   
   T   
    C    
   A   
    T    
    T    
   G   
   A   
   A   
   A   
   T   
    C    
   A   
   T   
    T    
   A   
   A   
    A    
   A   
    C    
    A    
   G   
   A   
    G    
   T   
   T   
   A   
    A    
    A    
    T    
   G   
   A   
   C   
    G    
   T   
    T    
    C    
    G    
    C    
    A    
    C    
   A   
    A    
    A    
   A   
   T   
   T   
   T   
   G   
   G   
   T   
   G   
   A   
   T   
    G    
    C    
   A   
   C   
   G   
   A   
   C   
   G   
    C    
   A   
   C   
   A   
   G   
   A   
   A   
    T    
   T   
   A   
    T    
   T   
   A   
   G   
   T   
    A    
   G   
   G   
   T   
   G   
   A   
    A    
   G   
   T   
   C   
    C    
   T   
    A    
   A   
   G   
   T   
   C   
   T   
    T    
   G   
   A   
   A   
   G   
   A   
   T   
   G   
   A   
   A   
   G   
   A   
   T   
    T    
   T   
    A    
   A   
   T   
    T    
   G   
   A   
   A   
   G   
   A   
   A   
   G   
   A   
   A   
   G   
   A   
   A   
    G    
   T   
   C   
   G   
   T   
    G    
   A   
   T   
   T   
   A   
   C   
    A    
    T    
   T   
    A    
   A   
   C   
    C    
   A   
   A   
    T    
   A   
   A   
    C    
   G   
   G   
    C    
   T   
   A   
    C    
   A   
   T   
    T    
   A   
   A   
    G    
   C   
   G   
    G    
    A    
   T   
   G   
   G   
    C    
   A   
   A   
   A   
    C    
    T    
    C    
    C    
   G   
   A   
   A   
   T   
   T   
   C   
    C    
   G   
    G    
   G   
   C   
    G    
   C   
   A   
   A   
   C   
   G   
    C    
    C    
   G   
    T    
   G   
   G   
    C    
   G   
   G   
    A    
   C   
   G   
   T   
   G   
   G   
    T    
   G   
   T   
    C    
   C   
   A   
   A   
   G   
   G   
    C    
   A   
   T   
   G   
   G   
   G   
    C    
    G    
   T   
    T    
   C   
   A   
    T    
    G    
   A   
   T   
   G   
   A   
    T    
   G   
   A   
    T    
   T   
   T   
   C   
   G   
   T   
    G    
   A   
   A   
    A    
   A   
   A   
    C    
   C   
   T   
    A    
    G    
   T   
   T   
   T   
   C   
   T   
   T   
   G   
    T    
   T   
   C   
    A    
   A   
   C   
    A    
   C   
   A   
    C    
   G   
   A   
    C    
   A   
   C   
    G    
   T   
   T   
    A    
   T   
   T   
   A   
   T   
   T   
    C    
   T   
   T   
   T   
   A   
   C   
   G   
   A   
   A   
    C    
   A   
    C    
    T    
   G   
   G   
    G    
   A   
   A   
   A   
   G   
   T   
    T    
   T   
   A   
    C    
   C   
   G   
    A    
   G   
   C   
   A   
   A   
   A   
   A   
   G   
   G   
    T    
   T   
   A   
    C    
   G   
   A   
   A   
   A   
   T   
    C    
   C   
   C   
    T    
   G   
   A   
    A    
   T   
   A   
   C   
   G   
   G   
    T    
    A    
   G   
    A    
   A   
   C   
   A   
   G   
   C   
    A    
   A   
   A   
   A   
   G   
   G   
    A    
   A   
   T   
    T    
   C   
   C   
   A   
    G    
   T   
    A    
   A   
   T   
    T    
   A   
   A   
    C    
   T   
   T   
    A    
   C   
   T   
    G    
   G   
   G   
    A    
   A   
   T   
    T    
   G   
   A   
    T    
   T   
   C   
    T    
    G    
    C    
    A    
   G   
   A   
   A   
   A   
    A    
    A    
   A   
   T   
    T    
   C   
   A   
    A    
   G   
   C   
    G    
   A   
   T   
    T    
   A   
   T   
   T   
    T    
   C   
    T    
   G   
   T   
    T    
    G    
    A    
   G   
   G   
   G   
    C    
    A    
   A   
   A   
   G   
   C   
    G    
   G   
   A   
   A   
   G   
    C    
    A    
   G   
   G   
    T    
    A    
   A   
    A    
   T   
   A   
    C    
    T    
   T   
    G    
   T   
   T   
   C   
   T   
   T   
    C    
   A   
   C   
    A    
   A   
   C   
    C    
    T    
    T    
    A    
    A    
   A   
   A   
   G   
   G   
    A    
   A   
   C   
    C    
   G   
   T   
    C    
   A   
   A   
   A   
   C   
   G   
    G    
   A   
   C   
    A    
    T    
   C   
    C    
   G   
   T   
    A    
   A   
   C   
    A    
   G   
   C   
    C    
   T   
   T   
    T    
    T    
   C   
   T   
   A   
   A   
    T    
   A   
   T   
    C    
   C   
   G   
    T    
   A   
   G   
    T    
   A   
   A   
   T   
   G   
   G   
    A    
    T    
   T   
    A    
   A   
   T   
    C    
   G   
   C   
    C    
   A   
   T   
   T   
    A    
   G   
    C    
   T   
   T   
    A    
   A   
   A   
   A   
   G   
   A   
   A   
   G   
   A   
    T    
   G   
   A   
    T    
   G   
   A   
    G    
    T    
   T   
    A    
    G    
   T   
    T    
   A   
   A   
    C    
   G   
   T   
    G    
    G    
   T   
   A   
    A    
    C    
   G   
   A   
   C   
    T    
    A    
   A   
    T    
   G   
   G   
    C    
    A    
   A   
    T    
    C    
    A    
    G    
   A   
   A   
    G    
   A   
   T   
    G    
   A   
   T   
    T    
   A   
   T   
    C    
   G   
   G   
    A    
   A   
   C   
   A   
   C   
   A   
   T   
    G    
    C    
    A    
   G   
   G   
    A    
   T   
   A   
   C   
   T   
   C   
   T   
   G   
   T   
    C    
   A   
   C   
    A    
   T   
   T   
    T    
    G    
   A   
    T    
   G   
   A   
    A    
    A    
    A    
    T    
    A    
   C   
    T    
   G   
    T    
    A    
   C   
   G   
    T    
   G   
   A   
   T   
   A   
   T   
   G   
   G   
   G   
    C    
   C   
   G   
    G    
   A   
   C   
   A   
   G   
   C   
    A    
   T   
   C   
    A    
   G   
   G   
    T    
   G   
   T   
    T    
    C    
   G   
    G    
   G   
   G   
    A    
   A   
   T   
    C    
   C   
   G   
    T    
    C    
   T   
    C    
   C   
   G   
    C    
   G   
   A   
    A    
    A    
   A   
    T    
   G   
   A   
    T    
   T   
    A    
    T    
   G   
   T   
    G    
   G   
   T   
    C    
   G   
   G   
    T    
    G    
   C   
   A   
   G   
   C   
    G    
    A    
   T   
    T    
    C    
   T   
   G   
    G    
   A   
    T    
   G   
    A    
   A   
    A    
   A   
    C    
    A    
   A   
   A   
   G   
   A   
   A   
   G   
   T   
    C    
    C    
   T   
    A    
   G   
   T   
    C    
   A   
   T   
    T    
   A   
   C   
    T    
   G   
   A   
   A   
   A   
   A   
    T    
   G   
   G   
   T   
   T   
   A   
    T    
   G   
   G   
    T    
   A   
   A   
    G    
   C   
   G   
    T    
   A   
   C   
    A    
   A   
   A   
   A   
   G   
    C    
    T    
   T   
   C   
    T    
   G   
   A   
    A    
   T   
   A   
   T   
   C   
   C   
   A   
   G   
   T   
    T    
   A   
   A   
    A    
   G   
   G   
    A    
   C   
   G   
    T    
   G   
   G   
    C    
   G   
   G   
   T   
   A   
   A   
   A   
   G   
   G   
    G    
   A   
   T   
    T    
   A   
   A   
    G    
   A   
   C   
    A    
    G    
   C   
    A    
   A   
   A   
    T    
   A   
   T   
   C   
   A   
   C   
   T   
   G   
   A   
    G    
   A   
   A   
   A   
   A   
   A   
   T   
   G   
   G   
   T   
   C   
   C   
   A   
    T    
   T   
   A   
   G   
   C   
    T    
   G   
   G   
    T    
   T   
   T   
    A    
   A   
   C   
   C   
   A   
   C   
    G    
   G   
   T   
    C    
    A    
    A    
   T   
   G   
   G   
    G    
    G    
   A   
   T   
   G   
   A   
   A   
   G   
   A   
    T    
   A   
   T   
   C   
    T    
   T   
    A    
    T    
   T   
    G    
   A   
   T   
    T    
   A   
   C   
    G    
    A    
   A   
    C    
   A   
   A   
   A   
   G   
   G   
    C    
   G   
   T   
    C    
   A   
   T   
   T   
   A   
   T   
   C   
   C   
   G   
    C    
   T   
   T   
    T    
   A   
   A   
   C   
   G   
   T   
    T    
    G    
    A    
    T    
    T    
   C   
   T   
   G   
   T   
   T   
   T   
   C   
   T   
   C   
   A   
   A   
   A   
   C   
    A    
   G   
   G   
   A   
   C   
   G   
    C    
    G    
   C   
   A   
   A   
   C   
   A   
   T   
   T   
    A    
   G   
   G   
    A    
   G   
   T   
    T    
   C   
   G   
   T   
   T   
   T   
    A    
   A   
   T   
   G   
   A   
    G    
    A    
   A   
   T   
   G   
   G   
   A   
   A   
   G   
    A    
    T    
   G   
    G    
    T    
    G    
   C   
    C    
   A   
   A   
   A   
   -   
   -   
   -   
   -   
   -   
   -   
   -   
   -   
   -   
   -   
   -   
   -   
   -   
   -   
   -   
   -   
   -   
   -   
   -   
   -   
   -   
   -   
   -   
   -   
   -   
   -   
   -   
   -   
   -   
   -   
   -   
   -   
   -   
   -   
   -   
   -   
   -   
   -   
   -   
   -   
   -   
   -   
   -   
   -   
   -   
   -   
   -   
   -   
   -   
   -   
   -   
   -   
   -   
   -   
   -   
   -   
   -   
   -   
   -   
   -   
   -   
   -   
   -   
   -   
   -   
   -   
   -   
   -   
   -   
   -   
   -   
   -   
 

 
   N54538   
   A   
   T   
   G   
   A   
   G   
   T   
   G   
   A   
   A   
   G   
   A   
   A   
   A   
   T   
   C   
   A   
   G   
   A   
   G   
   A   
   A   
   A   
   A   
   C   
   A   
   T   
   C   
   C   
   A   
   T   
   G   
   A   
   T   
   G   
   T   
   C   
   A   
   A   
   T   
   C   
   T   
   A   
   A   
   C   
   C   
   A   
   G   
   C   
   G   
   A   
   A   
   A   
   T   
   G   
   A   
   A   
   A   
   G   
   A   
   C   
   T   
   C   
   C   
   T   
   T   
   C   
   A   
   T   
   C   
   G   
   A   
   T   
   T   
   A   
   T   
   G   
   C   
   G   
   A   
   T   
   G   
   A   
   G   
   T   
   G   
   T   
   T   
   A   
   T   
   T   
   G   
   T   
   C   
   G   
   C   
   A   
   C   
   G   
   T   
   G   
   C   
   A   
   C   
   T   
   T   
   C   
   C   
   T   
   G   
   A   
   T   
   G   
   T   
   C   
   A   
   G   
   A   
   G   
   A   
   T   
   G   
   G   
   T   
   C   
   T   
   G   
   A   
   A   
   A   
   C   
   C   
   C   
   G   
   T   
   T   
   C   
   A   
   C   
   C   
   G   
   C   
   C   
   G   
   T   
   A   
   T   
   T   
   T   
   T   
   A   
   T   
   A   
   C   
   G   
   G   
   C   
   A   
   T   
   G   
   A   
   A   
   C   
   G   
   A   
   A   
   T   
   T   
   A   
   G   
   G   
   T   
   G   
   T   
   A   
   A   
   C   
   A   
   C   
   C   
   G   
   G   
   A   
   T   
   A   
   A   
   A   
   G   
   C   
   C   
   C   
   A   
   T   
   A   
   A   
   A   
   A   
   A   
   A   
   T   
   C   
   T   
   G   
   C   
   C   
   A   
   G   
   A   
   A   
   T   
   C   
   G   
   T   
   C   
   G   
   G   
   A   
   G   
   A   
   T   
   G   
   T   
   C   
   A   
   T   
   G   
   G   
   G   
   T   
   A   
   A   
   A   
   T   
   A   
   C   
   C   
   A   
   T   
   C   
   C   
   T   
   C   
   A   
   T   
   G   
   G   
   A   
   G   
   A   
   C   
   A   
   G   
   T   
   G   
   C   
   G   
   A   
   T   
   C   
   T   
   A   
   T   
   G   
   A   
   G   
   T   
   C   
   A   
   A   
   T   
   G   
   G   
   T   
   A   
   C   
   G   
   G   
   A   
   T   
   G   
   G   
   C   
   G   
   C   
   A   
   G   
   C   
   C   
   A   
   T   
   T   
   C   
   A   
   G   
   C   
   T   
   A   
   C   
   C   
   G   
   T   
   T   
   A   
   T   
   A   
   T   
   G   
   C   
   T   
   A   
   G   
   T   
   G   
   G   
   A   
   T   
   G   
   G   
   T   
   C   
   A   
   T   
   G   
   G   
   A   
   A   
   A   
   C   
   T   
   T   
   T   
   G   
   G   
   T   
   T   
   C   
   T   
   G   
   T   
   T   
   G   
   A   
   C   
   G   
   G   
   T   
   G   
   A   
   C   
   G   
   G   
   T   
   G   
   C   
   C   
   G   
   C   
   C   
   G   
   C   
   T   
   A   
   T   
   G   
   C   
   G   
   T   
   T   
   A   
   T   
   A   
   C   
   C   
   G   
   A   
   A   
   G   
   C   
   A   
   C   
   G   
   G   
   A   
   T   
   G   
   A   
   G   
   C   
   A   
   A   
   A   
   A   
   T   
   C   
   G   
   C   
   T   
   A   
   C   
   A   
   G   
   A   
   A   
   A   
   T   
   G   
   C   
   T   
   G   
   C   
   G   
   T   
   G   
   A   
   T   
   A   
   T   
   C   
   A   
   A   
   T   
   A   
   A   
   G   
   A   
   A   
   C   
   A   
   C   
   T   
   G   
   T   
   T   
   G   
   A   
   T   
   T   
   T   
   C   
   C   
   A   
   A   
   A   
   G   
   C   
   A   
   A   
   C   
   T   
   A   
   T   
   G   
   A   
   T   
   G   
   A   
   T   
   A   
   C   
   A   
   G   
   A   
   A   
   A   
   A   
   A   
   G   
   A   
   A   
   C   
   C   
   A   
   G   
   T   
   T   
   G   
   T   
   A   
   C   
   T   
   A   
   C   
   C   
   T   
   G   
   C   
   T   
   C   
   G   
   C   
   T   
   T   
   C   
   C   
   C   
   G   
   A   
   A   
   T   
   C   
   T   
   T   
   T   
   T   
   A   
   G   
   T   
   G   
   A   
   A   
   C   
   G   
   G   
   A   
   A   
   C   
   A   
   A   
   C   
   G   
   G   
   G   
   G   
   A   
   T   
   C   
   G   
   C   
   C   
   G   
   T   
   A   
   G   
   G   
   T   
   A   
   T   
   G   
   G   
   C   
   C   
   A   
   C   
   G   
   A   
   A   
   T   
   A   
   T   
   C   
   C   
   C   
   A   
   C   
   C   
   T   
   C   
   A   
   T   
   A   
   A   
   C   
   T   
   T   
   G   
   T   
   C   
   T   
   G   
   A   
   G   
   G   
   T   
   A   
   G   
   T   
   A   
   G   
   C   
   G   
   G   
   C   
   T   
   A   
   T   
   C   
   G   
   A   
   T   
   T   
   T   
   G   
   C   
   T   
   G   
   A   
   T   
   G   
   G   
   A   
   T   
   A   
   A   
   T   
   C   
   C   
   T   
   G   
   A   
   T   
   G   
   T   
   G   
   A   
   C   
   G   
   A   
   C   
   C   
   A   
   A   
   T   
   G   
   A   
   A   
   T   
   T   
   G   
   A   
   T   
   G   
   G   
   A   
   A   
   G   
   T   
   A   
   T   
   T   
   A   
   C   
   C   
   T   
   G   
   G   
   A   
   C   
   C   
   A   
   G   
   A   
   T   
   T   
   T   
   T   
   C   
   C   
   A   
   A   
   C   
   A   
   G   
   G   
   T   
   G   
   G   
   T   
   T   
   T   
   G   
   G   
   T   
   T   
   A   
   T   
   G   
   G   
   G   
   A   
   A   
   A   
   A   
   T   
   C   
   A   
   G   
   G   
    A    
   A   
   T   
    C    
   C   
   G   
    A    
    A    
   G   
   A   
   G   
   C   
    G    
   T   
   A   
   T   
   G   
   A   
   A   
   A   
   C   
    A    
   G   
   G   
    T    
   A   
   A   
    G    
   G   
   G   
   T   
   T   
   C   
    T    
   A   
   T   
   T   
   A   
   C   
    C    
   G   
   T   
    A    
   C   
   G   
   T   
   G   
   C   
   A   
   A   
   A   
   A   
   G   
   T   
   T   
   G   
   A   
    G    
    A    
   T   
    T    
   A   
    C    
   T   
   G   
   A   
    G    
   A   
   T   
   G   
    C    
   C   
    A    
   A   
   A   
   T   
   G   
   G   
    T    
   A   
   A   
   A   
   G   
   A   
   A   
   C   
   G   
    G    
   A   
   T   
    C    
    A    
   T   
    C    
   G   
   T   
    G    
   A   
   C   
    A    
   G   
   A   
   A   
   T   
   T   
    G    
   C   
   C   
   T   
   T   
   A   
   T   
   A   
   T   
   G   
   G   
   T   
    C    
   A   
   A   
   T   
   A   
   A   
   A   
   G   
   C   
    A    
   A   
   A   
   A   
   T   
   T   
    G    
   A   
   T   
    C    
   G   
   A   
   A   
   C   
   G   
    G    
   A   
   T   
   T   
   T   
   C   
   T   
   G   
   A   
    A    
    C    
   T   
    G    
   C   
   A   
   C   
   A   
   G   
   A   
   G   
   A   
    C    
   A   
   A   
    G    
   C   
   G   
    T    
   A   
   T   
    C    
   G   
   A   
   A   
   G   
   G   
    G    
   A   
   T   
    C    
   A   
   C   
    A    
   G   
   A   
   T   
   T   
   T   
   G   
   C   
   G   
   G   
   G   
   A   
    C    
   G   
   A   
   A   
   T   
   C   
   T   
   T   
   C   
    C    
   C   
   G   
   T   
   G   
   A   
    A    
   G   
   G   
    T    
   A   
   T   
   G   
   C   
   G   
    G    
   A   
   T   
    C    
   G   
   T   
   C   
   A   
   T   
    T    
   G   
   A   
    C    
    A    
   T   
    C    
   C   
   G   
   T   
   C   
   G   
    T    
   G   
   A   
    C    
   G   
    C    
    C    
   A   
   G   
    C    
   G   
   C   
    A    
   T   
   C   
    C    
   G   
   T   
   T   
    A    
   T   
    C    
   T   
   T   
    G    
   A   
   A   
    T    
   A   
   A   
   C   
   T   
   T   
    A    
   T   
   A   
    T    
   A   
   A   
   A   
    T    
   T   
   G   
   A   
   C   
    A    
    T    
   C   
    C    
   T   
   T   
    G    
   C   
   A   
    A    
   A   
    A    
    T    
   T   
   C   
    A    
   T   
   T   
    T    
   G   
   G   
    A    
   T   
   T   
    C    
   A   
   A   
    T    
   A   
   T   
   G   
   T   
   T   
    G    
   G   
   C   
    G    
   A   
   T   
    C    
   G   
   A   
   A   
   A   
   A   
   A   
   G   
   G   
    T    
   G   
   T   
    G    
   C   
   C   
    G    
   A   
   A   
   A   
    G    
   T   
   T   
   T   
   T   
    G    
   A   
   G   
    C    
   T   
   T   
   G   
   A   
   A   
   A   
   C   
    A    
    A    
   A   
   T   
    T    
    C    
   T   
    T    
   G   
   A   
   A   
   A   
   A   
    C    
   T   
   A   
    T    
    A    
   T   
    C    
   G   
   A   
    A    
   C   
   A   
    T    
   C   
   A   
    G    
    C    
    G    
    C    
    G    
   A   
   A   
   G   
   T   
   C   
   A   
   T   
    C    
   A   
   C   
    A    
    A    
   G   
    A    
   C   
   G   
   G   
   A   
   C   
    A    
    G    
   T   
    C    
   T   
   T   
   T   
   G   
   A   
    A    
   A   
   A   
   A   
    G    
   A   
    A    
   A   
   A   
    A    
   G   
   C   
   A   
   G   
   A   
   A   
   G   
   C   
   A   
    A    
   G   
    G    
   G   
   C   
    A    
   C   
   A   
    C    
   A   
   T   
    T    
   T   
   T   
   A   
   G   
   A   
   A   
   G   
   G   
   T   
    T    
   T   
    G    
   C   
   G   
    T    
   A   
   T   
   T   
   G   
   C   
    T    
   T   
   T   
    G    
   G   
   A   
   T   
   C   
   A   
   T   
   A   
   T   
   C   
    G    
   A   
   T   
   G   
   A   
   A   
   A   
   T   
    A    
   A   
   T   
   T   
   G   
   C   
    G    
   A   
   T   
   T   
   A   
   T   
    C    
   C   
   G   
    C    
   G   
   G   
    G    
   T   
   C   
    T    
    A    
   A   
   A   
   T   
   C   
   A   
   G   
   A   
   T   
   G   
   A   
    C    
   G   
   A   
    A    
   G   
   C   
   A   
   A   
   A   
   A   
   G   
   C   
    G    
   A   
    C    
    G    
    A    
   T   
    G    
   A   
   T   
    C    
   G   
   A   
   A   
   C   
   G   
    G    
   T   
   T   
    C    
   G   
   A   
    T    
   T   
   T   
    A    
   T   
   C   
    C    
   G   
   A   
   T   
   C   
   G   
    C    
   C   
   A   
    G    
    T    
   C   
    T    
   C   
   A   
   A   
   G   
   C   
    T    
   A   
   T   
    T    
   T   
   T   
   A   
   G   
   A   
   T   
   A   
   T   
   G   
   C   
   G   
   T   
   T   
   T   
    A    
   C   
   G   
    C    
   C   
   G   
    A    
   T   
   T   
    G    
   A   
   C   
    C    
   G   
   G   
    T    
   T   
   T   
    G    
   G   
   A   
   A   
   C   
   G   
    G    
   G   
   A   
    A    
   A   
   A   
   A   
   A   
   T   
    C    
   G   
   A   
    A    
   A   
   A   
   T   
   G   
   A   
   A   
   T   
   A   
    C    
   C   
   A   
   A   
   G   
   A   
    A    
   C   
   T   
    C    
   T   
   T   
    A    
   A   
    A    
   A   
    C    
   T   
    G    
   A   
   T   
   T   
   G   
    A    
    A    
   G   
   A   
    T    
   T   
   T   
    A    
    A    
    C    
    T    
   G   
   A   
   C   
   A   
   T   
   T   
    C    
   T   
    G    
   G   
   C   
    A    
    A    
   G   
    A    
   C   
   C   
    A    
   G   
   A   
    A    
   C   
   G   
    T    
   G   
   T   
    T    
   A   
    C    
    A    
   G   
   A   
   A   
   A   
   T   
    C    
   A   
   T   
    C    
   A   
   A   
    G    
   A   
    A    
    T    
   G   
   A   
    A    
   T   
   T   
   A   
    G    
    C    
    A    
   G   
   A   
   C   
    T    
   T   
    G    
    A    
    A    
    T    
    C    
    A    
   A   
    C    
    G    
   A   
   T   
   T   
   T   
   G   
   G   
   T   
   G   
   A   
   T   
    A    
    A    
   A   
   C   
   G   
   A   
   C   
   G   
    C    
   A   
   C   
   A   
   G   
   A   
   A   
    T    
   T   
   A   
    C    
   T   
   A   
   G   
   T   
    C    
   G   
   G   
   T   
   G   
   A   
    G    
   G   
   T   
   C   
    T    
   T   
    A    
   A   
   G   
   T   
   C   
   T   
    G    
   G   
   A   
   A   
   G   
   A   
   T   
   G   
   A   
   A   
   G   
   A   
   T   
    T    
   T   
    A    
   A   
   T   
    C    
   G   
   A   
   A   
   G   
   A   
   A   
   G   
   A   
   A   
   G   
   A   
   A   
    A    
   T   
   C   
   G   
   T   
    T    
   A   
   T   
   T   
   A   
   C   
    A    
    T    
   T   
    G    
   A   
   C   
    A    
   A   
   A   
    C    
   A   
   A   
    T    
   G   
   G   
    T    
   T   
   A   
    C    
   A   
   T   
    C    
   A   
   A   
    A    
   C   
   G   
    T    
    T    
   T   
   G   
   G   
    C    
   A   
   A   
   A   
    T    
    A    
    A    
    T    
   G   
   A   
   A   
   T   
   T   
   C   
    A    
   G   
    A    
   G   
   C   
    T    
   C   
   A   
   A   
   C   
   G   
    A    
    A    
   G   
    G    
   G   
   G   
    T    
   G   
   G   
    A    
   C   
   G   
   T   
   G   
   G   
    T    
   G   
   T   
    C    
   C   
   A   
   A   
   G   
   G   
    A    
   A   
   T   
   G   
   G   
   G   
    T    
    A    
   T   
    C    
   C   
   A   
    C    
    A    
   A   
   T   
   G   
   A   
    C    
   G   
   A   
    C    
   T   
   T   
   C   
   G   
   T   
    C    
   A   
   A   
    A    
   A   
   A   
    T    
   C   
   T   
    G    
    G    
   T   
   T   
   T   
   C   
   T   
   T   
   G   
    T    
   T   
   C   
    T    
   A   
   C   
    C    
   C   
   A   
    T    
   G   
   A   
    T    
   A   
   C   
    A    
   T   
   T   
    G    
   T   
   T   
   A   
   T   
   T   
    C    
   T   
   T   
   T   
   A   
   C   
   G   
   A   
   A   
    T    
   A   
    A    
    C    
   G   
   G   
    A    
   A   
   A   
   A   
   G   
   T   
    G    
   T   
   A   
    T    
   C   
   G   
    G    
   G   
   C   
   A   
   A   
   A   
   A   
   G   
   G   
    A    
   T   
   A   
    C    
   G   
   A   
   A   
   A   
   T   
    T    
   C   
   C   
    A    
   G   
   A   
    A    
   T   
   A   
   C   
   G   
   G   
    G    
    C    
   G   
    T    
   A   
   C   
   A   
   G   
   C   
    C    
   A   
   A   
   A   
   G   
   G   
    A    
   A   
   T   
    A    
   C   
   C   
   A   
    A    
   T   
    T    
   A   
   T   
    C    
   A   
   A   
    T    
   T   
   T   
    A    
   C   
   T   
    A    
   G   
   G   
    T    
   A   
   T   
    C    
   G   
   A   
    T    
   T   
   C   
    G    
    A    
    G    
    T    
   G   
   A   
   A   
   A   
    G    
    C    
   A   
   T   
    C    
   C   
   A   
    G    
   G   
   C   
    A    
   A   
   T   
    C    
   A   
   T   
   T   
    G    
   C   
    G    
   G   
   T   
    G    
    A    
    C    
   G   
   G   
   G   
    A    
    G    
   A   
   A   
   G   
   C   
    A    
   G   
   A   
   A   
   G   
    A    
    A    
   G   
   G   
    C    
    C    
   A   
    C    
   T   
   A   
    T    
    C    
   T   
    C    
   T   
   T   
   C   
   T   
   T   
    T    
   A   
   C   
    G    
   A   
   C   
    G    
    C    
    G    
    T    
    C    
   A   
   A   
   G   
   G   
    T    
   A   
   C   
    A    
   G   
   T   
    A    
   A   
   A   
   A   
   C   
   G   
    T    
   A   
   C   
    T    
    T    
   C   
    T    
   G   
   T   
    C    
   A   
   C   
    T    
   G   
   C   
    T    
   T   
   T   
    T    
    G    
   C   
   T   
   A   
   A   
    C    
   A   
   T   
    C    
   C   
   G   
    A    
   A   
   G   
    T    
   A   
   A   
   T   
   G   
   G   
    T    
    C    
   T   
    G    
   A   
   T   
    C    
   G   
   C   
    A    
   A   
   T   
   T   
    G    
   G   
    T    
   T   
   T   
    G    
   A   
   A   
   A   
   G   
   A   
   A   
   G   
   A   
    C    
   G   
   A   
    T    
   G   
   A   
    G    
    C    
   T   
    C    
    G    
   T   
    C    
   A   
   A   
    T    
   G   
   T   
    G    
    C    
   T   
   A   
    C    
    T    
   G   
   A   
   C   
    A    
    G    
   A   
    T    
   G   
   G   
    T    
    C    
   A   
    A    
    T    
    C    
    T    
   A   
   A   
    T    
   A   
   T   
    C    
   A   
   T   
    T    
   A   
   T   
    T    
   G   
   G   
    A    
   A   
   C   
   A   
   C   
   A   
   T   
    A    
    A    
    C    
   G   
   G   
    C    
   T   
   A   
   C   
   T   
   C   
   T   
   G   
   T   
    C    
   A   
   C   
    T    
   T   
   T   
    C    
    A    
   A   
    A    
   G   
   A   
    A    
    G    
    A    
    A    
    G    
   C   
    A    
   G   
    T    
    C    
   C   
   G   
    C    
   G   
   A   
   T   
   A   
   T   
   G   
   G   
   G   
    A    
   C   
   G   
    T    
   A   
   C   
   A   
   G   
   C   
    T    
   T   
   C   
    C    
   G   
   G   
    G    
   G   
   T   
    C    
    A    
   G   
    A    
   G   
   G   
    G    
   A   
   T   
    A    
   C   
   G   
    C    
    C    
   T   
    G    
   C   
   G   
    T    
   G   
   A   
    G    
    G    
   A   
    A    
   G   
   A   
    T    
   T   
    A    
    T    
   G   
   T   
    C    
   G   
   T   
    T    
   G   
   G   
    G    
    G    
   C   
   A   
   G   
   C   
    C    
    T    
   T   
    A    
    A    
   T   
   G   
    A    
   A   
    G    
   G   
    A    
   A   
    A    
   A   
    T    
    C    
   A   
   A   
   G   
   A   
   A   
   G   
   T   
    A    
    T    
   T   
    A    
   G   
   T   
    G    
   A   
   T   
    C    
   A   
   C   
    A    
   G   
   A   
   A   
   A   
   A   
    A    
   G   
   G   
   T   
   T   
   A   
    C    
   G   
   G   
    A    
   A   
   A   
    A    
   C   
   G   
    G    
   A   
   C   
    A    
   A   
   A   
   A   
   G   
    T    
    A    
   T   
   C   
    G    
   G   
   A   
    A    
   T   
   A   
   T   
   C   
   C   
   A   
   G   
   T   
    G    
   A   
   A   
    A    
   G   
   G   
    C    
   C   
   G   
    C    
   G   
   G   
    A    
   G   
   G   
   T   
   A   
   A   
   A   
   G   
   G   
    G    
   A   
   T   
    C    
   A   
   A   
    A    
   A   
   C   
    G    
    G    
   C   
    G    
   A   
   A   
    C    
   A   
   T   
   C   
   A   
   C   
   T   
   G   
   A   
    A    
   A   
   A   
   A   
   A   
   A   
   T   
   G   
   G   
   T   
   C   
   C   
   A   
    C    
   T   
   A   
   G   
   C   
    T    
   G   
   G   
    A    
   T   
   T   
    G    
   A   
   C   
   C   
   A   
   C   
    T    
   G   
   T   
    T    
    T    
    C    
   T   
   G   
   G   
    A    
    G    
   A   
   T   
   G   
   A   
   A   
   G   
   A   
    C    
   A   
   T   
   C   
    A    
   T   
    G    
    C    
   T   
    G    
   A   
   T   
    T    
   A   
   C   
    A    
    G    
   A   
    T    
   A   
   A   
   A   
   G   
   G   
    T    
   G   
   T   
    C    
   A   
   T   
   T   
   A   
   T   
   C   
   C   
   G   
    T    
   T   
   T   
    C    
   A   
   A   
   C   
   G   
   T   
    A    
    T    
    C    
    G    
    A    
   C   
   T   
   G   
   T   
   T   
   T   
   C   
   T   
   C   
   A   
   A   
   A   
   C   
    T    
   G   
   G   
   A   
   C   
   G   
    T    
    T    
   C   
   A   
   A   
   C   
   A   
   T   
   T   
    A    
   G   
   G   
    C    
   G   
   T   
    A    
   C   
   G   
   T   
   T   
   T   
    G    
   A   
   T   
   G   
   A   
    A    
    G    
   A   
   T   
   G   
   G   
   A   
   A   
   G   
    C    
    T    
   G   
    A    
    T    
    A    
   C   
    G    
   A   
   A   
   A   
   G   
   T   
   T   
   G   
   T   
   C   
   A   
   C   
   G   
   A   
   T   
   G   
   G   
   C   
   A   
   G   
   C   
   A   
   G   
   T   
   C   
   G   
   A   
   A   
   C   
   C   
   A   
   G   
   A   
   A   
   G   
   C   
   T   
   G   
   C   
   A   
   G   
   A   
   A   
   C   
   A   
   A   
   A   
   C   
   A   
   G   
   T   
   T   
   G   
   A   
   A   
   G   
   A   
   A   
   C   
   A   
   A   
   G   
   G   
   A   
   T   
   C   
   A   
   G   
   A   
   A   
   G   
   A   
   A   
   -   
   -   
   -   
 

 
   N59457F   
   A   
   T   
   G   
   A   
   G   
   T   
   G   
   A   
   A   
   G   
   A   
   A   
   A   
   T   
   T   
   A   
   A   
   A   
   G   
   A   
   A   
   A   
   A   
   C   
   A   
   T   
   T   
   C   
   A   
   A   
   G   
   A   
   C   
   G   
   T   
   C   
   A   
   A   
   T   
   C   
   T   
   G   
   A   
   C   
   C   
   A   
   G   
   C   
   G   
   A   
   A   
   A   
   T   
   G   
   A   
   A   
   A   
   G   
   A   
   A   
   T   
   C   
   T   
   T   
   T   
   C   
   A   
   T   
   T   
   G   
   A   
   C   
   T   
   A   
   C   
   G   
   C   
   A   
   A   
   T   
   G   
   A   
   G   
   T   
   G   
   T   
   T   
   A   
   T   
   C   
   G   
   T   
   A   
   G   
   C   
   C   
   C   
   G   
   C   
   G   
   C   
   G   
   T   
   T   
   A   
   C   
   C   
   T   
   G   
   A   
   C   
   G   
   T   
   T   
   C   
   G   
   T   
   G   
   A   
   C   
   G   
   G   
   T   
   T   
   T   
   A   
   A   
   A   
   A   
   C   
   C   
   T   
   G   
   T   
   T   
   C   
   A   
   T   
   C   
   G   
   C   
   C   
   G   
   A   
   A   
   T   
   C   
   T   
   T   
   A   
   T   
   A   
   T   
   G   
   G   
   A   
   A   
   T   
   G   
   A   
   A   
   C   
   G   
   A   
   A   
   T   
   T   
   A   
   G   
   G   
   G   
   G   
   T   
   A   
   A   
   C   
   C   
   C   
   C   
   T   
   G   
   A   
   T   
   A   
   A   
   A   
   C   
   C   
   A   
   C   
   A   
   C   
   A   
   A   
   A   
   A   
   A   
   A   
   T   
   C   
   A   
   G   
   C   
   C   
   C   
   G   
   G   
   A   
   T   
   T   
   G   
   T   
   T   
   G   
   G   
   G   
   G   
   A   
   T   
   G   
   T   
   T   
   A   
   T   
   G   
   G   
   G   
   T   
   A   
   A   
   A   
   T   
   A   
   T   
   C   
   A   
   C   
   C   
   C   
   C   
   C   
   A   
   T   
   G   
   G   
   G   
   G   
   A   
   C   
   A   
   G   
   T   
   G   
   C   
   G   
   A   
   T   
   T   
   T   
   A   
   C   
   G   
   A   
   A   
   T   
   C   
   A   
   A   
   T   
   G   
   G   
   T   
   G   
   C   
   G   
   G   
   A   
   T   
   G   
   G   
   C   
   A   
   C   
   A   
   A   
   C   
   C   
   T   
   T   
   T   
   T   
   A   
   G   
   T   
   T   
   A   
   T   
   C   
   G   
   G   
   G   
   C   
   T   
   A   
   T   
   G   
   T   
   T   
   A   
   G   
   T   
   T   
   G   
   A   
   C   
   G   
   G   
   C   
   C   
   A   
   C   
   G   
   G   
   A   
   A   
   A   
   C   
   T   
   T   
   C   
   G   
   G   
   T   
   T   
   C   
   T   
   G   
   T   
   C   
   G   
   A   
   T   
   G   
   G   
   T   
   G   
   A   
   C   
   G   
   G   
   C   
   G   
   C   
   T   
   G   
   C   
   C   
   G   
   C   
   T   
   A   
   T   
   G   
   C   
   G   
   T   
   T   
   A   
   T   
   A   
   C   
   C   
   G   
   A   
   A   
   G   
   C   
   A   
   C   
   G   
   T   
   A   
   T   
   G   
   A   
   G   
   T   
   A   
   A   
   A   
   A   
   T   
   T   
   G   
   C   
   T   
   T   
   T   
   A   
   G   
   A   
   A   
   A   
   T   
   G   
   C   
   T   
   A   
   C   
   G   
   A   
   G   
   A   
   T   
   A   
   T   
   T   
   A   
   A   
   T   
   A   
   A   
   A   
   A   
   A   
   T   
   A   
   C   
   A   
   G   
   T   
   C   
   G   
   A   
   T   
   T   
   T   
   C   
   C   
   A   
   A   
   G   
   G   
   A   
   A   
   A   
   C   
   T   
   A   
   T   
   G   
   A   
   T   
   G   
   A   
   T   
   T   
   C   
   A   
   G   
   A   
   A   
   C   
   A   
   A   
   G   
   A   
   A   
   C   
   C   
   A   
   G   
   T   
   A   
   G   
   T   
   T   
   T   
   T   
   A   
   C   
   C   
   T   
   G   
   C   
   T   
   C   
   G   
   T   
   T   
   T   
   T   
   C   
   C   
   A   
   A   
   A   
   C   
   T   
   T   
   A   
   C   
   T   
   A   
   G   
   T   
   T   
   A   
   A   
   C   
   G   
   G   
   A   
   A   
   C   
   A   
   A   
   C   
   G   
   G   
   G   
   G   
   A   
   T   
   T   
   G   
   C   
   G   
   G   
   T   
   G   
   G   
   G   
   G   
   A   
   T   
   G   
   G   
   C   
   A   
   A   
   C   
   A   
   A   
   A   
   T   
   A   
   T   
   T   
   C   
   C   
   A   
   C   
   C   
   A   
   C   
   A   
   C   
   A   
   A   
   T   
   T   
   T   
   A   
   A   
   G   
   T   
   G   
   A   
   A   
   G   
   T   
   G   
   A   
   T   
   T   
   G   
   A   
   T   
   G   
   C   
   A   
   A   
   C   
   A   
   A   
   G   
   T   
   T   
   T   
   A   
   T   
   T   
   G   
   A   
   T   
   G   
   G   
   A   
   C   
   A   
   A   
   T   
   C   
   C   
   T   
   G   
   A   
   T   
   G   
   T   
   A   
   A   
   C   
   G   
   A   
   C   
   G   
   A   
   A   
   T   
   G   
   A   
   A   
   T   
   T   
   G   
   A   
   T   
   G   
   G   
   A   
   A   
   G   
   T   
   G   
   T   
   T   
   A   
   C   
   C   
   T   
   G   
   G   
   A   
   C   
   C   
   A   
   G   
   A   
   T   
   T   
   T   
   T   
   C   
   C   
   A   
   A   
   C   
   A   
   G   
   G   
   C   
   G   
   G   
   T   
   T   
   T   
   A   
   G   
   T   
   G   
   A   
   T   
   G   
   G   
   G   
   G   
   A   
   A   
   A   
   T   
   C   
   A   
   G   
   G   
    G    
   A   
   T   
    T    
   C   
   G   
    C    
    C    
   G   
   A   
   G   
   C   
    A    
   T   
   A   
   T   
   G   
   A   
   A   
   A   
   C   
    G    
   G   
   G   
    G    
   A   
   A   
    A    
   G   
   G   
   T   
   T   
   C   
    G    
   A   
   T   
   T   
   A   
   C   
    A    
   G   
   T   
    T    
   C   
   G   
   T   
   G   
   C   
   A   
   A   
   A   
   A   
   G   
   T   
   T   
   G   
   A   
    A    
    T    
   T   
    G    
   A   
    C    
   T   
   G   
   A   
    A    
   A   
   T   
   G   
    C    
   C   
    G    
   A   
   A   
   T   
   G   
   G   
    A    
   A   
   A   
   A   
   G   
   A   
   A   
   C   
   G   
    T    
   A   
   T   
    T    
    T    
   T   
    A    
   G   
   T   
    A    
   A   
   C   
    T    
   G   
   A   
   A   
   T   
   T   
    G    
   C   
   C   
   T   
   T   
   A   
   T   
   A   
   T   
   G   
   G   
   T   
    G    
   A   
   A   
   T   
   A   
   A   
   A   
   G   
   C   
    C    
   A   
   A   
   A   
   T   
   T   
    A    
   A   
   T   
    C    
   G   
   A   
   A   
   C   
   G   
    A    
   A   
   T   
   T   
   T   
   C   
   T   
   G   
   A   
    A    
    T    
   T   
    A    
   C   
   A   
   C   
   A   
   G   
   A   
   G   
   A   
    T    
   A   
   A   
    A    
   C   
   G   
    A    
   A   
   T   
    T    
   G   
   A   
   A   
   G   
   G   
    A    
   A   
   T   
    T    
   A   
   C   
    T    
   G   
   A   
   T   
   T   
   T   
   G   
   C   
   G   
   G   
   G   
   A   
    T    
   G   
   A   
   A   
   T   
   C   
   T   
   T   
   C   
    T    
   C   
   G   
   T   
   G   
   A   
    A    
   G   
   G   
    C    
   A   
   T   
   G   
   C   
   G   
    G    
   A   
   T   
    T    
   G   
   T   
   C   
   A   
   T   
    C    
   G   
   A   
    T    
    G    
   T   
    T    
   C   
   G   
   T   
   C   
   G   
    A    
   G   
   A   
    T    
   G   
    T    
    G    
   A   
   G   
    T    
   G   
   C   
    C    
   T   
   C   
    T    
   G   
   T   
   T   
    G    
   T   
    G    
   T   
   T   
    A    
   A   
   A   
    C    
   A   
   A   
   C   
   T   
   T   
    G    
   T   
   A   
    C    
   A   
   A   
   A   
    A    
   T   
   G   
   A   
   C   
    T    
    G    
   C   
    C    
   T   
   T   
    A    
   C   
   A   
    A    
   A   
    C    
    A    
   T   
   C   
    T    
   T   
   T   
    T    
   G   
   G   
    T    
   T   
   T   
    T    
   A   
   A   
    C    
   A   
   T   
   G   
   T   
   T   
    A    
   G   
   C   
    C    
   A   
   T   
    T    
   G   
   A   
   A   
   A   
   A   
   A   
   G   
   G   
    T    
   G   
   T   
    A    
   C   
   C   
    G    
   A   
   A   
   A   
    A    
   T   
   T   
   T   
   T   
    A    
   A   
   G   
    C    
   T   
   T   
   G   
   A   
   A   
   A   
   C   
    G    
    T    
   A   
   T   
    T    
    T    
   T   
    A    
   G   
   A   
   A   
   A   
   A   
    C    
   T   
   A   
    C    
    G    
   T   
    C    
   G   
   A   
    G    
   C   
   A   
    T    
   C   
   A   
    A    
    A    
    A    
    A    
    G    
   A   
   A   
   G   
   T   
   C   
   A   
   T   
    T    
   A   
   C   
    T    
    C    
   G   
    C    
   C   
   G   
   G   
   A   
   C   
    G    
    A    
   T   
    T    
   T   
   T   
   T   
   G   
   A   
    C    
   A   
   A   
   A   
    A    
   A   
    C    
   A   
   A   
    A    
   G   
   C   
   A   
   G   
   A   
   A   
   G   
   C   
   A   
    C    
   G   
    G    
   G   
   C   
    A    
   C   
   A   
    T    
   A   
   T   
    T    
   T   
   T   
   A   
   G   
   A   
   A   
   G   
   G   
   T   
    C    
   T   
    C    
   C   
   G   
    A    
   A   
   T   
   T   
   G   
   C   
    C    
   T   
   T   
    A    
   G   
   A   
   T   
   C   
   A   
   T   
   A   
   T   
   C   
    G    
   A   
   T   
   G   
   A   
   A   
   A   
   T   
    C    
   A   
   T   
   T   
   G   
   C   
    C    
   A   
   T   
   T   
   A   
   T   
    C    
   C   
   G   
    T    
   G   
   G   
    A    
   T   
   C   
    A    
    C    
   A   
   A   
   T   
   C   
   A   
   G   
   A   
   T   
   G   
   A   
    C    
   G   
   A   
    A    
   G   
   C   
   A   
   A   
   A   
   A   
   G   
   C   
    A    
   A   
    C    
    G    
    T    
   T   
    A    
   A   
   T   
    T    
   G   
   A   
   A   
   C   
   G   
    C    
   T   
   T   
    T    
   G   
   A   
    A    
   T   
   T   
    T    
   T   
   C   
    A    
   G   
   A   
   T   
   C   
   G   
    T    
   C   
   A   
    A    
    G    
   C   
    G    
   C   
   A   
   A   
   G   
   C   
    G    
   A   
   T   
    T    
   T   
   T   
   A   
   G   
   A   
   T   
   A   
   T   
   G   
   C   
   G   
   T   
   T   
   T   
    A    
   C   
   G   
    C    
   C   
   G   
    T    
   T   
   T   
    A    
   A   
   C   
    A    
   G   
   G   
    C    
   T   
   T   
    A    
   G   
   A   
   A   
   C   
   G   
    C    
   G   
   A   
    C    
   A   
   A   
   A   
   A   
   T   
    T    
   G   
   A   
    A    
   A   
   A   
   T   
   G   
   A   
   A   
   T   
   A   
    T    
   C   
   A   
   A   
   G   
   A   
    G    
   C   
   T   
    A    
   T   
   T   
    G    
   A   
    A    
   A   
    T    
   T   
    C    
   A   
   T   
   T   
   G   
    C    
    G    
   G   
   A   
    C    
   T   
   T   
    A    
    G    
    A    
    G    
   G   
   A   
   C   
   A   
   T   
   T   
    T    
   T   
    A    
   G   
   C   
    C    
    C    
   G   
    C    
   C   
   C   
    A    
   G   
   A   
    A    
   C   
   G   
    C    
   G   
   T   
    C    
   A   
    T    
    T    
   G   
   A   
   A   
   A   
   T   
    C    
   A   
   T   
    T    
   A   
   A   
    A    
   A   
    C    
    A    
   G   
   A   
    G    
   T   
   T   
   A   
    A    
    A    
    T    
   G   
   A   
   C   
    G    
   T   
    T    
    C    
    G    
    C    
    A    
    C    
   A   
    A    
    A    
   A   
   T   
   T   
   T   
   G   
   G   
   T   
   G   
   A   
   T   
    G    
    C    
   A   
   C   
   G   
   A   
   C   
   G   
    C    
   A   
   C   
   A   
   G   
   A   
   A   
    T    
   T   
   A   
    T    
   T   
   A   
   G   
   T   
    A    
   G   
   G   
   T   
   G   
   A   
    A    
   G   
   T   
   C   
    T    
   T   
    A    
   A   
   G   
   T   
   C   
   T   
    T    
   G   
   A   
   A   
   G   
   A   
   T   
   G   
   A   
   A   
   G   
   A   
   T   
    T    
   T   
    A    
   A   
   T   
    C    
   G   
   A   
   A   
   G   
   A   
   A   
   G   
   A   
   A   
   G   
   A   
   A   
    G    
   T   
   C   
   G   
   T   
    G    
   A   
   T   
   T   
   A   
   C   
    A    
    T    
   T   
    A    
   A   
   C   
    C    
   A   
   A   
    T    
   A   
   A   
    C    
   G   
   G   
    C    
   T   
   A   
    C    
   A   
   T   
    T    
   A   
   A   
    G    
   C   
   G   
    G    
    A    
   T   
   G   
   G   
    C    
   A   
   A   
   A   
    C    
    T    
    C    
    T    
   G   
   A   
   A   
   T   
   T   
   C   
    C    
   G   
    G    
   G   
   C   
    G    
   C   
   A   
   A   
   C   
   G   
    C    
    C    
   G   
    T    
   G   
   G   
    C    
   G   
   G   
    A    
   C   
   G   
   T   
   G   
   G   
    T    
   G   
   T   
    C    
   C   
   A   
   A   
   G   
   G   
    C    
   A   
   T   
   G   
   G   
   G   
    C    
    G    
   T   
    T    
   C   
   A   
    T    
    G    
   A   
   T   
   G   
   A   
    T    
   G   
   A   
    T    
   T   
   T   
   C   
   G   
   T   
    G    
   A   
   A   
    A    
   A   
   A   
    C    
   C   
   T   
    A    
    G    
   T   
   T   
   T   
   C   
   T   
   T   
   G   
    T    
   T   
   C   
    A    
   A   
   C   
    A    
   C   
   A   
    C    
   G   
   A   
    C    
   A   
   C   
    G    
   T   
   T   
    A    
   T   
   T   
   A   
   T   
   T   
    C    
   T   
   T   
   T   
   A   
   C   
   G   
   A   
   A   
    C    
   A   
    C    
    T    
   G   
   G   
    G    
   A   
   A   
   A   
   G   
   T   
    T    
   T   
   A   
    C    
   C   
   G   
    A    
   G   
   C   
   A   
   A   
   A   
   A   
   G   
   G   
    G    
   T   
   A   
    C    
   G   
   A   
   A   
   A   
   T   
    C    
   C   
   C   
    T    
   G   
   A   
    A    
   T   
   A   
   C   
   G   
   G   
    T    
    A    
   G   
    A    
   A   
   C   
   A   
   G   
   C   
    A    
   A   
   A   
   A   
   G   
   G   
    A    
   A   
   T   
    T    
   C   
   C   
   A   
    G    
   T   
    G    
   A   
   T   
    T    
   A   
   A   
    C    
   T   
   T   
    A    
   C   
   T   
    G    
   G   
   G   
    A    
   A   
   T   
    T    
   G   
   A   
    T    
   T   
   C   
    T    
    G    
    C    
    A    
   G   
   A   
   A   
   A   
    A    
    A    
   A   
   T   
    T    
   C   
   A   
    A    
   G   
   C   
    G    
   A   
   T   
    T    
   A   
   T   
   T   
    T    
   C   
    T    
   G   
   T   
    T    
    G    
    A    
   G   
   G   
   G   
    C    
    A    
   A   
   A   
   G   
   C   
    G    
   G   
   A   
   A   
   G   
    C    
    A    
   G   
   G   
    T    
    A    
   A   
    A    
   T   
   A   
    C    
    T    
   T   
    G    
   T   
   T   
   C   
   T   
   T   
    C    
   A   
   C   
    A    
   A   
   C   
    C    
    T    
    T    
    A    
    A    
   A   
   A   
   G   
   G   
    A    
   A   
   C   
    C    
   G   
   T   
    C    
   A   
   A   
   A   
   C   
   G   
    G    
   A   
   C   
    A    
    G    
   C   
    C    
   G   
   T   
    A    
   A   
   C   
    A    
   G   
   C   
    C    
   T   
   T   
    T    
    T    
   C   
   T   
   A   
   A   
    T    
   A   
   T   
    C    
   C   
   G   
    T    
   A   
   G   
    T    
   A   
   A   
   T   
   G   
   G   
    A    
    T    
   T   
    A    
   A   
   T   
    C    
   G   
   C   
    C    
   A   
   T   
   T   
    A    
   G   
    C    
   T   
   T   
    A    
   A   
   A   
   A   
   G   
   A   
   A   
   G   
   A   
    T    
   G   
   A   
    T    
   G   
   A   
    G    
    T    
   T   
    A    
    G    
   T   
    T    
   A   
   A   
    C    
   G   
   T   
    G    
    G    
   T   
   A   
    A    
    C    
   G   
   A   
   C   
    T    
    A    
   A   
    T    
   G   
   G   
    C    
    A    
   A   
    T    
    C    
    A    
    G    
   A   
   A   
    G    
   A   
   T   
    G    
   A   
   T   
    T    
   A   
   T   
    C    
   G   
   G   
    A    
   A   
   C   
   A   
   C   
   A   
   T   
    G    
    C    
    A    
   G   
   G   
    A    
   T   
   A   
   C   
   T   
   C   
   T   
   G   
   T   
    C    
   A   
   C   
    A    
   T   
   T   
    T    
    G    
   A   
    T    
   G   
   A   
    A    
    A    
    A    
    T    
    A    
   C   
    T    
   G   
    T    
    A    
   C   
   G   
    T    
   G   
   A   
   T   
   A   
   T   
   G   
   G   
   G   
    C    
   C   
   G   
    G    
   A   
   C   
   A   
   G   
   C   
    A    
   T   
   C   
    A    
   G   
   G   
    T    
   G   
   T   
    T    
    C    
   G   
    G    
   G   
   G   
    A    
   A   
   T   
    C    
   C   
   G   
    T    
    C    
   T   
    C    
   C   
   G   
    C    
   G   
   A   
    A    
    A    
   A   
    T    
   G   
   A   
    T    
   T   
    A    
    T    
   G   
   T   
    G    
   G   
   T   
    C    
   G   
   G   
    C    
    G    
   C   
   A   
   G   
   C   
    G    
    A    
   T   
    T    
    C    
   T   
   G   
    G    
   A   
    T    
   G   
    A    
   A   
    A    
   A   
    T    
    A    
   A   
   A   
   G   
   A   
   A   
   G   
   T   
    C    
    C    
   T   
    A    
   G   
   T   
    C    
   A   
   T   
    T    
   A   
   C   
    T    
   G   
   A   
   A   
   A   
   A   
    T    
   G   
   G   
   T   
   T   
   A   
    T    
   G   
   G   
    T    
   A   
   A   
    G    
   C   
   G   
    T    
   A   
   C   
    A    
   A   
   A   
   A   
   G   
    C    
    T    
   T   
   C   
    T    
   G   
   A   
    A    
   T   
   A   
   T   
   C   
   C   
   A   
   G   
   T   
    T    
   A   
   A   
    A    
   G   
   G   
    A    
   C   
   G   
    T    
   G   
   G   
    C    
   G   
   G   
   T   
   A   
   A   
   A   
   G   
   G   
    G    
   A   
   T   
    T    
   A   
   A   
    G    
   A   
   C   
    A    
    G    
   C   
    A    
   A   
   A   
    T    
   A   
   T   
   C   
   A   
   C   
   T   
   G   
   A   
    G    
   A   
   A   
   A   
   A   
   A   
   T   
   G   
   G   
   T   
   C   
   C   
   A   
    T    
   T   
   A   
   G   
   C   
    T    
   G   
   G   
    T    
   T   
   T   
    A    
   A   
   C   
   C   
   A   
   C   
    G    
   G   
   T   
    C    
    A    
    A    
   T   
   G   
   G   
    G    
    G    
   A   
   T   
   G   
   A   
   A   
   G   
   A   
    T    
   A   
   T   
   C   
    T    
   T   
    A    
    T    
   T   
    G    
   A   
   T   
    T    
   A   
   C   
    G    
    A    
   A   
    C    
   A   
   A   
   A   
   G   
   G   
    C    
   G   
   T   
    C    
   A   
   T   
   T   
   A   
   T   
   C   
   C   
   G   
    C    
   T   
   T   
    T    
   A   
   A   
   C   
   G   
   T   
    T    
    G    
    A    
    T    
    T    
   C   
   T   
   G   
   T   
   T   
   T   
   C   
   T   
   C   
   A   
   A   
   A   
   C   
    A    
   G   
   G   
   A   
   C   
   G   
    C    
    G    
   C   
   A   
   A   
   C   
   A   
   T   
   T   
    A    
   G   
   G   
    G    
   G   
   T   
    T    
   C   
   G   
   T   
   T   
   T   
    A    
   A   
   T   
   G   
   A   
    G    
    A    
   A   
   T   
   G   
   G   
   A   
   A   
   G   
    A    
    T    
   G   
    G    
    T    
    G    
   C   
    C    
   A   
   A   
   A   
   -   
   -   
   -   
   -   
   -   
   -   
   -   
   -   
   -   
   -   
   -   
   -   
   -   
   -   
   -   
   -   
   -   
   -   
   -   
   -   
   -   
   -   
   -   
   -   
   -   
   -   
   -   
   -   
   -   
   -   
   -   
   -   
   -   
   -   
   -   
   -   
   -   
   -   
   -   
   -   
   -   
   -   
   -   
   -   
   -   
   -   
   -   
   -   
   -   
   -   
   -   
   -   
   -   
   -   
   -   
   -   
   -   
   -   
   -   
   -   
   -   
   -   
   -   
   -   
   -   
   -   
   -   
   -   
   -   
   -   
   -   
   -   
 

 
   N54551   
   A   
   T   
   G   
   A   
   G   
   T   
   G   
   A   
   A   
   G   
   A   
   A   
   A   
   T   
   C   
   A   
   G   
   A   
   G   
   A   
   A   
   A   
   A   
   C   
   A   
   T   
   C   
   C   
   A   
   T   
   G   
   A   
   T   
   G   
   T   
   C   
   A   
   A   
   T   
   C   
   T   
   A   
   A   
   C   
   C   
   A   
   G   
   C   
   G   
   A   
   A   
   A   
   T   
   G   
   A   
   A   
   A   
   G   
   A   
   C   
   T   
   C   
   C   
   T   
   T   
   C   
   A   
   T   
   C   
   G   
   A   
   T   
   T   
   A   
   T   
   G   
   C   
   G   
   A   
   T   
   G   
   A   
   G   
   T   
   G   
   T   
   T   
   A   
   T   
   T   
   G   
   T   
   C   
   G   
   C   
   A   
   C   
   G   
   T   
   G   
   C   
   A   
   C   
   T   
   T   
   C   
   C   
   T   
   G   
   A   
   T   
   G   
   T   
   C   
   A   
   G   
   A   
   G   
   A   
   T   
   G   
   G   
   T   
   C   
   T   
   G   
   A   
   A   
   A   
   C   
   C   
   C   
   G   
   T   
   T   
   C   
   A   
   C   
   C   
   G   
   C   
   C   
   G   
   T   
   A   
   T   
   T   
   T   
   T   
   A   
   T   
   A   
   C   
   G   
   G   
   C   
   A   
   T   
   G   
   A   
   A   
   C   
   G   
   A   
   A   
   T   
   T   
   A   
   G   
   G   
   T   
   G   
   T   
   A   
   A   
   C   
   A   
   C   
   C   
   G   
   G   
   A   
   T   
   A   
   A   
   A   
   G   
   C   
   C   
   C   
   A   
   T   
   A   
   A   
   A   
   A   
   A   
   A   
   T   
   C   
   T   
   G   
   C   
   C   
   A   
   G   
   A   
   A   
   T   
   C   
   G   
   T   
   C   
   G   
   G   
   A   
   G   
   A   
   T   
   G   
   T   
   C   
   A   
   T   
   G   
   G   
   G   
   T   
   A   
   A   
   A   
   T   
   A   
   C   
   C   
   A   
   T   
   C   
   C   
   T   
   C   
   A   
   T   
   G   
   G   
   A   
   G   
   A   
   C   
   A   
   G   
   T   
   G   
   C   
   G   
   A   
   T   
   C   
   T   
   A   
   T   
   G   
   A   
   G   
   T   
   C   
   A   
   A   
   T   
   G   
   G   
   T   
   A   
   C   
   G   
   G   
   A   
   T   
   G   
   G   
   C   
   G   
   C   
   A   
   G   
   C   
   C   
   A   
   T   
   T   
   C   
   A   
   G   
   C   
   T   
   A   
   C   
   C   
   G   
   T   
   T   
   A   
   T   
   A   
   T   
   G   
   C   
   T   
   A   
   G   
   T   
   G   
   G   
   A   
   T   
   G   
   G   
   T   
   C   
   A   
   T   
   G   
   G   
   A   
   A   
   A   
   C   
   T   
   T   
   T   
   G   
   G   
   T   
   T   
   C   
   T   
   G   
   T   
   T   
   G   
   A   
   C   
   G   
   G   
   T   
   G   
   A   
   C   
   G   
   G   
   T   
   G   
   C   
   C   
   G   
   C   
   C   
   G   
   C   
   T   
   A   
   T   
   G   
   C   
   G   
   T   
   T   
   A   
   T   
   A   
   C   
   C   
   G   
   A   
   A   
   G   
   C   
   A   
   C   
   G   
   G   
   A   
   T   
   G   
   A   
   G   
   C   
   A   
   A   
   A   
   A   
   T   
   C   
   G   
   C   
   T   
   A   
   C   
   A   
   G   
   A   
   A   
   A   
   T   
   G   
   C   
   T   
   G   
   C   
   G   
   T   
   G   
   A   
   T   
   A   
   T   
   C   
   A   
   A   
   T   
   A   
   A   
   G   
   A   
   A   
   C   
   A   
   C   
   T   
   G   
   T   
   T   
   G   
   A   
   T   
   T   
   T   
   C   
   C   
   A   
   A   
   A   
   G   
   C   
   A   
   A   
   C   
   T   
   A   
   T   
   G   
   A   
   T   
   G   
   A   
   T   
   A   
   C   
   A   
   G   
   A   
   A   
   A   
   A   
   A   
   G   
   A   
   A   
   C   
   C   
   A   
   G   
   T   
   T   
   G   
   T   
   A   
   C   
   T   
   A   
   C   
   C   
   T   
   G   
   C   
   T   
   C   
   G   
   C   
   T   
   T   
   C   
   C   
   C   
   G   
   A   
   A   
   T   
   C   
   T   
   T   
   T   
   T   
   A   
   G   
   T   
   G   
   A   
   A   
   C   
   G   
   G   
   A   
   A   
   C   
   A   
   A   
   C   
   G   
   G   
   G   
   G   
   A   
   T   
   C   
   G   
   C   
   C   
   G   
   T   
   A   
   G   
   G   
   T   
   A   
   T   
   G   
   G   
   C   
   C   
   A   
   C   
   G   
   A   
   A   
   T   
   A   
   T   
   C   
   C   
   C   
   A   
   C   
   C   
   T   
   C   
   A   
   T   
   A   
   A   
   C   
   T   
   T   
   G   
   T   
   C   
   T   
   G   
   A   
   G   
   G   
   T   
   A   
   G   
   T   
   A   
   G   
   C   
   G   
   G   
   C   
   T   
   A   
   T   
   C   
   G   
   A   
   T   
   T   
   T   
   G   
   C   
   T   
   G   
   A   
   T   
   G   
   G   
   A   
   T   
   A   
   A   
   T   
   C   
   C   
   T   
   G   
   A   
   T   
   G   
   T   
   G   
   A   
   C   
   G   
   A   
   C   
   C   
   A   
   A   
   T   
   G   
   A   
   A   
   T   
   T   
   G   
   A   
   T   
   G   
   G   
   A   
   A   
   G   
   T   
   A   
   T   
   T   
   A   
   C   
   C   
   T   
   G   
   G   
   A   
   C   
   C   
   A   
   G   
   A   
   T   
   T   
   T   
   T   
   C   
   C   
   A   
   A   
   C   
   A   
   G   
   G   
   T   
   G   
   G   
   T   
   T   
   T   
   G   
   G   
   T   
   T   
   A   
   T   
   G   
   G   
   G   
   A   
   A   
   A   
   A   
   T   
   C   
   A   
   G   
   G   
    A    
   A   
   T   
    C    
   C   
   G   
    A    
    A    
   G   
   A   
   G   
   C   
    G    
   T   
   A   
   T   
   G   
   A   
   A   
   A   
   C   
    A    
   G   
   G   
    T    
   A   
   A   
    G    
   G   
   G   
   T   
   T   
   C   
    T    
   A   
   T   
   T   
   A   
   C   
    C    
   G   
   T   
    A    
   C   
   G   
   T   
   G   
   C   
   A   
   A   
   A   
   A   
   G   
   T   
   T   
   G   
   A   
    G    
    A    
   T   
    T    
   A   
    C    
   T   
   G   
   A   
    G    
   A   
   T   
   G   
    C    
   C   
    A    
   A   
   A   
   T   
   G   
   G   
    T    
   A   
   A   
   A   
   G   
   A   
   A   
   C   
   G   
    G    
   A   
   T   
    C    
    A    
   T   
    C    
   G   
   T   
    G    
   A   
   C   
    A    
   G   
   A   
   A   
   T   
   T   
    G    
   C   
   C   
   T   
   T   
   A   
   T   
   A   
   T   
   G   
   G   
   T   
    C    
   A   
   A   
   T   
   A   
   A   
   A   
   G   
   C   
    A    
   A   
   A   
   A   
   T   
   T   
    G    
   A   
   T   
    C    
   G   
   A   
   A   
   C   
   G   
    G    
   A   
   T   
   T   
   T   
   C   
   T   
   G   
   A   
    A    
    C    
   T   
    G    
   C   
   A   
   C   
   A   
   G   
   A   
   G   
   A   
    C    
   A   
   A   
    G    
   C   
   G   
    T    
   A   
   T   
    C    
   G   
   A   
   A   
   G   
   G   
    G    
   A   
   T   
    C    
   A   
   C   
    A    
   G   
   A   
   T   
   T   
   T   
   G   
   C   
   G   
   G   
   G   
   A   
    C    
   G   
   A   
   A   
   T   
   C   
   T   
   T   
   C   
    C    
   C   
   G   
   T   
   G   
   A   
    A    
   G   
   G   
    T    
   A   
   T   
   G   
   C   
   G   
    G    
   A   
   T   
    C    
   G   
   T   
   C   
   A   
   T   
    T    
   G   
   A   
    C    
    A    
   T   
    C    
   C   
   G   
   T   
   C   
   G   
    T    
   G   
   A   
    C    
   G   
    C    
    C    
   A   
   G   
    C    
   G   
   C   
    A    
   T   
   C   
    C    
   G   
   T   
   T   
    A    
   T   
    C    
   T   
   T   
    G    
   A   
   A   
    T    
   A   
   A   
   C   
   T   
   T   
    A    
   T   
   A   
    T    
   A   
   A   
   A   
    T    
   T   
   G   
   A   
   C   
    A    
    T    
   C   
    C    
   T   
   T   
    G    
   C   
   A   
    A    
   A   
    A    
    T    
   T   
   C   
    A    
   T   
   T   
    T    
   G   
   G   
    A    
   T   
   T   
    C    
   A   
   A   
    T    
   A   
   T   
   G   
   T   
   T   
    G    
   G   
   C   
    G    
   A   
   T   
    C    
   G   
   A   
   A   
   A   
   A   
   A   
   G   
   G   
    T    
   G   
   T   
    G    
   C   
   C   
    G    
   A   
   A   
   A   
    G    
   T   
   T   
   T   
   T   
    G    
   A   
   G   
    C    
   T   
   T   
   G   
   A   
   A   
   A   
   C   
    A    
    A    
   A   
   T   
    T    
    C    
   T   
    T    
   G   
   A   
   A   
   A   
   A   
    C    
   T   
   A   
    T    
    A    
   T   
    C    
   G   
   A   
    A    
   C   
   A   
    T    
   C   
   A   
    G    
    C    
    G    
    C    
    G    
   A   
   A   
   G   
   T   
   C   
   A   
   T   
    C    
   A   
   C   
    A    
    A    
   G   
    A    
   C   
   G   
   G   
   A   
   C   
    A    
    G    
   T   
    C    
   T   
   T   
   T   
   G   
   A   
    A    
   A   
   A   
   A   
    G    
   A   
    A    
   A   
   A   
    A    
   G   
   C   
   A   
   G   
   A   
   A   
   G   
   C   
   A   
    A    
   G   
    G    
   G   
   C   
    A    
   C   
   A   
    C    
   A   
   T   
    T    
   T   
   T   
   A   
   G   
   A   
   A   
   G   
   G   
   T   
    T    
   T   
    G    
   C   
   G   
    T    
   A   
   T   
   T   
   G   
   C   
    T    
   T   
   T   
    G    
   G   
   A   
   T   
   C   
   A   
   T   
   A   
   T   
   C   
    G    
   A   
   T   
   G   
   A   
   A   
   A   
   T   
    A    
   A   
   T   
   T   
   G   
   C   
    G    
   A   
   T   
   T   
   A   
   T   
    C    
   C   
   G   
    C    
   G   
   G   
    G    
   T   
   C   
    T    
    A    
   A   
   A   
   T   
   C   
   A   
   G   
   A   
   T   
   G   
   A   
    C    
   G   
   A   
    A    
   G   
   C   
   A   
   A   
   A   
   A   
   G   
   C   
    G    
   A   
    C    
    G    
    A    
   T   
    G    
   A   
   T   
    C    
   G   
   A   
   A   
   C   
   G   
    G    
   T   
   T   
    C    
   G   
   A   
    T    
   T   
   T   
    A    
   T   
   C   
    C    
   G   
   A   
   T   
   C   
   G   
    C    
   C   
   A   
    G    
    T    
   C   
    T    
   C   
   A   
   A   
   G   
   C   
    T    
   A   
   T   
    T    
   T   
   T   
   A   
   G   
   A   
   T   
   A   
   T   
   G   
   C   
   G   
   T   
   T   
   T   
    A    
   C   
   G   
    C    
   C   
   G   
    A    
   T   
   T   
    G    
   A   
   C   
    C    
   G   
   G   
    T    
   T   
   T   
    G    
   G   
   A   
   A   
   C   
   G   
    G    
   G   
   A   
    A    
   A   
   A   
   A   
   A   
   T   
    C    
   G   
   A   
    A    
   A   
   A   
   T   
   G   
   A   
   A   
   T   
   A   
    C    
   C   
   A   
   A   
   G   
   A   
    A    
   C   
   T   
    C    
   T   
   T   
    A    
   A   
    A    
   A   
    C    
   T   
    G    
   A   
   T   
   T   
   G   
    A    
    A    
   G   
   A   
    T    
   T   
   T   
    A    
    A    
    C    
    T    
   G   
   A   
   C   
   A   
   T   
   T   
    C    
   T   
    G    
   G   
   C   
    A    
    A    
   G   
    A    
   C   
   C   
    A    
   G   
   A   
    A    
   C   
   G   
    T    
   G   
   T   
    T    
   A   
    C    
    A    
   G   
   A   
   A   
   A   
   T   
    C    
   A   
   T   
    C    
   A   
   A   
    G    
   A   
    A    
    T    
   G   
   A   
    A    
   T   
   T   
   A   
    G    
    C    
    A    
   G   
   A   
   C   
    T    
   T   
    G    
    A    
    A    
    T    
    C    
    A    
   A   
    C    
    G    
   A   
   T   
   T   
   T   
   G   
   G   
   T   
   G   
   A   
   T   
    A    
    A    
   A   
   C   
   G   
   A   
   C   
   G   
    C    
   A   
   C   
   A   
   G   
   A   
   A   
    T    
   T   
   A   
    C    
   T   
   A   
   G   
   T   
    C    
   G   
   G   
   T   
   G   
   A   
    G    
   G   
   T   
   C   
    T    
   T   
    A    
   A   
   G   
   T   
   C   
   T   
    G    
   G   
   A   
   A   
   G   
   A   
   T   
   G   
   A   
   A   
   G   
   A   
   T   
    T    
   T   
    A    
   A   
   T   
    C    
   G   
   A   
   A   
   G   
   A   
   A   
   G   
   A   
   A   
   G   
   A   
   A   
    A    
   T   
   C   
   G   
   T   
    T    
   A   
   T   
   T   
   A   
   C   
    A    
    T    
   T   
    G    
   A   
   C   
    A    
   A   
   A   
    C    
   A   
   A   
    T    
   G   
   G   
    T    
   T   
   A   
    C    
   A   
   T   
    C    
   A   
   A   
    A    
   C   
   G   
    T    
    T    
   T   
   G   
   G   
    C    
   A   
   A   
   A   
    T    
    A    
    A    
    T    
   G   
   A   
   A   
   T   
   T   
   C   
    A    
   G   
    A    
   G   
   C   
    T    
   C   
   A   
   A   
   C   
   G   
    A    
    A    
   G   
    G    
   G   
   G   
    T    
   G   
   G   
    A    
   C   
   G   
   T   
   G   
   G   
    T    
   G   
   T   
    C    
   C   
   A   
   A   
   G   
   G   
    A    
   A   
   T   
   G   
   G   
   G   
    T    
    A    
   T   
    C    
   C   
   A   
    C    
    A    
   A   
   T   
   G   
   A   
    C    
   G   
   A   
    C    
   T   
   T   
   C   
   G   
   T   
    C    
   A   
   A   
    A    
   A   
   A   
    T    
   C   
   T   
    G    
    G    
   T   
   T   
   T   
   C   
   T   
   T   
   G   
    T    
   T   
   C   
    T    
   A   
   C   
    C    
   C   
   A   
    T    
   G   
   A   
    T    
   A   
   C   
    A    
   T   
   T   
    G    
   T   
   T   
   A   
   T   
   T   
    C    
   T   
   T   
   T   
   A   
   C   
   G   
   A   
   A   
    T    
   A   
    A    
    C    
   G   
   G   
    A    
   A   
   A   
   A   
   G   
   T   
    G    
   T   
   A   
    T    
   C   
   G   
    G    
   G   
   C   
   A   
   A   
   A   
   A   
   G   
   G   
    A    
   T   
   A   
    C    
   G   
   A   
   A   
   A   
   T   
    T    
   C   
   C   
    A    
   G   
   A   
    A    
   T   
   A   
   C   
   G   
   G   
    G    
    C    
   G   
    T    
   A   
   C   
   A   
   G   
   C   
    C    
   A   
   A   
   A   
   G   
   G   
    A    
   A   
   T   
    A    
   C   
   C   
   A   
    A    
   T   
    T    
   A   
   T   
    C    
   A   
   A   
    T    
   T   
   T   
    A    
   C   
   T   
    A    
   G   
   G   
    T    
   A   
   T   
    C    
   G   
   A   
    T    
   T   
   C   
    G    
    A    
    G    
    T    
   G   
   A   
   A   
   A   
    G    
    C    
   A   
   T   
    C    
   C   
   A   
    G    
   G   
   C   
    A    
   A   
   T   
    C    
   A   
   T   
   T   
    G    
   C   
    G    
   G   
   T   
    G    
    A    
    C    
   G   
   G   
   G   
    A    
    G    
   A   
   A   
   G   
   C   
    A    
   G   
   A   
   A   
   G   
    A    
    A    
   G   
   G   
    C    
    C    
   A   
    C    
   T   
   A   
    T    
    C    
   T   
    C    
   T   
   T   
   C   
   T   
   T   
    T    
   A   
   C   
    G    
   A   
   C   
    G    
    C    
    G    
    T    
    C    
   A   
   A   
   G   
   G   
    T    
   A   
   C   
    A    
   G   
   T   
    A    
   A   
   A   
   A   
   C   
   G   
    T    
   A   
   C   
    T    
    T    
   C   
    T    
   G   
   T   
    C    
   A   
   C   
    T    
   G   
   C   
    T    
   T   
   T   
    T    
    G    
   C   
   T   
   A   
   A   
    C    
   A   
   T   
    C    
   C   
   G   
    A    
   A   
   G   
    T    
   A   
   A   
   T   
   G   
   G   
    T    
    C    
   T   
    G    
   A   
   T   
    C    
   G   
   C   
    A    
   A   
   T   
   T   
    G    
   G   
    T    
   T   
   T   
    G    
   A   
   A   
   A   
   G   
   A   
   A   
   G   
   A   
    C    
   G   
   A   
    T    
   G   
   A   
    G    
    C    
   T   
    C    
    G    
   T   
    C    
   A   
   A   
    T    
   G   
   T   
    G    
    C    
   T   
   A   
    C    
    T    
   G   
   A   
   C   
    A    
    G    
   A   
    T    
   G   
   G   
    T    
    C    
   A   
    A    
    T    
    C    
    T    
   A   
   A   
    T    
   A   
   T   
    C    
   A   
   T   
    T    
   A   
   T   
    T    
   G   
   G   
    A    
   A   
   C   
   A   
   C   
   A   
   T   
    A    
    A    
    C    
   G   
   G   
    C    
   T   
   A   
   C   
   T   
   C   
   T   
   G   
   T   
    C    
   A   
   C   
    T    
   T   
   T   
    C    
    A    
   A   
    A    
   G   
   A   
    A    
    G    
    A    
    A    
    G    
   C   
    A    
   G   
    T    
    C    
   C   
   G   
    C    
   G   
   A   
   T   
   A   
   T   
   G   
   G   
   G   
    A    
   C   
   G   
    T    
   A   
   C   
   A   
   G   
   C   
    T    
   T   
   C   
    C    
   G   
   G   
    G    
   G   
   T   
    C    
    A    
   G   
    A    
   G   
   G   
    A    
   A   
   T   
    A    
   C   
   G   
    C    
    C    
   T   
    G    
   C   
   G   
    T    
   G   
   A   
    G    
    G    
   A   
    A    
   G   
   A   
    T    
   T   
    A    
    T    
   G   
   T   
    C    
   G   
   T   
    T    
   G   
   G   
    G    
    G    
   C   
   A   
   G   
   C   
    C    
    T    
   T   
    A    
    A    
   T   
   G   
    A    
   A   
    G    
   G   
    A    
   A   
    A    
   A   
    T    
    C    
   A   
   A   
   G   
   A   
   A   
   G   
   T   
    A    
    T    
   T   
    A    
   G   
   T   
    G    
   A   
   T   
    C    
   A   
   C   
    A    
   G   
   A   
   A   
   A   
   A   
    A    
   G   
   G   
   T   
   T   
   A   
    C    
   G   
   G   
    A    
   A   
   A   
    A    
   C   
   G   
    G    
   A   
   C   
    A    
   A   
   A   
   A   
   G   
    T    
    A    
   T   
   C   
    G    
   G   
   A   
    A    
   T   
   A   
   T   
   C   
   C   
   A   
   G   
   T   
    G    
   A   
   A   
    A    
   G   
   G   
    C    
   C   
   G   
    C    
   G   
   G   
    A    
   G   
   G   
   T   
   A   
   A   
   A   
   G   
   G   
    G    
   A   
   T   
    C    
   A   
   A   
    A    
   A   
   C   
    G    
    G    
   C   
    G    
   A   
   A   
    C    
   A   
   T   
   C   
   A   
   C   
   T   
   G   
   A   
    A    
   A   
   A   
   A   
   A   
   A   
   T   
   G   
   G   
   T   
   C   
   C   
   A   
    C    
   T   
   A   
   G   
   C   
    T    
   G   
   G   
    A    
   T   
   T   
    G    
   A   
   C   
   C   
   A   
   C   
    T    
   G   
   T   
    T    
    T    
    C    
   T   
   G   
   G   
    A    
    G    
   A   
   T   
   G   
   A   
   A   
   G   
   A   
    C    
   A   
   T   
   C   
    A    
   T   
    G    
    C    
   T   
    G    
   A   
   T   
    T    
   A   
   C   
    A    
    G    
   A   
    T    
   A   
   A   
   A   
   G   
   G   
    T    
   G   
   T   
    C    
   A   
   T   
   T   
   A   
   T   
   C   
   C   
   G   
    T    
   T   
   T   
    C    
   A   
   A   
   C   
   G   
   T   
    A    
    T    
    C    
    G    
    A    
   C   
   T   
   G   
   T   
   T   
   T   
   C   
   T   
   C   
   A   
   A   
   A   
   C   
    T    
   G   
   G   
   A   
   C   
   G   
    T    
    T    
   C   
   A   
   A   
   C   
   A   
   T   
   T   
    A    
   G   
   G   
    C    
   G   
   T   
    A    
   C   
   G   
   T   
   T   
   T   
    G    
   A   
   T   
   G   
   A   
    A    
    G    
   A   
   T   
   G   
   G   
   A   
   A   
   G   
    C    
    T    
   G   
    A    
    T    
    A    
   C   
    G    
   A   
   A   
   A   
   G   
   T   
   T   
   G   
   T   
   C   
   A   
   C   
   G   
   A   
   T   
   G   
   G   
   C   
   A   
   G   
   C   
   A   
   G   
   T   
   C   
   G   
   A   
   A   
   C   
   C   
   A   
   G   
   A   
   A   
   G   
   C   
   T   
   G   
   C   
   A   
   G   
   A   
   A   
   C   
   A   
   A   
   A   
   C   
   A   
   G   
   T   
   T   
   G   
   A   
   A   
   G   
   A   
   A   
   C   
   A   
   A   
   G   
   G   
   A   
   T   
   C   
   A   
   G   
   A   
   A   
   G   
   A   
   A   
   -   
   -   
   -   
 

 
   N60027F   
   A   
   T   
   G   
   A   
   G   
   T   
   G   
   A   
   A   
   G   
   A   
   A   
   A   
   T   
   T   
   A   
   A   
   A   
   G   
   A   
   A   
   A   
   A   
   C   
   A   
   T   
   T   
   C   
   A   
   A   
   G   
   A   
   C   
   G   
   T   
   C   
   A   
   A   
   T   
   C   
   T   
   G   
   A   
   C   
   C   
   A   
   G   
   C   
   G   
   A   
   A   
   A   
   T   
   G   
   A   
   A   
   A   
   G   
   A   
   A   
   T   
   C   
   T   
   T   
   T   
   C   
   A   
   T   
   T   
   G   
   A   
   C   
   T   
   A   
   C   
   G   
   C   
   A   
   A   
   T   
   G   
   A   
   G   
   T   
   G   
   T   
   T   
   A   
   T   
   C   
   G   
   T   
   A   
   G   
   C   
   C   
   C   
   G   
   C   
   G   
   C   
   G   
   T   
   T   
   A   
   C   
   C   
   T   
   G   
   A   
   C   
   G   
   T   
   T   
   C   
   G   
   T   
   G   
   A   
   C   
   G   
   G   
   T   
   T   
   T   
   A   
   A   
   A   
   A   
   C   
   C   
   T   
   G   
   T   
   T   
   C   
   A   
   T   
   C   
   G   
   C   
   C   
   G   
   A   
   A   
   T   
   C   
   T   
   T   
   A   
   T   
   A   
   T   
   G   
   G   
   A   
   A   
   T   
   G   
   A   
   A   
   C   
   G   
   A   
   A   
   T   
   T   
   A   
   G   
   G   
   G   
   G   
   T   
   A   
   A   
   C   
   C   
   C   
   C   
   T   
   G   
   A   
   T   
   A   
   A   
   A   
   C   
   C   
   A   
   C   
   A   
   C   
   A   
   A   
   A   
   A   
   A   
   A   
   T   
   C   
   A   
   G   
   C   
   C   
   C   
   G   
   G   
   A   
   T   
   T   
   G   
   T   
   T   
   G   
   G   
   G   
   G   
   A   
   T   
   G   
   T   
   T   
   A   
   T   
   G   
   G   
   G   
   T   
   A   
   A   
   A   
   T   
   A   
   T   
   C   
   A   
   T   
   C   
   C   
   C   
   C   
   A   
   T   
   G   
   G   
   G   
   G   
   A   
   C   
   A   
   G   
   T   
   G   
   C   
   G   
   A   
   T   
   T   
   T   
   A   
   C   
   G   
   A   
   A   
   T   
   C   
   A   
   A   
   T   
   G   
   G   
   T   
   G   
   C   
   G   
   G   
   A   
   T   
   G   
   G   
   C   
   A   
   C   
   A   
   A   
   C   
   C   
   T   
   T   
   T   
   T   
   A   
   G   
   T   
   T   
   A   
   T   
   C   
   G   
   G   
   G   
   C   
   T   
   A   
   T   
   G   
   T   
   T   
   A   
   G   
   T   
   T   
   G   
   A   
   C   
   G   
   G   
   C   
   C   
   A   
   C   
   G   
   G   
   A   
   A   
   A   
   C   
   T   
   T   
   C   
   G   
   G   
   T   
   T   
   C   
   T   
   G   
   T   
   C   
   G   
   A   
   T   
   G   
   G   
   T   
   G   
   A   
   C   
   G   
   G   
   C   
   G   
   C   
   T   
   G   
   C   
   C   
   G   
   C   
   T   
   A   
   T   
   G   
   C   
   G   
   T   
   T   
   A   
   T   
   A   
   C   
   C   
   G   
   A   
   A   
   G   
   C   
   A   
   C   
   G   
   T   
   A   
   T   
   G   
   A   
   G   
   T   
   A   
   A   
   A   
   A   
   T   
   T   
   G   
   C   
   T   
   T   
   T   
   A   
   G   
   A   
   A   
   A   
   T   
   G   
   C   
   T   
   A   
   C   
   G   
   A   
   G   
   A   
   T   
   A   
   T   
   T   
   A   
   A   
   C   
   A   
   A   
   A   
   A   
   A   
   T   
   A   
   C   
   A   
   G   
   T   
   C   
   G   
   A   
   T   
   T   
   T   
   C   
   C   
   A   
   A   
   G   
   G   
   A   
   A   
   A   
   C   
   T   
   A   
   T   
   G   
   A   
   T   
   G   
   A   
   T   
   T   
   C   
   A   
   G   
   A   
   A   
   C   
   A   
   A   
   G   
   A   
   A   
   C   
   C   
   A   
   G   
   T   
   A   
   G   
   T   
   T   
   T   
   T   
   A   
   C   
   C   
   T   
   G   
   C   
   T   
   C   
   G   
   T   
   T   
   T   
   T   
   C   
   C   
   A   
   A   
   A   
   C   
   T   
   T   
   A   
   C   
   T   
   A   
   G   
   T   
   T   
   A   
   A   
   C   
   G   
   G   
   A   
   A   
   C   
   A   
   A   
   C   
   G   
   G   
   G   
   G   
   A   
   T   
   T   
   G   
   C   
   G   
   G   
   T   
   G   
   G   
   G   
   G   
   A   
   T   
   G   
   G   
   C   
   A   
   A   
   C   
   A   
   A   
   A   
   T   
   A   
   T   
   T   
   C   
   C   
   A   
   C   
   C   
   A   
   C   
   A   
   T   
   A   
   A   
   T   
   T   
   T   
   A   
   A   
   G   
   T   
   G   
   A   
   A   
   G   
   T   
   G   
   A   
   T   
   T   
   G   
   A   
   T   
   G   
   C   
   A   
   A   
   C   
   A   
   A   
   G   
   T   
   T   
   T   
   A   
   T   
   T   
   G   
   A   
   T   
   G   
   G   
   A   
   C   
   A   
   A   
   T   
   C   
   C   
   T   
   G   
   A   
   T   
   G   
   T   
   A   
   A   
   C   
   G   
   A   
   C   
   G   
   A   
   A   
   T   
   G   
   A   
   A   
   T   
   T   
   G   
   A   
   T   
   G   
   G   
   A   
   A   
   G   
   T   
   G   
   T   
   T   
   A   
   C   
   C   
   T   
   G   
   G   
   A   
   C   
   C   
   A   
   G   
   A   
   T   
   T   
   T   
   T   
   C   
   C   
   A   
   A   
   C   
   A   
   G   
   G   
   C   
   G   
   G   
   T   
   T   
   T   
   A   
   G   
   T   
   G   
   A   
   T   
   G   
   G   
   G   
   G   
   A   
   A   
   A   
   T   
   C   
   A   
   G   
   G   
    G    
   A   
   T   
    T    
   C   
   G   
    C    
    C    
   G   
   A   
   G   
   C   
    A    
   T   
   A   
   T   
   G   
   A   
   A   
   A   
   C   
    A    
   G   
   G   
    G    
   A   
   A   
    A    
   G   
   G   
   T   
   T   
   C   
    G    
   A   
   T   
   T   
   A   
   C   
    T    
   G   
   T   
    T    
   C   
   G   
   T   
   G   
   C   
   A   
   A   
   A   
   A   
   G   
   T   
   T   
   G   
   A   
    A    
    T    
   T   
    G    
   A   
    C    
   T   
   G   
   A   
    A    
   A   
   T   
   G   
    C    
   C   
    G    
   A   
   A   
   T   
   G   
   G   
    A    
   A   
   A   
   A   
   G   
   A   
   A   
   C   
   G   
    T    
   A   
   T   
    T    
    T    
   T   
    A    
   G   
   T   
    A    
   A   
   C   
    T    
   G   
   A   
   A   
   T   
   T   
    G    
   C   
   C   
   T   
   T   
   A   
   T   
   A   
   T   
   G   
   G   
   T   
    G    
   A   
   A   
   T   
   A   
   A   
   A   
   G   
   C   
    C    
   A   
   A   
   A   
   T   
   T   
    A    
   A   
   T   
    C    
   G   
   A   
   A   
   C   
   G   
    A    
   A   
   T   
   T   
   T   
   C   
   T   
   G   
   A   
    A    
    T    
   T   
    A    
   C   
   A   
   C   
   A   
   G   
   A   
   G   
   A   
    T    
   A   
   A   
    A    
   C   
   G   
    A    
   A   
   T   
    T    
   G   
   A   
   A   
   G   
   G   
    A    
   A   
   T   
    T    
   A   
   C   
    T    
   G   
   A   
   T   
   T   
   T   
   G   
   C   
   G   
   G   
   G   
   A   
    T    
   G   
   A   
   A   
   T   
   C   
   T   
   T   
   C   
    T    
   C   
   G   
   T   
   G   
   A   
    A    
   G   
   G   
    C    
   A   
   T   
   G   
   C   
   G   
    G    
   A   
   T   
    C    
   G   
   T   
   C   
   A   
   T   
    C    
   G   
   A   
    T    
    G    
   T   
    T    
   C   
   G   
   T   
   C   
   G   
    A    
   G   
   A   
    T    
   G   
    T    
    G    
   A   
   G   
    T    
   G   
   C   
    C    
   T   
   C   
    T    
   G   
   T   
   T   
    G    
   T   
    G    
   T   
   T   
    A    
   A   
   A   
    C    
   A   
   A   
   C   
   T   
   T   
    G    
   T   
   A   
    C    
   A   
   A   
   A   
    A    
   T   
   G   
   A   
   C   
    T    
    G    
   C   
    C    
   T   
   T   
    A    
   C   
   A   
    A    
   A   
    C    
    A    
   T   
   C   
    T    
   T   
   T   
    T    
   G   
   G   
    T    
   T   
   T   
    T    
   A   
   A   
    C    
   A   
   T   
   G   
   T   
   T   
    A    
   G   
   C   
    C    
   A   
   T   
    T    
   G   
   A   
   A   
   A   
   A   
   A   
   G   
   G   
    C    
   G   
   T   
    A    
   C   
   C   
    G    
   A   
   A   
   A   
    A    
   T   
   T   
   T   
   T   
    A    
   A   
   G   
    C    
   T   
   T   
   G   
   A   
   A   
   A   
   C   
    G    
    T    
   A   
   T   
    T    
    T    
   T   
    A    
   G   
   A   
   A   
   A   
   A   
    C    
   T   
   A   
    C    
    G    
   T   
    C    
   G   
   A   
    G    
   C   
   A   
    C    
   C   
   A   
    A    
    A    
    A    
    A    
    G    
   A   
   A   
   G   
   T   
   C   
   A   
   T   
    T    
   A   
   C   
    T    
    C    
   G   
    C    
   C   
   G   
   G   
   A   
   C   
    G    
    A    
   T   
    T    
   T   
   T   
   T   
   G   
   A   
    C    
   A   
   A   
   A   
    A    
   A   
    C    
   A   
   A   
    A    
   G   
   C   
   A   
   G   
   A   
   A   
   G   
   C   
   A   
    C    
   G   
    G    
   G   
   C   
    G    
   C   
   A   
    T    
   A   
   T   
    T    
   T   
   T   
   A   
   G   
   A   
   A   
   G   
   G   
   T   
    C    
   T   
    A    
   C   
   G   
    A    
   A   
   T   
   T   
   G   
   C   
    C    
   T   
   T   
    A    
   G   
   A   
   T   
   C   
   A   
   T   
   A   
   T   
   C   
    G    
   A   
   T   
   G   
   A   
   A   
   A   
   T   
    C    
   A   
   T   
   T   
   G   
   C   
    C    
   A   
   T   
   T   
   A   
   T   
    C    
   C   
   G   
    T    
   G   
   G   
    A    
   T   
   C   
    A    
    C    
   A   
   A   
   T   
   C   
   A   
   G   
   A   
   T   
   G   
   A   
    C    
   G   
   A   
    A    
   G   
   C   
   A   
   A   
   A   
   A   
   G   
   C   
    A    
   A   
    C    
    G    
    T    
   T   
    A    
   A   
   T   
    T    
   G   
   A   
   A   
   C   
   G   
    C    
   T   
   T   
    T    
   G   
   A   
    A    
   T   
   T   
    T    
   T   
   C   
    A    
   G   
   A   
   T   
   C   
   G   
    T    
   C   
   A   
    A    
    G    
   C   
    G    
   C   
   A   
   A   
   G   
   C   
    G    
   A   
   T   
    T    
   T   
   T   
   A   
   G   
   A   
   T   
   A   
   T   
   G   
   C   
   G   
   T   
   T   
   T   
    A    
   C   
   G   
    C    
   C   
   G   
    T    
   T   
   T   
    A    
   A   
   C   
    A    
   G   
   G   
    C    
   T   
   T   
    A    
   G   
   A   
   A   
   C   
   G   
    C    
   G   
   A   
    C    
   A   
   A   
   A   
   A   
   T   
    T    
   G   
   A   
    A    
   A   
   A   
   T   
   G   
   A   
   A   
   T   
   A   
    T    
   C   
   A   
   A   
   G   
   A   
    G    
   C   
   T   
    A    
   T   
   T   
    G    
   A   
    A    
   A   
    T    
   T   
    C    
   A   
   T   
   T   
   G   
    C    
    G    
   G   
   A   
    C    
   T   
   T   
    A    
    G    
    A    
    G    
   G   
   A   
   C   
   A   
   T   
   T   
    T    
   T   
    A    
   G   
   C   
    C    
    C    
   G   
    C    
   C   
   C   
    A    
   G   
   A   
    A    
   C   
   G   
    C    
   G   
   T   
    C    
   A   
    T    
    T    
   G   
   A   
   A   
   A   
   T   
    C    
   A   
   T   
    T    
   A   
   A   
    A    
   A   
    C    
    A    
   G   
   A   
    G    
   T   
   T   
   A   
    A    
    A    
    T    
   G   
   A   
   C   
    G    
   T   
    T    
    C    
    G    
    C    
    A    
    C    
   A   
    A    
    A    
   A   
   T   
   T   
   T   
   G   
   G   
   T   
   G   
   A   
   T   
    G    
    C    
   A   
   C   
   G   
   A   
   C   
   G   
    C    
   A   
   C   
   A   
   G   
   A   
   A   
    T    
   T   
   A   
    T    
   T   
   A   
   G   
   T   
    A    
   G   
   G   
   T   
   G   
   A   
    A    
   G   
   T   
   C   
    T    
   T   
    A    
   A   
   G   
   T   
   C   
   T   
    T    
   G   
   A   
   A   
   G   
   A   
   T   
   G   
   A   
   A   
   G   
   A   
   T   
    T    
   T   
    A    
   A   
   T   
    C    
   G   
   A   
   A   
   G   
   A   
   A   
   G   
   A   
   A   
   G   
   A   
   A   
    G    
   T   
   C   
   G   
   T   
    G    
   A   
   T   
   T   
   A   
   C   
    A    
    T    
   T   
    A    
   A   
   C   
    C    
   A   
   A   
    T    
   A   
   A   
    C    
   G   
   G   
    C    
   T   
   A   
    C    
   A   
   T   
    T    
   A   
   A   
    G    
   C   
   G   
    G    
    A    
   T   
   G   
   G   
    C    
   A   
   A   
   A   
    C    
    T    
    C    
    C    
   G   
   A   
   A   
   T   
   T   
   C   
    C    
   G   
    G    
   G   
   C   
    G    
   C   
   A   
   A   
   C   
   G   
    C    
    C    
   G   
    C    
   G   
   G   
    C    
   G   
   G   
    A    
   C   
   G   
   T   
   G   
   G   
    T    
   G   
   T   
    C    
   C   
   A   
   A   
   G   
   G   
    C    
   A   
   T   
   G   
   G   
   G   
    C    
    G    
   T   
    T    
   C   
   A   
    T    
    G    
   A   
   T   
   G   
   A   
    T    
   G   
   A   
    T    
   T   
   T   
   C   
   G   
   T   
    G    
   A   
   A   
    A    
   A   
   A   
    C    
   C   
   T   
    A    
    G    
   T   
   T   
   T   
   C   
   T   
   T   
   G   
    T    
   T   
   C   
    A    
   A   
   C   
    A    
   C   
   A   
    T    
   G   
   A   
    C    
   A   
   C   
    G    
   T   
   T   
    A    
   T   
   T   
   A   
   T   
   T   
    C    
   T   
   T   
   T   
   A   
   C   
   G   
   A   
   A   
    C    
   A   
    C    
    T    
   G   
   G   
    G    
   A   
   A   
   A   
   G   
   T   
    T    
   T   
   A   
    C    
   C   
   G   
    A    
   G   
   C   
   A   
   A   
   A   
   A   
   G   
   G   
    T    
   T   
   A   
    C    
   G   
   A   
   A   
   A   
   T   
    C    
   C   
   C   
    T    
   G   
   A   
    A    
   T   
   A   
   C   
   G   
   G   
    T    
    A    
   G   
    A    
   A   
   C   
   A   
   G   
   C   
    A    
   A   
   A   
   A   
   G   
   G   
    A    
   A   
   T   
    T    
   C   
   C   
   A   
    G    
   T   
    G    
   A   
   T   
    T    
   A   
   A   
    C    
   T   
   T   
    A    
   C   
   T   
    G    
   G   
   G   
    A    
   A   
   T   
    T    
   G   
   A   
    T    
   T   
   C   
    T    
    G    
    C    
    A    
   G   
   A   
   A   
   A   
    A    
    A    
   A   
   T   
    T    
   C   
   A   
    A    
   G   
   C   
    G    
   A   
   T   
    T    
   A   
   T   
   T   
    T    
   C   
    T    
   G   
   T   
    T    
    G    
    A    
   G   
   G   
   G   
    C    
    A    
   A   
   A   
   G   
   C   
    G    
   G   
   A   
   A   
   G   
    C    
    A    
   G   
   G   
    T    
    A    
   A   
    A    
   T   
   A   
    C    
    T    
   T   
    G    
   T   
   T   
   C   
   T   
   T   
    C    
   A   
   C   
    A    
   A   
   C   
    C    
    T    
    T    
    A    
    A    
   A   
   A   
   G   
   G   
    A    
   A   
   C   
    C    
   G   
   T   
    C    
   A   
   A   
   A   
   C   
   G   
    G    
   A   
   C   
    A    
    G    
   C   
    C    
   G   
   T   
    A    
   A   
   C   
    A    
   G   
   C   
    C    
   T   
   T   
    T    
    T    
   C   
   T   
   A   
   A   
    T    
   A   
   T   
    C    
   C   
   G   
    T    
   A   
   G   
    T    
   A   
   A   
   T   
   G   
   G   
    A    
    T    
   T   
    A    
   A   
   T   
    C    
   G   
   C   
    C    
   A   
   T   
   T   
    A    
   G   
    C    
   T   
   T   
    A    
   A   
   A   
   A   
   G   
   A   
   A   
   G   
   A   
    T    
   G   
   A   
    T    
   G   
   A   
    G    
    T    
   T   
    A    
    G    
   T   
    T    
   A   
   A   
    C    
   G   
   T   
    G    
    G    
   T   
   A   
    A    
    C    
   G   
   A   
   C   
    T    
    A    
   A   
    T    
   G   
   G   
    C    
    A    
   A   
    T    
    C    
    A    
    G    
   A   
   A   
    G    
   A   
   T   
    G    
   A   
   T   
    T    
   A   
   T   
    C    
   G   
   G   
    A    
   A   
   C   
   A   
   C   
   A   
   T   
    G    
    C    
    A    
   G   
   G   
    A    
   T   
   A   
   C   
   T   
   C   
   T   
   G   
   T   
    C    
   A   
   C   
    A    
   T   
   T   
    T    
    G    
   A   
    T    
   G   
   A   
    A    
    A    
    A    
    T    
    A    
   C   
    T    
   G   
    T    
    A    
   C   
   G   
    T    
   G   
   A   
   T   
   A   
   T   
   G   
   G   
   G   
    C    
   C   
   G   
    G    
   A   
   C   
   A   
   G   
   C   
    A    
   T   
   C   
    A    
   G   
   G   
    C    
   G   
   T   
    T    
    C    
   G   
    G    
   G   
   G   
    A    
   A   
   T   
    C    
   C   
   G   
    T    
    C    
   T   
    C    
   C   
   G   
    C    
   G   
   A   
    A    
    A    
   A   
    T    
   G   
   A   
    T    
   T   
    A    
    T    
   G   
   T   
    G    
   G   
   T   
    C    
   G   
   G   
    C    
    G    
   C   
   A   
   G   
   C   
    G    
    A    
   T   
    T    
    C    
   T   
   G   
    G    
   A   
    T    
   G   
    A    
   A   
    A    
   A   
    T    
    A    
   A   
   A   
   G   
   A   
   A   
   G   
   T   
    C    
    C    
   T   
    A    
   G   
   T   
    C    
   A   
   T   
    T    
   A   
   C   
    T    
   G   
   A   
   A   
   A   
   A   
    T    
   G   
   G   
   T   
   T   
   A   
    T    
   G   
   G   
    T    
   A   
   A   
    G    
   C   
   G   
    T    
   A   
   C   
    A    
   A   
   A   
   A   
   G   
    C    
    T    
   T   
   C   
    T    
   G   
   A   
    A    
   T   
   A   
   T   
   C   
   C   
   A   
   G   
   T   
    T    
   A   
   A   
    A    
   G   
   G   
    A    
   C   
   G   
    T    
   G   
   G   
    C    
   G   
   G   
   T   
   A   
   A   
   A   
   G   
   G   
    G    
   A   
   T   
    T    
   A   
   A   
    G    
   A   
   C   
    A    
    G    
   C   
    A    
   A   
   A   
    T    
   A   
   T   
   C   
   A   
   C   
   T   
   G   
   A   
    G    
   A   
   A   
   A   
   A   
   A   
   T   
   G   
   G   
   T   
   C   
   C   
   A   
    T    
   T   
   A   
   G   
   C   
    T    
   G   
   G   
    T    
   T   
   T   
    A    
   A   
   C   
   C   
   A   
   C   
    G    
   G   
   T   
    C    
    A    
    A    
   T   
   G   
   G   
    G    
    G    
   A   
   T   
   G   
   A   
   A   
   G   
   A   
    T    
   A   
   T   
   C   
    T    
   T   
    A    
    T    
   T   
    G    
   A   
   T   
    T    
   A   
   C   
    G    
    A    
   A   
    C    
   A   
   A   
   A   
   G   
   G   
    C    
   G   
   T   
    C    
   A   
   T   
   T   
   A   
   T   
   C   
   C   
   G   
    C    
   T   
   T   
    T    
   A   
   A   
   C   
   G   
   T   
    T    
    G    
    A    
    T    
    T    
   C   
   T   
   G   
   T   
   T   
   T   
   C   
   T   
   C   
   A   
   A   
   A   
   C   
    A    
   G   
   G   
   A   
   C   
   G   
    C    
    G    
   C   
   A   
   A   
   C   
   A   
   T   
   T   
    A    
   G   
   G   
    G    
   G   
   T   
    T    
   C   
   G   
   T   
   T   
   T   
    A    
   A   
   T   
   G   
   A   
    G    
    A    
   A   
   T   
   G   
   G   
   A   
   A   
   G   
    A    
    T    
   G   
    G    
    T    
    G    
   C   
    C    
   A   
   A   
   A   
   -   
   -   
   -   
   -   
   -   
   -   
   -   
   -   
   -   
   -   
   -   
   -   
   -   
   -   
   -   
   -   
   -   
   -   
   -   
   -   
   -   
   -   
   -   
   -   
   -   
   -   
   -   
   -   
   -   
   -   
   -   
   -   
   -   
   -   
   -   
   -   
   -   
   -   
   -   
   -   
   -   
   -   
   -   
   -   
   -   
   -   
   -   
   -   
   -   
   -   
   -   
   -   
   -   
   -   
   -   
   -   
   -   
   -   
   -   
   -   
   -   
   -   
   -   
   -   
   -   
   -   
   -   
   -   
   -   
   -   
   -   
   -   
 

 
   N59439F   
   A   
   T   
   G   
   A   
   G   
   T   
   G   
   A   
   A   
   G   
   A   
   A   
   A   
   T   
   C   
   A   
   G   
   A   
   G   
   A   
   A   
   A   
   A   
   C   
   A   
   T   
   C   
   C   
   A   
   T   
   G   
   A   
   T   
   G   
   T   
   C   
   A   
   A   
   T   
   C   
   T   
   A   
   A   
   C   
   C   
   A   
   G   
   C   
   G   
   A   
   A   
   A   
   T   
   G   
   A   
   A   
   A   
   G   
   A   
   C   
   T   
   C   
   C   
   T   
   T   
   C   
   A   
   T   
   C   
   G   
   A   
   T   
   T   
   A   
   T   
   G   
   C   
   G   
   A   
   T   
   G   
   A   
   G   
   T   
   G   
   T   
   T   
   A   
   T   
   T   
   G   
   T   
   C   
   G   
   C   
   A   
   C   
   G   
   T   
   G   
   C   
   A   
   C   
   T   
   T   
   C   
   C   
   T   
   G   
   A   
   T   
   G   
   T   
   C   
   A   
   G   
   A   
   G   
   A   
   T   
   G   
   G   
   T   
   C   
   T   
   G   
   A   
   A   
   A   
   C   
   C   
   C   
   G   
   T   
   T   
   C   
   A   
   T   
   C   
   G   
   C   
   C   
   G   
   T   
   A   
   T   
   T   
   T   
   T   
   A   
   T   
   A   
   C   
   G   
   G   
   C   
   A   
   T   
   G   
   A   
   A   
   C   
   G   
   A   
   A   
   T   
   T   
   A   
   G   
   G   
   T   
   G   
   T   
   A   
   A   
   C   
   A   
   C   
   C   
   G   
   G   
   A   
   T   
   A   
   A   
   A   
   G   
   C   
   C   
   C   
   A   
   T   
   A   
   A   
   A   
   A   
   A   
   A   
   T   
   C   
   T   
   G   
   C   
   C   
   A   
   G   
   A   
   A   
   T   
   C   
   G   
   T   
   C   
   G   
   G   
   A   
   G   
   A   
   T   
   G   
   T   
   C   
   A   
   T   
   G   
   G   
   G   
   T   
   A   
   A   
   A   
   T   
   A   
   C   
   C   
   A   
   T   
   C   
   C   
   T   
   C   
   A   
   T   
   G   
   G   
   A   
   G   
   A   
   C   
   A   
   G   
   T   
   G   
   C   
   G   
   A   
   T   
   C   
   T   
   A   
   T   
   G   
   A   
   G   
   T   
   C   
   A   
   A   
   T   
   G   
   G   
   T   
   A   
   C   
   G   
   G   
   A   
   T   
   G   
   G   
   C   
   A   
   C   
   A   
   G   
   C   
   C   
   A   
   T   
   T   
   C   
   A   
   G   
   C   
   T   
   A   
   C   
   C   
   G   
   T   
   T   
   A   
   T   
   A   
   T   
   G   
   C   
   T   
   A   
   G   
   T   
   G   
   G   
   A   
   T   
   G   
   G   
   T   
   C   
   A   
   T   
   G   
   G   
   A   
   A   
   A   
   C   
   T   
   T   
   T   
   G   
   G   
   T   
   T   
   C   
   T   
   G   
   T   
   T   
   G   
   A   
   C   
   G   
   G   
   T   
   G   
   A   
   C   
   G   
   G   
   T   
   G   
   C   
   C   
   G   
   C   
   C   
   G   
   C   
   T   
   A   
   T   
   G   
   C   
   G   
   T   
   T   
   A   
   T   
   A   
   C   
   C   
   G   
   A   
   A   
   G   
   C   
   A   
   C   
   G   
   G   
   A   
   T   
   G   
   A   
   G   
   C   
   A   
   A   
   A   
   A   
   T   
   C   
   G   
   C   
   T   
   A   
   C   
   A   
   G   
   A   
   A   
   A   
   T   
   G   
   C   
   T   
   G   
   C   
   G   
   T   
   G   
   A   
   T   
   A   
   T   
   C   
   A   
   A   
   T   
   A   
   A   
   G   
   A   
   A   
   C   
   A   
   C   
   T   
   G   
   T   
   T   
   G   
   A   
   T   
   T   
   T   
   C   
   C   
   A   
   A   
   A   
   G   
   C   
   A   
   A   
   C   
   T   
   A   
   T   
   G   
   A   
   T   
   G   
   A   
   T   
   A   
   C   
   A   
   G   
   A   
   A   
   A   
   A   
   A   
   G   
   A   
   A   
   C   
   C   
   A   
   G   
   T   
   T   
   G   
   T   
   A   
   C   
   T   
   A   
   C   
   C   
   T   
   G   
   C   
   T   
   C   
   G   
   C   
   T   
   T   
   C   
   C   
   C   
   G   
   A   
   A   
   T   
   C   
   T   
   T   
   T   
   T   
   A   
   G   
   T   
   G   
   A   
   A   
   C   
   G   
   G   
   A   
   A   
   C   
   A   
   A   
   C   
   G   
   G   
   G   
   G   
   A   
   T   
   C   
   G   
   C   
   C   
   G   
   T   
   A   
   G   
   G   
   T   
   A   
   T   
   G   
   G   
   C   
   C   
   A   
   C   
   G   
   A   
   A   
   T   
   A   
   T   
   C   
   C   
   C   
   A   
   C   
   C   
   T   
   C   
   A   
   T   
   A   
   A   
   C   
   T   
   T   
   G   
   T   
   C   
   T   
   G   
   A   
   G   
   G   
   T   
   A   
   G   
   T   
   A   
   G   
   C   
   G   
   G   
   C   
   T   
   A   
   T   
   C   
   G   
   A   
   T   
   T   
   T   
   A   
   C   
   T   
   G   
   A   
   T   
   G   
   G   
   A   
   T   
   A   
   A   
   T   
   C   
   C   
   T   
   G   
   A   
   T   
   G   
   T   
   G   
   A   
   C   
   G   
   A   
   C   
   C   
   A   
   A   
   T   
   G   
   A   
   A   
   T   
   T   
   G   
   A   
   T   
   G   
   G   
   A   
   A   
   G   
   T   
   A   
   T   
   T   
   A   
   C   
   C   
   T   
   G   
   G   
   A   
   C   
   C   
   A   
   G   
   A   
   T   
   T   
   T   
   T   
   C   
   C   
   A   
   A   
   C   
   A   
   G   
   G   
   T   
   G   
   G   
   T   
   T   
   T   
   G   
   G   
   T   
   T   
   A   
   T   
   G   
   G   
   G   
   G   
   A   
   A   
   A   
   T   
   C   
   A   
   G   
   G   
    A    
   A   
   T   
    C    
   C   
   G   
    A    
    A    
   G   
   A   
   G   
   C   
    G    
   T   
   A   
   T   
   G   
   A   
   A   
   A   
   C   
    A    
   G   
   G   
    T    
   A   
   A   
    G    
   G   
   G   
   T   
   T   
   C   
    T    
   A   
   T   
   T   
   A   
   C   
    C    
   G   
   T   
    A    
   C   
   G   
   T   
   G   
   C   
   A   
   A   
   A   
   A   
   G   
   T   
   T   
   G   
   A   
    G    
    A    
   T   
    T    
   A   
    C    
   T   
   G   
   A   
    G    
   A   
   T   
   G   
    C    
   C   
    A    
   A   
   A   
   T   
   G   
   G   
    T    
   A   
   A   
   A   
   G   
   A   
   A   
   C   
   G   
    G    
   A   
   T   
    C    
    A    
   T   
    C    
   G   
   T   
    G    
   A   
   C   
    A    
   G   
   A   
   A   
   T   
   T   
    G    
   C   
   C   
   T   
   T   
   A   
   T   
   A   
   T   
   G   
   G   
   T   
    C    
   A   
   A   
   T   
   A   
   A   
   A   
   G   
   C   
    A    
   A   
   A   
   A   
   T   
   T   
    G    
   A   
   T   
    C    
   G   
   A   
   A   
   C   
   G   
    G    
   A   
   T   
   T   
   T   
   C   
   T   
   G   
   A   
    A    
    C    
   T   
    G    
   C   
   A   
   C   
   A   
   G   
   A   
   G   
   A   
    C    
   A   
   A   
    G    
   C   
   G   
    T    
   A   
   T   
    C    
   G   
   A   
   A   
   G   
   G   
    G    
   A   
   T   
    C    
   A   
   C   
    A    
   G   
   A   
   T   
   T   
   T   
   G   
   C   
   G   
   G   
   G   
   A   
    C    
   G   
   A   
   A   
   T   
   C   
   T   
   T   
   C   
    C    
   C   
   G   
   T   
   G   
   A   
    A    
   G   
   G   
    T    
   A   
   T   
   G   
   C   
   G   
    G    
   A   
   T   
    C    
   G   
   T   
   C   
   A   
   T   
    T    
   G   
   A   
    C    
    A    
   T   
    C    
   C   
   G   
   T   
   C   
   G   
    T    
   G   
   A   
    C    
   G   
    C    
    C    
   A   
   G   
    C    
   G   
   C   
    A    
   T   
   C   
    C    
   G   
   T   
   T   
    A    
   T   
    C    
   T   
   T   
    G    
   A   
   A   
    T    
   A   
   A   
   C   
   T   
   T   
    A    
   T   
   A   
    T    
   A   
   A   
   A   
    T    
   T   
   G   
   A   
   C   
    A    
    T    
   C   
    C    
   T   
   T   
    G    
   C   
   A   
    A    
   A   
    A    
    T    
   T   
   C   
    A    
   T   
   T   
    T    
   G   
   G   
    A    
   T   
   T   
    C    
   A   
   A   
    T    
   A   
   T   
   G   
   T   
   T   
    G    
   G   
   C   
    G    
   A   
   T   
    C    
   G   
   A   
   A   
   A   
   A   
   A   
   G   
   G   
    T    
   G   
   T   
    G    
   C   
   C   
    G    
   A   
   A   
   A   
    G    
   T   
   T   
   T   
   T   
    G    
   A   
   G   
    C    
   T   
   T   
   G   
   A   
   A   
   A   
   C   
    A    
    A    
   A   
   T   
    C    
    C    
   T   
    T    
   G   
   A   
   A   
   A   
   A   
    C    
   T   
   A   
    T    
    A    
   T   
    C    
   G   
   A   
    A    
   C   
   A   
    T    
   C   
   A   
    A    
    C    
    G    
    C    
    G    
   A   
   A   
   G   
   T   
   C   
   A   
   T   
    C    
   A   
   C   
    A    
    C    
   G   
    A    
   C   
   G   
   G   
   A   
   C   
    G    
    G    
   T   
    C    
   T   
   T   
   T   
   G   
   A   
    A    
   A   
   A   
   A   
    G    
   A   
    A    
   A   
   A   
    A    
   G   
   C   
   A   
   G   
   A   
   A   
   G   
   C   
   A   
    A    
   G   
    A    
   G   
   C   
    A    
   C   
   A   
    C    
   A   
   T   
    C    
   T   
   T   
   A   
   G   
   A   
   A   
   G   
   G   
   T   
    T    
   T   
    G    
   C   
   G   
    T    
   A   
   T   
   T   
   G   
   C   
    T    
   T   
   T   
    G    
   G   
   A   
   T   
   C   
   A   
   T   
   A   
   T   
   C   
    G    
   A   
   T   
   G   
   A   
   A   
   A   
   T   
    C    
   A   
   T   
   T   
   G   
   C   
    G    
   A   
   T   
   T   
   A   
   T   
    C    
   C   
   G   
    C    
   G   
   G   
    G    
   T   
   C   
    T    
    A    
   A   
   A   
   T   
   C   
   A   
   G   
   A   
   T   
   G   
   A   
    C    
   G   
   A   
    G    
   G   
   C   
   A   
   A   
   A   
   A   
   G   
   C   
    A    
   A   
    C    
    A    
    A    
   T   
    G    
   A   
   T   
    C    
   G   
   A   
   A   
   C   
   G   
    G    
   T   
   T   
    C    
   G   
   A   
    T    
   T   
   T   
    A    
   T   
   C   
    C    
   G   
   A   
   T   
   C   
   G   
    C    
   C   
   A   
    G    
    T    
   C   
    T    
   C   
   A   
   A   
   G   
   C   
    T    
   A   
   T   
    T    
   T   
   T   
   A   
   G   
   A   
   T   
   A   
   T   
   G   
   C   
   G   
   T   
   T   
   T   
    A    
   C   
   G   
    C    
   C   
   G   
    A    
   T   
   T   
    G    
   A   
   C   
    C    
   G   
   G   
    T    
   T   
   T   
    G    
   G   
   A   
   A   
   C   
   G   
    G    
   G   
   A   
    A    
   A   
   A   
   A   
   A   
   T   
    C    
   G   
   A   
    A    
   A   
   A   
   T   
   G   
   A   
   A   
   T   
   A   
    C    
   C   
   A   
   A   
   G   
   A   
    A    
   C   
   T   
    C    
   T   
   T   
    A    
   A   
    A    
   A   
    C    
   T   
    G    
   A   
   T   
   T   
   G   
    A    
    A    
   G   
   A   
    T    
   T   
   T   
    A    
    A    
    C    
    T    
   G   
   A   
   C   
   A   
   T   
   T   
    C    
   T   
    G    
   G   
   C   
    A    
    A    
   G   
    A    
   C   
   C   
    A    
   G   
   A   
    A    
   C   
   G   
    T    
   G   
   T   
    T    
   A   
    C    
    A    
   G   
   A   
   A   
   A   
   T   
    C    
   A   
   T   
    C    
   A   
   A   
    G    
   A   
    A    
    T    
   G   
   A   
    A    
   T   
   T   
   A   
    G    
    C    
    A    
   G   
   A   
   C   
    T    
   T   
    G    
    A    
    A    
    T    
    C    
    A    
   A   
    C    
    G    
   A   
   T   
   T   
   T   
   G   
   G   
   T   
   G   
   A   
   T   
    A    
    A    
   A   
   C   
   G   
   A   
   C   
   G   
    C    
   A   
   C   
   A   
   G   
   A   
   A   
    T    
   T   
   A   
    C    
   T   
   A   
   G   
   T   
    C    
   G   
   G   
   T   
   G   
   A   
    G    
   G   
   T   
   C   
    T    
   T   
    A    
   A   
   G   
   T   
   C   
   T   
    G    
   G   
   A   
   A   
   G   
   A   
   T   
   G   
   A   
   A   
   G   
   A   
   T   
    T    
   T   
    A    
   A   
   T   
    C    
   G   
   A   
   A   
   G   
   A   
   A   
   G   
   A   
   A   
   G   
   A   
   A   
    A    
   T   
   C   
   G   
   T   
    T    
   A   
   T   
   T   
   A   
   C   
    A    
    T    
   T   
    G    
   A   
   C   
    A    
   A   
   A   
    C    
   A   
   A   
    T    
   G   
   G   
    T    
   T   
   A   
    C    
   A   
   T   
    C    
   A   
   A   
    A    
   C   
   G   
    T    
    T    
   T   
   G   
   G   
    C    
   A   
   A   
   A   
    T    
    A    
    A    
    T    
   G   
   A   
   A   
   T   
   T   
   C   
    A    
   G   
    A    
   G   
   C   
    T    
   C   
   A   
   A   
   C   
   G   
    A    
    A    
   G   
    G    
   G   
   G   
    T    
   G   
   G   
    A    
   C   
   G   
   T   
   G   
   G   
    T    
   G   
   T   
    C    
   C   
   A   
   A   
   G   
   G   
    A    
   A   
   T   
   G   
   G   
   G   
    T    
    A    
   T   
    C    
   C   
   A   
    C    
    A    
   A   
   T   
   G   
   A   
    C    
   G   
   A   
    C    
   T   
   T   
   C   
   G   
   T   
    C    
   A   
   A   
    A    
   A   
   A   
    T    
   C   
   T   
    G    
    G    
   T   
   T   
   T   
   C   
   T   
   T   
   G   
    T    
   T   
   C   
    T    
   A   
   C   
    C    
   C   
   A   
    T    
   G   
   A   
    T    
   A   
   C   
    A    
   T   
   T   
    G    
   T   
   T   
   A   
   T   
   T   
    C    
   T   
   T   
   T   
   A   
   C   
   G   
   A   
   A   
    T    
   A   
    A    
    C    
   G   
   G   
    A    
   A   
   A   
   A   
   G   
   T   
    G    
   T   
   A   
    T    
   C   
   G   
    G    
   G   
   C   
   A   
   A   
   A   
   A   
   G   
   G   
    A    
   T   
   A   
    C    
   G   
   A   
   A   
   A   
   T   
    T    
   C   
   C   
    A    
   G   
   A   
    A    
   T   
   A   
   C   
   G   
   G   
    G    
    C    
   G   
    T    
   A   
   C   
   A   
   G   
   C   
    C    
   A   
   A   
   A   
   G   
   G   
    A    
   A   
   T   
    C    
   C   
   C   
   A   
    A    
   T   
    C    
   A   
   T   
    C    
   A   
   A   
    T    
   T   
   T   
    A    
   C   
   T   
    A    
   G   
   G   
    T    
   A   
   T   
    C    
   G   
   A   
    T    
   T   
   C   
    G    
    A    
    G    
    T    
   G   
   A   
   A   
   A   
    G    
    C    
   A   
   T   
    C    
   C   
   A   
    G    
   G   
   C   
    A    
   A   
   T   
    C    
   A   
   T   
   T   
    G    
   C   
    G    
   G   
   T   
    G    
    A    
    C    
   G   
   G   
   G   
    A    
    G    
   A   
   A   
   G   
   C   
    A    
   G   
   A   
   A   
   G   
    A    
    A    
   G   
   G   
    C    
    C    
   A   
    C    
   T   
   A   
    T    
    C    
   T   
    C    
   T   
   T   
   C   
   T   
   T   
    T    
   A   
   C   
    G    
   A   
   C   
    G    
    C    
    G    
    T    
    C    
   A   
   A   
   G   
   G   
    T    
   A   
   C   
    A    
   G   
   T   
    A    
   A   
   A   
   A   
   C   
   G   
    T    
   A   
   C   
    T    
    T    
   C   
    T    
   G   
   T   
    C    
   A   
   C   
    T    
   G   
   C   
    T    
   T   
   T   
    T    
    G    
   C   
   T   
   A   
   A   
    C    
   A   
   T   
    C    
   C   
   G   
    A    
   A   
   G   
    T    
   A   
   A   
   T   
   G   
   G   
    T    
    C    
   T   
    G    
   A   
   T   
    C    
   G   
   C   
    A    
   A   
   T   
   T   
    G    
   G   
    T    
   T   
   T   
    G    
   A   
   A   
   A   
   G   
   A   
   A   
   G   
   A   
    C    
   G   
   A   
    T    
   G   
   A   
    G    
    C    
   T   
    C    
    G    
   T   
    C    
   A   
   A   
    T    
   G   
   T   
    G    
    C    
   T   
   A   
    C    
    T    
   G   
   A   
   C   
    A    
    G    
   A   
    T    
   G   
   G   
    T    
    C    
   A   
    A    
    T    
    C    
    T    
   A   
   A   
    T    
   A   
   T   
    C    
   A   
   T   
    T    
   A   
   T   
    T    
   G   
   G   
    A    
   A   
   C   
   A   
   C   
   A   
   T   
    A    
    A    
    C    
   G   
   G   
    C    
   T   
   A   
   C   
   T   
   C   
   T   
   G   
   T   
    C    
   A   
   C   
    T    
   T   
   T   
    C    
    A    
   A   
    A    
   G   
   A   
    A    
    G    
    A    
    A    
    G    
   C   
    A    
   G   
    T    
    C    
   C   
   G   
    C    
   G   
   A   
   T   
   A   
   T   
   G   
   G   
   G   
    A    
   C   
   G   
    T    
   A   
   C   
   A   
   G   
   C   
    T    
   T   
   C   
    C    
   G   
   G   
    G    
   G   
   T   
    C    
    A    
   G   
    A    
   G   
   G   
    G    
   A   
   T   
    A    
   C   
   G   
    C    
    C    
   T   
    G    
   C   
   G   
    T    
   G   
   A   
    G    
    G    
   A   
    A    
   G   
   A   
    T    
   T   
    A    
    T    
   G   
   T   
    C    
   G   
   T   
    T    
   G   
   G   
    G    
    G    
   C   
   A   
   G   
   C   
    C    
    T    
   T   
    A    
    A    
   T   
   G   
    A    
   A   
    G    
   G   
    A    
   A   
    A    
   A   
    T    
    C    
   A   
   A   
   G   
   A   
   A   
   G   
   T   
    A    
    T    
   T   
    A    
   G   
   T   
    G    
   A   
   T   
    C    
   A   
   C   
    A    
   G   
   A   
   A   
   A   
   A   
    A    
   G   
   G   
   T   
   T   
   A   
    C    
   G   
   G   
    A    
   A   
   A   
    A    
   C   
   G   
    G    
   A   
   C   
    A    
   A   
   A   
   A   
   G   
    T    
    A    
   T   
   C   
    G    
   G   
   A   
    A    
   T   
   A   
   T   
   C   
   C   
   A   
   G   
   T   
    G    
   A   
   A   
    A    
   G   
   G   
    C    
   C   
   G   
    C    
   G   
   G   
    A    
   G   
   G   
   T   
   A   
   A   
   A   
   G   
   G   
    G    
   A   
   T   
    C    
   A   
   A   
    A    
   A   
   C   
    G    
    G    
   C   
    G    
   A   
   A   
    C    
   A   
   T   
   C   
   A   
   C   
   T   
   G   
   A   
    A    
   A   
   A   
   A   
   A   
   A   
   T   
   G   
   G   
   T   
   C   
   C   
   A   
    C    
   T   
   A   
   G   
   C   
    T    
   G   
   G   
    A    
   T   
   T   
    G    
   A   
   C   
   C   
   A   
   C   
    T    
   G   
   T   
    T    
    T    
    C    
   T   
   G   
   G   
    A    
    G    
   A   
   T   
   G   
   A   
   A   
   G   
   A   
    C    
   A   
   T   
   C   
    A    
   T   
    G    
    C    
   T   
    G    
   A   
   T   
    T    
   A   
   C   
    A    
    G    
   A   
    T    
   A   
   A   
   A   
   G   
   G   
    T    
   G   
   T   
    C    
   A   
   T   
   T   
   A   
   T   
   C   
   C   
   G   
    T    
   T   
   T   
    C    
   A   
   A   
   C   
   G   
   T   
    A    
    T    
    C    
    G    
    A    
   C   
   T   
   G   
   T   
   T   
   T   
   C   
   T   
   C   
   A   
   A   
   A   
   C   
    T    
   G   
   G   
   A   
   C   
   G   
    T    
    T    
   C   
   A   
   A   
   C   
   A   
   T   
   T   
    A    
   G   
   G   
    C    
   G   
   T   
    A    
   C   
   G   
   T   
   T   
   T   
    G    
   A   
   T   
   G   
   A   
    A    
    G    
   A   
   T   
   G   
   G   
   A   
   A   
   G   
    C    
    T    
   G   
    A    
    T    
    A    
   C   
    G    
   A   
   A   
   A   
   G   
   T   
   T   
   G   
   T   
   C   
   A   
   C   
   G   
   A   
   T   
   G   
   G   
   C   
   A   
   G   
   C   
   A   
   G   
   T   
   C   
   G   
   A   
   A   
   C   
   C   
   A   
   G   
   A   
   A   
   G   
   C   
   T   
   G   
   C   
   A   
   G   
   A   
   A   
   C   
   A   
   A   
   A   
   C   
   A   
   G   
   T   
   T   
   G   
   A   
   A   
   G   
   A   
   A   
   C   
   A   
   A   
   G   
   G   
   A   
   T   
   C   
   A   
   G   
   A   
   A   
   G   
   A   
   A   
   -   
   -   
   -   
 

 
   N55072   
   A   
   T   
   G   
   A   
   G   
   T   
   G   
   A   
   A   
   G   
   A   
   A   
   A   
   T   
   C   
   A   
   G   
   A   
   G   
   A   
   A   
   A   
   A   
   C   
   A   
   T   
   C   
   C   
   A   
   T   
   G   
   A   
   T   
   G   
   T   
   C   
   A   
   A   
   T   
   C   
   T   
   A   
   A   
   C   
   C   
   A   
   G   
   C   
   G   
   A   
   A   
   A   
   T   
   G   
   A   
   A   
   A   
   G   
   A   
   C   
   T   
   C   
   C   
   T   
   T   
   C   
   A   
   T   
   C   
   G   
   A   
   T   
   T   
   A   
   T   
   G   
   C   
   G   
   A   
   T   
   G   
   A   
   G   
   T   
   G   
   T   
   T   
   A   
   T   
   T   
   G   
   T   
   C   
   G   
   C   
   A   
   C   
   G   
   T   
   G   
   C   
   A   
   C   
   T   
   T   
   C   
   C   
   T   
   G   
   A   
   T   
   G   
   T   
   C   
   A   
   G   
   A   
   G   
   A   
   T   
   G   
   G   
   T   
   C   
   T   
   G   
   A   
   A   
   A   
   C   
   C   
   C   
   G   
   T   
   T   
   C   
   A   
   T   
   C   
   G   
   C   
   C   
   G   
   T   
   A   
   T   
   T   
   T   
   T   
   A   
   T   
   A   
   C   
   G   
   G   
   C   
   A   
   T   
   G   
   A   
   A   
   C   
   G   
   A   
   A   
   T   
   T   
   A   
   G   
   G   
   T   
   G   
   T   
   A   
   A   
   C   
   A   
   C   
   C   
   G   
   G   
   A   
   T   
   A   
   A   
   A   
   G   
   C   
   C   
   C   
   A   
   T   
   A   
   A   
   A   
   A   
   A   
   A   
   T   
   C   
   T   
   G   
   C   
   C   
   A   
   G   
   A   
   A   
   T   
   C   
   G   
   T   
   C   
   G   
   G   
   A   
   G   
   A   
   T   
   G   
   T   
   C   
   A   
   T   
   G   
   G   
   G   
   T   
   A   
   A   
   A   
   T   
   A   
   C   
   C   
   A   
   T   
   C   
   C   
   T   
   C   
   A   
   T   
   G   
   G   
   A   
   G   
   A   
   C   
   A   
   G   
   T   
   G   
   C   
   G   
   A   
   T   
   C   
   T   
   A   
   T   
   G   
   A   
   G   
   T   
   C   
   A   
   A   
   T   
   G   
   G   
   T   
   A   
   C   
   G   
   G   
   A   
   T   
   G   
   G   
   C   
   A   
   C   
   A   
   G   
   C   
   C   
   A   
   T   
   T   
   C   
   A   
   G   
   C   
   T   
   A   
   C   
   C   
   G   
   T   
   T   
   A   
   T   
   A   
   T   
   G   
   C   
   T   
   A   
   G   
   T   
   G   
   G   
   A   
   T   
   G   
   G   
   T   
   C   
   A   
   T   
   G   
   G   
   A   
   A   
   A   
   C   
   T   
   T   
   T   
   G   
   G   
   T   
   T   
   C   
   T   
   G   
   T   
   T   
   G   
   A   
   C   
   G   
   G   
   T   
   G   
   A   
   C   
   G   
   G   
   T   
   G   
   C   
   C   
   G   
   C   
   C   
   G   
   C   
   T   
   A   
   T   
   G   
   C   
   G   
   T   
   T   
   A   
   T   
   A   
   C   
   C   
   G   
   A   
   A   
   G   
   C   
   A   
   C   
   G   
   G   
   A   
   T   
   G   
   A   
   G   
   C   
   A   
   A   
   A   
   A   
   T   
   C   
   G   
   C   
   T   
   A   
   C   
   A   
   G   
   A   
   A   
   A   
   T   
   G   
   C   
   T   
   G   
   C   
   G   
   T   
   G   
   A   
   T   
   A   
   T   
   C   
   A   
   A   
   T   
   A   
   A   
   G   
   A   
   A   
   C   
   A   
   C   
   T   
   G   
   T   
   T   
   G   
   A   
   T   
   T   
   T   
   C   
   C   
   A   
   A   
   A   
   G   
   C   
   A   
   A   
   C   
   T   
   A   
   T   
   G   
   A   
   T   
   G   
   A   
   T   
   A   
   C   
   A   
   G   
   A   
   A   
   A   
   A   
   A   
   G   
   A   
   A   
   C   
   C   
   A   
   G   
   T   
   T   
   G   
   T   
   A   
   C   
   T   
   A   
   C   
   C   
   T   
   G   
   C   
   T   
   C   
   G   
   C   
   T   
   T   
   C   
   C   
   C   
   G   
   A   
   A   
   T   
   C   
   T   
   T   
   T   
   T   
   A   
   G   
   T   
   G   
   A   
   A   
   C   
   G   
   G   
   A   
   A   
   C   
   A   
   A   
   C   
   G   
   G   
   G   
   G   
   A   
   T   
   C   
   G   
   C   
   C   
   G   
   T   
   A   
   G   
   G   
   T   
   A   
   T   
   G   
   G   
   C   
   C   
   A   
   C   
   G   
   A   
   A   
   T   
   A   
   T   
   C   
   C   
   C   
   A   
   C   
   C   
   T   
   C   
   A   
   T   
   A   
   A   
   C   
   T   
   T   
   G   
   T   
   C   
   T   
   G   
   A   
   G   
   G   
   T   
   A   
   G   
   T   
   A   
   G   
   C   
   G   
   G   
   C   
   T   
   A   
   T   
   C   
   G   
   A   
   T   
   T   
   T   
   A   
   C   
   T   
   G   
   A   
   T   
   G   
   G   
   A   
   T   
   A   
   A   
   T   
   C   
   C   
   T   
   G   
   A   
   T   
   G   
   T   
   G   
   A   
   C   
   G   
   A   
   C   
   C   
   A   
   A   
   T   
   G   
   A   
   A   
   T   
   T   
   G   
   A   
   T   
   G   
   G   
   A   
   A   
   G   
   T   
   A   
   T   
   T   
   A   
   C   
   C   
   T   
   G   
   G   
   A   
   C   
   C   
   A   
   G   
   A   
   T   
   T   
   T   
   T   
   C   
   C   
   A   
   A   
   C   
   A   
   G   
   G   
   T   
   G   
   G   
   T   
   T   
   T   
   G   
   G   
   T   
   T   
   A   
   T   
   G   
   G   
   G   
   G   
   A   
   A   
   A   
   T   
   C   
   A   
   G   
   G   
    A    
   A   
   T   
    C    
   C   
   G   
    A    
    A    
   G   
   A   
   G   
   C   
    G    
   T   
   A   
   T   
   G   
   A   
   A   
   A   
   C   
    A    
   G   
   G   
    T    
   A   
   A   
    G    
   G   
   G   
   T   
   T   
   C   
    T    
   A   
   T   
   T   
   A   
   C   
    C    
   G   
   T   
    A    
   C   
   G   
   T   
   G   
   C   
   A   
   A   
   A   
   A   
   G   
   T   
   T   
   G   
   A   
    G    
    A    
   T   
    T    
   A   
    C    
   T   
   G   
   A   
    G    
   A   
   T   
   G   
    C    
   C   
    A    
   A   
   A   
   T   
   G   
   G   
    T    
   A   
   A   
   A   
   G   
   A   
   A   
   C   
   G   
    G    
   A   
   T   
    C    
    A    
   T   
    C    
   G   
   T   
    G    
   A   
   C   
    A    
   G   
   A   
   A   
   T   
   T   
    G    
   C   
   C   
   T   
   T   
   A   
   T   
   A   
   T   
   G   
   G   
   T   
    C    
   A   
   A   
   T   
   A   
   A   
   A   
   G   
   C   
    A    
   A   
   A   
   A   
   T   
   T   
    G    
   A   
   T   
    C    
   G   
   A   
   A   
   C   
   G   
    G    
   A   
   T   
   T   
   T   
   C   
   T   
   G   
   A   
    A    
    C    
   T   
    G    
   C   
   A   
   C   
   A   
   G   
   A   
   G   
   A   
    C    
   A   
   A   
    G    
   C   
   G   
    T    
   A   
   T   
    C    
   G   
   A   
   A   
   G   
   G   
    G    
   A   
   T   
    C    
   A   
   C   
    A    
   G   
   A   
   T   
   T   
   T   
   G   
   C   
   G   
   G   
   G   
   A   
    C    
   G   
   A   
   A   
   T   
   C   
   T   
   T   
   C   
    C    
   C   
   G   
   T   
   G   
   A   
    A    
   G   
   G   
    T    
   A   
   T   
   G   
   C   
   G   
    G    
   A   
   T   
    C    
   G   
   T   
   C   
   A   
   T   
    T    
   G   
   A   
    C    
    A    
   T   
    C    
   C   
   G   
   T   
   C   
   G   
    T    
   G   
   A   
    C    
   G   
    C    
    C    
   A   
   G   
    C    
   G   
   C   
    A    
   T   
   C   
    C    
   G   
   T   
   T   
    A    
   T   
    C    
   T   
   T   
    G    
   A   
   A   
    T    
   A   
   A   
   C   
   T   
   T   
    A    
   T   
   A   
    T    
   A   
   A   
   A   
    T    
   T   
   G   
   A   
   C   
    A    
    T    
   C   
    C    
   T   
   T   
    G    
   C   
   A   
    A    
   A   
    A    
    T    
   T   
   C   
    A    
   T   
   T   
    T    
   G   
   G   
    A    
   T   
   T   
    C    
   A   
   A   
    T    
   A   
   T   
   G   
   T   
   T   
    G    
   G   
   C   
    G    
   A   
   T   
    C    
   G   
   A   
   A   
   A   
   A   
   A   
   G   
   G   
    T    
   G   
   T   
    G    
   C   
   C   
    G    
   A   
   A   
   A   
    G    
   T   
   T   
   T   
   T   
    G    
   A   
   G   
    C    
   T   
   T   
   G   
   A   
   A   
   A   
   C   
    A    
    A    
   A   
   T   
    C    
    C    
   T   
    T    
   G   
   A   
   A   
   A   
   A   
    C    
   T   
   A   
    T    
    A    
   T   
    C    
   G   
   A   
    A    
   C   
   A   
    T    
   C   
   A   
    A    
    C    
    G    
    C    
    G    
   A   
   A   
   G   
   T   
   C   
   A   
   T   
    C    
   A   
   C   
    A    
    C    
   G   
    A    
   C   
   G   
   G   
   A   
   C   
    G    
    G    
   T   
    C    
   T   
   T   
   T   
   G   
   A   
    A    
   A   
   A   
   A   
    G    
   A   
    A    
   A   
   A   
    A    
   G   
   C   
   A   
   G   
   A   
   A   
   G   
   C   
   A   
    A    
   G   
    A    
   G   
   C   
    A    
   C   
   A   
    C    
   A   
   T   
    C    
   T   
   T   
   A   
   G   
   A   
   A   
   G   
   G   
   T   
    T    
   T   
    G    
   C   
   G   
    T    
   A   
   T   
   T   
   G   
   C   
    T    
   T   
   T   
    G    
   G   
   A   
   T   
   C   
   A   
   T   
   A   
   T   
   C   
    G    
   A   
   T   
   G   
   A   
   A   
   A   
   T   
    C    
   A   
   T   
   T   
   G   
   C   
    G    
   A   
   T   
   T   
   A   
   T   
    C    
   C   
   G   
    C    
   G   
   G   
    G    
   T   
   C   
    T    
    A    
   A   
   A   
   T   
   C   
   A   
   G   
   A   
   T   
   G   
   A   
    C    
   G   
   A   
    G    
   G   
   C   
   A   
   A   
   A   
   A   
   G   
   C   
    A    
   A   
    C    
    A    
    A    
   T   
    G    
   A   
   T   
    C    
   G   
   A   
   A   
   C   
   G   
    G    
   T   
   T   
    C    
   G   
   A   
    T    
   T   
   T   
    A    
   T   
   C   
    C    
   G   
   A   
   T   
   C   
   G   
    C    
   C   
   A   
    G    
    T    
   C   
    T    
   C   
   A   
   A   
   G   
   C   
    T    
   A   
   T   
    T    
   T   
   T   
   A   
   G   
   A   
   T   
   A   
   T   
   G   
   C   
   G   
   T   
   T   
   T   
    A    
   C   
   G   
    C    
   C   
   G   
    A    
   T   
   T   
    G    
   A   
   C   
    C    
   G   
   G   
    T    
   T   
   T   
    G    
   G   
   A   
   A   
   C   
   G   
    G    
   G   
   A   
    A    
   A   
   A   
   A   
   A   
   T   
    C    
   G   
   A   
    A    
   A   
   A   
   T   
   G   
   A   
   A   
   T   
   A   
    C    
   C   
   A   
   A   
   G   
   A   
    A    
   C   
   T   
    C    
   T   
   T   
    A    
   A   
    A    
   A   
    C    
   T   
    G    
   A   
   T   
   T   
   G   
    A    
    A    
   G   
   A   
    T    
   T   
   T   
    A    
    A    
    C    
    T    
   G   
   A   
   C   
   A   
   T   
   T   
    C    
   T   
    G    
   G   
   C   
    A    
    A    
   G   
    A    
   C   
   C   
    A    
   G   
   A   
    A    
   C   
   G   
    T    
   G   
   T   
    T    
   A   
    C    
    A    
   G   
   A   
   A   
   A   
   T   
    C    
   A   
   T   
    C    
   A   
   A   
    G    
   A   
    A    
    T    
   G   
   A   
    A    
   T   
   T   
   A   
    G    
    C    
    A    
   G   
   A   
   C   
    T    
   T   
    G    
    A    
    A    
    T    
    C    
    A    
   A   
    C    
    G    
   A   
   T   
   T   
   T   
   G   
   G   
   T   
   G   
   A   
   T   
    A    
    A    
   A   
   C   
   G   
   A   
   C   
   G   
    C    
   A   
   C   
   A   
   G   
   A   
   A   
    T    
   T   
   A   
    C    
   T   
   A   
   G   
   T   
    C    
   G   
   G   
   T   
   G   
   A   
    G    
   G   
   T   
   C   
    T    
   T   
    A    
   A   
   G   
   T   
   C   
   T   
    G    
   G   
   A   
   A   
   G   
   A   
   T   
   G   
   A   
   A   
   G   
   A   
   T   
    T    
   T   
    A    
   A   
   T   
    C    
   G   
   A   
   A   
   G   
   A   
   A   
   G   
   A   
   A   
   G   
   A   
   A   
    A    
   T   
   C   
   G   
   T   
    T    
   A   
   T   
   T   
   A   
   C   
    A    
    T    
   T   
    G    
   A   
   C   
    A    
   A   
   A   
    C    
   A   
   A   
    T    
   G   
   G   
    T    
   T   
   A   
    C    
   A   
   T   
    C    
   A   
   A   
    A    
   C   
   G   
    T    
    T    
   T   
   G   
   G   
    C    
   A   
   A   
   A   
    T    
    A    
    A    
    T    
   G   
   A   
   A   
   T   
   T   
   C   
    A    
   G   
    A    
   G   
   C   
    T    
   C   
   A   
   A   
   C   
   G   
    A    
    A    
   G   
    G    
   G   
   G   
    T    
   G   
   G   
    A    
   C   
   G   
   T   
   G   
   G   
    T    
   G   
   T   
    C    
   C   
   A   
   A   
   G   
   G   
    A    
   A   
   T   
   G   
   G   
   G   
    T    
    A    
   T   
    C    
   C   
   A   
    C    
    A    
   A   
   T   
   G   
   A   
    C    
   G   
   A   
    C    
   T   
   T   
   C   
   G   
   T   
    C    
   A   
   A   
    A    
   A   
   A   
    T    
   C   
   T   
    G    
    G    
   T   
   T   
   T   
   C   
   T   
   T   
   G   
    T    
   T   
   C   
    T    
   A   
   C   
    C    
   C   
   A   
    T    
   G   
   A   
    T    
   A   
   C   
    A    
   T   
   T   
    G    
   T   
   T   
   A   
   T   
   T   
    C    
   T   
   T   
   T   
   A   
   C   
   G   
   A   
   A   
    T    
   A   
    A    
    C    
   G   
   G   
    A    
   A   
   A   
   A   
   G   
   T   
    G    
   T   
   A   
    T    
   C   
   G   
    G    
   G   
   C   
   A   
   A   
   A   
   A   
   G   
   G   
    A    
   T   
   A   
    C    
   G   
   A   
   A   
   A   
   T   
    T    
   C   
   C   
    A    
   G   
   A   
    A    
   T   
   A   
   C   
   G   
   G   
    G    
    C    
   G   
    T    
   A   
   C   
   A   
   G   
   C   
    C    
   A   
   A   
   A   
   G   
   G   
    A    
   A   
   T   
    C    
   C   
   C   
   A   
    A    
   T   
    C    
   A   
   T   
    C    
   A   
   A   
    T    
   T   
   T   
    A    
   C   
   T   
    A    
   G   
   G   
    T    
   A   
   T   
    C    
   G   
   A   
    T    
   T   
   C   
    G    
    A    
    G    
    T    
   G   
   A   
   A   
   A   
    G    
    C    
   A   
   T   
    C    
   C   
   A   
    G    
   G   
   C   
    A    
   A   
   T   
    C    
   A   
   T   
   T   
    G    
   C   
    G    
   G   
   T   
    G    
    A    
    C    
   G   
   G   
   G   
    A    
    G    
   A   
   A   
   G   
   C   
    A    
   G   
   A   
   A   
   G   
    A    
    A    
   G   
   G   
    C    
    C    
   A   
    C    
   T   
   A   
    T    
    C    
   T   
    C    
   T   
   T   
   C   
   T   
   T   
    T    
   A   
   C   
    G    
   A   
   C   
    G    
    C    
    G    
    T    
    C    
   A   
   A   
   G   
   G   
    T    
   A   
   C   
    A    
   G   
   T   
    A    
   A   
   A   
   A   
   C   
   G   
    T    
   A   
   C   
    T    
    T    
   C   
    T    
   G   
   T   
    C    
   A   
   C   
    T    
   G   
   C   
    T    
   T   
   T   
    T    
    G    
   C   
   T   
   A   
   A   
    C    
   A   
   T   
    C    
   C   
   G   
    A    
   A   
   G   
    T    
   A   
   A   
   T   
   G   
   G   
    T    
    C    
   T   
    G    
   A   
   T   
    C    
   G   
   C   
    A    
   A   
   T   
   T   
    G    
   G   
    T    
   T   
   T   
    G    
   A   
   A   
   A   
   G   
   A   
   A   
   G   
   A   
    C    
   G   
   A   
    T    
   G   
   A   
    G    
    C    
   T   
    C    
    G    
   T   
    C    
   A   
   A   
    T    
   G   
   T   
    G    
    C    
   T   
   A   
    C    
    T    
   G   
   A   
   C   
    A    
    G    
   A   
    T    
   G   
   G   
    T    
    C    
   A   
    A    
    T    
    C    
    T    
   A   
   A   
    T    
   A   
   T   
    C    
   A   
   T   
    T    
   A   
   T   
    T    
   G   
   G   
    A    
   A   
   C   
   A   
   C   
   A   
   T   
    A    
    A    
    C    
   G   
   G   
    C    
   T   
   A   
   C   
   T   
   C   
   T   
   G   
   T   
    C    
   A   
   C   
    T    
   T   
   T   
    C    
    A    
   A   
    A    
   G   
   A   
    A    
    G    
    A    
    A    
    G    
   C   
    A    
   G   
    T    
    C    
   C   
   G   
    C    
   G   
   A   
   T   
   A   
   T   
   G   
   G   
   G   
    A    
   C   
   G   
    T    
   A   
   C   
   A   
   G   
   C   
    T    
   T   
   C   
    C    
   G   
   G   
    G    
   G   
   T   
    C    
    A    
   G   
    A    
   G   
   G   
    G    
   A   
   T   
    A    
   C   
   G   
    C    
    C    
   T   
    G    
   C   
   G   
    T    
   G   
   A   
    G    
    G    
   A   
    A    
   G   
   A   
    T    
   T   
    A    
    T    
   G   
   T   
    C    
   G   
   T   
    T    
   G   
   G   
    G    
    A    
   C   
   A   
   G   
   C   
    C    
    T    
   T   
    A    
    A    
   T   
   G   
    A    
   A   
    G    
   G   
    A    
   A   
    A    
   A   
    T    
    C    
   A   
   A   
   G   
   A   
   A   
   G   
   T   
    A    
    T    
   T   
    A    
   G   
   T   
    G    
   A   
   T   
    C    
   A   
   C   
    A    
   G   
   A   
   A   
   A   
   A   
    A    
   G   
   G   
   T   
   T   
   A   
    C    
   G   
   G   
    A    
   A   
   A   
    A    
   C   
   G   
    G    
   A   
   C   
    A    
   A   
   A   
   A   
   G   
    T    
    A    
   T   
   C   
    G    
   G   
   A   
    A    
   T   
   A   
   T   
   C   
   C   
   A   
   G   
   T   
    G    
   A   
   A   
    A    
   G   
   G   
    C    
   C   
   G   
    C    
   G   
   G   
    A    
   G   
   G   
   T   
   A   
   A   
   A   
   G   
   G   
    G    
   A   
   T   
    C    
   A   
   A   
    A    
   A   
   C   
    G    
    G    
   C   
    G    
   A   
   A   
    C    
   A   
   T   
   C   
   A   
   C   
   T   
   G   
   A   
    A    
   A   
   A   
   A   
   A   
   A   
   T   
   G   
   G   
   T   
   C   
   C   
   A   
    C    
   T   
   A   
   G   
   C   
    T    
   G   
   G   
    A    
   T   
   T   
    G    
   A   
   C   
   C   
   A   
   C   
    T    
   G   
   T   
    T    
    T    
    C    
   T   
   G   
   G   
    A    
    G    
   A   
   T   
   G   
   A   
   A   
   G   
   A   
    C    
   A   
   T   
   C   
    A    
   T   
    G    
    C    
   T   
    G    
   A   
   T   
    T    
   A   
   C   
    A    
    G    
   A   
    T    
   A   
   A   
   A   
   G   
   G   
    T    
   G   
   T   
    C    
   A   
   T   
   T   
   A   
   T   
   C   
   C   
   G   
    T    
   T   
   T   
    C    
   A   
   A   
   C   
   G   
   T   
    A    
    T    
    C    
    G    
    A    
   C   
   T   
   G   
   T   
   T   
   T   
   C   
   T   
   C   
   A   
   A   
   A   
   C   
    T    
   G   
   G   
   A   
   C   
   G   
    T    
    T    
   C   
   A   
   A   
   C   
   A   
   T   
   T   
    A    
   G   
   G   
    C    
   G   
   T   
    A    
   C   
   G   
   T   
   T   
   T   
    G    
   A   
   T   
   G   
   A   
    A    
    G    
   A   
   T   
   G   
   G   
   A   
   A   
   G   
    C    
    T    
   G   
    A    
    T    
    A    
   C   
    G    
   A   
   A   
   A   
   G   
   T   
   T   
   G   
   T   
   C   
   A   
   C   
   G   
   A   
   T   
   G   
   G   
   C   
   A   
   G   
   C   
   A   
   G   
   T   
   C   
   G   
   A   
   A   
   C   
   C   
   A   
   G   
   A   
   A   
   G   
   C   
   T   
   G   
   C   
   A   
   G   
   A   
   A   
   C   
   A   
   A   
   A   
   C   
   A   
   G   
   T   
   T   
   G   
   A   
   A   
   G   
   A   
   A   
   C   
   A   
   A   
   G   
   G   
   A   
   T   
   C   
   A   
   G   
   A   
   A   
   G   
   A   
   A   
   -   
   -   
   -   
 

 
   N54550   
   A   
   T   
   G   
   A   
   G   
   T   
   G   
   A   
   A   
   G   
   A   
   A   
   A   
   T   
   C   
   A   
   G   
   A   
   G   
   A   
   A   
   A   
   A   
   C   
   A   
   T   
   C   
   C   
   A   
   T   
   G   
   A   
   T   
   G   
   T   
   C   
   A   
   A   
   T   
   C   
   T   
   A   
   A   
   C   
   C   
   A   
   G   
   C   
   G   
   A   
   A   
   A   
   T   
   G   
   A   
   A   
   A   
   G   
   A   
   C   
   T   
   C   
   C   
   T   
   T   
   C   
   A   
   T   
   C   
   G   
   A   
   T   
   T   
   A   
   T   
   G   
   C   
   G   
   A   
   T   
   G   
   A   
   G   
   T   
   G   
   T   
   T   
   A   
   T   
   T   
   G   
   T   
   C   
   G   
   C   
   A   
   C   
   G   
   T   
   G   
   C   
   A   
   C   
   T   
   T   
   C   
   C   
   T   
   G   
   A   
   T   
   G   
   T   
   C   
   A   
   G   
   A   
   G   
   A   
   T   
   G   
   G   
   T   
   C   
   T   
   G   
   A   
   A   
   A   
   C   
   C   
   C   
   G   
   T   
   T   
   C   
   A   
   C   
   C   
   G   
   C   
   C   
   G   
   T   
   A   
   T   
   T   
   T   
   T   
   A   
   T   
   A   
   C   
   G   
   G   
   C   
   A   
   T   
   G   
   A   
   A   
   C   
   G   
   A   
   A   
   T   
   T   
   A   
   G   
   G   
   T   
   G   
   T   
   A   
   A   
   C   
   A   
   C   
   C   
   G   
   G   
   A   
   T   
   A   
   A   
   A   
   G   
   C   
   C   
   C   
   A   
   T   
   A   
   A   
   A   
   A   
   A   
   A   
   T   
   C   
   T   
   G   
   C   
   C   
   A   
   G   
   A   
   A   
   T   
   C   
   G   
   T   
   C   
   G   
   G   
   A   
   G   
   A   
   T   
   G   
   T   
   C   
   A   
   T   
   G   
   G   
   G   
   T   
   A   
   A   
   A   
   T   
   A   
   C   
   C   
   A   
   T   
   C   
   C   
   T   
   C   
   A   
   T   
   G   
   G   
   A   
   G   
   A   
   C   
   A   
   G   
   T   
   G   
   C   
   G   
   A   
   T   
   C   
   T   
   A   
   T   
   G   
   A   
   G   
   T   
   C   
   A   
   A   
   T   
   G   
   G   
   T   
   A   
   C   
   G   
   G   
   A   
   T   
   G   
   G   
   C   
   G   
   C   
   A   
   G   
   C   
   C   
   A   
   T   
   T   
   C   
   A   
   G   
   C   
   T   
   A   
   C   
   C   
   G   
   T   
   T   
   A   
   T   
   A   
   T   
   G   
   C   
   T   
   A   
   G   
   T   
   G   
   G   
   A   
   T   
   G   
   G   
   T   
   C   
   A   
   T   
   G   
   G   
   A   
   A   
   A   
   C   
   T   
   T   
   T   
   G   
   G   
   T   
   T   
   C   
   T   
   G   
   T   
   T   
   G   
   A   
   C   
   G   
   G   
   T   
   G   
   A   
   C   
   G   
   G   
   T   
   G   
   C   
   C   
   G   
   C   
   C   
   G   
   C   
   T   
   A   
   T   
   G   
   C   
   G   
   T   
   T   
   A   
   T   
   A   
   C   
   C   
   G   
   A   
   A   
   G   
   C   
   A   
   C   
   G   
   G   
   A   
   T   
   G   
   A   
   G   
   C   
   A   
   A   
   A   
   A   
   T   
   C   
   G   
   C   
   T   
   A   
   C   
   A   
   G   
   A   
   A   
   A   
   T   
   G   
   C   
   T   
   G   
   C   
   G   
   T   
   G   
   A   
   T   
   A   
   T   
   C   
   A   
   A   
   T   
   A   
   A   
   G   
   A   
   A   
   C   
   A   
   C   
   T   
   G   
   T   
   T   
   G   
   A   
   T   
   T   
   T   
   C   
   C   
   A   
   A   
   A   
   G   
   C   
   A   
   A   
   C   
   T   
   A   
   T   
   G   
   A   
   T   
   G   
   A   
   T   
   A   
   C   
   A   
   G   
   A   
   A   
   A   
   A   
   A   
   G   
   A   
   A   
   C   
   C   
   A   
   G   
   T   
   T   
   G   
   T   
   A   
   C   
   T   
   A   
   C   
   C   
   T   
   G   
   C   
   T   
   C   
   G   
   C   
   T   
   T   
   C   
   C   
   C   
   G   
   A   
   A   
   T   
   C   
   T   
   T   
   T   
   T   
   A   
   G   
   T   
   G   
   A   
   A   
   C   
   G   
   G   
   A   
   A   
   C   
   A   
   A   
   C   
   G   
   G   
   G   
   G   
   A   
   T   
   C   
   G   
   C   
   C   
   G   
   T   
   A   
   G   
   G   
   T   
   A   
   T   
   G   
   G   
   C   
   C   
   A   
   C   
   G   
   A   
   A   
   T   
   A   
   T   
   C   
   C   
   C   
   A   
   C   
   C   
   T   
   C   
   A   
   T   
   A   
   A   
   C   
   T   
   T   
   G   
   T   
   C   
   T   
   G   
   A   
   G   
   G   
   T   
   A   
   G   
   T   
   A   
   G   
   C   
   G   
   G   
   C   
   T   
   A   
   T   
   C   
   G   
   A   
   T   
   T   
   T   
   G   
   C   
   T   
   G   
   A   
   T   
   G   
   G   
   A   
   T   
   A   
   A   
   T   
   C   
   C   
   T   
   G   
   A   
   T   
   G   
   T   
   G   
   A   
   C   
   G   
   A   
   C   
   C   
   A   
   A   
   T   
   G   
   A   
   A   
   T   
   T   
   G   
   A   
   T   
   G   
   G   
   A   
   A   
   G   
   T   
   A   
   T   
   T   
   A   
   C   
   C   
   T   
   G   
   G   
   A   
   C   
   C   
   A   
   G   
   A   
   T   
   T   
   T   
   T   
   C   
   C   
   A   
   A   
   C   
   A   
   G   
   G   
   T   
   G   
   G   
   T   
   T   
   T   
   G   
   G   
   T   
   T   
   A   
   T   
   G   
   G   
   G   
   A   
   A   
   A   
   A   
   T   
   C   
   A   
   G   
   G   
    A    
   A   
   T   
    C    
   C   
   G   
    A    
    A    
   G   
   A   
   G   
   C   
    G    
   T   
   A   
   T   
   G   
   A   
   A   
   A   
   C   
    A    
   G   
   G   
    T    
   A   
   A   
    G    
   G   
   G   
   T   
   T   
   C   
    T    
   A   
   T   
   T   
   A   
   C   
    C    
   G   
   T   
    A    
   C   
   G   
   T   
   G   
   C   
   A   
   A   
   A   
   A   
   G   
   T   
   T   
   G   
   A   
    G    
    A    
   T   
    T    
   A   
    C    
   T   
   G   
   A   
    G    
   A   
   T   
   G   
    C    
   C   
    A    
   A   
   A   
   T   
   G   
   G   
    T    
   A   
   A   
   A   
   G   
   A   
   A   
   C   
   G   
    G    
   A   
   T   
    C    
    A    
   T   
    C    
   G   
   T   
    G    
   A   
   C   
    A    
   G   
   A   
   A   
   T   
   T   
    G    
   C   
   C   
   T   
   T   
   A   
   T   
   A   
   T   
   G   
   G   
   T   
    C    
   A   
   A   
   T   
   A   
   A   
   A   
   G   
   C   
    A    
   A   
   A   
   A   
   T   
   T   
    G    
   A   
   T   
    C    
   G   
   A   
   A   
   C   
   G   
    G    
   A   
   T   
   T   
   T   
   C   
   T   
   G   
   A   
    A    
    C    
   T   
    G    
   C   
   A   
   C   
   A   
   G   
   A   
   G   
   A   
    C    
   A   
   A   
    G    
   C   
   G   
    T    
   A   
   T   
    C    
   G   
   A   
   A   
   G   
   G   
    G    
   A   
   T   
    C    
   A   
   C   
    A    
   G   
   A   
   T   
   T   
   T   
   G   
   C   
   G   
   G   
   G   
   A   
    C    
   G   
   A   
   A   
   T   
   C   
   T   
   T   
   C   
    C    
   C   
   G   
   T   
   G   
   A   
    A    
   G   
   G   
    T    
   A   
   T   
   G   
   C   
   G   
    G    
   A   
   T   
    C    
   G   
   T   
   C   
   A   
   T   
    T    
   G   
   A   
    C    
    A    
   T   
    C    
   C   
   G   
   T   
   C   
   G   
    T    
   G   
   A   
    C    
   G   
    C    
    C    
   A   
   G   
    C    
   G   
   C   
    A    
   T   
   C   
    C    
   G   
   T   
   T   
    A    
   T   
    C    
   T   
   T   
    G    
   A   
   A   
    T    
   A   
   A   
   C   
   T   
   T   
    A    
   T   
   A   
    T    
   A   
   A   
   A   
    T    
   T   
   G   
   A   
   C   
    A    
    T    
   C   
    C    
   T   
   T   
    G    
   C   
   A   
    A    
   A   
    A    
    T    
   T   
   C   
    A    
   T   
   T   
    T    
   G   
   G   
    A    
   T   
   T   
    C    
   A   
   A   
    T    
   A   
   T   
   G   
   T   
   T   
    G    
   G   
   C   
    G    
   A   
   T   
    C    
   G   
   A   
   A   
   A   
   A   
   A   
   G   
   G   
    T    
   G   
   T   
    G    
   C   
   C   
    G    
   A   
   A   
   A   
    G    
   T   
   T   
   T   
   T   
    G    
   A   
   G   
    C    
   T   
   T   
   G   
   A   
   A   
   A   
   C   
    A    
    A    
   A   
   T   
    T    
    C    
   T   
    T    
   G   
   A   
   A   
   A   
   A   
    C    
   T   
   A   
    T    
    A    
   T   
    C    
   G   
   A   
    A    
   C   
   A   
    T    
   C   
   A   
    G    
    C    
    G    
    C    
    G    
   A   
   A   
   G   
   T   
   C   
   A   
   T   
    C    
   A   
   C   
    A    
    A    
   G   
    A    
   C   
   G   
   G   
   A   
   C   
    A    
    G    
   T   
    C    
   T   
   T   
   T   
   G   
   A   
    A    
   A   
   A   
   A   
    G    
   A   
    A    
   A   
   A   
    A    
   G   
   C   
   A   
   G   
   A   
   A   
   G   
   C   
   A   
    A    
   G   
    G    
   G   
   C   
    A    
   C   
   A   
    C    
   A   
   T   
    T    
   T   
   T   
   A   
   G   
   A   
   A   
   G   
   G   
   T   
    T    
   T   
    G    
   C   
   G   
    T    
   A   
   T   
   T   
   G   
   C   
    T    
   T   
   T   
    G    
   G   
   A   
   T   
   C   
   A   
   T   
   A   
   T   
   C   
    G    
   A   
   T   
   G   
   A   
   A   
   A   
   T   
    A    
   A   
   T   
   T   
   G   
   C   
    G    
   A   
   T   
   T   
   A   
   T   
    C    
   C   
   G   
    C    
   G   
   G   
    G    
   T   
   C   
    T    
    A    
   A   
   A   
   T   
   C   
   A   
   G   
   A   
   T   
   G   
   A   
    C    
   G   
   A   
    A    
   G   
   C   
   A   
   A   
   A   
   A   
   G   
   C   
    G    
   A   
    C    
    G    
    A    
   T   
    G    
   A   
   T   
    C    
   G   
   A   
   A   
   C   
   G   
    G    
   T   
   T   
    C    
   G   
   A   
    T    
   T   
   T   
    A    
   T   
   C   
    C    
   G   
   A   
   T   
   C   
   G   
    C    
   C   
   A   
    G    
    T    
   C   
    T    
   C   
   A   
   A   
   G   
   C   
    T    
   A   
   T   
    T    
   T   
   T   
   A   
   G   
   A   
   T   
   A   
   T   
   G   
   C   
   G   
   T   
   T   
   T   
    A    
   C   
   G   
    C    
   C   
   G   
    A    
   T   
   T   
    G    
   A   
   C   
    C    
   G   
   G   
    T    
   T   
   T   
    G    
   G   
   A   
   A   
   C   
   G   
    G    
   G   
   A   
    A    
   A   
   A   
   A   
   A   
   T   
    C    
   G   
   A   
    A    
   A   
   A   
   T   
   G   
   A   
   A   
   T   
   A   
    C    
   C   
   A   
   A   
   G   
   A   
    A    
   C   
   T   
    C    
   T   
   T   
    A    
   A   
    A    
   A   
    C    
   T   
    G    
   A   
   T   
   T   
   G   
    A    
    A    
   G   
   A   
    T    
   T   
   T   
    A    
    A    
    C    
    T    
   G   
   A   
   C   
   A   
   T   
   T   
    C    
   T   
    G    
   G   
   C   
    A    
    A    
   G   
    A    
   C   
   C   
    A    
   G   
   A   
    A    
   C   
   G   
    T    
   G   
   T   
    T    
   A   
    C    
    A    
   G   
   A   
   A   
   A   
   T   
    C    
   A   
   T   
    C    
   A   
   A   
    G    
   A   
    A    
    T    
   G   
   A   
    A    
   T   
   T   
   A   
    G    
    C    
    A    
   G   
   A   
   C   
    T    
   T   
    G    
    A    
    A    
    T    
    C    
    A    
   A   
    C    
    G    
   A   
   T   
   T   
   T   
   G   
   G   
   T   
   G   
   A   
   T   
    A    
    A    
   A   
   C   
   G   
   A   
   C   
   G   
    C    
   A   
   C   
   A   
   G   
   A   
   A   
    T    
   T   
   A   
    C    
   T   
   A   
   G   
   T   
    C    
   G   
   G   
   T   
   G   
   A   
    G    
   G   
   T   
   C   
    T    
   T   
    A    
   A   
   G   
   T   
   C   
   T   
    G    
   G   
   A   
   A   
   G   
   A   
   T   
   G   
   A   
   A   
   G   
   A   
   T   
    T    
   T   
    A    
   A   
   T   
    C    
   G   
   A   
   A   
   G   
   A   
   A   
   G   
   A   
   A   
   G   
   A   
   A   
    A    
   T   
   C   
   G   
   T   
    T    
   A   
   T   
   T   
   A   
   C   
    A    
    T    
   T   
    G    
   A   
   C   
    A    
   A   
   A   
    C    
   A   
   A   
    T    
   G   
   G   
    T    
   T   
   A   
    C    
   A   
   T   
    C    
   A   
   A   
    A    
   C   
   G   
    T    
    T    
   T   
   G   
   G   
    C    
   A   
   A   
   A   
    T    
    A    
    A    
    T    
   G   
   A   
   A   
   T   
   T   
   C   
    A    
   G   
    A    
   G   
   C   
    T    
   C   
   A   
   A   
   C   
   G   
    A    
    A    
   G   
    G    
   G   
   G   
    T    
   G   
   G   
    A    
   C   
   G   
   T   
   G   
   G   
    T    
   G   
   T   
    C    
   C   
   A   
   A   
   G   
   G   
    A    
   A   
   T   
   G   
   G   
   G   
    T    
    A    
   T   
    C    
   C   
   A   
    C    
    A    
   A   
   T   
   G   
   A   
    C    
   G   
   A   
    C    
   T   
   T   
   C   
   G   
   T   
    C    
   A   
   A   
    A    
   A   
   A   
    T    
   C   
   T   
    G    
    G    
   T   
   T   
   T   
   C   
   T   
   T   
   G   
    T    
   T   
   C   
    T    
   A   
   C   
    C    
   C   
   A   
    T    
   G   
   A   
    T    
   A   
   C   
    A    
   T   
   T   
    G    
   T   
   T   
   A   
   T   
   T   
    C    
   T   
   T   
   T   
   A   
   C   
   G   
   A   
   A   
    T    
   A   
    A    
    C    
   G   
   G   
    A    
   A   
   A   
   A   
   G   
   T   
    G    
   T   
   A   
    T    
   C   
   G   
    G    
   G   
   C   
   A   
   A   
   A   
   A   
   G   
   G   
    A    
   T   
   A   
    C    
   G   
   A   
   A   
   A   
   T   
    T    
   C   
   C   
    A    
   G   
   A   
    A    
   T   
   A   
   C   
   G   
   G   
    G    
    C    
   G   
    T    
   A   
   C   
   A   
   G   
   C   
    C    
   A   
   A   
   A   
   G   
   G   
    A    
   A   
   T   
    A    
   C   
   C   
   A   
    A    
   T   
    T    
   A   
   T   
    C    
   A   
   A   
    T    
   T   
   T   
    A    
   C   
   T   
    A    
   G   
   G   
    T    
   A   
   T   
    C    
   G   
   A   
    T    
   T   
   C   
    G    
    A    
    G    
    T    
   G   
   A   
   A   
   A   
    G    
    C    
   A   
   T   
    C    
   C   
   A   
    G    
   G   
   C   
    A    
   A   
   T   
    C    
   A   
   T   
   T   
    G    
   C   
    G    
   G   
   T   
    G    
    A    
    C    
   G   
   G   
   G   
    A    
    G    
   A   
   A   
   G   
   C   
    A    
   G   
   A   
   A   
   G   
    A    
    A    
   G   
   G   
    C    
    C    
   A   
    C    
   T   
   A   
    T    
    C    
   T   
    C    
   T   
   T   
   C   
   T   
   T   
    T    
   A   
   C   
    G    
   A   
   C   
    G    
    C    
    G    
    T    
    C    
   A   
   A   
   G   
   G   
    T    
   A   
   C   
    A    
   G   
   T   
    A    
   A   
   A   
   A   
   C   
   G   
    T    
   A   
   C   
    T    
    T    
   C   
    T    
   G   
   T   
    C    
   A   
   C   
    T    
   G   
   C   
    T    
   T   
   T   
    T    
    G    
   C   
   T   
   A   
   A   
    C    
   A   
   T   
    C    
   C   
   G   
    A    
   A   
   G   
    T    
   A   
   A   
   T   
   G   
   G   
    T    
    C    
   T   
    G    
   A   
   T   
    C    
   G   
   C   
    A    
   A   
   T   
   T   
    G    
   G   
    T    
   T   
   T   
    G    
   A   
   A   
   A   
   G   
   A   
   A   
   G   
   A   
    C    
   G   
   A   
    T    
   G   
   A   
    G    
    C    
   T   
    C    
    G    
   T   
    C    
   A   
   A   
    T    
   G   
   T   
    G    
    C    
   T   
   A   
    C    
    T    
   G   
   A   
   C   
    A    
    G    
   A   
    T    
   G   
   G   
    T    
    C    
   A   
    A    
    T    
    C    
    T    
   A   
   A   
    T    
   A   
   T   
    C    
   A   
   T   
    T    
   A   
   T   
    T    
   G   
   G   
    A    
   A   
   C   
   A   
   C   
   A   
   T   
    A    
    A    
    C    
   G   
   G   
    C    
   T   
   A   
   C   
   T   
   C   
   T   
   G   
   T   
    C    
   A   
   C   
    T    
   T   
   T   
    C    
    A    
   A   
    A    
   G   
   A   
    A    
    G    
    A    
    A    
    G    
   C   
    A    
   G   
    T    
    C    
   C   
   G   
    C    
   G   
   A   
   T   
   A   
   T   
   G   
   G   
   G   
    A    
   C   
   G   
    T    
   A   
   C   
   A   
   G   
   C   
    T    
   T   
   C   
    C    
   G   
   G   
    G    
   G   
   T   
    C    
    A    
   G   
    A    
   G   
   G   
    G    
   A   
   T   
    A    
   C   
   G   
    C    
    C    
   T   
    G    
   C   
   G   
    T    
   G   
   A   
    G    
    G    
   A   
    A    
   G   
   A   
    T    
   T   
    A    
    T    
   G   
   T   
    C    
   G   
   T   
    T    
   G   
   G   
    G    
    G    
   C   
   A   
   G   
   C   
    C    
    T    
   T   
    A    
    A    
   T   
   G   
    A    
   A   
    G    
   G   
    A    
   A   
    A    
   A   
    T    
    C    
   A   
   A   
   G   
   A   
   A   
   G   
   T   
    A    
    T    
   T   
    A    
   G   
   T   
    G    
   A   
   T   
    C    
   A   
   C   
    A    
   G   
   A   
   A   
   A   
   A   
    A    
   G   
   G   
   T   
   T   
   A   
    C    
   G   
   G   
    A    
   A   
   A   
    A    
   C   
   G   
    G    
   A   
   C   
    A    
   A   
   A   
   A   
   G   
    T    
    A    
   T   
   C   
    G    
   G   
   A   
    A    
   T   
   A   
   T   
   C   
   C   
   A   
   G   
   T   
    G    
   A   
   A   
    A    
   G   
   G   
    C    
   C   
   G   
    C    
   G   
   G   
    A    
   G   
   G   
   T   
   A   
   A   
   A   
   G   
   G   
    G    
   A   
   T   
    C    
   A   
   A   
    A    
   A   
   C   
    G    
    G    
   C   
    G    
   A   
   A   
    C    
   A   
   T   
   C   
   A   
   C   
   T   
   G   
   A   
    A    
   A   
   A   
   A   
   A   
   A   
   T   
   G   
   G   
   T   
   C   
   C   
   A   
    C    
   T   
   A   
   G   
   C   
    T    
   G   
   G   
    A    
   T   
   T   
    G    
   A   
   C   
   C   
   A   
   C   
    T    
   G   
   T   
    T    
    T    
    C    
   T   
   G   
   G   
    A    
    G    
   A   
   T   
   G   
   A   
   A   
   G   
   A   
    C    
   A   
   T   
   C   
    A    
   T   
    G    
    C    
   T   
    G    
   A   
   T   
    T    
   A   
   C   
    A    
    G    
   A   
    T    
   A   
   A   
   A   
   G   
   G   
    T    
   G   
   T   
    C    
   A   
   T   
   T   
   A   
   T   
   C   
   C   
   G   
    T    
   T   
   T   
    C    
   A   
   A   
   C   
   G   
   T   
    A    
    T    
    C    
    G    
    A    
   C   
   T   
   G   
   T   
   T   
   T   
   C   
   T   
   C   
   A   
   A   
   A   
   C   
    T    
   G   
   G   
   A   
   C   
   G   
    T    
    T    
   C   
   A   
   A   
   C   
   A   
   T   
   T   
    A    
   G   
   G   
    C    
   G   
   T   
    A    
   C   
   G   
   T   
   T   
   T   
    G    
   A   
   T   
   G   
   A   
    A    
    G    
   A   
   T   
   G   
   G   
   A   
   A   
   G   
    C    
    T    
   G   
    A    
    T    
    A    
   C   
    G    
   A   
   A   
   A   
   G   
   T   
   T   
   G   
   T   
   C   
   A   
   C   
   G   
   A   
   T   
   G   
   G   
   C   
   A   
   G   
   C   
   A   
   G   
   T   
   C   
   G   
   A   
   A   
   C   
   C   
   A   
   G   
   A   
   A   
   G   
   C   
   T   
   G   
   C   
   A   
   G   
   A   
   A   
   C   
   A   
   A   
   A   
   C   
   A   
   G   
   T   
   T   
   G   
   A   
   A   
   G   
   A   
   A   
   C   
   A   
   A   
   G   
   G   
   A   
   T   
   C   
   A   
   G   
   A   
   A   
   G   
   A   
   A   
   -   
   -   
   -   
 

 
   N60114F   
   A   
   T   
   G   
   A   
   G   
   T   
   G   
   A   
   A   
   G   
   A   
   A   
   A   
   T   
   T   
   A   
   A   
   A   
   G   
   A   
   A   
   A   
   A   
   C   
   A   
   T   
   T   
   C   
   A   
   A   
   G   
   A   
   C   
   G   
   T   
   C   
   A   
   A   
   T   
   C   
   T   
   G   
   A   
   C   
   C   
   A   
   G   
   C   
   G   
   A   
   A   
   A   
   T   
   G   
   A   
   A   
   A   
   G   
   A   
   A   
   T   
   C   
   T   
   T   
   T   
   C   
   A   
   T   
   T   
   G   
   A   
   C   
   T   
   A   
   C   
   G   
   C   
   A   
   A   
   T   
   G   
   A   
   G   
   T   
   G   
   T   
   T   
   A   
   T   
   C   
   G   
   T   
   A   
   G   
   C   
   C   
   C   
   G   
   C   
   G   
   C   
   G   
   T   
   T   
   A   
   C   
   C   
   T   
   G   
   A   
   C   
   G   
   T   
   T   
   C   
   G   
   T   
   G   
   A   
   C   
   G   
   G   
   T   
   T   
   T   
   A   
   A   
   A   
   A   
   C   
   C   
   T   
   G   
   T   
   T   
   C   
   A   
   T   
   C   
   G   
   C   
   C   
   G   
   A   
   A   
   T   
   C   
   T   
   T   
   A   
   T   
   A   
   T   
   G   
   G   
   A   
   A   
   T   
   G   
   A   
   A   
   C   
   G   
   A   
   A   
   T   
   T   
   A   
   G   
   G   
   G   
   G   
   T   
   A   
   A   
   C   
   C   
   C   
   C   
   T   
   G   
   A   
   T   
   A   
   A   
   A   
   C   
   C   
   A   
   C   
   A   
   C   
   A   
   A   
   A   
   A   
   A   
   A   
   T   
   C   
   A   
   G   
   C   
   C   
   C   
   G   
   G   
   A   
   T   
   T   
   G   
   T   
   T   
   G   
   G   
   G   
   G   
   A   
   T   
   G   
   T   
   T   
   A   
   T   
   G   
   G   
   G   
   T   
   A   
   A   
   A   
   T   
   A   
   T   
   C   
   A   
   C   
   C   
   C   
   C   
   C   
   A   
   T   
   G   
   G   
   G   
   G   
   A   
   C   
   A   
   G   
   T   
   G   
   C   
   G   
   A   
   T   
   T   
   T   
   A   
   C   
   G   
   A   
   A   
   T   
   C   
   A   
   A   
   T   
   G   
   G   
   T   
   G   
   C   
   G   
   G   
   A   
   T   
   G   
   G   
   C   
   A   
   C   
   A   
   A   
   C   
   C   
   T   
   T   
   T   
   T   
   A   
   G   
   T   
   T   
   A   
   T   
   C   
   G   
   G   
   G   
   C   
   T   
   A   
   T   
   G   
   T   
   T   
   A   
   G   
   T   
   T   
   G   
   A   
   C   
   G   
   G   
   C   
   C   
   A   
   C   
   G   
   G   
   A   
   A   
   A   
   C   
   T   
   T   
   C   
   G   
   G   
   T   
   T   
   C   
   T   
   G   
   T   
   C   
   G   
   A   
   T   
   G   
   G   
   T   
   G   
   A   
   C   
   G   
   G   
   C   
   G   
   C   
   T   
   G   
   C   
   C   
   G   
   C   
   T   
   A   
   T   
   G   
   C   
   G   
   T   
   T   
   A   
   T   
   A   
   C   
   C   
   G   
   A   
   A   
   G   
   C   
   A   
   C   
   G   
   T   
   A   
   T   
   G   
   A   
   G   
   T   
   A   
   A   
   A   
   A   
   T   
   T   
   G   
   C   
   T   
   T   
   T   
   A   
   G   
   A   
   A   
   A   
   T   
   G   
   C   
   T   
   A   
   C   
   G   
   A   
   G   
   A   
   T   
   A   
   T   
   T   
   A   
   A   
   C   
   A   
   A   
   A   
   A   
   A   
   T   
   A   
   C   
   A   
   G   
   T   
   C   
   G   
   A   
   T   
   T   
   T   
   C   
   C   
   A   
   A   
   G   
   G   
   A   
   A   
   A   
   C   
   T   
   A   
   T   
   G   
   A   
   T   
   G   
   A   
   T   
   T   
   C   
   A   
   G   
   A   
   A   
   C   
   A   
   A   
   G   
   A   
   A   
   C   
   C   
   A   
   G   
   T   
   A   
   G   
   T   
   T   
   T   
   T   
   A   
   C   
   C   
   T   
   G   
   C   
   T   
   C   
   G   
   T   
   T   
   T   
   T   
   C   
   C   
   A   
   A   
   A   
   C   
   T   
   T   
   A   
   C   
   T   
   A   
   G   
   T   
   T   
   A   
   A   
   C   
   G   
   G   
   A   
   A   
   C   
   A   
   A   
   C   
   G   
   G   
   G   
   G   
   A   
   T   
   T   
   G   
   C   
   G   
   G   
   T   
   G   
   G   
   G   
   G   
   A   
   T   
   G   
   G   
   C   
   A   
   A   
   C   
   A   
   A   
   A   
   T   
   A   
   T   
   T   
   C   
   C   
   A   
   C   
   C   
   A   
   C   
   A   
   C   
   A   
   A   
   T   
   T   
   T   
   A   
   A   
   G   
   T   
   G   
   A   
   A   
   G   
   T   
   G   
   A   
   T   
   T   
   G   
   A   
   T   
   G   
   C   
   A   
   A   
   C   
   A   
   A   
   G   
   T   
   T   
   T   
   A   
   T   
   T   
   G   
   A   
   T   
   G   
   G   
   A   
   C   
   A   
   A   
   T   
   C   
   C   
   T   
   G   
   A   
   T   
   G   
   T   
   A   
   A   
   C   
   G   
   A   
   C   
   G   
   A   
   A   
   T   
   G   
   A   
   A   
   T   
   T   
   G   
   A   
   T   
   G   
   G   
   A   
   A   
   G   
   T   
   G   
   T   
   T   
   A   
   C   
   C   
   T   
   G   
   G   
   A   
   C   
   C   
   A   
   G   
   A   
   T   
   T   
   T   
   T   
   C   
   C   
   A   
   A   
   C   
   A   
   G   
   G   
   C   
   G   
   G   
   T   
   T   
   T   
   A   
   G   
   T   
   G   
   A   
   T   
   G   
   G   
   G   
   G   
   A   
   A   
   A   
   T   
   C   
   A   
   G   
   G   
    G    
   A   
   T   
    T    
   C   
   G   
    C    
    C    
   G   
   A   
   G   
   C   
    A    
   T   
   A   
   T   
   G   
   A   
   A   
   A   
   C   
    A    
   G   
   G   
    G    
   A   
   A   
    A    
   G   
   G   
   T   
   T   
   C   
    G    
   A   
   T   
   T   
   A   
   C   
    T    
   G   
   T   
    T    
   C   
   G   
   T   
   G   
   C   
   A   
   A   
   A   
   A   
   G   
   T   
   T   
   G   
   A   
    A    
    T    
   T   
    G    
   A   
    C    
   T   
   G   
   A   
    A    
   A   
   T   
   G   
    C    
   C   
    G    
   A   
   A   
   T   
   G   
   G   
    A    
   A   
   A   
   A   
   G   
   A   
   A   
   C   
   G   
    T    
   A   
   T   
    T    
    T    
   T   
    A    
   G   
   T   
    A    
   A   
   C   
    T    
   G   
   A   
   A   
   T   
   T   
    G    
   C   
   C   
   T   
   T   
   A   
   T   
   A   
   T   
   G   
   G   
   T   
    G    
   A   
   A   
   T   
   A   
   A   
   A   
   G   
   C   
    C    
   A   
   A   
   A   
   T   
   T   
    A    
   A   
   T   
    C    
   G   
   A   
   A   
   C   
   G   
    A    
   A   
   T   
   T   
   T   
   C   
   T   
   G   
   A   
    A    
    T    
   T   
    A    
   C   
   A   
   C   
   A   
   G   
   A   
   G   
   A   
    T    
   A   
   A   
    A    
   C   
   G   
    A    
   A   
   T   
    T    
   G   
   A   
   A   
   G   
   G   
    A    
   A   
   T   
    T    
   A   
   C   
    T    
   G   
   A   
   T   
   T   
   T   
   G   
   C   
   G   
   G   
   G   
   A   
    T    
   G   
   A   
   A   
   T   
   C   
   T   
   T   
   C   
    T    
   C   
   G   
   T   
   G   
   A   
    A    
   G   
   G   
    C    
   A   
   T   
   G   
   C   
   G   
    G    
   A   
   T   
    C    
   G   
   T   
   C   
   A   
   T   
    C    
   G   
   A   
    T    
    G    
   T   
    T    
   C   
   G   
   T   
   C   
   G   
    A    
   G   
   A   
    T    
   G   
    T    
    G    
   A   
   G   
    T    
   G   
   C   
    C    
   T   
   C   
    T    
   G   
   T   
   T   
    G    
   T   
    G    
   T   
   T   
    A    
   A   
   A   
    C    
   A   
   A   
   C   
   T   
   T   
    G    
   T   
   A   
    C    
   A   
   A   
   A   
    A    
   T   
   G   
   A   
   C   
    T    
    G    
   C   
    C    
   T   
   T   
    A    
   C   
   A   
    A    
   A   
    C    
    A    
   T   
   C   
    T    
   T   
   T   
    T    
   G   
   G   
    T    
   T   
   T   
    T    
   A   
   A   
    C    
   A   
   T   
   G   
   T   
   T   
    A    
   G   
   C   
    C    
   A   
   T   
    T    
   G   
   A   
   A   
   A   
   A   
   A   
   G   
   G   
    C    
   G   
   T   
    A    
   C   
   C   
    G    
   A   
   A   
   A   
    A    
   T   
   T   
   T   
   T   
    A    
   A   
   G   
    C    
   T   
   T   
   G   
   A   
   A   
   A   
   C   
    G    
    T    
   A   
   T   
    T    
    T    
   T   
    A    
   G   
   A   
   A   
   A   
   A   
    T    
   T   
   A   
    C    
    G    
   T   
    C    
   G   
   A   
    G    
   C   
   A   
    C    
   C   
   A   
    A    
    A    
    A    
    A    
    G    
   A   
   A   
   G   
   T   
   C   
   A   
   T   
    T    
   A   
   C   
    T    
    C    
   G   
    C    
   C   
   G   
   G   
   A   
   C   
    G    
    A    
   T   
    T    
   T   
   T   
   T   
   G   
   A   
    C    
   A   
   A   
   A   
    A    
   A   
    C    
   A   
   A   
    A    
   G   
   C   
   A   
   G   
   A   
   A   
   G   
   C   
   A   
    C    
   G   
    G    
   G   
   C   
    G    
   C   
   A   
    T    
   A   
   T   
    T    
   T   
   T   
   A   
   G   
   A   
   A   
   G   
   G   
   T   
    C    
   T   
    A    
   C   
   G   
    A    
   A   
   T   
   T   
   G   
   C   
    C    
   T   
   T   
    A    
   G   
   A   
   T   
   C   
   A   
   T   
   A   
   T   
   C   
    G    
   A   
   T   
   G   
   A   
   A   
   A   
   T   
    C    
   A   
   T   
   T   
   G   
   C   
    C    
   A   
   T   
   T   
   A   
   T   
    C    
   C   
   G   
    T    
   G   
   G   
    A    
   T   
   C   
    A    
    C    
   A   
   A   
   T   
   C   
   A   
   G   
   A   
   T   
   G   
   A   
    C    
   G   
   A   
    A    
   G   
   C   
   A   
   A   
   A   
   A   
   G   
   C   
    A    
   A   
    C    
    G    
    T    
   T   
    A    
   A   
   T   
    T    
   G   
   A   
   A   
   C   
   G   
    C    
   T   
   T   
    T    
   G   
   A   
    A    
   T   
   T   
    T    
   T   
   C   
    A    
   G   
   A   
   T   
   C   
   G   
    T    
   C   
   A   
    A    
    G    
   C   
    G    
   C   
   A   
   A   
   G   
   C   
    G    
   A   
   T   
    T    
   T   
   T   
   A   
   G   
   A   
   T   
   A   
   T   
   G   
   C   
   G   
   T   
   T   
   T   
    A    
   C   
   G   
    C    
   C   
   G   
    T    
   T   
   T   
    A    
   A   
   C   
    A    
   G   
   G   
    C    
   T   
   T   
    A    
   G   
   A   
   A   
   C   
   G   
    C    
   G   
   A   
    C    
   A   
   A   
   A   
   A   
   T   
    T    
   G   
   A   
    A    
   A   
   A   
   T   
   G   
   A   
   A   
   T   
   A   
    T    
   C   
   A   
   A   
   G   
   A   
    G    
   C   
   T   
    A    
   T   
   T   
    G    
   A   
    A    
   A   
    T    
   T   
    C    
   A   
   T   
   T   
   G   
    C    
    G    
   G   
   A   
    C    
   T   
   T   
    A    
    G    
    A    
    G    
   G   
   A   
   C   
   A   
   T   
   T   
    T    
   T   
    A    
   G   
   C   
    C    
    C    
   G   
    C    
   C   
   C   
    A    
   G   
   A   
    A    
   C   
   G   
    C    
   G   
   T   
    C    
   A   
    T    
    T    
   G   
   A   
   A   
   A   
   T   
    C    
   A   
   T   
    T    
   A   
   A   
    A    
   A   
    C    
    A    
   G   
   A   
    G    
   T   
   T   
   A   
    A    
    A    
    T    
   G   
   A   
   C   
    G    
   T   
    T    
    C    
    G    
    C    
    A    
    C    
   A   
    A    
    A    
   A   
   T   
   T   
   T   
   G   
   G   
   T   
   G   
   A   
   T   
    G    
    C    
   A   
   C   
   G   
   A   
   C   
   G   
    C    
   A   
   C   
   A   
   G   
   A   
   A   
    T    
   T   
   A   
    T    
   T   
   A   
   G   
   T   
    A    
   G   
   G   
   T   
   G   
   A   
    A    
   G   
   T   
   C   
    C    
   T   
    A    
   A   
   G   
   T   
   C   
   T   
    T    
   G   
   A   
   A   
   G   
   A   
   T   
   G   
   A   
   A   
   G   
   A   
   T   
    T    
   T   
    A    
   A   
   T   
    C    
   G   
   A   
   A   
   G   
   A   
   A   
   G   
   A   
   A   
   G   
   A   
   A   
    G    
   T   
   C   
   G   
   T   
    G    
   A   
   T   
   T   
   A   
   C   
    G    
    T    
   T   
    A    
   A   
   C   
    C    
   A   
   A   
    T    
   A   
   A   
    C    
   G   
   G   
    C    
   T   
   A   
    C    
   A   
   T   
    T    
   A   
   A   
    G    
   C   
   G   
    G    
    A    
   T   
   G   
   G   
    C    
   A   
   A   
   A   
    C    
    T    
    C    
    C    
   G   
   A   
   A   
   T   
   T   
   C   
    C    
   G   
    G    
   G   
   C   
    G    
   C   
   A   
   A   
   C   
   G   
    C    
    C    
   G   
    T    
   G   
   G   
    C    
   G   
   G   
    A    
   C   
   G   
   T   
   G   
   G   
    T    
   G   
   T   
    C    
   C   
   A   
   A   
   G   
   G   
    C    
   A   
   T   
   G   
   G   
   G   
    C    
    G    
   T   
    T    
   C   
   A   
    T    
    G    
   A   
   T   
   G   
   A   
    T    
   G   
   A   
    T    
   T   
   T   
   C   
   G   
   T   
    G    
   A   
   A   
    A    
   A   
   A   
    C    
   C   
   T   
    A    
    G    
   T   
   T   
   T   
   C   
   T   
   T   
   G   
    T    
   T   
   C   
    A    
   A   
   C   
    A    
   C   
   A   
    C    
   G   
   A   
    C    
   A   
   C   
    G    
   T   
   T   
    A    
   T   
   T   
   A   
   T   
   T   
    C    
   T   
   T   
   T   
   A   
   C   
   G   
   A   
   A   
    C    
   A   
    C    
    T    
   G   
   G   
    G    
   A   
   A   
   A   
   G   
   T   
    T    
   T   
   A   
    C    
   C   
   G   
    A    
   G   
   C   
   A   
   A   
   A   
   A   
   G   
   G   
    T    
   T   
   A   
    C    
   G   
   A   
   A   
   A   
   T   
    C    
   C   
   C   
    T    
   G   
   A   
    A    
   T   
   A   
   C   
   G   
   G   
    T    
    A    
   G   
    A    
   A   
   C   
   A   
   G   
   C   
    A    
   A   
   A   
   A   
   G   
   G   
    A    
   A   
   T   
    T    
   C   
   C   
   A   
    G    
   T   
    A    
   A   
   T   
    T    
   A   
   A   
    C    
   T   
   T   
    A    
   C   
   T   
    G    
   G   
   G   
    A    
   A   
   T   
    T    
   G   
   A   
    T    
   T   
   C   
    T    
    G    
    C    
    A    
   G   
   A   
   A   
   A   
    A    
    A    
   A   
   T   
    T    
   C   
   A   
    A    
   G   
   C   
    G    
   A   
   T   
    T    
   A   
   T   
   T   
    T    
   C   
    T    
   G   
   T   
    T    
    G    
    A    
   G   
   G   
   G   
    C    
    A    
   A   
   A   
   G   
   C   
    G    
   G   
   A   
   A   
   G   
    C    
    A    
   G   
   G   
    T    
    A    
   A   
    A    
   T   
   A   
    C    
    T    
   T   
    G    
   T   
   T   
   C   
   T   
   T   
    C    
   A   
   C   
    A    
   A   
   C   
    C    
    T    
    T    
    A    
    A    
   A   
   A   
   G   
   G   
    A    
   A   
   C   
    C    
   G   
   T   
    C    
   A   
   A   
   A   
   C   
   G   
    G    
   A   
   C   
    A    
    G    
   C   
    C    
   G   
   T   
    A    
   A   
   C   
    A    
   G   
   C   
    C    
   T   
   T   
    T    
    T    
   C   
   T   
   A   
   A   
    T    
   A   
   T   
    C    
   C   
   G   
    T    
   A   
   G   
    T    
   A   
   A   
   T   
   G   
   G   
    A    
    T    
   T   
    A    
   A   
   T   
    C    
   G   
   C   
    C    
   A   
   T   
   T   
    A    
   G   
    C    
   T   
   T   
    A    
   A   
   A   
   A   
   G   
   A   
   A   
   G   
   A   
    T    
   G   
   A   
    T    
   G   
   A   
    G    
    T    
   T   
    A    
    G    
   T   
    T    
   A   
   A   
    C    
   G   
   T   
    G    
    G    
   T   
   A   
    A    
    C    
   G   
   A   
   C   
    T    
    A    
   A   
    T    
   G   
   G   
    C    
    A    
   A   
    T    
    C    
    A    
    G    
   A   
   A   
    G    
   A   
   T   
    G    
   A   
   T   
    T    
   A   
   T   
    C    
   G   
   G   
    A    
   A   
   C   
   A   
   C   
   A   
   T   
    G    
    C    
    A    
   G   
   G   
    A    
   T   
   A   
   C   
   T   
   C   
   T   
   G   
   T   
    C    
   A   
   C   
    A    
   T   
   T   
    T    
    G    
   A   
    T    
   G   
   A   
    A    
    A    
    A    
    T    
    A    
   C   
    T    
   G   
    T    
    A    
   C   
   G   
    T    
   G   
   A   
   T   
   A   
   T   
   G   
   G   
   G   
    C    
   C   
   G   
    G    
   A   
   C   
   A   
   G   
   C   
    A    
   T   
   C   
    A    
   G   
   G   
    T    
   G   
   T   
    T    
    C    
   G   
    G    
   G   
   G   
    A    
   A   
   T   
    C    
   C   
   G   
    T    
    C    
   T   
    C    
   C   
   G   
    C    
   G   
   A   
    A    
    A    
   A   
    T    
   G   
   A   
    T    
   T   
    A    
    T    
   G   
   T   
    G    
   G   
   T   
    C    
   G   
   G   
    T    
    G    
   C   
   A   
   G   
   C   
    G    
    A    
   T   
    T    
    C    
   T   
   G   
    G    
   A   
    T    
   G   
    A    
   A   
    A    
   A   
    C    
    A    
   A   
   A   
   G   
   A   
   A   
   G   
   T   
    C    
    C    
   T   
    A    
   G   
   T   
    C    
   A   
   T   
    T    
   A   
   C   
    T    
   G   
   A   
   A   
   A   
   A   
    T    
   G   
   G   
   T   
   T   
   A   
    T    
   G   
   G   
    T    
   A   
   A   
    G    
   C   
   G   
    T    
   A   
   C   
    A    
   A   
   A   
   A   
   G   
    C    
    T    
   T   
   C   
    T    
   G   
   A   
    A    
   T   
   A   
   T   
   C   
   C   
   A   
   G   
   T   
    T    
   A   
   A   
    A    
   G   
   G   
    A    
   C   
   G   
    T    
   G   
   G   
    C    
   G   
   G   
   T   
   A   
   A   
   A   
   G   
   G   
    G    
   A   
   T   
    T    
   A   
   A   
    G    
   A   
   C   
    A    
    G    
   C   
    A    
   A   
   A   
    T    
   A   
   T   
   C   
   A   
   C   
   T   
   G   
   A   
    G    
   A   
   A   
   A   
   A   
   A   
   T   
   G   
   G   
   T   
   C   
   C   
   A   
    T    
   T   
   A   
   G   
   C   
    T    
   G   
   G   
    T    
   T   
   T   
    A    
   A   
   C   
   C   
   A   
   C   
    G    
   G   
   T   
    C    
    A    
    A    
   T   
   G   
   G   
    G    
    G    
   A   
   T   
   G   
   A   
   A   
   G   
   A   
    T    
   A   
   T   
   C   
    T    
   T   
    A    
    T    
   T   
    G    
   A   
   T   
    T    
   A   
   C   
    G    
    A    
   A   
    C    
   A   
   A   
   A   
   G   
   G   
    C    
   G   
   T   
    C    
   A   
   T   
   T   
   A   
   T   
   C   
   C   
   G   
    C    
   T   
   T   
    T    
   A   
   A   
   C   
   G   
   T   
    T    
    G    
    A    
    T    
    T    
   C   
   T   
   G   
   T   
   T   
   T   
   C   
   T   
   C   
   A   
   A   
   A   
   C   
    A    
   G   
   G   
   A   
   C   
   G   
    C    
    G    
   C   
   A   
   A   
   C   
   A   
   T   
   T   
    A    
   G   
   G   
    G    
   G   
   T   
    T    
   C   
   G   
   T   
   T   
   T   
    A    
   A   
   T   
   G   
   A   
    G    
    A    
   A   
   T   
   G   
   G   
   A   
   A   
   G   
    A    
    T    
   G   
    G    
    T    
    G    
   C   
    C    
   A   
   A   
   A   
   -   
   -   
   -   
   -   
   -   
   -   
   -   
   -   
   -   
   -   
   -   
   -   
   -   
   -   
   -   
   -   
   -   
   -   
   -   
   -   
   -   
   -   
   -   
   -   
   -   
   -   
   -   
   -   
   -   
   -   
   -   
   -   
   -   
   -   
   -   
   -   
   -   
   -   
   -   
   -   
   -   
   -   
   -   
   -   
   -   
   -   
   -   
   -   
   -   
   -   
   -   
   -   
   -   
   -   
   -   
   -   
   -   
   -   
   -   
   -   
   -   
   -   
   -   
   -   
   -   
   -   
   -   
   -   
   -   
   -   
   -   
   -   
 

 
   N52648   
   A   
   T   
   G   
   A   
   G   
   T   
   G   
   A   
   A   
   G   
   A   
   A   
   A   
   T   
   T   
   A   
   A   
   A   
   G   
   A   
   A   
   A   
   A   
   C   
   A   
   T   
   T   
   C   
   A   
   A   
   G   
   A   
   C   
   G   
   T   
   C   
   A   
   A   
   T   
   C   
   T   
   G   
   A   
   C   
   C   
   A   
   G   
   C   
   G   
   A   
   A   
   A   
   T   
   G   
   A   
   A   
   A   
   G   
   A   
   A   
   T   
   C   
   T   
   T   
   T   
   C   
   A   
   T   
   T   
   G   
   A   
   C   
   T   
   A   
   C   
   G   
   C   
   A   
   A   
   T   
   G   
   A   
   G   
   T   
   G   
   T   
   T   
   A   
   T   
   C   
   G   
   T   
   A   
   G   
   C   
   C   
   C   
   G   
   C   
   G   
   C   
   G   
   T   
   T   
   A   
   C   
   C   
   T   
   G   
   A   
   C   
   G   
   T   
   T   
   C   
   G   
   T   
   G   
   A   
   C   
   G   
   G   
   T   
   T   
   T   
   A   
   A   
   A   
   A   
   C   
   C   
   T   
   G   
   T   
   T   
   C   
   A   
   T   
   C   
   G   
   C   
   C   
   G   
   A   
   A   
   T   
   C   
   T   
   T   
   A   
   T   
   A   
   T   
   G   
   G   
   A   
   A   
   T   
   G   
   A   
   A   
   C   
   G   
   A   
   A   
   T   
   T   
   A   
   G   
   G   
   G   
   G   
   T   
   A   
   A   
   C   
   C   
   C   
   C   
   T   
   G   
   A   
   T   
   A   
   A   
   A   
   C   
   C   
   A   
   C   
   A   
   C   
   A   
   A   
   A   
   A   
   A   
   A   
   T   
   C   
   A   
   G   
   C   
   C   
   C   
   G   
   G   
   A   
   T   
   T   
   G   
   T   
   T   
   G   
   G   
   G   
   G   
   A   
   T   
   G   
   T   
   T   
   A   
   T   
   G   
   G   
   G   
   T   
   A   
   A   
   A   
   T   
   A   
   T   
   C   
   A   
   C   
   C   
   C   
   C   
   C   
   A   
   T   
   G   
   G   
   G   
   G   
   A   
   C   
   A   
   G   
   T   
   G   
   C   
   G   
   A   
   T   
   T   
   T   
   A   
   C   
   G   
   A   
   A   
   T   
   C   
   A   
   A   
   T   
   G   
   G   
   T   
   G   
   C   
   G   
   G   
   A   
   T   
   G   
   G   
   C   
   A   
   C   
   A   
   A   
   C   
   C   
   T   
   T   
   T   
   T   
   A   
   G   
   T   
   T   
   A   
   C   
   C   
   G   
   G   
   G   
   C   
   T   
   A   
   T   
   G   
   T   
   T   
   A   
   G   
   T   
   T   
   G   
   A   
   C   
   G   
   G   
   C   
   C   
   A   
   C   
   G   
   G   
   A   
   A   
   A   
   C   
   T   
   T   
   C   
   G   
   G   
   T   
   T   
   C   
   T   
   G   
   T   
   C   
   G   
   A   
   T   
   G   
   G   
   T   
   G   
   A   
   C   
   G   
   G   
   C   
   G   
   C   
   T   
   G   
   C   
   C   
   G   
   C   
   T   
   A   
   T   
   G   
   C   
   G   
   T   
   T   
   A   
   T   
   A   
   C   
   C   
   G   
   A   
   A   
   G   
   C   
   A   
   C   
   G   
   T   
   A   
   T   
   G   
   A   
   G   
   T   
   A   
   A   
   A   
   A   
   T   
   T   
   G   
   C   
   T   
   T   
   T   
   A   
   G   
   A   
   A   
   A   
   T   
   G   
   C   
   T   
   A   
   C   
   G   
   A   
   G   
   A   
   T   
   A   
   T   
   T   
   A   
   A   
   C   
   A   
   A   
   A   
   A   
   A   
   T   
   A   
   C   
   A   
   G   
   T   
   T   
   G   
   A   
   T   
   T   
   T   
   C   
   C   
   A   
   A   
   G   
   G   
   A   
   A   
   A   
   C   
   T   
   A   
   T   
   G   
   A   
   T   
   G   
   A   
   T   
   T   
   C   
   A   
   G   
   A   
   A   
   C   
   A   
   A   
   G   
   A   
   A   
   C   
   C   
   A   
   G   
   T   
   A   
   G   
   T   
   T   
   T   
   T   
   A   
   C   
   C   
   T   
   G   
   C   
   T   
   C   
   G   
   T   
   T   
   T   
   T   
   C   
   C   
   A   
   A   
   A   
   C   
   T   
   T   
   A   
   C   
   T   
   A   
   G   
   T   
   T   
   A   
   A   
   C   
   G   
   G   
   A   
   A   
   C   
   A   
   A   
   C   
   G   
   G   
   G   
   G   
   A   
   T   
   T   
   G   
   C   
   G   
   G   
   T   
   G   
   G   
   G   
   G   
   A   
   T   
   G   
   G   
   C   
   A   
   A   
   C   
   A   
   A   
   A   
   T   
   A   
   T   
   T   
   C   
   C   
   A   
   C   
   C   
   A   
   C   
   A   
   C   
   A   
   A   
   T   
   T   
   T   
   A   
   A   
   G   
   T   
   G   
   A   
   A   
   G   
   T   
   G   
   A   
   T   
   T   
   G   
   A   
   T   
   G   
   C   
   A   
   A   
   C   
   A   
   A   
   G   
   T   
   T   
   T   
   A   
   T   
   T   
   G   
   A   
   T   
   G   
   G   
   A   
   C   
   A   
   A   
   T   
   C   
   C   
   T   
   G   
   A   
   T   
   G   
   T   
   A   
   A   
   C   
   G   
   A   
   C   
   G   
   A   
   A   
   T   
   G   
   A   
   A   
   T   
   T   
   G   
   A   
   T   
   G   
   G   
   A   
   A   
   G   
   T   
   G   
   T   
   T   
   A   
   C   
   C   
   T   
   G   
   G   
   A   
   C   
   C   
   A   
   G   
   A   
   C   
   T   
   T   
   T   
   C   
   C   
   A   
   A   
   C   
   A   
   G   
   G   
   C   
   G   
   G   
   T   
   T   
   T   
   A   
   G   
   T   
   G   
   A   
   T   
   G   
   G   
   G   
   G   
   A   
   A   
   A   
   T   
   C   
   A   
   G   
   G   
    G    
   A   
   T   
    T    
   C   
   G   
    C    
    C    
   G   
   A   
   G   
   C   
    A    
   T   
   A   
   T   
   G   
   A   
   A   
   A   
   C   
    A    
   G   
   G   
    G    
   A   
   A   
    A    
   G   
   G   
   T   
   T   
   C   
    G    
   A   
   T   
   T   
   A   
   C   
    T    
   G   
   T   
    T    
   C   
   G   
   T   
   G   
   C   
   A   
   A   
   A   
   A   
   G   
   T   
   T   
   G   
   A   
    A    
    T    
   T   
    G    
   A   
    C    
   T   
   G   
   A   
    A    
   A   
   T   
   G   
    C    
   C   
    G    
   A   
   A   
   T   
   G   
   G   
    A    
   A   
   A   
   A   
   G   
   A   
   A   
   C   
   G   
    T    
   A   
   T   
    T    
    T    
   T   
    A    
   G   
   T   
    A    
   A   
   C   
    T    
   G   
   A   
   A   
   T   
   T   
    G    
   C   
   C   
   T   
   T   
   A   
   T   
   A   
   T   
   G   
   G   
   T   
    G    
   A   
   A   
   T   
   A   
   A   
   A   
   G   
   C   
    C    
   A   
   A   
   A   
   T   
   T   
    A    
   A   
   T   
    C    
   G   
   A   
   A   
   C   
   G   
    A    
   A   
   T   
   T   
   T   
   C   
   T   
   G   
   A   
    A    
    T    
   T   
    A    
   C   
   A   
   C   
   A   
   G   
   A   
   G   
   A   
    T    
   A   
   A   
    A    
   C   
   G   
    A    
   A   
   T   
    T    
   G   
   A   
   A   
   G   
   G   
    A    
   A   
   T   
    T    
   A   
   C   
    T    
   G   
   A   
   T   
   T   
   T   
   G   
   C   
   G   
   G   
   G   
   A   
    T    
   G   
   A   
   A   
   T   
   C   
   T   
   T   
   C   
    T    
   C   
   G   
   T   
   G   
   A   
    A    
   G   
   G   
    C    
   A   
   T   
   G   
   C   
   G   
    G    
   A   
   T   
    T    
   G   
   T   
   C   
   A   
   T   
    C    
   G   
   A   
    T    
    G    
   T   
    T    
   C   
   G   
   T   
   C   
   G   
    A    
   G   
   A   
    T    
   G   
    T    
    G    
   A   
   G   
    T    
   G   
   C   
    C    
   T   
   C   
    T    
   G   
   T   
   T   
    G    
   T   
    G    
   T   
   T   
    A    
   A   
   A   
    C    
   A   
   A   
   C   
   T   
   T   
    G    
   T   
   A   
    C    
   A   
   A   
   A   
    A    
   T   
   G   
   A   
   C   
    T    
    G    
   C   
    C    
   T   
   T   
    A    
   C   
   A   
    A    
   A   
    C    
    A    
   T   
   C   
    T    
   T   
   T   
    T    
   G   
   G   
    T    
   T   
   T   
    T    
   A   
   A   
    C    
   A   
   T   
   G   
   T   
   T   
    A    
   G   
   C   
    C    
   A   
   T   
    C    
   G   
   A   
   A   
   A   
   A   
   A   
   G   
   G   
    C    
   G   
   T   
    A    
   C   
   C   
    G    
   A   
   A   
   A   
    A    
   T   
   T   
   T   
   T   
    A    
   A   
   G   
    C    
   T   
   T   
   G   
   A   
   A   
   A   
   C   
    G    
    T    
   A   
   T   
    T    
    T    
   T   
    A    
   G   
   A   
   A   
   A   
   A   
    C    
   T   
   A   
    C    
    G    
   T   
    C    
   G   
   A   
    G    
   C   
   A   
    T    
   C   
   A   
    A    
    A    
    A    
    A    
    G    
   A   
   A   
   G   
   T   
   C   
   A   
   T   
    T    
   A   
   C   
    T    
    C    
   G   
    C    
   C   
   G   
   G   
   A   
   C   
    G    
    A    
   T   
    T    
   T   
   T   
   T   
   G   
   A   
    C    
   A   
   A   
   A   
    A    
   A   
    C    
   A   
   A   
    A    
   G   
   C   
   A   
   G   
   A   
   A   
   G   
   C   
   A   
    C    
   G   
    G    
   G   
   C   
    G    
   C   
   A   
    T    
   A   
   T   
    T    
   T   
   T   
   A   
   G   
   A   
   A   
   G   
   G   
   T   
    C    
   T   
    A    
   C   
   G   
    A    
   A   
   T   
   T   
   G   
   C   
    C    
   T   
   T   
    A    
   G   
   A   
   T   
   C   
   A   
   T   
   A   
   T   
   C   
    G    
   A   
   T   
   G   
   A   
   A   
   A   
   T   
    C    
   A   
   T   
   T   
   G   
   C   
    C    
   A   
   T   
   T   
   A   
   T   
    C    
   C   
   G   
    T    
   G   
   G   
    A    
   T   
   C   
    A    
    C    
   A   
   A   
   T   
   C   
   A   
   G   
   A   
   T   
   G   
   A   
    C    
   G   
   A   
    A    
   G   
   C   
   A   
   A   
   A   
   A   
   G   
   C   
    A    
   A   
    C    
    G    
    T    
   T   
    A    
   A   
   T   
    T    
   G   
   A   
   A   
   C   
   G   
    C    
   T   
   T   
    T    
   G   
   A   
    A    
   T   
   T   
    T    
   T   
   C   
    A    
   G   
   A   
   T   
   C   
   G   
    T    
   C   
   A   
    A    
    G    
   C   
    G    
   C   
   A   
   A   
   G   
   C   
    A    
   A   
   T   
    T    
   T   
   T   
   A   
   G   
   A   
   T   
   A   
   T   
   G   
   C   
   G   
   T   
   T   
   T   
    A    
   C   
   G   
    C    
   C   
   G   
    T    
   T   
   T   
    A    
   A   
   C   
    A    
   G   
   G   
    C    
   T   
   T   
    A    
   G   
   A   
   A   
   C   
   G   
    C    
   G   
   A   
    C    
   A   
   A   
   A   
   A   
   T   
    T    
   G   
   A   
    A    
   A   
   A   
   T   
   G   
   A   
   A   
   T   
   A   
    T    
   C   
   A   
   A   
   G   
   A   
    G    
   C   
   T   
    A    
   T   
   T   
    G    
   A   
    A    
   A   
    T    
   T   
    C    
   A   
   T   
   T   
   G   
    C    
    G    
   G   
   A   
    C    
   T   
   T   
    A    
    G    
    A    
    G    
   G   
   A   
   C   
   A   
   T   
   T   
    T    
   T   
    A    
   G   
   C   
    C    
    C    
   G   
    C    
   C   
   C   
    A    
   G   
   A   
    A    
   C   
   G   
    C    
   G   
   T   
    C    
   A   
    T    
    T    
   G   
   A   
   A   
   A   
   T   
    C    
   A   
   T   
    T    
   A   
   A   
    A    
   A   
    C    
    A    
   G   
   A   
    G    
   T   
   T   
   A   
    A    
    A    
    T    
   G   
   A   
   C   
    G    
   T   
    C    
    C    
    G    
    T    
    A    
    C    
   A   
    A    
    A    
   A   
   T   
   T   
   T   
   G   
   G   
   T   
   G   
   A   
   T   
    G    
    C    
   A   
   C   
   G   
   A   
   C   
   G   
    C    
   A   
   C   
   A   
   G   
   A   
   A   
    T    
   T   
   A   
    T    
   T   
   A   
   G   
   T   
    A    
   G   
   G   
   T   
   G   
   A   
    A    
   G   
   T   
   C   
    T    
   T   
    A    
   A   
   G   
   T   
   C   
   T   
    T    
   G   
   A   
   A   
   G   
   A   
   T   
   G   
   A   
   A   
   G   
   A   
   T   
    T    
   T   
    A    
   A   
   T   
    C    
   G   
   A   
   A   
   G   
   A   
   A   
   G   
   A   
   A   
   G   
   A   
   A   
    G    
   T   
   C   
   G   
   T   
    G    
   A   
   T   
   T   
   A   
   C   
    G    
    T    
   T   
    A    
   A   
   C   
    C    
   A   
   A   
    T    
   A   
   A   
    C    
   G   
   G   
    C    
   T   
   A   
    C    
   A   
   T   
    T    
   A   
   A   
    G    
   C   
   G   
    G    
    A    
   T   
   G   
   G   
    C    
   A   
   A   
   A   
    C    
    T    
    C    
    T    
   G   
   A   
   A   
   T   
   T   
   C   
    C    
   G   
    G    
   G   
   C   
    G    
   C   
   A   
   A   
   C   
   G   
    C    
    C    
   G   
    T    
   G   
   G   
    C    
   G   
   G   
    A    
   C   
   G   
   T   
   G   
   G   
    T    
   G   
   T   
    C    
   C   
   A   
   A   
   G   
   G   
    G    
   A   
   T   
   G   
   G   
   G   
    C    
    G    
   T   
    T    
   C   
   A   
    T    
    G    
   A   
   T   
   G   
   A   
    T    
   G   
   A   
    T    
   T   
   T   
   C   
   G   
   T   
    G    
   A   
   A   
    A    
   A   
   A   
    C    
   C   
   T   
    A    
    G    
   T   
   T   
   T   
   C   
   T   
   T   
   G   
    T    
   T   
   C   
    A    
   A   
   C   
    A    
   C   
   A   
    C    
   G   
   A   
    C    
   A   
   C   
    G    
   T   
   T   
    A    
   T   
   T   
   A   
   T   
   T   
    C    
   T   
   T   
   T   
   A   
   C   
   G   
   A   
   A   
    C    
   A   
    C    
    T    
   G   
   G   
    G    
   A   
   A   
   A   
   G   
   T   
    T    
   T   
   A   
    C    
   C   
   G   
    A    
   G   
   C   
   A   
   A   
   A   
   A   
   G   
   G   
    T    
   T   
   A   
    C    
   G   
   A   
   A   
   A   
   T   
    C    
   C   
   C   
    T    
   G   
   A   
    G    
   T   
   A   
   C   
   G   
   G   
    T    
    A    
   G   
    A    
   A   
   C   
   A   
   G   
   C   
    A    
   A   
   A   
   A   
   G   
   G   
    A    
   A   
   T   
    T    
   C   
   C   
   A   
    G    
   T   
    G    
   A   
   T   
    C    
   A   
   A   
    C    
   T   
   T   
    A    
   C   
   T   
    G    
   G   
   G   
    A    
   A   
   T   
    T    
   G   
   A   
    T    
   T   
   C   
    T    
    G    
    C    
    A    
   G   
   A   
   A   
   A   
    A    
    A    
   A   
   T   
    T    
   C   
   A   
    A    
   G   
   C   
    G    
   A   
   T   
    T    
   A   
   T   
   T   
    T    
   C   
    T    
   G   
   T   
    T    
    G    
    A    
   G   
   G   
   G   
    C    
    A    
   A   
   A   
   G   
   C   
    G    
   G   
   A   
   A   
   G   
    C    
    A    
   G   
   G   
    T    
    A    
   A   
    A    
   T   
   A   
    C    
    T    
   T   
    G    
   T   
   T   
   C   
   T   
   T   
    C    
   A   
   C   
    A    
   A   
   C   
    C    
    T    
    T    
    A    
    A    
   A   
   A   
   G   
   G   
    A    
   A   
   C   
    C    
   G   
   T   
    C    
   A   
   A   
   A   
   C   
   G   
    G    
   A   
   C   
    A    
    G    
   C   
    C    
   G   
   T   
    A    
   A   
   C   
    A    
   G   
   C   
    C    
   T   
   T   
    T    
    T    
   C   
   T   
   A   
   A   
    T    
   A   
   T   
    C    
   C   
   G   
    T    
   A   
   G   
    T    
   A   
   A   
   T   
   G   
   G   
    A    
    T    
   T   
    A    
   A   
   T   
    C    
   G   
   C   
    C    
   A   
   T   
   T   
    A    
   G   
    C    
   T   
   T   
    A    
   A   
   A   
   A   
   G   
   A   
   A   
   G   
   A   
    T    
   G   
   A   
    T    
   G   
   A   
    G    
    T    
   T   
    A    
    G    
   T   
    T    
   A   
   A   
    C    
   G   
   T   
    G    
    G    
   T   
   A   
    A    
    C    
   G   
   A   
   C   
    T    
    A    
   A   
    T    
   G   
   G   
    C    
    A    
   A   
    T    
    C    
    A    
    G    
   A   
   A   
    G    
   A   
   T   
    G    
   A   
   T   
    T    
   A   
   T   
    C    
   G   
   G   
    A    
   A   
   C   
   A   
   C   
   A   
   T   
    G    
    C    
    A    
   G   
   G   
    A    
   T   
   A   
   C   
   T   
   C   
   T   
   G   
   T   
    C    
   A   
   C   
    A    
   T   
   T   
    T    
    G    
   A   
    T    
   G   
   A   
    A    
    A    
    A    
    T    
    A    
   C   
    T    
   G   
    T    
    A    
   C   
   G   
    T    
   G   
   A   
   T   
   A   
   T   
   G   
   G   
   G   
    C    
   C   
   G   
    G    
   A   
   C   
   A   
   G   
   C   
    A    
   T   
   C   
    A    
   G   
   G   
    T    
   G   
   T   
    T    
    C    
   G   
    T    
   G   
   G   
    A    
   A   
   T   
    C    
   C   
   G   
    T    
    C    
   T   
    C    
   C   
   G   
    C    
   G   
   A   
    A    
    A    
   A   
    T    
   G   
   A   
    T    
   T   
    A    
    T    
   G   
   T   
    G    
   G   
   T   
    C    
   G   
   G   
    C    
    G    
   C   
   A   
   G   
   C   
    G    
    A    
   T   
    T    
    C    
   T   
   G   
    G    
   A   
    T    
   G   
    A    
   A   
    A    
   A   
    T    
    A    
   A   
   A   
   G   
   A   
   A   
   G   
   T   
    C    
    C    
   T   
    A    
   G   
   T   
    C    
   A   
   T   
    T    
   A   
   C   
    T    
   G   
   A   
   A   
   A   
   A   
    T    
   G   
   G   
   T   
   T   
   A   
    T    
   G   
   G   
    T    
   A   
   A   
    G    
   C   
   G   
    T    
   A   
   C   
    A    
   A   
   A   
   A   
   G   
    C    
    T    
   T   
   C   
    T    
   G   
   A   
    A    
   T   
   A   
   T   
   C   
   C   
   A   
   G   
   T   
    T    
   A   
   A   
    A    
   G   
   G   
    A    
   C   
   G   
    T    
   G   
   G   
    C    
   G   
   G   
   T   
   A   
   A   
   A   
   G   
   G   
    G    
   A   
   T   
    T    
   A   
   A   
    G    
   A   
   C   
    A    
    G    
   C   
    A    
   A   
   A   
    T    
   A   
   T   
   C   
   A   
   C   
   T   
   G   
   A   
    G    
   A   
   A   
   A   
   A   
   A   
   T   
   G   
   G   
   T   
   C   
   C   
   A   
    T    
   T   
   A   
   G   
   C   
    T    
   G   
   G   
    T    
   T   
   T   
    A    
   A   
   C   
   C   
   A   
   C   
    G    
   G   
   T   
    C    
    A    
    A    
   T   
   G   
   G   
    G    
    G    
   A   
   T   
   G   
   A   
   A   
   G   
   A   
    T    
   A   
   T   
   C   
    T    
   T   
    A    
    T    
   T   
    G    
   A   
   T   
    T    
   A   
   C   
    G    
    A    
   A   
    C    
   A   
   A   
   A   
   G   
   G   
    C    
   G   
   T   
    C    
   A   
   T   
   T   
   A   
   T   
   C   
   C   
   G   
    C    
   T   
   T   
    T    
   A   
   A   
   C   
   G   
   T   
    T    
    G    
    A    
    T    
    T    
   C   
   T   
   G   
   T   
   T   
   T   
   C   
   T   
   C   
   A   
   A   
   A   
   C   
    A    
   G   
   G   
   A   
   C   
   G   
    C    
    G    
   C   
   A   
   A   
   C   
   A   
   T   
   T   
    A    
   G   
   G   
    G    
   G   
   T   
    T    
   C   
   G   
   T   
   T   
   T   
    A    
   A   
   T   
   G   
   A   
    G    
    A    
   A   
   T   
   G   
   G   
   A   
   A   
   G   
    A    
    T    
   G   
    G    
    T    
    G    
   C   
    C    
   A   
   A   
   A   
   -   
   -   
   -   
   -   
   -   
   -   
   -   
   -   
   -   
   -   
   -   
   -   
   -   
   -   
   -   
   -   
   -   
   -   
   -   
   -   
   -   
   -   
   -   
   -   
   -   
   -   
   -   
   -   
   -   
   -   
   -   
   -   
   -   
   -   
   -   
   -   
   -   
   -   
   -   
   -   
   -   
   -   
   -   
   -   
   -   
   -   
   -   
   -   
   -   
   -   
   -   
   -   
   -   
   -   
   -   
   -   
   -   
   -   
   -   
   -   
   -   
   -   
   -   
   -   
   -   
   -   
   -   
   -   
   -   
   -   
   -   
   -   
 

 
   N54150   
   A   
   T   
   G   
   A   
   G   
   T   
   G   
   A   
   A   
   G   
   A   
   A   
   A   
   T   
   C   
   A   
   G   
   A   
   G   
   A   
   A   
   A   
   A   
   C   
   A   
   T   
   C   
   C   
   A   
   T   
   G   
   A   
   T   
   G   
   T   
   C   
   A   
   A   
   T   
   C   
   T   
   A   
   A   
   C   
   C   
   A   
   G   
   C   
   G   
   A   
   A   
   A   
   T   
   G   
   A   
   A   
   A   
   G   
   A   
   C   
   T   
   C   
   C   
   T   
   T   
   C   
   A   
   T   
   C   
   G   
   A   
   T   
   T   
   A   
   T   
   G   
   C   
   G   
   A   
   T   
   G   
   A   
   G   
   T   
   G   
   T   
   T   
   A   
   T   
   T   
   G   
   T   
   C   
   G   
   C   
   A   
   C   
   G   
   T   
   G   
   C   
   A   
   C   
   T   
   T   
   C   
   C   
   T   
   G   
   A   
   T   
   G   
   T   
   C   
   A   
   G   
   A   
   G   
   A   
   T   
   G   
   G   
   T   
   C   
   T   
   G   
   A   
   A   
   A   
   C   
   C   
   C   
   G   
   T   
   T   
   C   
   A   
   T   
   C   
   G   
   C   
   C   
   G   
   T   
   A   
   T   
   T   
   T   
   T   
   A   
   T   
   A   
   C   
   G   
   G   
   C   
   A   
   T   
   G   
   A   
   A   
   C   
   G   
   A   
   A   
   T   
   T   
   A   
   G   
   G   
   T   
   G   
   T   
   A   
   A   
   C   
   A   
   C   
   C   
   G   
   G   
   A   
   T   
   A   
   A   
   A   
   G   
   C   
   C   
   C   
   A   
   T   
   A   
   A   
   A   
   A   
   A   
   A   
   T   
   C   
   T   
   G   
   C   
   C   
   A   
   G   
   A   
   A   
   T   
   C   
   G   
   T   
   C   
   G   
   G   
   A   
   G   
   A   
   T   
   G   
   T   
   C   
   A   
   T   
   G   
   G   
   G   
   T   
   A   
   A   
   A   
   T   
   A   
   C   
   C   
   A   
   T   
   C   
   C   
   T   
   C   
   A   
   T   
   G   
   G   
   A   
   G   
   A   
   C   
   A   
   G   
   T   
   G   
   C   
   G   
   A   
   T   
   C   
   T   
   A   
   T   
   G   
   A   
   G   
   T   
   C   
   A   
   A   
   T   
   G   
   G   
   T   
   A   
   C   
   G   
   G   
   A   
   T   
   G   
   G   
   C   
   A   
   C   
   A   
   G   
   C   
   C   
   A   
   T   
   T   
   C   
   A   
   G   
   C   
   T   
   A   
   C   
   C   
   G   
   T   
   T   
   A   
   T   
   A   
   T   
   G   
   C   
   T   
   A   
   G   
   T   
   G   
   G   
   A   
   T   
   G   
   G   
   T   
   C   
   A   
   T   
   G   
   G   
   A   
   A   
   A   
   C   
   T   
   T   
   T   
   G   
   G   
   T   
   T   
   C   
   T   
   G   
   T   
   T   
   G   
   A   
   C   
   G   
   G   
   T   
   G   
   A   
   C   
   G   
   G   
   T   
   G   
   C   
   C   
   G   
   C   
   C   
   G   
   C   
   T   
   A   
   T   
   G   
   C   
   G   
   T   
   T   
   A   
   T   
   A   
   C   
   C   
   G   
   A   
   A   
   G   
   C   
   A   
   C   
   G   
   G   
   A   
   T   
   G   
   A   
   G   
   C   
   A   
   A   
   A   
   A   
   T   
   C   
   G   
   C   
   T   
   A   
   C   
   A   
   G   
   A   
   A   
   A   
   T   
   G   
   C   
   T   
   G   
   C   
   G   
   T   
   G   
   A   
   T   
   A   
   T   
   C   
   A   
   A   
   T   
   A   
   A   
   G   
   A   
   A   
   C   
   A   
   C   
   T   
   G   
   T   
   T   
   G   
   A   
   T   
   T   
   T   
   C   
   C   
   A   
   A   
   A   
   G   
   C   
   A   
   A   
   C   
   T   
   A   
   T   
   G   
   A   
   T   
   G   
   A   
   T   
   A   
   C   
   A   
   G   
   A   
   A   
   A   
   A   
   A   
   G   
   A   
   A   
   C   
   C   
   A   
   G   
   T   
   T   
   G   
   T   
   A   
   C   
   T   
   A   
   C   
   C   
   T   
   G   
   C   
   T   
   C   
   G   
   C   
   T   
   T   
   C   
   C   
   C   
   G   
   A   
   A   
   T   
   C   
   T   
   T   
   T   
   T   
   A   
   G   
   T   
   G   
   A   
   A   
   C   
   G   
   G   
   A   
   A   
   C   
   A   
   A   
   C   
   G   
   G   
   G   
   G   
   A   
   T   
   C   
   G   
   C   
   C   
   G   
   T   
   A   
   G   
   G   
   T   
   A   
   T   
   G   
   G   
   C   
   C   
   A   
   C   
   G   
   A   
   A   
   T   
   A   
   T   
   C   
   C   
   C   
   A   
   C   
   C   
   T   
   C   
   A   
   T   
   A   
   A   
   C   
   T   
   T   
   G   
   T   
   C   
   T   
   G   
   A   
   G   
   G   
   T   
   A   
   G   
   T   
   A   
   G   
   C   
   G   
   G   
   C   
   T   
   A   
   T   
   C   
   G   
   A   
   T   
   T   
   T   
   G   
   C   
   T   
   G   
   A   
   T   
   G   
   G   
   A   
   T   
   A   
   A   
   T   
   C   
   C   
   T   
   G   
   A   
   T   
   G   
   T   
   G   
   A   
   C   
   G   
   A   
   C   
   C   
   A   
   A   
   T   
   G   
   A   
   A   
   T   
   T   
   G   
   A   
   T   
   G   
   G   
   A   
   A   
   G   
   T   
   A   
   T   
   T   
   A   
   C   
   C   
   T   
   G   
   G   
   A   
   C   
   C   
   A   
   G   
   A   
   T   
   T   
   T   
   T   
   C   
   C   
   A   
   A   
   C   
   A   
   G   
   G   
   T   
   G   
   G   
   T   
   T   
   T   
   G   
   G   
   T   
   T   
   A   
   T   
   G   
   G   
   G   
   A   
   A   
   A   
   A   
   T   
   C   
   A   
   G   
   G   
    A    
   A   
   T   
    C    
   C   
   G   
    A    
    A    
   G   
   A   
   G   
   C   
    G    
   T   
   A   
   T   
   G   
   A   
   A   
   A   
   C   
    A    
   G   
   G   
    T    
   A   
   A   
    G    
   G   
   G   
   T   
   T   
   C   
    T    
   A   
   T   
   T   
   A   
   C   
    C    
   G   
   T   
    A    
   C   
   G   
   T   
   G   
   C   
   A   
   A   
   A   
   A   
   G   
   T   
   T   
   G   
   A   
    G    
    A    
   T   
    T    
   A   
    C    
   T   
   G   
   A   
    G    
   A   
   T   
   G   
    C    
   C   
    A    
   A   
   A   
   T   
   G   
   G   
    T    
   A   
   A   
   A   
   G   
   A   
   A   
   C   
   G   
    G    
   A   
   T   
    C    
    A    
   T   
    C    
   G   
   T   
    G    
   A   
   C   
    A    
   G   
   A   
   A   
   T   
   T   
    G    
   C   
   C   
   T   
   T   
   A   
   T   
   A   
   T   
   G   
   G   
   T   
    C    
   A   
   A   
   T   
   A   
   A   
   A   
   G   
   C   
    A    
   A   
   A   
   A   
   T   
   T   
    G    
   A   
   T   
    C    
   G   
   A   
   A   
   C   
   G   
    G    
   A   
   T   
   T   
   T   
   C   
   T   
   G   
   A   
    A    
    C    
   T   
    G    
   C   
   A   
   C   
   A   
   G   
   A   
   G   
   A   
    C    
   A   
   A   
    G    
   C   
   G   
    T    
   A   
   T   
    C    
   G   
   A   
   A   
   G   
   G   
    G    
   A   
   T   
    C    
   A   
   C   
    A    
   G   
   A   
   T   
   T   
   T   
   G   
   C   
   G   
   G   
   G   
   A   
    C    
   G   
   A   
   A   
   T   
   C   
   T   
   T   
   C   
    C    
   C   
   G   
   T   
   G   
   A   
    A    
   G   
   G   
    T    
   A   
   T   
   G   
   C   
   G   
    G    
   A   
   T   
    C    
   G   
   T   
   C   
   A   
   T   
    T    
   G   
   A   
    C    
    A    
   T   
    C    
   C   
   G   
   T   
   C   
   G   
    T    
   G   
   A   
    C    
   G   
    C    
    C    
   A   
   G   
    C    
   G   
   C   
    A    
   T   
   C   
    C    
   G   
   T   
   T   
    A    
   T   
    C    
   T   
   T   
    G    
   A   
   A   
    T    
   A   
   A   
   C   
   T   
   T   
    A    
   T   
   A   
    T    
   A   
   A   
   A   
    T    
   T   
   G   
   A   
   C   
    A    
    T    
   C   
    C    
   T   
   T   
    G    
   C   
   A   
    A    
   A   
    A    
    T    
   T   
   C   
    A    
   T   
   T   
    T    
   G   
   G   
    A    
   T   
   T   
    C    
   A   
   A   
    T    
   A   
   T   
   G   
   T   
   T   
    G    
   G   
   C   
    G    
   A   
   T   
    C    
   G   
   A   
   A   
   A   
   A   
   A   
   G   
   G   
    T    
   G   
   T   
    G    
   C   
   C   
    G    
   A   
   A   
   A   
    G    
   T   
   T   
   T   
   T   
    G    
   A   
   G   
    C    
   T   
   T   
   G   
   A   
   A   
   A   
   C   
    A    
    A    
   A   
   T   
    T    
    C    
   T   
    T    
   G   
   A   
   A   
   A   
   A   
    C    
   T   
   A   
    T    
    A    
   T   
    C    
   G   
   A   
    A    
   C   
   A   
    T    
   C   
   A   
    G    
    C    
    G    
    C    
    G    
   A   
   A   
   G   
   T   
   C   
   A   
   T   
    C    
   A   
   C   
    A    
    A    
   G   
    A    
   C   
   G   
   G   
   A   
   C   
    A    
    G    
   T   
    C    
   T   
   T   
   T   
   G   
   A   
    A    
   A   
   A   
   A   
    G    
   A   
    A    
   A   
   A   
    A    
   G   
   C   
   A   
   G   
   A   
   A   
   G   
   C   
   A   
    A    
   G   
    G    
   G   
   C   
    A    
   C   
   A   
    C    
   A   
   T   
    T    
   T   
   T   
   A   
   G   
   A   
   A   
   G   
   G   
   T   
    T    
   T   
    G    
   C   
   G   
    T    
   A   
   T   
   T   
   G   
   C   
    T    
   T   
   T   
    G    
   G   
   A   
   T   
   C   
   A   
   T   
   A   
   T   
   C   
    G    
   A   
   T   
   G   
   A   
   A   
   A   
   T   
    C    
   A   
   T   
   T   
   G   
   C   
    G    
   A   
   T   
   T   
   A   
   T   
    C    
   C   
   G   
    C    
   G   
   G   
    G    
   T   
   C   
    T    
    A    
   A   
   A   
   T   
   C   
   A   
   G   
   A   
   T   
   G   
   A   
    C    
   G   
   A   
    A    
   G   
   C   
   A   
   A   
   A   
   A   
   G   
   C   
    G    
   A   
    C    
    G    
    A    
   T   
    G    
   A   
   T   
    C    
   G   
   A   
   A   
   C   
   G   
    G    
   T   
   T   
    C    
   G   
   A   
    T    
   T   
   T   
    A    
   T   
   C   
    C    
   G   
   A   
   T   
   C   
   G   
    C    
   C   
   A   
    G    
    T    
   C   
    T    
   C   
   A   
   A   
   G   
   C   
    T    
   A   
   T   
    T    
   T   
   T   
   A   
   G   
   A   
   T   
   A   
   T   
   G   
   C   
   G   
   T   
   T   
   T   
    A    
   C   
   G   
    C    
   C   
   G   
    A    
   T   
   T   
    G    
   A   
   C   
    C    
   G   
   G   
    T    
   T   
   T   
    G    
   G   
   A   
   A   
   C   
   G   
    G    
   G   
   A   
    A    
   A   
   A   
   A   
   A   
   T   
    C    
   G   
   A   
    A    
   A   
   A   
   T   
   G   
   A   
   A   
   T   
   A   
    C    
   C   
   A   
   A   
   G   
   A   
    A    
   C   
   T   
    C    
   T   
   T   
    A    
   A   
    A    
   A   
    C    
   T   
    G    
   A   
   T   
   T   
   G   
    A    
    A    
   G   
   A   
    T    
   T   
   T   
    A    
    A    
    C    
    T    
   G   
   A   
   C   
   A   
   T   
   T   
    C    
   T   
    G    
   G   
   C   
    A    
    A    
   G   
    A    
   C   
   C   
    A    
   G   
   A   
    A    
   C   
   G   
    T    
   G   
   T   
    T    
   A   
    C    
    A    
   G   
   A   
   A   
   A   
   T   
    C    
   A   
   T   
    C    
   A   
   A   
    G    
   A   
    A    
    T    
   G   
   A   
    A    
   T   
   T   
   A   
    G    
    C    
    A    
   G   
   A   
   C   
    T    
   T   
    G    
    A    
    A    
    T    
    C    
    A    
   A   
    C    
    G    
   A   
   T   
   T   
   T   
   G   
   G   
   T   
   G   
   A   
   T   
    A    
    A    
   A   
   C   
   G   
   A   
   C   
   G   
    C    
   A   
   C   
   A   
   G   
   A   
   A   
    T    
   T   
   A   
    C    
   T   
   A   
   G   
   T   
    C    
   G   
   G   
   T   
   G   
   A   
    G    
   G   
   T   
   C   
    T    
   T   
    A    
   A   
   G   
   T   
   C   
   T   
    G    
   G   
   A   
   A   
   G   
   A   
   T   
   G   
   A   
   A   
   G   
   A   
   T   
    T    
   T   
    A    
   A   
   T   
    C    
   G   
   A   
   A   
   G   
   A   
   A   
   G   
   A   
   A   
   G   
   A   
   A   
    A    
   T   
   C   
   G   
   T   
    T    
   A   
   T   
   T   
   A   
   C   
    A    
    T    
   T   
    G    
   A   
   C   
    A    
   A   
   A   
    C    
   A   
   A   
    T    
   G   
   G   
    T    
   T   
   A   
    C    
   A   
   T   
    C    
   A   
   A   
    A    
   C   
   G   
    T    
    T    
   T   
   G   
   G   
    C    
   A   
   A   
   A   
    T    
    A    
    A    
    T    
   G   
   A   
   A   
   T   
   T   
   C   
    A    
   G   
    A    
   G   
   C   
    T    
   C   
   A   
   A   
   C   
   G   
    A    
    A    
   G   
    G    
   G   
   G   
    T    
   G   
   G   
    A    
   C   
   G   
   T   
   G   
   G   
    T    
   G   
   T   
    C    
   C   
   A   
   A   
   G   
   G   
    A    
   A   
   T   
   G   
   G   
   G   
    T    
    A    
   T   
    C    
   C   
   A   
    C    
    A    
   A   
   T   
   G   
   A   
    C    
   G   
   A   
    C    
   T   
   T   
   C   
   G   
   T   
    C    
   A   
   A   
    A    
   A   
   A   
    T    
   C   
   T   
    G    
    G    
   T   
   T   
   T   
   C   
   T   
   T   
   G   
    T    
   T   
   C   
    T    
   A   
   C   
    C    
   C   
   A   
    T    
   G   
   A   
    T    
   A   
   C   
    A    
   T   
   T   
    G    
   T   
   T   
   A   
   T   
   T   
    C    
   T   
   T   
   T   
   A   
   C   
   G   
   A   
   A   
    T    
   A   
    A    
    C    
   G   
   G   
    A    
   A   
   A   
   A   
   G   
   T   
    G    
   T   
   A   
    T    
   C   
   G   
    G    
   G   
   C   
   A   
   A   
   A   
   A   
   G   
   G   
    A    
   T   
   A   
    C    
   G   
   A   
   A   
   A   
   T   
    T    
   C   
   C   
    A    
   G   
   A   
    A    
   T   
   A   
   C   
   G   
   G   
    G    
    C    
   G   
    C    
   A   
   C   
   A   
   G   
   C   
    C    
   A   
   A   
   A   
   G   
   G   
    A    
   A   
   T   
    A    
   C   
   C   
   A   
    A    
   T   
    T    
   A   
   T   
    C    
   A   
   A   
    T    
   T   
   T   
    A    
   C   
   T   
    A    
   G   
   G   
    T    
   A   
   T   
    C    
   G   
   A   
    T    
   T   
   C   
    G    
    A    
    G    
    T    
   G   
   A   
   A   
   A   
    G    
    C    
   A   
   T   
    C    
   C   
   A   
    G    
   G   
   C   
    A    
   A   
   T   
    C    
   A   
   T   
   T   
    G    
   C   
    G    
   G   
   T   
    G    
    A    
    C    
   G   
   G   
   G   
    A    
    G    
   A   
   A   
   G   
   C   
    A    
   G   
   A   
   A   
   G   
    A    
    A    
   G   
   G   
    C    
    C    
   A   
    C    
   T   
   A   
    T    
    C    
   T   
    C    
   T   
   T   
   C   
   T   
   T   
    T    
   A   
   C   
    G    
   A   
   C   
    G    
    C    
    G    
    T    
    C    
   A   
   A   
   G   
   G   
    T    
   A   
   C   
    A    
   G   
   T   
    A    
   A   
   A   
   A   
   C   
   G   
    T    
   A   
   C   
    T    
    T    
   C   
    T    
   G   
   T   
    C    
   A   
   C   
    T    
   G   
   C   
    T    
   T   
   T   
    T    
    G    
   C   
   T   
   A   
   A   
    C    
   A   
   T   
    C    
   C   
   G   
    A    
   A   
   G   
    T    
   A   
   A   
   T   
   G   
   G   
    T    
    C    
   T   
    G    
   A   
   T   
    C    
   G   
   C   
    A    
   A   
   T   
   T   
    G    
   G   
    T    
   T   
   T   
    G    
   A   
   A   
   A   
   G   
   A   
   A   
   G   
   A   
    C    
   G   
   A   
    T    
   G   
   A   
    G    
    C    
   T   
    C    
    G    
   T   
    C    
   A   
   A   
    T    
   G   
   T   
    G    
    C    
   T   
   A   
    C    
    T    
   G   
   A   
   C   
    A    
    G    
   A   
    T    
   G   
   G   
    T    
    C    
   A   
    A    
    T    
    C    
    T    
   A   
   A   
    T    
   A   
   T   
    C    
   A   
   T   
    T    
   A   
   T   
    T    
   G   
   G   
    A    
   A   
   C   
   A   
   C   
   A   
   T   
    A    
    A    
    C    
   G   
   G   
    C    
   T   
   A   
   C   
   T   
   C   
   T   
   G   
   T   
    C    
   A   
   C   
    T    
   T   
   T   
    C    
    A    
   A   
    A    
   G   
   A   
    A    
    G    
    A    
    A    
    G    
   C   
    A    
   G   
    T    
    C    
   C   
   G   
    C    
   G   
   A   
   T   
   A   
   T   
   G   
   G   
   G   
    A    
   C   
   G   
    T    
   A   
   C   
   A   
   G   
   C   
    T    
   T   
   C   
    C    
   G   
   G   
    G    
   G   
   T   
    T    
    A    
   G   
    A    
   G   
   G   
    A    
   A   
   T   
    A    
   C   
   G   
    C    
    C    
   T   
    G    
   C   
   G   
    T    
   G   
   A   
    G    
    G    
   A   
    A    
   G   
   A   
    T    
   T   
    A    
    T    
   G   
   T   
    C    
   G   
   T   
    T    
   G   
   G   
    G    
    G    
   C   
   A   
   G   
   C   
    C    
    T    
   T   
    A    
    A    
   T   
   G   
    A    
   A   
    G    
   G   
    A    
   A   
    G    
   A   
    T    
    C    
   A   
   A   
   G   
   A   
   A   
   G   
   T   
    A    
    T    
   T   
    A    
   G   
   T   
    G    
   A   
   T   
    C    
   A   
   C   
    A    
   G   
   A   
   A   
   A   
   A   
    A    
   G   
   G   
   T   
   T   
   A   
    C    
   G   
   G   
    A    
   A   
   A   
    A    
   C   
   G   
    G    
   A   
   C   
    A    
   A   
   A   
   A   
   G   
    T    
    A    
   T   
   C   
    G    
   G   
   A   
    A    
   T   
   A   
   T   
   C   
   C   
   A   
   G   
   T   
    G    
   A   
   A   
    A    
   G   
   G   
    C    
   C   
   G   
    C    
   G   
   G   
    A    
   G   
   G   
   T   
   A   
   A   
   A   
   G   
   G   
    G    
   A   
   T   
    C    
   A   
   A   
    A    
   A   
   C   
    G    
    G    
   C   
    G    
   A   
   A   
    C    
   A   
   T   
   C   
   A   
   C   
   T   
   G   
   A   
    A    
   A   
   A   
   A   
   A   
   A   
   T   
   G   
   G   
   T   
   C   
   C   
   A   
    C    
   T   
   A   
   G   
   C   
    T    
   G   
   G   
    A    
   T   
   T   
    G    
   A   
   C   
   C   
   A   
   C   
    T    
   G   
   T   
    T    
    T    
    C    
   T   
   G   
   G   
    A    
    G    
   A   
   T   
   G   
   A   
   A   
   G   
   A   
    C    
   A   
   T   
   C   
    A    
   T   
    G    
    C    
   T   
    G    
   A   
   T   
    T    
   A   
   C   
    A    
    G    
   A   
    T    
   A   
   A   
   A   
   G   
   G   
    T    
   G   
   T   
    C    
   A   
   T   
   T   
   A   
   T   
   C   
   C   
   G   
    T    
   T   
   T   
    C    
   A   
   A   
   C   
   G   
   T   
    A    
    T    
    C    
    G    
    A    
   C   
   T   
   G   
   T   
   T   
   T   
   C   
   T   
   C   
   A   
   A   
   A   
   C   
    T    
   G   
   G   
   A   
   C   
   G   
    T    
    T    
   C   
   A   
   A   
   C   
   A   
   T   
   T   
    A    
   G   
   G   
    C    
   G   
   T   
    A    
   C   
   G   
   T   
   T   
   T   
    G    
   A   
   T   
   G   
   A   
    A    
    G    
   A   
   T   
   G   
   G   
   A   
   A   
   G   
    C    
    T    
   G   
    A    
    T    
    A    
   C   
    G    
   A   
   A   
   A   
   G   
   T   
   T   
   G   
   T   
   C   
   A   
   C   
   G   
   A   
   T   
   G   
   G   
   C   
   A   
   G   
   C   
   A   
   G   
   T   
   C   
   G   
   A   
   A   
   C   
   C   
   A   
   G   
   A   
   A   
   G   
   C   
   T   
   G   
   C   
   A   
   G   
   A   
   A   
   C   
   A   
   A   
   A   
   C   
   A   
   G   
   T   
   T   
   G   
   A   
   A   
   G   
   A   
   A   
   C   
   A   
   A   
   G   
   G   
   A   
   T   
   C   
   A   
   G   
   A   
   A   
   G   
   A   
   A   
   -   
   -   
   -   
 

 
   N60417F   
   A   
   T   
   G   
   A   
   G   
   T   
   G   
   A   
   A   
   G   
   A   
   A   
   A   
   T   
   C   
   A   
   G   
   A   
   G   
   A   
   A   
   A   
   A   
   C   
   A   
   T   
   C   
   C   
   A   
   T   
   G   
   A   
   T   
   G   
   T   
   C   
   A   
   A   
   T   
   C   
   T   
   A   
   A   
   C   
   C   
   A   
   G   
   C   
   G   
   A   
   A   
   A   
   T   
   G   
   A   
   A   
   A   
   G   
   A   
   C   
   T   
   C   
   C   
   T   
   T   
   C   
   A   
   T   
   C   
   G   
   A   
   T   
   T   
   A   
   T   
   G   
   C   
   G   
   A   
   T   
   G   
   A   
   G   
   T   
   G   
   T   
   T   
   A   
   T   
   T   
   G   
   T   
   T   
   G   
   C   
   A   
   C   
   G   
   T   
   G   
   C   
   A   
   C   
   T   
   G   
   C   
   C   
   T   
   G   
   A   
   T   
   G   
   T   
   C   
   A   
   G   
   A   
   G   
   A   
   T   
   G   
   G   
   T   
   C   
   T   
   G   
   A   
   A   
   A   
   C   
   C   
   C   
   G   
   T   
   T   
   C   
   A   
   T   
   C   
   G   
   C   
   C   
   G   
   T   
   A   
   T   
   T   
   T   
   T   
   A   
   T   
   A   
   C   
   G   
   G   
   C   
   A   
   T   
   G   
   A   
   A   
   C   
   G   
   A   
   A   
   T   
   T   
   A   
   G   
   G   
   T   
   G   
   T   
   G   
   A   
   C   
   A   
   C   
   C   
   G   
   G   
   A   
   T   
   A   
   A   
   A   
   G   
   C   
   C   
   C   
   A   
   T   
   A   
   A   
   A   
   A   
   A   
   A   
   T   
   C   
   T   
   G   
   C   
   C   
   A   
   G   
   A   
   A   
   T   
   C   
   G   
   T   
   C   
   G   
   G   
   A   
   G   
   A   
   T   
   G   
   T   
   C   
   A   
   T   
   G   
   G   
   G   
   T   
   A   
   A   
   A   
   T   
   A   
   C   
   C   
   A   
   T   
   C   
   C   
   T   
   C   
   A   
   T   
   G   
   G   
   G   
   G   
   A   
   T   
   A   
   G   
   T   
   G   
   C   
   A   
   A   
   T   
   C   
   T   
   A   
   T   
   G   
   A   
   G   
   T   
   C   
   A   
   A   
   T   
   G   
   G   
   T   
   A   
   C   
   G   
   G   
   A   
   T   
   G   
   G   
   C   
   G   
   C   
   A   
   G   
   C   
   C   
   A   
   T   
   T   
   T   
   A   
   G   
   C   
   T   
   A   
   C   
   C   
   G   
   T   
   T   
   A   
   T   
   A   
   T   
   G   
   C   
   T   
   A   
   G   
   T   
   G   
   G   
   A   
   T   
   G   
   G   
   T   
   C   
   A   
   T   
   G   
   G   
   A   
   A   
   A   
   C   
   T   
   T   
   T   
   G   
   G   
   T   
   T   
   C   
   T   
   G   
   T   
   T   
   G   
   A   
   C   
   G   
   G   
   T   
   G   
   A   
   C   
   G   
   G   
   T   
   G   
   C   
   C   
   G   
   C   
   T   
   G   
   C   
   T   
   A   
   T   
   G   
   C   
   G   
   T   
   T   
   A   
   T   
   A   
   C   
   C   
   G   
   A   
   A   
   G   
   C   
   A   
   C   
   G   
   G   
   A   
   T   
   G   
   A   
   G   
   C   
   A   
   A   
   A   
   A   
   T   
   C   
   G   
   C   
   T   
   A   
   C   
   A   
   G   
   A   
   A   
   A   
   T   
   G   
   C   
   T   
   G   
   C   
   G   
   T   
   G   
   A   
   T   
   A   
   T   
   C   
   A   
   A   
   T   
   A   
   A   
   G   
   A   
   A   
   T   
   A   
   C   
   T   
   G   
   T   
   G   
   G   
   A   
   T   
   T   
   T   
   C   
   C   
   A   
   A   
   A   
   G   
   C   
   A   
   A   
   C   
   T   
   A   
   T   
   G   
   A   
   T   
   G   
   A   
   C   
   A   
   C   
   A   
   G   
   A   
   A   
   A   
   A   
   A   
   G   
   A   
   A   
   C   
   C   
   C   
   G   
   T   
   T   
   G   
   T   
   A   
   T   
   T   
   A   
   C   
   C   
   T   
   G   
   C   
   T   
   C   
   G   
   C   
   T   
   T   
   C   
   C   
   C   
   T   
   A   
   A   
   T   
   C   
   T   
   T   
   T   
   T   
   G   
   G   
   T   
   G   
   A   
   A   
   C   
   G   
   G   
   A   
   A   
   C   
   G   
   A   
   C   
   G   
   G   
   G   
   G   
   A   
   T   
   C   
   G   
   C   
   C   
   G   
   T   
   G   
   G   
   G   
   T   
   A   
   T   
   G   
   G   
   C   
   A   
   A   
   C   
   G   
   A   
   A   
   T   
   A   
   T   
   C   
   C   
   C   
   A   
   C   
   C   
   T   
   C   
   A   
   T   
   A   
   A   
   T   
   T   
   T   
   G   
   T   
   C   
   T   
   G   
   A   
   G   
   G   
   T   
   A   
   G   
   T   
   A   
   G   
   C   
   G   
   G   
   C   
   T   
   A   
   T   
   C   
   G   
   A   
   T   
   T   
   T   
   G   
   C   
   T   
   G   
   A   
   T   
   G   
   G   
   A   
   T   
   A   
   A   
   T   
   C   
   C   
   T   
   G   
   A   
   T   
   G   
   T   
   G   
   A   
   C   
   G   
   A   
   C   
   C   
   A   
   A   
   T   
   G   
   A   
   A   
   T   
   T   
   G   
   A   
   T   
   G   
   G   
   A   
   A   
   G   
   T   
   A   
   T   
   T   
   A   
   C   
   C   
   T   
   G   
   G   
   A   
   C   
   C   
   A   
   G   
   A   
   T   
   T   
   T   
   T   
   C   
   C   
   A   
   A   
   C   
   A   
   G   
   G   
   T   
   G   
   G   
   T   
   T   
   T   
   G   
   G   
   T   
   C   
   A   
   T   
   G   
   G   
   G   
   A   
   A   
   A   
   A   
   T   
   C   
   A   
   G   
   G   
    A    
   A   
   T   
    C    
   C   
   G   
    A    
    A    
   G   
   A   
   G   
   C   
    G    
   T   
   A   
   T   
   G   
   A   
   A   
   A   
   C   
    A    
   G   
   G   
    T    
   A   
   A   
    A    
   G   
   G   
   T   
   T   
   C   
    T    
   A   
   T   
   T   
   A   
   C   
    C    
   G   
   T   
    A    
   C   
   G   
   T   
   G   
   C   
   A   
   A   
   A   
   A   
   G   
   T   
   T   
   G   
   A   
    G    
    A    
   T   
    C    
   A   
    C    
   T   
   G   
   A   
    G    
   A   
   T   
   G   
    C    
   C   
    A    
   A   
   A   
   T   
   G   
   G   
    C    
   A   
   A   
   A   
   G   
   A   
   A   
   C   
   G   
    G    
   A   
   T   
    C    
    C    
   T   
    C    
   G   
   T   
    G    
   A   
   C   
    G    
   G   
   A   
   A   
   T   
   T   
    A    
   C   
   C   
   T   
   T   
   A   
   T   
   A   
   T   
   G   
   G   
   T   
    C    
   A   
   A   
   T   
   A   
   A   
   A   
   G   
   C   
    A    
   A   
   A   
   A   
   T   
   T   
    G    
   A   
   T   
    T    
   G   
   A   
   A   
   C   
   G   
    G    
   A   
   T   
   T   
   T   
   C   
   T   
   G   
   A   
    G    
    T    
   T   
    G    
   C   
   A   
   C   
   A   
   G   
   A   
   G   
   A   
    C    
   A   
   A   
    G    
   C   
   G   
    T    
   A   
   T   
    C    
   G   
   A   
   A   
   G   
   G   
    A    
   A   
   T   
    T    
   A   
   C   
    G    
   G   
   A   
   T   
   T   
   T   
   G   
   C   
   G   
   G   
   G   
   A   
    T    
   G   
   A   
   A   
   T   
   C   
   T   
   T   
   C   
    C    
   C   
   G   
   T   
   G   
   A   
    A    
   G   
   G   
    C    
   A   
   T   
   G   
   C   
   G   
    A    
   A   
   T   
    T    
   G   
   T   
   C   
   A   
   T   
    T    
   G   
   A   
    C    
    G    
   T   
    T    
   C   
   G   
   T   
   C   
   G   
    T    
   G   
   A   
    C    
   G   
    C    
    C    
   A   
   G   
    C    
   G   
   C   
    A    
   T   
   C   
    A    
   G   
   T   
   T   
    A    
   T   
    T    
   T   
   T   
    G    
   A   
   A   
    T    
   A   
   A   
   C   
   T   
   T   
    A    
   T   
   A   
    C    
   A   
   A   
   A   
    T    
   T   
   G   
   A   
   C   
    A    
    T    
   C   
    C    
   T   
   T   
    G    
   C   
   A   
    G    
   A   
    A    
    T    
   T   
   C   
    A    
   T   
   T   
    C    
   G   
   G   
    A    
   T   
   T   
    C    
   A   
   A   
    T    
   A   
   T   
   G   
   T   
   T   
    G    
   G   
   C   
    A    
   A   
   T   
    C    
   G   
   A   
   A   
   A   
   A   
   A   
   G   
   G   
    C    
   G   
   T   
    G    
   C   
   C   
    G    
   A   
   A   
   A   
    G    
   T   
   T   
   T   
   T   
    A    
   A   
   G   
    T    
   T   
   T   
   G   
   A   
   A   
   A   
   C   
    A    
    A    
   A   
   T   
    C    
    C    
   T   
    T    
   G   
   A   
   A   
   A   
   A   
    T    
   T   
   A   
    T    
    A    
   T   
    C    
   G   
   A   
    A    
   C   
   A   
    T    
   C   
   A   
    A    
    C    
    G    
    C    
    G    
   A   
   A   
   G   
   T   
   C   
   A   
   T   
    C    
   A   
   C   
    A    
    C    
   G   
    A    
   C   
   G   
   G   
   A   
   C   
    G    
    G    
   T   
    C    
   T   
   T   
   T   
   G   
   A   
    A    
   A   
   A   
   A   
    G    
   A   
    A    
   A   
   A   
    A    
   G   
   C   
   A   
   G   
   A   
   A   
   G   
   C   
   A   
    A    
   G   
    A    
   G   
   C   
    A    
   C   
   A   
    C    
   A   
   T   
    C    
   T   
   T   
   A   
   G   
   A   
   A   
   G   
   G   
   T   
    T    
   T   
    G    
   C   
   G   
    T    
   A   
   T   
   T   
   G   
   C   
    T    
   T   
   T   
    G    
   G   
   A   
   T   
   C   
   A   
   T   
   A   
   T   
   C   
    G    
   A   
   T   
   G   
   A   
   A   
   A   
   T   
    C    
   A   
   T   
   T   
   G   
   C   
    G    
   A   
   T   
   T   
   A   
   T   
    C    
   C   
   G   
    C    
   G   
   G   
    G    
   T   
   C   
    T    
    A    
   A   
   A   
   T   
   C   
   A   
   G   
   A   
   T   
   G   
   A   
    C    
   G   
   A   
    A    
   G   
   C   
   A   
   A   
   A   
   A   
   G   
   C   
    A    
   A   
    C    
    A    
    A    
   T   
    G    
   A   
   T   
    C    
   G   
   A   
   A   
   C   
   G   
    G    
   T   
   T   
    C    
   G   
   A   
    T    
   T   
   T   
    A    
   T   
   C   
    C    
   G   
   A   
   T   
   C   
   G   
    C    
   C   
   A   
    A    
    T    
   C   
    A    
   C   
   A   
   A   
   G   
   C   
    T    
   A   
   T   
    C    
   T   
   T   
   A   
   G   
   A   
   T   
   A   
   T   
   G   
   C   
   G   
   T   
   T   
   T   
    G    
   C   
   G   
    T    
   C   
   G   
    A    
   T   
   T   
    G    
   A   
   C   
    C    
   G   
   G   
    T    
   T   
   T   
    G    
   G   
   A   
   A   
   C   
   G   
    G    
   G   
   A   
    A    
   A   
   A   
   A   
   A   
   T   
    C    
   G   
   A   
    A    
   A   
   A   
   T   
   G   
   A   
   A   
   T   
   A   
    T    
   C   
   A   
   A   
   G   
   A   
    A    
   C   
   T   
    A    
   T   
   T   
    G    
   A   
    A    
   A   
    C    
   T   
    G    
   A   
   T   
   T   
   G   
    A    
    A    
   G   
   A   
    T    
   T   
   T   
    G    
    A    
    C    
    T    
   G   
   A   
   C   
   A   
   T   
   T   
    C    
   T   
    G    
   G   
   C   
    A    
    A    
   G   
    A    
   C   
   C   
    C    
   G   
   A   
    A    
   C   
   G   
    T    
   G   
   T   
    C    
   A   
    C    
    A    
   G   
   A   
   A   
   A   
   T   
    C    
   A   
   T   
    C    
   A   
   A   
    G    
   A   
    A    
    T    
   G   
   A   
    A    
   T   
   T   
   A   
    G    
    C    
    A    
   G   
   A   
   C   
    T    
   T   
    G    
    A    
    A    
    T    
    C    
    A    
   A   
    C    
    G    
   A   
   T   
   T   
   T   
   G   
   G   
   T   
   G   
   A   
   T   
    A    
    A    
   A   
   C   
   G   
   A   
   C   
   G   
    C    
   A   
   C   
   A   
   G   
   A   
   A   
    T    
   T   
   A   
    C    
   T   
   A   
   G   
   T   
    C    
   G   
   G   
   T   
   G   
   A   
    G    
   G   
   T   
   C   
    T    
   T   
    A    
   A   
   G   
   T   
   C   
   T   
    A    
   G   
   A   
   A   
   G   
   A   
   T   
   G   
   A   
   A   
   G   
   A   
   T   
    T    
   T   
    G    
   A   
   T   
    T    
   G   
   A   
   A   
   G   
   A   
   A   
   G   
   A   
   A   
   G   
   A   
   A   
    A    
   T   
   C   
   G   
   T   
    T    
   A   
   T   
   T   
   A   
   C   
    A    
    T    
   T   
    G    
   A   
   C   
    A    
   A   
   A   
    C    
   A   
   A   
    T    
   G   
   G   
    T    
   T   
   A   
    T    
   A   
   T   
    C    
   A   
   A   
    A    
   C   
   G   
    T    
    T    
   T   
   G   
   G   
    C    
   A   
   A   
   A   
    T    
    A    
    A    
    T    
   G   
   A   
   A   
   T   
   T   
   C   
    A    
   G   
    A    
   G   
   C   
    T    
   C   
   A   
   A   
   C   
   G   
    A    
    A    
   G   
    A    
   G   
   G   
    C    
   G   
   G   
    G    
   C   
   G   
   T   
   G   
   G   
    T    
   G   
   T   
    C    
   C   
   A   
   A   
   G   
   G   
    A    
   A   
   T   
   G   
   G   
   G   
    T    
    A    
   T   
    C    
   C   
   A   
    C    
    A    
   A   
   T   
   G   
   A   
    C    
   G   
   A   
    C    
   T   
   T   
   C   
   G   
   T   
    C    
   A   
   A   
    G    
   A   
   A   
    T    
   C   
   T   
    A    
    G    
   T   
   T   
   T   
   C   
   T   
   T   
   G   
    T    
   T   
   C   
    T    
   A   
   C   
    C    
   C   
   A   
    T    
   G   
   A   
    T    
   A   
   C   
    A    
   T   
   T   
    G    
   T   
   T   
   A   
   T   
   T   
    C    
   T   
   T   
   T   
   A   
   C   
   G   
   A   
   A   
    T    
   A   
    A    
    C    
   G   
   G   
    A    
   A   
   A   
   A   
   G   
   T   
    G    
   T   
   A   
    T    
   C   
   G   
    G    
   G   
   C   
   A   
   A   
   A   
   A   
   G   
   G   
    A    
   T   
   A   
    C    
   G   
   A   
   A   
   A   
   T   
    T    
   C   
   C   
    A    
   G   
   A   
    A    
   T   
   A   
   C   
   G   
   G   
    G    
    C    
   G   
    T    
   A   
   C   
   A   
   G   
   C   
    C    
   A   
   A   
   A   
   G   
   G   
    A    
   A   
   T   
    C    
   C   
   C   
   A   
    A    
   T   
    C    
   A   
   T   
    C    
   A   
   A   
    T    
   T   
   T   
    G    
   C   
   T   
    G    
   G   
   G   
    T    
   A   
   T   
    C    
   G   
   A   
    C    
   T   
   C   
    G    
    A    
    G    
    T    
   G   
   A   
   A   
   A   
    G    
    C    
   A   
   T   
    C    
   C   
   A   
    A    
   G   
   C   
    A    
   A   
   T   
    T    
   A   
   T   
   T   
    G    
   C   
    G    
   G   
   T   
    G    
    A    
    C    
   G   
   G   
   G   
    A    
    G    
   A   
   A   
   G   
   C   
    A    
   G   
   A   
   A   
   G   
    A    
    A    
   G   
   G   
    C    
    C    
   A   
    C    
   T   
   A   
    T    
    C    
   T   
    T    
   T   
   T   
   C   
   T   
   T   
    T    
   A   
   C   
    G    
   A   
   C   
    G    
    C    
    G    
    T    
    C    
   A   
   A   
   G   
   G   
    T    
   A   
   C   
    A    
   G   
   T   
    T    
   A   
   A   
   A   
   C   
   G   
    T    
   A   
   C   
    G    
    T    
   C   
    T    
   G   
   T   
    C    
   A   
   C   
    T    
   G   
   C   
    T    
   T   
   T   
    C    
    G    
   C   
   T   
   A   
   A   
    C    
   A   
   T   
    T    
   C   
   G   
    A    
   A   
   G   
    C    
   A   
   A   
   T   
   G   
   G   
    C    
    C    
   T   
    G    
   A   
   T   
    T    
   G   
   C   
    A    
   A   
   T   
   T   
    G    
   G   
    T    
   T   
   T   
    G    
   A   
   A   
   A   
   G   
   A   
   A   
   G   
   A   
    T    
   G   
   A   
    C    
   G   
   A   
    A    
    T    
   T   
    A    
    G    
   T   
    C    
   A   
   A   
    T    
   G   
   T   
    T    
    C    
   T   
   A   
    T    
    T    
   G   
   A   
   C   
    A    
    G    
   A   
    C    
   G   
   G   
    A    
    C    
   A   
    G    
    T    
    C    
    A    
   A   
   A   
    T    
   A   
   T   
    C    
   A   
   T   
    C    
   A   
   T   
    T    
   G   
   G   
    C    
   A   
   C   
   A   
   C   
   A   
   T   
    A    
    A    
    C    
   G   
   G   
    C    
   T   
   A   
   C   
   T   
   C   
   T   
   G   
   T   
    G    
   A   
   C   
    C    
   T   
   T   
    C    
    A    
   A   
    A    
   G   
   A   
    A    
    G    
    A    
    A    
    G    
   C   
    A    
   G   
    T    
    C    
   C   
   G   
    C    
   G   
   A   
   T   
   A   
   T   
   G   
   G   
   G   
    A    
   C   
   G   
    T    
   A   
   C   
   A   
   G   
   C   
    T    
   T   
   C   
    C    
   G   
   G   
    C    
   G   
   T   
    C    
    A    
   G   
    A    
   G   
   G   
    G    
   A   
   T   
    A    
   C   
   G   
    T    
    C    
   T   
    G    
   C   
   G   
    T    
   G   
   A   
    G    
    G    
   A   
    A    
   G   
   A   
    T    
   T   
    A    
    C    
   G   
   T   
    C    
   G   
   T   
    T    
   G   
   G   
    G    
    G    
   C   
   A   
   G   
   C   
    C    
    T    
   T   
    G    
    A    
   T   
   G   
    A    
   A   
    G    
   G   
    A    
   A   
    G    
   A   
    C    
    C    
   A   
   A   
   G   
   A   
   A   
   G   
   T   
    A    
    C    
   T   
    G    
   G   
   T   
    G    
   A   
   T   
    T    
   A   
   C   
    A    
   G   
   A   
   A   
   A   
   A   
    A    
   G   
   G   
   T   
   T   
   A   
    C    
   G   
   G   
    A    
   A   
   A   
    A    
   C   
   G   
    G    
   A   
   C   
    C    
   A   
   A   
   A   
   G   
    T    
    A    
   T   
   C   
    G    
   G   
   A   
    A    
   T   
   A   
   T   
   C   
   C   
   A   
   G   
   T   
    G    
   A   
   A   
    A    
   G   
   G   
    C    
   C   
   G   
    C    
   G   
   G   
    A    
   G   
   G   
   T   
   A   
   A   
   A   
   G   
   G   
    G    
   A   
   T   
    C    
   A   
   A   
    A    
   A   
   C   
    G    
    G    
   C   
    G    
   A   
   A   
    T    
   A   
   T   
   C   
   A   
   C   
   T   
   G   
   A   
    A    
   A   
   A   
   A   
   A   
   A   
   T   
   G   
   G   
   T   
   C   
   C   
   A   
    C    
   T   
   A   
   G   
   C   
    C    
   G   
   G   
    A    
   T   
   T   
    G    
   A   
   C   
   C   
   A   
   C   
    T    
   G   
   T   
    T    
    T    
    C    
   T   
   G   
   G   
    A    
    A    
   A   
   T   
   G   
   A   
   A   
   G   
   A   
    C    
   A   
   T   
   C   
    A    
   T   
    G    
    C    
   T   
    G    
   A   
   T   
    T    
   A   
   C   
    A    
    G    
   A   
    T    
   A   
   A   
   A   
   G   
   G   
    T    
   G   
   T   
    G    
   A   
   T   
   T   
   A   
   T   
   C   
   C   
   G   
    T    
   T   
   T   
    C    
   A   
   A   
   C   
   G   
   T   
    A    
    T    
    C    
    G    
    A    
   C   
   T   
   G   
   T   
   T   
   T   
   C   
   T   
   C   
   A   
   A   
   A   
   C   
    T    
   G   
   G   
   A   
   C   
   G   
    T    
    T    
   C   
   A   
   A   
   C   
   A   
   T   
   T   
    G    
   G   
   G   
    T    
   G   
   T   
    A    
   C   
   G   
   T   
   T   
   T   
    G    
   A   
   T   
   G   
   A   
    A    
    G    
   A   
   T   
   G   
   G   
   A   
   A   
   G   
    C    
    T    
   G   
    A    
    T    
    A    
   C   
    G    
   A   
   A   
   A   
   G   
   T   
   C   
   G   
   T   
   C   
   A   
   C   
   A   
   A   
   T   
   G   
   -   
   -   
   -   
   -   
   -   
   -   
   -   
   -   
   -   
   -   
   -   
   -   
   -   
   -   
   -   
   -   
   -   
   -   
   -   
   -   
   -   
   -   
   -   
   -   
   -   
   -   
   -   
   -   
   -   
   -   
   -   
   -   
   -   
   -   
   -   
   -   
   -   
   -   
   -   
   -   
   -   
   -   
   -   
   -   
   -   
   -   
   -   
   -   
   -   
   -   
   -   
   -   
   -   
   -   
   -   
   -   
   -   
   -   
   -   
   -   
 

 
   N59836F   
   A   
   T   
   G   
   A   
   G   
   T   
   G   
   A   
   A   
   G   
   A   
   A   
   A   
   T   
   T   
   A   
   A   
   A   
   G   
   A   
   A   
   A   
   A   
   C   
   A   
   T   
   T   
   C   
   A   
   A   
   G   
   A   
   C   
   G   
   T   
   C   
   A   
   A   
   T   
   C   
   T   
   G   
   A   
   C   
   C   
   A   
   G   
   C   
   G   
   A   
   A   
   A   
   T   
   G   
   A   
   A   
   A   
   G   
   A   
   A   
   T   
   C   
   T   
   T   
   T   
   C   
   A   
   T   
   T   
   G   
   A   
   C   
   T   
   A   
   C   
   G   
   C   
   A   
   A   
   T   
   G   
   A   
   G   
   T   
   G   
   T   
   T   
   A   
   T   
   C   
   G   
   T   
   A   
   G   
   C   
   C   
   C   
   G   
   C   
   G   
   C   
   G   
   T   
   T   
   A   
   C   
   C   
   T   
   G   
   A   
   C   
   G   
   T   
   T   
   C   
   G   
   T   
   G   
   A   
   C   
   G   
   G   
   T   
   T   
   T   
   A   
   A   
   A   
   A   
   C   
   C   
   T   
   G   
   T   
   T   
   C   
   A   
   T   
   C   
   G   
   C   
   C   
   G   
   A   
   A   
   T   
   C   
   T   
   T   
   A   
   T   
   A   
   T   
   G   
   G   
   A   
   A   
   T   
   G   
   A   
   A   
   C   
   G   
   A   
   A   
   T   
   T   
   A   
   G   
   G   
   G   
   G   
   T   
   A   
   A   
   C   
   C   
   C   
   C   
   T   
   G   
   A   
   T   
   A   
   A   
   A   
   C   
   C   
   A   
   C   
   A   
   C   
   A   
   A   
   A   
   A   
   A   
   A   
   T   
   C   
   A   
   G   
   C   
   C   
   C   
   G   
   G   
   A   
   T   
   T   
   G   
   T   
   T   
   G   
   G   
   G   
   G   
   A   
   T   
   G   
   T   
   T   
   A   
   T   
   G   
   G   
   G   
   T   
   A   
   A   
   A   
   T   
   A   
   T   
   C   
   A   
   C   
   C   
   C   
   C   
   C   
   A   
   T   
   G   
   G   
   G   
   G   
   A   
   C   
   A   
   G   
   T   
   G   
   C   
   G   
   A   
   T   
   T   
   T   
   A   
   C   
   G   
   A   
   A   
   T   
   C   
   A   
   A   
   T   
   G   
   G   
   T   
   G   
   C   
   G   
   G   
   A   
   T   
   G   
   G   
   C   
   A   
   C   
   A   
   A   
   C   
   C   
   T   
   T   
   T   
   T   
   A   
   G   
   T   
   T   
   A   
   T   
   C   
   G   
   G   
   G   
   C   
   T   
   A   
   T   
   G   
   C   
   T   
   A   
   G   
   T   
   T   
   G   
   A   
   C   
   G   
   G   
   C   
   C   
   A   
   C   
   G   
   G   
   A   
   A   
   A   
   C   
   T   
   T   
   C   
   G   
   G   
   T   
   T   
   C   
   T   
   G   
   T   
   C   
   G   
   A   
   T   
   G   
   G   
   T   
   G   
   A   
   C   
   G   
   G   
   C   
   G   
   C   
   T   
   G   
   C   
   C   
   G   
   C   
   T   
   A   
   T   
   G   
   C   
   G   
   T   
   T   
   A   
   T   
   A   
   C   
   C   
   G   
   A   
   A   
   G   
   C   
   A   
   C   
   G   
   T   
   A   
   T   
   G   
   A   
   G   
   T   
   A   
   A   
   A   
   A   
   T   
   T   
   G   
   C   
   T   
   T   
   T   
   A   
   G   
   A   
   A   
   A   
   T   
   G   
   C   
   T   
   A   
   C   
   G   
   A   
   G   
   A   
   T   
   A   
   T   
   T   
   A   
   A   
   C   
   A   
   A   
   A   
   A   
   A   
   T   
   A   
   C   
   A   
   G   
   T   
   C   
   G   
   A   
   T   
   T   
   T   
   C   
   C   
   A   
   A   
   G   
   G   
   A   
   A   
   A   
   C   
   T   
   A   
   T   
   G   
   A   
   T   
   G   
   A   
   T   
   T   
   C   
   A   
   G   
   A   
   A   
   C   
   A   
   A   
   G   
   A   
   A   
   C   
   C   
   A   
   G   
   T   
   A   
   G   
   T   
   T   
   T   
   T   
   A   
   C   
   C   
   T   
   G   
   C   
   T   
   C   
   G   
   T   
   T   
   T   
   T   
   C   
   C   
   A   
   A   
   A   
   C   
   T   
   T   
   A   
   C   
   T   
   A   
   G   
   T   
   T   
   A   
   A   
   C   
   G   
   G   
   A   
   A   
   C   
   A   
   A   
   C   
   G   
   G   
   G   
   G   
   A   
   T   
   T   
   G   
   C   
   G   
   G   
   T   
   G   
   G   
   G   
   G   
   A   
   T   
   G   
   G   
   C   
   A   
   A   
   C   
   A   
   A   
   A   
   T   
   A   
   T   
   T   
   C   
   C   
   A   
   C   
   C   
   A   
   C   
   A   
   C   
   A   
   A   
   T   
   T   
   T   
   A   
   A   
   G   
   T   
   G   
   A   
   A   
   G   
   T   
   G   
   A   
   T   
   T   
   G   
   A   
   T   
   G   
   C   
   A   
   A   
   C   
   A   
   A   
   G   
   T   
   T   
   T   
   A   
   T   
   T   
   G   
   A   
   T   
   G   
   G   
   A   
   C   
   A   
   A   
   T   
   C   
   C   
   T   
   G   
   A   
   T   
   G   
   T   
   A   
   A   
   C   
   G   
   A   
   C   
   G   
   A   
   A   
   T   
   G   
   A   
   A   
   T   
   T   
   G   
   A   
   T   
   G   
   G   
   A   
   A   
   G   
   T   
   G   
   T   
   T   
   A   
   C   
   C   
   T   
   G   
   G   
   A   
   C   
   C   
   A   
   G   
   A   
   T   
   T   
   T   
   T   
   C   
   C   
   A   
   A   
   C   
   A   
   G   
   G   
   C   
   G   
   G   
   T   
   T   
   T   
   A   
   G   
   T   
   G   
   A   
   T   
   G   
   G   
   G   
   G   
   A   
   A   
   A   
   T   
   C   
   A   
   G   
   G   
    G    
   A   
   T   
    T    
   C   
   G   
    C    
    C    
   G   
   A   
   G   
   C   
    A    
   T   
   A   
   T   
   G   
   A   
   A   
   A   
   C   
    A    
   G   
   G   
    G    
   A   
   A   
    A    
   G   
   G   
   T   
   T   
   C   
    G    
   A   
   T   
   T   
   A   
   C   
    T    
   G   
   T   
    T    
   C   
   G   
   T   
   G   
   C   
   A   
   A   
   A   
   A   
   G   
   T   
   T   
   G   
   A   
    A    
    T    
   T   
    G    
   A   
    C    
   T   
   G   
   A   
    A    
   A   
   T   
   G   
    C    
   C   
    G    
   A   
   A   
   T   
   G   
   G   
    A    
   A   
   A   
   A   
   G   
   A   
   A   
   C   
   G   
    T    
   A   
   T   
    T    
    T    
   T   
    A    
   G   
   T   
    A    
   A   
   C   
    T    
   G   
   A   
   A   
   T   
   T   
    G    
   C   
   C   
   T   
   T   
   A   
   T   
   A   
   T   
   G   
   G   
   T   
    G    
   A   
   A   
   T   
   A   
   A   
   A   
   G   
   C   
    C    
   A   
   A   
   A   
   T   
   T   
    A    
   A   
   T   
    C    
   G   
   A   
   A   
   C   
   G   
    A    
   A   
   T   
   T   
   T   
   C   
   T   
   G   
   A   
    A    
    T    
   T   
    A    
   C   
   A   
   C   
   A   
   G   
   A   
   G   
   A   
    T    
   A   
   A   
    A    
   C   
   G   
    A    
   A   
   T   
    T    
   G   
   A   
   A   
   G   
   G   
    A    
   A   
   T   
    T    
   A   
   C   
    T    
   G   
   A   
   T   
   T   
   T   
   G   
   C   
   G   
   G   
   G   
   A   
    T    
   G   
   A   
   A   
   T   
   C   
   T   
   T   
   C   
    T    
   C   
   G   
   T   
   G   
   A   
    A    
   G   
   G   
    C    
   A   
   T   
   G   
   C   
   G   
    G    
   A   
   T   
    T    
   G   
   T   
   C   
   A   
   T   
    C    
   G   
   A   
    T    
    G    
   T   
    T    
   C   
   G   
   T   
   C   
   G   
    A    
   G   
   A   
    T    
   G   
    T    
    G    
   A   
   G   
    T    
   G   
   C   
    C    
   T   
   C   
    T    
   G   
   T   
   T   
    G    
   T   
    G    
   T   
   T   
    A    
   A   
   A   
    C    
   A   
   A   
   C   
   T   
   T   
    G    
   T   
   A   
    C    
   A   
   A   
   A   
    A    
   T   
   G   
   A   
   C   
    T    
    G    
   C   
    C    
   T   
   T   
    A    
   C   
   A   
    A    
   A   
    C    
    A    
   T   
   C   
    T    
   T   
   T   
    T    
   G   
   G   
    T    
   T   
   T   
    T    
   A   
   A   
    C    
   A   
   T   
   G   
   T   
   T   
    A    
   G   
   C   
    C    
   A   
   T   
    C    
   G   
   A   
   A   
   A   
   A   
   A   
   G   
   G   
    C    
   G   
   T   
    A    
   C   
   C   
    G    
   A   
   A   
   A   
    A    
   T   
   T   
   T   
   T   
    A    
   A   
   G   
    C    
   T   
   T   
   G   
   A   
   A   
   A   
   C   
    G    
    T    
   A   
   T   
    T    
    T    
   T   
    A    
   G   
   A   
   A   
   A   
   A   
    C    
   T   
   A   
    C    
    G    
   T   
    C    
   G   
   A   
    G    
   C   
   A   
    C    
   C   
   A   
    A    
    A    
    A    
    A    
    G    
   A   
   A   
   G   
   T   
   C   
   A   
   T   
    T    
   A   
   C   
    T    
    C    
   G   
    C    
   C   
   G   
   G   
   A   
   C   
    G    
    A    
   T   
    T    
   T   
   T   
   T   
   G   
   A   
    C    
   A   
   A   
   A   
    A    
   A   
    C    
   A   
   A   
    G    
   G   
   C   
   A   
   G   
   A   
   A   
   G   
   C   
   A   
    C    
   G   
    G    
   G   
   C   
    G    
   C   
   A   
    T    
   A   
   T   
    T    
   T   
   T   
   A   
   G   
   A   
   A   
   G   
   G   
   T   
    C    
   T   
    A    
   C   
   G   
    A    
   A   
   T   
   T   
   G   
   C   
    C    
   T   
   T   
    A    
   G   
   A   
   T   
   C   
   A   
   T   
   A   
   T   
   C   
    G    
   A   
   T   
   G   
   A   
   A   
   A   
   T   
    C    
   A   
   T   
   T   
   G   
   C   
    C    
   A   
   T   
   T   
   A   
   T   
    C    
   C   
   G   
    T    
   G   
   G   
    G    
   T   
   C   
    A    
    C    
   A   
   A   
   T   
   C   
   A   
   G   
   A   
   T   
   G   
   A   
    C    
   G   
   A   
    A    
   G   
   C   
   A   
   A   
   A   
   A   
   G   
   C   
    A    
   A   
    C    
    G    
    T    
   T   
    A    
   A   
   T   
    T    
   G   
   A   
   A   
   C   
   G   
    C    
   T   
   T   
    T    
   G   
   A   
    A    
   T   
   T   
    T    
   T   
   C   
    A    
   G   
   A   
   T   
   C   
   G   
    T    
   C   
   A   
    A    
    G    
   C   
    G    
   C   
   A   
   A   
   G   
   C   
    A    
   A   
   T   
    T    
   T   
   T   
   A   
   G   
   A   
   T   
   A   
   T   
   G   
   C   
   G   
   T   
   T   
   T   
    A    
   C   
   G   
    C    
   C   
   G   
    T    
   T   
   T   
    A    
   A   
   C   
    A    
   G   
   G   
    C    
   T   
   T   
    A    
   G   
   A   
   A   
   C   
   G   
    C    
   G   
   A   
    C    
   A   
   A   
   A   
   A   
   T   
    T    
   G   
   A   
    A    
   A   
   A   
   T   
   G   
   A   
   A   
   T   
   A   
    T    
   C   
   A   
   A   
   G   
   A   
    G    
   C   
   T   
    A    
   T   
   T   
    G    
   A   
    A    
   A   
    T    
   T   
    C    
   A   
   T   
   T   
   G   
    C    
    G    
   G   
   A   
    C    
   T   
   T   
    A    
    G    
    A    
    G    
   G   
   A   
   C   
   A   
   T   
   T   
    T    
   T   
    A    
   G   
   C   
    C    
    C    
   G   
    C    
   C   
   C   
    A    
   G   
   A   
    A    
   C   
   G   
    C    
   G   
   T   
    C    
   A   
    T    
    T    
   G   
   A   
   A   
   A   
   T   
    C    
   A   
   T   
    T    
   A   
   A   
    A    
   A   
    C    
    A    
   G   
   A   
    G    
   T   
   T   
   A   
    A    
    A    
    T    
   G   
   A   
   C   
    G    
   T   
    T    
    C    
    G    
    C    
    A    
    C    
   A   
    A    
    A    
   A   
   T   
   T   
   T   
   G   
   G   
   T   
   G   
   A   
   T   
    G    
    C    
   A   
   C   
   G   
   A   
   C   
   G   
    C    
   A   
   C   
   A   
   G   
   A   
   A   
    T    
   T   
   A   
    T    
   T   
   A   
   G   
   T   
    A    
   G   
   G   
   T   
   G   
   A   
    A    
   G   
   T   
   C   
    T    
   T   
    A    
   A   
   G   
   T   
   C   
   T   
    T    
   G   
   A   
   A   
   G   
   A   
   T   
   G   
   A   
   A   
   G   
   A   
   T   
    T    
   T   
    A    
   A   
   T   
    C    
   G   
   A   
   A   
   G   
   A   
   A   
   G   
   A   
   A   
   G   
   A   
   A   
    G    
   T   
   C   
   G   
   T   
    G    
   A   
   T   
   T   
   A   
   C   
    G    
    T    
   T   
    A    
   A   
   C   
    C    
   A   
   A   
    T    
   A   
   A   
    C    
   G   
   G   
    C    
   T   
   A   
    C    
   A   
   T   
    T    
   A   
   A   
    G    
   C   
   G   
    G    
    A    
   T   
   G   
   G   
    C    
   A   
   A   
   A   
    C    
    T    
    C    
    C    
   G   
   A   
   A   
   T   
   T   
   C   
    C    
   G   
    G    
   G   
   C   
    G    
   C   
   A   
   A   
   C   
   G   
    C    
    C    
   G   
    C    
   G   
   G   
    T    
   G   
   G   
    A    
   C   
   G   
   T   
   G   
   G   
    T    
   G   
   T   
    C    
   C   
   A   
   A   
   G   
   G   
    G    
   A   
   T   
   G   
   G   
   G   
    C    
    G    
   T   
    T    
   C   
   A   
    T    
    G    
   A   
   T   
   G   
   A   
    T    
   G   
   A   
    T    
   T   
   T   
   C   
   G   
   T   
    G    
   A   
   A   
    A    
   A   
   A   
    C    
   C   
   T   
    A    
    G    
   T   
   T   
   T   
   C   
   T   
   T   
   G   
    T    
   T   
   C   
    A    
   A   
   C   
    A    
   C   
   A   
    C    
   G   
   A   
    C    
   A   
   C   
    G    
   T   
   T   
    A    
   T   
   T   
   A   
   T   
   T   
    C    
   T   
   T   
   T   
   A   
   C   
   G   
   A   
   A   
    C    
   A   
    C    
    T    
   G   
   G   
    G    
   A   
   A   
   A   
   G   
   T   
    T    
   T   
   A   
    C    
   C   
   G   
    A    
   G   
   C   
   A   
   A   
   A   
   A   
   G   
   G   
    T    
   T   
   A   
    C    
   G   
   A   
   A   
   A   
   T   
    C    
   C   
   C   
    T    
   G   
   A   
    A    
   T   
   A   
   C   
   G   
   G   
    T    
    A    
   G   
    A    
   A   
   C   
   A   
   G   
   C   
    A    
   A   
   A   
   A   
   G   
   G   
    A    
   A   
   T   
    T    
   C   
   C   
   A   
    G    
   T   
    G    
   A   
   T   
    T    
   A   
   A   
    C    
   T   
   T   
    A    
   C   
   T   
    G    
   G   
   G   
    A    
   A   
   T   
    T    
   G   
   A   
    T    
   T   
   C   
    T    
    G    
    C    
    A    
   G   
   A   
   A   
   A   
    A    
    A    
   A   
   T   
    T    
   C   
   A   
    A    
   G   
   C   
    G    
   A   
   T   
    T    
   A   
   T   
   T   
    T    
   C   
    T    
   G   
   T   
    T    
    G    
    A    
   G   
   G   
   G   
    C    
    A    
   A   
   A   
   G   
   C   
    G    
   G   
   A   
   A   
   G   
    C    
    A    
   G   
   G   
    T    
    A    
   A   
    A    
   T   
   A   
    C    
    T    
   T   
    G    
   T   
   T   
   C   
   T   
   T   
    C    
   A   
   C   
    A    
   A   
   C   
    C    
    T    
    T    
    A    
    A    
   A   
   A   
   G   
   G   
    A    
   A   
   C   
    C    
   G   
   T   
    C    
   A   
   A   
   A   
   C   
   G   
    G    
   A   
   C   
    A    
    G    
   C   
    C    
   G   
   T   
    A    
   A   
   C   
    A    
   G   
   C   
    C    
   T   
   T   
    T    
    T    
   C   
   T   
   A   
   A   
    T    
   A   
   T   
    C    
   C   
   G   
    T    
   A   
   G   
    T    
   A   
   A   
   T   
   G   
   G   
    A    
    T    
   T   
    A    
   A   
   T   
    C    
   G   
   C   
    C    
   A   
   T   
   T   
    A    
   G   
    C    
   T   
   T   
    A    
   A   
   A   
   A   
   G   
   A   
   A   
   G   
   A   
    T    
   G   
   A   
    T    
   G   
   A   
    G    
    T    
   T   
    A    
    G    
   T   
    T    
   A   
   A   
    C    
   G   
   T   
    G    
    G    
   T   
   A   
    A    
    C    
   G   
   A   
   C   
    T    
    A    
   A   
    T    
   G   
   G   
    C    
    A    
   A   
    T    
    C    
    A    
    G    
   A   
   A   
    G    
   A   
   T   
    G    
   A   
   T   
    T    
   A   
   T   
    C    
   G   
   G   
    A    
   A   
   C   
   A   
   C   
   A   
   T   
    G    
    C    
    A    
   G   
   G   
    A    
   T   
   A   
   C   
   T   
   C   
   T   
   G   
   T   
    C    
   A   
   C   
    A    
   T   
   T   
    T    
    G    
   A   
    T    
   G   
   A   
    A    
    A    
    A    
    T    
    A    
   C   
    T    
   G   
    T    
    A    
   C   
   G   
    T    
   G   
   A   
   T   
   A   
   T   
   G   
   G   
   G   
    C    
   C   
   G   
    G    
   A   
   C   
   A   
   G   
   C   
    A    
   T   
   C   
    A    
   G   
   G   
    T    
   G   
   T   
    T    
    C    
   G   
    G    
   G   
   G   
    A    
   A   
   T   
    C    
   C   
   G   
    T    
    C    
   T   
    C    
   C   
   G   
    C    
   G   
   A   
    A    
    A    
   A   
    T    
   G   
   A   
    T    
   T   
    A    
    T    
   G   
   T   
    G    
   G   
   T   
    C    
   G   
   G   
    T    
    G    
   C   
   A   
   G   
   C   
    G    
    A    
   T   
    T    
    C    
   T   
   G   
    G    
   A   
    T    
   G   
    A    
   A   
    A    
   A   
    C    
    A    
   A   
   A   
   G   
   A   
   A   
   G   
   T   
    C    
    C    
   T   
    A    
   G   
   T   
    C    
   A   
   T   
    T    
   A   
   C   
    T    
   G   
   A   
   A   
   A   
   A   
    T    
   G   
   G   
   T   
   T   
   A   
    T    
   G   
   G   
    T    
   A   
   A   
    G    
   C   
   G   
    T    
   A   
   C   
    A    
   A   
   A   
   A   
   G   
    C    
    T    
   T   
   C   
    T    
   G   
   A   
    A    
   T   
   A   
   T   
   C   
   C   
   A   
   G   
   T   
    T    
   A   
   A   
    A    
   G   
   G   
    A    
   C   
   G   
    T    
   G   
   G   
    C    
   G   
   G   
   T   
   A   
   A   
   A   
   G   
   G   
    G    
   A   
   T   
    T    
   A   
   A   
    G    
   A   
   C   
    A    
    G    
   C   
    A    
   A   
   A   
    T    
   A   
   T   
   C   
   A   
   C   
   T   
   G   
   A   
    G    
   A   
   A   
   A   
   A   
   A   
   T   
   G   
   G   
   T   
   C   
   C   
   A   
    T    
   T   
   A   
   G   
   C   
    T    
   G   
   G   
    T    
   T   
   T   
    A    
   A   
   C   
   C   
   A   
   C   
    G    
   G   
   T   
    C    
    A    
    A    
   T   
   G   
   G   
    G    
    G    
   A   
   T   
   G   
   A   
   A   
   G   
   A   
    T    
   A   
   T   
   C   
    T    
   T   
    A    
    T    
   T   
    G    
   A   
   T   
    T    
   A   
   C   
    G    
    A    
   A   
    C    
   A   
   A   
   A   
   G   
   G   
    C    
   G   
   T   
    C    
   A   
   T   
   T   
   A   
   T   
   C   
   C   
   G   
    C    
   T   
   T   
    T    
   A   
   A   
   C   
   G   
   T   
    T    
    G    
    A    
    T    
    T    
   C   
   T   
   G   
   T   
   T   
   T   
   C   
   T   
   C   
   A   
   A   
   A   
   C   
    A    
   G   
   G   
   A   
   C   
   G   
    C    
    G    
   C   
   A   
   A   
   C   
   A   
   T   
   T   
    A    
   G   
   G   
    G    
   G   
   T   
    T    
   C   
   G   
   T   
   T   
   T   
    A    
   A   
   T   
   G   
   A   
    G    
    A    
   A   
   T   
   G   
   G   
   A   
   A   
   G   
    A    
    T    
   G   
    G    
    T    
    G    
   C   
    C    
   A   
   A   
   A   
   -   
   -   
   -   
   -   
   -   
   -   
   -   
   -   
   -   
   -   
   -   
   -   
   -   
   -   
   -   
   -   
   -   
   -   
   -   
   -   
   -   
   -   
   -   
   -   
   -   
   -   
   -   
   -   
   -   
   -   
   -   
   -   
   -   
   -   
   -   
   -   
   -   
   -   
   -   
   -   
   -   
   -   
   -   
   -   
   -   
   -   
   -   
   -   
   -   
   -   
   -   
   -   
   -   
   -   
   -   
   -   
   -   
   -   
   -   
   -   
   -   
   -   
   -   
   -   
   -   
   -   
   -   
   -   
   -   
   -   
   -   
   -   
 

 
   N59469F   
   A   
   T   
   G   
   A   
   G   
   T   
   G   
   A   
   A   
   G   
   A   
   A   
   A   
   T   
   C   
   A   
   G   
   A   
   G   
   A   
   A   
   A   
   A   
   C   
   A   
   T   
   C   
   C   
   A   
   T   
   G   
   A   
   T   
   G   
   T   
   C   
   A   
   A   
   T   
   C   
   T   
   A   
   A   
   C   
   C   
   A   
   G   
   C   
   G   
   A   
   A   
   A   
   T   
   G   
   A   
   A   
   A   
   G   
   A   
   C   
   T   
   C   
   C   
   T   
   T   
   C   
   A   
   T   
   C   
   G   
   A   
   T   
   T   
   A   
   T   
   G   
   C   
   G   
   A   
   T   
   G   
   A   
   G   
   T   
   G   
   T   
   T   
   A   
   T   
   T   
   G   
   T   
   C   
   G   
   C   
   A   
   C   
   G   
   T   
   G   
   C   
   A   
   C   
   T   
   T   
   C   
   C   
   T   
   G   
   A   
   T   
   G   
   T   
   C   
   A   
   G   
   A   
   G   
   A   
   T   
   G   
   G   
   T   
   C   
   T   
   G   
   A   
   A   
   A   
   C   
   C   
   C   
   G   
   T   
   T   
   C   
   A   
   T   
   C   
   G   
   C   
   C   
   G   
   T   
   A   
   T   
   T   
   T   
   T   
   A   
   T   
   A   
   C   
   G   
   G   
   C   
   A   
   T   
   G   
   A   
   A   
   C   
   G   
   A   
   A   
   T   
   T   
   A   
   G   
   G   
   T   
   G   
   T   
   A   
   A   
   C   
   A   
   C   
   C   
   G   
   G   
   A   
   T   
   A   
   A   
   A   
   G   
   C   
   C   
   C   
   A   
   T   
   A   
   A   
   A   
   A   
   A   
   A   
   T   
   C   
   T   
   G   
   C   
   C   
   A   
   G   
   A   
   A   
   T   
   C   
   G   
   T   
   C   
   G   
   G   
   A   
   G   
   A   
   T   
   G   
   T   
   C   
   A   
   T   
   G   
   G   
   G   
   T   
   A   
   A   
   A   
   T   
   A   
   C   
   C   
   A   
   T   
   C   
   C   
   T   
   C   
   A   
   T   
   G   
   G   
   A   
   G   
   A   
   C   
   A   
   G   
   T   
   G   
   C   
   G   
   A   
   T   
   C   
   T   
   A   
   T   
   G   
   A   
   G   
   T   
   C   
   A   
   A   
   T   
   G   
   G   
   T   
   A   
   C   
   G   
   G   
   A   
   T   
   G   
   G   
   C   
   A   
   C   
   A   
   G   
   C   
   C   
   A   
   T   
   T   
   C   
   A   
   G   
   C   
   T   
   A   
   C   
   C   
   G   
   T   
   T   
   A   
   T   
   A   
   T   
   G   
   C   
   T   
   A   
   G   
   T   
   G   
   G   
   A   
   T   
   G   
   G   
   C   
   C   
   A   
   T   
   G   
   G   
   A   
   A   
   A   
   C   
   T   
   T   
   T   
   G   
   G   
   T   
   T   
   C   
   T   
   G   
   T   
   T   
   G   
   A   
   C   
   G   
   G   
   T   
   G   
   A   
   C   
   G   
   G   
   T   
   G   
   C   
   C   
   G   
   C   
   T   
   G   
   C   
   T   
   A   
   T   
   G   
   C   
   G   
   T   
   T   
   A   
   T   
   A   
   C   
   C   
   G   
   A   
   A   
   G   
   C   
   A   
   C   
   G   
   G   
   A   
   T   
   G   
   A   
   G   
   T   
   A   
   A   
   A   
   A   
   T   
   C   
   G   
   C   
   T   
   A   
   C   
   A   
   G   
   A   
   A   
   A   
   T   
   G   
   C   
   T   
   G   
   C   
   G   
   T   
   G   
   A   
   T   
   A   
   T   
   C   
   A   
   A   
   T   
   A   
   A   
   G   
   A   
   A   
   C   
   A   
   C   
   T   
   G   
   T   
   T   
   G   
   A   
   T   
   T   
   T   
   C   
   C   
   A   
   A   
   A   
   G   
   C   
   A   
   A   
   C   
   T   
   A   
   T   
   G   
   A   
   T   
   G   
   A   
   T   
   A   
   C   
   A   
   G   
   A   
   A   
   A   
   A   
   A   
   G   
   A   
   A   
   C   
   C   
   A   
   G   
   T   
   T   
   G   
   T   
   A   
   C   
   T   
   A   
   C   
   C   
   T   
   G   
   C   
   T   
   C   
   G   
   C   
   T   
   T   
   C   
   C   
   C   
   G   
   A   
   A   
   T   
   C   
   T   
   T   
   T   
   T   
   A   
   G   
   T   
   G   
   A   
   A   
   C   
   G   
   G   
   A   
   A   
   C   
   A   
   A   
   C   
   G   
   G   
   G   
   G   
   A   
   T   
   C   
   G   
   C   
   C   
   G   
   T   
   A   
   G   
   G   
   T   
   A   
   T   
   G   
   G   
   C   
   C   
   A   
   C   
   G   
   A   
   A   
   T   
   A   
   T   
   C   
   C   
   C   
   A   
   C   
   C   
   T   
   C   
   A   
   T   
   A   
   A   
   C   
   T   
   T   
   G   
   T   
   C   
   T   
   G   
   A   
   G   
   G   
   T   
   A   
   G   
   T   
   A   
   G   
   C   
   G   
   G   
   C   
   T   
   A   
   T   
   C   
   G   
   A   
   T   
   T   
   T   
   G   
   C   
   T   
   G   
   A   
   T   
   G   
   G   
   A   
   T   
   A   
   A   
   T   
   C   
   C   
   T   
   G   
   A   
   T   
   G   
   T   
   G   
   A   
   C   
   G   
   A   
   C   
   C   
   A   
   A   
   T   
   G   
   A   
   A   
   T   
   T   
   G   
   A   
   T   
   G   
   G   
   A   
   A   
   G   
   T   
   A   
   T   
   T   
   A   
   C   
   C   
   T   
   G   
   G   
   A   
   C   
   C   
   A   
   G   
   A   
   T   
   T   
   T   
   T   
   C   
   C   
   A   
   A   
   C   
   A   
   G   
   G   
   T   
   G   
   G   
   T   
   T   
   T   
   G   
   G   
   T   
   T   
   A   
   T   
   G   
   G   
   G   
   A   
   A   
   A   
   A   
   T   
   C   
   A   
   G   
   G   
    A    
   A   
   T   
    C    
   C   
   G   
    A    
    A    
   G   
   A   
   G   
   C   
    G    
   T   
   A   
   T   
   G   
   A   
   A   
   A   
   C   
    A    
   G   
   G   
    T    
   A   
   A   
    G    
   G   
   G   
   T   
   T   
   C   
    T    
   A   
   T   
   T   
   A   
   C   
    C    
   G   
   T   
    A    
   C   
   G   
   T   
   G   
   C   
   A   
   A   
   A   
   A   
   G   
   T   
   T   
   G   
   A   
    G    
    A    
   T   
    T    
   A   
    C    
   T   
   G   
   A   
    G    
   A   
   T   
   G   
    C    
   C   
    A    
   A   
   A   
   T   
   G   
   G   
    T    
   A   
   A   
   A   
   G   
   A   
   A   
   C   
   G   
    G    
   A   
   T   
    C    
    A    
   T   
    C    
   G   
   T   
    G    
   A   
   C   
    A    
   G   
   A   
   A   
   T   
   T   
    G    
   C   
   C   
   T   
   T   
   A   
   T   
   A   
   T   
   G   
   G   
   T   
    C    
   A   
   A   
   T   
   A   
   A   
   A   
   G   
   C   
    A    
   A   
   A   
   A   
   T   
   T   
    G    
   A   
   T   
    C    
   G   
   A   
   A   
   C   
   G   
    G    
   A   
   T   
   T   
   T   
   C   
   T   
   G   
   A   
    A    
    C    
   T   
    G    
   C   
   A   
   C   
   A   
   G   
   A   
   G   
   A   
    C    
   A   
   A   
    G    
   C   
   G   
    T    
   A   
   T   
    C    
   G   
   A   
   A   
   G   
   G   
    G    
   A   
   T   
    C    
   A   
   C   
    A    
   G   
   A   
   T   
   T   
   T   
   G   
   C   
   G   
   G   
   G   
   A   
    C    
   G   
   A   
   A   
   T   
   C   
   T   
   T   
   C   
    C    
   C   
   G   
   T   
   G   
   A   
    A    
   G   
   G   
    T    
   A   
   T   
   G   
   C   
   G   
    G    
   A   
   T   
    C    
   G   
   T   
   C   
   A   
   T   
    T    
   G   
   A   
    C    
    A    
   T   
    C    
   C   
   G   
   T   
   C   
   G   
    T    
   G   
   A   
    C    
   G   
    C    
    C    
   A   
   G   
    C    
   G   
   C   
    A    
   T   
   C   
    C    
   G   
   T   
   T   
    A    
   T   
    C    
   T   
   T   
    G    
   A   
   A   
    T    
   A   
   A   
   C   
   T   
   T   
    A    
   T   
   A   
    T    
   A   
   A   
   A   
    T    
   T   
   G   
   A   
   C   
    A    
    T    
   C   
    C    
   T   
   T   
    G    
   C   
   A   
    A    
   A   
    A    
    T    
   T   
   C   
    A    
   T   
   T   
    T    
   G   
   G   
    A    
   T   
   T   
    C    
   A   
   A   
    T    
   A   
   T   
   G   
   T   
   T   
    G    
   G   
   C   
    G    
   A   
   T   
    C    
   G   
   A   
   A   
   A   
   A   
   A   
   G   
   G   
    T    
   G   
   T   
    G    
   C   
   C   
    G    
   A   
   A   
   A   
    G    
   T   
   T   
   T   
   T   
    G    
   A   
   G   
    C    
   T   
   T   
   G   
   A   
   A   
   A   
   C   
    A    
    A    
   A   
   T   
    T    
    C    
   T   
    T    
   G   
   A   
   A   
   A   
   A   
    C    
   T   
   A   
    T    
    A    
   T   
    C    
   G   
   A   
    A    
   C   
   A   
    T    
   C   
   A   
    G    
    C    
    G    
    C    
    G    
   A   
   A   
   G   
   T   
   C   
   A   
   T   
    C    
   A   
   C   
    A    
    A    
   G   
    A    
   C   
   G   
   G   
   A   
   C   
    A    
    G    
   T   
    C    
   T   
   T   
   T   
   G   
   A   
    A    
   A   
   A   
   A   
    G    
   A   
    A    
   A   
   A   
    A    
   G   
   C   
   A   
   G   
   A   
   A   
   G   
   C   
   A   
    A    
   G   
    G    
   G   
   C   
    A    
   C   
   A   
    C    
   A   
   T   
    T    
   T   
   T   
   A   
   G   
   A   
   A   
   G   
   G   
   T   
    T    
   T   
    G    
   C   
   G   
    T    
   A   
   T   
   T   
   G   
   C   
    T    
   T   
   T   
    G    
   G   
   A   
   T   
   C   
   A   
   T   
   A   
   T   
   C   
    G    
   A   
   T   
   G   
   A   
   A   
   A   
   T   
    A    
   A   
   T   
   T   
   G   
   C   
    G    
   A   
   T   
   T   
   A   
   T   
    C    
   C   
   G   
    C    
   G   
   G   
    G    
   T   
   C   
    T    
    A    
   A   
   A   
   T   
   C   
   A   
   G   
   A   
   T   
   G   
   A   
    C    
   G   
   A   
    A    
   G   
   C   
   A   
   A   
   A   
   A   
   G   
   C   
    G    
   A   
    C    
    G    
    A    
   T   
    G    
   A   
   T   
    C    
   G   
   A   
   A   
   C   
   G   
    G    
   T   
   T   
    C    
   G   
   A   
    T    
   T   
   T   
    A    
   T   
   C   
    C    
   G   
   A   
   T   
   C   
   G   
    C    
   C   
   A   
    G    
    T    
   C   
    T    
   C   
   A   
   A   
   G   
   C   
    T    
   A   
   T   
    T    
   T   
   T   
   A   
   G   
   A   
   T   
   A   
   T   
   G   
   C   
   G   
   T   
   T   
   T   
    A    
   C   
   G   
    C    
   C   
   G   
    A    
   T   
   T   
    G    
   A   
   C   
    C    
   G   
   G   
    T    
   T   
   T   
    G    
   G   
   A   
   A   
   C   
   G   
    G    
   G   
   A   
    A    
   A   
   A   
   A   
   A   
   T   
    C    
   G   
   A   
    A    
   A   
   A   
   T   
   G   
   A   
   A   
   T   
   A   
    C    
   C   
   A   
   A   
   G   
   A   
    A    
   C   
   T   
    C    
   T   
   T   
    A    
   A   
    A    
   A   
    C    
   T   
    G    
   A   
   T   
   T   
   G   
    A    
    A    
   G   
   A   
    T    
   T   
   T   
    A    
    A    
    C    
    T    
   G   
   A   
   C   
   A   
   T   
   T   
    C    
   T   
    G    
   G   
   C   
    A    
    A    
   G   
    A    
   C   
   C   
    A    
   G   
   A   
    A    
   C   
   G   
    T    
   G   
   T   
    T    
   A   
    C    
    A    
   G   
   A   
   A   
   A   
   T   
    C    
   A   
   T   
    C    
   A   
   A   
    G    
   A   
    A    
    T    
   G   
   A   
    A    
   T   
   T   
   A   
    G    
    C    
    A    
   G   
   A   
   C   
    T    
   T   
    A    
    A    
    A    
    T    
    C    
    A    
   A   
    C    
    G    
   A   
   T   
   T   
   T   
   G   
   G   
   T   
   G   
   A   
   T   
    A    
    A    
   A   
   C   
   G   
   A   
   C   
   G   
    C    
   A   
   C   
   A   
   G   
   A   
   A   
    T    
   T   
   A   
    C    
   T   
   A   
   G   
   T   
    C    
   G   
   G   
   T   
   G   
   A   
    G    
   G   
   T   
   C   
    T    
   T   
    A    
   A   
   G   
   T   
   C   
   T   
    G    
   G   
   A   
   A   
   G   
   A   
   T   
   G   
   A   
   A   
   G   
   A   
   T   
    T    
   T   
    A    
   A   
   T   
    C    
   G   
   A   
   A   
   G   
   A   
   A   
   G   
   A   
   A   
   G   
   A   
   A   
    A    
   T   
   C   
   G   
   T   
    T    
   A   
   T   
   T   
   A   
   C   
    A    
    T    
   T   
    G    
   A   
   C   
    A    
   A   
   A   
    C    
   A   
   A   
    T    
   G   
   G   
    T    
   T   
   A   
    C    
   A   
   T   
    C    
   A   
   A   
    A    
   C   
   G   
    T    
    T    
   T   
   G   
   G   
    C    
   A   
   A   
   A   
    T    
    A    
    A    
    T    
   G   
   A   
   A   
   T   
   T   
   C   
    A    
   G   
    A    
   G   
   C   
    T    
   C   
   A   
   A   
   C   
   G   
    A    
    A    
   G   
    G    
   G   
   G   
    T    
   G   
   G   
    A    
   C   
   G   
   T   
   G   
   G   
    T    
   G   
   T   
    C    
   C   
   A   
   A   
   G   
   G   
    A    
   A   
   T   
   G   
   G   
   G   
    T    
    A    
   T   
    C    
   C   
   A   
    C    
    A    
   A   
   T   
   G   
   A   
    C    
   G   
   A   
    C    
   T   
   T   
   C   
   G   
   T   
    C    
   A   
   A   
    A    
   A   
   A   
    T    
   C   
   T   
    G    
    G    
   T   
   T   
   T   
   C   
   T   
   T   
   G   
    C    
   T   
   C   
    T    
   A   
   C   
    C    
   C   
   A   
    T    
   G   
   A   
    T    
   A   
   C   
    A    
   T   
   T   
    G    
   T   
   T   
   A   
   T   
   T   
    C    
   T   
   T   
   T   
   A   
   C   
   G   
   A   
   A   
    T    
   A   
    A    
    C    
   G   
   G   
    A    
   A   
   A   
   A   
   G   
   T   
    G    
   T   
   A   
    T    
   C   
   G   
    G    
   G   
   C   
   A   
   A   
   A   
   A   
   G   
   G   
    A    
   T   
   A   
    C    
   G   
   A   
   A   
   A   
   T   
    T    
   C   
   C   
    A    
   G   
   A   
    A    
   T   
   A   
   C   
   G   
   G   
    G    
    C    
   G   
    C    
   A   
   C   
   A   
   G   
   C   
    C    
   A   
   A   
   A   
   G   
   G   
    A    
   A   
   T   
    A    
   C   
   C   
   A   
    A    
   T   
    T    
   A   
   T   
    C    
   A   
   A   
    T    
   T   
   T   
    A    
   C   
   T   
    A    
   G   
   G   
    T    
   A   
   T   
    C    
   G   
   A   
    T    
   T   
   C   
    G    
    A    
    G    
    T    
   G   
   A   
   A   
   A   
    G    
    C    
   A   
   T   
    C    
   C   
   A   
    G    
   G   
   C   
    A    
   A   
   T   
    C    
   A   
   T   
   T   
    G    
   C   
    G    
   G   
   T   
    G    
    A    
    C    
   G   
   G   
   G   
    A    
    G    
   A   
   A   
   G   
   C   
    A    
   G   
   A   
   A   
   G   
    A    
    A    
   G   
   G   
    C    
    C    
   A   
    C    
   T   
   A   
    T    
    C    
   T   
    C    
   T   
   T   
   C   
   T   
   T   
    T    
   A   
   C   
    G    
   A   
   C   
    G    
    C    
    G    
    T    
    C    
   A   
   A   
   G   
   G   
    T    
   A   
   C   
    A    
   G   
   T   
    A    
   A   
   A   
   A   
   C   
   G   
    T    
   A   
   C   
    T    
    T    
   C   
    T    
   G   
   T   
    C    
   A   
   C   
    T    
   G   
   C   
    T    
   T   
   T   
    T    
    G    
   C   
   T   
   A   
   A   
    C    
   A   
   T   
    C    
   C   
   G   
    A    
   A   
   G   
    T    
   A   
   A   
   T   
   G   
   G   
    T    
    C    
   T   
    G    
   A   
   T   
    C    
   G   
   C   
    A    
   A   
   T   
   T   
    G    
   G   
    T    
   T   
   T   
    G    
   A   
   A   
   A   
   G   
   A   
   A   
   G   
   A   
    C    
   G   
   A   
    T    
   G   
   A   
    G    
    C    
   T   
    C    
    G    
   T   
    C    
   A   
   A   
    T    
   G   
   T   
    G    
    C    
   T   
   A   
    C    
    T    
   G   
   A   
   C   
    A    
    G    
   A   
    T    
   G   
   G   
    T    
    C    
   A   
    A    
    T    
    C    
    T    
   A   
   A   
    T    
   A   
   T   
    C    
   A   
   T   
    T    
   A   
   T   
    T    
   G   
   G   
    A    
   A   
   C   
   A   
   C   
   A   
   T   
    A    
    A    
    C    
   G   
   G   
    C    
   T   
   A   
   C   
   T   
   C   
   T   
   G   
   T   
    C    
   A   
   C   
    T    
   T   
   T   
    C    
    A    
   A   
    A    
   G   
   A   
    A    
    G    
    A    
    A    
    G    
   C   
    A    
   G   
    T    
    C    
   C   
   G   
    C    
   G   
   A   
   T   
   A   
   T   
   G   
   G   
   G   
    A    
   C   
   G   
    T    
   A   
   C   
   A   
   G   
   C   
    T    
   T   
   C   
    C    
   G   
   G   
    G    
   G   
   T   
    C    
    A    
   G   
    A    
   G   
   G   
    A    
   A   
   T   
    A    
   C   
   G   
    C    
    C    
   T   
    G    
   C   
   G   
    T    
   G   
   A   
    G    
    G    
   A   
    A    
   G   
   A   
    T    
   T   
    A    
    T    
   G   
   T   
    C    
   G   
   T   
    T    
   G   
   G   
    G    
    G    
   C   
   A   
   G   
   C   
    C    
    T    
   T   
    A    
    A    
   T   
   G   
    A    
   A   
    G    
   G   
    A    
   A   
    G    
   A   
    T    
    C    
   A   
   A   
   G   
   A   
   A   
   G   
   T   
    A    
    T    
   T   
    A    
   G   
   T   
    G    
   A   
   T   
    C    
   A   
   C   
    A    
   G   
   A   
   A   
   A   
   A   
    A    
   G   
   G   
   T   
   T   
   A   
    C    
   G   
   G   
    A    
   A   
   A   
    A    
   C   
   G   
    G    
   A   
   C   
    A    
   A   
   A   
   A   
   G   
    T    
    A    
   T   
   C   
    G    
   G   
   A   
    A    
   T   
   A   
   T   
   C   
   C   
   A   
   G   
   T   
    G    
   A   
   A   
    G    
   G   
   G   
    C    
   C   
   G   
    C    
   G   
   G   
    A    
   G   
   G   
   T   
   A   
   A   
   A   
   G   
   G   
    G    
   A   
   T   
    C    
   A   
   A   
    A    
   A   
   C   
    G    
    G    
   C   
    G    
   A   
   A   
    C    
   A   
   T   
   C   
   A   
   C   
   T   
   G   
   A   
    A    
   A   
   A   
   A   
   A   
   A   
   T   
   G   
   G   
   T   
   C   
   C   
   A   
    C    
   T   
   A   
   G   
   C   
    T    
   G   
   G   
    A    
   T   
   T   
    G    
   A   
   C   
   C   
   A   
   C   
    T    
   G   
   T   
    T    
    T    
    C    
   T   
   G   
   G   
    A    
    G    
   A   
   T   
   G   
   A   
   A   
   G   
   A   
    C    
   A   
   T   
   C   
    A    
   T   
    G    
    C    
   T   
    G    
   A   
   T   
    T    
   A   
   C   
    A    
    G    
   A   
    T    
   A   
   A   
   A   
   G   
   G   
    T    
   G   
   T   
    C    
   A   
   T   
   T   
   A   
   T   
   C   
   C   
   G   
    T    
   T   
   T   
    C    
   A   
   A   
   C   
   G   
   T   
    A    
    T    
    C    
    G    
    A    
   C   
   T   
   G   
   T   
   T   
   T   
   C   
   T   
   C   
   A   
   A   
   A   
   C   
    T    
   G   
   G   
   A   
   C   
   G   
    T    
    T    
   C   
   A   
   A   
   C   
   A   
   T   
   T   
    A    
   G   
   G   
    C    
   G   
   T   
    A    
   C   
   G   
   T   
   T   
   T   
    G    
   A   
   T   
   G   
   A   
    A    
    G    
   A   
   T   
   G   
   G   
   A   
   A   
   G   
    C    
    T    
   G   
    A    
    T    
    A    
   C   
    G    
   A   
   A   
   A   
   G   
   T   
   T   
   G   
   T   
   C   
   A   
   C   
   G   
   A   
   T   
   G   
   G   
   C   
   A   
   G   
   C   
   A   
   G   
   T   
   C   
   G   
   A   
   A   
   C   
   C   
   A   
   G   
   A   
   A   
   G   
   C   
   T   
   G   
   C   
   A   
   G   
   A   
   A   
   C   
   A   
   A   
   A   
   C   
   A   
   G   
   T   
   T   
   G   
   A   
   A   
   G   
   A   
   A   
   C   
   A   
   A   
   G   
   G   
   A   
   T   
   C   
   A   
   G   
   A   
   A   
   G   
   A   
   A   
   -   
   -   
   -   
 

 
   N60519F   
   A   
   T   
   G   
   A   
   G   
   T   
   G   
   A   
   A   
   G   
   A   
   A   
   A   
   T   
   C   
   A   
   G   
   A   
   G   
   A   
   A   
   A   
   A   
   C   
   A   
   T   
   C   
   C   
   A   
   T   
   G   
   A   
   T   
   G   
   T   
   C   
   A   
   A   
   T   
   C   
   T   
   A   
   A   
   C   
   C   
   A   
   G   
   C   
   G   
   A   
   A   
   A   
   T   
   G   
   A   
   A   
   A   
   G   
   A   
   C   
   T   
   C   
   C   
   T   
   T   
   C   
   A   
   T   
   C   
   G   
   A   
   T   
   T   
   A   
   T   
   G   
   C   
   G   
   A   
   T   
   G   
   A   
   G   
   T   
   G   
   T   
   T   
   A   
   T   
   T   
   G   
   T   
   C   
   G   
   C   
   A   
   C   
   G   
   T   
   G   
   C   
   A   
   C   
   T   
   T   
   C   
   C   
   T   
   G   
   A   
   T   
   G   
   T   
   C   
   A   
   G   
   A   
   G   
   A   
   T   
   G   
   G   
   T   
   C   
   T   
   G   
   A   
   A   
   A   
   C   
   C   
   C   
   G   
   T   
   T   
   C   
   A   
   C   
   C   
   G   
   C   
   C   
   G   
   T   
   A   
   T   
   T   
   T   
   T   
   A   
   T   
   A   
   C   
   G   
   G   
   C   
   A   
   T   
   G   
   A   
   A   
   C   
   G   
   A   
   A   
   T   
   T   
   A   
   G   
   G   
   T   
   G   
   T   
   A   
   A   
   C   
   A   
   C   
   C   
   G   
   G   
   A   
   T   
   A   
   A   
   A   
   G   
   C   
   C   
   C   
   A   
   T   
   A   
   A   
   A   
   A   
   A   
   A   
   T   
   C   
   T   
   G   
   C   
   C   
   A   
   G   
   A   
   A   
   T   
   C   
   G   
   T   
   C   
   G   
   G   
   A   
   G   
   A   
   T   
   G   
   T   
   C   
   A   
   T   
   G   
   G   
   G   
   T   
   A   
   A   
   A   
   T   
   A   
   C   
   C   
   A   
   T   
   C   
   C   
   T   
   C   
   A   
   T   
   G   
   G   
   A   
   G   
   A   
   C   
   A   
   G   
   T   
   G   
   C   
   G   
   A   
   T   
   C   
   T   
   A   
   T   
   G   
   A   
   G   
   T   
   C   
   A   
   A   
   T   
   G   
   G   
   T   
   A   
   C   
   G   
   G   
   A   
   T   
   G   
   G   
   C   
   G   
   C   
   A   
   G   
   C   
   C   
   A   
   T   
   T   
   C   
   A   
   G   
   C   
   T   
   A   
   C   
   C   
   G   
   T   
   T   
   A   
   T   
   A   
   T   
   G   
   C   
   T   
   A   
   G   
   T   
   G   
   G   
   A   
   T   
   G   
   G   
   T   
   C   
   A   
   T   
   G   
   G   
   A   
   A   
   A   
   C   
   T   
   T   
   T   
   G   
   G   
   T   
   T   
   C   
   T   
   G   
   T   
   T   
   G   
   A   
   C   
   G   
   G   
   T   
   G   
   A   
   C   
   G   
   G   
   T   
   G   
   C   
   C   
   G   
   C   
   T   
   G   
   C   
   T   
   A   
   T   
   G   
   C   
   G   
   T   
   T   
   A   
   T   
   A   
   C   
   C   
   G   
   A   
   A   
   G   
   C   
   A   
   C   
   G   
   G   
   A   
   T   
   G   
   A   
   G   
   T   
   A   
   A   
   A   
   A   
   T   
   C   
   G   
   C   
   T   
   A   
   C   
   A   
   G   
   A   
   A   
   A   
   T   
   G   
   C   
   T   
   G   
   C   
   G   
   T   
   G   
   A   
   T   
   A   
   T   
   C   
   A   
   A   
   T   
   A   
   A   
   G   
   A   
   A   
   C   
   A   
   C   
   T   
   G   
   T   
   T   
   G   
   A   
   T   
   T   
   T   
   C   
   C   
   A   
   A   
   A   
   G   
   C   
   A   
   A   
   C   
   T   
   A   
   T   
   G   
   A   
   T   
   G   
   A   
   T   
   A   
   C   
   A   
   G   
   A   
   A   
   A   
   A   
   A   
   G   
   A   
   A   
   C   
   C   
   A   
   G   
   T   
   T   
   G   
   T   
   A   
   C   
   T   
   A   
   C   
   C   
   T   
   G   
   C   
   T   
   C   
   G   
   C   
   T   
   T   
   C   
   C   
   C   
   G   
   A   
   A   
   T   
   C   
   T   
   T   
   T   
   T   
   A   
   G   
   T   
   G   
   A   
   A   
   C   
   G   
   G   
   A   
   A   
   C   
   A   
   A   
   C   
   G   
   G   
   G   
   G   
   A   
   T   
   C   
   G   
   C   
   C   
   G   
   T   
   A   
   G   
   G   
   T   
   A   
   T   
   G   
   G   
   C   
   C   
   A   
   C   
   G   
   A   
   A   
   T   
   A   
   T   
   C   
   C   
   C   
   A   
   C   
   C   
   T   
   C   
   A   
   T   
   A   
   A   
   C   
   T   
   T   
   G   
   T   
   C   
   T   
   G   
   A   
   G   
   G   
   T   
   A   
   G   
   T   
   A   
   G   
   C   
   G   
   G   
   C   
   T   
   A   
   T   
   C   
   G   
   A   
   T   
   T   
   T   
   G   
   C   
   T   
   G   
   A   
   T   
   G   
   G   
   A   
   T   
   A   
   A   
   T   
   C   
   C   
   T   
   G   
   A   
   T   
   G   
   T   
   G   
   A   
   C   
   G   
   A   
   C   
   C   
   A   
   A   
   T   
   G   
   A   
   A   
   T   
   T   
   G   
   A   
   T   
   G   
   G   
   A   
   A   
   G   
   T   
   A   
   T   
   T   
   A   
   C   
   C   
   T   
   G   
   G   
   A   
   C   
   C   
   A   
   G   
   A   
   T   
   T   
   T   
   T   
   C   
   C   
   A   
   A   
   C   
   A   
   G   
   G   
   T   
   G   
   G   
   T   
   T   
   T   
   G   
   G   
   T   
   T   
   A   
   T   
   G   
   G   
   G   
   A   
   A   
   A   
   A   
   T   
   C   
   A   
   G   
   G   
    A    
   A   
   T   
    C    
   C   
   G   
    A    
    A    
   G   
   A   
   G   
   C   
    G    
   T   
   A   
   T   
   G   
   A   
   A   
   A   
   C   
    A    
   G   
   G   
    T    
   A   
   A   
    G    
   G   
   G   
   T   
   T   
   C   
    T    
   A   
   T   
   T   
   A   
   C   
    C    
   G   
   T   
    A    
   C   
   G   
   T   
   G   
   C   
   A   
   A   
   A   
   A   
   G   
   T   
   T   
   G   
   A   
    G    
    A    
   T   
    T    
   A   
    C    
   T   
   G   
   A   
    G    
   A   
   T   
   G   
    C    
   C   
    A    
   A   
   A   
   T   
   G   
   G   
    T    
   A   
   A   
   A   
   G   
   A   
   A   
   C   
   G   
    G    
   A   
   T   
    C    
    A    
   T   
    C    
   G   
   T   
    G    
   A   
   C   
    A    
   G   
   A   
   A   
   T   
   T   
    G    
   C   
   C   
   T   
   T   
   A   
   T   
   A   
   T   
   G   
   G   
   T   
    C    
   A   
   A   
   T   
   A   
   A   
   A   
   G   
   C   
    A    
   A   
   A   
   A   
   T   
   T   
    G    
   A   
   T   
    C    
   G   
   A   
   A   
   C   
   G   
    G    
   A   
   T   
   T   
   T   
   C   
   T   
   G   
   A   
    G    
    C    
   T   
    G    
   C   
   A   
   C   
   A   
   G   
   A   
   G   
   A   
    C    
   A   
   A   
    G    
   C   
   G   
    T    
   A   
   T   
    C    
   G   
   A   
   A   
   G   
   G   
    G    
   A   
   T   
    C    
   A   
   C   
    A    
   G   
   A   
   T   
   T   
   T   
   G   
   C   
   G   
   G   
   G   
   A   
    C    
   G   
   A   
   A   
   T   
   C   
   T   
   T   
   C   
    C    
   C   
   G   
   T   
   G   
   A   
    A    
   G   
   G   
    T    
   A   
   T   
   G   
   C   
   G   
    G    
   A   
   T   
    C    
   G   
   T   
   C   
   A   
   T   
    T    
   G   
   A   
    C    
    A    
   T   
    C    
   C   
   G   
   T   
   C   
   G   
    T    
   G   
   A   
    C    
   G   
    C    
    C    
   A   
   G   
    C    
   G   
   C   
    A    
   T   
   C   
    C    
   G   
   T   
   T   
    A    
   T   
    C    
   T   
   T   
    G    
   A   
   A   
    T    
   A   
   A   
   C   
   T   
   T   
    A    
   T   
   A   
    T    
   A   
   A   
   A   
    T    
   T   
   G   
   A   
   C   
    A    
    T    
   C   
    C    
   T   
   T   
    G    
   C   
   A   
    A    
   A   
    A    
    T    
   T   
   C   
    A    
   T   
   T   
    T    
   G   
   G   
    A    
   T   
   T   
    C    
   A   
   A   
    T    
   A   
   T   
   G   
   T   
   T   
    G    
   G   
   C   
    A    
   A   
   T   
    C    
   G   
   A   
   A   
   A   
   A   
   A   
   G   
   G   
    T    
   G   
   T   
    G    
   C   
   C   
    G    
   A   
   A   
   A   
    G    
   T   
   T   
   T   
   T   
    G    
   A   
   G   
    C    
   T   
   T   
   G   
   A   
   A   
   A   
   C   
    A    
    A    
   A   
   T   
    T    
    C    
   T   
    T    
   G   
   A   
   A   
   A   
   A   
    C    
   T   
   A   
    T    
    A    
   T   
    C    
   G   
   A   
    A    
   C   
   A   
    T    
   C   
   A   
    G    
    C    
    G    
    C    
    G    
   A   
   A   
   G   
   T   
   C   
   A   
   T   
    C    
   A   
   C   
    A    
    A    
   G   
    A    
   C   
   G   
   G   
   A   
   C   
    A    
    G    
   T   
    C    
   T   
   T   
   T   
   G   
   A   
    A    
   A   
   A   
   A   
    G    
   A   
    A    
   A   
   A   
    A    
   G   
   C   
   A   
   G   
   A   
   A   
   G   
   C   
   A   
    A    
   G   
    G    
   G   
   C   
    A    
   C   
   A   
    C    
   A   
   T   
    T    
   T   
   T   
   A   
   G   
   A   
   A   
   G   
   G   
   T   
    T    
   T   
    G    
   C   
   G   
    T    
   A   
   T   
   T   
   G   
   C   
    T    
   T   
   T   
    G    
   G   
   A   
   T   
   C   
   A   
   T   
   A   
   T   
   C   
    G    
   A   
   T   
   G   
   A   
   A   
   A   
   T   
    C    
   A   
   T   
   T   
   G   
   C   
    G    
   A   
   T   
   T   
   A   
   T   
    C    
   C   
   G   
    C    
   G   
   G   
    G    
   T   
   C   
    T    
    A    
   A   
   A   
   T   
   C   
   A   
   G   
   A   
   T   
   G   
   A   
    C    
   G   
   A   
    A    
   G   
   C   
   A   
   A   
   A   
   A   
   G   
   C   
    G    
   A   
    C    
    G    
    A    
   T   
    G    
   A   
   T   
    C    
   G   
   A   
   A   
   C   
   G   
    G    
   T   
   T   
    C    
   G   
   A   
    T    
   T   
   T   
    A    
   T   
   C   
    C    
   G   
   A   
   T   
   C   
   G   
    C    
   C   
   A   
    G    
    T    
   C   
    T    
   C   
   A   
   A   
   G   
   C   
    T    
   A   
   T   
    T    
   T   
   T   
   A   
   G   
   A   
   T   
   A   
   T   
   G   
   C   
   G   
   T   
   T   
   T   
    A    
   C   
   G   
    C    
   C   
   G   
    A    
   T   
   T   
    G    
   A   
   C   
    C    
   G   
   G   
    T    
   T   
   T   
    G    
   G   
   A   
   A   
   C   
   G   
    G    
   G   
   A   
    A    
   A   
   A   
   A   
   A   
   T   
    C    
   G   
   A   
    A    
   A   
   A   
   T   
   G   
   A   
   A   
   T   
   A   
    C    
   C   
   A   
   A   
   G   
   A   
    A    
   C   
   T   
    C    
   T   
   T   
    A    
   A   
    A    
   A   
    C    
   T   
    G    
   A   
   T   
   T   
   G   
    A    
    A    
   G   
   A   
    T    
   T   
   T   
    A    
    A    
    C    
    T    
   G   
   A   
   C   
   A   
   T   
   T   
    C    
   T   
    G    
   G   
   C   
    A    
    A    
   G   
    A    
   C   
   C   
    A    
   G   
   A   
    A    
   C   
   G   
    T    
   G   
   T   
    T    
   A   
    C    
    A    
   G   
   A   
   A   
   A   
   T   
    C    
   A   
   T   
    C    
   A   
   A   
    G    
   A   
    A    
    T    
   G   
   A   
    A    
   T   
   T   
   A   
    G    
    C    
    A    
   G   
   A   
   C   
    T    
   T   
    G    
    A    
    A    
    T    
    C    
    A    
   A   
    C    
    G    
   A   
   T   
   T   
   T   
   G   
   G   
   T   
   G   
   A   
   T   
    A    
    A    
   A   
   C   
   G   
   A   
   C   
   G   
    C    
   A   
   C   
   A   
   G   
   A   
   A   
    T    
   T   
   A   
    C    
   T   
   A   
   G   
   T   
    C    
   G   
   G   
   T   
   G   
   A   
    G    
   G   
   T   
   C   
    T    
   T   
    A    
   A   
   G   
   T   
   C   
   T   
    G    
   G   
   A   
   A   
   G   
   A   
   T   
   G   
   A   
   A   
   G   
   A   
   T   
    T    
   T   
    A    
   A   
   T   
    C    
   G   
   A   
   A   
   G   
   A   
   A   
   G   
   A   
   A   
   G   
   A   
   A   
    A    
   T   
   C   
   G   
   T   
    T    
   A   
   T   
   T   
   A   
   C   
    A    
    T    
   T   
    G    
   A   
   C   
    A    
   A   
   A   
    C    
   A   
   A   
    T    
   G   
   G   
    T    
   T   
   A   
    C    
   A   
   T   
    C    
   A   
   A   
    A    
   C   
   G   
    T    
    T    
   T   
   G   
   G   
    C    
   A   
   A   
   A   
    T    
    A    
    A    
    T    
   G   
   A   
   A   
   T   
   T   
   C   
    A    
   G   
    A    
   G   
   C   
    T    
   C   
   A   
   A   
   C   
   G   
    A    
    A    
   G   
    G    
   G   
   G   
    T    
   G   
   G   
    A    
   C   
   G   
   T   
   G   
   G   
    T    
   G   
   T   
    C    
   C   
   A   
   A   
   G   
   G   
    A    
   A   
   T   
   G   
   G   
   G   
    T    
    A    
   T   
    C    
   C   
   A   
    C    
    A    
   A   
   T   
   G   
   A   
    C    
   G   
   A   
    C    
   T   
   T   
   C   
   G   
   T   
    C    
   A   
   A   
    A    
   A   
   A   
    T    
   C   
   T   
    G    
    G    
   T   
   T   
   T   
   C   
   T   
   T   
   G   
    C    
   T   
   C   
    T    
   A   
   C   
    C    
   C   
   A   
    T    
   G   
   A   
    T    
   A   
   C   
    A    
   T   
   T   
    G    
   T   
   T   
   A   
   T   
   T   
    C    
   T   
   T   
   T   
   A   
   C   
   G   
   A   
   A   
    T    
   A   
    A    
    C    
   G   
   G   
    A    
   A   
   A   
   A   
   G   
   T   
    G    
   T   
   A   
    T    
   C   
   G   
    G    
   G   
   C   
   A   
   A   
   A   
   A   
   G   
   G   
    A    
   T   
   A   
    C    
   G   
   A   
   A   
   A   
   T   
    T    
   C   
   C   
    A    
   G   
   A   
    A    
   T   
   A   
   C   
   G   
   G   
    G    
    C    
   G   
    C    
   A   
   C   
   A   
   G   
   C   
    C    
   A   
   A   
   A   
   G   
   G   
    A    
   A   
   T   
    A    
   C   
   C   
   A   
    A    
   T   
    T    
   A   
   T   
    C    
   A   
   A   
    T    
   T   
   T   
    A    
   C   
   T   
    A    
   G   
   G   
    T    
   A   
   T   
    C    
   G   
   A   
    T    
   T   
   C   
    G    
    A    
    G    
    T    
   G   
   A   
   A   
   A   
    G    
    C    
   A   
   T   
    C    
   C   
   A   
    G    
   G   
   C   
    A    
   A   
   T   
    C    
   A   
   T   
   T   
    G    
   C   
    G    
   G   
   T   
    G    
    A    
    C    
   G   
   G   
   G   
    A    
    G    
   A   
   A   
   G   
   C   
    A    
   G   
   A   
   A   
   G   
    A    
    A    
   G   
   G   
    C    
    C    
   A   
    C    
   T   
   A   
    T    
    C    
   T   
    C    
   T   
   T   
   C   
   T   
   T   
    T    
   A   
   C   
    G    
   A   
   C   
    G    
    C    
    G    
    T    
    C    
   A   
   A   
   G   
   G   
    T    
   A   
   C   
    A    
   G   
   T   
    A    
   A   
   A   
   A   
   C   
   G   
    T    
   A   
   C   
    T    
    T    
   C   
    T    
   G   
   T   
    C    
   A   
   C   
    T    
   G   
   C   
    T    
   T   
   T   
    T    
    G    
   C   
   T   
   A   
   A   
    C    
   A   
   T   
    C    
   C   
   G   
    A    
   A   
   G   
    T    
   A   
   A   
   T   
   G   
   G   
    T    
    C    
   T   
    G    
   A   
   T   
    C    
   G   
   C   
    A    
   A   
   T   
   T   
    G    
   G   
    T    
   T   
   T   
    G    
   A   
   A   
   A   
   G   
   A   
   A   
   G   
   A   
    C    
   G   
   A   
    T    
   G   
   A   
    G    
    C    
   T   
    C    
    G    
   T   
    C    
   A   
   A   
    T    
   G   
   T   
    G    
    C    
   T   
   A   
    C    
    T    
   G   
   A   
   C   
    A    
    G    
   A   
    T    
   G   
   G   
    T    
    C    
   A   
    A    
    T    
    C    
    T    
   A   
   A   
    T    
   A   
   T   
    C    
   A   
   T   
    T    
   A   
   T   
    T    
   G   
   G   
    A    
   A   
   C   
   A   
   C   
   A   
   T   
    A    
    A    
    C    
   G   
   G   
    C    
   T   
   A   
   C   
   T   
   C   
   T   
   G   
   T   
    C    
   A   
   C   
    T    
   T   
   T   
    C    
    A    
   A   
    A    
   G   
   A   
    A    
    G    
    A    
    A    
    G    
   C   
    A    
   G   
    T    
    C    
   C   
   G   
    C    
   G   
   A   
   T   
   A   
   T   
   G   
   G   
   G   
    A    
   C   
   G   
    T    
   A   
   C   
   A   
   G   
   C   
    T    
   T   
   C   
    C    
   G   
   G   
    G    
   G   
   T   
    C    
    A    
   G   
    A    
   G   
   G   
    A    
   A   
   T   
    A    
   C   
   G   
    C    
    C    
   T   
    G    
   C   
   G   
    T    
   G   
   A   
    G    
    G    
   A   
    A    
   G   
   A   
    T    
   T   
    A    
    T    
   G   
   T   
    C    
   G   
   T   
    T    
   G   
   G   
    G    
    G    
   C   
   A   
   G   
   C   
    C    
    T    
   T   
    A    
    A    
   T   
   G   
    A    
   A   
    G    
   G   
    A    
   A   
    G    
   A   
    T    
    C    
   A   
   A   
   G   
   A   
   A   
   G   
   T   
    A    
    T    
   T   
    A    
   G   
   T   
    G    
   A   
   T   
    C    
   A   
   C   
    A    
   G   
   A   
   A   
   A   
   A   
    A    
   G   
   G   
   T   
   T   
   A   
    C    
   G   
   G   
    A    
   A   
   A   
    A    
   C   
   G   
    G    
   A   
   C   
    A    
   A   
   A   
   A   
   G   
    T    
    A    
   T   
   C   
    G    
   G   
   A   
    A    
   T   
   A   
   T   
   C   
   C   
   A   
   G   
   T   
    G    
   A   
   A   
    A    
   G   
   G   
    C    
   C   
   G   
    C    
   G   
   G   
    A    
   G   
   G   
   T   
   A   
   A   
   A   
   G   
   G   
    G    
   A   
   T   
    C    
   A   
   A   
    A    
   A   
   C   
    G    
    G    
   C   
    G    
   A   
   A   
    C    
   A   
   T   
   C   
   A   
   C   
   T   
   G   
   A   
    A    
   A   
   A   
   A   
   A   
   A   
   T   
   G   
   G   
   T   
   C   
   C   
   A   
    C    
   T   
   A   
   G   
   C   
    T    
   G   
   G   
    A    
   T   
   T   
    G    
   A   
   C   
   C   
   A   
   C   
    T    
   G   
   T   
    T    
    T    
    C    
   T   
   G   
   G   
    A    
    G    
   A   
   T   
   G   
   A   
   A   
   G   
   A   
    C    
   A   
   T   
   C   
    A    
   T   
    G    
    C    
   T   
    G    
   A   
   T   
    T    
   A   
   C   
    A    
    G    
   A   
    T    
   A   
   A   
   A   
   G   
   G   
    T    
   G   
   T   
    C    
   A   
   T   
   T   
   A   
   T   
   C   
   C   
   G   
    T    
   T   
   T   
    C    
   A   
   A   
   C   
   G   
   T   
    A    
    T    
    C    
    G    
    A    
   C   
   T   
   G   
   T   
   T   
   T   
   C   
   T   
   C   
   A   
   A   
   A   
   C   
    T    
   G   
   G   
   A   
   C   
   G   
    T    
    T    
   C   
   A   
   A   
   C   
   A   
   T   
   T   
    A    
   G   
   G   
    C    
   G   
   T   
    A    
   C   
   G   
   T   
   T   
   T   
    G    
   A   
   T   
   G   
   A   
    A    
    G    
   A   
   T   
   G   
   G   
   A   
   A   
   G   
    C    
    T    
   G   
    A    
    T    
    A    
   C   
    G    
   A   
   A   
   A   
   G   
   T   
   T   
   G   
   T   
   C   
   A   
   C   
   G   
   A   
   T   
   G   
   G   
   C   
   A   
   G   
   C   
   A   
   G   
   T   
   C   
   G   
   A   
   A   
   C   
   C   
   A   
   G   
   A   
   A   
   G   
   C   
   T   
   G   
   C   
   A   
   G   
   A   
   A   
   C   
   A   
   A   
   A   
   C   
   A   
   G   
   T   
   T   
   G   
   A   
   A   
   G   
   A   
   A   
   C   
   A   
   A   
   G   
   G   
   A   
   T   
   C   
   A   
   G   
   A   
   A   
   G   
   A   
   A   
   -   
   -   
   -   
 

 
   N55268   
   A   
   T   
   G   
   A   
   G   
   T   
   G   
   A   
   A   
   G   
   A   
   A   
   A   
   T   
   T   
   A   
   A   
   A   
   G   
   A   
   A   
   A   
   A   
   C   
   A   
   T   
   T   
   C   
   A   
   A   
   G   
   A   
   C   
   G   
   T   
   C   
   A   
   A   
   T   
   C   
   T   
   G   
   A   
   C   
   C   
   A   
   G   
   C   
   G   
   A   
   A   
   A   
   T   
   G   
   A   
   A   
   A   
   G   
   A   
   A   
   T   
   C   
   T   
   T   
   T   
   C   
   A   
   T   
   T   
   G   
   A   
   C   
   T   
   A   
   C   
   G   
   C   
   A   
   A   
   T   
   G   
   A   
   G   
   T   
   G   
   T   
   T   
   A   
   T   
   C   
   G   
   T   
   A   
   G   
   C   
   C   
   C   
   G   
   C   
   G   
   C   
   G   
   T   
   T   
   A   
   C   
   C   
   T   
   G   
   A   
   C   
   G   
   T   
   T   
   C   
   G   
   T   
   G   
   A   
   C   
   G   
   G   
   T   
   T   
   T   
   A   
   A   
   A   
   A   
   C   
   C   
   T   
   G   
   T   
   T   
   C   
   A   
   T   
   C   
   G   
   C   
   C   
   G   
   A   
   A   
   T   
   C   
   T   
   T   
   A   
   T   
   A   
   T   
   G   
   G   
   A   
   A   
   T   
   G   
   A   
   A   
   C   
   G   
   A   
   A   
   T   
   T   
   A   
   G   
   G   
   G   
   G   
   T   
   A   
   A   
   C   
   C   
   C   
   C   
   T   
   G   
   A   
   T   
   A   
   A   
   A   
   C   
   C   
   A   
   C   
   A   
   C   
   A   
   A   
   A   
   A   
   A   
   A   
   T   
   C   
   A   
   G   
   C   
   C   
   C   
   G   
   G   
   A   
   T   
   T   
   G   
   T   
   T   
   G   
   G   
   G   
   G   
   A   
   T   
   G   
   T   
   T   
   A   
   T   
   G   
   G   
   G   
   T   
   A   
   A   
   A   
   T   
   A   
   T   
   C   
   A   
   C   
   C   
   C   
   C   
   C   
   A   
   T   
   G   
   G   
   G   
   G   
   A   
   C   
   A   
   G   
   T   
   G   
   C   
   G   
   A   
   T   
   T   
   T   
   A   
   C   
   G   
   A   
   A   
   T   
   C   
   A   
   A   
   T   
   G   
   G   
   T   
   G   
   C   
   G   
   G   
   A   
   T   
   G   
   G   
   C   
   A   
   C   
   A   
   A   
   C   
   C   
   T   
   T   
   T   
   T   
   A   
   G   
   T   
   T   
   A   
   T   
   C   
   G   
   G   
   G   
   C   
   T   
   A   
   T   
   G   
   T   
   T   
   A   
   G   
   T   
   T   
   G   
   A   
   C   
   G   
   G   
   C   
   C   
   A   
   C   
   G   
   G   
   A   
   A   
   A   
   C   
   T   
   T   
   C   
   G   
   G   
   T   
   T   
   C   
   T   
   G   
   T   
   C   
   G   
   A   
   T   
   G   
   G   
   T   
   G   
   A   
   C   
   G   
   G   
   C   
   G   
   C   
   T   
   G   
   C   
   C   
   G   
   C   
   T   
   A   
   T   
   G   
   C   
   G   
   T   
   T   
   A   
   T   
   A   
   C   
   C   
   G   
   A   
   A   
   G   
   C   
   A   
   C   
   G   
   T   
   A   
   T   
   G   
   A   
   G   
   T   
   A   
   A   
   A   
   A   
   T   
   T   
   G   
   C   
   T   
   T   
   T   
   A   
   G   
   A   
   A   
   A   
   T   
   G   
   C   
   T   
   A   
   C   
   G   
   A   
   G   
   A   
   T   
   A   
   T   
   T   
   A   
   A   
   C   
   A   
   A   
   A   
   A   
   A   
   T   
   A   
   C   
   A   
   G   
   T   
   C   
   G   
   A   
   T   
   T   
   T   
   C   
   C   
   A   
   A   
   G   
   G   
   A   
   A   
   A   
   C   
   T   
   A   
   T   
   G   
   A   
   T   
   G   
   A   
   T   
   T   
   C   
   A   
   G   
   A   
   A   
   C   
   A   
   A   
   G   
   A   
   A   
   C   
   C   
   A   
   G   
   T   
   A   
   G   
   T   
   T   
   T   
   T   
   A   
   C   
   C   
   T   
   G   
   C   
   T   
   C   
   G   
   T   
   T   
   T   
   T   
   C   
   C   
   A   
   A   
   A   
   C   
   T   
   T   
   A   
   C   
   T   
   A   
   G   
   T   
   T   
   A   
   A   
   C   
   G   
   G   
   A   
   A   
   C   
   A   
   A   
   C   
   G   
   G   
   G   
   G   
   A   
   T   
   T   
   G   
   C   
   G   
   G   
   T   
   G   
   G   
   G   
   G   
   A   
   T   
   G   
   G   
   C   
   A   
   A   
   C   
   A   
   A   
   A   
   T   
   A   
   T   
   T   
   C   
   C   
   A   
   C   
   C   
   A   
   C   
   A   
   T   
   A   
   A   
   T   
   T   
   T   
   A   
   A   
   G   
   T   
   G   
   A   
   A   
   G   
   T   
   G   
   A   
   T   
   T   
   G   
   A   
   T   
   G   
   C   
   A   
   A   
   C   
   A   
   A   
   G   
   T   
   T   
   T   
   A   
   T   
   T   
   G   
   A   
   T   
   G   
   G   
   A   
   C   
   A   
   A   
   T   
   C   
   C   
   T   
   G   
   A   
   T   
   G   
   T   
   A   
   A   
   C   
   G   
   A   
   C   
   G   
   A   
   A   
   T   
   G   
   A   
   A   
   T   
   T   
   G   
   A   
   T   
   G   
   G   
   A   
   A   
   G   
   T   
   G   
   T   
   T   
   A   
   C   
   C   
   T   
   G   
   G   
   A   
   C   
   C   
   A   
   G   
   A   
   T   
   T   
   T   
   T   
   C   
   C   
   A   
   A   
   C   
   A   
   G   
   G   
   C   
   G   
   G   
   T   
   T   
   T   
   A   
   G   
   T   
   G   
   A   
   T   
   G   
   G   
   G   
   G   
   A   
   A   
   A   
   T   
   C   
   A   
   G   
   G   
    G    
   A   
   T   
    T    
   C   
   G   
    C    
    C    
   G   
   A   
   G   
   C   
    A    
   T   
   A   
   T   
   G   
   A   
   A   
   A   
   C   
    A    
   G   
   G   
    G    
   A   
   A   
    A    
   G   
   G   
   T   
   T   
   C   
    G    
   A   
   T   
   T   
   A   
   C   
    T    
   G   
   T   
    T    
   C   
   G   
   T   
   G   
   C   
   A   
   A   
   A   
   A   
   G   
   T   
   T   
   G   
   A   
    A    
    T    
   T   
    G    
   A   
    C    
   T   
   G   
   A   
    A    
   A   
   T   
   G   
    C    
   C   
    G    
   A   
   A   
   T   
   G   
   G   
    A    
   A   
   A   
   A   
   G   
   A   
   A   
   C   
   G   
    T    
   A   
   T   
    T    
    T    
   T   
    A    
   G   
   T   
    A    
   A   
   C   
    T    
   G   
   A   
   A   
   T   
   T   
    G    
   C   
   C   
   T   
   T   
   A   
   T   
   A   
   T   
   G   
   G   
   T   
    G    
   A   
   A   
   T   
   A   
   A   
   A   
   G   
   C   
    C    
   A   
   A   
   A   
   T   
   T   
    A    
   A   
   T   
    C    
   G   
   A   
   A   
   C   
   G   
    A    
   A   
   T   
   T   
   T   
   C   
   T   
   G   
   A   
    A    
    T    
   T   
    A    
   C   
   A   
   C   
   A   
   G   
   A   
   G   
   A   
    T    
   A   
   A   
    A    
   C   
   G   
    A    
   A   
   T   
    T    
   G   
   A   
   A   
   G   
   G   
    A    
   A   
   T   
    T    
   A   
   C   
    T    
   G   
   A   
   T   
   T   
   T   
   G   
   C   
   G   
   G   
   G   
   A   
    T    
   G   
   A   
   A   
   T   
   C   
   T   
   T   
   C   
    T    
   C   
   G   
   T   
   G   
   A   
    A    
   G   
   G   
    C    
   A   
   T   
   G   
   C   
   G   
    G    
   A   
   T   
    C    
   G   
   T   
   C   
   A   
   T   
    C    
   G   
   A   
    T    
    G    
   T   
    T    
   C   
   G   
   T   
   C   
   G   
    A    
   G   
   A   
    T    
   G   
    T    
    G    
   A   
   G   
    T    
   G   
   C   
    C    
   T   
   C   
    T    
   G   
   T   
   T   
    G    
   T   
    G    
   T   
   T   
    A    
   A   
   A   
    C    
   A   
   A   
   C   
   T   
   T   
    G    
   T   
   A   
    C    
   A   
   A   
   A   
    A    
   T   
   G   
   A   
   C   
    T    
    G    
   C   
    C    
   T   
   T   
    A    
   C   
   A   
    A    
   A   
    C    
    A    
   T   
   C   
    T    
   T   
   T   
    T    
   G   
   G   
    T    
   T   
   T   
    T    
   A   
   A   
    C    
   A   
   T   
   G   
   T   
   T   
    A    
   G   
   C   
    C    
   A   
   T   
    C    
   G   
   A   
   A   
   A   
   A   
   A   
   G   
   G   
    C    
   G   
   T   
    A    
   C   
   C   
    A    
   A   
   A   
   A   
    A    
   T   
   T   
   T   
   T   
    A    
   A   
   G   
    C    
   T   
   T   
   G   
   A   
   A   
   A   
   C   
    G    
    T    
   A   
   T   
    T    
    T    
   T   
    A    
   G   
   A   
   A   
   A   
   A   
    C    
   T   
   A   
    C    
    G    
   T   
    C    
   G   
   A   
    G    
   C   
   A   
    C    
   C   
   A   
    A    
    A    
    A    
    A    
    G    
   A   
   A   
   G   
   T   
   C   
   A   
   T   
    T    
   A   
   C   
    T    
    C    
   G   
    C    
   C   
   G   
   G   
   A   
   C   
    G    
    A    
   T   
    T    
   T   
   T   
   T   
   G   
   A   
    C    
   A   
   A   
   A   
    A    
   A   
    C    
   A   
   A   
    A    
   G   
   C   
   A   
   G   
   A   
   A   
   G   
   C   
   A   
    C    
   G   
    G    
   G   
   C   
    G    
   C   
   A   
    T    
   A   
   T   
    T    
   T   
   T   
   A   
   G   
   A   
   A   
   G   
   G   
   T   
    C    
   T   
    A    
   C   
   G   
    A    
   A   
   T   
   T   
   G   
   C   
    C    
   T   
   T   
    A    
   G   
   A   
   T   
   C   
   A   
   T   
   A   
   T   
   C   
    G    
   A   
   T   
   G   
   A   
   A   
   A   
   T   
    C    
   A   
   T   
   T   
   G   
   C   
    C    
   A   
   T   
   T   
   A   
   T   
    C    
   C   
   G   
    T    
   G   
   G   
    A    
   T   
   C   
    A    
    C    
   A   
   A   
   T   
   C   
   A   
   G   
   A   
   T   
   G   
   A   
    C    
   G   
   A   
    A    
   G   
   C   
   A   
   A   
   A   
   A   
   G   
   C   
    A    
   A   
    C    
    G    
    T    
   T   
    A    
   A   
   T   
    T    
   G   
   A   
   A   
   C   
   G   
    C    
   T   
   T   
    T    
   G   
   A   
    A    
   T   
   T   
    T    
   T   
   C   
    A    
   G   
   A   
   T   
   C   
   G   
    T    
   C   
   A   
    A    
    G    
   C   
    G    
   C   
   A   
   A   
   G   
   C   
    G    
   A   
   T   
    T    
   T   
   T   
   A   
   G   
   A   
   T   
   A   
   T   
   G   
   C   
   G   
   T   
   T   
   T   
    A    
   C   
   G   
    C    
   C   
   G   
    T    
   T   
   T   
    A    
   A   
   C   
    A    
   G   
   G   
    C    
   T   
   T   
    A    
   G   
   A   
   A   
   C   
   G   
    C    
   G   
   A   
    C    
   A   
   A   
   A   
   A   
   T   
    T    
   G   
   A   
    A    
   A   
   A   
   T   
   G   
   A   
   A   
   T   
   A   
    T    
   C   
   A   
   A   
   G   
   A   
    G    
   C   
   T   
    A    
   T   
   T   
    G    
   A   
    A    
   A   
    T    
   T   
    C    
   A   
   T   
   T   
   G   
    C    
    G    
   G   
   A   
    C    
   T   
   T   
    A    
    G    
    A    
    G    
   G   
   A   
   C   
   A   
   T   
   T   
    T    
   T   
    A    
   G   
   C   
    C    
    C    
   G   
    C    
   C   
   C   
    A    
   G   
   A   
    A    
   C   
   G   
    C    
   G   
   T   
    C    
   A   
    T    
    T    
   G   
   A   
   A   
   A   
   T   
    C    
   A   
   T   
    T    
   A   
   A   
    A    
   A   
    C    
    A    
   G   
   A   
    G    
   T   
   T   
   A   
    A    
    A    
    T    
   G   
   A   
   C   
    G    
   T   
    T    
    C    
    G    
    C    
    A    
    C    
   A   
    A    
    A    
   A   
   T   
   T   
   T   
   G   
   G   
   T   
   G   
   A   
   T   
    G    
    C    
   A   
   C   
   G   
   A   
   C   
   G   
    C    
   A   
   C   
   A   
   G   
   A   
   A   
    T    
   T   
   A   
    T    
   T   
   A   
   G   
   T   
    A    
   G   
   G   
   T   
   G   
   A   
    A    
   G   
   T   
   C   
    T    
   T   
    A    
   A   
   G   
   T   
   C   
   T   
    T    
   G   
   A   
   A   
   G   
   A   
   T   
   G   
   A   
   A   
   G   
   A   
   T   
    T    
   T   
    A    
   A   
   T   
    C    
   G   
   A   
   A   
   G   
   A   
   A   
   G   
   A   
   A   
   G   
   A   
   A   
    G    
   T   
   C   
   G   
   T   
    G    
   A   
   T   
   T   
   A   
   C   
    A    
    T    
   T   
    A    
   A   
   C   
    C    
   A   
   A   
    T    
   A   
   A   
    C    
   G   
   G   
    C    
   T   
   A   
    C    
   A   
   T   
    T    
   A   
   A   
    G    
   C   
   G   
    G    
    A    
   T   
   G   
   G   
    C    
   A   
   A   
   A   
    C    
    T    
    C    
    T    
   G   
   A   
   A   
   T   
   T   
   C   
    C    
   G   
    G    
   G   
   C   
    G    
   C   
   A   
   A   
   C   
   G   
    C    
    C    
   G   
    T    
   G   
   G   
    C    
   G   
   G   
    A    
   C   
   G   
   T   
   G   
   G   
    T    
   G   
   T   
    C    
   C   
   A   
   A   
   G   
   G   
    C    
   A   
   T   
   G   
   G   
   G   
    C    
    G    
   T   
    T    
   C   
   A   
    T    
    G    
   A   
   T   
   G   
   A   
    T    
   G   
   A   
    T    
   T   
   T   
   C   
   G   
   T   
    G    
   A   
   A   
    A    
   A   
   A   
    C    
   C   
   T   
    A    
    G    
   T   
   T   
   T   
   C   
   T   
   T   
   G   
    T    
   T   
   C   
    A    
   A   
   C   
    A    
   C   
   A   
    C    
   G   
   A   
    C    
   A   
   C   
    G    
   T   
   T   
    A    
   T   
   T   
   A   
   T   
   T   
    C    
   T   
   T   
   T   
   A   
   C   
   G   
   A   
   A   
    C    
   A   
    C    
    T    
   G   
   G   
    G    
   A   
   A   
   A   
   G   
   T   
    T    
   T   
   A   
    C    
   C   
   G   
    A    
   G   
   C   
   A   
   A   
   A   
   A   
   G   
   G   
    T    
   T   
   A   
    C    
   G   
   A   
   A   
   A   
   T   
    C    
   C   
   C   
    T    
   G   
   A   
    A    
   T   
   A   
   C   
   G   
   G   
    T    
    A    
   G   
    A    
   A   
   C   
   A   
   G   
   C   
    A    
   A   
   A   
   A   
   G   
   G   
    A    
   A   
   T   
    T    
   C   
   C   
   A   
    G    
   T   
    G    
   A   
   T   
    T    
   A   
   A   
    C    
   T   
   T   
    A    
   C   
   T   
    G    
   G   
   G   
    A    
   A   
   T   
    T    
   G   
   A   
    T    
   T   
   C   
    T    
    G    
    C    
    A    
   G   
   A   
   A   
   A   
    A    
    A    
   A   
   T   
    T    
   C   
   A   
    A    
   G   
   C   
    A    
   A   
   T   
    T    
   A   
   T   
   T   
    T    
   C   
    T    
   G   
   T   
    T    
    G    
    A    
   G   
   G   
   G   
    C    
    A    
   A   
   A   
   G   
   C   
    G    
   G   
   A   
   A   
   G   
    C    
    A    
   G   
   G   
    T    
    A    
   A   
    A    
   T   
   A   
    C    
    T    
   T   
    G    
   T   
   T   
   C   
   T   
   T   
    C    
   A   
   C   
    A    
   A   
   C   
    C    
    T    
    T    
    A    
    A    
   A   
   A   
   G   
   G   
    A    
   A   
   C   
    C    
   G   
   T   
    C    
   A   
   A   
   A   
   C   
   G   
    G    
   A   
   C   
    A    
    G    
   C   
    C    
   G   
   T   
    A    
   A   
   C   
    A    
   G   
   C   
    C    
   T   
   T   
    T    
    T    
   C   
   T   
   A   
   A   
    T    
   A   
   T   
    C    
   C   
   G   
    T    
   A   
   G   
    T    
   A   
   A   
   T   
   G   
   G   
    A    
    T    
   T   
    A    
   A   
   T   
    C    
   G   
   C   
    C    
   A   
   T   
   T   
    A    
   G   
    C    
   T   
   T   
    A    
   A   
   A   
   A   
   G   
   A   
   A   
   G   
   A   
    T    
   G   
   A   
    T    
   G   
   A   
    G    
    T    
   T   
    A    
    G    
   T   
    T    
   A   
   A   
    C    
   G   
   T   
    G    
    G    
   T   
   A   
    A    
    C    
   G   
   A   
   C   
    T    
    A    
   A   
    T    
   G   
   G   
    C    
    A    
   A   
    T    
    C    
    A    
    G    
   A   
   A   
    G    
   A   
   T   
    G    
   A   
   T   
    T    
   A   
   T   
    C    
   G   
   G   
    A    
   A   
   C   
   A   
   C   
   A   
   T   
    G    
    C    
    A    
   G   
   G   
    A    
   T   
   A   
   C   
   T   
   C   
   T   
   G   
   T   
    C    
   A   
   C   
    A    
   T   
   T   
    T    
    G    
   A   
    T    
   G   
   A   
    A    
    A    
    A    
    T    
    A    
   C   
    T    
   G   
    T    
    A    
   C   
   G   
    T    
   G   
   A   
   T   
   A   
   T   
   G   
   G   
   G   
    C    
   C   
   G   
    G    
   A   
   C   
   A   
   G   
   C   
    A    
   T   
   C   
    A    
   G   
   G   
    T    
   G   
   T   
    T    
    C    
   G   
    G    
   G   
   G   
    A    
   A   
   T   
    C    
   C   
   G   
    T    
    C    
   T   
    C    
   C   
   G   
    C    
   G   
   A   
    A    
    A    
   A   
    T    
   G   
   A   
    T    
   T   
    A    
    T    
   G   
   T   
    G    
   G   
   T   
    C    
   G   
   G   
    C    
    G    
   C   
   A   
   G   
   C   
    G    
    A    
   T   
    T    
    C    
   T   
   G   
    G    
   A   
    T    
   G   
    A    
   A   
    A    
   A   
    T    
    A    
   A   
   A   
   G   
   A   
   A   
   G   
   T   
    C    
    C    
   T   
    A    
   G   
   T   
    C    
   A   
   T   
    T    
   A   
   C   
    T    
   G   
   A   
   A   
   A   
   A   
    T    
   G   
   G   
   T   
   T   
   A   
    T    
   G   
   G   
    T    
   A   
   A   
    G    
   C   
   G   
    T    
   A   
   C   
    A    
   A   
   A   
   A   
   G   
    C    
    T    
   T   
   C   
    T    
   G   
   A   
    A    
   T   
   A   
   T   
   C   
   C   
   A   
   G   
   T   
    T    
   A   
   A   
    A    
   G   
   G   
    A    
   C   
   G   
    T    
   G   
   G   
    C    
   G   
   G   
   T   
   A   
   A   
   A   
   G   
   G   
    G    
   A   
   T   
    T    
   A   
   A   
    G    
   A   
   C   
    A    
    G    
   C   
    A    
   A   
   A   
    T    
   A   
   T   
   C   
   A   
   C   
   T   
   G   
   A   
    G    
   A   
   A   
   A   
   A   
   A   
   T   
   G   
   G   
   T   
   C   
   C   
   A   
    T    
   T   
   A   
   G   
   C   
    T    
   G   
   G   
    T    
   T   
   T   
    A    
   A   
   C   
   C   
   A   
   C   
    G    
   G   
   T   
    C    
    A    
    A    
   T   
   G   
   G   
    G    
    G    
   A   
   T   
   G   
   A   
   A   
   G   
   A   
    T    
   A   
   T   
   C   
    T    
   T   
    A    
    T    
   T   
    G    
   A   
   T   
    T    
   A   
   C   
    G    
    A    
   A   
    C    
   A   
   A   
   A   
   G   
   G   
    C    
   G   
   T   
    C    
   A   
   T   
   T   
   A   
   T   
   C   
   C   
   G   
    C    
   T   
   T   
    T    
   A   
   A   
   C   
   G   
   T   
    A    
    G    
    A    
    T    
    T    
   C   
   T   
   G   
   T   
   T   
   T   
   C   
   T   
   C   
   A   
   A   
   A   
   C   
    A    
   G   
   G   
   A   
   C   
   G   
    C    
    G    
   C   
   A   
   A   
   C   
   A   
   T   
   T   
    A    
   G   
   G   
    A    
   G   
   T   
    T    
   C   
   G   
   T   
   T   
   T   
    A    
   A   
   T   
   G   
   A   
    G    
    A    
   A   
   T   
   G   
   G   
   A   
   A   
   G   
    A    
    T    
   G   
    G    
    T    
    G    
   C   
    C    
   A   
   A   
   A   
   -   
   -   
   -   
   -   
   -   
   -   
   -   
   -   
   -   
   -   
   -   
   -   
   -   
   -   
   -   
   -   
   -   
   -   
   -   
   -   
   -   
   -   
   -   
   -   
   -   
   -   
   -   
   -   
   -   
   -   
   -   
   -   
   -   
   -   
   -   
   -   
   -   
   -   
   -   
   -   
   -   
   -   
   -   
   -   
   -   
   -   
   -   
   -   
   -   
   -   
   -   
   -   
   -   
   -   
   -   
   -   
   -   
   -   
   -   
   -   
   -   
   -   
   -   
   -   
   -   
   -   
   -   
   -   
   -   
   -   
   -   
   -   
 

 
   N54574   
   A   
   T   
   G   
   A   
   G   
   T   
   G   
   A   
   A   
   G   
   A   
   A   
   A   
   T   
   T   
   A   
   A   
   A   
   G   
   A   
   A   
   A   
   A   
   C   
   A   
   T   
   T   
   C   
   A   
   A   
   G   
   A   
   C   
   G   
   T   
   C   
   A   
   A   
   T   
   C   
   T   
   G   
   A   
   C   
   C   
   A   
   G   
   C   
   G   
   A   
   A   
   A   
   T   
   G   
   A   
   A   
   A   
   G   
   A   
   A   
   T   
   C   
   T   
   T   
   T   
   C   
   A   
   T   
   T   
   G   
   A   
   C   
   T   
   A   
   C   
   G   
   C   
   A   
   A   
   T   
   G   
   A   
   G   
   T   
   G   
   T   
   T   
   A   
   T   
   C   
   G   
   T   
   A   
   G   
   C   
   C   
   C   
   G   
   C   
   G   
   C   
   G   
   T   
   T   
   A   
   C   
   C   
   T   
   G   
   A   
   C   
   G   
   T   
   T   
   C   
   G   
   T   
   G   
   A   
   C   
   G   
   G   
   T   
   T   
   T   
   A   
   A   
   A   
   A   
   C   
   C   
   T   
   G   
   T   
   T   
   C   
   A   
   T   
   C   
   G   
   C   
   C   
   G   
   A   
   A   
   T   
   C   
   T   
   T   
   A   
   T   
   A   
   T   
   G   
   G   
   A   
   A   
   T   
   G   
   A   
   A   
   C   
   G   
   A   
   A   
   T   
   T   
   A   
   G   
   G   
   G   
   G   
   T   
   A   
   A   
   C   
   C   
   C   
   C   
   T   
   G   
   A   
   T   
   A   
   A   
   A   
   C   
   C   
   A   
   C   
   A   
   C   
   A   
   A   
   A   
   A   
   A   
   A   
   T   
   C   
   A   
   G   
   C   
   C   
   C   
   G   
   G   
   A   
   T   
   T   
   G   
   T   
   T   
   G   
   G   
   G   
   G   
   A   
   T   
   G   
   T   
   T   
   A   
   T   
   G   
   G   
   G   
   T   
   A   
   A   
   A   
   T   
   A   
   T   
   C   
   A   
   C   
   C   
   C   
   C   
   C   
   A   
   T   
   G   
   G   
   G   
   G   
   A   
   C   
   A   
   G   
   T   
   G   
   C   
   G   
   A   
   T   
   T   
   T   
   A   
   C   
   G   
   A   
   A   
   T   
   C   
   A   
   A   
   T   
   G   
   G   
   T   
   G   
   C   
   G   
   G   
   A   
   T   
   G   
   G   
   C   
   A   
   C   
   A   
   A   
   C   
   C   
   T   
   T   
   T   
   T   
   A   
   G   
   T   
   T   
   A   
   T   
   C   
   G   
   G   
   G   
   C   
   T   
   A   
   T   
   G   
   T   
   T   
   A   
   G   
   T   
   T   
   G   
   A   
   C   
   G   
   G   
   C   
   C   
   A   
   C   
   G   
   G   
   A   
   A   
   A   
   C   
   T   
   T   
   C   
   G   
   G   
   T   
   T   
   C   
   T   
   G   
   T   
   C   
   G   
   A   
   T   
   G   
   G   
   T   
   G   
   A   
   C   
   G   
   G   
   C   
   G   
   C   
   T   
   G   
   C   
   C   
   G   
   C   
   T   
   A   
   T   
   G   
   C   
   G   
   T   
   T   
   A   
   T   
   A   
   C   
   C   
   G   
   A   
   A   
   G   
   C   
   A   
   C   
   G   
   T   
   A   
   T   
   G   
   A   
   G   
   T   
   A   
   A   
   A   
   A   
   T   
   T   
   G   
   C   
   T   
   T   
   T   
   A   
   G   
   A   
   A   
   A   
   T   
   G   
   C   
   T   
   A   
   C   
   G   
   A   
   G   
   A   
   T   
   A   
   T   
   T   
   A   
   A   
   C   
   A   
   A   
   A   
   A   
   A   
   T   
   A   
   C   
   A   
   G   
   T   
   C   
   G   
   A   
   T   
   T   
   T   
   C   
   C   
   A   
   A   
   G   
   G   
   A   
   A   
   A   
   C   
   T   
   A   
   T   
   G   
   A   
   T   
   G   
   A   
   T   
   T   
   C   
   A   
   G   
   A   
   A   
   C   
   A   
   A   
   G   
   A   
   A   
   C   
   C   
   A   
   G   
   T   
   A   
   G   
   T   
   T   
   T   
   T   
   A   
   C   
   C   
   T   
   G   
   C   
   T   
   C   
   G   
   T   
   T   
   T   
   T   
   C   
   C   
   A   
   A   
   A   
   C   
   T   
   T   
   A   
   C   
   T   
   A   
   G   
   T   
   T   
   A   
   A   
   C   
   G   
   G   
   A   
   A   
   C   
   A   
   A   
   C   
   G   
   G   
   G   
   G   
   A   
   T   
   T   
   G   
   C   
   G   
   G   
   T   
   G   
   G   
   G   
   G   
   A   
   T   
   G   
   G   
   C   
   A   
   A   
   C   
   A   
   A   
   A   
   T   
   A   
   T   
   T   
   C   
   C   
   A   
   C   
   C   
   A   
   C   
   A   
   C   
   A   
   A   
   T   
   T   
   T   
   A   
   A   
   G   
   T   
   G   
   A   
   A   
   G   
   T   
   G   
   A   
   T   
   T   
   G   
   A   
   T   
   G   
   C   
   A   
   A   
   C   
   A   
   A   
   G   
   T   
   T   
   T   
   A   
   T   
   T   
   G   
   A   
   T   
   G   
   G   
   A   
   C   
   A   
   A   
   T   
   C   
   C   
   T   
   G   
   A   
   T   
   G   
   T   
   A   
   A   
   C   
   G   
   A   
   C   
   G   
   A   
   A   
   T   
   G   
   A   
   A   
   T   
   T   
   G   
   A   
   T   
   G   
   G   
   A   
   A   
   G   
   T   
   G   
   T   
   T   
   A   
   C   
   C   
   T   
   G   
   G   
   A   
   C   
   C   
   A   
   G   
   A   
   T   
   T   
   T   
   T   
   C   
   C   
   A   
   A   
   C   
   A   
   G   
   G   
   C   
   G   
   G   
   T   
   T   
   T   
   A   
   G   
   T   
   G   
   A   
   T   
   G   
   G   
   G   
   G   
   A   
   A   
   A   
   T   
   C   
   A   
   G   
   G   
    G    
   A   
   T   
    T    
   C   
   G   
    C    
    C    
   G   
   A   
   G   
   C   
    A    
   T   
   A   
   T   
   G   
   A   
   A   
   A   
   C   
    G    
   G   
   G   
    G    
   A   
   A   
    A    
   G   
   G   
   T   
   T   
   C   
    G    
   A   
   T   
   T   
   A   
   C   
    A    
   G   
   T   
    T    
   C   
   G   
   T   
   G   
   C   
   A   
   A   
   A   
   A   
   G   
   T   
   T   
   G   
   A   
    A    
    T    
   T   
    G    
   A   
    C    
   T   
   G   
   A   
    A    
   A   
   T   
   G   
    C    
   C   
    G    
   A   
   A   
   T   
   G   
   G   
    A    
   A   
   A   
   A   
   G   
   A   
   A   
   C   
   G   
    T    
   A   
   T   
    T    
    T    
   T   
    A    
   G   
   T   
    A    
   A   
   C   
    T    
   G   
   A   
   A   
   T   
   T   
    G    
   C   
   C   
   T   
   T   
   A   
   T   
   A   
   T   
   G   
   G   
   T   
    G    
   A   
   A   
   T   
   A   
   A   
   A   
   G   
   C   
    C    
   A   
   A   
   A   
   T   
   T   
    A    
   A   
   T   
    C    
   G   
   A   
   A   
   C   
   G   
    A    
   A   
   T   
   T   
   T   
   C   
   T   
   G   
   A   
    A    
    T    
   T   
    A    
   C   
   A   
   C   
   A   
   G   
   A   
   G   
   A   
    T    
   A   
   A   
    A    
   C   
   G   
    A    
   A   
   T   
    T    
   G   
   A   
   A   
   G   
   G   
    A    
   A   
   T   
    T    
   A   
   C   
    T    
   G   
   A   
   T   
   T   
   T   
   G   
   C   
   G   
   G   
   G   
   A   
    T    
   G   
   A   
   A   
   T   
   C   
   T   
   T   
   C   
    T    
   C   
   G   
   T   
   G   
   A   
    A    
   G   
   G   
    C    
   A   
   T   
   G   
   C   
   G   
    G    
   A   
   T   
    C    
   G   
   T   
   C   
   A   
   T   
    C    
   G   
   A   
    T    
    G    
   T   
    T    
   C   
   G   
   T   
   C   
   G   
    A    
   G   
   A   
    T    
   G   
    T    
    G    
   A   
   G   
    T    
   G   
   C   
    C    
   T   
   C   
    T    
   G   
   T   
   T   
    G    
   T   
    G    
   T   
   T   
    A    
   A   
   A   
    C    
   A   
   A   
   C   
   T   
   T   
    A    
   T   
   A   
    C    
   A   
   A   
   A   
    A    
   T   
   G   
   A   
   C   
    T    
    G    
   C   
    C    
   T   
   T   
    A    
   C   
   A   
    A    
   A   
    C    
    A    
   T   
   C   
    T    
   T   
   T   
    T    
   G   
   G   
    T    
   T   
   T   
    T    
   A   
   A   
    C    
   A   
   T   
   G   
   T   
   T   
    A    
   G   
   C   
    C    
   A   
   T   
    C    
   G   
   A   
   A   
   A   
   A   
   A   
   G   
   G   
    C    
   G   
   T   
    A    
   C   
   C   
    G    
   A   
   A   
   A   
    A    
   T   
   T   
   T   
   T   
    A    
   A   
   G   
    C    
   T   
   T   
   G   
   A   
   A   
   A   
   C   
    G    
    T    
   A   
   T   
    T    
    T    
   T   
    A    
   G   
   A   
   A   
   A   
   A   
    T    
   T   
   A   
    C    
    G    
   T   
    C    
   G   
   A   
    G    
   C   
   A   
    C    
   C   
   A   
    A    
    A    
    A    
    A    
    G    
   A   
   A   
   G   
   T   
   C   
   A   
   T   
    T    
   A   
   C   
    T    
    C    
   G   
    C    
   C   
   G   
   G   
   A   
   C   
    G    
    A    
   T   
    T    
   T   
   T   
   T   
   G   
   A   
    C    
   A   
   A   
   A   
    A    
   A   
    C    
   A   
   A   
    A    
   G   
   C   
   A   
   G   
   A   
   A   
   G   
   C   
   A   
    C    
   G   
    G    
   G   
   C   
    G    
   C   
   A   
    T    
   A   
   T   
    T    
   T   
   T   
   A   
   G   
   A   
   A   
   G   
   G   
   T   
    C    
   T   
    A    
   C   
   G   
    A    
   A   
   T   
   T   
   G   
   C   
    C    
   T   
   T   
    A    
   G   
   A   
   T   
   C   
   A   
   T   
   A   
   T   
   C   
    G    
   A   
   T   
   G   
   A   
   A   
   A   
   T   
    C    
   A   
   T   
   T   
   G   
   C   
    C    
   A   
   T   
   T   
   A   
   T   
    C    
   C   
   G   
    T    
   G   
   G   
    A    
   T   
   C   
    A    
    C    
   A   
   A   
   T   
   C   
   A   
   G   
   A   
   T   
   G   
   A   
    C    
   G   
   A   
    A    
   G   
   C   
   A   
   A   
   A   
   A   
   G   
   C   
    A    
   A   
    C    
    G    
    T    
   T   
    A    
   A   
   T   
    T    
   G   
   A   
   A   
   C   
   G   
    C    
   T   
   T   
    T    
   G   
   A   
    A    
   T   
   T   
    T    
   T   
   C   
    A    
   G   
   A   
   T   
   C   
   G   
    T    
   C   
   A   
    A    
    G    
   C   
    G    
   C   
   A   
   A   
   G   
   C   
    G    
   A   
   T   
    T    
   T   
   T   
   A   
   G   
   A   
   T   
   A   
   T   
   G   
   C   
   G   
   T   
   T   
   T   
    A    
   C   
   G   
    C    
   C   
   G   
    T    
   T   
   T   
    A    
   A   
   C   
    A    
   G   
   G   
    C    
   T   
   T   
    A    
   G   
   A   
   A   
   C   
   G   
    C    
   G   
   A   
    C    
   A   
   A   
   A   
   A   
   T   
    T    
   G   
   A   
    A    
   A   
   A   
   T   
   G   
   A   
   A   
   T   
   A   
    T    
   C   
   A   
   A   
   G   
   A   
    G    
   C   
   T   
    A    
   T   
   T   
    G    
   A   
    A    
   A   
    T    
   T   
    C    
   A   
   T   
   T   
   G   
    C    
    G    
   G   
   A   
    C    
   T   
   T   
    A    
    G    
    A    
    G    
   G   
   A   
   C   
   A   
   T   
   T   
    T    
   T   
    A    
   G   
   C   
    C    
    C    
   G   
    C    
   C   
   C   
    A    
   G   
   A   
    A    
   C   
   G   
    C    
   G   
   T   
    C    
   A   
    T    
    T    
   G   
   A   
   A   
   A   
   T   
    C    
   A   
   T   
    T    
   A   
   A   
    A    
   A   
    C    
    A    
   G   
   A   
    G    
   T   
   T   
   A   
    A    
    A    
    T    
   G   
   A   
   C   
    G    
   T   
    T    
    C    
    G    
    C    
    A    
    C    
   A   
    A    
    A    
   A   
   T   
   T   
   T   
   G   
   G   
   T   
   G   
   A   
   T   
    G    
    C    
   A   
   C   
   G   
   A   
   C   
   G   
    C    
   A   
   C   
   A   
   G   
   A   
   A   
    T    
   T   
   A   
    T    
   T   
   A   
   G   
   T   
    A    
   G   
   G   
   T   
   G   
   A   
    A    
   G   
   T   
   C   
    C    
   T   
    A    
   A   
   G   
   T   
   C   
   T   
    T    
   G   
   A   
   A   
   G   
   A   
   T   
   G   
   A   
   A   
   G   
   A   
   T   
    T    
   T   
    A    
   A   
   T   
    C    
   G   
   A   
   A   
   G   
   A   
   A   
   G   
   A   
   A   
   G   
   A   
   A   
    G    
   T   
   C   
   G   
   T   
    G    
   A   
   T   
   T   
   A   
   C   
    A    
    T    
   T   
    A    
   A   
   C   
    C    
   A   
   A   
    T    
   A   
   A   
    C    
   G   
   G   
    C    
   T   
   A   
    C    
   A   
   T   
    T    
   A   
   A   
    G    
   C   
   G   
    G    
    A    
   T   
   G   
   G   
    C    
   A   
   A   
   A   
    C    
    T    
    C    
    C    
   G   
   A   
   A   
   T   
   T   
   C   
    C    
   G   
    G    
   G   
   C   
    G    
   C   
   A   
   A   
   C   
   G   
    C    
    C    
   G   
    C    
   G   
   G   
    C    
   G   
   G   
    A    
   C   
   G   
   T   
   G   
   G   
    T    
   G   
   T   
    C    
   C   
   A   
   A   
   G   
   G   
    C    
   A   
   T   
   G   
   G   
   G   
    C    
    G    
   T   
    T    
   C   
   A   
    T    
    G    
   A   
   T   
   G   
   A   
    T    
   G   
   A   
    T    
   T   
   T   
   C   
   G   
   T   
    G    
   A   
   A   
    A    
   A   
   A   
    C    
   C   
   T   
    A    
    A    
   T   
   T   
   T   
   C   
   T   
   T   
   G   
    T    
   T   
   C   
    A    
   A   
   C   
    A    
   C   
   A   
    C    
   G   
   A   
    C    
   A   
   C   
    G    
   T   
   T   
    A    
   T   
   T   
   A   
   T   
   T   
    C    
   T   
   T   
   T   
   A   
   C   
   G   
   A   
   A   
    C    
   A   
    C    
    T    
   G   
   G   
    G    
   A   
   A   
   A   
   G   
   T   
    T    
   T   
   A   
    C    
   C   
   G   
    A    
   G   
   C   
   A   
   A   
   A   
   A   
   G   
   G   
    T    
   T   
   A   
    C    
   G   
   A   
   A   
   A   
   T   
    C    
   C   
   C   
    T    
   G   
   A   
    A    
   T   
   A   
   C   
   G   
   G   
    T    
    A    
   G   
    A    
   A   
   C   
   A   
   G   
   C   
    A    
   A   
   A   
   A   
   G   
   G   
    A    
   A   
   T   
    T    
   C   
   C   
   A   
    G    
   T   
    A    
   A   
   T   
    T    
   A   
   A   
    C    
   T   
   T   
    A    
   C   
   T   
    G    
   G   
   G   
    A    
   A   
   T   
    T    
   G   
   A   
    T    
   T   
   C   
    T    
    G    
    C    
    A    
   G   
   A   
   A   
   A   
    A    
    A    
   A   
   T   
    T    
   C   
   A   
    A    
   G   
   C   
    G    
   A   
   T   
    T    
   A   
   T   
   T   
    T    
   C   
    T    
   G   
   T   
    T    
    G    
    A    
   G   
   G   
   G   
    C    
    A    
   A   
   A   
   G   
   C   
    G    
   G   
   A   
   A   
   G   
    C    
    A    
   G   
   G   
    T    
    A    
   A   
    A    
   T   
   A   
    C    
    T    
   T   
    G    
   T   
   T   
   C   
   T   
   T   
    C    
   A   
   C   
    A    
   A   
   C   
    C    
    T    
    T    
    A    
    A    
   A   
   A   
   G   
   G   
    A    
   A   
   C   
    C    
   G   
   T   
    C    
   A   
   A   
   A   
   C   
   G   
    G    
   A   
   C   
    A    
    G    
   C   
    C    
   G   
   T   
    A    
   A   
   C   
    A    
   G   
   C   
    C    
   T   
   T   
    T    
    T    
   C   
   T   
   A   
   A   
    T    
   A   
   T   
    C    
   C   
   G   
    T    
   A   
   G   
    T    
   A   
   A   
   T   
   G   
   G   
    A    
    T    
   T   
    A    
   A   
   T   
    C    
   G   
   C   
    C    
   A   
   T   
   T   
    A    
   G   
    C    
   T   
   T   
    A    
   A   
   A   
   A   
   G   
   A   
   A   
   G   
   A   
    T    
   G   
   A   
    T    
   G   
   A   
    G    
    T    
   T   
    A    
    G    
   T   
    T    
   A   
   A   
    C    
   G   
   T   
    G    
    G    
   T   
   A   
    A    
    C    
   G   
   A   
   C   
    T    
    A    
   A   
    T    
   G   
   G   
    C    
    A    
   A   
    T    
    C    
    A    
    G    
   A   
   A   
    G    
   A   
   T   
    G    
   A   
   T   
    T    
   A   
   T   
    C    
   G   
   G   
    A    
   A   
   C   
   A   
   C   
   A   
   T   
    G    
    C    
    A    
   G   
   G   
    A    
   T   
   A   
   C   
   T   
   C   
   T   
   G   
   T   
    C    
   A   
   C   
    A    
   T   
   T   
    T    
    G    
   A   
    T    
   G   
   A   
    A    
    A    
    A    
    T    
    A    
   C   
    T    
   G   
    T    
    A    
   C   
   G   
    T    
   G   
   A   
   T   
   A   
   T   
   G   
   G   
   G   
    C    
   C   
   G   
    G    
   A   
   C   
   A   
   G   
   C   
    A    
   T   
   C   
    A    
   G   
   G   
    T    
   G   
   T   
    T    
    C    
   G   
    G    
   G   
   G   
    A    
   A   
   T   
    C    
   C   
   G   
    T    
    C    
   T   
    C    
   C   
   G   
    C    
   G   
   A   
    A    
    A    
   A   
    T    
   G   
   A   
    T    
   T   
    A    
    T    
   G   
   T   
    G    
   G   
   T   
    C    
   G   
   G   
    T    
    G    
   C   
   A   
   G   
   C   
    G    
    A    
   T   
    T    
    C    
   T   
   G   
    G    
   A   
    T    
   G   
    A    
   A   
    A    
   A   
    C    
    A    
   A   
   A   
   G   
   A   
   A   
   G   
   T   
    C    
    C    
   T   
    A    
   G   
   T   
    C    
   A   
   T   
    T    
   A   
   C   
    T    
   G   
   A   
   A   
   A   
   A   
    T    
   G   
   G   
   T   
   T   
   A   
    C    
   G   
   G   
    T    
   A   
   A   
    G    
   C   
   G   
    T    
   A   
   C   
    A    
   A   
   A   
   A   
   G   
    C    
    T    
   T   
   C   
    T    
   G   
   A   
    A    
   T   
   A   
   T   
   C   
   C   
   A   
   G   
   T   
    T    
   A   
   A   
    A    
   G   
   G   
    A    
   C   
   G   
    T    
   G   
   G   
    C    
   G   
   G   
   T   
   A   
   A   
   A   
   G   
   G   
    G    
   A   
   T   
    T    
   A   
   A   
    G    
   A   
   C   
    A    
    G    
   C   
    A    
   A   
   A   
    T    
   A   
   T   
   C   
   A   
   C   
   T   
   G   
   A   
    G    
   A   
   A   
   A   
   A   
   A   
   T   
   G   
   G   
   T   
   C   
   C   
   A   
    T    
   T   
   A   
   G   
   C   
    T    
   G   
   G   
    T    
   T   
   T   
    A    
   A   
   C   
   C   
   A   
   C   
    G    
   G   
   T   
    C    
    A    
    A    
   T   
   G   
   G   
    T    
    G    
   A   
   T   
   G   
   A   
   A   
   G   
   A   
    T    
   A   
   T   
   C   
    T    
   T   
    A    
    T    
   T   
    G    
   A   
   T   
    T    
   A   
   C   
    G    
    A    
   A   
    C    
   A   
   A   
   A   
   G   
   G   
    C    
   G   
   T   
    C    
   A   
   T   
   T   
   A   
   T   
   C   
   C   
   G   
    C    
   T   
   T   
    T    
   A   
   A   
   C   
   G   
   T   
    T    
    G    
    A    
    T    
    T    
   C   
   T   
   G   
   T   
   T   
   T   
   C   
   T   
   C   
   A   
   A   
   A   
   C   
    A    
   G   
   G   
   A   
   C   
   G   
    C    
    G    
   C   
   A   
   A   
   C   
   A   
   T   
   T   
    A    
   G   
   G   
    G    
   G   
   T   
    T    
   C   
   G   
   T   
   T   
   T   
    A    
   A   
   T   
   G   
   A   
    G    
    A    
   A   
   T   
   G   
   G   
   A   
   A   
   G   
    A    
    T    
   G   
    G    
    T    
    G    
   C   
    C    
   A   
   A   
   A   
   -   
   -   
   -   
   -   
   -   
   -   
   -   
   -   
   -   
   -   
   -   
   -   
   -   
   -   
   -   
   -   
   -   
   -   
   -   
   -   
   -   
   -   
   -   
   -   
   -   
   -   
   -   
   -   
   -   
   -   
   -   
   -   
   -   
   -   
   -   
   -   
   -   
   -   
   -   
   -   
   -   
   -   
   -   
   -   
   -   
   -   
   -   
   -   
   -   
   -   
   -   
   -   
   -   
   -   
   -   
   -   
   -   
   -   
   -   
   -   
   -   
   -   
   -   
   -   
   -   
   -   
   -   
   -   
   -   
   -   
   -   
   -   
 

 
   N59531F   
   A   
   T   
   G   
   A   
   G   
   T   
   G   
   A   
   A   
   G   
   A   
   A   
   A   
   T   
   C   
   A   
   G   
   A   
   G   
   A   
   A   
   A   
   A   
   C   
   A   
   T   
   C   
   C   
   A   
   T   
   G   
   A   
   T   
   G   
   T   
   C   
   A   
   A   
   T   
   C   
   T   
   A   
   A   
   C   
   C   
   A   
   G   
   C   
   G   
   A   
   A   
   A   
   T   
   G   
   A   
   A   
   A   
   G   
   A   
   C   
   T   
   C   
   C   
   T   
   T   
   C   
   A   
   T   
   C   
   G   
   A   
   T   
   T   
   A   
   T   
   G   
   C   
   G   
   A   
   T   
   G   
   A   
   G   
   T   
   G   
   T   
   T   
   A   
   T   
   T   
   G   
   T   
   C   
   G   
   C   
   A   
   C   
   G   
   T   
   G   
   C   
   A   
   C   
   T   
   G   
   C   
   C   
   T   
   G   
   A   
   T   
   G   
   T   
   C   
   A   
   G   
   A   
   G   
   A   
   T   
   G   
   G   
   T   
   C   
   T   
   G   
   A   
   A   
   A   
   C   
   C   
   C   
   G   
   T   
   T   
   C   
   A   
   T   
   C   
   G   
   C   
   C   
   G   
   T   
   A   
   T   
   T   
   T   
   T   
   A   
   T   
   A   
   C   
   G   
   G   
   C   
   A   
   T   
   G   
   A   
   A   
   C   
   G   
   A   
   A   
   T   
   T   
   A   
   G   
   G   
   T   
   G   
   T   
   G   
   A   
   C   
   A   
   C   
   C   
   G   
   G   
   A   
   T   
   A   
   A   
   A   
   G   
   C   
   C   
   C   
   A   
   T   
   A   
   A   
   A   
   A   
   A   
   A   
   T   
   C   
   T   
   G   
   C   
   C   
   A   
   G   
   A   
   A   
   T   
   C   
   G   
   T   
   C   
   G   
   G   
   A   
   G   
   A   
   T   
   G   
   T   
   C   
   A   
   T   
   G   
   G   
   G   
   T   
   A   
   A   
   A   
   T   
   A   
   C   
   C   
   A   
   T   
   C   
   C   
   T   
   C   
   A   
   T   
   G   
   G   
   G   
   G   
   A   
   T   
   A   
   G   
   T   
   G   
   C   
   A   
   A   
   T   
   C   
   T   
   A   
   T   
   G   
   A   
   G   
   T   
   C   
   A   
   A   
   T   
   G   
   G   
   T   
   A   
   C   
   G   
   G   
   A   
   T   
   G   
   G   
   C   
   G   
   C   
   A   
   G   
   C   
   C   
   A   
   T   
   T   
   T   
   A   
   G   
   C   
   T   
   A   
   C   
   C   
   G   
   T   
   T   
   A   
   T   
   A   
   T   
   G   
   C   
   T   
   A   
   G   
   T   
   G   
   G   
   A   
   T   
   G   
   G   
   T   
   C   
   A   
   T   
   G   
   G   
   A   
   A   
   A   
   C   
   T   
   T   
   T   
   G   
   G   
   T   
   T   
   C   
   T   
   G   
   T   
   T   
   G   
   A   
   C   
   G   
   G   
   T   
   G   
   A   
   C   
   G   
   G   
   T   
   G   
   C   
   C   
   G   
   C   
   T   
   G   
   C   
   T   
   A   
   T   
   G   
   C   
   G   
   T   
   T   
   A   
   T   
   A   
   C   
   C   
   G   
   A   
   A   
   G   
   C   
   A   
   C   
   G   
   G   
   A   
   T   
   G   
   A   
   G   
   C   
   A   
   A   
   A   
   A   
   T   
   C   
   G   
   C   
   T   
   A   
   C   
   A   
   G   
   A   
   A   
   A   
   T   
   G   
   C   
   T   
   G   
   C   
   G   
   T   
   G   
   A   
   T   
   A   
   T   
   C   
   A   
   A   
   T   
   A   
   A   
   G   
   A   
   A   
   C   
   A   
   C   
   T   
   G   
   T   
   T   
   G   
   A   
   C   
   T   
   T   
   C   
   C   
   A   
   A   
   A   
   G   
   C   
   A   
   A   
   C   
   T   
   A   
   T   
   G   
   A   
   T   
   G   
   A   
   C   
   A   
   C   
   A   
   G   
   A   
   A   
   A   
   A   
   A   
   G   
   A   
   A   
   C   
   C   
   C   
   G   
   T   
   T   
   G   
   T   
   A   
   T   
   T   
   A   
   C   
   C   
   T   
   G   
   C   
   T   
   C   
   G   
   C   
   T   
   T   
   C   
   C   
   C   
   T   
   A   
   A   
   T   
   C   
   T   
   T   
   T   
   T   
   G   
   G   
   T   
   G   
   A   
   A   
   C   
   G   
   G   
   A   
   A   
   C   
   G   
   A   
   C   
   G   
   G   
   G   
   G   
   A   
   T   
   C   
   G   
   C   
   C   
   G   
   T   
   A   
   G   
   G   
   T   
   A   
   T   
   G   
   G   
   C   
   A   
   A   
   C   
   G   
   A   
   A   
   T   
   A   
   T   
   C   
   C   
   C   
   A   
   C   
   C   
   T   
   C   
   A   
   T   
   A   
   A   
   T   
   T   
   T   
   G   
   T   
   C   
   T   
   G   
   A   
   G   
   G   
   T   
   A   
   G   
   T   
   A   
   G   
   C   
   G   
   G   
   C   
   T   
   A   
   T   
   C   
   G   
   A   
   T   
   T   
   T   
   G   
   C   
   T   
   G   
   A   
   T   
   G   
   G   
   A   
   T   
   A   
   A   
   T   
   C   
   C   
   T   
   G   
   A   
   T   
   G   
   T   
   G   
   A   
   C   
   G   
   A   
   C   
   C   
   A   
   A   
   T   
   G   
   A   
   A   
   T   
   T   
   G   
   A   
   T   
   G   
   G   
   A   
   A   
   G   
   T   
   A   
   T   
   T   
   A   
   C   
   C   
   T   
   G   
   G   
   A   
   C   
   C   
   A   
   G   
   A   
   T   
   T   
   T   
   T   
   C   
   C   
   A   
   A   
   C   
   A   
   G   
   G   
   T   
   G   
   G   
   C   
   T   
   T   
   G   
   G   
   T   
   C   
   A   
   T   
   G   
   G   
   G   
   A   
   A   
   A   
   A   
   T   
   C   
   A   
   G   
   G   
    A    
   A   
   T   
    C    
   C   
   G   
    A    
    A    
   G   
   A   
   G   
   C   
    G    
   T   
   A   
   T   
   G   
   A   
   A   
   A   
   C   
    A    
   G   
   G   
    T    
   A   
   A   
    A    
   G   
   G   
   T   
   T   
   C   
    T    
   A   
   T   
   T   
   A   
   C   
    C    
   G   
   T   
    A    
   C   
   G   
   T   
   G   
   C   
   A   
   A   
   A   
   A   
   G   
   T   
   T   
   G   
   A   
    G    
    A    
   T   
    C    
   A   
    C    
   T   
   G   
   A   
    G    
   A   
   T   
   G   
    C    
   C   
    A    
   A   
   A   
   T   
   G   
   G   
    C    
   A   
   A   
   A   
   G   
   A   
   A   
   C   
   G   
    G    
   A   
   T   
    C    
    C    
   T   
    C    
   G   
   T   
    G    
   A   
   C   
    G    
   G   
   A   
   A   
   T   
   T   
    A    
   C   
   C   
   T   
   T   
   A   
   T   
   A   
   T   
   G   
   G   
   T   
    C    
   A   
   A   
   T   
   A   
   A   
   A   
   G   
   C   
    A    
   A   
   A   
   A   
   T   
   T   
    G    
   A   
   T   
    T    
   G   
   A   
   A   
   C   
   G   
    G    
   A   
   T   
   T   
   T   
   C   
   T   
   G   
   A   
    G    
    T    
   T   
    G    
   C   
   A   
   C   
   A   
   G   
   A   
   G   
   A   
    C    
   A   
   A   
    G    
   C   
   G   
    T    
   A   
   T   
    C    
   G   
   A   
   A   
   G   
   G   
    A    
   A   
   T   
    T    
   A   
   C   
    G    
   G   
   A   
   T   
   T   
   T   
   G   
   C   
   G   
   G   
   G   
   A   
    T    
   G   
   A   
   A   
   T   
   C   
   T   
   T   
   C   
    C    
   C   
   G   
   T   
   G   
   A   
    A    
   G   
   G   
    C    
   A   
   T   
   G   
   C   
   G   
    A    
   A   
   T   
    T    
   G   
   T   
   C   
   A   
   T   
    T    
   G   
   A   
    C    
    G    
   T   
    T    
   C   
   G   
   T   
   C   
   G   
    T    
   G   
   A   
    C    
   G   
    C    
    C    
   A   
   G   
    C    
   G   
   C   
    A    
   T   
   C   
    A    
   G   
   T   
   T   
    A    
   T   
    T    
   T   
   T   
    G    
   A   
   A   
    T    
   A   
   A   
   C   
   T   
   T   
    A    
   T   
   A   
    C    
   A   
   A   
   A   
    T    
   T   
   G   
   A   
   C   
    A    
    T    
   C   
    C    
   T   
   T   
    G    
   C   
   A   
    G    
   A   
    A    
    T    
   T   
   C   
    A    
   T   
   T   
    T    
   G   
   G   
    A    
   T   
   T   
    C    
   A   
   A   
    T    
   A   
   T   
   G   
   T   
   T   
    G    
   G   
   C   
    A    
   A   
   T   
    C    
   G   
   A   
   A   
   A   
   A   
   A   
   G   
   G   
    C    
   G   
   T   
    G    
   C   
   C   
    G    
   A   
   A   
   A   
    G    
   T   
   T   
   T   
   T   
    A    
   A   
   G   
    T    
   T   
   T   
   G   
   A   
   A   
   A   
   C   
    A    
    A    
   A   
   T   
    C    
    C    
   T   
    T    
   G   
   A   
   A   
   A   
   A   
    C    
   T   
   A   
    T    
    A    
   T   
    C    
   G   
   A   
    A    
   C   
   A   
    T    
   C   
   A   
    A    
    C    
    G    
    C    
    G    
   A   
   A   
   G   
   T   
   C   
   A   
   T   
    C    
   A   
   C   
    A    
    C    
   G   
    A    
   C   
   G   
   G   
   A   
   C   
    G    
    G    
   T   
    C    
   T   
   T   
   T   
   G   
   A   
    A    
   A   
   A   
   A   
    G    
   A   
    A    
   A   
   A   
    A    
   G   
   C   
   A   
   G   
   A   
   A   
   G   
   C   
   A   
    A    
   G   
    A    
   G   
   C   
    A    
   C   
   A   
    C    
   A   
   T   
    C    
   T   
   T   
   A   
   G   
   A   
   A   
   G   
   G   
   T   
    T    
   T   
    G    
   C   
   G   
    T    
   A   
   T   
   T   
   G   
   C   
    T    
   T   
   T   
    G    
   G   
   A   
   T   
   C   
   A   
   T   
   A   
   T   
   C   
    G    
   A   
   T   
   G   
   A   
   A   
   A   
   T   
    C    
   A   
   T   
   T   
   G   
   C   
    G    
   A   
   T   
   T   
   A   
   T   
    C    
   C   
   G   
    C    
   G   
   G   
    G    
   T   
   C   
    T    
    A    
   A   
   A   
   T   
   C   
   A   
   G   
   A   
   T   
   G   
   A   
    C    
   G   
   A   
    A    
   G   
   C   
   A   
   A   
   A   
   A   
   G   
   C   
    A    
   A   
    C    
    A    
    A    
   T   
    G    
   A   
   T   
    C    
   G   
   A   
   A   
   C   
   G   
    G    
   T   
   T   
    C    
   G   
   A   
    T    
   T   
   T   
    A    
   T   
   C   
    C    
   G   
   A   
   T   
   C   
   G   
    C    
   C   
   A   
    A    
    T    
   C   
    A    
   C   
   A   
   A   
   G   
   C   
    T    
   A   
   T   
    C    
   T   
   T   
   A   
   G   
   A   
   T   
   A   
   T   
   G   
   C   
   G   
   T   
   T   
   T   
    G    
   C   
   G   
    T    
   C   
   G   
    A    
   T   
   T   
    G    
   A   
   C   
    C    
   G   
   G   
    T    
   T   
   T   
    G    
   G   
   A   
   A   
   C   
   G   
    G    
   G   
   A   
    A    
   A   
   A   
   A   
   A   
   T   
    C    
   G   
   A   
    A    
   A   
   A   
   T   
   G   
   A   
   A   
   T   
   A   
    C    
   C   
   A   
   A   
   G   
   A   
    A    
   C   
   T   
    A    
   T   
   T   
    G    
   A   
    A    
   A   
    C    
   T   
    G    
   A   
   T   
   T   
   G   
    A    
    A    
   G   
   A   
    T    
   T   
   T   
    G    
    A    
    C    
    T    
   G   
   A   
   C   
   A   
   T   
   T   
    C    
   T   
    G    
   G   
   C   
    A    
    A    
   G   
    A    
   C   
   C   
    C    
   G   
   A   
    A    
   C   
   G   
    T    
   G   
   T   
    C    
   A   
    C    
    A    
   G   
   A   
   A   
   A   
   T   
    C    
   A   
   T   
    C    
   A   
   A   
    G    
   A   
    A    
    T    
   G   
   A   
    A    
   T   
   T   
   A   
    G    
    C    
    A    
   G   
   A   
   C   
    T    
   T   
    G    
    A    
    A    
    T    
    C    
    A    
   A   
    C    
    G    
   A   
   T   
   T   
   T   
   G   
   G   
   T   
   G   
   A   
   T   
    A    
    A    
   A   
   C   
   G   
   A   
   C   
   G   
    C    
   A   
   C   
   A   
   G   
   A   
   A   
    T    
   T   
   A   
    C    
   T   
   A   
   G   
   T   
    C    
   G   
   G   
   T   
   G   
   A   
    G    
   G   
   T   
   C   
    T    
   T   
    A    
   A   
   G   
   T   
   C   
   T   
    A    
   G   
   A   
   A   
   G   
   A   
   T   
   G   
   A   
   A   
   G   
   A   
   T   
    T    
   T   
    G    
   A   
   T   
    T    
   G   
   A   
   A   
   G   
   A   
   A   
   G   
   A   
   A   
   G   
   A   
   A   
    A    
   T   
   C   
   G   
   T   
    T    
   A   
   T   
   T   
   A   
   C   
    A    
    T    
   T   
    G    
   A   
   C   
    A    
   A   
   A   
    C    
   A   
   A   
    T    
   G   
   G   
    T    
   T   
   A   
    T    
   A   
   T   
    C    
   A   
   A   
    A    
   C   
   G   
    T    
    T    
   T   
   G   
   G   
    C    
   A   
   A   
   A   
    T    
    A    
    A    
    T    
   G   
   A   
   A   
   T   
   T   
   C   
    A    
   G   
    A    
   G   
   C   
    T    
   C   
   A   
   A   
   C   
   G   
    A    
    A    
   G   
    A    
   G   
   G   
    C    
   G   
   G   
    G    
   C   
   G   
   T   
   G   
   G   
    T    
   G   
   T   
    C    
   C   
   A   
   A   
   G   
   G   
    A    
   A   
   T   
   G   
   G   
   G   
    T    
    A    
   T   
    C    
   C   
   A   
    C    
    A    
   A   
   T   
   G   
   A   
    C    
   G   
   A   
    C    
   T   
   T   
   C   
   G   
   T   
    C    
   A   
   A   
    G    
   A   
   A   
    T    
   C   
   T   
    A    
    G    
   T   
   T   
   T   
   C   
   T   
   T   
   G   
    T    
   T   
   C   
    T    
   A   
   C   
    C    
   C   
   A   
    T    
   G   
   A   
    T    
   A   
   C   
    A    
   T   
   T   
    G    
   T   
   T   
   A   
   T   
   T   
    C    
   T   
   T   
   T   
   A   
   C   
   G   
   A   
   A   
    T    
   A   
    A    
    C    
   G   
   G   
    A    
   A   
   A   
   A   
   G   
   T   
    G    
   T   
   A   
    T    
   C   
   G   
    G    
   G   
   C   
   A   
   A   
   A   
   A   
   G   
   G   
    A    
   T   
   A   
    C    
   G   
   A   
   A   
   A   
   T   
    T    
   C   
   C   
    A    
   G   
   A   
    A    
   T   
   A   
   C   
   G   
   G   
    G    
    C    
   G   
    T    
   A   
   C   
   A   
   G   
   C   
    C    
   A   
   A   
   A   
   G   
   G   
    A    
   A   
   T   
    C    
   C   
   C   
   A   
    A    
   T   
    C    
   A   
   T   
    C    
   A   
   A   
    T    
   T   
   T   
    G    
   C   
   T   
    G    
   G   
   G   
    T    
   A   
   T   
    C    
   G   
   A   
    C    
   T   
   C   
    G    
    A    
    G    
    T    
   G   
   A   
   A   
   A   
    G    
    C    
   A   
   T   
    C    
   C   
   A   
    A    
   G   
   C   
    A    
   A   
   T   
    T    
   A   
   T   
   T   
    G    
   C   
    G    
   G   
   T   
    G    
    A    
    C    
   G   
   G   
   G   
    A    
    G    
   A   
   A   
   G   
   C   
    A    
   G   
   A   
   A   
   G   
    A    
    A    
   G   
   G   
    C    
    C    
   A   
    C    
   T   
   A   
    T    
    C    
   T   
    C    
   T   
   T   
   C   
   T   
   T   
    T    
   A   
   C   
    G    
   A   
   C   
    G    
    C    
    G    
    T    
    C    
   A   
   A   
   G   
   G   
    T    
   A   
   C   
    A    
   G   
   T   
    T    
   A   
   A   
   A   
   C   
   G   
    T    
   A   
   C   
    G    
    T    
   C   
    T    
   G   
   T   
    C    
   A   
   C   
    T    
   G   
   C   
    T    
   T   
   T   
    C    
    G    
   C   
   T   
   A   
   A   
    C    
   A   
   T   
    C    
   C   
   G   
    A    
   A   
   G   
    C    
   A   
   A   
   T   
   G   
   G   
    C    
    C    
   T   
    G    
   A   
   T   
    T    
   G   
   C   
    A    
   A   
   T   
   T   
    G    
   G   
    T    
   T   
   T   
    G    
   A   
   A   
   A   
   G   
   A   
   A   
   G   
   A   
    T    
   G   
   A   
    C    
   G   
   A   
    A    
    T    
   T   
    A    
    G    
   T   
    C    
   A   
   A   
    T    
   G   
   T   
    T    
    C    
   T   
   A   
    T    
    T    
   G   
   A   
   C   
    A    
    G    
   A   
    C    
   G   
   G   
    A    
    C    
   A   
    G    
    T    
    C    
    A    
   A   
   A   
    T    
   A   
   T   
    C    
   A   
   T   
    C    
   A   
   T   
    T    
   G   
   G   
    C    
   A   
   C   
   A   
   C   
   A   
   T   
    A    
    A    
    C    
   G   
   G   
    T    
   T   
   A   
   C   
   T   
   C   
   T   
   G   
   T   
    G    
   A   
   C   
    C    
   T   
   T   
    C    
    A    
   A   
    A    
   G   
   A   
    A    
    G    
    A    
    A    
    G    
   C   
    A    
   G   
    T    
    C    
   C   
   G   
    C    
   G   
   A   
   T   
   A   
   T   
   G   
   G   
   G   
    A    
   C   
   G   
    T    
   A   
   C   
   A   
   G   
   C   
    T    
   T   
   C   
    C    
   G   
   G   
    C    
   G   
   T   
    C    
    A    
   G   
    A    
   G   
   G   
    G    
   A   
   T   
    A    
   C   
   G   
    T    
    C    
   T   
    G    
   C   
   G   
    T    
   G   
   A   
    G    
    G    
   A   
    A    
   G   
   A   
    T    
   T   
    A    
    C    
   G   
   T   
    C    
   G   
   T   
    T    
   G   
   G   
    G    
    G    
   C   
   A   
   G   
   C   
    C    
    C    
   T   
    G    
    A    
   T   
   G   
    A    
   A   
    G    
   G   
    A    
   A   
    G    
   A   
    C    
    C    
   A   
   A   
   G   
   A   
   A   
   G   
   T   
    A    
    C    
   T   
    A    
   G   
   T   
    G    
   A   
   T   
    T    
   A   
   C   
    A    
   G   
   A   
   A   
   A   
   A   
    A    
   G   
   G   
   T   
   T   
   A   
    C    
   G   
   G   
    A    
   A   
   A   
    A    
   C   
   G   
    G    
   A   
   C   
    C    
   A   
   A   
   A   
   G   
    T    
    A    
   T   
   C   
    G    
   G   
   A   
    A    
   T   
   A   
   T   
   C   
   C   
   A   
   G   
   T   
    G    
   A   
   A   
    A    
   G   
   G   
    C    
   C   
   G   
    C    
   G   
   G   
    A    
   G   
   G   
   T   
   A   
   A   
   A   
   G   
   G   
    A    
   A   
   T   
    C    
   A   
   A   
    A    
   A   
   C   
    G    
    G    
   C   
    G    
   A   
   A   
    T    
   A   
   T   
   C   
   A   
   C   
   T   
   G   
   A   
    A    
   A   
   A   
   A   
   A   
   A   
   T   
   G   
   G   
   T   
   C   
   C   
   A   
    C    
   T   
   A   
   G   
   C   
    C    
   G   
   G   
    A    
   T   
   T   
    G    
   A   
   C   
   C   
   A   
   C   
    T    
   G   
   T   
    T    
    T    
    C    
   T   
   G   
   G   
    A    
    A    
   A   
   T   
   G   
   A   
   A   
   G   
   A   
    C    
   A   
   T   
   C   
    A    
   T   
    G    
    C    
   T   
    G    
   A   
   T   
    T    
   A   
   C   
    A    
    G    
   A   
    T    
   A   
   A   
   A   
   G   
   G   
    T    
   G   
   T   
    G    
   A   
   T   
   T   
   A   
   T   
   C   
   C   
   G   
    T    
   T   
   T   
    C    
   A   
   A   
   C   
   G   
   T   
    A    
    T    
    C    
    G    
    A    
   C   
   T   
   G   
   T   
   T   
   T   
   C   
   T   
   C   
   A   
   A   
   A   
   C   
    T    
   G   
   G   
   A   
   C   
   G   
    T    
    T    
   C   
   A   
   A   
   C   
   A   
   T   
   T   
    G    
   G   
   G   
    T    
   G   
   T   
    A    
   C   
   G   
   T   
   T   
   T   
    G    
   A   
   T   
   G   
   A   
    A    
    G    
   A   
   T   
   G   
   G   
   A   
   A   
   G   
    C    
    T    
   G   
    A    
    T    
    A    
   C   
    G    
   A   
   A   
   A   
   G   
   T   
   C   
   G   
   T   
   C   
   A   
   C   
   A   
   A   
   T   
   G   
   -   
   -   
   -   
   -   
   -   
   -   
   -   
   -   
   -   
   -   
   -   
   -   
   -   
   -   
   -   
   -   
   -   
   -   
   -   
   -   
   -   
   -   
   -   
   -   
   -   
   -   
   -   
   -   
   -   
   -   
   -   
   -   
   -   
   -   
   -   
   -   
   -   
   -   
   -   
   -   
   -   
   -   
   -   
   -   
   -   
   -   
   -   
   -   
   -   
   -   
   -   
   -   
   -   
   -   
   -   
   -   
   -   
   -   
   -   
   -   
 

 
   N52649   
   A   
   T   
   G   
   A   
   G   
   T   
   G   
   A   
   A   
   G   
   A   
   A   
   A   
   T   
   C   
   A   
   G   
   A   
   G   
   A   
   A   
   A   
   A   
   C   
   A   
   T   
   C   
   C   
   A   
   T   
   G   
   A   
   T   
   G   
   T   
   C   
   A   
   A   
   T   
   C   
   T   
   A   
   A   
   C   
   C   
   A   
   G   
   C   
   G   
   A   
   A   
   A   
   T   
   G   
   A   
   A   
   A   
   G   
   A   
   C   
   T   
   C   
   C   
   T   
   T   
   C   
   A   
   T   
   C   
   G   
   A   
   T   
   T   
   A   
   T   
   G   
   C   
   G   
   A   
   T   
   G   
   A   
   G   
   T   
   G   
   T   
   T   
   A   
   T   
   T   
   G   
   T   
   C   
   G   
   C   
   A   
   C   
   G   
   T   
   G   
   C   
   A   
   C   
   T   
   T   
   C   
   C   
   T   
   G   
   A   
   T   
   G   
   T   
   C   
   A   
   G   
   A   
   G   
   A   
   T   
   G   
   G   
   T   
   C   
   T   
   G   
   A   
   A   
   A   
   C   
   C   
   C   
   G   
   T   
   T   
   C   
   A   
   T   
   C   
   G   
   C   
   C   
   G   
   T   
   A   
   T   
   T   
   T   
   T   
   A   
   T   
   A   
   C   
   G   
   G   
   C   
   A   
   T   
   G   
   A   
   A   
   C   
   G   
   A   
   A   
   T   
   T   
   A   
   G   
   G   
   T   
   G   
   T   
   A   
   A   
   C   
   A   
   C   
   C   
   G   
   G   
   A   
   T   
   A   
   A   
   A   
   G   
   C   
   C   
   C   
   A   
   T   
   A   
   A   
   A   
   A   
   A   
   A   
   T   
   C   
   T   
   G   
   C   
   C   
   A   
   G   
   A   
   A   
   T   
   C   
   G   
   T   
   C   
   G   
   G   
   A   
   G   
   A   
   T   
   G   
   T   
   C   
   A   
   T   
   G   
   G   
   G   
   T   
   A   
   A   
   A   
   T   
   A   
   C   
   C   
   A   
   T   
   C   
   C   
   T   
   C   
   A   
   T   
   G   
   G   
   A   
   G   
   A   
   C   
   A   
   G   
   T   
   G   
   C   
   G   
   A   
   T   
   C   
   T   
   A   
   T   
   G   
   A   
   G   
   T   
   C   
   A   
   A   
   T   
   G   
   G   
   T   
   A   
   C   
   G   
   G   
   A   
   T   
   G   
   G   
   C   
   G   
   C   
   A   
   G   
   C   
   C   
   A   
   T   
   T   
   C   
   A   
   G   
   C   
   T   
   A   
   C   
   C   
   G   
   T   
   T   
   A   
   T   
   A   
   T   
   G   
   C   
   T   
   A   
   G   
   T   
   G   
   G   
   A   
   T   
   G   
   G   
   T   
   C   
   A   
   T   
   G   
   G   
   A   
   A   
   A   
   C   
   T   
   T   
   T   
   G   
   G   
   T   
   T   
   C   
   T   
   G   
   T   
   T   
   G   
   A   
   C   
   G   
   G   
   T   
   G   
   A   
   C   
   G   
   G   
   T   
   G   
   C   
   C   
   G   
   C   
   T   
   G   
   C   
   T   
   A   
   T   
   G   
   C   
   G   
   T   
   T   
   A   
   T   
   A   
   C   
   C   
   G   
   A   
   A   
   G   
   C   
   A   
   C   
   G   
   G   
   A   
   T   
   G   
   A   
   G   
   T   
   A   
   A   
   A   
   A   
   T   
   C   
   G   
   C   
   T   
   A   
   C   
   A   
   G   
   A   
   A   
   A   
   T   
   G   
   C   
   T   
   G   
   C   
   G   
   T   
   G   
   A   
   T   
   A   
   T   
   C   
   A   
   A   
   T   
   A   
   A   
   G   
   A   
   A   
   C   
   A   
   C   
   T   
   G   
   T   
   T   
   G   
   A   
   T   
   T   
   T   
   C   
   C   
   A   
   A   
   A   
   G   
   C   
   A   
   A   
   C   
   T   
   A   
   T   
   G   
   A   
   T   
   G   
   A   
   T   
   A   
   C   
   A   
   G   
   A   
   A   
   A   
   A   
   A   
   G   
   A   
   A   
   C   
   C   
   A   
   G   
   T   
   T   
   G   
   T   
   A   
   C   
   T   
   A   
   C   
   C   
   T   
   G   
   C   
   T   
   C   
   G   
   C   
   T   
   T   
   C   
   C   
   C   
   G   
   A   
   A   
   T   
   C   
   T   
   T   
   T   
   T   
   A   
   G   
   T   
   G   
   A   
   A   
   C   
   G   
   G   
   A   
   A   
   C   
   A   
   A   
   C   
   G   
   G   
   G   
   G   
   A   
   T   
   C   
   G   
   C   
   C   
   G   
   T   
   A   
   G   
   G   
   T   
   A   
   T   
   G   
   G   
   C   
   C   
   A   
   C   
   G   
   A   
   A   
   T   
   A   
   T   
   C   
   C   
   C   
   A   
   C   
   C   
   T   
   C   
   A   
   T   
   A   
   A   
   C   
   T   
   T   
   G   
   T   
   C   
   T   
   G   
   A   
   G   
   G   
   T   
   A   
   G   
   T   
   A   
   G   
   C   
   G   
   G   
   C   
   T   
   A   
   T   
   C   
   G   
   A   
   T   
   T   
   T   
   G   
   C   
   T   
   G   
   A   
   T   
   G   
   G   
   A   
   T   
   A   
   A   
   T   
   C   
   C   
   T   
   G   
   A   
   T   
   G   
   T   
   G   
   A   
   C   
   G   
   A   
   C   
   C   
   A   
   A   
   T   
   G   
   A   
   A   
   T   
   T   
   G   
   A   
   T   
   G   
   G   
   A   
   A   
   G   
   T   
   A   
   T   
   T   
   A   
   C   
   C   
   T   
   G   
   G   
   A   
   C   
   C   
   A   
   G   
   A   
   T   
   T   
   T   
   T   
   C   
   C   
   A   
   A   
   C   
   A   
   G   
   G   
   T   
   G   
   G   
   T   
   T   
   T   
   G   
   G   
   T   
   T   
   A   
   T   
   G   
   G   
   G   
   A   
   A   
   A   
   A   
   T   
   C   
   A   
   G   
   G   
    A    
   A   
   T   
    C    
   C   
   G   
    A    
    A    
   G   
   A   
   G   
   C   
    G    
   T   
   A   
   T   
   G   
   A   
   A   
   A   
   C   
    A    
   G   
   G   
    T    
   A   
   A   
    G    
   G   
   G   
   T   
   T   
   C   
    T    
   A   
   T   
   T   
   A   
   C   
    C    
   G   
   T   
    A    
   C   
   G   
   T   
   G   
   C   
   A   
   A   
   A   
   A   
   G   
   T   
   T   
   G   
   A   
    G    
    A    
   T   
    T    
   A   
    C    
   T   
   G   
   A   
    G    
   A   
   T   
   G   
    C    
   C   
    A    
   A   
   A   
   T   
   G   
   G   
    T    
   A   
   A   
   A   
   G   
   A   
   A   
   C   
   G   
    G    
   A   
   T   
    C    
    A    
   T   
    C    
   G   
   T   
    G    
   A   
   C   
    A    
   G   
   A   
   A   
   T   
   T   
    G    
   C   
   C   
   T   
   T   
   A   
   T   
   A   
   T   
   G   
   G   
   T   
    C    
   A   
   A   
   T   
   A   
   A   
   A   
   G   
   C   
    A    
   A   
   A   
   A   
   T   
   T   
    G    
   A   
   T   
    C    
   G   
   A   
   A   
   C   
   G   
    G    
   A   
   T   
   T   
   T   
   C   
   T   
   G   
   A   
    A    
    C    
   T   
    G    
   C   
   A   
   C   
   A   
   G   
   A   
   G   
   A   
    C    
   A   
   A   
    G    
   C   
   G   
    T    
   A   
   T   
    C    
   G   
   A   
   A   
   G   
   G   
    G    
   A   
   T   
    C    
   A   
   C   
    A    
   G   
   A   
   T   
   T   
   T   
   G   
   C   
   G   
   G   
   G   
   A   
    C    
   G   
   A   
   A   
   T   
   C   
   T   
   T   
   C   
    C    
   C   
   G   
   T   
   G   
   A   
    A    
   G   
   G   
    T    
   A   
   T   
   G   
   C   
   G   
    G    
   A   
   T   
    C    
   G   
   T   
   C   
   A   
   T   
    T    
   G   
   A   
    C    
    A    
   T   
    C    
   C   
   G   
   T   
   C   
   G   
    T    
   G   
   A   
    C    
   G   
    C    
    C    
   A   
   G   
    C    
   G   
   C   
    A    
   T   
   C   
    T    
   G   
   T   
   T   
    A    
   T   
    C    
   T   
   T   
    G    
   A   
   A   
    T    
   A   
   A   
   C   
   T   
   T   
    A    
   T   
   A   
    T    
   A   
   A   
   A   
    T    
   T   
   G   
   A   
   C   
    A    
    T    
   C   
    C    
   T   
   T   
    G    
   C   
   A   
    A    
   A   
    A    
    T    
   T   
   C   
    A    
   T   
   T   
    T    
   G   
   G   
    A    
   T   
   T   
    C    
   A   
   A   
    T    
   A   
   T   
   G   
   T   
   T   
    G    
   G   
   C   
    G    
   A   
   T   
    C    
   G   
   A   
   A   
   A   
   A   
   A   
   G   
   G   
    T    
   G   
   T   
    G    
   C   
   C   
    G    
   A   
   A   
   A   
    G    
   T   
   T   
   T   
   T   
    G    
   A   
   G   
    C    
   T   
   T   
   G   
   A   
   A   
   A   
   C   
    A    
    A    
   A   
   T   
    T    
    C    
   T   
    T    
   G   
   A   
   A   
   A   
   A   
    C    
   T   
   A   
    T    
    A    
   T   
    C    
   G   
   A   
    A    
   C   
   A   
    T    
   C   
   A   
    G    
    C    
    G    
    C    
    G    
   A   
   A   
   G   
   T   
   C   
   A   
   T   
    C    
   A   
   C   
    A    
    A    
   G   
    A    
   C   
   G   
   G   
   A   
   C   
    A    
    G    
   T   
    C    
   T   
   T   
   T   
   G   
   A   
    A    
   A   
   A   
   A   
    G    
   A   
    A    
   A   
   A   
    A    
   G   
   C   
   A   
   G   
   A   
   A   
   G   
   C   
   A   
    A    
   G   
    G    
   G   
   C   
    A    
   C   
   A   
    C    
   A   
   T   
    T    
   T   
   T   
   A   
   G   
   A   
   A   
   G   
   G   
   T   
    T    
   T   
    G    
   C   
   G   
    T    
   A   
   T   
   T   
   G   
   C   
    T    
   T   
   T   
    G    
   G   
   A   
   T   
   C   
   A   
   T   
   A   
   T   
   C   
    G    
   A   
   T   
   G   
   A   
   A   
   A   
   T   
    A    
   A   
   T   
   T   
   G   
   C   
    G    
   A   
   T   
   T   
   A   
   T   
    C    
   C   
   G   
    C    
   G   
   G   
    G    
   T   
   C   
    T    
    A    
   A   
   A   
   T   
   C   
   A   
   G   
   A   
   T   
   G   
   A   
    C    
   G   
   A   
    A    
   G   
   C   
   A   
   A   
   A   
   A   
   G   
   C   
    G    
   A   
    C    
    G    
    A    
   T   
    G    
   A   
   T   
    C    
   G   
   A   
   A   
   C   
   G   
    G    
   T   
   T   
    C    
   G   
   A   
    T    
   T   
   T   
    A    
   T   
   C   
    C    
   G   
   A   
   T   
   C   
   G   
    C    
   C   
   A   
    G    
    T    
   C   
    T    
   C   
   A   
   A   
   G   
   C   
    T    
   A   
   T   
    T    
   T   
   T   
   A   
   G   
   A   
   T   
   A   
   T   
   G   
   C   
   G   
   T   
   T   
   T   
    A    
   C   
   G   
    C    
   C   
   G   
    A    
   T   
   T   
    G    
   A   
   C   
    C    
   G   
   G   
    T    
   T   
   T   
    G    
   G   
   A   
   A   
   C   
   G   
    G    
   G   
   A   
    A    
   A   
   A   
   A   
   A   
   T   
    C    
   G   
   A   
    A    
   A   
   A   
   T   
   G   
   A   
   A   
   T   
   A   
    C    
   C   
   A   
   A   
   G   
   A   
    A    
   C   
   T   
    C    
   T   
   T   
    A    
   A   
    A    
   A   
    C    
   T   
    G    
   A   
   T   
   T   
   G   
    A    
    A    
   G   
   A   
    T    
   T   
   T   
    A    
    A    
    C    
    T    
   G   
   A   
   C   
   A   
   T   
   T   
    C    
   T   
    G    
   G   
   C   
    A    
    A    
   G   
    A    
   C   
   C   
    A    
   G   
   A   
    A    
   C   
   G   
    T    
   G   
   T   
    T    
   A   
    C    
    A    
   G   
   A   
   A   
   A   
   T   
    C    
   A   
   T   
    C    
   A   
   A   
    G    
   A   
    A    
    T    
   G   
   A   
    A    
   T   
   T   
   A   
    G    
    C    
    A    
   G   
   A   
   C   
    T    
   T   
    G    
    A    
    A    
    T    
    C    
    A    
   A   
    C    
    G    
   A   
   T   
   T   
   T   
   G   
   G   
   T   
   G   
   A   
   T   
    A    
    A    
   A   
   C   
   G   
   A   
   C   
   G   
    C    
   A   
   C   
   A   
   G   
   A   
   A   
    T    
   T   
   A   
    C    
   T   
   A   
   G   
   T   
    C    
   G   
   G   
   T   
   G   
   A   
    G    
   G   
   T   
   C   
    T    
   T   
    A    
   A   
   G   
   T   
   C   
   T   
    G    
   G   
   A   
   A   
   G   
   A   
   T   
   G   
   A   
   A   
   G   
   A   
   T   
    T    
   T   
    A    
   A   
   T   
    C    
   G   
   A   
   A   
   G   
   A   
   A   
   G   
   A   
   A   
   G   
   A   
   A   
    A    
   T   
   C   
   G   
   T   
    T    
   A   
   T   
   T   
   A   
   C   
    A    
    T    
   T   
    G    
   A   
   C   
    A    
   A   
   A   
    C    
   A   
   A   
    T    
   G   
   G   
    T    
   T   
   A   
    C    
   A   
   T   
    C    
   A   
   A   
    A    
   C   
   G   
    T    
    T    
   T   
   G   
   G   
    C    
   A   
   A   
   A   
    T    
    A    
    A    
    T    
   G   
   A   
   A   
   T   
   T   
   C   
    A    
   G   
    A    
   G   
   C   
    T    
   C   
   A   
   A   
   C   
   G   
    A    
    A    
   G   
    G    
   G   
   G   
    T    
   G   
   G   
    A    
   C   
   G   
   T   
   G   
   G   
    T    
   G   
   T   
    C    
   C   
   A   
   A   
   G   
   G   
    A    
   A   
   T   
   G   
   G   
   G   
    T    
    A    
   T   
    C    
   C   
   A   
    C    
    A    
   A   
   T   
   G   
   A   
    C    
   G   
   A   
    C    
   T   
   T   
   C   
   G   
   T   
    C    
   A   
   A   
    A    
   A   
   A   
    T    
   C   
   T   
    G    
    G    
   T   
   T   
   T   
   C   
   T   
   T   
   G   
    T    
   T   
   C   
    T    
   A   
   C   
    C    
   C   
   A   
    T    
   G   
   A   
    T    
   A   
   C   
    A    
   T   
   T   
    G    
   T   
   T   
   A   
   T   
   T   
    C    
   T   
   T   
   T   
   A   
   C   
   G   
   A   
   A   
    T    
   A   
    A    
    C    
   G   
   G   
    A    
   A   
   A   
   A   
   G   
   T   
    G    
   T   
   A   
    T    
   C   
   G   
    G    
   G   
   C   
   A   
   A   
   A   
   A   
   G   
   G   
    A    
   T   
   A   
    C    
   G   
   A   
   A   
   A   
   T   
    T    
   C   
   C   
    A    
   G   
   A   
    A    
   T   
   A   
   C   
   G   
   G   
    G    
    C    
   G   
    C    
   A   
   C   
   A   
   G   
   C   
    C    
   A   
   A   
   A   
   G   
   G   
    A    
   A   
   T   
    A    
   C   
   C   
   A   
    A    
   T   
    T    
   A   
   T   
    C    
   A   
   A   
    T    
   T   
   T   
    A    
   C   
   T   
    A    
   G   
   G   
    T    
   A   
   T   
    C    
   G   
   A   
    T    
   T   
   C   
    G    
    A    
    G    
    T    
   G   
   A   
   A   
   A   
    G    
    C    
   A   
   T   
    C    
   C   
   A   
    G    
   G   
   C   
    A    
   A   
   T   
    C    
   A   
   T   
   T   
    G    
   C   
    G    
   G   
   T   
    G    
    A    
    C    
   G   
   G   
   G   
    A    
    G    
   A   
   A   
   G   
   C   
    A    
   G   
   A   
   A   
   G   
    A    
    A    
   G   
   G   
    C    
    C    
   A   
    C    
   T   
   A   
    T    
    C    
   T   
    C    
   T   
   T   
   C   
   T   
   T   
    T    
   A   
   C   
    G    
   A   
   C   
    G    
    C    
    G    
    T    
    C    
   A   
   A   
   G   
   G   
    T    
   A   
   C   
    A    
   G   
   T   
    A    
   A   
   A   
   A   
   C   
   G   
    T    
   A   
   C   
    T    
    T    
   C   
    T    
   G   
   T   
    C    
   A   
   C   
    T    
   G   
   C   
    T    
   T   
   T   
    T    
    G    
   C   
   T   
   A   
   A   
    C    
   A   
   T   
    C    
   C   
   G   
    A    
   A   
   G   
    T    
   A   
   A   
   T   
   G   
   G   
    T    
    C    
   T   
    G    
   A   
   T   
    C    
   G   
   C   
    A    
   A   
   T   
   T   
    G    
   G   
    T    
   T   
   T   
    G    
   A   
   A   
   A   
   G   
   A   
   A   
   G   
   A   
    C    
   G   
   A   
    T    
   G   
   A   
    G    
    C    
   T   
    C    
    G    
   T   
    C    
   A   
   A   
    T    
   G   
   T   
    G    
    C    
   T   
   A   
    C    
    T    
   G   
   A   
   C   
    A    
    G    
   A   
    T    
   G   
   G   
    T    
    C    
   A   
    A    
    T    
    C    
    T    
   A   
   A   
    T    
   A   
   T   
    C    
   A   
   T   
    T    
   A   
   T   
    T    
   G   
   G   
    A    
   A   
   C   
   A   
   C   
   A   
   T   
    A    
    A    
    C    
   G   
   G   
    C    
   T   
   A   
   C   
   T   
   C   
   T   
   G   
   T   
    C    
   A   
   C   
    T    
   T   
   T   
    C    
    A    
   A   
    A    
   G   
   A   
    A    
    G    
    A    
    A    
    G    
   C   
    A    
   G   
    T    
    C    
   C   
   G   
    C    
   G   
   A   
   T   
   A   
   T   
   G   
   G   
   G   
    A    
   C   
   G   
    T    
   A   
   C   
   A   
   G   
   C   
    T    
   T   
   C   
    C    
   G   
   G   
    G    
   G   
   T   
    C    
    A    
   G   
    A    
   G   
   G   
    G    
   A   
   T   
    A    
   C   
   G   
    C    
    C    
   T   
    G    
   C   
   G   
    T    
   G   
   A   
    G    
    G    
   A   
    A    
   G   
   A   
    T    
   T   
    A    
    T    
   G   
   T   
    C    
   G   
   T   
    T    
   G   
   G   
    G    
    G    
   C   
   A   
   G   
   C   
    C    
    T    
   T   
    A    
    A    
   T   
   G   
    A    
   A   
    G    
   G   
    A    
   A   
    A    
   A   
    T    
    C    
   A   
   A   
   G   
   A   
   A   
   G   
   T   
    A    
    T    
   T   
    A    
   G   
   T   
    G    
   A   
   T   
    C    
   A   
   C   
    A    
   G   
   A   
   A   
   A   
   A   
    A    
   G   
   G   
   T   
   T   
   A   
    C    
   G   
   G   
    A    
   A   
   A   
    A    
   C   
   G   
    G    
   A   
   C   
    A    
   A   
   A   
   A   
   G   
    T    
    A    
   T   
   C   
    G    
   G   
   A   
    A    
   T   
   A   
   T   
   C   
   C   
   A   
   G   
   T   
    G    
   A   
   A   
    A    
   G   
   G   
    C    
   C   
   G   
    C    
   G   
   G   
    A    
   G   
   G   
   T   
   A   
   A   
   A   
   G   
   G   
    G    
   A   
   T   
    C    
   A   
   A   
    A    
   A   
   C   
    G    
    G    
   C   
    G    
   A   
   A   
    C    
   A   
   T   
   C   
   A   
   C   
   T   
   G   
   A   
    A    
   A   
   A   
   A   
   A   
   A   
   T   
   G   
   G   
   T   
   C   
   C   
   A   
    C    
   T   
   A   
   G   
   C   
    T    
   G   
   G   
    A    
   T   
   T   
    G    
   A   
   C   
   C   
   A   
   C   
    T    
   G   
   T   
    T    
    T    
    C    
   T   
   G   
   G   
    A    
    G    
   A   
   T   
   G   
   A   
   A   
   G   
   A   
    C    
   A   
   T   
   C   
    A    
   T   
    G    
    C    
   T   
    G    
   A   
   T   
    T    
   A   
   C   
    A    
    G    
   A   
    T    
   A   
   A   
   A   
   G   
   G   
    T    
   G   
   T   
    C    
   A   
   T   
   T   
   A   
   T   
   C   
   C   
   G   
    T    
   T   
   T   
    C    
   A   
   A   
   C   
   G   
   T   
    A    
    T    
    C    
    G    
    A    
   C   
   T   
   G   
   T   
   T   
   T   
   C   
   T   
   C   
   A   
   A   
   A   
   C   
    T    
   G   
   G   
   A   
   C   
   G   
    T    
    T    
   C   
   A   
   A   
   C   
   A   
   T   
   T   
    A    
   G   
   G   
    C    
   G   
   T   
    A    
   C   
   G   
   T   
   T   
   T   
    G    
   A   
   T   
   G   
   A   
    A    
    G    
   A   
   T   
   G   
   G   
   A   
   A   
   G   
    C    
    T    
   G   
    A    
    T    
    A    
   C   
    G    
   A   
   A   
   A   
   G   
   T   
   T   
   G   
   T   
   C   
   A   
   C   
   G   
   A   
   T   
   G   
   G   
   C   
   A   
   G   
   C   
   A   
   G   
   T   
   C   
   G   
   A   
   A   
   C   
   C   
   A   
   G   
   A   
   A   
   G   
   C   
   T   
   G   
   C   
   A   
   G   
   A   
   A   
   C   
   A   
   A   
   A   
   C   
   A   
   G   
   T   
   T   
   G   
   A   
   A   
   G   
   A   
   A   
   C   
   A   
   A   
   G   
   G   
   A   
   T   
   C   
   A   
   G   
   A   
   A   
   G   
   A   
   A   
   -   
   -   
   -   
 

 
   N59492F   
   A   
   T   
   G   
   A   
   G   
   T   
   G   
   A   
   A   
   G   
   A   
   A   
   A   
   T   
   C   
   A   
   G   
   A   
   G   
   A   
   A   
   A   
   A   
   C   
   A   
   T   
   C   
   C   
   A   
   T   
   G   
   A   
   T   
   G   
   T   
   C   
   A   
   A   
   T   
   C   
   T   
   A   
   A   
   C   
   C   
   A   
   G   
   C   
   G   
   A   
   A   
   A   
   T   
   G   
   A   
   A   
   A   
   G   
   A   
   C   
   T   
   C   
   C   
   T   
   T   
   C   
   A   
   T   
   C   
   G   
   A   
   T   
   T   
   A   
   T   
   G   
   C   
   G   
   A   
   T   
   G   
   A   
   G   
   T   
   G   
   T   
   T   
   A   
   T   
   T   
   G   
   T   
   C   
   G   
   C   
   A   
   C   
   G   
   T   
   G   
   C   
   A   
   C   
   T   
   T   
   C   
   C   
   T   
   G   
   A   
   T   
   G   
   T   
   C   
   A   
   G   
   A   
   G   
   A   
   T   
   G   
   G   
   T   
   C   
   T   
   G   
   A   
   A   
   A   
   C   
   C   
   C   
   G   
   T   
   T   
   C   
   A   
   T   
   C   
   G   
   C   
   C   
   G   
   T   
   A   
   T   
   T   
   T   
   T   
   A   
   T   
   A   
   C   
   G   
   G   
   C   
   A   
   T   
   G   
   A   
   A   
   C   
   G   
   A   
   A   
   T   
   T   
   A   
   G   
   G   
   T   
   G   
   T   
   A   
   A   
   C   
   A   
   C   
   C   
   G   
   G   
   A   
   T   
   A   
   A   
   A   
   G   
   C   
   C   
   C   
   A   
   T   
   A   
   A   
   A   
   A   
   A   
   A   
   T   
   C   
   T   
   G   
   C   
   C   
   A   
   G   
   A   
   A   
   T   
   C   
   G   
   T   
   C   
   G   
   G   
   A   
   G   
   A   
   T   
   G   
   T   
   C   
   A   
   T   
   G   
   G   
   G   
   T   
   A   
   A   
   A   
   T   
   A   
   C   
   C   
   A   
   T   
   C   
   C   
   T   
   C   
   A   
   T   
   G   
   G   
   A   
   G   
   A   
   C   
   A   
   G   
   T   
   G   
   C   
   G   
   A   
   T   
   C   
   T   
   A   
   T   
   G   
   A   
   G   
   T   
   C   
   A   
   A   
   T   
   G   
   G   
   T   
   A   
   C   
   G   
   G   
   A   
   T   
   G   
   G   
   C   
   A   
   C   
   A   
   G   
   C   
   C   
   A   
   T   
   T   
   C   
   A   
   G   
   C   
   T   
   A   
   C   
   C   
   G   
   T   
   T   
   A   
   T   
   A   
   T   
   G   
   C   
   T   
   A   
   G   
   T   
   G   
   G   
   A   
   T   
   G   
   G   
   T   
   C   
   A   
   T   
   G   
   G   
   A   
   A   
   A   
   C   
   T   
   T   
   T   
   G   
   G   
   T   
   T   
   C   
   T   
   G   
   T   
   T   
   G   
   A   
   C   
   G   
   G   
   T   
   G   
   A   
   C   
   G   
   G   
   T   
   G   
   C   
   C   
   G   
   C   
   C   
   G   
   C   
   T   
   A   
   T   
   G   
   C   
   G   
   T   
   T   
   A   
   T   
   A   
   C   
   C   
   G   
   A   
   A   
   G   
   C   
   A   
   C   
   G   
   G   
   A   
   T   
   G   
   A   
   G   
   C   
   A   
   A   
   A   
   A   
   T   
   C   
   G   
   C   
   T   
   A   
   C   
   A   
   G   
   A   
   A   
   A   
   T   
   G   
   C   
   T   
   G   
   C   
   G   
   T   
   G   
   A   
   T   
   A   
   T   
   C   
   A   
   A   
   T   
   A   
   A   
   G   
   A   
   A   
   C   
   A   
   C   
   T   
   G   
   T   
   T   
   G   
   A   
   T   
   T   
   T   
   C   
   C   
   A   
   A   
   A   
   G   
   C   
   A   
   A   
   C   
   T   
   A   
   T   
   G   
   A   
   T   
   G   
   A   
   T   
   A   
   C   
   A   
   G   
   A   
   A   
   A   
   A   
   A   
   G   
   A   
   A   
   C   
   C   
   A   
   G   
   T   
   T   
   G   
   T   
   A   
   C   
   T   
   A   
   C   
   C   
   T   
   G   
   C   
   T   
   C   
   G   
   C   
   T   
   T   
   C   
   C   
   C   
   G   
   A   
   A   
   T   
   C   
   T   
   T   
   T   
   T   
   A   
   G   
   T   
   G   
   A   
   A   
   C   
   G   
   G   
   A   
   A   
   C   
   A   
   A   
   C   
   G   
   G   
   G   
   G   
   A   
   T   
   C   
   G   
   C   
   C   
   G   
   T   
   A   
   G   
   G   
   T   
   A   
   T   
   G   
   G   
   C   
   C   
   A   
   C   
   G   
   A   
   A   
   T   
   A   
   T   
   C   
   C   
   C   
   A   
   C   
   C   
   T   
   C   
   A   
   T   
   A   
   A   
   C   
   T   
   T   
   G   
   T   
   C   
   T   
   G   
   A   
   G   
   G   
   T   
   A   
   G   
   T   
   A   
   G   
   C   
   G   
   G   
   C   
   T   
   A   
   T   
   C   
   G   
   A   
   T   
   T   
   T   
   G   
   C   
   T   
   G   
   A   
   T   
   G   
   G   
   A   
   T   
   A   
   A   
   T   
   C   
   C   
   T   
   G   
   A   
   T   
   G   
   T   
   G   
   A   
   C   
   G   
   A   
   C   
   C   
   A   
   A   
   T   
   G   
   A   
   A   
   T   
   T   
   G   
   A   
   T   
   G   
   G   
   A   
   A   
   G   
   T   
   A   
   T   
   T   
   A   
   C   
   C   
   T   
   G   
   G   
   A   
   C   
   C   
   A   
   G   
   A   
   T   
   T   
   T   
   T   
   C   
   C   
   A   
   A   
   C   
   A   
   G   
   G   
   T   
   G   
   G   
   T   
   T   
   T   
   G   
   G   
   T   
   T   
   A   
   T   
   G   
   G   
   G   
   A   
   A   
   A   
   A   
   T   
   C   
   A   
   G   
   G   
    A    
   A   
   T   
    C    
   C   
   G   
    A    
    A    
   G   
   A   
   G   
   C   
    G    
   T   
   A   
   T   
   G   
   A   
   A   
   A   
   C   
    A    
   G   
   G   
    T    
   A   
   A   
    G    
   G   
   G   
   T   
   T   
   C   
    T    
   A   
   T   
   T   
   A   
   C   
    C    
   G   
   T   
    A    
   C   
   G   
   T   
   G   
   C   
   A   
   A   
   A   
   A   
   G   
   T   
   T   
   G   
   A   
    G    
    A    
   T   
    T    
   A   
    C    
   T   
   G   
   A   
    G    
   A   
   T   
   G   
    C    
   C   
    A    
   A   
   A   
   T   
   G   
   G   
    T    
   A   
   A   
   A   
   G   
   A   
   A   
   C   
   G   
    G    
   A   
   T   
    C    
    A    
   T   
    C    
   G   
   T   
    G    
   A   
   C   
    A    
   G   
   A   
   A   
   T   
   T   
    G    
   C   
   C   
   T   
   T   
   A   
   T   
   A   
   T   
   G   
   G   
   T   
    C    
   A   
   A   
   T   
   A   
   A   
   A   
   G   
   C   
    A    
   A   
   A   
   A   
   T   
   T   
    G    
   A   
   T   
    C    
   G   
   A   
   A   
   C   
   G   
    G    
   A   
   T   
   T   
   T   
   C   
   T   
   G   
   A   
    A    
    C    
   T   
    G    
   C   
   A   
   C   
   A   
   G   
   A   
   G   
   A   
    C    
   A   
   A   
    G    
   C   
   G   
    T    
   A   
   T   
    C    
   G   
   A   
   A   
   G   
   G   
    G    
   A   
   T   
    C    
   A   
   C   
    A    
   G   
   A   
   T   
   T   
   T   
   G   
   C   
   G   
   G   
   G   
   A   
    C    
   G   
   A   
   A   
   T   
   C   
   T   
   T   
   C   
    C    
   C   
   G   
   T   
   G   
   A   
    A    
   G   
   G   
    T    
   A   
   T   
   G   
   C   
   G   
    G    
   A   
   T   
    C    
   G   
   T   
   C   
   A   
   T   
    T    
   G   
   A   
    C    
    A    
   T   
    C    
   C   
   G   
   T   
   C   
   G   
    T    
   G   
   A   
    C    
   G   
    C    
    C    
   A   
   G   
    C    
   G   
   C   
    A    
   T   
   C   
    T    
   G   
   T   
   T   
    A    
   T   
    C    
   T   
   T   
    G    
   A   
   A   
    T    
   A   
   A   
   C   
   T   
   T   
    A    
   T   
   A   
    T    
   A   
   A   
   A   
    T    
   T   
   G   
   A   
   C   
    A    
    T    
   C   
    C    
   T   
   T   
    G    
   C   
   A   
    A    
   A   
    A    
    T    
   T   
   C   
    A    
   T   
   T   
    T    
   G   
   G   
    A    
   T   
   T   
    C    
   A   
   A   
    T    
   A   
   T   
   G   
   T   
   T   
    G    
   G   
   C   
    G    
   A   
   T   
    C    
   G   
   A   
   A   
   A   
   A   
   A   
   G   
   G   
    T    
   G   
   T   
    G    
   C   
   C   
    G    
   A   
   A   
   A   
    G    
   T   
   T   
   T   
   T   
    G    
   A   
   G   
    C    
   T   
   T   
   G   
   A   
   A   
   A   
   C   
    A    
    A    
   A   
   T   
    T    
    C    
   T   
    T    
   G   
   A   
   A   
   A   
   A   
    C    
   T   
   A   
    T    
    A    
   T   
    C    
   G   
   A   
    A    
   C   
   A   
    T    
   C   
   A   
    G    
    C    
    G    
    C    
    G    
   A   
   A   
   G   
   T   
   C   
   A   
   T   
    C    
   A   
   C   
    A    
    A    
   G   
    A    
   C   
   G   
   G   
   A   
   C   
    A    
    G    
   T   
    C    
   T   
   T   
   T   
   G   
   A   
    A    
   A   
   A   
   A   
    G    
   A   
    A    
   A   
   A   
    A    
   G   
   C   
   A   
   G   
   A   
   A   
   G   
   C   
   A   
    A    
   G   
    G    
   G   
   C   
    A    
   C   
   A   
    C    
   A   
   T   
    T    
   T   
   T   
   A   
   G   
   A   
   A   
   G   
   G   
   T   
    T    
   T   
    G    
   C   
   G   
    T    
   A   
   T   
   T   
   G   
   C   
    T    
   T   
   T   
    G    
   G   
   A   
   T   
   C   
   A   
   T   
   A   
   T   
   C   
    G    
   A   
   T   
   G   
   A   
   A   
   A   
   T   
    C    
   A   
   T   
   T   
   G   
   C   
    G    
   A   
   T   
   T   
   A   
   T   
    C    
   C   
   G   
    C    
   G   
   G   
    G    
   T   
   C   
    T    
    A    
   A   
   A   
   T   
   C   
   A   
   G   
   A   
   T   
   G   
   A   
    C    
   G   
   A   
    A    
   G   
   C   
   A   
   A   
   A   
   A   
   G   
   C   
    G    
   A   
    C    
    G    
    A    
   T   
    G    
   A   
   T   
    C    
   G   
   A   
   A   
   C   
   G   
    G    
   T   
   T   
    C    
   G   
   A   
    T    
   T   
   T   
    A    
   T   
   C   
    C    
   G   
   A   
   T   
   C   
   G   
    C    
   C   
   A   
    G    
    T    
   C   
    T    
   C   
   A   
   A   
   G   
   C   
    T    
   A   
   T   
    T    
   T   
   T   
   A   
   G   
   A   
   T   
   A   
   T   
   G   
   C   
   G   
   T   
   T   
   T   
    A    
   C   
   G   
    C    
   C   
   G   
    A    
   T   
   T   
    G    
   A   
   C   
    C    
   G   
   G   
    T    
   T   
   T   
    G    
   G   
   A   
   A   
   C   
   G   
    G    
   G   
   A   
    A    
   A   
   A   
   A   
   A   
   T   
    C    
   G   
   A   
    A    
   A   
   A   
   T   
   G   
   A   
   A   
   T   
   A   
    C    
   C   
   A   
   A   
   G   
   A   
    A    
   C   
   T   
    C    
   T   
   T   
    A    
   A   
    A    
   A   
    C    
   T   
    G    
   A   
   T   
   T   
   G   
    A    
    A    
   G   
   A   
    T    
   T   
   T   
    A    
    A    
    C    
    T    
   G   
   A   
   C   
   A   
   T   
   T   
    C    
   T   
    G    
   G   
   C   
    A    
    A    
   G   
    A    
   C   
   C   
    A    
   G   
   A   
    A    
   C   
   G   
    T    
   G   
   T   
    T    
   A   
    C    
    A    
   G   
   A   
   A   
   A   
   T   
    C    
   A   
   T   
    C    
   A   
   A   
    G    
   A   
    A    
    T    
   G   
   A   
    A    
   T   
   T   
   A   
    G    
    C    
    A    
   G   
   A   
   C   
    T    
   T   
    G    
    A    
    A    
    T    
    C    
    A    
   A   
    C    
    G    
   A   
   T   
   T   
   T   
   G   
   G   
   T   
   G   
   A   
   T   
    A    
    A    
   A   
   C   
   G   
   A   
   C   
   G   
    C    
   A   
   C   
   A   
   G   
   A   
   A   
    T    
   T   
   A   
    C    
   T   
   A   
   G   
   T   
    C    
   G   
   G   
   T   
   G   
   A   
    G    
   G   
   T   
   C   
    T    
   T   
    A    
   A   
   G   
   T   
   C   
   T   
    G    
   G   
   A   
   A   
   G   
   A   
   T   
   G   
   A   
   A   
   G   
   A   
   T   
    T    
   T   
    A    
   A   
   T   
    C    
   G   
   A   
   A   
   G   
   A   
   A   
   G   
   A   
   A   
   G   
   A   
   A   
    A    
   T   
   C   
   G   
   T   
    T    
   A   
   T   
   T   
   A   
   C   
    A    
    T    
   T   
    G    
   A   
   C   
    A    
   A   
   A   
    C    
   A   
   A   
    T    
   G   
   G   
    T    
   T   
   A   
    C    
   A   
   T   
    C    
   A   
   A   
    A    
   C   
   G   
    T    
    T    
   T   
   G   
   G   
    C    
   A   
   A   
   A   
    T    
    A    
    A    
    T    
   G   
   A   
   A   
   T   
   T   
   C   
    A    
   G   
    A    
   G   
   C   
    T    
   C   
   A   
   A   
   C   
   G   
    A    
    A    
   G   
    G    
   G   
   G   
    T    
   G   
   G   
    A    
   C   
   G   
   T   
   G   
   G   
    T    
   G   
   T   
    C    
   C   
   A   
   A   
   G   
   G   
    A    
   A   
   T   
   G   
   G   
   G   
    T    
    A    
   T   
    C    
   C   
   A   
    C    
    A    
   A   
   T   
   G   
   A   
    C    
   G   
   A   
    C    
   T   
   T   
   C   
   G   
   T   
    C    
   A   
   A   
    A    
   A   
   A   
    T    
   C   
   T   
    G    
    G    
   T   
   T   
   T   
   C   
   T   
   T   
   G   
    T    
   T   
   C   
    T    
   A   
   C   
    C    
   C   
   A   
    T    
   G   
   A   
    T    
   A   
   C   
    A    
   T   
   T   
    G    
   T   
   T   
   A   
   T   
   T   
    C    
   T   
   T   
   T   
   A   
   C   
   G   
   A   
   A   
    T    
   A   
    A    
    C    
   G   
   G   
    A    
   A   
   A   
   A   
   G   
   T   
    G    
   T   
   A   
    T    
   C   
   G   
    G    
   G   
   C   
   A   
   A   
   A   
   A   
   G   
   G   
    A    
   T   
   A   
    C    
   G   
   A   
   A   
   A   
   T   
    T    
   C   
   C   
    A    
   G   
   A   
    A    
   T   
   A   
   C   
   G   
   G   
    G    
    C    
   G   
    C    
   A   
   C   
   A   
   G   
   C   
    C    
   A   
   A   
   A   
   G   
   G   
    A    
   A   
   T   
    A    
   C   
   C   
   A   
    A    
   T   
    T    
   A   
   T   
    C    
   A   
   A   
    T    
   T   
   T   
    A    
   C   
   T   
    A    
   G   
   G   
    T    
   A   
   T   
    C    
   G   
   A   
    T    
   T   
   C   
    G    
    A    
    G    
    T    
   G   
   A   
   A   
   A   
    G    
    C    
   A   
   T   
    C    
   C   
   A   
    G    
   G   
   C   
    A    
   A   
   T   
    C    
   A   
   T   
   T   
    G    
   C   
    G    
   G   
   T   
    G    
    A    
    C    
   G   
   G   
   G   
    A    
    G    
   A   
   A   
   G   
   C   
    A    
   G   
   A   
   A   
   G   
    A    
    A    
   G   
   G   
    C    
    C    
   A   
    C    
   T   
   A   
    T    
    C    
   T   
    C    
   T   
   T   
   C   
   T   
   T   
    T    
   A   
   C   
    G    
   A   
   C   
    G    
    C    
    G    
    T    
    C    
   A   
   A   
   G   
   G   
    T    
   A   
   C   
    A    
   G   
   T   
    A    
   A   
   A   
   A   
   C   
   G   
    T    
   A   
   C   
    T    
    T    
   C   
    T    
   G   
   T   
    C    
   A   
   C   
    T    
   G   
   C   
    T    
   T   
   T   
    T    
    G    
   C   
   T   
   A   
   A   
    C    
   A   
   T   
    C    
   C   
   G   
    A    
   A   
   G   
    T    
   A   
   A   
   T   
   G   
   G   
    T    
    C    
   T   
    G    
   A   
   T   
    C    
   G   
   C   
    A    
   A   
   T   
   T   
    G    
   G   
    T    
   T   
   T   
    G    
   A   
   A   
   A   
   G   
   A   
   A   
   G   
   A   
    C    
   G   
   A   
    T    
   G   
   A   
    G    
    C    
   T   
    C    
    G    
   T   
    C    
   A   
   A   
    T    
   G   
   T   
    G    
    C    
   T   
   A   
    C    
    T    
   G   
   A   
   C   
    A    
    G    
   A   
    T    
   G   
   G   
    T    
    C    
   A   
    A    
    T    
    C    
    T    
   A   
   A   
    T    
   A   
   T   
    C    
   A   
   T   
    T    
   A   
   T   
    T    
   G   
   G   
    A    
   A   
   C   
   A   
   C   
   A   
   T   
    A    
    A    
    C    
   G   
   G   
    C    
   T   
   A   
   C   
   T   
   C   
   T   
   G   
   T   
    C    
   A   
   C   
    T    
   T   
   T   
    C    
    A    
   A   
    A    
   G   
   A   
    A    
    G    
    A    
    A    
    G    
   C   
    A    
   G   
    T    
    C    
   C   
   G   
    C    
   G   
   A   
   T   
   A   
   T   
   G   
   G   
   G   
    A    
   C   
   G   
    T    
   A   
   C   
   A   
   G   
   C   
    T    
   T   
   C   
    C    
   G   
   G   
    G    
   G   
   T   
    T    
    A    
   G   
    A    
   G   
   G   
    A    
   A   
   T   
    A    
   C   
   G   
    C    
    C    
   T   
    G    
   C   
   G   
    T    
   G   
   A   
    G    
    G    
   A   
    A    
   G   
   A   
    T    
   T   
    A    
    T    
   G   
   T   
    C    
   G   
   T   
    T    
   G   
   G   
    G    
    G    
   C   
   A   
   G   
   C   
    C    
    T    
   T   
    A    
    A    
   T   
   G   
    A    
   A   
    G    
   G   
    A    
   A   
    G    
   A   
    T    
    C    
   A   
   A   
   G   
   A   
   A   
   G   
   T   
    A    
    T    
   T   
    A    
   G   
   T   
    G    
   A   
   T   
    C    
   A   
   C   
    A    
   G   
   A   
   A   
   A   
   A   
    A    
   G   
   G   
   T   
   T   
   A   
    C    
   G   
   G   
    A    
   A   
   A   
    A    
   C   
   G   
    G    
   A   
   C   
    A    
   A   
   A   
   A   
   G   
    T    
    A    
   T   
   C   
    G    
   G   
   A   
    A    
   T   
   A   
   T   
   C   
   C   
   A   
   G   
   T   
    G    
   A   
   A   
    A    
   G   
   G   
    C    
   C   
   G   
    C    
   G   
   G   
    A    
   G   
   G   
   T   
   A   
   A   
   A   
   G   
   G   
    G    
   A   
   T   
    C    
   A   
   A   
    A    
   A   
   C   
    G    
    G    
   C   
    G    
   A   
   A   
    C    
   A   
   T   
   C   
   A   
   C   
   T   
   G   
   A   
    A    
   A   
   A   
   A   
   A   
   A   
   T   
   G   
   G   
   T   
   C   
   C   
   A   
    C    
   T   
   A   
   G   
   C   
    T    
   G   
   G   
    A    
   T   
   T   
    G    
   A   
   C   
   C   
   A   
   C   
    T    
   G   
   T   
    T    
    T    
    C    
   T   
   G   
   G   
    A    
    G    
   A   
   T   
   G   
   A   
   A   
   G   
   A   
    C    
   A   
   T   
   C   
    A    
   T   
    G    
    C    
   T   
    G    
   A   
   T   
    T    
   A   
   C   
    A    
    G    
   A   
    T    
   A   
   A   
   A   
   G   
   G   
    T    
   G   
   T   
    C    
   A   
   T   
   T   
   A   
   T   
   C   
   C   
   G   
    T    
   T   
   T   
    C    
   A   
   A   
   C   
   G   
   T   
    A    
    T    
    C    
    G    
    A    
   C   
   T   
   G   
   T   
   T   
   T   
   C   
   T   
   C   
   A   
   A   
   A   
   C   
    T    
   G   
   G   
   A   
   C   
   G   
    T    
    T    
   C   
   A   
   A   
   C   
   A   
   T   
   T   
    A    
   G   
   G   
    C    
   G   
   T   
    A    
   C   
   G   
   T   
   T   
   T   
    G    
   A   
   T   
   G   
   A   
    A    
    G    
   A   
   T   
   G   
   G   
   A   
   A   
   G   
    C    
    T    
   G   
    A    
    T    
    A    
   C   
    G    
   A   
   A   
   A   
   G   
   T   
   T   
   G   
   T   
   C   
   A   
   C   
   G   
   A   
   T   
   G   
   G   
   C   
   A   
   G   
   C   
   A   
   G   
   T   
   C   
   G   
   A   
   A   
   C   
   C   
   A   
   G   
   A   
   A   
   G   
   C   
   T   
   G   
   C   
   A   
   G   
   A   
   A   
   C   
   A   
   A   
   A   
   C   
   A   
   G   
   T   
   T   
   G   
   A   
   A   
   G   
   A   
   A   
   C   
   A   
   A   
   G   
   G   
   A   
   T   
   C   
   A   
   G   
   A   
   A   
   G   
   A   
   A   
   -   
   -   
   -   
 

 
   N59491F   
   A   
   T   
   G   
   A   
   G   
   T   
   G   
   A   
   A   
   G   
   A   
   A   
   A   
   T   
   T   
   A   
   A   
   A   
   G   
   A   
   A   
   A   
   A   
   C   
   A   
   T   
   T   
   C   
   A   
   A   
   G   
   A   
   C   
   G   
   T   
   C   
   A   
   A   
   T   
   C   
   T   
   G   
   A   
   C   
   C   
   A   
   G   
   C   
   G   
   A   
   A   
   A   
   T   
   G   
   A   
   A   
   A   
   G   
   A   
   A   
   T   
   C   
   T   
   T   
   T   
   C   
   A   
   T   
   T   
   G   
   A   
   C   
   T   
   A   
   C   
   G   
   C   
   A   
   A   
   T   
   G   
   A   
   G   
   T   
   G   
   T   
   T   
   A   
   T   
   C   
   G   
   T   
   A   
   G   
   C   
   C   
   C   
   G   
   C   
   G   
   C   
   G   
   T   
   T   
   A   
   C   
   C   
   T   
   G   
   A   
   C   
   G   
   T   
   T   
   C   
   G   
   T   
   G   
   A   
   C   
   G   
   G   
   T   
   T   
   T   
   A   
   A   
   A   
   A   
   C   
   C   
   T   
   G   
   T   
   T   
   C   
   A   
   T   
   C   
   G   
   C   
   C   
   G   
   A   
   A   
   T   
   C   
   T   
   T   
   A   
   T   
   A   
   T   
   G   
   G   
   A   
   A   
   T   
   G   
   A   
   A   
   C   
   G   
   A   
   A   
   T   
   T   
   A   
   G   
   G   
   G   
   G   
   T   
   A   
   A   
   C   
   C   
   C   
   C   
   T   
   G   
   A   
   T   
   A   
   A   
   G   
   C   
   C   
   A   
   C   
   A   
   C   
   A   
   A   
   A   
   A   
   A   
   A   
   T   
   C   
   A   
   G   
   C   
   C   
   C   
   G   
   G   
   A   
   T   
   T   
   G   
   T   
   T   
   G   
   G   
   G   
   G   
   A   
   T   
   G   
   T   
   T   
   A   
   T   
   G   
   G   
   G   
   T   
   A   
   A   
   A   
   T   
   A   
   T   
   C   
   A   
   C   
   C   
   C   
   C   
   C   
   A   
   T   
   G   
   G   
   G   
   G   
   A   
   C   
   A   
   G   
   T   
   G   
   C   
   G   
   A   
   T   
   T   
   T   
   A   
   C   
   G   
   A   
   A   
   T   
   C   
   A   
   A   
   T   
   G   
   G   
   T   
   G   
   C   
   G   
   G   
   A   
   T   
   G   
   G   
   C   
   A   
   C   
   A   
   A   
   C   
   C   
   T   
   T   
   T   
   T   
   A   
   G   
   T   
   T   
   A   
   T   
   C   
   G   
   G   
   G   
   C   
   T   
   A   
   T   
   G   
   T   
   T   
   A   
   G   
   T   
   T   
   G   
   A   
   C   
   G   
   G   
   C   
   C   
   A   
   C   
   G   
   G   
   A   
   A   
   A   
   C   
   T   
   T   
   C   
   G   
   G   
   T   
   T   
   C   
   T   
   G   
   T   
   C   
   G   
   A   
   T   
   G   
   G   
   T   
   G   
   A   
   C   
   G   
   G   
   C   
   G   
   C   
   T   
   G   
   C   
   C   
   G   
   C   
   T   
   A   
   T   
   G   
   C   
   G   
   T   
   T   
   A   
   T   
   A   
   C   
   C   
   G   
   A   
   A   
   G   
   C   
   A   
   C   
   G   
   T   
   A   
   T   
   G   
   A   
   G   
   T   
   A   
   A   
   A   
   A   
   T   
   T   
   G   
   C   
   T   
   T   
   T   
   A   
   G   
   A   
   A   
   A   
   T   
   G   
   C   
   T   
   A   
   C   
   G   
   A   
   G   
   A   
   T   
   A   
   T   
   T   
   A   
   A   
   C   
   A   
   A   
   A   
   A   
   A   
   T   
   A   
   C   
   A   
   G   
   T   
   C   
   G   
   A   
   T   
   T   
   T   
   C   
   C   
   A   
   A   
   G   
   G   
   A   
   A   
   A   
   C   
   T   
   A   
   T   
   G   
   A   
   T   
   G   
   A   
   T   
   T   
   C   
   A   
   G   
   A   
   A   
   C   
   A   
   A   
   G   
   A   
   A   
   C   
   C   
   A   
   G   
   T   
   A   
   G   
   T   
   T   
   T   
   T   
   A   
   C   
   C   
   T   
   G   
   C   
   T   
   C   
   G   
   T   
   T   
   T   
   T   
   C   
   C   
   A   
   A   
   A   
   C   
   T   
   T   
   A   
   C   
   T   
   A   
   G   
   T   
   T   
   A   
   A   
   C   
   G   
   G   
   A   
   A   
   C   
   A   
   A   
   C   
   G   
   G   
   G   
   G   
   A   
   T   
   T   
   G   
   C   
   G   
   G   
   T   
   G   
   G   
   G   
   G   
   A   
   T   
   G   
   G   
   C   
   A   
   A   
   C   
   A   
   A   
   A   
   T   
   A   
   T   
   T   
   C   
   C   
   A   
   C   
   C   
   A   
   C   
   A   
   C   
   A   
   A   
   T   
   T   
   T   
   A   
   A   
   G   
   T   
   G   
   A   
   A   
   G   
   T   
   G   
   A   
   T   
   T   
   G   
   A   
   T   
   G   
   C   
   A   
   A   
   C   
   A   
   A   
   G   
   T   
   T   
   T   
   A   
   T   
   T   
   G   
   A   
   T   
   G   
   G   
   A   
   C   
   A   
   A   
   T   
   C   
   C   
   T   
   G   
   A   
   T   
   G   
   T   
   A   
   A   
   C   
   G   
   A   
   C   
   G   
   A   
   A   
   T   
   G   
   A   
   A   
   T   
   T   
   G   
   A   
   T   
   G   
   G   
   A   
   A   
   G   
   T   
   G   
   T   
   T   
   A   
   C   
   C   
   T   
   G   
   G   
   A   
   C   
   C   
   A   
   G   
   A   
   T   
   T   
   T   
   T   
   C   
   C   
   A   
   A   
   C   
   A   
   G   
   G   
   C   
   G   
   G   
   T   
   T   
   T   
   A   
   G   
   T   
   G   
   A   
   T   
   G   
   G   
   G   
   G   
   A   
   A   
   A   
   T   
   C   
   A   
   G   
   G   
    G    
   A   
   T   
    T    
   C   
   G   
    C    
    C    
   G   
   A   
   G   
   C   
    A    
   T   
   A   
   T   
   G   
   A   
   A   
   A   
   C   
    G    
   G   
   G   
    G    
   A   
   A   
    A    
   G   
   G   
   T   
   T   
   C   
    G    
   A   
   T   
   T   
   A   
   C   
    A    
   G   
   T   
    T    
   C   
   G   
   T   
   G   
   C   
   A   
   A   
   A   
   A   
   G   
   T   
   T   
   G   
   A   
    A    
    T    
   T   
    G    
   A   
    C    
   T   
   G   
   A   
    A    
   A   
   T   
   G   
    C    
   C   
    G    
   A   
   A   
   T   
   G   
   G   
    A    
   A   
   A   
   A   
   G   
   A   
   A   
   C   
   G   
    T    
   A   
   T   
    T    
    T    
   T   
    A    
   G   
   T   
    A    
   A   
   C   
    T    
   G   
   A   
   A   
   T   
   T   
    G    
   C   
   C   
   T   
   T   
   A   
   T   
   A   
   T   
   G   
   G   
   T   
    G    
   A   
   A   
   T   
   A   
   A   
   A   
   G   
   C   
    C    
   A   
   A   
   A   
   T   
   T   
    A    
   A   
   T   
    C    
   G   
   A   
   A   
   C   
   G   
    A    
   A   
   T   
   T   
   T   
   C   
   T   
   G   
   A   
    A    
    T    
   T   
    A    
   C   
   A   
   C   
   A   
   G   
   A   
   G   
   A   
    T    
   A   
   A   
    A    
   C   
   G   
    A    
   A   
   T   
    T    
   G   
   A   
   A   
   G   
   G   
    A    
   A   
   T   
    T    
   A   
   C   
    T    
   G   
   A   
   T   
   T   
   T   
   G   
   C   
   G   
   G   
   G   
   A   
    T    
   G   
   A   
   A   
   T   
   C   
   T   
   T   
   C   
    T    
   C   
   G   
   T   
   G   
   A   
    A    
   G   
   G   
    C    
   A   
   T   
   G   
   C   
   G   
    G    
   A   
   T   
    T    
   G   
   T   
   C   
   A   
   T   
    C    
   G   
   A   
    T    
    G    
   T   
    T    
   C   
   G   
   T   
   C   
   G   
    A    
   G   
   A   
    T    
   G   
    T    
    G    
   A   
   G   
    T    
   G   
   C   
    C    
   T   
   C   
    T    
   G   
   T   
   T   
    G    
   T   
    G    
   T   
   T   
    A    
   A   
   A   
    C    
   A   
   A   
   C   
   T   
   T   
    G    
   T   
   A   
    C    
   A   
   A   
   A   
    A    
   T   
   G   
   A   
   C   
    T    
    G    
   C   
    C    
   T   
   T   
    A    
   C   
   A   
    A    
   A   
    C    
    A    
   T   
   C   
    T    
   T   
   T   
    T    
   G   
   G   
    T    
   T   
   T   
    T    
   A   
   A   
    C    
   A   
   T   
   G   
   T   
   T   
    A    
   G   
   C   
    C    
   A   
   T   
    T    
   G   
   A   
   A   
   A   
   A   
   A   
   G   
   G   
    T    
   G   
   T   
    A    
   C   
   C   
    G    
   A   
   A   
   A   
    A    
   T   
   T   
   T   
   T   
    A    
   A   
   G   
    C    
   T   
   T   
   G   
   A   
   A   
   A   
   C   
    G    
    T    
   A   
   T   
    T    
    T    
   T   
    A    
   G   
   A   
   A   
   A   
   A   
    C    
   T   
   A   
    C    
    G    
   T   
    C    
   G   
   A   
    G    
   C   
   A   
    T    
   C   
   A   
    A    
    A    
    A    
    A    
    G    
   A   
   A   
   G   
   T   
   C   
   A   
   T   
    T    
   A   
   C   
    T    
    C    
   G   
    C    
   C   
   G   
   G   
   A   
   C   
    G    
    A    
   T   
    T    
   T   
   T   
   T   
   G   
   A   
    C    
   A   
   A   
   A   
    A    
   A   
    C    
   A   
   A   
    A    
   G   
   C   
   A   
   G   
   A   
   A   
   G   
   C   
   A   
    C    
   G   
    G    
   G   
   C   
    G    
   C   
   A   
    T    
   A   
   T   
    T    
   T   
   T   
   A   
   G   
   A   
   A   
   G   
   G   
   T   
    C    
   T   
    C    
   C   
   G   
    A    
   A   
   T   
   T   
   G   
   C   
    C    
   T   
   T   
    A    
   G   
   A   
   T   
   C   
   A   
   T   
   A   
   T   
   C   
    G    
   A   
   T   
   G   
   A   
   A   
   A   
   T   
    C    
   A   
   T   
   T   
   G   
   C   
    C    
   A   
   T   
   T   
   A   
   T   
    C    
   C   
   G   
    T    
   G   
   G   
    A    
   T   
   C   
    A    
    C    
   A   
   A   
   T   
   C   
   A   
   G   
   A   
   T   
   G   
   A   
    C    
   G   
   A   
    A    
   G   
   C   
   A   
   A   
   A   
   A   
   G   
   C   
    A    
   A   
    C    
    G    
    T    
   T   
    A    
   A   
   T   
    T    
   G   
   A   
   A   
   C   
   G   
    C    
   T   
   T   
    T    
   G   
   A   
    A    
   T   
   T   
    T    
   T   
   C   
    A    
   G   
   A   
   T   
   C   
   G   
    T    
   C   
   A   
    A    
    G    
   C   
    G    
   C   
   A   
   A   
   G   
   C   
    G    
   A   
   T   
    T    
   T   
   T   
   A   
   G   
   A   
   T   
   A   
   T   
   G   
   C   
   G   
   T   
   T   
   T   
    A    
   C   
   G   
    C    
   C   
   G   
    T    
   T   
   T   
    A    
   A   
   C   
    A    
   G   
   G   
    C    
   T   
   T   
    A    
   G   
   A   
   A   
   C   
   G   
    C    
   G   
   A   
    C    
   A   
   A   
   A   
   A   
   T   
    T    
   G   
   A   
    A    
   A   
   A   
   T   
   G   
   A   
   A   
   T   
   A   
    T    
   C   
   A   
   A   
   G   
   A   
    G    
   C   
   T   
    A    
   T   
   T   
    G    
   A   
    A    
   A   
    T    
   T   
    C    
   A   
   T   
   T   
   G   
    C    
    G    
   G   
   A   
    C    
   T   
   T   
    A    
    G    
    A    
    G    
   G   
   A   
   C   
   A   
   T   
   T   
    T    
   T   
    A    
   G   
   C   
    C    
    C    
   G   
    C    
   C   
   C   
    A    
   G   
   A   
    A    
   C   
   G   
    C    
   G   
   T   
    C    
   A   
    T    
    T    
   G   
   A   
   A   
   A   
   T   
    C    
   A   
   T   
    T    
   A   
   A   
    A    
   A   
    C    
    A    
   G   
   A   
    G    
   T   
   T   
   A   
    A    
    A    
    T    
   G   
   A   
   C   
    G    
   T   
    T    
    C    
    G    
    C    
    A    
    C    
   A   
    A    
    A    
   A   
   T   
   T   
   T   
   G   
   G   
   T   
   G   
   A   
   T   
    G    
    C    
   A   
   C   
   G   
   A   
   C   
   G   
    C    
   A   
   C   
   A   
   G   
   A   
   A   
    T    
   T   
   A   
    T    
   T   
   A   
   G   
   T   
    A    
   G   
   G   
   T   
   G   
   A   
    A    
   G   
   T   
   C   
    T    
   T   
    A    
   A   
   G   
   T   
   C   
   T   
    T    
   G   
   A   
   A   
   G   
   A   
   T   
   G   
   A   
   A   
   G   
   A   
   T   
    T    
   T   
    A    
   A   
   T   
    C    
   G   
   A   
   A   
   G   
   A   
   A   
   G   
   A   
   A   
   G   
   A   
   A   
    G    
   T   
   C   
   G   
   T   
    G    
   A   
   T   
   T   
   A   
   C   
    A    
    T    
   T   
    A    
   A   
   C   
    C    
   A   
   A   
    T    
   A   
   A   
    C    
   G   
   G   
    C    
   T   
   A   
    C    
   A   
   T   
    T    
   A   
   A   
    G    
   C   
   G   
    G    
    A    
   T   
   G   
   G   
    C    
   A   
   A   
   A   
    C    
    T    
    C    
    T    
   G   
   A   
   A   
   T   
   T   
   C   
    C    
   G   
    G    
   G   
   C   
    G    
   C   
   A   
   A   
   C   
   G   
    C    
    C    
   G   
    T    
   G   
   G   
    C    
   G   
   G   
    A    
   C   
   G   
   T   
   G   
   G   
    T    
   G   
   T   
    C    
   C   
   A   
   A   
   G   
   G   
    C    
   A   
   T   
   G   
   G   
   G   
    C    
    G    
   T   
    T    
   C   
   A   
    T    
    G    
   A   
   T   
   G   
   A   
    T    
   G   
   A   
    T    
   T   
   T   
   C   
   G   
   T   
    G    
   A   
   A   
    A    
   A   
   A   
    A    
   C   
   T   
    A    
    G    
   T   
   T   
   T   
   C   
   T   
   T   
   G   
    T    
   T   
   C   
    A    
   A   
   C   
    A    
   C   
   A   
    C    
   G   
   A   
    C    
   A   
   C   
    G    
   T   
   T   
    A    
   T   
   T   
   A   
   T   
   T   
    C    
   T   
   T   
   T   
   A   
   C   
   G   
   A   
   A   
    C    
   A   
    C    
    T    
   G   
   G   
    G    
   A   
   A   
   A   
   G   
   T   
    T    
   T   
   A   
    C    
   C   
   G   
    A    
   G   
   C   
   A   
   A   
   A   
   A   
   G   
   G   
    T    
   T   
   A   
    C    
   G   
   A   
   A   
   A   
   T   
    C    
   C   
   C   
    T    
   G   
   A   
    A    
   T   
   A   
   C   
   G   
   G   
    T    
    A    
   G   
    A    
   A   
   C   
   A   
   G   
   C   
    A    
   A   
   A   
   A   
   G   
   G   
    A    
   A   
   T   
    T    
   C   
   C   
   A   
    G    
   T   
    G    
   A   
   T   
    T    
   A   
   A   
    C    
   T   
   T   
    A    
   C   
   T   
    G    
   G   
   G   
    A    
   A   
   T   
    T    
   G   
   A   
    T    
   T   
   C   
    T    
    G    
    C    
    A    
   G   
   A   
   A   
   A   
    A    
    A    
   A   
   T   
    T    
   C   
   A   
    A    
   G   
   C   
    G    
   A   
   T   
    T    
   A   
   T   
   T   
    T    
   C   
    T    
   G   
   T   
    T    
    G    
    A    
   G   
   G   
   G   
    C    
    A    
   A   
   A   
   G   
   C   
    G    
   G   
   A   
   A   
   G   
    C    
    A    
   G   
   G   
    T    
    A    
   A   
    A    
   T   
   A   
    C    
    T    
   T   
    G    
   T   
   T   
   C   
   T   
   T   
    C    
   A   
   C   
    A    
   A   
   C   
    C    
    T    
    T    
    A    
    A    
   A   
   A   
   G   
   G   
    A    
   A   
   C   
    C    
   G   
   T   
    C    
   A   
   A   
   A   
   C   
   G   
    G    
   A   
   C   
    A    
    G    
   C   
    C    
   G   
   T   
    A    
   A   
   C   
    A    
   G   
   C   
    C    
   T   
   T   
    T    
    T    
   C   
   T   
   A   
   A   
    T    
   A   
   T   
    C    
   C   
   G   
    T    
   A   
   G   
    T    
   A   
   A   
   T   
   G   
   G   
    T    
    T    
   T   
    A    
   A   
   T   
    C    
   G   
   C   
    C    
   A   
   T   
   T   
    A    
   G   
    C    
   T   
   T   
    A    
   A   
   A   
   A   
   G   
   A   
   A   
   G   
   A   
    T    
   G   
   A   
    T    
   G   
   A   
    G    
    T    
   T   
    A    
    G    
   T   
    T    
   A   
   A   
    C    
   G   
   T   
    G    
    G    
   T   
   A   
    A    
    C    
   G   
   A   
   C   
    T    
    A    
   A   
    T    
   G   
   G   
    C    
    A    
   A   
    T    
    C    
    A    
    G    
   A   
   A   
    G    
   A   
   T   
    G    
   A   
   T   
    T    
   A   
   T   
    C    
   G   
   G   
    A    
   A   
   C   
   A   
   C   
   A   
   T   
    G    
    C    
    A    
   G   
   G   
    A    
   T   
   A   
   C   
   T   
   C   
   T   
   G   
   T   
    C    
   A   
   C   
    A    
   T   
   T   
    T    
    G    
   A   
    T    
   G   
   A   
    A    
    A    
    A    
    T    
    A    
   C   
    T    
   G   
    T    
    A    
   C   
   G   
    T    
   G   
   A   
   T   
   A   
   T   
   G   
   G   
   G   
    C    
   C   
   G   
    G    
   A   
   C   
   A   
   G   
   C   
    A    
   T   
   C   
    C    
   G   
   G   
    T    
   G   
   T   
    T    
    C    
   G   
    G    
   G   
   G   
    A    
   A   
   T   
    C    
   C   
   G   
    T    
    C    
   T   
    C    
   C   
   G   
    C    
   G   
   A   
    A    
    A    
   A   
    T    
   G   
   A   
    T    
   T   
    A    
    T    
   G   
   T   
    G    
   G   
   T   
    C    
   G   
   G   
    C    
    G    
   C   
   A   
   G   
   C   
    G    
    A    
   T   
    T    
    C    
   T   
   G   
    G    
   A   
    T    
   G   
    A    
   A   
    A    
   A   
    T    
    A    
   A   
   A   
   G   
   A   
   A   
   G   
   T   
    C    
    C    
   T   
    A    
   G   
   T   
    C    
   A   
   T   
    T    
   A   
   C   
    T    
   G   
   A   
   A   
   A   
   A   
    T    
   G   
   G   
   T   
   T   
   A   
    T    
   G   
   G   
    T    
   A   
   A   
    G    
   C   
   G   
    T    
   A   
   C   
    A    
   A   
   A   
   A   
   G   
    C    
    T    
   T   
   C   
    T    
   G   
   A   
    A    
   T   
   A   
   T   
   C   
   C   
   A   
   G   
   T   
    T    
   A   
   A   
    A    
   G   
   G   
    A    
   C   
   G   
    T    
   G   
   G   
    C    
   G   
   G   
   T   
   A   
   A   
   A   
   G   
   G   
    G    
   A   
   T   
    T    
   A   
   A   
    G    
   A   
   C   
    A    
    G    
   C   
    A    
   A   
   A   
    T    
   A   
   T   
   C   
   A   
   C   
   T   
   G   
   A   
    G    
   A   
   A   
   A   
   A   
   A   
   T   
   G   
   G   
   T   
   C   
   C   
   A   
    T    
   T   
   A   
   G   
   C   
    T    
   G   
   G   
    T    
   T   
   T   
    A    
   A   
   C   
   C   
   A   
   C   
    G    
   G   
   T   
    C    
    A    
    A    
   T   
   G   
   G   
    G    
    G    
   A   
   T   
   G   
   A   
   A   
   G   
   A   
    T    
   A   
   T   
   C   
    T    
   T   
    A    
    T    
   T   
    G    
   A   
   T   
    T    
   A   
   C   
    G    
    A    
   A   
    C    
   A   
   A   
   A   
   G   
   G   
    C    
   G   
   T   
    C    
   A   
   T   
   T   
   A   
   T   
   C   
   C   
   G   
    C    
   T   
   T   
    T    
   A   
   A   
   C   
   G   
   T   
    T    
    G    
    A    
    T    
    T    
   C   
   T   
   G   
   T   
   T   
   T   
   C   
   T   
   C   
   A   
   A   
   A   
   C   
    A    
   G   
   G   
   A   
   C   
   G   
    C    
    G    
   C   
   A   
   A   
   C   
   A   
   T   
   T   
    A    
   G   
   G   
    G    
   G   
   T   
    T    
   C   
   G   
   T   
   T   
   T   
    A    
   A   
   T   
   G   
   A   
    G    
    A    
   A   
   T   
   G   
   G   
   A   
   A   
   G   
    A    
    T    
   G   
    G    
    T    
    G    
   C   
    C    
   A   
   A   
   A   
   -   
   -   
   -   
   -   
   -   
   -   
   -   
   -   
   -   
   -   
   -   
   -   
   -   
   -   
   -   
   -   
   -   
   -   
   -   
   -   
   -   
   -   
   -   
   -   
   -   
   -   
   -   
   -   
   -   
   -   
   -   
   -   
   -   
   -   
   -   
   -   
   -   
   -   
   -   
   -   
   -   
   -   
   -   
   -   
   -   
   -   
   -   
   -   
   -   
   -   
   -   
   -   
   -   
   -   
   -   
   -   
   -   
   -   
   -   
   -   
   -   
   -   
   -   
   -   
   -   
   -   
   -   
   -   
   -   
   -   
   -   
   -   
 

 
   N54588   
   A   
   T   
   G   
   A   
   G   
   T   
   G   
   A   
   A   
   G   
   A   
   A   
   A   
   T   
   T   
   A   
   A   
   A   
   G   
   A   
   A   
   A   
   A   
   C   
   A   
   T   
   T   
   C   
   A   
   A   
   G   
   A   
   C   
   G   
   T   
   C   
   A   
   A   
   T   
   C   
   T   
   G   
   A   
   C   
   C   
   A   
   G   
   C   
   G   
   A   
   A   
   A   
   T   
   G   
   A   
   A   
   A   
   G   
   A   
   A   
   T   
   C   
   T   
   T   
   T   
   C   
   A   
   T   
   T   
   G   
   A   
   C   
   T   
   A   
   C   
   G   
   C   
   A   
   A   
   T   
   G   
   A   
   G   
   T   
   G   
   T   
   T   
   A   
   T   
   C   
   G   
   T   
   A   
   G   
   C   
   C   
   C   
   G   
   C   
   G   
   C   
   G   
   T   
   T   
   A   
   C   
   C   
   T   
   G   
   A   
   C   
   G   
   T   
   T   
   C   
   G   
   T   
   G   
   A   
   C   
   G   
   G   
   T   
   T   
   T   
   A   
   A   
   A   
   A   
   C   
   C   
   T   
   G   
   T   
   T   
   C   
   A   
   T   
   C   
   G   
   C   
   C   
   G   
   A   
   A   
   T   
   C   
   T   
   T   
   A   
   T   
   A   
   T   
   G   
   G   
   A   
   A   
   T   
   G   
   A   
   A   
   C   
   G   
   A   
   A   
   T   
   T   
   A   
   G   
   G   
   G   
   G   
   T   
   A   
   A   
   C   
   C   
   C   
   C   
   T   
   G   
   A   
   T   
   A   
   A   
   A   
   C   
   C   
   A   
   C   
   A   
   C   
   A   
   A   
   A   
   A   
   A   
   A   
   T   
   C   
   A   
   G   
   C   
   C   
   C   
   G   
   G   
   A   
   T   
   T   
   G   
   T   
   T   
   G   
   G   
   G   
   G   
   A   
   T   
   G   
   T   
   T   
   A   
   T   
   G   
   G   
   G   
   T   
   A   
   A   
   A   
   T   
   A   
   T   
   C   
   A   
   C   
   C   
   C   
   C   
   C   
   A   
   T   
   G   
   G   
   G   
   G   
   A   
   C   
   A   
   G   
   T   
   G   
   C   
   G   
   A   
   T   
   T   
   T   
   A   
   C   
   G   
   A   
   A   
   T   
   C   
   A   
   A   
   T   
   G   
   G   
   T   
   G   
   C   
   G   
   G   
   A   
   T   
   G   
   G   
   C   
   A   
   C   
   A   
   A   
   C   
   C   
   T   
   T   
   T   
   T   
   A   
   G   
   T   
   T   
   A   
   T   
   C   
   G   
   A   
   G   
   C   
   T   
   A   
   T   
   G   
   T   
   T   
   A   
   G   
   T   
   T   
   G   
   A   
   C   
   G   
   G   
   C   
   C   
   A   
   C   
   G   
   G   
   A   
   A   
   A   
   C   
   T   
   T   
   C   
   G   
   G   
   T   
   T   
   C   
   T   
   G   
   T   
   C   
   G   
   A   
   T   
   G   
   G   
   T   
   G   
   A   
   C   
   G   
   G   
   C   
   G   
   C   
   T   
   G   
   C   
   C   
   G   
   C   
   T   
   A   
   T   
   G   
   C   
   G   
   T   
   T   
   A   
   T   
   A   
   C   
   C   
   G   
   A   
   A   
   G   
   C   
   A   
   C   
   G   
   T   
   A   
   T   
   G   
   A   
   G   
   T   
   A   
   A   
   A   
   A   
   T   
   T   
   G   
   C   
   T   
   T   
   T   
   A   
   G   
   A   
   A   
   A   
   T   
   G   
   C   
   T   
   A   
   C   
   G   
   A   
   G   
   A   
   T   
   A   
   T   
   T   
   A   
   A   
   C   
   A   
   A   
   A   
   A   
   A   
   T   
   A   
   C   
   A   
   G   
   T   
   C   
   G   
   A   
   T   
   T   
   T   
   C   
   C   
   A   
   A   
   G   
   G   
   A   
   A   
   A   
   C   
   T   
   A   
   T   
   G   
   A   
   T   
   G   
   A   
   T   
   T   
   C   
   A   
   G   
   A   
   A   
   C   
   A   
   A   
   G   
   A   
   A   
   C   
   C   
   A   
   G   
   T   
   A   
   G   
   T   
   T   
   T   
   T   
   A   
   C   
   C   
   T   
   G   
   C   
   T   
   C   
   G   
   T   
   T   
   T   
   T   
   C   
   C   
   A   
   A   
   A   
   C   
   T   
   T   
   A   
   C   
   T   
   A   
   G   
   T   
   T   
   A   
   A   
   C   
   G   
   G   
   A   
   A   
   C   
   A   
   A   
   C   
   G   
   G   
   G   
   G   
   A   
   T   
   T   
   G   
   C   
   G   
   G   
   T   
   G   
   G   
   G   
   G   
   A   
   T   
   G   
   G   
   C   
   A   
   A   
   C   
   A   
   A   
   A   
   T   
   A   
   T   
   T   
   C   
   C   
   A   
   C   
   C   
   A   
   C   
   A   
   C   
   A   
   A   
   T   
   T   
   T   
   A   
   A   
   G   
   T   
   G   
   A   
   A   
   G   
   T   
   G   
   A   
   T   
   T   
   G   
   A   
   T   
   G   
   C   
   A   
   A   
   C   
   A   
   A   
   G   
   T   
   T   
   T   
   A   
   T   
   T   
   G   
   A   
   T   
   G   
   G   
   A   
   C   
   A   
   A   
   T   
   C   
   C   
   T   
   G   
   A   
   T   
   G   
   T   
   A   
   A   
   C   
   G   
   A   
   C   
   G   
   A   
   A   
   T   
   G   
   A   
   A   
   T   
   T   
   G   
   A   
   T   
   G   
   G   
   A   
   A   
   G   
   T   
   G   
   T   
   T   
   A   
   C   
   C   
   T   
   G   
   G   
   A   
   C   
   C   
   A   
   G   
   A   
   T   
   T   
   T   
   T   
   C   
   C   
   A   
   A   
   C   
   A   
   G   
   G   
   C   
   G   
   G   
   T   
   T   
   T   
   A   
   G   
   T   
   G   
   A   
   T   
   G   
   G   
   G   
   G   
   A   
   A   
   A   
   T   
   C   
   A   
   G   
   G   
    G    
   A   
   T   
    T    
   C   
   G   
    C    
    C    
   G   
   A   
   G   
   C   
    A    
   T   
   A   
   T   
   G   
   A   
   A   
   A   
   C   
    A    
   G   
   G   
    G    
   A   
   A   
    A    
   G   
   G   
   T   
   T   
   C   
    G    
   A   
   T   
   T   
   A   
   C   
    T    
   G   
   T   
    T    
   C   
   G   
   T   
   G   
   C   
   A   
   A   
   A   
   A   
   G   
   T   
   T   
   G   
   A   
    A    
    T    
   T   
    G    
   A   
    C    
   T   
   G   
   A   
    A    
   A   
   T   
   G   
    C    
   C   
    G    
   A   
   A   
   T   
   G   
   G   
    A    
   A   
   A   
   A   
   G   
   A   
   A   
   C   
   G   
    T    
   A   
   T   
    T    
    T    
   T   
    A    
   G   
   T   
    A    
   A   
   C   
    T    
   G   
   A   
   A   
   T   
   T   
    G    
   C   
   C   
   T   
   T   
   A   
   T   
   A   
   T   
   G   
   G   
   T   
    G    
   A   
   A   
   T   
   A   
   A   
   A   
   G   
   C   
    C    
   A   
   A   
   A   
   T   
   T   
    A    
   A   
   T   
    C    
   G   
   A   
   A   
   C   
   G   
    A    
   A   
   T   
   T   
   T   
   C   
   T   
   G   
   A   
    A    
    T    
   T   
    A    
   C   
   A   
   C   
   A   
   G   
   A   
   G   
   A   
    T    
   A   
   A   
    A    
   C   
   G   
    A    
   A   
   T   
    T    
   G   
   A   
   A   
   G   
   G   
    A    
   A   
   T   
    T    
   A   
   C   
    T    
   G   
   A   
   T   
   T   
   T   
   G   
   C   
   G   
   G   
   G   
   A   
    T    
   G   
   A   
   A   
   T   
   C   
   T   
   T   
   C   
    T    
   C   
   G   
   T   
   G   
   A   
    A    
   G   
   G   
    C    
   A   
   T   
   G   
   C   
   G   
    G    
   A   
   T   
    C    
   G   
   T   
   C   
   A   
   T   
    C    
   G   
   A   
    T    
    G    
   T   
    T    
   C   
   G   
   T   
   C   
   G   
    A    
   G   
   A   
    T    
   G   
    T    
    G    
   A   
   G   
    T    
   G   
   C   
    C    
   T   
   C   
    T    
   G   
   T   
   T   
    G    
   T   
    G    
   T   
   T   
    A    
   A   
   A   
    C    
   A   
   A   
   C   
   T   
   T   
    G    
   T   
   A   
    C    
   A   
   A   
   A   
    A    
   T   
   G   
   A   
   C   
    T    
    G    
   C   
    C    
   T   
   T   
    A    
   C   
   A   
    A    
   A   
    C    
    A    
   T   
   C   
    T    
   T   
   T   
    T    
   G   
   G   
    T    
   T   
   T   
    T    
   A   
   A   
    C    
   A   
   T   
   G   
   T   
   T   
    A    
   G   
   C   
    C    
   A   
   T   
    C    
   G   
   A   
   A   
   A   
   A   
   A   
   G   
   G   
    C    
   G   
   T   
    A    
   C   
   C   
    G    
   A   
   A   
   A   
    A    
   T   
   T   
   T   
   T   
    A    
   A   
   G   
    C    
   T   
   T   
   G   
   A   
   A   
   A   
   C   
    G    
    T    
   A   
   T   
    T    
    T    
   T   
    A    
   G   
   A   
   A   
   A   
   A   
    C    
   T   
   A   
    C    
    G    
   T   
    C    
   G   
   A   
    G    
   C   
   A   
    C    
   C   
   A   
    A    
    A    
    A    
    A    
    G    
   A   
   A   
   G   
   T   
   C   
   A   
   T   
    T    
   A   
   C   
    T    
    C    
   G   
    C    
   C   
   G   
   G   
   A   
   C   
    G    
    A    
   T   
    T    
   T   
   T   
   T   
   G   
   A   
    C    
   A   
   A   
   A   
    A    
   A   
    C    
   A   
   A   
    A    
   G   
   C   
   A   
   G   
   A   
   A   
   G   
   C   
   A   
    C    
   G   
    G    
   G   
   C   
    G    
   C   
   A   
    T    
   A   
   T   
    T    
   T   
   T   
   A   
   G   
   A   
   A   
   G   
   G   
   T   
    C    
   T   
    A    
   C   
   G   
    A    
   A   
   T   
   T   
   G   
   C   
    C    
   T   
   T   
    A    
   G   
   A   
   T   
   C   
   A   
   T   
   A   
   T   
   C   
    G    
   A   
   T   
   G   
   A   
   A   
   A   
   T   
    C    
   A   
   T   
   T   
   G   
   C   
    C    
   A   
   T   
   T   
   A   
   T   
    C    
   C   
   G   
    T    
   G   
   G   
    G    
   T   
   C   
    A    
    C    
   A   
   A   
   T   
   C   
   A   
   G   
   A   
   T   
   G   
   A   
    C    
   G   
   A   
    A    
   G   
   C   
   A   
   A   
   A   
   A   
   G   
   C   
    A    
   A   
    C    
    G    
    T    
   T   
    A    
   A   
   T   
    T    
   G   
   A   
   A   
   C   
   G   
    C    
   T   
   T   
    T    
   G   
   A   
    A    
   T   
   T   
    T    
   T   
   C   
    A    
   G   
   A   
   T   
   C   
   G   
    T    
   C   
   A   
    A    
    G    
   C   
    G    
   C   
   A   
   A   
   G   
   C   
    G    
   A   
   T   
    T    
   T   
   T   
   A   
   G   
   A   
   T   
   A   
   T   
   G   
   C   
   G   
   T   
   T   
   T   
    A    
   C   
   G   
    C    
   C   
   G   
    T    
   T   
   T   
    A    
   A   
   C   
    A    
   G   
   G   
    C    
   T   
   T   
    A    
   G   
   A   
   A   
   C   
   G   
    C    
   G   
   A   
    C    
   A   
   A   
   A   
   A   
   T   
    T    
   G   
   A   
    A    
   A   
   A   
   T   
   G   
   A   
   A   
   T   
   A   
    T    
   C   
   A   
   A   
   G   
   A   
    G    
   C   
   T   
    A    
   T   
   T   
    G    
   A   
    A    
   A   
    T    
   T   
    C    
   A   
   T   
   T   
   G   
    C    
    G    
   G   
   A   
    C    
   T   
   T   
    A    
    G    
    A    
    G    
   G   
   A   
   C   
   A   
   T   
   T   
    T    
   T   
    A    
   G   
   C   
    C    
    C    
   G   
    C    
   C   
   C   
    A    
   G   
   A   
    A    
   C   
   G   
    C    
   G   
   T   
    C    
   A   
    T    
    T    
   G   
   A   
   A   
   A   
   T   
    C    
   A   
   T   
    T    
   A   
   A   
    A    
   A   
    C    
    A    
   G   
   A   
    G    
   T   
   T   
   A   
    A    
    A    
    T    
   G   
   A   
   C   
    G    
   T   
    T    
    C    
    G    
    C    
    A    
    C    
   A   
    A    
    A    
   A   
   T   
   T   
   T   
   G   
   G   
   T   
   G   
   A   
   T   
    G    
    C    
   A   
   C   
   G   
   A   
   C   
   G   
    C    
   A   
   C   
   A   
   G   
   A   
   A   
    T    
   T   
   A   
    T    
   T   
   A   
   G   
   T   
    A    
   G   
   G   
   T   
   G   
   A   
    A    
   G   
   T   
   C   
    C    
   T   
    A    
   A   
   G   
   T   
   C   
   T   
    T    
   G   
   A   
   A   
   G   
   A   
   T   
   G   
   A   
   A   
   G   
   A   
   T   
    T    
   T   
    A    
   A   
   T   
    C    
   G   
   A   
   A   
   G   
   A   
   A   
   G   
   A   
   A   
   G   
   A   
   A   
    G    
   T   
   C   
   G   
   T   
    G    
   A   
   T   
   T   
   A   
   C   
    G    
    T    
   T   
    A    
   A   
   C   
    C    
   A   
   A   
    T    
   A   
   A   
    C    
   G   
   G   
    C    
   T   
   A   
    C    
   A   
   T   
    T    
   A   
   A   
    G    
   C   
   G   
    G    
    A    
   T   
   G   
   G   
    C    
   A   
   A   
   A   
    C    
    T    
    C    
    C    
   G   
   A   
   A   
   T   
   T   
   C   
    C    
   G   
    G    
   G   
   C   
    G    
   C   
   A   
   A   
   C   
   G   
    C    
    C    
   G   
    T    
   G   
   G   
    C    
   G   
   G   
    A    
   C   
   G   
   T   
   G   
   G   
    T    
   G   
   T   
    C    
   C   
   A   
   A   
   G   
   G   
    C    
   A   
   T   
   G   
   G   
   G   
    C    
    G    
   T   
    T    
   C   
   A   
    T    
    G    
   A   
   T   
   G   
   A   
    T    
   G   
   A   
    T    
   T   
   T   
   C   
   G   
   T   
    G    
   A   
   A   
    A    
   A   
   A   
    C    
   C   
   T   
    A    
    G    
   T   
   T   
   T   
   C   
   T   
   T   
   G   
    T    
   T   
   C   
    A    
   A   
   C   
    A    
   C   
   A   
    C    
   G   
   A   
    C    
   A   
   C   
    G    
   T   
   T   
    A    
   T   
   T   
   A   
   T   
   T   
    C    
   T   
   T   
   T   
   A   
   C   
   G   
   A   
   A   
    C    
   A   
    C    
    T    
   G   
   G   
    G    
   A   
   A   
   A   
   G   
   T   
    T    
   T   
   A   
    C    
   C   
   G   
    A    
   G   
   C   
   A   
   A   
   A   
   A   
   G   
   G   
    T    
   T   
   A   
    C    
   G   
   A   
   A   
   A   
   T   
    C    
   C   
   C   
    T    
   G   
   A   
    A    
   T   
   A   
   C   
   G   
   G   
    T    
    A    
   G   
    A    
   A   
   C   
   A   
   G   
   C   
    A    
   A   
   A   
   A   
   G   
   G   
    A    
   A   
   T   
    T    
   C   
   C   
   A   
    G    
   T   
    G    
   A   
   T   
    T    
   A   
   A   
    C    
   T   
   T   
    A    
   C   
   T   
    G    
   G   
   G   
    A    
   A   
   T   
    T    
   G   
   A   
    T    
   T   
   C   
    T    
    G    
    C    
    A    
   G   
   A   
   A   
   A   
    A    
    A    
   A   
   T   
    T    
   C   
   A   
    A    
   G   
   C   
    G    
   A   
   T   
    T    
   A   
   T   
   T   
    T    
   C   
    T    
   G   
   T   
    T    
    G    
    A    
   G   
   G   
   G   
    C    
    A    
   A   
   A   
   G   
   C   
    G    
   G   
   A   
   A   
   G   
    C    
    A    
   G   
   G   
    T    
    A    
   A   
    A    
   T   
   A   
    C    
    T    
   T   
    G    
   T   
   T   
   C   
   T   
   T   
    C    
   A   
   C   
    A    
   A   
   C   
    C    
    T    
    T    
    A    
    A    
   A   
   A   
   G   
   G   
    A    
   A   
   C   
    C    
   G   
   T   
    C    
   A   
   A   
   A   
   C   
   G   
    G    
   A   
   C   
    A    
    G    
   C   
    C    
   G   
   T   
    A    
   A   
   C   
    A    
   G   
   C   
    C    
   T   
   T   
    T    
    T    
   C   
   T   
   A   
   A   
    T    
   A   
   T   
    C    
   C   
   G   
    T    
   A   
   G   
    T    
   A   
   A   
   T   
   G   
   G   
    A    
    T    
   T   
    A    
   A   
   T   
    C    
   G   
   C   
    C    
   A   
   T   
   T   
    A    
   G   
    C    
   T   
   T   
    A    
   A   
   A   
   A   
   G   
   A   
   A   
   G   
   A   
    T    
   G   
   A   
    T    
   G   
   A   
    G    
    T    
   T   
    A    
    G    
   T   
    T    
   A   
   A   
    C    
   G   
   T   
    G    
    G    
   T   
   A   
    A    
    C    
   G   
   A   
   C   
    T    
    A    
   A   
    T    
   G   
   G   
    C    
    A    
   A   
    T    
    C    
    A    
    G    
   A   
   A   
    G    
   A   
   T   
    G    
   A   
   T   
    T    
   A   
   T   
    C    
   G   
   G   
    A    
   A   
   C   
   A   
   C   
   A   
   T   
    G    
    C    
    A    
   G   
   G   
    A    
   T   
   A   
   C   
   T   
   C   
   T   
   G   
   T   
    C    
   A   
   C   
    A    
   T   
   T   
    T    
    G    
   A   
    T    
   G   
   A   
    A    
    A    
    A    
    T    
    A    
   C   
    T    
   G   
    T    
    A    
   C   
   G   
    T    
   G   
   A   
   T   
   A   
   T   
   G   
   G   
   G   
    C    
   C   
   G   
    G    
   A   
   C   
   A   
   G   
   C   
    A    
   T   
   C   
    A    
   G   
   G   
    C    
   G   
   T   
    T    
    C    
   G   
    G    
   G   
   G   
    A    
   A   
   T   
    C    
   C   
   G   
    T    
    C    
   T   
    C    
   C   
   G   
    C    
   G   
   A   
    A    
    A    
   A   
    T    
   G   
   A   
    T    
   T   
    A    
    T    
   G   
   T   
    G    
   G   
   T   
    C    
   G   
   G   
    T    
    G    
   C   
   A   
   G   
   C   
    G    
    A    
   T   
    T    
    C    
   T   
   G   
    G    
   A   
    T    
   G   
    A    
   A   
    A    
   A   
    T    
    A    
   A   
   A   
   G   
   A   
   A   
   G   
   T   
    C    
    C    
   T   
    A    
   G   
   T   
    C    
   A   
   T   
    T    
   A   
   C   
    T    
   G   
   A   
   A   
   A   
   A   
    T    
   G   
   G   
   T   
   T   
   A   
    T    
   G   
   G   
    T    
   A   
   A   
    G    
   C   
   G   
    T    
   A   
   C   
    A    
   A   
   A   
   A   
   G   
    C    
    T    
   T   
   C   
    T    
   G   
   A   
    A    
   T   
   A   
   T   
   C   
   C   
   A   
   G   
   T   
    T    
   A   
   A   
    A    
   G   
   G   
    A    
   C   
   G   
    T    
   G   
   G   
    C    
   G   
   G   
   T   
   A   
   A   
   A   
   G   
   G   
    G    
   A   
   T   
    T    
   A   
   A   
    G    
   A   
   C   
    A    
    G    
   C   
    A    
   A   
   A   
    T    
   A   
   T   
   C   
   A   
   C   
   T   
   G   
   A   
    G    
   A   
   A   
   A   
   A   
   A   
   T   
   G   
   G   
   T   
   C   
   C   
   A   
    T    
   T   
   A   
   G   
   C   
    T    
   G   
   G   
    T    
   T   
   T   
    A    
   A   
   C   
   C   
   A   
   C   
    G    
   G   
   T   
    C    
    A    
    A    
   T   
   G   
   G   
    G    
    G    
   A   
   T   
   G   
   A   
   A   
   G   
   A   
    T    
   A   
   T   
   C   
    T    
   T   
    G    
    T    
   T   
    G    
   A   
   T   
    T    
   A   
   C   
    G    
    A    
   A   
    C    
   A   
   A   
   A   
   G   
   G   
    C    
   G   
   T   
    C    
   A   
   T   
   T   
   A   
   T   
   C   
   C   
   G   
    C    
   T   
   T   
    T    
   A   
   A   
   C   
   G   
   T   
    A    
    G    
    A    
    T    
    T    
   C   
   T   
   G   
   T   
   T   
   T   
   C   
   T   
   C   
   A   
   A   
   A   
   C   
    A    
   G   
   G   
   A   
   C   
   G   
    C    
    G    
   C   
   A   
   A   
   C   
   A   
   T   
   T   
    A    
   G   
   G   
    G    
   G   
   T   
    T    
   C   
   G   
   T   
   T   
   T   
    A    
   A   
   T   
   G   
   A   
    G    
    A    
   A   
   T   
   G   
   G   
   A   
   A   
   G   
    A    
    T    
   G   
    G    
    T    
    G    
   C   
    C    
   A   
   A   
   A   
   -   
   -   
   -   
   -   
   -   
   -   
   -   
   -   
   -   
   -   
   -   
   -   
   -   
   -   
   -   
   -   
   -   
   -   
   -   
   -   
   -   
   -   
   -   
   -   
   -   
   -   
   -   
   -   
   -   
   -   
   -   
   -   
   -   
   -   
   -   
   -   
   -   
   -   
   -   
   -   
   -   
   -   
   -   
   -   
   -   
   -   
   -   
   -   
   -   
   -   
   -   
   -   
   -   
   -   
   -   
   -   
   -   
   -   
   -   
   -   
   -   
   -   
   -   
   -   
   -   
   -   
   -   
   -   
   -   
   -   
   -   
   -   
 

 
   N53548   
   A   
   T   
   G   
   A   
   G   
   T   
   G   
   A   
   A   
   G   
   A   
   A   
   A   
   T   
   C   
   A   
   G   
   A   
   G   
   A   
   A   
   A   
   A   
   C   
   A   
   T   
   C   
   C   
   A   
   T   
   G   
   A   
   T   
   G   
   T   
   C   
   A   
   A   
   T   
   C   
   T   
   A   
   A   
   C   
   C   
   A   
   G   
   C   
   G   
   A   
   A   
   A   
   T   
   G   
   A   
   A   
   A   
   G   
   A   
   C   
   T   
   C   
   C   
   T   
   T   
   C   
   A   
   T   
   C   
   G   
   A   
   T   
   T   
   A   
   T   
   G   
   C   
   G   
   A   
   T   
   G   
   A   
   G   
   T   
   G   
   T   
   T   
   A   
   T   
   T   
   G   
   T   
   C   
   G   
   C   
   A   
   C   
   G   
   T   
   G   
   C   
   A   
   C   
   T   
   T   
   C   
   C   
   T   
   G   
   A   
   T   
   G   
   T   
   C   
   A   
   G   
   A   
   G   
   A   
   T   
   G   
   G   
   T   
   C   
   T   
   G   
   A   
   A   
   A   
   C   
   C   
   C   
   G   
   T   
   T   
   C   
   A   
   T   
   C   
   G   
   C   
   C   
   G   
   T   
   A   
   T   
   T   
   T   
   T   
   A   
   T   
   A   
   C   
   G   
   G   
   C   
   A   
   T   
   G   
   A   
   A   
   C   
   G   
   A   
   A   
   T   
   T   
   A   
   G   
   G   
   T   
   G   
   T   
   G   
   A   
   C   
   A   
   C   
   C   
   G   
   G   
   A   
   T   
   A   
   A   
   A   
   G   
   C   
   C   
   C   
   A   
   T   
   A   
   A   
   A   
   A   
   A   
   A   
   T   
   C   
   T   
   G   
   C   
   C   
   A   
   G   
   A   
   A   
   T   
   C   
   G   
   T   
   C   
   G   
   G   
   A   
   G   
   A   
   T   
   G   
   T   
   C   
   A   
   T   
   G   
   G   
   G   
   T   
   A   
   A   
   A   
   T   
   A   
   C   
   C   
   A   
   T   
   C   
   C   
   T   
   C   
   A   
   T   
   G   
   G   
   A   
   G   
   A   
   C   
   A   
   G   
   T   
   G   
   C   
   G   
   A   
   T   
   C   
   T   
   A   
   T   
   G   
   A   
   G   
   T   
   C   
   A   
   A   
   T   
   G   
   G   
   T   
   A   
   C   
   G   
   G   
   A   
   T   
   G   
   G   
   C   
   G   
   C   
   A   
   G   
   C   
   C   
   A   
   T   
   T   
   C   
   A   
   G   
   C   
   T   
   A   
   C   
   C   
   G   
   T   
   T   
   A   
   T   
   A   
   T   
   G   
   C   
   T   
   A   
   G   
   T   
   G   
   G   
   A   
   T   
   G   
   G   
   T   
   C   
   A   
   T   
   G   
   G   
   A   
   A   
   A   
   C   
   T   
   T   
   T   
   G   
   G   
   T   
   T   
   C   
   T   
   G   
   T   
   T   
   G   
   A   
   C   
   G   
   G   
   T   
   G   
   A   
   C   
   G   
   G   
   T   
   G   
   C   
   C   
   G   
   C   
   C   
   G   
   C   
   T   
   A   
   T   
   G   
   C   
   G   
   T   
   T   
   A   
   T   
   A   
   C   
   C   
   G   
   A   
   A   
   G   
   C   
   A   
   C   
   G   
   G   
   A   
   T   
   G   
   A   
   G   
   C   
   A   
   A   
   A   
   A   
   T   
   C   
   G   
   C   
   T   
   A
[truncated: 2,134,414 more chars]
