# Supplementary material for: sraX: A Novel Comprehensive Resistome Analysis Tool
Source: Front Microbiol. 2020 Feb 5;11:52. doi: 10.3389/fmicb.2020.00052 (PMC7025521; doi:10.3389/fmicb.2020.00052)
Supplement: Supplementary file 4 [file Data_Sheet_3.ZIP › eatAv/eatAv_dna.html]

# eatAv

|  |
| --- |
|  |

|  |
| --- |
|  |

|  |  |
| --- | --- |
| **Definition:** | Mutated form of the wildtype eatA ABC-F subfamily protein isolated from Enterococcus faecium conferring resistance to lincosamides streptogramin A's and pleuromutilins (LSaP phenotype). |

|  |
| --- |
|  |

|  |
| --- |
|  |

|  |  |  |
| --- | --- | --- |
| **Reference organism:** | *Enterococcus faecium* | [NCBI, CARD] |

|  |
| --- |
|  |

|  |
| --- |
|  |

|  |  |
| --- | --- |
| **Summary Data** | || **Parameter** | **Value_(n)_** || Total number of genomes | 99 |
| MSA length | 1506 |
| Total number of SNPs | 157 |
| SNP density (# SNPs/MSA length) | 0.104 |

|  |
| --- |
|  |

|  |
| --- |
|  |

|  |
| --- |
|  |

|  |  |  |  |  |  |  |  |  |  |  |  |  |  |  |  |  |  |  |  |  |  |  |  |  |  |  |  |  |  |  |  |  |  |  |  |  |  |  |  |  |  |  |  |  |  |  |  |  |  |  |  |  |  |  |  |  |  |  |  |  |  |  |  |  |  |  |  |  |  |  |  |  |  |  |  |  |  |  |  |  |  |  |  |  |  |  |  |  |  |  |  |  |  |  |  |  |  |  |  |  |  |  |  |  |  |  |  |  |  |  |  |  |  |  |  |  |  |  |  |  |  |  |  |  |  |  |  |  |  |  |  |  |  |  |  |  |  |  |  |  |  |  |  |  |  |  |  |  |  |  |  |  |  |  |  |  |  |  |  |  |  |  |  |  |  |  |  |  |  |  |  |  |  |  |  |  |  |  |  |  |  |  |  |  |  |  |  |  |  |  |  |  |  |  |  |  |  |  |  |  |  |  |  |  |  |  |  |  |  |  |  |  |  |  |  |  |  |  |  |  |  |  |  |  |  |  |  |  |  |  |  |  |  |  |  |  |  |  |  |  |  |  |  |  |  |  |  |  |  |  |  |  |  |  |  |  |  |  |  |  |  |  |  |  |  |  |  |  |  |  |  |  |  |  |  |  |  |  |  |  |  |  |  |  |  |  |  |  |  |  |  |  |  |  |  |  |  |  |  |  |  |  |  |  |  |  |  |  |  |  |  |  |  |  |  |  |  |  |  |  |  |  |  |  |  |  |  |  |  |  |  |  |  |  |  |  |  |  |  |  |  |  |  |  |  |  |  |  |  |  |  |  |  |  |  |  |  |  |  |  |  |  |  |  |  |  |  |  |  |  |  |  |  |  |  |  |  |  |  |  |  |  |  |  |  |  |  |  |  |  |  |  |  |  |  |  |  |  |  |  |  |  |  |  |  |  |  |  |  |  |  |  |  |  |  |  |  |  |  |  |  |  |  |  |  |  |  |  |  |  |  |  |  |  |  |  |  |  |  |  |  |  |  |  |  |  |  |  |  |  |  |  |  |  |  |  |  |  |  |  |  |  |  |  |  |  |  |  |  |  |  |  |  |  |  |  |  |  |  |  |  |  |  |  |  |  |  |  |  |  |  |  |  |  |  |  |  |  |  |  |  |  |  |  |  |  |  |  |  |  |  |  |  |  |  |  |  |  |  |  |  |  |  |  |  |  |  |  |  |  |  |  |  |  |  |  |  |  |  |  |  |  |  |  |  |  |  |  |  |  |  |  |  |  |  |  |  |  |  |  |  |  |  |  |  |  |  |  |  |  |  |  |  |  |  |  |  |  |  |  |  |  |  |  |  |  |  |  |  |  |  |  |  |  |  |  |  |  |  |  |  |  |  |  |  |  |  |  |  |  |  |  |  |  |  |  |  |  |  |  |  |  |  |  |  |  |  |  |  |  |  |  |  |  |  |  |  |  |  |  |  |  |  |  |  |  |  |  |  |  |  |  |  |  |  |  |  |  |  |  |  |  |  |  |  |  |  |  |  |  |  |  |  |  |  |  |  |  |  |  |  |  |  |  |  |  |  |  |  |  |  |  |  |  |  |  |  |  |  |  |  |  |  |  |  |  |  |  |  |  |  |  |  |  |  |  |  |  |  |  |  |  |  |  |  |  |  |  |  |  |  |  |  |  |  |  |  |  |  |  |  |  |  |  |  |  |  |  |  |  |  |  |  |  |  |  |  |  |  |  |  |  |  |  |  |  |  |  |  |  |  |  |  |  |  |  |  |  |  |  |  |  |  |  |  |  |  |  |  |  |  |  |  |  |  |  |  |  |  |  |  |  |  |  |  |  |  |  |  |  |  |  |  |  |  |  |  |  |  |  |  |  |  |  |  |  |  |  |  |  |  |  |  |  |  |  |  |  |  |  |  |  |  |  |  |  |  |  |  |  |  |  |  |  |  |  |  |  |  |  |  |  |  |  |  |  |  |  |  |  |  |  |  |  |  |  |  |  |  |  |  |  |  |  |  |  |  |  |  |  |  |  |  |  |  |  |  |  |  |  |  |  |  |  |  |  |  |  |  |  |  |  |  |  |  |  |  |  |  |  |  |  |  |  |  |  |  |  |  |  |  |  |  |  |  |  |  |  |  |  |  |  |  |  |  |  |  |  |  |  |  |  |  |  |  |  |  |  |  |  |  |  |  |  |  |  |  |  |  |  |  |  |  |  |  |  |  |  |  |  |  |  |  |  |  |  |  |  |  |  |  |  |  |  |  |  |  |  |  |  |  |  |  |  |  |  |  |  |  |  |  |  |  |  |  |  |  |  |  |  |  |  |  |  |  |  |  |  |  |  |  |  |  |  |  |  |  |  |  |  |  |  |  |  |  |  |  |  |  |  |  |  |  |  |  |  |  |  |  |  |  |  |  |  |  |  |  |  |  |  |  |  |  |  |  |  |  |  |  |  |  |  |  |  |  |  |  |  |  |  |  |  |  |  |  |  |  |  |  |  |  |  |  |  |  |  |  |  |  |  |  |  |  |  |  |  |  |  |  |  |  |  |  |  |  |  |  |  |  |  |  |  |  |  |  |  |  |  |  |  |  |  |  |  |  |  |  |  |  |  |  |  |  |  |  |  |  |  |  |  |  |  |  |  |  |  |  |  |  |  |  |  |  |  |  |  |  |  |  |  |  |  |  |  |  |  |  |  |  |  |  |  |  |  |  |  |  |  |  |  |  |  |  |  |  |  |  |  |  |  |  |  |  |  |  |  |  |  |  |  |  |  |  |  |  |  |  |  |  |  |  |  |  |  |  |  |  |  |  |  |  |  |  |  |  |  |  |  |  |  |  |  |  |  |  |  |  |  |  |  |  |  |  |  |  |  |  |  |  |  |  |  |  |  |  |  |  |  |  |  |  |  |  |  |  |  |  |  |  |  |  |  |  |  |  |  |  |  |  |  |  |  |  |  |  |  |  |  |  |  |  |  |  |  |  |  |  |  |  |  |  |  |  |  |  |  |  |  |  |  |  |  |  |  |  |  |  |  |  |  |  |  |  |  |  |  |  |  |  |  |  |  |  |  |  |  |  |  |  |  |  |  |  |  |  |  |  |  |  |  |  |  |  |  |  |  |  |  |  |  |  |  |  |  |  |  |  |  |  |  |  |  |  |  |  |  |  |  |  |  |  |  |  |  |  |  |  |  |  |  |  |  |  |  |  |  |  |  |  |  |  |  |  |  |  |  |  |  |  |  |  |  |  |  |  |  |  |  |  |  |  |  |  |  |  |  |  |  |  |  |  |  |  |  |  |  |  |  |  |  |  |  |  |  |  |  |  |  |  |  |  |  |  |  |  |  |  |  |  |  |  |  |  |  |  |  |  |  |  |  |  |  |  |  |  |  |  |  |  |  |  |  |  |  |  |  |  |
| --- | --- | --- | --- | --- | --- | --- | --- | --- | --- | --- | --- | --- | --- | --- | --- | --- | --- | --- | --- | --- | --- | --- | --- | --- | --- | --- | --- | --- | --- | --- | --- | --- | --- | --- | --- | --- | --- | --- | --- | --- | --- | --- | --- | --- | --- | --- | --- | --- | --- | --- | --- | --- | --- | --- | --- | --- | --- | --- | --- | --- | --- | --- | --- | --- | --- | --- | --- | --- | --- | --- | --- | --- | --- | --- | --- | --- | --- | --- | --- | --- | --- | --- | --- | --- | --- | --- | --- | --- | --- | --- | --- | --- | --- | --- | --- | --- | --- | --- | --- | --- | --- | --- | --- | --- | --- | --- | --- | --- | --- | --- | --- | --- | --- | --- | --- | --- | --- | --- | --- | --- | --- | --- | --- | --- | --- | --- | --- | --- | --- | --- | --- | --- | --- | --- | --- | --- | --- | --- | --- | --- | --- | --- | --- | --- | --- | --- | --- | --- | --- | --- | --- | --- | --- | --- | --- | --- | --- | --- | --- | --- | --- | --- | --- | --- | --- | --- | --- | --- | --- | --- | --- | --- | --- | --- | --- | --- | --- | --- | --- | --- | --- | --- | --- | --- | --- | --- | --- | --- | --- | --- | --- | --- | --- | --- | --- | --- | --- | --- | --- | --- | --- | --- | --- | --- | --- | --- | --- | --- | --- | --- | --- | --- | --- | --- | --- | --- | --- | --- | --- | --- | --- | --- | --- | --- | --- | --- | --- | --- | --- | --- | --- | --- | --- | --- | --- | --- | --- | --- | --- | --- | --- | --- | --- | --- | --- | --- | --- | --- | --- | --- | --- | --- | --- | --- | --- | --- | --- | --- | --- | --- | --- | --- | --- | --- | --- | --- | --- | --- | --- | --- | --- | --- | --- | --- | --- | --- | --- | --- | --- | --- | --- | --- | --- | --- | --- | --- | --- | --- | --- | --- | --- | --- | --- | --- | --- | --- | --- | --- | --- | --- | --- | --- | --- | --- | --- | --- | --- | --- | --- | --- | --- | --- | --- | --- | --- | --- | --- | --- | --- | --- | --- | --- | --- | --- | --- | --- | --- | --- | --- | --- | --- | --- | --- | --- | --- | --- | --- | --- | --- | --- | --- | --- | --- | --- | --- | --- | --- | --- | --- | --- | --- | --- | --- | --- | --- | --- | --- | --- | --- | --- | --- | --- | --- | --- | --- | --- | --- | --- | --- | --- | --- | --- | --- | --- | --- | --- | --- | --- | --- | --- | --- | --- | --- | --- | --- | --- | --- | --- | --- | --- | --- | --- | --- | --- | --- | --- | --- | --- | --- | --- | --- | --- | --- | --- | --- | --- | --- | --- | --- | --- | --- | --- | --- | --- | --- | --- | --- | --- | --- | --- | --- | --- | --- | --- | --- | --- | --- | --- | --- | --- | --- | --- | --- | --- | --- | --- | --- | --- | --- | --- | --- | --- | --- | --- | --- | --- | --- | --- | --- | --- | --- | --- | --- | --- | --- | --- | --- | --- | --- | --- | --- | --- | --- | --- | --- | --- | --- | --- | --- | --- | --- | --- | --- | --- | --- | --- | --- | --- | --- | --- | --- | --- | --- | --- | --- | --- | --- | --- | --- | --- | --- | --- | --- | --- | --- | --- | --- | --- | --- | --- | --- | --- | --- | --- | --- | --- | --- | --- | --- | --- | --- | --- | --- | --- | --- | --- | --- | --- | --- | --- | --- | --- | --- | --- | --- | --- | --- | --- | --- | --- | --- | --- | --- | --- | --- | --- | --- | --- | --- | --- | --- | --- | --- | --- | --- | --- | --- | --- | --- | --- | --- | --- | --- | --- | --- | --- | --- | --- | --- | --- | --- | --- | --- | --- | --- | --- | --- | --- | --- | --- | --- | --- | --- | --- | --- | --- | --- | --- | --- | --- | --- | --- | --- | --- | --- | --- | --- | --- | --- | --- | --- | --- | --- | --- | --- | --- | --- | --- | --- | --- | --- | --- | --- | --- | --- | --- | --- | --- | --- | --- | --- | --- | --- | --- | --- | --- | --- | --- | --- | --- | --- | --- | --- | --- | --- | --- | --- | --- | --- | --- | --- | --- | --- | --- | --- | --- | --- | --- | --- | --- | --- | --- | --- | --- | --- | --- | --- | --- | --- | --- | --- | --- | --- | --- | --- | --- | --- | --- | --- | --- | --- | --- | --- | --- | --- | --- | --- | --- | --- | --- | --- | --- | --- | --- | --- | --- | --- | --- | --- | --- | --- | --- | --- | --- | --- | --- | --- | --- | --- | --- | --- | --- | --- | --- | --- | --- | --- | --- | --- | --- | --- | --- | --- | --- | --- | --- | --- | --- | --- | --- | --- | --- | --- | --- | --- | --- | --- | --- | --- | --- | --- | --- | --- | --- | --- | --- | --- | --- | --- | --- | --- | --- | --- | --- | --- | --- | --- | --- | --- | --- | --- | --- | --- | --- | --- | --- | --- | --- | --- | --- | --- | --- | --- | --- | --- | --- | --- | --- | --- | --- | --- | --- | --- | --- | --- | --- | --- | --- | --- | --- | --- | --- | --- | --- | --- | --- | --- | --- | --- | --- | --- | --- | --- | --- | --- | --- | --- | --- | --- | --- | --- | --- | --- | --- | --- | --- | --- | --- | --- | --- | --- | --- | --- | --- | --- | --- | --- | --- | --- | --- | --- | --- | --- | --- | --- | --- | --- | --- | --- | --- | --- | --- | --- | --- | --- | --- | --- | --- | --- | --- | --- | --- | --- | --- | --- | --- | --- | --- | --- | --- | --- | --- | --- | --- | --- | --- | --- | --- | --- | --- | --- | --- | --- | --- | --- | --- | --- | --- | --- | --- | --- | --- | --- | --- | --- | --- | --- | --- | --- | --- | --- | --- | --- | --- | --- | --- | --- | --- | --- | --- | --- | --- | --- | --- | --- | --- | --- | --- | --- | --- | --- | --- | --- | --- | --- | --- | --- | --- | --- | --- | --- | --- | --- | --- | --- | --- | --- | --- | --- | --- | --- | --- | --- | --- | --- | --- | --- | --- | --- | --- | --- | --- | --- | --- | --- | --- | --- | --- | --- | --- | --- | --- | --- | --- | --- | --- | --- | --- | --- | --- | --- | --- | --- | --- | --- | --- | --- | --- | --- | --- | --- | --- | --- | --- | --- | --- | --- | --- | --- | --- | --- | --- | --- | --- | --- | --- | --- | --- | --- | --- | --- | --- | --- | --- | --- | --- | --- | --- | --- | --- | --- | --- | --- | --- | --- | --- | --- | --- | --- | --- | --- | --- | --- | --- | --- | --- | --- | --- | --- | --- | --- | --- | --- | --- | --- | --- | --- | --- | --- | --- | --- | --- | --- | --- | --- | --- | --- | --- | --- | --- | --- | --- | --- | --- | --- | --- | --- | --- | --- | --- | --- | --- | --- | --- | --- | --- | --- | --- | --- | --- | --- | --- | --- | --- | --- | --- | --- | --- | --- | --- | --- | --- | --- | --- | --- | --- | --- | --- | --- | --- | --- | --- | --- | --- | --- | --- | --- | --- | --- | --- | --- | --- | --- | --- | --- | --- | --- | --- | --- | --- | --- | --- | --- | --- | --- | --- | --- | --- | --- | --- | --- | --- | --- | --- | --- | --- | --- | --- | --- | --- | --- | --- | --- | --- | --- | --- | --- | --- | --- | --- | --- | --- | --- | --- | --- | --- | --- | --- | --- | --- | --- | --- | --- | --- | --- | --- | --- | --- | --- | --- | --- | --- | --- | --- | --- | --- | --- | --- | --- | --- | --- | --- | --- | --- | --- | --- | --- | --- | --- | --- | --- | --- | --- | --- | --- | --- | --- | --- | --- | --- | --- | --- | --- | --- | --- | --- | --- | --- | --- | --- | --- | --- | --- | --- | --- | --- | --- | --- | --- | --- | --- | --- | --- | --- | --- | --- | --- | --- | --- | --- | --- | --- | --- | --- | --- | --- | --- | --- | --- | --- | --- | --- | --- | --- | --- | --- | --- | --- | --- | --- | --- | --- | --- | --- | --- | --- | --- | --- | --- | --- | --- | --- | --- | --- | --- | --- | --- | --- | --- | --- | --- | --- | --- | --- | --- | --- | --- | --- | --- | --- | --- | --- | --- | --- | --- | --- | --- | --- | --- | --- | --- | --- | --- | --- | --- | --- | --- | --- | --- | --- | --- | --- | --- | --- | --- | --- | --- | --- | --- | --- | --- | --- | --- | --- | --- | --- | --- | --- | --- | --- | --- | --- | --- | --- | --- | --- | --- | --- | --- | --- | --- | --- | --- | --- | --- | --- | --- | --- | --- | --- | --- | --- | --- | --- | --- | --- | --- | --- | --- | --- | --- | --- | --- | --- | --- | --- | --- | --- | --- | --- | --- | --- | --- | --- | --- | --- | --- | --- | --- | --- | --- | --- | --- | --- | --- | --- | --- | --- | --- | --- | --- | --- | --- | --- | --- | --- | --- | --- | --- | --- | --- | --- | --- | --- | --- | --- | --- | --- | --- | --- | --- | --- | --- | --- | --- | --- | --- | --- | --- | --- | --- | --- | --- | --- | --- | --- | --- | --- | --- | --- | --- | --- | --- | --- | --- | --- | --- | --- | --- | --- | --- | --- | --- | --- | --- | --- | --- | --- | --- | --- | --- | --- | --- | --- | --- | --- | --- | --- | --- | --- | --- | --- | --- | --- | --- | --- | --- | --- | --- | --- | --- | --- | --- | --- | --- | --- | --- | --- | --- | --- | --- | --- | --- | --- | --- | --- | --- | --- | --- | --- | --- | --- | --- | --- | --- | --- | --- | --- | --- | --- | --- | --- | --- | --- | --- | --- | --- | --- | --- | --- | --- | --- | --- | --- | --- | --- | --- | --- | --- | --- | --- | --- | --- | --- | --- | --- | --- | --- | --- | --- | --- | --- | --- | --- | --- | --- | --- | --- | --- | --- | --- | --- | --- | --- | --- | --- | --- | --- | --- | --- | --- | --- | --- | --- | --- | --- | --- |
|  || **Enterococcus faecium (AGQ48857.1)** | **A** | **T** | **G** | **T** | **C** | **T** | **A** | **A** | **A** | **A** | **T** | **C** | **G** | **A** | **A** | **A** | **T** | **A** | **A** | **A** | **A** | **A** | **A** | **T** | **C** | **T** | **G** | **A** | **C** | **A** | **T** | **T** | **C** | **G** | **G** | **C** | **T** | **A** | **C** | **G** | **A** | **C** | **A** | **G** | **C** | **C** | **A** | **A** | **G** | **G** | **C** | **A** | **C** | **A** | **T** | **T** | **A** | **T** | **T** | **A** | **T** | **T** | **T** | **G** | **A** | **A** | **C** | **A** | **A** | **G** | **C** | **A** | **A** | **A** | **T** | **C** | **T** | **A** | **A** | **A** | **T** | **T** | **T** | **T** | **G** | **A** | **C** | **A** | **C** | **A** | **C** | **A** | **A** | **T** | **G** | **G** | **A** | **A** | **A** | **C** | **T** | **A** | **G** | **G** | **A** | **C** | **T** | **T** | **A** | **T** | **C** | **G** | **G** | **A** | **C** | **G** | **A** | **A** | **A** | **C** | **G** | **G** | **T** | **C** | **G** | **A** | **G** | **G** | **A** | **A** | **A** | **G** | **A** | **C** | **A** | **A** | **C** | **T** | **T** | **T** | **A** | **C** | **T** | **G** | **A** | **A** | **T** | **A** | **T** | **T** | **C** | **T** | **A** | **C** | **A** | **A** | **A** | **A** | **C** | **A** | **A** | **A** | **C** | **T** | **A** | **C** | **C** | **T** | **T** | **A** | **T** | **C** | **A** | **A** | **G** | **G** | **G** | **C** | **A** | **A** | **G** | **T** | **A** | **A** | **T** | **C** | **C** | **A** | **T** | **C** | **A** | **G** | **C** | **A** | **A** | **G** | **A** | **A** | **T** | **T** | **T** | **G** | **C** | **C** | **T** | **A** | **T** | **T** | **T** | **C** | **C** | **C** | **G** | **C** | **A** | **A** | **C** | **A** | **G** | **A** | **C** | **A** | **A** | **A** | **A** | **G** | **A** | **T** | **A** | **A** | **A** | **G** | **A** | **A** | **C** | **G** | **T** | **T** | **T** | **A** | **A** | **C** | **C** | **T** | **A** | **T** | **T** | **A** | **C** | **G** | **T** | **G** | **T** | **T** | **A** | **A** | **A** | **T** | **G** | **A** | **T** | **A** | **T** | **T** | **A** | **C** | **G** | **G** | **A** | **T** | **T** | **T** | **T** | **G** | **A** | **G** | **A** | **T** | **A** | **T** | **G** | **G** | **G** | **A** | **A** | **A** | **T** | **C** | **G** | **A** | **A** | **A** | **G** | **A** | **G** | **A** | **G** | **C** | **T** | **C** | **C** | **A** | **A** | **T** | **T** | **G** | **A** | **T** | **G** | **C** | **A** | **A** | **A** | **C** | **A** | **G** | **A** | **T** | **C** | **C** | **T** | **G** | **A** | **A** | **A** | **T** | **C** | **T** | **T** | **A** | **T** | **G** | **G** | **A** | **G** | **A** | **G** | **A** | **A** | **T** | **T** | **C** | **A** | **G** | **C** | **A** | **C** | **A** | **C** | **T** | **A** | **T** | **C** | **G** | **G** | **G** | **G** | **G** | **G** | **A** | **G** | **A** | **G** | **A** | **A** | **G** | **A** | **C** | **A** | **A** | **A** | **A** | **G** | **T** | **C** | **C** | **T** | **A** | **C** | **T** | **G** | **G** | **C** | **A** | **C** | **T** | **T** | **T** | **T** | **A** | **T** | **T** | **T** | **G** | **T** | **G** | **G** | **A** | **T** | **G** | **A** | **C** | **A** | **C** | **T** | **C** | **A** | **T** | **T** | **T** | **C** | **C** | **C** | **G** | **T** | **T** | **A** | **A** | **T** | **C** | **G** | **A** | **T** | **G** | **A** | **A** | **C** | **C** | **A** | **A** | **C** | **G** | **A** | **A** | **T** | **C** | **A** | **T** | **T** | **T** | **G** | **G** | **A** | **-** | **-** | **-** | **T** | **A** | **T** | **C** | **T** | **C** | **T** | **G** | **G** | **T** | **A** | **G** | **A** | **A** | **A** | **A** | **C** | **A** | **A** | **G** | **T** | **A** | **G** | **C** | **G** | **G** | **C** | **T** | **T** | **A** | **T** | **T** | **T** | **G** | **A** | **A** | **A** | **A** | **A** | **G** | **A** | **A** | **A** | **A** | **A** | **A** | **C** | **A** | **A** | **G** | **G** | **C** | **T** | **T** | **C** | **A** | **T** | **C** | **G** | **T** | **G** | **G** | **T** | **C** | **A** | **G** | **C** | **C** | **A** | **T** | **G** | **A** | **C** | **C** | **G** | **G** | **G** | **G** | **A** | **T** | **T** | **T** | **A** | **T** | **C** | **G** | **A** | **T** | **G** | **A** | **A** | **G** | **T** | **A** | **G** | **T** | **G** | **G** | **A** | **C** | **C** | **A** | **T** | **G** | **T** | **T** | **T** | **T** | **A** | **G** | **C** | **A** | **A** | **T** | **C** | **G** | **A** | **A** | **A** | **A** | **A** | **A** | **G** | **T** | **C** | **A** | **A** | **C** | **T** | **G** | **G** | **A** | **A** | **C** | **T** | **T** | **T** | **A** | **T** | **C** | **A** | **A** | **G** | **G** | **G** | **A** | **A** | **T** | **T** | **T** | **C** | **T** | **C** | **T** | **A** | **T** | **C** | **T** | **A** | **T** | **G** | **A** | **A** | **G** | **A** | **A** | **C** | **A** | **G** | **A** | **A** | **A** | **A** | **A** | **A** | **C** | **T** | **T** | **C** | **G** | **T** | **G** | **A** | **T** | **G** | **A** | **A** | **T** | **T** | **T** | **G** | **A** | **A** | **A** | **T** | **G** | **G** | **C** | **T** | **C** | **A** | **A** | **A** | **A** | **T** | **G** | **A** | **A** | **A** | **A** | **A** | **T** | **T** | **G** | **A** | **A** | **A** | **A** | **A** | **A** | **G** | **A** | **A** | **G** | **T** | **C** | **A** | **G** | **T** | **A** | **G** | **G** | **C** | **T** | **A** | **A** | **A** | **G** | **A** | **A** | **A** | **A** | **C** | **A** | **G** | **C** | **A** | **G** | **C** | **T** | **G** | **A** | **A** | **A** | **A** | **A** | **G** | **C** | **C** | **G** | **A** | **A** | **T** | **G** | **G** | **T** | **C** | **T** | **C** | **G** | **T** | **T** | **C** | **C** | **C** | **G** | **A** | **G** | **A** | **A** | **G** | **G** | **A** | **G** | **A** | **T** | **A** | **A** | **A** | **A** | **C** | **A** | **A** | **A** | **G** | **A** | **A** | **A** | **C** | **A** | **A** | **G** | **T** | **C** | **G** | **G** | **A** | **T** | **T** | **C** | **A** | **T** | **C** | **G** | **A** | **T** | **A** | **C** | **T** | **G** | **A** | **A** | **T** | **C** | **T** | **A** | **G** | **A** | **C** | **G** | **A** | **G** | **T** | **G** | **A** | **A** | **T** | **A** | **A** | **A** | **G** | **G** | **A** | **G** | **C** | **A** | **G** | **T** | **G** | **G** | **G** | **T** | **G** | **C** | **T** | **G** | **A** | **T** | **G** | **C** | **T** | **G** | **C** | **A** | **C** | **G** | **G** | **A** | **C** | **G** | **A** | **T** | **G** | **A** | **A** | **A** | **C** | **G** | **A** | **T** | **C** | **C** | **A** | **A** | **A** | **G** | **C** | **A** | **A** | **T** | **C** | **G** | **T** | **G** | **A** | **A** | **T** | **C** | **G** | **G** | **A** | **T** | **G** | **G** | **A** | **G** | **A** | **C** | **C** | **C** | **A** | **G** | **A** | **T** | **C** | **A** | **G** | **C** | **G** | **A** | **G** | **A** | **A** | **A** | **G** | **A** | **A** | **A** | **A** | **A** | **C** | **T** | **A** | **T** | **T** | **A** | **A** | **A** | **A** | **G** | **A** | **T** | **A** | **T** | **C** | **G** | **A** | **A** | **T** | **A** | **T** | **A** | **T** | **C** | **G** | **A** | **T** | **T** | **C** | **T** | **T** | **T** | **G** | **A** | **C** | **G** | **A** | **T** | **G** | **A** | **A** | **T** | **A** | **G** | **C** | **C** | **A** | **A** | **G** | **C** | **G** | **T** | **C** | **T** | **C** | **A** | **C** | **C** | **A** | **T** | **A** | **A** | **G** | **C** | **G** | **A** | **C** | **T** | **T** | **T** | **T** | **A** | **A** | **G** | **C** | **G** | **T** | **A** | **G** | **A** | **A** | **G** | **A** | **T** | **C** | **T** | **T** | **C** | **A** | **A** | **T** | **T** | **A** | **G** | **G** | **G** | **T** | **A** | **T** | **G** | **A** | **A** | **A** | **A** | **T** | **C** | **T** | **G** | **T** | **T** | **A** | **T** | **T** | **C** | **G** | **A** | **G** | **C** | **C** | **A** | **A** | **T** | **T** | **C** | **A** | **T** | **T** | **T** | **T** | **A** | **C** | **A** | **A** | **T** | **C** | **G** | **A** | **G** | **C** | **C** | **T** | **C** | **A** | **T** | **C** | **A** | **G** | **C** | **G** | **G** | **G** | **T** | **G** | **G** | **C** | **G** | **A** | **T** | **T** | **T** | **C** | **A** | **G** | **G** | **T** | **C** | **C** | **T** | **A** | **A** | **C** | **G** | **G** | **T** | **G** | **C** | **A** | **G** | **G** | **A** | **A** | **A** | **G** | **T** | **C** | **A** | **T** | **C** | **C** | **A** | **T** | **T** | **A** | **T** | **C** | **C** | **A** | **T** | **T** | **A** | **T** | **C** | **T** | **T** | **C** | **T** | **G** | **G** | **G** | **G** | **G** | **C** | **A** | **T** | **T** | **C** | **A** | **A** | **C** | **G** | **G** | **C** | **A** | **A** | **G** | **G** | **T** | **T** | **A** | **T** | **A** | **G** | **G** | **A** | **G** | **A** | **A** | **A** | **A** | **A** | **T** | **A** | **C** | **C** | **A** | **G** | **C** | **C** | **A** | **A** | **A** | **A** | **C** | **A** | **T** | **C** | **T** | **G** | **A** | **G** | **C** | **A** | **T** | **T** | **A** | **G** | **T** | **T** | **A** | **T** | **G** | **C** | **A** | **A** | **G** | **C** | **C** | **A** | **A** | **A** | **A** | **T** | **T** | **A** | **T** | **G** | **A** | **A** | **G** | **A** | **C** | **A** | **A** | **T** | **C** | **G** | **A** | **G** | **G** | **A** | **A** | **C** | **G** | **T** | **T** | **G** | **G** | **C** | **G** | **G** | **A** | **A** | **T** | **T** | **T** | **G** | **C** | **A** | **G** | **A** | **G** | **A** | **A** | **A** | **A** | **A** | **C** | **C** | **A** | **A** | **G** | **T** | **A** | **G** | **A** | **C** | **T** | **A** | **C** | **C** | **A** | **A** | **G** | **C** | **A** | **T** | **T** | **T** | **T** | **T** | **G** | **A** | **A** | **C** | **A** | **A** | **C** | **C** | **T** | **C** | **C** | **G** | **A** | **A** | **A** | **G** | **C** | **T** | **T** | **G** | **G** | **G** | **A** | **T** | **G** | **G** | **A** | **A** | **A** | **G** | **A** | **G** | **A** | **T** | **G** | **T** | **T** | **T** | **T** | **T** | **C** | **A** | **T** | **A** | **A** | **C** | **A** | **A** | **G** | **A** | **T** | **C** | **G** | **A** | **G** | **C** | **A** | **G** | **A** | **T** | **G** | **A** | **G** | **T** | **A** | **T** | **G** | **G** | **G** | **C** | **C** | **A** | **A** | **C** | **G** | **G** | **A** | **A** | **A** | **A** | **A** | **A** | **G** | **T** | **G** | **G** | **A** | **A** | **T** | **T** | **G** | **G** | **C** | **T** | **A** | **A** | **A** | **T** | **C** | **T** | **T** | **T** | **A** | **T** | **C** | **A** | **C** | **A** | **G** | **C** | **C** | **A** | **G** | **C** | **T** | **G** | **A** | **A** | **C** | **T** | **A** | **T** | **A** | **T** | **A** | **T** | **A** | **T** | **G** | **G** | **G** | **A** | **T** | **G** | **A** | **A** | **C** | **C** | **A** | **T** | **T** | **G** | **A** | **A** | **T** | **T** | **A** | **T** | **T** | **T** | **G** | **G** | **A** | **T** | **G** | **T** | **C** | **T** | **T** | **C** | **A** | **A** | **T** | **C** | **A** | **A** | **G** | **A** | **A** | **C** | **A** | **A** | **T** | **T** | **A** | **G** | **A** | **A** | **C** | **A** | **A** | **C** | **T** | **G** | **A** | **T** | **C** | **T** | **T** | **G** | **A** | **A** | **C** | **G** | **T** | **G** | **A** | **A** | **A** | **C** | **C** | **T** | **G** | **C** | **C** | **A** | **T** | **G** | **T** | **T** | **A** | **C** | **T** | **A** | **G** | **T** | **G** | **G** | **A** | **A** | **C** | **A** | **T** | **G** | **A** | **T** | **C** | **A** | **A** | **A** | **C** | **C** | **T** | **T** | **C** | **C** | **T** | **G** | **G** | **A** | **T** | **A** | **A** | **A** | **G** | **T** | **A** | **T** | **C** | **T** | **A** | **C** | **T** | **G** | **A** | **G** | **A** | **T** | **T** | **A** | **T** | **T** | **T** | **C** | **T** | **C** | **T** | **T** | **G** | **A** | **G** | **A** | **G** | **A** | **A** | **T** | **C** | **T** | **A** | **A** |
| **N52629** | **A** | **T** | **G** | **T** | **C** | **T** | **A** | **A** | **A** | **A** | **T** | **C** | **G** | **A** | **A** | **A** | **T** | **A** | **A** | **A** | **A** | **A** | **A** | **T** | **C** | **T** | **G** | **A** | **C** | **A** | **T** | **T** | **C** | **G** | **G** | **C** | **T** | **A** | **C** | **G** | **A** | **C** | **A** | **G** | **C** | **C** | **A** | **A** | **G** | **G** | **C** | **A** | **C** | **A** | **T** | **T** | **A** | **T** | **T** | **A** | **T** | **T** | **T** | **G** | **A** | **A** | **C** | **A** | **A** | **G** | **C** | **A** | **A** | **A** | **T** | **C** | **T** | **A** | **A** | **A** | **T** | **T** | **T** | **T** | **G** | **A** | **C** | **A** | **C** | **A** | **C** | **A** | **A** | **T** | **G** | **G** | **A** | **A** | **A** | **C** | **T** | **A** | **G** | **G** | **A** | **C** | **T** | **T** | **A** | **T** | **C** | **A** | **G** | **A** | **C** | **G** | **A** | **A** | **A** | **C** | **G** | **G** | **T** | **C** | **G** | **A** | **G** | **G** | **A** | **A** | **A** | **G** | **A** | **C** | **A** | **A** | **C** | **T** | **T** | **T** | **A** | **C** | **T** | **G** | **A** | **A** | **T** | **A** | **T** | **T** | **C** | **T** | **A** | **C** | **A** | **A** | **A** | **A** | **C** | **A** | **A** | **A** | **C** | **T** | **A** | **C** | **C** | **T** | **T** | **A** | **T** | **C** | **A** | **A** | **G** | **G** | **G** | **C** | **A** | **A** | **G** | **T** | **A** | **A** | **T** | **C** | **C** | **A** | **T** | **C** | **A** | **G** | **C** | **A** | **A** | **G** | **A** | **A** | **T** | **T** | **T** | **G** | **T** | **C** | **T** | **A** | **T** | **T** | **T** | **C** | **C** | **C** | **G** | **C** | **A** | **A** | **C** | **A** | **G** | **A** | **C** | **A** | **A** | **A** | **A** | **G** | **A** | **T** | **A** | **A** | **A** | **G** | **A** | **A** | **C** | **G** | **T** | **T** | **T** | **A** | **A** | **C** | **C** | **T** | **A** | **T** | **T** | **A** | **C** | **G** | **T** | **G** | **T** | **T** | **A** | **A** | **A** | **T** | **G** | **A** | **T** | **A** | **T** | **T** | **A** | **C** | **G** | **G** | **A** | **T** | **T** | **T** | **T** | **G** | **A** | **G** | **A** | **T** | **A** | **T** | **G** | **G** | **G** | **A** | **A** | **A** | **T** | **C** | **G** | **A** | **A** | **A** | **G** | **A** | **G** | **A** | **G** | **C** | **T** | **C** | **C** | **A** | **A** | **T** | **T** | **T** | **A** | **T** | **G** | **C** | **A** | **A** | **A** | **C** | **A** | **G** | **A** | **T** | **C** | **C** | **T** | **G** | **A** | **A** | **A** | **T** | **C** | **T** | **T** | **A** | **T** | **G** | **G** | **A** | **G** | **A** | **G** | **A** | **A** | **T** | **T** | **C** | **A** | **G** | **C** | **A** | **C** | **A** | **C** | **T** | **A** | **T** | **C** | **G** | **G** | **G** | **G** | **G** | **G** | **A** | **G** | **A** | **G** | **A** | **A** | **G** | **A** | **C** | **A** | **A** | **A** | **A** | **G** | **T** | **C** | **C** | **T** | **A** | **T** | **T** | **G** | **G** | **C** | **A** | **C** | **T** | **T** | **T** | **T** | **A** | **T** | **T** | **T** | **G** | **T** | **G** | **G** | **A** | **T** | **G** | **A** | **C** | **A** | **C** | **T** | **C** | **A** | **T** | **T** | **T** | **C** | **C** | **C** | **G** | **T** | **T** | **A** | **A** | **T** | **C** | **G** | **A** | **T** | **G** | **A** | **A** | **C** | **C** | **A** | **A** | **C** | **G** | **A** | **A** | **T** | **C** | **A** | **T** | **T** | **T** | **G** | **G** | **A** | **-** | **-** | **-** | **T** | **A** | **T** | **C** | **T** | **C** | **T** | **G** | **G** | **T** | **A** | **G** | **A** | **A** | **A** | **A** | **C** | **A** | **A** | **G** | **T** | **A** | **G** | **C** | **G** | **G** | **C** | **T** | **T** | **A** | **T** | **T** | **T** | **G** | **A** | **A** | **A** | **A** | **A** | **G** | **A** | **A** | **A** | **A** | **A** | **A** | **C** | **A** | **A** | **G** | **G** | **C** | **T** | **T** | **C** | **A** | **T** | **C** | **G** | **T** | **G** | **G** | **T** | **C** | **A** | **G** | **C** | **C** | **A** | **T** | **G** | **A** | **C** | **C** | **G** | **G** | **G** | **G** | **A** | **T** | **T** | **T** | **A** | **T** | **C** | **G** | **A** | **T** | **G** | **A** | **A** | **G** | **T** | **A** | **G** | **T** | **G** | **G** | **A** | **C** | **C** | **A** | **T** | **G** | **T** | **T** | **T** | **T** | **A** | **G** | **C** | **A** | **A** | **T** | **C** | **G** | **A** | **A** | **A** | **A** | **A** | **A** | **G** | **T** | **C** | **A** | **A** | **C** | **T** | **G** | **G** | **A** | **A** | **C** | **T** | **T** | **T** | **A** | **T** | **C** | **A** | **A** | **G** | **G** | **G** | **A** | **A** | **T** | **T** | **T** | **C** | **T** | **C** | **T** | **A** | **T** | **C** | **T** | **A** | **T** | **G** | **A** | **A** | **G** | **A** | **A** | **C** | **A** | **G** | **A** | **A** | **A** | **A** | **A** | **A** | **C** | **T** | **T** | **C** | **G** | **T** | **G** | **A** | **T** | **G** | **A** | **A** | **T** | **T** | **T** | **G** | **A** | **A** | **A** | **T** | **G** | **G** | **C** | **T** | **C** | **A** | **A** | **A** | **A** | **T** | **G** | **A** | **A** | **A** | **A** | **A** | **T** | **T** | **G** | **A** | **A** | **A** | **A** | **A** | **A** | **G** | **A** | **A** | **G** | **T** | **C** | **A** | **G** | **T** | **A** | **G** | **G** | **C** | **T** | **A** | **A** | **A** | **G** | **A** | **A** | **A** | **A** | **C** | **A** | **G** | **C** | **A** | **G** | **C** | **T** | **G** | **A** | **A** | **A** | **A** | **A** | **G** | **C** | **C** | **G** | **A** | **A** | **T** | **G** | **G** | **T** | **C** | **T** | **C** | **G** | **T** | **T** | **C** | **C** | **C** | **G** | **A** | **G** | **A** | **A** | **G** | **G** | **A** | **G** | **A** | **T** | **A** | **A** | **A** | **A** | **C** | **A** | **A** | **A** | **G** | **A** | **A** | **A** | **C** | **A** | **A** | **G** | **T** | **C** | **G** | **G** | **A** | **T** | **T** | **C** | **A** | **T** | **C** | **G** | **A** | **T** | **A** | **C** | **T** | **G** | **A** | **A** | **T** | **C** | **T** | **A** | **G** | **A** | **C** | **G** | **A** | **G** | **T** | **G** | **A** | **A** | **T** | **A** | **A** | **A** | **G** | **G** | **A** | **G** | **C** | **A** | **G** | **T** | **G** | **G** | **G** | **T** | **G** | **C** | **T** | **G** | **A** | **T** | **G** | **C** | **T** | **G** | **C** | **A** | **C** | **G** | **G** | **G** | **C** | **G** | **A** | **T** | **G** | **A** | **A** | **A** | **C** | **G** | **A** | **T** | **C** | **C** | **A** | **A** | **A** | **G** | **C** | **A** | **A** | **T** | **C** | **G** | **T** | **G** | **A** | **A** | **T** | **C** | **G** | **G** | **A** | **T** | **G** | **G** | **A** | **G** | **A** | **C** | **C** | **C** | **A** | **G** | **A** | **T** | **C** | **A** | **G** | **C** | **G** | **A** | **G** | **A** | **A** | **A** | **G** | **A** | **A** | **A** | **A** | **A** | **C** | **T** | **A** | **T** | **T** | **A** | **A** | **A** | **A** | **G** | **A** | **T** | **A** | **T** | **C** | **G** | **A** | **A** | **T** | **A** | **T** | **A** | **T** | **C** | **G** | **A** | **T** | **T** | **C** | **T** | **T** | **T** | **G** | **A** | **C** | **G** | **A** | **T** | **G** | **A** | **A** | **T** | **A** | **G** | **C** | **C** | **A** | **A** | **G** | **C** | **G** | **T** | **C** | **T** | **C** | **A** | **C** | **C** | **A** | **T** | **A** | **A** | **G** | **C** | **G** | **A** | **C** | **T** | **T** | **T** | **T** | **A** | **A** | **G** | **C** | **G** | **T** | **A** | **G** | **A** | **A** | **G** | **A** | **T** | **C** | **T** | **T** | **C** | **A** | **A** | **T** | **T** | **A** | **G** | **G** | **G** | **T** | **A** | **T** | **G** | **A** | **A** | **A** | **A** | **T** | **C** | **T** | **G** | **T** | **T** | **A** | **T** | **T** | **C** | **G** | **A** | **G** | **C** | **C** | **A** | **A** | **T** | **T** | **C** | **A** | **T** | **T** | **T** | **T** | **A** | **C** | **A** | **A** | **T** | **C** | **G** | **A** | **G** | **C** | **C** | **T** | **C** | **A** | **T** | **C** | **A** | **G** | **C** | **G** | **G** | **G** | **T** | **G** | **G** | **C** | **G** | **A** | **T** | **T** | **T** | **C** | **A** | **G** | **G** | **T** | **C** | **C** | **T** | **A** | **A** | **C** | **G** | **G** | **T** | **G** | **C** | **A** | **G** | **G** | **A** | **A** | **A** | **G** | **T** | **C** | **A** | **T** | **C** | **C** | **A** | **T** | **T** | **A** | **T** | **C** | **C** | **A** | **T** | **T** | **A** | **T** | **C** | **T** | **T** | **C** | **T** | **G** | **G** | **G** | **G** | **G** | **C** | **A** | **T** | **T** | **C** | **A** | **A** | **C** | **G** | **G** | **C** | **A** | **A** | **G** | **G** | **T** | **T** | **A** | **T** | **T** | **G** | **G** | **A** | **G** | **A** | **A** | **A** | **A** | **A** | **T** | **C** | **C** | **C** | **A** | **G** | **C** | **C** | **A** | **A** | **A** | **A** | **C** | **A** | **T** | **C** | **T** | **G** | **A** | **G** | **C** | **A** | **T** | **T** | **A** | **G** | **T** | **T** | **A** | **T** | **G** | **T** | **A** | **C** | **G** | **C** | **C** | **A** | **A** | **A** | **A** | **T** | **T** | **A** | **T** | **G** | **A** | **A** | **G** | **A** | **C** | **A** | **A** | **T** | **C** | **G** | **A** | **G** | **G** | **A** | **A** | **C** | **G** | **T** | **T** | **G** | **G** | **C** | **G** | **G** | **A** | **A** | **T** | **T** | **T** | **G** | **C** | **A** | **G** | **A** | **G** | **A** | **A** | **A** | **A** | **A** | **C** | **C** | **A** | **A** | **G** | **T** | **A** | **G** | **A** | **C** | **T** | **A** | **C** | **C** | **A** | **A** | **G** | **C** | **A** | **T** | **T** | **T** | **T** | **T** | **G** | **A** | **A** | **C** | **A** | **A** | **C** | **C** | **T** | **C** | **C** | **G** | **A** | **A** | **A** | **G** | **C** | **T** | **T** | **G** | **G** | **G** | **A** | **T** | **G** | **G** | **A** | **A** | **A** | **G** | **A** | **G** | **A** | **T** | **G** | **T** | **T** | **T** | **T** | **T** | **C** | **A** | **T** | **A** | **A** | **C** | **A** | **A** | **G** | **A** | **T** | **T** | **G** | **A** | **G** | **C** | **A** | **G** | **A** | **T** | **G** | **A** | **G** | **T** | **A** | **T** | **G** | **G** | **G** | **C** | **C** | **A** | **A** | **C** | **G** | **G** | **A** | **A** | **A** | **A** | **A** | **A** | **G** | **T** | **G** | **G** | **A** | **A** | **T** | **T** | **G** | **G** | **C** | **T** | **A** | **A** | **A** | **T** | **C** | **T** | **T** | **T** | **A** | **T** | **C** | **A** | **C** | **A** | **A** | **C** | **C** | **A** | **G** | **C** | **T** | **G** | **A** | **A** | **C** | **T** | **A** | **T** | **A** | **T** | **A** | **T** | **A** | **T** | **G** | **G** | **G** | **A** | **T** | **G** | **A** | **A** | **C** | **C** | **A** | **T** | **T** | **G** | **A** | **A** | **T** | **T** | **A** | **T** | **T** | **T** | **G** | **G** | **A** | **T** | **G** | **T** | **C** | **T** | **T** | **C** | **A** | **A** | **T** | **C** | **A** | **A** | **G** | **A** | **A** | **C** | **A** | **A** | **T** | **T** | **A** | **G** | **A** | **A** | **C** | **A** | **A** | **C** | **T** | **G** | **A** | **T** | **C** | **T** | **T** | **G** | **A** | **A** | **C** | **G** | **T** | **G** | **A** | **A** | **A** | **C** | **C** | **T** | **G** | **C** | **C** | **A** | **T** | **G** | **T** | **T** | **A** | **C** | **T** | **G** | **G** | **T** | **G** | **G** | **A** | **A** | **C** | **A** | **T** | **G** | **A** | **T** | **C** | **A** | **A** | **A** | **C** | **C** | **T** | **T** | **C** | **C** | **T** | **G** | **G** | **A** | **T** | **A** | **A** | **A** | **G** | **T** | **A** | **T** | **C** | **T** | **A** | **C** | **T** | **G** | **A** | **G** | **A** | **T** | **T** | **A** | **T** | **T** | **T** | **C** | **T** | **C** | **T** | **T** | **G** | **A** | **G** | **A** | **A** | **A** | **A** | **T** | **C** | **-** | **-** | **-** |
| **N52824** | **A** | **T** | **G** | **T** | **C** | **T** | **A** | **A** | **A** | **A** | **T** | **C** | **G** | **A** | **A** | **A** | **T** | **A** | **A** | **A** | **A** | **A** | **A** | **T** | **C** | **T** | **G** | **A** | **C** | **A** | **T** | **T** | **C** | **G** | **G** | **C** | **T** | **A** | **C** | **G** | **A** | **C** | **A** | **G** | **C** | **C** | **A** | **A** | **G** | **G** | **C** | **A** | **C** | **A** | **T** | **T** | **A** | **T** | **T** | **A** | **T** | **T** | **T** | **G** | **A** | **A** | **C** | **A** | **A** | **G** | **C** | **A** | **A** | **A** | **T** | **C** | **T** | **A** | **A** | **A** | **T** | **T** | **T** | **T** | **G** | **A** | **C** | **A** | **C** | **A** | **C** | **A** | **A** | **T** | **G** | **G** | **A** | **A** | **A** | **C** | **T** | **A** | **G** | **G** | **A** | **C** | **T** | **T** | **A** | **T** | **C** | **G** | **G** | **A** | **C** | **G** | **A** | **A** | **A** | **C** | **G** | **G** | **T** | **C** | **G** | **A** | **G** | **G** | **A** | **A** | **A** | **G** | **A** | **C** | **A** | **A** | **C** | **T** | **T** | **T** | **A** | **C** | **T** | **G** | **A** | **A** | **T** | **A** | **T** | **T** | **C** | **T** | **A** | **C** | **A** | **A** | **A** | **A** | **C** | **A** | **A** | **A** | **C** | **T** | **A** | **C** | **C** | **T** | **T** | **A** | **T** | **C** | **A** | **A** | **G** | **G** | **G** | **C** | **A** | **A** | **G** | **T** | **A** | **A** | **T** | **C** | **C** | **A** | **T** | **C** | **A** | **G** | **C** | **A** | **A** | **G** | **A** | **A** | **T** | **T** | **T** | **G** | **T** | **C** | **T** | **A** | **T** | **T** | **T** | **C** | **C** | **C** | **G** | **C** | **A** | **G** | **C** | **A** | **A** | **A** | **C** | **A** | **A** | **A** | **A** | **G** | **A** | **T** | **A** | **A** | **A** | **G** | **A** | **G** | **C** | **G** | **T** | **T** | **T** | **A** | **A** | **C** | **C** | **T** | **A** | **T** | **T** | **A** | **T** | **G** | **T** | **G** | **T** | **T** | **A** | **A** | **A** | **T** | **G** | **A** | **G** | **A** | **T** | **T** | **A** | **C** | **G** | **G** | **A** | **T** | **T** | **T** | **T** | **G** | **A** | **G** | **A** | **T** | **A** | **T** | **G** | **G** | **G** | **A** | **A** | **A** | **T** | **T** | **G** | **A** | **A** | **A** | **G** | **A** | **G** | **A** | **G** | **C** | **T** | **C** | **C** | **A** | **A** | **C** | **T** | **G** | **A** | **T** | **G** | **C** | **A** | **A** | **A** | **C** | **A** | **G** | **A** | **T** | **C** | **C** | **T** | **G** | **A** | **A** | **A** | **T** | **C** | **T** | **T** | **A** | **T** | **G** | **G** | **A** | **G** | **A** | **G** | **A** | **A** | **T** | **T** | **C** | **A** | **G** | **C** | **A** | **C** | **A** | **C** | **T** | **A** | **T** | **C** | **A** | **G** | **G** | **A** | **G** | **G** | **A** | **G** | **A** | **G** | **A** | **A** | **G** | **A** | **C** | **A** | **A** | **A** | **A** | **G** | **T** | **C** | **C** | **T** | **A** | **C** | **T** | **G** | **G** | **C** | **A** | **C** | **T** | **T** | **T** | **T** | **A** | **T** | **T** | **T** | **G** | **T** | **G** | **G** | **A** | **T** | **G** | **A** | **C** | **A** | **C** | **T** | **C** | **A** | **T** | **T** | **T** | **C** | **C** | **C** | **G** | **T** | **T** | **A** | **A** | **T** | **C** | **G** | **A** | **T** | **G** | **A** | **A** | **C** | **C** | **A** | **A** | **C** | **G** | **A** | **A** | **T** | **C** | **A** | **T** | **T** | **T** | **G** | **G** | **A** | **-** | **-** | **-** | **T** | **A** | **T** | **C** | **T** | **C** | **T** | **G** | **G** | **C** | **A** | **G** | **A** | **A** | **A** | **A** | **C** | **A** | **A** | **G** | **T** | **A** | **G** | **C** | **G** | **G** | **C** | **T** | **T** | **A** | **C** | **T** | **T** | **G** | **A** | **A** | **A** | **A** | **A** | **G** | **A** | **A** | **A** | **A** | **A** | **A** | **C** | **A** | **A** | **G** | **G** | **C** | **T** | **T** | **C** | **A** | **T** | **C** | **G** | **T** | **G** | **G** | **T** | **C** | **A** | **G** | **C** | **C** | **A** | **T** | **G** | **A** | **C** | **C** | **G** | **G** | **G** | **G** | **A** | **T** | **T** | **T** | **A** | **T** | **C** | **G** | **A** | **T** | **G** | **A** | **A** | **G** | **T** | **A** | **G** | **T** | **G** | **G** | **A** | **C** | **C** | **A** | **T** | **G** | **T** | **T** | **T** | **T** | **A** | **G** | **C** | **A** | **A** | **T** | **C** | **G** | **A** | **A** | **A** | **A** | **A** | **A** | **G** | **T** | **C** | **A** | **A** | **C** | **T** | **G** | **G** | **A** | **A** | **C** | **T** | **T** | **T** | **A** | **T** | **C** | **A** | **A** | **G** | **G** | **G** | **A** | **A** | **T** | **T** | **T** | **C** | **T** | **C** | **T** | **A** | **T** | **C** | **T** | **A** | **T** | **G** | **A** | **A** | **G** | **A** | **A** | **C** | **A** | **G** | **A** | **A** | **A** | **A** | **A** | **A** | **C** | **T** | **T** | **C** | **G** | **T** | **G** | **A** | **T** | **G** | **A** | **A** | **T** | **T** | **T** | **G** | **A** | **A** | **A** | **T** | **G** | **G** | **C** | **T** | **C** | **A** | **A** | **A** | **A** | **T** | **G** | **A** | **A** | **A** | **A** | **A** | **T** | **T** | **G** | **A** | **A** | **A** | **A** | **A** | **A** | **G** | **A** | **A** | **G** | **T** | **C** | **A** | **G** | **T** | **A** | **G** | **G** | **C** | **T** | **A** | **A** | **A** | **G** | **A** | **A** | **A** | **A** | **C** | **A** | **G** | **C** | **A** | **G** | **C** | **T** | **G** | **A** | **A** | **A** | **A** | **A** | **G** | **C** | **C** | **G** | **A** | **A** | **T** | **G** | **G** | **T** | **C** | **T** | **C** | **G** | **T** | **T** | **C** | **C** | **C** | **G** | **A** | **G** | **A** | **A** | **G** | **G** | **A** | **G** | **A** | **T** | **A** | **A** | **A** | **A** | **C** | **A** | **A** | **A** | **G** | **A** | **A** | **A** | **C** | **A** | **A** | **G** | **T** | **C** | **G** | **G** | **A** | **T** | **T** | **C** | **A** | **T** | **C** | **G** | **A** | **T** | **A** | **C** | **T** | **G** | **A** | **A** | **T** | **C** | **T** | **A** | **G** | **A** | **C** | **G** | **A** | **G** | **T** | **G** | **A** | **A** | **T** | **A** | **A** | **A** | **G** | **G** | **A** | **G** | **C** | **A** | **G** | **T** | **G** | **G** | **G** | **T** | **G** | **C** | **T** | **G** | **A** | **T** | **G** | **C** | **T** | **G** | **C** | **A** | **C** | **G** | **G** | **A** | **C** | **G** | **A** | **T** | **G** | **A** | **A** | **A** | **C** | **G** | **A** | **T** | **C** | **C** | **A** | **A** | **A** | **G** | **C** | **A** | **A** | **T** | **C** | **G** | **T** | **G** | **A** | **A** | **T** | **C** | **G** | **G** | **A** | **T** | **G** | **G** | **A** | **G** | **A** | **C** | **C** | **C** | **A** | **G** | **A** | **T** | **C** | **A** | **G** | **C** | **G** | **A** | **G** | **A** | **A** | **A** | **G** | **A** | **A** | **A** | **A** | **A** | **C** | **T** | **A** | **T** | **T** | **G** | **A** | **A** | **A** | **G** | **A** | **T** | **A** | **T** | **C** | **G** | **A** | **A** | **T** | **A** | **T** | **A** | **T** | **C** | **G** | **A** | **T** | **T** | **C** | **T** | **T** | **T** | **G** | **A** | **C** | **G** | **A** | **T** | **G** | **A** | **A** | **T** | **A** | **G** | **C** | **C** | **A** | **A** | **G** | **C** | **G** | **T** | **C** | **T** | **C** | **A** | **C** | **C** | **A** | **T** | **A** | **A** | **G** | **C** | **G** | **A** | **C** | **T** | **T** | **T** | **T** | **A** | **A** | **G** | **C** | **G** | **T** | **A** | **G** | **A** | **A** | **G** | **A** | **T** | **C** | **T** | **T** | **C** | **A** | **A** | **T** | **T** | **A** | **G** | **G** | **G** | **T** | **A** | **T** | **G** | **A** | **G** | **A** | **A** | **T** | **C** | **T** | **G** | **T** | **T** | **A** | **T** | **T** | **C** | **G** | **A** | **G** | **C** | **C** | **A** | **A** | **T** | **T** | **C** | **A** | **T** | **T** | **T** | **T** | **A** | **C** | **A** | **A** | **T** | **C** | **G** | **A** | **G** | **C** | **C** | **T** | **C** | **A** | **T** | **C** | **A** | **G** | **C** | **G** | **G** | **G** | **T** | **G** | **G** | **C** | **G** | **A** | **T** | **T** | **T** | **C** | **A** | **G** | **G** | **T** | **C** | **C** | **T** | **A** | **A** | **C** | **G** | **G** | **T** | **G** | **C** | **A** | **G** | **G** | **A** | **A** | **A** | **G** | **T** | **C** | **A** | **T** | **C** | **C** | **A** | **T** | **T** | **A** | **T** | **C** | **C** | **A** | **T** | **T** | **A** | **T** | **C** | **T** | **T** | **C** | **T** | **G** | **G** | **G** | **G** | **G** | **C** | **A** | **T** | **T** | **C** | **A** | **A** | **C** | **G** | **G** | **C** | **A** | **A** | **G** | **G** | **T** | **T** | **A** | **T** | **A** | **G** | **G** | **A** | **G** | **A** | **A** | **A** | **A** | **A** | **T** | **C** | **C** | **C** | **A** | **G** | **C** | **C** | **A** | **A** | **A** | **A** | **C** | **A** | **T** | **C** | **T** | **G** | **A** | **G** | **C** | **A** | **T** | **T** | **A** | **G** | **T** | **T** | **A** | **T** | **G** | **T** | **A** | **C** | **G** | **C** | **C** | **A** | **A** | **A** | **A** | **T** | **T** | **A** | **T** | **G** | **A** | **A** | **G** | **A** | **C** | **A** | **A** | **T** | **C** | **G** | **A** | **G** | **G** | **A** | **A** | **C** | **G** | **T** | **T** | **G** | **G** | **C** | **G** | **G** | **A** | **A** | **T** | **T** | **T** | **G** | **C** | **A** | **G** | **A** | **G** | **A** | **A** | **A** | **A** | **A** | **C** | **C** | **A** | **A** | **G** | **T** | **A** | **G** | **A** | **C** | **T** | **A** | **C** | **C** | **A** | **A** | **G** | **C** | **A** | **T** | **T** | **T** | **T** | **T** | **G** | **A** | **A** | **C** | **A** | **A** | **C** | **C** | **T** | **C** | **C** | **G** | **A** | **A** | **A** | **G** | **C** | **T** | **T** | **G** | **G** | **G** | **A** | **T** | **G** | **G** | **A** | **A** | **A** | **G** | **A** | **G** | **A** | **T** | **G** | **T** | **T** | **T** | **T** | **T** | **C** | **A** | **T** | **A** | **A** | **C** | **A** | **A** | **G** | **A** | **T** | **C** | **G** | **A** | **G** | **C** | **A** | **G** | **A** | **T** | **G** | **A** | **G** | **T** | **A** | **T** | **G** | **G** | **G** | **C** | **C** | **A** | **A** | **C** | **G** | **G** | **A** | **A** | **A** | **A** | **A** | **A** | **G** | **T** | **G** | **G** | **A** | **A** | **T** | **T** | **G** | **G** | **C** | **T** | **A** | **A** | **A** | **T** | **C** | **T** | **T** | **T** | **A** | **T** | **C** | **A** | **C** | **A** | **G** | **C** | **C** | **A** | **G** | **C** | **T** | **G** | **A** | **A** | **C** | **T** | **A** | **T** | **A** | **T** | **A** | **T** | **A** | **T** | **G** | **G** | **G** | **A** | **T** | **G** | **A** | **A** | **C** | **C** | **A** | **T** | **T** | **G** | **A** | **A** | **T** | **T** | **A** | **T** | **T** | **T** | **G** | **G** | **A** | **T** | **G** | **T** | **C** | **T** | **T** | **C** | **A** | **A** | **T** | **C** | **A** | **A** | **G** | **A** | **A** | **C** | **A** | **A** | **T** | **T** | **A** | **G** | **A** | **A** | **C** | **A** | **A** | **C** | **T** | **G** | **A** | **T** | **C** | **T** | **T** | **G** | **A** | **A** | **C** | **G** | **T** | **G** | **A** | **A** | **A** | **C** | **C** | **T** | **G** | **C** | **C** | **A** | **T** | **G** | **T** | **T** | **A** | **C** | **T** | **A** | **G** | **T** | **G** | **G** | **A** | **A** | **C** | **A** | **T** | **G** | **A** | **T** | **C** | **A** | **A** | **A** | **C** | **C** | **T** | **T** | **C** | **C** | **T** | **G** | **G** | **A** | **T** | **A** | **A** | **A** | **G** | **T** | **A** | **T** | **C** | **T** | **A** | **C** | **T** | **G** | **A** | **G** | **A** | **T** | **T** | **A** | **T** | **T** | **T** | **C** | **T** | **C** | **T** | **T** | **G** | **A** | **G** | **A** | **A** | **A** | **A** | **T** | **C** | **-** | **-** | **-** |
| **N59559F** | **A** | **T** | **G** | **T** | **C** | **T** | **A** | **A** | **A** | **A** | **T** | **C** | **G** | **A** | **A** | **A** | **T** | **A** | **A** | **A** | **A** | **A** | **A** | **T** | **C** | **T** | **G** | **A** | **C** | **A** | **T** | **T** | **C** | **G** | **G** | **C** | **T** | **A** | **C** | **G** | **A** | **C** | **A** | **G** | **C** | **C** | **A** | **A** | **G** | **G** | **C** | **A** | **C** | **A** | **T** | **T** | **A** | **T** | **T** | **A** | **T** | **T** | **T** | **G** | **A** | **A** | **C** | **A** | **A** | **G** | **C** | **A** | **A** | **A** | **T** | **C** | **T** | **A** | **A** | **A** | **T** | **T** | **T** | **T** | **G** | **A** | **C** | **A** | **C** | **A** | **C** | **A** | **A** | **T** | **G** | **G** | **A** | **A** | **A** | **C** | **T** | **A** | **G** | **G** | **A** | **C** | **T** | **T** | **A** | **T** | **C** | **G** | **G** | **A** | **C** | **G** | **A** | **A** | **A** | **C** | **G** | **G** | **T** | **C** | **G** | **A** | **G** | **G** | **A** | **A** | **A** | **G** | **A** | **C** | **A** | **A** | **C** | **T** | **T** | **T** | **A** | **C** | **T** | **G** | **A** | **A** | **T** | **A** | **T** | **T** | **C** | **T** | **A** | **C** | **A** | **A** | **A** | **A** | **C** | **A** | **A** | **A** | **C** | **T** | **A** | **C** | **C** | **T** | **T** | **A** | **T** | **C** | **A** | **A** | **G** | **G** | **G** | **C** | **A** | **A** | **G** | **T** | **A** | **A** | **T** | **C** | **C** | **A** | **T** | **C** | **A** | **G** | **C** | **A** | **A** | **G** | **A** | **A** | **T** | **T** | **T** | **G** | **T** | **C** | **T** | **A** | **T** | **T** | **T** | **C** | **C** | **C** | **G** | **C** | **A** | **A** | **C** | **A** | **G** | **A** | **C** | **A** | **A** | **A** | **A** | **G** | **A** | **T** | **A** | **A** | **A** | **G** | **A** | **A** | **C** | **G** | **T** | **T** | **T** | **A** | **A** | **C** | **C** | **T** | **A** | **T** | **T** | **A** | **C** | **G** | **T** | **G** | **T** | **T** | **A** | **A** | **A** | **T** | **G** | **A** | **T** | **A** | **T** | **T** | **A** | **C** | **G** | **G** | **A** | **T** | **T** | **T** | **T** | **G** | **A** | **G** | **A** | **T** | **A** | **T** | **G** | **G** | **G** | **A** | **A** | **A** | **T** | **C** | **G** | **A** | **A** | **A** | **G** | **A** | **G** | **A** | **G** | **C** | **T** | **C** | **C** | **A** | **A** | **T** | **T** | **G** | **A** | **T** | **A** | **C** | **A** | **A** | **A** | **C** | **A** | **G** | **A** | **T** | **C** | **C** | **T** | **G** | **A** | **A** | **A** | **T** | **C** | **T** | **T** | **A** | **T** | **G** | **G** | **A** | **G** | **A** | **G** | **A** | **A** | **T** | **T** | **C** | **A** | **G** | **C** | **A** | **C** | **A** | **C** | **T** | **A** | **T** | **C** | **G** | **G** | **G** | **G** | **G** | **G** | **A** | **G** | **A** | **G** | **A** | **A** | **G** | **A** | **C** | **A** | **A** | **A** | **A** | **G** | **T** | **C** | **C** | **T** | **A** | **C** | **T** | **G** | **G** | **C** | **A** | **C** | **T** | **T** | **T** | **T** | **A** | **T** | **T** | **T** | **G** | **T** | **G** | **G** | **A** | **T** | **G** | **A** | **C** | **A** | **C** | **T** | **C** | **A** | **T** | **T** | **T** | **C** | **C** | **C** | **G** | **T** | **T** | **A** | **A** | **T** | **C** | **G** | **A** | **T** | **G** | **A** | **A** | **C** | **C** | **A** | **A** | **C** | **G** | **A** | **A** | **T** | **C** | **A** | **T** | **T** | **T** | **G** | **G** | **A** | **-** | **-** | **-** | **T** | **A** | **T** | **C** | **T** | **C** | **T** | **G** | **G** | **T** | **A** | **G** | **A** | **A** | **A** | **A** | **C** | **A** | **A** | **G** | **T** | **A** | **G** | **C** | **G** | **G** | **C** | **T** | **T** | **A** | **T** | **T** | **T** | **G** | **A** | **A** | **A** | **A** | **A** | **G** | **A** | **A** | **A** | **A** | **A** | **A** | **C** | **A** | **A** | **G** | **G** | **C** | **T** | **T** | **C** | **A** | **T** | **C** | **G** | **T** | **G** | **G** | **T** | **C** | **A** | **G** | **C** | **C** | **A** | **T** | **G** | **A** | **C** | **C** | **G** | **G** | **G** | **G** | **A** | **T** | **T** | **T** | **A** | **T** | **C** | **G** | **A** | **T** | **G** | **A** | **A** | **G** | **T** | **A** | **G** | **T** | **G** | **G** | **A** | **C** | **C** | **A** | **T** | **G** | **T** | **T** | **T** | **T** | **A** | **G** | **C** | **A** | **A** | **T** | **C** | **G** | **A** | **A** | **A** | **A** | **A** | **A** | **G** | **T** | **C** | **A** | **A** | **C** | **T** | **G** | **G** | **A** | **A** | **C** | **T** | **T** | **T** | **A** | **T** | **C** | **A** | **A** | **G** | **G** | **G** | **A** | **A** | **T** | **T** | **T** | **C** | **T** | **C** | **T** | **A** | **T** | **C** | **T** | **A** | **T** | **G** | **A** | **A** | **G** | **A** | **A** | **C** | **A** | **G** | **A** | **A** | **A** | **A** | **A** | **A** | **C** | **T** | **T** | **C** | **G** | **T** | **G** | **A** | **T** | **G** | **A** | **A** | **T** | **T** | **T** | **G** | **A** | **A** | **A** | **T** | **G** | **G** | **C** | **T** | **C** | **A** | **A** | **A** | **A** | **T** | **G** | **A** | **A** | **A** | **A** | **A** | **T** | **T** | **G** | **A** | **A** | **A** | **A** | **A** | **A** | **G** | **A** | **A** | **G** | **T** | **C** | **A** | **G** | **T** | **A** | **G** | **G** | **C** | **T** | **A** | **A** | **A** | **G** | **A** | **A** | **A** | **A** | **C** | **A** | **G** | **C** | **A** | **G** | **C** | **T** | **G** | **A** | **A** | **A** | **A** | **A** | **G** | **C** | **C** | **G** | **A** | **A** | **T** | **G** | **G** | **T** | **C** | **T** | **C** | **G** | **T** | **T** | **C** | **C** | **C** | **G** | **A** | **G** | **A** | **A** | **G** | **G** | **A** | **G** | **A** | **T** | **A** | **A** | **A** | **A** | **C** | **A** | **A** | **A** | **G** | **A** | **A** | **A** | **C** | **A** | **A** | **G** | **T** | **C** | **G** | **G** | **A** | **T** | **T** | **C** | **A** | **T** | **C** | **G** | **A** | **T** | **A** | **C** | **T** | **G** | **A** | **A** | **T** | **C** | **T** | **A** | **G** | **A** | **C** | **G** | **A** | **G** | **T** | **G** | **A** | **A** | **T** | **A** | **A** | **A** | **G** | **G** | **A** | **G** | **C** | **A** | **G** | **T** | **G** | **G** | **G** | **T** | **G** | **C** | **T** | **G** | **A** | **T** | **G** | **C** | **T** | **G** | **C** | **A** | **C** | **G** | **G** | **A** | **C** | **G** | **A** | **T** | **G** | **A** | **A** | **A** | **C** | **G** | **A** | **T** | **C** | **C** | **A** | **A** | **A** | **G** | **C** | **A** | **A** | **T** | **C** | **G** | **T** | **G** | **A** | **A** | **T** | **C** | **G** | **G** | **A** | **T** | **G** | **G** | **A** | **G** | **A** | **C** | **C** | **C** | **A** | **G** | **A** | **T** | **C** | **A** | **G** | **C** | **G** | **A** | **G** | **A** | **A** | **A** | **G** | **A** | **A** | **A** | **A** | **A** | **C** | **T** | **A** | **T** | **T** | **A** | **A** | **A** | **A** | **G** | **A** | **T** | **A** | **T** | **C** | **G** | **A** | **A** | **T** | **A** | **T** | **A** | **T** | **C** | **G** | **A** | **T** | **T** | **C** | **T** | **T** | **T** | **G** | **A** | **C** | **G** | **A** | **T** | **G** | **A** | **A** | **T** | **A** | **G** | **C** | **C** | **A** | **A** | **G** | **C** | **G** | **T** | **C** | **T** | **C** | **A** | **C** | **C** | **A** | **T** | **A** | **A** | **G** | **C** | **G** | **A** | **C** | **T** | **T** | **T** | **T** | **A** | **A** | **G** | **C** | **G** | **T** | **A** | **G** | **A** | **A** | **G** | **A** | **T** | **C** | **T** | **T** | **C** | **A** | **A** | **T** | **T** | **A** | **G** | **G** | **G** | **T** | **A** | **T** | **G** | **A** | **A** | **A** | **A** | **T** | **C** | **T** | **G** | **T** | **T** | **A** | **T** | **T** | **C** | **G** | **A** | **G** | **C** | **C** | **A** | **A** | **T** | **T** | **C** | **A** | **T** | **T** | **T** | **T** | **A** | **C** | **A** | **A** | **T** | **C** | **G** | **A** | **G** | **C** | **C** | **T** | **C** | **A** | **T** | **C** | **A** | **G** | **C** | **G** | **G** | **G** | **T** | **G** | **G** | **C** | **G** | **A** | **T** | **T** | **T** | **C** | **A** | **G** | **G** | **T** | **C** | **C** | **T** | **A** | **A** | **C** | **G** | **G** | **T** | **G** | **C** | **A** | **G** | **G** | **A** | **A** | **A** | **G** | **T** | **C** | **A** | **T** | **C** | **C** | **A** | **T** | **T** | **A** | **T** | **C** | **C** | **A** | **T** | **T** | **A** | **T** | **C** | **T** | **T** | **C** | **T** | **G** | **T** | **G** | **G** | **G** | **C** | **A** | **T** | **T** | **C** | **A** | **A** | **C** | **G** | **G** | **C** | **A** | **A** | **G** | **G** | **T** | **T** | **A** | **T** | **A** | **G** | **G** | **A** | **G** | **A** | **A** | **A** | **A** | **A** | **T** | **C** | **C** | **C** | **A** | **G** | **C** | **C** | **A** | **A** | **A** | **A** | **C** | **A** | **T** | **C** | **T** | **G** | **A** | **G** | **C** | **A** | **T** | **T** | **A** | **G** | **T** | **T** | **A** | **T** | **G** | **T** | **A** | **C** | **G** | **C** | **C** | **A** | **A** | **A** | **A** | **T** | **T** | **A** | **T** | **G** | **A** | **A** | **G** | **A** | **C** | **A** | **A** | **T** | **C** | **G** | **A** | **G** | **G** | **A** | **A** | **C** | **G** | **T** | **T** | **G** | **G** | **C** | **G** | **G** | **A** | **A** | **T** | **T** | **T** | **G** | **C** | **A** | **G** | **A** | **G** | **A** | **A** | **A** | **A** | **A** | **C** | **C** | **A** | **A** | **G** | **T** | **A** | **G** | **A** | **C** | **T** | **A** | **C** | **C** | **A** | **A** | **G** | **C** | **A** | **T** | **T** | **T** | **T** | **T** | **G** | **A** | **A** | **C** | **A** | **A** | **C** | **C** | **T** | **C** | **C** | **G** | **A** | **A** | **A** | **G** | **C** | **T** | **T** | **G** | **G** | **G** | **A** | **T** | **G** | **G** | **A** | **A** | **A** | **G** | **A** | **G** | **A** | **T** | **G** | **T** | **T** | **T** | **T** | **T** | **C** | **A** | **T** | **A** | **A** | **C** | **A** | **A** | **G** | **A** | **T** | **T** | **G** | **A** | **G** | **C** | **A** | **G** | **A** | **T** | **G** | **A** | **G** | **T** | **A** | **T** | **G** | **G** | **G** | **C** | **C** | **A** | **A** | **C** | **G** | **G** | **A** | **A** | **A** | **A** | **A** | **A** | **G** | **T** | **G** | **G** | **A** | **A** | **T** | **T** | **G** | **G** | **C** | **T** | **A** | **A** | **A** | **T** | **C** | **T** | **T** | **T** | **A** | **T** | **C** | **A** | **C** | **A** | **G** | **C** | **C** | **A** | **G** | **C** | **T** | **G** | **A** | **A** | **C** | **T** | **A** | **T** | **A** | **T** | **A** | **T** | **A** | **T** | **G** | **G** | **G** | **A** | **T** | **G** | **A** | **A** | **C** | **C** | **A** | **T** | **T** | **G** | **A** | **A** | **T** | **T** | **A** | **T** | **T** | **T** | **G** | **G** | **A** | **T** | **G** | **T** | **C** | **T** | **T** | **C** | **A** | **A** | **T** | **C** | **A** | **A** | **G** | **A** | **A** | **C** | **A** | **A** | **T** | **T** | **A** | **G** | **A** | **A** | **C** | **A** | **A** | **C** | **T** | **G** | **A** | **T** | **C** | **T** | **T** | **G** | **A** | **A** | **C** | **G** | **T** | **G** | **A** | **A** | **A** | **C** | **C** | **T** | **G** | **C** | **C** | **A** | **T** | **G** | **T** | **T** | **A** | **C** | **T** | **G** | **G** | **T** | **G** | **G** | **A** | **A** | **C** | **A** | **T** | **G** | **A** | **T** | **C** | **A** | **A** | **A** | **C** | **C** | **T** | **T** | **C** | **C** | **T** | **G** | **G** | **A** | **T** | **A** | **A** | **A** | **G** | **T** | **A** | **T** | **C** | **T** | **A** | **C** | **T** | **G** | **A** | **G** | **A** | **T** | **T** | **A** | **T** | **T** | **T** | **C** | **T** | **C** | **T** | **T** | **G** | **A** | **G** | **A** | **A** | **A** | **A** | **T** | **C** | **-** | **-** | **-** |
| **N55655** | **A** | **T** | **G** | **T** | **C** | **T** | **A** | **A** | **A** | **A** | **T** | **C** | **G** | **A** | **A** | **A** | **T** | **A** | **A** | **A** | **A** | **A** | **A** | **T** | **C** | **T** | **G** | **A** | **C** | **A** | **T** | **T** | **C** | **G** | **G** | **C** | **T** | **A** | **C** | **G** | **A** | **C** | **A** | **G** | **C** | **C** | **A** | **A** | **G** | **G** | **C** | **A** | **C** | **A** | **T** | **T** | **A** | **T** | **T** | **A** | **T** | **T** | **T** | **G** | **A** | **A** | **C** | **A** | **A** | **G** | **C** | **A** | **A** | **A** | **T** | **C** | **T** | **A** | **A** | **A** | **T** | **T** | **T** | **T** | **G** | **A** | **C** | **A** | **C** | **A** | **C** | **A** | **A** | **T** | **G** | **G** | **A** | **A** | **A** | **C** | **T** | **A** | **G** | **G** | **A** | **C** | **T** | **T** | **A** | **T** | **C** | **G** | **G** | **A** | **C** | **G** | **A** | **A** | **A** | **C** | **G** | **G** | **T** | **C** | **G** | **A** | **G** | **G** | **A** | **A** | **A** | **G** | **A** | **C** | **A** | **A** | **C** | **T** | **T** | **T** | **A** | **C** | **T** | **G** | **A** | **A** | **T** | **A** | **T** | **T** | **C** | **T** | **A** | **C** | **A** | **A** | **A** | **A** | **C** | **A** | **A** | **A** | **C** | **T** | **A** | **C** | **C** | **T** | **T** | **A** | **T** | **C** | **A** | **A** | **G** | **G** | **G** | **C** | **A** | **A** | **G** | **T** | **A** | **A** | **T** | **C** | **C** | **A** | **T** | **C** | **A** | **G** | **C** | **A** | **A** | **G** | **A** | **A** | **T** | **T** | **T** | **G** | **T** | **C** | **T** | **A** | **T** | **T** | **T** | **C** | **C** | **C** | **G** | **C** | **A** | **G** | **C** | **A** | **A** | **A** | **C** | **A** | **A** | **A** | **A** | **G** | **A** | **T** | **A** | **A** | **A** | **G** | **A** | **G** | **C** | **G** | **T** | **T** | **T** | **A** | **A** | **C** | **C** | **T** | **A** | **T** | **T** | **A** | **T** | **G** | **T** | **G** | **T** | **T** | **A** | **A** | **A** | **T** | **G** | **A** | **G** | **A** | **T** | **T** | **A** | **C** | **G** | **G** | **A** | **T** | **T** | **T** | **T** | **G** | **A** | **G** | **A** | **T** | **A** | **T** | **G** | **G** | **G** | **A** | **A** | **A** | **T** | **T** | **G** | **A** | **A** | **A** | **G** | **A** | **G** | **A** | **G** | **C** | **T** | **C** | **C** | **A** | **A** | **C** | **T** | **G** | **A** | **T** | **G** | **C** | **A** | **A** | **A** | **C** | **A** | **G** | **A** | **T** | **C** | **C** | **T** | **G** | **A** | **A** | **A** | **T** | **C** | **T** | **T** | **A** | **T** | **G** | **G** | **A** | **G** | **A** | **G** | **A** | **A** | **T** | **T** | **C** | **A** | **G** | **C** | **A** | **C** | **A** | **C** | **T** | **A** | **T** | **C** | **A** | **G** | **G** | **A** | **G** | **G** | **A** | **G** | **A** | **G** | **A** | **A** | **G** | **A** | **C** | **A** | **A** | **A** | **A** | **G** | **T** | **C** | **C** | **T** | **A** | **C** | **T** | **G** | **G** | **C** | **A** | **C** | **T** | **T** | **T** | **T** | **A** | **T** | **T** | **T** | **G** | **T** | **G** | **G** | **A** | **T** | **G** | **A** | **C** | **A** | **C** | **T** | **C** | **A** | **T** | **T** | **T** | **C** | **C** | **C** | **G** | **T** | **T** | **A** | **A** | **T** | **C** | **G** | **A** | **T** | **G** | **A** | **A** | **C** | **C** | **A** | **A** | **C** | **G** | **A** | **A** | **T** | **C** | **A** | **T** | **T** | **T** | **G** | **G** | **A** | **-** | **-** | **-** | **T** | **A** | **T** | **C** | **T** | **C** | **T** | **G** | **A** | **T** | **A** | **G** | **A** | **A** | **A** | **A** | **C** | **A** | **A** | **G** | **T** | **A** | **G** | **C** | **G** | **G** | **C** | **T** | **T** | **A** | **C** | **T** | **T** | **G** | **A** | **A** | **A** | **A** | **A** | **G** | **A** | **A** | **A** | **A** | **A** | **A** | **C** | **A** | **A** | **G** | **G** | **C** | **T** | **T** | **C** | **A** | **T** | **C** | **G** | **T** | **G** | **G** | **T** | **C** | **A** | **G** | **C** | **C** | **A** | **T** | **G** | **A** | **C** | **C** | **G** | **G** | **G** | **G** | **A** | **T** | **T** | **T** | **A** | **T** | **C** | **G** | **A** | **T** | **G** | **A** | **A** | **G** | **T** | **A** | **G** | **T** | **G** | **G** | **A** | **C** | **C** | **A** | **T** | **G** | **T** | **T** | **T** | **T** | **A** | **G** | **C** | **A** | **A** | **T** | **C** | **G** | **A** | **A** | **A** | **A** | **A** | **A** | **G** | **T** | **C** | **A** | **A** | **C** | **T** | **G** | **G** | **A** | **A** | **C** | **T** | **T** | **T** | **A** | **T** | **C** | **A** | **A** | **G** | **G** | **G** | **A** | **A** | **T** | **T** | **T** | **C** | **T** | **C** | **T** | **A** | **T** | **C** | **T** | **A** | **T** | **G** | **A** | **A** | **G** | **A** | **A** | **C** | **A** | **G** | **A** | **A** | **A** | **A** | **A** | **A** | **C** | **T** | **T** | **C** | **G** | **T** | **G** | **A** | **T** | **G** | **A** | **A** | **T** | **T** | **T** | **G** | **A** | **A** | **A** | **T** | **A** | **G** | **C** | **T** | **C** | **A** | **A** | **A** | **A** | **T** | **G** | **A** | **A** | **A** | **A** | **A** | **T** | **T** | **G** | **A** | **A** | **A** | **A** | **A** | **A** | **G** | **A** | **A** | **G** | **T** | **C** | **A** | **G** | **T** | **A** | **G** | **G** | **C** | **T** | **A** | **A** | **A** | **G** | **A** | **A** | **A** | **A** | **C** | **A** | **G** | **C** | **A** | **G** | **C** | **T** | **G** | **A** | **A** | **A** | **A** | **A** | **G** | **C** | **C** | **G** | **A** | **A** | **T** | **G** | **G** | **T** | **C** | **T** | **C** | **G** | **T** | **T** | **C** | **C** | **C** | **G** | **A** | **G** | **A** | **A** | **G** | **G** | **A** | **G** | **A** | **T** | **A** | **A** | **A** | **A** | **C** | **A** | **A** | **A** | **G** | **A** | **A** | **A** | **C** | **A** | **A** | **G** | **T** | **C** | **G** | **G** | **A** | **T** | **T** | **C** | **A** | **T** | **C** | **G** | **A** | **T** | **A** | **C** | **T** | **G** | **A** | **A** | **T** | **C** | **T** | **A** | **G** | **A** | **C** | **G** | **A** | **G** | **T** | **G** | **A** | **A** | **T** | **A** | **A** | **A** | **G** | **G** | **A** | **G** | **C** | **A** | **G** | **T** | **G** | **G** | **G** | **T** | **G** | **C** | **T** | **G** | **A** | **T** | **G** | **C** | **T** | **G** | **C** | **A** | **C** | **G** | **G** | **A** | **C** | **G** | **A** | **T** | **G** | **A** | **A** | **A** | **C** | **G** | **A** | **T** | **C** | **C** | **A** | **A** | **A** | **G** | **C** | **A** | **A** | **T** | **C** | **G** | **T** | **G** | **A** | **A** | **T** | **C** | **G** | **G** | **A** | **T** | **G** | **G** | **A** | **G** | **A** | **C** | **C** | **C** | **A** | **G** | **A** | **T** | **C** | **A** | **G** | **C** | **G** | **A** | **G** | **A** | **A** | **A** | **G** | **A** | **A** | **A** | **A** | **A** | **C** | **T** | **A** | **T** | **T** | **A** | **A** | **A** | **A** | **G** | **A** | **T** | **A** | **T** | **C** | **G** | **A** | **A** | **T** | **A** | **T** | **A** | **T** | **C** | **G** | **A** | **T** | **T** | **C** | **T** | **T** | **T** | **G** | **A** | **C** | **G** | **A** | **T** | **G** | **A** | **A** | **T** | **A** | **G** | **C** | **C** | **A** | **A** | **G** | **C** | **G** | **T** | **C** | **T** | **C** | **A** | **C** | **C** | **A** | **T** | **A** | **A** | **G** | **C** | **G** | **A** | **C** | **T** | **T** | **T** | **T** | **A** | **A** | **G** | **C** | **G** | **T** | **A** | **G** | **A** | **A** | **G** | **A** | **T** | **C** | **T** | **T** | **C** | **A** | **A** | **T** | **T** | **A** | **G** | **G** | **G** | **T** | **A** | **T** | **G** | **A** | **A** | **A** | **A** | **T** | **C** | **T** | **G** | **T** | **T** | **A** | **T** | **T** | **C** | **G** | **A** | **G** | **C** | **C** | **A** | **A** | **T** | **T** | **C** | **A** | **T** | **T** | **T** | **T** | **A** | **C** | **A** | **A** | **T** | **C** | **G** | **A** | **G** | **C** | **C** | **T** | **C** | **A** | **T** | **C** | **A** | **G** | **C** | **G** | **G** | **G** | **T** | **G** | **G** | **C** | **G** | **A** | **T** | **T** | **T** | **C** | **A** | **G** | **G** | **T** | **C** | **C** | **T** | **A** | **A** | **C** | **G** | **G** | **T** | **G** | **C** | **A** | **G** | **G** | **A** | **A** | **A** | **G** | **T** | **C** | **A** | **T** | **C** | **C** | **A** | **T** | **T** | **A** | **T** | **C** | **C** | **A** | **T** | **T** | **A** | **T** | **C** | **T** | **T** | **C** | **T** | **G** | **G** | **G** | **G** | **G** | **C** | **A** | **T** | **T** | **C** | **A** | **A** | **C** | **G** | **G** | **C** | **A** | **A** | **G** | **G** | **T** | **T** | **A** | **T** | **A** | **G** | **G** | **A** | **G** | **A** | **A** | **A** | **A** | **A** | **T** | **C** | **C** | **C** | **A** | **G** | **C** | **C** | **A** | **A** | **A** | **A** | **C** | **A** | **T** | **C** | **T** | **G** | **A** | **G** | **C** | **A** | **T** | **T** | **A** | **G** | **T** | **T** | **A** | **T** | **G** | **T** | **A** | **C** | **G** | **C** | **C** | **A** | **A** | **A** | **A** | **T** | **T** | **A** | **T** | **G** | **A** | **A** | **G** | **A** | **C** | **A** | **A** | **T** | **C** | **G** | **A** | **G** | **G** | **A** | **A** | **C** | **G** | **T** | **T** | **G** | **G** | **C** | **G** | **G** | **A** | **A** | **T** | **T** | **T** | **G** | **C** | **A** | **G** | **A** | **G** | **A** | **A** | **A** | **A** | **A** | **C** | **C** | **A** | **A** | **G** | **T** | **A** | **G** | **A** | **C** | **T** | **A** | **C** | **C** | **A** | **A** | **G** | **C** | **A** | **T** | **T** | **T** | **T** | **T** | **G** | **A** | **A** | **C** | **A** | **A** | **C** | **C** | **T** | **C** | **C** | **G** | **A** | **A** | **A** | **G** | **C** | **T** | **T** | **G** | **G** | **G** | **A** | **T** | **G** | **G** | **A** | **A** | **A** | **G** | **A** | **G** | **A** | **T** | **G** | **T** | **T** | **T** | **T** | **T** | **C** | **A** | **T** | **A** | **A** | **C** | **A** | **A** | **G** | **A** | **T** | **C** | **G** | **A** | **G** | **C** | **A** | **G** | **A** | **T** | **G** | **A** | **G** | **T** | **A** | **T** | **G** | **G** | **G** | **C** | **C** | **A** | **A** | **C** | **G** | **G** | **A** | **A** | **A** | **A** | **A** | **A** | **G** | **T** | **G** | **G** | **A** | **A** | **T** | **T** | **G** | **G** | **C** | **T** | **A** | **A** | **A** | **T** | **C** | **T** | **T** | **T** | **A** | **T** | **C** | **A** | **C** | **A** | **G** | **C** | **C** | **A** | **G** | **C** | **T** | **G** | **A** | **A** | **C** | **T** | **A** | **T** | **A** | **T** | **A** | **T** | **A** | **T** | **G** | **G** | **G** | **A** | **T** | **G** | **A** | **A** | **C** | **C** | **A** | **T** | **T** | **G** | **A** | **A** | **T** | **T** | **A** | **T** | **T** | **T** | **G** | **G** | **A** | **T** | **G** | **T** | **C** | **T** | **T** | **C** | **A** | **A** | **T** | **C** | **A** | **A** | **G** | **A** | **A** | **C** | **A** | **A** | **T** | **T** | **A** | **G** | **A** | **A** | **C** | **A** | **A** | **C** | **T** | **G** | **A** | **T** | **C** | **T** | **T** | **G** | **A** | **A** | **C** | **G** | **T** | **G** | **A** | **A** | **A** | **C** | **C** | **T** | **G** | **C** | **C** | **A** | **T** | **G** | **T** | **T** | **A** | **C** | **T** | **A** | **G** | **T** | **G** | **G** | **A** | **A** | **C** | **A** | **T** | **G** | **A** | **T** | **C** | **A** | **A** | **A** | **C** | **C** | **T** | **T** | **C** | **C** | **T** | **G** | **G** | **A** | **T** | **A** | **A** | **A** | **G** | **T** | **A** | **T** | **C** | **T** | **A** | **C** | **T** | **G** | **A** | **G** | **A** | **T** | **T** | **A** | **T** | **T** | **T** | **C** | **T** | **C** | **T** | **T** | **G** | **A** | **G** | **A** | **A** | **A** | **A** | **T** | **C** | **-** | **-** | **-** |
| **N53559** | **A** | **T** | **G** | **T** | **C** | **T** | **A** | **A** | **A** | **A** | **T** | **C** | **G** | **A** | **A** | **A** | **T** | **A** | **A** | **A** | **A** | **A** | **A** | **T** | **C** | **T** | **G** | **A** | **C** | **A** | **T** | **T** | **C** | **G** | **G** | **C** | **T** | **A** | **C** | **G** | **A** | **C** | **A** | **G** | **C** | **C** | **A** | **A** | **G** | **G** | **C** | **A** | **C** | **A** | **T** | **T** | **A** | **T** | **T** | **A** | **T** | **T** | **T** | **G** | **A** | **A** | **C** | **A** | **A** | **G** | **C** | **A** | **A** | **A** | **T** | **C** | **T** | **A** | **A** | **A** | **T** | **T** | **T** | **T** | **G** | **A** | **C** | **A** | **C** | **A** | **C** | **A** | **A** | **T** | **G** | **G** | **A** | **A** | **A** | **C** | **T** | **A** | **G** | **G** | **A** | **C** | **T** | **T** | **A** | **T** | **C** | **G** | **G** | **A** | **C** | **G** | **A** | **A** | **A** | **C** | **G** | **G** | **T** | **C** | **G** | **A** | **G** | **G** | **A** | **A** | **A** | **G** | **A** | **C** | **A** | **A** | **C** | **T** | **T** | **T** | **A** | **C** | **T** | **G** | **A** | **A** | **T** | **A** | **T** | **T** | **C** | **T** | **A** | **C** | **A** | **A** | **A** | **A** | **C** | **A** | **A** | **A** | **C** | **T** | **A** | **C** | **C** | **T** | **T** | **A** | **T** | **C** | **A** | **A** | **G** | **G** | **G** | **C** | **A** | **A** | **G** | **T** | **A** | **A** | **T** | **C** | **C** | **A** | **T** | **C** | **A** | **G** | **C** | **A** | **A** | **G** | **A** | **A** | **T** | **T** | **T** | **G** | **T** | **C** | **T** | **A** | **T** | **T** | **T** | **C** | **C** | **C** | **G** | **C** | **A** | **G** | **C** | **A** | **A** | **A** | **C** | **A** | **A** | **A** | **A** | **G** | **A** | **T** | **A** | **A** | **A** | **G** | **A** | **G** | **C** | **G** | **T** | **T** | **T** | **A** | **A** | **C** | **C** | **T** | **A** | **T** | **T** | **A** | **T** | **G** | **T** | **G** | **T** | **T** | **A** | **A** | **A** | **T** | **G** | **A** | **G** | **A** | **T** | **T** | **A** | **C** | **G** | **G** | **A** | **T** | **T** | **T** | **T** | **G** | **A** | **G** | **A** | **T** | **A** | **T** | **G** | **G** | **G** | **A** | **A** | **A** | **T** | **T** | **G** | **A** | **A** | **A** | **G** | **A** | **G** | **A** | **G** | **C** | **T** | **C** | **C** | **A** | **A** | **C** | **T** | **G** | **A** | **T** | **G** | **C** | **A** | **A** | **A** | **C** | **A** | **G** | **A** | **T** | **C** | **C** | **T** | **G** | **A** | **A** | **A** | **T** | **C** | **T** | **T** | **A** | **T** | **G** | **G** | **A** | **G** | **A** | **G** | **A** | **A** | **T** | **T** | **C** | **A** | **G** | **C** | **A** | **C** | **A** | **C** | **T** | **A** | **T** | **C** | **A** | **G** | **G** | **A** | **G** | **G** | **A** | **G** | **A** | **G** | **A** | **A** | **G** | **A** | **C** | **A** | **A** | **A** | **A** | **G** | **T** | **C** | **C** | **T** | **A** | **C** | **T** | **G** | **G** | **C** | **A** | **C** | **T** | **T** | **T** | **T** | **A** | **T** | **T** | **T** | **G** | **T** | **G** | **G** | **A** | **T** | **G** | **A** | **C** | **A** | **C** | **T** | **C** | **A** | **T** | **T** | **T** | **C** | **C** | **C** | **G** | **T** | **T** | **A** | **A** | **T** | **C** | **G** | **A** | **T** | **G** | **A** | **A** | **C** | **C** | **A** | **A** | **C** | **G** | **A** | **A** | **T** | **C** | **A** | **T** | **T** | **T** | **G** | **G** | **A** | **-** | **-** | **-** | **T** | **A** | **T** | **C** | **T** | **C** | **T** | **G** | **G** | **C** | **A** | **G** | **A** | **A** | **A** | **A** | **C** | **A** | **A** | **G** | **T** | **A** | **G** | **C** | **G** | **G** | **C** | **T** | **T** | **A** | **C** | **T** | **T** | **G** | **A** | **A** | **A** | **A** | **A** | **G** | **A** | **A** | **A** | **A** | **A** | **A** | **C** | **A** | **A** | **G** | **G** | **C** | **T** | **T** | **C** | **A** | **T** | **C** | **G** | **T** | **G** | **G** | **T** | **C** | **A** | **G** | **C** | **C** | **A** | **T** | **G** | **A** | **C** | **C** | **G** | **G** | **G** | **G** | **A** | **T** | **T** | **T** | **A** | **T** | **C** | **G** | **A** | **T** | **G** | **A** | **A** | **G** | **T** | **A** | **G** | **T** | **G** | **G** | **A** | **C** | **C** | **A** | **T** | **G** | **T** | **T** | **T** | **T** | **A** | **G** | **C** | **A** | **A** | **T** | **C** | **G** | **A** | **A** | **A** | **A** | **A** | **A** | **G** | **T** | **C** | **A** | **A** | **C** | **T** | **G** | **G** | **A** | **A** | **C** | **T** | **T** | **T** | **A** | **T** | **C** | **A** | **A** | **G** | **G** | **G** | **A** | **A** | **T** | **T** | **T** | **C** | **T** | **C** | **T** | **A** | **T** | **C** | **T** | **A** | **T** | **G** | **A** | **A** | **G** | **A** | **A** | **C** | **A** | **G** | **A** | **A** | **A** | **A** | **A** | **A** | **C** | **T** | **T** | **C** | **G** | **T** | **G** | **A** | **T** | **G** | **A** | **A** | **T** | **T** | **T** | **G** | **A** | **A** | **A** | **T** | **G** | **G** | **C** | **T** | **C** | **A** | **A** | **A** | **A** | **T** | **G** | **A** | **A** | **A** | **A** | **A** | **T** | **T** | **G** | **A** | **A** | **A** | **A** | **A** | **A** | **G** | **A** | **A** | **G** | **T** | **C** | **A** | **G** | **T** | **A** | **G** | **G** | **C** | **T** | **A** | **A** | **A** | **G** | **A** | **A** | **A** | **A** | **C** | **A** | **G** | **C** | **A** | **G** | **C** | **T** | **G** | **A** | **A** | **A** | **A** | **A** | **G** | **C** | **C** | **G** | **A** | **A** | **T** | **G** | **G** | **T** | **C** | **T** | **C** | **G** | **T** | **T** | **C** | **C** | **C** | **G** | **A** | **G** | **A** | **A** | **G** | **G** | **A** | **G** | **A** | **T** | **A** | **A** | **A** | **A** | **C** | **A** | **A** | **A** | **G** | **A** | **A** | **A** | **C** | **A** | **A** | **G** | **T** | **C** | **G** | **G** | **A** | **T** | **T** | **C** | **A** | **T** | **C** | **G** | **A** | **T** | **A** | **C** | **T** | **G** | **A** | **A** | **T** | **C** | **T** | **A** | **G** | **A** | **C** | **G** | **A** | **G** | **T** | **G** | **A** | **A** | **T** | **A** | **A** | **A** | **G** | **G** | **A** | **G** | **C** | **A** | **G** | **T** | **G** | **G** | **G** | **T** | **G** | **C** | **T** | **G** | **A** | **T** | **G** | **C** | **T** | **G** | **C** | **A** | **C** | **G** | **G** | **A** | **C** | **G** | **A** | **T** | **G** | **A** | **A** | **A** | **C** | **G** | **A** | **T** | **C** | **C** | **A** | **A** | **A** | **G** | **C** | **A** | **A** | **T** | **C** | **G** | **T** | **G** | **A** | **A** | **T** | **C** | **G** | **G** | **A** | **T** | **G** | **G** | **A** | **G** | **A** | **C** | **C** | **C** | **A** | **G** | **A** | **T** | **C** | **A** | **G** | **C** | **G** | **A** | **G** | **A** | **A** | **A** | **G** | **A** | **A** | **A** | **A** | **A** | **C** | **T** | **A** | **T** | **T** | **G** | **A** | **A** | **A** | **G** | **A** | **T** | **A** | **T** | **C** | **G** | **A** | **A** | **T** | **A** | **T** | **A** | **T** | **C** | **G** | **A** | **T** | **T** | **C** | **T** | **T** | **T** | **G** | **A** | **C** | **G** | **A** | **T** | **G** | **A** | **A** | **T** | **A** | **G** | **C** | **C** | **A** | **A** | **G** | **C** | **G** | **T** | **C** | **T** | **C** | **A** | **C** | **C** | **A** | **T** | **A** | **A** | **G** | **C** | **G** | **A** | **C** | **T** | **T** | **T** | **T** | **A** | **A** | **G** | **C** | **G** | **T** | **A** | **G** | **A** | **A** | **G** | **A** | **T** | **C** | **T** | **T** | **C** | **A** | **A** | **T** | **T** | **A** | **G** | **G** | **G** | **T** | **A** | **T** | **G** | **A** | **G** | **A** | **A** | **T** | **C** | **T** | **G** | **T** | **T** | **A** | **T** | **T** | **C** | **G** | **A** | **G** | **C** | **C** | **A** | **A** | **T** | **T** | **C** | **A** | **T** | **T** | **T** | **T** | **A** | **C** | **A** | **A** | **T** | **C** | **G** | **A** | **G** | **C** | **C** | **T** | **C** | **A** | **T** | **C** | **A** | **G** | **C** | **G** | **G** | **G** | **T** | **G** | **G** | **C** | **G** | **A** | **T** | **T** | **T** | **C** | **A** | **G** | **G** | **T** | **C** | **C** | **T** | **A** | **A** | **C** | **G** | **G** | **T** | **G** | **C** | **A** | **G** | **G** | **A** | **A** | **A** | **G** | **T** | **C** | **A** | **T** | **C** | **C** | **A** | **T** | **T** | **A** | **T** | **C** | **C** | **A** | **T** | **T** | **A** | **T** | **C** | **T** | **T** | **C** | **T** | **G** | **G** | **G** | **G** | **G** | **C** | **A** | **T** | **T** | **C** | **A** | **A** | **C** | **G** | **G** | **C** | **A** | **A** | **G** | **G** | **T** | **T** | **A** | **T** | **A** | **G** | **G** | **A** | **G** | **A** | **A** | **A** | **A** | **A** | **T** | **C** | **C** | **C** | **A** | **G** | **C** | **C** | **A** | **A** | **A** | **A** | **C** | **A** | **T** | **C** | **T** | **G** | **A** | **G** | **C** | **A** | **T** | **T** | **A** | **G** | **T** | **T** | **A** | **T** | **G** | **T** | **A** | **C** | **G** | **C** | **C** | **A** | **A** | **A** | **A** | **T** | **T** | **A** | **T** | **G** | **A** | **A** | **G** | **A** | **C** | **A** | **A** | **T** | **C** | **G** | **A** | **G** | **G** | **A** | **A** | **C** | **G** | **T** | **T** | **G** | **G** | **C** | **G** | **G** | **A** | **A** | **T** | **T** | **T** | **G** | **C** | **A** | **G** | **A** | **G** | **A** | **A** | **A** | **A** | **A** | **C** | **C** | **A** | **A** | **G** | **T** | **A** | **G** | **A** | **C** | **T** | **A** | **C** | **C** | **A** | **A** | **G** | **C** | **A** | **T** | **T** | **T** | **T** | **T** | **G** | **A** | **A** | **C** | **A** | **A** | **C** | **C** | **T** | **C** | **C** | **G** | **A** | **A** | **A** | **G** | **C** | **T** | **T** | **G** | **G** | **G** | **A** | **T** | **G** | **G** | **A** | **A** | **A** | **G** | **A** | **G** | **A** | **T** | **G** | **T** | **T** | **T** | **T** | **T** | **C** | **A** | **T** | **A** | **A** | **C** | **A** | **A** | **G** | **A** | **T** | **C** | **G** | **A** | **G** | **C** | **A** | **G** | **A** | **T** | **G** | **A** | **G** | **T** | **A** | **T** | **G** | **G** | **G** | **C** | **C** | **A** | **A** | **C** | **G** | **G** | **A** | **A** | **A** | **A** | **A** | **A** | **G** | **T** | **G** | **G** | **A** | **A** | **T** | **T** | **G** | **G** | **C** | **T** | **A** | **A** | **A** | **T** | **C** | **T** | **T** | **T** | **A** | **T** | **C** | **A** | **C** | **A** | **G** | **C** | **C** | **A** | **G** | **C** | **T** | **G** | **A** | **A** | **C** | **T** | **A** | **T** | **A** | **T** | **A** | **T** | **A** | **T** | **G** | **G** | **G** | **A** | **T** | **G** | **A** | **A** | **C** | **C** | **A** | **T** | **T** | **G** | **A** | **A** | **T** | **T** | **A** | **T** | **T** | **T** | **G** | **G** | **A** | **T** | **G** | **T** | **C** | **T** | **T** | **C** | **A** | **A** | **T** | **C** | **A** | **A** | **G** | **A** | **A** | **C** | **A** | **A** | **T** | **T** | **A** | **G** | **A** | **A** | **C** | **A** | **A** | **C** | **T** | **G** | **A** | **T** | **C** | **T** | **T** | **G** | **A** | **A** | **C** | **G** | **T** | **G** | **A** | **A** | **A** | **C** | **C** | **T** | **G** | **C** | **C** | **A** | **T** | **G** | **T** | **T** | **A** | **C** | **T** | **A** | **G** | **T** | **G** | **G** | **A** | **A** | **C** | **A** | **T** | **G** | **A** | **T** | **C** | **A** | **A** | **A** | **C** | **C** | **T** | **T** | **C** | **C** | **T** | **G** | **G** | **A** | **T** | **A** | **A** | **A** | **G** | **T** | **A** | **T** | **C** | **T** | **A** | **C** | **T** | **G** | **A** | **G** | **A** | **T** | **T** | **A** | **T** | **T** | **T** | **C** | **T** | **C** | **T** | **T** | **G** | **A** | **G** | **A** | **A** | **A** | **A** | **T** | **C** | **-** | **-** | **-** |
| **N55229** | **A** | **T** | **G** | **T** | **C** | **T** | **A** | **A** | **A** | **A** | **T** | **C** | **G** | **A** | **A** | **A** | **T** | **A** | **A** | **A** | **A** | **A** | **A** | **T** | **C** | **T** | **G** | **A** | **C** | **A** | **T** | **T** | **C** | **G** | **G** | **C** | **T** | **A** | **C** | **G** | **A** | **C** | **A** | **G** | **C** | **C** | **A** | **A** | **G** | **G** | **C** | **A** | **C** | **A** | **T** | **T** | **A** | **T** | **T** | **A** | **T** | **T** | **T** | **G** | **A** | **A** | **C** | **A** | **A** | **G** | **C** | **A** | **A** | **A** | **T** | **C** | **T** | **A** | **A** | **A** | **T** | **T** | **T** | **T** | **G** | **A** | **C** | **A** | **C** | **A** | **C** | **A** | **A** | **T** | **G** | **G** | **A** | **A** | **A** | **C** | **T** | **A** | **G** | **G** | **A** | **C** | **T** | **T** | **A** | **T** | **C** | **G** | **G** | **A** | **C** | **G** | **A** | **A** | **A** | **C** | **G** | **G** | **T** | **C** | **G** | **A** | **G** | **G** | **A** | **A** | **A** | **G** | **A** | **C** | **A** | **A** | **C** | **T** | **T** | **T** | **A** | **C** | **T** | **G** | **A** | **A** | **T** | **A** | **T** | **T** | **C** | **T** | **A** | **C** | **A** | **A** | **A** | **A** | **C** | **A** | **A** | **A** | **C** | **T** | **A** | **C** | **C** | **T** | **T** | **A** | **T** | **C** | **A** | **A** | **G** | **G** | **G** | **C** | **A** | **A** | **G** | **T** | **A** | **A** | **T** | **C** | **C** | **A** | **T** | **C** | **A** | **G** | **C** | **A** | **A** | **G** | **A** | **A** | **T** | **T** | **T** | **G** | **T** | **C** | **T** | **A** | **T** | **T** | **T** | **C** | **C** | **C** | **G** | **C** | **A** | **A** | **C** | **A** | **G** | **A** | **C** | **A** | **A** | **A** | **A** | **G** | **A** | **T** | **A** | **A** | **A** | **G** | **A** | **A** | **C** | **G** | **T** | **T** | **T** | **A** | **A** | **C** | **C** | **T** | **A** | **T** | **T** | **A** | **C** | **G** | **T** | **G** | **T** | **T** | **A** | **A** | **A** | **T** | **G** | **A** | **T** | **A** | **T** | **T** | **A** | **C** | **G** | **G** | **A** | **T** | **T** | **T** | **T** | **G** | **A** | **G** | **A** | **T** | **A** | **T** | **G** | **G** | **G** | **A** | **A** | **A** | **T** | **C** | **G** | **A** | **A** | **A** | **G** | **A** | **G** | **A** | **G** | **C** | **T** | **C** | **C** | **A** | **A** | **T** | **T** | **G** | **A** | **T** | **G** | **C** | **A** | **A** | **A** | **C** | **A** | **G** | **A** | **T** | **C** | **C** | **T** | **G** | **A** | **A** | **A** | **T** | **C** | **T** | **T** | **A** | **T** | **G** | **G** | **A** | **G** | **A** | **G** | **A** | **A** | **T** | **T** | **C** | **A** | **G** | **C** | **A** | **C** | **A** | **C** | **T** | **A** | **T** | **C** | **A** | **G** | **G** | **G** | **G** | **G** | **A** | **G** | **A** | **G** | **A** | **A** | **G** | **A** | **C** | **A** | **A** | **A** | **A** | **G** | **T** | **C** | **C** | **T** | **A** | **C** | **T** | **G** | **G** | **C** | **A** | **C** | **T** | **T** | **T** | **T** | **A** | **T** | **T** | **T** | **G** | **T** | **G** | **G** | **A** | **T** | **G** | **A** | **C** | **A** | **C** | **T** | **C** | **A** | **T** | **T** | **T** | **C** | **C** | **C** | **G** | **T** | **T** | **A** | **A** | **T** | **C** | **G** | **A** | **T** | **G** | **A** | **A** | **C** | **C** | **A** | **A** | **C** | **G** | **A** | **A** | **T** | **C** | **A** | **T** | **T** | **T** | **G** | **G** | **A** | **-** | **-** | **-** | **T** | **A** | **T** | **C** | **T** | **C** | **T** | **G** | **G** | **T** | **A** | **G** | **A** | **A** | **A** | **A** | **C** | **A** | **A** | **G** | **T** | **A** | **G** | **C** | **G** | **G** | **C** | **T** | **T** | **A** | **T** | **T** | **T** | **G** | **A** | **A** | **A** | **A** | **A** | **G** | **A** | **A** | **A** | **A** | **A** | **A** | **C** | **A** | **A** | **G** | **G** | **C** | **T** | **T** | **C** | **A** | **T** | **C** | **G** | **T** | **G** | **G** | **T** | **C** | **A** | **G** | **C** | **C** | **A** | **T** | **G** | **A** | **C** | **C** | **G** | **G** | **G** | **G** | **A** | **T** | **T** | **T** | **A** | **T** | **C** | **G** | **A** | **T** | **G** | **A** | **A** | **G** | **T** | **A** | **G** | **T** | **G** | **G** | **A** | **C** | **C** | **A** | **T** | **G** | **T** | **T** | **T** | **T** | **A** | **G** | **C** | **A** | **A** | **T** | **C** | **G** | **A** | **A** | **A** | **A** | **A** | **A** | **G** | **T** | **C** | **A** | **A** | **C** | **T** | **G** | **G** | **A** | **A** | **C** | **T** | **T** | **T** | **A** | **T** | **C** | **A** | **A** | **G** | **G** | **G** | **A** | **A** | **T** | **T** | **T** | **C** | **T** | **C** | **T** | **A** | **T** | **C** | **T** | **A** | **T** | **G** | **A** | **A** | **G** | **A** | **A** | **C** | **A** | **G** | **A** | **A** | **A** | **A** | **A** | **A** | **C** | **T** | **T** | **C** | **G** | **T** | **G** | **A** | **T** | **G** | **A** | **A** | **T** | **T** | **T** | **G** | **A** | **A** | **A** | **T** | **G** | **G** | **C** | **T** | **C** | **A** | **A** | **A** | **A** | **T** | **G** | **A** | **A** | **A** | **A** | **A** | **T** | **T** | **G** | **A** | **A** | **A** | **A** | **A** | **A** | **G** | **A** | **A** | **G** | **T** | **C** | **A** | **G** | **T** | **A** | **G** | **G** | **C** | **T** | **A** | **A** | **A** | **G** | **A** | **A** | **A** | **A** | **C** | **A** | **G** | **C** | **A** | **G** | **C** | **T** | **G** | **A** | **A** | **A** | **A** | **A** | **G** | **C** | **C** | **G** | **A** | **A** | **T** | **G** | **G** | **T** | **C** | **T** | **C** | **G** | **T** | **T** | **C** | **C** | **C** | **G** | **A** | **G** | **A** | **A** | **G** | **G** | **A** | **G** | **A** | **T** | **A** | **A** | **A** | **A** | **C** | **A** | **A** | **A** | **G** | **A** | **A** | **A** | **C** | **A** | **A** | **G** | **T** | **C** | **G** | **G** | **A** | **T** | **T** | **C** | **A** | **T** | **C** | **G** | **A** | **T** | **A** | **C** | **T** | **G** | **A** | **A** | **T** | **C** | **T** | **A** | **G** | **A** | **C** | **G** | **A** | **G** | **T** | **G** | **A** | **A** | **T** | **A** | **A** | **A** | **G** | **G** | **A** | **G** | **C** | **A** | **G** | **T** | **G** | **G** | **G** | **T** | **G** | **C** | **T** | **G** | **A** | **T** | **G** | **C** | **T** | **G** | **C** | **A** | **C** | **G** | **G** | **A** | **C** | **G** | **A** | **T** | **G** | **A** | **A** | **A** | **C** | **G** | **A** | **T** | **C** | **C** | **A** | **A** | **A** | **G** | **C** | **A** | **A** | **T** | **C** | **G** | **T** | **G** | **A** | **A** | **T** | **C** | **G** | **G** | **A** | **T** | **G** | **G** | **A** | **G** | **A** | **C** | **C** | **C** | **A** | **G** | **A** | **T** | **C** | **A** | **G** | **C** | **G** | **A** | **G** | **A** | **A** | **A** | **G** | **A** | **A** | **A** | **A** | **A** | **C** | **T** | **A** | **T** | **T** | **A** | **A** | **A** | **A** | **G** | **A** | **T** | **A** | **T** | **C** | **G** | **A** | **A** | **T** | **A** | **T** | **A** | **T** | **C** | **G** | **A** | **T** | **T** | **C** | **T** | **T** | **T** | **G** | **A** | **C** | **G** | **A** | **T** | **G** | **A** | **A** | **T** | **A** | **G** | **C** | **C** | **A** | **A** | **G** | **C** | **G** | **T** | **C** | **T** | **C** | **A** | **C** | **C** | **A** | **T** | **A** | **A** | **G** | **C** | **G** | **A** | **C** | **T** | **T** | **T** | **T** | **A** | **A** | **G** | **C** | **G** | **T** | **A** | **G** | **A** | **A** | **G** | **A** | **T** | **C** | **T** | **T** | **C** | **A** | **A** | **T** | **T** | **A** | **G** | **G** | **G** | **T** | **A** | **T** | **G** | **A** | **A** | **A** | **A** | **T** | **C** | **T** | **G** | **T** | **T** | **A** | **T** | **T** | **C** | **G** | **A** | **G** | **C** | **C** | **A** | **A** | **T** | **T** | **C** | **A** | **T** | **T** | **T** | **T** | **A** | **C** | **A** | **A** | **T** | **C** | **G** | **A** | **G** | **C** | **C** | **T** | **C** | **A** | **T** | **C** | **A** | **G** | **C** | **G** | **G** | **G** | **T** | **G** | **G** | **C** | **G** | **A** | **T** | **T** | **T** | **C** | **A** | **G** | **G** | **T** | **C** | **C** | **T** | **A** | **A** | **C** | **G** | **G** | **T** | **G** | **C** | **A** | **G** | **G** | **A** | **A** | **A** | **G** | **T** | **C** | **A** | **T** | **C** | **C** | **A** | **T** | **T** | **A** | **T** | **C** | **C** | **A** | **T** | **T** | **A** | **T** | **C** | **T** | **T** | **C** | **T** | **G** | **G** | **G** | **G** | **G** | **C** | **A** | **T** | **T** | **C** | **A** | **A** | **C** | **G** | **G** | **C** | **A** | **A** | **G** | **G** | **T** | **T** | **A** | **T** | **A** | **G** | **G** | **A** | **G** | **A** | **A** | **A** | **A** | **A** | **T** | **C** | **C** | **C** | **A** | **G** | **C** | **C** | **A** | **A** | **A** | **A** | **C** | **A** | **T** | **C** | **T** | **G** | **A** | **G** | **C** | **A** | **T** | **T** | **A** | **G** | **T** | **T** | **A** | **T** | **G** | **T** | **A** | **C** | **G** | **C** | **C** | **A** | **A** | **A** | **A** | **T** | **T** | **A** | **T** | **G** | **A** | **A** | **G** | **A** | **C** | **A** | **A** | **T** | **C** | **G** | **A** | **G** | **G** | **A** | **A** | **C** | **G** | **T** | **T** | **G** | **G** | **C** | **G** | **G** | **A** | **A** | **T** | **T** | **T** | **G** | **C** | **A** | **G** | **A** | **G** | **A** | **A** | **A** | **A** | **A** | **C** | **C** | **A** | **A** | **G** | **T** | **A** | **G** | **A** | **C** | **T** | **A** | **C** | **C** | **A** | **A** | **G** | **C** | **A** | **T** | **T** | **T** | **T** | **T** | **G** | **A** | **A** | **C** | **A** | **A** | **C** | **C** | **T** | **C** | **C** | **G** | **A** | **A** | **A** | **G** | **C** | **T** | **T** | **G** | **G** | **G** | **A** | **T** | **G** | **G** | **A** | **A** | **A** | **G** | **A** | **G** | **A** | **T** | **G** | **T** | **T** | **T** | **T** | **T** | **C** | **A** | **T** | **A** | **A** | **C** | **A** | **A** | **G** | **A** | **T** | **C** | **G** | **A** | **G** | **C** | **A** | **G** | **A** | **T** | **G** | **A** | **G** | **T** | **A** | **T** | **G** | **G** | **G** | **C** | **C** | **A** | **A** | **C** | **G** | **G** | **A** | **A** | **A** | **A** | **A** | **A** | **G** | **T** | **G** | **G** | **A** | **A** | **T** | **T** | **G** | **G** | **C** | **T** | **A** | **A** | **A** | **T** | **C** | **T** | **T** | **T** | **A** | **T** | **C** | **A** | **C** | **A** | **G** | **C** | **C** | **A** | **G** | **C** | **T** | **G** | **A** | **A** | **C** | **T** | **A** | **T** | **A** | **T** | **A** | **T** | **A** | **T** | **G** | **G** | **G** | **A** | **T** | **G** | **A** | **A** | **C** | **C** | **A** | **T** | **T** | **G** | **A** | **A** | **T** | **T** | **A** | **T** | **T** | **T** | **G** | **G** | **A** | **T** | **G** | **T** | **C** | **T** | **T** | **C** | **A** | **A** | **T** | **C** | **A** | **A** | **G** | **A** | **A** | **C** | **A** | **A** | **T** | **T** | **A** | **G** | **A** | **A** | **C** | **A** | **A** | **C** | **T** | **G** | **A** | **T** | **C** | **T** | **T** | **G** | **A** | **A** | **C** | **G** | **T** | **G** | **A** | **A** | **A** | **C** | **C** | **T** | **G** | **C** | **C** | **A** | **T** | **G** | **T** | **T** | **A** | **C** | **T** | **A** | **G** | **T** | **G** | **G** | **A** | **A** | **C** | **A** | **T** | **G** | **A** | **T** | **C** | **A** | **A** | **A** | **C** | **C** | **T** | **T** | **C** | **C** | **T** | **G** | **G** | **A** | **T** | **A** | **A** | **A** | **G** | **T** | **A** | **T** | **C** | **T** | **A** | **C** | **T** | **G** | **A** | **G** | **A** | **T** | **T** | **A** | **T** | **T** | **T** | **C** | **T** | **C** | **T** | **T** | **G** | **A** | **G** | **A** | **A** | **A** | **A** | **T** | **C** | **-** | **-** | **-** |
| **N54070** | **A** | **T** | **G** | **T** | **C** | **T** | **A** | **A** | **A** | **A** | **T** | **C** | **G** | **A** | **A** | **A** | **T** | **A** | **A** | **A** | **A** | **A** | **A** | **T** | **C** | **T** | **G** | **A** | **C** | **A** | **T** | **T** | **C** | **G** | **G** | **C** | **T** | **A** | **C** | **G** | **A** | **C** | **A** | **G** | **C** | **C** | **A** | **A** | **G** | **G** | **C** | **A** | **C** | **A** | **T** | **T** | **A** | **T** | **T** | **A** | **T** | **T** | **T** | **G** | **A** | **A** | **C** | **A** | **A** | **G** | **C** | **A** | **A** | **A** | **T** | **C** | **T** | **A** | **A** | **A** | **T** | **T** | **T** | **T** | **G** | **A** | **C** | **A** | **C** | **A** | **C** | **A** | **A** | **T** | **G** | **G** | **A** | **A** | **A** | **C** | **T** | **A** | **G** | **G** | **A** | **C** | **T** | **T** | **A** | **T** | **C** | **G** | **G** | **A** | **C** | **G** | **A** | **A** | **A** | **C** | **G** | **G** | **T** | **C** | **G** | **A** | **G** | **G** | **A** | **A** | **A** | **G** | **A** | **C** | **A** | **A** | **C** | **T** | **T** | **T** | **A** | **C** | **T** | **G** | **A** | **A** | **T** | **A** | **T** | **T** | **C** | **T** | **A** | **C** | **A** | **A** | **A** | **A** | **C** | **A** | **A** | **A** | **C** | **T** | **A** | **C** | **C** | **T** | **T** | **A** | **T** | **C** | **A** | **A** | **G** | **G** | **G** | **C** | **A** | **A** | **G** | **T** | **A** | **A** | **T** | **C** | **C** | **A** | **T** | **C** | **A** | **G** | **C** | **A** | **A** | **G** | **A** | **A** | **T** | **T** | **T** | **G** | **T** | **C** | **T** | **A** | **T** | **T** | **T** | **C** | **C** | **C** | **G** | **C** | **A** | **G** | **C** | **A** | **A** | **A** | **C** | **A** | **A** | **A** | **A** | **G** | **A** | **T** | **A** | **A** | **A** | **G** | **A** | **G** | **C** | **G** | **T** | **T** | **T** | **A** | **A** | **C** | **C** | **T** | **A** | **T** | **T** | **A** | **T** | **G** | **T** | **G** | **T** | **T** | **A** | **A** | **A** | **T** | **G** | **A** | **G** | **A** | **T** | **T** | **A** | **C** | **G** | **G** | **A** | **T** | **T** | **T** | **T** | **G** | **A** | **G** | **A** | **T** | **A** | **T** | **G** | **G** | **G** | **A** | **A** | **A** | **T** | **T** | **G** | **A** | **A** | **A** | **G** | **A** | **G** | **A** | **G** | **C** | **T** | **C** | **C** | **A** | **A** | **C** | **T** | **G** | **A** | **T** | **G** | **C** | **A** | **A** | **A** | **C** | **A** | **G** | **A** | **T** | **C** | **C** | **T** | **G** | **A** | **A** | **A** | **T** | **C** | **T** | **T** | **A** | **T** | **G** | **G** | **A** | **G** | **A** | **G** | **A** | **A** | **T** | **T** | **C** | **A** | **G** | **C** | **A** | **C** | **A** | **C** | **T** | **A** | **T** | **C** | **A** | **G** | **G** | **A** | **G** | **G** | **A** | **G** | **A** | **G** | **A** | **A** | **G** | **A** | **C** | **A** | **A** | **A** | **A** | **G** | **T** | **C** | **C** | **T** | **A** | **C** | **T** | **G** | **G** | **C** | **A** | **C** | **T** | **T** | **T** | **T** | **A** | **T** | **T** | **T** | **G** | **T** | **G** | **G** | **A** | **T** | **G** | **A** | **C** | **A** | **C** | **T** | **C** | **A** | **T** | **T** | **T** | **C** | **C** | **C** | **G** | **T** | **T** | **A** | **A** | **T** | **C** | **G** | **A** | **T** | **G** | **A** | **A** | **C** | **C** | **A** | **A** | **C** | **G** | **A** | **A** | **T** | **C** | **A** | **T** | **T** | **T** | **G** | **G** | **A** | **-** | **-** | **-** | **T** | **A** | **T** | **C** | **T** | **C** | **T** | **G** | **A** | **T** | **A** | **G** | **A** | **A** | **A** | **A** | **C** | **A** | **A** | **G** | **T** | **A** | **G** | **C** | **G** | **G** | **C** | **T** | **T** | **A** | **C** | **T** | **T** | **G** | **A** | **A** | **A** | **A** | **A** | **G** | **A** | **A** | **A** | **A** | **A** | **A** | **C** | **A** | **A** | **G** | **G** | **C** | **T** | **T** | **C** | **A** | **T** | **C** | **G** | **T** | **G** | **G** | **T** | **C** | **A** | **G** | **C** | **C** | **A** | **T** | **G** | **A** | **C** | **C** | **G** | **G** | **G** | **G** | **A** | **T** | **T** | **T** | **A** | **T** | **C** | **G** | **A** | **T** | **G** | **A** | **A** | **G** | **T** | **A** | **G** | **T** | **G** | **G** | **A** | **C** | **C** | **A** | **T** | **G** | **T** | **T** | **T** | **T** | **A** | **G** | **C** | **A** | **A** | **T** | **C** | **G** | **A** | **A** | **A** | **A** | **A** | **A** | **G** | **T** | **C** | **A** | **A** | **C** | **T** | **G** | **G** | **A** | **A** | **C** | **T** | **T** | **T** | **A** | **T** | **C** | **A** | **A** | **G** | **G** | **G** | **A** | **A** | **T** | **T** | **T** | **C** | **T** | **C** | **T** | **A** | **T** | **C** | **T** | **A** | **T** | **G** | **A** | **A** | **G** | **A** | **A** | **C** | **A** | **G** | **A** | **A** | **A** | **A** | **A** | **A** | **C** | **T** | **T** | **C** | **G** | **T** | **G** | **A** | **T** | **G** | **A** | **A** | **T** | **T** | **T** | **G** | **A** | **A** | **A** | **T** | **A** | **G** | **C** | **T** | **C** | **A** | **A** | **A** | **A** | **T** | **G** | **A** | **A** | **A** | **A** | **A** | **T** | **T** | **G** | **A** | **A** | **A** | **A** | **A** | **A** | **G** | **A** | **A** | **G** | **T** | **C** | **A** | **G** | **T** | **A** | **G** | **G** | **C** | **T** | **A** | **A** | **A** | **G** | **A** | **A** | **A** | **A** | **C** | **A** | **G** | **C** | **A** | **G** | **C** | **T** | **G** | **A** | **A** | **A** | **A** | **A** | **G** | **C** | **C** | **G** | **A** | **A** | **T** | **G** | **G** | **T** | **C** | **T** | **C** | **G** | **T** | **T** | **C** | **C** | **C** | **G** | **A** | **G** | **A** | **A** | **G** | **G** | **A** | **G** | **A** | **T** | **A** | **A** | **A** | **A** | **C** | **A** | **A** | **A** | **G** | **A** | **A** | **A** | **C** | **A** | **A** | **G** | **T** | **C** | **G** | **G** | **A** | **T** | **T** | **C** | **A** | **T** | **C** | **G** | **A** | **T** | **A** | **C** | **T** | **G** | **A** | **A** | **T** | **C** | **T** | **A** | **G** | **A** | **C** | **G** | **A** | **G** | **T** | **G** | **A** | **A** | **T** | **A** | **A** | **A** | **G** | **G** | **A** | **G** | **C** | **A** | **G** | **T** | **G** | **G** | **G** | **T** | **G** | **C** | **T** | **G** | **A** | **T** | **G** | **C** | **T** | **G** | **C** | **A** | **C** | **G** | **G** | **A** | **C** | **G** | **A** | **T** | **G** | **A** | **A** | **A** | **C** | **G** | **A** | **T** | **C** | **C** | **A** | **A** | **A** | **G** | **C** | **A** | **A** | **T** | **C** | **G** | **T** | **G** | **A** | **A** | **T** | **C** | **G** | **G** | **A** | **T** | **G** | **G** | **A** | **G** | **A** | **C** | **C** | **C** | **A** | **G** | **A** | **T** | **C** | **A** | **G** | **C** | **G** | **A** | **G** | **A** | **A** | **A** | **G** | **A** | **A** | **A** | **A** | **A** | **C** | **T** | **A** | **T** | **T** | **A** | **A** | **A** | **A** | **G** | **A** | **T** | **A** | **T** | **C** | **G** | **A** | **A** | **T** | **A** | **T** | **A** | **T** | **C** | **G** | **A** | **T** | **T** | **C** | **T** | **T** | **T** | **G** | **A** | **C** | **G** | **A** | **T** | **G** | **A** | **A** | **T** | **A** | **G** | **C** | **C** | **A** | **A** | **G** | **C** | **G** | **T** | **C** | **T** | **C** | **A** | **C** | **C** | **A** | **T** | **A** | **A** | **G** | **C** | **G** | **A** | **C** | **T** | **T** | **T** | **T** | **A** | **A** | **G** | **C** | **G** | **T** | **A** | **G** | **A** | **A** | **G** | **A** | **T** | **C** | **T** | **T** | **C** | **A** | **A** | **T** | **T** | **A** | **G** | **G** | **G** | **T** | **A** | **T** | **G** | **A** | **A** | **A** | **A** | **T** | **C** | **T** | **G** | **T** | **T** | **A** | **T** | **T** | **C** | **G** | **A** | **G** | **C** | **C** | **A** | **A** | **T** | **T** | **C** | **A** | **T** | **T** | **T** | **T** | **A** | **C** | **A** | **A** | **T** | **C** | **G** | **A** | **G** | **C** | **C** | **T** | **C** | **A** | **T** | **C** | **A** | **G** | **C** | **G** | **G** | **G** | **T** | **G** | **G** | **C** | **G** | **A** | **T** | **T** | **T** | **C** | **A** | **G** | **G** | **T** | **C** | **C** | **T** | **A** | **A** | **C** | **G** | **G** | **T** | **G** | **C** | **A** | **G** | **G** | **A** | **A** | **A** | **G** | **T** | **C** | **A** | **T** | **C** | **C** | **A** | **T** | **T** | **A** | **T** | **C** | **C** | **A** | **T** | **T** | **A** | **T** | **C** | **T** | **T** | **C** | **T** | **G** | **G** | **G** | **G** | **G** | **C** | **A** | **T** | **T** | **C** | **A** | **A** | **C** | **G** | **G** | **C** | **A** | **A** | **G** | **G** | **T** | **T** | **A** | **T** | **A** | **G** | **G** | **A** | **G** | **A** | **A** | **A** | **A** | **A** | **T** | **C** | **C** | **C** | **A** | **G** | **C** | **C** | **A** | **A** | **A** | **A** | **C** | **A** | **T** | **C** | **T** | **G** | **A** | **G** | **C** | **A** | **T** | **T** | **A** | **G** | **T** | **T** | **A** | **T** | **G** | **T** | **A** | **C** | **G** | **C** | **C** | **A** | **A** | **A** | **A** | **T** | **T** | **A** | **T** | **G** | **A** | **A** | **G** | **A** | **C** | **A** | **A** | **T** | **C** | **G** | **A** | **G** | **G** | **A** | **A** | **C** | **G** | **T** | **T** | **G** | **G** | **C** | **G** | **G** | **A** | **A** | **T** | **T** | **T** | **G** | **C** | **A** | **G** | **A** | **G** | **A** | **A** | **A** | **A** | **A** | **C** | **C** | **A** | **A** | **G** | **T** | **A** | **G** | **A** | **C** | **T** | **A** | **C** | **C** | **A** | **A** | **G** | **C** | **A** | **T** | **T** | **T** | **T** | **T** | **G** | **A** | **A** | **C** | **A** | **A** | **C** | **C** | **T** | **C** | **C** | **G** | **A** | **A** | **A** | **G** | **C** | **T** | **T** | **G** | **G** | **G** | **A** | **T** | **G** | **G** | **A** | **A** | **A** | **G** | **A** | **G** | **A** | **T** | **G** | **T** | **T** | **T** | **T** | **T** | **C** | **A** | **T** | **A** | **A** | **C** | **A** | **A** | **G** | **A** | **T** | **C** | **G** | **A** | **G** | **C** | **A** | **G** | **A** | **T** | **G** | **A** | **G** | **T** | **A** | **T** | **G** | **G** | **G** | **C** | **C** | **A** | **A** | **C** | **G** | **G** | **A** | **A** | **A** | **A** | **A** | **A** | **G** | **T** | **G** | **G** | **A** | **A** | **T** | **T** | **G** | **G** | **C** | **T** | **A** | **A** | **A** | **T** | **C** | **T** | **T** | **T** | **A** | **T** | **C** | **A** | **C** | **A** | **G** | **C** | **C** | **A** | **G** | **C** | **T** | **G** | **A** | **A** | **C** | **T** | **A** | **T** | **A** | **T** | **A** | **T** | **A** | **T** | **G** | **G** | **G** | **A** | **T** | **G** | **A** | **A** | **C** | **C** | **A** | **T** | **T** | **G** | **A** | **A** | **T** | **T** | **A** | **T** | **T** | **T** | **G** | **G** | **A** | **T** | **G** | **T** | **C** | **T** | **T** | **C** | **A** | **A** | **T** | **C** | **A** | **A** | **G** | **A** | **A** | **C** | **A** | **A** | **T** | **T** | **A** | **G** | **A** | **A** | **C** | **A** | **A** | **C** | **T** | **G** | **A** | **T** | **C** | **T** | **T** | **G** | **A** | **A** | **C** | **G** | **T** | **G** | **A** | **A** | **A** | **C** | **C** | **T** | **G** | **C** | **C** | **A** | **T** | **G** | **T** | **T** | **A** | **C** | **T** | **A** | **G** | **T** | **G** | **G** | **A** | **A** | **C** | **A** | **T** | **G** | **A** | **T** | **C** | **A** | **A** | **A** | **C** | **C** | **T** | **T** | **C** | **C** | **T** | **G** | **G** | **A** | **T** | **A** | **A** | **A** | **G** | **T** | **A** | **T** | **C** | **T** | **A** | **C** | **T** | **G** | **A** | **G** | **A** | **T** | **T** | **A** | **T** | **T** | **T** | **C** | **T** | **C** | **T** | **T** | **G** | **A** | **G** | **A** | **A** | **A** | **A** | **T** | **C** | **-** | **-** | **-** |
| **N52829** | **A** | **T** | **G** | **T** | **C** | **T** | **A** | **A** | **A** | **A** | **T** | **C** | **G** | **A** | **A** | **A** | **T** | **A** | **A** | **A** | **A** | **A** | **A** | **T** | **C** | **T** | **G** | **A** | **C** | **A** | **T** | **T** | **C** | **G** | **G** | **C** | **T** | **A** | **C** | **G** | **A** | **C** | **A** | **G** | **C** | **C** | **A** | **A** | **G** | **G** | **C** | **A** | **C** | **A** | **T** | **T** | **A** | **T** | **T** | **A** | **T** | **T** | **T** | **G** | **A** | **A** | **C** | **A** | **A** | **G** | **C** | **A** | **A** | **A** | **T** | **C** | **T** | **A** | **A** | **A** | **T** | **T** | **T** | **T** | **G** | **A** | **C** | **A** | **C** | **A** | **C** | **A** | **A** | **T** | **G** | **G** | **A** | **A** | **A** | **C** | **T** | **A** | **G** | **G** | **A** | **C** | **T** | **T** | **A** | **T** | **C** | **G** | **G** | **A** | **C** | **G** | **A** | **A** | **A** | **C** | **G** | **G** | **T** | **C** | **G** | **A** | **G** | **G** | **A** | **A** | **A** | **G** | **A** | **C** | **A** | **A** | **C** | **T** | **T** | **T** | **A** | **C** | **T** | **G** | **A** | **A** | **T** | **A** | **T** | **T** | **C** | **T** | **A** | **C** | **A** | **A** | **A** | **A** | **C** | **A** | **A** | **A** | **C** | **T** | **A** | **C** | **C** | **T** | **T** | **A** | **T** | **C** | **A** | **A** | **G** | **G** | **G** | **C** | **A** | **A** | **G** | **T** | **A** | **A** | **T** | **C** | **C** | **A** | **T** | **C** | **A** | **G** | **C** | **A** | **A** | **G** | **A** | **A** | **T** | **T** | **T** | **G** | **T** | **C** | **T** | **A** | **T** | **T** | **T** | **C** | **C** | **C** | **G** | **C** | **A** | **A** | **C** | **A** | **G** | **A** | **C** | **A** | **A** | **A** | **A** | **G** | **A** | **T** | **A** | **A** | **A** | **G** | **A** | **A** | **C** | **G** | **T** | **T** | **T** | **A** | **A** | **C** | **C** | **T** | **A** | **T** | **T** | **A** | **C** | **G** | **T** | **G** | **T** | **T** | **A** | **A** | **A** | **T** | **G** | **A** | **T** | **A** | **T** | **T** | **A** | **C** | **G** | **G** | **A** | **T** | **T** | **T** | **T** | **G** | **A** | **G** | **A** | **T** | **A** | **T** | **G** | **G** | **G** | **A** | **A** | **A** | **T** | **C** | **G** | **A** | **A** | **A** | **G** | **A** | **G** | **A** | **G** | **C** | **T** | **C** | **C** | **A** | **A** | **T** | **T** | **G** | **A** | **T** | **G** | **C** | **A** | **A** | **A** | **C** | **A** | **G** | **A** | **T** | **C** | **C** | **T** | **G** | **A** | **A** | **A** | **T** | **C** | **T** | **T** | **A** | **T** | **G** | **G** | **A** | **G** | **A** | **G** | **A** | **A** | **T** | **T** | **C** | **A** | **G** | **C** | **A** | **C** | **A** | **C** | **T** | **A** | **T** | **C** | **G** | **G** | **G** | **G** | **G** | **G** | **A** | **G** | **A** | **G** | **A** | **A** | **G** | **A** | **C** | **A** | **A** | **A** | **A** | **G** | **T** | **C** | **C** | **T** | **A** | **C** | **T** | **G** | **G** | **C** | **A** | **C** | **T** | **T** | **T** | **T** | **A** | **T** | **T** | **T** | **G** | **T** | **G** | **G** | **A** | **T** | **G** | **A** | **C** | **A** | **C** | **T** | **C** | **A** | **T** | **T** | **T** | **T** | **C** | **C** | **G** | **T** | **T** | **A** | **A** | **T** | **C** | **G** | **A** | **T** | **G** | **A** | **A** | **C** | **C** | **A** | **A** | **C** | **G** | **A** | **A** | **T** | **C** | **A** | **T** | **T** | **T** | **G** | **G** | **A** | **-** | **-** | **-** | **T** | **A** | **T** | **C** | **T** | **C** | **T** | **G** | **G** | **T** | **A** | **G** | **A** | **A** | **A** | **A** | **C** | **A** | **A** | **G** | **T** | **A** | **G** | **C** | **G** | **G** | **C** | **T** | **T** | **A** | **T** | **T** | **T** | **G** | **A** | **A** | **A** | **A** | **A** | **G** | **A** | **A** | **A** | **A** | **A** | **A** | **C** | **A** | **A** | **G** | **G** | **C** | **T** | **T** | **C** | **A** | **T** | **C** | **G** | **T** | **G** | **G** | **T** | **C** | **A** | **G** | **C** | **C** | **A** | **T** | **G** | **A** | **C** | **C** | **G** | **G** | **G** | **G** | **A** | **T** | **T** | **T** | **A** | **T** | **C** | **G** | **A** | **T** | **G** | **A** | **A** | **G** | **T** | **A** | **G** | **T** | **G** | **G** | **A** | **C** | **C** | **A** | **T** | **G** | **T** | **T** | **T** | **T** | **A** | **G** | **C** | **A** | **A** | **T** | **C** | **G** | **A** | **A** | **A** | **A** | **A** | **A** | **G** | **T** | **C** | **A** | **A** | **C** | **T** | **G** | **G** | **A** | **A** | **C** | **T** | **T** | **T** | **A** | **T** | **C** | **A** | **A** | **G** | **G** | **G** | **A** | **A** | **T** | **T** | **T** | **C** | **T** | **C** | **T** | **A** | **T** | **C** | **T** | **A** | **T** | **G** | **A** | **A** | **G** | **A** | **A** | **C** | **A** | **G** | **A** | **A** | **A** | **A** | **A** | **A** | **C** | **T** | **T** | **C** | **G** | **T** | **G** | **A** | **T** | **G** | **A** | **A** | **T** | **T** | **T** | **G** | **A** | **A** | **A** | **T** | **G** | **G** | **C** | **T** | **C** | **A** | **A** | **A** | **A** | **T** | **G** | **A** | **A** | **A** | **A** | **A** | **T** | **T** | **G** | **A** | **A** | **A** | **A** | **A** | **A** | **G** | **A** | **A** | **G** | **T** | **C** | **A** | **G** | **C** | **A** | **G** | **G** | **C** | **T** | **A** | **A** | **A** | **G** | **A** | **C** | **A** | **A** | **C** | **A** | **G** | **C** | **A** | **G** | **C** | **T** | **G** | **A** | **A** | **A** | **A** | **A** | **G** | **C** | **C** | **G** | **A** | **A** | **T** | **G** | **G** | **T** | **C** | **T** | **C** | **G** | **T** | **T** | **C** | **C** | **C** | **G** | **A** | **G** | **A** | **A** | **G** | **G** | **A** | **G** | **A** | **T** | **A** | **A** | **A** | **A** | **C** | **A** | **A** | **A** | **G** | **A** | **A** | **A** | **C** | **A** | **A** | **G** | **T** | **C** | **G** | **G** | **A** | **C** | **T** | **C** | **A** | **T** | **C** | **G** | **A** | **T** | **A** | **C** | **T** | **G** | **A** | **A** | **T** | **C** | **T** | **A** | **G** | **A** | **C** | **G** | **A** | **G** | **T** | **G** | **A** | **A** | **T** | **A** | **A** | **A** | **G** | **G** | **A** | **G** | **C** | **A** | **G** | **T** | **G** | **G** | **G** | **T** | **G** | **C** | **T** | **G** | **A** | **T** | **G** | **C** | **T** | **G** | **C** | **A** | **C** | **G** | **G** | **A** | **C** | **G** | **A** | **T** | **G** | **A** | **A** | **A** | **C** | **G** | **A** | **T** | **C** | **C** | **A** | **A** | **A** | **G** | **C** | **A** | **A** | **T** | **C** | **G** | **T** | **G** | **A** | **A** | **T** | **C** | **G** | **G** | **A** | **T** | **G** | **G** | **A** | **G** | **A** | **C** | **C** | **C** | **A** | **G** | **A** | **T** | **C** | **A** | **G** | **C** | **G** | **A** | **G** | **A** | **A** | **A** | **G** | **A** | **A** | **A** | **A** | **A** | **C** | **T** | **A** | **T** | **T** | **A** | **A** | **A** | **A** | **G** | **A** | **T** | **A** | **T** | **C** | **G** | **A** | **A** | **T** | **A** | **T** | **A** | **T** | **C** | **G** | **A** | **T** | **T** | **C** | **T** | **T** | **T** | **G** | **A** | **C** | **G** | **A** | **T** | **G** | **A** | **A** | **T** | **A** | **G** | **C** | **C** | **A** | **A** | **G** | **C** | **G** | **T** | **C** | **T** | **C** | **A** | **C** | **C** | **A** | **T** | **A** | **A** | **G** | **C** | **G** | **A** | **C** | **T** | **T** | **T** | **T** | **A** | **A** | **G** | **C** | **G** | **T** | **A** | **G** | **A** | **A** | **G** | **A** | **T** | **C** | **T** | **T** | **C** | **A** | **A** | **T** | **T** | **A** | **G** | **G** | **G** | **T** | **A** | **T** | **G** | **A** | **A** | **A** | **A** | **T** | **C** | **T** | **G** | **T** | **T** | **A** | **T** | **T** | **C** | **G** | **A** | **G** | **C** | **C** | **A** | **A** | **T** | **T** | **C** | **A** | **T** | **T** | **T** | **T** | **A** | **C** | **A** | **A** | **T** | **C** | **G** | **A** | **G** | **C** | **C** | **T** | **C** | **A** | **T** | **C** | **A** | **G** | **C** | **G** | **G** | **G** | **T** | **G** | **G** | **C** | **G** | **A** | **T** | **T** | **T** | **C** | **A** | **G** | **G** | **T** | **C** | **C** | **T** | **A** | **A** | **C** | **G** | **G** | **T** | **G** | **C** | **A** | **G** | **G** | **A** | **A** | **A** | **G** | **T** | **C** | **A** | **T** | **C** | **C** | **A** | **T** | **T** | **A** | **T** | **C** | **C** | **A** | **T** | **T** | **A** | **T** | **C** | **T** | **T** | **C** | **T** | **G** | **G** | **G** | **G** | **T** | **C** | **A** | **T** | **T** | **C** | **A** | **A** | **C** | **G** | **G** | **C** | **A** | **A** | **G** | **G** | **T** | **T** | **A** | **T** | **A** | **G** | **G** | **A** | **G** | **A** | **A** | **A** | **A** | **A** | **T** | **C** | **C** | **C** | **A** | **G** | **C** | **C** | **A** | **A** | **A** | **A** | **C** | **A** | **T** | **C** | **T** | **G** | **A** | **G** | **C** | **A** | **T** | **T** | **A** | **G** | **T** | **T** | **A** | **T** | **G** | **T** | **A** | **C** | **G** | **C** | **C** | **A** | **A** | **A** | **A** | **T** | **T** | **A** | **T** | **G** | **A** | **A** | **G** | **A** | **C** | **A** | **A** | **T** | **C** | **G** | **A** | **G** | **G** | **A** | **A** | **C** | **G** | **T** | **T** | **G** | **G** | **C** | **G** | **G** | **A** | **A** | **T** | **T** | **T** | **G** | **C** | **A** | **G** | **A** | **G** | **A** | **A** | **A** | **A** | **A** | **C** | **C** | **A** | **A** | **G** | **T** | **A** | **G** | **A** | **C** | **T** | **A** | **C** | **C** | **A** | **A** | **G** | **C** | **A** | **T** | **T** | **T** | **T** | **T** | **G** | **A** | **A** | **C** | **A** | **A** | **C** | **C** | **T** | **C** | **C** | **G** | **A** | **A** | **A** | **G** | **C** | **T** | **T** | **G** | **G** | **G** | **A** | **T** | **G** | **G** | **A** | **A** | **A** | **G** | **A** | **G** | **A** | **T** | **G** | **T** | **T** | **T** | **T** | **T** | **C** | **A** | **T** | **A** | **A** | **C** | **A** | **A** | **G** | **A** | **T** | **T** | **G** | **A** | **G** | **C** | **A** | **G** | **A** | **T** | **G** | **A** | **G** | **T** | **A** | **T** | **G** | **G** | **G** | **C** | **C** | **A** | **A** | **C** | **G** | **G** | **A** | **A** | **A** | **A** | **A** | **A** | **G** | **T** | **G** | **G** | **A** | **A** | **T** | **T** | **G** | **G** | **C** | **T** | **A** | **A** | **A** | **T** | **C** | **T** | **T** | **T** | **A** | **T** | **C** | **A** | **C** | **A** | **G** | **C** | **C** | **A** | **G** | **C** | **T** | **G** | **A** | **A** | **C** | **T** | **A** | **T** | **A** | **T** | **A** | **T** | **A** | **T** | **G** | **G** | **G** | **A** | **T** | **G** | **A** | **A** | **C** | **C** | **A** | **T** | **T** | **G** | **A** | **A** | **T** | **T** | **A** | **T** | **T** | **T** | **G** | **G** | **A** | **T** | **G** | **T** | **C** | **T** | **T** | **C** | **A** | **A** | **T** | **C** | **A** | **A** | **G** | **A** | **A** | **C** | **A** | **A** | **T** | **T** | **A** | **G** | **A** | **A** | **C** | **A** | **A** | **C** | **T** | **G** | **A** | **T** | **C** | **T** | **T** | **G** | **A** | **A** | **C** | **G** | **T** | **G** | **A** | **A** | **A** | **C** | **C** | **T** | **G** | **C** | **C** | **A** | **T** | **G** | **T** | **T** | **A** | **C** | **T** | **G** | **G** | **T** | **G** | **G** | **A** | **A** | **C** | **A** | **T** | **G** | **A** | **T** | **C** | **A** | **A** | **A** | **C** | **C** | **T** | **T** | **C** | **C** | **T** | **G** | **G** | **A** | **T** | **A** | **A** | **A** | **G** | **T** | **A** | **T** | **C** | **T** | **A** | **C** | **T** | **G** | **A** | **G** | **A** | **T** | **T** | **A** | **T** | **T** | **T** | **C** | **T** | **C** | **T** | **T** | **G** | **A** | **G** | **A** | **A** | **A** | **A** | **T** | **C** | **-** | **-** | **-** |
| **N55279** | **A** | **T** | **G** | **T** | **C** | **T** | **A** | **A** | **A** | **A** | **T** | **C** | **G** | **A** | **A** | **A** | **T** | **A** | **A** | **A** | **A** | **A** | **A** | **T** | **C** | **T** | **G** | **A** | **C** | **A** | **T** | **T** | **C** | **G** | **G** | **C** | **T** | **A** | **T** | **G** | **A** | **C** | **A** | **G** | **C** | **C** | **A** | **A** | **G** | **G** | **C** | **A** | **C** | **A** | **T** | **T** | **A** | **T** | **T** | **A** | **T** | **T** | **T** | **G** | **A** | **A** | **C** | **A** | **A** | **G** | **C** | **C** | **A** | **A** | **T** | **C** | **T** | **A** | **A** | **A** | **C** | **T** | **T** | **T** | **G** | **A** | **C** | **A** | **C** | **A** | **C** | **A** | **A** | **T** | **G** | **G** | **A** | **A** | **A** | **C** | **T** | **A** | **G** | **G** | **A** | **C** | **T** | **T** | **A** | **T** | **C** | **G** | **G** | **A** | **C** | **G** | **A** | **A** | **A** | **C** | **G** | **G** | **T** | **C** | **G** | **A** | **G** | **G** | **A** | **A** | **A** | **G** | **A** | **C** | **A** | **A** | **C** | **T** | **T** | **T** | **A** | **C** | **T** | **G** | **A** | **A** | **T** | **A** | **T** | **C** | **C** | **T** | **G** | **C** | **A** | **A** | **A** | **A** | **C** | **A** | **A** | **A** | **C** | **T** | **C** | **C** | **C** | **T** | **T** | **A** | **T** | **C** | **A** | **A** | **G** | **G** | **G** | **C** | **A** | **A** | **G** | **T** | **G** | **A** | **T** | **C** | **C** | **A** | **T** | **C** | **A** | **G** | **C** | **A** | **A** | **G** | **C** | **G** | **T** | **T** | **T** | **G** | **T** | **C** | **T** | **A** | **T** | **T** | **T** | **C** | **C** | **C** | **G** | **C** | **A** | **G** | **C** | **A** | **A** | **A** | **C** | **A** | **A** | **A** | **A** | **G** | **A** | **T** | **A** | **A** | **A** | **G** | **A** | **G** | **C** | **G** | **T** | **T** | **T** | **G** | **A** | **C** | **C** | **T** | **A** | **T** | **T** | **A** | **T** | **G** | **T** | **G** | **T** | **T** | **A** | **A** | **A** | **T** | **G** | **A** | **G** | **A** | **T** | **T** | **A** | **C** | **G** | **G** | **A** | **T** | **T** | **T** | **T** | **G** | **A** | **G** | **A** | **T** | **A** | **T** | **G** | **G** | **G** | **A** | **A** | **A** | **T** | **T** | **G** | **A** | **A** | **A** | **G** | **A** | **G** | **A** | **G** | **C** | **T** | **C** | **C** | **A** | **A** | **C** | **T** | **G** | **A** | **T** | **G** | **C** | **A** | **A** | **A** | **C** | **A** | **G** | **A** | **T** | **C** | **C** | **T** | **G** | **A** | **A** | **A** | **T** | **C** | **T** | **T** | **A** | **T** | **G** | **G** | **A** | **G** | **A** | **G** | **A** | **A** | **T** | **T** | **C** | **A** | **G** | **C** | **A** | **C** | **A** | **C** | **T** | **A** | **T** | **C** | **A** | **G** | **G** | **A** | **G** | **G** | **A** | **G** | **A** | **G** | **A** | **A** | **G** | **A** | **C** | **A** | **A** | **A** | **A** | **G** | **T** | **C** | **C** | **T** | **A** | **C** | **T** | **G** | **G** | **C** | **A** | **C** | **T** | **C** | **T** | **T** | **A** | **T** | **T** | **T** | **G** | **T** | **G** | **G** | **A** | **T** | **G** | **A** | **C** | **A** | **C** | **T** | **C** | **A** | **T** | **T** | **T** | **C** | **C** | **C** | **G** | **T** | **T** | **A** | **A** | **T** | **C** | **G** | **A** | **T** | **G** | **A** | **A** | **C** | **C** | **A** | **A** | **C** | **G** | **A** | **A** | **T** | **C** | **A** | **T** | **T** | **T** | **G** | **G** | **A** | **-** | **-** | **-** | **T** | **A** | **T** | **C** | **T** | **C** | **T** | **G** | **G** | **C** | **A** | **G** | **A** | **A** | **A** | **A** | **C** | **A** | **A** | **G** | **T** | **A** | **G** | **C** | **G** | **G** | **C** | **T** | **T** | **A** | **C** | **T** | **T** | **G** | **A** | **A** | **A** | **A** | **A** | **G** | **A** | **A** | **A** | **A** | **A** | **A** | **C** | **A** | **A** | **G** | **G** | **A** | **T** | **T** | **C** | **A** | **T** | **C** | **G** | **T** | **G** | **G** | **T** | **G** | **A** | **G** | **T** | **C** | **A** | **T** | **G** | **A** | **T** | **C** | **G** | **G** | **G** | **G** | **A** | **T** | **T** | **T** | **A** | **T** | **C** | **G** | **A** | **T** | **G** | **A** | **A** | **G** | **T** | **A** | **G** | **T** | **G** | **G** | **A** | **C** | **C** | **A** | **T** | **G** | **T** | **T** | **T** | **T** | **A** | **G** | **C** | **A** | **A** | **T** | **C** | **G** | **A** | **A** | **A** | **A** | **G** | **A** | **G** | **C** | **C** | **A** | **A** | **C** | **T** | **G** | **G** | **A** | **A** | **C** | **T** | **T** | **T** | **A** | **T** | **C** | **A** | **A** | **G** | **G** | **G** | **A** | **A** | **T** | **T** | **T** | **C** | **T** | **C** | **T** | **A** | **T** | **C** | **T** | **A** | **T** | **G** | **A** | **A** | **G** | **A** | **A** | **C** | **A** | **G** | **A** | **A** | **A** | **A** | **A** | **A** | **C** | **T** | **T** | **C** | **G** | **T** | **G** | **A** | **T** | **G** | **A** | **A** | **T** | **T** | **T** | **G** | **A** | **A** | **A** | **T** | **A** | **G** | **C** | **T** | **A** | **A** | **A** | **A** | **A** | **T** | **G** | **A** | **A** | **A** | **A** | **A** | **T** | **T** | **G** | **A** | **A** | **A** | **A** | **A** | **A** | **G** | **A** | **A** | **G** | **T** | **C** | **A** | **G** | **C** | **A** | **G** | **G** | **C** | **T** | **A** | **A** | **A** | **G** | **A** | **A** | **A** | **A** | **C** | **A** | **G** | **C** | **A** | **G** | **C** | **T** | **G** | **A** | **A** | **A** | **A** | **A** | **G** | **C** | **A** | **G** | **A** | **A** | **T** | **G** | **G** | **T** | **C** | **T** | **C** | **G** | **T** | **T** | **C** | **C** | **C** | **G** | **A** | **G** | **A** | **A** | **G** | **G** | **A** | **G** | **A** | **C** | **A** | **A** | **A** | **A** | **C** | **A** | **A** | **A** | **G** | **A** | **A** | **A** | **A** | **A** | **A** | **G** | **T** | **C** | **G** | **G** | **A** | **T** | **T** | **C** | **A** | **T** | **C** | **G** | **A** | **T** | **A** | **C** | **G** | **G** | **A** | **A** | **T** | **C** | **T** | **A** | **G** | **G** | **C** | **G** | **A** | **G** | **T** | **G** | **A** | **A** | **T** | **A** | **A** | **A** | **G** | **G** | **A** | **G** | **C** | **A** | **G** | **T** | **A** | **G** | **G** | **C** | **G** | **C** | **T** | **G** | **A** | **T** | **G** | **C** | **T** | **G** | **C** | **A** | **C** | **G** | **G** | **A** | **C** | **G** | **A** | **T** | **G** | **A** | **A** | **A** | **C** | **G** | **A** | **T** | **C** | **T** | **A** | **A** | **A** | **G** | **C** | **A** | **A** | **T** | **C** | **G** | **T** | **G** | **A** | **A** | **T** | **C** | **G** | **G** | **A** | **T** | **G** | **G** | **A** | **A** | **A** | **C** | **C** | **C** | **A** | **G** | **A** | **T** | **C** | **A** | **G** | **T** | **G** | **A** | **G** | **A** | **A** | **A** | **G** | **A** | **A** | **A** | **A** | **A** | **T** | **T** | **A** | **T** | **T** | **A** | **A** | **A** | **A** | **G** | **A** | **T** | **A** | **T** | **C** | **G** | **A** | **G** | **T** | **A** | **T** | **A** | **T** | **C** | **G** | **A** | **T** | **G** | **C** | **T** | **T** | **T** | **G** | **A** | **C** | **G** | **A** | **T** | **G** | **A** | **A** | **T** | **A** | **G** | **C** | **C** | **A** | **A** | **G** | **T** | **G** | **T** | **C** | **T** | **C** | **A** | **T** | **C** | **A** | **T** | **A** | **A** | **A** | **C** | **G** | **G** | **C** | **T** | **G** | **T** | **T** | **A** | **A** | **G** | **C** | **G** | **T** | **A** | **G** | **A** | **A** | **A** | **A** | **T** | **C** | **T** | **T** | **C** | **A** | **A** | **T** | **T** | **A** | **G** | **G** | **G** | **T** | **A** | **T** | **G** | **A** | **A** | **A** | **A** | **T** | **C** | **T** | **G** | **T** | **T** | **A** | **T** | **T** | **C** | **G** | **A** | **G** | **C** | **C** | **A** | **G** | **T** | **T** | **C** | **A** | **T** | **T** | **T** | **T** | **A** | **C** | **G** | **A** | **T** | **C** | **G** | **A** | **A** | **C** | **C** | **T** | **C** | **A** | **T** | **C** | **A** | **G** | **C** | **G** | **G** | **G** | **T** | **G** | **G** | **C** | **A** | **A** | **T** | **T** | **T** | **C** | **A** | **G** | **G** | **T** | **C** | **C** | **T** | **A** | **A** | **C** | **G** | **G** | **C** | **G** | **C** | **G** | **G** | **G** | **A** | **A** | **A** | **G** | **T** | **C** | **A** | **T** | **C** | **C** | **A** | **T** | **T** | **A** | **T** | **C** | **C** | **A** | **T** | **T** | **A** | **T** | **C** | **T** | **T** | **C** | **T** | **G** | **G** | **G** | **G** | **A** | **C** | **A** | **T** | **T** | **C** | **A** | **A** | **C** | **G** | **G** | **C** | **A** | **A** | **G** | **G** | **T** | **T** | **G** | **T** | **A** | **G** | **G** | **A** | **G** | **A** | **A** | **A** | **A** | **A** | **T** | **C** | **C** | **C** | **A** | **G** | **C** | **C** | **C** | **A** | **A** | **A** | **C** | **A** | **T** | **T** | **T** | **G** | **A** | **C** | **C** | **A** | **T** | **T** | **A** | **G** | **T** | **T** | **A** | **C** | **G** | **T** | **A** | **C** | **G** | **C** | **C** | **A** | **A** | **A** | **A** | **T** | **T** | **A** | **T** | **G** | **A** | **A** | **G** | **A** | **C** | **A** | **A** | **T** | **C** | **G** | **A** | **G** | **G** | **G** | **A** | **C** | **A** | **T** | **T** | **G** | **G** | **C** | **G** | **G** | **A** | **A** | **T** | **T** | **C** | **G** | **C** | **G** | **A** | **A** | **G** | **A** | **A** | **A** | **A** | **A** | **C** | **C** | **G** | **A** | **G** | **T** | **A** | **G** | **A** | **C** | **T** | **A** | **T** | **C** | **A** | **A** | **G** | **C** | **A** | **T** | **T** | **T** | **T** | **T** | **G** | **A** | **A** | **T** | **A** | **A** | **T** | **C** | **T** | **C** | **C** | **G** | **A** | **A** | **A** | **G** | **C** | **T** | **T** | **G** | **G** | **G** | **A** | **T** | **G** | **G** | **A** | **A** | **A** | **G** | **A** | **G** | **A** | **T** | **G** | **T** | **T** | **T** | **T** | **T** | **C** | **A** | **T** | **A** | **A** | **T** | **A** | **A** | **A** | **A** | **T** | **C** | **G** | **A** | **A** | **C** | **A** | **G** | **A** | **T** | **G** | **A** | **G** | **T** | **A** | **T** | **G** | **G** | **G** | **A** | **C** | **A** | **A** | **C** | **G** | **G** | **A** | **A** | **A** | **A** | **A** | **A** | **G** | **T** | **G** | **G** | **A** | **A** | **T** | **T** | **G** | **G** | **C** | **T** | **A** | **A** | **A** | **T** | **C** | **T** | **T** | **T** | **A** | **T** | **C** | **C** | **C** | **A** | **A** | **C** | **C** | **A** | **G** | **C** | **T** | **G** | **A** | **G** | **C** | **T** | **C** | **T** | **A** | **T** | **A** | **T** | **A** | **T** | **G** | **G** | **G** | **A** | **T** | **G** | **A** | **A** | **C** | **C** | **A** | **T** | **T** | **G** | **A** | **A** | **T** | **T** | **A** | **T** | **T** | **T** | **G** | **G** | **A** | **T** | **G** | **T** | **C** | **T** | **T** | **C** | **A** | **A** | **T** | **C** | **A** | **A** | **G** | **A** | **A** | **C** | **A** | **A** | **T** | **T** | **A** | **G** | **A** | **A** | **C** | **A** | **A** | **C** | **T** | **G** | **A** | **T** | **T** | **T** | **T** | **G** | **A** | **A** | **C** | **G** | **T** | **G** | **A** | **A** | **A** | **C** | **C** | **T** | **G** | **C** | **T** | **A** | **T** | **G** | **T** | **T** | **A** | **C** | **T** | **A** | **G** | **T** | **G** | **G** | **A** | **A** | **C** | **A** | **T** | **G** | **A** | **T** | **C** | **A** | **A** | **A** | **C** | **C** | **T** | **T** | **T** | **C** | **T** | **A** | **G** | **A** | **T** | **A** | **A** | **A** | **G** | **T** | **A** | **T** | **C** | **T** | **A** | **C** | **T** | **G** | **A** | **G** | **A** | **T** | **T** | **A** | **T** | **T** | **T** | **C** | **T** | **C** | **T** | **T** | **G** | **A** | **G** | **A** | **A** | **A** | **A** | **T** | **C** | **-** | **-** | **-** |
| **N60045F** | **A** | **T** | **G** | **T** | **C** | **T** | **A** | **A** | **A** | **A** | **T** | **C** | **G** | **A** | **A** | **A** | **T** | **A** | **A** | **A** | **A** | **A** | **A** | **T** | **C** | **T** | **G** | **A** | **C** | **A** | **T** | **T** | **C** | **G** | **G** | **C** | **T** | **A** | **C** | **G** | **A** | **C** | **A** | **G** | **C** | **C** | **A** | **A** | **G** | **G** | **C** | **A** | **C** | **A** | **T** | **T** | **A** | **T** | **T** | **A** | **T** | **T** | **T** | **G** | **A** | **A** | **C** | **A** | **A** | **G** | **C** | **A** | **A** | **A** | **T** | **C** | **T** | **A** | **A** | **A** | **T** | **T** | **T** | **T** | **G** | **A** | **C** | **A** | **T** | **A** | **C** | **A** | **A** | **T** | **G** | **G** | **A** | **A** | **A** | **C** | **T** | **A** | **G** | **G** | **A** | **C** | **T** | **T** | **A** | **T** | **C** | **G** | **G** | **A** | **C** | **G** | **A** | **A** | **A** | **C** | **G** | **G** | **T** | **C** | **G** | **A** | **G** | **G** | **A** | **A** | **A** | **G** | **A** | **C** | **A** | **A** | **C** | **T** | **T** | **T** | **A** | **C** | **T** | **G** | **A** | **A** | **T** | **A** | **T** | **T** | **C** | **T** | **A** | **C** | **A** | **A** | **A** | **A** | **C** | **A** | **A** | **A** | **C** | **T** | **A** | **C** | **C** | **T** | **T** | **A** | **T** | **C** | **A** | **A** | **G** | **G** | **G** | **C** | **A** | **A** | **G** | **T** | **A** | **A** | **T** | **C** | **C** | **A** | **T** | **C** | **A** | **G** | **C** | **A** | **A** | **G** | **A** | **A** | **T** | **T** | **T** | **G** | **T** | **C** | **T** | **A** | **T** | **T** | **T** | **C** | **C** | **C** | **G** | **C** | **A** | **A** | **C** | **A** | **G** | **A** | **C** | **A** | **A** | **A** | **A** | **G** | **A** | **T** | **A** | **A** | **A** | **G** | **A** | **A** | **C** | **G** | **T** | **T** | **T** | **A** | **A** | **C** | **C** | **T** | **A** | **T** | **T** | **A** | **C** | **G** | **T** | **G** | **T** | **T** | **A** | **A** | **A** | **T** | **G** | **A** | **T** | **A** | **T** | **T** | **A** | **C** | **G** | **G** | **A** | **T** | **T** | **T** | **T** | **G** | **A** | **G** | **A** | **T** | **A** | **T** | **G** | **G** | **G** | **A** | **A** | **A** | **T** | **C** | **G** | **A** | **A** | **A** | **G** | **A** | **G** | **A** | **G** | **C** | **T** | **C** | **C** | **A** | **A** | **T** | **T** | **G** | **A** | **T** | **G** | **C** | **A** | **A** | **A** | **C** | **A** | **G** | **A** | **T** | **C** | **C** | **T** | **G** | **A** | **A** | **A** | **T** | **C** | **T** | **T** | **A** | **T** | **G** | **G** | **A** | **G** | **A** | **G** | **A** | **A** | **T** | **T** | **C** | **A** | **G** | **C** | **A** | **C** | **A** | **C** | **T** | **A** | **T** | **C** | **A** | **G** | **G** | **G** | **G** | **G** | **A** | **G** | **A** | **G** | **A** | **A** | **G** | **A** | **C** | **A** | **A** | **A** | **A** | **G** | **T** | **C** | **C** | **T** | **A** | **C** | **T** | **G** | **G** | **C** | **A** | **C** | **T** | **T** | **T** | **T** | **A** | **T** | **T** | **T** | **G** | **T** | **G** | **G** | **A** | **T** | **G** | **A** | **C** | **A** | **C** | **T** | **C** | **A** | **T** | **T** | **T** | **C** | **C** | **C** | **G** | **T** | **T** | **A** | **A** | **T** | **C** | **G** | **A** | **T** | **G** | **A** | **A** | **C** | **C** | **A** | **A** | **C** | **G** | **A** | **A** | **T** | **C** | **A** | **T** | **T** | **T** | **G** | **G** | **A** | **-** | **-** | **-** | **T** | **A** | **T** | **C** | **T** | **C** | **T** | **G** | **G** | **T** | **A** | **G** | **A** | **A** | **A** | **A** | **C** | **A** | **A** | **G** | **T** | **A** | **G** | **C** | **G** | **G** | **C** | **T** | **T** | **A** | **T** | **T** | **T** | **G** | **A** | **A** | **A** | **A** | **A** | **G** | **A** | **A** | **A** | **A** | **A** | **A** | **C** | **A** | **A** | **G** | **G** | **C** | **T** | **T** | **C** | **A** | **T** | **C** | **G** | **T** | **G** | **G** | **T** | **C** | **A** | **G** | **C** | **C** | **A** | **T** | **G** | **A** | **C** | **C** | **G** | **G** | **G** | **G** | **A** | **T** | **T** | **T** | **A** | **T** | **C** | **G** | **A** | **T** | **G** | **A** | **A** | **G** | **T** | **A** | **G** | **T** | **G** | **G** | **A** | **C** | **C** | **A** | **T** | **G** | **T** | **T** | **T** | **T** | **A** | **G** | **C** | **A** | **A** | **T** | **C** | **G** | **A** | **A** | **A** | **A** | **A** | **A** | **G** | **T** | **C** | **A** | **A** | **C** | **T** | **G** | **G** | **A** | **A** | **C** | **T** | **T** | **T** | **A** | **T** | **C** | **A** | **A** | **G** | **G** | **G** | **A** | **A** | **T** | **T** | **T** | **C** | **T** | **C** | **T** | **A** | **T** | **C** | **T** | **A** | **T** | **G** | **A** | **A** | **G** | **A** | **A** | **C** | **A** | **G** | **A** | **A** | **A** | **A** | **A** | **A** | **C** | **T** | **T** | **C** | **G** | **T** | **G** | **A** | **T** | **G** | **A** | **A** | **T** | **T** | **T** | **G** | **A** | **A** | **A** | **T** | **G** | **G** | **C** | **T** | **C** | **A** | **A** | **A** | **A** | **T** | **G** | **A** | **A** | **A** | **A** | **A** | **T** | **T** | **G** | **A** | **A** | **A** | **A** | **A** | **A** | **G** | **A** | **A** | **G** | **T** | **C** | **A** | **G** | **T** | **A** | **G** | **G** | **C** | **T** | **A** | **A** | **A** | **G** | **A** | **A** | **A** | **A** | **C** | **A** | **G** | **C** | **A** | **G** | **C** | **T** | **G** | **A** | **A** | **A** | **A** | **A** | **G** | **C** | **C** | **G** | **A** | **A** | **T** | **G** | **G** | **T** | **C** | **T** | **C** | **G** | **T** | **T** | **C** | **C** | **C** | **G** | **A** | **G** | **A** | **A** | **G** | **G** | **A** | **G** | **A** | **T** | **A** | **A** | **A** | **A** | **C** | **A** | **A** | **A** | **G** | **A** | **A** | **A** | **C** | **A** | **A** | **G** | **T** | **C** | **G** | **G** | **A** | **T** | **T** | **C** | **A** | **T** | **C** | **G** | **A** | **T** | **A** | **C** | **T** | **G** | **A** | **A** | **T** | **C** | **T** | **A** | **G** | **A** | **C** | **G** | **A** | **G** | **T** | **G** | **A** | **A** | **T** | **A** | **A** | **A** | **G** | **G** | **A** | **G** | **C** | **A** | **G** | **T** | **G** | **G** | **G** | **T** | **G** | **C** | **T** | **G** | **A** | **T** | **G** | **C** | **T** | **G** | **C** | **A** | **C** | **G** | **G** | **A** | **C** | **G** | **A** | **T** | **G** | **A** | **A** | **A** | **C** | **G** | **A** | **T** | **C** | **C** | **A** | **A** | **A** | **G** | **C** | **A** | **A** | **T** | **C** | **G** | **T** | **G** | **A** | **A** | **T** | **C** | **G** | **G** | **A** | **T** | **G** | **G** | **A** | **G** | **A** | **C** | **C** | **C** | **A** | **G** | **A** | **T** | **C** | **A** | **G** | **C** | **G** | **A** | **G** | **A** | **A** | **A** | **G** | **A** | **A** | **A** | **A** | **A** | **C** | **T** | **A** | **T** | **T** | **A** | **A** | **A** | **A** | **G** | **A** | **T** | **A** | **T** | **C** | **G** | **A** | **A** | **T** | **A** | **T** | **A** | **T** | **C** | **G** | **A** | **T** | **T** | **C** | **T** | **T** | **T** | **G** | **A** | **C** | **G** | **A** | **T** | **G** | **A** | **A** | **T** | **A** | **G** | **T** | **C** | **A** | **A** | **G** | **C** | **G** | **T** | **C** | **T** | **C** | **A** | **C** | **C** | **A** | **T** | **A** | **A** | **G** | **C** | **G** | **A** | **C** | **T** | **T** | **T** | **T** | **A** | **A** | **G** | **C** | **G** | **T** | **A** | **G** | **A** | **A** | **G** | **A** | **T** | **C** | **T** | **T** | **C** | **A** | **A** | **T** | **T** | **A** | **G** | **G** | **G** | **T** | **A** | **T** | **G** | **A** | **A** | **A** | **A** | **T** | **C** | **T** | **G** | **T** | **T** | **A** | **T** | **T** | **C** | **G** | **A** | **G** | **C** | **C** | **A** | **A** | **T** | **T** | **C** | **A** | **T** | **T** | **T** | **T** | **A** | **C** | **A** | **A** | **T** | **C** | **G** | **A** | **G** | **C** | **C** | **T** | **C** | **A** | **T** | **C** | **A** | **G** | **C** | **G** | **G** | **G** | **T** | **G** | **G** | **C** | **G** | **A** | **T** | **T** | **T** | **C** | **A** | **G** | **G** | **T** | **C** | **C** | **T** | **A** | **A** | **C** | **G** | **G** | **T** | **G** | **C** | **A** | **G** | **G** | **A** | **A** | **A** | **G** | **T** | **C** | **A** | **T** | **C** | **C** | **A** | **T** | **T** | **A** | **T** | **C** | **C** | **A** | **T** | **T** | **A** | **T** | **C** | **T** | **T** | **C** | **T** | **G** | **G** | **G** | **G** | **G** | **C** | **A** | **T** | **T** | **C** | **A** | **A** | **C** | **G** | **G** | **C** | **A** | **A** | **G** | **G** | **T** | **T** | **A** | **T** | **A** | **G** | **G** | **A** | **G** | **A** | **A** | **A** | **A** | **A** | **T** | **C** | **C** | **C** | **A** | **G** | **C** | **C** | **A** | **A** | **A** | **A** | **C** | **A** | **T** | **C** | **T** | **G** | **A** | **G** | **C** | **A** | **T** | **T** | **A** | **G** | **T** | **T** | **A** | **T** | **G** | **T** | **A** | **C** | **G** | **C** | **C** | **A** | **A** | **A** | **A** | **T** | **T** | **A** | **T** | **G** | **A** | **A** | **G** | **A** | **C** | **A** | **A** | **T** | **C** | **G** | **A** | **G** | **G** | **A** | **A** | **C** | **G** | **T** | **T** | **G** | **G** | **C** | **G** | **G** | **A** | **A** | **T** | **T** | **T** | **G** | **C** | **A** | **G** | **A** | **G** | **A** | **A** | **A** | **A** | **A** | **C** | **C** | **A** | **A** | **G** | **T** | **A** | **G** | **A** | **C** | **T** | **A** | **C** | **C** | **A** | **A** | **G** | **C** | **A** | **T** | **T** | **T** | **T** | **T** | **G** | **A** | **A** | **C** | **A** | **A** | **C** | **C** | **T** | **C** | **C** | **G** | **A** | **A** | **A** | **G** | **C** | **T** | **T** | **G** | **G** | **G** | **A** | **T** | **G** | **G** | **A** | **A** | **A** | **G** | **A** | **G** | **A** | **T** | **G** | **T** | **T** | **T** | **T** | **T** | **C** | **A** | **T** | **A** | **A** | **C** | **A** | **A** | **G** | **A** | **T** | **C** | **G** | **A** | **G** | **C** | **A** | **G** | **A** | **T** | **G** | **A** | **G** | **T** | **A** | **T** | **G** | **G** | **G** | **T** | **C** | **A** | **A** | **C** | **G** | **G** | **A** | **A** | **A** | **A** | **A** | **A** | **G** | **T** | **G** | **G** | **A** | **A** | **T** | **T** | **G** | **G** | **C** | **T** | **A** | **A** | **A** | **T** | **C** | **T** | **T** | **T** | **A** | **T** | **C** | **A** | **C** | **A** | **G** | **C** | **C** | **A** | **G** | **C** | **T** | **G** | **A** | **A** | **C** | **T** | **A** | **T** | **A** | **T** | **A** | **T** | **A** | **T** | **G** | **G** | **G** | **A** | **T** | **G** | **A** | **A** | **C** | **C** | **A** | **T** | **T** | **G** | **A** | **A** | **T** | **T** | **A** | **T** | **T** | **T** | **G** | **G** | **A** | **T** | **G** | **T** | **C** | **T** | **T** | **C** | **A** | **A** | **T** | **C** | **A** | **A** | **G** | **A** | **A** | **C** | **A** | **A** | **T** | **T** | **A** | **G** | **A** | **A** | **C** | **A** | **A** | **C** | **T** | **G** | **A** | **T** | **C** | **T** | **T** | **G** | **A** | **A** | **C** | **G** | **T** | **G** | **A** | **A** | **A** | **C** | **C** | **T** | **G** | **C** | **C** | **A** | **T** | **G** | **T** | **T** | **A** | **C** | **T** | **A** | **G** | **T** | **G** | **G** | **A** | **A** | **C** | **A** | **T** | **G** | **A** | **T** | **C** | **A** | **A** | **A** | **C** | **C** | **T** | **T** | **C** | **C** | **T** | **G** | **G** | **A** | **T** | **A** | **A** | **A** | **G** | **T** | **A** | **T** | **C** | **T** | **A** | **C** | **T** | **G** | **A** | **G** | **A** | **T** | **T** | **A** | **T** | **T** | **T** | **C** | **T** | **C** | **T** | **T** | **G** | **A** | **G** | **A** | **A** | **A** | **A** | **T** | **C** | **-** | **-** | **-** |
| **N60260F** | **A** | **T** | **G** | **T** | **C** | **T** | **A** | **A** | **A** | **A** | **T** | **C** | **G** | **A** | **A** | **A** | **T** | **A** | **A** | **A** | **A** | **A** | **A** | **T** | **C** | **T** | **G** | **A** | **C** | **A** | **T** | **T** | **C** | **G** | **G** | **C** | **T** | **A** | **C** | **G** | **A** | **C** | **A** | **G** | **C** | **C** | **A** | **A** | **G** | **G** | **C** | **A** | **C** | **A** | **T** | **T** | **A** | **T** | **T** | **A** | **T** | **T** | **T** | **G** | **A** | **A** | **C** | **A** | **A** | **G** | **C** | **A** | **A** | **A** | **T** | **C** | **T** | **A** | **A** | **A** | **T** | **T** | **T** | **T** | **G** | **A** | **C** | **A** | **C** | **A** | **C** | **A** | **A** | **T** | **G** | **G** | **A** | **A** | **A** | **C** | **T** | **A** | **G** | **G** | **A** | **C** | **T** | **T** | **A** | **T** | **C** | **G** | **G** | **A** | **C** | **G** | **A** | **A** | **A** | **C** | **G** | **G** | **T** | **C** | **G** | **A** | **G** | **G** | **A** | **A** | **A** | **G** | **A** | **C** | **A** | **A** | **C** | **T** | **T** | **T** | **A** | **C** | **T** | **G** | **A** | **A** | **T** | **A** | **T** | **T** | **C** | **T** | **A** | **C** | **A** | **A** | **A** | **A** | **C** | **A** | **A** | **A** | **C** | **T** | **A** | **C** | **C** | **T** | **T** | **A** | **T** | **C** | **A** | **A** | **G** | **G** | **G** | **C** | **A** | **A** | **G** | **T** | **A** | **A** | **T** | **C** | **C** | **A** | **T** | **C** | **A** | **G** | **C** | **A** | **A** | **G** | **A** | **A** | **T** | **T** | **T** | **G** | **T** | **C** | **T** | **A** | **T** | **T** | **T** | **C** | **C** | **C** | **G** | **C** | **A** | **G** | **C** | **A** | **A** | **A** | **C** | **A** | **A** | **A** | **A** | **G** | **A** | **T** | **A** | **A** | **A** | **G** | **A** | **G** | **C** | **G** | **T** | **T** | **T** | **A** | **A** | **C** | **C** | **T** | **A** | **T** | **T** | **A** | **T** | **G** | **T** | **G** | **T** | **T** | **A** | **A** | **A** | **T** | **G** | **A** | **G** | **A** | **T** | **T** | **A** | **C** | **G** | **G** | **A** | **T** | **T** | **T** | **T** | **G** | **A** | **G** | **A** | **T** | **A** | **T** | **G** | **G** | **G** | **A** | **A** | **A** | **T** | **T** | **G** | **A** | **A** | **A** | **G** | **A** | **G** | **A** | **G** | **C** | **T** | **C** | **C** | **A** | **A** | **C** | **T** | **G** | **A** | **T** | **G** | **C** | **A** | **A** | **A** | **C** | **A** | **G** | **A** | **T** | **C** | **C** | **T** | **G** | **A** | **A** | **A** | **T** | **C** | **T** | **T** | **A** | **T** | **G** | **G** | **A** | **G** | **A** | **G** | **A** | **A** | **T** | **T** | **C** | **A** | **G** | **C** | **A** | **C** | **A** | **C** | **T** | **A** | **T** | **C** | **A** | **G** | **G** | **A** | **G** | **G** | **A** | **G** | **A** | **G** | **A** | **A** | **G** | **A** | **C** | **A** | **A** | **A** | **A** | **G** | **T** | **C** | **C** | **T** | **A** | **C** | **T** | **G** | **G** | **C** | **A** | **C** | **T** | **T** | **T** | **T** | **A** | **T** | **T** | **T** | **G** | **T** | **G** | **G** | **A** | **T** | **G** | **A** | **C** | **A** | **C** | **T** | **C** | **A** | **T** | **T** | **T** | **C** | **C** | **C** | **G** | **T** | **T** | **A** | **A** | **T** | **C** | **G** | **A** | **T** | **G** | **A** | **A** | **C** | **C** | **A** | **A** | **C** | **G** | **A** | **A** | **T** | **C** | **A** | **T** | **T** | **T** | **G** | **G** | **A** | **-** | **-** | **-** | **T** | **A** | **T** | **C** | **T** | **C** | **T** | **G** | **G** | **C** | **A** | **G** | **A** | **A** | **A** | **A** | **C** | **A** | **A** | **G** | **T** | **A** | **G** | **C** | **G** | **G** | **C** | **T** | **T** | **A** | **C** | **T** | **T** | **G** | **A** | **A** | **A** | **A** | **A** | **G** | **A** | **A** | **A** | **A** | **A** | **A** | **C** | **A** | **A** | **G** | **G** | **C** | **T** | **T** | **C** | **A** | **T** | **C** | **G** | **T** | **G** | **G** | **T** | **C** | **A** | **G** | **C** | **C** | **A** | **T** | **G** | **A** | **C** | **C** | **G** | **G** | **G** | **G** | **A** | **T** | **T** | **T** | **A** | **T** | **C** | **G** | **A** | **T** | **G** | **A** | **A** | **G** | **T** | **A** | **G** | **T** | **G** | **G** | **A** | **C** | **C** | **A** | **T** | **G** | **T** | **T** | **T** | **T** | **A** | **G** | **C** | **A** | **A** | **T** | **C** | **G** | **A** | **A** | **A** | **A** | **A** | **A** | **G** | **T** | **C** | **A** | **A** | **C** | **T** | **G** | **G** | **A** | **A** | **C** | **T** | **T** | **T** | **A** | **T** | **C** | **A** | **A** | **G** | **G** | **G** | **A** | **A** | **T** | **T** | **T** | **C** | **T** | **C** | **T** | **A** | **T** | **C** | **T** | **A** | **T** | **G** | **A** | **A** | **G** | **A** | **A** | **C** | **A** | **G** | **A** | **A** | **A** | **A** | **A** | **A** | **C** | **T** | **T** | **C** | **G** | **T** | **G** | **A** | **T** | **G** | **A** | **A** | **T** | **T** | **T** | **G** | **A** | **A** | **A** | **T** | **A** | **G** | **C** | **T** | **C** | **A** | **A** | **A** | **A** | **T** | **G** | **A** | **A** | **A** | **A** | **A** | **T** | **T** | **G** | **A** | **A** | **A** | **A** | **A** | **A** | **G** | **A** | **A** | **G** | **T** | **C** | **A** | **G** | **T** | **A** | **G** | **G** | **C** | **T** | **A** | **A** | **A** | **G** | **A** | **A** | **A** | **A** | **C** | **A** | **G** | **C** | **A** | **G** | **C** | **T** | **G** | **A** | **A** | **A** | **A** | **A** | **G** | **C** | **C** | **G** | **A** | **A** | **T** | **G** | **G** | **T** | **C** | **T** | **C** | **G** | **T** | **T** | **C** | **C** | **C** | **G** | **A** | **G** | **A** | **A** | **G** | **G** | **A** | **G** | **A** | **T** | **A** | **A** | **A** | **A** | **C** | **A** | **A** | **A** | **G** | **A** | **A** | **A** | **C** | **A** | **A** | **G** | **T** | **C** | **G** | **G** | **A** | **T** | **T** | **C** | **A** | **T** | **C** | **G** | **A** | **T** | **A** | **C** | **T** | **G** | **A** | **A** | **T** | **C** | **T** | **A** | **G** | **A** | **C** | **G** | **A** | **G** | **T** | **G** | **A** | **A** | **T** | **A** | **A** | **A** | **G** | **G** | **A** | **G** | **C** | **A** | **G** | **T** | **G** | **G** | **G** | **T** | **G** | **C** | **T** | **G** | **A** | **T** | **G** | **C** | **T** | **G** | **C** | **A** | **C** | **G** | **G** | **A** | **C** | **G** | **A** | **T** | **G** | **A** | **A** | **A** | **C** | **G** | **A** | **T** | **C** | **C** | **A** | **A** | **A** | **G** | **C** | **A** | **A** | **T** | **C** | **G** | **T** | **G** | **A** | **A** | **T** | **C** | **G** | **G** | **A** | **T** | **G** | **G** | **A** | **G** | **A** | **C** | **C** | **C** | **A** | **G** | **A** | **T** | **C** | **A** | **G** | **C** | **G** | **A** | **G** | **A** | **A** | **A** | **G** | **A** | **A** | **A** | **A** | **A** | **C** | **T** | **A** | **T** | **T** | **A** | **A** | **A** | **A** | **G** | **A** | **T** | **A** | **T** | **C** | **G** | **A** | **A** | **T** | **A** | **T** | **A** | **T** | **C** | **G** | **A** | **T** | **T** | **C** | **T** | **T** | **T** | **G** | **A** | **C** | **G** | **A** | **T** | **G** | **A** | **A** | **T** | **A** | **G** | **C** | **C** | **A** | **A** | **G** | **C** | **G** | **T** | **C** | **T** | **C** | **A** | **C** | **C** | **A** | **T** | **A** | **A** | **G** | **C** | **G** | **A** | **C** | **T** | **T** | **T** | **T** | **A** | **A** | **G** | **C** | **G** | **T** | **A** | **G** | **A** | **A** | **G** | **A** | **T** | **C** | **T** | **T** | **C** | **A** | **A** | **T** | **T** | **A** | **G** | **G** | **G** | **T** | **A** | **T** | **G** | **A** | **A** | **A** | **A** | **T** | **C** | **T** | **G** | **T** | **T** | **A** | **T** | **T** | **C** | **G** | **A** | **G** | **C** | **C** | **A** | **A** | **T** | **T** | **C** | **A** | **T** | **T** | **T** | **T** | **A** | **C** | **A** | **A** | **T** | **C** | **G** | **A** | **G** | **C** | **C** | **T** | **C** | **A** | **T** | **C** | **A** | **G** | **C** | **G** | **G** | **G** | **T** | **G** | **G** | **C** | **G** | **A** | **T** | **T** | **T** | **C** | **A** | **G** | **G** | **T** | **C** | **C** | **T** | **A** | **A** | **C** | **G** | **G** | **T** | **G** | **C** | **A** | **G** | **G** | **A** | **A** | **A** | **G** | **T** | **C** | **A** | **T** | **C** | **C** | **A** | **T** | **T** | **A** | **T** | **C** | **C** | **A** | **T** | **T** | **A** | **T** | **C** | **T** | **T** | **C** | **T** | **G** | **G** | **G** | **G** | **G** | **C** | **A** | **T** | **T** | **C** | **A** | **A** | **C** | **G** | **G** | **C** | **A** | **A** | **G** | **G** | **T** | **T** | **A** | **T** | **A** | **G** | **G** | **A** | **G** | **A** | **A** | **A** | **A** | **A** | **T** | **C** | **C** | **C** | **A** | **G** | **C** | **C** | **A** | **A** | **A** | **A** | **C** | **A** | **T** | **C** | **T** | **G** | **A** | **G** | **C** | **A** | **T** | **T** | **A** | **G** | **T** | **T** | **A** | **T** | **G** | **T** | **A** | **C** | **G** | **C** | **C** | **A** | **A** | **A** | **A** | **T** | **T** | **A** | **T** | **G** | **A** | **A** | **G** | **A** | **C** | **A** | **A** | **T** | **C** | **G** | **A** | **G** | **G** | **A** | **A** | **C** | **G** | **T** | **T** | **G** | **G** | **C** | **G** | **G** | **A** | **A** | **T** | **T** | **T** | **G** | **C** | **A** | **G** | **A** | **G** | **A** | **A** | **A** | **A** | **A** | **C** | **C** | **A** | **A** | **G** | **T** | **A** | **G** | **A** | **C** | **T** | **A** | **C** | **C** | **A** | **A** | **G** | **C** | **A** | **T** | **T** | **T** | **T** | **T** | **G** | **A** | **A** | **C** | **A** | **A** | **C** | **C** | **T** | **C** | **C** | **G** | **A** | **A** | **A** | **G** | **C** | **T** | **T** | **G** | **G** | **G** | **A** | **T** | **G** | **G** | **A** | **A** | **A** | **G** | **A** | **G** | **A** | **T** | **G** | **T** | **T** | **T** | **T** | **T** | **C** | **A** | **T** | **A** | **A** | **C** | **A** | **A** | **G** | **A** | **T** | **C** | **G** | **A** | **G** | **C** | **A** | **G** | **A** | **T** | **G** | **A** | **G** | **T** | **A** | **T** | **G** | **G** | **G** | **C** | **C** | **A** | **A** | **C** | **G** | **G** | **A** | **A** | **A** | **A** | **A** | **A** | **G** | **T** | **G** | **G** | **A** | **A** | **T** | **T** | **G** | **G** | **C** | **T** | **A** | **A** | **A** | **T** | **C** | **T** | **T** | **T** | **A** | **T** | **C** | **A** | **C** | **A** | **G** | **C** | **C** | **A** | **G** | **C** | **T** | **G** | **A** | **A** | **C** | **T** | **A** | **T** | **A** | **T** | **A** | **T** | **A** | **T** | **G** | **G** | **G** | **A** | **T** | **G** | **A** | **A** | **C** | **C** | **A** | **T** | **T** | **G** | **A** | **A** | **T** | **T** | **A** | **T** | **T** | **T** | **G** | **G** | **A** | **T** | **G** | **T** | **C** | **T** | **T** | **C** | **A** | **A** | **T** | **C** | **A** | **A** | **G** | **A** | **A** | **C** | **A** | **A** | **T** | **T** | **A** | **G** | **A** | **A** | **C** | **A** | **A** | **C** | **T** | **G** | **A** | **T** | **C** | **T** | **T** | **G** | **A** | **A** | **C** | **G** | **T** | **G** | **A** | **A** | **A** | **C** | **C** | **T** | **G** | **C** | **C** | **A** | **T** | **G** | **T** | **T** | **A** | **C** | **T** | **A** | **G** | **T** | **G** | **G** | **A** | **A** | **C** | **A** | **T** | **G** | **A** | **T** | **C** | **A** | **A** | **A** | **C** | **C** | **T** | **T** | **C** | **C** | **T** | **G** | **G** | **A** | **T** | **A** | **A** | **A** | **G** | **T** | **A** | **T** | **C** | **T** | **A** | **C** | **T** | **G** | **A** | **G** | **A** | **T** | **T** | **A** | **T** | **T** | **T** | **C** | **T** | **C** | **T** | **T** | **G** | **A** | **G** | **A** | **A** | **A** | **A** | **T** | **C** | **-** | **-** | **-** |
| **N54107** | **A** | **T** | **G** | **T** | **C** | **T** | **A** | **A** | **A** | **A** | **T** | **C** | **G** | **A** | **A** | **A** | **T** | **A** | **A** | **A** | **A** | **A** | **A** | **T** | **C** | **T** | **G** | **A** | **C** | **A** | **T** | **T** | **C** | **G** | **G** | **C** | **T** | **A** | **C** | **G** | **A** | **C** | **A** | **G** | **C** | **C** | **A** | **A** | **G** | **G** | **C** | **A** | **C** | **A** | **T** | **T** | **A** | **T** | **T** | **A** | **T** | **T** | **T** | **G** | **A** | **A** | **C** | **A** | **A** | **G** | **C** | **A** | **A** | **A** | **T** | **C** | **T** | **A** | **A** | **A** | **T** | **T** | **T** | **T** | **G** | **A** | **C** | **A** | **C** | **A** | **C** | **A** | **A** | **T** | **G** | **G** | **A** | **A** | **A** | **C** | **T** | **A** | **G** | **G** | **A** | **C** | **T** | **T** | **A** | **T** | **C** | **G** | **G** | **A** | **C** | **G** | **A** | **A** | **A** | **C** | **G** | **G** | **T** | **C** | **G** | **A** | **G** | **G** | **A** | **A** | **A** | **G** | **A** | **C** | **A** | **A** | **C** | **T** | **T** | **T** | **A** | **C** | **T** | **G** | **A** | **A** | **T** | **A** | **T** | **T** | **C** | **T** | **A** | **C** | **A** | **A** | **A** | **A** | **C** | **A** | **A** | **A** | **C** | **T** | **A** | **C** | **C** | **T** | **T** | **A** | **T** | **C** | **A** | **A** | **G** | **G** | **G** | **C** | **A** | **A** | **G** | **T** | **A** | **A** | **T** | **C** | **C** | **A** | **T** | **C** | **A** | **G** | **C** | **A** | **A** | **G** | **A** | **A** | **T** | **T** | **T** | **G** | **T** | **C** | **T** | **A** | **T** | **T** | **T** | **C** | **C** | **C** | **G** | **C** | **A** | **G** | **C** | **A** | **A** | **A** | **C** | **A** | **A** | **A** | **A** | **G** | **A** | **T** | **A** | **A** | **A** | **G** | **A** | **G** | **C** | **G** | **T** | **T** | **T** | **A** | **A** | **C** | **C** | **T** | **A** | **T** | **T** | **A** | **T** | **G** | **T** | **G** | **T** | **T** | **A** | **A** | **A** | **T** | **G** | **A** | **G** | **A** | **T** | **T** | **A** | **C** | **G** | **G** | **A** | **T** | **T** | **T** | **T** | **G** | **A** | **G** | **A** | **T** | **A** | **T** | **G** | **G** | **G** | **A** | **A** | **A** | **T** | **T** | **G** | **A** | **A** | **A** | **G** | **A** | **G** | **A** | **G** | **C** | **T** | **C** | **C** | **A** | **A** | **C** | **T** | **G** | **A** | **T** | **G** | **C** | **A** | **A** | **A** | **C** | **A** | **G** | **A** | **T** | **C** | **C** | **T** | **G** | **A** | **A** | **A** | **T** | **C** | **T** | **T** | **A** | **T** | **G** | **G** | **A** | **G** | **A** | **G** | **A** | **A** | **T** | **T** | **C** | **A** | **G** | **C** | **A** | **C** | **A** | **C** | **T** | **A** | **T** | **C** | **A** | **G** | **G** | **A** | **G** | **G** | **A** | **G** | **A** | **G** | **A** | **A** | **G** | **A** | **C** | **A** | **A** | **A** | **A** | **G** | **T** | **C** | **C** | **T** | **A** | **C** | **T** | **G** | **G** | **C** | **A** | **C** | **T** | **T** | **T** | **T** | **A** | **T** | **T** | **T** | **G** | **T** | **G** | **G** | **A** | **T** | **G** | **A** | **C** | **A** | **C** | **T** | **C** | **A** | **T** | **T** | **T** | **C** | **C** | **C** | **G** | **T** | **T** | **A** | **A** | **T** | **C** | **G** | **A** | **T** | **G** | **A** | **A** | **C** | **C** | **A** | **A** | **C** | **G** | **A** | **A** | **T** | **C** | **A** | **T** | **T** | **T** | **G** | **G** | **A** | **-** | **-** | **-** | **T** | **A** | **T** | **C** | **T** | **C** | **T** | **G** | **G** | **C** | **A** | **G** | **A** | **A** | **A** | **A** | **C** | **A** | **A** | **G** | **T** | **A** | **G** | **C** | **G** | **G** | **C** | **T** | **T** | **A** | **C** | **T** | **T** | **G** | **A** | **A** | **A** | **A** | **A** | **G** | **A** | **A** | **A** | **A** | **A** | **A** | **C** | **A** | **A** | **G** | **G** | **C** | **T** | **T** | **C** | **A** | **T** | **C** | **G** | **T** | **G** | **G** | **T** | **C** | **A** | **G** | **C** | **C** | **A** | **T** | **G** | **A** | **C** | **C** | **G** | **G** | **G** | **G** | **A** | **T** | **T** | **T** | **A** | **T** | **C** | **G** | **A** | **T** | **G** | **A** | **A** | **G** | **T** | **A** | **G** | **T** | **G** | **G** | **A** | **C** | **C** | **A** | **T** | **G** | **T** | **T** | **T** | **T** | **A** | **G** | **C** | **A** | **A** | **T** | **C** | **G** | **A** | **A** | **A** | **A** | **A** | **A** | **G** | **T** | **C** | **A** | **A** | **C** | **T** | **G** | **G** | **A** | **A** | **C** | **T** | **T** | **T** | **A** | **T** | **C** | **A** | **A** | **G** | **G** | **G** | **A** | **A** | **T** | **T** | **T** | **C** | **T** | **C** | **T** | **A** | **T** | **C** | **T** | **A** | **T** | **G** | **A** | **A** | **G** | **A** | **A** | **C** | **A** | **G** | **A** | **A** | **A** | **A** | **A** | **A** | **C** | **T** | **T** | **C** | **G** | **T** | **G** | **A** | **T** | **G** | **A** | **A** | **T** | **T** | **T** | **G** | **A** | **A** | **A** | **T** | **A** | **G** | **C** | **T** | **C** | **A** | **A** | **A** | **A** | **T** | **G** | **A** | **A** | **A** | **A** | **A** | **T** | **T** | **G** | **A** | **A** | **A** | **A** | **A** | **A** | **G** | **A** | **A** | **G** | **T** | **C** | **A** | **G** | **T** | **A** | **G** | **G** | **C** | **T** | **A** | **A** | **A** | **G** | **A** | **A** | **A** | **A** | **C** | **A** | **G** | **C** | **A** | **G** | **C** | **T** | **G** | **A** | **A** | **A** | **A** | **A** | **G** | **C** | **C** | **G** | **A** | **A** | **T** | **G** | **G** | **T** | **C** | **T** | **C** | **G** | **T** | **T** | **C** | **C** | **C** | **G** | **A** | **G** | **A** | **A** | **G** | **G** | **A** | **G** | **A** | **T** | **A** | **A** | **A** | **A** | **C** | **A** | **A** | **A** | **G** | **A** | **A** | **A** | **C** | **A** | **A** | **G** | **T** | **C** | **G** | **G** | **A** | **T** | **T** | **C** | **A** | **T** | **C** | **G** | **A** | **T** | **A** | **C** | **T** | **G** | **A** | **A** | **T** | **C** | **T** | **A** | **G** | **A** | **C** | **G** | **A** | **G** | **T** | **G** | **A** | **A** | **T** | **A** | **A** | **A** | **G** | **G** | **A** | **G** | **C** | **A** | **G** | **T** | **G** | **G** | **G** | **T** | **G** | **C** | **T** | **G** | **A** | **T** | **G** | **C** | **T** | **G** | **C** | **A** | **C** | **G** | **G** | **A** | **C** | **G** | **A** | **T** | **G** | **A** | **A** | **A** | **C** | **G** | **A** | **T** | **C** | **C** | **A** | **A** | **A** | **G** | **C** | **A** | **A** | **T** | **C** | **G** | **T** | **G** | **A** | **A** | **T** | **C** | **G** | **G** | **A** | **T** | **G** | **G** | **A** | **G** | **A** | **C** | **C** | **C** | **A** | **G** | **A** | **T** | **C** | **A** | **G** | **C** | **G** | **A** | **G** | **A** | **A** | **A** | **G** | **A** | **A** | **A** | **A** | **A** | **C** | **T** | **A** | **T** | **T** | **A** | **A** | **A** | **A** | **G** | **A** | **T** | **A** | **T** | **C** | **G** | **A** | **A** | **T** | **A** | **T** | **A** | **T** | **C** | **G** | **A** | **T** | **T** | **C** | **T** | **T** | **T** | **G** | **A** | **C** | **G** | **A** | **T** | **G** | **A** | **A** | **T** | **A** | **G** | **C** | **C** | **A** | **A** | **G** | **C** | **G** | **T** | **C** | **T** | **C** | **A** | **C** | **C** | **A** | **T** | **A** | **A** | **G** | **C** | **G** | **A** | **C** | **T** | **T** | **T** | **T** | **A** | **A** | **G** | **C** | **G** | **T** | **A** | **G** | **A** | **A** | **G** | **A** | **T** | **C** | **T** | **T** | **C** | **A** | **A** | **T** | **T** | **A** | **G** | **G** | **G** | **T** | **A** | **T** | **G** | **A** | **A** | **A** | **A** | **T** | **C** | **T** | **G** | **T** | **T** | **A** | **T** | **T** | **C** | **G** | **A** | **G** | **C** | **C** | **A** | **A** | **T** | **T** | **C** | **A** | **T** | **T** | **T** | **T** | **A** | **C** | **A** | **A** | **T** | **C** | **G** | **A** | **G** | **C** | **C** | **T** | **C** | **A** | **T** | **C** | **A** | **G** | **C** | **G** | **G** | **G** | **T** | **G** | **G** | **C** | **G** | **A** | **T** | **T** | **T** | **C** | **A** | **C** | **G** | **T** | **C** | **C** | **T** | **A** | **A** | **C** | **G** | **G** | **T** | **G** | **C** | **A** | **G** | **G** | **A** | **A** | **A** | **G** | **T** | **C** | **A** | **T** | **C** | **C** | **A** | **T** | **T** | **A** | **T** | **C** | **C** | **A** | **T** | **T** | **A** | **T** | **C** | **T** | **T** | **C** | **T** | **G** | **G** | **G** | **G** | **G** | **C** | **A** | **T** | **T** | **C** | **A** | **A** | **C** | **G** | **G** | **C** | **A** | **A** | **G** | **G** | **T** | **T** | **A** | **T** | **A** | **G** | **G** | **A** | **G** | **A** | **A** | **A** | **A** | **A** | **T** | **C** | **C** | **C** | **A** | **G** | **C** | **C** | **A** | **A** | **A** | **A** | **C** | **A** | **T** | **C** | **T** | **G** | **A** | **G** | **C** | **A** | **T** | **T** | **A** | **G** | **T** | **T** | **A** | **T** | **G** | **T** | **A** | **C** | **G** | **C** | **C** | **A** | **A** | **A** | **A** | **T** | **T** | **A** | **T** | **G** | **A** | **A** | **G** | **A** | **C** | **A** | **A** | **T** | **C** | **G** | **A** | **G** | **G** | **A** | **A** | **C** | **G** | **T** | **T** | **G** | **G** | **C** | **G** | **G** | **A** | **A** | **T** | **T** | **T** | **G** | **C** | **A** | **G** | **A** | **G** | **A** | **A** | **A** | **A** | **A** | **C** | **C** | **A** | **A** | **G** | **T** | **A** | **G** | **A** | **C** | **T** | **A** | **C** | **C** | **A** | **A** | **G** | **C** | **A** | **T** | **T** | **T** | **T** | **T** | **G** | **A** | **A** | **C** | **A** | **A** | **C** | **C** | **T** | **C** | **C** | **G** | **A** | **A** | **A** | **G** | **C** | **T** | **T** | **G** | **G** | **G** | **A** | **T** | **G** | **G** | **A** | **A** | **A** | **G** | **A** | **G** | **A** | **T** | **G** | **T** | **T** | **T** | **T** | **T** | **C** | **A** | **T** | **A** | **A** | **C** | **A** | **A** | **G** | **A** | **T** | **C** | **G** | **A** | **G** | **C** | **A** | **G** | **A** | **T** | **G** | **A** | **G** | **T** | **A** | **T** | **G** | **G** | **G** | **C** | **C** | **A** | **A** | **C** | **G** | **G** | **A** | **A** | **A** | **A** | **A** | **A** | **G** | **T** | **G** | **G** | **A** | **A** | **T** | **T** | **G** | **G** | **C** | **T** | **A** | **A** | **A** | **T** | **C** | **T** | **T** | **T** | **A** | **T** | **C** | **A** | **C** | **A** | **G** | **C** | **C** | **A** | **G** | **C** | **T** | **G** | **A** | **A** | **C** | **T** | **A** | **T** | **A** | **T** | **A** | **T** | **A** | **T** | **G** | **G** | **G** | **A** | **T** | **G** | **A** | **A** | **C** | **C** | **A** | **T** | **T** | **G** | **A** | **A** | **T** | **T** | **A** | **T** | **T** | **T** | **G** | **G** | **A** | **T** | **G** | **T** | **C** | **T** | **T** | **C** | **A** | **A** | **T** | **C** | **A** | **A** | **G** | **A** | **A** | **C** | **A** | **A** | **T** | **T** | **A** | **G** | **A** | **A** | **C** | **A** | **A** | **C** | **T** | **G** | **A** | **T** | **C** | **T** | **T** | **G** | **A** | **A** | **C** | **G** | **T** | **G** | **A** | **A** | **A** | **C** | **C** | **T** | **G** | **C** | **C** | **A** | **T** | **G** | **T** | **T** | **A** | **C** | **T** | **A** | **G** | **T** | **G** | **G** | **A** | **A** | **C** | **A** | **T** | **G** | **A** | **T** | **C** | **A** | **A** | **A** | **C** | **C** | **T** | **T** | **C** | **C** | **T** | **G** | **G** | **A** | **T** | **A** | **A** | **A** | **G** | **T** | **A** | **T** | **C** | **T** | **A** | **C** | **T** | **G** | **A** | **G** | **A** | **T** | **T** | **A** | **T** | **T** | **T** | **C** | **T** | **C** | **T** | **T** | **G** | **A** | **G** | **A** | **A** | **A** | **A** | **T** | **C** | **-** | **-** | **-** |
| **N59788F** | **A** | **T** | **G** | **T** | **C** | **T** | **A** | **A** | **A** | **A** | **T** | **C** | **G** | **A** | **A** | **A** | **T** | **A** | **A** | **A** | **A** | **A** | **A** | **T** | **C** | **T** | **G** | **A** | **C** | **A** | **T** | **T** | **C** | **G** | **G** | **C** | **T** | **A** | **C** | **G** | **A** | **C** | **A** | **G** | **C** | **C** | **A** | **A** | **G** | **G** | **C** | **A** | **C** | **A** | **T** | **T** | **A** | **T** | **T** | **A** | **T** | **T** | **T** | **G** | **A** | **A** | **C** | **A** | **A** | **G** | **C** | **A** | **A** | **A** | **T** | **C** | **T** | **A** | **A** | **A** | **T** | **T** | **T** | **T** | **G** | **A** | **C** | **A** | **C** | **A** | **C** | **A** | **A** | **T** | **G** | **G** | **A** | **A** | **A** | **C** | **T** | **A** | **G** | **G** | **A** | **C** | **T** | **T** | **A** | **T** | **C** | **G** | **G** | **A** | **C** | **G** | **A** | **A** | **A** | **C** | **G** | **G** | **T** | **C** | **G** | **A** | **G** | **G** | **A** | **A** | **A** | **G** | **A** | **C** | **A** | **A** | **C** | **T** | **T** | **T** | **A** | **C** | **T** | **G** | **A** | **A** | **T** | **A** | **T** | **T** | **C** | **T** | **A** | **C** | **A** | **A** | **A** | **A** | **C** | **A** | **A** | **A** | **C** | **T** | **A** | **C** | **C** | **T** | **T** | **A** | **T** | **C** | **A** | **A** | **G** | **G** | **G** | **C** | **A** | **A** | **G** | **T** | **A** | **A** | **T** | **C** | **C** | **A** | **T** | **C** | **A** | **G** | **C** | **A** | **A** | **G** | **A** | **A** | **T** | **T** | **T** | **G** | **T** | **C** | **T** | **A** | **T** | **T** | **T** | **C** | **C** | **C** | **G** | **C** | **A** | **A** | **C** | **A** | **G** | **A** | **C** | **A** | **A** | **A** | **A** | **G** | **A** | **T** | **A** | **A** | **A** | **G** | **A** | **A** | **C** | **G** | **T** | **T** | **T** | **A** | **A** | **C** | **C** | **T** | **A** | **T** | **T** | **A** | **C** | **G** | **T** | **G** | **T** | **T** | **A** | **A** | **A** | **T** | **G** | **A** | **T** | **A** | **T** | **T** | **A** | **C** | **G** | **G** | **A** | **T** | **T** | **T** | **T** | **G** | **A** | **G** | **A** | **T** | **A** | **T** | **G** | **G** | **G** | **A** | **A** | **A** | **T** | **C** | **G** | **A** | **A** | **A** | **G** | **A** | **G** | **A** | **G** | **C** | **T** | **C** | **C** | **A** | **A** | **T** | **T** | **G** | **A** | **T** | **G** | **C** | **A** | **A** | **A** | **C** | **A** | **G** | **A** | **T** | **C** | **C** | **T** | **G** | **A** | **A** | **A** | **T** | **C** | **T** | **T** | **A** | **T** | **G** | **G** | **A** | **G** | **A** | **G** | **A** | **A** | **T** | **T** | **C** | **A** | **G** | **C** | **A** | **C** | **A** | **C** | **T** | **A** | **T** | **C** | **G** | **G** | **G** | **G** | **G** | **G** | **A** | **G** | **A** | **G** | **A** | **A** | **G** | **A** | **C** | **A** | **A** | **A** | **A** | **G** | **T** | **C** | **C** | **T** | **A** | **C** | **T** | **G** | **G** | **C** | **A** | **C** | **T** | **T** | **T** | **T** | **A** | **T** | **T** | **T** | **G** | **T** | **G** | **G** | **A** | **T** | **G** | **A** | **C** | **A** | **C** | **T** | **C** | **A** | **T** | **T** | **T** | **C** | **C** | **C** | **G** | **T** | **T** | **A** | **A** | **T** | **C** | **G** | **A** | **T** | **G** | **A** | **A** | **C** | **C** | **A** | **A** | **C** | **G** | **A** | **A** | **T** | **C** | **A** | **T** | **T** | **T** | **G** | **G** | **A** | **-** | **-** | **-** | **T** | **A** | **T** | **C** | **T** | **C** | **T** | **G** | **G** | **T** | **A** | **G** | **A** | **A** | **A** | **A** | **C** | **A** | **A** | **G** | **T** | **A** | **G** | **C** | **G** | **G** | **C** | **T** | **T** | **A** | **T** | **T** | **T** | **G** | **A** | **A** | **A** | **A** | **A** | **G** | **A** | **A** | **A** | **A** | **A** | **A** | **C** | **A** | **A** | **G** | **G** | **C** | **T** | **T** | **C** | **A** | **T** | **C** | **G** | **T** | **G** | **G** | **T** | **C** | **A** | **G** | **C** | **C** | **A** | **T** | **G** | **A** | **C** | **C** | **G** | **G** | **G** | **G** | **A** | **T** | **T** | **T** | **A** | **T** | **C** | **G** | **A** | **T** | **G** | **A** | **A** | **G** | **T** | **A** | **G** | **T** | **G** | **G** | **A** | **C** | **C** | **A** | **T** | **G** | **T** | **T** | **T** | **T** | **A** | **G** | **C** | **A** | **A** | **T** | **C** | **G** | **A** | **A** | **A** | **A** | **A** | **A** | **G** | **T** | **C** | **A** | **A** | **C** | **T** | **G** | **G** | **A** | **A** | **C** | **T** | **T** | **T** | **A** | **T** | **C** | **A** | **A** | **G** | **G** | **G** | **A** | **A** | **T** | **T** | **T** | **C** | **T** | **C** | **T** | **A** | **T** | **C** | **T** | **A** | **T** | **G** | **A** | **A** | **G** | **A** | **A** | **C** | **A** | **G** | **A** | **A** | **A** | **A** | **A** | **A** | **C** | **T** | **T** | **C** | **G** | **T** | **G** | **A** | **T** | **G** | **A** | **A** | **T** | **T** | **T** | **G** | **A** | **A** | **A** | **T** | **G** | **G** | **C** | **T** | **C** | **A** | **A** | **A** | **A** | **T** | **G** | **A** | **A** | **A** | **A** | **A** | **T** | **T** | **G** | **A** | **A** | **A** | **A** | **A** | **A** | **G** | **A** | **A** | **G** | **T** | **C** | **A** | **G** | **T** | **A** | **G** | **G** | **C** | **T** | **A** | **A** | **A** | **G** | **A** | **A** | **A** | **A** | **C** | **A** | **G** | **C** | **A** | **G** | **C** | **T** | **G** | **A** | **A** | **A** | **A** | **A** | **G** | **C** | **C** | **G** | **A** | **A** | **T** | **G** | **G** | **T** | **C** | **T** | **C** | **G** | **T** | **T** | **C** | **C** | **C** | **G** | **A** | **G** | **A** | **A** | **G** | **G** | **A** | **G** | **A** | **T** | **A** | **A** | **A** | **A** | **C** | **A** | **A** | **A** | **G** | **A** | **A** | **A** | **C** | **A** | **A** | **G** | **T** | **C** | **G** | **G** | **A** | **T** | **T** | **C** | **A** | **T** | **C** | **G** | **A** | **T** | **A** | **C** | **T** | **G** | **A** | **A** | **T** | **C** | **T** | **A** | **G** | **A** | **C** | **G** | **A** | **G** | **T** | **G** | **A** | **A** | **T** | **A** | **A** | **A** | **G** | **G** | **A** | **G** | **C** | **A** | **G** | **T** | **G** | **G** | **G** | **T** | **G** | **C** | **T** | **G** | **A** | **T** | **G** | **C** | **T** | **G** | **C** | **A** | **C** | **G** | **G** | **A** | **C** | **G** | **A** | **T** | **G** | **A** | **A** | **A** | **C** | **G** | **A** | **T** | **C** | **C** | **A** | **A** | **A** | **G** | **C** | **A** | **A** | **T** | **C** | **G** | **T** | **G** | **A** | **A** | **T** | **C** | **G** | **G** | **A** | **T** | **G** | **G** | **A** | **G** | **A** | **C** | **C** | **C** | **A** | **G** | **A** | **T** | **C** | **A** | **G** | **C** | **G** | **A** | **G** | **A** | **A** | **A** | **G** | **A** | **A** | **A** | **A** | **A** | **T** | **T** | **A** | **T** | **T** | **A** | **A** | **A** | **A** | **G** | **A** | **T** | **A** | **T** | **C** | **G** | **A** | **A** | **T** | **A** | **T** | **A** | **T** | **C** | **G** | **A** | **T** | **T** | **C** | **T** | **T** | **T** | **G** | **A** | **C** | **G** | **A** | **T** | **G** | **A** | **A** | **T** | **A** | **G** | **C** | **C** | **A** | **A** | **G** | **C** | **G** | **T** | **C** | **T** | **C** | **A** | **C** | **C** | **A** | **T** | **A** | **A** | **G** | **C** | **G** | **A** | **C** | **T** | **T** | **T** | **T** | **A** | **A** | **G** | **C** | **G** | **T** | **A** | **G** | **A** | **A** | **G** | **A** | **T** | **C** | **T** | **T** | **C** | **A** | **A** | **T** | **T** | **A** | **G** | **G** | **G** | **T** | **A** | **T** | **G** | **A** | **A** | **A** | **A** | **T** | **C** | **T** | **G** | **T** | **T** | **A** | **T** | **T** | **C** | **G** | **A** | **G** | **C** | **C** | **A** | **A** | **T** | **T** | **C** | **A** | **T** | **T** | **T** | **T** | **A** | **C** | **A** | **A** | **T** | **C** | **G** | **A** | **G** | **C** | **C** | **T** | **C** | **A** | **T** | **C** | **A** | **G** | **C** | **G** | **G** | **G** | **T** | **G** | **G** | **C** | **G** | **A** | **T** | **T** | **T** | **C** | **A** | **G** | **G** | **T** | **C** | **C** | **T** | **A** | **A** | **C** | **G** | **G** | **T** | **G** | **C** | **A** | **G** | **G** | **A** | **A** | **A** | **G** | **T** | **C** | **A** | **T** | **C** | **C** | **A** | **T** | **T** | **A** | **T** | **C** | **C** | **A** | **T** | **T** | **A** | **T** | **C** | **T** | **T** | **C** | **T** | **G** | **G** | **G** | **G** | **G** | **C** | **A** | **T** | **T** | **C** | **A** | **A** | **C** | **G** | **G** | **C** | **A** | **A** | **G** | **G** | **T** | **T** | **A** | **T** | **A** | **G** | **G** | **A** | **G** | **A** | **A** | **A** | **A** | **A** | **T** | **C** | **C** | **C** | **A** | **G** | **C** | **C** | **A** | **A** | **A** | **A** | **C** | **A** | **T** | **C** | **T** | **G** | **A** | **G** | **C** | **A** | **T** | **T** | **A** | **G** | **T** | **T** | **A** | **T** | **G** | **T** | **A** | **C** | **G** | **C** | **C** | **A** | **A** | **A** | **A** | **T** | **T** | **A** | **T** | **G** | **A** | **A** | **G** | **A** | **C** | **A** | **A** | **T** | **C** | **G** | **A** | **G** | **G** | **A** | **A** | **C** | **G** | **T** | **T** | **G** | **G** | **C** | **G** | **G** | **A** | **A** | **T** | **T** | **T** | **G** | **C** | **A** | **G** | **A** | **G** | **A** | **A** | **A** | **A** | **A** | **C** | **C** | **A** | **A** | **G** | **T** | **A** | **G** | **A** | **C** | **T** | **A** | **C** | **C** | **A** | **A** | **G** | **C** | **A** | **T** | **T** | **T** | **T** | **T** | **G** | **A** | **A** | **C** | **A** | **A** | **C** | **C** | **T** | **C** | **C** | **G** | **A** | **A** | **A** | **G** | **C** | **T** | **T** | **G** | **G** | **G** | **A** | **T** | **G** | **G** | **A** | **A** | **A** | **G** | **A** | **G** | **A** | **T** | **G** | **T** | **T** | **T** | **T** | **T** | **C** | **A** | **T** | **A** | **A** | **C** | **A** | **A** | **G** | **A** | **T** | **C** | **G** | **A** | **G** | **C** | **A** | **G** | **A** | **T** | **G** | **A** | **G** | **T** | **A** | **T** | **G** | **G** | **G** | **C** | **C** | **A** | **A** | **C** | **G** | **G** | **A** | **A** | **A** | **A** | **A** | **A** | **G** | **T** | **G** | **G** | **A** | **A** | **T** | **T** | **G** | **G** | **C** | **T** | **A** | **A** | **A** | **T** | **C** | **T** | **T** | **T** | **A** | **T** | **C** | **A** | **C** | **A** | **G** | **C** | **C** | **A** | **G** | **C** | **T** | **G** | **A** | **A** | **C** | **T** | **A** | **T** | **A** | **T** | **A** | **T** | **A** | **T** | **G** | **G** | **G** | **A** | **T** | **G** | **A** | **A** | **C** | **C** | **A** | **T** | **T** | **G** | **A** | **A** | **T** | **T** | **A** | **T** | **T** | **T** | **G** | **G** | **A** | **T** | **G** | **T** | **C** | **T** | **T** | **C** | **A** | **A** | **T** | **C** | **A** | **A** | **G** | **A** | **A** | **C** | **A** | **A** | **T** | **T** | **A** | **G** | **A** | **A** | **C** | **A** | **A** | **C** | **T** | **G** | **A** | **T** | **C** | **T** | **T** | **G** | **A** | **A** | **C** | **G** | **T** | **G** | **A** | **A** | **A** | **C** | **C** | **T** | **G** | **C** | **C** | **A** | **T** | **G** | **T** | **T** | **A** | **C** | **T** | **A** | **G** | **T** | **G** | **G** | **A** | **A** | **C** | **A** | **T** | **G** | **A** | **T** | **C** | **A** | **A** | **A** | **C** | **C** | **T** | **T** | **C** | **C** | **T** | **G** | **G** | **A** | **T** | **A** | **A** | **A** | **G** | **T** | **A** | **T** | **C** | **T** | **A** | **C** | **T** | **G** | **A** | **G** | **A** | **T** | **T** | **A** | **T** | **T** | **T** | **C** | **T** | **C** | **T** | **T** | **G** | **A** | **G** | **A** | **A** | **A** | **A** | **T** | **C** | **-** | **-** | **-** |
| **N53805** | **A** | **T** | **G** | **T** | **C** | **T** | **A** | **A** | **A** | **A** | **T** | **C** | **G** | **A** | **A** | **A** | **T** | **A** | **A** | **A** | **A** | **A** | **A** | **T** | **C** | **T** | **G** | **A** | **C** | **A** | **T** | **T** | **C** | **G** | **G** | **C** | **T** | **A** | **C** | **G** | **A** | **C** | **A** | **G** | **C** | **C** | **A** | **A** | **G** | **G** | **C** | **A** | **C** | **A** | **T** | **T** | **A** | **T** | **T** | **A** | **T** | **T** | **T** | **G** | **A** | **A** | **C** | **A** | **A** | **G** | **C** | **A** | **A** | **A** | **T** | **C** | **T** | **A** | **A** | **A** | **T** | **T** | **T** | **T** | **G** | **A** | **C** | **A** | **C** | **A** | **C** | **A** | **A** | **T** | **G** | **G** | **A** | **A** | **A** | **C** | **T** | **A** | **G** | **G** | **A** | **C** | **T** | **T** | **A** | **T** | **C** | **G** | **G** | **A** | **C** | **G** | **A** | **A** | **A** | **C** | **G** | **G** | **T** | **C** | **G** | **A** | **G** | **G** | **A** | **A** | **A** | **G** | **A** | **C** | **A** | **A** | **C** | **T** | **T** | **T** | **A** | **C** | **T** | **G** | **A** | **A** | **T** | **A** | **T** | **T** | **C** | **T** | **A** | **C** | **A** | **A** | **A** | **A** | **C** | **A** | **A** | **A** | **C** | **T** | **A** | **C** | **C** | **T** | **T** | **A** | **T** | **C** | **A** | **A** | **G** | **G** | **G** | **C** | **A** | **A** | **G** | **T** | **A** | **A** | **T** | **C** | **C** | **A** | **T** | **C** | **A** | **G** | **C** | **A** | **A** | **G** | **A** | **A** | **T** | **T** | **T** | **G** | **T** | **C** | **T** | **A** | **T** | **T** | **T** | **C** | **C** | **C** | **G** | **C** | **A** | **G** | **C** | **A** | **A** | **A** | **C** | **A** | **A** | **A** | **A** | **G** | **A** | **T** | **A** | **A** | **A** | **G** | **A** | **G** | **C** | **G** | **T** | **T** | **T** | **A** | **A** | **C** | **C** | **T** | **A** | **T** | **T** | **A** | **T** | **G** | **T** | **G** | **T** | **T** | **A** | **A** | **A** | **T** | **G** | **A** | **G** | **A** | **T** | **T** | **A** | **C** | **G** | **G** | **A** | **T** | **T** | **T** | **T** | **G** | **A** | **G** | **A** | **T** | **A** | **T** | **G** | **G** | **G** | **A** | **A** | **A** | **T** | **T** | **G** | **A** | **A** | **A** | **G** | **A** | **G** | **A** | **G** | **C** | **T** | **C** | **C** | **A** | **A** | **C** | **T** | **G** | **A** | **T** | **G** | **C** | **A** | **A** | **A** | **C** | **A** | **G** | **A** | **T** | **C** | **C** | **T** | **G** | **A** | **A** | **A** | **T** | **C** | **T** | **T** | **A** | **T** | **G** | **G** | **A** | **G** | **A** | **G** | **A** | **A** | **T** | **T** | **C** | **A** | **G** | **C** | **A** | **C** | **A** | **C** | **T** | **A** | **T** | **C** | **A** | **G** | **G** | **A** | **G** | **G** | **A** | **G** | **A** | **G** | **A** | **A** | **G** | **A** | **C** | **A** | **A** | **A** | **A** | **G** | **T** | **C** | **C** | **T** | **A** | **C** | **T** | **G** | **G** | **C** | **A** | **C** | **T** | **T** | **T** | **T** | **A** | **T** | **T** | **T** | **G** | **T** | **G** | **G** | **A** | **T** | **G** | **A** | **C** | **A** | **C** | **T** | **C** | **A** | **T** | **T** | **T** | **C** | **C** | **C** | **G** | **T** | **T** | **A** | **A** | **T** | **C** | **G** | **A** | **T** | **G** | **A** | **A** | **C** | **C** | **A** | **A** | **C** | **G** | **A** | **A** | **T** | **C** | **A** | **T** | **T** | **T** | **G** | **G** | **A** | **-** | **-** | **-** | **T** | **A** | **T** | **C** | **T** | **C** | **T** | **G** | **G** | **C** | **A** | **G** | **A** | **A** | **A** | **A** | **C** | **A** | **A** | **G** | **T** | **A** | **G** | **C** | **G** | **G** | **C** | **T** | **T** | **A** | **C** | **T** | **T** | **G** | **A** | **A** | **A** | **A** | **A** | **G** | **A** | **A** | **A** | **A** | **A** | **A** | **C** | **A** | **A** | **G** | **G** | **C** | **T** | **T** | **C** | **A** | **T** | **C** | **G** | **T** | **G** | **G** | **T** | **C** | **A** | **G** | **C** | **C** | **A** | **T** | **G** | **A** | **C** | **C** | **G** | **G** | **G** | **G** | **A** | **T** | **T** | **T** | **A** | **T** | **C** | **G** | **A** | **T** | **G** | **A** | **A** | **G** | **T** | **A** | **G** | **T** | **G** | **G** | **A** | **C** | **C** | **A** | **T** | **G** | **T** | **T** | **T** | **T** | **A** | **G** | **C** | **A** | **A** | **T** | **C** | **G** | **A** | **A** | **A** | **A** | **A** | **A** | **G** | **T** | **C** | **A** | **A** | **C** | **T** | **G** | **G** | **A** | **A** | **C** | **T** | **T** | **T** | **A** | **T** | **C** | **A** | **A** | **G** | **G** | **G** | **A** | **A** | **T** | **T** | **T** | **C** | **T** | **C** | **T** | **A** | **T** | **C** | **T** | **A** | **T** | **G** | **A** | **A** | **G** | **A** | **A** | **C** | **A** | **G** | **A** | **A** | **A** | **A** | **A** | **A** | **C** | **T** | **T** | **C** | **G** | **T** | **G** | **A** | **T** | **G** | **A** | **A** | **T** | **T** | **T** | **G** | **A** | **A** | **A** | **T** | **A** | **G** | **C** | **T** | **C** | **A** | **A** | **A** | **A** | **T** | **G** | **A** | **A** | **A** | **A** | **A** | **T** | **T** | **G** | **A** | **A** | **A** | **A** | **A** | **A** | **G** | **A** | **A** | **G** | **T** | **C** | **A** | **G** | **T** | **A** | **G** | **G** | **C** | **T** | **A** | **A** | **A** | **G** | **A** | **A** | **A** | **A** | **C** | **A** | **G** | **C** | **A** | **G** | **C** | **T** | **G** | **A** | **A** | **A** | **A** | **A** | **G** | **C** | **C** | **G** | **A** | **A** | **T** | **G** | **G** | **T** | **C** | **T** | **C** | **G** | **T** | **T** | **C** | **C** | **C** | **G** | **A** | **G** | **A** | **A** | **G** | **G** | **A** | **G** | **A** | **T** | **A** | **A** | **A** | **A** | **C** | **A** | **A** | **A** | **G** | **A** | **A** | **A** | **C** | **A** | **A** | **G** | **T** | **C** | **G** | **G** | **A** | **T** | **T** | **C** | **A** | **T** | **C** | **G** | **A** | **T** | **A** | **C** | **T** | **G** | **A** | **A** | **T** | **C** | **T** | **A** | **G** | **A** | **C** | **G** | **A** | **G** | **T** | **G** | **A** | **A** | **T** | **A** | **A** | **A** | **G** | **G** | **A** | **G** | **C** | **A** | **G** | **T** | **G** | **G** | **G** | **T** | **G** | **C** | **T** | **G** | **A** | **T** | **G** | **C** | **T** | **G** | **C** | **A** | **C** | **G** | **G** | **A** | **C** | **G** | **A** | **T** | **G** | **A** | **A** | **A** | **C** | **G** | **A** | **T** | **C** | **C** | **A** | **A** | **A** | **G** | **C** | **A** | **A** | **T** | **C** | **G** | **T** | **G** | **A** | **A** | **T** | **C** | **G** | **G** | **A** | **T** | **G** | **G** | **A** | **G** | **A** | **C** | **C** | **C** | **A** | **G** | **A** | **T** | **C** | **A** | **G** | **C** | **G** | **A** | **G** | **A** | **A** | **A** | **G** | **A** | **A** | **A** | **A** | **A** | **C** | **T** | **A** | **T** | **T** | **A** | **A** | **A** | **A** | **G** | **A** | **T** | **A** | **T** | **C** | **G** | **A** | **A** | **T** | **A** | **T** | **A** | **T** | **C** | **G** | **A** | **T** | **T** | **C** | **T** | **T** | **T** | **G** | **A** | **C** | **G** | **A** | **T** | **G** | **A** | **A** | **T** | **A** | **G** | **C** | **C** | **A** | **A** | **G** | **C** | **G** | **T** | **C** | **T** | **C** | **A** | **C** | **C** | **A** | **T** | **A** | **A** | **G** | **C** | **G** | **A** | **C** | **T** | **T** | **T** | **T** | **A** | **A** | **G** | **C** | **G** | **T** | **A** | **G** | **A** | **A** | **G** | **A** | **T** | **C** | **T** | **T** | **C** | **A** | **A** | **T** | **T** | **A** | **G** | **G** | **G** | **T** | **A** | **T** | **G** | **A** | **A** | **A** | **A** | **T** | **C** | **T** | **G** | **T** | **T** | **A** | **T** | **T** | **C** | **G** | **A** | **G** | **C** | **C** | **A** | **A** | **T** | **T** | **C** | **A** | **T** | **T** | **T** | **T** | **A** | **C** | **A** | **A** | **T** | **C** | **G** | **A** | **G** | **C** | **C** | **T** | **C** | **A** | **T** | **C** | **A** | **G** | **C** | **G** | **G** | **G** | **T** | **G** | **G** | **C** | **G** | **A** | **T** | **T** | **T** | **C** | **A** | **G** | **G** | **T** | **C** | **C** | **T** | **A** | **A** | **C** | **G** | **G** | **T** | **G** | **C** | **A** | **G** | **G** | **A** | **A** | **A** | **G** | **T** | **C** | **A** | **T** | **C** | **C** | **A** | **T** | **T** | **A** | **T** | **C** | **C** | **A** | **T** | **T** | **A** | **T** | **C** | **T** | **T** | **C** | **T** | **G** | **G** | **G** | **G** | **G** | **C** | **A** | **T** | **T** | **C** | **A** | **A** | **C** | **G** | **G** | **C** | **A** | **A** | **G** | **G** | **T** | **T** | **A** | **T** | **A** | **G** | **G** | **A** | **G** | **A** | **A** | **A** | **A** | **A** | **T** | **C** | **C** | **C** | **A** | **G** | **C** | **C** | **A** | **A** | **A** | **A** | **C** | **A** | **T** | **C** | **T** | **G** | **A** | **G** | **C** | **A** | **T** | **T** | **A** | **G** | **T** | **T** | **A** | **T** | **G** | **T** | **A** | **C** | **G** | **C** | **C** | **A** | **A** | **A** | **A** | **T** | **T** | **A** | **T** | **G** | **A** | **A** | **G** | **A** | **C** | **A** | **A** | **T** | **C** | **G** | **A** | **G** | **G** | **A** | **A** | **C** | **G** | **T** | **T** | **G** | **G** | **C** | **G** | **G** | **A** | **A** | **T** | **T** | **T** | **G** | **C** | **A** | **G** | **A** | **G** | **A** | **A** | **A** | **A** | **A** | **C** | **C** | **A** | **A** | **G** | **T** | **A** | **G** | **A** | **C** | **T** | **A** | **C** | **C** | **A** | **A** | **G** | **C** | **A** | **T** | **T** | **T** | **T** | **T** | **G** | **A** | **A** | **C** | **A** | **A** | **C** | **C** | **T** | **C** | **C** | **G** | **A** | **A** | **A** | **G** | **C** | **T** | **T** | **G** | **G** | **G** | **A** | **T** | **G** | **G** | **A** | **A** | **A** | **G** | **A** | **G** | **A** | **T** | **G** | **T** | **T** | **T** | **T** | **T** | **C** | **A** | **T** | **A** | **A** | **C** | **A** | **A** | **G** | **A** | **T** | **C** | **G** | **A** | **G** | **C** | **A** | **G** | **A** | **T** | **G** | **A** | **G** | **T** | **A** | **T** | **G** | **G** | **G** | **C** | **C** | **A** | **A** | **C** | **G** | **G** | **A** | **A** | **A** | **A** | **A** | **A** | **G** | **T** | **G** | **G** | **A** | **A** | **T** | **T** | **G** | **G** | **C** | **T** | **A** | **A** | **A** | **T** | **C** | **T** | **T** | **T** | **A** | **T** | **C** | **A** | **C** | **A** | **G** | **C** | **C** | **A** | **G** | **C** | **T** | **G** | **A** | **A** | **C** | **T** | **A** | **T** | **A** | **T** | **A** | **T** | **A** | **T** | **G** | **G** | **G** | **A** | **T** | **G** | **A** | **A** | **C** | **C** | **A** | **T** | **T** | **G** | **A** | **A** | **T** | **T** | **A** | **T** | **T** | **T** | **G** | **G** | **A** | **T** | **G** | **T** | **C** | **T** | **T** | **C** | **A** | **A** | **T** | **C** | **A** | **A** | **G** | **A** | **A** | **C** | **A** | **A** | **T** | **T** | **A** | **G** | **A** | **A** | **C** | **A** | **A** | **C** | **T** | **G** | **A** | **T** | **C** | **T** | **T** | **G** | **A** | **A** | **C** | **G** | **T** | **G** | **A** | **A** | **A** | **C** | **C** | **T** | **G** | **C** | **C** | **A** | **T** | **G** | **T** | **T** | **A** | **C** | **T** | **A** | **G** | **T** | **G** | **G** | **A** | **A** | **C** | **A** | **T** | **G** | **A** | **T** | **C** | **A** | **A** | **A** | **C** | **C** | **T** | **T** | **C** | **C** | **T** | **G** | **G** | **A** | **T** | **A** | **A** | **A** | **G** | **T** | **A** | **T** | **C** | **T** | **A** | **C** | **T** | **G** | **A** | **G** | **A** | **T** | **T** | **A** | **T** | **T** | **T** | **C** | **T** | **C** | **T** | **T** | **G** | **A** | **G** | **A** | **A** | **A** | **A** | **T** | **C** | **-** | **-** | **-** |
| **N60425F** | **A** | **T** | **G** | **T** | **C** | **T** | **A** | **A** | **A** | **A** | **T** | **C** | **G** | **A** | **A** | **A** | **T** | **A** | **A** | **A** | **A** | **A** | **A** | **T** | **C** | **T** | **G** | **A** | **C** | **A** | **T** | **T** | **C** | **G** | **G** | **C** | **T** | **A** | **C** | **G** | **A** | **C** | **A** | **G** | **C** | **C** | **A** | **A** | **G** | **G** | **C** | **A** | **C** | **A** | **T** | **T** | **A** | **T** | **T** | **A** | **T** | **T** | **T** | **G** | **A** | **A** | **C** | **A** | **A** | **G** | **C** | **A** | **A** | **A** | **T** | **C** | **T** | **A** | **A** | **A** | **T** | **T** | **T** | **T** | **G** | **A** | **C** | **A** | **T** | **A** | **C** | **A** | **A** | **T** | **G** | **G** | **A** | **A** | **A** | **C** | **T** | **A** | **G** | **G** | **A** | **C** | **T** | **T** | **A** | **T** | **C** | **G** | **G** | **A** | **C** | **G** | **A** | **A** | **A** | **C** | **G** | **G** | **T** | **C** | **G** | **A** | **G** | **G** | **A** | **A** | **A** | **G** | **A** | **C** | **A** | **A** | **C** | **T** | **T** | **T** | **A** | **C** | **T** | **G** | **A** | **A** | **T** | **A** | **T** | **T** | **C** | **T** | **A** | **C** | **A** | **A** | **A** | **A** | **C** | **A** | **A** | **A** | **C** | **T** | **A** | **C** | **C** | **T** | **T** | **A** | **T** | **C** | **A** | **A** | **G** | **G** | **G** | **C** | **A** | **A** | **G** | **T** | **A** | **A** | **T** | **C** | **C** | **A** | **T** | **C** | **A** | **G** | **C** | **A** | **A** | **G** | **A** | **A** | **T** | **T** | **T** | **G** | **T** | **C** | **T** | **A** | **T** | **T** | **T** | **C** | **C** | **C** | **G** | **C** | **A** | **A** | **C** | **A** | **G** | **A** | **C** | **A** | **A** | **A** | **A** | **G** | **A** | **T** | **A** | **A** | **A** | **G** | **A** | **A** | **C** | **G** | **T** | **T** | **T** | **A** | **A** | **C** | **C** | **T** | **A** | **T** | **T** | **A** | **C** | **G** | **T** | **G** | **T** | **T** | **A** | **A** | **A** | **T** | **G** | **A** | **T** | **A** | **T** | **T** | **A** | **C** | **G** | **G** | **A** | **T** | **T** | **T** | **T** | **G** | **A** | **G** | **A** | **T** | **A** | **T** | **G** | **G** | **G** | **A** | **A** | **A** | **T** | **C** | **G** | **A** | **A** | **A** | **G** | **A** | **G** | **A** | **G** | **C** | **T** | **C** | **C** | **A** | **A** | **T** | **T** | **G** | **A** | **T** | **G** | **C** | **A** | **A** | **A** | **C** | **A** | **G** | **A** | **T** | **C** | **C** | **T** | **G** | **A** | **A** | **A** | **T** | **C** | **T** | **T** | **A** | **T** | **G** | **G** | **A** | **G** | **A** | **G** | **A** | **A** | **T** | **T** | **C** | **A** | **G** | **C** | **A** | **C** | **A** | **C** | **T** | **A** | **T** | **C** | **A** | **G** | **G** | **G** | **G** | **G** | **A** | **G** | **A** | **G** | **A** | **A** | **G** | **A** | **C** | **A** | **A** | **A** | **A** | **G** | **T** | **C** | **C** | **T** | **A** | **C** | **T** | **G** | **G** | **C** | **A** | **C** | **T** | **T** | **T** | **T** | **A** | **T** | **T** | **T** | **G** | **T** | **G** | **G** | **A** | **T** | **G** | **A** | **C** | **A** | **C** | **T** | **C** | **A** | **T** | **T** | **T** | **C** | **C** | **C** | **G** | **T** | **T** | **A** | **A** | **T** | **C** | **G** | **A** | **T** | **G** | **A** | **A** | **C** | **C** | **A** | **A** | **C** | **G** | **A** | **A** | **T** | **C** | **A** | **T** | **T** | **T** | **G** | **G** | **A** | **-** | **-** | **-** | **T** | **A** | **T** | **C** | **T** | **C** | **T** | **G** | **G** | **T** | **A** | **G** | **A** | **A** | **A** | **A** | **C** | **A** | **A** | **G** | **T** | **A** | **G** | **C** | **G** | **G** | **C** | **T** | **T** | **A** | **T** | **T** | **T** | **G** | **A** | **A** | **A** | **A** | **A** | **G** | **A** | **A** | **A** | **A** | **A** | **A** | **C** | **A** | **A** | **G** | **G** | **C** | **T** | **T** | **C** | **A** | **T** | **C** | **G** | **T** | **G** | **G** | **T** | **C** | **A** | **G** | **C** | **C** | **A** | **T** | **G** | **A** | **C** | **C** | **G** | **G** | **G** | **G** | **A** | **T** | **T** | **T** | **A** | **T** | **C** | **G** | **A** | **T** | **G** | **A** | **A** | **G** | **T** | **A** | **G** | **T** | **G** | **G** | **A** | **C** | **C** | **A** | **T** | **G** | **T** | **T** | **T** | **T** | **A** | **G** | **C** | **A** | **A** | **T** | **C** | **G** | **A** | **A** | **A** | **A** | **A** | **A** | **G** | **T** | **C** | **A** | **A** | **C** | **T** | **G** | **G** | **A** | **A** | **C** | **T** | **T** | **T** | **A** | **T** | **C** | **A** | **A** | **G** | **G** | **G** | **A** | **A** | **T** | **T** | **T** | **C** | **T** | **C** | **T** | **A** | **T** | **C** | **T** | **A** | **T** | **G** | **A** | **A** | **G** | **A** | **A** | **C** | **A** | **G** | **A** | **A** | **A** | **A** | **A** | **A** | **C** | **T** | **T** | **C** | **G** | **T** | **G** | **A** | **T** | **G** | **A** | **A** | **T** | **T** | **T** | **G** | **A** | **A** | **A** | **T** | **G** | **G** | **C** | **T** | **C** | **A** | **A** | **A** | **A** | **T** | **G** | **A** | **A** | **A** | **A** | **A** | **T** | **T** | **G** | **A** | **A** | **A** | **A** | **A** | **A** | **G** | **A** | **A** | **G** | **T** | **C** | **A** | **G** | **T** | **A** | **G** | **G** | **C** | **T** | **A** | **A** | **A** | **G** | **A** | **A** | **A** | **A** | **C** | **A** | **G** | **C** | **A** | **G** | **C** | **T** | **G** | **A** | **A** | **A** | **A** | **A** | **G** | **C** | **C** | **G** | **A** | **A** | **T** | **G** | **G** | **T** | **C** | **T** | **C** | **G** | **T** | **T** | **C** | **C** | **C** | **G** | **A** | **G** | **A** | **A** | **G** | **G** | **A** | **G** | **A** | **T** | **A** | **A** | **A** | **A** | **C** | **A** | **A** | **A** | **G** | **A** | **A** | **A** | **C** | **A** | **A** | **G** | **T** | **C** | **G** | **G** | **A** | **T** | **T** | **C** | **A** | **T** | **C** | **G** | **A** | **T** | **A** | **C** | **T** | **G** | **A** | **A** | **T** | **C** | **T** | **A** | **G** | **A** | **C** | **G** | **A** | **G** | **T** | **G** | **A** | **A** | **T** | **A** | **A** | **A** | **G** | **G** | **A** | **G** | **C** | **A** | **G** | **T** | **G** | **G** | **G** | **T** | **G** | **C** | **T** | **G** | **A** | **T** | **G** | **C** | **T** | **G** | **C** | **A** | **C** | **G** | **G** | **A** | **C** | **G** | **A** | **T** | **G** | **A** | **A** | **A** | **C** | **G** | **A** | **T** | **C** | **C** | **A** | **A** | **A** | **G** | **C** | **A** | **A** | **T** | **C** | **G** | **T** | **G** | **A** | **A** | **T** | **C** | **G** | **G** | **A** | **T** | **G** | **G** | **A** | **G** | **A** | **C** | **C** | **C** | **A** | **G** | **A** | **T** | **C** | **A** | **G** | **C** | **G** | **A** | **G** | **A** | **A** | **A** | **G** | **A** | **A** | **A** | **A** | **A** | **C** | **T** | **A** | **T** | **T** | **A** | **A** | **A** | **A** | **G** | **A** | **T** | **A** | **T** | **C** | **G** | **A** | **A** | **T** | **A** | **T** | **A** | **T** | **C** | **G** | **A** | **T** | **T** | **C** | **T** | **T** | **T** | **G** | **A** | **C** | **G** | **A** | **T** | **G** | **A** | **A** | **T** | **A** | **G** | **T** | **C** | **A** | **A** | **G** | **C** | **G** | **T** | **C** | **T** | **C** | **A** | **C** | **C** | **A** | **T** | **A** | **A** | **G** | **C** | **G** | **A** | **C** | **T** | **T** | **T** | **T** | **A** | **A** | **G** | **C** | **G** | **T** | **A** | **G** | **A** | **A** | **G** | **A** | **T** | **C** | **T** | **T** | **C** | **A** | **A** | **T** | **T** | **A** | **G** | **G** | **G** | **T** | **A** | **T** | **G** | **A** | **A** | **A** | **A** | **T** | **C** | **T** | **G** | **T** | **T** | **A** | **T** | **T** | **C** | **G** | **A** | **G** | **C** | **C** | **A** | **A** | **T** | **T** | **C** | **A** | **T** | **T** | **T** | **T** | **A** | **C** | **A** | **A** | **T** | **C** | **G** | **A** | **G** | **C** | **C** | **T** | **C** | **A** | **T** | **C** | **A** | **G** | **C** | **G** | **G** | **G** | **T** | **G** | **G** | **C** | **G** | **A** | **T** | **T** | **T** | **C** | **A** | **G** | **G** | **T** | **C** | **C** | **T** | **A** | **A** | **C** | **G** | **G** | **T** | **G** | **C** | **A** | **G** | **G** | **A** | **A** | **A** | **G** | **T** | **C** | **A** | **T** | **C** | **C** | **A** | **T** | **T** | **A** | **T** | **C** | **C** | **A** | **T** | **T** | **A** | **T** | **C** | **T** | **T** | **C** | **T** | **G** | **G** | **G** | **G** | **G** | **C** | **A** | **T** | **T** | **C** | **A** | **A** | **C** | **G** | **G** | **C** | **A** | **A** | **G** | **G** | **T** | **T** | **A** | **T** | **A** | **G** | **G** | **A** | **G** | **A** | **A** | **A** | **A** | **A** | **T** | **C** | **C** | **C** | **A** | **G** | **C** | **C** | **A** | **A** | **A** | **A** | **C** | **A** | **T** | **C** | **T** | **G** | **A** | **G** | **C** | **A** | **T** | **T** | **A** | **G** | **T** | **T** | **A** | **T** | **G** | **T** | **A** | **C** | **G** | **C** | **C** | **A** | **A** | **A** | **A** | **T** | **T** | **A** | **T** | **G** | **A** | **A** | **G** | **A** | **C** | **A** | **A** | **T** | **C** | **G** | **A** | **G** | **G** | **A** | **A** | **C** | **G** | **T** | **T** | **G** | **G** | **C** | **G** | **G** | **A** | **A** | **T** | **T** | **T** | **G** | **C** | **A** | **G** | **A** | **G** | **A** | **A** | **A** | **A** | **A** | **C** | **C** | **A** | **A** | **G** | **T** | **A** | **G** | **A** | **C** | **T** | **A** | **C** | **C** | **A** | **A** | **G** | **C** | **A** | **T** | **T** | **T** | **T** | **T** | **G** | **A** | **A** | **C** | **A** | **A** | **C** | **C** | **T** | **C** | **C** | **G** | **A** | **A** | **A** | **G** | **C** | **T** | **T** | **G** | **G** | **G** | **A** | **T** | **G** | **G** | **A** | **A** | **A** | **G** | **A** | **G** | **A** | **T** | **G** | **T** | **T** | **T** | **T** | **T** | **C** | **A** | **T** | **A** | **A** | **C** | **A** | **A** | **G** | **A** | **T** | **C** | **G** | **A** | **G** | **C** | **A** | **G** | **A** | **T** | **G** | **A** | **G** | **T** | **A** | **T** | **G** | **G** | **G** | **T** | **C** | **A** | **A** | **C** | **G** | **G** | **A** | **A** | **A** | **A** | **A** | **A** | **G** | **T** | **G** | **G** | **A** | **A** | **T** | **T** | **G** | **G** | **C** | **T** | **A** | **A** | **A** | **T** | **C** | **T** | **T** | **T** | **A** | **T** | **C** | **A** | **C** | **A** | **G** | **C** | **C** | **A** | **G** | **C** | **T** | **G** | **A** | **A** | **C** | **T** | **A** | **T** | **A** | **T** | **A** | **T** | **A** | **T** | **G** | **G** | **G** | **A** | **T** | **G** | **A** | **A** | **C** | **C** | **A** | **T** | **T** | **G** | **A** | **A** | **T** | **T** | **A** | **T** | **T** | **T** | **G** | **G** | **A** | **T** | **G** | **T** | **C** | **T** | **T** | **C** | **A** | **A** | **T** | **C** | **A** | **A** | **G** | **A** | **A** | **C** | **A** | **A** | **T** | **T** | **A** | **G** | **A** | **A** | **C** | **A** | **A** | **C** | **T** | **G** | **A** | **T** | **C** | **T** | **T** | **G** | **A** | **A** | **C** | **G** | **T** | **G** | **A** | **A** | **A** | **C** | **C** | **T** | **G** | **C** | **C** | **A** | **T** | **G** | **T** | **T** | **A** | **C** | **T** | **A** | **G** | **T** | **G** | **G** | **A** | **A** | **C** | **A** | **T** | **G** | **A** | **T** | **C** | **A** | **A** | **A** | **C** | **C** | **T** | **T** | **C** | **C** | **T** | **G** | **G** | **A** | **T** | **A** | **A** | **A** | **G** | **T** | **A** | **T** | **C** | **T** | **A** | **C** | **T** | **G** | **A** | **G** | **A** | **T** | **T** | **A** | **T** | **T** | **T** | **C** | **T** | **C** | **T** | **T** | **G** | **A** | **G** | **A** | **A** | **A** | **A** | **T** | **C** | **-** | **-** | **-** |
| **N55072** | **A** | **T** | **G** | **T** | **C** | **T** | **A** | **A** | **A** | **A** | **T** | **C** | **G** | **A** | **A** | **A** | **T** | **A** | **A** | **A** | **A** | **A** | **A** | **T** | **C** | **T** | **G** | **A** | **C** | **A** | **T** | **T** | **C** | **G** | **G** | **C** | **T** | **A** | **C** | **G** | **A** | **C** | **A** | **G** | **C** | **C** | **A** | **A** | **G** | **G** | **C** | **A** | **C** | **A** | **T** | **T** | **A** | **T** | **T** | **A** | **T** | **T** | **T** | **G** | **A** | **A** | **C** | **A** | **A** | **G** | **C** | **A** | **A** | **A** | **T** | **C** | **T** | **A** | **A** | **A** | **T** | **T** | **T** | **T** | **G** | **A** | **C** | **A** | **C** | **A** | **C** | **A** | **A** | **T** | **G** | **G** | **A** | **A** | **A** | **C** | **T** | **A** | **G** | **G** | **A** | **C** | **T** | **T** | **A** | **T** | **C** | **G** | **G** | **A** | **C** | **G** | **A** | **A** | **A** | **C** | **G** | **G** | **T** | **C** | **G** | **A** | **G** | **G** | **A** | **A** | **A** | **G** | **A** | **C** | **A** | **A** | **C** | **T** | **T** | **T** | **A** | **C** | **T** | **G** | **A** | **A** | **T** | **A** | **T** | **T** | **C** | **T** | **A** | **C** | **A** | **A** | **A** | **A** | **C** | **A** | **A** | **A** | **C** | **T** | **A** | **C** | **C** | **T** | **T** | **A** | **T** | **C** | **A** | **A** | **G** | **G** | **G** | **C** | **A** | **A** | **G** | **T** | **A** | **A** | **T** | **C** | **C** | **A** | **T** | **C** | **A** | **G** | **C** | **A** | **A** | **G** | **A** | **A** | **T** | **T** | **T** | **G** | **C** | **C** | **T** | **A** | **T** | **T** | **T** | **C** | **C** | **C** | **G** | **C** | **A** | **A** | **C** | **A** | **G** | **A** | **C** | **A** | **A** | **A** | **A** | **G** | **A** | **T** | **A** | **A** | **A** | **G** | **A** | **A** | **C** | **G** | **T** | **T** | **T** | **A** | **A** | **C** | **C** | **T** | **A** | **T** | **T** | **A** | **C** | **G** | **T** | **G** | **T** | **T** | **A** | **A** | **A** | **T** | **G** | **A** | **T** | **A** | **T** | **T** | **A** | **C** | **G** | **G** | **A** | **T** | **T** | **T** | **T** | **G** | **A** | **G** | **A** | **T** | **A** | **T** | **G** | **G** | **G** | **A** | **A** | **A** | **T** | **C** | **G** | **A** | **A** | **A** | **G** | **A** | **G** | **A** | **G** | **C** | **T** | **C** | **C** | **A** | **A** | **T** | **T** | **G** | **A** | **T** | **G** | **C** | **A** | **A** | **A** | **C** | **A** | **G** | **A** | **T** | **C** | **C** | **T** | **G** | **A** | **A** | **A** | **T** | **C** | **T** | **T** | **A** | **T** | **G** | **G** | **A** | **G** | **A** | **G** | **A** | **A** | **T** | **T** | **C** | **A** | **G** | **C** | **A** | **C** | **A** | **C** | **T** | **A** | **T** | **C** | **G** | **G** | **G** | **G** | **G** | **G** | **A** | **G** | **A** | **G** | **A** | **A** | **G** | **A** | **C** | **A** | **A** | **A** | **A** | **G** | **T** | **C** | **C** | **T** | **A** | **C** | **T** | **G** | **G** | **C** | **A** | **C** | **T** | **T** | **T** | **T** | **A** | **T** | **T** | **T** | **G** | **T** | **G** | **G** | **A** | **T** | **G** | **A** | **C** | **A** | **C** | **T** | **C** | **A** | **T** | **T** | **T** | **C** | **C** | **C** | **G** | **T** | **T** | **A** | **A** | **T** | **C** | **G** | **A** | **T** | **G** | **A** | **A** | **C** | **C** | **A** | **A** | **C** | **G** | **A** | **A** | **T** | **C** | **A** | **T** | **T** | **T** | **G** | **G** | **A** | **-** | **-** | **-** | **T** | **A** | **T** | **C** | **T** | **C** | **T** | **G** | **G** | **T** | **A** | **G** | **A** | **A** | **A** | **A** | **C** | **A** | **A** | **G** | **T** | **A** | **G** | **C** | **G** | **G** | **C** | **T** | **T** | **A** | **T** | **T** | **T** | **G** | **A** | **A** | **A** | **A** | **A** | **G** | **A** | **A** | **A** | **A** | **A** | **A** | **C** | **A** | **A** | **G** | **G** | **C** | **T** | **T** | **C** | **A** | **T** | **C** | **G** | **T** | **G** | **G** | **T** | **C** | **A** | **G** | **C** | **C** | **A** | **T** | **G** | **A** | **C** | **C** | **G** | **G** | **G** | **G** | **A** | **T** | **T** | **T** | **A** | **T** | **C** | **G** | **A** | **T** | **G** | **A** | **A** | **G** | **T** | **A** | **G** | **T** | **G** | **G** | **A** | **C** | **C** | **A** | **T** | **G** | **T** | **T** | **T** | **T** | **A** | **G** | **C** | **A** | **A** | **T** | **C** | **G** | **A** | **A** | **A** | **A** | **A** | **A** | **G** | **T** | **C** | **A** | **A** | **C** | **T** | **G** | **G** | **A** | **A** | **C** | **T** | **T** | **T** | **A** | **T** | **C** | **A** | **A** | **G** | **G** | **G** | **A** | **A** | **T** | **T** | **T** | **C** | **T** | **C** | **T** | **A** | **T** | **C** | **T** | **A** | **T** | **G** | **A** | **A** | **G** | **A** | **A** | **C** | **A** | **G** | **A** | **A** | **A** | **A** | **A** | **A** | **C** | **T** | **T** | **C** | **G** | **T** | **G** | **A** | **T** | **G** | **A** | **A** | **T** | **T** | **T** | **G** | **A** | **A** | **A** | **T** | **G** | **G** | **C** | **T** | **C** | **A** | **A** | **A** | **A** | **T** | **G** | **A** | **A** | **A** | **A** | **A** | **T** | **T** | **G** | **A** | **A** | **A** | **A** | **A** | **A** | **G** | **A** | **A** | **G** | **T** | **C** | **A** | **G** | **T** | **A** | **G** | **G** | **C** | **T** | **A** | **A** | **A** | **G** | **A** | **A** | **A** | **A** | **C** | **A** | **G** | **C** | **A** | **G** | **C** | **T** | **G** | **A** | **A** | **A** | **A** | **A** | **G** | **C** | **C** | **G** | **A** | **A** | **T** | **G** | **G** | **T** | **C** | **T** | **C** | **G** | **T** | **T** | **C** | **C** | **C** | **G** | **A** | **G** | **A** | **A** | **G** | **G** | **A** | **G** | **A** | **T** | **A** | **A** | **A** | **A** | **C** | **A** | **A** | **A** | **G** | **A** | **A** | **A** | **C** | **A** | **A** | **G** | **T** | **C** | **G** | **G** | **A** | **T** | **T** | **C** | **A** | **T** | **C** | **G** | **A** | **T** | **A** | **C** | **T** | **G** | **A** | **A** | **T** | **C** | **T** | **A** | **G** | **A** | **C** | **G** | **A** | **G** | **T** | **G** | **A** | **A** | **T** | **A** | **A** | **A** | **G** | **G** | **A** | **G** | **C** | **A** | **G** | **T** | **G** | **G** | **G** | **T** | **G** | **C** | **T** | **G** | **A** | **T** | **G** | **C** | **T** | **G** | **C** | **A** | **C** | **G** | **G** | **A** | **C** | **G** | **A** | **T** | **G** | **A** | **A** | **A** | **C** | **G** | **A** | **T** | **C** | **C** | **A** | **A** | **A** | **G** | **C** | **A** | **A** | **T** | **C** | **G** | **T** | **G** | **A** | **A** | **T** | **C** | **G** | **G** | **A** | **T** | **G** | **G** | **A** | **G** | **A** | **C** | **C** | **C** | **A** | **G** | **A** | **T** | **C** | **A** | **G** | **C** | **G** | **A** | **G** | **A** | **A** | **A** | **G** | **A** | **A** | **A** | **A** | **A** | **C** | **T** | **A** | **T** | **T** | **A** | **A** | **A** | **A** | **G** | **A** | **T** | **A** | **T** | **C** | **G** | **A** | **A** | **T** | **A** | **T** | **A** | **T** | **C** | **G** | **A** | **T** | **T** | **C** | **T** | **T** | **T** | **G** | **A** | **C** | **G** | **A** | **T** | **G** | **A** | **A** | **T** | **A** | **G** | **C** | **C** | **A** | **A** | **G** | **C** | **G** | **T** | **C** | **T** | **C** | **A** | **C** | **C** | **A** | **T** | **A** | **A** | **G** | **C** | **G** | **A** | **C** | **T** | **T** | **T** | **T** | **A** | **A** | **G** | **C** | **G** | **T** | **A** | **G** | **A** | **A** | **G** | **A** | **T** | **C** | **T** | **T** | **C** | **A** | **A** | **T** | **T** | **A** | **G** | **G** | **G** | **T** | **A** | **T** | **G** | **A** | **A** | **A** | **A** | **T** | **C** | **T** | **G** | **T** | **T** | **A** | **T** | **T** | **C** | **G** | **A** | **G** | **C** | **C** | **A** | **A** | **T** | **T** | **C** | **A** | **T** | **T** | **T** | **T** | **A** | **C** | **A** | **A** | **T** | **C** | **G** | **A** | **G** | **C** | **C** | **T** | **C** | **A** | **T** | **C** | **A** | **G** | **C** | **G** | **G** | **G** | **T** | **G** | **G** | **C** | **G** | **A** | **T** | **T** | **T** | **C** | **A** | **G** | **G** | **T** | **C** | **C** | **T** | **A** | **A** | **C** | **G** | **G** | **T** | **G** | **C** | **A** | **G** | **G** | **A** | **A** | **A** | **G** | **T** | **C** | **A** | **T** | **C** | **C** | **A** | **T** | **T** | **A** | **T** | **C** | **C** | **A** | **T** | **T** | **A** | **T** | **C** | **T** | **T** | **C** | **T** | **G** | **G** | **G** | **G** | **G** | **C** | **A** | **T** | **T** | **C** | **A** | **A** | **C** | **G** | **G** | **C** | **A** | **A** | **G** | **G** | **T** | **T** | **A** | **T** | **A** | **G** | **G** | **A** | **G** | **A** | **A** | **A** | **A** | **A** | **T** | **A** | **C** | **C** | **A** | **G** | **C** | **C** | **A** | **A** | **A** | **A** | **C** | **A** | **T** | **C** | **T** | **G** | **A** | **G** | **C** | **A** | **T** | **T** | **A** | **G** | **T** | **T** | **A** | **T** | **G** | **C** | **A** | **A** | **G** | **C** | **C** | **A** | **A** | **A** | **A** | **T** | **T** | **A** | **T** | **G** | **A** | **A** | **G** | **A** | **C** | **A** | **A** | **T** | **C** | **G** | **A** | **G** | **G** | **A** | **A** | **C** | **G** | **T** | **T** | **G** | **G** | **C** | **G** | **G** | **A** | **A** | **T** | **T** | **T** | **G** | **C** | **A** | **G** | **A** | **G** | **A** | **A** | **A** | **A** | **A** | **C** | **C** | **A** | **A** | **G** | **T** | **A** | **G** | **A** | **C** | **T** | **A** | **C** | **C** | **A** | **A** | **G** | **C** | **A** | **T** | **T** | **T** | **T** | **T** | **G** | **A** | **A** | **C** | **A** | **A** | **C** | **C** | **T** | **C** | **C** | **G** | **A** | **A** | **A** | **G** | **C** | **T** | **T** | **G** | **G** | **G** | **A** | **T** | **G** | **G** | **A** | **A** | **A** | **G** | **A** | **G** | **A** | **T** | **G** | **T** | **T** | **T** | **T** | **T** | **C** | **A** | **T** | **A** | **A** | **C** | **A** | **A** | **G** | **A** | **T** | **C** | **G** | **A** | **G** | **C** | **A** | **G** | **A** | **T** | **G** | **A** | **G** | **T** | **A** | **T** | **G** | **G** | **G** | **C** | **C** | **A** | **A** | **C** | **G** | **G** | **A** | **A** | **A** | **A** | **A** | **A** | **G** | **T** | **G** | **G** | **A** | **A** | **T** | **T** | **G** | **G** | **C** | **T** | **A** | **A** | **A** | **T** | **C** | **T** | **T** | **T** | **A** | **T** | **C** | **A** | **C** | **A** | **G** | **C** | **C** | **A** | **G** | **C** | **T** | **G** | **A** | **A** | **C** | **T** | **A** | **T** | **A** | **T** | **A** | **T** | **A** | **T** | **G** | **G** | **G** | **A** | **T** | **G** | **A** | **A** | **C** | **C** | **A** | **T** | **T** | **G** | **A** | **A** | **T** | **T** | **A** | **T** | **T** | **T** | **G** | **G** | **A** | **T** | **G** | **T** | **C** | **T** | **T** | **C** | **A** | **A** | **T** | **C** | **A** | **A** | **G** | **A** | **A** | **C** | **A** | **A** | **T** | **T** | **A** | **G** | **A** | **A** | **C** | **A** | **A** | **C** | **T** | **G** | **A** | **T** | **C** | **T** | **T** | **G** | **A** | **A** | **C** | **G** | **T** | **G** | **A** | **A** | **A** | **C** | **C** | **T** | **G** | **C** | **C** | **A** | **T** | **G** | **T** | **T** | **A** | **C** | **T** | **A** | **G** | **T** | **G** | **G** | **A** | **A** | **C** | **A** | **T** | **G** | **A** | **T** | **C** | **A** | **A** | **A** | **C** | **C** | **T** | **T** | **C** | **C** | **T** | **G** | **G** | **A** | **T** | **A** | **A** | **A** | **G** | **T** | **A** | **T** | **C** | **T** | **A** | **C** | **T** | **G** | **A** | **G** | **A** | **T** | **T** | **A** | **T** | **T** | **T** | **C** | **T** | **C** | **T** | **T** | **G** | **A** | **G** | **A** | **G** | **A** | **A** | **T** | **C** | **-** | **-** | **-** |
| **N54546** | **A** | **T** | **G** | **T** | **C** | **T** | **A** | **A** | **A** | **A** | **T** | **C** | **G** | **A** | **A** | **A** | **T** | **A** | **A** | **A** | **A** | **A** | **A** | **T** | **C** | **T** | **G** | **A** | **C** | **A** | **T** | **T** | **C** | **G** | **G** | **C** | **T** | **A** | **C** | **G** | **A** | **C** | **A** | **G** | **C** | **C** | **A** | **A** | **G** | **G** | **C** | **A** | **C** | **A** | **T** | **T** | **A** | **T** | **T** | **A** | **T** | **T** | **T** | **G** | **A** | **A** | **C** | **A** | **A** | **G** | **C** | **A** | **A** | **A** | **T** | **C** | **T** | **A** | **A** | **A** | **T** | **T** | **T** | **T** | **G** | **A** | **C** | **A** | **C** | **A** | **C** | **A** | **A** | **T** | **G** | **G** | **A** | **A** | **A** | **C** | **T** | **A** | **G** | **G** | **A** | **C** | **T** | **T** | **A** | **T** | **C** | **G** | **G** | **A** | **C** | **G** | **A** | **A** | **A** | **C** | **G** | **G** | **T** | **C** | **G** | **A** | **G** | **G** | **A** | **A** | **A** | **G** | **A** | **C** | **A** | **A** | **C** | **T** | **T** | **T** | **A** | **C** | **T** | **G** | **A** | **A** | **T** | **A** | **T** | **T** | **C** | **T** | **A** | **C** | **A** | **A** | **A** | **A** | **C** | **A** | **A** | **A** | **C** | **T** | **A** | **C** | **C** | **T** | **T** | **A** | **T** | **C** | **A** | **A** | **G** | **G** | **G** | **C** | **A** | **A** | **G** | **T** | **A** | **A** | **T** | **C** | **C** | **A** | **T** | **C** | **A** | **G** | **C** | **A** | **A** | **G** | **A** | **A** | **T** | **T** | **T** | **G** | **T** | **C** | **T** | **A** | **T** | **T** | **T** | **C** | **C** | **C** | **G** | **C** | **A** | **A** | **C** | **A** | **G** | **A** | **C** | **A** | **A** | **A** | **A** | **G** | **A** | **T** | **A** | **A** | **A** | **G** | **A** | **A** | **C** | **G** | **T** | **T** | **T** | **A** | **A** | **C** | **C** | **T** | **A** | **T** | **T** | **A** | **C** | **G** | **T** | **G** | **T** | **T** | **A** | **A** | **A** | **T** | **G** | **A** | **T** | **A** | **T** | **T** | **A** | **C** | **G** | **G** | **A** | **T** | **T** | **T** | **T** | **G** | **A** | **G** | **A** | **C** | **A** | **T** | **G** | **G** | **G** | **A** | **A** | **A** | **T** | **C** | **G** | **A** | **A** | **A** | **G** | **A** | **G** | **A** | **G** | **C** | **T** | **C** | **C** | **A** | **A** | **T** | **T** | **T** | **A** | **T** | **G** | **C** | **A** | **A** | **A** | **C** | **A** | **G** | **A** | **T** | **C** | **C** | **T** | **G** | **A** | **A** | **A** | **T** | **C** | **T** | **T** | **A** | **T** | **G** | **G** | **A** | **G** | **A** | **G** | **A** | **A** | **T** | **T** | **C** | **A** | **G** | **C** | **A** | **C** | **A** | **C** | **T** | **A** | **T** | **C** | **G** | **G** | **G** | **G** | **G** | **G** | **A** | **G** | **A** | **G** | **A** | **A** | **G** | **A** | **C** | **A** | **A** | **A** | **A** | **G** | **T** | **C** | **C** | **T** | **A** | **T** | **T** | **G** | **G** | **C** | **A** | **C** | **T** | **T** | **T** | **T** | **A** | **T** | **T** | **T** | **G** | **T** | **G** | **G** | **A** | **T** | **G** | **A** | **C** | **A** | **C** | **T** | **C** | **A** | **T** | **T** | **T** | **C** | **C** | **C** | **G** | **T** | **T** | **A** | **A** | **T** | **C** | **G** | **A** | **T** | **G** | **A** | **A** | **C** | **C** | **A** | **A** | **C** | **G** | **A** | **A** | **T** | **C** | **A** | **T** | **T** | **T** | **G** | **G** | **A** | **-** | **-** | **-** | **T** | **A** | **T** | **C** | **T** | **C** | **T** | **G** | **G** | **T** | **A** | **G** | **A** | **A** | **A** | **A** | **C** | **A** | **A** | **G** | **T** | **A** | **G** | **C** | **G** | **G** | **C** | **T** | **T** | **A** | **T** | **T** | **T** | **G** | **A** | **A** | **A** | **A** | **A** | **G** | **A** | **A** | **A** | **A** | **A** | **A** | **C** | **A** | **A** | **G** | **G** | **C** | **T** | **T** | **C** | **A** | **T** | **C** | **G** | **T** | **G** | **G** | **T** | **C** | **A** | **G** | **C** | **C** | **A** | **T** | **G** | **A** | **C** | **C** | **G** | **G** | **G** | **G** | **A** | **T** | **T** | **T** | **A** | **T** | **C** | **G** | **A** | **T** | **G** | **A** | **A** | **G** | **T** | **A** | **G** | **T** | **G** | **G** | **A** | **C** | **C** | **A** | **T** | **G** | **T** | **T** | **T** | **T** | **A** | **G** | **C** | **A** | **A** | **T** | **C** | **G** | **A** | **A** | **A** | **A** | **A** | **A** | **G** | **T** | **C** | **A** | **A** | **C** | **T** | **G** | **G** | **A** | **A** | **C** | **T** | **T** | **T** | **A** | **T** | **C** | **A** | **A** | **G** | **G** | **G** | **A** | **A** | **T** | **T** | **T** | **C** | **T** | **C** | **T** | **A** | **T** | **C** | **T** | **A** | **T** | **G** | **A** | **A** | **G** | **A** | **A** | **C** | **A** | **G** | **A** | **A** | **A** | **A** | **A** | **A** | **C** | **T** | **T** | **C** | **G** | **T** | **G** | **A** | **T** | **G** | **A** | **A** | **T** | **T** | **T** | **G** | **A** | **A** | **A** | **T** | **G** | **G** | **C** | **T** | **C** | **A** | **A** | **A** | **A** | **T** | **G** | **A** | **A** | **A** | **A** | **A** | **T** | **T** | **G** | **A** | **A** | **A** | **A** | **A** | **A** | **G** | **A** | **A** | **G** | **T** | **C** | **A** | **G** | **T** | **A** | **G** | **G** | **C** | **T** | **A** | **A** | **A** | **G** | **A** | **A** | **A** | **A** | **C** | **A** | **G** | **C** | **A** | **G** | **C** | **T** | **G** | **A** | **A** | **A** | **A** | **A** | **G** | **C** | **C** | **G** | **A** | **A** | **T** | **G** | **G** | **T** | **C** | **T** | **C** | **G** | **T** | **T** | **C** | **C** | **C** | **G** | **A** | **G** | **A** | **A** | **G** | **G** | **A** | **G** | **A** | **T** | **A** | **A** | **A** | **A** | **C** | **A** | **A** | **A** | **G** | **A** | **A** | **A** | **C** | **A** | **A** | **G** | **T** | **C** | **G** | **G** | **A** | **T** | **T** | **C** | **A** | **T** | **C** | **G** | **A** | **T** | **A** | **C** | **T** | **G** | **A** | **A** | **T** | **C** | **T** | **A** | **G** | **A** | **C** | **G** | **A** | **G** | **T** | **G** | **A** | **A** | **T** | **A** | **A** | **A** | **G** | **G** | **A** | **G** | **C** | **A** | **G** | **T** | **G** | **G** | **G** | **T** | **G** | **C** | **T** | **G** | **A** | **T** | **G** | **C** | **T** | **G** | **C** | **A** | **C** | **G** | **G** | **A** | **C** | **G** | **A** | **T** | **G** | **A** | **A** | **A** | **C** | **G** | **A** | **T** | **C** | **C** | **A** | **A** | **A** | **G** | **C** | **A** | **A** | **T** | **C** | **G** | **T** | **G** | **A** | **A** | **T** | **C** | **G** | **G** | **A** | **T** | **G** | **G** | **A** | **G** | **A** | **C** | **C** | **C** | **A** | **G** | **A** | **T** | **C** | **A** | **G** | **C** | **G** | **A** | **G** | **A** | **A** | **A** | **G** | **A** | **A** | **A** | **A** | **A** | **C** | **T** | **A** | **T** | **T** | **A** | **A** | **A** | **A** | **G** | **A** | **T** | **A** | **T** | **C** | **G** | **A** | **A** | **T** | **A** | **T** | **A** | **T** | **C** | **G** | **A** | **T** | **T** | **C** | **T** | **T** | **T** | **G** | **A** | **C** | **G** | **A** | **T** | **G** | **A** | **A** | **T** | **A** | **G** | **C** | **C** | **A** | **A** | **G** | **C** | **G** | **T** | **C** | **T** | **C** | **A** | **C** | **C** | **A** | **T** | **A** | **A** | **G** | **C** | **G** | **A** | **C** | **T** | **T** | **T** | **T** | **A** | **A** | **G** | **C** | **G** | **T** | **A** | **G** | **A** | **A** | **G** | **A** | **T** | **C** | **T** | **T** | **C** | **A** | **A** | **T** | **T** | **A** | **G** | **G** | **G** | **T** | **A** | **T** | **G** | **A** | **A** | **A** | **A** | **T** | **C** | **T** | **G** | **T** | **T** | **A** | **T** | **T** | **C** | **G** | **A** | **G** | **C** | **C** | **A** | **A** | **T** | **T** | **C** | **A** | **T** | **T** | **T** | **T** | **A** | **C** | **A** | **A** | **T** | **C** | **G** | **A** | **G** | **C** | **C** | **T** | **A** | **A** | **T** | **C** | **A** | **G** | **C** | **G** | **G** | **G** | **T** | **G** | **G** | **C** | **G** | **A** | **T** | **T** | **T** | **C** | **A** | **G** | **G** | **T** | **C** | **C** | **T** | **A** | **A** | **C** | **G** | **G** | **T** | **G** | **C** | **A** | **G** | **G** | **A** | **A** | **A** | **G** | **T** | **C** | **A** | **T** | **C** | **C** | **A** | **T** | **T** | **A** | **T** | **C** | **C** | **A** | **T** | **T** | **A** | **T** | **C** | **T** | **T** | **C** | **T** | **G** | **G** | **G** | **G** | **G** | **C** | **A** | **T** | **T** | **C** | **A** | **A** | **C** | **G** | **G** | **C** | **A** | **A** | **G** | **G** | **T** | **T** | **A** | **T** | **A** | **G** | **G** | **A** | **G** | **A** | **A** | **A** | **A** | **A** | **T** | **A** | **C** | **C** | **A** | **G** | **C** | **C** | **A** | **A** | **A** | **A** | **C** | **A** | **T** | **C** | **T** | **G** | **A** | **G** | **C** | **A** | **T** | **T** | **A** | **G** | **T** | **T** | **A** | **T** | **G** | **T** | **A** | **C** | **G** | **C** | **C** | **A** | **A** | **A** | **A** | **T** | **T** | **A** | **T** | **G** | **A** | **A** | **G** | **A** | **C** | **A** | **A** | **T** | **C** | **G** | **A** | **G** | **G** | **A** | **A** | **C** | **G** | **T** | **T** | **G** | **G** | **C** | **G** | **G** | **A** | **A** | **T** | **T** | **T** | **G** | **C** | **A** | **G** | **A** | **G** | **A** | **A** | **A** | **A** | **A** | **C** | **C** | **A** | **A** | **G** | **T** | **A** | **G** | **A** | **C** | **T** | **A** | **C** | **C** | **A** | **A** | **G** | **C** | **A** | **T** | **T** | **T** | **T** | **T** | **G** | **A** | **A** | **C** | **A** | **A** | **C** | **C** | **T** | **C** | **C** | **G** | **A** | **A** | **A** | **G** | **C** | **T** | **T** | **G** | **G** | **G** | **A** | **T** | **G** | **G** | **A** | **A** | **A** | **G** | **A** | **G** | **A** | **T** | **G** | **T** | **T** | **T** | **T** | **T** | **C** | **A** | **T** | **A** | **A** | **C** | **A** | **A** | **G** | **A** | **T** | **C** | **G** | **A** | **G** | **C** | **A** | **G** | **A** | **T** | **G** | **A** | **G** | **T** | **A** | **T** | **G** | **G** | **G** | **C** | **C** | **A** | **A** | **C** | **G** | **G** | **A** | **A** | **A** | **A** | **A** | **A** | **G** | **T** | **G** | **G** | **A** | **A** | **T** | **T** | **G** | **G** | **C** | **T** | **A** | **A** | **A** | **T** | **C** | **T** | **T** | **T** | **A** | **T** | **C** | **A** | **C** | **A** | **G** | **C** | **C** | **A** | **G** | **C** | **T** | **G** | **A** | **A** | **C** | **T** | **A** | **T** | **A** | **T** | **A** | **T** | **A** | **T** | **G** | **G** | **G** | **A** | **T** | **G** | **A** | **A** | **C** | **C** | **A** | **T** | **T** | **G** | **A** | **A** | **T** | **T** | **A** | **T** | **T** | **T** | **G** | **G** | **A** | **T** | **G** | **T** | **C** | **T** | **T** | **C** | **A** | **A** | **T** | **C** | **A** | **A** | **G** | **A** | **A** | **C** | **A** | **A** | **T** | **T** | **A** | **G** | **A** | **A** | **C** | **A** | **A** | **C** | **T** | **G** | **A** | **T** | **C** | **T** | **T** | **G** | **A** | **A** | **C** | **G** | **T** | **G** | **A** | **A** | **A** | **C** | **C** | **T** | **G** | **C** | **C** | **A** | **T** | **G** | **T** | **T** | **A** | **C** | **T** | **A** | **G** | **T** | **G** | **G** | **A** | **A** | **C** | **A** | **T** | **G** | **A** | **T** | **C** | **A** | **A** | **A** | **C** | **C** | **T** | **T** | **C** | **C** | **T** | **G** | **G** | **A** | **T** | **A** | **A** | **A** | **G** | **T** | **A** | **T** | **C** | **T** | **A** | **C** | **T** | **G** | **A** | **G** | **A** | **T** | **T** | **A** | **T** | **T** | **T** | **C** | **T** | **C** | **T** | **T** | **G** | **A** | **G** | **A** | **A** | **A** | **A** | **T** | **C** | **-** | **-** | **-** |
| **N59624F** | **A** | **T** | **G** | **T** | **C** | **T** | **A** | **A** | **A** | **A** | **T** | **C** | **G** | **A** | **A** | **A** | **T** | **A** | **A** | **A** | **A** | **A** | **A** | **T** | **C** | **T** | **G** | **A** | **C** | **A** | **T** | **T** | **C** | **G** | **G** | **C** | **T** | **A** | **T** | **G** | **A** | **C** | **A** | **G** | **C** | **C** | **A** | **A** | **G** | **G** | **C** | **A** | **C** | **A** | **T** | **T** | **A** | **T** | **T** | **A** | **T** | **T** | **T** | **G** | **A** | **A** | **C** | **A** | **A** | **G** | **C** | **C** | **A** | **A** | **T** | **C** | **T** | **A** | **A** | **A** | **C** | **T** | **T** | **T** | **G** | **A** | **C** | **A** | **C** | **A** | **C** | **A** | **A** | **T** | **G** | **G** | **A** | **A** | **A** | **C** | **T** | **A** | **G** | **G** | **A** | **C** | **T** | **T** | **A** | **T** | **C** | **G** | **G** | **A** | **C** | **G** | **A** | **A** | **A** | **C** | **G** | **G** | **T** | **C** | **G** | **A** | **G** | **G** | **A** | **A** | **A** | **G** | **A** | **C** | **A** | **A** | **C** | **T** | **T** | **T** | **A** | **C** | **T** | **G** | **A** | **A** | **T** | **A** | **T** | **C** | **C** | **T** | **G** | **C** | **A** | **A** | **A** | **A** | **C** | **A** | **A** | **A** | **C** | **T** | **C** | **C** | **C** | **T** | **T** | **A** | **T** | **C** | **A** | **A** | **G** | **G** | **G** | **C** | **A** | **A** | **G** | **T** | **G** | **A** | **T** | **C** | **C** | **A** | **T** | **C** | **A** | **G** | **C** | **A** | **A** | **G** | **C** | **G** | **T** | **T** | **T** | **G** | **T** | **C** | **T** | **A** | **T** | **T** | **T** | **C** | **C** | **C** | **G** | **C** | **A** | **G** | **C** | **A** | **A** | **A** | **C** | **A** | **A** | **A** | **A** | **G** | **A** | **T** | **A** | **A** | **A** | **G** | **A** | **G** | **C** | **G** | **T** | **T** | **T** | **G** | **A** | **C** | **C** | **T** | **A** | **T** | **T** | **A** | **T** | **G** | **T** | **G** | **T** | **T** | **A** | **A** | **A** | **T** | **G** | **A** | **G** | **A** | **T** | **T** | **A** | **T** | **G** | **G** | **A** | **T** | **T** | **T** | **T** | **G** | **A** | **G** | **A** | **T** | **A** | **T** | **G** | **G** | **G** | **A** | **A** | **A** | **T** | **T** | **G** | **A** | **A** | **A** | **G** | **A** | **G** | **A** | **G** | **C** | **T** | **C** | **C** | **A** | **A** | **C** | **T** | **G** | **A** | **T** | **G** | **C** | **A** | **A** | **A** | **C** | **A** | **G** | **A** | **T** | **C** | **C** | **T** | **G** | **A** | **A** | **A** | **T** | **C** | **T** | **T** | **A** | **T** | **G** | **G** | **A** | **G** | **A** | **G** | **A** | **A** | **T** | **T** | **C** | **A** | **G** | **C** | **A** | **C** | **A** | **C** | **T** | **A** | **T** | **C** | **A** | **G** | **G** | **A** | **G** | **G** | **A** | **G** | **A** | **G** | **A** | **A** | **G** | **A** | **C** | **A** | **A** | **A** | **A** | **G** | **T** | **C** | **C** | **T** | **A** | **C** | **T** | **G** | **G** | **C** | **A** | **C** | **T** | **C** | **T** | **T** | **A** | **T** | **T** | **T** | **G** | **T** | **G** | **G** | **A** | **T** | **G** | **A** | **C** | **A** | **C** | **T** | **C** | **A** | **T** | **T** | **T** | **C** | **C** | **C** | **G** | **T** | **T** | **A** | **A** | **T** | **C** | **G** | **A** | **T** | **G** | **A** | **A** | **C** | **C** | **A** | **A** | **C** | **G** | **A** | **A** | **T** | **C** | **A** | **T** | **T** | **T** | **G** | **G** | **A** | **-** | **-** | **-** | **T** | **A** | **T** | **C** | **T** | **C** | **T** | **G** | **G** | **C** | **A** | **G** | **A** | **A** | **A** | **A** | **C** | **A** | **A** | **G** | **T** | **A** | **G** | **C** | **G** | **G** | **C** | **T** | **T** | **A** | **C** | **T** | **T** | **G** | **A** | **A** | **A** | **A** | **A** | **G** | **A** | **A** | **A** | **A** | **A** | **A** | **C** | **A** | **A** | **G** | **G** | **A** | **T** | **T** | **C** | **A** | **T** | **C** | **G** | **T** | **G** | **G** | **T** | **G** | **A** | **G** | **T** | **C** | **A** | **T** | **G** | **A** | **T** | **C** | **G** | **G** | **G** | **G** | **A** | **T** | **T** | **T** | **A** | **T** | **C** | **G** | **A** | **T** | **G** | **A** | **A** | **G** | **T** | **A** | **G** | **T** | **G** | **G** | **A** | **C** | **C** | **A** | **T** | **G** | **T** | **T** | **T** | **T** | **A** | **G** | **C** | **A** | **A** | **T** | **C** | **G** | **A** | **A** | **A** | **A** | **G** | **A** | **G** | **C** | **C** | **A** | **A** | **C** | **T** | **G** | **G** | **A** | **A** | **C** | **T** | **T** | **T** | **A** | **T** | **C** | **A** | **A** | **G** | **G** | **G** | **A** | **A** | **T** | **T** | **T** | **C** | **T** | **C** | **T** | **A** | **T** | **C** | **T** | **A** | **T** | **G** | **A** | **A** | **G** | **A** | **A** | **C** | **A** | **G** | **A** | **A** | **A** | **A** | **A** | **A** | **C** | **T** | **T** | **C** | **G** | **T** | **G** | **A** | **T** | **G** | **A** | **A** | **T** | **T** | **T** | **G** | **A** | **A** | **A** | **T** | **A** | **G** | **C** | **T** | **A** | **A** | **A** | **A** | **A** | **T** | **G** | **A** | **A** | **A** | **A** | **A** | **T** | **T** | **G** | **A** | **A** | **A** | **A** | **A** | **A** | **G** | **A** | **A** | **G** | **T** | **C** | **A** | **G** | **C** | **A** | **G** | **G** | **C** | **T** | **A** | **A** | **A** | **G** | **A** | **A** | **A** | **A** | **C** | **A** | **G** | **C** | **A** | **G** | **C** | **T** | **G** | **A** | **A** | **A** | **A** | **A** | **G** | **C** | **A** | **G** | **A** | **A** | **T** | **G** | **G** | **T** | **C** | **T** | **C** | **G** | **T** | **T** | **C** | **C** | **C** | **G** | **A** | **G** | **A** | **A** | **G** | **G** | **A** | **G** | **A** | **C** | **A** | **A** | **A** | **A** | **C** | **A** | **A** | **A** | **G** | **A** | **A** | **A** | **A** | **A** | **A** | **G** | **T** | **C** | **G** | **G** | **A** | **T** | **T** | **C** | **A** | **T** | **C** | **G** | **A** | **T** | **A** | **C** | **G** | **G** | **A** | **A** | **T** | **C** | **T** | **A** | **G** | **G** | **C** | **G** | **A** | **G** | **T** | **G** | **A** | **A** | **T** | **A** | **A** | **A** | **G** | **G** | **A** | **G** | **C** | **A** | **G** | **T** | **A** | **G** | **G** | **C** | **G** | **C** | **T** | **G** | **A** | **T** | **G** | **C** | **T** | **G** | **C** | **A** | **C** | **G** | **G** | **A** | **C** | **G** | **A** | **T** | **G** | **A** | **A** | **A** | **C** | **G** | **A** | **T** | **C** | **T** | **A** | **A** | **A** | **G** | **C** | **A** | **A** | **T** | **C** | **G** | **T** | **G** | **A** | **A** | **T** | **C** | **G** | **G** | **A** | **T** | **G** | **G** | **A** | **A** | **A** | **C** | **C** | **C** | **A** | **G** | **A** | **T** | **C** | **A** | **G** | **T** | **G** | **A** | **G** | **A** | **A** | **A** | **G** | **A** | **A** | **A** | **A** | **A** | **T** | **T** | **A** | **T** | **T** | **A** | **A** | **A** | **A** | **G** | **A** | **T** | **A** | **T** | **C** | **G** | **A** | **G** | **T** | **A** | **T** | **A** | **T** | **C** | **G** | **A** | **T** | **G** | **C** | **T** | **T** | **T** | **G** | **A** | **C** | **G** | **A** | **T** | **G** | **A** | **A** | **T** | **A** | **G** | **C** | **C** | **A** | **A** | **G** | **T** | **G** | **T** | **C** | **T** | **C** | **A** | **T** | **C** | **A** | **T** | **A** | **A** | **A** | **C** | **G** | **G** | **C** | **T** | **G** | **T** | **T** | **A** | **A** | **G** | **C** | **G** | **T** | **A** | **G** | **A** | **A** | **A** | **A** | **T** | **C** | **T** | **T** | **C** | **A** | **A** | **T** | **T** | **A** | **G** | **G** | **G** | **T** | **A** | **T** | **G** | **A** | **A** | **A** | **A** | **T** | **C** | **T** | **G** | **T** | **T** | **A** | **T** | **T** | **C** | **G** | **A** | **G** | **C** | **C** | **A** | **G** | **T** | **T** | **C** | **A** | **T** | **T** | **T** | **T** | **A** | **C** | **G** | **A** | **T** | **C** | **G** | **A** | **A** | **C** | **C** | **T** | **C** | **A** | **T** | **C** | **A** | **G** | **C** | **G** | **G** | **G** | **T** | **G** | **G** | **C** | **A** | **A** | **T** | **T** | **T** | **C** | **A** | **G** | **G** | **T** | **C** | **C** | **T** | **A** | **A** | **C** | **G** | **G** | **C** | **G** | **C** | **G** | **G** | **G** | **A** | **A** | **A** | **G** | **T** | **C** | **A** | **T** | **C** | **C** | **A** | **T** | **T** | **A** | **T** | **C** | **C** | **A** | **T** | **T** | **A** | **T** | **C** | **T** | **T** | **C** | **T** | **G** | **G** | **G** | **G** | **A** | **C** | **A** | **T** | **T** | **C** | **A** | **A** | **C** | **G** | **G** | **C** | **A** | **A** | **G** | **G** | **T** | **T** | **G** | **T** | **A** | **G** | **G** | **A** | **G** | **A** | **A** | **A** | **A** | **A** | **T** | **C** | **C** | **C** | **A** | **G** | **C** | **C** | **C** | **A** | **A** | **A** | **C** | **A** | **T** | **T** | **T** | **G** | **A** | **C** | **C** | **A** | **T** | **T** | **A** | **G** | **T** | **T** | **A** | **C** | **G** | **T** | **A** | **C** | **G** | **C** | **C** | **A** | **A** | **A** | **A** | **T** | **T** | **A** | **T** | **G** | **A** | **A** | **G** | **A** | **C** | **A** | **A** | **T** | **C** | **G** | **A** | **G** | **G** | **G** | **A** | **C** | **A** | **T** | **T** | **G** | **G** | **C** | **G** | **G** | **A** | **A** | **T** | **T** | **C** | **G** | **C** | **G** | **A** | **A** | **G** | **A** | **A** | **A** | **A** | **A** | **C** | **C** | **G** | **A** | **G** | **T** | **A** | **G** | **A** | **C** | **T** | **A** | **T** | **C** | **A** | **A** | **G** | **C** | **A** | **T** | **T** | **T** | **T** | **T** | **G** | **A** | **A** | **T** | **A** | **A** | **T** | **C** | **T** | **C** | **C** | **G** | **A** | **A** | **A** | **G** | **C** | **T** | **T** | **G** | **G** | **G** | **A** | **T** | **G** | **G** | **A** | **A** | **A** | **G** | **A** | **G** | **A** | **T** | **G** | **T** | **T** | **T** | **T** | **T** | **C** | **A** | **T** | **A** | **A** | **T** | **A** | **A** | **A** | **A** | **T** | **C** | **G** | **A** | **A** | **C** | **A** | **G** | **A** | **T** | **G** | **A** | **G** | **T** | **A** | **T** | **G** | **G** | **G** | **A** | **C** | **A** | **A** | **C** | **G** | **G** | **A** | **A** | **A** | **A** | **A** | **A** | **G** | **T** | **G** | **G** | **A** | **A** | **T** | **T** | **G** | **G** | **C** | **T** | **A** | **A** | **A** | **T** | **C** | **T** | **T** | **T** | **A** | **T** | **C** | **C** | **C** | **A** | **A** | **C** | **C** | **A** | **G** | **C** | **T** | **G** | **A** | **G** | **C** | **T** | **C** | **T** | **A** | **T** | **A** | **T** | **A** | **T** | **G** | **G** | **G** | **A** | **T** | **G** | **A** | **A** | **C** | **C** | **A** | **T** | **T** | **G** | **A** | **A** | **T** | **T** | **A** | **T** | **T** | **T** | **G** | **G** | **A** | **T** | **G** | **T** | **C** | **T** | **T** | **C** | **A** | **A** | **T** | **C** | **A** | **A** | **G** | **A** | **A** | **C** | **A** | **A** | **T** | **T** | **A** | **G** | **A** | **A** | **C** | **A** | **A** | **C** | **T** | **G** | **A** | **T** | **T** | **T** | **T** | **G** | **A** | **A** | **C** | **G** | **T** | **G** | **A** | **A** | **A** | **C** | **C** | **T** | **G** | **C** | **T** | **A** | **T** | **G** | **T** | **T** | **A** | **C** | **T** | **A** | **G** | **T** | **G** | **G** | **A** | **A** | **C** | **A** | **T** | **G** | **A** | **T** | **C** | **A** | **A** | **A** | **C** | **C** | **T** | **T** | **T** | **C** | **T** | **A** | **G** | **A** | **T** | **A** | **A** | **A** | **G** | **T** | **A** | **T** | **C** | **T** | **A** | **C** | **T** | **G** | **A** | **G** | **A** | **T** | **T** | **A** | **T** | **T** | **T** | **C** | **T** | **C** | **T** | **T** | **G** | **A** | **G** | **A** | **A** | **A** | **A** | **T** | **C** | **-** | **-** | **-** |
| **N60299F** | **A** | **T** | **G** | **T** | **C** | **T** | **A** | **A** | **A** | **A** | **T** | **C** | **G** | **A** | **A** | **A** | **T** | **A** | **A** | **A** | **A** | **A** | **A** | **T** | **C** | **T** | **G** | **A** | **C** | **A** | **T** | **T** | **C** | **G** | **G** | **C** | **T** | **A** | **C** | **G** | **A** | **C** | **A** | **G** | **C** | **C** | **A** | **A** | **G** | **G** | **C** | **A** | **C** | **A** | **T** | **T** | **A** | **T** | **T** | **A** | **T** | **T** | **T** | **G** | **A** | **A** | **C** | **A** | **A** | **G** | **C** | **A** | **A** | **A** | **T** | **C** | **T** | **A** | **A** | **A** | **T** | **T** | **T** | **T** | **G** | **A** | **C** | **A** | **C** | **A** | **C** | **A** | **A** | **T** | **G** | **G** | **A** | **A** | **A** | **C** | **T** | **A** | **G** | **G** | **A** | **C** | **T** | **T** | **A** | **T** | **C** | **G** | **G** | **A** | **C** | **G** | **A** | **A** | **A** | **C** | **G** | **G** | **T** | **C** | **G** | **A** | **G** | **G** | **A** | **A** | **A** | **G** | **A** | **C** | **A** | **A** | **C** | **T** | **T** | **T** | **A** | **C** | **T** | **G** | **A** | **A** | **T** | **A** | **T** | **T** | **C** | **T** | **A** | **C** | **A** | **A** | **A** | **A** | **C** | **A** | **A** | **A** | **C** | **T** | **A** | **C** | **C** | **T** | **T** | **A** | **T** | **C** | **A** | **A** | **G** | **G** | **G** | **C** | **A** | **A** | **G** | **T** | **A** | **A** | **T** | **C** | **C** | **A** | **T** | **C** | **A** | **G** | **C** | **A** | **A** | **G** | **A** | **A** | **T** | **T** | **T** | **G** | **T** | **C** | **T** | **A** | **T** | **T** | **T** | **C** | **C** | **C** | **G** | **C** | **A** | **G** | **C** | **A** | **A** | **A** | **C** | **A** | **A** | **A** | **A** | **G** | **A** | **T** | **A** | **A** | **A** | **G** | **A** | **G** | **C** | **G** | **T** | **T** | **T** | **A** | **A** | **C** | **C** | **T** | **A** | **T** | **T** | **A** | **T** | **G** | **T** | **G** | **T** | **T** | **A** | **A** | **A** | **T** | **G** | **A** | **G** | **A** | **T** | **T** | **A** | **C** | **G** | **G** | **A** | **T** | **T** | **T** | **T** | **G** | **A** | **G** | **A** | **T** | **A** | **T** | **G** | **G** | **G** | **A** | **A** | **A** | **T** | **T** | **G** | **A** | **A** | **A** | **G** | **A** | **G** | **A** | **G** | **C** | **T** | **C** | **C** | **A** | **A** | **C** | **T** | **G** | **A** | **T** | **G** | **C** | **A** | **A** | **A** | **C** | **A** | **G** | **A** | **T** | **C** | **C** | **T** | **G** | **A** | **A** | **A** | **T** | **C** | **T** | **T** | **A** | **T** | **G** | **G** | **A** | **G** | **A** | **G** | **A** | **A** | **T** | **T** | **C** | **A** | **G** | **C** | **A** | **C** | **A** | **C** | **T** | **A** | **T** | **C** | **A** | **G** | **G** | **A** | **G** | **G** | **A** | **G** | **A** | **G** | **A** | **A** | **G** | **A** | **C** | **A** | **A** | **A** | **A** | **G** | **T** | **C** | **C** | **T** | **A** | **C** | **T** | **G** | **G** | **C** | **A** | **C** | **T** | **T** | **T** | **T** | **A** | **T** | **T** | **T** | **G** | **T** | **G** | **G** | **A** | **T** | **G** | **A** | **C** | **A** | **C** | **T** | **C** | **A** | **T** | **T** | **T** | **C** | **C** | **C** | **G** | **T** | **T** | **A** | **A** | **T** | **C** | **G** | **A** | **T** | **G** | **A** | **A** | **C** | **C** | **A** | **A** | **C** | **G** | **A** | **A** | **T** | **C** | **A** | **T** | **T** | **T** | **G** | **G** | **A** | **-** | **-** | **-** | **T** | **A** | **T** | **C** | **T** | **C** | **T** | **G** | **G** | **C** | **A** | **G** | **A** | **A** | **A** | **A** | **C** | **A** | **A** | **G** | **T** | **A** | **G** | **C** | **G** | **G** | **C** | **T** | **T** | **A** | **C** | **T** | **T** | **G** | **A** | **A** | **A** | **A** | **A** | **G** | **A** | **A** | **A** | **A** | **A** | **A** | **C** | **A** | **A** | **G** | **G** | **C** | **T** | **T** | **C** | **A** | **T** | **C** | **G** | **T** | **G** | **G** | **T** | **C** | **A** | **G** | **C** | **C** | **A** | **T** | **G** | **A** | **C** | **C** | **G** | **G** | **G** | **G** | **A** | **T** | **T** | **T** | **A** | **T** | **C** | **G** | **A** | **T** | **G** | **A** | **A** | **G** | **T** | **A** | **G** | **T** | **G** | **G** | **A** | **C** | **C** | **A** | **T** | **G** | **T** | **T** | **T** | **T** | **A** | **G** | **C** | **A** | **A** | **T** | **C** | **G** | **A** | **A** | **A** | **A** | **A** | **A** | **G** | **T** | **C** | **A** | **A** | **C** | **T** | **G** | **G** | **A** | **A** | **C** | **T** | **T** | **T** | **A** | **T** | **C** | **A** | **A** | **G** | **G** | **G** | **A** | **A** | **T** | **T** | **T** | **C** | **T** | **C** | **T** | **A** | **T** | **C** | **T** | **A** | **T** | **G** | **A** | **A** | **G** | **A** | **A** | **C** | **A** | **G** | **A** | **A** | **A** | **A** | **A** | **A** | **C** | **T** | **T** | **C** | **G** | **T** | **G** | **A** | **T** | **G** | **A** | **A** | **T** | **T** | **T** | **G** | **A** | **A** | **A** | **T** | **G** | **G** | **C** | **T** | **C** | **A** | **A** | **A** | **A** | **T** | **G** | **A** | **A** | **A** | **A** | **A** | **T** | **T** | **G** | **A** | **A** | **A** | **A** | **A** | **A** | **G** | **A** | **A** | **G** | **T** | **C** | **A** | **G** | **T** | **A** | **G** | **G** | **C** | **T** | **A** | **A** | **A** | **G** | **A** | **A** | **A** | **A** | **C** | **A** | **G** | **C** | **A** | **G** | **C** | **T** | **G** | **A** | **A** | **A** | **A** | **A** | **G** | **C** | **C** | **G** | **A** | **A** | **T** | **G** | **G** | **T** | **C** | **T** | **C** | **G** | **T** | **T** | **C** | **C** | **C** | **G** | **A** | **G** | **A** | **A** | **G** | **G** | **A** | **G** | **A** | **T** | **A** | **A** | **A** | **A** | **C** | **A** | **A** | **A** | **G** | **A** | **A** | **A** | **C** | **A** | **A** | **G** | **T** | **C** | **G** | **G** | **A** | **T** | **T** | **C** | **A** | **T** | **C** | **G** | **A** | **T** | **A** | **C** | **T** | **G** | **A** | **A** | **T** | **C** | **T** | **A** | **G** | **A** | **C** | **G** | **A** | **G** | **T** | **G** | **A** | **A** | **T** | **A** | **A** | **A** | **G** | **G** | **A** | **G** | **C** | **A** | **G** | **T** | **G** | **G** | **G** | **T** | **G** | **C** | **T** | **G** | **A** | **T** | **G** | **C** | **T** | **G** | **C** | **A** | **C** | **G** | **G** | **A** | **C** | **G** | **A** | **T** | **G** | **A** | **A** | **A** | **C** | **G** | **A** | **T** | **C** | **C** | **A** | **A** | **A** | **G** | **C** | **A** | **A** | **T** | **C** | **G** | **T** | **G** | **A** | **A** | **T** | **C** | **G** | **G** | **A** | **T** | **G** | **G** | **A** | **G** | **A** | **C** | **C** | **C** | **A** | **G** | **A** | **T** | **C** | **A** | **G** | **C** | **G** | **A** | **G** | **A** | **A** | **A** | **G** | **A** | **A** | **A** | **A** | **A** | **C** | **T** | **A** | **T** | **T** | **G** | **A** | **A** | **A** | **G** | **A** | **T** | **A** | **T** | **C** | **G** | **A** | **A** | **T** | **A** | **T** | **A** | **T** | **C** | **G** | **A** | **T** | **T** | **C** | **T** | **T** | **T** | **G** | **A** | **C** | **G** | **A** | **T** | **G** | **A** | **A** | **T** | **A** | **G** | **C** | **C** | **A** | **A** | **G** | **C** | **G** | **T** | **C** | **T** | **C** | **A** | **C** | **C** | **A** | **T** | **A** | **A** | **G** | **C** | **G** | **A** | **C** | **T** | **T** | **T** | **T** | **A** | **A** | **G** | **C** | **G** | **T** | **A** | **G** | **A** | **A** | **G** | **A** | **T** | **C** | **T** | **T** | **C** | **A** | **A** | **T** | **T** | **A** | **G** | **G** | **G** | **T** | **A** | **T** | **G** | **A** | **G** | **A** | **A** | **T** | **C** | **T** | **G** | **T** | **T** | **A** | **T** | **T** | **C** | **G** | **A** | **G** | **C** | **C** | **A** | **A** | **T** | **T** | **C** | **A** | **T** | **T** | **T** | **T** | **A** | **C** | **A** | **A** | **T** | **C** | **G** | **A** | **G** | **C** | **C** | **T** | **C** | **A** | **T** | **C** | **A** | **G** | **C** | **G** | **G** | **G** | **T** | **G** | **G** | **C** | **G** | **A** | **T** | **T** | **T** | **C** | **A** | **G** | **G** | **T** | **C** | **C** | **T** | **A** | **A** | **C** | **G** | **G** | **T** | **G** | **C** | **A** | **G** | **G** | **A** | **A** | **A** | **G** | **T** | **C** | **A** | **T** | **C** | **C** | **A** | **T** | **T** | **A** | **T** | **C** | **C** | **A** | **T** | **T** | **A** | **T** | **C** | **T** | **T** | **C** | **T** | **G** | **G** | **G** | **G** | **G** | **C** | **A** | **T** | **T** | **C** | **A** | **A** | **C** | **G** | **G** | **C** | **A** | **A** | **G** | **G** | **T** | **T** | **A** | **T** | **A** | **G** | **G** | **A** | **G** | **A** | **A** | **A** | **A** | **A** | **T** | **C** | **C** | **C** | **A** | **G** | **C** | **C** | **A** | **A** | **A** | **A** | **C** | **A** | **T** | **C** | **T** | **G** | **A** | **G** | **C** | **A** | **T** | **T** | **A** | **G** | **T** | **T** | **A** | **T** | **G** | **T** | **A** | **C** | **G** | **C** | **C** | **A** | **A** | **A** | **A** | **T** | **T** | **A** | **T** | **G** | **A** | **A** | **G** | **A** | **C** | **A** | **A** | **T** | **C** | **G** | **A** | **G** | **G** | **A** | **A** | **C** | **G** | **T** | **T** | **G** | **G** | **C** | **G** | **G** | **A** | **A** | **T** | **T** | **T** | **G** | **C** | **A** | **G** | **A** | **G** | **A** | **A** | **A** | **A** | **A** | **C** | **C** | **A** | **A** | **G** | **T** | **A** | **G** | **A** | **C** | **T** | **A** | **C** | **C** | **A** | **A** | **G** | **C** | **A** | **T** | **T** | **T** | **T** | **T** | **G** | **A** | **A** | **C** | **A** | **A** | **C** | **C** | **T** | **C** | **C** | **G** | **A** | **A** | **A** | **G** | **C** | **T** | **T** | **G** | **G** | **G** | **A** | **T** | **G** | **G** | **A** | **A** | **A** | **G** | **A** | **G** | **A** | **T** | **G** | **T** | **T** | **T** | **T** | **T** | **C** | **A** | **T** | **A** | **A** | **C** | **A** | **A** | **G** | **A** | **T** | **C** | **G** | **A** | **G** | **C** | **A** | **G** | **A** | **T** | **G** | **A** | **G** | **T** | **A** | **T** | **G** | **G** | **G** | **C** | **C** | **A** | **A** | **C** | **G** | **G** | **A** | **A** | **A** | **A** | **A** | **A** | **G** | **T** | **G** | **G** | **A** | **A** | **T** | **T** | **G** | **G** | **C** | **T** | **A** | **A** | **A** | **T** | **C** | **T** | **T** | **T** | **A** | **T** | **C** | **A** | **C** | **A** | **G** | **C** | **C** | **A** | **G** | **C** | **T** | **G** | **A** | **A** | **C** | **T** | **A** | **T** | **A** | **T** | **A** | **T** | **A** | **T** | **G** | **G** | **G** | **A** | **T** | **G** | **A** | **A** | **C** | **C** | **A** | **T** | **T** | **G** | **A** | **A** | **T** | **T** | **A** | **T** | **T** | **T** | **G** | **G** | **A** | **T** | **G** | **T** | **C** | **T** | **T** | **C** | **A** | **A** | **T** | **C** | **A** | **A** | **G** | **A** | **A** | **C** | **A** | **A** | **T** | **T** | **A** | **G** | **A** | **A** | **C** | **A** | **A** | **C** | **T** | **G** | **A** | **T** | **C** | **T** | **T** | **G** | **A** | **A** | **C** | **G** | **T** | **G** | **A** | **A** | **A** | **C** | **C** | **T** | **G** | **C** | **C** | **A** | **T** | **G** | **T** | **T** | **A** | **C** | **T** | **A** | **G** | **T** | **G** | **G** | **A** | **A** | **C** | **A** | **T** | **G** | **A** | **T** | **C** | **A** | **A** | **A** | **C** | **C** | **T** | **T** | **C** | **C** | **T** | **G** | **G** | **A** | **T** | **A** | **A** | **A** | **G** | **T** | **A** | **T** | **C** | **T** | **A** | **C** | **T** | **G** | **A** | **G** | **A** | **T** | **T** | **A** | **T** | **T** | **T** | **C** | **T** | **C** | **T** | **T** | **G** | **A** | **G** | **A** | **A** | **A** | **A** | **T** | **C** | **-** | **-** | **-** |
| **N60336F** | **A** | **T** | **G** | **T** | **C** | **T** | **A** | **A** | **A** | **A** | **T** | **C** | **G** | **A** | **A** | **A** | **T** | **A** | **A** | **A** | **A** | **A** | **A** | **T** | **C** | **T** | **G** | **A** | **C** | **A** | **T** | **T** | **C** | **G** | **G** | **C** | **T** | **A** | **C** | **G** | **A** | **C** | **A** | **G** | **C** | **C** | **A** | **A** | **G** | **G** | **C** | **A** | **C** | **A** | **T** | **T** | **A** | **T** | **T** | **A** | **T** | **T** | **T** | **G** | **A** | **A** | **C** | **A** | **A** | **G** | **C** | **A** | **A** | **A** | **T** | **C** | **T** | **A** | **A** | **A** | **T** | **T** | **T** | **T** | **G** | **A** | **C** | **A** | **C** | **A** | **C** | **A** | **A** | **T** | **G** | **G** | **A** | **A** | **A** | **C** | **T** | **A** | **G** | **G** | **A** | **C** | **T** | **T** | **A** | **T** | **C** | **G** | **G** | **A** | **C** | **G** | **A** | **A** | **A** | **C** | **G** | **G** | **T** | **C** | **G** | **A** | **G** | **G** | **A** | **A** | **A** | **G** | **A** | **C** | **A** | **A** | **C** | **T** | **T** | **T** | **A** | **C** | **T** | **G** | **A** | **A** | **T** | **A** | **T** | **T** | **C** | **T** | **A** | **C** | **A** | **A** | **A** | **A** | **C** | **A** | **A** | **A** | **C** | **T** | **A** | **C** | **C** | **T** | **T** | **A** | **T** | **C** | **A** | **A** | **G** | **G** | **G** | **C** | **A** | **A** | **G** | **T** | **A** | **A** | **T** | **C** | **C** | **A** | **T** | **C** | **A** | **G** | **C** | **A** | **A** | **G** | **A** | **A** | **T** | **T** | **T** | **G** | **C** | **C** | **T** | **A** | **T** | **T** | **T** | **C** | **C** | **C** | **G** | **C** | **A** | **A** | **C** | **A** | **G** | **A** | **C** | **A** | **A** | **A** | **A** | **G** | **A** | **T** | **A** | **A** | **A** | **G** | **A** | **A** | **C** | **G** | **T** | **T** | **T** | **A** | **A** | **C** | **C** | **T** | **A** | **T** | **T** | **A** | **C** | **G** | **T** | **G** | **T** | **T** | **A** | **A** | **A** | **T** | **G** | **A** | **T** | **A** | **T** | **T** | **A** | **C** | **G** | **G** | **A** | **T** | **T** | **T** | **T** | **G** | **A** | **G** | **A** | **T** | **A** | **T** | **G** | **G** | **G** | **A** | **A** | **A** | **T** | **C** | **G** | **A** | **A** | **A** | **G** | **A** | **G** | **A** | **G** | **C** | **T** | **C** | **C** | **A** | **A** | **T** | **T** | **G** | **A** | **T** | **G** | **C** | **A** | **A** | **A** | **C** | **A** | **G** | **A** | **T** | **C** | **C** | **T** | **G** | **A** | **A** | **A** | **T** | **C** | **T** | **T** | **A** | **T** | **G** | **G** | **A** | **G** | **A** | **G** | **A** | **A** | **T** | **T** | **C** | **A** | **G** | **C** | **A** | **C** | **A** | **C** | **T** | **A** | **T** | **C** | **G** | **G** | **G** | **G** | **G** | **G** | **A** | **G** | **A** | **G** | **A** | **A** | **G** | **A** | **C** | **A** | **A** | **A** | **A** | **G** | **T** | **C** | **C** | **T** | **A** | **C** | **T** | **G** | **G** | **C** | **A** | **C** | **T** | **T** | **T** | **T** | **A** | **T** | **T** | **T** | **G** | **T** | **G** | **G** | **A** | **T** | **G** | **A** | **C** | **A** | **C** | **T** | **C** | **A** | **T** | **T** | **T** | **C** | **C** | **C** | **G** | **T** | **T** | **A** | **A** | **T** | **C** | **G** | **A** | **T** | **G** | **A** | **A** | **C** | **C** | **A** | **A** | **C** | **G** | **A** | **A** | **T** | **C** | **A** | **T** | **T** | **T** | **G** | **G** | **A** | **-** | **-** | **-** | **T** | **A** | **T** | **C** | **T** | **C** | **T** | **G** | **G** | **T** | **A** | **G** | **A** | **A** | **A** | **A** | **C** | **A** | **A** | **G** | **T** | **A** | **G** | **C** | **G** | **G** | **C** | **T** | **T** | **A** | **T** | **T** | **T** | **G** | **A** | **A** | **A** | **A** | **A** | **G** | **A** | **A** | **A** | **A** | **A** | **A** | **C** | **A** | **A** | **G** | **G** | **C** | **T** | **T** | **C** | **A** | **T** | **C** | **G** | **T** | **G** | **G** | **T** | **C** | **A** | **G** | **C** | **C** | **A** | **T** | **G** | **A** | **C** | **C** | **G** | **G** | **G** | **G** | **A** | **T** | **T** | **T** | **A** | **T** | **C** | **G** | **A** | **T** | **G** | **A** | **A** | **G** | **T** | **A** | **G** | **T** | **G** | **G** | **A** | **C** | **C** | **A** | **T** | **G** | **T** | **T** | **T** | **T** | **A** | **G** | **C** | **A** | **A** | **T** | **C** | **G** | **A** | **A** | **A** | **A** | **A** | **A** | **G** | **T** | **C** | **A** | **A** | **C** | **T** | **G** | **G** | **A** | **A** | **C** | **T** | **T** | **T** | **A** | **T** | **C** | **A** | **A** | **G** | **G** | **G** | **A** | **A** | **T** | **T** | **T** | **C** | **T** | **C** | **T** | **A** | **T** | **C** | **T** | **A** | **T** | **G** | **A** | **A** | **G** | **A** | **A** | **C** | **A** | **G** | **A** | **A** | **A** | **A** | **A** | **A** | **C** | **T** | **T** | **C** | **G** | **T** | **G** | **A** | **T** | **G** | **A** | **A** | **T** | **T** | **T** | **G** | **A** | **A** | **A** | **T** | **G** | **G** | **C** | **T** | **C** | **A** | **A** | **A** | **A** | **T** | **G** | **A** | **A** | **A** | **A** | **A** | **T** | **T** | **G** | **A** | **A** | **A** | **A** | **A** | **A** | **G** | **A** | **A** | **G** | **T** | **C** | **A** | **G** | **T** | **A** | **G** | **G** | **C** | **T** | **A** | **A** | **A** | **G** | **A** | **A** | **A** | **A** | **C** | **A** | **G** | **C** | **A** | **G** | **C** | **T** | **G** | **A** | **A** | **A** | **A** | **A** | **G** | **C** | **C** | **G** | **A** | **A** | **T** | **G** | **G** | **T** | **C** | **T** | **C** | **G** | **T** | **T** | **C** | **C** | **C** | **G** | **A** | **G** | **A** | **A** | **G** | **G** | **A** | **G** | **A** | **T** | **A** | **A** | **A** | **A** | **C** | **A** | **A** | **A** | **G** | **A** | **A** | **A** | **C** | **A** | **A** | **G** | **T** | **C** | **G** | **G** | **A** | **T** | **T** | **C** | **A** | **T** | **C** | **G** | **A** | **T** | **A** | **C** | **T** | **G** | **A** | **A** | **T** | **C** | **T** | **A** | **G** | **A** | **C** | **G** | **A** | **G** | **T** | **G** | **A** | **A** | **T** | **A** | **A** | **A** | **G** | **G** | **A** | **G** | **C** | **A** | **G** | **T** | **G** | **G** | **G** | **T** | **G** | **C** | **T** | **G** | **A** | **T** | **G** | **C** | **T** | **G** | **C** | **A** | **C** | **G** | **G** | **A** | **C** | **G** | **A** | **T** | **G** | **A** | **A** | **A** | **C** | **G** | **A** | **T** | **C** | **C** | **A** | **A** | **A** | **G** | **C** | **A** | **A** | **T** | **C** | **G** | **T** | **G** | **A** | **A** | **T** | **C** | **G** | **G** | **A** | **T** | **G** | **G** | **A** | **G** | **A** | **C** | **C** | **C** | **A** | **G** | **A** | **T** | **C** | **A** | **G** | **C** | **G** | **A** | **G** | **A** | **A** | **A** | **G** | **A** | **A** | **A** | **A** | **A** | **C** | **T** | **A** | **T** | **T** | **A** | **A** | **A** | **A** | **G** | **A** | **T** | **A** | **T** | **C** | **G** | **A** | **A** | **T** | **A** | **T** | **A** | **T** | **C** | **G** | **A** | **T** | **T** | **C** | **T** | **T** | **T** | **G** | **A** | **C** | **G** | **A** | **T** | **G** | **A** | **A** | **T** | **A** | **G** | **C** | **C** | **A** | **A** | **G** | **C** | **G** | **T** | **C** | **T** | **C** | **A** | **C** | **C** | **A** | **T** | **A** | **A** | **G** | **C** | **G** | **A** | **C** | **T** | **T** | **T** | **T** | **A** | **A** | **G** | **C** | **G** | **T** | **A** | **G** | **A** | **A** | **G** | **A** | **T** | **C** | **T** | **T** | **C** | **A** | **A** | **T** | **T** | **A** | **G** | **G** | **G** | **T** | **A** | **T** | **G** | **A** | **A** | **A** | **A** | **T** | **C** | **T** | **G** | **T** | **T** | **A** | **T** | **T** | **C** | **G** | **A** | **G** | **C** | **C** | **A** | **A** | **T** | **T** | **C** | **A** | **T** | **T** | **T** | **T** | **A** | **C** | **A** | **A** | **T** | **C** | **G** | **A** | **G** | **C** | **C** | **T** | **C** | **A** | **T** | **C** | **A** | **G** | **C** | **G** | **G** | **G** | **T** | **G** | **G** | **C** | **G** | **A** | **T** | **T** | **T** | **C** | **A** | **G** | **G** | **T** | **C** | **C** | **T** | **A** | **A** | **C** | **G** | **G** | **T** | **G** | **C** | **A** | **G** | **G** | **A** | **A** | **A** | **G** | **T** | **C** | **A** | **T** | **C** | **C** | **A** | **T** | **T** | **A** | **T** | **C** | **C** | **A** | **T** | **T** | **A** | **T** | **C** | **T** | **T** | **C** | **T** | **G** | **G** | **G** | **G** | **G** | **C** | **A** | **T** | **T** | **C** | **A** | **A** | **C** | **G** | **G** | **C** | **A** | **A** | **G** | **G** | **T** | **T** | **A** | **T** | **A** | **G** | **G** | **A** | **G** | **A** | **A** | **A** | **A** | **A** | **T** | **A** | **C** | **C** | **A** | **G** | **C** | **C** | **A** | **A** | **A** | **A** | **C** | **A** | **T** | **C** | **T** | **G** | **A** | **G** | **C** | **A** | **T** | **T** | **A** | **G** | **T** | **T** | **A** | **T** | **G** | **C** | **A** | **A** | **G** | **C** | **C** | **A** | **A** | **A** | **A** | **T** | **T** | **A** | **T** | **G** | **A** | **A** | **G** | **A** | **C** | **A** | **A** | **T** | **C** | **G** | **A** | **G** | **G** | **A** | **A** | **C** | **G** | **T** | **T** | **G** | **G** | **C** | **G** | **G** | **A** | **A** | **T** | **T** | **T** | **G** | **C** | **A** | **G** | **A** | **G** | **A** | **A** | **A** | **A** | **A** | **C** | **C** | **A** | **A** | **G** | **T** | **A** | **G** | **A** | **C** | **T** | **A** | **C** | **C** | **A** | **A** | **G** | **C** | **A** | **T** | **T** | **T** | **T** | **T** | **G** | **A** | **A** | **C** | **A** | **A** | **C** | **C** | **T** | **C** | **C** | **G** | **A** | **A** | **A** | **G** | **C** | **T** | **T** | **G** | **G** | **G** | **A** | **T** | **G** | **G** | **A** | **A** | **A** | **G** | **A** | **G** | **A** | **T** | **G** | **T** | **T** | **T** | **T** | **T** | **C** | **A** | **T** | **A** | **A** | **C** | **A** | **A** | **G** | **A** | **T** | **C** | **G** | **A** | **G** | **C** | **A** | **G** | **A** | **T** | **G** | **A** | **G** | **T** | **A** | **T** | **G** | **G** | **G** | **C** | **C** | **A** | **A** | **C** | **G** | **G** | **A** | **A** | **A** | **A** | **A** | **A** | **G** | **T** | **G** | **G** | **A** | **A** | **T** | **T** | **G** | **G** | **C** | **T** | **A** | **A** | **A** | **T** | **C** | **T** | **T** | **T** | **A** | **T** | **C** | **A** | **C** | **A** | **G** | **C** | **C** | **A** | **G** | **C** | **T** | **G** | **A** | **A** | **C** | **T** | **A** | **T** | **A** | **T** | **A** | **C** | **A** | **T** | **G** | **G** | **G** | **A** | **T** | **G** | **A** | **A** | **C** | **C** | **A** | **T** | **T** | **G** | **A** | **A** | **T** | **T** | **A** | **T** | **T** | **T** | **G** | **G** | **A** | **T** | **G** | **T** | **C** | **T** | **T** | **C** | **A** | **A** | **T** | **C** | **A** | **A** | **G** | **A** | **A** | **C** | **A** | **A** | **T** | **T** | **A** | **G** | **A** | **A** | **C** | **A** | **A** | **C** | **T** | **G** | **A** | **T** | **C** | **T** | **T** | **G** | **A** | **A** | **C** | **G** | **T** | **G** | **A** | **A** | **A** | **C** | **C** | **T** | **G** | **C** | **C** | **A** | **T** | **G** | **T** | **T** | **A** | **C** | **T** | **A** | **G** | **T** | **G** | **G** | **A** | **A** | **C** | **A** | **T** | **G** | **A** | **T** | **C** | **A** | **A** | **A** | **C** | **C** | **T** | **T** | **C** | **C** | **T** | **G** | **G** | **A** | **T** | **A** | **A** | **A** | **G** | **T** | **A** | **T** | **C** | **T** | **A** | **C** | **T** | **G** | **G** | **G** | **A** | **T** | **T** | **A** | **T** | **T** | **T** | **C** | **T** | **C** | **T** | **T** | **G** | **A** | **G** | **A** | **G** | **A** | **A** | **T** | **C** | **-** | **-** | **-** |
| **N54551** | **A** | **T** | **G** | **T** | **C** | **T** | **A** | **A** | **A** | **A** | **T** | **C** | **G** | **A** | **A** | **A** | **T** | **A** | **A** | **A** | **A** | **A** | **A** | **T** | **C** | **T** | **G** | **A** | **C** | **A** | **T** | **T** | **C** | **G** | **G** | **C** | **T** | **A** | **C** | **G** | **A** | **C** | **A** | **G** | **C** | **C** | **A** | **A** | **G** | **G** | **C** | **A** | **C** | **A** | **T** | **T** | **A** | **T** | **T** | **A** | **T** | **T** | **T** | **G** | **A** | **A** | **C** | **A** | **A** | **G** | **C** | **A** | **A** | **A** | **T** | **C** | **T** | **A** | **A** | **A** | **T** | **T** | **T** | **T** | **G** | **A** | **C** | **A** | **C** | **A** | **C** | **A** | **A** | **T** | **G** | **G** | **A** | **A** | **A** | **C** | **T** | **A** | **G** | **G** | **A** | **C** | **T** | **T** | **A** | **T** | **C** | **G** | **G** | **A** | **C** | **G** | **A** | **A** | **A** | **C** | **G** | **G** | **T** | **C** | **G** | **A** | **G** | **G** | **A** | **A** | **A** | **G** | **A** | **C** | **A** | **A** | **C** | **T** | **T** | **T** | **A** | **C** | **T** | **G** | **A** | **A** | **T** | **A** | **T** | **T** | **C** | **T** | **A** | **C** | **A** | **A** | **A** | **A** | **C** | **A** | **A** | **A** | **C** | **T** | **A** | **C** | **C** | **T** | **T** | **A** | **T** | **C** | **A** | **A** | **G** | **G** | **G** | **C** | **A** | **A** | **G** | **T** | **A** | **A** | **T** | **C** | **C** | **A** | **T** | **C** | **A** | **G** | **C** | **A** | **A** | **G** | **A** | **A** | **T** | **T** | **T** | **G** | **T** | **C** | **T** | **A** | **T** | **T** | **T** | **C** | **C** | **C** | **G** | **C** | **A** | **A** | **C** | **A** | **G** | **A** | **C** | **A** | **A** | **A** | **A** | **G** | **A** | **T** | **A** | **A** | **A** | **G** | **A** | **A** | **C** | **G** | **T** | **T** | **T** | **A** | **A** | **C** | **C** | **T** | **A** | **T** | **T** | **A** | **C** | **G** | **T** | **G** | **T** | **T** | **A** | **A** | **A** | **T** | **G** | **A** | **T** | **A** | **T** | **T** | **A** | **C** | **G** | **G** | **A** | **T** | **T** | **T** | **T** | **G** | **A** | **G** | **A** | **T** | **A** | **T** | **G** | **G** | **G** | **A** | **A** | **A** | **T** | **C** | **G** | **A** | **A** | **A** | **G** | **A** | **G** | **A** | **G** | **C** | **T** | **C** | **C** | **A** | **A** | **T** | **T** | **G** | **A** | **T** | **G** | **C** | **A** | **A** | **A** | **C** | **A** | **G** | **A** | **T** | **C** | **C** | **T** | **G** | **A** | **A** | **A** | **T** | **C** | **T** | **T** | **A** | **T** | **G** | **G** | **A** | **G** | **A** | **G** | **A** | **A** | **T** | **T** | **C** | **A** | **G** | **C** | **A** | **C** | **A** | **C** | **T** | **A** | **T** | **C** | **A** | **G** | **G** | **G** | **G** | **G** | **A** | **G** | **A** | **G** | **A** | **A** | **G** | **A** | **C** | **A** | **A** | **A** | **A** | **G** | **T** | **C** | **C** | **T** | **A** | **C** | **T** | **G** | **G** | **C** | **A** | **C** | **T** | **T** | **T** | **T** | **A** | **T** | **T** | **T** | **G** | **T** | **G** | **G** | **A** | **T** | **G** | **A** | **C** | **A** | **C** | **T** | **C** | **A** | **T** | **T** | **T** | **C** | **C** | **C** | **G** | **T** | **T** | **A** | **A** | **T** | **C** | **G** | **A** | **T** | **G** | **A** | **A** | **C** | **C** | **A** | **A** | **C** | **G** | **A** | **A** | **T** | **C** | **A** | **T** | **T** | **T** | **G** | **G** | **A** | **-** | **-** | **-** | **T** | **A** | **T** | **C** | **T** | **C** | **T** | **G** | **G** | **T** | **A** | **G** | **A** | **A** | **A** | **A** | **C** | **A** | **A** | **G** | **T** | **A** | **G** | **C** | **G** | **G** | **C** | **T** | **T** | **A** | **T** | **T** | **T** | **G** | **A** | **A** | **A** | **A** | **A** | **G** | **A** | **A** | **A** | **A** | **A** | **A** | **C** | **A** | **A** | **G** | **G** | **C** | **T** | **T** | **C** | **A** | **T** | **C** | **G** | **T** | **G** | **G** | **T** | **C** | **A** | **G** | **C** | **C** | **A** | **T** | **G** | **A** | **C** | **C** | **G** | **G** | **G** | **G** | **A** | **T** | **T** | **T** | **A** | **T** | **C** | **G** | **A** | **T** | **G** | **A** | **A** | **G** | **T** | **A** | **G** | **T** | **G** | **G** | **A** | **C** | **C** | **A** | **T** | **G** | **T** | **T** | **T** | **T** | **A** | **G** | **C** | **A** | **A** | **T** | **C** | **G** | **A** | **A** | **A** | **A** | **A** | **A** | **G** | **T** | **C** | **A** | **A** | **C** | **T** | **G** | **G** | **A** | **A** | **C** | **T** | **T** | **T** | **A** | **T** | **C** | **A** | **A** | **G** | **G** | **G** | **A** | **A** | **T** | **T** | **T** | **C** | **T** | **C** | **T** | **A** | **T** | **C** | **T** | **A** | **T** | **G** | **A** | **A** | **G** | **A** | **A** | **C** | **A** | **G** | **A** | **A** | **A** | **A** | **A** | **A** | **C** | **T** | **T** | **C** | **G** | **T** | **G** | **A** | **T** | **G** | **A** | **A** | **T** | **T** | **T** | **G** | **A** | **A** | **A** | **T** | **G** | **G** | **C** | **T** | **C** | **A** | **A** | **A** | **A** | **T** | **G** | **A** | **A** | **A** | **A** | **A** | **T** | **T** | **G** | **A** | **A** | **A** | **A** | **A** | **A** | **G** | **A** | **A** | **G** | **T** | **C** | **A** | **G** | **T** | **A** | **G** | **G** | **C** | **T** | **A** | **A** | **A** | **G** | **A** | **A** | **A** | **A** | **C** | **A** | **G** | **C** | **A** | **G** | **C** | **T** | **G** | **A** | **A** | **A** | **A** | **A** | **G** | **C** | **C** | **G** | **A** | **A** | **T** | **G** | **G** | **T** | **C** | **T** | **C** | **G** | **T** | **T** | **C** | **C** | **C** | **G** | **A** | **G** | **A** | **A** | **G** | **G** | **A** | **G** | **A** | **T** | **A** | **A** | **A** | **A** | **C** | **A** | **A** | **A** | **G** | **A** | **A** | **A** | **C** | **A** | **A** | **G** | **T** | **C** | **G** | **G** | **A** | **T** | **T** | **C** | **A** | **T** | **C** | **G** | **A** | **T** | **A** | **C** | **T** | **G** | **A** | **A** | **T** | **C** | **T** | **A** | **G** | **A** | **C** | **G** | **A** | **G** | **T** | **G** | **A** | **A** | **T** | **A** | **A** | **A** | **G** | **G** | **A** | **G** | **C** | **A** | **G** | **T** | **G** | **G** | **G** | **T** | **G** | **C** | **T** | **G** | **A** | **T** | **G** | **C** | **T** | **G** | **C** | **A** | **C** | **G** | **G** | **A** | **C** | **G** | **A** | **T** | **G** | **A** | **A** | **A** | **C** | **G** | **A** | **T** | **C** | **C** | **A** | **A** | **A** | **G** | **C** | **A** | **A** | **T** | **C** | **G** | **T** | **G** | **A** | **A** | **T** | **C** | **G** | **G** | **A** | **T** | **G** | **G** | **A** | **G** | **A** | **C** | **C** | **C** | **A** | **G** | **A** | **T** | **C** | **A** | **G** | **C** | **G** | **A** | **G** | **A** | **A** | **A** | **G** | **A** | **A** | **A** | **A** | **A** | **C** | **T** | **A** | **T** | **T** | **G** | **A** | **A** | **A** | **G** | **A** | **T** | **A** | **T** | **C** | **G** | **A** | **A** | **T** | **A** | **T** | **A** | **T** | **C** | **G** | **A** | **T** | **T** | **C** | **T** | **T** | **T** | **G** | **A** | **C** | **G** | **A** | **T** | **G** | **A** | **A** | **T** | **A** | **G** | **C** | **C** | **A** | **A** | **G** | **C** | **G** | **T** | **C** | **T** | **C** | **A** | **C** | **C** | **A** | **T** | **A** | **A** | **G** | **C** | **G** | **A** | **C** | **T** | **T** | **T** | **T** | **A** | **A** | **G** | **C** | **G** | **T** | **A** | **G** | **A** | **A** | **G** | **A** | **T** | **C** | **T** | **T** | **C** | **A** | **A** | **T** | **T** | **A** | **G** | **G** | **G** | **T** | **A** | **T** | **G** | **A** | **G** | **A** | **A** | **T** | **C** | **T** | **G** | **T** | **T** | **A** | **T** | **T** | **C** | **G** | **A** | **G** | **C** | **C** | **A** | **A** | **T** | **T** | **C** | **A** | **T** | **T** | **T** | **T** | **A** | **C** | **A** | **A** | **T** | **C** | **G** | **A** | **G** | **C** | **C** | **T** | **C** | **A** | **T** | **C** | **A** | **G** | **C** | **G** | **G** | **G** | **T** | **G** | **G** | **C** | **G** | **A** | **T** | **T** | **T** | **C** | **A** | **G** | **G** | **T** | **C** | **C** | **T** | **A** | **A** | **C** | **G** | **G** | **T** | **G** | **C** | **A** | **G** | **G** | **A** | **A** | **A** | **G** | **T** | **C** | **A** | **T** | **C** | **C** | **A** | **T** | **T** | **A** | **T** | **C** | **C** | **A** | **T** | **T** | **A** | **T** | **C** | **T** | **T** | **C** | **T** | **G** | **G** | **G** | **G** | **G** | **C** | **A** | **T** | **T** | **C** | **A** | **A** | **C** | **G** | **G** | **C** | **A** | **A** | **G** | **G** | **T** | **T** | **A** | **T** | **A** | **G** | **G** | **A** | **G** | **A** | **A** | **A** | **A** | **A** | **T** | **C** | **C** | **C** | **A** | **G** | **C** | **C** | **A** | **A** | **A** | **A** | **C** | **A** | **T** | **C** | **T** | **G** | **A** | **G** | **C** | **A** | **T** | **T** | **A** | **G** | **T** | **T** | **A** | **T** | **G** | **T** | **A** | **C** | **G** | **C** | **C** | **A** | **A** | **A** | **A** | **T** | **T** | **A** | **T** | **G** | **A** | **A** | **G** | **A** | **C** | **A** | **A** | **T** | **C** | **G** | **A** | **G** | **G** | **A** | **A** | **C** | **G** | **T** | **T** | **G** | **G** | **C** | **G** | **G** | **A** | **A** | **T** | **T** | **T** | **G** | **C** | **A** | **G** | **A** | **G** | **A** | **A** | **A** | **A** | **A** | **C** | **C** | **A** | **A** | **G** | **T** | **A** | **G** | **A** | **C** | **T** | **A** | **C** | **C** | **A** | **A** | **G** | **C** | **A** | **T** | **T** | **T** | **T** | **T** | **G** | **A** | **A** | **C** | **A** | **A** | **C** | **C** | **T** | **C** | **C** | **G** | **A** | **A** | **A** | **G** | **C** | **T** | **T** | **G** | **G** | **G** | **A** | **T** | **G** | **G** | **A** | **A** | **A** | **G** | **A** | **G** | **A** | **T** | **G** | **T** | **T** | **T** | **T** | **T** | **C** | **A** | **T** | **A** | **A** | **C** | **A** | **A** | **G** | **A** | **T** | **C** | **G** | **A** | **G** | **C** | **A** | **G** | **A** | **T** | **G** | **A** | **G** | **T** | **A** | **T** | **G** | **G** | **G** | **C** | **C** | **A** | **A** | **C** | **G** | **G** | **A** | **A** | **A** | **A** | **A** | **A** | **G** | **T** | **G** | **G** | **A** | **A** | **T** | **T** | **G** | **G** | **C** | **T** | **A** | **A** | **A** | **T** | **C** | **T** | **T** | **T** | **A** | **T** | **C** | **A** | **C** | **A** | **G** | **C** | **C** | **A** | **G** | **C** | **T** | **G** | **A** | **A** | **C** | **T** | **A** | **T** | **A** | **T** | **A** | **T** | **A** | **T** | **G** | **G** | **G** | **A** | **T** | **G** | **A** | **A** | **C** | **C** | **A** | **T** | **T** | **G** | **A** | **A** | **T** | **T** | **A** | **T** | **T** | **T** | **G** | **G** | **A** | **T** | **G** | **T** | **C** | **T** | **T** | **C** | **A** | **A** | **T** | **C** | **A** | **A** | **G** | **A** | **A** | **C** | **A** | **A** | **T** | **T** | **A** | **G** | **A** | **A** | **C** | **A** | **A** | **C** | **T** | **G** | **A** | **T** | **C** | **T** | **T** | **G** | **A** | **A** | **C** | **G** | **T** | **G** | **A** | **A** | **A** | **C** | **C** | **T** | **G** | **C** | **C** | **A** | **T** | **G** | **T** | **T** | **A** | **C** | **T** | **A** | **G** | **T** | **G** | **G** | **A** | **A** | **C** | **A** | **T** | **G** | **A** | **T** | **C** | **A** | **A** | **A** | **C** | **C** | **T** | **T** | **C** | **C** | **T** | **G** | **G** | **A** | **T** | **A** | **A** | **A** | **G** | **T** | **A** | **T** | **C** | **T** | **A** | **C** | **T** | **G** | **A** | **G** | **A** | **T** | **T** | **A** | **T** | **T** | **T** | **C** | **T** | **C** | **T** | **T** | **G** | **A** | **G** | **A** | **A** | **A** | **A** | **T** | **C** | **-** | **-** | **-** |
| **N52656** | **A** | **T** | **G** | **T** | **C** | **T** | **A** | **A** | **A** | **A** | **T** | **C** | **G** | **A** | **A** | **A** | **T** | **A** | **A** | **A** | **A** | **A** | **A** | **T** | **C** | **T** | **G** | **A** | **C** | **A** | **T** | **T** | **C** | **G** | **G** | **C** | **T** | **A** | **C** | **G** | **A** | **C** | **A** | **G** | **C** | **C** | **A** | **A** | **G** | **G** | **C** | **A** | **C** | **A** | **T** | **T** | **A** | **T** | **T** | **A** | **T** | **T** | **T** | **G** | **A** | **A** | **C** | **A** | **A** | **G** | **C** | **A** | **A** | **A** | **T** | **C** | **T** | **A** | **A** | **A** | **T** | **T** | **T** | **T** | **G** | **A** | **C** | **A** | **C** | **A** | **C** | **A** | **A** | **T** | **G** | **G** | **A** | **A** | **A** | **C** | **T** | **A** | **G** | **G** | **A** | **C** | **T** | **T** | **A** | **T** | **C** | **G** | **G** | **A** | **C** | **G** | **A** | **A** | **A** | **C** | **G** | **G** | **T** | **C** | **G** | **A** | **G** | **G** | **A** | **A** | **A** | **G** | **A** | **C** | **A** | **A** | **C** | **T** | **T** | **T** | **A** | **C** | **T** | **G** | **A** | **A** | **T** | **A** | **T** | **T** | **C** | **T** | **A** | **C** | **A** | **A** | **A** | **A** | **C** | **A** | **A** | **A** | **C** | **T** | **A** | **C** | **C** | **T** | **T** | **A** | **T** | **C** | **A** | **A** | **G** | **G** | **G** | **C** | **A** | **A** | **G** | **T** | **A** | **A** | **T** | **C** | **C** | **A** | **T** | **C** | **A** | **G** | **C** | **A** | **A** | **G** | **A** | **A** | **T** | **T** | **T** | **G** | **T** | **C** | **T** | **A** | **T** | **T** | **T** | **C** | **C** | **C** | **G** | **C** | **A** | **A** | **C** | **A** | **G** | **A** | **C** | **A** | **A** | **A** | **A** | **G** | **A** | **T** | **A** | **A** | **A** | **G** | **A** | **A** | **C** | **G** | **T** | **T** | **T** | **A** | **A** | **C** | **C** | **T** | **A** | **T** | **T** | **A** | **C** | **G** | **T** | **G** | **T** | **T** | **A** | **A** | **A** | **T** | **G** | **A** | **T** | **A** | **T** | **T** | **A** | **C** | **G** | **G** | **A** | **T** | **T** | **T** | **T** | **G** | **A** | **G** | **A** | **T** | **A** | **T** | **G** | **G** | **G** | **A** | **A** | **A** | **T** | **C** | **G** | **A** | **A** | **A** | **G** | **A** | **G** | **A** | **G** | **C** | **T** | **C** | **C** | **A** | **A** | **T** | **T** | **G** | **A** | **T** | **G** | **C** | **A** | **A** | **A** | **C** | **A** | **G** | **A** | **T** | **C** | **C** | **T** | **G** | **A** | **A** | **A** | **T** | **C** | **T** | **T** | **A** | **T** | **G** | **G** | **A** | **G** | **A** | **G** | **A** | **A** | **T** | **T** | **C** | **A** | **G** | **C** | **A** | **C** | **A** | **C** | **T** | **A** | **T** | **C** | **G** | **G** | **G** | **G** | **G** | **G** | **A** | **G** | **A** | **G** | **A** | **A** | **G** | **A** | **C** | **A** | **A** | **A** | **A** | **G** | **T** | **C** | **C** | **T** | **A** | **C** | **T** | **G** | **G** | **C** | **A** | **C** | **T** | **T** | **T** | **T** | **A** | **T** | **T** | **T** | **G** | **T** | **G** | **G** | **A** | **T** | **G** | **A** | **C** | **A** | **C** | **T** | **C** | **A** | **T** | **T** | **T** | **C** | **C** | **C** | **G** | **T** | **T** | **A** | **A** | **T** | **C** | **G** | **A** | **T** | **G** | **A** | **A** | **C** | **C** | **A** | **A** | **C** | **G** | **A** | **A** | **T** | **C** | **A** | **T** | **T** | **T** | **G** | **G** | **A** | **-** | **-** | **-** | **T** | **A** | **T** | **C** | **T** | **C** | **T** | **G** | **G** | **T** | **A** | **G** | **A** | **A** | **A** | **A** | **C** | **A** | **A** | **G** | **T** | **A** | **G** | **C** | **G** | **G** | **C** | **T** | **T** | **A** | **T** | **T** | **T** | **G** | **A** | **A** | **A** | **A** | **A** | **G** | **A** | **A** | **A** | **A** | **A** | **A** | **C** | **A** | **A** | **G** | **G** | **C** | **T** | **T** | **C** | **A** | **T** | **C** | **G** | **T** | **G** | **G** | **T** | **C** | **A** | **G** | **C** | **C** | **A** | **T** | **G** | **A** | **C** | **C** | **G** | **G** | **G** | **G** | **A** | **T** | **T** | **T** | **A** | **T** | **C** | **G** | **A** | **T** | **G** | **A** | **A** | **G** | **T** | **A** | **G** | **T** | **G** | **G** | **A** | **C** | **C** | **A** | **T** | **G** | **T** | **T** | **T** | **T** | **A** | **G** | **C** | **A** | **A** | **T** | **C** | **G** | **A** | **A** | **A** | **A** | **A** | **A** | **G** | **T** | **C** | **A** | **A** | **C** | **T** | **G** | **G** | **A** | **A** | **C** | **T** | **T** | **T** | **A** | **T** | **C** | **A** | **A** | **G** | **G** | **G** | **A** | **A** | **T** | **T** | **T** | **C** | **T** | **C** | **T** | **A** | **T** | **C** | **T** | **A** | **T** | **G** | **A** | **A** | **G** | **A** | **A** | **C** | **A** | **G** | **A** | **A** | **A** | **A** | **A** | **A** | **C** | **T** | **T** | **C** | **G** | **T** | **G** | **A** | **T** | **G** | **A** | **A** | **T** | **T** | **T** | **G** | **A** | **A** | **A** | **T** | **G** | **G** | **C** | **T** | **C** | **A** | **A** | **A** | **A** | **T** | **G** | **A** | **A** | **A** | **A** | **A** | **T** | **T** | **G** | **A** | **A** | **A** | **A** | **A** | **A** | **G** | **A** | **A** | **G** | **T** | **C** | **A** | **G** | **T** | **A** | **G** | **G** | **C** | **T** | **A** | **A** | **A** | **G** | **A** | **A** | **A** | **A** | **C** | **A** | **G** | **C** | **A** | **G** | **C** | **T** | **G** | **A** | **A** | **A** | **A** | **A** | **G** | **C** | **C** | **G** | **A** | **A** | **T** | **G** | **G** | **T** | **C** | **T** | **C** | **G** | **T** | **T** | **C** | **C** | **C** | **G** | **A** | **G** | **A** | **A** | **G** | **G** | **A** | **G** | **A** | **T** | **A** | **A** | **A** | **A** | **C** | **A** | **A** | **A** | **G** | **A** | **A** | **A** | **C** | **A** | **A** | **G** | **T** | **C** | **G** | **G** | **A** | **T** | **T** | **C** | **A** | **T** | **C** | **G** | **A** | **T** | **A** | **C** | **T** | **G** | **A** | **A** | **T** | **C** | **T** | **A** | **G** | **A** | **C** | **G** | **A** | **G** | **T** | **G** | **A** | **A** | **T** | **A** | **A** | **A** | **G** | **G** | **A** | **G** | **C** | **A** | **G** | **T** | **G** | **G** | **G** | **T** | **G** | **C** | **T** | **G** | **A** | **T** | **G** | **C** | **T** | **G** | **C** | **A** | **C** | **G** | **G** | **A** | **C** | **G** | **A** | **T** | **G** | **A** | **A** | **A** | **C** | **G** | **A** | **T** | **C** | **C** | **A** | **A** | **A** | **G** | **C** | **A** | **A** | **T** | **C** | **G** | **T** | **G** | **A** | **A** | **T** | **C** | **G** | **G** | **A** | **T** | **G** | **G** | **A** | **G** | **A** | **C** | **C** | **C** | **A** | **G** | **A** | **T** | **C** | **A** | **G** | **C** | **G** | **A** | **G** | **A** | **A** | **A** | **G** | **A** | **A** | **A** | **A** | **A** | **T** | **T** | **A** | **T** | **T** | **A** | **A** | **A** | **A** | **G** | **A** | **T** | **A** | **T** | **C** | **G** | **A** | **A** | **T** | **A** | **T** | **A** | **T** | **C** | **G** | **A** | **T** | **T** | **C** | **T** | **T** | **T** | **G** | **A** | **C** | **G** | **A** | **T** | **G** | **A** | **A** | **T** | **A** | **G** | **C** | **C** | **A** | **A** | **G** | **C** | **G** | **T** | **C** | **T** | **C** | **A** | **C** | **C** | **A** | **T** | **A** | **A** | **G** | **C** | **G** | **A** | **C** | **T** | **T** | **T** | **T** | **A** | **A** | **G** | **C** | **G** | **T** | **A** | **G** | **A** | **A** | **G** | **A** | **T** | **C** | **T** | **T** | **C** | **A** | **A** | **T** | **T** | **A** | **G** | **G** | **G** | **T** | **A** | **T** | **G** | **A** | **A** | **A** | **A** | **T** | **C** | **T** | **G** | **T** | **T** | **A** | **T** | **T** | **C** | **G** | **A** | **G** | **C** | **C** | **A** | **A** | **T** | **T** | **C** | **A** | **T** | **T** | **T** | **T** | **A** | **C** | **A** | **A** | **T** | **C** | **G** | **A** | **G** | **C** | **C** | **T** | **C** | **A** | **T** | **C** | **A** | **G** | **C** | **G** | **G** | **G** | **T** | **G** | **G** | **C** | **G** | **A** | **T** | **T** | **T** | **C** | **A** | **G** | **G** | **T** | **C** | **C** | **T** | **A** | **A** | **C** | **G** | **G** | **T** | **G** | **C** | **A** | **G** | **G** | **A** | **A** | **A** | **G** | **T** | **C** | **A** | **T** | **C** | **C** | **A** | **T** | **T** | **A** | **T** | **C** | **C** | **A** | **T** | **T** | **A** | **T** | **C** | **T** | **T** | **C** | **T** | **G** | **G** | **G** | **G** | **G** | **C** | **A** | **T** | **T** | **C** | **A** | **A** | **C** | **G** | **G** | **C** | **A** | **A** | **G** | **G** | **T** | **T** | **A** | **T** | **A** | **G** | **G** | **A** | **G** | **A** | **A** | **A** | **A** | **A** | **T** | **C** | **C** | **C** | **A** | **G** | **C** | **C** | **A** | **A** | **A** | **A** | **C** | **A** | **T** | **C** | **T** | **T** | **A** | **G** | **C** | **A** | **T** | **T** | **A** | **G** | **T** | **T** | **A** | **T** | **G** | **T** | **A** | **C** | **G** | **C** | **C** | **A** | **A** | **A** | **A** | **T** | **T** | **A** | **T** | **G** | **A** | **A** | **G** | **A** | **C** | **A** | **A** | **T** | **C** | **G** | **A** | **G** | **G** | **A** | **A** | **C** | **G** | **T** | **T** | **G** | **G** | **C** | **G** | **G** | **A** | **A** | **T** | **T** | **T** | **G** | **C** | **A** | **G** | **A** | **G** | **A** | **A** | **A** | **A** | **A** | **C** | **C** | **A** | **A** | **G** | **T** | **A** | **G** | **A** | **C** | **T** | **A** | **C** | **C** | **A** | **A** | **G** | **C** | **A** | **T** | **T** | **T** | **T** | **T** | **G** | **A** | **A** | **C** | **A** | **A** | **C** | **C** | **T** | **C** | **C** | **G** | **A** | **A** | **A** | **G** | **C** | **T** | **T** | **G** | **G** | **G** | **A** | **T** | **G** | **G** | **A** | **A** | **A** | **G** | **A** | **G** | **A** | **T** | **G** | **T** | **T** | **T** | **T** | **T** | **C** | **A** | **T** | **A** | **A** | **C** | **A** | **A** | **G** | **A** | **T** | **C** | **G** | **A** | **G** | **C** | **A** | **G** | **A** | **T** | **G** | **A** | **G** | **T** | **A** | **T** | **G** | **G** | **G** | **C** | **C** | **A** | **A** | **C** | **G** | **G** | **A** | **A** | **A** | **A** | **A** | **A** | **G** | **T** | **G** | **G** | **A** | **A** | **T** | **T** | **G** | **G** | **C** | **T** | **A** | **A** | **A** | **T** | **C** | **T** | **T** | **T** | **A** | **T** | **C** | **A** | **C** | **A** | **G** | **C** | **C** | **A** | **G** | **C** | **T** | **G** | **A** | **A** | **C** | **T** | **A** | **T** | **A** | **T** | **A** | **T** | **A** | **T** | **G** | **G** | **G** | **A** | **T** | **G** | **A** | **A** | **C** | **C** | **A** | **T** | **T** | **G** | **A** | **A** | **T** | **T** | **A** | **T** | **T** | **T** | **G** | **G** | **A** | **T** | **G** | **T** | **C** | **T** | **T** | **C** | **A** | **A** | **T** | **C** | **A** | **A** | **G** | **A** | **A** | **C** | **A** | **A** | **T** | **T** | **A** | **G** | **A** | **A** | **C** | **A** | **A** | **C** | **T** | **G** | **A** | **T** | **C** | **T** | **T** | **G** | **A** | **A** | **C** | **G** | **T** | **G** | **A** | **A** | **A** | **C** | **C** | **T** | **G** | **C** | **C** | **A** | **T** | **G** | **T** | **T** | **A** | **C** | **T** | **A** | **G** | **T** | **G** | **G** | **A** | **A** | **C** | **A** | **T** | **G** | **A** | **T** | **C** | **A** | **A** | **A** | **C** | **C** | **T** | **T** | **C** | **C** | **T** | **G** | **G** | **A** | **T** | **A** | **A** | **A** | **G** | **T** | **A** | **T** | **C** | **T** | **A** | **C** | **T** | **G** | **A** | **G** | **A** | **T** | **T** | **A** | **T** | **T** | **T** | **C** | **T** | **C** | **T** | **T** | **G** | **A** | **G** | **A** | **A** | **A** | **A** | **T** | **C** | **-** | **-** | **-** |
| **N59517F** | **A** | **T** | **G** | **T** | **C** | **T** | **A** | **A** | **A** | **A** | **T** | **C** | **G** | **A** | **A** | **A** | **T** | **A** | **A** | **A** | **A** | **A** | **A** | **T** | **C** | **T** | **G** | **A** | **C** | **A** | **T** | **T** | **C** | **G** | **G** | **C** | **T** | **A** | **C** | **G** | **A** | **C** | **A** | **G** | **C** | **C** | **A** | **A** | **G** | **G** | **C** | **A** | **C** | **A** | **T** | **T** | **A** | **T** | **T** | **A** | **T** | **T** | **T** | **G** | **A** | **A** | **C** | **A** | **A** | **G** | **C** | **A** | **A** | **A** | **T** | **C** | **T** | **A** | **A** | **A** | **T** | **T** | **T** | **T** | **G** | **A** | **C** | **A** | **C** | **A** | **C** | **A** | **A** | **T** | **G** | **G** | **A** | **A** | **A** | **C** | **T** | **A** | **G** | **G** | **A** | **C** | **T** | **T** | **A** | **T** | **C** | **G** | **G** | **A** | **C** | **G** | **A** | **A** | **A** | **C** | **G** | **G** | **T** | **C** | **G** | **A** | **G** | **G** | **A** | **A** | **A** | **G** | **A** | **C** | **A** | **A** | **C** | **T** | **T** | **T** | **A** | **C** | **T** | **G** | **A** | **A** | **T** | **A** | **T** | **T** | **C** | **T** | **A** | **C** | **A** | **A** | **A** | **A** | **C** | **A** | **A** | **A** | **C** | **T** | **A** | **C** | **C** | **T** | **T** | **A** | **T** | **C** | **A** | **A** | **G** | **G** | **G** | **C** | **A** | **A** | **G** | **T** | **A** | **A** | **T** | **C** | **C** | **A** | **T** | **C** | **A** | **G** | **C** | **A** | **A** | **G** | **A** | **A** | **T** | **T** | **T** | **G** | **C** | **C** | **T** | **A** | **T** | **T** | **T** | **C** | **C** | **C** | **G** | **C** | **A** | **A** | **C** | **A** | **G** | **A** | **C** | **A** | **A** | **A** | **A** | **G** | **A** | **T** | **A** | **A** | **A** | **G** | **A** | **A** | **C** | **G** | **T** | **T** | **T** | **A** | **A** | **C** | **C** | **T** | **A** | **T** | **T** | **A** | **C** | **G** | **T** | **G** | **T** | **T** | **A** | **A** | **A** | **T** | **G** | **A** | **T** | **A** | **T** | **T** | **A** | **C** | **G** | **G** | **A** | **T** | **T** | **T** | **T** | **G** | **A** | **G** | **A** | **T** | **A** | **T** | **G** | **G** | **G** | **A** | **A** | **A** | **T** | **C** | **G** | **A** | **A** | **A** | **G** | **A** | **G** | **A** | **G** | **C** | **T** | **C** | **C** | **A** | **A** | **T** | **T** | **G** | **A** | **T** | **G** | **C** | **A** | **A** | **A** | **C** | **A** | **G** | **A** | **T** | **C** | **C** | **T** | **G** | **A** | **A** | **A** | **T** | **C** | **T** | **T** | **A** | **T** | **G** | **G** | **A** | **G** | **A** | **G** | **A** | **A** | **T** | **T** | **C** | **A** | **G** | **C** | **A** | **C** | **A** | **C** | **T** | **A** | **T** | **C** | **G** | **G** | **G** | **G** | **G** | **G** | **A** | **G** | **A** | **G** | **A** | **A** | **G** | **A** | **C** | **A** | **A** | **A** | **A** | **G** | **T** | **C** | **C** | **T** | **A** | **C** | **T** | **G** | **G** | **C** | **A** | **C** | **T** | **T** | **T** | **T** | **A** | **T** | **T** | **T** | **G** | **T** | **G** | **G** | **A** | **T** | **G** | **A** | **C** | **A** | **C** | **T** | **C** | **A** | **T** | **T** | **T** | **C** | **C** | **C** | **G** | **T** | **T** | **A** | **A** | **T** | **C** | **G** | **A** | **T** | **G** | **A** | **A** | **C** | **C** | **A** | **A** | **C** | **G** | **A** | **A** | **T** | **C** | **A** | **T** | **T** | **T** | **G** | **G** | **A** | **-** | **-** | **-** | **T** | **A** | **T** | **C** | **T** | **C** | **T** | **G** | **G** | **T** | **A** | **G** | **A** | **A** | **A** | **A** | **C** | **A** | **A** | **G** | **T** | **A** | **G** | **C** | **G** | **G** | **C** | **T** | **T** | **A** | **T** | **T** | **T** | **G** | **A** | **A** | **A** | **A** | **A** | **G** | **A** | **A** | **A** | **A** | **A** | **A** | **C** | **A** | **A** | **G** | **G** | **C** | **T** | **T** | **C** | **A** | **T** | **C** | **G** | **T** | **G** | **G** | **T** | **C** | **A** | **G** | **C** | **C** | **A** | **T** | **G** | **A** | **C** | **C** | **G** | **G** | **G** | **G** | **A** | **T** | **T** | **T** | **A** | **T** | **C** | **G** | **A** | **T** | **G** | **A** | **A** | **G** | **T** | **A** | **G** | **T** | **G** | **G** | **A** | **C** | **C** | **A** | **T** | **G** | **T** | **T** | **T** | **T** | **A** | **G** | **C** | **A** | **A** | **T** | **C** | **G** | **A** | **A** | **A** | **A** | **A** | **A** | **G** | **T** | **C** | **A** | **A** | **C** | **T** | **G** | **G** | **A** | **A** | **C** | **T** | **T** | **T** | **A** | **T** | **C** | **A** | **A** | **G** | **G** | **G** | **A** | **A** | **T** | **T** | **T** | **C** | **T** | **C** | **T** | **A** | **T** | **C** | **T** | **A** | **T** | **G** | **A** | **A** | **G** | **A** | **A** | **C** | **A** | **G** | **A** | **A** | **A** | **A** | **A** | **A** | **C** | **T** | **T** | **C** | **G** | **T** | **G** | **A** | **T** | **G** | **A** | **A** | **T** | **T** | **T** | **G** | **A** | **A** | **A** | **T** | **G** | **G** | **C** | **T** | **C** | **A** | **A** | **A** | **A** | **T** | **G** | **A** | **A** | **A** | **A** | **A** | **T** | **T** | **G** | **A** | **A** | **A** | **A** | **A** | **A** | **G** | **A** | **A** | **G** | **T** | **C** | **A** | **G** | **T** | **A** | **G** | **G** | **C** | **T** | **A** | **A** | **A** | **G** | **A** | **A** | **A** | **A** | **C** | **A** | **G** | **C** | **A** | **G** | **C** | **T** | **G** | **A** | **A** | **A** | **A** | **A** | **G** | **C** | **C** | **G** | **A** | **A** | **T** | **G** | **G** | **T** | **C** | **T** | **C** | **G** | **T** | **T** | **C** | **C** | **C** | **G** | **A** | **G** | **A** | **A** | **G** | **G** | **A** | **G** | **A** | **T** | **A** | **A** | **A** | **A** | **C** | **A** | **A** | **A** | **G** | **A** | **A** | **A** | **C** | **A** | **A** | **G** | **T** | **C** | **G** | **G** | **A** | **T** | **T** | **C** | **A** | **T** | **C** | **G** | **A** | **T** | **A** | **C** | **T** | **G** | **A** | **A** | **T** | **C** | **T** | **A** | **G** | **A** | **C** | **G** | **A** | **G** | **T** | **G** | **A** | **A** | **T** | **A** | **A** | **A** | **G** | **G** | **A** | **G** | **C** | **A** | **G** | **T** | **G** | **G** | **G** | **T** | **G** | **C** | **T** | **G** | **A** | **T** | **G** | **C** | **T** | **G** | **C** | **A** | **C** | **G** | **G** | **A** | **C** | **G** | **A** | **T** | **G** | **A** | **A** | **A** | **C** | **G** | **A** | **T** | **C** | **C** | **A** | **A** | **A** | **G** | **C** | **A** | **A** | **T** | **C** | **G** | **T** | **G** | **A** | **A** | **T** | **C** | **G** | **G** | **A** | **T** | **G** | **G** | **A** | **G** | **A** | **C** | **C** | **C** | **A** | **G** | **A** | **T** | **C** | **A** | **G** | **C** | **G** | **A** | **G** | **A** | **A** | **A** | **G** | **A** | **A** | **A** | **A** | **A** | **C** | **T** | **A** | **T** | **T** | **A** | **A** | **A** | **A** | **G** | **A** | **T** | **A** | **T** | **C** | **G** | **A** | **A** | **T** | **A** | **T** | **A** | **T** | **C** | **G** | **A** | **T** | **T** | **C** | **T** | **T** | **T** | **G** | **A** | **C** | **G** | **A** | **T** | **G** | **A** | **A** | **T** | **A** | **G** | **C** | **C** | **A** | **A** | **G** | **C** | **G** | **T** | **C** | **T** | **C** | **A** | **C** | **C** | **A** | **T** | **A** | **A** | **G** | **C** | **G** | **A** | **C** | **T** | **T** | **T** | **T** | **A** | **A** | **G** | **C** | **G** | **T** | **A** | **G** | **A** | **A** | **G** | **A** | **T** | **C** | **T** | **T** | **C** | **A** | **A** | **T** | **T** | **A** | **G** | **G** | **G** | **T** | **A** | **T** | **G** | **A** | **A** | **A** | **A** | **T** | **C** | **T** | **G** | **T** | **T** | **A** | **T** | **T** | **C** | **G** | **A** | **G** | **C** | **C** | **A** | **A** | **T** | **T** | **C** | **A** | **T** | **T** | **T** | **T** | **A** | **C** | **A** | **A** | **T** | **C** | **G** | **A** | **G** | **C** | **C** | **T** | **C** | **A** | **T** | **C** | **A** | **G** | **C** | **G** | **G** | **G** | **T** | **G** | **G** | **C** | **G** | **A** | **T** | **T** | **T** | **C** | **A** | **G** | **G** | **T** | **C** | **C** | **T** | **A** | **A** | **C** | **G** | **G** | **T** | **G** | **C** | **A** | **G** | **G** | **A** | **A** | **A** | **G** | **T** | **C** | **A** | **T** | **C** | **C** | **A** | **T** | **T** | **A** | **T** | **C** | **C** | **A** | **T** | **T** | **A** | **T** | **C** | **T** | **T** | **C** | **T** | **G** | **G** | **G** | **G** | **G** | **C** | **A** | **T** | **T** | **C** | **A** | **A** | **C** | **G** | **G** | **C** | **A** | **A** | **G** | **G** | **T** | **T** | **A** | **T** | **A** | **G** | **G** | **A** | **G** | **A** | **A** | **A** | **A** | **A** | **T** | **A** | **C** | **C** | **A** | **G** | **C** | **C** | **A** | **A** | **A** | **A** | **C** | **A** | **T** | **C** | **T** | **G** | **A** | **G** | **C** | **A** | **T** | **T** | **A** | **G** | **T** | **T** | **A** | **T** | **G** | **C** | **A** | **A** | **G** | **C** | **C** | **A** | **A** | **A** | **A** | **T** | **T** | **A** | **T** | **G** | **A** | **A** | **G** | **A** | **C** | **A** | **A** | **T** | **C** | **G** | **A** | **G** | **G** | **A** | **A** | **C** | **G** | **T** | **T** | **G** | **G** | **C** | **G** | **G** | **A** | **A** | **T** | **T** | **T** | **G** | **C** | **A** | **G** | **A** | **G** | **A** | **A** | **A** | **A** | **A** | **C** | **C** | **A** | **A** | **G** | **T** | **A** | **G** | **A** | **C** | **T** | **A** | **C** | **C** | **A** | **A** | **G** | **C** | **A** | **T** | **T** | **T** | **T** | **T** | **G** | **A** | **A** | **C** | **A** | **A** | **C** | **C** | **T** | **C** | **C** | **G** | **A** | **A** | **A** | **G** | **C** | **T** | **T** | **G** | **G** | **G** | **A** | **T** | **G** | **G** | **A** | **A** | **A** | **G** | **A** | **G** | **A** | **T** | **G** | **T** | **T** | **T** | **T** | **T** | **C** | **A** | **T** | **A** | **A** | **C** | **A** | **A** | **G** | **A** | **T** | **T** | **G** | **A** | **G** | **C** | **A** | **G** | **A** | **T** | **G** | **A** | **G** | **T** | **A** | **T** | **G** | **G** | **G** | **C** | **C** | **A** | **A** | **C** | **G** | **G** | **A** | **A** | **A** | **A** | **A** | **A** | **G** | **T** | **G** | **G** | **A** | **A** | **T** | **T** | **G** | **G** | **C** | **T** | **A** | **A** | **A** | **T** | **C** | **T** | **T** | **T** | **A** | **T** | **C** | **A** | **C** | **A** | **G** | **C** | **C** | **A** | **G** | **C** | **T** | **G** | **A** | **A** | **C** | **T** | **A** | **T** | **A** | **T** | **A** | **T** | **A** | **T** | **G** | **G** | **G** | **A** | **T** | **G** | **A** | **A** | **C** | **C** | **A** | **T** | **T** | **G** | **A** | **A** | **T** | **T** | **A** | **T** | **T** | **T** | **G** | **G** | **A** | **T** | **G** | **T** | **C** | **T** | **T** | **C** | **A** | **A** | **T** | **C** | **A** | **A** | **G** | **A** | **A** | **C** | **A** | **A** | **T** | **T** | **A** | **G** | **A** | **A** | **C** | **A** | **A** | **C** | **T** | **G** | **A** | **T** | **C** | **T** | **T** | **G** | **A** | **A** | **C** | **G** | **T** | **G** | **A** | **A** | **A** | **C** | **C** | **T** | **G** | **C** | **C** | **A** | **T** | **G** | **T** | **T** | **A** | **C** | **T** | **A** | **G** | **T** | **G** | **G** | **A** | **A** | **C** | **A** | **T** | **G** | **A** | **T** | **C** | **A** | **A** | **A** | **C** | **C** | **T** | **T** | **C** | **C** | **T** | **G** | **G** | **A** | **T** | **A** | **A** | **A** | **G** | **T** | **A** | **T** | **C** | **T** | **A** | **C** | **T** | **G** | **A** | **G** | **A** | **T** | **T** | **A** | **T** | **T** | **T** | **C** | **T** | **C** | **T** | **T** | **G** | **A** | **G** | **A** | **G** | **A** | **A** | **T** | **C** | **-** | **-** | **-** |
| **N60417F** | **A** | **T** | **G** | **T** | **C** | **T** | **A** | **A** | **A** | **A** | **T** | **C** | **G** | **A** | **A** | **A** | **T** | **A** | **A** | **A** | **A** | **A** | **A** | **T** | **C** | **T** | **G** | **A** | **C** | **A** | **T** | **T** | **C** | **G** | **G** | **C** | **T** | **A** | **C** | **G** | **A** | **C** | **A** | **G** | **C** | **C** | **A** | **A** | **G** | **G** | **C** | **A** | **C** | **A** | **T** | **T** | **A** | **T** | **T** | **A** | **T** | **T** | **T** | **G** | **A** | **A** | **C** | **A** | **A** | **G** | **C** | **A** | **A** | **A** | **T** | **C** | **T** | **A** | **A** | **A** | **T** | **T** | **T** | **T** | **G** | **A** | **C** | **A** | **C** | **A** | **C** | **A** | **A** | **T** | **G** | **G** | **A** | **A** | **A** | **C** | **T** | **A** | **G** | **G** | **A** | **C** | **T** | **T** | **A** | **T** | **C** | **G** | **G** | **A** | **C** | **G** | **A** | **A** | **A** | **C** | **G** | **G** | **T** | **C** | **G** | **A** | **G** | **G** | **A** | **A** | **A** | **G** | **A** | **C** | **A** | **A** | **C** | **T** | **T** | **T** | **A** | **C** | **T** | **G** | **A** | **A** | **T** | **A** | **T** | **T** | **C** | **T** | **A** | **C** | **A** | **A** | **A** | **A** | **C** | **A** | **A** | **A** | **C** | **T** | **A** | **C** | **C** | **T** | **T** | **A** | **T** | **C** | **A** | **A** | **G** | **G** | **G** | **C** | **A** | **A** | **G** | **T** | **A** | **A** | **T** | **C** | **C** | **A** | **T** | **C** | **A** | **G** | **C** | **A** | **A** | **G** | **A** | **A** | **T** | **T** | **T** | **G** | **T** | **C** | **T** | **A** | **T** | **T** | **T** | **C** | **C** | **C** | **G** | **C** | **A** | **A** | **C** | **A** | **G** | **A** | **C** | **A** | **A** | **A** | **A** | **G** | **A** | **T** | **A** | **A** | **A** | **G** | **A** | **A** | **C** | **G** | **T** | **T** | **T** | **A** | **A** | **C** | **C** | **T** | **A** | **T** | **T** | **A** | **C** | **G** | **T** | **G** | **T** | **T** | **A** | **A** | **A** | **T** | **G** | **A** | **T** | **A** | **T** | **T** | **A** | **C** | **G** | **G** | **A** | **T** | **T** | **T** | **T** | **G** | **A** | **G** | **A** | **T** | **A** | **T** | **G** | **G** | **G** | **A** | **A** | **A** | **T** | **C** | **G** | **A** | **A** | **A** | **G** | **A** | **G** | **A** | **G** | **C** | **T** | **C** | **C** | **A** | **A** | **T** | **T** | **G** | **A** | **T** | **G** | **C** | **A** | **A** | **A** | **C** | **A** | **G** | **A** | **T** | **C** | **C** | **T** | **G** | **A** | **A** | **A** | **T** | **C** | **T** | **T** | **A** | **T** | **G** | **G** | **A** | **G** | **A** | **G** | **A** | **A** | **T** | **T** | **C** | **A** | **G** | **C** | **A** | **C** | **A** | **C** | **T** | **A** | **T** | **C** | **G** | **G** | **G** | **G** | **G** | **G** | **A** | **G** | **A** | **G** | **A** | **A** | **G** | **A** | **C** | **A** | **A** | **A** | **A** | **G** | **T** | **C** | **C** | **T** | **A** | **C** | **T** | **G** | **G** | **C** | **A** | **C** | **T** | **T** | **T** | **T** | **A** | **T** | **T** | **T** | **G** | **T** | **G** | **G** | **A** | **T** | **G** | **A** | **C** | **A** | **C** | **T** | **C** | **A** | **T** | **T** | **T** | **C** | **C** | **C** | **G** | **T** | **T** | **A** | **A** | **T** | **C** | **G** | **A** | **T** | **G** | **A** | **A** | **C** | **C** | **A** | **A** | **C** | **G** | **A** | **A** | **T** | **C** | **A** | **T** | **T** | **T** | **G** | **G** | **A** | **-** | **-** | **-** | **T** | **A** | **T** | **C** | **T** | **C** | **T** | **G** | **G** | **T** | **A** | **G** | **A** | **A** | **A** | **A** | **C** | **A** | **A** | **G** | **T** | **A** | **G** | **C** | **G** | **G** | **C** | **T** | **T** | **A** | **T** | **T** | **T** | **G** | **A** | **A** | **A** | **A** | **A** | **G** | **A** | **A** | **A** | **A** | **A** | **A** | **C** | **A** | **A** | **G** | **G** | **C** | **T** | **T** | **C** | **A** | **T** | **C** | **G** | **T** | **G** | **G** | **T** | **C** | **A** | **G** | **C** | **C** | **A** | **T** | **G** | **A** | **C** | **C** | **G** | **G** | **G** | **G** | **A** | **T** | **T** | **T** | **A** | **T** | **C** | **G** | **A** | **T** | **G** | **A** | **A** | **G** | **T** | **A** | **G** | **T** | **G** | **G** | **A** | **C** | **C** | **A** | **T** | **G** | **T** | **T** | **T** | **T** | **A** | **G** | **C** | **A** | **A** | **T** | **C** | **G** | **A** | **A** | **A** | **A** | **A** | **A** | **G** | **T** | **C** | **A** | **A** | **C** | **T** | **G** | **G** | **A** | **A** | **C** | **T** | **T** | **T** | **A** | **T** | **C** | **A** | **A** | **G** | **G** | **G** | **A** | **A** | **T** | **T** | **T** | **C** | **T** | **C** | **T** | **A** | **T** | **C** | **T** | **A** | **T** | **G** | **A** | **A** | **G** | **A** | **A** | **C** | **A** | **G** | **A** | **A** | **A** | **A** | **A** | **A** | **C** | **T** | **T** | **C** | **G** | **T** | **G** | **A** | **T** | **G** | **A** | **A** | **T** | **T** | **T** | **G** | **A** | **A** | **A** | **T** | **G** | **G** | **C** | **T** | **C** | **A** | **A** | **A** | **A** | **T** | **G** | **A** | **A** | **A** | **A** | **A** | **T** | **T** | **G** | **A** | **A** | **A** | **A** | **A** | **A** | **G** | **A** | **A** | **G** | **T** | **C** | **A** | **G** | **T** | **A** | **G** | **G** | **C** | **T** | **A** | **A** | **A** | **G** | **A** | **A** | **A** | **A** | **C** | **A** | **G** | **C** | **A** | **G** | **C** | **T** | **G** | **A** | **A** | **A** | **A** | **A** | **G** | **C** | **C** | **G** | **A** | **A** | **T** | **G** | **G** | **T** | **C** | **T** | **C** | **G** | **T** | **T** | **C** | **C** | **C** | **G** | **A** | **G** | **A** | **A** | **G** | **G** | **A** | **G** | **A** | **T** | **A** | **A** | **A** | **A** | **C** | **A** | **A** | **A** | **G** | **A** | **A** | **A** | **C** | **A** | **A** | **G** | **T** | **C** | **G** | **G** | **A** | **T** | **T** | **C** | **A** | **T** | **C** | **G** | **A** | **T** | **A** | **C** | **T** | **G** | **A** | **A** | **T** | **C** | **T** | **A** | **G** | **A** | **C** | **G** | **A** | **G** | **T** | **G** | **A** | **A** | **T** | **A** | **A** | **A** | **G** | **G** | **A** | **G** | **C** | **A** | **G** | **T** | **G** | **G** | **G** | **T** | **G** | **C** | **T** | **G** | **A** | **T** | **G** | **C** | **T** | **G** | **C** | **A** | **C** | **G** | **G** | **A** | **C** | **G** | **A** | **T** | **G** | **A** | **A** | **A** | **C** | **G** | **A** | **T** | **C** | **C** | **A** | **A** | **A** | **G** | **C** | **A** | **A** | **T** | **C** | **G** | **T** | **G** | **A** | **A** | **T** | **C** | **G** | **G** | **A** | **T** | **G** | **G** | **A** | **G** | **A** | **C** | **C** | **C** | **A** | **G** | **A** | **T** | **C** | **A** | **G** | **C** | **G** | **A** | **G** | **A** | **A** | **A** | **G** | **A** | **A** | **A** | **A** | **A** | **T** | **T** | **A** | **T** | **T** | **A** | **A** | **A** | **A** | **G** | **A** | **T** | **A** | **T** | **C** | **G** | **A** | **A** | **T** | **A** | **T** | **A** | **T** | **C** | **G** | **A** | **T** | **T** | **C** | **T** | **T** | **T** | **G** | **A** | **C** | **G** | **A** | **T** | **G** | **A** | **A** | **T** | **A** | **G** | **C** | **C** | **A** | **A** | **G** | **C** | **G** | **T** | **C** | **T** | **C** | **A** | **C** | **C** | **A** | **T** | **A** | **A** | **G** | **C** | **G** | **A** | **C** | **T** | **T** | **T** | **T** | **A** | **A** | **G** | **C** | **G** | **T** | **A** | **G** | **A** | **A** | **G** | **A** | **T** | **C** | **T** | **T** | **C** | **A** | **A** | **T** | **T** | **A** | **G** | **G** | **G** | **T** | **A** | **T** | **G** | **A** | **A** | **A** | **A** | **T** | **C** | **T** | **G** | **T** | **T** | **A** | **T** | **T** | **C** | **G** | **A** | **G** | **C** | **C** | **A** | **A** | **T** | **T** | **C** | **A** | **T** | **T** | **T** | **T** | **A** | **C** | **A** | **A** | **T** | **C** | **G** | **A** | **G** | **C** | **C** | **T** | **C** | **A** | **T** | **C** | **A** | **G** | **C** | **G** | **G** | **G** | **T** | **G** | **G** | **C** | **G** | **A** | **T** | **T** | **T** | **C** | **A** | **G** | **G** | **T** | **C** | **C** | **T** | **A** | **A** | **C** | **G** | **G** | **T** | **G** | **C** | **A** | **G** | **G** | **A** | **A** | **A** | **G** | **T** | **C** | **A** | **T** | **C** | **C** | **A** | **T** | **T** | **A** | **T** | **C** | **C** | **A** | **T** | **T** | **A** | **T** | **C** | **T** | **T** | **C** | **T** | **G** | **G** | **G** | **G** | **G** | **C** | **A** | **T** | **T** | **C** | **A** | **A** | **C** | **G** | **G** | **C** | **A** | **A** | **G** | **G** | **T** | **T** | **A** | **T** | **A** | **G** | **G** | **A** | **G** | **A** | **A** | **A** | **A** | **A** | **T** | **C** | **C** | **C** | **A** | **G** | **C** | **C** | **A** | **A** | **A** | **A** | **C** | **A** | **T** | **C** | **T** | **T** | **A** | **G** | **C** | **A** | **T** | **T** | **A** | **G** | **T** | **T** | **A** | **T** | **G** | **T** | **A** | **C** | **G** | **C** | **C** | **A** | **A** | **A** | **A** | **T** | **T** | **A** | **T** | **G** | **A** | **A** | **G** | **A** | **C** | **A** | **A** | **T** | **C** | **G** | **A** | **G** | **G** | **A** | **A** | **C** | **G** | **T** | **T** | **G** | **G** | **C** | **G** | **G** | **A** | **A** | **T** | **T** | **T** | **G** | **C** | **A** | **G** | **A** | **G** | **A** | **A** | **A** | **A** | **A** | **C** | **C** | **A** | **A** | **G** | **T** | **A** | **G** | **A** | **C** | **T** | **A** | **C** | **C** | **A** | **A** | **G** | **C** | **A** | **T** | **T** | **T** | **T** | **T** | **G** | **A** | **A** | **C** | **A** | **A** | **C** | **C** | **T** | **C** | **C** | **G** | **A** | **A** | **A** | **G** | **C** | **T** | **T** | **G** | **G** | **G** | **A** | **T** | **G** | **G** | **A** | **A** | **A** | **G** | **A** | **G** | **A** | **T** | **G** | **T** | **T** | **T** | **T** | **T** | **C** | **A** | **T** | **A** | **A** | **C** | **A** | **A** | **G** | **A** | **T** | **C** | **G** | **A** | **G** | **C** | **A** | **G** | **A** | **T** | **G** | **A** | **G** | **T** | **A** | **T** | **G** | **G** | **G** | **C** | **C** | **A** | **A** | **C** | **G** | **G** | **A** | **A** | **A** | **A** | **A** | **A** | **G** | **T** | **G** | **G** | **A** | **A** | **T** | **T** | **G** | **G** | **C** | **T** | **A** | **A** | **A** | **T** | **C** | **T** | **T** | **T** | **A** | **T** | **C** | **A** | **C** | **A** | **G** | **C** | **C** | **A** | **G** | **C** | **T** | **G** | **A** | **A** | **C** | **T** | **A** | **T** | **A** | **T** | **A** | **T** | **A** | **T** | **G** | **G** | **G** | **A** | **T** | **G** | **A** | **A** | **C** | **C** | **A** | **T** | **T** | **G** | **A** | **A** | **T** | **T** | **A** | **T** | **T** | **T** | **G** | **G** | **A** | **T** | **G** | **T** | **C** | **T** | **T** | **C** | **A** | **A** | **T** | **C** | **A** | **A** | **G** | **A** | **A** | **C** | **A** | **A** | **T** | **T** | **A** | **G** | **A** | **A** | **C** | **A** | **A** | **C** | **T** | **G** | **A** | **T** | **C** | **T** | **T** | **G** | **A** | **A** | **C** | **G** | **T** | **G** | **A** | **A** | **A** | **C** | **C** | **T** | **G** | **C** | **C** | **A** | **T** | **G** | **T** | **T** | **A** | **C** | **T** | **A** | **G** | **T** | **G** | **G** | **A** | **A** | **C** | **A** | **T** | **G** | **A** | **T** | **C** | **A** | **A** | **A** | **C** | **C** | **T** | **T** | **C** | **C** | **T** | **G** | **G** | **A** | **T** | **A** | **A** | **A** | **G** | **T** | **A** | **T** | **C** | **T** | **A** | **C** | **T** | **G** | **A** | **G** | **A** | **T** | **T** | **A** | **T** | **T** | **T** | **C** | **T** | **C** | **T** | **T** | **G** | **A** | **G** | **A** | **A** | **A** | **A** | **T** | **C** | **-** | **-** | **-** |
| **N52459** | **A** | **T** | **G** | **T** | **C** | **T** | **A** | **A** | **A** | **A** | **T** | **C** | **G** | **A** | **A** | **A** | **T** | **A** | **A** | **A** | **A** | **A** | **A** | **T** | **C** | **T** | **G** | **A** | **C** | **A** | **T** | **T** | **C** | **G** | **G** | **C** | **T** | **A** | **C** | **G** | **A** | **C** | **A** | **G** | **C** | **C** | **A** | **A** | **G** | **G** | **C** | **A** | **C** | **A** | **T** | **T** | **A** | **T** | **T** | **A** | **T** | **T** | **T** | **G** | **A** | **A** | **C** | **A** | **A** | **G** | **C** | **A** | **A** | **A** | **T** | **C** | **T** | **A** | **A** | **A** | **T** | **T** | **T** | **T** | **G** | **A** | **C** | **A** | **C** | **A** | **C** | **A** | **A** | **T** | **G** | **G** | **A** | **A** | **A** | **C** | **T** | **A** | **G** | **G** | **A** | **C** | **T** | **T** | **A** | **T** | **C** | **G** | **G** | **A** | **C** | **G** | **A** | **A** | **A** | **C** | **G** | **G** | **T** | **C** | **G** | **A** | **G** | **G** | **A** | **A** | **A** | **G** | **A** | **C** | **A** | **A** | **C** | **T** | **T** | **T** | **A** | **C** | **T** | **G** | **A** | **A** | **T** | **A** | **T** | **T** | **C** | **T** | **A** | **C** | **A** | **A** | **A** | **A** | **C** | **A** | **A** | **A** | **C** | **T** | **A** | **C** | **C** | **T** | **T** | **A** | **T** | **C** | **A** | **A** | **G** | **G** | **G** | **C** | **A** | **A** | **G** | **T** | **A** | **A** | **T** | **C** | **C** | **A** | **T** | **C** | **A** | **G** | **C** | **A** | **A** | **G** | **A** | **A** | **T** | **T** | **T** | **G** | **T** | **C** | **T** | **A** | **T** | **T** | **T** | **C** | **C** | **C** | **G** | **C** | **A** | **G** | **C** | **A** | **A** | **A** | **C** | **A** | **A** | **A** | **A** | **G** | **A** | **T** | **A** | **A** | **A** | **G** | **A** | **G** | **C** | **G** | **T** | **T** | **T** | **A** | **A** | **C** | **C** | **T** | **A** | **T** | **T** | **A** | **T** | **G** | **T** | **G** | **T** | **T** | **A** | **A** | **A** | **T** | **G** | **A** | **G** | **A** | **T** | **T** | **A** | **C** | **G** | **G** | **A** | **T** | **T** | **T** | **T** | **G** | **A** | **G** | **A** | **T** | **A** | **T** | **G** | **G** | **G** | **A** | **A** | **A** | **T** | **T** | **G** | **A** | **A** | **A** | **G** | **A** | **G** | **A** | **G** | **C** | **T** | **C** | **C** | **A** | **A** | **C** | **T** | **G** | **A** | **T** | **G** | **C** | **A** | **A** | **A** | **C** | **A** | **G** | **A** | **T** | **C** | **C** | **T** | **G** | **A** | **A** | **A** | **T** | **C** | **T** | **T** | **A** | **T** | **G** | **G** | **A** | **G** | **A** | **G** | **A** | **A** | **T** | **T** | **C** | **A** | **G** | **C** | **A** | **C** | **A** | **C** | **T** | **A** | **T** | **C** | **A** | **G** | **G** | **A** | **G** | **G** | **A** | **G** | **A** | **G** | **A** | **A** | **G** | **A** | **C** | **A** | **A** | **A** | **A** | **G** | **T** | **C** | **C** | **T** | **A** | **C** | **T** | **G** | **G** | **C** | **A** | **C** | **T** | **T** | **T** | **T** | **A** | **T** | **T** | **T** | **G** | **T** | **G** | **G** | **A** | **T** | **G** | **A** | **C** | **A** | **C** | **T** | **C** | **A** | **T** | **T** | **T** | **C** | **C** | **C** | **G** | **T** | **T** | **A** | **A** | **T** | **C** | **G** | **A** | **T** | **G** | **A** | **A** | **C** | **C** | **A** | **A** | **C** | **G** | **A** | **A** | **T** | **C** | **A** | **T** | **T** | **T** | **G** | **G** | **A** | **-** | **-** | **-** | **T** | **A** | **T** | **C** | **T** | **C** | **T** | **G** | **G** | **C** | **A** | **G** | **A** | **A** | **A** | **A** | **C** | **A** | **A** | **G** | **T** | **A** | **G** | **C** | **G** | **G** | **C** | **T** | **T** | **A** | **C** | **T** | **T** | **G** | **A** | **A** | **A** | **A** | **A** | **G** | **A** | **A** | **A** | **A** | **A** | **A** | **C** | **A** | **A** | **G** | **G** | **C** | **T** | **T** | **C** | **A** | **T** | **C** | **G** | **T** | **G** | **G** | **T** | **C** | **A** | **G** | **C** | **C** | **A** | **T** | **G** | **A** | **C** | **C** | **G** | **G** | **G** | **G** | **A** | **T** | **T** | **T** | **A** | **T** | **C** | **G** | **A** | **T** | **G** | **A** | **A** | **G** | **T** | **A** | **G** | **T** | **G** | **G** | **A** | **C** | **C** | **A** | **T** | **G** | **T** | **T** | **T** | **T** | **A** | **G** | **C** | **A** | **A** | **T** | **C** | **G** | **A** | **A** | **A** | **A** | **A** | **A** | **G** | **T** | **C** | **A** | **A** | **C** | **T** | **G** | **G** | **A** | **A** | **C** | **T** | **T** | **T** | **A** | **T** | **C** | **A** | **A** | **G** | **G** | **G** | **A** | **A** | **T** | **T** | **T** | **C** | **T** | **C** | **T** | **A** | **T** | **C** | **T** | **A** | **T** | **G** | **A** | **A** | **G** | **A** | **A** | **C** | **A** | **G** | **A** | **A** | **A** | **A** | **A** | **A** | **C** | **T** | **T** | **C** | **G** | **T** | **G** | **A** | **T** | **G** | **A** | **A** | **T** | **T** | **T** | **G** | **A** | **A** | **A** | **T** | **A** | **G** | **C** | **T** | **C** | **A** | **A** | **A** | **A** | **T** | **G** | **A** | **A** | **A** | **A** | **A** | **T** | **T** | **G** | **A** | **A** | **A** | **A** | **A** | **A** | **G** | **A** | **A** | **G** | **T** | **C** | **A** | **G** | **T** | **A** | **G** | **G** | **C** | **T** | **A** | **A** | **A** | **G** | **A** | **A** | **A** | **A** | **C** | **A** | **G** | **C** | **A** | **G** | **C** | **T** | **G** | **A** | **A** | **A** | **A** | **A** | **G** | **C** | **C** | **G** | **A** | **A** | **T** | **G** | **G** | **T** | **C** | **T** | **C** | **G** | **T** | **T** | **C** | **C** | **C** | **G** | **A** | **G** | **A** | **A** | **G** | **G** | **A** | **G** | **A** | **T** | **A** | **A** | **A** | **A** | **C** | **A** | **A** | **A** | **G** | **A** | **A** | **A** | **C** | **A** | **A** | **G** | **T** | **C** | **G** | **G** | **A** | **T** | **T** | **C** | **A** | **T** | **C** | **G** | **A** | **T** | **A** | **C** | **T** | **G** | **A** | **A** | **T** | **C** | **T** | **A** | **G** | **A** | **C** | **G** | **A** | **G** | **T** | **G** | **A** | **A** | **T** | **A** | **A** | **A** | **G** | **G** | **A** | **G** | **C** | **A** | **G** | **T** | **G** | **G** | **G** | **T** | **G** | **C** | **T** | **G** | **A** | **T** | **G** | **C** | **T** | **G** | **C** | **A** | **C** | **G** | **G** | **A** | **C** | **G** | **A** | **T** | **G** | **A** | **A** | **A** | **C** | **G** | **A** | **T** | **C** | **C** | **A** | **A** | **A** | **G** | **C** | **A** | **A** | **T** | **C** | **G** | **T** | **G** | **A** | **A** | **T** | **C** | **G** | **G** | **A** | **T** | **G** | **G** | **A** | **G** | **A** | **C** | **C** | **C** | **A** | **G** | **A** | **T** | **C** | **A** | **G** | **C** | **G** | **A** | **G** | **A** | **A** | **A** | **G** | **A** | **A** | **A** | **A** | **A** | **C** | **T** | **A** | **T** | **T** | **A** | **A** | **A** | **A** | **G** | **A** | **T** | **A** | **T** | **C** | **G** | **A** | **A** | **T** | **A** | **T** | **A** | **T** | **C** | **G** | **A** | **T** | **T** | **C** | **T** | **T** | **T** | **G** | **A** | **C** | **G** | **A** | **T** | **G** | **A** | **A** | **T** | **A** | **G** | **C** | **C** | **A** | **A** | **G** | **C** | **G** | **T** | **C** | **T** | **C** | **A** | **C** | **C** | **A** | **T** | **A** | **A** | **G** | **C** | **G** | **A** | **C** | **T** | **T** | **T** | **T** | **A** | **A** | **G** | **C** | **G** | **T** | **A** | **G** | **A** | **A** | **G** | **A** | **T** | **C** | **T** | **T** | **C** | **A** | **A** | **T** | **T** | **A** | **G** | **G** | **G** | **T** | **A** | **T** | **G** | **A** | **A** | **A** | **A** | **T** | **C** | **T** | **G** | **T** | **T** | **A** | **T** | **T** | **C** | **G** | **A** | **G** | **C** | **C** | **A** | **A** | **T** | **T** | **C** | **A** | **T** | **T** | **T** | **T** | **A** | **C** | **A** | **A** | **T** | **C** | **G** | **A** | **G** | **C** | **C** | **T** | **C** | **A** | **T** | **C** | **A** | **G** | **C** | **G** | **G** | **G** | **T** | **G** | **G** | **C** | **G** | **A** | **T** | **T** | **T** | **C** | **A** | **G** | **G** | **T** | **C** | **C** | **T** | **A** | **A** | **C** | **G** | **G** | **T** | **G** | **C** | **A** | **G** | **G** | **A** | **A** | **A** | **G** | **T** | **C** | **A** | **T** | **C** | **C** | **A** | **T** | **T** | **A** | **T** | **C** | **C** | **A** | **T** | **T** | **A** | **T** | **C** | **T** | **T** | **C** | **T** | **G** | **G** | **G** | **G** | **G** | **C** | **A** | **T** | **T** | **C** | **A** | **A** | **C** | **G** | **G** | **C** | **A** | **A** | **G** | **G** | **T** | **T** | **A** | **T** | **A** | **G** | **G** | **A** | **G** | **A** | **A** | **A** | **A** | **A** | **T** | **C** | **C** | **C** | **A** | **G** | **C** | **C** | **A** | **A** | **A** | **A** | **C** | **A** | **T** | **C** | **T** | **G** | **A** | **G** | **C** | **A** | **T** | **T** | **A** | **G** | **T** | **T** | **A** | **T** | **G** | **T** | **A** | **C** | **G** | **C** | **C** | **A** | **A** | **A** | **A** | **T** | **T** | **A** | **T** | **G** | **A** | **A** | **G** | **A** | **C** | **A** | **A** | **T** | **C** | **G** | **A** | **G** | **G** | **A** | **A** | **C** | **G** | **T** | **T** | **G** | **G** | **C** | **G** | **G** | **A** | **A** | **T** | **T** | **T** | **G** | **C** | **A** | **G** | **A** | **G** | **A** | **A** | **A** | **A** | **A** | **C** | **C** | **A** | **A** | **G** | **T** | **A** | **G** | **A** | **C** | **T** | **A** | **C** | **C** | **A** | **A** | **G** | **C** | **A** | **T** | **T** | **T** | **T** | **T** | **G** | **A** | **A** | **C** | **A** | **A** | **C** | **C** | **T** | **C** | **C** | **G** | **A** | **A** | **A** | **G** | **C** | **T** | **T** | **G** | **G** | **G** | **A** | **T** | **G** | **G** | **A** | **A** | **A** | **G** | **A** | **G** | **A** | **T** | **G** | **T** | **T** | **T** | **T** | **T** | **C** | **A** | **T** | **A** | **A** | **C** | **A** | **A** | **G** | **A** | **T** | **C** | **G** | **A** | **G** | **C** | **A** | **G** | **A** | **T** | **G** | **A** | **G** | **T** | **A** | **T** | **G** | **G** | **G** | **C** | **C** | **A** | **A** | **C** | **G** | **G** | **A** | **A** | **A** | **A** | **A** | **A** | **G** | **T** | **G** | **G** | **A** | **A** | **T** | **T** | **G** | **G** | **C** | **T** | **A** | **A** | **A** | **T** | **C** | **T** | **T** | **T** | **A** | **T** | **C** | **A** | **C** | **A** | **G** | **C** | **C** | **A** | **G** | **C** | **T** | **G** | **A** | **A** | **C** | **T** | **A** | **T** | **A** | **T** | **A** | **T** | **A** | **T** | **G** | **G** | **G** | **A** | **T** | **G** | **A** | **A** | **C** | **C** | **A** | **T** | **T** | **G** | **A** | **A** | **T** | **T** | **A** | **T** | **T** | **T** | **G** | **G** | **A** | **T** | **G** | **T** | **C** | **T** | **T** | **C** | **A** | **A** | **T** | **C** | **A** | **A** | **G** | **A** | **A** | **C** | **A** | **A** | **T** | **T** | **A** | **G** | **A** | **A** | **C** | **A** | **A** | **C** | **T** | **G** | **A** | **T** | **C** | **T** | **T** | **G** | **A** | **A** | **C** | **G** | **T** | **G** | **A** | **A** | **A** | **C** | **C** | **T** | **G** | **C** | **C** | **A** | **T** | **G** | **T** | **T** | **A** | **C** | **T** | **A** | **G** | **T** | **G** | **G** | **A** | **A** | **C** | **A** | **T** | **G** | **A** | **T** | **C** | **A** | **A** | **A** | **C** | **C** | **T** | **T** | **C** | **C** | **T** | **G** | **G** | **A** | **T** | **A** | **A** | **A** | **G** | **T** | **A** | **T** | **C** | **T** | **A** | **C** | **T** | **G** | **A** | **G** | **A** | **T** | **T** | **A** | **T** | **T** | **T** | **C** | **T** | **C** | **T** | **T** | **G** | **A** | **G** | **A** | **A** | **A** | **A** | **T** | **C** | **-** | **-** | **-** |
| **N59439F** | **A** | **T** | **G** | **T** | **C** | **T** | **A** | **A** | **A** | **A** | **T** | **C** | **G** | **A** | **A** | **A** | **T** | **A** | **A** | **A** | **A** | **A** | **A** | **T** | **C** | **T** | **G** | **A** | **C** | **A** | **T** | **T** | **C** | **G** | **G** | **C** | **T** | **A** | **C** | **G** | **A** | **C** | **A** | **G** | **C** | **C** | **A** | **A** | **G** | **G** | **C** | **A** | **C** | **A** | **T** | **T** | **A** | **T** | **T** | **A** | **T** | **T** | **T** | **G** | **A** | **A** | **C** | **A** | **A** | **G** | **C** | **A** | **A** | **A** | **T** | **C** | **T** | **A** | **A** | **A** | **T** | **T** | **T** | **T** | **G** | **A** | **C** | **A** | **C** | **A** | **C** | **A** | **A** | **T** | **G** | **G** | **A** | **A** | **A** | **C** | **T** | **A** | **G** | **G** | **A** | **C** | **T** | **T** | **A** | **T** | **C** | **G** | **G** | **A** | **C** | **G** | **A** | **A** | **A** | **C** | **G** | **G** | **T** | **C** | **G** | **A** | **G** | **G** | **A** | **A** | **A** | **G** | **A** | **C** | **A** | **A** | **C** | **T** | **T** | **T** | **A** | **C** | **T** | **G** | **A** | **A** | **T** | **A** | **T** | **T** | **C** | **T** | **A** | **C** | **A** | **A** | **A** | **A** | **C** | **A** | **A** | **A** | **C** | **T** | **A** | **C** | **C** | **T** | **T** | **A** | **T** | **C** | **A** | **A** | **G** | **G** | **G** | **C** | **A** | **A** | **G** | **T** | **A** | **A** | **T** | **C** | **C** | **A** | **T** | **C** | **A** | **G** | **C** | **A** | **A** | **G** | **A** | **A** | **T** | **T** | **T** | **G** | **C** | **C** | **T** | **A** | **T** | **T** | **T** | **C** | **C** | **C** | **G** | **C** | **A** | **A** | **C** | **A** | **G** | **A** | **C** | **A** | **A** | **A** | **A** | **G** | **A** | **T** | **A** | **A** | **A** | **G** | **A** | **A** | **C** | **G** | **T** | **T** | **T** | **A** | **A** | **C** | **C** | **T** | **A** | **T** | **T** | **A** | **C** | **G** | **T** | **G** | **T** | **T** | **A** | **A** | **A** | **T** | **G** | **A** | **T** | **A** | **T** | **T** | **A** | **C** | **G** | **G** | **A** | **T** | **T** | **T** | **T** | **G** | **A** | **G** | **A** | **T** | **A** | **T** | **G** | **G** | **G** | **A** | **A** | **A** | **T** | **C** | **G** | **A** | **A** | **A** | **G** | **A** | **G** | **A** | **G** | **C** | **T** | **C** | **C** | **A** | **A** | **T** | **T** | **G** | **A** | **T** | **G** | **C** | **A** | **A** | **A** | **C** | **A** | **G** | **A** | **T** | **C** | **C** | **T** | **G** | **A** | **A** | **A** | **T** | **C** | **T** | **T** | **A** | **T** | **G** | **G** | **A** | **G** | **A** | **G** | **A** | **A** | **T** | **T** | **C** | **A** | **G** | **C** | **A** | **C** | **A** | **C** | **T** | **A** | **T** | **C** | **G** | **G** | **G** | **G** | **G** | **G** | **A** | **G** | **A** | **G** | **A** | **A** | **G** | **A** | **C** | **A** | **A** | **A** | **A** | **G** | **T** | **C** | **C** | **T** | **A** | **C** | **T** | **G** | **G** | **C** | **A** | **C** | **T** | **T** | **T** | **T** | **A** | **T** | **T** | **T** | **G** | **T** | **G** | **G** | **A** | **T** | **G** | **A** | **C** | **A** | **C** | **T** | **C** | **A** | **T** | **T** | **T** | **C** | **C** | **C** | **G** | **T** | **T** | **A** | **A** | **T** | **C** | **G** | **A** | **T** | **G** | **A** | **A** | **C** | **C** | **A** | **A** | **C** | **G** | **A** | **A** | **T** | **C** | **A** | **T** | **T** | **T** | **G** | **G** | **A** | **-** | **-** | **-** | **T** | **A** | **T** | **C** | **T** | **C** | **T** | **G** | **G** | **T** | **A** | **G** | **A** | **A** | **A** | **A** | **C** | **A** | **A** | **G** | **G** | **A** | **G** | **C** | **T** | **G** | **C** | **T** | **T** | **A** | **T** | **T** | **T** | **G** | **A** | **A** | **A** | **A** | **A** | **G** | **A** | **A** | **A** | **A** | **A** | **A** | **C** | **A** | **A** | **G** | **G** | **C** | **T** | **T** | **C** | **A** | **T** | **C** | **G** | **T** | **G** | **G** | **T** | **C** | **A** | **G** | **C** | **C** | **A** | **T** | **G** | **A** | **C** | **C** | **G** | **G** | **G** | **G** | **A** | **T** | **T** | **T** | **A** | **T** | **C** | **G** | **A** | **T** | **G** | **A** | **A** | **G** | **T** | **A** | **G** | **T** | **G** | **G** | **A** | **C** | **C** | **A** | **T** | **G** | **T** | **T** | **T** | **T** | **A** | **G** | **C** | **A** | **A** | **T** | **C** | **G** | **A** | **A** | **A** | **A** | **A** | **A** | **G** | **T** | **C** | **A** | **A** | **C** | **T** | **G** | **G** | **A** | **A** | **C** | **T** | **T** | **T** | **A** | **T** | **C** | **A** | **A** | **G** | **G** | **G** | **A** | **A** | **T** | **T** | **T** | **C** | **T** | **C** | **T** | **A** | **T** | **C** | **T** | **A** | **T** | **G** | **A** | **A** | **G** | **A** | **A** | **C** | **A** | **G** | **A** | **A** | **A** | **A** | **A** | **A** | **C** | **T** | **T** | **C** | **G** | **T** | **G** | **A** | **T** | **G** | **A** | **A** | **T** | **T** | **T** | **G** | **A** | **A** | **A** | **T** | **G** | **G** | **C** | **T** | **C** | **A** | **A** | **A** | **A** | **T** | **G** | **A** | **A** | **A** | **A** | **A** | **T** | **T** | **G** | **A** | **A** | **A** | **A** | **A** | **A** | **G** | **A** | **A** | **G** | **T** | **C** | **A** | **G** | **T** | **A** | **G** | **G** | **C** | **T** | **A** | **A** | **A** | **G** | **A** | **A** | **A** | **A** | **C** | **A** | **G** | **C** | **A** | **G** | **C** | **T** | **G** | **A** | **A** | **A** | **A** | **A** | **G** | **C** | **C** | **G** | **A** | **A** | **T** | **G** | **G** | **T** | **C** | **T** | **C** | **G** | **T** | **T** | **C** | **C** | **C** | **G** | **A** | **G** | **A** | **A** | **G** | **G** | **A** | **G** | **A** | **T** | **A** | **A** | **A** | **A** | **C** | **A** | **A** | **A** | **G** | **A** | **A** | **A** | **C** | **A** | **A** | **G** | **T** | **C** | **G** | **G** | **A** | **T** | **T** | **C** | **A** | **T** | **C** | **G** | **A** | **T** | **A** | **C** | **T** | **G** | **A** | **A** | **T** | **C** | **T** | **A** | **G** | **A** | **C** | **G** | **A** | **G** | **T** | **G** | **A** | **A** | **T** | **A** | **A** | **A** | **G** | **G** | **A** | **G** | **C** | **A** | **G** | **T** | **G** | **G** | **G** | **T** | **G** | **C** | **T** | **G** | **A** | **T** | **G** | **C** | **T** | **G** | **C** | **A** | **C** | **G** | **G** | **A** | **C** | **G** | **A** | **T** | **G** | **A** | **A** | **A** | **C** | **G** | **A** | **T** | **C** | **C** | **A** | **A** | **A** | **G** | **C** | **A** | **A** | **T** | **C** | **G** | **T** | **G** | **A** | **A** | **T** | **C** | **G** | **G** | **A** | **T** | **G** | **G** | **A** | **G** | **A** | **C** | **C** | **C** | **A** | **G** | **A** | **T** | **C** | **A** | **G** | **C** | **G** | **A** | **G** | **A** | **A** | **A** | **G** | **A** | **A** | **A** | **A** | **A** | **C** | **T** | **A** | **T** | **T** | **A** | **A** | **A** | **A** | **G** | **A** | **T** | **A** | **T** | **C** | **G** | **A** | **A** | **T** | **A** | **T** | **A** | **T** | **C** | **G** | **A** | **T** | **T** | **C** | **T** | **T** | **T** | **G** | **A** | **C** | **G** | **A** | **T** | **G** | **A** | **A** | **T** | **A** | **G** | **C** | **C** | **A** | **A** | **G** | **C** | **G** | **T** | **C** | **T** | **C** | **A** | **C** | **C** | **A** | **T** | **A** | **A** | **G** | **C** | **G** | **A** | **C** | **T** | **T** | **T** | **T** | **A** | **A** | **G** | **C** | **G** | **T** | **A** | **G** | **A** | **A** | **G** | **A** | **T** | **C** | **T** | **T** | **C** | **A** | **A** | **T** | **T** | **A** | **G** | **G** | **G** | **T** | **A** | **T** | **G** | **A** | **A** | **A** | **A** | **T** | **C** | **T** | **G** | **T** | **T** | **A** | **T** | **T** | **C** | **G** | **A** | **G** | **C** | **C** | **A** | **A** | **T** | **T** | **C** | **A** | **T** | **T** | **T** | **T** | **A** | **C** | **A** | **A** | **T** | **C** | **G** | **A** | **G** | **C** | **C** | **T** | **C** | **A** | **T** | **C** | **A** | **G** | **C** | **G** | **G** | **G** | **T** | **G** | **G** | **C** | **G** | **A** | **T** | **T** | **T** | **C** | **A** | **G** | **G** | **T** | **C** | **C** | **T** | **A** | **A** | **C** | **G** | **G** | **T** | **G** | **C** | **A** | **G** | **G** | **A** | **A** | **A** | **G** | **T** | **C** | **A** | **T** | **C** | **C** | **A** | **T** | **T** | **A** | **T** | **C** | **C** | **A** | **T** | **T** | **A** | **T** | **C** | **T** | **T** | **C** | **T** | **G** | **G** | **G** | **G** | **G** | **C** | **A** | **T** | **T** | **C** | **A** | **A** | **C** | **G** | **G** | **C** | **A** | **A** | **G** | **G** | **T** | **T** | **A** | **T** | **A** | **G** | **G** | **A** | **G** | **A** | **A** | **A** | **A** | **A** | **T** | **A** | **C** | **C** | **A** | **G** | **C** | **C** | **A** | **A** | **A** | **A** | **C** | **A** | **T** | **C** | **T** | **G** | **A** | **G** | **C** | **A** | **T** | **T** | **A** | **G** | **T** | **T** | **A** | **T** | **G** | **C** | **A** | **A** | **G** | **C** | **C** | **A** | **A** | **A** | **A** | **T** | **T** | **A** | **T** | **G** | **A** | **A** | **G** | **A** | **C** | **A** | **A** | **T** | **C** | **G** | **A** | **G** | **G** | **A** | **A** | **C** | **G** | **T** | **T** | **G** | **G** | **C** | **G** | **G** | **A** | **A** | **T** | **T** | **T** | **G** | **C** | **A** | **G** | **A** | **G** | **A** | **A** | **A** | **A** | **A** | **C** | **C** | **A** | **A** | **G** | **T** | **A** | **G** | **A** | **C** | **T** | **A** | **C** | **C** | **A** | **A** | **G** | **C** | **A** | **T** | **T** | **T** | **T** | **T** | **G** | **A** | **A** | **C** | **A** | **A** | **C** | **C** | **T** | **C** | **C** | **G** | **A** | **A** | **A** | **G** | **C** | **T** | **T** | **G** | **G** | **G** | **A** | **T** | **G** | **G** | **A** | **A** | **A** | **G** | **A** | **G** | **A** | **T** | **G** | **T** | **T** | **T** | **T** | **T** | **C** | **A** | **T** | **A** | **A** | **C** | **A** | **A** | **G** | **A** | **T** | **C** | **G** | **A** | **G** | **C** | **A** | **G** | **A** | **T** | **G** | **A** | **G** | **T** | **A** | **T** | **G** | **G** | **G** | **C** | **C** | **A** | **A** | **C** | **G** | **G** | **A** | **A** | **A** | **A** | **A** | **A** | **G** | **T** | **G** | **G** | **A** | **A** | **T** | **T** | **G** | **G** | **C** | **T** | **A** | **A** | **A** | **T** | **C** | **T** | **T** | **T** | **A** | **T** | **C** | **A** | **C** | **A** | **G** | **C** | **C** | **A** | **G** | **C** | **T** | **G** | **A** | **A** | **C** | **T** | **A** | **T** | **A** | **T** | **A** | **C** | **A** | **T** | **G** | **G** | **G** | **A** | **T** | **G** | **A** | **A** | **C** | **C** | **A** | **T** | **T** | **G** | **A** | **A** | **T** | **T** | **A** | **T** | **T** | **T** | **G** | **G** | **A** | **T** | **G** | **T** | **C** | **T** | **T** | **C** | **A** | **A** | **T** | **C** | **A** | **A** | **G** | **A** | **A** | **C** | **A** | **A** | **T** | **T** | **A** | **G** | **A** | **A** | **C** | **A** | **A** | **C** | **T** | **G** | **A** | **T** | **C** | **T** | **T** | **G** | **A** | **A** | **C** | **G** | **T** | **G** | **A** | **A** | **A** | **C** | **C** | **T** | **G** | **C** | **C** | **A** | **T** | **G** | **T** | **T** | **A** | **C** | **T** | **A** | **G** | **T** | **G** | **G** | **A** | **A** | **C** | **A** | **T** | **G** | **A** | **T** | **C** | **A** | **A** | **A** | **C** | **C** | **T** | **T** | **C** | **C** | **T** | **G** | **G** | **A** | **T** | **A** | **A** | **A** | **G** | **T** | **A** | **T** | **C** | **T** | **A** | **C** | **T** | **G** | **A** | **G** | **A** | **T** | **T** | **A** | **T** | **T** | **T** | **C** | **T** | **C** | **T** | **T** | **G** | **A** | **G** | **A** | **G** | **A** | **A** | **T** | **C** | **-** | **-** | **-** |
| **N55317** | **A** | **T** | **G** | **T** | **C** | **T** | **A** | **A** | **A** | **A** | **T** | **C** | **G** | **A** | **A** | **A** | **T** | **A** | **A** | **A** | **A** | **A** | **A** | **T** | **C** | **T** | **G** | **A** | **C** | **A** | **T** | **T** | **C** | **G** | **G** | **C** | **T** | **A** | **C** | **G** | **A** | **C** | **A** | **G** | **C** | **C** | **A** | **A** | **G** | **G** | **C** | **A** | **C** | **A** | **T** | **T** | **A** | **T** | **T** | **A** | **T** | **T** | **T** | **G** | **A** | **A** | **C** | **A** | **A** | **G** | **C** | **A** | **A** | **A** | **T** | **C** | **T** | **A** | **A** | **A** | **T** | **T** | **T** | **T** | **G** | **A** | **C** | **A** | **C** | **A** | **C** | **A** | **A** | **T** | **G** | **G** | **A** | **A** | **A** | **C** | **T** | **A** | **G** | **G** | **A** | **C** | **T** | **T** | **A** | **T** | **C** | **G** | **G** | **A** | **C** | **G** | **A** | **A** | **A** | **C** | **G** | **G** | **T** | **C** | **G** | **A** | **G** | **G** | **A** | **A** | **A** | **G** | **A** | **C** | **A** | **A** | **C** | **T** | **T** | **T** | **A** | **C** | **T** | **G** | **A** | **A** | **T** | **A** | **T** | **T** | **C** | **T** | **A** | **C** | **A** | **A** | **A** | **A** | **C** | **A** | **A** | **A** | **C** | **T** | **A** | **C** | **C** | **T** | **T** | **A** | **T** | **C** | **A** | **A** | **G** | **G** | **G** | **C** | **A** | **A** | **G** | **T** | **A** | **A** | **T** | **C** | **C** | **A** | **T** | **C** | **A** | **G** | **C** | **A** | **A** | **G** | **A** | **A** | **T** | **T** | **T** | **G** | **T** | **C** | **T** | **A** | **T** | **T** | **T** | **C** | **C** | **C** | **G** | **C** | **A** | **A** | **C** | **A** | **G** | **A** | **C** | **A** | **A** | **A** | **A** | **G** | **A** | **T** | **A** | **A** | **A** | **G** | **A** | **A** | **C** | **G** | **T** | **T** | **T** | **A** | **A** | **C** | **C** | **T** | **A** | **T** | **T** | **A** | **C** | **G** | **T** | **G** | **T** | **T** | **A** | **A** | **A** | **T** | **G** | **A** | **T** | **A** | **T** | **T** | **A** | **C** | **G** | **G** | **A** | **T** | **T** | **T** | **T** | **G** | **A** | **G** | **A** | **T** | **A** | **T** | **G** | **G** | **G** | **A** | **A** | **A** | **T** | **C** | **G** | **A** | **A** | **A** | **G** | **A** | **G** | **A** | **G** | **C** | **T** | **C** | **C** | **A** | **A** | **T** | **T** | **T** | **A** | **T** | **G** | **C** | **A** | **A** | **A** | **C** | **A** | **G** | **A** | **T** | **C** | **C** | **T** | **G** | **A** | **A** | **A** | **T** | **C** | **T** | **T** | **A** | **T** | **G** | **G** | **A** | **G** | **A** | **G** | **A** | **A** | **T** | **T** | **C** | **A** | **G** | **C** | **A** | **C** | **A** | **C** | **T** | **A** | **T** | **C** | **G** | **G** | **G** | **G** | **G** | **G** | **A** | **G** | **A** | **G** | **A** | **A** | **G** | **A** | **C** | **A** | **A** | **A** | **A** | **G** | **T** | **C** | **C** | **T** | **A** | **T** | **T** | **G** | **G** | **C** | **A** | **C** | **T** | **T** | **T** | **T** | **A** | **T** | **T** | **T** | **G** | **T** | **G** | **G** | **A** | **T** | **G** | **A** | **C** | **A** | **C** | **T** | **C** | **A** | **T** | **T** | **T** | **C** | **C** | **C** | **G** | **T** | **T** | **A** | **A** | **T** | **C** | **G** | **A** | **T** | **G** | **A** | **A** | **C** | **C** | **A** | **A** | **C** | **G** | **A** | **A** | **T** | **C** | **A** | **T** | **T** | **T** | **G** | **G** | **A** | **-** | **-** | **-** | **T** | **A** | **T** | **C** | **T** | **C** | **T** | **G** | **G** | **T** | **A** | **G** | **A** | **A** | **A** | **A** | **C** | **A** | **A** | **G** | **T** | **A** | **G** | **C** | **G** | **G** | **C** | **T** | **T** | **A** | **T** | **T** | **T** | **G** | **A** | **A** | **A** | **A** | **A** | **G** | **A** | **A** | **A** | **A** | **A** | **A** | **C** | **A** | **A** | **G** | **G** | **C** | **T** | **T** | **C** | **A** | **T** | **C** | **G** | **T** | **G** | **G** | **T** | **C** | **A** | **G** | **C** | **C** | **A** | **T** | **G** | **A** | **C** | **C** | **G** | **G** | **G** | **G** | **A** | **T** | **T** | **T** | **A** | **T** | **C** | **G** | **A** | **T** | **G** | **A** | **A** | **G** | **T** | **A** | **G** | **T** | **G** | **G** | **A** | **C** | **C** | **A** | **T** | **G** | **T** | **T** | **T** | **T** | **A** | **G** | **C** | **A** | **A** | **T** | **C** | **G** | **A** | **A** | **A** | **A** | **A** | **A** | **G** | **T** | **C** | **A** | **A** | **C** | **T** | **G** | **G** | **A** | **A** | **C** | **T** | **T** | **T** | **A** | **T** | **C** | **A** | **A** | **G** | **G** | **G** | **A** | **A** | **T** | **T** | **T** | **C** | **T** | **C** | **T** | **A** | **T** | **C** | **T** | **A** | **T** | **G** | **A** | **A** | **G** | **A** | **A** | **C** | **A** | **G** | **A** | **A** | **A** | **A** | **A** | **A** | **C** | **T** | **T** | **C** | **G** | **T** | **G** | **A** | **T** | **G** | **A** | **A** | **T** | **T** | **T** | **G** | **A** | **A** | **A** | **T** | **G** | **G** | **C** | **T** | **C** | **A** | **A** | **A** | **A** | **T** | **G** | **A** | **A** | **A** | **A** | **A** | **T** | **T** | **G** | **A** | **A** | **A** | **A** | **A** | **A** | **G** | **A** | **A** | **G** | **T** | **C** | **A** | **G** | **T** | **A** | **G** | **G** | **C** | **T** | **A** | **A** | **A** | **G** | **A** | **A** | **A** | **A** | **C** | **A** | **G** | **C** | **A** | **G** | **C** | **T** | **G** | **A** | **A** | **A** | **A** | **A** | **G** | **C** | **C** | **G** | **A** | **A** | **T** | **G** | **G** | **T** | **C** | **T** | **C** | **G** | **T** | **T** | **C** | **C** | **C** | **G** | **A** | **G** | **A** | **A** | **G** | **G** | **A** | **G** | **A** | **T** | **A** | **A** | **A** | **A** | **C** | **A** | **A** | **A** | **G** | **A** | **A** | **A** | **C** | **A** | **A** | **G** | **T** | **C** | **G** | **G** | **A** | **T** | **T** | **C** | **A** | **T** | **C** | **G** | **A** | **T** | **A** | **C** | **T** | **G** | **A** | **A** | **T** | **C** | **T** | **A** | **G** | **A** | **C** | **G** | **A** | **G** | **T** | **G** | **A** | **A** | **T** | **A** | **A** | **A** | **G** | **G** | **A** | **G** | **C** | **A** | **G** | **T** | **G** | **G** | **G** | **T** | **G** | **C** | **T** | **G** | **A** | **T** | **G** | **C** | **T** | **G** | **C** | **A** | **C** | **G** | **G** | **G** | **C** | **G** | **A** | **T** | **G** | **A** | **A** | **A** | **C** | **G** | **A** | **T** | **C** | **C** | **A** | **A** | **A** | **G** | **C** | **A** | **A** | **T** | **C** | **G** | **T** | **G** | **A** | **A** | **T** | **C** | **G** | **G** | **A** | **T** | **G** | **G** | **A** | **G** | **A** | **C** | **C** | **C** | **A** | **G** | **A** | **T** | **C** | **A** | **G** | **C** | **G** | **A** | **G** | **A** | **A** | **A** | **G** | **A** | **A** | **A** | **A** | **A** | **C** | **T** | **A** | **T** | **T** | **A** | **A** | **A** | **A** | **G** | **A** | **T** | **A** | **T** | **C** | **G** | **A** | **A** | **T** | **A** | **T** | **A** | **T** | **C** | **G** | **A** | **T** | **T** | **C** | **T** | **T** | **T** | **G** | **A** | **C** | **G** | **A** | **T** | **G** | **A** | **A** | **T** | **A** | **G** | **C** | **C** | **A** | **A** | **G** | **C** | **G** | **T** | **C** | **T** | **C** | **A** | **C** | **C** | **A** | **T** | **A** | **A** | **G** | **C** | **G** | **A** | **C** | **T** | **T** | **T** | **T** | **A** | **A** | **G** | **C** | **G** | **T** | **A** | **G** | **A** | **A** | **G** | **A** | **T** | **C** | **T** | **T** | **C** | **A** | **A** | **T** | **T** | **A** | **G** | **G** | **G** | **T** | **A** | **T** | **G** | **A** | **A** | **A** | **A** | **T** | **C** | **T** | **G** | **T** | **T** | **A** | **T** | **T** | **C** | **G** | **A** | **G** | **C** | **C** | **A** | **A** | **T** | **T** | **C** | **A** | **T** | **T** | **T** | **T** | **A** | **C** | **A** | **A** | **T** | **C** | **G** | **A** | **G** | **C** | **C** | **T** | **C** | **A** | **T** | **C** | **A** | **G** | **C** | **G** | **G** | **G** | **T** | **G** | **G** | **C** | **G** | **A** | **T** | **T** | **T** | **C** | **A** | **G** | **G** | **T** | **C** | **C** | **T** | **A** | **A** | **C** | **G** | **G** | **T** | **G** | **C** | **A** | **G** | **G** | **A** | **A** | **A** | **G** | **T** | **C** | **A** | **T** | **C** | **C** | **A** | **T** | **T** | **A** | **T** | **C** | **C** | **A** | **T** | **T** | **A** | **T** | **C** | **T** | **T** | **C** | **T** | **G** | **G** | **G** | **G** | **G** | **C** | **A** | **T** | **T** | **C** | **A** | **A** | **C** | **G** | **G** | **C** | **A** | **A** | **G** | **G** | **T** | **T** | **A** | **T** | **A** | **G** | **G** | **A** | **G** | **A** | **A** | **A** | **A** | **A** | **T** | **C** | **C** | **C** | **A** | **G** | **C** | **C** | **A** | **A** | **A** | **A** | **C** | **A** | **T** | **C** | **T** | **G** | **A** | **G** | **C** | **A** | **T** | **T** | **A** | **G** | **T** | **T** | **A** | **T** | **G** | **T** | **A** | **C** | **G** | **C** | **C** | **A** | **A** | **A** | **A** | **T** | **T** | **A** | **T** | **G** | **A** | **A** | **G** | **A** | **C** | **A** | **A** | **T** | **C** | **G** | **A** | **G** | **G** | **A** | **A** | **C** | **G** | **T** | **T** | **G** | **G** | **C** | **G** | **G** | **A** | **A** | **T** | **T** | **T** | **G** | **C** | **A** | **G** | **A** | **G** | **A** | **A** | **A** | **A** | **A** | **C** | **C** | **A** | **A** | **G** | **T** | **A** | **G** | **A** | **C** | **T** | **A** | **C** | **C** | **A** | **A** | **G** | **C** | **A** | **T** | **T** | **T** | **T** | **T** | **G** | **A** | **A** | **C** | **A** | **A** | **C** | **C** | **T** | **C** | **C** | **G** | **A** | **A** | **A** | **G** | **C** | **T** | **T** | **G** | **G** | **G** | **A** | **T** | **G** | **G** | **A** | **A** | **A** | **G** | **A** | **G** | **A** | **T** | **G** | **T** | **T** | **T** | **T** | **T** | **C** | **A** | **T** | **A** | **A** | **C** | **A** | **A** | **G** | **A** | **T** | **T** | **G** | **A** | **G** | **C** | **A** | **G** | **A** | **T** | **G** | **A** | **G** | **T** | **A** | **T** | **G** | **G** | **G** | **C** | **C** | **A** | **A** | **C** | **G** | **G** | **A** | **A** | **A** | **A** | **A** | **A** | **G** | **T** | **G** | **G** | **A** | **A** | **T** | **T** | **G** | **G** | **C** | **T** | **A** | **A** | **A** | **T** | **C** | **T** | **T** | **T** | **A** | **T** | **C** | **A** | **C** | **A** | **G** | **C** | **C** | **A** | **G** | **C** | **T** | **G** | **A** | **A** | **C** | **T** | **A** | **T** | **A** | **T** | **A** | **T** | **A** | **T** | **G** | **G** | **G** | **A** | **T** | **G** | **A** | **A** | **C** | **C** | **A** | **T** | **T** | **G** | **A** | **A** | **T** | **T** | **A** | **T** | **T** | **T** | **G** | **G** | **A** | **T** | **G** | **T** | **C** | **T** | **T** | **C** | **A** | **A** | **T** | **C** | **A** | **A** | **G** | **A** | **A** | **C** | **A** | **A** | **T** | **T** | **A** | **G** | **A** | **A** | **C** | **A** | **A** | **C** | **T** | **G** | **A** | **T** | **C** | **T** | **T** | **G** | **A** | **A** | **C** | **G** | **T** | **G** | **A** | **A** | **A** | **C** | **C** | **T** | **G** | **C** | **C** | **A** | **T** | **G** | **T** | **T** | **A** | **C** | **T** | **G** | **G** | **T** | **G** | **G** | **A** | **A** | **C** | **A** | **T** | **G** | **A** | **T** | **C** | **A** | **A** | **A** | **C** | **C** | **T** | **T** | **C** | **C** | **T** | **G** | **G** | **A** | **T** | **A** | **A** | **A** | **G** | **T** | **A** | **T** | **C** | **T** | **A** | **C** | **T** | **G** | **A** | **G** | **A** | **T** | **T** | **A** | **T** | **T** | **T** | **C** | **T** | **C** | **T** | **T** | **G** | **A** | **G** | **A** | **A** | **A** | **A** | **T** | **C** | **-** | **-** | **-** |
| **N55728** | **A** | **T** | **G** | **T** | **C** | **T** | **A** | **A** | **A** | **A** | **T** | **C** | **G** | **A** | **A** | **A** | **T** | **A** | **A** | **A** | **A** | **A** | **A** | **T** | **C** | **T** | **G** | **A** | **C** | **A** | **T** | **T** | **C** | **G** | **G** | **C** | **T** | **A** | **C** | **G** | **A** | **C** | **A** | **G** | **C** | **C** | **A** | **A** | **G** | **G** | **C** | **A** | **C** | **A** | **T** | **T** | **A** | **T** | **T** | **A** | **T** | **T** | **T** | **G** | **A** | **A** | **C** | **A** | **A** | **G** | **C** | **A** | **A** | **A** | **T** | **C** | **T** | **A** | **A** | **A** | **T** | **T** | **T** | **T** | **G** | **A** | **C** | **A** | **C** | **A** | **C** | **A** | **A** | **T** | **G** | **G** | **A** | **A** | **A** | **C** | **T** | **A** | **G** | **G** | **A** | **C** | **T** | **T** | **A** | **T** | **C** | **G** | **G** | **A** | **C** | **G** | **A** | **A** | **A** | **C** | **G** | **G** | **T** | **C** | **G** | **A** | **G** | **G** | **A** | **A** | **A** | **G** | **A** | **C** | **A** | **A** | **C** | **T** | **T** | **T** | **A** | **C** | **T** | **G** | **A** | **A** | **T** | **A** | **T** | **T** | **C** | **T** | **A** | **C** | **A** | **A** | **A** | **A** | **C** | **A** | **A** | **A** | **C** | **T** | **A** | **C** | **C** | **T** | **T** | **A** | **T** | **C** | **A** | **A** | **G** | **G** | **G** | **C** | **A** | **A** | **G** | **T** | **A** | **A** | **T** | **C** | **C** | **A** | **T** | **C** | **A** | **G** | **C** | **A** | **A** | **G** | **A** | **A** | **T** | **T** | **T** | **G** | **T** | **C** | **T** | **A** | **T** | **T** | **T** | **C** | **C** | **C** | **G** | **C** | **A** | **G** | **C** | **A** | **A** | **A** | **C** | **A** | **A** | **A** | **A** | **G** | **A** | **T** | **A** | **A** | **A** | **G** | **A** | **G** | **C** | **G** | **T** | **T** | **T** | **A** | **A** | **C** | **C** | **T** | **A** | **T** | **T** | **A** | **T** | **G** | **T** | **G** | **T** | **T** | **A** | **A** | **A** | **T** | **G** | **A** | **G** | **A** | **T** | **T** | **A** | **C** | **G** | **G** | **A** | **T** | **T** | **T** | **T** | **G** | **A** | **G** | **A** | **T** | **A** | **T** | **G** | **G** | **G** | **A** | **A** | **A** | **T** | **T** | **G** | **A** | **A** | **A** | **G** | **A** | **G** | **A** | **G** | **C** | **T** | **C** | **C** | **A** | **A** | **C** | **T** | **G** | **A** | **T** | **G** | **C** | **A** | **A** | **A** | **C** | **A** | **G** | **A** | **T** | **C** | **C** | **T** | **G** | **A** | **A** | **A** | **T** | **C** | **T** | **T** | **A** | **T** | **G** | **G** | **A** | **G** | **A** | **G** | **A** | **A** | **T** | **T** | **C** | **A** | **G** | **C** | **A** | **C** | **A** | **C** | **T** | **A** | **T** | **C** | **A** | **G** | **G** | **A** | **G** | **G** | **A** | **G** | **A** | **G** | **A** | **A** | **G** | **A** | **C** | **A** | **A** | **A** | **A** | **G** | **T** | **C** | **C** | **T** | **A** | **C** | **T** | **G** | **G** | **C** | **A** | **C** | **T** | **T** | **T** | **T** | **A** | **T** | **T** | **T** | **G** | **T** | **G** | **G** | **A** | **T** | **G** | **A** | **C** | **A** | **C** | **T** | **C** | **A** | **T** | **T** | **T** | **C** | **C** | **C** | **G** | **T** | **T** | **A** | **A** | **T** | **C** | **G** | **A** | **T** | **G** | **A** | **A** | **C** | **C** | **A** | **A** | **C** | **G** | **A** | **A** | **T** | **C** | **A** | **T** | **T** | **T** | **G** | **G** | **A** | **-** | **-** | **-** | **T** | **A** | **T** | **C** | **T** | **C** | **T** | **G** | **G** | **C** | **A** | **G** | **A** | **A** | **A** | **A** | **C** | **A** | **A** | **G** | **T** | **A** | **G** | **C** | **G** | **G** | **C** | **T** | **T** | **A** | **C** | **T** | **T** | **G** | **A** | **A** | **A** | **A** | **A** | **G** | **A** | **A** | **A** | **A** | **A** | **A** | **C** | **A** | **A** | **G** | **G** | **C** | **T** | **T** | **C** | **A** | **T** | **C** | **G** | **T** | **G** | **G** | **T** | **C** | **A** | **G** | **C** | **C** | **A** | **T** | **G** | **A** | **C** | **C** | **G** | **G** | **G** | **G** | **A** | **T** | **T** | **T** | **A** | **T** | **C** | **G** | **A** | **T** | **G** | **A** | **A** | **G** | **T** | **A** | **G** | **T** | **G** | **G** | **A** | **C** | **C** | **A** | **T** | **G** | **T** | **T** | **T** | **T** | **A** | **G** | **C** | **A** | **A** | **T** | **C** | **G** | **A** | **A** | **A** | **A** | **A** | **A** | **G** | **T** | **C** | **A** | **A** | **C** | **T** | **G** | **G** | **A** | **A** | **C** | **T** | **T** | **T** | **A** | **T** | **C** | **A** | **A** | **G** | **G** | **G** | **A** | **A** | **T** | **T** | **T** | **C** | **T** | **C** | **T** | **A** | **T** | **C** | **T** | **A** | **T** | **G** | **A** | **A** | **G** | **A** | **A** | **C** | **A** | **G** | **A** | **A** | **A** | **A** | **A** | **A** | **C** | **T** | **T** | **C** | **G** | **T** | **G** | **A** | **T** | **G** | **A** | **A** | **T** | **T** | **T** | **G** | **A** | **A** | **A** | **T** | **G** | **G** | **C** | **T** | **C** | **A** | **A** | **A** | **A** | **T** | **G** | **A** | **A** | **A** | **A** | **A** | **T** | **T** | **G** | **A** | **A** | **A** | **A** | **A** | **A** | **G** | **A** | **A** | **G** | **T** | **C** | **A** | **G** | **T** | **A** | **G** | **G** | **C** | **T** | **A** | **A** | **A** | **G** | **A** | **A** | **A** | **A** | **C** | **A** | **G** | **C** | **A** | **G** | **C** | **T** | **G** | **A** | **A** | **A** | **A** | **A** | **G** | **C** | **C** | **G** | **A** | **A** | **T** | **G** | **G** | **T** | **C** | **T** | **C** | **G** | **T** | **T** | **C** | **C** | **C** | **G** | **A** | **G** | **A** | **A** | **G** | **G** | **A** | **G** | **A** | **T** | **A** | **A** | **A** | **A** | **C** | **A** | **A** | **A** | **G** | **A** | **A** | **A** | **C** | **A** | **A** | **G** | **T** | **C** | **G** | **G** | **A** | **T** | **T** | **C** | **A** | **T** | **C** | **G** | **A** | **T** | **A** | **C** | **T** | **G** | **A** | **A** | **T** | **C** | **T** | **A** | **G** | **A** | **C** | **G** | **A** | **G** | **T** | **G** | **A** | **A** | **T** | **A** | **A** | **A** | **G** | **G** | **A** | **G** | **C** | **A** | **G** | **T** | **G** | **G** | **G** | **T** | **G** | **C** | **T** | **G** | **A** | **T** | **G** | **C** | **T** | **G** | **C** | **A** | **C** | **G** | **G** | **A** | **C** | **G** | **A** | **T** | **G** | **A** | **A** | **A** | **C** | **G** | **A** | **T** | **C** | **C** | **A** | **A** | **A** | **G** | **C** | **A** | **A** | **T** | **C** | **G** | **T** | **G** | **A** | **A** | **T** | **C** | **G** | **G** | **A** | **T** | **G** | **G** | **A** | **G** | **A** | **C** | **C** | **C** | **A** | **G** | **A** | **T** | **C** | **A** | **G** | **C** | **G** | **A** | **G** | **A** | **A** | **A** | **G** | **A** | **A** | **A** | **A** | **A** | **C** | **T** | **A** | **T** | **T** | **G** | **A** | **A** | **A** | **G** | **A** | **T** | **A** | **T** | **C** | **G** | **A** | **A** | **T** | **A** | **T** | **A** | **T** | **C** | **G** | **A** | **T** | **T** | **C** | **T** | **T** | **T** | **G** | **A** | **C** | **G** | **A** | **T** | **G** | **A** | **A** | **T** | **A** | **G** | **C** | **C** | **A** | **A** | **G** | **C** | **G** | **T** | **C** | **T** | **C** | **A** | **C** | **C** | **A** | **T** | **A** | **A** | **G** | **C** | **G** | **A** | **C** | **T** | **T** | **T** | **T** | **A** | **A** | **G** | **C** | **G** | **T** | **A** | **G** | **A** | **A** | **G** | **A** | **T** | **C** | **T** | **T** | **C** | **A** | **A** | **T** | **T** | **A** | **G** | **G** | **G** | **T** | **A** | **T** | **G** | **A** | **G** | **A** | **A** | **T** | **C** | **T** | **G** | **T** | **T** | **A** | **T** | **T** | **C** | **G** | **A** | **G** | **C** | **C** | **A** | **A** | **T** | **T** | **C** | **A** | **T** | **T** | **T** | **T** | **A** | **C** | **A** | **A** | **T** | **C** | **G** | **A** | **G** | **C** | **C** | **T** | **C** | **A** | **T** | **C** | **A** | **G** | **C** | **G** | **G** | **G** | **T** | **G** | **G** | **C** | **G** | **A** | **T** | **T** | **T** | **C** | **A** | **G** | **G** | **T** | **C** | **C** | **T** | **A** | **A** | **C** | **G** | **G** | **T** | **G** | **C** | **A** | **G** | **G** | **A** | **A** | **A** | **G** | **T** | **C** | **A** | **T** | **C** | **C** | **A** | **T** | **T** | **A** | **T** | **C** | **C** | **A** | **T** | **T** | **A** | **T** | **C** | **T** | **T** | **C** | **T** | **G** | **G** | **G** | **G** | **G** | **C** | **A** | **T** | **T** | **C** | **A** | **A** | **C** | **G** | **G** | **C** | **A** | **A** | **G** | **G** | **T** | **T** | **A** | **T** | **A** | **G** | **G** | **A** | **G** | **A** | **A** | **A** | **A** | **A** | **T** | **C** | **C** | **C** | **A** | **G** | **C** | **C** | **A** | **A** | **A** | **A** | **C** | **A** | **T** | **C** | **T** | **G** | **A** | **G** | **C** | **A** | **T** | **T** | **A** | **G** | **T** | **T** | **A** | **T** | **G** | **T** | **A** | **C** | **G** | **C** | **C** | **A** | **A** | **A** | **A** | **T** | **T** | **A** | **T** | **G** | **A** | **A** | **G** | **A** | **C** | **A** | **A** | **T** | **C** | **G** | **A** | **G** | **G** | **A** | **A** | **C** | **G** | **T** | **T** | **G** | **G** | **C** | **G** | **G** | **A** | **A** | **T** | **T** | **T** | **G** | **C** | **A** | **G** | **A** | **G** | **A** | **A** | **A** | **A** | **A** | **C** | **C** | **A** | **A** | **G** | **T** | **A** | **G** | **A** | **C** | **T** | **A** | **C** | **C** | **A** | **A** | **G** | **C** | **A** | **T** | **T** | **T** | **T** | **T** | **G** | **A** | **A** | **C** | **A** | **A** | **C** | **C** | **T** | **C** | **C** | **G** | **A** | **A** | **A** | **G** | **C** | **T** | **T** | **G** | **G** | **G** | **A** | **T** | **G** | **G** | **A** | **A** | **A** | **G** | **A** | **G** | **A** | **T** | **G** | **T** | **T** | **T** | **T** | **T** | **C** | **A** | **T** | **A** | **A** | **C** | **A** | **A** | **G** | **A** | **T** | **C** | **G** | **A** | **G** | **C** | **A** | **G** | **A** | **T** | **G** | **A** | **G** | **T** | **A** | **T** | **G** | **G** | **G** | **C** | **C** | **A** | **A** | **C** | **G** | **G** | **A** | **A** | **A** | **A** | **A** | **A** | **G** | **T** | **G** | **G** | **A** | **A** | **T** | **T** | **G** | **G** | **C** | **T** | **A** | **A** | **A** | **T** | **C** | **T** | **T** | **T** | **A** | **T** | **C** | **A** | **C** | **A** | **G** | **C** | **C** | **A** | **G** | **C** | **T** | **G** | **A** | **A** | **C** | **T** | **A** | **T** | **A** | **T** | **A** | **T** | **A** | **T** | **G** | **G** | **G** | **A** | **T** | **G** | **A** | **A** | **C** | **C** | **A** | **T** | **T** | **G** | **A** | **A** | **T** | **T** | **A** | **T** | **T** | **T** | **G** | **G** | **A** | **T** | **G** | **T** | **C** | **T** | **T** | **C** | **A** | **A** | **T** | **C** | **A** | **A** | **G** | **A** | **A** | **C** | **A** | **A** | **T** | **T** | **A** | **G** | **A** | **A** | **C** | **A** | **A** | **C** | **T** | **G** | **A** | **T** | **C** | **T** | **T** | **G** | **A** | **A** | **C** | **G** | **T** | **G** | **A** | **A** | **A** | **C** | **C** | **T** | **G** | **C** | **C** | **A** | **T** | **G** | **T** | **T** | **A** | **C** | **T** | **A** | **G** | **T** | **G** | **G** | **A** | **A** | **C** | **A** | **T** | **G** | **A** | **T** | **C** | **A** | **A** | **A** | **C** | **C** | **T** | **T** | **C** | **C** | **T** | **G** | **G** | **A** | **T** | **A** | **A** | **A** | **G** | **T** | **A** | **T** | **C** | **T** | **A** | **C** | **T** | **G** | **A** | **G** | **A** | **T** | **T** | **A** | **T** | **T** | **T** | **C** | **T** | **C** | **T** | **T** | **G** | **A** | **G** | **A** | **A** | **A** | **A** | **T** | **C** | **-** | **-** | **-** |
| **N59653F** | **A** | **T** | **G** | **T** | **C** | **T** | **A** | **A** | **A** | **A** | **T** | **C** | **G** | **A** | **A** | **A** | **T** | **A** | **A** | **A** | **A** | **A** | **A** | **T** | **C** | **T** | **G** | **A** | **C** | **A** | **T** | **T** | **C** | **G** | **G** | **C** | **T** | **A** | **C** | **G** | **A** | **C** | **A** | **G** | **C** | **C** | **A** | **A** | **G** | **G** | **C** | **A** | **C** | **A** | **T** | **T** | **A** | **T** | **T** | **A** | **T** | **T** | **T** | **G** | **A** | **A** | **C** | **A** | **A** | **G** | **C** | **A** | **A** | **A** | **T** | **C** | **T** | **A** | **A** | **A** | **T** | **T** | **T** | **T** | **G** | **A** | **C** | **A** | **C** | **A** | **C** | **A** | **A** | **T** | **G** | **G** | **A** | **A** | **A** | **C** | **T** | **A** | **G** | **G** | **A** | **C** | **T** | **T** | **A** | **T** | **C** | **G** | **G** | **A** | **C** | **G** | **A** | **A** | **A** | **C** | **G** | **G** | **T** | **C** | **G** | **A** | **G** | **G** | **A** | **A** | **A** | **G** | **A** | **C** | **A** | **A** | **C** | **T** | **T** | **T** | **A** | **C** | **T** | **G** | **A** | **A** | **T** | **A** | **T** | **T** | **C** | **T** | **A** | **C** | **A** | **A** | **A** | **A** | **C** | **A** | **A** | **A** | **C** | **T** | **A** | **C** | **C** | **T** | **T** | **A** | **T** | **C** | **A** | **A** | **G** | **G** | **G** | **C** | **A** | **A** | **G** | **T** | **A** | **A** | **T** | **C** | **C** | **A** | **T** | **C** | **A** | **G** | **C** | **A** | **A** | **G** | **A** | **A** | **T** | **T** | **T** | **G** | **T** | **C** | **T** | **A** | **T** | **T** | **T** | **C** | **C** | **C** | **G** | **C** | **A** | **A** | **C** | **A** | **G** | **A** | **C** | **A** | **A** | **A** | **A** | **G** | **A** | **T** | **A** | **A** | **A** | **G** | **A** | **A** | **C** | **G** | **T** | **T** | **T** | **A** | **A** | **C** | **C** | **T** | **A** | **T** | **T** | **A** | **C** | **G** | **T** | **G** | **T** | **T** | **A** | **A** | **A** | **T** | **G** | **A** | **T** | **A** | **T** | **T** | **A** | **C** | **G** | **G** | **A** | **T** | **T** | **T** | **T** | **G** | **A** | **G** | **A** | **T** | **A** | **T** | **G** | **G** | **G** | **A** | **A** | **A** | **T** | **C** | **G** | **A** | **A** | **A** | **G** | **A** | **G** | **A** | **G** | **C** | **T** | **C** | **C** | **A** | **A** | **T** | **T** | **G** | **A** | **T** | **G** | **C** | **A** | **A** | **A** | **C** | **A** | **G** | **A** | **T** | **C** | **C** | **T** | **G** | **A** | **A** | **A** | **T** | **C** | **T** | **T** | **A** | **T** | **G** | **G** | **A** | **G** | **A** | **G** | **A** | **A** | **T** | **T** | **C** | **A** | **G** | **C** | **A** | **C** | **A** | **C** | **T** | **A** | **T** | **C** | **G** | **G** | **G** | **G** | **G** | **G** | **A** | **G** | **A** | **G** | **A** | **A** | **G** | **A** | **C** | **A** | **A** | **A** | **A** | **G** | **T** | **C** | **C** | **T** | **A** | **C** | **T** | **G** | **G** | **C** | **A** | **C** | **T** | **T** | **T** | **T** | **A** | **T** | **T** | **T** | **G** | **T** | **G** | **G** | **A** | **T** | **G** | **A** | **C** | **A** | **C** | **T** | **C** | **A** | **T** | **T** | **T** | **C** | **C** | **C** | **G** | **T** | **T** | **A** | **A** | **T** | **C** | **G** | **A** | **T** | **G** | **A** | **A** | **C** | **C** | **A** | **A** | **C** | **G** | **A** | **A** | **T** | **C** | **A** | **T** | **T** | **T** | **G** | **G** | **A** | **-** | **-** | **-** | **T** | **A** | **T** | **C** | **T** | **C** | **T** | **G** | **G** | **T** | **A** | **G** | **A** | **A** | **A** | **A** | **C** | **A** | **A** | **G** | **T** | **A** | **G** | **C** | **G** | **G** | **C** | **T** | **T** | **A** | **T** | **T** | **T** | **G** | **A** | **A** | **A** | **A** | **A** | **G** | **A** | **A** | **A** | **A** | **A** | **A** | **C** | **A** | **A** | **G** | **G** | **C** | **T** | **T** | **C** | **A** | **T** | **C** | **G** | **T** | **G** | **G** | **T** | **C** | **A** | **G** | **C** | **C** | **A** | **T** | **G** | **A** | **C** | **C** | **G** | **G** | **G** | **G** | **A** | **T** | **T** | **T** | **A** | **T** | **C** | **G** | **A** | **T** | **G** | **A** | **A** | **G** | **T** | **A** | **G** | **T** | **G** | **G** | **A** | **C** | **C** | **A** | **T** | **G** | **T** | **T** | **T** | **T** | **A** | **G** | **C** | **A** | **A** | **T** | **C** | **G** | **A** | **A** | **A** | **A** | **A** | **A** | **G** | **T** | **C** | **A** | **A** | **C** | **T** | **G** | **G** | **A** | **A** | **C** | **T** | **T** | **T** | **A** | **T** | **C** | **A** | **A** | **G** | **G** | **G** | **A** | **A** | **T** | **T** | **T** | **C** | **T** | **C** | **T** | **A** | **T** | **C** | **T** | **A** | **T** | **G** | **A** | **A** | **G** | **A** | **A** | **C** | **A** | **G** | **A** | **A** | **A** | **A** | **A** | **A** | **C** | **T** | **T** | **C** | **G** | **T** | **G** | **A** | **T** | **G** | **A** | **A** | **T** | **T** | **T** | **G** | **A** | **A** | **A** | **T** | **G** | **G** | **C** | **T** | **C** | **A** | **A** | **A** | **A** | **T** | **G** | **A** | **A** | **A** | **A** | **A** | **T** | **T** | **G** | **A** | **A** | **A** | **A** | **A** | **A** | **G** | **A** | **A** | **G** | **T** | **C** | **A** | **G** | **T** | **A** | **G** | **G** | **C** | **T** | **A** | **A** | **A** | **G** | **A** | **A** | **A** | **A** | **C** | **A** | **G** | **C** | **A** | **G** | **C** | **T** | **G** | **A** | **A** | **A** | **A** | **A** | **G** | **C** | **C** | **G** | **A** | **A** | **T** | **G** | **G** | **T** | **C** | **T** | **C** | **G** | **T** | **T** | **C** | **C** | **C** | **G** | **A** | **G** | **A** | **A** | **G** | **G** | **A** | **G** | **A** | **T** | **A** | **A** | **A** | **A** | **C** | **A** | **A** | **A** | **G** | **A** | **A** | **A** | **C** | **A** | **A** | **G** | **T** | **C** | **G** | **G** | **A** | **T** | **T** | **C** | **A** | **T** | **C** | **G** | **A** | **T** | **A** | **C** | **T** | **G** | **A** | **A** | **T** | **C** | **T** | **A** | **G** | **A** | **C** | **G** | **A** | **G** | **T** | **G** | **A** | **A** | **T** | **A** | **A** | **A** | **G** | **G** | **A** | **G** | **C** | **A** | **G** | **T** | **G** | **G** | **G** | **T** | **G** | **C** | **T** | **G** | **A** | **T** | **G** | **C** | **T** | **G** | **C** | **A** | **C** | **G** | **G** | **A** | **C** | **G** | **A** | **T** | **G** | **A** | **A** | **A** | **C** | **G** | **A** | **T** | **C** | **C** | **A** | **A** | **A** | **G** | **C** | **A** | **A** | **T** | **C** | **G** | **T** | **G** | **A** | **A** | **T** | **C** | **G** | **G** | **A** | **T** | **G** | **G** | **A** | **G** | **A** | **C** | **C** | **C** | **A** | **G** | **A** | **T** | **C** | **A** | **G** | **C** | **G** | **A** | **G** | **A** | **A** | **A** | **G** | **A** | **A** | **A** | **A** | **A** | **T** | **T** | **A** | **T** | **T** | **A** | **A** | **A** | **A** | **G** | **A** | **T** | **A** | **T** | **C** | **G** | **A** | **A** | **T** | **A** | **T** | **A** | **T** | **C** | **G** | **A** | **T** | **T** | **C** | **T** | **T** | **T** | **G** | **A** | **C** | **G** | **A** | **T** | **G** | **A** | **A** | **T** | **A** | **G** | **C** | **C** | **A** | **A** | **G** | **C** | **G** | **T** | **C** | **T** | **C** | **A** | **C** | **C** | **A** | **T** | **A** | **A** | **G** | **C** | **G** | **A** | **C** | **T** | **T** | **T** | **T** | **A** | **A** | **G** | **C** | **G** | **T** | **A** | **G** | **A** | **A** | **G** | **A** | **T** | **C** | **T** | **T** | **C** | **A** | **A** | **T** | **T** | **A** | **G** | **G** | **G** | **T** | **A** | **T** | **G** | **A** | **A** | **A** | **A** | **T** | **C** | **T** | **G** | **T** | **T** | **A** | **T** | **T** | **C** | **G** | **A** | **G** | **C** | **C** | **A** | **A** | **T** | **T** | **C** | **A** | **T** | **T** | **T** | **T** | **A** | **C** | **A** | **A** | **T** | **C** | **G** | **A** | **G** | **C** | **C** | **T** | **C** | **A** | **T** | **C** | **A** | **G** | **C** | **G** | **G** | **G** | **T** | **G** | **G** | **C** | **G** | **A** | **T** | **T** | **T** | **C** | **A** | **G** | **G** | **T** | **C** | **C** | **T** | **A** | **A** | **C** | **G** | **G** | **T** | **G** | **C** | **A** | **G** | **G** | **A** | **A** | **A** | **G** | **T** | **C** | **A** | **T** | **C** | **C** | **A** | **T** | **T** | **A** | **T** | **C** | **C** | **A** | **T** | **T** | **A** | **T** | **C** | **T** | **T** | **C** | **T** | **G** | **G** | **G** | **G** | **G** | **C** | **A** | **T** | **T** | **C** | **A** | **A** | **C** | **G** | **G** | **C** | **A** | **A** | **G** | **G** | **T** | **T** | **A** | **T** | **A** | **G** | **G** | **A** | **G** | **A** | **A** | **A** | **A** | **A** | **T** | **C** | **C** | **C** | **A** | **G** | **C** | **C** | **A** | **A** | **A** | **A** | **C** | **A** | **T** | **C** | **T** | **T** | **A** | **G** | **C** | **A** | **T** | **T** | **A** | **G** | **T** | **T** | **A** | **T** | **G** | **T** | **A** | **C** | **G** | **C** | **C** | **A** | **A** | **A** | **A** | **T** | **T** | **A** | **T** | **G** | **A** | **A** | **G** | **A** | **C** | **A** | **A** | **T** | **C** | **G** | **A** | **G** | **G** | **A** | **A** | **C** | **G** | **T** | **T** | **G** | **G** | **C** | **G** | **G** | **A** | **A** | **T** | **T** | **T** | **G** | **C** | **A** | **G** | **A** | **G** | **A** | **A** | **A** | **A** | **A** | **C** | **C** | **A** | **A** | **G** | **T** | **A** | **G** | **A** | **C** | **T** | **A** | **C** | **C** | **A** | **A** | **G** | **C** | **A** | **T** | **T** | **T** | **T** | **T** | **G** | **A** | **A** | **C** | **A** | **A** | **C** | **C** | **T** | **C** | **C** | **G** | **A** | **A** | **A** | **G** | **C** | **T** | **T** | **G** | **G** | **G** | **A** | **T** | **G** | **G** | **A** | **A** | **A** | **G** | **A** | **G** | **A** | **T** | **G** | **T** | **T** | **T** | **T** | **T** | **C** | **A** | **T** | **A** | **A** | **C** | **A** | **A** | **G** | **A** | **T** | **C** | **G** | **A** | **G** | **C** | **A** | **G** | **A** | **T** | **G** | **A** | **G** | **T** | **A** | **T** | **G** | **G** | **G** | **C** | **C** | **A** | **A** | **C** | **G** | **G** | **A** | **A** | **A** | **A** | **A** | **A** | **G** | **T** | **G** | **G** | **A** | **A** | **T** | **T** | **G** | **G** | **C** | **T** | **A** | **A** | **A** | **T** | **C** | **T** | **T** | **T** | **A** | **T** | **C** | **A** | **C** | **A** | **G** | **C** | **C** | **A** | **G** | **C** | **T** | **G** | **A** | **A** | **C** | **T** | **A** | **T** | **A** | **T** | **A** | **T** | **A** | **T** | **G** | **G** | **G** | **A** | **T** | **G** | **A** | **A** | **C** | **C** | **A** | **T** | **T** | **G** | **A** | **A** | **T** | **T** | **A** | **T** | **T** | **T** | **G** | **G** | **A** | **T** | **G** | **T** | **C** | **T** | **T** | **C** | **A** | **A** | **T** | **C** | **A** | **A** | **G** | **A** | **A** | **C** | **A** | **A** | **T** | **T** | **A** | **G** | **A** | **A** | **C** | **A** | **A** | **C** | **T** | **G** | **A** | **T** | **C** | **T** | **T** | **G** | **A** | **A** | **C** | **G** | **T** | **G** | **A** | **A** | **A** | **C** | **C** | **T** | **G** | **C** | **C** | **A** | **T** | **G** | **T** | **T** | **A** | **C** | **T** | **A** | **G** | **T** | **G** | **G** | **A** | **A** | **C** | **A** | **T** | **G** | **A** | **T** | **C** | **A** | **A** | **A** | **C** | **C** | **T** | **T** | **C** | **C** | **T** | **G** | **G** | **A** | **T** | **A** | **A** | **A** | **G** | **T** | **A** | **T** | **C** | **T** | **A** | **C** | **T** | **G** | **A** | **G** | **A** | **T** | **T** | **A** | **T** | **T** | **T** | **C** | **T** | **C** | **T** | **T** | **G** | **A** | **G** | **A** | **A** | **A** | **A** | **T** | **C** | **-** | **-** | **-** |
| **N59891F** | **A** | **T** | **G** | **T** | **C** | **T** | **A** | **A** | **A** | **A** | **T** | **C** | **G** | **A** | **A** | **A** | **T** | **A** | **A** | **A** | **A** | **A** | **A** | **T** | **C** | **T** | **G** | **A** | **C** | **A** | **T** | **T** | **C** | **G** | **G** | **C** | **T** | **A** | **C** | **G** | **A** | **C** | **A** | **G** | **C** | **C** | **A** | **A** | **G** | **G** | **C** | **A** | **C** | **A** | **T** | **T** | **A** | **T** | **T** | **A** | **T** | **T** | **T** | **G** | **A** | **A** | **C** | **A** | **A** | **G** | **C** | **A** | **A** | **A** | **T** | **C** | **T** | **A** | **A** | **A** | **T** | **T** | **T** | **T** | **G** | **A** | **C** | **A** | **C** | **A** | **C** | **A** | **A** | **T** | **G** | **G** | **A** | **A** | **A** | **C** | **T** | **A** | **G** | **G** | **A** | **C** | **T** | **T** | **A** | **T** | **C** | **G** | **G** | **A** | **C** | **G** | **A** | **A** | **A** | **C** | **G** | **G** | **T** | **C** | **G** | **A** | **G** | **G** | **A** | **A** | **A** | **G** | **A** | **C** | **A** | **A** | **C** | **T** | **T** | **T** | **A** | **C** | **T** | **G** | **A** | **A** | **T** | **A** | **T** | **T** | **C** | **T** | **A** | **C** | **A** | **A** | **A** | **A** | **C** | **A** | **A** | **A** | **C** | **T** | **A** | **C** | **C** | **T** | **T** | **A** | **T** | **C** | **A** | **A** | **G** | **G** | **G** | **C** | **A** | **A** | **G** | **T** | **A** | **A** | **T** | **C** | **C** | **A** | **T** | **C** | **A** | **G** | **C** | **A** | **A** | **G** | **A** | **A** | **T** | **T** | **T** | **G** | **C** | **C** | **T** | **A** | **T** | **T** | **T** | **C** | **C** | **C** | **G** | **C** | **A** | **A** | **C** | **A** | **G** | **A** | **C** | **A** | **A** | **A** | **A** | **G** | **A** | **T** | **A** | **A** | **A** | **G** | **A** | **A** | **C** | **G** | **T** | **T** | **T** | **A** | **A** | **C** | **C** | **T** | **A** | **T** | **T** | **A** | **C** | **G** | **T** | **G** | **T** | **T** | **A** | **A** | **A** | **T** | **G** | **A** | **T** | **A** | **T** | **T** | **A** | **C** | **G** | **G** | **A** | **T** | **T** | **T** | **T** | **G** | **A** | **G** | **A** | **T** | **A** | **T** | **G** | **G** | **G** | **A** | **A** | **A** | **T** | **C** | **G** | **A** | **A** | **A** | **G** | **A** | **G** | **A** | **G** | **C** | **T** | **C** | **C** | **A** | **A** | **T** | **T** | **G** | **A** | **T** | **G** | **C** | **A** | **A** | **A** | **C** | **A** | **G** | **A** | **T** | **C** | **C** | **T** | **G** | **A** | **A** | **A** | **T** | **C** | **T** | **T** | **A** | **T** | **G** | **G** | **A** | **G** | **A** | **G** | **A** | **A** | **T** | **T** | **C** | **A** | **G** | **C** | **A** | **C** | **A** | **C** | **T** | **A** | **T** | **C** | **G** | **G** | **G** | **G** | **G** | **G** | **A** | **G** | **A** | **G** | **A** | **A** | **G** | **A** | **C** | **A** | **A** | **A** | **A** | **G** | **T** | **C** | **C** | **T** | **A** | **C** | **T** | **G** | **G** | **C** | **A** | **C** | **T** | **T** | **T** | **T** | **A** | **T** | **T** | **T** | **G** | **T** | **G** | **G** | **A** | **T** | **G** | **A** | **C** | **A** | **C** | **T** | **C** | **A** | **T** | **T** | **T** | **C** | **C** | **C** | **G** | **T** | **T** | **A** | **A** | **T** | **C** | **G** | **A** | **T** | **G** | **A** | **A** | **C** | **C** | **A** | **A** | **C** | **G** | **A** | **A** | **T** | **C** | **A** | **T** | **T** | **T** | **G** | **G** | **A** | **-** | **-** | **-** | **T** | **A** | **T** | **C** | **T** | **C** | **T** | **G** | **G** | **T** | **A** | **G** | **A** | **A** | **A** | **A** | **C** | **A** | **A** | **G** | **T** | **A** | **G** | **C** | **G** | **G** | **C** | **T** | **T** | **A** | **T** | **T** | **T** | **G** | **A** | **A** | **A** | **A** | **A** | **G** | **A** | **A** | **A** | **A** | **A** | **A** | **C** | **A** | **A** | **G** | **G** | **C** | **T** | **T** | **C** | **A** | **T** | **C** | **G** | **T** | **G** | **G** | **T** | **C** | **A** | **G** | **C** | **C** | **A** | **T** | **G** | **A** | **C** | **C** | **G** | **G** | **G** | **G** | **A** | **T** | **T** | **T** | **A** | **T** | **C** | **G** | **A** | **T** | **G** | **A** | **A** | **G** | **T** | **A** | **G** | **T** | **G** | **G** | **A** | **C** | **C** | **A** | **T** | **G** | **T** | **T** | **T** | **T** | **A** | **G** | **C** | **A** | **A** | **T** | **C** | **G** | **A** | **A** | **A** | **A** | **A** | **A** | **G** | **T** | **C** | **A** | **A** | **C** | **T** | **G** | **G** | **A** | **A** | **C** | **T** | **T** | **T** | **A** | **T** | **C** | **A** | **A** | **G** | **G** | **G** | **A** | **A** | **T** | **T** | **T** | **C** | **T** | **C** | **T** | **A** | **T** | **C** | **T** | **A** | **T** | **G** | **A** | **A** | **G** | **A** | **A** | **C** | **A** | **T** | **A** | **A** | **A** | **A** | **A** | **A** | **C** | **T** | **T** | **C** | **G** | **T** | **G** | **A** | **T** | **G** | **A** | **A** | **T** | **T** | **T** | **G** | **A** | **A** | **A** | **T** | **G** | **G** | **C** | **T** | **C** | **A** | **A** | **A** | **A** | **T** | **G** | **A** | **A** | **A** | **A** | **A** | **T** | **T** | **G** | **A** | **A** | **A** | **A** | **A** | **A** | **G** | **A** | **A** | **G** | **T** | **C** | **A** | **G** | **T** | **A** | **G** | **G** | **C** | **T** | **A** | **A** | **A** | **G** | **A** | **A** | **A** | **A** | **C** | **A** | **G** | **C** | **A** | **G** | **C** | **T** | **G** | **A** | **A** | **A** | **A** | **A** | **G** | **C** | **C** | **G** | **A** | **A** | **T** | **G** | **G** | **T** | **C** | **T** | **C** | **G** | **T** | **T** | **C** | **C** | **C** | **G** | **A** | **G** | **A** | **A** | **G** | **G** | **A** | **G** | **A** | **T** | **A** | **A** | **A** | **A** | **C** | **A** | **A** | **A** | **G** | **A** | **A** | **A** | **C** | **A** | **A** | **G** | **T** | **C** | **G** | **G** | **A** | **T** | **T** | **C** | **A** | **T** | **C** | **G** | **A** | **T** | **A** | **C** | **T** | **G** | **A** | **A** | **T** | **C** | **T** | **A** | **G** | **A** | **C** | **G** | **A** | **G** | **T** | **G** | **A** | **A** | **T** | **A** | **A** | **A** | **G** | **G** | **A** | **G** | **C** | **A** | **G** | **T** | **G** | **G** | **G** | **T** | **G** | **C** | **T** | **G** | **A** | **T** | **G** | **C** | **T** | **G** | **C** | **A** | **C** | **G** | **G** | **A** | **C** | **G** | **A** | **T** | **G** | **A** | **A** | **A** | **C** | **G** | **A** | **T** | **C** | **C** | **A** | **A** | **A** | **G** | **C** | **A** | **A** | **T** | **C** | **G** | **T** | **G** | **A** | **A** | **T** | **C** | **G** | **G** | **A** | **T** | **G** | **G** | **A** | **G** | **A** | **C** | **C** | **C** | **A** | **G** | **A** | **T** | **C** | **A** | **G** | **C** | **G** | **A** | **G** | **A** | **A** | **A** | **G** | **A** | **A** | **A** | **A** | **A** | **C** | **T** | **A** | **T** | **T** | **A** | **A** | **A** | **A** | **G** | **A** | **T** | **A** | **T** | **C** | **G** | **A** | **A** | **T** | **A** | **T** | **A** | **T** | **C** | **G** | **A** | **T** | **T** | **C** | **T** | **T** | **T** | **G** | **A** | **C** | **G** | **A** | **T** | **G** | **A** | **A** | **T** | **A** | **G** | **C** | **C** | **A** | **A** | **G** | **C** | **G** | **T** | **C** | **T** | **C** | **A** | **C** | **C** | **A** | **T** | **A** | **A** | **G** | **C** | **G** | **A** | **C** | **T** | **T** | **T** | **T** | **A** | **A** | **G** | **C** | **G** | **T** | **A** | **G** | **A** | **A** | **G** | **A** | **T** | **C** | **T** | **T** | **C** | **A** | **A** | **T** | **T** | **A** | **G** | **G** | **G** | **T** | **A** | **T** | **G** | **A** | **A** | **A** | **A** | **T** | **C** | **T** | **G** | **T** | **T** | **A** | **T** | **T** | **C** | **G** | **A** | **G** | **C** | **C** | **A** | **A** | **T** | **T** | **C** | **A** | **T** | **T** | **T** | **T** | **A** | **C** | **A** | **A** | **T** | **C** | **G** | **A** | **G** | **C** | **C** | **T** | **C** | **A** | **T** | **C** | **A** | **G** | **C** | **G** | **G** | **G** | **T** | **G** | **G** | **C** | **G** | **A** | **T** | **T** | **T** | **C** | **A** | **G** | **G** | **T** | **C** | **C** | **T** | **A** | **A** | **C** | **G** | **G** | **T** | **G** | **C** | **A** | **G** | **G** | **A** | **A** | **A** | **G** | **T** | **C** | **A** | **T** | **C** | **C** | **A** | **T** | **T** | **A** | **T** | **C** | **C** | **A** | **T** | **T** | **A** | **T** | **C** | **T** | **T** | **C** | **T** | **G** | **G** | **G** | **G** | **G** | **C** | **A** | **T** | **T** | **C** | **A** | **A** | **C** | **G** | **G** | **C** | **A** | **A** | **G** | **G** | **T** | **T** | **A** | **T** | **A** | **G** | **G** | **A** | **G** | **A** | **A** | **A** | **A** | **A** | **T** | **A** | **C** | **C** | **A** | **G** | **C** | **C** | **A** | **A** | **A** | **A** | **C** | **A** | **T** | **C** | **T** | **G** | **A** | **G** | **C** | **A** | **T** | **T** | **A** | **G** | **T** | **T** | **A** | **T** | **G** | **C** | **A** | **A** | **G** | **C** | **C** | **A** | **A** | **A** | **A** | **T** | **T** | **A** | **T** | **G** | **A** | **A** | **G** | **A** | **C** | **A** | **A** | **T** | **C** | **G** | **A** | **G** | **G** | **A** | **A** | **C** | **G** | **T** | **T** | **G** | **G** | **C** | **G** | **G** | **A** | **A** | **T** | **T** | **T** | **G** | **C** | **A** | **G** | **A** | **G** | **A** | **A** | **A** | **A** | **A** | **C** | **C** | **A** | **A** | **G** | **T** | **A** | **G** | **A** | **C** | **T** | **A** | **C** | **C** | **A** | **A** | **G** | **C** | **A** | **T** | **T** | **T** | **T** | **T** | **G** | **A** | **A** | **C** | **A** | **A** | **C** | **C** | **T** | **C** | **C** | **G** | **A** | **A** | **A** | **G** | **C** | **T** | **T** | **G** | **G** | **G** | **A** | **T** | **G** | **G** | **A** | **A** | **A** | **G** | **A** | **G** | **A** | **T** | **G** | **T** | **T** | **T** | **T** | **T** | **C** | **A** | **T** | **A** | **A** | **C** | **A** | **A** | **G** | **A** | **T** | **C** | **G** | **A** | **G** | **C** | **A** | **G** | **A** | **T** | **G** | **A** | **G** | **T** | **A** | **T** | **G** | **G** | **G** | **C** | **C** | **A** | **A** | **C** | **G** | **G** | **A** | **A** | **A** | **A** | **A** | **A** | **G** | **T** | **G** | **G** | **A** | **A** | **T** | **T** | **G** | **G** | **C** | **T** | **A** | **A** | **A** | **T** | **C** | **T** | **T** | **T** | **A** | **T** | **C** | **A** | **C** | **A** | **G** | **C** | **C** | **A** | **G** | **C** | **T** | **G** | **A** | **A** | **C** | **T** | **A** | **T** | **A** | **T** | **A** | **C** | **A** | **T** | **G** | **G** | **G** | **A** | **T** | **G** | **A** | **A** | **C** | **C** | **A** | **T** | **T** | **G** | **A** | **A** | **T** | **T** | **A** | **T** | **T** | **T** | **G** | **G** | **A** | **T** | **G** | **T** | **C** | **T** | **T** | **C** | **A** | **A** | **T** | **C** | **A** | **A** | **G** | **A** | **A** | **C** | **A** | **A** | **T** | **T** | **A** | **G** | **A** | **A** | **C** | **A** | **A** | **C** | **T** | **G** | **A** | **T** | **C** | **T** | **T** | **G** | **A** | **A** | **C** | **G** | **T** | **G** | **A** | **A** | **A** | **C** | **C** | **T** | **G** | **C** | **C** | **A** | **T** | **G** | **T** | **T** | **A** | **C** | **T** | **A** | **G** | **T** | **G** | **G** | **A** | **A** | **C** | **A** | **T** | **G** | **A** | **T** | **C** | **A** | **A** | **A** | **C** | **C** | **T** | **T** | **C** | **C** | **T** | **G** | **G** | **A** | **T** | **A** | **A** | **A** | **G** | **T** | **A** | **T** | **C** | **T** | **A** | **C** | **T** | **G** | **A** | **G** | **A** | **T** | **T** | **A** | **T** | **T** | **T** | **C** | **T** | **C** | **T** | **T** | **G** | **A** | **G** | **A** | **G** | **A** | **A** | **T** | **C** | **-** | **-** | **-** |
| **N54567** | **A** | **T** | **G** | **T** | **C** | **T** | **A** | **A** | **A** | **A** | **T** | **C** | **G** | **A** | **A** | **A** | **T** | **A** | **A** | **A** | **A** | **A** | **A** | **T** | **C** | **T** | **G** | **A** | **C** | **A** | **T** | **T** | **C** | **G** | **G** | **C** | **T** | **A** | **C** | **G** | **A** | **C** | **A** | **G** | **C** | **C** | **A** | **A** | **G** | **G** | **C** | **A** | **C** | **A** | **T** | **T** | **A** | **T** | **T** | **A** | **T** | **T** | **T** | **G** | **A** | **A** | **C** | **A** | **A** | **G** | **C** | **A** | **A** | **A** | **T** | **C** | **T** | **A** | **A** | **A** | **T** | **T** | **T** | **T** | **G** | **A** | **C** | **A** | **C** | **A** | **C** | **A** | **A** | **T** | **G** | **G** | **A** | **A** | **A** | **C** | **T** | **A** | **G** | **G** | **A** | **C** | **T** | **T** | **A** | **T** | **C** | **G** | **G** | **A** | **C** | **G** | **A** | **A** | **A** | **C** | **G** | **G** | **T** | **C** | **G** | **A** | **G** | **G** | **A** | **A** | **A** | **G** | **A** | **C** | **A** | **A** | **C** | **T** | **T** | **T** | **A** | **C** | **T** | **G** | **A** | **A** | **T** | **A** | **T** | **T** | **C** | **T** | **A** | **C** | **A** | **A** | **A** | **A** | **C** | **A** | **A** | **A** | **C** | **T** | **A** | **C** | **C** | **T** | **T** | **A** | **T** | **C** | **A** | **A** | **G** | **G** | **G** | **C** | **A** | **A** | **G** | **T** | **A** | **A** | **T** | **C** | **C** | **A** | **T** | **C** | **A** | **G** | **C** | **A** | **A** | **G** | **A** | **A** | **T** | **T** | **T** | **G** | **T** | **C** | **T** | **A** | **T** | **T** | **T** | **C** | **C** | **C** | **G** | **C** | **A** | **A** | **C** | **A** | **G** | **A** | **C** | **A** | **A** | **A** | **A** | **G** | **A** | **T** | **A** | **A** | **A** | **G** | **A** | **A** | **C** | **G** | **T** | **T** | **T** | **A** | **A** | **C** | **C** | **T** | **A** | **T** | **T** | **A** | **C** | **G** | **T** | **G** | **T** | **T** | **A** | **A** | **A** | **T** | **G** | **A** | **T** | **A** | **T** | **T** | **A** | **C** | **G** | **G** | **A** | **T** | **T** | **T** | **T** | **G** | **A** | **G** | **A** | **T** | **A** | **T** | **G** | **G** | **G** | **A** | **A** | **A** | **T** | **C** | **G** | **A** | **A** | **A** | **G** | **A** | **G** | **A** | **G** | **C** | **T** | **C** | **C** | **A** | **A** | **T** | **T** | **G** | **A** | **T** | **G** | **C** | **A** | **A** | **A** | **C** | **A** | **G** | **A** | **T** | **C** | **C** | **T** | **G** | **A** | **A** | **A** | **T** | **C** | **T** | **T** | **A** | **T** | **G** | **G** | **A** | **G** | **A** | **G** | **A** | **A** | **T** | **T** | **C** | **A** | **G** | **C** | **A** | **C** | **A** | **C** | **T** | **A** | **T** | **C** | **A** | **G** | **G** | **G** | **G** | **G** | **A** | **G** | **A** | **G** | **A** | **A** | **G** | **A** | **C** | **A** | **A** | **A** | **A** | **G** | **T** | **C** | **C** | **T** | **A** | **C** | **T** | **G** | **G** | **C** | **A** | **C** | **T** | **T** | **T** | **T** | **A** | **T** | **T** | **T** | **G** | **T** | **G** | **G** | **A** | **T** | **G** | **A** | **C** | **A** | **C** | **T** | **C** | **A** | **T** | **T** | **T** | **C** | **C** | **C** | **G** | **T** | **T** | **A** | **A** | **T** | **C** | **G** | **A** | **T** | **G** | **A** | **A** | **C** | **C** | **A** | **A** | **C** | **G** | **A** | **A** | **T** | **C** | **A** | **T** | **T** | **T** | **G** | **G** | **A** | **-** | **-** | **-** | **T** | **A** | **T** | **C** | **T** | **C** | **T** | **G** | **G** | **T** | **A** | **G** | **A** | **A** | **A** | **A** | **C** | **A** | **A** | **G** | **T** | **A** | **G** | **C** | **G** | **G** | **C** | **T** | **T** | **A** | **T** | **T** | **T** | **G** | **A** | **A** | **A** | **A** | **A** | **G** | **A** | **A** | **A** | **A** | **A** | **A** | **C** | **A** | **A** | **G** | **G** | **C** | **T** | **T** | **C** | **A** | **T** | **C** | **G** | **T** | **G** | **G** | **T** | **C** | **A** | **G** | **C** | **C** | **A** | **T** | **G** | **A** | **C** | **C** | **G** | **G** | **G** | **G** | **A** | **T** | **T** | **T** | **A** | **T** | **C** | **G** | **A** | **T** | **G** | **A** | **A** | **G** | **T** | **A** | **G** | **T** | **G** | **G** | **A** | **C** | **C** | **A** | **T** | **G** | **T** | **T** | **T** | **T** | **A** | **G** | **C** | **A** | **A** | **T** | **C** | **G** | **A** | **A** | **A** | **A** | **A** | **A** | **G** | **T** | **C** | **A** | **A** | **C** | **T** | **G** | **G** | **A** | **A** | **C** | **T** | **T** | **T** | **A** | **T** | **C** | **A** | **A** | **G** | **G** | **G** | **A** | **A** | **T** | **T** | **T** | **C** | **T** | **C** | **T** | **A** | **T** | **C** | **T** | **A** | **T** | **G** | **A** | **A** | **G** | **A** | **A** | **C** | **A** | **G** | **A** | **A** | **A** | **A** | **A** | **A** | **C** | **T** | **T** | **C** | **G** | **T** | **G** | **A** | **T** | **G** | **A** | **A** | **T** | **T** | **T** | **G** | **A** | **A** | **A** | **T** | **G** | **G** | **C** | **T** | **C** | **A** | **A** | **A** | **A** | **T** | **G** | **A** | **A** | **A** | **A** | **A** | **T** | **T** | **G** | **A** | **A** | **A** | **A** | **A** | **A** | **G** | **A** | **A** | **G** | **T** | **C** | **A** | **G** | **T** | **A** | **G** | **G** | **C** | **T** | **A** | **A** | **A** | **G** | **A** | **A** | **A** | **A** | **C** | **A** | **G** | **C** | **A** | **G** | **C** | **T** | **G** | **A** | **A** | **A** | **A** | **A** | **G** | **C** | **C** | **G** | **A** | **A** | **T** | **G** | **G** | **T** | **C** | **T** | **C** | **G** | **T** | **T** | **C** | **C** | **C** | **G** | **A** | **G** | **A** | **A** | **G** | **G** | **A** | **G** | **A** | **T** | **A** | **A** | **A** | **A** | **C** | **A** | **A** | **A** | **G** | **A** | **A** | **A** | **C** | **A** | **A** | **G** | **T** | **C** | **G** | **G** | **A** | **T** | **T** | **C** | **A** | **T** | **C** | **G** | **A** | **T** | **A** | **C** | **T** | **G** | **A** | **A** | **T** | **C** | **T** | **A** | **G** | **A** | **C** | **G** | **A** | **G** | **T** | **G** | **A** | **A** | **T** | **A** | **A** | **A** | **G** | **G** | **A** | **G** | **C** | **A** | **G** | **T** | **G** | **G** | **G** | **T** | **G** | **C** | **T** | **G** | **A** | **T** | **G** | **C** | **T** | **G** | **C** | **A** | **C** | **G** | **G** | **A** | **C** | **G** | **A** | **T** | **G** | **A** | **A** | **A** | **C** | **G** | **A** | **T** | **C** | **C** | **A** | **A** | **A** | **G** | **C** | **A** | **A** | **T** | **C** | **G** | **T** | **G** | **A** | **A** | **T** | **C** | **G** | **G** | **A** | **T** | **G** | **G** | **A** | **G** | **A** | **C** | **C** | **C** | **A** | **G** | **A** | **T** | **C** | **A** | **G** | **C** | **G** | **A** | **G** | **A** | **A** | **A** | **G** | **A** | **A** | **A** | **A** | **A** | **C** | **T** | **A** | **T** | **T** | **G** | **A** | **A** | **A** | **G** | **A** | **T** | **A** | **T** | **C** | **G** | **A** | **A** | **T** | **A** | **T** | **A** | **T** | **C** | **G** | **A** | **T** | **T** | **C** | **T** | **T** | **T** | **G** | **A** | **C** | **G** | **A** | **T** | **G** | **A** | **A** | **T** | **A** | **G** | **C** | **C** | **A** | **A** | **G** | **C** | **G** | **T** | **C** | **T** | **C** | **A** | **C** | **C** | **A** | **T** | **A** | **A** | **G** | **C** | **G** | **A** | **C** | **T** | **T** | **T** | **T** | **A** | **A** | **G** | **C** | **G** | **T** | **A** | **G** | **A** | **A** | **G** | **A** | **T** | **C** | **T** | **T** | **C** | **A** | **A** | **T** | **T** | **A** | **G** | **G** | **G** | **T** | **A** | **T** | **G** | **A** | **G** | **A** | **A** | **T** | **C** | **T** | **G** | **T** | **T** | **A** | **T** | **T** | **C** | **G** | **A** | **G** | **C** | **C** | **A** | **A** | **T** | **T** | **C** | **A** | **T** | **T** | **T** | **T** | **A** | **C** | **A** | **A** | **T** | **C** | **G** | **A** | **G** | **C** | **C** | **T** | **C** | **A** | **T** | **C** | **A** | **G** | **C** | **G** | **G** | **G** | **T** | **G** | **G** | **C** | **G** | **A** | **T** | **T** | **T** | **C** | **A** | **G** | **G** | **T** | **C** | **C** | **T** | **A** | **A** | **C** | **G** | **G** | **T** | **G** | **C** | **A** | **G** | **G** | **A** | **A** | **A** | **G** | **T** | **C** | **A** | **T** | **C** | **C** | **A** | **T** | **T** | **A** | **T** | **C** | **C** | **A** | **T** | **T** | **A** | **T** | **C** | **T** | **T** | **C** | **T** | **G** | **G** | **G** | **G** | **G** | **C** | **A** | **T** | **T** | **C** | **A** | **A** | **C** | **G** | **G** | **C** | **A** | **A** | **G** | **G** | **T** | **T** | **A** | **T** | **A** | **G** | **G** | **A** | **G** | **A** | **A** | **A** | **A** | **A** | **T** | **C** | **C** | **C** | **A** | **G** | **C** | **C** | **A** | **A** | **A** | **A** | **C** | **A** | **T** | **C** | **T** | **G** | **A** | **G** | **C** | **A** | **T** | **T** | **A** | **G** | **T** | **T** | **A** | **T** | **G** | **T** | **A** | **C** | **G** | **C** | **C** | **A** | **A** | **A** | **A** | **T** | **T** | **A** | **T** | **G** | **A** | **A** | **G** | **A** | **C** | **A** | **A** | **T** | **C** | **G** | **A** | **G** | **G** | **A** | **A** | **C** | **G** | **T** | **T** | **G** | **G** | **C** | **G** | **G** | **A** | **A** | **T** | **T** | **T** | **G** | **C** | **A** | **G** | **A** | **G** | **A** | **A** | **A** | **A** | **A** | **C** | **C** | **A** | **A** | **G** | **T** | **A** | **G** | **A** | **C** | **T** | **A** | **C** | **C** | **A** | **A** | **G** | **C** | **A** | **T** | **T** | **T** | **T** | **T** | **G** | **A** | **A** | **C** | **A** | **A** | **C** | **C** | **T** | **C** | **C** | **G** | **A** | **A** | **A** | **G** | **C** | **T** | **T** | **G** | **G** | **G** | **A** | **T** | **G** | **G** | **A** | **A** | **A** | **G** | **A** | **G** | **A** | **T** | **G** | **T** | **T** | **T** | **T** | **T** | **C** | **A** | **T** | **A** | **A** | **C** | **A** | **A** | **G** | **A** | **T** | **C** | **G** | **A** | **G** | **C** | **A** | **G** | **A** | **T** | **G** | **A** | **G** | **T** | **A** | **T** | **G** | **G** | **G** | **C** | **C** | **A** | **A** | **C** | **G** | **G** | **A** | **A** | **A** | **A** | **A** | **A** | **G** | **T** | **G** | **G** | **A** | **A** | **T** | **T** | **G** | **G** | **C** | **T** | **A** | **A** | **A** | **T** | **C** | **T** | **T** | **T** | **A** | **T** | **C** | **A** | **C** | **A** | **G** | **C** | **C** | **A** | **G** | **C** | **T** | **G** | **A** | **A** | **C** | **T** | **A** | **T** | **A** | **T** | **A** | **T** | **A** | **T** | **G** | **G** | **G** | **A** | **T** | **G** | **A** | **A** | **C** | **C** | **A** | **T** | **T** | **G** | **A** | **A** | **T** | **T** | **A** | **T** | **T** | **T** | **G** | **G** | **A** | **T** | **G** | **T** | **C** | **T** | **T** | **C** | **A** | **A** | **T** | **C** | **A** | **A** | **G** | **A** | **A** | **C** | **A** | **A** | **T** | **T** | **A** | **G** | **A** | **A** | **C** | **A** | **A** | **C** | **T** | **G** | **A** | **T** | **C** | **T** | **T** | **G** | **A** | **A** | **C** | **G** | **T** | **G** | **A** | **A** | **A** | **C** | **C** | **T** | **G** | **C** | **C** | **A** | **T** | **G** | **T** | **T** | **A** | **C** | **T** | **A** | **G** | **T** | **G** | **G** | **A** | **A** | **C** | **A** | **T** | **G** | **A** | **T** | **C** | **A** | **A** | **A** | **C** | **C** | **T** | **T** | **C** | **C** | **T** | **G** | **G** | **A** | **T** | **A** | **A** | **A** | **G** | **T** | **A** | **T** | **C** | **T** | **A** | **C** | **T** | **G** | **A** | **G** | **A** | **T** | **T** | **A** | **T** | **T** | **T** | **C** | **T** | **C** | **T** | **T** | **G** | **A** | **G** | **A** | **A** | **A** | **A** | **T** | **C** | **-** | **-** | **-** |
| **N52777** | **A** | **T** | **G** | **T** | **C** | **T** | **A** | **A** | **A** | **A** | **T** | **C** | **G** | **A** | **A** | **A** | **T** | **A** | **A** | **A** | **A** | **A** | **A** | **T** | **C** | **T** | **G** | **A** | **C** | **A** | **T** | **T** | **C** | **G** | **G** | **C** | **T** | **A** | **C** | **G** | **A** | **C** | **A** | **G** | **C** | **C** | **A** | **A** | **G** | **G** | **C** | **A** | **C** | **A** | **T** | **T** | **A** | **T** | **T** | **A** | **T** | **T** | **T** | **G** | **A** | **A** | **C** | **A** | **A** | **G** | **C** | **A** | **A** | **A** | **T** | **C** | **T** | **A** | **A** | **A** | **T** | **T** | **T** | **T** | **G** | **A** | **C** | **A** | **C** | **A** | **C** | **A** | **A** | **T** | **G** | **G** | **A** | **A** | **A** | **C** | **T** | **A** | **G** | **G** | **A** | **C** | **T** | **T** | **A** | **T** | **C** | **G** | **G** | **A** | **C** | **G** | **A** | **A** | **A** | **C** | **G** | **G** | **T** | **C** | **G** | **A** | **G** | **G** | **A** | **A** | **A** | **G** | **A** | **C** | **A** | **A** | **C** | **T** | **T** | **T** | **A** | **C** | **T** | **G** | **A** | **A** | **T** | **A** | **T** | **T** | **C** | **T** | **A** | **C** | **A** | **A** | **A** | **A** | **C** | **A** | **A** | **A** | **C** | **T** | **A** | **C** | **C** | **T** | **T** | **A** | **T** | **C** | **A** | **A** | **G** | **G** | **G** | **C** | **A** | **A** | **G** | **T** | **A** | **A** | **T** | **C** | **C** | **A** | **T** | **C** | **A** | **G** | **C** | **A** | **A** | **G** | **A** | **A** | **T** | **T** | **T** | **G** | **T** | **C** | **T** | **A** | **T** | **T** | **T** | **C** | **C** | **C** | **G** | **C** | **A** | **A** | **C** | **A** | **G** | **A** | **C** | **A** | **A** | **A** | **A** | **G** | **A** | **T** | **A** | **A** | **A** | **G** | **A** | **A** | **C** | **G** | **T** | **T** | **T** | **A** | **A** | **C** | **C** | **T** | **A** | **T** | **T** | **A** | **C** | **G** | **T** | **G** | **T** | **T** | **A** | **A** | **A** | **T** | **G** | **A** | **T** | **A** | **T** | **T** | **A** | **C** | **G** | **G** | **A** | **T** | **T** | **T** | **T** | **G** | **A** | **G** | **A** | **T** | **A** | **T** | **G** | **G** | **G** | **A** | **A** | **A** | **T** | **C** | **G** | **A** | **A** | **A** | **G** | **A** | **G** | **A** | **G** | **C** | **T** | **C** | **C** | **A** | **A** | **T** | **T** | **G** | **A** | **T** | **G** | **C** | **A** | **A** | **A** | **C** | **A** | **G** | **A** | **T** | **C** | **C** | **T** | **G** | **A** | **A** | **A** | **T** | **C** | **T** | **T** | **A** | **T** | **G** | **G** | **A** | **G** | **A** | **G** | **A** | **A** | **T** | **T** | **C** | **A** | **G** | **C** | **A** | **C** | **A** | **C** | **T** | **A** | **T** | **C** | **G** | **G** | **G** | **G** | **G** | **G** | **A** | **G** | **A** | **G** | **A** | **A** | **G** | **A** | **C** | **A** | **A** | **A** | **A** | **G** | **T** | **C** | **C** | **T** | **A** | **T** | **T** | **G** | **G** | **C** | **A** | **C** | **T** | **T** | **T** | **T** | **A** | **T** | **T** | **T** | **G** | **T** | **G** | **G** | **A** | **T** | **G** | **A** | **C** | **A** | **C** | **T** | **C** | **A** | **T** | **T** | **T** | **C** | **C** | **C** | **G** | **T** | **T** | **A** | **A** | **T** | **C** | **G** | **A** | **T** | **G** | **A** | **A** | **C** | **C** | **A** | **A** | **C** | **G** | **A** | **A** | **T** | **C** | **A** | **T** | **T** | **T** | **G** | **G** | **A** | **-** | **-** | **-** | **T** | **A** | **T** | **C** | **T** | **C** | **T** | **G** | **G** | **T** | **A** | **G** | **A** | **A** | **A** | **A** | **C** | **A** | **A** | **G** | **T** | **A** | **G** | **C** | **G** | **G** | **C** | **T** | **T** | **A** | **T** | **T** | **T** | **G** | **A** | **A** | **A** | **A** | **A** | **G** | **A** | **A** | **A** | **A** | **A** | **A** | **C** | **A** | **A** | **G** | **G** | **C** | **T** | **T** | **C** | **A** | **T** | **C** | **G** | **T** | **G** | **G** | **T** | **C** | **A** | **G** | **C** | **C** | **A** | **T** | **G** | **A** | **C** | **C** | **G** | **G** | **G** | **G** | **A** | **T** | **T** | **T** | **A** | **T** | **C** | **G** | **A** | **T** | **G** | **A** | **A** | **G** | **T** | **A** | **G** | **T** | **G** | **G** | **A** | **C** | **C** | **A** | **T** | **G** | **T** | **T** | **T** | **T** | **A** | **G** | **C** | **A** | **A** | **T** | **C** | **G** | **A** | **A** | **A** | **A** | **A** | **A** | **G** | **T** | **C** | **A** | **A** | **C** | **T** | **G** | **G** | **A** | **A** | **C** | **T** | **T** | **T** | **A** | **T** | **C** | **A** | **A** | **G** | **G** | **G** | **A** | **A** | **T** | **T** | **T** | **C** | **T** | **C** | **T** | **A** | **T** | **C** | **T** | **A** | **T** | **G** | **A** | **A** | **G** | **A** | **A** | **C** | **A** | **G** | **A** | **A** | **A** | **A** | **A** | **A** | **C** | **T** | **T** | **C** | **G** | **T** | **G** | **A** | **T** | **G** | **A** | **A** | **T** | **T** | **T** | **G** | **A** | **A** | **A** | **T** | **G** | **G** | **C** | **T** | **C** | **A** | **A** | **A** | **A** | **T** | **G** | **A** | **A** | **A** | **A** | **A** | **T** | **T** | **G** | **A** | **A** | **A** | **A** | **A** | **A** | **G** | **A** | **A** | **G** | **T** | **C** | **A** | **G** | **T** | **A** | **G** | **G** | **C** | **T** | **A** | **A** | **A** | **G** | **A** | **A** | **A** | **A** | **C** | **A** | **G** | **C** | **A** | **G** | **C** | **T** | **G** | **A** | **A** | **A** | **A** | **A** | **G** | **C** | **C** | **G** | **A** | **A** | **T** | **G** | **G** | **T** | **C** | **T** | **C** | **G** | **T** | **T** | **C** | **C** | **C** | **G** | **A** | **G** | **A** | **A** | **G** | **G** | **A** | **G** | **A** | **T** | **A** | **A** | **A** | **A** | **C** | **A** | **A** | **A** | **G** | **A** | **A** | **A** | **C** | **A** | **A** | **G** | **T** | **C** | **G** | **G** | **A** | **T** | **T** | **C** | **A** | **T** | **C** | **G** | **A** | **T** | **A** | **C** | **T** | **G** | **A** | **A** | **T** | **C** | **T** | **A** | **G** | **A** | **C** | **G** | **A** | **G** | **T** | **G** | **A** | **A** | **T** | **A** | **A** | **A** | **G** | **G** | **A** | **G** | **C** | **A** | **G** | **T** | **G** | **G** | **G** | **T** | **G** | **C** | **T** | **G** | **A** | **T** | **G** | **C** | **T** | **G** | **C** | **A** | **C** | **G** | **G** | **A** | **C** | **G** | **A** | **T** | **G** | **A** | **A** | **A** | **C** | **G** | **A** | **T** | **C** | **C** | **A** | **A** | **A** | **G** | **C** | **A** | **A** | **T** | **C** | **G** | **T** | **G** | **A** | **A** | **T** | **C** | **G** | **G** | **A** | **T** | **G** | **G** | **A** | **G** | **A** | **C** | **C** | **C** | **A** | **G** | **A** | **T** | **C** | **A** | **G** | **C** | **G** | **A** | **G** | **A** | **A** | **A** | **G** | **A** | **A** | **A** | **A** | **A** | **C** | **T** | **A** | **T** | **T** | **A** | **A** | **A** | **A** | **G** | **A** | **T** | **A** | **T** | **C** | **G** | **A** | **A** | **T** | **A** | **T** | **A** | **T** | **C** | **G** | **A** | **T** | **T** | **C** | **T** | **T** | **T** | **G** | **A** | **C** | **G** | **A** | **T** | **G** | **A** | **A** | **T** | **A** | **G** | **C** | **C** | **A** | **A** | **G** | **C** | **G** | **T** | **C** | **T** | **C** | **A** | **C** | **C** | **A** | **T** | **A** | **A** | **G** | **C** | **G** | **A** | **C** | **T** | **T** | **T** | **T** | **A** | **A** | **G** | **C** | **G** | **T** | **A** | **G** | **A** | **A** | **G** | **A** | **T** | **C** | **T** | **T** | **C** | **A** | **A** | **T** | **T** | **A** | **G** | **G** | **G** | **T** | **A** | **T** | **G** | **A** | **A** | **A** | **A** | **T** | **C** | **T** | **G** | **T** | **T** | **A** | **T** | **T** | **C** | **G** | **A** | **G** | **C** | **C** | **A** | **A** | **T** | **T** | **C** | **A** | **T** | **T** | **T** | **T** | **A** | **C** | **A** | **A** | **T** | **C** | **G** | **A** | **G** | **C** | **C** | **T** | **C** | **A** | **T** | **C** | **A** | **G** | **C** | **G** | **G** | **G** | **T** | **G** | **G** | **C** | **G** | **A** | **T** | **T** | **T** | **C** | **A** | **G** | **G** | **T** | **C** | **C** | **T** | **A** | **A** | **C** | **G** | **G** | **T** | **G** | **C** | **A** | **G** | **G** | **A** | **A** | **A** | **G** | **T** | **C** | **A** | **T** | **C** | **C** | **A** | **T** | **T** | **A** | **T** | **C** | **C** | **A** | **T** | **T** | **A** | **T** | **C** | **T** | **T** | **C** | **T** | **G** | **G** | **G** | **G** | **G** | **C** | **A** | **T** | **T** | **C** | **A** | **A** | **C** | **G** | **G** | **C** | **A** | **A** | **G** | **G** | **T** | **T** | **A** | **T** | **A** | **G** | **G** | **A** | **G** | **A** | **A** | **A** | **A** | **A** | **T** | **C** | **C** | **C** | **A** | **G** | **C** | **C** | **A** | **A** | **A** | **A** | **C** | **A** | **T** | **C** | **T** | **G** | **A** | **G** | **C** | **A** | **T** | **T** | **A** | **G** | **T** | **T** | **A** | **T** | **G** | **T** | **A** | **C** | **G** | **C** | **C** | **A** | **A** | **A** | **A** | **T** | **T** | **A** | **T** | **G** | **A** | **A** | **G** | **A** | **C** | **A** | **A** | **T** | **C** | **G** | **A** | **G** | **G** | **A** | **A** | **C** | **G** | **T** | **T** | **G** | **G** | **C** | **G** | **G** | **A** | **A** | **T** | **T** | **T** | **G** | **C** | **A** | **G** | **A** | **G** | **A** | **A** | **A** | **A** | **A** | **C** | **C** | **A** | **A** | **G** | **T** | **A** | **G** | **A** | **C** | **T** | **A** | **C** | **C** | **A** | **A** | **G** | **C** | **A** | **T** | **T** | **T** | **T** | **T** | **G** | **A** | **A** | **C** | **A** | **A** | **C** | **C** | **T** | **C** | **C** | **G** | **A** | **A** | **A** | **G** | **C** | **T** | **T** | **G** | **G** | **G** | **A** | **T** | **G** | **G** | **A** | **A** | **A** | **G** | **A** | **G** | **A** | **T** | **G** | **T** | **T** | **T** | **T** | **T** | **C** | **A** | **T** | **A** | **A** | **C** | **A** | **A** | **G** | **A** | **T** | **T** | **G** | **A** | **G** | **C** | **A** | **G** | **A** | **T** | **G** | **A** | **G** | **T** | **A** | **T** | **G** | **G** | **G** | **C** | **C** | **A** | **A** | **C** | **G** | **G** | **A** | **A** | **A** | **A** | **A** | **A** | **G** | **T** | **G** | **G** | **A** | **A** | **T** | **T** | **G** | **G** | **C** | **T** | **A** | **A** | **A** | **T** | **C** | **T** | **T** | **T** | **A** | **T** | **C** | **A** | **C** | **A** | **G** | **C** | **C** | **A** | **G** | **C** | **T** | **G** | **A** | **A** | **C** | **T** | **A** | **T** | **A** | **T** | **A** | **T** | **A** | **T** | **G** | **G** | **G** | **A** | **T** | **G** | **A** | **A** | **C** | **C** | **A** | **T** | **T** | **G** | **A** | **A** | **T** | **T** | **A** | **T** | **T** | **T** | **G** | **G** | **A** | **T** | **G** | **T** | **C** | **T** | **T** | **C** | **A** | **A** | **T** | **C** | **A** | **A** | **G** | **A** | **A** | **C** | **A** | **A** | **T** | **T** | **A** | **G** | **A** | **A** | **C** | **A** | **A** | **C** | **T** | **G** | **A** | **T** | **C** | **T** | **T** | **G** | **A** | **A** | **C** | **G** | **T** | **G** | **A** | **A** | **A** | **C** | **C** | **T** | **G** | **C** | **C** | **A** | **T** | **G** | **T** | **T** | **A** | **C** | **T** | **G** | **G** | **T** | **G** | **G** | **A** | **A** | **C** | **A** | **T** | **G** | **A** | **T** | **C** | **A** | **A** | **A** | **C** | **C** | **T** | **T** | **C** | **C** | **T** | **G** | **G** | **A** | **T** | **A** | **A** | **A** | **G** | **T** | **A** | **T** | **C** | **T** | **A** | **C** | **T** | **G** | **A** | **G** | **A** | **T** | **T** | **A** | **T** | **T** | **T** | **C** | **T** | **C** | **T** | **T** | **G** | **A** | **G** | **A** | **A** | **A** | **A** | **T** | **C** | **-** | **-** | **-** |
| **N54550** | **A** | **T** | **G** | **T** | **C** | **T** | **A** | **A** | **A** | **A** | **T** | **C** | **G** | **A** | **A** | **A** | **T** | **A** | **A** | **A** | **A** | **A** | **A** | **T** | **C** | **T** | **G** | **A** | **C** | **A** | **T** | **T** | **C** | **G** | **G** | **C** | **T** | **A** | **C** | **G** | **A** | **C** | **A** | **G** | **C** | **C** | **A** | **A** | **G** | **G** | **C** | **A** | **C** | **A** | **T** | **T** | **A** | **T** | **T** | **A** | **T** | **T** | **T** | **G** | **A** | **A** | **C** | **A** | **A** | **G** | **C** | **A** | **A** | **A** | **T** | **C** | **T** | **A** | **A** | **A** | **T** | **T** | **T** | **T** | **G** | **A** | **C** | **A** | **C** | **A** | **C** | **A** | **A** | **T** | **G** | **G** | **A** | **A** | **A** | **C** | **T** | **A** | **G** | **G** | **A** | **C** | **T** | **T** | **A** | **T** | **C** | **G** | **G** | **A** | **C** | **G** | **A** | **A** | **A** | **C** | **G** | **G** | **T** | **C** | **G** | **A** | **G** | **G** | **A** | **A** | **A** | **G** | **A** | **C** | **A** | **A** | **C** | **T** | **T** | **T** | **A** | **C** | **T** | **G** | **A** | **A** | **T** | **A** | **T** | **T** | **C** | **T** | **A** | **C** | **A** | **A** | **A** | **A** | **C** | **A** | **A** | **A** | **C** | **T** | **A** | **C** | **C** | **T** | **T** | **A** | **T** | **C** | **A** | **A** | **G** | **G** | **G** | **C** | **A** | **A** | **G** | **T** | **A** | **A** | **T** | **C** | **C** | **A** | **T** | **C** | **A** | **G** | **C** | **A** | **A** | **G** | **A** | **A** | **T** | **T** | **T** | **G** | **T** | **C** | **T** | **A** | **T** | **T** | **T** | **C** | **C** | **C** | **G** | **C** | **A** | **G** | **C** | **A** | **A** | **A** | **C** | **A** | **A** | **A** | **A** | **G** | **A** | **T** | **A** | **A** | **A** | **G** | **A** | **G** | **C** | **G** | **T** | **T** | **T** | **A** | **A** | **C** | **C** | **T** | **A** | **T** | **T** | **A** | **T** | **G** | **T** | **G** | **T** | **T** | **A** | **A** | **A** | **T** | **G** | **A** | **G** | **A** | **T** | **T** | **A** | **C** | **G** | **G** | **A** | **T** | **T** | **T** | **T** | **G** | **A** | **G** | **A** | **T** | **A** | **T** | **G** | **G** | **G** | **A** | **A** | **A** | **T** | **T** | **G** | **A** | **A** | **A** | **G** | **A** | **G** | **A** | **G** | **C** | **T** | **C** | **C** | **A** | **A** | **C** | **T** | **G** | **A** | **T** | **G** | **C** | **A** | **A** | **A** | **C** | **A** | **G** | **A** | **T** | **C** | **C** | **T** | **G** | **A** | **A** | **A** | **T** | **C** | **T** | **T** | **A** | **T** | **G** | **G** | **A** | **G** | **A** | **G** | **A** | **A** | **T** | **T** | **C** | **A** | **G** | **C** | **A** | **C** | **A** | **C** | **T** | **A** | **T** | **C** | **A** | **G** | **G** | **A** | **G** | **G** | **A** | **G** | **A** | **G** | **A** | **A** | **G** | **A** | **C** | **A** | **A** | **A** | **A** | **G** | **T** | **C** | **C** | **T** | **A** | **C** | **T** | **G** | **G** | **C** | **A** | **C** | **T** | **T** | **T** | **T** | **A** | **T** | **T** | **T** | **G** | **T** | **G** | **G** | **A** | **T** | **G** | **A** | **C** | **A** | **C** | **T** | **C** | **A** | **T** | **T** | **T** | **C** | **C** | **C** | **G** | **T** | **T** | **A** | **A** | **T** | **C** | **G** | **A** | **T** | **G** | **A** | **A** | **C** | **C** | **A** | **A** | **C** | **G** | **A** | **A** | **T** | **C** | **A** | **T** | **T** | **T** | **G** | **G** | **A** | **-** | **-** | **-** | **T** | **A** | **T** | **C** | **T** | **C** | **T** | **G** | **A** | **T** | **A** | **G** | **A** | **A** | **A** | **A** | **C** | **A** | **A** | **G** | **T** | **A** | **G** | **C** | **G** | **G** | **C** | **T** | **T** | **A** | **C** | **T** | **T** | **G** | **A** | **A** | **A** | **A** | **A** | **G** | **A** | **A** | **A** | **A** | **A** | **A** | **C** | **A** | **A** | **G** | **G** | **C** | **T** | **T** | **C** | **A** | **T** | **C** | **G** | **T** | **G** | **G** | **T** | **C** | **A** | **G** | **C** | **C** | **A** | **T** | **G** | **A** | **C** | **C** | **G** | **G** | **G** | **G** | **A** | **T** | **T** | **T** | **A** | **T** | **C** | **G** | **A** | **T** | **G** | **A** | **A** | **G** | **T** | **A** | **G** | **T** | **G** | **G** | **A** | **C** | **C** | **A** | **T** | **G** | **T** | **T** | **T** | **T** | **A** | **G** | **C** | **A** | **A** | **T** | **C** | **G** | **A** | **A** | **A** | **A** | **A** | **A** | **G** | **T** | **C** | **A** | **A** | **C** | **T** | **G** | **G** | **A** | **A** | **C** | **T** | **T** | **T** | **A** | **T** | **C** | **A** | **A** | **G** | **G** | **G** | **A** | **A** | **T** | **T** | **T** | **C** | **T** | **C** | **T** | **A** | **T** | **C** | **T** | **A** | **T** | **G** | **A** | **A** | **G** | **A** | **A** | **C** | **A** | **G** | **A** | **A** | **A** | **A** | **A** | **A** | **C** | **T** | **T** | **C** | **G** | **T** | **G** | **A** | **T** | **G** | **A** | **A** | **T** | **T** | **T** | **G** | **A** | **A** | **A** | **T** | **A** | **G** | **C** | **T** | **C** | **A** | **A** | **A** | **A** | **T** | **G** | **A** | **A** | **A** | **A** | **A** | **T** | **T** | **G** | **A** | **A** | **A** | **A** | **A** | **A** | **G** | **A** | **A** | **G** | **T** | **C** | **A** | **G** | **T** | **A** | **G** | **G** | **C** | **T** | **A** | **A** | **A** | **G** | **A** | **A** | **A** | **A** | **C** | **A** | **G** | **C** | **A** | **G** | **C** | **T** | **G** | **A** | **A** | **A** | **A** | **A** | **G** | **C** | **C** | **G** | **A** | **A** | **T** | **G** | **G** | **T** | **C** | **T** | **C** | **G** | **T** | **T** | **C** | **C** | **C** | **G** | **A** | **G** | **A** | **A** | **G** | **G** | **A** | **G** | **A** | **T** | **A** | **A** | **A** | **A** | **C** | **A** | **A** | **A** | **G** | **A** | **A** | **A** | **C** | **A** | **A** | **G** | **T** | **C** | **G** | **G** | **A** | **T** | **T** | **C** | **A** | **T** | **C** | **G** | **A** | **T** | **A** | **C** | **T** | **G** | **A** | **A** | **T** | **C** | **T** | **A** | **G** | **A** | **C** | **G** | **A** | **G** | **T** | **G** | **A** | **A** | **T** | **A** | **A** | **A** | **G** | **G** | **A** | **G** | **C** | **A** | **G** | **T** | **G** | **G** | **G** | **T** | **G** | **C** | **T** | **G** | **A** | **T** | **G** | **C** | **T** | **G** | **C** | **A** | **C** | **G** | **G** | **A** | **C** | **G** | **A** | **T** | **G** | **A** | **A** | **A** | **C** | **G** | **A** | **T** | **C** | **C** | **A** | **A** | **A** | **G** | **C** | **A** | **A** | **T** | **C** | **G** | **T** | **G** | **A** | **A** | **T** | **C** | **G** | **G** | **A** | **T** | **G** | **G** | **A** | **G** | **A** | **C** | **C** | **C** | **A** | **G** | **A** | **T** | **C** | **A** | **G** | **C** | **G** | **A** | **G** | **A** | **A** | **A** | **G** | **A** | **A** | **A** | **A** | **A** | **C** | **T** | **A** | **T** | **T** | **A** | **A** | **A** | **A** | **G** | **A** | **T** | **A** | **T** | **C** | **G** | **A** | **A** | **T** | **A** | **T** | **A** | **T** | **C** | **G** | **A** | **T** | **T** | **C** | **T** | **T** | **T** | **G** | **A** | **C** | **G** | **A** | **T** | **G** | **A** | **A** | **T** | **A** | **G** | **C** | **C** | **A** | **A** | **G** | **C** | **G** | **T** | **C** | **T** | **C** | **A** | **C** | **C** | **A** | **T** | **A** | **A** | **G** | **C** | **G** | **A** | **C** | **T** | **T** | **T** | **T** | **A** | **A** | **G** | **C** | **G** | **T** | **A** | **G** | **A** | **A** | **G** | **A** | **T** | **C** | **T** | **T** | **C** | **A** | **A** | **T** | **T** | **A** | **G** | **G** | **G** | **T** | **A** | **T** | **G** | **A** | **A** | **A** | **A** | **T** | **C** | **T** | **G** | **T** | **T** | **A** | **T** | **T** | **C** | **G** | **A** | **G** | **C** | **C** | **A** | **A** | **T** | **T** | **C** | **A** | **T** | **T** | **T** | **T** | **A** | **C** | **A** | **A** | **T** | **C** | **G** | **A** | **G** | **C** | **C** | **T** | **C** | **A** | **T** | **C** | **A** | **G** | **C** | **G** | **G** | **G** | **T** | **G** | **G** | **C** | **G** | **A** | **T** | **T** | **T** | **C** | **A** | **G** | **G** | **T** | **C** | **C** | **T** | **A** | **A** | **C** | **G** | **G** | **T** | **G** | **C** | **A** | **G** | **G** | **A** | **A** | **A** | **G** | **T** | **C** | **A** | **T** | **C** | **C** | **A** | **T** | **T** | **A** | **T** | **C** | **C** | **A** | **T** | **T** | **A** | **T** | **C** | **T** | **T** | **C** | **T** | **G** | **G** | **G** | **G** | **G** | **C** | **A** | **T** | **T** | **C** | **A** | **A** | **C** | **G** | **G** | **C** | **A** | **A** | **G** | **G** | **T** | **T** | **A** | **T** | **A** | **G** | **G** | **A** | **G** | **A** | **A** | **A** | **A** | **A** | **T** | **C** | **C** | **C** | **A** | **G** | **C** | **C** | **A** | **A** | **A** | **A** | **C** | **A** | **T** | **C** | **T** | **G** | **A** | **G** | **C** | **A** | **T** | **T** | **A** | **G** | **T** | **T** | **A** | **T** | **G** | **T** | **A** | **C** | **G** | **C** | **C** | **A** | **A** | **A** | **A** | **T** | **T** | **A** | **T** | **G** | **A** | **A** | **G** | **A** | **C** | **A** | **A** | **T** | **C** | **G** | **A** | **G** | **G** | **A** | **A** | **C** | **G** | **T** | **T** | **G** | **G** | **C** | **G** | **G** | **A** | **A** | **T** | **T** | **T** | **G** | **C** | **A** | **G** | **A** | **G** | **A** | **A** | **A** | **A** | **A** | **C** | **C** | **A** | **A** | **G** | **T** | **A** | **G** | **A** | **C** | **T** | **A** | **C** | **C** | **A** | **A** | **G** | **C** | **A** | **T** | **T** | **T** | **T** | **T** | **G** | **A** | **A** | **C** | **A** | **A** | **C** | **C** | **T** | **C** | **C** | **G** | **A** | **A** | **A** | **G** | **C** | **T** | **T** | **G** | **G** | **G** | **A** | **T** | **G** | **G** | **A** | **A** | **A** | **G** | **A** | **G** | **A** | **T** | **G** | **T** | **T** | **T** | **T** | **T** | **C** | **A** | **T** | **A** | **A** | **C** | **A** | **A** | **G** | **A** | **T** | **C** | **G** | **A** | **G** | **C** | **A** | **G** | **A** | **T** | **G** | **A** | **G** | **T** | **A** | **T** | **G** | **G** | **G** | **C** | **C** | **A** | **A** | **C** | **G** | **G** | **A** | **A** | **A** | **A** | **A** | **A** | **G** | **T** | **G** | **G** | **A** | **A** | **T** | **T** | **G** | **G** | **C** | **T** | **A** | **A** | **A** | **T** | **C** | **T** | **T** | **T** | **A** | **T** | **C** | **A** | **C** | **A** | **G** | **C** | **C** | **A** | **G** | **C** | **T** | **G** | **A** | **A** | **C** | **T** | **A** | **T** | **A** | **T** | **A** | **T** | **A** | **T** | **G** | **G** | **G** | **A** | **T** | **G** | **A** | **A** | **C** | **C** | **A** | **T** | **T** | **G** | **A** | **A** | **T** | **T** | **A** | **T** | **T** | **T** | **G** | **G** | **A** | **T** | **G** | **T** | **C** | **T** | **T** | **C** | **A** | **A** | **T** | **C** | **A** | **A** | **G** | **A** | **A** | **C** | **A** | **A** | **T** | **T** | **A** | **G** | **A** | **A** | **C** | **A** | **A** | **C** | **T** | **G** | **A** | **T** | **C** | **T** | **T** | **G** | **A** | **A** | **C** | **G** | **T** | **G** | **A** | **A** | **A** | **C** | **C** | **T** | **G** | **C** | **C** | **A** | **T** | **G** | **T** | **T** | **A** | **C** | **T** | **A** | **G** | **T** | **G** | **G** | **A** | **A** | **C** | **A** | **T** | **G** | **A** | **T** | **C** | **A** | **A** | **A** | **C** | **C** | **T** | **T** | **C** | **C** | **T** | **G** | **G** | **A** | **T** | **A** | **A** | **A** | **G** | **T** | **A** | **T** | **C** | **T** | **A** | **C** | **T** | **G** | **A** | **G** | **A** | **T** | **T** | **A** | **T** | **T** | **T** | **C** | **T** | **C** | **T** | **T** | **G** | **A** | **G** | **A** | **A** | **A** | **A** | **T** | **C** | **-** | **-** | **-** |
| **N54150** | **A** | **T** | **G** | **T** | **C** | **T** | **A** | **A** | **A** | **A** | **T** | **C** | **G** | **A** | **A** | **A** | **T** | **A** | **A** | **A** | **A** | **A** | **A** | **T** | **C** | **T** | **G** | **A** | **C** | **A** | **T** | **T** | **C** | **G** | **G** | **C** | **T** | **A** | **C** | **G** | **A** | **C** | **A** | **G** | **C** | **C** | **A** | **A** | **G** | **G** | **C** | **A** | **C** | **A** | **T** | **T** | **A** | **T** | **T** | **A** | **T** | **T** | **T** | **G** | **A** | **A** | **C** | **A** | **A** | **G** | **C** | **A** | **A** | **A** | **T** | **C** | **T** | **A** | **A** | **A** | **T** | **T** | **T** | **T** | **G** | **A** | **C** | **A** | **C** | **A** | **C** | **A** | **A** | **T** | **G** | **G** | **A** | **A** | **A** | **C** | **T** | **A** | **G** | **G** | **A** | **C** | **T** | **T** | **A** | **T** | **C** | **G** | **G** | **A** | **C** | **G** | **A** | **A** | **A** | **C** | **G** | **G** | **T** | **C** | **G** | **A** | **G** | **G** | **A** | **A** | **A** | **G** | **A** | **C** | **A** | **A** | **C** | **T** | **T** | **T** | **A** | **C** | **T** | **G** | **A** | **A** | **T** | **A** | **T** | **T** | **C** | **T** | **A** | **C** | **A** | **A** | **A** | **A** | **C** | **A** | **A** | **A** | **C** | **T** | **A** | **C** | **C** | **T** | **T** | **A** | **T** | **C** | **A** | **A** | **G** | **G** | **G** | **C** | **A** | **A** | **G** | **T** | **A** | **A** | **T** | **C** | **C** | **A** | **T** | **C** | **A** | **G** | **C** | **A** | **A** | **G** | **A** | **A** | **T** | **T** | **T** | **G** | **T** | **C** | **T** | **A** | **T** | **T** | **T** | **C** | **C** | **C** | **G** | **C** | **A** | **A** | **C** | **A** | **G** | **A** | **C** | **A** | **A** | **A** | **A** | **G** | **A** | **T** | **A** | **A** | **A** | **G** | **A** | **A** | **C** | **G** | **T** | **T** | **T** | **A** | **A** | **C** | **C** | **T** | **A** | **T** | **T** | **A** | **C** | **G** | **T** | **G** | **T** | **T** | **A** | **A** | **A** | **T** | **G** | **A** | **T** | **A** | **T** | **T** | **A** | **C** | **G** | **G** | **A** | **T** | **T** | **T** | **T** | **G** | **A** | **G** | **A** | **T** | **A** | **T** | **G** | **G** | **G** | **A** | **A** | **A** | **T** | **C** | **G** | **A** | **A** | **A** | **G** | **A** | **G** | **A** | **G** | **C** | **T** | **C** | **C** | **A** | **A** | **T** | **T** | **G** | **A** | **T** | **G** | **C** | **A** | **A** | **A** | **C** | **A** | **G** | **A** | **T** | **C** | **C** | **T** | **G** | **A** | **A** | **A** | **T** | **C** | **T** | **T** | **A** | **T** | **G** | **G** | **A** | **G** | **A** | **G** | **A** | **A** | **T** | **T** | **C** | **A** | **G** | **C** | **A** | **C** | **A** | **C** | **T** | **A** | **T** | **C** | **A** | **G** | **G** | **G** | **G** | **G** | **A** | **G** | **A** | **G** | **A** | **A** | **G** | **A** | **C** | **A** | **A** | **A** | **A** | **G** | **T** | **C** | **C** | **T** | **A** | **C** | **T** | **G** | **G** | **C** | **A** | **C** | **T** | **T** | **T** | **T** | **A** | **T** | **T** | **T** | **G** | **T** | **G** | **G** | **A** | **T** | **G** | **A** | **C** | **A** | **C** | **T** | **C** | **A** | **T** | **T** | **T** | **C** | **C** | **C** | **G** | **T** | **T** | **A** | **A** | **T** | **C** | **G** | **A** | **T** | **G** | **A** | **A** | **C** | **C** | **A** | **A** | **C** | **G** | **A** | **A** | **T** | **C** | **A** | **T** | **T** | **T** | **G** | **G** | **A** | **-** | **-** | **-** | **T** | **A** | **T** | **C** | **T** | **C** | **T** | **G** | **G** | **T** | **A** | **G** | **A** | **A** | **A** | **A** | **C** | **A** | **A** | **G** | **T** | **A** | **G** | **C** | **G** | **G** | **C** | **T** | **T** | **A** | **T** | **T** | **T** | **G** | **A** | **A** | **A** | **A** | **A** | **G** | **A** | **A** | **A** | **A** | **A** | **A** | **C** | **A** | **A** | **G** | **G** | **C** | **T** | **T** | **C** | **A** | **T** | **C** | **G** | **T** | **G** | **G** | **T** | **C** | **A** | **G** | **C** | **C** | **A** | **T** | **G** | **A** | **C** | **C** | **G** | **G** | **G** | **G** | **A** | **T** | **T** | **T** | **A** | **T** | **C** | **G** | **A** | **T** | **G** | **A** | **A** | **G** | **T** | **A** | **G** | **T** | **G** | **G** | **A** | **C** | **C** | **A** | **T** | **G** | **T** | **T** | **T** | **T** | **A** | **G** | **C** | **A** | **A** | **T** | **C** | **G** | **A** | **A** | **A** | **A** | **A** | **A** | **G** | **T** | **C** | **A** | **A** | **C** | **T** | **G** | **G** | **A** | **A** | **C** | **T** | **T** | **T** | **A** | **T** | **C** | **A** | **A** | **G** | **G** | **G** | **A** | **A** | **T** | **T** | **T** | **C** | **T** | **C** | **T** | **A** | **T** | **C** | **T** | **A** | **T** | **G** | **A** | **A** | **G** | **A** | **A** | **C** | **A** | **G** | **A** | **A** | **A** | **A** | **A** | **A** | **C** | **T** | **T** | **C** | **G** | **T** | **G** | **A** | **T** | **G** | **A** | **A** | **T** | **T** | **T** | **G** | **A** | **A** | **A** | **T** | **G** | **G** | **C** | **T** | **C** | **A** | **A** | **A** | **A** | **T** | **G** | **A** | **A** | **A** | **A** | **A** | **T** | **T** | **G** | **A** | **A** | **A** | **A** | **A** | **A** | **G** | **A** | **A** | **G** | **T** | **C** | **A** | **G** | **T** | **A** | **G** | **G** | **C** | **T** | **A** | **A** | **A** | **G** | **A** | **A** | **A** | **A** | **C** | **A** | **G** | **C** | **A** | **G** | **C** | **T** | **G** | **A** | **A** | **A** | **A** | **A** | **G** | **C** | **C** | **G** | **A** | **A** | **T** | **G** | **G** | **T** | **C** | **T** | **C** | **G** | **T** | **T** | **C** | **C** | **C** | **G** | **A** | **G** | **A** | **A** | **G** | **G** | **A** | **G** | **A** | **T** | **A** | **A** | **A** | **A** | **C** | **A** | **A** | **A** | **G** | **A** | **A** | **A** | **C** | **A** | **A** | **G** | **T** | **C** | **G** | **G** | **A** | **T** | **T** | **C** | **A** | **T** | **C** | **G** | **A** | **T** | **A** | **C** | **T** | **G** | **A** | **A** | **T** | **C** | **T** | **A** | **G** | **A** | **C** | **G** | **A** | **G** | **T** | **G** | **A** | **A** | **T** | **A** | **A** | **A** | **G** | **G** | **A** | **G** | **C** | **A** | **G** | **T** | **G** | **G** | **G** | **T** | **G** | **C** | **T** | **G** | **A** | **T** | **G** | **C** | **T** | **G** | **C** | **A** | **C** | **G** | **G** | **A** | **C** | **G** | **A** | **T** | **G** | **A** | **A** | **A** | **C** | **G** | **A** | **T** | **C** | **C** | **A** | **A** | **A** | **G** | **C** | **A** | **A** | **T** | **C** | **G** | **T** | **G** | **A** | **A** | **T** | **C** | **G** | **G** | **A** | **T** | **G** | **G** | **A** | **G** | **A** | **C** | **C** | **C** | **A** | **G** | **A** | **T** | **C** | **A** | **G** | **C** | **G** | **A** | **G** | **A** | **A** | **A** | **G** | **A** | **A** | **A** | **A** | **A** | **C** | **T** | **A** | **T** | **T** | **A** | **A** | **A** | **A** | **G** | **A** | **T** | **A** | **T** | **C** | **G** | **A** | **A** | **T** | **A** | **T** | **A** | **T** | **C** | **G** | **A** | **T** | **T** | **C** | **T** | **T** | **T** | **G** | **A** | **C** | **G** | **A** | **T** | **G** | **A** | **A** | **T** | **A** | **G** | **C** | **C** | **A** | **A** | **G** | **C** | **G** | **T** | **C** | **T** | **C** | **A** | **C** | **C** | **A** | **T** | **A** | **A** | **G** | **C** | **G** | **A** | **C** | **T** | **T** | **T** | **T** | **A** | **A** | **G** | **C** | **G** | **T** | **A** | **G** | **A** | **A** | **G** | **A** | **T** | **C** | **T** | **T** | **C** | **A** | **A** | **T** | **T** | **A** | **G** | **G** | **G** | **T** | **A** | **T** | **G** | **A** | **A** | **A** | **A** | **T** | **C** | **T** | **G** | **T** | **T** | **A** | **T** | **T** | **C** | **G** | **A** | **G** | **C** | **C** | **A** | **A** | **T** | **T** | **C** | **A** | **T** | **T** | **T** | **T** | **A** | **C** | **A** | **A** | **T** | **C** | **G** | **A** | **G** | **C** | **C** | **T** | **C** | **A** | **T** | **C** | **A** | **G** | **C** | **G** | **G** | **G** | **T** | **G** | **G** | **C** | **G** | **A** | **T** | **T** | **T** | **C** | **A** | **G** | **G** | **T** | **C** | **C** | **T** | **A** | **A** | **C** | **G** | **G** | **T** | **G** | **C** | **A** | **G** | **G** | **A** | **A** | **A** | **G** | **T** | **C** | **A** | **T** | **C** | **C** | **A** | **T** | **T** | **A** | **T** | **C** | **C** | **A** | **T** | **T** | **A** | **T** | **C** | **T** | **T** | **C** | **T** | **G** | **G** | **G** | **G** | **G** | **C** | **A** | **T** | **T** | **C** | **A** | **A** | **C** | **G** | **G** | **C** | **A** | **A** | **G** | **G** | **T** | **T** | **A** | **T** | **A** | **G** | **G** | **A** | **G** | **A** | **A** | **A** | **A** | **A** | **T** | **C** | **C** | **C** | **A** | **G** | **C** | **C** | **A** | **A** | **A** | **A** | **C** | **A** | **T** | **C** | **T** | **G** | **A** | **G** | **C** | **A** | **T** | **T** | **A** | **G** | **T** | **T** | **A** | **T** | **G** | **T** | **A** | **C** | **G** | **C** | **C** | **A** | **A** | **A** | **A** | **T** | **T** | **A** | **T** | **G** | **A** | **A** | **G** | **A** | **C** | **A** | **A** | **T** | **C** | **G** | **A** | **G** | **G** | **A** | **A** | **C** | **G** | **T** | **T** | **G** | **G** | **C** | **G** | **G** | **A** | **A** | **T** | **T** | **T** | **G** | **C** | **A** | **G** | **A** | **G** | **A** | **A** | **A** | **A** | **A** | **C** | **C** | **A** | **A** | **G** | **T** | **A** | **G** | **A** | **C** | **T** | **A** | **C** | **C** | **A** | **A** | **G** | **C** | **A** | **T** | **T** | **T** | **T** | **T** | **G** | **A** | **A** | **C** | **A** | **A** | **C** | **C** | **T** | **C** | **C** | **G** | **A** | **A** | **A** | **G** | **C** | **T** | **T** | **G** | **G** | **G** | **A** | **T** | **G** | **G** | **A** | **A** | **A** | **G** | **A** | **G** | **A** | **T** | **G** | **T** | **T** | **T** | **T** | **T** | **C** | **A** | **T** | **A** | **A** | **C** | **A** | **A** | **G** | **A** | **T** | **C** | **G** | **A** | **G** | **C** | **A** | **G** | **A** | **T** | **G** | **A** | **G** | **T** | **A** | **T** | **G** | **G** | **G** | **C** | **C** | **A** | **A** | **C** | **G** | **G** | **A** | **A** | **A** | **A** | **A** | **A** | **G** | **T** | **G** | **G** | **A** | **A** | **T** | **T** | **G** | **G** | **C** | **T** | **A** | **A** | **A** | **T** | **C** | **T** | **T** | **T** | **A** | **T** | **C** | **A** | **C** | **A** | **G** | **C** | **C** | **A** | **G** | **C** | **T** | **G** | **A** | **A** | **C** | **T** | **A** | **T** | **A** | **T** | **A** | **T** | **A** | **T** | **G** | **G** | **G** | **A** | **T** | **G** | **A** | **A** | **C** | **C** | **A** | **T** | **T** | **G** | **A** | **A** | **T** | **T** | **A** | **T** | **T** | **T** | **G** | **G** | **A** | **T** | **G** | **T** | **C** | **T** | **T** | **C** | **A** | **A** | **T** | **C** | **A** | **A** | **G** | **A** | **A** | **C** | **A** | **A** | **T** | **T** | **A** | **G** | **A** | **A** | **C** | **A** | **A** | **C** | **T** | **G** | **A** | **T** | **C** | **T** | **T** | **G** | **A** | **A** | **C** | **G** | **T** | **G** | **A** | **A** | **A** | **C** | **C** | **T** | **G** | **C** | **C** | **A** | **T** | **G** | **T** | **T** | **A** | **C** | **T** | **A** | **G** | **T** | **G** | **G** | **A** | **A** | **C** | **A** | **T** | **G** | **A** | **T** | **C** | **A** | **A** | **A** | **C** | **C** | **T** | **T** | **C** | **C** | **T** | **G** | **G** | **A** | **T** | **A** | **A** | **A** | **G** | **T** | **A** | **T** | **C** | **T** | **A** | **C** | **T** | **G** | **A** | **G** | **A** | **T** | **T** | **A** | **T** | **T** | **T** | **C** | **T** | **C** | **T** | **T** | **G** | **A** | **G** | **A** | **A** | **A** | **A** | **T** | **C** | **-** | **-** | **-** |
| **N60357F** | **A** | **T** | **G** | **T** | **C** | **T** | **A** | **A** | **A** | **A** | **T** | **C** | **G** | **A** | **A** | **A** | **T** | **A** | **A** | **A** | **A** | **A** | **A** | **T** | **C** | **T** | **G** | **A** | **C** | **A** | **T** | **T** | **C** | **G** | **G** | **C** | **T** | **A** | **C** | **G** | **A** | **C** | **A** | **G** | **C** | **C** | **A** | **A** | **G** | **G** | **C** | **A** | **C** | **A** | **T** | **T** | **A** | **T** | **T** | **A** | **T** | **T** | **T** | **G** | **A** | **A** | **C** | **A** | **A** | **G** | **C** | **A** | **A** | **A** | **T** | **C** | **T** | **A** | **A** | **A** | **T** | **T** | **T** | **T** | **G** | **A** | **C** | **A** | **C** | **A** | **C** | **A** | **A** | **T** | **G** | **G** | **A** | **A** | **A** | **C** | **T** | **A** | **G** | **G** | **A** | **C** | **T** | **T** | **A** | **T** | **C** | **G** | **G** | **A** | **C** | **G** | **A** | **A** | **A** | **C** | **G** | **G** | **T** | **C** | **G** | **A** | **G** | **G** | **A** | **A** | **A** | **G** | **A** | **C** | **A** | **A** | **C** | **T** | **T** | **T** | **A** | **C** | **T** | **G** | **A** | **A** | **T** | **A** | **T** | **T** | **C** | **T** | **A** | **C** | **A** | **A** | **A** | **A** | **C** | **A** | **A** | **A** | **C** | **T** | **A** | **C** | **C** | **T** | **T** | **A** | **T** | **C** | **A** | **A** | **G** | **G** | **G** | **C** | **A** | **A** | **G** | **T** | **A** | **A** | **T** | **C** | **C** | **A** | **T** | **C** | **A** | **G** | **C** | **A** | **A** | **G** | **A** | **A** | **T** | **T** | **T** | **G** | **T** | **C** | **T** | **A** | **T** | **T** | **T** | **C** | **C** | **C** | **G** | **C** | **A** | **A** | **C** | **A** | **G** | **A** | **C** | **A** | **A** | **A** | **A** | **G** | **A** | **T** | **A** | **A** | **A** | **G** | **A** | **A** | **C** | **G** | **T** | **T** | **T** | **A** | **A** | **C** | **C** | **T** | **A** | **T** | **T** | **A** | **C** | **G** | **T** | **G** | **T** | **T** | **A** | **A** | **A** | **T** | **G** | **A** | **T** | **A** | **T** | **T** | **A** | **C** | **G** | **G** | **A** | **T** | **T** | **T** | **T** | **G** | **A** | **G** | **A** | **T** | **A** | **T** | **G** | **G** | **G** | **A** | **A** | **A** | **T** | **C** | **G** | **A** | **A** | **A** | **G** | **A** | **G** | **A** | **G** | **C** | **T** | **C** | **C** | **A** | **A** | **T** | **T** | **G** | **A** | **T** | **G** | **C** | **A** | **A** | **A** | **C** | **A** | **G** | **A** | **T** | **C** | **C** | **T** | **G** | **A** | **A** | **A** | **T** | **C** | **T** | **T** | **A** | **T** | **G** | **G** | **A** | **G** | **A** | **G** | **A** | **A** | **T** | **T** | **C** | **A** | **G** | **C** | **A** | **C** | **A** | **C** | **T** | **A** | **T** | **C** | **G** | **G** | **G** | **G** | **G** | **G** | **A** | **G** | **A** | **G** | **A** | **A** | **G** | **A** | **C** | **A** | **A** | **A** | **A** | **G** | **T** | **C** | **C** | **T** | **A** | **C** | **T** | **G** | **G** | **C** | **A** | **C** | **T** | **T** | **T** | **T** | **A** | **T** | **T** | **T** | **G** | **T** | **G** | **G** | **A** | **T** | **G** | **A** | **C** | **A** | **C** | **T** | **C** | **A** | **T** | **T** | **T** | **T** | **C** | **C** | **G** | **T** | **T** | **A** | **A** | **T** | **C** | **G** | **A** | **T** | **G** | **A** | **A** | **C** | **C** | **A** | **A** | **C** | **G** | **A** | **A** | **T** | **C** | **A** | **T** | **T** | **T** | **G** | **G** | **A** | **-** | **-** | **-** | **T** | **A** | **T** | **C** | **T** | **C** | **T** | **G** | **G** | **T** | **A** | **G** | **A** | **A** | **A** | **A** | **C** | **A** | **A** | **G** | **T** | **A** | **G** | **C** | **G** | **G** | **C** | **T** | **T** | **A** | **T** | **T** | **T** | **G** | **A** | **A** | **A** | **A** | **A** | **G** | **A** | **A** | **A** | **A** | **A** | **A** | **C** | **A** | **A** | **G** | **G** | **C** | **T** | **T** | **C** | **A** | **T** | **C** | **G** | **T** | **G** | **G** | **T** | **C** | **A** | **G** | **C** | **C** | **A** | **T** | **G** | **A** | **C** | **C** | **G** | **G** | **G** | **G** | **A** | **T** | **T** | **T** | **A** | **T** | **C** | **G** | **A** | **T** | **G** | **A** | **A** | **G** | **T** | **A** | **G** | **T** | **G** | **G** | **A** | **C** | **C** | **A** | **T** | **G** | **T** | **T** | **T** | **T** | **A** | **G** | **C** | **A** | **A** | **T** | **C** | **G** | **A** | **A** | **A** | **A** | **A** | **A** | **G** | **T** | **C** | **A** | **A** | **C** | **T** | **G** | **G** | **A** | **A** | **C** | **T** | **T** | **T** | **A** | **T** | **C** | **A** | **A** | **G** | **G** | **G** | **A** | **A** | **T** | **T** | **T** | **C** | **T** | **C** | **T** | **A** | **T** | **C** | **T** | **A** | **T** | **G** | **A** | **A** | **G** | **A** | **A** | **C** | **A** | **G** | **A** | **G** | **A** | **A** | **A** | **A** | **C** | **T** | **T** | **C** | **G** | **T** | **G** | **A** | **T** | **G** | **A** | **A** | **T** | **T** | **T** | **G** | **A** | **A** | **A** | **T** | **G** | **G** | **C** | **T** | **C** | **A** | **A** | **A** | **A** | **T** | **G** | **A** | **A** | **A** | **A** | **A** | **T** | **T** | **G** | **A** | **A** | **A** | **A** | **A** | **A** | **G** | **A** | **A** | **G** | **T** | **C** | **A** | **G** | **C** | **A** | **G** | **G** | **C** | **T** | **A** | **A** | **A** | **G** | **A** | **C** | **A** | **A** | **C** | **A** | **G** | **C** | **A** | **G** | **C** | **T** | **G** | **A** | **A** | **A** | **A** | **A** | **G** | **C** | **C** | **G** | **A** | **A** | **T** | **G** | **G** | **T** | **C** | **T** | **C** | **G** | **T** | **T** | **C** | **C** | **C** | **G** | **A** | **G** | **A** | **A** | **G** | **G** | **A** | **G** | **A** | **T** | **A** | **A** | **A** | **A** | **C** | **A** | **A** | **A** | **G** | **A** | **A** | **A** | **C** | **A** | **A** | **G** | **T** | **C** | **G** | **G** | **A** | **C** | **T** | **C** | **A** | **T** | **C** | **G** | **A** | **T** | **A** | **C** | **T** | **G** | **A** | **A** | **T** | **C** | **T** | **A** | **G** | **A** | **C** | **G** | **A** | **G** | **T** | **G** | **A** | **A** | **T** | **A** | **A** | **A** | **G** | **G** | **A** | **G** | **C** | **A** | **G** | **T** | **G** | **G** | **G** | **T** | **G** | **C** | **T** | **G** | **A** | **T** | **G** | **C** | **T** | **G** | **C** | **A** | **C** | **G** | **G** | **A** | **C** | **G** | **A** | **T** | **G** | **A** | **A** | **A** | **C** | **G** | **A** | **T** | **C** | **C** | **A** | **A** | **A** | **G** | **C** | **A** | **A** | **T** | **C** | **G** | **T** | **G** | **A** | **A** | **T** | **C** | **G** | **G** | **A** | **T** | **G** | **G** | **A** | **G** | **A** | **C** | **C** | **C** | **A** | **G** | **A** | **T** | **C** | **A** | **G** | **C** | **G** | **A** | **G** | **A** | **A** | **A** | **G** | **A** | **A** | **A** | **A** | **A** | **C** | **T** | **A** | **T** | **T** | **A** | **A** | **A** | **A** | **G** | **A** | **T** | **A** | **T** | **C** | **G** | **A** | **A** | **T** | **A** | **T** | **A** | **T** | **C** | **G** | **A** | **T** | **T** | **C** | **T** | **T** | **T** | **G** | **A** | **C** | **G** | **A** | **T** | **G** | **A** | **A** | **T** | **A** | **G** | **C** | **C** | **A** | **A** | **G** | **C** | **G** | **T** | **C** | **T** | **C** | **A** | **C** | **C** | **A** | **T** | **A** | **A** | **G** | **C** | **G** | **A** | **C** | **T** | **T** | **T** | **T** | **A** | **A** | **G** | **C** | **G** | **T** | **A** | **G** | **A** | **A** | **G** | **A** | **T** | **C** | **T** | **T** | **C** | **A** | **A** | **T** | **T** | **A** | **G** | **G** | **G** | **T** | **A** | **T** | **G** | **A** | **A** | **A** | **A** | **T** | **C** | **T** | **G** | **T** | **T** | **A** | **T** | **T** | **C** | **G** | **A** | **G** | **C** | **C** | **A** | **A** | **T** | **T** | **C** | **A** | **T** | **T** | **T** | **T** | **A** | **C** | **A** | **A** | **T** | **C** | **G** | **A** | **G** | **C** | **C** | **T** | **C** | **A** | **T** | **C** | **A** | **G** | **C** | **G** | **G** | **G** | **T** | **G** | **G** | **C** | **G** | **A** | **T** | **T** | **T** | **C** | **A** | **G** | **G** | **T** | **C** | **C** | **T** | **A** | **A** | **C** | **G** | **G** | **T** | **G** | **C** | **A** | **G** | **G** | **A** | **A** | **A** | **G** | **T** | **C** | **A** | **T** | **C** | **C** | **A** | **T** | **T** | **A** | **T** | **C** | **C** | **A** | **T** | **T** | **A** | **T** | **C** | **T** | **T** | **C** | **T** | **G** | **G** | **G** | **G** | **T** | **C** | **A** | **T** | **T** | **C** | **A** | **A** | **C** | **G** | **G** | **C** | **A** | **A** | **G** | **G** | **T** | **T** | **A** | **T** | **A** | **G** | **G** | **A** | **G** | **A** | **A** | **A** | **A** | **A** | **T** | **C** | **C** | **C** | **A** | **G** | **C** | **C** | **A** | **A** | **A** | **A** | **C** | **A** | **T** | **C** | **T** | **G** | **A** | **G** | **C** | **A** | **T** | **T** | **A** | **G** | **T** | **T** | **A** | **T** | **G** | **T** | **A** | **C** | **G** | **C** | **C** | **A** | **A** | **A** | **A** | **T** | **T** | **A** | **T** | **G** | **A** | **A** | **G** | **A** | **C** | **A** | **A** | **T** | **C** | **G** | **A** | **G** | **G** | **A** | **A** | **C** | **G** | **T** | **T** | **G** | **G** | **C** | **G** | **G** | **A** | **A** | **T** | **T** | **T** | **G** | **C** | **A** | **G** | **A** | **G** | **A** | **A** | **A** | **A** | **A** | **C** | **C** | **A** | **A** | **G** | **T** | **A** | **G** | **A** | **C** | **T** | **A** | **C** | **C** | **A** | **A** | **G** | **C** | **A** | **T** | **T** | **T** | **T** | **T** | **G** | **A** | **A** | **C** | **A** | **A** | **C** | **C** | **T** | **C** | **C** | **G** | **A** | **A** | **A** | **G** | **C** | **T** | **T** | **G** | **G** | **G** | **A** | **T** | **G** | **G** | **A** | **A** | **A** | **G** | **A** | **G** | **A** | **T** | **G** | **T** | **T** | **T** | **T** | **T** | **C** | **A** | **T** | **A** | **A** | **C** | **A** | **A** | **G** | **A** | **T** | **T** | **G** | **A** | **G** | **C** | **A** | **G** | **A** | **T** | **G** | **A** | **G** | **T** | **A** | **T** | **G** | **G** | **G** | **C** | **C** | **A** | **A** | **C** | **G** | **G** | **A** | **A** | **A** | **A** | **A** | **A** | **G** | **T** | **G** | **G** | **A** | **A** | **T** | **T** | **G** | **G** | **C** | **T** | **A** | **A** | **A** | **T** | **C** | **T** | **T** | **T** | **A** | **T** | **C** | **A** | **C** | **A** | **G** | **C** | **C** | **A** | **G** | **C** | **T** | **G** | **A** | **A** | **C** | **T** | **A** | **T** | **A** | **T** | **A** | **T** | **A** | **T** | **G** | **G** | **G** | **A** | **T** | **G** | **A** | **A** | **C** | **C** | **A** | **T** | **T** | **G** | **A** | **A** | **T** | **T** | **A** | **T** | **T** | **T** | **G** | **G** | **A** | **T** | **G** | **T** | **C** | **T** | **T** | **C** | **A** | **A** | **T** | **C** | **A** | **A** | **G** | **A** | **A** | **C** | **A** | **A** | **T** | **T** | **A** | **G** | **A** | **A** | **C** | **A** | **A** | **C** | **T** | **G** | **A** | **T** | **C** | **T** | **T** | **G** | **A** | **A** | **C** | **G** | **T** | **G** | **A** | **A** | **A** | **C** | **C** | **T** | **G** | **C** | **C** | **A** | **T** | **G** | **T** | **T** | **A** | **C** | **T** | **G** | **G** | **T** | **G** | **G** | **A** | **A** | **C** | **A** | **T** | **G** | **A** | **T** | **C** | **A** | **A** | **A** | **C** | **C** | **T** | **T** | **C** | **C** | **T** | **G** | **G** | **A** | **T** | **A** | **A** | **A** | **G** | **T** | **A** | **T** | **C** | **T** | **A** | **C** | **T** | **G** | **A** | **G** | **A** | **T** | **T** | **A** | **T** | **T** | **T** | **C** | **T** | **C** | **T** | **T** | **G** | **A** | **G** | **A** | **A** | **A** | **A** | **T** | **C** | **-** | **-** | **-** |
| **N59504F** | **A** | **T** | **G** | **T** | **C** | **T** | **A** | **A** | **A** | **A** | **T** | **C** | **G** | **A** | **A** | **A** | **T** | **A** | **A** | **A** | **A** | **A** | **A** | **T** | **C** | **T** | **G** | **A** | **C** | **A** | **T** | **T** | **C** | **G** | **G** | **C** | **T** | **A** | **C** | **G** | **A** | **C** | **A** | **G** | **C** | **C** | **A** | **A** | **G** | **G** | **C** | **A** | **C** | **A** | **T** | **T** | **A** | **T** | **T** | **A** | **T** | **T** | **T** | **G** | **A** | **A** | **C** | **A** | **A** | **G** | **C** | **A** | **A** | **A** | **T** | **C** | **T** | **A** | **A** | **A** | **T** | **T** | **T** | **T** | **G** | **A** | **C** | **A** | **C** | **A** | **C** | **A** | **A** | **T** | **G** | **G** | **A** | **A** | **A** | **C** | **T** | **A** | **G** | **G** | **A** | **C** | **T** | **T** | **A** | **T** | **C** | **G** | **G** | **A** | **C** | **G** | **A** | **A** | **A** | **C** | **G** | **G** | **T** | **C** | **G** | **A** | **G** | **G** | **A** | **A** | **A** | **G** | **A** | **C** | **A** | **A** | **C** | **T** | **T** | **T** | **A** | **C** | **T** | **G** | **A** | **A** | **T** | **A** | **T** | **T** | **C** | **T** | **A** | **C** | **A** | **A** | **A** | **A** | **C** | **A** | **A** | **A** | **C** | **T** | **A** | **C** | **C** | **T** | **T** | **A** | **T** | **C** | **A** | **A** | **G** | **G** | **G** | **C** | **A** | **A** | **G** | **T** | **A** | **A** | **T** | **C** | **C** | **A** | **T** | **C** | **A** | **G** | **C** | **A** | **A** | **G** | **A** | **A** | **T** | **T** | **T** | **G** | **C** | **C** | **T** | **A** | **T** | **T** | **T** | **C** | **C** | **C** | **G** | **C** | **A** | **A** | **C** | **A** | **G** | **A** | **C** | **A** | **A** | **A** | **A** | **G** | **A** | **T** | **A** | **A** | **A** | **G** | **A** | **A** | **C** | **G** | **T** | **T** | **T** | **A** | **A** | **C** | **C** | **T** | **A** | **T** | **T** | **A** | **C** | **G** | **T** | **G** | **T** | **T** | **A** | **A** | **A** | **T** | **G** | **A** | **T** | **A** | **T** | **T** | **A** | **C** | **G** | **G** | **A** | **T** | **T** | **T** | **T** | **G** | **A** | **G** | **A** | **T** | **A** | **T** | **G** | **G** | **G** | **A** | **A** | **A** | **T** | **C** | **G** | **A** | **A** | **A** | **G** | **A** | **G** | **A** | **G** | **C** | **T** | **C** | **C** | **A** | **A** | **T** | **T** | **G** | **A** | **T** | **G** | **C** | **A** | **A** | **A** | **C** | **A** | **G** | **A** | **T** | **C** | **C** | **T** | **G** | **A** | **A** | **A** | **T** | **C** | **T** | **T** | **A** | **T** | **G** | **G** | **A** | **G** | **A** | **G** | **A** | **A** | **T** | **T** | **C** | **A** | **G** | **C** | **A** | **C** | **A** | **C** | **T** | **A** | **T** | **C** | **G** | **G** | **G** | **G** | **G** | **G** | **A** | **G** | **A** | **G** | **A** | **A** | **G** | **A** | **C** | **A** | **A** | **A** | **A** | **G** | **T** | **C** | **C** | **T** | **A** | **C** | **T** | **G** | **G** | **C** | **A** | **C** | **T** | **T** | **T** | **T** | **A** | **T** | **T** | **T** | **G** | **T** | **G** | **G** | **A** | **T** | **G** | **A** | **C** | **A** | **C** | **T** | **C** | **A** | **T** | **T** | **T** | **C** | **C** | **C** | **G** | **T** | **T** | **A** | **A** | **T** | **C** | **G** | **A** | **T** | **G** | **A** | **A** | **C** | **C** | **A** | **A** | **C** | **G** | **A** | **A** | **T** | **C** | **A** | **T** | **T** | **T** | **G** | **G** | **A** | **-** | **-** | **-** | **T** | **A** | **T** | **C** | **T** | **C** | **T** | **G** | **G** | **T** | **A** | **G** | **A** | **A** | **A** | **A** | **C** | **A** | **A** | **G** | **T** | **A** | **G** | **C** | **G** | **G** | **C** | **T** | **T** | **A** | **T** | **T** | **T** | **G** | **A** | **A** | **A** | **A** | **A** | **G** | **A** | **A** | **A** | **A** | **A** | **A** | **C** | **A** | **A** | **G** | **G** | **C** | **T** | **T** | **C** | **A** | **T** | **C** | **G** | **T** | **G** | **G** | **T** | **C** | **A** | **G** | **C** | **C** | **A** | **T** | **G** | **A** | **C** | **C** | **G** | **G** | **G** | **G** | **A** | **T** | **T** | **T** | **A** | **T** | **C** | **G** | **A** | **T** | **G** | **A** | **A** | **G** | **T** | **A** | **G** | **T** | **G** | **G** | **A** | **C** | **C** | **A** | **T** | **G** | **T** | **T** | **T** | **T** | **A** | **G** | **C** | **A** | **A** | **T** | **C** | **G** | **A** | **A** | **A** | **A** | **A** | **A** | **G** | **T** | **C** | **A** | **A** | **C** | **T** | **G** | **G** | **A** | **A** | **C** | **T** | **T** | **T** | **A** | **T** | **C** | **A** | **A** | **G** | **G** | **G** | **A** | **A** | **T** | **T** | **T** | **C** | **T** | **C** | **T** | **A** | **T** | **C** | **T** | **A** | **T** | **G** | **A** | **A** | **G** | **A** | **A** | **C** | **A** | **G** | **A** | **A** | **A** | **A** | **A** | **A** | **C** | **T** | **T** | **C** | **G** | **T** | **G** | **A** | **T** | **G** | **A** | **A** | **T** | **T** | **T** | **G** | **A** | **A** | **A** | **T** | **G** | **G** | **C** | **T** | **C** | **A** | **A** | **A** | **A** | **T** | **G** | **A** | **A** | **A** | **A** | **A** | **T** | **T** | **G** | **A** | **A** | **A** | **A** | **A** | **A** | **G** | **A** | **A** | **G** | **T** | **C** | **A** | **G** | **T** | **A** | **G** | **G** | **C** | **T** | **A** | **A** | **A** | **G** | **A** | **A** | **A** | **A** | **C** | **A** | **G** | **C** | **A** | **G** | **C** | **T** | **G** | **A** | **A** | **A** | **A** | **A** | **G** | **C** | **C** | **G** | **A** | **A** | **T** | **G** | **G** | **T** | **C** | **T** | **C** | **G** | **T** | **T** | **C** | **C** | **C** | **G** | **A** | **G** | **A** | **A** | **G** | **G** | **A** | **G** | **A** | **T** | **A** | **A** | **A** | **A** | **C** | **A** | **A** | **A** | **G** | **A** | **A** | **A** | **C** | **A** | **A** | **G** | **T** | **C** | **G** | **G** | **A** | **T** | **T** | **C** | **A** | **T** | **C** | **G** | **A** | **T** | **A** | **C** | **T** | **G** | **A** | **A** | **T** | **C** | **T** | **A** | **G** | **A** | **C** | **G** | **A** | **G** | **T** | **G** | **A** | **A** | **T** | **A** | **A** | **A** | **G** | **G** | **A** | **G** | **C** | **A** | **G** | **T** | **G** | **G** | **G** | **T** | **G** | **C** | **T** | **G** | **A** | **T** | **G** | **C** | **T** | **G** | **C** | **A** | **C** | **G** | **G** | **A** | **C** | **G** | **A** | **T** | **G** | **A** | **A** | **A** | **C** | **G** | **A** | **T** | **C** | **C** | **A** | **A** | **A** | **G** | **C** | **A** | **A** | **T** | **C** | **G** | **T** | **G** | **A** | **A** | **T** | **C** | **G** | **G** | **A** | **T** | **G** | **G** | **A** | **G** | **A** | **C** | **C** | **C** | **A** | **G** | **A** | **T** | **C** | **A** | **G** | **C** | **G** | **A** | **G** | **A** | **A** | **A** | **G** | **A** | **A** | **A** | **A** | **A** | **C** | **T** | **A** | **T** | **T** | **A** | **A** | **A** | **A** | **G** | **A** | **T** | **A** | **T** | **C** | **G** | **A** | **A** | **T** | **A** | **T** | **A** | **T** | **C** | **G** | **A** | **T** | **T** | **C** | **T** | **T** | **T** | **G** | **A** | **C** | **G** | **A** | **T** | **G** | **A** | **A** | **T** | **A** | **G** | **C** | **C** | **A** | **A** | **G** | **C** | **G** | **T** | **C** | **T** | **C** | **A** | **C** | **C** | **A** | **T** | **A** | **A** | **G** | **C** | **G** | **A** | **C** | **T** | **T** | **T** | **T** | **A** | **A** | **G** | **C** | **G** | **T** | **A** | **G** | **A** | **A** | **G** | **A** | **T** | **C** | **T** | **T** | **C** | **A** | **A** | **T** | **T** | **A** | **G** | **G** | **G** | **T** | **A** | **T** | **G** | **A** | **A** | **A** | **A** | **T** | **C** | **T** | **G** | **T** | **T** | **A** | **T** | **T** | **C** | **G** | **A** | **G** | **C** | **C** | **A** | **A** | **T** | **T** | **C** | **A** | **T** | **T** | **T** | **T** | **A** | **C** | **A** | **A** | **T** | **C** | **G** | **A** | **G** | **C** | **C** | **T** | **C** | **A** | **T** | **C** | **A** | **G** | **C** | **G** | **G** | **G** | **T** | **G** | **G** | **C** | **G** | **A** | **T** | **T** | **T** | **C** | **A** | **G** | **G** | **T** | **C** | **C** | **T** | **A** | **A** | **C** | **G** | **G** | **T** | **G** | **C** | **A** | **G** | **G** | **A** | **A** | **A** | **G** | **T** | **C** | **A** | **T** | **C** | **C** | **A** | **T** | **T** | **A** | **T** | **C** | **C** | **A** | **T** | **T** | **A** | **T** | **C** | **T** | **T** | **C** | **T** | **G** | **G** | **G** | **G** | **G** | **C** | **A** | **T** | **T** | **C** | **A** | **A** | **C** | **G** | **G** | **C** | **A** | **A** | **G** | **G** | **T** | **T** | **A** | **T** | **A** | **G** | **G** | **A** | **G** | **A** | **A** | **A** | **A** | **A** | **T** | **A** | **C** | **C** | **A** | **G** | **C** | **C** | **A** | **A** | **A** | **A** | **C** | **A** | **T** | **C** | **T** | **G** | **A** | **G** | **C** | **A** | **T** | **T** | **A** | **G** | **T** | **T** | **A** | **T** | **G** | **C** | **A** | **A** | **G** | **C** | **C** | **A** | **A** | **A** | **A** | **T** | **T** | **A** | **T** | **G** | **A** | **A** | **G** | **A** | **C** | **A** | **A** | **T** | **C** | **G** | **A** | **G** | **G** | **A** | **A** | **C** | **G** | **T** | **T** | **G** | **G** | **C** | **G** | **G** | **A** | **A** | **T** | **T** | **T** | **G** | **C** | **A** | **G** | **A** | **G** | **A** | **A** | **A** | **A** | **A** | **C** | **C** | **A** | **A** | **G** | **T** | **A** | **G** | **A** | **C** | **T** | **A** | **C** | **C** | **A** | **A** | **G** | **C** | **A** | **T** | **T** | **T** | **T** | **T** | **G** | **A** | **A** | **C** | **A** | **A** | **C** | **C** | **T** | **C** | **C** | **G** | **A** | **A** | **A** | **G** | **C** | **T** | **T** | **G** | **G** | **G** | **A** | **T** | **G** | **G** | **A** | **A** | **A** | **G** | **A** | **G** | **A** | **T** | **G** | **T** | **T** | **T** | **T** | **T** | **C** | **A** | **T** | **A** | **A** | **C** | **A** | **A** | **G** | **A** | **T** | **C** | **G** | **A** | **G** | **C** | **A** | **G** | **A** | **T** | **G** | **A** | **G** | **T** | **A** | **T** | **G** | **G** | **G** | **C** | **C** | **A** | **A** | **C** | **G** | **G** | **A** | **A** | **A** | **A** | **A** | **A** | **G** | **T** | **G** | **G** | **A** | **A** | **T** | **T** | **G** | **G** | **C** | **T** | **A** | **A** | **A** | **T** | **C** | **T** | **T** | **T** | **A** | **T** | **C** | **A** | **C** | **A** | **G** | **C** | **C** | **A** | **G** | **C** | **T** | **G** | **A** | **A** | **C** | **T** | **A** | **T** | **A** | **T** | **A** | **C** | **A** | **T** | **G** | **G** | **G** | **A** | **T** | **G** | **A** | **A** | **C** | **C** | **A** | **T** | **T** | **G** | **A** | **A** | **T** | **T** | **A** | **T** | **T** | **T** | **G** | **G** | **A** | **T** | **G** | **T** | **C** | **T** | **T** | **C** | **A** | **A** | **T** | **C** | **A** | **A** | **G** | **A** | **A** | **C** | **A** | **A** | **T** | **T** | **A** | **G** | **A** | **A** | **C** | **A** | **A** | **C** | **T** | **G** | **A** | **T** | **C** | **T** | **T** | **G** | **A** | **A** | **C** | **G** | **T** | **G** | **A** | **A** | **A** | **C** | **C** | **T** | **G** | **C** | **C** | **A** | **T** | **G** | **T** | **T** | **A** | **C** | **T** | **A** | **G** | **T** | **G** | **G** | **A** | **A** | **C** | **A** | **T** | **G** | **A** | **T** | **C** | **A** | **A** | **A** | **C** | **C** | **T** | **T** | **C** | **C** | **T** | **G** | **G** | **A** | **T** | **A** | **A** | **A** | **G** | **T** | **A** | **T** | **C** | **T** | **A** | **C** | **T** | **G** | **A** | **G** | **A** | **T** | **T** | **A** | **T** | **T** | **T** | **C** | **T** | **C** | **T** | **T** | **G** | **A** | **G** | **A** | **G** | **A** | **A** | **T** | **C** | **-** | **-** | **-** |
| **N59947F** | **A** | **T** | **G** | **T** | **C** | **T** | **A** | **A** | **A** | **A** | **T** | **C** | **G** | **A** | **A** | **A** | **T** | **A** | **A** | **A** | **A** | **A** | **A** | **T** | **C** | **T** | **G** | **A** | **C** | **A** | **T** | **T** | **C** | **G** | **G** | **C** | **T** | **A** | **C** | **G** | **A** | **C** | **A** | **G** | **C** | **C** | **A** | **A** | **G** | **G** | **C** | **A** | **C** | **A** | **T** | **T** | **A** | **T** | **T** | **A** | **T** | **T** | **T** | **G** | **A** | **A** | **C** | **A** | **A** | **G** | **C** | **A** | **A** | **A** | **T** | **C** | **T** | **A** | **A** | **A** | **T** | **T** | **T** | **T** | **G** | **A** | **C** | **A** | **C** | **A** | **C** | **A** | **A** | **T** | **G** | **G** | **A** | **A** | **A** | **C** | **T** | **A** | **G** | **G** | **A** | **C** | **T** | **T** | **A** | **T** | **C** | **G** | **G** | **A** | **C** | **G** | **A** | **A** | **A** | **C** | **G** | **G** | **T** | **C** | **G** | **A** | **G** | **G** | **A** | **A** | **A** | **G** | **A** | **C** | **A** | **A** | **C** | **T** | **T** | **T** | **A** | **C** | **T** | **G** | **A** | **A** | **T** | **A** | **T** | **T** | **C** | **T** | **A** | **C** | **A** | **A** | **A** | **A** | **C** | **A** | **A** | **A** | **C** | **T** | **A** | **C** | **C** | **T** | **T** | **A** | **T** | **C** | **A** | **A** | **G** | **G** | **G** | **C** | **A** | **A** | **G** | **T** | **A** | **A** | **T** | **C** | **C** | **A** | **T** | **C** | **A** | **G** | **C** | **A** | **A** | **G** | **A** | **A** | **T** | **T** | **T** | **G** | **T** | **C** | **T** | **A** | **T** | **T** | **T** | **C** | **C** | **C** | **G** | **C** | **A** | **A** | **C** | **A** | **G** | **A** | **C** | **A** | **A** | **A** | **A** | **G** | **A** | **T** | **A** | **A** | **A** | **G** | **A** | **A** | **C** | **G** | **T** | **T** | **T** | **A** | **A** | **C** | **C** | **T** | **A** | **T** | **T** | **A** | **C** | **G** | **T** | **G** | **T** | **T** | **A** | **A** | **A** | **T** | **G** | **A** | **T** | **A** | **T** | **T** | **A** | **C** | **G** | **G** | **A** | **T** | **T** | **T** | **T** | **G** | **A** | **G** | **A** | **T** | **A** | **T** | **G** | **G** | **G** | **A** | **A** | **A** | **T** | **C** | **G** | **A** | **A** | **A** | **G** | **A** | **G** | **A** | **G** | **C** | **T** | **C** | **C** | **A** | **A** | **T** | **T** | **G** | **A** | **T** | **G** | **C** | **A** | **A** | **A** | **C** | **A** | **G** | **A** | **T** | **C** | **C** | **T** | **G** | **A** | **A** | **A** | **T** | **C** | **T** | **T** | **A** | **T** | **G** | **G** | **A** | **G** | **A** | **G** | **A** | **A** | **T** | **T** | **C** | **A** | **G** | **C** | **A** | **C** | **A** | **C** | **T** | **A** | **T** | **C** | **A** | **G** | **G** | **G** | **G** | **G** | **A** | **G** | **A** | **G** | **A** | **A** | **G** | **A** | **C** | **A** | **A** | **A** | **A** | **G** | **T** | **C** | **C** | **T** | **A** | **C** | **T** | **G** | **G** | **C** | **A** | **C** | **T** | **T** | **T** | **T** | **A** | **T** | **T** | **T** | **G** | **T** | **G** | **G** | **A** | **T** | **G** | **A** | **C** | **A** | **C** | **T** | **C** | **A** | **T** | **T** | **T** | **C** | **C** | **C** | **G** | **T** | **T** | **A** | **A** | **T** | **C** | **G** | **A** | **T** | **G** | **A** | **A** | **C** | **C** | **A** | **A** | **C** | **G** | **A** | **A** | **T** | **C** | **A** | **T** | **T** | **T** | **G** | **G** | **A** | **-** | **-** | **-** | **T** | **A** | **T** | **C** | **T** | **C** | **T** | **G** | **G** | **T** | **A** | **G** | **A** | **A** | **A** | **A** | **C** | **A** | **A** | **G** | **T** | **A** | **G** | **C** | **G** | **G** | **C** | **T** | **T** | **A** | **T** | **T** | **T** | **G** | **A** | **A** | **A** | **A** | **A** | **G** | **A** | **A** | **A** | **A** | **A** | **A** | **C** | **A** | **A** | **G** | **G** | **C** | **T** | **T** | **C** | **A** | **T** | **C** | **G** | **T** | **G** | **G** | **T** | **C** | **A** | **G** | **C** | **C** | **A** | **T** | **G** | **A** | **C** | **C** | **G** | **G** | **G** | **G** | **A** | **T** | **T** | **T** | **A** | **T** | **C** | **G** | **A** | **T** | **G** | **A** | **A** | **G** | **T** | **A** | **G** | **T** | **G** | **G** | **A** | **C** | **C** | **A** | **T** | **G** | **T** | **T** | **T** | **T** | **A** | **G** | **C** | **A** | **A** | **T** | **C** | **G** | **A** | **A** | **A** | **A** | **A** | **A** | **G** | **T** | **C** | **A** | **A** | **C** | **T** | **G** | **G** | **A** | **A** | **C** | **T** | **T** | **T** | **A** | **T** | **C** | **A** | **A** | **G** | **G** | **G** | **A** | **A** | **T** | **T** | **T** | **C** | **T** | **C** | **T** | **A** | **T** | **C** | **T** | **A** | **T** | **G** | **A** | **A** | **G** | **A** | **A** | **C** | **A** | **G** | **A** | **A** | **A** | **A** | **A** | **A** | **C** | **T** | **T** | **C** | **G** | **T** | **G** | **A** | **T** | **G** | **A** | **A** | **T** | **T** | **T** | **G** | **A** | **A** | **A** | **T** | **G** | **G** | **C** | **T** | **C** | **A** | **A** | **A** | **A** | **T** | **G** | **A** | **A** | **A** | **A** | **A** | **T** | **T** | **G** | **A** | **A** | **A** | **A** | **A** | **A** | **G** | **A** | **A** | **G** | **T** | **C** | **A** | **G** | **T** | **A** | **G** | **G** | **C** | **T** | **A** | **A** | **A** | **G** | **A** | **A** | **A** | **A** | **C** | **A** | **G** | **C** | **A** | **G** | **C** | **T** | **G** | **A** | **A** | **A** | **A** | **A** | **G** | **C** | **C** | **G** | **A** | **A** | **T** | **G** | **G** | **T** | **C** | **T** | **C** | **G** | **T** | **T** | **C** | **C** | **C** | **G** | **A** | **G** | **A** | **A** | **G** | **G** | **A** | **G** | **A** | **T** | **A** | **A** | **A** | **A** | **C** | **A** | **A** | **A** | **G** | **A** | **A** | **A** | **C** | **A** | **A** | **G** | **T** | **C** | **G** | **G** | **A** | **T** | **T** | **C** | **A** | **T** | **C** | **G** | **A** | **T** | **A** | **C** | **T** | **G** | **A** | **A** | **T** | **C** | **T** | **A** | **G** | **A** | **C** | **G** | **A** | **G** | **T** | **G** | **A** | **A** | **T** | **A** | **A** | **A** | **G** | **G** | **A** | **G** | **C** | **A** | **G** | **T** | **G** | **G** | **G** | **T** | **G** | **C** | **T** | **G** | **A** | **T** | **G** | **C** | **T** | **G** | **C** | **A** | **C** | **G** | **G** | **A** | **C** | **G** | **A** | **T** | **G** | **A** | **A** | **A** | **C** | **G** | **A** | **T** | **C** | **C** | **A** | **A** | **A** | **G** | **C** | **A** | **A** | **T** | **C** | **G** | **T** | **G** | **A** | **A** | **T** | **C** | **G** | **G** | **A** | **T** | **G** | **G** | **A** | **G** | **A** | **C** | **C** | **C** | **A** | **G** | **A** | **T** | **C** | **A** | **G** | **C** | **G** | **A** | **G** | **A** | **A** | **A** | **G** | **A** | **A** | **A** | **A** | **A** | **C** | **T** | **A** | **T** | **T** | **A** | **A** | **A** | **A** | **G** | **A** | **T** | **A** | **T** | **C** | **G** | **A** | **A** | **T** | **A** | **T** | **A** | **T** | **C** | **G** | **A** | **T** | **T** | **C** | **T** | **T** | **T** | **G** | **A** | **C** | **G** | **A** | **T** | **G** | **A** | **A** | **T** | **A** | **G** | **C** | **C** | **A** | **A** | **G** | **C** | **G** | **T** | **C** | **T** | **C** | **A** | **C** | **C** | **A** | **T** | **A** | **A** | **G** | **C** | **G** | **A** | **C** | **T** | **T** | **T** | **T** | **A** | **A** | **G** | **C** | **G** | **T** | **A** | **G** | **A** | **A** | **G** | **A** | **T** | **C** | **T** | **T** | **C** | **A** | **A** | **T** | **T** | **A** | **G** | **G** | **G** | **T** | **A** | **T** | **G** | **A** | **A** | **A** | **A** | **T** | **C** | **T** | **G** | **T** | **T** | **A** | **T** | **T** | **C** | **G** | **A** | **G** | **C** | **C** | **A** | **A** | **T** | **T** | **C** | **A** | **T** | **T** | **T** | **T** | **A** | **C** | **A** | **A** | **T** | **C** | **G** | **A** | **G** | **C** | **C** | **T** | **C** | **A** | **T** | **C** | **A** | **G** | **C** | **G** | **G** | **G** | **T** | **G** | **G** | **C** | **G** | **A** | **T** | **T** | **T** | **C** | **A** | **G** | **G** | **A** | **C** | **C** | **T** | **A** | **A** | **C** | **G** | **G** | **T** | **G** | **C** | **A** | **G** | **G** | **A** | **A** | **A** | **G** | **T** | **C** | **A** | **T** | **C** | **C** | **A** | **T** | **T** | **A** | **T** | **C** | **C** | **A** | **T** | **T** | **A** | **T** | **C** | **T** | **T** | **C** | **T** | **G** | **G** | **G** | **G** | **G** | **C** | **A** | **T** | **T** | **C** | **A** | **A** | **C** | **G** | **G** | **C** | **A** | **A** | **G** | **G** | **T** | **T** | **A** | **T** | **A** | **G** | **G** | **A** | **G** | **A** | **A** | **A** | **A** | **A** | **T** | **C** | **C** | **C** | **A** | **G** | **C** | **C** | **A** | **A** | **A** | **A** | **C** | **A** | **T** | **C** | **T** | **G** | **A** | **G** | **C** | **A** | **T** | **T** | **A** | **G** | **T** | **T** | **A** | **T** | **G** | **T** | **A** | **C** | **G** | **C** | **C** | **A** | **A** | **A** | **A** | **T** | **T** | **A** | **T** | **G** | **A** | **A** | **G** | **A** | **C** | **A** | **A** | **T** | **C** | **G** | **A** | **G** | **G** | **A** | **A** | **C** | **G** | **T** | **T** | **G** | **G** | **C** | **G** | **G** | **A** | **A** | **T** | **T** | **T** | **G** | **C** | **A** | **G** | **A** | **G** | **A** | **A** | **A** | **A** | **A** | **C** | **C** | **A** | **A** | **G** | **T** | **A** | **G** | **A** | **C** | **T** | **A** | **C** | **C** | **A** | **A** | **G** | **C** | **A** | **T** | **T** | **T** | **T** | **T** | **G** | **A** | **A** | **C** | **A** | **A** | **C** | **C** | **T** | **C** | **C** | **G** | **A** | **A** | **A** | **G** | **C** | **T** | **T** | **G** | **G** | **G** | **A** | **T** | **G** | **G** | **A** | **A** | **A** | **G** | **A** | **G** | **A** | **T** | **G** | **T** | **T** | **T** | **T** | **T** | **C** | **A** | **T** | **A** | **A** | **C** | **A** | **A** | **G** | **A** | **T** | **C** | **G** | **A** | **G** | **C** | **A** | **G** | **A** | **T** | **G** | **A** | **G** | **T** | **A** | **T** | **G** | **G** | **G** | **C** | **C** | **A** | **A** | **C** | **G** | **G** | **A** | **A** | **A** | **A** | **A** | **A** | **G** | **T** | **G** | **G** | **A** | **A** | **T** | **T** | **G** | **G** | **C** | **T** | **A** | **A** | **A** | **T** | **C** | **T** | **T** | **T** | **A** | **T** | **C** | **A** | **C** | **A** | **G** | **C** | **C** | **A** | **G** | **C** | **T** | **G** | **A** | **A** | **C** | **T** | **A** | **T** | **A** | **T** | **A** | **T** | **A** | **T** | **G** | **G** | **G** | **A** | **T** | **G** | **A** | **A** | **C** | **C** | **A** | **T** | **T** | **G** | **A** | **A** | **T** | **T** | **A** | **T** | **T** | **T** | **G** | **G** | **A** | **T** | **G** | **T** | **C** | **T** | **T** | **C** | **A** | **A** | **T** | **C** | **A** | **A** | **G** | **A** | **A** | **C** | **A** | **A** | **T** | **T** | **A** | **G** | **A** | **A** | **C** | **A** | **A** | **C** | **T** | **G** | **A** | **T** | **C** | **T** | **T** | **G** | **A** | **A** | **C** | **G** | **T** | **G** | **A** | **A** | **A** | **C** | **C** | **T** | **G** | **C** | **C** | **A** | **T** | **G** | **T** | **T** | **A** | **C** | **T** | **A** | **G** | **T** | **G** | **G** | **A** | **A** | **C** | **A** | **T** | **G** | **A** | **T** | **C** | **A** | **A** | **A** | **C** | **C** | **T** | **T** | **C** | **C** | **T** | **G** | **G** | **A** | **T** | **A** | **A** | **A** | **G** | **T** | **A** | **T** | **C** | **T** | **A** | **C** | **T** | **G** | **A** | **G** | **A** | **T** | **T** | **A** | **T** | **T** | **T** | **C** | **T** | **C** | **T** | **T** | **G** | **A** | **G** | **A** | **A** | **A** | **A** | **T** | **C** | **-** | **-** | **-** |
| **N52732** | **A** | **T** | **G** | **T** | **C** | **T** | **A** | **A** | **A** | **A** | **T** | **C** | **G** | **A** | **A** | **A** | **T** | **A** | **A** | **A** | **A** | **A** | **A** | **T** | **C** | **T** | **G** | **A** | **C** | **A** | **T** | **T** | **C** | **G** | **G** | **C** | **T** | **A** | **C** | **G** | **A** | **C** | **A** | **G** | **C** | **C** | **A** | **A** | **G** | **G** | **C** | **A** | **C** | **A** | **T** | **T** | **A** | **T** | **T** | **A** | **T** | **T** | **T** | **G** | **A** | **A** | **C** | **A** | **A** | **G** | **C** | **A** | **A** | **A** | **T** | **C** | **T** | **A** | **A** | **A** | **T** | **T** | **T** | **T** | **G** | **A** | **C** | **A** | **C** | **A** | **C** | **A** | **A** | **T** | **G** | **G** | **A** | **A** | **A** | **C** | **T** | **A** | **G** | **G** | **A** | **C** | **T** | **T** | **A** | **T** | **C** | **G** | **G** | **A** | **C** | **G** | **A** | **A** | **A** | **C** | **G** | **G** | **T** | **C** | **G** | **A** | **G** | **G** | **A** | **A** | **A** | **G** | **A** | **C** | **A** | **A** | **C** | **T** | **T** | **T** | **A** | **C** | **T** | **G** | **A** | **A** | **T** | **A** | **T** | **T** | **C** | **T** | **A** | **C** | **A** | **A** | **A** | **A** | **C** | **A** | **A** | **A** | **C** | **T** | **A** | **C** | **C** | **T** | **T** | **A** | **T** | **C** | **A** | **A** | **G** | **G** | **G** | **C** | **A** | **A** | **G** | **T** | **A** | **A** | **T** | **C** | **C** | **A** | **T** | **C** | **A** | **G** | **C** | **A** | **A** | **G** | **A** | **A** | **T** | **T** | **T** | **G** | **T** | **C** | **T** | **A** | **T** | **T** | **T** | **C** | **C** | **C** | **G** | **C** | **A** | **A** | **C** | **A** | **G** | **A** | **C** | **A** | **A** | **A** | **A** | **G** | **A** | **T** | **A** | **A** | **A** | **G** | **A** | **A** | **C** | **G** | **T** | **T** | **T** | **A** | **A** | **C** | **C** | **T** | **A** | **T** | **T** | **A** | **C** | **G** | **T** | **G** | **T** | **T** | **A** | **A** | **A** | **T** | **G** | **A** | **T** | **A** | **T** | **T** | **A** | **C** | **G** | **G** | **A** | **T** | **T** | **T** | **T** | **G** | **A** | **G** | **A** | **T** | **A** | **T** | **G** | **G** | **G** | **A** | **A** | **A** | **T** | **C** | **G** | **A** | **A** | **A** | **G** | **A** | **G** | **A** | **G** | **C** | **T** | **C** | **C** | **A** | **A** | **T** | **T** | **G** | **A** | **T** | **A** | **C** | **A** | **A** | **A** | **C** | **A** | **G** | **A** | **T** | **C** | **C** | **T** | **G** | **A** | **A** | **A** | **T** | **C** | **T** | **T** | **A** | **T** | **G** | **G** | **A** | **G** | **A** | **G** | **A** | **A** | **T** | **T** | **C** | **A** | **G** | **C** | **A** | **C** | **A** | **C** | **T** | **A** | **T** | **C** | **G** | **G** | **G** | **G** | **G** | **G** | **A** | **G** | **A** | **G** | **A** | **A** | **G** | **A** | **C** | **A** | **A** | **A** | **A** | **G** | **T** | **C** | **C** | **T** | **A** | **C** | **T** | **G** | **G** | **C** | **A** | **C** | **T** | **T** | **T** | **T** | **A** | **T** | **T** | **T** | **G** | **T** | **G** | **G** | **A** | **T** | **G** | **A** | **C** | **A** | **C** | **T** | **C** | **A** | **T** | **T** | **T** | **C** | **C** | **C** | **G** | **T** | **T** | **A** | **A** | **T** | **C** | **G** | **A** | **T** | **G** | **A** | **A** | **C** | **C** | **A** | **A** | **C** | **G** | **A** | **A** | **T** | **C** | **A** | **T** | **T** | **T** | **G** | **G** | **A** | **-** | **-** | **-** | **T** | **A** | **T** | **C** | **T** | **C** | **T** | **G** | **G** | **T** | **A** | **G** | **A** | **A** | **A** | **A** | **C** | **A** | **A** | **G** | **T** | **A** | **G** | **C** | **G** | **G** | **C** | **T** | **T** | **A** | **T** | **T** | **T** | **G** | **A** | **A** | **A** | **A** | **A** | **G** | **A** | **A** | **A** | **A** | **A** | **A** | **C** | **A** | **A** | **G** | **G** | **C** | **T** | **T** | **C** | **A** | **T** | **C** | **G** | **T** | **G** | **G** | **T** | **C** | **A** | **G** | **C** | **C** | **A** | **T** | **G** | **A** | **C** | **C** | **G** | **G** | **G** | **G** | **A** | **T** | **T** | **T** | **A** | **T** | **C** | **G** | **A** | **T** | **G** | **A** | **A** | **G** | **T** | **A** | **G** | **T** | **G** | **G** | **A** | **C** | **C** | **A** | **T** | **G** | **T** | **T** | **T** | **T** | **A** | **G** | **C** | **A** | **A** | **T** | **C** | **G** | **A** | **A** | **A** | **A** | **A** | **A** | **G** | **T** | **C** | **A** | **A** | **C** | **T** | **G** | **G** | **A** | **A** | **C** | **T** | **T** | **T** | **A** | **T** | **C** | **A** | **A** | **G** | **G** | **G** | **A** | **A** | **T** | **T** | **T** | **C** | **T** | **C** | **T** | **A** | **T** | **C** | **T** | **A** | **T** | **G** | **A** | **A** | **G** | **A** | **A** | **C** | **A** | **G** | **A** | **A** | **A** | **A** | **A** | **A** | **C** | **T** | **T** | **C** | **G** | **T** | **G** | **A** | **T** | **G** | **A** | **A** | **T** | **T** | **T** | **G** | **A** | **A** | **A** | **T** | **G** | **G** | **C** | **T** | **C** | **A** | **A** | **A** | **A** | **T** | **G** | **A** | **A** | **A** | **A** | **A** | **T** | **T** | **G** | **A** | **A** | **A** | **A** | **A** | **A** | **G** | **A** | **A** | **G** | **T** | **C** | **A** | **G** | **T** | **A** | **G** | **G** | **C** | **T** | **A** | **A** | **A** | **G** | **A** | **A** | **A** | **A** | **C** | **A** | **G** | **C** | **A** | **G** | **C** | **T** | **G** | **A** | **A** | **A** | **A** | **A** | **G** | **C** | **C** | **G** | **A** | **A** | **T** | **G** | **G** | **T** | **C** | **T** | **C** | **G** | **T** | **T** | **C** | **C** | **C** | **G** | **A** | **G** | **A** | **A** | **G** | **G** | **A** | **G** | **A** | **T** | **A** | **A** | **A** | **A** | **C** | **A** | **A** | **A** | **G** | **A** | **A** | **A** | **C** | **A** | **A** | **G** | **T** | **C** | **G** | **G** | **A** | **T** | **T** | **C** | **A** | **T** | **C** | **G** | **A** | **T** | **A** | **C** | **T** | **G** | **A** | **A** | **T** | **C** | **T** | **A** | **G** | **A** | **C** | **G** | **A** | **G** | **T** | **G** | **A** | **A** | **T** | **A** | **A** | **A** | **G** | **G** | **A** | **G** | **C** | **A** | **G** | **T** | **G** | **G** | **G** | **T** | **G** | **C** | **T** | **G** | **A** | **T** | **G** | **C** | **T** | **G** | **C** | **A** | **C** | **G** | **G** | **A** | **C** | **G** | **A** | **T** | **G** | **A** | **A** | **A** | **C** | **G** | **A** | **T** | **C** | **C** | **A** | **A** | **A** | **G** | **C** | **A** | **A** | **T** | **C** | **G** | **T** | **G** | **A** | **A** | **T** | **C** | **G** | **G** | **A** | **T** | **G** | **G** | **A** | **G** | **A** | **C** | **C** | **C** | **A** | **G** | **A** | **T** | **C** | **A** | **G** | **C** | **G** | **A** | **G** | **A** | **A** | **A** | **G** | **A** | **A** | **A** | **A** | **A** | **C** | **T** | **A** | **T** | **T** | **A** | **A** | **A** | **A** | **G** | **A** | **T** | **A** | **T** | **C** | **G** | **A** | **A** | **T** | **A** | **T** | **A** | **T** | **C** | **G** | **A** | **T** | **T** | **C** | **T** | **T** | **T** | **G** | **A** | **C** | **G** | **A** | **T** | **G** | **A** | **A** | **T** | **A** | **G** | **C** | **C** | **A** | **A** | **G** | **C** | **G** | **T** | **C** | **T** | **C** | **A** | **C** | **C** | **A** | **T** | **A** | **A** | **G** | **C** | **G** | **A** | **C** | **T** | **T** | **T** | **T** | **A** | **A** | **G** | **C** | **G** | **T** | **A** | **G** | **A** | **A** | **G** | **A** | **T** | **C** | **T** | **T** | **C** | **A** | **A** | **T** | **T** | **A** | **G** | **G** | **G** | **T** | **A** | **T** | **G** | **A** | **A** | **A** | **A** | **T** | **C** | **T** | **G** | **T** | **T** | **A** | **T** | **T** | **C** | **G** | **A** | **G** | **C** | **C** | **A** | **A** | **T** | **T** | **C** | **A** | **T** | **T** | **T** | **T** | **A** | **C** | **A** | **A** | **T** | **C** | **G** | **A** | **G** | **C** | **C** | **T** | **C** | **A** | **T** | **C** | **A** | **G** | **C** | **G** | **G** | **G** | **T** | **G** | **G** | **C** | **G** | **A** | **T** | **T** | **T** | **C** | **A** | **G** | **G** | **T** | **C** | **C** | **T** | **A** | **A** | **C** | **G** | **G** | **T** | **G** | **C** | **A** | **G** | **G** | **A** | **A** | **A** | **G** | **T** | **C** | **A** | **T** | **C** | **C** | **A** | **T** | **T** | **A** | **T** | **C** | **C** | **A** | **T** | **T** | **A** | **T** | **C** | **T** | **T** | **C** | **T** | **G** | **G** | **G** | **G** | **G** | **C** | **A** | **T** | **T** | **C** | **A** | **A** | **C** | **G** | **G** | **C** | **A** | **A** | **G** | **G** | **T** | **T** | **A** | **T** | **A** | **G** | **G** | **A** | **G** | **A** | **A** | **A** | **A** | **A** | **T** | **C** | **C** | **C** | **A** | **G** | **C** | **C** | **A** | **A** | **A** | **A** | **C** | **A** | **T** | **C** | **T** | **G** | **A** | **G** | **C** | **A** | **T** | **T** | **A** | **G** | **T** | **T** | **A** | **T** | **G** | **T** | **A** | **C** | **G** | **C** | **C** | **A** | **A** | **A** | **A** | **T** | **T** | **A** | **T** | **G** | **A** | **A** | **G** | **A** | **T** | **A** | **A** | **T** | **C** | **G** | **A** | **G** | **G** | **A** | **A** | **C** | **G** | **T** | **T** | **G** | **G** | **C** | **G** | **G** | **A** | **A** | **T** | **T** | **T** | **G** | **C** | **A** | **G** | **A** | **G** | **A** | **A** | **A** | **A** | **A** | **C** | **C** | **A** | **A** | **G** | **T** | **A** | **G** | **A** | **C** | **T** | **A** | **C** | **C** | **A** | **A** | **G** | **C** | **A** | **T** | **T** | **T** | **T** | **T** | **G** | **A** | **A** | **C** | **A** | **A** | **C** | **C** | **T** | **C** | **C** | **G** | **A** | **A** | **A** | **G** | **C** | **T** | **T** | **G** | **G** | **G** | **A** | **T** | **G** | **G** | **A** | **A** | **A** | **G** | **A** | **G** | **A** | **T** | **G** | **T** | **T** | **T** | **T** | **T** | **C** | **A** | **T** | **A** | **A** | **C** | **A** | **A** | **G** | **A** | **T** | **T** | **G** | **A** | **G** | **C** | **A** | **G** | **A** | **T** | **G** | **A** | **G** | **T** | **A** | **T** | **G** | **G** | **G** | **C** | **C** | **A** | **A** | **C** | **G** | **G** | **A** | **A** | **A** | **A** | **A** | **A** | **G** | **T** | **G** | **G** | **A** | **A** | **T** | **T** | **G** | **G** | **C** | **T** | **A** | **A** | **A** | **T** | **C** | **T** | **T** | **T** | **A** | **T** | **C** | **A** | **C** | **A** | **G** | **C** | **C** | **A** | **G** | **C** | **T** | **G** | **A** | **A** | **C** | **T** | **A** | **T** | **A** | **T** | **A** | **T** | **A** | **T** | **G** | **G** | **G** | **A** | **T** | **G** | **A** | **A** | **C** | **C** | **A** | **T** | **T** | **G** | **A** | **A** | **T** | **T** | **A** | **T** | **T** | **T** | **G** | **G** | **A** | **T** | **G** | **T** | **C** | **T** | **T** | **C** | **A** | **A** | **T** | **C** | **A** | **A** | **G** | **A** | **A** | **C** | **A** | **A** | **T** | **T** | **A** | **G** | **A** | **A** | **C** | **A** | **A** | **C** | **T** | **G** | **A** | **T** | **C** | **T** | **T** | **G** | **A** | **A** | **C** | **G** | **T** | **G** | **A** | **A** | **A** | **C** | **C** | **T** | **G** | **C** | **C** | **A** | **T** | **G** | **T** | **T** | **A** | **C** | **T** | **G** | **G** | **T** | **G** | **G** | **A** | **A** | **C** | **A** | **T** | **G** | **A** | **T** | **C** | **A** | **A** | **A** | **C** | **C** | **T** | **T** | **C** | **C** | **T** | **G** | **G** | **A** | **T** | **A** | **A** | **A** | **G** | **T** | **A** | **T** | **C** | **T** | **A** | **C** | **T** | **G** | **A** | **G** | **A** | **T** | **T** | **A** | **T** | **T** | **T** | **C** | **T** | **C** | **T** | **T** | **G** | **A** | **G** | **A** | **A** | **A** | **A** | **T** | **C** | **-** | **-** | **-** |
| **N60463F** | **A** | **T** | **G** | **T** | **C** | **T** | **A** | **A** | **A** | **A** | **T** | **C** | **G** | **A** | **A** | **A** | **T** | **A** | **A** | **A** | **A** | **A** | **A** | **T** | **C** | **T** | **G** | **A** | **A** | **A** | **T** | **T** | **C** | **G** | **G** | **C** | **T** | **A** | **C** | **G** | **A** | **C** | **A** | **G** | **C** | **C** | **A** | **A** | **G** | **G** | **C** | **A** | **C** | **A** | **T** | **T** | **A** | **T** | **T** | **A** | **T** | **T** | **T** | **G** | **A** | **A** | **C** | **A** | **A** | **G** | **C** | **A** | **A** | **A** | **T** | **C** | **T** | **A** | **A** | **A** | **T** | **T** | **T** | **T** | **G** | **A** | **C** | **A** | **C** | **A** | **C** | **A** | **A** | **T** | **G** | **G** | **A** | **A** | **A** | **C** | **T** | **A** | **G** | **G** | **A** | **C** | **T** | **T** | **A** | **T** | **C** | **G** | **G** | **A** | **C** | **G** | **A** | **A** | **A** | **C** | **G** | **G** | **T** | **C** | **G** | **A** | **G** | **G** | **A** | **A** | **A** | **G** | **A** | **C** | **A** | **A** | **C** | **T** | **T** | **T** | **A** | **C** | **T** | **G** | **A** | **A** | **T** | **A** | **T** | **T** | **C** | **T** | **A** | **C** | **A** | **A** | **A** | **A** | **T** | **A** | **A** | **A** | **C** | **T** | **A** | **C** | **C** | **T** | **T** | **A** | **T** | **C** | **A** | **A** | **G** | **G** | **G** | **C** | **A** | **A** | **G** | **T** | **A** | **A** | **T** | **C** | **C** | **A** | **T** | **C** | **A** | **G** | **C** | **A** | **A** | **G** | **A** | **A** | **T** | **T** | **T** | **G** | **T** | **C** | **T** | **A** | **T** | **T** | **T** | **C** | **C** | **C** | **G** | **C** | **A** | **A** | **C** | **A** | **G** | **A** | **C** | **A** | **A** | **A** | **A** | **G** | **A** | **T** | **A** | **A** | **A** | **G** | **A** | **A** | **C** | **G** | **T** | **T** | **T** | **A** | **A** | **C** | **C** | **T** | **A** | **T** | **T** | **A** | **C** | **G** | **T** | **G** | **T** | **T** | **A** | **A** | **A** | **T** | **G** | **A** | **T** | **A** | **T** | **T** | **A** | **C** | **G** | **G** | **A** | **T** | **T** | **T** | **T** | **G** | **A** | **G** | **A** | **T** | **A** | **T** | **G** | **G** | **G** | **A** | **A** | **A** | **T** | **C** | **G** | **A** | **A** | **A** | **G** | **A** | **G** | **A** | **G** | **C** | **T** | **C** | **C** | **A** | **A** | **T** | **T** | **G** | **A** | **T** | **G** | **C** | **A** | **A** | **A** | **C** | **A** | **G** | **A** | **T** | **C** | **C** | **T** | **G** | **A** | **A** | **A** | **T** | **C** | **T** | **T** | **A** | **T** | **G** | **G** | **A** | **G** | **A** | **G** | **A** | **A** | **T** | **T** | **C** | **A** | **G** | **C** | **A** | **C** | **A** | **C** | **T** | **A** | **T** | **C** | **G** | **G** | **G** | **G** | **G** | **G** | **A** | **G** | **A** | **G** | **A** | **A** | **G** | **A** | **C** | **A** | **A** | **A** | **A** | **G** | **T** | **C** | **C** | **T** | **A** | **C** | **T** | **G** | **G** | **C** | **A** | **C** | **T** | **T** | **T** | **T** | **A** | **T** | **T** | **T** | **G** | **T** | **G** | **G** | **A** | **T** | **G** | **A** | **C** | **A** | **C** | **T** | **C** | **A** | **T** | **T** | **T** | **C** | **C** | **C** | **G** | **T** | **T** | **A** | **A** | **T** | **C** | **G** | **A** | **T** | **G** | **A** | **A** | **C** | **C** | **A** | **A** | **C** | **G** | **A** | **A** | **T** | **C** | **A** | **T** | **T** | **T** | **G** | **G** | **A** | **-** | **-** | **-** | **T** | **A** | **T** | **C** | **T** | **C** | **T** | **G** | **A** | **T** | **A** | **G** | **A** | **A** | **A** | **A** | **C** | **A** | **A** | **G** | **T** | **A** | **G** | **C** | **G** | **G** | **C** | **T** | **T** | **A** | **T** | **T** | **T** | **G** | **A** | **A** | **A** | **A** | **A** | **G** | **A** | **A** | **A** | **A** | **A** | **A** | **C** | **A** | **A** | **G** | **G** | **C** | **T** | **T** | **C** | **A** | **T** | **C** | **G** | **T** | **G** | **G** | **T** | **C** | **A** | **G** | **C** | **C** | **A** | **T** | **G** | **A** | **C** | **C** | **G** | **G** | **G** | **G** | **A** | **T** | **T** | **T** | **A** | **T** | **C** | **G** | **A** | **T** | **G** | **A** | **A** | **G** | **T** | **A** | **G** | **T** | **G** | **G** | **A** | **C** | **C** | **A** | **T** | **G** | **T** | **T** | **T** | **T** | **A** | **G** | **C** | **A** | **A** | **T** | **C** | **G** | **A** | **A** | **A** | **A** | **A** | **A** | **G** | **T** | **C** | **A** | **A** | **C** | **T** | **G** | **G** | **A** | **A** | **C** | **T** | **T** | **T** | **A** | **T** | **C** | **A** | **A** | **G** | **G** | **G** | **A** | **A** | **T** | **T** | **T** | **C** | **T** | **C** | **T** | **A** | **T** | **C** | **T** | **A** | **T** | **G** | **A** | **A** | **G** | **A** | **A** | **C** | **A** | **G** | **A** | **A** | **A** | **A** | **A** | **A** | **C** | **T** | **T** | **C** | **G** | **T** | **G** | **A** | **T** | **G** | **A** | **A** | **T** | **T** | **T** | **G** | **A** | **A** | **A** | **T** | **G** | **G** | **C** | **T** | **C** | **A** | **A** | **A** | **A** | **T** | **G** | **A** | **A** | **A** | **A** | **A** | **T** | **T** | **G** | **A** | **A** | **A** | **A** | **A** | **A** | **G** | **A** | **A** | **G** | **T** | **C** | **A** | **G** | **T** | **A** | **G** | **G** | **C** | **T** | **A** | **A** | **A** | **G** | **A** | **A** | **A** | **A** | **C** | **A** | **G** | **C** | **A** | **G** | **C** | **T** | **G** | **A** | **A** | **A** | **A** | **A** | **G** | **C** | **C** | **G** | **A** | **A** | **T** | **G** | **G** | **T** | **C** | **T** | **C** | **G** | **T** | **T** | **C** | **C** | **C** | **G** | **A** | **G** | **A** | **A** | **G** | **G** | **A** | **G** | **A** | **T** | **A** | **A** | **A** | **A** | **C** | **A** | **A** | **A** | **G** | **A** | **A** | **A** | **C** | **A** | **A** | **G** | **T** | **C** | **G** | **G** | **A** | **T** | **T** | **C** | **A** | **T** | **C** | **G** | **A** | **T** | **A** | **C** | **T** | **G** | **A** | **A** | **T** | **C** | **T** | **A** | **G** | **A** | **C** | **G** | **A** | **G** | **T** | **G** | **A** | **A** | **T** | **A** | **A** | **A** | **G** | **G** | **A** | **G** | **C** | **A** | **G** | **T** | **G** | **G** | **G** | **T** | **G** | **C** | **T** | **G** | **A** | **T** | **G** | **C** | **T** | **G** | **C** | **A** | **C** | **G** | **G** | **A** | **C** | **G** | **A** | **T** | **G** | **A** | **A** | **A** | **C** | **G** | **A** | **T** | **C** | **C** | **A** | **A** | **A** | **G** | **C** | **A** | **A** | **T** | **C** | **G** | **T** | **G** | **A** | **A** | **T** | **C** | **G** | **G** | **A** | **T** | **G** | **G** | **A** | **G** | **A** | **C** | **C** | **C** | **A** | **G** | **A** | **T** | **C** | **A** | **G** | **C** | **G** | **A** | **G** | **A** | **A** | **A** | **G** | **A** | **A** | **A** | **A** | **A** | **C** | **T** | **A** | **T** | **T** | **A** | **A** | **A** | **A** | **G** | **A** | **T** | **A** | **T** | **C** | **G** | **A** | **A** | **T** | **A** | **T** | **A** | **T** | **C** | **G** | **A** | **T** | **T** | **C** | **T** | **T** | **T** | **G** | **A** | **C** | **G** | **A** | **T** | **G** | **A** | **A** | **T** | **A** | **G** | **C** | **C** | **A** | **A** | **G** | **C** | **G** | **T** | **C** | **T** | **C** | **A** | **C** | **C** | **A** | **T** | **A** | **A** | **G** | **C** | **G** | **A** | **C** | **T** | **T** | **T** | **T** | **A** | **A** | **G** | **C** | **G** | **T** | **A** | **G** | **A** | **A** | **G** | **A** | **T** | **C** | **T** | **T** | **C** | **A** | **A** | **T** | **T** | **A** | **G** | **G** | **G** | **T** | **A** | **T** | **G** | **A** | **A** | **A** | **A** | **T** | **C** | **T** | **G** | **T** | **T** | **A** | **T** | **T** | **C** | **G** | **A** | **G** | **C** | **C** | **A** | **A** | **T** | **T** | **C** | **A** | **T** | **T** | **T** | **T** | **A** | **C** | **A** | **A** | **T** | **C** | **G** | **A** | **G** | **C** | **C** | **T** | **C** | **A** | **T** | **C** | **A** | **G** | **C** | **G** | **G** | **G** | **T** | **G** | **G** | **C** | **G** | **A** | **T** | **T** | **T** | **C** | **A** | **G** | **G** | **T** | **C** | **C** | **T** | **A** | **A** | **C** | **G** | **G** | **T** | **G** | **C** | **A** | **G** | **G** | **A** | **A** | **A** | **G** | **T** | **C** | **A** | **T** | **C** | **C** | **A** | **T** | **T** | **A** | **T** | **C** | **C** | **A** | **T** | **T** | **A** | **T** | **C** | **T** | **T** | **C** | **T** | **G** | **G** | **G** | **G** | **G** | **C** | **A** | **T** | **T** | **C** | **A** | **A** | **C** | **G** | **G** | **C** | **A** | **A** | **G** | **G** | **T** | **T** | **A** | **T** | **A** | **G** | **G** | **A** | **G** | **A** | **A** | **A** | **A** | **A** | **T** | **A** | **C** | **C** | **A** | **G** | **C** | **C** | **A** | **A** | **A** | **A** | **C** | **A** | **T** | **C** | **T** | **G** | **A** | **G** | **C** | **A** | **T** | **T** | **A** | **G** | **T** | **T** | **A** | **T** | **G** | **T** | **A** | **C** | **G** | **C** | **C** | **A** | **A** | **A** | **A** | **T** | **T** | **A** | **T** | **G** | **A** | **A** | **G** | **A** | **C** | **A** | **A** | **T** | **C** | **G** | **A** | **G** | **G** | **A** | **A** | **C** | **G** | **T** | **T** | **G** | **G** | **C** | **G** | **G** | **A** | **A** | **T** | **T** | **T** | **G** | **C** | **A** | **G** | **A** | **G** | **A** | **A** | **A** | **A** | **A** | **C** | **C** | **A** | **A** | **G** | **T** | **A** | **G** | **A** | **C** | **T** | **A** | **C** | **C** | **A** | **A** | **G** | **C** | **A** | **T** | **T** | **T** | **T** | **T** | **G** | **A** | **A** | **C** | **A** | **A** | **C** | **C** | **T** | **C** | **C** | **G** | **A** | **A** | **A** | **G** | **C** | **T** | **T** | **G** | **G** | **G** | **A** | **T** | **G** | **G** | **A** | **A** | **A** | **G** | **A** | **G** | **A** | **T** | **G** | **T** | **T** | **T** | **T** | **T** | **C** | **A** | **T** | **A** | **A** | **C** | **A** | **A** | **G** | **A** | **T** | **C** | **G** | **A** | **G** | **C** | **A** | **G** | **A** | **T** | **G** | **A** | **G** | **T** | **A** | **T** | **G** | **G** | **G** | **C** | **C** | **A** | **A** | **C** | **G** | **G** | **A** | **A** | **A** | **A** | **A** | **A** | **G** | **T** | **G** | **G** | **A** | **A** | **T** | **T** | **G** | **G** | **C** | **T** | **A** | **A** | **A** | **T** | **C** | **T** | **T** | **T** | **A** | **T** | **C** | **A** | **C** | **A** | **G** | **C** | **C** | **A** | **G** | **C** | **T** | **G** | **A** | **A** | **C** | **T** | **A** | **T** | **A** | **T** | **A** | **T** | **A** | **T** | **G** | **G** | **G** | **A** | **T** | **G** | **A** | **A** | **C** | **C** | **A** | **T** | **T** | **G** | **A** | **A** | **T** | **T** | **A** | **T** | **T** | **T** | **G** | **G** | **A** | **T** | **G** | **T** | **C** | **T** | **T** | **C** | **A** | **A** | **T** | **C** | **A** | **A** | **G** | **A** | **A** | **C** | **A** | **A** | **T** | **T** | **A** | **G** | **A** | **A** | **C** | **A** | **A** | **C** | **T** | **G** | **A** | **T** | **C** | **T** | **T** | **G** | **A** | **A** | **C** | **G** | **T** | **G** | **A** | **A** | **A** | **C** | **C** | **T** | **G** | **C** | **C** | **A** | **T** | **G** | **T** | **T** | **A** | **C** | **T** | **A** | **G** | **T** | **G** | **G** | **A** | **A** | **C** | **A** | **T** | **G** | **A** | **T** | **C** | **A** | **A** | **A** | **C** | **C** | **T** | **T** | **C** | **C** | **T** | **G** | **G** | **A** | **T** | **A** | **A** | **A** | **G** | **T** | **A** | **T** | **C** | **T** | **A** | **C** | **T** | **G** | **A** | **G** | **A** | **T** | **T** | **A** | **T** | **T** | **T** | **C** | **T** | **C** | **T** | **T** | **G** | **A** | **G** | **A** | **A** | **A** | **A** | **T** | **C** | **-** | **-** | **-** |
| **N52769** | **A** | **T** | **G** | **T** | **C** | **T** | **A** | **A** | **A** | **A** | **T** | **C** | **G** | **A** | **A** | **A** | **T** | **A** | **A** | **A** | **A** | **A** | **A** | **T** | **C** | **T** | **G** | **A** | **C** | **A** | **T** | **T** | **C** | **G** | **G** | **C** | **T** | **A** | **C** | **G** | **A** | **C** | **A** | **G** | **C** | **C** | **A** | **A** | **G** | **G** | **C** | **A** | **C** | **A** | **T** | **T** | **A** | **T** | **T** | **A** | **T** | **T** | **T** | **G** | **A** | **A** | **C** | **A** | **A** | **G** | **C** | **A** | **A** | **A** | **T** | **C** | **T** | **A** | **A** | **A** | **T** | **T** | **T** | **T** | **G** | **A** | **C** | **A** | **C** | **A** | **C** | **A** | **A** | **T** | **G** | **G** | **A** | **A** | **A** | **C** | **T** | **A** | **G** | **G** | **A** | **C** | **T** | **T** | **A** | **T** | **C** | **G** | **G** | **A** | **C** | **G** | **A** | **A** | **A** | **C** | **G** | **G** | **T** | **C** | **G** | **A** | **G** | **G** | **A** | **A** | **A** | **G** | **A** | **C** | **A** | **A** | **C** | **T** | **T** | **T** | **A** | **C** | **T** | **G** | **A** | **A** | **T** | **A** | **T** | **T** | **C** | **T** | **A** | **C** | **A** | **A** | **A** | **A** | **C** | **A** | **A** | **A** | **C** | **T** | **A** | **C** | **C** | **T** | **T** | **A** | **T** | **C** | **A** | **A** | **G** | **G** | **G** | **C** | **A** | **A** | **G** | **T** | **A** | **A** | **T** | **C** | **C** | **A** | **T** | **C** | **A** | **G** | **C** | **A** | **A** | **G** | **A** | **A** | **T** | **T** | **T** | **G** | **T** | **C** | **T** | **A** | **T** | **T** | **T** | **C** | **C** | **C** | **G** | **C** | **A** | **A** | **C** | **A** | **G** | **A** | **C** | **A** | **A** | **A** | **A** | **G** | **A** | **T** | **A** | **A** | **A** | **G** | **A** | **A** | **C** | **G** | **T** | **T** | **T** | **A** | **A** | **C** | **C** | **T** | **A** | **T** | **T** | **A** | **C** | **G** | **T** | **G** | **T** | **T** | **A** | **A** | **A** | **T** | **G** | **A** | **T** | **A** | **T** | **T** | **A** | **C** | **G** | **G** | **A** | **T** | **T** | **T** | **T** | **G** | **A** | **G** | **A** | **T** | **A** | **T** | **G** | **G** | **G** | **A** | **A** | **A** | **T** | **C** | **G** | **A** | **A** | **A** | **G** | **A** | **G** | **A** | **G** | **C** | **T** | **C** | **C** | **A** | **A** | **T** | **T** | **T** | **A** | **T** | **G** | **C** | **A** | **A** | **A** | **C** | **A** | **G** | **A** | **T** | **C** | **C** | **T** | **G** | **A** | **A** | **A** | **T** | **C** | **T** | **T** | **A** | **T** | **G** | **G** | **A** | **G** | **A** | **G** | **A** | **A** | **T** | **T** | **C** | **A** | **G** | **C** | **A** | **C** | **A** | **C** | **T** | **A** | **T** | **C** | **G** | **G** | **G** | **G** | **G** | **G** | **A** | **G** | **A** | **G** | **A** | **A** | **G** | **A** | **C** | **A** | **A** | **A** | **A** | **G** | **T** | **C** | **C** | **T** | **A** | **T** | **T** | **G** | **G** | **C** | **A** | **C** | **T** | **T** | **T** | **T** | **A** | **T** | **T** | **T** | **G** | **T** | **G** | **G** | **A** | **T** | **G** | **A** | **C** | **A** | **C** | **T** | **C** | **A** | **T** | **T** | **T** | **C** | **C** | **C** | **G** | **T** | **T** | **A** | **A** | **T** | **C** | **G** | **A** | **T** | **G** | **A** | **A** | **C** | **C** | **A** | **A** | **C** | **G** | **A** | **A** | **T** | **C** | **A** | **T** | **T** | **T** | **G** | **G** | **A** | **-** | **-** | **-** | **T** | **A** | **T** | **C** | **T** | **C** | **T** | **G** | **G** | **T** | **A** | **G** | **A** | **A** | **A** | **A** | **C** | **A** | **A** | **G** | **T** | **A** | **G** | **C** | **G** | **G** | **C** | **T** | **T** | **A** | **T** | **T** | **T** | **G** | **A** | **A** | **A** | **A** | **A** | **G** | **A** | **A** | **A** | **A** | **A** | **A** | **C** | **A** | **A** | **G** | **G** | **C** | **T** | **T** | **C** | **A** | **T** | **C** | **G** | **T** | **G** | **G** | **T** | **C** | **A** | **G** | **C** | **C** | **A** | **T** | **G** | **A** | **C** | **C** | **G** | **G** | **G** | **G** | **A** | **T** | **T** | **T** | **A** | **T** | **C** | **G** | **A** | **T** | **G** | **A** | **A** | **G** | **T** | **A** | **G** | **T** | **G** | **G** | **A** | **C** | **C** | **A** | **T** | **G** | **T** | **T** | **T** | **T** | **A** | **G** | **C** | **A** | **A** | **T** | **C** | **G** | **A** | **A** | **A** | **A** | **A** | **A** | **G** | **T** | **C** | **A** | **A** | **C** | **T** | **G** | **G** | **A** | **A** | **C** | **T** | **T** | **T** | **A** | **T** | **C** | **A** | **A** | **G** | **G** | **G** | **A** | **A** | **T** | **T** | **T** | **C** | **T** | **C** | **T** | **A** | **T** | **C** | **T** | **A** | **T** | **G** | **A** | **A** | **G** | **A** | **A** | **C** | **A** | **G** | **A** | **A** | **A** | **A** | **A** | **A** | **C** | **T** | **T** | **C** | **G** | **T** | **G** | **A** | **T** | **G** | **A** | **A** | **T** | **T** | **T** | **G** | **A** | **A** | **A** | **T** | **G** | **G** | **C** | **T** | **C** | **A** | **A** | **A** | **A** | **T** | **G** | **A** | **A** | **A** | **A** | **A** | **T** | **T** | **G** | **A** | **A** | **A** | **A** | **A** | **A** | **G** | **A** | **A** | **G** | **T** | **C** | **A** | **G** | **T** | **A** | **G** | **G** | **C** | **T** | **A** | **A** | **A** | **G** | **A** | **A** | **A** | **A** | **C** | **A** | **G** | **C** | **A** | **G** | **C** | **T** | **G** | **A** | **A** | **A** | **A** | **A** | **G** | **C** | **T** | **G** | **A** | **A** | **T** | **G** | **G** | **T** | **C** | **T** | **C** | **G** | **T** | **T** | **C** | **C** | **C** | **G** | **A** | **G** | **A** | **A** | **G** | **G** | **A** | **G** | **A** | **T** | **A** | **A** | **A** | **A** | **C** | **A** | **A** | **A** | **G** | **A** | **A** | **A** | **C** | **A** | **A** | **G** | **T** | **C** | **G** | **G** | **A** | **T** | **T** | **C** | **A** | **T** | **C** | **G** | **A** | **T** | **A** | **C** | **T** | **G** | **A** | **A** | **T** | **C** | **T** | **A** | **G** | **A** | **C** | **G** | **A** | **G** | **T** | **G** | **A** | **A** | **T** | **A** | **A** | **A** | **G** | **G** | **A** | **G** | **C** | **A** | **G** | **T** | **G** | **G** | **G** | **T** | **G** | **C** | **T** | **G** | **A** | **T** | **G** | **C** | **T** | **G** | **C** | **A** | **C** | **G** | **G** | **G** | **C** | **G** | **A** | **T** | **G** | **A** | **A** | **A** | **C** | **G** | **A** | **T** | **C** | **C** | **A** | **A** | **A** | **G** | **C** | **A** | **A** | **T** | **C** | **G** | **T** | **G** | **A** | **A** | **T** | **C** | **G** | **G** | **A** | **T** | **G** | **G** | **A** | **G** | **A** | **C** | **C** | **C** | **A** | **G** | **A** | **T** | **C** | **A** | **G** | **C** | **G** | **A** | **G** | **A** | **A** | **A** | **G** | **A** | **A** | **A** | **A** | **A** | **C** | **T** | **A** | **T** | **T** | **A** | **A** | **A** | **A** | **G** | **A** | **T** | **A** | **T** | **C** | **G** | **A** | **A** | **T** | **A** | **T** | **A** | **T** | **C** | **G** | **A** | **T** | **T** | **C** | **T** | **T** | **T** | **G** | **A** | **C** | **G** | **A** | **T** | **G** | **A** | **A** | **T** | **A** | **G** | **C** | **C** | **A** | **A** | **G** | **C** | **G** | **T** | **C** | **T** | **C** | **A** | **C** | **C** | **A** | **T** | **A** | **A** | **G** | **C** | **G** | **A** | **C** | **T** | **T** | **T** | **T** | **A** | **A** | **G** | **C** | **G** | **T** | **A** | **G** | **A** | **A** | **G** | **A** | **T** | **C** | **T** | **T** | **C** | **A** | **A** | **T** | **T** | **A** | **G** | **G** | **G** | **T** | **A** | **T** | **G** | **A** | **A** | **A** | **A** | **T** | **C** | **T** | **G** | **T** | **T** | **A** | **T** | **T** | **C** | **G** | **A** | **G** | **C** | **C** | **A** | **A** | **T** | **T** | **C** | **A** | **T** | **T** | **T** | **T** | **A** | **C** | **A** | **A** | **T** | **C** | **G** | **A** | **G** | **C** | **C** | **T** | **C** | **A** | **T** | **C** | **A** | **G** | **C** | **G** | **G** | **G** | **T** | **G** | **G** | **C** | **G** | **A** | **T** | **T** | **T** | **C** | **A** | **G** | **G** | **T** | **C** | **C** | **T** | **A** | **A** | **C** | **G** | **G** | **T** | **G** | **C** | **A** | **G** | **G** | **A** | **A** | **A** | **G** | **T** | **C** | **A** | **T** | **C** | **C** | **A** | **T** | **T** | **A** | **T** | **C** | **C** | **A** | **T** | **T** | **A** | **T** | **C** | **T** | **T** | **C** | **T** | **G** | **G** | **G** | **G** | **G** | **C** | **A** | **T** | **T** | **C** | **A** | **A** | **C** | **G** | **G** | **C** | **A** | **A** | **G** | **G** | **T** | **T** | **A** | **T** | **A** | **G** | **G** | **A** | **G** | **A** | **A** | **A** | **A** | **A** | **T** | **C** | **C** | **C** | **A** | **G** | **C** | **C** | **A** | **A** | **A** | **A** | **C** | **A** | **T** | **C** | **T** | **G** | **A** | **G** | **C** | **A** | **T** | **T** | **A** | **G** | **T** | **T** | **A** | **T** | **G** | **T** | **A** | **C** | **G** | **C** | **C** | **A** | **A** | **A** | **A** | **T** | **T** | **A** | **T** | **G** | **A** | **A** | **G** | **A** | **C** | **A** | **A** | **T** | **C** | **G** | **A** | **G** | **G** | **A** | **A** | **C** | **G** | **T** | **T** | **G** | **G** | **C** | **G** | **G** | **A** | **A** | **T** | **T** | **T** | **G** | **C** | **A** | **G** | **A** | **G** | **A** | **A** | **A** | **A** | **A** | **C** | **C** | **A** | **A** | **G** | **T** | **A** | **G** | **A** | **C** | **T** | **A** | **C** | **C** | **A** | **A** | **G** | **C** | **A** | **T** | **T** | **T** | **T** | **T** | **G** | **A** | **A** | **C** | **A** | **A** | **C** | **C** | **T** | **C** | **C** | **G** | **A** | **A** | **A** | **G** | **C** | **T** | **T** | **G** | **G** | **G** | **A** | **T** | **G** | **G** | **A** | **A** | **A** | **G** | **A** | **G** | **A** | **T** | **G** | **T** | **T** | **T** | **T** | **T** | **C** | **A** | **T** | **A** | **A** | **C** | **A** | **A** | **G** | **A** | **T** | **T** | **G** | **A** | **G** | **C** | **A** | **G** | **A** | **T** | **G** | **A** | **G** | **T** | **A** | **T** | **G** | **G** | **G** | **C** | **C** | **A** | **A** | **C** | **G** | **G** | **A** | **A** | **A** | **A** | **A** | **A** | **G** | **T** | **G** | **G** | **A** | **A** | **T** | **T** | **G** | **G** | **C** | **T** | **A** | **A** | **A** | **T** | **C** | **T** | **T** | **T** | **A** | **T** | **C** | **A** | **C** | **A** | **G** | **C** | **C** | **A** | **G** | **C** | **T** | **G** | **A** | **A** | **C** | **T** | **A** | **T** | **A** | **T** | **A** | **T** | **A** | **T** | **G** | **G** | **G** | **A** | **T** | **G** | **A** | **A** | **C** | **C** | **A** | **T** | **T** | **G** | **A** | **A** | **T** | **T** | **A** | **T** | **T** | **T** | **G** | **G** | **A** | **T** | **G** | **T** | **C** | **T** | **T** | **C** | **A** | **A** | **T** | **C** | **A** | **A** | **G** | **A** | **A** | **C** | **A** | **A** | **T** | **T** | **A** | **G** | **A** | **A** | **C** | **A** | **A** | **C** | **T** | **G** | **A** | **T** | **C** | **T** | **T** | **G** | **A** | **A** | **C** | **G** | **T** | **G** | **A** | **A** | **A** | **C** | **C** | **T** | **G** | **C** | **C** | **A** | **T** | **G** | **T** | **T** | **A** | **C** | **T** | **G** | **G** | **T** | **G** | **G** | **A** | **A** | **C** | **A** | **T** | **G** | **A** | **T** | **C** | **A** | **A** | **A** | **C** | **C** | **T** | **T** | **C** | **C** | **T** | **G** | **G** | **A** | **T** | **A** | **A** | **A** | **G** | **T** | **A** | **T** | **C** | **T** | **A** | **C** | **T** | **G** | **A** | **G** | **A** | **T** | **T** | **A** | **T** | **T** | **T** | **C** | **T** | **C** | **T** | **T** | **G** | **A** | **G** | **A** | **A** | **A** | **A** | **T** | **C** | **-** | **-** | **-** |
| **N60449F** | **A** | **T** | **G** | **T** | **C** | **T** | **A** | **A** | **A** | **A** | **T** | **C** | **G** | **A** | **A** | **A** | **T** | **A** | **A** | **A** | **A** | **A** | **A** | **T** | **C** | **T** | **G** | **A** | **C** | **A** | **T** | **T** | **C** | **G** | **G** | **C** | **T** | **A** | **C** | **G** | **A** | **C** | **A** | **G** | **C** | **C** | **A** | **A** | **G** | **G** | **C** | **A** | **C** | **A** | **T** | **T** | **A** | **T** | **T** | **A** | **T** | **T** | **T** | **G** | **A** | **A** | **C** | **A** | **A** | **G** | **C** | **A** | **A** | **A** | **T** | **C** | **T** | **A** | **A** | **A** | **T** | **T** | **T** | **T** | **G** | **A** | **C** | **A** | **C** | **A** | **C** | **A** | **A** | **T** | **G** | **G** | **A** | **A** | **A** | **C** | **T** | **A** | **G** | **G** | **A** | **C** | **T** | **T** | **A** | **T** | **C** | **G** | **G** | **A** | **C** | **G** | **A** | **A** | **A** | **C** | **G** | **G** | **T** | **C** | **G** | **A** | **G** | **G** | **A** | **A** | **A** | **G** | **A** | **C** | **A** | **A** | **C** | **T** | **T** | **T** | **A** | **C** | **T** | **G** | **A** | **A** | **T** | **A** | **T** | **T** | **C** | **T** | **A** | **C** | **A** | **A** | **A** | **A** | **C** | **A** | **A** | **A** | **C** | **T** | **A** | **C** | **C** | **T** | **T** | **A** | **T** | **C** | **A** | **A** | **G** | **G** | **G** | **C** | **A** | **A** | **G** | **T** | **A** | **A** | **T** | **C** | **C** | **A** | **T** | **C** | **A** | **G** | **C** | **A** | **A** | **G** | **A** | **A** | **T** | **T** | **T** | **G** | **C** | **C** | **T** | **A** | **T** | **T** | **T** | **C** | **C** | **C** | **G** | **C** | **A** | **A** | **C** | **A** | **G** | **A** | **C** | **A** | **A** | **A** | **A** | **G** | **A** | **T** | **A** | **A** | **A** | **G** | **A** | **A** | **C** | **G** | **T** | **T** | **T** | **A** | **A** | **C** | **C** | **T** | **A** | **T** | **T** | **A** | **C** | **G** | **T** | **G** | **T** | **T** | **A** | **A** | **A** | **T** | **G** | **A** | **T** | **A** | **T** | **T** | **A** | **C** | **G** | **G** | **A** | **T** | **T** | **T** | **T** | **G** | **A** | **G** | **A** | **T** | **A** | **T** | **G** | **G** | **G** | **A** | **A** | **A** | **T** | **C** | **G** | **A** | **A** | **A** | **G** | **A** | **G** | **A** | **G** | **C** | **T** | **C** | **C** | **A** | **A** | **T** | **T** | **G** | **A** | **T** | **G** | **C** | **A** | **A** | **A** | **C** | **A** | **G** | **A** | **T** | **C** | **C** | **T** | **G** | **A** | **A** | **A** | **T** | **C** | **T** | **T** | **A** | **T** | **G** | **G** | **A** | **G** | **A** | **G** | **A** | **A** | **T** | **T** | **C** | **A** | **G** | **C** | **A** | **C** | **A** | **C** | **T** | **A** | **T** | **C** | **G** | **G** | **G** | **G** | **G** | **G** | **A** | **G** | **A** | **G** | **A** | **A** | **G** | **A** | **C** | **A** | **A** | **A** | **A** | **G** | **T** | **C** | **C** | **T** | **A** | **C** | **T** | **G** | **G** | **C** | **A** | **C** | **T** | **T** | **T** | **T** | **A** | **T** | **T** | **T** | **G** | **T** | **G** | **G** | **A** | **T** | **G** | **A** | **C** | **A** | **C** | **T** | **C** | **A** | **T** | **T** | **T** | **C** | **C** | **C** | **G** | **T** | **T** | **A** | **A** | **T** | **C** | **G** | **A** | **T** | **G** | **A** | **A** | **C** | **C** | **A** | **A** | **C** | **G** | **A** | **A** | **T** | **C** | **A** | **T** | **T** | **T** | **G** | **G** | **A** | **-** | **-** | **-** | **T** | **A** | **T** | **C** | **T** | **C** | **T** | **G** | **G** | **T** | **A** | **G** | **A** | **A** | **A** | **A** | **C** | **A** | **A** | **G** | **T** | **A** | **G** | **C** | **G** | **G** | **C** | **T** | **T** | **A** | **T** | **T** | **T** | **G** | **A** | **A** | **A** | **A** | **A** | **G** | **A** | **A** | **A** | **A** | **A** | **A** | **C** | **A** | **A** | **G** | **G** | **C** | **T** | **T** | **C** | **A** | **T** | **C** | **G** | **T** | **G** | **G** | **T** | **C** | **A** | **G** | **C** | **C** | **A** | **T** | **G** | **A** | **C** | **C** | **G** | **G** | **G** | **G** | **A** | **T** | **T** | **T** | **A** | **T** | **C** | **G** | **A** | **T** | **G** | **A** | **A** | **G** | **T** | **A** | **G** | **T** | **G** | **G** | **A** | **C** | **C** | **A** | **T** | **G** | **T** | **T** | **T** | **T** | **A** | **G** | **C** | **A** | **A** | **T** | **C** | **G** | **A** | **A** | **A** | **A** | **A** | **A** | **G** | **T** | **C** | **A** | **A** | **C** | **T** | **G** | **G** | **A** | **A** | **C** | **T** | **T** | **T** | **A** | **T** | **C** | **A** | **A** | **G** | **G** | **G** | **A** | **A** | **T** | **T** | **T** | **C** | **T** | **C** | **T** | **A** | **T** | **C** | **T** | **A** | **T** | **G** | **A** | **A** | **G** | **A** | **A** | **C** | **A** | **G** | **A** | **A** | **A** | **A** | **A** | **A** | **C** | **T** | **T** | **C** | **G** | **T** | **G** | **A** | **T** | **G** | **A** | **A** | **T** | **T** | **T** | **G** | **A** | **A** | **A** | **T** | **G** | **G** | **C** | **T** | **C** | **A** | **A** | **A** | **A** | **T** | **G** | **A** | **A** | **A** | **A** | **A** | **T** | **T** | **G** | **A** | **A** | **A** | **A** | **A** | **A** | **G** | **A** | **A** | **G** | **T** | **C** | **A** | **G** | **T** | **A** | **G** | **G** | **C** | **T** | **A** | **A** | **A** | **G** | **A** | **A** | **A** | **A** | **C** | **A** | **G** | **C** | **A** | **G** | **C** | **T** | **G** | **A** | **A** | **A** | **A** | **A** | **G** | **C** | **C** | **G** | **A** | **A** | **T** | **G** | **G** | **T** | **C** | **T** | **C** | **G** | **T** | **T** | **C** | **C** | **C** | **G** | **A** | **G** | **A** | **A** | **G** | **G** | **A** | **G** | **A** | **T** | **A** | **A** | **A** | **A** | **C** | **A** | **A** | **A** | **G** | **A** | **A** | **A** | **C** | **A** | **A** | **G** | **T** | **C** | **G** | **G** | **A** | **T** | **T** | **C** | **A** | **T** | **C** | **G** | **A** | **T** | **A** | **C** | **T** | **G** | **A** | **A** | **T** | **C** | **T** | **A** | **G** | **A** | **C** | **G** | **A** | **G** | **T** | **G** | **A** | **A** | **T** | **A** | **A** | **A** | **G** | **G** | **A** | **G** | **C** | **A** | **G** | **T** | **G** | **G** | **G** | **T** | **G** | **C** | **T** | **G** | **A** | **T** | **G** | **C** | **T** | **G** | **C** | **A** | **C** | **G** | **G** | **A** | **C** | **G** | **A** | **T** | **G** | **A** | **A** | **A** | **C** | **G** | **A** | **T** | **C** | **C** | **A** | **A** | **A** | **G** | **C** | **A** | **A** | **T** | **C** | **G** | **T** | **G** | **A** | **A** | **T** | **C** | **G** | **G** | **A** | **T** | **G** | **G** | **A** | **G** | **A** | **C** | **C** | **C** | **A** | **G** | **A** | **T** | **C** | **A** | **G** | **C** | **G** | **A** | **G** | **A** | **A** | **A** | **G** | **A** | **A** | **A** | **A** | **A** | **C** | **T** | **A** | **T** | **T** | **A** | **A** | **A** | **A** | **G** | **A** | **T** | **A** | **T** | **C** | **G** | **A** | **A** | **T** | **A** | **T** | **A** | **T** | **C** | **G** | **A** | **T** | **T** | **C** | **T** | **T** | **T** | **G** | **A** | **C** | **G** | **A** | **T** | **G** | **A** | **A** | **T** | **A** | **G** | **C** | **C** | **A** | **A** | **G** | **C** | **G** | **T** | **C** | **T** | **C** | **A** | **C** | **C** | **A** | **T** | **A** | **A** | **G** | **C** | **G** | **A** | **C** | **T** | **T** | **T** | **T** | **A** | **A** | **G** | **C** | **G** | **T** | **A** | **G** | **A** | **A** | **G** | **A** | **T** | **C** | **T** | **T** | **C** | **A** | **A** | **T** | **T** | **A** | **G** | **G** | **G** | **T** | **A** | **T** | **G** | **A** | **A** | **A** | **A** | **T** | **C** | **T** | **G** | **T** | **T** | **A** | **T** | **T** | **C** | **G** | **A** | **G** | **C** | **C** | **A** | **A** | **T** | **T** | **C** | **A** | **T** | **T** | **T** | **T** | **A** | **C** | **A** | **A** | **T** | **C** | **G** | **A** | **G** | **C** | **C** | **T** | **C** | **A** | **T** | **C** | **A** | **G** | **C** | **G** | **G** | **G** | **T** | **G** | **G** | **C** | **G** | **A** | **T** | **T** | **T** | **C** | **A** | **G** | **G** | **T** | **C** | **C** | **T** | **A** | **A** | **C** | **G** | **G** | **T** | **G** | **C** | **A** | **G** | **G** | **A** | **A** | **A** | **G** | **T** | **C** | **A** | **T** | **C** | **C** | **A** | **T** | **T** | **A** | **T** | **C** | **C** | **A** | **T** | **T** | **A** | **T** | **C** | **T** | **T** | **C** | **T** | **G** | **G** | **G** | **G** | **G** | **C** | **A** | **T** | **T** | **C** | **A** | **A** | **C** | **G** | **G** | **C** | **A** | **A** | **G** | **G** | **T** | **T** | **A** | **T** | **A** | **G** | **G** | **A** | **G** | **A** | **A** | **A** | **A** | **A** | **T** | **A** | **C** | **C** | **A** | **G** | **C** | **C** | **A** | **A** | **A** | **A** | **C** | **A** | **T** | **C** | **T** | **G** | **A** | **G** | **C** | **A** | **T** | **T** | **A** | **G** | **T** | **T** | **A** | **T** | **G** | **C** | **A** | **A** | **G** | **C** | **C** | **A** | **A** | **A** | **A** | **T** | **T** | **A** | **T** | **G** | **A** | **A** | **G** | **A** | **C** | **A** | **A** | **T** | **C** | **G** | **A** | **G** | **G** | **A** | **A** | **C** | **G** | **T** | **T** | **G** | **G** | **C** | **G** | **G** | **A** | **A** | **T** | **T** | **T** | **G** | **C** | **A** | **G** | **A** | **G** | **A** | **A** | **A** | **A** | **A** | **C** | **C** | **A** | **A** | **G** | **T** | **A** | **G** | **A** | **C** | **T** | **A** | **C** | **C** | **A** | **A** | **G** | **C** | **A** | **T** | **T** | **T** | **T** | **T** | **G** | **A** | **A** | **C** | **A** | **A** | **C** | **C** | **T** | **C** | **C** | **G** | **A** | **A** | **A** | **G** | **C** | **T** | **T** | **G** | **G** | **G** | **A** | **T** | **G** | **G** | **A** | **A** | **A** | **G** | **A** | **G** | **A** | **T** | **G** | **T** | **T** | **T** | **T** | **T** | **C** | **A** | **T** | **A** | **A** | **C** | **A** | **A** | **G** | **A** | **T** | **C** | **G** | **A** | **G** | **C** | **A** | **G** | **A** | **T** | **G** | **A** | **G** | **T** | **A** | **T** | **G** | **G** | **G** | **C** | **C** | **A** | **A** | **C** | **G** | **G** | **A** | **A** | **A** | **A** | **A** | **A** | **G** | **T** | **G** | **G** | **A** | **A** | **T** | **T** | **G** | **G** | **C** | **T** | **A** | **A** | **A** | **T** | **C** | **T** | **T** | **T** | **A** | **T** | **C** | **A** | **C** | **A** | **G** | **C** | **C** | **A** | **G** | **C** | **T** | **G** | **A** | **A** | **C** | **T** | **A** | **T** | **A** | **T** | **A** | **C** | **A** | **T** | **G** | **G** | **G** | **A** | **T** | **G** | **A** | **A** | **C** | **C** | **A** | **T** | **T** | **G** | **A** | **A** | **T** | **T** | **A** | **T** | **T** | **T** | **G** | **G** | **A** | **T** | **G** | **T** | **C** | **T** | **T** | **C** | **A** | **A** | **T** | **C** | **A** | **A** | **G** | **A** | **A** | **C** | **A** | **A** | **T** | **T** | **A** | **G** | **A** | **A** | **C** | **A** | **A** | **C** | **T** | **G** | **A** | **T** | **C** | **T** | **T** | **G** | **A** | **A** | **C** | **G** | **T** | **G** | **A** | **A** | **A** | **C** | **C** | **T** | **G** | **C** | **C** | **A** | **T** | **G** | **T** | **T** | **A** | **C** | **T** | **A** | **G** | **T** | **G** | **G** | **A** | **A** | **C** | **A** | **T** | **G** | **A** | **T** | **C** | **A** | **A** | **A** | **C** | **C** | **T** | **T** | **C** | **C** | **T** | **G** | **G** | **A** | **T** | **A** | **A** | **A** | **G** | **T** | **A** | **T** | **C** | **T** | **A** | **C** | **T** | **G** | **A** | **G** | **A** | **T** | **T** | **A** | **T** | **T** | **T** | **C** | **T** | **C** | **T** | **T** | **G** | **A** | **G** | **A** | **G** | **A** | **A** | **T** | **C** | **-** | **-** | **-** |
| **N54152** | **A** | **T** | **G** | **T** | **C** | **T** | **A** | **A** | **A** | **A** | **T** | **C** | **G** | **A** | **A** | **A** | **T** | **A** | **A** | **A** | **A** | **A** | **A** | **T** | **C** | **T** | **G** | **A** | **C** | **A** | **T** | **T** | **C** | **G** | **G** | **C** | **T** | **A** | **C** | **G** | **A** | **C** | **A** | **G** | **C** | **C** | **A** | **A** | **G** | **G** | **C** | **A** | **C** | **A** | **T** | **T** | **A** | **T** | **T** | **A** | **T** | **T** | **T** | **G** | **A** | **A** | **C** | **A** | **A** | **G** | **C** | **A** | **A** | **A** | **T** | **C** | **T** | **A** | **A** | **A** | **T** | **T** | **T** | **T** | **G** | **A** | **C** | **A** | **C** | **A** | **C** | **A** | **A** | **T** | **G** | **G** | **A** | **A** | **A** | **C** | **T** | **A** | **G** | **G** | **A** | **C** | **T** | **T** | **A** | **T** | **C** | **G** | **G** | **A** | **C** | **G** | **A** | **A** | **A** | **C** | **G** | **G** | **T** | **C** | **G** | **A** | **G** | **G** | **A** | **A** | **A** | **G** | **A** | **C** | **A** | **A** | **C** | **T** | **T** | **T** | **A** | **C** | **T** | **G** | **A** | **A** | **T** | **A** | **T** | **T** | **C** | **T** | **A** | **C** | **A** | **A** | **A** | **A** | **C** | **A** | **A** | **A** | **C** | **T** | **A** | **C** | **C** | **T** | **T** | **A** | **T** | **C** | **A** | **A** | **G** | **G** | **G** | **C** | **A** | **A** | **G** | **T** | **A** | **A** | **T** | **C** | **C** | **A** | **T** | **C** | **A** | **G** | **C** | **A** | **A** | **G** | **A** | **A** | **T** | **T** | **T** | **G** | **T** | **C** | **T** | **A** | **T** | **T** | **T** | **C** | **C** | **C** | **G** | **C** | **A** | **A** | **C** | **A** | **G** | **A** | **C** | **A** | **A** | **A** | **A** | **G** | **A** | **T** | **A** | **A** | **A** | **G** | **A** | **A** | **C** | **G** | **T** | **T** | **T** | **A** | **A** | **C** | **C** | **T** | **A** | **T** | **T** | **A** | **C** | **G** | **T** | **G** | **T** | **T** | **A** | **A** | **A** | **T** | **G** | **A** | **T** | **A** | **T** | **T** | **A** | **C** | **G** | **G** | **A** | **T** | **T** | **T** | **T** | **G** | **A** | **G** | **A** | **T** | **A** | **T** | **G** | **G** | **G** | **A** | **A** | **A** | **T** | **C** | **G** | **A** | **A** | **A** | **G** | **A** | **G** | **A** | **G** | **C** | **T** | **C** | **C** | **A** | **A** | **T** | **T** | **G** | **A** | **T** | **G** | **C** | **A** | **A** | **A** | **C** | **A** | **G** | **A** | **T** | **C** | **C** | **T** | **G** | **A** | **A** | **A** | **T** | **C** | **T** | **T** | **A** | **T** | **G** | **G** | **A** | **G** | **A** | **G** | **A** | **A** | **T** | **T** | **C** | **A** | **G** | **C** | **A** | **C** | **A** | **C** | **T** | **A** | **T** | **C** | **G** | **G** | **G** | **G** | **G** | **G** | **A** | **G** | **A** | **G** | **A** | **A** | **G** | **A** | **C** | **A** | **A** | **A** | **A** | **G** | **T** | **C** | **C** | **T** | **A** | **T** | **T** | **G** | **G** | **C** | **A** | **C** | **T** | **T** | **T** | **T** | **A** | **T** | **T** | **T** | **G** | **T** | **G** | **G** | **A** | **T** | **G** | **A** | **C** | **A** | **C** | **T** | **C** | **A** | **T** | **T** | **T** | **C** | **C** | **C** | **G** | **T** | **T** | **A** | **A** | **T** | **C** | **G** | **A** | **T** | **G** | **A** | **A** | **C** | **C** | **A** | **A** | **C** | **G** | **A** | **A** | **T** | **C** | **A** | **T** | **T** | **T** | **G** | **G** | **A** | **-** | **-** | **-** | **T** | **A** | **T** | **C** | **T** | **C** | **T** | **G** | **G** | **T** | **A** | **G** | **A** | **A** | **A** | **A** | **C** | **A** | **A** | **G** | **T** | **A** | **G** | **C** | **G** | **G** | **C** | **T** | **T** | **A** | **T** | **T** | **T** | **G** | **A** | **A** | **A** | **A** | **A** | **G** | **A** | **A** | **A** | **A** | **A** | **A** | **C** | **A** | **A** | **G** | **G** | **C** | **T** | **T** | **C** | **A** | **T** | **C** | **G** | **T** | **G** | **G** | **T** | **C** | **A** | **G** | **C** | **C** | **A** | **T** | **G** | **A** | **C** | **C** | **G** | **G** | **G** | **G** | **A** | **T** | **T** | **T** | **A** | **T** | **C** | **G** | **A** | **T** | **G** | **A** | **A** | **G** | **T** | **A** | **G** | **T** | **G** | **G** | **A** | **C** | **C** | **A** | **T** | **G** | **T** | **T** | **T** | **T** | **A** | **G** | **C** | **A** | **A** | **T** | **C** | **G** | **A** | **A** | **A** | **A** | **A** | **A** | **G** | **T** | **C** | **A** | **A** | **C** | **T** | **G** | **G** | **A** | **A** | **C** | **T** | **T** | **T** | **A** | **T** | **C** | **A** | **A** | **G** | **G** | **G** | **A** | **A** | **T** | **T** | **T** | **C** | **T** | **C** | **T** | **A** | **T** | **C** | **T** | **A** | **T** | **G** | **A** | **A** | **G** | **A** | **A** | **C** | **A** | **G** | **A** | **A** | **A** | **A** | **A** | **A** | **C** | **T** | **T** | **C** | **G** | **T** | **G** | **A** | **T** | **G** | **A** | **A** | **T** | **T** | **T** | **G** | **A** | **A** | **A** | **T** | **G** | **G** | **C** | **T** | **C** | **A** | **A** | **A** | **A** | **T** | **G** | **A** | **A** | **A** | **A** | **A** | **T** | **T** | **G** | **A** | **A** | **A** | **A** | **A** | **A** | **G** | **A** | **A** | **G** | **T** | **C** | **A** | **G** | **T** | **A** | **G** | **G** | **C** | **T** | **A** | **A** | **A** | **G** | **A** | **A** | **A** | **A** | **C** | **A** | **G** | **C** | **A** | **G** | **C** | **T** | **G** | **A** | **A** | **A** | **A** | **A** | **G** | **C** | **C** | **G** | **A** | **A** | **T** | **G** | **G** | **T** | **C** | **T** | **C** | **G** | **T** | **T** | **C** | **C** | **C** | **G** | **A** | **G** | **A** | **A** | **G** | **G** | **A** | **G** | **A** | **T** | **A** | **A** | **A** | **A** | **C** | **A** | **A** | **A** | **G** | **A** | **A** | **A** | **C** | **A** | **A** | **G** | **T** | **C** | **G** | **G** | **A** | **T** | **T** | **C** | **A** | **T** | **C** | **G** | **A** | **T** | **A** | **C** | **T** | **G** | **A** | **A** | **T** | **C** | **T** | **A** | **G** | **A** | **C** | **G** | **A** | **G** | **T** | **G** | **A** | **A** | **T** | **A** | **A** | **A** | **G** | **G** | **A** | **G** | **C** | **A** | **G** | **T** | **G** | **G** | **G** | **T** | **G** | **C** | **T** | **G** | **A** | **T** | **G** | **C** | **T** | **G** | **C** | **A** | **C** | **G** | **G** | **A** | **C** | **G** | **A** | **T** | **G** | **A** | **A** | **A** | **C** | **G** | **A** | **T** | **C** | **C** | **A** | **A** | **A** | **G** | **C** | **A** | **A** | **T** | **C** | **G** | **T** | **G** | **A** | **A** | **T** | **C** | **G** | **G** | **A** | **T** | **G** | **G** | **A** | **G** | **A** | **C** | **C** | **C** | **A** | **G** | **A** | **T** | **C** | **A** | **G** | **C** | **G** | **A** | **G** | **A** | **A** | **A** | **G** | **A** | **A** | **A** | **A** | **A** | **C** | **T** | **A** | **T** | **T** | **A** | **A** | **A** | **A** | **G** | **A** | **T** | **A** | **T** | **C** | **G** | **A** | **A** | **T** | **A** | **T** | **A** | **T** | **C** | **G** | **A** | **T** | **T** | **C** | **T** | **T** | **T** | **G** | **A** | **C** | **G** | **A** | **T** | **G** | **A** | **A** | **T** | **A** | **G** | **C** | **C** | **A** | **A** | **G** | **C** | **G** | **T** | **C** | **T** | **C** | **A** | **C** | **C** | **A** | **T** | **A** | **A** | **G** | **C** | **G** | **A** | **C** | **T** | **T** | **T** | **T** | **A** | **A** | **G** | **C** | **G** | **T** | **A** | **G** | **A** | **A** | **G** | **A** | **T** | **C** | **T** | **T** | **C** | **A** | **A** | **T** | **T** | **A** | **G** | **G** | **G** | **T** | **A** | **T** | **G** | **A** | **A** | **A** | **A** | **T** | **C** | **T** | **G** | **T** | **T** | **A** | **T** | **T** | **C** | **G** | **A** | **G** | **C** | **C** | **A** | **A** | **T** | **T** | **C** | **A** | **T** | **T** | **T** | **T** | **A** | **C** | **A** | **A** | **T** | **C** | **G** | **A** | **G** | **C** | **C** | **T** | **C** | **A** | **T** | **C** | **A** | **G** | **C** | **G** | **G** | **G** | **T** | **G** | **G** | **C** | **G** | **A** | **T** | **T** | **T** | **C** | **A** | **G** | **G** | **T** | **C** | **C** | **T** | **A** | **A** | **C** | **G** | **G** | **T** | **G** | **C** | **A** | **G** | **G** | **A** | **A** | **A** | **G** | **T** | **C** | **A** | **T** | **C** | **C** | **A** | **T** | **T** | **A** | **T** | **C** | **C** | **A** | **T** | **T** | **A** | **T** | **C** | **T** | **T** | **C** | **T** | **G** | **G** | **G** | **G** | **G** | **C** | **A** | **T** | **T** | **C** | **A** | **A** | **C** | **G** | **G** | **C** | **A** | **A** | **G** | **G** | **T** | **T** | **A** | **T** | **A** | **G** | **G** | **A** | **G** | **A** | **A** | **A** | **A** | **A** | **T** | **C** | **C** | **C** | **A** | **G** | **C** | **C** | **A** | **A** | **A** | **A** | **C** | **A** | **T** | **C** | **T** | **G** | **A** | **G** | **C** | **A** | **T** | **T** | **A** | **G** | **T** | **T** | **A** | **T** | **G** | **T** | **A** | **C** | **G** | **C** | **C** | **A** | **A** | **A** | **A** | **T** | **T** | **A** | **T** | **G** | **A** | **A** | **G** | **A** | **C** | **A** | **A** | **T** | **C** | **G** | **A** | **G** | **G** | **A** | **A** | **C** | **G** | **T** | **T** | **G** | **G** | **C** | **G** | **G** | **A** | **A** | **T** | **T** | **T** | **G** | **C** | **A** | **G** | **A** | **G** | **A** | **A** | **A** | **A** | **A** | **C** | **C** | **A** | **A** | **G** | **T** | **A** | **G** | **A** | **C** | **T** | **A** | **C** | **C** | **A** | **A** | **G** | **C** | **A** | **T** | **T** | **T** | **T** | **T** | **G** | **A** | **A** | **C** | **A** | **A** | **C** | **C** | **T** | **C** | **C** | **G** | **A** | **A** | **A** | **G** | **C** | **T** | **T** | **G** | **G** | **G** | **A** | **T** | **G** | **G** | **A** | **A** | **A** | **G** | **A** | **G** | **A** | **T** | **G** | **T** | **T** | **T** | **T** | **T** | **C** | **A** | **T** | **A** | **A** | **C** | **A** | **A** | **G** | **A** | **T** | **T** | **G** | **A** | **G** | **C** | **A** | **G** | **A** | **T** | **G** | **A** | **G** | **T** | **A** | **T** | **G** | **G** | **G** | **C** | **C** | **A** | **A** | **C** | **G** | **G** | **A** | **A** | **A** | **A** | **A** | **A** | **G** | **T** | **G** | **G** | **A** | **A** | **T** | **T** | **G** | **G** | **C** | **T** | **A** | **A** | **A** | **T** | **C** | **T** | **T** | **T** | **A** | **T** | **C** | **A** | **C** | **A** | **G** | **C** | **C** | **A** | **G** | **C** | **T** | **G** | **A** | **A** | **C** | **T** | **A** | **T** | **A** | **T** | **A** | **T** | **A** | **T** | **G** | **G** | **G** | **A** | **T** | **G** | **A** | **A** | **C** | **C** | **A** | **T** | **T** | **G** | **A** | **A** | **T** | **T** | **A** | **T** | **T** | **T** | **G** | **G** | **A** | **T** | **G** | **T** | **C** | **T** | **T** | **C** | **A** | **A** | **T** | **C** | **A** | **A** | **G** | **A** | **A** | **C** | **A** | **A** | **T** | **T** | **A** | **G** | **A** | **A** | **C** | **A** | **A** | **C** | **T** | **G** | **A** | **T** | **C** | **T** | **T** | **G** | **A** | **A** | **C** | **G** | **T** | **G** | **A** | **A** | **A** | **C** | **C** | **T** | **G** | **C** | **C** | **A** | **T** | **G** | **T** | **T** | **A** | **C** | **T** | **G** | **G** | **T** | **G** | **G** | **A** | **A** | **C** | **A** | **T** | **G** | **A** | **T** | **C** | **A** | **A** | **A** | **C** | **C** | **T** | **T** | **C** | **C** | **T** | **G** | **G** | **A** | **T** | **A** | **A** | **A** | **G** | **T** | **A** | **T** | **C** | **T** | **A** | **C** | **T** | **G** | **A** | **G** | **A** | **T** | **T** | **A** | **T** | **T** | **T** | **C** | **T** | **C** | **T** | **T** | **G** | **A** | **G** | **A** | **A** | **A** | **A** | **T** | **C** | **-** | **-** | **-** |
| **N54155** | **A** | **T** | **G** | **T** | **C** | **T** | **A** | **A** | **A** | **A** | **T** | **C** | **G** | **A** | **A** | **A** | **T** | **A** | **A** | **A** | **A** | **A** | **A** | **T** | **C** | **T** | **G** | **A** | **C** | **A** | **T** | **T** | **C** | **G** | **G** | **C** | **T** | **A** | **C** | **G** | **A** | **C** | **A** | **G** | **C** | **C** | **A** | **A** | **G** | **G** | **C** | **A** | **C** | **A** | **T** | **T** | **A** | **T** | **T** | **A** | **T** | **T** | **T** | **G** | **A** | **A** | **C** | **A** | **A** | **G** | **C** | **A** | **A** | **A** | **T** | **C** | **T** | **A** | **A** | **A** | **T** | **T** | **T** | **T** | **G** | **A** | **C** | **A** | **C** | **A** | **C** | **A** | **A** | **T** | **G** | **G** | **A** | **A** | **A** | **C** | **T** | **A** | **G** | **G** | **A** | **C** | **T** | **T** | **A** | **T** | **C** | **G** | **G** | **A** | **C** | **G** | **A** | **A** | **A** | **C** | **G** | **G** | **T** | **C** | **G** | **A** | **G** | **G** | **A** | **A** | **A** | **G** | **A** | **C** | **A** | **A** | **C** | **T** | **T** | **T** | **A** | **C** | **T** | **G** | **A** | **A** | **T** | **A** | **T** | **T** | **C** | **T** | **A** | **C** | **A** | **A** | **A** | **A** | **C** | **A** | **A** | **A** | **C** | **T** | **A** | **C** | **C** | **T** | **T** | **A** | **T** | **C** | **A** | **A** | **G** | **G** | **G** | **C** | **A** | **A** | **G** | **T** | **A** | **A** | **T** | **C** | **C** | **A** | **T** | **C** | **A** | **G** | **C** | **A** | **A** | **G** | **A** | **A** | **T** | **T** | **T** | **G** | **T** | **C** | **T** | **A** | **T** | **T** | **T** | **C** | **C** | **C** | **G** | **C** | **A** | **G** | **C** | **A** | **A** | **A** | **C** | **A** | **A** | **A** | **A** | **G** | **A** | **T** | **A** | **A** | **A** | **G** | **A** | **G** | **C** | **G** | **T** | **T** | **T** | **A** | **A** | **C** | **C** | **T** | **A** | **T** | **T** | **A** | **T** | **G** | **T** | **G** | **T** | **T** | **A** | **A** | **A** | **T** | **G** | **A** | **G** | **A** | **T** | **T** | **A** | **C** | **G** | **G** | **A** | **T** | **T** | **T** | **T** | **G** | **A** | **G** | **A** | **T** | **A** | **T** | **G** | **G** | **G** | **A** | **A** | **A** | **T** | **T** | **G** | **A** | **A** | **A** | **G** | **A** | **G** | **A** | **G** | **C** | **T** | **C** | **C** | **A** | **A** | **C** | **T** | **G** | **A** | **T** | **G** | **C** | **A** | **A** | **A** | **C** | **A** | **G** | **A** | **T** | **C** | **C** | **T** | **G** | **A** | **A** | **A** | **T** | **C** | **T** | **T** | **A** | **T** | **G** | **G** | **A** | **G** | **A** | **G** | **A** | **A** | **T** | **T** | **C** | **A** | **G** | **C** | **A** | **C** | **A** | **C** | **T** | **A** | **T** | **C** | **A** | **G** | **G** | **A** | **G** | **G** | **A** | **G** | **A** | **G** | **A** | **A** | **G** | **A** | **C** | **A** | **A** | **A** | **A** | **G** | **T** | **C** | **C** | **T** | **A** | **C** | **T** | **G** | **G** | **C** | **A** | **C** | **T** | **T** | **T** | **T** | **A** | **T** | **T** | **T** | **G** | **T** | **G** | **G** | **A** | **T** | **G** | **A** | **C** | **A** | **C** | **T** | **C** | **A** | **T** | **T** | **T** | **C** | **C** | **C** | **G** | **T** | **T** | **A** | **A** | **T** | **C** | **G** | **A** | **T** | **G** | **A** | **A** | **C** | **C** | **A** | **A** | **C** | **G** | **A** | **A** | **T** | **C** | **A** | **T** | **T** | **T** | **G** | **G** | **A** | **-** | **-** | **-** | **T** | **A** | **T** | **C** | **T** | **C** | **T** | **G** | **G** | **C** | **A** | **G** | **A** | **A** | **A** | **A** | **C** | **A** | **A** | **G** | **T** | **A** | **G** | **C** | **G** | **G** | **C** | **T** | **T** | **A** | **C** | **T** | **T** | **G** | **A** | **A** | **A** | **A** | **A** | **G** | **A** | **A** | **A** | **A** | **A** | **A** | **C** | **A** | **A** | **G** | **G** | **C** | **T** | **T** | **C** | **A** | **T** | **C** | **G** | **T** | **G** | **G** | **T** | **C** | **A** | **G** | **C** | **C** | **A** | **T** | **G** | **A** | **C** | **C** | **G** | **G** | **G** | **G** | **A** | **T** | **T** | **T** | **A** | **T** | **C** | **G** | **A** | **T** | **G** | **A** | **A** | **G** | **T** | **A** | **G** | **T** | **G** | **G** | **A** | **C** | **C** | **A** | **T** | **G** | **T** | **T** | **T** | **T** | **A** | **G** | **C** | **A** | **A** | **T** | **C** | **G** | **A** | **A** | **A** | **A** | **A** | **A** | **G** | **T** | **C** | **A** | **A** | **C** | **T** | **G** | **G** | **A** | **A** | **C** | **T** | **T** | **T** | **A** | **T** | **C** | **A** | **A** | **G** | **G** | **G** | **A** | **A** | **T** | **T** | **T** | **C** | **T** | **C** | **T** | **A** | **T** | **C** | **T** | **A** | **T** | **G** | **A** | **A** | **G** | **A** | **A** | **C** | **A** | **G** | **A** | **A** | **A** | **A** | **A** | **A** | **C** | **T** | **T** | **C** | **G** | **T** | **G** | **A** | **T** | **G** | **A** | **A** | **T** | **T** | **T** | **G** | **A** | **A** | **A** | **T** | **G** | **G** | **C** | **T** | **C** | **A** | **A** | **A** | **A** | **T** | **G** | **A** | **A** | **A** | **A** | **A** | **T** | **T** | **G** | **A** | **A** | **A** | **A** | **A** | **A** | **G** | **A** | **A** | **G** | **T** | **C** | **A** | **G** | **T** | **A** | **G** | **G** | **C** | **T** | **A** | **A** | **A** | **G** | **A** | **A** | **A** | **A** | **C** | **A** | **G** | **C** | **A** | **G** | **C** | **T** | **G** | **A** | **A** | **A** | **A** | **A** | **G** | **C** | **C** | **G** | **A** | **A** | **T** | **G** | **G** | **T** | **C** | **T** | **C** | **G** | **T** | **T** | **C** | **C** | **C** | **G** | **A** | **G** | **A** | **A** | **G** | **G** | **A** | **G** | **A** | **T** | **A** | **A** | **A** | **A** | **C** | **A** | **A** | **A** | **G** | **A** | **A** | **A** | **C** | **A** | **A** | **G** | **T** | **C** | **G** | **G** | **A** | **T** | **T** | **C** | **A** | **T** | **C** | **G** | **A** | **T** | **A** | **C** | **T** | **G** | **A** | **A** | **T** | **C** | **T** | **A** | **G** | **A** | **C** | **G** | **A** | **G** | **T** | **G** | **A** | **A** | **T** | **A** | **A** | **A** | **G** | **G** | **A** | **G** | **C** | **A** | **G** | **T** | **G** | **G** | **G** | **T** | **G** | **C** | **T** | **G** | **A** | **T** | **G** | **C** | **T** | **G** | **C** | **A** | **C** | **G** | **G** | **A** | **C** | **G** | **A** | **T** | **G** | **A** | **A** | **A** | **C** | **G** | **A** | **T** | **C** | **C** | **A** | **A** | **A** | **G** | **C** | **A** | **A** | **T** | **C** | **G** | **T** | **G** | **A** | **A** | **T** | **C** | **G** | **G** | **A** | **T** | **G** | **G** | **A** | **G** | **A** | **C** | **C** | **C** | **A** | **G** | **A** | **T** | **C** | **A** | **G** | **C** | **G** | **A** | **G** | **A** | **A** | **A** | **G** | **A** | **A** | **A** | **A** | **A** | **C** | **T** | **A** | **T** | **T** | **G** | **A** | **A** | **A** | **G** | **A** | **T** | **A** | **T** | **C** | **G** | **A** | **A** | **T** | **A** | **T** | **A** | **T** | **C** | **G** | **A** | **T** | **T** | **C** | **T** | **T** | **T** | **G** | **A** | **C** | **G** | **A** | **T** | **G** | **A** | **A** | **T** | **A** | **G** | **C** | **C** | **A** | **A** | **G** | **C** | **G** | **T** | **C** | **T** | **C** | **A** | **C** | **C** | **A** | **T** | **A** | **A** | **G** | **C** | **G** | **A** | **C** | **T** | **T** | **T** | **T** | **A** | **A** | **G** | **C** | **G** | **T** | **A** | **G** | **A** | **A** | **G** | **A** | **T** | **C** | **T** | **T** | **C** | **A** | **A** | **T** | **T** | **A** | **G** | **G** | **G** | **T** | **A** | **T** | **G** | **A** | **G** | **A** | **A** | **T** | **C** | **T** | **G** | **T** | **T** | **A** | **T** | **T** | **C** | **G** | **A** | **G** | **C** | **C** | **A** | **A** | **T** | **T** | **C** | **A** | **T** | **T** | **T** | **T** | **A** | **C** | **A** | **A** | **T** | **C** | **G** | **A** | **G** | **C** | **C** | **T** | **C** | **A** | **T** | **C** | **A** | **G** | **C** | **G** | **G** | **G** | **T** | **G** | **G** | **C** | **G** | **A** | **T** | **T** | **T** | **C** | **A** | **G** | **G** | **T** | **C** | **C** | **T** | **A** | **A** | **C** | **G** | **G** | **T** | **G** | **C** | **A** | **G** | **G** | **A** | **A** | **A** | **G** | **T** | **C** | **A** | **T** | **C** | **C** | **A** | **T** | **T** | **A** | **T** | **C** | **C** | **A** | **T** | **T** | **A** | **T** | **C** | **T** | **T** | **C** | **T** | **G** | **G** | **G** | **G** | **G** | **C** | **A** | **T** | **T** | **C** | **A** | **A** | **C** | **G** | **G** | **C** | **A** | **A** | **G** | **G** | **T** | **T** | **A** | **T** | **A** | **G** | **G** | **A** | **G** | **A** | **A** | **A** | **A** | **A** | **T** | **C** | **C** | **C** | **A** | **G** | **C** | **C** | **A** | **A** | **A** | **A** | **C** | **A** | **T** | **C** | **T** | **G** | **A** | **G** | **C** | **A** | **T** | **T** | **A** | **G** | **T** | **T** | **A** | **T** | **G** | **T** | **A** | **C** | **G** | **C** | **C** | **A** | **A** | **A** | **A** | **T** | **T** | **A** | **T** | **G** | **A** | **A** | **G** | **A** | **C** | **A** | **A** | **T** | **C** | **G** | **A** | **G** | **G** | **A** | **A** | **C** | **G** | **T** | **T** | **G** | **G** | **C** | **G** | **G** | **A** | **A** | **T** | **T** | **T** | **G** | **C** | **A** | **G** | **A** | **G** | **A** | **A** | **A** | **A** | **A** | **C** | **C** | **A** | **A** | **G** | **T** | **A** | **G** | **A** | **C** | **T** | **A** | **C** | **C** | **A** | **A** | **G** | **C** | **A** | **T** | **T** | **T** | **T** | **T** | **G** | **A** | **A** | **C** | **A** | **A** | **C** | **C** | **T** | **C** | **C** | **G** | **A** | **A** | **A** | **G** | **C** | **T** | **T** | **G** | **G** | **G** | **A** | **T** | **G** | **G** | **A** | **A** | **A** | **G** | **A** | **G** | **A** | **T** | **G** | **T** | **T** | **T** | **T** | **T** | **C** | **A** | **T** | **A** | **A** | **C** | **A** | **A** | **G** | **A** | **T** | **C** | **G** | **A** | **G** | **C** | **A** | **G** | **A** | **T** | **G** | **A** | **G** | **T** | **A** | **T** | **G** | **G** | **G** | **C** | **C** | **A** | **A** | **C** | **G** | **G** | **A** | **A** | **A** | **A** | **A** | **A** | **G** | **T** | **G** | **G** | **A** | **A** | **T** | **T** | **G** | **G** | **C** | **T** | **A** | **A** | **A** | **T** | **C** | **T** | **T** | **T** | **A** | **T** | **C** | **A** | **C** | **A** | **G** | **C** | **C** | **A** | **G** | **C** | **T** | **G** | **A** | **A** | **C** | **T** | **A** | **T** | **A** | **T** | **A** | **T** | **A** | **T** | **G** | **G** | **G** | **A** | **T** | **G** | **A** | **A** | **C** | **C** | **A** | **T** | **T** | **G** | **A** | **A** | **T** | **T** | **A** | **T** | **T** | **T** | **G** | **G** | **A** | **T** | **G** | **T** | **C** | **T** | **T** | **C** | **A** | **A** | **T** | **C** | **A** | **A** | **G** | **A** | **A** | **C** | **A** | **A** | **T** | **T** | **A** | **G** | **A** | **A** | **C** | **A** | **A** | **C** | **T** | **G** | **A** | **T** | **C** | **T** | **T** | **G** | **A** | **A** | **C** | **G** | **T** | **G** | **A** | **A** | **A** | **C** | **C** | **T** | **G** | **C** | **C** | **A** | **T** | **G** | **T** | **T** | **A** | **C** | **T** | **A** | **G** | **T** | **G** | **G** | **A** | **A** | **C** | **A** | **T** | **G** | **A** | **T** | **C** | **A** | **A** | **A** | **C** | **C** | **T** | **T** | **C** | **C** | **T** | **G** | **G** | **A** | **T** | **A** | **A** | **A** | **G** | **T** | **A** | **T** | **C** | **T** | **A** | **C** | **T** | **G** | **A** | **G** | **A** | **T** | **T** | **A** | **T** | **T** | **T** | **C** | **T** | **C** | **T** | **T** | **G** | **A** | **G** | **A** | **A** | **A** | **A** | **T** | **C** | **-** | **-** | **-** |
| **N59745F** | **A** | **T** | **G** | **T** | **C** | **T** | **A** | **A** | **A** | **A** | **T** | **C** | **G** | **A** | **A** | **A** | **T** | **A** | **A** | **A** | **A** | **A** | **A** | **T** | **C** | **T** | **G** | **A** | **C** | **A** | **T** | **T** | **C** | **G** | **G** | **C** | **T** | **A** | **T** | **G** | **A** | **C** | **A** | **G** | **C** | **C** | **A** | **A** | **G** | **G** | **C** | **A** | **C** | **A** | **T** | **T** | **A** | **T** | **T** | **A** | **T** | **T** | **T** | **G** | **A** | **A** | **C** | **A** | **A** | **G** | **C** | **C** | **A** | **A** | **T** | **C** | **T** | **A** | **A** | **A** | **C** | **T** | **T** | **T** | **G** | **A** | **C** | **A** | **C** | **A** | **C** | **A** | **A** | **T** | **G** | **G** | **A** | **A** | **A** | **C** | **T** | **A** | **G** | **G** | **A** | **C** | **T** | **T** | **A** | **T** | **C** | **G** | **G** | **A** | **C** | **G** | **A** | **A** | **A** | **C** | **G** | **G** | **T** | **C** | **G** | **A** | **G** | **G** | **A** | **A** | **A** | **G** | **A** | **C** | **A** | **A** | **C** | **T** | **T** | **T** | **A** | **C** | **T** | **G** | **A** | **A** | **T** | **A** | **T** | **C** | **C** | **T** | **G** | **C** | **A** | **A** | **A** | **A** | **C** | **A** | **A** | **A** | **C** | **T** | **C** | **C** | **C** | **T** | **T** | **A** | **T** | **C** | **A** | **A** | **G** | **G** | **G** | **C** | **A** | **A** | **G** | **T** | **G** | **A** | **T** | **C** | **C** | **A** | **T** | **C** | **A** | **G** | **C** | **A** | **A** | **G** | **C** | **G** | **T** | **T** | **T** | **G** | **T** | **C** | **T** | **A** | **T** | **T** | **T** | **C** | **C** | **C** | **G** | **C** | **A** | **G** | **C** | **A** | **A** | **A** | **C** | **A** | **A** | **A** | **A** | **G** | **A** | **T** | **A** | **A** | **A** | **G** | **A** | **G** | **C** | **G** | **T** | **T** | **T** | **G** | **A** | **C** | **C** | **T** | **A** | **T** | **T** | **A** | **T** | **G** | **T** | **G** | **T** | **T** | **A** | **A** | **A** | **T** | **G** | **A** | **G** | **A** | **T** | **T** | **A** | **T** | **G** | **G** | **A** | **T** | **T** | **T** | **T** | **G** | **A** | **G** | **A** | **T** | **A** | **T** | **G** | **G** | **G** | **A** | **A** | **A** | **T** | **T** | **G** | **A** | **A** | **A** | **G** | **A** | **G** | **A** | **G** | **C** | **T** | **C** | **C** | **A** | **A** | **C** | **T** | **G** | **A** | **T** | **G** | **C** | **A** | **A** | **A** | **C** | **A** | **G** | **A** | **T** | **C** | **C** | **T** | **G** | **A** | **A** | **A** | **T** | **C** | **T** | **T** | **A** | **T** | **G** | **G** | **A** | **G** | **A** | **G** | **A** | **A** | **T** | **T** | **C** | **A** | **G** | **C** | **A** | **C** | **A** | **C** | **T** | **A** | **T** | **C** | **A** | **G** | **G** | **A** | **G** | **G** | **A** | **G** | **A** | **G** | **A** | **A** | **G** | **A** | **C** | **A** | **A** | **A** | **A** | **G** | **T** | **C** | **C** | **T** | **A** | **C** | **T** | **G** | **G** | **C** | **A** | **C** | **T** | **C** | **T** | **T** | **A** | **T** | **T** | **T** | **G** | **T** | **G** | **G** | **A** | **T** | **G** | **A** | **C** | **A** | **C** | **T** | **C** | **A** | **T** | **T** | **T** | **C** | **C** | **C** | **G** | **T** | **T** | **A** | **A** | **T** | **C** | **G** | **A** | **T** | **G** | **A** | **A** | **C** | **C** | **A** | **A** | **C** | **G** | **A** | **A** | **T** | **C** | **A** | **T** | **T** | **T** | **G** | **G** | **A** | **-** | **-** | **-** | **T** | **A** | **T** | **C** | **T** | **C** | **T** | **G** | **G** | **C** | **A** | **G** | **A** | **A** | **A** | **A** | **C** | **A** | **A** | **G** | **T** | **A** | **G** | **C** | **G** | **G** | **C** | **T** | **T** | **A** | **C** | **T** | **T** | **G** | **A** | **A** | **A** | **A** | **A** | **G** | **A** | **A** | **A** | **A** | **A** | **A** | **C** | **A** | **A** | **G** | **G** | **A** | **T** | **T** | **C** | **A** | **T** | **C** | **G** | **T** | **G** | **G** | **T** | **G** | **A** | **G** | **T** | **C** | **A** | **T** | **G** | **A** | **T** | **C** | **G** | **G** | **G** | **G** | **A** | **T** | **T** | **T** | **A** | **T** | **C** | **G** | **A** | **T** | **G** | **A** | **A** | **G** | **T** | **A** | **G** | **T** | **G** | **G** | **A** | **C** | **C** | **A** | **T** | **G** | **T** | **T** | **T** | **T** | **A** | **G** | **C** | **A** | **A** | **T** | **C** | **G** | **A** | **A** | **A** | **A** | **G** | **A** | **G** | **C** | **C** | **A** | **A** | **C** | **T** | **G** | **G** | **A** | **A** | **C** | **T** | **T** | **T** | **A** | **T** | **C** | **A** | **A** | **G** | **G** | **G** | **A** | **A** | **T** | **T** | **T** | **C** | **T** | **C** | **T** | **A** | **T** | **C** | **T** | **A** | **T** | **G** | **A** | **A** | **G** | **A** | **A** | **C** | **A** | **G** | **A** | **A** | **A** | **A** | **A** | **A** | **C** | **T** | **T** | **C** | **G** | **T** | **G** | **A** | **T** | **G** | **A** | **A** | **T** | **T** | **T** | **G** | **A** | **A** | **A** | **T** | **A** | **G** | **C** | **T** | **A** | **A** | **A** | **A** | **A** | **T** | **G** | **A** | **A** | **A** | **A** | **A** | **T** | **T** | **G** | **A** | **A** | **A** | **A** | **A** | **A** | **G** | **A** | **A** | **G** | **T** | **C** | **A** | **A** | **C** | **A** | **G** | **G** | **C** | **T** | **A** | **A** | **A** | **G** | **A** | **A** | **A** | **A** | **C** | **A** | **G** | **C** | **A** | **G** | **C** | **T** | **G** | **A** | **A** | **A** | **A** | **A** | **G** | **C** | **A** | **G** | **A** | **A** | **T** | **G** | **G** | **T** | **C** | **T** | **C** | **G** | **T** | **T** | **C** | **C** | **C** | **G** | **A** | **G** | **A** | **A** | **G** | **G** | **A** | **G** | **A** | **C** | **A** | **A** | **A** | **A** | **C** | **A** | **A** | **A** | **G** | **A** | **A** | **A** | **A** | **A** | **A** | **G** | **T** | **C** | **G** | **G** | **A** | **T** | **T** | **C** | **A** | **T** | **C** | **G** | **A** | **T** | **A** | **C** | **G** | **G** | **A** | **A** | **T** | **C** | **T** | **A** | **G** | **G** | **C** | **G** | **A** | **G** | **T** | **G** | **A** | **A** | **T** | **A** | **A** | **A** | **G** | **G** | **A** | **G** | **C** | **A** | **G** | **T** | **A** | **G** | **G** | **C** | **G** | **C** | **T** | **G** | **A** | **T** | **G** | **C** | **T** | **G** | **C** | **A** | **C** | **G** | **G** | **A** | **C** | **G** | **A** | **T** | **G** | **A** | **A** | **A** | **C** | **G** | **A** | **T** | **C** | **T** | **A** | **A** | **A** | **G** | **C** | **A** | **A** | **T** | **C** | **G** | **T** | **G** | **A** | **A** | **T** | **C** | **G** | **G** | **A** | **T** | **G** | **G** | **A** | **A** | **A** | **C** | **C** | **C** | **A** | **G** | **A** | **T** | **C** | **G** | **G** | **T** | **G** | **A** | **G** | **A** | **A** | **A** | **G** | **A** | **A** | **A** | **A** | **A** | **T** | **T** | **A** | **T** | **T** | **A** | **A** | **A** | **A** | **G** | **A** | **T** | **A** | **T** | **C** | **G** | **A** | **G** | **T** | **A** | **T** | **A** | **T** | **C** | **G** | **A** | **T** | **G** | **C** | **T** | **T** | **T** | **G** | **A** | **C** | **G** | **A** | **T** | **G** | **A** | **A** | **T** | **A** | **G** | **C** | **C** | **A** | **A** | **G** | **T** | **G** | **T** | **C** | **T** | **C** | **A** | **T** | **C** | **A** | **T** | **A** | **A** | **A** | **C** | **G** | **G** | **C** | **T** | **G** | **T** | **T** | **A** | **A** | **G** | **C** | **G** | **T** | **A** | **G** | **A** | **A** | **A** | **A** | **T** | **C** | **T** | **T** | **C** | **A** | **A** | **T** | **T** | **A** | **G** | **G** | **G** | **T** | **A** | **T** | **G** | **A** | **A** | **A** | **A** | **T** | **C** | **T** | **G** | **T** | **T** | **A** | **T** | **T** | **C** | **G** | **A** | **G** | **C** | **C** | **A** | **G** | **T** | **T** | **C** | **A** | **T** | **T** | **T** | **T** | **A** | **C** | **G** | **A** | **T** | **C** | **G** | **A** | **A** | **C** | **C** | **T** | **C** | **A** | **T** | **C** | **A** | **G** | **C** | **G** | **G** | **G** | **T** | **G** | **G** | **C** | **A** | **A** | **T** | **T** | **T** | **C** | **A** | **G** | **G** | **T** | **C** | **C** | **T** | **A** | **A** | **C** | **G** | **G** | **C** | **G** | **C** | **G** | **G** | **G** | **A** | **A** | **A** | **G** | **T** | **C** | **A** | **T** | **C** | **C** | **A** | **T** | **T** | **A** | **T** | **C** | **C** | **A** | **T** | **T** | **A** | **T** | **C** | **T** | **T** | **C** | **T** | **G** | **G** | **G** | **G** | **A** | **C** | **A** | **T** | **T** | **C** | **A** | **A** | **C** | **G** | **G** | **C** | **A** | **A** | **G** | **G** | **T** | **T** | **G** | **T** | **A** | **G** | **G** | **A** | **G** | **A** | **A** | **A** | **A** | **A** | **T** | **C** | **C** | **C** | **A** | **G** | **C** | **C** | **C** | **A** | **A** | **A** | **C** | **A** | **T** | **T** | **T** | **G** | **A** | **C** | **C** | **A** | **T** | **T** | **A** | **G** | **T** | **T** | **A** | **C** | **G** | **T** | **A** | **C** | **G** | **C** | **C** | **A** | **A** | **A** | **A** | **T** | **T** | **A** | **T** | **G** | **A** | **A** | **G** | **A** | **C** | **A** | **A** | **T** | **C** | **G** | **A** | **G** | **G** | **G** | **A** | **C** | **A** | **T** | **T** | **G** | **G** | **C** | **G** | **G** | **A** | **A** | **T** | **T** | **C** | **G** | **C** | **G** | **G** | **A** | **G** | **A** | **A** | **A** | **A** | **A** | **C** | **C** | **G** | **A** | **G** | **T** | **A** | **G** | **A** | **C** | **T** | **A** | **T** | **C** | **A** | **A** | **G** | **C** | **A** | **T** | **T** | **T** | **T** | **T** | **G** | **A** | **A** | **T** | **A** | **A** | **T** | **C** | **T** | **C** | **C** | **G** | **A** | **A** | **A** | **G** | **C** | **T** | **T** | **G** | **G** | **G** | **A** | **T** | **G** | **G** | **A** | **A** | **A** | **G** | **A** | **G** | **A** | **T** | **G** | **T** | **T** | **T** | **T** | **T** | **C** | **A** | **T** | **A** | **A** | **T** | **A** | **A** | **A** | **A** | **T** | **C** | **G** | **A** | **A** | **C** | **A** | **G** | **A** | **T** | **G** | **A** | **G** | **T** | **A** | **T** | **G** | **G** | **G** | **A** | **C** | **A** | **A** | **C** | **G** | **G** | **A** | **A** | **A** | **A** | **A** | **A** | **G** | **T** | **G** | **G** | **A** | **A** | **T** | **T** | **G** | **G** | **C** | **T** | **A** | **A** | **A** | **T** | **C** | **T** | **T** | **T** | **A** | **T** | **C** | **C** | **C** | **A** | **A** | **C** | **C** | **A** | **G** | **C** | **T** | **G** | **A** | **G** | **C** | **T** | **C** | **T** | **A** | **T** | **A** | **T** | **A** | **T** | **G** | **G** | **G** | **A** | **T** | **G** | **A** | **A** | **C** | **C** | **A** | **T** | **T** | **G** | **A** | **A** | **C** | **T** | **A** | **T** | **T** | **T** | **G** | **G** | **A** | **T** | **G** | **T** | **C** | **T** | **T** | **C** | **A** | **A** | **T** | **C** | **A** | **A** | **G** | **A** | **A** | **C** | **A** | **A** | **T** | **T** | **A** | **G** | **A** | **A** | **C** | **A** | **A** | **C** | **T** | **G** | **A** | **T** | **T** | **T** | **T** | **G** | **A** | **A** | **C** | **G** | **T** | **G** | **A** | **A** | **A** | **C** | **C** | **T** | **G** | **C** | **T** | **A** | **T** | **G** | **T** | **T** | **A** | **C** | **T** | **A** | **G** | **T** | **G** | **G** | **A** | **A** | **C** | **A** | **T** | **G** | **A** | **T** | **C** | **A** | **A** | **A** | **C** | **C** | **T** | **T** | **T** | **C** | **T** | **A** | **G** | **A** | **T** | **A** | **A** | **A** | **G** | **T** | **A** | **T** | **C** | **T** | **A** | **C** | **T** | **G** | **A** | **G** | **A** | **T** | **T** | **A** | **T** | **T** | **T** | **C** | **T** | **C** | **T** | **T** | **G** | **A** | **G** | **A** | **A** | **A** | **A** | **T** | **C** | **-** | **-** | **-** |
| **N53548** | **A** | **T** | **G** | **T** | **C** | **T** | **A** | **A** | **A** | **A** | **T** | **C** | **G** | **A** | **A** | **A** | **T** | **A** | **A** | **A** | **A** | **A** | **A** | **T** | **C** | **T** | **G** | **A** | **C** | **A** | **C** | **T** | **C** | **G** | **G** | **C** | **T** | **A** | **C** | **G** | **A** | **C** | **A** | **G** | **C** | **C** | **A** | **A** | **G** | **G** | **C** | **A** | **C** | **A** | **T** | **T** | **A** | **T** | **T** | **A** | **T** | **T** | **T** | **G** | **A** | **A** | **C** | **A** | **A** | **G** | **C** | **A** | **A** | **A** | **T** | **C** | **T** | **A** | **A** | **A** | **T** | **T** | **T** | **T** | **G** | **A** | **C** | **A** | **C** | **A** | **C** | **A** | **A** | **T** | **G** | **G** | **A** | **A** | **A** | **C** | **T** | **A** | **G** | **G** | **A** | **C** | **T** | **T** | **A** | **T** | **C** | **G** | **G** | **A** | **C** | **G** | **A** | **A** | **A** | **C** | **G** | **G** | **T** | **C** | **G** | **A** | **G** | **G** | **A** | **A** | **A** | **G** | **A** | **C** | **A** | **A** | **C** | **T** | **T** | **T** | **A** | **C** | **T** | **G** | **A** | **A** | **T** | **A** | **T** | **T** | **C** | **T** | **A** | **C** | **A** | **A** | **A** | **A** | **C** | **A** | **A** | **A** | **C** | **T** | **A** | **C** | **C** | **T** | **T** | **A** | **T** | **C** | **A** | **A** | **G** | **G** | **G** | **C** | **A** | **A** | **G** | **T** | **A** | **A** | **T** | **C** | **C** | **A** | **T** | **C** | **A** | **G** | **C** | **A** | **A** | **G** | **A** | **A** | **T** | **T** | **T** | **G** | **T** | **C** | **T** | **A** | **T** | **T** | **T** | **C** | **C** | **C** | **G** | **C** | **A** | **A** | **C** | **A** | **G** | **A** | **C** | **A** | **A** | **A** | **A** | **G** | **A** | **T** | **A** | **A** | **A** | **G** | **A** | **A** | **C** | **G** | **T** | **T** | **T** | **A** | **A** | **C** | **C** | **T** | **A** | **T** | **T** | **A** | **C** | **G** | **T** | **G** | **T** | **T** | **A** | **A** | **A** | **T** | **G** | **A** | **T** | **A** | **T** | **T** | **A** | **C** | **G** | **G** | **G** | **T** | **T** | **T** | **T** | **G** | **A** | **G** | **A** | **T** | **A** | **T** | **G** | **G** | **G** | **A** | **A** | **A** | **T** | **C** | **G** | **A** | **A** | **A** | **G** | **A** | **G** | **A** | **G** | **C** | **T** | **C** | **C** | **A** | **A** | **T** | **T** | **G** | **A** | **T** | **G** | **C** | **A** | **A** | **A** | **C** | **A** | **G** | **A** | **T** | **C** | **C** | **T** | **G** | **A** | **A** | **A** | **T** | **C** | **T** | **T** | **A** | **T** | **G** | **G** | **A** | **G** | **A** | **G** | **A** | **A** | **T** | **T** | **C** | **A** | **G** | **C** | **A** | **C** | **A** | **C** | **T** | **A** | **T** | **C** | **A** | **G** | **G** | **G** | **G** | **G** | **A** | **G** | **A** | **G** | **A** | **A** | **G** | **A** | **C** | **A** | **A** | **A** | **A** | **G** | **T** | **C** | **C** | **T** | **A** | **C** | **T** | **G** | **G** | **C** | **A** | **C** | **T** | **T** | **T** | **T** | **A** | **T** | **T** | **T** | **G** | **T** | **G** | **G** | **A** | **T** | **G** | **A** | **C** | **A** | **C** | **T** | **C** | **A** | **T** | **T** | **T** | **C** | **C** | **C** | **G** | **T** | **T** | **A** | **A** | **T** | **C** | **G** | **A** | **T** | **G** | **A** | **A** | **C** | **C** | **A** | **A** | **C** | **G** | **A** | **A** | **T** | **C** | **A** | **T** | **T** | **T** | **G** | **G** | **A** | **-** | **-** | **-** | **T** | **A** | **T** | **C** | **T** | **C** | **T** | **G** | **G** | **T** | **A** | **G** | **A** | **A** | **A** | **A** | **C** | **A** | **A** | **G** | **T** | **A** | **G** | **C** | **G** | **G** | **C** | **T** | **T** | **A** | **T** | **T** | **T** | **G** | **A** | **A** | **A** | **A** | **A** | **G** | **A** | **A** | **A** | **A** | **A** | **A** | **C** | **A** | **A** | **G** | **G** | **C** | **T** | **T** | **C** | **A** | **T** | **C** | **G** | **T** | **G** | **G** | **T** | **C** | **A** | **G** | **C** | **C** | **A** | **T** | **G** | **A** | **C** | **C** | **G** | **G** | **G** | **G** | **A** | **T** | **T** | **T** | **A** | **T** | **C** | **G** | **A** | **T** | **G** | **A** | **A** | **G** | **T** | **A** | **G** | **T** | **G** | **G** | **A** | **C** | **C** | **A** | **T** | **G** | **T** | **T** | **T** | **T** | **A** | **G** | **C** | **A** | **A** | **T** | **C** | **G** | **A** | **A** | **A** | **A** | **A** | **A** | **G** | **T** | **C** | **A** | **A** | **C** | **T** | **G** | **G** | **A** | **A** | **C** | **T** | **T** | **T** | **A** | **T** | **C** | **A** | **A** | **G** | **G** | **G** | **A** | **A** | **T** | **T** | **T** | **C** | **T** | **C** | **T** | **A** | **T** | **C** | **T** | **A** | **T** | **G** | **A** | **A** | **G** | **A** | **A** | **C** | **A** | **G** | **A** | **A** | **A** | **A** | **A** | **A** | **C** | **T** | **T** | **C** | **G** | **T** | **G** | **A** | **T** | **G** | **A** | **A** | **T** | **T** | **T** | **G** | **A** | **A** | **A** | **T** | **G** | **G** | **C** | **T** | **C** | **A** | **A** | **A** | **A** | **T** | **G** | **A** | **A** | **A** | **A** | **A** | **T** | **T** | **G** | **A** | **A** | **A** | **A** | **A** | **A** | **G** | **A** | **A** | **G** | **T** | **C** | **A** | **G** | **T** | **A** | **G** | **G** | **C** | **T** | **A** | **A** | **A** | **G** | **A** | **A** | **A** | **A** | **C** | **A** | **G** | **C** | **A** | **G** | **C** | **T** | **G** | **A** | **A** | **A** | **A** | **A** | **G** | **C** | **C** | **G** | **A** | **A** | **T** | **G** | **G** | **T** | **C** | **T** | **C** | **G** | **T** | **T** | **C** | **C** | **C** | **G** | **A** | **G** | **A** | **A** | **G** | **G** | **A** | **G** | **A** | **T** | **A** | **A** | **A** | **A** | **C** | **A** | **A** | **A** | **G** | **A** | **A** | **A** | **C** | **A** | **A** | **G** | **T** | **C** | **G** | **G** | **A** | **T** | **T** | **C** | **A** | **T** | **C** | **G** | **A** | **T** | **A** | **C** | **T** | **G** | **A** | **A** | **T** | **C** | **T** | **A** | **G** | **A** | **C** | **G** | **A** | **G** | **T** | **G** | **A** | **A** | **T** | **A** | **A** | **A** | **G** | **G** | **A** | **G** | **C** | **A** | **G** | **T** | **G** | **G** | **G** | **T** | **G** | **C** | **T** | **G** | **A** | **T** | **G** | **C** | **T** | **G** | **C** | **A** | **C** | **G** | **G** | **A** | **C** | **G** | **A** | **T** | **G** | **A** | **A** | **A** | **C** | **G** | **A** | **T** | **C** | **C** | **A** | **A** | **A** | **G** | **C** | **A** | **A** | **T** | **C** | **G** | **T** | **G** | **A** | **A** | **T** | **C** | **G** | **G** | **A** | **T** | **G** | **G** | **A** | **G** | **A** | **C** | **C** | **C** | **A** | **G** | **A** | **T** | **C** | **A** | **G** | **C** | **G** | **A** | **G** | **A** | **A** | **A** | **G** | **A** | **A** | **A** | **A** | **A** | **C** | **T** | **A** | **T** | **T** | **A** | **A** | **A** | **A** | **G** | **A** | **T** | **A** | **T** | **C** | **G** | **A** | **A** | **T** | **A** | **T** | **A** | **T** | **C** | **G** | **A** | **T** | **T** | **C** | **T** | **T** | **T** | **G** | **A** | **C** | **G** | **A** | **T** | **G** | **A** | **A** | **T** | **A** | **G** | **C** | **C** | **A** | **A** | **G** | **C** | **G** | **T** | **C** | **T** | **C** | **A** | **C** | **C** | **A** | **T** | **A** | **A** | **G** | **C** | **G** | **A** | **C** | **T** | **T** | **T** | **T** | **A** | **A** | **G** | **C** | **G** | **T** | **A** | **G** | **A** | **A** | **G** | **A** | **T** | **C** | **T** | **T** | **C** | **A** | **A** | **T** | **T** | **A** | **G** | **G** | **G** | **T** | **A** | **T** | **G** | **A** | **A** | **A** | **A** | **T** | **C** | **T** | **G** | **T** | **T** | **A** | **T** | **T** | **C** | **G** | **A** | **G** | **C** | **C** | **A** | **A** | **T** | **T** | **C** | **A** | **T** | **T** | **T** | **T** | **A** | **C** | **A** | **A** | **T** | **C** | **G** | **A** | **G** | **C** | **C** | **T** | **C** | **A** | **T** | **C** | **A** | **G** | **C** | **G** | **G** | **G** | **T** | **G** | **G** | **C** | **G** | **A** | **T** | **T** | **T** | **C** | **A** | **G** | **G** | **T** | **C** | **C** | **T** | **A** | **A** | **C** | **G** | **G** | **T** | **G** | **C** | **A** | **G** | **G** | **A** | **A** | **A** | **G** | **T** | **C** | **A** | **T** | **C** | **C** | **A** | **T** | **T** | **A** | **T** | **C** | **C** | **A** | **T** | **T** | **A** | **T** | **C** | **T** | **T** | **C** | **T** | **G** | **G** | **G** | **G** | **G** | **C** | **A** | **T** | **T** | **C** | **A** | **A** | **C** | **G** | **G** | **C** | **A** | **A** | **G** | **G** | **T** | **T** | **A** | **T** | **A** | **G** | **G** | **A** | **G** | **A** | **A** | **A** | **A** | **A** | **T** | **C** | **C** | **C** | **A** | **G** | **C** | **C** | **A** | **A** | **A** | **A** | **C** | **A** | **T** | **C** | **T** | **G** | **A** | **G** | **C** | **A** | **T** | **T** | **A** | **G** | **T** | **T** | **A** | **T** | **G** | **T** | **A** | **C** | **G** | **C** | **C** | **A** | **A** | **A** | **A** | **T** | **T** | **A** | **T** | **G** | **A** | **A** | **G** | **A** | **C** | **A** | **A** | **T** | **C** | **G** | **A** | **G** | **G** | **A** | **A** | **C** | **G** | **T** | **T** | **G** | **G** | **C** | **G** | **G** | **A** | **A** | **T** | **T** | **T** | **G** | **C** | **A** | **G** | **A** | **G** | **A** | **A** | **A** | **A** | **A** | **C** | **C** | **A** | **A** | **G** | **T** | **A** | **G** | **A** | **C** | **T** | **A** | **C** | **C** | **A** | **A** | **G** | **C** | **A** | **T** | **T** | **T** | **T** | **T** | **G** | **A** | **A** | **C** | **A** | **A** | **C** | **C** | **T** | **C** | **C** | **G** | **A** | **A** | **A** | **G** | **C** | **T** | **T** | **G** | **G** | **G** | **A** | **T** | **G** | **G** | **A** | **A** | **A** | **G** | **A** | **G** | **A** | **T** | **G** | **T** | **T** | **T** | **T** | **T** | **C** | **A** | **T** | **A** | **A** | **C** | **A** | **A** | **G** | **A** | **T** | **T** | **G** | **A** | **G** | **C** | **A** | **G** | **A** | **T** | **G** | **A** | **G** | **T** | **A** | **T** | **G** | **G** | **G** | **C** | **C** | **A** | **A** | **C** | **G** | **G** | **A** | **A** | **A** | **A** | **A** | **A** | **G** | **T** | **G** | **G** | **A** | **A** | **T** | **T** | **G** | **G** | **C** | **T** | **A** | **A** | **A** | **T** | **C** | **T** | **T** | **T** | **A** | **T** | **C** | **A** | **C** | **A** | **G** | **C** | **C** | **A** | **G** | **C** | **T** | **G** | **A** | **A** | **C** | **T** | **A** | **T** | **A** | **T** | **A** | **T** | **A** | **T** | **G** | **G** | **G** | **A** | **T** | **G** | **A** | **A** | **C** | **C** | **A** | **T** | **T** | **G** | **A** | **A** | **T** | **T** | **A** | **T** | **T** | **T** | **G** | **G** | **A** | **T** | **G** | **T** | **C** | **T** | **T** | **C** | **A** | **A** | **T** | **C** | **A** | **A** | **G** | **A** | **A** | **C** | **A** | **A** | **T** | **T** | **A** | **G** | **A** | **A** | **C** | **A** | **A** | **C** | **T** | **G** | **A** | **T** | **C** | **T** | **T** | **G** | **A** | **A** | **C** | **G** | **T** | **G** | **A** | **A** | **A** | **C** | **C** | **T** | **G** | **C** | **C** | **A** | **T** | **G** | **T** | **T** | **A** | **C** | **T** | **G** | **G** | **T** | **G** | **G** | **A** | **A** | **C** | **A** | **T** | **G** | **A** | **T** | **C** | **A** | **A** | **A** | **C** | **C** | **T** | **T** | **C** | **C** | **T** | **G** | **G** | **A** | **T** | **A** | **A** | **A** | **G** | **T** | **A** | **T** | **C** | **T** | **A** | **C** | **T** | **G** | **A** | **G** | **A** | **T** | **T** | **A** | **T** | **T** | **T** | **C** | **T** | **C** | **T** | **T** | **G** | **A** | **G** | **A** | **A** | **A** | **A** | **T** | **C** | **-** | **-** | **-** |
| **N52702** | **A** | **T** | **G** | **T** | **C** | **T** | **A** | **A** | **A** | **A** | **T** | **C** | **G** | **A** | **A** | **A** | **T** | **A** | **A** | **A** | **A** | **A** | **A** | **T** | **C** | **T** | **G** | **A** | **C** | **A** | **T** | **T** | **C** | **G** | **G** | **C** | **T** | **A** | **C** | **G** | **A** | **C** | **A** | **G** | **C** | **C** | **A** | **A** | **G** | **G** | **C** | **A** | **C** | **A** | **T** | **T** | **A** | **T** | **T** | **A** | **T** | **T** | **T** | **G** | **A** | **A** | **C** | **A** | **A** | **G** | **C** | **A** | **A** | **A** | **T** | **C** | **T** | **A** | **A** | **A** | **T** | **T** | **T** | **T** | **G** | **A** | **C** | **A** | **C** | **A** | **C** | **A** | **A** | **T** | **G** | **G** | **A** | **A** | **A** | **C** | **T** | **A** | **G** | **G** | **A** | **C** | **T** | **T** | **A** | **T** | **C** | **G** | **G** | **A** | **C** | **G** | **A** | **A** | **A** | **C** | **G** | **G** | **T** | **C** | **G** | **A** | **G** | **G** | **A** | **A** | **A** | **G** | **A** | **C** | **A** | **A** | **C** | **T** | **T** | **T** | **A** | **C** | **T** | **G** | **A** | **A** | **T** | **A** | **T** | **T** | **C** | **T** | **A** | **C** | **A** | **A** | **A** | **A** | **C** | **A** | **A** | **A** | **C** | **T** | **A** | **C** | **C** | **T** | **T** | **A** | **T** | **C** | **A** | **A** | **G** | **G** | **G** | **C** | **A** | **A** | **G** | **T** | **A** | **A** | **T** | **C** | **C** | **A** | **T** | **C** | **A** | **G** | **C** | **A** | **A** | **G** | **A** | **A** | **T** | **T** | **T** | **G** | **T** | **C** | **T** | **A** | **T** | **T** | **T** | **C** | **C** | **C** | **G** | **C** | **A** | **G** | **C** | **A** | **A** | **A** | **C** | **A** | **A** | **A** | **A** | **G** | **A** | **T** | **A** | **A** | **A** | **G** | **A** | **G** | **C** | **G** | **T** | **T** | **T** | **A** | **A** | **C** | **C** | **T** | **A** | **T** | **T** | **A** | **T** | **G** | **T** | **G** | **T** | **T** | **A** | **A** | **A** | **T** | **G** | **A** | **G** | **A** | **T** | **T** | **A** | **C** | **G** | **G** | **A** | **T** | **T** | **T** | **T** | **G** | **A** | **G** | **A** | **T** | **A** | **T** | **G** | **G** | **G** | **A** | **A** | **A** | **T** | **T** | **G** | **A** | **A** | **A** | **G** | **A** | **G** | **A** | **G** | **C** | **T** | **C** | **C** | **A** | **A** | **C** | **T** | **G** | **A** | **T** | **G** | **C** | **A** | **A** | **A** | **C** | **A** | **G** | **A** | **T** | **C** | **C** | **T** | **G** | **A** | **A** | **A** | **T** | **C** | **T** | **T** | **A** | **T** | **G** | **G** | **A** | **G** | **A** | **G** | **A** | **A** | **T** | **T** | **C** | **A** | **G** | **C** | **A** | **C** | **A** | **C** | **T** | **A** | **T** | **C** | **A** | **G** | **G** | **A** | **G** | **G** | **A** | **G** | **A** | **G** | **A** | **A** | **G** | **A** | **C** | **A** | **A** | **A** | **A** | **G** | **T** | **C** | **C** | **T** | **A** | **C** | **T** | **G** | **G** | **C** | **A** | **C** | **T** | **T** | **T** | **T** | **A** | **T** | **T** | **T** | **G** | **T** | **G** | **G** | **A** | **T** | **G** | **A** | **C** | **A** | **C** | **T** | **C** | **A** | **T** | **T** | **T** | **C** | **C** | **C** | **G** | **T** | **T** | **A** | **A** | **T** | **C** | **G** | **A** | **T** | **G** | **A** | **A** | **C** | **C** | **A** | **A** | **C** | **G** | **A** | **A** | **T** | **C** | **A** | **T** | **T** | **T** | **G** | **G** | **A** | **-** | **-** | **-** | **T** | **A** | **T** | **C** | **T** | **C** | **T** | **G** | **G** | **C** | **A** | **G** | **A** | **A** | **A** | **A** | **C** | **A** | **A** | **G** | **T** | **A** | **G** | **C** | **G** | **G** | **C** | **T** | **T** | **A** | **C** | **T** | **T** | **G** | **A** | **A** | **A** | **A** | **A** | **G** | **A** | **A** | **A** | **A** | **A** | **A** | **C** | **A** | **A** | **G** | **G** | **C** | **T** | **T** | **C** | **A** | **T** | **C** | **G** | **T** | **G** | **G** | **T** | **C** | **A** | **G** | **C** | **C** | **A** | **T** | **G** | **A** | **C** | **C** | **G** | **G** | **G** | **G** | **A** | **T** | **T** | **T** | **A** | **T** | **C** | **G** | **A** | **T** | **G** | **A** | **A** | **G** | **T** | **A** | **G** | **T** | **G** | **G** | **A** | **C** | **C** | **A** | **T** | **G** | **T** | **T** | **T** | **T** | **A** | **G** | **C** | **A** | **A** | **T** | **C** | **G** | **A** | **A** | **A** | **A** | **A** | **A** | **G** | **T** | **C** | **A** | **A** | **C** | **T** | **G** | **G** | **A** | **A** | **C** | **T** | **T** | **T** | **A** | **T** | **C** | **A** | **A** | **G** | **G** | **G** | **A** | **A** | **T** | **T** | **T** | **C** | **T** | **C** | **T** | **A** | **T** | **C** | **T** | **A** | **T** | **G** | **A** | **A** | **G** | **A** | **A** | **C** | **A** | **G** | **A** | **A** | **A** | **A** | **A** | **A** | **C** | **T** | **T** | **C** | **G** | **T** | **G** | **A** | **T** | **G** | **A** | **A** | **T** | **T** | **T** | **G** | **A** | **A** | **A** | **T** | **A** | **G** | **C** | **T** | **C** | **A** | **A** | **A** | **A** | **T** | **G** | **A** | **A** | **A** | **A** | **A** | **T** | **T** | **G** | **A** | **A** | **A** | **A** | **A** | **A** | **G** | **A** | **A** | **G** | **T** | **C** | **A** | **G** | **T** | **A** | **G** | **G** | **C** | **T** | **A** | **A** | **A** | **G** | **A** | **A** | **A** | **A** | **C** | **A** | **G** | **C** | **A** | **G** | **C** | **T** | **G** | **A** | **A** | **A** | **A** | **A** | **G** | **C** | **C** | **G** | **A** | **A** | **T** | **G** | **G** | **T** | **C** | **T** | **C** | **G** | **T** | **T** | **C** | **C** | **C** | **G** | **A** | **G** | **A** | **A** | **G** | **G** | **A** | **G** | **A** | **T** | **A** | **A** | **A** | **A** | **C** | **A** | **A** | **A** | **G** | **A** | **A** | **A** | **C** | **A** | **A** | **G** | **T** | **C** | **G** | **G** | **A** | **T** | **T** | **C** | **A** | **T** | **C** | **G** | **A** | **T** | **A** | **C** | **T** | **G** | **A** | **A** | **T** | **C** | **T** | **A** | **G** | **A** | **C** | **G** | **A** | **G** | **T** | **G** | **A** | **A** | **T** | **A** | **A** | **A** | **G** | **G** | **A** | **G** | **C** | **A** | **G** | **T** | **G** | **G** | **G** | **T** | **G** | **C** | **T** | **G** | **A** | **T** | **G** | **C** | **T** | **G** | **C** | **A** | **C** | **G** | **G** | **A** | **C** | **G** | **A** | **T** | **G** | **A** | **A** | **A** | **C** | **G** | **A** | **T** | **C** | **C** | **A** | **A** | **A** | **G** | **C** | **A** | **A** | **T** | **C** | **G** | **T** | **G** | **A** | **A** | **T** | **C** | **G** | **G** | **A** | **T** | **G** | **G** | **A** | **G** | **A** | **C** | **C** | **C** | **A** | **G** | **A** | **T** | **C** | **A** | **G** | **C** | **G** | **A** | **G** | **A** | **A** | **A** | **G** | **A** | **A** | **A** | **A** | **A** | **C** | **T** | **A** | **T** | **T** | **A** | **A** | **A** | **A** | **G** | **A** | **T** | **A** | **T** | **C** | **G** | **A** | **A** | **T** | **A** | **T** | **A** | **T** | **C** | **G** | **A** | **T** | **T** | **C** | **T** | **T** | **T** | **G** | **A** | **C** | **G** | **A** | **T** | **G** | **A** | **A** | **T** | **A** | **G** | **C** | **C** | **A** | **A** | **G** | **C** | **G** | **T** | **C** | **T** | **C** | **A** | **C** | **C** | **A** | **T** | **A** | **A** | **G** | **C** | **G** | **A** | **C** | **T** | **T** | **T** | **T** | **A** | **A** | **G** | **C** | **G** | **T** | **A** | **G** | **A** | **A** | **G** | **A** | **T** | **C** | **T** | **T** | **C** | **A** | **A** | **T** | **T** | **A** | **G** | **G** | **G** | **T** | **A** | **T** | **G** | **A** | **A** | **A** | **A** | **T** | **C** | **T** | **G** | **T** | **T** | **A** | **T** | **T** | **C** | **G** | **A** | **G** | **C** | **C** | **A** | **A** | **T** | **T** | **C** | **A** | **T** | **T** | **T** | **T** | **A** | **C** | **A** | **A** | **T** | **C** | **G** | **A** | **G** | **C** | **C** | **T** | **C** | **A** | **T** | **C** | **A** | **G** | **C** | **G** | **G** | **G** | **T** | **G** | **G** | **C** | **G** | **A** | **T** | **T** | **T** | **C** | **A** | **G** | **G** | **T** | **C** | **C** | **T** | **A** | **A** | **C** | **G** | **G** | **T** | **G** | **C** | **A** | **G** | **G** | **A** | **A** | **A** | **G** | **T** | **C** | **A** | **T** | **C** | **C** | **A** | **T** | **T** | **A** | **T** | **C** | **C** | **A** | **T** | **T** | **A** | **T** | **C** | **T** | **T** | **C** | **T** | **G** | **G** | **G** | **G** | **G** | **C** | **A** | **T** | **T** | **C** | **A** | **A** | **C** | **G** | **G** | **C** | **A** | **A** | **G** | **G** | **T** | **T** | **A** | **T** | **A** | **G** | **G** | **A** | **G** | **A** | **A** | **A** | **A** | **A** | **T** | **C** | **C** | **C** | **A** | **G** | **C** | **C** | **A** | **A** | **A** | **A** | **C** | **A** | **T** | **C** | **T** | **G** | **A** | **G** | **C** | **A** | **T** | **T** | **A** | **G** | **T** | **T** | **A** | **T** | **G** | **T** | **A** | **C** | **G** | **C** | **C** | **A** | **A** | **A** | **A** | **T** | **T** | **A** | **T** | **G** | **A** | **A** | **G** | **A** | **C** | **A** | **A** | **T** | **C** | **G** | **A** | **G** | **G** | **A** | **A** | **C** | **G** | **T** | **T** | **G** | **G** | **C** | **G** | **G** | **A** | **A** | **T** | **T** | **T** | **G** | **C** | **A** | **G** | **A** | **G** | **A** | **A** | **A** | **A** | **A** | **C** | **C** | **A** | **A** | **G** | **T** | **A** | **G** | **A** | **C** | **T** | **A** | **C** | **C** | **A** | **A** | **G** | **C** | **A** | **T** | **T** | **T** | **T** | **T** | **G** | **A** | **A** | **C** | **A** | **A** | **C** | **C** | **T** | **C** | **C** | **G** | **A** | **A** | **A** | **G** | **C** | **T** | **T** | **G** | **G** | **G** | **A** | **T** | **G** | **G** | **A** | **A** | **A** | **G** | **A** | **G** | **A** | **T** | **G** | **T** | **T** | **T** | **T** | **T** | **C** | **A** | **T** | **A** | **A** | **C** | **A** | **A** | **G** | **A** | **T** | **C** | **G** | **A** | **G** | **C** | **A** | **G** | **A** | **T** | **G** | **A** | **G** | **T** | **A** | **T** | **G** | **G** | **G** | **C** | **C** | **A** | **A** | **C** | **G** | **G** | **A** | **A** | **A** | **A** | **A** | **A** | **G** | **T** | **G** | **G** | **A** | **A** | **T** | **T** | **G** | **G** | **C** | **T** | **A** | **A** | **A** | **T** | **C** | **T** | **T** | **T** | **A** | **T** | **C** | **A** | **C** | **A** | **G** | **C** | **C** | **A** | **G** | **C** | **T** | **G** | **A** | **A** | **C** | **T** | **A** | **T** | **A** | **T** | **A** | **T** | **A** | **T** | **G** | **G** | **G** | **A** | **T** | **G** | **A** | **A** | **C** | **C** | **A** | **T** | **T** | **G** | **A** | **A** | **T** | **T** | **A** | **T** | **T** | **T** | **G** | **G** | **A** | **T** | **G** | **T** | **C** | **T** | **T** | **C** | **A** | **A** | **T** | **C** | **A** | **A** | **G** | **A** | **A** | **C** | **A** | **A** | **T** | **T** | **A** | **G** | **A** | **A** | **C** | **A** | **A** | **C** | **T** | **G** | **A** | **T** | **C** | **T** | **T** | **G** | **A** | **A** | **C** | **G** | **T** | **G** | **A** | **A** | **A** | **C** | **C** | **T** | **G** | **C** | **C** | **A** | **T** | **G** | **T** | **T** | **A** | **C** | **T** | **A** | **G** | **T** | **G** | **G** | **A** | **A** | **C** | **A** | **T** | **G** | **A** | **T** | **C** | **A** | **A** | **A** | **C** | **C** | **T** | **T** | **C** | **C** | **T** | **G** | **G** | **A** | **T** | **A** | **A** | **A** | **G** | **T** | **A** | **T** | **C** | **T** | **A** | **C** | **T** | **G** | **A** | **G** | **A** | **T** | **T** | **A** | **T** | **T** | **T** | **C** | **T** | **C** | **T** | **T** | **G** | **A** | **G** | **A** | **A** | **A** | **A** | **T** | **C** | **-** | **-** | **-** |
| **N60363F** | **A** | **T** | **G** | **T** | **C** | **T** | **A** | **A** | **A** | **A** | **T** | **C** | **G** | **A** | **A** | **A** | **T** | **A** | **A** | **A** | **A** | **A** | **A** | **T** | **C** | **T** | **G** | **A** | **C** | **A** | **T** | **T** | **C** | **G** | **G** | **C** | **T** | **A** | **C** | **G** | **A** | **C** | **A** | **G** | **C** | **C** | **A** | **A** | **G** | **G** | **C** | **A** | **C** | **A** | **T** | **T** | **A** | **T** | **T** | **A** | **T** | **T** | **T** | **G** | **A** | **A** | **C** | **A** | **A** | **G** | **C** | **A** | **A** | **A** | **T** | **C** | **T** | **A** | **A** | **A** | **T** | **T** | **T** | **T** | **G** | **A** | **C** | **A** | **C** | **A** | **C** | **A** | **A** | **T** | **G** | **G** | **A** | **A** | **A** | **C** | **T** | **A** | **G** | **G** | **A** | **C** | **T** | **T** | **A** | **T** | **C** | **G** | **G** | **A** | **C** | **G** | **A** | **A** | **A** | **C** | **G** | **G** | **T** | **C** | **G** | **A** | **G** | **G** | **A** | **A** | **A** | **G** | **A** | **C** | **A** | **A** | **C** | **T** | **T** | **T** | **A** | **C** | **T** | **G** | **A** | **A** | **T** | **A** | **T** | **T** | **C** | **T** | **A** | **C** | **A** | **A** | **A** | **A** | **C** | **A** | **A** | **A** | **C** | **T** | **A** | **C** | **C** | **T** | **T** | **A** | **T** | **C** | **A** | **A** | **G** | **G** | **G** | **C** | **A** | **A** | **G** | **T** | **A** | **A** | **T** | **C** | **C** | **A** | **T** | **C** | **A** | **G** | **C** | **A** | **A** | **G** | **A** | **A** | **T** | **T** | **T** | **G** | **C** | **C** | **T** | **A** | **T** | **T** | **T** | **C** | **C** | **C** | **G** | **C** | **A** | **A** | **C** | **A** | **G** | **A** | **C** | **A** | **A** | **A** | **A** | **G** | **A** | **T** | **A** | **A** | **A** | **G** | **A** | **A** | **C** | **G** | **T** | **T** | **T** | **A** | **A** | **C** | **C** | **T** | **A** | **T** | **T** | **A** | **C** | **G** | **T** | **G** | **T** | **T** | **A** | **A** | **A** | **T** | **G** | **A** | **T** | **A** | **T** | **T** | **A** | **C** | **G** | **G** | **A** | **T** | **T** | **T** | **T** | **G** | **A** | **G** | **A** | **T** | **A** | **T** | **G** | **G** | **G** | **A** | **A** | **A** | **T** | **C** | **G** | **A** | **A** | **A** | **G** | **A** | **G** | **A** | **G** | **C** | **T** | **C** | **C** | **A** | **A** | **T** | **T** | **G** | **A** | **T** | **G** | **C** | **A** | **A** | **A** | **C** | **A** | **G** | **A** | **T** | **C** | **C** | **T** | **G** | **A** | **A** | **A** | **T** | **C** | **T** | **T** | **A** | **T** | **G** | **G** | **A** | **G** | **A** | **G** | **A** | **A** | **T** | **T** | **C** | **A** | **G** | **C** | **A** | **C** | **A** | **C** | **T** | **A** | **T** | **C** | **G** | **G** | **G** | **G** | **G** | **G** | **A** | **G** | **A** | **G** | **A** | **A** | **G** | **A** | **C** | **A** | **A** | **A** | **A** | **G** | **T** | **C** | **C** | **T** | **A** | **C** | **T** | **G** | **G** | **C** | **A** | **C** | **T** | **T** | **T** | **T** | **A** | **T** | **T** | **T** | **G** | **T** | **G** | **G** | **A** | **T** | **G** | **A** | **C** | **A** | **C** | **T** | **C** | **A** | **T** | **T** | **T** | **C** | **C** | **C** | **G** | **T** | **T** | **A** | **A** | **T** | **C** | **G** | **A** | **T** | **G** | **A** | **A** | **C** | **C** | **A** | **A** | **C** | **G** | **A** | **A** | **T** | **C** | **A** | **T** | **T** | **T** | **G** | **G** | **A** | **-** | **-** | **-** | **T** | **A** | **T** | **C** | **T** | **C** | **T** | **G** | **G** | **T** | **A** | **G** | **A** | **A** | **A** | **A** | **C** | **A** | **A** | **G** | **T** | **A** | **G** | **C** | **G** | **G** | **C** | **T** | **T** | **A** | **T** | **T** | **T** | **G** | **A** | **A** | **A** | **A** | **A** | **G** | **A** | **A** | **A** | **A** | **A** | **A** | **C** | **A** | **A** | **G** | **G** | **C** | **T** | **T** | **C** | **A** | **T** | **C** | **G** | **T** | **G** | **G** | **T** | **C** | **A** | **G** | **C** | **C** | **A** | **T** | **G** | **A** | **C** | **C** | **G** | **G** | **G** | **G** | **A** | **T** | **T** | **T** | **A** | **T** | **C** | **G** | **A** | **T** | **G** | **A** | **A** | **G** | **T** | **A** | **G** | **T** | **G** | **G** | **A** | **C** | **C** | **A** | **T** | **G** | **T** | **T** | **T** | **T** | **A** | **G** | **C** | **A** | **A** | **T** | **C** | **G** | **A** | **A** | **A** | **A** | **A** | **A** | **G** | **T** | **C** | **A** | **A** | **C** | **T** | **G** | **G** | **A** | **A** | **C** | **T** | **T** | **T** | **A** | **T** | **C** | **A** | **A** | **G** | **G** | **G** | **A** | **A** | **T** | **T** | **T** | **C** | **T** | **C** | **T** | **A** | **T** | **C** | **T** | **A** | **T** | **G** | **A** | **A** | **G** | **A** | **A** | **C** | **A** | **G** | **A** | **A** | **A** | **A** | **A** | **A** | **C** | **T** | **T** | **C** | **G** | **T** | **G** | **A** | **T** | **G** | **A** | **A** | **T** | **T** | **T** | **G** | **A** | **A** | **A** | **T** | **G** | **G** | **C** | **T** | **C** | **A** | **A** | **A** | **A** | **T** | **G** | **A** | **A** | **A** | **A** | **A** | **T** | **T** | **G** | **A** | **A** | **A** | **A** | **A** | **A** | **G** | **A** | **A** | **G** | **T** | **C** | **A** | **G** | **T** | **A** | **G** | **G** | **C** | **T** | **A** | **A** | **A** | **G** | **A** | **A** | **A** | **A** | **C** | **A** | **G** | **C** | **A** | **G** | **C** | **T** | **G** | **A** | **A** | **A** | **A** | **A** | **G** | **C** | **C** | **G** | **A** | **A** | **T** | **G** | **G** | **T** | **C** | **T** | **C** | **G** | **T** | **T** | **C** | **C** | **C** | **G** | **A** | **G** | **A** | **A** | **G** | **G** | **A** | **G** | **A** | **T** | **A** | **A** | **A** | **A** | **C** | **A** | **A** | **A** | **G** | **A** | **A** | **A** | **C** | **A** | **A** | **G** | **T** | **C** | **G** | **G** | **A** | **T** | **T** | **C** | **A** | **T** | **C** | **G** | **A** | **T** | **A** | **C** | **T** | **G** | **A** | **A** | **T** | **C** | **T** | **A** | **G** | **A** | **C** | **G** | **A** | **G** | **T** | **G** | **A** | **A** | **T** | **A** | **A** | **A** | **G** | **G** | **A** | **G** | **C** | **A** | **G** | **T** | **G** | **G** | **G** | **T** | **G** | **C** | **T** | **G** | **A** | **T** | **G** | **C** | **T** | **G** | **C** | **A** | **C** | **G** | **G** | **A** | **C** | **G** | **A** | **T** | **G** | **A** | **A** | **A** | **C** | **G** | **A** | **T** | **C** | **C** | **A** | **A** | **A** | **G** | **C** | **A** | **A** | **T** | **C** | **G** | **T** | **G** | **A** | **A** | **T** | **C** | **G** | **G** | **A** | **T** | **G** | **G** | **A** | **G** | **A** | **C** | **C** | **C** | **A** | **G** | **A** | **T** | **C** | **A** | **G** | **C** | **G** | **A** | **G** | **A** | **A** | **A** | **G** | **A** | **A** | **A** | **A** | **A** | **C** | **T** | **A** | **T** | **T** | **A** | **A** | **A** | **A** | **G** | **A** | **T** | **A** | **T** | **C** | **G** | **A** | **A** | **T** | **A** | **T** | **A** | **T** | **C** | **G** | **A** | **T** | **T** | **C** | **T** | **T** | **T** | **G** | **A** | **C** | **G** | **A** | **T** | **G** | **A** | **A** | **T** | **A** | **G** | **C** | **C** | **A** | **A** | **G** | **C** | **G** | **T** | **C** | **T** | **C** | **A** | **C** | **C** | **A** | **T** | **A** | **A** | **G** | **C** | **G** | **A** | **C** | **T** | **T** | **T** | **T** | **A** | **A** | **G** | **C** | **G** | **T** | **A** | **G** | **A** | **A** | **G** | **A** | **T** | **C** | **T** | **T** | **C** | **A** | **A** | **T** | **T** | **A** | **G** | **G** | **G** | **T** | **A** | **T** | **G** | **A** | **A** | **A** | **A** | **T** | **C** | **T** | **G** | **T** | **T** | **A** | **T** | **T** | **C** | **G** | **A** | **G** | **C** | **C** | **A** | **A** | **T** | **T** | **C** | **A** | **T** | **T** | **T** | **T** | **A** | **C** | **A** | **A** | **T** | **C** | **G** | **A** | **G** | **C** | **C** | **T** | **C** | **A** | **T** | **C** | **A** | **G** | **C** | **G** | **G** | **G** | **T** | **G** | **G** | **C** | **G** | **A** | **T** | **T** | **T** | **C** | **A** | **G** | **G** | **T** | **C** | **C** | **T** | **A** | **A** | **C** | **G** | **G** | **T** | **G** | **C** | **A** | **G** | **G** | **A** | **A** | **A** | **G** | **T** | **C** | **A** | **T** | **C** | **C** | **A** | **T** | **T** | **A** | **T** | **C** | **C** | **A** | **T** | **T** | **A** | **T** | **C** | **T** | **T** | **C** | **T** | **G** | **G** | **G** | **G** | **G** | **C** | **A** | **T** | **T** | **C** | **A** | **A** | **C** | **G** | **G** | **C** | **A** | **A** | **G** | **G** | **T** | **T** | **A** | **T** | **A** | **G** | **G** | **A** | **G** | **A** | **A** | **A** | **A** | **A** | **T** | **A** | **C** | **C** | **A** | **G** | **C** | **C** | **A** | **A** | **A** | **A** | **C** | **A** | **T** | **C** | **T** | **G** | **A** | **G** | **C** | **A** | **T** | **T** | **A** | **G** | **T** | **T** | **A** | **T** | **G** | **C** | **A** | **A** | **G** | **C** | **C** | **A** | **A** | **A** | **A** | **T** | **T** | **A** | **T** | **G** | **A** | **A** | **G** | **A** | **C** | **A** | **A** | **T** | **C** | **G** | **A** | **G** | **G** | **A** | **A** | **C** | **G** | **T** | **T** | **G** | **G** | **C** | **G** | **G** | **A** | **A** | **T** | **T** | **T** | **G** | **C** | **A** | **G** | **A** | **G** | **A** | **A** | **A** | **A** | **A** | **C** | **C** | **A** | **A** | **G** | **T** | **A** | **G** | **A** | **C** | **T** | **A** | **C** | **C** | **A** | **A** | **G** | **C** | **A** | **T** | **T** | **T** | **T** | **T** | **G** | **A** | **A** | **C** | **A** | **A** | **C** | **C** | **T** | **C** | **C** | **G** | **A** | **A** | **A** | **G** | **C** | **T** | **T** | **G** | **G** | **G** | **A** | **T** | **G** | **G** | **A** | **A** | **A** | **G** | **A** | **G** | **A** | **T** | **G** | **T** | **T** | **T** | **T** | **T** | **C** | **A** | **T** | **A** | **A** | **C** | **A** | **A** | **G** | **A** | **T** | **C** | **G** | **A** | **G** | **C** | **A** | **G** | **A** | **T** | **G** | **A** | **G** | **T** | **A** | **T** | **G** | **G** | **G** | **C** | **C** | **A** | **A** | **C** | **G** | **G** | **A** | **A** | **A** | **A** | **A** | **A** | **G** | **T** | **G** | **G** | **A** | **A** | **T** | **T** | **G** | **G** | **C** | **T** | **A** | **A** | **A** | **T** | **C** | **T** | **T** | **T** | **A** | **T** | **C** | **A** | **C** | **A** | **G** | **C** | **C** | **A** | **G** | **C** | **T** | **G** | **A** | **A** | **C** | **T** | **A** | **T** | **A** | **T** | **A** | **C** | **A** | **T** | **G** | **G** | **G** | **A** | **T** | **G** | **A** | **A** | **C** | **C** | **A** | **T** | **T** | **G** | **A** | **A** | **T** | **T** | **A** | **T** | **T** | **T** | **G** | **G** | **A** | **T** | **G** | **T** | **C** | **T** | **T** | **C** | **A** | **A** | **T** | **C** | **A** | **A** | **G** | **A** | **A** | **C** | **A** | **A** | **T** | **T** | **A** | **G** | **A** | **A** | **C** | **A** | **A** | **C** | **T** | **G** | **A** | **T** | **C** | **T** | **T** | **G** | **A** | **A** | **C** | **G** | **T** | **G** | **A** | **A** | **A** | **C** | **C** | **T** | **G** | **C** | **C** | **A** | **T** | **G** | **T** | **T** | **A** | **C** | **T** | **A** | **G** | **T** | **G** | **G** | **A** | **A** | **C** | **A** | **T** | **G** | **A** | **T** | **C** | **A** | **A** | **A** | **C** | **C** | **T** | **T** | **C** | **C** | **T** | **G** | **G** | **A** | **T** | **A** | **A** | **A** | **G** | **T** | **A** | **T** | **C** | **T** | **A** | **C** | **T** | **G** | **A** | **G** | **A** | **T** | **T** | **A** | **T** | **T** | **T** | **C** | **T** | **C** | **T** | **T** | **G** | **A** | **G** | **A** | **G** | **A** | **A** | **T** | **C** | **-** | **-** | **-** |
| **N53702** | **A** | **T** | **G** | **T** | **C** | **T** | **A** | **A** | **A** | **A** | **T** | **C** | **G** | **A** | **A** | **A** | **T** | **A** | **A** | **A** | **A** | **A** | **A** | **T** | **C** | **T** | **G** | **A** | **C** | **A** | **T** | **T** | **C** | **G** | **G** | **C** | **T** | **A** | **C** | **G** | **A** | **C** | **A** | **G** | **C** | **C** | **A** | **A** | **G** | **G** | **C** | **A** | **C** | **A** | **T** | **T** | **A** | **T** | **T** | **A** | **T** | **T** | **T** | **G** | **A** | **A** | **C** | **A** | **A** | **G** | **C** | **A** | **A** | **A** | **T** | **C** | **T** | **A** | **A** | **A** | **T** | **T** | **T** | **T** | **G** | **A** | **C** | **A** | **C** | **A** | **C** | **A** | **A** | **T** | **G** | **G** | **A** | **A** | **A** | **C** | **T** | **A** | **G** | **G** | **A** | **C** | **T** | **T** | **A** | **T** | **C** | **G** | **G** | **A** | **C** | **G** | **A** | **A** | **A** | **C** | **G** | **G** | **T** | **C** | **G** | **A** | **G** | **G** | **A** | **A** | **A** | **G** | **A** | **C** | **A** | **A** | **C** | **T** | **T** | **T** | **A** | **C** | **T** | **G** | **A** | **A** | **T** | **A** | **T** | **T** | **C** | **T** | **A** | **C** | **A** | **A** | **A** | **A** | **C** | **A** | **A** | **A** | **C** | **T** | **A** | **C** | **C** | **T** | **T** | **A** | **T** | **C** | **A** | **A** | **G** | **G** | **G** | **C** | **A** | **A** | **G** | **T** | **A** | **A** | **T** | **C** | **C** | **A** | **T** | **C** | **A** | **G** | **C** | **A** | **A** | **G** | **A** | **A** | **T** | **T** | **T** | **G** | **T** | **C** | **T** | **A** | **T** | **T** | **T** | **C** | **C** | **C** | **G** | **C** | **A** | **G** | **C** | **A** | **A** | **A** | **C** | **A** | **A** | **A** | **A** | **G** | **A** | **T** | **A** | **A** | **A** | **G** | **A** | **G** | **C** | **G** | **T** | **T** | **T** | **A** | **A** | **C** | **C** | **T** | **A** | **T** | **T** | **A** | **T** | **G** | **T** | **G** | **T** | **T** | **A** | **A** | **A** | **T** | **G** | **A** | **G** | **A** | **T** | **T** | **A** | **C** | **G** | **G** | **A** | **T** | **T** | **T** | **T** | **G** | **A** | **G** | **A** | **T** | **A** | **T** | **G** | **G** | **G** | **A** | **A** | **A** | **T** | **T** | **G** | **A** | **A** | **A** | **G** | **A** | **G** | **A** | **G** | **C** | **T** | **C** | **C** | **A** | **A** | **C** | **T** | **G** | **A** | **T** | **G** | **C** | **A** | **A** | **A** | **C** | **A** | **G** | **A** | **T** | **C** | **C** | **T** | **G** | **A** | **A** | **A** | **T** | **C** | **T** | **T** | **A** | **T** | **G** | **G** | **A** | **G** | **A** | **G** | **A** | **A** | **T** | **T** | **C** | **A** | **G** | **C** | **A** | **C** | **A** | **C** | **T** | **A** | **T** | **C** | **A** | **G** | **G** | **A** | **G** | **G** | **A** | **G** | **A** | **G** | **A** | **A** | **G** | **A** | **C** | **A** | **A** | **A** | **A** | **G** | **T** | **C** | **C** | **T** | **A** | **C** | **T** | **G** | **G** | **C** | **A** | **C** | **T** | **T** | **T** | **T** | **A** | **T** | **T** | **T** | **G** | **T** | **G** | **G** | **A** | **T** | **G** | **A** | **C** | **A** | **C** | **T** | **C** | **A** | **T** | **T** | **T** | **C** | **C** | **C** | **G** | **T** | **T** | **A** | **A** | **T** | **C** | **G** | **A** | **T** | **G** | **A** | **A** | **C** | **C** | **A** | **A** | **C** | **G** | **A** | **A** | **T** | **C** | **A** | **T** | **T** | **T** | **G** | **G** | **A** | **-** | **-** | **-** | **T** | **A** | **T** | **C** | **T** | **C** | **T** | **G** | **G** | **C** | **A** | **G** | **A** | **A** | **A** | **A** | **C** | **A** | **A** | **G** | **T** | **A** | **G** | **C** | **G** | **G** | **C** | **T** | **T** | **A** | **C** | **T** | **T** | **G** | **A** | **A** | **A** | **A** | **A** | **G** | **A** | **A** | **A** | **A** | **A** | **A** | **C** | **A** | **A** | **G** | **G** | **C** | **T** | **T** | **C** | **A** | **T** | **C** | **G** | **T** | **G** | **G** | **T** | **C** | **A** | **G** | **C** | **C** | **A** | **T** | **G** | **A** | **C** | **C** | **G** | **G** | **G** | **G** | **A** | **T** | **T** | **T** | **A** | **T** | **C** | **G** | **A** | **T** | **G** | **A** | **A** | **G** | **T** | **A** | **G** | **T** | **G** | **G** | **A** | **C** | **C** | **A** | **T** | **G** | **T** | **T** | **T** | **T** | **A** | **G** | **C** | **A** | **A** | **T** | **C** | **G** | **A** | **A** | **A** | **A** | **A** | **A** | **G** | **T** | **C** | **A** | **A** | **C** | **T** | **G** | **G** | **A** | **A** | **C** | **T** | **T** | **T** | **A** | **T** | **C** | **A** | **A** | **G** | **G** | **G** | **A** | **A** | **T** | **T** | **T** | **C** | **T** | **C** | **T** | **A** | **T** | **C** | **T** | **A** | **T** | **G** | **A** | **A** | **G** | **A** | **A** | **C** | **A** | **G** | **A** | **A** | **A** | **A** | **A** | **A** | **C** | **T** | **T** | **C** | **G** | **T** | **G** | **A** | **T** | **G** | **A** | **A** | **T** | **T** | **T** | **G** | **A** | **A** | **A** | **T** | **A** | **G** | **C** | **T** | **C** | **A** | **A** | **A** | **A** | **T** | **G** | **A** | **A** | **A** | **A** | **A** | **T** | **T** | **G** | **A** | **A** | **A** | **A** | **A** | **A** | **G** | **A** | **A** | **G** | **T** | **C** | **A** | **G** | **T** | **A** | **G** | **G** | **C** | **T** | **A** | **A** | **A** | **G** | **A** | **A** | **A** | **A** | **C** | **A** | **G** | **C** | **A** | **G** | **C** | **T** | **G** | **A** | **A** | **A** | **A** | **A** | **G** | **C** | **C** | **G** | **A** | **A** | **T** | **G** | **G** | **T** | **C** | **T** | **C** | **G** | **T** | **T** | **C** | **C** | **C** | **G** | **A** | **G** | **A** | **A** | **G** | **G** | **A** | **G** | **A** | **T** | **A** | **A** | **A** | **A** | **C** | **A** | **A** | **A** | **G** | **A** | **A** | **A** | **C** | **A** | **A** | **G** | **T** | **C** | **G** | **G** | **A** | **T** | **T** | **C** | **A** | **T** | **C** | **G** | **A** | **T** | **A** | **C** | **T** | **G** | **A** | **A** | **T** | **C** | **T** | **A** | **G** | **A** | **C** | **G** | **A** | **G** | **T** | **G** | **A** | **A** | **T** | **A** | **A** | **A** | **G** | **G** | **A** | **G** | **C** | **A** | **G** | **T** | **G** | **G** | **G** | **T** | **G** | **C** | **T** | **G** | **A** | **T** | **G** | **C** | **T** | **G** | **C** | **A** | **C** | **G** | **G** | **A** | **C** | **G** | **A** | **T** | **G** | **A** | **A** | **A** | **C** | **G** | **A** | **T** | **C** | **C** | **A** | **A** | **A** | **G** | **C** | **A** | **A** | **T** | **C** | **G** | **T** | **G** | **A** | **A** | **T** | **C** | **G** | **G** | **A** | **T** | **G** | **G** | **A** | **G** | **A** | **C** | **C** | **C** | **A** | **G** | **A** | **T** | **C** | **A** | **G** | **C** | **G** | **A** | **G** | **A** | **A** | **A** | **G** | **A** | **A** | **A** | **A** | **A** | **C** | **T** | **A** | **T** | **T** | **A** | **A** | **A** | **A** | **G** | **A** | **T** | **A** | **T** | **C** | **G** | **A** | **A** | **T** | **A** | **T** | **A** | **T** | **C** | **G** | **A** | **T** | **T** | **C** | **T** | **T** | **T** | **G** | **A** | **C** | **G** | **A** | **T** | **G** | **A** | **A** | **T** | **A** | **G** | **C** | **C** | **A** | **A** | **G** | **C** | **G** | **T** | **C** | **T** | **C** | **A** | **C** | **C** | **A** | **T** | **A** | **A** | **G** | **C** | **G** | **A** | **C** | **T** | **T** | **T** | **T** | **A** | **A** | **G** | **C** | **G** | **T** | **A** | **G** | **A** | **A** | **G** | **A** | **T** | **C** | **T** | **T** | **C** | **A** | **A** | **T** | **T** | **A** | **G** | **G** | **G** | **T** | **A** | **T** | **G** | **A** | **A** | **A** | **A** | **T** | **C** | **T** | **G** | **T** | **T** | **A** | **T** | **T** | **C** | **G** | **A** | **G** | **C** | **C** | **A** | **A** | **T** | **T** | **C** | **A** | **T** | **T** | **T** | **T** | **A** | **C** | **A** | **A** | **T** | **C** | **G** | **A** | **G** | **C** | **C** | **T** | **C** | **A** | **T** | **C** | **A** | **G** | **C** | **G** | **G** | **G** | **T** | **G** | **G** | **C** | **G** | **A** | **T** | **T** | **T** | **C** | **A** | **G** | **G** | **T** | **C** | **C** | **T** | **A** | **A** | **C** | **G** | **G** | **T** | **G** | **C** | **A** | **G** | **G** | **A** | **A** | **A** | **G** | **T** | **C** | **A** | **T** | **C** | **C** | **A** | **T** | **T** | **A** | **T** | **C** | **C** | **A** | **T** | **T** | **A** | **T** | **C** | **T** | **T** | **C** | **T** | **G** | **G** | **G** | **G** | **G** | **C** | **A** | **T** | **T** | **C** | **A** | **A** | **C** | **G** | **G** | **C** | **A** | **A** | **G** | **G** | **T** | **T** | **A** | **T** | **A** | **G** | **G** | **A** | **G** | **A** | **A** | **A** | **A** | **A** | **T** | **C** | **C** | **C** | **A** | **G** | **C** | **C** | **A** | **A** | **A** | **A** | **C** | **A** | **T** | **C** | **T** | **G** | **A** | **G** | **C** | **A** | **T** | **T** | **A** | **G** | **T** | **T** | **A** | **T** | **G** | **T** | **A** | **C** | **G** | **C** | **C** | **A** | **A** | **A** | **A** | **T** | **T** | **A** | **T** | **G** | **A** | **A** | **G** | **A** | **C** | **A** | **A** | **T** | **C** | **G** | **A** | **G** | **G** | **A** | **A** | **C** | **G** | **T** | **T** | **G** | **G** | **C** | **G** | **G** | **A** | **A** | **T** | **T** | **T** | **G** | **C** | **A** | **G** | **A** | **G** | **A** | **A** | **A** | **A** | **A** | **C** | **C** | **A** | **A** | **G** | **T** | **A** | **G** | **A** | **C** | **T** | **A** | **C** | **C** | **A** | **A** | **G** | **C** | **A** | **T** | **T** | **T** | **T** | **T** | **G** | **A** | **A** | **C** | **A** | **A** | **C** | **C** | **T** | **C** | **C** | **G** | **A** | **A** | **A** | **G** | **C** | **T** | **T** | **G** | **G** | **G** | **A** | **T** | **G** | **G** | **A** | **A** | **A** | **G** | **A** | **G** | **A** | **T** | **G** | **T** | **T** | **T** | **T** | **T** | **C** | **A** | **T** | **A** | **A** | **C** | **A** | **A** | **G** | **A** | **T** | **C** | **G** | **A** | **G** | **C** | **A** | **G** | **A** | **T** | **G** | **A** | **G** | **T** | **A** | **T** | **G** | **G** | **G** | **C** | **C** | **A** | **A** | **C** | **G** | **G** | **A** | **A** | **A** | **A** | **A** | **A** | **G** | **T** | **G** | **G** | **A** | **A** | **T** | **T** | **G** | **G** | **C** | **T** | **A** | **A** | **A** | **T** | **C** | **T** | **T** | **T** | **A** | **T** | **C** | **A** | **C** | **A** | **G** | **C** | **C** | **A** | **G** | **C** | **T** | **G** | **A** | **A** | **C** | **T** | **A** | **T** | **A** | **T** | **A** | **T** | **A** | **T** | **G** | **G** | **G** | **A** | **T** | **G** | **A** | **A** | **C** | **C** | **A** | **T** | **T** | **G** | **A** | **A** | **T** | **T** | **A** | **T** | **T** | **T** | **G** | **G** | **A** | **T** | **G** | **T** | **C** | **T** | **T** | **C** | **A** | **A** | **T** | **C** | **A** | **A** | **G** | **A** | **A** | **C** | **A** | **A** | **T** | **T** | **A** | **G** | **A** | **A** | **C** | **A** | **A** | **C** | **T** | **G** | **A** | **T** | **C** | **T** | **T** | **G** | **A** | **A** | **C** | **G** | **T** | **G** | **A** | **A** | **A** | **C** | **C** | **T** | **G** | **C** | **C** | **A** | **T** | **G** | **T** | **T** | **A** | **C** | **T** | **A** | **G** | **T** | **G** | **G** | **A** | **A** | **C** | **A** | **T** | **G** | **A** | **T** | **C** | **A** | **A** | **A** | **C** | **C** | **T** | **T** | **C** | **C** | **T** | **G** | **G** | **A** | **T** | **A** | **A** | **A** | **G** | **T** | **A** | **T** | **C** | **T** | **A** | **C** | **T** | **G** | **A** | **G** | **A** | **T** | **T** | **A** | **T** | **T** | **T** | **C** | **T** | **C** | **T** | **T** | **G** | **A** | **G** | **A** | **A** | **A** | **A** | **T** | **C** | **-** | **-** | **-** |
| **N60519F** | **A** | **T** | **G** | **T** | **C** | **T** | **A** | **A** | **A** | **A** | **T** | **C** | **G** | **A** | **A** | **A** | **T** | **A** | **A** | **A** | **A** | **A** | **A** | **T** | **C** | **T** | **G** | **A** | **C** | **A** | **T** | **T** | **C** | **G** | **G** | **C** | **T** | **A** | **C** | **G** | **A** | **C** | **A** | **G** | **C** | **C** | **A** | **A** | **G** | **G** | **C** | **A** | **C** | **A** | **T** | **T** | **A** | **T** | **T** | **A** | **T** | **T** | **T** | **G** | **A** | **A** | **C** | **A** | **A** | **G** | **C** | **A** | **A** | **A** | **T** | **C** | **T** | **A** | **A** | **A** | **T** | **T** | **T** | **T** | **G** | **A** | **C** | **A** | **C** | **A** | **C** | **A** | **A** | **T** | **G** | **G** | **A** | **A** | **A** | **C** | **T** | **A** | **G** | **G** | **A** | **C** | **T** | **T** | **A** | **T** | **C** | **G** | **G** | **A** | **C** | **G** | **A** | **A** | **A** | **C** | **G** | **G** | **T** | **C** | **G** | **A** | **G** | **G** | **A** | **A** | **A** | **G** | **A** | **C** | **A** | **A** | **C** | **T** | **T** | **T** | **A** | **C** | **T** | **G** | **A** | **A** | **T** | **A** | **T** | **T** | **C** | **T** | **A** | **C** | **A** | **A** | **A** | **A** | **C** | **A** | **A** | **A** | **C** | **T** | **A** | **C** | **C** | **T** | **T** | **A** | **T** | **C** | **A** | **A** | **G** | **G** | **G** | **C** | **A** | **A** | **G** | **T** | **A** | **A** | **T** | **C** | **C** | **A** | **T** | **C** | **A** | **G** | **C** | **A** | **A** | **G** | **A** | **A** | **T** | **T** | **T** | **G** | **C** | **C** | **T** | **A** | **T** | **T** | **T** | **C** | **C** | **C** | **G** | **C** | **A** | **A** | **C** | **A** | **G** | **A** | **C** | **A** | **A** | **A** | **A** | **G** | **A** | **T** | **A** | **A** | **A** | **G** | **A** | **A** | **C** | **G** | **T** | **T** | **T** | **A** | **A** | **C** | **C** | **T** | **A** | **T** | **T** | **A** | **C** | **G** | **T** | **G** | **T** | **T** | **A** | **A** | **A** | **T** | **G** | **A** | **T** | **A** | **T** | **T** | **A** | **C** | **G** | **G** | **A** | **T** | **T** | **T** | **T** | **G** | **A** | **G** | **A** | **T** | **A** | **T** | **G** | **G** | **G** | **A** | **A** | **A** | **T** | **C** | **G** | **A** | **A** | **A** | **G** | **A** | **G** | **A** | **G** | **C** | **T** | **C** | **C** | **A** | **A** | **T** | **T** | **G** | **A** | **T** | **G** | **C** | **A** | **A** | **A** | **C** | **A** | **G** | **A** | **T** | **C** | **C** | **T** | **G** | **A** | **A** | **A** | **T** | **C** | **T** | **T** | **A** | **T** | **G** | **G** | **A** | **G** | **A** | **G** | **A** | **A** | **T** | **T** | **C** | **A** | **G** | **C** | **A** | **C** | **A** | **C** | **T** | **A** | **T** | **C** | **G** | **G** | **G** | **G** | **G** | **G** | **A** | **G** | **A** | **G** | **A** | **A** | **G** | **A** | **C** | **A** | **A** | **A** | **A** | **G** | **T** | **C** | **C** | **T** | **A** | **C** | **T** | **G** | **G** | **C** | **A** | **C** | **T** | **T** | **T** | **T** | **A** | **T** | **T** | **T** | **G** | **T** | **G** | **G** | **A** | **T** | **G** | **A** | **C** | **A** | **C** | **T** | **C** | **A** | **T** | **T** | **T** | **C** | **C** | **C** | **G** | **T** | **T** | **A** | **A** | **T** | **C** | **G** | **A** | **T** | **G** | **A** | **A** | **C** | **C** | **A** | **A** | **C** | **G** | **A** | **A** | **T** | **C** | **A** | **T** | **T** | **T** | **G** | **G** | **A** | **-** | **-** | **-** | **T** | **A** | **T** | **C** | **T** | **C** | **T** | **G** | **G** | **T** | **A** | **G** | **A** | **A** | **A** | **A** | **C** | **A** | **A** | **G** | **T** | **A** | **G** | **C** | **G** | **G** | **C** | **T** | **T** | **A** | **T** | **T** | **T** | **G** | **A** | **A** | **A** | **A** | **A** | **G** | **A** | **A** | **A** | **A** | **A** | **A** | **C** | **A** | **A** | **G** | **G** | **C** | **T** | **T** | **C** | **A** | **T** | **C** | **G** | **T** | **G** | **G** | **T** | **C** | **A** | **G** | **C** | **C** | **A** | **T** | **G** | **A** | **C** | **C** | **G** | **G** | **G** | **G** | **A** | **T** | **T** | **T** | **A** | **T** | **C** | **G** | **A** | **T** | **G** | **A** | **A** | **G** | **T** | **A** | **G** | **T** | **G** | **G** | **A** | **C** | **C** | **A** | **T** | **G** | **T** | **T** | **T** | **T** | **A** | **G** | **C** | **A** | **A** | **T** | **C** | **G** | **A** | **A** | **A** | **A** | **A** | **A** | **G** | **T** | **C** | **A** | **A** | **C** | **T** | **G** | **G** | **A** | **A** | **C** | **T** | **T** | **T** | **A** | **T** | **C** | **A** | **A** | **G** | **G** | **G** | **A** | **A** | **T** | **T** | **T** | **C** | **T** | **C** | **T** | **A** | **T** | **C** | **T** | **A** | **T** | **G** | **A** | **A** | **G** | **A** | **A** | **C** | **A** | **G** | **A** | **A** | **A** | **A** | **A** | **A** | **C** | **T** | **T** | **C** | **G** | **T** | **G** | **A** | **T** | **G** | **A** | **A** | **T** | **T** | **T** | **G** | **A** | **A** | **A** | **T** | **G** | **G** | **C** | **T** | **C** | **A** | **A** | **A** | **A** | **T** | **G** | **A** | **A** | **A** | **A** | **A** | **T** | **T** | **G** | **A** | **A** | **A** | **A** | **A** | **A** | **G** | **A** | **A** | **G** | **T** | **C** | **A** | **G** | **T** | **A** | **G** | **G** | **C** | **T** | **A** | **A** | **A** | **G** | **A** | **A** | **A** | **A** | **C** | **A** | **G** | **C** | **A** | **G** | **C** | **T** | **G** | **A** | **A** | **A** | **A** | **A** | **G** | **C** | **C** | **G** | **A** | **A** | **T** | **G** | **G** | **T** | **C** | **T** | **C** | **G** | **T** | **T** | **C** | **C** | **C** | **G** | **A** | **G** | **A** | **A** | **G** | **G** | **A** | **G** | **A** | **T** | **A** | **A** | **A** | **A** | **C** | **A** | **A** | **A** | **G** | **A** | **A** | **A** | **C** | **A** | **A** | **G** | **T** | **C** | **G** | **G** | **A** | **T** | **T** | **C** | **A** | **T** | **C** | **G** | **A** | **T** | **A** | **C** | **T** | **G** | **A** | **A** | **T** | **C** | **T** | **A** | **G** | **A** | **C** | **G** | **A** | **G** | **T** | **G** | **A** | **A** | **T** | **A** | **A** | **A** | **G** | **G** | **A** | **G** | **C** | **A** | **G** | **T** | **G** | **G** | **G** | **T** | **G** | **C** | **T** | **G** | **A** | **T** | **G** | **C** | **T** | **G** | **C** | **A** | **C** | **G** | **G** | **A** | **C** | **G** | **A** | **T** | **G** | **A** | **A** | **A** | **C** | **G** | **A** | **T** | **C** | **C** | **A** | **A** | **A** | **G** | **C** | **A** | **A** | **T** | **C** | **G** | **T** | **G** | **A** | **A** | **T** | **C** | **G** | **G** | **A** | **T** | **G** | **G** | **A** | **G** | **A** | **C** | **C** | **C** | **A** | **G** | **A** | **T** | **C** | **A** | **G** | **C** | **G** | **A** | **G** | **A** | **A** | **A** | **G** | **A** | **A** | **A** | **A** | **A** | **C** | **T** | **A** | **T** | **T** | **A** | **A** | **A** | **A** | **G** | **A** | **T** | **A** | **T** | **C** | **G** | **A** | **A** | **T** | **A** | **T** | **A** | **T** | **C** | **G** | **A** | **T** | **T** | **C** | **T** | **T** | **T** | **G** | **A** | **C** | **G** | **A** | **T** | **G** | **A** | **A** | **T** | **A** | **G** | **C** | **C** | **A** | **A** | **G** | **C** | **G** | **T** | **C** | **T** | **C** | **A** | **C** | **C** | **A** | **T** | **A** | **A** | **G** | **C** | **G** | **A** | **C** | **T** | **T** | **T** | **T** | **A** | **A** | **G** | **C** | **G** | **T** | **A** | **G** | **A** | **A** | **G** | **A** | **T** | **C** | **T** | **T** | **C** | **A** | **A** | **T** | **T** | **A** | **G** | **G** | **G** | **T** | **A** | **T** | **G** | **A** | **A** | **A** | **A** | **T** | **C** | **T** | **G** | **T** | **T** | **A** | **T** | **T** | **C** | **G** | **A** | **G** | **C** | **C** | **A** | **A** | **T** | **T** | **C** | **A** | **T** | **T** | **T** | **T** | **A** | **C** | **A** | **A** | **T** | **C** | **G** | **A** | **G** | **C** | **C** | **T** | **C** | **A** | **T** | **C** | **A** | **G** | **C** | **G** | **G** | **G** | **T** | **G** | **G** | **C** | **G** | **A** | **T** | **T** | **T** | **C** | **A** | **G** | **G** | **T** | **C** | **C** | **T** | **A** | **A** | **C** | **G** | **G** | **T** | **G** | **C** | **A** | **G** | **G** | **A** | **A** | **A** | **G** | **T** | **C** | **A** | **T** | **C** | **C** | **A** | **T** | **T** | **A** | **T** | **C** | **C** | **A** | **T** | **T** | **A** | **T** | **C** | **T** | **T** | **C** | **T** | **G** | **G** | **G** | **G** | **G** | **C** | **A** | **T** | **T** | **C** | **A** | **A** | **C** | **G** | **G** | **C** | **A** | **A** | **G** | **G** | **T** | **T** | **A** | **T** | **A** | **G** | **G** | **A** | **G** | **A** | **A** | **A** | **A** | **A** | **T** | **A** | **C** | **C** | **A** | **G** | **C** | **C** | **A** | **A** | **A** | **A** | **C** | **A** | **T** | **C** | **T** | **G** | **A** | **G** | **C** | **A** | **T** | **T** | **A** | **G** | **T** | **T** | **A** | **T** | **G** | **C** | **A** | **A** | **G** | **C** | **C** | **A** | **A** | **A** | **A** | **T** | **T** | **A** | **T** | **G** | **A** | **A** | **G** | **A** | **C** | **A** | **A** | **T** | **C** | **G** | **A** | **G** | **G** | **A** | **A** | **C** | **G** | **T** | **T** | **G** | **G** | **C** | **G** | **G** | **A** | **A** | **T** | **T** | **T** | **G** | **C** | **A** | **G** | **A** | **G** | **A** | **A** | **A** | **A** | **A** | **C** | **C** | **A** | **A** | **G** | **T** | **A** | **G** | **A** | **C** | **T** | **A** | **C** | **C** | **A** | **A** | **G** | **C** | **A** | **T** | **T** | **T** | **T** | **T** | **G** | **A** | **A** | **C** | **A** | **A** | **C** | **C** | **T** | **C** | **C** | **G** | **A** | **A** | **A** | **G** | **C** | **T** | **T** | **G** | **G** | **G** | **A** | **T** | **G** | **G** | **A** | **A** | **A** | **G** | **A** | **G** | **A** | **T** | **G** | **T** | **T** | **T** | **T** | **T** | **C** | **A** | **T** | **A** | **A** | **C** | **A** | **A** | **G** | **A** | **T** | **C** | **G** | **A** | **G** | **C** | **A** | **G** | **A** | **T** | **G** | **A** | **G** | **T** | **A** | **T** | **G** | **G** | **G** | **C** | **C** | **A** | **A** | **C** | **G** | **G** | **A** | **A** | **A** | **A** | **A** | **A** | **G** | **T** | **G** | **G** | **A** | **A** | **T** | **T** | **G** | **G** | **C** | **T** | **A** | **A** | **A** | **T** | **C** | **T** | **T** | **T** | **A** | **T** | **C** | **A** | **C** | **A** | **G** | **C** | **C** | **A** | **G** | **C** | **T** | **G** | **A** | **A** | **C** | **T** | **A** | **T** | **A** | **T** | **A** | **C** | **A** | **T** | **G** | **G** | **G** | **A** | **T** | **G** | **A** | **A** | **C** | **C** | **A** | **T** | **T** | **G** | **A** | **A** | **T** | **T** | **A** | **T** | **T** | **T** | **G** | **G** | **A** | **T** | **G** | **T** | **C** | **T** | **T** | **C** | **A** | **A** | **T** | **C** | **A** | **A** | **G** | **A** | **A** | **C** | **A** | **A** | **T** | **T** | **A** | **G** | **A** | **A** | **C** | **A** | **A** | **C** | **T** | **G** | **A** | **T** | **C** | **T** | **T** | **G** | **A** | **A** | **C** | **G** | **T** | **G** | **A** | **A** | **A** | **C** | **C** | **T** | **G** | **C** | **C** | **A** | **T** | **G** | **T** | **T** | **A** | **C** | **T** | **A** | **G** | **T** | **G** | **G** | **A** | **A** | **C** | **A** | **T** | **G** | **A** | **T** | **C** | **A** | **A** | **A** | **C** | **C** | **T** | **T** | **C** | **C** | **T** | **G** | **G** | **A** | **T** | **A** | **A** | **A** | **G** | **T** | **A** | **T** | **C** | **T** | **A** | **C** | **T** | **G** | **A** | **G** | **A** | **T** | **T** | **A** | **T** | **T** | **T** | **C** | **T** | **C** | **T** | **T** | **G** | **A** | **G** | **A** | **G** | **A** | **A** | **T** | **C** | **-** | **-** | **-** |
| **N59754F** | **A** | **T** | **G** | **T** | **C** | **T** | **A** | **A** | **A** | **A** | **T** | **C** | **G** | **A** | **A** | **A** | **T** | **A** | **A** | **A** | **A** | **A** | **A** | **T** | **C** | **T** | **G** | **A** | **A** | **A** | **T** | **T** | **C** | **G** | **G** | **C** | **T** | **A** | **C** | **G** | **A** | **C** | **A** | **G** | **C** | **C** | **A** | **A** | **G** | **G** | **C** | **A** | **C** | **A** | **T** | **T** | **A** | **T** | **T** | **A** | **T** | **T** | **T** | **G** | **A** | **A** | **C** | **A** | **A** | **G** | **C** | **A** | **A** | **A** | **T** | **C** | **T** | **A** | **A** | **A** | **T** | **T** | **T** | **T** | **G** | **A** | **C** | **A** | **C** | **A** | **C** | **A** | **A** | **T** | **G** | **G** | **A** | **A** | **A** | **C** | **T** | **A** | **G** | **G** | **A** | **C** | **T** | **T** | **A** | **T** | **C** | **G** | **G** | **A** | **C** | **G** | **A** | **A** | **A** | **C** | **G** | **G** | **T** | **C** | **G** | **A** | **G** | **G** | **A** | **A** | **A** | **G** | **A** | **C** | **A** | **A** | **C** | **T** | **T** | **T** | **A** | **C** | **T** | **G** | **A** | **A** | **T** | **A** | **T** | **T** | **C** | **T** | **A** | **C** | **A** | **A** | **A** | **A** | **T** | **A** | **A** | **A** | **C** | **T** | **A** | **C** | **C** | **T** | **T** | **A** | **T** | **C** | **A** | **A** | **G** | **G** | **G** | **C** | **A** | **A** | **G** | **T** | **A** | **A** | **T** | **C** | **C** | **A** | **T** | **C** | **A** | **G** | **C** | **A** | **A** | **G** | **A** | **A** | **T** | **T** | **T** | **G** | **T** | **C** | **T** | **A** | **T** | **T** | **T** | **C** | **C** | **C** | **G** | **C** | **A** | **A** | **C** | **A** | **G** | **A** | **C** | **A** | **A** | **A** | **A** | **G** | **A** | **T** | **A** | **A** | **A** | **G** | **A** | **A** | **C** | **G** | **T** | **T** | **T** | **A** | **A** | **C** | **C** | **T** | **A** | **T** | **T** | **A** | **C** | **G** | **T** | **G** | **T** | **T** | **A** | **A** | **A** | **T** | **G** | **A** | **T** | **A** | **T** | **T** | **A** | **C** | **G** | **G** | **A** | **T** | **T** | **T** | **T** | **G** | **A** | **G** | **A** | **T** | **A** | **T** | **G** | **G** | **G** | **A** | **A** | **A** | **T** | **C** | **G** | **A** | **A** | **A** | **G** | **A** | **G** | **A** | **G** | **C** | **T** | **C** | **C** | **A** | **A** | **T** | **T** | **G** | **A** | **T** | **G** | **C** | **A** | **A** | **A** | **C** | **A** | **G** | **A** | **T** | **C** | **C** | **T** | **G** | **A** | **A** | **A** | **T** | **C** | **T** | **T** | **A** | **T** | **G** | **G** | **A** | **G** | **A** | **G** | **A** | **A** | **T** | **T** | **C** | **A** | **G** | **C** | **A** | **C** | **A** | **C** | **T** | **A** | **T** | **C** | **G** | **G** | **G** | **G** | **G** | **G** | **A** | **G** | **A** | **G** | **A** | **A** | **G** | **A** | **C** | **A** | **A** | **A** | **A** | **G** | **T** | **C** | **C** | **T** | **A** | **C** | **T** | **G** | **G** | **C** | **A** | **C** | **T** | **T** | **T** | **T** | **A** | **T** | **T** | **T** | **G** | **T** | **G** | **G** | **A** | **T** | **G** | **A** | **C** | **A** | **C** | **T** | **C** | **A** | **T** | **T** | **T** | **C** | **C** | **C** | **G** | **T** | **T** | **A** | **A** | **T** | **C** | **G** | **A** | **T** | **G** | **A** | **A** | **C** | **C** | **A** | **A** | **C** | **G** | **A** | **A** | **T** | **C** | **A** | **T** | **T** | **T** | **G** | **G** | **A** | **-** | **-** | **-** | **T** | **A** | **T** | **C** | **T** | **C** | **T** | **G** | **A** | **T** | **A** | **G** | **A** | **A** | **A** | **A** | **C** | **A** | **A** | **G** | **T** | **A** | **G** | **C** | **G** | **G** | **C** | **T** | **T** | **A** | **T** | **T** | **T** | **G** | **A** | **A** | **A** | **A** | **A** | **G** | **A** | **A** | **A** | **A** | **A** | **A** | **C** | **A** | **A** | **G** | **G** | **C** | **T** | **T** | **C** | **A** | **T** | **C** | **G** | **T** | **G** | **G** | **T** | **C** | **A** | **G** | **C** | **C** | **A** | **T** | **G** | **A** | **C** | **C** | **G** | **G** | **G** | **G** | **A** | **T** | **T** | **T** | **A** | **T** | **C** | **G** | **A** | **T** | **G** | **A** | **A** | **G** | **T** | **A** | **G** | **T** | **G** | **G** | **A** | **C** | **C** | **A** | **T** | **G** | **T** | **T** | **T** | **T** | **A** | **G** | **C** | **A** | **A** | **T** | **C** | **G** | **A** | **A** | **A** | **A** | **A** | **A** | **G** | **T** | **C** | **A** | **A** | **C** | **T** | **G** | **G** | **A** | **A** | **C** | **T** | **T** | **T** | **A** | **T** | **C** | **A** | **A** | **G** | **G** | **G** | **A** | **A** | **T** | **T** | **T** | **C** | **T** | **C** | **T** | **A** | **T** | **C** | **T** | **A** | **T** | **G** | **A** | **A** | **G** | **A** | **A** | **C** | **A** | **G** | **A** | **A** | **A** | **A** | **A** | **A** | **C** | **T** | **T** | **C** | **G** | **T** | **G** | **A** | **T** | **G** | **A** | **A** | **T** | **T** | **T** | **G** | **A** | **A** | **A** | **T** | **G** | **G** | **C** | **T** | **C** | **A** | **A** | **A** | **A** | **T** | **G** | **A** | **A** | **A** | **A** | **A** | **T** | **T** | **G** | **A** | **A** | **A** | **A** | **A** | **A** | **G** | **A** | **A** | **G** | **T** | **C** | **A** | **G** | **T** | **A** | **G** | **G** | **C** | **T** | **A** | **A** | **A** | **G** | **A** | **A** | **A** | **A** | **C** | **A** | **G** | **C** | **A** | **G** | **C** | **T** | **G** | **A** | **A** | **A** | **A** | **A** | **G** | **C** | **C** | **G** | **A** | **A** | **T** | **G** | **G** | **T** | **C** | **T** | **C** | **G** | **T** | **T** | **C** | **C** | **C** | **G** | **A** | **G** | **A** | **A** | **G** | **G** | **A** | **G** | **A** | **T** | **A** | **A** | **A** | **A** | **C** | **A** | **A** | **A** | **G** | **A** | **A** | **A** | **C** | **A** | **A** | **G** | **T** | **C** | **G** | **G** | **A** | **T** | **T** | **C** | **A** | **T** | **C** | **G** | **A** | **T** | **A** | **C** | **T** | **G** | **A** | **A** | **T** | **C** | **T** | **A** | **G** | **A** | **C** | **G** | **A** | **G** | **T** | **G** | **A** | **A** | **T** | **A** | **A** | **A** | **G** | **G** | **A** | **G** | **C** | **A** | **G** | **T** | **G** | **G** | **G** | **T** | **G** | **C** | **T** | **G** | **A** | **T** | **G** | **C** | **T** | **G** | **C** | **A** | **C** | **G** | **G** | **A** | **C** | **G** | **A** | **T** | **G** | **A** | **A** | **A** | **C** | **G** | **A** | **T** | **C** | **C** | **A** | **A** | **A** | **G** | **C** | **A** | **A** | **T** | **C** | **G** | **T** | **G** | **A** | **A** | **T** | **C** | **G** | **G** | **A** | **T** | **G** | **G** | **A** | **G** | **A** | **C** | **C** | **C** | **A** | **G** | **A** | **T** | **C** | **A** | **G** | **C** | **G** | **A** | **G** | **A** | **A** | **A** | **G** | **A** | **A** | **A** | **A** | **A** | **C** | **T** | **A** | **T** | **T** | **A** | **A** | **A** | **A** | **G** | **A** | **T** | **A** | **T** | **C** | **G** | **A** | **A** | **T** | **A** | **T** | **A** | **T** | **C** | **G** | **A** | **T** | **T** | **C** | **T** | **T** | **T** | **G** | **A** | **C** | **G** | **A** | **T** | **G** | **A** | **A** | **T** | **A** | **G** | **C** | **C** | **A** | **A** | **G** | **C** | **G** | **T** | **C** | **T** | **C** | **A** | **C** | **C** | **A** | **T** | **A** | **A** | **G** | **C** | **G** | **A** | **C** | **T** | **T** | **T** | **T** | **A** | **A** | **G** | **C** | **G** | **T** | **A** | **G** | **A** | **A** | **G** | **A** | **T** | **C** | **T** | **T** | **C** | **A** | **A** | **T** | **T** | **A** | **G** | **G** | **G** | **T** | **A** | **T** | **G** | **A** | **A** | **A** | **A** | **T** | **C** | **T** | **G** | **T** | **T** | **A** | **T** | **T** | **C** | **G** | **A** | **G** | **C** | **C** | **A** | **A** | **T** | **T** | **C** | **A** | **T** | **T** | **T** | **T** | **A** | **C** | **A** | **A** | **T** | **C** | **G** | **A** | **G** | **C** | **C** | **T** | **C** | **A** | **T** | **C** | **A** | **G** | **C** | **G** | **G** | **G** | **T** | **G** | **G** | **C** | **G** | **A** | **T** | **T** | **T** | **C** | **A** | **G** | **G** | **T** | **C** | **C** | **T** | **A** | **A** | **C** | **G** | **G** | **T** | **G** | **C** | **A** | **G** | **G** | **A** | **A** | **A** | **G** | **T** | **C** | **A** | **T** | **C** | **C** | **A** | **T** | **T** | **A** | **T** | **C** | **C** | **A** | **T** | **T** | **A** | **T** | **C** | **T** | **T** | **C** | **T** | **G** | **G** | **G** | **G** | **G** | **C** | **A** | **T** | **T** | **C** | **A** | **A** | **C** | **G** | **G** | **C** | **A** | **A** | **G** | **G** | **T** | **T** | **A** | **T** | **A** | **G** | **G** | **A** | **G** | **A** | **A** | **A** | **A** | **A** | **T** | **A** | **C** | **C** | **A** | **G** | **C** | **C** | **A** | **A** | **A** | **A** | **C** | **A** | **T** | **C** | **T** | **G** | **A** | **G** | **C** | **A** | **T** | **T** | **A** | **G** | **T** | **T** | **A** | **T** | **G** | **T** | **A** | **C** | **G** | **C** | **C** | **A** | **A** | **A** | **A** | **T** | **T** | **A** | **T** | **G** | **A** | **A** | **G** | **A** | **C** | **A** | **A** | **T** | **C** | **G** | **A** | **G** | **G** | **A** | **A** | **C** | **G** | **T** | **T** | **G** | **G** | **C** | **G** | **G** | **A** | **A** | **T** | **T** | **T** | **G** | **C** | **A** | **G** | **A** | **G** | **A** | **A** | **A** | **A** | **A** | **C** | **C** | **A** | **A** | **G** | **T** | **A** | **G** | **A** | **C** | **T** | **A** | **C** | **C** | **A** | **A** | **G** | **C** | **A** | **T** | **T** | **T** | **T** | **T** | **G** | **A** | **A** | **C** | **A** | **A** | **C** | **C** | **T** | **C** | **C** | **G** | **A** | **A** | **A** | **G** | **C** | **T** | **T** | **G** | **G** | **G** | **A** | **T** | **G** | **G** | **A** | **A** | **A** | **G** | **A** | **G** | **A** | **T** | **G** | **T** | **T** | **T** | **T** | **T** | **C** | **A** | **T** | **A** | **A** | **C** | **A** | **A** | **G** | **A** | **T** | **C** | **G** | **A** | **G** | **C** | **A** | **G** | **A** | **T** | **G** | **A** | **G** | **T** | **A** | **T** | **G** | **G** | **G** | **C** | **C** | **A** | **A** | **C** | **G** | **G** | **A** | **A** | **A** | **A** | **A** | **A** | **G** | **T** | **G** | **G** | **A** | **A** | **T** | **T** | **G** | **G** | **C** | **T** | **A** | **A** | **A** | **T** | **C** | **T** | **T** | **T** | **A** | **T** | **C** | **A** | **C** | **A** | **G** | **C** | **C** | **A** | **G** | **C** | **T** | **G** | **A** | **A** | **C** | **T** | **A** | **T** | **A** | **T** | **A** | **T** | **A** | **T** | **G** | **G** | **G** | **A** | **T** | **G** | **A** | **A** | **C** | **C** | **A** | **T** | **T** | **G** | **A** | **A** | **T** | **T** | **A** | **T** | **T** | **T** | **G** | **G** | **A** | **T** | **G** | **T** | **C** | **T** | **T** | **C** | **A** | **A** | **T** | **C** | **A** | **A** | **G** | **A** | **A** | **C** | **A** | **A** | **T** | **T** | **A** | **G** | **A** | **A** | **C** | **A** | **A** | **C** | **T** | **G** | **A** | **T** | **C** | **T** | **T** | **G** | **A** | **A** | **C** | **G** | **T** | **G** | **A** | **A** | **A** | **C** | **C** | **T** | **G** | **C** | **C** | **A** | **T** | **G** | **T** | **T** | **A** | **C** | **T** | **A** | **G** | **T** | **G** | **G** | **A** | **A** | **C** | **A** | **T** | **G** | **A** | **T** | **C** | **A** | **A** | **A** | **C** | **C** | **T** | **T** | **C** | **C** | **T** | **G** | **G** | **A** | **T** | **A** | **A** | **A** | **G** | **T** | **A** | **T** | **C** | **T** | **A** | **C** | **T** | **G** | **A** | **G** | **A** | **T** | **T** | **A** | **T** | **T** | **T** | **C** | **T** | **C** | **T** | **T** | **G** | **A** | **G** | **A** | **A** | **A** | **A** | **T** | **C** | **-** | **-** | **-** |
| **N55299** | **A** | **T** | **G** | **T** | **C** | **T** | **A** | **A** | **A** | **A** | **T** | **C** | **G** | **A** | **A** | **A** | **T** | **A** | **A** | **A** | **A** | **A** | **A** | **T** | **C** | **T** | **G** | **A** | **C** | **A** | **T** | **T** | **C** | **G** | **G** | **C** | **T** | **A** | **C** | **G** | **A** | **C** | **A** | **G** | **C** | **C** | **A** | **A** | **G** | **G** | **C** | **A** | **C** | **A** | **T** | **T** | **A** | **T** | **T** | **A** | **T** | **T** | **T** | **G** | **A** | **A** | **C** | **A** | **A** | **G** | **C** | **A** | **A** | **A** | **T** | **C** | **T** | **A** | **A** | **A** | **T** | **T** | **T** | **T** | **G** | **A** | **C** | **A** | **C** | **A** | **C** | **A** | **A** | **T** | **G** | **G** | **A** | **A** | **A** | **C** | **T** | **A** | **G** | **G** | **A** | **C** | **T** | **T** | **A** | **T** | **C** | **G** | **G** | **A** | **C** | **G** | **A** | **A** | **A** | **C** | **G** | **G** | **T** | **C** | **G** | **A** | **G** | **G** | **A** | **A** | **A** | **G** | **A** | **C** | **A** | **A** | **C** | **T** | **T** | **T** | **A** | **C** | **T** | **G** | **A** | **A** | **T** | **A** | **T** | **T** | **C** | **T** | **A** | **C** | **A** | **A** | **A** | **A** | **C** | **A** | **A** | **A** | **C** | **T** | **A** | **C** | **C** | **T** | **T** | **A** | **T** | **C** | **A** | **A** | **G** | **G** | **G** | **C** | **A** | **A** | **G** | **T** | **A** | **A** | **T** | **C** | **C** | **A** | **T** | **C** | **A** | **G** | **C** | **A** | **A** | **G** | **A** | **A** | **T** | **T** | **T** | **G** | **C** | **C** | **T** | **A** | **T** | **T** | **T** | **C** | **C** | **C** | **G** | **C** | **A** | **A** | **C** | **A** | **G** | **A** | **C** | **A** | **A** | **A** | **A** | **G** | **A** | **T** | **A** | **A** | **A** | **G** | **A** | **A** | **C** | **G** | **T** | **T** | **T** | **A** | **A** | **C** | **C** | **T** | **A** | **T** | **T** | **A** | **C** | **G** | **T** | **G** | **T** | **T** | **A** | **A** | **A** | **T** | **G** | **A** | **T** | **A** | **T** | **T** | **A** | **C** | **G** | **G** | **A** | **T** | **T** | **T** | **T** | **G** | **A** | **G** | **A** | **T** | **A** | **T** | **G** | **G** | **G** | **A** | **A** | **A** | **T** | **C** | **G** | **A** | **A** | **A** | **G** | **A** | **G** | **A** | **G** | **C** | **T** | **C** | **C** | **A** | **A** | **T** | **T** | **G** | **A** | **T** | **G** | **C** | **A** | **A** | **A** | **C** | **A** | **G** | **A** | **T** | **C** | **C** | **T** | **G** | **A** | **A** | **A** | **T** | **C** | **T** | **T** | **A** | **T** | **G** | **G** | **A** | **G** | **A** | **G** | **A** | **A** | **T** | **T** | **C** | **A** | **G** | **C** | **A** | **C** | **A** | **C** | **T** | **A** | **T** | **C** | **G** | **G** | **G** | **G** | **G** | **G** | **A** | **G** | **A** | **G** | **A** | **A** | **G** | **A** | **C** | **A** | **A** | **A** | **A** | **G** | **T** | **C** | **C** | **T** | **A** | **C** | **T** | **G** | **G** | **C** | **A** | **C** | **T** | **T** | **T** | **T** | **A** | **T** | **T** | **T** | **G** | **T** | **G** | **G** | **A** | **T** | **G** | **A** | **C** | **A** | **C** | **T** | **C** | **A** | **T** | **T** | **T** | **C** | **C** | **C** | **G** | **T** | **T** | **A** | **A** | **T** | **C** | **G** | **A** | **T** | **G** | **A** | **A** | **C** | **C** | **A** | **A** | **C** | **G** | **A** | **A** | **T** | **C** | **A** | **T** | **T** | **T** | **G** | **G** | **A** | **-** | **-** | **-** | **T** | **A** | **T** | **C** | **T** | **C** | **T** | **G** | **G** | **T** | **A** | **G** | **A** | **A** | **A** | **A** | **C** | **A** | **A** | **G** | **T** | **A** | **G** | **C** | **G** | **G** | **C** | **T** | **T** | **A** | **T** | **T** | **T** | **G** | **A** | **A** | **A** | **A** | **A** | **G** | **A** | **A** | **A** | **A** | **A** | **A** | **C** | **A** | **A** | **G** | **G** | **C** | **T** | **T** | **C** | **A** | **T** | **C** | **G** | **T** | **G** | **G** | **T** | **C** | **A** | **G** | **C** | **C** | **A** | **T** | **G** | **A** | **C** | **C** | **G** | **G** | **G** | **G** | **A** | **T** | **T** | **T** | **A** | **T** | **C** | **G** | **A** | **T** | **G** | **A** | **A** | **G** | **T** | **A** | **G** | **T** | **G** | **G** | **A** | **C** | **C** | **A** | **T** | **G** | **T** | **T** | **T** | **T** | **A** | **G** | **C** | **A** | **A** | **T** | **C** | **G** | **A** | **A** | **A** | **A** | **A** | **A** | **G** | **T** | **C** | **A** | **A** | **C** | **T** | **G** | **G** | **A** | **A** | **C** | **T** | **T** | **T** | **A** | **T** | **C** | **A** | **A** | **G** | **G** | **G** | **A** | **A** | **T** | **T** | **T** | **C** | **T** | **C** | **T** | **A** | **T** | **C** | **T** | **A** | **T** | **G** | **A** | **A** | **G** | **A** | **A** | **C** | **A** | **G** | **A** | **A** | **A** | **A** | **A** | **A** | **C** | **T** | **T** | **C** | **G** | **T** | **G** | **A** | **T** | **G** | **A** | **A** | **T** | **T** | **T** | **G** | **A** | **A** | **A** | **T** | **G** | **G** | **C** | **T** | **C** | **A** | **A** | **A** | **A** | **T** | **G** | **A** | **A** | **A** | **A** | **A** | **T** | **T** | **G** | **A** | **A** | **A** | **A** | **A** | **A** | **G** | **A** | **A** | **G** | **T** | **C** | **A** | **G** | **T** | **A** | **G** | **G** | **C** | **T** | **A** | **A** | **A** | **G** | **A** | **A** | **A** | **A** | **C** | **A** | **G** | **C** | **A** | **G** | **C** | **T** | **G** | **A** | **A** | **A** | **A** | **A** | **G** | **C** | **C** | **G** | **A** | **A** | **T** | **G** | **G** | **T** | **C** | **T** | **C** | **G** | **T** | **T** | **C** | **C** | **C** | **G** | **A** | **G** | **A** | **A** | **G** | **G** | **A** | **G** | **A** | **T** | **A** | **A** | **A** | **A** | **C** | **A** | **A** | **A** | **G** | **A** | **A** | **A** | **C** | **A** | **A** | **G** | **T** | **C** | **G** | **G** | **A** | **T** | **T** | **C** | **A** | **T** | **C** | **G** | **A** | **T** | **A** | **C** | **T** | **G** | **A** | **A** | **T** | **C** | **T** | **A** | **G** | **A** | **C** | **G** | **A** | **G** | **T** | **G** | **A** | **A** | **T** | **A** | **A** | **A** | **G** | **G** | **A** | **G** | **C** | **A** | **G** | **T** | **G** | **G** | **G** | **T** | **G** | **C** | **T** | **G** | **A** | **T** | **G** | **C** | **T** | **G** | **C** | **A** | **C** | **G** | **G** | **A** | **C** | **G** | **A** | **T** | **G** | **A** | **A** | **A** | **C** | **G** | **A** | **T** | **C** | **C** | **A** | **A** | **A** | **G** | **C** | **A** | **A** | **T** | **C** | **G** | **T** | **G** | **A** | **A** | **T** | **C** | **G** | **G** | **A** | **T** | **G** | **G** | **A** | **G** | **A** | **C** | **C** | **C** | **A** | **G** | **A** | **T** | **C** | **A** | **G** | **C** | **G** | **A** | **G** | **A** | **A** | **A** | **G** | **A** | **A** | **A** | **A** | **A** | **C** | **T** | **A** | **T** | **T** | **A** | **A** | **A** | **A** | **G** | **A** | **T** | **A** | **T** | **C** | **G** | **A** | **A** | **T** | **A** | **T** | **A** | **T** | **C** | **G** | **A** | **T** | **T** | **C** | **T** | **T** | **T** | **G** | **A** | **C** | **G** | **A** | **T** | **G** | **A** | **A** | **T** | **A** | **G** | **C** | **C** | **A** | **A** | **G** | **C** | **G** | **T** | **C** | **T** | **C** | **A** | **C** | **C** | **A** | **T** | **A** | **A** | **G** | **C** | **G** | **A** | **C** | **T** | **T** | **T** | **T** | **A** | **A** | **G** | **C** | **G** | **T** | **A** | **G** | **A** | **A** | **G** | **A** | **T** | **C** | **T** | **T** | **C** | **A** | **A** | **T** | **T** | **A** | **G** | **G** | **G** | **T** | **A** | **T** | **G** | **A** | **A** | **A** | **A** | **T** | **C** | **T** | **G** | **T** | **T** | **A** | **T** | **T** | **C** | **G** | **A** | **G** | **C** | **C** | **A** | **A** | **T** | **T** | **C** | **A** | **T** | **T** | **T** | **T** | **A** | **C** | **A** | **A** | **T** | **C** | **G** | **A** | **G** | **C** | **C** | **T** | **C** | **A** | **T** | **C** | **A** | **G** | **C** | **G** | **G** | **G** | **T** | **G** | **G** | **C** | **G** | **A** | **T** | **T** | **T** | **C** | **A** | **G** | **G** | **T** | **C** | **C** | **T** | **A** | **A** | **C** | **G** | **G** | **T** | **G** | **C** | **A** | **G** | **G** | **A** | **A** | **A** | **G** | **T** | **C** | **A** | **T** | **C** | **C** | **A** | **T** | **T** | **A** | **T** | **C** | **C** | **A** | **T** | **T** | **A** | **T** | **C** | **T** | **T** | **C** | **T** | **G** | **G** | **G** | **G** | **G** | **C** | **A** | **T** | **T** | **C** | **A** | **A** | **C** | **G** | **G** | **C** | **A** | **A** | **G** | **G** | **T** | **T** | **A** | **T** | **A** | **G** | **G** | **A** | **G** | **A** | **A** | **A** | **A** | **A** | **T** | **A** | **C** | **C** | **A** | **G** | **C** | **C** | **A** | **A** | **A** | **A** | **C** | **A** | **T** | **C** | **T** | **G** | **A** | **G** | **C** | **A** | **T** | **T** | **A** | **G** | **T** | **T** | **A** | **T** | **G** | **C** | **A** | **A** | **G** | **C** | **C** | **A** | **A** | **A** | **A** | **T** | **T** | **A** | **T** | **G** | **A** | **A** | **G** | **A** | **C** | **A** | **A** | **T** | **C** | **G** | **A** | **G** | **G** | **A** | **A** | **C** | **G** | **T** | **T** | **G** | **G** | **C** | **G** | **G** | **A** | **A** | **T** | **T** | **T** | **G** | **C** | **A** | **G** | **A** | **G** | **A** | **A** | **A** | **A** | **A** | **C** | **C** | **A** | **A** | **G** | **T** | **A** | **G** | **A** | **C** | **T** | **A** | **C** | **C** | **A** | **A** | **G** | **C** | **A** | **T** | **T** | **T** | **T** | **T** | **G** | **A** | **A** | **C** | **A** | **A** | **C** | **C** | **T** | **C** | **C** | **G** | **A** | **A** | **A** | **G** | **C** | **T** | **T** | **G** | **G** | **G** | **A** | **T** | **G** | **G** | **A** | **A** | **A** | **G** | **A** | **G** | **A** | **T** | **G** | **T** | **T** | **T** | **T** | **T** | **C** | **A** | **T** | **A** | **A** | **C** | **A** | **A** | **G** | **A** | **T** | **C** | **G** | **A** | **G** | **C** | **A** | **G** | **A** | **T** | **G** | **A** | **G** | **T** | **A** | **T** | **G** | **G** | **G** | **C** | **C** | **A** | **A** | **C** | **G** | **G** | **A** | **A** | **A** | **A** | **A** | **A** | **G** | **T** | **G** | **G** | **A** | **A** | **T** | **T** | **G** | **G** | **C** | **T** | **A** | **A** | **A** | **T** | **C** | **T** | **T** | **T** | **A** | **T** | **C** | **A** | **C** | **A** | **G** | **C** | **C** | **A** | **G** | **C** | **T** | **G** | **A** | **A** | **C** | **T** | **A** | **T** | **A** | **T** | **A** | **C** | **A** | **T** | **G** | **G** | **G** | **A** | **T** | **G** | **A** | **A** | **C** | **C** | **A** | **T** | **T** | **G** | **A** | **A** | **T** | **T** | **A** | **T** | **T** | **T** | **G** | **G** | **A** | **T** | **G** | **T** | **C** | **T** | **T** | **C** | **A** | **A** | **T** | **C** | **A** | **A** | **G** | **A** | **A** | **C** | **A** | **A** | **T** | **T** | **A** | **G** | **A** | **A** | **C** | **A** | **A** | **C** | **T** | **G** | **A** | **T** | **C** | **T** | **T** | **G** | **A** | **A** | **C** | **G** | **T** | **G** | **A** | **A** | **A** | **C** | **C** | **T** | **G** | **C** | **C** | **A** | **T** | **G** | **T** | **T** | **A** | **C** | **T** | **A** | **G** | **T** | **G** | **G** | **A** | **A** | **C** | **A** | **T** | **G** | **A** | **T** | **C** | **A** | **A** | **A** | **C** | **C** | **T** | **T** | **C** | **C** | **T** | **G** | **G** | **A** | **T** | **A** | **A** | **A** | **G** | **T** | **A** | **T** | **C** | **T** | **A** | **C** | **T** | **G** | **A** | **G** | **A** | **T** | **T** | **A** | **T** | **T** | **T** | **C** | **T** | **C** | **T** | **T** | **G** | **A** | **G** | **A** | **G** | **A** | **A** | **T** | **C** | **-** | **-** | **-** |
| **N52643** | **A** | **T** | **G** | **T** | **C** | **T** | **A** | **A** | **A** | **A** | **T** | **C** | **G** | **A** | **A** | **A** | **T** | **A** | **A** | **A** | **A** | **A** | **A** | **T** | **C** | **T** | **G** | **A** | **C** | **A** | **T** | **T** | **C** | **G** | **G** | **C** | **T** | **A** | **C** | **G** | **A** | **C** | **A** | **G** | **C** | **C** | **A** | **A** | **G** | **G** | **C** | **A** | **C** | **A** | **T** | **T** | **A** | **T** | **T** | **A** | **T** | **T** | **T** | **G** | **A** | **A** | **C** | **A** | **A** | **G** | **C** | **A** | **A** | **A** | **T** | **C** | **T** | **A** | **A** | **A** | **T** | **T** | **T** | **T** | **G** | **A** | **C** | **A** | **C** | **A** | **C** | **A** | **A** | **T** | **G** | **G** | **A** | **A** | **A** | **C** | **T** | **A** | **G** | **G** | **A** | **C** | **T** | **T** | **A** | **T** | **C** | **G** | **G** | **A** | **C** | **G** | **A** | **A** | **A** | **C** | **G** | **G** | **T** | **C** | **G** | **A** | **G** | **G** | **A** | **A** | **A** | **G** | **A** | **C** | **A** | **A** | **C** | **T** | **T** | **T** | **A** | **C** | **T** | **G** | **A** | **A** | **T** | **A** | **T** | **T** | **C** | **T** | **A** | **C** | **A** | **A** | **A** | **A** | **C** | **A** | **A** | **A** | **C** | **T** | **A** | **C** | **C** | **T** | **T** | **A** | **T** | **C** | **A** | **A** | **G** | **G** | **G** | **C** | **A** | **A** | **G** | **T** | **A** | **A** | **T** | **C** | **C** | **A** | **T** | **C** | **A** | **G** | **C** | **A** | **A** | **G** | **A** | **A** | **T** | **T** | **T** | **G** | **T** | **C** | **T** | **A** | **T** | **T** | **T** | **C** | **C** | **C** | **G** | **C** | **A** | **A** | **C** | **A** | **G** | **A** | **C** | **A** | **A** | **A** | **A** | **G** | **A** | **T** | **A** | **A** | **A** | **G** | **A** | **A** | **C** | **G** | **T** | **T** | **T** | **A** | **A** | **C** | **C** | **T** | **A** | **T** | **T** | **A** | **C** | **G** | **T** | **G** | **T** | **T** | **A** | **A** | **A** | **T** | **G** | **A** | **T** | **A** | **T** | **T** | **A** | **C** | **G** | **G** | **A** | **T** | **T** | **T** | **T** | **G** | **A** | **G** | **A** | **T** | **A** | **T** | **G** | **G** | **G** | **A** | **A** | **A** | **T** | **C** | **G** | **A** | **A** | **A** | **G** | **A** | **G** | **A** | **G** | **C** | **T** | **C** | **C** | **A** | **A** | **T** | **T** | **G** | **A** | **T** | **G** | **C** | **A** | **A** | **A** | **C** | **A** | **G** | **A** | **T** | **C** | **C** | **T** | **G** | **A** | **A** | **A** | **T** | **C** | **T** | **T** | **A** | **T** | **G** | **G** | **A** | **G** | **A** | **G** | **A** | **A** | **T** | **T** | **C** | **A** | **G** | **C** | **A** | **C** | **A** | **C** | **T** | **A** | **T** | **C** | **A** | **G** | **G** | **G** | **G** | **G** | **A** | **G** | **A** | **G** | **A** | **A** | **G** | **A** | **C** | **A** | **A** | **A** | **A** | **G** | **T** | **C** | **C** | **T** | **A** | **C** | **T** | **G** | **G** | **C** | **A** | **C** | **T** | **T** | **T** | **T** | **A** | **T** | **T** | **T** | **G** | **T** | **G** | **G** | **A** | **T** | **G** | **A** | **C** | **A** | **C** | **T** | **C** | **A** | **T** | **T** | **T** | **C** | **C** | **C** | **G** | **T** | **T** | **A** | **A** | **T** | **C** | **G** | **A** | **T** | **G** | **A** | **A** | **C** | **C** | **A** | **A** | **C** | **G** | **A** | **A** | **T** | **C** | **A** | **T** | **T** | **T** | **G** | **G** | **A** | **-** | **-** | **-** | **T** | **A** | **T** | **C** | **T** | **C** | **T** | **G** | **G** | **T** | **A** | **G** | **A** | **A** | **A** | **A** | **C** | **A** | **A** | **G** | **T** | **A** | **G** | **C** | **G** | **G** | **C** | **T** | **T** | **A** | **T** | **T** | **T** | **G** | **A** | **A** | **A** | **A** | **A** | **G** | **A** | **A** | **A** | **A** | **A** | **A** | **C** | **A** | **A** | **G** | **G** | **C** | **T** | **T** | **C** | **A** | **T** | **C** | **G** | **T** | **G** | **G** | **T** | **C** | **A** | **G** | **C** | **C** | **A** | **T** | **G** | **A** | **C** | **C** | **G** | **G** | **G** | **G** | **A** | **T** | **T** | **T** | **A** | **T** | **C** | **G** | **A** | **T** | **G** | **A** | **A** | **G** | **T** | **A** | **G** | **T** | **G** | **G** | **A** | **C** | **C** | **A** | **T** | **G** | **T** | **T** | **T** | **T** | **A** | **G** | **C** | **A** | **A** | **T** | **C** | **G** | **A** | **A** | **A** | **A** | **A** | **A** | **G** | **T** | **C** | **A** | **A** | **C** | **T** | **G** | **G** | **A** | **A** | **C** | **T** | **T** | **T** | **A** | **T** | **C** | **A** | **A** | **G** | **G** | **A** | **A** | **A** | **T** | **T** | **T** | **C** | **T** | **C** | **T** | **A** | **T** | **C** | **T** | **A** | **T** | **G** | **A** | **A** | **G** | **A** | **A** | **C** | **A** | **G** | **A** | **A** | **A** | **A** | **A** | **A** | **C** | **T** | **T** | **C** | **G** | **T** | **G** | **A** | **T** | **G** | **A** | **A** | **T** | **T** | **T** | **G** | **A** | **A** | **A** | **T** | **G** | **G** | **C** | **T** | **C** | **A** | **A** | **A** | **A** | **T** | **G** | **A** | **A** | **A** | **A** | **A** | **T** | **T** | **G** | **A** | **A** | **A** | **A** | **A** | **A** | **G** | **A** | **A** | **G** | **T** | **C** | **A** | **G** | **T** | **A** | **G** | **G** | **C** | **T** | **A** | **A** | **A** | **G** | **A** | **A** | **A** | **A** | **C** | **A** | **G** | **C** | **A** | **G** | **C** | **T** | **G** | **A** | **A** | **A** | **A** | **A** | **G** | **C** | **C** | **G** | **A** | **A** | **T** | **G** | **G** | **T** | **C** | **T** | **C** | **G** | **T** | **T** | **C** | **C** | **C** | **G** | **A** | **G** | **A** | **A** | **G** | **G** | **A** | **G** | **A** | **T** | **A** | **A** | **A** | **A** | **C** | **A** | **A** | **A** | **G** | **A** | **A** | **A** | **C** | **A** | **A** | **G** | **T** | **C** | **G** | **G** | **A** | **T** | **T** | **C** | **A** | **T** | **C** | **G** | **A** | **T** | **A** | **C** | **T** | **G** | **A** | **A** | **T** | **C** | **T** | **A** | **G** | **A** | **C** | **G** | **A** | **G** | **T** | **G** | **A** | **A** | **T** | **A** | **A** | **A** | **G** | **G** | **A** | **G** | **C** | **A** | **G** | **T** | **G** | **G** | **G** | **T** | **G** | **C** | **T** | **G** | **A** | **T** | **G** | **C** | **T** | **G** | **C** | **A** | **C** | **G** | **G** | **A** | **C** | **G** | **A** | **T** | **G** | **A** | **A** | **A** | **C** | **G** | **A** | **T** | **C** | **C** | **A** | **A** | **A** | **G** | **C** | **A** | **A** | **T** | **C** | **G** | **T** | **G** | **A** | **A** | **T** | **C** | **G** | **G** | **A** | **T** | **G** | **G** | **A** | **G** | **A** | **C** | **C** | **C** | **A** | **G** | **A** | **T** | **C** | **A** | **G** | **C** | **G** | **A** | **G** | **A** | **A** | **A** | **G** | **A** | **A** | **A** | **A** | **A** | **C** | **T** | **A** | **T** | **T** | **A** | **A** | **A** | **A** | **G** | **A** | **T** | **A** | **T** | **C** | **G** | **A** | **A** | **T** | **A** | **T** | **A** | **T** | **C** | **G** | **A** | **T** | **T** | **C** | **T** | **T** | **T** | **G** | **A** | **C** | **G** | **A** | **T** | **G** | **A** | **A** | **T** | **A** | **G** | **C** | **C** | **A** | **A** | **G** | **C** | **G** | **T** | **C** | **T** | **C** | **A** | **C** | **C** | **A** | **T** | **A** | **A** | **G** | **C** | **G** | **A** | **C** | **T** | **T** | **T** | **T** | **A** | **A** | **G** | **C** | **G** | **T** | **A** | **G** | **A** | **A** | **G** | **A** | **T** | **C** | **T** | **T** | **C** | **A** | **A** | **T** | **T** | **A** | **G** | **G** | **G** | **T** | **A** | **T** | **G** | **A** | **A** | **A** | **A** | **T** | **C** | **T** | **G** | **T** | **T** | **A** | **T** | **T** | **C** | **G** | **A** | **G** | **C** | **C** | **A** | **A** | **T** | **T** | **C** | **A** | **T** | **T** | **T** | **T** | **A** | **C** | **A** | **A** | **T** | **C** | **G** | **A** | **G** | **C** | **C** | **T** | **C** | **A** | **T** | **C** | **A** | **G** | **C** | **G** | **G** | **G** | **T** | **G** | **G** | **C** | **G** | **A** | **T** | **T** | **T** | **C** | **A** | **G** | **G** | **A** | **C** | **C** | **T** | **A** | **A** | **C** | **G** | **G** | **T** | **G** | **C** | **A** | **G** | **G** | **A** | **A** | **A** | **G** | **T** | **C** | **A** | **T** | **C** | **C** | **A** | **T** | **T** | **A** | **T** | **C** | **C** | **A** | **T** | **T** | **A** | **T** | **C** | **T** | **T** | **C** | **T** | **G** | **G** | **G** | **G** | **G** | **C** | **A** | **T** | **T** | **C** | **A** | **A** | **C** | **G** | **G** | **C** | **A** | **A** | **G** | **G** | **T** | **T** | **A** | **T** | **A** | **G** | **G** | **A** | **G** | **A** | **A** | **A** | **A** | **A** | **T** | **C** | **C** | **C** | **A** | **G** | **C** | **C** | **A** | **A** | **A** | **A** | **C** | **A** | **T** | **C** | **T** | **G** | **A** | **G** | **C** | **A** | **T** | **T** | **A** | **G** | **T** | **T** | **A** | **T** | **G** | **T** | **A** | **C** | **G** | **C** | **C** | **A** | **A** | **A** | **A** | **T** | **T** | **A** | **T** | **G** | **A** | **A** | **G** | **A** | **C** | **A** | **A** | **T** | **C** | **G** | **A** | **G** | **G** | **A** | **A** | **C** | **G** | **T** | **T** | **G** | **G** | **C** | **G** | **G** | **A** | **A** | **T** | **T** | **T** | **G** | **C** | **A** | **G** | **A** | **G** | **A** | **A** | **A** | **A** | **A** | **C** | **C** | **A** | **A** | **G** | **T** | **A** | **G** | **A** | **C** | **T** | **A** | **C** | **C** | **A** | **A** | **G** | **C** | **A** | **T** | **T** | **T** | **T** | **T** | **G** | **A** | **A** | **C** | **A** | **A** | **C** | **C** | **T** | **C** | **C** | **G** | **A** | **A** | **A** | **G** | **C** | **T** | **T** | **G** | **G** | **G** | **A** | **T** | **G** | **G** | **A** | **A** | **A** | **G** | **A** | **G** | **A** | **T** | **G** | **T** | **T** | **T** | **T** | **T** | **C** | **A** | **T** | **A** | **A** | **C** | **A** | **A** | **G** | **A** | **T** | **C** | **G** | **A** | **G** | **C** | **A** | **G** | **A** | **T** | **G** | **A** | **G** | **T** | **A** | **T** | **G** | **G** | **G** | **C** | **C** | **A** | **A** | **C** | **G** | **G** | **A** | **A** | **A** | **A** | **A** | **A** | **G** | **T** | **G** | **G** | **A** | **A** | **T** | **T** | **G** | **G** | **C** | **T** | **A** | **A** | **A** | **T** | **C** | **T** | **T** | **T** | **A** | **T** | **C** | **A** | **C** | **A** | **G** | **C** | **C** | **A** | **G** | **C** | **T** | **G** | **A** | **A** | **C** | **T** | **A** | **T** | **A** | **T** | **A** | **T** | **A** | **T** | **G** | **G** | **G** | **A** | **T** | **G** | **A** | **A** | **C** | **C** | **A** | **T** | **T** | **G** | **A** | **A** | **T** | **T** | **A** | **T** | **T** | **T** | **G** | **G** | **A** | **T** | **G** | **T** | **C** | **T** | **T** | **C** | **A** | **A** | **T** | **C** | **A** | **A** | **G** | **A** | **A** | **C** | **A** | **A** | **T** | **T** | **A** | **G** | **A** | **A** | **C** | **A** | **A** | **C** | **T** | **G** | **A** | **T** | **C** | **T** | **T** | **G** | **A** | **A** | **C** | **G** | **T** | **G** | **A** | **A** | **A** | **C** | **C** | **T** | **G** | **C** | **C** | **A** | **T** | **G** | **T** | **T** | **A** | **C** | **T** | **A** | **G** | **T** | **G** | **G** | **A** | **A** | **C** | **A** | **T** | **G** | **A** | **T** | **C** | **A** | **A** | **A** | **C** | **C** | **T** | **T** | **C** | **C** | **T** | **G** | **G** | **A** | **T** | **A** | **A** | **A** | **G** | **T** | **A** | **T** | **C** | **T** | **A** | **C** | **T** | **G** | **A** | **G** | **A** | **T** | **T** | **A** | **T** | **T** | **T** | **C** | **T** | **C** | **T** | **T** | **G** | **A** | **G** | **A** | **A** | **A** | **A** | **T** | **C** | **-** | **-** | **-** |
| **N59435F** | **A** | **T** | **G** | **T** | **C** | **T** | **A** | **A** | **A** | **A** | **T** | **C** | **G** | **A** | **A** | **A** | **T** | **A** | **A** | **A** | **A** | **A** | **A** | **T** | **C** | **T** | **G** | **A** | **C** | **A** | **T** | **T** | **C** | **G** | **G** | **C** | **T** | **A** | **C** | **G** | **A** | **C** | **A** | **G** | **C** | **C** | **A** | **A** | **G** | **G** | **C** | **A** | **C** | **A** | **T** | **T** | **A** | **T** | **T** | **A** | **T** | **T** | **T** | **G** | **A** | **A** | **C** | **A** | **A** | **G** | **C** | **A** | **A** | **A** | **T** | **C** | **T** | **A** | **A** | **A** | **T** | **T** | **T** | **T** | **G** | **A** | **C** | **A** | **C** | **A** | **C** | **A** | **A** | **T** | **G** | **G** | **A** | **A** | **A** | **C** | **T** | **A** | **G** | **G** | **A** | **C** | **T** | **T** | **A** | **T** | **C** | **G** | **G** | **A** | **C** | **G** | **A** | **A** | **A** | **C** | **G** | **G** | **T** | **C** | **G** | **A** | **G** | **G** | **A** | **A** | **A** | **G** | **A** | **C** | **A** | **A** | **C** | **T** | **T** | **T** | **A** | **C** | **T** | **G** | **A** | **A** | **T** | **A** | **T** | **T** | **C** | **T** | **A** | **C** | **A** | **A** | **A** | **A** | **C** | **A** | **A** | **A** | **C** | **T** | **A** | **C** | **C** | **T** | **T** | **A** | **T** | **C** | **A** | **A** | **G** | **G** | **G** | **C** | **A** | **A** | **G** | **T** | **A** | **A** | **T** | **C** | **C** | **A** | **T** | **C** | **A** | **G** | **C** | **A** | **A** | **G** | **A** | **A** | **T** | **T** | **T** | **G** | **T** | **C** | **T** | **A** | **T** | **T** | **T** | **C** | **C** | **C** | **G** | **C** | **A** | **A** | **C** | **A** | **G** | **A** | **C** | **A** | **A** | **A** | **A** | **G** | **A** | **T** | **A** | **A** | **A** | **G** | **A** | **A** | **C** | **G** | **T** | **T** | **T** | **A** | **A** | **C** | **C** | **T** | **A** | **T** | **T** | **A** | **C** | **G** | **T** | **G** | **T** | **T** | **A** | **A** | **A** | **T** | **G** | **A** | **T** | **A** | **T** | **T** | **A** | **C** | **G** | **G** | **A** | **T** | **T** | **T** | **T** | **G** | **A** | **G** | **A** | **T** | **A** | **T** | **G** | **G** | **G** | **A** | **A** | **A** | **T** | **C** | **G** | **A** | **A** | **A** | **G** | **A** | **G** | **A** | **G** | **C** | **T** | **C** | **C** | **A** | **A** | **T** | **T** | **G** | **A** | **T** | **G** | **C** | **A** | **A** | **A** | **C** | **A** | **G** | **A** | **T** | **C** | **C** | **T** | **G** | **A** | **A** | **A** | **T** | **C** | **T** | **T** | **A** | **T** | **G** | **G** | **A** | **G** | **A** | **G** | **A** | **A** | **T** | **T** | **C** | **A** | **G** | **C** | **A** | **C** | **A** | **C** | **T** | **A** | **T** | **C** | **G** | **G** | **G** | **G** | **G** | **G** | **A** | **G** | **A** | **G** | **A** | **A** | **G** | **A** | **C** | **A** | **A** | **A** | **A** | **G** | **T** | **C** | **C** | **T** | **A** | **C** | **T** | **G** | **G** | **C** | **A** | **C** | **T** | **T** | **T** | **T** | **A** | **T** | **T** | **T** | **G** | **T** | **G** | **G** | **A** | **T** | **G** | **A** | **C** | **A** | **C** | **T** | **C** | **A** | **T** | **T** | **T** | **C** | **C** | **C** | **G** | **T** | **T** | **A** | **A** | **T** | **C** | **G** | **A** | **T** | **G** | **A** | **A** | **C** | **C** | **A** | **A** | **C** | **G** | **A** | **A** | **T** | **C** | **A** | **T** | **T** | **T** | **G** | **G** | **A** | **-** | **-** | **-** | **T** | **A** | **T** | **C** | **T** | **C** | **T** | **G** | **G** | **T** | **A** | **G** | **A** | **A** | **A** | **A** | **C** | **A** | **A** | **G** | **T** | **A** | **G** | **C** | **G** | **G** | **C** | **T** | **T** | **A** | **T** | **T** | **T** | **G** | **A** | **A** | **A** | **A** | **A** | **G** | **A** | **A** | **A** | **A** | **A** | **A** | **C** | **A** | **A** | **G** | **G** | **C** | **T** | **T** | **C** | **A** | **T** | **C** | **G** | **T** | **G** | **G** | **T** | **C** | **A** | **G** | **C** | **C** | **A** | **T** | **G** | **A** | **C** | **C** | **G** | **G** | **G** | **G** | **A** | **T** | **T** | **T** | **A** | **T** | **C** | **G** | **A** | **T** | **G** | **A** | **A** | **G** | **T** | **A** | **G** | **T** | **G** | **G** | **A** | **C** | **C** | **A** | **T** | **G** | **T** | **T** | **T** | **T** | **A** | **G** | **C** | **A** | **A** | **T** | **C** | **G** | **A** | **A** | **A** | **A** | **A** | **A** | **G** | **T** | **C** | **A** | **A** | **C** | **T** | **G** | **G** | **A** | **A** | **C** | **T** | **T** | **T** | **A** | **T** | **C** | **A** | **A** | **G** | **G** | **G** | **A** | **A** | **T** | **T** | **T** | **C** | **T** | **C** | **T** | **A** | **T** | **C** | **T** | **A** | **T** | **G** | **A** | **A** | **G** | **A** | **A** | **C** | **A** | **G** | **A** | **A** | **A** | **A** | **A** | **A** | **C** | **T** | **T** | **C** | **G** | **T** | **G** | **A** | **T** | **G** | **A** | **A** | **T** | **T** | **T** | **G** | **A** | **A** | **A** | **T** | **G** | **G** | **C** | **T** | **C** | **A** | **A** | **A** | **A** | **T** | **G** | **A** | **A** | **A** | **A** | **A** | **T** | **T** | **G** | **A** | **A** | **A** | **A** | **A** | **A** | **G** | **A** | **A** | **G** | **T** | **C** | **A** | **G** | **T** | **A** | **G** | **G** | **C** | **T** | **A** | **A** | **A** | **G** | **A** | **A** | **A** | **A** | **C** | **A** | **G** | **C** | **A** | **G** | **C** | **T** | **G** | **A** | **A** | **A** | **A** | **A** | **G** | **C** | **C** | **G** | **A** | **A** | **T** | **G** | **G** | **T** | **C** | **T** | **C** | **G** | **T** | **T** | **C** | **C** | **C** | **G** | **A** | **G** | **A** | **A** | **G** | **G** | **A** | **G** | **A** | **T** | **A** | **A** | **A** | **A** | **C** | **A** | **A** | **A** | **G** | **A** | **A** | **A** | **C** | **A** | **A** | **G** | **T** | **C** | **G** | **G** | **A** | **T** | **T** | **C** | **A** | **T** | **C** | **G** | **A** | **T** | **A** | **C** | **T** | **G** | **A** | **A** | **T** | **C** | **T** | **A** | **G** | **A** | **C** | **G** | **A** | **G** | **T** | **G** | **A** | **A** | **T** | **A** | **A** | **A** | **G** | **G** | **A** | **G** | **C** | **A** | **G** | **T** | **G** | **G** | **G** | **T** | **G** | **C** | **T** | **G** | **A** | **T** | **G** | **C** | **T** | **G** | **C** | **A** | **C** | **G** | **G** | **A** | **C** | **G** | **A** | **T** | **G** | **A** | **A** | **A** | **C** | **G** | **A** | **T** | **C** | **C** | **A** | **A** | **A** | **G** | **C** | **A** | **A** | **T** | **C** | **G** | **T** | **G** | **A** | **A** | **T** | **C** | **G** | **G** | **A** | **T** | **G** | **G** | **A** | **G** | **A** | **C** | **C** | **C** | **A** | **G** | **A** | **T** | **C** | **A** | **G** | **C** | **G** | **A** | **G** | **A** | **A** | **A** | **G** | **A** | **A** | **A** | **A** | **A** | **C** | **T** | **A** | **T** | **T** | **A** | **A** | **A** | **A** | **G** | **A** | **T** | **A** | **T** | **C** | **G** | **A** | **A** | **T** | **A** | **T** | **A** | **T** | **C** | **G** | **A** | **T** | **T** | **C** | **T** | **T** | **T** | **G** | **A** | **C** | **G** | **A** | **T** | **G** | **A** | **A** | **T** | **A** | **G** | **C** | **C** | **A** | **A** | **G** | **C** | **G** | **T** | **C** | **T** | **C** | **A** | **C** | **C** | **A** | **T** | **A** | **A** | **G** | **C** | **G** | **A** | **C** | **T** | **T** | **T** | **T** | **A** | **A** | **G** | **C** | **G** | **T** | **A** | **G** | **A** | **A** | **G** | **A** | **T** | **C** | **T** | **T** | **C** | **A** | **A** | **T** | **T** | **A** | **G** | **G** | **G** | **T** | **A** | **T** | **G** | **A** | **A** | **A** | **A** | **T** | **C** | **T** | **G** | **T** | **T** | **A** | **T** | **T** | **C** | **G** | **A** | **G** | **C** | **C** | **A** | **A** | **T** | **T** | **C** | **A** | **T** | **T** | **T** | **T** | **A** | **C** | **A** | **A** | **T** | **C** | **G** | **A** | **G** | **C** | **C** | **T** | **C** | **A** | **T** | **C** | **A** | **G** | **C** | **G** | **G** | **G** | **T** | **G** | **G** | **C** | **G** | **A** | **T** | **T** | **T** | **C** | **A** | **G** | **G** | **T** | **C** | **C** | **T** | **A** | **A** | **C** | **G** | **G** | **T** | **G** | **C** | **A** | **G** | **G** | **A** | **A** | **A** | **G** | **T** | **C** | **A** | **T** | **C** | **C** | **A** | **T** | **T** | **A** | **T** | **C** | **C** | **A** | **T** | **T** | **A** | **T** | **C** | **T** | **T** | **C** | **T** | **G** | **G** | **G** | **G** | **G** | **C** | **A** | **T** | **T** | **C** | **A** | **A** | **C** | **G** | **G** | **C** | **A** | **A** | **G** | **G** | **T** | **T** | **A** | **T** | **A** | **G** | **G** | **A** | **G** | **A** | **A** | **A** | **A** | **A** | **T** | **C** | **C** | **C** | **A** | **G** | **C** | **C** | **A** | **A** | **A** | **A** | **C** | **A** | **T** | **C** | **T** | **G** | **A** | **G** | **C** | **A** | **T** | **T** | **A** | **G** | **T** | **T** | **A** | **T** | **G** | **T** | **A** | **C** | **G** | **C** | **C** | **A** | **A** | **A** | **A** | **T** | **T** | **A** | **T** | **G** | **A** | **A** | **G** | **A** | **C** | **A** | **A** | **T** | **C** | **G** | **A** | **G** | **G** | **A** | **A** | **C** | **G** | **T** | **T** | **G** | **G** | **C** | **G** | **G** | **A** | **A** | **T** | **T** | **T** | **G** | **C** | **A** | **G** | **A** | **G** | **A** | **A** | **A** | **A** | **A** | **C** | **C** | **A** | **A** | **G** | **T** | **A** | **G** | **A** | **C** | **T** | **A** | **C** | **C** | **A** | **A** | **G** | **C** | **A** | **T** | **T** | **T** | **T** | **T** | **G** | **A** | **A** | **C** | **A** | **A** | **C** | **C** | **T** | **C** | **C** | **G** | **A** | **A** | **A** | **G** | **C** | **T** | **T** | **G** | **G** | **G** | **A** | **T** | **G** | **G** | **A** | **A** | **A** | **G** | **A** | **G** | **A** | **T** | **G** | **T** | **T** | **T** | **T** | **T** | **C** | **A** | **T** | **A** | **A** | **C** | **A** | **A** | **G** | **A** | **T** | **C** | **G** | **A** | **G** | **C** | **A** | **G** | **A** | **T** | **G** | **A** | **G** | **T** | **A** | **T** | **G** | **G** | **G** | **C** | **C** | **A** | **A** | **C** | **G** | **G** | **A** | **A** | **A** | **A** | **A** | **A** | **G** | **T** | **G** | **G** | **A** | **A** | **T** | **T** | **G** | **G** | **C** | **T** | **A** | **A** | **A** | **T** | **C** | **T** | **T** | **T** | **A** | **T** | **C** | **A** | **C** | **A** | **G** | **C** | **C** | **A** | **G** | **C** | **T** | **G** | **A** | **A** | **C** | **T** | **A** | **T** | **A** | **T** | **A** | **T** | **A** | **T** | **G** | **G** | **G** | **A** | **T** | **G** | **A** | **A** | **C** | **C** | **A** | **T** | **T** | **G** | **A** | **A** | **T** | **T** | **A** | **T** | **T** | **T** | **G** | **G** | **A** | **T** | **G** | **T** | **C** | **T** | **T** | **C** | **A** | **A** | **T** | **C** | **A** | **A** | **G** | **A** | **A** | **C** | **A** | **A** | **T** | **T** | **A** | **G** | **A** | **A** | **C** | **A** | **A** | **C** | **T** | **G** | **A** | **T** | **C** | **T** | **T** | **G** | **A** | **A** | **C** | **G** | **T** | **G** | **A** | **A** | **A** | **C** | **C** | **T** | **G** | **C** | **C** | **A** | **T** | **G** | **T** | **T** | **A** | **C** | **T** | **A** | **G** | **C** | **G** | **G** | **A** | **A** | **C** | **A** | **T** | **G** | **A** | **T** | **C** | **A** | **A** | **A** | **C** | **C** | **T** | **T** | **C** | **C** | **T** | **G** | **G** | **A** | **T** | **A** | **A** | **A** | **G** | **T** | **A** | **T** | **C** | **T** | **A** | **C** | **T** | **G** | **A** | **G** | **A** | **T** | **T** | **A** | **T** | **T** | **T** | **C** | **T** | **C** | **T** | **T** | **G** | **A** | **G** | **A** | **A** | **A** | **A** | **T** | **C** | **-** | **-** | **-** |
| **N59567F** | **A** | **T** | **G** | **T** | **C** | **T** | **A** | **A** | **A** | **A** | **T** | **C** | **G** | **A** | **A** | **A** | **T** | **A** | **A** | **A** | **A** | **A** | **A** | **T** | **C** | **T** | **G** | **A** | **C** | **A** | **T** | **T** | **C** | **G** | **G** | **C** | **T** | **A** | **C** | **G** | **A** | **C** | **A** | **G** | **C** | **C** | **A** | **A** | **G** | **G** | **C** | **A** | **C** | **A** | **T** | **T** | **A** | **T** | **T** | **A** | **T** | **T** | **T** | **G** | **A** | **A** | **C** | **A** | **A** | **G** | **C** | **A** | **A** | **A** | **T** | **C** | **T** | **A** | **A** | **A** | **T** | **T** | **T** | **T** | **G** | **A** | **C** | **A** | **C** | **A** | **C** | **A** | **A** | **T** | **G** | **G** | **A** | **A** | **A** | **C** | **T** | **A** | **G** | **G** | **A** | **C** | **T** | **T** | **A** | **T** | **C** | **G** | **G** | **A** | **C** | **G** | **A** | **A** | **A** | **C** | **G** | **G** | **T** | **C** | **G** | **A** | **G** | **G** | **A** | **A** | **A** | **G** | **A** | **C** | **A** | **A** | **C** | **T** | **T** | **T** | **A** | **C** | **T** | **G** | **A** | **A** | **T** | **A** | **T** | **T** | **C** | **T** | **A** | **C** | **A** | **A** | **A** | **A** | **T** | **A** | **A** | **A** | **C** | **T** | **A** | **C** | **C** | **T** | **T** | **A** | **T** | **C** | **A** | **A** | **G** | **G** | **G** | **C** | **A** | **A** | **G** | **T** | **A** | **A** | **T** | **C** | **C** | **A** | **T** | **C** | **A** | **G** | **C** | **A** | **A** | **G** | **A** | **A** | **T** | **T** | **T** | **G** | **T** | **C** | **T** | **A** | **T** | **T** | **T** | **C** | **C** | **C** | **G** | **C** | **A** | **A** | **C** | **A** | **G** | **A** | **C** | **A** | **A** | **A** | **A** | **G** | **A** | **T** | **A** | **A** | **A** | **G** | **A** | **A** | **C** | **G** | **T** | **T** | **T** | **A** | **A** | **C** | **C** | **T** | **A** | **T** | **T** | **A** | **C** | **G** | **T** | **G** | **T** | **T** | **A** | **A** | **A** | **T** | **G** | **A** | **G** | **A** | **T** | **T** | **A** | **C** | **G** | **G** | **A** | **T** | **T** | **T** | **T** | **G** | **A** | **G** | **A** | **T** | **A** | **T** | **G** | **G** | **G** | **A** | **A** | **A** | **T** | **T** | **G** | **A** | **A** | **A** | **G** | **A** | **G** | **A** | **G** | **C** | **T** | **C** | **C** | **A** | **A** | **T** | **T** | **G** | **A** | **T** | **G** | **C** | **A** | **A** | **A** | **C** | **A** | **G** | **A** | **T** | **C** | **C** | **T** | **G** | **A** | **A** | **A** | **T** | **C** | **T** | **T** | **A** | **T** | **G** | **G** | **A** | **G** | **A** | **G** | **A** | **A** | **T** | **T** | **C** | **A** | **G** | **C** | **A** | **C** | **A** | **C** | **T** | **A** | **T** | **C** | **G** | **G** | **G** | **G** | **G** | **G** | **A** | **G** | **A** | **G** | **A** | **A** | **G** | **A** | **C** | **A** | **A** | **A** | **A** | **G** | **T** | **C** | **C** | **T** | **A** | **C** | **T** | **G** | **G** | **C** | **A** | **C** | **T** | **T** | **T** | **T** | **A** | **T** | **T** | **T** | **G** | **T** | **G** | **G** | **A** | **T** | **G** | **A** | **C** | **A** | **C** | **T** | **C** | **A** | **T** | **T** | **T** | **C** | **C** | **C** | **G** | **T** | **T** | **A** | **A** | **T** | **C** | **G** | **A** | **T** | **G** | **A** | **A** | **C** | **C** | **A** | **A** | **C** | **G** | **A** | **A** | **T** | **C** | **A** | **T** | **T** | **T** | **G** | **G** | **A** | **-** | **-** | **-** | **T** | **A** | **T** | **C** | **T** | **C** | **T** | **G** | **G** | **T** | **A** | **G** | **A** | **A** | **A** | **A** | **C** | **A** | **A** | **G** | **T** | **A** | **G** | **C** | **G** | **G** | **C** | **T** | **T** | **A** | **T** | **T** | **T** | **G** | **A** | **A** | **A** | **A** | **A** | **G** | **A** | **A** | **A** | **A** | **A** | **A** | **C** | **A** | **A** | **G** | **G** | **C** | **T** | **T** | **C** | **A** | **T** | **C** | **G** | **T** | **G** | **G** | **T** | **C** | **A** | **G** | **C** | **C** | **A** | **T** | **G** | **A** | **C** | **C** | **G** | **G** | **G** | **G** | **A** | **T** | **T** | **T** | **A** | **T** | **C** | **G** | **A** | **T** | **G** | **A** | **A** | **G** | **T** | **A** | **G** | **T** | **G** | **G** | **A** | **C** | **C** | **A** | **T** | **G** | **T** | **T** | **T** | **T** | **A** | **G** | **C** | **A** | **A** | **T** | **C** | **G** | **A** | **A** | **A** | **A** | **A** | **A** | **G** | **T** | **C** | **A** | **A** | **C** | **T** | **G** | **G** | **A** | **A** | **C** | **T** | **T** | **T** | **A** | **T** | **C** | **A** | **A** | **G** | **G** | **G** | **A** | **A** | **T** | **T** | **T** | **C** | **T** | **C** | **T** | **A** | **T** | **C** | **T** | **A** | **T** | **G** | **A** | **A** | **G** | **A** | **A** | **C** | **A** | **G** | **A** | **A** | **A** | **A** | **A** | **A** | **C** | **T** | **T** | **C** | **G** | **T** | **G** | **A** | **T** | **G** | **A** | **A** | **T** | **T** | **T** | **G** | **A** | **A** | **A** | **T** | **G** | **G** | **C** | **T** | **C** | **A** | **A** | **A** | **A** | **T** | **G** | **A** | **A** | **A** | **A** | **A** | **T** | **T** | **G** | **A** | **A** | **A** | **A** | **A** | **A** | **G** | **A** | **A** | **G** | **T** | **C** | **A** | **G** | **T** | **A** | **G** | **G** | **C** | **T** | **A** | **A** | **A** | **G** | **A** | **A** | **A** | **A** | **C** | **A** | **G** | **C** | **A** | **G** | **C** | **T** | **G** | **A** | **A** | **A** | **A** | **A** | **G** | **C** | **C** | **G** | **A** | **A** | **T** | **G** | **G** | **T** | **C** | **T** | **C** | **G** | **T** | **T** | **C** | **C** | **C** | **G** | **A** | **G** | **A** | **A** | **G** | **G** | **A** | **G** | **A** | **T** | **A** | **A** | **A** | **A** | **C** | **A** | **A** | **A** | **G** | **A** | **A** | **A** | **C** | **A** | **A** | **G** | **T** | **C** | **G** | **G** | **A** | **T** | **T** | **C** | **A** | **T** | **C** | **G** | **A** | **T** | **A** | **C** | **T** | **G** | **A** | **A** | **T** | **C** | **T** | **A** | **G** | **A** | **C** | **G** | **A** | **G** | **T** | **G** | **A** | **A** | **T** | **A** | **A** | **A** | **G** | **G** | **A** | **G** | **C** | **A** | **G** | **T** | **G** | **G** | **G** | **T** | **G** | **C** | **T** | **G** | **A** | **T** | **G** | **C** | **T** | **G** | **C** | **A** | **C** | **G** | **G** | **A** | **C** | **G** | **A** | **T** | **G** | **A** | **A** | **A** | **C** | **G** | **A** | **T** | **C** | **C** | **A** | **A** | **A** | **G** | **C** | **A** | **A** | **T** | **C** | **G** | **T** | **G** | **A** | **A** | **T** | **C** | **G** | **G** | **A** | **T** | **G** | **G** | **A** | **G** | **A** | **C** | **C** | **C** | **A** | **G** | **A** | **T** | **C** | **A** | **G** | **C** | **G** | **A** | **G** | **A** | **A** | **A** | **G** | **A** | **A** | **A** | **A** | **A** | **C** | **T** | **A** | **T** | **T** | **A** | **A** | **A** | **A** | **G** | **A** | **T** | **A** | **T** | **C** | **G** | **A** | **A** | **T** | **A** | **T** | **A** | **T** | **C** | **G** | **A** | **T** | **T** | **C** | **T** | **T** | **T** | **G** | **A** | **C** | **G** | **A** | **T** | **G** | **A** | **A** | **T** | **A** | **G** | **C** | **C** | **A** | **A** | **G** | **C** | **G** | **T** | **C** | **T** | **C** | **A** | **C** | **C** | **A** | **T** | **A** | **A** | **G** | **C** | **G** | **A** | **C** | **T** | **T** | **T** | **T** | **A** | **A** | **G** | **C** | **G** | **T** | **A** | **G** | **A** | **A** | **G** | **A** | **T** | **C** | **T** | **T** | **C** | **A** | **A** | **T** | **T** | **A** | **G** | **G** | **G** | **T** | **A** | **T** | **G** | **A** | **A** | **A** | **A** | **T** | **C** | **T** | **G** | **T** | **T** | **A** | **T** | **T** | **C** | **G** | **A** | **G** | **C** | **C** | **A** | **A** | **T** | **T** | **C** | **A** | **T** | **T** | **T** | **T** | **A** | **C** | **A** | **A** | **T** | **C** | **G** | **A** | **G** | **C** | **C** | **T** | **C** | **A** | **T** | **C** | **A** | **G** | **C** | **G** | **G** | **G** | **T** | **G** | **G** | **C** | **G** | **A** | **T** | **T** | **T** | **C** | **A** | **G** | **G** | **T** | **C** | **C** | **T** | **A** | **A** | **C** | **G** | **G** | **T** | **G** | **C** | **A** | **G** | **G** | **A** | **A** | **A** | **G** | **T** | **C** | **A** | **T** | **C** | **C** | **A** | **T** | **T** | **A** | **T** | **C** | **C** | **A** | **T** | **T** | **A** | **T** | **C** | **T** | **T** | **C** | **T** | **G** | **G** | **G** | **G** | **G** | **C** | **A** | **C** | **T** | **C** | **A** | **A** | **C** | **G** | **G** | **C** | **A** | **A** | **G** | **G** | **T** | **T** | **A** | **T** | **A** | **G** | **G** | **A** | **G** | **A** | **A** | **A** | **A** | **A** | **T** | **C** | **C** | **C** | **A** | **G** | **C** | **C** | **A** | **A** | **A** | **A** | **C** | **A** | **T** | **C** | **T** | **G** | **A** | **G** | **C** | **A** | **T** | **T** | **A** | **G** | **T** | **T** | **A** | **T** | **G** | **T** | **A** | **C** | **G** | **C** | **C** | **A** | **A** | **A** | **A** | **T** | **T** | **A** | **T** | **G** | **A** | **A** | **G** | **A** | **C** | **A** | **A** | **T** | **C** | **G** | **A** | **G** | **G** | **A** | **A** | **C** | **G** | **T** | **T** | **G** | **G** | **C** | **G** | **G** | **A** | **A** | **T** | **T** | **T** | **G** | **C** | **A** | **G** | **A** | **G** | **A** | **A** | **A** | **A** | **A** | **C** | **C** | **A** | **A** | **G** | **T** | **A** | **G** | **A** | **C** | **T** | **A** | **C** | **C** | **A** | **A** | **G** | **C** | **A** | **T** | **T** | **T** | **T** | **T** | **G** | **A** | **A** | **C** | **A** | **A** | **C** | **C** | **T** | **C** | **C** | **G** | **A** | **A** | **A** | **G** | **C** | **T** | **T** | **G** | **G** | **G** | **A** | **T** | **G** | **G** | **A** | **A** | **A** | **G** | **A** | **G** | **A** | **T** | **G** | **T** | **T** | **T** | **T** | **T** | **C** | **A** | **T** | **A** | **A** | **C** | **A** | **A** | **G** | **A** | **T** | **C** | **G** | **A** | **G** | **C** | **A** | **G** | **A** | **T** | **G** | **A** | **G** | **T** | **A** | **T** | **G** | **G** | **G** | **C** | **C** | **A** | **A** | **C** | **G** | **G** | **A** | **A** | **A** | **A** | **A** | **A** | **G** | **T** | **G** | **G** | **A** | **A** | **T** | **T** | **G** | **G** | **C** | **T** | **A** | **A** | **A** | **T** | **C** | **T** | **T** | **T** | **A** | **T** | **C** | **A** | **C** | **A** | **G** | **C** | **C** | **A** | **G** | **C** | **T** | **G** | **A** | **A** | **C** | **T** | **A** | **T** | **A** | **T** | **A** | **T** | **A** | **T** | **G** | **G** | **G** | **A** | **T** | **G** | **A** | **A** | **C** | **C** | **A** | **T** | **T** | **G** | **A** | **A** | **T** | **T** | **A** | **T** | **T** | **T** | **G** | **G** | **A** | **T** | **G** | **T** | **C** | **T** | **T** | **C** | **A** | **A** | **T** | **C** | **A** | **A** | **G** | **A** | **A** | **C** | **A** | **A** | **T** | **T** | **A** | **G** | **A** | **A** | **C** | **A** | **A** | **C** | **T** | **G** | **A** | **T** | **C** | **T** | **T** | **G** | **A** | **A** | **C** | **G** | **T** | **G** | **A** | **A** | **A** | **C** | **C** | **T** | **G** | **C** | **C** | **A** | **T** | **G** | **T** | **T** | **A** | **C** | **T** | **A** | **G** | **T** | **G** | **G** | **A** | **A** | **C** | **A** | **T** | **G** | **A** | **T** | **C** | **A** | **A** | **A** | **C** | **C** | **T** | **T** | **C** | **C** | **T** | **G** | **G** | **A** | **T** | **A** | **A** | **A** | **G** | **T** | **A** | **T** | **C** | **T** | **A** | **C** | **T** | **G** | **A** | **G** | **A** | **T** | **T** | **A** | **T** | **T** | **T** | **C** | **T** | **C** | **T** | **T** | **G** | **A** | **G** | **A** | **A** | **A** | **A** | **T** | **C** | **-** | **-** | **-** |
| **N53533** | **A** | **T** | **G** | **T** | **C** | **T** | **A** | **A** | **A** | **A** | **T** | **C** | **G** | **A** | **A** | **A** | **T** | **A** | **A** | **A** | **A** | **A** | **A** | **T** | **C** | **T** | **G** | **A** | **C** | **A** | **T** | **T** | **C** | **G** | **G** | **C** | **T** | **A** | **C** | **G** | **A** | **C** | **A** | **G** | **C** | **C** | **A** | **A** | **G** | **G** | **C** | **A** | **C** | **A** | **T** | **T** | **A** | **T** | **T** | **A** | **T** | **T** | **T** | **G** | **A** | **A** | **C** | **A** | **A** | **G** | **C** | **A** | **A** | **A** | **T** | **C** | **T** | **A** | **A** | **A** | **T** | **T** | **T** | **T** | **G** | **A** | **C** | **A** | **C** | **A** | **C** | **A** | **A** | **T** | **G** | **G** | **A** | **A** | **A** | **C** | **T** | **A** | **G** | **G** | **A** | **C** | **T** | **T** | **A** | **T** | **C** | **G** | **G** | **A** | **C** | **G** | **A** | **A** | **A** | **C** | **G** | **G** | **T** | **C** | **G** | **A** | **G** | **G** | **A** | **A** | **A** | **G** | **A** | **C** | **A** | **A** | **C** | **T** | **T** | **T** | **A** | **C** | **T** | **G** | **A** | **A** | **T** | **A** | **T** | **T** | **C** | **T** | **A** | **C** | **A** | **A** | **A** | **A** | **C** | **A** | **A** | **A** | **C** | **T** | **A** | **C** | **C** | **T** | **T** | **A** | **T** | **C** | **A** | **A** | **G** | **G** | **G** | **C** | **A** | **A** | **G** | **T** | **A** | **A** | **T** | **C** | **C** | **A** | **T** | **C** | **A** | **G** | **C** | **A** | **A** | **G** | **A** | **A** | **T** | **T** | **T** | **G** | **C** | **C** | **T** | **A** | **T** | **T** | **T** | **C** | **C** | **C** | **G** | **C** | **A** | **A** | **C** | **A** | **G** | **A** | **C** | **A** | **A** | **A** | **A** | **G** | **A** | **T** | **A** | **A** | **A** | **G** | **A** | **A** | **C** | **G** | **T** | **T** | **T** | **A** | **A** | **C** | **C** | **T** | **A** | **T** | **T** | **A** | **C** | **G** | **T** | **G** | **T** | **T** | **A** | **A** | **A** | **T** | **G** | **A** | **T** | **A** | **T** | **T** | **A** | **C** | **G** | **G** | **A** | **T** | **T** | **T** | **T** | **G** | **A** | **G** | **A** | **T** | **A** | **T** | **G** | **G** | **G** | **A** | **A** | **A** | **T** | **C** | **G** | **A** | **A** | **A** | **G** | **A** | **G** | **A** | **G** | **C** | **T** | **C** | **C** | **A** | **A** | **T** | **T** | **G** | **A** | **T** | **G** | **C** | **A** | **A** | **A** | **C** | **A** | **G** | **A** | **T** | **C** | **C** | **T** | **G** | **A** | **A** | **A** | **T** | **C** | **T** | **T** | **A** | **T** | **G** | **G** | **A** | **G** | **A** | **G** | **A** | **A** | **T** | **T** | **C** | **A** | **G** | **C** | **A** | **C** | **A** | **C** | **T** | **A** | **T** | **C** | **G** | **G** | **G** | **G** | **G** | **G** | **A** | **G** | **A** | **G** | **A** | **A** | **G** | **A** | **C** | **A** | **A** | **A** | **A** | **G** | **T** | **C** | **C** | **T** | **A** | **C** | **T** | **G** | **G** | **C** | **A** | **C** | **T** | **T** | **T** | **T** | **A** | **T** | **T** | **T** | **G** | **T** | **G** | **G** | **A** | **T** | **G** | **A** | **C** | **A** | **C** | **T** | **C** | **A** | **T** | **T** | **T** | **C** | **C** | **C** | **G** | **T** | **T** | **A** | **A** | **T** | **C** | **G** | **A** | **T** | **G** | **A** | **A** | **C** | **C** | **A** | **A** | **C** | **G** | **A** | **A** | **T** | **C** | **A** | **T** | **T** | **T** | **G** | **G** | **A** | **-** | **-** | **-** | **T** | **A** | **T** | **C** | **T** | **C** | **T** | **G** | **G** | **T** | **A** | **G** | **A** | **A** | **A** | **A** | **C** | **A** | **A** | **G** | **T** | **A** | **G** | **C** | **G** | **G** | **C** | **T** | **T** | **A** | **T** | **T** | **T** | **G** | **A** | **A** | **A** | **A** | **A** | **G** | **A** | **A** | **A** | **A** | **A** | **A** | **C** | **A** | **A** | **G** | **G** | **C** | **T** | **T** | **C** | **A** | **T** | **C** | **G** | **T** | **G** | **G** | **T** | **C** | **A** | **G** | **C** | **C** | **A** | **T** | **G** | **A** | **C** | **C** | **G** | **G** | **G** | **G** | **A** | **T** | **T** | **T** | **A** | **T** | **C** | **G** | **A** | **T** | **G** | **A** | **A** | **G** | **T** | **A** | **G** | **T** | **G** | **G** | **A** | **C** | **C** | **A** | **T** | **G** | **T** | **T** | **T** | **T** | **A** | **G** | **C** | **A** | **A** | **T** | **C** | **G** | **A** | **A** | **A** | **A** | **A** | **A** | **G** | **T** | **C** | **A** | **A** | **C** | **T** | **G** | **G** | **A** | **A** | **C** | **T** | **T** | **T** | **A** | **T** | **C** | **A** | **A** | **G** | **G** | **G** | **A** | **A** | **T** | **T** | **T** | **C** | **T** | **C** | **T** | **A** | **T** | **C** | **T** | **A** | **T** | **G** | **A** | **A** | **G** | **A** | **A** | **C** | **A** | **G** | **A** | **A** | **A** | **A** | **A** | **A** | **C** | **T** | **T** | **C** | **G** | **T** | **G** | **A** | **T** | **G** | **A** | **A** | **T** | **T** | **T** | **G** | **A** | **A** | **A** | **T** | **G** | **G** | **C** | **T** | **C** | **A** | **A** | **A** | **A** | **T** | **G** | **A** | **A** | **A** | **A** | **A** | **T** | **T** | **G** | **A** | **A** | **A** | **A** | **A** | **A** | **G** | **A** | **A** | **G** | **T** | **C** | **A** | **G** | **T** | **A** | **G** | **G** | **C** | **T** | **A** | **A** | **A** | **G** | **A** | **A** | **A** | **A** | **C** | **A** | **G** | **C** | **A** | **G** | **C** | **T** | **G** | **A** | **A** | **A** | **A** | **A** | **G** | **C** | **C** | **G** | **A** | **A** | **T** | **G** | **G** | **T** | **C** | **T** | **C** | **G** | **T** | **T** | **C** | **C** | **C** | **G** | **A** | **G** | **A** | **A** | **G** | **G** | **A** | **G** | **A** | **T** | **A** | **A** | **A** | **A** | **C** | **A** | **A** | **A** | **G** | **A** | **A** | **A** | **C** | **A** | **A** | **G** | **T** | **C** | **G** | **G** | **A** | **T** | **T** | **C** | **A** | **T** | **C** | **G** | **A** | **T** | **A** | **C** | **T** | **G** | **A** | **A** | **T** | **C** | **T** | **A** | **G** | **A** | **C** | **G** | **A** | **G** | **T** | **G** | **A** | **A** | **T** | **A** | **A** | **A** | **G** | **G** | **A** | **G** | **C** | **A** | **G** | **T** | **G** | **G** | **G** | **T** | **G** | **C** | **T** | **G** | **A** | **T** | **G** | **C** | **T** | **G** | **C** | **A** | **C** | **G** | **G** | **A** | **C** | **G** | **A** | **T** | **G** | **A** | **A** | **A** | **C** | **G** | **A** | **T** | **C** | **C** | **A** | **A** | **A** | **G** | **C** | **A** | **A** | **T** | **C** | **G** | **T** | **G** | **A** | **A** | **T** | **C** | **G** | **G** | **A** | **T** | **G** | **G** | **A** | **G** | **A** | **C** | **C** | **C** | **A** | **G** | **A** | **T** | **C** | **A** | **G** | **C** | **G** | **A** | **G** | **A** | **A** | **A** | **G** | **A** | **A** | **A** | **A** | **A** | **C** | **T** | **A** | **T** | **T** | **A** | **A** | **A** | **A** | **G** | **A** | **T** | **A** | **T** | **C** | **G** | **A** | **A** | **T** | **A** | **T** | **A** | **T** | **C** | **G** | **A** | **T** | **T** | **C** | **T** | **T** | **T** | **G** | **A** | **C** | **G** | **A** | **T** | **G** | **A** | **A** | **T** | **A** | **G** | **C** | **C** | **A** | **A** | **G** | **C** | **G** | **T** | **C** | **T** | **C** | **A** | **C** | **C** | **A** | **T** | **A** | **A** | **G** | **C** | **G** | **A** | **C** | **T** | **T** | **T** | **T** | **A** | **A** | **G** | **C** | **G** | **T** | **A** | **G** | **A** | **A** | **G** | **A** | **T** | **C** | **T** | **T** | **C** | **A** | **A** | **T** | **T** | **A** | **G** | **G** | **G** | **T** | **A** | **T** | **G** | **A** | **A** | **A** | **A** | **T** | **C** | **T** | **G** | **T** | **T** | **A** | **T** | **T** | **C** | **G** | **A** | **G** | **C** | **C** | **A** | **A** | **T** | **T** | **C** | **A** | **T** | **T** | **T** | **T** | **A** | **C** | **A** | **A** | **T** | **C** | **G** | **A** | **G** | **C** | **C** | **T** | **C** | **A** | **T** | **C** | **A** | **G** | **C** | **G** | **G** | **G** | **T** | **G** | **G** | **C** | **G** | **A** | **T** | **T** | **T** | **C** | **A** | **G** | **G** | **T** | **C** | **C** | **T** | **A** | **A** | **C** | **G** | **G** | **T** | **G** | **C** | **A** | **G** | **G** | **A** | **A** | **A** | **G** | **T** | **C** | **A** | **T** | **C** | **C** | **A** | **T** | **T** | **A** | **T** | **C** | **C** | **A** | **T** | **T** | **A** | **T** | **C** | **T** | **T** | **C** | **T** | **G** | **G** | **G** | **G** | **G** | **C** | **A** | **T** | **T** | **C** | **A** | **A** | **C** | **G** | **G** | **C** | **A** | **A** | **G** | **G** | **T** | **T** | **A** | **T** | **A** | **G** | **G** | **A** | **G** | **A** | **A** | **A** | **A** | **A** | **T** | **A** | **C** | **C** | **A** | **G** | **C** | **C** | **A** | **A** | **A** | **A** | **C** | **A** | **T** | **C** | **T** | **G** | **A** | **G** | **C** | **A** | **T** | **T** | **A** | **G** | **T** | **T** | **A** | **T** | **G** | **C** | **A** | **A** | **G** | **C** | **C** | **A** | **A** | **A** | **A** | **T** | **T** | **A** | **T** | **G** | **A** | **A** | **G** | **A** | **C** | **A** | **A** | **T** | **C** | **G** | **A** | **G** | **G** | **A** | **A** | **C** | **G** | **T** | **T** | **G** | **G** | **C** | **G** | **G** | **A** | **A** | **T** | **T** | **T** | **G** | **C** | **A** | **G** | **A** | **G** | **A** | **A** | **A** | **A** | **A** | **C** | **C** | **A** | **A** | **G** | **T** | **A** | **G** | **A** | **C** | **T** | **A** | **C** | **C** | **A** | **A** | **G** | **C** | **A** | **T** | **T** | **T** | **T** | **T** | **G** | **A** | **A** | **C** | **A** | **A** | **C** | **C** | **T** | **C** | **C** | **G** | **A** | **A** | **A** | **G** | **C** | **T** | **T** | **G** | **G** | **G** | **A** | **T** | **G** | **G** | **A** | **A** | **A** | **G** | **A** | **G** | **A** | **T** | **G** | **T** | **T** | **T** | **T** | **T** | **C** | **A** | **T** | **A** | **A** | **C** | **A** | **A** | **G** | **A** | **T** | **C** | **G** | **A** | **G** | **C** | **A** | **G** | **A** | **T** | **G** | **A** | **G** | **T** | **A** | **T** | **A** | **G** | **G** | **C** | **C** | **A** | **A** | **C** | **G** | **G** | **A** | **A** | **A** | **A** | **A** | **A** | **G** | **T** | **G** | **G** | **A** | **A** | **T** | **T** | **G** | **G** | **C** | **T** | **A** | **A** | **A** | **T** | **C** | **T** | **T** | **T** | **A** | **T** | **C** | **A** | **C** | **A** | **G** | **C** | **C** | **A** | **G** | **C** | **T** | **G** | **A** | **A** | **C** | **T** | **A** | **T** | **A** | **T** | **A** | **C** | **A** | **T** | **G** | **G** | **G** | **A** | **T** | **G** | **A** | **A** | **C** | **C** | **A** | **T** | **T** | **G** | **A** | **A** | **T** | **T** | **A** | **T** | **T** | **T** | **G** | **G** | **A** | **T** | **G** | **T** | **C** | **T** | **T** | **C** | **A** | **A** | **T** | **C** | **A** | **A** | **G** | **A** | **A** | **C** | **A** | **A** | **T** | **T** | **A** | **G** | **A** | **A** | **C** | **A** | **A** | **C** | **T** | **G** | **A** | **T** | **C** | **T** | **T** | **G** | **A** | **A** | **C** | **G** | **T** | **G** | **A** | **A** | **A** | **C** | **C** | **T** | **G** | **C** | **C** | **A** | **T** | **G** | **T** | **T** | **A** | **C** | **T** | **A** | **G** | **T** | **G** | **G** | **A** | **A** | **C** | **A** | **T** | **G** | **A** | **T** | **C** | **A** | **A** | **A** | **C** | **C** | **T** | **T** | **C** | **C** | **T** | **G** | **G** | **A** | **T** | **A** | **A** | **A** | **G** | **T** | **A** | **T** | **C** | **T** | **A** | **C** | **T** | **G** | **A** | **G** | **A** | **T** | **T** | **A** | **T** | **T** | **T** | **C** | **T** | **C** | **T** | **T** | **G** | **A** | **G** | **A** | **G** | **A** | **A** | **T** | **C** | **-** | **-** | **-** |
| **N54671** | **A** | **T** | **G** | **T** | **C** | **T** | **A** | **A** | **A** | **A** | **T** | **C** | **G** | **A** | **A** | **A** | **T** | **A** | **A** | **A** | **A** | **A** | **A** | **T** | **C** | **T** | **G** | **A** | **C** | **A** | **T** | **T** | **C** | **G** | **G** | **C** | **T** | **A** | **C** | **G** | **A** | **C** | **A** | **G** | **C** | **C** | **A** | **A** | **G** | **G** | **C** | **A** | **C** | **A** | **T** | **T** | **A** | **T** | **T** | **A** | **T** | **T** | **T** | **G** | **A** | **A** | **C** | **A** | **A** | **G** | **C** | **A** | **A** | **A** | **T** | **C** | **T** | **A** | **A** | **A** | **T** | **T** | **T** | **T** | **G** | **A** | **C** | **A** | **C** | **A** | **C** | **A** | **A** | **T** | **G** | **G** | **A** | **A** | **A** | **C** | **T** | **A** | **G** | **G** | **A** | **C** | **T** | **T** | **A** | **T** | **C** | **G** | **G** | **A** | **C** | **G** | **A** | **A** | **A** | **C** | **G** | **G** | **T** | **C** | **G** | **A** | **G** | **G** | **A** | **A** | **A** | **G** | **A** | **C** | **A** | **A** | **C** | **T** | **T** | **T** | **A** | **C** | **T** | **G** | **A** | **A** | **T** | **A** | **T** | **T** | **C** | **T** | **A** | **C** | **A** | **A** | **A** | **A** | **C** | **A** | **A** | **A** | **C** | **T** | **A** | **C** | **C** | **T** | **T** | **A** | **T** | **C** | **A** | **A** | **G** | **G** | **G** | **C** | **A** | **A** | **G** | **T** | **A** | **A** | **T** | **C** | **C** | **A** | **T** | **C** | **A** | **G** | **C** | **A** | **A** | **G** | **A** | **A** | **T** | **T** | **T** | **G** | **T** | **C** | **T** | **A** | **T** | **T** | **T** | **C** | **C** | **C** | **G** | **C** | **A** | **G** | **C** | **A** | **A** | **A** | **C** | **A** | **A** | **A** | **A** | **G** | **A** | **T** | **A** | **A** | **A** | **G** | **A** | **G** | **C** | **G** | **T** | **T** | **T** | **A** | **A** | **C** | **C** | **T** | **A** | **T** | **T** | **A** | **T** | **G** | **T** | **G** | **T** | **T** | **A** | **A** | **A** | **T** | **G** | **A** | **G** | **A** | **T** | **T** | **A** | **C** | **G** | **G** | **A** | **T** | **T** | **T** | **T** | **G** | **A** | **G** | **A** | **T** | **A** | **T** | **G** | **G** | **G** | **A** | **A** | **A** | **T** | **T** | **G** | **A** | **A** | **A** | **G** | **A** | **G** | **A** | **G** | **C** | **T** | **C** | **C** | **A** | **A** | **C** | **T** | **G** | **A** | **T** | **G** | **C** | **A** | **A** | **A** | **C** | **A** | **G** | **A** | **T** | **C** | **C** | **T** | **G** | **A** | **A** | **A** | **T** | **C** | **T** | **T** | **A** | **T** | **G** | **G** | **A** | **G** | **A** | **G** | **A** | **A** | **T** | **T** | **C** | **A** | **G** | **C** | **A** | **C** | **A** | **C** | **T** | **A** | **T** | **C** | **A** | **G** | **G** | **A** | **G** | **G** | **A** | **G** | **A** | **G** | **A** | **A** | **G** | **A** | **C** | **A** | **A** | **A** | **A** | **G** | **T** | **C** | **C** | **T** | **A** | **C** | **T** | **G** | **G** | **C** | **A** | **C** | **T** | **T** | **T** | **T** | **A** | **T** | **T** | **T** | **G** | **T** | **G** | **G** | **A** | **T** | **G** | **A** | **C** | **A** | **C** | **T** | **C** | **A** | **T** | **T** | **T** | **C** | **C** | **C** | **G** | **T** | **T** | **A** | **A** | **T** | **C** | **G** | **A** | **T** | **G** | **A** | **A** | **C** | **C** | **A** | **A** | **C** | **G** | **A** | **A** | **T** | **C** | **A** | **T** | **T** | **T** | **G** | **G** | **A** | **-** | **-** | **-** | **T** | **A** | **T** | **C** | **T** | **C** | **T** | **G** | **G** | **C** | **A** | **G** | **A** | **A** | **A** | **A** | **C** | **A** | **A** | **G** | **T** | **A** | **G** | **C** | **G** | **G** | **C** | **T** | **T** | **A** | **C** | **T** | **T** | **G** | **A** | **A** | **A** | **A** | **A** | **G** | **A** | **A** | **A** | **A** | **A** | **A** | **C** | **A** | **A** | **G** | **G** | **C** | **T** | **T** | **C** | **A** | **T** | **C** | **G** | **T** | **G** | **G** | **T** | **C** | **A** | **G** | **C** | **C** | **A** | **T** | **G** | **A** | **C** | **C** | **G** | **G** | **G** | **G** | **A** | **T** | **T** | **T** | **A** | **T** | **C** | **G** | **A** | **T** | **G** | **A** | **A** | **G** | **T** | **A** | **G** | **T** | **G** | **G** | **A** | **C** | **C** | **A** | **T** | **G** | **T** | **T** | **T** | **T** | **A** | **G** | **C** | **A** | **A** | **T** | **C** | **G** | **A** | **A** | **A** | **A** | **A** | **A** | **G** | **T** | **C** | **A** | **A** | **C** | **T** | **G** | **G** | **A** | **A** | **C** | **T** | **T** | **T** | **A** | **T** | **C** | **A** | **A** | **G** | **G** | **G** | **A** | **A** | **T** | **T** | **T** | **C** | **T** | **C** | **T** | **A** | **T** | **C** | **T** | **A** | **T** | **G** | **A** | **A** | **G** | **A** | **A** | **C** | **A** | **G** | **A** | **A** | **A** | **A** | **A** | **A** | **C** | **T** | **T** | **C** | **G** | **T** | **G** | **A** | **T** | **G** | **A** | **A** | **T** | **T** | **T** | **G** | **A** | **A** | **A** | **T** | **G** | **G** | **C** | **T** | **C** | **A** | **A** | **A** | **A** | **T** | **G** | **A** | **A** | **A** | **A** | **A** | **T** | **T** | **G** | **A** | **A** | **A** | **A** | **A** | **A** | **G** | **A** | **A** | **G** | **T** | **C** | **A** | **G** | **T** | **A** | **G** | **G** | **C** | **T** | **A** | **A** | **A** | **G** | **A** | **A** | **A** | **A** | **C** | **A** | **G** | **C** | **A** | **G** | **C** | **T** | **G** | **A** | **A** | **A** | **A** | **A** | **G** | **C** | **C** | **G** | **A** | **A** | **T** | **G** | **G** | **T** | **C** | **T** | **C** | **G** | **T** | **T** | **C** | **C** | **C** | **G** | **A** | **G** | **A** | **A** | **G** | **G** | **A** | **G** | **A** | **T** | **A** | **A** | **A** | **A** | **C** | **A** | **A** | **A** | **G** | **A** | **A** | **A** | **C** | **A** | **A** | **G** | **T** | **C** | **G** | **G** | **A** | **T** | **T** | **C** | **A** | **T** | **C** | **G** | **A** | **T** | **A** | **C** | **T** | **G** | **A** | **A** | **T** | **C** | **T** | **A** | **G** | **A** | **C** | **G** | **A** | **G** | **T** | **G** | **A** | **A** | **T** | **A** | **A** | **A** | **G** | **G** | **A** | **G** | **C** | **A** | **G** | **T** | **G** | **G** | **G** | **T** | **G** | **C** | **T** | **G** | **A** | **T** | **G** | **C** | **T** | **G** | **C** | **A** | **C** | **G** | **G** | **A** | **C** | **G** | **A** | **T** | **G** | **A** | **A** | **A** | **C** | **G** | **A** | **T** | **C** | **C** | **A** | **A** | **A** | **G** | **C** | **A** | **A** | **T** | **C** | **G** | **T** | **G** | **A** | **A** | **T** | **C** | **G** | **G** | **A** | **T** | **G** | **G** | **A** | **G** | **A** | **C** | **C** | **C** | **A** | **G** | **A** | **T** | **C** | **A** | **G** | **C** | **G** | **A** | **G** | **A** | **A** | **A** | **G** | **A** | **A** | **A** | **A** | **A** | **C** | **T** | **A** | **T** | **T** | **G** | **A** | **A** | **A** | **G** | **A** | **T** | **A** | **T** | **C** | **G** | **A** | **A** | **T** | **A** | **T** | **A** | **T** | **C** | **G** | **A** | **T** | **T** | **C** | **T** | **T** | **T** | **G** | **A** | **C** | **G** | **A** | **T** | **G** | **A** | **A** | **T** | **A** | **G** | **C** | **C** | **A** | **A** | **G** | **C** | **G** | **T** | **C** | **T** | **C** | **A** | **C** | **C** | **A** | **T** | **A** | **A** | **G** | **C** | **G** | **A** | **C** | **T** | **T** | **T** | **T** | **A** | **A** | **G** | **C** | **G** | **T** | **A** | **G** | **A** | **A** | **G** | **A** | **T** | **C** | **T** | **T** | **C** | **A** | **A** | **T** | **T** | **A** | **G** | **G** | **G** | **T** | **A** | **T** | **G** | **A** | **G** | **A** | **A** | **T** | **C** | **T** | **G** | **T** | **T** | **A** | **T** | **T** | **C** | **G** | **A** | **G** | **C** | **C** | **A** | **A** | **T** | **T** | **C** | **A** | **T** | **T** | **T** | **T** | **A** | **C** | **A** | **A** | **T** | **C** | **G** | **A** | **G** | **C** | **C** | **T** | **C** | **A** | **T** | **C** | **A** | **G** | **C** | **G** | **G** | **G** | **T** | **G** | **G** | **C** | **G** | **A** | **T** | **T** | **T** | **C** | **A** | **G** | **G** | **T** | **C** | **C** | **T** | **A** | **A** | **C** | **G** | **G** | **T** | **G** | **C** | **A** | **G** | **G** | **A** | **A** | **A** | **G** | **T** | **C** | **A** | **T** | **C** | **C** | **A** | **T** | **T** | **A** | **T** | **C** | **C** | **A** | **T** | **T** | **A** | **T** | **C** | **T** | **T** | **C** | **T** | **G** | **G** | **G** | **G** | **G** | **C** | **A** | **T** | **T** | **C** | **A** | **A** | **C** | **G** | **G** | **C** | **A** | **A** | **G** | **G** | **T** | **T** | **A** | **T** | **A** | **G** | **G** | **A** | **G** | **A** | **A** | **A** | **A** | **A** | **T** | **C** | **C** | **C** | **A** | **G** | **C** | **C** | **A** | **A** | **A** | **A** | **C** | **A** | **T** | **C** | **T** | **G** | **A** | **G** | **C** | **A** | **T** | **T** | **A** | **G** | **T** | **T** | **A** | **T** | **G** | **T** | **A** | **C** | **G** | **C** | **C** | **A** | **A** | **A** | **A** | **T** | **T** | **A** | **T** | **G** | **A** | **A** | **G** | **A** | **C** | **A** | **A** | **T** | **C** | **G** | **A** | **G** | **G** | **A** | **A** | **C** | **G** | **T** | **T** | **G** | **G** | **C** | **G** | **G** | **A** | **A** | **T** | **T** | **T** | **G** | **C** | **A** | **G** | **A** | **G** | **A** | **A** | **A** | **A** | **A** | **C** | **C** | **A** | **A** | **G** | **T** | **A** | **G** | **A** | **C** | **T** | **A** | **C** | **C** | **A** | **A** | **G** | **C** | **A** | **T** | **T** | **T** | **T** | **T** | **G** | **A** | **A** | **C** | **A** | **A** | **C** | **C** | **T** | **C** | **C** | **G** | **A** | **A** | **A** | **G** | **C** | **T** | **T** | **G** | **G** | **G** | **A** | **T** | **G** | **G** | **A** | **A** | **A** | **G** | **A** | **G** | **A** | **T** | **G** | **T** | **T** | **T** | **T** | **T** | **C** | **A** | **T** | **A** | **A** | **C** | **A** | **A** | **G** | **A** | **T** | **C** | **G** | **A** | **G** | **C** | **A** | **G** | **A** | **T** | **G** | **A** | **G** | **T** | **A** | **T** | **G** | **G** | **G** | **C** | **C** | **A** | **A** | **C** | **G** | **G** | **A** | **A** | **A** | **A** | **A** | **A** | **G** | **T** | **G** | **G** | **A** | **A** | **T** | **T** | **G** | **G** | **C** | **T** | **A** | **A** | **A** | **T** | **C** | **T** | **T** | **T** | **A** | **T** | **C** | **A** | **C** | **A** | **G** | **C** | **C** | **A** | **G** | **C** | **T** | **G** | **A** | **A** | **C** | **T** | **A** | **T** | **A** | **T** | **A** | **T** | **A** | **T** | **G** | **G** | **G** | **A** | **T** | **G** | **A** | **A** | **C** | **C** | **A** | **T** | **T** | **G** | **A** | **A** | **T** | **T** | **A** | **T** | **T** | **T** | **G** | **G** | **A** | **T** | **G** | **T** | **C** | **T** | **T** | **C** | **A** | **A** | **T** | **C** | **A** | **A** | **G** | **A** | **A** | **C** | **A** | **A** | **T** | **T** | **A** | **G** | **A** | **A** | **C** | **A** | **A** | **C** | **T** | **G** | **A** | **T** | **C** | **T** | **T** | **G** | **A** | **A** | **C** | **G** | **T** | **G** | **A** | **A** | **A** | **C** | **C** | **T** | **G** | **C** | **C** | **A** | **T** | **G** | **T** | **T** | **A** | **C** | **T** | **A** | **G** | **T** | **G** | **G** | **A** | **A** | **C** | **A** | **T** | **G** | **A** | **T** | **C** | **A** | **A** | **A** | **C** | **C** | **T** | **T** | **C** | **C** | **T** | **G** | **G** | **A** | **T** | **A** | **A** | **A** | **G** | **T** | **A** | **T** | **C** | **T** | **A** | **C** | **T** | **G** | **A** | **G** | **A** | **T** | **T** | **A** | **T** | **T** | **T** | **C** | **T** | **C** | **T** | **T** | **G** | **A** | **G** | **A** | **A** | **A** | **A** | **T** | **C** | **-** | **-** | **-** |
| **N60252F** | **A** | **T** | **G** | **T** | **C** | **T** | **A** | **A** | **A** | **A** | **T** | **C** | **G** | **A** | **A** | **A** | **T** | **A** | **A** | **A** | **A** | **A** | **A** | **T** | **C** | **T** | **G** | **A** | **C** | **A** | **T** | **T** | **C** | **G** | **G** | **C** | **T** | **A** | **C** | **G** | **A** | **C** | **A** | **G** | **C** | **C** | **A** | **A** | **G** | **G** | **C** | **A** | **C** | **A** | **T** | **T** | **A** | **T** | **T** | **A** | **T** | **T** | **T** | **G** | **A** | **A** | **C** | **A** | **A** | **G** | **C** | **A** | **A** | **A** | **T** | **C** | **T** | **A** | **A** | **A** | **T** | **T** | **T** | **T** | **G** | **A** | **C** | **A** | **C** | **A** | **C** | **A** | **A** | **T** | **G** | **G** | **A** | **A** | **A** | **C** | **T** | **A** | **G** | **G** | **A** | **C** | **T** | **T** | **A** | **T** | **C** | **G** | **G** | **A** | **C** | **G** | **A** | **A** | **A** | **C** | **G** | **G** | **T** | **C** | **G** | **A** | **G** | **G** | **A** | **A** | **A** | **G** | **A** | **C** | **A** | **A** | **C** | **T** | **T** | **T** | **A** | **C** | **T** | **G** | **A** | **A** | **T** | **A** | **T** | **T** | **C** | **T** | **A** | **C** | **A** | **A** | **A** | **A** | **C** | **A** | **A** | **A** | **C** | **T** | **A** | **C** | **C** | **T** | **T** | **A** | **T** | **C** | **A** | **A** | **G** | **G** | **G** | **C** | **A** | **A** | **G** | **T** | **A** | **A** | **T** | **C** | **C** | **A** | **T** | **C** | **A** | **G** | **C** | **A** | **A** | **G** | **A** | **A** | **T** | **T** | **T** | **G** | **C** | **C** | **T** | **A** | **T** | **T** | **T** | **C** | **C** | **C** | **G** | **C** | **A** | **A** | **C** | **A** | **G** | **A** | **C** | **A** | **A** | **A** | **A** | **G** | **A** | **T** | **A** | **A** | **A** | **G** | **A** | **A** | **C** | **G** | **T** | **T** | **T** | **A** | **A** | **C** | **C** | **T** | **A** | **T** | **T** | **A** | **C** | **G** | **T** | **G** | **T** | **T** | **A** | **A** | **A** | **T** | **G** | **A** | **T** | **A** | **T** | **T** | **A** | **C** | **G** | **G** | **A** | **T** | **T** | **T** | **T** | **G** | **A** | **G** | **A** | **T** | **A** | **T** | **G** | **G** | **G** | **A** | **A** | **A** | **T** | **C** | **G** | **A** | **A** | **A** | **G** | **A** | **G** | **A** | **G** | **C** | **T** | **C** | **C** | **A** | **A** | **T** | **T** | **G** | **A** | **T** | **G** | **C** | **A** | **A** | **A** | **C** | **A** | **G** | **A** | **T** | **C** | **C** | **T** | **G** | **A** | **A** | **A** | **T** | **C** | **T** | **T** | **A** | **T** | **G** | **G** | **A** | **G** | **A** | **G** | **A** | **A** | **T** | **T** | **C** | **A** | **G** | **C** | **A** | **C** | **A** | **C** | **T** | **A** | **T** | **C** | **G** | **G** | **G** | **G** | **G** | **G** | **A** | **G** | **A** | **G** | **A** | **A** | **G** | **A** | **C** | **A** | **A** | **A** | **A** | **G** | **T** | **C** | **C** | **T** | **A** | **C** | **T** | **G** | **G** | **C** | **A** | **C** | **T** | **T** | **T** | **T** | **A** | **T** | **T** | **T** | **G** | **T** | **G** | **G** | **A** | **T** | **G** | **A** | **C** | **A** | **C** | **T** | **C** | **A** | **T** | **T** | **T** | **C** | **C** | **C** | **G** | **T** | **T** | **A** | **A** | **T** | **C** | **G** | **A** | **T** | **G** | **A** | **A** | **C** | **C** | **A** | **A** | **C** | **G** | **A** | **A** | **T** | **C** | **A** | **T** | **T** | **T** | **G** | **G** | **A** | **-** | **-** | **-** | **T** | **A** | **T** | **C** | **T** | **C** | **T** | **G** | **G** | **T** | **A** | **G** | **A** | **A** | **A** | **A** | **C** | **A** | **A** | **G** | **T** | **A** | **G** | **C** | **G** | **G** | **C** | **T** | **T** | **A** | **T** | **T** | **T** | **G** | **A** | **A** | **A** | **A** | **A** | **G** | **A** | **A** | **A** | **A** | **A** | **A** | **C** | **A** | **A** | **G** | **G** | **C** | **T** | **T** | **C** | **A** | **T** | **C** | **G** | **T** | **G** | **G** | **T** | **C** | **A** | **G** | **C** | **C** | **A** | **T** | **G** | **A** | **C** | **C** | **G** | **G** | **G** | **G** | **A** | **T** | **T** | **T** | **A** | **T** | **C** | **G** | **A** | **T** | **G** | **A** | **A** | **G** | **T** | **A** | **G** | **T** | **G** | **G** | **A** | **C** | **C** | **A** | **T** | **G** | **T** | **T** | **T** | **T** | **A** | **G** | **C** | **A** | **A** | **T** | **C** | **G** | **A** | **A** | **A** | **A** | **A** | **A** | **G** | **T** | **C** | **A** | **A** | **C** | **T** | **G** | **G** | **A** | **A** | **C** | **T** | **T** | **T** | **A** | **T** | **C** | **A** | **A** | **G** | **G** | **G** | **A** | **A** | **T** | **T** | **T** | **C** | **T** | **C** | **T** | **A** | **T** | **C** | **T** | **A** | **T** | **G** | **A** | **A** | **G** | **A** | **A** | **C** | **A** | **G** | **A** | **A** | **A** | **A** | **A** | **A** | **C** | **T** | **T** | **C** | **G** | **T** | **G** | **A** | **T** | **G** | **A** | **A** | **T** | **T** | **T** | **G** | **A** | **A** | **A** | **T** | **G** | **G** | **C** | **T** | **C** | **A** | **A** | **A** | **A** | **T** | **G** | **A** | **A** | **A** | **A** | **A** | **T** | **T** | **G** | **A** | **A** | **A** | **A** | **A** | **A** | **G** | **A** | **A** | **G** | **T** | **C** | **A** | **G** | **T** | **A** | **G** | **G** | **C** | **T** | **A** | **A** | **A** | **G** | **A** | **A** | **A** | **A** | **C** | **A** | **G** | **C** | **A** | **G** | **C** | **T** | **G** | **A** | **A** | **A** | **A** | **A** | **G** | **C** | **C** | **G** | **A** | **A** | **T** | **G** | **G** | **T** | **C** | **T** | **C** | **G** | **T** | **T** | **C** | **C** | **C** | **G** | **A** | **G** | **A** | **A** | **G** | **G** | **A** | **G** | **A** | **T** | **A** | **A** | **A** | **A** | **C** | **A** | **A** | **A** | **G** | **A** | **A** | **A** | **C** | **A** | **A** | **G** | **T** | **C** | **G** | **G** | **A** | **T** | **T** | **C** | **A** | **T** | **C** | **G** | **A** | **T** | **A** | **C** | **T** | **G** | **A** | **A** | **T** | **C** | **T** | **A** | **G** | **A** | **C** | **G** | **A** | **G** | **T** | **G** | **A** | **A** | **T** | **A** | **A** | **A** | **G** | **G** | **A** | **G** | **C** | **A** | **G** | **T** | **G** | **G** | **G** | **T** | **G** | **C** | **T** | **G** | **A** | **T** | **G** | **C** | **T** | **G** | **C** | **A** | **C** | **G** | **G** | **A** | **C** | **G** | **A** | **T** | **G** | **A** | **A** | **A** | **C** | **G** | **A** | **T** | **C** | **C** | **A** | **A** | **A** | **G** | **C** | **A** | **A** | **T** | **C** | **G** | **T** | **G** | **A** | **A** | **T** | **C** | **G** | **G** | **A** | **T** | **G** | **G** | **A** | **G** | **A** | **C** | **C** | **C** | **A** | **G** | **A** | **T** | **C** | **A** | **G** | **C** | **G** | **A** | **G** | **A** | **A** | **A** | **G** | **A** | **A** | **A** | **A** | **A** | **C** | **T** | **A** | **T** | **T** | **A** | **A** | **A** | **A** | **G** | **A** | **T** | **A** | **T** | **C** | **G** | **A** | **A** | **T** | **A** | **T** | **A** | **T** | **C** | **G** | **A** | **T** | **T** | **C** | **T** | **T** | **T** | **G** | **A** | **C** | **G** | **A** | **T** | **G** | **A** | **A** | **T** | **A** | **G** | **C** | **C** | **A** | **A** | **G** | **C** | **G** | **T** | **C** | **T** | **C** | **A** | **C** | **C** | **A** | **T** | **A** | **A** | **G** | **C** | **G** | **A** | **C** | **T** | **T** | **T** | **T** | **A** | **A** | **G** | **C** | **G** | **T** | **A** | **G** | **A** | **A** | **G** | **A** | **T** | **C** | **T** | **T** | **C** | **A** | **A** | **T** | **T** | **A** | **G** | **G** | **G** | **T** | **A** | **T** | **G** | **A** | **A** | **A** | **A** | **T** | **C** | **T** | **G** | **T** | **T** | **A** | **T** | **T** | **C** | **G** | **A** | **G** | **C** | **C** | **A** | **A** | **T** | **T** | **C** | **A** | **T** | **T** | **T** | **T** | **A** | **C** | **A** | **A** | **T** | **C** | **G** | **A** | **G** | **C** | **C** | **T** | **C** | **A** | **T** | **C** | **A** | **G** | **C** | **G** | **G** | **G** | **T** | **G** | **G** | **C** | **G** | **A** | **T** | **T** | **T** | **C** | **A** | **G** | **G** | **T** | **C** | **C** | **T** | **A** | **A** | **C** | **G** | **G** | **T** | **G** | **C** | **A** | **G** | **G** | **A** | **A** | **A** | **G** | **T** | **C** | **A** | **T** | **C** | **C** | **A** | **T** | **T** | **A** | **T** | **C** | **C** | **A** | **T** | **T** | **A** | **T** | **C** | **T** | **T** | **C** | **T** | **G** | **G** | **G** | **G** | **G** | **C** | **A** | **T** | **T** | **C** | **A** | **A** | **C** | **G** | **G** | **C** | **A** | **A** | **G** | **G** | **T** | **T** | **A** | **T** | **A** | **G** | **G** | **A** | **G** | **A** | **A** | **A** | **A** | **A** | **T** | **A** | **C** | **C** | **A** | **G** | **C** | **C** | **A** | **A** | **A** | **A** | **C** | **A** | **T** | **C** | **T** | **G** | **A** | **G** | **C** | **A** | **T** | **T** | **A** | **G** | **T** | **T** | **A** | **T** | **G** | **C** | **A** | **A** | **G** | **C** | **C** | **A** | **A** | **A** | **A** | **T** | **T** | **A** | **T** | **G** | **A** | **A** | **G** | **A** | **C** | **A** | **A** | **T** | **C** | **G** | **A** | **G** | **G** | **A** | **A** | **C** | **G** | **T** | **T** | **G** | **G** | **C** | **G** | **G** | **A** | **A** | **T** | **T** | **T** | **G** | **C** | **A** | **G** | **A** | **G** | **A** | **A** | **A** | **A** | **A** | **C** | **C** | **A** | **A** | **G** | **T** | **A** | **G** | **A** | **C** | **T** | **A** | **C** | **C** | **A** | **A** | **G** | **C** | **A** | **T** | **T** | **T** | **T** | **T** | **G** | **A** | **A** | **C** | **A** | **A** | **C** | **C** | **T** | **C** | **C** | **G** | **A** | **A** | **A** | **G** | **C** | **T** | **T** | **G** | **G** | **G** | **A** | **T** | **G** | **G** | **A** | **A** | **A** | **G** | **A** | **G** | **A** | **T** | **G** | **T** | **T** | **T** | **T** | **T** | **C** | **A** | **T** | **A** | **A** | **C** | **A** | **A** | **G** | **A** | **T** | **C** | **G** | **A** | **G** | **C** | **A** | **G** | **A** | **T** | **G** | **A** | **G** | **T** | **A** | **T** | **G** | **G** | **G** | **C** | **C** | **A** | **A** | **C** | **G** | **G** | **A** | **A** | **A** | **A** | **A** | **A** | **G** | **T** | **G** | **G** | **A** | **A** | **T** | **T** | **G** | **G** | **C** | **T** | **A** | **A** | **A** | **T** | **C** | **T** | **T** | **T** | **A** | **T** | **C** | **A** | **C** | **A** | **G** | **C** | **C** | **A** | **G** | **C** | **T** | **G** | **A** | **A** | **C** | **T** | **A** | **T** | **A** | **T** | **A** | **C** | **A** | **T** | **G** | **G** | **G** | **A** | **T** | **G** | **A** | **A** | **C** | **C** | **A** | **T** | **T** | **G** | **A** | **A** | **T** | **T** | **A** | **T** | **T** | **T** | **G** | **G** | **A** | **T** | **G** | **T** | **C** | **T** | **T** | **C** | **A** | **A** | **T** | **C** | **A** | **A** | **G** | **A** | **A** | **C** | **A** | **A** | **T** | **T** | **A** | **G** | **A** | **A** | **C** | **A** | **A** | **C** | **T** | **G** | **A** | **T** | **C** | **T** | **T** | **G** | **A** | **A** | **C** | **G** | **T** | **G** | **A** | **A** | **A** | **C** | **C** | **T** | **G** | **C** | **C** | **A** | **T** | **G** | **T** | **T** | **A** | **C** | **T** | **A** | **G** | **T** | **G** | **G** | **A** | **A** | **C** | **A** | **T** | **G** | **A** | **T** | **C** | **A** | **A** | **A** | **C** | **C** | **T** | **T** | **C** | **C** | **T** | **G** | **G** | **A** | **T** | **A** | **A** | **A** | **G** | **T** | **A** | **T** | **C** | **T** | **A** | **C** | **T** | **G** | **A** | **G** | **A** | **T** | **T** | **A** | **T** | **T** | **T** | **C** | **T** | **C** | **T** | **T** | **G** | **A** | **G** | **A** | **G** | **A** | **A** | **T** | **C** | **-** | **-** | **-** |
| **N59469F** | **A** | **T** | **G** | **T** | **C** | **T** | **A** | **A** | **A** | **A** | **T** | **C** | **G** | **A** | **A** | **A** | **T** | **A** | **A** | **A** | **A** | **A** | **A** | **T** | **C** | **T** | **G** | **A** | **C** | **A** | **T** | **T** | **C** | **G** | **G** | **C** | **T** | **A** | **C** | **G** | **A** | **C** | **A** | **G** | **C** | **C** | **A** | **A** | **G** | **G** | **C** | **A** | **C** | **A** | **T** | **T** | **A** | **T** | **T** | **A** | **T** | **T** | **T** | **G** | **A** | **A** | **C** | **A** | **A** | **G** | **C** | **A** | **A** | **A** | **T** | **C** | **T** | **A** | **A** | **A** | **T** | **T** | **T** | **T** | **G** | **A** | **C** | **A** | **T** | **A** | **C** | **A** | **A** | **T** | **G** | **G** | **A** | **A** | **A** | **C** | **T** | **A** | **G** | **G** | **A** | **C** | **T** | **T** | **A** | **T** | **C** | **G** | **G** | **A** | **C** | **G** | **A** | **A** | **A** | **C** | **G** | **G** | **T** | **C** | **G** | **A** | **G** | **G** | **A** | **A** | **A** | **G** | **A** | **C** | **A** | **A** | **C** | **T** | **T** | **T** | **A** | **C** | **T** | **G** | **A** | **A** | **T** | **A** | **T** | **T** | **C** | **T** | **A** | **C** | **A** | **A** | **A** | **A** | **C** | **A** | **A** | **A** | **C** | **T** | **A** | **C** | **C** | **T** | **T** | **A** | **T** | **C** | **A** | **A** | **G** | **G** | **G** | **C** | **A** | **A** | **G** | **T** | **A** | **A** | **T** | **C** | **C** | **A** | **T** | **C** | **A** | **G** | **C** | **A** | **A** | **G** | **A** | **A** | **T** | **T** | **T** | **G** | **T** | **C** | **T** | **A** | **T** | **T** | **T** | **C** | **C** | **C** | **G** | **C** | **A** | **A** | **C** | **A** | **G** | **A** | **C** | **A** | **A** | **A** | **A** | **G** | **A** | **T** | **A** | **A** | **A** | **G** | **A** | **A** | **C** | **G** | **T** | **T** | **T** | **A** | **A** | **C** | **C** | **T** | **A** | **T** | **T** | **A** | **C** | **G** | **T** | **G** | **T** | **T** | **A** | **A** | **A** | **T** | **G** | **A** | **T** | **A** | **T** | **T** | **A** | **C** | **G** | **G** | **A** | **T** | **T** | **T** | **T** | **G** | **A** | **G** | **A** | **T** | **A** | **T** | **G** | **G** | **G** | **A** | **A** | **A** | **T** | **C** | **G** | **A** | **A** | **A** | **G** | **A** | **G** | **A** | **G** | **C** | **T** | **C** | **C** | **A** | **A** | **T** | **T** | **G** | **A** | **T** | **G** | **C** | **A** | **A** | **A** | **C** | **A** | **G** | **A** | **T** | **C** | **C** | **T** | **G** | **A** | **A** | **A** | **T** | **C** | **T** | **T** | **A** | **T** | **G** | **G** | **A** | **G** | **A** | **G** | **A** | **A** | **T** | **T** | **C** | **A** | **G** | **C** | **A** | **C** | **A** | **C** | **T** | **A** | **T** | **C** | **A** | **G** | **G** | **G** | **G** | **G** | **A** | **G** | **A** | **G** | **A** | **A** | **G** | **A** | **C** | **A** | **A** | **A** | **A** | **G** | **T** | **C** | **C** | **T** | **A** | **C** | **T** | **G** | **G** | **C** | **A** | **C** | **T** | **T** | **T** | **T** | **A** | **T** | **T** | **T** | **G** | **T** | **G** | **G** | **A** | **T** | **G** | **A** | **C** | **A** | **C** | **T** | **C** | **A** | **T** | **T** | **T** | **C** | **C** | **C** | **G** | **T** | **T** | **A** | **A** | **T** | **C** | **G** | **A** | **T** | **G** | **A** | **A** | **C** | **C** | **A** | **A** | **C** | **G** | **A** | **A** | **T** | **C** | **A** | **T** | **T** | **T** | **G** | **G** | **A** | **-** | **-** | **-** | **T** | **A** | **T** | **C** | **T** | **C** | **T** | **G** | **G** | **T** | **A** | **G** | **A** | **A** | **A** | **A** | **C** | **A** | **A** | **G** | **T** | **A** | **G** | **C** | **G** | **G** | **C** | **T** | **T** | **A** | **T** | **T** | **T** | **G** | **A** | **A** | **A** | **A** | **A** | **G** | **A** | **A** | **A** | **A** | **A** | **A** | **C** | **A** | **A** | **G** | **G** | **C** | **T** | **T** | **C** | **A** | **T** | **C** | **G** | **T** | **G** | **G** | **T** | **C** | **A** | **G** | **C** | **C** | **A** | **T** | **G** | **A** | **C** | **C** | **G** | **G** | **G** | **G** | **A** | **T** | **T** | **T** | **A** | **T** | **C** | **G** | **A** | **T** | **G** | **A** | **A** | **G** | **T** | **A** | **G** | **T** | **G** | **G** | **A** | **C** | **C** | **A** | **T** | **G** | **T** | **T** | **T** | **T** | **A** | **G** | **C** | **A** | **A** | **T** | **C** | **G** | **A** | **A** | **A** | **A** | **A** | **A** | **G** | **T** | **C** | **A** | **A** | **C** | **T** | **G** | **G** | **A** | **A** | **C** | **T** | **T** | **T** | **A** | **T** | **C** | **A** | **A** | **G** | **G** | **G** | **A** | **A** | **T** | **T** | **T** | **C** | **T** | **C** | **T** | **A** | **T** | **C** | **T** | **A** | **T** | **G** | **A** | **A** | **G** | **A** | **A** | **C** | **A** | **G** | **A** | **A** | **A** | **A** | **A** | **A** | **C** | **T** | **T** | **C** | **G** | **T** | **G** | **A** | **T** | **G** | **A** | **A** | **T** | **T** | **T** | **G** | **A** | **A** | **A** | **T** | **G** | **G** | **C** | **T** | **C** | **A** | **A** | **A** | **A** | **T** | **G** | **A** | **A** | **A** | **A** | **A** | **T** | **T** | **G** | **A** | **A** | **A** | **A** | **A** | **A** | **G** | **A** | **A** | **G** | **T** | **C** | **A** | **G** | **T** | **A** | **G** | **G** | **C** | **T** | **A** | **A** | **A** | **G** | **A** | **A** | **A** | **A** | **C** | **A** | **G** | **C** | **A** | **G** | **C** | **T** | **G** | **A** | **A** | **A** | **A** | **A** | **G** | **C** | **C** | **G** | **A** | **A** | **T** | **G** | **G** | **T** | **C** | **T** | **C** | **G** | **T** | **T** | **C** | **C** | **C** | **G** | **A** | **G** | **A** | **A** | **G** | **G** | **A** | **G** | **A** | **T** | **A** | **A** | **A** | **A** | **C** | **A** | **A** | **A** | **G** | **A** | **A** | **A** | **C** | **A** | **A** | **G** | **T** | **C** | **G** | **G** | **A** | **T** | **T** | **C** | **A** | **T** | **C** | **G** | **A** | **T** | **A** | **C** | **T** | **G** | **A** | **A** | **T** | **C** | **T** | **A** | **G** | **A** | **C** | **G** | **A** | **G** | **T** | **G** | **A** | **A** | **T** | **A** | **A** | **A** | **G** | **G** | **A** | **G** | **C** | **A** | **G** | **T** | **G** | **G** | **G** | **T** | **G** | **C** | **T** | **G** | **A** | **T** | **G** | **C** | **T** | **G** | **C** | **A** | **C** | **G** | **G** | **A** | **C** | **G** | **A** | **T** | **G** | **A** | **A** | **A** | **C** | **G** | **A** | **T** | **C** | **C** | **A** | **A** | **A** | **G** | **C** | **A** | **A** | **T** | **C** | **G** | **T** | **G** | **A** | **A** | **T** | **C** | **G** | **G** | **A** | **T** | **G** | **G** | **A** | **G** | **A** | **C** | **C** | **C** | **A** | **G** | **A** | **T** | **C** | **A** | **G** | **C** | **G** | **A** | **G** | **A** | **A** | **A** | **G** | **A** | **A** | **A** | **A** | **A** | **C** | **T** | **A** | **T** | **T** | **A** | **A** | **A** | **A** | **G** | **A** | **T** | **A** | **T** | **C** | **G** | **A** | **A** | **T** | **A** | **T** | **A** | **T** | **C** | **G** | **A** | **T** | **T** | **C** | **T** | **T** | **T** | **G** | **A** | **C** | **G** | **A** | **T** | **G** | **A** | **A** | **T** | **A** | **G** | **T** | **C** | **A** | **A** | **G** | **C** | **G** | **T** | **C** | **T** | **C** | **A** | **C** | **C** | **A** | **T** | **A** | **A** | **G** | **C** | **G** | **A** | **C** | **T** | **T** | **T** | **T** | **A** | **A** | **G** | **C** | **G** | **T** | **A** | **G** | **A** | **A** | **G** | **A** | **T** | **C** | **T** | **T** | **C** | **A** | **A** | **T** | **T** | **A** | **G** | **G** | **G** | **T** | **A** | **T** | **G** | **A** | **A** | **A** | **A** | **T** | **C** | **T** | **G** | **T** | **T** | **A** | **T** | **T** | **C** | **G** | **A** | **G** | **C** | **C** | **A** | **A** | **T** | **T** | **C** | **A** | **T** | **T** | **T** | **T** | **A** | **C** | **A** | **A** | **T** | **C** | **G** | **A** | **G** | **C** | **C** | **T** | **C** | **A** | **T** | **C** | **A** | **G** | **C** | **G** | **G** | **G** | **T** | **G** | **G** | **C** | **G** | **A** | **T** | **T** | **T** | **C** | **A** | **G** | **G** | **T** | **C** | **C** | **T** | **A** | **A** | **C** | **G** | **G** | **T** | **G** | **C** | **A** | **G** | **G** | **A** | **A** | **A** | **G** | **T** | **C** | **A** | **T** | **C** | **C** | **A** | **T** | **T** | **A** | **T** | **C** | **C** | **A** | **T** | **T** | **A** | **T** | **C** | **T** | **T** | **C** | **T** | **G** | **G** | **G** | **G** | **G** | **C** | **A** | **T** | **T** | **C** | **A** | **A** | **C** | **G** | **G** | **C** | **A** | **A** | **G** | **G** | **T** | **T** | **A** | **T** | **A** | **G** | **G** | **A** | **G** | **A** | **A** | **A** | **A** | **A** | **T** | **C** | **C** | **C** | **A** | **G** | **C** | **C** | **A** | **A** | **A** | **A** | **C** | **A** | **T** | **C** | **T** | **G** | **A** | **G** | **C** | **A** | **T** | **T** | **A** | **G** | **T** | **T** | **A** | **T** | **G** | **T** | **A** | **C** | **G** | **C** | **C** | **A** | **A** | **A** | **A** | **T** | **T** | **A** | **T** | **G** | **A** | **A** | **G** | **A** | **C** | **A** | **A** | **T** | **C** | **G** | **A** | **G** | **G** | **A** | **A** | **C** | **G** | **T** | **T** | **G** | **G** | **C** | **G** | **G** | **A** | **A** | **T** | **T** | **T** | **G** | **C** | **A** | **G** | **A** | **G** | **A** | **A** | **A** | **A** | **A** | **C** | **C** | **A** | **A** | **G** | **T** | **A** | **G** | **A** | **C** | **T** | **A** | **C** | **C** | **A** | **A** | **G** | **C** | **A** | **T** | **T** | **T** | **T** | **T** | **G** | **A** | **A** | **C** | **A** | **A** | **C** | **C** | **T** | **C** | **C** | **G** | **A** | **A** | **A** | **G** | **C** | **T** | **T** | **G** | **G** | **G** | **A** | **T** | **G** | **G** | **A** | **A** | **A** | **G** | **A** | **G** | **A** | **T** | **G** | **T** | **T** | **T** | **T** | **T** | **C** | **A** | **T** | **A** | **A** | **C** | **A** | **A** | **G** | **A** | **T** | **C** | **G** | **A** | **G** | **C** | **A** | **G** | **A** | **T** | **G** | **A** | **G** | **T** | **A** | **T** | **G** | **G** | **G** | **T** | **C** | **A** | **A** | **C** | **G** | **G** | **A** | **A** | **A** | **A** | **A** | **A** | **G** | **T** | **G** | **G** | **A** | **A** | **T** | **T** | **G** | **G** | **C** | **T** | **A** | **A** | **A** | **T** | **C** | **T** | **T** | **T** | **A** | **T** | **C** | **A** | **C** | **A** | **G** | **C** | **C** | **A** | **G** | **C** | **T** | **G** | **A** | **A** | **C** | **T** | **A** | **T** | **A** | **T** | **A** | **T** | **A** | **T** | **G** | **G** | **G** | **A** | **T** | **G** | **A** | **A** | **C** | **C** | **A** | **T** | **T** | **G** | **A** | **A** | **T** | **T** | **A** | **T** | **T** | **T** | **G** | **G** | **A** | **T** | **G** | **T** | **C** | **T** | **T** | **C** | **A** | **A** | **T** | **C** | **A** | **A** | **G** | **A** | **A** | **C** | **A** | **A** | **T** | **T** | **A** | **G** | **A** | **A** | **C** | **A** | **A** | **C** | **T** | **G** | **A** | **T** | **C** | **T** | **T** | **G** | **A** | **A** | **C** | **G** | **T** | **G** | **A** | **A** | **A** | **C** | **C** | **T** | **G** | **C** | **C** | **A** | **T** | **G** | **T** | **T** | **A** | **C** | **T** | **A** | **G** | **T** | **G** | **G** | **A** | **A** | **C** | **A** | **T** | **G** | **A** | **T** | **C** | **A** | **A** | **A** | **C** | **C** | **T** | **T** | **C** | **C** | **T** | **G** | **G** | **A** | **T** | **A** | **A** | **A** | **G** | **T** | **A** | **T** | **C** | **T** | **A** | **C** | **T** | **G** | **A** | **G** | **A** | **T** | **T** | **A** | **T** | **T** | **T** | **C** | **T** | **C** | **T** | **T** | **G** | **A** | **G** | **A** | **A** | **A** | **A** | **T** | **C** | **-** | **-** | **-** |
| **N59613F** | **A** | **T** | **G** | **T** | **C** | **T** | **A** | **A** | **A** | **A** | **T** | **C** | **G** | **A** | **A** | **A** | **T** | **A** | **A** | **A** | **A** | **A** | **A** | **T** | **C** | **T** | **G** | **A** | **C** | **A** | **T** | **T** | **C** | **G** | **G** | **C** | **T** | **A** | **T** | **G** | **A** | **C** | **A** | **G** | **C** | **C** | **A** | **A** | **G** | **G** | **C** | **A** | **C** | **A** | **T** | **T** | **A** | **T** | **T** | **A** | **T** | **T** | **T** | **G** | **A** | **A** | **C** | **A** | **A** | **G** | **C** | **C** | **A** | **A** | **T** | **C** | **T** | **A** | **A** | **A** | **C** | **T** | **T** | **T** | **G** | **A** | **C** | **A** | **C** | **A** | **C** | **A** | **A** | **T** | **G** | **G** | **A** | **A** | **A** | **C** | **T** | **A** | **G** | **G** | **A** | **C** | **T** | **T** | **A** | **T** | **C** | **G** | **G** | **A** | **C** | **G** | **A** | **A** | **A** | **C** | **G** | **G** | **T** | **C** | **G** | **A** | **G** | **G** | **A** | **A** | **A** | **G** | **A** | **C** | **A** | **A** | **C** | **T** | **T** | **T** | **A** | **C** | **T** | **G** | **A** | **A** | **T** | **A** | **T** | **C** | **C** | **T** | **G** | **C** | **A** | **A** | **A** | **A** | **C** | **A** | **A** | **A** | **C** | **T** | **C** | **C** | **C** | **T** | **T** | **A** | **T** | **C** | **A** | **A** | **G** | **G** | **G** | **C** | **A** | **A** | **G** | **T** | **G** | **A** | **T** | **C** | **C** | **A** | **T** | **C** | **A** | **G** | **C** | **A** | **A** | **G** | **C** | **G** | **T** | **T** | **T** | **G** | **T** | **C** | **T** | **A** | **T** | **T** | **T** | **C** | **C** | **C** | **G** | **C** | **A** | **G** | **C** | **A** | **A** | **A** | **C** | **A** | **A** | **A** | **A** | **G** | **A** | **T** | **A** | **A** | **A** | **G** | **A** | **G** | **C** | **G** | **T** | **T** | **T** | **G** | **A** | **C** | **C** | **T** | **A** | **T** | **T** | **A** | **T** | **G** | **T** | **G** | **T** | **T** | **A** | **A** | **A** | **T** | **G** | **A** | **G** | **A** | **T** | **T** | **A** | **C** | **G** | **G** | **A** | **T** | **T** | **T** | **T** | **G** | **A** | **G** | **A** | **T** | **A** | **T** | **G** | **G** | **G** | **A** | **A** | **A** | **T** | **T** | **G** | **A** | **A** | **A** | **G** | **A** | **G** | **A** | **G** | **C** | **T** | **C** | **C** | **A** | **A** | **C** | **T** | **G** | **A** | **T** | **G** | **C** | **A** | **A** | **A** | **C** | **A** | **G** | **A** | **T** | **C** | **C** | **T** | **G** | **A** | **A** | **A** | **T** | **C** | **T** | **T** | **A** | **T** | **G** | **G** | **A** | **G** | **A** | **G** | **A** | **A** | **T** | **T** | **C** | **A** | **G** | **C** | **A** | **C** | **A** | **C** | **T** | **A** | **T** | **C** | **A** | **G** | **G** | **A** | **G** | **G** | **A** | **G** | **A** | **G** | **A** | **A** | **G** | **A** | **C** | **A** | **A** | **A** | **A** | **G** | **T** | **C** | **C** | **T** | **A** | **C** | **T** | **G** | **G** | **C** | **A** | **C** | **T** | **C** | **T** | **T** | **A** | **T** | **T** | **T** | **G** | **T** | **G** | **G** | **A** | **T** | **G** | **A** | **C** | **A** | **C** | **T** | **C** | **A** | **T** | **T** | **T** | **C** | **C** | **C** | **G** | **T** | **T** | **A** | **A** | **T** | **C** | **G** | **A** | **T** | **G** | **A** | **A** | **C** | **C** | **A** | **A** | **C** | **G** | **A** | **A** | **T** | **C** | **A** | **T** | **T** | **T** | **G** | **G** | **A** | **-** | **-** | **-** | **T** | **A** | **T** | **C** | **T** | **C** | **T** | **G** | **G** | **C** | **A** | **G** | **A** | **A** | **A** | **A** | **C** | **A** | **A** | **G** | **T** | **A** | **G** | **C** | **G** | **G** | **C** | **T** | **T** | **A** | **C** | **T** | **T** | **G** | **A** | **A** | **A** | **A** | **A** | **G** | **A** | **A** | **A** | **A** | **A** | **A** | **C** | **A** | **A** | **G** | **G** | **A** | **T** | **T** | **C** | **A** | **T** | **C** | **G** | **T** | **G** | **G** | **T** | **G** | **A** | **G** | **T** | **C** | **A** | **T** | **G** | **A** | **T** | **C** | **G** | **G** | **G** | **G** | **A** | **T** | **T** | **T** | **A** | **T** | **C** | **G** | **A** | **T** | **G** | **A** | **A** | **G** | **T** | **A** | **G** | **T** | **G** | **G** | **A** | **C** | **C** | **A** | **T** | **G** | **T** | **T** | **T** | **T** | **A** | **G** | **C** | **A** | **A** | **T** | **C** | **G** | **A** | **A** | **A** | **A** | **G** | **A** | **G** | **C** | **C** | **A** | **A** | **C** | **T** | **G** | **G** | **A** | **A** | **C** | **T** | **T** | **T** | **A** | **T** | **C** | **A** | **A** | **G** | **G** | **G** | **A** | **A** | **T** | **T** | **T** | **C** | **T** | **C** | **T** | **A** | **T** | **C** | **T** | **A** | **T** | **G** | **A** | **A** | **G** | **A** | **A** | **C** | **A** | **G** | **A** | **A** | **A** | **A** | **A** | **A** | **C** | **T** | **T** | **C** | **G** | **T** | **G** | **A** | **T** | **G** | **A** | **A** | **T** | **T** | **T** | **G** | **A** | **A** | **A** | **T** | **A** | **G** | **C** | **T** | **A** | **A** | **A** | **A** | **A** | **T** | **G** | **A** | **A** | **A** | **A** | **A** | **T** | **T** | **G** | **A** | **A** | **A** | **A** | **A** | **A** | **G** | **A** | **A** | **G** | **T** | **C** | **A** | **G** | **C** | **A** | **G** | **G** | **C** | **T** | **A** | **A** | **A** | **G** | **A** | **A** | **A** | **A** | **C** | **A** | **G** | **C** | **A** | **G** | **C** | **T** | **G** | **A** | **A** | **A** | **A** | **A** | **G** | **C** | **A** | **G** | **A** | **A** | **T** | **G** | **G** | **T** | **C** | **T** | **C** | **G** | **T** | **T** | **C** | **C** | **C** | **G** | **A** | **G** | **A** | **A** | **G** | **G** | **A** | **G** | **A** | **C** | **A** | **A** | **A** | **A** | **C** | **A** | **A** | **A** | **G** | **A** | **A** | **A** | **A** | **A** | **A** | **G** | **T** | **C** | **G** | **G** | **A** | **T** | **T** | **C** | **A** | **T** | **C** | **G** | **A** | **T** | **A** | **C** | **G** | **G** | **A** | **A** | **T** | **C** | **T** | **A** | **G** | **G** | **C** | **G** | **A** | **G** | **T** | **G** | **A** | **A** | **T** | **A** | **A** | **A** | **G** | **G** | **A** | **G** | **C** | **A** | **G** | **T** | **A** | **G** | **G** | **C** | **G** | **C** | **T** | **G** | **A** | **T** | **G** | **C** | **T** | **G** | **C** | **A** | **C** | **G** | **G** | **A** | **C** | **G** | **A** | **T** | **G** | **A** | **A** | **A** | **C** | **G** | **A** | **T** | **C** | **T** | **A** | **A** | **A** | **G** | **C** | **A** | **A** | **T** | **C** | **G** | **T** | **G** | **A** | **A** | **T** | **C** | **G** | **G** | **A** | **T** | **G** | **G** | **A** | **A** | **A** | **C** | **C** | **C** | **A** | **G** | **A** | **T** | **C** | **A** | **G** | **T** | **G** | **A** | **G** | **A** | **A** | **A** | **G** | **A** | **A** | **A** | **A** | **A** | **T** | **T** | **A** | **T** | **T** | **A** | **A** | **A** | **A** | **G** | **A** | **T** | **A** | **T** | **C** | **G** | **A** | **G** | **T** | **A** | **T** | **A** | **T** | **C** | **G** | **A** | **T** | **G** | **C** | **T** | **T** | **T** | **G** | **A** | **C** | **G** | **A** | **T** | **G** | **A** | **A** | **T** | **A** | **G** | **C** | **C** | **A** | **A** | **G** | **T** | **G** | **T** | **C** | **T** | **C** | **A** | **T** | **C** | **A** | **T** | **A** | **A** | **A** | **C** | **G** | **G** | **C** | **T** | **G** | **T** | **T** | **A** | **A** | **G** | **C** | **G** | **T** | **A** | **G** | **A** | **A** | **A** | **A** | **T** | **C** | **T** | **T** | **C** | **A** | **A** | **T** | **T** | **A** | **G** | **G** | **G** | **T** | **A** | **T** | **G** | **A** | **A** | **A** | **A** | **T** | **C** | **T** | **G** | **T** | **T** | **A** | **T** | **T** | **C** | **G** | **A** | **G** | **C** | **C** | **A** | **G** | **T** | **T** | **C** | **A** | **T** | **T** | **T** | **T** | **A** | **C** | **G** | **A** | **T** | **C** | **G** | **A** | **A** | **C** | **C** | **T** | **C** | **A** | **T** | **C** | **A** | **A** | **C** | **G** | **G** | **G** | **T** | **G** | **G** | **C** | **A** | **A** | **T** | **T** | **T** | **C** | **A** | **G** | **G** | **T** | **C** | **C** | **T** | **A** | **A** | **C** | **G** | **G** | **C** | **G** | **C** | **G** | **G** | **G** | **A** | **A** | **A** | **G** | **T** | **C** | **A** | **T** | **C** | **C** | **A** | **T** | **T** | **A** | **T** | **C** | **C** | **A** | **T** | **T** | **A** | **T** | **C** | **T** | **T** | **C** | **T** | **G** | **G** | **G** | **G** | **A** | **C** | **A** | **T** | **T** | **C** | **A** | **A** | **C** | **G** | **G** | **C** | **A** | **A** | **G** | **G** | **T** | **T** | **G** | **T** | **A** | **G** | **G** | **A** | **G** | **A** | **A** | **A** | **A** | **A** | **T** | **C** | **C** | **C** | **A** | **G** | **C** | **C** | **C** | **A** | **A** | **A** | **C** | **A** | **T** | **T** | **T** | **G** | **A** | **C** | **C** | **A** | **T** | **T** | **A** | **G** | **T** | **T** | **A** | **C** | **G** | **T** | **A** | **C** | **G** | **C** | **C** | **A** | **A** | **A** | **A** | **T** | **T** | **A** | **T** | **G** | **A** | **A** | **G** | **A** | **C** | **A** | **A** | **T** | **C** | **G** | **A** | **G** | **G** | **G** | **A** | **C** | **A** | **T** | **T** | **G** | **G** | **C** | **G** | **G** | **A** | **A** | **T** | **T** | **C** | **G** | **C** | **G** | **A** | **A** | **G** | **A** | **A** | **A** | **A** | **A** | **C** | **C** | **G** | **A** | **G** | **T** | **A** | **G** | **A** | **C** | **T** | **A** | **T** | **C** | **A** | **A** | **G** | **C** | **A** | **T** | **T** | **T** | **T** | **T** | **G** | **A** | **A** | **T** | **A** | **A** | **T** | **C** | **T** | **C** | **C** | **G** | **A** | **A** | **A** | **G** | **C** | **T** | **T** | **G** | **G** | **G** | **A** | **T** | **G** | **G** | **A** | **A** | **A** | **G** | **A** | **G** | **A** | **T** | **G** | **T** | **T** | **T** | **T** | **T** | **C** | **A** | **T** | **A** | **A** | **T** | **A** | **A** | **A** | **A** | **T** | **C** | **G** | **A** | **A** | **C** | **A** | **G** | **A** | **T** | **G** | **A** | **G** | **T** | **A** | **T** | **G** | **G** | **G** | **A** | **C** | **A** | **A** | **C** | **G** | **G** | **A** | **A** | **A** | **A** | **A** | **A** | **G** | **T** | **G** | **G** | **A** | **A** | **T** | **T** | **G** | **G** | **C** | **T** | **A** | **A** | **A** | **T** | **C** | **T** | **T** | **T** | **A** | **T** | **C** | **C** | **C** | **A** | **A** | **C** | **C** | **A** | **G** | **C** | **T** | **G** | **A** | **G** | **C** | **T** | **C** | **T** | **A** | **T** | **A** | **T** | **A** | **T** | **G** | **G** | **G** | **A** | **T** | **G** | **A** | **A** | **C** | **C** | **A** | **T** | **T** | **G** | **A** | **A** | **T** | **T** | **A** | **T** | **T** | **T** | **G** | **G** | **A** | **T** | **G** | **T** | **C** | **T** | **T** | **C** | **A** | **A** | **T** | **C** | **A** | **A** | **G** | **A** | **A** | **C** | **A** | **A** | **T** | **T** | **A** | **G** | **A** | **A** | **C** | **A** | **A** | **C** | **T** | **G** | **A** | **T** | **T** | **T** | **T** | **G** | **A** | **A** | **C** | **G** | **T** | **G** | **A** | **A** | **A** | **C** | **C** | **T** | **G** | **C** | **T** | **A** | **T** | **G** | **T** | **T** | **A** | **C** | **T** | **A** | **G** | **T** | **G** | **G** | **A** | **A** | **C** | **A** | **T** | **G** | **A** | **T** | **C** | **A** | **A** | **A** | **C** | **C** | **T** | **T** | **T** | **C** | **T** | **A** | **G** | **A** | **T** | **A** | **A** | **A** | **G** | **T** | **A** | **T** | **C** | **T** | **A** | **C** | **T** | **G** | **A** | **G** | **A** | **T** | **T** | **A** | **T** | **T** | **T** | **C** | **T** | **C** | **T** | **T** | **G** | **A** | **G** | **A** | **A** | **A** | **A** | **T** | **C** | **-** | **-** | **-** |
| **N53655** | **A** | **T** | **G** | **T** | **C** | **T** | **A** | **A** | **A** | **A** | **T** | **C** | **G** | **A** | **A** | **A** | **T** | **A** | **A** | **A** | **A** | **A** | **A** | **T** | **C** | **T** | **G** | **A** | **C** | **A** | **T** | **T** | **C** | **G** | **G** | **C** | **T** | **A** | **C** | **G** | **A** | **C** | **A** | **G** | **C** | **C** | **A** | **A** | **G** | **G** | **C** | **A** | **C** | **A** | **T** | **T** | **A** | **T** | **T** | **A** | **T** | **T** | **T** | **G** | **A** | **A** | **C** | **A** | **A** | **G** | **C** | **A** | **A** | **A** | **T** | **C** | **T** | **A** | **A** | **A** | **T** | **T** | **T** | **T** | **G** | **A** | **C** | **A** | **C** | **A** | **C** | **A** | **A** | **T** | **G** | **G** | **A** | **A** | **A** | **C** | **T** | **A** | **G** | **G** | **A** | **C** | **T** | **T** | **A** | **T** | **C** | **G** | **G** | **A** | **C** | **G** | **A** | **A** | **A** | **C** | **G** | **G** | **T** | **C** | **G** | **A** | **G** | **G** | **A** | **A** | **A** | **G** | **A** | **C** | **A** | **A** | **C** | **T** | **T** | **T** | **A** | **C** | **T** | **G** | **A** | **A** | **T** | **A** | **T** | **T** | **C** | **T** | **A** | **C** | **A** | **A** | **A** | **A** | **C** | **A** | **A** | **A** | **C** | **T** | **A** | **C** | **C** | **T** | **T** | **A** | **T** | **C** | **A** | **A** | **G** | **G** | **G** | **C** | **A** | **A** | **G** | **T** | **A** | **A** | **T** | **C** | **C** | **A** | **T** | **C** | **A** | **G** | **C** | **A** | **A** | **G** | **A** | **A** | **T** | **T** | **T** | **G** | **T** | **C** | **T** | **A** | **T** | **T** | **T** | **C** | **C** | **C** | **G** | **C** | **A** | **A** | **C** | **A** | **G** | **A** | **C** | **A** | **A** | **A** | **A** | **G** | **A** | **T** | **A** | **A** | **A** | **G** | **A** | **A** | **C** | **G** | **T** | **T** | **T** | **A** | **A** | **C** | **C** | **T** | **A** | **T** | **T** | **A** | **C** | **G** | **T** | **G** | **T** | **T** | **A** | **A** | **A** | **T** | **G** | **A** | **T** | **A** | **T** | **T** | **A** | **C** | **G** | **G** | **A** | **T** | **T** | **T** | **T** | **G** | **A** | **G** | **A** | **T** | **A** | **T** | **G** | **G** | **G** | **A** | **A** | **A** | **T** | **C** | **G** | **A** | **A** | **A** | **G** | **A** | **G** | **A** | **G** | **C** | **T** | **C** | **C** | **A** | **A** | **T** | **T** | **G** | **A** | **T** | **G** | **C** | **A** | **A** | **A** | **C** | **A** | **G** | **A** | **T** | **C** | **C** | **T** | **G** | **A** | **A** | **A** | **T** | **C** | **T** | **T** | **A** | **T** | **G** | **G** | **A** | **G** | **A** | **G** | **A** | **A** | **T** | **T** | **C** | **A** | **G** | **C** | **A** | **C** | **A** | **C** | **T** | **A** | **T** | **C** | **A** | **G** | **G** | **G** | **G** | **G** | **A** | **G** | **A** | **G** | **A** | **A** | **G** | **A** | **C** | **A** | **A** | **A** | **A** | **G** | **T** | **C** | **C** | **T** | **A** | **C** | **T** | **G** | **G** | **C** | **A** | **C** | **T** | **T** | **T** | **T** | **A** | **T** | **T** | **T** | **G** | **T** | **G** | **G** | **A** | **T** | **G** | **A** | **C** | **A** | **C** | **T** | **C** | **A** | **T** | **T** | **T** | **C** | **C** | **C** | **G** | **T** | **T** | **A** | **A** | **T** | **C** | **G** | **A** | **T** | **G** | **A** | **A** | **C** | **C** | **A** | **A** | **C** | **G** | **A** | **A** | **T** | **C** | **A** | **T** | **T** | **T** | **G** | **G** | **A** | **-** | **-** | **-** | **T** | **A** | **T** | **C** | **T** | **C** | **T** | **G** | **G** | **T** | **A** | **G** | **A** | **A** | **A** | **A** | **C** | **A** | **A** | **G** | **T** | **A** | **G** | **C** | **G** | **G** | **C** | **T** | **T** | **A** | **T** | **T** | **T** | **G** | **A** | **A** | **A** | **A** | **A** | **G** | **A** | **A** | **A** | **A** | **A** | **A** | **C** | **A** | **A** | **G** | **G** | **C** | **T** | **T** | **C** | **A** | **T** | **C** | **G** | **T** | **G** | **G** | **T** | **C** | **A** | **G** | **C** | **C** | **A** | **T** | **G** | **A** | **C** | **C** | **G** | **G** | **G** | **G** | **A** | **T** | **T** | **T** | **A** | **T** | **C** | **G** | **A** | **T** | **G** | **A** | **A** | **G** | **T** | **A** | **G** | **T** | **G** | **G** | **A** | **C** | **C** | **A** | **T** | **G** | **T** | **T** | **T** | **T** | **A** | **G** | **C** | **A** | **A** | **T** | **C** | **G** | **A** | **A** | **A** | **A** | **A** | **A** | **G** | **T** | **C** | **A** | **A** | **C** | **T** | **G** | **G** | **A** | **A** | **C** | **T** | **T** | **T** | **A** | **T** | **C** | **A** | **A** | **G** | **G** | **G** | **A** | **A** | **T** | **T** | **T** | **C** | **T** | **C** | **T** | **A** | **T** | **C** | **T** | **A** | **T** | **G** | **A** | **A** | **G** | **A** | **A** | **C** | **A** | **G** | **A** | **A** | **A** | **A** | **A** | **A** | **C** | **T** | **T** | **C** | **G** | **T** | **G** | **A** | **T** | **G** | **A** | **A** | **T** | **T** | **T** | **G** | **A** | **A** | **A** | **T** | **G** | **G** | **C** | **T** | **C** | **A** | **A** | **A** | **A** | **T** | **G** | **A** | **A** | **A** | **A** | **A** | **T** | **T** | **G** | **A** | **A** | **A** | **A** | **A** | **A** | **G** | **A** | **A** | **G** | **T** | **C** | **A** | **G** | **T** | **A** | **G** | **G** | **C** | **T** | **A** | **A** | **A** | **G** | **A** | **A** | **A** | **A** | **C** | **A** | **G** | **C** | **A** | **G** | **C** | **T** | **G** | **A** | **A** | **A** | **A** | **A** | **G** | **C** | **C** | **G** | **A** | **A** | **T** | **G** | **G** | **T** | **C** | **T** | **C** | **G** | **T** | **T** | **C** | **C** | **C** | **G** | **A** | **G** | **A** | **A** | **G** | **G** | **A** | **G** | **A** | **T** | **A** | **A** | **A** | **A** | **C** | **A** | **A** | **A** | **G** | **A** | **A** | **A** | **C** | **A** | **A** | **G** | **T** | **C** | **G** | **G** | **A** | **T** | **T** | **C** | **A** | **T** | **C** | **G** | **A** | **T** | **A** | **C** | **T** | **G** | **A** | **A** | **T** | **C** | **T** | **A** | **G** | **A** | **C** | **G** | **A** | **G** | **T** | **G** | **A** | **A** | **T** | **A** | **A** | **A** | **G** | **G** | **A** | **G** | **C** | **A** | **G** | **T** | **G** | **G** | **G** | **T** | **G** | **C** | **T** | **G** | **A** | **T** | **G** | **C** | **T** | **G** | **C** | **A** | **C** | **G** | **G** | **A** | **C** | **G** | **A** | **T** | **G** | **A** | **A** | **A** | **C** | **G** | **A** | **T** | **C** | **C** | **A** | **A** | **A** | **G** | **C** | **A** | **A** | **T** | **C** | **G** | **T** | **G** | **A** | **A** | **T** | **C** | **G** | **G** | **A** | **T** | **G** | **G** | **A** | **G** | **A** | **C** | **C** | **C** | **A** | **G** | **A** | **T** | **C** | **A** | **G** | **C** | **G** | **A** | **G** | **A** | **A** | **A** | **G** | **A** | **A** | **A** | **A** | **A** | **C** | **T** | **A** | **T** | **T** | **G** | **A** | **A** | **A** | **G** | **A** | **T** | **A** | **T** | **C** | **G** | **A** | **A** | **T** | **A** | **T** | **A** | **T** | **C** | **G** | **A** | **T** | **T** | **C** | **T** | **T** | **T** | **G** | **A** | **C** | **G** | **A** | **T** | **G** | **A** | **A** | **T** | **A** | **G** | **C** | **C** | **A** | **A** | **G** | **C** | **G** | **T** | **C** | **T** | **C** | **A** | **C** | **C** | **A** | **T** | **A** | **A** | **G** | **C** | **G** | **A** | **C** | **T** | **T** | **T** | **T** | **A** | **A** | **G** | **C** | **G** | **T** | **A** | **G** | **A** | **A** | **G** | **A** | **T** | **C** | **T** | **T** | **C** | **A** | **A** | **T** | **T** | **A** | **G** | **G** | **G** | **T** | **A** | **T** | **G** | **A** | **G** | **A** | **A** | **T** | **C** | **T** | **G** | **T** | **T** | **A** | **T** | **T** | **C** | **G** | **A** | **G** | **C** | **C** | **A** | **A** | **T** | **T** | **C** | **A** | **T** | **T** | **T** | **T** | **A** | **C** | **A** | **A** | **T** | **C** | **G** | **A** | **G** | **C** | **C** | **T** | **C** | **A** | **T** | **C** | **A** | **G** | **C** | **G** | **G** | **G** | **T** | **G** | **G** | **C** | **G** | **A** | **T** | **T** | **T** | **C** | **A** | **G** | **G** | **T** | **C** | **C** | **T** | **A** | **A** | **C** | **G** | **G** | **T** | **G** | **C** | **A** | **G** | **G** | **A** | **A** | **A** | **G** | **T** | **C** | **A** | **T** | **C** | **C** | **A** | **T** | **T** | **A** | **T** | **C** | **C** | **A** | **T** | **T** | **A** | **T** | **C** | **T** | **T** | **C** | **T** | **G** | **G** | **G** | **G** | **G** | **C** | **A** | **T** | **T** | **C** | **A** | **A** | **C** | **G** | **G** | **C** | **A** | **A** | **G** | **G** | **T** | **T** | **A** | **T** | **A** | **G** | **G** | **A** | **G** | **A** | **A** | **A** | **A** | **A** | **T** | **C** | **C** | **C** | **A** | **G** | **C** | **C** | **A** | **A** | **A** | **A** | **C** | **A** | **T** | **C** | **T** | **G** | **A** | **G** | **C** | **A** | **T** | **T** | **A** | **G** | **T** | **T** | **A** | **T** | **G** | **T** | **A** | **C** | **G** | **C** | **C** | **A** | **A** | **A** | **A** | **T** | **T** | **A** | **T** | **G** | **A** | **A** | **G** | **A** | **C** | **A** | **A** | **T** | **C** | **G** | **A** | **G** | **G** | **A** | **A** | **C** | **G** | **T** | **T** | **G** | **G** | **C** | **G** | **G** | **A** | **A** | **T** | **T** | **T** | **G** | **C** | **A** | **G** | **A** | **G** | **A** | **A** | **A** | **A** | **A** | **C** | **C** | **A** | **A** | **G** | **T** | **A** | **G** | **A** | **C** | **T** | **A** | **C** | **C** | **A** | **A** | **G** | **C** | **A** | **T** | **T** | **T** | **T** | **T** | **G** | **A** | **A** | **C** | **A** | **A** | **C** | **C** | **T** | **C** | **C** | **G** | **A** | **A** | **A** | **G** | **C** | **T** | **T** | **G** | **G** | **G** | **A** | **T** | **G** | **G** | **A** | **A** | **A** | **G** | **A** | **G** | **A** | **T** | **G** | **T** | **T** | **T** | **T** | **T** | **C** | **A** | **T** | **A** | **A** | **C** | **A** | **A** | **G** | **A** | **T** | **C** | **G** | **A** | **G** | **C** | **A** | **G** | **A** | **T** | **G** | **A** | **G** | **T** | **A** | **T** | **G** | **G** | **G** | **C** | **C** | **A** | **A** | **C** | **G** | **G** | **A** | **A** | **A** | **A** | **A** | **A** | **G** | **T** | **G** | **G** | **A** | **A** | **T** | **T** | **G** | **G** | **C** | **T** | **A** | **A** | **A** | **T** | **C** | **T** | **T** | **T** | **A** | **T** | **C** | **A** | **C** | **A** | **G** | **C** | **C** | **A** | **G** | **C** | **T** | **G** | **A** | **A** | **C** | **T** | **A** | **T** | **A** | **T** | **A** | **T** | **A** | **T** | **G** | **G** | **G** | **A** | **T** | **G** | **A** | **A** | **C** | **C** | **A** | **T** | **T** | **G** | **A** | **A** | **T** | **T** | **A** | **T** | **T** | **T** | **G** | **G** | **A** | **T** | **G** | **T** | **C** | **T** | **T** | **C** | **A** | **A** | **T** | **C** | **A** | **A** | **G** | **A** | **A** | **C** | **A** | **A** | **T** | **T** | **A** | **G** | **A** | **A** | **C** | **A** | **A** | **C** | **T** | **G** | **A** | **T** | **C** | **T** | **T** | **G** | **A** | **A** | **C** | **G** | **T** | **G** | **A** | **A** | **A** | **C** | **C** | **T** | **G** | **C** | **C** | **A** | **T** | **G** | **T** | **T** | **A** | **C** | **T** | **A** | **G** | **T** | **G** | **G** | **A** | **A** | **C** | **A** | **T** | **G** | **A** | **T** | **C** | **A** | **A** | **A** | **C** | **C** | **T** | **T** | **C** | **C** | **T** | **G** | **G** | **A** | **T** | **A** | **A** | **A** | **G** | **T** | **A** | **T** | **C** | **T** | **A** | **C** | **T** | **G** | **A** | **G** | **A** | **T** | **T** | **A** | **T** | **T** | **T** | **C** | **T** | **C** | **T** | **T** | **G** | **A** | **G** | **A** | **A** | **A** | **A** | **T** | **C** | **-** | **-** | **-** |
| **N59779F** | **A** | **T** | **G** | **T** | **C** | **T** | **A** | **A** | **A** | **A** | **T** | **C** | **G** | **A** | **A** | **A** | **T** | **A** | **A** | **A** | **A** | **A** | **A** | **T** | **C** | **T** | **G** | **A** | **C** | **A** | **T** | **T** | **C** | **G** | **G** | **C** | **T** | **A** | **C** | **G** | **A** | **C** | **A** | **G** | **C** | **C** | **A** | **A** | **G** | **G** | **C** | **A** | **C** | **A** | **T** | **T** | **A** | **T** | **T** | **A** | **T** | **T** | **T** | **G** | **A** | **A** | **C** | **A** | **A** | **G** | **C** | **A** | **A** | **A** | **T** | **C** | **T** | **A** | **A** | **A** | **T** | **T** | **T** | **T** | **G** | **A** | **C** | **A** | **C** | **A** | **C** | **A** | **A** | **T** | **G** | **G** | **A** | **A** | **A** | **C** | **T** | **A** | **G** | **G** | **A** | **C** | **T** | **T** | **A** | **T** | **C** | **G** | **G** | **A** | **C** | **G** | **A** | **A** | **A** | **C** | **G** | **G** | **T** | **C** | **G** | **A** | **G** | **G** | **A** | **A** | **A** | **G** | **A** | **C** | **A** | **A** | **C** | **T** | **T** | **T** | **A** | **C** | **T** | **G** | **A** | **A** | **T** | **A** | **T** | **T** | **C** | **T** | **A** | **C** | **A** | **A** | **A** | **A** | **C** | **A** | **A** | **A** | **C** | **T** | **A** | **C** | **C** | **T** | **T** | **A** | **T** | **C** | **A** | **A** | **G** | **G** | **G** | **C** | **A** | **A** | **G** | **T** | **A** | **A** | **T** | **C** | **C** | **A** | **T** | **C** | **A** | **G** | **C** | **A** | **A** | **G** | **A** | **A** | **T** | **T** | **T** | **G** | **C** | **C** | **T** | **A** | **T** | **T** | **T** | **C** | **C** | **C** | **G** | **C** | **A** | **A** | **C** | **A** | **G** | **A** | **C** | **A** | **A** | **A** | **A** | **G** | **A** | **T** | **A** | **A** | **A** | **G** | **A** | **A** | **C** | **G** | **T** | **T** | **T** | **A** | **A** | **C** | **C** | **T** | **A** | **T** | **T** | **A** | **C** | **G** | **T** | **G** | **T** | **T** | **A** | **A** | **A** | **T** | **G** | **A** | **T** | **A** | **T** | **T** | **A** | **C** | **G** | **G** | **A** | **T** | **T** | **T** | **T** | **G** | **A** | **G** | **A** | **T** | **A** | **T** | **G** | **G** | **G** | **A** | **A** | **A** | **T** | **C** | **G** | **A** | **A** | **A** | **G** | **A** | **G** | **A** | **G** | **C** | **T** | **C** | **C** | **A** | **A** | **T** | **T** | **G** | **A** | **T** | **G** | **C** | **A** | **A** | **A** | **C** | **A** | **G** | **A** | **T** | **C** | **C** | **T** | **G** | **A** | **A** | **A** | **T** | **C** | **T** | **T** | **A** | **T** | **G** | **G** | **A** | **G** | **A** | **G** | **A** | **A** | **T** | **T** | **C** | **A** | **G** | **C** | **A** | **C** | **A** | **C** | **T** | **A** | **T** | **C** | **G** | **G** | **G** | **G** | **G** | **G** | **A** | **G** | **A** | **G** | **A** | **A** | **G** | **A** | **C** | **A** | **A** | **A** | **A** | **G** | **T** | **C** | **C** | **T** | **A** | **C** | **T** | **G** | **G** | **C** | **A** | **C** | **T** | **T** | **T** | **T** | **A** | **T** | **T** | **T** | **G** | **T** | **G** | **G** | **A** | **T** | **G** | **A** | **C** | **A** | **C** | **T** | **C** | **A** | **T** | **T** | **T** | **C** | **C** | **C** | **G** | **T** | **T** | **A** | **A** | **T** | **C** | **G** | **A** | **T** | **G** | **A** | **A** | **C** | **C** | **A** | **A** | **C** | **G** | **A** | **A** | **T** | **C** | **A** | **T** | **T** | **T** | **G** | **G** | **A** | **-** | **-** | **-** | **T** | **A** | **T** | **C** | **T** | **C** | **T** | **G** | **G** | **T** | **A** | **G** | **A** | **A** | **A** | **A** | **C** | **A** | **A** | **G** | **T** | **A** | **G** | **C** | **G** | **G** | **C** | **T** | **T** | **A** | **T** | **T** | **T** | **G** | **A** | **A** | **A** | **A** | **A** | **G** | **A** | **A** | **A** | **A** | **A** | **A** | **C** | **A** | **A** | **G** | **G** | **C** | **T** | **T** | **C** | **A** | **T** | **C** | **G** | **T** | **G** | **G** | **T** | **C** | **A** | **G** | **C** | **C** | **A** | **T** | **G** | **A** | **C** | **C** | **G** | **G** | **G** | **G** | **A** | **T** | **T** | **T** | **A** | **T** | **C** | **G** | **A** | **T** | **G** | **A** | **A** | **G** | **T** | **A** | **G** | **T** | **G** | **G** | **A** | **C** | **C** | **A** | **T** | **G** | **T** | **T** | **T** | **T** | **A** | **G** | **C** | **A** | **A** | **T** | **C** | **G** | **A** | **A** | **A** | **A** | **A** | **A** | **G** | **T** | **C** | **A** | **A** | **C** | **T** | **G** | **G** | **A** | **A** | **C** | **T** | **T** | **T** | **A** | **T** | **C** | **A** | **A** | **G** | **G** | **G** | **A** | **A** | **T** | **T** | **T** | **C** | **T** | **C** | **T** | **A** | **T** | **C** | **T** | **A** | **T** | **G** | **A** | **A** | **G** | **A** | **A** | **C** | **A** | **G** | **A** | **A** | **A** | **A** | **A** | **A** | **C** | **T** | **T** | **C** | **G** | **T** | **G** | **A** | **T** | **G** | **A** | **A** | **T** | **T** | **T** | **G** | **A** | **A** | **A** | **T** | **G** | **G** | **C** | **T** | **C** | **A** | **A** | **A** | **A** | **T** | **G** | **A** | **A** | **A** | **A** | **A** | **T** | **T** | **G** | **A** | **A** | **A** | **A** | **A** | **A** | **G** | **A** | **A** | **G** | **T** | **C** | **A** | **G** | **T** | **A** | **G** | **G** | **C** | **T** | **A** | **A** | **A** | **G** | **A** | **A** | **A** | **A** | **C** | **A** | **G** | **C** | **A** | **G** | **C** | **T** | **G** | **A** | **A** | **A** | **A** | **A** | **G** | **C** | **C** | **G** | **A** | **A** | **T** | **G** | **G** | **T** | **C** | **T** | **C** | **G** | **T** | **T** | **C** | **C** | **C** | **G** | **A** | **G** | **A** | **A** | **G** | **G** | **A** | **G** | **A** | **T** | **A** | **A** | **A** | **A** | **C** | **A** | **A** | **A** | **G** | **A** | **A** | **A** | **C** | **A** | **A** | **G** | **T** | **C** | **G** | **G** | **A** | **T** | **T** | **C** | **A** | **T** | **C** | **G** | **A** | **T** | **A** | **C** | **T** | **G** | **A** | **A** | **T** | **C** | **T** | **A** | **G** | **A** | **C** | **G** | **A** | **G** | **T** | **G** | **A** | **A** | **T** | **A** | **A** | **A** | **G** | **G** | **A** | **G** | **C** | **A** | **G** | **T** | **G** | **G** | **G** | **T** | **G** | **C** | **T** | **G** | **A** | **T** | **G** | **C** | **T** | **G** | **C** | **A** | **C** | **G** | **G** | **A** | **C** | **G** | **A** | **T** | **G** | **A** | **A** | **A** | **C** | **G** | **A** | **T** | **C** | **C** | **A** | **A** | **A** | **G** | **C** | **A** | **A** | **T** | **C** | **G** | **T** | **G** | **A** | **A** | **T** | **C** | **G** | **G** | **A** | **T** | **G** | **G** | **A** | **G** | **A** | **C** | **C** | **C** | **A** | **G** | **A** | **T** | **C** | **A** | **G** | **C** | **G** | **A** | **G** | **A** | **A** | **A** | **G** | **A** | **A** | **A** | **A** | **A** | **C** | **T** | **A** | **T** | **T** | **A** | **A** | **A** | **A** | **G** | **A** | **T** | **A** | **T** | **C** | **G** | **A** | **A** | **T** | **A** | **T** | **A** | **T** | **C** | **G** | **A** | **T** | **T** | **C** | **T** | **T** | **T** | **G** | **A** | **C** | **G** | **A** | **T** | **G** | **A** | **A** | **T** | **A** | **G** | **C** | **C** | **A** | **A** | **G** | **C** | **G** | **T** | **C** | **T** | **C** | **A** | **C** | **C** | **A** | **T** | **A** | **A** | **G** | **C** | **G** | **A** | **C** | **T** | **T** | **T** | **T** | **A** | **A** | **G** | **C** | **G** | **T** | **A** | **G** | **A** | **A** | **G** | **A** | **T** | **C** | **T** | **T** | **C** | **A** | **A** | **T** | **T** | **A** | **G** | **G** | **G** | **T** | **A** | **T** | **G** | **A** | **A** | **A** | **A** | **T** | **C** | **T** | **G** | **T** | **T** | **A** | **T** | **T** | **C** | **G** | **A** | **G** | **C** | **C** | **A** | **A** | **T** | **T** | **C** | **A** | **T** | **T** | **T** | **T** | **A** | **C** | **A** | **A** | **T** | **C** | **G** | **A** | **G** | **C** | **C** | **T** | **C** | **A** | **T** | **C** | **A** | **G** | **C** | **G** | **G** | **G** | **T** | **G** | **G** | **C** | **G** | **A** | **T** | **T** | **T** | **C** | **A** | **G** | **G** | **T** | **C** | **C** | **T** | **A** | **A** | **C** | **G** | **G** | **T** | **G** | **C** | **A** | **G** | **G** | **A** | **A** | **A** | **G** | **T** | **C** | **A** | **T** | **C** | **C** | **A** | **T** | **T** | **A** | **T** | **C** | **C** | **A** | **T** | **T** | **A** | **T** | **C** | **T** | **T** | **C** | **T** | **G** | **G** | **G** | **G** | **G** | **C** | **A** | **T** | **T** | **C** | **A** | **A** | **C** | **G** | **G** | **C** | **A** | **A** | **G** | **G** | **T** | **T** | **A** | **T** | **A** | **G** | **G** | **A** | **G** | **A** | **A** | **A** | **A** | **A** | **T** | **A** | **C** | **C** | **A** | **G** | **C** | **C** | **A** | **A** | **A** | **A** | **C** | **A** | **T** | **C** | **T** | **G** | **A** | **G** | **C** | **A** | **T** | **T** | **A** | **G** | **T** | **T** | **A** | **T** | **G** | **C** | **A** | **A** | **G** | **C** | **C** | **A** | **A** | **A** | **A** | **T** | **T** | **A** | **T** | **G** | **A** | **A** | **G** | **A** | **C** | **A** | **A** | **T** | **C** | **G** | **A** | **G** | **G** | **A** | **A** | **C** | **G** | **T** | **T** | **G** | **G** | **C** | **G** | **G** | **A** | **A** | **T** | **T** | **T** | **G** | **C** | **A** | **G** | **A** | **G** | **A** | **A** | **A** | **A** | **A** | **C** | **C** | **A** | **A** | **G** | **T** | **A** | **G** | **A** | **C** | **T** | **A** | **C** | **C** | **A** | **A** | **G** | **C** | **A** | **T** | **T** | **T** | **T** | **T** | **G** | **A** | **A** | **C** | **A** | **A** | **C** | **C** | **T** | **C** | **C** | **G** | **A** | **A** | **A** | **G** | **C** | **T** | **T** | **G** | **G** | **G** | **A** | **T** | **G** | **G** | **A** | **A** | **A** | **G** | **A** | **G** | **A** | **T** | **G** | **T** | **T** | **T** | **T** | **T** | **C** | **A** | **T** | **A** | **A** | **C** | **A** | **A** | **G** | **A** | **T** | **C** | **G** | **A** | **G** | **C** | **A** | **G** | **A** | **T** | **G** | **A** | **G** | **T** | **A** | **T** | **G** | **G** | **G** | **C** | **C** | **A** | **A** | **C** | **G** | **G** | **A** | **A** | **A** | **A** | **A** | **A** | **G** | **T** | **G** | **G** | **A** | **A** | **T** | **T** | **G** | **G** | **C** | **T** | **A** | **A** | **A** | **T** | **C** | **T** | **T** | **T** | **A** | **T** | **C** | **A** | **C** | **A** | **G** | **C** | **C** | **A** | **G** | **C** | **T** | **G** | **A** | **A** | **C** | **T** | **A** | **T** | **A** | **T** | **A** | **C** | **A** | **T** | **G** | **G** | **G** | **A** | **T** | **G** | **A** | **A** | **C** | **C** | **A** | **T** | **T** | **G** | **A** | **A** | **T** | **T** | **A** | **T** | **T** | **T** | **G** | **G** | **A** | **T** | **G** | **T** | **C** | **T** | **T** | **C** | **A** | **A** | **T** | **C** | **A** | **A** | **G** | **A** | **A** | **C** | **A** | **A** | **T** | **T** | **A** | **G** | **A** | **A** | **C** | **A** | **A** | **C** | **T** | **G** | **A** | **T** | **C** | **T** | **T** | **G** | **A** | **A** | **C** | **G** | **T** | **G** | **A** | **A** | **A** | **C** | **C** | **T** | **G** | **C** | **C** | **A** | **T** | **G** | **T** | **T** | **A** | **C** | **T** | **A** | **G** | **T** | **G** | **G** | **A** | **A** | **C** | **A** | **T** | **G** | **A** | **T** | **C** | **A** | **A** | **A** | **C** | **C** | **T** | **T** | **C** | **C** | **T** | **G** | **G** | **A** | **T** | **A** | **A** | **A** | **G** | **T** | **A** | **T** | **C** | **T** | **A** | **C** | **T** | **G** | **A** | **G** | **A** | **T** | **T** | **A** | **T** | **T** | **T** | **C** | **T** | **C** | **T** | **T** | **G** | **A** | **G** | **A** | **G** | **A** | **A** | **T** | **C** | **-** | **-** | **-** |
| **N54538** | **A** | **T** | **G** | **T** | **C** | **T** | **A** | **A** | **A** | **A** | **T** | **C** | **G** | **A** | **A** | **A** | **T** | **A** | **A** | **A** | **A** | **A** | **A** | **T** | **C** | **T** | **G** | **A** | **C** | **A** | **T** | **T** | **C** | **G** | **G** | **C** | **T** | **A** | **C** | **G** | **A** | **C** | **A** | **G** | **C** | **C** | **A** | **A** | **G** | **G** | **C** | **A** | **C** | **A** | **T** | **T** | **A** | **T** | **T** | **A** | **T** | **T** | **T** | **G** | **A** | **A** | **C** | **A** | **A** | **G** | **C** | **A** | **A** | **A** | **T** | **C** | **T** | **A** | **A** | **A** | **T** | **T** | **T** | **T** | **G** | **A** | **C** | **A** | **C** | **A** | **C** | **A** | **A** | **T** | **G** | **G** | **A** | **A** | **A** | **C** | **T** | **A** | **G** | **G** | **A** | **C** | **T** | **T** | **A** | **T** | **C** | **G** | **G** | **A** | **C** | **G** | **A** | **A** | **A** | **C** | **G** | **G** | **T** | **C** | **G** | **A** | **G** | **G** | **A** | **A** | **A** | **G** | **A** | **C** | **A** | **A** | **C** | **T** | **T** | **T** | **A** | **C** | **T** | **G** | **A** | **A** | **T** | **A** | **T** | **T** | **C** | **T** | **A** | **C** | **A** | **A** | **A** | **A** | **C** | **A** | **A** | **A** | **C** | **T** | **A** | **C** | **C** | **T** | **T** | **A** | **T** | **C** | **A** | **A** | **G** | **G** | **G** | **C** | **A** | **A** | **G** | **T** | **A** | **A** | **T** | **C** | **C** | **A** | **T** | **C** | **A** | **G** | **C** | **A** | **A** | **G** | **A** | **A** | **T** | **T** | **T** | **G** | **T** | **C** | **T** | **A** | **T** | **T** | **T** | **C** | **C** | **C** | **G** | **C** | **A** | **G** | **C** | **A** | **A** | **A** | **C** | **A** | **A** | **A** | **A** | **G** | **A** | **T** | **A** | **A** | **A** | **G** | **A** | **G** | **C** | **G** | **T** | **T** | **T** | **A** | **A** | **C** | **C** | **T** | **A** | **T** | **T** | **A** | **T** | **G** | **T** | **G** | **T** | **T** | **A** | **A** | **A** | **T** | **G** | **A** | **G** | **A** | **T** | **T** | **A** | **C** | **G** | **G** | **A** | **T** | **T** | **T** | **T** | **G** | **A** | **G** | **A** | **T** | **A** | **T** | **G** | **G** | **G** | **A** | **A** | **A** | **T** | **T** | **G** | **A** | **A** | **A** | **G** | **A** | **G** | **A** | **G** | **C** | **T** | **C** | **C** | **A** | **A** | **C** | **T** | **G** | **A** | **T** | **G** | **C** | **A** | **A** | **A** | **C** | **A** | **G** | **A** | **T** | **C** | **C** | **T** | **G** | **A** | **A** | **A** | **T** | **C** | **T** | **T** | **A** | **T** | **G** | **G** | **A** | **G** | **A** | **G** | **A** | **A** | **T** | **T** | **C** | **A** | **G** | **C** | **A** | **C** | **A** | **C** | **T** | **A** | **T** | **C** | **A** | **G** | **G** | **A** | **G** | **G** | **A** | **G** | **A** | **G** | **A** | **A** | **G** | **A** | **C** | **A** | **A** | **A** | **A** | **G** | **T** | **C** | **C** | **T** | **A** | **C** | **T** | **G** | **G** | **C** | **A** | **C** | **T** | **T** | **T** | **T** | **A** | **T** | **T** | **T** | **G** | **T** | **G** | **G** | **A** | **T** | **G** | **A** | **C** | **A** | **C** | **T** | **C** | **A** | **T** | **T** | **T** | **C** | **C** | **C** | **G** | **T** | **T** | **A** | **A** | **T** | **C** | **G** | **A** | **T** | **G** | **A** | **A** | **C** | **C** | **A** | **A** | **C** | **G** | **A** | **A** | **T** | **C** | **A** | **T** | **T** | **T** | **G** | **G** | **A** | **-** | **-** | **-** | **T** | **A** | **T** | **C** | **T** | **C** | **T** | **G** | **G** | **C** | **A** | **G** | **A** | **A** | **A** | **A** | **C** | **A** | **A** | **G** | **T** | **A** | **G** | **C** | **G** | **G** | **C** | **T** | **T** | **A** | **C** | **T** | **T** | **G** | **A** | **A** | **A** | **A** | **A** | **G** | **A** | **A** | **A** | **A** | **A** | **A** | **C** | **A** | **A** | **G** | **G** | **C** | **T** | **T** | **C** | **A** | **T** | **C** | **G** | **T** | **G** | **G** | **T** | **C** | **A** | **G** | **C** | **C** | **A** | **T** | **G** | **A** | **C** | **C** | **G** | **G** | **G** | **G** | **A** | **T** | **T** | **T** | **A** | **T** | **C** | **G** | **A** | **T** | **G** | **A** | **A** | **G** | **T** | **A** | **G** | **T** | **G** | **G** | **A** | **C** | **C** | **A** | **T** | **G** | **T** | **T** | **T** | **T** | **A** | **G** | **C** | **A** | **A** | **T** | **C** | **G** | **A** | **A** | **A** | **A** | **A** | **A** | **G** | **T** | **C** | **A** | **A** | **C** | **T** | **G** | **G** | **A** | **A** | **C** | **T** | **T** | **T** | **A** | **T** | **C** | **A** | **A** | **G** | **G** | **G** | **A** | **A** | **T** | **T** | **T** | **C** | **T** | **C** | **T** | **A** | **T** | **C** | **T** | **A** | **T** | **G** | **A** | **A** | **G** | **A** | **A** | **C** | **A** | **G** | **A** | **A** | **A** | **A** | **A** | **A** | **C** | **T** | **T** | **C** | **G** | **T** | **G** | **A** | **T** | **G** | **A** | **A** | **T** | **T** | **T** | **G** | **A** | **A** | **A** | **T** | **A** | **G** | **C** | **T** | **C** | **A** | **A** | **A** | **A** | **T** | **G** | **A** | **A** | **A** | **A** | **A** | **T** | **T** | **G** | **A** | **A** | **A** | **A** | **A** | **A** | **G** | **A** | **A** | **G** | **T** | **C** | **A** | **G** | **T** | **A** | **G** | **G** | **C** | **T** | **A** | **A** | **A** | **G** | **A** | **A** | **A** | **A** | **C** | **A** | **G** | **C** | **A** | **G** | **C** | **T** | **G** | **A** | **A** | **A** | **A** | **A** | **G** | **C** | **C** | **G** | **A** | **A** | **T** | **G** | **G** | **T** | **C** | **T** | **C** | **G** | **T** | **T** | **C** | **C** | **C** | **G** | **A** | **G** | **A** | **A** | **G** | **G** | **A** | **G** | **A** | **T** | **A** | **A** | **A** | **A** | **C** | **A** | **A** | **A** | **G** | **A** | **A** | **A** | **C** | **A** | **A** | **G** | **T** | **C** | **G** | **G** | **A** | **T** | **T** | **C** | **A** | **T** | **C** | **G** | **A** | **T** | **A** | **C** | **T** | **G** | **A** | **A** | **T** | **C** | **T** | **A** | **G** | **A** | **C** | **G** | **A** | **G** | **T** | **G** | **A** | **A** | **T** | **A** | **A** | **A** | **G** | **G** | **A** | **G** | **C** | **A** | **G** | **T** | **G** | **G** | **G** | **T** | **G** | **C** | **T** | **G** | **A** | **T** | **G** | **C** | **T** | **G** | **C** | **A** | **C** | **G** | **G** | **A** | **C** | **G** | **A** | **T** | **G** | **A** | **A** | **A** | **C** | **G** | **A** | **T** | **C** | **C** | **A** | **A** | **A** | **G** | **C** | **A** | **A** | **T** | **C** | **G** | **T** | **G** | **A** | **A** | **T** | **C** | **G** | **G** | **A** | **T** | **G** | **G** | **A** | **G** | **A** | **C** | **C** | **C** | **A** | **G** | **A** | **T** | **C** | **A** | **G** | **C** | **G** | **A** | **G** | **A** | **A** | **A** | **G** | **A** | **A** | **A** | **A** | **A** | **C** | **T** | **A** | **T** | **T** | **A** | **A** | **A** | **A** | **G** | **A** | **T** | **A** | **T** | **C** | **G** | **A** | **A** | **T** | **A** | **T** | **A** | **T** | **C** | **G** | **A** | **T** | **T** | **C** | **T** | **T** | **T** | **G** | **A** | **C** | **G** | **A** | **T** | **G** | **A** | **A** | **T** | **A** | **G** | **C** | **C** | **A** | **A** | **G** | **C** | **G** | **T** | **C** | **T** | **C** | **A** | **C** | **C** | **A** | **T** | **A** | **A** | **G** | **C** | **G** | **A** | **C** | **T** | **T** | **T** | **T** | **A** | **A** | **G** | **C** | **G** | **T** | **A** | **G** | **A** | **A** | **G** | **A** | **T** | **C** | **T** | **T** | **C** | **A** | **A** | **T** | **T** | **A** | **G** | **G** | **G** | **T** | **A** | **T** | **G** | **A** | **A** | **A** | **A** | **T** | **C** | **T** | **G** | **T** | **T** | **A** | **T** | **T** | **C** | **G** | **A** | **G** | **C** | **C** | **A** | **A** | **T** | **T** | **C** | **A** | **T** | **T** | **T** | **T** | **A** | **C** | **A** | **A** | **T** | **C** | **G** | **A** | **G** | **C** | **C** | **T** | **C** | **A** | **T** | **C** | **A** | **G** | **C** | **G** | **G** | **G** | **T** | **G** | **G** | **C** | **G** | **A** | **T** | **T** | **T** | **C** | **A** | **G** | **G** | **T** | **C** | **C** | **T** | **A** | **A** | **C** | **G** | **G** | **T** | **G** | **C** | **A** | **G** | **G** | **A** | **A** | **A** | **G** | **T** | **C** | **A** | **T** | **C** | **C** | **A** | **T** | **T** | **A** | **T** | **C** | **C** | **A** | **T** | **T** | **A** | **T** | **C** | **T** | **T** | **C** | **T** | **G** | **G** | **G** | **G** | **G** | **C** | **A** | **T** | **T** | **C** | **A** | **A** | **C** | **G** | **G** | **C** | **A** | **A** | **G** | **G** | **T** | **T** | **A** | **T** | **A** | **G** | **G** | **A** | **G** | **A** | **A** | **A** | **A** | **A** | **T** | **C** | **C** | **C** | **A** | **G** | **C** | **C** | **A** | **A** | **A** | **A** | **C** | **A** | **T** | **C** | **T** | **G** | **A** | **G** | **C** | **A** | **T** | **T** | **A** | **G** | **T** | **T** | **A** | **T** | **G** | **T** | **A** | **C** | **G** | **C** | **C** | **A** | **A** | **A** | **A** | **T** | **T** | **A** | **T** | **G** | **A** | **A** | **G** | **A** | **C** | **A** | **A** | **T** | **C** | **G** | **A** | **G** | **G** | **A** | **A** | **C** | **G** | **T** | **T** | **G** | **G** | **C** | **G** | **G** | **A** | **A** | **T** | **T** | **T** | **G** | **C** | **A** | **G** | **A** | **G** | **A** | **A** | **A** | **A** | **A** | **C** | **C** | **A** | **A** | **G** | **T** | **A** | **G** | **A** | **C** | **T** | **A** | **C** | **C** | **A** | **A** | **G** | **C** | **A** | **T** | **T** | **T** | **T** | **T** | **G** | **A** | **A** | **C** | **A** | **A** | **C** | **C** | **T** | **C** | **C** | **G** | **A** | **A** | **A** | **G** | **C** | **T** | **T** | **G** | **G** | **G** | **A** | **T** | **G** | **G** | **A** | **A** | **A** | **G** | **A** | **G** | **A** | **T** | **G** | **T** | **T** | **T** | **T** | **T** | **C** | **A** | **T** | **A** | **A** | **C** | **A** | **A** | **G** | **A** | **T** | **C** | **G** | **A** | **G** | **C** | **A** | **G** | **A** | **T** | **G** | **A** | **G** | **T** | **A** | **T** | **G** | **G** | **G** | **C** | **C** | **A** | **A** | **C** | **G** | **G** | **A** | **A** | **A** | **A** | **A** | **A** | **G** | **T** | **G** | **G** | **A** | **A** | **T** | **T** | **G** | **G** | **C** | **T** | **A** | **A** | **A** | **T** | **C** | **T** | **T** | **T** | **A** | **T** | **C** | **A** | **C** | **A** | **G** | **C** | **C** | **A** | **G** | **C** | **T** | **G** | **A** | **A** | **C** | **T** | **A** | **T** | **A** | **T** | **A** | **T** | **A** | **T** | **G** | **G** | **G** | **A** | **T** | **G** | **A** | **A** | **C** | **C** | **A** | **T** | **T** | **G** | **A** | **A** | **T** | **T** | **A** | **T** | **T** | **T** | **G** | **G** | **A** | **T** | **G** | **T** | **C** | **T** | **T** | **C** | **A** | **A** | **T** | **C** | **A** | **A** | **G** | **A** | **A** | **C** | **A** | **A** | **T** | **T** | **A** | **G** | **A** | **A** | **C** | **A** | **A** | **C** | **T** | **G** | **A** | **T** | **C** | **T** | **T** | **G** | **A** | **A** | **C** | **G** | **T** | **G** | **A** | **A** | **A** | **C** | **C** | **T** | **G** | **C** | **C** | **A** | **T** | **G** | **T** | **T** | **A** | **C** | **T** | **A** | **G** | **T** | **G** | **G** | **A** | **A** | **C** | **A** | **T** | **G** | **A** | **T** | **C** | **A** | **A** | **A** | **C** | **C** | **T** | **T** | **C** | **C** | **T** | **G** | **G** | **A** | **T** | **A** | **A** | **A** | **G** | **T** | **A** | **T** | **C** | **T** | **A** | **C** | **T** | **G** | **A** | **G** | **A** | **T** | **T** | **A** | **T** | **T** | **T** | **C** | **T** | **C** | **T** | **T** | **G** | **A** | **G** | **A** | **A** | **A** | **A** | **T** | **C** | **-** | **-** | **-** |
| **N60061F** | **A** | **T** | **G** | **T** | **C** | **T** | **A** | **A** | **A** | **A** | **T** | **C** | **G** | **A** | **A** | **A** | **T** | **A** | **A** | **A** | **A** | **A** | **A** | **T** | **C** | **T** | **G** | **A** | **C** | **A** | **T** | **T** | **C** | **G** | **G** | **C** | **T** | **A** | **C** | **G** | **A** | **C** | **A** | **G** | **C** | **C** | **A** | **A** | **G** | **G** | **C** | **A** | **C** | **A** | **T** | **T** | **A** | **T** | **T** | **A** | **T** | **T** | **T** | **G** | **A** | **A** | **C** | **A** | **A** | **G** | **C** | **A** | **A** | **A** | **T** | **C** | **T** | **A** | **A** | **A** | **T** | **T** | **T** | **T** | **G** | **A** | **C** | **A** | **T** | **A** | **C** | **A** | **A** | **T** | **G** | **G** | **A** | **A** | **A** | **C** | **T** | **A** | **G** | **G** | **A** | **C** | **T** | **T** | **A** | **T** | **C** | **G** | **G** | **A** | **C** | **G** | **A** | **A** | **A** | **C** | **G** | **G** | **T** | **C** | **G** | **A** | **G** | **G** | **A** | **A** | **A** | **G** | **A** | **C** | **A** | **A** | **C** | **T** | **T** | **T** | **A** | **C** | **T** | **G** | **A** | **A** | **T** | **A** | **T** | **T** | **C** | **T** | **A** | **C** | **A** | **A** | **A** | **A** | **C** | **A** | **A** | **A** | **C** | **T** | **A** | **C** | **C** | **T** | **T** | **A** | **T** | **C** | **A** | **A** | **G** | **G** | **G** | **C** | **A** | **A** | **G** | **T** | **A** | **A** | **T** | **C** | **C** | **A** | **T** | **C** | **A** | **G** | **C** | **A** | **A** | **G** | **A** | **A** | **T** | **T** | **T** | **G** | **T** | **C** | **T** | **A** | **T** | **T** | **T** | **C** | **C** | **C** | **G** | **C** | **A** | **A** | **C** | **A** | **G** | **A** | **C** | **A** | **A** | **A** | **A** | **G** | **A** | **T** | **A** | **A** | **A** | **G** | **A** | **A** | **C** | **G** | **T** | **T** | **T** | **A** | **A** | **C** | **C** | **T** | **A** | **T** | **T** | **A** | **C** | **G** | **T** | **G** | **T** | **T** | **A** | **A** | **A** | **T** | **G** | **A** | **T** | **A** | **T** | **T** | **A** | **C** | **G** | **G** | **A** | **T** | **T** | **T** | **T** | **G** | **A** | **G** | **A** | **T** | **A** | **T** | **G** | **G** | **G** | **A** | **A** | **A** | **T** | **C** | **G** | **A** | **A** | **A** | **G** | **A** | **G** | **A** | **G** | **C** | **T** | **C** | **C** | **A** | **A** | **T** | **T** | **G** | **A** | **T** | **G** | **C** | **A** | **A** | **A** | **C** | **A** | **G** | **A** | **T** | **C** | **C** | **T** | **G** | **A** | **A** | **A** | **T** | **C** | **T** | **T** | **A** | **T** | **G** | **G** | **A** | **G** | **A** | **G** | **A** | **A** | **T** | **T** | **C** | **A** | **G** | **C** | **A** | **C** | **A** | **C** | **T** | **A** | **T** | **C** | **A** | **G** | **G** | **G** | **G** | **G** | **A** | **G** | **A** | **G** | **A** | **A** | **G** | **A** | **C** | **A** | **A** | **A** | **A** | **G** | **T** | **C** | **C** | **T** | **A** | **C** | **T** | **G** | **G** | **C** | **A** | **C** | **T** | **T** | **T** | **T** | **A** | **T** | **T** | **T** | **G** | **T** | **G** | **G** | **A** | **T** | **G** | **A** | **C** | **A** | **C** | **T** | **C** | **A** | **T** | **T** | **T** | **C** | **C** | **C** | **G** | **T** | **T** | **A** | **A** | **T** | **C** | **G** | **A** | **T** | **G** | **A** | **A** | **C** | **C** | **A** | **A** | **C** | **G** | **A** | **A** | **T** | **C** | **A** | **T** | **T** | **T** | **G** | **G** | **A** | **-** | **-** | **-** | **T** | **A** | **T** | **C** | **T** | **C** | **T** | **G** | **G** | **T** | **A** | **G** | **A** | **A** | **A** | **A** | **C** | **A** | **A** | **G** | **T** | **A** | **G** | **C** | **G** | **G** | **C** | **T** | **T** | **A** | **T** | **T** | **T** | **G** | **A** | **A** | **A** | **A** | **A** | **G** | **A** | **A** | **A** | **A** | **A** | **A** | **C** | **A** | **A** | **G** | **G** | **C** | **T** | **T** | **C** | **A** | **T** | **C** | **G** | **T** | **G** | **G** | **T** | **C** | **A** | **G** | **C** | **C** | **A** | **T** | **G** | **A** | **C** | **C** | **G** | **G** | **G** | **G** | **A** | **T** | **T** | **T** | **A** | **T** | **C** | **G** | **A** | **T** | **G** | **A** | **A** | **G** | **T** | **A** | **G** | **T** | **G** | **G** | **A** | **C** | **C** | **A** | **T** | **G** | **T** | **T** | **T** | **T** | **A** | **G** | **C** | **A** | **A** | **T** | **C** | **G** | **A** | **A** | **A** | **A** | **A** | **A** | **G** | **T** | **C** | **A** | **A** | **C** | **T** | **G** | **G** | **A** | **A** | **C** | **T** | **T** | **T** | **A** | **T** | **C** | **A** | **A** | **G** | **G** | **G** | **A** | **A** | **T** | **T** | **T** | **C** | **T** | **C** | **T** | **A** | **T** | **C** | **T** | **A** | **T** | **G** | **A** | **A** | **G** | **A** | **A** | **C** | **A** | **G** | **A** | **A** | **A** | **A** | **A** | **A** | **C** | **T** | **T** | **C** | **G** | **T** | **G** | **A** | **T** | **G** | **A** | **A** | **T** | **T** | **T** | **G** | **A** | **A** | **A** | **T** | **G** | **G** | **C** | **T** | **C** | **A** | **A** | **A** | **A** | **T** | **G** | **A** | **A** | **A** | **A** | **A** | **T** | **T** | **G** | **A** | **A** | **A** | **A** | **A** | **A** | **G** | **A** | **A** | **G** | **T** | **C** | **A** | **G** | **T** | **A** | **G** | **G** | **C** | **T** | **A** | **A** | **A** | **G** | **A** | **A** | **A** | **A** | **C** | **A** | **G** | **C** | **A** | **G** | **C** | **T** | **G** | **A** | **A** | **A** | **A** | **A** | **G** | **C** | **C** | **G** | **A** | **A** | **T** | **G** | **G** | **T** | **C** | **T** | **C** | **G** | **T** | **T** | **C** | **C** | **C** | **G** | **A** | **G** | **A** | **A** | **G** | **G** | **A** | **G** | **A** | **T** | **A** | **A** | **A** | **A** | **C** | **A** | **A** | **A** | **G** | **A** | **A** | **A** | **C** | **A** | **A** | **G** | **T** | **C** | **G** | **G** | **A** | **T** | **T** | **C** | **A** | **T** | **C** | **G** | **A** | **T** | **A** | **C** | **T** | **G** | **A** | **A** | **T** | **C** | **T** | **A** | **G** | **A** | **C** | **G** | **A** | **G** | **T** | **G** | **A** | **A** | **T** | **A** | **A** | **A** | **G** | **G** | **A** | **G** | **C** | **A** | **G** | **T** | **G** | **G** | **G** | **T** | **G** | **C** | **T** | **G** | **A** | **T** | **G** | **C** | **T** | **G** | **C** | **A** | **C** | **G** | **G** | **A** | **C** | **G** | **A** | **T** | **G** | **A** | **A** | **A** | **C** | **G** | **A** | **T** | **C** | **C** | **A** | **A** | **A** | **G** | **C** | **A** | **A** | **T** | **C** | **G** | **T** | **G** | **A** | **A** | **T** | **C** | **G** | **G** | **A** | **T** | **G** | **G** | **A** | **G** | **A** | **C** | **C** | **C** | **A** | **G** | **A** | **T** | **C** | **A** | **G** | **C** | **G** | **A** | **G** | **A** | **A** | **A** | **G** | **A** | **A** | **A** | **A** | **A** | **C** | **T** | **A** | **T** | **T** | **A** | **A** | **A** | **A** | **G** | **A** | **T** | **A** | **T** | **C** | **G** | **A** | **A** | **T** | **A** | **T** | **A** | **T** | **C** | **G** | **A** | **T** | **T** | **C** | **T** | **T** | **T** | **G** | **A** | **C** | **G** | **A** | **T** | **G** | **A** | **A** | **T** | **A** | **G** | **T** | **C** | **A** | **A** | **G** | **C** | **G** | **T** | **C** | **T** | **C** | **A** | **C** | **C** | **A** | **T** | **A** | **A** | **G** | **C** | **G** | **A** | **C** | **T** | **T** | **T** | **T** | **A** | **A** | **G** | **C** | **G** | **T** | **A** | **G** | **A** | **A** | **G** | **A** | **T** | **C** | **T** | **T** | **C** | **A** | **A** | **T** | **T** | **A** | **G** | **G** | **G** | **T** | **A** | **T** | **G** | **A** | **A** | **A** | **A** | **T** | **C** | **T** | **G** | **T** | **T** | **A** | **T** | **T** | **C** | **G** | **A** | **G** | **C** | **C** | **A** | **A** | **T** | **T** | **C** | **A** | **T** | **T** | **T** | **T** | **A** | **C** | **A** | **A** | **T** | **C** | **G** | **A** | **G** | **C** | **C** | **T** | **C** | **A** | **T** | **C** | **A** | **G** | **C** | **G** | **G** | **G** | **T** | **G** | **G** | **C** | **G** | **A** | **T** | **T** | **T** | **C** | **A** | **G** | **G** | **T** | **C** | **C** | **T** | **A** | **A** | **C** | **G** | **G** | **T** | **G** | **C** | **A** | **G** | **G** | **A** | **A** | **A** | **G** | **T** | **C** | **A** | **T** | **C** | **C** | **A** | **T** | **T** | **A** | **T** | **C** | **C** | **A** | **T** | **T** | **A** | **T** | **C** | **T** | **T** | **C** | **T** | **G** | **G** | **G** | **G** | **G** | **C** | **A** | **T** | **T** | **C** | **A** | **A** | **C** | **G** | **G** | **C** | **A** | **A** | **G** | **G** | **T** | **T** | **A** | **T** | **A** | **G** | **G** | **A** | **G** | **A** | **A** | **A** | **A** | **A** | **T** | **C** | **C** | **C** | **A** | **G** | **C** | **C** | **A** | **A** | **A** | **A** | **C** | **A** | **T** | **C** | **T** | **G** | **A** | **G** | **C** | **A** | **T** | **T** | **A** | **G** | **T** | **T** | **A** | **T** | **G** | **T** | **A** | **C** | **G** | **C** | **C** | **A** | **A** | **A** | **A** | **T** | **T** | **A** | **T** | **G** | **A** | **A** | **G** | **A** | **C** | **A** | **A** | **T** | **C** | **G** | **A** | **G** | **G** | **A** | **A** | **C** | **G** | **T** | **T** | **G** | **G** | **C** | **G** | **G** | **A** | **A** | **T** | **T** | **T** | **G** | **C** | **A** | **G** | **A** | **G** | **A** | **A** | **A** | **A** | **A** | **C** | **C** | **A** | **A** | **G** | **T** | **A** | **G** | **A** | **C** | **T** | **A** | **C** | **C** | **A** | **A** | **G** | **C** | **A** | **T** | **T** | **T** | **T** | **T** | **G** | **A** | **A** | **C** | **A** | **A** | **C** | **C** | **T** | **C** | **C** | **G** | **A** | **A** | **A** | **G** | **C** | **T** | **T** | **G** | **G** | **G** | **A** | **T** | **G** | **G** | **A** | **A** | **A** | **G** | **A** | **G** | **A** | **T** | **G** | **T** | **T** | **T** | **T** | **T** | **C** | **A** | **T** | **A** | **A** | **C** | **A** | **A** | **G** | **A** | **T** | **C** | **G** | **A** | **G** | **C** | **A** | **G** | **A** | **T** | **G** | **A** | **G** | **T** | **A** | **T** | **G** | **G** | **G** | **T** | **C** | **A** | **A** | **C** | **G** | **G** | **A** | **A** | **A** | **A** | **A** | **A** | **G** | **T** | **G** | **G** | **A** | **A** | **T** | **T** | **G** | **G** | **C** | **T** | **A** | **A** | **A** | **T** | **C** | **T** | **T** | **T** | **A** | **T** | **C** | **A** | **C** | **A** | **G** | **C** | **C** | **A** | **G** | **C** | **T** | **G** | **A** | **A** | **C** | **T** | **A** | **T** | **A** | **T** | **A** | **T** | **A** | **T** | **G** | **G** | **G** | **A** | **T** | **G** | **A** | **A** | **C** | **C** | **A** | **T** | **T** | **G** | **A** | **A** | **T** | **T** | **A** | **T** | **T** | **T** | **G** | **G** | **A** | **T** | **G** | **T** | **C** | **T** | **T** | **C** | **A** | **A** | **T** | **C** | **A** | **A** | **G** | **A** | **A** | **C** | **A** | **A** | **T** | **T** | **A** | **G** | **A** | **A** | **C** | **A** | **A** | **C** | **T** | **G** | **A** | **T** | **C** | **T** | **T** | **G** | **A** | **A** | **C** | **G** | **T** | **G** | **A** | **A** | **A** | **C** | **C** | **T** | **G** | **C** | **C** | **A** | **T** | **G** | **T** | **T** | **A** | **C** | **T** | **A** | **G** | **T** | **G** | **G** | **A** | **A** | **C** | **A** | **T** | **G** | **A** | **T** | **C** | **A** | **A** | **A** | **C** | **C** | **T** | **T** | **C** | **C** | **T** | **G** | **G** | **A** | **T** | **A** | **A** | **A** | **G** | **T** | **A** | **T** | **C** | **T** | **A** | **C** | **T** | **G** | **A** | **G** | **A** | **T** | **T** | **A** | **T** | **T** | **T** | **C** | **T** | **C** | **T** | **T** | **G** | **A** | **G** | **A** | **A** | **A** | **A** | **T** | **C** | **-** | **-** | **-** |
| **N52690** | **A** | **T** | **G** | **T** | **C** | **T** | **A** | **A** | **A** | **A** | **T** | **C** | **G** | **A** | **A** | **A** | **T** | **A** | **A** | **A** | **A** | **A** | **A** | **T** | **C** | **T** | **G** | **A** | **C** | **A** | **T** | **T** | **C** | **G** | **G** | **C** | **T** | **A** | **C** | **G** | **A** | **C** | **A** | **G** | **C** | **C** | **A** | **A** | **G** | **G** | **C** | **A** | **C** | **A** | **T** | **T** | **A** | **T** | **T** | **A** | **T** | **T** | **T** | **G** | **A** | **A** | **C** | **A** | **A** | **G** | **C** | **A** | **A** | **A** | **T** | **C** | **T** | **A** | **A** | **A** | **T** | **T** | **T** | **T** | **G** | **A** | **C** | **A** | **C** | **A** | **C** | **A** | **A** | **T** | **G** | **G** | **A** | **A** | **A** | **C** | **T** | **A** | **G** | **G** | **A** | **C** | **T** | **T** | **A** | **T** | **C** | **G** | **G** | **A** | **C** | **G** | **A** | **A** | **A** | **C** | **G** | **G** | **T** | **C** | **G** | **A** | **G** | **G** | **A** | **A** | **A** | **G** | **A** | **C** | **A** | **A** | **C** | **T** | **T** | **T** | **A** | **C** | **T** | **G** | **A** | **A** | **T** | **A** | **T** | **T** | **C** | **T** | **A** | **C** | **A** | **A** | **A** | **A** | **C** | **A** | **A** | **A** | **C** | **T** | **A** | **C** | **C** | **T** | **T** | **A** | **T** | **C** | **A** | **A** | **G** | **G** | **G** | **C** | **A** | **A** | **G** | **T** | **A** | **A** | **T** | **C** | **C** | **A** | **T** | **C** | **A** | **G** | **C** | **A** | **A** | **G** | **A** | **A** | **T** | **T** | **T** | **G** | **T** | **C** | **T** | **A** | **T** | **T** | **T** | **C** | **C** | **C** | **G** | **C** | **A** | **A** | **C** | **A** | **G** | **A** | **C** | **A** | **A** | **A** | **A** | **G** | **A** | **T** | **A** | **A** | **A** | **G** | **A** | **A** | **C** | **G** | **T** | **T** | **T** | **A** | **A** | **C** | **C** | **T** | **A** | **T** | **T** | **A** | **C** | **G** | **T** | **G** | **T** | **T** | **A** | **A** | **A** | **T** | **G** | **A** | **T** | **A** | **T** | **T** | **A** | **C** | **G** | **G** | **A** | **T** | **T** | **T** | **T** | **G** | **A** | **G** | **A** | **T** | **A** | **T** | **G** | **G** | **G** | **A** | **A** | **A** | **T** | **C** | **G** | **A** | **A** | **A** | **G** | **A** | **G** | **A** | **G** | **C** | **T** | **C** | **C** | **A** | **A** | **T** | **T** | **G** | **A** | **T** | **G** | **C** | **A** | **A** | **A** | **C** | **A** | **G** | **A** | **T** | **C** | **C** | **T** | **G** | **A** | **A** | **A** | **T** | **C** | **T** | **T** | **A** | **T** | **G** | **G** | **A** | **G** | **A** | **G** | **A** | **A** | **T** | **T** | **C** | **A** | **G** | **C** | **A** | **C** | **A** | **C** | **T** | **A** | **T** | **C** | **G** | **G** | **G** | **G** | **G** | **G** | **A** | **G** | **A** | **G** | **A** | **A** | **G** | **A** | **C** | **A** | **A** | **A** | **A** | **G** | **T** | **C** | **C** | **T** | **A** | **C** | **T** | **G** | **G** | **C** | **A** | **C** | **T** | **T** | **T** | **T** | **A** | **T** | **T** | **T** | **G** | **T** | **G** | **G** | **A** | **T** | **G** | **A** | **C** | **A** | **C** | **T** | **C** | **A** | **T** | **T** | **T** | **C** | **C** | **C** | **G** | **T** | **T** | **A** | **A** | **T** | **C** | **G** | **A** | **T** | **G** | **A** | **A** | **C** | **C** | **A** | **A** | **C** | **G** | **A** | **A** | **T** | **C** | **A** | **T** | **T** | **T** | **G** | **G** | **A** | **-** | **-** | **-** | **T** | **A** | **T** | **C** | **T** | **C** | **T** | **G** | **G** | **T** | **A** | **G** | **A** | **A** | **A** | **A** | **C** | **A** | **A** | **G** | **T** | **A** | **G** | **C** | **G** | **G** | **C** | **T** | **T** | **A** | **T** | **T** | **T** | **G** | **A** | **A** | **A** | **A** | **A** | **G** | **A** | **A** | **A** | **A** | **A** | **A** | **C** | **A** | **A** | **G** | **G** | **C** | **T** | **T** | **C** | **A** | **T** | **C** | **G** | **T** | **G** | **G** | **T** | **C** | **A** | **G** | **C** | **C** | **A** | **T** | **G** | **A** | **C** | **C** | **G** | **G** | **G** | **G** | **A** | **T** | **T** | **T** | **A** | **T** | **C** | **G** | **A** | **T** | **G** | **A** | **A** | **G** | **T** | **A** | **G** | **T** | **G** | **G** | **A** | **C** | **C** | **A** | **T** | **G** | **T** | **T** | **T** | **T** | **A** | **G** | **C** | **A** | **A** | **T** | **C** | **G** | **A** | **A** | **A** | **A** | **A** | **A** | **G** | **T** | **C** | **A** | **A** | **C** | **T** | **G** | **G** | **A** | **A** | **C** | **T** | **T** | **T** | **A** | **T** | **C** | **A** | **A** | **G** | **G** | **G** | **A** | **A** | **T** | **T** | **T** | **C** | **T** | **C** | **T** | **A** | **T** | **C** | **T** | **A** | **T** | **G** | **A** | **A** | **G** | **A** | **A** | **C** | **A** | **G** | **A** | **A** | **A** | **A** | **A** | **A** | **C** | **T** | **T** | **C** | **G** | **T** | **G** | **A** | **T** | **G** | **A** | **A** | **T** | **T** | **T** | **G** | **A** | **A** | **A** | **T** | **G** | **G** | **C** | **T** | **C** | **A** | **A** | **A** | **A** | **T** | **G** | **A** | **A** | **A** | **A** | **A** | **T** | **T** | **G** | **A** | **A** | **A** | **A** | **A** | **A** | **G** | **A** | **A** | **G** | **T** | **C** | **A** | **G** | **T** | **A** | **G** | **G** | **C** | **T** | **A** | **A** | **A** | **G** | **A** | **A** | **A** | **A** | **C** | **A** | **G** | **C** | **A** | **G** | **C** | **T** | **G** | **A** | **A** | **A** | **A** | **A** | **G** | **C** | **C** | **G** | **A** | **A** | **T** | **G** | **G** | **T** | **C** | **T** | **C** | **G** | **T** | **T** | **C** | **C** | **C** | **G** | **A** | **G** | **A** | **A** | **G** | **G** | **A** | **G** | **A** | **T** | **A** | **A** | **A** | **A** | **C** | **A** | **A** | **A** | **G** | **A** | **A** | **A** | **C** | **A** | **A** | **G** | **T** | **C** | **G** | **G** | **A** | **T** | **T** | **C** | **A** | **T** | **C** | **G** | **A** | **T** | **A** | **C** | **T** | **G** | **A** | **A** | **T** | **C** | **T** | **A** | **G** | **A** | **C** | **G** | **A** | **G** | **T** | **G** | **A** | **A** | **T** | **A** | **A** | **A** | **G** | **G** | **A** | **G** | **C** | **A** | **G** | **T** | **G** | **G** | **G** | **T** | **G** | **C** | **T** | **G** | **A** | **T** | **G** | **C** | **T** | **G** | **C** | **A** | **C** | **G** | **G** | **A** | **C** | **G** | **A** | **T** | **G** | **A** | **A** | **A** | **C** | **G** | **A** | **T** | **C** | **C** | **A** | **A** | **A** | **G** | **C** | **A** | **A** | **T** | **C** | **G** | **T** | **G** | **A** | **A** | **T** | **C** | **G** | **G** | **A** | **T** | **G** | **G** | **A** | **G** | **A** | **C** | **C** | **C** | **A** | **G** | **A** | **T** | **C** | **A** | **G** | **C** | **G** | **A** | **G** | **A** | **A** | **A** | **G** | **A** | **A** | **A** | **A** | **A** | **C** | **T** | **A** | **T** | **T** | **A** | **A** | **A** | **A** | **G** | **A** | **T** | **A** | **T** | **C** | **G** | **A** | **A** | **T** | **A** | **T** | **A** | **T** | **C** | **G** | **A** | **T** | **T** | **C** | **T** | **T** | **T** | **G** | **A** | **C** | **G** | **A** | **T** | **G** | **A** | **A** | **T** | **A** | **G** | **C** | **C** | **A** | **A** | **G** | **C** | **G** | **T** | **C** | **T** | **C** | **A** | **C** | **C** | **A** | **T** | **A** | **A** | **G** | **C** | **G** | **A** | **C** | **T** | **T** | **T** | **T** | **A** | **A** | **G** | **C** | **G** | **T** | **A** | **G** | **A** | **A** | **G** | **A** | **T** | **C** | **T** | **T** | **C** | **A** | **A** | **T** | **T** | **A** | **G** | **G** | **G** | **T** | **A** | **T** | **G** | **G** | **A** | **A** | **A** | **T** | **C** | **T** | **G** | **T** | **T** | **A** | **T** | **T** | **C** | **G** | **A** | **G** | **C** | **C** | **A** | **A** | **T** | **T** | **C** | **A** | **T** | **T** | **T** | **T** | **A** | **C** | **A** | **A** | **T** | **C** | **G** | **A** | **G** | **C** | **C** | **T** | **C** | **A** | **T** | **C** | **A** | **G** | **C** | **G** | **G** | **G** | **T** | **G** | **G** | **C** | **G** | **A** | **T** | **T** | **T** | **C** | **A** | **G** | **G** | **T** | **C** | **C** | **T** | **A** | **A** | **C** | **G** | **G** | **T** | **G** | **C** | **A** | **G** | **G** | **A** | **A** | **A** | **G** | **T** | **C** | **A** | **T** | **C** | **C** | **A** | **T** | **T** | **A** | **T** | **C** | **C** | **A** | **T** | **T** | **A** | **T** | **C** | **T** | **T** | **C** | **T** | **G** | **G** | **G** | **G** | **G** | **C** | **A** | **T** | **T** | **C** | **A** | **A** | **C** | **G** | **G** | **C** | **A** | **A** | **G** | **G** | **T** | **T** | **A** | **T** | **A** | **G** | **G** | **A** | **G** | **A** | **A** | **A** | **A** | **A** | **T** | **C** | **C** | **C** | **A** | **G** | **C** | **C** | **A** | **A** | **A** | **A** | **C** | **A** | **T** | **C** | **T** | **G** | **A** | **G** | **C** | **A** | **T** | **T** | **A** | **G** | **T** | **T** | **A** | **T** | **G** | **T** | **A** | **C** | **G** | **C** | **C** | **A** | **A** | **A** | **A** | **T** | **T** | **A** | **T** | **G** | **A** | **A** | **G** | **A** | **C** | **A** | **A** | **T** | **C** | **G** | **A** | **G** | **G** | **A** | **A** | **C** | **G** | **T** | **T** | **G** | **G** | **C** | **G** | **G** | **A** | **A** | **T** | **T** | **T** | **G** | **C** | **A** | **G** | **A** | **G** | **A** | **A** | **A** | **A** | **A** | **C** | **C** | **A** | **A** | **G** | **T** | **A** | **G** | **A** | **C** | **T** | **A** | **C** | **C** | **A** | **A** | **G** | **C** | **A** | **T** | **T** | **T** | **T** | **T** | **G** | **A** | **A** | **C** | **A** | **A** | **C** | **C** | **T** | **C** | **C** | **G** | **A** | **A** | **A** | **G** | **C** | **T** | **T** | **G** | **G** | **G** | **A** | **T** | **G** | **G** | **A** | **A** | **A** | **G** | **A** | **G** | **A** | **T** | **G** | **T** | **T** | **T** | **T** | **T** | **C** | **A** | **T** | **A** | **A** | **C** | **A** | **A** | **G** | **A** | **T** | **T** | **G** | **A** | **G** | **C** | **A** | **G** | **A** | **T** | **G** | **A** | **G** | **T** | **A** | **T** | **G** | **G** | **G** | **C** | **C** | **A** | **A** | **C** | **G** | **G** | **A** | **A** | **A** | **A** | **A** | **A** | **G** | **T** | **G** | **G** | **A** | **A** | **T** | **T** | **G** | **G** | **C** | **T** | **A** | **A** | **A** | **T** | **C** | **T** | **T** | **T** | **A** | **T** | **C** | **A** | **C** | **A** | **G** | **C** | **C** | **A** | **G** | **C** | **T** | **G** | **A** | **A** | **C** | **T** | **A** | **T** | **A** | **T** | **A** | **T** | **A** | **T** | **G** | **G** | **G** | **A** | **T** | **G** | **A** | **A** | **C** | **C** | **A** | **T** | **T** | **G** | **A** | **A** | **T** | **T** | **A** | **T** | **T** | **T** | **G** | **G** | **A** | **T** | **G** | **T** | **C** | **T** | **T** | **C** | **A** | **A** | **T** | **C** | **A** | **A** | **G** | **A** | **A** | **C** | **A** | **A** | **T** | **T** | **A** | **G** | **A** | **A** | **C** | **A** | **A** | **C** | **T** | **G** | **A** | **T** | **C** | **T** | **T** | **G** | **A** | **A** | **C** | **G** | **T** | **G** | **A** | **A** | **A** | **C** | **C** | **T** | **G** | **C** | **C** | **A** | **T** | **G** | **T** | **T** | **A** | **C** | **T** | **A** | **G** | **T** | **G** | **G** | **A** | **A** | **C** | **A** | **T** | **G** | **A** | **T** | **C** | **A** | **A** | **A** | **C** | **C** | **T** | **T** | **C** | **C** | **T** | **G** | **G** | **A** | **T** | **A** | **A** | **A** | **G** | **T** | **A** | **T** | **C** | **T** | **A** | **C** | **T** | **G** | **A** | **G** | **A** | **T** | **T** | **A** | **T** | **T** | **T** | **C** | **T** | **C** | **T** | **T** | **G** | **A** | **G** | **A** | **A** | **A** | **A** | **T** | **C** | **-** | **-** | **-** |
| **N52636** | **A** | **T** | **G** | **T** | **C** | **T** | **A** | **A** | **A** | **A** | **T** | **C** | **G** | **A** | **A** | **A** | **T** | **A** | **A** | **A** | **A** | **A** | **A** | **T** | **C** | **T** | **G** | **A** | **C** | **A** | **T** | **T** | **C** | **G** | **G** | **C** | **T** | **A** | **C** | **G** | **A** | **C** | **A** | **G** | **C** | **C** | **A** | **A** | **G** | **G** | **C** | **A** | **C** | **A** | **T** | **T** | **A** | **T** | **T** | **A** | **T** | **T** | **T** | **G** | **A** | **A** | **C** | **A** | **A** | **G** | **C** | **A** | **A** | **A** | **T** | **C** | **T** | **A** | **A** | **A** | **T** | **T** | **T** | **T** | **G** | **A** | **C** | **A** | **C** | **A** | **C** | **A** | **A** | **T** | **G** | **G** | **A** | **A** | **A** | **C** | **T** | **A** | **G** | **G** | **A** | **C** | **T** | **T** | **A** | **T** | **C** | **G** | **G** | **A** | **C** | **G** | **A** | **A** | **A** | **C** | **G** | **G** | **T** | **C** | **G** | **A** | **G** | **G** | **A** | **A** | **A** | **G** | **A** | **C** | **A** | **A** | **C** | **T** | **T** | **T** | **A** | **C** | **T** | **G** | **A** | **A** | **T** | **A** | **T** | **T** | **C** | **T** | **A** | **C** | **A** | **A** | **A** | **A** | **C** | **A** | **A** | **A** | **C** | **T** | **A** | **C** | **C** | **T** | **T** | **A** | **T** | **C** | **A** | **A** | **G** | **G** | **G** | **C** | **A** | **A** | **G** | **T** | **A** | **A** | **T** | **C** | **C** | **A** | **T** | **C** | **A** | **G** | **C** | **A** | **A** | **G** | **A** | **A** | **T** | **T** | **T** | **G** | **T** | **C** | **T** | **A** | **T** | **T** | **T** | **C** | **C** | **C** | **G** | **C** | **A** | **A** | **C** | **A** | **G** | **A** | **C** | **A** | **A** | **A** | **A** | **G** | **A** | **T** | **A** | **A** | **A** | **G** | **A** | **A** | **C** | **G** | **T** | **T** | **T** | **A** | **A** | **C** | **C** | **T** | **A** | **T** | **T** | **A** | **C** | **G** | **T** | **G** | **T** | **T** | **A** | **A** | **A** | **T** | **G** | **A** | **T** | **A** | **T** | **T** | **A** | **C** | **G** | **G** | **A** | **T** | **T** | **T** | **T** | **G** | **A** | **G** | **A** | **T** | **A** | **T** | **G** | **G** | **G** | **A** | **A** | **A** | **T** | **C** | **G** | **A** | **A** | **A** | **G** | **A** | **G** | **A** | **G** | **C** | **T** | **C** | **C** | **A** | **A** | **T** | **T** | **G** | **A** | **T** | **G** | **C** | **A** | **A** | **A** | **C** | **A** | **G** | **A** | **T** | **C** | **C** | **T** | **G** | **A** | **A** | **A** | **T** | **C** | **T** | **T** | **A** | **T** | **G** | **G** | **A** | **G** | **A** | **G** | **A** | **A** | **T** | **T** | **C** | **A** | **G** | **C** | **A** | **C** | **A** | **C** | **T** | **A** | **T** | **C** | **A** | **G** | **G** | **G** | **G** | **G** | **A** | **G** | **A** | **G** | **A** | **A** | **G** | **A** | **C** | **A** | **A** | **A** | **A** | **G** | **T** | **C** | **C** | **T** | **A** | **C** | **T** | **G** | **G** | **C** | **A** | **C** | **T** | **T** | **T** | **T** | **A** | **T** | **T** | **T** | **G** | **T** | **G** | **G** | **A** | **T** | **G** | **A** | **C** | **A** | **C** | **T** | **C** | **A** | **T** | **T** | **T** | **C** | **C** | **C** | **G** | **T** | **T** | **A** | **A** | **T** | **C** | **G** | **A** | **T** | **G** | **A** | **A** | **C** | **C** | **A** | **A** | **C** | **G** | **A** | **A** | **T** | **C** | **A** | **T** | **T** | **T** | **G** | **G** | **A** | **-** | **-** | **-** | **T** | **A** | **T** | **C** | **T** | **C** | **T** | **G** | **G** | **T** | **A** | **G** | **A** | **A** | **A** | **A** | **C** | **A** | **A** | **G** | **T** | **A** | **G** | **C** | **G** | **G** | **C** | **T** | **T** | **A** | **T** | **T** | **T** | **G** | **A** | **A** | **A** | **A** | **A** | **G** | **A** | **A** | **A** | **A** | **A** | **A** | **C** | **A** | **A** | **G** | **G** | **C** | **T** | **T** | **C** | **A** | **T** | **C** | **G** | **T** | **G** | **G** | **T** | **C** | **A** | **G** | **C** | **C** | **A** | **T** | **G** | **A** | **C** | **C** | **G** | **G** | **G** | **G** | **A** | **T** | **T** | **T** | **A** | **T** | **C** | **G** | **A** | **T** | **G** | **A** | **A** | **G** | **T** | **A** | **G** | **T** | **G** | **G** | **A** | **C** | **C** | **A** | **T** | **G** | **T** | **T** | **T** | **T** | **A** | **G** | **C** | **A** | **A** | **T** | **C** | **G** | **A** | **A** | **A** | **A** | **A** | **A** | **G** | **T** | **C** | **A** | **A** | **C** | **T** | **G** | **G** | **A** | **A** | **C** | **T** | **T** | **T** | **A** | **T** | **C** | **A** | **A** | **G** | **G** | **G** | **A** | **A** | **T** | **T** | **T** | **C** | **T** | **C** | **T** | **A** | **T** | **C** | **T** | **A** | **T** | **G** | **A** | **A** | **G** | **A** | **A** | **C** | **A** | **G** | **A** | **A** | **A** | **A** | **A** | **A** | **C** | **T** | **T** | **C** | **G** | **T** | **G** | **A** | **T** | **G** | **A** | **A** | **T** | **T** | **T** | **G** | **A** | **A** | **A** | **T** | **G** | **G** | **C** | **T** | **C** | **A** | **A** | **A** | **A** | **T** | **G** | **A** | **A** | **A** | **A** | **A** | **T** | **T** | **G** | **A** | **A** | **A** | **A** | **A** | **A** | **G** | **A** | **A** | **G** | **T** | **C** | **A** | **G** | **T** | **A** | **G** | **G** | **C** | **T** | **A** | **A** | **A** | **G** | **A** | **A** | **A** | **A** | **C** | **A** | **G** | **C** | **A** | **G** | **C** | **T** | **G** | **A** | **A** | **A** | **A** | **A** | **G** | **C** | **C** | **G** | **A** | **A** | **T** | **G** | **G** | **T** | **C** | **T** | **C** | **G** | **T** | **T** | **C** | **C** | **C** | **G** | **A** | **G** | **A** | **A** | **G** | **G** | **A** | **G** | **A** | **T** | **A** | **A** | **A** | **A** | **C** | **A** | **A** | **A** | **G** | **A** | **A** | **A** | **C** | **A** | **A** | **G** | **T** | **C** | **G** | **G** | **A** | **T** | **T** | **C** | **A** | **T** | **C** | **G** | **A** | **T** | **A** | **C** | **T** | **G** | **A** | **A** | **T** | **C** | **T** | **A** | **G** | **A** | **C** | **G** | **A** | **G** | **T** | **G** | **A** | **A** | **T** | **A** | **A** | **A** | **G** | **G** | **A** | **G** | **C** | **A** | **G** | **T** | **G** | **G** | **G** | **T** | **G** | **C** | **T** | **G** | **A** | **T** | **G** | **C** | **T** | **G** | **C** | **A** | **C** | **G** | **G** | **A** | **C** | **G** | **A** | **T** | **G** | **A** | **A** | **A** | **C** | **G** | **A** | **T** | **C** | **C** | **A** | **A** | **A** | **G** | **C** | **A** | **A** | **T** | **C** | **G** | **T** | **G** | **A** | **A** | **T** | **C** | **G** | **G** | **A** | **T** | **G** | **G** | **A** | **G** | **A** | **C** | **C** | **C** | **A** | **G** | **A** | **T** | **C** | **A** | **G** | **C** | **G** | **A** | **G** | **A** | **A** | **A** | **G** | **A** | **A** | **A** | **A** | **A** | **C** | **T** | **A** | **T** | **T** | **A** | **A** | **A** | **A** | **G** | **A** | **T** | **A** | **T** | **C** | **G** | **A** | **A** | **T** | **A** | **T** | **A** | **T** | **C** | **G** | **A** | **T** | **T** | **C** | **T** | **T** | **T** | **G** | **A** | **C** | **G** | **A** | **T** | **G** | **A** | **A** | **T** | **A** | **G** | **C** | **C** | **A** | **A** | **G** | **C** | **G** | **T** | **C** | **T** | **C** | **A** | **C** | **C** | **A** | **T** | **A** | **A** | **G** | **C** | **G** | **A** | **C** | **T** | **T** | **T** | **T** | **A** | **A** | **G** | **C** | **G** | **T** | **A** | **G** | **A** | **A** | **G** | **A** | **T** | **C** | **T** | **T** | **C** | **A** | **A** | **T** | **T** | **A** | **G** | **G** | **G** | **T** | **A** | **T** | **G** | **A** | **A** | **A** | **A** | **T** | **C** | **T** | **G** | **T** | **T** | **A** | **T** | **T** | **C** | **G** | **A** | **G** | **C** | **C** | **A** | **A** | **T** | **T** | **C** | **A** | **T** | **T** | **T** | **T** | **A** | **C** | **A** | **A** | **T** | **C** | **G** | **A** | **G** | **C** | **C** | **T** | **C** | **A** | **T** | **C** | **A** | **G** | **C** | **G** | **G** | **G** | **T** | **G** | **G** | **C** | **G** | **A** | **T** | **T** | **T** | **C** | **A** | **G** | **G** | **T** | **C** | **C** | **T** | **A** | **A** | **C** | **G** | **G** | **T** | **G** | **C** | **A** | **G** | **G** | **A** | **A** | **A** | **G** | **T** | **C** | **A** | **T** | **C** | **C** | **A** | **T** | **T** | **A** | **T** | **C** | **C** | **A** | **T** | **T** | **A** | **T** | **C** | **T** | **T** | **C** | **T** | **G** | **G** | **G** | **G** | **G** | **C** | **A** | **T** | **T** | **C** | **A** | **A** | **C** | **G** | **G** | **C** | **A** | **A** | **G** | **G** | **T** | **T** | **A** | **T** | **A** | **G** | **G** | **A** | **G** | **A** | **A** | **A** | **A** | **A** | **T** | **C** | **C** | **C** | **A** | **G** | **C** | **C** | **A** | **A** | **A** | **A** | **C** | **A** | **T** | **C** | **T** | **G** | **A** | **G** | **C** | **A** | **T** | **T** | **A** | **G** | **T** | **T** | **A** | **T** | **G** | **T** | **A** | **C** | **G** | **C** | **C** | **A** | **A** | **A** | **A** | **T** | **T** | **A** | **T** | **G** | **A** | **A** | **G** | **A** | **C** | **A** | **A** | **T** | **C** | **G** | **A** | **G** | **G** | **A** | **A** | **C** | **G** | **T** | **T** | **G** | **G** | **C** | **G** | **G** | **A** | **A** | **T** | **T** | **T** | **G** | **C** | **A** | **G** | **A** | **G** | **A** | **A** | **A** | **A** | **A** | **C** | **C** | **A** | **A** | **G** | **T** | **A** | **G** | **A** | **C** | **T** | **A** | **C** | **C** | **A** | **A** | **G** | **C** | **A** | **T** | **T** | **T** | **T** | **T** | **G** | **A** | **A** | **C** | **A** | **A** | **C** | **C** | **T** | **C** | **C** | **G** | **A** | **A** | **A** | **G** | **C** | **T** | **T** | **G** | **G** | **G** | **A** | **T** | **G** | **G** | **A** | **A** | **A** | **G** | **A** | **G** | **A** | **T** | **G** | **T** | **T** | **T** | **T** | **T** | **C** | **A** | **T** | **A** | **A** | **C** | **A** | **A** | **G** | **A** | **T** | **C** | **G** | **A** | **G** | **C** | **A** | **G** | **A** | **T** | **G** | **A** | **G** | **T** | **A** | **T** | **G** | **G** | **G** | **C** | **C** | **A** | **A** | **C** | **G** | **G** | **A** | **A** | **A** | **A** | **A** | **A** | **G** | **T** | **G** | **G** | **A** | **A** | **T** | **T** | **G** | **G** | **C** | **T** | **A** | **A** | **A** | **T** | **C** | **T** | **T** | **T** | **A** | **T** | **C** | **A** | **C** | **A** | **G** | **C** | **C** | **A** | **G** | **C** | **T** | **G** | **A** | **A** | **C** | **T** | **A** | **T** | **A** | **T** | **A** | **T** | **A** | **T** | **G** | **G** | **G** | **A** | **T** | **G** | **A** | **A** | **C** | **C** | **A** | **T** | **T** | **G** | **A** | **A** | **T** | **T** | **A** | **T** | **T** | **T** | **G** | **G** | **A** | **T** | **G** | **T** | **C** | **T** | **T** | **C** | **A** | **A** | **T** | **C** | **A** | **A** | **G** | **A** | **A** | **C** | **A** | **A** | **T** | **T** | **A** | **G** | **A** | **A** | **C** | **A** | **A** | **C** | **T** | **G** | **A** | **T** | **C** | **T** | **T** | **G** | **A** | **A** | **C** | **G** | **T** | **G** | **A** | **A** | **A** | **C** | **C** | **T** | **G** | **C** | **C** | **A** | **T** | **G** | **T** | **T** | **A** | **C** | **T** | **A** | **G** | **T** | **G** | **G** | **A** | **A** | **C** | **A** | **T** | **G** | **A** | **T** | **C** | **A** | **A** | **A** | **C** | **C** | **T** | **T** | **C** | **C** | **T** | **G** | **G** | **A** | **T** | **A** | **A** | **A** | **G** | **T** | **A** | **T** | **C** | **T** | **A** | **C** | **T** | **G** | **A** | **G** | **A** | **T** | **T** | **A** | **T** | **T** | **T** | **C** | **T** | **C** | **T** | **T** | **G** | **A** | **G** | **A** | **A** | **A** | **A** | **T** | **C** | **-** | **-** | **-** |
| **N54519** | **A** | **T** | **G** | **T** | **C** | **T** | **A** | **A** | **A** | **A** | **T** | **C** | **G** | **A** | **A** | **A** | **T** | **A** | **A** | **A** | **A** | **A** | **A** | **T** | **C** | **T** | **G** | **A** | **C** | **A** | **T** | **T** | **C** | **G** | **G** | **C** | **T** | **A** | **C** | **G** | **A** | **C** | **A** | **G** | **C** | **C** | **A** | **A** | **G** | **G** | **C** | **A** | **C** | **A** | **T** | **T** | **A** | **T** | **T** | **A** | **T** | **T** | **T** | **G** | **A** | **A** | **C** | **A** | **A** | **G** | **C** | **A** | **A** | **A** | **T** | **C** | **T** | **A** | **A** | **A** | **T** | **T** | **T** | **T** | **G** | **A** | **C** | **A** | **C** | **A** | **C** | **A** | **A** | **T** | **G** | **G** | **A** | **A** | **A** | **C** | **T** | **A** | **G** | **G** | **A** | **C** | **T** | **T** | **A** | **T** | **C** | **G** | **G** | **A** | **C** | **G** | **A** | **A** | **A** | **C** | **G** | **G** | **T** | **C** | **G** | **A** | **G** | **G** | **A** | **A** | **A** | **G** | **A** | **C** | **A** | **A** | **C** | **T** | **T** | **T** | **A** | **C** | **T** | **G** | **A** | **A** | **T** | **A** | **T** | **T** | **C** | **T** | **A** | **C** | **A** | **A** | **A** | **A** | **C** | **A** | **A** | **A** | **C** | **T** | **A** | **C** | **C** | **T** | **T** | **A** | **T** | **C** | **A** | **A** | **G** | **G** | **G** | **C** | **A** | **A** | **G** | **T** | **A** | **A** | **T** | **C** | **C** | **A** | **T** | **C** | **A** | **G** | **C** | **A** | **A** | **G** | **A** | **A** | **T** | **T** | **T** | **G** | **T** | **C** | **T** | **A** | **T** | **T** | **T** | **C** | **C** | **C** | **G** | **C** | **A** | **A** | **C** | **A** | **G** | **A** | **C** | **A** | **A** | **A** | **A** | **G** | **A** | **T** | **A** | **A** | **A** | **G** | **A** | **A** | **C** | **G** | **T** | **T** | **T** | **A** | **A** | **C** | **C** | **T** | **A** | **T** | **T** | **A** | **C** | **G** | **T** | **G** | **T** | **T** | **A** | **A** | **A** | **T** | **G** | **A** | **T** | **A** | **T** | **T** | **A** | **C** | **G** | **G** | **A** | **T** | **T** | **T** | **T** | **G** | **A** | **G** | **A** | **T** | **A** | **T** | **G** | **G** | **G** | **A** | **A** | **A** | **T** | **C** | **G** | **A** | **A** | **A** | **G** | **A** | **G** | **A** | **G** | **C** | **T** | **C** | **C** | **A** | **A** | **T** | **T** | **G** | **A** | **T** | **A** | **C** | **A** | **A** | **A** | **C** | **A** | **G** | **A** | **T** | **C** | **C** | **T** | **G** | **A** | **A** | **A** | **T** | **C** | **T** | **T** | **A** | **T** | **G** | **G** | **A** | **G** | **A** | **G** | **A** | **A** | **T** | **T** | **C** | **A** | **G** | **C** | **A** | **C** | **A** | **C** | **T** | **A** | **T** | **C** | **G** | **G** | **G** | **G** | **G** | **G** | **A** | **G** | **A** | **G** | **A** | **A** | **G** | **A** | **C** | **A** | **A** | **A** | **A** | **G** | **T** | **C** | **C** | **T** | **A** | **C** | **T** | **G** | **G** | **C** | **A** | **C** | **T** | **T** | **T** | **T** | **A** | **T** | **T** | **T** | **G** | **T** | **G** | **G** | **A** | **T** | **G** | **A** | **C** | **A** | **C** | **T** | **C** | **A** | **T** | **T** | **T** | **C** | **C** | **C** | **G** | **T** | **T** | **A** | **A** | **T** | **C** | **G** | **A** | **T** | **G** | **A** | **A** | **C** | **C** | **A** | **A** | **C** | **G** | **A** | **A** | **T** | **C** | **A** | **T** | **T** | **T** | **G** | **G** | **A** | **-** | **-** | **-** | **T** | **A** | **T** | **C** | **T** | **C** | **T** | **G** | **G** | **T** | **A** | **G** | **A** | **A** | **A** | **A** | **C** | **A** | **A** | **G** | **T** | **A** | **G** | **C** | **G** | **G** | **C** | **T** | **T** | **A** | **T** | **T** | **T** | **G** | **A** | **A** | **A** | **A** | **A** | **G** | **A** | **A** | **A** | **A** | **A** | **A** | **C** | **A** | **A** | **G** | **G** | **C** | **T** | **T** | **C** | **A** | **T** | **C** | **G** | **T** | **G** | **G** | **T** | **C** | **A** | **G** | **C** | **C** | **A** | **T** | **G** | **A** | **C** | **C** | **G** | **G** | **G** | **G** | **A** | **T** | **T** | **T** | **A** | **T** | **C** | **G** | **A** | **T** | **G** | **A** | **A** | **G** | **T** | **A** | **G** | **T** | **G** | **G** | **A** | **C** | **C** | **A** | **T** | **G** | **T** | **T** | **T** | **T** | **A** | **G** | **C** | **A** | **A** | **T** | **C** | **G** | **A** | **A** | **A** | **A** | **A** | **A** | **G** | **T** | **C** | **A** | **A** | **C** | **T** | **G** | **G** | **A** | **A** | **C** | **T** | **T** | **T** | **A** | **T** | **C** | **A** | **A** | **G** | **G** | **G** | **A** | **A** | **T** | **T** | **T** | **C** | **T** | **C** | **T** | **A** | **T** | **C** | **T** | **A** | **T** | **G** | **A** | **A** | **G** | **A** | **A** | **C** | **A** | **G** | **A** | **A** | **A** | **A** | **A** | **A** | **C** | **T** | **T** | **C** | **G** | **T** | **G** | **A** | **T** | **G** | **A** | **A** | **T** | **T** | **T** | **G** | **A** | **A** | **A** | **T** | **G** | **G** | **C** | **T** | **C** | **A** | **A** | **A** | **A** | **T** | **G** | **A** | **A** | **A** | **A** | **A** | **T** | **T** | **G** | **A** | **A** | **A** | **A** | **A** | **A** | **G** | **A** | **A** | **G** | **T** | **C** | **A** | **G** | **T** | **A** | **G** | **G** | **C** | **T** | **A** | **A** | **A** | **G** | **A** | **A** | **A** | **A** | **C** | **A** | **G** | **C** | **A** | **G** | **C** | **T** | **G** | **A** | **A** | **A** | **A** | **A** | **G** | **C** | **C** | **G** | **A** | **A** | **T** | **G** | **G** | **T** | **C** | **T** | **C** | **G** | **T** | **T** | **C** | **C** | **C** | **G** | **A** | **G** | **A** | **A** | **G** | **G** | **A** | **G** | **A** | **T** | **A** | **A** | **A** | **A** | **C** | **A** | **A** | **A** | **G** | **A** | **A** | **A** | **C** | **A** | **A** | **G** | **T** | **C** | **G** | **G** | **A** | **T** | **T** | **C** | **A** | **T** | **C** | **G** | **A** | **T** | **A** | **C** | **T** | **G** | **A** | **A** | **T** | **C** | **T** | **A** | **G** | **A** | **C** | **G** | **A** | **G** | **T** | **G** | **A** | **A** | **T** | **A** | **A** | **A** | **G** | **G** | **A** | **G** | **C** | **A** | **G** | **T** | **G** | **G** | **G** | **T** | **G** | **C** | **T** | **G** | **A** | **T** | **G** | **C** | **T** | **G** | **C** | **A** | **C** | **G** | **G** | **A** | **C** | **G** | **A** | **T** | **G** | **A** | **A** | **A** | **C** | **G** | **A** | **T** | **C** | **C** | **A** | **A** | **A** | **G** | **C** | **A** | **A** | **T** | **C** | **G** | **T** | **G** | **A** | **A** | **T** | **C** | **G** | **G** | **A** | **T** | **G** | **G** | **A** | **G** | **A** | **C** | **C** | **C** | **A** | **G** | **A** | **T** | **C** | **A** | **G** | **C** | **G** | **A** | **G** | **A** | **A** | **A** | **G** | **A** | **A** | **A** | **A** | **A** | **C** | **T** | **A** | **T** | **T** | **A** | **A** | **A** | **A** | **G** | **A** | **T** | **A** | **T** | **C** | **G** | **A** | **A** | **T** | **A** | **T** | **A** | **T** | **C** | **G** | **A** | **T** | **T** | **C** | **T** | **T** | **T** | **G** | **A** | **C** | **G** | **A** | **T** | **G** | **A** | **A** | **T** | **A** | **G** | **C** | **C** | **A** | **A** | **G** | **C** | **G** | **T** | **C** | **T** | **C** | **A** | **C** | **C** | **A** | **T** | **A** | **A** | **G** | **C** | **G** | **A** | **C** | **T** | **T** | **T** | **T** | **A** | **A** | **G** | **C** | **G** | **T** | **A** | **G** | **A** | **A** | **G** | **A** | **T** | **C** | **T** | **T** | **C** | **A** | **A** | **T** | **T** | **A** | **G** | **G** | **G** | **T** | **A** | **T** | **G** | **A** | **A** | **A** | **A** | **T** | **C** | **T** | **G** | **T** | **T** | **A** | **T** | **T** | **C** | **G** | **A** | **G** | **C** | **C** | **A** | **A** | **T** | **T** | **C** | **A** | **T** | **T** | **T** | **T** | **A** | **C** | **A** | **A** | **T** | **C** | **G** | **A** | **G** | **C** | **C** | **T** | **C** | **A** | **T** | **C** | **A** | **G** | **C** | **G** | **G** | **G** | **T** | **G** | **G** | **C** | **G** | **A** | **T** | **T** | **T** | **C** | **A** | **G** | **G** | **T** | **C** | **C** | **T** | **A** | **A** | **C** | **G** | **G** | **T** | **G** | **C** | **A** | **G** | **G** | **A** | **A** | **A** | **G** | **T** | **C** | **A** | **T** | **C** | **C** | **A** | **T** | **T** | **A** | **T** | **C** | **C** | **A** | **T** | **T** | **A** | **T** | **C** | **T** | **T** | **C** | **T** | **G** | **G** | **G** | **G** | **G** | **C** | **A** | **T** | **T** | **C** | **A** | **A** | **C** | **G** | **G** | **C** | **A** | **A** | **G** | **G** | **T** | **T** | **A** | **T** | **A** | **G** | **G** | **A** | **G** | **A** | **A** | **A** | **A** | **A** | **T** | **C** | **C** | **C** | **A** | **G** | **C** | **C** | **A** | **A** | **A** | **A** | **C** | **A** | **T** | **C** | **T** | **G** | **A** | **G** | **C** | **A** | **T** | **T** | **A** | **G** | **T** | **T** | **A** | **T** | **G** | **T** | **A** | **C** | **G** | **C** | **C** | **A** | **A** | **A** | **A** | **T** | **T** | **A** | **T** | **G** | **A** | **A** | **G** | **A** | **T** | **A** | **A** | **T** | **C** | **G** | **A** | **G** | **G** | **A** | **A** | **C** | **G** | **T** | **T** | **G** | **G** | **C** | **G** | **G** | **A** | **A** | **T** | **T** | **T** | **G** | **C** | **A** | **G** | **A** | **G** | **A** | **A** | **A** | **A** | **A** | **C** | **C** | **A** | **A** | **G** | **T** | **A** | **G** | **A** | **C** | **T** | **A** | **C** | **C** | **A** | **A** | **G** | **C** | **A** | **T** | **T** | **T** | **T** | **T** | **G** | **A** | **A** | **C** | **A** | **A** | **C** | **C** | **T** | **C** | **C** | **G** | **A** | **A** | **A** | **G** | **C** | **T** | **T** | **G** | **G** | **G** | **A** | **T** | **G** | **G** | **A** | **A** | **A** | **G** | **A** | **G** | **A** | **T** | **G** | **T** | **T** | **T** | **T** | **T** | **C** | **A** | **T** | **A** | **A** | **C** | **A** | **A** | **G** | **A** | **T** | **T** | **G** | **A** | **G** | **C** | **A** | **G** | **A** | **T** | **G** | **A** | **G** | **T** | **A** | **T** | **G** | **G** | **G** | **C** | **C** | **A** | **A** | **C** | **G** | **G** | **A** | **A** | **A** | **A** | **A** | **A** | **G** | **T** | **G** | **G** | **A** | **A** | **T** | **T** | **G** | **G** | **C** | **T** | **A** | **A** | **A** | **T** | **C** | **T** | **T** | **T** | **A** | **T** | **C** | **A** | **C** | **A** | **G** | **C** | **C** | **A** | **G** | **C** | **T** | **G** | **A** | **A** | **C** | **T** | **A** | **T** | **A** | **T** | **A** | **T** | **A** | **T** | **G** | **G** | **G** | **A** | **T** | **G** | **A** | **A** | **C** | **C** | **A** | **T** | **T** | **G** | **A** | **A** | **T** | **T** | **A** | **T** | **T** | **T** | **G** | **G** | **A** | **T** | **G** | **T** | **C** | **T** | **T** | **C** | **A** | **A** | **T** | **C** | **A** | **A** | **G** | **A** | **A** | **C** | **A** | **A** | **T** | **T** | **A** | **G** | **A** | **A** | **C** | **A** | **A** | **C** | **T** | **G** | **A** | **T** | **C** | **T** | **T** | **G** | **A** | **A** | **C** | **G** | **T** | **G** | **A** | **A** | **A** | **C** | **C** | **T** | **G** | **C** | **C** | **A** | **T** | **G** | **T** | **T** | **A** | **C** | **T** | **G** | **G** | **T** | **G** | **G** | **A** | **A** | **C** | **A** | **T** | **G** | **A** | **T** | **C** | **A** | **A** | **A** | **C** | **C** | **T** | **T** | **C** | **C** | **T** | **G** | **G** | **A** | **T** | **A** | **A** | **A** | **G** | **T** | **A** | **T** | **C** | **T** | **A** | **C** | **T** | **G** | **A** | **G** | **A** | **T** | **T** | **A** | **T** | **T** | **T** | **C** | **T** | **C** | **T** | **T** | **G** | **A** | **G** | **A** | **A** | **A** | **A** | **T** | **C** | **-** | **-** | **-** |
| **N54560** | **A** | **T** | **G** | **T** | **C** | **T** | **A** | **A** | **A** | **A** | **T** | **C** | **G** | **A** | **A** | **A** | **T** | **A** | **A** | **A** | **A** | **A** | **A** | **T** | **C** | **T** | **G** | **A** | **C** | **A** | **T** | **T** | **C** | **G** | **G** | **C** | **T** | **A** | **C** | **G** | **A** | **C** | **A** | **G** | **C** | **C** | **A** | **A** | **G** | **G** | **C** | **A** | **C** | **A** | **T** | **T** | **A** | **T** | **T** | **A** | **T** | **T** | **T** | **G** | **A** | **A** | **C** | **A** | **A** | **G** | **C** | **A** | **A** | **A** | **T** | **C** | **T** | **A** | **A** | **A** | **T** | **T** | **T** | **T** | **G** | **A** | **C** | **A** | **T** | **A** | **C** | **A** | **A** | **T** | **G** | **G** | **A** | **A** | **A** | **C** | **T** | **A** | **G** | **G** | **A** | **C** | **T** | **T** | **A** | **T** | **C** | **G** | **G** | **A** | **C** | **G** | **A** | **A** | **A** | **C** | **G** | **G** | **T** | **C** | **G** | **A** | **G** | **G** | **A** | **A** | **A** | **G** | **A** | **C** | **A** | **A** | **C** | **T** | **T** | **T** | **A** | **C** | **T** | **G** | **A** | **A** | **T** | **A** | **T** | **T** | **C** | **T** | **A** | **C** | **A** | **A** | **A** | **A** | **C** | **A** | **A** | **A** | **C** | **T** | **A** | **C** | **C** | **T** | **T** | **A** | **T** | **C** | **A** | **A** | **G** | **G** | **G** | **C** | **A** | **A** | **G** | **T** | **A** | **A** | **T** | **C** | **C** | **A** | **T** | **C** | **A** | **G** | **C** | **A** | **A** | **G** | **A** | **A** | **T** | **T** | **T** | **G** | **T** | **C** | **T** | **A** | **T** | **T** | **T** | **C** | **C** | **C** | **G** | **C** | **A** | **A** | **C** | **A** | **G** | **A** | **C** | **A** | **A** | **A** | **A** | **G** | **A** | **T** | **A** | **A** | **A** | **G** | **A** | **A** | **C** | **G** | **T** | **T** | **T** | **A** | **A** | **C** | **C** | **T** | **A** | **T** | **T** | **A** | **C** | **G** | **T** | **G** | **T** | **T** | **A** | **A** | **A** | **T** | **G** | **A** | **T** | **A** | **T** | **T** | **A** | **C** | **G** | **G** | **A** | **T** | **T** | **T** | **T** | **G** | **A** | **G** | **A** | **T** | **A** | **T** | **G** | **G** | **G** | **A** | **A** | **A** | **T** | **C** | **G** | **A** | **A** | **A** | **G** | **A** | **G** | **A** | **G** | **C** | **T** | **C** | **C** | **A** | **A** | **T** | **T** | **G** | **A** | **T** | **G** | **C** | **A** | **A** | **A** | **C** | **A** | **G** | **A** | **T** | **C** | **C** | **T** | **G** | **A** | **A** | **A** | **T** | **C** | **T** | **T** | **A** | **T** | **G** | **G** | **A** | **G** | **A** | **G** | **A** | **A** | **T** | **T** | **C** | **A** | **G** | **C** | **A** | **C** | **A** | **C** | **T** | **A** | **T** | **C** | **A** | **G** | **G** | **G** | **G** | **G** | **A** | **G** | **A** | **G** | **A** | **A** | **G** | **A** | **C** | **A** | **A** | **A** | **A** | **G** | **T** | **C** | **C** | **T** | **A** | **C** | **T** | **G** | **G** | **C** | **A** | **C** | **T** | **T** | **T** | **T** | **A** | **T** | **T** | **T** | **G** | **T** | **G** | **G** | **A** | **T** | **G** | **A** | **C** | **A** | **C** | **T** | **C** | **A** | **T** | **T** | **T** | **C** | **C** | **C** | **G** | **T** | **T** | **A** | **A** | **T** | **C** | **G** | **A** | **T** | **G** | **A** | **A** | **C** | **C** | **A** | **A** | **C** | **G** | **A** | **A** | **T** | **C** | **A** | **T** | **T** | **T** | **G** | **G** | **A** | **-** | **-** | **-** | **T** | **A** | **T** | **C** | **T** | **C** | **T** | **G** | **G** | **T** | **A** | **G** | **A** | **A** | **A** | **A** | **C** | **A** | **A** | **G** | **T** | **A** | **G** | **C** | **G** | **G** | **C** | **T** | **T** | **A** | **T** | **T** | **T** | **G** | **A** | **A** | **A** | **A** | **A** | **G** | **A** | **A** | **A** | **A** | **A** | **A** | **C** | **A** | **A** | **G** | **G** | **C** | **T** | **T** | **C** | **A** | **T** | **C** | **G** | **T** | **G** | **G** | **T** | **C** | **A** | **G** | **C** | **C** | **A** | **T** | **G** | **A** | **C** | **C** | **G** | **G** | **G** | **G** | **A** | **T** | **T** | **T** | **A** | **T** | **C** | **G** | **A** | **T** | **G** | **A** | **A** | **G** | **T** | **A** | **G** | **T** | **G** | **G** | **A** | **C** | **C** | **A** | **T** | **G** | **T** | **T** | **T** | **T** | **A** | **G** | **C** | **A** | **A** | **T** | **C** | **G** | **A** | **A** | **A** | **A** | **A** | **A** | **G** | **T** | **C** | **A** | **A** | **C** | **T** | **G** | **G** | **A** | **A** | **C** | **T** | **T** | **T** | **A** | **T** | **C** | **A** | **A** | **G** | **G** | **G** | **A** | **A** | **T** | **T** | **T** | **C** | **T** | **C** | **T** | **A** | **T** | **C** | **T** | **A** | **T** | **G** | **A** | **A** | **G** | **A** | **A** | **C** | **A** | **G** | **A** | **A** | **A** | **A** | **A** | **A** | **C** | **T** | **T** | **C** | **G** | **T** | **G** | **A** | **T** | **G** | **A** | **A** | **T** | **T** | **T** | **G** | **A** | **A** | **A** | **T** | **G** | **G** | **C** | **T** | **C** | **A** | **A** | **A** | **A** | **T** | **G** | **A** | **A** | **A** | **A** | **A** | **T** | **T** | **G** | **A** | **A** | **A** | **A** | **A** | **A** | **G** | **A** | **A** | **G** | **T** | **C** | **A** | **G** | **T** | **A** | **G** | **G** | **C** | **T** | **A** | **A** | **A** | **G** | **A** | **A** | **A** | **A** | **C** | **A** | **G** | **C** | **A** | **G** | **C** | **T** | **G** | **A** | **A** | **A** | **A** | **A** | **G** | **C** | **C** | **G** | **A** | **A** | **T** | **G** | **G** | **T** | **C** | **T** | **C** | **G** | **T** | **T** | **C** | **C** | **C** | **G** | **A** | **G** | **A** | **A** | **G** | **G** | **A** | **G** | **A** | **T** | **A** | **A** | **A** | **A** | **C** | **A** | **A** | **A** | **G** | **A** | **A** | **A** | **C** | **A** | **A** | **G** | **T** | **C** | **G** | **G** | **A** | **T** | **T** | **C** | **A** | **T** | **C** | **G** | **A** | **T** | **A** | **C** | **T** | **G** | **A** | **A** | **T** | **C** | **T** | **A** | **G** | **A** | **C** | **G** | **A** | **G** | **T** | **G** | **A** | **A** | **T** | **A** | **A** | **A** | **G** | **G** | **A** | **G** | **C** | **A** | **G** | **T** | **G** | **G** | **G** | **T** | **G** | **C** | **T** | **G** | **A** | **T** | **G** | **C** | **T** | **G** | **C** | **A** | **C** | **G** | **G** | **A** | **C** | **G** | **A** | **T** | **G** | **A** | **A** | **A** | **C** | **G** | **A** | **T** | **C** | **C** | **A** | **A** | **A** | **G** | **C** | **A** | **A** | **T** | **C** | **G** | **T** | **G** | **A** | **A** | **T** | **C** | **G** | **G** | **A** | **T** | **G** | **G** | **A** | **G** | **A** | **C** | **C** | **C** | **A** | **G** | **A** | **T** | **C** | **A** | **G** | **C** | **G** | **A** | **G** | **A** | **A** | **A** | **G** | **A** | **A** | **A** | **A** | **A** | **C** | **T** | **A** | **T** | **T** | **A** | **A** | **A** | **A** | **G** | **A** | **T** | **A** | **T** | **C** | **G** | **A** | **A** | **T** | **A** | **T** | **A** | **T** | **C** | **G** | **A** | **T** | **T** | **C** | **T** | **T** | **T** | **G** | **A** | **C** | **G** | **A** | **T** | **G** | **A** | **A** | **T** | **A** | **G** | **T** | **C** | **A** | **A** | **G** | **C** | **G** | **T** | **C** | **T** | **C** | **A** | **C** | **C** | **A** | **T** | **A** | **A** | **G** | **C** | **G** | **A** | **C** | **T** | **T** | **T** | **T** | **A** | **A** | **G** | **C** | **G** | **T** | **A** | **G** | **A** | **A** | **G** | **A** | **T** | **C** | **T** | **T** | **C** | **A** | **A** | **T** | **T** | **A** | **G** | **G** | **G** | **T** | **A** | **T** | **G** | **A** | **A** | **A** | **A** | **T** | **C** | **T** | **G** | **T** | **T** | **A** | **T** | **T** | **C** | **G** | **A** | **G** | **C** | **C** | **A** | **A** | **T** | **T** | **C** | **A** | **T** | **T** | **T** | **T** | **A** | **C** | **A** | **A** | **T** | **C** | **G** | **A** | **G** | **C** | **C** | **T** | **C** | **A** | **T** | **C** | **A** | **G** | **C** | **G** | **G** | **G** | **T** | **G** | **G** | **C** | **G** | **A** | **T** | **T** | **T** | **C** | **A** | **G** | **G** | **T** | **C** | **C** | **T** | **A** | **A** | **C** | **G** | **G** | **T** | **G** | **C** | **A** | **G** | **G** | **A** | **A** | **A** | **G** | **T** | **C** | **A** | **T** | **C** | **C** | **A** | **T** | **T** | **A** | **T** | **C** | **C** | **A** | **T** | **T** | **A** | **T** | **C** | **T** | **T** | **C** | **T** | **G** | **G** | **G** | **G** | **G** | **C** | **A** | **T** | **T** | **C** | **A** | **A** | **C** | **G** | **G** | **C** | **A** | **A** | **G** | **G** | **T** | **T** | **A** | **T** | **A** | **G** | **G** | **A** | **G** | **A** | **A** | **A** | **A** | **A** | **T** | **C** | **C** | **C** | **A** | **G** | **C** | **C** | **A** | **A** | **A** | **A** | **C** | **A** | **T** | **C** | **T** | **G** | **A** | **G** | **C** | **A** | **T** | **T** | **A** | **G** | **T** | **T** | **A** | **T** | **G** | **T** | **A** | **C** | **G** | **C** | **C** | **A** | **A** | **A** | **A** | **T** | **T** | **A** | **T** | **G** | **A** | **A** | **G** | **A** | **C** | **A** | **A** | **T** | **C** | **G** | **A** | **G** | **G** | **A** | **A** | **C** | **G** | **T** | **T** | **G** | **G** | **C** | **G** | **G** | **A** | **A** | **T** | **T** | **T** | **G** | **C** | **A** | **G** | **A** | **G** | **A** | **A** | **A** | **A** | **A** | **C** | **C** | **A** | **A** | **G** | **T** | **A** | **G** | **A** | **C** | **T** | **A** | **C** | **C** | **A** | **A** | **G** | **C** | **A** | **T** | **T** | **T** | **T** | **T** | **G** | **A** | **A** | **C** | **A** | **A** | **C** | **C** | **T** | **C** | **C** | **G** | **A** | **A** | **A** | **G** | **C** | **T** | **T** | **G** | **G** | **G** | **A** | **T** | **G** | **G** | **A** | **A** | **A** | **G** | **A** | **G** | **A** | **T** | **G** | **T** | **T** | **T** | **T** | **T** | **C** | **A** | **T** | **A** | **A** | **C** | **A** | **A** | **G** | **A** | **T** | **C** | **G** | **A** | **G** | **C** | **A** | **G** | **A** | **T** | **G** | **A** | **G** | **T** | **A** | **T** | **G** | **G** | **G** | **T** | **C** | **A** | **A** | **C** | **G** | **G** | **A** | **A** | **A** | **A** | **A** | **A** | **G** | **T** | **G** | **G** | **A** | **A** | **T** | **T** | **G** | **G** | **C** | **T** | **A** | **A** | **A** | **T** | **C** | **T** | **T** | **T** | **A** | **T** | **C** | **A** | **C** | **A** | **G** | **C** | **C** | **A** | **G** | **C** | **T** | **G** | **A** | **A** | **C** | **T** | **A** | **T** | **A** | **T** | **A** | **T** | **A** | **T** | **G** | **G** | **G** | **A** | **T** | **G** | **A** | **A** | **C** | **C** | **A** | **T** | **T** | **G** | **A** | **A** | **T** | **T** | **A** | **T** | **T** | **T** | **G** | **G** | **A** | **T** | **G** | **T** | **C** | **T** | **T** | **C** | **A** | **A** | **T** | **C** | **A** | **A** | **G** | **A** | **A** | **C** | **A** | **A** | **T** | **T** | **A** | **G** | **A** | **A** | **C** | **A** | **A** | **C** | **T** | **G** | **A** | **T** | **C** | **T** | **T** | **G** | **A** | **A** | **C** | **G** | **T** | **G** | **A** | **A** | **A** | **C** | **C** | **T** | **G** | **C** | **C** | **A** | **T** | **G** | **T** | **T** | **A** | **C** | **T** | **A** | **G** | **T** | **G** | **G** | **A** | **A** | **C** | **A** | **T** | **G** | **A** | **T** | **C** | **A** | **A** | **A** | **C** | **C** | **T** | **T** | **C** | **C** | **T** | **G** | **G** | **A** | **T** | **A** | **A** | **A** | **G** | **T** | **A** | **T** | **C** | **T** | **A** | **C** | **T** | **G** | **A** | **G** | **A** | **T** | **T** | **A** | **T** | **T** | **T** | **C** | **T** | **C** | **T** | **T** | **G** | **A** | **G** | **A** | **A** | **A** | **A** | **T** | **C** | **-** | **-** | **-** |
| **N60200F** | **A** | **T** | **G** | **T** | **C** | **T** | **A** | **A** | **A** | **A** | **T** | **C** | **G** | **A** | **A** | **A** | **T** | **A** | **A** | **A** | **A** | **A** | **A** | **T** | **C** | **T** | **G** | **A** | **C** | **A** | **T** | **T** | **C** | **G** | **G** | **C** | **T** | **A** | **C** | **G** | **A** | **C** | **A** | **G** | **C** | **C** | **A** | **A** | **G** | **G** | **C** | **A** | **C** | **A** | **T** | **T** | **A** | **T** | **T** | **A** | **T** | **T** | **T** | **G** | **A** | **A** | **C** | **A** | **A** | **G** | **C** | **A** | **A** | **T** | **T** | **C** | **T** | **A** | **A** | **A** | **T** | **T** | **T** | **T** | **G** | **A** | **C** | **A** | **C** | **A** | **C** | **A** | **A** | **T** | **G** | **G** | **A** | **A** | **A** | **C** | **T** | **A** | **G** | **G** | **A** | **C** | **T** | **T** | **A** | **T** | **C** | **G** | **G** | **A** | **C** | **G** | **A** | **A** | **A** | **C** | **G** | **G** | **T** | **C** | **G** | **A** | **G** | **G** | **A** | **A** | **A** | **G** | **A** | **C** | **A** | **A** | **C** | **T** | **T** | **T** | **A** | **C** | **T** | **G** | **A** | **A** | **T** | **A** | **T** | **T** | **C** | **T** | **A** | **C** | **A** | **A** | **A** | **A** | **C** | **A** | **A** | **A** | **C** | **T** | **A** | **C** | **C** | **T** | **T** | **A** | **T** | **C** | **A** | **A** | **G** | **G** | **G** | **C** | **A** | **A** | **G** | **T** | **A** | **A** | **T** | **C** | **C** | **A** | **T** | **C** | **A** | **G** | **C** | **A** | **A** | **G** | **A** | **A** | **T** | **T** | **T** | **G** | **T** | **C** | **T** | **A** | **T** | **T** | **T** | **C** | **C** | **C** | **G** | **C** | **A** | **A** | **C** | **A** | **G** | **A** | **C** | **A** | **A** | **A** | **A** | **G** | **A** | **T** | **A** | **A** | **A** | **G** | **A** | **A** | **C** | **G** | **T** | **T** | **T** | **A** | **A** | **C** | **C** | **T** | **A** | **T** | **T** | **A** | **C** | **G** | **T** | **G** | **T** | **T** | **A** | **A** | **A** | **T** | **G** | **A** | **T** | **A** | **T** | **T** | **A** | **C** | **G** | **G** | **A** | **T** | **T** | **T** | **T** | **G** | **A** | **G** | **A** | **T** | **A** | **T** | **G** | **G** | **G** | **A** | **A** | **A** | **T** | **C** | **G** | **A** | **A** | **A** | **G** | **A** | **G** | **A** | **G** | **C** | **T** | **C** | **C** | **A** | **A** | **T** | **T** | **G** | **A** | **T** | **G** | **C** | **A** | **A** | **A** | **C** | **A** | **G** | **A** | **T** | **C** | **C** | **T** | **G** | **A** | **A** | **A** | **T** | **C** | **T** | **T** | **A** | **T** | **G** | **G** | **A** | **G** | **A** | **G** | **A** | **A** | **T** | **T** | **C** | **A** | **G** | **C** | **A** | **C** | **A** | **C** | **T** | **A** | **T** | **C** | **G** | **G** | **G** | **G** | **G** | **G** | **A** | **G** | **A** | **G** | **A** | **A** | **G** | **A** | **C** | **A** | **A** | **A** | **A** | **G** | **T** | **C** | **C** | **T** | **A** | **C** | **T** | **G** | **G** | **C** | **A** | **C** | **T** | **T** | **T** | **T** | **A** | **T** | **T** | **T** | **G** | **T** | **G** | **G** | **A** | **T** | **G** | **A** | **C** | **A** | **C** | **T** | **C** | **A** | **T** | **T** | **T** | **T** | **C** | **C** | **G** | **T** | **T** | **A** | **A** | **T** | **C** | **G** | **A** | **T** | **G** | **A** | **A** | **C** | **C** | **A** | **A** | **C** | **G** | **A** | **A** | **T** | **C** | **A** | **T** | **T** | **T** | **G** | **G** | **A** | **-** | **-** | **-** | **T** | **A** | **T** | **C** | **T** | **C** | **T** | **G** | **G** | **T** | **A** | **G** | **A** | **A** | **A** | **A** | **C** | **A** | **A** | **G** | **T** | **A** | **G** | **C** | **G** | **G** | **C** | **T** | **T** | **A** | **T** | **T** | **T** | **G** | **A** | **A** | **A** | **A** | **A** | **G** | **A** | **A** | **A** | **A** | **A** | **A** | **C** | **A** | **A** | **G** | **G** | **C** | **T** | **T** | **C** | **A** | **T** | **C** | **G** | **T** | **G** | **G** | **T** | **C** | **A** | **G** | **C** | **C** | **A** | **T** | **G** | **A** | **C** | **C** | **G** | **G** | **G** | **G** | **A** | **T** | **T** | **T** | **A** | **T** | **C** | **G** | **A** | **T** | **G** | **A** | **A** | **G** | **T** | **A** | **G** | **T** | **G** | **G** | **A** | **C** | **C** | **A** | **T** | **G** | **T** | **T** | **T** | **T** | **A** | **G** | **C** | **A** | **A** | **T** | **C** | **G** | **A** | **A** | **A** | **A** | **A** | **A** | **G** | **T** | **C** | **A** | **A** | **C** | **T** | **G** | **G** | **A** | **A** | **C** | **T** | **T** | **T** | **A** | **T** | **C** | **A** | **A** | **G** | **G** | **G** | **A** | **A** | **T** | **T** | **T** | **C** | **T** | **C** | **T** | **A** | **T** | **C** | **T** | **A** | **T** | **G** | **A** | **A** | **G** | **A** | **A** | **C** | **A** | **G** | **A** | **A** | **A** | **A** | **A** | **A** | **C** | **T** | **T** | **C** | **G** | **T** | **G** | **A** | **T** | **G** | **A** | **A** | **T** | **T** | **T** | **G** | **A** | **A** | **A** | **T** | **G** | **G** | **C** | **T** | **C** | **A** | **A** | **A** | **A** | **T** | **G** | **A** | **A** | **A** | **A** | **A** | **T** | **T** | **G** | **A** | **A** | **A** | **A** | **A** | **A** | **G** | **A** | **A** | **G** | **T** | **C** | **A** | **G** | **C** | **A** | **G** | **G** | **C** | **T** | **A** | **A** | **A** | **G** | **A** | **C** | **A** | **A** | **C** | **A** | **G** | **C** | **A** | **G** | **C** | **T** | **G** | **A** | **A** | **A** | **A** | **A** | **G** | **C** | **C** | **G** | **A** | **A** | **T** | **G** | **G** | **T** | **C** | **T** | **C** | **G** | **T** | **T** | **C** | **C** | **C** | **G** | **A** | **G** | **A** | **A** | **G** | **G** | **A** | **G** | **A** | **T** | **A** | **A** | **A** | **A** | **C** | **A** | **A** | **A** | **G** | **A** | **A** | **A** | **C** | **A** | **A** | **G** | **T** | **C** | **G** | **G** | **A** | **C** | **T** | **C** | **A** | **T** | **C** | **G** | **A** | **T** | **A** | **C** | **T** | **G** | **A** | **A** | **T** | **C** | **T** | **A** | **G** | **A** | **C** | **G** | **A** | **G** | **T** | **G** | **A** | **A** | **T** | **A** | **A** | **A** | **G** | **G** | **A** | **G** | **C** | **A** | **G** | **T** | **G** | **G** | **G** | **T** | **G** | **C** | **T** | **G** | **A** | **T** | **G** | **C** | **T** | **G** | **C** | **A** | **C** | **G** | **G** | **A** | **C** | **G** | **A** | **T** | **G** | **A** | **A** | **A** | **C** | **G** | **A** | **T** | **C** | **C** | **A** | **A** | **A** | **G** | **C** | **A** | **A** | **T** | **C** | **G** | **T** | **G** | **A** | **A** | **T** | **C** | **G** | **G** | **A** | **T** | **G** | **G** | **A** | **G** | **A** | **C** | **C** | **C** | **A** | **G** | **A** | **T** | **C** | **A** | **G** | **C** | **G** | **A** | **G** | **A** | **A** | **A** | **G** | **A** | **A** | **A** | **A** | **A** | **C** | **T** | **A** | **T** | **T** | **A** | **A** | **A** | **A** | **G** | **A** | **T** | **A** | **T** | **C** | **G** | **A** | **A** | **T** | **A** | **T** | **A** | **T** | **C** | **G** | **A** | **T** | **T** | **C** | **T** | **T** | **T** | **G** | **A** | **C** | **G** | **A** | **T** | **G** | **A** | **A** | **T** | **A** | **G** | **C** | **C** | **A** | **A** | **G** | **C** | **G** | **T** | **C** | **T** | **C** | **A** | **C** | **C** | **A** | **T** | **A** | **A** | **G** | **C** | **G** | **A** | **C** | **T** | **T** | **T** | **T** | **A** | **A** | **G** | **C** | **G** | **T** | **A** | **G** | **A** | **A** | **G** | **A** | **T** | **C** | **T** | **T** | **C** | **A** | **A** | **T** | **T** | **A** | **G** | **G** | **G** | **T** | **A** | **T** | **G** | **A** | **A** | **A** | **A** | **T** | **C** | **T** | **G** | **T** | **T** | **A** | **T** | **T** | **C** | **G** | **A** | **G** | **C** | **C** | **A** | **A** | **T** | **T** | **C** | **A** | **T** | **T** | **T** | **T** | **A** | **C** | **A** | **A** | **T** | **C** | **G** | **A** | **G** | **C** | **C** | **T** | **C** | **A** | **T** | **C** | **A** | **G** | **C** | **G** | **G** | **G** | **T** | **G** | **G** | **C** | **G** | **A** | **T** | **T** | **T** | **C** | **A** | **G** | **G** | **T** | **C** | **C** | **T** | **A** | **A** | **C** | **G** | **G** | **T** | **G** | **C** | **A** | **G** | **G** | **A** | **A** | **A** | **G** | **T** | **C** | **A** | **T** | **C** | **C** | **A** | **T** | **T** | **A** | **T** | **C** | **C** | **A** | **T** | **T** | **A** | **T** | **C** | **T** | **T** | **C** | **T** | **G** | **G** | **G** | **G** | **T** | **C** | **A** | **T** | **T** | **C** | **A** | **A** | **C** | **G** | **G** | **C** | **A** | **A** | **G** | **G** | **T** | **T** | **A** | **T** | **A** | **G** | **G** | **A** | **G** | **A** | **A** | **A** | **A** | **A** | **T** | **C** | **C** | **C** | **A** | **G** | **C** | **C** | **A** | **A** | **A** | **A** | **C** | **A** | **T** | **C** | **T** | **G** | **A** | **G** | **C** | **A** | **T** | **T** | **A** | **G** | **T** | **T** | **A** | **T** | **G** | **T** | **A** | **C** | **G** | **C** | **C** | **A** | **A** | **A** | **A** | **T** | **T** | **A** | **T** | **G** | **A** | **A** | **G** | **A** | **C** | **A** | **A** | **T** | **C** | **G** | **A** | **G** | **G** | **A** | **A** | **C** | **G** | **T** | **T** | **G** | **G** | **C** | **G** | **G** | **A** | **A** | **T** | **T** | **T** | **G** | **C** | **A** | **G** | **A** | **G** | **A** | **A** | **A** | **A** | **A** | **C** | **C** | **A** | **A** | **G** | **T** | **A** | **G** | **A** | **C** | **T** | **A** | **C** | **C** | **A** | **A** | **G** | **C** | **A** | **T** | **T** | **T** | **T** | **T** | **G** | **A** | **A** | **C** | **A** | **A** | **C** | **C** | **T** | **C** | **C** | **G** | **A** | **A** | **A** | **G** | **C** | **T** | **T** | **G** | **G** | **G** | **A** | **T** | **G** | **G** | **A** | **A** | **A** | **G** | **A** | **G** | **A** | **T** | **G** | **T** | **T** | **T** | **T** | **T** | **C** | **A** | **T** | **A** | **A** | **C** | **A** | **A** | **G** | **A** | **T** | **T** | **G** | **A** | **G** | **C** | **A** | **G** | **A** | **T** | **G** | **A** | **G** | **T** | **A** | **T** | **G** | **G** | **G** | **C** | **C** | **A** | **A** | **C** | **G** | **G** | **A** | **A** | **A** | **A** | **A** | **A** | **G** | **T** | **G** | **G** | **A** | **A** | **T** | **T** | **G** | **G** | **C** | **T** | **A** | **A** | **A** | **T** | **C** | **T** | **T** | **T** | **A** | **T** | **C** | **A** | **C** | **A** | **G** | **C** | **C** | **A** | **G** | **C** | **T** | **G** | **A** | **A** | **C** | **T** | **A** | **T** | **A** | **T** | **A** | **T** | **A** | **T** | **G** | **G** | **G** | **A** | **T** | **G** | **A** | **A** | **C** | **C** | **A** | **T** | **T** | **G** | **A** | **A** | **T** | **T** | **A** | **T** | **T** | **T** | **G** | **G** | **A** | **T** | **G** | **T** | **C** | **T** | **T** | **C** | **A** | **A** | **T** | **C** | **A** | **A** | **G** | **A** | **A** | **C** | **A** | **A** | **T** | **T** | **A** | **G** | **A** | **A** | **C** | **A** | **A** | **C** | **T** | **G** | **A** | **T** | **C** | **T** | **T** | **G** | **A** | **A** | **C** | **G** | **T** | **G** | **A** | **A** | **A** | **C** | **C** | **T** | **G** | **C** | **C** | **A** | **T** | **G** | **T** | **T** | **A** | **C** | **T** | **G** | **G** | **T** | **G** | **G** | **A** | **A** | **C** | **A** | **T** | **G** | **A** | **T** | **C** | **A** | **A** | **A** | **C** | **C** | **T** | **T** | **C** | **C** | **T** | **G** | **G** | **A** | **T** | **A** | **A** | **A** | **G** | **T** | **A** | **T** | **C** | **T** | **A** | **C** | **T** | **G** | **A** | **G** | **A** | **T** | **T** | **A** | **T** | **T** | **T** | **C** | **T** | **C** | **T** | **T** | **G** | **A** | **G** | **A** | **A** | **A** | **A** | **T** | **C** | **-** | **-** | **-** |
| **N55290** | **A** | **T** | **G** | **T** | **C** | **T** | **A** | **A** | **A** | **A** | **T** | **C** | **G** | **A** | **A** | **A** | **T** | **A** | **A** | **A** | **A** | **A** | **A** | **T** | **C** | **T** | **G** | **A** | **C** | **A** | **T** | **T** | **C** | **G** | **G** | **C** | **T** | **A** | **C** | **G** | **A** | **C** | **A** | **G** | **C** | **C** | **A** | **A** | **G** | **G** | **C** | **A** | **C** | **A** | **T** | **T** | **A** | **T** | **T** | **A** | **T** | **T** | **T** | **G** | **A** | **A** | **C** | **A** | **A** | **G** | **C** | **A** | **A** | **A** | **T** | **C** | **T** | **A** | **A** | **A** | **T** | **T** | **T** | **T** | **G** | **A** | **C** | **A** | **C** | **A** | **C** | **A** | **A** | **T** | **G** | **G** | **A** | **A** | **A** | **C** | **T** | **A** | **G** | **G** | **A** | **C** | **T** | **T** | **A** | **T** | **C** | **G** | **G** | **A** | **C** | **G** | **A** | **A** | **A** | **C** | **G** | **G** | **T** | **C** | **G** | **A** | **G** | **G** | **A** | **A** | **A** | **G** | **A** | **C** | **A** | **A** | **C** | **T** | **T** | **T** | **A** | **C** | **T** | **G** | **A** | **A** | **T** | **A** | **T** | **T** | **C** | **T** | **A** | **C** | **A** | **A** | **A** | **A** | **C** | **A** | **A** | **A** | **C** | **T** | **A** | **C** | **C** | **T** | **T** | **A** | **T** | **C** | **A** | **A** | **G** | **G** | **G** | **C** | **A** | **A** | **G** | **T** | **A** | **A** | **T** | **C** | **C** | **A** | **T** | **C** | **A** | **G** | **C** | **A** | **A** | **G** | **A** | **A** | **T** | **T** | **T** | **G** | **T** | **C** | **T** | **A** | **T** | **T** | **T** | **C** | **C** | **C** | **G** | **C** | **A** | **A** | **C** | **A** | **G** | **A** | **C** | **A** | **A** | **A** | **A** | **G** | **A** | **T** | **A** | **A** | **A** | **G** | **A** | **A** | **C** | **G** | **T** | **T** | **T** | **A** | **A** | **C** | **C** | **T** | **A** | **T** | **T** | **A** | **C** | **G** | **T** | **G** | **T** | **T** | **A** | **A** | **A** | **T** | **G** | **A** | **T** | **A** | **T** | **T** | **A** | **C** | **G** | **G** | **A** | **T** | **T** | **T** | **T** | **G** | **A** | **G** | **A** | **T** | **A** | **T** | **G** | **G** | **G** | **A** | **A** | **A** | **T** | **C** | **G** | **A** | **A** | **A** | **G** | **A** | **G** | **A** | **G** | **C** | **T** | **C** | **C** | **A** | **A** | **T** | **T** | **T** | **A** | **T** | **G** | **C** | **A** | **A** | **A** | **C** | **A** | **G** | **A** | **T** | **C** | **C** | **T** | **G** | **A** | **A** | **A** | **T** | **C** | **T** | **T** | **A** | **T** | **G** | **G** | **A** | **G** | **A** | **G** | **A** | **A** | **T** | **T** | **C** | **A** | **G** | **C** | **A** | **C** | **A** | **C** | **T** | **A** | **T** | **C** | **G** | **G** | **G** | **G** | **G** | **G** | **A** | **G** | **A** | **G** | **A** | **A** | **G** | **A** | **C** | **A** | **A** | **A** | **A** | **G** | **T** | **C** | **C** | **T** | **A** | **T** | **T** | **G** | **G** | **C** | **A** | **C** | **T** | **T** | **T** | **T** | **A** | **T** | **T** | **T** | **G** | **T** | **G** | **G** | **A** | **T** | **G** | **A** | **C** | **A** | **C** | **T** | **C** | **A** | **T** | **T** | **T** | **C** | **C** | **C** | **G** | **T** | **T** | **A** | **A** | **T** | **C** | **G** | **A** | **T** | **G** | **A** | **A** | **C** | **C** | **A** | **A** | **C** | **G** | **A** | **A** | **T** | **C** | **A** | **T** | **T** | **T** | **G** | **G** | **A** | **-** | **-** | **-** | **T** | **A** | **T** | **C** | **T** | **C** | **T** | **G** | **G** | **T** | **A** | **G** | **A** | **A** | **A** | **A** | **C** | **A** | **A** | **G** | **T** | **A** | **G** | **C** | **G** | **G** | **C** | **T** | **T** | **A** | **T** | **T** | **T** | **G** | **A** | **A** | **A** | **A** | **A** | **G** | **A** | **A** | **A** | **A** | **A** | **A** | **C** | **A** | **A** | **G** | **G** | **C** | **T** | **T** | **C** | **A** | **T** | **C** | **G** | **T** | **G** | **G** | **T** | **C** | **A** | **G** | **C** | **C** | **A** | **T** | **G** | **A** | **C** | **C** | **G** | **G** | **G** | **G** | **A** | **T** | **T** | **T** | **A** | **T** | **C** | **G** | **A** | **T** | **G** | **A** | **A** | **G** | **T** | **A** | **G** | **T** | **G** | **G** | **A** | **C** | **C** | **A** | **T** | **G** | **T** | **T** | **T** | **T** | **A** | **G** | **C** | **A** | **A** | **T** | **C** | **G** | **A** | **A** | **A** | **A** | **A** | **A** | **G** | **T** | **C** | **A** | **A** | **C** | **T** | **G** | **G** | **A** | **A** | **C** | **T** | **T** | **T** | **A** | **T** | **C** | **A** | **A** | **G** | **G** | **G** | **A** | **A** | **T** | **T** | **T** | **C** | **T** | **C** | **T** | **A** | **T** | **C** | **T** | **A** | **T** | **G** | **A** | **A** | **G** | **A** | **A** | **C** | **A** | **G** | **A** | **A** | **A** | **A** | **A** | **A** | **C** | **T** | **T** | **C** | **G** | **T** | **G** | **A** | **T** | **G** | **A** | **A** | **T** | **T** | **T** | **G** | **A** | **A** | **A** | **T** | **G** | **G** | **C** | **T** | **C** | **A** | **A** | **A** | **A** | **T** | **G** | **A** | **A** | **A** | **A** | **A** | **T** | **T** | **G** | **A** | **A** | **A** | **A** | **A** | **A** | **G** | **A** | **A** | **G** | **T** | **C** | **A** | **G** | **T** | **A** | **G** | **G** | **C** | **T** | **A** | **A** | **A** | **G** | **A** | **A** | **A** | **A** | **C** | **A** | **G** | **C** | **A** | **G** | **C** | **T** | **G** | **A** | **A** | **A** | **A** | **A** | **G** | **C** | **C** | **G** | **A** | **A** | **T** | **G** | **G** | **T** | **C** | **T** | **C** | **G** | **T** | **T** | **C** | **C** | **C** | **G** | **A** | **G** | **A** | **A** | **G** | **G** | **A** | **G** | **A** | **T** | **A** | **A** | **A** | **A** | **C** | **A** | **A** | **A** | **G** | **A** | **A** | **A** | **C** | **A** | **A** | **G** | **T** | **C** | **G** | **G** | **A** | **T** | **T** | **C** | **A** | **T** | **C** | **G** | **A** | **T** | **A** | **C** | **T** | **G** | **A** | **A** | **T** | **C** | **T** | **A** | **G** | **A** | **C** | **G** | **A** | **G** | **T** | **G** | **A** | **A** | **T** | **A** | **A** | **A** | **G** | **G** | **A** | **G** | **C** | **A** | **G** | **T** | **G** | **G** | **G** | **T** | **G** | **C** | **T** | **G** | **A** | **T** | **G** | **C** | **T** | **G** | **C** | **A** | **C** | **G** | **G** | **G** | **C** | **G** | **A** | **T** | **G** | **A** | **A** | **A** | **C** | **G** | **A** | **T** | **C** | **C** | **A** | **A** | **A** | **G** | **C** | **A** | **A** | **T** | **C** | **G** | **T** | **G** | **A** | **A** | **T** | **C** | **G** | **G** | **A** | **T** | **G** | **G** | **A** | **G** | **A** | **C** | **C** | **C** | **A** | **G** | **A** | **T** | **C** | **A** | **G** | **C** | **G** | **A** | **G** | **A** | **A** | **A** | **G** | **A** | **A** | **A** | **A** | **A** | **C** | **T** | **A** | **T** | **T** | **A** | **A** | **A** | **A** | **G** | **A** | **T** | **A** | **T** | **C** | **G** | **A** | **A** | **T** | **A** | **T** | **A** | **T** | **C** | **G** | **A** | **T** | **T** | **C** | **T** | **T** | **T** | **G** | **A** | **C** | **G** | **A** | **T** | **G** | **A** | **A** | **T** | **A** | **G** | **C** | **C** | **A** | **A** | **G** | **C** | **G** | **T** | **C** | **T** | **C** | **A** | **C** | **C** | **A** | **T** | **A** | **A** | **G** | **C** | **G** | **A** | **C** | **T** | **T** | **T** | **T** | **A** | **A** | **G** | **C** | **G** | **T** | **A** | **G** | **A** | **A** | **G** | **A** | **T** | **C** | **T** | **T** | **C** | **A** | **A** | **T** | **T** | **A** | **G** | **G** | **G** | **T** | **A** | **T** | **G** | **A** | **A** | **A** | **A** | **T** | **C** | **T** | **G** | **T** | **T** | **A** | **T** | **T** | **C** | **G** | **A** | **G** | **C** | **C** | **A** | **A** | **T** | **T** | **C** | **A** | **T** | **T** | **T** | **T** | **A** | **C** | **A** | **A** | **T** | **C** | **G** | **A** | **G** | **C** | **C** | **T** | **C** | **A** | **T** | **C** | **A** | **G** | **C** | **G** | **G** | **G** | **T** | **G** | **G** | **C** | **G** | **A** | **T** | **T** | **T** | **C** | **A** | **G** | **G** | **T** | **C** | **C** | **T** | **A** | **A** | **C** | **G** | **G** | **T** | **G** | **C** | **A** | **G** | **G** | **A** | **A** | **A** | **G** | **T** | **C** | **A** | **T** | **C** | **C** | **A** | **T** | **T** | **A** | **T** | **C** | **C** | **A** | **T** | **T** | **A** | **T** | **C** | **T** | **T** | **C** | **T** | **G** | **G** | **G** | **G** | **G** | **C** | **A** | **T** | **T** | **C** | **A** | **A** | **C** | **G** | **G** | **C** | **A** | **A** | **G** | **G** | **T** | **T** | **A** | **T** | **A** | **G** | **G** | **A** | **G** | **A** | **A** | **A** | **A** | **A** | **T** | **C** | **C** | **C** | **A** | **G** | **C** | **C** | **A** | **A** | **A** | **A** | **C** | **A** | **T** | **C** | **T** | **G** | **A** | **G** | **C** | **A** | **T** | **T** | **A** | **G** | **T** | **T** | **A** | **T** | **G** | **T** | **A** | **C** | **G** | **C** | **C** | **A** | **A** | **A** | **A** | **T** | **T** | **A** | **T** | **G** | **A** | **A** | **G** | **A** | **C** | **A** | **A** | **T** | **C** | **G** | **A** | **G** | **G** | **A** | **A** | **C** | **G** | **T** | **T** | **G** | **G** | **C** | **G** | **G** | **A** | **A** | **T** | **T** | **T** | **G** | **C** | **A** | **G** | **A** | **G** | **A** | **A** | **A** | **A** | **A** | **C** | **C** | **A** | **A** | **G** | **T** | **A** | **G** | **A** | **C** | **T** | **A** | **C** | **C** | **A** | **A** | **G** | **C** | **A** | **T** | **T** | **T** | **T** | **T** | **G** | **A** | **A** | **C** | **A** | **A** | **C** | **C** | **T** | **C** | **C** | **G** | **A** | **A** | **A** | **G** | **C** | **T** | **T** | **G** | **G** | **G** | **A** | **T** | **G** | **G** | **A** | **A** | **A** | **G** | **A** | **G** | **A** | **T** | **G** | **T** | **T** | **T** | **T** | **T** | **C** | **A** | **T** | **A** | **A** | **C** | **A** | **A** | **G** | **A** | **T** | **T** | **G** | **A** | **G** | **C** | **A** | **G** | **A** | **T** | **G** | **A** | **G** | **T** | **A** | **T** | **G** | **G** | **G** | **C** | **C** | **A** | **A** | **C** | **G** | **G** | **A** | **A** | **A** | **A** | **A** | **A** | **G** | **T** | **G** | **G** | **A** | **A** | **T** | **T** | **G** | **G** | **C** | **T** | **A** | **A** | **A** | **T** | **C** | **T** | **T** | **T** | **A** | **T** | **C** | **A** | **C** | **A** | **G** | **C** | **C** | **A** | **G** | **C** | **T** | **G** | **A** | **A** | **C** | **T** | **A** | **T** | **A** | **T** | **A** | **T** | **A** | **T** | **G** | **G** | **G** | **A** | **T** | **G** | **A** | **A** | **C** | **C** | **A** | **T** | **T** | **G** | **A** | **A** | **T** | **T** | **A** | **T** | **T** | **T** | **G** | **G** | **A** | **T** | **G** | **T** | **C** | **T** | **T** | **C** | **A** | **A** | **T** | **C** | **A** | **A** | **G** | **A** | **A** | **C** | **A** | **A** | **T** | **T** | **A** | **G** | **A** | **A** | **C** | **A** | **A** | **C** | **T** | **G** | **A** | **T** | **C** | **T** | **T** | **G** | **A** | **A** | **C** | **G** | **T** | **G** | **A** | **A** | **A** | **C** | **C** | **T** | **G** | **C** | **C** | **A** | **T** | **G** | **T** | **T** | **A** | **C** | **T** | **G** | **G** | **T** | **G** | **G** | **A** | **A** | **C** | **A** | **T** | **G** | **A** | **T** | **C** | **A** | **A** | **A** | **C** | **C** | **T** | **T** | **C** | **C** | **T** | **G** | **G** | **A** | **T** | **A** | **A** | **A** | **G** | **T** | **A** | **T** | **C** | **T** | **A** | **C** | **T** | **G** | **A** | **G** | **A** | **T** | **T** | **A** | **T** | **T** | **T** | **C** | **T** | **C** | **T** | **T** | **G** | **A** | **G** | **A** | **A** | **A** | **A** | **T** | **C** | **-** | **-** | **-** |
| **N59998F** | **A** | **T** | **G** | **T** | **C** | **T** | **A** | **A** | **A** | **A** | **T** | **C** | **G** | **A** | **A** | **A** | **T** | **A** | **A** | **A** | **A** | **A** | **A** | **T** | **C** | **T** | **G** | **A** | **C** | **A** | **T** | **T** | **C** | **G** | **G** | **C** | **T** | **A** | **C** | **G** | **A** | **C** | **A** | **G** | **C** | **C** | **A** | **A** | **G** | **G** | **C** | **A** | **C** | **A** | **T** | **T** | **A** | **T** | **T** | **A** | **T** | **T** | **T** | **G** | **A** | **A** | **C** | **A** | **A** | **G** | **C** | **A** | **A** | **A** | **T** | **C** | **T** | **A** | **A** | **A** | **T** | **T** | **T** | **T** | **G** | **A** | **C** | **A** | **C** | **A** | **C** | **A** | **A** | **T** | **G** | **G** | **A** | **A** | **A** | **C** | **T** | **A** | **G** | **G** | **A** | **C** | **T** | **T** | **A** | **T** | **C** | **G** | **G** | **A** | **C** | **G** | **A** | **A** | **A** | **C** | **G** | **G** | **T** | **C** | **G** | **A** | **G** | **G** | **A** | **A** | **A** | **G** | **A** | **C** | **A** | **A** | **C** | **T** | **T** | **T** | **A** | **C** | **T** | **G** | **A** | **A** | **T** | **A** | **T** | **T** | **C** | **T** | **A** | **C** | **A** | **A** | **A** | **A** | **C** | **A** | **A** | **A** | **C** | **T** | **A** | **C** | **C** | **T** | **T** | **A** | **T** | **C** | **A** | **A** | **G** | **G** | **G** | **C** | **A** | **A** | **G** | **T** | **A** | **A** | **T** | **C** | **C** | **A** | **T** | **C** | **A** | **G** | **C** | **A** | **A** | **G** | **A** | **A** | **T** | **T** | **T** | **G** | **C** | **C** | **T** | **A** | **T** | **T** | **T** | **C** | **C** | **C** | **G** | **C** | **A** | **A** | **C** | **A** | **G** | **A** | **C** | **A** | **A** | **A** | **A** | **G** | **A** | **T** | **A** | **A** | **A** | **G** | **A** | **A** | **C** | **G** | **T** | **T** | **T** | **A** | **A** | **C** | **C** | **T** | **A** | **T** | **T** | **A** | **C** | **G** | **T** | **G** | **T** | **T** | **A** | **A** | **A** | **T** | **G** | **A** | **T** | **A** | **T** | **T** | **A** | **C** | **G** | **G** | **A** | **T** | **T** | **T** | **T** | **G** | **A** | **G** | **A** | **T** | **A** | **T** | **G** | **G** | **G** | **A** | **A** | **A** | **T** | **C** | **G** | **A** | **A** | **A** | **G** | **A** | **G** | **A** | **G** | **C** | **T** | **C** | **C** | **A** | **A** | **T** | **T** | **G** | **A** | **T** | **G** | **C** | **A** | **A** | **A** | **C** | **A** | **G** | **A** | **T** | **C** | **C** | **T** | **G** | **A** | **A** | **A** | **T** | **C** | **T** | **T** | **A** | **T** | **G** | **G** | **A** | **G** | **A** | **G** | **A** | **A** | **T** | **T** | **C** | **A** | **G** | **C** | **A** | **C** | **A** | **C** | **T** | **A** | **T** | **C** | **G** | **G** | **G** | **G** | **G** | **G** | **A** | **G** | **A** | **G** | **A** | **A** | **G** | **A** | **C** | **A** | **A** | **A** | **A** | **G** | **T** | **C** | **C** | **T** | **A** | **C** | **T** | **G** | **G** | **C** | **A** | **C** | **T** | **T** | **T** | **T** | **A** | **T** | **T** | **T** | **G** | **T** | **G** | **G** | **A** | **T** | **G** | **A** | **C** | **A** | **C** | **T** | **C** | **A** | **T** | **T** | **T** | **C** | **C** | **C** | **G** | **T** | **T** | **A** | **A** | **T** | **C** | **G** | **A** | **T** | **G** | **A** | **A** | **C** | **C** | **A** | **A** | **C** | **G** | **A** | **A** | **T** | **C** | **A** | **T** | **T** | **T** | **G** | **G** | **A** | **-** | **-** | **-** | **T** | **A** | **T** | **C** | **T** | **C** | **T** | **G** | **G** | **T** | **A** | **G** | **A** | **A** | **A** | **A** | **C** | **A** | **A** | **G** | **T** | **A** | **G** | **C** | **G** | **G** | **C** | **T** | **T** | **A** | **T** | **T** | **T** | **G** | **A** | **A** | **A** | **A** | **A** | **G** | **A** | **A** | **A** | **A** | **A** | **A** | **C** | **A** | **A** | **G** | **G** | **C** | **T** | **T** | **C** | **A** | **T** | **C** | **G** | **T** | **G** | **G** | **T** | **C** | **A** | **G** | **C** | **C** | **A** | **T** | **G** | **A** | **C** | **C** | **G** | **G** | **G** | **G** | **A** | **T** | **T** | **T** | **A** | **T** | **C** | **G** | **A** | **T** | **G** | **A** | **A** | **G** | **T** | **A** | **G** | **T** | **G** | **G** | **A** | **C** | **C** | **A** | **T** | **G** | **T** | **T** | **T** | **T** | **A** | **G** | **C** | **A** | **A** | **T** | **C** | **G** | **A** | **A** | **A** | **A** | **A** | **A** | **G** | **T** | **C** | **A** | **A** | **C** | **T** | **G** | **G** | **A** | **A** | **C** | **T** | **T** | **T** | **A** | **T** | **C** | **A** | **A** | **G** | **G** | **G** | **A** | **A** | **T** | **T** | **T** | **C** | **T** | **C** | **T** | **A** | **T** | **C** | **T** | **A** | **T** | **G** | **A** | **A** | **G** | **A** | **A** | **C** | **A** | **G** | **A** | **A** | **A** | **A** | **A** | **A** | **C** | **T** | **T** | **C** | **G** | **T** | **G** | **A** | **T** | **G** | **A** | **A** | **T** | **T** | **T** | **G** | **A** | **A** | **A** | **T** | **G** | **G** | **C** | **T** | **C** | **A** | **A** | **A** | **A** | **T** | **G** | **A** | **A** | **A** | **A** | **A** | **T** | **T** | **G** | **A** | **A** | **A** | **A** | **A** | **A** | **G** | **A** | **A** | **G** | **T** | **C** | **A** | **G** | **T** | **A** | **G** | **G** | **C** | **T** | **A** | **A** | **A** | **G** | **A** | **A** | **A** | **A** | **C** | **A** | **G** | **C** | **A** | **G** | **C** | **T** | **G** | **A** | **A** | **A** | **A** | **A** | **G** | **C** | **C** | **G** | **A** | **A** | **T** | **G** | **G** | **T** | **C** | **T** | **C** | **G** | **T** | **T** | **C** | **C** | **C** | **G** | **A** | **G** | **A** | **A** | **G** | **G** | **A** | **G** | **A** | **T** | **A** | **A** | **A** | **A** | **C** | **A** | **A** | **A** | **G** | **A** | **A** | **A** | **C** | **A** | **A** | **G** | **T** | **C** | **G** | **G** | **A** | **T** | **T** | **C** | **A** | **T** | **C** | **G** | **A** | **T** | **A** | **C** | **T** | **G** | **A** | **A** | **T** | **C** | **T** | **A** | **G** | **A** | **C** | **G** | **A** | **G** | **T** | **G** | **A** | **A** | **T** | **A** | **A** | **A** | **G** | **G** | **A** | **G** | **C** | **A** | **G** | **T** | **G** | **G** | **G** | **T** | **G** | **C** | **T** | **G** | **A** | **T** | **G** | **C** | **T** | **G** | **C** | **A** | **C** | **G** | **G** | **A** | **C** | **G** | **A** | **T** | **G** | **A** | **A** | **A** | **C** | **G** | **A** | **T** | **C** | **C** | **A** | **A** | **A** | **G** | **C** | **A** | **A** | **T** | **C** | **G** | **T** | **G** | **A** | **A** | **T** | **C** | **G** | **G** | **A** | **T** | **G** | **G** | **A** | **G** | **A** | **C** | **C** | **C** | **A** | **G** | **A** | **T** | **C** | **A** | **G** | **C** | **G** | **A** | **G** | **A** | **A** | **A** | **G** | **A** | **A** | **A** | **A** | **A** | **C** | **T** | **A** | **T** | **T** | **A** | **A** | **A** | **A** | **G** | **A** | **T** | **A** | **T** | **C** | **G** | **A** | **A** | **T** | **A** | **T** | **A** | **T** | **C** | **G** | **A** | **T** | **T** | **C** | **T** | **T** | **T** | **G** | **A** | **C** | **G** | **A** | **T** | **G** | **A** | **A** | **T** | **A** | **G** | **C** | **C** | **A** | **A** | **G** | **C** | **G** | **T** | **C** | **T** | **C** | **A** | **C** | **C** | **A** | **T** | **A** | **A** | **G** | **C** | **G** | **A** | **C** | **T** | **T** | **T** | **T** | **A** | **A** | **G** | **C** | **G** | **T** | **A** | **G** | **A** | **A** | **G** | **A** | **T** | **C** | **T** | **T** | **C** | **A** | **A** | **T** | **T** | **A** | **G** | **G** | **G** | **T** | **A** | **T** | **G** | **A** | **A** | **A** | **A** | **T** | **C** | **T** | **G** | **T** | **T** | **A** | **T** | **T** | **C** | **G** | **A** | **G** | **C** | **C** | **A** | **A** | **T** | **T** | **C** | **A** | **T** | **T** | **T** | **T** | **A** | **C** | **A** | **A** | **T** | **C** | **G** | **A** | **G** | **C** | **C** | **T** | **C** | **A** | **T** | **C** | **A** | **G** | **C** | **G** | **G** | **G** | **T** | **G** | **G** | **C** | **G** | **A** | **T** | **T** | **T** | **C** | **A** | **G** | **G** | **T** | **C** | **C** | **T** | **A** | **A** | **C** | **G** | **G** | **T** | **G** | **C** | **A** | **G** | **G** | **A** | **A** | **A** | **G** | **T** | **C** | **A** | **T** | **C** | **C** | **A** | **T** | **T** | **A** | **T** | **C** | **C** | **A** | **T** | **T** | **A** | **T** | **C** | **T** | **T** | **C** | **T** | **G** | **G** | **G** | **G** | **G** | **C** | **A** | **T** | **T** | **C** | **A** | **A** | **C** | **G** | **G** | **C** | **A** | **A** | **G** | **G** | **T** | **T** | **A** | **T** | **A** | **G** | **G** | **A** | **G** | **A** | **A** | **A** | **A** | **A** | **T** | **A** | **C** | **C** | **A** | **G** | **C** | **C** | **A** | **A** | **A** | **A** | **C** | **A** | **T** | **C** | **T** | **G** | **A** | **G** | **C** | **A** | **T** | **T** | **A** | **G** | **T** | **T** | **A** | **T** | **G** | **C** | **A** | **A** | **G** | **C** | **C** | **A** | **A** | **A** | **A** | **T** | **T** | **A** | **T** | **G** | **A** | **A** | **G** | **A** | **C** | **A** | **A** | **T** | **C** | **G** | **A** | **G** | **G** | **A** | **A** | **C** | **G** | **T** | **T** | **G** | **G** | **C** | **G** | **G** | **A** | **A** | **T** | **T** | **T** | **G** | **C** | **A** | **G** | **A** | **G** | **A** | **A** | **A** | **A** | **A** | **C** | **C** | **A** | **A** | **G** | **T** | **A** | **G** | **A** | **C** | **T** | **A** | **C** | **C** | **A** | **A** | **G** | **C** | **A** | **T** | **T** | **T** | **T** | **T** | **G** | **A** | **A** | **C** | **A** | **A** | **C** | **C** | **T** | **C** | **C** | **G** | **A** | **A** | **A** | **G** | **C** | **T** | **T** | **G** | **G** | **G** | **A** | **T** | **G** | **G** | **A** | **A** | **A** | **G** | **A** | **G** | **A** | **T** | **G** | **T** | **T** | **T** | **T** | **T** | **C** | **A** | **T** | **A** | **A** | **C** | **A** | **A** | **G** | **A** | **T** | **C** | **G** | **A** | **G** | **C** | **A** | **G** | **A** | **T** | **G** | **A** | **G** | **T** | **A** | **T** | **G** | **G** | **G** | **C** | **C** | **A** | **A** | **C** | **G** | **G** | **A** | **A** | **A** | **A** | **A** | **A** | **G** | **T** | **G** | **G** | **A** | **A** | **T** | **T** | **G** | **G** | **C** | **T** | **A** | **A** | **A** | **T** | **C** | **T** | **T** | **T** | **A** | **T** | **C** | **A** | **C** | **A** | **G** | **C** | **C** | **A** | **G** | **C** | **T** | **G** | **A** | **A** | **C** | **T** | **A** | **T** | **A** | **T** | **A** | **C** | **A** | **T** | **G** | **G** | **G** | **A** | **T** | **G** | **A** | **A** | **C** | **C** | **A** | **T** | **T** | **G** | **A** | **A** | **T** | **T** | **A** | **T** | **T** | **T** | **G** | **G** | **A** | **T** | **G** | **T** | **C** | **T** | **T** | **C** | **A** | **A** | **T** | **C** | **A** | **A** | **G** | **A** | **A** | **C** | **A** | **A** | **T** | **T** | **A** | **G** | **A** | **A** | **C** | **A** | **A** | **C** | **T** | **G** | **A** | **T** | **C** | **T** | **T** | **G** | **A** | **A** | **C** | **G** | **T** | **G** | **A** | **A** | **A** | **C** | **C** | **T** | **G** | **C** | **C** | **A** | **T** | **G** | **T** | **T** | **A** | **C** | **T** | **A** | **G** | **T** | **G** | **G** | **A** | **A** | **C** | **A** | **T** | **G** | **A** | **T** | **C** | **A** | **A** | **A** | **C** | **C** | **T** | **T** | **C** | **C** | **T** | **G** | **G** | **A** | **T** | **A** | **A** | **A** | **G** | **T** | **A** | **T** | **C** | **T** | **A** | **C** | **T** | **G** | **A** | **G** | **A** | **T** | **T** | **A** | **T** | **T** | **T** | **C** | **T** | **C** | **T** | **T** | **G** | **A** | **G** | **A** | **G** | **A** | **A** | **T** | **C** | **-** | **-** | **-** |
| **N54190** | **A** | **T** | **G** | **T** | **C** | **T** | **A** | **A** | **A** | **A** | **T** | **C** | **G** | **A** | **A** | **A** | **T** | **A** | **A** | **A** | **A** | **A** | **A** | **T** | **C** | **T** | **G** | **A** | **C** | **A** | **T** | **T** | **C** | **G** | **G** | **C** | **T** | **A** | **C** | **G** | **A** | **C** | **A** | **G** | **C** | **C** | **A** | **A** | **G** | **G** | **C** | **A** | **C** | **A** | **T** | **T** | **A** | **T** | **T** | **A** | **T** | **T** | **T** | **G** | **A** | **A** | **C** | **A** | **A** | **G** | **C** | **A** | **A** | **T** | **T** | **C** | **T** | **A** | **A** | **A** | **T** | **T** | **T** | **T** | **G** | **A** | **C** | **A** | **C** | **A** | **C** | **A** | **A** | **T** | **G** | **G** | **A** | **A** | **A** | **C** | **T** | **A** | **G** | **G** | **A** | **C** | **T** | **T** | **A** | **T** | **C** | **G** | **G** | **A** | **C** | **G** | **A** | **A** | **A** | **C** | **G** | **G** | **T** | **C** | **G** | **A** | **G** | **G** | **A** | **A** | **A** | **G** | **A** | **C** | **A** | **A** | **C** | **T** | **T** | **T** | **A** | **C** | **T** | **G** | **A** | **A** | **T** | **A** | **T** | **T** | **C** | **T** | **A** | **C** | **A** | **A** | **A** | **A** | **C** | **A** | **A** | **A** | **C** | **T** | **A** | **C** | **C** | **T** | **T** | **A** | **T** | **C** | **A** | **A** | **G** | **G** | **G** | **C** | **A** | **A** | **G** | **T** | **A** | **A** | **T** | **C** | **C** | **A** | **T** | **C** | **A** | **G** | **C** | **A** | **A** | **G** | **A** | **A** | **T** | **T** | **T** | **G** | **T** | **C** | **T** | **A** | **T** | **T** | **T** | **C** | **C** | **C** | **G** | **C** | **A** | **A** | **C** | **A** | **G** | **A** | **C** | **A** | **A** | **A** | **A** | **G** | **A** | **T** | **A** | **A** | **A** | **G** | **A** | **A** | **C** | **G** | **T** | **T** | **T** | **A** | **A** | **C** | **C** | **T** | **A** | **T** | **T** | **A** | **C** | **G** | **T** | **G** | **T** | **T** | **A** | **A** | **A** | **T** | **G** | **A** | **T** | **A** | **T** | **T** | **A** | **C** | **G** | **G** | **A** | **T** | **T** | **T** | **T** | **G** | **A** | **G** | **A** | **T** | **A** | **T** | **G** | **G** | **G** | **A** | **A** | **A** | **T** | **C** | **G** | **A** | **A** | **A** | **G** | **A** | **G** | **A** | **G** | **C** | **T** | **C** | **C** | **A** | **A** | **T** | **T** | **G** | **A** | **T** | **G** | **C** | **A** | **A** | **A** | **C** | **A** | **G** | **A** | **T** | **C** | **C** | **T** | **G** | **A** | **A** | **A** | **T** | **C** | **T** | **T** | **A** | **T** | **G** | **G** | **A** | **G** | **A** | **G** | **A** | **A** | **T** | **T** | **C** | **A** | **G** | **C** | **A** | **C** | **A** | **C** | **T** | **A** | **T** | **C** | **G** | **G** | **G** | **G** | **G** | **G** | **A** | **G** | **A** | **G** | **A** | **A** | **G** | **A** | **C** | **A** | **A** | **A** | **A** | **G** | **T** | **C** | **C** | **T** | **A** | **C** | **T** | **G** | **G** | **C** | **A** | **C** | **T** | **T** | **T** | **T** | **A** | **T** | **T** | **T** | **G** | **T** | **G** | **G** | **A** | **T** | **G** | **A** | **C** | **A** | **C** | **T** | **C** | **A** | **T** | **T** | **T** | **T** | **C** | **C** | **G** | **T** | **T** | **A** | **A** | **T** | **C** | **G** | **A** | **T** | **G** | **A** | **A** | **C** | **C** | **A** | **A** | **C** | **G** | **A** | **A** | **T** | **C** | **A** | **T** | **T** | **T** | **G** | **G** | **A** | **-** | **-** | **-** | **T** | **A** | **T** | **C** | **T** | **C** | **T** | **G** | **G** | **T** | **A** | **G** | **A** | **A** | **A** | **A** | **C** | **A** | **A** | **G** | **T** | **A** | **G** | **C** | **G** | **G** | **C** | **T** | **T** | **A** | **T** | **T** | **T** | **G** | **A** | **A** | **A** | **A** | **A** | **G** | **A** | **A** | **A** | **A** | **A** | **A** | **C** | **A** | **A** | **G** | **G** | **C** | **T** | **T** | **C** | **A** | **T** | **C** | **G** | **T** | **G** | **G** | **T** | **C** | **A** | **G** | **C** | **C** | **A** | **T** | **G** | **A** | **C** | **C** | **G** | **G** | **G** | **G** | **A** | **T** | **T** | **T** | **A** | **T** | **C** | **G** | **A** | **T** | **G** | **A** | **A** | **G** | **T** | **A** | **G** | **T** | **G** | **G** | **A** | **C** | **C** | **A** | **T** | **G** | **T** | **T** | **T** | **T** | **A** | **G** | **C** | **A** | **A** | **T** | **C** | **G** | **A** | **A** | **A** | **A** | **A** | **A** | **G** | **T** | **C** | **A** | **A** | **C** | **T** | **G** | **G** | **A** | **A** | **C** | **T** | **T** | **T** | **A** | **T** | **C** | **A** | **A** | **G** | **G** | **G** | **A** | **A** | **T** | **T** | **T** | **C** | **T** | **C** | **T** | **A** | **T** | **C** | **T** | **A** | **T** | **G** | **A** | **A** | **G** | **A** | **A** | **C** | **A** | **G** | **A** | **A** | **A** | **A** | **A** | **A** | **C** | **T** | **T** | **C** | **G** | **T** | **G** | **A** | **T** | **G** | **A** | **A** | **T** | **T** | **T** | **G** | **A** | **A** | **A** | **T** | **G** | **G** | **C** | **T** | **C** | **A** | **A** | **A** | **A** | **T** | **G** | **A** | **A** | **A** | **A** | **A** | **T** | **T** | **G** | **A** | **A** | **A** | **A** | **A** | **A** | **G** | **A** | **A** | **G** | **T** | **C** | **A** | **G** | **C** | **A** | **G** | **G** | **C** | **T** | **A** | **A** | **A** | **G** | **A** | **C** | **A** | **A** | **C** | **A** | **G** | **C** | **A** | **G** | **C** | **T** | **G** | **A** | **A** | **A** | **A** | **A** | **G** | **C** | **C** | **G** | **A** | **A** | **T** | **G** | **G** | **T** | **C** | **T** | **C** | **G** | **T** | **T** | **C** | **C** | **C** | **G** | **A** | **G** | **A** | **A** | **G** | **G** | **A** | **G** | **A** | **T** | **A** | **A** | **A** | **A** | **C** | **A** | **A** | **A** | **G** | **A** | **A** | **A** | **C** | **A** | **A** | **G** | **T** | **C** | **G** | **G** | **A** | **C** | **T** | **C** | **A** | **T** | **C** | **G** | **A** | **T** | **A** | **C** | **T** | **G** | **A** | **A** | **T** | **C** | **T** | **A** | **G** | **A** | **C** | **G** | **A** | **G** | **T** | **G** | **A** | **A** | **T** | **A** | **A** | **A** | **G** | **G** | **A** | **G** | **C** | **A** | **G** | **T** | **G** | **G** | **G** | **T** | **G** | **C** | **T** | **G** | **A** | **T** | **G** | **C** | **T** | **G** | **C** | **A** | **C** | **G** | **G** | **A** | **C** | **G** | **A** | **T** | **G** | **A** | **A** | **A** | **C** | **G** | **A** | **T** | **C** | **C** | **A** | **A** | **A** | **G** | **C** | **A** | **A** | **T** | **C** | **G** | **T** | **G** | **A** | **A** | **T** | **C** | **G** | **G** | **A** | **T** | **G** | **G** | **A** | **G** | **A** | **C** | **C** | **C** | **A** | **G** | **A** | **T** | **C** | **A** | **G** | **C** | **G** | **A** | **G** | **A** | **A** | **A** | **G** | **A** | **A** | **A** | **A** | **A** | **C** | **T** | **A** | **T** | **T** | **A** | **A** | **A** | **A** | **G** | **A** | **T** | **A** | **T** | **C** | **G** | **A** | **A** | **T** | **A** | **T** | **A** | **T** | **C** | **G** | **A** | **T** | **T** | **C** | **T** | **T** | **T** | **G** | **A** | **C** | **G** | **A** | **T** | **G** | **A** | **A** | **T** | **A** | **G** | **C** | **C** | **A** | **A** | **G** | **C** | **G** | **T** | **C** | **T** | **C** | **A** | **C** | **C** | **A** | **T** | **A** | **A** | **G** | **C** | **G** | **A** | **C** | **T** | **T** | **T** | **T** | **A** | **A** | **G** | **C** | **G** | **T** | **A** | **G** | **A** | **A** | **G** | **A** | **T** | **C** | **T** | **T** | **C** | **A** | **A** | **T** | **T** | **A** | **G** | **G** | **G** | **T** | **A** | **T** | **G** | **A** | **A** | **A** | **A** | **T** | **C** | **T** | **G** | **T** | **T** | **A** | **T** | **T** | **C** | **G** | **A** | **G** | **C** | **C** | **A** | **A** | **T** | **T** | **C** | **A** | **T** | **T** | **T** | **T** | **A** | **C** | **A** | **A** | **T** | **C** | **G** | **A** | **G** | **C** | **C** | **T** | **C** | **A** | **T** | **C** | **A** | **G** | **C** | **G** | **G** | **G** | **T** | **G** | **G** | **C** | **G** | **A** | **T** | **T** | **T** | **C** | **A** | **G** | **G** | **T** | **C** | **C** | **T** | **A** | **A** | **C** | **G** | **G** | **T** | **G** | **C** | **A** | **G** | **G** | **A** | **A** | **A** | **G** | **T** | **C** | **A** | **T** | **C** | **C** | **A** | **T** | **T** | **A** | **T** | **C** | **C** | **A** | **T** | **T** | **A** | **T** | **C** | **T** | **T** | **C** | **T** | **G** | **G** | **G** | **G** | **T** | **C** | **A** | **T** | **T** | **C** | **A** | **A** | **C** | **G** | **G** | **C** | **A** | **A** | **G** | **G** | **T** | **T** | **A** | **T** | **A** | **G** | **G** | **A** | **G** | **A** | **A** | **A** | **A** | **A** | **T** | **C** | **C** | **C** | **A** | **G** | **C** | **C** | **A** | **A** | **A** | **A** | **C** | **A** | **T** | **C** | **T** | **G** | **A** | **G** | **C** | **A** | **T** | **T** | **A** | **G** | **T** | **T** | **A** | **T** | **G** | **T** | **A** | **C** | **G** | **C** | **C** | **A** | **A** | **A** | **A** | **T** | **T** | **A** | **T** | **G** | **A** | **A** | **G** | **A** | **C** | **A** | **A** | **T** | **C** | **G** | **A** | **G** | **G** | **A** | **A** | **C** | **G** | **T** | **T** | **G** | **G** | **C** | **G** | **G** | **A** | **A** | **T** | **T** | **T** | **G** | **C** | **A** | **G** | **A** | **G** | **A** | **A** | **A** | **A** | **A** | **C** | **C** | **A** | **A** | **G** | **T** | **A** | **G** | **A** | **C** | **T** | **A** | **C** | **C** | **A** | **A** | **G** | **C** | **A** | **T** | **T** | **T** | **T** | **T** | **G** | **A** | **A** | **C** | **A** | **A** | **C** | **C** | **T** | **C** | **C** | **G** | **A** | **A** | **A** | **G** | **C** | **T** | **T** | **G** | **G** | **G** | **A** | **T** | **G** | **G** | **A** | **A** | **A** | **G** | **A** | **G** | **A** | **T** | **G** | **T** | **T** | **T** | **T** | **T** | **C** | **A** | **T** | **A** | **A** | **C** | **A** | **A** | **G** | **A** | **T** | **T** | **G** | **A** | **G** | **C** | **A** | **G** | **A** | **T** | **G** | **A** | **G** | **T** | **A** | **T** | **G** | **G** | **G** | **C** | **C** | **A** | **A** | **C** | **G** | **G** | **A** | **A** | **A** | **A** | **A** | **A** | **G** | **T** | **G** | **G** | **A** | **A** | **T** | **T** | **G** | **G** | **C** | **T** | **A** | **A** | **A** | **T** | **C** | **T** | **T** | **T** | **A** | **T** | **C** | **A** | **C** | **A** | **G** | **C** | **C** | **A** | **G** | **C** | **T** | **G** | **A** | **A** | **C** | **T** | **A** | **T** | **A** | **T** | **A** | **T** | **A** | **T** | **G** | **G** | **G** | **A** | **T** | **G** | **A** | **A** | **C** | **C** | **A** | **T** | **T** | **G** | **A** | **A** | **T** | **T** | **A** | **T** | **T** | **T** | **G** | **G** | **A** | **T** | **G** | **T** | **C** | **T** | **T** | **C** | **A** | **A** | **T** | **C** | **A** | **A** | **G** | **A** | **A** | **C** | **A** | **A** | **T** | **T** | **A** | **G** | **A** | **A** | **C** | **A** | **A** | **C** | **T** | **G** | **A** | **T** | **C** | **T** | **T** | **G** | **A** | **A** | **C** | **G** | **T** | **G** | **A** | **A** | **A** | **C** | **C** | **T** | **G** | **C** | **C** | **A** | **T** | **G** | **T** | **T** | **A** | **C** | **T** | **G** | **G** | **T** | **G** | **G** | **A** | **A** | **C** | **A** | **T** | **G** | **A** | **T** | **C** | **A** | **A** | **A** | **C** | **C** | **T** | **T** | **C** | **C** | **T** | **G** | **G** | **A** | **T** | **A** | **A** | **A** | **G** | **T** | **A** | **T** | **C** | **T** | **A** | **C** | **T** | **G** | **A** | **G** | **A** | **T** | **T** | **A** | **T** | **T** | **T** | **C** | **T** | **C** | **T** | **T** | **G** | **A** | **G** | **A** | **A** | **A** | **A** | **T** | **C** | **-** | **-** | **-** |
| **N60190F** | **A** | **T** | **G** | **T** | **C** | **T** | **A** | **A** | **A** | **A** | **T** | **C** | **G** | **A** | **A** | **A** | **T** | **A** | **A** | **A** | **A** | **A** | **A** | **T** | **C** | **T** | **G** | **A** | **C** | **A** | **T** | **T** | **C** | **G** | **G** | **C** | **T** | **A** | **C** | **G** | **A** | **C** | **A** | **G** | **C** | **C** | **A** | **A** | **G** | **G** | **C** | **A** | **C** | **A** | **T** | **T** | **A** | **T** | **T** | **A** | **T** | **T** | **T** | **G** | **A** | **A** | **C** | **A** | **A** | **G** | **C** | **A** | **A** | **A** | **T** | **C** | **T** | **A** | **A** | **A** | **T** | **T** | **T** | **T** | **G** | **A** | **C** | **A** | **C** | **A** | **C** | **A** | **A** | **T** | **G** | **G** | **A** | **A** | **A** | **C** | **T** | **A** | **G** | **G** | **A** | **C** | **T** | **T** | **A** | **T** | **C** | **G** | **G** | **A** | **C** | **G** | **A** | **A** | **A** | **C** | **G** | **G** | **T** | **C** | **G** | **A** | **G** | **G** | **A** | **A** | **A** | **G** | **A** | **C** | **A** | **A** | **C** | **T** | **T** | **T** | **A** | **C** | **T** | **G** | **A** | **A** | **T** | **A** | **T** | **T** | **C** | **T** | **A** | **C** | **A** | **A** | **A** | **A** | **C** | **A** | **A** | **A** | **C** | **T** | **A** | **C** | **C** | **T** | **T** | **A** | **T** | **C** | **A** | **A** | **G** | **G** | **G** | **C** | **A** | **A** | **G** | **T** | **A** | **A** | **T** | **C** | **C** | **A** | **T** | **C** | **A** | **G** | **C** | **A** | **A** | **G** | **A** | **A** | **T** | **T** | **T** | **G** | **T** | **C** | **T** | **A** | **T** | **T** | **T** | **C** | **C** | **C** | **G** | **C** | **A** | **A** | **C** | **A** | **G** | **A** | **C** | **A** | **A** | **A** | **A** | **G** | **A** | **T** | **A** | **A** | **A** | **G** | **A** | **A** | **C** | **G** | **T** | **T** | **T** | **A** | **A** | **C** | **C** | **T** | **A** | **T** | **T** | **A** | **C** | **G** | **T** | **G** | **T** | **T** | **A** | **A** | **A** | **T** | **G** | **A** | **T** | **A** | **T** | **T** | **A** | **C** | **G** | **G** | **A** | **T** | **T** | **T** | **T** | **G** | **A** | **G** | **A** | **T** | **A** | **T** | **G** | **G** | **G** | **A** | **A** | **A** | **T** | **C** | **G** | **A** | **A** | **A** | **G** | **A** | **G** | **A** | **G** | **C** | **T** | **C** | **C** | **A** | **A** | **T** | **T** | **T** | **A** | **T** | **G** | **C** | **A** | **A** | **A** | **C** | **A** | **G** | **A** | **T** | **C** | **C** | **T** | **G** | **A** | **A** | **A** | **T** | **C** | **T** | **T** | **A** | **T** | **G** | **G** | **A** | **G** | **A** | **G** | **A** | **A** | **T** | **T** | **C** | **A** | **G** | **C** | **A** | **C** | **A** | **C** | **T** | **A** | **T** | **C** | **G** | **G** | **G** | **G** | **G** | **G** | **A** | **G** | **A** | **G** | **A** | **A** | **G** | **A** | **C** | **A** | **A** | **A** | **A** | **G** | **T** | **C** | **C** | **T** | **A** | **T** | **T** | **G** | **G** | **C** | **A** | **C** | **T** | **T** | **T** | **T** | **A** | **T** | **T** | **T** | **G** | **T** | **G** | **G** | **A** | **T** | **G** | **A** | **C** | **A** | **C** | **T** | **C** | **A** | **T** | **T** | **T** | **C** | **C** | **C** | **G** | **T** | **T** | **A** | **A** | **T** | **C** | **G** | **A** | **T** | **G** | **A** | **A** | **C** | **C** | **A** | **A** | **C** | **G** | **A** | **A** | **T** | **C** | **A** | **T** | **T** | **T** | **G** | **G** | **A** | **T** | **A** | **T** | **T** | **A** | **T** | **C** | **T** | **C** | **T** | **G** | **G** | **T** | **A** | **G** | **A** | **A** | **A** | **A** | **C** | **A** | **A** | **G** | **T** | **A** | **G** | **C** | **G** | **G** | **C** | **T** | **T** | **A** | **T** | **T** | **T** | **G** | **A** | **A** | **A** | **A** | **A** | **G** | **A** | **A** | **A** | **A** | **A** | **A** | **C** | **A** | **A** | **G** | **G** | **C** | **T** | **T** | **C** | **A** | **T** | **C** | **G** | **T** | **G** | **G** | **T** | **C** | **A** | **G** | **C** | **C** | **A** | **T** | **G** | **A** | **C** | **C** | **G** | **G** | **G** | **G** | **A** | **T** | **T** | **T** | **A** | **T** | **C** | **G** | **A** | **T** | **G** | **A** | **A** | **G** | **T** | **A** | **G** | **T** | **G** | **G** | **A** | **C** | **C** | **A** | **T** | **G** | **T** | **T** | **T** | **T** | **A** | **G** | **C** | **A** | **A** | **T** | **C** | **G** | **A** | **A** | **A** | **A** | **A** | **A** | **G** | **T** | **C** | **A** | **A** | **C** | **T** | **G** | **G** | **A** | **A** | **C** | **T** | **T** | **T** | **A** | **T** | **C** | **A** | **A** | **G** | **G** | **G** | **A** | **A** | **T** | **T** | **T** | **C** | **T** | **C** | **T** | **A** | **T** | **C** | **T** | **A** | **T** | **G** | **A** | **A** | **G** | **A** | **A** | **C** | **A** | **G** | **A** | **A** | **A** | **A** | **A** | **A** | **C** | **T** | **T** | **C** | **G** | **T** | **G** | **A** | **T** | **G** | **A** | **A** | **T** | **T** | **T** | **G** | **A** | **A** | **A** | **T** | **G** | **G** | **C** | **T** | **C** | **A** | **A** | **A** | **A** | **T** | **G** | **A** | **A** | **A** | **A** | **A** | **T** | **T** | **G** | **A** | **A** | **A** | **A** | **A** | **A** | **G** | **A** | **A** | **G** | **T** | **C** | **A** | **G** | **T** | **A** | **G** | **G** | **C** | **T** | **A** | **A** | **A** | **G** | **A** | **A** | **A** | **A** | **C** | **A** | **G** | **C** | **A** | **G** | **C** | **T** | **G** | **A** | **A** | **A** | **A** | **A** | **G** | **C** | **C** | **G** | **A** | **A** | **T** | **G** | **G** | **T** | **C** | **T** | **C** | **G** | **T** | **T** | **C** | **C** | **C** | **G** | **A** | **G** | **A** | **A** | **G** | **G** | **A** | **G** | **A** | **T** | **A** | **A** | **A** | **A** | **C** | **A** | **A** | **A** | **G** | **A** | **A** | **A** | **C** | **A** | **A** | **G** | **T** | **C** | **G** | **G** | **A** | **T** | **T** | **C** | **A** | **T** | **C** | **G** | **A** | **T** | **A** | **C** | **T** | **G** | **A** | **A** | **T** | **C** | **T** | **A** | **G** | **A** | **C** | **G** | **A** | **G** | **T** | **G** | **A** | **A** | **T** | **A** | **A** | **A** | **G** | **G** | **A** | **G** | **C** | **A** | **G** | **T** | **G** | **G** | **G** | **T** | **G** | **C** | **T** | **G** | **A** | **T** | **G** | **C** | **T** | **G** | **C** | **A** | **C** | **G** | **G** | **A** | **C** | **G** | **A** | **T** | **G** | **A** | **A** | **A** | **C** | **G** | **A** | **T** | **C** | **C** | **A** | **A** | **A** | **G** | **C** | **A** | **A** | **T** | **C** | **G** | **T** | **G** | **A** | **A** | **T** | **C** | **G** | **G** | **A** | **T** | **G** | **G** | **A** | **G** | **A** | **C** | **C** | **C** | **A** | **G** | **A** | **T** | **C** | **A** | **G** | **C** | **G** | **A** | **G** | **A** | **A** | **A** | **G** | **A** | **A** | **A** | **A** | **A** | **C** | **T** | **A** | **T** | **T** | **A** | **A** | **A** | **A** | **G** | **A** | **T** | **A** | **T** | **C** | **G** | **A** | **A** | **T** | **A** | **T** | **A** | **T** | **C** | **G** | **A** | **T** | **T** | **C** | **T** | **T** | **T** | **G** | **A** | **C** | **G** | **A** | **T** | **G** | **A** | **A** | **T** | **A** | **G** | **C** | **C** | **A** | **A** | **G** | **C** | **G** | **T** | **C** | **T** | **C** | **A** | **C** | **C** | **A** | **T** | **A** | **A** | **G** | **C** | **G** | **A** | **C** | **T** | **T** | **T** | **T** | **A** | **A** | **G** | **C** | **G** | **T** | **A** | **G** | **A** | **A** | **G** | **A** | **T** | **C** | **T** | **T** | **C** | **A** | **A** | **T** | **T** | **A** | **G** | **G** | **G** | **T** | **A** | **T** | **G** | **A** | **A** | **A** | **A** | **T** | **C** | **T** | **G** | **T** | **T** | **A** | **T** | **T** | **C** | **G** | **A** | **G** | **C** | **C** | **A** | **A** | **T** | **T** | **C** | **A** | **T** | **T** | **T** | **T** | **A** | **C** | **A** | **A** | **T** | **C** | **G** | **A** | **G** | **C** | **C** | **T** | **C** | **A** | **T** | **C** | **A** | **G** | **C** | **G** | **G** | **G** | **T** | **G** | **G** | **C** | **G** | **A** | **T** | **T** | **T** | **C** | **A** | **G** | **G** | **T** | **C** | **C** | **T** | **A** | **A** | **C** | **G** | **G** | **T** | **G** | **C** | **A** | **G** | **G** | **A** | **A** | **A** | **G** | **T** | **C** | **A** | **T** | **C** | **C** | **A** | **T** | **T** | **A** | **T** | **C** | **C** | **A** | **T** | **T** | **A** | **T** | **C** | **T** | **T** | **C** | **T** | **G** | **G** | **G** | **G** | **G** | **C** | **A** | **T** | **T** | **C** | **A** | **A** | **C** | **G** | **G** | **C** | **A** | **A** | **G** | **G** | **T** | **T** | **A** | **T** | **A** | **G** | **G** | **A** | **G** | **A** | **A** | **A** | **A** | **A** | **T** | **C** | **C** | **C** | **A** | **G** | **C** | **C** | **A** | **A** | **A** | **A** | **C** | **A** | **T** | **C** | **T** | **G** | **A** | **G** | **C** | **A** | **T** | **T** | **A** | **G** | **T** | **T** | **A** | **T** | **G** | **T** | **A** | **C** | **G** | **C** | **C** | **A** | **A** | **A** | **A** | **T** | **T** | **A** | **T** | **G** | **A** | **A** | **G** | **A** | **C** | **A** | **A** | **T** | **C** | **G** | **A** | **G** | **G** | **A** | **A** | **C** | **G** | **T** | **T** | **G** | **G** | **C** | **G** | **G** | **A** | **A** | **T** | **T** | **T** | **G** | **C** | **A** | **G** | **A** | **G** | **A** | **A** | **A** | **A** | **A** | **C** | **C** | **A** | **A** | **G** | **T** | **A** | **G** | **A** | **C** | **T** | **A** | **C** | **C** | **A** | **A** | **G** | **C** | **A** | **T** | **T** | **T** | **T** | **T** | **G** | **A** | **A** | **C** | **A** | **A** | **C** | **C** | **T** | **C** | **C** | **G** | **A** | **A** | **A** | **G** | **C** | **T** | **T** | **G** | **G** | **G** | **A** | **T** | **G** | **G** | **A** | **A** | **A** | **G** | **A** | **G** | **A** | **T** | **G** | **T** | **T** | **T** | **T** | **T** | **C** | **A** | **T** | **A** | **A** | **C** | **A** | **A** | **G** | **A** | **T** | **T** | **G** | **A** | **G** | **C** | **A** | **G** | **A** | **T** | **G** | **A** | **G** | **T** | **A** | **T** | **G** | **G** | **G** | **C** | **C** | **A** | **A** | **C** | **G** | **G** | **A** | **A** | **A** | **A** | **A** | **A** | **G** | **T** | **G** | **G** | **A** | **A** | **T** | **T** | **G** | **G** | **C** | **T** | **A** | **A** | **A** | **T** | **C** | **T** | **T** | **T** | **A** | **T** | **C** | **A** | **C** | **A** | **G** | **C** | **C** | **A** | **G** | **C** | **T** | **G** | **A** | **A** | **C** | **T** | **A** | **T** | **A** | **T** | **A** | **T** | **A** | **T** | **G** | **G** | **G** | **A** | **T** | **G** | **A** | **A** | **C** | **C** | **A** | **T** | **T** | **G** | **A** | **A** | **T** | **T** | **A** | **T** | **T** | **T** | **G** | **G** | **A** | **T** | **G** | **T** | **C** | **T** | **T** | **C** | **A** | **A** | **T** | **C** | **A** | **A** | **G** | **A** | **A** | **C** | **A** | **A** | **T** | **T** | **A** | **G** | **A** | **A** | **C** | **A** | **A** | **C** | **T** | **G** | **A** | **T** | **C** | **T** | **T** | **G** | **A** | **A** | **C** | **G** | **T** | **G** | **A** | **A** | **A** | **C** | **C** | **T** | **G** | **C** | **C** | **A** | **T** | **G** | **T** | **T** | **A** | **C** | **T** | **G** | **G** | **T** | **G** | **G** | **A** | **A** | **C** | **A** | **T** | **G** | **A** | **T** | **C** | **A** | **A** | **A** | **C** | **C** | **T** | **T** | **C** | **C** | **T** | **G** | **G** | **A** | **T** | **A** | **A** | **A** | **G** | **T** | **A** | **T** | **C** | **T** | **A** | **C** | **T** | **G** | **A** | **G** | **A** | **T** | **T** | **A** | **T** | **T** | **T** | **C** | **T** | **C** | **T** | **T** | **G** | **A** | **G** | **A** | **A** | **A** | **A** | **T** | **C** | **-** | **-** | **-** |
| **N60490F** | **A** | **T** | **G** | **T** | **C** | **T** | **A** | **A** | **A** | **A** | **T** | **C** | **G** | **A** | **A** | **A** | **T** | **A** | **A** | **A** | **A** | **A** | **A** | **T** | **C** | **T** | **G** | **A** | **C** | **A** | **T** | **T** | **C** | **G** | **G** | **C** | **T** | **A** | **C** | **G** | **A** | **C** | **A** | **G** | **C** | **C** | **A** | **A** | **G** | **G** | **C** | **A** | **C** | **A** | **T** | **T** | **A** | **T** | **T** | **A** | **T** | **T** | **T** | **G** | **A** | **A** | **C** | **A** | **A** | **G** | **C** | **A** | **A** | **A** | **T** | **C** | **T** | **A** | **A** | **A** | **T** | **T** | **T** | **T** | **G** | **A** | **C** | **A** | **C** | **A** | **C** | **A** | **A** | **T** | **G** | **G** | **A** | **A** | **A** | **C** | **T** | **A** | **G** | **G** | **G** | **C** | **T** | **T** | **A** | **T** | **T** | **G** | **G** | **A** | **C** | **G** | **A** | **A** | **A** | **C** | **G** | **G** | **T** | **C** | **G** | **A** | **G** | **G** | **A** | **A** | **A** | **G** | **A** | **C** | **A** | **A** | **C** | **T** | **T** | **T** | **A** | **C** | **T** | **G** | **A** | **A** | **T** | **A** | **T** | **T** | **C** | **T** | **A** | **C** | **A** | **A** | **A** | **A** | **C** | **A** | **A** | **A** | **C** | **T** | **A** | **C** | **C** | **T** | **T** | **A** | **T** | **C** | **A** | **A** | **G** | **G** | **G** | **C** | **A** | **A** | **G** | **T** | **A** | **A** | **T** | **C** | **C** | **A** | **T** | **C** | **A** | **G** | **C** | **A** | **A** | **G** | **A** | **A** | **T** | **T** | **T** | **G** | **T** | **C** | **T** | **A** | **T** | **T** | **T** | **C** | **C** | **C** | **G** | **C** | **A** | **A** | **C** | **A** | **G** | **A** | **C** | **A** | **A** | **A** | **A** | **G** | **A** | **T** | **A** | **A** | **A** | **G** | **A** | **A** | **C** | **G** | **T** | **T** | **T** | **A** | **A** | **C** | **C** | **T** | **A** | **T** | **T** | **A** | **C** | **G** | **T** | **G** | **T** | **T** | **A** | **A** | **A** | **T** | **G** | **A** | **T** | **A** | **T** | **T** | **A** | **C** | **G** | **G** | **A** | **T** | **T** | **T** | **T** | **G** | **A** | **G** | **A** | **T** | **A** | **T** | **G** | **G** | **G** | **A** | **A** | **A** | **T** | **C** | **G** | **A** | **A** | **A** | **G** | **A** | **G** | **A** | **G** | **C** | **T** | **C** | **C** | **A** | **A** | **T** | **T** | **G** | **A** | **T** | **G** | **C** | **A** | **A** | **A** | **C** | **A** | **G** | **A** | **T** | **C** | **C** | **T** | **G** | **A** | **A** | **A** | **T** | **C** | **T** | **T** | **A** | **T** | **G** | **G** | **A** | **G** | **A** | **G** | **A** | **A** | **T** | **T** | **C** | **A** | **G** | **C** | **A** | **C** | **A** | **C** | **T** | **A** | **T** | **C** | **G** | **G** | **G** | **G** | **G** | **G** | **A** | **G** | **A** | **G** | **A** | **A** | **G** | **A** | **C** | **A** | **A** | **A** | **A** | **G** | **T** | **C** | **C** | **T** | **A** | **C** | **T** | **G** | **G** | **C** | **A** | **C** | **T** | **T** | **T** | **T** | **A** | **T** | **T** | **T** | **G** | **T** | **G** | **G** | **A** | **T** | **G** | **A** | **C** | **A** | **C** | **T** | **C** | **A** | **T** | **T** | **T** | **C** | **C** | **C** | **G** | **T** | **T** | **A** | **A** | **T** | **C** | **G** | **A** | **T** | **G** | **A** | **A** | **C** | **C** | **A** | **A** | **C** | **G** | **A** | **A** | **T** | **C** | **A** | **T** | **T** | **T** | **G** | **G** | **A** | **-** | **-** | **-** | **T** | **A** | **T** | **C** | **T** | **C** | **T** | **G** | **G** | **T** | **A** | **G** | **A** | **A** | **A** | **A** | **C** | **A** | **A** | **G** | **T** | **A** | **G** | **C** | **G** | **G** | **C** | **T** | **T** | **A** | **T** | **T** | **T** | **G** | **A** | **A** | **A** | **A** | **A** | **G** | **A** | **A** | **A** | **A** | **A** | **A** | **C** | **A** | **A** | **G** | **G** | **C** | **T** | **T** | **C** | **A** | **T** | **C** | **G** | **T** | **G** | **G** | **T** | **C** | **A** | **G** | **C** | **C** | **A** | **T** | **G** | **A** | **C** | **C** | **G** | **G** | **G** | **G** | **A** | **T** | **T** | **T** | **A** | **T** | **C** | **G** | **A** | **T** | **G** | **A** | **A** | **G** | **T** | **A** | **G** | **T** | **G** | **G** | **A** | **C** | **C** | **A** | **T** | **G** | **T** | **T** | **T** | **T** | **A** | **G** | **C** | **A** | **A** | **T** | **C** | **G** | **A** | **A** | **A** | **A** | **A** | **A** | **G** | **T** | **C** | **A** | **A** | **C** | **T** | **G** | **G** | **A** | **A** | **C** | **T** | **T** | **T** | **A** | **T** | **C** | **A** | **A** | **G** | **G** | **G** | **A** | **A** | **T** | **T** | **T** | **C** | **T** | **C** | **T** | **A** | **T** | **C** | **T** | **A** | **T** | **G** | **A** | **A** | **G** | **A** | **A** | **C** | **A** | **G** | **A** | **A** | **A** | **A** | **A** | **A** | **C** | **T** | **T** | **C** | **G** | **T** | **G** | **A** | **T** | **G** | **A** | **A** | **T** | **T** | **T** | **G** | **A** | **A** | **A** | **T** | **G** | **G** | **C** | **T** | **C** | **A** | **A** | **A** | **A** | **T** | **G** | **A** | **A** | **A** | **A** | **A** | **T** | **T** | **G** | **A** | **A** | **A** | **A** | **A** | **A** | **G** | **A** | **A** | **G** | **T** | **C** | **A** | **G** | **T** | **A** | **G** | **G** | **C** | **T** | **A** | **A** | **A** | **G** | **A** | **A** | **A** | **A** | **C** | **A** | **G** | **C** | **A** | **G** | **C** | **T** | **G** | **A** | **A** | **A** | **A** | **A** | **G** | **C** | **C** | **G** | **A** | **A** | **T** | **G** | **G** | **T** | **C** | **T** | **C** | **G** | **T** | **T** | **C** | **C** | **C** | **G** | **A** | **G** | **A** | **A** | **G** | **G** | **A** | **G** | **A** | **T** | **A** | **A** | **A** | **A** | **C** | **A** | **A** | **A** | **G** | **A** | **A** | **A** | **C** | **A** | **A** | **G** | **T** | **C** | **G** | **G** | **A** | **T** | **T** | **C** | **A** | **T** | **C** | **G** | **A** | **T** | **A** | **C** | **T** | **G** | **A** | **A** | **T** | **C** | **T** | **A** | **G** | **A** | **C** | **G** | **A** | **G** | **T** | **G** | **A** | **A** | **T** | **A** | **A** | **A** | **G** | **G** | **A** | **G** | **C** | **A** | **G** | **T** | **G** | **G** | **G** | **T** | **G** | **C** | **T** | **G** | **A** | **T** | **G** | **C** | **T** | **G** | **C** | **A** | **C** | **G** | **G** | **A** | **C** | **G** | **A** | **T** | **G** | **A** | **A** | **A** | **C** | **G** | **A** | **T** | **C** | **C** | **A** | **A** | **A** | **G** | **C** | **A** | **A** | **T** | **C** | **G** | **T** | **G** | **A** | **A** | **T** | **C** | **G** | **G** | **A** | **T** | **A** | **G** | **A** | **G** | **A** | **C** | **C** | **C** | **A** | **G** | **A** | **T** | **C** | **A** | **G** | **C** | **G** | **A** | **G** | **A** | **A** | **A** | **G** | **A** | **A** | **A** | **A** | **A** | **C** | **T** | **A** | **T** | **T** | **A** | **A** | **A** | **A** | **G** | **A** | **T** | **A** | **T** | **C** | **G** | **A** | **A** | **T** | **A** | **T** | **A** | **T** | **C** | **G** | **A** | **T** | **T** | **C** | **T** | **T** | **T** | **G** | **A** | **C** | **G** | **A** | **T** | **G** | **A** | **A** | **T** | **A** | **G** | **C** | **C** | **A** | **A** | **G** | **C** | **G** | **T** | **C** | **T** | **C** | **A** | **C** | **C** | **A** | **T** | **A** | **A** | **G** | **C** | **G** | **A** | **C** | **T** | **T** | **T** | **T** | **A** | **A** | **G** | **C** | **G** | **T** | **A** | **G** | **A** | **A** | **G** | **A** | **T** | **C** | **T** | **T** | **C** | **A** | **A** | **T** | **T** | **A** | **G** | **G** | **G** | **T** | **A** | **T** | **G** | **A** | **A** | **A** | **A** | **T** | **C** | **T** | **G** | **T** | **T** | **A** | **T** | **T** | **C** | **G** | **A** | **G** | **C** | **C** | **A** | **A** | **T** | **T** | **C** | **A** | **T** | **T** | **T** | **T** | **A** | **C** | **A** | **A** | **T** | **C** | **G** | **A** | **G** | **C** | **C** | **T** | **C** | **A** | **T** | **C** | **A** | **G** | **C** | **G** | **G** | **G** | **T** | **G** | **G** | **C** | **G** | **A** | **T** | **T** | **T** | **C** | **A** | **G** | **G** | **T** | **C** | **C** | **T** | **A** | **A** | **C** | **G** | **G** | **T** | **G** | **C** | **A** | **G** | **G** | **A** | **A** | **A** | **G** | **T** | **C** | **A** | **T** | **C** | **C** | **A** | **T** | **T** | **A** | **T** | **C** | **C** | **A** | **T** | **T** | **A** | **T** | **C** | **T** | **T** | **C** | **T** | **G** | **G** | **G** | **G** | **G** | **C** | **A** | **T** | **T** | **C** | **A** | **A** | **C** | **G** | **G** | **C** | **A** | **A** | **G** | **G** | **T** | **T** | **A** | **T** | **A** | **G** | **G** | **A** | **G** | **A** | **A** | **A** | **A** | **A** | **T** | **C** | **C** | **C** | **A** | **G** | **C** | **C** | **A** | **A** | **A** | **A** | **C** | **A** | **T** | **C** | **T** | **G** | **A** | **G** | **C** | **A** | **T** | **T** | **A** | **G** | **T** | **T** | **A** | **T** | **G** | **T** | **A** | **C** | **G** | **C** | **C** | **A** | **A** | **A** | **A** | **T** | **T** | **A** | **T** | **G** | **A** | **A** | **G** | **A** | **C** | **A** | **A** | **T** | **C** | **G** | **A** | **G** | **G** | **A** | **A** | **C** | **G** | **T** | **T** | **G** | **G** | **C** | **G** | **G** | **A** | **A** | **T** | **T** | **T** | **G** | **C** | **A** | **G** | **A** | **G** | **A** | **A** | **A** | **A** | **A** | **C** | **C** | **A** | **A** | **G** | **T** | **A** | **G** | **A** | **C** | **T** | **A** | **C** | **C** | **A** | **A** | **G** | **C** | **A** | **T** | **T** | **T** | **T** | **T** | **G** | **A** | **A** | **C** | **A** | **A** | **C** | **C** | **T** | **C** | **C** | **G** | **A** | **A** | **A** | **G** | **C** | **T** | **T** | **G** | **G** | **G** | **A** | **T** | **G** | **G** | **A** | **A** | **A** | **G** | **A** | **G** | **A** | **T** | **G** | **T** | **T** | **T** | **T** | **T** | **C** | **A** | **T** | **A** | **A** | **C** | **A** | **A** | **G** | **A** | **T** | **T** | **G** | **A** | **G** | **C** | **A** | **G** | **A** | **T** | **G** | **A** | **G** | **T** | **A** | **T** | **G** | **G** | **G** | **C** | **C** | **A** | **A** | **C** | **G** | **G** | **A** | **A** | **A** | **A** | **A** | **A** | **G** | **T** | **G** | **G** | **A** | **A** | **T** | **T** | **G** | **G** | **C** | **T** | **A** | **A** | **A** | **T** | **C** | **T** | **T** | **T** | **A** | **T** | **C** | **A** | **C** | **A** | **G** | **C** | **C** | **A** | **G** | **C** | **T** | **G** | **A** | **A** | **C** | **T** | **A** | **T** | **A** | **T** | **A** | **T** | **A** | **T** | **G** | **G** | **G** | **A** | **T** | **G** | **A** | **A** | **C** | **C** | **A** | **T** | **T** | **G** | **A** | **A** | **T** | **T** | **A** | **T** | **T** | **T** | **G** | **G** | **A** | **T** | **G** | **T** | **C** | **T** | **T** | **C** | **A** | **A** | **T** | **C** | **A** | **A** | **G** | **A** | **A** | **C** | **A** | **A** | **T** | **T** | **A** | **G** | **A** | **A** | **C** | **A** | **A** | **C** | **T** | **G** | **A** | **T** | **C** | **T** | **T** | **G** | **A** | **A** | **C** | **G** | **T** | **G** | **A** | **A** | **A** | **C** | **C** | **T** | **G** | **C** | **C** | **A** | **T** | **G** | **T** | **T** | **A** | **C** | **T** | **G** | **G** | **T** | **G** | **G** | **A** | **A** | **C** | **A** | **T** | **G** | **A** | **T** | **C** | **A** | **A** | **A** | **C** | **C** | **T** | **T** | **C** | **C** | **T** | **G** | **G** | **A** | **T** | **A** | **A** | **A** | **G** | **T** | **A** | **T** | **C** | **T** | **A** | **C** | **T** | **G** | **A** | **G** | **A** | **T** | **T** | **A** | **T** | **T** | **T** | **C** | **T** | **C** | **T** | **T** | **G** | **A** | **G** | **A** | **A** | **A** | **A** | **T** | **C** | **-** | **-** | **-** |
| **N59542F** | **A** | **T** | **G** | **T** | **C** | **T** | **A** | **A** | **A** | **A** | **T** | **C** | **G** | **A** | **A** | **A** | **T** | **A** | **A** | **A** | **A** | **A** | **A** | **T** | **C** | **T** | **G** | **A** | **C** | **A** | **T** | **T** | **C** | **G** | **G** | **C** | **T** | **A** | **C** | **G** | **A** | **C** | **A** | **G** | **C** | **C** | **A** | **A** | **G** | **G** | **C** | **A** | **C** | **A** | **T** | **T** | **A** | **T** | **T** | **A** | **T** | **T** | **T** | **G** | **A** | **A** | **C** | **A** | **A** | **G** | **C** | **A** | **A** | **A** | **T** | **C** | **T** | **A** | **A** | **A** | **T** | **T** | **T** | **T** | **G** | **A** | **C** | **A** | **C** | **A** | **C** | **A** | **A** | **T** | **G** | **G** | **A** | **A** | **A** | **C** | **T** | **A** | **G** | **G** | **A** | **C** | **T** | **T** | **A** | **T** | **C** | **G** | **G** | **A** | **C** | **G** | **A** | **A** | **A** | **C** | **G** | **G** | **T** | **C** | **G** | **A** | **G** | **G** | **A** | **A** | **A** | **G** | **A** | **C** | **A** | **A** | **C** | **T** | **T** | **T** | **A** | **C** | **T** | **G** | **A** | **A** | **T** | **A** | **T** | **T** | **C** | **T** | **A** | **C** | **A** | **A** | **A** | **A** | **C** | **A** | **A** | **A** | **C** | **T** | **A** | **C** | **C** | **T** | **T** | **A** | **T** | **C** | **A** | **A** | **G** | **G** | **G** | **C** | **A** | **A** | **G** | **T** | **A** | **A** | **T** | **C** | **C** | **A** | **T** | **C** | **A** | **G** | **C** | **A** | **A** | **G** | **A** | **A** | **T** | **T** | **T** | **G** | **C** | **C** | **T** | **A** | **T** | **T** | **T** | **C** | **C** | **C** | **G** | **C** | **A** | **A** | **C** | **A** | **G** | **A** | **C** | **A** | **A** | **A** | **A** | **G** | **A** | **T** | **A** | **A** | **A** | **G** | **A** | **A** | **C** | **G** | **T** | **T** | **T** | **A** | **A** | **C** | **C** | **T** | **A** | **T** | **T** | **A** | **C** | **G** | **T** | **G** | **T** | **T** | **A** | **A** | **A** | **T** | **G** | **A** | **T** | **A** | **T** | **T** | **A** | **C** | **G** | **G** | **A** | **T** | **T** | **T** | **T** | **G** | **A** | **G** | **A** | **T** | **A** | **T** | **G** | **G** | **G** | **A** | **A** | **A** | **T** | **C** | **G** | **A** | **A** | **A** | **G** | **A** | **G** | **A** | **G** | **C** | **T** | **C** | **C** | **A** | **A** | **T** | **T** | **G** | **A** | **T** | **G** | **C** | **A** | **A** | **A** | **C** | **A** | **G** | **A** | **T** | **C** | **C** | **T** | **G** | **A** | **A** | **A** | **T** | **C** | **T** | **T** | **A** | **T** | **G** | **G** | **A** | **G** | **A** | **G** | **A** | **A** | **T** | **T** | **C** | **A** | **G** | **C** | **A** | **C** | **A** | **C** | **T** | **A** | **T** | **C** | **G** | **G** | **G** | **G** | **G** | **G** | **A** | **G** | **A** | **G** | **A** | **A** | **G** | **A** | **C** | **A** | **A** | **A** | **A** | **G** | **T** | **C** | **C** | **T** | **A** | **C** | **T** | **G** | **G** | **C** | **A** | **C** | **T** | **T** | **T** | **T** | **A** | **T** | **T** | **T** | **G** | **T** | **G** | **G** | **A** | **T** | **G** | **A** | **C** | **A** | **C** | **T** | **C** | **A** | **T** | **T** | **T** | **C** | **C** | **C** | **G** | **T** | **T** | **A** | **A** | **T** | **C** | **G** | **A** | **T** | **G** | **A** | **A** | **C** | **C** | **A** | **A** | **C** | **G** | **A** | **A** | **T** | **C** | **A** | **T** | **T** | **T** | **G** | **G** | **A** | **-** | **-** | **-** | **T** | **A** | **T** | **C** | **T** | **C** | **T** | **G** | **G** | **T** | **A** | **G** | **A** | **A** | **A** | **A** | **C** | **A** | **A** | **G** | **T** | **A** | **G** | **C** | **G** | **G** | **C** | **T** | **T** | **A** | **T** | **T** | **T** | **G** | **A** | **A** | **A** | **A** | **A** | **G** | **A** | **A** | **A** | **A** | **A** | **A** | **C** | **A** | **A** | **G** | **G** | **C** | **T** | **T** | **C** | **A** | **T** | **C** | **G** | **T** | **G** | **G** | **T** | **C** | **A** | **G** | **C** | **C** | **A** | **T** | **G** | **A** | **C** | **C** | **G** | **G** | **G** | **G** | **A** | **T** | **T** | **T** | **A** | **T** | **C** | **G** | **A** | **T** | **G** | **A** | **A** | **G** | **T** | **A** | **G** | **T** | **G** | **G** | **A** | **C** | **C** | **A** | **T** | **G** | **T** | **T** | **T** | **T** | **A** | **G** | **C** | **A** | **A** | **T** | **C** | **G** | **A** | **A** | **A** | **A** | **A** | **A** | **G** | **T** | **C** | **A** | **A** | **C** | **T** | **G** | **G** | **A** | **A** | **C** | **T** | **T** | **T** | **A** | **T** | **C** | **A** | **A** | **G** | **G** | **G** | **A** | **A** | **T** | **T** | **T** | **C** | **T** | **C** | **T** | **A** | **T** | **C** | **T** | **A** | **T** | **G** | **A** | **A** | **G** | **A** | **A** | **C** | **A** | **G** | **A** | **A** | **A** | **A** | **A** | **A** | **C** | **T** | **T** | **C** | **G** | **T** | **G** | **A** | **T** | **G** | **A** | **A** | **T** | **T** | **T** | **G** | **A** | **A** | **A** | **T** | **G** | **G** | **C** | **T** | **C** | **A** | **A** | **A** | **A** | **T** | **G** | **A** | **A** | **A** | **A** | **A** | **T** | **T** | **G** | **A** | **A** | **A** | **A** | **A** | **A** | **G** | **A** | **A** | **G** | **T** | **C** | **A** | **G** | **T** | **A** | **G** | **G** | **C** | **T** | **A** | **A** | **A** | **G** | **A** | **A** | **A** | **A** | **C** | **A** | **G** | **C** | **A** | **G** | **C** | **T** | **G** | **A** | **A** | **A** | **A** | **A** | **G** | **C** | **C** | **G** | **A** | **A** | **T** | **G** | **G** | **T** | **C** | **T** | **C** | **G** | **T** | **T** | **C** | **C** | **C** | **G** | **A** | **G** | **A** | **A** | **G** | **G** | **A** | **G** | **A** | **T** | **A** | **A** | **A** | **A** | **C** | **A** | **A** | **A** | **G** | **A** | **A** | **A** | **C** | **A** | **A** | **G** | **T** | **C** | **G** | **G** | **A** | **T** | **T** | **C** | **A** | **T** | **C** | **G** | **A** | **T** | **A** | **C** | **T** | **G** | **A** | **A** | **T** | **C** | **T** | **A** | **G** | **A** | **C** | **G** | **A** | **G** | **T** | **G** | **A** | **A** | **T** | **A** | **A** | **A** | **G** | **G** | **A** | **G** | **C** | **A** | **G** | **T** | **G** | **G** | **G** | **T** | **G** | **C** | **T** | **G** | **A** | **T** | **G** | **C** | **T** | **G** | **C** | **A** | **C** | **G** | **G** | **A** | **C** | **G** | **A** | **T** | **G** | **A** | **A** | **A** | **C** | **G** | **A** | **T** | **C** | **C** | **A** | **A** | **A** | **G** | **C** | **A** | **A** | **T** | **C** | **G** | **T** | **G** | **A** | **A** | **T** | **C** | **G** | **G** | **A** | **T** | **G** | **G** | **A** | **G** | **A** | **C** | **C** | **C** | **A** | **G** | **A** | **T** | **C** | **A** | **G** | **C** | **G** | **A** | **G** | **A** | **A** | **A** | **G** | **A** | **A** | **A** | **A** | **A** | **C** | **T** | **A** | **T** | **T** | **A** | **A** | **A** | **A** | **G** | **A** | **T** | **A** | **T** | **C** | **G** | **A** | **A** | **T** | **A** | **T** | **A** | **T** | **C** | **G** | **A** | **T** | **T** | **C** | **T** | **T** | **T** | **G** | **A** | **C** | **G** | **A** | **T** | **G** | **A** | **A** | **T** | **A** | **G** | **C** | **C** | **A** | **A** | **G** | **C** | **G** | **T** | **C** | **T** | **C** | **A** | **C** | **C** | **A** | **T** | **A** | **A** | **G** | **C** | **G** | **A** | **C** | **T** | **T** | **T** | **T** | **A** | **A** | **G** | **C** | **G** | **T** | **A** | **G** | **A** | **A** | **G** | **A** | **T** | **C** | **T** | **T** | **C** | **A** | **A** | **T** | **T** | **A** | **G** | **G** | **G** | **T** | **A** | **T** | **G** | **A** | **A** | **A** | **A** | **T** | **C** | **T** | **G** | **T** | **T** | **A** | **T** | **T** | **C** | **G** | **A** | **G** | **C** | **C** | **A** | **A** | **T** | **T** | **C** | **A** | **T** | **T** | **T** | **T** | **A** | **C** | **A** | **A** | **T** | **C** | **G** | **A** | **G** | **C** | **C** | **T** | **C** | **A** | **T** | **C** | **A** | **G** | **C** | **G** | **G** | **G** | **T** | **G** | **G** | **C** | **G** | **A** | **T** | **T** | **T** | **C** | **A** | **G** | **G** | **T** | **C** | **C** | **T** | **A** | **A** | **C** | **G** | **G** | **T** | **G** | **C** | **A** | **G** | **G** | **A** | **A** | **A** | **G** | **T** | **C** | **A** | **T** | **C** | **C** | **A** | **T** | **T** | **A** | **T** | **C** | **C** | **A** | **T** | **T** | **A** | **T** | **C** | **T** | **T** | **C** | **T** | **G** | **G** | **G** | **G** | **G** | **C** | **A** | **T** | **T** | **C** | **A** | **A** | **C** | **G** | **G** | **C** | **A** | **A** | **G** | **G** | **T** | **T** | **A** | **T** | **A** | **G** | **G** | **A** | **G** | **A** | **A** | **A** | **A** | **A** | **T** | **A** | **C** | **C** | **A** | **G** | **C** | **C** | **A** | **A** | **A** | **A** | **C** | **A** | **T** | **C** | **T** | **G** | **A** | **G** | **C** | **A** | **T** | **T** | **A** | **G** | **T** | **T** | **A** | **T** | **G** | **C** | **A** | **A** | **G** | **C** | **C** | **A** | **A** | **A** | **A** | **T** | **T** | **A** | **T** | **G** | **A** | **A** | **G** | **A** | **C** | **A** | **A** | **T** | **C** | **G** | **A** | **G** | **G** | **A** | **A** | **C** | **G** | **T** | **T** | **G** | **G** | **C** | **G** | **G** | **A** | **A** | **T** | **T** | **T** | **G** | **C** | **A** | **G** | **A** | **G** | **A** | **A** | **A** | **A** | **A** | **C** | **C** | **A** | **A** | **G** | **T** | **A** | **G** | **A** | **C** | **T** | **A** | **C** | **C** | **A** | **A** | **G** | **C** | **A** | **T** | **T** | **T** | **T** | **T** | **G** | **A** | **A** | **C** | **A** | **A** | **C** | **C** | **T** | **C** | **C** | **G** | **A** | **A** | **A** | **G** | **C** | **T** | **T** | **G** | **G** | **G** | **A** | **T** | **G** | **G** | **A** | **A** | **A** | **G** | **A** | **G** | **A** | **T** | **G** | **T** | **T** | **T** | **T** | **T** | **C** | **A** | **T** | **A** | **A** | **C** | **A** | **A** | **G** | **A** | **T** | **C** | **G** | **A** | **G** | **C** | **A** | **G** | **A** | **T** | **G** | **A** | **G** | **T** | **A** | **T** | **G** | **G** | **G** | **C** | **C** | **A** | **A** | **C** | **G** | **G** | **A** | **A** | **A** | **A** | **A** | **A** | **G** | **T** | **G** | **G** | **A** | **A** | **T** | **T** | **G** | **G** | **C** | **T** | **A** | **A** | **A** | **T** | **C** | **T** | **T** | **T** | **A** | **T** | **C** | **A** | **C** | **A** | **G** | **C** | **C** | **A** | **G** | **C** | **T** | **G** | **A** | **A** | **C** | **T** | **A** | **T** | **A** | **T** | **A** | **T** | **A** | **T** | **G** | **G** | **G** | **A** | **T** | **G** | **A** | **A** | **C** | **C** | **A** | **T** | **T** | **G** | **A** | **A** | **T** | **T** | **A** | **T** | **T** | **T** | **G** | **G** | **A** | **T** | **G** | **T** | **C** | **T** | **T** | **C** | **A** | **A** | **T** | **C** | **A** | **A** | **G** | **A** | **A** | **C** | **A** | **A** | **T** | **T** | **A** | **G** | **A** | **A** | **C** | **A** | **A** | **C** | **T** | **G** | **A** | **T** | **C** | **T** | **T** | **G** | **A** | **A** | **C** | **G** | **T** | **G** | **A** | **A** | **A** | **C** | **C** | **T** | **G** | **C** | **C** | **A** | **T** | **G** | **T** | **T** | **A** | **C** | **T** | **A** | **G** | **T** | **G** | **G** | **A** | **A** | **C** | **A** | **T** | **G** | **A** | **T** | **C** | **A** | **A** | **A** | **C** | **C** | **T** | **T** | **C** | **C** | **T** | **G** | **G** | **A** | **T** | **A** | **A** | **A** | **G** | **T** | **A** | **T** | **C** | **T** | **A** | **C** | **T** | **G** | **A** | **G** | **A** | **T** | **T** | **A** | **T** | **T** | **T** | **C** | **T** | **C** | **T** | **T** | **G** | **A** | **G** | **A** | **G** | **A** | **A** | **T** | **C** | **-** | **-** | **-** |
| **N59711F** | **A** | **T** | **G** | **T** | **C** | **T** | **A** | **A** | **A** | **A** | **T** | **C** | **G** | **A** | **A** | **A** | **T** | **A** | **A** | **A** | **A** | **A** | **A** | **T** | **C** | **T** | **G** | **A** | **C** | **A** | **T** | **T** | **C** | **G** | **G** | **C** | **T** | **A** | **T** | **G** | **A** | **C** | **A** | **G** | **C** | **C** | **A** | **A** | **G** | **G** | **C** | **A** | **C** | **A** | **T** | **T** | **A** | **T** | **T** | **A** | **T** | **T** | **T** | **G** | **A** | **A** | **C** | **A** | **A** | **G** | **C** | **C** | **A** | **A** | **T** | **C** | **T** | **A** | **A** | **A** | **C** | **T** | **T** | **T** | **G** | **A** | **C** | **A** | **C** | **A** | **C** | **A** | **A** | **T** | **G** | **G** | **A** | **A** | **A** | **C** | **T** | **A** | **G** | **G** | **A** | **C** | **T** | **T** | **A** | **T** | **C** | **G** | **G** | **A** | **C** | **G** | **A** | **A** | **A** | **C** | **G** | **G** | **T** | **C** | **G** | **A** | **G** | **G** | **A** | **A** | **A** | **G** | **A** | **C** | **A** | **A** | **C** | **T** | **T** | **T** | **A** | **C** | **T** | **G** | **A** | **A** | **T** | **A** | **T** | **C** | **C** | **T** | **G** | **C** | **A** | **A** | **A** | **A** | **C** | **A** | **A** | **A** | **C** | **T** | **C** | **C** | **C** | **T** | **T** | **A** | **T** | **C** | **A** | **A** | **G** | **G** | **G** | **C** | **A** | **A** | **G** | **T** | **G** | **A** | **T** | **C** | **C** | **A** | **T** | **C** | **A** | **G** | **C** | **A** | **A** | **G** | **C** | **G** | **T** | **T** | **T** | **G** | **T** | **C** | **T** | **A** | **T** | **T** | **T** | **C** | **C** | **C** | **G** | **C** | **A** | **G** | **C** | **A** | **A** | **A** | **C** | **A** | **A** | **A** | **A** | **G** | **A** | **T** | **A** | **A** | **A** | **G** | **A** | **G** | **C** | **G** | **T** | **T** | **T** | **G** | **A** | **C** | **C** | **T** | **A** | **T** | **T** | **A** | **T** | **G** | **T** | **G** | **T** | **T** | **A** | **A** | **A** | **T** | **G** | **A** | **G** | **A** | **T** | **T** | **A** | **C** | **G** | **G** | **A** | **T** | **T** | **T** | **T** | **G** | **A** | **G** | **A** | **T** | **A** | **T** | **G** | **G** | **G** | **A** | **A** | **A** | **T** | **T** | **G** | **A** | **A** | **A** | **G** | **A** | **G** | **A** | **G** | **C** | **T** | **C** | **C** | **A** | **A** | **C** | **T** | **G** | **A** | **T** | **G** | **C** | **A** | **A** | **A** | **C** | **A** | **G** | **A** | **T** | **C** | **C** | **T** | **G** | **A** | **A** | **A** | **T** | **C** | **T** | **T** | **A** | **T** | **G** | **G** | **A** | **G** | **A** | **G** | **A** | **A** | **T** | **T** | **C** | **A** | **G** | **C** | **A** | **C** | **A** | **C** | **T** | **A** | **T** | **C** | **A** | **G** | **G** | **A** | **G** | **G** | **A** | **G** | **A** | **G** | **A** | **A** | **G** | **A** | **C** | **A** | **A** | **A** | **A** | **G** | **T** | **C** | **C** | **T** | **A** | **C** | **T** | **G** | **G** | **C** | **A** | **C** | **T** | **C** | **T** | **T** | **A** | **T** | **T** | **T** | **G** | **T** | **G** | **G** | **A** | **T** | **G** | **A** | **C** | **A** | **C** | **T** | **C** | **A** | **T** | **T** | **T** | **C** | **C** | **C** | **G** | **T** | **T** | **A** | **A** | **T** | **C** | **G** | **A** | **T** | **G** | **A** | **A** | **C** | **C** | **A** | **A** | **C** | **G** | **A** | **A** | **T** | **C** | **A** | **T** | **T** | **T** | **G** | **G** | **A** | **-** | **-** | **-** | **T** | **A** | **T** | **C** | **T** | **C** | **T** | **G** | **G** | **C** | **A** | **G** | **A** | **A** | **A** | **A** | **C** | **A** | **A** | **G** | **T** | **A** | **G** | **C** | **G** | **G** | **C** | **T** | **T** | **A** | **C** | **T** | **T** | **G** | **A** | **A** | **A** | **A** | **A** | **G** | **A** | **A** | **A** | **A** | **A** | **A** | **C** | **A** | **A** | **G** | **G** | **A** | **T** | **T** | **C** | **A** | **T** | **C** | **G** | **T** | **G** | **G** | **T** | **G** | **A** | **G** | **T** | **C** | **A** | **T** | **G** | **A** | **T** | **C** | **G** | **G** | **G** | **G** | **A** | **T** | **T** | **T** | **A** | **T** | **C** | **G** | **A** | **T** | **G** | **A** | **A** | **G** | **T** | **A** | **G** | **T** | **G** | **G** | **A** | **C** | **C** | **A** | **T** | **G** | **T** | **T** | **T** | **T** | **A** | **G** | **C** | **A** | **A** | **T** | **C** | **G** | **A** | **A** | **A** | **A** | **G** | **A** | **G** | **C** | **C** | **A** | **A** | **C** | **T** | **G** | **G** | **A** | **A** | **C** | **T** | **T** | **T** | **A** | **T** | **C** | **A** | **A** | **G** | **G** | **G** | **A** | **A** | **T** | **T** | **T** | **C** | **T** | **C** | **T** | **A** | **T** | **C** | **T** | **A** | **T** | **G** | **A** | **A** | **G** | **A** | **A** | **C** | **A** | **G** | **A** | **A** | **A** | **A** | **A** | **A** | **C** | **T** | **T** | **C** | **G** | **T** | **G** | **A** | **T** | **G** | **A** | **A** | **T** | **T** | **T** | **G** | **A** | **A** | **A** | **T** | **A** | **G** | **C** | **T** | **A** | **A** | **A** | **A** | **A** | **T** | **G** | **A** | **A** | **A** | **A** | **A** | **T** | **T** | **G** | **A** | **A** | **A** | **A** | **A** | **A** | **G** | **A** | **A** | **G** | **T** | **C** | **A** | **G** | **C** | **A** | **G** | **G** | **C** | **T** | **A** | **A** | **A** | **G** | **A** | **A** | **A** | **A** | **C** | **A** | **G** | **C** | **A** | **G** | **C** | **T** | **G** | **A** | **A** | **A** | **A** | **A** | **G** | **C** | **A** | **G** | **A** | **A** | **T** | **G** | **G** | **T** | **C** | **T** | **C** | **G** | **T** | **T** | **C** | **C** | **C** | **G** | **A** | **G** | **A** | **A** | **G** | **G** | **A** | **G** | **A** | **C** | **A** | **A** | **A** | **A** | **C** | **A** | **A** | **A** | **G** | **A** | **A** | **A** | **A** | **A** | **A** | **G** | **T** | **C** | **G** | **G** | **A** | **T** | **T** | **C** | **A** | **T** | **C** | **G** | **A** | **T** | **A** | **C** | **G** | **G** | **A** | **A** | **T** | **C** | **T** | **A** | **G** | **G** | **C** | **G** | **A** | **G** | **T** | **G** | **A** | **A** | **T** | **A** | **A** | **A** | **G** | **G** | **A** | **G** | **C** | **A** | **G** | **T** | **A** | **G** | **G** | **C** | **G** | **C** | **T** | **G** | **A** | **T** | **G** | **C** | **T** | **G** | **C** | **A** | **C** | **G** | **G** | **A** | **C** | **G** | **A** | **T** | **G** | **A** | **A** | **A** | **C** | **G** | **A** | **T** | **C** | **T** | **A** | **A** | **A** | **G** | **C** | **A** | **A** | **T** | **C** | **G** | **T** | **G** | **A** | **A** | **T** | **C** | **G** | **G** | **A** | **T** | **G** | **G** | **A** | **A** | **A** | **C** | **C** | **C** | **A** | **G** | **A** | **T** | **C** | **A** | **G** | **T** | **G** | **A** | **G** | **A** | **A** | **A** | **G** | **A** | **A** | **A** | **A** | **A** | **T** | **T** | **A** | **T** | **T** | **A** | **A** | **A** | **A** | **G** | **A** | **T** | **A** | **T** | **C** | **G** | **A** | **G** | **T** | **A** | **T** | **A** | **T** | **C** | **G** | **A** | **T** | **G** | **C** | **T** | **T** | **T** | **G** | **A** | **C** | **G** | **A** | **T** | **G** | **A** | **A** | **T** | **A** | **G** | **C** | **C** | **A** | **A** | **G** | **T** | **G** | **T** | **C** | **T** | **C** | **A** | **T** | **C** | **A** | **T** | **A** | **A** | **A** | **C** | **G** | **G** | **C** | **T** | **G** | **T** | **T** | **A** | **A** | **G** | **C** | **G** | **T** | **A** | **G** | **A** | **A** | **A** | **A** | **T** | **C** | **T** | **T** | **C** | **A** | **A** | **T** | **T** | **A** | **G** | **G** | **G** | **T** | **A** | **T** | **G** | **A** | **A** | **A** | **A** | **T** | **C** | **T** | **G** | **T** | **T** | **A** | **T** | **T** | **C** | **G** | **A** | **G** | **C** | **C** | **A** | **G** | **T** | **T** | **C** | **A** | **T** | **T** | **T** | **T** | **A** | **C** | **G** | **A** | **T** | **C** | **G** | **A** | **A** | **C** | **C** | **T** | **C** | **A** | **T** | **C** | **A** | **G** | **C** | **G** | **G** | **G** | **T** | **G** | **G** | **C** | **A** | **A** | **T** | **T** | **T** | **C** | **A** | **G** | **G** | **T** | **C** | **C** | **T** | **A** | **A** | **C** | **G** | **G** | **C** | **G** | **C** | **G** | **G** | **G** | **A** | **A** | **A** | **G** | **T** | **C** | **A** | **T** | **C** | **C** | **A** | **T** | **T** | **A** | **T** | **C** | **C** | **A** | **T** | **T** | **A** | **T** | **C** | **T** | **T** | **C** | **T** | **G** | **G** | **G** | **G** | **A** | **C** | **A** | **T** | **T** | **C** | **A** | **A** | **C** | **G** | **G** | **C** | **A** | **A** | **G** | **G** | **T** | **T** | **G** | **T** | **A** | **G** | **G** | **A** | **G** | **A** | **A** | **A** | **A** | **A** | **T** | **C** | **C** | **C** | **A** | **G** | **C** | **C** | **C** | **A** | **A** | **A** | **C** | **A** | **T** | **T** | **T** | **G** | **A** | **C** | **C** | **A** | **T** | **T** | **A** | **G** | **T** | **T** | **A** | **C** | **G** | **T** | **A** | **C** | **G** | **C** | **C** | **A** | **A** | **A** | **A** | **T** | **T** | **A** | **T** | **G** | **A** | **A** | **G** | **A** | **C** | **A** | **A** | **T** | **C** | **G** | **A** | **G** | **G** | **G** | **A** | **C** | **A** | **T** | **T** | **G** | **G** | **C** | **G** | **G** | **A** | **A** | **T** | **T** | **C** | **G** | **C** | **G** | **A** | **A** | **G** | **A** | **A** | **A** | **A** | **A** | **C** | **C** | **G** | **A** | **G** | **T** | **A** | **G** | **A** | **C** | **T** | **A** | **T** | **C** | **A** | **A** | **G** | **C** | **A** | **T** | **T** | **T** | **T** | **T** | **G** | **A** | **A** | **T** | **A** | **A** | **T** | **C** | **T** | **C** | **C** | **G** | **A** | **A** | **A** | **G** | **C** | **T** | **T** | **G** | **G** | **G** | **A** | **T** | **G** | **G** | **A** | **A** | **A** | **G** | **A** | **G** | **A** | **T** | **G** | **T** | **T** | **T** | **T** | **T** | **C** | **A** | **T** | **A** | **A** | **T** | **A** | **A** | **A** | **A** | **T** | **C** | **G** | **A** | **A** | **C** | **A** | **G** | **A** | **T** | **G** | **A** | **G** | **T** | **A** | **T** | **G** | **G** | **G** | **A** | **C** | **A** | **A** | **C** | **G** | **G** | **A** | **A** | **A** | **A** | **A** | **A** | **G** | **T** | **G** | **G** | **A** | **A** | **T** | **T** | **G** | **G** | **C** | **T** | **A** | **A** | **A** | **T** | **C** | **T** | **T** | **T** | **A** | **T** | **C** | **C** | **C** | **A** | **A** | **C** | **C** | **A** | **G** | **C** | **T** | **G** | **A** | **G** | **C** | **T** | **C** | **T** | **A** | **T** | **A** | **T** | **A** | **T** | **G** | **G** | **G** | **A** | **T** | **G** | **A** | **A** | **C** | **C** | **A** | **T** | **T** | **G** | **A** | **A** | **T** | **T** | **A** | **T** | **T** | **T** | **G** | **G** | **A** | **T** | **G** | **T** | **C** | **T** | **T** | **C** | **A** | **A** | **T** | **C** | **A** | **A** | **G** | **A** | **A** | **C** | **A** | **A** | **T** | **T** | **A** | **G** | **A** | **A** | **C** | **A** | **A** | **C** | **T** | **G** | **A** | **T** | **T** | **T** | **T** | **G** | **A** | **A** | **C** | **G** | **T** | **G** | **A** | **A** | **A** | **C** | **C** | **T** | **G** | **C** | **T** | **A** | **T** | **G** | **T** | **T** | **A** | **C** | **T** | **A** | **G** | **T** | **G** | **G** | **A** | **A** | **C** | **A** | **T** | **G** | **A** | **T** | **C** | **A** | **A** | **A** | **C** | **C** | **T** | **T** | **T** | **C** | **T** | **A** | **G** | **A** | **T** | **A** | **A** | **A** | **G** | **T** | **A** | **T** | **C** | **T** | **A** | **C** | **T** | **G** | **A** | **G** | **A** | **T** | **T** | **A** | **T** | **T** | **T** | **C** | **T** | **C** | **T** | **T** | **G** | **A** | **G** | **A** | **A** | **A** | **A** | **T** | **C** | **-** | **-** | **-** |
| **N60324F** | **A** | **T** | **G** | **T** | **C** | **T** | **A** | **A** | **A** | **A** | **T** | **C** | **G** | **A** | **A** | **A** | **T** | **A** | **A** | **A** | **A** | **A** | **A** | **T** | **C** | **T** | **G** | **A** | **C** | **A** | **T** | **T** | **C** | **G** | **G** | **C** | **T** | **A** | **C** | **G** | **A** | **C** | **A** | **G** | **C** | **C** | **A** | **A** | **G** | **G** | **C** | **A** | **C** | **A** | **T** | **T** | **A** | **T** | **T** | **A** | **T** | **T** | **T** | **G** | **A** | **A** | **C** | **A** | **A** | **G** | **C** | **A** | **A** | **A** | **T** | **C** | **T** | **A** | **A** | **A** | **T** | **T** | **T** | **T** | **G** | **A** | **C** | **A** | **C** | **A** | **C** | **A** | **A** | **T** | **G** | **G** | **A** | **A** | **A** | **C** | **T** | **A** | **G** | **G** | **A** | **C** | **T** | **T** | **A** | **T** | **C** | **G** | **G** | **A** | **C** | **G** | **A** | **A** | **A** | **C** | **G** | **G** | **T** | **C** | **G** | **A** | **G** | **G** | **A** | **A** | **A** | **G** | **A** | **C** | **A** | **A** | **C** | **T** | **T** | **T** | **A** | **C** | **T** | **G** | **A** | **A** | **T** | **A** | **T** | **T** | **C** | **T** | **A** | **C** | **A** | **A** | **A** | **A** | **C** | **A** | **A** | **A** | **C** | **T** | **A** | **C** | **C** | **T** | **T** | **A** | **T** | **C** | **A** | **A** | **G** | **G** | **G** | **C** | **A** | **A** | **G** | **T** | **A** | **A** | **T** | **C** | **C** | **A** | **T** | **C** | **A** | **G** | **C** | **A** | **A** | **G** | **A** | **A** | **T** | **T** | **T** | **G** | **T** | **C** | **T** | **A** | **T** | **T** | **T** | **C** | **C** | **C** | **G** | **C** | **A** | **G** | **C** | **A** | **A** | **A** | **C** | **A** | **A** | **A** | **A** | **G** | **A** | **T** | **A** | **A** | **A** | **G** | **A** | **G** | **C** | **G** | **T** | **T** | **T** | **A** | **A** | **C** | **C** | **T** | **A** | **T** | **T** | **A** | **T** | **G** | **T** | **G** | **T** | **T** | **A** | **A** | **A** | **T** | **G** | **A** | **G** | **A** | **T** | **T** | **A** | **C** | **G** | **G** | **A** | **T** | **T** | **T** | **T** | **G** | **A** | **G** | **A** | **T** | **A** | **T** | **G** | **G** | **G** | **A** | **A** | **A** | **T** | **T** | **G** | **A** | **A** | **A** | **G** | **A** | **G** | **A** | **G** | **C** | **T** | **C** | **C** | **A** | **A** | **C** | **T** | **G** | **A** | **T** | **G** | **C** | **A** | **A** | **A** | **C** | **A** | **G** | **A** | **T** | **C** | **C** | **T** | **G** | **A** | **A** | **A** | **T** | **C** | **T** | **T** | **A** | **T** | **G** | **G** | **A** | **G** | **A** | **G** | **A** | **A** | **T** | **T** | **C** | **A** | **G** | **C** | **A** | **C** | **A** | **C** | **T** | **A** | **T** | **C** | **A** | **G** | **G** | **A** | **G** | **G** | **A** | **G** | **A** | **G** | **A** | **A** | **G** | **A** | **C** | **A** | **A** | **A** | **A** | **G** | **T** | **C** | **C** | **T** | **A** | **C** | **T** | **G** | **G** | **C** | **A** | **C** | **T** | **T** | **T** | **T** | **A** | **T** | **T** | **T** | **G** | **T** | **G** | **G** | **A** | **T** | **G** | **A** | **C** | **A** | **C** | **T** | **C** | **A** | **T** | **T** | **T** | **C** | **C** | **C** | **G** | **T** | **T** | **A** | **A** | **T** | **C** | **G** | **A** | **T** | **G** | **A** | **A** | **C** | **C** | **A** | **A** | **C** | **G** | **A** | **A** | **T** | **C** | **A** | **T** | **T** | **T** | **G** | **G** | **A** | **-** | **-** | **-** | **T** | **A** | **T** | **C** | **T** | **C** | **T** | **G** | **G** | **C** | **A** | **G** | **A** | **A** | **A** | **A** | **C** | **A** | **A** | **G** | **T** | **A** | **G** | **C** | **G** | **G** | **C** | **T** | **T** | **A** | **C** | **T** | **T** | **G** | **A** | **A** | **A** | **A** | **A** | **G** | **A** | **A** | **A** | **A** | **A** | **A** | **C** | **A** | **A** | **G** | **G** | **C** | **T** | **T** | **C** | **A** | **T** | **C** | **G** | **T** | **G** | **G** | **T** | **C** | **A** | **G** | **C** | **C** | **A** | **T** | **G** | **A** | **C** | **C** | **G** | **G** | **G** | **G** | **A** | **T** | **T** | **T** | **A** | **T** | **C** | **G** | **A** | **T** | **G** | **A** | **A** | **G** | **T** | **A** | **G** | **T** | **G** | **G** | **A** | **C** | **C** | **A** | **T** | **G** | **T** | **T** | **T** | **T** | **A** | **G** | **C** | **A** | **A** | **T** | **C** | **G** | **A** | **A** | **A** | **A** | **A** | **A** | **G** | **T** | **C** | **A** | **A** | **C** | **T** | **G** | **G** | **A** | **A** | **C** | **T** | **T** | **T** | **A** | **T** | **C** | **A** | **A** | **G** | **G** | **G** | **A** | **A** | **T** | **T** | **T** | **C** | **T** | **C** | **T** | **A** | **T** | **C** | **T** | **A** | **T** | **G** | **A** | **A** | **G** | **A** | **A** | **C** | **A** | **G** | **A** | **A** | **A** | **A** | **A** | **A** | **C** | **T** | **T** | **C** | **G** | **T** | **G** | **A** | **T** | **G** | **A** | **A** | **T** | **T** | **T** | **G** | **A** | **A** | **A** | **T** | **G** | **G** | **C** | **T** | **C** | **A** | **A** | **A** | **A** | **T** | **G** | **A** | **A** | **A** | **A** | **A** | **T** | **T** | **G** | **A** | **A** | **A** | **A** | **A** | **A** | **G** | **A** | **A** | **G** | **T** | **C** | **A** | **G** | **T** | **A** | **G** | **G** | **C** | **T** | **A** | **A** | **A** | **G** | **A** | **A** | **A** | **A** | **C** | **A** | **G** | **C** | **A** | **G** | **C** | **T** | **G** | **A** | **A** | **A** | **A** | **A** | **G** | **C** | **C** | **G** | **A** | **A** | **T** | **G** | **G** | **T** | **C** | **T** | **C** | **G** | **T** | **T** | **C** | **C** | **C** | **G** | **A** | **G** | **A** | **A** | **G** | **G** | **A** | **G** | **A** | **T** | **A** | **A** | **A** | **A** | **C** | **A** | **A** | **A** | **G** | **A** | **A** | **A** | **C** | **A** | **A** | **G** | **T** | **C** | **G** | **G** | **A** | **T** | **T** | **C** | **A** | **T** | **C** | **G** | **A** | **T** | **A** | **C** | **T** | **G** | **A** | **A** | **T** | **C** | **T** | **A** | **G** | **A** | **C** | **G** | **A** | **G** | **T** | **G** | **A** | **A** | **T** | **A** | **A** | **A** | **G** | **G** | **A** | **G** | **C** | **A** | **G** | **T** | **G** | **G** | **G** | **T** | **G** | **C** | **T** | **G** | **A** | **T** | **G** | **C** | **T** | **G** | **C** | **A** | **C** | **G** | **G** | **A** | **C** | **G** | **A** | **T** | **G** | **A** | **A** | **A** | **C** | **G** | **A** | **T** | **C** | **C** | **A** | **A** | **A** | **G** | **C** | **A** | **A** | **T** | **C** | **G** | **T** | **G** | **A** | **A** | **T** | **C** | **G** | **G** | **A** | **T** | **G** | **G** | **A** | **G** | **A** | **C** | **C** | **C** | **A** | **G** | **A** | **T** | **C** | **A** | **G** | **C** | **G** | **A** | **G** | **A** | **A** | **A** | **G** | **A** | **A** | **A** | **A** | **A** | **C** | **T** | **A** | **T** | **T** | **G** | **A** | **A** | **A** | **G** | **A** | **T** | **A** | **T** | **C** | **G** | **A** | **A** | **T** | **A** | **T** | **A** | **T** | **C** | **G** | **A** | **T** | **T** | **C** | **T** | **T** | **T** | **G** | **A** | **C** | **G** | **A** | **T** | **G** | **A** | **A** | **T** | **A** | **G** | **C** | **C** | **A** | **A** | **G** | **C** | **G** | **T** | **C** | **T** | **C** | **A** | **C** | **C** | **A** | **T** | **A** | **A** | **G** | **C** | **G** | **A** | **C** | **T** | **T** | **T** | **T** | **A** | **A** | **G** | **C** | **G** | **T** | **A** | **G** | **A** | **A** | **G** | **A** | **T** | **C** | **T** | **T** | **C** | **A** | **A** | **T** | **T** | **A** | **G** | **G** | **G** | **T** | **A** | **T** | **G** | **A** | **G** | **A** | **A** | **T** | **C** | **T** | **G** | **T** | **T** | **A** | **T** | **T** | **C** | **G** | **A** | **G** | **C** | **C** | **A** | **A** | **T** | **T** | **C** | **A** | **T** | **T** | **T** | **T** | **A** | **C** | **A** | **A** | **T** | **C** | **G** | **A** | **G** | **C** | **C** | **T** | **C** | **A** | **T** | **C** | **A** | **G** | **C** | **G** | **G** | **G** | **T** | **G** | **G** | **C** | **G** | **A** | **T** | **T** | **T** | **C** | **A** | **G** | **G** | **T** | **C** | **C** | **T** | **A** | **A** | **C** | **G** | **G** | **T** | **G** | **C** | **A** | **G** | **G** | **A** | **A** | **A** | **G** | **T** | **C** | **A** | **T** | **C** | **C** | **A** | **T** | **T** | **A** | **T** | **C** | **C** | **A** | **T** | **T** | **A** | **T** | **C** | **T** | **T** | **C** | **T** | **G** | **G** | **G** | **G** | **G** | **C** | **A** | **T** | **T** | **C** | **A** | **A** | **C** | **G** | **G** | **C** | **A** | **A** | **G** | **G** | **T** | **T** | **A** | **T** | **A** | **G** | **G** | **A** | **G** | **A** | **A** | **A** | **A** | **A** | **T** | **C** | **C** | **C** | **A** | **G** | **C** | **C** | **A** | **A** | **A** | **A** | **C** | **A** | **T** | **C** | **T** | **G** | **A** | **G** | **C** | **A** | **T** | **T** | **A** | **G** | **T** | **T** | **A** | **T** | **G** | **T** | **A** | **C** | **G** | **C** | **C** | **A** | **A** | **A** | **A** | **T** | **T** | **A** | **T** | **G** | **A** | **A** | **G** | **A** | **C** | **A** | **A** | **T** | **C** | **G** | **A** | **G** | **G** | **A** | **A** | **C** | **G** | **T** | **T** | **G** | **G** | **C** | **G** | **G** | **A** | **A** | **T** | **T** | **T** | **G** | **C** | **A** | **G** | **A** | **G** | **A** | **A** | **A** | **A** | **A** | **C** | **C** | **A** | **A** | **G** | **T** | **A** | **G** | **A** | **C** | **T** | **A** | **C** | **C** | **A** | **A** | **G** | **C** | **A** | **T** | **T** | **T** | **T** | **T** | **G** | **A** | **A** | **C** | **A** | **A** | **C** | **C** | **T** | **C** | **C** | **G** | **A** | **A** | **A** | **G** | **C** | **T** | **T** | **G** | **G** | **G** | **A** | **T** | **G** | **G** | **A** | **A** | **A** | **G** | **A** | **G** | **A** | **T** | **G** | **T** | **T** | **T** | **T** | **T** | **C** | **A** | **T** | **A** | **A** | **C** | **A** | **A** | **G** | **A** | **T** | **C** | **G** | **A** | **G** | **C** | **A** | **G** | **A** | **T** | **G** | **A** | **G** | **T** | **A** | **T** | **G** | **G** | **G** | **C** | **C** | **A** | **A** | **C** | **G** | **G** | **A** | **A** | **A** | **A** | **A** | **A** | **G** | **T** | **G** | **G** | **A** | **A** | **T** | **T** | **G** | **G** | **C** | **T** | **A** | **A** | **A** | **T** | **C** | **T** | **T** | **T** | **A** | **T** | **C** | **A** | **C** | **A** | **G** | **C** | **C** | **A** | **G** | **C** | **T** | **G** | **A** | **A** | **C** | **T** | **A** | **T** | **A** | **T** | **A** | **T** | **A** | **T** | **G** | **G** | **G** | **A** | **T** | **G** | **A** | **A** | **C** | **C** | **A** | **T** | **T** | **G** | **A** | **A** | **T** | **T** | **A** | **T** | **T** | **T** | **G** | **G** | **A** | **T** | **G** | **T** | **C** | **T** | **T** | **C** | **A** | **A** | **T** | **C** | **A** | **A** | **G** | **A** | **A** | **C** | **A** | **A** | **T** | **T** | **A** | **G** | **A** | **A** | **C** | **A** | **A** | **C** | **T** | **G** | **A** | **T** | **C** | **T** | **T** | **G** | **A** | **A** | **C** | **G** | **T** | **G** | **A** | **A** | **A** | **C** | **C** | **T** | **G** | **C** | **C** | **A** | **T** | **G** | **T** | **T** | **A** | **C** | **T** | **A** | **G** | **T** | **G** | **G** | **A** | **A** | **C** | **A** | **T** | **G** | **A** | **T** | **C** | **A** | **A** | **A** | **C** | **C** | **T** | **T** | **C** | **C** | **T** | **G** | **G** | **A** | **T** | **A** | **A** | **A** | **G** | **T** | **A** | **T** | **C** | **T** | **A** | **C** | **T** | **G** | **A** | **G** | **A** | **T** | **T** | **A** | **T** | **T** | **T** | **C** | **T** | **C** | **T** | **T** | **G** | **A** | **G** | **A** | **A** | **A** | **A** | **T** | **C** | **-** | **-** | **-** |
| **N53804** | **A** | **T** | **G** | **T** | **C** | **T** | **A** | **A** | **A** | **A** | **T** | **C** | **G** | **A** | **A** | **A** | **T** | **A** | **A** | **A** | **A** | **A** | **A** | **T** | **C** | **T** | **G** | **A** | **C** | **A** | **T** | **T** | **C** | **G** | **G** | **C** | **T** | **A** | **C** | **G** | **A** | **C** | **A** | **G** | **C** | **C** | **A** | **A** | **G** | **G** | **C** | **A** | **C** | **A** | **T** | **T** | **A** | **T** | **T** | **A** | **T** | **T** | **T** | **G** | **A** | **A** | **C** | **A** | **A** | **G** | **C** | **A** | **A** | **A** | **T** | **C** | **T** | **A** | **A** | **A** | **T** | **T** | **T** | **T** | **G** | **A** | **C** | **A** | **C** | **A** | **C** | **A** | **A** | **T** | **G** | **G** | **A** | **A** | **A** | **C** | **T** | **A** | **G** | **G** | **A** | **C** | **T** | **T** | **A** | **T** | **C** | **G** | **G** | **A** | **C** | **G** | **A** | **A** | **A** | **C** | **G** | **G** | **T** | **C** | **G** | **A** | **G** | **G** | **A** | **A** | **A** | **G** | **A** | **C** | **A** | **A** | **C** | **T** | **T** | **T** | **A** | **C** | **T** | **G** | **A** | **A** | **T** | **A** | **T** | **T** | **C** | **T** | **A** | **C** | **A** | **A** | **A** | **A** | **C** | **A** | **A** | **A** | **C** | **T** | **A** | **C** | **C** | **T** | **T** | **A** | **T** | **C** | **A** | **A** | **G** | **G** | **G** | **C** | **A** | **A** | **G** | **T** | **A** | **A** | **T** | **C** | **C** | **A** | **T** | **C** | **A** | **G** | **C** | **A** | **A** | **G** | **A** | **A** | **T** | **T** | **T** | **G** | **T** | **C** | **T** | **A** | **T** | **T** | **T** | **C** | **C** | **C** | **G** | **C** | **A** | **A** | **C** | **A** | **G** | **A** | **C** | **A** | **A** | **A** | **A** | **G** | **A** | **T** | **A** | **A** | **A** | **G** | **A** | **A** | **T** | **G** | **T** | **T** | **T** | **A** | **A** | **C** | **C** | **T** | **A** | **T** | **T** | **A** | **C** | **G** | **T** | **G** | **T** | **T** | **A** | **A** | **A** | **T** | **G** | **A** | **T** | **A** | **T** | **T** | **A** | **C** | **G** | **G** | **A** | **T** | **T** | **T** | **T** | **G** | **A** | **G** | **A** | **T** | **A** | **T** | **G** | **G** | **G** | **A** | **A** | **A** | **T** | **C** | **G** | **A** | **A** | **A** | **G** | **A** | **G** | **A** | **G** | **C** | **T** | **C** | **C** | **A** | **A** | **T** | **T** | **G** | **A** | **T** | **G** | **C** | **A** | **A** | **A** | **C** | **A** | **G** | **A** | **T** | **C** | **C** | **T** | **G** | **A** | **A** | **A** | **T** | **C** | **T** | **T** | **A** | **T** | **G** | **G** | **A** | **G** | **A** | **G** | **A** | **A** | **T** | **T** | **C** | **A** | **G** | **C** | **A** | **C** | **A** | **C** | **T** | **A** | **T** | **C** | **A** | **G** | **G** | **G** | **G** | **G** | **A** | **G** | **A** | **G** | **A** | **A** | **G** | **A** | **C** | **A** | **A** | **A** | **A** | **G** | **T** | **C** | **C** | **T** | **A** | **C** | **T** | **G** | **G** | **C** | **A** | **C** | **T** | **T** | **T** | **T** | **A** | **T** | **T** | **T** | **G** | **T** | **G** | **G** | **A** | **T** | **G** | **A** | **C** | **A** | **C** | **T** | **C** | **A** | **T** | **T** | **T** | **C** | **C** | **C** | **G** | **T** | **T** | **A** | **A** | **T** | **C** | **G** | **A** | **T** | **G** | **A** | **A** | **C** | **C** | **A** | **A** | **C** | **G** | **A** | **A** | **T** | **C** | **A** | **T** | **T** | **T** | **G** | **G** | **A** | **-** | **-** | **-** | **T** | **A** | **T** | **C** | **T** | **C** | **T** | **G** | **G** | **T** | **A** | **G** | **A** | **A** | **A** | **A** | **C** | **A** | **A** | **G** | **T** | **A** | **G** | **C** | **G** | **G** | **C** | **T** | **T** | **A** | **T** | **T** | **T** | **G** | **A** | **A** | **A** | **A** | **A** | **G** | **A** | **A** | **A** | **A** | **A** | **A** | **C** | **A** | **A** | **G** | **G** | **C** | **T** | **T** | **C** | **A** | **T** | **C** | **G** | **T** | **G** | **G** | **T** | **C** | **A** | **G** | **C** | **C** | **A** | **T** | **G** | **A** | **C** | **C** | **G** | **G** | **G** | **G** | **A** | **T** | **T** | **T** | **A** | **T** | **C** | **G** | **A** | **T** | **G** | **A** | **A** | **G** | **T** | **A** | **G** | **T** | **G** | **G** | **A** | **C** | **C** | **A** | **T** | **G** | **T** | **T** | **T** | **T** | **A** | **G** | **C** | **A** | **A** | **T** | **C** | **G** | **A** | **A** | **A** | **A** | **A** | **A** | **G** | **T** | **C** | **A** | **A** | **C** | **T** | **G** | **G** | **A** | **A** | **C** | **T** | **T** | **T** | **A** | **T** | **C** | **A** | **A** | **G** | **G** | **G** | **A** | **A** | **T** | **T** | **T** | **C** | **T** | **C** | **T** | **A** | **T** | **C** | **T** | **A** | **T** | **G** | **A** | **A** | **G** | **A** | **A** | **C** | **A** | **G** | **A** | **A** | **A** | **A** | **A** | **A** | **C** | **T** | **T** | **C** | **G** | **T** | **G** | **A** | **T** | **G** | **A** | **A** | **T** | **T** | **T** | **G** | **A** | **A** | **A** | **T** | **G** | **G** | **C** | **T** | **C** | **A** | **A** | **A** | **A** | **T** | **G** | **A** | **A** | **A** | **A** | **A** | **T** | **T** | **G** | **A** | **A** | **A** | **A** | **A** | **A** | **G** | **A** | **A** | **G** | **T** | **C** | **A** | **G** | **T** | **A** | **G** | **G** | **C** | **T** | **A** | **A** | **A** | **G** | **A** | **A** | **A** | **A** | **C** | **A** | **G** | **C** | **A** | **G** | **C** | **T** | **G** | **A** | **A** | **A** | **A** | **A** | **G** | **C** | **C** | **G** | **A** | **A** | **T** | **G** | **G** | **T** | **C** | **T** | **C** | **G** | **T** | **T** | **C** | **C** | **C** | **G** | **A** | **G** | **A** | **A** | **G** | **G** | **A** | **G** | **A** | **T** | **A** | **A** | **A** | **A** | **C** | **A** | **A** | **A** | **G** | **A** | **A** | **A** | **C** | **A** | **A** | **G** | **T** | **C** | **G** | **G** | **A** | **T** | **T** | **C** | **A** | **T** | **C** | **G** | **A** | **T** | **A** | **C** | **T** | **G** | **A** | **A** | **T** | **C** | **T** | **A** | **G** | **A** | **C** | **G** | **A** | **G** | **T** | **G** | **A** | **A** | **T** | **A** | **A** | **A** | **G** | **G** | **A** | **G** | **C** | **A** | **G** | **T** | **G** | **G** | **G** | **T** | **G** | **C** | **T** | **G** | **A** | **T** | **G** | **C** | **T** | **G** | **C** | **A** | **C** | **G** | **G** | **A** | **C** | **G** | **A** | **T** | **G** | **A** | **A** | **A** | **C** | **G** | **A** | **T** | **C** | **C** | **A** | **A** | **A** | **G** | **C** | **A** | **A** | **T** | **C** | **G** | **T** | **G** | **A** | **A** | **T** | **C** | **G** | **G** | **A** | **T** | **G** | **G** | **A** | **G** | **A** | **C** | **C** | **C** | **A** | **G** | **A** | **T** | **C** | **A** | **G** | **C** | **G** | **A** | **G** | **A** | **A** | **A** | **G** | **A** | **A** | **A** | **A** | **A** | **C** | **T** | **A** | **T** | **T** | **A** | **A** | **A** | **A** | **G** | **A** | **T** | **A** | **T** | **C** | **G** | **A** | **A** | **T** | **A** | **T** | **A** | **T** | **C** | **G** | **A** | **T** | **T** | **C** | **T** | **T** | **T** | **G** | **A** | **C** | **G** | **A** | **T** | **G** | **A** | **A** | **T** | **A** | **G** | **C** | **C** | **A** | **A** | **G** | **C** | **G** | **T** | **C** | **T** | **C** | **A** | **C** | **C** | **A** | **T** | **A** | **A** | **G** | **C** | **G** | **A** | **C** | **T** | **T** | **T** | **T** | **A** | **A** | **G** | **C** | **G** | **T** | **A** | **G** | **A** | **A** | **G** | **A** | **T** | **C** | **T** | **T** | **C** | **A** | **A** | **T** | **T** | **A** | **G** | **G** | **G** | **T** | **A** | **T** | **G** | **A** | **A** | **A** | **A** | **T** | **C** | **T** | **G** | **T** | **T** | **A** | **T** | **T** | **C** | **G** | **A** | **G** | **C** | **C** | **A** | **A** | **T** | **T** | **C** | **A** | **T** | **T** | **T** | **T** | **A** | **C** | **A** | **A** | **T** | **C** | **G** | **A** | **G** | **C** | **C** | **T** | **C** | **A** | **T** | **C** | **A** | **G** | **C** | **G** | **G** | **G** | **T** | **G** | **G** | **C** | **G** | **A** | **T** | **T** | **T** | **C** | **A** | **G** | **G** | **A** | **C** | **C** | **T** | **A** | **A** | **C** | **G** | **G** | **T** | **G** | **C** | **A** | **G** | **G** | **A** | **A** | **A** | **G** | **T** | **C** | **A** | **T** | **C** | **C** | **A** | **T** | **T** | **A** | **T** | **C** | **C** | **A** | **T** | **T** | **A** | **T** | **C** | **T** | **T** | **C** | **T** | **G** | **G** | **G** | **G** | **G** | **C** | **A** | **T** | **T** | **C** | **A** | **A** | **C** | **G** | **G** | **C** | **A** | **A** | **G** | **G** | **T** | **T** | **A** | **T** | **A** | **G** | **G** | **A** | **G** | **A** | **A** | **A** | **A** | **A** | **T** | **C** | **C** | **C** | **A** | **G** | **C** | **C** | **A** | **A** | **A** | **A** | **C** | **A** | **T** | **C** | **T** | **G** | **A** | **G** | **C** | **A** | **T** | **T** | **A** | **G** | **T** | **T** | **A** | **T** | **G** | **T** | **A** | **C** | **G** | **C** | **C** | **A** | **A** | **A** | **A** | **T** | **T** | **A** | **T** | **G** | **A** | **A** | **G** | **A** | **C** | **A** | **A** | **T** | **C** | **G** | **A** | **G** | **G** | **A** | **A** | **C** | **G** | **T** | **T** | **G** | **G** | **C** | **G** | **G** | **A** | **A** | **T** | **T** | **T** | **G** | **C** | **A** | **G** | **A** | **G** | **A** | **A** | **A** | **A** | **A** | **C** | **C** | **A** | **A** | **G** | **T** | **A** | **G** | **A** | **C** | **T** | **A** | **C** | **C** | **A** | **A** | **G** | **C** | **A** | **T** | **T** | **T** | **T** | **T** | **G** | **A** | **A** | **C** | **A** | **A** | **C** | **C** | **T** | **C** | **C** | **G** | **A** | **A** | **A** | **G** | **C** | **T** | **T** | **G** | **G** | **G** | **A** | **T** | **G** | **G** | **A** | **A** | **A** | **G** | **A** | **G** | **A** | **T** | **G** | **T** | **T** | **T** | **T** | **T** | **C** | **A** | **T** | **A** | **A** | **C** | **A** | **A** | **G** | **A** | **T** | **C** | **G** | **A** | **G** | **C** | **A** | **G** | **A** | **T** | **G** | **A** | **G** | **T** | **A** | **T** | **G** | **G** | **G** | **C** | **C** | **A** | **A** | **C** | **G** | **G** | **A** | **A** | **A** | **A** | **A** | **A** | **G** | **T** | **G** | **G** | **A** | **A** | **T** | **T** | **G** | **G** | **C** | **T** | **A** | **A** | **A** | **T** | **C** | **T** | **T** | **T** | **A** | **T** | **C** | **A** | **C** | **A** | **G** | **C** | **C** | **A** | **G** | **C** | **T** | **G** | **A** | **A** | **C** | **T** | **A** | **T** | **A** | **T** | **A** | **T** | **A** | **T** | **G** | **G** | **G** | **A** | **T** | **G** | **A** | **A** | **C** | **C** | **A** | **T** | **T** | **G** | **A** | **A** | **T** | **T** | **A** | **T** | **T** | **T** | **G** | **G** | **A** | **T** | **G** | **T** | **C** | **T** | **T** | **C** | **A** | **A** | **T** | **C** | **A** | **A** | **G** | **A** | **A** | **C** | **A** | **A** | **T** | **T** | **A** | **G** | **A** | **A** | **C** | **A** | **A** | **C** | **T** | **G** | **A** | **T** | **C** | **T** | **T** | **G** | **A** | **A** | **C** | **G** | **T** | **G** | **A** | **A** | **A** | **C** | **C** | **T** | **G** | **C** | **C** | **A** | **T** | **G** | **T** | **T** | **A** | **C** | **T** | **A** | **G** | **T** | **G** | **G** | **A** | **A** | **C** | **A** | **T** | **G** | **A** | **T** | **C** | **A** | **A** | **A** | **C** | **C** | **T** | **T** | **C** | **C** | **T** | **G** | **G** | **A** | **T** | **A** | **A** | **A** | **G** | **T** | **A** | **T** | **C** | **T** | **A** | **C** | **T** | **G** | **A** | **G** | **A** | **T** | **T** | **A** | **T** | **T** | **T** | **C** | **T** | **C** | **T** | **T** | **G** | **A** | **G** | **A** | **A** | **A** | **A** | **T** | **C** | **-** | **-** | **-** |
| **N53479** | **A** | **T** | **G** | **T** | **C** | **T** | **A** | **A** | **A** | **A** | **T** | **C** | **G** | **A** | **A** | **A** | **T** | **A** | **A** | **A** | **A** | **A** | **A** | **T** | **C** | **T** | **G** | **A** | **C** | **A** | **T** | **T** | **C** | **G** | **G** | **C** | **T** | **A** | **C** | **G** | **A** | **C** | **A** | **G** | **C** | **C** | **A** | **A** | **G** | **G** | **C** | **A** | **C** | **A** | **T** | **T** | **A** | **T** | **T** | **A** | **T** | **T** | **T** | **G** | **A** | **A** | **C** | **A** | **A** | **G** | **C** | **A** | **A** | **A** | **T** | **C** | **T** | **A** | **A** | **A** | **T** | **T** | **T** | **T** | **G** | **A** | **C** | **A** | **C** | **A** | **C** | **A** | **A** | **T** | **G** | **G** | **A** | **A** | **A** | **C** | **T** | **A** | **G** | **G** | **A** | **C** | **T** | **T** | **A** | **T** | **C** | **G** | **G** | **A** | **C** | **G** | **A** | **A** | **A** | **C** | **G** | **G** | **T** | **C** | **G** | **A** | **G** | **G** | **A** | **A** | **A** | **G** | **A** | **C** | **A** | **A** | **C** | **T** | **T** | **T** | **A** | **C** | **T** | **G** | **A** | **A** | **T** | **A** | **T** | **T** | **C** | **T** | **A** | **C** | **A** | **A** | **A** | **A** | **C** | **A** | **A** | **A** | **C** | **T** | **A** | **C** | **C** | **T** | **T** | **A** | **T** | **C** | **A** | **A** | **G** | **G** | **G** | **C** | **A** | **A** | **G** | **T** | **A** | **A** | **T** | **C** | **C** | **A** | **T** | **C** | **A** | **G** | **C** | **A** | **A** | **G** | **A** | **A** | **T** | **T** | **T** | **G** | **C** | **C** | **T** | **A** | **T** | **T** | **T** | **C** | **C** | **C** | **G** | **C** | **A** | **A** | **C** | **A** | **G** | **A** | **C** | **A** | **A** | **A** | **A** | **G** | **A** | **T** | **A** | **A** | **A** | **G** | **A** | **A** | **T** | **G** | **T** | **T** | **T** | **A** | **A** | **C** | **C** | **T** | **A** | **T** | **T** | **A** | **C** | **G** | **T** | **G** | **T** | **T** | **A** | **A** | **A** | **T** | **G** | **A** | **T** | **A** | **T** | **T** | **A** | **C** | **G** | **G** | **A** | **T** | **T** | **T** | **T** | **G** | **A** | **G** | **A** | **T** | **A** | **T** | **G** | **G** | **G** | **A** | **A** | **A** | **T** | **C** | **G** | **A** | **A** | **A** | **G** | **A** | **G** | **A** | **G** | **C** | **T** | **C** | **C** | **A** | **A** | **T** | **T** | **G** | **A** | **T** | **G** | **C** | **A** | **A** | **A** | **C** | **A** | **G** | **A** | **T** | **C** | **C** | **T** | **G** | **A** | **A** | **A** | **T** | **C** | **T** | **T** | **A** | **T** | **G** | **G** | **A** | **G** | **A** | **G** | **A** | **A** | **T** | **T** | **C** | **A** | **G** | **C** | **A** | **C** | **A** | **C** | **T** | **A** | **T** | **C** | **G** | **G** | **G** | **G** | **G** | **G** | **A** | **G** | **A** | **G** | **A** | **A** | **G** | **A** | **C** | **A** | **A** | **A** | **A** | **G** | **T** | **C** | **C** | **T** | **A** | **C** | **T** | **G** | **G** | **C** | **A** | **C** | **T** | **T** | **T** | **T** | **A** | **T** | **T** | **T** | **G** | **T** | **G** | **G** | **A** | **T** | **G** | **A** | **C** | **A** | **C** | **T** | **C** | **A** | **T** | **T** | **T** | **C** | **C** | **C** | **G** | **T** | **T** | **A** | **A** | **T** | **C** | **G** | **A** | **T** | **G** | **A** | **A** | **C** | **C** | **A** | **A** | **C** | **G** | **A** | **A** | **T** | **C** | **A** | **T** | **T** | **T** | **G** | **G** | **A** | **-** | **-** | **-** | **T** | **A** | **T** | **C** | **T** | **C** | **T** | **G** | **G** | **T** | **A** | **G** | **A** | **A** | **A** | **A** | **C** | **A** | **A** | **G** | **T** | **A** | **G** | **C** | **G** | **G** | **C** | **T** | **T** | **A** | **T** | **T** | **T** | **G** | **A** | **A** | **A** | **A** | **A** | **G** | **A** | **A** | **A** | **A** | **A** | **A** | **C** | **A** | **A** | **G** | **G** | **C** | **T** | **T** | **C** | **A** | **T** | **C** | **G** | **T** | **G** | **G** | **T** | **C** | **A** | **G** | **C** | **C** | **A** | **T** | **G** | **A** | **C** | **C** | **G** | **G** | **G** | **G** | **A** | **T** | **T** | **T** | **A** | **T** | **C** | **G** | **A** | **T** | **G** | **A** | **A** | **G** | **T** | **A** | **G** | **T** | **G** | **G** | **A** | **C** | **C** | **A** | **T** | **G** | **T** | **T** | **T** | **T** | **A** | **G** | **C** | **A** | **A** | **T** | **C** | **G** | **A** | **A** | **A** | **A** | **A** | **A** | **G** | **T** | **C** | **A** | **A** | **C** | **T** | **G** | **G** | **A** | **A** | **C** | **T** | **T** | **T** | **A** | **T** | **C** | **A** | **A** | **G** | **G** | **G** | **A** | **A** | **T** | **T** | **T** | **C** | **T** | **C** | **T** | **A** | **T** | **C** | **T** | **A** | **T** | **G** | **A** | **A** | **G** | **A** | **A** | **C** | **A** | **G** | **A** | **A** | **A** | **A** | **A** | **A** | **C** | **T** | **T** | **C** | **G** | **T** | **G** | **A** | **T** | **G** | **A** | **A** | **T** | **T** | **T** | **G** | **A** | **A** | **A** | **T** | **G** | **G** | **C** | **T** | **C** | **A** | **A** | **A** | **A** | **T** | **G** | **A** | **A** | **A** | **A** | **A** | **T** | **T** | **G** | **A** | **A** | **A** | **A** | **A** | **A** | **G** | **A** | **A** | **G** | **T** | **C** | **A** | **G** | **T** | **A** | **G** | **G** | **C** | **T** | **A** | **A** | **A** | **G** | **A** | **A** | **A** | **A** | **C** | **A** | **G** | **C** | **A** | **G** | **C** | **T** | **G** | **A** | **A** | **A** | **A** | **A** | **G** | **C** | **C** | **G** | **A** | **A** | **T** | **G** | **G** | **T** | **C** | **T** | **C** | **G** | **T** | **T** | **C** | **C** | **C** | **G** | **A** | **G** | **A** | **A** | **G** | **G** | **A** | **G** | **A** | **T** | **A** | **A** | **A** | **A** | **C** | **A** | **A** | **A** | **G** | **A** | **A** | **A** | **C** | **A** | **A** | **G** | **T** | **T** | **G** | **G** | **A** | **T** | **T** | **C** | **A** | **T** | **C** | **G** | **A** | **T** | **A** | **C** | **T** | **G** | **A** | **A** | **T** | **C** | **T** | **A** | **G** | **A** | **C** | **G** | **A** | **G** | **T** | **G** | **A** | **A** | **T** | **A** | **A** | **A** | **G** | **G** | **A** | **G** | **C** | **A** | **G** | **T** | **G** | **G** | **G** | **T** | **G** | **C** | **T** | **G** | **A** | **T** | **G** | **C** | **T** | **G** | **C** | **A** | **C** | **G** | **G** | **A** | **C** | **G** | **A** | **T** | **G** | **A** | **A** | **A** | **C** | **G** | **A** | **T** | **C** | **C** | **A** | **A** | **A** | **G** | **C** | **A** | **A** | **T** | **C** | **G** | **T** | **G** | **A** | **A** | **T** | **C** | **G** | **G** | **A** | **T** | **G** | **G** | **A** | **G** | **A** | **C** | **C** | **C** | **A** | **G** | **A** | **T** | **C** | **A** | **G** | **C** | **G** | **A** | **G** | **A** | **A** | **A** | **G** | **A** | **A** | **A** | **A** | **A** | **C** | **T** | **A** | **T** | **T** | **A** | **A** | **A** | **A** | **G** | **A** | **T** | **A** | **T** | **C** | **G** | **A** | **A** | **T** | **A** | **T** | **A** | **T** | **C** | **G** | **A** | **T** | **T** | **C** | **T** | **T** | **T** | **G** | **A** | **C** | **G** | **A** | **T** | **G** | **A** | **A** | **T** | **A** | **G** | **C** | **C** | **A** | **A** | **G** | **C** | **G** | **T** | **C** | **T** | **C** | **A** | **C** | **C** | **A** | **T** | **A** | **A** | **G** | **C** | **G** | **A** | **C** | **T** | **T** | **T** | **T** | **A** | **A** | **G** | **C** | **G** | **T** | **A** | **G** | **A** | **A** | **G** | **A** | **T** | **C** | **T** | **T** | **C** | **A** | **A** | **T** | **T** | **A** | **G** | **G** | **G** | **T** | **A** | **T** | **G** | **A** | **A** | **A** | **A** | **T** | **C** | **T** | **G** | **T** | **T** | **A** | **T** | **T** | **C** | **G** | **A** | **G** | **C** | **C** | **A** | **A** | **T** | **T** | **C** | **A** | **T** | **T** | **T** | **T** | **A** | **C** | **A** | **A** | **T** | **C** | **G** | **A** | **G** | **C** | **C** | **T** | **C** | **A** | **T** | **C** | **A** | **G** | **C** | **G** | **G** | **G** | **T** | **G** | **G** | **C** | **G** | **A** | **T** | **T** | **T** | **C** | **A** | **G** | **G** | **T** | **C** | **C** | **T** | **A** | **A** | **C** | **G** | **G** | **T** | **G** | **C** | **A** | **G** | **G** | **A** | **A** | **A** | **G** | **T** | **C** | **A** | **T** | **C** | **C** | **A** | **T** | **T** | **A** | **T** | **C** | **C** | **A** | **T** | **T** | **A** | **T** | **C** | **T** | **T** | **C** | **T** | **G** | **G** | **G** | **G** | **G** | **C** | **A** | **T** | **T** | **C** | **A** | **A** | **C** | **G** | **G** | **C** | **A** | **A** | **G** | **G** | **T** | **T** | **A** | **T** | **A** | **G** | **G** | **A** | **G** | **A** | **A** | **A** | **A** | **A** | **T** | **A** | **C** | **C** | **A** | **G** | **C** | **C** | **A** | **A** | **A** | **A** | **C** | **A** | **T** | **C** | **T** | **G** | **A** | **G** | **C** | **A** | **T** | **T** | **A** | **G** | **T** | **T** | **A** | **T** | **G** | **C** | **A** | **A** | **G** | **C** | **C** | **A** | **A** | **A** | **A** | **T** | **T** | **A** | **T** | **G** | **A** | **A** | **G** | **A** | **C** | **A** | **A** | **T** | **C** | **G** | **A** | **G** | **G** | **A** | **A** | **C** | **G** | **T** | **T** | **G** | **G** | **C** | **G** | **G** | **A** | **A** | **T** | **T** | **T** | **G** | **C** | **A** | **G** | **A** | **G** | **A** | **A** | **A** | **A** | **A** | **C** | **C** | **A** | **A** | **G** | **T** | **A** | **G** | **A** | **C** | **T** | **A** | **C** | **C** | **A** | **A** | **G** | **C** | **A** | **T** | **T** | **T** | **T** | **T** | **G** | **A** | **A** | **C** | **A** | **A** | **C** | **C** | **T** | **C** | **C** | **G** | **A** | **A** | **A** | **G** | **C** | **T** | **T** | **G** | **G** | **G** | **A** | **T** | **G** | **G** | **A** | **A** | **A** | **G** | **A** | **G** | **A** | **T** | **G** | **T** | **T** | **T** | **T** | **T** | **C** | **A** | **T** | **A** | **A** | **C** | **A** | **A** | **G** | **A** | **T** | **C** | **G** | **A** | **G** | **C** | **A** | **G** | **A** | **T** | **G** | **A** | **G** | **T** | **A** | **T** | **G** | **G** | **G** | **C** | **C** | **A** | **A** | **C** | **G** | **G** | **A** | **A** | **A** | **A** | **A** | **A** | **G** | **T** | **G** | **G** | **A** | **A** | **T** | **T** | **G** | **G** | **C** | **T** | **A** | **A** | **A** | **T** | **C** | **T** | **T** | **T** | **A** | **T** | **C** | **A** | **C** | **A** | **G** | **C** | **C** | **A** | **G** | **C** | **T** | **G** | **A** | **A** | **C** | **T** | **A** | **T** | **A** | **T** | **A** | **C** | **A** | **T** | **G** | **G** | **G** | **A** | **T** | **G** | **A** | **A** | **C** | **C** | **A** | **T** | **T** | **G** | **A** | **A** | **T** | **T** | **A** | **T** | **T** | **T** | **G** | **G** | **A** | **T** | **G** | **T** | **C** | **T** | **T** | **C** | **A** | **A** | **T** | **C** | **A** | **A** | **G** | **A** | **A** | **C** | **A** | **A** | **T** | **T** | **A** | **G** | **A** | **A** | **C** | **A** | **A** | **C** | **T** | **G** | **A** | **T** | **C** | **T** | **T** | **G** | **A** | **A** | **C** | **G** | **T** | **G** | **A** | **A** | **A** | **C** | **C** | **T** | **G** | **C** | **C** | **A** | **T** | **G** | **T** | **T** | **A** | **C** | **T** | **A** | **G** | **T** | **G** | **G** | **A** | **A** | **C** | **A** | **T** | **G** | **A** | **T** | **C** | **A** | **A** | **A** | **C** | **C** | **T** | **T** | **C** | **C** | **T** | **G** | **G** | **A** | **T** | **A** | **A** | **A** | **G** | **T** | **A** | **T** | **C** | **T** | **A** | **C** | **T** | **G** | **A** | **G** | **A** | **T** | **T** | **A** | **T** | **T** | **T** | **C** | **T** | **C** | **T** | **T** | **G** | **A** | **G** | **A** | **G** | **A** | **A** | **T** | **C** | **-** | **-** | **-** |
| **N59589F** | **A** | **T** | **G** | **T** | **C** | **T** | **A** | **A** | **A** | **A** | **T** | **C** | **G** | **A** | **A** | **A** | **T** | **A** | **A** | **A** | **A** | **A** | **A** | **T** | **C** | **T** | **G** | **A** | **C** | **A** | **T** | **T** | **C** | **G** | **G** | **C** | **T** | **A** | **T** | **G** | **A** | **C** | **A** | **G** | **C** | **C** | **A** | **A** | **G** | **G** | **C** | **A** | **C** | **A** | **T** | **T** | **A** | **T** | **T** | **A** | **T** | **T** | **T** | **G** | **A** | **A** | **C** | **A** | **A** | **G** | **C** | **C** | **A** | **A** | **T** | **C** | **T** | **A** | **A** | **A** | **C** | **T** | **T** | **T** | **G** | **A** | **C** | **A** | **C** | **A** | **C** | **A** | **A** | **T** | **G** | **G** | **A** | **A** | **A** | **C** | **T** | **A** | **G** | **G** | **A** | **C** | **T** | **T** | **A** | **T** | **C** | **G** | **G** | **A** | **C** | **G** | **A** | **A** | **A** | **C** | **G** | **G** | **T** | **C** | **G** | **A** | **G** | **G** | **A** | **A** | **A** | **G** | **A** | **C** | **A** | **A** | **C** | **T** | **T** | **T** | **A** | **C** | **T** | **G** | **A** | **A** | **T** | **A** | **T** | **C** | **C** | **T** | **G** | **C** | **A** | **A** | **A** | **A** | **C** | **A** | **A** | **A** | **C** | **T** | **C** | **C** | **C** | **T** | **T** | **A** | **T** | **C** | **A** | **A** | **G** | **G** | **G** | **C** | **A** | **A** | **G** | **T** | **G** | **A** | **T** | **C** | **C** | **A** | **T** | **C** | **A** | **G** | **C** | **A** | **A** | **G** | **C** | **G** | **T** | **T** | **T** | **G** | **T** | **C** | **T** | **A** | **T** | **T** | **T** | **C** | **C** | **C** | **G** | **C** | **A** | **G** | **C** | **A** | **A** | **A** | **C** | **A** | **A** | **A** | **A** | **G** | **A** | **T** | **A** | **A** | **A** | **G** | **A** | **G** | **C** | **G** | **T** | **T** | **T** | **G** | **A** | **C** | **C** | **T** | **A** | **T** | **T** | **A** | **T** | **G** | **T** | **G** | **T** | **T** | **A** | **A** | **A** | **T** | **G** | **A** | **G** | **A** | **T** | **T** | **A** | **T** | **G** | **G** | **A** | **T** | **T** | **T** | **T** | **G** | **A** | **G** | **A** | **T** | **A** | **T** | **G** | **G** | **G** | **A** | **A** | **A** | **T** | **T** | **G** | **A** | **A** | **A** | **G** | **A** | **G** | **A** | **G** | **C** | **T** | **C** | **C** | **A** | **A** | **C** | **T** | **G** | **A** | **T** | **G** | **C** | **A** | **A** | **A** | **C** | **A** | **G** | **A** | **T** | **C** | **C** | **T** | **G** | **A** | **A** | **A** | **T** | **C** | **T** | **T** | **A** | **T** | **G** | **G** | **A** | **G** | **A** | **G** | **A** | **A** | **T** | **T** | **C** | **A** | **G** | **C** | **A** | **C** | **A** | **C** | **T** | **A** | **T** | **C** | **A** | **G** | **G** | **A** | **G** | **G** | **A** | **G** | **A** | **G** | **A** | **A** | **G** | **A** | **C** | **A** | **A** | **A** | **A** | **G** | **T** | **C** | **C** | **T** | **A** | **C** | **T** | **G** | **G** | **C** | **A** | **C** | **T** | **C** | **T** | **T** | **A** | **T** | **T** | **T** | **G** | **T** | **G** | **G** | **A** | **T** | **G** | **A** | **C** | **A** | **C** | **T** | **C** | **A** | **T** | **T** | **T** | **C** | **C** | **C** | **G** | **T** | **T** | **A** | **A** | **T** | **C** | **G** | **A** | **T** | **G** | **A** | **A** | **C** | **C** | **A** | **A** | **C** | **G** | **A** | **A** | **T** | **C** | **A** | **T** | **T** | **T** | **G** | **G** | **A** | **-** | **-** | **-** | **T** | **A** | **T** | **C** | **T** | **C** | **T** | **G** | **G** | **C** | **A** | **G** | **A** | **A** | **A** | **A** | **C** | **A** | **A** | **G** | **T** | **A** | **G** | **C** | **G** | **G** | **C** | **T** | **T** | **A** | **C** | **T** | **T** | **G** | **A** | **A** | **A** | **A** | **A** | **G** | **A** | **A** | **A** | **A** | **A** | **A** | **C** | **A** | **A** | **G** | **G** | **A** | **T** | **T** | **C** | **A** | **T** | **C** | **G** | **T** | **G** | **G** | **T** | **G** | **A** | **G** | **T** | **C** | **A** | **T** | **G** | **A** | **T** | **C** | **G** | **G** | **G** | **G** | **A** | **T** | **T** | **T** | **A** | **T** | **C** | **G** | **A** | **T** | **G** | **A** | **A** | **G** | **T** | **A** | **G** | **T** | **G** | **G** | **A** | **C** | **C** | **A** | **T** | **G** | **T** | **T** | **T** | **T** | **A** | **G** | **C** | **A** | **A** | **T** | **C** | **G** | **A** | **A** | **A** | **A** | **G** | **A** | **G** | **C** | **C** | **A** | **A** | **C** | **T** | **G** | **G** | **A** | **A** | **C** | **T** | **T** | **T** | **A** | **T** | **C** | **A** | **A** | **G** | **G** | **G** | **A** | **A** | **T** | **T** | **T** | **C** | **T** | **C** | **T** | **A** | **T** | **C** | **T** | **A** | **T** | **G** | **A** | **A** | **G** | **A** | **A** | **C** | **A** | **G** | **A** | **A** | **A** | **A** | **A** | **A** | **C** | **T** | **T** | **C** | **G** | **T** | **G** | **A** | **T** | **G** | **A** | **A** | **T** | **T** | **T** | **G** | **A** | **A** | **A** | **T** | **A** | **G** | **C** | **T** | **A** | **A** | **A** | **A** | **A** | **T** | **G** | **A** | **A** | **A** | **A** | **A** | **T** | **T** | **G** | **A** | **A** | **A** | **A** | **A** | **A** | **G** | **A** | **A** | **G** | **T** | **C** | **A** | **G** | **C** | **A** | **G** | **G** | **C** | **T** | **A** | **A** | **A** | **G** | **A** | **A** | **A** | **A** | **C** | **A** | **G** | **C** | **A** | **G** | **C** | **T** | **G** | **A** | **A** | **A** | **A** | **A** | **G** | **C** | **A** | **G** | **A** | **A** | **T** | **G** | **G** | **T** | **C** | **T** | **C** | **G** | **T** | **T** | **C** | **C** | **C** | **G** | **A** | **G** | **A** | **A** | **G** | **G** | **A** | **G** | **A** | **C** | **A** | **A** | **A** | **A** | **C** | **A** | **A** | **A** | **G** | **A** | **A** | **A** | **A** | **A** | **A** | **G** | **T** | **C** | **G** | **G** | **A** | **T** | **T** | **C** | **A** | **T** | **C** | **G** | **A** | **T** | **A** | **C** | **G** | **G** | **A** | **A** | **T** | **C** | **T** | **A** | **G** | **G** | **C** | **G** | **A** | **G** | **T** | **G** | **A** | **A** | **T** | **A** | **A** | **A** | **G** | **G** | **A** | **G** | **C** | **A** | **G** | **T** | **A** | **G** | **G** | **C** | **G** | **C** | **T** | **G** | **A** | **T** | **G** | **C** | **T** | **G** | **C** | **A** | **C** | **G** | **G** | **A** | **C** | **G** | **A** | **T** | **G** | **A** | **A** | **A** | **C** | **G** | **A** | **T** | **C** | **T** | **A** | **A** | **A** | **G** | **C** | **A** | **A** | **T** | **C** | **G** | **T** | **G** | **A** | **A** | **T** | **C** | **G** | **G** | **A** | **T** | **G** | **G** | **A** | **A** | **A** | **C** | **C** | **C** | **A** | **G** | **A** | **T** | **C** | **A** | **G** | **T** | **G** | **A** | **G** | **A** | **A** | **A** | **G** | **A** | **A** | **A** | **A** | **A** | **T** | **T** | **A** | **T** | **T** | **A** | **A** | **A** | **A** | **G** | **A** | **T** | **A** | **T** | **C** | **G** | **A** | **G** | **T** | **A** | **T** | **A** | **T** | **C** | **G** | **A** | **T** | **G** | **C** | **T** | **T** | **T** | **G** | **A** | **C** | **G** | **A** | **T** | **G** | **A** | **A** | **T** | **A** | **G** | **C** | **C** | **A** | **A** | **G** | **T** | **G** | **T** | **C** | **T** | **C** | **A** | **T** | **C** | **A** | **T** | **A** | **A** | **A** | **C** | **G** | **G** | **C** | **T** | **G** | **T** | **T** | **A** | **A** | **G** | **C** | **G** | **T** | **A** | **G** | **A** | **A** | **A** | **A** | **T** | **C** | **T** | **T** | **C** | **A** | **A** | **T** | **T** | **A** | **G** | **G** | **G** | **T** | **A** | **T** | **G** | **A** | **A** | **A** | **A** | **T** | **C** | **T** | **G** | **T** | **T** | **A** | **T** | **T** | **C** | **G** | **A** | **G** | **C** | **C** | **A** | **G** | **T** | **T** | **C** | **A** | **T** | **T** | **T** | **T** | **A** | **C** | **G** | **A** | **T** | **C** | **G** | **A** | **A** | **C** | **C** | **T** | **C** | **A** | **T** | **C** | **A** | **G** | **C** | **G** | **G** | **G** | **T** | **G** | **G** | **C** | **A** | **A** | **T** | **T** | **T** | **C** | **A** | **G** | **G** | **T** | **C** | **C** | **T** | **A** | **A** | **C** | **G** | **G** | **C** | **G** | **C** | **G** | **G** | **G** | **A** | **A** | **A** | **G** | **T** | **C** | **A** | **T** | **C** | **C** | **A** | **T** | **T** | **A** | **T** | **C** | **C** | **A** | **T** | **T** | **A** | **T** | **C** | **T** | **T** | **C** | **T** | **G** | **G** | **G** | **G** | **A** | **C** | **A** | **T** | **T** | **C** | **A** | **A** | **C** | **G** | **G** | **C** | **A** | **A** | **G** | **G** | **T** | **T** | **G** | **T** | **A** | **G** | **G** | **A** | **G** | **A** | **A** | **A** | **A** | **A** | **T** | **C** | **C** | **C** | **A** | **G** | **C** | **C** | **C** | **A** | **A** | **A** | **C** | **A** | **T** | **T** | **T** | **G** | **A** | **C** | **C** | **A** | **T** | **T** | **A** | **G** | **T** | **T** | **A** | **C** | **G** | **T** | **A** | **C** | **G** | **C** | **C** | **A** | **A** | **A** | **A** | **T** | **T** | **A** | **T** | **G** | **A** | **A** | **G** | **A** | **C** | **A** | **A** | **T** | **C** | **G** | **A** | **G** | **G** | **G** | **A** | **C** | **A** | **T** | **T** | **G** | **G** | **C** | **G** | **G** | **A** | **A** | **T** | **T** | **C** | **G** | **C** | **G** | **A** | **A** | **G** | **A** | **A** | **A** | **A** | **A** | **C** | **C** | **G** | **A** | **G** | **T** | **A** | **G** | **A** | **C** | **T** | **A** | **T** | **C** | **A** | **A** | **G** | **C** | **A** | **T** | **T** | **T** | **T** | **T** | **G** | **A** | **A** | **T** | **A** | **A** | **T** | **C** | **T** | **C** | **C** | **G** | **A** | **A** | **A** | **G** | **C** | **T** | **T** | **G** | **G** | **G** | **A** | **T** | **G** | **G** | **A** | **A** | **A** | **G** | **A** | **G** | **A** | **T** | **G** | **T** | **T** | **T** | **T** | **T** | **C** | **A** | **T** | **A** | **A** | **T** | **A** | **A** | **A** | **A** | **T** | **C** | **G** | **A** | **A** | **C** | **A** | **G** | **A** | **T** | **G** | **A** | **G** | **T** | **A** | **T** | **G** | **G** | **G** | **A** | **C** | **A** | **A** | **C** | **G** | **G** | **A** | **A** | **A** | **A** | **A** | **A** | **G** | **T** | **G** | **G** | **A** | **A** | **T** | **T** | **G** | **G** | **C** | **T** | **A** | **A** | **A** | **T** | **C** | **T** | **T** | **T** | **A** | **T** | **C** | **C** | **C** | **A** | **A** | **C** | **C** | **A** | **G** | **C** | **T** | **G** | **A** | **G** | **C** | **T** | **C** | **T** | **A** | **T** | **A** | **T** | **A** | **T** | **G** | **G** | **G** | **A** | **T** | **G** | **A** | **A** | **C** | **C** | **A** | **T** | **T** | **G** | **A** | **A** | **T** | **T** | **A** | **T** | **T** | **T** | **G** | **G** | **A** | **T** | **G** | **T** | **C** | **T** | **T** | **C** | **A** | **A** | **T** | **C** | **A** | **A** | **G** | **A** | **A** | **C** | **A** | **A** | **T** | **T** | **A** | **G** | **A** | **A** | **C** | **A** | **A** | **C** | **T** | **G** | **A** | **T** | **T** | **T** | **T** | **G** | **A** | **A** | **C** | **G** | **T** | **G** | **A** | **A** | **A** | **C** | **C** | **T** | **G** | **C** | **T** | **A** | **T** | **G** | **T** | **T** | **A** | **C** | **T** | **A** | **G** | **T** | **G** | **G** | **A** | **A** | **C** | **A** | **T** | **G** | **A** | **T** | **C** | **A** | **A** | **A** | **C** | **C** | **T** | **T** | **T** | **C** | **T** | **A** | **G** | **A** | **T** | **A** | **A** | **A** | **G** | **T** | **A** | **T** | **C** | **T** | **A** | **C** | **T** | **G** | **A** | **G** | **A** | **T** | **T** | **A** | **T** | **T** | **T** | **C** | **T** | **C** | **T** | **T** | **G** | **A** | **G** | **A** | **A** | **A** | **A** | **T** | **C** | **-** | **-** | **-** |
| **N53439** | **A** | **T** | **G** | **T** | **C** | **T** | **A** | **A** | **A** | **A** | **T** | **C** | **G** | **A** | **A** | **A** | **T** | **A** | **A** | **A** | **A** | **A** | **A** | **T** | **C** | **T** | **G** | **A** | **C** | **A** | **T** | **T** | **C** | **G** | **G** | **C** | **T** | **A** | **C** | **G** | **A** | **C** | **A** | **G** | **C** | **C** | **A** | **A** | **G** | **G** | **C** | **A** | **C** | **A** | **T** | **T** | **A** | **T** | **T** | **A** | **T** | **T** | **T** | **G** | **A** | **A** | **C** | **A** | **A** | **G** | **C** | **A** | **A** | **A** | **T** | **C** | **T** | **A** | **A** | **A** | **T** | **T** | **T** | **T** | **G** | **A** | **C** | **A** | **C** | **A** | **C** | **A** | **A** | **T** | **G** | **G** | **A** | **A** | **A** | **C** | **T** | **A** | **G** | **G** | **A** | **C** | **T** | **T** | **A** | **T** | **C** | **G** | **G** | **A** | **C** | **G** | **A** | **A** | **A** | **C** | **G** | **G** | **T** | **C** | **G** | **A** | **G** | **G** | **A** | **A** | **A** | **G** | **A** | **C** | **A** | **A** | **C** | **T** | **T** | **T** | **A** | **C** | **T** | **G** | **A** | **A** | **T** | **A** | **T** | **T** | **C** | **T** | **A** | **C** | **A** | **A** | **A** | **A** | **C** | **A** | **A** | **A** | **C** | **T** | **A** | **C** | **C** | **T** | **T** | **A** | **T** | **C** | **A** | **A** | **G** | **G** | **G** | **C** | **A** | **A** | **G** | **T** | **A** | **A** | **T** | **C** | **C** | **A** | **T** | **C** | **A** | **G** | **C** | **A** | **A** | **G** | **A** | **A** | **T** | **T** | **T** | **G** | **T** | **C** | **T** | **A** | **T** | **T** | **T** | **C** | **C** | **C** | **G** | **C** | **A** | **G** | **C** | **A** | **A** | **A** | **C** | **A** | **A** | **A** | **A** | **G** | **A** | **T** | **A** | **A** | **A** | **G** | **A** | **G** | **C** | **G** | **T** | **T** | **T** | **A** | **A** | **C** | **C** | **T** | **A** | **T** | **T** | **A** | **T** | **G** | **T** | **G** | **T** | **T** | **A** | **A** | **A** | **T** | **G** | **A** | **G** | **A** | **T** | **T** | **A** | **C** | **G** | **G** | **A** | **T** | **T** | **T** | **T** | **G** | **A** | **G** | **A** | **T** | **A** | **T** | **G** | **G** | **G** | **A** | **A** | **A** | **T** | **T** | **G** | **A** | **A** | **A** | **G** | **A** | **G** | **A** | **G** | **C** | **T** | **C** | **C** | **A** | **A** | **C** | **T** | **G** | **A** | **T** | **G** | **C** | **A** | **A** | **A** | **C** | **A** | **G** | **A** | **T** | **C** | **C** | **T** | **G** | **A** | **A** | **A** | **T** | **C** | **T** | **T** | **A** | **T** | **G** | **G** | **A** | **G** | **A** | **G** | **A** | **A** | **T** | **T** | **C** | **A** | **G** | **C** | **A** | **C** | **A** | **C** | **T** | **A** | **T** | **C** | **A** | **G** | **G** | **A** | **G** | **G** | **A** | **G** | **A** | **G** | **A** | **A** | **G** | **A** | **C** | **A** | **A** | **A** | **A** | **G** | **T** | **C** | **C** | **T** | **A** | **C** | **T** | **G** | **G** | **C** | **A** | **C** | **T** | **T** | **T** | **T** | **A** | **T** | **T** | **T** | **G** | **T** | **G** | **G** | **A** | **T** | **G** | **A** | **C** | **A** | **C** | **T** | **C** | **A** | **T** | **T** | **T** | **C** | **C** | **C** | **G** | **T** | **T** | **A** | **A** | **T** | **C** | **G** | **A** | **T** | **G** | **A** | **A** | **C** | **C** | **A** | **A** | **C** | **G** | **A** | **A** | **T** | **C** | **A** | **T** | **T** | **T** | **G** | **G** | **A** | **-** | **-** | **-** | **T** | **A** | **T** | **C** | **T** | **C** | **T** | **G** | **G** | **C** | **A** | **G** | **A** | **A** | **A** | **A** | **C** | **A** | **A** | **G** | **T** | **A** | **G** | **C** | **G** | **G** | **C** | **T** | **T** | **A** | **C** | **T** | **T** | **G** | **A** | **A** | **A** | **A** | **A** | **G** | **A** | **A** | **A** | **A** | **A** | **A** | **C** | **A** | **A** | **G** | **G** | **C** | **T** | **T** | **C** | **A** | **T** | **C** | **G** | **T** | **G** | **G** | **T** | **C** | **A** | **G** | **C** | **C** | **A** | **T** | **G** | **A** | **C** | **C** | **G** | **G** | **G** | **G** | **A** | **T** | **T** | **T** | **A** | **T** | **C** | **G** | **A** | **T** | **G** | **A** | **A** | **G** | **T** | **A** | **G** | **T** | **G** | **G** | **A** | **C** | **C** | **A** | **T** | **G** | **T** | **T** | **T** | **T** | **A** | **G** | **C** | **A** | **A** | **T** | **C** | **G** | **A** | **A** | **A** | **A** | **A** | **A** | **G** | **T** | **C** | **A** | **A** | **C** | **T** | **G** | **G** | **A** | **A** | **C** | **T** | **T** | **T** | **A** | **T** | **C** | **A** | **A** | **G** | **G** | **G** | **A** | **A** | **T** | **T** | **T** | **C** | **T** | **C** | **T** | **A** | **T** | **C** | **T** | **A** | **T** | **G** | **A** | **A** | **G** | **A** | **A** | **C** | **A** | **G** | **A** | **A** | **A** | **A** | **A** | **A** | **C** | **T** | **T** | **C** | **G** | **T** | **G** | **A** | **T** | **G** | **A** | **A** | **T** | **T** | **T** | **G** | **A** | **A** | **A** | **T** | **G** | **G** | **C** | **T** | **C** | **A** | **A** | **A** | **A** | **T** | **G** | **A** | **A** | **A** | **A** | **A** | **T** | **T** | **G** | **A** | **A** | **A** | **A** | **A** | **A** | **G** | **A** | **A** | **G** | **T** | **C** | **A** | **G** | **T** | **A** | **G** | **G** | **C** | **T** | **A** | **A** | **A** | **G** | **A** | **A** | **A** | **A** | **C** | **A** | **G** | **C** | **A** | **G** | **C** | **T** | **G** | **A** | **A** | **A** | **A** | **A** | **G** | **C** | **C** | **G** | **A** | **A** | **T** | **G** | **G** | **T** | **C** | **T** | **C** | **G** | **T** | **T** | **C** | **C** | **C** | **G** | **A** | **G** | **A** | **A** | **G** | **G** | **A** | **G** | **A** | **T** | **A** | **A** | **A** | **A** | **C** | **A** | **A** | **A** | **G** | **A** | **A** | **A** | **C** | **A** | **A** | **G** | **T** | **C** | **G** | **G** | **A** | **T** | **T** | **C** | **A** | **T** | **C** | **G** | **A** | **T** | **A** | **C** | **T** | **G** | **A** | **A** | **T** | **C** | **T** | **A** | **G** | **A** | **C** | **G** | **A** | **G** | **T** | **G** | **A** | **A** | **T** | **A** | **A** | **A** | **G** | **G** | **A** | **G** | **C** | **A** | **G** | **T** | **G** | **G** | **G** | **T** | **G** | **C** | **T** | **G** | **A** | **T** | **G** | **C** | **T** | **G** | **C** | **A** | **C** | **G** | **G** | **A** | **C** | **G** | **A** | **T** | **G** | **A** | **A** | **A** | **C** | **G** | **A** | **T** | **C** | **C** | **A** | **A** | **A** | **G** | **C** | **A** | **A** | **T** | **C** | **G** | **T** | **G** | **A** | **A** | **T** | **C** | **G** | **G** | **A** | **T** | **G** | **G** | **A** | **G** | **A** | **C** | **C** | **C** | **A** | **G** | **A** | **T** | **C** | **A** | **G** | **C** | **G** | **A** | **G** | **A** | **A** | **A** | **G** | **A** | **A** | **A** | **A** | **A** | **C** | **T** | **A** | **T** | **T** | **G** | **A** | **A** | **A** | **G** | **A** | **T** | **A** | **T** | **C** | **G** | **A** | **A** | **T** | **A** | **T** | **A** | **T** | **C** | **G** | **A** | **T** | **T** | **C** | **T** | **T** | **T** | **G** | **A** | **C** | **G** | **A** | **T** | **G** | **A** | **A** | **T** | **A** | **G** | **C** | **C** | **A** | **A** | **G** | **C** | **G** | **T** | **C** | **T** | **C** | **A** | **C** | **C** | **A** | **T** | **A** | **A** | **G** | **C** | **G** | **A** | **C** | **T** | **T** | **T** | **T** | **A** | **A** | **G** | **C** | **G** | **T** | **A** | **G** | **A** | **A** | **G** | **A** | **T** | **C** | **T** | **T** | **C** | **A** | **A** | **T** | **T** | **A** | **G** | **G** | **G** | **T** | **A** | **T** | **G** | **A** | **G** | **A** | **A** | **T** | **C** | **T** | **G** | **T** | **T** | **A** | **T** | **T** | **C** | **G** | **A** | **G** | **C** | **C** | **A** | **A** | **T** | **T** | **C** | **A** | **T** | **T** | **T** | **T** | **A** | **C** | **A** | **A** | **T** | **C** | **G** | **A** | **G** | **C** | **C** | **T** | **C** | **A** | **T** | **C** | **A** | **G** | **C** | **G** | **G** | **G** | **T** | **G** | **G** | **C** | **G** | **A** | **T** | **T** | **T** | **C** | **A** | **G** | **G** | **T** | **C** | **C** | **T** | **A** | **A** | **C** | **G** | **G** | **T** | **G** | **C** | **A** | **G** | **G** | **A** | **A** | **A** | **G** | **T** | **C** | **A** | **T** | **C** | **C** | **A** | **T** | **T** | **A** | **T** | **C** | **C** | **A** | **T** | **T** | **A** | **T** | **C** | **T** | **T** | **C** | **T** | **G** | **G** | **G** | **G** | **G** | **C** | **A** | **T** | **T** | **C** | **A** | **A** | **C** | **G** | **G** | **C** | **A** | **A** | **G** | **G** | **T** | **T** | **A** | **T** | **A** | **G** | **G** | **A** | **G** | **A** | **A** | **A** | **A** | **A** | **T** | **C** | **C** | **C** | **A** | **G** | **C** | **C** | **A** | **A** | **A** | **A** | **C** | **A** | **T** | **C** | **T** | **G** | **A** | **G** | **C** | **A** | **T** | **T** | **A** | **G** | **T** | **T** | **A** | **T** | **G** | **T** | **A** | **C** | **G** | **C** | **C** | **A** | **A** | **A** | **A** | **T** | **T** | **A** | **T** | **G** | **A** | **A** | **G** | **A** | **C** | **A** | **A** | **T** | **C** | **G** | **A** | **G** | **G** | **A** | **A** | **C** | **G** | **T** | **T** | **G** | **G** | **C** | **G** | **G** | **A** | **A** | **T** | **T** | **T** | **G** | **C** | **A** | **G** | **A** | **G** | **A** | **A** | **A** | **A** | **A** | **C** | **C** | **A** | **A** | **G** | **T** | **A** | **G** | **A** | **C** | **T** | **A** | **C** | **C** | **A** | **A** | **G** | **C** | **A** | **T** | **T** | **T** | **T** | **T** | **G** | **A** | **A** | **C** | **A** | **A** | **C** | **C** | **T** | **C** | **C** | **G** | **A** | **A** | **A** | **G** | **C** | **T** | **T** | **G** | **G** | **G** | **A** | **T** | **G** | **G** | **A** | **A** | **A** | **G** | **A** | **G** | **A** | **T** | **G** | **T** | **T** | **T** | **T** | **T** | **C** | **A** | **T** | **A** | **A** | **C** | **A** | **A** | **G** | **A** | **T** | **C** | **G** | **A** | **G** | **C** | **A** | **G** | **A** | **T** | **G** | **A** | **G** | **T** | **A** | **T** | **G** | **G** | **G** | **C** | **C** | **A** | **A** | **C** | **G** | **G** | **A** | **A** | **A** | **A** | **A** | **A** | **G** | **T** | **G** | **G** | **A** | **A** | **T** | **T** | **G** | **G** | **C** | **T** | **A** | **A** | **A** | **T** | **C** | **T** | **T** | **T** | **A** | **T** | **C** | **A** | **C** | **A** | **G** | **C** | **C** | **A** | **G** | **C** | **T** | **G** | **A** | **A** | **C** | **T** | **A** | **T** | **A** | **T** | **A** | **T** | **A** | **T** | **G** | **G** | **G** | **A** | **T** | **G** | **A** | **A** | **C** | **C** | **A** | **T** | **T** | **G** | **A** | **A** | **T** | **T** | **A** | **T** | **T** | **T** | **G** | **G** | **A** | **T** | **G** | **T** | **C** | **T** | **T** | **C** | **A** | **A** | **T** | **C** | **A** | **A** | **G** | **A** | **A** | **C** | **A** | **A** | **T** | **T** | **A** | **G** | **A** | **A** | **C** | **A** | **A** | **C** | **T** | **G** | **A** | **T** | **C** | **T** | **T** | **G** | **A** | **A** | **C** | **G** | **T** | **G** | **A** | **A** | **A** | **C** | **C** | **T** | **G** | **C** | **C** | **A** | **T** | **G** | **T** | **T** | **A** | **C** | **T** | **A** | **G** | **T** | **G** | **G** | **A** | **A** | **C** | **A** | **T** | **G** | **A** | **T** | **C** | **A** | **A** | **A** | **C** | **C** | **T** | **T** | **C** | **C** | **T** | **G** | **G** | **A** | **T** | **A** | **A** | **A** | **G** | **T** | **A** | **T** | **C** | **T** | **A** | **C** | **T** | **G** | **A** | **G** | **A** | **T** | **T** | **A** | **T** | **T** | **T** | **C** | **T** | **C** | **T** | **T** | **G** | **A** | **G** | **A** | **A** | **A** | **A** | **T** | **C** | **-** | **-** | **-** |
| **N59492F** | **A** | **T** | **G** | **T** | **C** | **T** | **A** | **A** | **A** | **A** | **T** | **C** | **G** | **A** | **A** | **A** | **T** | **A** | **A** | **A** | **A** | **A** | **A** | **T** | **C** | **T** | **G** | **A** | **C** | **A** | **T** | **T** | **C** | **G** | **G** | **C** | **T** | **A** | **C** | **G** | **A** | **C** | **A** | **G** | **C** | **C** | **A** | **A** | **G** | **G** | **C** | **A** | **C** | **A** | **T** | **T** | **A** | **T** | **T** | **A** | **T** | **T** | **T** | **G** | **A** | **A** | **C** | **A** | **A** | **G** | **C** | **A** | **A** | **A** | **T** | **C** | **T** | **A** | **A** | **A** | **T** | **T** | **T** | **T** | **G** | **A** | **C** | **A** | **C** | **A** | **C** | **A** | **A** | **T** | **G** | **G** | **A** | **A** | **A** | **C** | **T** | **A** | **G** | **G** | **A** | **C** | **T** | **T** | **A** | **T** | **C** | **G** | **G** | **A** | **C** | **G** | **A** | **A** | **A** | **C** | **G** | **G** | **T** | **C** | **G** | **A** | **G** | **G** | **A** | **A** | **A** | **G** | **A** | **C** | **A** | **A** | **C** | **T** | **T** | **T** | **A** | **C** | **T** | **G** | **A** | **A** | **T** | **A** | **T** | **T** | **C** | **T** | **A** | **C** | **A** | **A** | **A** | **A** | **C** | **A** | **A** | **A** | **C** | **T** | **A** | **C** | **C** | **T** | **T** | **A** | **T** | **C** | **A** | **A** | **G** | **G** | **G** | **C** | **A** | **A** | **G** | **T** | **A** | **A** | **T** | **C** | **C** | **A** | **T** | **C** | **A** | **G** | **C** | **A** | **A** | **G** | **A** | **A** | **T** | **T** | **T** | **G** | **T** | **C** | **T** | **A** | **T** | **T** | **T** | **C** | **C** | **C** | **G** | **C** | **A** | **A** | **C** | **A** | **A** | **A** | **C** | **A** | **A** | **A** | **A** | **G** | **A** | **T** | **A** | **A** | **A** | **G** | **A** | **A** | **C** | **G** | **T** | **T** | **T** | **A** | **A** | **C** | **C** | **T** | **A** | **T** | **T** | **A** | **C** | **G** | **T** | **G** | **T** | **T** | **A** | **A** | **A** | **T** | **G** | **A** | **T** | **A** | **T** | **T** | **A** | **C** | **G** | **G** | **A** | **T** | **T** | **T** | **T** | **G** | **A** | **G** | **A** | **T** | **A** | **T** | **G** | **G** | **G** | **A** | **A** | **A** | **T** | **C** | **G** | **A** | **A** | **A** | **G** | **A** | **G** | **A** | **G** | **C** | **T** | **C** | **C** | **A** | **A** | **T** | **T** | **G** | **A** | **T** | **G** | **C** | **A** | **A** | **A** | **C** | **A** | **G** | **A** | **T** | **C** | **C** | **T** | **G** | **A** | **A** | **A** | **T** | **C** | **T** | **T** | **A** | **T** | **G** | **G** | **A** | **G** | **A** | **G** | **A** | **A** | **T** | **T** | **C** | **A** | **G** | **C** | **A** | **C** | **A** | **C** | **T** | **A** | **T** | **C** | **G** | **G** | **G** | **G** | **G** | **G** | **A** | **G** | **A** | **G** | **A** | **A** | **G** | **A** | **C** | **A** | **A** | **A** | **A** | **G** | **T** | **C** | **C** | **T** | **A** | **C** | **T** | **G** | **G** | **C** | **A** | **C** | **T** | **T** | **T** | **T** | **A** | **T** | **T** | **T** | **G** | **T** | **G** | **G** | **A** | **T** | **G** | **A** | **C** | **A** | **C** | **T** | **C** | **A** | **T** | **T** | **T** | **C** | **C** | **C** | **G** | **T** | **T** | **A** | **A** | **T** | **C** | **G** | **A** | **T** | **G** | **A** | **A** | **C** | **C** | **A** | **A** | **C** | **G** | **A** | **A** | **T** | **C** | **A** | **T** | **T** | **T** | **G** | **G** | **A** | **-** | **-** | **-** | **T** | **A** | **T** | **C** | **T** | **C** | **T** | **G** | **G** | **T** | **A** | **G** | **A** | **A** | **A** | **A** | **C** | **A** | **A** | **G** | **T** | **A** | **G** | **C** | **G** | **G** | **C** | **T** | **T** | **A** | **T** | **T** | **T** | **G** | **A** | **A** | **A** | **A** | **A** | **G** | **A** | **A** | **A** | **A** | **A** | **A** | **C** | **A** | **A** | **G** | **G** | **C** | **T** | **T** | **C** | **A** | **T** | **C** | **G** | **T** | **G** | **G** | **T** | **C** | **A** | **G** | **C** | **C** | **A** | **T** | **G** | **A** | **C** | **C** | **G** | **G** | **G** | **G** | **A** | **T** | **T** | **T** | **A** | **T** | **C** | **G** | **A** | **T** | **G** | **A** | **A** | **G** | **T** | **A** | **G** | **T** | **G** | **G** | **A** | **C** | **C** | **A** | **T** | **G** | **T** | **T** | **T** | **T** | **A** | **G** | **C** | **A** | **A** | **T** | **C** | **G** | **A** | **A** | **A** | **A** | **A** | **A** | **G** | **T** | **C** | **A** | **A** | **C** | **T** | **G** | **G** | **A** | **A** | **C** | **T** | **T** | **T** | **A** | **T** | **C** | **A** | **A** | **G** | **G** | **G** | **A** | **A** | **T** | **T** | **T** | **C** | **T** | **C** | **T** | **A** | **T** | **C** | **T** | **A** | **T** | **G** | **A** | **A** | **G** | **A** | **A** | **C** | **A** | **G** | **A** | **A** | **A** | **G** | **A** | **A** | **C** | **T** | **T** | **C** | **G** | **T** | **G** | **A** | **T** | **G** | **A** | **A** | **T** | **T** | **T** | **G** | **A** | **A** | **A** | **T** | **G** | **G** | **C** | **T** | **C** | **A** | **A** | **A** | **A** | **T** | **G** | **A** | **A** | **A** | **A** | **A** | **T** | **T** | **G** | **A** | **A** | **A** | **A** | **A** | **A** | **G** | **A** | **A** | **G** | **T** | **C** | **A** | **G** | **T** | **A** | **G** | **G** | **C** | **T** | **A** | **A** | **A** | **G** | **A** | **A** | **A** | **A** | **C** | **A** | **G** | **C** | **A** | **G** | **C** | **T** | **G** | **A** | **A** | **A** | **A** | **A** | **G** | **C** | **C** | **G** | **A** | **A** | **T** | **G** | **G** | **T** | **C** | **T** | **C** | **G** | **T** | **T** | **C** | **C** | **C** | **G** | **A** | **G** | **A** | **A** | **G** | **G** | **A** | **G** | **A** | **T** | **A** | **A** | **A** | **A** | **C** | **A** | **A** | **A** | **G** | **A** | **A** | **A** | **C** | **A** | **A** | **G** | **T** | **C** | **G** | **G** | **A** | **T** | **T** | **C** | **A** | **T** | **C** | **G** | **A** | **T** | **A** | **C** | **T** | **G** | **A** | **A** | **T** | **C** | **T** | **A** | **G** | **A** | **C** | **G** | **A** | **G** | **T** | **G** | **A** | **A** | **T** | **A** | **A** | **A** | **G** | **G** | **A** | **G** | **C** | **A** | **G** | **T** | **G** | **G** | **G** | **T** | **G** | **C** | **T** | **G** | **A** | **T** | **G** | **C** | **T** | **G** | **C** | **A** | **C** | **G** | **G** | **A** | **C** | **G** | **A** | **T** | **G** | **A** | **A** | **A** | **C** | **G** | **A** | **T** | **C** | **C** | **A** | **A** | **A** | **G** | **C** | **A** | **A** | **T** | **C** | **G** | **T** | **G** | **A** | **A** | **T** | **C** | **G** | **G** | **A** | **T** | **G** | **G** | **A** | **G** | **A** | **C** | **C** | **C** | **A** | **G** | **A** | **T** | **C** | **A** | **G** | **C** | **G** | **A** | **G** | **A** | **A** | **A** | **G** | **A** | **A** | **A** | **A** | **A** | **C** | **T** | **A** | **T** | **T** | **A** | **A** | **A** | **A** | **G** | **A** | **T** | **A** | **T** | **C** | **G** | **A** | **A** | **T** | **A** | **T** | **A** | **T** | **C** | **G** | **A** | **T** | **T** | **C** | **T** | **T** | **T** | **G** | **A** | **C** | **G** | **A** | **T** | **G** | **A** | **A** | **T** | **A** | **G** | **C** | **C** | **A** | **A** | **G** | **C** | **G** | **T** | **C** | **T** | **C** | **A** | **C** | **C** | **A** | **T** | **A** | **A** | **G** | **C** | **G** | **A** | **C** | **T** | **T** | **T** | **T** | **A** | **A** | **G** | **C** | **G** | **T** | **A** | **G** | **A** | **A** | **G** | **A** | **T** | **C** | **T** | **T** | **C** | **A** | **A** | **T** | **T** | **A** | **G** | **G** | **G** | **T** | **A** | **T** | **G** | **A** | **A** | **A** | **A** | **T** | **C** | **T** | **G** | **T** | **T** | **A** | **T** | **T** | **C** | **G** | **A** | **G** | **C** | **C** | **A** | **A** | **T** | **T** | **C** | **A** | **T** | **T** | **T** | **T** | **A** | **C** | **A** | **A** | **T** | **C** | **G** | **A** | **G** | **C** | **C** | **T** | **C** | **A** | **T** | **C** | **A** | **G** | **C** | **G** | **G** | **G** | **T** | **G** | **G** | **C** | **G** | **A** | **T** | **T** | **T** | **C** | **A** | **G** | **G** | **T** | **C** | **C** | **T** | **A** | **A** | **C** | **G** | **G** | **T** | **G** | **C** | **A** | **G** | **G** | **A** | **A** | **A** | **G** | **T** | **C** | **A** | **T** | **C** | **C** | **A** | **T** | **T** | **A** | **T** | **C** | **C** | **A** | **T** | **T** | **A** | **T** | **C** | **T** | **T** | **C** | **T** | **G** | **G** | **G** | **G** | **G** | **C** | **A** | **T** | **T** | **C** | **A** | **A** | **C** | **G** | **G** | **C** | **A** | **A** | **G** | **G** | **T** | **T** | **A** | **T** | **A** | **G** | **G** | **A** | **G** | **A** | **A** | **A** | **A** | **A** | **T** | **C** | **C** | **C** | **A** | **G** | **C** | **C** | **A** | **A** | **A** | **A** | **C** | **A** | **T** | **C** | **T** | **G** | **A** | **G** | **C** | **A** | **T** | **T** | **A** | **G** | **T** | **T** | **A** | **T** | **G** | **T** | **A** | **C** | **G** | **C** | **C** | **A** | **A** | **A** | **A** | **T** | **T** | **A** | **T** | **G** | **A** | **A** | **G** | **A** | **C** | **A** | **A** | **T** | **C** | **G** | **A** | **G** | **G** | **A** | **A** | **C** | **G** | **T** | **T** | **G** | **G** | **C** | **G** | **G** | **A** | **A** | **T** | **T** | **T** | **G** | **C** | **A** | **G** | **A** | **G** | **A** | **A** | **A** | **A** | **A** | **C** | **C** | **A** | **A** | **G** | **T** | **A** | **G** | **A** | **C** | **T** | **A** | **C** | **C** | **A** | **A** | **G** | **C** | **A** | **T** | **T** | **T** | **T** | **T** | **G** | **A** | **A** | **C** | **A** | **A** | **C** | **C** | **T** | **C** | **C** | **G** | **A** | **A** | **A** | **G** | **C** | **T** | **T** | **G** | **G** | **G** | **A** | **T** | **G** | **G** | **A** | **A** | **A** | **G** | **A** | **G** | **A** | **T** | **G** | **T** | **T** | **T** | **T** | **T** | **C** | **A** | **T** | **A** | **A** | **C** | **A** | **A** | **G** | **A** | **T** | **T** | **G** | **A** | **G** | **C** | **A** | **G** | **A** | **T** | **G** | **A** | **G** | **T** | **A** | **T** | **G** | **G** | **G** | **C** | **C** | **A** | **A** | **C** | **G** | **G** | **A** | **A** | **A** | **A** | **A** | **A** | **G** | **T** | **G** | **G** | **A** | **A** | **T** | **T** | **G** | **G** | **C** | **T** | **A** | **A** | **A** | **T** | **C** | **T** | **T** | **T** | **A** | **T** | **C** | **A** | **C** | **A** | **G** | **C** | **C** | **A** | **G** | **C** | **T** | **G** | **A** | **A** | **C** | **T** | **A** | **T** | **A** | **T** | **A** | **T** | **A** | **T** | **G** | **G** | **G** | **A** | **T** | **G** | **A** | **A** | **C** | **C** | **A** | **T** | **T** | **G** | **A** | **A** | **T** | **T** | **A** | **T** | **T** | **T** | **G** | **G** | **A** | **T** | **G** | **T** | **C** | **T** | **T** | **C** | **A** | **A** | **T** | **C** | **A** | **A** | **G** | **A** | **A** | **C** | **A** | **A** | **T** | **T** | **A** | **G** | **A** | **A** | **C** | **A** | **A** | **C** | **T** | **G** | **A** | **T** | **C** | **T** | **T** | **G** | **A** | **A** | **C** | **G** | **T** | **G** | **A** | **A** | **A** | **C** | **C** | **T** | **G** | **C** | **C** | **A** | **T** | **G** | **T** | **T** | **A** | **C** | **T** | **G** | **G** | **T** | **G** | **G** | **A** | **A** | **C** | **A** | **T** | **G** | **A** | **T** | **C** | **A** | **A** | **A** | **C** | **C** | **T** | **T** | **C** | **C** | **T** | **G** | **G** | **A** | **T** | **A** | **A** | **A** | **G** | **T** | **A** | **T** | **C** | **T** | **A** | **C** | **T** | **G** | **A** | **G** | **A** | **T** | **T** | **A** | **T** | **T** | **T** | **C** | **T** | **C** | **T** | **T** | **G** | **A** | **G** | **A** | **A** | **A** | **A** | **T** | **C** | **-** | **-** | **-** |
| **N59951F** | **A** | **T** | **G** | **T** | **C** | **T** | **A** | **A** | **A** | **A** | **T** | **C** | **G** | **A** | **A** | **A** | **T** | **A** | **A** | **A** | **A** | **A** | **A** | **T** | **C** | **T** | **G** | **A** | **C** | **A** | **T** | **T** | **C** | **G** | **G** | **C** | **T** | **A** | **C** | **G** | **A** | **C** | **A** | **G** | **C** | **C** | **A** | **A** | **G** | **G** | **C** | **A** | **C** | **A** | **T** | **T** | **A** | **T** | **T** | **A** | **T** | **T** | **T** | **G** | **A** | **A** | **C** | **A** | **A** | **G** | **C** | **A** | **A** | **A** | **T** | **C** | **T** | **A** | **A** | **A** | **T** | **T** | **T** | **T** | **G** | **A** | **C** | **A** | **T** | **A** | **C** | **A** | **A** | **T** | **G** | **G** | **A** | **A** | **A** | **C** | **T** | **A** | **G** | **G** | **A** | **C** | **T** | **T** | **A** | **T** | **C** | **G** | **G** | **A** | **C** | **G** | **A** | **A** | **A** | **C** | **G** | **G** | **T** | **C** | **G** | **A** | **G** | **G** | **A** | **A** | **A** | **G** | **A** | **C** | **A** | **A** | **C** | **T** | **T** | **T** | **A** | **C** | **T** | **G** | **A** | **A** | **T** | **A** | **T** | **T** | **C** | **T** | **A** | **C** | **A** | **A** | **A** | **A** | **C** | **A** | **A** | **A** | **C** | **T** | **A** | **C** | **C** | **T** | **T** | **A** | **T** | **C** | **A** | **A** | **G** | **G** | **G** | **C** | **A** | **A** | **G** | **T** | **A** | **A** | **T** | **C** | **C** | **A** | **T** | **C** | **A** | **G** | **C** | **A** | **A** | **G** | **A** | **A** | **T** | **T** | **T** | **G** | **T** | **C** | **T** | **A** | **T** | **T** | **T** | **C** | **C** | **C** | **G** | **C** | **A** | **A** | **C** | **A** | **G** | **A** | **C** | **A** | **A** | **A** | **A** | **G** | **A** | **T** | **A** | **A** | **A** | **G** | **A** | **A** | **C** | **G** | **T** | **T** | **T** | **A** | **A** | **C** | **C** | **T** | **A** | **T** | **T** | **A** | **C** | **G** | **T** | **G** | **T** | **T** | **A** | **A** | **A** | **T** | **G** | **A** | **T** | **A** | **T** | **T** | **A** | **C** | **G** | **G** | **A** | **T** | **T** | **T** | **T** | **G** | **A** | **G** | **A** | **T** | **A** | **T** | **G** | **G** | **G** | **A** | **A** | **A** | **T** | **C** | **G** | **A** | **A** | **A** | **G** | **A** | **G** | **A** | **G** | **C** | **T** | **C** | **C** | **A** | **A** | **T** | **T** | **G** | **A** | **T** | **G** | **C** | **A** | **A** | **A** | **C** | **A** | **G** | **A** | **T** | **C** | **C** | **T** | **G** | **A** | **A** | **A** | **T** | **C** | **T** | **T** | **A** | **T** | **G** | **G** | **A** | **G** | **A** | **G** | **A** | **A** | **T** | **T** | **C** | **A** | **G** | **C** | **A** | **C** | **A** | **C** | **T** | **A** | **T** | **C** | **A** | **G** | **G** | **G** | **G** | **G** | **A** | **G** | **A** | **G** | **A** | **A** | **G** | **A** | **C** | **A** | **A** | **A** | **A** | **G** | **T** | **C** | **C** | **T** | **A** | **C** | **T** | **G** | **G** | **C** | **A** | **C** | **T** | **T** | **T** | **T** | **A** | **T** | **T** | **T** | **G** | **T** | **G** | **G** | **A** | **T** | **G** | **A** | **C** | **A** | **C** | **T** | **C** | **A** | **T** | **T** | **T** | **C** | **C** | **C** | **G** | **T** | **T** | **A** | **A** | **T** | **C** | **G** | **A** | **T** | **G** | **A** | **A** | **C** | **C** | **A** | **A** | **C** | **G** | **A** | **A** | **T** | **C** | **A** | **T** | **T** | **T** | **A** | **G** | **A** | **-** | **-** | **-** | **T** | **A** | **T** | **C** | **T** | **C** | **T** | **G** | **G** | **T** | **A** | **G** | **A** | **A** | **A** | **A** | **C** | **A** | **A** | **G** | **T** | **A** | **G** | **C** | **G** | **G** | **C** | **T** | **T** | **A** | **T** | **T** | **T** | **G** | **A** | **A** | **A** | **A** | **A** | **G** | **A** | **A** | **A** | **A** | **A** | **A** | **C** | **A** | **A** | **G** | **G** | **C** | **T** | **T** | **C** | **A** | **T** | **C** | **G** | **T** | **G** | **G** | **T** | **C** | **A** | **G** | **C** | **C** | **A** | **T** | **G** | **A** | **C** | **C** | **G** | **G** | **G** | **G** | **A** | **T** | **T** | **T** | **A** | **T** | **C** | **G** | **A** | **T** | **G** | **A** | **A** | **G** | **T** | **A** | **G** | **T** | **G** | **G** | **A** | **C** | **C** | **A** | **T** | **G** | **T** | **T** | **T** | **T** | **A** | **G** | **C** | **A** | **A** | **T** | **C** | **G** | **A** | **A** | **A** | **A** | **A** | **A** | **G** | **T** | **C** | **A** | **A** | **C** | **T** | **G** | **G** | **A** | **A** | **C** | **T** | **T** | **T** | **A** | **T** | **C** | **A** | **A** | **G** | **G** | **G** | **A** | **A** | **T** | **T** | **T** | **C** | **T** | **C** | **T** | **A** | **T** | **C** | **T** | **A** | **T** | **G** | **A** | **A** | **G** | **A** | **A** | **C** | **A** | **G** | **A** | **A** | **A** | **A** | **A** | **A** | **C** | **T** | **T** | **C** | **G** | **T** | **G** | **A** | **T** | **G** | **A** | **A** | **T** | **T** | **T** | **G** | **A** | **A** | **A** | **T** | **G** | **G** | **C** | **T** | **C** | **A** | **A** | **A** | **A** | **T** | **G** | **A** | **A** | **A** | **A** | **A** | **T** | **T** | **G** | **A** | **A** | **A** | **A** | **A** | **A** | **G** | **A** | **A** | **G** | **T** | **C** | **A** | **G** | **T** | **A** | **G** | **G** | **C** | **T** | **A** | **A** | **A** | **G** | **A** | **A** | **A** | **A** | **C** | **A** | **G** | **C** | **A** | **G** | **C** | **T** | **G** | **A** | **A** | **A** | **A** | **A** | **G** | **C** | **C** | **G** | **A** | **A** | **T** | **G** | **G** | **T** | **C** | **T** | **C** | **G** | **T** | **T** | **C** | **C** | **C** | **G** | **A** | **G** | **A** | **A** | **G** | **G** | **A** | **G** | **A** | **T** | **A** | **A** | **A** | **A** | **C** | **A** | **A** | **A** | **G** | **A** | **A** | **A** | **C** | **A** | **A** | **G** | **T** | **C** | **G** | **G** | **A** | **T** | **T** | **C** | **A** | **T** | **C** | **G** | **A** | **T** | **A** | **C** | **T** | **G** | **A** | **A** | **T** | **C** | **T** | **A** | **G** | **A** | **C** | **G** | **A** | **G** | **T** | **G** | **A** | **A** | **T** | **A** | **A** | **A** | **G** | **G** | **A** | **G** | **C** | **A** | **G** | **T** | **G** | **G** | **G** | **T** | **G** | **C** | **T** | **G** | **A** | **T** | **G** | **C** | **T** | **G** | **C** | **A** | **C** | **G** | **G** | **A** | **C** | **G** | **A** | **T** | **G** | **A** | **A** | **A** | **C** | **G** | **A** | **T** | **C** | **C** | **A** | **A** | **A** | **G** | **C** | **A** | **A** | **T** | **C** | **G** | **T** | **G** | **A** | **A** | **T** | **C** | **G** | **G** | **A** | **T** | **G** | **G** | **A** | **G** | **A** | **C** | **C** | **C** | **A** | **G** | **A** | **T** | **C** | **A** | **G** | **C** | **G** | **A** | **G** | **A** | **A** | **A** | **G** | **A** | **A** | **A** | **A** | **A** | **C** | **T** | **A** | **T** | **T** | **A** | **A** | **A** | **A** | **G** | **A** | **T** | **A** | **T** | **C** | **G** | **A** | **A** | **T** | **A** | **T** | **A** | **T** | **C** | **G** | **A** | **T** | **T** | **C** | **T** | **T** | **T** | **G** | **A** | **C** | **G** | **A** | **T** | **G** | **A** | **A** | **T** | **A** | **G** | **T** | **C** | **A** | **A** | **G** | **C** | **G** | **T** | **C** | **T** | **C** | **A** | **C** | **C** | **A** | **T** | **A** | **A** | **G** | **C** | **G** | **A** | **C** | **T** | **T** | **T** | **T** | **A** | **A** | **G** | **C** | **G** | **T** | **A** | **G** | **A** | **A** | **G** | **A** | **T** | **C** | **T** | **T** | **C** | **A** | **A** | **T** | **T** | **A** | **G** | **G** | **G** | **T** | **A** | **T** | **G** | **A** | **A** | **A** | **A** | **T** | **C** | **T** | **G** | **T** | **T** | **A** | **T** | **T** | **C** | **G** | **A** | **G** | **C** | **C** | **A** | **A** | **T** | **T** | **C** | **A** | **T** | **T** | **T** | **T** | **A** | **C** | **A** | **A** | **T** | **C** | **G** | **A** | **G** | **C** | **C** | **T** | **C** | **A** | **T** | **C** | **A** | **G** | **C** | **G** | **G** | **G** | **T** | **G** | **G** | **C** | **G** | **A** | **T** | **T** | **T** | **C** | **A** | **G** | **G** | **T** | **C** | **C** | **T** | **A** | **A** | **C** | **G** | **G** | **T** | **G** | **C** | **A** | **G** | **G** | **A** | **A** | **A** | **G** | **T** | **C** | **A** | **T** | **C** | **C** | **A** | **T** | **T** | **A** | **T** | **C** | **C** | **A** | **T** | **T** | **A** | **T** | **C** | **T** | **T** | **C** | **T** | **G** | **G** | **G** | **G** | **G** | **C** | **A** | **T** | **T** | **C** | **A** | **A** | **C** | **G** | **G** | **C** | **A** | **A** | **G** | **G** | **T** | **T** | **A** | **T** | **A** | **G** | **G** | **A** | **G** | **A** | **A** | **A** | **A** | **A** | **T** | **C** | **C** | **C** | **A** | **G** | **C** | **C** | **A** | **A** | **A** | **A** | **C** | **A** | **T** | **C** | **T** | **G** | **A** | **G** | **C** | **A** | **T** | **T** | **A** | **G** | **T** | **T** | **A** | **T** | **G** | **T** | **A** | **C** | **G** | **C** | **C** | **A** | **A** | **A** | **A** | **T** | **T** | **A** | **T** | **G** | **A** | **A** | **G** | **A** | **C** | **A** | **A** | **T** | **C** | **G** | **A** | **G** | **G** | **A** | **A** | **C** | **G** | **T** | **T** | **G** | **G** | **C** | **G** | **G** | **A** | **A** | **T** | **T** | **T** | **G** | **C** | **A** | **G** | **A** | **G** | **A** | **A** | **A** | **A** | **A** | **C** | **C** | **A** | **A** | **G** | **T** | **A** | **G** | **A** | **C** | **T** | **A** | **C** | **C** | **A** | **A** | **G** | **C** | **A** | **T** | **T** | **T** | **T** | **T** | **G** | **A** | **A** | **C** | **A** | **A** | **C** | **C** | **T** | **C** | **C** | **G** | **A** | **A** | **A** | **G** | **C** | **T** | **T** | **G** | **G** | **G** | **A** | **T** | **G** | **G** | **A** | **A** | **A** | **G** | **A** | **G** | **A** | **T** | **G** | **T** | **T** | **T** | **T** | **T** | **C** | **A** | **T** | **A** | **A** | **C** | **A** | **A** | **G** | **A** | **T** | **C** | **G** | **A** | **G** | **C** | **A** | **G** | **A** | **T** | **G** | **A** | **G** | **T** | **A** | **T** | **G** | **G** | **G** | **T** | **C** | **A** | **A** | **C** | **G** | **G** | **A** | **A** | **A** | **A** | **A** | **A** | **G** | **T** | **G** | **G** | **A** | **A** | **T** | **T** | **G** | **G** | **C** | **T** | **A** | **A** | **A** | **T** | **C** | **T** | **T** | **T** | **A** | **T** | **C** | **A** | **C** | **A** | **G** | **C** | **C** | **A** | **G** | **C** | **T** | **G** | **A** | **A** | **C** | **T** | **A** | **T** | **A** | **T** | **A** | **T** | **A** | **T** | **G** | **G** | **G** | **A** | **T** | **G** | **A** | **A** | **C** | **C** | **A** | **T** | **T** | **G** | **A** | **A** | **T** | **T** | **A** | **T** | **T** | **T** | **G** | **G** | **A** | **T** | **G** | **T** | **C** | **T** | **T** | **C** | **A** | **A** | **T** | **C** | **A** | **A** | **G** | **A** | **A** | **C** | **A** | **A** | **T** | **T** | **A** | **G** | **A** | **A** | **C** | **A** | **A** | **C** | **T** | **G** | **A** | **T** | **C** | **T** | **T** | **G** | **A** | **A** | **C** | **G** | **T** | **G** | **A** | **A** | **A** | **C** | **C** | **T** | **G** | **C** | **C** | **A** | **T** | **G** | **T** | **T** | **A** | **C** | **T** | **A** | **G** | **T** | **G** | **G** | **A** | **A** | **C** | **A** | **T** | **G** | **A** | **T** | **C** | **A** | **A** | **A** | **C** | **C** | **T** | **T** | **C** | **C** | **T** | **G** | **G** | **A** | **T** | **A** | **A** | **A** | **G** | **T** | **A** | **T** | **C** | **T** | **A** | **C** | **T** | **G** | **A** | **G** | **A** | **T** | **T** | **A** | **T** | **T** | **T** | **C** | **T** | **C** | **T** | **T** | **G** | **A** | **G** | **A** | **A** | **A** | **A** | **T** | **C** | **-** | **-** | **-** |
| **N54035** | **A** | **T** | **G** | **T** | **C** | **T** | **A** | **A** | **A** | **A** | **T** | **C** | **G** | **A** | **A** | **A** | **T** | **A** | **A** | **A** | **A** | **A** | **A** | **T** | **C** | **T** | **G** | **A** | **C** | **A** | **T** | **T** | **C** | **G** | **G** | **C** | **T** | **A** | **C** | **G** | **A** | **C** | **A** | **G** | **C** | **C** | **A** | **A** | **G** | **G** | **C** | **A** | **C** | **A** | **T** | **T** | **A** | **T** | **T** | **A** | **T** | **T** | **T** | **G** | **A** | **A** | **C** | **A** | **A** | **G** | **C** | **A** | **A** | **A** | **T** | **C** | **T** | **A** | **A** | **A** | **T** | **T** | **T** | **T** | **G** | **A** | **C** | **A** | **T** | **A** | **C** | **A** | **A** | **T** | **G** | **G** | **A** | **A** | **A** | **C** | **T** | **A** | **G** | **G** | **A** | **C** | **T** | **T** | **A** | **T** | **C** | **G** | **G** | **A** | **C** | **G** | **A** | **A** | **A** | **C** | **G** | **G** | **T** | **C** | **G** | **A** | **G** | **G** | **A** | **A** | **A** | **G** | **A** | **C** | **A** | **A** | **C** | **T** | **T** | **T** | **A** | **C** | **T** | **G** | **A** | **A** | **T** | **A** | **T** | **T** | **C** | **T** | **A** | **C** | **A** | **A** | **A** | **A** | **C** | **A** | **A** | **A** | **C** | **T** | **A** | **C** | **C** | **T** | **T** | **A** | **T** | **C** | **A** | **A** | **G** | **G** | **G** | **C** | **A** | **A** | **G** | **T** | **A** | **A** | **T** | **C** | **C** | **A** | **T** | **C** | **A** | **G** | **C** | **A** | **A** | **G** | **A** | **A** | **T** | **T** | **T** | **G** | **T** | **C** | **T** | **A** | **T** | **T** | **T** | **C** | **C** | **C** | **G** | **C** | **A** | **A** | **C** | **A** | **G** | **A** | **C** | **A** | **A** | **A** | **A** | **G** | **A** | **T** | **A** | **A** | **A** | **G** | **A** | **A** | **C** | **G** | **T** | **T** | **T** | **A** | **A** | **C** | **C** | **T** | **A** | **T** | **T** | **A** | **C** | **G** | **T** | **G** | **T** | **T** | **A** | **A** | **A** | **T** | **G** | **A** | **T** | **A** | **T** | **T** | **A** | **C** | **G** | **G** | **A** | **T** | **T** | **T** | **T** | **G** | **A** | **G** | **A** | **T** | **A** | **T** | **G** | **G** | **G** | **A** | **A** | **A** | **T** | **C** | **G** | **A** | **A** | **A** | **G** | **A** | **G** | **A** | **G** | **C** | **T** | **C** | **C** | **A** | **A** | **T** | **T** | **G** | **A** | **T** | **G** | **C** | **A** | **A** | **A** | **C** | **A** | **G** | **A** | **T** | **C** | **C** | **T** | **G** | **A** | **A** | **A** | **T** | **C** | **T** | **T** | **A** | **T** | **G** | **G** | **A** | **G** | **A** | **G** | **A** | **A** | **T** | **T** | **C** | **A** | **G** | **C** | **A** | **C** | **A** | **C** | **T** | **A** | **T** | **C** | **A** | **G** | **G** | **G** | **G** | **G** | **A** | **G** | **A** | **G** | **A** | **A** | **G** | **A** | **C** | **A** | **A** | **A** | **A** | **G** | **T** | **C** | **C** | **T** | **A** | **C** | **T** | **G** | **G** | **C** | **A** | **C** | **T** | **T** | **T** | **T** | **A** | **T** | **T** | **T** | **G** | **T** | **G** | **G** | **A** | **T** | **G** | **A** | **C** | **A** | **C** | **T** | **C** | **A** | **T** | **T** | **T** | **C** | **C** | **C** | **G** | **T** | **T** | **A** | **A** | **T** | **C** | **G** | **A** | **T** | **G** | **A** | **A** | **C** | **C** | **A** | **A** | **C** | **G** | **A** | **A** | **T** | **C** | **A** | **T** | **T** | **T** | **G** | **G** | **A** | **-** | **-** | **-** | **T** | **A** | **T** | **C** | **T** | **C** | **T** | **G** | **G** | **T** | **A** | **G** | **A** | **A** | **A** | **A** | **C** | **A** | **A** | **G** | **T** | **A** | **G** | **C** | **G** | **G** | **C** | **T** | **T** | **A** | **T** | **T** | **T** | **G** | **A** | **A** | **A** | **A** | **A** | **G** | **A** | **A** | **A** | **A** | **A** | **A** | **C** | **A** | **A** | **G** | **G** | **C** | **T** | **T** | **C** | **A** | **T** | **C** | **G** | **T** | **G** | **G** | **T** | **C** | **A** | **G** | **C** | **C** | **A** | **T** | **G** | **A** | **C** | **C** | **G** | **G** | **G** | **G** | **A** | **T** | **T** | **T** | **A** | **T** | **C** | **G** | **A** | **T** | **G** | **A** | **A** | **G** | **T** | **A** | **G** | **T** | **G** | **G** | **A** | **C** | **C** | **A** | **T** | **G** | **T** | **T** | **T** | **T** | **A** | **G** | **C** | **A** | **A** | **T** | **C** | **G** | **A** | **A** | **A** | **A** | **A** | **A** | **G** | **T** | **C** | **A** | **A** | **C** | **T** | **G** | **G** | **A** | **A** | **C** | **T** | **T** | **T** | **A** | **T** | **C** | **A** | **A** | **G** | **G** | **G** | **A** | **A** | **T** | **T** | **T** | **C** | **T** | **C** | **T** | **A** | **T** | **C** | **T** | **A** | **T** | **G** | **A** | **A** | **G** | **A** | **A** | **C** | **A** | **G** | **A** | **A** | **A** | **A** | **A** | **A** | **C** | **T** | **T** | **C** | **G** | **T** | **G** | **A** | **T** | **G** | **A** | **A** | **T** | **T** | **T** | **G** | **A** | **A** | **A** | **T** | **G** | **G** | **C** | **T** | **C** | **A** | **A** | **A** | **A** | **T** | **G** | **A** | **A** | **A** | **A** | **A** | **T** | **T** | **G** | **A** | **A** | **A** | **A** | **A** | **A** | **G** | **A** | **A** | **G** | **T** | **C** | **A** | **G** | **T** | **A** | **G** | **G** | **C** | **T** | **A** | **A** | **A** | **G** | **A** | **A** | **A** | **A** | **C** | **A** | **G** | **C** | **A** | **G** | **C** | **T** | **G** | **A** | **A** | **A** | **A** | **A** | **G** | **C** | **C** | **G** | **A** | **A** | **T** | **G** | **G** | **T** | **C** | **T** | **C** | **G** | **T** | **T** | **C** | **C** | **C** | **G** | **A** | **G** | **A** | **A** | **G** | **G** | **A** | **G** | **A** | **T** | **A** | **A** | **A** | **A** | **C** | **A** | **A** | **A** | **G** | **A** | **A** | **A** | **C** | **A** | **A** | **G** | **T** | **C** | **G** | **G** | **A** | **T** | **T** | **C** | **A** | **T** | **C** | **G** | **A** | **T** | **A** | **C** | **T** | **G** | **A** | **A** | **T** | **C** | **T** | **A** | **G** | **A** | **C** | **G** | **A** | **G** | **T** | **G** | **A** | **A** | **T** | **A** | **A** | **A** | **G** | **G** | **A** | **G** | **C** | **A** | **G** | **T** | **G** | **G** | **G** | **T** | **G** | **C** | **T** | **G** | **A** | **T** | **G** | **C** | **T** | **G** | **C** | **A** | **C** | **G** | **G** | **A** | **C** | **G** | **A** | **T** | **G** | **A** | **A** | **A** | **C** | **G** | **A** | **T** | **C** | **C** | **A** | **A** | **A** | **G** | **C** | **A** | **A** | **T** | **C** | **G** | **T** | **G** | **A** | **A** | **T** | **C** | **G** | **G** | **A** | **T** | **G** | **G** | **A** | **G** | **A** | **C** | **C** | **C** | **A** | **G** | **A** | **T** | **C** | **A** | **G** | **C** | **G** | **A** | **G** | **A** | **A** | **A** | **G** | **A** | **A** | **A** | **A** | **A** | **C** | **T** | **A** | **T** | **T** | **A** | **A** | **A** | **A** | **G** | **A** | **T** | **A** | **T** | **C** | **G** | **A** | **A** | **T** | **A** | **T** | **A** | **T** | **C** | **G** | **A** | **T** | **T** | **C** | **T** | **T** | **T** | **G** | **A** | **C** | **G** | **A** | **T** | **G** | **A** | **A** | **T** | **A** | **G** | **T** | **C** | **A** | **A** | **G** | **C** | **G** | **T** | **C** | **T** | **C** | **A** | **C** | **C** | **A** | **T** | **A** | **A** | **G** | **C** | **G** | **A** | **C** | **T** | **T** | **T** | **T** | **A** | **A** | **G** | **C** | **G** | **T** | **A** | **G** | **A** | **A** | **G** | **A** | **T** | **C** | **T** | **T** | **C** | **A** | **A** | **T** | **T** | **A** | **G** | **G** | **G** | **T** | **A** | **T** | **G** | **A** | **A** | **A** | **A** | **T** | **C** | **T** | **G** | **T** | **T** | **A** | **T** | **T** | **C** | **G** | **A** | **G** | **C** | **C** | **A** | **A** | **T** | **T** | **C** | **A** | **T** | **T** | **T** | **T** | **A** | **C** | **A** | **A** | **T** | **C** | **G** | **A** | **G** | **C** | **C** | **T** | **C** | **A** | **T** | **C** | **A** | **G** | **C** | **G** | **G** | **G** | **T** | **G** | **G** | **C** | **G** | **A** | **T** | **T** | **T** | **C** | **A** | **G** | **G** | **T** | **C** | **C** | **T** | **A** | **A** | **C** | **G** | **G** | **T** | **G** | **C** | **A** | **G** | **G** | **A** | **A** | **A** | **G** | **T** | **C** | **A** | **T** | **C** | **C** | **A** | **T** | **T** | **A** | **T** | **C** | **C** | **A** | **T** | **T** | **A** | **T** | **C** | **T** | **T** | **C** | **T** | **G** | **G** | **G** | **G** | **G** | **C** | **A** | **T** | **T** | **C** | **A** | **A** | **C** | **G** | **G** | **C** | **A** | **A** | **G** | **G** | **T** | **T** | **A** | **T** | **A** | **G** | **G** | **A** | **G** | **A** | **A** | **A** | **A** | **A** | **T** | **C** | **C** | **C** | **A** | **G** | **C** | **C** | **A** | **A** | **A** | **A** | **C** | **A** | **T** | **C** | **T** | **G** | **A** | **G** | **C** | **A** | **T** | **T** | **A** | **G** | **T** | **T** | **A** | **T** | **G** | **T** | **A** | **C** | **G** | **C** | **C** | **A** | **A** | **A** | **A** | **T** | **T** | **A** | **T** | **G** | **A** | **A** | **G** | **A** | **C** | **A** | **A** | **T** | **C** | **G** | **A** | **G** | **G** | **A** | **A** | **C** | **G** | **T** | **T** | **G** | **G** | **C** | **G** | **G** | **A** | **A** | **T** | **T** | **T** | **G** | **C** | **A** | **G** | **A** | **G** | **A** | **A** | **A** | **A** | **A** | **C** | **C** | **A** | **A** | **G** | **T** | **A** | **G** | **A** | **C** | **T** | **A** | **C** | **C** | **A** | **A** | **G** | **C** | **A** | **T** | **T** | **T** | **T** | **T** | **G** | **A** | **A** | **C** | **A** | **A** | **C** | **C** | **T** | **C** | **C** | **G** | **A** | **A** | **A** | **G** | **C** | **T** | **T** | **G** | **G** | **G** | **A** | **T** | **G** | **G** | **A** | **A** | **A** | **G** | **A** | **G** | **A** | **T** | **G** | **T** | **T** | **T** | **T** | **T** | **C** | **A** | **T** | **A** | **A** | **C** | **A** | **A** | **G** | **A** | **T** | **C** | **G** | **A** | **G** | **C** | **A** | **G** | **A** | **T** | **G** | **A** | **G** | **T** | **A** | **T** | **G** | **G** | **G** | **T** | **C** | **A** | **A** | **C** | **G** | **G** | **A** | **A** | **A** | **A** | **A** | **A** | **G** | **T** | **G** | **G** | **A** | **A** | **T** | **T** | **G** | **G** | **C** | **T** | **A** | **A** | **A** | **T** | **C** | **T** | **T** | **T** | **A** | **T** | **C** | **A** | **C** | **A** | **G** | **C** | **C** | **A** | **G** | **C** | **T** | **G** | **A** | **A** | **C** | **T** | **A** | **T** | **A** | **T** | **A** | **T** | **A** | **T** | **G** | **G** | **G** | **A** | **T** | **G** | **A** | **A** | **C** | **C** | **A** | **T** | **T** | **G** | **A** | **A** | **T** | **T** | **A** | **T** | **T** | **T** | **G** | **G** | **A** | **T** | **G** | **T** | **C** | **T** | **T** | **C** | **A** | **A** | **T** | **C** | **A** | **A** | **G** | **A** | **A** | **C** | **A** | **A** | **T** | **T** | **A** | **G** | **A** | **A** | **C** | **A** | **A** | **C** | **T** | **G** | **A** | **T** | **C** | **T** | **T** | **G** | **A** | **A** | **C** | **G** | **T** | **G** | **A** | **A** | **A** | **C** | **C** | **T** | **G** | **C** | **C** | **A** | **T** | **G** | **T** | **T** | **A** | **C** | **T** | **A** | **G** | **T** | **G** | **G** | **A** | **A** | **C** | **A** | **T** | **G** | **A** | **T** | **C** | **A** | **A** | **A** | **C** | **C** | **T** | **T** | **C** | **C** | **T** | **G** | **G** | **A** | **T** | **A** | **A** | **A** | **G** | **T** | **A** | **T** | **C** | **T** | **A** | **C** | **T** | **G** | **A** | **G** | **A** | **T** | **T** | **A** | **T** | **T** | **T** | **C** | **T** | **C** | **T** | **T** | **G** | **A** | **G** | **A** | **A** | **A** | **A** | **T** | **C** | **-** | **-** | **-** |
| **N59484F** | **A** | **T** | **G** | **T** | **C** | **T** | **A** | **A** | **A** | **A** | **T** | **C** | **G** | **A** | **A** | **A** | **T** | **A** | **A** | **A** | **A** | **A** | **A** | **T** | **C** | **T** | **G** | **A** | **C** | **A** | **T** | **T** | **C** | **G** | **G** | **C** | **T** | **A** | **C** | **G** | **A** | **C** | **A** | **G** | **C** | **C** | **A** | **A** | **G** | **G** | **C** | **A** | **C** | **A** | **T** | **T** | **A** | **T** | **T** | **A** | **T** | **T** | **T** | **G** | **A** | **A** | **C** | **A** | **A** | **G** | **C** | **A** | **A** | **A** | **T** | **C** | **T** | **A** | **A** | **A** | **T** | **T** | **T** | **T** | **G** | **A** | **C** | **A** | **C** | **A** | **C** | **A** | **A** | **T** | **G** | **G** | **A** | **A** | **A** | **C** | **T** | **A** | **G** | **G** | **A** | **C** | **T** | **T** | **A** | **T** | **C** | **G** | **G** | **A** | **C** | **G** | **A** | **A** | **A** | **C** | **G** | **G** | **T** | **C** | **G** | **A** | **G** | **G** | **A** | **A** | **A** | **G** | **A** | **C** | **A** | **A** | **C** | **T** | **T** | **T** | **A** | **C** | **T** | **G** | **A** | **A** | **T** | **A** | **T** | **T** | **C** | **T** | **A** | **C** | **A** | **A** | **A** | **A** | **T** | **A** | **A** | **A** | **C** | **T** | **A** | **C** | **C** | **T** | **T** | **A** | **T** | **C** | **A** | **A** | **G** | **G** | **G** | **C** | **A** | **A** | **G** | **T** | **A** | **A** | **T** | **C** | **C** | **A** | **T** | **C** | **A** | **G** | **C** | **A** | **A** | **G** | **A** | **A** | **T** | **T** | **T** | **G** | **T** | **C** | **T** | **A** | **T** | **T** | **T** | **C** | **C** | **C** | **G** | **C** | **A** | **A** | **C** | **A** | **G** | **A** | **C** | **A** | **A** | **A** | **A** | **G** | **A** | **T** | **A** | **A** | **A** | **G** | **A** | **A** | **C** | **G** | **T** | **T** | **T** | **A** | **A** | **C** | **C** | **T** | **A** | **T** | **T** | **A** | **C** | **G** | **T** | **G** | **T** | **T** | **A** | **A** | **A** | **T** | **G** | **A** | **G** | **A** | **T** | **T** | **A** | **C** | **G** | **G** | **A** | **T** | **T** | **T** | **T** | **G** | **A** | **G** | **A** | **T** | **A** | **T** | **G** | **G** | **G** | **A** | **A** | **A** | **T** | **T** | **G** | **A** | **A** | **A** | **G** | **A** | **G** | **A** | **G** | **C** | **T** | **C** | **C** | **A** | **A** | **T** | **T** | **G** | **A** | **T** | **G** | **C** | **A** | **A** | **A** | **C** | **A** | **G** | **A** | **T** | **C** | **C** | **T** | **G** | **A** | **A** | **A** | **T** | **C** | **T** | **T** | **A** | **T** | **G** | **G** | **A** | **G** | **A** | **G** | **A** | **A** | **T** | **T** | **C** | **A** | **G** | **C** | **A** | **C** | **A** | **C** | **T** | **A** | **T** | **C** | **G** | **G** | **G** | **G** | **G** | **G** | **A** | **G** | **A** | **G** | **A** | **A** | **G** | **A** | **C** | **A** | **A** | **A** | **A** | **G** | **T** | **C** | **C** | **T** | **A** | **C** | **T** | **G** | **G** | **C** | **A** | **C** | **T** | **T** | **T** | **T** | **A** | **T** | **T** | **T** | **G** | **T** | **G** | **G** | **A** | **T** | **G** | **A** | **C** | **A** | **C** | **T** | **C** | **A** | **T** | **T** | **T** | **C** | **C** | **C** | **G** | **T** | **T** | **A** | **A** | **T** | **C** | **G** | **A** | **T** | **G** | **A** | **A** | **C** | **C** | **A** | **A** | **C** | **G** | **A** | **A** | **T** | **C** | **A** | **T** | **T** | **T** | **G** | **G** | **A** | **-** | **-** | **-** | **T** | **A** | **T** | **C** | **T** | **C** | **T** | **G** | **G** | **T** | **A** | **G** | **A** | **A** | **A** | **A** | **C** | **A** | **A** | **G** | **T** | **A** | **G** | **C** | **G** | **G** | **C** | **T** | **T** | **A** | **T** | **T** | **T** | **G** | **A** | **A** | **A** | **A** | **A** | **G** | **A** | **A** | **A** | **A** | **A** | **A** | **C** | **A** | **A** | **G** | **G** | **C** | **T** | **T** | **C** | **A** | **T** | **C** | **G** | **T** | **G** | **G** | **T** | **C** | **A** | **G** | **C** | **C** | **A** | **T** | **G** | **A** | **C** | **C** | **G** | **G** | **G** | **G** | **A** | **T** | **T** | **T** | **A** | **T** | **C** | **G** | **A** | **T** | **G** | **A** | **A** | **G** | **T** | **A** | **G** | **T** | **G** | **G** | **A** | **C** | **C** | **A** | **T** | **G** | **T** | **T** | **T** | **T** | **A** | **G** | **C** | **A** | **A** | **T** | **C** | **G** | **A** | **A** | **A** | **A** | **A** | **A** | **G** | **T** | **C** | **A** | **A** | **C** | **T** | **G** | **G** | **A** | **A** | **C** | **T** | **T** | **T** | **A** | **T** | **C** | **A** | **A** | **G** | **G** | **G** | **A** | **A** | **T** | **T** | **T** | **C** | **T** | **C** | **T** | **A** | **T** | **C** | **T** | **A** | **T** | **G** | **A** | **A** | **G** | **A** | **A** | **C** | **A** | **G** | **A** | **A** | **A** | **A** | **A** | **A** | **C** | **T** | **T** | **C** | **G** | **T** | **G** | **A** | **T** | **G** | **A** | **A** | **T** | **T** | **T** | **G** | **A** | **A** | **A** | **T** | **G** | **G** | **C** | **T** | **C** | **A** | **A** | **A** | **A** | **T** | **G** | **A** | **A** | **A** | **A** | **A** | **T** | **T** | **G** | **A** | **A** | **A** | **A** | **A** | **A** | **G** | **A** | **A** | **G** | **T** | **C** | **A** | **G** | **T** | **A** | **G** | **G** | **C** | **T** | **A** | **A** | **A** | **G** | **A** | **A** | **A** | **A** | **C** | **A** | **G** | **C** | **A** | **G** | **C** | **T** | **G** | **A** | **A** | **A** | **A** | **A** | **G** | **C** | **C** | **G** | **A** | **A** | **T** | **G** | **G** | **T** | **C** | **T** | **C** | **G** | **T** | **T** | **C** | **C** | **C** | **G** | **A** | **G** | **A** | **A** | **G** | **G** | **A** | **G** | **A** | **T** | **A** | **A** | **A** | **A** | **C** | **A** | **A** | **A** | **G** | **A** | **A** | **A** | **C** | **A** | **A** | **G** | **T** | **C** | **G** | **G** | **A** | **T** | **T** | **C** | **A** | **T** | **C** | **G** | **A** | **T** | **A** | **C** | **T** | **G** | **A** | **A** | **T** | **C** | **T** | **A** | **G** | **A** | **C** | **G** | **A** | **G** | **T** | **G** | **A** | **A** | **T** | **A** | **A** | **A** | **G** | **G** | **A** | **G** | **C** | **A** | **G** | **T** | **G** | **G** | **G** | **T** | **G** | **C** | **T** | **G** | **A** | **T** | **G** | **C** | **T** | **G** | **C** | **A** | **C** | **G** | **G** | **A** | **C** | **G** | **A** | **T** | **G** | **A** | **A** | **A** | **C** | **G** | **A** | **T** | **C** | **C** | **A** | **A** | **A** | **G** | **C** | **A** | **A** | **T** | **C** | **G** | **T** | **G** | **A** | **A** | **T** | **C** | **G** | **G** | **A** | **T** | **G** | **G** | **A** | **G** | **A** | **C** | **C** | **C** | **A** | **G** | **A** | **T** | **C** | **A** | **G** | **C** | **G** | **A** | **G** | **A** | **A** | **A** | **G** | **A** | **A** | **A** | **A** | **A** | **C** | **T** | **A** | **T** | **T** | **A** | **A** | **A** | **A** | **G** | **A** | **T** | **A** | **T** | **C** | **G** | **A** | **A** | **T** | **A** | **T** | **A** | **T** | **C** | **G** | **A** | **T** | **T** | **C** | **T** | **T** | **T** | **G** | **A** | **C** | **G** | **A** | **T** | **G** | **A** | **A** | **T** | **A** | **G** | **C** | **C** | **A** | **A** | **G** | **C** | **G** | **T** | **C** | **T** | **C** | **A** | **C** | **C** | **A** | **T** | **A** | **A** | **G** | **C** | **G** | **A** | **C** | **T** | **T** | **T** | **T** | **A** | **A** | **G** | **C** | **G** | **T** | **A** | **G** | **A** | **A** | **G** | **A** | **T** | **C** | **T** | **T** | **C** | **A** | **A** | **T** | **T** | **A** | **G** | **G** | **G** | **T** | **A** | **T** | **G** | **A** | **A** | **A** | **A** | **T** | **C** | **T** | **G** | **T** | **T** | **A** | **T** | **T** | **C** | **G** | **A** | **G** | **C** | **C** | **A** | **A** | **T** | **T** | **C** | **A** | **T** | **T** | **T** | **T** | **A** | **C** | **A** | **A** | **T** | **C** | **G** | **A** | **G** | **C** | **C** | **T** | **C** | **A** | **T** | **C** | **A** | **G** | **C** | **G** | **G** | **G** | **T** | **G** | **G** | **C** | **G** | **A** | **T** | **T** | **T** | **C** | **A** | **G** | **G** | **T** | **C** | **C** | **T** | **A** | **A** | **C** | **G** | **G** | **T** | **G** | **C** | **A** | **G** | **G** | **A** | **A** | **A** | **G** | **T** | **C** | **A** | **T** | **C** | **C** | **A** | **T** | **T** | **A** | **T** | **C** | **C** | **A** | **T** | **T** | **A** | **T** | **C** | **T** | **T** | **C** | **T** | **G** | **G** | **G** | **G** | **G** | **C** | **A** | **T** | **T** | **C** | **A** | **A** | **C** | **G** | **G** | **C** | **A** | **A** | **G** | **G** | **T** | **T** | **A** | **T** | **A** | **G** | **G** | **A** | **G** | **A** | **A** | **A** | **A** | **A** | **T** | **C** | **C** | **C** | **A** | **G** | **C** | **C** | **A** | **A** | **A** | **A** | **C** | **A** | **T** | **C** | **T** | **G** | **A** | **G** | **C** | **A** | **T** | **T** | **A** | **G** | **T** | **T** | **A** | **T** | **G** | **T** | **A** | **C** | **G** | **C** | **C** | **A** | **A** | **A** | **A** | **T** | **T** | **A** | **T** | **G** | **A** | **A** | **G** | **A** | **C** | **A** | **A** | **T** | **C** | **G** | **A** | **G** | **G** | **A** | **A** | **C** | **G** | **T** | **T** | **G** | **G** | **C** | **G** | **G** | **A** | **A** | **T** | **T** | **T** | **G** | **C** | **A** | **G** | **A** | **G** | **A** | **A** | **A** | **A** | **A** | **C** | **C** | **A** | **A** | **G** | **T** | **A** | **G** | **A** | **C** | **T** | **A** | **C** | **C** | **A** | **A** | **G** | **C** | **A** | **T** | **T** | **T** | **T** | **T** | **G** | **A** | **A** | **C** | **A** | **A** | **C** | **C** | **T** | **C** | **C** | **G** | **A** | **A** | **A** | **G** | **C** | **T** | **T** | **G** | **G** | **G** | **A** | **T** | **G** | **G** | **A** | **A** | **A** | **G** | **A** | **G** | **A** | **T** | **G** | **T** | **T** | **T** | **T** | **T** | **C** | **A** | **T** | **A** | **A** | **C** | **A** | **A** | **G** | **A** | **T** | **C** | **G** | **A** | **G** | **C** | **A** | **G** | **A** | **T** | **G** | **A** | **G** | **T** | **A** | **T** | **G** | **G** | **G** | **C** | **C** | **A** | **A** | **C** | **G** | **G** | **A** | **A** | **A** | **A** | **A** | **A** | **G** | **T** | **G** | **G** | **A** | **A** | **T** | **T** | **G** | **G** | **C** | **T** | **A** | **A** | **A** | **T** | **C** | **T** | **T** | **T** | **A** | **T** | **C** | **A** | **C** | **A** | **G** | **C** | **C** | **A** | **G** | **C** | **T** | **G** | **A** | **A** | **C** | **T** | **A** | **T** | **A** | **T** | **A** | **T** | **A** | **T** | **G** | **G** | **G** | **A** | **T** | **G** | **A** | **A** | **C** | **C** | **A** | **T** | **T** | **G** | **A** | **A** | **T** | **T** | **A** | **T** | **T** | **T** | **G** | **G** | **A** | **T** | **G** | **T** | **C** | **T** | **T** | **C** | **A** | **A** | **T** | **C** | **A** | **A** | **G** | **A** | **A** | **C** | **A** | **A** | **T** | **T** | **A** | **G** | **A** | **A** | **C** | **A** | **A** | **C** | **T** | **G** | **A** | **T** | **C** | **T** | **T** | **G** | **A** | **A** | **C** | **G** | **T** | **G** | **A** | **A** | **A** | **C** | **C** | **T** | **G** | **C** | **C** | **A** | **T** | **G** | **T** | **T** | **A** | **C** | **T** | **A** | **G** | **T** | **G** | **G** | **A** | **A** | **C** | **A** | **T** | **G** | **A** | **T** | **C** | **A** | **A** | **A** | **C** | **C** | **T** | **T** | **C** | **C** | **T** | **G** | **G** | **A** | **T** | **A** | **A** | **A** | **G** | **T** | **A** | **T** | **C** | **T** | **A** | **C** | **T** | **G** | **A** | **G** | **A** | **T** | **T** | **A** | **T** | **T** | **T** | **C** | **T** | **C** | **T** | **T** | **G** | **A** | **G** | **A** | **A** | **A** | **A** | **T** | **C** | **-** | **-** | **-** |
| **N59907F** | **A** | **T** | **G** | **T** | **C** | **T** | **A** | **A** | **A** | **A** | **T** | **C** | **G** | **A** | **A** | **A** | **T** | **A** | **A** | **A** | **A** | **A** | **A** | **T** | **C** | **T** | **G** | **A** | **C** | **A** | **T** | **T** | **C** | **G** | **G** | **C** | **T** | **A** | **C** | **G** | **A** | **C** | **A** | **G** | **C** | **C** | **A** | **A** | **G** | **G** | **C** | **A** | **C** | **A** | **T** | **T** | **A** | **T** | **T** | **A** | **T** | **T** | **T** | **G** | **A** | **A** | **C** | **A** | **A** | **G** | **C** | **A** | **A** | **A** | **T** | **C** | **T** | **A** | **A** | **A** | **T** | **T** | **T** | **T** | **G** | **A** | **C** | **A** | **T** | **A** | **C** | **A** | **A** | **T** | **G** | **G** | **A** | **A** | **A** | **C** | **T** | **A** | **G** | **G** | **A** | **C** | **T** | **T** | **A** | **T** | **C** | **G** | **G** | **A** | **C** | **G** | **A** | **A** | **A** | **C** | **G** | **G** | **T** | **C** | **G** | **A** | **G** | **G** | **A** | **A** | **A** | **G** | **A** | **C** | **A** | **A** | **C** | **T** | **T** | **T** | **A** | **C** | **T** | **G** | **A** | **A** | **T** | **A** | **T** | **T** | **C** | **T** | **A** | **C** | **A** | **A** | **A** | **A** | **C** | **A** | **A** | **A** | **C** | **T** | **A** | **C** | **C** | **T** | **T** | **A** | **T** | **C** | **A** | **A** | **G** | **G** | **G** | **C** | **A** | **A** | **G** | **T** | **A** | **A** | **T** | **C** | **C** | **A** | **T** | **C** | **A** | **G** | **C** | **A** | **A** | **G** | **A** | **A** | **T** | **T** | **T** | **G** | **T** | **C** | **T** | **A** | **T** | **T** | **T** | **C** | **C** | **C** | **G** | **C** | **A** | **A** | **C** | **A** | **G** | **A** | **C** | **A** | **A** | **A** | **A** | **G** | **A** | **T** | **A** | **A** | **A** | **G** | **A** | **A** | **C** | **G** | **T** | **T** | **T** | **A** | **A** | **C** | **C** | **T** | **A** | **T** | **T** | **A** | **C** | **G** | **T** | **G** | **T** | **T** | **A** | **A** | **A** | **T** | **G** | **A** | **T** | **A** | **T** | **T** | **A** | **C** | **G** | **G** | **A** | **T** | **T** | **T** | **T** | **G** | **A** | **G** | **A** | **T** | **A** | **T** | **G** | **G** | **G** | **A** | **A** | **A** | **T** | **C** | **G** | **A** | **A** | **A** | **G** | **A** | **G** | **A** | **G** | **C** | **T** | **C** | **C** | **A** | **A** | **T** | **T** | **G** | **A** | **T** | **G** | **C** | **A** | **A** | **A** | **C** | **A** | **G** | **A** | **T** | **C** | **C** | **T** | **G** | **A** | **A** | **A** | **T** | **C** | **T** | **T** | **A** | **T** | **G** | **G** | **A** | **G** | **A** | **G** | **A** | **A** | **T** | **T** | **C** | **A** | **G** | **C** | **A** | **C** | **A** | **C** | **T** | **A** | **T** | **C** | **A** | **G** | **G** | **G** | **G** | **G** | **A** | **G** | **A** | **G** | **A** | **A** | **G** | **A** | **C** | **A** | **A** | **A** | **A** | **G** | **T** | **C** | **C** | **T** | **A** | **C** | **T** | **G** | **G** | **C** | **A** | **C** | **T** | **T** | **T** | **T** | **A** | **T** | **T** | **T** | **G** | **T** | **G** | **G** | **A** | **T** | **G** | **A** | **C** | **A** | **C** | **T** | **C** | **A** | **T** | **T** | **T** | **C** | **C** | **C** | **G** | **T** | **T** | **A** | **A** | **T** | **C** | **G** | **A** | **T** | **G** | **A** | **A** | **C** | **C** | **A** | **A** | **C** | **G** | **A** | **A** | **T** | **C** | **A** | **T** | **T** | **T** | **G** | **G** | **A** | **-** | **-** | **-** | **T** | **A** | **T** | **C** | **T** | **C** | **T** | **G** | **G** | **T** | **A** | **G** | **A** | **A** | **A** | **A** | **C** | **A** | **A** | **G** | **T** | **A** | **G** | **C** | **G** | **G** | **C** | **T** | **T** | **A** | **T** | **T** | **T** | **G** | **A** | **A** | **A** | **A** | **A** | **G** | **A** | **A** | **A** | **A** | **A** | **A** | **C** | **A** | **A** | **G** | **G** | **C** | **T** | **T** | **C** | **A** | **T** | **C** | **G** | **T** | **G** | **G** | **T** | **C** | **A** | **G** | **C** | **C** | **A** | **T** | **G** | **A** | **C** | **C** | **G** | **G** | **G** | **G** | **A** | **T** | **T** | **T** | **A** | **T** | **C** | **G** | **A** | **T** | **G** | **A** | **A** | **G** | **T** | **A** | **G** | **T** | **G** | **G** | **A** | **C** | **C** | **A** | **T** | **G** | **T** | **T** | **T** | **T** | **A** | **G** | **C** | **A** | **A** | **T** | **C** | **G** | **A** | **A** | **A** | **A** | **A** | **A** | **G** | **T** | **C** | **A** | **A** | **C** | **T** | **G** | **G** | **A** | **A** | **C** | **T** | **T** | **T** | **A** | **T** | **C** | **A** | **A** | **G** | **G** | **G** | **A** | **A** | **T** | **T** | **T** | **C** | **T** | **C** | **T** | **A** | **T** | **C** | **T** | **A** | **T** | **G** | **A** | **A** | **G** | **A** | **A** | **C** | **A** | **G** | **A** | **A** | **A** | **A** | **A** | **A** | **C** | **T** | **T** | **C** | **G** | **T** | **G** | **A** | **T** | **G** | **A** | **A** | **T** | **T** | **T** | **G** | **A** | **A** | **A** | **T** | **G** | **G** | **C** | **T** | **C** | **A** | **A** | **A** | **A** | **T** | **G** | **A** | **A** | **A** | **A** | **A** | **T** | **T** | **G** | **A** | **A** | **A** | **A** | **A** | **A** | **G** | **A** | **A** | **G** | **T** | **C** | **A** | **G** | **T** | **A** | **G** | **G** | **C** | **T** | **A** | **A** | **A** | **G** | **A** | **A** | **A** | **A** | **C** | **A** | **G** | **C** | **A** | **G** | **C** | **T** | **G** | **A** | **A** | **A** | **A** | **A** | **G** | **C** | **C** | **G** | **A** | **A** | **T** | **G** | **G** | **T** | **C** | **T** | **C** | **G** | **T** | **T** | **C** | **C** | **C** | **G** | **A** | **G** | **A** | **A** | **G** | **G** | **A** | **G** | **A** | **T** | **A** | **A** | **A** | **A** | **C** | **A** | **A** | **A** | **G** | **A** | **A** | **A** | **C** | **A** | **A** | **G** | **T** | **C** | **G** | **G** | **A** | **T** | **T** | **C** | **A** | **T** | **C** | **G** | **A** | **T** | **A** | **C** | **T** | **G** | **A** | **A** | **T** | **C** | **T** | **A** | **G** | **A** | **C** | **G** | **A** | **G** | **T** | **G** | **A** | **A** | **T** | **A** | **A** | **A** | **G** | **G** | **A** | **G** | **C** | **A** | **G** | **T** | **G** | **G** | **G** | **T** | **G** | **C** | **T** | **G** | **A** | **T** | **G** | **C** | **T** | **G** | **C** | **A** | **C** | **G** | **G** | **A** | **C** | **G** | **A** | **T** | **G** | **A** | **A** | **A** | **C** | **G** | **A** | **T** | **C** | **C** | **A** | **A** | **A** | **G** | **C** | **A** | **A** | **T** | **C** | **G** | **T** | **G** | **A** | **A** | **T** | **C** | **G** | **G** | **A** | **T** | **G** | **G** | **A** | **G** | **A** | **C** | **C** | **C** | **A** | **G** | **A** | **T** | **C** | **A** | **G** | **C** | **G** | **A** | **G** | **A** | **A** | **A** | **G** | **A** | **A** | **A** | **A** | **A** | **C** | **T** | **A** | **T** | **T** | **A** | **A** | **A** | **A** | **G** | **A** | **T** | **A** | **T** | **C** | **G** | **A** | **A** | **T** | **A** | **T** | **A** | **T** | **C** | **G** | **A** | **T** | **T** | **C** | **T** | **T** | **T** | **G** | **A** | **C** | **G** | **A** | **T** | **G** | **A** | **A** | **T** | **A** | **G** | **T** | **C** | **A** | **A** | **G** | **C** | **G** | **T** | **C** | **T** | **C** | **A** | **C** | **C** | **A** | **T** | **A** | **A** | **G** | **C** | **G** | **A** | **C** | **T** | **T** | **T** | **T** | **A** | **A** | **G** | **C** | **G** | **T** | **A** | **G** | **A** | **A** | **G** | **A** | **T** | **C** | **T** | **T** | **C** | **A** | **A** | **T** | **T** | **A** | **G** | **G** | **G** | **T** | **A** | **T** | **G** | **A** | **A** | **A** | **A** | **T** | **C** | **T** | **G** | **T** | **T** | **A** | **T** | **T** | **C** | **G** | **A** | **G** | **C** | **C** | **A** | **A** | **T** | **T** | **C** | **A** | **T** | **T** | **T** | **T** | **A** | **C** | **A** | **A** | **T** | **C** | **G** | **A** | **G** | **C** | **C** | **T** | **C** | **A** | **T** | **C** | **A** | **G** | **C** | **G** | **G** | **G** | **T** | **G** | **G** | **C** | **G** | **A** | **T** | **T** | **T** | **C** | **A** | **G** | **G** | **T** | **C** | **C** | **T** | **A** | **A** | **C** | **G** | **G** | **T** | **G** | **C** | **A** | **G** | **G** | **A** | **A** | **A** | **G** | **T** | **C** | **A** | **T** | **C** | **C** | **A** | **T** | **T** | **A** | **T** | **C** | **C** | **A** | **T** | **T** | **A** | **T** | **C** | **T** | **T** | **C** | **T** | **G** | **G** | **G** | **G** | **G** | **C** | **A** | **T** | **T** | **C** | **A** | **A** | **C** | **G** | **G** | **C** | **A** | **A** | **G** | **G** | **T** | **T** | **A** | **T** | **A** | **G** | **G** | **A** | **G** | **A** | **A** | **A** | **A** | **A** | **T** | **C** | **C** | **C** | **A** | **G** | **C** | **C** | **A** | **A** | **A** | **A** | **C** | **A** | **T** | **C** | **T** | **G** | **A** | **G** | **C** | **A** | **T** | **T** | **A** | **G** | **T** | **T** | **A** | **T** | **G** | **T** | **A** | **C** | **G** | **C** | **C** | **A** | **A** | **A** | **A** | **T** | **T** | **A** | **T** | **G** | **A** | **A** | **G** | **A** | **C** | **A** | **A** | **T** | **C** | **G** | **A** | **G** | **G** | **A** | **A** | **C** | **G** | **T** | **T** | **G** | **G** | **C** | **G** | **G** | **A** | **A** | **T** | **T** | **T** | **G** | **C** | **A** | **G** | **A** | **G** | **A** | **A** | **A** | **A** | **A** | **C** | **C** | **A** | **A** | **G** | **T** | **A** | **G** | **A** | **C** | **T** | **A** | **C** | **C** | **A** | **A** | **G** | **C** | **A** | **T** | **T** | **T** | **T** | **T** | **G** | **A** | **A** | **C** | **A** | **A** | **C** | **C** | **T** | **C** | **C** | **G** | **A** | **A** | **A** | **G** | **C** | **T** | **T** | **G** | **G** | **G** | **A** | **T** | **G** | **G** | **A** | **A** | **A** | **G** | **A** | **G** | **A** | **T** | **G** | **T** | **T** | **T** | **T** | **T** | **C** | **A** | **T** | **A** | **A** | **C** | **A** | **A** | **G** | **A** | **T** | **C** | **G** | **A** | **G** | **C** | **A** | **G** | **A** | **T** | **G** | **A** | **G** | **T** | **A** | **T** | **G** | **G** | **G** | **T** | **C** | **A** | **A** | **C** | **G** | **G** | **A** | **A** | **A** | **A** | **A** | **A** | **G** | **T** | **G** | **G** | **A** | **A** | **T** | **T** | **G** | **G** | **C** | **T** | **A** | **A** | **A** | **T** | **C** | **T** | **T** | **T** | **A** | **T** | **C** | **A** | **C** | **A** | **G** | **C** | **C** | **A** | **G** | **C** | **T** | **G** | **A** | **A** | **C** | **T** | **A** | **T** | **A** | **T** | **A** | **T** | **A** | **T** | **G** | **G** | **G** | **A** | **T** | **G** | **A** | **A** | **C** | **C** | **A** | **T** | **T** | **G** | **A** | **A** | **T** | **T** | **A** | **T** | **T** | **T** | **G** | **G** | **A** | **T** | **G** | **T** | **C** | **T** | **T** | **C** | **A** | **A** | **T** | **C** | **A** | **A** | **G** | **A** | **A** | **C** | **A** | **A** | **T** | **T** | **A** | **G** | **A** | **A** | **C** | **A** | **A** | **C** | **T** | **G** | **A** | **T** | **C** | **T** | **T** | **G** | **A** | **A** | **C** | **G** | **T** | **G** | **A** | **A** | **A** | **C** | **C** | **T** | **G** | **C** | **C** | **A** | **T** | **G** | **T** | **T** | **A** | **C** | **T** | **A** | **G** | **T** | **G** | **G** | **A** | **A** | **C** | **A** | **T** | **G** | **A** | **T** | **C** | **A** | **A** | **A** | **C** | **C** | **T** | **T** | **C** | **C** | **T** | **G** | **G** | **A** | **T** | **A** | **A** | **A** | **G** | **T** | **A** | **T** | **C** | **T** | **A** | **C** | **T** | **G** | **A** | **G** | **A** | **T** | **T** | **A** | **T** | **T** | **T** | **C** | **T** | **C** | **T** | **T** | **G** | **A** | **G** | **A** | **A** | **A** | **A** | **T** | **C** | **-** | **-** | **-** |
| **N60001F** | **A** | **T** | **G** | **T** | **C** | **T** | **A** | **A** | **A** | **A** | **T** | **C** | **G** | **A** | **A** | **A** | **T** | **A** | **A** | **A** | **A** | **A** | **A** | **T** | **C** | **T** | **G** | **A** | **C** | **A** | **T** | **T** | **C** | **G** | **G** | **C** | **T** | **A** | **T** | **G** | **A** | **C** | **A** | **G** | **C** | **C** | **A** | **A** | **G** | **G** | **C** | **A** | **C** | **A** | **T** | **T** | **A** | **T** | **T** | **A** | **T** | **T** | **T** | **G** | **A** | **A** | **C** | **A** | **A** | **G** | **C** | **C** | **A** | **A** | **T** | **C** | **T** | **A** | **A** | **A** | **C** | **T** | **T** | **T** | **G** | **A** | **C** | **A** | **C** | **A** | **C** | **A** | **A** | **T** | **G** | **G** | **A** | **A** | **A** | **C** | **T** | **A** | **G** | **G** | **A** | **C** | **T** | **T** | **A** | **T** | **C** | **G** | **G** | **A** | **C** | **G** | **A** | **A** | **A** | **C** | **G** | **G** | **T** | **C** | **G** | **A** | **G** | **G** | **A** | **A** | **A** | **G** | **A** | **C** | **A** | **A** | **C** | **T** | **T** | **T** | **A** | **C** | **T** | **G** | **A** | **A** | **T** | **A** | **T** | **C** | **C** | **T** | **G** | **C** | **A** | **A** | **A** | **A** | **C** | **A** | **A** | **A** | **C** | **T** | **C** | **C** | **C** | **G** | **T** | **A** | **T** | **C** | **A** | **A** | **G** | **G** | **G** | **C** | **A** | **A** | **G** | **T** | **G** | **A** | **T** | **C** | **C** | **A** | **T** | **C** | **A** | **G** | **C** | **A** | **A** | **G** | **C** | **G** | **T** | **T** | **T** | **G** | **T** | **C** | **T** | **A** | **T** | **T** | **T** | **C** | **C** | **C** | **G** | **C** | **A** | **G** | **C** | **A** | **A** | **A** | **C** | **A** | **A** | **A** | **A** | **G** | **A** | **T** | **A** | **A** | **A** | **G** | **A** | **G** | **C** | **G** | **T** | **T** | **T** | **G** | **A** | **C** | **C** | **T** | **A** | **T** | **T** | **A** | **T** | **G** | **T** | **G** | **T** | **T** | **A** | **A** | **A** | **T** | **G** | **A** | **G** | **A** | **T** | **T** | **A** | **T** | **G** | **G** | **A** | **T** | **T** | **T** | **T** | **G** | **A** | **G** | **A** | **T** | **A** | **T** | **G** | **G** | **G** | **A** | **A** | **A** | **T** | **T** | **G** | **A** | **A** | **A** | **G** | **A** | **G** | **A** | **G** | **C** | **T** | **C** | **C** | **A** | **A** | **C** | **T** | **G** | **A** | **T** | **G** | **C** | **A** | **A** | **A** | **C** | **A** | **G** | **A** | **T** | **C** | **C** | **T** | **G** | **A** | **A** | **A** | **T** | **C** | **T** | **T** | **A** | **T** | **G** | **G** | **A** | **G** | **A** | **G** | **A** | **A** | **T** | **T** | **C** | **A** | **G** | **C** | **A** | **C** | **A** | **C** | **T** | **A** | **T** | **C** | **A** | **G** | **G** | **A** | **G** | **G** | **A** | **G** | **A** | **G** | **A** | **A** | **G** | **A** | **C** | **A** | **A** | **A** | **A** | **G** | **T** | **C** | **C** | **T** | **A** | **C** | **T** | **G** | **G** | **C** | **A** | **C** | **T** | **C** | **T** | **T** | **A** | **T** | **T** | **T** | **G** | **T** | **G** | **G** | **A** | **T** | **G** | **A** | **C** | **A** | **C** | **T** | **C** | **A** | **T** | **T** | **T** | **C** | **C** | **C** | **G** | **T** | **T** | **A** | **A** | **T** | **C** | **G** | **A** | **T** | **G** | **A** | **A** | **C** | **C** | **A** | **A** | **C** | **G** | **A** | **A** | **T** | **C** | **A** | **T** | **T** | **T** | **G** | **G** | **A** | **-** | **-** | **-** | **T** | **A** | **T** | **C** | **T** | **C** | **T** | **G** | **G** | **C** | **A** | **G** | **A** | **A** | **A** | **A** | **C** | **A** | **A** | **G** | **T** | **A** | **G** | **C** | **G** | **G** | **C** | **T** | **T** | **A** | **C** | **T** | **T** | **G** | **A** | **A** | **A** | **A** | **A** | **G** | **A** | **A** | **A** | **A** | **A** | **A** | **C** | **A** | **A** | **G** | **G** | **A** | **T** | **T** | **C** | **A** | **T** | **C** | **G** | **T** | **G** | **G** | **T** | **G** | **A** | **G** | **T** | **C** | **A** | **T** | **G** | **A** | **T** | **C** | **G** | **G** | **G** | **G** | **A** | **T** | **T** | **T** | **A** | **T** | **C** | **G** | **A** | **T** | **G** | **A** | **A** | **A** | **T** | **A** | **G** | **T** | **G** | **G** | **A** | **C** | **C** | **A** | **T** | **G** | **T** | **T** | **T** | **T** | **A** | **G** | **C** | **A** | **A** | **T** | **C** | **G** | **A** | **A** | **A** | **A** | **G** | **A** | **G** | **C** | **C** | **A** | **A** | **C** | **T** | **G** | **G** | **A** | **A** | **C** | **T** | **T** | **T** | **A** | **T** | **C** | **A** | **A** | **G** | **G** | **G** | **A** | **A** | **T** | **T** | **T** | **C** | **T** | **C** | **T** | **A** | **T** | **C** | **T** | **A** | **T** | **G** | **A** | **A** | **G** | **A** | **A** | **C** | **A** | **G** | **A** | **A** | **A** | **A** | **A** | **A** | **C** | **T** | **T** | **C** | **G** | **T** | **G** | **A** | **T** | **G** | **A** | **A** | **T** | **T** | **T** | **G** | **A** | **A** | **A** | **T** | **A** | **G** | **C** | **T** | **A** | **A** | **A** | **A** | **A** | **T** | **G** | **A** | **A** | **A** | **A** | **A** | **T** | **T** | **G** | **A** | **A** | **A** | **A** | **A** | **A** | **G** | **A** | **A** | **G** | **T** | **C** | **A** | **G** | **C** | **A** | **G** | **G** | **C** | **T** | **A** | **A** | **A** | **G** | **A** | **A** | **A** | **A** | **C** | **A** | **G** | **C** | **A** | **G** | **C** | **T** | **G** | **A** | **A** | **A** | **A** | **A** | **G** | **C** | **A** | **G** | **A** | **A** | **T** | **G** | **G** | **T** | **C** | **T** | **C** | **G** | **T** | **T** | **C** | **C** | **C** | **G** | **A** | **G** | **A** | **A** | **G** | **G** | **A** | **G** | **A** | **C** | **A** | **A** | **A** | **A** | **C** | **A** | **A** | **A** | **G** | **A** | **A** | **A** | **A** | **A** | **A** | **G** | **T** | **C** | **G** | **G** | **A** | **T** | **T** | **C** | **A** | **T** | **C** | **G** | **A** | **T** | **A** | **C** | **G** | **G** | **A** | **A** | **T** | **C** | **T** | **A** | **G** | **G** | **C** | **G** | **A** | **G** | **T** | **G** | **A** | **A** | **T** | **A** | **A** | **A** | **G** | **G** | **A** | **G** | **C** | **A** | **G** | **T** | **A** | **G** | **G** | **C** | **G** | **C** | **T** | **G** | **A** | **T** | **G** | **C** | **T** | **G** | **C** | **A** | **C** | **G** | **G** | **A** | **C** | **G** | **A** | **T** | **G** | **A** | **A** | **A** | **C** | **G** | **A** | **T** | **C** | **T** | **A** | **A** | **A** | **G** | **C** | **A** | **A** | **T** | **C** | **G** | **T** | **G** | **A** | **A** | **T** | **C** | **G** | **G** | **A** | **T** | **G** | **G** | **A** | **A** | **A** | **C** | **C** | **C** | **A** | **G** | **A** | **T** | **C** | **A** | **G** | **T** | **G** | **A** | **G** | **A** | **A** | **A** | **G** | **A** | **A** | **A** | **A** | **A** | **T** | **T** | **A** | **T** | **T** | **A** | **A** | **A** | **A** | **G** | **A** | **T** | **A** | **T** | **C** | **G** | **A** | **G** | **T** | **A** | **T** | **A** | **T** | **C** | **G** | **A** | **T** | **G** | **C** | **T** | **T** | **T** | **G** | **A** | **C** | **G** | **A** | **T** | **G** | **A** | **A** | **T** | **A** | **G** | **C** | **C** | **A** | **A** | **G** | **T** | **G** | **T** | **C** | **T** | **C** | **A** | **T** | **C** | **A** | **T** | **A** | **A** | **A** | **C** | **G** | **G** | **C** | **T** | **G** | **T** | **T** | **A** | **A** | **G** | **C** | **G** | **T** | **A** | **G** | **A** | **A** | **A** | **A** | **T** | **C** | **T** | **T** | **C** | **A** | **A** | **T** | **T** | **A** | **G** | **G** | **G** | **T** | **A** | **T** | **G** | **A** | **A** | **A** | **A** | **T** | **C** | **T** | **G** | **T** | **T** | **A** | **T** | **T** | **C** | **G** | **A** | **G** | **C** | **C** | **A** | **G** | **T** | **T** | **C** | **A** | **T** | **T** | **T** | **T** | **A** | **C** | **G** | **A** | **T** | **C** | **G** | **A** | **A** | **C** | **C** | **T** | **C** | **A** | **T** | **C** | **A** | **G** | **C** | **G** | **G** | **G** | **T** | **G** | **G** | **C** | **A** | **A** | **T** | **T** | **T** | **C** | **A** | **G** | **G** | **T** | **C** | **C** | **T** | **A** | **A** | **C** | **G** | **G** | **C** | **G** | **T** | **G** | **G** | **G** | **A** | **A** | **A** | **G** | **T** | **C** | **A** | **T** | **C** | **C** | **A** | **T** | **T** | **A** | **T** | **C** | **C** | **A** | **T** | **T** | **A** | **T** | **C** | **T** | **T** | **C** | **T** | **G** | **G** | **G** | **G** | **A** | **C** | **A** | **T** | **T** | **C** | **A** | **A** | **C** | **G** | **G** | **C** | **A** | **A** | **G** | **G** | **T** | **T** | **G** | **T** | **A** | **G** | **G** | **A** | **G** | **A** | **A** | **A** | **A** | **A** | **T** | **C** | **C** | **C** | **A** | **G** | **C** | **C** | **C** | **A** | **A** | **A** | **C** | **A** | **T** | **T** | **T** | **G** | **A** | **C** | **C** | **A** | **T** | **T** | **A** | **G** | **T** | **T** | **A** | **C** | **G** | **T** | **A** | **C** | **G** | **C** | **C** | **A** | **A** | **A** | **A** | **T** | **T** | **A** | **T** | **G** | **A** | **A** | **G** | **A** | **C** | **A** | **A** | **T** | **C** | **G** | **A** | **G** | **G** | **G** | **A** | **C** | **A** | **T** | **T** | **G** | **G** | **C** | **G** | **G** | **A** | **A** | **T** | **T** | **C** | **G** | **C** | **G** | **G** | **A** | **G** | **A** | **A** | **A** | **A** | **A** | **C** | **C** | **G** | **A** | **G** | **T** | **A** | **G** | **A** | **C** | **T** | **A** | **T** | **C** | **A** | **A** | **G** | **C** | **A** | **T** | **T** | **T** | **T** | **T** | **G** | **A** | **A** | **T** | **A** | **A** | **T** | **C** | **T** | **C** | **C** | **G** | **A** | **A** | **A** | **G** | **C** | **T** | **T** | **G** | **G** | **G** | **A** | **T** | **G** | **G** | **A** | **A** | **A** | **G** | **A** | **G** | **A** | **T** | **G** | **T** | **T** | **T** | **T** | **T** | **C** | **A** | **T** | **A** | **A** | **T** | **A** | **A** | **A** | **A** | **T** | **C** | **G** | **A** | **A** | **C** | **A** | **G** | **A** | **T** | **G** | **A** | **G** | **T** | **A** | **T** | **G** | **G** | **G** | **A** | **C** | **A** | **A** | **C** | **G** | **G** | **A** | **A** | **A** | **A** | **A** | **A** | **G** | **T** | **G** | **G** | **A** | **A** | **T** | **T** | **G** | **G** | **C** | **T** | **A** | **A** | **A** | **T** | **C** | **T** | **T** | **T** | **A** | **T** | **C** | **C** | **C** | **A** | **A** | **C** | **C** | **A** | **G** | **C** | **T** | **G** | **A** | **G** | **C** | **T** | **C** | **T** | **A** | **T** | **A** | **T** | **A** | **T** | **G** | **G** | **G** | **A** | **T** | **G** | **A** | **A** | **C** | **C** | **A** | **T** | **T** | **G** | **A** | **A** | **T** | **T** | **A** | **T** | **T** | **T** | **G** | **G** | **A** | **T** | **G** | **T** | **C** | **T** | **T** | **C** | **A** | **A** | **T** | **C** | **A** | **A** | **G** | **A** | **A** | **C** | **A** | **A** | **T** | **T** | **A** | **G** | **A** | **A** | **C** | **A** | **A** | **C** | **T** | **G** | **A** | **T** | **T** | **T** | **T** | **G** | **A** | **A** | **C** | **G** | **T** | **G** | **A** | **A** | **A** | **C** | **C** | **T** | **G** | **C** | **T** | **A** | **T** | **G** | **T** | **T** | **A** | **C** | **T** | **A** | **G** | **T** | **G** | **G** | **A** | **A** | **C** | **A** | **T** | **G** | **A** | **T** | **C** | **A** | **A** | **A** | **C** | **C** | **T** | **T** | **T** | **C** | **T** | **A** | **G** | **A** | **T** | **A** | **A** | **A** | **G** | **T** | **A** | **T** | **C** | **T** | **A** | **C** | **T** | **G** | **A** | **G** | **A** | **T** | **T** | **A** | **T** | **T** | **T** | **C** | **T** | **C** | **T** | **T** | **G** | **A** | **G** | **A** | **A** | **A** | **A** | **T** | **C** | **-** | **-** | **-** |
| **N52649** | **A** | **T** | **G** | **T** | **C** | **T** | **A** | **A** | **A** | **A** | **T** | **C** | **G** | **A** | **A** | **A** | **T** | **A** | **A** | **A** | **A** | **A** | **A** | **T** | **C** | **T** | **G** | **A** | **C** | **A** | **T** | **T** | **C** | **G** | **G** | **C** | **T** | **A** | **C** | **G** | **A** | **C** | **A** | **G** | **C** | **C** | **A** | **A** | **G** | **G** | **C** | **A** | **C** | **A** | **T** | **T** | **A** | **T** | **T** | **A** | **T** | **T** | **T** | **G** | **A** | **A** | **C** | **A** | **A** | **G** | **C** | **A** | **A** | **A** | **T** | **C** | **T** | **A** | **A** | **A** | **T** | **T** | **T** | **T** | **G** | **A** | **C** | **A** | **C** | **A** | **C** | **A** | **A** | **T** | **G** | **G** | **A** | **A** | **A** | **C** | **T** | **A** | **G** | **G** | **A** | **C** | **T** | **T** | **A** | **T** | **C** | **G** | **G** | **A** | **C** | **G** | **A** | **A** | **A** | **C** | **G** | **G** | **T** | **C** | **G** | **A** | **G** | **G** | **A** | **A** | **A** | **G** | **A** | **C** | **A** | **A** | **C** | **T** | **T** | **T** | **A** | **C** | **T** | **G** | **A** | **A** | **T** | **A** | **T** | **T** | **C** | **T** | **A** | **C** | **A** | **A** | **A** | **A** | **C** | **A** | **A** | **A** | **C** | **T** | **A** | **C** | **C** | **T** | **T** | **A** | **T** | **C** | **A** | **A** | **G** | **G** | **G** | **C** | **A** | **A** | **G** | **T** | **A** | **A** | **T** | **C** | **C** | **A** | **T** | **C** | **A** | **G** | **C** | **A** | **A** | **G** | **A** | **A** | **T** | **T** | **T** | **G** | **T** | **C** | **T** | **A** | **T** | **T** | **T** | **C** | **C** | **C** | **G** | **C** | **A** | **A** | **C** | **A** | **G** | **A** | **C** | **A** | **A** | **A** | **A** | **G** | **A** | **T** | **A** | **A** | **A** | **G** | **A** | **A** | **C** | **G** | **T** | **T** | **T** | **A** | **A** | **C** | **C** | **T** | **A** | **T** | **T** | **A** | **C** | **G** | **T** | **G** | **T** | **T** | **A** | **A** | **A** | **T** | **G** | **A** | **T** | **A** | **T** | **T** | **A** | **C** | **G** | **G** | **A** | **T** | **T** | **T** | **T** | **G** | **A** | **G** | **A** | **T** | **A** | **T** | **G** | **G** | **G** | **A** | **A** | **A** | **T** | **C** | **G** | **A** | **A** | **A** | **G** | **A** | **G** | **A** | **G** | **C** | **T** | **C** | **C** | **A** | **A** | **T** | **T** | **G** | **A** | **T** | **G** | **C** | **A** | **A** | **A** | **C** | **A** | **G** | **A** | **T** | **C** | **C** | **T** | **G** | **A** | **A** | **A** | **T** | **C** | **T** | **T** | **A** | **T** | **G** | **G** | **A** | **G** | **A** | **G** | **A** | **A** | **T** | **T** | **C** | **A** | **G** | **C** | **A** | **C** | **A** | **C** | **T** | **A** | **T** | **C** | **G** | **G** | **G** | **G** | **G** | **G** | **A** | **G** | **A** | **G** | **A** | **A** | **G** | **A** | **C** | **A** | **A** | **A** | **A** | **G** | **T** | **C** | **C** | **T** | **A** | **C** | **T** | **G** | **G** | **C** | **A** | **C** | **T** | **T** | **T** | **T** | **A** | **T** | **T** | **T** | **G** | **T** | **G** | **G** | **A** | **T** | **G** | **A** | **C** | **A** | **C** | **T** | **C** | **A** | **T** | **T** | **T** | **C** | **C** | **C** | **G** | **T** | **T** | **A** | **A** | **T** | **C** | **G** | **A** | **T** | **G** | **A** | **A** | **C** | **C** | **A** | **A** | **C** | **G** | **A** | **A** | **T** | **C** | **A** | **T** | **T** | **T** | **G** | **G** | **A** | **-** | **-** | **-** | **T** | **A** | **T** | **C** | **T** | **C** | **T** | **G** | **G** | **T** | **A** | **G** | **A** | **A** | **A** | **A** | **C** | **A** | **A** | **G** | **T** | **A** | **G** | **C** | **G** | **G** | **C** | **T** | **T** | **A** | **T** | **T** | **T** | **G** | **A** | **A** | **A** | **A** | **A** | **G** | **A** | **A** | **A** | **A** | **A** | **A** | **C** | **A** | **A** | **G** | **G** | **C** | **T** | **T** | **C** | **A** | **T** | **C** | **G** | **T** | **G** | **G** | **T** | **C** | **A** | **G** | **C** | **C** | **A** | **T** | **G** | **A** | **C** | **C** | **G** | **G** | **G** | **G** | **A** | **T** | **T** | **T** | **A** | **T** | **C** | **G** | **A** | **T** | **G** | **A** | **A** | **G** | **T** | **A** | **G** | **T** | **G** | **G** | **A** | **C** | **C** | **A** | **T** | **G** | **T** | **T** | **T** | **T** | **A** | **G** | **C** | **A** | **A** | **T** | **C** | **G** | **A** | **A** | **A** | **A** | **A** | **A** | **G** | **T** | **C** | **A** | **A** | **C** | **T** | **G** | **G** | **A** | **A** | **C** | **T** | **T** | **T** | **A** | **T** | **C** | **A** | **A** | **G** | **G** | **G** | **A** | **A** | **T** | **T** | **T** | **C** | **T** | **C** | **T** | **A** | **T** | **C** | **T** | **A** | **T** | **G** | **A** | **A** | **G** | **A** | **A** | **C** | **A** | **G** | **A** | **A** | **A** | **A** | **A** | **A** | **C** | **T** | **T** | **C** | **G** | **T** | **G** | **A** | **T** | **G** | **A** | **A** | **T** | **T** | **T** | **G** | **A** | **A** | **A** | **T** | **G** | **G** | **C** | **T** | **C** | **A** | **A** | **A** | **A** | **T** | **G** | **A** | **A** | **A** | **A** | **A** | **T** | **T** | **G** | **A** | **A** | **A** | **A** | **A** | **A** | **G** | **A** | **A** | **G** | **T** | **C** | **A** | **G** | **T** | **A** | **G** | **G** | **C** | **T** | **A** | **A** | **A** | **G** | **A** | **A** | **A** | **A** | **C** | **A** | **G** | **C** | **A** | **G** | **C** | **T** | **G** | **A** | **A** | **A** | **A** | **A** | **G** | **C** | **C** | **G** | **A** | **A** | **T** | **G** | **G** | **T** | **C** | **T** | **C** | **G** | **T** | **T** | **C** | **C** | **C** | **G** | **A** | **G** | **A** | **A** | **G** | **G** | **A** | **G** | **A** | **T** | **A** | **A** | **A** | **A** | **C** | **A** | **A** | **A** | **G** | **A** | **A** | **A** | **C** | **A** | **A** | **G** | **T** | **C** | **G** | **G** | **A** | **T** | **T** | **C** | **A** | **T** | **C** | **G** | **A** | **T** | **A** | **C** | **T** | **G** | **A** | **A** | **T** | **C** | **T** | **A** | **G** | **A** | **C** | **G** | **A** | **G** | **T** | **G** | **A** | **A** | **T** | **A** | **A** | **A** | **G** | **G** | **A** | **G** | **C** | **A** | **G** | **T** | **G** | **G** | **G** | **T** | **G** | **C** | **T** | **G** | **A** | **T** | **G** | **C** | **T** | **G** | **C** | **A** | **C** | **G** | **G** | **A** | **C** | **G** | **A** | **T** | **G** | **A** | **A** | **A** | **C** | **G** | **A** | **T** | **C** | **C** | **A** | **A** | **A** | **G** | **C** | **A** | **A** | **T** | **C** | **G** | **T** | **G** | **A** | **A** | **T** | **C** | **G** | **G** | **A** | **T** | **G** | **G** | **A** | **G** | **A** | **C** | **C** | **C** | **A** | **G** | **A** | **T** | **C** | **A** | **G** | **C** | **G** | **A** | **G** | **A** | **A** | **A** | **G** | **A** | **A** | **A** | **A** | **A** | **C** | **T** | **A** | **T** | **T** | **A** | **A** | **A** | **A** | **G** | **A** | **T** | **A** | **T** | **C** | **G** | **A** | **A** | **T** | **A** | **T** | **A** | **T** | **C** | **G** | **A** | **T** | **T** | **C** | **T** | **T** | **T** | **G** | **A** | **C** | **G** | **A** | **T** | **G** | **A** | **A** | **T** | **A** | **G** | **C** | **C** | **A** | **A** | **G** | **C** | **G** | **T** | **C** | **T** | **C** | **A** | **C** | **C** | **A** | **T** | **A** | **A** | **G** | **C** | **G** | **A** | **C** | **T** | **T** | **T** | **T** | **A** | **A** | **G** | **C** | **G** | **T** | **A** | **G** | **A** | **A** | **G** | **A** | **T** | **C** | **T** | **T** | **C** | **A** | **A** | **T** | **T** | **A** | **G** | **G** | **G** | **T** | **A** | **T** | **G** | **A** | **A** | **A** | **A** | **T** | **C** | **T** | **G** | **T** | **T** | **A** | **T** | **T** | **C** | **G** | **A** | **G** | **C** | **C** | **A** | **A** | **T** | **T** | **C** | **A** | **T** | **T** | **T** | **T** | **A** | **C** | **A** | **A** | **T** | **C** | **G** | **A** | **G** | **C** | **C** | **T** | **C** | **A** | **T** | **C** | **A** | **G** | **C** | **G** | **G** | **G** | **T** | **G** | **G** | **C** | **G** | **A** | **T** | **T** | **T** | **C** | **A** | **G** | **G** | **T** | **C** | **C** | **T** | **A** | **A** | **C** | **G** | **G** | **T** | **G** | **C** | **A** | **G** | **G** | **A** | **A** | **A** | **G** | **T** | **C** | **A** | **T** | **C** | **C** | **A** | **T** | **T** | **A** | **T** | **C** | **C** | **A** | **T** | **T** | **A** | **T** | **C** | **T** | **T** | **C** | **T** | **G** | **G** | **G** | **G** | **G** | **C** | **A** | **T** | **T** | **C** | **A** | **A** | **C** | **G** | **G** | **C** | **A** | **A** | **G** | **G** | **T** | **T** | **A** | **T** | **A** | **G** | **G** | **A** | **G** | **A** | **A** | **A** | **A** | **A** | **T** | **C** | **C** | **C** | **A** | **G** | **C** | **C** | **A** | **A** | **A** | **A** | **C** | **A** | **T** | **C** | **T** | **G** | **A** | **G** | **C** | **A** | **T** | **T** | **A** | **G** | **T** | **T** | **A** | **T** | **G** | **T** | **A** | **C** | **G** | **C** | **C** | **A** | **A** | **A** | **A** | **T** | **T** | **A** | **T** | **G** | **A** | **A** | **G** | **A** | **C** | **A** | **A** | **T** | **C** | **G** | **A** | **G** | **G** | **A** | **A** | **C** | **G** | **T** | **T** | **G** | **G** | **C** | **G** | **G** | **A** | **A** | **T** | **T** | **T** | **G** | **C** | **A** | **G** | **A** | **G** | **A** | **A** | **A** | **A** | **A** | **C** | **C** | **A** | **A** | **G** | **T** | **A** | **G** | **A** | **C** | **T** | **A** | **C** | **C** | **A** | **A** | **G** | **C** | **A** | **T** | **T** | **T** | **T** | **T** | **G** | **A** | **A** | **C** | **A** | **A** | **C** | **C** | **T** | **C** | **C** | **G** | **A** | **A** | **A** | **G** | **C** | **T** | **T** | **G** | **G** | **G** | **A** | **T** | **G** | **G** | **A** | **A** | **A** | **G** | **A** | **G** | **A** | **T** | **G** | **T** | **T** | **T** | **T** | **T** | **C** | **A** | **T** | **A** | **A** | **C** | **A** | **A** | **G** | **A** | **T** | **T** | **G** | **A** | **G** | **C** | **A** | **G** | **A** | **T** | **G** | **A** | **G** | **T** | **A** | **T** | **G** | **G** | **G** | **C** | **C** | **A** | **A** | **C** | **G** | **G** | **A** | **A** | **A** | **A** | **A** | **A** | **G** | **T** | **G** | **G** | **A** | **A** | **T** | **T** | **G** | **G** | **C** | **T** | **A** | **A** | **A** | **T** | **C** | **T** | **T** | **T** | **A** | **T** | **C** | **A** | **C** | **A** | **G** | **C** | **C** | **A** | **G** | **C** | **T** | **G** | **A** | **A** | **C** | **T** | **A** | **T** | **A** | **T** | **A** | **T** | **A** | **T** | **G** | **G** | **G** | **A** | **T** | **G** | **A** | **A** | **C** | **C** | **A** | **T** | **T** | **G** | **A** | **A** | **T** | **T** | **A** | **T** | **T** | **T** | **G** | **G** | **A** | **T** | **G** | **T** | **C** | **T** | **T** | **C** | **A** | **A** | **T** | **C** | **A** | **A** | **G** | **A** | **A** | **C** | **A** | **A** | **T** | **T** | **A** | **G** | **A** | **A** | **C** | **A** | **A** | **C** | **T** | **G** | **A** | **T** | **C** | **T** | **T** | **G** | **A** | **A** | **C** | **G** | **T** | **G** | **A** | **A** | **A** | **C** | **C** | **T** | **G** | **C** | **C** | **A** | **T** | **G** | **T** | **T** | **A** | **C** | **T** | **G** | **G** | **T** | **G** | **G** | **A** | **A** | **C** | **A** | **T** | **G** | **A** | **T** | **C** | **A** | **A** | **A** | **C** | **C** | **T** | **T** | **C** | **C** | **T** | **G** | **G** | **A** | **T** | **A** | **A** | **A** | **G** | **T** | **A** | **T** | **C** | **T** | **A** | **C** | **T** | **G** | **A** | **G** | **A** | **T** | **T** | **A** | **T** | **T** | **T** | **C** | **T** | **C** | **T** | **T** | **G** | **A** | **G** | **A** | **A** | **A** | **A** | **T** | **C** | **-** | **-** | **-** |
| **N59503F** | **A** | **T** | **G** | **T** | **C** | **T** | **A** | **A** | **A** | **A** | **T** | **C** | **G** | **A** | **A** | **A** | **T** | **A** | **A** | **A** | **A** | **A** | **A** | **T** | **C** | **T** | **G** | **A** | **C** | **A** | **T** | **T** | **C** | **G** | **G** | **C** | **T** | **A** | **C** | **G** | **A** | **C** | **A** | **G** | **C** | **C** | **A** | **A** | **G** | **G** | **C** | **A** | **C** | **A** | **T** | **T** | **A** | **T** | **T** | **A** | **T** | **T** | **T** | **G** | **A** | **A** | **C** | **A** | **A** | **G** | **C** | **A** | **A** | **A** | **T** | **C** | **T** | **A** | **A** | **A** | **T** | **T** | **T** | **T** | **G** | **A** | **C** | **A** | **C** | **A** | **C** | **A** | **A** | **T** | **G** | **G** | **A** | **A** | **A** | **C** | **T** | **A** | **G** | **G** | **G** | **A** | **T** | **T** | **A** | **T** | **T** | **G** | **G** | **A** | **C** | **G** | **A** | **A** | **A** | **C** | **G** | **G** | **T** | **C** | **G** | **A** | **G** | **G** | **A** | **A** | **A** | **G** | **A** | **C** | **A** | **A** | **C** | **T** | **T** | **T** | **A** | **C** | **T** | **G** | **A** | **A** | **T** | **A** | **T** | **T** | **C** | **T** | **A** | **C** | **A** | **A** | **A** | **A** | **C** | **A** | **A** | **A** | **C** | **T** | **A** | **C** | **C** | **T** | **T** | **A** | **T** | **C** | **A** | **A** | **G** | **G** | **G** | **C** | **A** | **A** | **G** | **T** | **A** | **A** | **T** | **C** | **C** | **A** | **T** | **C** | **A** | **G** | **C** | **A** | **A** | **G** | **A** | **A** | **T** | **T** | **T** | **G** | **T** | **C** | **T** | **A** | **T** | **T** | **T** | **C** | **C** | **C** | **G** | **C** | **A** | **A** | **C** | **A** | **G** | **A** | **C** | **A** | **A** | **A** | **A** | **G** | **A** | **T** | **A** | **A** | **A** | **G** | **A** | **A** | **C** | **G** | **T** | **T** | **T** | **A** | **A** | **C** | **C** | **T** | **A** | **T** | **T** | **A** | **C** | **G** | **T** | **G** | **T** | **T** | **A** | **A** | **A** | **T** | **G** | **A** | **T** | **A** | **T** | **T** | **A** | **C** | **G** | **G** | **A** | **T** | **T** | **T** | **T** | **G** | **A** | **G** | **A** | **T** | **A** | **T** | **G** | **G** | **G** | **A** | **A** | **A** | **T** | **C** | **G** | **A** | **A** | **A** | **G** | **A** | **G** | **A** | **G** | **C** | **T** | **C** | **C** | **A** | **A** | **T** | **T** | **G** | **A** | **T** | **G** | **C** | **A** | **A** | **A** | **C** | **A** | **G** | **A** | **T** | **C** | **C** | **T** | **G** | **A** | **A** | **A** | **T** | **C** | **T** | **T** | **A** | **T** | **G** | **G** | **A** | **G** | **A** | **G** | **A** | **A** | **T** | **T** | **C** | **A** | **G** | **C** | **A** | **C** | **A** | **C** | **T** | **A** | **T** | **C** | **G** | **G** | **G** | **G** | **G** | **G** | **A** | **G** | **A** | **G** | **A** | **A** | **G** | **A** | **C** | **A** | **A** | **A** | **A** | **G** | **T** | **C** | **C** | **T** | **A** | **C** | **T** | **G** | **G** | **C** | **A** | **C** | **T** | **T** | **T** | **T** | **A** | **T** | **T** | **T** | **G** | **T** | **G** | **G** | **A** | **T** | **G** | **A** | **C** | **A** | **C** | **T** | **C** | **A** | **T** | **T** | **T** | **C** | **C** | **C** | **G** | **T** | **T** | **A** | **A** | **T** | **C** | **G** | **A** | **T** | **G** | **A** | **A** | **C** | **C** | **A** | **A** | **C** | **G** | **A** | **A** | **T** | **C** | **A** | **T** | **T** | **T** | **G** | **G** | **A** | **-** | **-** | **-** | **T** | **A** | **T** | **C** | **T** | **C** | **T** | **G** | **G** | **T** | **A** | **G** | **A** | **A** | **A** | **A** | **C** | **A** | **A** | **G** | **T** | **A** | **G** | **C** | **G** | **G** | **C** | **T** | **T** | **A** | **T** | **T** | **T** | **G** | **A** | **A** | **A** | **A** | **A** | **G** | **A** | **A** | **A** | **A** | **A** | **A** | **C** | **A** | **A** | **G** | **G** | **C** | **T** | **T** | **C** | **A** | **T** | **C** | **G** | **T** | **G** | **G** | **T** | **C** | **A** | **G** | **C** | **C** | **A** | **T** | **G** | **A** | **C** | **C** | **G** | **G** | **G** | **G** | **A** | **T** | **T** | **T** | **A** | **T** | **C** | **G** | **A** | **T** | **G** | **A** | **A** | **G** | **T** | **A** | **G** | **T** | **G** | **G** | **A** | **C** | **C** | **A** | **T** | **G** | **T** | **T** | **T** | **T** | **A** | **G** | **C** | **A** | **A** | **T** | **C** | **G** | **A** | **A** | **A** | **A** | **A** | **A** | **G** | **T** | **C** | **A** | **A** | **C** | **T** | **G** | **G** | **A** | **A** | **C** | **T** | **T** | **T** | **A** | **T** | **C** | **A** | **A** | **G** | **G** | **G** | **A** | **A** | **T** | **T** | **T** | **C** | **T** | **C** | **T** | **A** | **T** | **C** | **T** | **A** | **T** | **G** | **A** | **A** | **G** | **A** | **A** | **C** | **A** | **G** | **A** | **A** | **A** | **A** | **A** | **A** | **C** | **T** | **T** | **C** | **G** | **T** | **G** | **A** | **T** | **G** | **A** | **A** | **T** | **T** | **T** | **G** | **A** | **A** | **A** | **T** | **G** | **G** | **C** | **T** | **C** | **A** | **A** | **A** | **A** | **T** | **G** | **A** | **A** | **A** | **A** | **A** | **T** | **T** | **G** | **A** | **A** | **A** | **A** | **A** | **A** | **G** | **A** | **A** | **G** | **T** | **C** | **A** | **G** | **T** | **A** | **G** | **G** | **C** | **T** | **A** | **A** | **A** | **G** | **A** | **A** | **A** | **A** | **C** | **A** | **G** | **C** | **A** | **G** | **C** | **T** | **G** | **A** | **A** | **A** | **A** | **A** | **G** | **C** | **C** | **G** | **A** | **A** | **T** | **G** | **G** | **T** | **C** | **T** | **C** | **G** | **T** | **T** | **C** | **C** | **C** | **G** | **A** | **G** | **A** | **A** | **G** | **G** | **A** | **G** | **A** | **T** | **A** | **A** | **A** | **A** | **C** | **A** | **A** | **A** | **G** | **A** | **A** | **A** | **C** | **A** | **A** | **G** | **T** | **C** | **G** | **G** | **A** | **T** | **T** | **C** | **A** | **T** | **C** | **G** | **A** | **T** | **A** | **C** | **T** | **G** | **A** | **A** | **T** | **C** | **T** | **A** | **G** | **A** | **C** | **G** | **A** | **G** | **T** | **G** | **A** | **A** | **T** | **A** | **A** | **A** | **G** | **G** | **A** | **G** | **C** | **A** | **G** | **T** | **G** | **G** | **G** | **T** | **G** | **C** | **T** | **G** | **A** | **T** | **G** | **C** | **T** | **G** | **C** | **A** | **C** | **G** | **G** | **A** | **C** | **G** | **A** | **T** | **G** | **A** | **A** | **A** | **C** | **G** | **A** | **T** | **C** | **C** | **A** | **A** | **A** | **G** | **C** | **A** | **A** | **T** | **C** | **G** | **T** | **G** | **A** | **A** | **T** | **C** | **G** | **G** | **A** | **T** | **A** | **G** | **A** | **G** | **A** | **C** | **C** | **C** | **A** | **G** | **A** | **T** | **C** | **A** | **G** | **C** | **G** | **A** | **G** | **A** | **A** | **A** | **G** | **A** | **A** | **A** | **A** | **A** | **C** | **T** | **A** | **T** | **T** | **A** | **A** | **A** | **A** | **G** | **A** | **T** | **A** | **T** | **C** | **G** | **A** | **A** | **T** | **A** | **T** | **A** | **T** | **C** | **G** | **A** | **T** | **T** | **C** | **T** | **T** | **T** | **G** | **A** | **C** | **G** | **A** | **T** | **G** | **A** | **A** | **T** | **A** | **G** | **C** | **C** | **A** | **A** | **G** | **C** | **G** | **T** | **C** | **T** | **C** | **A** | **C** | **C** | **A** | **T** | **A** | **A** | **G** | **C** | **G** | **A** | **C** | **T** | **T** | **T** | **T** | **A** | **A** | **G** | **C** | **G** | **T** | **A** | **G** | **A** | **A** | **G** | **A** | **T** | **C** | **T** | **T** | **C** | **A** | **A** | **T** | **T** | **A** | **G** | **G** | **G** | **T** | **A** | **T** | **G** | **A** | **A** | **A** | **A** | **T** | **C** | **T** | **G** | **T** | **T** | **A** | **T** | **T** | **C** | **G** | **A** | **G** | **C** | **C** | **A** | **A** | **T** | **T** | **C** | **A** | **T** | **T** | **T** | **T** | **A** | **C** | **A** | **A** | **T** | **C** | **G** | **A** | **G** | **C** | **C** | **T** | **C** | **A** | **T** | **C** | **A** | **G** | **C** | **G** | **G** | **G** | **T** | **G** | **G** | **C** | **G** | **A** | **T** | **T** | **T** | **C** | **A** | **G** | **G** | **T** | **C** | **C** | **T** | **A** | **A** | **C** | **G** | **G** | **T** | **G** | **C** | **A** | **G** | **G** | **A** | **A** | **A** | **G** | **T** | **C** | **A** | **T** | **C** | **C** | **A** | **T** | **T** | **A** | **T** | **C** | **C** | **A** | **T** | **T** | **A** | **T** | **C** | **T** | **T** | **C** | **T** | **G** | **G** | **G** | **G** | **G** | **C** | **A** | **T** | **T** | **C** | **A** | **A** | **C** | **G** | **G** | **C** | **A** | **A** | **G** | **G** | **T** | **T** | **A** | **T** | **A** | **G** | **G** | **A** | **G** | **A** | **A** | **A** | **A** | **A** | **T** | **C** | **C** | **C** | **A** | **G** | **C** | **C** | **A** | **A** | **A** | **A** | **C** | **A** | **T** | **C** | **T** | **G** | **A** | **G** | **C** | **A** | **T** | **T** | **A** | **G** | **T** | **T** | **A** | **T** | **G** | **T** | **A** | **C** | **G** | **C** | **C** | **A** | **A** | **A** | **A** | **T** | **T** | **A** | **T** | **G** | **A** | **A** | **G** | **A** | **C** | **A** | **A** | **T** | **C** | **G** | **A** | **G** | **G** | **A** | **A** | **C** | **G** | **T** | **T** | **G** | **G** | **C** | **G** | **G** | **A** | **A** | **T** | **T** | **T** | **G** | **C** | **A** | **G** | **A** | **G** | **A** | **A** | **A** | **A** | **A** | **C** | **C** | **A** | **A** | **G** | **T** | **A** | **G** | **A** | **C** | **T** | **A** | **C** | **C** | **A** | **A** | **G** | **C** | **A** | **T** | **T** | **T** | **T** | **T** | **G** | **A** | **A** | **C** | **A** | **A** | **C** | **C** | **T** | **C** | **C** | **G** | **A** | **A** | **A** | **G** | **C** | **T** | **T** | **G** | **G** | **G** | **A** | **T** | **G** | **G** | **A** | **A** | **A** | **G** | **A** | **G** | **A** | **T** | **G** | **T** | **T** | **T** | **T** | **T** | **C** | **A** | **T** | **A** | **A** | **C** | **A** | **A** | **G** | **A** | **T** | **T** | **G** | **A** | **G** | **C** | **A** | **G** | **A** | **T** | **G** | **A** | **G** | **T** | **A** | **T** | **G** | **G** | **G** | **C** | **C** | **A** | **A** | **C** | **G** | **G** | **A** | **A** | **A** | **A** | **A** | **A** | **G** | **T** | **G** | **G** | **A** | **A** | **T** | **T** | **G** | **G** | **C** | **T** | **A** | **A** | **A** | **T** | **C** | **T** | **T** | **T** | **A** | **T** | **C** | **A** | **C** | **A** | **G** | **C** | **C** | **A** | **G** | **C** | **T** | **G** | **A** | **A** | **C** | **T** | **A** | **T** | **A** | **T** | **A** | **T** | **A** | **T** | **G** | **G** | **G** | **A** | **T** | **G** | **A** | **A** | **C** | **C** | **A** | **T** | **T** | **G** | **A** | **A** | **T** | **T** | **A** | **T** | **T** | **T** | **G** | **G** | **A** | **T** | **G** | **T** | **C** | **T** | **T** | **C** | **A** | **A** | **T** | **C** | **A** | **A** | **G** | **A** | **A** | **C** | **A** | **A** | **T** | **T** | **A** | **G** | **A** | **A** | **C** | **A** | **A** | **C** | **T** | **G** | **A** | **T** | **C** | **T** | **T** | **G** | **A** | **A** | **C** | **G** | **T** | **G** | **A** | **A** | **A** | **C** | **C** | **T** | **G** | **C** | **C** | **A** | **T** | **G** | **T** | **T** | **A** | **C** | **T** | **G** | **G** | **T** | **G** | **G** | **A** | **A** | **C** | **A** | **T** | **G** | **A** | **T** | **C** | **A** | **A** | **A** | **C** | **C** | **T** | **T** | **C** | **C** | **T** | **G** | **G** | **A** | **T** | **A** | **A** | **A** | **G** | **T** | **A** | **T** | **C** | **T** | **A** | **C** | **T** | **G** | **A** | **G** | **A** | **T** | **T** | **A** | **T** | **T** | **T** | **C** | **T** | **C** | **T** | **T** | **G** | **A** | **G** | **A** | **A** | **A** | **A** | **T** | **C** | **-** | **-** | **-** |
| **N55337** | **A** | **T** | **G** | **T** | **C** | **T** | **A** | **A** | **A** | **A** | **T** | **C** | **G** | **A** | **A** | **A** | **T** | **A** | **A** | **A** | **A** | **A** | **A** | **T** | **C** | **T** | **G** | **A** | **C** | **A** | **T** | **T** | **C** | **G** | **G** | **C** | **T** | **A** | **C** | **G** | **A** | **C** | **A** | **G** | **C** | **C** | **A** | **A** | **G** | **G** | **C** | **A** | **C** | **A** | **T** | **T** | **A** | **T** | **T** | **A** | **T** | **T** | **T** | **G** | **A** | **A** | **C** | **A** | **A** | **G** | **C** | **A** | **A** | **A** | **T** | **C** | **T** | **A** | **A** | **A** | **T** | **T** | **T** | **T** | **G** | **A** | **C** | **A** | **C** | **A** | **C** | **A** | **A** | **T** | **G** | **G** | **A** | **A** | **A** | **C** | **T** | **A** | **G** | **G** | **A** | **C** | **T** | **T** | **A** | **T** | **C** | **G** | **G** | **A** | **C** | **G** | **A** | **A** | **A** | **C** | **G** | **G** | **T** | **C** | **G** | **A** | **G** | **G** | **A** | **A** | **A** | **G** | **A** | **C** | **A** | **A** | **C** | **T** | **T** | **T** | **A** | **C** | **T** | **G** | **A** | **A** | **T** | **A** | **T** | **T** | **C** | **T** | **A** | **C** | **A** | **A** | **A** | **A** | **C** | **A** | **A** | **A** | **C** | **T** | **A** | **C** | **C** | **T** | **T** | **A** | **T** | **C** | **A** | **A** | **G** | **G** | **G** | **C** | **A** | **A** | **G** | **T** | **A** | **A** | **T** | **C** | **C** | **A** | **T** | **C** | **A** | **G** | **C** | **A** | **A** | **G** | **A** | **A** | **T** | **T** | **T** | **G** | **T** | **C** | **T** | **A** | **T** | **T** | **T** | **C** | **C** | **C** | **G** | **C** | **A** | **G** | **C** | **A** | **A** | **A** | **C** | **A** | **A** | **A** | **A** | **G** | **A** | **T** | **A** | **A** | **A** | **G** | **A** | **G** | **C** | **G** | **T** | **T** | **T** | **A** | **A** | **C** | **C** | **T** | **A** | **T** | **T** | **A** | **T** | **G** | **T** | **G** | **T** | **T** | **A** | **A** | **A** | **T** | **G** | **A** | **G** | **A** | **T** | **T** | **A** | **C** | **G** | **G** | **A** | **T** | **T** | **T** | **T** | **G** | **A** | **G** | **A** | **T** | **A** | **T** | **G** | **G** | **G** | **A** | **A** | **A** | **T** | **T** | **G** | **A** | **A** | **A** | **G** | **A** | **G** | **A** | **G** | **C** | **T** | **C** | **C** | **A** | **A** | **C** | **T** | **G** | **A** | **T** | **G** | **C** | **A** | **A** | **A** | **C** | **A** | **G** | **A** | **T** | **C** | **C** | **T** | **G** | **A** | **A** | **A** | **T** | **C** | **T** | **T** | **A** | **T** | **G** | **G** | **A** | **G** | **A** | **G** | **A** | **A** | **T** | **T** | **C** | **A** | **G** | **C** | **A** | **C** | **A** | **C** | **T** | **A** | **T** | **C** | **A** | **G** | **G** | **A** | **G** | **G** | **A** | **G** | **A** | **G** | **A** | **A** | **G** | **A** | **C** | **A** | **A** | **A** | **A** | **G** | **T** | **C** | **C** | **T** | **A** | **C** | **T** | **G** | **G** | **C** | **A** | **C** | **T** | **T** | **T** | **T** | **A** | **T** | **T** | **T** | **G** | **T** | **G** | **G** | **A** | **T** | **G** | **A** | **C** | **A** | **C** | **T** | **C** | **A** | **T** | **T** | **T** | **C** | **C** | **C** | **G** | **T** | **T** | **A** | **A** | **T** | **C** | **G** | **A** | **T** | **G** | **A** | **A** | **C** | **C** | **A** | **A** | **C** | **G** | **A** | **A** | **T** | **C** | **A** | **T** | **T** | **T** | **G** | **G** | **A** | **-** | **-** | **-** | **T** | **A** | **T** | **C** | **T** | **C** | **T** | **G** | **G** | **C** | **A** | **G** | **A** | **A** | **A** | **A** | **C** | **A** | **A** | **G** | **T** | **A** | **G** | **C** | **G** | **G** | **C** | **T** | **T** | **A** | **C** | **T** | **T** | **G** | **A** | **A** | **A** | **A** | **A** | **G** | **A** | **A** | **A** | **A** | **A** | **A** | **C** | **A** | **A** | **G** | **G** | **C** | **T** | **T** | **C** | **A** | **T** | **C** | **G** | **T** | **G** | **G** | **T** | **C** | **A** | **G** | **C** | **C** | **A** | **T** | **G** | **A** | **C** | **C** | **G** | **G** | **G** | **G** | **A** | **T** | **T** | **T** | **A** | **T** | **C** | **G** | **A** | **T** | **G** | **A** | **A** | **G** | **T** | **A** | **G** | **T** | **G** | **G** | **A** | **C** | **C** | **A** | **T** | **G** | **T** | **T** | **T** | **T** | **A** | **G** | **C** | **A** | **A** | **T** | **C** | **G** | **A** | **A** | **A** | **A** | **A** | **A** | **G** | **T** | **C** | **A** | **A** | **C** | **T** | **G** | **G** | **A** | **A** | **C** | **T** | **T** | **T** | **A** | **T** | **C** | **A** | **A** | **G** | **G** | **G** | **A** | **A** | **T** | **T** | **T** | **C** | **T** | **C** | **T** | **A** | **T** | **C** | **T** | **A** | **T** | **G** | **A** | **A** | **G** | **A** | **A** | **C** | **A** | **G** | **A** | **A** | **A** | **A** | **A** | **A** | **C** | **T** | **T** | **C** | **G** | **T** | **G** | **A** | **T** | **G** | **A** | **A** | **T** | **T** | **T** | **G** | **A** | **A** | **A** | **T** | **G** | **G** | **C** | **T** | **C** | **A** | **A** | **A** | **A** | **T** | **G** | **A** | **A** | **A** | **A** | **A** | **T** | **T** | **G** | **A** | **A** | **A** | **A** | **A** | **A** | **G** | **A** | **A** | **G** | **T** | **C** | **A** | **G** | **T** | **A** | **G** | **G** | **C** | **T** | **A** | **A** | **A** | **G** | **A** | **A** | **A** | **A** | **C** | **A** | **G** | **C** | **A** | **G** | **C** | **T** | **G** | **A** | **A** | **A** | **A** | **A** | **G** | **C** | **C** | **G** | **A** | **A** | **T** | **G** | **G** | **T** | **C** | **T** | **C** | **G** | **T** | **T** | **C** | **C** | **C** | **G** | **A** | **G** | **A** | **A** | **G** | **G** | **A** | **G** | **A** | **T** | **A** | **A** | **A** | **A** | **C** | **A** | **A** | **A** | **G** | **A** | **A** | **A** | **C** | **A** | **A** | **G** | **T** | **C** | **G** | **G** | **A** | **T** | **T** | **C** | **A** | **T** | **C** | **G** | **A** | **T** | **A** | **C** | **T** | **G** | **A** | **A** | **T** | **C** | **T** | **A** | **G** | **A** | **C** | **G** | **A** | **G** | **T** | **G** | **A** | **A** | **T** | **A** | **A** | **A** | **G** | **G** | **A** | **G** | **C** | **A** | **G** | **T** | **G** | **G** | **G** | **T** | **G** | **C** | **T** | **G** | **A** | **T** | **G** | **C** | **T** | **G** | **C** | **A** | **C** | **G** | **G** | **A** | **C** | **G** | **A** | **T** | **G** | **A** | **A** | **A** | **C** | **G** | **A** | **T** | **C** | **C** | **A** | **A** | **A** | **G** | **C** | **A** | **A** | **T** | **C** | **G** | **T** | **G** | **A** | **A** | **T** | **C** | **G** | **G** | **A** | **T** | **G** | **G** | **A** | **G** | **A** | **C** | **C** | **C** | **A** | **G** | **A** | **T** | **C** | **A** | **G** | **C** | **G** | **A** | **G** | **A** | **A** | **A** | **G** | **A** | **A** | **A** | **A** | **A** | **C** | **T** | **A** | **T** | **T** | **G** | **A** | **A** | **A** | **G** | **A** | **T** | **A** | **T** | **C** | **G** | **A** | **A** | **T** | **A** | **T** | **A** | **T** | **C** | **G** | **A** | **T** | **T** | **C** | **T** | **T** | **T** | **G** | **A** | **C** | **G** | **A** | **T** | **G** | **A** | **A** | **T** | **A** | **G** | **C** | **C** | **A** | **A** | **G** | **C** | **G** | **T** | **C** | **T** | **C** | **A** | **C** | **C** | **A** | **T** | **A** | **A** | **G** | **C** | **G** | **A** | **C** | **T** | **T** | **T** | **T** | **A** | **A** | **G** | **C** | **G** | **T** | **A** | **G** | **A** | **A** | **G** | **A** | **T** | **C** | **T** | **T** | **C** | **A** | **A** | **T** | **T** | **A** | **G** | **G** | **G** | **T** | **A** | **T** | **G** | **A** | **G** | **A** | **A** | **T** | **C** | **T** | **G** | **T** | **T** | **A** | **T** | **T** | **C** | **G** | **A** | **G** | **C** | **C** | **A** | **A** | **T** | **T** | **C** | **A** | **T** | **T** | **T** | **T** | **A** | **C** | **A** | **A** | **T** | **C** | **G** | **A** | **G** | **C** | **C** | **T** | **C** | **A** | **T** | **C** | **A** | **G** | **C** | **G** | **G** | **G** | **T** | **G** | **G** | **C** | **G** | **A** | **T** | **T** | **T** | **C** | **A** | **G** | **G** | **T** | **C** | **C** | **T** | **A** | **A** | **C** | **G** | **G** | **T** | **G** | **C** | **A** | **G** | **G** | **A** | **A** | **A** | **G** | **T** | **C** | **A** | **T** | **C** | **C** | **A** | **T** | **T** | **A** | **T** | **C** | **C** | **A** | **T** | **T** | **A** | **T** | **C** | **T** | **T** | **C** | **T** | **G** | **G** | **G** | **G** | **G** | **C** | **A** | **T** | **T** | **C** | **A** | **A** | **C** | **G** | **G** | **C** | **A** | **A** | **G** | **G** | **T** | **T** | **A** | **T** | **A** | **G** | **G** | **A** | **G** | **A** | **A** | **A** | **A** | **A** | **T** | **C** | **C** | **C** | **A** | **G** | **C** | **C** | **A** | **A** | **A** | **A** | **C** | **A** | **T** | **C** | **T** | **G** | **A** | **G** | **C** | **A** | **T** | **T** | **A** | **G** | **T** | **T** | **A** | **T** | **G** | **T** | **A** | **C** | **G** | **C** | **C** | **A** | **A** | **A** | **A** | **T** | **T** | **A** | **T** | **G** | **A** | **A** | **G** | **A** | **C** | **A** | **A** | **T** | **C** | **G** | **A** | **G** | **G** | **A** | **A** | **C** | **G** | **T** | **T** | **G** | **G** | **C** | **G** | **G** | **A** | **A** | **T** | **T** | **T** | **G** | **C** | **A** | **G** | **A** | **G** | **A** | **A** | **A** | **A** | **A** | **C** | **C** | **A** | **A** | **G** | **T** | **A** | **G** | **A** | **C** | **T** | **A** | **C** | **C** | **A** | **A** | **G** | **C** | **A** | **T** | **T** | **T** | **T** | **T** | **G** | **A** | **A** | **C** | **A** | **A** | **C** | **C** | **T** | **C** | **C** | **G** | **A** | **A** | **A** | **G** | **C** | **T** | **T** | **G** | **G** | **G** | **A** | **T** | **G** | **G** | **A** | **A** | **A** | **G** | **A** | **G** | **A** | **T** | **G** | **T** | **T** | **T** | **T** | **T** | **C** | **A** | **T** | **A** | **A** | **C** | **A** | **A** | **G** | **A** | **T** | **C** | **G** | **A** | **G** | **C** | **A** | **G** | **A** | **T** | **G** | **A** | **G** | **T** | **A** | **T** | **G** | **G** | **G** | **C** | **C** | **A** | **A** | **C** | **G** | **G** | **A** | **A** | **A** | **A** | **A** | **A** | **G** | **T** | **G** | **G** | **A** | **A** | **T** | **T** | **G** | **G** | **C** | **T** | **A** | **A** | **A** | **T** | **C** | **T** | **T** | **T** | **A** | **T** | **C** | **A** | **C** | **A** | **G** | **C** | **C** | **A** | **G** | **C** | **T** | **G** | **A** | **A** | **C** | **T** | **A** | **T** | **A** | **T** | **A** | **T** | **A** | **T** | **G** | **G** | **G** | **A** | **T** | **G** | **A** | **A** | **C** | **C** | **A** | **T** | **T** | **G** | **A** | **A** | **T** | **T** | **A** | **T** | **T** | **T** | **G** | **G** | **A** | **T** | **G** | **T** | **C** | **T** | **T** | **C** | **A** | **A** | **T** | **C** | **A** | **A** | **G** | **A** | **A** | **C** | **A** | **A** | **T** | **T** | **A** | **G** | **A** | **A** | **C** | **A** | **A** | **C** | **T** | **G** | **A** | **T** | **C** | **T** | **T** | **G** | **A** | **A** | **C** | **G** | **T** | **G** | **A** | **A** | **A** | **C** | **C** | **T** | **G** | **C** | **C** | **A** | **T** | **G** | **T** | **T** | **A** | **C** | **T** | **A** | **G** | **T** | **G** | **G** | **A** | **A** | **C** | **A** | **T** | **G** | **A** | **T** | **C** | **A** | **A** | **A** | **C** | **C** | **T** | **T** | **C** | **C** | **T** | **G** | **G** | **A** | **T** | **A** | **A** | **A** | **G** | **T** | **A** | **T** | **C** | **T** | **A** | **C** | **T** | **G** | **A** | **G** | **A** | **T** | **T** | **A** | **T** | **T** | **T** | **C** | **T** | **C** | **T** | **T** | **G** | **A** | **G** | **A** | **A** | **A** | **A** | **T** | **C** | **-** | **-** | **-** |
| **N60048F** | **A** | **T** | **G** | **T** | **C** | **T** | **A** | **A** | **A** | **A** | **T** | **C** | **G** | **A** | **A** | **A** | **T** | **A** | **A** | **A** | **A** | **A** | **A** | **T** | **C** | **T** | **G** | **A** | **C** | **A** | **T** | **T** | **C** | **G** | **G** | **C** | **T** | **A** | **C** | **G** | **A** | **C** | **A** | **G** | **C** | **C** | **A** | **A** | **G** | **G** | **C** | **A** | **C** | **A** | **T** | **T** | **A** | **T** | **T** | **A** | **T** | **T** | **T** | **G** | **A** | **A** | **C** | **A** | **A** | **G** | **C** | **A** | **A** | **A** | **T** | **C** | **T** | **A** | **A** | **A** | **T** | **T** | **T** | **T** | **G** | **A** | **C** | **A** | **T** | **A** | **C** | **A** | **A** | **T** | **G** | **G** | **A** | **A** | **A** | **C** | **T** | **A** | **G** | **G** | **A** | **C** | **T** | **T** | **A** | **T** | **C** | **G** | **G** | **A** | **C** | **G** | **A** | **A** | **A** | **C** | **G** | **G** | **T** | **C** | **G** | **A** | **G** | **G** | **A** | **A** | **A** | **G** | **A** | **C** | **A** | **A** | **C** | **T** | **T** | **T** | **A** | **C** | **T** | **G** | **A** | **A** | **T** | **A** | **T** | **T** | **C** | **T** | **A** | **C** | **A** | **A** | **A** | **A** | **C** | **A** | **A** | **A** | **C** | **T** | **A** | **C** | **C** | **T** | **T** | **A** | **T** | **C** | **A** | **A** | **G** | **G** | **G** | **C** | **A** | **A** | **G** | **T** | **A** | **A** | **T** | **C** | **C** | **A** | **T** | **C** | **A** | **G** | **C** | **A** | **A** | **G** | **A** | **A** | **T** | **T** | **T** | **G** | **T** | **C** | **T** | **A** | **T** | **T** | **T** | **C** | **C** | **C** | **G** | **C** | **A** | **A** | **C** | **A** | **G** | **A** | **C** | **A** | **A** | **A** | **A** | **G** | **A** | **T** | **A** | **A** | **A** | **G** | **A** | **A** | **C** | **G** | **T** | **T** | **T** | **A** | **A** | **C** | **C** | **T** | **A** | **T** | **T** | **A** | **C** | **G** | **T** | **G** | **T** | **T** | **A** | **A** | **A** | **T** | **G** | **A** | **T** | **A** | **T** | **T** | **A** | **C** | **G** | **G** | **A** | **T** | **T** | **T** | **T** | **G** | **A** | **G** | **A** | **T** | **A** | **T** | **G** | **G** | **G** | **A** | **A** | **A** | **T** | **C** | **G** | **A** | **A** | **A** | **G** | **A** | **G** | **A** | **G** | **C** | **T** | **C** | **C** | **A** | **A** | **T** | **T** | **G** | **A** | **T** | **G** | **C** | **A** | **A** | **A** | **C** | **A** | **G** | **A** | **T** | **C** | **C** | **T** | **G** | **A** | **A** | **A** | **T** | **C** | **T** | **T** | **A** | **T** | **G** | **G** | **A** | **G** | **A** | **G** | **A** | **A** | **T** | **T** | **C** | **A** | **G** | **C** | **A** | **C** | **A** | **C** | **T** | **A** | **T** | **C** | **A** | **G** | **G** | **G** | **G** | **G** | **A** | **G** | **A** | **G** | **A** | **A** | **G** | **A** | **C** | **A** | **A** | **A** | **A** | **G** | **T** | **C** | **C** | **T** | **A** | **C** | **T** | **G** | **G** | **C** | **A** | **C** | **T** | **T** | **T** | **T** | **A** | **T** | **T** | **T** | **G** | **T** | **G** | **G** | **A** | **T** | **G** | **A** | **C** | **A** | **C** | **T** | **C** | **A** | **T** | **T** | **T** | **C** | **C** | **C** | **G** | **T** | **T** | **A** | **A** | **T** | **C** | **G** | **A** | **T** | **G** | **A** | **A** | **C** | **C** | **A** | **A** | **C** | **G** | **A** | **A** | **T** | **C** | **A** | **T** | **T** | **T** | **G** | **G** | **A** | **-** | **-** | **-** | **T** | **A** | **T** | **C** | **T** | **C** | **T** | **G** | **G** | **T** | **A** | **G** | **A** | **A** | **A** | **A** | **C** | **A** | **A** | **G** | **T** | **A** | **G** | **C** | **G** | **G** | **C** | **T** | **T** | **A** | **T** | **T** | **T** | **G** | **A** | **A** | **A** | **A** | **A** | **G** | **A** | **A** | **A** | **A** | **A** | **A** | **C** | **A** | **A** | **G** | **G** | **C** | **T** | **T** | **C** | **A** | **T** | **C** | **G** | **T** | **G** | **G** | **T** | **C** | **A** | **G** | **C** | **C** | **A** | **T** | **G** | **A** | **C** | **C** | **G** | **G** | **G** | **G** | **A** | **T** | **T** | **T** | **A** | **T** | **C** | **G** | **A** | **T** | **G** | **A** | **A** | **G** | **T** | **A** | **G** | **T** | **G** | **G** | **A** | **C** | **C** | **A** | **T** | **G** | **T** | **T** | **T** | **T** | **A** | **G** | **C** | **A** | **A** | **T** | **C** | **G** | **A** | **A** | **A** | **A** | **A** | **A** | **G** | **T** | **C** | **A** | **A** | **C** | **T** | **G** | **G** | **A** | **A** | **C** | **T** | **T** | **T** | **A** | **T** | **C** | **A** | **A** | **G** | **G** | **G** | **A** | **A** | **T** | **T** | **T** | **C** | **T** | **C** | **T** | **A** | **T** | **C** | **T** | **A** | **T** | **G** | **A** | **A** | **G** | **A** | **A** | **C** | **A** | **G** | **A** | **A** | **A** | **A** | **A** | **A** | **C** | **T** | **T** | **C** | **G** | **T** | **G** | **A** | **T** | **G** | **A** | **A** | **T** | **T** | **T** | **G** | **A** | **A** | **A** | **T** | **G** | **G** | **C** | **T** | **C** | **A** | **A** | **A** | **A** | **T** | **G** | **A** | **A** | **A** | **A** | **A** | **T** | **T** | **G** | **A** | **A** | **A** | **A** | **A** | **A** | **G** | **A** | **A** | **G** | **T** | **C** | **A** | **G** | **T** | **A** | **G** | **G** | **C** | **T** | **A** | **A** | **A** | **G** | **A** | **A** | **A** | **A** | **C** | **A** | **G** | **C** | **A** | **G** | **C** | **T** | **G** | **A** | **A** | **A** | **A** | **A** | **G** | **C** | **C** | **G** | **A** | **A** | **T** | **G** | **G** | **T** | **C** | **T** | **C** | **G** | **T** | **T** | **C** | **C** | **C** | **G** | **A** | **G** | **A** | **A** | **G** | **G** | **A** | **G** | **A** | **T** | **A** | **A** | **A** | **A** | **C** | **A** | **A** | **A** | **G** | **A** | **A** | **A** | **C** | **A** | **A** | **G** | **T** | **C** | **G** | **G** | **A** | **T** | **T** | **C** | **A** | **T** | **C** | **G** | **A** | **T** | **A** | **C** | **T** | **G** | **A** | **A** | **T** | **C** | **T** | **A** | **G** | **A** | **C** | **G** | **A** | **G** | **T** | **G** | **A** | **A** | **T** | **A** | **A** | **A** | **G** | **G** | **A** | **G** | **C** | **A** | **G** | **T** | **G** | **G** | **G** | **T** | **G** | **C** | **T** | **G** | **A** | **T** | **G** | **C** | **T** | **G** | **C** | **A** | **C** | **G** | **G** | **A** | **C** | **G** | **A** | **T** | **G** | **A** | **A** | **A** | **C** | **G** | **A** | **T** | **C** | **C** | **A** | **A** | **A** | **G** | **C** | **A** | **A** | **T** | **C** | **G** | **T** | **G** | **A** | **A** | **T** | **C** | **G** | **G** | **A** | **T** | **G** | **G** | **A** | **G** | **A** | **C** | **C** | **C** | **A** | **G** | **A** | **T** | **C** | **A** | **G** | **C** | **G** | **A** | **G** | **A** | **A** | **A** | **G** | **A** | **A** | **A** | **A** | **A** | **C** | **T** | **A** | **T** | **T** | **A** | **A** | **A** | **A** | **G** | **A** | **T** | **A** | **T** | **C** | **G** | **A** | **A** | **T** | **A** | **T** | **A** | **T** | **C** | **G** | **A** | **T** | **T** | **C** | **T** | **T** | **T** | **G** | **A** | **C** | **G** | **A** | **T** | **G** | **A** | **A** | **T** | **A** | **G** | **T** | **C** | **A** | **A** | **G** | **C** | **G** | **T** | **C** | **T** | **C** | **A** | **C** | **C** | **A** | **T** | **A** | **A** | **G** | **C** | **G** | **A** | **C** | **T** | **T** | **T** | **T** | **A** | **A** | **G** | **C** | **G** | **T** | **A** | **G** | **A** | **A** | **G** | **A** | **T** | **C** | **T** | **T** | **C** | **A** | **A** | **T** | **T** | **A** | **G** | **G** | **G** | **T** | **A** | **T** | **G** | **A** | **A** | **A** | **A** | **T** | **C** | **T** | **G** | **T** | **T** | **A** | **T** | **T** | **C** | **G** | **A** | **G** | **C** | **C** | **A** | **A** | **T** | **T** | **C** | **A** | **T** | **T** | **T** | **T** | **A** | **C** | **A** | **A** | **T** | **C** | **G** | **A** | **G** | **C** | **C** | **T** | **C** | **A** | **T** | **C** | **A** | **G** | **C** | **G** | **G** | **G** | **T** | **G** | **G** | **C** | **G** | **A** | **T** | **T** | **T** | **C** | **A** | **G** | **G** | **T** | **C** | **C** | **T** | **A** | **A** | **C** | **G** | **G** | **T** | **G** | **C** | **A** | **G** | **G** | **A** | **A** | **A** | **G** | **T** | **C** | **A** | **T** | **C** | **C** | **A** | **T** | **T** | **A** | **T** | **C** | **C** | **A** | **T** | **T** | **A** | **T** | **C** | **T** | **T** | **C** | **T** | **G** | **G** | **G** | **G** | **G** | **C** | **A** | **T** | **T** | **C** | **A** | **A** | **C** | **G** | **G** | **C** | **A** | **A** | **G** | **G** | **T** | **T** | **A** | **T** | **A** | **G** | **G** | **A** | **G** | **A** | **A** | **A** | **A** | **A** | **T** | **C** | **C** | **C** | **A** | **G** | **C** | **C** | **A** | **A** | **A** | **A** | **C** | **A** | **T** | **C** | **T** | **G** | **A** | **G** | **C** | **A** | **T** | **T** | **A** | **G** | **T** | **T** | **A** | **T** | **G** | **T** | **A** | **C** | **G** | **C** | **C** | **A** | **A** | **A** | **A** | **T** | **T** | **A** | **T** | **G** | **A** | **A** | **G** | **A** | **C** | **A** | **A** | **T** | **C** | **G** | **A** | **G** | **G** | **A** | **A** | **C** | **G** | **T** | **T** | **G** | **G** | **C** | **G** | **G** | **A** | **A** | **T** | **T** | **T** | **G** | **C** | **A** | **G** | **A** | **G** | **A** | **A** | **A** | **A** | **A** | **C** | **C** | **A** | **A** | **G** | **T** | **A** | **G** | **A** | **C** | **T** | **A** | **C** | **C** | **A** | **A** | **G** | **C** | **A** | **T** | **T** | **T** | **T** | **T** | **G** | **A** | **A** | **C** | **A** | **A** | **C** | **C** | **T** | **C** | **C** | **G** | **A** | **A** | **A** | **G** | **C** | **T** | **T** | **G** | **G** | **G** | **A** | **T** | **G** | **G** | **A** | **A** | **A** | **G** | **A** | **G** | **A** | **T** | **G** | **T** | **T** | **T** | **T** | **T** | **C** | **A** | **T** | **A** | **A** | **C** | **A** | **A** | **G** | **A** | **T** | **C** | **G** | **A** | **G** | **C** | **A** | **G** | **A** | **T** | **G** | **A** | **G** | **T** | **A** | **T** | **G** | **G** | **G** | **T** | **C** | **A** | **A** | **C** | **G** | **G** | **A** | **A** | **A** | **A** | **A** | **A** | **G** | **T** | **G** | **G** | **A** | **A** | **T** | **T** | **G** | **G** | **C** | **T** | **A** | **A** | **A** | **T** | **C** | **T** | **T** | **T** | **A** | **T** | **C** | **A** | **C** | **A** | **G** | **C** | **C** | **A** | **G** | **C** | **T** | **G** | **A** | **A** | **C** | **T** | **A** | **T** | **A** | **T** | **A** | **T** | **A** | **T** | **G** | **G** | **G** | **A** | **T** | **G** | **A** | **A** | **C** | **C** | **A** | **T** | **T** | **G** | **A** | **A** | **T** | **T** | **A** | **T** | **T** | **T** | **G** | **G** | **A** | **T** | **G** | **T** | **C** | **T** | **T** | **C** | **A** | **A** | **T** | **C** | **A** | **A** | **G** | **A** | **A** | **C** | **A** | **A** | **T** | **T** | **A** | **G** | **A** | **A** | **C** | **A** | **A** | **C** | **T** | **G** | **A** | **T** | **C** | **T** | **T** | **G** | **A** | **A** | **C** | **G** | **T** | **G** | **A** | **A** | **A** | **C** | **C** | **T** | **G** | **C** | **C** | **A** | **T** | **G** | **T** | **T** | **A** | **C** | **T** | **A** | **G** | **T** | **G** | **G** | **A** | **A** | **C** | **A** | **T** | **G** | **A** | **T** | **C** | **A** | **A** | **A** | **C** | **C** | **T** | **T** | **C** | **C** | **T** | **G** | **G** | **A** | **T** | **A** | **A** | **A** | **G** | **T** | **A** | **T** | **C** | **T** | **A** | **C** | **T** | **G** | **A** | **G** | **A** | **T** | **T** | **A** | **T** | **T** | **T** | **C** | **T** | **C** | **T** | **T** | **G** | **A** | **G** | **A** | **A** | **A** | **A** | **T** | **C** | **-** | **-** | **-** |
| **N60365F** | **A** | **T** | **G** | **T** | **C** | **T** | **A** | **A** | **A** | **A** | **T** | **C** | **G** | **A** | **A** | **A** | **T** | **A** | **A** | **A** | **A** | **A** | **A** | **T** | **C** | **T** | **G** | **A** | **C** | **A** | **T** | **T** | **C** | **G** | **G** | **C** | **T** | **A** | **C** | **G** | **A** | **C** | **A** | **G** | **C** | **C** | **A** | **A** | **G** | **G** | **C** | **A** | **C** | **A** | **T** | **T** | **A** | **T** | **T** | **A** | **T** | **T** | **T** | **G** | **A** | **A** | **C** | **A** | **A** | **G** | **C** | **A** | **A** | **A** | **T** | **C** | **T** | **A** | **A** | **A** | **T** | **T** | **T** | **T** | **G** | **A** | **C** | **A** | **C** | **A** | **C** | **A** | **A** | **T** | **G** | **G** | **A** | **A** | **A** | **C** | **T** | **A** | **G** | **G** | **A** | **C** | **T** | **T** | **A** | **T** | **C** | **G** | **G** | **A** | **C** | **G** | **A** | **A** | **A** | **C** | **G** | **G** | **T** | **C** | **G** | **A** | **G** | **G** | **A** | **A** | **A** | **G** | **A** | **C** | **A** | **A** | **C** | **T** | **T** | **T** | **A** | **C** | **T** | **G** | **A** | **A** | **T** | **A** | **T** | **T** | **C** | **T** | **A** | **C** | **A** | **A** | **A** | **A** | **C** | **A** | **A** | **A** | **C** | **T** | **A** | **C** | **C** | **T** | **T** | **A** | **T** | **C** | **A** | **A** | **G** | **G** | **G** | **C** | **A** | **A** | **G** | **T** | **A** | **A** | **T** | **C** | **C** | **A** | **T** | **C** | **A** | **G** | **C** | **A** | **A** | **G** | **A** | **A** | **T** | **T** | **T** | **G** | **C** | **C** | **T** | **A** | **T** | **T** | **T** | **C** | **C** | **C** | **G** | **C** | **A** | **A** | **C** | **A** | **G** | **A** | **C** | **A** | **A** | **A** | **A** | **G** | **A** | **T** | **A** | **A** | **A** | **G** | **A** | **A** | **C** | **G** | **T** | **T** | **T** | **A** | **A** | **C** | **C** | **T** | **A** | **T** | **T** | **A** | **C** | **G** | **T** | **G** | **T** | **T** | **A** | **A** | **A** | **T** | **G** | **A** | **T** | **A** | **T** | **T** | **A** | **C** | **G** | **G** | **A** | **T** | **T** | **T** | **T** | **G** | **A** | **G** | **A** | **T** | **A** | **T** | **G** | **G** | **G** | **A** | **A** | **A** | **T** | **C** | **G** | **A** | **A** | **A** | **G** | **A** | **G** | **A** | **G** | **C** | **T** | **C** | **C** | **A** | **A** | **T** | **T** | **G** | **A** | **T** | **G** | **C** | **A** | **A** | **A** | **C** | **A** | **G** | **A** | **T** | **C** | **C** | **T** | **G** | **A** | **A** | **A** | **T** | **C** | **T** | **T** | **A** | **T** | **G** | **G** | **A** | **G** | **A** | **G** | **A** | **A** | **T** | **T** | **C** | **A** | **G** | **C** | **A** | **C** | **A** | **C** | **T** | **A** | **T** | **C** | **G** | **G** | **G** | **G** | **G** | **G** | **A** | **G** | **A** | **G** | **A** | **A** | **G** | **A** | **C** | **A** | **A** | **A** | **A** | **G** | **T** | **C** | **C** | **T** | **A** | **C** | **T** | **G** | **G** | **C** | **A** | **C** | **T** | **T** | **T** | **T** | **A** | **T** | **T** | **T** | **G** | **T** | **G** | **G** | **A** | **T** | **G** | **A** | **C** | **A** | **C** | **T** | **C** | **A** | **T** | **T** | **T** | **C** | **C** | **T** | **G** | **T** | **T** | **A** | **A** | **T** | **C** | **G** | **A** | **T** | **G** | **A** | **A** | **C** | **C** | **A** | **A** | **C** | **G** | **A** | **A** | **T** | **C** | **A** | **T** | **T** | **T** | **G** | **G** | **A** | **-** | **-** | **-** | **T** | **A** | **T** | **C** | **T** | **C** | **T** | **G** | **G** | **T** | **A** | **G** | **A** | **A** | **A** | **A** | **C** | **A** | **A** | **G** | **T** | **A** | **G** | **C** | **G** | **G** | **C** | **T** | **T** | **A** | **T** | **T** | **T** | **G** | **A** | **A** | **A** | **A** | **A** | **G** | **A** | **A** | **A** | **A** | **A** | **A** | **C** | **A** | **A** | **G** | **G** | **C** | **T** | **T** | **C** | **A** | **T** | **C** | **G** | **T** | **G** | **G** | **T** | **C** | **A** | **G** | **C** | **C** | **A** | **T** | **G** | **A** | **C** | **C** | **G** | **G** | **G** | **G** | **A** | **T** | **T** | **T** | **A** | **T** | **C** | **G** | **A** | **T** | **G** | **A** | **A** | **G** | **T** | **A** | **G** | **T** | **G** | **G** | **A** | **C** | **C** | **A** | **T** | **G** | **T** | **T** | **T** | **T** | **A** | **G** | **C** | **A** | **A** | **T** | **C** | **G** | **A** | **C** | **A** | **A** | **A** | **A** | **G** | **T** | **C** | **A** | **A** | **C** | **T** | **G** | **G** | **A** | **A** | **C** | **T** | **T** | **T** | **A** | **T** | **C** | **A** | **A** | **G** | **G** | **G** | **A** | **A** | **T** | **T** | **T** | **C** | **T** | **C** | **T** | **A** | **T** | **C** | **T** | **A** | **T** | **G** | **A** | **A** | **G** | **A** | **A** | **C** | **A** | **G** | **A** | **A** | **A** | **A** | **A** | **A** | **C** | **T** | **T** | **C** | **G** | **T** | **G** | **A** | **T** | **G** | **A** | **A** | **T** | **T** | **T** | **G** | **A** | **A** | **A** | **T** | **G** | **G** | **C** | **T** | **C** | **A** | **A** | **A** | **A** | **T** | **G** | **A** | **A** | **A** | **A** | **A** | **T** | **T** | **G** | **A** | **A** | **A** | **A** | **A** | **A** | **G** | **A** | **A** | **G** | **T** | **C** | **A** | **G** | **T** | **A** | **G** | **G** | **C** | **T** | **A** | **A** | **A** | **G** | **A** | **A** | **A** | **A** | **C** | **A** | **G** | **C** | **A** | **G** | **C** | **T** | **G** | **A** | **A** | **A** | **A** | **A** | **G** | **C** | **C** | **G** | **A** | **A** | **T** | **G** | **G** | **T** | **C** | **T** | **C** | **G** | **T** | **T** | **C** | **C** | **C** | **G** | **A** | **G** | **A** | **A** | **G** | **G** | **A** | **G** | **A** | **T** | **A** | **A** | **A** | **A** | **C** | **A** | **A** | **A** | **G** | **A** | **A** | **A** | **C** | **A** | **A** | **G** | **T** | **C** | **G** | **G** | **A** | **T** | **T** | **C** | **A** | **T** | **C** | **G** | **A** | **T** | **A** | **C** | **T** | **G** | **A** | **A** | **T** | **C** | **T** | **A** | **G** | **A** | **C** | **G** | **A** | **G** | **T** | **G** | **A** | **A** | **T** | **A** | **A** | **A** | **G** | **G** | **A** | **G** | **C** | **A** | **G** | **T** | **G** | **G** | **G** | **T** | **G** | **C** | **T** | **G** | **A** | **T** | **G** | **C** | **T** | **G** | **C** | **A** | **C** | **G** | **G** | **A** | **C** | **G** | **A** | **T** | **G** | **A** | **A** | **A** | **C** | **G** | **A** | **T** | **C** | **C** | **A** | **A** | **A** | **G** | **C** | **A** | **A** | **T** | **C** | **G** | **T** | **G** | **A** | **A** | **T** | **C** | **G** | **G** | **A** | **T** | **G** | **G** | **A** | **G** | **A** | **C** | **C** | **C** | **A** | **G** | **A** | **T** | **C** | **A** | **G** | **C** | **G** | **A** | **G** | **A** | **A** | **A** | **G** | **A** | **A** | **A** | **A** | **A** | **C** | **T** | **A** | **T** | **T** | **A** | **A** | **A** | **A** | **G** | **A** | **T** | **A** | **T** | **C** | **G** | **A** | **A** | **T** | **A** | **T** | **A** | **T** | **C** | **G** | **A** | **T** | **T** | **C** | **T** | **T** | **T** | **G** | **A** | **C** | **G** | **A** | **T** | **G** | **A** | **A** | **T** | **A** | **G** | **C** | **C** | **A** | **A** | **G** | **C** | **G** | **T** | **C** | **T** | **C** | **A** | **C** | **C** | **A** | **T** | **A** | **A** | **G** | **C** | **G** | **A** | **C** | **T** | **T** | **T** | **T** | **A** | **A** | **G** | **C** | **G** | **T** | **A** | **G** | **A** | **A** | **G** | **A** | **T** | **C** | **T** | **T** | **C** | **A** | **A** | **T** | **T** | **A** | **G** | **G** | **G** | **T** | **A** | **T** | **G** | **A** | **A** | **A** | **A** | **T** | **C** | **T** | **G** | **T** | **T** | **A** | **T** | **T** | **C** | **G** | **A** | **G** | **C** | **C** | **A** | **A** | **T** | **T** | **C** | **A** | **T** | **T** | **T** | **T** | **A** | **C** | **A** | **A** | **T** | **C** | **G** | **A** | **G** | **C** | **C** | **T** | **C** | **A** | **T** | **C** | **A** | **G** | **C** | **G** | **G** | **G** | **T** | **G** | **G** | **C** | **G** | **A** | **T** | **T** | **T** | **C** | **A** | **G** | **G** | **T** | **C** | **C** | **T** | **A** | **A** | **C** | **G** | **G** | **T** | **G** | **C** | **A** | **G** | **G** | **A** | **A** | **A** | **G** | **T** | **C** | **A** | **T** | **C** | **C** | **A** | **T** | **T** | **A** | **T** | **C** | **C** | **A** | **T** | **T** | **A** | **T** | **C** | **T** | **T** | **C** | **T** | **G** | **G** | **G** | **G** | **G** | **C** | **A** | **T** | **T** | **C** | **A** | **A** | **C** | **G** | **G** | **C** | **A** | **A** | **G** | **G** | **T** | **T** | **A** | **T** | **A** | **G** | **G** | **A** | **G** | **A** | **A** | **A** | **A** | **A** | **T** | **A** | **C** | **C** | **A** | **G** | **C** | **C** | **A** | **A** | **A** | **A** | **C** | **A** | **T** | **C** | **T** | **G** | **A** | **G** | **C** | **A** | **T** | **T** | **A** | **G** | **T** | **T** | **A** | **T** | **G** | **C** | **A** | **A** | **G** | **C** | **C** | **A** | **A** | **A** | **A** | **T** | **T** | **A** | **T** | **G** | **A** | **A** | **G** | **A** | **C** | **A** | **A** | **T** | **C** | **G** | **A** | **G** | **G** | **A** | **A** | **C** | **G** | **T** | **T** | **G** | **G** | **C** | **G** | **G** | **A** | **A** | **T** | **T** | **T** | **G** | **C** | **A** | **G** | **A** | **G** | **A** | **A** | **A** | **A** | **A** | **C** | **C** | **A** | **A** | **G** | **T** | **A** | **G** | **A** | **C** | **T** | **A** | **C** | **C** | **A** | **A** | **G** | **C** | **A** | **T** | **T** | **T** | **T** | **T** | **G** | **A** | **A** | **C** | **A** | **A** | **C** | **C** | **T** | **C** | **C** | **G** | **A** | **A** | **A** | **G** | **C** | **T** | **T** | **G** | **G** | **G** | **A** | **T** | **G** | **G** | **A** | **A** | **A** | **G** | **A** | **G** | **A** | **T** | **G** | **T** | **T** | **T** | **T** | **T** | **C** | **A** | **T** | **A** | **A** | **C** | **A** | **A** | **G** | **A** | **T** | **C** | **G** | **A** | **G** | **C** | **A** | **G** | **A** | **T** | **G** | **A** | **G** | **T** | **A** | **T** | **G** | **G** | **G** | **C** | **C** | **A** | **A** | **C** | **G** | **G** | **A** | **A** | **A** | **A** | **A** | **A** | **G** | **T** | **G** | **G** | **A** | **A** | **T** | **T** | **G** | **G** | **C** | **T** | **A** | **A** | **A** | **T** | **C** | **T** | **T** | **T** | **A** | **T** | **C** | **A** | **C** | **A** | **G** | **C** | **C** | **A** | **G** | **C** | **T** | **G** | **A** | **A** | **C** | **T** | **A** | **T** | **A** | **T** | **A** | **T** | **A** | **T** | **G** | **G** | **G** | **A** | **T** | **G** | **A** | **A** | **C** | **C** | **A** | **T** | **T** | **G** | **A** | **A** | **T** | **T** | **A** | **T** | **T** | **T** | **G** | **G** | **A** | **T** | **G** | **T** | **C** | **T** | **T** | **C** | **A** | **A** | **T** | **C** | **A** | **A** | **G** | **A** | **A** | **C** | **A** | **A** | **T** | **T** | **A** | **G** | **A** | **A** | **C** | **A** | **A** | **C** | **T** | **G** | **A** | **T** | **C** | **T** | **T** | **G** | **A** | **A** | **C** | **G** | **T** | **G** | **A** | **A** | **A** | **C** | **C** | **T** | **G** | **C** | **C** | **A** | **T** | **G** | **T** | **T** | **A** | **C** | **T** | **A** | **G** | **T** | **G** | **G** | **A** | **A** | **C** | **A** | **T** | **G** | **A** | **T** | **C** | **A** | **A** | **A** | **C** | **C** | **T** | **T** | **C** | **C** | **T** | **G** | **G** | **A** | **T** | **A** | **A** | **A** | **G** | **T** | **A** | **T** | **C** | **T** | **A** | **C** | **T** | **G** | **A** | **G** | **A** | **T** | **T** | **A** | **T** | **T** | **T** | **C** | **T** | **C** | **T** | **T** | **G** | **A** | **G** | **A** | **G** | **A** | **A** | **T** | **C** | **-** | **-** | **-** |
| **N59943F** | **A** | **T** | **G** | **T** | **C** | **T** | **A** | **A** | **A** | **A** | **T** | **C** | **G** | **A** | **A** | **A** | **T** | **A** | **A** | **A** | **A** | **A** | **A** | **T** | **C** | **T** | **G** | **A** | **C** | **A** | **T** | **T** | **C** | **G** | **G** | **C** | **T** | **A** | **C** | **G** | **A** | **C** | **A** | **G** | **C** | **C** | **A** | **A** | **G** | **G** | **C** | **A** | **C** | **A** | **T** | **T** | **A** | **T** | **T** | **A** | **T** | **T** | **T** | **G** | **A** | **A** | **C** | **A** | **A** | **G** | **C** | **A** | **A** | **A** | **T** | **C** | **T** | **A** | **A** | **A** | **T** | **T** | **T** | **T** | **G** | **A** | **C** | **A** | **C** | **A** | **C** | **A** | **A** | **T** | **G** | **G** | **A** | **A** | **A** | **C** | **T** | **A** | **G** | **G** | **A** | **C** | **T** | **T** | **A** | **T** | **C** | **G** | **G** | **A** | **C** | **G** | **A** | **A** | **A** | **C** | **G** | **G** | **T** | **C** | **G** | **A** | **G** | **G** | **A** | **A** | **A** | **G** | **A** | **C** | **A** | **A** | **C** | **T** | **T** | **T** | **A** | **C** | **T** | **G** | **A** | **A** | **T** | **A** | **T** | **T** | **C** | **T** | **A** | **C** | **A** | **A** | **A** | **A** | **C** | **A** | **A** | **A** | **C** | **T** | **A** | **C** | **C** | **T** | **T** | **A** | **T** | **C** | **A** | **A** | **G** | **G** | **G** | **C** | **A** | **A** | **G** | **T** | **A** | **A** | **T** | **C** | **C** | **A** | **T** | **C** | **A** | **G** | **C** | **A** | **A** | **G** | **A** | **A** | **T** | **T** | **T** | **G** | **T** | **C** | **T** | **A** | **T** | **T** | **T** | **C** | **C** | **C** | **G** | **C** | **A** | **A** | **C** | **A** | **G** | **A** | **C** | **A** | **A** | **A** | **A** | **G** | **A** | **T** | **A** | **A** | **A** | **G** | **A** | **A** | **C** | **G** | **T** | **T** | **T** | **A** | **A** | **C** | **C** | **T** | **A** | **T** | **T** | **A** | **C** | **G** | **T** | **G** | **T** | **T** | **A** | **A** | **A** | **T** | **G** | **A** | **T** | **A** | **T** | **T** | **A** | **C** | **G** | **G** | **A** | **T** | **T** | **T** | **T** | **G** | **A** | **G** | **A** | **T** | **A** | **T** | **G** | **G** | **G** | **A** | **A** | **A** | **T** | **C** | **G** | **A** | **A** | **A** | **G** | **A** | **G** | **A** | **G** | **C** | **T** | **C** | **C** | **A** | **A** | **T** | **T** | **G** | **A** | **T** | **G** | **C** | **A** | **A** | **A** | **C** | **A** | **G** | **A** | **T** | **C** | **C** | **T** | **G** | **A** | **A** | **A** | **T** | **C** | **T** | **T** | **A** | **T** | **G** | **G** | **A** | **G** | **A** | **G** | **A** | **A** | **T** | **T** | **C** | **A** | **G** | **C** | **A** | **C** | **A** | **C** | **T** | **A** | **T** | **C** | **G** | **G** | **G** | **G** | **G** | **G** | **A** | **G** | **A** | **G** | **A** | **A** | **G** | **A** | **C** | **A** | **A** | **A** | **A** | **G** | **T** | **C** | **C** | **T** | **A** | **C** | **T** | **G** | **G** | **C** | **A** | **C** | **T** | **T** | **T** | **T** | **A** | **T** | **T** | **T** | **G** | **T** | **G** | **G** | **A** | **T** | **G** | **A** | **C** | **A** | **C** | **T** | **C** | **A** | **T** | **T** | **T** | **C** | **C** | **C** | **G** | **T** | **T** | **A** | **A** | **T** | **C** | **G** | **A** | **T** | **G** | **A** | **A** | **C** | **C** | **A** | **A** | **C** | **G** | **A** | **A** | **T** | **C** | **A** | **T** | **T** | **T** | **G** | **G** | **A** | **-** | **-** | **-** | **T** | **A** | **T** | **C** | **T** | **C** | **T** | **G** | **G** | **T** | **A** | **G** | **A** | **A** | **A** | **A** | **C** | **A** | **A** | **G** | **T** | **A** | **G** | **C** | **G** | **G** | **C** | **T** | **T** | **A** | **T** | **T** | **T** | **G** | **A** | **A** | **A** | **A** | **A** | **G** | **A** | **A** | **A** | **A** | **A** | **A** | **C** | **A** | **A** | **G** | **G** | **C** | **T** | **T** | **C** | **A** | **T** | **C** | **G** | **T** | **G** | **G** | **T** | **C** | **A** | **G** | **C** | **C** | **A** | **T** | **G** | **A** | **C** | **C** | **G** | **G** | **G** | **G** | **A** | **T** | **T** | **T** | **A** | **T** | **C** | **G** | **A** | **T** | **G** | **A** | **A** | **G** | **T** | **A** | **G** | **T** | **G** | **G** | **A** | **C** | **C** | **A** | **T** | **G** | **T** | **T** | **T** | **T** | **A** | **G** | **C** | **A** | **A** | **T** | **C** | **G** | **A** | **A** | **A** | **A** | **A** | **A** | **G** | **T** | **C** | **A** | **A** | **C** | **T** | **G** | **G** | **A** | **A** | **C** | **T** | **T** | **T** | **A** | **T** | **C** | **A** | **A** | **G** | **G** | **G** | **A** | **A** | **T** | **T** | **T** | **C** | **T** | **C** | **T** | **A** | **T** | **C** | **T** | **A** | **T** | **G** | **A** | **A** | **G** | **A** | **A** | **C** | **A** | **G** | **A** | **A** | **A** | **A** | **A** | **A** | **C** | **T** | **T** | **C** | **G** | **T** | **G** | **A** | **T** | **G** | **A** | **A** | **T** | **T** | **T** | **G** | **A** | **A** | **A** | **T** | **G** | **G** | **C** | **T** | **C** | **A** | **A** | **A** | **A** | **T** | **G** | **A** | **A** | **A** | **A** | **A** | **T** | **T** | **G** | **A** | **A** | **A** | **A** | **A** | **A** | **G** | **A** | **A** | **G** | **T** | **C** | **A** | **G** | **T** | **A** | **G** | **G** | **C** | **T** | **A** | **A** | **A** | **G** | **A** | **A** | **A** | **A** | **C** | **A** | **G** | **C** | **A** | **G** | **C** | **T** | **G** | **A** | **A** | **A** | **A** | **A** | **G** | **C** | **C** | **G** | **A** | **A** | **T** | **G** | **G** | **T** | **C** | **T** | **C** | **G** | **T** | **T** | **C** | **C** | **C** | **G** | **A** | **G** | **A** | **A** | **G** | **G** | **A** | **G** | **A** | **T** | **A** | **A** | **A** | **A** | **C** | **A** | **A** | **A** | **G** | **A** | **A** | **A** | **C** | **A** | **A** | **G** | **T** | **C** | **G** | **G** | **A** | **T** | **T** | **C** | **A** | **T** | **C** | **G** | **A** | **T** | **A** | **C** | **T** | **G** | **A** | **A** | **T** | **C** | **T** | **A** | **G** | **A** | **C** | **G** | **A** | **G** | **T** | **G** | **A** | **A** | **T** | **A** | **A** | **A** | **G** | **G** | **A** | **G** | **C** | **A** | **G** | **T** | **G** | **G** | **G** | **T** | **G** | **C** | **T** | **G** | **A** | **T** | **G** | **C** | **T** | **G** | **C** | **A** | **C** | **G** | **G** | **A** | **C** | **G** | **A** | **T** | **G** | **A** | **A** | **A** | **C** | **G** | **A** | **T** | **C** | **C** | **A** | **A** | **A** | **G** | **C** | **A** | **A** | **T** | **C** | **G** | **T** | **G** | **A** | **A** | **T** | **C** | **G** | **G** | **A** | **T** | **G** | **G** | **A** | **G** | **A** | **C** | **C** | **C** | **A** | **G** | **A** | **T** | **C** | **A** | **G** | **C** | **G** | **A** | **G** | **A** | **A** | **A** | **G** | **A** | **A** | **A** | **A** | **A** | **T** | **T** | **A** | **T** | **T** | **A** | **A** | **A** | **A** | **G** | **A** | **T** | **A** | **T** | **C** | **G** | **A** | **A** | **T** | **A** | **T** | **A** | **T** | **C** | **G** | **A** | **T** | **T** | **C** | **T** | **T** | **T** | **G** | **A** | **C** | **G** | **A** | **T** | **G** | **A** | **A** | **T** | **A** | **G** | **C** | **C** | **A** | **A** | **G** | **C** | **G** | **T** | **C** | **T** | **C** | **A** | **C** | **C** | **A** | **T** | **A** | **A** | **G** | **C** | **G** | **A** | **C** | **T** | **T** | **T** | **T** | **A** | **A** | **G** | **C** | **G** | **T** | **A** | **G** | **A** | **A** | **G** | **A** | **T** | **C** | **T** | **T** | **C** | **A** | **A** | **T** | **T** | **A** | **G** | **G** | **G** | **T** | **A** | **T** | **G** | **A** | **A** | **A** | **A** | **T** | **C** | **T** | **G** | **T** | **T** | **A** | **T** | **T** | **C** | **G** | **A** | **G** | **C** | **C** | **A** | **A** | **T** | **T** | **C** | **A** | **T** | **T** | **T** | **T** | **A** | **C** | **A** | **A** | **T** | **C** | **G** | **A** | **G** | **C** | **C** | **T** | **C** | **A** | **T** | **C** | **A** | **G** | **C** | **G** | **G** | **G** | **T** | **G** | **G** | **C** | **G** | **A** | **T** | **T** | **T** | **C** | **A** | **G** | **G** | **T** | **C** | **C** | **T** | **A** | **A** | **C** | **G** | **G** | **T** | **G** | **C** | **A** | **G** | **G** | **A** | **A** | **A** | **G** | **T** | **C** | **A** | **T** | **C** | **C** | **A** | **T** | **T** | **A** | **T** | **C** | **C** | **A** | **T** | **T** | **A** | **T** | **C** | **T** | **T** | **C** | **T** | **G** | **G** | **G** | **G** | **G** | **C** | **A** | **T** | **T** | **C** | **A** | **A** | **C** | **G** | **G** | **C** | **A** | **A** | **G** | **G** | **T** | **T** | **A** | **T** | **A** | **G** | **G** | **A** | **G** | **A** | **A** | **A** | **A** | **A** | **T** | **C** | **C** | **C** | **A** | **G** | **C** | **C** | **A** | **A** | **A** | **A** | **C** | **A** | **T** | **C** | **T** | **T** | **A** | **G** | **C** | **A** | **T** | **T** | **A** | **G** | **T** | **T** | **A** | **T** | **G** | **T** | **A** | **C** | **G** | **C** | **C** | **A** | **A** | **A** | **A** | **T** | **T** | **A** | **T** | **G** | **A** | **A** | **G** | **A** | **C** | **A** | **A** | **T** | **C** | **G** | **A** | **G** | **G** | **A** | **A** | **C** | **G** | **T** | **T** | **G** | **G** | **C** | **G** | **G** | **A** | **A** | **T** | **T** | **T** | **G** | **C** | **A** | **G** | **A** | **G** | **A** | **A** | **A** | **A** | **A** | **C** | **C** | **A** | **A** | **G** | **T** | **A** | **G** | **A** | **C** | **T** | **A** | **C** | **C** | **A** | **A** | **G** | **C** | **A** | **T** | **T** | **T** | **T** | **T** | **G** | **A** | **A** | **C** | **A** | **A** | **C** | **C** | **T** | **C** | **C** | **G** | **A** | **A** | **A** | **G** | **C** | **T** | **T** | **G** | **G** | **G** | **A** | **T** | **G** | **G** | **A** | **A** | **A** | **G** | **A** | **G** | **A** | **T** | **G** | **T** | **T** | **T** | **T** | **T** | **C** | **A** | **T** | **A** | **A** | **C** | **A** | **A** | **G** | **A** | **T** | **C** | **G** | **A** | **G** | **C** | **A** | **G** | **A** | **T** | **G** | **A** | **G** | **T** | **A** | **T** | **G** | **G** | **G** | **C** | **C** | **A** | **A** | **C** | **G** | **G** | **A** | **A** | **A** | **A** | **A** | **A** | **G** | **T** | **G** | **G** | **A** | **A** | **T** | **T** | **G** | **G** | **C** | **T** | **A** | **A** | **A** | **T** | **C** | **T** | **T** | **T** | **A** | **T** | **C** | **A** | **C** | **A** | **G** | **C** | **C** | **A** | **G** | **C** | **T** | **G** | **A** | **A** | **C** | **T** | **A** | **T** | **A** | **T** | **A** | **T** | **A** | **T** | **G** | **G** | **G** | **A** | **T** | **G** | **A** | **A** | **C** | **C** | **A** | **T** | **T** | **G** | **A** | **A** | **T** | **T** | **A** | **T** | **T** | **T** | **G** | **G** | **A** | **T** | **G** | **T** | **C** | **T** | **T** | **C** | **A** | **A** | **T** | **C** | **A** | **A** | **G** | **A** | **A** | **C** | **A** | **A** | **T** | **T** | **A** | **G** | **A** | **A** | **C** | **A** | **A** | **C** | **T** | **G** | **A** | **T** | **C** | **T** | **T** | **G** | **A** | **A** | **C** | **G** | **T** | **G** | **A** | **A** | **A** | **C** | **C** | **T** | **G** | **C** | **C** | **A** | **T** | **G** | **T** | **T** | **A** | **C** | **T** | **A** | **G** | **T** | **G** | **G** | **A** | **A** | **C** | **A** | **T** | **G** | **A** | **T** | **C** | **A** | **A** | **A** | **C** | **C** | **T** | **T** | **C** | **C** | **T** | **G** | **G** | **A** | **T** | **A** | **A** | **A** | **G** | **T** | **A** | **T** | **C** | **T** | **A** | **C** | **T** | **G** | **A** | **G** | **A** | **T** | **T** | **A** | **T** | **T** | **T** | **C** | **T** | **C** | **T** | **T** | **G** | **A** | **G** | **A** | **A** | **A** | **A** | **T** | **C** | **-** | **-** | **-** |
| **N52751** | **A** | **T** | **G** | **T** | **C** | **T** | **A** | **A** | **A** | **A** | **T** | **C** | **G** | **A** | **A** | **A** | **T** | **A** | **A** | **A** | **A** | **A** | **A** | **T** | **C** | **T** | **G** | **A** | **C** | **A** | **T** | **T** | **C** | **G** | **G** | **C** | **T** | **A** | **C** | **G** | **A** | **C** | **A** | **G** | **C** | **C** | **A** | **A** | **G** | **G** | **C** | **A** | **C** | **A** | **T** | **T** | **A** | **T** | **T** | **A** | **T** | **T** | **T** | **G** | **A** | **A** | **C** | **A** | **A** | **G** | **C** | **A** | **A** | **A** | **T** | **C** | **T** | **A** | **A** | **A** | **T** | **T** | **T** | **T** | **G** | **A** | **C** | **A** | **C** | **A** | **C** | **A** | **A** | **T** | **G** | **G** | **A** | **A** | **A** | **C** | **T** | **A** | **G** | **G** | **A** | **C** | **T** | **T** | **A** | **T** | **C** | **G** | **G** | **A** | **C** | **G** | **A** | **A** | **A** | **C** | **G** | **G** | **T** | **C** | **G** | **A** | **G** | **G** | **A** | **A** | **A** | **G** | **A** | **C** | **A** | **A** | **C** | **T** | **T** | **T** | **A** | **C** | **T** | **G** | **A** | **A** | **T** | **A** | **T** | **T** | **C** | **T** | **A** | **C** | **A** | **A** | **A** | **A** | **C** | **A** | **A** | **A** | **C** | **T** | **A** | **C** | **C** | **T** | **T** | **A** | **T** | **C** | **A** | **A** | **G** | **G** | **G** | **C** | **A** | **A** | **G** | **T** | **A** | **A** | **T** | **C** | **C** | **A** | **T** | **C** | **A** | **G** | **C** | **A** | **A** | **G** | **A** | **A** | **T** | **T** | **T** | **G** | **T** | **C** | **T** | **A** | **T** | **T** | **T** | **C** | **C** | **C** | **G** | **C** | **A** | **G** | **C** | **A** | **A** | **A** | **C** | **A** | **A** | **A** | **A** | **G** | **A** | **T** | **A** | **A** | **A** | **G** | **A** | **G** | **C** | **G** | **T** | **T** | **T** | **A** | **A** | **C** | **C** | **T** | **A** | **T** | **T** | **A** | **T** | **G** | **T** | **G** | **T** | **T** | **A** | **A** | **A** | **T** | **G** | **A** | **G** | **A** | **T** | **T** | **A** | **C** | **G** | **G** | **A** | **T** | **T** | **T** | **T** | **G** | **A** | **G** | **A** | **T** | **A** | **T** | **G** | **G** | **G** | **A** | **A** | **A** | **T** | **T** | **G** | **A** | **A** | **A** | **G** | **A** | **G** | **A** | **G** | **C** | **T** | **C** | **C** | **A** | **A** | **C** | **T** | **G** | **A** | **T** | **G** | **C** | **A** | **A** | **A** | **C** | **A** | **G** | **A** | **T** | **C** | **C** | **T** | **G** | **A** | **A** | **A** | **T** | **C** | **T** | **T** | **A** | **T** | **G** | **G** | **A** | **G** | **A** | **G** | **A** | **A** | **T** | **T** | **C** | **A** | **G** | **C** | **A** | **C** | **A** | **C** | **T** | **A** | **T** | **C** | **A** | **G** | **G** | **A** | **G** | **G** | **A** | **G** | **A** | **G** | **A** | **A** | **G** | **A** | **C** | **A** | **A** | **A** | **A** | **G** | **T** | **C** | **C** | **T** | **A** | **C** | **T** | **G** | **G** | **C** | **A** | **C** | **T** | **T** | **T** | **T** | **A** | **T** | **T** | **T** | **G** | **T** | **G** | **G** | **A** | **T** | **G** | **A** | **C** | **A** | **C** | **T** | **C** | **A** | **T** | **T** | **T** | **C** | **C** | **C** | **G** | **T** | **T** | **A** | **A** | **T** | **C** | **G** | **A** | **T** | **G** | **A** | **A** | **C** | **C** | **A** | **A** | **C** | **G** | **A** | **A** | **T** | **C** | **A** | **T** | **T** | **T** | **G** | **G** | **A** | **-** | **-** | **-** | **T** | **A** | **T** | **C** | **T** | **C** | **T** | **G** | **A** | **T** | **A** | **G** | **A** | **A** | **A** | **A** | **C** | **A** | **A** | **G** | **T** | **A** | **G** | **C** | **G** | **G** | **C** | **T** | **T** | **A** | **C** | **T** | **T** | **G** | **A** | **A** | **A** | **A** | **A** | **G** | **A** | **A** | **A** | **A** | **A** | **A** | **C** | **A** | **A** | **G** | **G** | **C** | **T** | **T** | **C** | **A** | **T** | **C** | **G** | **T** | **G** | **G** | **T** | **C** | **A** | **G** | **C** | **C** | **A** | **T** | **G** | **A** | **C** | **C** | **G** | **G** | **G** | **G** | **A** | **T** | **T** | **T** | **A** | **T** | **C** | **G** | **A** | **T** | **G** | **A** | **A** | **G** | **T** | **A** | **G** | **T** | **G** | **G** | **A** | **C** | **C** | **A** | **T** | **G** | **T** | **T** | **T** | **T** | **A** | **G** | **C** | **A** | **A** | **T** | **C** | **G** | **A** | **A** | **A** | **A** | **A** | **A** | **G** | **T** | **C** | **A** | **A** | **C** | **T** | **G** | **G** | **A** | **A** | **C** | **T** | **T** | **T** | **A** | **T** | **C** | **A** | **A** | **G** | **G** | **G** | **A** | **A** | **T** | **T** | **T** | **C** | **T** | **C** | **T** | **A** | **T** | **C** | **T** | **A** | **T** | **G** | **A** | **A** | **G** | **A** | **A** | **C** | **A** | **G** | **A** | **A** | **A** | **A** | **A** | **A** | **C** | **T** | **T** | **C** | **G** | **T** | **G** | **A** | **T** | **G** | **A** | **A** | **T** | **T** | **T** | **G** | **A** | **A** | **A** | **T** | **A** | **G** | **C** | **T** | **C** | **A** | **A** | **A** | **A** | **T** | **G** | **A** | **A** | **A** | **A** | **A** | **T** | **T** | **G** | **A** | **A** | **A** | **A** | **A** | **A** | **G** | **A** | **A** | **G** | **T** | **C** | **A** | **G** | **T** | **A** | **G** | **G** | **C** | **T** | **A** | **A** | **A** | **G** | **A** | **A** | **A** | **A** | **C** | **A** | **G** | **C** | **A** | **G** | **C** | **T** | **G** | **A** | **A** | **A** | **A** | **A** | **G** | **C** | **C** | **G** | **A** | **A** | **T** | **G** | **G** | **T** | **C** | **T** | **C** | **G** | **T** | **T** | **C** | **C** | **C** | **G** | **A** | **G** | **A** | **A** | **G** | **G** | **A** | **G** | **A** | **T** | **A** | **A** | **A** | **A** | **C** | **A** | **A** | **A** | **G** | **A** | **A** | **A** | **C** | **A** | **A** | **G** | **T** | **C** | **G** | **G** | **A** | **T** | **T** | **C** | **A** | **T** | **C** | **G** | **A** | **T** | **A** | **C** | **T** | **G** | **A** | **A** | **T** | **C** | **T** | **A** | **G** | **A** | **C** | **G** | **A** | **G** | **T** | **G** | **A** | **A** | **T** | **A** | **A** | **A** | **G** | **G** | **A** | **G** | **C** | **A** | **G** | **T** | **G** | **G** | **G** | **T** | **G** | **C** | **T** | **G** | **A** | **T** | **G** | **C** | **T** | **G** | **C** | **A** | **C** | **G** | **G** | **A** | **C** | **G** | **A** | **T** | **G** | **A** | **A** | **A** | **C** | **G** | **A** | **T** | **C** | **C** | **A** | **A** | **A** | **G** | **C** | **A** | **A** | **T** | **C** | **G** | **T** | **G** | **A** | **A** | **T** | **C** | **G** | **G** | **A** | **T** | **G** | **G** | **A** | **G** | **A** | **C** | **C** | **C** | **A** | **G** | **A** | **T** | **C** | **A** | **G** | **C** | **G** | **A** | **G** | **A** | **A** | **A** | **G** | **A** | **A** | **A** | **A** | **A** | **C** | **T** | **A** | **T** | **T** | **A** | **A** | **A** | **A** | **G** | **A** | **T** | **A** | **T** | **C** | **G** | **A** | **A** | **T** | **A** | **T** | **A** | **T** | **C** | **G** | **A** | **T** | **T** | **C** | **T** | **T** | **T** | **G** | **A** | **C** | **G** | **A** | **T** | **G** | **A** | **A** | **T** | **A** | **G** | **C** | **C** | **A** | **A** | **G** | **C** | **G** | **T** | **C** | **T** | **C** | **A** | **C** | **C** | **A** | **T** | **A** | **A** | **G** | **C** | **G** | **A** | **C** | **T** | **T** | **T** | **T** | **A** | **A** | **G** | **C** | **G** | **T** | **A** | **G** | **A** | **A** | **G** | **A** | **T** | **C** | **T** | **T** | **C** | **A** | **A** | **T** | **T** | **A** | **G** | **G** | **G** | **T** | **A** | **T** | **G** | **A** | **A** | **A** | **A** | **T** | **C** | **T** | **G** | **T** | **T** | **A** | **T** | **T** | **C** | **G** | **A** | **G** | **C** | **C** | **A** | **A** | **T** | **T** | **C** | **A** | **T** | **T** | **T** | **T** | **A** | **C** | **A** | **A** | **T** | **C** | **G** | **A** | **G** | **C** | **C** | **T** | **C** | **A** | **T** | **C** | **A** | **G** | **C** | **G** | **G** | **G** | **T** | **G** | **G** | **C** | **G** | **A** | **T** | **T** | **T** | **C** | **A** | **G** | **G** | **T** | **C** | **C** | **T** | **A** | **A** | **C** | **G** | **G** | **T** | **G** | **C** | **A** | **G** | **G** | **A** | **A** | **A** | **G** | **T** | **C** | **A** | **T** | **C** | **C** | **A** | **T** | **T** | **A** | **T** | **C** | **C** | **A** | **T** | **T** | **A** | **T** | **C** | **T** | **T** | **C** | **T** | **G** | **G** | **G** | **G** | **G** | **C** | **A** | **T** | **T** | **C** | **A** | **A** | **C** | **G** | **G** | **C** | **A** | **A** | **G** | **G** | **T** | **T** | **A** | **T** | **A** | **G** | **G** | **A** | **G** | **A** | **A** | **A** | **A** | **A** | **T** | **C** | **C** | **C** | **A** | **G** | **C** | **C** | **A** | **A** | **A** | **A** | **C** | **A** | **T** | **C** | **T** | **G** | **A** | **G** | **C** | **A** | **T** | **T** | **A** | **G** | **T** | **T** | **A** | **T** | **G** | **T** | **A** | **C** | **G** | **C** | **C** | **A** | **A** | **A** | **A** | **T** | **T** | **A** | **T** | **G** | **A** | **A** | **G** | **A** | **C** | **A** | **A** | **T** | **C** | **G** | **A** | **G** | **G** | **A** | **A** | **C** | **G** | **T** | **T** | **G** | **G** | **C** | **G** | **G** | **A** | **A** | **T** | **T** | **T** | **G** | **C** | **A** | **G** | **A** | **G** | **A** | **A** | **A** | **A** | **A** | **C** | **C** | **A** | **A** | **G** | **T** | **A** | **G** | **A** | **C** | **T** | **A** | **C** | **C** | **A** | **A** | **G** | **C** | **A** | **T** | **T** | **T** | **T** | **T** | **G** | **A** | **A** | **C** | **A** | **A** | **C** | **C** | **T** | **C** | **C** | **G** | **A** | **A** | **A** | **G** | **C** | **T** | **T** | **G** | **G** | **G** | **A** | **T** | **G** | **G** | **A** | **A** | **A** | **G** | **A** | **G** | **A** | **T** | **G** | **T** | **T** | **T** | **T** | **T** | **C** | **A** | **T** | **A** | **A** | **C** | **A** | **A** | **G** | **A** | **T** | **C** | **G** | **A** | **G** | **C** | **A** | **G** | **A** | **T** | **G** | **A** | **G** | **T** | **A** | **T** | **G** | **G** | **G** | **C** | **C** | **A** | **A** | **C** | **G** | **G** | **A** | **A** | **A** | **A** | **A** | **A** | **G** | **T** | **G** | **G** | **A** | **A** | **T** | **T** | **G** | **G** | **C** | **T** | **A** | **A** | **A** | **T** | **C** | **T** | **T** | **T** | **A** | **T** | **C** | **A** | **C** | **A** | **G** | **C** | **C** | **A** | **G** | **C** | **T** | **G** | **A** | **A** | **C** | **T** | **A** | **T** | **A** | **T** | **A** | **T** | **A** | **T** | **G** | **G** | **G** | **A** | **T** | **G** | **A** | **A** | **C** | **C** | **A** | **T** | **T** | **G** | **A** | **A** | **T** | **T** | **A** | **T** | **T** | **T** | **G** | **G** | **A** | **T** | **G** | **T** | **C** | **T** | **T** | **C** | **A** | **A** | **T** | **C** | **A** | **A** | **G** | **A** | **A** | **C** | **A** | **A** | **T** | **T** | **A** | **G** | **A** | **A** | **C** | **A** | **A** | **C** | **T** | **G** | **A** | **T** | **C** | **T** | **T** | **G** | **A** | **A** | **C** | **G** | **T** | **G** | **A** | **A** | **A** | **C** | **C** | **T** | **G** | **C** | **C** | **A** | **T** | **G** | **T** | **T** | **A** | **C** | **T** | **A** | **G** | **T** | **G** | **G** | **A** | **A** | **C** | **A** | **T** | **G** | **A** | **T** | **C** | **A** | **A** | **A** | **C** | **C** | **T** | **T** | **C** | **C** | **T** | **G** | **G** | **A** | **T** | **A** | **A** | **A** | **G** | **T** | **A** | **T** | **C** | **T** | **A** | **C** | **T** | **G** | **A** | **G** | **A** | **T** | **T** | **A** | **T** | **T** | **T** | **C** | **T** | **C** | **T** | **T** | **G** | **A** | **G** | **A** | **A** | **A** | **A** | **T** | **C** | **-** | **-** | **-** |
| **N55077** | **A** | **T** | **G** | **T** | **C** | **T** | **A** | **A** | **A** | **A** | **T** | **C** | **G** | **A** | **A** | **A** | **T** | **A** | **A** | **A** | **A** | **A** | **A** | **T** | **C** | **T** | **G** | **A** | **C** | **A** | **C** | **T** | **C** | **G** | **G** | **C** | **T** | **A** | **C** | **G** | **A** | **C** | **A** | **G** | **C** | **C** | **A** | **A** | **G** | **G** | **C** | **A** | **C** | **A** | **T** | **T** | **A** | **T** | **T** | **A** | **T** | **T** | **T** | **G** | **A** | **A** | **C** | **A** | **A** | **G** | **C** | **A** | **A** | **A** | **T** | **C** | **T** | **A** | **A** | **A** | **T** | **T** | **T** | **T** | **G** | **A** | **C** | **A** | **C** | **A** | **C** | **A** | **A** | **T** | **G** | **G** | **A** | **A** | **A** | **C** | **T** | **A** | **G** | **G** | **A** | **C** | **T** | **T** | **A** | **T** | **C** | **G** | **G** | **A** | **C** | **G** | **A** | **A** | **A** | **C** | **G** | **G** | **T** | **C** | **G** | **A** | **G** | **G** | **A** | **A** | **A** | **G** | **A** | **C** | **A** | **A** | **C** | **T** | **T** | **T** | **A** | **C** | **T** | **G** | **A** | **A** | **T** | **A** | **T** | **T** | **C** | **T** | **A** | **C** | **A** | **A** | **A** | **A** | **C** | **A** | **A** | **A** | **C** | **T** | **A** | **C** | **C** | **T** | **T** | **A** | **T** | **C** | **A** | **A** | **G** | **G** | **G** | **C** | **A** | **A** | **G** | **T** | **A** | **A** | **T** | **C** | **C** | **A** | **T** | **C** | **A** | **G** | **C** | **A** | **A** | **G** | **A** | **A** | **T** | **T** | **T** | **G** | **T** | **C** | **T** | **A** | **T** | **T** | **T** | **C** | **C** | **C** | **G** | **C** | **A** | **A** | **C** | **A** | **G** | **A** | **C** | **A** | **A** | **A** | **A** | **G** | **A** | **T** | **A** | **A** | **A** | **G** | **A** | **A** | **C** | **G** | **T** | **T** | **T** | **A** | **A** | **C** | **C** | **T** | **A** | **T** | **T** | **A** | **C** | **G** | **T** | **G** | **T** | **T** | **A** | **A** | **A** | **T** | **G** | **A** | **T** | **A** | **T** | **T** | **A** | **C** | **G** | **G** | **G** | **T** | **T** | **T** | **T** | **G** | **A** | **G** | **A** | **T** | **A** | **T** | **G** | **G** | **G** | **A** | **A** | **A** | **T** | **C** | **G** | **A** | **A** | **A** | **G** | **A** | **G** | **A** | **G** | **C** | **T** | **C** | **C** | **A** | **A** | **T** | **T** | **G** | **A** | **T** | **G** | **C** | **A** | **A** | **A** | **C** | **A** | **G** | **A** | **T** | **C** | **C** | **T** | **G** | **A** | **A** | **A** | **T** | **C** | **T** | **T** | **A** | **T** | **G** | **G** | **A** | **G** | **A** | **G** | **A** | **A** | **T** | **T** | **C** | **A** | **G** | **C** | **A** | **C** | **A** | **C** | **T** | **A** | **T** | **C** | **A** | **G** | **G** | **G** | **G** | **G** | **A** | **G** | **A** | **G** | **A** | **A** | **G** | **A** | **C** | **A** | **A** | **A** | **A** | **G** | **T** | **C** | **C** | **T** | **A** | **C** | **T** | **G** | **G** | **C** | **A** | **C** | **T** | **T** | **T** | **T** | **A** | **T** | **T** | **T** | **G** | **T** | **G** | **G** | **A** | **T** | **G** | **A** | **C** | **A** | **C** | **T** | **C** | **A** | **T** | **T** | **T** | **C** | **C** | **C** | **G** | **T** | **T** | **A** | **A** | **T** | **C** | **G** | **A** | **T** | **G** | **A** | **A** | **C** | **C** | **A** | **A** | **C** | **G** | **A** | **A** | **T** | **C** | **A** | **T** | **T** | **T** | **G** | **G** | **A** | **-** | **-** | **-** | **T** | **A** | **T** | **C** | **T** | **C** | **T** | **G** | **G** | **T** | **A** | **G** | **A** | **A** | **A** | **A** | **C** | **A** | **A** | **G** | **T** | **A** | **G** | **C** | **G** | **G** | **C** | **T** | **T** | **A** | **T** | **T** | **T** | **G** | **A** | **A** | **A** | **A** | **A** | **G** | **A** | **A** | **A** | **A** | **A** | **A** | **C** | **A** | **A** | **G** | **G** | **C** | **T** | **T** | **C** | **A** | **T** | **C** | **G** | **T** | **G** | **G** | **T** | **C** | **A** | **G** | **C** | **C** | **A** | **T** | **G** | **A** | **C** | **C** | **G** | **G** | **G** | **G** | **A** | **T** | **T** | **T** | **A** | **T** | **C** | **G** | **A** | **T** | **G** | **A** | **A** | **G** | **T** | **A** | **G** | **T** | **G** | **G** | **A** | **C** | **C** | **A** | **T** | **G** | **T** | **T** | **T** | **T** | **A** | **G** | **C** | **A** | **A** | **T** | **C** | **G** | **A** | **A** | **A** | **A** | **A** | **A** | **G** | **T** | **C** | **A** | **A** | **C** | **T** | **G** | **G** | **A** | **A** | **C** | **T** | **T** | **T** | **A** | **T** | **C** | **A** | **A** | **G** | **G** | **G** | **A** | **A** | **T** | **T** | **T** | **C** | **T** | **C** | **T** | **A** | **T** | **C** | **T** | **A** | **T** | **G** | **A** | **A** | **G** | **A** | **A** | **C** | **A** | **G** | **A** | **A** | **A** | **A** | **A** | **A** | **C** | **T** | **T** | **C** | **G** | **T** | **G** | **A** | **T** | **G** | **A** | **A** | **T** | **T** | **T** | **G** | **A** | **A** | **A** | **T** | **G** | **G** | **C** | **T** | **C** | **A** | **A** | **A** | **A** | **T** | **G** | **A** | **A** | **A** | **A** | **A** | **T** | **T** | **G** | **A** | **A** | **A** | **A** | **A** | **A** | **G** | **A** | **A** | **G** | **T** | **C** | **A** | **G** | **T** | **A** | **G** | **G** | **C** | **T** | **A** | **A** | **A** | **G** | **A** | **A** | **A** | **A** | **C** | **A** | **G** | **C** | **A** | **G** | **C** | **T** | **G** | **A** | **A** | **A** | **A** | **A** | **G** | **C** | **C** | **G** | **A** | **A** | **T** | **G** | **G** | **T** | **C** | **T** | **C** | **G** | **T** | **T** | **C** | **C** | **C** | **G** | **A** | **G** | **A** | **A** | **G** | **G** | **A** | **G** | **A** | **T** | **A** | **A** | **A** | **A** | **C** | **A** | **A** | **A** | **G** | **A** | **A** | **A** | **C** | **A** | **A** | **G** | **T** | **C** | **G** | **G** | **A** | **T** | **T** | **C** | **A** | **T** | **C** | **G** | **A** | **T** | **A** | **C** | **T** | **G** | **A** | **A** | **T** | **C** | **T** | **A** | **G** | **A** | **C** | **G** | **A** | **G** | **T** | **G** | **A** | **A** | **T** | **A** | **A** | **A** | **G** | **G** | **A** | **G** | **C** | **A** | **G** | **T** | **G** | **G** | **G** | **T** | **G** | **C** | **T** | **G** | **A** | **T** | **G** | **C** | **T** | **G** | **C** | **A** | **C** | **G** | **G** | **A** | **C** | **G** | **A** | **T** | **G** | **A** | **A** | **A** | **C** | **G** | **A** | **T** | **C** | **C** | **A** | **A** | **A** | **G** | **C** | **A** | **A** | **T** | **C** | **G** | **T** | **G** | **A** | **A** | **T** | **C** | **G** | **G** | **A** | **T** | **G** | **G** | **A** | **G** | **A** | **C** | **C** | **C** | **A** | **G** | **A** | **T** | **C** | **A** | **G** | **C** | **G** | **A** | **G** | **A** | **A** | **A** | **G** | **A** | **A** | **A** | **A** | **A** | **C** | **T** | **A** | **T** | **T** | **A** | **A** | **A** | **A** | **G** | **A** | **T** | **A** | **T** | **C** | **G** | **A** | **A** | **T** | **A** | **T** | **A** | **T** | **C** | **G** | **A** | **T** | **T** | **C** | **T** | **T** | **T** | **G** | **A** | **C** | **G** | **A** | **T** | **G** | **A** | **A** | **T** | **A** | **G** | **C** | **C** | **A** | **A** | **G** | **C** | **G** | **T** | **C** | **T** | **C** | **A** | **C** | **C** | **A** | **T** | **A** | **A** | **G** | **C** | **G** | **A** | **C** | **T** | **T** | **T** | **T** | **A** | **A** | **G** | **C** | **G** | **T** | **A** | **G** | **A** | **A** | **G** | **A** | **T** | **C** | **T** | **T** | **C** | **A** | **A** | **T** | **T** | **A** | **G** | **G** | **G** | **T** | **A** | **T** | **G** | **A** | **A** | **A** | **A** | **T** | **C** | **T** | **G** | **T** | **T** | **A** | **T** | **T** | **C** | **G** | **A** | **G** | **C** | **C** | **A** | **A** | **T** | **T** | **C** | **A** | **T** | **T** | **T** | **T** | **A** | **C** | **A** | **A** | **T** | **C** | **G** | **A** | **G** | **C** | **C** | **T** | **C** | **A** | **T** | **C** | **A** | **G** | **C** | **G** | **G** | **G** | **T** | **G** | **G** | **C** | **G** | **A** | **T** | **T** | **T** | **C** | **A** | **G** | **G** | **T** | **C** | **C** | **T** | **A** | **A** | **C** | **G** | **G** | **T** | **G** | **C** | **A** | **G** | **G** | **A** | **A** | **A** | **G** | **T** | **C** | **A** | **T** | **C** | **C** | **A** | **T** | **T** | **A** | **T** | **C** | **C** | **A** | **T** | **T** | **A** | **T** | **C** | **T** | **T** | **C** | **T** | **G** | **G** | **G** | **G** | **G** | **C** | **A** | **T** | **T** | **C** | **A** | **A** | **C** | **G** | **G** | **C** | **A** | **A** | **G** | **G** | **T** | **T** | **A** | **T** | **A** | **G** | **G** | **A** | **G** | **A** | **A** | **A** | **A** | **A** | **T** | **C** | **C** | **C** | **A** | **G** | **C** | **C** | **A** | **A** | **A** | **A** | **C** | **A** | **T** | **C** | **T** | **G** | **A** | **G** | **C** | **A** | **T** | **T** | **A** | **G** | **T** | **T** | **A** | **T** | **G** | **T** | **A** | **C** | **G** | **C** | **C** | **A** | **A** | **A** | **A** | **T** | **T** | **A** | **T** | **G** | **A** | **A** | **G** | **A** | **C** | **A** | **A** | **T** | **C** | **G** | **A** | **G** | **G** | **A** | **A** | **C** | **G** | **T** | **T** | **G** | **G** | **C** | **G** | **G** | **A** | **A** | **T** | **T** | **T** | **G** | **C** | **A** | **G** | **A** | **G** | **A** | **A** | **A** | **A** | **A** | **C** | **C** | **A** | **A** | **G** | **T** | **A** | **G** | **A** | **C** | **T** | **A** | **C** | **C** | **A** | **A** | **G** | **C** | **A** | **T** | **T** | **T** | **T** | **T** | **G** | **A** | **A** | **C** | **A** | **A** | **C** | **C** | **T** | **C** | **C** | **G** | **A** | **A** | **A** | **G** | **C** | **T** | **T** | **G** | **G** | **G** | **A** | **T** | **G** | **G** | **A** | **A** | **A** | **G** | **A** | **G** | **A** | **T** | **G** | **T** | **T** | **T** | **T** | **T** | **C** | **A** | **T** | **A** | **A** | **C** | **A** | **A** | **G** | **A** | **T** | **T** | **G** | **A** | **G** | **C** | **A** | **G** | **A** | **T** | **G** | **A** | **G** | **T** | **A** | **T** | **G** | **G** | **G** | **C** | **C** | **A** | **A** | **C** | **G** | **G** | **A** | **A** | **A** | **A** | **A** | **A** | **G** | **T** | **G** | **G** | **A** | **A** | **T** | **T** | **G** | **G** | **C** | **T** | **A** | **A** | **A** | **T** | **C** | **T** | **T** | **T** | **A** | **T** | **C** | **A** | **C** | **A** | **G** | **C** | **C** | **A** | **G** | **C** | **T** | **G** | **A** | **A** | **C** | **T** | **A** | **T** | **A** | **T** | **A** | **T** | **A** | **T** | **G** | **G** | **G** | **A** | **T** | **G** | **A** | **A** | **C** | **C** | **A** | **T** | **T** | **G** | **A** | **A** | **T** | **T** | **A** | **T** | **T** | **T** | **G** | **G** | **A** | **T** | **G** | **T** | **C** | **T** | **T** | **C** | **A** | **A** | **T** | **C** | **A** | **A** | **G** | **A** | **A** | **C** | **A** | **A** | **T** | **T** | **A** | **G** | **A** | **A** | **C** | **A** | **A** | **C** | **T** | **G** | **A** | **T** | **C** | **T** | **T** | **G** | **A** | **A** | **C** | **G** | **T** | **G** | **A** | **A** | **A** | **C** | **C** | **T** | **G** | **C** | **C** | **A** | **T** | **G** | **T** | **T** | **A** | **C** | **T** | **G** | **G** | **T** | **G** | **G** | **A** | **A** | **C** | **A** | **T** | **G** | **A** | **T** | **C** | **A** | **A** | **A** | **C** | **C** | **T** | **T** | **C** | **C** | **T** | **G** | **G** | **A** | **T** | **A** | **A** | **A** | **G** | **T** | **A** | **T** | **C** | **T** | **A** | **C** | **T** | **G** | **A** | **G** | **A** | **T** | **T** | **A** | **T** | **T** | **T** | **C** | **T** | **C** | **T** | **T** | **G** | **A** | **G** | **A** | **A** | **A** | **A** | **T** | **C** | **-** | **-** | **-** |
| **N60123F** | **A** | **T** | **G** | **T** | **C** | **T** | **A** | **A** | **A** | **A** | **T** | **C** | **G** | **A** | **A** | **A** | **T** | **A** | **A** | **A** | **A** | **A** | **A** | **T** | **C** | **T** | **G** | **A** | **C** | **A** | **T** | **T** | **C** | **G** | **G** | **C** | **T** | **A** | **C** | **G** | **A** | **C** | **A** | **G** | **C** | **C** | **A** | **A** | **G** | **G** | **C** | **A** | **C** | **A** | **T** | **T** | **A** | **T** | **T** | **A** | **T** | **T** | **T** | **G** | **A** | **A** | **C** | **A** | **A** | **G** | **C** | **A** | **A** | **A** | **T** | **C** | **T** | **A** | **A** | **A** | **T** | **T** | **T** | **T** | **G** | **A** | **C** | **A** | **C** | **A** | **C** | **A** | **A** | **T** | **G** | **G** | **A** | **A** | **A** | **C** | **T** | **A** | **G** | **G** | **A** | **C** | **T** | **T** | **A** | **T** | **C** | **G** | **G** | **A** | **C** | **G** | **A** | **A** | **A** | **C** | **G** | **G** | **T** | **C** | **G** | **A** | **G** | **G** | **A** | **A** | **A** | **G** | **A** | **C** | **A** | **A** | **C** | **T** | **T** | **T** | **A** | **C** | **T** | **G** | **A** | **A** | **T** | **A** | **T** | **T** | **C** | **T** | **A** | **C** | **A** | **A** | **A** | **A** | **C** | **A** | **A** | **A** | **C** | **T** | **A** | **C** | **C** | **T** | **T** | **A** | **T** | **C** | **A** | **A** | **G** | **G** | **G** | **C** | **A** | **A** | **G** | **T** | **A** | **A** | **T** | **C** | **C** | **A** | **T** | **C** | **A** | **G** | **C** | **A** | **A** | **G** | **A** | **A** | **T** | **T** | **T** | **G** | **T** | **C** | **T** | **A** | **T** | **T** | **T** | **C** | **C** | **C** | **G** | **C** | **A** | **A** | **C** | **A** | **A** | **A** | **C** | **A** | **A** | **A** | **A** | **G** | **A** | **T** | **A** | **A** | **A** | **G** | **A** | **A** | **C** | **G** | **T** | **T** | **T** | **A** | **A** | **C** | **C** | **T** | **A** | **T** | **T** | **A** | **C** | **G** | **T** | **G** | **T** | **T** | **A** | **A** | **A** | **T** | **G** | **A** | **T** | **A** | **T** | **T** | **A** | **C** | **G** | **G** | **A** | **T** | **T** | **T** | **T** | **G** | **A** | **G** | **A** | **T** | **A** | **T** | **G** | **G** | **G** | **A** | **A** | **A** | **T** | **C** | **G** | **A** | **A** | **A** | **G** | **A** | **G** | **A** | **G** | **C** | **T** | **C** | **C** | **A** | **A** | **T** | **T** | **G** | **A** | **T** | **G** | **C** | **A** | **A** | **A** | **C** | **A** | **G** | **A** | **T** | **C** | **C** | **T** | **G** | **A** | **A** | **A** | **T** | **C** | **T** | **T** | **A** | **T** | **G** | **G** | **A** | **G** | **A** | **G** | **A** | **A** | **T** | **T** | **C** | **A** | **G** | **C** | **A** | **C** | **A** | **C** | **T** | **A** | **T** | **C** | **G** | **G** | **G** | **G** | **G** | **G** | **A** | **G** | **A** | **G** | **A** | **A** | **G** | **A** | **C** | **A** | **A** | **A** | **A** | **G** | **T** | **C** | **C** | **T** | **A** | **C** | **T** | **G** | **G** | **C** | **A** | **C** | **T** | **T** | **T** | **T** | **A** | **T** | **T** | **T** | **G** | **T** | **G** | **G** | **A** | **T** | **G** | **A** | **C** | **A** | **C** | **T** | **C** | **A** | **T** | **T** | **T** | **C** | **C** | **C** | **G** | **T** | **T** | **A** | **A** | **T** | **C** | **G** | **A** | **T** | **G** | **A** | **A** | **C** | **C** | **A** | **A** | **C** | **G** | **A** | **A** | **T** | **C** | **A** | **T** | **T** | **T** | **G** | **G** | **A** | **-** | **-** | **-** | **T** | **A** | **T** | **C** | **T** | **C** | **T** | **G** | **G** | **T** | **A** | **G** | **A** | **A** | **A** | **A** | **C** | **A** | **A** | **G** | **T** | **A** | **G** | **C** | **G** | **G** | **C** | **T** | **T** | **A** | **T** | **T** | **T** | **G** | **A** | **A** | **A** | **A** | **A** | **G** | **A** | **A** | **A** | **A** | **A** | **A** | **C** | **A** | **A** | **G** | **G** | **C** | **T** | **T** | **C** | **A** | **T** | **C** | **G** | **T** | **G** | **G** | **T** | **C** | **A** | **G** | **C** | **C** | **A** | **T** | **G** | **A** | **C** | **C** | **G** | **G** | **G** | **G** | **A** | **T** | **T** | **T** | **A** | **T** | **C** | **G** | **A** | **T** | **G** | **A** | **A** | **G** | **T** | **A** | **G** | **T** | **G** | **G** | **A** | **C** | **C** | **A** | **T** | **G** | **T** | **T** | **T** | **T** | **A** | **G** | **C** | **A** | **A** | **T** | **C** | **G** | **A** | **A** | **A** | **A** | **A** | **A** | **G** | **T** | **C** | **A** | **A** | **C** | **T** | **G** | **G** | **A** | **A** | **C** | **T** | **T** | **T** | **A** | **T** | **C** | **A** | **A** | **G** | **G** | **G** | **A** | **A** | **T** | **T** | **T** | **C** | **T** | **C** | **T** | **A** | **T** | **C** | **T** | **A** | **T** | **G** | **A** | **A** | **G** | **A** | **A** | **C** | **A** | **G** | **A** | **A** | **A** | **G** | **A** | **A** | **C** | **T** | **T** | **C** | **G** | **T** | **G** | **A** | **T** | **G** | **A** | **A** | **T** | **T** | **T** | **G** | **A** | **A** | **A** | **T** | **G** | **G** | **C** | **T** | **C** | **A** | **A** | **A** | **A** | **T** | **G** | **A** | **A** | **A** | **A** | **A** | **T** | **T** | **G** | **A** | **A** | **A** | **A** | **A** | **A** | **G** | **A** | **A** | **G** | **T** | **C** | **A** | **G** | **T** | **A** | **G** | **G** | **C** | **T** | **A** | **A** | **A** | **G** | **A** | **A** | **A** | **A** | **C** | **A** | **G** | **C** | **A** | **G** | **C** | **T** | **G** | **A** | **A** | **A** | **A** | **A** | **G** | **C** | **C** | **G** | **A** | **A** | **T** | **G** | **G** | **T** | **C** | **T** | **C** | **G** | **T** | **T** | **C** | **C** | **C** | **G** | **A** | **G** | **A** | **A** | **G** | **G** | **A** | **G** | **A** | **T** | **A** | **A** | **A** | **A** | **C** | **A** | **A** | **A** | **G** | **A** | **A** | **A** | **C** | **A** | **A** | **G** | **T** | **C** | **G** | **G** | **A** | **T** | **T** | **C** | **A** | **T** | **C** | **G** | **A** | **T** | **A** | **C** | **T** | **G** | **A** | **A** | **T** | **C** | **T** | **A** | **G** | **A** | **C** | **G** | **A** | **G** | **T** | **G** | **A** | **A** | **T** | **A** | **A** | **A** | **G** | **G** | **A** | **G** | **C** | **A** | **G** | **T** | **G** | **G** | **G** | **T** | **G** | **C** | **T** | **G** | **A** | **T** | **G** | **C** | **T** | **G** | **C** | **A** | **C** | **G** | **G** | **A** | **C** | **G** | **A** | **T** | **G** | **A** | **A** | **A** | **C** | **G** | **A** | **T** | **C** | **C** | **A** | **A** | **A** | **G** | **C** | **A** | **A** | **T** | **C** | **G** | **T** | **G** | **A** | **A** | **T** | **C** | **G** | **G** | **A** | **T** | **G** | **G** | **A** | **G** | **A** | **C** | **C** | **C** | **A** | **G** | **A** | **T** | **C** | **A** | **G** | **C** | **G** | **A** | **G** | **A** | **A** | **A** | **G** | **A** | **A** | **A** | **A** | **A** | **C** | **T** | **A** | **T** | **T** | **A** | **A** | **A** | **A** | **G** | **A** | **T** | **A** | **T** | **C** | **G** | **A** | **A** | **T** | **A** | **T** | **A** | **T** | **C** | **G** | **A** | **T** | **T** | **C** | **T** | **T** | **T** | **G** | **A** | **C** | **G** | **A** | **T** | **G** | **A** | **A** | **T** | **A** | **G** | **C** | **C** | **A** | **A** | **G** | **C** | **G** | **T** | **C** | **T** | **C** | **A** | **C** | **C** | **A** | **T** | **A** | **A** | **G** | **C** | **G** | **A** | **C** | **T** | **T** | **T** | **T** | **A** | **A** | **G** | **C** | **G** | **T** | **A** | **G** | **A** | **A** | **G** | **A** | **T** | **C** | **T** | **T** | **C** | **A** | **A** | **T** | **T** | **A** | **G** | **G** | **G** | **T** | **A** | **T** | **G** | **A** | **A** | **A** | **A** | **T** | **C** | **T** | **G** | **T** | **T** | **A** | **T** | **T** | **C** | **G** | **A** | **G** | **C** | **C** | **A** | **A** | **T** | **T** | **C** | **A** | **T** | **T** | **T** | **T** | **A** | **C** | **A** | **A** | **T** | **C** | **G** | **A** | **G** | **C** | **C** | **T** | **C** | **A** | **T** | **C** | **A** | **G** | **C** | **G** | **G** | **G** | **T** | **G** | **G** | **C** | **G** | **A** | **T** | **T** | **T** | **C** | **A** | **G** | **G** | **T** | **C** | **C** | **T** | **A** | **A** | **C** | **G** | **G** | **T** | **G** | **C** | **A** | **G** | **G** | **A** | **A** | **A** | **G** | **T** | **C** | **A** | **T** | **C** | **C** | **A** | **T** | **T** | **A** | **T** | **C** | **C** | **A** | **T** | **T** | **A** | **T** | **C** | **T** | **T** | **C** | **T** | **G** | **G** | **G** | **G** | **G** | **C** | **A** | **T** | **T** | **C** | **A** | **A** | **C** | **G** | **G** | **C** | **A** | **A** | **G** | **G** | **T** | **T** | **A** | **T** | **A** | **G** | **G** | **A** | **G** | **A** | **A** | **A** | **A** | **A** | **T** | **C** | **C** | **C** | **A** | **G** | **C** | **C** | **A** | **A** | **A** | **A** | **C** | **A** | **T** | **C** | **T** | **G** | **A** | **G** | **C** | **A** | **T** | **T** | **A** | **G** | **T** | **T** | **A** | **T** | **G** | **T** | **A** | **C** | **G** | **C** | **C** | **A** | **A** | **A** | **A** | **T** | **T** | **A** | **T** | **G** | **A** | **A** | **G** | **A** | **C** | **A** | **A** | **T** | **C** | **G** | **A** | **G** | **G** | **A** | **A** | **C** | **G** | **T** | **T** | **G** | **G** | **C** | **G** | **G** | **A** | **A** | **T** | **T** | **T** | **G** | **C** | **A** | **G** | **A** | **G** | **A** | **A** | **A** | **A** | **A** | **C** | **C** | **A** | **A** | **G** | **T** | **A** | **G** | **A** | **C** | **T** | **A** | **C** | **C** | **A** | **A** | **G** | **C** | **A** | **T** | **T** | **T** | **T** | **T** | **G** | **A** | **A** | **C** | **A** | **A** | **C** | **C** | **T** | **C** | **C** | **G** | **A** | **A** | **A** | **G** | **C** | **T** | **T** | **G** | **G** | **G** | **A** | **T** | **G** | **G** | **A** | **A** | **A** | **G** | **A** | **G** | **A** | **T** | **G** | **T** | **T** | **T** | **T** | **T** | **C** | **A** | **T** | **A** | **A** | **C** | **A** | **A** | **G** | **A** | **T** | **T** | **G** | **A** | **G** | **C** | **A** | **G** | **A** | **T** | **G** | **A** | **G** | **T** | **A** | **T** | **G** | **G** | **G** | **C** | **C** | **A** | **A** | **C** | **G** | **G** | **A** | **A** | **A** | **A** | **A** | **A** | **G** | **T** | **G** | **G** | **A** | **A** | **T** | **T** | **G** | **G** | **C** | **T** | **A** | **A** | **A** | **T** | **C** | **T** | **T** | **T** | **A** | **T** | **C** | **A** | **C** | **A** | **G** | **C** | **C** | **A** | **G** | **C** | **T** | **G** | **A** | **A** | **C** | **T** | **A** | **T** | **A** | **T** | **A** | **T** | **A** | **T** | **G** | **G** | **G** | **A** | **T** | **G** | **A** | **A** | **C** | **C** | **A** | **T** | **T** | **G** | **A** | **A** | **T** | **T** | **A** | **T** | **T** | **T** | **G** | **G** | **A** | **T** | **G** | **T** | **C** | **T** | **T** | **C** | **A** | **A** | **T** | **C** | **A** | **A** | **G** | **A** | **A** | **C** | **A** | **A** | **T** | **T** | **A** | **G** | **A** | **A** | **C** | **A** | **A** | **C** | **T** | **G** | **A** | **T** | **C** | **T** | **T** | **G** | **A** | **A** | **C** | **G** | **T** | **G** | **A** | **A** | **A** | **C** | **C** | **T** | **G** | **C** | **C** | **A** | **T** | **G** | **T** | **T** | **A** | **C** | **T** | **G** | **G** | **T** | **G** | **G** | **A** | **A** | **C** | **A** | **T** | **G** | **A** | **T** | **C** | **A** | **A** | **A** | **C** | **C** | **T** | **T** | **C** | **C** | **T** | **G** | **G** | **A** | **T** | **A** | **A** | **A** | **G** | **T** | **A** | **T** | **C** | **T** | **A** | **C** | **T** | **G** | **A** | **G** | **A** | **T** | **T** | **A** | **T** | **T** | **T** | **C** | **T** | **C** | **T** | **T** | **G** | **A** | **G** | **A** | **A** | **A** | **A** | **T** | **C** | **-** | **-** | **-** |
| **N53595** | **A** | **T** | **G** | **T** | **C** | **T** | **A** | **A** | **A** | **A** | **T** | **C** | **G** | **A** | **A** | **A** | **T** | **A** | **A** | **A** | **A** | **A** | **A** | **T** | **C** | **T** | **G** | **A** | **C** | **A** | **T** | **T** | **C** | **G** | **G** | **C** | **T** | **A** | **C** | **G** | **A** | **C** | **A** | **G** | **C** | **C** | **A** | **A** | **G** | **G** | **C** | **A** | **C** | **A** | **T** | **T** | **A** | **T** | **T** | **A** | **T** | **T** | **T** | **G** | **A** | **A** | **C** | **A** | **A** | **G** | **C** | **A** | **A** | **A** | **T** | **C** | **T** | **A** | **A** | **A** | **T** | **T** | **T** | **T** | **G** | **A** | **C** | **A** | **C** | **A** | **C** | **A** | **A** | **T** | **G** | **G** | **A** | **A** | **A** | **C** | **T** | **A** | **G** | **G** | **A** | **C** | **T** | **T** | **A** | **T** | **C** | **G** | **G** | **A** | **C** | **G** | **A** | **A** | **A** | **C** | **G** | **G** | **T** | **C** | **G** | **A** | **G** | **G** | **A** | **A** | **A** | **G** | **A** | **C** | **A** | **A** | **C** | **T** | **T** | **T** | **A** | **C** | **T** | **G** | **A** | **A** | **T** | **A** | **T** | **T** | **C** | **T** | **A** | **C** | **A** | **A** | **A** | **A** | **C** | **A** | **A** | **A** | **C** | **T** | **A** | **C** | **C** | **T** | **T** | **A** | **T** | **C** | **A** | **A** | **G** | **G** | **G** | **C** | **A** | **A** | **G** | **T** | **A** | **A** | **T** | **C** | **C** | **A** | **T** | **C** | **A** | **G** | **C** | **A** | **A** | **G** | **A** | **A** | **T** | **T** | **T** | **G** | **T** | **C** | **T** | **A** | **T** | **T** | **T** | **C** | **C** | **C** | **G** | **C** | **A** | **G** | **C** | **A** | **A** | **A** | **C** | **A** | **A** | **A** | **A** | **G** | **A** | **T** | **A** | **A** | **A** | **G** | **A** | **G** | **C** | **G** | **T** | **T** | **T** | **A** | **A** | **C** | **C** | **T** | **A** | **T** | **T** | **A** | **T** | **G** | **T** | **G** | **T** | **T** | **A** | **A** | **A** | **T** | **G** | **A** | **G** | **A** | **T** | **T** | **A** | **C** | **G** | **G** | **A** | **T** | **T** | **T** | **T** | **G** | **A** | **G** | **A** | **T** | **A** | **T** | **G** | **G** | **G** | **A** | **A** | **A** | **T** | **T** | **G** | **A** | **A** | **A** | **G** | **A** | **G** | **A** | **G** | **C** | **T** | **C** | **C** | **A** | **A** | **C** | **T** | **G** | **A** | **T** | **G** | **C** | **A** | **A** | **A** | **C** | **A** | **G** | **A** | **T** | **C** | **C** | **T** | **G** | **A** | **A** | **A** | **T** | **C** | **T** | **T** | **A** | **T** | **G** | **G** | **A** | **G** | **A** | **G** | **A** | **A** | **T** | **T** | **C** | **A** | **G** | **C** | **A** | **C** | **A** | **C** | **T** | **A** | **T** | **C** | **A** | **G** | **G** | **A** | **G** | **G** | **A** | **G** | **A** | **G** | **A** | **A** | **G** | **A** | **C** | **A** | **A** | **A** | **A** | **G** | **T** | **C** | **C** | **T** | **A** | **C** | **T** | **G** | **G** | **C** | **A** | **C** | **T** | **T** | **T** | **T** | **A** | **T** | **T** | **T** | **G** | **T** | **G** | **G** | **A** | **T** | **G** | **A** | **C** | **A** | **C** | **T** | **C** | **A** | **T** | **T** | **T** | **C** | **C** | **C** | **G** | **T** | **T** | **A** | **A** | **T** | **C** | **G** | **A** | **T** | **G** | **A** | **A** | **C** | **C** | **A** | **A** | **C** | **G** | **A** | **A** | **T** | **C** | **A** | **T** | **T** | **T** | **G** | **G** | **A** | **-** | **-** | **-** | **T** | **A** | **T** | **C** | **T** | **C** | **T** | **G** | **G** | **C** | **A** | **G** | **A** | **A** | **A** | **A** | **C** | **A** | **A** | **G** | **T** | **A** | **G** | **C** | **G** | **G** | **C** | **T** | **T** | **A** | **C** | **T** | **T** | **G** | **A** | **A** | **A** | **A** | **A** | **G** | **A** | **A** | **A** | **A** | **A** | **A** | **C** | **A** | **A** | **G** | **G** | **C** | **T** | **T** | **C** | **A** | **T** | **C** | **G** | **T** | **G** | **G** | **T** | **C** | **A** | **G** | **C** | **C** | **A** | **T** | **G** | **A** | **C** | **C** | **G** | **G** | **G** | **G** | **A** | **T** | **T** | **T** | **A** | **T** | **C** | **G** | **A** | **T** | **G** | **A** | **A** | **G** | **T** | **A** | **G** | **T** | **G** | **G** | **A** | **C** | **C** | **A** | **T** | **G** | **T** | **T** | **T** | **T** | **A** | **G** | **C** | **A** | **A** | **T** | **C** | **G** | **A** | **A** | **A** | **A** | **A** | **A** | **G** | **T** | **C** | **A** | **A** | **C** | **T** | **G** | **G** | **A** | **A** | **C** | **T** | **T** | **T** | **A** | **T** | **C** | **A** | **A** | **G** | **G** | **G** | **A** | **A** | **T** | **T** | **T** | **C** | **T** | **C** | **T** | **A** | **T** | **C** | **T** | **A** | **T** | **G** | **A** | **A** | **G** | **A** | **A** | **C** | **A** | **G** | **A** | **A** | **A** | **A** | **A** | **A** | **C** | **T** | **T** | **C** | **G** | **T** | **G** | **A** | **T** | **G** | **A** | **A** | **T** | **T** | **T** | **G** | **A** | **A** | **A** | **T** | **G** | **G** | **C** | **T** | **C** | **A** | **A** | **A** | **A** | **T** | **G** | **A** | **A** | **A** | **A** | **A** | **T** | **T** | **G** | **A** | **A** | **A** | **A** | **A** | **A** | **G** | **A** | **A** | **G** | **T** | **C** | **A** | **G** | **T** | **A** | **G** | **G** | **C** | **T** | **A** | **A** | **A** | **G** | **A** | **A** | **A** | **A** | **C** | **A** | **G** | **C** | **A** | **G** | **C** | **T** | **G** | **A** | **A** | **A** | **A** | **A** | **G** | **C** | **C** | **G** | **A** | **A** | **T** | **G** | **G** | **T** | **C** | **T** | **C** | **G** | **T** | **T** | **C** | **C** | **C** | **G** | **A** | **G** | **A** | **A** | **G** | **G** | **A** | **G** | **A** | **T** | **A** | **A** | **A** | **A** | **C** | **A** | **A** | **A** | **G** | **A** | **A** | **A** | **C** | **A** | **A** | **G** | **T** | **C** | **G** | **G** | **A** | **T** | **T** | **C** | **A** | **T** | **C** | **G** | **A** | **T** | **A** | **C** | **T** | **G** | **A** | **A** | **T** | **C** | **T** | **A** | **G** | **A** | **C** | **G** | **A** | **G** | **T** | **G** | **A** | **A** | **T** | **A** | **A** | **A** | **G** | **G** | **A** | **G** | **C** | **A** | **G** | **T** | **G** | **G** | **G** | **T** | **G** | **C** | **T** | **G** | **A** | **T** | **G** | **C** | **T** | **G** | **C** | **A** | **C** | **G** | **G** | **A** | **C** | **G** | **A** | **T** | **G** | **A** | **A** | **A** | **C** | **G** | **A** | **T** | **C** | **C** | **A** | **A** | **A** | **G** | **C** | **A** | **A** | **T** | **C** | **G** | **T** | **G** | **A** | **A** | **T** | **C** | **G** | **G** | **A** | **T** | **G** | **G** | **A** | **G** | **A** | **C** | **C** | **C** | **A** | **G** | **A** | **T** | **C** | **A** | **G** | **C** | **G** | **A** | **G** | **A** | **A** | **A** | **G** | **A** | **A** | **A** | **A** | **A** | **C** | **T** | **A** | **T** | **T** | **G** | **A** | **A** | **A** | **G** | **A** | **T** | **A** | **T** | **C** | **G** | **A** | **A** | **T** | **A** | **T** | **A** | **T** | **C** | **G** | **A** | **T** | **T** | **C** | **T** | **T** | **T** | **G** | **A** | **C** | **G** | **A** | **T** | **G** | **A** | **A** | **T** | **A** | **G** | **C** | **C** | **A** | **A** | **G** | **C** | **G** | **T** | **C** | **T** | **C** | **A** | **C** | **C** | **A** | **T** | **A** | **A** | **G** | **C** | **G** | **A** | **C** | **T** | **T** | **T** | **T** | **A** | **A** | **G** | **C** | **G** | **T** | **A** | **G** | **A** | **A** | **G** | **A** | **T** | **C** | **T** | **T** | **C** | **A** | **A** | **T** | **T** | **A** | **G** | **G** | **G** | **T** | **A** | **T** | **G** | **A** | **G** | **A** | **A** | **T** | **C** | **T** | **G** | **T** | **T** | **A** | **T** | **T** | **C** | **G** | **A** | **G** | **C** | **C** | **A** | **A** | **T** | **T** | **C** | **A** | **T** | **T** | **T** | **T** | **A** | **C** | **A** | **A** | **T** | **C** | **G** | **A** | **G** | **C** | **C** | **T** | **C** | **A** | **T** | **C** | **A** | **G** | **C** | **G** | **G** | **G** | **T** | **G** | **G** | **C** | **G** | **A** | **T** | **T** | **T** | **C** | **A** | **G** | **G** | **T** | **C** | **C** | **T** | **A** | **A** | **C** | **G** | **G** | **T** | **G** | **C** | **A** | **G** | **G** | **A** | **A** | **A** | **G** | **T** | **C** | **A** | **T** | **C** | **C** | **A** | **T** | **T** | **A** | **T** | **C** | **C** | **A** | **T** | **T** | **A** | **T** | **C** | **T** | **T** | **C** | **T** | **G** | **G** | **G** | **G** | **G** | **C** | **A** | **T** | **T** | **C** | **A** | **A** | **C** | **G** | **G** | **C** | **A** | **A** | **G** | **G** | **T** | **T** | **A** | **T** | **A** | **G** | **G** | **A** | **G** | **A** | **A** | **A** | **A** | **A** | **T** | **C** | **C** | **C** | **A** | **G** | **C** | **C** | **A** | **A** | **A** | **A** | **C** | **A** | **T** | **C** | **T** | **G** | **A** | **G** | **C** | **A** | **T** | **T** | **A** | **G** | **T** | **T** | **A** | **T** | **G** | **T** | **A** | **C** | **G** | **C** | **C** | **A** | **A** | **A** | **A** | **T** | **T** | **A** | **T** | **G** | **A** | **A** | **G** | **A** | **C** | **A** | **A** | **T** | **C** | **G** | **A** | **G** | **G** | **A** | **A** | **C** | **G** | **T** | **T** | **G** | **G** | **C** | **G** | **G** | **A** | **A** | **T** | **T** | **T** | **G** | **C** | **A** | **G** | **A** | **G** | **A** | **A** | **A** | **A** | **A** | **C** | **C** | **A** | **A** | **G** | **T** | **A** | **G** | **A** | **C** | **T** | **A** | **C** | **C** | **A** | **A** | **G** | **C** | **A** | **T** | **T** | **T** | **T** | **T** | **G** | **A** | **A** | **C** | **A** | **A** | **C** | **C** | **T** | **C** | **C** | **G** | **A** | **A** | **A** | **G** | **C** | **T** | **T** | **G** | **G** | **G** | **A** | **T** | **G** | **G** | **A** | **A** | **A** | **G** | **A** | **G** | **A** | **T** | **G** | **T** | **T** | **T** | **T** | **T** | **C** | **A** | **T** | **A** | **A** | **C** | **A** | **A** | **G** | **A** | **T** | **C** | **G** | **A** | **G** | **C** | **A** | **G** | **A** | **T** | **G** | **A** | **G** | **T** | **A** | **T** | **G** | **G** | **G** | **C** | **C** | **A** | **A** | **C** | **G** | **G** | **A** | **A** | **A** | **A** | **A** | **A** | **G** | **T** | **G** | **G** | **A** | **A** | **T** | **T** | **G** | **G** | **C** | **T** | **A** | **A** | **A** | **T** | **C** | **T** | **T** | **T** | **A** | **T** | **C** | **A** | **C** | **A** | **G** | **C** | **C** | **A** | **G** | **C** | **T** | **G** | **A** | **A** | **C** | **T** | **A** | **T** | **A** | **T** | **A** | **T** | **A** | **T** | **G** | **G** | **G** | **A** | **T** | **G** | **A** | **A** | **C** | **C** | **A** | **T** | **T** | **G** | **A** | **A** | **T** | **T** | **A** | **T** | **T** | **T** | **G** | **G** | **A** | **T** | **G** | **T** | **C** | **T** | **T** | **C** | **A** | **A** | **T** | **C** | **A** | **A** | **G** | **A** | **A** | **C** | **A** | **A** | **T** | **T** | **A** | **G** | **A** | **A** | **C** | **A** | **A** | **C** | **T** | **G** | **A** | **T** | **C** | **T** | **T** | **G** | **A** | **A** | **C** | **G** | **T** | **G** | **A** | **A** | **A** | **C** | **C** | **T** | **G** | **C** | **C** | **A** | **T** | **G** | **T** | **T** | **A** | **C** | **T** | **A** | **G** | **T** | **G** | **G** | **A** | **A** | **C** | **A** | **T** | **G** | **A** | **T** | **C** | **A** | **A** | **A** | **C** | **C** | **T** | **T** | **C** | **C** | **T** | **G** | **G** | **A** | **T** | **A** | **A** | **A** | **G** | **T** | **A** | **T** | **C** | **T** | **A** | **C** | **T** | **G** | **A** | **G** | **A** | **T** | **T** | **A** | **T** | **T** | **T** | **C** | **T** | **C** | **T** | **T** | **G** | **A** | **G** | **A** | **A** | **A** | **A** | **T** | **C** | **-** | **-** | **-** |
| **N54599** | **A** | **T** | **G** | **T** | **C** | **T** | **A** | **A** | **A** | **A** | **T** | **C** | **G** | **A** | **A** | **A** | **T** | **A** | **A** | **A** | **A** | **A** | **A** | **T** | **C** | **T** | **G** | **A** | **C** | **A** | **T** | **T** | **C** | **G** | **G** | **C** | **T** | **A** | **C** | **G** | **A** | **C** | **A** | **G** | **C** | **C** | **A** | **A** | **G** | **G** | **C** | **A** | **C** | **A** | **T** | **T** | **A** | **T** | **T** | **A** | **T** | **T** | **T** | **G** | **A** | **A** | **C** | **A** | **A** | **G** | **C** | **A** | **A** | **A** | **T** | **C** | **T** | **A** | **A** | **A** | **T** | **T** | **T** | **T** | **G** | **A** | **C** | **A** | **C** | **A** | **C** | **A** | **A** | **T** | **G** | **G** | **A** | **A** | **A** | **C** | **T** | **A** | **G** | **G** | **A** | **C** | **T** | **T** | **A** | **T** | **C** | **G** | **G** | **A** | **C** | **G** | **A** | **A** | **A** | **C** | **G** | **G** | **T** | **C** | **G** | **A** | **G** | **G** | **A** | **A** | **A** | **G** | **A** | **C** | **A** | **A** | **C** | **T** | **T** | **T** | **A** | **C** | **T** | **G** | **A** | **A** | **T** | **A** | **T** | **T** | **C** | **T** | **A** | **C** | **A** | **A** | **A** | **A** | **C** | **A** | **A** | **A** | **C** | **T** | **A** | **C** | **C** | **T** | **T** | **A** | **T** | **C** | **A** | **A** | **G** | **G** | **G** | **C** | **A** | **A** | **G** | **T** | **A** | **A** | **T** | **C** | **C** | **A** | **T** | **C** | **A** | **G** | **C** | **A** | **A** | **G** | **A** | **A** | **T** | **T** | **T** | **G** | **T** | **C** | **T** | **A** | **T** | **T** | **T** | **C** | **C** | **C** | **G** | **C** | **A** | **A** | **C** | **A** | **G** | **A** | **C** | **A** | **A** | **A** | **A** | **G** | **A** | **T** | **A** | **A** | **A** | **G** | **A** | **A** | **C** | **G** | **T** | **T** | **T** | **A** | **A** | **C** | **C** | **T** | **A** | **T** | **T** | **A** | **C** | **G** | **T** | **G** | **T** | **T** | **A** | **A** | **A** | **T** | **G** | **A** | **T** | **A** | **T** | **T** | **A** | **C** | **G** | **G** | **A** | **T** | **T** | **T** | **T** | **G** | **A** | **G** | **A** | **T** | **A** | **T** | **G** | **G** | **G** | **A** | **A** | **A** | **T** | **C** | **G** | **A** | **A** | **A** | **G** | **A** | **G** | **A** | **G** | **C** | **T** | **C** | **C** | **A** | **A** | **T** | **T** | **G** | **A** | **T** | **G** | **C** | **A** | **A** | **A** | **C** | **A** | **G** | **A** | **T** | **C** | **C** | **T** | **G** | **A** | **A** | **A** | **T** | **C** | **T** | **T** | **A** | **T** | **G** | **G** | **A** | **G** | **A** | **G** | **A** | **A** | **T** | **T** | **C** | **A** | **G** | **C** | **A** | **C** | **A** | **C** | **T** | **A** | **T** | **C** | **G** | **G** | **G** | **G** | **G** | **G** | **A** | **G** | **A** | **G** | **A** | **A** | **G** | **A** | **C** | **A** | **A** | **A** | **A** | **G** | **T** | **C** | **C** | **T** | **A** | **C** | **T** | **G** | **G** | **C** | **A** | **C** | **T** | **T** | **T** | **T** | **A** | **T** | **T** | **T** | **G** | **T** | **G** | **G** | **A** | **T** | **G** | **A** | **C** | **A** | **C** | **T** | **C** | **A** | **T** | **T** | **T** | **C** | **C** | **C** | **G** | **T** | **T** | **A** | **A** | **T** | **C** | **G** | **A** | **T** | **G** | **A** | **A** | **C** | **C** | **A** | **A** | **C** | **G** | **A** | **A** | **T** | **C** | **A** | **T** | **T** | **T** | **G** | **G** | **A** | **-** | **-** | **-** | **T** | **A** | **T** | **C** | **T** | **C** | **T** | **G** | **G** | **T** | **A** | **G** | **A** | **A** | **A** | **A** | **C** | **A** | **A** | **G** | **T** | **A** | **G** | **C** | **G** | **G** | **C** | **T** | **T** | **A** | **T** | **T** | **T** | **G** | **A** | **A** | **A** | **A** | **A** | **G** | **A** | **A** | **A** | **A** | **A** | **A** | **C** | **A** | **A** | **G** | **G** | **C** | **T** | **T** | **C** | **A** | **T** | **C** | **G** | **T** | **G** | **G** | **T** | **C** | **A** | **G** | **C** | **C** | **A** | **T** | **G** | **A** | **C** | **C** | **G** | **G** | **G** | **G** | **A** | **T** | **T** | **T** | **A** | **T** | **C** | **G** | **A** | **T** | **G** | **A** | **A** | **G** | **T** | **A** | **G** | **T** | **G** | **G** | **A** | **C** | **C** | **A** | **T** | **G** | **T** | **T** | **T** | **T** | **A** | **G** | **C** | **A** | **A** | **T** | **C** | **G** | **A** | **A** | **A** | **A** | **A** | **A** | **G** | **T** | **C** | **A** | **A** | **C** | **T** | **G** | **G** | **A** | **A** | **C** | **T** | **T** | **T** | **A** | **T** | **C** | **A** | **A** | **G** | **G** | **G** | **A** | **A** | **T** | **T** | **T** | **C** | **T** | **C** | **T** | **A** | **T** | **C** | **T** | **A** | **T** | **G** | **A** | **A** | **G** | **A** | **A** | **C** | **A** | **G** | **A** | **A** | **A** | **A** | **A** | **A** | **C** | **T** | **T** | **C** | **G** | **T** | **G** | **A** | **T** | **G** | **A** | **A** | **T** | **T** | **T** | **G** | **A** | **A** | **A** | **T** | **G** | **G** | **C** | **T** | **C** | **A** | **A** | **A** | **A** | **T** | **G** | **A** | **A** | **A** | **A** | **A** | **T** | **T** | **G** | **A** | **A** | **A** | **A** | **A** | **A** | **G** | **A** | **A** | **G** | **T** | **C** | **A** | **G** | **T** | **A** | **G** | **G** | **C** | **T** | **A** | **A** | **A** | **G** | **A** | **A** | **A** | **A** | **C** | **A** | **G** | **C** | **A** | **G** | **C** | **T** | **G** | **A** | **A** | **A** | **A** | **A** | **G** | **C** | **C** | **G** | **A** | **A** | **T** | **G** | **G** | **T** | **C** | **T** | **C** | **G** | **T** | **T** | **C** | **C** | **C** | **G** | **A** | **G** | **A** | **A** | **G** | **G** | **A** | **G** | **A** | **T** | **A** | **A** | **A** | **A** | **C** | **A** | **A** | **A** | **G** | **A** | **A** | **A** | **C** | **A** | **A** | **G** | **T** | **C** | **G** | **G** | **A** | **T** | **T** | **C** | **A** | **T** | **C** | **G** | **A** | **T** | **A** | **C** | **T** | **G** | **A** | **A** | **T** | **C** | **T** | **A** | **G** | **A** | **C** | **G** | **A** | **G** | **T** | **G** | **A** | **A** | **T** | **A** | **A** | **A** | **G** | **G** | **A** | **G** | **C** | **A** | **G** | **T** | **G** | **G** | **G** | **T** | **G** | **C** | **T** | **G** | **A** | **T** | **G** | **C** | **T** | **G** | **C** | **A** | **C** | **G** | **G** | **A** | **C** | **G** | **A** | **T** | **G** | **A** | **A** | **A** | **C** | **G** | **A** | **T** | **C** | **C** | **A** | **A** | **A** | **G** | **C** | **A** | **A** | **T** | **C** | **G** | **T** | **G** | **A** | **A** | **T** | **C** | **G** | **G** | **A** | **T** | **G** | **G** | **A** | **G** | **A** | **C** | **C** | **C** | **A** | **G** | **A** | **T** | **C** | **A** | **G** | **C** | **G** | **A** | **G** | **A** | **A** | **A** | **G** | **A** | **A** | **A** | **A** | **A** | **C** | **T** | **A** | **T** | **T** | **A** | **A** | **A** | **A** | **G** | **A** | **T** | **A** | **T** | **C** | **G** | **A** | **A** | **T** | **A** | **T** | **A** | **T** | **C** | **G** | **A** | **T** | **T** | **C** | **T** | **T** | **T** | **G** | **A** | **C** | **G** | **A** | **T** | **G** | **A** | **A** | **T** | **A** | **G** | **C** | **C** | **A** | **A** | **G** | **C** | **G** | **T** | **C** | **T** | **C** | **A** | **C** | **C** | **A** | **T** | **A** | **A** | **G** | **C** | **G** | **A** | **C** | **T** | **T** | **T** | **T** | **A** | **A** | **G** | **C** | **G** | **T** | **A** | **G** | **A** | **A** | **G** | **A** | **T** | **C** | **T** | **T** | **C** | **A** | **A** | **T** | **T** | **A** | **G** | **G** | **G** | **T** | **A** | **T** | **G** | **A** | **A** | **A** | **A** | **T** | **C** | **T** | **G** | **T** | **T** | **A** | **T** | **T** | **C** | **G** | **A** | **G** | **C** | **C** | **A** | **A** | **T** | **T** | **C** | **A** | **T** | **T** | **T** | **T** | **A** | **C** | **A** | **A** | **T** | **C** | **G** | **A** | **G** | **C** | **C** | **T** | **C** | **A** | **T** | **C** | **A** | **G** | **C** | **G** | **G** | **G** | **T** | **G** | **G** | **C** | **G** | **A** | **T** | **T** | **T** | **C** | **A** | **G** | **G** | **T** | **C** | **C** | **T** | **A** | **A** | **C** | **G** | **G** | **T** | **G** | **C** | **A** | **G** | **G** | **A** | **A** | **A** | **G** | **T** | **C** | **A** | **T** | **C** | **C** | **A** | **T** | **T** | **A** | **T** | **C** | **C** | **A** | **T** | **T** | **A** | **T** | **C** | **T** | **T** | **C** | **T** | **G** | **G** | **G** | **G** | **G** | **C** | **A** | **T** | **T** | **C** | **A** | **A** | **C** | **G** | **G** | **C** | **A** | **A** | **G** | **G** | **T** | **T** | **A** | **T** | **A** | **G** | **G** | **A** | **G** | **A** | **A** | **A** | **A** | **A** | **T** | **C** | **C** | **C** | **A** | **G** | **C** | **C** | **A** | **A** | **A** | **A** | **C** | **A** | **T** | **C** | **T** | **G** | **A** | **G** | **C** | **A** | **T** | **T** | **A** | **G** | **T** | **T** | **A** | **T** | **G** | **T** | **A** | **C** | **G** | **C** | **C** | **A** | **A** | **A** | **A** | **T** | **T** | **A** | **T** | **G** | **A** | **A** | **G** | **A** | **C** | **A** | **A** | **T** | **C** | **G** | **A** | **G** | **G** | **A** | **A** | **C** | **G** | **T** | **T** | **G** | **G** | **C** | **G** | **G** | **A** | **A** | **T** | **T** | **T** | **G** | **C** | **A** | **G** | **A** | **G** | **A** | **A** | **A** | **A** | **A** | **C** | **C** | **A** | **A** | **G** | **T** | **A** | **G** | **A** | **C** | **T** | **A** | **C** | **C** | **A** | **A** | **G** | **C** | **A** | **T** | **T** | **T** | **T** | **T** | **G** | **A** | **A** | **C** | **A** | **A** | **C** | **C** | **T** | **C** | **C** | **G** | **A** | **A** | **A** | **G** | **C** | **T** | **T** | **G** | **G** | **G** | **A** | **T** | **G** | **G** | **A** | **A** | **A** | **G** | **A** | **G** | **A** | **T** | **G** | **T** | **T** | **T** | **T** | **T** | **C** | **A** | **T** | **A** | **A** | **C** | **A** | **A** | **G** | **A** | **T** | **T** | **G** | **A** | **G** | **C** | **A** | **G** | **A** | **T** | **G** | **A** | **G** | **T** | **A** | **T** | **G** | **G** | **G** | **C** | **C** | **A** | **A** | **C** | **G** | **G** | **A** | **A** | **A** | **A** | **A** | **A** | **G** | **T** | **G** | **G** | **A** | **A** | **T** | **T** | **G** | **G** | **C** | **T** | **A** | **A** | **A** | **T** | **C** | **T** | **T** | **T** | **A** | **T** | **C** | **A** | **C** | **A** | **G** | **C** | **C** | **A** | **G** | **C** | **T** | **G** | **A** | **A** | **C** | **T** | **A** | **T** | **A** | **T** | **A** | **T** | **A** | **T** | **G** | **G** | **G** | **A** | **T** | **G** | **A** | **A** | **C** | **C** | **A** | **T** | **T** | **G** | **A** | **A** | **T** | **T** | **A** | **T** | **T** | **T** | **G** | **G** | **A** | **T** | **G** | **T** | **C** | **T** | **T** | **C** | **A** | **A** | **T** | **C** | **A** | **A** | **G** | **A** | **A** | **C** | **A** | **A** | **T** | **T** | **A** | **G** | **A** | **A** | **C** | **A** | **A** | **C** | **T** | **G** | **A** | **T** | **C** | **T** | **T** | **G** | **A** | **A** | **C** | **G** | **T** | **G** | **A** | **A** | **A** | **C** | **C** | **T** | **G** | **C** | **C** | **A** | **T** | **G** | **T** | **T** | **A** | **C** | **T** | **G** | **G** | **T** | **G** | **G** | **A** | **A** | **C** | **A** | **T** | **G** | **A** | **T** | **C** | **A** | **A** | **A** | **C** | **C** | **T** | **T** | **C** | **C** | **T** | **G** | **G** | **A** | **T** | **A** | **A** | **A** | **G** | **T** | **A** | **T** | **C** | **T** | **A** | **C** | **T** | **G** | **A** | **G** | **A** | **T** | **T** | **A** | **T** | **T** | **T** | **C** | **T** | **C** | **T** | **T** | **G** | **A** | **G** | **A** | **A** | **A** | **A** | **T** | **C** | **-** | **-** | **-** |
| **N59531F** | **A** | **T** | **G** | **T** | **C** | **T** | **A** | **A** | **A** | **A** | **T** | **C** | **G** | **A** | **A** | **A** | **T** | **A** | **A** | **A** | **A** | **A** | **A** | **T** | **C** | **T** | **G** | **A** | **C** | **A** | **T** | **T** | **C** | **G** | **G** | **C** | **T** | **A** | **T** | **G** | **A** | **C** | **A** | **G** | **C** | **C** | **A** | **A** | **G** | **G** | **C** | **A** | **C** | **A** | **T** | **T** | **A** | **T** | **T** | **A** | **T** | **T** | **T** | **G** | **A** | **A** | **C** | **A** | **A** | **G** | **C** | **C** | **A** | **A** | **T** | **C** | **T** | **A** | **A** | **A** | **C** | **T** | **T** | **T** | **G** | **A** | **C** | **A** | **C** | **C** | **C** | **A** | **A** | **T** | **G** | **G** | **A** | **A** | **A** | **C** | **T** | **A** | **G** | **G** | **A** | **C** | **T** | **T** | **A** | **T** | **C** | **G** | **G** | **A** | **C** | **G** | **A** | **A** | **A** | **C** | **G** | **G** | **T** | **C** | **G** | **A** | **G** | **G** | **A** | **A** | **A** | **G** | **A** | **C** | **A** | **A** | **C** | **T** | **T** | **T** | **A** | **C** | **T** | **G** | **A** | **A** | **T** | **A** | **T** | **C** | **C** | **T** | **G** | **C** | **A** | **A** | **A** | **A** | **C** | **A** | **A** | **A** | **C** | **T** | **C** | **C** | **C** | **T** | **T** | **A** | **C** | **C** | **A** | **A** | **G** | **G** | **G** | **C** | **A** | **A** | **G** | **T** | **G** | **A** | **T** | **C** | **C** | **A** | **T** | **C** | **A** | **G** | **C** | **A** | **A** | **G** | **C** | **G** | **T** | **T** | **T** | **G** | **T** | **C** | **T** | **A** | **T** | **T** | **T** | **C** | **C** | **C** | **G** | **C** | **A** | **G** | **C** | **A** | **A** | **A** | **C** | **A** | **A** | **A** | **A** | **G** | **A** | **T** | **A** | **A** | **A** | **G** | **A** | **G** | **C** | **G** | **T** | **T** | **T** | **G** | **A** | **C** | **C** | **T** | **A** | **T** | **T** | **A** | **T** | **G** | **T** | **G** | **T** | **T** | **A** | **A** | **A** | **T** | **G** | **A** | **G** | **A** | **T** | **T** | **A** | **C** | **G** | **G** | **A** | **T** | **T** | **T** | **T** | **G** | **A** | **G** | **A** | **T** | **A** | **T** | **G** | **G** | **G** | **A** | **A** | **A** | **T** | **T** | **G** | **A** | **A** | **A** | **G** | **A** | **G** | **A** | **G** | **C** | **T** | **C** | **C** | **A** | **A** | **C** | **T** | **G** | **A** | **T** | **G** | **C** | **A** | **A** | **A** | **C** | **A** | **G** | **A** | **T** | **C** | **C** | **T** | **G** | **A** | **A** | **A** | **T** | **C** | **T** | **T** | **A** | **T** | **G** | **G** | **A** | **G** | **A** | **G** | **A** | **A** | **T** | **T** | **C** | **A** | **G** | **C** | **A** | **C** | **A** | **C** | **T** | **A** | **T** | **C** | **A** | **G** | **G** | **A** | **G** | **G** | **A** | **G** | **A** | **G** | **A** | **A** | **G** | **A** | **C** | **A** | **A** | **A** | **A** | **G** | **T** | **C** | **C** | **T** | **A** | **C** | **T** | **G** | **G** | **C** | **A** | **C** | **T** | **C** | **T** | **T** | **A** | **T** | **T** | **T** | **G** | **T** | **G** | **G** | **A** | **T** | **G** | **A** | **C** | **A** | **C** | **T** | **C** | **A** | **T** | **T** | **T** | **C** | **C** | **C** | **G** | **T** | **T** | **A** | **A** | **T** | **C** | **G** | **A** | **T** | **G** | **A** | **A** | **C** | **C** | **A** | **A** | **C** | **G** | **A** | **A** | **T** | **C** | **A** | **T** | **T** | **T** | **G** | **G** | **A** | **-** | **-** | **-** | **T** | **A** | **T** | **C** | **T** | **C** | **T** | **G** | **G** | **C** | **A** | **G** | **A** | **A** | **A** | **A** | **C** | **A** | **A** | **G** | **T** | **A** | **G** | **C** | **G** | **G** | **C** | **T** | **T** | **A** | **T** | **T** | **T** | **G** | **A** | **A** | **A** | **A** | **A** | **G** | **A** | **A** | **A** | **A** | **A** | **A** | **C** | **A** | **A** | **G** | **G** | **A** | **T** | **T** | **C** | **A** | **T** | **C** | **G** | **T** | **G** | **G** | **T** | **G** | **A** | **G** | **T** | **C** | **A** | **T** | **G** | **A** | **T** | **C** | **G** | **G** | **G** | **G** | **A** | **T** | **T** | **T** | **A** | **T** | **C** | **G** | **A** | **T** | **G** | **A** | **A** | **G** | **T** | **A** | **G** | **T** | **G** | **G** | **A** | **C** | **C** | **A** | **T** | **G** | **T** | **T** | **T** | **T** | **A** | **G** | **T** | **A** | **A** | **T** | **C** | **G** | **A** | **A** | **A** | **A** | **G** | **A** | **G** | **C** | **C** | **A** | **A** | **C** | **T** | **G** | **G** | **A** | **A** | **C** | **T** | **T** | **T** | **A** | **T** | **C** | **A** | **A** | **G** | **G** | **G** | **A** | **A** | **T** | **T** | **T** | **C** | **T** | **C** | **T** | **A** | **T** | **C** | **T** | **A** | **T** | **G** | **A** | **A** | **G** | **A** | **A** | **C** | **A** | **G** | **A** | **A** | **A** | **A** | **A** | **A** | **C** | **T** | **T** | **C** | **G** | **T** | **G** | **A** | **T** | **G** | **A** | **A** | **T** | **T** | **T** | **G** | **A** | **A** | **A** | **T** | **A** | **G** | **C** | **T** | **A** | **A** | **A** | **A** | **A** | **T** | **G** | **A** | **A** | **A** | **A** | **A** | **T** | **T** | **G** | **A** | **A** | **A** | **A** | **A** | **A** | **G** | **A** | **A** | **G** | **T** | **C** | **A** | **G** | **C** | **A** | **G** | **G** | **C** | **T** | **A** | **A** | **A** | **G** | **A** | **A** | **A** | **A** | **C** | **A** | **G** | **C** | **G** | **G** | **C** | **A** | **G** | **A** | **A** | **A** | **A** | **A** | **G** | **C** | **A** | **G** | **A** | **A** | **T** | **G** | **G** | **T** | **C** | **T** | **C** | **G** | **T** | **T** | **C** | **C** | **C** | **G** | **T** | **G** | **A** | **A** | **G** | **G** | **A** | **G** | **A** | **C** | **A** | **A** | **A** | **A** | **C** | **A** | **A** | **A** | **G** | **A** | **A** | **A** | **A** | **A** | **A** | **G** | **T** | **C** | **G** | **G** | **A** | **T** | **T** | **C** | **A** | **T** | **C** | **G** | **A** | **T** | **A** | **C** | **G** | **G** | **A** | **A** | **T** | **C** | **T** | **A** | **G** | **G** | **C** | **G** | **A** | **G** | **T** | **G** | **A** | **A** | **T** | **A** | **A** | **A** | **G** | **G** | **A** | **G** | **C** | **A** | **G** | **T** | **A** | **G** | **G** | **C** | **G** | **C** | **T** | **G** | **A** | **T** | **G** | **C** | **T** | **G** | **C** | **A** | **C** | **G** | **G** | **A** | **C** | **G** | **A** | **T** | **G** | **A** | **A** | **A** | **C** | **G** | **A** | **T** | **C** | **T** | **A** | **A** | **A** | **G** | **C** | **A** | **A** | **T** | **C** | **G** | **T** | **G** | **A** | **A** | **T** | **C** | **G** | **G** | **A** | **T** | **G** | **G** | **A** | **A** | **A** | **C** | **C** | **C** | **A** | **G** | **A** | **T** | **C** | **A** | **G** | **T** | **G** | **A** | **G** | **A** | **A** | **A** | **G** | **A** | **A** | **A** | **A** | **A** | **T** | **T** | **A** | **T** | **T** | **A** | **A** | **A** | **A** | **G** | **A** | **T** | **A** | **T** | **C** | **G** | **A** | **G** | **T** | **A** | **T** | **A** | **T** | **C** | **G** | **A** | **T** | **G** | **C** | **T** | **T** | **T** | **G** | **A** | **C** | **G** | **A** | **T** | **G** | **A** | **A** | **T** | **A** | **G** | **C** | **C** | **A** | **A** | **G** | **T** | **G** | **T** | **C** | **T** | **C** | **A** | **T** | **C** | **A** | **T** | **A** | **A** | **A** | **C** | **G** | **G** | **C** | **T** | **G** | **T** | **T** | **A** | **A** | **G** | **C** | **G** | **T** | **A** | **G** | **A** | **A** | **A** | **A** | **T** | **C** | **T** | **T** | **C** | **A** | **A** | **T** | **T** | **A** | **G** | **G** | **G** | **T** | **A** | **T** | **G** | **A** | **A** | **A** | **A** | **T** | **C** | **T** | **G** | **T** | **T** | **A** | **T** | **T** | **C** | **G** | **A** | **G** | **C** | **C** | **A** | **G** | **T** | **T** | **C** | **A** | **T** | **T** | **T** | **T** | **A** | **C** | **G** | **A** | **T** | **C** | **G** | **A** | **A** | **C** | **C** | **A** | **C** | **A** | **T** | **C** | **A** | **G** | **C** | **G** | **G** | **G** | **T** | **G** | **G** | **C** | **A** | **A** | **T** | **T** | **T** | **C** | **A** | **G** | **G** | **C** | **C** | **C** | **T** | **A** | **A** | **C** | **G** | **G** | **C** | **G** | **C** | **A** | **G** | **G** | **A** | **A** | **A** | **G** | **T** | **C** | **A** | **T** | **C** | **C** | **A** | **T** | **T** | **A** | **T** | **C** | **C** | **A** | **T** | **T** | **A** | **T** | **C** | **T** | **T** | **C** | **T** | **G** | **G** | **G** | **A** | **A** | **C** | **A** | **T** | **T** | **C** | **A** | **A** | **C** | **G** | **G** | **C** | **A** | **A** | **G** | **G** | **T** | **T** | **G** | **T** | **A** | **G** | **G** | **A** | **G** | **A** | **A** | **A** | **A** | **A** | **T** | **C** | **C** | **C** | **A** | **G** | **C** | **C** | **C** | **A** | **A** | **A** | **C** | **A** | **T** | **T** | **T** | **G** | **A** | **C** | **C** | **A** | **T** | **T** | **A** | **G** | **T** | **T** | **A** | **C** | **G** | **T** | **A** | **C** | **G** | **C** | **C** | **A** | **A** | **A** | **A** | **T** | **T** | **A** | **T** | **G** | **A** | **A** | **G** | **A** | **C** | **A** | **A** | **T** | **C** | **G** | **A** | **G** | **G** | **G** | **A** | **C** | **G** | **T** | **T** | **G** | **G** | **C** | **G** | **G** | **A** | **A** | **T** | **T** | **C** | **G** | **C** | **G** | **G** | **A** | **G** | **A** | **A** | **A** | **A** | **A** | **C** | **C** | **G** | **A** | **G** | **T** | **A** | **G** | **A** | **C** | **T** | **A** | **C** | **C** | **A** | **A** | **G** | **C** | **A** | **T** | **T** | **T** | **T** | **T** | **G** | **A** | **A** | **T** | **A** | **A** | **T** | **C** | **T** | **C** | **C** | **G** | **A** | **A** | **A** | **G** | **C** | **T** | **T** | **G** | **G** | **G** | **A** | **T** | **G** | **G** | **A** | **A** | **A** | **G** | **A** | **G** | **A** | **T** | **G** | **T** | **T** | **T** | **T** | **T** | **C** | **A** | **T** | **A** | **A** | **C** | **A** | **A** | **A** | **A** | **T** | **C** | **G** | **A** | **A** | **C** | **A** | **G** | **A** | **T** | **G** | **A** | **G** | **T** | **A** | **T** | **G** | **G** | **G** | **A** | **C** | **A** | **A** | **C** | **G** | **G** | **A** | **A** | **A** | **A** | **A** | **A** | **G** | **T** | **G** | **G** | **A** | **A** | **T** | **T** | **G** | **G** | **C** | **T** | **A** | **A** | **A** | **T** | **C** | **T** | **T** | **T** | **A** | **T** | **C** | **A** | **C** | **A** | **A** | **C** | **C** | **A** | **G** | **C** | **T** | **G** | **A** | **G** | **C** | **T** | **A** | **T** | **A** | **T** | **A** | **T** | **A** | **T** | **G** | **G** | **G** | **A** | **T** | **G** | **A** | **A** | **C** | **C** | **A** | **T** | **T** | **G** | **A** | **A** | **T** | **T** | **A** | **T** | **T** | **T** | **G** | **G** | **A** | **T** | **G** | **T** | **C** | **T** | **T** | **C** | **A** | **A** | **T** | **C** | **A** | **A** | **G** | **A** | **A** | **C** | **A** | **A** | **T** | **T** | **A** | **G** | **A** | **A** | **C** | **A** | **A** | **C** | **T** | **G** | **A** | **T** | **T** | **T** | **T** | **G** | **A** | **A** | **C** | **G** | **T** | **G** | **A** | **A** | **A** | **C** | **C** | **T** | **G** | **C** | **T** | **A** | **T** | **G** | **T** | **T** | **A** | **C** | **T** | **A** | **G** | **T** | **G** | **G** | **A** | **A** | **C** | **A** | **T** | **G** | **A** | **T** | **C** | **A** | **A** | **A** | **C** | **C** | **T** | **T** | **T** | **C** | **T** | **A** | **G** | **A** | **T** | **A** | **A** | **A** | **G** | **T** | **A** | **T** | **C** | **T** | **A** | **C** | **T** | **G** | **A** | **G** | **A** | **T** | **T** | **A** | **T** | **T** | **T** | **C** | **T** | **C** | **T** | **T** | **G** | **A** | **G** | **A** | **A** | **A** | **A** | **T** | **C** | **-** | **-** | **-** |
 **SNP position** | . | . | . | . | . | . | . | . | . | . | . | . | . | . | . | . | . | . | . | . | . | . | . | . | . | . | . | . | 29 | . | 31 | . | . | . | . | . | . | . | 39 | . | . | . | . | . | . | . | . | . | . | . | . | . | . | . | . | . | . | . | . | . | . | . | . | . | . | . | . | . | . | . | . | 72 | . | 74 | . | . | . | . | . | . | 81 | . | . | . | . | . | . | . | 89 | 90 | . | . | . | . | . | . | . | . | . | . | . | . | . | . | 105 | 106 | . | . | . | . | 111 | 112 | . | . | . | . | . | . | . | . | . | . | . | . | . | . | . | . | . | . | . | . | . | . | . | . | . | . | . | . | . | . | . | . | . | . | . | . | . | 150 | . | . | 153 | . | . | . | . | . | 159 | . | . | . | . | . | 165 | . | . | 168 | . | . | 171 | . | . | . | . | . | . | . | . | . | . | . | 183 | . | . | . | . | . | . | . | . | . | . | . | . | . | 197 | 198 | . | . | . | . | 203 | . | . | . | . | . | . | . | . | . | . | . | . | 216 | . | . | 219 | . | . | . | . | . | . | . | . | . | . | . | . | . | . | 234 | 235 | . | . | . | . | 240 | . | . | . | . | . | . | . | . | 249 | . | . | . | . | . | . | . | . | . | . | . | 261 | . | . | . | . | 266 | . | . | 269 | . | . | . | . | . | . | . | . | 278 | . | . | . | . | . | . | . | . | . | 288 | . | . | . | . | . | . | . | . | . | . | . | . | . | . | . | 304 | . | 306 | . | . | 309 | . | . | . | . | . | . | . | . | . | . | . | . | . | . | . | . | . | . | . | . | . | . | . | . | . | . | . | . | . | . | . | . | . | . | . | . | . | . | . | . | . | . | . | . | 354 | . | . | 357 | . | . | . | . | . | . | . | . | . | . | . | . | . | . | . | . | . | . | . | . | . | 379 | . | . | . | . | . | . | . | 387 | . | . | . | . | . | . | . | . | . | . | . | . | . | . | . | . | . | . | . | . | . | . | . | 411 | . | 413 | . | . | . | . | . | . | . | . | . | . | . | . | . | . | . | . | . | . | . | . | . | . | . | . | . | . | . | 441 | . | . | . | . | . | . | . | . | . | . | . | . | . | 455 | 456 | . | . | . | . | . | . | . | . | . | . | 467 | . | . | . | 471 | . | . | . | . | . | 477 | . | . | . | . | . | . | . | . | . | . | . | . | . | . | . | . | . | . | . | . | 498 | . | . | . | . | . | . | . | . | . | . | . | 510 | . | . | 513 | . | . | . | . | . | 519 | . | . | . | . | . | . | . | . | . | . | . | . | . | . | . | . | . | . | 538 | . | . | . | . | . | . | . | . | . | . | . | . | . | . | . | . | . | . | 557 | . | . | . | . | . | . | 564 | . | . | 567 | . | . | 570 | . | . | . | . | . | . | . | . | . | . | . | . | . | . | . | . | . | . | . | . | 591 | . | . | . | . | . | . | . | . | . | . | . | . | . | . | . | . | . | . | . | . | . | . | . | 615 | . | 617 | . | 619 | . | . | . | . | . | . | . | . | . | . | . | . | . | . | . | . | . | . | . | . | . | . | 642 | . | . | . | 646 | . | . | . | . | . | . | . | . | . | . | . | . | . | . | . | . | . | . | . | . | . | . | . | . | . | . | . | 674 | 675 | . | . | . | . | . | . | . | . | . | . | 686 | . | . | . | . | . | . | 693 | . | . | 696 | . | . | . | . | . | . | . | . | 705 | . | . | . | . | . | . | . | . | . | . | . | . | . | . | . | . | . | 723 | . | . | . | . | . | . | . | . | 732 | . | . | . | . | . | . | . | . | . | . | . | . | 745 | . | . | . | . | 750 | . | . | . | 754 | . | . | . | . | . | . | . | . | . | . | 765 | . | . | . | . | . | . | . | . | 774 | . | . | . | . | . | . | . | . | . | . | . | . | . | . | . | . | . | . | . | . | 795 | . | . | 798 | . | . | . | . | . | . | . | . | . | . | . | . | . | . | . | 814 | . | . | . | . | . | . | . | . | . | . | . | . | . | 828 | . | . | . | . | . | . | . | . | . | . | . | . | . | . | . | . | . | . | . | . | 849 | . | . | 852 | . | . | . | . | . | . | . | . | . | 862 | . | 864 | . | . | . | . | . | . | . | . | . | . | . | . | 877 | . | . | . | . | 882 | . | . | . | . | . | . | . | . | . | . | . | 894 | . | . | . | . | . | . | . | . | . | 904 | . | . | . | . | . | . | . | . | . | . | . | . | . | . | . | . | 921 | . | . | . | . | 926 | . | . | . | . | . | . | 933 | . | . | . | . | . | 939 | . | . | 942 | . | . | 945 | . | . | . | . | . | . | . | . | . | . | . | . | 958 | . | . | . | . | . | . | . | . | . | . | . | . | . | . | . | . | . | . | 977 | 978 | . | . | . | . | . | . | . | . | . | . | . | . | . | . | . | . | . | . | 997 | . | . | . | . | . | . | . | . | . | . | 1008 | . | . | . | . | . | 1014 | . | . | 1017 | 1018 | . | . | . | . | 1023 | . | . | . | . | . | . | . | . | 1032 | . | . | . | . | . | . | 1039 | . | 1041 | . | . | . | . | . | . | . | . | 1050 | . | 1052 | 1053 | . | . | . | . | . | . | . | . | . | . | . | . | . | . | . | . | . | . | . | . | . | . | . | . | . | . | . | . | . | . | 1084 | . | 1086 | 1087 | . | . | 1090 | . | . | . | . | . | . | . | . | . | . | . | . | . | . | 1105 | . | 1107 | . | . | . | . | . | . | . | . | . | . | 1118 | . | . | . | . | . | . | 1125 | . | . | . | . | . | . | 1132 | . | 1134 | . | 1136 | . | . | . | . | . | . | . | . | . | 1146 | . | 1148 | . | 1150 | . | . | . | . | . | . | . | . | . | . | . | . | . | . | . | . | 1167 | . | . | . | . | . | . | . | . | 1176 | . | . | 1179 | . | . | . | . | . | . | . | . | . | . | . | 1191 | . | . | 1194 | 1195 | . | . | . | . | . | . | . | . | . | 1205 | . | . | . | . | . | . | . | . | . | 1215 | . | . | . | . | . | . | . | . | . | . | . | . | . | . | 1230 | . | . | 1233 | . | . | . | . | . | . | . | . | . | . | . | . | . | . | . | . | . | . | . | . | . | . | . | . | . | . | . | . | . | . | . | . | . | . | . | . | . | . | 1272 | . | . | 1275 | . | . | 1278 | . | . | 1281 | . | . | . | . | . | . | . | . | . | . | . | 1293 | . | . | 1296 | . | . | . | . | . | . | . | . | . | . | . | . | . | . | . | . | . | . | . | . | . | . | . | . | . | . | . | . | . | . | . | . | . | . | . | 1332 | . | . | 1335 | . | . | . | . | . | . | . | . | 1344 | . | . | 1347 | . | . | . | . | 1352 | . | . | . | . | . | . | . | . | . | . | . | . | . | . | . | . | . | . | 1371 | . | . | . | . | . | . | . | . | . | . | . | . | . | . | . | . | . | . | . | . | . | . | . | . | . | . | . | . | . | . | . | . | . | . | . | . | . | . | . | . | . | 1413 | . | . | . | . | . | . | . | . | . | . | . | . | . | . | . | . | . | 1431 | . | . | . | . | . | . | . | . | 1440 | . | 1442 | . | . | . | . | . | . | . | . | . | . | . | . | . | . | . | . | . | . | 1461 | . | . | 1464 | . | . | . | . | . | . | . | . | . | . | . | . | . | . | . | . | 1481 | . | . | . | . | . | . | . | . | . | . | . | . | . | . | . | . | . | 1499 | . | . | . | . | . | . | . |

|  |
| --- |
|  |

|  |  |
| --- | --- |
|  |

|  |  |
| --- | --- |
| **Legend** | |
  | **Reference Sequence** |

  |
|  | **Detected SNPs** |
|  |  |
