# Supplementary material for: sraX: A Novel Comprehensive Resistome Analysis Tool
Source: Front Microbiol. 2020 Feb 5;11:52. doi: 10.3389/fmicb.2020.00052 (PMC7025521; doi:10.3389/fmicb.2020.00052)
Supplement: Supplementary file 4 [file Data_Sheet_3.ZIP › cls/cls_Efaecalis_dna.html]

# cls

|  |
| --- |
|  |

|  |
| --- |
|  |

|  |  |
| --- | --- |
| **Definition:** | Cls or cardiolipin synthase is an inner membrane protein involved in membrane synthesis and phosopholipid metabolism with mutations to the gene being capable of conferring daptomycin resistance. |

|  |
| --- |
|  |

|  |
| --- |
|  |

|  |  |  |
| --- | --- | --- |
| **Reference organism:** | *Enterococcus faecalis OG1RF* | [NCBI, CARD] |

|  |
| --- |
|  |

|  |
| --- |
|  |

|  |  |
| --- | --- |
| **Summary Data** | || **Parameter** | **Value_(n)_** || Total number of genomes | 97 |
| MSA length | 1446 |
| Total number of SNPs | 84 |
| SNP density (# SNPs/MSA length) | 0.058 |

|  |
| --- |
|  |

|  |
| --- |
|  |

|  |
| --- |
|  |

|  |  |  |  |  |  |  |  |  |  |  |  |  |  |  |  |  |  |  |  |  |  |  |  |  |  |  |  |  |  |  |  |  |  |  |  |  |  |  |  |  |  |  |  |  |  |  |  |  |  |  |  |  |  |  |  |  |  |  |  |  |  |  |  |  |  |  |  |  |  |  |  |  |  |  |  |  |  |  |  |  |  |  |  |  |  |  |  |  |  |  |  |  |  |  |  |  |  |  |  |  |  |  |  |  |  |  |  |  |  |  |  |  |  |  |  |  |  |  |  |  |  |  |  |  |  |  |  |  |  |  |  |  |  |  |  |  |  |  |  |  |  |  |  |  |  |  |  |  |  |  |  |  |  |  |  |  |  |  |  |  |  |  |  |  |  |  |  |  |  |  |  |  |  |  |  |  |  |  |  |  |  |  |  |  |  |  |  |  |  |  |  |  |  |  |  |  |  |  |  |  |  |  |  |  |  |  |  |  |  |  |  |  |  |  |  |  |  |  |  |  |  |  |  |  |  |  |  |  |  |  |  |  |  |  |  |  |  |  |  |  |  |  |  |  |  |  |  |  |  |  |  |  |  |  |  |  |  |  |  |  |  |  |  |  |  |  |  |  |  |  |  |  |  |  |  |  |  |  |  |  |  |  |  |  |  |  |  |  |  |  |  |  |  |  |  |  |  |  |  |  |  |  |  |  |  |  |  |  |  |  |  |  |  |  |  |  |  |  |  |  |  |  |  |  |  |  |  |  |  |  |  |  |  |  |  |  |  |  |  |  |  |  |  |  |  |  |  |  |  |  |  |  |  |  |  |  |  |  |  |  |  |  |  |  |  |  |  |  |  |  |  |  |  |  |  |  |  |  |  |  |  |  |  |  |  |  |  |  |  |  |  |  |  |  |  |  |  |  |  |  |  |  |  |  |  |  |  |  |  |  |  |  |  |  |  |  |  |  |  |  |  |  |  |  |  |  |  |  |  |  |  |  |  |  |  |  |  |  |  |  |  |  |  |  |  |  |  |  |  |  |  |  |  |  |  |  |  |  |  |  |  |  |  |  |  |  |  |  |  |  |  |  |  |  |  |  |  |  |  |  |  |  |  |  |  |  |  |  |  |  |  |  |  |  |  |  |  |  |  |  |  |  |  |  |  |  |  |  |  |  |  |  |  |  |  |  |  |  |  |  |  |  |  |  |  |  |  |  |  |  |  |  |  |  |  |  |  |  |  |  |  |  |  |  |  |  |  |  |  |  |  |  |  |  |  |  |  |  |  |  |  |  |  |  |  |  |  |  |  |  |  |  |  |  |  |  |  |  |  |  |  |  |  |  |  |  |  |  |  |  |  |  |  |  |  |  |  |  |  |  |  |  |  |  |  |  |  |  |  |  |  |  |  |  |  |  |  |  |  |  |  |  |  |  |  |  |  |  |  |  |  |  |  |  |  |  |  |  |  |  |  |  |  |  |  |  |  |  |  |  |  |  |  |  |  |  |  |  |  |  |  |  |  |  |  |  |  |  |  |  |  |  |  |  |  |  |  |  |  |  |  |  |  |  |  |  |  |  |  |  |  |  |  |  |  |  |  |  |  |  |  |  |  |  |  |  |  |  |  |  |  |  |  |  |  |  |  |  |  |  |  |  |  |  |  |  |  |  |  |  |  |  |  |  |  |  |  |  |  |  |  |  |  |  |  |  |  |  |  |  |  |  |  |  |  |  |  |  |  |  |  |  |  |  |  |  |  |  |  |  |  |  |  |  |  |  |  |  |  |  |  |  |  |  |  |  |  |  |  |  |  |  |  |  |  |  |  |  |  |  |  |  |  |  |  |  |  |  |  |  |  |  |  |  |  |  |  |  |  |  |  |  |  |  |  |  |  |  |  |  |  |  |  |  |  |  |  |  |  |  |  |  |  |  |  |  |  |  |  |  |  |  |  |  |  |  |  |  |  |  |  |  |  |  |  |  |  |  |  |  |  |  |  |  |  |  |  |  |  |  |  |  |  |  |  |  |  |  |  |  |  |  |  |  |  |  |  |  |  |  |  |  |  |  |  |  |  |  |  |  |  |  |  |  |  |  |  |  |  |  |  |  |  |  |  |  |  |  |  |  |  |  |  |  |  |  |  |  |  |  |  |  |  |  |  |  |  |  |  |  |  |  |  |  |  |  |  |  |  |  |  |  |  |  |  |  |  |  |  |  |  |  |  |  |  |  |  |  |  |  |  |  |  |  |  |  |  |  |  |  |  |  |  |  |  |  |  |  |  |  |  |  |  |  |  |  |  |  |  |  |  |  |  |  |  |  |  |  |  |  |  |  |  |  |  |  |  |  |  |  |  |  |  |  |  |  |  |  |  |  |  |  |  |  |  |  |  |  |  |  |  |  |  |  |  |  |  |  |  |  |  |  |  |  |  |  |  |  |  |  |  |  |  |  |  |  |  |  |  |  |  |  |  |  |  |  |  |  |  |  |  |  |  |  |  |  |  |  |  |  |  |  |  |  |  |  |  |  |  |  |  |  |  |  |  |  |  |  |  |  |  |  |  |  |  |  |  |  |  |  |  |  |  |  |  |  |  |  |  |  |  |  |  |  |  |  |  |  |  |  |  |  |  |  |  |  |  |  |  |  |  |  |  |  |  |  |  |  |  |  |  |  |  |  |  |  |  |  |  |  |  |  |  |  |  |  |  |  |  |  |  |  |  |  |  |  |  |  |  |  |  |  |  |  |  |  |  |  |  |  |  |  |  |  |  |  |  |  |  |  |  |  |  |  |  |  |  |  |  |  |  |  |  |  |  |  |  |  |  |  |  |  |  |  |  |  |  |  |  |  |  |  |  |  |  |  |  |  |  |  |  |  |  |  |  |  |  |  |  |  |  |  |  |  |  |  |  |  |  |  |  |  |  |  |  |  |  |  |  |  |  |  |  |  |  |  |  |  |  |  |  |  |  |  |  |  |  |  |  |  |  |  |  |  |  |  |  |  |  |  |  |  |  |  |  |  |  |  |  |  |  |  |  |  |  |  |  |  |  |  |  |  |  |  |  |  |  |  |  |  |  |  |  |  |  |  |  |  |  |  |  |  |  |  |  |  |  |  |  |  |  |  |  |  |  |  |  |  |  |  |  |  |  |  |  |  |  |  |  |  |  |  |  |  |  |  |  |  |  |  |  |  |  |  |  |  |  |  |  |  |  |  |  |  |  |  |  |  |  |  |  |  |  |  |  |  |  |  |  |  |  |  |  |  |  |  |  |  |  |  |  |  |  |  |  |  |  |
| --- | --- | --- | --- | --- | --- | --- | --- | --- | --- | --- | --- | --- | --- | --- | --- | --- | --- | --- | --- | --- | --- | --- | --- | --- | --- | --- | --- | --- | --- | --- | --- | --- | --- | --- | --- | --- | --- | --- | --- | --- | --- | --- | --- | --- | --- | --- | --- | --- | --- | --- | --- | --- | --- | --- | --- | --- | --- | --- | --- | --- | --- | --- | --- | --- | --- | --- | --- | --- | --- | --- | --- | --- | --- | --- | --- | --- | --- | --- | --- | --- | --- | --- | --- | --- | --- | --- | --- | --- | --- | --- | --- | --- | --- | --- | --- | --- | --- | --- | --- | --- | --- | --- | --- | --- | --- | --- | --- | --- | --- | --- | --- | --- | --- | --- | --- | --- | --- | --- | --- | --- | --- | --- | --- | --- | --- | --- | --- | --- | --- | --- | --- | --- | --- | --- | --- | --- | --- | --- | --- | --- | --- | --- | --- | --- | --- | --- | --- | --- | --- | --- | --- | --- | --- | --- | --- | --- | --- | --- | --- | --- | --- | --- | --- | --- | --- | --- | --- | --- | --- | --- | --- | --- | --- | --- | --- | --- | --- | --- | --- | --- | --- | --- | --- | --- | --- | --- | --- | --- | --- | --- | --- | --- | --- | --- | --- | --- | --- | --- | --- | --- | --- | --- | --- | --- | --- | --- | --- | --- | --- | --- | --- | --- | --- | --- | --- | --- | --- | --- | --- | --- | --- | --- | --- | --- | --- | --- | --- | --- | --- | --- | --- | --- | --- | --- | --- | --- | --- | --- | --- | --- | --- | --- | --- | --- | --- | --- | --- | --- | --- | --- | --- | --- | --- | --- | --- | --- | --- | --- | --- | --- | --- | --- | --- | --- | --- | --- | --- | --- | --- | --- | --- | --- | --- | --- | --- | --- | --- | --- | --- | --- | --- | --- | --- | --- | --- | --- | --- | --- | --- | --- | --- | --- | --- | --- | --- | --- | --- | --- | --- | --- | --- | --- | --- | --- | --- | --- | --- | --- | --- | --- | --- | --- | --- | --- | --- | --- | --- | --- | --- | --- | --- | --- | --- | --- | --- | --- | --- | --- | --- | --- | --- | --- | --- | --- | --- | --- | --- | --- | --- | --- | --- | --- | --- | --- | --- | --- | --- | --- | --- | --- | --- | --- | --- | --- | --- | --- | --- | --- | --- | --- | --- | --- | --- | --- | --- | --- | --- | --- | --- | --- | --- | --- | --- | --- | --- | --- | --- | --- | --- | --- | --- | --- | --- | --- | --- | --- | --- | --- | --- | --- | --- | --- | --- | --- | --- | --- | --- | --- | --- | --- | --- | --- | --- | --- | --- | --- | --- | --- | --- | --- | --- | --- | --- | --- | --- | --- | --- | --- | --- | --- | --- | --- | --- | --- | --- | --- | --- | --- | --- | --- | --- | --- | --- | --- | --- | --- | --- | --- | --- | --- | --- | --- | --- | --- | --- | --- | --- | --- | --- | --- | --- | --- | --- | --- | --- | --- | --- | --- | --- | --- | --- | --- | --- | --- | --- | --- | --- | --- | --- | --- | --- | --- | --- | --- | --- | --- | --- | --- | --- | --- | --- | --- | --- | --- | --- | --- | --- | --- | --- | --- | --- | --- | --- | --- | --- | --- | --- | --- | --- | --- | --- | --- | --- | --- | --- | --- | --- | --- | --- | --- | --- | --- | --- | --- | --- | --- | --- | --- | --- | --- | --- | --- | --- | --- | --- | --- | --- | --- | --- | --- | --- | --- | --- | --- | --- | --- | --- | --- | --- | --- | --- | --- | --- | --- | --- | --- | --- | --- | --- | --- | --- | --- | --- | --- | --- | --- | --- | --- | --- | --- | --- | --- | --- | --- | --- | --- | --- | --- | --- | --- | --- | --- | --- | --- | --- | --- | --- | --- | --- | --- | --- | --- | --- | --- | --- | --- | --- | --- | --- | --- | --- | --- | --- | --- | --- | --- | --- | --- | --- | --- | --- | --- | --- | --- | --- | --- | --- | --- | --- | --- | --- | --- | --- | --- | --- | --- | --- | --- | --- | --- | --- | --- | --- | --- | --- | --- | --- | --- | --- | --- | --- | --- | --- | --- | --- | --- | --- | --- | --- | --- | --- | --- | --- | --- | --- | --- | --- | --- | --- | --- | --- | --- | --- | --- | --- | --- | --- | --- | --- | --- | --- | --- | --- | --- | --- | --- | --- | --- | --- | --- | --- | --- | --- | --- | --- | --- | --- | --- | --- | --- | --- | --- | --- | --- | --- | --- | --- | --- | --- | --- | --- | --- | --- | --- | --- | --- | --- | --- | --- | --- | --- | --- | --- | --- | --- | --- | --- | --- | --- | --- | --- | --- | --- | --- | --- | --- | --- | --- | --- | --- | --- | --- | --- | --- | --- | --- | --- | --- | --- | --- | --- | --- | --- | --- | --- | --- | --- | --- | --- | --- | --- | --- | --- | --- | --- | --- | --- | --- | --- | --- | --- | --- | --- | --- | --- | --- | --- | --- | --- | --- | --- | --- | --- | --- | --- | --- | --- | --- | --- | --- | --- | --- | --- | --- | --- | --- | --- | --- | --- | --- | --- | --- | --- | --- | --- | --- | --- | --- | --- | --- | --- | --- | --- | --- | --- | --- | --- | --- | --- | --- | --- | --- | --- | --- | --- | --- | --- | --- | --- | --- | --- | --- | --- | --- | --- | --- | --- | --- | --- | --- | --- | --- | --- | --- | --- | --- | --- | --- | --- | --- | --- | --- | --- | --- | --- | --- | --- | --- | --- | --- | --- | --- | --- | --- | --- | --- | --- | --- | --- | --- | --- | --- | --- | --- | --- | --- | --- | --- | --- | --- | --- | --- | --- | --- | --- | --- | --- | --- | --- | --- | --- | --- | --- | --- | --- | --- | --- | --- | --- | --- | --- | --- | --- | --- | --- | --- | --- | --- | --- | --- | --- | --- | --- | --- | --- | --- | --- | --- | --- | --- | --- | --- | --- | --- | --- | --- | --- | --- | --- | --- | --- | --- | --- | --- | --- | --- | --- | --- | --- | --- | --- | --- | --- | --- | --- | --- | --- | --- | --- | --- | --- | --- | --- | --- | --- | --- | --- | --- | --- | --- | --- | --- | --- | --- | --- | --- | --- | --- | --- | --- | --- | --- | --- | --- | --- | --- | --- | --- | --- | --- | --- | --- | --- | --- | --- | --- | --- | --- | --- | --- | --- | --- | --- | --- | --- | --- | --- | --- | --- | --- | --- | --- | --- | --- | --- | --- | --- | --- | --- | --- | --- | --- | --- | --- | --- | --- | --- | --- | --- | --- | --- | --- | --- | --- | --- | --- | --- | --- | --- | --- | --- | --- | --- | --- | --- | --- | --- | --- | --- | --- | --- | --- | --- | --- | --- | --- | --- | --- | --- | --- | --- | --- | --- | --- | --- | --- | --- | --- | --- | --- | --- | --- | --- | --- | --- | --- | --- | --- | --- | --- | --- | --- | --- | --- | --- | --- | --- | --- | --- | --- | --- | --- | --- | --- | --- | --- | --- | --- | --- | --- | --- | --- | --- | --- | --- | --- | --- | --- | --- | --- | --- | --- | --- | --- | --- | --- | --- | --- | --- | --- | --- | --- | --- | --- | --- | --- | --- | --- | --- | --- | --- | --- | --- | --- | --- | --- | --- | --- | --- | --- | --- | --- | --- | --- | --- | --- | --- | --- | --- | --- | --- | --- | --- | --- | --- | --- | --- | --- | --- | --- | --- | --- | --- | --- | --- | --- | --- | --- | --- | --- | --- | --- | --- | --- | --- | --- | --- | --- | --- | --- | --- | --- | --- | --- | --- | --- | --- | --- | --- | --- | --- | --- | --- | --- | --- | --- | --- | --- | --- | --- | --- | --- | --- | --- | --- | --- | --- | --- | --- | --- | --- | --- | --- | --- | --- | --- | --- | --- | --- | --- | --- | --- | --- | --- | --- | --- | --- | --- | --- | --- | --- | --- | --- | --- | --- | --- | --- | --- | --- | --- | --- | --- | --- | --- | --- | --- | --- | --- | --- | --- | --- | --- | --- | --- | --- | --- | --- | --- | --- | --- | --- | --- | --- | --- | --- | --- | --- | --- | --- | --- | --- | --- | --- | --- | --- | --- | --- | --- | --- | --- | --- | --- | --- | --- | --- | --- | --- | --- | --- | --- | --- | --- | --- | --- | --- | --- | --- | --- | --- | --- | --- | --- | --- | --- | --- | --- | --- | --- | --- | --- | --- | --- | --- | --- | --- | --- | --- | --- | --- | --- | --- | --- | --- | --- | --- | --- | --- | --- | --- | --- | --- | --- | --- | --- | --- | --- | --- | --- | --- | --- | --- | --- | --- | --- | --- | --- | --- | --- | --- | --- | --- | --- | --- | --- | --- | --- | --- | --- | --- | --- | --- | --- | --- | --- | --- | --- | --- | --- | --- | --- | --- | --- | --- | --- | --- | --- | --- | --- | --- | --- | --- | --- | --- | --- | --- | --- | --- | --- | --- | --- | --- | --- | --- | --- | --- | --- | --- | --- | --- | --- | --- | --- | --- | --- | --- | --- | --- | --- | --- | --- | --- | --- | --- | --- | --- | --- | --- | --- | --- | --- | --- | --- | --- | --- | --- | --- | --- | --- | --- | --- | --- | --- | --- | --- | --- | --- | --- | --- | --- | --- | --- | --- | --- | --- | --- | --- | --- | --- | --- | --- | --- | --- | --- | --- | --- | --- | --- | --- | --- | --- | --- | --- | --- | --- | --- | --- | --- | --- | --- | --- | --- | --- | --- | --- | --- | --- | --- |
|  || **Enterococcus faecalis OG1RF (AEA93051.1)** | **T** | **T** | **G** | **A** | **T** | **T** | **C** | **T** | **T** | **A** | **G** | **C** | **G** | **T** | **C** | **T** | **T** | **A** | **A** | **C** | **A** | **G** | **T** | **T** | **A** | **T** | **T** | **T** | **A** | **T** | **T** | **T** | **T** | **A** | **T** | **T** | **A** | **A** | **T** | **G** | **C** | **A** | **A** | **T** | **T** | **A** | **T** | **T** | **G** | **C** | **G** | **G** | **G** | **A** | **A** | **T** | **T** | **A** | **C** | **C** | **A** | **T** | **T** | **T** | **T** | **G** | **T** | **T** | **A** | **A** | **A** | **A** | **C** | **C** | **T** | **C** | **G** | **A** | **G** | **A** | **T** | **G** | **T** | **A** | **G** | **C** | **A** | **G** | **C** | **A** | **A** | **T** | **T** | **T** | **G** | **G** | **G** | **C** | **A** | **T** | **G** | **G** | **C** | **T** | **A** | **C** | **T** | **C** | **G** | **T** | **A** | **C** | **T** | **G** | **A** | **T** | **T** | **G** | **C** | **T** | **C** | **T** | **A** | **C** | **C** | **C** | **G** | **T** | **T** | **T** | **T** | **T** | **G** | **G** | **T** | **T** | **T** | **T** | **T** | **T** | **C** | **T** | **T** | **A** | **T** | **A** | **T** | **T** | **T** | **A** | **T** | **T** | **C** | **T** | **T** | **T** | **G** | **G** | **T** | **C** | **G** | **C** | **G** | **G** | **T** | **T** | **T** | **A** | **A** | **C** | **C** | **G** | **A** | **C** | **A** | **A** | **A** | **A** | **A** | **G** | **A** | **A** | **A** | **T** | **T** | **T** | **T** | **A** | **T** | **T** | **T** | **G** | **C** | **A** | **A** | **C** | **A** | **A** | **A** | **G** | **T** | **G** | **A** | **T** | **T** | **T** | **G** | **C** | **G** | **G** | **G** | **A** | **A** | **T** | **T** | **A** | **G** | **A** | **G** | **A** | **A** | **T** | **T** | **T** | **T** | **C** | **A** | **G** | **A** | **A** | **T** | **T** | **T** | **C** | **C** | **A** | **A** | **G** | **A** | **A** | **G** | **A** | **A** | **A** | **G** | **T** | **T** | **T** | **T** | **G** | **A** | **A** | **C** | **T** | **T** | **T** | **A** | **T** | **A** | **G** | **T** | **C** | **A** | **A** | **A** | **A** | **G** | **A** | **T** | **G** | **C** | **C** | **G** | **A** | **C** | **G** | **G** | **A** | **G** | **G** | **A** | **G** | **C** | **A** | **A** | **C** | **A** | **A** | **C** | **A** | **G** | **T** | **T** | **T** | **A** | **C** | **A** | **G** | **A** | **C** | **T** | **T** | **T** | **T** | **T** | **T** | **T** | **C** | **C** | **T** | **C** | **G** | **T** | **T** | **A** | **A** | **A** | **T** | **A** | **G** | **G** | **A** | **T** | **G** | **C** | **C** | **T** | **C** | **T** | **G** | **A** | **C** | **T** | **A** | **A** | **G** | **A** | **A** | **A** | **A** | **A** | **T** | **G** | **A** | **T** | **G** | **T** | **T** | **G** | **A** | **A** | **A** | **T** | **T** | **T** | **T** | **T** | **A** | **C** | **G** | **G** | **A** | **T** | **G** | **G** | **T** | **A** | **C** | **G** | **G** | **A** | **A** | **A** | **A** | **A** | **T** | **T** | **C** | **A** | **A** | **T** | **G** | **C** | **G** | **T** | **T** | **G** | **A** | **T** | **G** | **G** | **C** | **G** | **G** | **A** | **T** | **A** | **T** | **A** | **A** | **A** | **A** | **A** | **A** | **A** | **G** | **C** | **T** | **C** | **A** | **G** | **C** | **A** | **C** | **T** | **C** | **C** | **A** | **T** | **T** | **C** | **A** | **T** | **A** | **T** | **T** | **G** | **A** | **A** | **T** | **A** | **T** | **T** | **A** | **T** | **G** | **C** | **C** | **T** | **T** | **T** | **G** | **T** | **A** | **A** | **C** | **G** | **G** | **A** | **T** | **C** | **A** | **T** | **A** | **T** | **C** | **G** | **G** | **T** | **A** | **C** | **C** | **A** | **A** | **A** | **A** | **T** | **T** | **T** | **T** | **G** | **A** | **A** | **C** | **T** | **T** | **A** | **T** | **T** | **A** | **G** | **A** | **A** | **G** | **A** | **A** | **A** | **A** | **G** | **G** | **C** | **G** | **G** | **C** | **T** | **G** | **A** | **A** | **G** | **G** | **G** | **G** | **T** | **T** | **G** | **A** | **A** | **G** | **T** | **G** | **C** | **G** | **G** | **T** | **T** | **G** | **C** | **T** | **T** | **T** | **A** | **C** | **G** | **A** | **T** | **G** | **C** | **C** | **T** | **T** | **T** | **G** | **G** | **C** | **T** | **C** | **A** | **A** | **A** | **G** | **G** | **G** | **C** | **A** | **C** | **C** | **A** | **A** | **G** | **G** | **T** | **T** | **C** | **A** | **T** | **C** | **A** | **T** | **T** | **T** | **G** | **A** | **A** | **T** | **G** | **A** | **A** | **T** | **T** | **G** | **A** | **A** | **A** | **A** | **A** | **A** | **A** | **A** | **C** | **G** | **G** | **T** | **G** | **G** | **T** | **T** | **T** | **T** | **G** | **T** | **C** | **C** | **A** | **A** | **A** | **C** | **G** | **T** | **T** | **T** | **A** | **T** | **T** | **A** | **C** | **T** | **T** | **C** | **T** | **C** | **A** | **A** | **A** | **A** | **A** | **G** | **C** | **A** | **C** | **T** | **T** | **T** | **T** | **G** | **A** | **A** | **G** | **T** | **T** | **T** | **C** | **G** | **T** | **T** | **T** | **G** | **A** | **A** | **T** | **T** | **A** | **T** | **C** | **A** | **T** | **G** | **A** | **T** | **C** | **A** | **C** | **C** | **G** | **G** | **A** | **A** | **A** | **A** | **T** | **T** | **G** | **T** | **T** | **G** | **T** | **T** | **A** | **T** | **T** | **G** | **A** | **C** | **G** | **G** | **A** | **A** | **A** | **A** | **G** | **T** | **A** | **G** | **G** | **C** | **T** | **A** | **C** | **A** | **T** | **T** | **G** | **G** | **C** | **G** | **G** | **C** | **T** | **T** | **T** | **A** | **A** | **T** | **G** | **T** | **T** | **G** | **C** | **C** | **G** | **A** | **T** | **C** | **A** | **A** | **T** | **A** | **T** | **G** | **C** | **C** | **G** | **G** | **A** | **A** | **C** | **G** | **A** | **C** | **T** | **A** | **A** | **A** | **A** | **A** | **G** | **T** | **T** | **T** | **G** | **G** | **C** | **T** | **A** | **T** | **T** | **G** | **G** | **C** | **G** | **G** | **G** | **A** | **T** | **A** | **C** | **A** | **C** | **A** | **T** | **T** | **T** | **A** | **C** | **G** | **G** | **A** | **T** | **T** | **C** | **A** | **A** | **G** | **G** | **G** | **C** | **C** | **A** | **G** | **C** | **A** | **A** | **C** | **C** | **T** | **C** | **A** | **T** | **T** | **A** | **C** | **T** | **G** | **C** | **A** | **A** | **A** | **T** | **G** | **C** | **G** | **T** | **T** | **T** | **T** | **T** | **T** | **A** | **A** | **T** | **G** | **G** | **A** | **T** | **T** | **G** | **G** | **A** | **A** | **C** | **G** | **T** | **C** | **T** | **C** | **T** | **T** | **C** | **C** | **C** | **C** | **C** | **G** | **A** | **G** | **A** | **A** | **A** | **A** | **A** | **T** | **C** | **G** | **T** | **G** | **T** | **G** | **G** | **C** | **G** | **T** | **A** | **T** | **C** | **A** | **A** | **T** | **T** | **G** | **G** | **A** | **T** | **T** | **A** | **T** | **T** | **T** | **C** | **T** | **T** | **T** | **A** | **A** | **A** | **C** | **T** | **T** | **G** | **A** | **A** | **G** | **C** | **A** | **T** | **T** | **G** | **G** | **T** | **G** | **C** | **C** | **A** | **G** | **A** | **G** | **G** | **C** | **A** | **A** | **A** | **T** | **A** | **C** | **A** | **T** | **C** | **C** | **A** | **T** | **T** | **C** | **A** | **G** | **A** | **T** | **G** | **A** | **T** | **T** | **G** | **C** | **C** | **A** | **G** | **T** | **G** | **G** | **T** | **C** | **C** | **T** | **A** | **A** | **C** | **A** | **G** | **T** | **G** | **A** | **C** | **C** | **G** | **T** | **G** | **A** | **A** | **C** | **A** | **A** | **A** | **T** | **T** | **A** | **A** | **A** | **T** | **T** | **G** | **G** | **C** | **C** | **T** | **T** | **T** | **A** | **T** | **T** | **A** | **A** | **A** | **T** | **T** | **G** | **A** | **T** | **T** | **A** | **C** | **T** | **T** | **C** | **T** | **G** | **C** | **C** | **A** | **A** | **G** | **A** | **A** | **A** | **A** | **G** | **A** | **G** | **T** | **C** | **T** | **G** | **G** | **A** | **T** | **T** | **C** | **A** | **A** | **A** | **C** | **G** | **C** | **C** | **G** | **T** | **A** | **T** | **T** | **T** | **A** | **G** | **T** | **T** | **C** | **C** | **T** | **G** | **A** | **T** | **G** | **A** | **T** | **A** | **G** | **T** | **G** | **T** | **C** | **T** | **T** | **G** | **G** | **C** | **T** | **G** | **C** | **T** | **T** | **T** | **A** | **A** | **A** | **G** | **G** | **T** | **T** | **G** | **C** | **T** | **G** | **C** | **G** | **G** | **C** | **T** | **T** | **C** | **A** | **G** | **G** | **A** | **G** | **T** | **A** | **G** | **A** | **T** | **G** | **T** | **T** | **A** | **A** | **A** | **A** | **T** | **T** | **A** | **T** | **G** | **A** | **T** | **T** | **C** | **C** | **A** | **G** | **A** | **T** | **A** | **A** | **G** | **C** | **C** | **C** | **G** | **A** | **C** | **C** | **A** | **T** | **C** | **C** | **G** | **T** | **T** | **T** | **A** | **T** | **T** | **T** | **A** | **T** | **C** | **G** | **A** | **G** | **C** | **A** | **A** | **C** | **A** | **C** | **A** | **G** | **T** | **A** | **T** | **T** | **A** | **C** | **G** | **G** | **C** | **C** | **G** | **C** | **T** | **T** | **A** | **T** | **T** | **G** | **A** | **T** | **G** | **A** | **A** | **A** | **G** | **A** | **A** | **A** | **A** | **T** | **A** | **T** | **T** | **G** | **A** | **A** | **A** | **T** | **T** | **T** | **T** | **A** | **A** | **T** | **T** | **T** | **A** | **T** | **A** | **A** | **C** | **G** | **G** | **T** | **G** | **G** | **T** | **T** | **T** | **C** | **T** | **T** | **A** | **C** | **A** | **T** | **G** | **C** | **G** | **A** | **A** | **A** | **A** | **C** | **A** | **A** | **T** | **G** | **A** | **T** | **T** | **A** | **T** | **G** | **G** | **A** | **T** | **G** | **A** | **T** | **G** | **A** | **A** | **G** | **T** | **C** | **T** | **G** | **C** | **A** | **C** | **A** | **G** | **T** | **T** | **G** | **G** | **T** | **T** | **C** | **A** | **G** | **C** | **C** | **A** | **A** | **T** | **C** | **A** | **A** | **G** | **A** | **T** | **A** | **T** | **C** | **C** | **G** | **A** | **A** | **G** | **T** | **T** | **A** | **C** | **A** | **A** | **A** | **T** | **T** | **A** | **A** | **A** | **C** | **T** | **T** | **T** | **G** | **A** | **A** | **G** | **C** | **A** | **A** | **A** | **T** | **G** | **C** | **T** | **G** | **T** | **G** | **T** | **T** | **A** | **T** | **A** | **T** | **G** | **A** | **T** | **A** | **A** | **A** | **A** | **A** | **A** | **A** | **T** | **C** | **A** | **T** | **T** | **G** | **A** | **T** | **C** | **A** | **A** | **T** | **T** | **A** | **G** | **A** | **A** | **G** | **C** | **A** | **A** | **T** | **T** | **T** | **T** | **C** | **T** | **T** | **A** | **G** | **A** | **A** | **G** | **A** | **T** | **C** | **G** | **A** | **A** | **A** | **A** | **A** | **A** | **A** | **T** | **G** | **T** | **A** | **C** | **A** | **A** | **C** | **A** | **A** | **T** | **G** | **A** | **C** | **T** | **C** | **C** | **A** | **G** | **A** | **A** | **G** | **T** | **T** | **G** | **T** | **T** | **C** | **G** | **T** | **G** | **A** | **C** | **A** | **T** | **G** | **T** | **C** | **A** | **A** | **A** | **A** | **T** | **G** | **G** | **T** | **T** | **G** | **A** | **T** | **T** | **T** | **T** | **T** | **A** | **A** | **A** | **C** | **A** | **A** | **C** | **A** | **A** | **A** | **T** | **T** | **T** | **C** | **A** | **C** | **G** | **A** | **T** | **T** | **A** | **T** | **T** | **T** | **T** | **C** | **A** | **C** | **C** | **A** | **A** | **T** | **T** | **C** | **T** | **T** | **T** | **A** | **A** |
| **N60280F** | **T** | **T** | **G** | **A** | **T** | **T** | **C** | **T** | **T** | **A** | **G** | **C** | **G** | **T** | **C** | **T** | **T** | **A** | **A** | **C** | **A** | **G** | **T** | **T** | **A** | **T** | **T** | **T** | **A** | **T** | **T** | **T** | **T** | **A** | **T** | **T** | **A** | **A** | **T** | **G** | **C** | **A** | **A** | **T** | **T** | **A** | **T** | **T** | **G** | **C** | **G** | **G** | **G** | **A** | **A** | **T** | **T** | **A** | **C** | **C** | **A** | **T** | **T** | **T** | **T** | **G** | **T** | **T** | **A** | **A** | **A** | **A** | **C** | **C** | **T** | **C** | **G** | **A** | **G** | **A** | **T** | **G** | **T** | **A** | **G** | **C** | **A** | **G** | **C** | **A** | **A** | **T** | **T** | **T** | **G** | **G** | **G** | **C** | **A** | **T** | **G** | **G** | **C** | **T** | **A** | **C** | **T** | **C** | **G** | **T** | **A** | **C** | **T** | **G** | **A** | **T** | **T** | **G** | **C** | **T** | **C** | **T** | **A** | **C** | **C** | **C** | **G** | **T** | **T** | **T** | **T** | **T** | **G** | **G** | **T** | **T** | **T** | **T** | **T** | **T** | **C** | **T** | **T** | **A** | **T** | **A** | **T** | **T** | **T** | **A** | **T** | **T** | **C** | **T** | **T** | **T** | **G** | **G** | **G** | **C** | **G** | **C** | **G** | **G** | **T** | **T** | **T** | **A** | **A** | **C** | **C** | **G** | **A** | **C** | **A** | **A** | **A** | **A** | **A** | **G** | **A** | **A** | **A** | **T** | **T** | **T** | **T** | **A** | **T** | **T** | **T** | **G** | **C** | **A** | **A** | **C** | **A** | **A** | **A** | **G** | **T** | **G** | **A** | **T** | **T** | **T** | **G** | **C** | **G** | **G** | **G** | **A** | **A** | **T** | **T** | **A** | **G** | **A** | **G** | **A** | **A** | **T** | **T** | **T** | **T** | **C** | **A** | **G** | **A** | **A** | **T** | **T** | **T** | **C** | **C** | **A** | **A** | **G** | **A** | **A** | **G** | **A** | **A** | **A** | **G** | **T** | **T** | **T** | **T** | **G** | **A** | **A** | **C** | **T** | **T** | **T** | **A** | **T** | **A** | **G** | **T** | **C** | **A** | **A** | **A** | **A** | **G** | **A** | **T** | **G** | **C** | **C** | **G** | **A** | **C** | **G** | **G** | **A** | **G** | **G** | **A** | **G** | **C** | **A** | **A** | **C** | **A** | **A** | **C** | **A** | **G** | **T** | **T** | **T** | **A** | **C** | **A** | **G** | **A** | **C** | **T** | **T** | **T** | **T** | **T** | **T** | **T** | **C** | **C** | **T** | **C** | **G** | **T** | **T** | **A** | **A** | **A** | **T** | **A** | **G** | **G** | **A** | **T** | **G** | **C** | **C** | **T** | **C** | **T** | **G** | **A** | **C** | **T** | **A** | **A** | **G** | **A** | **A** | **A** | **A** | **A** | **T** | **G** | **A** | **T** | **G** | **T** | **T** | **G** | **A** | **A** | **A** | **T** | **T** | **T** | **T** | **T** | **A** | **C** | **G** | **G** | **A** | **T** | **G** | **G** | **T** | **A** | **C** | **G** | **G** | **A** | **A** | **A** | **A** | **A** | **T** | **T** | **C** | **A** | **A** | **T** | **G** | **C** | **G** | **T** | **T** | **G** | **A** | **T** | **G** | **G** | **C** | **G** | **G** | **A** | **C** | **A** | **T** | **A** | **A** | **A** | **A** | **A** | **A** | **A** | **G** | **C** | **T** | **C** | **A** | **G** | **C** | **A** | **T** | **T** | **C** | **C** | **A** | **T** | **T** | **C** | **A** | **T** | **A** | **T** | **T** | **G** | **A** | **A** | **T** | **A** | **T** | **T** | **A** | **T** | **G** | **C** | **C** | **T** | **T** | **T** | **G** | **T** | **A** | **A** | **C** | **G** | **G** | **A** | **T** | **C** | **A** | **T** | **A** | **T** | **C** | **G** | **G** | **T** | **A** | **C** | **C** | **A** | **A** | **A** | **A** | **T** | **T** | **T** | **T** | **G** | **A** | **A** | **C** | **T** | **T** | **A** | **T** | **T** | **A** | **G** | **A** | **A** | **G** | **A** | **A** | **A** | **A** | **G** | **G** | **C** | **G** | **G** | **C** | **T** | **G** | **A** | **A** | **G** | **G** | **G** | **G** | **T** | **T** | **G** | **A** | **A** | **G** | **T** | **G** | **C** | **G** | **G** | **T** | **T** | **G** | **C** | **T** | **T** | **T** | **A** | **C** | **G** | **A** | **T** | **G** | **C** | **C** | **T** | **T** | **T** | **G** | **G** | **C** | **T** | **C** | **A** | **A** | **A** | **G** | **G** | **G** | **C** | **A** | **C** | **C** | **A** | **A** | **G** | **G** | **T** | **T** | **C** | **A** | **T** | **C** | **A** | **T** | **T** | **T** | **G** | **A** | **A** | **T** | **G** | **A** | **A** | **T** | **T** | **G** | **A** | **A** | **A** | **A** | **A** | **A** | **A** | **A** | **C** | **G** | **G** | **C** | **G** | **G** | **T** | **T** | **T** | **T** | **G** | **T** | **C** | **C** | **A** | **A** | **A** | **C** | **G** | **T** | **T** | **T** | **A** | **T** | **T** | **A** | **C** | **T** | **T** | **C** | **T** | **C** | **A** | **A** | **A** | **A** | **A** | **G** | **C** | **A** | **C** | **T** | **T** | **T** | **T** | **G** | **A** | **A** | **G** | **T** | **T** | **T** | **C** | **G** | **T** | **T** | **T** | **G** | **A** | **A** | **T** | **T** | **A** | **T** | **C** | **A** | **T** | **G** | **A** | **T** | **C** | **A** | **C** | **C** | **G** | **G** | **A** | **A** | **A** | **A** | **T** | **T** | **G** | **T** | **T** | **G** | **T** | **T** | **A** | **T** | **T** | **G** | **A** | **T** | **G** | **G** | **G** | **A** | **A** | **A** | **G** | **T** | **A** | **G** | **G** | **C** | **T** | **A** | **C** | **A** | **T** | **T** | **G** | **G** | **C** | **G** | **G** | **C** | **T** | **T** | **T** | **A** | **A** | **T** | **G** | **T** | **T** | **G** | **C** | **C** | **G** | **A** | **T** | **C** | **A** | **A** | **T** | **A** | **T** | **G** | **C** | **T** | **G** | **G** | **A** | **A** | **C** | **G** | **A** | **C** | **T** | **A** | **A** | **A** | **A** | **A** | **G** | **T** | **T** | **T** | **G** | **G** | **C** | **T** | **A** | **T** | **T** | **G** | **G** | **C** | **G** | **G** | **G** | **A** | **T** | **A** | **C** | **A** | **C** | **A** | **T** | **T** | **T** | **A** | **C** | **G** | **G** | **A** | **T** | **T** | **C** | **A** | **A** | **G** | **G** | **G** | **C** | **C** | **A** | **G** | **C** | **A** | **G** | **C** | **C** | **T** | **C** | **A** | **T** | **T** | **A** | **C** | **T** | **G** | **C** | **A** | **A** | **A** | **T** | **G** | **C** | **G** | **T** | **T** | **T** | **T** | **T** | **T** | **A** | **A** | **T** | **G** | **G** | **A** | **T** | **T** | **G** | **G** | **A** | **A** | **C** | **G** | **T** | **C** | **T** | **C** | **T** | **T** | **C** | **C** | **C** | **C** | **T** | **G** | **A** | **G** | **A** | **A** | **A** | **A** | **A** | **T** | **C** | **G** | **T** | **G** | **T** | **G** | **G** | **C** | **G** | **T** | **A** | **T** | **C** | **A** | **A** | **T** | **T** | **G** | **G** | **A** | **T** | **T** | **A** | **T** | **T** | **T** | **C** | **T** | **T** | **T** | **A** | **A** | **A** | **C** | **T** | **T** | **G** | **A** | **A** | **G** | **C** | **G** | **T** | **T** | **G** | **G** | **T** | **G** | **C** | **C** | **A** | **G** | **A** | **G** | **G** | **C** | **A** | **A** | **A** | **T** | **A** | **C** | **A** | **T** | **C** | **C** | **A** | **T** | **T** | **C** | **A** | **G** | **A** | **T** | **G** | **A** | **T** | **T** | **G** | **C** | **C** | **A** | **G** | **T** | **G** | **G** | **T** | **C** | **C** | **T** | **A** | **A** | **T** | **A** | **G** | **T** | **G** | **A** | **C** | **C** | **G** | **T** | **G** | **A** | **A** | **C** | **A** | **A** | **A** | **T** | **T** | **A** | **A** | **A** | **T** | **T** | **G** | **G** | **C** | **C** | **T** | **T** | **T** | **A** | **T** | **T** | **A** | **A** | **A** | **T** | **T** | **G** | **A** | **T** | **T** | **A** | **C** | **T** | **T** | **C** | **T** | **G** | **C** | **C** | **A** | **A** | **G** | **A** | **A** | **A** | **A** | **G** | **A** | **G** | **T** | **C** | **T** | **G** | **G** | **A** | **T** | **T** | **C** | **A** | **A** | **A** | **C** | **G** | **C** | **C** | **G** | **T** | **A** | **T** | **T** | **T** | **A** | **G** | **T** | **T** | **C** | **C** | **T** | **G** | **A** | **T** | **G** | **A** | **T** | **A** | **G** | **T** | **G** | **T** | **C** | **T** | **T** | **G** | **G** | **C** | **G** | **G** | **C** | **T** | **T** | **T** | **A** | **A** | **A** | **G** | **G** | **T** | **T** | **G** | **C** | **T** | **G** | **C** | **G** | **G** | **C** | **T** | **T** | **C** | **A** | **G** | **G** | **A** | **G** | **T** | **A** | **G** | **A** | **T** | **G** | **T** | **T** | **A** | **A** | **A** | **A** | **T** | **T** | **A** | **T** | **G** | **A** | **T** | **T** | **C** | **C** | **A** | **G** | **A** | **T** | **A** | **A** | **G** | **C** | **C** | **C** | **G** | **A** | **C** | **C** | **A** | **T** | **C** | **C** | **G** | **T** | **T** | **T** | **A** | **T** | **T** | **T** | **A** | **T** | **C** | **G** | **A** | **G** | **C** | **A** | **A** | **C** | **A** | **C** | **A** | **G** | **T** | **A** | **T** | **T** | **A** | **C** | **G** | **G** | **G** | **C** | **G** | **C** | **T** | **T** | **G** | **T** | **T** | **G** | **A** | **T** | **G** | **A** | **A** | **A** | **G** | **A** | **A** | **A** | **A** | **T** | **A** | **T** | **T** | **G** | **A** | **A** | **A** | **T** | **T** | **T** | **T** | **A** | **A** | **T** | **T** | **T** | **A** | **T** | **A** | **A** | **C** | **G** | **G** | **T** | **G** | **G** | **T** | **T** | **T** | **C** | **T** | **T** | **A** | **C** | **A** | **T** | **G** | **C** | **G** | **A** | **A** | **A** | **A** | **C** | **A** | **A** | **T** | **G** | **A** | **T** | **T** | **A** | **T** | **G** | **G** | **A** | **T** | **G** | **A** | **T** | **G** | **A** | **A** | **G** | **T** | **C** | **T** | **G** | **C** | **A** | **C** | **A** | **G** | **T** | **A** | **G** | **G** | **T** | **T** | **C** | **A** | **G** | **C** | **C** | **A** | **A** | **T** | **C** | **A** | **A** | **G** | **A** | **T** | **A** | **T** | **C** | **C** | **G** | **A** | **A** | **G** | **T** | **T** | **A** | **C** | **A** | **A** | **A** | **T** | **T** | **A** | **A** | **A** | **C** | **T** | **T** | **T** | **G** | **A** | **A** | **G** | **C** | **A** | **A** | **A** | **T** | **G** | **C** | **T** | **G** | **T** | **G** | **T** | **T** | **A** | **T** | **A** | **T** | **G** | **A** | **T** | **A** | **A** | **A** | **A** | **A** | **A** | **A** | **T** | **C** | **A** | **T** | **T** | **G** | **A** | **T** | **C** | **A** | **A** | **T** | **T** | **A** | **G** | **A** | **A** | **G** | **C** | **A** | **A** | **T** | **T** | **T** | **T** | **C** | **T** | **T** | **A** | **G** | **A** | **A** | **G** | **A** | **T** | **C** | **G** | **A** | **A** | **A** | **A** | **A** | **A** | **A** | **T** | **G** | **T** | **A** | **C** | **A** | **A** | **C** | **A** | **A** | **T** | **G** | **A** | **C** | **T** | **C** | **C** | **A** | **G** | **A** | **A** | **G** | **T** | **T** | **G** | **T** | **T** | **C** | **G** | **C** | **G** | **A** | **C** | **A** | **T** | **G** | **T** | **C** | **A** | **A** | **A** | **A** | **T** | **G** | **G** | **T** | **T** | **G** | **A** | **T** | **T** | **T** | **T** | **T** | **A** | **A** | **A** | **C** | **A** | **A** | **C** | **A** | **A** | **A** | **T** | **T** | **T** | **C** | **A** | **C** | **G** | **A** | **T** | **T** | **A** | **T** | **T** | **C** | **T** | **C** | **A** | **C** | **C** | **A** | **A** | **T** | **T** | **C** | **T** | **T** | **-** | **-** | **-** |
| **N52755** | **T** | **T** | **G** | **A** | **T** | **T** | **C** | **T** | **T** | **A** | **G** | **C** | **G** | **T** | **C** | **T** | **T** | **A** | **A** | **C** | **A** | **G** | **T** | **T** | **A** | **T** | **T** | **T** | **A** | **T** | **T** | **T** | **T** | **A** | **T** | **T** | **A** | **A** | **T** | **G** | **C** | **A** | **A** | **T** | **T** | **A** | **T** | **T** | **G** | **C** | **G** | **G** | **G** | **A** | **A** | **T** | **T** | **A** | **C** | **C** | **A** | **T** | **T** | **T** | **T** | **G** | **T** | **T** | **A** | **A** | **A** | **A** | **C** | **C** | **T** | **C** | **G** | **A** | **G** | **A** | **T** | **G** | **T** | **A** | **G** | **C** | **A** | **G** | **C** | **A** | **A** | **T** | **T** | **T** | **G** | **G** | **G** | **C** | **A** | **T** | **G** | **G** | **C** | **T** | **A** | **C** | **T** | **C** | **G** | **T** | **A** | **C** | **T** | **G** | **A** | **T** | **T** | **G** | **C** | **T** | **C** | **T** | **A** | **C** | **C** | **C** | **G** | **T** | **T** | **T** | **T** | **T** | **G** | **G** | **T** | **T** | **T** | **T** | **T** | **T** | **C** | **T** | **T** | **A** | **T** | **A** | **T** | **T** | **T** | **A** | **T** | **T** | **C** | **T** | **T** | **T** | **G** | **G** | **T** | **C** | **G** | **A** | **G** | **G** | **G** | **T** | **T** | **A** | **A** | **C** | **C** | **G** | **A** | **C** | **A** | **A** | **A** | **A** | **A** | **G** | **A** | **A** | **A** | **T** | **T** | **T** | **T** | **A** | **T** | **T** | **T** | **G** | **C** | **A** | **A** | **C** | **A** | **A** | **A** | **G** | **T** | **G** | **A** | **T** | **T** | **T** | **A** | **C** | **G** | **G** | **G** | **A** | **A** | **T** | **T** | **A** | **G** | **A** | **G** | **A** | **A** | **T** | **T** | **T** | **T** | **C** | **A** | **G** | **A** | **A** | **T** | **T** | **T** | **C** | **C** | **A** | **A** | **G** | **A** | **A** | **G** | **A** | **A** | **A** | **G** | **T** | **T** | **T** | **T** | **G** | **A** | **A** | **C** | **T** | **T** | **T** | **A** | **T** | **A** | **G** | **T** | **C** | **A** | **A** | **A** | **A** | **G** | **A** | **T** | **G** | **C** | **C** | **G** | **A** | **C** | **G** | **G** | **A** | **G** | **G** | **A** | **G** | **C** | **A** | **A** | **C** | **A** | **A** | **C** | **A** | **G** | **T** | **T** | **T** | **A** | **C** | **A** | **G** | **A** | **C** | **T** | **T** | **T** | **T** | **T** | **T** | **T** | **C** | **C** | **T** | **C** | **G** | **T** | **T** | **A** | **A** | **A** | **T** | **A** | **G** | **G** | **A** | **T** | **G** | **C** | **C** | **T** | **C** | **T** | **G** | **A** | **C** | **T** | **A** | **A** | **G** | **A** | **A** | **A** | **A** | **A** | **T** | **G** | **A** | **T** | **G** | **T** | **T** | **G** | **A** | **A** | **A** | **T** | **T** | **T** | **T** | **T** | **A** | **C** | **G** | **G** | **A** | **T** | **G** | **G** | **T** | **A** | **C** | **G** | **G** | **A** | **A** | **A** | **A** | **A** | **T** | **T** | **C** | **A** | **A** | **T** | **G** | **C** | **G** | **T** | **T** | **G** | **A** | **T** | **G** | **G** | **C** | **G** | **G** | **A** | **C** | **A** | **T** | **A** | **A** | **A** | **A** | **A** | **A** | **A** | **G** | **C** | **T** | **C** | **A** | **G** | **C** | **A** | **C** | **T** | **C** | **T** | **A** | **T** | **T** | **C** | **A** | **T** | **A** | **T** | **T** | **G** | **A** | **A** | **T** | **A** | **T** | **T** | **A** | **T** | **G** | **C** | **C** | **T** | **T** | **T** | **G** | **T** | **A** | **A** | **C** | **G** | **G** | **A** | **T** | **C** | **A** | **T** | **A** | **T** | **C** | **G** | **G** | **C** | **A** | **C** | **C** | **A** | **A** | **A** | **A** | **T** | **T** | **T** | **T** | **G** | **A** | **A** | **C** | **T** | **T** | **A** | **T** | **T** | **A** | **G** | **A** | **A** | **G** | **A** | **A** | **A** | **A** | **G** | **G** | **C** | **G** | **G** | **C** | **T** | **G** | **A** | **A** | **G** | **G** | **G** | **G** | **T** | **T** | **G** | **A** | **A** | **G** | **T** | **G** | **C** | **G** | **G** | **T** | **T** | **G** | **C** | **T** | **T** | **T** | **A** | **C** | **G** | **A** | **C** | **G** | **C** | **C** | **T** | **T** | **T** | **G** | **G** | **C** | **T** | **C** | **A** | **A** | **A** | **G** | **G** | **G** | **C** | **A** | **C** | **C** | **A** | **A** | **G** | **G** | **T** | **T** | **C** | **A** | **T** | **C** | **A** | **T** | **T** | **T** | **G** | **A** | **A** | **T** | **G** | **A** | **A** | **T** | **T** | **G** | **A** | **A** | **A** | **A** | **A** | **A** | **A** | **A** | **C** | **G** | **G** | **T** | **G** | **G** | **T** | **T** | **T** | **T** | **G** | **T** | **C** | **C** | **A** | **A** | **A** | **C** | **G** | **T** | **T** | **T** | **A** | **T** | **T** | **A** | **C** | **T** | **T** | **C** | **T** | **C** | **A** | **A** | **A** | **A** | **A** | **G** | **C** | **A** | **C** | **T** | **T** | **T** | **T** | **G** | **A** | **A** | **G** | **T** | **T** | **T** | **C** | **G** | **T** | **T** | **T** | **G** | **A** | **A** | **T** | **T** | **A** | **T** | **C** | **A** | **T** | **G** | **A** | **T** | **C** | **A** | **C** | **C** | **G** | **G** | **A** | **A** | **A** | **A** | **T** | **T** | **G** | **T** | **T** | **G** | **T** | **T** | **A** | **T** | **T** | **G** | **A** | **C** | **G** | **G** | **A** | **A** | **A** | **A** | **G** | **T** | **A** | **G** | **G** | **C** | **T** | **A** | **C** | **A** | **T** | **T** | **G** | **G** | **C** | **G** | **G** | **C** | **T** | **T** | **T** | **A** | **A** | **T** | **G** | **T** | **T** | **G** | **C** | **C** | **G** | **A** | **T** | **C** | **A** | **A** | **T** | **A** | **T** | **G** | **C** | **C** | **G** | **G** | **A** | **A** | **C** | **G** | **A** | **C** | **T** | **A** | **A** | **A** | **A** | **A** | **G** | **T** | **T** | **T** | **G** | **G** | **C** | **T** | **A** | **T** | **T** | **G** | **G** | **C** | **G** | **G** | **G** | **A** | **T** | **A** | **C** | **A** | **C** | **A** | **T** | **T** | **T** | **A** | **C** | **G** | **G** | **A** | **T** | **T** | **C** | **A** | **A** | **G** | **G** | **G** | **C** | **C** | **A** | **G** | **C** | **A** | **G** | **C** | **C** | **T** | **C** | **A** | **T** | **T** | **A** | **C** | **T** | **G** | **C** | **A** | **A** | **A** | **T** | **G** | **C** | **G** | **T** | **T** | **T** | **T** | **T** | **T** | **A** | **A** | **T** | **G** | **G** | **A** | **T** | **T** | **G** | **G** | **A** | **A** | **C** | **G** | **T** | **C** | **T** | **C** | **T** | **T** | **C** | **C** | **C** | **C** | **C** | **G** | **A** | **G** | **A** | **A** | **A** | **A** | **A** | **T** | **C** | **G** | **T** | **G** | **T** | **G** | **G** | **C** | **G** | **T** | **A** | **T** | **C** | **A** | **A** | **T** | **T** | **G** | **G** | **A** | **T** | **T** | **A** | **T** | **T** | **T** | **C** | **T** | **T** | **T** | **A** | **A** | **A** | **C** | **T** | **T** | **G** | **A** | **A** | **G** | **C** | **A** | **T** | **T** | **G** | **G** | **T** | **G** | **C** | **C** | **A** | **G** | **A** | **G** | **G** | **C** | **A** | **A** | **A** | **T** | **A** | **C** | **A** | **T** | **C** | **C** | **A** | **T** | **T** | **C** | **A** | **G** | **A** | **T** | **G** | **A** | **T** | **T** | **G** | **C** | **C** | **A** | **G** | **T** | **G** | **G** | **T** | **C** | **C** | **T** | **A** | **A** | **C** | **A** | **G** | **T** | **G** | **A** | **C** | **C** | **G** | **T** | **G** | **A** | **A** | **C** | **A** | **A** | **A** | **T** | **T** | **A** | **A** | **A** | **T** | **T** | **G** | **G** | **C** | **C** | **T** | **T** | **T** | **A** | **T** | **T** | **A** | **A** | **A** | **T** | **T** | **G** | **A** | **T** | **T** | **A** | **C** | **T** | **T** | **C** | **T** | **G** | **C** | **C** | **A** | **A** | **G** | **A** | **A** | **A** | **A** | **G** | **A** | **G** | **T** | **C** | **T** | **G** | **G** | **A** | **T** | **T** | **C** | **A** | **A** | **A** | **C** | **G** | **C** | **C** | **G** | **T** | **A** | **T** | **T** | **T** | **A** | **G** | **T** | **T** | **C** | **C** | **T** | **G** | **A** | **T** | **G** | **A** | **T** | **A** | **G** | **T** | **G** | **T** | **C** | **T** | **T** | **G** | **G** | **C** | **T** | **G** | **C** | **T** | **T** | **T** | **A** | **A** | **A** | **A** | **G** | **T** | **T** | **G** | **C** | **T** | **G** | **C** | **G** | **G** | **C** | **T** | **T** | **C** | **A** | **G** | **G** | **A** | **G** | **T** | **A** | **G** | **A** | **T** | **G** | **T** | **T** | **A** | **A** | **A** | **A** | **T** | **T** | **A** | **T** | **G** | **A** | **T** | **T** | **C** | **C** | **A** | **G** | **A** | **T** | **A** | **A** | **G** | **C** | **C** | **C** | **G** | **A** | **C** | **C** | **A** | **T** | **C** | **C** | **G** | **T** | **T** | **T** | **A** | **T** | **T** | **T** | **A** | **T** | **C** | **G** | **A** | **G** | **C** | **A** | **A** | **C** | **A** | **C** | **A** | **G** | **T** | **A** | **T** | **T** | **A** | **T** | **G** | **G** | **C** | **C** | **G** | **C** | **T** | **T** | **A** | **T** | **T** | **G** | **A** | **T** | **G** | **A** | **A** | **A** | **G** | **A** | **A** | **A** | **A** | **T** | **A** | **T** | **T** | **G** | **A** | **A** | **A** | **T** | **T** | **T** | **T** | **A** | **A** | **T** | **T** | **T** | **A** | **T** | **A** | **A** | **C** | **G** | **G** | **T** | **G** | **G** | **T** | **T** | **T** | **C** | **T** | **T** | **A** | **C** | **A** | **T** | **G** | **C** | **G** | **A** | **A** | **A** | **A** | **C** | **A** | **A** | **T** | **G** | **A** | **T** | **T** | **A** | **T** | **G** | **G** | **A** | **T** | **G** | **A** | **T** | **G** | **A** | **A** | **G** | **T** | **C** | **T** | **G** | **C** | **A** | **C** | **A** | **G** | **T** | **T** | **G** | **G** | **T** | **T** | **C** | **A** | **G** | **C** | **C** | **A** | **A** | **T** | **C** | **A** | **A** | **G** | **A** | **T** | **A** | **T** | **C** | **C** | **G** | **A** | **A** | **G** | **T** | **T** | **A** | **C** | **A** | **A** | **A** | **T** | **T** | **A** | **A** | **A** | **C** | **T** | **T** | **T** | **G** | **A** | **A** | **G** | **C** | **A** | **A** | **A** | **T** | **G** | **C** | **T** | **G** | **T** | **G** | **T** | **T** | **A** | **T** | **A** | **T** | **G** | **A** | **T** | **A** | **A** | **A** | **A** | **A** | **A** | **A** | **T** | **C** | **A** | **T** | **T** | **G** | **A** | **T** | **C** | **A** | **A** | **T** | **T** | **A** | **G** | **A** | **A** | **G** | **C** | **A** | **A** | **T** | **T** | **T** | **T** | **C** | **T** | **T** | **A** | **G** | **A** | **A** | **G** | **A** | **T** | **C** | **G** | **A** | **A** | **A** | **A** | **A** | **A** | **A** | **T** | **G** | **T** | **A** | **C** | **A** | **A** | **C** | **A** | **A** | **T** | **G** | **A** | **C** | **T** | **C** | **C** | **A** | **G** | **A** | **A** | **G** | **T** | **T** | **G** | **T** | **T** | **C** | **G** | **C** | **G** | **A** | **C** | **A** | **T** | **G** | **T** | **C** | **A** | **A** | **A** | **A** | **T** | **G** | **G** | **T** | **T** | **G** | **A** | **T** | **T** | **T** | **T** | **T** | **A** | **A** | **A** | **C** | **A** | **A** | **C** | **A** | **A** | **A** | **T** | **T** | **T** | **C** | **A** | **C** | **G** | **A** | **T** | **T** | **A** | **T** | **T** | **T** | **T** | **C** | **A** | **C** | **C** | **A** | **A** | **T** | **T** | **C** | **T** | **T** | **-** | **-** | **-** |
| **N60392F** | **T** | **T** | **G** | **A** | **T** | **T** | **C** | **T** | **T** | **A** | **G** | **C** | **G** | **T** | **C** | **T** | **T** | **A** | **A** | **C** | **A** | **G** | **T** | **T** | **A** | **T** | **T** | **T** | **A** | **T** | **T** | **T** | **T** | **A** | **T** | **T** | **A** | **A** | **T** | **G** | **C** | **A** | **A** | **T** | **T** | **A** | **T** | **T** | **G** | **C** | **G** | **G** | **G** | **A** | **A** | **T** | **T** | **A** | **C** | **C** | **A** | **T** | **T** | **T** | **T** | **G** | **T** | **T** | **A** | **A** | **A** | **A** | **C** | **C** | **T** | **C** | **G** | **A** | **G** | **A** | **T** | **G** | **T** | **A** | **G** | **C** | **A** | **G** | **C** | **A** | **A** | **T** | **T** | **T** | **G** | **G** | **G** | **C** | **A** | **T** | **G** | **G** | **C** | **T** | **A** | **C** | **T** | **C** | **G** | **T** | **A** | **C** | **T** | **G** | **A** | **T** | **T** | **G** | **C** | **T** | **C** | **T** | **A** | **C** | **C** | **C** | **G** | **T** | **T** | **T** | **T** | **T** | **G** | **G** | **T** | **T** | **T** | **T** | **T** | **T** | **C** | **T** | **T** | **A** | **T** | **A** | **T** | **T** | **T** | **A** | **T** | **T** | **C** | **T** | **T** | **T** | **G** | **G** | **G** | **C** | **G** | **C** | **G** | **G** | **T** | **T** | **T** | **A** | **A** | **C** | **C** | **G** | **A** | **C** | **A** | **A** | **A** | **A** | **A** | **G** | **A** | **A** | **A** | **T** | **T** | **T** | **T** | **A** | **T** | **T** | **T** | **G** | **C** | **A** | **A** | **C** | **A** | **A** | **A** | **G** | **T** | **G** | **A** | **T** | **T** | **T** | **G** | **C** | **G** | **G** | **G** | **A** | **A** | **T** | **T** | **A** | **G** | **A** | **G** | **A** | **A** | **T** | **T** | **T** | **T** | **C** | **A** | **G** | **A** | **A** | **T** | **T** | **T** | **C** | **C** | **A** | **A** | **G** | **A** | **A** | **G** | **A** | **A** | **A** | **G** | **T** | **T** | **T** | **T** | **G** | **A** | **A** | **C** | **T** | **T** | **T** | **A** | **T** | **A** | **G** | **T** | **C** | **A** | **A** | **A** | **A** | **G** | **A** | **T** | **G** | **C** | **C** | **G** | **A** | **C** | **G** | **G** | **A** | **G** | **G** | **A** | **G** | **C** | **A** | **A** | **C** | **A** | **A** | **C** | **A** | **G** | **T** | **T** | **T** | **A** | **C** | **A** | **G** | **A** | **C** | **T** | **T** | **T** | **T** | **T** | **T** | **T** | **C** | **C** | **T** | **C** | **G** | **T** | **T** | **A** | **A** | **A** | **T** | **A** | **G** | **G** | **A** | **T** | **G** | **C** | **C** | **T** | **C** | **T** | **G** | **A** | **C** | **T** | **A** | **A** | **G** | **A** | **A** | **A** | **A** | **A** | **T** | **G** | **A** | **T** | **G** | **T** | **T** | **G** | **A** | **A** | **A** | **T** | **T** | **T** | **T** | **T** | **A** | **C** | **G** | **G** | **A** | **T** | **G** | **G** | **T** | **A** | **C** | **G** | **G** | **A** | **A** | **A** | **A** | **A** | **T** | **T** | **C** | **A** | **A** | **T** | **G** | **C** | **G** | **T** | **T** | **G** | **A** | **T** | **G** | **G** | **C** | **G** | **G** | **A** | **C** | **A** | **T** | **A** | **A** | **A** | **A** | **A** | **A** | **A** | **G** | **C** | **T** | **C** | **A** | **G** | **C** | **A** | **C** | **T** | **C** | **C** | **A** | **T** | **T** | **C** | **A** | **T** | **A** | **T** | **T** | **G** | **A** | **A** | **T** | **A** | **T** | **T** | **A** | **T** | **G** | **C** | **C** | **T** | **T** | **T** | **G** | **T** | **A** | **A** | **C** | **G** | **G** | **A** | **T** | **C** | **A** | **T** | **A** | **T** | **C** | **G** | **G** | **C** | **A** | **C** | **C** | **A** | **A** | **A** | **A** | **T** | **T** | **T** | **T** | **G** | **A** | **A** | **C** | **T** | **T** | **A** | **T** | **T** | **A** | **G** | **A** | **A** | **G** | **A** | **A** | **A** | **A** | **G** | **G** | **C** | **G** | **G** | **C** | **T** | **G** | **A** | **A** | **G** | **G** | **G** | **G** | **T** | **T** | **G** | **A** | **A** | **G** | **T** | **G** | **C** | **G** | **G** | **T** | **T** | **G** | **C** | **T** | **T** | **T** | **A** | **C** | **G** | **A** | **T** | **G** | **C** | **C** | **T** | **T** | **T** | **G** | **G** | **C** | **T** | **C** | **A** | **A** | **A** | **G** | **G** | **G** | **C** | **A** | **C** | **C** | **A** | **A** | **G** | **G** | **T** | **T** | **C** | **A** | **T** | **C** | **A** | **T** | **T** | **T** | **G** | **A** | **A** | **T** | **G** | **A** | **A** | **T** | **T** | **G** | **A** | **A** | **A** | **A** | **A** | **A** | **A** | **A** | **C** | **G** | **G** | **T** | **G** | **G** | **T** | **T** | **T** | **T** | **G** | **T** | **C** | **C** | **A** | **A** | **A** | **C** | **G** | **T** | **T** | **T** | **A** | **T** | **T** | **A** | **C** | **T** | **T** | **C** | **T** | **C** | **A** | **A** | **A** | **A** | **A** | **G** | **C** | **A** | **C** | **T** | **T** | **T** | **T** | **G** | **A** | **A** | **G** | **T** | **T** | **T** | **C** | **G** | **T** | **T** | **T** | **G** | **A** | **A** | **T** | **T** | **A** | **T** | **C** | **A** | **T** | **G** | **A** | **T** | **C** | **A** | **C** | **C** | **G** | **G** | **A** | **A** | **A** | **A** | **T** | **T** | **G** | **T** | **T** | **G** | **T** | **T** | **A** | **T** | **T** | **G** | **A** | **C** | **G** | **G** | **A** | **A** | **A** | **A** | **G** | **T** | **A** | **G** | **G** | **C** | **T** | **A** | **C** | **A** | **T** | **T** | **G** | **G** | **C** | **G** | **G** | **C** | **T** | **T** | **T** | **A** | **A** | **T** | **G** | **T** | **T** | **G** | **C** | **C** | **G** | **A** | **T** | **C** | **A** | **A** | **T** | **A** | **T** | **G** | **C** | **T** | **G** | **G** | **A** | **A** | **C** | **G** | **A** | **C** | **T** | **A** | **A** | **A** | **A** | **A** | **G** | **T** | **T** | **T** | **G** | **G** | **C** | **T** | **A** | **T** | **T** | **G** | **G** | **C** | **G** | **G** | **G** | **A** | **T** | **A** | **C** | **G** | **C** | **A** | **T** | **T** | **T** | **A** | **C** | **G** | **G** | **A** | **T** | **T** | **C** | **A** | **A** | **G** | **G** | **G** | **C** | **C** | **A** | **G** | **C** | **A** | **G** | **C** | **C** | **T** | **C** | **A** | **T** | **T** | **A** | **C** | **T** | **G** | **C** | **A** | **A** | **A** | **T** | **G** | **C** | **G** | **T** | **T** | **T** | **T** | **T** | **T** | **A** | **A** | **T** | **G** | **G** | **A** | **T** | **T** | **G** | **G** | **A** | **A** | **C** | **G** | **T** | **C** | **T** | **C** | **T** | **T** | **C** | **C** | **C** | **C** | **C** | **G** | **A** | **G** | **A** | **A** | **A** | **A** | **A** | **T** | **C** | **G** | **T** | **G** | **T** | **G** | **G** | **C** | **G** | **T** | **A** | **T** | **C** | **A** | **A** | **T** | **T** | **G** | **G** | **A** | **T** | **T** | **A** | **T** | **T** | **T** | **C** | **T** | **T** | **T** | **A** | **A** | **A** | **C** | **T** | **T** | **G** | **A** | **A** | **G** | **C** | **A** | **T** | **T** | **G** | **G** | **T** | **G** | **C** | **C** | **A** | **G** | **A** | **G** | **G** | **C** | **A** | **A** | **A** | **T** | **A** | **C** | **A** | **T** | **C** | **C** | **A** | **T** | **T** | **C** | **A** | **G** | **A** | **T** | **G** | **A** | **T** | **T** | **G** | **C** | **C** | **A** | **G** | **T** | **G** | **G** | **T** | **C** | **C** | **T** | **A** | **A** | **C** | **A** | **G** | **T** | **G** | **A** | **C** | **C** | **G** | **T** | **G** | **A** | **A** | **C** | **A** | **A** | **A** | **T** | **T** | **A** | **A** | **A** | **T** | **T** | **G** | **G** | **C** | **C** | **T** | **T** | **T** | **A** | **T** | **T** | **A** | **A** | **A** | **T** | **T** | **G** | **A** | **T** | **T** | **A** | **C** | **T** | **T** | **C** | **T** | **G** | **C** | **C** | **A** | **A** | **G** | **A** | **A** | **A** | **A** | **G** | **A** | **G** | **T** | **C** | **T** | **G** | **G** | **A** | **T** | **T** | **C** | **A** | **A** | **A** | **C** | **G** | **C** | **C** | **G** | **T** | **A** | **T** | **T** | **T** | **A** | **G** | **T** | **T** | **C** | **C** | **T** | **G** | **A** | **T** | **G** | **A** | **T** | **A** | **G** | **T** | **G** | **T** | **C** | **T** | **T** | **G** | **G** | **C** | **T** | **G** | **C** | **T** | **T** | **T** | **A** | **A** | **A** | **G** | **G** | **T** | **T** | **G** | **C** | **T** | **G** | **C** | **G** | **G** | **C** | **T** | **T** | **C** | **A** | **G** | **G** | **A** | **G** | **T** | **A** | **G** | **A** | **T** | **G** | **T** | **T** | **A** | **A** | **A** | **A** | **T** | **T** | **A** | **T** | **G** | **A** | **T** | **T** | **C** | **C** | **A** | **G** | **A** | **T** | **A** | **A** | **G** | **C** | **C** | **C** | **G** | **A** | **C** | **C** | **A** | **T** | **C** | **C** | **G** | **T** | **T** | **T** | **A** | **T** | **T** | **T** | **A** | **T** | **C** | **G** | **A** | **G** | **C** | **A** | **A** | **C** | **A** | **C** | **A** | **G** | **T** | **A** | **T** | **T** | **A** | **C** | **G** | **G** | **C** | **C** | **G** | **C** | **T** | **T** | **A** | **T** | **T** | **G** | **A** | **T** | **G** | **A** | **A** | **A** | **G** | **A** | **A** | **A** | **A** | **T** | **A** | **T** | **T** | **G** | **A** | **A** | **A** | **T** | **T** | **T** | **T** | **A** | **A** | **T** | **T** | **T** | **A** | **T** | **A** | **A** | **C** | **G** | **G** | **T** | **G** | **G** | **T** | **T** | **T** | **C** | **T** | **T** | **A** | **C** | **A** | **T** | **G** | **C** | **G** | **A** | **A** | **A** | **A** | **C** | **A** | **A** | **T** | **G** | **A** | **T** | **T** | **A** | **T** | **G** | **G** | **A** | **T** | **G** | **A** | **T** | **G** | **A** | **A** | **G** | **T** | **C** | **T** | **G** | **C** | **A** | **C** | **A** | **G** | **T** | **T** | **G** | **G** | **T** | **T** | **C** | **A** | **G** | **C** | **C** | **A** | **A** | **T** | **C** | **A** | **A** | **G** | **A** | **T** | **A** | **T** | **C** | **C** | **G** | **A** | **A** | **G** | **T** | **T** | **A** | **C** | **A** | **A** | **A** | **T** | **T** | **A** | **A** | **A** | **C** | **T** | **T** | **T** | **G** | **A** | **A** | **G** | **C** | **A** | **A** | **A** | **T** | **G** | **C** | **T** | **G** | **T** | **G** | **T** | **T** | **A** | **T** | **A** | **T** | **G** | **A** | **T** | **A** | **A** | **A** | **A** | **A** | **A** | **A** | **T** | **C** | **A** | **T** | **T** | **G** | **A** | **T** | **C** | **A** | **A** | **T** | **T** | **A** | **G** | **A** | **A** | **G** | **C** | **A** | **A** | **T** | **T** | **T** | **T** | **C** | **T** | **T** | **A** | **G** | **A** | **A** | **G** | **A** | **T** | **C** | **G** | **A** | **A** | **A** | **A** | **A** | **A** | **A** | **T** | **G** | **T** | **A** | **C** | **A** | **A** | **C** | **A** | **A** | **T** | **G** | **A** | **C** | **T** | **C** | **C** | **A** | **G** | **A** | **A** | **G** | **T** | **T** | **G** | **T** | **T** | **C** | **G** | **C** | **G** | **A** | **C** | **A** | **T** | **G** | **T** | **C** | **A** | **A** | **A** | **A** | **T** | **G** | **G** | **T** | **T** | **G** | **A** | **T** | **T** | **T** | **T** | **T** | **A** | **A** | **A** | **C** | **A** | **A** | **C** | **A** | **A** | **A** | **T** | **T** | **T** | **C** | **A** | **C** | **G** | **A** | **T** | **T** | **A** | **T** | **T** | **T** | **T** | **C** | **A** | **C** | **C** | **A** | **A** | **T** | **T** | **C** | **T** | **T** | **-** | **-** | **-** |
| **N52648** | **T** | **T** | **G** | **A** | **T** | **T** | **C** | **T** | **T** | **A** | **G** | **C** | **G** | **T** | **C** | **T** | **T** | **A** | **A** | **C** | **A** | **G** | **T** | **T** | **A** | **T** | **T** | **T** | **A** | **T** | **T** | **T** | **T** | **A** | **T** | **T** | **A** | **A** | **T** | **G** | **C** | **A** | **A** | **T** | **T** | **A** | **T** | **T** | **G** | **C** | **G** | **G** | **G** | **A** | **A** | **T** | **T** | **A** | **C** | **C** | **A** | **T** | **T** | **T** | **T** | **G** | **T** | **T** | **A** | **A** | **A** | **A** | **C** | **C** | **T** | **C** | **G** | **A** | **G** | **A** | **T** | **G** | **T** | **A** | **G** | **C** | **A** | **G** | **C** | **A** | **A** | **T** | **T** | **T** | **G** | **G** | **G** | **C** | **A** | **T** | **G** | **G** | **C** | **T** | **A** | **C** | **T** | **C** | **G** | **T** | **A** | **C** | **T** | **G** | **A** | **T** | **T** | **G** | **C** | **T** | **C** | **T** | **A** | **C** | **C** | **C** | **G** | **T** | **T** | **T** | **T** | **T** | **G** | **G** | **T** | **T** | **T** | **T** | **T** | **T** | **C** | **T** | **T** | **A** | **T** | **A** | **T** | **T** | **T** | **A** | **T** | **T** | **C** | **T** | **T** | **T** | **G** | **G** | **G** | **C** | **G** | **C** | **G** | **G** | **T** | **T** | **T** | **A** | **A** | **C** | **C** | **G** | **A** | **C** | **A** | **A** | **A** | **A** | **A** | **G** | **A** | **A** | **A** | **T** | **T** | **T** | **T** | **A** | **T** | **T** | **T** | **G** | **C** | **A** | **A** | **C** | **A** | **A** | **A** | **G** | **T** | **G** | **A** | **T** | **T** | **T** | **A** | **C** | **G** | **G** | **G** | **A** | **A** | **T** | **T** | **A** | **G** | **A** | **G** | **A** | **A** | **T** | **T** | **T** | **T** | **C** | **A** | **G** | **A** | **A** | **C** | **T** | **T** | **C** | **C** | **A** | **A** | **G** | **A** | **A** | **G** | **A** | **A** | **A** | **G** | **T** | **T** | **T** | **T** | **G** | **A** | **A** | **C** | **T** | **T** | **T** | **A** | **T** | **A** | **G** | **T** | **C** | **A** | **A** | **A** | **A** | **G** | **A** | **T** | **G** | **C** | **C** | **G** | **A** | **C** | **G** | **G** | **A** | **G** | **G** | **A** | **G** | **C** | **A** | **A** | **C** | **A** | **A** | **C** | **A** | **G** | **T** | **T** | **T** | **A** | **C** | **A** | **G** | **A** | **C** | **T** | **T** | **T** | **T** | **T** | **T** | **T** | **C** | **C** | **T** | **C** | **G** | **T** | **T** | **A** | **A** | **A** | **T** | **A** | **G** | **G** | **A** | **T** | **G** | **C** | **C** | **C** | **C** | **T** | **G** | **A** | **C** | **T** | **A** | **A** | **G** | **A** | **A** | **A** | **A** | **A** | **T** | **G** | **A** | **T** | **G** | **T** | **T** | **G** | **A** | **A** | **A** | **T** | **T** | **T** | **T** | **T** | **A** | **C** | **G** | **G** | **A** | **T** | **G** | **G** | **T** | **A** | **C** | **G** | **G** | **A** | **A** | **A** | **A** | **A** | **T** | **T** | **C** | **A** | **A** | **T** | **G** | **C** | **G** | **T** | **T** | **G** | **A** | **T** | **G** | **G** | **C** | **G** | **G** | **A** | **C** | **A** | **T** | **A** | **A** | **A** | **A** | **A** | **A** | **A** | **G** | **C** | **T** | **C** | **A** | **G** | **C** | **A** | **C** | **T** | **C** | **C** | **A** | **T** | **T** | **C** | **A** | **T** | **A** | **T** | **T** | **G** | **A** | **A** | **T** | **A** | **T** | **T** | **A** | **T** | **G** | **C** | **C** | **T** | **T** | **T** | **G** | **T** | **A** | **A** | **C** | **G** | **G** | **A** | **T** | **C** | **A** | **T** | **A** | **T** | **C** | **G** | **G** | **T** | **A** | **C** | **C** | **A** | **A** | **A** | **A** | **T** | **T** | **T** | **T** | **G** | **A** | **A** | **C** | **T** | **T** | **A** | **T** | **T** | **A** | **G** | **A** | **A** | **G** | **A** | **A** | **A** | **A** | **G** | **G** | **C** | **G** | **G** | **C** | **T** | **G** | **A** | **A** | **G** | **G** | **G** | **G** | **T** | **T** | **G** | **A** | **A** | **G** | **T** | **G** | **C** | **G** | **G** | **T** | **T** | **G** | **C** | **T** | **T** | **T** | **A** | **C** | **G** | **A** | **T** | **G** | **C** | **C** | **T** | **T** | **T** | **G** | **G** | **C** | **T** | **C** | **A** | **A** | **A** | **G** | **G** | **G** | **C** | **A** | **C** | **C** | **A** | **A** | **G** | **G** | **T** | **T** | **C** | **A** | **T** | **C** | **A** | **T** | **T** | **T** | **G** | **A** | **A** | **T** | **G** | **A** | **A** | **T** | **T** | **G** | **A** | **A** | **A** | **A** | **A** | **A** | **A** | **A** | **C** | **G** | **G** | **C** | **G** | **G** | **T** | **T** | **T** | **T** | **G** | **T** | **C** | **C** | **A** | **A** | **A** | **C** | **G** | **T** | **T** | **T** | **A** | **T** | **T** | **A** | **C** | **T** | **T** | **C** | **T** | **C** | **A** | **A** | **A** | **A** | **A** | **G** | **C** | **A** | **C** | **T** | **T** | **T** | **T** | **G** | **A** | **A** | **G** | **T** | **T** | **T** | **C** | **G** | **T** | **T** | **T** | **G** | **A** | **A** | **T** | **T** | **A** | **T** | **C** | **A** | **T** | **G** | **A** | **T** | **C** | **A** | **C** | **C** | **G** | **G** | **A** | **A** | **A** | **A** | **T** | **T** | **G** | **T** | **T** | **G** | **T** | **T** | **A** | **T** | **T** | **G** | **A** | **T** | **G** | **G** | **G** | **A** | **A** | **A** | **G** | **T** | **A** | **G** | **G** | **C** | **T** | **A** | **C** | **A** | **T** | **T** | **G** | **G** | **C** | **G** | **G** | **C** | **T** | **T** | **T** | **A** | **A** | **T** | **G** | **T** | **T** | **G** | **C** | **C** | **G** | **A** | **T** | **C** | **A** | **A** | **T** | **A** | **T** | **G** | **C** | **C** | **G** | **G** | **A** | **A** | **C** | **G** | **A** | **C** | **T** | **A** | **A** | **A** | **A** | **A** | **G** | **T** | **T** | **T** | **G** | **G** | **C** | **T** | **A** | **T** | **T** | **G** | **G** | **C** | **G** | **G** | **G** | **A** | **T** | **A** | **C** | **A** | **C** | **A** | **T** | **T** | **T** | **A** | **C** | **G** | **G** | **A** | **T** | **T** | **C** | **A** | **A** | **G** | **G** | **G** | **C** | **C** | **A** | **G** | **C** | **A** | **G** | **C** | **C** | **T** | **C** | **A** | **T** | **T** | **A** | **C** | **T** | **G** | **C** | **A** | **A** | **A** | **T** | **G** | **C** | **G** | **T** | **T** | **T** | **T** | **T** | **T** | **A** | **A** | **T** | **G** | **G** | **A** | **T** | **T** | **G** | **G** | **A** | **A** | **C** | **G** | **T** | **C** | **T** | **C** | **T** | **T** | **C** | **C** | **C** | **C** | **T** | **G** | **A** | **G** | **A** | **A** | **A** | **A** | **A** | **T** | **C** | **G** | **T** | **G** | **T** | **G** | **G** | **C** | **G** | **T** | **A** | **T** | **C** | **A** | **A** | **T** | **T** | **G** | **G** | **A** | **T** | **T** | **A** | **T** | **T** | **T** | **C** | **T** | **T** | **T** | **A** | **A** | **A** | **C** | **T** | **T** | **G** | **A** | **A** | **G** | **C** | **G** | **T** | **T** | **G** | **G** | **T** | **G** | **C** | **C** | **A** | **G** | **A** | **G** | **G** | **C** | **A** | **A** | **A** | **T** | **A** | **C** | **A** | **T** | **C** | **C** | **A** | **T** | **T** | **C** | **A** | **G** | **A** | **T** | **G** | **A** | **T** | **T** | **G** | **C** | **C** | **A** | **G** | **T** | **G** | **G** | **T** | **C** | **C** | **T** | **A** | **A** | **T** | **A** | **G** | **T** | **G** | **A** | **C** | **C** | **G** | **T** | **G** | **A** | **A** | **C** | **A** | **A** | **A** | **T** | **T** | **A** | **A** | **A** | **T** | **T** | **G** | **G** | **C** | **T** | **T** | **T** | **T** | **A** | **T** | **T** | **A** | **A** | **A** | **T** | **T** | **G** | **A** | **T** | **T** | **A** | **C** | **T** | **T** | **C** | **T** | **G** | **C** | **C** | **A** | **A** | **G** | **A** | **A** | **A** | **A** | **G** | **A** | **G** | **T** | **C** | **T** | **G** | **G** | **A** | **T** | **T** | **C** | **A** | **A** | **A** | **C** | **G** | **C** | **C** | **G** | **T** | **A** | **T** | **T** | **T** | **A** | **G** | **T** | **T** | **C** | **C** | **T** | **G** | **A** | **T** | **G** | **A** | **T** | **A** | **G** | **T** | **G** | **T** | **C** | **T** | **T** | **G** | **G** | **C** | **G** | **G** | **C** | **T** | **T** | **T** | **A** | **A** | **A** | **G** | **G** | **T** | **T** | **G** | **C** | **T** | **G** | **C** | **G** | **G** | **C** | **T** | **T** | **C** | **A** | **G** | **G** | **A** | **G** | **T** | **A** | **G** | **A** | **T** | **G** | **T** | **T** | **A** | **A** | **A** | **A** | **T** | **T** | **A** | **T** | **G** | **A** | **T** | **T** | **C** | **C** | **A** | **G** | **A** | **T** | **A** | **A** | **G** | **C** | **C** | **C** | **G** | **A** | **C** | **C** | **A** | **T** | **C** | **C** | **G** | **T** | **T** | **T** | **A** | **T** | **T** | **T** | **A** | **T** | **C** | **G** | **A** | **G** | **C** | **A** | **A** | **C** | **A** | **C** | **A** | **G** | **T** | **A** | **T** | **T** | **A** | **C** | **G** | **G** | **G** | **C** | **G** | **C** | **T** | **T** | **G** | **T** | **T** | **G** | **A** | **T** | **G** | **A** | **A** | **A** | **G** | **A** | **A** | **A** | **A** | **T** | **A** | **T** | **T** | **G** | **A** | **A** | **A** | **T** | **T** | **T** | **T** | **A** | **A** | **T** | **T** | **T** | **A** | **T** | **A** | **A** | **C** | **G** | **G** | **T** | **G** | **G** | **T** | **T** | **T** | **C** | **T** | **T** | **A** | **C** | **A** | **T** | **G** | **C** | **G** | **A** | **A** | **A** | **A** | **C** | **A** | **A** | **T** | **G** | **A** | **T** | **T** | **A** | **T** | **G** | **G** | **A** | **T** | **G** | **A** | **T** | **G** | **A** | **A** | **G** | **T** | **C** | **T** | **G** | **C** | **A** | **C** | **A** | **G** | **T** | **A** | **G** | **G** | **T** | **T** | **C** | **A** | **G** | **C** | **C** | **A** | **A** | **T** | **C** | **A** | **A** | **G** | **A** | **T** | **A** | **T** | **C** | **C** | **G** | **A** | **A** | **G** | **T** | **T** | **A** | **C** | **A** | **A** | **A** | **T** | **T** | **A** | **A** | **A** | **C** | **T** | **T** | **T** | **G** | **A** | **A** | **G** | **C** | **A** | **A** | **A** | **T** | **G** | **C** | **T** | **G** | **T** | **G** | **T** | **T** | **A** | **T** | **A** | **T** | **G** | **A** | **T** | **A** | **A** | **A** | **A** | **A** | **A** | **A** | **T** | **C** | **A** | **T** | **T** | **G** | **A** | **T** | **C** | **A** | **A** | **T** | **T** | **A** | **G** | **A** | **A** | **G** | **C** | **A** | **A** | **T** | **T** | **T** | **T** | **C** | **T** | **T** | **A** | **G** | **A** | **A** | **G** | **A** | **T** | **C** | **G** | **A** | **A** | **A** | **A** | **A** | **A** | **A** | **T** | **G** | **T** | **A** | **C** | **A** | **A** | **C** | **A** | **A** | **T** | **G** | **A** | **C** | **T** | **C** | **C** | **A** | **G** | **A** | **A** | **G** | **T** | **T** | **G** | **T** | **T** | **C** | **G** | **C** | **G** | **A** | **C** | **A** | **T** | **G** | **T** | **C** | **A** | **A** | **A** | **A** | **T** | **G** | **G** | **T** | **T** | **G** | **A** | **T** | **T** | **T** | **T** | **T** | **A** | **A** | **A** | **C** | **A** | **A** | **C** | **A** | **A** | **A** | **T** | **T** | **T** | **C** | **A** | **C** | **G** | **A** | **T** | **T** | **A** | **T** | **T** | **C** | **T** | **C** | **A** | **C** | **C** | **A** | **A** | **T** | **T** | **C** | **T** | **T** | **-** | **-** | **-** |
| **N60114F** | **T** | **T** | **G** | **A** | **T** | **T** | **C** | **T** | **T** | **A** | **G** | **C** | **G** | **T** | **C** | **T** | **T** | **A** | **A** | **C** | **A** | **G** | **T** | **T** | **A** | **T** | **T** | **T** | **A** | **T** | **T** | **T** | **T** | **A** | **T** | **T** | **A** | **A** | **T** | **G** | **C** | **A** | **A** | **T** | **T** | **A** | **T** | **T** | **G** | **C** | **G** | **G** | **G** | **A** | **A** | **T** | **T** | **A** | **C** | **C** | **A** | **T** | **T** | **T** | **T** | **G** | **T** | **T** | **A** | **A** | **A** | **A** | **C** | **C** | **T** | **C** | **G** | **A** | **G** | **A** | **T** | **G** | **T** | **A** | **G** | **C** | **A** | **G** | **C** | **A** | **A** | **T** | **T** | **T** | **G** | **G** | **G** | **C** | **A** | **T** | **G** | **G** | **C** | **T** | **A** | **C** | **T** | **C** | **G** | **T** | **A** | **C** | **T** | **G** | **A** | **T** | **T** | **G** | **C** | **T** | **C** | **T** | **A** | **C** | **C** | **C** | **G** | **T** | **T** | **T** | **T** | **T** | **G** | **G** | **T** | **T** | **T** | **T** | **T** | **T** | **C** | **T** | **T** | **A** | **T** | **A** | **T** | **T** | **T** | **A** | **T** | **T** | **C** | **T** | **T** | **T** | **G** | **G** | **G** | **C** | **G** | **C** | **G** | **G** | **T** | **T** | **T** | **A** | **A** | **C** | **C** | **G** | **A** | **C** | **A** | **A** | **A** | **A** | **A** | **G** | **A** | **A** | **A** | **T** | **T** | **T** | **T** | **A** | **T** | **T** | **T** | **G** | **C** | **A** | **A** | **C** | **A** | **A** | **A** | **G** | **T** | **G** | **A** | **T** | **T** | **T** | **A** | **C** | **G** | **G** | **G** | **A** | **A** | **T** | **T** | **A** | **G** | **A** | **G** | **A** | **A** | **T** | **T** | **T** | **C** | **C** | **A** | **G** | **A** | **A** | **T** | **T** | **T** | **C** | **C** | **A** | **A** | **G** | **A** | **A** | **G** | **A** | **A** | **A** | **G** | **T** | **T** | **T** | **T** | **G** | **A** | **A** | **C** | **T** | **T** | **T** | **A** | **T** | **A** | **G** | **T** | **C** | **A** | **A** | **A** | **A** | **G** | **A** | **T** | **G** | **C** | **C** | **G** | **A** | **C** | **G** | **G** | **A** | **G** | **G** | **A** | **G** | **C** | **A** | **A** | **C** | **A** | **A** | **C** | **A** | **G** | **T** | **T** | **T** | **A** | **C** | **A** | **G** | **A** | **C** | **T** | **T** | **T** | **T** | **T** | **T** | **T** | **C** | **C** | **T** | **C** | **G** | **T** | **T** | **A** | **A** | **A** | **T** | **A** | **G** | **G** | **A** | **T** | **G** | **C** | **C** | **T** | **C** | **T** | **G** | **A** | **C** | **T** | **A** | **A** | **G** | **A** | **A** | **A** | **A** | **A** | **T** | **G** | **A** | **T** | **G** | **T** | **T** | **G** | **A** | **A** | **A** | **T** | **T** | **T** | **T** | **T** | **A** | **C** | **G** | **G** | **A** | **T** | **G** | **G** | **T** | **A** | **C** | **G** | **G** | **A** | **A** | **A** | **A** | **A** | **T** | **T** | **C** | **A** | **A** | **T** | **G** | **C** | **G** | **T** | **T** | **G** | **A** | **T** | **G** | **G** | **C** | **G** | **G** | **A** | **C** | **A** | **T** | **A** | **A** | **A** | **A** | **A** | **A** | **A** | **G** | **C** | **T** | **C** | **A** | **G** | **C** | **A** | **C** | **T** | **C** | **C** | **A** | **T** | **T** | **C** | **A** | **T** | **A** | **T** | **T** | **G** | **A** | **A** | **T** | **A** | **T** | **T** | **A** | **T** | **G** | **C** | **C** | **T** | **T** | **T** | **G** | **T** | **A** | **A** | **C** | **G** | **G** | **A** | **T** | **C** | **A** | **T** | **A** | **T** | **C** | **G** | **G** | **C** | **A** | **C** | **C** | **A** | **A** | **A** | **A** | **T** | **T** | **T** | **T** | **G** | **A** | **A** | **C** | **T** | **T** | **A** | **T** | **T** | **A** | **G** | **A** | **A** | **G** | **A** | **A** | **A** | **A** | **G** | **G** | **C** | **G** | **G** | **C** | **T** | **G** | **A** | **A** | **G** | **G** | **G** | **G** | **T** | **T** | **G** | **A** | **A** | **G** | **T** | **G** | **C** | **G** | **G** | **T** | **T** | **G** | **C** | **T** | **T** | **T** | **A** | **C** | **G** | **A** | **T** | **G** | **C** | **C** | **T** | **T** | **T** | **G** | **G** | **C** | **T** | **C** | **A** | **A** | **A** | **G** | **G** | **G** | **C** | **A** | **C** | **C** | **A** | **A** | **G** | **G** | **T** | **T** | **C** | **A** | **T** | **C** | **A** | **T** | **T** | **T** | **G** | **A** | **A** | **T** | **G** | **A** | **A** | **T** | **T** | **G** | **A** | **A** | **A** | **A** | **A** | **A** | **A** | **A** | **C** | **G** | **G** | **C** | **G** | **G** | **T** | **T** | **T** | **T** | **G** | **T** | **C** | **C** | **A** | **A** | **A** | **C** | **A** | **T** | **T** | **T** | **A** | **T** | **T** | **A** | **C** | **T** | **T** | **C** | **T** | **C** | **A** | **A** | **A** | **A** | **A** | **G** | **C** | **A** | **C** | **T** | **T** | **T** | **T** | **G** | **A** | **A** | **G** | **T** | **T** | **T** | **C** | **G** | **T** | **T** | **T** | **G** | **A** | **A** | **T** | **T** | **A** | **T** | **C** | **A** | **T** | **G** | **A** | **T** | **C** | **A** | **C** | **C** | **G** | **G** | **A** | **A** | **A** | **A** | **T** | **T** | **G** | **T** | **T** | **G** | **T** | **T** | **A** | **T** | **T** | **G** | **A** | **C** | **G** | **G** | **A** | **A** | **A** | **A** | **G** | **T** | **A** | **G** | **G** | **C** | **T** | **A** | **C** | **A** | **T** | **T** | **G** | **G** | **C** | **G** | **G** | **C** | **T** | **T** | **T** | **A** | **A** | **T** | **G** | **T** | **T** | **G** | **C** | **C** | **G** | **A** | **T** | **C** | **A** | **A** | **T** | **A** | **T** | **G** | **C** | **C** | **G** | **G** | **A** | **A** | **C** | **G** | **A** | **C** | **T** | **A** | **A** | **A** | **A** | **A** | **G** | **T** | **T** | **T** | **G** | **G** | **C** | **T** | **A** | **T** | **T** | **G** | **G** | **C** | **G** | **G** | **G** | **A** | **T** | **A** | **C** | **G** | **C** | **A** | **T** | **T** | **T** | **A** | **C** | **G** | **G** | **A** | **T** | **T** | **C** | **A** | **A** | **G** | **G** | **G** | **C** | **C** | **A** | **G** | **C** | **A** | **G** | **C** | **C** | **T** | **C** | **A** | **T** | **T** | **A** | **C** | **T** | **G** | **C** | **A** | **A** | **A** | **T** | **G** | **C** | **G** | **T** | **T** | **T** | **T** | **T** | **T** | **A** | **A** | **T** | **G** | **G** | **A** | **T** | **T** | **G** | **G** | **A** | **A** | **C** | **G** | **T** | **C** | **T** | **C** | **T** | **T** | **C** | **C** | **C** | **C** | **C** | **G** | **A** | **G** | **A** | **A** | **A** | **A** | **A** | **T** | **C** | **G** | **T** | **G** | **T** | **G** | **G** | **C** | **G** | **T** | **A** | **T** | **C** | **A** | **A** | **T** | **T** | **G** | **G** | **A** | **T** | **T** | **A** | **T** | **T** | **T** | **C** | **T** | **T** | **T** | **A** | **A** | **A** | **C** | **T** | **T** | **G** | **A** | **A** | **G** | **C** | **A** | **T** | **T** | **G** | **G** | **T** | **G** | **C** | **C** | **A** | **G** | **A** | **G** | **G** | **C** | **A** | **A** | **A** | **T** | **A** | **C** | **A** | **T** | **C** | **C** | **A** | **T** | **T** | **C** | **A** | **G** | **A** | **T** | **G** | **A** | **T** | **T** | **G** | **C** | **C** | **A** | **G** | **T** | **G** | **G** | **T** | **C** | **C** | **T** | **A** | **A** | **C** | **A** | **G** | **T** | **G** | **A** | **C** | **C** | **G** | **T** | **G** | **A** | **A** | **C** | **A** | **A** | **A** | **T** | **T** | **A** | **A** | **A** | **T** | **T** | **G** | **G** | **C** | **C** | **T** | **T** | **T** | **A** | **T** | **T** | **A** | **A** | **A** | **T** | **T** | **G** | **A** | **T** | **T** | **A** | **C** | **T** | **T** | **C** | **T** | **G** | **C** | **C** | **A** | **A** | **G** | **A** | **A** | **A** | **A** | **G** | **A** | **G** | **T** | **C** | **T** | **G** | **G** | **A** | **T** | **T** | **C** | **A** | **A** | **A** | **C** | **G** | **C** | **C** | **G** | **T** | **A** | **T** | **T** | **T** | **A** | **G** | **T** | **T** | **C** | **C** | **T** | **G** | **A** | **T** | **G** | **A** | **T** | **A** | **G** | **T** | **G** | **T** | **C** | **T** | **T** | **G** | **G** | **C** | **T** | **G** | **C** | **T** | **T** | **T** | **A** | **A** | **A** | **G** | **G** | **T** | **T** | **G** | **C** | **T** | **G** | **C** | **G** | **G** | **C** | **T** | **T** | **C** | **A** | **G** | **G** | **A** | **G** | **T** | **A** | **G** | **A** | **T** | **G** | **T** | **T** | **A** | **A** | **A** | **A** | **T** | **T** | **A** | **T** | **G** | **A** | **T** | **T** | **C** | **C** | **A** | **G** | **A** | **T** | **A** | **A** | **G** | **C** | **C** | **C** | **G** | **A** | **C** | **C** | **A** | **T** | **C** | **C** | **G** | **T** | **T** | **T** | **A** | **T** | **T** | **T** | **A** | **T** | **C** | **G** | **A** | **G** | **C** | **A** | **A** | **C** | **A** | **C** | **A** | **G** | **T** | **A** | **T** | **T** | **A** | **C** | **G** | **G** | **C** | **C** | **G** | **C** | **T** | **T** | **A** | **T** | **T** | **G** | **A** | **T** | **G** | **A** | **A** | **A** | **G** | **A** | **A** | **A** | **A** | **T** | **A** | **T** | **T** | **G** | **A** | **A** | **A** | **T** | **T** | **T** | **T** | **A** | **A** | **T** | **T** | **T** | **A** | **T** | **A** | **A** | **C** | **G** | **G** | **T** | **G** | **G** | **T** | **T** | **T** | **C** | **T** | **T** | **A** | **C** | **A** | **T** | **G** | **C** | **G** | **A** | **A** | **A** | **A** | **C** | **A** | **A** | **T** | **G** | **A** | **T** | **T** | **A** | **T** | **G** | **G** | **A** | **T** | **G** | **A** | **T** | **G** | **A** | **A** | **G** | **T** | **C** | **T** | **G** | **C** | **A** | **C** | **A** | **G** | **T** | **T** | **G** | **G** | **T** | **T** | **C** | **A** | **G** | **C** | **C** | **A** | **A** | **T** | **C** | **A** | **A** | **G** | **A** | **T** | **A** | **T** | **C** | **C** | **G** | **A** | **A** | **G** | **T** | **T** | **A** | **C** | **A** | **A** | **A** | **T** | **T** | **A** | **A** | **A** | **C** | **T** | **T** | **T** | **G** | **A** | **A** | **G** | **C** | **A** | **A** | **A** | **T** | **G** | **C** | **T** | **G** | **T** | **G** | **T** | **T** | **A** | **T** | **A** | **T** | **G** | **A** | **T** | **A** | **A** | **A** | **A** | **A** | **A** | **A** | **T** | **C** | **A** | **T** | **T** | **G** | **A** | **T** | **C** | **A** | **A** | **T** | **T** | **A** | **G** | **A** | **A** | **G** | **C** | **A** | **A** | **T** | **T** | **T** | **T** | **C** | **T** | **T** | **A** | **G** | **A** | **A** | **G** | **A** | **T** | **C** | **G** | **A** | **A** | **A** | **A** | **A** | **A** | **A** | **T** | **G** | **T** | **A** | **C** | **A** | **A** | **C** | **A** | **A** | **T** | **G** | **A** | **C** | **T** | **C** | **C** | **A** | **G** | **A** | **A** | **G** | **T** | **T** | **G** | **T** | **T** | **C** | **G** | **C** | **G** | **A** | **C** | **A** | **T** | **G** | **T** | **C** | **A** | **A** | **A** | **A** | **T** | **G** | **G** | **T** | **T** | **G** | **A** | **T** | **T** | **T** | **T** | **T** | **A** | **A** | **A** | **C** | **A** | **A** | **C** | **A** | **A** | **A** | **T** | **T** | **T** | **C** | **A** | **C** | **G** | **A** | **T** | **T** | **A** | **T** | **T** | **T** | **T** | **C** | **A** | **C** | **C** | **A** | **A** | **T** | **T** | **C** | **T** | **T** | **-** | **-** | **-** |
| **N52467** | **T** | **T** | **G** | **A** | **T** | **T** | **C** | **T** | **T** | **A** | **G** | **T** | **G** | **T** | **C** | **T** | **T** | **A** | **A** | **C** | **A** | **G** | **T** | **T** | **A** | **T** | **T** | **T** | **A** | **T** | **T** | **T** | **T** | **A** | **T** | **T** | **A** | **A** | **T** | **G** | **C** | **A** | **A** | **T** | **T** | **A** | **T** | **T** | **G** | **C** | **G** | **G** | **G** | **A** | **A** | **T** | **T** | **A** | **C** | **C** | **A** | **T** | **C** | **T** | **T** | **G** | **T** | **T** | **A** | **A** | **A** | **A** | **C** | **C** | **T** | **C** | **G** | **A** | **G** | **A** | **T** | **G** | **T** | **A** | **G** | **C** | **G** | **G** | **C** | **A** | **A** | **T** | **T** | **T** | **G** | **G** | **G** | **C** | **A** | **T** | **G** | **G** | **T** | **T** | **A** | **C** | **T** | **C** | **G** | **T** | **A** | **C** | **T** | **G** | **A** | **T** | **T** | **G** | **C** | **T** | **C** | **T** | **A** | **C** | **C** | **C** | **G** | **T** | **T** | **T** | **T** | **T** | **G** | **G** | **T** | **T** | **T** | **T** | **T** | **T** | **C** | **T** | **T** | **A** | **T** | **A** | **T** | **T** | **T** | **A** | **T** | **T** | **C** | **T** | **T** | **T** | **G** | **G** | **T** | **C** | **G** | **A** | **G** | **G** | **C** | **T** | **T** | **G** | **A** | **C** | **C** | **G** | **A** | **C** | **A** | **A** | **A** | **A** | **A** | **G** | **A** | **A** | **A** | **T** | **T** | **T** | **T** | **A** | **T** | **T** | **T** | **G** | **C** | **A** | **A** | **C** | **A** | **A** | **A** | **G** | **T** | **G** | **A** | **T** | **T** | **T** | **A** | **C** | **G** | **G** | **G** | **A** | **A** | **T** | **T** | **A** | **G** | **A** | **G** | **A** | **A** | **T** | **T** | **T** | **T** | **C** | **A** | **G** | **A** | **A** | **T** | **T** | **T** | **C** | **C** | **A** | **A** | **G** | **A** | **A** | **G** | **A** | **A** | **A** | **G** | **T** | **T** | **T** | **T** | **G** | **A** | **A** | **C** | **T** | **T** | **T** | **A** | **T** | **A** | **G** | **T** | **C** | **A** | **A** | **A** | **A** | **G** | **A** | **T** | **G** | **C** | **C** | **G** | **A** | **C** | **G** | **G** | **A** | **G** | **G** | **A** | **G** | **C** | **A** | **A** | **C** | **A** | **G** | **C** | **A** | **G** | **T** | **T** | **T** | **A** | **C** | **A** | **G** | **A** | **C** | **T** | **T** | **T** | **T** | **T** | **C** | **T** | **C** | **C** | **T** | **C** | **G** | **T** | **T** | **A** | **A** | **A** | **T** | **A** | **G** | **G** | **A** | **T** | **G** | **C** | **C** | **T** | **C** | **T** | **G** | **A** | **C** | **T** | **A** | **A** | **G** | **A** | **A** | **A** | **A** | **A** | **T** | **G** | **A** | **C** | **G** | **T** | **T** | **G** | **A** | **G** | **A** | **T** | **T** | **T** | **T** | **T** | **A** | **C** | **G** | **G** | **A** | **T** | **G** | **G** | **T** | **A** | **T** | **G** | **G** | **A** | **A** | **A** | **A** | **A** | **T** | **T** | **C** | **A** | **A** | **T** | **G** | **C** | **G** | **T** | **T** | **G** | **A** | **T** | **G** | **G** | **C** | **G** | **G** | **A** | **C** | **A** | **T** | **A** | **A** | **A** | **A** | **A** | **A** | **A** | **G** | **C** | **T** | **C** | **A** | **G** | **C** | **A** | **T** | **T** | **C** | **C** | **A** | **T** | **T** | **C** | **A** | **T** | **A** | **T** | **T** | **G** | **A** | **A** | **T** | **A** | **T** | **T** | **A** | **T** | **G** | **C** | **C** | **T** | **T** | **T** | **G** | **T** | **A** | **A** | **C** | **G** | **G** | **A** | **T** | **C** | **A** | **T** | **A** | **T** | **C** | **G** | **G** | **C** | **A** | **C** | **C** | **A** | **A** | **A** | **A** | **T** | **T** | **T** | **T** | **G** | **A** | **A** | **C** | **T** | **T** | **A** | **T** | **T** | **A** | **G** | **A** | **A** | **G** | **A** | **A** | **A** | **A** | **G** | **G** | **C** | **G** | **G** | **C** | **T** | **G** | **A** | **A** | **G** | **G** | **G** | **G** | **T** | **T** | **G** | **A** | **A** | **G** | **T** | **G** | **C** | **G** | **G** | **T** | **T** | **G** | **C** | **T** | **T** | **T** | **A** | **C** | **G** | **A** | **T** | **G** | **C** | **C** | **T** | **T** | **T** | **G** | **G** | **C** | **T** | **C** | **A** | **A** | **A** | **G** | **G** | **G** | **C** | **A** | **C** | **C** | **A** | **A** | **G** | **G** | **T** | **T** | **C** | **A** | **T** | **C** | **A** | **T** | **T** | **T** | **G** | **A** | **A** | **T** | **G** | **A** | **A** | **T** | **T** | **G** | **A** | **A** | **A** | **A** | **A** | **A** | **A** | **A** | **C** | **G** | **G** | **C** | **G** | **G** | **T** | **T** | **T** | **T** | **G** | **T** | **C** | **C** | **A** | **A** | **A** | **C** | **G** | **T** | **T** | **T** | **A** | **T** | **T** | **A** | **C** | **T** | **T** | **C** | **T** | **C** | **A** | **A** | **A** | **A** | **A** | **G** | **C** | **A** | **C** | **T** | **C** | **T** | **T** | **G** | **A** | **A** | **G** | **T** | **T** | **T** | **C** | **G** | **T** | **T** | **T** | **G** | **A** | **A** | **T** | **T** | **A** | **T** | **C** | **A** | **T** | **G** | **A** | **T** | **C** | **A** | **C** | **C** | **G** | **G** | **A** | **A** | **A** | **A** | **T** | **T** | **G** | **T** | **C** | **G** | **T** | **T** | **A** | **T** | **T** | **G** | **A** | **C** | **G** | **G** | **A** | **A** | **A** | **A** | **G** | **T** | **A** | **G** | **G** | **C** | **T** | **A** | **C** | **A** | **T** | **T** | **G** | **G** | **T** | **G** | **G** | **T** | **T** | **T** | **T** | **A** | **A** | **T** | **G** | **T** | **T** | **G** | **C** | **C** | **G** | **A** | **T** | **C** | **A** | **A** | **T** | **A** | **T** | **G** | **C** | **T** | **G** | **G** | **A** | **A** | **C** | **G** | **A** | **C** | **T** | **A** | **A** | **A** | **A** | **A** | **G** | **T** | **T** | **T** | **G** | **G** | **C** | **T** | **A** | **T** | **T** | **G** | **G** | **C** | **G** | **G** | **G** | **A** | **T** | **A** | **C** | **G** | **C** | **A** | **T** | **T** | **T** | **A** | **C** | **G** | **G** | **A** | **T** | **T** | **C** | **A** | **A** | **G** | **G** | **G** | **C** | **C** | **A** | **G** | **C** | **A** | **G** | **C** | **C** | **T** | **C** | **A** | **T** | **T** | **A** | **C** | **T** | **G** | **C** | **A** | **A** | **A** | **T** | **G** | **C** | **G** | **T** | **T** | **T** | **T** | **T** | **T** | **A** | **A** | **T** | **G** | **G** | **A** | **T** | **T** | **G** | **G** | **A** | **A** | **C** | **G** | **T** | **C** | **T** | **C** | **T** | **T** | **C** | **C** | **C** | **C** | **T** | **G** | **A** | **G** | **A** | **A** | **A** | **A** | **A** | **T** | **C** | **G** | **T** | **G** | **T** | **G** | **G** | **C** | **G** | **T** | **A** | **T** | **C** | **A** | **A** | **T** | **T** | **G** | **G** | **A** | **T** | **T** | **A** | **T** | **T** | **T** | **C** | **T** | **T** | **T** | **A** | **A** | **A** | **C** | **T** | **T** | **G** | **A** | **A** | **G** | **C** | **G** | **T** | **T** | **G** | **G** | **T** | **G** | **C** | **C** | **A** | **G** | **A** | **G** | **G** | **C** | **A** | **A** | **A** | **T** | **A** | **C** | **A** | **T** | **C** | **C** | **A** | **T** | **T** | **C** | **A** | **G** | **A** | **T** | **G** | **A** | **T** | **T** | **G** | **C** | **A** | **A** | **G** | **T** | **G** | **G** | **A** | **C** | **C** | **T** | **A** | **A** | **C** | **A** | **G** | **T** | **G** | **A** | **C** | **C** | **G** | **T** | **G** | **A** | **A** | **C** | **A** | **A** | **A** | **T** | **T** | **A** | **A** | **A** | **T** | **T** | **G** | **G** | **C** | **C** | **T** | **T** | **T** | **A** | **T** | **T** | **A** | **A** | **A** | **T** | **T** | **G** | **A** | **T** | **T** | **A** | **C** | **T** | **T** | **C** | **T** | **G** | **C** | **C** | **A** | **A** | **G** | **A** | **A** | **A** | **A** | **G** | **A** | **G** | **T** | **C** | **T** | **G** | **G** | **A** | **T** | **T** | **C** | **A** | **A** | **A** | **C** | **G** | **C** | **C** | **G** | **T** | **A** | **T** | **T** | **T** | **A** | **G** | **T** | **T** | **C** | **C** | **T** | **G** | **A** | **T** | **G** | **A** | **T** | **A** | **G** | **T** | **G** | **T** | **C** | **T** | **T** | **G** | **G** | **C** | **T** | **G** | **C** | **T** | **T** | **T** | **A** | **A** | **A** | **G** | **G** | **T** | **T** | **G** | **C** | **T** | **G** | **C** | **G** | **G** | **C** | **T** | **T** | **C** | **A** | **G** | **G** | **A** | **G** | **T** | **A** | **G** | **A** | **T** | **G** | **T** | **T** | **A** | **A** | **A** | **A** | **T** | **T** | **A** | **T** | **G** | **A** | **T** | **T** | **C** | **C** | **A** | **G** | **A** | **T** | **A** | **A** | **G** | **C** | **C** | **C** | **G** | **A** | **C** | **C** | **A** | **T** | **C** | **C** | **G** | **T** | **T** | **T** | **A** | **T** | **T** | **T** | **A** | **T** | **C** | **G** | **A** | **G** | **C** | **A** | **A** | **C** | **A** | **C** | **A** | **G** | **T** | **A** | **T** | **T** | **A** | **C** | **G** | **G** | **C** | **C** | **G** | **C** | **T** | **T** | **G** | **T** | **T** | **A** | **A** | **T** | **G** | **A** | **A** | **A** | **G** | **A** | **A** | **A** | **A** | **T** | **A** | **T** | **T** | **G** | **A** | **A** | **A** | **T** | **T** | **T** | **T** | **A** | **A** | **T** | **T** | **T** | **A** | **T** | **A** | **A** | **C** | **G** | **G** | **T** | **G** | **G** | **T** | **T** | **T** | **C** | **T** | **T** | **A** | **C** | **A** | **T** | **G** | **C** | **G** | **A** | **A** | **A** | **A** | **C** | **A** | **A** | **T** | **G** | **A** | **T** | **T** | **A** | **T** | **G** | **G** | **A** | **T** | **G** | **A** | **T** | **G** | **A** | **A** | **G** | **T** | **C** | **T** | **G** | **C** | **A** | **C** | **A** | **G** | **T** | **T** | **G** | **G** | **T** | **T** | **C** | **A** | **G** | **C** | **T** | **A** | **A** | **T** | **C** | **A** | **A** | **G** | **A** | **T** | **A** | **T** | **C** | **C** | **G** | **A** | **A** | **G** | **T** | **T** | **A** | **C** | **A** | **A** | **A** | **T** | **T** | **A** | **A** | **A** | **C** | **T** | **T** | **T** | **G** | **A** | **A** | **G** | **C** | **A** | **A** | **A** | **T** | **G** | **C** | **T** | **G** | **T** | **G** | **T** | **T** | **A** | **T** | **A** | **T** | **G** | **A** | **T** | **A** | **A** | **A** | **A** | **A** | **A** | **A** | **T** | **C** | **A** | **T** | **T** | **G** | **A** | **T** | **C** | **A** | **A** | **T** | **T** | **A** | **G** | **A** | **A** | **G** | **C** | **A** | **A** | **T** | **T** | **T** | **T** | **C** | **T** | **T** | **A** | **G** | **A** | **A** | **G** | **A** | **T** | **C** | **G** | **A** | **A** | **A** | **A** | **A** | **A** | **A** | **T** | **G** | **T** | **A** | **C** | **A** | **A** | **C** | **A** | **A** | **T** | **G** | **A** | **C** | **T** | **C** | **C** | **A** | **G** | **A** | **A** | **G** | **T** | **T** | **G** | **T** | **T** | **C** | **G** | **C** | **G** | **A** | **C** | **A** | **T** | **G** | **T** | **C** | **A** | **A** | **A** | **A** | **T** | **G** | **G** | **T** | **T** | **G** | **A** | **T** | **T** | **T** | **T** | **T** | **A** | **A** | **A** | **C** | **A** | **A** | **C** | **A** | **A** | **A** | **T** | **T** | **T** | **C** | **A** | **C** | **G** | **A** | **T** | **T** | **A** | **T** | **T** | **T** | **T** | **C** | **A** | **C** | **C** | **A** | **A** | **T** | **T** | **C** | **T** | **T** | **-** | **-** | **-** |
| **N59584F** | **T** | **T** | **G** | **A** | **T** | **T** | **C** | **T** | **T** | **A** | **G** | **C** | **G** | **T** | **C** | **T** | **T** | **A** | **A** | **C** | **A** | **G** | **T** | **T** | **A** | **T** | **T** | **T** | **A** | **T** | **T** | **T** | **T** | **A** | **T** | **T** | **A** | **A** | **T** | **G** | **C** | **A** | **A** | **T** | **T** | **A** | **T** | **T** | **G** | **C** | **G** | **G** | **G** | **A** | **A** | **T** | **T** | **A** | **C** | **C** | **A** | **T** | **T** | **T** | **T** | **G** | **T** | **T** | **A** | **A** | **A** | **A** | **C** | **C** | **T** | **C** | **G** | **A** | **G** | **A** | **T** | **G** | **T** | **A** | **G** | **C** | **A** | **G** | **C** | **A** | **A** | **T** | **T** | **T** | **G** | **G** | **G** | **C** | **A** | **T** | **G** | **G** | **C** | **T** | **A** | **C** | **T** | **C** | **G** | **T** | **A** | **C** | **T** | **G** | **A** | **T** | **T** | **G** | **C** | **T** | **C** | **T** | **A** | **C** | **C** | **C** | **G** | **T** | **T** | **T** | **T** | **T** | **G** | **G** | **T** | **T** | **T** | **T** | **T** | **T** | **C** | **T** | **T** | **A** | **T** | **A** | **T** | **T** | **T** | **A** | **T** | **T** | **C** | **T** | **T** | **T** | **G** | **G** | **G** | **C** | **G** | **C** | **G** | **G** | **T** | **T** | **T** | **A** | **A** | **C** | **C** | **G** | **A** | **C** | **A** | **A** | **A** | **A** | **A** | **G** | **A** | **A** | **A** | **T** | **T** | **T** | **T** | **A** | **T** | **T** | **T** | **G** | **C** | **A** | **A** | **C** | **A** | **A** | **A** | **G** | **T** | **G** | **A** | **T** | **T** | **T** | **A** | **C** | **G** | **G** | **G** | **A** | **A** | **T** | **T** | **A** | **G** | **A** | **G** | **A** | **A** | **T** | **T** | **T** | **C** | **C** | **A** | **G** | **A** | **A** | **T** | **T** | **T** | **C** | **C** | **A** | **A** | **G** | **A** | **A** | **G** | **A** | **A** | **A** | **G** | **T** | **T** | **T** | **T** | **G** | **A** | **A** | **C** | **T** | **T** | **T** | **A** | **T** | **A** | **G** | **T** | **C** | **A** | **A** | **A** | **A** | **G** | **A** | **T** | **G** | **C** | **C** | **G** | **A** | **C** | **G** | **G** | **A** | **G** | **G** | **A** | **G** | **C** | **A** | **A** | **C** | **A** | **A** | **C** | **A** | **G** | **T** | **T** | **T** | **A** | **C** | **A** | **G** | **A** | **C** | **T** | **T** | **T** | **T** | **T** | **T** | **T** | **C** | **C** | **T** | **C** | **G** | **T** | **T** | **A** | **A** | **A** | **T** | **A** | **G** | **G** | **A** | **T** | **G** | **C** | **C** | **C** | **C** | **T** | **G** | **A** | **C** | **T** | **A** | **A** | **G** | **A** | **A** | **A** | **A** | **A** | **T** | **G** | **A** | **T** | **G** | **T** | **T** | **G** | **A** | **A** | **A** | **T** | **T** | **T** | **T** | **T** | **A** | **C** | **G** | **G** | **A** | **T** | **G** | **G** | **T** | **A** | **C** | **G** | **G** | **A** | **A** | **A** | **A** | **A** | **T** | **T** | **C** | **A** | **A** | **T** | **G** | **C** | **G** | **T** | **T** | **G** | **A** | **T** | **G** | **G** | **C** | **G** | **G** | **A** | **C** | **A** | **T** | **A** | **A** | **A** | **A** | **A** | **A** | **A** | **G** | **C** | **T** | **C** | **A** | **G** | **C** | **A** | **C** | **T** | **C** | **C** | **A** | **T** | **T** | **C** | **A** | **T** | **A** | **T** | **T** | **G** | **A** | **A** | **T** | **A** | **T** | **T** | **A** | **T** | **G** | **C** | **C** | **T** | **T** | **T** | **G** | **T** | **A** | **A** | **C** | **G** | **G** | **A** | **T** | **C** | **A** | **T** | **A** | **T** | **C** | **G** | **G** | **T** | **A** | **C** | **C** | **A** | **A** | **A** | **A** | **T** | **T** | **T** | **T** | **G** | **A** | **A** | **C** | **T** | **T** | **A** | **T** | **T** | **A** | **G** | **A** | **A** | **G** | **A** | **A** | **A** | **A** | **G** | **G** | **C** | **G** | **G** | **C** | **T** | **G** | **A** | **A** | **G** | **G** | **G** | **G** | **T** | **T** | **G** | **A** | **A** | **G** | **T** | **G** | **C** | **G** | **G** | **T** | **T** | **G** | **C** | **T** | **T** | **T** | **A** | **C** | **G** | **A** | **T** | **G** | **C** | **C** | **T** | **T** | **T** | **G** | **G** | **C** | **T** | **C** | **A** | **A** | **A** | **G** | **G** | **G** | **C** | **A** | **C** | **C** | **A** | **A** | **G** | **G** | **T** | **T** | **C** | **A** | **T** | **C** | **A** | **T** | **T** | **T** | **G** | **A** | **A** | **T** | **G** | **A** | **A** | **T** | **T** | **G** | **A** | **A** | **A** | **A** | **A** | **A** | **A** | **A** | **C** | **G** | **G** | **T** | **G** | **G** | **T** | **T** | **T** | **T** | **G** | **T** | **C** | **C** | **A** | **A** | **A** | **C** | **G** | **T** | **T** | **T** | **A** | **T** | **T** | **A** | **C** | **T** | **T** | **C** | **T** | **C** | **A** | **A** | **A** | **A** | **A** | **G** | **C** | **A** | **C** | **T** | **T** | **T** | **T** | **G** | **A** | **A** | **G** | **T** | **T** | **T** | **C** | **G** | **T** | **T** | **T** | **G** | **A** | **A** | **T** | **T** | **A** | **T** | **C** | **A** | **T** | **G** | **A** | **T** | **C** | **A** | **C** | **C** | **G** | **G** | **A** | **A** | **A** | **A** | **T** | **T** | **G** | **T** | **T** | **G** | **T** | **T** | **A** | **T** | **T** | **G** | **A** | **C** | **G** | **G** | **A** | **A** | **A** | **A** | **G** | **T** | **A** | **G** | **G** | **C** | **T** | **A** | **C** | **A** | **T** | **T** | **G** | **G** | **C** | **G** | **G** | **C** | **T** | **T** | **T** | **A** | **A** | **T** | **G** | **T** | **T** | **G** | **C** | **C** | **G** | **A** | **T** | **C** | **A** | **A** | **T** | **A** | **T** | **G** | **C** | **C** | **G** | **G** | **A** | **A** | **C** | **G** | **A** | **C** | **T** | **A** | **A** | **A** | **A** | **A** | **G** | **T** | **T** | **T** | **G** | **G** | **C** | **T** | **A** | **T** | **T** | **G** | **G** | **C** | **G** | **G** | **G** | **A** | **T** | **A** | **C** | **A** | **C** | **A** | **T** | **T** | **T** | **A** | **C** | **G** | **G** | **A** | **T** | **T** | **C** | **A** | **A** | **G** | **G** | **G** | **C** | **C** | **A** | **G** | **C** | **A** | **G** | **C** | **C** | **T** | **C** | **A** | **T** | **T** | **A** | **C** | **T** | **G** | **C** | **A** | **A** | **A** | **T** | **G** | **C** | **G** | **T** | **T** | **T** | **T** | **T** | **T** | **A** | **A** | **T** | **G** | **G** | **A** | **T** | **T** | **G** | **G** | **A** | **A** | **C** | **G** | **T** | **C** | **T** | **C** | **T** | **T** | **C** | **C** | **C** | **C** | **C** | **G** | **A** | **G** | **A** | **A** | **A** | **A** | **A** | **T** | **C** | **G** | **T** | **G** | **T** | **G** | **G** | **C** | **G** | **T** | **A** | **T** | **C** | **A** | **A** | **T** | **T** | **G** | **G** | **A** | **T** | **T** | **A** | **T** | **T** | **T** | **C** | **T** | **T** | **T** | **A** | **A** | **A** | **C** | **T** | **T** | **G** | **A** | **A** | **G** | **C** | **A** | **T** | **T** | **G** | **G** | **T** | **G** | **C** | **C** | **A** | **G** | **A** | **G** | **G** | **C** | **A** | **A** | **A** | **T** | **A** | **C** | **A** | **T** | **C** | **C** | **A** | **T** | **T** | **C** | **A** | **G** | **A** | **T** | **G** | **A** | **T** | **T** | **G** | **C** | **C** | **A** | **G** | **T** | **G** | **G** | **T** | **C** | **C** | **T** | **A** | **A** | **C** | **A** | **G** | **T** | **G** | **A** | **C** | **C** | **G** | **T** | **G** | **A** | **A** | **C** | **A** | **A** | **A** | **T** | **T** | **A** | **A** | **A** | **T** | **T** | **G** | **G** | **C** | **C** | **T** | **T** | **T** | **A** | **T** | **T** | **A** | **A** | **A** | **T** | **T** | **G** | **A** | **T** | **T** | **A** | **C** | **T** | **T** | **C** | **T** | **G** | **C** | **C** | **A** | **A** | **G** | **A** | **A** | **A** | **A** | **G** | **A** | **G** | **T** | **C** | **T** | **G** | **G** | **A** | **T** | **T** | **C** | **A** | **A** | **A** | **C** | **G** | **C** | **C** | **G** | **T** | **A** | **T** | **T** | **T** | **A** | **G** | **T** | **T** | **C** | **C** | **T** | **G** | **A** | **T** | **G** | **A** | **C** | **A** | **G** | **T** | **G** | **T** | **C** | **T** | **T** | **G** | **G** | **C** | **T** | **G** | **C** | **T** | **T** | **T** | **A** | **A** | **A** | **G** | **G** | **T** | **T** | **G** | **C** | **T** | **G** | **C** | **G** | **G** | **C** | **T** | **T** | **C** | **A** | **G** | **G** | **A** | **G** | **T** | **A** | **G** | **A** | **T** | **G** | **T** | **T** | **A** | **A** | **A** | **A** | **T** | **T** | **A** | **T** | **G** | **A** | **T** | **T** | **C** | **C** | **A** | **G** | **A** | **T** | **A** | **A** | **G** | **C** | **C** | **C** | **G** | **A** | **C** | **C** | **A** | **T** | **C** | **C** | **G** | **T** | **T** | **T** | **A** | **T** | **T** | **T** | **A** | **T** | **C** | **G** | **A** | **G** | **C** | **A** | **A** | **C** | **A** | **C** | **A** | **G** | **T** | **A** | **T** | **T** | **A** | **C** | **G** | **G** | **C** | **C** | **G** | **C** | **T** | **T** | **A** | **T** | **T** | **G** | **A** | **T** | **G** | **A** | **A** | **A** | **G** | **A** | **A** | **A** | **A** | **T** | **A** | **T** | **T** | **G** | **A** | **A** | **A** | **T** | **T** | **T** | **T** | **A** | **A** | **T** | **T** | **T** | **A** | **T** | **A** | **A** | **C** | **G** | **G** | **T** | **G** | **G** | **T** | **T** | **T** | **C** | **T** | **T** | **A** | **C** | **A** | **T** | **G** | **C** | **G** | **A** | **A** | **A** | **A** | **C** | **A** | **A** | **T** | **G** | **A** | **T** | **T** | **A** | **T** | **G** | **G** | **A** | **T** | **G** | **A** | **T** | **G** | **A** | **A** | **G** | **T** | **C** | **T** | **G** | **C** | **A** | **C** | **A** | **G** | **T** | **T** | **G** | **G** | **T** | **T** | **C** | **A** | **G** | **C** | **C** | **A** | **A** | **T** | **C** | **A** | **A** | **G** | **A** | **T** | **A** | **T** | **C** | **C** | **G** | **A** | **A** | **G** | **T** | **T** | **A** | **C** | **A** | **A** | **A** | **T** | **T** | **A** | **A** | **A** | **C** | **T** | **T** | **T** | **G** | **A** | **A** | **G** | **C** | **A** | **A** | **A** | **T** | **G** | **C** | **T** | **G** | **T** | **G** | **T** | **T** | **A** | **T** | **A** | **T** | **G** | **A** | **T** | **A** | **A** | **A** | **A** | **A** | **A** | **A** | **T** | **C** | **A** | **T** | **T** | **G** | **A** | **T** | **C** | **A** | **A** | **T** | **T** | **A** | **G** | **A** | **A** | **G** | **C** | **A** | **A** | **T** | **T** | **T** | **T** | **C** | **T** | **T** | **A** | **G** | **A** | **A** | **G** | **A** | **T** | **C** | **G** | **A** | **A** | **A** | **A** | **A** | **A** | **A** | **T** | **G** | **T** | **A** | **C** | **A** | **A** | **C** | **A** | **A** | **T** | **G** | **A** | **C** | **T** | **C** | **C** | **A** | **G** | **A** | **A** | **G** | **T** | **T** | **G** | **T** | **T** | **C** | **G** | **C** | **G** | **A** | **C** | **A** | **T** | **G** | **T** | **C** | **A** | **A** | **A** | **A** | **T** | **G** | **G** | **T** | **T** | **G** | **A** | **T** | **T** | **T** | **T** | **T** | **A** | **A** | **A** | **C** | **A** | **A** | **C** | **A** | **A** | **A** | **T** | **T** | **T** | **C** | **A** | **C** | **G** | **A** | **T** | **T** | **A** | **T** | **T** | **T** | **T** | **C** | **A** | **C** | **C** | **A** | **A** | **T** | **T** | **C** | **T** | **T** | **-** | **-** | **-** |
| **N52724** | **T** | **T** | **G** | **A** | **T** | **T** | **C** | **T** | **T** | **A** | **G** | **C** | **G** | **T** | **C** | **T** | **T** | **A** | **A** | **C** | **A** | **G** | **T** | **T** | **A** | **T** | **T** | **T** | **A** | **T** | **T** | **T** | **T** | **A** | **T** | **T** | **A** | **A** | **T** | **G** | **C** | **A** | **A** | **T** | **T** | **A** | **T** | **T** | **G** | **C** | **G** | **G** | **G** | **A** | **A** | **T** | **T** | **A** | **C** | **C** | **A** | **T** | **T** | **T** | **T** | **G** | **T** | **T** | **A** | **A** | **A** | **A** | **C** | **C** | **T** | **C** | **G** | **A** | **G** | **A** | **T** | **G** | **T** | **A** | **G** | **C** | **A** | **G** | **C** | **A** | **A** | **T** | **T** | **T** | **G** | **G** | **G** | **C** | **A** | **T** | **G** | **G** | **C** | **T** | **A** | **C** | **T** | **C** | **G** | **T** | **A** | **C** | **T** | **G** | **A** | **T** | **T** | **G** | **C** | **T** | **C** | **T** | **A** | **C** | **C** | **C** | **G** | **T** | **T** | **T** | **T** | **T** | **G** | **G** | **T** | **T** | **T** | **T** | **T** | **T** | **C** | **T** | **T** | **A** | **T** | **A** | **T** | **T** | **T** | **A** | **T** | **T** | **C** | **T** | **T** | **T** | **G** | **G** | **G** | **C** | **G** | **C** | **G** | **G** | **T** | **T** | **T** | **A** | **A** | **C** | **C** | **G** | **A** | **C** | **A** | **A** | **A** | **A** | **A** | **G** | **A** | **A** | **A** | **T** | **T** | **T** | **T** | **A** | **T** | **T** | **T** | **G** | **C** | **A** | **A** | **C** | **A** | **A** | **A** | **G** | **T** | **G** | **A** | **T** | **T** | **T** | **A** | **C** | **G** | **G** | **G** | **A** | **A** | **T** | **T** | **A** | **G** | **A** | **G** | **A** | **A** | **T** | **T** | **T** | **T** | **C** | **A** | **G** | **A** | **A** | **C** | **T** | **T** | **C** | **C** | **A** | **A** | **G** | **A** | **A** | **G** | **A** | **A** | **A** | **G** | **T** | **T** | **T** | **T** | **G** | **A** | **A** | **C** | **T** | **T** | **T** | **A** | **T** | **A** | **G** | **T** | **C** | **A** | **A** | **A** | **A** | **G** | **A** | **T** | **G** | **C** | **C** | **G** | **A** | **C** | **G** | **G** | **A** | **G** | **G** | **A** | **G** | **C** | **A** | **A** | **C** | **A** | **A** | **C** | **A** | **G** | **T** | **T** | **T** | **A** | **C** | **A** | **G** | **A** | **C** | **T** | **T** | **T** | **T** | **T** | **T** | **T** | **C** | **C** | **T** | **C** | **G** | **T** | **T** | **A** | **A** | **A** | **T** | **A** | **G** | **G** | **A** | **T** | **G** | **C** | **C** | **T** | **C** | **T** | **G** | **A** | **C** | **T** | **A** | **A** | **G** | **A** | **A** | **A** | **A** | **A** | **T** | **G** | **A** | **T** | **G** | **T** | **T** | **G** | **A** | **A** | **A** | **T** | **T** | **T** | **T** | **T** | **A** | **C** | **G** | **G** | **A** | **T** | **G** | **G** | **T** | **A** | **C** | **G** | **G** | **A** | **A** | **A** | **A** | **A** | **T** | **T** | **C** | **A** | **A** | **T** | **G** | **C** | **G** | **T** | **T** | **G** | **A** | **T** | **G** | **G** | **C** | **G** | **G** | **A** | **C** | **A** | **T** | **A** | **A** | **A** | **A** | **A** | **A** | **A** | **G** | **C** | **T** | **C** | **A** | **G** | **C** | **A** | **C** | **T** | **C** | **C** | **A** | **T** | **T** | **C** | **A** | **T** | **A** | **T** | **T** | **G** | **A** | **A** | **T** | **A** | **T** | **T** | **A** | **T** | **G** | **C** | **C** | **T** | **T** | **T** | **G** | **T** | **A** | **A** | **C** | **G** | **G** | **A** | **T** | **C** | **A** | **T** | **A** | **T** | **C** | **G** | **G** | **T** | **A** | **C** | **C** | **A** | **A** | **A** | **A** | **T** | **T** | **T** | **T** | **G** | **A** | **A** | **C** | **T** | **T** | **A** | **T** | **T** | **A** | **G** | **A** | **A** | **G** | **A** | **A** | **A** | **A** | **G** | **G** | **C** | **G** | **G** | **C** | **T** | **G** | **A** | **A** | **G** | **G** | **G** | **G** | **T** | **T** | **G** | **A** | **A** | **G** | **T** | **G** | **C** | **G** | **G** | **T** | **T** | **G** | **C** | **T** | **T** | **T** | **A** | **C** | **G** | **A** | **T** | **G** | **C** | **C** | **T** | **T** | **T** | **G** | **G** | **C** | **T** | **C** | **A** | **A** | **A** | **G** | **G** | **G** | **C** | **A** | **C** | **C** | **A** | **A** | **G** | **G** | **T** | **T** | **C** | **A** | **T** | **C** | **A** | **T** | **T** | **T** | **G** | **A** | **A** | **T** | **G** | **A** | **A** | **T** | **T** | **G** | **A** | **A** | **A** | **A** | **A** | **A** | **A** | **A** | **C** | **G** | **G** | **C** | **G** | **G** | **T** | **T** | **T** | **T** | **G** | **T** | **C** | **C** | **A** | **A** | **A** | **C** | **G** | **T** | **T** | **T** | **A** | **T** | **T** | **A** | **C** | **T** | **T** | **C** | **T** | **C** | **A** | **A** | **A** | **A** | **A** | **G** | **C** | **A** | **C** | **T** | **T** | **T** | **T** | **G** | **A** | **A** | **G** | **T** | **T** | **T** | **C** | **G** | **T** | **T** | **T** | **G** | **A** | **A** | **T** | **T** | **A** | **T** | **C** | **A** | **T** | **G** | **A** | **T** | **C** | **A** | **C** | **C** | **G** | **G** | **A** | **A** | **A** | **A** | **T** | **T** | **G** | **T** | **T** | **G** | **T** | **T** | **A** | **T** | **T** | **G** | **A** | **T** | **G** | **G** | **G** | **A** | **A** | **A** | **G** | **T** | **A** | **G** | **G** | **C** | **T** | **A** | **C** | **A** | **T** | **T** | **G** | **G** | **C** | **G** | **G** | **C** | **T** | **T** | **T** | **A** | **A** | **T** | **G** | **T** | **T** | **G** | **C** | **C** | **G** | **A** | **T** | **C** | **A** | **A** | **T** | **A** | **T** | **G** | **C** | **T** | **G** | **G** | **A** | **A** | **C** | **G** | **A** | **C** | **T** | **A** | **A** | **A** | **A** | **A** | **G** | **T** | **T** | **T** | **G** | **G** | **C** | **T** | **A** | **T** | **T** | **G** | **G** | **C** | **G** | **G** | **G** | **A** | **T** | **A** | **C** | **A** | **C** | **A** | **T** | **T** | **T** | **A** | **C** | **G** | **G** | **A** | **T** | **T** | **C** | **A** | **A** | **G** | **G** | **G** | **C** | **C** | **A** | **G** | **C** | **A** | **G** | **C** | **C** | **T** | **C** | **A** | **T** | **T** | **A** | **C** | **T** | **G** | **C** | **A** | **A** | **A** | **T** | **G** | **C** | **G** | **T** | **T** | **T** | **T** | **T** | **T** | **A** | **A** | **T** | **G** | **G** | **A** | **T** | **T** | **G** | **G** | **A** | **A** | **C** | **G** | **T** | **C** | **T** | **C** | **T** | **T** | **C** | **C** | **C** | **C** | **C** | **G** | **A** | **G** | **A** | **A** | **A** | **A** | **A** | **T** | **C** | **G** | **T** | **G** | **T** | **G** | **G** | **C** | **G** | **T** | **A** | **T** | **C** | **A** | **A** | **T** | **T** | **G** | **G** | **A** | **T** | **T** | **A** | **T** | **T** | **T** | **C** | **T** | **T** | **T** | **A** | **A** | **A** | **C** | **T** | **T** | **G** | **A** | **A** | **G** | **C** | **A** | **T** | **T** | **G** | **G** | **T** | **T** | **C** | **C** | **A** | **G** | **A** | **G** | **G** | **C** | **A** | **A** | **A** | **T** | **A** | **C** | **A** | **T** | **C** | **C** | **A** | **T** | **T** | **C** | **A** | **G** | **A** | **T** | **G** | **A** | **T** | **T** | **G** | **C** | **C** | **A** | **G** | **T** | **G** | **G** | **T** | **C** | **C** | **T** | **A** | **A** | **T** | **A** | **G** | **T** | **G** | **A** | **C** | **C** | **G** | **T** | **G** | **A** | **A** | **C** | **A** | **A** | **A** | **T** | **T** | **A** | **A** | **A** | **T** | **T** | **G** | **G** | **C** | **C** | **T** | **T** | **T** | **A** | **T** | **T** | **A** | **A** | **A** | **T** | **T** | **G** | **A** | **T** | **T** | **A** | **C** | **T** | **T** | **C** | **T** | **G** | **C** | **C** | **A** | **A** | **G** | **A** | **A** | **A** | **A** | **G** | **A** | **G** | **T** | **C** | **T** | **G** | **G** | **A** | **T** | **T** | **C** | **A** | **A** | **A** | **C** | **A** | **C** | **C** | **G** | **T** | **A** | **T** | **T** | **T** | **A** | **G** | **T** | **T** | **C** | **C** | **T** | **G** | **A** | **T** | **G** | **A** | **T** | **A** | **G** | **T** | **G** | **T** | **C** | **T** | **T** | **G** | **G** | **C** | **G** | **G** | **C** | **T** | **T** | **T** | **A** | **A** | **A** | **G** | **G** | **T** | **T** | **G** | **C** | **T** | **G** | **C** | **G** | **G** | **C** | **T** | **T** | **C** | **A** | **G** | **G** | **A** | **G** | **T** | **A** | **G** | **A** | **T** | **G** | **T** | **T** | **A** | **A** | **A** | **A** | **T** | **T** | **A** | **T** | **G** | **A** | **T** | **T** | **C** | **C** | **A** | **G** | **A** | **T** | **A** | **A** | **G** | **C** | **C** | **C** | **G** | **A** | **C** | **C** | **A** | **T** | **C** | **C** | **G** | **T** | **T** | **T** | **A** | **T** | **T** | **T** | **A** | **T** | **C** | **G** | **A** | **G** | **C** | **A** | **A** | **C** | **A** | **C** | **A** | **G** | **T** | **A** | **T** | **T** | **A** | **C** | **G** | **G** | **G** | **C** | **G** | **C** | **T** | **T** | **G** | **T** | **T** | **G** | **A** | **T** | **G** | **A** | **A** | **A** | **G** | **A** | **A** | **A** | **A** | **T** | **A** | **T** | **T** | **G** | **A** | **A** | **A** | **T** | **T** | **T** | **T** | **A** | **A** | **T** | **T** | **T** | **A** | **T** | **A** | **A** | **C** | **G** | **G** | **T** | **G** | **G** | **T** | **T** | **T** | **C** | **T** | **T** | **A** | **C** | **A** | **T** | **G** | **C** | **G** | **A** | **A** | **A** | **A** | **C** | **A** | **A** | **T** | **G** | **A** | **T** | **T** | **A** | **T** | **G** | **G** | **A** | **T** | **G** | **A** | **T** | **G** | **A** | **A** | **G** | **T** | **C** | **T** | **G** | **C** | **A** | **C** | **A** | **G** | **T** | **T** | **G** | **G** | **T** | **T** | **C** | **A** | **G** | **C** | **C** | **A** | **A** | **T** | **C** | **A** | **A** | **G** | **A** | **T** | **A** | **T** | **C** | **C** | **G** | **A** | **A** | **G** | **T** | **T** | **A** | **C** | **A** | **A** | **A** | **T** | **T** | **A** | **A** | **A** | **C** | **T** | **T** | **T** | **G** | **A** | **A** | **G** | **C** | **A** | **A** | **A** | **T** | **G** | **C** | **T** | **G** | **T** | **G** | **T** | **T** | **A** | **T** | **A** | **T** | **G** | **A** | **T** | **A** | **A** | **A** | **A** | **A** | **A** | **A** | **T** | **C** | **A** | **T** | **T** | **G** | **A** | **T** | **C** | **A** | **A** | **T** | **T** | **A** | **G** | **A** | **A** | **G** | **C** | **A** | **A** | **T** | **T** | **T** | **T** | **C** | **T** | **T** | **A** | **G** | **A** | **A** | **G** | **A** | **T** | **C** | **G** | **A** | **A** | **A** | **A** | **A** | **A** | **A** | **T** | **G** | **T** | **A** | **C** | **A** | **A** | **C** | **A** | **A** | **T** | **G** | **A** | **C** | **T** | **C** | **C** | **A** | **G** | **A** | **A** | **G** | **T** | **T** | **G** | **T** | **T** | **C** | **G** | **C** | **G** | **A** | **C** | **A** | **T** | **G** | **T** | **C** | **A** | **A** | **A** | **A** | **T** | **G** | **G** | **T** | **T** | **G** | **A** | **T** | **T** | **T** | **T** | **T** | **A** | **A** | **A** | **C** | **A** | **A** | **C** | **A** | **A** | **A** | **T** | **T** | **T** | **C** | **A** | **C** | **G** | **A** | **T** | **T** | **A** | **T** | **T** | **C** | **T** | **C** | **A** | **C** | **C** | **A** | **A** | **T** | **T** | **C** | **T** | **T** | **-** | **-** | **-** |
| **N53766** | **T** | **T** | **G** | **A** | **T** | **T** | **C** | **T** | **T** | **A** | **G** | **C** | **G** | **T** | **C** | **T** | **T** | **A** | **A** | **C** | **A** | **G** | **T** | **T** | **A** | **T** | **T** | **T** | **A** | **T** | **T** | **T** | **T** | **A** | **T** | **T** | **A** | **A** | **T** | **G** | **C** | **A** | **A** | **T** | **T** | **A** | **T** | **T** | **G** | **C** | **G** | **G** | **G** | **A** | **A** | **T** | **T** | **A** | **C** | **C** | **A** | **T** | **T** | **T** | **T** | **G** | **T** | **T** | **A** | **A** | **A** | **A** | **C** | **C** | **T** | **C** | **G** | **A** | **G** | **A** | **T** | **G** | **T** | **A** | **G** | **C** | **A** | **G** | **C** | **A** | **A** | **T** | **T** | **T** | **G** | **G** | **G** | **C** | **A** | **T** | **G** | **G** | **C** | **T** | **A** | **C** | **T** | **A** | **G** | **T** | **A** | **C** | **T** | **G** | **A** | **T** | **T** | **G** | **C** | **T** | **C** | **T** | **A** | **C** | **C** | **C** | **G** | **T** | **T** | **T** | **T** | **T** | **G** | **G** | **T** | **T** | **T** | **T** | **T** | **T** | **C** | **T** | **T** | **A** | **T** | **A** | **T** | **T** | **T** | **A** | **T** | **T** | **C** | **T** | **T** | **T** | **G** | **G** | **G** | **C** | **G** | **C** | **G** | **G** | **T** | **T** | **T** | **A** | **A** | **C** | **C** | **G** | **A** | **C** | **A** | **A** | **A** | **A** | **A** | **G** | **A** | **A** | **A** | **T** | **T** | **T** | **T** | **A** | **T** | **T** | **T** | **G** | **C** | **A** | **A** | **C** | **A** | **A** | **A** | **G** | **T** | **G** | **A** | **T** | **T** | **T** | **A** | **C** | **G** | **G** | **G** | **A** | **A** | **T** | **T** | **A** | **G** | **A** | **G** | **A** | **A** | **T** | **T** | **T** | **C** | **C** | **A** | **G** | **A** | **A** | **T** | **T** | **T** | **C** | **C** | **A** | **A** | **G** | **A** | **A** | **G** | **A** | **A** | **A** | **G** | **T** | **T** | **T** | **T** | **G** | **A** | **A** | **C** | **T** | **T** | **T** | **A** | **T** | **A** | **G** | **T** | **C** | **A** | **A** | **A** | **A** | **G** | **A** | **T** | **G** | **C** | **C** | **G** | **A** | **C** | **G** | **G** | **A** | **G** | **G** | **A** | **G** | **C** | **A** | **A** | **C** | **A** | **A** | **C** | **A** | **G** | **T** | **T** | **T** | **A** | **C** | **A** | **G** | **A** | **C** | **T** | **T** | **T** | **T** | **T** | **T** | **T** | **C** | **C** | **T** | **C** | **G** | **T** | **T** | **A** | **A** | **A** | **T** | **A** | **G** | **G** | **A** | **T** | **G** | **C** | **C** | **T** | **C** | **T** | **G** | **A** | **C** | **T** | **A** | **A** | **G** | **A** | **A** | **A** | **A** | **A** | **T** | **G** | **A** | **T** | **G** | **T** | **T** | **G** | **A** | **A** | **A** | **T** | **T** | **T** | **T** | **T** | **A** | **C** | **G** | **G** | **A** | **T** | **G** | **G** | **T** | **A** | **C** | **G** | **G** | **A** | **A** | **A** | **A** | **A** | **T** | **T** | **C** | **A** | **A** | **T** | **G** | **C** | **G** | **T** | **T** | **G** | **A** | **T** | **G** | **G** | **C** | **G** | **G** | **A** | **C** | **A** | **T** | **A** | **A** | **A** | **A** | **A** | **A** | **A** | **G** | **C** | **T** | **C** | **A** | **G** | **C** | **A** | **C** | **T** | **C** | **C** | **A** | **T** | **T** | **C** | **A** | **T** | **A** | **T** | **T** | **G** | **A** | **A** | **T** | **A** | **T** | **T** | **A** | **T** | **G** | **C** | **C** | **T** | **T** | **T** | **G** | **T** | **A** | **A** | **C** | **G** | **G** | **A** | **T** | **C** | **A** | **T** | **A** | **T** | **C** | **G** | **G** | **C** | **A** | **C** | **C** | **A** | **A** | **A** | **A** | **T** | **T** | **T** | **T** | **G** | **A** | **A** | **C** | **T** | **T** | **A** | **T** | **T** | **A** | **G** | **A** | **A** | **G** | **A** | **A** | **A** | **A** | **G** | **G** | **C** | **G** | **G** | **C** | **T** | **G** | **A** | **A** | **G** | **G** | **G** | **G** | **T** | **T** | **G** | **A** | **A** | **G** | **T** | **G** | **C** | **G** | **G** | **T** | **T** | **G** | **C** | **T** | **T** | **T** | **A** | **C** | **G** | **A** | **T** | **G** | **C** | **C** | **T** | **T** | **T** | **G** | **G** | **C** | **T** | **C** | **A** | **A** | **A** | **G** | **G** | **G** | **C** | **A** | **C** | **C** | **A** | **A** | **G** | **G** | **T** | **T** | **C** | **A** | **T** | **C** | **A** | **T** | **T** | **T** | **G** | **A** | **A** | **T** | **G** | **A** | **A** | **T** | **T** | **G** | **A** | **A** | **A** | **A** | **A** | **A** | **A** | **A** | **C** | **G** | **G** | **C** | **G** | **G** | **T** | **T** | **T** | **T** | **G** | **T** | **C** | **C** | **A** | **A** | **A** | **C** | **A** | **T** | **T** | **T** | **A** | **T** | **T** | **A** | **C** | **T** | **T** | **C** | **T** | **C** | **A** | **A** | **A** | **A** | **A** | **G** | **C** | **A** | **C** | **T** | **T** | **T** | **T** | **G** | **A** | **A** | **G** | **T** | **T** | **T** | **C** | **G** | **T** | **T** | **T** | **G** | **A** | **A** | **T** | **T** | **A** | **T** | **C** | **A** | **T** | **G** | **A** | **T** | **C** | **A** | **C** | **C** | **G** | **G** | **A** | **A** | **A** | **A** | **T** | **T** | **G** | **T** | **T** | **G** | **T** | **T** | **A** | **T** | **T** | **G** | **A** | **C** | **G** | **G** | **A** | **A** | **A** | **A** | **G** | **T** | **A** | **G** | **G** | **C** | **T** | **A** | **C** | **A** | **T** | **T** | **G** | **G** | **C** | **G** | **G** | **C** | **T** | **T** | **T** | **A** | **A** | **T** | **G** | **T** | **T** | **G** | **C** | **C** | **G** | **A** | **T** | **C** | **A** | **A** | **T** | **A** | **T** | **G** | **C** | **C** | **G** | **G** | **A** | **A** | **C** | **G** | **A** | **C** | **T** | **A** | **A** | **A** | **A** | **A** | **G** | **T** | **T** | **T** | **G** | **G** | **C** | **T** | **A** | **T** | **T** | **G** | **G** | **C** | **G** | **G** | **G** | **A** | **T** | **A** | **C** | **G** | **C** | **A** | **T** | **T** | **T** | **A** | **C** | **G** | **G** | **A** | **T** | **T** | **C** | **A** | **A** | **G** | **G** | **G** | **C** | **C** | **A** | **G** | **C** | **A** | **G** | **C** | **C** | **T** | **C** | **A** | **T** | **T** | **A** | **C** | **T** | **G** | **C** | **A** | **A** | **A** | **T** | **G** | **C** | **G** | **T** | **T** | **T** | **T** | **T** | **T** | **A** | **A** | **T** | **G** | **G** | **A** | **T** | **T** | **G** | **G** | **A** | **A** | **C** | **G** | **T** | **C** | **T** | **C** | **T** | **T** | **C** | **C** | **C** | **C** | **C** | **G** | **A** | **G** | **A** | **A** | **A** | **A** | **A** | **T** | **C** | **G** | **T** | **G** | **T** | **G** | **G** | **C** | **G** | **T** | **A** | **T** | **C** | **A** | **A** | **T** | **T** | **G** | **G** | **A** | **T** | **T** | **A** | **T** | **T** | **T** | **C** | **T** | **T** | **T** | **A** | **A** | **A** | **C** | **T** | **T** | **G** | **A** | **A** | **G** | **C** | **A** | **T** | **T** | **G** | **G** | **T** | **G** | **C** | **C** | **A** | **G** | **A** | **G** | **G** | **C** | **A** | **A** | **A** | **T** | **A** | **C** | **A** | **T** | **C** | **C** | **A** | **T** | **T** | **C** | **A** | **G** | **A** | **T** | **G** | **A** | **T** | **T** | **G** | **C** | **C** | **A** | **G** | **T** | **G** | **G** | **T** | **C** | **C** | **T** | **A** | **A** | **C** | **A** | **G** | **T** | **G** | **A** | **C** | **C** | **G** | **T** | **G** | **A** | **A** | **C** | **A** | **A** | **A** | **T** | **T** | **A** | **A** | **A** | **T** | **T** | **G** | **G** | **C** | **C** | **T** | **T** | **T** | **A** | **T** | **T** | **A** | **A** | **A** | **T** | **T** | **G** | **A** | **T** | **T** | **A** | **C** | **T** | **T** | **C** | **T** | **G** | **C** | **C** | **A** | **A** | **G** | **A** | **A** | **A** | **A** | **G** | **A** | **G** | **T** | **C** | **T** | **G** | **G** | **A** | **T** | **T** | **C** | **A** | **A** | **A** | **C** | **G** | **C** | **C** | **G** | **T** | **A** | **T** | **T** | **T** | **A** | **G** | **T** | **T** | **C** | **C** | **T** | **G** | **A** | **T** | **G** | **A** | **T** | **A** | **G** | **T** | **G** | **T** | **C** | **T** | **T** | **G** | **G** | **C** | **T** | **G** | **C** | **T** | **T** | **T** | **A** | **A** | **A** | **G** | **G** | **T** | **T** | **G** | **C** | **T** | **G** | **C** | **G** | **G** | **C** | **T** | **T** | **C** | **A** | **G** | **G** | **A** | **G** | **T** | **A** | **G** | **A** | **T** | **G** | **T** | **T** | **A** | **A** | **A** | **A** | **T** | **T** | **A** | **T** | **G** | **A** | **T** | **T** | **C** | **C** | **A** | **G** | **A** | **T** | **A** | **A** | **G** | **C** | **C** | **C** | **G** | **A** | **C** | **C** | **A** | **T** | **C** | **C** | **G** | **T** | **T** | **T** | **A** | **T** | **T** | **T** | **A** | **T** | **C** | **G** | **A** | **G** | **C** | **A** | **A** | **C** | **A** | **C** | **A** | **G** | **T** | **A** | **T** | **T** | **A** | **C** | **G** | **G** | **C** | **C** | **G** | **C** | **T** | **T** | **A** | **T** | **T** | **G** | **A** | **T** | **G** | **A** | **A** | **A** | **G** | **A** | **A** | **A** | **A** | **T** | **A** | **T** | **T** | **G** | **A** | **A** | **A** | **T** | **T** | **T** | **T** | **A** | **A** | **T** | **T** | **T** | **A** | **T** | **A** | **A** | **C** | **G** | **G** | **T** | **G** | **G** | **T** | **T** | **T** | **C** | **T** | **T** | **A** | **C** | **A** | **T** | **G** | **C** | **G** | **A** | **A** | **A** | **A** | **C** | **A** | **A** | **T** | **G** | **A** | **T** | **T** | **A** | **T** | **G** | **G** | **A** | **T** | **G** | **A** | **T** | **G** | **A** | **A** | **G** | **T** | **C** | **T** | **G** | **C** | **A** | **C** | **A** | **G** | **T** | **T** | **G** | **G** | **T** | **T** | **C** | **A** | **G** | **C** | **C** | **A** | **A** | **T** | **C** | **A** | **A** | **G** | **A** | **T** | **A** | **T** | **C** | **C** | **G** | **A** | **A** | **G** | **T** | **T** | **A** | **C** | **A** | **A** | **A** | **T** | **T** | **A** | **A** | **A** | **C** | **T** | **T** | **T** | **G** | **A** | **A** | **G** | **C** | **A** | **A** | **A** | **T** | **G** | **C** | **T** | **G** | **T** | **G** | **T** | **T** | **A** | **T** | **A** | **T** | **G** | **A** | **T** | **A** | **A** | **A** | **A** | **A** | **A** | **A** | **T** | **C** | **A** | **T** | **T** | **G** | **A** | **T** | **C** | **A** | **A** | **T** | **T** | **A** | **G** | **A** | **A** | **G** | **C** | **A** | **A** | **T** | **T** | **T** | **T** | **C** | **T** | **T** | **A** | **G** | **A** | **A** | **G** | **A** | **T** | **C** | **G** | **A** | **A** | **A** | **A** | **A** | **A** | **A** | **T** | **G** | **T** | **A** | **C** | **A** | **A** | **C** | **A** | **A** | **T** | **G** | **A** | **C** | **T** | **C** | **C** | **A** | **G** | **A** | **A** | **G** | **T** | **T** | **G** | **T** | **T** | **C** | **G** | **C** | **G** | **A** | **T** | **A** | **T** | **G** | **T** | **C** | **A** | **A** | **A** | **A** | **T** | **G** | **G** | **T** | **T** | **G** | **A** | **T** | **T** | **T** | **T** | **T** | **A** | **A** | **A** | **C** | **A** | **A** | **C** | **A** | **A** | **A** | **T** | **T** | **T** | **C** | **A** | **C** | **G** | **A** | **T** | **T** | **A** | **T** | **T** | **T** | **T** | **C** | **A** | **C** | **C** | **A** | **A** | **T** | **T** | **C** | **T** | **T** | **-** | **-** | **-** |
| **N59599F** | **T** | **T** | **G** | **A** | **T** | **T** | **C** | **T** | **T** | **A** | **G** | **C** | **G** | **T** | **C** | **T** | **T** | **A** | **A** | **C** | **A** | **G** | **T** | **T** | **A** | **T** | **T** | **T** | **A** | **T** | **T** | **T** | **T** | **A** | **T** | **T** | **A** | **A** | **T** | **G** | **C** | **A** | **A** | **T** | **T** | **A** | **T** | **T** | **G** | **C** | **G** | **G** | **G** | **A** | **A** | **T** | **T** | **A** | **C** | **C** | **A** | **T** | **T** | **T** | **T** | **G** | **T** | **T** | **A** | **A** | **A** | **A** | **C** | **C** | **T** | **C** | **G** | **A** | **G** | **A** | **T** | **G** | **T** | **A** | **G** | **C** | **A** | **G** | **C** | **A** | **A** | **T** | **T** | **T** | **G** | **G** | **G** | **C** | **A** | **T** | **G** | **G** | **C** | **T** | **A** | **C** | **T** | **C** | **G** | **T** | **A** | **C** | **T** | **G** | **A** | **T** | **T** | **G** | **C** | **T** | **C** | **T** | **A** | **C** | **C** | **C** | **G** | **T** | **T** | **T** | **T** | **T** | **G** | **G** | **T** | **T** | **T** | **T** | **T** | **T** | **C** | **T** | **T** | **A** | **T** | **A** | **T** | **T** | **T** | **A** | **T** | **T** | **C** | **T** | **T** | **T** | **G** | **G** | **G** | **C** | **G** | **C** | **G** | **G** | **T** | **T** | **T** | **A** | **A** | **C** | **C** | **G** | **A** | **C** | **A** | **A** | **A** | **A** | **A** | **G** | **A** | **A** | **A** | **T** | **T** | **T** | **T** | **A** | **T** | **T** | **T** | **G** | **C** | **A** | **A** | **C** | **A** | **A** | **A** | **G** | **T** | **G** | **A** | **T** | **T** | **T** | **G** | **C** | **G** | **G** | **G** | **A** | **A** | **T** | **T** | **A** | **G** | **A** | **G** | **A** | **A** | **T** | **T** | **T** | **T** | **C** | **A** | **G** | **A** | **A** | **T** | **T** | **T** | **C** | **C** | **A** | **A** | **G** | **A** | **A** | **G** | **A** | **A** | **A** | **G** | **T** | **T** | **T** | **T** | **G** | **A** | **A** | **C** | **T** | **T** | **T** | **A** | **T** | **A** | **G** | **T** | **C** | **A** | **A** | **A** | **A** | **G** | **A** | **T** | **G** | **C** | **C** | **G** | **A** | **C** | **G** | **G** | **A** | **G** | **G** | **A** | **G** | **C** | **A** | **A** | **C** | **A** | **A** | **C** | **A** | **G** | **T** | **T** | **T** | **A** | **C** | **A** | **G** | **A** | **C** | **T** | **T** | **T** | **T** | **T** | **T** | **T** | **C** | **C** | **T** | **C** | **G** | **T** | **T** | **A** | **A** | **A** | **T** | **A** | **G** | **G** | **A** | **T** | **G** | **C** | **C** | **T** | **C** | **T** | **G** | **A** | **C** | **T** | **A** | **A** | **G** | **A** | **A** | **A** | **A** | **A** | **T** | **G** | **A** | **T** | **G** | **T** | **T** | **G** | **A** | **A** | **A** | **T** | **T** | **T** | **T** | **T** | **A** | **C** | **G** | **G** | **A** | **T** | **G** | **G** | **T** | **A** | **C** | **G** | **G** | **A** | **A** | **A** | **A** | **A** | **T** | **T** | **C** | **A** | **A** | **T** | **G** | **C** | **G** | **T** | **T** | **G** | **A** | **T** | **G** | **G** | **T** | **G** | **G** | **A** | **C** | **A** | **T** | **A** | **A** | **A** | **A** | **A** | **A** | **A** | **G** | **C** | **T** | **C** | **A** | **G** | **C** | **A** | **C** | **T** | **C** | **C** | **A** | **T** | **T** | **C** | **A** | **T** | **A** | **T** | **T** | **G** | **A** | **A** | **T** | **A** | **T** | **T** | **A** | **T** | **G** | **C** | **C** | **T** | **T** | **T** | **G** | **T** | **A** | **A** | **C** | **G** | **G** | **A** | **T** | **C** | **A** | **T** | **A** | **T** | **C** | **G** | **G** | **C** | **A** | **C** | **C** | **A** | **A** | **A** | **A** | **T** | **T** | **T** | **T** | **G** | **A** | **A** | **C** | **T** | **T** | **A** | **T** | **T** | **A** | **G** | **A** | **A** | **G** | **A** | **A** | **A** | **A** | **G** | **G** | **C** | **G** | **G** | **C** | **T** | **G** | **A** | **A** | **G** | **G** | **G** | **G** | **T** | **T** | **G** | **A** | **A** | **G** | **T** | **G** | **C** | **G** | **G** | **T** | **T** | **G** | **C** | **T** | **T** | **T** | **A** | **C** | **G** | **A** | **T** | **G** | **C** | **C** | **T** | **T** | **T** | **G** | **G** | **C** | **T** | **C** | **A** | **A** | **A** | **G** | **G** | **G** | **C** | **A** | **C** | **C** | **A** | **A** | **G** | **G** | **T** | **T** | **C** | **A** | **T** | **C** | **A** | **T** | **T** | **T** | **G** | **A** | **A** | **T** | **G** | **A** | **A** | **T** | **T** | **G** | **A** | **A** | **A** | **A** | **A** | **A** | **A** | **A** | **C** | **G** | **G** | **T** | **G** | **G** | **T** | **T** | **T** | **T** | **G** | **T** | **C** | **C** | **A** | **A** | **A** | **C** | **G** | **T** | **T** | **T** | **A** | **T** | **T** | **A** | **C** | **T** | **T** | **C** | **T** | **C** | **A** | **A** | **A** | **A** | **A** | **G** | **C** | **A** | **C** | **T** | **T** | **T** | **T** | **G** | **A** | **A** | **G** | **T** | **T** | **T** | **C** | **G** | **T** | **T** | **T** | **G** | **A** | **A** | **T** | **T** | **A** | **T** | **C** | **A** | **T** | **G** | **A** | **T** | **C** | **A** | **C** | **C** | **G** | **G** | **A** | **A** | **A** | **A** | **T** | **T** | **G** | **T** | **T** | **G** | **T** | **T** | **A** | **T** | **T** | **G** | **A** | **C** | **G** | **G** | **A** | **A** | **A** | **A** | **G** | **T** | **A** | **G** | **G** | **C** | **T** | **A** | **C** | **A** | **T** | **T** | **G** | **G** | **C** | **G** | **G** | **C** | **T** | **T** | **T** | **A** | **A** | **T** | **G** | **T** | **T** | **G** | **C** | **C** | **G** | **A** | **T** | **C** | **A** | **A** | **T** | **A** | **T** | **G** | **C** | **C** | **G** | **G** | **A** | **A** | **C** | **G** | **A** | **C** | **T** | **A** | **A** | **A** | **A** | **A** | **G** | **T** | **T** | **T** | **G** | **G** | **C** | **T** | **A** | **T** | **T** | **G** | **G** | **C** | **G** | **G** | **G** | **A** | **T** | **A** | **C** | **A** | **C** | **A** | **T** | **T** | **T** | **A** | **C** | **G** | **G** | **A** | **T** | **T** | **C** | **A** | **A** | **G** | **G** | **G** | **C** | **C** | **A** | **G** | **C** | **A** | **G** | **C** | **C** | **T** | **C** | **A** | **T** | **T** | **A** | **C** | **T** | **G** | **C** | **A** | **A** | **A** | **T** | **G** | **C** | **G** | **T** | **T** | **T** | **T** | **T** | **T** | **A** | **A** | **T** | **G** | **G** | **A** | **T** | **T** | **G** | **G** | **A** | **A** | **C** | **G** | **T** | **C** | **T** | **C** | **T** | **T** | **C** | **C** | **C** | **C** | **C** | **G** | **A** | **G** | **A** | **A** | **A** | **A** | **A** | **T** | **C** | **G** | **T** | **G** | **T** | **G** | **G** | **C** | **G** | **T** | **A** | **T** | **C** | **A** | **A** | **T** | **T** | **G** | **G** | **A** | **T** | **T** | **A** | **T** | **T** | **T** | **C** | **T** | **T** | **T** | **A** | **A** | **A** | **C** | **T** | **T** | **G** | **A** | **A** | **G** | **C** | **A** | **T** | **T** | **G** | **G** | **T** | **G** | **C** | **C** | **A** | **G** | **A** | **G** | **G** | **C** | **A** | **A** | **A** | **T** | **A** | **C** | **A** | **T** | **C** | **C** | **A** | **T** | **T** | **C** | **A** | **G** | **A** | **T** | **G** | **A** | **T** | **T** | **G** | **C** | **C** | **A** | **G** | **T** | **G** | **G** | **T** | **C** | **C** | **T** | **A** | **A** | **C** | **A** | **G** | **T** | **G** | **A** | **C** | **C** | **G** | **T** | **G** | **A** | **A** | **C** | **A** | **A** | **A** | **T** | **T** | **A** | **A** | **A** | **T** | **T** | **G** | **G** | **C** | **C** | **T** | **T** | **T** | **A** | **T** | **T** | **A** | **A** | **A** | **T** | **T** | **G** | **A** | **T** | **T** | **A** | **C** | **T** | **T** | **C** | **T** | **G** | **C** | **C** | **A** | **A** | **G** | **A** | **A** | **A** | **A** | **G** | **A** | **G** | **T** | **C** | **T** | **G** | **G** | **A** | **T** | **T** | **C** | **A** | **A** | **A** | **C** | **G** | **C** | **C** | **G** | **T** | **A** | **T** | **T** | **T** | **A** | **G** | **T** | **T** | **C** | **C** | **T** | **G** | **A** | **T** | **G** | **A** | **C** | **A** | **G** | **T** | **G** | **T** | **C** | **T** | **T** | **G** | **G** | **C** | **T** | **G** | **C** | **T** | **T** | **T** | **A** | **A** | **A** | **G** | **G** | **T** | **T** | **G** | **C** | **T** | **G** | **C** | **G** | **G** | **C** | **T** | **T** | **C** | **A** | **G** | **G** | **A** | **G** | **T** | **A** | **G** | **A** | **T** | **G** | **T** | **T** | **A** | **A** | **A** | **A** | **T** | **T** | **A** | **T** | **G** | **A** | **T** | **T** | **C** | **C** | **A** | **G** | **A** | **T** | **A** | **A** | **G** | **C** | **C** | **C** | **G** | **A** | **C** | **C** | **A** | **T** | **C** | **C** | **G** | **T** | **T** | **T** | **A** | **T** | **T** | **T** | **A** | **T** | **C** | **G** | **A** | **G** | **C** | **A** | **A** | **C** | **A** | **C** | **A** | **G** | **T** | **A** | **T** | **T** | **A** | **C** | **G** | **G** | **C** | **C** | **G** | **C** | **T** | **T** | **A** | **T** | **T** | **G** | **A** | **T** | **G** | **A** | **A** | **A** | **G** | **A** | **A** | **A** | **A** | **T** | **A** | **T** | **T** | **G** | **A** | **A** | **A** | **T** | **T** | **T** | **T** | **A** | **A** | **T** | **T** | **T** | **A** | **T** | **A** | **A** | **C** | **G** | **G** | **T** | **G** | **G** | **T** | **T** | **T** | **C** | **T** | **T** | **A** | **C** | **A** | **T** | **G** | **C** | **G** | **A** | **A** | **A** | **A** | **C** | **A** | **A** | **T** | **G** | **A** | **T** | **T** | **A** | **T** | **G** | **G** | **A** | **T** | **G** | **A** | **T** | **G** | **A** | **A** | **G** | **T** | **C** | **T** | **G** | **C** | **A** | **C** | **A** | **G** | **T** | **T** | **G** | **G** | **T** | **T** | **C** | **A** | **G** | **C** | **C** | **A** | **A** | **T** | **C** | **A** | **A** | **G** | **A** | **T** | **A** | **T** | **C** | **C** | **G** | **A** | **A** | **G** | **T** | **T** | **A** | **C** | **A** | **A** | **A** | **T** | **T** | **A** | **A** | **A** | **C** | **T** | **T** | **T** | **G** | **A** | **A** | **G** | **C** | **A** | **A** | **A** | **T** | **G** | **C** | **T** | **G** | **T** | **G** | **T** | **T** | **A** | **T** | **A** | **T** | **G** | **A** | **T** | **A** | **A** | **A** | **A** | **A** | **A** | **A** | **T** | **C** | **A** | **T** | **T** | **G** | **A** | **T** | **C** | **A** | **A** | **T** | **T** | **A** | **G** | **A** | **A** | **G** | **C** | **A** | **A** | **T** | **T** | **T** | **T** | **C** | **T** | **T** | **A** | **G** | **A** | **A** | **G** | **A** | **T** | **C** | **G** | **A** | **A** | **A** | **A** | **A** | **A** | **A** | **T** | **G** | **T** | **A** | **C** | **A** | **A** | **C** | **A** | **A** | **T** | **G** | **A** | **C** | **T** | **C** | **C** | **A** | **G** | **A** | **A** | **G** | **T** | **T** | **G** | **T** | **T** | **C** | **G** | **C** | **G** | **A** | **C** | **A** | **T** | **G** | **T** | **C** | **A** | **A** | **A** | **A** | **T** | **G** | **G** | **T** | **T** | **G** | **A** | **T** | **T** | **T** | **T** | **T** | **A** | **A** | **A** | **C** | **A** | **A** | **C** | **A** | **A** | **A** | **T** | **T** | **T** | **C** | **A** | **C** | **G** | **A** | **T** | **T** | **A** | **T** | **T** | **T** | **T** | **C** | **A** | **C** | **C** | **A** | **A** | **T** | **T** | **C** | **T** | **T** | **-** | **-** | **-** |
| **N59550F** | **T** | **T** | **G** | **A** | **T** | **T** | **C** | **T** | **T** | **A** | **G** | **C** | **G** | **T** | **C** | **T** | **T** | **A** | **A** | **C** | **A** | **G** | **T** | **T** | **A** | **T** | **T** | **T** | **A** | **T** | **T** | **T** | **T** | **A** | **T** | **T** | **A** | **A** | **T** | **G** | **C** | **A** | **A** | **T** | **T** | **A** | **T** | **T** | **G** | **C** | **G** | **G** | **G** | **A** | **A** | **T** | **T** | **A** | **C** | **C** | **A** | **T** | **T** | **T** | **T** | **G** | **T** | **T** | **A** | **A** | **A** | **A** | **C** | **C** | **T** | **C** | **G** | **A** | **G** | **A** | **T** | **G** | **T** | **A** | **G** | **C** | **A** | **G** | **C** | **A** | **A** | **T** | **T** | **T** | **G** | **G** | **G** | **C** | **A** | **T** | **G** | **G** | **C** | **T** | **A** | **C** | **T** | **C** | **G** | **T** | **A** | **C** | **T** | **G** | **A** | **T** | **T** | **G** | **C** | **T** | **C** | **T** | **A** | **C** | **C** | **C** | **G** | **T** | **T** | **T** | **T** | **T** | **G** | **G** | **T** | **T** | **T** | **T** | **T** | **T** | **C** | **T** | **T** | **A** | **T** | **A** | **T** | **T** | **T** | **A** | **T** | **T** | **C** | **T** | **T** | **T** | **G** | **G** | **T** | **C** | **G** | **A** | **G** | **G** | **G** | **T** | **T** | **A** | **A** | **C** | **C** | **G** | **A** | **C** | **A** | **A** | **A** | **A** | **A** | **G** | **A** | **A** | **A** | **T** | **T** | **T** | **T** | **A** | **T** | **T** | **T** | **G** | **C** | **A** | **A** | **C** | **A** | **A** | **A** | **G** | **T** | **G** | **A** | **T** | **T** | **T** | **A** | **C** | **G** | **G** | **G** | **A** | **A** | **T** | **T** | **A** | **G** | **A** | **G** | **A** | **A** | **T** | **T** | **T** | **T** | **C** | **A** | **G** | **A** | **A** | **T** | **T** | **T** | **C** | **C** | **A** | **A** | **G** | **A** | **A** | **G** | **A** | **A** | **A** | **G** | **T** | **T** | **T** | **T** | **G** | **A** | **A** | **C** | **T** | **T** | **T** | **A** | **T** | **A** | **G** | **T** | **C** | **A** | **A** | **A** | **A** | **G** | **A** | **T** | **G** | **C** | **C** | **G** | **A** | **C** | **G** | **G** | **A** | **G** | **G** | **A** | **G** | **C** | **A** | **A** | **C** | **A** | **A** | **C** | **A** | **G** | **T** | **T** | **T** | **A** | **C** | **A** | **G** | **A** | **C** | **T** | **T** | **T** | **T** | **T** | **T** | **T** | **C** | **C** | **T** | **C** | **G** | **T** | **T** | **A** | **A** | **A** | **T** | **A** | **G** | **G** | **A** | **T** | **G** | **C** | **C** | **T** | **C** | **T** | **G** | **A** | **C** | **T** | **A** | **A** | **G** | **A** | **A** | **A** | **A** | **A** | **T** | **G** | **A** | **T** | **G** | **T** | **T** | **G** | **A** | **A** | **A** | **T** | **T** | **T** | **T** | **T** | **A** | **C** | **G** | **G** | **A** | **T** | **G** | **G** | **T** | **A** | **C** | **G** | **G** | **A** | **A** | **A** | **A** | **A** | **T** | **T** | **C** | **A** | **A** | **T** | **G** | **C** | **G** | **T** | **T** | **G** | **A** | **T** | **G** | **G** | **C** | **G** | **G** | **A** | **C** | **A** | **T** | **A** | **A** | **A** | **A** | **A** | **A** | **A** | **G** | **C** | **T** | **C** | **A** | **G** | **C** | **A** | **C** | **T** | **C** | **T** | **A** | **T** | **T** | **C** | **A** | **T** | **A** | **T** | **T** | **G** | **A** | **A** | **T** | **A** | **T** | **T** | **A** | **T** | **G** | **C** | **C** | **T** | **T** | **T** | **G** | **T** | **A** | **A** | **C** | **G** | **G** | **A** | **T** | **C** | **A** | **T** | **A** | **T** | **C** | **G** | **G** | **C** | **A** | **C** | **C** | **A** | **A** | **A** | **A** | **T** | **T** | **T** | **T** | **G** | **A** | **A** | **C** | **T** | **T** | **A** | **T** | **T** | **A** | **G** | **A** | **A** | **G** | **A** | **A** | **A** | **A** | **G** | **G** | **C** | **G** | **G** | **C** | **T** | **G** | **A** | **A** | **G** | **G** | **G** | **G** | **T** | **T** | **G** | **A** | **A** | **G** | **T** | **G** | **C** | **G** | **G** | **T** | **T** | **G** | **C** | **T** | **T** | **T** | **A** | **C** | **G** | **A** | **T** | **G** | **C** | **C** | **T** | **T** | **T** | **G** | **G** | **C** | **T** | **C** | **A** | **A** | **A** | **G** | **G** | **G** | **C** | **A** | **C** | **C** | **A** | **A** | **G** | **G** | **T** | **T** | **C** | **A** | **T** | **C** | **A** | **T** | **T** | **T** | **G** | **A** | **A** | **T** | **G** | **A** | **A** | **T** | **T** | **G** | **A** | **A** | **A** | **A** | **A** | **A** | **A** | **A** | **C** | **G** | **G** | **C** | **G** | **G** | **T** | **T** | **T** | **T** | **G** | **T** | **C** | **C** | **A** | **A** | **A** | **C** | **G** | **T** | **T** | **T** | **A** | **T** | **T** | **A** | **C** | **T** | **T** | **C** | **T** | **C** | **A** | **A** | **A** | **A** | **A** | **G** | **C** | **A** | **C** | **T** | **T** | **T** | **T** | **G** | **A** | **A** | **G** | **T** | **T** | **T** | **C** | **G** | **T** | **T** | **T** | **G** | **A** | **A** | **T** | **T** | **A** | **T** | **C** | **A** | **T** | **G** | **A** | **T** | **C** | **A** | **C** | **C** | **G** | **G** | **A** | **A** | **A** | **A** | **T** | **T** | **G** | **T** | **T** | **G** | **T** | **T** | **A** | **T** | **T** | **G** | **A** | **C** | **G** | **G** | **A** | **A** | **A** | **A** | **G** | **T** | **A** | **G** | **G** | **C** | **T** | **A** | **C** | **A** | **T** | **T** | **G** | **G** | **C** | **G** | **G** | **C** | **T** | **T** | **T** | **A** | **A** | **T** | **G** | **T** | **T** | **G** | **C** | **C** | **G** | **A** | **T** | **C** | **A** | **A** | **T** | **A** | **T** | **G** | **C** | **C** | **G** | **G** | **A** | **A** | **C** | **G** | **A** | **C** | **T** | **A** | **A** | **A** | **A** | **A** | **G** | **T** | **T** | **T** | **G** | **G** | **C** | **T** | **A** | **T** | **T** | **G** | **G** | **C** | **G** | **G** | **G** | **A** | **T** | **A** | **C** | **A** | **C** | **A** | **T** | **T** | **T** | **A** | **C** | **G** | **G** | **A** | **T** | **T** | **C** | **A** | **A** | **G** | **G** | **G** | **C** | **C** | **A** | **G** | **C** | **A** | **G** | **C** | **C** | **T** | **C** | **A** | **T** | **T** | **A** | **C** | **T** | **G** | **C** | **A** | **A** | **A** | **T** | **G** | **C** | **G** | **T** | **T** | **T** | **T** | **T** | **T** | **A** | **A** | **T** | **G** | **G** | **A** | **T** | **T** | **G** | **G** | **A** | **A** | **C** | **G** | **T** | **C** | **T** | **C** | **T** | **T** | **C** | **C** | **C** | **C** | **C** | **G** | **A** | **G** | **A** | **A** | **A** | **A** | **A** | **T** | **C** | **G** | **T** | **G** | **T** | **G** | **G** | **C** | **G** | **T** | **A** | **T** | **C** | **A** | **A** | **T** | **T** | **G** | **G** | **A** | **T** | **T** | **A** | **T** | **T** | **T** | **C** | **T** | **T** | **T** | **A** | **A** | **A** | **C** | **T** | **T** | **G** | **A** | **A** | **G** | **C** | **A** | **T** | **T** | **G** | **G** | **T** | **G** | **C** | **C** | **A** | **G** | **A** | **G** | **G** | **C** | **A** | **A** | **A** | **T** | **A** | **C** | **A** | **T** | **C** | **C** | **A** | **T** | **T** | **C** | **A** | **G** | **A** | **T** | **G** | **A** | **T** | **T** | **G** | **C** | **C** | **A** | **G** | **T** | **G** | **G** | **T** | **C** | **C** | **T** | **A** | **A** | **C** | **A** | **G** | **T** | **G** | **A** | **C** | **C** | **G** | **T** | **G** | **A** | **A** | **C** | **A** | **A** | **A** | **T** | **T** | **A** | **A** | **A** | **T** | **T** | **G** | **G** | **C** | **C** | **T** | **T** | **T** | **A** | **T** | **T** | **A** | **A** | **A** | **T** | **T** | **G** | **A** | **T** | **T** | **A** | **C** | **T** | **T** | **C** | **T** | **G** | **C** | **C** | **A** | **A** | **G** | **A** | **A** | **A** | **A** | **G** | **A** | **G** | **T** | **C** | **T** | **G** | **G** | **A** | **T** | **T** | **C** | **A** | **A** | **A** | **C** | **G** | **C** | **C** | **G** | **T** | **A** | **T** | **T** | **T** | **A** | **G** | **T** | **T** | **C** | **C** | **T** | **G** | **A** | **T** | **G** | **A** | **T** | **A** | **G** | **T** | **G** | **T** | **C** | **T** | **T** | **G** | **G** | **C** | **T** | **G** | **C** | **T** | **T** | **T** | **A** | **A** | **A** | **G** | **G** | **T** | **T** | **G** | **C** | **T** | **G** | **C** | **G** | **G** | **C** | **T** | **T** | **C** | **A** | **G** | **G** | **A** | **G** | **T** | **A** | **G** | **A** | **T** | **G** | **T** | **T** | **A** | **A** | **A** | **A** | **T** | **T** | **A** | **T** | **G** | **A** | **T** | **T** | **C** | **C** | **A** | **G** | **A** | **T** | **A** | **A** | **G** | **C** | **C** | **C** | **G** | **A** | **C** | **C** | **A** | **T** | **C** | **C** | **G** | **T** | **T** | **T** | **A** | **T** | **T** | **T** | **A** | **T** | **C** | **G** | **A** | **G** | **C** | **A** | **A** | **C** | **A** | **C** | **A** | **G** | **T** | **A** | **T** | **T** | **A** | **C** | **G** | **G** | **C** | **C** | **G** | **C** | **T** | **T** | **A** | **T** | **T** | **G** | **A** | **T** | **G** | **A** | **A** | **A** | **G** | **A** | **A** | **A** | **A** | **T** | **A** | **T** | **T** | **G** | **A** | **A** | **A** | **T** | **T** | **T** | **T** | **A** | **A** | **T** | **T** | **T** | **A** | **T** | **A** | **A** | **C** | **G** | **G** | **T** | **G** | **G** | **T** | **T** | **T** | **C** | **T** | **T** | **A** | **C** | **A** | **T** | **G** | **C** | **G** | **A** | **A** | **A** | **A** | **C** | **A** | **A** | **T** | **G** | **A** | **T** | **T** | **A** | **T** | **G** | **G** | **A** | **T** | **G** | **A** | **T** | **G** | **A** | **A** | **G** | **T** | **C** | **T** | **G** | **C** | **A** | **C** | **A** | **G** | **T** | **T** | **G** | **G** | **T** | **T** | **C** | **A** | **G** | **C** | **C** | **A** | **A** | **T** | **C** | **A** | **A** | **G** | **A** | **T** | **A** | **T** | **C** | **C** | **G** | **A** | **A** | **G** | **T** | **T** | **A** | **C** | **A** | **A** | **A** | **T** | **T** | **A** | **A** | **A** | **C** | **T** | **T** | **T** | **G** | **A** | **A** | **G** | **C** | **A** | **A** | **A** | **T** | **G** | **C** | **T** | **G** | **T** | **G** | **T** | **T** | **A** | **T** | **A** | **T** | **G** | **A** | **T** | **A** | **A** | **A** | **A** | **A** | **A** | **A** | **T** | **C** | **A** | **T** | **T** | **G** | **A** | **T** | **C** | **A** | **A** | **T** | **T** | **A** | **G** | **A** | **A** | **G** | **C** | **A** | **A** | **T** | **T** | **T** | **T** | **C** | **T** | **T** | **A** | **G** | **A** | **A** | **G** | **A** | **T** | **C** | **G** | **A** | **A** | **A** | **A** | **A** | **A** | **A** | **T** | **G** | **T** | **A** | **C** | **A** | **A** | **C** | **A** | **A** | **T** | **G** | **A** | **C** | **T** | **C** | **C** | **A** | **G** | **A** | **A** | **G** | **T** | **T** | **G** | **T** | **T** | **C** | **G** | **C** | **G** | **A** | **C** | **A** | **T** | **G** | **T** | **C** | **A** | **A** | **A** | **A** | **T** | **G** | **G** | **T** | **T** | **G** | **A** | **T** | **T** | **T** | **T** | **T** | **A** | **A** | **A** | **C** | **A** | **A** | **C** | **A** | **A** | **A** | **T** | **T** | **T** | **C** | **A** | **C** | **G** | **A** | **T** | **T** | **A** | **T** | **T** | **T** | **T** | **C** | **A** | **C** | **C** | **A** | **A** | **T** | **T** | **C** | **T** | **T** | **-** | **-** | **-** |
| **N59592F** | **T** | **T** | **G** | **A** | **T** | **T** | **C** | **T** | **T** | **A** | **G** | **C** | **G** | **T** | **C** | **T** | **T** | **A** | **A** | **C** | **A** | **G** | **T** | **T** | **A** | **T** | **T** | **T** | **A** | **T** | **T** | **T** | **T** | **A** | **T** | **T** | **A** | **A** | **T** | **G** | **C** | **A** | **A** | **T** | **T** | **A** | **T** | **T** | **G** | **C** | **G** | **G** | **G** | **A** | **A** | **T** | **T** | **A** | **C** | **C** | **A** | **T** | **T** | **T** | **T** | **G** | **T** | **T** | **A** | **A** | **A** | **A** | **C** | **C** | **T** | **C** | **G** | **A** | **G** | **A** | **T** | **G** | **T** | **A** | **G** | **C** | **A** | **G** | **C** | **A** | **A** | **T** | **T** | **T** | **G** | **G** | **G** | **C** | **A** | **T** | **G** | **G** | **C** | **T** | **A** | **C** | **T** | **C** | **G** | **T** | **A** | **C** | **T** | **G** | **A** | **T** | **T** | **G** | **C** | **T** | **C** | **T** | **A** | **C** | **C** | **C** | **G** | **T** | **T** | **T** | **T** | **T** | **G** | **G** | **T** | **T** | **T** | **T** | **T** | **T** | **C** | **T** | **T** | **A** | **T** | **A** | **T** | **T** | **T** | **A** | **T** | **T** | **C** | **T** | **T** | **T** | **G** | **G** | **G** | **C** | **G** | **C** | **G** | **G** | **T** | **T** | **T** | **A** | **A** | **C** | **C** | **G** | **A** | **C** | **A** | **A** | **A** | **A** | **A** | **G** | **A** | **A** | **A** | **T** | **T** | **T** | **T** | **A** | **T** | **T** | **T** | **G** | **C** | **A** | **A** | **C** | **A** | **A** | **A** | **G** | **T** | **G** | **A** | **T** | **T** | **T** | **G** | **C** | **G** | **G** | **G** | **A** | **A** | **T** | **T** | **A** | **G** | **A** | **G** | **A** | **A** | **T** | **T** | **T** | **T** | **C** | **A** | **G** | **A** | **A** | **T** | **T** | **T** | **C** | **C** | **A** | **A** | **G** | **A** | **A** | **G** | **A** | **A** | **A** | **G** | **T** | **T** | **T** | **T** | **G** | **A** | **A** | **C** | **T** | **T** | **T** | **A** | **T** | **A** | **G** | **T** | **C** | **A** | **A** | **A** | **A** | **G** | **A** | **T** | **G** | **C** | **C** | **G** | **A** | **C** | **G** | **G** | **A** | **G** | **G** | **A** | **G** | **C** | **A** | **A** | **C** | **A** | **A** | **C** | **A** | **G** | **T** | **T** | **T** | **A** | **C** | **A** | **G** | **A** | **C** | **T** | **T** | **T** | **T** | **T** | **T** | **T** | **C** | **C** | **T** | **C** | **G** | **T** | **T** | **A** | **A** | **A** | **T** | **A** | **G** | **G** | **A** | **T** | **G** | **C** | **C** | **T** | **C** | **T** | **G** | **A** | **C** | **T** | **A** | **A** | **G** | **A** | **A** | **A** | **A** | **A** | **T** | **G** | **A** | **T** | **G** | **T** | **T** | **G** | **A** | **A** | **A** | **T** | **T** | **T** | **T** | **T** | **A** | **C** | **G** | **G** | **A** | **T** | **G** | **G** | **T** | **A** | **C** | **G** | **G** | **A** | **A** | **A** | **A** | **A** | **T** | **T** | **C** | **A** | **A** | **T** | **G** | **C** | **G** | **T** | **T** | **G** | **A** | **T** | **G** | **G** | **C** | **G** | **G** | **A** | **C** | **A** | **T** | **A** | **A** | **A** | **A** | **A** | **A** | **A** | **G** | **C** | **T** | **C** | **A** | **G** | **C** | **A** | **T** | **T** | **C** | **C** | **A** | **T** | **T** | **C** | **A** | **T** | **A** | **T** | **T** | **G** | **A** | **A** | **T** | **A** | **T** | **T** | **A** | **T** | **G** | **C** | **C** | **T** | **T** | **T** | **G** | **T** | **A** | **A** | **C** | **G** | **G** | **A** | **T** | **C** | **A** | **T** | **A** | **T** | **C** | **G** | **G** | **T** | **A** | **C** | **C** | **A** | **A** | **A** | **A** | **T** | **T** | **T** | **T** | **G** | **A** | **A** | **C** | **T** | **T** | **A** | **T** | **T** | **A** | **G** | **A** | **A** | **G** | **A** | **A** | **A** | **A** | **G** | **G** | **C** | **G** | **G** | **C** | **T** | **G** | **A** | **A** | **G** | **G** | **G** | **G** | **T** | **T** | **G** | **A** | **A** | **G** | **T** | **G** | **C** | **G** | **G** | **T** | **T** | **G** | **C** | **T** | **T** | **T** | **A** | **C** | **G** | **A** | **T** | **G** | **C** | **C** | **T** | **T** | **T** | **G** | **G** | **C** | **T** | **C** | **A** | **A** | **A** | **G** | **G** | **G** | **C** | **A** | **C** | **C** | **A** | **A** | **G** | **G** | **T** | **T** | **C** | **A** | **T** | **C** | **A** | **T** | **T** | **T** | **G** | **A** | **A** | **T** | **G** | **A** | **A** | **T** | **T** | **G** | **A** | **A** | **A** | **A** | **A** | **A** | **A** | **A** | **C** | **G** | **G** | **C** | **G** | **G** | **T** | **T** | **T** | **T** | **G** | **T** | **C** | **C** | **A** | **A** | **A** | **C** | **G** | **T** | **T** | **T** | **A** | **T** | **T** | **A** | **C** | **T** | **T** | **C** | **T** | **C** | **A** | **A** | **A** | **A** | **A** | **G** | **C** | **A** | **C** | **T** | **T** | **T** | **T** | **G** | **A** | **A** | **G** | **T** | **T** | **T** | **C** | **G** | **T** | **T** | **T** | **G** | **A** | **A** | **T** | **T** | **A** | **T** | **C** | **A** | **T** | **G** | **A** | **T** | **C** | **A** | **C** | **C** | **G** | **G** | **A** | **A** | **A** | **A** | **T** | **T** | **G** | **T** | **T** | **G** | **T** | **T** | **A** | **T** | **T** | **G** | **A** | **T** | **G** | **G** | **G** | **A** | **A** | **A** | **G** | **T** | **A** | **G** | **G** | **C** | **T** | **A** | **C** | **A** | **T** | **T** | **G** | **G** | **C** | **G** | **G** | **C** | **T** | **T** | **T** | **A** | **A** | **T** | **G** | **T** | **T** | **G** | **C** | **C** | **G** | **A** | **T** | **C** | **A** | **A** | **T** | **A** | **T** | **G** | **C** | **T** | **G** | **G** | **A** | **A** | **C** | **G** | **A** | **C** | **T** | **A** | **A** | **A** | **A** | **A** | **G** | **T** | **T** | **T** | **G** | **G** | **C** | **T** | **A** | **T** | **T** | **G** | **G** | **C** | **G** | **G** | **G** | **A** | **T** | **A** | **C** | **A** | **C** | **A** | **T** | **T** | **T** | **A** | **C** | **G** | **G** | **A** | **T** | **T** | **C** | **A** | **A** | **G** | **G** | **G** | **C** | **C** | **A** | **G** | **C** | **A** | **G** | **C** | **C** | **T** | **C** | **A** | **T** | **T** | **A** | **C** | **T** | **G** | **C** | **A** | **A** | **A** | **T** | **G** | **C** | **G** | **T** | **T** | **T** | **T** | **T** | **T** | **A** | **A** | **T** | **G** | **G** | **A** | **T** | **T** | **G** | **G** | **A** | **A** | **C** | **G** | **T** | **C** | **T** | **C** | **T** | **T** | **C** | **C** | **C** | **C** | **T** | **G** | **A** | **G** | **A** | **A** | **A** | **A** | **A** | **T** | **C** | **G** | **T** | **G** | **T** | **G** | **G** | **C** | **G** | **T** | **A** | **T** | **C** | **A** | **A** | **T** | **T** | **G** | **G** | **A** | **T** | **T** | **A** | **T** | **T** | **T** | **C** | **T** | **T** | **T** | **A** | **A** | **A** | **C** | **T** | **T** | **G** | **A** | **A** | **G** | **C** | **G** | **T** | **T** | **G** | **G** | **T** | **G** | **C** | **C** | **A** | **G** | **A** | **G** | **G** | **C** | **A** | **A** | **A** | **T** | **A** | **C** | **A** | **T** | **C** | **C** | **A** | **T** | **T** | **C** | **A** | **G** | **A** | **T** | **G** | **A** | **T** | **T** | **G** | **C** | **C** | **A** | **G** | **T** | **G** | **G** | **T** | **C** | **C** | **T** | **A** | **A** | **T** | **A** | **G** | **T** | **G** | **A** | **C** | **C** | **G** | **T** | **G** | **A** | **A** | **C** | **A** | **A** | **A** | **T** | **T** | **A** | **A** | **A** | **T** | **T** | **G** | **G** | **C** | **C** | **T** | **T** | **T** | **A** | **T** | **T** | **A** | **A** | **A** | **T** | **T** | **G** | **A** | **T** | **T** | **A** | **C** | **T** | **T** | **C** | **T** | **G** | **C** | **C** | **A** | **A** | **G** | **A** | **A** | **A** | **A** | **G** | **A** | **G** | **T** | **C** | **T** | **G** | **G** | **A** | **T** | **T** | **C** | **A** | **A** | **A** | **C** | **G** | **C** | **C** | **G** | **T** | **A** | **T** | **T** | **T** | **A** | **G** | **T** | **T** | **C** | **C** | **T** | **G** | **A** | **T** | **G** | **A** | **T** | **A** | **G** | **T** | **G** | **T** | **C** | **T** | **T** | **G** | **G** | **C** | **G** | **G** | **C** | **T** | **T** | **T** | **A** | **A** | **A** | **G** | **G** | **T** | **T** | **G** | **C** | **T** | **G** | **C** | **G** | **G** | **C** | **T** | **T** | **C** | **A** | **G** | **G** | **A** | **G** | **T** | **A** | **G** | **A** | **T** | **G** | **T** | **T** | **A** | **A** | **A** | **A** | **T** | **T** | **A** | **T** | **G** | **A** | **T** | **T** | **C** | **C** | **A** | **G** | **A** | **T** | **A** | **A** | **G** | **C** | **C** | **C** | **G** | **A** | **C** | **C** | **A** | **T** | **C** | **C** | **G** | **T** | **T** | **T** | **A** | **T** | **T** | **T** | **A** | **T** | **C** | **G** | **A** | **G** | **C** | **A** | **A** | **C** | **A** | **C** | **A** | **G** | **T** | **A** | **T** | **T** | **A** | **C** | **G** | **G** | **G** | **C** | **G** | **C** | **T** | **T** | **G** | **T** | **T** | **G** | **A** | **T** | **G** | **A** | **A** | **A** | **G** | **A** | **A** | **A** | **A** | **T** | **A** | **T** | **T** | **G** | **A** | **A** | **A** | **T** | **T** | **T** | **T** | **A** | **A** | **T** | **T** | **T** | **A** | **T** | **A** | **A** | **C** | **G** | **G** | **T** | **G** | **G** | **T** | **T** | **T** | **C** | **T** | **T** | **A** | **C** | **A** | **T** | **G** | **C** | **G** | **A** | **A** | **A** | **A** | **C** | **A** | **A** | **T** | **G** | **A** | **T** | **T** | **A** | **T** | **G** | **G** | **A** | **T** | **G** | **A** | **T** | **G** | **A** | **A** | **G** | **T** | **C** | **T** | **G** | **C** | **A** | **C** | **A** | **G** | **T** | **A** | **G** | **G** | **T** | **T** | **C** | **A** | **G** | **C** | **C** | **A** | **A** | **T** | **C** | **A** | **A** | **G** | **A** | **T** | **A** | **T** | **C** | **C** | **G** | **A** | **A** | **G** | **T** | **T** | **A** | **C** | **A** | **A** | **A** | **T** | **T** | **A** | **A** | **A** | **C** | **T** | **T** | **T** | **G** | **A** | **A** | **G** | **C** | **A** | **A** | **A** | **T** | **G** | **C** | **T** | **G** | **T** | **G** | **T** | **T** | **A** | **T** | **A** | **T** | **G** | **A** | **T** | **A** | **A** | **A** | **A** | **A** | **A** | **A** | **T** | **C** | **A** | **T** | **T** | **G** | **A** | **T** | **C** | **A** | **A** | **T** | **T** | **A** | **G** | **A** | **A** | **G** | **C** | **A** | **A** | **T** | **T** | **T** | **T** | **C** | **T** | **T** | **A** | **G** | **A** | **A** | **G** | **A** | **T** | **C** | **G** | **A** | **A** | **A** | **A** | **A** | **A** | **A** | **T** | **G** | **T** | **A** | **C** | **A** | **A** | **C** | **A** | **A** | **T** | **G** | **A** | **C** | **T** | **C** | **C** | **A** | **G** | **A** | **A** | **G** | **T** | **T** | **G** | **T** | **T** | **C** | **G** | **C** | **G** | **A** | **C** | **A** | **T** | **G** | **T** | **C** | **A** | **A** | **A** | **A** | **T** | **G** | **G** | **T** | **T** | **G** | **A** | **T** | **T** | **T** | **T** | **T** | **A** | **A** | **A** | **C** | **A** | **A** | **C** | **A** | **A** | **A** | **T** | **T** | **T** | **C** | **A** | **C** | **G** | **A** | **T** | **T** | **A** | **T** | **T** | **C** | **T** | **C** | **A** | **C** | **C** | **A** | **A** | **T** | **T** | **C** | **T** | **T** | **-** | **-** | **-** |
| **N53457** | **T** | **T** | **G** | **A** | **T** | **T** | **C** | **T** | **T** | **A** | **G** | **C** | **G** | **T** | **C** | **T** | **T** | **A** | **A** | **C** | **A** | **G** | **T** | **T** | **A** | **T** | **T** | **T** | **A** | **T** | **T** | **T** | **T** | **A** | **T** | **T** | **A** | **A** | **T** | **G** | **C** | **A** | **A** | **T** | **T** | **A** | **T** | **T** | **G** | **C** | **G** | **G** | **G** | **A** | **A** | **T** | **T** | **A** | **C** | **C** | **A** | **T** | **T** | **T** | **T** | **G** | **T** | **T** | **A** | **A** | **A** | **A** | **C** | **C** | **T** | **C** | **G** | **A** | **G** | **A** | **T** | **G** | **T** | **A** | **G** | **C** | **A** | **G** | **C** | **A** | **A** | **T** | **T** | **T** | **G** | **G** | **G** | **C** | **A** | **T** | **G** | **G** | **C** | **T** | **A** | **C** | **T** | **C** | **G** | **T** | **A** | **C** | **T** | **G** | **A** | **T** | **T** | **G** | **C** | **T** | **C** | **T** | **A** | **C** | **C** | **C** | **G** | **T** | **T** | **T** | **T** | **T** | **G** | **G** | **T** | **T** | **T** | **T** | **T** | **T** | **C** | **T** | **T** | **A** | **T** | **A** | **T** | **T** | **T** | **A** | **T** | **T** | **C** | **T** | **T** | **T** | **G** | **G** | **G** | **C** | **G** | **C** | **G** | **G** | **T** | **T** | **T** | **A** | **A** | **C** | **C** | **G** | **A** | **C** | **A** | **A** | **A** | **A** | **A** | **G** | **A** | **A** | **A** | **T** | **T** | **T** | **T** | **A** | **T** | **T** | **T** | **G** | **C** | **A** | **A** | **C** | **A** | **A** | **A** | **G** | **T** | **G** | **A** | **T** | **T** | **T** | **G** | **C** | **G** | **G** | **G** | **A** | **A** | **T** | **T** | **A** | **G** | **A** | **G** | **A** | **A** | **T** | **T** | **T** | **T** | **C** | **A** | **G** | **A** | **A** | **T** | **T** | **T** | **C** | **C** | **A** | **A** | **G** | **A** | **A** | **G** | **A** | **A** | **A** | **G** | **T** | **T** | **T** | **T** | **G** | **A** | **A** | **C** | **T** | **T** | **T** | **A** | **T** | **A** | **G** | **T** | **C** | **A** | **A** | **A** | **A** | **G** | **A** | **T** | **G** | **C** | **C** | **G** | **A** | **C** | **G** | **G** | **A** | **G** | **G** | **A** | **G** | **C** | **A** | **A** | **C** | **A** | **A** | **C** | **A** | **G** | **T** | **T** | **T** | **A** | **C** | **A** | **G** | **A** | **C** | **T** | **T** | **T** | **T** | **T** | **T** | **T** | **C** | **C** | **T** | **C** | **G** | **T** | **T** | **A** | **A** | **A** | **T** | **A** | **G** | **G** | **A** | **T** | **G** | **C** | **C** | **T** | **C** | **T** | **G** | **A** | **C** | **T** | **A** | **A** | **G** | **A** | **A** | **A** | **A** | **A** | **T** | **G** | **A** | **T** | **G** | **T** | **T** | **G** | **A** | **A** | **A** | **T** | **T** | **T** | **T** | **T** | **A** | **C** | **G** | **G** | **A** | **T** | **G** | **G** | **T** | **A** | **C** | **G** | **G** | **A** | **A** | **A** | **A** | **A** | **T** | **T** | **C** | **A** | **A** | **T** | **G** | **C** | **G** | **T** | **T** | **G** | **A** | **T** | **G** | **G** | **C** | **A** | **G** | **A** | **C** | **A** | **T** | **A** | **A** | **A** | **A** | **A** | **A** | **A** | **G** | **C** | **T** | **C** | **A** | **G** | **C** | **A** | **C** | **T** | **C** | **C** | **A** | **T** | **T** | **C** | **A** | **T** | **A** | **T** | **T** | **G** | **A** | **A** | **T** | **A** | **T** | **T** | **A** | **T** | **G** | **C** | **C** | **T** | **T** | **T** | **G** | **T** | **A** | **A** | **C** | **G** | **G** | **A** | **T** | **C** | **A** | **T** | **A** | **T** | **C** | **G** | **G** | **T** | **A** | **C** | **C** | **A** | **A** | **A** | **A** | **T** | **T** | **T** | **T** | **G** | **A** | **A** | **C** | **T** | **T** | **A** | **T** | **T** | **A** | **G** | **A** | **A** | **G** | **A** | **A** | **A** | **A** | **G** | **G** | **C** | **G** | **G** | **C** | **T** | **G** | **A** | **A** | **G** | **G** | **G** | **G** | **T** | **T** | **G** | **A** | **A** | **G** | **T** | **G** | **C** | **G** | **G** | **T** | **T** | **G** | **C** | **T** | **T** | **T** | **A** | **C** | **G** | **A** | **T** | **G** | **C** | **C** | **T** | **T** | **T** | **G** | **G** | **C** | **T** | **C** | **A** | **A** | **A** | **G** | **G** | **G** | **T** | **A** | **C** | **C** | **A** | **A** | **G** | **G** | **T** | **T** | **C** | **A** | **T** | **C** | **A** | **T** | **T** | **T** | **G** | **A** | **A** | **T** | **G** | **A** | **A** | **T** | **T** | **G** | **A** | **A** | **A** | **A** | **A** | **A** | **A** | **A** | **C** | **G** | **G** | **C** | **G** | **G** | **T** | **T** | **T** | **T** | **G** | **T** | **C** | **C** | **A** | **A** | **A** | **C** | **G** | **T** | **T** | **T** | **A** | **T** | **T** | **A** | **C** | **T** | **T** | **C** | **T** | **C** | **A** | **A** | **A** | **A** | **A** | **G** | **C** | **A** | **C** | **T** | **T** | **T** | **T** | **G** | **A** | **A** | **G** | **T** | **T** | **T** | **C** | **G** | **T** | **T** | **T** | **G** | **A** | **A** | **T** | **T** | **A** | **T** | **C** | **A** | **T** | **G** | **A** | **T** | **C** | **A** | **C** | **C** | **G** | **G** | **A** | **A** | **A** | **A** | **T** | **T** | **G** | **T** | **T** | **G** | **T** | **T** | **A** | **T** | **T** | **G** | **A** | **C** | **G** | **G** | **A** | **A** | **A** | **A** | **G** | **T** | **A** | **G** | **G** | **C** | **T** | **A** | **C** | **A** | **T** | **T** | **G** | **G** | **C** | **G** | **G** | **C** | **T** | **T** | **T** | **A** | **A** | **T** | **G** | **T** | **T** | **G** | **C** | **C** | **G** | **A** | **T** | **C** | **A** | **A** | **T** | **A** | **T** | **G** | **C** | **C** | **G** | **G** | **A** | **A** | **C** | **G** | **A** | **C** | **T** | **A** | **A** | **A** | **A** | **A** | **G** | **T** | **T** | **T** | **G** | **G** | **C** | **T** | **A** | **T** | **T** | **G** | **G** | **C** | **G** | **G** | **G** | **A** | **T** | **A** | **C** | **A** | **C** | **A** | **T** | **T** | **T** | **A** | **C** | **G** | **G** | **A** | **T** | **T** | **C** | **A** | **A** | **G** | **G** | **G** | **C** | **C** | **A** | **G** | **C** | **A** | **G** | **C** | **C** | **T** | **C** | **A** | **T** | **T** | **A** | **C** | **T** | **G** | **C** | **A** | **A** | **A** | **T** | **G** | **C** | **G** | **T** | **T** | **T** | **T** | **T** | **T** | **A** | **A** | **T** | **G** | **G** | **A** | **T** | **T** | **G** | **G** | **A** | **A** | **C** | **G** | **T** | **C** | **T** | **C** | **T** | **T** | **C** | **C** | **C** | **C** | **C** | **G** | **A** | **G** | **A** | **A** | **A** | **A** | **A** | **T** | **C** | **G** | **T** | **G** | **T** | **G** | **G** | **C** | **G** | **T** | **A** | **T** | **C** | **A** | **A** | **T** | **T** | **G** | **G** | **A** | **T** | **T** | **A** | **T** | **T** | **T** | **C** | **T** | **T** | **T** | **A** | **A** | **A** | **C** | **T** | **T** | **G** | **A** | **A** | **G** | **C** | **A** | **T** | **T** | **G** | **G** | **T** | **G** | **C** | **C** | **A** | **G** | **A** | **G** | **G** | **C** | **A** | **A** | **A** | **T** | **A** | **C** | **A** | **T** | **C** | **C** | **A** | **T** | **T** | **C** | **A** | **G** | **A** | **T** | **G** | **A** | **T** | **T** | **G** | **C** | **C** | **A** | **G** | **T** | **G** | **G** | **T** | **C** | **C** | **T** | **A** | **A** | **C** | **A** | **G** | **T** | **G** | **A** | **C** | **C** | **G** | **T** | **G** | **A** | **A** | **C** | **A** | **A** | **A** | **T** | **T** | **A** | **A** | **A** | **T** | **T** | **G** | **G** | **C** | **C** | **T** | **T** | **T** | **A** | **T** | **T** | **A** | **A** | **A** | **T** | **T** | **G** | **A** | **T** | **T** | **A** | **C** | **T** | **T** | **C** | **T** | **G** | **C** | **C** | **A** | **A** | **G** | **A** | **A** | **A** | **A** | **G** | **A** | **G** | **T** | **C** | **T** | **G** | **G** | **A** | **T** | **T** | **C** | **A** | **A** | **A** | **C** | **G** | **C** | **C** | **G** | **T** | **A** | **T** | **T** | **T** | **A** | **G** | **T** | **T** | **C** | **C** | **T** | **G** | **A** | **T** | **G** | **A** | **C** | **A** | **G** | **T** | **G** | **T** | **C** | **T** | **T** | **G** | **G** | **C** | **T** | **G** | **C** | **T** | **T** | **T** | **A** | **A** | **A** | **G** | **G** | **T** | **T** | **G** | **C** | **T** | **G** | **C** | **G** | **G** | **C** | **T** | **T** | **C** | **A** | **G** | **G** | **A** | **G** | **T** | **A** | **G** | **A** | **T** | **G** | **T** | **T** | **A** | **A** | **A** | **A** | **T** | **T** | **A** | **T** | **G** | **A** | **T** | **T** | **C** | **C** | **A** | **G** | **A** | **T** | **A** | **A** | **G** | **C** | **C** | **C** | **G** | **A** | **C** | **C** | **A** | **T** | **C** | **C** | **G** | **T** | **T** | **T** | **A** | **T** | **T** | **T** | **A** | **T** | **C** | **G** | **A** | **G** | **C** | **A** | **A** | **C** | **A** | **C** | **A** | **G** | **T** | **A** | **T** | **T** | **A** | **C** | **G** | **G** | **C** | **C** | **G** | **C** | **T** | **T** | **A** | **T** | **T** | **G** | **A** | **T** | **G** | **A** | **A** | **A** | **G** | **A** | **A** | **A** | **A** | **T** | **A** | **T** | **T** | **G** | **A** | **A** | **A** | **T** | **T** | **T** | **T** | **A** | **A** | **T** | **T** | **T** | **A** | **T** | **A** | **A** | **C** | **G** | **G** | **T** | **G** | **G** | **T** | **T** | **T** | **C** | **T** | **T** | **A** | **C** | **A** | **T** | **G** | **C** | **G** | **A** | **A** | **A** | **A** | **C** | **A** | **A** | **T** | **G** | **A** | **T** | **T** | **A** | **T** | **G** | **G** | **A** | **T** | **G** | **A** | **T** | **G** | **A** | **A** | **G** | **T** | **C** | **T** | **G** | **C** | **A** | **C** | **A** | **G** | **T** | **T** | **G** | **G** | **T** | **T** | **C** | **A** | **G** | **C** | **C** | **A** | **A** | **T** | **C** | **A** | **A** | **G** | **A** | **T** | **A** | **T** | **C** | **C** | **G** | **A** | **A** | **G** | **T** | **T** | **A** | **C** | **A** | **A** | **A** | **T** | **T** | **A** | **A** | **A** | **C** | **T** | **T** | **T** | **G** | **A** | **A** | **G** | **C** | **A** | **A** | **A** | **T** | **G** | **C** | **T** | **G** | **T** | **G** | **T** | **T** | **A** | **T** | **A** | **T** | **G** | **A** | **T** | **A** | **A** | **A** | **A** | **A** | **A** | **A** | **T** | **C** | **A** | **T** | **T** | **G** | **A** | **T** | **C** | **A** | **A** | **T** | **T** | **A** | **G** | **A** | **A** | **G** | **C** | **A** | **A** | **T** | **T** | **T** | **T** | **C** | **T** | **T** | **A** | **G** | **A** | **A** | **G** | **A** | **T** | **C** | **G** | **A** | **A** | **A** | **A** | **A** | **A** | **A** | **T** | **G** | **T** | **A** | **C** | **A** | **A** | **C** | **A** | **A** | **T** | **G** | **A** | **C** | **T** | **C** | **C** | **A** | **G** | **A** | **A** | **G** | **T** | **T** | **G** | **T** | **T** | **C** | **G** | **T** | **G** | **A** | **C** | **A** | **T** | **G** | **T** | **C** | **A** | **A** | **A** | **A** | **T** | **G** | **G** | **T** | **T** | **G** | **A** | **T** | **T** | **T** | **T** | **T** | **A** | **A** | **A** | **C** | **A** | **A** | **C** | **A** | **A** | **A** | **T** | **T** | **T** | **C** | **A** | **C** | **G** | **A** | **T** | **T** | **A** | **T** | **T** | **T** | **T** | **C** | **A** | **C** | **C** | **A** | **A** | **T** | **T** | **C** | **T** | **T** | **-** | **-** | **-** |
| **N53434** | **T** | **T** | **G** | **A** | **T** | **T** | **C** | **T** | **T** | **A** | **G** | **C** | **G** | **T** | **C** | **T** | **T** | **A** | **A** | **C** | **A** | **G** | **T** | **T** | **A** | **T** | **T** | **T** | **A** | **T** | **T** | **T** | **T** | **A** | **T** | **T** | **A** | **A** | **T** | **G** | **C** | **A** | **A** | **T** | **T** | **A** | **T** | **T** | **G** | **C** | **G** | **G** | **G** | **A** | **A** | **T** | **T** | **A** | **C** | **C** | **A** | **T** | **T** | **T** | **T** | **G** | **T** | **T** | **A** | **A** | **A** | **A** | **C** | **C** | **T** | **C** | **G** | **A** | **G** | **A** | **T** | **G** | **T** | **A** | **G** | **C** | **A** | **G** | **C** | **A** | **A** | **T** | **T** | **T** | **G** | **G** | **G** | **C** | **A** | **T** | **G** | **G** | **C** | **T** | **A** | **C** | **T** | **C** | **G** | **T** | **A** | **C** | **T** | **G** | **A** | **T** | **T** | **G** | **C** | **T** | **C** | **T** | **A** | **C** | **C** | **C** | **G** | **T** | **T** | **T** | **T** | **T** | **G** | **G** | **T** | **T** | **T** | **T** | **T** | **T** | **C** | **T** | **T** | **A** | **T** | **A** | **T** | **T** | **T** | **A** | **T** | **T** | **C** | **T** | **T** | **T** | **G** | **G** | **G** | **C** | **G** | **C** | **G** | **G** | **T** | **T** | **T** | **A** | **A** | **C** | **C** | **G** | **A** | **C** | **A** | **A** | **A** | **A** | **A** | **G** | **A** | **A** | **A** | **T** | **T** | **T** | **T** | **A** | **T** | **T** | **T** | **G** | **C** | **A** | **A** | **C** | **A** | **A** | **A** | **G** | **T** | **G** | **A** | **T** | **T** | **T** | **A** | **C** | **G** | **G** | **G** | **A** | **A** | **T** | **T** | **A** | **G** | **A** | **G** | **A** | **A** | **T** | **T** | **T** | **C** | **C** | **A** | **G** | **A** | **A** | **T** | **T** | **T** | **C** | **C** | **A** | **A** | **G** | **A** | **A** | **G** | **A** | **A** | **A** | **G** | **T** | **T** | **T** | **T** | **G** | **A** | **A** | **C** | **T** | **T** | **T** | **A** | **T** | **A** | **G** | **T** | **C** | **A** | **A** | **A** | **A** | **G** | **A** | **T** | **G** | **C** | **C** | **G** | **A** | **C** | **G** | **G** | **A** | **G** | **G** | **A** | **G** | **C** | **A** | **A** | **C** | **A** | **A** | **C** | **A** | **G** | **T** | **T** | **T** | **A** | **C** | **A** | **G** | **A** | **C** | **T** | **T** | **T** | **T** | **T** | **T** | **T** | **C** | **C** | **T** | **C** | **G** | **T** | **T** | **A** | **A** | **A** | **T** | **A** | **G** | **G** | **A** | **T** | **G** | **C** | **C** | **T** | **C** | **T** | **G** | **A** | **C** | **T** | **A** | **A** | **G** | **A** | **A** | **A** | **A** | **A** | **T** | **G** | **A** | **T** | **G** | **T** | **T** | **G** | **A** | **A** | **A** | **T** | **T** | **T** | **T** | **T** | **A** | **C** | **G** | **G** | **A** | **T** | **G** | **G** | **T** | **A** | **C** | **G** | **G** | **A** | **A** | **A** | **A** | **A** | **T** | **T** | **C** | **A** | **A** | **T** | **G** | **C** | **G** | **T** | **T** | **G** | **A** | **T** | **G** | **G** | **C** | **G** | **G** | **A** | **T** | **A** | **T** | **A** | **A** | **A** | **A** | **A** | **A** | **A** | **G** | **C** | **T** | **C** | **A** | **G** | **C** | **A** | **C** | **T** | **C** | **T** | **A** | **T** | **T** | **C** | **A** | **T** | **A** | **T** | **T** | **G** | **A** | **A** | **T** | **A** | **T** | **T** | **A** | **T** | **G** | **C** | **C** | **T** | **T** | **T** | **G** | **T** | **A** | **A** | **C** | **G** | **G** | **A** | **T** | **C** | **A** | **T** | **A** | **T** | **C** | **G** | **G** | **T** | **A** | **C** | **C** | **A** | **A** | **A** | **A** | **T** | **T** | **T** | **T** | **G** | **A** | **A** | **C** | **T** | **T** | **A** | **T** | **T** | **A** | **G** | **A** | **A** | **G** | **A** | **A** | **A** | **A** | **G** | **G** | **C** | **G** | **G** | **C** | **T** | **G** | **A** | **A** | **G** | **G** | **G** | **G** | **T** | **T** | **G** | **A** | **A** | **G** | **T** | **G** | **C** | **G** | **G** | **T** | **T** | **G** | **C** | **T** | **T** | **T** | **A** | **C** | **G** | **A** | **T** | **G** | **C** | **C** | **T** | **T** | **T** | **G** | **G** | **C** | **T** | **C** | **A** | **A** | **A** | **G** | **G** | **G** | **C** | **A** | **C** | **C** | **A** | **A** | **G** | **G** | **T** | **T** | **C** | **A** | **T** | **C** | **A** | **T** | **T** | **T** | **G** | **A** | **A** | **T** | **G** | **A** | **A** | **T** | **T** | **G** | **A** | **A** | **A** | **A** | **A** | **A** | **A** | **A** | **C** | **G** | **G** | **C** | **G** | **G** | **T** | **T** | **T** | **T** | **G** | **T** | **C** | **C** | **A** | **A** | **A** | **C** | **G** | **T** | **T** | **T** | **A** | **T** | **T** | **A** | **C** | **T** | **T** | **C** | **T** | **C** | **A** | **A** | **A** | **A** | **A** | **G** | **C** | **A** | **C** | **T** | **T** | **T** | **T** | **G** | **A** | **A** | **G** | **T** | **T** | **T** | **C** | **G** | **T** | **T** | **T** | **G** | **A** | **A** | **T** | **T** | **A** | **T** | **C** | **A** | **T** | **G** | **A** | **T** | **C** | **A** | **C** | **C** | **G** | **G** | **A** | **A** | **A** | **A** | **T** | **T** | **G** | **T** | **T** | **G** | **T** | **T** | **A** | **T** | **T** | **G** | **A** | **C** | **G** | **G** | **A** | **A** | **A** | **A** | **G** | **T** | **A** | **G** | **G** | **C** | **T** | **A** | **C** | **A** | **T** | **T** | **G** | **G** | **C** | **G** | **G** | **C** | **T** | **T** | **T** | **A** | **A** | **T** | **G** | **T** | **T** | **G** | **C** | **C** | **G** | **A** | **T** | **C** | **A** | **A** | **T** | **A** | **T** | **G** | **C** | **T** | **G** | **G** | **A** | **A** | **C** | **G** | **A** | **C** | **T** | **A** | **A** | **A** | **A** | **A** | **G** | **T** | **T** | **T** | **G** | **G** | **C** | **T** | **A** | **T** | **T** | **G** | **G** | **C** | **G** | **G** | **G** | **A** | **T** | **A** | **C** | **A** | **C** | **A** | **T** | **T** | **T** | **A** | **C** | **G** | **G** | **A** | **T** | **T** | **C** | **A** | **A** | **G** | **G** | **G** | **C** | **C** | **A** | **G** | **C** | **A** | **G** | **C** | **C** | **T** | **C** | **A** | **T** | **T** | **A** | **C** | **T** | **G** | **C** | **A** | **A** | **A** | **T** | **G** | **C** | **G** | **T** | **T** | **T** | **T** | **T** | **T** | **A** | **A** | **T** | **G** | **G** | **A** | **T** | **T** | **G** | **G** | **A** | **A** | **C** | **G** | **T** | **C** | **T** | **C** | **T** | **T** | **C** | **C** | **C** | **C** | **C** | **G** | **A** | **G** | **A** | **A** | **A** | **A** | **A** | **T** | **C** | **G** | **T** | **G** | **T** | **G** | **G** | **C** | **G** | **T** | **A** | **T** | **C** | **A** | **A** | **T** | **T** | **G** | **G** | **A** | **T** | **T** | **A** | **T** | **T** | **T** | **C** | **T** | **T** | **T** | **A** | **A** | **A** | **C** | **T** | **T** | **G** | **A** | **A** | **G** | **C** | **A** | **T** | **T** | **G** | **G** | **T** | **G** | **C** | **C** | **A** | **G** | **A** | **G** | **G** | **C** | **A** | **A** | **A** | **T** | **A** | **C** | **A** | **T** | **C** | **C** | **A** | **T** | **T** | **C** | **A** | **G** | **A** | **T** | **G** | **A** | **T** | **T** | **G** | **C** | **C** | **A** | **G** | **T** | **G** | **G** | **T** | **C** | **C** | **T** | **A** | **A** | **C** | **A** | **G** | **T** | **G** | **A** | **C** | **C** | **G** | **T** | **G** | **A** | **A** | **C** | **A** | **A** | **A** | **T** | **T** | **A** | **A** | **A** | **T** | **T** | **G** | **G** | **C** | **C** | **T** | **T** | **T** | **A** | **T** | **T** | **A** | **A** | **A** | **T** | **T** | **G** | **A** | **T** | **T** | **A** | **C** | **T** | **T** | **C** | **T** | **G** | **C** | **C** | **A** | **A** | **G** | **A** | **A** | **A** | **A** | **G** | **A** | **G** | **T** | **C** | **T** | **G** | **G** | **A** | **T** | **T** | **C** | **A** | **A** | **A** | **C** | **G** | **C** | **C** | **G** | **T** | **A** | **T** | **T** | **T** | **A** | **G** | **T** | **T** | **C** | **C** | **T** | **G** | **A** | **T** | **G** | **A** | **T** | **A** | **G** | **T** | **G** | **T** | **C** | **T** | **T** | **G** | **G** | **C** | **T** | **G** | **C** | **T** | **T** | **T** | **A** | **A** | **A** | **G** | **G** | **T** | **T** | **G** | **C** | **T** | **G** | **C** | **G** | **G** | **C** | **T** | **T** | **C** | **A** | **G** | **G** | **A** | **G** | **T** | **A** | **G** | **A** | **T** | **G** | **T** | **T** | **A** | **A** | **A** | **A** | **T** | **T** | **A** | **T** | **G** | **A** | **T** | **T** | **C** | **C** | **A** | **G** | **A** | **T** | **A** | **A** | **G** | **C** | **C** | **C** | **G** | **A** | **C** | **C** | **A** | **T** | **C** | **C** | **G** | **T** | **T** | **T** | **A** | **T** | **T** | **T** | **A** | **T** | **C** | **G** | **A** | **G** | **C** | **A** | **A** | **C** | **A** | **C** | **A** | **G** | **T** | **A** | **T** | **T** | **A** | **C** | **G** | **G** | **C** | **C** | **G** | **C** | **T** | **T** | **A** | **T** | **T** | **G** | **A** | **T** | **G** | **A** | **A** | **A** | **G** | **A** | **A** | **A** | **A** | **T** | **A** | **T** | **T** | **G** | **A** | **A** | **A** | **T** | **T** | **T** | **T** | **A** | **A** | **T** | **T** | **T** | **A** | **T** | **A** | **A** | **C** | **G** | **G** | **T** | **G** | **G** | **T** | **T** | **T** | **C** | **T** | **T** | **A** | **C** | **A** | **T** | **G** | **C** | **G** | **A** | **A** | **A** | **A** | **C** | **A** | **A** | **T** | **G** | **A** | **T** | **T** | **A** | **T** | **G** | **G** | **A** | **T** | **G** | **A** | **T** | **G** | **A** | **A** | **G** | **T** | **C** | **T** | **G** | **C** | **A** | **C** | **A** | **G** | **T** | **T** | **G** | **G** | **T** | **T** | **C** | **A** | **G** | **C** | **C** | **A** | **A** | **T** | **C** | **A** | **A** | **G** | **A** | **T** | **A** | **T** | **C** | **C** | **G** | **A** | **A** | **G** | **T** | **T** | **A** | **C** | **A** | **A** | **A** | **T** | **T** | **A** | **A** | **A** | **C** | **T** | **T** | **T** | **G** | **A** | **A** | **G** | **C** | **A** | **A** | **A** | **T** | **G** | **C** | **T** | **G** | **T** | **G** | **T** | **T** | **A** | **T** | **A** | **T** | **G** | **A** | **T** | **A** | **A** | **A** | **A** | **A** | **A** | **A** | **T** | **C** | **A** | **T** | **T** | **G** | **A** | **T** | **C** | **A** | **A** | **T** | **T** | **A** | **G** | **A** | **A** | **G** | **C** | **A** | **A** | **T** | **T** | **T** | **T** | **C** | **T** | **T** | **A** | **G** | **A** | **A** | **G** | **A** | **T** | **C** | **G** | **A** | **A** | **A** | **A** | **A** | **A** | **A** | **T** | **G** | **T** | **A** | **C** | **A** | **A** | **C** | **A** | **A** | **T** | **G** | **A** | **C** | **T** | **C** | **C** | **A** | **G** | **A** | **A** | **G** | **T** | **T** | **G** | **T** | **T** | **C** | **G** | **T** | **G** | **A** | **C** | **A** | **T** | **G** | **T** | **C** | **A** | **A** | **A** | **A** | **T** | **G** | **G** | **T** | **T** | **G** | **A** | **T** | **T** | **T** | **T** | **T** | **A** | **A** | **A** | **C** | **A** | **A** | **C** | **A** | **A** | **A** | **T** | **T** | **T** | **C** | **A** | **C** | **G** | **A** | **T** | **T** | **A** | **T** | **T** | **T** | **T** | **C** | **A** | **C** | **C** | **A** | **A** | **T** | **T** | **C** | **T** | **T** | **-** | **-** | **-** |
| **N55724** | **T** | **T** | **G** | **A** | **T** | **T** | **C** | **T** | **T** | **A** | **G** | **C** | **G** | **T** | **C** | **T** | **T** | **A** | **A** | **C** | **A** | **G** | **T** | **T** | **A** | **T** | **T** | **T** | **A** | **T** | **T** | **T** | **T** | **A** | **T** | **T** | **A** | **A** | **T** | **G** | **C** | **A** | **A** | **T** | **T** | **A** | **T** | **T** | **G** | **C** | **G** | **G** | **G** | **A** | **A** | **T** | **T** | **A** | **C** | **C** | **A** | **T** | **T** | **T** | **T** | **G** | **T** | **T** | **A** | **A** | **A** | **A** | **C** | **C** | **T** | **C** | **G** | **A** | **G** | **A** | **T** | **G** | **T** | **A** | **G** | **C** | **A** | **G** | **C** | **A** | **A** | **T** | **T** | **T** | **G** | **G** | **G** | **C** | **A** | **T** | **G** | **G** | **C** | **T** | **A** | **C** | **T** | **C** | **G** | **T** | **A** | **C** | **T** | **G** | **A** | **T** | **T** | **G** | **C** | **T** | **C** | **T** | **A** | **C** | **C** | **C** | **G** | **T** | **T** | **T** | **T** | **T** | **G** | **G** | **T** | **T** | **T** | **T** | **T** | **T** | **C** | **T** | **T** | **A** | **T** | **A** | **T** | **T** | **T** | **A** | **T** | **T** | **C** | **T** | **T** | **T** | **G** | **G** | **G** | **C** | **G** | **C** | **G** | **G** | **T** | **T** | **T** | **A** | **A** | **C** | **C** | **G** | **A** | **C** | **A** | **A** | **A** | **A** | **A** | **G** | **A** | **A** | **A** | **T** | **T** | **T** | **T** | **A** | **T** | **T** | **T** | **G** | **C** | **A** | **A** | **C** | **A** | **A** | **A** | **G** | **T** | **G** | **A** | **T** | **T** | **T** | **G** | **C** | **G** | **G** | **G** | **A** | **A** | **T** | **T** | **A** | **G** | **A** | **G** | **A** | **A** | **T** | **T** | **T** | **T** | **C** | **A** | **G** | **A** | **A** | **T** | **T** | **T** | **C** | **C** | **A** | **A** | **G** | **A** | **A** | **G** | **A** | **A** | **A** | **G** | **T** | **T** | **T** | **T** | **G** | **A** | **A** | **C** | **T** | **T** | **T** | **A** | **T** | **A** | **G** | **T** | **C** | **A** | **A** | **A** | **A** | **G** | **A** | **T** | **G** | **C** | **C** | **G** | **A** | **C** | **G** | **G** | **A** | **G** | **G** | **A** | **G** | **C** | **A** | **A** | **C** | **A** | **A** | **C** | **A** | **G** | **T** | **T** | **T** | **A** | **C** | **A** | **G** | **A** | **C** | **T** | **T** | **T** | **T** | **T** | **T** | **T** | **C** | **C** | **T** | **C** | **G** | **T** | **T** | **A** | **A** | **A** | **T** | **A** | **G** | **G** | **A** | **T** | **A** | **C** | **C** | **T** | **C** | **T** | **G** | **A** | **C** | **T** | **A** | **A** | **G** | **A** | **A** | **A** | **A** | **A** | **T** | **G** | **A** | **T** | **G** | **T** | **T** | **G** | **A** | **A** | **A** | **T** | **T** | **T** | **T** | **T** | **A** | **C** | **G** | **G** | **A** | **T** | **G** | **G** | **T** | **A** | **C** | **G** | **G** | **A** | **A** | **A** | **A** | **A** | **T** | **T** | **C** | **A** | **A** | **T** | **G** | **C** | **G** | **T** | **T** | **G** | **A** | **T** | **G** | **G** | **C** | **A** | **G** | **A** | **C** | **A** | **T** | **A** | **A** | **A** | **A** | **A** | **A** | **A** | **G** | **C** | **T** | **C** | **A** | **G** | **C** | **A** | **C** | **T** | **C** | **C** | **A** | **T** | **T** | **C** | **A** | **T** | **A** | **T** | **T** | **G** | **A** | **A** | **T** | **A** | **T** | **T** | **A** | **T** | **G** | **C** | **C** | **T** | **T** | **T** | **G** | **T** | **A** | **A** | **C** | **G** | **G** | **A** | **T** | **C** | **A** | **T** | **A** | **T** | **C** | **G** | **G** | **T** | **A** | **C** | **C** | **A** | **A** | **A** | **A** | **T** | **T** | **T** | **T** | **G** | **A** | **A** | **C** | **T** | **T** | **A** | **T** | **T** | **A** | **G** | **A** | **A** | **G** | **A** | **A** | **A** | **A** | **G** | **G** | **C** | **G** | **G** | **C** | **T** | **G** | **A** | **A** | **G** | **G** | **G** | **G** | **T** | **T** | **G** | **A** | **A** | **G** | **T** | **G** | **C** | **G** | **G** | **T** | **T** | **G** | **C** | **T** | **T** | **T** | **A** | **C** | **G** | **A** | **T** | **G** | **C** | **C** | **T** | **T** | **T** | **G** | **G** | **C** | **T** | **C** | **A** | **A** | **A** | **G** | **G** | **G** | **C** | **A** | **C** | **C** | **A** | **A** | **G** | **G** | **T** | **T** | **C** | **A** | **T** | **C** | **A** | **T** | **T** | **T** | **G** | **A** | **A** | **T** | **G** | **A** | **A** | **T** | **T** | **G** | **A** | **A** | **A** | **A** | **A** | **A** | **A** | **A** | **C** | **G** | **G** | **C** | **G** | **G** | **T** | **T** | **T** | **T** | **G** | **T** | **C** | **C** | **A** | **A** | **A** | **C** | **G** | **T** | **T** | **T** | **A** | **T** | **T** | **A** | **C** | **T** | **T** | **C** | **T** | **C** | **A** | **A** | **A** | **A** | **A** | **G** | **C** | **A** | **C** | **T** | **T** | **T** | **T** | **G** | **A** | **A** | **G** | **T** | **T** | **T** | **C** | **G** | **T** | **T** | **T** | **G** | **A** | **A** | **T** | **T** | **A** | **T** | **C** | **A** | **T** | **G** | **A** | **T** | **C** | **A** | **C** | **C** | **G** | **G** | **A** | **A** | **A** | **A** | **T** | **T** | **G** | **T** | **T** | **G** | **T** | **T** | **A** | **T** | **T** | **G** | **A** | **C** | **G** | **G** | **G** | **A** | **A** | **A** | **G** | **T** | **A** | **G** | **G** | **C** | **T** | **A** | **C** | **A** | **T** | **T** | **G** | **G** | **C** | **G** | **G** | **C** | **T** | **T** | **T** | **A** | **A** | **T** | **G** | **T** | **T** | **G** | **C** | **C** | **G** | **A** | **T** | **C** | **A** | **A** | **T** | **A** | **T** | **G** | **C** | **C** | **G** | **G** | **A** | **A** | **C** | **G** | **A** | **C** | **T** | **A** | **A** | **A** | **A** | **A** | **G** | **T** | **T** | **T** | **G** | **G** | **C** | **T** | **A** | **T** | **T** | **G** | **G** | **C** | **G** | **G** | **G** | **A** | **T** | **A** | **C** | **G** | **C** | **A** | **T** | **T** | **T** | **A** | **C** | **G** | **G** | **A** | **T** | **T** | **C** | **A** | **A** | **G** | **G** | **G** | **C** | **C** | **A** | **G** | **C** | **A** | **G** | **C** | **C** | **T** | **C** | **A** | **T** | **T** | **A** | **C** | **T** | **G** | **C** | **A** | **A** | **A** | **T** | **G** | **C** | **G** | **T** | **T** | **T** | **T** | **T** | **T** | **A** | **A** | **T** | **G** | **G** | **A** | **T** | **T** | **G** | **G** | **A** | **A** | **C** | **G** | **T** | **C** | **T** | **C** | **T** | **T** | **C** | **C** | **C** | **C** | **C** | **G** | **A** | **G** | **A** | **A** | **A** | **A** | **A** | **T** | **C** | **G** | **T** | **G** | **T** | **G** | **G** | **C** | **G** | **T** | **A** | **T** | **C** | **A** | **A** | **T** | **T** | **G** | **G** | **A** | **T** | **T** | **A** | **T** | **T** | **T** | **C** | **T** | **T** | **T** | **A** | **A** | **A** | **C** | **T** | **T** | **G** | **A** | **A** | **G** | **C** | **A** | **T** | **T** | **G** | **G** | **T** | **G** | **C** | **C** | **A** | **G** | **A** | **G** | **G** | **C** | **A** | **A** | **A** | **T** | **A** | **C** | **A** | **T** | **C** | **C** | **A** | **T** | **T** | **C** | **A** | **G** | **A** | **T** | **G** | **A** | **T** | **T** | **G** | **C** | **C** | **A** | **G** | **T** | **G** | **G** | **T** | **C** | **C** | **T** | **A** | **A** | **C** | **A** | **G** | **T** | **G** | **A** | **C** | **C** | **G** | **T** | **G** | **A** | **A** | **C** | **A** | **A** | **A** | **T** | **T** | **A** | **A** | **A** | **T** | **T** | **G** | **G** | **C** | **C** | **T** | **T** | **T** | **A** | **T** | **T** | **A** | **A** | **A** | **T** | **T** | **G** | **A** | **T** | **T** | **A** | **C** | **T** | **T** | **C** | **T** | **G** | **C** | **C** | **A** | **A** | **G** | **A** | **A** | **A** | **A** | **G** | **A** | **G** | **T** | **C** | **T** | **G** | **G** | **A** | **T** | **T** | **C** | **A** | **A** | **A** | **C** | **G** | **C** | **C** | **G** | **T** | **A** | **T** | **T** | **T** | **A** | **G** | **T** | **T** | **C** | **C** | **T** | **G** | **A** | **T** | **G** | **A** | **T** | **A** | **G** | **T** | **G** | **T** | **C** | **T** | **T** | **G** | **G** | **C** | **G** | **G** | **C** | **T** | **T** | **T** | **A** | **A** | **A** | **G** | **G** | **T** | **T** | **G** | **C** | **T** | **G** | **C** | **G** | **G** | **C** | **T** | **T** | **C** | **A** | **G** | **G** | **A** | **G** | **T** | **A** | **G** | **A** | **T** | **G** | **T** | **T** | **A** | **A** | **A** | **A** | **T** | **T** | **A** | **T** | **G** | **A** | **T** | **T** | **C** | **C** | **A** | **G** | **A** | **T** | **A** | **A** | **G** | **C** | **C** | **C** | **G** | **A** | **C** | **C** | **A** | **T** | **C** | **C** | **G** | **T** | **T** | **T** | **A** | **T** | **T** | **T** | **A** | **T** | **C** | **G** | **A** | **G** | **C** | **A** | **A** | **C** | **A** | **C** | **A** | **G** | **T** | **A** | **T** | **T** | **A** | **C** | **G** | **G** | **C** | **C** | **G** | **C** | **T** | **T** | **G** | **T** | **T** | **G** | **A** | **T** | **G** | **A** | **A** | **A** | **G** | **A** | **A** | **A** | **A** | **T** | **A** | **T** | **T** | **G** | **A** | **A** | **A** | **T** | **T** | **T** | **T** | **A** | **A** | **T** | **T** | **T** | **A** | **T** | **A** | **A** | **C** | **G** | **G** | **T** | **G** | **G** | **T** | **T** | **T** | **C** | **T** | **T** | **A** | **C** | **A** | **T** | **G** | **C** | **G** | **A** | **A** | **A** | **A** | **C** | **A** | **A** | **T** | **G** | **A** | **T** | **T** | **A** | **T** | **G** | **G** | **A** | **T** | **G** | **A** | **T** | **G** | **A** | **A** | **G** | **T** | **C** | **T** | **G** | **C** | **A** | **C** | **A** | **G** | **T** | **T** | **G** | **G** | **T** | **T** | **C** | **A** | **G** | **C** | **C** | **A** | **A** | **T** | **C** | **A** | **A** | **G** | **A** | **T** | **A** | **T** | **C** | **C** | **G** | **A** | **A** | **G** | **T** | **T** | **A** | **C** | **A** | **A** | **A** | **T** | **T** | **A** | **A** | **A** | **C** | **T** | **T** | **T** | **G** | **A** | **A** | **G** | **C** | **A** | **A** | **A** | **T** | **G** | **C** | **T** | **G** | **T** | **G** | **T** | **T** | **A** | **T** | **A** | **T** | **G** | **A** | **T** | **A** | **A** | **A** | **A** | **A** | **A** | **A** | **T** | **C** | **A** | **T** | **T** | **G** | **A** | **T** | **C** | **A** | **A** | **T** | **T** | **A** | **G** | **A** | **A** | **G** | **C** | **A** | **A** | **T** | **T** | **T** | **T** | **C** | **T** | **T** | **A** | **G** | **A** | **A** | **G** | **A** | **T** | **C** | **G** | **A** | **A** | **A** | **A** | **A** | **A** | **A** | **T** | **G** | **T** | **A** | **C** | **A** | **A** | **C** | **A** | **A** | **T** | **G** | **A** | **C** | **T** | **C** | **C** | **A** | **G** | **A** | **A** | **G** | **T** | **T** | **G** | **T** | **T** | **C** | **G** | **C** | **G** | **A** | **C** | **A** | **T** | **G** | **T** | **C** | **A** | **A** | **A** | **A** | **T** | **G** | **G** | **T** | **T** | **G** | **A** | **T** | **T** | **T** | **T** | **T** | **A** | **A** | **A** | **C** | **A** | **A** | **C** | **A** | **A** | **A** | **T** | **T** | **T** | **C** | **A** | **C** | **G** | **A** | **T** | **T** | **A** | **T** | **T** | **T** | **T** | **C** | **A** | **C** | **C** | **A** | **A** | **T** | **T** | **C** | **T** | **T** | **-** | **-** | **-** |
| **N60027F** | **T** | **T** | **G** | **A** | **T** | **T** | **C** | **T** | **T** | **A** | **G** | **C** | **G** | **T** | **C** | **T** | **T** | **A** | **A** | **C** | **A** | **G** | **T** | **T** | **A** | **T** | **T** | **T** | **A** | **T** | **T** | **T** | **T** | **A** | **T** | **T** | **A** | **A** | **T** | **G** | **C** | **A** | **A** | **T** | **T** | **A** | **T** | **T** | **G** | **C** | **G** | **G** | **G** | **A** | **A** | **T** | **T** | **A** | **C** | **C** | **A** | **T** | **T** | **T** | **T** | **G** | **T** | **T** | **A** | **A** | **A** | **A** | **C** | **C** | **T** | **C** | **G** | **A** | **G** | **A** | **T** | **G** | **T** | **A** | **G** | **C** | **A** | **G** | **C** | **A** | **A** | **T** | **T** | **T** | **G** | **G** | **G** | **C** | **A** | **T** | **G** | **G** | **C** | **T** | **A** | **C** | **T** | **C** | **G** | **T** | **A** | **C** | **T** | **G** | **A** | **T** | **T** | **G** | **C** | **T** | **C** | **T** | **A** | **C** | **C** | **C** | **G** | **T** | **T** | **T** | **T** | **T** | **G** | **G** | **T** | **T** | **T** | **T** | **T** | **T** | **C** | **T** | **T** | **A** | **T** | **A** | **T** | **T** | **T** | **A** | **T** | **T** | **C** | **T** | **T** | **T** | **G** | **G** | **T** | **C** | **G** | **C** | **G** | **G** | **T** | **T** | **T** | **A** | **A** | **C** | **C** | **G** | **A** | **C** | **A** | **A** | **A** | **A** | **A** | **G** | **A** | **A** | **A** | **T** | **T** | **T** | **T** | **A** | **T** | **T** | **T** | **G** | **C** | **A** | **A** | **C** | **A** | **A** | **A** | **G** | **T** | **G** | **A** | **T** | **T** | **T** | **G** | **C** | **G** | **G** | **G** | **A** | **A** | **T** | **T** | **A** | **G** | **A** | **G** | **A** | **A** | **T** | **T** | **T** | **T** | **C** | **A** | **G** | **A** | **A** | **T** | **T** | **T** | **C** | **C** | **A** | **A** | **G** | **A** | **A** | **G** | **A** | **A** | **A** | **G** | **T** | **T** | **T** | **T** | **G** | **A** | **A** | **C** | **T** | **T** | **T** | **A** | **T** | **A** | **G** | **T** | **C** | **A** | **A** | **A** | **A** | **G** | **A** | **T** | **G** | **C** | **C** | **G** | **A** | **C** | **G** | **G** | **A** | **G** | **G** | **A** | **G** | **C** | **A** | **A** | **C** | **A** | **A** | **C** | **A** | **G** | **T** | **T** | **T** | **A** | **C** | **A** | **G** | **A** | **C** | **T** | **T** | **T** | **T** | **T** | **T** | **T** | **C** | **C** | **T** | **C** | **G** | **T** | **T** | **A** | **A** | **A** | **T** | **A** | **G** | **G** | **A** | **T** | **G** | **C** | **C** | **T** | **C** | **T** | **G** | **A** | **C** | **T** | **A** | **A** | **G** | **A** | **A** | **A** | **A** | **A** | **T** | **G** | **A** | **T** | **G** | **T** | **T** | **G** | **A** | **A** | **A** | **T** | **T** | **T** | **T** | **T** | **A** | **C** | **G** | **G** | **A** | **T** | **G** | **G** | **T** | **A** | **C** | **G** | **G** | **A** | **A** | **A** | **A** | **A** | **T** | **T** | **C** | **A** | **A** | **T** | **G** | **C** | **G** | **T** | **T** | **G** | **A** | **T** | **G** | **G** | **C** | **G** | **G** | **A** | **C** | **A** | **T** | **A** | **A** | **A** | **A** | **A** | **A** | **A** | **G** | **C** | **T** | **C** | **A** | **G** | **C** | **A** | **C** | **T** | **C** | **C** | **A** | **T** | **T** | **C** | **A** | **T** | **A** | **T** | **T** | **G** | **A** | **A** | **T** | **A** | **T** | **T** | **A** | **T** | **G** | **C** | **C** | **T** | **T** | **T** | **G** | **T** | **A** | **A** | **C** | **G** | **G** | **A** | **T** | **C** | **A** | **T** | **A** | **T** | **C** | **G** | **G** | **C** | **A** | **C** | **C** | **A** | **A** | **A** | **A** | **T** | **T** | **T** | **T** | **G** | **A** | **A** | **C** | **T** | **T** | **A** | **T** | **T** | **A** | **G** | **A** | **A** | **G** | **A** | **A** | **A** | **A** | **G** | **G** | **C** | **G** | **G** | **C** | **T** | **G** | **A** | **A** | **G** | **G** | **G** | **G** | **T** | **T** | **G** | **A** | **A** | **G** | **T** | **G** | **C** | **G** | **G** | **T** | **T** | **G** | **C** | **T** | **T** | **T** | **A** | **C** | **G** | **A** | **T** | **G** | **C** | **C** | **T** | **T** | **T** | **G** | **G** | **C** | **T** | **C** | **A** | **A** | **A** | **G** | **G** | **G** | **C** | **A** | **C** | **C** | **A** | **A** | **G** | **G** | **T** | **T** | **C** | **A** | **T** | **C** | **A** | **T** | **T** | **T** | **G** | **A** | **A** | **T** | **G** | **A** | **A** | **T** | **T** | **G** | **A** | **A** | **A** | **A** | **A** | **A** | **A** | **A** | **C** | **G** | **G** | **T** | **G** | **G** | **T** | **T** | **T** | **T** | **G** | **T** | **C** | **C** | **A** | **A** | **A** | **C** | **G** | **T** | **T** | **T** | **A** | **T** | **T** | **A** | **C** | **T** | **T** | **C** | **T** | **C** | **A** | **A** | **A** | **A** | **A** | **G** | **C** | **A** | **C** | **T** | **T** | **T** | **T** | **G** | **A** | **A** | **G** | **T** | **T** | **T** | **C** | **G** | **T** | **T** | **T** | **G** | **A** | **A** | **T** | **T** | **A** | **T** | **C** | **A** | **T** | **G** | **A** | **T** | **C** | **A** | **C** | **C** | **G** | **G** | **A** | **A** | **A** | **A** | **T** | **T** | **G** | **T** | **T** | **G** | **T** | **T** | **A** | **T** | **T** | **G** | **A** | **C** | **G** | **G** | **A** | **A** | **A** | **A** | **G** | **T** | **A** | **G** | **G** | **C** | **T** | **A** | **C** | **A** | **T** | **T** | **G** | **G** | **C** | **G** | **G** | **C** | **T** | **T** | **T** | **A** | **A** | **T** | **G** | **T** | **T** | **G** | **C** | **C** | **G** | **A** | **T** | **C** | **A** | **A** | **T** | **A** | **T** | **G** | **C** | **T** | **G** | **G** | **A** | **A** | **C** | **G** | **A** | **C** | **T** | **A** | **A** | **A** | **A** | **A** | **G** | **T** | **T** | **T** | **G** | **G** | **C** | **T** | **A** | **T** | **T** | **G** | **G** | **C** | **G** | **G** | **G** | **A** | **T** | **A** | **C** | **G** | **C** | **A** | **T** | **T** | **T** | **A** | **C** | **G** | **G** | **A** | **T** | **T** | **C** | **A** | **A** | **G** | **G** | **G** | **C** | **C** | **A** | **G** | **C** | **A** | **G** | **C** | **C** | **T** | **C** | **A** | **T** | **T** | **A** | **C** | **T** | **G** | **C** | **A** | **A** | **A** | **T** | **G** | **C** | **G** | **T** | **T** | **T** | **T** | **T** | **T** | **A** | **A** | **T** | **G** | **G** | **A** | **T** | **T** | **G** | **G** | **A** | **A** | **C** | **G** | **T** | **C** | **T** | **C** | **T** | **T** | **C** | **C** | **C** | **C** | **C** | **G** | **A** | **G** | **A** | **A** | **A** | **A** | **A** | **T** | **C** | **G** | **T** | **G** | **T** | **G** | **G** | **C** | **G** | **T** | **A** | **T** | **C** | **A** | **A** | **T** | **T** | **G** | **G** | **A** | **T** | **T** | **A** | **T** | **T** | **T** | **C** | **T** | **T** | **T** | **A** | **A** | **A** | **C** | **T** | **T** | **G** | **A** | **A** | **G** | **C** | **A** | **T** | **T** | **G** | **G** | **T** | **G** | **C** | **C** | **A** | **G** | **A** | **G** | **G** | **C** | **A** | **A** | **A** | **T** | **A** | **C** | **A** | **T** | **C** | **C** | **A** | **T** | **T** | **C** | **A** | **G** | **A** | **T** | **G** | **A** | **T** | **T** | **G** | **C** | **C** | **A** | **G** | **T** | **G** | **G** | **T** | **C** | **C** | **T** | **A** | **A** | **C** | **A** | **G** | **T** | **G** | **A** | **C** | **C** | **G** | **T** | **G** | **A** | **A** | **C** | **A** | **A** | **A** | **T** | **T** | **A** | **A** | **A** | **T** | **T** | **G** | **G** | **C** | **C** | **T** | **T** | **T** | **A** | **T** | **T** | **A** | **A** | **A** | **T** | **T** | **G** | **A** | **T** | **T** | **A** | **C** | **T** | **T** | **C** | **T** | **G** | **C** | **C** | **A** | **A** | **G** | **A** | **A** | **A** | **A** | **G** | **A** | **G** | **T** | **C** | **T** | **G** | **G** | **A** | **T** | **T** | **C** | **A** | **A** | **A** | **C** | **G** | **C** | **C** | **G** | **T** | **A** | **T** | **T** | **T** | **A** | **G** | **T** | **T** | **C** | **C** | **T** | **G** | **A** | **T** | **G** | **A** | **T** | **A** | **G** | **T** | **G** | **T** | **C** | **T** | **T** | **G** | **G** | **C** | **T** | **G** | **C** | **T** | **T** | **T** | **A** | **A** | **A** | **G** | **G** | **T** | **T** | **G** | **C** | **T** | **G** | **C** | **G** | **G** | **C** | **T** | **T** | **C** | **A** | **G** | **G** | **A** | **G** | **T** | **A** | **G** | **A** | **T** | **G** | **T** | **T** | **A** | **A** | **A** | **A** | **T** | **T** | **A** | **T** | **G** | **A** | **T** | **T** | **C** | **C** | **A** | **G** | **A** | **T** | **A** | **A** | **G** | **C** | **C** | **C** | **G** | **A** | **C** | **C** | **A** | **T** | **C** | **C** | **G** | **T** | **T** | **T** | **A** | **T** | **T** | **T** | **A** | **T** | **C** | **G** | **A** | **G** | **C** | **A** | **A** | **C** | **A** | **C** | **A** | **G** | **T** | **A** | **T** | **T** | **A** | **C** | **G** | **G** | **C** | **C** | **G** | **C** | **T** | **T** | **A** | **T** | **T** | **G** | **A** | **T** | **G** | **A** | **A** | **A** | **G** | **A** | **A** | **A** | **A** | **T** | **A** | **T** | **T** | **G** | **A** | **A** | **A** | **T** | **T** | **T** | **T** | **A** | **A** | **T** | **T** | **T** | **A** | **T** | **A** | **A** | **C** | **G** | **G** | **T** | **G** | **G** | **T** | **T** | **T** | **C** | **T** | **T** | **A** | **C** | **A** | **T** | **G** | **C** | **G** | **A** | **A** | **A** | **A** | **C** | **A** | **A** | **T** | **G** | **A** | **T** | **T** | **A** | **T** | **G** | **G** | **A** | **T** | **G** | **A** | **T** | **G** | **A** | **A** | **G** | **T** | **C** | **T** | **G** | **C** | **A** | **C** | **A** | **G** | **T** | **T** | **G** | **G** | **T** | **T** | **C** | **A** | **G** | **C** | **C** | **A** | **A** | **C** | **C** | **A** | **A** | **G** | **A** | **T** | **A** | **T** | **C** | **C** | **G** | **A** | **A** | **G** | **T** | **T** | **A** | **C** | **A** | **A** | **A** | **T** | **T** | **A** | **A** | **A** | **C** | **T** | **T** | **T** | **G** | **A** | **A** | **G** | **C** | **A** | **A** | **A** | **T** | **G** | **C** | **T** | **G** | **T** | **G** | **T** | **T** | **A** | **T** | **A** | **T** | **G** | **A** | **T** | **A** | **A** | **A** | **A** | **A** | **A** | **A** | **T** | **C** | **A** | **T** | **T** | **G** | **A** | **T** | **C** | **A** | **A** | **T** | **T** | **A** | **G** | **A** | **A** | **G** | **C** | **A** | **A** | **T** | **T** | **T** | **T** | **C** | **T** | **T** | **A** | **G** | **A** | **A** | **G** | **A** | **T** | **C** | **G** | **A** | **A** | **A** | **A** | **A** | **A** | **A** | **T** | **G** | **T** | **A** | **C** | **A** | **A** | **C** | **A** | **A** | **T** | **G** | **A** | **C** | **T** | **C** | **C** | **A** | **G** | **A** | **A** | **G** | **T** | **T** | **G** | **T** | **T** | **C** | **G** | **C** | **G** | **A** | **C** | **A** | **T** | **G** | **T** | **C** | **A** | **A** | **A** | **A** | **T** | **G** | **G** | **T** | **T** | **G** | **A** | **T** | **T** | **T** | **T** | **T** | **A** | **A** | **A** | **C** | **A** | **A** | **C** | **A** | **A** | **A** | **T** | **T** | **T** | **C** | **A** | **C** | **G** | **A** | **T** | **T** | **A** | **T** | **T** | **T** | **T** | **C** | **A** | **C** | **C** | **A** | **A** | **T** | **T** | **C** | **T** | **T** | **-** | **-** | **-** |
| **N53420** | **T** | **T** | **G** | **A** | **T** | **T** | **C** | **T** | **T** | **A** | **G** | **T** | **G** | **T** | **C** | **T** | **T** | **A** | **A** | **C** | **A** | **G** | **T** | **T** | **A** | **T** | **T** | **T** | **A** | **T** | **T** | **T** | **T** | **A** | **T** | **T** | **A** | **A** | **T** | **G** | **C** | **A** | **A** | **T** | **T** | **A** | **T** | **T** | **G** | **C** | **G** | **G** | **G** | **A** | **A** | **T** | **T** | **A** | **C** | **C** | **A** | **T** | **C** | **T** | **T** | **G** | **T** | **T** | **A** | **A** | **A** | **A** | **C** | **C** | **T** | **C** | **G** | **A** | **G** | **A** | **T** | **G** | **T** | **A** | **G** | **C** | **G** | **G** | **C** | **A** | **A** | **T** | **T** | **T** | **G** | **G** | **G** | **C** | **A** | **T** | **G** | **G** | **T** | **T** | **A** | **C** | **T** | **C** | **G** | **T** | **A** | **C** | **T** | **G** | **A** | **T** | **T** | **G** | **C** | **T** | **C** | **T** | **A** | **C** | **C** | **C** | **G** | **T** | **T** | **T** | **T** | **T** | **G** | **G** | **T** | **T** | **T** | **T** | **T** | **T** | **C** | **T** | **T** | **A** | **T** | **A** | **T** | **T** | **T** | **A** | **T** | **T** | **C** | **T** | **T** | **T** | **G** | **G** | **T** | **C** | **G** | **A** | **G** | **G** | **C** | **T** | **T** | **G** | **A** | **C** | **C** | **G** | **A** | **C** | **A** | **A** | **A** | **A** | **A** | **G** | **A** | **A** | **A** | **T** | **T** | **T** | **T** | **A** | **T** | **T** | **T** | **G** | **C** | **A** | **A** | **C** | **A** | **A** | **A** | **G** | **T** | **G** | **A** | **T** | **T** | **T** | **A** | **C** | **G** | **G** | **G** | **A** | **A** | **T** | **T** | **A** | **G** | **A** | **G** | **A** | **A** | **T** | **T** | **T** | **T** | **C** | **A** | **G** | **A** | **A** | **T** | **T** | **T** | **C** | **C** | **A** | **A** | **G** | **A** | **A** | **G** | **A** | **A** | **A** | **G** | **T** | **T** | **T** | **T** | **G** | **A** | **A** | **C** | **T** | **T** | **T** | **A** | **T** | **A** | **G** | **T** | **C** | **A** | **A** | **A** | **A** | **G** | **A** | **T** | **G** | **C** | **C** | **G** | **A** | **C** | **G** | **G** | **A** | **G** | **G** | **A** | **G** | **C** | **A** | **A** | **C** | **A** | **G** | **C** | **A** | **G** | **T** | **T** | **T** | **A** | **C** | **A** | **G** | **A** | **C** | **T** | **T** | **T** | **T** | **T** | **C** | **T** | **C** | **C** | **T** | **C** | **G** | **T** | **T** | **A** | **A** | **A** | **T** | **A** | **G** | **G** | **A** | **T** | **G** | **C** | **C** | **T** | **C** | **T** | **G** | **A** | **C** | **T** | **A** | **A** | **G** | **A** | **A** | **A** | **A** | **A** | **T** | **G** | **A** | **C** | **G** | **T** | **T** | **G** | **A** | **G** | **A** | **T** | **T** | **T** | **T** | **T** | **A** | **C** | **G** | **G** | **A** | **T** | **G** | **G** | **T** | **A** | **T** | **G** | **G** | **A** | **A** | **A** | **A** | **A** | **T** | **T** | **C** | **A** | **A** | **T** | **G** | **C** | **G** | **T** | **T** | **G** | **A** | **T** | **G** | **G** | **C** | **G** | **G** | **A** | **C** | **A** | **T** | **A** | **A** | **A** | **A** | **A** | **A** | **A** | **G** | **C** | **T** | **C** | **A** | **G** | **C** | **A** | **T** | **T** | **C** | **C** | **A** | **T** | **T** | **C** | **A** | **T** | **A** | **T** | **T** | **G** | **A** | **A** | **T** | **A** | **T** | **T** | **A** | **T** | **G** | **C** | **C** | **T** | **T** | **T** | **G** | **T** | **A** | **A** | **C** | **G** | **G** | **A** | **T** | **C** | **A** | **T** | **A** | **T** | **C** | **G** | **G** | **C** | **A** | **C** | **C** | **A** | **A** | **A** | **A** | **T** | **T** | **T** | **T** | **G** | **A** | **A** | **C** | **T** | **T** | **A** | **T** | **T** | **A** | **G** | **A** | **A** | **G** | **A** | **A** | **A** | **A** | **G** | **G** | **C** | **G** | **G** | **C** | **T** | **G** | **A** | **A** | **G** | **G** | **G** | **G** | **T** | **T** | **G** | **A** | **A** | **G** | **T** | **G** | **C** | **G** | **G** | **T** | **T** | **G** | **C** | **T** | **T** | **T** | **A** | **C** | **G** | **A** | **T** | **G** | **C** | **C** | **T** | **T** | **T** | **G** | **G** | **C** | **T** | **C** | **A** | **A** | **A** | **G** | **G** | **G** | **C** | **A** | **C** | **C** | **A** | **A** | **G** | **G** | **T** | **T** | **C** | **A** | **T** | **C** | **A** | **T** | **T** | **T** | **G** | **A** | **A** | **T** | **G** | **A** | **A** | **T** | **T** | **G** | **A** | **A** | **A** | **A** | **A** | **A** | **A** | **A** | **C** | **G** | **G** | **C** | **G** | **G** | **T** | **T** | **T** | **T** | **G** | **T** | **C** | **C** | **A** | **A** | **A** | **C** | **G** | **T** | **T** | **T** | **A** | **T** | **T** | **A** | **C** | **T** | **T** | **C** | **T** | **C** | **A** | **A** | **A** | **A** | **A** | **G** | **C** | **A** | **C** | **T** | **C** | **T** | **T** | **G** | **A** | **A** | **G** | **T** | **T** | **T** | **C** | **G** | **T** | **T** | **T** | **G** | **A** | **A** | **T** | **T** | **A** | **T** | **C** | **A** | **T** | **G** | **A** | **T** | **C** | **A** | **C** | **C** | **G** | **G** | **A** | **A** | **A** | **A** | **T** | **T** | **G** | **T** | **C** | **G** | **T** | **T** | **A** | **T** | **T** | **G** | **A** | **C** | **G** | **G** | **A** | **A** | **A** | **A** | **G** | **T** | **A** | **G** | **G** | **C** | **T** | **A** | **C** | **A** | **T** | **T** | **G** | **G** | **T** | **G** | **G** | **T** | **T** | **T** | **T** | **A** | **A** | **T** | **G** | **T** | **T** | **G** | **C** | **C** | **G** | **A** | **T** | **C** | **A** | **A** | **T** | **A** | **T** | **G** | **C** | **T** | **G** | **G** | **A** | **A** | **C** | **G** | **A** | **C** | **T** | **A** | **A** | **A** | **A** | **A** | **G** | **T** | **T** | **T** | **G** | **G** | **C** | **T** | **A** | **T** | **T** | **G** | **G** | **C** | **G** | **G** | **G** | **A** | **T** | **A** | **C** | **G** | **C** | **A** | **T** | **T** | **T** | **A** | **C** | **G** | **G** | **A** | **T** | **T** | **C** | **A** | **A** | **G** | **G** | **G** | **C** | **C** | **A** | **G** | **C** | **A** | **G** | **C** | **C** | **T** | **C** | **A** | **T** | **T** | **A** | **C** | **T** | **G** | **C** | **A** | **A** | **A** | **T** | **G** | **C** | **G** | **T** | **T** | **T** | **T** | **T** | **T** | **A** | **A** | **T** | **G** | **G** | **A** | **T** | **T** | **G** | **G** | **A** | **A** | **C** | **G** | **T** | **C** | **T** | **C** | **T** | **T** | **C** | **C** | **C** | **C** | **T** | **G** | **A** | **G** | **A** | **A** | **A** | **A** | **A** | **T** | **C** | **G** | **T** | **G** | **T** | **G** | **G** | **C** | **G** | **T** | **A** | **T** | **C** | **A** | **A** | **T** | **T** | **G** | **G** | **A** | **T** | **T** | **A** | **T** | **T** | **T** | **C** | **T** | **T** | **T** | **A** | **A** | **A** | **C** | **T** | **T** | **G** | **A** | **A** | **G** | **C** | **G** | **T** | **T** | **G** | **G** | **T** | **G** | **C** | **C** | **A** | **G** | **A** | **G** | **G** | **C** | **A** | **A** | **A** | **T** | **A** | **C** | **A** | **T** | **C** | **C** | **A** | **T** | **T** | **C** | **A** | **G** | **A** | **T** | **G** | **A** | **T** | **T** | **G** | **C** | **A** | **A** | **G** | **T** | **G** | **G** | **A** | **C** | **C** | **T** | **A** | **A** | **C** | **A** | **G** | **T** | **G** | **A** | **C** | **C** | **G** | **T** | **G** | **A** | **A** | **C** | **A** | **A** | **A** | **T** | **T** | **A** | **A** | **A** | **T** | **T** | **G** | **G** | **C** | **C** | **T** | **T** | **T** | **A** | **T** | **T** | **A** | **A** | **A** | **T** | **T** | **G** | **A** | **T** | **T** | **A** | **C** | **T** | **T** | **C** | **T** | **G** | **C** | **C** | **A** | **A** | **G** | **A** | **A** | **A** | **A** | **G** | **A** | **G** | **T** | **C** | **T** | **G** | **G** | **A** | **T** | **T** | **C** | **A** | **A** | **A** | **C** | **G** | **C** | **C** | **G** | **T** | **A** | **T** | **T** | **T** | **A** | **G** | **T** | **T** | **C** | **C** | **T** | **G** | **A** | **T** | **G** | **A** | **T** | **A** | **G** | **T** | **G** | **T** | **C** | **T** | **T** | **G** | **G** | **C** | **T** | **G** | **C** | **T** | **T** | **T** | **A** | **A** | **A** | **G** | **G** | **T** | **T** | **G** | **C** | **T** | **G** | **C** | **G** | **G** | **C** | **T** | **T** | **C** | **A** | **G** | **G** | **A** | **G** | **T** | **A** | **G** | **A** | **T** | **G** | **T** | **T** | **A** | **A** | **A** | **A** | **T** | **T** | **A** | **T** | **G** | **A** | **T** | **T** | **C** | **C** | **A** | **G** | **A** | **T** | **A** | **A** | **G** | **C** | **C** | **C** | **G** | **A** | **C** | **C** | **A** | **T** | **C** | **C** | **G** | **T** | **T** | **T** | **A** | **T** | **T** | **T** | **A** | **T** | **C** | **G** | **A** | **G** | **C** | **A** | **A** | **C** | **A** | **C** | **A** | **G** | **T** | **A** | **T** | **T** | **A** | **C** | **G** | **G** | **C** | **C** | **G** | **C** | **T** | **T** | **G** | **T** | **T** | **A** | **A** | **T** | **G** | **A** | **A** | **A** | **G** | **A** | **A** | **A** | **A** | **T** | **A** | **T** | **T** | **G** | **A** | **A** | **A** | **T** | **T** | **T** | **T** | **A** | **A** | **T** | **T** | **T** | **A** | **T** | **A** | **A** | **C** | **G** | **G** | **T** | **G** | **G** | **T** | **T** | **T** | **C** | **T** | **T** | **A** | **C** | **A** | **T** | **G** | **C** | **G** | **A** | **A** | **A** | **A** | **C** | **A** | **A** | **T** | **G** | **A** | **T** | **T** | **A** | **T** | **G** | **G** | **A** | **T** | **G** | **A** | **T** | **G** | **A** | **A** | **G** | **T** | **C** | **T** | **G** | **C** | **A** | **C** | **A** | **G** | **T** | **T** | **G** | **G** | **T** | **T** | **C** | **A** | **G** | **C** | **T** | **A** | **A** | **T** | **C** | **A** | **A** | **G** | **A** | **T** | **A** | **T** | **C** | **C** | **G** | **A** | **A** | **G** | **T** | **T** | **A** | **C** | **A** | **A** | **A** | **T** | **T** | **A** | **A** | **A** | **C** | **T** | **T** | **T** | **G** | **A** | **A** | **G** | **C** | **A** | **A** | **A** | **T** | **G** | **C** | **T** | **G** | **T** | **G** | **T** | **T** | **A** | **T** | **A** | **T** | **G** | **A** | **T** | **A** | **A** | **A** | **A** | **A** | **A** | **A** | **T** | **C** | **A** | **T** | **T** | **G** | **A** | **T** | **C** | **A** | **A** | **T** | **T** | **A** | **G** | **A** | **A** | **G** | **C** | **A** | **A** | **T** | **T** | **T** | **T** | **C** | **T** | **T** | **A** | **G** | **A** | **A** | **G** | **A** | **T** | **C** | **G** | **A** | **A** | **A** | **A** | **A** | **A** | **A** | **T** | **G** | **T** | **A** | **C** | **A** | **A** | **C** | **A** | **A** | **T** | **G** | **A** | **C** | **T** | **C** | **C** | **A** | **G** | **A** | **A** | **G** | **T** | **T** | **G** | **T** | **T** | **C** | **G** | **C** | **G** | **A** | **C** | **A** | **T** | **G** | **T** | **C** | **A** | **A** | **A** | **A** | **T** | **G** | **G** | **T** | **T** | **G** | **A** | **T** | **T** | **T** | **T** | **T** | **A** | **A** | **A** | **C** | **A** | **A** | **C** | **A** | **A** | **A** | **T** | **T** | **T** | **C** | **A** | **C** | **G** | **A** | **T** | **T** | **A** | **T** | **T** | **T** | **T** | **C** | **A** | **C** | **C** | **A** | **A** | **T** | **T** | **C** | **T** | **T** | **-** | **-** | **-** |
| **N53761** | **T** | **T** | **G** | **A** | **T** | **T** | **C** | **T** | **T** | **A** | **G** | **C** | **G** | **T** | **C** | **T** | **T** | **A** | **A** | **C** | **A** | **G** | **T** | **T** | **A** | **T** | **T** | **T** | **A** | **T** | **T** | **T** | **T** | **A** | **T** | **T** | **A** | **A** | **T** | **G** | **C** | **A** | **A** | **T** | **T** | **A** | **T** | **T** | **G** | **C** | **G** | **G** | **G** | **A** | **A** | **T** | **T** | **A** | **C** | **C** | **A** | **T** | **T** | **T** | **T** | **G** | **T** | **T** | **A** | **A** | **A** | **A** | **C** | **C** | **T** | **C** | **G** | **A** | **G** | **A** | **T** | **G** | **T** | **A** | **G** | **C** | **A** | **G** | **C** | **A** | **A** | **T** | **T** | **T** | **G** | **G** | **G** | **C** | **A** | **T** | **G** | **G** | **C** | **T** | **A** | **C** | **T** | **C** | **G** | **T** | **A** | **C** | **T** | **G** | **A** | **T** | **T** | **G** | **C** | **T** | **C** | **T** | **A** | **C** | **C** | **C** | **G** | **T** | **T** | **T** | **T** | **T** | **G** | **G** | **T** | **T** | **T** | **T** | **T** | **T** | **C** | **T** | **T** | **A** | **T** | **A** | **T** | **T** | **T** | **A** | **T** | **T** | **C** | **T** | **T** | **T** | **G** | **G** | **T** | **C** | **G** | **C** | **G** | **G** | **T** | **T** | **T** | **A** | **A** | **C** | **C** | **G** | **A** | **C** | **A** | **A** | **A** | **A** | **A** | **G** | **A** | **A** | **A** | **T** | **T** | **T** | **T** | **A** | **T** | **T** | **T** | **G** | **C** | **A** | **A** | **C** | **A** | **A** | **A** | **G** | **T** | **G** | **A** | **T** | **T** | **T** | **G** | **C** | **G** | **G** | **G** | **A** | **A** | **T** | **T** | **A** | **G** | **A** | **G** | **A** | **A** | **T** | **T** | **T** | **T** | **C** | **A** | **G** | **A** | **A** | **T** | **T** | **T** | **C** | **C** | **A** | **A** | **G** | **A** | **A** | **G** | **A** | **A** | **A** | **G** | **T** | **T** | **T** | **T** | **G** | **A** | **A** | **C** | **T** | **T** | **T** | **A** | **T** | **A** | **G** | **T** | **C** | **A** | **A** | **A** | **A** | **G** | **A** | **T** | **G** | **C** | **C** | **G** | **A** | **C** | **G** | **G** | **A** | **G** | **G** | **A** | **G** | **C** | **A** | **A** | **C** | **A** | **A** | **C** | **A** | **G** | **T** | **T** | **T** | **A** | **C** | **A** | **G** | **A** | **C** | **T** | **T** | **T** | **T** | **T** | **T** | **T** | **C** | **C** | **T** | **C** | **G** | **T** | **T** | **A** | **A** | **A** | **T** | **A** | **G** | **G** | **A** | **T** | **G** | **C** | **C** | **T** | **C** | **T** | **G** | **A** | **C** | **T** | **A** | **A** | **G** | **A** | **A** | **A** | **A** | **A** | **T** | **G** | **A** | **T** | **G** | **T** | **T** | **G** | **A** | **A** | **A** | **T** | **T** | **T** | **T** | **T** | **A** | **C** | **G** | **G** | **A** | **T** | **G** | **G** | **T** | **A** | **C** | **G** | **G** | **A** | **A** | **A** | **A** | **A** | **T** | **T** | **C** | **A** | **A** | **T** | **G** | **C** | **G** | **T** | **T** | **G** | **A** | **T** | **G** | **G** | **C** | **A** | **G** | **A** | **C** | **A** | **T** | **A** | **A** | **A** | **A** | **A** | **A** | **A** | **G** | **C** | **T** | **C** | **A** | **G** | **C** | **A** | **C** | **T** | **C** | **C** | **A** | **T** | **T** | **C** | **A** | **T** | **A** | **T** | **T** | **G** | **A** | **A** | **T** | **A** | **T** | **T** | **A** | **T** | **G** | **C** | **C** | **T** | **T** | **T** | **G** | **T** | **A** | **A** | **C** | **G** | **G** | **A** | **T** | **C** | **A** | **T** | **A** | **T** | **C** | **G** | **G** | **T** | **A** | **C** | **C** | **A** | **A** | **A** | **A** | **T** | **T** | **T** | **T** | **G** | **A** | **A** | **C** | **T** | **T** | **A** | **T** | **T** | **A** | **G** | **A** | **A** | **G** | **A** | **A** | **A** | **A** | **G** | **G** | **C** | **G** | **G** | **C** | **T** | **G** | **A** | **A** | **G** | **G** | **G** | **G** | **T** | **T** | **G** | **A** | **A** | **G** | **T** | **G** | **C** | **G** | **G** | **T** | **T** | **G** | **C** | **T** | **T** | **T** | **A** | **C** | **G** | **A** | **T** | **G** | **C** | **C** | **T** | **T** | **T** | **G** | **G** | **C** | **T** | **C** | **A** | **A** | **A** | **G** | **G** | **G** | **T** | **A** | **C** | **C** | **A** | **A** | **G** | **G** | **T** | **T** | **C** | **A** | **T** | **C** | **A** | **T** | **T** | **T** | **G** | **A** | **A** | **T** | **G** | **A** | **A** | **T** | **T** | **G** | **A** | **A** | **A** | **A** | **A** | **A** | **A** | **A** | **C** | **G** | **G** | **C** | **G** | **G** | **T** | **T** | **T** | **T** | **G** | **T** | **C** | **C** | **A** | **A** | **A** | **C** | **G** | **T** | **T** | **T** | **A** | **T** | **T** | **A** | **C** | **T** | **T** | **C** | **T** | **C** | **A** | **A** | **A** | **A** | **A** | **G** | **C** | **A** | **C** | **T** | **T** | **T** | **T** | **G** | **A** | **A** | **G** | **T** | **T** | **T** | **C** | **G** | **T** | **T** | **T** | **G** | **A** | **A** | **T** | **T** | **A** | **T** | **C** | **A** | **T** | **G** | **A** | **T** | **C** | **A** | **C** | **C** | **G** | **G** | **A** | **A** | **A** | **A** | **T** | **T** | **G** | **T** | **T** | **G** | **T** | **T** | **A** | **T** | **T** | **G** | **A** | **C** | **G** | **G** | **A** | **A** | **A** | **A** | **G** | **T** | **A** | **G** | **G** | **C** | **T** | **A** | **C** | **A** | **T** | **T** | **G** | **G** | **C** | **G** | **G** | **C** | **T** | **T** | **T** | **A** | **A** | **T** | **G** | **T** | **T** | **G** | **C** | **C** | **G** | **A** | **T** | **C** | **A** | **A** | **T** | **A** | **T** | **G** | **C** | **C** | **G** | **G** | **A** | **A** | **C** | **G** | **A** | **C** | **T** | **A** | **A** | **A** | **A** | **A** | **G** | **T** | **T** | **T** | **G** | **G** | **C** | **T** | **A** | **T** | **T** | **G** | **G** | **C** | **G** | **G** | **G** | **A** | **T** | **A** | **C** | **A** | **C** | **A** | **T** | **T** | **T** | **A** | **C** | **G** | **G** | **A** | **T** | **T** | **C** | **A** | **A** | **G** | **G** | **G** | **C** | **C** | **A** | **G** | **C** | **A** | **G** | **C** | **C** | **T** | **C** | **A** | **T** | **T** | **A** | **C** | **T** | **G** | **C** | **A** | **A** | **A** | **T** | **G** | **C** | **G** | **T** | **T** | **T** | **T** | **T** | **T** | **A** | **A** | **T** | **G** | **G** | **A** | **T** | **T** | **G** | **G** | **A** | **A** | **C** | **G** | **T** | **C** | **T** | **C** | **T** | **T** | **C** | **C** | **C** | **C** | **C** | **G** | **A** | **G** | **A** | **A** | **A** | **A** | **A** | **T** | **C** | **G** | **T** | **G** | **T** | **G** | **G** | **C** | **G** | **T** | **A** | **T** | **C** | **A** | **A** | **T** | **T** | **G** | **G** | **A** | **T** | **T** | **A** | **T** | **T** | **T** | **C** | **T** | **T** | **T** | **A** | **A** | **A** | **C** | **T** | **T** | **G** | **A** | **A** | **G** | **C** | **A** | **T** | **T** | **G** | **G** | **T** | **G** | **C** | **C** | **A** | **G** | **A** | **G** | **G** | **C** | **A** | **A** | **A** | **T** | **A** | **C** | **A** | **T** | **C** | **C** | **A** | **T** | **T** | **C** | **A** | **G** | **A** | **T** | **G** | **A** | **T** | **T** | **G** | **C** | **C** | **A** | **G** | **T** | **G** | **G** | **T** | **C** | **C** | **T** | **A** | **A** | **C** | **A** | **G** | **T** | **G** | **A** | **C** | **C** | **G** | **T** | **G** | **A** | **A** | **C** | **A** | **A** | **A** | **T** | **T** | **A** | **A** | **A** | **T** | **T** | **G** | **G** | **C** | **C** | **T** | **T** | **T** | **A** | **T** | **T** | **A** | **A** | **A** | **T** | **T** | **G** | **A** | **T** | **T** | **A** | **C** | **T** | **T** | **C** | **T** | **G** | **C** | **C** | **A** | **A** | **G** | **A** | **A** | **A** | **A** | **G** | **A** | **G** | **T** | **C** | **T** | **G** | **G** | **A** | **T** | **T** | **C** | **A** | **A** | **A** | **C** | **G** | **C** | **C** | **G** | **T** | **A** | **T** | **T** | **T** | **A** | **G** | **T** | **T** | **C** | **C** | **T** | **G** | **A** | **T** | **G** | **A** | **C** | **A** | **G** | **T** | **G** | **T** | **C** | **T** | **T** | **G** | **G** | **C** | **T** | **G** | **C** | **T** | **T** | **T** | **A** | **A** | **A** | **G** | **G** | **T** | **T** | **G** | **C** | **T** | **G** | **C** | **G** | **G** | **C** | **T** | **T** | **C** | **A** | **G** | **G** | **A** | **G** | **T** | **A** | **G** | **A** | **T** | **G** | **T** | **T** | **A** | **A** | **A** | **A** | **T** | **T** | **A** | **T** | **G** | **A** | **T** | **T** | **C** | **C** | **A** | **G** | **A** | **T** | **A** | **A** | **G** | **C** | **C** | **C** | **G** | **A** | **C** | **C** | **A** | **T** | **C** | **C** | **G** | **T** | **T** | **T** | **A** | **T** | **T** | **T** | **A** | **T** | **C** | **G** | **A** | **G** | **C** | **A** | **A** | **C** | **A** | **C** | **A** | **G** | **T** | **A** | **T** | **T** | **A** | **C** | **G** | **G** | **C** | **C** | **G** | **C** | **T** | **T** | **A** | **T** | **T** | **G** | **A** | **T** | **G** | **A** | **A** | **A** | **G** | **A** | **A** | **A** | **A** | **T** | **A** | **T** | **T** | **G** | **A** | **A** | **A** | **T** | **T** | **T** | **T** | **A** | **A** | **T** | **T** | **T** | **A** | **T** | **A** | **A** | **C** | **G** | **G** | **T** | **G** | **G** | **T** | **T** | **T** | **C** | **T** | **T** | **A** | **C** | **A** | **T** | **G** | **C** | **G** | **A** | **A** | **A** | **A** | **C** | **A** | **A** | **T** | **G** | **A** | **T** | **T** | **A** | **T** | **G** | **G** | **A** | **T** | **G** | **A** | **T** | **G** | **A** | **A** | **G** | **T** | **C** | **T** | **G** | **C** | **A** | **C** | **A** | **G** | **T** | **T** | **G** | **G** | **T** | **T** | **C** | **A** | **G** | **C** | **C** | **A** | **A** | **T** | **C** | **A** | **A** | **G** | **A** | **T** | **A** | **T** | **C** | **C** | **G** | **A** | **A** | **G** | **T** | **T** | **A** | **C** | **A** | **A** | **A** | **T** | **T** | **A** | **A** | **A** | **C** | **T** | **T** | **T** | **G** | **A** | **A** | **G** | **C** | **A** | **A** | **A** | **T** | **G** | **C** | **T** | **G** | **T** | **G** | **T** | **T** | **A** | **T** | **A** | **T** | **G** | **A** | **T** | **A** | **A** | **A** | **A** | **A** | **A** | **A** | **T** | **C** | **A** | **T** | **T** | **G** | **A** | **T** | **C** | **A** | **A** | **T** | **T** | **A** | **G** | **A** | **A** | **G** | **C** | **A** | **A** | **T** | **T** | **T** | **T** | **C** | **T** | **T** | **A** | **G** | **A** | **A** | **G** | **A** | **T** | **C** | **G** | **A** | **A** | **A** | **A** | **A** | **A** | **A** | **T** | **G** | **T** | **A** | **C** | **A** | **A** | **C** | **A** | **A** | **T** | **G** | **A** | **C** | **T** | **C** | **C** | **A** | **G** | **A** | **A** | **G** | **T** | **T** | **G** | **T** | **T** | **C** | **G** | **C** | **G** | **A** | **C** | **A** | **T** | **G** | **T** | **C** | **A** | **A** | **A** | **A** | **T** | **G** | **G** | **T** | **T** | **G** | **A** | **T** | **T** | **T** | **T** | **T** | **A** | **A** | **A** | **C** | **A** | **A** | **C** | **A** | **A** | **A** | **T** | **T** | **T** | **C** | **A** | **C** | **G** | **A** | **T** | **T** | **A** | **T** | **T** | **T** | **T** | **C** | **A** | **C** | **C** | **A** | **A** | **T** | **T** | **C** | **T** | **T** | **-** | **-** | **-** |
| **N59600F** | **T** | **T** | **G** | **A** | **T** | **T** | **C** | **T** | **T** | **A** | **G** | **C** | **G** | **T** | **C** | **T** | **T** | **A** | **A** | **C** | **A** | **G** | **T** | **T** | **A** | **T** | **T** | **T** | **A** | **T** | **T** | **T** | **T** | **A** | **T** | **T** | **A** | **A** | **T** | **G** | **C** | **A** | **A** | **T** | **T** | **A** | **T** | **T** | **G** | **C** | **G** | **G** | **G** | **A** | **A** | **T** | **T** | **A** | **C** | **C** | **A** | **T** | **T** | **T** | **T** | **G** | **T** | **T** | **A** | **A** | **A** | **A** | **C** | **C** | **T** | **C** | **G** | **A** | **G** | **A** | **T** | **G** | **T** | **A** | **G** | **C** | **A** | **G** | **C** | **A** | **A** | **T** | **T** | **T** | **G** | **G** | **G** | **C** | **A** | **T** | **G** | **G** | **C** | **T** | **A** | **C** | **T** | **C** | **G** | **T** | **A** | **C** | **T** | **G** | **A** | **T** | **T** | **G** | **C** | **T** | **C** | **T** | **A** | **C** | **C** | **C** | **G** | **T** | **T** | **T** | **T** | **T** | **G** | **G** | **T** | **T** | **T** | **T** | **T** | **T** | **C** | **T** | **T** | **A** | **T** | **A** | **T** | **T** | **T** | **A** | **T** | **T** | **C** | **T** | **T** | **T** | **G** | **G** | **G** | **C** | **G** | **C** | **G** | **G** | **T** | **T** | **T** | **A** | **A** | **C** | **C** | **G** | **A** | **C** | **A** | **A** | **A** | **A** | **A** | **G** | **A** | **A** | **A** | **T** | **T** | **T** | **T** | **A** | **T** | **T** | **T** | **G** | **C** | **A** | **A** | **C** | **A** | **A** | **A** | **G** | **T** | **G** | **A** | **T** | **T** | **T** | **G** | **C** | **G** | **G** | **G** | **A** | **A** | **T** | **T** | **A** | **G** | **A** | **G** | **A** | **A** | **T** | **T** | **T** | **T** | **C** | **A** | **G** | **A** | **A** | **T** | **T** | **T** | **C** | **C** | **A** | **A** | **G** | **A** | **A** | **G** | **A** | **A** | **A** | **G** | **T** | **T** | **T** | **T** | **G** | **A** | **A** | **C** | **T** | **T** | **T** | **A** | **T** | **A** | **G** | **T** | **C** | **A** | **A** | **A** | **A** | **G** | **A** | **T** | **G** | **C** | **C** | **G** | **A** | **C** | **G** | **G** | **A** | **G** | **G** | **A** | **G** | **C** | **A** | **A** | **C** | **A** | **A** | **C** | **A** | **G** | **T** | **T** | **T** | **A** | **C** | **A** | **G** | **A** | **C** | **T** | **T** | **T** | **T** | **T** | **T** | **T** | **C** | **C** | **T** | **C** | **G** | **T** | **T** | **G** | **A** | **A** | **T** | **A** | **G** | **G** | **A** | **T** | **G** | **C** | **C** | **T** | **C** | **T** | **G** | **A** | **C** | **T** | **A** | **A** | **G** | **A** | **A** | **A** | **A** | **A** | **T** | **G** | **A** | **T** | **G** | **T** | **T** | **C** | **A** | **A** | **A** | **T** | **T** | **T** | **T** | **T** | **A** | **C** | **G** | **G** | **A** | **T** | **G** | **G** | **T** | **A** | **C** | **G** | **G** | **A** | **A** | **A** | **A** | **A** | **T** | **T** | **C** | **A** | **A** | **T** | **G** | **C** | **G** | **T** | **T** | **G** | **A** | **T** | **G** | **G** | **C** | **G** | **G** | **A** | **C** | **A** | **T** | **A** | **A** | **A** | **A** | **A** | **A** | **A** | **G** | **C** | **T** | **C** | **A** | **G** | **C** | **A** | **C** | **T** | **C** | **C** | **A** | **T** | **T** | **C** | **A** | **T** | **A** | **T** | **T** | **G** | **A** | **A** | **T** | **A** | **T** | **T** | **A** | **T** | **G** | **C** | **C** | **T** | **T** | **T** | **G** | **T** | **A** | **A** | **C** | **G** | **G** | **A** | **T** | **C** | **A** | **T** | **A** | **T** | **C** | **G** | **G** | **C** | **A** | **C** | **C** | **A** | **A** | **A** | **A** | **T** | **T** | **T** | **T** | **G** | **A** | **A** | **C** | **T** | **T** | **A** | **T** | **T** | **A** | **G** | **A** | **A** | **G** | **A** | **A** | **A** | **A** | **G** | **G** | **C** | **G** | **G** | **C** | **T** | **G** | **A** | **A** | **G** | **G** | **G** | **G** | **T** | **T** | **G** | **A** | **A** | **G** | **T** | **G** | **C** | **G** | **G** | **T** | **T** | **G** | **C** | **T** | **T** | **T** | **A** | **C** | **G** | **A** | **T** | **G** | **C** | **C** | **T** | **T** | **T** | **G** | **G** | **C** | **T** | **C** | **A** | **A** | **A** | **G** | **G** | **G** | **C** | **A** | **C** | **C** | **A** | **A** | **G** | **G** | **T** | **T** | **C** | **A** | **T** | **C** | **A** | **T** | **T** | **T** | **G** | **A** | **A** | **T** | **G** | **A** | **A** | **T** | **T** | **G** | **A** | **A** | **A** | **A** | **A** | **A** | **A** | **A** | **C** | **G** | **G** | **T** | **G** | **G** | **T** | **T** | **T** | **T** | **G** | **T** | **C** | **C** | **A** | **A** | **A** | **C** | **G** | **T** | **T** | **T** | **A** | **T** | **T** | **A** | **C** | **T** | **T** | **C** | **T** | **C** | **A** | **A** | **A** | **A** | **A** | **G** | **C** | **A** | **C** | **T** | **T** | **T** | **T** | **G** | **A** | **A** | **G** | **T** | **T** | **T** | **C** | **G** | **T** | **T** | **T** | **G** | **A** | **A** | **T** | **T** | **A** | **T** | **C** | **A** | **T** | **G** | **A** | **T** | **C** | **A** | **C** | **C** | **G** | **G** | **A** | **A** | **A** | **A** | **T** | **T** | **G** | **T** | **T** | **G** | **T** | **T** | **A** | **T** | **T** | **G** | **A** | **C** | **G** | **G** | **A** | **A** | **A** | **A** | **G** | **T** | **A** | **G** | **G** | **C** | **T** | **A** | **C** | **A** | **T** | **T** | **G** | **G** | **C** | **G** | **G** | **C** | **T** | **T** | **T** | **A** | **A** | **T** | **G** | **T** | **T** | **G** | **C** | **C** | **G** | **A** | **T** | **C** | **A** | **A** | **T** | **A** | **T** | **G** | **C** | **T** | **G** | **G** | **A** | **A** | **C** | **G** | **A** | **C** | **T** | **A** | **A** | **A** | **A** | **A** | **G** | **T** | **T** | **T** | **G** | **G** | **C** | **T** | **A** | **T** | **T** | **G** | **G** | **C** | **G** | **G** | **G** | **A** | **T** | **A** | **C** | **G** | **C** | **A** | **T** | **T** | **T** | **A** | **C** | **G** | **G** | **A** | **T** | **T** | **C** | **A** | **A** | **G** | **G** | **G** | **C** | **C** | **A** | **G** | **C** | **A** | **G** | **C** | **C** | **T** | **C** | **A** | **T** | **T** | **A** | **C** | **T** | **G** | **C** | **A** | **A** | **A** | **T** | **G** | **C** | **G** | **T** | **T** | **T** | **T** | **T** | **T** | **A** | **A** | **T** | **G** | **G** | **A** | **T** | **T** | **G** | **G** | **A** | **A** | **C** | **G** | **T** | **C** | **T** | **C** | **T** | **T** | **C** | **C** | **C** | **C** | **C** | **G** | **A** | **G** | **A** | **A** | **A** | **A** | **A** | **T** | **C** | **G** | **T** | **G** | **T** | **G** | **G** | **C** | **G** | **T** | **A** | **T** | **C** | **A** | **A** | **T** | **T** | **G** | **G** | **A** | **T** | **T** | **A** | **T** | **T** | **T** | **C** | **T** | **T** | **T** | **A** | **A** | **A** | **C** | **T** | **T** | **G** | **A** | **A** | **G** | **C** | **A** | **T** | **T** | **G** | **G** | **T** | **G** | **C** | **C** | **A** | **G** | **A** | **G** | **G** | **C** | **A** | **A** | **A** | **T** | **A** | **C** | **A** | **T** | **C** | **C** | **A** | **T** | **T** | **C** | **A** | **G** | **A** | **T** | **G** | **A** | **T** | **T** | **G** | **C** | **C** | **A** | **G** | **T** | **G** | **G** | **T** | **C** | **C** | **T** | **A** | **A** | **C** | **A** | **G** | **T** | **G** | **A** | **C** | **C** | **G** | **T** | **G** | **A** | **A** | **C** | **A** | **A** | **A** | **T** | **T** | **A** | **A** | **A** | **T** | **T** | **G** | **G** | **C** | **C** | **T** | **T** | **T** | **A** | **T** | **T** | **A** | **A** | **A** | **T** | **T** | **G** | **A** | **T** | **T** | **A** | **C** | **T** | **T** | **C** | **T** | **G** | **C** | **C** | **A** | **A** | **G** | **A** | **A** | **A** | **A** | **G** | **A** | **G** | **T** | **C** | **T** | **G** | **G** | **A** | **T** | **T** | **C** | **A** | **A** | **A** | **C** | **G** | **C** | **C** | **G** | **T** | **A** | **T** | **T** | **T** | **A** | **G** | **T** | **T** | **C** | **C** | **T** | **G** | **A** | **T** | **G** | **A** | **T** | **A** | **G** | **T** | **G** | **T** | **C** | **T** | **T** | **G** | **G** | **C** | **T** | **G** | **C** | **T** | **T** | **T** | **A** | **A** | **A** | **G** | **G** | **T** | **T** | **G** | **C** | **T** | **G** | **C** | **G** | **G** | **C** | **T** | **T** | **C** | **A** | **G** | **G** | **A** | **G** | **T** | **A** | **G** | **A** | **T** | **G** | **T** | **T** | **A** | **A** | **A** | **A** | **T** | **T** | **A** | **T** | **G** | **A** | **T** | **T** | **C** | **C** | **A** | **G** | **A** | **T** | **A** | **A** | **G** | **C** | **C** | **C** | **G** | **A** | **C** | **C** | **A** | **T** | **C** | **C** | **G** | **T** | **T** | **T** | **A** | **T** | **T** | **T** | **A** | **T** | **C** | **G** | **A** | **G** | **C** | **A** | **A** | **C** | **A** | **C** | **A** | **G** | **T** | **A** | **T** | **T** | **A** | **C** | **G** | **G** | **C** | **C** | **G** | **C** | **T** | **T** | **A** | **T** | **T** | **G** | **A** | **T** | **G** | **A** | **A** | **A** | **G** | **A** | **A** | **A** | **A** | **T** | **A** | **T** | **T** | **G** | **A** | **A** | **A** | **T** | **T** | **T** | **T** | **A** | **A** | **T** | **T** | **T** | **A** | **T** | **A** | **A** | **C** | **G** | **G** | **T** | **G** | **G** | **T** | **T** | **T** | **C** | **T** | **T** | **A** | **C** | **A** | **T** | **G** | **C** | **G** | **A** | **A** | **A** | **A** | **C** | **A** | **A** | **T** | **G** | **A** | **T** | **T** | **A** | **T** | **G** | **G** | **A** | **T** | **G** | **A** | **T** | **G** | **A** | **A** | **G** | **T** | **C** | **T** | **G** | **C** | **A** | **C** | **A** | **G** | **T** | **T** | **G** | **G** | **T** | **T** | **C** | **A** | **G** | **C** | **C** | **A** | **A** | **T** | **C** | **A** | **A** | **G** | **A** | **T** | **A** | **T** | **C** | **C** | **G** | **A** | **A** | **G** | **T** | **T** | **A** | **C** | **A** | **A** | **A** | **T** | **T** | **A** | **A** | **A** | **C** | **T** | **T** | **T** | **G** | **A** | **A** | **G** | **C** | **A** | **A** | **A** | **T** | **G** | **C** | **T** | **G** | **T** | **G** | **T** | **T** | **A** | **T** | **A** | **T** | **G** | **A** | **T** | **A** | **A** | **A** | **A** | **A** | **A** | **A** | **T** | **C** | **A** | **T** | **T** | **G** | **A** | **T** | **C** | **A** | **A** | **T** | **T** | **A** | **G** | **A** | **A** | **G** | **C** | **A** | **A** | **T** | **T** | **T** | **T** | **C** | **T** | **T** | **A** | **G** | **A** | **A** | **G** | **A** | **T** | **C** | **G** | **A** | **A** | **A** | **A** | **A** | **A** | **A** | **T** | **G** | **T** | **A** | **C** | **A** | **A** | **C** | **A** | **A** | **T** | **G** | **A** | **C** | **T** | **C** | **C** | **A** | **G** | **A** | **A** | **G** | **T** | **T** | **G** | **T** | **T** | **C** | **G** | **C** | **G** | **A** | **C** | **A** | **T** | **G** | **T** | **C** | **A** | **A** | **A** | **A** | **T** | **G** | **G** | **T** | **T** | **G** | **A** | **T** | **T** | **T** | **T** | **T** | **A** | **A** | **A** | **C** | **A** | **A** | **C** | **A** | **A** | **A** | **T** | **T** | **T** | **C** | **A** | **C** | **G** | **A** | **T** | **T** | **A** | **T** | **T** | **T** | **T** | **C** | **A** | **C** | **C** | **A** | **A** | **T** | **T** | **C** | **T** | **T** | **-** | **-** | **-** |
| **N59457F** | **T** | **T** | **G** | **A** | **T** | **T** | **C** | **T** | **T** | **A** | **G** | **C** | **G** | **T** | **C** | **T** | **T** | **A** | **A** | **C** | **A** | **G** | **T** | **T** | **A** | **T** | **T** | **T** | **A** | **T** | **T** | **T** | **T** | **A** | **T** | **T** | **A** | **A** | **T** | **G** | **C** | **A** | **A** | **T** | **T** | **A** | **T** | **T** | **G** | **C** | **G** | **G** | **G** | **A** | **A** | **T** | **T** | **A** | **C** | **C** | **A** | **T** | **T** | **T** | **T** | **G** | **T** | **T** | **A** | **A** | **A** | **A** | **C** | **C** | **T** | **C** | **G** | **A** | **G** | **A** | **T** | **G** | **T** | **A** | **G** | **C** | **A** | **G** | **C** | **A** | **A** | **T** | **T** | **T** | **G** | **G** | **G** | **C** | **A** | **T** | **G** | **G** | **C** | **T** | **A** | **C** | **T** | **C** | **G** | **T** | **A** | **C** | **T** | **G** | **A** | **T** | **T** | **G** | **C** | **T** | **C** | **T** | **A** | **C** | **C** | **C** | **G** | **T** | **T** | **T** | **T** | **T** | **G** | **G** | **T** | **T** | **T** | **T** | **T** | **T** | **C** | **T** | **T** | **A** | **T** | **A** | **T** | **T** | **T** | **A** | **T** | **T** | **C** | **T** | **T** | **T** | **G** | **G** | **G** | **C** | **G** | **C** | **G** | **G** | **T** | **T** | **T** | **A** | **A** | **C** | **C** | **G** | **A** | **C** | **A** | **A** | **A** | **A** | **A** | **G** | **A** | **A** | **A** | **T** | **T** | **T** | **T** | **A** | **T** | **T** | **T** | **G** | **C** | **A** | **A** | **C** | **A** | **A** | **A** | **G** | **T** | **G** | **A** | **T** | **T** | **T** | **G** | **C** | **G** | **G** | **G** | **A** | **A** | **T** | **T** | **A** | **G** | **A** | **G** | **A** | **A** | **T** | **T** | **T** | **T** | **C** | **A** | **G** | **A** | **A** | **T** | **T** | **T** | **C** | **C** | **A** | **A** | **G** | **A** | **A** | **G** | **A** | **A** | **A** | **G** | **T** | **T** | **T** | **T** | **G** | **A** | **A** | **C** | **T** | **T** | **T** | **A** | **T** | **A** | **G** | **T** | **C** | **A** | **A** | **A** | **A** | **G** | **A** | **T** | **G** | **C** | **C** | **G** | **A** | **C** | **G** | **G** | **A** | **G** | **G** | **A** | **G** | **C** | **A** | **A** | **C** | **A** | **A** | **C** | **A** | **G** | **T** | **T** | **T** | **A** | **C** | **A** | **G** | **A** | **C** | **T** | **T** | **T** | **T** | **T** | **T** | **T** | **C** | **C** | **T** | **C** | **G** | **T** | **T** | **A** | **A** | **A** | **T** | **A** | **G** | **G** | **A** | **T** | **G** | **C** | **C** | **T** | **C** | **T** | **G** | **A** | **C** | **T** | **A** | **A** | **G** | **A** | **A** | **A** | **A** | **A** | **T** | **G** | **A** | **T** | **G** | **T** | **T** | **G** | **A** | **A** | **A** | **T** | **T** | **T** | **T** | **T** | **A** | **C** | **G** | **G** | **A** | **T** | **G** | **G** | **T** | **A** | **C** | **G** | **G** | **A** | **A** | **A** | **A** | **A** | **T** | **T** | **C** | **A** | **A** | **T** | **G** | **C** | **G** | **T** | **T** | **G** | **A** | **T** | **G** | **G** | **C** | **G** | **G** | **A** | **C** | **A** | **T** | **A** | **A** | **A** | **A** | **A** | **A** | **A** | **G** | **C** | **T** | **C** | **A** | **G** | **C** | **A** | **C** | **T** | **C** | **C** | **A** | **T** | **T** | **C** | **A** | **T** | **A** | **T** | **T** | **G** | **A** | **A** | **T** | **A** | **T** | **T** | **A** | **T** | **G** | **C** | **C** | **T** | **T** | **T** | **G** | **T** | **A** | **A** | **C** | **G** | **G** | **A** | **T** | **C** | **A** | **T** | **A** | **T** | **C** | **G** | **G** | **C** | **A** | **C** | **C** | **A** | **A** | **A** | **A** | **T** | **T** | **T** | **T** | **G** | **A** | **A** | **C** | **T** | **T** | **A** | **T** | **T** | **A** | **G** | **A** | **A** | **G** | **A** | **A** | **A** | **A** | **G** | **G** | **C** | **G** | **G** | **C** | **T** | **G** | **A** | **A** | **G** | **G** | **G** | **G** | **T** | **T** | **G** | **A** | **A** | **G** | **T** | **G** | **C** | **G** | **G** | **T** | **T** | **G** | **C** | **T** | **T** | **T** | **A** | **C** | **G** | **A** | **T** | **G** | **C** | **C** | **T** | **T** | **T** | **G** | **G** | **C** | **T** | **C** | **A** | **A** | **A** | **G** | **G** | **G** | **C** | **A** | **C** | **C** | **A** | **A** | **G** | **G** | **T** | **T** | **C** | **A** | **T** | **C** | **A** | **T** | **T** | **T** | **G** | **A** | **A** | **T** | **G** | **A** | **A** | **T** | **T** | **G** | **A** | **A** | **A** | **A** | **A** | **A** | **A** | **A** | **C** | **G** | **G** | **C** | **G** | **G** | **T** | **T** | **T** | **T** | **G** | **T** | **C** | **C** | **A** | **A** | **A** | **C** | **A** | **T** | **T** | **T** | **A** | **T** | **T** | **A** | **C** | **T** | **T** | **C** | **T** | **C** | **A** | **A** | **A** | **A** | **A** | **G** | **C** | **A** | **C** | **T** | **T** | **T** | **T** | **G** | **A** | **A** | **G** | **T** | **T** | **T** | **C** | **G** | **T** | **T** | **T** | **G** | **A** | **A** | **T** | **T** | **A** | **T** | **C** | **A** | **T** | **G** | **A** | **T** | **C** | **A** | **C** | **C** | **G** | **G** | **A** | **A** | **A** | **A** | **T** | **T** | **G** | **T** | **T** | **G** | **T** | **T** | **A** | **T** | **T** | **G** | **A** | **C** | **G** | **G** | **A** | **A** | **A** | **A** | **G** | **T** | **A** | **G** | **G** | **C** | **T** | **A** | **C** | **A** | **T** | **T** | **G** | **G** | **C** | **G** | **G** | **C** | **T** | **T** | **T** | **A** | **A** | **T** | **G** | **T** | **T** | **G** | **C** | **C** | **G** | **A** | **T** | **C** | **A** | **A** | **T** | **A** | **T** | **G** | **C** | **T** | **G** | **G** | **A** | **A** | **C** | **G** | **A** | **C** | **T** | **A** | **A** | **A** | **A** | **A** | **G** | **T** | **T** | **T** | **G** | **G** | **C** | **T** | **A** | **T** | **T** | **G** | **G** | **C** | **G** | **G** | **G** | **A** | **T** | **A** | **C** | **G** | **C** | **A** | **T** | **T** | **T** | **A** | **C** | **G** | **G** | **A** | **T** | **T** | **C** | **A** | **A** | **G** | **G** | **G** | **C** | **C** | **A** | **G** | **C** | **A** | **G** | **C** | **C** | **T** | **C** | **A** | **T** | **T** | **A** | **C** | **T** | **G** | **C** | **A** | **A** | **A** | **T** | **G** | **C** | **G** | **T** | **T** | **T** | **T** | **T** | **T** | **A** | **A** | **T** | **G** | **G** | **A** | **T** | **T** | **G** | **G** | **A** | **A** | **C** | **G** | **T** | **C** | **T** | **C** | **T** | **T** | **C** | **C** | **C** | **C** | **C** | **G** | **A** | **G** | **A** | **A** | **A** | **A** | **A** | **T** | **C** | **G** | **T** | **G** | **T** | **G** | **G** | **C** | **G** | **T** | **A** | **T** | **C** | **A** | **A** | **T** | **T** | **G** | **G** | **A** | **T** | **T** | **A** | **T** | **T** | **T** | **C** | **T** | **T** | **T** | **A** | **A** | **A** | **C** | **T** | **T** | **G** | **A** | **A** | **G** | **C** | **A** | **T** | **T** | **G** | **G** | **T** | **G** | **C** | **C** | **A** | **G** | **A** | **G** | **G** | **C** | **A** | **A** | **A** | **T** | **A** | **C** | **A** | **T** | **C** | **C** | **A** | **T** | **T** | **C** | **A** | **G** | **A** | **T** | **G** | **A** | **T** | **T** | **G** | **C** | **C** | **A** | **G** | **T** | **G** | **G** | **T** | **C** | **C** | **T** | **A** | **A** | **C** | **A** | **G** | **T** | **G** | **A** | **C** | **C** | **G** | **T** | **G** | **A** | **A** | **C** | **A** | **A** | **A** | **T** | **T** | **A** | **A** | **A** | **T** | **T** | **G** | **G** | **C** | **C** | **T** | **T** | **T** | **A** | **T** | **T** | **A** | **A** | **A** | **T** | **T** | **G** | **A** | **T** | **T** | **A** | **C** | **T** | **T** | **C** | **T** | **G** | **C** | **C** | **A** | **A** | **G** | **A** | **A** | **A** | **A** | **G** | **A** | **G** | **T** | **C** | **T** | **G** | **G** | **A** | **T** | **T** | **C** | **A** | **A** | **A** | **C** | **G** | **C** | **C** | **G** | **T** | **A** | **T** | **T** | **T** | **A** | **G** | **T** | **T** | **C** | **C** | **T** | **G** | **A** | **T** | **G** | **A** | **T** | **A** | **G** | **T** | **G** | **T** | **C** | **T** | **T** | **G** | **G** | **C** | **T** | **G** | **C** | **T** | **T** | **T** | **A** | **A** | **A** | **G** | **G** | **T** | **T** | **G** | **C** | **T** | **G** | **C** | **G** | **G** | **C** | **T** | **T** | **C** | **A** | **G** | **G** | **A** | **G** | **T** | **A** | **G** | **A** | **T** | **G** | **T** | **T** | **A** | **A** | **A** | **A** | **T** | **T** | **A** | **T** | **G** | **A** | **T** | **T** | **C** | **C** | **A** | **G** | **A** | **T** | **A** | **A** | **G** | **C** | **C** | **C** | **G** | **A** | **C** | **C** | **A** | **T** | **C** | **C** | **G** | **T** | **T** | **T** | **A** | **T** | **T** | **T** | **A** | **T** | **C** | **G** | **A** | **G** | **C** | **A** | **A** | **C** | **A** | **C** | **A** | **G** | **T** | **A** | **T** | **T** | **A** | **C** | **G** | **G** | **C** | **C** | **G** | **C** | **T** | **T** | **A** | **T** | **T** | **G** | **A** | **T** | **G** | **A** | **A** | **A** | **G** | **A** | **A** | **A** | **A** | **T** | **A** | **T** | **T** | **G** | **A** | **A** | **A** | **T** | **T** | **T** | **T** | **A** | **A** | **T** | **T** | **T** | **A** | **T** | **A** | **A** | **C** | **G** | **G** | **T** | **G** | **G** | **T** | **T** | **T** | **C** | **T** | **T** | **A** | **C** | **A** | **T** | **G** | **C** | **G** | **A** | **A** | **A** | **A** | **C** | **A** | **A** | **T** | **G** | **A** | **T** | **T** | **A** | **T** | **G** | **G** | **A** | **T** | **G** | **A** | **T** | **G** | **A** | **A** | **G** | **T** | **C** | **T** | **G** | **C** | **A** | **C** | **A** | **G** | **T** | **T** | **G** | **G** | **T** | **T** | **C** | **A** | **G** | **C** | **C** | **A** | **A** | **T** | **C** | **A** | **A** | **G** | **A** | **T** | **A** | **T** | **C** | **C** | **G** | **A** | **A** | **G** | **T** | **T** | **A** | **C** | **A** | **A** | **A** | **T** | **T** | **A** | **A** | **A** | **C** | **T** | **T** | **T** | **G** | **A** | **A** | **G** | **C** | **A** | **A** | **A** | **T** | **G** | **C** | **T** | **G** | **T** | **G** | **T** | **T** | **A** | **T** | **A** | **T** | **G** | **A** | **T** | **A** | **A** | **A** | **A** | **A** | **A** | **A** | **T** | **C** | **A** | **T** | **T** | **G** | **A** | **T** | **C** | **A** | **A** | **T** | **T** | **A** | **G** | **A** | **A** | **G** | **C** | **A** | **A** | **T** | **T** | **T** | **T** | **C** | **T** | **T** | **A** | **G** | **A** | **A** | **G** | **A** | **T** | **C** | **G** | **A** | **A** | **A** | **A** | **A** | **A** | **A** | **T** | **G** | **T** | **A** | **C** | **A** | **A** | **C** | **A** | **A** | **T** | **G** | **A** | **C** | **T** | **C** | **C** | **A** | **G** | **A** | **A** | **G** | **T** | **T** | **G** | **T** | **T** | **C** | **G** | **C** | **G** | **A** | **C** | **A** | **T** | **G** | **T** | **C** | **A** | **A** | **A** | **A** | **T** | **G** | **G** | **T** | **T** | **G** | **A** | **T** | **T** | **T** | **T** | **T** | **A** | **A** | **A** | **C** | **A** | **A** | **C** | **A** | **A** | **A** | **T** | **T** | **T** | **C** | **A** | **C** | **G** | **A** | **T** | **T** | **A** | **T** | **T** | **T** | **T** | **C** | **A** | **C** | **C** | **A** | **A** | **T** | **T** | **C** | **T** | **T** | **-** | **-** | **-** |
| **N55185** | **T** | **T** | **G** | **A** | **T** | **T** | **C** | **T** | **T** | **A** | **G** | **C** | **G** | **T** | **C** | **T** | **T** | **A** | **A** | **C** | **A** | **G** | **T** | **T** | **A** | **T** | **T** | **T** | **A** | **T** | **T** | **T** | **T** | **A** | **T** | **T** | **A** | **A** | **T** | **G** | **C** | **A** | **A** | **T** | **T** | **A** | **T** | **T** | **G** | **C** | **G** | **G** | **G** | **A** | **A** | **T** | **T** | **A** | **C** | **C** | **A** | **T** | **T** | **T** | **T** | **G** | **T** | **T** | **A** | **A** | **A** | **A** | **C** | **C** | **T** | **C** | **G** | **A** | **G** | **A** | **T** | **G** | **T** | **A** | **G** | **C** | **A** | **G** | **C** | **A** | **A** | **T** | **T** | **T** | **G** | **G** | **G** | **C** | **A** | **T** | **G** | **G** | **C** | **T** | **A** | **C** | **T** | **C** | **G** | **T** | **A** | **C** | **T** | **G** | **A** | **T** | **T** | **G** | **C** | **T** | **C** | **T** | **A** | **C** | **C** | **C** | **G** | **T** | **T** | **T** | **T** | **T** | **G** | **G** | **T** | **T** | **T** | **T** | **T** | **T** | **C** | **T** | **T** | **A** | **T** | **A** | **T** | **T** | **T** | **A** | **T** | **T** | **C** | **T** | **T** | **T** | **G** | **G** | **G** | **C** | **G** | **C** | **G** | **G** | **T** | **T** | **T** | **A** | **A** | **C** | **C** | **G** | **A** | **C** | **A** | **A** | **A** | **A** | **A** | **G** | **A** | **A** | **A** | **T** | **T** | **T** | **T** | **A** | **T** | **T** | **T** | **G** | **C** | **A** | **A** | **C** | **A** | **A** | **A** | **G** | **T** | **G** | **A** | **T** | **T** | **T** | **A** | **C** | **G** | **G** | **G** | **A** | **A** | **T** | **T** | **A** | **G** | **A** | **G** | **A** | **A** | **T** | **T** | **T** | **T** | **C** | **A** | **G** | **A** | **A** | **C** | **T** | **T** | **C** | **C** | **A** | **A** | **G** | **A** | **A** | **G** | **A** | **A** | **A** | **G** | **T** | **T** | **T** | **T** | **G** | **A** | **A** | **C** | **T** | **T** | **T** | **A** | **T** | **A** | **G** | **T** | **C** | **A** | **A** | **A** | **A** | **G** | **A** | **T** | **G** | **C** | **C** | **G** | **A** | **C** | **G** | **G** | **A** | **G** | **G** | **A** | **G** | **C** | **A** | **A** | **C** | **A** | **A** | **C** | **A** | **G** | **T** | **T** | **T** | **A** | **C** | **A** | **G** | **A** | **C** | **T** | **T** | **T** | **T** | **T** | **T** | **T** | **C** | **C** | **T** | **C** | **G** | **T** | **T** | **A** | **A** | **A** | **T** | **A** | **G** | **G** | **A** | **T** | **G** | **C** | **C** | **T** | **C** | **T** | **G** | **A** | **C** | **T** | **A** | **A** | **G** | **A** | **A** | **A** | **A** | **A** | **T** | **G** | **A** | **T** | **G** | **T** | **T** | **G** | **A** | **A** | **A** | **T** | **T** | **T** | **T** | **T** | **A** | **C** | **G** | **G** | **A** | **T** | **G** | **G** | **T** | **A** | **C** | **G** | **G** | **A** | **A** | **A** | **A** | **A** | **T** | **T** | **C** | **A** | **A** | **T** | **G** | **C** | **G** | **T** | **T** | **G** | **A** | **T** | **G** | **G** | **C** | **G** | **G** | **A** | **C** | **A** | **T** | **A** | **A** | **A** | **A** | **A** | **A** | **A** | **G** | **C** | **T** | **C** | **A** | **G** | **C** | **A** | **C** | **T** | **C** | **C** | **A** | **T** | **T** | **C** | **A** | **T** | **A** | **T** | **T** | **G** | **A** | **A** | **T** | **A** | **T** | **T** | **A** | **T** | **G** | **C** | **C** | **T** | **T** | **T** | **G** | **T** | **A** | **A** | **C** | **G** | **G** | **A** | **T** | **C** | **A** | **T** | **A** | **T** | **C** | **G** | **G** | **T** | **A** | **C** | **C** | **A** | **A** | **A** | **A** | **T** | **T** | **T** | **T** | **G** | **A** | **A** | **C** | **T** | **T** | **A** | **T** | **T** | **A** | **G** | **A** | **A** | **G** | **A** | **A** | **A** | **A** | **G** | **G** | **C** | **G** | **G** | **C** | **T** | **G** | **A** | **A** | **G** | **G** | **G** | **G** | **T** | **T** | **G** | **A** | **A** | **G** | **T** | **G** | **C** | **G** | **G** | **T** | **T** | **G** | **C** | **T** | **T** | **T** | **A** | **C** | **G** | **A** | **T** | **G** | **C** | **C** | **T** | **T** | **T** | **G** | **G** | **C** | **T** | **C** | **A** | **A** | **A** | **G** | **G** | **G** | **C** | **A** | **C** | **C** | **A** | **A** | **G** | **G** | **T** | **T** | **C** | **A** | **T** | **C** | **A** | **T** | **T** | **T** | **G** | **A** | **A** | **T** | **G** | **A** | **A** | **T** | **T** | **G** | **A** | **A** | **A** | **A** | **A** | **A** | **A** | **A** | **C** | **G** | **G** | **C** | **G** | **G** | **T** | **T** | **T** | **T** | **G** | **T** | **C** | **C** | **A** | **A** | **A** | **C** | **G** | **T** | **T** | **T** | **A** | **T** | **T** | **A** | **C** | **T** | **T** | **C** | **T** | **C** | **A** | **A** | **A** | **A** | **A** | **G** | **C** | **A** | **C** | **T** | **T** | **T** | **T** | **G** | **A** | **A** | **G** | **T** | **T** | **T** | **C** | **G** | **T** | **T** | **T** | **G** | **A** | **A** | **T** | **T** | **A** | **T** | **C** | **A** | **T** | **G** | **A** | **T** | **C** | **A** | **C** | **C** | **G** | **G** | **A** | **A** | **A** | **A** | **T** | **T** | **G** | **T** | **T** | **G** | **T** | **T** | **A** | **T** | **T** | **G** | **A** | **T** | **G** | **G** | **G** | **A** | **A** | **A** | **G** | **T** | **A** | **G** | **G** | **C** | **T** | **A** | **C** | **A** | **T** | **T** | **G** | **G** | **C** | **G** | **G** | **C** | **T** | **T** | **T** | **A** | **A** | **T** | **G** | **T** | **T** | **G** | **C** | **C** | **G** | **A** | **T** | **C** | **A** | **A** | **T** | **A** | **T** | **G** | **C** | **T** | **G** | **G** | **A** | **A** | **C** | **G** | **A** | **C** | **T** | **A** | **A** | **A** | **A** | **A** | **G** | **T** | **T** | **T** | **G** | **G** | **C** | **T** | **A** | **T** | **T** | **G** | **G** | **C** | **G** | **G** | **G** | **A** | **T** | **A** | **C** | **A** | **C** | **A** | **T** | **T** | **T** | **A** | **C** | **G** | **G** | **A** | **T** | **T** | **C** | **A** | **A** | **G** | **G** | **G** | **C** | **C** | **A** | **G** | **C** | **A** | **G** | **C** | **C** | **T** | **C** | **A** | **T** | **T** | **A** | **C** | **T** | **G** | **C** | **A** | **A** | **A** | **T** | **G** | **C** | **G** | **T** | **T** | **T** | **T** | **T** | **T** | **A** | **A** | **T** | **G** | **G** | **A** | **T** | **T** | **G** | **G** | **A** | **A** | **C** | **G** | **T** | **C** | **T** | **C** | **T** | **T** | **C** | **C** | **C** | **C** | **C** | **G** | **A** | **G** | **A** | **A** | **A** | **A** | **A** | **T** | **C** | **G** | **T** | **G** | **T** | **G** | **G** | **C** | **G** | **T** | **A** | **T** | **C** | **A** | **A** | **T** | **T** | **G** | **G** | **A** | **T** | **T** | **A** | **T** | **T** | **T** | **C** | **T** | **T** | **T** | **A** | **A** | **A** | **C** | **T** | **T** | **G** | **A** | **A** | **G** | **C** | **A** | **T** | **T** | **G** | **G** | **T** | **T** | **C** | **C** | **A** | **G** | **A** | **G** | **G** | **C** | **A** | **A** | **A** | **T** | **A** | **C** | **A** | **T** | **C** | **C** | **A** | **T** | **T** | **C** | **A** | **G** | **A** | **T** | **G** | **A** | **T** | **T** | **G** | **C** | **C** | **A** | **G** | **T** | **G** | **G** | **T** | **C** | **C** | **T** | **A** | **A** | **T** | **A** | **G** | **T** | **G** | **A** | **C** | **C** | **G** | **T** | **G** | **A** | **A** | **C** | **A** | **A** | **A** | **T** | **T** | **A** | **A** | **A** | **T** | **T** | **G** | **G** | **C** | **C** | **T** | **T** | **T** | **A** | **T** | **T** | **A** | **A** | **A** | **T** | **T** | **G** | **A** | **T** | **T** | **A** | **C** | **T** | **T** | **C** | **T** | **G** | **C** | **C** | **A** | **A** | **G** | **A** | **A** | **A** | **A** | **G** | **A** | **G** | **T** | **C** | **T** | **G** | **G** | **A** | **T** | **T** | **C** | **A** | **A** | **A** | **C** | **A** | **C** | **C** | **G** | **T** | **A** | **T** | **T** | **T** | **A** | **G** | **T** | **T** | **C** | **C** | **T** | **G** | **A** | **T** | **G** | **A** | **T** | **A** | **G** | **T** | **G** | **T** | **C** | **T** | **T** | **G** | **G** | **C** | **G** | **G** | **C** | **T** | **T** | **T** | **A** | **A** | **A** | **G** | **G** | **T** | **T** | **G** | **C** | **T** | **G** | **C** | **G** | **G** | **C** | **T** | **T** | **C** | **A** | **G** | **G** | **A** | **G** | **T** | **A** | **G** | **A** | **T** | **G** | **T** | **T** | **A** | **A** | **A** | **A** | **T** | **T** | **A** | **T** | **G** | **A** | **T** | **T** | **C** | **C** | **A** | **G** | **A** | **T** | **A** | **A** | **G** | **C** | **C** | **C** | **G** | **A** | **C** | **C** | **A** | **T** | **C** | **C** | **G** | **T** | **T** | **T** | **A** | **T** | **T** | **T** | **A** | **T** | **C** | **G** | **A** | **G** | **C** | **A** | **A** | **C** | **A** | **C** | **A** | **G** | **T** | **A** | **T** | **T** | **A** | **C** | **G** | **G** | **G** | **C** | **G** | **C** | **T** | **T** | **G** | **T** | **T** | **G** | **A** | **T** | **G** | **A** | **A** | **A** | **G** | **A** | **A** | **A** | **A** | **T** | **A** | **T** | **T** | **G** | **A** | **A** | **A** | **T** | **T** | **T** | **T** | **A** | **A** | **T** | **T** | **T** | **A** | **T** | **A** | **A** | **C** | **G** | **G** | **T** | **G** | **G** | **T** | **T** | **T** | **C** | **T** | **T** | **A** | **C** | **A** | **T** | **G** | **C** | **G** | **A** | **A** | **A** | **A** | **C** | **A** | **A** | **T** | **G** | **A** | **T** | **T** | **A** | **T** | **G** | **G** | **A** | **T** | **G** | **A** | **T** | **G** | **A** | **A** | **G** | **T** | **C** | **T** | **G** | **C** | **A** | **C** | **A** | **G** | **T** | **T** | **G** | **G** | **T** | **T** | **C** | **A** | **G** | **C** | **C** | **A** | **A** | **T** | **C** | **A** | **A** | **G** | **A** | **T** | **A** | **T** | **C** | **C** | **G** | **A** | **A** | **G** | **T** | **T** | **A** | **C** | **A** | **A** | **A** | **T** | **T** | **A** | **A** | **A** | **C** | **T** | **T** | **T** | **G** | **A** | **A** | **G** | **C** | **A** | **A** | **A** | **T** | **G** | **C** | **T** | **G** | **T** | **G** | **T** | **T** | **A** | **T** | **A** | **T** | **G** | **A** | **T** | **A** | **A** | **A** | **A** | **A** | **A** | **A** | **T** | **C** | **A** | **T** | **T** | **G** | **A** | **T** | **C** | **A** | **A** | **T** | **T** | **A** | **G** | **A** | **A** | **G** | **C** | **A** | **A** | **T** | **T** | **T** | **T** | **C** | **T** | **T** | **A** | **G** | **A** | **A** | **G** | **A** | **T** | **C** | **G** | **A** | **A** | **A** | **A** | **A** | **A** | **A** | **T** | **G** | **T** | **A** | **C** | **A** | **A** | **C** | **A** | **A** | **T** | **G** | **A** | **C** | **T** | **C** | **C** | **A** | **G** | **A** | **A** | **G** | **T** | **T** | **G** | **T** | **T** | **C** | **G** | **C** | **G** | **A** | **C** | **A** | **T** | **G** | **T** | **C** | **A** | **A** | **A** | **A** | **T** | **G** | **G** | **T** | **T** | **G** | **A** | **T** | **T** | **T** | **T** | **T** | **A** | **A** | **A** | **C** | **A** | **A** | **C** | **A** | **A** | **A** | **T** | **T** | **T** | **C** | **A** | **C** | **G** | **A** | **T** | **T** | **A** | **T** | **T** | **C** | **T** | **C** | **A** | **C** | **C** | **A** | **A** | **T** | **T** | **C** | **T** | **T** | **-** | **-** | **-** |
| **N59847F** | **T** | **T** | **G** | **A** | **T** | **T** | **C** | **T** | **T** | **A** | **G** | **C** | **G** | **T** | **C** | **T** | **T** | **A** | **A** | **C** | **A** | **G** | **T** | **T** | **A** | **T** | **T** | **T** | **A** | **T** | **T** | **T** | **T** | **A** | **T** | **T** | **A** | **A** | **T** | **G** | **C** | **A** | **A** | **T** | **T** | **A** | **T** | **T** | **G** | **C** | **G** | **G** | **G** | **A** | **A** | **T** | **T** | **A** | **C** | **C** | **A** | **T** | **T** | **T** | **T** | **G** | **T** | **T** | **A** | **A** | **A** | **A** | **C** | **C** | **T** | **C** | **G** | **A** | **G** | **A** | **T** | **G** | **T** | **A** | **G** | **C** | **A** | **G** | **C** | **A** | **A** | **T** | **T** | **T** | **G** | **G** | **G** | **C** | **A** | **T** | **G** | **G** | **C** | **T** | **A** | **C** | **T** | **C** | **G** | **T** | **A** | **C** | **T** | **G** | **A** | **T** | **T** | **G** | **C** | **T** | **C** | **T** | **A** | **C** | **C** | **C** | **G** | **T** | **T** | **T** | **T** | **T** | **G** | **G** | **T** | **T** | **T** | **T** | **T** | **T** | **C** | **T** | **T** | **A** | **T** | **A** | **T** | **T** | **T** | **A** | **T** | **T** | **C** | **T** | **T** | **T** | **G** | **G** | **T** | **C** | **G** | **A** | **G** | **G** | **T** | **T** | **T** | **A** | **A** | **C** | **C** | **G** | **A** | **C** | **A** | **A** | **A** | **A** | **A** | **G** | **A** | **A** | **A** | **T** | **T** | **T** | **T** | **A** | **T** | **T** | **T** | **G** | **C** | **A** | **A** | **C** | **A** | **A** | **A** | **G** | **T** | **G** | **A** | **T** | **T** | **T** | **A** | **C** | **G** | **G** | **G** | **A** | **A** | **T** | **T** | **A** | **G** | **A** | **G** | **A** | **A** | **T** | **T** | **T** | **C** | **C** | **A** | **G** | **A** | **A** | **T** | **T** | **T** | **C** | **C** | **A** | **A** | **G** | **A** | **A** | **G** | **A** | **A** | **A** | **G** | **T** | **T** | **T** | **T** | **G** | **A** | **A** | **C** | **T** | **T** | **T** | **A** | **T** | **A** | **G** | **T** | **C** | **A** | **A** | **A** | **A** | **G** | **A** | **T** | **G** | **C** | **C** | **G** | **A** | **C** | **G** | **G** | **A** | **G** | **G** | **A** | **G** | **C** | **A** | **A** | **C** | **A** | **A** | **C** | **A** | **G** | **T** | **T** | **T** | **A** | **C** | **A** | **G** | **A** | **C** | **T** | **T** | **T** | **T** | **T** | **T** | **T** | **C** | **C** | **T** | **C** | **G** | **T** | **T** | **A** | **A** | **A** | **T** | **A** | **G** | **G** | **A** | **T** | **G** | **C** | **C** | **T** | **C** | **T** | **G** | **A** | **C** | **T** | **A** | **A** | **G** | **A** | **A** | **A** | **A** | **A** | **T** | **G** | **A** | **T** | **G** | **T** | **T** | **G** | **A** | **A** | **A** | **T** | **T** | **T** | **T** | **T** | **A** | **C** | **G** | **G** | **A** | **T** | **G** | **G** | **T** | **A** | **C** | **G** | **G** | **A** | **A** | **A** | **A** | **A** | **T** | **T** | **C** | **A** | **A** | **T** | **G** | **C** | **G** | **T** | **T** | **G** | **A** | **T** | **G** | **G** | **C** | **G** | **G** | **A** | **T** | **A** | **T** | **A** | **A** | **A** | **A** | **A** | **A** | **A** | **G** | **C** | **T** | **C** | **A** | **G** | **C** | **A** | **C** | **T** | **C** | **C** | **A** | **T** | **T** | **C** | **A** | **T** | **A** | **T** | **T** | **G** | **A** | **A** | **T** | **A** | **T** | **T** | **A** | **T** | **G** | **C** | **C** | **T** | **T** | **T** | **G** | **T** | **A** | **A** | **C** | **G** | **G** | **A** | **T** | **C** | **A** | **T** | **A** | **T** | **C** | **G** | **G** | **C** | **A** | **C** | **C** | **A** | **A** | **A** | **A** | **T** | **T** | **T** | **T** | **G** | **A** | **A** | **C** | **T** | **T** | **A** | **T** | **T** | **A** | **G** | **A** | **A** | **G** | **A** | **A** | **A** | **A** | **G** | **G** | **C** | **G** | **G** | **C** | **T** | **G** | **A** | **A** | **G** | **G** | **G** | **G** | **T** | **T** | **G** | **A** | **A** | **G** | **T** | **G** | **C** | **G** | **G** | **T** | **T** | **G** | **C** | **T** | **T** | **T** | **A** | **C** | **G** | **A** | **T** | **G** | **C** | **C** | **T** | **T** | **T** | **G** | **G** | **C** | **T** | **C** | **A** | **A** | **A** | **G** | **G** | **G** | **C** | **A** | **C** | **C** | **A** | **A** | **G** | **G** | **T** | **T** | **C** | **A** | **T** | **C** | **A** | **T** | **T** | **T** | **G** | **A** | **A** | **T** | **G** | **A** | **A** | **T** | **T** | **G** | **A** | **A** | **A** | **A** | **A** | **A** | **A** | **A** | **C** | **G** | **G** | **T** | **G** | **G** | **T** | **T** | **T** | **T** | **G** | **T** | **C** | **C** | **A** | **A** | **A** | **C** | **G** | **T** | **T** | **T** | **A** | **T** | **T** | **A** | **C** | **T** | **T** | **C** | **T** | **C** | **A** | **A** | **A** | **A** | **A** | **G** | **C** | **A** | **C** | **T** | **T** | **T** | **T** | **G** | **A** | **A** | **G** | **T** | **T** | **T** | **C** | **G** | **T** | **T** | **T** | **G** | **A** | **A** | **T** | **T** | **A** | **T** | **C** | **A** | **T** | **G** | **A** | **T** | **C** | **A** | **C** | **C** | **G** | **G** | **A** | **A** | **A** | **A** | **T** | **T** | **G** | **T** | **T** | **G** | **T** | **T** | **A** | **T** | **T** | **G** | **A** | **C** | **G** | **G** | **A** | **A** | **A** | **A** | **G** | **T** | **A** | **G** | **G** | **C** | **T** | **A** | **C** | **A** | **T** | **T** | **G** | **G** | **C** | **G** | **G** | **C** | **T** | **T** | **T** | **A** | **A** | **T** | **G** | **T** | **T** | **G** | **C** | **C** | **G** | **A** | **T** | **C** | **A** | **A** | **T** | **A** | **T** | **G** | **C** | **C** | **G** | **G** | **A** | **A** | **C** | **G** | **A** | **C** | **T** | **A** | **A** | **A** | **A** | **A** | **G** | **T** | **T** | **T** | **G** | **G** | **C** | **T** | **A** | **T** | **T** | **G** | **G** | **C** | **G** | **G** | **G** | **A** | **T** | **A** | **C** | **A** | **C** | **A** | **T** | **T** | **T** | **A** | **C** | **G** | **G** | **A** | **T** | **T** | **C** | **A** | **A** | **G** | **G** | **G** | **C** | **C** | **A** | **G** | **C** | **A** | **G** | **C** | **C** | **T** | **C** | **A** | **T** | **T** | **A** | **C** | **T** | **G** | **C** | **A** | **A** | **A** | **T** | **G** | **C** | **G** | **T** | **T** | **T** | **T** | **T** | **T** | **A** | **A** | **T** | **G** | **G** | **A** | **T** | **T** | **G** | **G** | **A** | **A** | **C** | **G** | **T** | **C** | **T** | **C** | **T** | **T** | **C** | **C** | **C** | **C** | **C** | **G** | **A** | **G** | **A** | **A** | **A** | **A** | **A** | **T** | **C** | **G** | **T** | **G** | **T** | **G** | **G** | **C** | **G** | **T** | **A** | **T** | **C** | **A** | **A** | **T** | **T** | **G** | **G** | **A** | **T** | **T** | **A** | **T** | **T** | **T** | **C** | **T** | **T** | **T** | **A** | **A** | **A** | **C** | **T** | **T** | **G** | **A** | **A** | **G** | **C** | **A** | **T** | **T** | **G** | **G** | **T** | **G** | **C** | **C** | **A** | **G** | **A** | **G** | **G** | **C** | **A** | **A** | **A** | **T** | **A** | **C** | **A** | **T** | **C** | **C** | **A** | **T** | **T** | **C** | **A** | **G** | **A** | **T** | **G** | **A** | **T** | **T** | **G** | **C** | **C** | **A** | **G** | **T** | **G** | **G** | **T** | **C** | **C** | **T** | **A** | **A** | **C** | **A** | **G** | **T** | **G** | **A** | **C** | **C** | **G** | **T** | **G** | **A** | **A** | **C** | **A** | **A** | **A** | **T** | **T** | **A** | **A** | **A** | **T** | **T** | **G** | **G** | **C** | **C** | **T** | **T** | **T** | **A** | **T** | **T** | **A** | **A** | **A** | **T** | **T** | **G** | **A** | **T** | **T** | **A** | **C** | **T** | **T** | **C** | **T** | **G** | **C** | **C** | **A** | **A** | **G** | **A** | **A** | **A** | **A** | **G** | **A** | **G** | **T** | **C** | **T** | **G** | **G** | **A** | **T** | **T** | **C** | **A** | **A** | **A** | **C** | **G** | **C** | **C** | **G** | **T** | **A** | **T** | **T** | **T** | **A** | **G** | **T** | **T** | **C** | **C** | **T** | **G** | **A** | **T** | **G** | **A** | **T** | **A** | **G** | **T** | **G** | **T** | **C** | **T** | **T** | **G** | **G** | **C** | **T** | **G** | **C** | **T** | **T** | **T** | **A** | **A** | **A** | **G** | **G** | **T** | **T** | **G** | **C** | **T** | **G** | **C** | **G** | **G** | **C** | **T** | **T** | **C** | **A** | **G** | **G** | **A** | **G** | **T** | **A** | **G** | **A** | **T** | **G** | **T** | **T** | **A** | **A** | **A** | **A** | **T** | **T** | **A** | **T** | **G** | **A** | **T** | **T** | **C** | **C** | **A** | **G** | **A** | **T** | **A** | **A** | **G** | **C** | **C** | **C** | **G** | **A** | **C** | **C** | **A** | **T** | **C** | **C** | **G** | **T** | **T** | **T** | **A** | **T** | **T** | **T** | **A** | **T** | **C** | **G** | **A** | **G** | **C** | **A** | **A** | **C** | **A** | **C** | **A** | **G** | **T** | **A** | **T** | **T** | **A** | **C** | **G** | **G** | **C** | **C** | **G** | **C** | **T** | **T** | **A** | **T** | **T** | **G** | **A** | **T** | **G** | **A** | **A** | **A** | **G** | **A** | **A** | **A** | **A** | **T** | **A** | **T** | **T** | **G** | **A** | **A** | **A** | **T** | **T** | **T** | **T** | **A** | **A** | **T** | **T** | **T** | **A** | **T** | **A** | **A** | **C** | **G** | **G** | **T** | **G** | **G** | **T** | **T** | **T** | **C** | **T** | **T** | **A** | **C** | **A** | **T** | **G** | **C** | **G** | **A** | **A** | **A** | **A** | **C** | **A** | **A** | **T** | **G** | **A** | **T** | **T** | **A** | **T** | **G** | **G** | **A** | **T** | **G** | **A** | **T** | **G** | **A** | **A** | **G** | **T** | **C** | **T** | **G** | **C** | **A** | **C** | **A** | **G** | **T** | **T** | **G** | **G** | **T** | **T** | **C** | **A** | **G** | **C** | **C** | **A** | **A** | **T** | **C** | **A** | **A** | **G** | **A** | **T** | **A** | **T** | **C** | **C** | **G** | **A** | **A** | **G** | **T** | **T** | **A** | **C** | **A** | **A** | **A** | **T** | **T** | **A** | **A** | **A** | **C** | **T** | **T** | **T** | **G** | **A** | **A** | **G** | **C** | **A** | **A** | **A** | **T** | **G** | **C** | **T** | **G** | **T** | **G** | **T** | **T** | **A** | **T** | **A** | **T** | **G** | **A** | **T** | **A** | **A** | **A** | **A** | **A** | **A** | **A** | **T** | **C** | **A** | **T** | **T** | **G** | **A** | **T** | **C** | **A** | **A** | **T** | **T** | **A** | **G** | **A** | **A** | **G** | **C** | **A** | **A** | **T** | **T** | **T** | **T** | **C** | **T** | **T** | **A** | **G** | **A** | **A** | **G** | **A** | **T** | **C** | **G** | **A** | **A** | **A** | **A** | **A** | **A** | **A** | **T** | **G** | **T** | **A** | **C** | **A** | **A** | **C** | **A** | **A** | **T** | **G** | **A** | **C** | **T** | **C** | **C** | **A** | **G** | **A** | **A** | **G** | **T** | **T** | **G** | **T** | **T** | **C** | **G** | **C** | **G** | **A** | **C** | **A** | **T** | **G** | **T** | **C** | **A** | **A** | **A** | **A** | **T** | **G** | **G** | **T** | **T** | **G** | **A** | **T** | **T** | **T** | **T** | **T** | **A** | **A** | **A** | **C** | **A** | **A** | **C** | **A** | **A** | **A** | **T** | **T** | **T** | **C** | **A** | **C** | **G** | **A** | **T** | **T** | **A** | **T** | **T** | **T** | **T** | **C** | **A** | **C** | **C** | **A** | **A** | **T** | **T** | **C** | **T** | **T** | **-** | **-** | **-** |
| **N59963F** | **T** | **T** | **G** | **A** | **T** | **T** | **C** | **T** | **T** | **A** | **G** | **C** | **G** | **T** | **C** | **T** | **T** | **A** | **A** | **C** | **A** | **G** | **T** | **T** | **A** | **T** | **T** | **T** | **A** | **T** | **T** | **T** | **T** | **A** | **T** | **T** | **A** | **A** | **T** | **G** | **C** | **A** | **A** | **T** | **T** | **A** | **T** | **T** | **G** | **C** | **G** | **G** | **G** | **A** | **A** | **T** | **T** | **A** | **C** | **C** | **A** | **T** | **T** | **T** | **T** | **G** | **T** | **T** | **A** | **A** | **A** | **A** | **C** | **C** | **T** | **C** | **G** | **A** | **G** | **A** | **T** | **G** | **T** | **A** | **G** | **C** | **A** | **G** | **C** | **A** | **A** | **T** | **T** | **T** | **G** | **G** | **G** | **C** | **A** | **T** | **G** | **G** | **C** | **T** | **A** | **C** | **T** | **C** | **G** | **T** | **A** | **C** | **T** | **G** | **A** | **T** | **T** | **G** | **C** | **T** | **C** | **T** | **A** | **C** | **C** | **C** | **G** | **T** | **T** | **T** | **T** | **T** | **G** | **G** | **T** | **T** | **T** | **T** | **T** | **T** | **C** | **T** | **T** | **A** | **T** | **A** | **T** | **T** | **T** | **A** | **T** | **T** | **C** | **T** | **T** | **T** | **G** | **G** | **G** | **C** | **G** | **C** | **G** | **G** | **T** | **T** | **T** | **A** | **A** | **C** | **C** | **G** | **A** | **C** | **A** | **A** | **A** | **A** | **A** | **G** | **A** | **A** | **A** | **T** | **T** | **T** | **T** | **A** | **T** | **T** | **T** | **G** | **C** | **A** | **A** | **C** | **A** | **A** | **A** | **G** | **T** | **G** | **A** | **T** | **T** | **T** | **G** | **C** | **G** | **G** | **G** | **A** | **A** | **T** | **T** | **A** | **G** | **A** | **G** | **A** | **A** | **T** | **T** | **T** | **T** | **C** | **A** | **G** | **A** | **A** | **T** | **T** | **T** | **C** | **C** | **A** | **A** | **G** | **A** | **A** | **G** | **A** | **A** | **A** | **G** | **T** | **T** | **T** | **T** | **G** | **A** | **A** | **C** | **T** | **T** | **T** | **A** | **T** | **A** | **G** | **T** | **C** | **A** | **A** | **A** | **A** | **G** | **A** | **T** | **G** | **C** | **C** | **G** | **A** | **C** | **G** | **G** | **A** | **G** | **G** | **A** | **G** | **C** | **A** | **C** | **C** | **A** | **A** | **C** | **A** | **G** | **T** | **T** | **T** | **A** | **C** | **A** | **G** | **A** | **C** | **T** | **T** | **T** | **T** | **T** | **T** | **T** | **C** | **C** | **T** | **C** | **G** | **T** | **T** | **G** | **A** | **A** | **T** | **A** | **G** | **G** | **A** | **T** | **G** | **C** | **C** | **T** | **C** | **T** | **G** | **A** | **C** | **T** | **A** | **A** | **G** | **A** | **A** | **A** | **A** | **A** | **T** | **G** | **A** | **T** | **G** | **T** | **T** | **C** | **A** | **A** | **A** | **T** | **T** | **T** | **T** | **T** | **A** | **C** | **G** | **G** | **A** | **T** | **G** | **G** | **T** | **A** | **C** | **G** | **G** | **A** | **A** | **A** | **A** | **A** | **T** | **T** | **C** | **A** | **A** | **T** | **G** | **C** | **G** | **T** | **T** | **G** | **A** | **T** | **G** | **G** | **C** | **G** | **G** | **A** | **C** | **A** | **T** | **A** | **A** | **A** | **A** | **A** | **A** | **A** | **G** | **C** | **T** | **C** | **A** | **G** | **C** | **A** | **C** | **T** | **C** | **C** | **A** | **T** | **T** | **C** | **A** | **T** | **A** | **T** | **T** | **G** | **A** | **A** | **T** | **A** | **T** | **T** | **A** | **T** | **G** | **C** | **C** | **T** | **T** | **T** | **G** | **T** | **A** | **A** | **C** | **G** | **G** | **A** | **T** | **C** | **A** | **T** | **A** | **T** | **C** | **G** | **G** | **C** | **A** | **C** | **C** | **A** | **A** | **A** | **A** | **T** | **T** | **T** | **T** | **G** | **A** | **A** | **C** | **T** | **T** | **A** | **T** | **T** | **A** | **G** | **A** | **A** | **G** | **A** | **A** | **A** | **A** | **G** | **G** | **C** | **G** | **G** | **C** | **T** | **G** | **A** | **A** | **G** | **G** | **G** | **G** | **T** | **T** | **G** | **A** | **A** | **G** | **T** | **G** | **C** | **G** | **G** | **T** | **T** | **G** | **C** | **T** | **T** | **T** | **A** | **C** | **G** | **A** | **T** | **G** | **C** | **C** | **T** | **T** | **T** | **G** | **G** | **C** | **T** | **C** | **A** | **A** | **A** | **G** | **G** | **G** | **C** | **A** | **C** | **C** | **A** | **A** | **G** | **G** | **T** | **T** | **C** | **A** | **T** | **C** | **A** | **T** | **T** | **T** | **G** | **A** | **A** | **T** | **G** | **A** | **A** | **T** | **T** | **G** | **A** | **A** | **A** | **A** | **A** | **A** | **A** | **A** | **C** | **G** | **G** | **T** | **G** | **G** | **T** | **T** | **T** | **T** | **G** | **T** | **C** | **C** | **A** | **A** | **A** | **C** | **G** | **T** | **T** | **T** | **A** | **T** | **T** | **A** | **C** | **T** | **T** | **C** | **T** | **C** | **A** | **A** | **A** | **A** | **A** | **G** | **C** | **A** | **C** | **T** | **T** | **T** | **T** | **G** | **A** | **A** | **G** | **T** | **T** | **T** | **C** | **G** | **T** | **T** | **T** | **G** | **A** | **A** | **T** | **T** | **A** | **T** | **C** | **A** | **T** | **G** | **A** | **T** | **C** | **A** | **C** | **C** | **G** | **G** | **A** | **A** | **A** | **A** | **T** | **T** | **G** | **T** | **T** | **G** | **T** | **T** | **A** | **T** | **T** | **G** | **A** | **C** | **G** | **G** | **A** | **A** | **A** | **A** | **G** | **T** | **A** | **G** | **G** | **C** | **T** | **A** | **C** | **A** | **T** | **T** | **G** | **G** | **C** | **G** | **G** | **C** | **T** | **T** | **T** | **A** | **A** | **T** | **G** | **T** | **T** | **G** | **C** | **C** | **G** | **A** | **T** | **C** | **A** | **A** | **T** | **A** | **T** | **G** | **C** | **C** | **G** | **G** | **A** | **A** | **C** | **G** | **A** | **C** | **T** | **A** | **A** | **A** | **A** | **A** | **G** | **T** | **T** | **T** | **G** | **G** | **C** | **T** | **A** | **T** | **T** | **G** | **G** | **C** | **G** | **G** | **G** | **A** | **T** | **A** | **C** | **A** | **C** | **A** | **T** | **T** | **T** | **A** | **C** | **G** | **G** | **A** | **T** | **T** | **C** | **A** | **A** | **G** | **G** | **G** | **C** | **C** | **A** | **G** | **C** | **A** | **G** | **C** | **C** | **T** | **C** | **A** | **T** | **T** | **A** | **C** | **T** | **G** | **C** | **A** | **A** | **A** | **T** | **G** | **C** | **G** | **T** | **T** | **T** | **T** | **T** | **T** | **A** | **A** | **T** | **G** | **G** | **A** | **T** | **T** | **G** | **G** | **A** | **A** | **C** | **G** | **T** | **C** | **T** | **C** | **T** | **T** | **C** | **C** | **C** | **C** | **C** | **G** | **A** | **G** | **A** | **A** | **A** | **A** | **A** | **T** | **C** | **G** | **T** | **G** | **T** | **G** | **G** | **C** | **G** | **T** | **A** | **T** | **C** | **A** | **A** | **T** | **T** | **G** | **G** | **A** | **T** | **T** | **A** | **T** | **T** | **T** | **C** | **T** | **T** | **T** | **A** | **A** | **A** | **C** | **T** | **T** | **G** | **A** | **A** | **G** | **C** | **A** | **T** | **T** | **G** | **G** | **T** | **G** | **C** | **C** | **A** | **G** | **A** | **G** | **G** | **C** | **A** | **A** | **A** | **T** | **A** | **C** | **A** | **T** | **C** | **C** | **A** | **T** | **T** | **C** | **A** | **G** | **A** | **T** | **G** | **A** | **T** | **T** | **G** | **C** | **C** | **A** | **G** | **T** | **G** | **G** | **T** | **C** | **C** | **T** | **A** | **A** | **C** | **A** | **G** | **T** | **G** | **A** | **C** | **C** | **G** | **T** | **G** | **A** | **A** | **C** | **A** | **A** | **A** | **T** | **T** | **A** | **A** | **A** | **T** | **T** | **G** | **G** | **C** | **C** | **T** | **T** | **T** | **A** | **T** | **T** | **A** | **A** | **A** | **T** | **T** | **G** | **A** | **T** | **T** | **A** | **C** | **T** | **T** | **C** | **T** | **G** | **C** | **C** | **A** | **A** | **G** | **A** | **A** | **A** | **A** | **G** | **A** | **G** | **T** | **C** | **T** | **G** | **G** | **A** | **T** | **T** | **C** | **A** | **A** | **A** | **C** | **G** | **C** | **C** | **G** | **T** | **A** | **T** | **T** | **T** | **A** | **G** | **T** | **T** | **C** | **C** | **T** | **G** | **A** | **T** | **G** | **A** | **T** | **A** | **G** | **T** | **G** | **T** | **C** | **T** | **T** | **G** | **G** | **C** | **T** | **G** | **C** | **T** | **T** | **T** | **A** | **A** | **A** | **G** | **G** | **T** | **T** | **G** | **C** | **T** | **G** | **C** | **G** | **G** | **C** | **T** | **T** | **C** | **A** | **G** | **G** | **A** | **G** | **T** | **A** | **G** | **A** | **T** | **G** | **T** | **T** | **A** | **A** | **A** | **A** | **T** | **T** | **A** | **T** | **G** | **A** | **T** | **T** | **C** | **C** | **A** | **G** | **A** | **T** | **A** | **A** | **G** | **C** | **C** | **C** | **G** | **A** | **C** | **C** | **A** | **T** | **C** | **C** | **G** | **T** | **T** | **T** | **A** | **T** | **T** | **T** | **A** | **T** | **C** | **G** | **A** | **G** | **C** | **A** | **A** | **C** | **A** | **C** | **A** | **G** | **T** | **A** | **T** | **T** | **A** | **C** | **G** | **G** | **C** | **C** | **G** | **C** | **T** | **T** | **A** | **T** | **T** | **G** | **A** | **T** | **G** | **A** | **A** | **A** | **G** | **A** | **A** | **A** | **A** | **T** | **A** | **T** | **T** | **G** | **A** | **A** | **A** | **T** | **T** | **T** | **T** | **A** | **A** | **T** | **T** | **T** | **A** | **T** | **A** | **A** | **C** | **G** | **G** | **T** | **G** | **G** | **T** | **T** | **T** | **C** | **T** | **T** | **A** | **C** | **A** | **T** | **G** | **C** | **G** | **A** | **A** | **A** | **A** | **C** | **A** | **A** | **T** | **G** | **A** | **T** | **T** | **A** | **T** | **G** | **G** | **A** | **T** | **G** | **A** | **T** | **G** | **A** | **A** | **G** | **T** | **C** | **T** | **G** | **C** | **A** | **C** | **A** | **G** | **T** | **T** | **G** | **G** | **T** | **T** | **C** | **A** | **G** | **C** | **C** | **A** | **A** | **T** | **C** | **A** | **A** | **G** | **A** | **T** | **A** | **T** | **C** | **C** | **G** | **A** | **A** | **G** | **T** | **T** | **A** | **C** | **A** | **A** | **A** | **T** | **T** | **A** | **A** | **A** | **C** | **T** | **T** | **T** | **G** | **A** | **A** | **G** | **C** | **A** | **A** | **A** | **T** | **G** | **C** | **T** | **G** | **T** | **G** | **T** | **T** | **A** | **T** | **A** | **T** | **G** | **A** | **T** | **A** | **A** | **A** | **A** | **A** | **A** | **A** | **T** | **C** | **A** | **T** | **T** | **G** | **A** | **T** | **C** | **A** | **A** | **T** | **T** | **A** | **G** | **A** | **A** | **G** | **C** | **A** | **A** | **T** | **T** | **T** | **T** | **C** | **T** | **T** | **A** | **G** | **A** | **A** | **G** | **A** | **T** | **C** | **G** | **A** | **A** | **A** | **A** | **A** | **A** | **A** | **T** | **G** | **T** | **A** | **C** | **A** | **A** | **C** | **A** | **A** | **T** | **G** | **A** | **C** | **T** | **C** | **C** | **A** | **G** | **A** | **A** | **G** | **T** | **T** | **G** | **T** | **T** | **C** | **G** | **C** | **G** | **A** | **C** | **A** | **T** | **G** | **T** | **C** | **A** | **A** | **A** | **A** | **T** | **G** | **G** | **T** | **T** | **G** | **A** | **T** | **T** | **T** | **T** | **T** | **A** | **A** | **A** | **C** | **A** | **A** | **C** | **A** | **A** | **A** | **T** | **T** | **T** | **C** | **A** | **C** | **G** | **A** | **T** | **T** | **A** | **T** | **T** | **T** | **T** | **C** | **A** | **C** | **C** | **A** | **A** | **T** | **T** | **C** | **T** | **T** | **-** | **-** | **-** |
| **N53519** | **T** | **T** | **G** | **A** | **T** | **T** | **C** | **T** | **T** | **A** | **G** | **C** | **G** | **T** | **C** | **T** | **T** | **A** | **A** | **C** | **A** | **G** | **T** | **T** | **A** | **T** | **T** | **T** | **A** | **T** | **T** | **T** | **T** | **A** | **T** | **T** | **A** | **A** | **T** | **G** | **C** | **A** | **A** | **T** | **T** | **A** | **T** | **T** | **G** | **C** | **G** | **G** | **G** | **A** | **A** | **T** | **T** | **A** | **C** | **C** | **A** | **T** | **T** | **T** | **T** | **G** | **T** | **T** | **A** | **A** | **A** | **A** | **C** | **C** | **T** | **C** | **G** | **A** | **G** | **A** | **T** | **G** | **T** | **A** | **G** | **C** | **A** | **G** | **C** | **A** | **A** | **T** | **T** | **T** | **G** | **G** | **G** | **C** | **A** | **T** | **G** | **G** | **C** | **T** | **A** | **C** | **T** | **C** | **G** | **T** | **A** | **C** | **T** | **G** | **A** | **T** | **T** | **G** | **C** | **T** | **C** | **T** | **A** | **C** | **C** | **C** | **G** | **T** | **T** | **T** | **T** | **T** | **G** | **G** | **T** | **T** | **T** | **T** | **T** | **T** | **C** | **T** | **T** | **A** | **T** | **A** | **T** | **T** | **T** | **A** | **T** | **T** | **C** | **T** | **T** | **T** | **G** | **G** | **G** | **C** | **G** | **C** | **G** | **G** | **T** | **T** | **T** | **A** | **A** | **C** | **C** | **G** | **A** | **C** | **A** | **A** | **A** | **A** | **A** | **G** | **A** | **A** | **A** | **T** | **T** | **T** | **T** | **A** | **T** | **T** | **T** | **G** | **C** | **A** | **A** | **C** | **A** | **A** | **A** | **G** | **T** | **G** | **A** | **T** | **T** | **T** | **A** | **C** | **G** | **G** | **G** | **A** | **A** | **T** | **T** | **A** | **G** | **A** | **G** | **A** | **A** | **T** | **T** | **T** | **C** | **C** | **A** | **G** | **A** | **A** | **T** | **T** | **T** | **C** | **C** | **A** | **A** | **G** | **A** | **A** | **G** | **A** | **A** | **A** | **G** | **T** | **T** | **T** | **T** | **G** | **A** | **A** | **C** | **T** | **T** | **T** | **A** | **T** | **A** | **G** | **T** | **C** | **A** | **A** | **A** | **A** | **G** | **A** | **T** | **G** | **C** | **C** | **G** | **A** | **C** | **G** | **G** | **A** | **G** | **G** | **A** | **G** | **C** | **A** | **A** | **C** | **A** | **A** | **C** | **A** | **G** | **T** | **T** | **T** | **A** | **C** | **A** | **G** | **A** | **C** | **T** | **T** | **T** | **T** | **T** | **T** | **T** | **C** | **C** | **T** | **C** | **G** | **T** | **T** | **A** | **A** | **A** | **T** | **A** | **G** | **G** | **A** | **T** | **G** | **C** | **C** | **T** | **C** | **T** | **G** | **A** | **C** | **T** | **A** | **A** | **G** | **A** | **A** | **A** | **A** | **A** | **T** | **G** | **A** | **T** | **G** | **T** | **T** | **G** | **A** | **A** | **A** | **T** | **T** | **T** | **T** | **T** | **A** | **C** | **G** | **G** | **A** | **T** | **G** | **G** | **T** | **A** | **C** | **G** | **G** | **A** | **A** | **A** | **A** | **A** | **T** | **T** | **C** | **A** | **A** | **T** | **G** | **C** | **G** | **T** | **T** | **G** | **A** | **T** | **G** | **G** | **C** | **G** | **G** | **A** | **C** | **A** | **T** | **A** | **A** | **A** | **A** | **A** | **A** | **A** | **G** | **C** | **T** | **C** | **A** | **G** | **C** | **A** | **C** | **T** | **C** | **C** | **A** | **T** | **T** | **C** | **A** | **T** | **A** | **T** | **T** | **G** | **A** | **A** | **T** | **A** | **T** | **T** | **A** | **T** | **G** | **C** | **C** | **T** | **T** | **T** | **G** | **T** | **A** | **A** | **C** | **G** | **G** | **A** | **T** | **C** | **A** | **T** | **A** | **T** | **C** | **G** | **G** | **C** | **A** | **C** | **C** | **A** | **A** | **A** | **A** | **T** | **T** | **T** | **T** | **G** | **A** | **A** | **C** | **T** | **T** | **A** | **T** | **T** | **A** | **G** | **A** | **A** | **G** | **A** | **A** | **A** | **A** | **G** | **G** | **C** | **G** | **G** | **C** | **T** | **G** | **A** | **A** | **G** | **G** | **G** | **G** | **T** | **T** | **G** | **A** | **A** | **G** | **T** | **G** | **C** | **G** | **G** | **T** | **T** | **G** | **C** | **T** | **T** | **T** | **A** | **C** | **G** | **A** | **T** | **G** | **C** | **C** | **T** | **T** | **T** | **G** | **G** | **C** | **T** | **C** | **A** | **A** | **A** | **G** | **G** | **G** | **C** | **A** | **C** | **C** | **A** | **A** | **G** | **G** | **T** | **T** | **C** | **A** | **T** | **C** | **A** | **T** | **T** | **T** | **G** | **A** | **A** | **T** | **G** | **A** | **A** | **T** | **T** | **G** | **A** | **A** | **A** | **A** | **A** | **A** | **A** | **A** | **C** | **G** | **G** | **C** | **G** | **G** | **T** | **T** | **T** | **T** | **G** | **T** | **C** | **C** | **A** | **A** | **A** | **C** | **A** | **T** | **T** | **T** | **A** | **T** | **T** | **A** | **C** | **T** | **T** | **C** | **T** | **C** | **A** | **A** | **A** | **A** | **A** | **G** | **C** | **A** | **C** | **T** | **T** | **T** | **T** | **G** | **A** | **A** | **G** | **T** | **T** | **T** | **C** | **G** | **T** | **T** | **T** | **G** | **A** | **A** | **T** | **T** | **A** | **T** | **C** | **A** | **T** | **G** | **A** | **T** | **C** | **A** | **C** | **C** | **G** | **G** | **A** | **A** | **A** | **A** | **T** | **T** | **G** | **T** | **T** | **G** | **T** | **T** | **A** | **T** | **T** | **G** | **A** | **C** | **G** | **G** | **A** | **A** | **A** | **A** | **G** | **T** | **A** | **G** | **G** | **C** | **T** | **A** | **C** | **A** | **T** | **T** | **G** | **G** | **C** | **G** | **G** | **C** | **T** | **T** | **T** | **A** | **A** | **T** | **G** | **T** | **T** | **G** | **C** | **C** | **G** | **A** | **T** | **C** | **A** | **A** | **T** | **A** | **T** | **G** | **C** | **C** | **G** | **G** | **A** | **A** | **C** | **G** | **A** | **C** | **T** | **A** | **A** | **A** | **A** | **A** | **G** | **T** | **T** | **T** | **G** | **G** | **C** | **T** | **A** | **T** | **T** | **G** | **G** | **C** | **G** | **G** | **G** | **A** | **T** | **A** | **C** | **G** | **C** | **A** | **T** | **T** | **T** | **A** | **C** | **G** | **G** | **A** | **T** | **T** | **C** | **A** | **A** | **G** | **G** | **G** | **C** | **C** | **A** | **G** | **C** | **A** | **G** | **C** | **C** | **T** | **C** | **A** | **T** | **T** | **A** | **C** | **T** | **G** | **C** | **A** | **A** | **A** | **T** | **G** | **C** | **G** | **T** | **T** | **T** | **T** | **T** | **T** | **A** | **A** | **T** | **G** | **G** | **A** | **T** | **T** | **G** | **G** | **A** | **A** | **C** | **G** | **T** | **C** | **T** | **C** | **T** | **T** | **C** | **C** | **C** | **C** | **C** | **G** | **A** | **G** | **A** | **A** | **A** | **A** | **A** | **T** | **C** | **G** | **T** | **G** | **T** | **G** | **G** | **C** | **G** | **T** | **A** | **T** | **C** | **A** | **A** | **T** | **T** | **G** | **G** | **A** | **T** | **T** | **A** | **T** | **T** | **T** | **C** | **T** | **T** | **T** | **A** | **A** | **A** | **C** | **T** | **T** | **G** | **A** | **A** | **G** | **C** | **A** | **T** | **T** | **G** | **G** | **T** | **G** | **C** | **C** | **A** | **G** | **A** | **G** | **G** | **C** | **A** | **A** | **A** | **T** | **A** | **C** | **A** | **T** | **C** | **C** | **A** | **T** | **T** | **C** | **A** | **G** | **A** | **T** | **G** | **A** | **T** | **T** | **G** | **C** | **C** | **A** | **G** | **T** | **G** | **G** | **T** | **C** | **C** | **T** | **A** | **A** | **C** | **A** | **G** | **T** | **G** | **A** | **C** | **C** | **G** | **T** | **G** | **A** | **A** | **C** | **A** | **A** | **A** | **T** | **T** | **A** | **A** | **A** | **T** | **T** | **G** | **G** | **C** | **C** | **T** | **T** | **T** | **A** | **T** | **T** | **A** | **A** | **A** | **T** | **T** | **G** | **A** | **T** | **T** | **A** | **C** | **T** | **T** | **C** | **T** | **G** | **C** | **C** | **A** | **A** | **G** | **A** | **A** | **A** | **A** | **G** | **A** | **G** | **T** | **C** | **T** | **G** | **G** | **A** | **T** | **T** | **C** | **A** | **A** | **A** | **C** | **G** | **C** | **C** | **G** | **T** | **A** | **T** | **T** | **T** | **A** | **G** | **T** | **T** | **C** | **C** | **T** | **G** | **A** | **T** | **G** | **A** | **T** | **A** | **G** | **T** | **G** | **T** | **C** | **T** | **T** | **G** | **G** | **C** | **T** | **G** | **C** | **T** | **T** | **T** | **A** | **A** | **A** | **G** | **G** | **T** | **T** | **G** | **C** | **T** | **G** | **C** | **G** | **G** | **C** | **T** | **T** | **C** | **A** | **G** | **G** | **A** | **G** | **T** | **A** | **G** | **A** | **T** | **G** | **T** | **T** | **A** | **A** | **A** | **A** | **T** | **T** | **A** | **T** | **G** | **A** | **T** | **T** | **C** | **C** | **A** | **G** | **A** | **T** | **A** | **A** | **G** | **C** | **C** | **C** | **G** | **A** | **C** | **C** | **A** | **T** | **C** | **C** | **G** | **T** | **T** | **T** | **A** | **T** | **T** | **T** | **A** | **T** | **C** | **G** | **A** | **G** | **C** | **A** | **A** | **C** | **A** | **C** | **A** | **G** | **T** | **A** | **T** | **T** | **A** | **T** | **G** | **G** | **C** | **C** | **G** | **C** | **T** | **T** | **A** | **T** | **T** | **G** | **A** | **T** | **G** | **A** | **A** | **A** | **G** | **A** | **A** | **A** | **A** | **T** | **A** | **T** | **T** | **G** | **A** | **A** | **A** | **T** | **T** | **T** | **T** | **A** | **A** | **T** | **T** | **T** | **A** | **T** | **A** | **A** | **C** | **G** | **G** | **T** | **G** | **G** | **T** | **T** | **T** | **C** | **T** | **T** | **A** | **C** | **A** | **T** | **G** | **C** | **G** | **A** | **A** | **A** | **A** | **C** | **A** | **A** | **T** | **G** | **A** | **T** | **T** | **A** | **T** | **G** | **G** | **A** | **T** | **G** | **A** | **T** | **G** | **A** | **A** | **G** | **T** | **C** | **T** | **G** | **T** | **A** | **C** | **A** | **G** | **T** | **T** | **G** | **G** | **T** | **T** | **C** | **A** | **G** | **C** | **C** | **A** | **A** | **T** | **C** | **A** | **A** | **G** | **A** | **T** | **A** | **T** | **C** | **C** | **G** | **A** | **A** | **G** | **T** | **T** | **A** | **C** | **A** | **A** | **A** | **T** | **T** | **A** | **A** | **A** | **C** | **T** | **T** | **T** | **G** | **A** | **A** | **G** | **C** | **A** | **A** | **A** | **T** | **G** | **C** | **T** | **G** | **T** | **G** | **T** | **T** | **A** | **T** | **A** | **T** | **G** | **A** | **T** | **A** | **A** | **A** | **A** | **A** | **A** | **A** | **T** | **C** | **A** | **T** | **T** | **G** | **A** | **T** | **C** | **A** | **A** | **T** | **T** | **A** | **G** | **A** | **A** | **G** | **C** | **A** | **A** | **T** | **T** | **T** | **T** | **C** | **T** | **T** | **A** | **G** | **A** | **A** | **G** | **A** | **T** | **C** | **G** | **A** | **A** | **A** | **A** | **A** | **A** | **A** | **T** | **G** | **T** | **A** | **C** | **A** | **A** | **C** | **A** | **A** | **T** | **G** | **A** | **C** | **A** | **C** | **C** | **A** | **G** | **A** | **A** | **G** | **T** | **T** | **G** | **T** | **T** | **C** | **G** | **A** | **G** | **A** | **C** | **A** | **T** | **G** | **T** | **C** | **C** | **A** | **A** | **A** | **T** | **G** | **G** | **T** | **T** | **G** | **A** | **T** | **T** | **T** | **T** | **T** | **A** | **A** | **A** | **C** | **A** | **A** | **C** | **A** | **A** | **A** | **T** | **T** | **T** | **C** | **A** | **C** | **G** | **A** | **T** | **T** | **A** | **T** | **T** | **T** | **T** | **C** | **C** | **C** | **C** | **A** | **A** | **T** | **T** | **C** | **T** | **T** | **-** | **-** | **-** |
| **N54121** | **T** | **T** | **G** | **A** | **T** | **T** | **C** | **T** | **T** | **A** | **G** | **C** | **G** | **T** | **C** | **T** | **T** | **A** | **A** | **C** | **A** | **G** | **T** | **T** | **A** | **T** | **T** | **T** | **A** | **T** | **T** | **T** | **T** | **A** | **T** | **T** | **A** | **A** | **T** | **G** | **C** | **A** | **A** | **T** | **T** | **A** | **T** | **T** | **G** | **C** | **G** | **G** | **G** | **A** | **A** | **T** | **T** | **A** | **C** | **C** | **A** | **T** | **T** | **T** | **T** | **G** | **T** | **T** | **A** | **A** | **A** | **A** | **C** | **C** | **T** | **C** | **G** | **A** | **G** | **A** | **T** | **G** | **T** | **A** | **G** | **C** | **A** | **G** | **C** | **A** | **A** | **T** | **T** | **T** | **G** | **G** | **G** | **C** | **A** | **T** | **G** | **G** | **C** | **T** | **A** | **C** | **T** | **C** | **G** | **T** | **A** | **C** | **T** | **G** | **A** | **T** | **T** | **G** | **C** | **T** | **C** | **T** | **A** | **C** | **C** | **C** | **G** | **T** | **T** | **T** | **T** | **T** | **G** | **G** | **T** | **T** | **T** | **T** | **T** | **T** | **C** | **T** | **T** | **A** | **T** | **A** | **T** | **T** | **T** | **A** | **T** | **T** | **C** | **T** | **T** | **T** | **G** | **G** | **G** | **C** | **G** | **C** | **G** | **G** | **T** | **T** | **T** | **A** | **A** | **C** | **C** | **G** | **A** | **C** | **A** | **A** | **A** | **A** | **A** | **G** | **A** | **A** | **A** | **T** | **T** | **T** | **T** | **A** | **T** | **T** | **T** | **G** | **C** | **A** | **A** | **C** | **A** | **A** | **A** | **G** | **T** | **G** | **A** | **T** | **T** | **T** | **G** | **C** | **G** | **G** | **G** | **A** | **A** | **T** | **T** | **A** | **G** | **A** | **G** | **A** | **A** | **T** | **T** | **T** | **T** | **C** | **A** | **G** | **A** | **A** | **T** | **T** | **T** | **C** | **C** | **A** | **A** | **G** | **A** | **A** | **G** | **A** | **A** | **A** | **G** | **T** | **T** | **T** | **T** | **G** | **A** | **A** | **C** | **T** | **T** | **T** | **A** | **T** | **A** | **G** | **T** | **C** | **A** | **A** | **A** | **A** | **G** | **A** | **T** | **G** | **C** | **C** | **G** | **A** | **C** | **G** | **G** | **A** | **G** | **G** | **A** | **G** | **C** | **A** | **A** | **C** | **A** | **A** | **C** | **A** | **G** | **T** | **T** | **T** | **A** | **C** | **A** | **G** | **A** | **C** | **T** | **T** | **T** | **T** | **T** | **T** | **T** | **C** | **C** | **T** | **C** | **G** | **T** | **T** | **G** | **A** | **A** | **T** | **A** | **G** | **G** | **A** | **T** | **G** | **C** | **C** | **T** | **C** | **T** | **G** | **A** | **C** | **T** | **A** | **A** | **G** | **A** | **A** | **A** | **A** | **A** | **T** | **G** | **A** | **T** | **G** | **T** | **T** | **C** | **A** | **A** | **A** | **T** | **T** | **T** | **T** | **T** | **A** | **C** | **G** | **G** | **A** | **T** | **G** | **G** | **T** | **A** | **C** | **G** | **G** | **A** | **A** | **A** | **A** | **A** | **T** | **T** | **C** | **A** | **A** | **T** | **G** | **C** | **G** | **T** | **T** | **G** | **A** | **T** | **G** | **G** | **C** | **G** | **G** | **A** | **C** | **A** | **T** | **A** | **A** | **A** | **A** | **A** | **A** | **A** | **G** | **C** | **T** | **C** | **A** | **G** | **C** | **A** | **C** | **T** | **C** | **C** | **A** | **T** | **T** | **C** | **A** | **T** | **A** | **T** | **T** | **G** | **A** | **A** | **T** | **A** | **T** | **T** | **A** | **T** | **G** | **C** | **C** | **T** | **T** | **T** | **G** | **T** | **A** | **A** | **C** | **G** | **G** | **A** | **T** | **C** | **A** | **T** | **A** | **T** | **C** | **G** | **G** | **C** | **A** | **C** | **C** | **A** | **A** | **A** | **A** | **T** | **T** | **T** | **T** | **G** | **A** | **A** | **C** | **T** | **T** | **A** | **T** | **T** | **A** | **G** | **A** | **A** | **G** | **A** | **A** | **A** | **A** | **G** | **G** | **C** | **G** | **G** | **C** | **T** | **G** | **A** | **A** | **G** | **G** | **G** | **G** | **T** | **T** | **G** | **A** | **A** | **G** | **T** | **G** | **C** | **G** | **G** | **T** | **T** | **G** | **C** | **T** | **T** | **T** | **A** | **C** | **G** | **A** | **T** | **G** | **C** | **C** | **T** | **T** | **T** | **G** | **G** | **C** | **T** | **C** | **A** | **A** | **A** | **G** | **G** | **G** | **C** | **A** | **C** | **C** | **A** | **A** | **G** | **G** | **T** | **T** | **C** | **A** | **T** | **C** | **A** | **T** | **T** | **T** | **G** | **A** | **A** | **T** | **G** | **A** | **A** | **T** | **T** | **G** | **A** | **A** | **A** | **A** | **A** | **A** | **A** | **A** | **C** | **G** | **G** | **T** | **G** | **G** | **T** | **T** | **T** | **T** | **G** | **T** | **C** | **C** | **A** | **A** | **A** | **C** | **G** | **T** | **T** | **T** | **A** | **T** | **T** | **A** | **C** | **T** | **T** | **C** | **T** | **C** | **A** | **A** | **A** | **A** | **A** | **G** | **C** | **A** | **C** | **T** | **T** | **T** | **T** | **G** | **A** | **A** | **G** | **T** | **T** | **T** | **C** | **G** | **T** | **T** | **T** | **G** | **A** | **A** | **T** | **T** | **A** | **T** | **C** | **A** | **T** | **G** | **A** | **T** | **C** | **A** | **C** | **C** | **G** | **G** | **A** | **A** | **A** | **A** | **T** | **T** | **G** | **T** | **T** | **G** | **T** | **T** | **A** | **T** | **T** | **G** | **A** | **C** | **G** | **G** | **A** | **A** | **A** | **A** | **G** | **T** | **A** | **G** | **G** | **C** | **T** | **A** | **C** | **A** | **T** | **T** | **G** | **G** | **C** | **G** | **G** | **C** | **T** | **T** | **T** | **A** | **A** | **T** | **G** | **T** | **T** | **G** | **C** | **C** | **G** | **A** | **T** | **C** | **A** | **A** | **T** | **A** | **T** | **G** | **C** | **T** | **G** | **G** | **A** | **A** | **C** | **G** | **A** | **C** | **T** | **A** | **A** | **A** | **A** | **A** | **G** | **T** | **T** | **T** | **G** | **G** | **C** | **T** | **A** | **T** | **T** | **G** | **G** | **C** | **G** | **G** | **G** | **A** | **T** | **A** | **C** | **G** | **C** | **A** | **T** | **T** | **T** | **A** | **C** | **G** | **G** | **A** | **T** | **T** | **C** | **A** | **A** | **G** | **G** | **G** | **C** | **C** | **A** | **G** | **C** | **A** | **G** | **C** | **C** | **T** | **C** | **A** | **T** | **T** | **A** | **C** | **T** | **G** | **C** | **A** | **A** | **A** | **T** | **G** | **C** | **G** | **T** | **T** | **T** | **T** | **T** | **T** | **A** | **A** | **T** | **G** | **G** | **A** | **T** | **T** | **G** | **G** | **A** | **A** | **C** | **G** | **T** | **C** | **T** | **C** | **T** | **T** | **C** | **C** | **C** | **C** | **C** | **G** | **A** | **G** | **A** | **A** | **A** | **A** | **A** | **T** | **C** | **G** | **T** | **G** | **T** | **G** | **G** | **C** | **G** | **T** | **A** | **T** | **C** | **A** | **A** | **T** | **T** | **G** | **G** | **A** | **T** | **T** | **A** | **T** | **T** | **T** | **C** | **T** | **T** | **T** | **A** | **A** | **A** | **C** | **T** | **T** | **G** | **A** | **A** | **G** | **C** | **A** | **T** | **T** | **G** | **G** | **T** | **G** | **C** | **C** | **A** | **G** | **A** | **G** | **G** | **C** | **A** | **A** | **A** | **T** | **A** | **C** | **A** | **T** | **C** | **C** | **A** | **T** | **T** | **C** | **A** | **G** | **A** | **T** | **G** | **A** | **T** | **T** | **G** | **C** | **C** | **A** | **G** | **T** | **G** | **G** | **T** | **C** | **C** | **T** | **A** | **A** | **C** | **A** | **G** | **T** | **G** | **A** | **C** | **C** | **G** | **T** | **G** | **A** | **A** | **C** | **A** | **A** | **A** | **T** | **T** | **A** | **A** | **A** | **T** | **T** | **G** | **G** | **C** | **C** | **T** | **T** | **T** | **A** | **T** | **T** | **A** | **A** | **A** | **T** | **T** | **G** | **A** | **T** | **T** | **A** | **C** | **T** | **T** | **C** | **T** | **G** | **C** | **C** | **A** | **A** | **G** | **A** | **A** | **A** | **A** | **G** | **A** | **G** | **T** | **C** | **T** | **G** | **G** | **A** | **T** | **T** | **C** | **A** | **A** | **A** | **C** | **G** | **C** | **C** | **G** | **T** | **A** | **T** | **T** | **T** | **A** | **G** | **T** | **T** | **C** | **C** | **T** | **G** | **A** | **T** | **G** | **A** | **T** | **A** | **G** | **T** | **G** | **T** | **C** | **T** | **T** | **G** | **G** | **C** | **T** | **G** | **C** | **T** | **T** | **T** | **A** | **A** | **A** | **G** | **G** | **T** | **T** | **G** | **C** | **T** | **G** | **C** | **G** | **G** | **C** | **T** | **T** | **C** | **A** | **G** | **G** | **A** | **G** | **T** | **A** | **G** | **A** | **T** | **G** | **T** | **T** | **A** | **A** | **A** | **A** | **T** | **T** | **A** | **T** | **G** | **A** | **T** | **T** | **C** | **C** | **A** | **G** | **A** | **T** | **A** | **A** | **G** | **C** | **C** | **C** | **G** | **A** | **C** | **C** | **A** | **T** | **C** | **C** | **G** | **T** | **T** | **T** | **A** | **T** | **T** | **T** | **A** | **T** | **C** | **G** | **A** | **G** | **C** | **A** | **A** | **C** | **A** | **C** | **A** | **G** | **T** | **A** | **T** | **T** | **A** | **C** | **G** | **G** | **C** | **C** | **G** | **C** | **T** | **T** | **A** | **T** | **T** | **G** | **A** | **T** | **G** | **A** | **A** | **A** | **G** | **A** | **A** | **A** | **A** | **T** | **A** | **T** | **T** | **G** | **A** | **A** | **A** | **T** | **T** | **T** | **T** | **A** | **A** | **T** | **T** | **T** | **A** | **T** | **A** | **A** | **C** | **G** | **G** | **T** | **G** | **G** | **T** | **T** | **T** | **C** | **T** | **T** | **A** | **C** | **A** | **T** | **G** | **C** | **G** | **A** | **A** | **A** | **A** | **C** | **A** | **A** | **T** | **G** | **A** | **T** | **T** | **A** | **T** | **G** | **G** | **A** | **T** | **G** | **A** | **T** | **G** | **A** | **A** | **G** | **T** | **C** | **T** | **G** | **C** | **A** | **C** | **A** | **G** | **T** | **T** | **G** | **G** | **T** | **T** | **C** | **A** | **G** | **C** | **C** | **A** | **A** | **T** | **C** | **A** | **A** | **G** | **A** | **T** | **A** | **T** | **C** | **C** | **G** | **A** | **A** | **G** | **T** | **T** | **A** | **C** | **A** | **A** | **A** | **T** | **T** | **A** | **A** | **A** | **C** | **T** | **T** | **T** | **G** | **A** | **A** | **G** | **C** | **A** | **A** | **A** | **T** | **G** | **C** | **T** | **G** | **T** | **G** | **T** | **T** | **A** | **T** | **A** | **T** | **G** | **A** | **T** | **A** | **A** | **A** | **A** | **A** | **A** | **A** | **T** | **C** | **A** | **T** | **T** | **G** | **A** | **T** | **C** | **A** | **A** | **T** | **T** | **A** | **G** | **A** | **A** | **G** | **C** | **A** | **A** | **T** | **T** | **T** | **T** | **C** | **T** | **T** | **A** | **G** | **A** | **A** | **G** | **A** | **T** | **C** | **G** | **A** | **A** | **A** | **A** | **A** | **A** | **A** | **T** | **G** | **T** | **A** | **C** | **A** | **A** | **C** | **A** | **A** | **T** | **G** | **A** | **C** | **T** | **C** | **C** | **A** | **G** | **A** | **A** | **G** | **T** | **T** | **G** | **T** | **T** | **C** | **G** | **C** | **G** | **A** | **C** | **A** | **T** | **G** | **T** | **C** | **A** | **A** | **A** | **A** | **T** | **G** | **G** | **T** | **T** | **G** | **A** | **T** | **T** | **T** | **T** | **T** | **A** | **A** | **A** | **C** | **A** | **A** | **C** | **A** | **A** | **A** | **T** | **T** | **T** | **C** | **A** | **C** | **G** | **A** | **T** | **T** | **A** | **T** | **T** | **T** | **T** | **C** | **A** | **C** | **C** | **A** | **A** | **T** | **T** | **C** | **T** | **T** | **-** | **-** | **-** |
| **N52773** | **T** | **T** | **G** | **A** | **T** | **T** | **C** | **T** | **T** | **A** | **G** | **C** | **G** | **T** | **C** | **T** | **T** | **A** | **A** | **C** | **A** | **G** | **T** | **T** | **A** | **T** | **T** | **T** | **A** | **T** | **T** | **T** | **T** | **A** | **T** | **T** | **A** | **A** | **T** | **G** | **C** | **A** | **A** | **T** | **T** | **A** | **T** | **T** | **G** | **C** | **G** | **G** | **G** | **A** | **A** | **T** | **T** | **A** | **C** | **C** | **A** | **T** | **T** | **T** | **T** | **G** | **T** | **T** | **A** | **A** | **A** | **A** | **C** | **C** | **T** | **C** | **G** | **A** | **G** | **A** | **T** | **G** | **T** | **A** | **G** | **C** | **A** | **G** | **C** | **A** | **A** | **T** | **T** | **T** | **G** | **G** | **G** | **C** | **A** | **T** | **G** | **G** | **C** | **T** | **A** | **C** | **T** | **C** | **G** | **T** | **A** | **C** | **T** | **G** | **A** | **T** | **T** | **G** | **C** | **T** | **C** | **T** | **A** | **C** | **C** | **C** | **G** | **T** | **T** | **T** | **T** | **T** | **G** | **G** | **T** | **T** | **T** | **T** | **T** | **T** | **C** | **T** | **T** | **A** | **T** | **A** | **T** | **T** | **T** | **A** | **T** | **T** | **C** | **T** | **T** | **T** | **G** | **G** | **T** | **C** | **G** | **A** | **G** | **G** | **G** | **T** | **T** | **A** | **A** | **C** | **C** | **G** | **A** | **C** | **A** | **A** | **A** | **A** | **A** | **G** | **A** | **A** | **A** | **T** | **T** | **T** | **T** | **A** | **T** | **T** | **T** | **G** | **C** | **A** | **A** | **C** | **A** | **A** | **A** | **G** | **T** | **G** | **A** | **T** | **T** | **T** | **A** | **C** | **G** | **G** | **G** | **A** | **A** | **T** | **T** | **A** | **G** | **A** | **G** | **A** | **A** | **T** | **T** | **T** | **C** | **C** | **A** | **G** | **A** | **A** | **T** | **T** | **T** | **C** | **C** | **A** | **A** | **G** | **A** | **A** | **G** | **A** | **A** | **A** | **G** | **T** | **T** | **T** | **T** | **G** | **A** | **A** | **C** | **T** | **T** | **T** | **A** | **T** | **A** | **G** | **T** | **C** | **A** | **A** | **A** | **A** | **G** | **A** | **T** | **G** | **C** | **C** | **G** | **A** | **C** | **G** | **G** | **A** | **G** | **G** | **A** | **G** | **C** | **A** | **A** | **C** | **A** | **A** | **C** | **A** | **G** | **T** | **T** | **T** | **A** | **C** | **A** | **G** | **A** | **C** | **T** | **T** | **T** | **T** | **T** | **T** | **T** | **C** | **C** | **T** | **C** | **G** | **T** | **T** | **A** | **A** | **A** | **T** | **A** | **G** | **G** | **A** | **T** | **G** | **C** | **C** | **T** | **C** | **T** | **G** | **A** | **C** | **T** | **A** | **A** | **G** | **A** | **A** | **A** | **A** | **A** | **T** | **G** | **A** | **T** | **G** | **T** | **T** | **G** | **A** | **A** | **A** | **T** | **T** | **T** | **T** | **T** | **A** | **C** | **G** | **G** | **A** | **T** | **G** | **G** | **T** | **A** | **C** | **G** | **G** | **A** | **A** | **A** | **A** | **A** | **T** | **T** | **C** | **A** | **A** | **T** | **G** | **C** | **G** | **T** | **T** | **G** | **A** | **T** | **G** | **G** | **C** | **G** | **G** | **A** | **C** | **A** | **T** | **A** | **A** | **A** | **A** | **A** | **A** | **A** | **G** | **C** | **T** | **C** | **A** | **G** | **C** | **A** | **C** | **T** | **C** | **C** | **A** | **T** | **T** | **C** | **A** | **T** | **A** | **T** | **T** | **G** | **A** | **A** | **T** | **A** | **T** | **T** | **A** | **T** | **G** | **C** | **C** | **T** | **T** | **T** | **G** | **T** | **A** | **A** | **C** | **G** | **G** | **A** | **T** | **C** | **A** | **T** | **A** | **T** | **C** | **G** | **G** | **C** | **A** | **C** | **C** | **A** | **A** | **A** | **A** | **T** | **T** | **T** | **T** | **G** | **A** | **A** | **C** | **T** | **T** | **A** | **T** | **T** | **A** | **G** | **A** | **A** | **G** | **A** | **A** | **A** | **A** | **G** | **G** | **C** | **G** | **G** | **C** | **T** | **G** | **A** | **A** | **G** | **G** | **G** | **G** | **T** | **T** | **G** | **A** | **A** | **G** | **T** | **G** | **C** | **G** | **G** | **T** | **T** | **G** | **C** | **T** | **T** | **T** | **A** | **C** | **G** | **A** | **T** | **G** | **C** | **C** | **T** | **T** | **T** | **G** | **G** | **C** | **T** | **C** | **A** | **A** | **A** | **G** | **G** | **G** | **C** | **A** | **C** | **C** | **A** | **A** | **G** | **G** | **T** | **T** | **C** | **A** | **T** | **C** | **A** | **T** | **T** | **T** | **G** | **A** | **A** | **T** | **G** | **A** | **A** | **T** | **T** | **G** | **A** | **A** | **A** | **A** | **A** | **A** | **A** | **A** | **C** | **G** | **G** | **C** | **G** | **G** | **T** | **T** | **T** | **T** | **G** | **T** | **C** | **C** | **A** | **A** | **A** | **C** | **A** | **T** | **T** | **T** | **A** | **T** | **T** | **A** | **C** | **T** | **T** | **C** | **T** | **C** | **A** | **A** | **A** | **A** | **A** | **G** | **C** | **A** | **C** | **T** | **T** | **T** | **T** | **G** | **A** | **A** | **G** | **T** | **T** | **T** | **C** | **G** | **T** | **T** | **T** | **G** | **A** | **A** | **T** | **T** | **A** | **T** | **C** | **A** | **T** | **G** | **A** | **T** | **C** | **A** | **C** | **C** | **G** | **G** | **A** | **A** | **A** | **A** | **T** | **T** | **G** | **T** | **T** | **G** | **T** | **T** | **A** | **T** | **T** | **G** | **A** | **C** | **G** | **G** | **A** | **A** | **A** | **A** | **G** | **T** | **A** | **G** | **G** | **C** | **T** | **A** | **C** | **A** | **T** | **T** | **G** | **G** | **C** | **G** | **G** | **C** | **T** | **T** | **T** | **A** | **A** | **T** | **G** | **T** | **T** | **G** | **C** | **C** | **G** | **A** | **T** | **C** | **A** | **A** | **T** | **A** | **T** | **G** | **C** | **C** | **G** | **G** | **A** | **A** | **C** | **G** | **A** | **C** | **T** | **A** | **A** | **A** | **A** | **A** | **G** | **T** | **T** | **T** | **G** | **G** | **C** | **T** | **A** | **T** | **T** | **G** | **G** | **C** | **G** | **G** | **G** | **A** | **T** | **A** | **C** | **G** | **C** | **A** | **T** | **T** | **T** | **A** | **C** | **G** | **G** | **A** | **T** | **T** | **C** | **A** | **A** | **G** | **G** | **G** | **C** | **C** | **A** | **G** | **C** | **A** | **G** | **C** | **C** | **T** | **C** | **A** | **T** | **T** | **A** | **C** | **T** | **G** | **C** | **A** | **A** | **A** | **T** | **G** | **C** | **G** | **T** | **T** | **T** | **T** | **T** | **T** | **A** | **A** | **T** | **G** | **G** | **A** | **T** | **T** | **G** | **G** | **A** | **A** | **C** | **G** | **T** | **C** | **T** | **C** | **T** | **T** | **C** | **C** | **C** | **C** | **C** | **G** | **A** | **G** | **A** | **A** | **A** | **A** | **A** | **T** | **C** | **G** | **T** | **G** | **T** | **G** | **G** | **C** | **G** | **T** | **A** | **T** | **C** | **A** | **A** | **T** | **T** | **G** | **G** | **A** | **T** | **T** | **A** | **T** | **T** | **T** | **C** | **T** | **T** | **T** | **A** | **A** | **A** | **C** | **T** | **T** | **G** | **A** | **A** | **G** | **C** | **A** | **T** | **T** | **G** | **G** | **T** | **G** | **C** | **C** | **A** | **G** | **A** | **G** | **G** | **C** | **A** | **A** | **A** | **T** | **A** | **C** | **A** | **T** | **C** | **C** | **A** | **T** | **T** | **C** | **A** | **G** | **A** | **T** | **G** | **A** | **T** | **T** | **G** | **C** | **C** | **A** | **G** | **T** | **G** | **G** | **T** | **C** | **C** | **T** | **A** | **A** | **C** | **A** | **G** | **T** | **G** | **A** | **C** | **C** | **G** | **T** | **G** | **A** | **A** | **C** | **A** | **A** | **A** | **T** | **T** | **A** | **A** | **A** | **T** | **T** | **G** | **G** | **C** | **C** | **T** | **T** | **T** | **A** | **T** | **T** | **A** | **A** | **A** | **T** | **T** | **G** | **A** | **T** | **T** | **A** | **C** | **T** | **T** | **C** | **T** | **G** | **C** | **C** | **A** | **A** | **G** | **A** | **A** | **A** | **A** | **G** | **A** | **G** | **T** | **C** | **T** | **G** | **G** | **A** | **T** | **T** | **C** | **A** | **A** | **A** | **C** | **G** | **C** | **C** | **G** | **T** | **A** | **T** | **T** | **T** | **A** | **G** | **T** | **T** | **C** | **C** | **T** | **G** | **A** | **T** | **G** | **A** | **T** | **A** | **G** | **T** | **G** | **T** | **C** | **T** | **T** | **G** | **G** | **C** | **T** | **G** | **C** | **T** | **T** | **T** | **A** | **A** | **A** | **G** | **G** | **T** | **T** | **G** | **C** | **T** | **G** | **C** | **G** | **G** | **C** | **T** | **T** | **C** | **A** | **G** | **G** | **A** | **G** | **T** | **A** | **G** | **A** | **T** | **G** | **T** | **T** | **A** | **A** | **A** | **A** | **T** | **T** | **A** | **T** | **G** | **A** | **T** | **T** | **C** | **C** | **A** | **G** | **A** | **T** | **A** | **A** | **G** | **C** | **C** | **C** | **G** | **A** | **C** | **C** | **A** | **T** | **C** | **C** | **G** | **T** | **T** | **T** | **A** | **T** | **T** | **T** | **A** | **T** | **C** | **G** | **A** | **G** | **C** | **A** | **A** | **C** | **A** | **C** | **A** | **G** | **T** | **A** | **T** | **T** | **A** | **C** | **G** | **G** | **C** | **C** | **G** | **C** | **T** | **T** | **A** | **T** | **T** | **G** | **A** | **T** | **G** | **A** | **A** | **A** | **G** | **A** | **A** | **A** | **A** | **T** | **A** | **T** | **T** | **G** | **A** | **A** | **A** | **T** | **T** | **T** | **T** | **A** | **A** | **T** | **T** | **T** | **A** | **T** | **A** | **A** | **C** | **G** | **G** | **T** | **G** | **G** | **T** | **T** | **T** | **C** | **T** | **T** | **A** | **C** | **A** | **T** | **G** | **C** | **A** | **A** | **A** | **A** | **A** | **C** | **A** | **A** | **T** | **G** | **A** | **T** | **T** | **A** | **T** | **G** | **G** | **A** | **T** | **G** | **A** | **T** | **G** | **A** | **A** | **G** | **T** | **C** | **T** | **G** | **C** | **A** | **C** | **A** | **G** | **T** | **T** | **G** | **G** | **T** | **T** | **C** | **A** | **G** | **C** | **C** | **A** | **A** | **T** | **C** | **A** | **A** | **G** | **A** | **T** | **A** | **T** | **C** | **C** | **G** | **A** | **A** | **G** | **T** | **T** | **A** | **C** | **A** | **A** | **A** | **T** | **T** | **A** | **A** | **A** | **C** | **T** | **T** | **T** | **G** | **A** | **A** | **G** | **C** | **A** | **A** | **A** | **T** | **G** | **C** | **T** | **G** | **T** | **G** | **T** | **T** | **A** | **T** | **A** | **T** | **G** | **A** | **T** | **A** | **A** | **A** | **A** | **A** | **A** | **A** | **T** | **C** | **A** | **T** | **T** | **G** | **A** | **T** | **C** | **A** | **A** | **T** | **T** | **A** | **G** | **A** | **A** | **G** | **C** | **A** | **A** | **T** | **T** | **T** | **T** | **C** | **T** | **T** | **A** | **G** | **A** | **A** | **G** | **A** | **T** | **C** | **G** | **A** | **A** | **A** | **A** | **A** | **A** | **A** | **T** | **G** | **T** | **A** | **C** | **A** | **A** | **C** | **A** | **A** | **T** | **G** | **A** | **C** | **T** | **C** | **C** | **A** | **G** | **A** | **A** | **G** | **T** | **T** | **G** | **T** | **T** | **C** | **G** | **C** | **G** | **A** | **T** | **A** | **T** | **G** | **T** | **C** | **A** | **A** | **A** | **A** | **T** | **G** | **G** | **T** | **T** | **G** | **A** | **T** | **T** | **T** | **T** | **T** | **A** | **A** | **A** | **C** | **A** | **A** | **C** | **A** | **A** | **A** | **T** | **T** | **T** | **C** | **A** | **C** | **G** | **A** | **T** | **T** | **A** | **T** | **T** | **T** | **T** | **C** | **A** | **C** | **C** | **A** | **A** | **T** | **T** | **C** | **T** | **T** | **-** | **-** | **-** |
| **N59555F** | **T** | **T** | **G** | **A** | **T** | **T** | **C** | **T** | **T** | **A** | **G** | **C** | **G** | **T** | **C** | **T** | **T** | **A** | **A** | **C** | **A** | **G** | **T** | **T** | **A** | **T** | **T** | **T** | **A** | **T** | **T** | **T** | **T** | **A** | **T** | **T** | **A** | **A** | **T** | **G** | **C** | **A** | **A** | **T** | **T** | **A** | **T** | **T** | **G** | **C** | **G** | **G** | **G** | **A** | **A** | **T** | **T** | **A** | **C** | **C** | **A** | **T** | **T** | **T** | **T** | **G** | **T** | **T** | **A** | **A** | **A** | **A** | **C** | **C** | **T** | **C** | **G** | **A** | **G** | **A** | **T** | **G** | **T** | **A** | **G** | **C** | **A** | **G** | **C** | **A** | **A** | **T** | **T** | **T** | **G** | **G** | **G** | **C** | **A** | **T** | **G** | **G** | **C** | **T** | **A** | **C** | **T** | **C** | **G** | **T** | **A** | **C** | **T** | **G** | **A** | **T** | **T** | **G** | **C** | **T** | **C** | **T** | **A** | **C** | **C** | **C** | **G** | **T** | **T** | **T** | **T** | **T** | **G** | **G** | **T** | **T** | **T** | **T** | **T** | **T** | **C** | **T** | **T** | **A** | **T** | **A** | **T** | **T** | **T** | **A** | **T** | **T** | **C** | **T** | **T** | **T** | **G** | **G** | **G** | **C** | **G** | **C** | **G** | **G** | **T** | **T** | **T** | **A** | **A** | **C** | **C** | **G** | **A** | **C** | **A** | **A** | **A** | **A** | **A** | **G** | **A** | **A** | **A** | **T** | **T** | **T** | **T** | **A** | **T** | **T** | **T** | **G** | **C** | **A** | **A** | **C** | **A** | **A** | **A** | **G** | **T** | **G** | **A** | **T** | **T** | **T** | **A** | **C** | **G** | **G** | **G** | **A** | **A** | **T** | **T** | **A** | **G** | **A** | **G** | **A** | **A** | **T** | **T** | **T** | **T** | **C** | **A** | **G** | **A** | **A** | **C** | **T** | **T** | **C** | **C** | **A** | **A** | **G** | **A** | **A** | **G** | **A** | **A** | **A** | **G** | **T** | **T** | **T** | **T** | **G** | **A** | **A** | **C** | **T** | **T** | **T** | **A** | **T** | **A** | **G** | **T** | **C** | **A** | **A** | **A** | **A** | **G** | **A** | **T** | **G** | **C** | **C** | **G** | **A** | **C** | **G** | **G** | **A** | **G** | **G** | **A** | **G** | **C** | **A** | **A** | **C** | **A** | **A** | **C** | **A** | **G** | **T** | **T** | **T** | **A** | **C** | **A** | **G** | **A** | **C** | **T** | **T** | **T** | **T** | **T** | **T** | **T** | **C** | **C** | **T** | **C** | **G** | **T** | **T** | **A** | **A** | **A** | **T** | **A** | **G** | **G** | **A** | **T** | **G** | **C** | **C** | **C** | **C** | **T** | **G** | **A** | **C** | **T** | **A** | **A** | **G** | **A** | **A** | **A** | **A** | **A** | **T** | **G** | **A** | **T** | **G** | **T** | **T** | **G** | **A** | **A** | **A** | **T** | **T** | **T** | **T** | **T** | **A** | **C** | **G** | **G** | **A** | **T** | **G** | **G** | **T** | **A** | **C** | **G** | **G** | **A** | **A** | **A** | **A** | **A** | **T** | **T** | **C** | **A** | **A** | **T** | **G** | **C** | **G** | **T** | **T** | **G** | **A** | **T** | **G** | **G** | **C** | **G** | **G** | **A** | **C** | **A** | **T** | **A** | **A** | **A** | **A** | **A** | **A** | **A** | **G** | **C** | **T** | **C** | **A** | **G** | **C** | **A** | **C** | **T** | **C** | **C** | **A** | **T** | **T** | **C** | **A** | **T** | **A** | **T** | **T** | **G** | **A** | **A** | **T** | **A** | **T** | **T** | **A** | **T** | **G** | **C** | **C** | **T** | **T** | **T** | **G** | **T** | **A** | **A** | **C** | **G** | **G** | **A** | **T** | **C** | **A** | **T** | **A** | **T** | **C** | **G** | **G** | **T** | **A** | **C** | **C** | **A** | **A** | **A** | **A** | **T** | **T** | **T** | **T** | **G** | **A** | **A** | **C** | **T** | **T** | **A** | **T** | **T** | **A** | **G** | **A** | **A** | **G** | **A** | **A** | **A** | **A** | **G** | **G** | **C** | **G** | **G** | **C** | **T** | **G** | **A** | **A** | **G** | **G** | **G** | **G** | **T** | **T** | **G** | **A** | **A** | **G** | **T** | **G** | **C** | **G** | **G** | **T** | **T** | **G** | **C** | **T** | **T** | **T** | **A** | **C** | **G** | **A** | **T** | **G** | **C** | **C** | **T** | **T** | **T** | **G** | **G** | **C** | **T** | **C** | **A** | **A** | **A** | **G** | **G** | **G** | **C** | **A** | **C** | **C** | **A** | **A** | **G** | **G** | **T** | **T** | **C** | **A** | **T** | **C** | **A** | **T** | **T** | **T** | **G** | **A** | **A** | **T** | **G** | **A** | **A** | **T** | **T** | **G** | **A** | **A** | **A** | **A** | **A** | **A** | **A** | **A** | **C** | **G** | **G** | **C** | **G** | **G** | **T** | **T** | **T** | **T** | **G** | **T** | **C** | **C** | **A** | **A** | **A** | **C** | **G** | **T** | **T** | **T** | **A** | **T** | **T** | **A** | **C** | **T** | **T** | **C** | **T** | **C** | **A** | **A** | **A** | **A** | **A** | **G** | **C** | **A** | **C** | **T** | **T** | **T** | **T** | **G** | **A** | **A** | **G** | **T** | **T** | **T** | **C** | **G** | **T** | **T** | **T** | **G** | **A** | **A** | **T** | **T** | **A** | **T** | **C** | **A** | **T** | **G** | **A** | **T** | **C** | **A** | **C** | **C** | **G** | **G** | **A** | **A** | **A** | **A** | **T** | **T** | **G** | **T** | **T** | **G** | **T** | **T** | **A** | **T** | **T** | **G** | **A** | **T** | **G** | **G** | **G** | **A** | **A** | **A** | **G** | **T** | **A** | **G** | **G** | **C** | **T** | **A** | **C** | **A** | **T** | **T** | **G** | **G** | **C** | **G** | **G** | **C** | **T** | **T** | **T** | **A** | **A** | **T** | **G** | **T** | **T** | **G** | **C** | **C** | **G** | **A** | **T** | **C** | **A** | **A** | **T** | **A** | **T** | **G** | **C** | **T** | **G** | **G** | **A** | **A** | **C** | **G** | **A** | **C** | **T** | **A** | **A** | **A** | **A** | **A** | **G** | **T** | **T** | **T** | **G** | **G** | **C** | **T** | **A** | **T** | **T** | **G** | **G** | **C** | **G** | **G** | **G** | **A** | **T** | **A** | **C** | **A** | **C** | **A** | **T** | **T** | **T** | **A** | **C** | **G** | **G** | **A** | **T** | **T** | **C** | **A** | **A** | **G** | **G** | **G** | **C** | **C** | **A** | **G** | **C** | **A** | **G** | **C** | **C** | **T** | **C** | **A** | **T** | **T** | **A** | **C** | **T** | **G** | **C** | **A** | **A** | **A** | **T** | **G** | **C** | **G** | **T** | **T** | **T** | **T** | **T** | **T** | **A** | **A** | **T** | **G** | **G** | **A** | **T** | **T** | **G** | **G** | **A** | **A** | **C** | **G** | **T** | **C** | **T** | **C** | **T** | **T** | **C** | **C** | **C** | **C** | **T** | **G** | **A** | **G** | **A** | **A** | **A** | **A** | **A** | **T** | **C** | **G** | **T** | **G** | **T** | **G** | **G** | **C** | **G** | **T** | **A** | **T** | **C** | **A** | **A** | **T** | **T** | **G** | **G** | **A** | **T** | **T** | **A** | **T** | **T** | **T** | **C** | **T** | **T** | **T** | **A** | **A** | **A** | **C** | **T** | **T** | **G** | **A** | **A** | **G** | **C** | **G** | **T** | **T** | **G** | **G** | **T** | **G** | **C** | **C** | **A** | **G** | **A** | **G** | **G** | **C** | **A** | **A** | **A** | **T** | **A** | **C** | **A** | **T** | **C** | **C** | **A** | **T** | **T** | **C** | **A** | **G** | **A** | **T** | **G** | **A** | **T** | **T** | **G** | **C** | **C** | **A** | **G** | **T** | **G** | **G** | **T** | **C** | **C** | **T** | **A** | **A** | **T** | **A** | **G** | **T** | **G** | **A** | **C** | **C** | **G** | **T** | **G** | **A** | **A** | **C** | **A** | **A** | **A** | **T** | **T** | **A** | **A** | **A** | **T** | **T** | **G** | **G** | **C** | **C** | **T** | **T** | **T** | **A** | **T** | **T** | **A** | **A** | **A** | **T** | **T** | **G** | **A** | **T** | **T** | **A** | **C** | **T** | **T** | **C** | **T** | **G** | **C** | **C** | **A** | **A** | **G** | **A** | **A** | **A** | **A** | **G** | **A** | **G** | **T** | **C** | **T** | **G** | **G** | **A** | **T** | **T** | **C** | **A** | **A** | **A** | **C** | **G** | **C** | **C** | **G** | **T** | **A** | **T** | **T** | **T** | **A** | **G** | **T** | **T** | **C** | **C** | **T** | **G** | **A** | **T** | **G** | **A** | **T** | **A** | **G** | **T** | **G** | **T** | **C** | **T** | **T** | **G** | **G** | **C** | **G** | **G** | **C** | **T** | **T** | **T** | **A** | **A** | **A** | **G** | **G** | **T** | **T** | **G** | **C** | **T** | **G** | **C** | **G** | **G** | **C** | **T** | **T** | **C** | **A** | **G** | **G** | **A** | **G** | **T** | **A** | **G** | **A** | **T** | **G** | **T** | **T** | **A** | **A** | **A** | **A** | **T** | **T** | **A** | **T** | **G** | **A** | **T** | **T** | **C** | **C** | **A** | **G** | **A** | **T** | **A** | **A** | **G** | **C** | **C** | **C** | **G** | **A** | **C** | **C** | **A** | **T** | **C** | **C** | **G** | **T** | **T** | **T** | **A** | **T** | **T** | **T** | **A** | **T** | **C** | **G** | **A** | **G** | **C** | **A** | **A** | **C** | **A** | **C** | **A** | **G** | **T** | **A** | **T** | **T** | **A** | **C** | **G** | **G** | **G** | **C** | **G** | **C** | **T** | **T** | **G** | **T** | **T** | **G** | **A** | **T** | **G** | **A** | **A** | **A** | **G** | **A** | **A** | **A** | **A** | **T** | **A** | **T** | **T** | **G** | **A** | **A** | **A** | **T** | **T** | **T** | **T** | **A** | **A** | **T** | **T** | **T** | **A** | **T** | **A** | **A** | **C** | **G** | **G** | **T** | **G** | **G** | **T** | **T** | **T** | **C** | **T** | **T** | **A** | **C** | **A** | **T** | **G** | **C** | **G** | **A** | **A** | **A** | **A** | **C** | **A** | **A** | **T** | **G** | **A** | **T** | **T** | **A** | **T** | **G** | **G** | **A** | **T** | **G** | **A** | **T** | **G** | **A** | **A** | **G** | **T** | **C** | **T** | **G** | **C** | **A** | **C** | **A** | **G** | **T** | **A** | **G** | **G** | **T** | **T** | **C** | **A** | **G** | **C** | **C** | **A** | **A** | **T** | **C** | **A** | **A** | **G** | **A** | **T** | **A** | **T** | **C** | **C** | **G** | **A** | **A** | **G** | **T** | **T** | **A** | **C** | **A** | **A** | **A** | **T** | **T** | **A** | **A** | **A** | **C** | **T** | **T** | **T** | **G** | **A** | **A** | **G** | **C** | **A** | **A** | **A** | **T** | **G** | **C** | **T** | **G** | **T** | **G** | **T** | **T** | **A** | **T** | **A** | **T** | **G** | **A** | **T** | **A** | **A** | **A** | **A** | **A** | **A** | **A** | **T** | **C** | **A** | **T** | **T** | **G** | **A** | **T** | **C** | **A** | **A** | **T** | **T** | **A** | **G** | **A** | **A** | **G** | **C** | **A** | **A** | **T** | **T** | **T** | **T** | **C** | **T** | **T** | **A** | **G** | **A** | **A** | **G** | **A** | **T** | **C** | **G** | **A** | **A** | **A** | **A** | **A** | **A** | **A** | **T** | **G** | **T** | **A** | **C** | **A** | **A** | **C** | **A** | **A** | **T** | **G** | **A** | **C** | **T** | **C** | **C** | **A** | **G** | **A** | **A** | **G** | **T** | **T** | **G** | **T** | **T** | **C** | **G** | **C** | **G** | **A** | **C** | **A** | **T** | **G** | **T** | **C** | **A** | **A** | **A** | **A** | **T** | **G** | **G** | **T** | **T** | **G** | **A** | **T** | **T** | **T** | **T** | **T** | **A** | **A** | **A** | **C** | **A** | **A** | **C** | **A** | **A** | **A** | **T** | **T** | **T** | **C** | **A** | **C** | **G** | **A** | **T** | **T** | **A** | **T** | **T** | **C** | **T** | **C** | **A** | **C** | **C** | **A** | **A** | **T** | **T** | **C** | **T** | **T** | **-** | **-** | **-** |
| **N59454F** | **T** | **T** | **G** | **A** | **T** | **T** | **C** | **T** | **T** | **A** | **G** | **C** | **G** | **T** | **C** | **T** | **T** | **A** | **A** | **C** | **A** | **G** | **T** | **T** | **A** | **T** | **T** | **T** | **A** | **T** | **T** | **T** | **T** | **A** | **T** | **T** | **A** | **A** | **T** | **G** | **C** | **A** | **A** | **T** | **T** | **A** | **T** | **T** | **G** | **C** | **G** | **G** | **G** | **A** | **A** | **T** | **T** | **A** | **C** | **C** | **A** | **T** | **T** | **T** | **T** | **G** | **T** | **T** | **A** | **A** | **A** | **A** | **C** | **C** | **T** | **C** | **G** | **A** | **G** | **A** | **T** | **G** | **T** | **A** | **G** | **C** | **A** | **G** | **C** | **A** | **A** | **T** | **T** | **T** | **G** | **G** | **G** | **C** | **A** | **T** | **G** | **G** | **C** | **T** | **A** | **C** | **T** | **C** | **G** | **T** | **A** | **C** | **T** | **G** | **A** | **T** | **T** | **G** | **C** | **T** | **C** | **T** | **A** | **C** | **C** | **C** | **G** | **T** | **T** | **T** | **T** | **T** | **G** | **G** | **T** | **T** | **T** | **T** | **T** | **T** | **C** | **T** | **T** | **A** | **T** | **A** | **T** | **T** | **T** | **A** | **T** | **T** | **C** | **T** | **T** | **T** | **G** | **G** | **G** | **C** | **G** | **C** | **G** | **G** | **T** | **T** | **T** | **A** | **A** | **C** | **C** | **G** | **A** | **C** | **A** | **A** | **A** | **A** | **A** | **G** | **A** | **A** | **A** | **T** | **T** | **T** | **T** | **A** | **T** | **T** | **T** | **G** | **C** | **A** | **A** | **C** | **A** | **A** | **A** | **G** | **T** | **G** | **A** | **T** | **T** | **T** | **A** | **C** | **G** | **G** | **G** | **A** | **A** | **T** | **T** | **A** | **G** | **A** | **G** | **A** | **A** | **T** | **T** | **T** | **C** | **C** | **A** | **G** | **A** | **A** | **T** | **T** | **T** | **C** | **C** | **A** | **A** | **G** | **A** | **A** | **G** | **A** | **A** | **A** | **G** | **T** | **T** | **T** | **T** | **G** | **A** | **A** | **C** | **T** | **T** | **T** | **A** | **T** | **A** | **G** | **T** | **C** | **A** | **A** | **A** | **A** | **G** | **A** | **T** | **G** | **C** | **C** | **G** | **A** | **C** | **G** | **G** | **A** | **G** | **G** | **A** | **G** | **C** | **A** | **A** | **C** | **A** | **A** | **C** | **A** | **G** | **T** | **T** | **T** | **A** | **C** | **A** | **G** | **A** | **C** | **T** | **T** | **T** | **T** | **T** | **T** | **T** | **C** | **C** | **T** | **C** | **G** | **T** | **T** | **A** | **A** | **A** | **T** | **A** | **G** | **G** | **A** | **T** | **G** | **C** | **C** | **T** | **C** | **T** | **G** | **A** | **C** | **T** | **A** | **A** | **G** | **A** | **A** | **A** | **A** | **A** | **T** | **G** | **A** | **T** | **G** | **T** | **T** | **G** | **A** | **A** | **A** | **T** | **T** | **T** | **T** | **T** | **A** | **C** | **G** | **G** | **A** | **T** | **G** | **G** | **T** | **A** | **C** | **G** | **G** | **A** | **A** | **A** | **A** | **A** | **T** | **T** | **C** | **A** | **A** | **T** | **G** | **C** | **G** | **T** | **T** | **G** | **A** | **T** | **G** | **G** | **C** | **G** | **G** | **A** | **C** | **A** | **T** | **A** | **A** | **A** | **A** | **A** | **A** | **A** | **G** | **C** | **T** | **C** | **A** | **G** | **C** | **A** | **C** | **T** | **C** | **C** | **A** | **T** | **T** | **C** | **A** | **T** | **A** | **T** | **T** | **G** | **A** | **A** | **T** | **A** | **T** | **T** | **A** | **T** | **G** | **C** | **C** | **T** | **T** | **T** | **G** | **T** | **A** | **A** | **C** | **G** | **G** | **A** | **T** | **C** | **A** | **T** | **A** | **T** | **C** | **G** | **G** | **T** | **A** | **C** | **C** | **A** | **A** | **A** | **A** | **T** | **T** | **T** | **T** | **G** | **A** | **A** | **C** | **T** | **T** | **A** | **T** | **T** | **A** | **G** | **A** | **A** | **G** | **A** | **A** | **A** | **A** | **G** | **G** | **C** | **G** | **G** | **C** | **T** | **G** | **A** | **A** | **G** | **G** | **G** | **G** | **T** | **T** | **G** | **A** | **A** | **G** | **T** | **G** | **C** | **G** | **G** | **T** | **T** | **G** | **C** | **T** | **T** | **T** | **A** | **C** | **G** | **A** | **T** | **G** | **C** | **C** | **T** | **T** | **T** | **G** | **G** | **C** | **T** | **C** | **A** | **A** | **A** | **G** | **G** | **G** | **C** | **A** | **C** | **C** | **A** | **A** | **G** | **G** | **T** | **T** | **C** | **A** | **T** | **C** | **A** | **T** | **T** | **T** | **G** | **A** | **A** | **T** | **G** | **A** | **A** | **T** | **T** | **G** | **A** | **A** | **A** | **A** | **A** | **A** | **A** | **A** | **C** | **G** | **G** | **T** | **G** | **G** | **T** | **T** | **T** | **T** | **G** | **T** | **C** | **C** | **A** | **A** | **A** | **C** | **G** | **T** | **T** | **T** | **A** | **T** | **T** | **A** | **C** | **T** | **T** | **C** | **T** | **C** | **A** | **A** | **A** | **A** | **A** | **G** | **C** | **A** | **C** | **T** | **T** | **T** | **T** | **G** | **A** | **A** | **G** | **T** | **T** | **T** | **C** | **G** | **T** | **T** | **T** | **G** | **A** | **A** | **T** | **T** | **A** | **T** | **C** | **A** | **T** | **G** | **A** | **T** | **C** | **A** | **C** | **C** | **G** | **G** | **A** | **A** | **A** | **A** | **T** | **T** | **G** | **T** | **T** | **G** | **T** | **T** | **A** | **T** | **T** | **G** | **A** | **C** | **G** | **G** | **A** | **A** | **A** | **A** | **G** | **T** | **A** | **G** | **G** | **C** | **T** | **A** | **C** | **A** | **T** | **T** | **G** | **G** | **C** | **G** | **G** | **C** | **T** | **T** | **T** | **A** | **A** | **T** | **G** | **T** | **T** | **G** | **C** | **C** | **G** | **A** | **T** | **C** | **A** | **A** | **T** | **A** | **T** | **G** | **C** | **T** | **G** | **G** | **A** | **A** | **C** | **G** | **A** | **C** | **T** | **A** | **A** | **A** | **A** | **A** | **G** | **T** | **T** | **T** | **G** | **G** | **C** | **T** | **A** | **T** | **T** | **G** | **G** | **C** | **G** | **G** | **G** | **A** | **T** | **A** | **C** | **G** | **C** | **A** | **T** | **T** | **T** | **A** | **C** | **G** | **G** | **A** | **T** | **T** | **C** | **A** | **A** | **G** | **G** | **G** | **C** | **C** | **A** | **G** | **C** | **A** | **G** | **C** | **C** | **T** | **C** | **A** | **T** | **T** | **A** | **C** | **T** | **G** | **C** | **A** | **A** | **A** | **T** | **G** | **C** | **G** | **T** | **T** | **T** | **T** | **T** | **T** | **A** | **A** | **T** | **G** | **G** | **A** | **T** | **T** | **G** | **G** | **A** | **A** | **C** | **G** | **T** | **C** | **T** | **C** | **T** | **T** | **C** | **C** | **C** | **C** | **C** | **G** | **A** | **G** | **A** | **A** | **A** | **A** | **A** | **T** | **C** | **A** | **T** | **G** | **T** | **G** | **G** | **C** | **G** | **T** | **A** | **T** | **C** | **A** | **A** | **T** | **T** | **G** | **G** | **A** | **T** | **T** | **A** | **T** | **T** | **T** | **C** | **T** | **T** | **T** | **A** | **A** | **A** | **C** | **T** | **T** | **G** | **A** | **A** | **G** | **C** | **A** | **T** | **T** | **G** | **G** | **T** | **G** | **C** | **C** | **A** | **G** | **A** | **G** | **G** | **C** | **A** | **A** | **A** | **T** | **A** | **C** | **A** | **T** | **C** | **C** | **A** | **T** | **T** | **C** | **A** | **G** | **A** | **T** | **G** | **A** | **T** | **T** | **G** | **C** | **C** | **A** | **G** | **T** | **G** | **G** | **T** | **C** | **C** | **T** | **A** | **A** | **C** | **A** | **G** | **T** | **G** | **A** | **C** | **C** | **G** | **T** | **G** | **A** | **A** | **C** | **A** | **A** | **A** | **T** | **T** | **A** | **A** | **A** | **T** | **T** | **G** | **G** | **C** | **C** | **T** | **T** | **T** | **A** | **T** | **T** | **A** | **A** | **A** | **T** | **T** | **G** | **A** | **T** | **T** | **A** | **C** | **T** | **T** | **C** | **T** | **G** | **C** | **C** | **A** | **A** | **G** | **A** | **A** | **A** | **A** | **G** | **A** | **G** | **T** | **C** | **T** | **G** | **G** | **A** | **T** | **T** | **C** | **A** | **A** | **A** | **C** | **G** | **C** | **C** | **G** | **T** | **A** | **T** | **T** | **T** | **A** | **G** | **T** | **T** | **C** | **C** | **T** | **G** | **A** | **T** | **G** | **A** | **T** | **A** | **G** | **T** | **G** | **T** | **C** | **T** | **T** | **G** | **G** | **C** | **T** | **G** | **C** | **T** | **T** | **T** | **A** | **A** | **A** | **G** | **G** | **T** | **T** | **G** | **C** | **T** | **G** | **C** | **G** | **G** | **C** | **T** | **T** | **C** | **A** | **G** | **G** | **A** | **G** | **T** | **A** | **G** | **A** | **T** | **G** | **T** | **T** | **A** | **A** | **A** | **A** | **T** | **T** | **A** | **T** | **G** | **A** | **T** | **T** | **C** | **C** | **A** | **G** | **A** | **T** | **A** | **A** | **G** | **C** | **C** | **C** | **G** | **A** | **C** | **C** | **A** | **T** | **C** | **C** | **G** | **T** | **T** | **T** | **A** | **T** | **T** | **T** | **A** | **T** | **C** | **G** | **A** | **G** | **C** | **A** | **A** | **C** | **A** | **C** | **A** | **G** | **T** | **A** | **T** | **T** | **A** | **C** | **G** | **G** | **C** | **C** | **G** | **C** | **T** | **T** | **A** | **T** | **T** | **G** | **A** | **T** | **G** | **A** | **A** | **A** | **G** | **A** | **A** | **A** | **A** | **T** | **A** | **T** | **T** | **G** | **A** | **A** | **A** | **T** | **T** | **T** | **T** | **A** | **A** | **T** | **T** | **T** | **A** | **T** | **A** | **A** | **C** | **G** | **G** | **T** | **G** | **G** | **T** | **T** | **T** | **C** | **T** | **T** | **A** | **C** | **A** | **T** | **G** | **C** | **G** | **A** | **A** | **A** | **A** | **C** | **A** | **A** | **T** | **G** | **A** | **T** | **T** | **A** | **T** | **G** | **G** | **A** | **T** | **G** | **A** | **T** | **G** | **A** | **A** | **G** | **T** | **C** | **T** | **G** | **T** | **A** | **C** | **A** | **G** | **T** | **T** | **G** | **G** | **T** | **T** | **C** | **A** | **G** | **C** | **C** | **A** | **A** | **T** | **C** | **A** | **A** | **G** | **A** | **T** | **A** | **T** | **C** | **C** | **G** | **A** | **A** | **G** | **T** | **T** | **A** | **C** | **A** | **A** | **A** | **T** | **T** | **A** | **A** | **A** | **C** | **T** | **T** | **T** | **G** | **A** | **A** | **G** | **C** | **A** | **A** | **A** | **T** | **G** | **C** | **T** | **G** | **T** | **G** | **T** | **T** | **A** | **T** | **A** | **T** | **G** | **A** | **T** | **A** | **A** | **A** | **A** | **A** | **A** | **A** | **T** | **C** | **A** | **T** | **T** | **G** | **A** | **T** | **C** | **A** | **A** | **T** | **T** | **A** | **G** | **A** | **A** | **G** | **C** | **A** | **A** | **T** | **T** | **T** | **T** | **C** | **T** | **T** | **A** | **G** | **A** | **A** | **G** | **A** | **T** | **C** | **G** | **A** | **A** | **A** | **A** | **A** | **A** | **A** | **T** | **G** | **T** | **A** | **C** | **A** | **A** | **C** | **A** | **A** | **T** | **G** | **A** | **C** | **A** | **C** | **C** | **A** | **G** | **A** | **A** | **G** | **T** | **T** | **G** | **T** | **T** | **C** | **G** | **A** | **G** | **A** | **C** | **A** | **T** | **G** | **T** | **C** | **C** | **A** | **A** | **A** | **T** | **G** | **G** | **T** | **T** | **G** | **A** | **T** | **T** | **T** | **T** | **T** | **A** | **A** | **A** | **C** | **A** | **A** | **C** | **A** | **A** | **A** | **T** | **T** | **T** | **C** | **A** | **C** | **G** | **A** | **T** | **T** | **A** | **T** | **T** | **T** | **T** | **C** | **C** | **C** | **C** | **A** | **A** | **T** | **T** | **C** | **T** | **T** | **-** | **-** | **-** |
| **N53418** | **T** | **T** | **G** | **A** | **T** | **T** | **C** | **T** | **T** | **A** | **G** | **C** | **G** | **T** | **C** | **T** | **T** | **A** | **A** | **C** | **A** | **G** | **T** | **T** | **A** | **T** | **T** | **T** | **A** | **T** | **T** | **T** | **T** | **A** | **T** | **T** | **A** | **A** | **T** | **G** | **C** | **A** | **A** | **T** | **T** | **A** | **T** | **T** | **G** | **C** | **G** | **G** | **G** | **A** | **A** | **T** | **T** | **A** | **C** | **C** | **A** | **T** | **T** | **T** | **T** | **G** | **T** | **T** | **A** | **A** | **A** | **A** | **C** | **C** | **T** | **C** | **G** | **A** | **G** | **A** | **T** | **G** | **T** | **A** | **G** | **C** | **A** | **G** | **C** | **A** | **A** | **T** | **T** | **T** | **G** | **G** | **G** | **C** | **A** | **T** | **G** | **G** | **C** | **T** | **A** | **C** | **T** | **C** | **G** | **T** | **A** | **C** | **T** | **G** | **A** | **T** | **T** | **G** | **C** | **T** | **C** | **T** | **A** | **C** | **C** | **C** | **G** | **T** | **T** | **T** | **T** | **T** | **G** | **G** | **T** | **T** | **T** | **T** | **T** | **T** | **C** | **T** | **T** | **A** | **T** | **A** | **T** | **T** | **T** | **A** | **T** | **T** | **C** | **T** | **T** | **T** | **G** | **G** | **G** | **C** | **G** | **C** | **G** | **G** | **T** | **T** | **T** | **A** | **A** | **C** | **C** | **G** | **A** | **C** | **A** | **A** | **A** | **A** | **A** | **G** | **A** | **A** | **A** | **T** | **T** | **T** | **T** | **A** | **T** | **T** | **T** | **G** | **C** | **A** | **A** | **C** | **A** | **A** | **A** | **G** | **T** | **G** | **A** | **T** | **T** | **T** | **A** | **C** | **G** | **G** | **G** | **A** | **A** | **T** | **T** | **A** | **G** | **A** | **G** | **A** | **A** | **T** | **T** | **T** | **C** | **C** | **A** | **G** | **A** | **A** | **T** | **T** | **T** | **C** | **C** | **A** | **A** | **G** | **A** | **A** | **G** | **A** | **A** | **A** | **G** | **T** | **T** | **T** | **T** | **G** | **A** | **A** | **C** | **T** | **T** | **T** | **A** | **T** | **A** | **G** | **T** | **C** | **A** | **A** | **A** | **A** | **G** | **A** | **T** | **G** | **C** | **C** | **G** | **A** | **C** | **G** | **G** | **A** | **G** | **G** | **A** | **G** | **C** | **A** | **A** | **C** | **A** | **A** | **C** | **A** | **G** | **T** | **T** | **T** | **A** | **C** | **A** | **G** | **A** | **C** | **T** | **T** | **T** | **T** | **T** | **T** | **T** | **C** | **C** | **T** | **C** | **G** | **T** | **T** | **A** | **A** | **A** | **T** | **A** | **G** | **G** | **A** | **T** | **G** | **C** | **C** | **T** | **C** | **T** | **G** | **A** | **C** | **T** | **A** | **A** | **G** | **A** | **A** | **A** | **A** | **A** | **T** | **G** | **A** | **T** | **G** | **T** | **T** | **G** | **A** | **A** | **A** | **T** | **T** | **T** | **T** | **T** | **A** | **C** | **G** | **G** | **A** | **T** | **G** | **G** | **T** | **A** | **C** | **G** | **G** | **A** | **A** | **A** | **A** | **A** | **T** | **T** | **C** | **A** | **A** | **T** | **G** | **C** | **G** | **T** | **T** | **G** | **A** | **T** | **G** | **G** | **C** | **G** | **G** | **A** | **C** | **A** | **T** | **A** | **A** | **A** | **A** | **A** | **A** | **A** | **G** | **C** | **T** | **C** | **A** | **G** | **C** | **A** | **C** | **T** | **C** | **C** | **A** | **T** | **T** | **C** | **A** | **T** | **A** | **T** | **T** | **G** | **A** | **A** | **T** | **A** | **T** | **T** | **A** | **T** | **G** | **C** | **C** | **T** | **T** | **T** | **G** | **T** | **A** | **A** | **C** | **G** | **G** | **A** | **T** | **C** | **A** | **T** | **A** | **T** | **C** | **G** | **G** | **C** | **A** | **C** | **C** | **A** | **A** | **A** | **A** | **T** | **T** | **T** | **T** | **G** | **A** | **A** | **C** | **T** | **T** | **A** | **T** | **T** | **A** | **G** | **A** | **A** | **G** | **A** | **A** | **A** | **A** | **G** | **G** | **C** | **G** | **G** | **C** | **T** | **G** | **A** | **A** | **G** | **G** | **G** | **G** | **T** | **T** | **G** | **A** | **A** | **G** | **T** | **G** | **C** | **G** | **G** | **T** | **T** | **G** | **C** | **T** | **T** | **T** | **A** | **C** | **G** | **A** | **T** | **G** | **C** | **C** | **T** | **T** | **T** | **G** | **G** | **C** | **T** | **C** | **A** | **A** | **A** | **G** | **G** | **G** | **C** | **A** | **C** | **C** | **A** | **A** | **G** | **G** | **T** | **T** | **C** | **A** | **T** | **C** | **A** | **T** | **T** | **T** | **G** | **A** | **A** | **T** | **G** | **A** | **A** | **T** | **T** | **G** | **A** | **A** | **A** | **A** | **A** | **A** | **A** | **A** | **C** | **G** | **G** | **C** | **G** | **G** | **T** | **T** | **T** | **T** | **G** | **T** | **C** | **C** | **A** | **A** | **A** | **C** | **A** | **T** | **T** | **T** | **A** | **T** | **T** | **A** | **C** | **T** | **T** | **C** | **T** | **C** | **A** | **A** | **A** | **A** | **A** | **G** | **C** | **A** | **C** | **T** | **T** | **T** | **T** | **G** | **A** | **A** | **G** | **T** | **T** | **T** | **C** | **G** | **T** | **T** | **T** | **G** | **A** | **A** | **T** | **T** | **A** | **T** | **C** | **A** | **T** | **G** | **A** | **T** | **C** | **A** | **C** | **C** | **G** | **G** | **A** | **A** | **A** | **A** | **T** | **T** | **G** | **T** | **T** | **G** | **T** | **T** | **A** | **T** | **T** | **G** | **A** | **C** | **G** | **G** | **A** | **A** | **A** | **A** | **G** | **T** | **A** | **G** | **G** | **C** | **T** | **A** | **C** | **A** | **T** | **T** | **G** | **G** | **C** | **G** | **G** | **C** | **T** | **T** | **T** | **A** | **A** | **T** | **G** | **T** | **T** | **G** | **C** | **C** | **G** | **A** | **T** | **C** | **A** | **A** | **T** | **A** | **T** | **G** | **C** | **C** | **G** | **G** | **A** | **A** | **C** | **G** | **A** | **C** | **T** | **A** | **A** | **A** | **A** | **A** | **G** | **T** | **T** | **T** | **G** | **G** | **C** | **T** | **A** | **T** | **T** | **G** | **G** | **C** | **G** | **G** | **G** | **A** | **T** | **A** | **C** | **G** | **C** | **A** | **T** | **T** | **T** | **A** | **C** | **G** | **G** | **A** | **T** | **T** | **C** | **A** | **A** | **G** | **G** | **G** | **C** | **C** | **A** | **G** | **C** | **A** | **G** | **C** | **C** | **T** | **C** | **A** | **T** | **T** | **A** | **C** | **T** | **G** | **C** | **A** | **A** | **A** | **T** | **G** | **C** | **G** | **T** | **T** | **T** | **T** | **T** | **T** | **A** | **A** | **T** | **G** | **G** | **A** | **T** | **T** | **G** | **G** | **A** | **A** | **C** | **G** | **T** | **C** | **T** | **C** | **T** | **T** | **C** | **C** | **C** | **C** | **C** | **G** | **A** | **G** | **A** | **A** | **A** | **A** | **A** | **T** | **C** | **G** | **T** | **G** | **T** | **G** | **G** | **C** | **G** | **T** | **A** | **T** | **C** | **A** | **A** | **T** | **T** | **G** | **G** | **A** | **T** | **T** | **A** | **T** | **T** | **T** | **C** | **T** | **T** | **T** | **A** | **A** | **A** | **C** | **T** | **T** | **G** | **A** | **A** | **G** | **C** | **A** | **T** | **T** | **G** | **G** | **T** | **G** | **C** | **C** | **A** | **G** | **A** | **G** | **G** | **C** | **A** | **A** | **A** | **T** | **A** | **C** | **A** | **T** | **C** | **C** | **A** | **T** | **T** | **C** | **A** | **G** | **A** | **T** | **G** | **A** | **T** | **T** | **G** | **C** | **C** | **A** | **G** | **T** | **G** | **G** | **T** | **C** | **C** | **T** | **A** | **A** | **C** | **A** | **G** | **T** | **G** | **A** | **C** | **C** | **G** | **T** | **G** | **A** | **A** | **C** | **A** | **A** | **A** | **T** | **T** | **A** | **A** | **A** | **T** | **T** | **G** | **G** | **C** | **C** | **T** | **T** | **T** | **A** | **T** | **T** | **A** | **A** | **A** | **T** | **T** | **G** | **A** | **T** | **T** | **A** | **C** | **T** | **T** | **C** | **T** | **G** | **C** | **C** | **A** | **A** | **G** | **A** | **A** | **A** | **A** | **G** | **A** | **G** | **T** | **C** | **T** | **G** | **G** | **A** | **T** | **T** | **C** | **A** | **A** | **A** | **C** | **G** | **C** | **C** | **G** | **T** | **A** | **T** | **T** | **T** | **A** | **G** | **T** | **T** | **C** | **C** | **T** | **G** | **A** | **T** | **G** | **A** | **T** | **A** | **G** | **T** | **G** | **T** | **C** | **T** | **T** | **G** | **G** | **C** | **T** | **G** | **C** | **T** | **T** | **T** | **A** | **A** | **A** | **G** | **G** | **T** | **T** | **G** | **C** | **T** | **G** | **C** | **G** | **G** | **C** | **T** | **T** | **C** | **A** | **G** | **G** | **A** | **G** | **T** | **A** | **G** | **A** | **T** | **G** | **T** | **T** | **A** | **A** | **A** | **A** | **T** | **T** | **A** | **T** | **G** | **A** | **T** | **T** | **C** | **C** | **A** | **G** | **A** | **T** | **A** | **A** | **G** | **C** | **C** | **C** | **G** | **A** | **C** | **C** | **A** | **T** | **C** | **C** | **G** | **T** | **T** | **T** | **A** | **T** | **T** | **T** | **A** | **T** | **C** | **G** | **A** | **G** | **C** | **A** | **A** | **C** | **A** | **C** | **A** | **G** | **T** | **A** | **T** | **T** | **A** | **C** | **G** | **G** | **C** | **C** | **G** | **C** | **T** | **T** | **A** | **T** | **T** | **G** | **A** | **T** | **G** | **A** | **A** | **A** | **G** | **A** | **A** | **A** | **A** | **T** | **A** | **T** | **T** | **G** | **A** | **A** | **A** | **T** | **T** | **T** | **T** | **A** | **A** | **T** | **T** | **T** | **A** | **T** | **A** | **A** | **C** | **G** | **G** | **T** | **G** | **G** | **T** | **T** | **T** | **C** | **T** | **T** | **A** | **C** | **A** | **T** | **G** | **C** | **G** | **A** | **A** | **A** | **A** | **C** | **A** | **A** | **T** | **G** | **A** | **T** | **T** | **A** | **T** | **G** | **G** | **A** | **T** | **G** | **A** | **T** | **G** | **A** | **A** | **G** | **T** | **C** | **T** | **G** | **C** | **A** | **C** | **A** | **G** | **T** | **T** | **G** | **G** | **T** | **T** | **C** | **A** | **G** | **C** | **C** | **A** | **A** | **T** | **C** | **A** | **A** | **G** | **A** | **T** | **A** | **T** | **C** | **C** | **G** | **A** | **A** | **G** | **T** | **T** | **A** | **C** | **A** | **A** | **A** | **T** | **T** | **A** | **A** | **A** | **C** | **T** | **T** | **T** | **G** | **A** | **A** | **G** | **C** | **A** | **A** | **A** | **T** | **G** | **C** | **T** | **G** | **T** | **G** | **T** | **T** | **A** | **T** | **A** | **T** | **G** | **A** | **T** | **A** | **A** | **A** | **A** | **A** | **A** | **A** | **T** | **C** | **A** | **T** | **T** | **G** | **A** | **T** | **C** | **A** | **A** | **T** | **T** | **A** | **G** | **A** | **A** | **G** | **C** | **A** | **A** | **T** | **T** | **T** | **T** | **C** | **T** | **T** | **A** | **G** | **A** | **A** | **G** | **A** | **T** | **C** | **G** | **A** | **A** | **A** | **A** | **A** | **A** | **A** | **T** | **G** | **T** | **A** | **C** | **A** | **A** | **C** | **A** | **A** | **T** | **G** | **A** | **C** | **T** | **C** | **C** | **A** | **G** | **A** | **A** | **G** | **T** | **T** | **G** | **T** | **T** | **C** | **G** | **C** | **G** | **A** | **C** | **A** | **T** | **G** | **T** | **C** | **A** | **A** | **A** | **A** | **T** | **G** | **G** | **T** | **T** | **G** | **A** | **T** | **T** | **T** | **T** | **T** | **A** | **A** | **A** | **C** | **A** | **A** | **C** | **A** | **A** | **A** | **T** | **T** | **T** | **C** | **A** | **C** | **G** | **A** | **T** | **T** | **A** | **T** | **T** | **T** | **T** | **C** | **A** | **C** | **C** | **A** | **A** | **T** | **T** | **C** | **T** | **T** | **-** | **-** | **-** |
| **N59862F** | **T** | **T** | **G** | **A** | **T** | **T** | **C** | **T** | **T** | **A** | **G** | **C** | **G** | **T** | **C** | **T** | **T** | **A** | **A** | **C** | **A** | **G** | **T** | **T** | **A** | **T** | **T** | **T** | **A** | **T** | **T** | **T** | **T** | **A** | **T** | **T** | **A** | **A** | **T** | **G** | **C** | **A** | **A** | **T** | **T** | **A** | **T** | **T** | **G** | **C** | **G** | **G** | **G** | **A** | **A** | **T** | **T** | **A** | **C** | **C** | **A** | **T** | **T** | **T** | **T** | **G** | **T** | **T** | **A** | **A** | **A** | **A** | **C** | **C** | **T** | **C** | **G** | **A** | **G** | **A** | **T** | **G** | **T** | **A** | **G** | **C** | **A** | **G** | **C** | **A** | **A** | **T** | **T** | **T** | **G** | **G** | **G** | **C** | **A** | **T** | **G** | **G** | **C** | **T** | **A** | **C** | **T** | **C** | **G** | **T** | **A** | **C** | **T** | **G** | **A** | **T** | **T** | **G** | **C** | **T** | **C** | **T** | **A** | **C** | **C** | **C** | **G** | **T** | **T** | **T** | **T** | **T** | **G** | **G** | **T** | **T** | **T** | **T** | **T** | **T** | **C** | **T** | **T** | **A** | **T** | **A** | **T** | **T** | **T** | **A** | **T** | **T** | **C** | **T** | **T** | **T** | **G** | **G** | **T** | **C** | **G** | **A** | **G** | **G** | **T** | **T** | **T** | **A** | **A** | **C** | **C** | **G** | **A** | **C** | **A** | **A** | **A** | **A** | **A** | **G** | **A** | **A** | **A** | **T** | **T** | **T** | **T** | **A** | **T** | **T** | **T** | **G** | **C** | **A** | **A** | **C** | **A** | **A** | **A** | **G** | **T** | **G** | **A** | **T** | **T** | **T** | **A** | **C** | **G** | **G** | **G** | **A** | **A** | **T** | **T** | **A** | **G** | **A** | **G** | **A** | **A** | **T** | **T** | **T** | **C** | **C** | **A** | **G** | **A** | **A** | **T** | **T** | **T** | **C** | **C** | **A** | **A** | **G** | **A** | **A** | **G** | **A** | **A** | **A** | **G** | **T** | **T** | **T** | **T** | **G** | **A** | **A** | **C** | **T** | **T** | **T** | **A** | **T** | **A** | **G** | **T** | **C** | **A** | **A** | **A** | **A** | **G** | **A** | **T** | **G** | **C** | **C** | **G** | **A** | **C** | **G** | **G** | **A** | **G** | **G** | **A** | **G** | **C** | **A** | **A** | **C** | **A** | **A** | **C** | **A** | **G** | **T** | **T** | **T** | **A** | **C** | **A** | **G** | **A** | **C** | **T** | **T** | **T** | **T** | **T** | **T** | **T** | **C** | **C** | **T** | **C** | **G** | **T** | **T** | **A** | **A** | **A** | **T** | **A** | **G** | **G** | **A** | **T** | **G** | **C** | **C** | **T** | **C** | **T** | **G** | **A** | **C** | **T** | **A** | **A** | **G** | **A** | **A** | **A** | **A** | **A** | **T** | **G** | **A** | **T** | **G** | **T** | **T** | **G** | **A** | **A** | **A** | **T** | **T** | **T** | **T** | **T** | **A** | **C** | **G** | **G** | **A** | **T** | **G** | **G** | **T** | **A** | **C** | **G** | **G** | **A** | **A** | **A** | **A** | **A** | **T** | **T** | **C** | **A** | **A** | **T** | **G** | **C** | **G** | **T** | **T** | **G** | **A** | **T** | **G** | **G** | **C** | **G** | **G** | **A** | **T** | **A** | **T** | **A** | **A** | **A** | **A** | **A** | **A** | **A** | **G** | **C** | **T** | **C** | **A** | **G** | **C** | **A** | **C** | **T** | **C** | **C** | **A** | **T** | **T** | **C** | **A** | **T** | **A** | **T** | **T** | **G** | **A** | **A** | **T** | **A** | **T** | **T** | **A** | **T** | **G** | **C** | **C** | **T** | **T** | **T** | **G** | **T** | **A** | **A** | **C** | **G** | **G** | **A** | **T** | **C** | **A** | **T** | **A** | **T** | **C** | **G** | **G** | **C** | **A** | **C** | **C** | **A** | **A** | **A** | **A** | **T** | **T** | **T** | **T** | **G** | **A** | **A** | **C** | **T** | **T** | **A** | **T** | **T** | **A** | **G** | **A** | **A** | **G** | **A** | **A** | **A** | **A** | **G** | **G** | **C** | **G** | **G** | **C** | **T** | **G** | **A** | **A** | **G** | **G** | **G** | **G** | **T** | **T** | **G** | **A** | **A** | **G** | **T** | **G** | **C** | **G** | **G** | **T** | **T** | **G** | **C** | **T** | **T** | **T** | **A** | **C** | **G** | **A** | **T** | **G** | **C** | **C** | **T** | **T** | **T** | **G** | **G** | **C** | **T** | **C** | **A** | **A** | **A** | **G** | **G** | **G** | **C** | **A** | **C** | **C** | **A** | **A** | **G** | **G** | **T** | **T** | **C** | **A** | **T** | **C** | **A** | **T** | **T** | **T** | **G** | **A** | **A** | **T** | **G** | **A** | **A** | **T** | **T** | **G** | **A** | **A** | **A** | **A** | **A** | **A** | **A** | **A** | **C** | **G** | **G** | **C** | **G** | **G** | **T** | **T** | **T** | **T** | **G** | **T** | **C** | **C** | **A** | **A** | **A** | **C** | **A** | **T** | **T** | **T** | **A** | **T** | **T** | **A** | **C** | **T** | **T** | **C** | **T** | **C** | **A** | **A** | **A** | **A** | **A** | **G** | **C** | **A** | **C** | **T** | **T** | **T** | **T** | **G** | **A** | **A** | **G** | **T** | **T** | **T** | **C** | **G** | **T** | **T** | **T** | **G** | **A** | **A** | **T** | **T** | **A** | **T** | **C** | **A** | **T** | **G** | **A** | **T** | **C** | **A** | **C** | **C** | **G** | **G** | **A** | **A** | **A** | **A** | **T** | **T** | **G** | **T** | **T** | **G** | **T** | **T** | **A** | **T** | **T** | **G** | **A** | **C** | **G** | **G** | **A** | **A** | **A** | **A** | **G** | **T** | **A** | **G** | **G** | **C** | **T** | **A** | **C** | **A** | **T** | **T** | **G** | **G** | **C** | **G** | **G** | **C** | **T** | **T** | **T** | **A** | **A** | **T** | **G** | **T** | **T** | **G** | **C** | **C** | **G** | **A** | **T** | **C** | **A** | **A** | **T** | **A** | **T** | **G** | **C** | **T** | **G** | **G** | **A** | **A** | **C** | **G** | **A** | **C** | **T** | **A** | **A** | **A** | **A** | **A** | **G** | **T** | **T** | **T** | **G** | **G** | **C** | **T** | **A** | **T** | **T** | **G** | **G** | **C** | **G** | **G** | **G** | **A** | **T** | **A** | **C** | **G** | **C** | **A** | **T** | **T** | **T** | **A** | **C** | **G** | **G** | **A** | **T** | **T** | **C** | **A** | **A** | **G** | **G** | **G** | **C** | **C** | **A** | **G** | **C** | **A** | **G** | **C** | **C** | **T** | **C** | **A** | **T** | **T** | **A** | **C** | **T** | **G** | **C** | **A** | **A** | **A** | **T** | **G** | **C** | **G** | **T** | **T** | **T** | **T** | **T** | **T** | **A** | **A** | **T** | **G** | **G** | **A** | **T** | **T** | **G** | **G** | **A** | **A** | **C** | **G** | **T** | **C** | **T** | **C** | **T** | **T** | **C** | **C** | **C** | **C** | **C** | **G** | **A** | **G** | **A** | **A** | **A** | **A** | **A** | **T** | **C** | **G** | **T** | **G** | **T** | **G** | **G** | **C** | **G** | **T** | **A** | **T** | **C** | **A** | **A** | **T** | **T** | **G** | **G** | **A** | **T** | **T** | **A** | **T** | **T** | **T** | **C** | **T** | **T** | **T** | **A** | **A** | **A** | **C** | **T** | **T** | **G** | **A** | **A** | **G** | **C** | **A** | **T** | **T** | **G** | **G** | **T** | **G** | **C** | **C** | **A** | **G** | **A** | **G** | **G** | **C** | **A** | **A** | **A** | **T** | **A** | **C** | **A** | **T** | **C** | **C** | **A** | **T** | **T** | **C** | **A** | **G** | **A** | **T** | **G** | **A** | **T** | **T** | **G** | **C** | **C** | **A** | **G** | **T** | **G** | **G** | **T** | **C** | **C** | **T** | **A** | **A** | **C** | **A** | **G** | **T** | **G** | **A** | **C** | **C** | **G** | **T** | **G** | **A** | **A** | **C** | **A** | **A** | **A** | **T** | **T** | **A** | **A** | **A** | **T** | **T** | **G** | **G** | **C** | **C** | **T** | **T** | **T** | **A** | **T** | **T** | **A** | **A** | **A** | **T** | **T** | **G** | **A** | **T** | **T** | **A** | **C** | **T** | **T** | **C** | **T** | **G** | **C** | **C** | **A** | **A** | **G** | **A** | **A** | **A** | **A** | **G** | **A** | **G** | **T** | **C** | **T** | **G** | **G** | **A** | **T** | **T** | **C** | **A** | **A** | **A** | **C** | **G** | **C** | **C** | **G** | **T** | **A** | **T** | **T** | **T** | **A** | **G** | **T** | **T** | **C** | **C** | **T** | **G** | **A** | **T** | **G** | **A** | **T** | **A** | **G** | **T** | **G** | **T** | **C** | **T** | **T** | **G** | **G** | **C** | **T** | **G** | **C** | **T** | **T** | **T** | **A** | **A** | **A** | **G** | **G** | **T** | **T** | **G** | **C** | **T** | **G** | **C** | **G** | **G** | **C** | **T** | **T** | **C** | **A** | **G** | **G** | **A** | **G** | **T** | **A** | **G** | **A** | **T** | **G** | **T** | **T** | **A** | **A** | **A** | **A** | **T** | **T** | **A** | **T** | **G** | **A** | **T** | **T** | **C** | **C** | **A** | **G** | **A** | **T** | **A** | **A** | **G** | **C** | **C** | **C** | **G** | **A** | **C** | **C** | **A** | **T** | **C** | **C** | **G** | **T** | **T** | **T** | **A** | **T** | **T** | **T** | **A** | **T** | **C** | **G** | **A** | **G** | **C** | **A** | **A** | **C** | **A** | **C** | **A** | **G** | **T** | **A** | **T** | **T** | **A** | **C** | **G** | **G** | **C** | **C** | **G** | **C** | **T** | **T** | **A** | **T** | **T** | **G** | **A** | **T** | **G** | **A** | **A** | **A** | **G** | **A** | **A** | **A** | **A** | **T** | **A** | **T** | **T** | **G** | **A** | **A** | **A** | **T** | **T** | **T** | **T** | **A** | **A** | **T** | **T** | **T** | **A** | **T** | **A** | **A** | **C** | **G** | **G** | **T** | **G** | **G** | **T** | **T** | **T** | **C** | **T** | **T** | **A** | **C** | **A** | **T** | **G** | **C** | **G** | **A** | **A** | **A** | **A** | **C** | **A** | **A** | **T** | **G** | **A** | **T** | **T** | **A** | **T** | **G** | **G** | **A** | **T** | **G** | **A** | **T** | **G** | **A** | **A** | **G** | **T** | **C** | **T** | **G** | **T** | **A** | **C** | **A** | **G** | **T** | **T** | **G** | **G** | **T** | **T** | **C** | **A** | **G** | **C** | **C** | **A** | **A** | **T** | **C** | **A** | **A** | **G** | **A** | **T** | **A** | **T** | **C** | **C** | **G** | **A** | **A** | **G** | **T** | **T** | **A** | **C** | **A** | **A** | **A** | **T** | **T** | **A** | **A** | **A** | **C** | **T** | **T** | **T** | **G** | **A** | **A** | **G** | **C** | **A** | **A** | **A** | **T** | **G** | **C** | **T** | **G** | **T** | **G** | **T** | **T** | **A** | **T** | **A** | **T** | **G** | **A** | **T** | **A** | **A** | **A** | **A** | **A** | **A** | **A** | **T** | **C** | **A** | **T** | **T** | **G** | **A** | **T** | **C** | **A** | **A** | **T** | **T** | **A** | **G** | **A** | **A** | **G** | **C** | **A** | **A** | **T** | **T** | **T** | **T** | **C** | **T** | **T** | **A** | **G** | **A** | **A** | **G** | **A** | **T** | **C** | **G** | **A** | **A** | **A** | **A** | **A** | **A** | **A** | **T** | **G** | **T** | **A** | **C** | **A** | **A** | **C** | **A** | **A** | **T** | **G** | **A** | **C** | **T** | **C** | **C** | **A** | **G** | **A** | **A** | **G** | **T** | **T** | **G** | **T** | **T** | **C** | **G** | **C** | **G** | **A** | **C** | **A** | **T** | **G** | **T** | **C** | **A** | **A** | **A** | **A** | **T** | **G** | **G** | **T** | **T** | **G** | **A** | **T** | **T** | **T** | **T** | **T** | **A** | **A** | **A** | **C** | **A** | **A** | **C** | **A** | **A** | **A** | **T** | **T** | **T** | **C** | **A** | **C** | **G** | **A** | **T** | **T** | **A** | **T** | **T** | **T** | **T** | **C** | **A** | **C** | **C** | **A** | **A** | **T** | **T** | **C** | **T** | **T** | **-** | **-** | **-** |
| **N52789** | **T** | **T** | **G** | **A** | **T** | **T** | **C** | **T** | **T** | **A** | **G** | **C** | **G** | **T** | **C** | **T** | **T** | **A** | **A** | **C** | **A** | **G** | **T** | **T** | **A** | **T** | **T** | **T** | **A** | **T** | **T** | **T** | **T** | **A** | **T** | **T** | **A** | **A** | **T** | **G** | **C** | **A** | **A** | **T** | **T** | **A** | **T** | **T** | **G** | **C** | **G** | **G** | **G** | **A** | **A** | **T** | **T** | **A** | **C** | **C** | **A** | **T** | **T** | **T** | **T** | **G** | **T** | **T** | **A** | **A** | **A** | **A** | **C** | **C** | **T** | **C** | **G** | **A** | **G** | **A** | **T** | **G** | **T** | **A** | **G** | **C** | **A** | **G** | **C** | **A** | **A** | **T** | **T** | **T** | **G** | **G** | **G** | **C** | **A** | **T** | **G** | **G** | **C** | **T** | **A** | **C** | **T** | **C** | **G** | **T** | **A** | **C** | **T** | **G** | **A** | **T** | **T** | **G** | **C** | **T** | **C** | **T** | **A** | **C** | **C** | **C** | **G** | **T** | **T** | **T** | **T** | **T** | **G** | **G** | **T** | **T** | **T** | **T** | **T** | **T** | **C** | **T** | **T** | **A** | **T** | **A** | **T** | **T** | **T** | **A** | **T** | **T** | **C** | **T** | **T** | **T** | **G** | **G** | **G** | **C** | **G** | **C** | **G** | **G** | **T** | **T** | **T** | **A** | **A** | **C** | **C** | **G** | **A** | **C** | **A** | **A** | **A** | **A** | **A** | **G** | **A** | **A** | **A** | **T** | **T** | **T** | **T** | **A** | **T** | **T** | **T** | **G** | **C** | **A** | **A** | **C** | **A** | **A** | **A** | **G** | **T** | **G** | **A** | **T** | **T** | **T** | **A** | **C** | **G** | **G** | **G** | **A** | **A** | **T** | **T** | **A** | **G** | **A** | **G** | **A** | **A** | **T** | **T** | **T** | **T** | **C** | **A** | **G** | **A** | **A** | **C** | **T** | **T** | **C** | **C** | **A** | **A** | **G** | **A** | **A** | **G** | **A** | **A** | **A** | **G** | **T** | **T** | **T** | **T** | **G** | **A** | **A** | **C** | **T** | **T** | **T** | **A** | **T** | **A** | **G** | **T** | **C** | **A** | **A** | **A** | **A** | **G** | **A** | **T** | **G** | **C** | **C** | **G** | **A** | **C** | **G** | **G** | **A** | **G** | **G** | **A** | **G** | **C** | **A** | **A** | **C** | **A** | **A** | **C** | **A** | **G** | **T** | **T** | **T** | **A** | **C** | **A** | **G** | **A** | **C** | **T** | **T** | **T** | **T** | **T** | **T** | **T** | **C** | **C** | **T** | **C** | **G** | **T** | **T** | **A** | **A** | **A** | **T** | **A** | **G** | **G** | **A** | **T** | **G** | **C** | **C** | **C** | **C** | **T** | **G** | **A** | **C** | **T** | **A** | **A** | **G** | **A** | **A** | **A** | **A** | **A** | **T** | **G** | **A** | **T** | **G** | **T** | **T** | **G** | **A** | **A** | **A** | **T** | **T** | **T** | **T** | **T** | **A** | **C** | **G** | **G** | **A** | **T** | **G** | **G** | **T** | **A** | **C** | **G** | **G** | **A** | **A** | **A** | **A** | **A** | **T** | **T** | **C** | **A** | **A** | **T** | **G** | **C** | **G** | **T** | **T** | **G** | **A** | **T** | **G** | **G** | **C** | **G** | **G** | **A** | **C** | **A** | **T** | **A** | **A** | **A** | **A** | **A** | **A** | **A** | **G** | **C** | **T** | **C** | **A** | **G** | **C** | **A** | **C** | **T** | **C** | **C** | **A** | **T** | **T** | **C** | **A** | **T** | **A** | **T** | **T** | **G** | **A** | **A** | **T** | **A** | **T** | **T** | **A** | **T** | **G** | **C** | **C** | **T** | **T** | **T** | **G** | **T** | **A** | **A** | **C** | **G** | **G** | **A** | **T** | **C** | **A** | **T** | **A** | **T** | **C** | **G** | **G** | **T** | **A** | **C** | **C** | **A** | **A** | **A** | **A** | **T** | **T** | **T** | **T** | **G** | **A** | **A** | **C** | **T** | **T** | **A** | **T** | **T** | **A** | **G** | **A** | **A** | **G** | **A** | **A** | **A** | **A** | **G** | **G** | **C** | **G** | **G** | **C** | **T** | **G** | **A** | **A** | **G** | **G** | **G** | **G** | **T** | **T** | **G** | **A** | **A** | **G** | **T** | **G** | **C** | **G** | **G** | **T** | **T** | **G** | **C** | **T** | **T** | **T** | **A** | **C** | **G** | **A** | **T** | **G** | **C** | **C** | **T** | **T** | **T** | **G** | **G** | **C** | **T** | **C** | **A** | **A** | **A** | **G** | **G** | **G** | **C** | **A** | **C** | **C** | **A** | **A** | **G** | **G** | **T** | **T** | **C** | **A** | **T** | **C** | **A** | **T** | **T** | **T** | **G** | **A** | **A** | **T** | **G** | **A** | **A** | **T** | **T** | **G** | **A** | **A** | **A** | **A** | **A** | **A** | **A** | **A** | **C** | **G** | **G** | **C** | **G** | **G** | **T** | **T** | **T** | **T** | **G** | **T** | **C** | **C** | **A** | **A** | **A** | **C** | **G** | **T** | **T** | **T** | **A** | **T** | **T** | **A** | **C** | **T** | **T** | **C** | **T** | **C** | **A** | **A** | **A** | **A** | **A** | **G** | **C** | **A** | **C** | **T** | **T** | **T** | **T** | **G** | **A** | **A** | **G** | **T** | **T** | **T** | **C** | **G** | **T** | **T** | **T** | **G** | **A** | **A** | **T** | **T** | **A** | **T** | **C** | **A** | **T** | **G** | **A** | **T** | **C** | **A** | **C** | **C** | **G** | **G** | **A** | **A** | **A** | **A** | **T** | **T** | **G** | **T** | **T** | **G** | **T** | **T** | **A** | **T** | **T** | **G** | **A** | **T** | **G** | **G** | **G** | **A** | **A** | **A** | **G** | **T** | **A** | **G** | **G** | **C** | **T** | **A** | **C** | **A** | **T** | **T** | **G** | **G** | **C** | **G** | **G** | **C** | **T** | **T** | **T** | **A** | **A** | **T** | **G** | **T** | **T** | **G** | **C** | **C** | **G** | **A** | **T** | **C** | **A** | **A** | **T** | **A** | **T** | **G** | **C** | **C** | **G** | **G** | **A** | **A** | **C** | **G** | **A** | **C** | **T** | **A** | **A** | **A** | **A** | **A** | **G** | **T** | **T** | **T** | **G** | **G** | **C** | **T** | **A** | **T** | **T** | **G** | **G** | **C** | **G** | **G** | **G** | **A** | **T** | **A** | **C** | **A** | **C** | **A** | **T** | **T** | **T** | **A** | **C** | **G** | **G** | **A** | **T** | **T** | **C** | **A** | **A** | **G** | **G** | **G** | **C** | **C** | **A** | **G** | **C** | **A** | **G** | **C** | **C** | **T** | **C** | **A** | **T** | **T** | **A** | **C** | **T** | **G** | **C** | **A** | **A** | **A** | **T** | **G** | **C** | **G** | **T** | **T** | **T** | **T** | **T** | **T** | **A** | **A** | **T** | **G** | **G** | **A** | **T** | **T** | **G** | **G** | **A** | **A** | **C** | **G** | **T** | **C** | **T** | **C** | **T** | **T** | **C** | **C** | **C** | **C** | **T** | **G** | **A** | **G** | **A** | **A** | **A** | **A** | **A** | **T** | **C** | **G** | **T** | **G** | **T** | **G** | **G** | **C** | **G** | **T** | **A** | **T** | **C** | **A** | **A** | **T** | **T** | **G** | **G** | **A** | **T** | **T** | **A** | **T** | **T** | **T** | **C** | **T** | **T** | **T** | **A** | **A** | **A** | **C** | **T** | **T** | **G** | **A** | **A** | **G** | **C** | **G** | **T** | **T** | **G** | **G** | **T** | **G** | **C** | **C** | **A** | **G** | **A** | **G** | **G** | **C** | **A** | **A** | **A** | **T** | **A** | **C** | **A** | **T** | **C** | **C** | **A** | **T** | **T** | **C** | **A** | **G** | **A** | **T** | **G** | **A** | **T** | **T** | **G** | **C** | **C** | **A** | **G** | **T** | **G** | **G** | **T** | **C** | **C** | **T** | **A** | **A** | **T** | **A** | **G** | **T** | **G** | **A** | **C** | **C** | **G** | **T** | **G** | **A** | **A** | **C** | **A** | **A** | **A** | **T** | **T** | **A** | **A** | **A** | **T** | **T** | **G** | **G** | **C** | **T** | **T** | **T** | **T** | **A** | **T** | **T** | **A** | **A** | **A** | **T** | **T** | **G** | **A** | **T** | **T** | **A** | **C** | **T** | **T** | **C** | **T** | **G** | **C** | **C** | **A** | **A** | **G** | **A** | **A** | **A** | **A** | **G** | **A** | **G** | **T** | **C** | **T** | **G** | **G** | **A** | **T** | **T** | **C** | **A** | **A** | **A** | **C** | **G** | **C** | **C** | **G** | **T** | **A** | **T** | **T** | **T** | **A** | **G** | **T** | **T** | **C** | **C** | **T** | **G** | **A** | **T** | **G** | **A** | **T** | **A** | **G** | **T** | **G** | **T** | **C** | **T** | **T** | **G** | **G** | **C** | **G** | **G** | **C** | **T** | **T** | **T** | **A** | **A** | **A** | **G** | **G** | **T** | **T** | **G** | **C** | **T** | **G** | **C** | **G** | **G** | **C** | **T** | **T** | **C** | **A** | **G** | **G** | **A** | **G** | **T** | **A** | **G** | **A** | **T** | **G** | **T** | **T** | **A** | **A** | **A** | **A** | **T** | **T** | **A** | **T** | **G** | **A** | **T** | **T** | **C** | **C** | **A** | **G** | **A** | **T** | **A** | **A** | **G** | **C** | **C** | **C** | **G** | **A** | **C** | **C** | **A** | **T** | **C** | **C** | **G** | **T** | **T** | **T** | **A** | **T** | **T** | **T** | **A** | **T** | **C** | **G** | **A** | **G** | **C** | **A** | **A** | **C** | **A** | **C** | **A** | **G** | **T** | **A** | **T** | **T** | **A** | **C** | **G** | **G** | **G** | **C** | **G** | **C** | **T** | **T** | **G** | **T** | **T** | **G** | **A** | **T** | **G** | **A** | **A** | **A** | **G** | **A** | **A** | **A** | **A** | **T** | **A** | **T** | **T** | **G** | **A** | **A** | **A** | **T** | **T** | **T** | **T** | **A** | **A** | **T** | **T** | **T** | **A** | **T** | **A** | **A** | **C** | **G** | **G** | **T** | **G** | **G** | **T** | **T** | **T** | **C** | **T** | **T** | **A** | **C** | **A** | **T** | **G** | **C** | **G** | **A** | **A** | **A** | **A** | **C** | **A** | **A** | **T** | **G** | **A** | **T** | **T** | **A** | **T** | **G** | **G** | **A** | **T** | **G** | **A** | **T** | **G** | **A** | **A** | **G** | **T** | **C** | **T** | **G** | **C** | **A** | **C** | **A** | **G** | **T** | **A** | **G** | **G** | **T** | **T** | **C** | **A** | **G** | **C** | **C** | **A** | **A** | **T** | **C** | **A** | **A** | **G** | **A** | **T** | **A** | **T** | **C** | **C** | **G** | **A** | **A** | **G** | **T** | **T** | **A** | **C** | **A** | **A** | **A** | **T** | **T** | **A** | **A** | **A** | **C** | **T** | **T** | **T** | **G** | **A** | **A** | **G** | **C** | **A** | **A** | **A** | **T** | **G** | **C** | **T** | **G** | **T** | **G** | **T** | **T** | **A** | **T** | **A** | **T** | **G** | **A** | **T** | **A** | **A** | **A** | **A** | **A** | **A** | **A** | **T** | **C** | **A** | **T** | **T** | **G** | **A** | **T** | **C** | **A** | **A** | **T** | **T** | **A** | **G** | **A** | **A** | **G** | **C** | **A** | **A** | **T** | **T** | **T** | **T** | **C** | **T** | **T** | **A** | **G** | **A** | **A** | **G** | **A** | **T** | **C** | **G** | **A** | **A** | **A** | **A** | **A** | **A** | **A** | **T** | **G** | **T** | **A** | **C** | **A** | **A** | **C** | **A** | **A** | **T** | **G** | **A** | **C** | **T** | **C** | **C** | **A** | **G** | **A** | **A** | **G** | **T** | **T** | **G** | **T** | **T** | **C** | **G** | **C** | **G** | **A** | **C** | **A** | **T** | **G** | **T** | **C** | **A** | **A** | **A** | **A** | **T** | **G** | **G** | **T** | **T** | **G** | **A** | **T** | **T** | **T** | **T** | **T** | **A** | **A** | **A** | **C** | **A** | **A** | **C** | **A** | **A** | **A** | **T** | **T** | **T** | **C** | **A** | **C** | **G** | **A** | **T** | **T** | **A** | **T** | **T** | **C** | **T** | **C** | **A** | **C** | **C** | **A** | **A** | **T** | **T** | **C** | **T** | **T** | **-** | **-** | **-** |
| **N55725** | **T** | **T** | **G** | **A** | **T** | **T** | **C** | **T** | **T** | **A** | **G** | **C** | **G** | **T** | **C** | **T** | **T** | **A** | **A** | **C** | **A** | **G** | **T** | **T** | **A** | **T** | **T** | **T** | **A** | **T** | **T** | **T** | **T** | **A** | **T** | **T** | **A** | **A** | **T** | **G** | **C** | **A** | **A** | **T** | **T** | **A** | **T** | **T** | **G** | **C** | **G** | **G** | **G** | **A** | **A** | **T** | **T** | **A** | **C** | **C** | **A** | **T** | **T** | **T** | **T** | **G** | **T** | **T** | **A** | **A** | **A** | **A** | **C** | **C** | **T** | **C** | **G** | **A** | **G** | **A** | **T** | **G** | **T** | **A** | **G** | **C** | **A** | **G** | **C** | **A** | **A** | **T** | **T** | **T** | **G** | **G** | **G** | **C** | **A** | **T** | **G** | **G** | **C** | **T** | **A** | **C** | **T** | **C** | **G** | **T** | **A** | **C** | **T** | **G** | **A** | **T** | **T** | **G** | **C** | **T** | **C** | **T** | **A** | **C** | **C** | **C** | **G** | **T** | **T** | **T** | **T** | **T** | **G** | **G** | **T** | **T** | **T** | **T** | **T** | **T** | **C** | **T** | **T** | **A** | **T** | **A** | **T** | **T** | **T** | **A** | **T** | **T** | **C** | **T** | **T** | **T** | **G** | **G** | **T** | **C** | **G** | **A** | **G** | **G** | **T** | **T** | **T** | **A** | **A** | **C** | **C** | **G** | **A** | **C** | **A** | **A** | **A** | **A** | **A** | **G** | **A** | **A** | **A** | **T** | **T** | **T** | **T** | **A** | **T** | **T** | **T** | **G** | **C** | **A** | **A** | **C** | **A** | **A** | **A** | **G** | **T** | **G** | **A** | **T** | **T** | **T** | **A** | **C** | **G** | **G** | **G** | **A** | **A** | **T** | **T** | **A** | **G** | **A** | **G** | **A** | **A** | **T** | **T** | **T** | **C** | **C** | **A** | **G** | **A** | **A** | **T** | **T** | **T** | **C** | **C** | **A** | **A** | **G** | **A** | **A** | **G** | **A** | **A** | **A** | **G** | **T** | **T** | **T** | **T** | **G** | **A** | **A** | **C** | **T** | **T** | **T** | **A** | **T** | **A** | **G** | **T** | **C** | **A** | **A** | **A** | **A** | **G** | **A** | **T** | **G** | **C** | **C** | **G** | **A** | **C** | **G** | **G** | **A** | **G** | **G** | **A** | **G** | **C** | **A** | **A** | **C** | **A** | **A** | **C** | **A** | **G** | **T** | **T** | **T** | **A** | **C** | **A** | **G** | **A** | **C** | **T** | **T** | **T** | **T** | **T** | **T** | **T** | **C** | **C** | **T** | **C** | **G** | **T** | **T** | **A** | **A** | **A** | **T** | **A** | **G** | **G** | **A** | **T** | **G** | **C** | **C** | **T** | **C** | **T** | **G** | **A** | **C** | **T** | **A** | **A** | **G** | **A** | **A** | **A** | **A** | **A** | **T** | **G** | **A** | **T** | **G** | **T** | **T** | **G** | **A** | **A** | **A** | **T** | **T** | **T** | **T** | **T** | **A** | **C** | **G** | **G** | **A** | **T** | **G** | **G** | **T** | **A** | **C** | **G** | **G** | **A** | **A** | **A** | **A** | **A** | **T** | **T** | **C** | **A** | **A** | **T** | **G** | **C** | **G** | **T** | **T** | **G** | **A** | **T** | **G** | **G** | **C** | **G** | **G** | **A** | **T** | **A** | **T** | **A** | **A** | **A** | **A** | **A** | **A** | **A** | **G** | **C** | **T** | **C** | **A** | **G** | **C** | **A** | **C** | **T** | **C** | **C** | **A** | **T** | **T** | **C** | **A** | **T** | **A** | **T** | **T** | **G** | **A** | **A** | **T** | **A** | **T** | **T** | **A** | **T** | **G** | **C** | **C** | **T** | **T** | **T** | **G** | **T** | **A** | **A** | **C** | **G** | **G** | **A** | **T** | **C** | **A** | **T** | **A** | **T** | **C** | **G** | **G** | **C** | **A** | **C** | **C** | **A** | **A** | **A** | **A** | **T** | **T** | **T** | **T** | **G** | **A** | **A** | **C** | **T** | **T** | **A** | **T** | **T** | **A** | **G** | **A** | **A** | **G** | **A** | **A** | **A** | **A** | **G** | **G** | **C** | **G** | **G** | **C** | **T** | **G** | **A** | **A** | **G** | **G** | **G** | **G** | **T** | **T** | **G** | **A** | **A** | **G** | **T** | **G** | **C** | **G** | **G** | **T** | **T** | **G** | **C** | **T** | **T** | **T** | **A** | **C** | **G** | **A** | **T** | **G** | **C** | **C** | **T** | **T** | **T** | **G** | **G** | **C** | **T** | **C** | **A** | **A** | **A** | **G** | **G** | **G** | **C** | **A** | **C** | **C** | **A** | **A** | **G** | **G** | **T** | **T** | **C** | **A** | **T** | **C** | **A** | **T** | **T** | **T** | **G** | **A** | **A** | **T** | **G** | **A** | **A** | **T** | **T** | **G** | **A** | **A** | **A** | **A** | **A** | **A** | **A** | **A** | **C** | **G** | **G** | **T** | **G** | **G** | **T** | **T** | **T** | **T** | **G** | **T** | **C** | **C** | **A** | **A** | **A** | **C** | **G** | **T** | **T** | **T** | **A** | **T** | **T** | **A** | **C** | **T** | **T** | **C** | **T** | **C** | **A** | **A** | **A** | **A** | **A** | **G** | **C** | **A** | **C** | **T** | **T** | **T** | **T** | **G** | **A** | **A** | **G** | **T** | **T** | **T** | **C** | **G** | **T** | **T** | **T** | **G** | **A** | **A** | **T** | **T** | **A** | **T** | **C** | **A** | **T** | **G** | **A** | **T** | **C** | **A** | **C** | **C** | **G** | **G** | **A** | **A** | **A** | **A** | **T** | **T** | **G** | **T** | **T** | **G** | **T** | **T** | **A** | **T** | **T** | **G** | **A** | **C** | **G** | **G** | **A** | **A** | **A** | **A** | **G** | **T** | **A** | **G** | **G** | **C** | **T** | **A** | **C** | **A** | **T** | **T** | **G** | **G** | **C** | **G** | **G** | **C** | **T** | **T** | **T** | **A** | **A** | **T** | **G** | **T** | **T** | **G** | **C** | **C** | **G** | **A** | **T** | **C** | **A** | **A** | **T** | **A** | **T** | **G** | **C** | **C** | **G** | **G** | **A** | **A** | **C** | **G** | **A** | **C** | **T** | **A** | **A** | **A** | **A** | **A** | **G** | **T** | **T** | **T** | **G** | **G** | **C** | **T** | **A** | **T** | **T** | **G** | **G** | **C** | **G** | **G** | **G** | **A** | **T** | **A** | **C** | **A** | **C** | **A** | **T** | **T** | **T** | **A** | **C** | **G** | **G** | **A** | **T** | **T** | **C** | **A** | **A** | **G** | **G** | **G** | **C** | **C** | **A** | **G** | **C** | **A** | **G** | **C** | **C** | **T** | **C** | **A** | **T** | **T** | **A** | **C** | **T** | **G** | **C** | **A** | **A** | **A** | **T** | **G** | **C** | **G** | **T** | **T** | **T** | **T** | **T** | **T** | **A** | **A** | **T** | **G** | **G** | **A** | **T** | **T** | **G** | **G** | **A** | **A** | **C** | **G** | **T** | **C** | **T** | **C** | **T** | **T** | **C** | **C** | **C** | **C** | **C** | **G** | **A** | **G** | **A** | **A** | **A** | **A** | **A** | **T** | **C** | **G** | **T** | **G** | **T** | **G** | **G** | **C** | **G** | **T** | **A** | **T** | **C** | **A** | **A** | **T** | **T** | **G** | **G** | **A** | **T** | **T** | **A** | **T** | **T** | **T** | **C** | **T** | **T** | **T** | **A** | **A** | **A** | **C** | **T** | **T** | **G** | **A** | **A** | **G** | **C** | **A** | **T** | **T** | **G** | **G** | **T** | **G** | **C** | **C** | **A** | **G** | **A** | **G** | **G** | **C** | **A** | **A** | **A** | **T** | **A** | **C** | **A** | **T** | **C** | **C** | **A** | **T** | **T** | **C** | **A** | **G** | **A** | **T** | **G** | **A** | **T** | **T** | **G** | **C** | **C** | **A** | **G** | **T** | **G** | **G** | **T** | **C** | **C** | **T** | **A** | **A** | **C** | **A** | **G** | **T** | **G** | **A** | **C** | **C** | **G** | **T** | **G** | **A** | **A** | **C** | **A** | **A** | **A** | **T** | **T** | **A** | **A** | **A** | **T** | **T** | **G** | **G** | **C** | **C** | **T** | **T** | **T** | **A** | **T** | **T** | **A** | **A** | **A** | **T** | **T** | **G** | **A** | **T** | **T** | **A** | **C** | **T** | **T** | **C** | **T** | **G** | **C** | **C** | **A** | **A** | **G** | **A** | **A** | **A** | **A** | **G** | **A** | **G** | **T** | **C** | **T** | **G** | **G** | **A** | **T** | **T** | **C** | **A** | **A** | **A** | **C** | **G** | **C** | **C** | **G** | **T** | **A** | **T** | **T** | **T** | **A** | **G** | **T** | **T** | **C** | **C** | **T** | **G** | **A** | **T** | **G** | **A** | **T** | **A** | **G** | **T** | **G** | **T** | **C** | **T** | **T** | **G** | **G** | **C** | **T** | **G** | **C** | **T** | **T** | **T** | **A** | **A** | **A** | **G** | **G** | **T** | **T** | **G** | **C** | **T** | **G** | **C** | **G** | **G** | **C** | **T** | **T** | **C** | **A** | **G** | **G** | **A** | **G** | **T** | **A** | **G** | **A** | **T** | **G** | **T** | **T** | **A** | **A** | **A** | **A** | **T** | **T** | **A** | **T** | **G** | **A** | **T** | **T** | **C** | **C** | **A** | **G** | **A** | **T** | **A** | **A** | **G** | **C** | **C** | **C** | **G** | **A** | **C** | **C** | **A** | **T** | **C** | **C** | **G** | **T** | **T** | **T** | **A** | **T** | **T** | **T** | **A** | **T** | **C** | **G** | **A** | **G** | **C** | **A** | **A** | **C** | **A** | **C** | **A** | **G** | **T** | **A** | **T** | **T** | **A** | **C** | **G** | **G** | **C** | **C** | **G** | **C** | **T** | **T** | **A** | **T** | **T** | **G** | **A** | **T** | **G** | **A** | **A** | **A** | **G** | **A** | **A** | **A** | **A** | **T** | **A** | **T** | **T** | **G** | **A** | **A** | **A** | **T** | **T** | **T** | **T** | **A** | **A** | **T** | **T** | **T** | **A** | **T** | **A** | **A** | **C** | **G** | **G** | **T** | **G** | **G** | **T** | **T** | **T** | **C** | **T** | **T** | **A** | **C** | **A** | **T** | **G** | **C** | **G** | **A** | **A** | **A** | **A** | **C** | **A** | **A** | **T** | **G** | **A** | **T** | **T** | **A** | **T** | **G** | **G** | **A** | **T** | **G** | **A** | **T** | **G** | **A** | **A** | **G** | **T** | **C** | **T** | **G** | **C** | **A** | **C** | **A** | **G** | **T** | **T** | **G** | **G** | **T** | **T** | **C** | **A** | **G** | **C** | **C** | **A** | **A** | **T** | **C** | **A** | **A** | **G** | **A** | **T** | **A** | **T** | **C** | **C** | **G** | **A** | **A** | **G** | **T** | **T** | **A** | **C** | **A** | **A** | **A** | **T** | **T** | **A** | **A** | **A** | **C** | **T** | **T** | **T** | **G** | **A** | **A** | **G** | **C** | **A** | **A** | **A** | **T** | **G** | **C** | **T** | **G** | **T** | **G** | **T** | **T** | **A** | **T** | **A** | **T** | **G** | **A** | **T** | **A** | **A** | **A** | **A** | **A** | **A** | **A** | **T** | **C** | **A** | **T** | **T** | **G** | **A** | **T** | **C** | **A** | **A** | **T** | **T** | **A** | **G** | **A** | **A** | **G** | **C** | **A** | **A** | **T** | **T** | **T** | **T** | **C** | **T** | **T** | **A** | **G** | **A** | **A** | **G** | **A** | **T** | **C** | **G** | **A** | **A** | **A** | **A** | **A** | **A** | **A** | **T** | **G** | **T** | **A** | **C** | **A** | **A** | **C** | **A** | **A** | **T** | **G** | **A** | **C** | **T** | **C** | **C** | **A** | **G** | **A** | **A** | **G** | **T** | **T** | **G** | **T** | **T** | **C** | **G** | **C** | **G** | **A** | **C** | **A** | **T** | **G** | **T** | **C** | **A** | **A** | **A** | **A** | **T** | **G** | **G** | **T** | **T** | **G** | **A** | **T** | **T** | **T** | **T** | **T** | **A** | **A** | **A** | **C** | **A** | **A** | **C** | **A** | **A** | **A** | **T** | **T** | **T** | **C** | **A** | **C** | **G** | **A** | **T** | **T** | **A** | **T** | **T** | **T** | **T** | **C** | **A** | **C** | **C** | **A** | **A** | **T** | **T** | **C** | **T** | **T** | **-** | **-** | **-** |
| **N53453** | **T** | **T** | **G** | **A** | **T** | **T** | **C** | **T** | **T** | **A** | **G** | **C** | **G** | **T** | **C** | **T** | **T** | **A** | **A** | **C** | **A** | **G** | **T** | **T** | **A** | **T** | **T** | **T** | **A** | **T** | **T** | **T** | **T** | **A** | **T** | **T** | **A** | **A** | **T** | **G** | **C** | **A** | **A** | **T** | **T** | **A** | **T** | **T** | **G** | **C** | **G** | **G** | **G** | **A** | **A** | **T** | **T** | **A** | **C** | **C** | **A** | **T** | **T** | **T** | **T** | **G** | **T** | **T** | **A** | **A** | **A** | **A** | **C** | **C** | **T** | **C** | **G** | **A** | **G** | **A** | **T** | **G** | **T** | **A** | **G** | **C** | **A** | **G** | **C** | **A** | **A** | **T** | **T** | **T** | **G** | **G** | **G** | **C** | **A** | **T** | **G** | **G** | **C** | **T** | **A** | **C** | **T** | **C** | **G** | **T** | **A** | **C** | **T** | **G** | **A** | **T** | **T** | **G** | **C** | **T** | **C** | **T** | **A** | **C** | **C** | **C** | **G** | **T** | **T** | **T** | **T** | **T** | **G** | **G** | **T** | **T** | **T** | **T** | **T** | **T** | **C** | **T** | **T** | **A** | **T** | **A** | **T** | **T** | **T** | **A** | **T** | **T** | **C** | **T** | **T** | **T** | **G** | **G** | **G** | **C** | **G** | **C** | **G** | **G** | **T** | **T** | **T** | **A** | **A** | **C** | **C** | **G** | **A** | **C** | **A** | **A** | **A** | **A** | **A** | **G** | **A** | **A** | **A** | **T** | **T** | **T** | **T** | **A** | **T** | **T** | **T** | **G** | **C** | **A** | **A** | **C** | **A** | **A** | **A** | **G** | **T** | **G** | **A** | **T** | **T** | **T** | **A** | **C** | **G** | **G** | **G** | **A** | **A** | **T** | **T** | **A** | **G** | **A** | **G** | **A** | **A** | **T** | **T** | **T** | **C** | **C** | **A** | **G** | **A** | **A** | **T** | **T** | **T** | **C** | **C** | **A** | **A** | **G** | **A** | **A** | **G** | **A** | **A** | **A** | **G** | **T** | **T** | **T** | **T** | **G** | **A** | **A** | **C** | **T** | **T** | **T** | **A** | **T** | **A** | **G** | **T** | **C** | **T** | **A** | **A** | **A** | **G** | **A** | **T** | **G** | **C** | **C** | **G** | **A** | **C** | **G** | **G** | **A** | **G** | **G** | **A** | **G** | **C** | **A** | **A** | **C** | **A** | **A** | **C** | **A** | **G** | **T** | **T** | **T** | **A** | **C** | **A** | **G** | **A** | **C** | **T** | **T** | **T** | **T** | **T** | **T** | **T** | **C** | **C** | **T** | **C** | **G** | **T** | **T** | **A** | **A** | **A** | **T** | **A** | **G** | **G** | **A** | **T** | **G** | **C** | **C** | **C** | **C** | **T** | **G** | **A** | **C** | **T** | **A** | **A** | **G** | **A** | **A** | **A** | **A** | **A** | **T** | **G** | **A** | **T** | **G** | **T** | **T** | **G** | **A** | **A** | **A** | **T** | **T** | **T** | **T** | **T** | **A** | **C** | **G** | **G** | **A** | **T** | **G** | **G** | **T** | **A** | **C** | **G** | **G** | **A** | **A** | **A** | **A** | **A** | **T** | **T** | **C** | **A** | **A** | **T** | **G** | **C** | **G** | **T** | **T** | **G** | **A** | **T** | **G** | **G** | **C** | **G** | **G** | **A** | **C** | **A** | **T** | **A** | **A** | **A** | **A** | **A** | **A** | **A** | **G** | **C** | **T** | **C** | **A** | **G** | **C** | **A** | **C** | **T** | **C** | **C** | **A** | **T** | **T** | **C** | **A** | **T** | **A** | **T** | **T** | **G** | **A** | **A** | **T** | **A** | **T** | **T** | **A** | **T** | **G** | **C** | **C** | **T** | **T** | **T** | **G** | **T** | **A** | **A** | **C** | **G** | **G** | **A** | **T** | **C** | **A** | **T** | **A** | **T** | **C** | **G** | **G** | **T** | **A** | **C** | **C** | **A** | **A** | **A** | **A** | **T** | **T** | **T** | **T** | **G** | **A** | **A** | **C** | **T** | **T** | **A** | **T** | **T** | **A** | **G** | **A** | **A** | **G** | **A** | **A** | **A** | **A** | **G** | **G** | **C** | **G** | **G** | **C** | **T** | **G** | **A** | **A** | **G** | **G** | **G** | **G** | **T** | **T** | **G** | **A** | **A** | **G** | **T** | **G** | **C** | **G** | **G** | **T** | **T** | **G** | **C** | **T** | **T** | **T** | **A** | **C** | **G** | **A** | **T** | **G** | **C** | **C** | **T** | **T** | **T** | **G** | **G** | **C** | **T** | **C** | **A** | **A** | **A** | **G** | **G** | **G** | **C** | **A** | **C** | **C** | **A** | **A** | **G** | **G** | **T** | **T** | **C** | **A** | **T** | **C** | **A** | **T** | **T** | **T** | **G** | **A** | **A** | **T** | **G** | **A** | **A** | **T** | **T** | **G** | **A** | **A** | **A** | **A** | **A** | **A** | **A** | **A** | **C** | **G** | **G** | **C** | **G** | **G** | **T** | **T** | **T** | **T** | **G** | **T** | **C** | **C** | **A** | **A** | **A** | **C** | **G** | **T** | **T** | **T** | **A** | **T** | **T** | **A** | **C** | **T** | **T** | **C** | **T** | **C** | **A** | **A** | **A** | **A** | **A** | **G** | **C** | **A** | **C** | **T** | **T** | **T** | **T** | **G** | **A** | **A** | **G** | **T** | **T** | **T** | **C** | **G** | **T** | **T** | **T** | **G** | **A** | **A** | **T** | **T** | **A** | **T** | **C** | **A** | **T** | **G** | **A** | **T** | **C** | **A** | **C** | **C** | **G** | **G** | **A** | **A** | **A** | **A** | **T** | **T** | **G** | **T** | **T** | **G** | **T** | **T** | **A** | **T** | **T** | **G** | **A** | **C** | **G** | **G** | **A** | **A** | **A** | **A** | **G** | **T** | **A** | **G** | **G** | **C** | **T** | **A** | **C** | **A** | **T** | **T** | **G** | **G** | **C** | **G** | **G** | **C** | **T** | **T** | **T** | **A** | **A** | **T** | **G** | **T** | **T** | **G** | **C** | **C** | **G** | **A** | **T** | **C** | **A** | **A** | **T** | **A** | **T** | **G** | **C** | **C** | **G** | **G** | **A** | **A** | **C** | **G** | **A** | **C** | **T** | **A** | **A** | **A** | **A** | **A** | **G** | **T** | **T** | **T** | **G** | **G** | **C** | **T** | **A** | **T** | **T** | **G** | **G** | **C** | **G** | **G** | **G** | **A** | **T** | **A** | **C** | **A** | **C** | **A** | **T** | **T** | **T** | **A** | **C** | **G** | **G** | **A** | **T** | **T** | **C** | **A** | **A** | **G** | **G** | **G** | **C** | **C** | **A** | **G** | **C** | **A** | **G** | **C** | **C** | **T** | **C** | **A** | **T** | **T** | **A** | **C** | **T** | **G** | **C** | **A** | **A** | **A** | **T** | **G** | **C** | **G** | **T** | **T** | **T** | **T** | **T** | **T** | **A** | **A** | **T** | **G** | **G** | **A** | **T** | **T** | **G** | **G** | **A** | **A** | **C** | **G** | **T** | **C** | **T** | **C** | **T** | **T** | **C** | **C** | **C** | **C** | **C** | **G** | **A** | **G** | **A** | **A** | **A** | **A** | **A** | **T** | **C** | **G** | **T** | **G** | **T** | **G** | **G** | **C** | **G** | **T** | **A** | **T** | **C** | **A** | **A** | **T** | **T** | **G** | **G** | **A** | **T** | **T** | **A** | **T** | **T** | **T** | **C** | **T** | **T** | **T** | **A** | **A** | **A** | **C** | **T** | **T** | **G** | **A** | **A** | **G** | **C** | **A** | **T** | **T** | **G** | **G** | **T** | **G** | **C** | **C** | **A** | **G** | **A** | **G** | **G** | **C** | **A** | **A** | **A** | **T** | **A** | **C** | **A** | **T** | **C** | **C** | **A** | **T** | **T** | **C** | **A** | **G** | **A** | **T** | **G** | **A** | **T** | **T** | **G** | **C** | **C** | **A** | **G** | **T** | **G** | **G** | **T** | **C** | **C** | **T** | **A** | **A** | **C** | **A** | **G** | **T** | **G** | **A** | **C** | **C** | **G** | **T** | **G** | **A** | **A** | **C** | **A** | **A** | **A** | **T** | **T** | **A** | **A** | **A** | **T** | **T** | **G** | **G** | **C** | **C** | **T** | **T** | **T** | **A** | **T** | **T** | **A** | **A** | **A** | **T** | **T** | **G** | **A** | **T** | **T** | **A** | **C** | **T** | **T** | **C** | **T** | **G** | **C** | **C** | **A** | **A** | **G** | **A** | **A** | **A** | **A** | **G** | **A** | **G** | **T** | **C** | **T** | **G** | **G** | **A** | **T** | **T** | **C** | **A** | **A** | **A** | **C** | **G** | **C** | **C** | **G** | **T** | **A** | **T** | **T** | **T** | **A** | **G** | **T** | **T** | **C** | **C** | **T** | **G** | **A** | **T** | **G** | **A** | **T** | **A** | **G** | **T** | **G** | **T** | **C** | **T** | **T** | **G** | **G** | **C** | **T** | **G** | **C** | **T** | **T** | **T** | **A** | **A** | **A** | **G** | **G** | **T** | **T** | **G** | **C** | **T** | **G** | **C** | **G** | **G** | **C** | **T** | **T** | **C** | **A** | **G** | **G** | **A** | **G** | **T** | **A** | **G** | **A** | **T** | **G** | **T** | **T** | **A** | **A** | **A** | **A** | **T** | **T** | **A** | **T** | **G** | **A** | **T** | **T** | **C** | **C** | **A** | **G** | **A** | **T** | **A** | **A** | **G** | **C** | **C** | **C** | **G** | **A** | **C** | **C** | **A** | **T** | **C** | **C** | **G** | **T** | **T** | **T** | **A** | **T** | **T** | **T** | **A** | **T** | **C** | **G** | **A** | **G** | **C** | **A** | **A** | **C** | **A** | **C** | **A** | **G** | **T** | **A** | **T** | **T** | **A** | **C** | **G** | **G** | **C** | **C** | **G** | **C** | **T** | **T** | **A** | **T** | **T** | **G** | **A** | **T** | **G** | **A** | **A** | **A** | **G** | **A** | **A** | **A** | **A** | **T** | **A** | **T** | **T** | **G** | **A** | **A** | **A** | **T** | **T** | **T** | **T** | **A** | **A** | **T** | **T** | **T** | **A** | **T** | **A** | **A** | **C** | **G** | **G** | **T** | **G** | **G** | **T** | **T** | **T** | **C** | **T** | **T** | **A** | **C** | **A** | **T** | **G** | **C** | **G** | **A** | **A** | **A** | **A** | **C** | **A** | **A** | **T** | **G** | **A** | **T** | **T** | **A** | **T** | **G** | **G** | **A** | **T** | **G** | **A** | **T** | **G** | **A** | **A** | **G** | **T** | **C** | **T** | **G** | **C** | **A** | **C** | **A** | **G** | **T** | **T** | **G** | **G** | **T** | **T** | **C** | **A** | **G** | **C** | **C** | **A** | **A** | **T** | **C** | **A** | **A** | **G** | **A** | **T** | **A** | **T** | **C** | **C** | **G** | **A** | **A** | **G** | **T** | **T** | **A** | **C** | **A** | **A** | **A** | **T** | **T** | **A** | **A** | **A** | **C** | **T** | **T** | **T** | **G** | **A** | **A** | **G** | **C** | **A** | **A** | **A** | **T** | **G** | **C** | **T** | **G** | **T** | **G** | **T** | **T** | **A** | **T** | **A** | **T** | **G** | **A** | **T** | **A** | **A** | **A** | **A** | **A** | **A** | **A** | **T** | **C** | **A** | **T** | **T** | **G** | **A** | **T** | **C** | **A** | **A** | **T** | **T** | **A** | **G** | **A** | **A** | **G** | **C** | **A** | **A** | **T** | **T** | **T** | **T** | **C** | **T** | **T** | **A** | **G** | **A** | **A** | **G** | **A** | **T** | **C** | **G** | **A** | **A** | **A** | **A** | **A** | **A** | **A** | **T** | **G** | **T** | **A** | **C** | **A** | **A** | **C** | **A** | **A** | **T** | **G** | **A** | **C** | **T** | **C** | **C** | **A** | **G** | **A** | **A** | **G** | **T** | **T** | **G** | **T** | **T** | **C** | **G** | **C** | **G** | **A** | **C** | **A** | **T** | **G** | **T** | **C** | **A** | **A** | **A** | **A** | **T** | **G** | **G** | **T** | **T** | **G** | **A** | **T** | **T** | **T** | **T** | **T** | **A** | **A** | **A** | **C** | **A** | **G** | **C** | **A** | **A** | **A** | **T** | **T** | **T** | **C** | **A** | **C** | **G** | **A** | **T** | **T** | **A** | **T** | **T** | **T** | **T** | **C** | **A** | **C** | **C** | **A** | **A** | **T** | **T** | **C** | **T** | **T** | **-** | **-** | **-** |
| **N59508F** | **T** | **T** | **G** | **A** | **T** | **T** | **C** | **T** | **T** | **A** | **G** | **C** | **G** | **T** | **C** | **T** | **T** | **A** | **A** | **C** | **A** | **G** | **T** | **T** | **A** | **T** | **T** | **T** | **A** | **T** | **T** | **T** | **T** | **A** | **T** | **T** | **A** | **A** | **T** | **G** | **C** | **A** | **A** | **T** | **T** | **A** | **T** | **T** | **G** | **C** | **G** | **G** | **G** | **A** | **A** | **T** | **T** | **A** | **C** | **C** | **A** | **T** | **T** | **T** | **T** | **G** | **T** | **T** | **A** | **A** | **A** | **A** | **C** | **C** | **T** | **C** | **G** | **A** | **G** | **A** | **T** | **G** | **T** | **A** | **G** | **C** | **A** | **G** | **C** | **A** | **A** | **T** | **T** | **T** | **G** | **G** | **G** | **C** | **A** | **T** | **G** | **G** | **C** | **T** | **A** | **C** | **T** | **C** | **G** | **T** | **A** | **C** | **T** | **G** | **A** | **T** | **T** | **G** | **C** | **T** | **C** | **T** | **A** | **C** | **C** | **C** | **G** | **T** | **T** | **T** | **T** | **T** | **G** | **G** | **T** | **T** | **T** | **T** | **T** | **T** | **C** | **T** | **T** | **A** | **T** | **A** | **T** | **T** | **T** | **A** | **T** | **T** | **C** | **T** | **T** | **T** | **G** | **G** | **G** | **C** | **G** | **C** | **G** | **G** | **T** | **T** | **T** | **A** | **A** | **C** | **C** | **G** | **A** | **C** | **A** | **A** | **A** | **A** | **A** | **G** | **A** | **A** | **A** | **T** | **T** | **T** | **T** | **A** | **T** | **T** | **T** | **G** | **C** | **A** | **A** | **C** | **A** | **A** | **A** | **G** | **T** | **G** | **A** | **T** | **T** | **T** | **G** | **C** | **G** | **G** | **G** | **A** | **A** | **T** | **T** | **A** | **G** | **A** | **G** | **A** | **A** | **T** | **T** | **T** | **T** | **C** | **A** | **G** | **A** | **A** | **T** | **T** | **T** | **C** | **C** | **A** | **A** | **G** | **A** | **A** | **G** | **A** | **A** | **A** | **G** | **T** | **T** | **T** | **T** | **G** | **A** | **A** | **C** | **T** | **T** | **T** | **A** | **T** | **A** | **G** | **T** | **C** | **A** | **A** | **A** | **A** | **G** | **A** | **T** | **G** | **C** | **C** | **G** | **A** | **C** | **G** | **G** | **A** | **G** | **G** | **A** | **G** | **C** | **A** | **A** | **C** | **A** | **A** | **C** | **A** | **G** | **T** | **T** | **T** | **A** | **C** | **A** | **G** | **A** | **C** | **T** | **T** | **T** | **T** | **T** | **T** | **T** | **C** | **C** | **T** | **C** | **G** | **T** | **T** | **G** | **A** | **A** | **T** | **A** | **G** | **G** | **A** | **T** | **G** | **C** | **C** | **T** | **C** | **T** | **G** | **A** | **C** | **T** | **A** | **A** | **G** | **A** | **A** | **A** | **A** | **A** | **T** | **G** | **A** | **T** | **G** | **T** | **T** | **C** | **A** | **A** | **A** | **T** | **T** | **T** | **T** | **T** | **A** | **C** | **G** | **G** | **A** | **T** | **G** | **G** | **T** | **A** | **C** | **G** | **G** | **A** | **A** | **A** | **A** | **A** | **T** | **T** | **C** | **A** | **A** | **T** | **G** | **C** | **G** | **T** | **T** | **G** | **A** | **T** | **G** | **G** | **C** | **G** | **G** | **A** | **C** | **A** | **T** | **A** | **A** | **A** | **A** | **A** | **A** | **A** | **G** | **C** | **T** | **C** | **A** | **G** | **C** | **A** | **C** | **T** | **C** | **C** | **A** | **T** | **T** | **C** | **A** | **T** | **A** | **T** | **T** | **G** | **A** | **A** | **T** | **A** | **T** | **T** | **A** | **T** | **G** | **C** | **C** | **T** | **T** | **T** | **G** | **T** | **A** | **A** | **C** | **G** | **G** | **A** | **T** | **C** | **A** | **T** | **A** | **T** | **C** | **G** | **G** | **C** | **A** | **C** | **C** | **A** | **A** | **A** | **A** | **T** | **T** | **T** | **T** | **G** | **A** | **A** | **C** | **T** | **T** | **A** | **T** | **T** | **A** | **G** | **A** | **A** | **G** | **A** | **A** | **A** | **A** | **G** | **G** | **C** | **G** | **G** | **C** | **T** | **G** | **A** | **A** | **G** | **G** | **G** | **G** | **T** | **T** | **G** | **A** | **A** | **G** | **T** | **G** | **C** | **G** | **G** | **T** | **T** | **G** | **C** | **T** | **T** | **T** | **A** | **C** | **G** | **A** | **T** | **G** | **C** | **C** | **T** | **T** | **T** | **G** | **G** | **C** | **T** | **C** | **A** | **A** | **A** | **G** | **G** | **G** | **C** | **A** | **C** | **C** | **A** | **A** | **G** | **G** | **T** | **T** | **C** | **A** | **T** | **C** | **A** | **T** | **T** | **T** | **G** | **A** | **A** | **T** | **G** | **A** | **A** | **T** | **T** | **G** | **A** | **A** | **A** | **A** | **A** | **A** | **A** | **A** | **C** | **G** | **G** | **T** | **G** | **G** | **T** | **T** | **T** | **T** | **G** | **T** | **C** | **C** | **A** | **A** | **A** | **C** | **G** | **T** | **T** | **T** | **A** | **T** | **T** | **A** | **C** | **T** | **T** | **C** | **T** | **C** | **A** | **A** | **A** | **A** | **A** | **G** | **C** | **A** | **C** | **T** | **T** | **T** | **T** | **G** | **A** | **A** | **G** | **T** | **T** | **T** | **C** | **G** | **T** | **T** | **T** | **G** | **A** | **A** | **T** | **T** | **A** | **T** | **C** | **A** | **T** | **G** | **A** | **T** | **C** | **A** | **C** | **C** | **G** | **G** | **A** | **A** | **A** | **A** | **T** | **T** | **G** | **T** | **T** | **G** | **T** | **T** | **A** | **T** | **T** | **G** | **A** | **C** | **G** | **G** | **A** | **A** | **A** | **A** | **G** | **T** | **A** | **G** | **G** | **C** | **T** | **A** | **C** | **A** | **T** | **T** | **G** | **G** | **C** | **G** | **G** | **C** | **T** | **T** | **T** | **A** | **A** | **T** | **G** | **T** | **T** | **G** | **C** | **C** | **G** | **A** | **T** | **C** | **A** | **A** | **T** | **A** | **T** | **G** | **C** | **C** | **G** | **G** | **A** | **A** | **C** | **G** | **A** | **C** | **T** | **A** | **A** | **A** | **A** | **A** | **G** | **T** | **T** | **T** | **G** | **G** | **C** | **T** | **A** | **T** | **T** | **G** | **G** | **C** | **G** | **G** | **G** | **A** | **T** | **A** | **C** | **A** | **C** | **A** | **T** | **T** | **T** | **A** | **C** | **G** | **G** | **A** | **T** | **T** | **C** | **A** | **A** | **G** | **G** | **G** | **C** | **C** | **A** | **G** | **C** | **A** | **G** | **C** | **C** | **T** | **C** | **A** | **T** | **T** | **A** | **C** | **T** | **G** | **C** | **A** | **A** | **A** | **T** | **G** | **C** | **G** | **T** | **T** | **T** | **T** | **T** | **T** | **A** | **A** | **T** | **G** | **G** | **A** | **T** | **T** | **G** | **G** | **A** | **A** | **C** | **G** | **T** | **C** | **T** | **C** | **T** | **T** | **C** | **C** | **C** | **C** | **C** | **G** | **A** | **G** | **A** | **A** | **A** | **A** | **A** | **T** | **C** | **G** | **T** | **G** | **T** | **G** | **G** | **C** | **G** | **T** | **A** | **T** | **C** | **A** | **A** | **T** | **T** | **G** | **G** | **A** | **T** | **T** | **A** | **T** | **T** | **T** | **C** | **T** | **T** | **T** | **A** | **A** | **A** | **C** | **T** | **T** | **G** | **A** | **A** | **G** | **C** | **A** | **T** | **T** | **G** | **G** | **T** | **G** | **C** | **C** | **A** | **G** | **A** | **G** | **G** | **C** | **A** | **A** | **A** | **T** | **A** | **C** | **A** | **T** | **C** | **C** | **A** | **T** | **T** | **C** | **A** | **G** | **A** | **T** | **G** | **A** | **T** | **T** | **G** | **C** | **C** | **A** | **G** | **T** | **G** | **G** | **T** | **C** | **C** | **T** | **A** | **A** | **C** | **A** | **G** | **T** | **G** | **A** | **C** | **C** | **G** | **T** | **G** | **A** | **A** | **C** | **A** | **A** | **A** | **T** | **T** | **A** | **A** | **A** | **T** | **T** | **G** | **G** | **C** | **C** | **T** | **T** | **T** | **A** | **T** | **T** | **A** | **A** | **A** | **T** | **T** | **G** | **A** | **T** | **T** | **A** | **C** | **T** | **T** | **C** | **T** | **G** | **C** | **C** | **A** | **A** | **G** | **A** | **A** | **A** | **A** | **G** | **A** | **G** | **T** | **C** | **T** | **G** | **G** | **A** | **T** | **T** | **C** | **A** | **A** | **A** | **C** | **G** | **C** | **C** | **G** | **T** | **A** | **T** | **T** | **T** | **A** | **G** | **T** | **T** | **C** | **C** | **T** | **G** | **A** | **T** | **G** | **A** | **T** | **A** | **G** | **T** | **G** | **T** | **C** | **T** | **T** | **G** | **G** | **C** | **T** | **G** | **C** | **T** | **T** | **T** | **A** | **A** | **A** | **G** | **G** | **T** | **T** | **G** | **C** | **T** | **G** | **C** | **G** | **G** | **C** | **T** | **T** | **C** | **A** | **G** | **G** | **A** | **G** | **T** | **A** | **G** | **A** | **T** | **G** | **T** | **T** | **A** | **A** | **A** | **A** | **T** | **T** | **A** | **T** | **G** | **A** | **T** | **T** | **C** | **C** | **A** | **G** | **A** | **T** | **A** | **A** | **G** | **C** | **C** | **C** | **G** | **A** | **C** | **C** | **A** | **T** | **C** | **C** | **G** | **T** | **T** | **T** | **A** | **T** | **T** | **T** | **A** | **T** | **C** | **G** | **A** | **G** | **C** | **A** | **A** | **C** | **A** | **C** | **A** | **G** | **T** | **A** | **T** | **T** | **A** | **C** | **G** | **G** | **C** | **C** | **G** | **C** | **T** | **T** | **A** | **T** | **T** | **G** | **A** | **T** | **G** | **A** | **A** | **A** | **G** | **A** | **A** | **A** | **A** | **T** | **A** | **T** | **T** | **G** | **A** | **A** | **A** | **T** | **T** | **T** | **T** | **A** | **A** | **T** | **T** | **T** | **A** | **T** | **A** | **A** | **C** | **G** | **G** | **T** | **G** | **G** | **T** | **T** | **T** | **C** | **T** | **T** | **A** | **C** | **A** | **T** | **G** | **C** | **G** | **A** | **A** | **A** | **A** | **C** | **A** | **A** | **T** | **G** | **A** | **T** | **T** | **A** | **T** | **G** | **G** | **A** | **T** | **G** | **A** | **T** | **G** | **A** | **A** | **G** | **T** | **C** | **T** | **G** | **C** | **A** | **C** | **A** | **G** | **T** | **T** | **G** | **G** | **T** | **T** | **C** | **A** | **G** | **C** | **C** | **A** | **A** | **T** | **C** | **A** | **A** | **G** | **A** | **T** | **A** | **T** | **C** | **C** | **G** | **A** | **A** | **G** | **T** | **T** | **A** | **C** | **A** | **A** | **A** | **T** | **T** | **A** | **A** | **A** | **C** | **T** | **T** | **T** | **G** | **A** | **A** | **G** | **C** | **A** | **A** | **A** | **T** | **G** | **C** | **T** | **G** | **T** | **G** | **T** | **T** | **A** | **T** | **A** | **T** | **G** | **A** | **T** | **A** | **A** | **A** | **A** | **A** | **A** | **A** | **T** | **C** | **A** | **T** | **T** | **G** | **A** | **T** | **C** | **A** | **A** | **T** | **T** | **A** | **G** | **A** | **A** | **G** | **C** | **A** | **A** | **T** | **T** | **T** | **T** | **C** | **T** | **T** | **A** | **G** | **A** | **A** | **G** | **A** | **T** | **C** | **G** | **A** | **A** | **A** | **A** | **A** | **A** | **A** | **T** | **G** | **T** | **A** | **C** | **A** | **A** | **C** | **A** | **A** | **T** | **G** | **A** | **C** | **T** | **C** | **C** | **A** | **G** | **A** | **A** | **G** | **T** | **T** | **G** | **T** | **T** | **C** | **G** | **C** | **G** | **A** | **C** | **A** | **T** | **G** | **T** | **C** | **A** | **A** | **A** | **A** | **T** | **G** | **G** | **T** | **T** | **G** | **A** | **T** | **T** | **T** | **T** | **T** | **A** | **A** | **A** | **C** | **A** | **A** | **C** | **A** | **A** | **A** | **T** | **T** | **T** | **C** | **A** | **C** | **G** | **A** | **T** | **T** | **A** | **T** | **T** | **T** | **T** | **C** | **A** | **C** | **C** | **A** | **A** | **T** | **T** | **C** | **T** | **T** | **-** | **-** | **-** |
| **N59836F** | **T** | **T** | **G** | **A** | **T** | **T** | **C** | **T** | **T** | **A** | **G** | **C** | **G** | **T** | **C** | **T** | **T** | **A** | **A** | **C** | **A** | **G** | **T** | **T** | **A** | **T** | **T** | **T** | **A** | **T** | **T** | **T** | **T** | **A** | **T** | **T** | **A** | **A** | **T** | **G** | **C** | **A** | **A** | **T** | **T** | **A** | **T** | **T** | **G** | **C** | **G** | **G** | **G** | **A** | **A** | **T** | **T** | **A** | **C** | **C** | **A** | **T** | **T** | **T** | **T** | **G** | **T** | **T** | **A** | **A** | **A** | **A** | **C** | **C** | **T** | **C** | **G** | **A** | **G** | **A** | **T** | **G** | **T** | **A** | **G** | **C** | **A** | **G** | **C** | **A** | **A** | **T** | **T** | **T** | **G** | **G** | **G** | **C** | **A** | **T** | **G** | **G** | **C** | **T** | **A** | **C** | **T** | **C** | **G** | **T** | **A** | **C** | **T** | **G** | **A** | **T** | **T** | **G** | **C** | **T** | **C** | **T** | **A** | **C** | **C** | **C** | **G** | **T** | **T** | **T** | **T** | **T** | **G** | **G** | **T** | **T** | **T** | **T** | **T** | **T** | **C** | **T** | **T** | **A** | **T** | **A** | **T** | **T** | **T** | **A** | **T** | **T** | **C** | **T** | **T** | **T** | **G** | **G** | **G** | **C** | **G** | **C** | **G** | **G** | **T** | **T** | **T** | **A** | **A** | **C** | **C** | **G** | **A** | **C** | **A** | **A** | **A** | **A** | **A** | **G** | **A** | **A** | **A** | **T** | **T** | **T** | **T** | **A** | **T** | **T** | **T** | **G** | **C** | **A** | **A** | **C** | **A** | **A** | **A** | **G** | **T** | **G** | **A** | **T** | **T** | **T** | **A** | **C** | **G** | **G** | **G** | **A** | **A** | **T** | **T** | **A** | **G** | **A** | **G** | **A** | **A** | **T** | **T** | **T** | **C** | **C** | **A** | **G** | **A** | **A** | **T** | **T** | **T** | **C** | **C** | **A** | **A** | **G** | **A** | **A** | **G** | **A** | **A** | **A** | **G** | **T** | **T** | **T** | **T** | **G** | **A** | **A** | **C** | **T** | **T** | **T** | **A** | **T** | **A** | **G** | **T** | **C** | **A** | **A** | **A** | **A** | **G** | **A** | **T** | **G** | **C** | **C** | **G** | **A** | **C** | **G** | **G** | **A** | **G** | **G** | **A** | **G** | **C** | **A** | **A** | **C** | **A** | **A** | **C** | **A** | **G** | **T** | **T** | **T** | **A** | **C** | **A** | **G** | **A** | **C** | **T** | **T** | **T** | **T** | **T** | **T** | **T** | **C** | **C** | **T** | **C** | **G** | **T** | **T** | **A** | **A** | **A** | **T** | **A** | **G** | **G** | **A** | **T** | **G** | **C** | **C** | **T** | **C** | **T** | **G** | **A** | **C** | **T** | **A** | **A** | **G** | **A** | **A** | **A** | **A** | **A** | **T** | **G** | **A** | **T** | **G** | **T** | **T** | **G** | **A** | **A** | **A** | **T** | **T** | **T** | **T** | **T** | **A** | **C** | **G** | **G** | **A** | **T** | **G** | **G** | **T** | **A** | **C** | **G** | **G** | **A** | **A** | **A** | **A** | **A** | **T** | **T** | **C** | **A** | **A** | **T** | **G** | **C** | **G** | **T** | **T** | **G** | **A** | **T** | **G** | **G** | **C** | **G** | **G** | **A** | **T** | **A** | **T** | **A** | **A** | **A** | **A** | **A** | **A** | **A** | **G** | **C** | **T** | **C** | **A** | **G** | **C** | **A** | **C** | **T** | **C** | **T** | **A** | **T** | **T** | **C** | **A** | **T** | **A** | **T** | **T** | **G** | **A** | **A** | **T** | **A** | **T** | **T** | **A** | **T** | **G** | **C** | **C** | **T** | **T** | **T** | **G** | **T** | **A** | **A** | **C** | **G** | **G** | **A** | **T** | **C** | **A** | **T** | **A** | **T** | **C** | **G** | **G** | **T** | **A** | **C** | **C** | **A** | **A** | **A** | **A** | **T** | **T** | **T** | **T** | **G** | **A** | **A** | **C** | **T** | **T** | **A** | **T** | **T** | **A** | **G** | **A** | **A** | **G** | **A** | **A** | **A** | **A** | **G** | **G** | **C** | **G** | **G** | **C** | **T** | **G** | **A** | **A** | **G** | **G** | **G** | **G** | **T** | **T** | **G** | **A** | **A** | **G** | **T** | **G** | **C** | **G** | **G** | **T** | **T** | **G** | **C** | **T** | **T** | **T** | **A** | **C** | **G** | **A** | **T** | **G** | **C** | **C** | **T** | **T** | **T** | **G** | **G** | **C** | **T** | **C** | **A** | **A** | **A** | **G** | **G** | **G** | **C** | **A** | **C** | **C** | **A** | **A** | **G** | **G** | **T** | **T** | **C** | **A** | **T** | **C** | **A** | **T** | **T** | **T** | **G** | **A** | **A** | **T** | **G** | **A** | **A** | **T** | **T** | **G** | **A** | **A** | **A** | **A** | **A** | **A** | **A** | **A** | **C** | **G** | **G** | **C** | **G** | **G** | **T** | **T** | **T** | **T** | **G** | **T** | **C** | **C** | **A** | **A** | **A** | **C** | **G** | **T** | **T** | **T** | **A** | **T** | **T** | **A** | **C** | **T** | **T** | **C** | **T** | **C** | **A** | **A** | **A** | **A** | **A** | **G** | **C** | **A** | **C** | **T** | **T** | **T** | **T** | **G** | **A** | **A** | **G** | **T** | **T** | **T** | **C** | **G** | **T** | **T** | **T** | **G** | **A** | **A** | **T** | **T** | **A** | **T** | **C** | **A** | **T** | **G** | **A** | **T** | **C** | **A** | **C** | **C** | **G** | **G** | **A** | **A** | **A** | **A** | **T** | **T** | **G** | **T** | **T** | **G** | **T** | **T** | **A** | **T** | **T** | **G** | **A** | **C** | **G** | **G** | **A** | **A** | **A** | **A** | **G** | **T** | **A** | **G** | **G** | **C** | **T** | **A** | **C** | **A** | **T** | **T** | **G** | **G** | **C** | **G** | **G** | **C** | **T** | **T** | **T** | **A** | **A** | **T** | **G** | **T** | **T** | **G** | **C** | **C** | **G** | **A** | **T** | **C** | **A** | **A** | **T** | **A** | **T** | **G** | **C** | **T** | **G** | **G** | **A** | **A** | **C** | **G** | **A** | **C** | **T** | **A** | **A** | **A** | **A** | **A** | **G** | **T** | **T** | **T** | **G** | **G** | **C** | **T** | **A** | **T** | **T** | **G** | **G** | **C** | **G** | **G** | **G** | **A** | **T** | **A** | **C** | **A** | **C** | **A** | **T** | **T** | **T** | **A** | **C** | **G** | **G** | **A** | **T** | **T** | **C** | **A** | **A** | **G** | **G** | **G** | **C** | **C** | **A** | **G** | **C** | **A** | **G** | **C** | **C** | **T** | **C** | **A** | **T** | **T** | **A** | **C** | **T** | **G** | **C** | **A** | **A** | **A** | **T** | **G** | **C** | **G** | **T** | **T** | **T** | **T** | **T** | **T** | **A** | **A** | **T** | **G** | **G** | **A** | **T** | **T** | **G** | **G** | **A** | **A** | **C** | **G** | **T** | **C** | **T** | **C** | **T** | **T** | **C** | **C** | **C** | **C** | **C** | **G** | **A** | **G** | **A** | **A** | **A** | **A** | **A** | **T** | **C** | **G** | **T** | **G** | **T** | **G** | **G** | **C** | **G** | **T** | **A** | **T** | **C** | **A** | **A** | **T** | **T** | **G** | **G** | **A** | **T** | **T** | **A** | **T** | **T** | **T** | **C** | **T** | **T** | **T** | **A** | **A** | **A** | **C** | **T** | **T** | **G** | **A** | **A** | **G** | **C** | **A** | **T** | **T** | **G** | **G** | **T** | **G** | **C** | **C** | **A** | **G** | **A** | **G** | **G** | **C** | **A** | **A** | **A** | **T** | **A** | **C** | **A** | **T** | **C** | **C** | **A** | **T** | **T** | **C** | **A** | **G** | **A** | **T** | **G** | **A** | **T** | **T** | **G** | **C** | **C** | **A** | **G** | **T** | **G** | **G** | **T** | **C** | **C** | **T** | **A** | **A** | **C** | **A** | **G** | **T** | **G** | **A** | **C** | **C** | **G** | **T** | **G** | **A** | **A** | **C** | **A** | **A** | **A** | **T** | **T** | **A** | **A** | **A** | **T** | **T** | **G** | **G** | **C** | **C** | **T** | **T** | **T** | **A** | **T** | **T** | **A** | **A** | **A** | **T** | **T** | **G** | **A** | **T** | **T** | **A** | **C** | **T** | **T** | **C** | **T** | **G** | **C** | **C** | **A** | **A** | **G** | **A** | **A** | **A** | **A** | **G** | **A** | **G** | **T** | **C** | **T** | **G** | **G** | **A** | **T** | **T** | **C** | **A** | **A** | **A** | **C** | **G** | **C** | **C** | **G** | **T** | **A** | **T** | **T** | **T** | **A** | **G** | **T** | **T** | **C** | **C** | **T** | **G** | **A** | **T** | **G** | **A** | **T** | **A** | **G** | **T** | **G** | **T** | **C** | **T** | **T** | **G** | **G** | **C** | **T** | **G** | **C** | **T** | **T** | **T** | **A** | **A** | **A** | **G** | **G** | **T** | **T** | **G** | **C** | **T** | **G** | **C** | **G** | **G** | **C** | **T** | **T** | **C** | **A** | **G** | **G** | **A** | **G** | **T** | **A** | **G** | **A** | **T** | **G** | **T** | **T** | **A** | **A** | **A** | **A** | **T** | **T** | **A** | **T** | **G** | **A** | **T** | **T** | **C** | **C** | **A** | **G** | **A** | **T** | **A** | **A** | **G** | **C** | **C** | **C** | **G** | **A** | **C** | **C** | **A** | **T** | **C** | **C** | **G** | **T** | **T** | **T** | **A** | **T** | **T** | **T** | **A** | **T** | **C** | **G** | **A** | **G** | **C** | **A** | **A** | **C** | **A** | **C** | **A** | **G** | **T** | **A** | **T** | **T** | **A** | **C** | **G** | **G** | **C** | **C** | **G** | **C** | **T** | **T** | **A** | **T** | **T** | **G** | **A** | **T** | **G** | **A** | **A** | **A** | **G** | **A** | **A** | **A** | **A** | **T** | **A** | **T** | **T** | **G** | **A** | **A** | **A** | **T** | **T** | **T** | **T** | **A** | **A** | **T** | **T** | **T** | **A** | **T** | **A** | **A** | **C** | **G** | **G** | **T** | **G** | **G** | **T** | **T** | **T** | **C** | **T** | **T** | **A** | **C** | **A** | **T** | **G** | **C** | **G** | **A** | **A** | **A** | **A** | **C** | **A** | **A** | **T** | **G** | **A** | **T** | **T** | **A** | **T** | **G** | **G** | **A** | **T** | **G** | **A** | **T** | **G** | **A** | **A** | **G** | **T** | **C** | **T** | **G** | **C** | **A** | **C** | **A** | **G** | **T** | **T** | **G** | **G** | **T** | **T** | **C** | **A** | **G** | **C** | **C** | **A** | **A** | **T** | **C** | **A** | **A** | **G** | **A** | **T** | **A** | **T** | **C** | **C** | **G** | **A** | **A** | **G** | **T** | **T** | **A** | **C** | **A** | **A** | **A** | **T** | **T** | **A** | **A** | **A** | **C** | **T** | **T** | **T** | **G** | **A** | **A** | **G** | **C** | **A** | **A** | **A** | **T** | **G** | **C** | **T** | **G** | **T** | **G** | **T** | **T** | **A** | **T** | **A** | **T** | **G** | **A** | **T** | **A** | **A** | **A** | **A** | **A** | **A** | **A** | **T** | **C** | **A** | **T** | **T** | **G** | **A** | **T** | **C** | **A** | **A** | **T** | **T** | **A** | **G** | **A** | **A** | **G** | **C** | **A** | **A** | **T** | **T** | **T** | **T** | **C** | **T** | **T** | **A** | **G** | **A** | **A** | **G** | **A** | **T** | **C** | **G** | **A** | **A** | **A** | **A** | **A** | **A** | **A** | **T** | **G** | **T** | **A** | **C** | **A** | **A** | **C** | **A** | **A** | **T** | **G** | **A** | **C** | **T** | **C** | **C** | **A** | **G** | **A** | **A** | **G** | **T** | **T** | **G** | **T** | **T** | **C** | **G** | **T** | **G** | **A** | **C** | **A** | **T** | **G** | **T** | **C** | **A** | **A** | **A** | **A** | **T** | **G** | **G** | **T** | **T** | **G** | **A** | **T** | **T** | **T** | **T** | **T** | **A** | **A** | **A** | **C** | **A** | **A** | **C** | **A** | **A** | **A** | **T** | **T** | **T** | **C** | **A** | **C** | **G** | **A** | **T** | **T** | **A** | **T** | **T** | **T** | **T** | **C** | **A** | **C** | **C** | **A** | **A** | **T** | **T** | **C** | **T** | **T** | **-** | **-** | **-** |
| **N60440F** | **T** | **T** | **G** | **A** | **T** | **T** | **C** | **T** | **T** | **A** | **G** | **T** | **G** | **T** | **C** | **T** | **T** | **A** | **A** | **C** | **A** | **G** | **T** | **T** | **A** | **T** | **T** | **T** | **A** | **T** | **T** | **T** | **T** | **A** | **T** | **T** | **A** | **A** | **T** | **G** | **C** | **A** | **A** | **T** | **T** | **A** | **T** | **T** | **G** | **C** | **G** | **G** | **G** | **A** | **A** | **T** | **T** | **A** | **C** | **C** | **A** | **T** | **T** | **T** | **T** | **G** | **T** | **T** | **A** | **A** | **A** | **A** | **C** | **C** | **T** | **C** | **G** | **A** | **G** | **A** | **T** | **G** | **T** | **A** | **G** | **C** | **A** | **G** | **C** | **A** | **A** | **T** | **T** | **T** | **G** | **G** | **G** | **C** | **A** | **T** | **G** | **G** | **C** | **T** | **A** | **C** | **T** | **C** | **G** | **T** | **A** | **C** | **T** | **G** | **A** | **T** | **T** | **G** | **C** | **T** | **C** | **T** | **A** | **C** | **C** | **C** | **G** | **T** | **T** | **T** | **T** | **T** | **G** | **G** | **T** | **T** | **T** | **T** | **T** | **T** | **C** | **T** | **T** | **A** | **T** | **A** | **T** | **T** | **T** | **A** | **T** | **T** | **C** | **T** | **T** | **T** | **G** | **G** | **G** | **C** | **G** | **C** | **G** | **G** | **T** | **T** | **T** | **A** | **A** | **C** | **C** | **G** | **A** | **C** | **A** | **A** | **A** | **A** | **A** | **G** | **A** | **A** | **A** | **T** | **T** | **T** | **T** | **A** | **T** | **T** | **T** | **G** | **C** | **A** | **A** | **C** | **A** | **A** | **A** | **G** | **T** | **G** | **A** | **T** | **T** | **T** | **G** | **C** | **G** | **G** | **G** | **A** | **A** | **T** | **T** | **A** | **G** | **A** | **G** | **A** | **A** | **T** | **T** | **T** | **T** | **C** | **A** | **G** | **A** | **A** | **T** | **T** | **T** | **C** | **C** | **A** | **A** | **G** | **A** | **A** | **G** | **A** | **A** | **A** | **G** | **T** | **T** | **T** | **T** | **G** | **A** | **A** | **C** | **T** | **T** | **T** | **A** | **T** | **A** | **G** | **T** | **C** | **A** | **A** | **A** | **A** | **G** | **A** | **T** | **G** | **C** | **C** | **G** | **A** | **C** | **G** | **G** | **A** | **G** | **G** | **A** | **G** | **C** | **A** | **A** | **C** | **A** | **A** | **C** | **A** | **G** | **T** | **T** | **T** | **A** | **C** | **A** | **G** | **A** | **C** | **T** | **T** | **T** | **T** | **T** | **T** | **T** | **C** | **C** | **T** | **C** | **G** | **T** | **T** | **A** | **A** | **A** | **T** | **A** | **G** | **G** | **A** | **T** | **G** | **C** | **C** | **T** | **C** | **T** | **G** | **A** | **C** | **T** | **A** | **A** | **G** | **A** | **A** | **A** | **A** | **A** | **T** | **G** | **A** | **T** | **G** | **T** | **T** | **G** | **A** | **A** | **A** | **T** | **T** | **T** | **T** | **T** | **A** | **C** | **G** | **G** | **A** | **T** | **G** | **G** | **T** | **A** | **C** | **G** | **G** | **A** | **A** | **A** | **A** | **A** | **T** | **T** | **C** | **A** | **A** | **T** | **G** | **C** | **G** | **T** | **T** | **G** | **A** | **T** | **G** | **G** | **C** | **G** | **G** | **A** | **C** | **A** | **T** | **A** | **A** | **A** | **A** | **A** | **A** | **A** | **G** | **C** | **T** | **C** | **A** | **G** | **C** | **A** | **C** | **T** | **C** | **C** | **A** | **T** | **T** | **C** | **A** | **T** | **A** | **T** | **T** | **G** | **A** | **A** | **T** | **A** | **T** | **T** | **A** | **T** | **G** | **C** | **C** | **T** | **T** | **T** | **G** | **T** | **A** | **A** | **C** | **G** | **G** | **A** | **T** | **C** | **A** | **T** | **A** | **T** | **C** | **G** | **G** | **C** | **A** | **C** | **C** | **A** | **A** | **A** | **A** | **T** | **T** | **T** | **T** | **G** | **A** | **A** | **C** | **T** | **T** | **A** | **T** | **T** | **A** | **G** | **A** | **A** | **G** | **A** | **A** | **A** | **A** | **G** | **G** | **C** | **G** | **G** | **C** | **T** | **G** | **A** | **A** | **G** | **G** | **G** | **G** | **T** | **T** | **G** | **A** | **A** | **G** | **T** | **G** | **C** | **G** | **G** | **T** | **T** | **G** | **C** | **T** | **T** | **T** | **A** | **C** | **G** | **A** | **T** | **G** | **C** | **C** | **T** | **T** | **T** | **G** | **G** | **C** | **T** | **C** | **A** | **A** | **A** | **G** | **G** | **G** | **C** | **A** | **C** | **C** | **A** | **A** | **G** | **G** | **T** | **T** | **C** | **A** | **T** | **C** | **A** | **T** | **T** | **T** | **G** | **A** | **A** | **T** | **G** | **A** | **A** | **T** | **T** | **G** | **A** | **A** | **A** | **A** | **A** | **A** | **A** | **A** | **C** | **G** | **G** | **C** | **G** | **G** | **T** | **T** | **T** | **T** | **G** | **T** | **C** | **C** | **A** | **A** | **A** | **C** | **A** | **T** | **T** | **T** | **A** | **T** | **T** | **A** | **C** | **T** | **T** | **C** | **T** | **C** | **A** | **A** | **A** | **A** | **A** | **G** | **C** | **A** | **C** | **T** | **T** | **T** | **T** | **G** | **A** | **A** | **G** | **T** | **T** | **T** | **C** | **G** | **T** | **T** | **T** | **G** | **A** | **A** | **T** | **T** | **A** | **T** | **C** | **A** | **T** | **G** | **A** | **T** | **C** | **A** | **C** | **C** | **G** | **G** | **A** | **A** | **A** | **A** | **T** | **T** | **G** | **T** | **T** | **G** | **T** | **T** | **A** | **T** | **T** | **G** | **A** | **C** | **G** | **G** | **A** | **A** | **A** | **A** | **G** | **T** | **A** | **G** | **G** | **C** | **T** | **A** | **C** | **A** | **T** | **T** | **G** | **G** | **C** | **G** | **G** | **C** | **T** | **T** | **T** | **A** | **A** | **T** | **G** | **T** | **T** | **G** | **C** | **C** | **G** | **A** | **T** | **C** | **A** | **A** | **T** | **A** | **T** | **G** | **C** | **C** | **G** | **G** | **A** | **A** | **C** | **G** | **A** | **C** | **T** | **A** | **A** | **A** | **A** | **A** | **G** | **T** | **T** | **T** | **G** | **G** | **C** | **T** | **A** | **T** | **T** | **G** | **G** | **C** | **G** | **G** | **G** | **A** | **T** | **A** | **C** | **G** | **C** | **A** | **T** | **T** | **T** | **A** | **C** | **G** | **G** | **A** | **T** | **T** | **C** | **A** | **A** | **G** | **G** | **G** | **C** | **C** | **A** | **G** | **C** | **A** | **G** | **C** | **C** | **T** | **C** | **A** | **T** | **T** | **A** | **C** | **T** | **G** | **C** | **A** | **A** | **A** | **T** | **G** | **C** | **G** | **T** | **T** | **T** | **T** | **T** | **T** | **A** | **A** | **T** | **G** | **G** | **A** | **T** | **T** | **G** | **G** | **A** | **A** | **C** | **G** | **T** | **C** | **T** | **C** | **T** | **T** | **C** | **C** | **C** | **C** | **C** | **G** | **A** | **G** | **A** | **A** | **A** | **A** | **A** | **T** | **C** | **G** | **T** | **G** | **T** | **G** | **G** | **C** | **G** | **T** | **A** | **T** | **C** | **A** | **A** | **T** | **T** | **G** | **G** | **A** | **T** | **T** | **A** | **T** | **T** | **T** | **C** | **T** | **T** | **T** | **A** | **A** | **A** | **C** | **T** | **T** | **G** | **A** | **A** | **G** | **C** | **A** | **T** | **T** | **G** | **G** | **T** | **G** | **C** | **C** | **A** | **G** | **A** | **G** | **G** | **C** | **A** | **A** | **A** | **T** | **A** | **C** | **A** | **T** | **C** | **C** | **A** | **T** | **T** | **C** | **A** | **G** | **A** | **T** | **G** | **A** | **T** | **T** | **G** | **C** | **C** | **A** | **G** | **T** | **G** | **G** | **T** | **C** | **C** | **T** | **A** | **A** | **C** | **A** | **G** | **T** | **G** | **A** | **C** | **C** | **G** | **T** | **G** | **A** | **A** | **C** | **A** | **A** | **A** | **T** | **T** | **A** | **A** | **A** | **T** | **T** | **G** | **G** | **C** | **C** | **T** | **T** | **T** | **A** | **T** | **T** | **A** | **A** | **A** | **T** | **T** | **G** | **A** | **T** | **T** | **A** | **C** | **T** | **T** | **C** | **T** | **G** | **C** | **C** | **A** | **A** | **G** | **A** | **A** | **A** | **A** | **G** | **A** | **G** | **T** | **C** | **T** | **G** | **G** | **A** | **T** | **T** | **C** | **A** | **A** | **A** | **C** | **G** | **C** | **C** | **G** | **T** | **A** | **T** | **T** | **T** | **A** | **G** | **T** | **T** | **C** | **C** | **T** | **G** | **A** | **T** | **G** | **A** | **T** | **A** | **G** | **T** | **G** | **T** | **C** | **T** | **T** | **G** | **G** | **C** | **T** | **G** | **C** | **T** | **T** | **T** | **A** | **A** | **A** | **G** | **G** | **T** | **T** | **G** | **C** | **T** | **G** | **C** | **G** | **G** | **C** | **T** | **T** | **C** | **A** | **G** | **G** | **A** | **G** | **T** | **A** | **G** | **A** | **T** | **G** | **T** | **T** | **A** | **A** | **A** | **A** | **T** | **T** | **A** | **T** | **G** | **A** | **T** | **T** | **C** | **C** | **A** | **G** | **A** | **T** | **A** | **A** | **G** | **C** | **C** | **C** | **G** | **A** | **C** | **C** | **A** | **T** | **C** | **C** | **G** | **T** | **T** | **T** | **A** | **T** | **T** | **T** | **A** | **T** | **C** | **G** | **A** | **G** | **C** | **A** | **A** | **C** | **A** | **C** | **A** | **G** | **T** | **A** | **T** | **T** | **A** | **C** | **G** | **G** | **C** | **C** | **G** | **C** | **T** | **T** | **A** | **T** | **T** | **G** | **A** | **T** | **G** | **A** | **A** | **A** | **G** | **A** | **A** | **A** | **A** | **T** | **A** | **T** | **T** | **G** | **A** | **A** | **A** | **T** | **T** | **T** | **T** | **A** | **A** | **T** | **T** | **T** | **A** | **T** | **A** | **A** | **C** | **G** | **G** | **T** | **G** | **G** | **T** | **T** | **T** | **C** | **T** | **T** | **A** | **C** | **A** | **T** | **G** | **C** | **G** | **A** | **A** | **A** | **A** | **C** | **A** | **A** | **T** | **G** | **A** | **T** | **T** | **A** | **T** | **G** | **G** | **A** | **T** | **G** | **A** | **T** | **G** | **A** | **A** | **G** | **T** | **C** | **T** | **G** | **C** | **A** | **C** | **A** | **G** | **T** | **T** | **G** | **G** | **T** | **T** | **C** | **A** | **G** | **C** | **C** | **A** | **A** | **T** | **C** | **A** | **A** | **G** | **A** | **T** | **A** | **T** | **C** | **C** | **G** | **A** | **A** | **G** | **T** | **T** | **A** | **C** | **A** | **A** | **A** | **T** | **T** | **A** | **A** | **A** | **C** | **T** | **T** | **T** | **G** | **A** | **A** | **G** | **C** | **A** | **A** | **A** | **T** | **G** | **C** | **T** | **G** | **T** | **G** | **T** | **T** | **A** | **T** | **A** | **T** | **G** | **A** | **T** | **A** | **A** | **A** | **A** | **A** | **A** | **A** | **T** | **C** | **A** | **T** | **T** | **G** | **A** | **T** | **C** | **A** | **A** | **T** | **T** | **A** | **G** | **A** | **A** | **G** | **C** | **A** | **A** | **T** | **T** | **T** | **T** | **C** | **T** | **T** | **A** | **G** | **A** | **A** | **G** | **A** | **T** | **C** | **G** | **A** | **A** | **A** | **A** | **A** | **A** | **A** | **T** | **G** | **T** | **A** | **C** | **A** | **A** | **C** | **A** | **A** | **T** | **G** | **A** | **C** | **T** | **C** | **C** | **A** | **G** | **A** | **A** | **G** | **T** | **T** | **G** | **T** | **T** | **C** | **G** | **C** | **G** | **A** | **C** | **A** | **T** | **G** | **T** | **C** | **A** | **A** | **A** | **A** | **T** | **G** | **G** | **T** | **T** | **G** | **A** | **T** | **T** | **T** | **T** | **T** | **A** | **A** | **A** | **C** | **A** | **A** | **C** | **A** | **A** | **A** | **T** | **T** | **T** | **C** | **A** | **C** | **G** | **A** | **T** | **T** | **A** | **T** | **T** | **T** | **T** | **C** | **A** | **C** | **C** | **A** | **A** | **T** | **T** | **C** | **T** | **T** | **-** | **-** | **-** |
| **N52662** | **T** | **T** | **G** | **A** | **T** | **T** | **C** | **T** | **T** | **A** | **G** | **T** | **G** | **T** | **C** | **T** | **T** | **A** | **A** | **C** | **A** | **G** | **T** | **T** | **A** | **T** | **T** | **T** | **A** | **T** | **T** | **T** | **T** | **A** | **T** | **T** | **A** | **A** | **T** | **G** | **C** | **A** | **A** | **T** | **T** | **A** | **T** | **T** | **G** | **C** | **G** | **G** | **G** | **A** | **A** | **T** | **T** | **A** | **C** | **C** | **A** | **T** | **C** | **T** | **T** | **G** | **T** | **T** | **A** | **A** | **A** | **A** | **C** | **C** | **T** | **C** | **G** | **A** | **G** | **A** | **T** | **G** | **T** | **A** | **G** | **C** | **G** | **G** | **C** | **A** | **A** | **T** | **T** | **T** | **G** | **G** | **G** | **C** | **A** | **T** | **G** | **G** | **T** | **T** | **A** | **C** | **T** | **C** | **G** | **T** | **A** | **C** | **T** | **G** | **A** | **T** | **T** | **G** | **C** | **T** | **C** | **T** | **A** | **C** | **C** | **C** | **G** | **T** | **T** | **T** | **T** | **T** | **G** | **G** | **T** | **T** | **T** | **T** | **T** | **T** | **C** | **T** | **T** | **A** | **T** | **A** | **T** | **T** | **T** | **A** | **T** | **T** | **C** | **T** | **T** | **T** | **G** | **G** | **T** | **C** | **G** | **A** | **G** | **G** | **C** | **T** | **T** | **G** | **A** | **C** | **C** | **G** | **A** | **C** | **A** | **A** | **A** | **A** | **A** | **G** | **A** | **A** | **A** | **T** | **T** | **T** | **T** | **A** | **T** | **T** | **T** | **G** | **C** | **A** | **A** | **C** | **A** | **A** | **A** | **G** | **T** | **G** | **A** | **T** | **T** | **T** | **A** | **C** | **G** | **G** | **G** | **A** | **A** | **T** | **T** | **A** | **G** | **A** | **G** | **A** | **A** | **T** | **T** | **T** | **T** | **C** | **A** | **G** | **A** | **A** | **T** | **T** | **T** | **C** | **C** | **A** | **A** | **G** | **A** | **A** | **G** | **A** | **A** | **A** | **G** | **T** | **T** | **T** | **T** | **G** | **A** | **A** | **C** | **T** | **T** | **T** | **A** | **T** | **A** | **G** | **T** | **C** | **A** | **A** | **A** | **A** | **G** | **A** | **T** | **G** | **C** | **C** | **G** | **A** | **C** | **G** | **G** | **A** | **G** | **G** | **A** | **G** | **C** | **A** | **A** | **C** | **A** | **G** | **C** | **A** | **G** | **T** | **T** | **T** | **A** | **C** | **A** | **G** | **A** | **C** | **T** | **T** | **T** | **T** | **T** | **C** | **T** | **C** | **C** | **T** | **C** | **G** | **T** | **T** | **A** | **A** | **A** | **T** | **A** | **G** | **G** | **A** | **T** | **G** | **C** | **C** | **T** | **C** | **T** | **G** | **A** | **C** | **T** | **A** | **A** | **G** | **A** | **A** | **A** | **A** | **A** | **T** | **G** | **A** | **C** | **G** | **T** | **T** | **G** | **A** | **G** | **A** | **T** | **T** | **T** | **T** | **T** | **A** | **C** | **G** | **G** | **A** | **T** | **G** | **G** | **T** | **A** | **T** | **G** | **G** | **A** | **A** | **A** | **A** | **A** | **T** | **T** | **C** | **A** | **A** | **T** | **G** | **C** | **G** | **T** | **T** | **G** | **A** | **T** | **G** | **G** | **C** | **G** | **G** | **A** | **C** | **A** | **T** | **A** | **A** | **A** | **A** | **A** | **A** | **A** | **G** | **C** | **T** | **C** | **A** | **G** | **C** | **A** | **T** | **T** | **C** | **C** | **A** | **T** | **T** | **C** | **A** | **T** | **A** | **T** | **T** | **G** | **A** | **A** | **T** | **A** | **T** | **T** | **A** | **T** | **G** | **C** | **C** | **T** | **T** | **T** | **G** | **T** | **A** | **A** | **C** | **G** | **G** | **A** | **T** | **C** | **A** | **T** | **A** | **T** | **C** | **G** | **G** | **C** | **A** | **C** | **C** | **A** | **A** | **A** | **A** | **T** | **T** | **T** | **T** | **G** | **A** | **A** | **C** | **T** | **T** | **A** | **T** | **T** | **A** | **G** | **A** | **A** | **G** | **A** | **A** | **A** | **A** | **G** | **G** | **C** | **G** | **G** | **C** | **T** | **G** | **A** | **A** | **G** | **G** | **G** | **G** | **T** | **T** | **G** | **A** | **A** | **G** | **T** | **G** | **C** | **G** | **G** | **T** | **T** | **G** | **C** | **T** | **T** | **T** | **A** | **C** | **G** | **A** | **T** | **G** | **C** | **C** | **T** | **T** | **T** | **G** | **G** | **C** | **T** | **C** | **A** | **A** | **A** | **G** | **G** | **G** | **C** | **A** | **C** | **C** | **A** | **A** | **G** | **G** | **T** | **T** | **C** | **A** | **T** | **C** | **A** | **T** | **T** | **T** | **G** | **A** | **A** | **T** | **G** | **A** | **A** | **T** | **T** | **G** | **A** | **A** | **A** | **A** | **A** | **A** | **A** | **A** | **C** | **G** | **G** | **C** | **G** | **G** | **T** | **T** | **T** | **T** | **G** | **T** | **C** | **C** | **A** | **A** | **A** | **C** | **G** | **T** | **T** | **T** | **A** | **T** | **T** | **A** | **C** | **T** | **T** | **C** | **T** | **C** | **A** | **A** | **A** | **A** | **A** | **G** | **C** | **A** | **C** | **T** | **C** | **T** | **T** | **G** | **A** | **A** | **G** | **T** | **T** | **T** | **C** | **G** | **T** | **T** | **T** | **G** | **A** | **A** | **T** | **T** | **A** | **T** | **C** | **A** | **T** | **G** | **A** | **T** | **C** | **A** | **C** | **C** | **G** | **G** | **A** | **A** | **A** | **A** | **T** | **T** | **G** | **T** | **C** | **G** | **T** | **T** | **A** | **T** | **T** | **G** | **A** | **C** | **G** | **G** | **A** | **A** | **A** | **A** | **G** | **T** | **A** | **G** | **G** | **C** | **T** | **A** | **C** | **A** | **T** | **T** | **G** | **G** | **T** | **G** | **G** | **T** | **T** | **T** | **T** | **A** | **A** | **T** | **G** | **T** | **T** | **G** | **C** | **C** | **G** | **A** | **T** | **C** | **A** | **A** | **T** | **A** | **T** | **G** | **C** | **T** | **G** | **G** | **A** | **A** | **C** | **G** | **A** | **C** | **T** | **A** | **A** | **A** | **A** | **A** | **G** | **T** | **T** | **T** | **G** | **G** | **C** | **T** | **A** | **T** | **T** | **G** | **G** | **C** | **G** | **G** | **G** | **A** | **T** | **A** | **C** | **G** | **C** | **A** | **T** | **T** | **T** | **A** | **C** | **G** | **G** | **A** | **T** | **T** | **C** | **A** | **A** | **G** | **G** | **G** | **C** | **C** | **A** | **G** | **C** | **A** | **G** | **C** | **C** | **T** | **C** | **A** | **T** | **T** | **A** | **C** | **T** | **G** | **C** | **A** | **A** | **A** | **T** | **G** | **C** | **G** | **T** | **T** | **T** | **T** | **T** | **T** | **A** | **A** | **T** | **G** | **G** | **A** | **T** | **T** | **G** | **G** | **A** | **A** | **C** | **G** | **T** | **C** | **T** | **C** | **T** | **T** | **C** | **C** | **C** | **C** | **T** | **G** | **A** | **G** | **A** | **A** | **A** | **A** | **A** | **T** | **C** | **G** | **T** | **G** | **T** | **G** | **G** | **C** | **G** | **T** | **A** | **T** | **C** | **A** | **A** | **T** | **T** | **G** | **G** | **A** | **T** | **T** | **A** | **T** | **T** | **T** | **C** | **T** | **T** | **T** | **A** | **A** | **A** | **C** | **T** | **T** | **G** | **A** | **A** | **G** | **C** | **G** | **T** | **T** | **G** | **G** | **T** | **G** | **C** | **C** | **A** | **G** | **A** | **G** | **G** | **C** | **A** | **A** | **A** | **T** | **A** | **C** | **A** | **T** | **C** | **C** | **A** | **T** | **T** | **C** | **A** | **G** | **A** | **T** | **G** | **A** | **T** | **T** | **G** | **C** | **A** | **A** | **G** | **T** | **G** | **G** | **A** | **C** | **C** | **T** | **A** | **A** | **C** | **A** | **G** | **T** | **G** | **A** | **C** | **C** | **G** | **T** | **G** | **A** | **A** | **C** | **A** | **A** | **A** | **T** | **T** | **A** | **A** | **A** | **T** | **T** | **G** | **G** | **C** | **C** | **T** | **T** | **T** | **A** | **T** | **T** | **A** | **A** | **A** | **T** | **T** | **G** | **A** | **T** | **T** | **A** | **C** | **T** | **T** | **C** | **T** | **G** | **C** | **C** | **A** | **A** | **G** | **A** | **A** | **A** | **A** | **G** | **A** | **G** | **T** | **C** | **T** | **G** | **G** | **A** | **T** | **T** | **C** | **A** | **A** | **A** | **C** | **G** | **C** | **C** | **G** | **T** | **A** | **T** | **T** | **T** | **A** | **G** | **T** | **T** | **C** | **C** | **T** | **G** | **A** | **T** | **G** | **A** | **T** | **A** | **G** | **T** | **G** | **T** | **C** | **T** | **T** | **G** | **G** | **C** | **T** | **G** | **C** | **T** | **T** | **T** | **A** | **A** | **A** | **G** | **G** | **T** | **T** | **G** | **C** | **T** | **G** | **C** | **G** | **G** | **C** | **T** | **T** | **C** | **A** | **G** | **G** | **A** | **G** | **T** | **A** | **G** | **A** | **T** | **G** | **T** | **T** | **A** | **A** | **A** | **A** | **T** | **T** | **A** | **T** | **G** | **A** | **T** | **T** | **C** | **C** | **A** | **G** | **A** | **T** | **A** | **A** | **G** | **C** | **C** | **C** | **G** | **A** | **C** | **C** | **A** | **T** | **C** | **C** | **G** | **T** | **T** | **T** | **A** | **T** | **T** | **T** | **A** | **T** | **C** | **G** | **A** | **G** | **C** | **A** | **A** | **C** | **A** | **C** | **A** | **G** | **T** | **A** | **T** | **T** | **A** | **C** | **G** | **G** | **C** | **C** | **G** | **C** | **T** | **T** | **G** | **T** | **T** | **A** | **A** | **T** | **G** | **A** | **A** | **A** | **G** | **A** | **A** | **A** | **A** | **T** | **A** | **T** | **T** | **G** | **A** | **A** | **A** | **T** | **T** | **T** | **T** | **A** | **A** | **T** | **T** | **T** | **A** | **T** | **A** | **A** | **C** | **G** | **G** | **T** | **G** | **G** | **T** | **T** | **T** | **C** | **T** | **T** | **A** | **C** | **A** | **T** | **G** | **C** | **G** | **A** | **A** | **A** | **A** | **C** | **A** | **A** | **T** | **G** | **A** | **T** | **T** | **A** | **T** | **G** | **G** | **A** | **T** | **G** | **A** | **T** | **G** | **A** | **A** | **G** | **T** | **C** | **T** | **G** | **C** | **A** | **C** | **A** | **G** | **T** | **T** | **G** | **G** | **T** | **T** | **C** | **A** | **G** | **C** | **T** | **A** | **A** | **T** | **C** | **A** | **A** | **G** | **A** | **T** | **A** | **T** | **C** | **C** | **G** | **A** | **A** | **G** | **T** | **T** | **A** | **C** | **A** | **A** | **A** | **T** | **T** | **A** | **A** | **A** | **C** | **T** | **T** | **T** | **G** | **A** | **A** | **G** | **C** | **A** | **A** | **A** | **T** | **G** | **C** | **T** | **G** | **T** | **G** | **T** | **T** | **A** | **T** | **A** | **T** | **G** | **A** | **T** | **A** | **A** | **A** | **A** | **A** | **A** | **A** | **T** | **C** | **A** | **T** | **T** | **G** | **A** | **T** | **C** | **A** | **A** | **T** | **T** | **A** | **G** | **A** | **A** | **G** | **C** | **A** | **A** | **T** | **T** | **T** | **T** | **C** | **T** | **T** | **A** | **G** | **A** | **A** | **G** | **A** | **T** | **C** | **G** | **A** | **A** | **A** | **A** | **A** | **A** | **A** | **T** | **G** | **T** | **A** | **C** | **A** | **A** | **C** | **A** | **A** | **T** | **G** | **A** | **C** | **T** | **C** | **C** | **A** | **G** | **A** | **A** | **G** | **T** | **T** | **G** | **T** | **T** | **C** | **G** | **C** | **G** | **A** | **C** | **A** | **T** | **G** | **T** | **C** | **A** | **A** | **A** | **A** | **T** | **G** | **G** | **T** | **T** | **G** | **A** | **T** | **T** | **T** | **T** | **T** | **A** | **A** | **A** | **C** | **A** | **A** | **C** | **A** | **A** | **A** | **T** | **T** | **T** | **C** | **A** | **C** | **G** | **A** | **T** | **T** | **A** | **T** | **T** | **T** | **T** | **C** | **A** | **C** | **C** | **A** | **A** | **T** | **T** | **C** | **T** | **T** | **-** | **-** | **-** |
| **N54553** | **T** | **T** | **G** | **A** | **T** | **T** | **C** | **T** | **T** | **A** | **G** | **C** | **G** | **T** | **C** | **T** | **T** | **A** | **A** | **C** | **A** | **G** | **T** | **T** | **A** | **T** | **T** | **T** | **A** | **T** | **T** | **T** | **T** | **A** | **T** | **T** | **A** | **A** | **T** | **G** | **C** | **A** | **A** | **T** | **T** | **A** | **T** | **T** | **G** | **C** | **G** | **G** | **G** | **A** | **A** | **T** | **T** | **A** | **C** | **C** | **A** | **T** | **T** | **T** | **T** | **G** | **T** | **T** | **A** | **A** | **A** | **A** | **C** | **C** | **T** | **C** | **G** | **A** | **G** | **A** | **T** | **G** | **T** | **A** | **G** | **C** | **A** | **G** | **C** | **A** | **A** | **T** | **T** | **T** | **G** | **G** | **G** | **C** | **A** | **T** | **G** | **G** | **C** | **T** | **A** | **C** | **T** | **C** | **G** | **T** | **A** | **C** | **T** | **G** | **A** | **T** | **T** | **G** | **C** | **T** | **C** | **T** | **A** | **C** | **C** | **C** | **G** | **T** | **T** | **T** | **T** | **T** | **G** | **G** | **T** | **T** | **T** | **T** | **T** | **T** | **C** | **T** | **T** | **A** | **T** | **A** | **T** | **T** | **T** | **A** | **T** | **T** | **C** | **T** | **T** | **T** | **G** | **G** | **G** | **C** | **G** | **C** | **G** | **G** | **T** | **T** | **T** | **A** | **A** | **C** | **C** | **G** | **A** | **C** | **A** | **A** | **A** | **A** | **A** | **G** | **A** | **A** | **A** | **T** | **T** | **T** | **T** | **A** | **T** | **T** | **T** | **G** | **C** | **A** | **A** | **C** | **A** | **A** | **A** | **G** | **T** | **G** | **A** | **T** | **T** | **T** | **G** | **C** | **G** | **G** | **G** | **A** | **A** | **T** | **T** | **A** | **G** | **A** | **G** | **A** | **A** | **T** | **T** | **T** | **T** | **C** | **A** | **G** | **A** | **A** | **T** | **T** | **T** | **C** | **C** | **A** | **A** | **G** | **A** | **A** | **G** | **A** | **A** | **A** | **G** | **T** | **T** | **T** | **T** | **G** | **A** | **A** | **C** | **T** | **T** | **T** | **A** | **T** | **A** | **G** | **T** | **C** | **A** | **A** | **A** | **A** | **G** | **A** | **T** | **G** | **C** | **C** | **G** | **A** | **C** | **G** | **G** | **A** | **G** | **G** | **A** | **G** | **C** | **A** | **A** | **C** | **A** | **A** | **C** | **A** | **G** | **T** | **T** | **T** | **A** | **C** | **A** | **G** | **A** | **C** | **T** | **T** | **T** | **T** | **T** | **T** | **T** | **C** | **C** | **T** | **C** | **G** | **T** | **T** | **G** | **A** | **A** | **T** | **A** | **G** | **G** | **A** | **T** | **G** | **C** | **C** | **T** | **C** | **T** | **G** | **A** | **C** | **T** | **A** | **A** | **G** | **A** | **A** | **A** | **A** | **A** | **T** | **G** | **A** | **T** | **G** | **T** | **T** | **C** | **A** | **A** | **A** | **T** | **T** | **T** | **T** | **T** | **A** | **C** | **G** | **G** | **A** | **T** | **G** | **G** | **T** | **A** | **C** | **G** | **G** | **A** | **A** | **A** | **A** | **A** | **T** | **T** | **C** | **A** | **A** | **T** | **G** | **C** | **G** | **T** | **T** | **G** | **A** | **T** | **G** | **G** | **C** | **G** | **G** | **A** | **C** | **A** | **T** | **A** | **A** | **A** | **A** | **A** | **A** | **A** | **G** | **C** | **T** | **C** | **A** | **G** | **C** | **A** | **C** | **T** | **C** | **C** | **A** | **T** | **T** | **C** | **A** | **T** | **A** | **T** | **T** | **G** | **A** | **A** | **T** | **A** | **T** | **T** | **A** | **T** | **G** | **C** | **C** | **T** | **T** | **T** | **G** | **T** | **A** | **A** | **C** | **G** | **G** | **A** | **T** | **C** | **A** | **T** | **A** | **T** | **C** | **G** | **G** | **C** | **A** | **C** | **C** | **A** | **A** | **A** | **A** | **T** | **T** | **T** | **T** | **G** | **A** | **A** | **C** | **T** | **T** | **A** | **T** | **T** | **A** | **G** | **A** | **A** | **G** | **A** | **A** | **A** | **A** | **G** | **G** | **C** | **G** | **G** | **C** | **T** | **G** | **A** | **A** | **G** | **G** | **G** | **G** | **T** | **T** | **G** | **A** | **A** | **G** | **T** | **G** | **C** | **G** | **G** | **T** | **T** | **G** | **C** | **T** | **T** | **T** | **A** | **C** | **G** | **A** | **T** | **G** | **C** | **C** | **T** | **T** | **T** | **G** | **G** | **C** | **T** | **C** | **A** | **A** | **A** | **G** | **G** | **G** | **C** | **A** | **C** | **C** | **A** | **A** | **G** | **G** | **T** | **T** | **C** | **A** | **T** | **C** | **A** | **T** | **T** | **T** | **G** | **A** | **A** | **T** | **G** | **A** | **A** | **T** | **T** | **G** | **A** | **A** | **A** | **A** | **A** | **A** | **A** | **A** | **C** | **G** | **G** | **C** | **G** | **G** | **T** | **T** | **T** | **T** | **G** | **T** | **C** | **C** | **A** | **A** | **A** | **C** | **A** | **T** | **T** | **T** | **A** | **T** | **T** | **A** | **C** | **T** | **T** | **C** | **T** | **C** | **A** | **A** | **A** | **A** | **A** | **G** | **C** | **A** | **C** | **T** | **T** | **T** | **T** | **G** | **A** | **A** | **G** | **T** | **T** | **T** | **C** | **G** | **T** | **T** | **T** | **G** | **A** | **A** | **T** | **T** | **A** | **T** | **C** | **A** | **T** | **G** | **A** | **T** | **C** | **A** | **C** | **C** | **G** | **G** | **A** | **A** | **A** | **A** | **T** | **T** | **G** | **T** | **T** | **G** | **T** | **T** | **A** | **T** | **T** | **G** | **A** | **C** | **G** | **G** | **A** | **A** | **A** | **A** | **G** | **T** | **A** | **G** | **G** | **C** | **T** | **A** | **C** | **A** | **T** | **T** | **G** | **G** | **C** | **G** | **G** | **C** | **T** | **T** | **T** | **A** | **A** | **T** | **G** | **T** | **T** | **G** | **C** | **C** | **G** | **A** | **T** | **C** | **A** | **A** | **T** | **A** | **T** | **G** | **C** | **T** | **G** | **G** | **A** | **A** | **C** | **G** | **A** | **C** | **T** | **A** | **A** | **A** | **A** | **A** | **G** | **T** | **T** | **T** | **G** | **G** | **C** | **T** | **A** | **T** | **T** | **G** | **G** | **C** | **G** | **G** | **G** | **A** | **T** | **A** | **C** | **A** | **C** | **A** | **T** | **T** | **T** | **A** | **C** | **G** | **G** | **A** | **T** | **T** | **C** | **A** | **A** | **G** | **G** | **G** | **C** | **C** | **A** | **G** | **C** | **A** | **G** | **C** | **C** | **T** | **C** | **A** | **T** | **T** | **A** | **C** | **T** | **G** | **C** | **A** | **A** | **A** | **T** | **G** | **C** | **G** | **T** | **T** | **T** | **T** | **T** | **T** | **A** | **A** | **T** | **G** | **G** | **A** | **T** | **T** | **G** | **G** | **A** | **A** | **C** | **G** | **T** | **C** | **T** | **C** | **T** | **T** | **C** | **C** | **C** | **C** | **C** | **G** | **A** | **G** | **A** | **A** | **A** | **A** | **A** | **T** | **C** | **G** | **T** | **G** | **T** | **G** | **G** | **C** | **G** | **T** | **A** | **T** | **C** | **A** | **A** | **T** | **T** | **G** | **G** | **A** | **T** | **T** | **A** | **T** | **T** | **T** | **C** | **T** | **T** | **T** | **A** | **A** | **A** | **C** | **T** | **T** | **G** | **A** | **A** | **G** | **C** | **A** | **T** | **T** | **G** | **G** | **T** | **G** | **C** | **C** | **A** | **G** | **A** | **G** | **G** | **C** | **A** | **A** | **A** | **T** | **A** | **C** | **A** | **T** | **C** | **C** | **A** | **T** | **T** | **C** | **A** | **G** | **A** | **T** | **G** | **A** | **T** | **T** | **G** | **C** | **C** | **A** | **G** | **T** | **G** | **G** | **T** | **C** | **C** | **T** | **A** | **A** | **C** | **A** | **G** | **T** | **G** | **A** | **C** | **C** | **G** | **T** | **G** | **A** | **A** | **C** | **A** | **A** | **A** | **T** | **T** | **A** | **A** | **A** | **T** | **T** | **G** | **G** | **C** | **C** | **T** | **T** | **T** | **A** | **T** | **T** | **A** | **A** | **A** | **T** | **T** | **G** | **A** | **T** | **T** | **A** | **C** | **T** | **T** | **C** | **T** | **G** | **C** | **C** | **A** | **A** | **G** | **A** | **A** | **A** | **A** | **G** | **A** | **G** | **T** | **C** | **T** | **G** | **G** | **A** | **T** | **T** | **C** | **A** | **A** | **A** | **C** | **G** | **C** | **C** | **G** | **T** | **A** | **T** | **T** | **T** | **A** | **G** | **T** | **T** | **C** | **C** | **T** | **G** | **A** | **T** | **G** | **A** | **T** | **A** | **G** | **T** | **G** | **T** | **C** | **T** | **T** | **G** | **G** | **C** | **T** | **G** | **C** | **T** | **T** | **T** | **A** | **A** | **A** | **A** | **G** | **T** | **T** | **G** | **C** | **T** | **G** | **C** | **G** | **G** | **C** | **T** | **T** | **C** | **A** | **G** | **G** | **A** | **G** | **T** | **A** | **G** | **A** | **T** | **G** | **T** | **T** | **A** | **A** | **A** | **A** | **T** | **T** | **A** | **T** | **G** | **A** | **T** | **T** | **C** | **C** | **A** | **G** | **A** | **T** | **A** | **A** | **G** | **C** | **C** | **C** | **G** | **A** | **C** | **C** | **A** | **T** | **C** | **C** | **G** | **T** | **T** | **T** | **A** | **T** | **T** | **T** | **A** | **T** | **C** | **G** | **A** | **G** | **C** | **A** | **A** | **C** | **A** | **C** | **A** | **G** | **T** | **A** | **T** | **T** | **A** | **C** | **G** | **G** | **C** | **C** | **G** | **C** | **T** | **T** | **A** | **T** | **T** | **G** | **A** | **T** | **G** | **A** | **A** | **A** | **G** | **A** | **A** | **A** | **A** | **T** | **A** | **T** | **T** | **G** | **A** | **A** | **A** | **T** | **T** | **T** | **T** | **A** | **A** | **T** | **T** | **T** | **A** | **T** | **A** | **A** | **C** | **G** | **G** | **T** | **G** | **G** | **T** | **T** | **T** | **C** | **T** | **T** | **A** | **C** | **A** | **T** | **G** | **C** | **G** | **A** | **A** | **A** | **A** | **C** | **A** | **A** | **T** | **G** | **A** | **T** | **T** | **A** | **T** | **G** | **G** | **A** | **T** | **G** | **A** | **T** | **G** | **A** | **A** | **G** | **T** | **C** | **T** | **G** | **C** | **A** | **C** | **A** | **G** | **T** | **T** | **G** | **G** | **T** | **T** | **C** | **A** | **G** | **C** | **C** | **A** | **A** | **T** | **C** | **A** | **A** | **G** | **A** | **T** | **A** | **T** | **C** | **C** | **G** | **A** | **A** | **G** | **T** | **T** | **A** | **C** | **A** | **A** | **A** | **T** | **T** | **A** | **A** | **A** | **C** | **T** | **T** | **T** | **G** | **A** | **A** | **G** | **C** | **A** | **A** | **A** | **T** | **G** | **C** | **T** | **G** | **T** | **G** | **T** | **T** | **A** | **T** | **A** | **T** | **G** | **A** | **T** | **A** | **A** | **A** | **A** | **A** | **A** | **A** | **T** | **C** | **A** | **T** | **T** | **G** | **A** | **T** | **C** | **A** | **A** | **T** | **T** | **A** | **G** | **A** | **A** | **G** | **C** | **A** | **A** | **T** | **T** | **T** | **T** | **C** | **T** | **T** | **A** | **G** | **A** | **A** | **G** | **A** | **T** | **C** | **G** | **A** | **A** | **A** | **A** | **A** | **A** | **A** | **T** | **G** | **T** | **A** | **C** | **A** | **A** | **C** | **A** | **A** | **T** | **G** | **A** | **C** | **T** | **C** | **C** | **A** | **G** | **A** | **A** | **G** | **T** | **T** | **G** | **T** | **T** | **C** | **G** | **C** | **G** | **A** | **C** | **A** | **T** | **G** | **T** | **C** | **A** | **A** | **A** | **A** | **T** | **G** | **G** | **T** | **T** | **G** | **A** | **T** | **T** | **T** | **T** | **T** | **A** | **A** | **A** | **C** | **A** | **A** | **C** | **A** | **A** | **A** | **T** | **T** | **T** | **C** | **A** | **C** | **G** | **A** | **T** | **T** | **A** | **T** | **T** | **T** | **T** | **C** | **A** | **C** | **C** | **A** | **A** | **T** | **T** | **C** | **T** | **T** | **-** | **-** | **-** |
| **N53661** | **T** | **T** | **G** | **A** | **T** | **T** | **C** | **T** | **T** | **A** | **G** | **C** | **G** | **T** | **C** | **T** | **T** | **A** | **A** | **C** | **A** | **G** | **T** | **T** | **A** | **T** | **T** | **T** | **A** | **T** | **T** | **T** | **T** | **A** | **T** | **T** | **A** | **A** | **T** | **G** | **C** | **A** | **A** | **T** | **T** | **A** | **T** | **T** | **G** | **C** | **G** | **G** | **G** | **A** | **A** | **T** | **T** | **A** | **C** | **C** | **A** | **T** | **T** | **T** | **T** | **G** | **T** | **T** | **A** | **A** | **A** | **A** | **C** | **C** | **T** | **C** | **G** | **A** | **G** | **A** | **T** | **G** | **T** | **A** | **G** | **C** | **A** | **G** | **C** | **A** | **A** | **T** | **T** | **T** | **G** | **G** | **G** | **C** | **A** | **T** | **G** | **G** | **C** | **T** | **A** | **C** | **T** | **C** | **G** | **T** | **A** | **C** | **T** | **G** | **A** | **T** | **T** | **G** | **C** | **T** | **C** | **T** | **A** | **C** | **C** | **C** | **G** | **T** | **T** | **T** | **T** | **T** | **G** | **G** | **T** | **T** | **T** | **T** | **T** | **T** | **C** | **T** | **T** | **A** | **T** | **A** | **T** | **T** | **T** | **A** | **T** | **T** | **C** | **T** | **T** | **T** | **G** | **G** | **G** | **C** | **G** | **C** | **G** | **G** | **T** | **T** | **T** | **A** | **A** | **C** | **C** | **G** | **A** | **C** | **A** | **A** | **A** | **A** | **A** | **G** | **A** | **A** | **A** | **T** | **T** | **T** | **T** | **A** | **T** | **T** | **T** | **G** | **C** | **A** | **A** | **C** | **A** | **A** | **A** | **G** | **T** | **G** | **A** | **T** | **T** | **T** | **G** | **C** | **G** | **G** | **G** | **A** | **A** | **T** | **T** | **A** | **G** | **A** | **G** | **A** | **A** | **T** | **T** | **T** | **T** | **C** | **A** | **G** | **A** | **A** | **T** | **T** | **T** | **C** | **C** | **A** | **A** | **G** | **A** | **A** | **G** | **A** | **A** | **A** | **G** | **T** | **T** | **T** | **T** | **G** | **A** | **A** | **C** | **T** | **T** | **T** | **A** | **T** | **A** | **G** | **T** | **C** | **A** | **A** | **A** | **A** | **G** | **A** | **T** | **G** | **C** | **C** | **G** | **A** | **C** | **G** | **G** | **A** | **G** | **G** | **A** | **G** | **C** | **A** | **A** | **C** | **A** | **A** | **C** | **A** | **G** | **T** | **T** | **T** | **A** | **C** | **A** | **G** | **A** | **C** | **T** | **T** | **T** | **T** | **T** | **T** | **T** | **C** | **C** | **T** | **C** | **G** | **T** | **T** | **G** | **A** | **A** | **T** | **A** | **G** | **G** | **A** | **T** | **G** | **C** | **C** | **T** | **C** | **T** | **G** | **A** | **C** | **T** | **A** | **A** | **G** | **A** | **A** | **A** | **A** | **A** | **T** | **G** | **A** | **T** | **G** | **T** | **T** | **C** | **A** | **A** | **A** | **T** | **T** | **T** | **T** | **T** | **A** | **C** | **G** | **G** | **A** | **T** | **G** | **G** | **T** | **A** | **C** | **G** | **G** | **A** | **A** | **A** | **A** | **A** | **T** | **T** | **C** | **A** | **A** | **T** | **G** | **C** | **G** | **T** | **T** | **G** | **A** | **T** | **G** | **G** | **C** | **G** | **G** | **A** | **C** | **A** | **T** | **A** | **A** | **A** | **A** | **A** | **A** | **A** | **G** | **C** | **T** | **C** | **A** | **G** | **C** | **A** | **C** | **T** | **C** | **C** | **A** | **T** | **T** | **C** | **A** | **T** | **A** | **T** | **T** | **G** | **A** | **A** | **T** | **A** | **T** | **T** | **A** | **T** | **G** | **C** | **C** | **T** | **T** | **T** | **G** | **T** | **A** | **A** | **C** | **G** | **G** | **A** | **T** | **C** | **A** | **T** | **A** | **T** | **C** | **G** | **G** | **C** | **A** | **C** | **C** | **A** | **A** | **A** | **A** | **T** | **T** | **T** | **T** | **G** | **A** | **A** | **C** | **T** | **T** | **A** | **T** | **T** | **A** | **G** | **A** | **A** | **G** | **A** | **A** | **A** | **A** | **G** | **G** | **C** | **G** | **G** | **C** | **T** | **G** | **A** | **A** | **G** | **G** | **G** | **G** | **T** | **T** | **G** | **A** | **A** | **G** | **T** | **G** | **C** | **G** | **G** | **T** | **T** | **G** | **C** | **T** | **T** | **T** | **A** | **C** | **G** | **A** | **T** | **G** | **C** | **C** | **T** | **T** | **T** | **G** | **G** | **C** | **T** | **C** | **A** | **A** | **A** | **G** | **G** | **G** | **C** | **A** | **C** | **C** | **A** | **A** | **G** | **G** | **T** | **T** | **C** | **A** | **T** | **C** | **A** | **T** | **T** | **T** | **G** | **A** | **A** | **T** | **G** | **A** | **A** | **T** | **T** | **G** | **A** | **A** | **A** | **A** | **A** | **A** | **A** | **A** | **C** | **G** | **G** | **T** | **G** | **G** | **T** | **T** | **T** | **T** | **G** | **T** | **C** | **C** | **A** | **A** | **A** | **C** | **G** | **T** | **T** | **T** | **A** | **T** | **T** | **A** | **C** | **T** | **T** | **C** | **T** | **C** | **A** | **A** | **A** | **A** | **A** | **G** | **C** | **A** | **C** | **T** | **T** | **T** | **T** | **G** | **A** | **A** | **G** | **T** | **T** | **T** | **C** | **G** | **T** | **T** | **T** | **G** | **A** | **A** | **T** | **T** | **A** | **T** | **C** | **A** | **T** | **G** | **A** | **T** | **C** | **A** | **C** | **C** | **G** | **G** | **A** | **A** | **A** | **A** | **T** | **T** | **G** | **T** | **T** | **G** | **T** | **T** | **A** | **T** | **T** | **G** | **A** | **C** | **G** | **G** | **A** | **A** | **A** | **A** | **G** | **T** | **A** | **G** | **G** | **C** | **T** | **A** | **C** | **A** | **T** | **T** | **G** | **G** | **C** | **G** | **G** | **C** | **T** | **T** | **T** | **A** | **A** | **T** | **G** | **T** | **T** | **G** | **C** | **C** | **G** | **A** | **T** | **C** | **A** | **A** | **T** | **A** | **T** | **G** | **C** | **C** | **G** | **G** | **A** | **A** | **C** | **G** | **A** | **C** | **T** | **A** | **A** | **A** | **A** | **A** | **G** | **T** | **T** | **T** | **G** | **G** | **C** | **T** | **A** | **T** | **T** | **G** | **G** | **C** | **G** | **G** | **G** | **A** | **T** | **A** | **C** | **A** | **C** | **A** | **T** | **T** | **T** | **A** | **C** | **G** | **G** | **A** | **T** | **T** | **C** | **A** | **A** | **G** | **G** | **G** | **C** | **C** | **A** | **G** | **C** | **A** | **G** | **C** | **C** | **T** | **C** | **A** | **T** | **T** | **A** | **C** | **T** | **G** | **C** | **A** | **A** | **A** | **T** | **G** | **C** | **G** | **T** | **T** | **T** | **T** | **T** | **T** | **A** | **A** | **T** | **G** | **G** | **A** | **T** | **T** | **G** | **G** | **A** | **A** | **C** | **G** | **T** | **C** | **T** | **C** | **T** | **T** | **C** | **C** | **C** | **C** | **C** | **G** | **A** | **G** | **A** | **A** | **A** | **A** | **A** | **T** | **C** | **G** | **T** | **G** | **T** | **G** | **G** | **C** | **G** | **T** | **A** | **T** | **C** | **A** | **A** | **T** | **T** | **G** | **G** | **A** | **T** | **T** | **A** | **T** | **T** | **T** | **C** | **T** | **T** | **T** | **A** | **A** | **A** | **C** | **T** | **T** | **G** | **A** | **A** | **G** | **C** | **A** | **T** | **T** | **G** | **G** | **T** | **G** | **C** | **C** | **A** | **G** | **A** | **G** | **G** | **C** | **A** | **A** | **A** | **T** | **A** | **C** | **A** | **T** | **C** | **C** | **A** | **T** | **T** | **C** | **A** | **G** | **A** | **T** | **G** | **A** | **T** | **T** | **G** | **C** | **C** | **A** | **G** | **T** | **G** | **G** | **T** | **C** | **C** | **T** | **A** | **A** | **C** | **A** | **G** | **T** | **G** | **A** | **C** | **C** | **G** | **T** | **G** | **A** | **A** | **C** | **A** | **A** | **A** | **T** | **T** | **A** | **A** | **A** | **T** | **T** | **G** | **G** | **C** | **C** | **T** | **T** | **T** | **A** | **T** | **T** | **A** | **A** | **A** | **T** | **T** | **G** | **A** | **T** | **T** | **A** | **C** | **T** | **T** | **C** | **T** | **G** | **C** | **C** | **A** | **A** | **G** | **A** | **A** | **A** | **A** | **G** | **A** | **G** | **T** | **C** | **T** | **G** | **G** | **A** | **T** | **T** | **C** | **A** | **A** | **A** | **C** | **G** | **C** | **C** | **G** | **T** | **A** | **T** | **T** | **T** | **A** | **G** | **T** | **T** | **C** | **C** | **T** | **G** | **A** | **T** | **G** | **A** | **T** | **A** | **G** | **T** | **G** | **T** | **C** | **T** | **T** | **G** | **G** | **C** | **T** | **G** | **C** | **T** | **T** | **T** | **A** | **A** | **A** | **G** | **G** | **T** | **T** | **G** | **C** | **T** | **G** | **C** | **G** | **G** | **C** | **T** | **T** | **C** | **A** | **G** | **G** | **A** | **G** | **T** | **A** | **G** | **A** | **T** | **G** | **T** | **T** | **A** | **A** | **A** | **A** | **T** | **T** | **A** | **T** | **G** | **A** | **T** | **T** | **C** | **C** | **A** | **G** | **A** | **T** | **A** | **A** | **G** | **C** | **C** | **C** | **G** | **A** | **C** | **C** | **A** | **T** | **C** | **C** | **G** | **T** | **T** | **T** | **A** | **T** | **T** | **T** | **A** | **T** | **C** | **G** | **A** | **G** | **C** | **A** | **A** | **C** | **A** | **C** | **A** | **G** | **T** | **A** | **T** | **T** | **A** | **C** | **G** | **G** | **C** | **C** | **G** | **C** | **T** | **T** | **G** | **T** | **T** | **G** | **A** | **T** | **G** | **A** | **A** | **A** | **G** | **A** | **A** | **A** | **A** | **T** | **A** | **T** | **T** | **G** | **A** | **A** | **A** | **T** | **T** | **T** | **T** | **A** | **A** | **T** | **T** | **T** | **A** | **T** | **A** | **A** | **C** | **G** | **G** | **T** | **G** | **G** | **T** | **T** | **T** | **C** | **T** | **T** | **A** | **C** | **A** | **T** | **G** | **C** | **G** | **A** | **A** | **A** | **A** | **C** | **A** | **A** | **T** | **G** | **A** | **T** | **T** | **A** | **T** | **G** | **G** | **A** | **T** | **G** | **A** | **T** | **G** | **A** | **A** | **G** | **T** | **C** | **T** | **G** | **C** | **A** | **C** | **A** | **G** | **T** | **A** | **G** | **G** | **T** | **T** | **C** | **A** | **G** | **C** | **C** | **A** | **A** | **T** | **C** | **A** | **A** | **G** | **A** | **T** | **A** | **T** | **C** | **C** | **G** | **A** | **A** | **G** | **T** | **T** | **A** | **C** | **A** | **A** | **A** | **T** | **T** | **A** | **A** | **A** | **C** | **T** | **T** | **T** | **G** | **A** | **A** | **G** | **C** | **A** | **A** | **A** | **T** | **G** | **C** | **T** | **G** | **T** | **G** | **T** | **T** | **A** | **T** | **A** | **T** | **G** | **A** | **T** | **A** | **A** | **A** | **A** | **A** | **A** | **A** | **T** | **C** | **A** | **T** | **T** | **G** | **A** | **T** | **C** | **A** | **A** | **T** | **T** | **A** | **G** | **A** | **A** | **G** | **C** | **A** | **A** | **T** | **T** | **T** | **T** | **C** | **T** | **T** | **A** | **G** | **A** | **A** | **G** | **A** | **T** | **C** | **G** | **A** | **A** | **A** | **A** | **A** | **A** | **A** | **T** | **G** | **T** | **A** | **C** | **A** | **A** | **C** | **A** | **A** | **T** | **G** | **A** | **C** | **T** | **C** | **C** | **A** | **G** | **A** | **A** | **G** | **T** | **T** | **G** | **T** | **T** | **C** | **G** | **C** | **G** | **A** | **C** | **A** | **T** | **G** | **T** | **C** | **C** | **A** | **A** | **A** | **T** | **G** | **G** | **T** | **T** | **G** | **A** | **T** | **T** | **T** | **T** | **T** | **A** | **A** | **A** | **C** | **A** | **A** | **C** | **A** | **A** | **A** | **T** | **T** | **T** | **C** | **A** | **C** | **G** | **A** | **T** | **T** | **A** | **T** | **T** | **T** | **T** | **C** | **A** | **C** | **C** | **A** | **A** | **T** | **T** | **C** | **T** | **T** | **-** | **-** | **-** |
| **N55181** | **T** | **T** | **G** | **A** | **T** | **T** | **C** | **T** | **T** | **A** | **G** | **C** | **G** | **T** | **C** | **T** | **T** | **A** | **A** | **C** | **A** | **G** | **T** | **T** | **A** | **T** | **T** | **T** | **A** | **T** | **T** | **T** | **T** | **A** | **T** | **T** | **A** | **A** | **T** | **G** | **C** | **A** | **A** | **T** | **T** | **A** | **T** | **T** | **G** | **C** | **G** | **G** | **G** | **A** | **A** | **T** | **T** | **A** | **C** | **C** | **A** | **T** | **T** | **T** | **T** | **G** | **T** | **T** | **A** | **A** | **A** | **A** | **C** | **C** | **T** | **C** | **G** | **A** | **G** | **A** | **T** | **G** | **T** | **A** | **G** | **C** | **A** | **G** | **C** | **A** | **A** | **T** | **T** | **T** | **G** | **G** | **G** | **C** | **A** | **T** | **G** | **G** | **C** | **T** | **A** | **C** | **T** | **C** | **G** | **T** | **A** | **C** | **T** | **G** | **A** | **T** | **T** | **G** | **C** | **T** | **C** | **T** | **A** | **C** | **C** | **C** | **G** | **T** | **T** | **T** | **T** | **T** | **G** | **G** | **T** | **T** | **T** | **T** | **T** | **T** | **C** | **T** | **T** | **A** | **T** | **A** | **T** | **T** | **T** | **A** | **T** | **T** | **C** | **T** | **T** | **T** | **G** | **G** | **G** | **C** | **G** | **C** | **G** | **G** | **T** | **T** | **T** | **A** | **A** | **C** | **C** | **G** | **A** | **C** | **A** | **A** | **A** | **A** | **A** | **G** | **A** | **A** | **A** | **T** | **T** | **T** | **T** | **A** | **T** | **T** | **T** | **G** | **C** | **A** | **A** | **C** | **A** | **A** | **A** | **G** | **T** | **G** | **A** | **T** | **T** | **T** | **A** | **C** | **G** | **G** | **G** | **A** | **A** | **T** | **T** | **A** | **G** | **A** | **G** | **A** | **A** | **T** | **T** | **T** | **T** | **C** | **A** | **G** | **A** | **A** | **C** | **T** | **T** | **C** | **C** | **A** | **A** | **G** | **A** | **A** | **G** | **A** | **A** | **A** | **G** | **T** | **T** | **T** | **T** | **G** | **A** | **A** | **C** | **T** | **T** | **T** | **A** | **T** | **A** | **G** | **T** | **C** | **A** | **A** | **A** | **A** | **G** | **A** | **T** | **G** | **C** | **C** | **G** | **A** | **C** | **G** | **G** | **A** | **G** | **G** | **A** | **G** | **C** | **A** | **A** | **C** | **A** | **A** | **C** | **A** | **G** | **T** | **T** | **T** | **A** | **C** | **A** | **G** | **A** | **C** | **T** | **T** | **T** | **T** | **T** | **T** | **T** | **C** | **C** | **T** | **C** | **G** | **T** | **T** | **A** | **A** | **A** | **T** | **A** | **G** | **G** | **A** | **T** | **G** | **C** | **C** | **C** | **C** | **T** | **G** | **A** | **C** | **T** | **A** | **A** | **G** | **A** | **A** | **A** | **A** | **A** | **T** | **G** | **A** | **T** | **G** | **T** | **T** | **G** | **A** | **A** | **A** | **T** | **T** | **T** | **T** | **T** | **A** | **C** | **G** | **G** | **A** | **T** | **G** | **G** | **T** | **A** | **C** | **G** | **G** | **A** | **A** | **A** | **A** | **A** | **T** | **T** | **C** | **A** | **A** | **T** | **G** | **C** | **G** | **T** | **T** | **G** | **A** | **T** | **G** | **G** | **C** | **G** | **G** | **A** | **C** | **A** | **T** | **A** | **A** | **A** | **A** | **A** | **A** | **A** | **G** | **C** | **T** | **C** | **A** | **G** | **C** | **A** | **C** | **T** | **C** | **C** | **A** | **T** | **T** | **C** | **A** | **T** | **A** | **T** | **T** | **G** | **A** | **A** | **T** | **A** | **T** | **T** | **A** | **T** | **G** | **C** | **C** | **T** | **T** | **T** | **G** | **T** | **A** | **A** | **C** | **G** | **G** | **A** | **T** | **C** | **A** | **T** | **A** | **T** | **C** | **G** | **G** | **T** | **A** | **C** | **C** | **A** | **A** | **A** | **A** | **T** | **T** | **T** | **T** | **G** | **A** | **A** | **C** | **T** | **T** | **A** | **T** | **T** | **A** | **G** | **A** | **A** | **G** | **A** | **A** | **A** | **A** | **G** | **G** | **C** | **G** | **G** | **C** | **T** | **G** | **A** | **A** | **G** | **G** | **G** | **G** | **T** | **T** | **G** | **A** | **A** | **G** | **T** | **G** | **C** | **G** | **G** | **T** | **T** | **G** | **C** | **T** | **T** | **T** | **A** | **C** | **G** | **A** | **T** | **G** | **C** | **C** | **T** | **T** | **T** | **G** | **G** | **C** | **T** | **C** | **A** | **A** | **A** | **G** | **G** | **G** | **C** | **A** | **C** | **C** | **A** | **A** | **G** | **G** | **T** | **T** | **C** | **A** | **T** | **C** | **A** | **T** | **T** | **T** | **G** | **A** | **A** | **T** | **G** | **A** | **A** | **T** | **T** | **G** | **A** | **A** | **A** | **A** | **A** | **A** | **A** | **A** | **C** | **G** | **G** | **C** | **G** | **G** | **T** | **T** | **T** | **T** | **G** | **T** | **C** | **C** | **A** | **A** | **A** | **C** | **G** | **T** | **T** | **T** | **A** | **T** | **T** | **A** | **C** | **T** | **T** | **C** | **T** | **C** | **A** | **A** | **A** | **A** | **A** | **G** | **C** | **A** | **C** | **T** | **T** | **T** | **T** | **G** | **A** | **A** | **G** | **T** | **T** | **T** | **C** | **G** | **T** | **T** | **T** | **G** | **A** | **A** | **T** | **T** | **A** | **T** | **C** | **A** | **T** | **G** | **A** | **T** | **C** | **A** | **C** | **C** | **G** | **G** | **A** | **A** | **A** | **A** | **T** | **T** | **G** | **T** | **T** | **G** | **T** | **T** | **A** | **T** | **T** | **G** | **A** | **T** | **G** | **G** | **G** | **A** | **A** | **A** | **G** | **T** | **A** | **G** | **G** | **C** | **T** | **A** | **C** | **A** | **T** | **T** | **G** | **G** | **C** | **G** | **G** | **C** | **T** | **T** | **T** | **A** | **A** | **T** | **G** | **T** | **T** | **G** | **C** | **C** | **G** | **A** | **T** | **C** | **A** | **A** | **T** | **A** | **T** | **G** | **C** | **C** | **G** | **G** | **A** | **A** | **C** | **G** | **A** | **C** | **T** | **A** | **A** | **A** | **A** | **A** | **G** | **T** | **T** | **T** | **G** | **G** | **C** | **T** | **A** | **T** | **T** | **G** | **G** | **C** | **G** | **G** | **G** | **A** | **T** | **A** | **C** | **A** | **C** | **A** | **T** | **T** | **T** | **A** | **C** | **G** | **G** | **A** | **T** | **T** | **C** | **A** | **A** | **G** | **G** | **G** | **C** | **C** | **A** | **G** | **C** | **A** | **G** | **C** | **C** | **T** | **C** | **A** | **T** | **T** | **A** | **C** | **T** | **G** | **C** | **A** | **A** | **A** | **T** | **G** | **C** | **G** | **T** | **T** | **T** | **T** | **T** | **T** | **A** | **A** | **T** | **G** | **G** | **A** | **T** | **T** | **G** | **G** | **A** | **A** | **C** | **G** | **T** | **C** | **T** | **C** | **T** | **T** | **C** | **C** | **C** | **C** | **T** | **G** | **A** | **G** | **A** | **A** | **A** | **A** | **A** | **T** | **C** | **G** | **T** | **G** | **T** | **G** | **G** | **C** | **G** | **T** | **A** | **T** | **C** | **A** | **A** | **T** | **T** | **G** | **G** | **A** | **T** | **T** | **A** | **T** | **T** | **T** | **C** | **T** | **T** | **T** | **A** | **A** | **A** | **C** | **T** | **T** | **G** | **A** | **A** | **G** | **C** | **G** | **T** | **T** | **G** | **G** | **T** | **G** | **C** | **C** | **A** | **G** | **A** | **G** | **G** | **C** | **A** | **A** | **A** | **T** | **A** | **C** | **A** | **T** | **C** | **C** | **A** | **T** | **T** | **C** | **A** | **G** | **A** | **T** | **G** | **A** | **T** | **T** | **G** | **C** | **C** | **A** | **G** | **T** | **G** | **G** | **T** | **C** | **C** | **T** | **A** | **A** | **T** | **A** | **G** | **T** | **G** | **A** | **C** | **C** | **G** | **T** | **G** | **A** | **A** | **C** | **A** | **A** | **A** | **T** | **T** | **A** | **A** | **A** | **T** | **T** | **G** | **G** | **C** | **T** | **T** | **T** | **T** | **A** | **T** | **T** | **A** | **A** | **A** | **T** | **T** | **G** | **A** | **T** | **T** | **A** | **C** | **T** | **T** | **C** | **T** | **G** | **C** | **C** | **A** | **A** | **G** | **A** | **A** | **A** | **A** | **G** | **A** | **G** | **T** | **C** | **T** | **G** | **G** | **A** | **T** | **T** | **C** | **A** | **A** | **A** | **C** | **G** | **C** | **C** | **G** | **T** | **A** | **T** | **T** | **T** | **A** | **G** | **T** | **T** | **C** | **C** | **T** | **G** | **A** | **T** | **G** | **A** | **T** | **A** | **G** | **T** | **G** | **T** | **C** | **T** | **T** | **G** | **G** | **C** | **G** | **G** | **C** | **T** | **T** | **T** | **A** | **A** | **A** | **G** | **G** | **T** | **T** | **G** | **C** | **T** | **G** | **C** | **G** | **G** | **C** | **T** | **T** | **C** | **A** | **G** | **G** | **A** | **G** | **T** | **A** | **G** | **A** | **T** | **G** | **T** | **T** | **A** | **A** | **A** | **A** | **T** | **T** | **A** | **T** | **G** | **A** | **T** | **T** | **C** | **C** | **A** | **G** | **A** | **T** | **A** | **A** | **G** | **C** | **C** | **C** | **G** | **A** | **C** | **C** | **A** | **T** | **C** | **C** | **G** | **T** | **T** | **T** | **A** | **T** | **T** | **T** | **A** | **T** | **C** | **G** | **A** | **G** | **C** | **A** | **A** | **C** | **A** | **C** | **A** | **G** | **T** | **A** | **T** | **T** | **A** | **C** | **G** | **G** | **G** | **C** | **G** | **C** | **T** | **T** | **G** | **T** | **T** | **G** | **A** | **T** | **G** | **A** | **A** | **A** | **G** | **A** | **A** | **A** | **A** | **T** | **A** | **T** | **T** | **G** | **A** | **A** | **A** | **T** | **T** | **T** | **T** | **A** | **A** | **T** | **T** | **T** | **A** | **T** | **A** | **A** | **C** | **G** | **G** | **T** | **G** | **G** | **T** | **T** | **T** | **C** | **T** | **T** | **A** | **C** | **A** | **T** | **G** | **C** | **G** | **A** | **A** | **A** | **A** | **C** | **A** | **A** | **T** | **G** | **A** | **T** | **T** | **A** | **T** | **G** | **G** | **A** | **T** | **G** | **A** | **T** | **G** | **A** | **A** | **G** | **T** | **C** | **T** | **G** | **C** | **A** | **C** | **A** | **G** | **T** | **A** | **G** | **G** | **T** | **T** | **C** | **A** | **G** | **C** | **C** | **A** | **A** | **T** | **C** | **A** | **A** | **G** | **A** | **T** | **A** | **T** | **C** | **C** | **G** | **A** | **A** | **G** | **T** | **T** | **A** | **C** | **A** | **A** | **A** | **T** | **T** | **A** | **A** | **A** | **C** | **T** | **T** | **T** | **G** | **A** | **A** | **G** | **C** | **A** | **A** | **A** | **T** | **G** | **C** | **T** | **G** | **T** | **G** | **T** | **T** | **A** | **T** | **A** | **T** | **G** | **A** | **T** | **A** | **A** | **A** | **A** | **A** | **A** | **A** | **T** | **C** | **A** | **T** | **T** | **G** | **A** | **T** | **C** | **A** | **A** | **T** | **T** | **A** | **G** | **A** | **A** | **G** | **C** | **A** | **A** | **T** | **T** | **T** | **T** | **C** | **T** | **T** | **A** | **G** | **A** | **A** | **G** | **A** | **T** | **C** | **G** | **A** | **A** | **A** | **A** | **A** | **A** | **A** | **T** | **G** | **T** | **A** | **C** | **A** | **A** | **C** | **A** | **A** | **T** | **G** | **A** | **C** | **T** | **C** | **C** | **A** | **G** | **A** | **A** | **G** | **T** | **T** | **G** | **T** | **T** | **C** | **G** | **C** | **G** | **A** | **C** | **A** | **T** | **G** | **T** | **C** | **A** | **A** | **A** | **A** | **T** | **G** | **G** | **T** | **T** | **G** | **A** | **T** | **T** | **T** | **T** | **T** | **A** | **A** | **A** | **C** | **A** | **A** | **C** | **A** | **A** | **A** | **T** | **T** | **T** | **C** | **A** | **C** | **G** | **A** | **T** | **T** | **A** | **T** | **T** | **C** | **T** | **C** | **A** | **C** | **C** | **A** | **A** | **T** | **T** | **C** | **T** | **T** | **-** | **-** | **-** |
| **N54588** | **T** | **T** | **G** | **A** | **T** | **T** | **C** | **T** | **T** | **A** | **G** | **C** | **G** | **T** | **C** | **T** | **T** | **A** | **A** | **C** | **A** | **G** | **T** | **T** | **A** | **T** | **T** | **T** | **A** | **T** | **T** | **T** | **T** | **A** | **T** | **T** | **A** | **A** | **T** | **G** | **C** | **A** | **A** | **T** | **T** | **A** | **T** | **T** | **G** | **C** | **G** | **G** | **G** | **A** | **A** | **T** | **T** | **A** | **C** | **C** | **A** | **T** | **T** | **T** | **T** | **G** | **T** | **T** | **A** | **A** | **A** | **A** | **C** | **C** | **T** | **C** | **G** | **A** | **G** | **A** | **T** | **G** | **T** | **A** | **G** | **C** | **A** | **G** | **C** | **A** | **A** | **T** | **T** | **T** | **G** | **G** | **G** | **C** | **A** | **T** | **G** | **G** | **C** | **T** | **A** | **C** | **T** | **C** | **G** | **T** | **A** | **C** | **T** | **G** | **A** | **T** | **T** | **G** | **C** | **T** | **C** | **T** | **A** | **C** | **C** | **C** | **G** | **T** | **T** | **T** | **T** | **T** | **G** | **G** | **T** | **T** | **T** | **T** | **T** | **T** | **C** | **T** | **T** | **A** | **T** | **A** | **T** | **T** | **T** | **A** | **T** | **T** | **C** | **T** | **T** | **T** | **G** | **G** | **T** | **C** | **G** | **A** | **G** | **G** | **T** | **T** | **T** | **A** | **A** | **C** | **C** | **G** | **A** | **C** | **A** | **A** | **A** | **A** | **A** | **G** | **A** | **A** | **A** | **T** | **T** | **T** | **T** | **A** | **T** | **T** | **T** | **G** | **C** | **A** | **A** | **C** | **A** | **A** | **A** | **G** | **T** | **G** | **A** | **T** | **T** | **T** | **A** | **C** | **G** | **G** | **G** | **A** | **A** | **T** | **T** | **A** | **G** | **A** | **G** | **A** | **A** | **T** | **T** | **T** | **C** | **C** | **A** | **G** | **A** | **A** | **T** | **T** | **T** | **C** | **C** | **A** | **A** | **G** | **A** | **A** | **G** | **A** | **A** | **A** | **G** | **T** | **T** | **T** | **T** | **G** | **A** | **A** | **C** | **T** | **T** | **T** | **A** | **T** | **A** | **G** | **T** | **C** | **A** | **A** | **A** | **A** | **G** | **A** | **T** | **G** | **C** | **C** | **G** | **A** | **C** | **G** | **G** | **A** | **G** | **G** | **A** | **G** | **C** | **A** | **A** | **C** | **A** | **A** | **C** | **A** | **G** | **T** | **T** | **T** | **A** | **C** | **A** | **G** | **A** | **C** | **T** | **T** | **T** | **T** | **T** | **T** | **T** | **C** | **C** | **T** | **C** | **G** | **T** | **T** | **A** | **A** | **A** | **T** | **A** | **G** | **G** | **A** | **T** | **G** | **C** | **C** | **T** | **C** | **T** | **G** | **A** | **C** | **T** | **A** | **A** | **G** | **A** | **A** | **A** | **A** | **A** | **T** | **G** | **A** | **T** | **G** | **T** | **T** | **G** | **A** | **A** | **A** | **T** | **T** | **T** | **T** | **T** | **A** | **C** | **G** | **G** | **A** | **T** | **G** | **G** | **T** | **A** | **C** | **G** | **G** | **A** | **A** | **A** | **A** | **A** | **T** | **T** | **C** | **A** | **A** | **T** | **G** | **C** | **G** | **T** | **T** | **G** | **A** | **T** | **G** | **G** | **C** | **G** | **G** | **A** | **T** | **A** | **T** | **A** | **A** | **A** | **A** | **A** | **A** | **A** | **G** | **C** | **T** | **C** | **A** | **G** | **C** | **A** | **C** | **T** | **C** | **C** | **A** | **T** | **T** | **C** | **A** | **T** | **A** | **T** | **T** | **G** | **A** | **A** | **T** | **A** | **T** | **T** | **A** | **T** | **G** | **C** | **C** | **T** | **T** | **T** | **G** | **T** | **A** | **A** | **C** | **G** | **G** | **A** | **T** | **C** | **A** | **T** | **A** | **T** | **C** | **G** | **G** | **C** | **A** | **C** | **C** | **A** | **A** | **A** | **A** | **T** | **T** | **T** | **T** | **G** | **A** | **A** | **C** | **T** | **T** | **A** | **T** | **T** | **A** | **G** | **A** | **A** | **G** | **A** | **A** | **A** | **A** | **G** | **G** | **C** | **G** | **G** | **C** | **T** | **G** | **A** | **A** | **G** | **G** | **G** | **G** | **T** | **T** | **G** | **A** | **A** | **G** | **T** | **G** | **C** | **G** | **G** | **T** | **T** | **G** | **C** | **T** | **T** | **T** | **A** | **C** | **G** | **A** | **T** | **G** | **C** | **C** | **T** | **T** | **T** | **G** | **G** | **C** | **T** | **C** | **A** | **A** | **A** | **G** | **G** | **G** | **C** | **A** | **C** | **C** | **A** | **A** | **G** | **G** | **T** | **T** | **C** | **A** | **T** | **C** | **A** | **T** | **T** | **T** | **G** | **A** | **A** | **T** | **G** | **A** | **A** | **T** | **T** | **G** | **A** | **A** | **A** | **A** | **A** | **A** | **A** | **A** | **C** | **G** | **G** | **C** | **G** | **G** | **T** | **T** | **T** | **T** | **G** | **T** | **C** | **C** | **A** | **A** | **A** | **C** | **G** | **T** | **T** | **T** | **A** | **T** | **T** | **A** | **C** | **T** | **T** | **C** | **T** | **C** | **A** | **A** | **A** | **A** | **A** | **G** | **C** | **A** | **C** | **T** | **T** | **T** | **T** | **G** | **A** | **A** | **G** | **T** | **T** | **T** | **C** | **G** | **T** | **T** | **T** | **G** | **A** | **A** | **T** | **T** | **A** | **T** | **C** | **A** | **T** | **G** | **A** | **T** | **C** | **A** | **C** | **C** | **G** | **G** | **A** | **A** | **A** | **A** | **T** | **T** | **G** | **T** | **T** | **G** | **T** | **T** | **A** | **T** | **T** | **G** | **A** | **C** | **G** | **G** | **A** | **A** | **A** | **A** | **G** | **T** | **A** | **G** | **G** | **C** | **T** | **A** | **C** | **A** | **T** | **T** | **G** | **G** | **C** | **G** | **G** | **C** | **T** | **T** | **T** | **A** | **A** | **T** | **G** | **T** | **T** | **G** | **C** | **C** | **G** | **A** | **T** | **C** | **A** | **A** | **T** | **A** | **T** | **G** | **C** | **C** | **G** | **G** | **A** | **A** | **C** | **G** | **A** | **C** | **T** | **A** | **A** | **A** | **A** | **A** | **G** | **T** | **T** | **T** | **G** | **G** | **C** | **T** | **A** | **T** | **T** | **G** | **G** | **C** | **G** | **G** | **G** | **A** | **T** | **A** | **C** | **A** | **C** | **A** | **T** | **T** | **T** | **A** | **C** | **G** | **G** | **A** | **T** | **T** | **C** | **A** | **A** | **G** | **G** | **G** | **C** | **C** | **A** | **G** | **C** | **A** | **G** | **C** | **C** | **T** | **C** | **A** | **T** | **T** | **A** | **C** | **T** | **G** | **C** | **A** | **A** | **A** | **T** | **G** | **C** | **G** | **T** | **T** | **T** | **T** | **T** | **T** | **A** | **A** | **T** | **G** | **G** | **A** | **T** | **T** | **G** | **G** | **A** | **A** | **C** | **G** | **T** | **C** | **T** | **C** | **T** | **T** | **C** | **C** | **C** | **C** | **C** | **G** | **A** | **G** | **A** | **A** | **A** | **A** | **A** | **T** | **C** | **G** | **T** | **G** | **T** | **G** | **G** | **C** | **G** | **T** | **A** | **T** | **C** | **A** | **A** | **T** | **T** | **G** | **G** | **A** | **T** | **T** | **A** | **T** | **T** | **T** | **C** | **T** | **T** | **T** | **A** | **A** | **A** | **C** | **T** | **T** | **G** | **A** | **A** | **G** | **C** | **A** | **T** | **T** | **G** | **G** | **T** | **G** | **C** | **C** | **A** | **G** | **A** | **G** | **G** | **C** | **A** | **A** | **A** | **T** | **A** | **C** | **A** | **T** | **C** | **C** | **A** | **T** | **T** | **C** | **A** | **G** | **A** | **T** | **G** | **A** | **T** | **T** | **G** | **C** | **C** | **A** | **G** | **T** | **G** | **G** | **T** | **C** | **C** | **T** | **A** | **A** | **C** | **A** | **G** | **T** | **G** | **A** | **C** | **C** | **G** | **T** | **G** | **A** | **A** | **C** | **A** | **A** | **A** | **T** | **T** | **A** | **A** | **A** | **T** | **T** | **G** | **G** | **C** | **C** | **T** | **T** | **T** | **A** | **T** | **T** | **A** | **A** | **A** | **T** | **T** | **G** | **A** | **T** | **T** | **A** | **C** | **T** | **T** | **C** | **T** | **G** | **C** | **C** | **A** | **A** | **G** | **A** | **A** | **A** | **A** | **G** | **A** | **G** | **T** | **C** | **T** | **G** | **G** | **A** | **T** | **T** | **C** | **A** | **A** | **A** | **C** | **G** | **C** | **C** | **G** | **T** | **A** | **T** | **T** | **T** | **A** | **G** | **T** | **T** | **C** | **C** | **T** | **G** | **A** | **T** | **G** | **A** | **C** | **A** | **G** | **T** | **G** | **T** | **C** | **T** | **T** | **G** | **G** | **C** | **T** | **G** | **C** | **T** | **T** | **T** | **A** | **A** | **A** | **G** | **G** | **T** | **T** | **G** | **C** | **T** | **G** | **C** | **G** | **G** | **C** | **T** | **T** | **C** | **A** | **G** | **G** | **A** | **G** | **T** | **A** | **G** | **A** | **T** | **G** | **T** | **T** | **A** | **A** | **A** | **A** | **T** | **T** | **A** | **T** | **G** | **A** | **T** | **T** | **C** | **C** | **A** | **G** | **A** | **T** | **A** | **A** | **G** | **C** | **C** | **C** | **G** | **A** | **C** | **C** | **A** | **T** | **C** | **C** | **G** | **T** | **T** | **T** | **A** | **T** | **T** | **T** | **A** | **T** | **C** | **G** | **A** | **G** | **C** | **A** | **A** | **C** | **A** | **C** | **A** | **G** | **T** | **A** | **T** | **T** | **A** | **C** | **G** | **G** | **C** | **C** | **G** | **C** | **T** | **T** | **A** | **T** | **T** | **G** | **A** | **T** | **G** | **A** | **A** | **A** | **G** | **A** | **A** | **A** | **A** | **T** | **A** | **T** | **T** | **G** | **A** | **A** | **A** | **T** | **T** | **T** | **T** | **A** | **A** | **T** | **T** | **T** | **A** | **T** | **A** | **A** | **C** | **G** | **G** | **T** | **G** | **G** | **T** | **T** | **T** | **C** | **T** | **T** | **A** | **C** | **A** | **T** | **G** | **C** | **G** | **A** | **A** | **A** | **A** | **C** | **A** | **A** | **T** | **G** | **A** | **T** | **T** | **A** | **T** | **G** | **G** | **A** | **T** | **G** | **A** | **T** | **G** | **A** | **A** | **G** | **T** | **C** | **T** | **G** | **C** | **A** | **C** | **A** | **G** | **T** | **T** | **G** | **G** | **T** | **T** | **C** | **A** | **G** | **C** | **C** | **A** | **A** | **T** | **C** | **A** | **A** | **G** | **A** | **T** | **A** | **T** | **C** | **C** | **G** | **A** | **A** | **G** | **T** | **T** | **A** | **C** | **A** | **A** | **A** | **T** | **T** | **A** | **A** | **A** | **C** | **T** | **T** | **T** | **G** | **A** | **A** | **G** | **C** | **A** | **A** | **A** | **T** | **G** | **C** | **T** | **G** | **T** | **G** | **T** | **T** | **A** | **T** | **A** | **T** | **G** | **A** | **T** | **A** | **A** | **A** | **A** | **A** | **A** | **A** | **T** | **C** | **A** | **T** | **T** | **G** | **A** | **T** | **C** | **A** | **A** | **T** | **T** | **A** | **G** | **A** | **A** | **G** | **C** | **A** | **A** | **T** | **T** | **T** | **T** | **C** | **T** | **T** | **A** | **G** | **A** | **A** | **G** | **A** | **T** | **C** | **G** | **A** | **A** | **A** | **A** | **A** | **A** | **A** | **T** | **G** | **T** | **A** | **C** | **A** | **A** | **C** | **A** | **A** | **T** | **G** | **A** | **C** | **T** | **C** | **C** | **A** | **G** | **A** | **A** | **G** | **T** | **T** | **G** | **T** | **T** | **C** | **G** | **C** | **G** | **A** | **C** | **A** | **T** | **G** | **T** | **C** | **A** | **A** | **A** | **A** | **T** | **G** | **G** | **T** | **T** | **G** | **A** | **T** | **T** | **T** | **T** | **T** | **A** | **A** | **A** | **C** | **A** | **A** | **C** | **A** | **A** | **A** | **T** | **T** | **T** | **C** | **A** | **C** | **G** | **A** | **T** | **T** | **A** | **T** | **T** | **T** | **T** | **C** | **A** | **C** | **C** | **A** | **A** | **T** | **T** | **C** | **T** | **T** | **-** | **-** | **-** |
| **N60100F** | **T** | **T** | **G** | **A** | **T** | **T** | **C** | **T** | **T** | **A** | **G** | **C** | **G** | **T** | **C** | **T** | **T** | **A** | **A** | **C** | **A** | **G** | **T** | **T** | **A** | **T** | **T** | **T** | **A** | **T** | **T** | **T** | **T** | **A** | **T** | **T** | **A** | **A** | **T** | **G** | **C** | **A** | **A** | **T** | **T** | **A** | **T** | **T** | **G** | **C** | **G** | **G** | **G** | **A** | **A** | **T** | **T** | **A** | **C** | **C** | **A** | **T** | **T** | **T** | **T** | **G** | **T** | **T** | **A** | **A** | **A** | **A** | **C** | **C** | **T** | **C** | **G** | **A** | **G** | **A** | **T** | **G** | **T** | **A** | **G** | **C** | **A** | **G** | **C** | **A** | **A** | **T** | **T** | **T** | **G** | **G** | **G** | **C** | **A** | **T** | **G** | **G** | **C** | **T** | **A** | **C** | **T** | **C** | **G** | **T** | **A** | **C** | **T** | **G** | **A** | **T** | **T** | **G** | **C** | **T** | **C** | **T** | **A** | **C** | **C** | **C** | **G** | **T** | **T** | **T** | **T** | **T** | **G** | **G** | **T** | **T** | **T** | **T** | **T** | **T** | **C** | **T** | **T** | **A** | **T** | **A** | **T** | **T** | **T** | **A** | **T** | **T** | **C** | **T** | **T** | **T** | **G** | **G** | **G** | **C** | **G** | **C** | **G** | **G** | **T** | **T** | **T** | **A** | **A** | **C** | **C** | **G** | **A** | **C** | **A** | **A** | **A** | **A** | **A** | **G** | **A** | **A** | **A** | **T** | **T** | **T** | **T** | **A** | **T** | **T** | **T** | **G** | **C** | **A** | **A** | **C** | **A** | **A** | **A** | **G** | **T** | **G** | **A** | **T** | **T** | **T** | **A** | **C** | **G** | **G** | **G** | **A** | **A** | **T** | **T** | **A** | **G** | **A** | **G** | **A** | **A** | **T** | **T** | **T** | **C** | **C** | **A** | **G** | **A** | **A** | **T** | **T** | **T** | **C** | **C** | **A** | **A** | **G** | **A** | **A** | **G** | **A** | **A** | **A** | **G** | **T** | **T** | **T** | **T** | **G** | **A** | **A** | **C** | **T** | **T** | **T** | **A** | **T** | **A** | **G** | **T** | **C** | **A** | **A** | **A** | **A** | **G** | **A** | **T** | **G** | **C** | **C** | **G** | **A** | **C** | **G** | **G** | **A** | **G** | **G** | **A** | **G** | **C** | **A** | **A** | **C** | **A** | **A** | **C** | **A** | **G** | **T** | **T** | **T** | **A** | **C** | **A** | **G** | **A** | **C** | **T** | **T** | **T** | **T** | **T** | **T** | **T** | **C** | **C** | **T** | **C** | **G** | **T** | **T** | **A** | **A** | **A** | **T** | **A** | **G** | **G** | **A** | **T** | **G** | **C** | **C** | **T** | **C** | **T** | **G** | **A** | **C** | **T** | **A** | **A** | **G** | **A** | **A** | **A** | **A** | **A** | **T** | **G** | **A** | **T** | **G** | **T** | **T** | **G** | **A** | **A** | **A** | **T** | **T** | **T** | **T** | **T** | **A** | **C** | **G** | **G** | **A** | **T** | **G** | **G** | **T** | **A** | **C** | **G** | **G** | **A** | **A** | **A** | **A** | **A** | **T** | **T** | **C** | **A** | **A** | **T** | **G** | **C** | **G** | **T** | **T** | **G** | **A** | **T** | **G** | **G** | **C** | **G** | **G** | **A** | **C** | **A** | **T** | **A** | **A** | **A** | **A** | **A** | **A** | **A** | **G** | **C** | **T** | **C** | **A** | **G** | **C** | **A** | **C** | **T** | **C** | **C** | **A** | **T** | **T** | **C** | **A** | **T** | **A** | **T** | **T** | **G** | **A** | **A** | **T** | **A** | **T** | **T** | **A** | **T** | **G** | **C** | **C** | **T** | **T** | **T** | **G** | **T** | **A** | **A** | **C** | **G** | **G** | **A** | **T** | **C** | **A** | **T** | **A** | **T** | **C** | **G** | **G** | **T** | **A** | **C** | **C** | **A** | **A** | **A** | **A** | **T** | **T** | **T** | **T** | **G** | **A** | **A** | **C** | **T** | **T** | **A** | **T** | **T** | **A** | **G** | **A** | **A** | **G** | **A** | **A** | **A** | **A** | **G** | **G** | **C** | **G** | **G** | **C** | **T** | **G** | **A** | **A** | **G** | **G** | **G** | **G** | **T** | **T** | **G** | **A** | **A** | **G** | **T** | **G** | **C** | **G** | **G** | **T** | **T** | **G** | **C** | **T** | **T** | **T** | **A** | **C** | **G** | **A** | **T** | **G** | **C** | **C** | **T** | **T** | **T** | **G** | **G** | **C** | **T** | **C** | **A** | **A** | **A** | **G** | **G** | **G** | **C** | **A** | **C** | **C** | **A** | **A** | **G** | **G** | **T** | **T** | **C** | **A** | **T** | **C** | **A** | **T** | **T** | **T** | **G** | **A** | **A** | **T** | **G** | **A** | **A** | **T** | **T** | **G** | **A** | **A** | **A** | **A** | **A** | **A** | **A** | **A** | **C** | **G** | **G** | **C** | **G** | **G** | **T** | **T** | **T** | **T** | **G** | **T** | **C** | **C** | **A** | **A** | **A** | **C** | **G** | **T** | **T** | **T** | **A** | **T** | **T** | **A** | **C** | **T** | **T** | **C** | **T** | **C** | **A** | **A** | **A** | **A** | **A** | **G** | **C** | **A** | **C** | **T** | **T** | **T** | **T** | **G** | **A** | **A** | **G** | **T** | **T** | **T** | **C** | **G** | **T** | **T** | **T** | **G** | **A** | **A** | **T** | **T** | **A** | **T** | **C** | **A** | **T** | **G** | **A** | **T** | **C** | **A** | **C** | **C** | **G** | **G** | **A** | **A** | **A** | **A** | **T** | **T** | **G** | **T** | **T** | **G** | **T** | **T** | **A** | **T** | **T** | **G** | **A** | **T** | **G** | **G** | **G** | **A** | **A** | **A** | **G** | **T** | **A** | **G** | **G** | **C** | **T** | **A** | **C** | **A** | **T** | **T** | **G** | **G** | **C** | **G** | **G** | **C** | **T** | **T** | **T** | **A** | **A** | **T** | **G** | **T** | **T** | **G** | **C** | **C** | **G** | **A** | **T** | **C** | **A** | **A** | **T** | **A** | **T** | **G** | **C** | **T** | **G** | **G** | **A** | **A** | **C** | **G** | **A** | **C** | **T** | **A** | **A** | **A** | **A** | **A** | **G** | **T** | **T** | **T** | **G** | **G** | **C** | **T** | **A** | **T** | **T** | **G** | **G** | **C** | **G** | **G** | **G** | **A** | **T** | **A** | **C** | **A** | **C** | **A** | **T** | **T** | **T** | **A** | **C** | **G** | **G** | **A** | **T** | **T** | **C** | **A** | **A** | **G** | **G** | **G** | **C** | **C** | **A** | **G** | **C** | **A** | **G** | **C** | **C** | **T** | **C** | **A** | **T** | **T** | **A** | **C** | **T** | **G** | **C** | **A** | **A** | **A** | **T** | **G** | **C** | **G** | **T** | **T** | **T** | **T** | **T** | **T** | **A** | **A** | **T** | **G** | **G** | **A** | **T** | **T** | **G** | **G** | **A** | **A** | **C** | **G** | **T** | **C** | **T** | **C** | **T** | **T** | **C** | **C** | **C** | **C** | **C** | **G** | **A** | **G** | **A** | **A** | **A** | **A** | **A** | **T** | **C** | **G** | **T** | **G** | **T** | **G** | **G** | **C** | **G** | **T** | **A** | **T** | **C** | **A** | **A** | **T** | **T** | **G** | **G** | **A** | **T** | **T** | **A** | **T** | **T** | **T** | **C** | **T** | **T** | **T** | **A** | **A** | **A** | **C** | **T** | **T** | **G** | **A** | **A** | **G** | **C** | **A** | **T** | **T** | **G** | **G** | **T** | **T** | **C** | **C** | **A** | **G** | **A** | **G** | **G** | **C** | **A** | **A** | **A** | **T** | **A** | **C** | **A** | **T** | **C** | **C** | **A** | **T** | **T** | **C** | **A** | **G** | **A** | **T** | **G** | **A** | **T** | **T** | **G** | **C** | **C** | **A** | **G** | **T** | **G** | **G** | **T** | **C** | **C** | **T** | **A** | **A** | **T** | **A** | **G** | **T** | **G** | **A** | **C** | **C** | **G** | **T** | **G** | **A** | **A** | **C** | **A** | **A** | **A** | **T** | **T** | **A** | **A** | **A** | **T** | **T** | **G** | **G** | **C** | **C** | **T** | **T** | **T** | **A** | **T** | **T** | **A** | **A** | **A** | **T** | **T** | **G** | **A** | **T** | **T** | **A** | **C** | **T** | **T** | **C** | **T** | **G** | **C** | **C** | **A** | **A** | **G** | **A** | **A** | **A** | **A** | **G** | **A** | **G** | **T** | **C** | **T** | **G** | **G** | **A** | **T** | **T** | **C** | **A** | **A** | **A** | **C** | **A** | **C** | **C** | **G** | **T** | **A** | **T** | **T** | **T** | **A** | **G** | **T** | **T** | **C** | **C** | **T** | **G** | **A** | **T** | **G** | **A** | **T** | **A** | **G** | **T** | **G** | **T** | **C** | **T** | **T** | **G** | **G** | **C** | **G** | **G** | **C** | **T** | **T** | **T** | **A** | **A** | **A** | **G** | **G** | **T** | **T** | **G** | **C** | **T** | **G** | **C** | **G** | **G** | **C** | **T** | **T** | **C** | **A** | **G** | **G** | **A** | **G** | **T** | **A** | **G** | **A** | **T** | **G** | **T** | **T** | **A** | **A** | **A** | **A** | **T** | **T** | **A** | **T** | **G** | **A** | **T** | **T** | **C** | **C** | **A** | **G** | **A** | **T** | **A** | **A** | **G** | **C** | **C** | **C** | **G** | **A** | **C** | **C** | **A** | **T** | **C** | **C** | **G** | **T** | **T** | **T** | **A** | **T** | **T** | **T** | **A** | **T** | **C** | **G** | **A** | **G** | **C** | **A** | **A** | **C** | **A** | **C** | **A** | **G** | **T** | **A** | **T** | **T** | **A** | **C** | **G** | **G** | **G** | **C** | **G** | **C** | **T** | **T** | **G** | **T** | **T** | **G** | **A** | **T** | **G** | **A** | **A** | **A** | **G** | **A** | **A** | **A** | **A** | **T** | **A** | **T** | **T** | **G** | **A** | **A** | **A** | **T** | **T** | **T** | **T** | **A** | **A** | **T** | **T** | **T** | **A** | **T** | **A** | **A** | **C** | **G** | **G** | **T** | **G** | **G** | **T** | **T** | **T** | **C** | **T** | **T** | **A** | **C** | **A** | **T** | **G** | **C** | **G** | **A** | **A** | **A** | **A** | **C** | **A** | **A** | **T** | **G** | **A** | **T** | **T** | **A** | **T** | **G** | **G** | **A** | **T** | **G** | **A** | **T** | **G** | **A** | **A** | **G** | **T** | **C** | **T** | **G** | **C** | **A** | **C** | **A** | **G** | **T** | **T** | **G** | **G** | **T** | **T** | **C** | **A** | **G** | **C** | **C** | **A** | **A** | **T** | **C** | **A** | **A** | **G** | **A** | **T** | **A** | **T** | **C** | **C** | **G** | **A** | **A** | **G** | **T** | **T** | **A** | **C** | **A** | **A** | **A** | **T** | **T** | **A** | **A** | **A** | **C** | **T** | **T** | **T** | **G** | **A** | **A** | **G** | **C** | **A** | **A** | **A** | **T** | **G** | **C** | **T** | **G** | **T** | **G** | **T** | **T** | **A** | **T** | **A** | **T** | **G** | **A** | **T** | **A** | **A** | **A** | **A** | **A** | **A** | **A** | **T** | **C** | **A** | **T** | **T** | **G** | **A** | **T** | **C** | **A** | **A** | **T** | **T** | **A** | **G** | **A** | **A** | **G** | **C** | **A** | **A** | **T** | **T** | **T** | **T** | **C** | **T** | **T** | **A** | **G** | **A** | **A** | **G** | **A** | **T** | **C** | **G** | **A** | **A** | **A** | **A** | **A** | **A** | **A** | **T** | **G** | **T** | **A** | **C** | **A** | **A** | **C** | **A** | **A** | **T** | **G** | **A** | **C** | **T** | **C** | **C** | **A** | **G** | **A** | **A** | **G** | **T** | **T** | **G** | **T** | **T** | **C** | **G** | **C** | **G** | **A** | **C** | **A** | **T** | **G** | **T** | **C** | **A** | **A** | **A** | **A** | **T** | **G** | **G** | **T** | **T** | **G** | **A** | **T** | **T** | **T** | **T** | **T** | **A** | **A** | **A** | **C** | **A** | **A** | **C** | **A** | **A** | **A** | **T** | **T** | **T** | **C** | **A** | **C** | **G** | **A** | **T** | **T** | **A** | **T** | **T** | **C** | **T** | **C** | **A** | **C** | **C** | **A** | **A** | **T** | **T** | **C** | **T** | **T** | **-** | **-** | **-** |
| **N59458F** | **T** | **T** | **G** | **A** | **T** | **T** | **C** | **T** | **T** | **A** | **G** | **C** | **G** | **T** | **C** | **T** | **T** | **A** | **A** | **C** | **A** | **G** | **T** | **T** | **A** | **T** | **T** | **T** | **A** | **T** | **T** | **T** | **T** | **A** | **T** | **T** | **A** | **A** | **T** | **G** | **C** | **A** | **A** | **T** | **T** | **A** | **T** | **T** | **G** | **C** | **G** | **G** | **G** | **A** | **A** | **T** | **T** | **A** | **C** | **C** | **A** | **T** | **T** | **T** | **T** | **G** | **T** | **T** | **A** | **A** | **A** | **A** | **C** | **C** | **T** | **C** | **G** | **A** | **G** | **A** | **T** | **G** | **T** | **A** | **G** | **C** | **A** | **G** | **C** | **A** | **A** | **T** | **T** | **T** | **G** | **G** | **G** | **C** | **A** | **T** | **G** | **G** | **C** | **T** | **A** | **C** | **T** | **C** | **G** | **T** | **A** | **C** | **T** | **G** | **A** | **T** | **T** | **G** | **C** | **T** | **C** | **T** | **A** | **C** | **C** | **C** | **G** | **T** | **T** | **T** | **T** | **T** | **G** | **G** | **T** | **T** | **T** | **T** | **T** | **T** | **C** | **T** | **T** | **A** | **T** | **A** | **T** | **T** | **T** | **A** | **T** | **T** | **C** | **T** | **T** | **T** | **G** | **G** | **G** | **C** | **G** | **C** | **G** | **G** | **T** | **T** | **T** | **A** | **A** | **C** | **C** | **G** | **A** | **C** | **A** | **A** | **A** | **A** | **A** | **G** | **A** | **A** | **A** | **T** | **T** | **T** | **T** | **A** | **T** | **T** | **T** | **G** | **C** | **A** | **A** | **C** | **A** | **A** | **A** | **G** | **T** | **G** | **A** | **T** | **T** | **T** | **A** | **C** | **G** | **G** | **G** | **A** | **A** | **T** | **T** | **A** | **G** | **A** | **G** | **A** | **A** | **T** | **T** | **T** | **C** | **C** | **A** | **G** | **A** | **A** | **T** | **T** | **T** | **C** | **C** | **A** | **A** | **G** | **A** | **A** | **G** | **A** | **A** | **A** | **G** | **T** | **T** | **T** | **T** | **G** | **A** | **A** | **C** | **T** | **T** | **T** | **A** | **T** | **A** | **G** | **T** | **C** | **A** | **A** | **A** | **A** | **G** | **A** | **T** | **G** | **C** | **C** | **G** | **A** | **C** | **G** | **G** | **A** | **G** | **G** | **A** | **G** | **C** | **A** | **A** | **C** | **A** | **A** | **C** | **A** | **G** | **T** | **T** | **T** | **A** | **C** | **A** | **G** | **A** | **C** | **T** | **T** | **T** | **T** | **T** | **T** | **T** | **C** | **C** | **T** | **C** | **G** | **T** | **T** | **A** | **A** | **A** | **T** | **A** | **G** | **G** | **A** | **T** | **G** | **C** | **C** | **T** | **C** | **T** | **G** | **A** | **C** | **T** | **A** | **A** | **G** | **A** | **A** | **A** | **A** | **A** | **T** | **G** | **A** | **T** | **G** | **T** | **T** | **G** | **A** | **A** | **A** | **T** | **T** | **T** | **T** | **T** | **A** | **C** | **G** | **G** | **A** | **T** | **G** | **G** | **T** | **A** | **C** | **G** | **G** | **A** | **A** | **A** | **A** | **A** | **T** | **T** | **C** | **A** | **A** | **T** | **G** | **C** | **G** | **T** | **T** | **G** | **A** | **T** | **G** | **G** | **C** | **G** | **G** | **A** | **C** | **A** | **T** | **A** | **A** | **A** | **A** | **A** | **A** | **A** | **G** | **C** | **T** | **C** | **A** | **G** | **C** | **A** | **C** | **T** | **C** | **C** | **A** | **T** | **T** | **C** | **A** | **T** | **A** | **T** | **T** | **G** | **A** | **A** | **T** | **A** | **T** | **T** | **A** | **T** | **G** | **C** | **C** | **T** | **T** | **T** | **G** | **T** | **A** | **A** | **C** | **G** | **G** | **A** | **T** | **C** | **A** | **T** | **A** | **T** | **C** | **G** | **G** | **T** | **A** | **C** | **C** | **A** | **A** | **A** | **A** | **T** | **T** | **T** | **T** | **G** | **A** | **A** | **C** | **T** | **T** | **A** | **T** | **T** | **A** | **G** | **A** | **A** | **G** | **A** | **A** | **A** | **A** | **G** | **G** | **C** | **G** | **G** | **C** | **T** | **G** | **A** | **A** | **G** | **G** | **G** | **G** | **T** | **T** | **G** | **A** | **A** | **G** | **T** | **G** | **C** | **G** | **G** | **T** | **T** | **G** | **C** | **T** | **T** | **T** | **A** | **C** | **G** | **A** | **T** | **G** | **C** | **C** | **T** | **T** | **T** | **G** | **G** | **C** | **T** | **C** | **A** | **A** | **A** | **G** | **G** | **G** | **C** | **A** | **C** | **C** | **A** | **A** | **G** | **G** | **T** | **T** | **C** | **A** | **T** | **C** | **A** | **T** | **T** | **T** | **G** | **A** | **A** | **T** | **G** | **A** | **A** | **T** | **T** | **G** | **A** | **A** | **A** | **A** | **A** | **A** | **A** | **A** | **C** | **G** | **G** | **T** | **G** | **G** | **T** | **T** | **T** | **T** | **G** | **T** | **C** | **C** | **A** | **A** | **A** | **C** | **G** | **T** | **T** | **T** | **A** | **T** | **T** | **A** | **C** | **T** | **T** | **C** | **T** | **C** | **A** | **A** | **A** | **A** | **A** | **G** | **C** | **A** | **C** | **T** | **T** | **T** | **T** | **G** | **A** | **A** | **G** | **T** | **T** | **T** | **C** | **G** | **T** | **T** | **T** | **G** | **A** | **A** | **T** | **T** | **A** | **T** | **C** | **A** | **T** | **G** | **A** | **T** | **C** | **A** | **C** | **C** | **G** | **G** | **A** | **A** | **A** | **A** | **T** | **T** | **G** | **T** | **T** | **G** | **T** | **T** | **A** | **T** | **T** | **G** | **A** | **C** | **G** | **G** | **A** | **A** | **A** | **A** | **G** | **T** | **A** | **G** | **G** | **C** | **T** | **A** | **C** | **A** | **T** | **T** | **G** | **G** | **C** | **G** | **G** | **C** | **T** | **T** | **T** | **A** | **A** | **T** | **G** | **T** | **T** | **G** | **C** | **C** | **G** | **A** | **T** | **C** | **A** | **A** | **T** | **A** | **T** | **G** | **C** | **T** | **G** | **G** | **A** | **A** | **C** | **G** | **A** | **C** | **T** | **A** | **A** | **A** | **A** | **A** | **G** | **T** | **T** | **T** | **G** | **G** | **C** | **T** | **A** | **T** | **T** | **G** | **G** | **C** | **G** | **G** | **G** | **A** | **T** | **A** | **C** | **G** | **C** | **A** | **T** | **T** | **T** | **A** | **C** | **G** | **G** | **A** | **T** | **T** | **C** | **A** | **A** | **G** | **G** | **G** | **C** | **C** | **A** | **G** | **C** | **A** | **G** | **C** | **C** | **T** | **C** | **A** | **T** | **T** | **A** | **C** | **T** | **G** | **C** | **A** | **A** | **A** | **T** | **G** | **C** | **G** | **T** | **T** | **T** | **T** | **T** | **T** | **A** | **A** | **T** | **G** | **G** | **A** | **T** | **T** | **G** | **G** | **A** | **A** | **C** | **G** | **T** | **C** | **T** | **C** | **T** | **T** | **C** | **C** | **C** | **C** | **C** | **G** | **A** | **G** | **A** | **A** | **A** | **A** | **A** | **T** | **C** | **A** | **T** | **G** | **T** | **G** | **G** | **C** | **G** | **T** | **A** | **T** | **C** | **A** | **A** | **T** | **T** | **G** | **G** | **A** | **T** | **T** | **A** | **T** | **T** | **T** | **C** | **T** | **T** | **T** | **A** | **A** | **A** | **C** | **T** | **T** | **G** | **A** | **A** | **G** | **C** | **A** | **T** | **T** | **G** | **G** | **T** | **G** | **C** | **C** | **A** | **G** | **A** | **G** | **G** | **C** | **A** | **A** | **A** | **T** | **A** | **C** | **A** | **T** | **C** | **C** | **A** | **T** | **T** | **C** | **A** | **G** | **A** | **T** | **G** | **A** | **T** | **T** | **G** | **C** | **C** | **A** | **G** | **T** | **G** | **G** | **T** | **C** | **C** | **T** | **A** | **A** | **C** | **A** | **G** | **T** | **G** | **A** | **C** | **C** | **G** | **T** | **G** | **A** | **A** | **C** | **A** | **A** | **A** | **T** | **T** | **A** | **A** | **A** | **T** | **T** | **G** | **G** | **C** | **C** | **T** | **T** | **T** | **A** | **T** | **T** | **A** | **A** | **A** | **T** | **T** | **G** | **A** | **T** | **T** | **A** | **C** | **T** | **T** | **C** | **T** | **G** | **C** | **C** | **A** | **A** | **G** | **A** | **A** | **A** | **A** | **G** | **A** | **G** | **T** | **C** | **T** | **G** | **G** | **A** | **T** | **T** | **C** | **A** | **A** | **A** | **C** | **G** | **C** | **C** | **G** | **T** | **A** | **T** | **T** | **T** | **A** | **G** | **T** | **T** | **C** | **C** | **T** | **G** | **A** | **T** | **G** | **A** | **T** | **A** | **G** | **T** | **G** | **T** | **C** | **T** | **T** | **G** | **G** | **C** | **T** | **G** | **C** | **T** | **T** | **T** | **A** | **A** | **A** | **G** | **G** | **T** | **T** | **G** | **C** | **T** | **G** | **C** | **G** | **G** | **C** | **T** | **T** | **C** | **A** | **G** | **G** | **A** | **G** | **T** | **A** | **G** | **A** | **T** | **G** | **T** | **T** | **A** | **A** | **A** | **A** | **T** | **T** | **A** | **T** | **G** | **A** | **T** | **T** | **C** | **C** | **A** | **G** | **A** | **T** | **A** | **A** | **G** | **C** | **C** | **C** | **G** | **A** | **C** | **C** | **A** | **T** | **C** | **C** | **G** | **T** | **T** | **T** | **A** | **T** | **T** | **T** | **A** | **T** | **C** | **G** | **A** | **G** | **C** | **A** | **A** | **C** | **A** | **C** | **A** | **G** | **T** | **A** | **T** | **T** | **A** | **C** | **G** | **G** | **C** | **C** | **G** | **C** | **T** | **T** | **A** | **T** | **T** | **G** | **A** | **T** | **G** | **A** | **A** | **A** | **G** | **A** | **A** | **A** | **A** | **T** | **A** | **T** | **T** | **G** | **A** | **A** | **A** | **T** | **T** | **T** | **T** | **A** | **A** | **T** | **T** | **T** | **A** | **T** | **A** | **A** | **C** | **G** | **G** | **T** | **G** | **G** | **T** | **T** | **T** | **C** | **T** | **T** | **A** | **C** | **A** | **T** | **G** | **C** | **G** | **A** | **A** | **A** | **A** | **C** | **A** | **A** | **T** | **G** | **A** | **T** | **T** | **A** | **T** | **G** | **G** | **A** | **T** | **G** | **A** | **T** | **G** | **A** | **A** | **G** | **T** | **C** | **T** | **G** | **T** | **A** | **C** | **A** | **G** | **T** | **T** | **G** | **G** | **T** | **T** | **C** | **A** | **G** | **C** | **C** | **A** | **A** | **T** | **C** | **A** | **A** | **G** | **A** | **T** | **A** | **T** | **C** | **C** | **G** | **A** | **A** | **G** | **T** | **T** | **A** | **C** | **A** | **A** | **A** | **T** | **T** | **A** | **A** | **A** | **C** | **T** | **T** | **T** | **G** | **A** | **A** | **G** | **C** | **A** | **A** | **A** | **T** | **G** | **C** | **T** | **G** | **T** | **G** | **T** | **T** | **A** | **T** | **A** | **T** | **G** | **A** | **T** | **A** | **A** | **A** | **A** | **A** | **A** | **A** | **T** | **C** | **A** | **T** | **T** | **G** | **A** | **T** | **C** | **A** | **A** | **T** | **T** | **A** | **G** | **A** | **A** | **G** | **C** | **A** | **A** | **T** | **T** | **T** | **T** | **C** | **T** | **T** | **A** | **G** | **A** | **A** | **G** | **A** | **T** | **C** | **G** | **A** | **A** | **A** | **A** | **A** | **A** | **A** | **T** | **G** | **T** | **A** | **C** | **A** | **A** | **C** | **A** | **A** | **T** | **G** | **A** | **C** | **A** | **C** | **C** | **A** | **G** | **A** | **A** | **G** | **T** | **T** | **G** | **T** | **T** | **C** | **G** | **A** | **G** | **A** | **C** | **A** | **T** | **G** | **T** | **C** | **C** | **A** | **A** | **A** | **T** | **G** | **G** | **T** | **T** | **G** | **A** | **T** | **T** | **T** | **T** | **T** | **A** | **A** | **A** | **C** | **A** | **A** | **C** | **A** | **A** | **A** | **T** | **T** | **T** | **C** | **A** | **C** | **G** | **A** | **T** | **T** | **A** | **T** | **T** | **T** | **T** | **C** | **C** | **C** | **C** | **A** | **A** | **T** | **T** | **C** | **T** | **T** | **-** | **-** | **-** |
| **N59455F** | **T** | **T** | **G** | **A** | **T** | **T** | **C** | **T** | **T** | **A** | **G** | **C** | **G** | **T** | **C** | **T** | **T** | **A** | **A** | **C** | **A** | **G** | **T** | **T** | **A** | **T** | **T** | **T** | **A** | **T** | **T** | **T** | **T** | **A** | **T** | **T** | **A** | **A** | **T** | **G** | **C** | **A** | **A** | **T** | **T** | **A** | **T** | **T** | **G** | **C** | **G** | **G** | **G** | **A** | **A** | **T** | **T** | **A** | **C** | **C** | **A** | **T** | **T** | **T** | **T** | **G** | **T** | **T** | **A** | **A** | **A** | **A** | **C** | **C** | **T** | **C** | **G** | **A** | **G** | **A** | **T** | **G** | **T** | **A** | **G** | **C** | **A** | **G** | **C** | **A** | **A** | **T** | **T** | **T** | **G** | **G** | **G** | **C** | **A** | **T** | **G** | **G** | **C** | **T** | **A** | **C** | **T** | **C** | **G** | **T** | **A** | **C** | **T** | **G** | **A** | **T** | **T** | **G** | **C** | **T** | **C** | **T** | **A** | **C** | **C** | **C** | **G** | **T** | **T** | **T** | **T** | **T** | **G** | **G** | **T** | **T** | **T** | **T** | **T** | **T** | **C** | **T** | **T** | **A** | **T** | **A** | **T** | **T** | **T** | **A** | **T** | **T** | **C** | **T** | **T** | **T** | **G** | **G** | **G** | **C** | **G** | **C** | **G** | **G** | **T** | **T** | **T** | **A** | **A** | **C** | **C** | **G** | **A** | **C** | **A** | **A** | **A** | **A** | **A** | **G** | **A** | **A** | **A** | **T** | **T** | **T** | **T** | **A** | **T** | **T** | **T** | **G** | **C** | **A** | **A** | **C** | **A** | **A** | **A** | **G** | **T** | **G** | **A** | **T** | **T** | **T** | **A** | **C** | **G** | **G** | **G** | **A** | **A** | **T** | **T** | **A** | **G** | **A** | **G** | **A** | **A** | **T** | **T** | **T** | **T** | **C** | **A** | **G** | **A** | **A** | **C** | **T** | **T** | **C** | **C** | **A** | **A** | **G** | **A** | **A** | **G** | **A** | **A** | **A** | **G** | **T** | **T** | **T** | **T** | **G** | **A** | **A** | **C** | **T** | **T** | **T** | **A** | **T** | **A** | **G** | **T** | **C** | **A** | **A** | **A** | **A** | **G** | **A** | **T** | **G** | **C** | **C** | **G** | **A** | **C** | **G** | **G** | **A** | **G** | **G** | **A** | **G** | **C** | **A** | **A** | **C** | **A** | **A** | **C** | **A** | **G** | **T** | **T** | **T** | **A** | **C** | **A** | **G** | **A** | **C** | **T** | **T** | **T** | **T** | **T** | **T** | **T** | **C** | **C** | **T** | **C** | **G** | **T** | **T** | **A** | **A** | **A** | **T** | **A** | **G** | **G** | **A** | **T** | **G** | **C** | **C** | **T** | **C** | **T** | **G** | **A** | **C** | **T** | **A** | **A** | **G** | **A** | **A** | **A** | **A** | **A** | **T** | **G** | **A** | **T** | **G** | **T** | **T** | **G** | **A** | **A** | **A** | **T** | **T** | **T** | **T** | **T** | **A** | **C** | **G** | **G** | **A** | **T** | **G** | **G** | **T** | **A** | **C** | **G** | **G** | **A** | **A** | **A** | **A** | **A** | **T** | **T** | **C** | **A** | **A** | **T** | **G** | **C** | **G** | **T** | **T** | **G** | **A** | **T** | **G** | **G** | **C** | **G** | **G** | **A** | **C** | **A** | **T** | **A** | **A** | **A** | **A** | **A** | **A** | **A** | **G** | **C** | **T** | **C** | **A** | **G** | **C** | **A** | **C** | **T** | **C** | **C** | **A** | **T** | **T** | **C** | **A** | **T** | **A** | **T** | **T** | **G** | **A** | **A** | **T** | **A** | **T** | **T** | **A** | **T** | **G** | **C** | **C** | **T** | **T** | **T** | **G** | **T** | **A** | **A** | **C** | **G** | **G** | **A** | **T** | **C** | **A** | **T** | **A** | **T** | **C** | **G** | **G** | **T** | **A** | **C** | **C** | **A** | **A** | **A** | **A** | **T** | **T** | **T** | **T** | **G** | **A** | **A** | **C** | **T** | **T** | **A** | **T** | **T** | **A** | **G** | **A** | **A** | **G** | **A** | **A** | **A** | **A** | **G** | **G** | **C** | **G** | **G** | **C** | **T** | **G** | **A** | **A** | **G** | **G** | **G** | **G** | **T** | **T** | **G** | **A** | **A** | **G** | **T** | **G** | **C** | **G** | **G** | **T** | **T** | **G** | **C** | **T** | **T** | **T** | **A** | **C** | **G** | **A** | **T** | **G** | **C** | **C** | **T** | **T** | **T** | **G** | **G** | **C** | **T** | **C** | **A** | **A** | **A** | **G** | **G** | **G** | **C** | **A** | **C** | **C** | **A** | **A** | **G** | **G** | **T** | **T** | **C** | **A** | **T** | **C** | **A** | **T** | **T** | **T** | **G** | **A** | **A** | **T** | **G** | **A** | **A** | **T** | **T** | **G** | **A** | **A** | **A** | **A** | **A** | **A** | **A** | **A** | **C** | **G** | **G** | **C** | **G** | **G** | **T** | **T** | **T** | **T** | **G** | **T** | **C** | **C** | **A** | **A** | **A** | **C** | **G** | **T** | **T** | **T** | **A** | **T** | **T** | **A** | **C** | **T** | **T** | **C** | **T** | **C** | **A** | **A** | **A** | **A** | **A** | **G** | **C** | **A** | **C** | **T** | **T** | **T** | **T** | **G** | **A** | **A** | **G** | **T** | **T** | **T** | **C** | **G** | **T** | **T** | **T** | **G** | **A** | **A** | **T** | **T** | **A** | **T** | **C** | **A** | **T** | **G** | **A** | **T** | **C** | **A** | **C** | **C** | **G** | **G** | **A** | **A** | **A** | **A** | **T** | **T** | **G** | **T** | **T** | **G** | **T** | **T** | **A** | **T** | **T** | **G** | **A** | **T** | **G** | **G** | **G** | **A** | **A** | **A** | **G** | **T** | **A** | **G** | **G** | **C** | **T** | **A** | **C** | **A** | **T** | **T** | **G** | **G** | **C** | **G** | **G** | **C** | **T** | **T** | **T** | **A** | **A** | **T** | **G** | **T** | **T** | **G** | **C** | **C** | **G** | **A** | **T** | **C** | **A** | **A** | **T** | **A** | **T** | **G** | **C** | **T** | **G** | **G** | **A** | **A** | **C** | **G** | **A** | **C** | **T** | **A** | **A** | **A** | **A** | **A** | **G** | **T** | **T** | **T** | **G** | **G** | **C** | **T** | **A** | **T** | **T** | **G** | **G** | **C** | **G** | **G** | **G** | **A** | **T** | **A** | **C** | **A** | **C** | **A** | **T** | **T** | **T** | **A** | **C** | **G** | **G** | **A** | **T** | **T** | **C** | **A** | **A** | **G** | **G** | **G** | **C** | **C** | **A** | **G** | **C** | **A** | **G** | **C** | **C** | **T** | **C** | **A** | **T** | **T** | **A** | **C** | **T** | **G** | **C** | **A** | **A** | **A** | **T** | **G** | **C** | **G** | **T** | **T** | **T** | **T** | **T** | **T** | **A** | **A** | **T** | **G** | **G** | **A** | **T** | **T** | **G** | **G** | **A** | **A** | **C** | **G** | **T** | **C** | **T** | **C** | **T** | **T** | **C** | **C** | **C** | **C** | **C** | **G** | **A** | **G** | **A** | **A** | **A** | **A** | **A** | **T** | **C** | **G** | **T** | **G** | **T** | **G** | **G** | **C** | **G** | **T** | **A** | **T** | **C** | **A** | **A** | **T** | **T** | **G** | **G** | **A** | **T** | **T** | **A** | **T** | **T** | **T** | **C** | **T** | **T** | **T** | **A** | **A** | **A** | **C** | **T** | **T** | **G** | **A** | **A** | **G** | **C** | **A** | **T** | **T** | **G** | **G** | **T** | **T** | **C** | **C** | **A** | **G** | **A** | **G** | **G** | **C** | **A** | **A** | **A** | **T** | **A** | **C** | **A** | **T** | **C** | **C** | **A** | **T** | **T** | **C** | **A** | **G** | **A** | **T** | **G** | **A** | **T** | **T** | **G** | **C** | **C** | **A** | **G** | **T** | **G** | **G** | **T** | **C** | **C** | **T** | **A** | **A** | **T** | **A** | **G** | **T** | **G** | **A** | **C** | **C** | **G** | **T** | **G** | **A** | **A** | **C** | **A** | **A** | **A** | **T** | **T** | **A** | **A** | **A** | **T** | **T** | **G** | **G** | **C** | **C** | **T** | **T** | **T** | **A** | **T** | **T** | **A** | **A** | **A** | **T** | **T** | **G** | **A** | **T** | **T** | **A** | **C** | **T** | **T** | **C** | **T** | **G** | **C** | **C** | **A** | **A** | **G** | **A** | **A** | **A** | **A** | **G** | **A** | **G** | **T** | **C** | **T** | **G** | **G** | **A** | **T** | **T** | **C** | **A** | **A** | **A** | **C** | **A** | **C** | **C** | **G** | **T** | **A** | **T** | **T** | **T** | **A** | **G** | **T** | **T** | **C** | **C** | **T** | **G** | **A** | **T** | **G** | **A** | **T** | **A** | **G** | **T** | **G** | **T** | **C** | **T** | **T** | **G** | **G** | **C** | **G** | **G** | **C** | **T** | **T** | **T** | **A** | **A** | **A** | **G** | **G** | **T** | **T** | **G** | **C** | **T** | **G** | **C** | **G** | **G** | **C** | **T** | **T** | **C** | **A** | **G** | **G** | **A** | **G** | **T** | **A** | **G** | **A** | **T** | **G** | **T** | **T** | **A** | **A** | **A** | **A** | **T** | **T** | **A** | **T** | **G** | **A** | **T** | **T** | **C** | **C** | **A** | **G** | **A** | **T** | **A** | **A** | **G** | **C** | **C** | **C** | **G** | **A** | **C** | **C** | **A** | **T** | **C** | **C** | **G** | **T** | **T** | **T** | **A** | **T** | **T** | **T** | **A** | **T** | **C** | **G** | **A** | **G** | **C** | **A** | **A** | **C** | **A** | **C** | **A** | **G** | **T** | **A** | **T** | **T** | **A** | **C** | **G** | **G** | **G** | **C** | **G** | **C** | **T** | **T** | **G** | **T** | **T** | **G** | **A** | **T** | **G** | **A** | **A** | **A** | **G** | **A** | **A** | **A** | **A** | **T** | **A** | **T** | **T** | **G** | **A** | **A** | **A** | **T** | **T** | **T** | **T** | **A** | **A** | **T** | **T** | **T** | **A** | **T** | **A** | **A** | **C** | **G** | **G** | **T** | **G** | **G** | **T** | **T** | **T** | **C** | **T** | **T** | **A** | **C** | **A** | **T** | **G** | **C** | **G** | **A** | **A** | **A** | **A** | **C** | **A** | **A** | **T** | **G** | **A** | **T** | **T** | **A** | **T** | **G** | **G** | **A** | **T** | **G** | **A** | **T** | **G** | **A** | **A** | **G** | **T** | **C** | **T** | **G** | **C** | **A** | **C** | **A** | **G** | **T** | **T** | **G** | **G** | **T** | **T** | **C** | **A** | **G** | **C** | **C** | **A** | **A** | **T** | **C** | **A** | **A** | **G** | **A** | **T** | **A** | **T** | **C** | **C** | **G** | **A** | **A** | **G** | **T** | **T** | **A** | **C** | **A** | **A** | **A** | **T** | **T** | **A** | **A** | **A** | **C** | **T** | **T** | **T** | **G** | **A** | **A** | **G** | **C** | **A** | **A** | **A** | **T** | **G** | **C** | **T** | **G** | **T** | **G** | **T** | **T** | **A** | **T** | **A** | **T** | **G** | **A** | **T** | **A** | **A** | **A** | **A** | **A** | **A** | **A** | **T** | **C** | **A** | **T** | **T** | **G** | **A** | **T** | **C** | **A** | **A** | **T** | **T** | **A** | **G** | **A** | **A** | **G** | **C** | **A** | **A** | **T** | **T** | **T** | **T** | **C** | **T** | **T** | **A** | **G** | **A** | **A** | **G** | **A** | **T** | **C** | **G** | **A** | **A** | **A** | **A** | **A** | **A** | **A** | **T** | **G** | **T** | **A** | **C** | **A** | **A** | **C** | **A** | **A** | **T** | **G** | **A** | **C** | **T** | **C** | **C** | **A** | **G** | **A** | **A** | **G** | **T** | **T** | **G** | **T** | **T** | **C** | **G** | **C** | **G** | **A** | **C** | **A** | **T** | **G** | **T** | **C** | **A** | **A** | **A** | **A** | **T** | **G** | **G** | **T** | **T** | **G** | **A** | **T** | **T** | **T** | **T** | **T** | **A** | **A** | **A** | **C** | **A** | **A** | **C** | **A** | **A** | **A** | **T** | **T** | **T** | **C** | **A** | **C** | **G** | **A** | **T** | **T** | **A** | **T** | **T** | **C** | **T** | **C** | **A** | **C** | **C** | **A** | **A** | **T** | **T** | **C** | **T** | **T** | **-** | **-** | **-** |
| **N52734** | **T** | **T** | **G** | **A** | **T** | **T** | **C** | **T** | **T** | **A** | **G** | **C** | **G** | **T** | **C** | **T** | **T** | **A** | **A** | **C** | **A** | **G** | **T** | **T** | **A** | **T** | **T** | **T** | **A** | **T** | **T** | **T** | **T** | **A** | **T** | **T** | **A** | **A** | **T** | **G** | **C** | **A** | **A** | **T** | **T** | **A** | **T** | **T** | **G** | **C** | **G** | **G** | **G** | **A** | **A** | **T** | **T** | **A** | **C** | **C** | **A** | **T** | **T** | **T** | **T** | **G** | **T** | **T** | **A** | **A** | **A** | **A** | **C** | **C** | **T** | **C** | **G** | **A** | **G** | **A** | **T** | **G** | **T** | **A** | **G** | **C** | **A** | **G** | **C** | **A** | **A** | **T** | **T** | **T** | **G** | **G** | **G** | **C** | **A** | **T** | **G** | **G** | **C** | **T** | **A** | **C** | **T** | **C** | **G** | **T** | **A** | **C** | **T** | **G** | **A** | **T** | **T** | **G** | **C** | **T** | **C** | **T** | **A** | **C** | **C** | **C** | **G** | **T** | **T** | **T** | **T** | **T** | **G** | **G** | **T** | **T** | **T** | **T** | **T** | **T** | **C** | **T** | **T** | **A** | **T** | **A** | **T** | **T** | **T** | **A** | **T** | **T** | **C** | **T** | **T** | **T** | **G** | **G** | **G** | **C** | **G** | **C** | **G** | **G** | **T** | **T** | **T** | **A** | **A** | **C** | **C** | **G** | **A** | **C** | **A** | **A** | **A** | **A** | **A** | **G** | **A** | **A** | **A** | **T** | **T** | **T** | **T** | **A** | **T** | **T** | **T** | **G** | **C** | **A** | **A** | **C** | **A** | **A** | **A** | **G** | **T** | **G** | **A** | **T** | **T** | **T** | **A** | **C** | **G** | **G** | **G** | **A** | **A** | **T** | **T** | **A** | **G** | **A** | **G** | **A** | **A** | **T** | **T** | **T** | **C** | **C** | **A** | **G** | **A** | **A** | **T** | **T** | **T** | **C** | **C** | **A** | **A** | **G** | **A** | **A** | **G** | **A** | **A** | **A** | **G** | **T** | **T** | **T** | **T** | **G** | **A** | **A** | **C** | **T** | **T** | **T** | **A** | **T** | **A** | **G** | **T** | **C** | **A** | **A** | **A** | **A** | **G** | **A** | **T** | **G** | **C** | **C** | **G** | **A** | **C** | **G** | **G** | **A** | **G** | **G** | **A** | **G** | **C** | **A** | **A** | **C** | **A** | **A** | **C** | **A** | **G** | **T** | **T** | **T** | **A** | **C** | **A** | **G** | **A** | **C** | **T** | **T** | **T** | **T** | **T** | **T** | **T** | **C** | **C** | **T** | **C** | **G** | **T** | **T** | **A** | **A** | **A** | **T** | **A** | **G** | **G** | **A** | **T** | **G** | **C** | **C** | **T** | **C** | **T** | **G** | **A** | **C** | **T** | **A** | **A** | **G** | **A** | **A** | **A** | **A** | **A** | **T** | **G** | **A** | **T** | **G** | **T** | **T** | **G** | **A** | **A** | **A** | **T** | **T** | **T** | **T** | **T** | **A** | **C** | **G** | **G** | **A** | **T** | **G** | **G** | **T** | **A** | **C** | **G** | **G** | **A** | **A** | **A** | **A** | **A** | **T** | **T** | **C** | **A** | **A** | **T** | **G** | **C** | **G** | **T** | **T** | **G** | **A** | **T** | **G** | **G** | **C** | **G** | **G** | **A** | **C** | **A** | **T** | **A** | **A** | **A** | **A** | **A** | **A** | **A** | **G** | **C** | **T** | **C** | **A** | **G** | **C** | **A** | **C** | **T** | **C** | **C** | **A** | **T** | **T** | **C** | **A** | **T** | **A** | **T** | **T** | **G** | **A** | **A** | **T** | **A** | **T** | **T** | **A** | **T** | **G** | **C** | **C** | **T** | **T** | **T** | **G** | **T** | **A** | **A** | **C** | **G** | **G** | **A** | **T** | **C** | **A** | **T** | **A** | **T** | **C** | **G** | **G** | **C** | **A** | **C** | **C** | **A** | **A** | **A** | **A** | **T** | **T** | **T** | **T** | **G** | **A** | **A** | **C** | **T** | **T** | **A** | **T** | **T** | **A** | **G** | **A** | **A** | **G** | **A** | **A** | **A** | **A** | **G** | **G** | **C** | **G** | **G** | **C** | **T** | **G** | **A** | **A** | **G** | **G** | **G** | **G** | **T** | **T** | **G** | **A** | **A** | **G** | **T** | **G** | **C** | **G** | **G** | **T** | **T** | **G** | **C** | **T** | **T** | **T** | **A** | **C** | **G** | **A** | **T** | **G** | **C** | **C** | **T** | **T** | **T** | **G** | **G** | **C** | **T** | **C** | **A** | **A** | **A** | **G** | **G** | **G** | **C** | **A** | **C** | **C** | **A** | **A** | **G** | **G** | **T** | **T** | **C** | **A** | **T** | **C** | **A** | **T** | **T** | **T** | **G** | **A** | **A** | **T** | **G** | **A** | **A** | **T** | **T** | **G** | **A** | **A** | **A** | **A** | **A** | **A** | **A** | **A** | **C** | **G** | **G** | **C** | **G** | **G** | **T** | **T** | **T** | **T** | **G** | **T** | **C** | **C** | **A** | **A** | **A** | **C** | **A** | **T** | **T** | **T** | **A** | **T** | **T** | **A** | **C** | **T** | **T** | **C** | **T** | **C** | **A** | **A** | **A** | **A** | **A** | **G** | **C** | **A** | **C** | **T** | **T** | **T** | **T** | **G** | **A** | **A** | **G** | **T** | **T** | **T** | **C** | **G** | **T** | **T** | **T** | **G** | **A** | **A** | **T** | **T** | **A** | **T** | **C** | **A** | **T** | **G** | **A** | **T** | **C** | **A** | **C** | **C** | **G** | **G** | **A** | **A** | **A** | **A** | **T** | **T** | **G** | **T** | **T** | **G** | **T** | **T** | **A** | **T** | **T** | **G** | **A** | **C** | **G** | **G** | **A** | **A** | **A** | **A** | **G** | **T** | **A** | **G** | **G** | **C** | **T** | **A** | **C** | **A** | **T** | **T** | **G** | **G** | **C** | **G** | **G** | **C** | **T** | **T** | **T** | **A** | **A** | **T** | **G** | **T** | **T** | **G** | **C** | **C** | **G** | **A** | **T** | **C** | **A** | **A** | **T** | **A** | **T** | **G** | **C** | **C** | **G** | **G** | **A** | **A** | **C** | **G** | **A** | **C** | **T** | **A** | **A** | **A** | **A** | **A** | **G** | **T** | **T** | **T** | **G** | **G** | **C** | **T** | **A** | **T** | **T** | **G** | **G** | **C** | **G** | **G** | **G** | **A** | **T** | **A** | **C** | **G** | **C** | **A** | **T** | **T** | **T** | **A** | **C** | **G** | **G** | **A** | **T** | **T** | **C** | **A** | **A** | **G** | **G** | **G** | **C** | **C** | **A** | **G** | **C** | **A** | **G** | **C** | **C** | **T** | **C** | **A** | **T** | **T** | **A** | **C** | **T** | **G** | **C** | **A** | **A** | **A** | **T** | **G** | **C** | **G** | **T** | **T** | **T** | **T** | **T** | **T** | **A** | **A** | **T** | **G** | **G** | **A** | **T** | **T** | **G** | **G** | **A** | **A** | **C** | **G** | **T** | **C** | **T** | **C** | **T** | **T** | **C** | **C** | **C** | **C** | **C** | **G** | **A** | **G** | **A** | **A** | **A** | **A** | **A** | **T** | **C** | **G** | **T** | **G** | **T** | **G** | **G** | **C** | **G** | **T** | **A** | **T** | **C** | **A** | **A** | **T** | **T** | **G** | **G** | **A** | **T** | **T** | **A** | **T** | **T** | **T** | **C** | **T** | **T** | **T** | **A** | **A** | **A** | **C** | **T** | **T** | **G** | **A** | **A** | **G** | **C** | **A** | **T** | **T** | **G** | **G** | **T** | **G** | **C** | **C** | **A** | **G** | **A** | **G** | **G** | **C** | **A** | **A** | **A** | **T** | **A** | **C** | **A** | **T** | **C** | **C** | **A** | **T** | **T** | **C** | **A** | **G** | **A** | **T** | **G** | **A** | **T** | **T** | **G** | **C** | **C** | **A** | **G** | **T** | **G** | **G** | **T** | **C** | **C** | **T** | **A** | **A** | **C** | **A** | **G** | **T** | **G** | **A** | **C** | **C** | **G** | **T** | **G** | **A** | **A** | **C** | **A** | **A** | **A** | **T** | **T** | **A** | **A** | **A** | **T** | **T** | **G** | **G** | **C** | **C** | **T** | **T** | **T** | **A** | **T** | **T** | **A** | **A** | **A** | **T** | **T** | **G** | **A** | **T** | **T** | **A** | **C** | **T** | **T** | **C** | **T** | **G** | **C** | **C** | **A** | **A** | **G** | **A** | **A** | **A** | **A** | **G** | **A** | **G** | **T** | **C** | **T** | **G** | **G** | **A** | **T** | **T** | **C** | **A** | **A** | **A** | **C** | **G** | **C** | **C** | **G** | **T** | **A** | **T** | **T** | **T** | **A** | **G** | **T** | **T** | **C** | **C** | **T** | **G** | **A** | **T** | **G** | **A** | **T** | **A** | **G** | **T** | **G** | **T** | **C** | **T** | **T** | **G** | **G** | **C** | **T** | **G** | **C** | **T** | **T** | **T** | **A** | **A** | **A** | **G** | **G** | **T** | **T** | **G** | **C** | **T** | **G** | **C** | **G** | **G** | **C** | **T** | **T** | **C** | **A** | **G** | **G** | **A** | **G** | **T** | **A** | **G** | **A** | **T** | **G** | **T** | **T** | **A** | **A** | **A** | **A** | **T** | **T** | **A** | **T** | **G** | **A** | **T** | **T** | **C** | **C** | **A** | **G** | **A** | **T** | **A** | **A** | **G** | **C** | **C** | **C** | **G** | **A** | **C** | **C** | **A** | **T** | **C** | **C** | **G** | **T** | **T** | **T** | **A** | **T** | **T** | **T** | **A** | **T** | **C** | **G** | **A** | **G** | **C** | **A** | **A** | **C** | **A** | **C** | **A** | **G** | **T** | **A** | **T** | **T** | **A** | **T** | **G** | **G** | **C** | **C** | **G** | **C** | **T** | **T** | **A** | **T** | **T** | **G** | **A** | **T** | **G** | **A** | **A** | **A** | **G** | **A** | **A** | **A** | **A** | **T** | **A** | **T** | **T** | **G** | **A** | **A** | **A** | **T** | **T** | **T** | **T** | **A** | **A** | **T** | **T** | **T** | **A** | **T** | **A** | **A** | **C** | **G** | **G** | **T** | **G** | **G** | **T** | **T** | **T** | **C** | **T** | **T** | **A** | **C** | **A** | **T** | **G** | **C** | **G** | **A** | **A** | **A** | **A** | **C** | **A** | **A** | **T** | **G** | **A** | **T** | **T** | **A** | **T** | **G** | **G** | **A** | **T** | **G** | **A** | **T** | **G** | **A** | **A** | **G** | **T** | **C** | **T** | **G** | **T** | **A** | **C** | **A** | **G** | **T** | **T** | **G** | **G** | **T** | **T** | **C** | **A** | **G** | **C** | **C** | **A** | **A** | **T** | **C** | **A** | **A** | **G** | **A** | **T** | **A** | **T** | **C** | **C** | **G** | **A** | **A** | **G** | **T** | **T** | **A** | **C** | **A** | **A** | **A** | **T** | **T** | **A** | **A** | **A** | **C** | **T** | **T** | **T** | **G** | **A** | **A** | **G** | **C** | **A** | **A** | **A** | **T** | **G** | **C** | **T** | **G** | **T** | **G** | **T** | **T** | **A** | **T** | **A** | **T** | **G** | **A** | **T** | **A** | **A** | **A** | **A** | **A** | **A** | **A** | **T** | **C** | **A** | **T** | **T** | **G** | **A** | **T** | **C** | **A** | **A** | **T** | **T** | **A** | **G** | **A** | **A** | **G** | **C** | **A** | **A** | **T** | **T** | **T** | **T** | **C** | **T** | **T** | **A** | **G** | **A** | **A** | **G** | **A** | **T** | **C** | **G** | **A** | **A** | **A** | **A** | **A** | **A** | **A** | **T** | **G** | **T** | **A** | **C** | **A** | **A** | **C** | **A** | **A** | **T** | **G** | **A** | **C** | **A** | **C** | **C** | **A** | **G** | **A** | **A** | **G** | **T** | **T** | **G** | **T** | **T** | **C** | **G** | **A** | **G** | **A** | **C** | **A** | **T** | **G** | **T** | **C** | **C** | **A** | **A** | **A** | **T** | **G** | **G** | **T** | **T** | **G** | **A** | **T** | **T** | **T** | **T** | **T** | **A** | **A** | **A** | **C** | **A** | **A** | **C** | **A** | **A** | **A** | **T** | **T** | **T** | **C** | **A** | **C** | **G** | **A** | **T** | **T** | **A** | **T** | **T** | **T** | **T** | **C** | **C** | **C** | **C** | **A** | **A** | **T** | **T** | **C** | **T** | **T** | **-** | **-** | **-** |
| **N59462F** | **T** | **T** | **G** | **A** | **T** | **T** | **C** | **T** | **T** | **A** | **G** | **C** | **G** | **T** | **C** | **T** | **T** | **A** | **A** | **C** | **A** | **G** | **T** | **T** | **A** | **T** | **T** | **T** | **A** | **T** | **T** | **T** | **T** | **A** | **T** | **T** | **A** | **A** | **T** | **G** | **C** | **A** | **A** | **T** | **T** | **A** | **T** | **T** | **G** | **C** | **G** | **G** | **G** | **A** | **A** | **T** | **T** | **A** | **C** | **C** | **A** | **T** | **T** | **T** | **T** | **G** | **T** | **T** | **A** | **A** | **A** | **A** | **C** | **C** | **T** | **C** | **G** | **A** | **G** | **A** | **T** | **G** | **T** | **A** | **G** | **C** | **A** | **G** | **C** | **A** | **A** | **T** | **T** | **T** | **G** | **G** | **G** | **C** | **A** | **T** | **G** | **G** | **C** | **T** | **A** | **C** | **T** | **C** | **G** | **T** | **A** | **C** | **T** | **G** | **A** | **T** | **T** | **G** | **C** | **T** | **C** | **T** | **A** | **C** | **C** | **C** | **G** | **T** | **T** | **T** | **T** | **T** | **G** | **G** | **T** | **T** | **T** | **T** | **T** | **T** | **C** | **T** | **T** | **A** | **T** | **A** | **T** | **T** | **T** | **A** | **T** | **T** | **C** | **T** | **T** | **T** | **G** | **G** | **G** | **C** | **G** | **C** | **G** | **G** | **T** | **T** | **T** | **A** | **A** | **C** | **C** | **G** | **A** | **C** | **A** | **A** | **A** | **A** | **A** | **G** | **A** | **A** | **A** | **T** | **T** | **T** | **T** | **A** | **T** | **T** | **T** | **G** | **C** | **A** | **A** | **C** | **A** | **A** | **A** | **G** | **T** | **G** | **A** | **T** | **T** | **T** | **G** | **C** | **G** | **G** | **G** | **A** | **A** | **T** | **T** | **A** | **G** | **A** | **G** | **A** | **A** | **T** | **T** | **T** | **T** | **C** | **A** | **G** | **A** | **A** | **T** | **T** | **T** | **C** | **C** | **A** | **A** | **G** | **A** | **A** | **G** | **A** | **A** | **A** | **G** | **T** | **T** | **T** | **T** | **G** | **A** | **A** | **C** | **T** | **T** | **T** | **A** | **T** | **A** | **G** | **T** | **C** | **A** | **A** | **A** | **A** | **G** | **A** | **T** | **G** | **C** | **C** | **G** | **A** | **C** | **G** | **G** | **A** | **G** | **G** | **A** | **G** | **C** | **A** | **A** | **C** | **A** | **A** | **C** | **A** | **G** | **T** | **T** | **T** | **A** | **C** | **A** | **G** | **A** | **C** | **T** | **T** | **T** | **T** | **T** | **T** | **T** | **C** | **C** | **T** | **C** | **G** | **T** | **T** | **A** | **A** | **A** | **T** | **A** | **G** | **G** | **A** | **T** | **G** | **C** | **C** | **T** | **C** | **T** | **G** | **A** | **C** | **T** | **A** | **A** | **G** | **A** | **A** | **A** | **A** | **A** | **T** | **G** | **A** | **T** | **G** | **T** | **T** | **G** | **A** | **A** | **A** | **T** | **T** | **T** | **T** | **T** | **A** | **C** | **G** | **G** | **A** | **T** | **G** | **G** | **T** | **A** | **C** | **G** | **G** | **A** | **A** | **A** | **A** | **A** | **T** | **T** | **C** | **A** | **A** | **T** | **G** | **C** | **G** | **T** | **T** | **G** | **A** | **T** | **G** | **G** | **C** | **A** | **G** | **A** | **C** | **A** | **T** | **A** | **A** | **A** | **A** | **A** | **A** | **A** | **G** | **C** | **T** | **C** | **A** | **G** | **C** | **A** | **C** | **T** | **C** | **C** | **A** | **T** | **T** | **C** | **A** | **T** | **A** | **T** | **T** | **G** | **A** | **A** | **T** | **A** | **T** | **T** | **A** | **T** | **G** | **C** | **C** | **T** | **T** | **T** | **G** | **T** | **A** | **A** | **C** | **G** | **G** | **A** | **T** | **C** | **A** | **T** | **A** | **T** | **C** | **G** | **G** | **T** | **A** | **C** | **C** | **A** | **A** | **A** | **A** | **T** | **T** | **T** | **T** | **G** | **A** | **A** | **C** | **T** | **T** | **A** | **T** | **T** | **A** | **G** | **A** | **A** | **G** | **A** | **A** | **A** | **A** | **G** | **G** | **C** | **G** | **G** | **C** | **T** | **G** | **A** | **A** | **G** | **G** | **G** | **G** | **T** | **T** | **G** | **A** | **A** | **G** | **T** | **G** | **C** | **G** | **G** | **T** | **T** | **G** | **C** | **T** | **T** | **T** | **A** | **C** | **G** | **A** | **T** | **G** | **C** | **C** | **T** | **T** | **T** | **G** | **G** | **C** | **T** | **C** | **A** | **A** | **A** | **G** | **G** | **G** | **C** | **A** | **C** | **C** | **A** | **A** | **G** | **G** | **T** | **T** | **C** | **A** | **T** | **C** | **A** | **T** | **T** | **T** | **G** | **A** | **A** | **T** | **G** | **A** | **A** | **T** | **T** | **G** | **A** | **A** | **A** | **A** | **A** | **A** | **A** | **A** | **C** | **G** | **G** | **C** | **G** | **G** | **T** | **T** | **T** | **T** | **G** | **T** | **C** | **C** | **A** | **A** | **A** | **C** | **G** | **T** | **T** | **T** | **A** | **T** | **T** | **A** | **C** | **T** | **T** | **C** | **T** | **C** | **A** | **A** | **A** | **A** | **A** | **G** | **C** | **A** | **C** | **T** | **T** | **T** | **T** | **G** | **A** | **A** | **G** | **T** | **T** | **T** | **C** | **G** | **T** | **T** | **T** | **G** | **A** | **A** | **T** | **T** | **A** | **T** | **C** | **A** | **T** | **G** | **A** | **T** | **C** | **A** | **C** | **C** | **G** | **G** | **A** | **A** | **A** | **A** | **T** | **T** | **G** | **T** | **T** | **G** | **T** | **T** | **A** | **T** | **T** | **G** | **A** | **C** | **G** | **G** | **G** | **A** | **A** | **A** | **G** | **T** | **A** | **G** | **G** | **C** | **T** | **A** | **C** | **A** | **T** | **T** | **G** | **G** | **C** | **G** | **G** | **C** | **T** | **T** | **T** | **A** | **A** | **T** | **G** | **T** | **T** | **G** | **C** | **C** | **G** | **A** | **T** | **C** | **A** | **A** | **T** | **A** | **T** | **G** | **C** | **C** | **G** | **G** | **A** | **A** | **C** | **G** | **A** | **C** | **T** | **A** | **A** | **A** | **A** | **A** | **G** | **T** | **T** | **T** | **G** | **G** | **C** | **T** | **A** | **T** | **T** | **G** | **G** | **C** | **G** | **G** | **G** | **A** | **T** | **A** | **C** | **G** | **C** | **A** | **T** | **T** | **T** | **A** | **C** | **G** | **G** | **A** | **T** | **T** | **C** | **A** | **A** | **G** | **G** | **G** | **C** | **C** | **A** | **G** | **C** | **A** | **G** | **C** | **C** | **T** | **C** | **A** | **T** | **T** | **A** | **C** | **T** | **G** | **C** | **A** | **A** | **A** | **T** | **G** | **C** | **G** | **T** | **T** | **T** | **T** | **T** | **T** | **A** | **A** | **T** | **G** | **G** | **A** | **T** | **T** | **G** | **G** | **A** | **A** | **C** | **G** | **T** | **C** | **T** | **C** | **T** | **T** | **C** | **C** | **C** | **C** | **C** | **G** | **A** | **G** | **A** | **A** | **A** | **A** | **A** | **T** | **C** | **G** | **T** | **G** | **T** | **G** | **G** | **C** | **G** | **T** | **A** | **T** | **C** | **A** | **A** | **T** | **T** | **G** | **G** | **A** | **T** | **T** | **A** | **T** | **T** | **T** | **C** | **T** | **T** | **T** | **A** | **A** | **A** | **C** | **T** | **T** | **G** | **A** | **A** | **G** | **C** | **A** | **T** | **T** | **G** | **G** | **T** | **G** | **C** | **C** | **A** | **G** | **A** | **G** | **G** | **C** | **A** | **A** | **A** | **T** | **A** | **C** | **A** | **T** | **C** | **C** | **A** | **T** | **T** | **C** | **A** | **G** | **A** | **T** | **G** | **A** | **T** | **T** | **G** | **C** | **C** | **A** | **G** | **T** | **G** | **G** | **T** | **C** | **C** | **T** | **A** | **A** | **C** | **A** | **G** | **T** | **G** | **A** | **C** | **C** | **G** | **T** | **G** | **A** | **A** | **C** | **A** | **A** | **A** | **T** | **T** | **A** | **A** | **A** | **T** | **T** | **G** | **G** | **C** | **C** | **T** | **T** | **T** | **A** | **T** | **T** | **A** | **A** | **A** | **T** | **T** | **G** | **A** | **T** | **T** | **A** | **C** | **T** | **T** | **C** | **T** | **G** | **C** | **C** | **A** | **A** | **G** | **A** | **A** | **A** | **A** | **G** | **A** | **G** | **T** | **C** | **T** | **G** | **G** | **A** | **T** | **T** | **C** | **A** | **A** | **A** | **C** | **G** | **C** | **C** | **G** | **T** | **A** | **T** | **T** | **T** | **A** | **G** | **T** | **T** | **C** | **C** | **T** | **G** | **A** | **T** | **G** | **A** | **T** | **A** | **G** | **T** | **G** | **T** | **C** | **T** | **T** | **G** | **G** | **C** | **G** | **G** | **C** | **T** | **T** | **T** | **A** | **A** | **A** | **G** | **G** | **T** | **T** | **G** | **C** | **T** | **G** | **C** | **G** | **G** | **C** | **T** | **T** | **C** | **A** | **G** | **G** | **A** | **G** | **T** | **A** | **G** | **A** | **T** | **G** | **T** | **T** | **A** | **A** | **A** | **A** | **T** | **T** | **A** | **T** | **G** | **A** | **T** | **T** | **C** | **C** | **A** | **G** | **A** | **T** | **A** | **A** | **G** | **C** | **C** | **C** | **G** | **A** | **C** | **C** | **A** | **T** | **C** | **C** | **G** | **T** | **T** | **T** | **A** | **T** | **T** | **T** | **A** | **T** | **C** | **G** | **A** | **G** | **C** | **A** | **A** | **C** | **A** | **C** | **A** | **G** | **T** | **A** | **T** | **T** | **A** | **C** | **G** | **G** | **C** | **C** | **G** | **C** | **T** | **T** | **G** | **T** | **T** | **G** | **A** | **T** | **G** | **A** | **A** | **A** | **G** | **A** | **A** | **A** | **A** | **T** | **A** | **T** | **T** | **G** | **A** | **A** | **A** | **T** | **T** | **T** | **T** | **A** | **A** | **T** | **T** | **T** | **A** | **T** | **A** | **A** | **C** | **G** | **G** | **T** | **G** | **G** | **T** | **T** | **T** | **C** | **T** | **T** | **A** | **C** | **A** | **T** | **G** | **C** | **G** | **A** | **A** | **A** | **A** | **C** | **A** | **A** | **T** | **G** | **A** | **T** | **T** | **A** | **T** | **G** | **G** | **A** | **T** | **G** | **A** | **T** | **G** | **A** | **A** | **G** | **T** | **C** | **T** | **G** | **C** | **A** | **C** | **A** | **G** | **T** | **T** | **G** | **G** | **T** | **T** | **C** | **A** | **G** | **C** | **C** | **A** | **A** | **T** | **C** | **A** | **A** | **G** | **A** | **T** | **A** | **T** | **C** | **C** | **G** | **A** | **A** | **G** | **T** | **T** | **A** | **C** | **A** | **A** | **A** | **T** | **T** | **A** | **A** | **A** | **C** | **T** | **T** | **T** | **G** | **A** | **A** | **G** | **C** | **A** | **A** | **A** | **T** | **G** | **C** | **T** | **G** | **T** | **G** | **T** | **T** | **A** | **T** | **A** | **T** | **G** | **A** | **T** | **A** | **A** | **A** | **A** | **A** | **A** | **A** | **T** | **C** | **A** | **T** | **T** | **G** | **A** | **T** | **C** | **A** | **A** | **T** | **T** | **A** | **G** | **A** | **A** | **G** | **C** | **A** | **A** | **T** | **T** | **T** | **T** | **C** | **T** | **T** | **A** | **G** | **A** | **A** | **G** | **A** | **T** | **C** | **G** | **A** | **A** | **A** | **A** | **A** | **A** | **A** | **T** | **G** | **T** | **A** | **C** | **A** | **A** | **C** | **A** | **A** | **T** | **G** | **A** | **C** | **T** | **C** | **C** | **A** | **G** | **A** | **A** | **G** | **T** | **T** | **G** | **T** | **T** | **C** | **G** | **C** | **G** | **A** | **C** | **A** | **T** | **G** | **T** | **C** | **A** | **A** | **A** | **A** | **T** | **G** | **G** | **T** | **T** | **G** | **A** | **T** | **T** | **T** | **T** | **T** | **A** | **A** | **A** | **C** | **A** | **A** | **C** | **A** | **A** | **A** | **T** | **T** | **T** | **C** | **A** | **C** | **G** | **A** | **T** | **T** | **A** | **T** | **T** | **T** | **T** | **C** | **A** | **C** | **C** | **A** | **A** | **T** | **T** | **C** | **T** | **T** | **-** | **-** | **-** |
| **N52469** | **T** | **T** | **G** | **A** | **T** | **T** | **C** | **T** | **T** | **A** | **G** | **C** | **G** | **T** | **C** | **T** | **T** | **A** | **A** | **C** | **A** | **G** | **T** | **T** | **A** | **T** | **T** | **T** | **A** | **T** | **T** | **T** | **T** | **A** | **T** | **T** | **A** | **A** | **T** | **G** | **C** | **A** | **A** | **T** | **T** | **A** | **T** | **T** | **G** | **C** | **G** | **G** | **G** | **A** | **A** | **T** | **T** | **A** | **C** | **C** | **A** | **T** | **T** | **T** | **T** | **G** | **T** | **T** | **A** | **A** | **A** | **A** | **C** | **C** | **T** | **C** | **G** | **A** | **G** | **A** | **T** | **G** | **T** | **A** | **G** | **C** | **A** | **G** | **C** | **A** | **A** | **T** | **T** | **T** | **G** | **G** | **G** | **C** | **A** | **T** | **G** | **G** | **C** | **T** | **A** | **C** | **T** | **C** | **G** | **T** | **A** | **C** | **T** | **G** | **A** | **T** | **T** | **G** | **C** | **T** | **C** | **T** | **A** | **C** | **C** | **C** | **G** | **T** | **T** | **T** | **T** | **T** | **G** | **G** | **T** | **T** | **T** | **T** | **T** | **T** | **C** | **T** | **T** | **A** | **T** | **A** | **T** | **T** | **T** | **A** | **T** | **T** | **C** | **T** | **T** | **T** | **G** | **G** | **G** | **C** | **G** | **C** | **G** | **G** | **T** | **T** | **T** | **A** | **A** | **C** | **C** | **G** | **A** | **C** | **A** | **A** | **A** | **A** | **A** | **G** | **A** | **A** | **A** | **T** | **T** | **T** | **T** | **A** | **T** | **T** | **T** | **G** | **C** | **A** | **A** | **C** | **A** | **A** | **A** | **G** | **T** | **G** | **A** | **T** | **T** | **T** | **A** | **C** | **G** | **G** | **G** | **A** | **A** | **T** | **T** | **A** | **G** | **A** | **G** | **A** | **A** | **T** | **T** | **T** | **C** | **C** | **A** | **G** | **A** | **A** | **T** | **T** | **T** | **C** | **C** | **A** | **A** | **G** | **A** | **A** | **G** | **A** | **A** | **A** | **G** | **T** | **T** | **T** | **T** | **G** | **A** | **A** | **C** | **T** | **T** | **T** | **A** | **T** | **A** | **G** | **T** | **C** | **T** | **A** | **A** | **A** | **G** | **A** | **T** | **G** | **C** | **C** | **G** | **A** | **C** | **G** | **G** | **A** | **G** | **G** | **A** | **G** | **C** | **A** | **A** | **C** | **A** | **A** | **C** | **A** | **G** | **T** | **T** | **T** | **A** | **C** | **A** | **G** | **A** | **C** | **T** | **T** | **T** | **T** | **T** | **T** | **T** | **C** | **C** | **T** | **C** | **G** | **T** | **T** | **A** | **A** | **A** | **T** | **A** | **G** | **G** | **A** | **T** | **G** | **C** | **C** | **C** | **C** | **T** | **G** | **A** | **C** | **T** | **A** | **A** | **G** | **A** | **A** | **A** | **A** | **A** | **T** | **G** | **A** | **T** | **G** | **T** | **T** | **G** | **A** | **A** | **A** | **T** | **T** | **T** | **T** | **T** | **A** | **C** | **G** | **G** | **A** | **T** | **G** | **G** | **T** | **A** | **C** | **G** | **G** | **A** | **A** | **A** | **A** | **A** | **T** | **T** | **C** | **A** | **A** | **T** | **G** | **C** | **G** | **T** | **T** | **G** | **A** | **T** | **G** | **G** | **C** | **G** | **G** | **A** | **C** | **A** | **T** | **A** | **A** | **A** | **A** | **A** | **A** | **A** | **G** | **C** | **T** | **C** | **A** | **G** | **C** | **A** | **C** | **T** | **C** | **C** | **A** | **T** | **T** | **C** | **A** | **T** | **A** | **T** | **T** | **G** | **A** | **A** | **T** | **A** | **T** | **T** | **A** | **T** | **G** | **C** | **C** | **T** | **T** | **T** | **G** | **T** | **A** | **A** | **C** | **G** | **G** | **A** | **T** | **C** | **A** | **T** | **A** | **T** | **C** | **G** | **G** | **T** | **A** | **C** | **C** | **A** | **A** | **A** | **A** | **T** | **T** | **T** | **T** | **G** | **A** | **A** | **C** | **T** | **T** | **A** | **T** | **T** | **A** | **G** | **A** | **A** | **G** | **A** | **A** | **A** | **A** | **G** | **G** | **C** | **G** | **G** | **C** | **T** | **G** | **A** | **A** | **G** | **G** | **G** | **G** | **T** | **T** | **G** | **A** | **A** | **G** | **T** | **G** | **C** | **G** | **G** | **T** | **T** | **G** | **C** | **T** | **T** | **T** | **A** | **C** | **G** | **A** | **T** | **G** | **C** | **C** | **T** | **T** | **T** | **G** | **G** | **C** | **T** | **C** | **A** | **A** | **A** | **G** | **G** | **G** | **C** | **A** | **C** | **C** | **A** | **A** | **G** | **G** | **T** | **T** | **C** | **A** | **T** | **C** | **A** | **T** | **T** | **T** | **G** | **A** | **A** | **T** | **G** | **A** | **A** | **T** | **T** | **G** | **A** | **A** | **A** | **A** | **A** | **A** | **A** | **A** | **C** | **G** | **G** | **C** | **G** | **G** | **T** | **T** | **T** | **T** | **G** | **T** | **C** | **C** | **A** | **A** | **A** | **C** | **G** | **T** | **T** | **T** | **A** | **T** | **T** | **A** | **C** | **T** | **T** | **C** | **T** | **C** | **A** | **A** | **A** | **A** | **A** | **G** | **C** | **A** | **C** | **T** | **T** | **T** | **T** | **G** | **A** | **A** | **G** | **T** | **T** | **T** | **C** | **G** | **T** | **T** | **T** | **G** | **A** | **A** | **T** | **T** | **A** | **T** | **C** | **A** | **T** | **G** | **A** | **T** | **C** | **A** | **C** | **C** | **G** | **G** | **A** | **A** | **A** | **A** | **T** | **T** | **G** | **T** | **T** | **G** | **T** | **T** | **A** | **T** | **T** | **G** | **A** | **C** | **G** | **G** | **A** | **A** | **A** | **A** | **G** | **T** | **A** | **G** | **G** | **C** | **T** | **A** | **C** | **A** | **T** | **T** | **G** | **G** | **C** | **G** | **G** | **C** | **T** | **T** | **T** | **A** | **A** | **T** | **G** | **T** | **T** | **G** | **C** | **C** | **G** | **A** | **T** | **C** | **A** | **A** | **T** | **A** | **T** | **G** | **C** | **C** | **G** | **G** | **A** | **A** | **C** | **G** | **A** | **C** | **T** | **A** | **A** | **A** | **A** | **A** | **G** | **T** | **T** | **T** | **G** | **G** | **C** | **T** | **A** | **T** | **T** | **G** | **G** | **C** | **G** | **G** | **G** | **A** | **T** | **A** | **C** | **A** | **C** | **A** | **T** | **T** | **T** | **A** | **C** | **G** | **G** | **A** | **T** | **T** | **C** | **A** | **A** | **G** | **G** | **G** | **C** | **C** | **A** | **G** | **C** | **A** | **G** | **C** | **C** | **T** | **C** | **A** | **T** | **T** | **A** | **C** | **T** | **G** | **C** | **A** | **A** | **A** | **T** | **G** | **C** | **G** | **T** | **T** | **T** | **T** | **T** | **T** | **A** | **A** | **T** | **G** | **G** | **A** | **T** | **T** | **G** | **G** | **A** | **A** | **C** | **G** | **T** | **C** | **T** | **C** | **T** | **T** | **C** | **C** | **C** | **C** | **C** | **G** | **A** | **G** | **A** | **A** | **A** | **A** | **A** | **T** | **C** | **G** | **T** | **G** | **T** | **G** | **G** | **C** | **G** | **T** | **A** | **T** | **C** | **A** | **A** | **T** | **T** | **G** | **G** | **A** | **T** | **T** | **A** | **T** | **T** | **T** | **C** | **T** | **T** | **T** | **A** | **A** | **A** | **C** | **T** | **T** | **G** | **A** | **A** | **G** | **C** | **A** | **T** | **T** | **G** | **G** | **T** | **G** | **C** | **C** | **A** | **G** | **A** | **G** | **G** | **C** | **A** | **A** | **A** | **T** | **A** | **C** | **A** | **T** | **C** | **C** | **A** | **T** | **T** | **C** | **A** | **G** | **A** | **T** | **G** | **A** | **T** | **T** | **G** | **C** | **C** | **A** | **G** | **T** | **G** | **G** | **T** | **C** | **C** | **T** | **A** | **A** | **C** | **A** | **G** | **T** | **G** | **A** | **C** | **C** | **G** | **T** | **G** | **A** | **A** | **C** | **A** | **A** | **A** | **T** | **T** | **A** | **A** | **A** | **T** | **T** | **G** | **G** | **C** | **C** | **T** | **T** | **T** | **A** | **T** | **T** | **A** | **A** | **A** | **T** | **T** | **G** | **A** | **T** | **T** | **A** | **C** | **T** | **T** | **C** | **T** | **G** | **C** | **C** | **A** | **A** | **G** | **A** | **A** | **A** | **A** | **G** | **A** | **G** | **T** | **C** | **T** | **G** | **G** | **A** | **T** | **T** | **C** | **A** | **A** | **A** | **C** | **G** | **C** | **C** | **G** | **T** | **A** | **T** | **T** | **T** | **A** | **G** | **T** | **T** | **C** | **C** | **T** | **G** | **A** | **T** | **G** | **A** | **T** | **A** | **G** | **T** | **G** | **T** | **C** | **T** | **T** | **G** | **G** | **C** | **T** | **G** | **C** | **T** | **T** | **T** | **A** | **A** | **A** | **G** | **G** | **T** | **T** | **G** | **C** | **T** | **G** | **C** | **G** | **G** | **C** | **T** | **T** | **C** | **A** | **G** | **G** | **A** | **G** | **T** | **A** | **G** | **A** | **T** | **G** | **T** | **T** | **A** | **A** | **A** | **A** | **T** | **T** | **A** | **T** | **G** | **A** | **T** | **T** | **C** | **C** | **A** | **G** | **A** | **T** | **A** | **A** | **G** | **C** | **C** | **C** | **G** | **A** | **C** | **C** | **A** | **T** | **C** | **C** | **G** | **T** | **T** | **T** | **A** | **T** | **T** | **T** | **A** | **T** | **C** | **G** | **A** | **G** | **C** | **A** | **A** | **C** | **A** | **C** | **A** | **G** | **T** | **A** | **T** | **T** | **A** | **C** | **G** | **G** | **C** | **C** | **G** | **C** | **T** | **T** | **A** | **T** | **T** | **G** | **A** | **T** | **G** | **A** | **A** | **A** | **G** | **A** | **A** | **A** | **A** | **T** | **A** | **T** | **T** | **G** | **A** | **A** | **A** | **T** | **T** | **T** | **T** | **A** | **A** | **T** | **T** | **T** | **A** | **T** | **A** | **A** | **C** | **G** | **G** | **T** | **G** | **G** | **T** | **T** | **T** | **C** | **T** | **T** | **A** | **C** | **A** | **T** | **G** | **C** | **G** | **A** | **A** | **A** | **A** | **C** | **A** | **A** | **T** | **G** | **A** | **T** | **T** | **A** | **T** | **G** | **G** | **A** | **T** | **G** | **A** | **T** | **G** | **A** | **A** | **G** | **T** | **C** | **T** | **G** | **C** | **A** | **C** | **A** | **G** | **T** | **T** | **G** | **G** | **T** | **T** | **C** | **A** | **G** | **C** | **C** | **A** | **A** | **T** | **C** | **A** | **A** | **G** | **A** | **T** | **A** | **T** | **C** | **C** | **G** | **A** | **A** | **G** | **T** | **T** | **A** | **C** | **A** | **A** | **A** | **T** | **T** | **A** | **A** | **A** | **C** | **T** | **T** | **T** | **G** | **A** | **A** | **G** | **C** | **A** | **A** | **A** | **T** | **G** | **C** | **T** | **G** | **T** | **G** | **T** | **T** | **A** | **T** | **A** | **T** | **G** | **A** | **T** | **A** | **A** | **A** | **A** | **A** | **A** | **A** | **T** | **C** | **A** | **T** | **T** | **G** | **A** | **T** | **C** | **A** | **A** | **T** | **T** | **A** | **G** | **A** | **A** | **G** | **C** | **A** | **A** | **T** | **T** | **T** | **T** | **C** | **T** | **T** | **A** | **G** | **A** | **A** | **G** | **A** | **T** | **C** | **G** | **A** | **A** | **A** | **A** | **A** | **A** | **A** | **T** | **G** | **T** | **A** | **C** | **A** | **A** | **C** | **A** | **A** | **T** | **G** | **A** | **C** | **T** | **C** | **C** | **A** | **G** | **A** | **A** | **G** | **T** | **T** | **G** | **T** | **T** | **C** | **G** | **C** | **G** | **A** | **C** | **A** | **T** | **G** | **T** | **C** | **A** | **A** | **A** | **A** | **T** | **G** | **G** | **T** | **T** | **G** | **A** | **T** | **T** | **T** | **T** | **T** | **A** | **A** | **A** | **C** | **A** | **G** | **C** | **A** | **A** | **A** | **T** | **T** | **T** | **C** | **A** | **C** | **G** | **A** | **T** | **T** | **A** | **T** | **T** | **T** | **T** | **C** | **A** | **C** | **C** | **A** | **A** | **T** | **T** | **C** | **T** | **T** | **-** | **-** | **-** |
| **N59609F** | **T** | **T** | **G** | **A** | **T** | **T** | **C** | **T** | **T** | **A** | **G** | **C** | **G** | **T** | **C** | **T** | **T** | **A** | **A** | **C** | **A** | **G** | **T** | **T** | **A** | **T** | **T** | **T** | **A** | **T** | **T** | **T** | **T** | **A** | **T** | **T** | **A** | **A** | **T** | **G** | **C** | **A** | **A** | **T** | **T** | **A** | **T** | **T** | **G** | **C** | **G** | **G** | **G** | **A** | **A** | **T** | **T** | **A** | **C** | **C** | **A** | **T** | **T** | **T** | **T** | **G** | **T** | **T** | **A** | **A** | **A** | **A** | **C** | **C** | **T** | **C** | **G** | **A** | **G** | **A** | **T** | **G** | **T** | **A** | **G** | **C** | **A** | **G** | **C** | **A** | **A** | **T** | **T** | **T** | **G** | **G** | **G** | **C** | **A** | **T** | **G** | **G** | **C** | **T** | **A** | **C** | **T** | **C** | **G** | **T** | **A** | **C** | **T** | **G** | **A** | **T** | **T** | **G** | **C** | **T** | **C** | **T** | **A** | **C** | **C** | **C** | **G** | **T** | **T** | **T** | **T** | **T** | **G** | **G** | **T** | **T** | **T** | **T** | **T** | **T** | **C** | **T** | **T** | **A** | **T** | **A** | **T** | **T** | **T** | **A** | **T** | **T** | **C** | **T** | **T** | **T** | **G** | **G** | **G** | **C** | **G** | **C** | **G** | **G** | **T** | **T** | **T** | **A** | **A** | **C** | **C** | **G** | **A** | **C** | **A** | **A** | **A** | **A** | **A** | **G** | **A** | **A** | **A** | **T** | **T** | **T** | **T** | **A** | **T** | **T** | **T** | **G** | **C** | **A** | **A** | **C** | **A** | **A** | **A** | **G** | **T** | **G** | **A** | **T** | **T** | **T** | **G** | **C** | **G** | **G** | **G** | **A** | **A** | **T** | **T** | **A** | **G** | **A** | **G** | **A** | **A** | **T** | **T** | **T** | **T** | **C** | **A** | **G** | **A** | **A** | **T** | **T** | **T** | **C** | **C** | **A** | **A** | **G** | **A** | **A** | **G** | **A** | **A** | **A** | **G** | **T** | **T** | **T** | **T** | **G** | **A** | **A** | **C** | **T** | **T** | **T** | **A** | **T** | **A** | **G** | **T** | **C** | **A** | **A** | **A** | **A** | **G** | **A** | **T** | **G** | **C** | **C** | **G** | **A** | **C** | **G** | **G** | **A** | **G** | **G** | **A** | **G** | **C** | **A** | **A** | **C** | **A** | **A** | **C** | **A** | **G** | **T** | **T** | **T** | **A** | **C** | **A** | **G** | **A** | **C** | **T** | **T** | **T** | **T** | **T** | **T** | **T** | **C** | **C** | **T** | **C** | **G** | **T** | **T** | **A** | **A** | **A** | **T** | **A** | **G** | **G** | **A** | **T** | **G** | **C** | **C** | **T** | **C** | **T** | **G** | **A** | **C** | **T** | **A** | **A** | **G** | **A** | **A** | **A** | **A** | **A** | **T** | **G** | **A** | **T** | **G** | **T** | **T** | **G** | **A** | **A** | **A** | **T** | **T** | **T** | **T** | **T** | **A** | **C** | **G** | **G** | **A** | **T** | **G** | **G** | **T** | **A** | **C** | **G** | **G** | **A** | **A** | **A** | **A** | **A** | **T** | **T** | **C** | **A** | **A** | **T** | **G** | **C** | **G** | **T** | **T** | **G** | **A** | **T** | **G** | **G** | **C** | **G** | **G** | **A** | **C** | **A** | **T** | **A** | **A** | **A** | **A** | **A** | **A** | **A** | **G** | **C** | **T** | **C** | **A** | **G** | **C** | **A** | **C** | **T** | **C** | **C** | **A** | **T** | **T** | **C** | **A** | **T** | **A** | **T** | **T** | **G** | **A** | **A** | **T** | **A** | **T** | **T** | **A** | **T** | **G** | **C** | **C** | **T** | **T** | **T** | **G** | **T** | **A** | **A** | **C** | **G** | **G** | **A** | **T** | **C** | **A** | **T** | **A** | **T** | **C** | **G** | **G** | **C** | **A** | **C** | **C** | **A** | **A** | **A** | **A** | **T** | **T** | **T** | **T** | **G** | **A** | **A** | **C** | **T** | **T** | **A** | **T** | **T** | **A** | **G** | **A** | **A** | **G** | **A** | **A** | **A** | **A** | **G** | **G** | **C** | **G** | **G** | **C** | **T** | **G** | **A** | **A** | **G** | **G** | **G** | **G** | **T** | **T** | **G** | **A** | **A** | **G** | **T** | **G** | **C** | **G** | **G** | **T** | **T** | **G** | **C** | **T** | **T** | **T** | **A** | **C** | **G** | **A** | **T** | **G** | **C** | **C** | **T** | **T** | **T** | **G** | **G** | **C** | **T** | **C** | **A** | **A** | **A** | **G** | **G** | **G** | **C** | **A** | **C** | **C** | **A** | **A** | **G** | **G** | **T** | **T** | **C** | **A** | **T** | **C** | **A** | **T** | **T** | **T** | **G** | **A** | **A** | **T** | **G** | **A** | **A** | **T** | **T** | **G** | **A** | **A** | **A** | **A** | **A** | **A** | **A** | **A** | **C** | **G** | **G** | **T** | **G** | **G** | **T** | **T** | **T** | **T** | **G** | **T** | **C** | **C** | **A** | **A** | **A** | **C** | **G** | **T** | **T** | **T** | **A** | **T** | **T** | **A** | **C** | **T** | **T** | **C** | **T** | **C** | **A** | **A** | **A** | **A** | **A** | **G** | **C** | **A** | **C** | **T** | **T** | **T** | **T** | **G** | **A** | **A** | **G** | **T** | **T** | **T** | **C** | **G** | **T** | **T** | **T** | **G** | **A** | **A** | **T** | **T** | **A** | **T** | **C** | **A** | **T** | **G** | **A** | **T** | **C** | **A** | **C** | **C** | **G** | **G** | **A** | **A** | **A** | **A** | **T** | **T** | **G** | **T** | **T** | **G** | **T** | **T** | **A** | **T** | **T** | **G** | **A** | **C** | **G** | **G** | **A** | **A** | **A** | **A** | **G** | **T** | **A** | **G** | **G** | **C** | **T** | **A** | **C** | **A** | **T** | **T** | **G** | **G** | **C** | **G** | **G** | **C** | **T** | **T** | **T** | **A** | **A** | **T** | **G** | **T** | **T** | **G** | **C** | **C** | **G** | **A** | **T** | **C** | **A** | **A** | **T** | **A** | **T** | **G** | **C** | **C** | **G** | **G** | **A** | **A** | **C** | **G** | **A** | **C** | **T** | **A** | **A** | **A** | **A** | **A** | **G** | **T** | **T** | **T** | **G** | **G** | **C** | **T** | **A** | **T** | **T** | **G** | **G** | **C** | **G** | **G** | **G** | **A** | **T** | **A** | **C** | **A** | **C** | **A** | **T** | **T** | **T** | **A** | **C** | **G** | **G** | **A** | **T** | **T** | **C** | **A** | **A** | **G** | **G** | **G** | **C** | **C** | **A** | **G** | **C** | **A** | **G** | **C** | **C** | **T** | **C** | **A** | **T** | **T** | **A** | **C** | **T** | **G** | **C** | **A** | **A** | **A** | **T** | **G** | **C** | **G** | **T** | **T** | **T** | **T** | **T** | **T** | **A** | **A** | **T** | **G** | **G** | **A** | **T** | **T** | **G** | **G** | **A** | **A** | **C** | **G** | **T** | **C** | **T** | **C** | **T** | **T** | **C** | **C** | **C** | **C** | **C** | **G** | **A** | **G** | **A** | **A** | **A** | **A** | **A** | **T** | **C** | **G** | **T** | **G** | **T** | **G** | **G** | **C** | **G** | **T** | **A** | **T** | **C** | **A** | **A** | **T** | **T** | **G** | **G** | **A** | **T** | **T** | **A** | **T** | **T** | **T** | **C** | **T** | **T** | **T** | **A** | **A** | **A** | **C** | **T** | **T** | **G** | **A** | **A** | **G** | **C** | **A** | **T** | **T** | **G** | **G** | **T** | **G** | **C** | **C** | **A** | **G** | **A** | **G** | **G** | **C** | **A** | **A** | **A** | **T** | **A** | **C** | **A** | **T** | **C** | **C** | **A** | **T** | **T** | **C** | **A** | **G** | **A** | **T** | **G** | **A** | **T** | **T** | **G** | **C** | **C** | **A** | **G** | **T** | **G** | **G** | **T** | **C** | **C** | **T** | **A** | **A** | **C** | **A** | **G** | **T** | **G** | **A** | **C** | **C** | **G** | **T** | **G** | **A** | **A** | **C** | **A** | **A** | **A** | **T** | **T** | **A** | **A** | **A** | **T** | **T** | **G** | **G** | **C** | **C** | **T** | **T** | **T** | **A** | **T** | **T** | **A** | **A** | **A** | **T** | **T** | **G** | **A** | **T** | **T** | **A** | **C** | **T** | **T** | **C** | **T** | **G** | **C** | **C** | **A** | **A** | **G** | **A** | **A** | **A** | **A** | **G** | **A** | **G** | **T** | **C** | **T** | **G** | **G** | **A** | **T** | **T** | **C** | **A** | **A** | **A** | **C** | **G** | **C** | **C** | **G** | **T** | **A** | **T** | **T** | **T** | **A** | **G** | **T** | **T** | **C** | **C** | **T** | **G** | **A** | **T** | **G** | **A** | **C** | **A** | **G** | **T** | **G** | **T** | **C** | **T** | **T** | **G** | **G** | **C** | **T** | **G** | **C** | **T** | **T** | **T** | **A** | **A** | **A** | **G** | **G** | **T** | **T** | **G** | **C** | **T** | **G** | **C** | **G** | **G** | **C** | **T** | **T** | **C** | **A** | **G** | **G** | **A** | **G** | **T** | **A** | **G** | **A** | **T** | **G** | **T** | **T** | **A** | **A** | **A** | **A** | **T** | **T** | **A** | **T** | **G** | **A** | **T** | **T** | **C** | **C** | **A** | **G** | **A** | **T** | **A** | **A** | **G** | **C** | **C** | **C** | **G** | **A** | **C** | **C** | **A** | **T** | **C** | **C** | **G** | **T** | **T** | **T** | **A** | **T** | **T** | **T** | **A** | **T** | **C** | **G** | **A** | **G** | **C** | **A** | **A** | **C** | **A** | **C** | **A** | **G** | **T** | **A** | **T** | **T** | **A** | **C** | **G** | **G** | **C** | **C** | **G** | **C** | **T** | **T** | **A** | **T** | **T** | **G** | **A** | **T** | **G** | **A** | **A** | **A** | **G** | **A** | **A** | **A** | **A** | **T** | **A** | **T** | **T** | **G** | **A** | **A** | **A** | **T** | **T** | **T** | **T** | **A** | **A** | **T** | **T** | **T** | **A** | **T** | **A** | **A** | **C** | **G** | **G** | **T** | **G** | **G** | **T** | **T** | **T** | **C** | **T** | **T** | **A** | **C** | **A** | **T** | **G** | **C** | **G** | **A** | **A** | **A** | **A** | **C** | **A** | **A** | **T** | **G** | **A** | **T** | **T** | **A** | **T** | **G** | **G** | **A** | **T** | **G** | **A** | **T** | **G** | **A** | **A** | **G** | **T** | **C** | **T** | **G** | **C** | **A** | **C** | **A** | **G** | **T** | **T** | **G** | **G** | **T** | **T** | **C** | **A** | **G** | **C** | **C** | **A** | **A** | **T** | **C** | **A** | **A** | **G** | **A** | **T** | **A** | **T** | **C** | **C** | **G** | **A** | **A** | **G** | **T** | **T** | **A** | **C** | **A** | **A** | **A** | **T** | **T** | **A** | **A** | **A** | **C** | **T** | **T** | **T** | **G** | **A** | **A** | **G** | **C** | **A** | **A** | **A** | **T** | **G** | **C** | **T** | **G** | **T** | **G** | **T** | **T** | **A** | **T** | **A** | **T** | **G** | **A** | **T** | **A** | **A** | **A** | **A** | **A** | **A** | **A** | **T** | **C** | **A** | **T** | **T** | **G** | **A** | **T** | **C** | **A** | **A** | **T** | **T** | **A** | **G** | **A** | **A** | **G** | **C** | **A** | **A** | **T** | **T** | **T** | **T** | **C** | **T** | **T** | **A** | **G** | **A** | **A** | **G** | **A** | **T** | **C** | **G** | **A** | **A** | **A** | **A** | **A** | **A** | **A** | **T** | **G** | **T** | **A** | **C** | **A** | **A** | **C** | **A** | **A** | **T** | **G** | **A** | **C** | **T** | **C** | **C** | **A** | **G** | **A** | **A** | **G** | **T** | **T** | **G** | **T** | **T** | **C** | **G** | **C** | **G** | **A** | **C** | **A** | **T** | **G** | **T** | **C** | **A** | **A** | **A** | **A** | **T** | **G** | **G** | **T** | **T** | **G** | **A** | **T** | **T** | **T** | **T** | **T** | **A** | **A** | **A** | **C** | **A** | **A** | **C** | **A** | **A** | **A** | **T** | **T** | **T** | **C** | **A** | **C** | **G** | **A** | **T** | **T** | **A** | **T** | **T** | **T** | **T** | **C** | **A** | **C** | **C** | **A** | **A** | **T** | **T** | **C** | **T** | **T** | **-** | **-** | **-** |
| **N59591F** | **T** | **T** | **G** | **A** | **T** | **T** | **C** | **T** | **T** | **A** | **G** | **C** | **G** | **T** | **C** | **T** | **T** | **A** | **A** | **C** | **A** | **G** | **T** | **T** | **A** | **T** | **T** | **T** | **A** | **T** | **T** | **T** | **T** | **A** | **T** | **T** | **A** | **A** | **T** | **G** | **C** | **A** | **A** | **T** | **T** | **A** | **T** | **T** | **G** | **C** | **G** | **G** | **G** | **A** | **A** | **T** | **T** | **A** | **C** | **C** | **A** | **T** | **T** | **T** | **T** | **G** | **T** | **T** | **A** | **A** | **A** | **A** | **C** | **C** | **T** | **C** | **G** | **A** | **G** | **A** | **T** | **G** | **T** | **A** | **G** | **C** | **A** | **G** | **C** | **A** | **A** | **T** | **T** | **T** | **G** | **G** | **G** | **C** | **A** | **T** | **G** | **G** | **C** | **T** | **A** | **C** | **T** | **C** | **G** | **T** | **A** | **C** | **T** | **G** | **A** | **T** | **T** | **G** | **C** | **T** | **C** | **T** | **A** | **C** | **C** | **C** | **G** | **T** | **T** | **T** | **T** | **T** | **G** | **G** | **T** | **T** | **T** | **T** | **T** | **T** | **C** | **T** | **T** | **A** | **T** | **A** | **T** | **T** | **T** | **A** | **T** | **T** | **C** | **T** | **T** | **T** | **G** | **G** | **G** | **C** | **G** | **C** | **G** | **G** | **T** | **T** | **T** | **A** | **A** | **C** | **C** | **G** | **A** | **C** | **A** | **A** | **A** | **A** | **A** | **G** | **A** | **A** | **A** | **T** | **T** | **T** | **T** | **A** | **T** | **T** | **T** | **G** | **C** | **A** | **A** | **C** | **A** | **A** | **A** | **G** | **T** | **G** | **A** | **T** | **T** | **T** | **A** | **C** | **G** | **G** | **G** | **A** | **A** | **T** | **T** | **A** | **G** | **A** | **G** | **A** | **A** | **T** | **T** | **T** | **C** | **C** | **A** | **G** | **A** | **A** | **T** | **T** | **T** | **C** | **C** | **A** | **A** | **G** | **A** | **A** | **G** | **A** | **A** | **A** | **G** | **T** | **T** | **T** | **T** | **G** | **A** | **A** | **C** | **T** | **T** | **T** | **A** | **T** | **A** | **G** | **T** | **C** | **A** | **A** | **A** | **A** | **G** | **A** | **T** | **G** | **C** | **C** | **G** | **A** | **C** | **G** | **G** | **A** | **G** | **G** | **A** | **G** | **C** | **A** | **A** | **C** | **A** | **A** | **C** | **A** | **G** | **T** | **T** | **T** | **A** | **C** | **A** | **G** | **A** | **C** | **T** | **T** | **T** | **T** | **T** | **T** | **T** | **C** | **C** | **T** | **C** | **G** | **T** | **T** | **A** | **A** | **A** | **T** | **A** | **G** | **G** | **A** | **T** | **G** | **C** | **C** | **T** | **C** | **T** | **G** | **A** | **C** | **T** | **A** | **A** | **G** | **A** | **A** | **A** | **A** | **A** | **T** | **G** | **A** | **T** | **G** | **T** | **T** | **G** | **A** | **A** | **A** | **T** | **T** | **T** | **T** | **T** | **A** | **C** | **G** | **G** | **A** | **T** | **G** | **G** | **T** | **A** | **C** | **G** | **G** | **A** | **A** | **A** | **A** | **A** | **T** | **T** | **C** | **A** | **A** | **T** | **G** | **C** | **G** | **T** | **T** | **G** | **A** | **T** | **G** | **G** | **C** | **G** | **G** | **A** | **T** | **A** | **T** | **A** | **A** | **A** | **A** | **A** | **A** | **A** | **G** | **C** | **T** | **C** | **A** | **G** | **C** | **A** | **C** | **T** | **C** | **T** | **A** | **T** | **T** | **C** | **A** | **T** | **A** | **T** | **T** | **G** | **A** | **A** | **T** | **A** | **T** | **T** | **A** | **T** | **G** | **C** | **C** | **T** | **T** | **T** | **G** | **T** | **A** | **A** | **C** | **G** | **G** | **A** | **T** | **C** | **A** | **T** | **A** | **T** | **C** | **G** | **G** | **T** | **A** | **C** | **C** | **A** | **A** | **A** | **A** | **T** | **T** | **T** | **T** | **G** | **A** | **A** | **C** | **T** | **T** | **A** | **T** | **T** | **A** | **G** | **A** | **A** | **G** | **A** | **A** | **A** | **A** | **G** | **G** | **C** | **G** | **G** | **C** | **T** | **G** | **A** | **A** | **G** | **G** | **G** | **G** | **T** | **T** | **G** | **A** | **A** | **G** | **T** | **G** | **C** | **G** | **G** | **T** | **T** | **G** | **C** | **T** | **T** | **T** | **A** | **C** | **G** | **A** | **T** | **G** | **C** | **C** | **T** | **T** | **T** | **G** | **G** | **C** | **T** | **C** | **A** | **A** | **A** | **G** | **G** | **G** | **C** | **A** | **C** | **C** | **A** | **A** | **G** | **G** | **T** | **T** | **C** | **A** | **T** | **C** | **A** | **T** | **T** | **T** | **G** | **A** | **A** | **T** | **G** | **A** | **A** | **T** | **T** | **G** | **A** | **A** | **A** | **A** | **A** | **A** | **A** | **A** | **C** | **G** | **G** | **T** | **G** | **G** | **T** | **T** | **T** | **T** | **G** | **T** | **C** | **C** | **A** | **A** | **A** | **C** | **G** | **T** | **T** | **T** | **A** | **T** | **T** | **A** | **C** | **T** | **T** | **C** | **T** | **C** | **A** | **A** | **A** | **A** | **A** | **G** | **C** | **A** | **C** | **T** | **T** | **T** | **T** | **G** | **A** | **A** | **G** | **T** | **T** | **T** | **C** | **G** | **T** | **T** | **T** | **G** | **A** | **A** | **T** | **T** | **A** | **T** | **C** | **A** | **T** | **G** | **A** | **T** | **C** | **A** | **C** | **C** | **G** | **G** | **A** | **A** | **A** | **A** | **T** | **T** | **G** | **T** | **T** | **G** | **T** | **T** | **A** | **T** | **T** | **G** | **A** | **C** | **G** | **G** | **A** | **A** | **A** | **A** | **G** | **T** | **A** | **G** | **G** | **C** | **T** | **A** | **C** | **A** | **T** | **T** | **G** | **G** | **C** | **G** | **G** | **C** | **T** | **T** | **T** | **A** | **A** | **T** | **G** | **T** | **T** | **G** | **C** | **C** | **G** | **A** | **T** | **C** | **A** | **A** | **T** | **A** | **T** | **G** | **C** | **C** | **G** | **G** | **A** | **A** | **C** | **G** | **A** | **C** | **T** | **A** | **A** | **A** | **A** | **A** | **G** | **T** | **T** | **T** | **G** | **G** | **C** | **T** | **A** | **T** | **T** | **G** | **G** | **C** | **G** | **G** | **G** | **A** | **T** | **A** | **C** | **A** | **C** | **A** | **T** | **T** | **T** | **A** | **C** | **G** | **G** | **A** | **T** | **T** | **C** | **A** | **A** | **G** | **G** | **G** | **C** | **C** | **A** | **G** | **C** | **A** | **G** | **C** | **C** | **T** | **C** | **A** | **T** | **T** | **A** | **C** | **T** | **G** | **C** | **A** | **A** | **A** | **T** | **G** | **C** | **G** | **T** | **T** | **T** | **T** | **T** | **T** | **A** | **A** | **T** | **G** | **G** | **A** | **T** | **T** | **G** | **G** | **A** | **A** | **C** | **G** | **T** | **C** | **T** | **C** | **T** | **T** | **C** | **C** | **C** | **C** | **C** | **G** | **A** | **G** | **A** | **A** | **A** | **A** | **A** | **T** | **C** | **G** | **T** | **G** | **T** | **G** | **G** | **C** | **G** | **T** | **A** | **T** | **C** | **A** | **A** | **T** | **T** | **G** | **G** | **A** | **T** | **T** | **A** | **T** | **T** | **T** | **C** | **T** | **T** | **T** | **A** | **A** | **A** | **C** | **T** | **T** | **G** | **A** | **A** | **G** | **C** | **G** | **T** | **T** | **G** | **G** | **T** | **G** | **C** | **C** | **A** | **G** | **A** | **G** | **G** | **C** | **A** | **A** | **A** | **T** | **A** | **C** | **A** | **T** | **C** | **C** | **A** | **T** | **T** | **C** | **A** | **G** | **A** | **T** | **G** | **A** | **T** | **T** | **G** | **C** | **C** | **A** | **G** | **T** | **G** | **G** | **T** | **C** | **C** | **T** | **A** | **A** | **T** | **A** | **G** | **T** | **G** | **A** | **C** | **C** | **G** | **T** | **G** | **A** | **A** | **C** | **A** | **A** | **A** | **T** | **T** | **A** | **A** | **A** | **T** | **T** | **G** | **G** | **C** | **C** | **T** | **T** | **T** | **A** | **T** | **T** | **A** | **A** | **A** | **T** | **T** | **G** | **A** | **T** | **T** | **A** | **C** | **T** | **T** | **C** | **T** | **G** | **C** | **C** | **A** | **A** | **G** | **A** | **A** | **A** | **A** | **G** | **A** | **G** | **T** | **C** | **T** | **G** | **G** | **A** | **T** | **T** | **C** | **A** | **A** | **A** | **C** | **G** | **C** | **C** | **G** | **T** | **A** | **T** | **T** | **T** | **A** | **G** | **T** | **T** | **C** | **C** | **T** | **G** | **A** | **T** | **G** | **A** | **T** | **A** | **G** | **T** | **G** | **T** | **C** | **T** | **T** | **G** | **G** | **C** | **G** | **G** | **C** | **T** | **T** | **T** | **A** | **A** | **A** | **G** | **G** | **T** | **T** | **G** | **C** | **T** | **G** | **C** | **G** | **G** | **C** | **T** | **T** | **C** | **A** | **G** | **G** | **A** | **G** | **T** | **A** | **G** | **A** | **T** | **G** | **T** | **T** | **A** | **A** | **A** | **A** | **T** | **T** | **A** | **T** | **G** | **A** | **T** | **T** | **C** | **C** | **A** | **G** | **A** | **T** | **A** | **A** | **G** | **C** | **C** | **C** | **G** | **A** | **C** | **C** | **A** | **T** | **C** | **C** | **G** | **T** | **T** | **T** | **A** | **T** | **T** | **T** | **A** | **T** | **C** | **G** | **A** | **G** | **C** | **A** | **A** | **C** | **A** | **C** | **A** | **G** | **T** | **A** | **T** | **T** | **A** | **C** | **G** | **G** | **C** | **C** | **G** | **C** | **T** | **T** | **A** | **T** | **T** | **G** | **A** | **T** | **G** | **A** | **A** | **A** | **G** | **A** | **A** | **A** | **A** | **T** | **A** | **T** | **T** | **G** | **A** | **A** | **A** | **T** | **T** | **T** | **T** | **A** | **A** | **T** | **T** | **T** | **A** | **T** | **A** | **A** | **C** | **G** | **G** | **T** | **G** | **G** | **T** | **T** | **T** | **C** | **T** | **T** | **A** | **C** | **A** | **T** | **G** | **C** | **G** | **A** | **A** | **A** | **A** | **C** | **A** | **A** | **T** | **G** | **A** | **T** | **T** | **A** | **T** | **G** | **G** | **A** | **T** | **G** | **A** | **T** | **G** | **A** | **A** | **G** | **T** | **C** | **T** | **G** | **C** | **A** | **C** | **A** | **G** | **T** | **T** | **G** | **G** | **T** | **T** | **C** | **A** | **G** | **C** | **C** | **A** | **A** | **T** | **C** | **A** | **A** | **G** | **A** | **T** | **A** | **T** | **C** | **C** | **G** | **A** | **A** | **G** | **T** | **T** | **A** | **C** | **A** | **A** | **A** | **T** | **T** | **A** | **A** | **A** | **C** | **T** | **T** | **T** | **G** | **A** | **A** | **G** | **C** | **A** | **A** | **A** | **T** | **G** | **C** | **T** | **G** | **T** | **G** | **T** | **T** | **A** | **T** | **A** | **T** | **G** | **A** | **T** | **A** | **A** | **A** | **A** | **A** | **A** | **A** | **T** | **C** | **A** | **T** | **T** | **G** | **A** | **T** | **C** | **A** | **A** | **T** | **T** | **A** | **G** | **A** | **A** | **G** | **C** | **A** | **A** | **T** | **T** | **T** | **T** | **C** | **T** | **T** | **A** | **G** | **A** | **A** | **G** | **A** | **T** | **C** | **G** | **A** | **A** | **A** | **A** | **A** | **A** | **A** | **T** | **G** | **T** | **A** | **C** | **A** | **A** | **C** | **A** | **A** | **T** | **G** | **A** | **C** | **T** | **C** | **C** | **A** | **G** | **A** | **A** | **G** | **T** | **T** | **G** | **T** | **T** | **C** | **G** | **C** | **G** | **A** | **C** | **A** | **T** | **G** | **T** | **C** | **A** | **A** | **A** | **A** | **T** | **G** | **G** | **T** | **T** | **G** | **A** | **T** | **T** | **T** | **T** | **T** | **A** | **A** | **A** | **C** | **A** | **A** | **C** | **A** | **A** | **A** | **T** | **T** | **T** | **C** | **A** | **C** | **G** | **A** | **T** | **T** | **A** | **T** | **T** | **T** | **T** | **C** | **A** | **C** | **C** | **A** | **A** | **T** | **T** | **C** | **T** | **T** | **-** | **-** | **-** |
| **N59910F** | **T** | **T** | **G** | **A** | **T** | **T** | **C** | **T** | **T** | **A** | **G** | **C** | **G** | **T** | **C** | **T** | **T** | **A** | **A** | **C** | **A** | **G** | **T** | **T** | **A** | **T** | **T** | **T** | **A** | **T** | **T** | **T** | **T** | **A** | **T** | **T** | **A** | **A** | **T** | **G** | **C** | **A** | **A** | **T** | **T** | **A** | **T** | **T** | **G** | **C** | **G** | **G** | **G** | **A** | **A** | **T** | **T** | **A** | **C** | **C** | **A** | **T** | **T** | **T** | **T** | **G** | **T** | **T** | **A** | **A** | **A** | **A** | **C** | **C** | **T** | **C** | **G** | **A** | **G** | **A** | **T** | **G** | **T** | **A** | **G** | **C** | **A** | **G** | **C** | **A** | **A** | **T** | **T** | **T** | **G** | **G** | **G** | **C** | **A** | **T** | **G** | **G** | **C** | **T** | **A** | **C** | **T** | **C** | **G** | **T** | **A** | **C** | **T** | **G** | **A** | **T** | **T** | **G** | **C** | **T** | **C** | **T** | **A** | **C** | **C** | **C** | **G** | **T** | **T** | **T** | **T** | **T** | **G** | **G** | **T** | **T** | **T** | **T** | **T** | **T** | **C** | **T** | **T** | **A** | **T** | **A** | **T** | **T** | **T** | **A** | **T** | **T** | **C** | **T** | **T** | **T** | **G** | **G** | **T** | **C** | **G** | **A** | **G** | **G** | **G** | **T** | **T** | **A** | **A** | **C** | **C** | **G** | **A** | **C** | **A** | **A** | **A** | **A** | **A** | **G** | **A** | **A** | **A** | **T** | **T** | **T** | **T** | **A** | **T** | **T** | **T** | **G** | **C** | **A** | **A** | **C** | **A** | **A** | **A** | **G** | **T** | **G** | **A** | **T** | **T** | **T** | **A** | **C** | **G** | **G** | **G** | **A** | **A** | **T** | **T** | **A** | **G** | **A** | **G** | **A** | **A** | **T** | **T** | **T** | **T** | **C** | **A** | **G** | **A** | **A** | **T** | **T** | **T** | **C** | **C** | **A** | **A** | **G** | **A** | **A** | **G** | **A** | **A** | **A** | **G** | **T** | **T** | **T** | **T** | **G** | **A** | **A** | **C** | **T** | **T** | **T** | **A** | **T** | **A** | **G** | **T** | **C** | **A** | **A** | **A** | **A** | **G** | **A** | **T** | **G** | **C** | **C** | **G** | **A** | **C** | **G** | **G** | **A** | **G** | **G** | **A** | **G** | **C** | **A** | **A** | **C** | **A** | **A** | **C** | **A** | **G** | **T** | **T** | **T** | **A** | **C** | **A** | **G** | **A** | **C** | **T** | **T** | **T** | **T** | **T** | **T** | **T** | **C** | **C** | **T** | **C** | **G** | **T** | **T** | **A** | **A** | **A** | **T** | **A** | **G** | **G** | **A** | **T** | **G** | **C** | **C** | **T** | **C** | **T** | **G** | **A** | **C** | **T** | **A** | **A** | **G** | **A** | **A** | **A** | **A** | **A** | **T** | **G** | **A** | **T** | **G** | **T** | **T** | **G** | **A** | **A** | **A** | **T** | **T** | **T** | **T** | **T** | **A** | **C** | **G** | **G** | **A** | **T** | **G** | **G** | **T** | **A** | **C** | **G** | **G** | **A** | **A** | **A** | **A** | **A** | **T** | **T** | **C** | **A** | **A** | **T** | **G** | **C** | **G** | **T** | **T** | **G** | **A** | **T** | **G** | **G** | **C** | **G** | **G** | **A** | **C** | **A** | **T** | **A** | **A** | **A** | **A** | **A** | **A** | **A** | **G** | **C** | **T** | **C** | **A** | **G** | **C** | **A** | **C** | **T** | **C** | **T** | **A** | **T** | **T** | **C** | **A** | **T** | **A** | **T** | **T** | **G** | **A** | **A** | **T** | **A** | **T** | **T** | **A** | **T** | **G** | **C** | **C** | **T** | **T** | **T** | **G** | **T** | **A** | **A** | **C** | **G** | **G** | **A** | **T** | **C** | **A** | **T** | **A** | **T** | **C** | **G** | **G** | **C** | **A** | **C** | **C** | **A** | **A** | **A** | **A** | **T** | **T** | **T** | **T** | **G** | **A** | **A** | **C** | **T** | **T** | **A** | **T** | **T** | **A** | **G** | **A** | **A** | **G** | **A** | **A** | **A** | **A** | **G** | **G** | **C** | **G** | **G** | **C** | **T** | **G** | **A** | **A** | **G** | **G** | **G** | **G** | **T** | **T** | **G** | **A** | **A** | **G** | **T** | **G** | **C** | **G** | **G** | **T** | **T** | **G** | **C** | **T** | **T** | **T** | **A** | **C** | **G** | **A** | **T** | **G** | **C** | **C** | **T** | **T** | **T** | **G** | **G** | **C** | **T** | **C** | **A** | **A** | **A** | **G** | **G** | **G** | **C** | **A** | **C** | **C** | **A** | **A** | **G** | **G** | **T** | **T** | **C** | **A** | **T** | **C** | **A** | **T** | **T** | **T** | **G** | **A** | **A** | **T** | **G** | **A** | **A** | **T** | **T** | **G** | **A** | **A** | **A** | **A** | **A** | **A** | **A** | **A** | **C** | **G** | **G** | **C** | **G** | **G** | **T** | **T** | **T** | **T** | **G** | **T** | **C** | **C** | **A** | **A** | **A** | **C** | **G** | **T** | **T** | **T** | **A** | **T** | **T** | **A** | **C** | **T** | **T** | **C** | **T** | **C** | **A** | **A** | **A** | **A** | **A** | **G** | **C** | **A** | **C** | **T** | **T** | **T** | **T** | **G** | **A** | **A** | **G** | **T** | **T** | **T** | **C** | **G** | **T** | **T** | **T** | **G** | **A** | **A** | **T** | **T** | **A** | **T** | **C** | **A** | **T** | **G** | **A** | **T** | **C** | **A** | **C** | **C** | **G** | **G** | **A** | **A** | **A** | **A** | **T** | **T** | **G** | **T** | **T** | **G** | **T** | **T** | **A** | **T** | **T** | **G** | **A** | **C** | **G** | **G** | **A** | **A** | **A** | **A** | **G** | **T** | **A** | **G** | **G** | **C** | **T** | **A** | **C** | **A** | **T** | **T** | **G** | **G** | **C** | **G** | **G** | **C** | **T** | **T** | **T** | **A** | **A** | **T** | **G** | **T** | **T** | **G** | **C** | **C** | **G** | **A** | **T** | **C** | **A** | **A** | **T** | **A** | **T** | **G** | **C** | **C** | **G** | **G** | **A** | **A** | **C** | **G** | **A** | **C** | **T** | **A** | **A** | **A** | **A** | **A** | **G** | **T** | **T** | **T** | **G** | **G** | **C** | **T** | **A** | **T** | **T** | **G** | **G** | **C** | **G** | **G** | **G** | **A** | **T** | **A** | **C** | **A** | **C** | **A** | **T** | **T** | **T** | **A** | **C** | **G** | **G** | **A** | **T** | **T** | **C** | **A** | **A** | **G** | **G** | **G** | **C** | **C** | **A** | **G** | **C** | **A** | **G** | **C** | **C** | **T** | **C** | **A** | **T** | **T** | **A** | **C** | **T** | **G** | **C** | **A** | **A** | **A** | **T** | **G** | **C** | **G** | **T** | **T** | **T** | **T** | **T** | **T** | **A** | **A** | **T** | **G** | **G** | **A** | **T** | **T** | **G** | **G** | **A** | **A** | **C** | **G** | **T** | **C** | **T** | **C** | **T** | **T** | **C** | **C** | **C** | **C** | **C** | **G** | **A** | **G** | **A** | **A** | **A** | **A** | **A** | **T** | **C** | **G** | **T** | **G** | **T** | **G** | **G** | **C** | **G** | **T** | **A** | **T** | **C** | **A** | **A** | **T** | **T** | **G** | **G** | **A** | **T** | **T** | **A** | **T** | **T** | **T** | **C** | **T** | **T** | **T** | **A** | **A** | **A** | **C** | **T** | **T** | **G** | **A** | **A** | **G** | **C** | **A** | **T** | **T** | **G** | **G** | **T** | **G** | **C** | **C** | **A** | **G** | **A** | **G** | **G** | **C** | **A** | **A** | **A** | **T** | **A** | **C** | **A** | **T** | **C** | **C** | **A** | **T** | **T** | **C** | **A** | **G** | **A** | **T** | **G** | **A** | **T** | **T** | **G** | **C** | **C** | **A** | **G** | **T** | **G** | **G** | **T** | **C** | **C** | **T** | **A** | **A** | **C** | **A** | **G** | **T** | **G** | **A** | **C** | **C** | **G** | **T** | **G** | **A** | **A** | **C** | **A** | **A** | **A** | **T** | **T** | **A** | **A** | **A** | **T** | **T** | **G** | **G** | **C** | **C** | **T** | **T** | **T** | **A** | **T** | **T** | **A** | **A** | **A** | **T** | **T** | **G** | **A** | **T** | **T** | **A** | **C** | **T** | **T** | **C** | **T** | **G** | **C** | **C** | **A** | **A** | **G** | **A** | **A** | **A** | **A** | **G** | **A** | **G** | **T** | **C** | **T** | **G** | **G** | **A** | **T** | **T** | **C** | **A** | **A** | **A** | **C** | **G** | **C** | **C** | **G** | **T** | **A** | **T** | **T** | **T** | **A** | **G** | **T** | **T** | **C** | **C** | **T** | **G** | **A** | **T** | **G** | **A** | **T** | **A** | **G** | **T** | **G** | **T** | **C** | **T** | **T** | **G** | **G** | **C** | **T** | **G** | **C** | **T** | **T** | **T** | **A** | **A** | **A** | **G** | **G** | **T** | **T** | **G** | **C** | **T** | **G** | **C** | **G** | **G** | **C** | **T** | **T** | **C** | **A** | **G** | **G** | **A** | **G** | **T** | **A** | **G** | **A** | **T** | **G** | **T** | **T** | **A** | **A** | **A** | **A** | **T** | **T** | **A** | **T** | **G** | **A** | **T** | **T** | **C** | **C** | **A** | **G** | **A** | **T** | **A** | **A** | **G** | **C** | **C** | **C** | **G** | **A** | **C** | **C** | **A** | **T** | **C** | **C** | **G** | **T** | **T** | **T** | **A** | **T** | **T** | **T** | **A** | **T** | **C** | **G** | **A** | **G** | **C** | **A** | **A** | **C** | **A** | **C** | **A** | **G** | **T** | **A** | **T** | **T** | **A** | **C** | **G** | **G** | **C** | **C** | **G** | **C** | **T** | **T** | **A** | **T** | **T** | **G** | **A** | **T** | **G** | **A** | **A** | **A** | **G** | **A** | **A** | **A** | **A** | **T** | **A** | **T** | **T** | **G** | **A** | **A** | **A** | **T** | **T** | **T** | **T** | **A** | **A** | **T** | **T** | **T** | **A** | **T** | **A** | **A** | **C** | **G** | **G** | **T** | **G** | **G** | **T** | **T** | **T** | **C** | **T** | **T** | **A** | **C** | **A** | **T** | **G** | **C** | **G** | **A** | **A** | **A** | **A** | **C** | **A** | **A** | **T** | **G** | **A** | **T** | **T** | **A** | **T** | **G** | **G** | **A** | **T** | **G** | **A** | **T** | **G** | **A** | **A** | **G** | **T** | **C** | **T** | **G** | **C** | **A** | **C** | **A** | **G** | **T** | **T** | **G** | **G** | **T** | **T** | **C** | **A** | **G** | **C** | **C** | **A** | **A** | **T** | **C** | **A** | **A** | **G** | **A** | **T** | **A** | **T** | **C** | **C** | **G** | **A** | **A** | **G** | **T** | **T** | **A** | **C** | **A** | **A** | **A** | **T** | **T** | **A** | **A** | **A** | **C** | **T** | **T** | **T** | **G** | **A** | **A** | **G** | **C** | **A** | **A** | **A** | **T** | **G** | **C** | **T** | **G** | **T** | **G** | **T** | **T** | **A** | **T** | **A** | **T** | **G** | **A** | **T** | **A** | **A** | **A** | **A** | **A** | **A** | **A** | **T** | **C** | **A** | **T** | **T** | **G** | **A** | **T** | **C** | **A** | **A** | **T** | **T** | **A** | **G** | **A** | **A** | **G** | **C** | **A** | **A** | **T** | **T** | **T** | **T** | **C** | **T** | **T** | **A** | **G** | **A** | **A** | **G** | **A** | **T** | **C** | **G** | **A** | **A** | **A** | **A** | **A** | **A** | **A** | **T** | **G** | **T** | **A** | **C** | **A** | **A** | **C** | **A** | **A** | **T** | **G** | **A** | **C** | **T** | **C** | **C** | **A** | **G** | **A** | **A** | **G** | **T** | **T** | **G** | **T** | **T** | **C** | **G** | **C** | **G** | **A** | **C** | **A** | **T** | **G** | **T** | **C** | **A** | **A** | **A** | **A** | **T** | **G** | **G** | **T** | **T** | **G** | **A** | **T** | **T** | **T** | **T** | **T** | **A** | **A** | **A** | **C** | **A** | **A** | **C** | **A** | **A** | **A** | **T** | **T** | **T** | **C** | **A** | **C** | **G** | **A** | **T** | **T** | **A** | **T** | **T** | **T** | **T** | **C** | **A** | **C** | **C** | **A** | **A** | **T** | **T** | **C** | **T** | **T** | **-** | **-** | **-** |
| **N60268F** | **T** | **T** | **G** | **A** | **T** | **T** | **C** | **T** | **T** | **A** | **G** | **C** | **G** | **T** | **C** | **T** | **T** | **A** | **A** | **C** | **A** | **G** | **T** | **T** | **A** | **T** | **T** | **T** | **A** | **T** | **T** | **T** | **T** | **A** | **T** | **T** | **A** | **A** | **T** | **G** | **C** | **A** | **A** | **T** | **T** | **A** | **T** | **T** | **G** | **C** | **G** | **G** | **G** | **A** | **A** | **T** | **T** | **A** | **C** | **C** | **A** | **T** | **T** | **T** | **T** | **G** | **T** | **T** | **A** | **A** | **A** | **A** | **C** | **C** | **T** | **C** | **G** | **A** | **G** | **A** | **T** | **G** | **T** | **A** | **G** | **C** | **A** | **G** | **C** | **A** | **A** | **T** | **T** | **T** | **G** | **G** | **G** | **C** | **A** | **T** | **G** | **G** | **C** | **T** | **A** | **C** | **T** | **C** | **G** | **T** | **A** | **C** | **T** | **G** | **A** | **T** | **T** | **G** | **C** | **T** | **C** | **T** | **A** | **C** | **C** | **C** | **G** | **T** | **T** | **T** | **T** | **T** | **G** | **G** | **T** | **T** | **T** | **T** | **T** | **T** | **C** | **T** | **T** | **A** | **T** | **A** | **T** | **T** | **T** | **A** | **T** | **T** | **C** | **T** | **T** | **T** | **G** | **G** | **T** | **C** | **G** | **A** | **G** | **G** | **G** | **T** | **T** | **A** | **A** | **C** | **C** | **G** | **A** | **C** | **A** | **A** | **A** | **A** | **A** | **G** | **A** | **A** | **A** | **T** | **T** | **T** | **T** | **A** | **T** | **T** | **T** | **G** | **C** | **A** | **A** | **C** | **A** | **A** | **A** | **G** | **T** | **G** | **A** | **T** | **T** | **T** | **A** | **C** | **G** | **G** | **G** | **A** | **A** | **T** | **T** | **A** | **G** | **A** | **G** | **A** | **A** | **T** | **T** | **T** | **T** | **C** | **A** | **G** | **A** | **A** | **T** | **T** | **T** | **C** | **C** | **A** | **A** | **G** | **A** | **A** | **G** | **A** | **A** | **A** | **G** | **T** | **T** | **T** | **T** | **G** | **A** | **A** | **C** | **T** | **T** | **T** | **A** | **T** | **A** | **G** | **T** | **C** | **A** | **A** | **A** | **A** | **G** | **A** | **T** | **G** | **C** | **C** | **G** | **A** | **C** | **G** | **G** | **A** | **G** | **G** | **A** | **G** | **C** | **A** | **A** | **C** | **A** | **A** | **C** | **A** | **G** | **T** | **T** | **T** | **A** | **C** | **A** | **G** | **A** | **C** | **T** | **T** | **T** | **T** | **T** | **T** | **T** | **C** | **C** | **T** | **C** | **G** | **T** | **T** | **A** | **A** | **A** | **T** | **A** | **G** | **G** | **A** | **T** | **G** | **C** | **C** | **T** | **C** | **T** | **G** | **A** | **C** | **T** | **A** | **A** | **G** | **A** | **A** | **A** | **A** | **A** | **T** | **G** | **A** | **T** | **G** | **T** | **T** | **G** | **A** | **A** | **A** | **T** | **T** | **T** | **T** | **T** | **A** | **C** | **G** | **G** | **A** | **T** | **G** | **G** | **T** | **A** | **C** | **G** | **G** | **A** | **A** | **A** | **A** | **A** | **T** | **T** | **C** | **A** | **A** | **T** | **G** | **C** | **G** | **T** | **T** | **G** | **A** | **T** | **G** | **G** | **C** | **G** | **G** | **A** | **C** | **A** | **T** | **A** | **A** | **A** | **A** | **A** | **A** | **A** | **G** | **C** | **T** | **C** | **A** | **G** | **C** | **A** | **C** | **T** | **C** | **T** | **A** | **T** | **T** | **C** | **A** | **T** | **A** | **T** | **T** | **G** | **A** | **A** | **T** | **A** | **T** | **T** | **A** | **T** | **G** | **C** | **C** | **T** | **T** | **T** | **G** | **T** | **A** | **A** | **C** | **G** | **G** | **A** | **T** | **C** | **A** | **T** | **A** | **T** | **C** | **G** | **G** | **C** | **A** | **C** | **C** | **A** | **A** | **A** | **A** | **T** | **T** | **T** | **T** | **G** | **A** | **A** | **C** | **T** | **T** | **A** | **T** | **T** | **A** | **G** | **A** | **A** | **G** | **A** | **A** | **A** | **A** | **G** | **G** | **C** | **G** | **G** | **C** | **T** | **G** | **A** | **A** | **G** | **G** | **G** | **G** | **T** | **T** | **G** | **A** | **A** | **G** | **T** | **G** | **C** | **G** | **G** | **T** | **T** | **G** | **C** | **T** | **T** | **T** | **A** | **C** | **G** | **A** | **T** | **G** | **C** | **C** | **T** | **T** | **T** | **G** | **G** | **C** | **T** | **C** | **A** | **A** | **A** | **G** | **G** | **G** | **C** | **A** | **C** | **C** | **A** | **A** | **G** | **G** | **T** | **T** | **C** | **A** | **T** | **C** | **A** | **T** | **T** | **T** | **G** | **A** | **A** | **T** | **G** | **A** | **A** | **T** | **T** | **G** | **A** | **A** | **A** | **A** | **A** | **A** | **A** | **A** | **C** | **G** | **G** | **C** | **G** | **G** | **T** | **T** | **T** | **T** | **G** | **T** | **C** | **C** | **A** | **A** | **A** | **C** | **G** | **T** | **T** | **T** | **A** | **T** | **T** | **A** | **C** | **T** | **T** | **C** | **T** | **C** | **A** | **A** | **A** | **A** | **A** | **G** | **C** | **A** | **C** | **T** | **T** | **T** | **T** | **G** | **A** | **A** | **G** | **T** | **T** | **T** | **C** | **G** | **T** | **T** | **T** | **G** | **A** | **A** | **T** | **T** | **A** | **T** | **C** | **A** | **T** | **G** | **A** | **T** | **C** | **A** | **C** | **C** | **G** | **G** | **A** | **A** | **A** | **A** | **T** | **T** | **G** | **T** | **T** | **G** | **T** | **T** | **A** | **T** | **T** | **G** | **A** | **C** | **G** | **G** | **A** | **A** | **A** | **A** | **G** | **T** | **A** | **G** | **G** | **C** | **T** | **A** | **C** | **A** | **T** | **T** | **G** | **G** | **C** | **G** | **G** | **C** | **T** | **T** | **T** | **A** | **A** | **T** | **G** | **T** | **T** | **G** | **C** | **C** | **G** | **A** | **T** | **C** | **A** | **A** | **T** | **A** | **T** | **G** | **C** | **C** | **G** | **G** | **A** | **A** | **C** | **G** | **A** | **C** | **T** | **A** | **A** | **A** | **A** | **A** | **G** | **T** | **T** | **T** | **G** | **G** | **C** | **T** | **A** | **T** | **T** | **G** | **G** | **C** | **G** | **G** | **G** | **A** | **T** | **A** | **C** | **A** | **C** | **A** | **T** | **T** | **T** | **A** | **C** | **G** | **G** | **A** | **T** | **T** | **C** | **A** | **A** | **G** | **G** | **G** | **C** | **C** | **A** | **G** | **C** | **A** | **G** | **C** | **C** | **T** | **C** | **A** | **T** | **T** | **A** | **C** | **T** | **G** | **C** | **A** | **A** | **A** | **T** | **G** | **C** | **G** | **T** | **T** | **T** | **T** | **T** | **T** | **A** | **A** | **T** | **G** | **G** | **A** | **T** | **T** | **G** | **G** | **A** | **A** | **C** | **G** | **T** | **C** | **T** | **C** | **T** | **T** | **C** | **C** | **C** | **C** | **C** | **G** | **A** | **G** | **A** | **A** | **A** | **A** | **A** | **T** | **C** | **G** | **T** | **G** | **T** | **G** | **G** | **C** | **G** | **T** | **A** | **T** | **C** | **A** | **A** | **T** | **T** | **G** | **G** | **A** | **T** | **T** | **A** | **T** | **T** | **T** | **C** | **T** | **T** | **T** | **A** | **A** | **A** | **C** | **T** | **T** | **G** | **A** | **A** | **G** | **C** | **A** | **T** | **T** | **G** | **G** | **T** | **G** | **C** | **C** | **A** | **G** | **A** | **G** | **G** | **C** | **A** | **A** | **A** | **T** | **A** | **C** | **A** | **T** | **C** | **C** | **A** | **T** | **T** | **C** | **A** | **G** | **A** | **T** | **G** | **A** | **T** | **T** | **G** | **C** | **C** | **A** | **G** | **T** | **G** | **G** | **T** | **C** | **C** | **T** | **A** | **A** | **C** | **A** | **G** | **T** | **G** | **A** | **C** | **C** | **G** | **T** | **G** | **A** | **A** | **C** | **A** | **A** | **A** | **T** | **T** | **A** | **A** | **A** | **T** | **T** | **G** | **G** | **C** | **C** | **T** | **T** | **T** | **A** | **T** | **T** | **A** | **A** | **A** | **T** | **T** | **G** | **A** | **T** | **T** | **A** | **C** | **T** | **T** | **C** | **T** | **G** | **C** | **C** | **A** | **A** | **G** | **A** | **A** | **A** | **A** | **G** | **A** | **G** | **T** | **C** | **T** | **G** | **G** | **A** | **T** | **T** | **C** | **A** | **A** | **A** | **C** | **G** | **C** | **C** | **G** | **T** | **A** | **T** | **T** | **T** | **A** | **G** | **T** | **T** | **C** | **C** | **T** | **G** | **A** | **T** | **G** | **A** | **T** | **A** | **G** | **T** | **G** | **T** | **C** | **T** | **T** | **G** | **G** | **C** | **T** | **G** | **C** | **T** | **T** | **T** | **A** | **A** | **A** | **G** | **G** | **T** | **T** | **G** | **C** | **T** | **G** | **C** | **G** | **G** | **C** | **T** | **T** | **C** | **A** | **G** | **G** | **A** | **G** | **T** | **A** | **G** | **A** | **T** | **G** | **T** | **T** | **A** | **A** | **A** | **A** | **T** | **T** | **A** | **T** | **G** | **A** | **T** | **T** | **C** | **C** | **A** | **G** | **A** | **T** | **A** | **A** | **G** | **C** | **C** | **C** | **G** | **A** | **C** | **C** | **A** | **T** | **C** | **C** | **G** | **T** | **T** | **T** | **A** | **T** | **T** | **T** | **A** | **T** | **C** | **G** | **A** | **G** | **C** | **A** | **A** | **C** | **A** | **C** | **A** | **G** | **T** | **A** | **T** | **T** | **A** | **C** | **G** | **G** | **C** | **C** | **G** | **C** | **T** | **T** | **A** | **T** | **T** | **G** | **A** | **T** | **G** | **A** | **A** | **A** | **G** | **A** | **A** | **A** | **A** | **T** | **A** | **T** | **T** | **G** | **A** | **A** | **A** | **T** | **T** | **T** | **T** | **A** | **A** | **T** | **T** | **T** | **A** | **T** | **A** | **A** | **C** | **G** | **G** | **T** | **G** | **G** | **T** | **T** | **T** | **C** | **T** | **T** | **A** | **C** | **A** | **T** | **G** | **C** | **G** | **A** | **A** | **A** | **A** | **C** | **A** | **A** | **T** | **G** | **A** | **T** | **T** | **A** | **T** | **G** | **G** | **A** | **T** | **G** | **A** | **T** | **G** | **A** | **A** | **G** | **T** | **C** | **T** | **G** | **C** | **A** | **C** | **A** | **G** | **T** | **T** | **G** | **G** | **T** | **T** | **C** | **A** | **G** | **C** | **C** | **A** | **A** | **T** | **C** | **A** | **A** | **G** | **A** | **T** | **A** | **T** | **C** | **C** | **G** | **A** | **A** | **G** | **T** | **T** | **A** | **C** | **A** | **A** | **A** | **T** | **T** | **A** | **A** | **A** | **C** | **T** | **T** | **T** | **G** | **A** | **A** | **G** | **C** | **A** | **A** | **A** | **T** | **G** | **C** | **T** | **G** | **T** | **G** | **T** | **T** | **A** | **T** | **A** | **T** | **G** | **A** | **T** | **A** | **A** | **A** | **A** | **A** | **A** | **A** | **T** | **C** | **A** | **T** | **T** | **G** | **A** | **T** | **C** | **A** | **A** | **T** | **T** | **A** | **G** | **A** | **A** | **G** | **C** | **A** | **A** | **T** | **T** | **T** | **T** | **C** | **T** | **T** | **A** | **G** | **A** | **A** | **G** | **A** | **T** | **C** | **G** | **A** | **A** | **A** | **A** | **A** | **A** | **A** | **T** | **G** | **T** | **A** | **C** | **A** | **A** | **C** | **A** | **A** | **T** | **G** | **A** | **C** | **T** | **C** | **C** | **A** | **G** | **A** | **A** | **G** | **T** | **T** | **G** | **T** | **T** | **C** | **G** | **C** | **G** | **A** | **C** | **A** | **T** | **G** | **T** | **C** | **A** | **A** | **A** | **A** | **T** | **G** | **G** | **T** | **T** | **G** | **A** | **T** | **T** | **T** | **T** | **T** | **A** | **A** | **A** | **C** | **A** | **A** | **C** | **A** | **A** | **A** | **T** | **T** | **T** | **C** | **A** | **C** | **G** | **A** | **T** | **T** | **A** | **T** | **T** | **T** | **T** | **C** | **A** | **C** | **C** | **A** | **A** | **T** | **T** | **C** | **T** | **T** | **-** | **-** | **-** |
| **N54576** | **T** | **T** | **G** | **A** | **T** | **T** | **C** | **T** | **T** | **A** | **G** | **C** | **G** | **T** | **C** | **T** | **T** | **A** | **A** | **C** | **A** | **G** | **T** | **T** | **A** | **T** | **T** | **T** | **A** | **T** | **T** | **T** | **T** | **A** | **T** | **T** | **A** | **A** | **T** | **G** | **C** | **A** | **A** | **T** | **T** | **A** | **T** | **T** | **G** | **C** | **G** | **G** | **G** | **A** | **A** | **T** | **T** | **A** | **C** | **C** | **A** | **T** | **T** | **T** | **T** | **G** | **T** | **T** | **A** | **A** | **A** | **A** | **C** | **C** | **T** | **C** | **G** | **A** | **G** | **A** | **T** | **G** | **T** | **A** | **G** | **C** | **A** | **G** | **C** | **A** | **A** | **T** | **T** | **T** | **G** | **G** | **G** | **C** | **A** | **T** | **G** | **G** | **C** | **T** | **A** | **C** | **T** | **C** | **G** | **T** | **A** | **C** | **T** | **G** | **A** | **T** | **T** | **G** | **C** | **T** | **C** | **T** | **A** | **C** | **C** | **C** | **G** | **T** | **T** | **T** | **T** | **T** | **G** | **G** | **T** | **T** | **T** | **T** | **T** | **T** | **C** | **T** | **T** | **A** | **T** | **A** | **T** | **T** | **T** | **A** | **T** | **T** | **C** | **T** | **T** | **T** | **G** | **G** | **G** | **C** | **G** | **C** | **G** | **G** | **T** | **T** | **T** | **A** | **A** | **C** | **C** | **G** | **A** | **C** | **A** | **A** | **A** | **A** | **A** | **G** | **A** | **A** | **A** | **T** | **T** | **T** | **T** | **A** | **T** | **T** | **T** | **G** | **C** | **A** | **A** | **C** | **A** | **A** | **A** | **G** | **T** | **G** | **A** | **T** | **T** | **T** | **G** | **C** | **G** | **G** | **G** | **A** | **A** | **T** | **T** | **A** | **G** | **A** | **G** | **A** | **A** | **T** | **T** | **T** | **T** | **C** | **A** | **G** | **A** | **A** | **T** | **T** | **T** | **C** | **C** | **A** | **A** | **G** | **A** | **A** | **G** | **A** | **A** | **A** | **G** | **T** | **T** | **T** | **T** | **G** | **A** | **A** | **C** | **T** | **T** | **T** | **A** | **T** | **A** | **G** | **T** | **C** | **A** | **A** | **A** | **A** | **G** | **A** | **T** | **G** | **C** | **C** | **G** | **A** | **C** | **G** | **G** | **A** | **G** | **G** | **A** | **G** | **C** | **A** | **A** | **C** | **A** | **A** | **C** | **A** | **G** | **T** | **T** | **T** | **A** | **C** | **A** | **G** | **A** | **C** | **T** | **T** | **T** | **T** | **T** | **T** | **T** | **C** | **C** | **T** | **C** | **G** | **T** | **T** | **G** | **A** | **A** | **T** | **A** | **G** | **G** | **A** | **T** | **G** | **C** | **C** | **T** | **C** | **T** | **G** | **A** | **C** | **T** | **A** | **A** | **G** | **A** | **A** | **A** | **A** | **A** | **T** | **G** | **A** | **T** | **G** | **T** | **T** | **C** | **A** | **A** | **A** | **T** | **T** | **T** | **T** | **T** | **A** | **C** | **G** | **G** | **A** | **T** | **G** | **G** | **T** | **A** | **C** | **G** | **G** | **A** | **A** | **A** | **A** | **A** | **T** | **T** | **C** | **A** | **A** | **T** | **G** | **C** | **G** | **T** | **T** | **G** | **A** | **T** | **G** | **G** | **C** | **G** | **G** | **A** | **C** | **A** | **T** | **A** | **A** | **A** | **A** | **A** | **A** | **A** | **G** | **C** | **T** | **C** | **A** | **G** | **C** | **A** | **C** | **T** | **C** | **C** | **A** | **T** | **T** | **C** | **A** | **T** | **A** | **T** | **T** | **G** | **A** | **A** | **T** | **A** | **T** | **T** | **A** | **T** | **G** | **C** | **C** | **T** | **T** | **T** | **G** | **T** | **A** | **A** | **C** | **G** | **G** | **A** | **T** | **C** | **A** | **T** | **A** | **T** | **C** | **G** | **G** | **C** | **A** | **C** | **C** | **A** | **A** | **A** | **A** | **T** | **T** | **T** | **T** | **G** | **A** | **A** | **C** | **T** | **T** | **A** | **T** | **T** | **A** | **G** | **A** | **A** | **G** | **A** | **A** | **A** | **A** | **G** | **G** | **C** | **G** | **G** | **C** | **T** | **G** | **A** | **A** | **G** | **G** | **G** | **G** | **T** | **T** | **G** | **A** | **A** | **G** | **T** | **G** | **C** | **G** | **G** | **T** | **T** | **G** | **C** | **T** | **T** | **T** | **A** | **C** | **G** | **A** | **T** | **G** | **C** | **C** | **T** | **T** | **T** | **G** | **G** | **C** | **T** | **C** | **A** | **A** | **A** | **G** | **G** | **G** | **C** | **A** | **C** | **C** | **A** | **A** | **G** | **G** | **T** | **T** | **C** | **A** | **T** | **C** | **A** | **T** | **T** | **T** | **G** | **A** | **A** | **T** | **G** | **A** | **A** | **T** | **T** | **G** | **A** | **A** | **A** | **A** | **A** | **A** | **A** | **A** | **C** | **G** | **G** | **T** | **G** | **G** | **T** | **T** | **T** | **T** | **G** | **T** | **C** | **C** | **A** | **A** | **A** | **C** | **G** | **T** | **T** | **T** | **A** | **T** | **T** | **A** | **C** | **T** | **T** | **C** | **T** | **C** | **A** | **A** | **A** | **A** | **A** | **G** | **C** | **A** | **C** | **T** | **T** | **T** | **T** | **G** | **A** | **A** | **G** | **T** | **T** | **T** | **C** | **G** | **T** | **T** | **T** | **G** | **A** | **A** | **T** | **T** | **A** | **T** | **C** | **A** | **T** | **G** | **A** | **T** | **C** | **A** | **C** | **C** | **G** | **G** | **A** | **A** | **A** | **A** | **T** | **T** | **G** | **T** | **T** | **G** | **T** | **T** | **A** | **T** | **T** | **G** | **A** | **C** | **G** | **G** | **A** | **A** | **A** | **A** | **G** | **T** | **A** | **G** | **G** | **C** | **T** | **A** | **C** | **A** | **T** | **T** | **G** | **G** | **C** | **G** | **G** | **C** | **T** | **T** | **T** | **A** | **A** | **T** | **G** | **T** | **T** | **G** | **C** | **C** | **G** | **A** | **T** | **C** | **A** | **A** | **T** | **A** | **T** | **G** | **C** | **C** | **G** | **G** | **A** | **A** | **C** | **G** | **A** | **C** | **T** | **A** | **A** | **A** | **A** | **A** | **G** | **T** | **T** | **T** | **G** | **G** | **C** | **T** | **A** | **T** | **T** | **G** | **G** | **C** | **G** | **G** | **G** | **A** | **T** | **A** | **C** | **A** | **C** | **A** | **T** | **T** | **T** | **A** | **C** | **G** | **G** | **A** | **T** | **T** | **C** | **A** | **A** | **G** | **G** | **G** | **C** | **C** | **A** | **G** | **C** | **A** | **G** | **C** | **C** | **T** | **C** | **A** | **T** | **T** | **A** | **C** | **T** | **G** | **C** | **A** | **A** | **A** | **T** | **G** | **C** | **G** | **T** | **T** | **T** | **T** | **T** | **T** | **A** | **A** | **T** | **G** | **G** | **A** | **T** | **T** | **G** | **G** | **A** | **A** | **C** | **G** | **T** | **C** | **T** | **C** | **T** | **T** | **C** | **C** | **C** | **C** | **C** | **G** | **A** | **G** | **A** | **A** | **A** | **A** | **A** | **T** | **C** | **G** | **T** | **G** | **T** | **G** | **G** | **C** | **G** | **T** | **A** | **T** | **C** | **A** | **A** | **T** | **T** | **G** | **G** | **A** | **T** | **T** | **A** | **T** | **T** | **T** | **C** | **T** | **T** | **T** | **A** | **A** | **A** | **C** | **T** | **T** | **G** | **A** | **A** | **G** | **C** | **A** | **T** | **T** | **G** | **G** | **T** | **G** | **C** | **C** | **A** | **G** | **A** | **G** | **G** | **C** | **A** | **A** | **A** | **T** | **A** | **C** | **A** | **T** | **C** | **C** | **A** | **T** | **T** | **C** | **A** | **G** | **A** | **T** | **G** | **A** | **T** | **T** | **G** | **C** | **C** | **A** | **G** | **T** | **G** | **G** | **T** | **C** | **C** | **T** | **A** | **A** | **C** | **A** | **G** | **T** | **G** | **A** | **C** | **C** | **G** | **T** | **G** | **A** | **A** | **C** | **A** | **A** | **A** | **T** | **T** | **A** | **A** | **A** | **T** | **T** | **G** | **G** | **C** | **C** | **T** | **T** | **T** | **A** | **T** | **T** | **A** | **A** | **A** | **T** | **T** | **G** | **A** | **T** | **T** | **A** | **C** | **T** | **T** | **C** | **T** | **G** | **C** | **C** | **A** | **A** | **G** | **A** | **A** | **A** | **A** | **G** | **A** | **G** | **T** | **C** | **T** | **G** | **G** | **A** | **T** | **T** | **C** | **A** | **A** | **A** | **C** | **G** | **C** | **C** | **G** | **T** | **A** | **T** | **T** | **T** | **A** | **G** | **T** | **T** | **C** | **C** | **T** | **G** | **A** | **T** | **G** | **A** | **T** | **A** | **G** | **T** | **G** | **T** | **C** | **T** | **T** | **G** | **G** | **C** | **T** | **G** | **C** | **T** | **T** | **T** | **A** | **A** | **A** | **G** | **G** | **T** | **T** | **G** | **C** | **T** | **G** | **C** | **G** | **G** | **C** | **T** | **T** | **C** | **A** | **G** | **G** | **A** | **G** | **T** | **A** | **G** | **A** | **T** | **G** | **T** | **T** | **A** | **A** | **A** | **A** | **T** | **T** | **A** | **T** | **G** | **A** | **T** | **T** | **C** | **C** | **A** | **G** | **A** | **T** | **A** | **A** | **G** | **C** | **C** | **C** | **G** | **A** | **C** | **C** | **A** | **T** | **C** | **C** | **G** | **T** | **T** | **T** | **A** | **T** | **T** | **T** | **A** | **T** | **C** | **G** | **A** | **G** | **C** | **A** | **A** | **C** | **A** | **C** | **A** | **G** | **T** | **A** | **T** | **T** | **A** | **C** | **G** | **G** | **C** | **C** | **G** | **C** | **T** | **T** | **G** | **T** | **T** | **G** | **A** | **T** | **G** | **A** | **A** | **A** | **G** | **A** | **A** | **A** | **A** | **T** | **A** | **T** | **T** | **G** | **A** | **A** | **A** | **T** | **T** | **T** | **T** | **A** | **A** | **T** | **T** | **T** | **A** | **T** | **A** | **A** | **C** | **G** | **G** | **T** | **G** | **G** | **T** | **T** | **T** | **C** | **T** | **T** | **A** | **C** | **A** | **T** | **G** | **C** | **G** | **A** | **A** | **A** | **A** | **C** | **A** | **A** | **T** | **G** | **A** | **T** | **T** | **A** | **T** | **G** | **G** | **A** | **T** | **G** | **A** | **T** | **G** | **A** | **A** | **G** | **T** | **C** | **T** | **G** | **C** | **A** | **C** | **A** | **G** | **T** | **A** | **G** | **G** | **T** | **T** | **C** | **A** | **G** | **C** | **C** | **A** | **A** | **T** | **C** | **A** | **A** | **G** | **A** | **T** | **A** | **T** | **C** | **C** | **G** | **A** | **A** | **G** | **T** | **T** | **A** | **C** | **A** | **A** | **A** | **T** | **T** | **A** | **A** | **A** | **C** | **T** | **T** | **T** | **G** | **A** | **A** | **G** | **C** | **A** | **A** | **A** | **T** | **G** | **C** | **T** | **G** | **T** | **G** | **T** | **T** | **A** | **T** | **A** | **T** | **G** | **A** | **T** | **A** | **A** | **A** | **A** | **A** | **A** | **A** | **T** | **C** | **A** | **T** | **T** | **G** | **A** | **T** | **C** | **A** | **A** | **T** | **T** | **A** | **G** | **A** | **A** | **G** | **C** | **A** | **A** | **T** | **T** | **T** | **T** | **C** | **T** | **T** | **A** | **G** | **A** | **A** | **G** | **A** | **T** | **C** | **G** | **A** | **A** | **A** | **A** | **A** | **A** | **A** | **T** | **G** | **T** | **A** | **C** | **A** | **A** | **C** | **A** | **A** | **T** | **G** | **A** | **C** | **T** | **C** | **C** | **A** | **G** | **A** | **A** | **G** | **T** | **T** | **G** | **T** | **T** | **C** | **G** | **C** | **G** | **A** | **C** | **A** | **T** | **G** | **T** | **C** | **C** | **A** | **A** | **A** | **T** | **G** | **G** | **T** | **T** | **G** | **A** | **T** | **T** | **T** | **T** | **T** | **A** | **A** | **A** | **C** | **A** | **A** | **C** | **A** | **A** | **A** | **T** | **T** | **T** | **C** | **A** | **C** | **G** | **A** | **T** | **T** | **A** | **T** | **T** | **T** | **T** | **C** | **A** | **C** | **C** | **A** | **A** | **T** | **T** | **C** | **T** | **T** | **-** | **-** | **-** |
| **N54574** | **T** | **T** | **G** | **A** | **T** | **T** | **C** | **T** | **T** | **A** | **G** | **C** | **G** | **T** | **C** | **T** | **T** | **A** | **A** | **C** | **A** | **G** | **T** | **T** | **A** | **T** | **T** | **T** | **A** | **T** | **T** | **T** | **T** | **A** | **T** | **T** | **A** | **A** | **T** | **G** | **C** | **A** | **A** | **T** | **T** | **A** | **T** | **T** | **G** | **C** | **G** | **G** | **G** | **A** | **A** | **T** | **T** | **A** | **C** | **C** | **A** | **T** | **T** | **T** | **T** | **G** | **T** | **T** | **A** | **A** | **A** | **A** | **C** | **C** | **T** | **C** | **G** | **A** | **G** | **A** | **T** | **G** | **T** | **A** | **G** | **C** | **A** | **G** | **C** | **A** | **A** | **T** | **T** | **T** | **G** | **G** | **G** | **C** | **A** | **T** | **G** | **G** | **C** | **T** | **A** | **C** | **T** | **C** | **G** | **T** | **A** | **C** | **T** | **G** | **A** | **T** | **T** | **G** | **C** | **T** | **C** | **T** | **A** | **C** | **C** | **C** | **G** | **T** | **T** | **T** | **T** | **T** | **G** | **G** | **T** | **T** | **T** | **T** | **T** | **T** | **C** | **T** | **T** | **A** | **T** | **A** | **T** | **T** | **T** | **A** | **T** | **T** | **C** | **T** | **T** | **T** | **G** | **G** | **T** | **C** | **G** | **C** | **G** | **G** | **T** | **T** | **T** | **A** | **A** | **C** | **C** | **G** | **A** | **C** | **A** | **A** | **A** | **A** | **A** | **G** | **A** | **A** | **A** | **T** | **T** | **T** | **T** | **A** | **T** | **T** | **T** | **G** | **C** | **A** | **A** | **C** | **A** | **A** | **A** | **G** | **T** | **G** | **A** | **T** | **T** | **T** | **G** | **C** | **G** | **G** | **G** | **A** | **A** | **T** | **T** | **A** | **G** | **A** | **G** | **A** | **A** | **T** | **T** | **T** | **T** | **C** | **A** | **G** | **A** | **A** | **T** | **T** | **T** | **C** | **C** | **A** | **A** | **G** | **A** | **A** | **G** | **A** | **A** | **A** | **G** | **T** | **T** | **T** | **T** | **G** | **A** | **A** | **C** | **T** | **T** | **T** | **A** | **T** | **A** | **G** | **T** | **C** | **A** | **A** | **A** | **A** | **G** | **A** | **T** | **G** | **C** | **C** | **G** | **A** | **C** | **G** | **G** | **A** | **G** | **G** | **A** | **G** | **C** | **A** | **A** | **C** | **A** | **A** | **C** | **A** | **G** | **T** | **T** | **T** | **A** | **C** | **A** | **G** | **A** | **C** | **T** | **T** | **T** | **T** | **T** | **T** | **T** | **C** | **C** | **T** | **C** | **G** | **T** | **T** | **A** | **A** | **A** | **T** | **A** | **G** | **G** | **A** | **T** | **G** | **C** | **C** | **T** | **C** | **T** | **G** | **A** | **C** | **T** | **A** | **A** | **G** | **A** | **A** | **A** | **A** | **A** | **T** | **G** | **A** | **T** | **G** | **T** | **T** | **G** | **A** | **A** | **A** | **T** | **T** | **T** | **T** | **T** | **A** | **C** | **G** | **G** | **A** | **T** | **G** | **G** | **T** | **A** | **C** | **G** | **G** | **A** | **A** | **A** | **A** | **A** | **T** | **T** | **C** | **A** | **A** | **T** | **G** | **C** | **G** | **T** | **T** | **G** | **A** | **T** | **G** | **G** | **C** | **G** | **G** | **A** | **T** | **A** | **T** | **A** | **A** | **A** | **A** | **A** | **A** | **A** | **G** | **C** | **T** | **C** | **A** | **G** | **C** | **A** | **C** | **T** | **C** | **T** | **A** | **T** | **T** | **C** | **A** | **T** | **A** | **T** | **T** | **G** | **A** | **A** | **T** | **A** | **T** | **T** | **A** | **T** | **G** | **C** | **C** | **T** | **T** | **T** | **G** | **T** | **A** | **A** | **C** | **G** | **G** | **A** | **T** | **C** | **A** | **T** | **A** | **T** | **C** | **G** | **G** | **T** | **A** | **C** | **C** | **A** | **A** | **A** | **A** | **T** | **T** | **T** | **T** | **G** | **A** | **A** | **C** | **T** | **T** | **A** | **T** | **T** | **A** | **G** | **A** | **A** | **G** | **A** | **A** | **A** | **A** | **G** | **G** | **C** | **G** | **G** | **C** | **T** | **G** | **A** | **A** | **G** | **G** | **G** | **G** | **T** | **T** | **G** | **A** | **A** | **G** | **T** | **G** | **C** | **G** | **G** | **T** | **T** | **G** | **C** | **T** | **T** | **T** | **A** | **C** | **G** | **A** | **T** | **G** | **C** | **C** | **T** | **T** | **T** | **G** | **G** | **C** | **T** | **C** | **A** | **A** | **A** | **G** | **G** | **G** | **C** | **A** | **C** | **C** | **A** | **A** | **G** | **G** | **T** | **T** | **C** | **A** | **T** | **C** | **A** | **T** | **T** | **T** | **G** | **A** | **A** | **T** | **G** | **A** | **A** | **T** | **T** | **G** | **A** | **A** | **A** | **A** | **A** | **A** | **A** | **A** | **C** | **G** | **G** | **T** | **G** | **G** | **T** | **T** | **T** | **T** | **G** | **T** | **C** | **C** | **A** | **A** | **A** | **C** | **G** | **T** | **T** | **T** | **A** | **T** | **T** | **A** | **C** | **T** | **T** | **C** | **T** | **C** | **A** | **A** | **A** | **A** | **A** | **G** | **C** | **A** | **C** | **T** | **T** | **T** | **T** | **G** | **A** | **A** | **G** | **T** | **T** | **T** | **C** | **G** | **T** | **T** | **T** | **G** | **A** | **A** | **T** | **T** | **A** | **T** | **C** | **A** | **T** | **G** | **A** | **T** | **C** | **A** | **C** | **C** | **G** | **G** | **A** | **A** | **A** | **A** | **T** | **T** | **G** | **T** | **T** | **G** | **T** | **T** | **A** | **T** | **T** | **G** | **A** | **C** | **G** | **G** | **A** | **A** | **A** | **A** | **G** | **T** | **A** | **G** | **G** | **C** | **T** | **A** | **C** | **A** | **T** | **T** | **G** | **G** | **C** | **G** | **G** | **C** | **T** | **T** | **T** | **A** | **A** | **T** | **G** | **T** | **T** | **G** | **C** | **C** | **G** | **A** | **T** | **C** | **A** | **A** | **T** | **A** | **T** | **G** | **C** | **T** | **G** | **G** | **A** | **A** | **C** | **G** | **A** | **C** | **T** | **A** | **A** | **A** | **A** | **A** | **G** | **T** | **T** | **T** | **G** | **G** | **C** | **T** | **A** | **T** | **T** | **G** | **G** | **C** | **G** | **G** | **G** | **A** | **T** | **A** | **C** | **G** | **C** | **A** | **T** | **T** | **T** | **A** | **C** | **G** | **G** | **A** | **T** | **T** | **C** | **A** | **A** | **G** | **G** | **G** | **C** | **C** | **A** | **G** | **C** | **A** | **G** | **C** | **C** | **T** | **C** | **A** | **T** | **T** | **A** | **C** | **T** | **G** | **C** | **A** | **A** | **A** | **T** | **G** | **C** | **G** | **T** | **T** | **T** | **T** | **T** | **T** | **A** | **A** | **T** | **G** | **G** | **A** | **T** | **T** | **G** | **G** | **A** | **A** | **C** | **G** | **T** | **C** | **T** | **C** | **T** | **T** | **C** | **C** | **C** | **C** | **C** | **G** | **A** | **G** | **A** | **A** | **A** | **A** | **A** | **T** | **C** | **G** | **T** | **G** | **T** | **G** | **G** | **C** | **G** | **T** | **A** | **T** | **C** | **A** | **A** | **T** | **T** | **G** | **G** | **A** | **T** | **T** | **A** | **T** | **T** | **T** | **C** | **T** | **T** | **T** | **A** | **A** | **A** | **C** | **T** | **T** | **G** | **A** | **A** | **G** | **C** | **A** | **T** | **T** | **G** | **G** | **T** | **G** | **C** | **C** | **A** | **G** | **A** | **G** | **G** | **C** | **A** | **A** | **A** | **T** | **A** | **C** | **A** | **T** | **C** | **C** | **A** | **T** | **T** | **C** | **A** | **G** | **A** | **T** | **G** | **A** | **T** | **T** | **G** | **C** | **C** | **A** | **G** | **T** | **G** | **G** | **T** | **C** | **C** | **T** | **A** | **A** | **C** | **A** | **G** | **T** | **G** | **A** | **C** | **C** | **G** | **T** | **G** | **A** | **A** | **C** | **A** | **A** | **A** | **T** | **T** | **A** | **A** | **A** | **T** | **T** | **G** | **G** | **C** | **C** | **T** | **T** | **T** | **A** | **T** | **T** | **A** | **A** | **A** | **T** | **T** | **G** | **A** | **T** | **T** | **A** | **C** | **T** | **T** | **C** | **T** | **G** | **C** | **C** | **A** | **A** | **G** | **A** | **A** | **A** | **A** | **G** | **A** | **G** | **T** | **C** | **T** | **G** | **G** | **A** | **T** | **T** | **C** | **A** | **A** | **A** | **C** | **G** | **C** | **C** | **G** | **T** | **A** | **T** | **T** | **T** | **A** | **G** | **T** | **T** | **C** | **C** | **T** | **G** | **A** | **T** | **G** | **A** | **T** | **A** | **G** | **T** | **G** | **T** | **C** | **T** | **T** | **G** | **G** | **C** | **T** | **G** | **C** | **T** | **T** | **T** | **A** | **A** | **A** | **G** | **G** | **T** | **T** | **G** | **C** | **T** | **G** | **C** | **G** | **G** | **C** | **T** | **T** | **C** | **A** | **G** | **G** | **A** | **G** | **T** | **A** | **G** | **A** | **T** | **G** | **T** | **T** | **A** | **A** | **A** | **A** | **T** | **T** | **A** | **T** | **G** | **A** | **T** | **T** | **C** | **C** | **A** | **G** | **A** | **T** | **A** | **A** | **G** | **C** | **C** | **C** | **G** | **A** | **C** | **C** | **A** | **T** | **C** | **C** | **G** | **T** | **T** | **T** | **A** | **T** | **T** | **T** | **A** | **T** | **C** | **G** | **A** | **G** | **C** | **A** | **A** | **C** | **A** | **C** | **A** | **G** | **T** | **A** | **T** | **T** | **A** | **C** | **G** | **G** | **C** | **C** | **G** | **C** | **T** | **T** | **A** | **T** | **T** | **G** | **A** | **T** | **G** | **A** | **A** | **A** | **G** | **A** | **A** | **A** | **A** | **T** | **A** | **T** | **T** | **G** | **A** | **A** | **A** | **T** | **T** | **T** | **T** | **A** | **A** | **T** | **T** | **T** | **A** | **T** | **A** | **A** | **C** | **G** | **G** | **T** | **G** | **G** | **T** | **T** | **T** | **C** | **T** | **T** | **A** | **C** | **A** | **T** | **G** | **C** | **G** | **A** | **A** | **A** | **A** | **C** | **A** | **A** | **T** | **G** | **A** | **T** | **T** | **A** | **T** | **G** | **G** | **A** | **T** | **G** | **A** | **T** | **G** | **A** | **A** | **G** | **T** | **C** | **T** | **G** | **C** | **A** | **C** | **A** | **G** | **T** | **T** | **G** | **G** | **T** | **T** | **C** | **A** | **G** | **C** | **C** | **A** | **A** | **T** | **C** | **A** | **A** | **G** | **A** | **T** | **A** | **T** | **C** | **C** | **G** | **A** | **A** | **G** | **T** | **T** | **A** | **C** | **A** | **A** | **A** | **T** | **T** | **A** | **A** | **A** | **C** | **T** | **T** | **T** | **G** | **A** | **A** | **G** | **C** | **A** | **A** | **A** | **T** | **G** | **C** | **T** | **G** | **T** | **G** | **T** | **T** | **A** | **T** | **A** | **T** | **G** | **A** | **T** | **A** | **A** | **A** | **A** | **A** | **A** | **A** | **T** | **C** | **A** | **T** | **T** | **G** | **A** | **T** | **C** | **A** | **A** | **T** | **T** | **A** | **G** | **A** | **A** | **G** | **C** | **A** | **A** | **T** | **T** | **T** | **T** | **T** | **T** | **T** | **A** | **G** | **A** | **A** | **G** | **A** | **T** | **C** | **G** | **A** | **A** | **A** | **A** | **A** | **A** | **A** | **T** | **G** | **T** | **A** | **C** | **A** | **A** | **C** | **A** | **A** | **T** | **G** | **A** | **C** | **T** | **C** | **C** | **A** | **G** | **A** | **A** | **G** | **T** | **T** | **G** | **T** | **T** | **C** | **G** | **C** | **G** | **A** | **C** | **A** | **T** | **G** | **T** | **C** | **A** | **A** | **A** | **A** | **T** | **G** | **G** | **T** | **T** | **G** | **A** | **T** | **T** | **T** | **T** | **T** | **A** | **A** | **A** | **C** | **A** | **A** | **C** | **A** | **A** | **A** | **T** | **T** | **T** | **C** | **A** | **C** | **G** | **A** | **T** | **T** | **A** | **T** | **T** | **T** | **T** | **C** | **A** | **C** | **C** | **A** | **A** | **T** | **T** | **C** | **T** | **T** | **-** | **-** | **-** |
| **N59491F** | **T** | **T** | **G** | **A** | **T** | **T** | **C** | **T** | **T** | **A** | **G** | **C** | **G** | **T** | **C** | **T** | **T** | **A** | **A** | **C** | **A** | **G** | **T** | **T** | **A** | **T** | **T** | **T** | **A** | **T** | **T** | **T** | **T** | **A** | **T** | **T** | **A** | **A** | **T** | **G** | **C** | **A** | **A** | **T** | **T** | **A** | **T** | **T** | **G** | **C** | **G** | **G** | **G** | **A** | **A** | **T** | **T** | **A** | **C** | **C** | **A** | **T** | **T** | **T** | **T** | **G** | **T** | **T** | **A** | **A** | **A** | **A** | **C** | **C** | **T** | **C** | **G** | **A** | **G** | **A** | **T** | **G** | **T** | **A** | **G** | **C** | **A** | **G** | **C** | **A** | **A** | **T** | **T** | **T** | **G** | **G** | **G** | **C** | **A** | **T** | **G** | **G** | **C** | **T** | **A** | **C** | **T** | **C** | **G** | **T** | **A** | **C** | **T** | **G** | **A** | **T** | **T** | **G** | **C** | **T** | **C** | **T** | **A** | **C** | **C** | **C** | **G** | **T** | **T** | **T** | **T** | **T** | **G** | **G** | **T** | **T** | **T** | **T** | **T** | **T** | **C** | **T** | **T** | **A** | **T** | **A** | **T** | **T** | **T** | **A** | **T** | **T** | **C** | **T** | **T** | **T** | **G** | **G** | **G** | **C** | **G** | **C** | **G** | **G** | **T** | **T** | **T** | **A** | **A** | **C** | **C** | **G** | **A** | **C** | **A** | **A** | **A** | **A** | **A** | **G** | **A** | **A** | **A** | **T** | **T** | **T** | **T** | **A** | **T** | **T** | **T** | **G** | **C** | **A** | **A** | **C** | **A** | **A** | **A** | **G** | **T** | **G** | **A** | **T** | **T** | **T** | **A** | **C** | **G** | **G** | **G** | **A** | **A** | **T** | **T** | **A** | **G** | **A** | **G** | **A** | **A** | **T** | **T** | **T** | **T** | **C** | **A** | **G** | **A** | **A** | **C** | **T** | **T** | **C** | **C** | **A** | **A** | **G** | **A** | **A** | **G** | **A** | **A** | **A** | **G** | **T** | **T** | **T** | **T** | **G** | **A** | **A** | **C** | **T** | **T** | **T** | **A** | **T** | **A** | **G** | **T** | **C** | **A** | **A** | **A** | **A** | **G** | **A** | **T** | **G** | **C** | **C** | **G** | **A** | **C** | **G** | **G** | **A** | **G** | **G** | **A** | **G** | **C** | **A** | **A** | **C** | **A** | **A** | **C** | **A** | **G** | **T** | **T** | **T** | **A** | **C** | **A** | **G** | **A** | **C** | **T** | **T** | **T** | **T** | **T** | **T** | **T** | **C** | **C** | **T** | **C** | **G** | **T** | **T** | **A** | **A** | **A** | **T** | **A** | **G** | **G** | **A** | **T** | **G** | **C** | **C** | **T** | **C** | **T** | **G** | **A** | **C** | **T** | **A** | **A** | **G** | **A** | **A** | **A** | **A** | **A** | **T** | **G** | **A** | **T** | **G** | **T** | **T** | **G** | **A** | **A** | **A** | **T** | **T** | **T** | **T** | **T** | **A** | **C** | **G** | **G** | **A** | **T** | **G** | **G** | **T** | **A** | **C** | **G** | **G** | **A** | **A** | **A** | **A** | **A** | **T** | **T** | **C** | **A** | **A** | **T** | **G** | **C** | **G** | **T** | **T** | **G** | **A** | **T** | **G** | **G** | **C** | **G** | **G** | **A** | **C** | **A** | **T** | **A** | **A** | **A** | **A** | **A** | **A** | **A** | **G** | **C** | **T** | **C** | **A** | **G** | **C** | **A** | **C** | **T** | **C** | **C** | **A** | **T** | **T** | **C** | **A** | **T** | **A** | **T** | **T** | **G** | **A** | **A** | **T** | **A** | **T** | **T** | **A** | **T** | **G** | **C** | **C** | **T** | **T** | **T** | **G** | **T** | **A** | **A** | **C** | **G** | **G** | **A** | **T** | **C** | **A** | **T** | **A** | **T** | **C** | **G** | **G** | **T** | **A** | **C** | **C** | **A** | **A** | **A** | **A** | **T** | **T** | **T** | **T** | **G** | **A** | **A** | **C** | **T** | **T** | **A** | **T** | **T** | **A** | **G** | **A** | **A** | **G** | **A** | **A** | **A** | **A** | **G** | **G** | **C** | **G** | **G** | **C** | **T** | **G** | **A** | **A** | **G** | **G** | **G** | **G** | **T** | **T** | **G** | **A** | **A** | **G** | **T** | **G** | **C** | **G** | **G** | **T** | **T** | **G** | **C** | **T** | **T** | **T** | **A** | **C** | **G** | **A** | **T** | **G** | **C** | **C** | **T** | **T** | **T** | **G** | **G** | **C** | **T** | **C** | **A** | **A** | **A** | **G** | **G** | **G** | **C** | **A** | **C** | **C** | **A** | **A** | **G** | **G** | **T** | **T** | **C** | **A** | **T** | **C** | **A** | **T** | **T** | **T** | **G** | **A** | **A** | **T** | **G** | **A** | **A** | **T** | **T** | **G** | **A** | **A** | **A** | **A** | **A** | **A** | **A** | **A** | **C** | **G** | **G** | **C** | **G** | **G** | **T** | **T** | **T** | **T** | **G** | **T** | **C** | **C** | **A** | **A** | **A** | **C** | **G** | **T** | **T** | **T** | **A** | **T** | **T** | **A** | **C** | **T** | **T** | **C** | **T** | **C** | **A** | **A** | **A** | **A** | **A** | **G** | **C** | **A** | **C** | **T** | **T** | **T** | **T** | **G** | **A** | **A** | **G** | **T** | **T** | **T** | **C** | **G** | **T** | **T** | **T** | **G** | **A** | **A** | **T** | **T** | **A** | **T** | **C** | **A** | **T** | **G** | **A** | **T** | **C** | **A** | **C** | **C** | **G** | **G** | **A** | **A** | **A** | **A** | **T** | **T** | **G** | **T** | **T** | **G** | **T** | **T** | **A** | **T** | **T** | **G** | **A** | **T** | **G** | **G** | **G** | **A** | **A** | **A** | **G** | **T** | **A** | **G** | **G** | **C** | **T** | **A** | **C** | **A** | **T** | **T** | **G** | **G** | **C** | **G** | **G** | **C** | **T** | **T** | **T** | **A** | **A** | **T** | **G** | **T** | **T** | **G** | **C** | **C** | **G** | **A** | **T** | **C** | **A** | **A** | **T** | **A** | **T** | **G** | **C** | **T** | **G** | **G** | **A** | **A** | **C** | **G** | **A** | **C** | **T** | **A** | **A** | **A** | **A** | **A** | **G** | **T** | **T** | **T** | **G** | **G** | **C** | **T** | **A** | **T** | **T** | **G** | **G** | **C** | **G** | **G** | **G** | **A** | **T** | **A** | **C** | **A** | **C** | **A** | **T** | **T** | **T** | **A** | **C** | **G** | **G** | **A** | **T** | **T** | **C** | **A** | **A** | **G** | **G** | **G** | **C** | **C** | **A** | **G** | **C** | **A** | **G** | **C** | **C** | **T** | **C** | **A** | **T** | **T** | **A** | **C** | **T** | **G** | **C** | **A** | **A** | **A** | **T** | **G** | **C** | **G** | **T** | **T** | **T** | **T** | **T** | **T** | **A** | **A** | **T** | **G** | **G** | **A** | **T** | **T** | **G** | **G** | **A** | **A** | **C** | **G** | **T** | **C** | **T** | **C** | **T** | **T** | **C** | **C** | **C** | **C** | **C** | **G** | **A** | **G** | **A** | **A** | **A** | **A** | **A** | **T** | **C** | **G** | **T** | **G** | **T** | **G** | **G** | **C** | **G** | **T** | **A** | **T** | **C** | **A** | **A** | **T** | **T** | **G** | **G** | **A** | **T** | **T** | **A** | **T** | **T** | **T** | **C** | **T** | **T** | **T** | **A** | **A** | **A** | **C** | **T** | **T** | **G** | **A** | **A** | **G** | **C** | **A** | **T** | **T** | **G** | **G** | **T** | **T** | **C** | **C** | **A** | **G** | **A** | **G** | **G** | **C** | **A** | **A** | **A** | **T** | **A** | **C** | **A** | **T** | **C** | **C** | **A** | **T** | **T** | **C** | **A** | **G** | **A** | **T** | **G** | **A** | **T** | **T** | **G** | **C** | **C** | **A** | **G** | **T** | **G** | **G** | **T** | **C** | **C** | **T** | **A** | **A** | **T** | **A** | **G** | **T** | **G** | **A** | **C** | **C** | **G** | **T** | **G** | **A** | **A** | **C** | **A** | **A** | **A** | **T** | **T** | **A** | **A** | **A** | **T** | **T** | **G** | **G** | **C** | **C** | **T** | **T** | **T** | **A** | **T** | **T** | **A** | **A** | **A** | **T** | **T** | **G** | **A** | **T** | **T** | **A** | **C** | **T** | **T** | **C** | **T** | **G** | **C** | **C** | **A** | **A** | **G** | **A** | **A** | **A** | **A** | **G** | **A** | **G** | **T** | **C** | **T** | **G** | **G** | **A** | **T** | **T** | **C** | **A** | **A** | **A** | **C** | **A** | **C** | **C** | **G** | **T** | **A** | **T** | **T** | **T** | **A** | **G** | **T** | **T** | **C** | **C** | **T** | **G** | **A** | **T** | **G** | **A** | **T** | **A** | **G** | **T** | **G** | **T** | **C** | **T** | **T** | **G** | **G** | **C** | **G** | **G** | **C** | **T** | **T** | **T** | **A** | **A** | **A** | **G** | **G** | **T** | **T** | **G** | **C** | **T** | **G** | **C** | **G** | **G** | **C** | **T** | **T** | **C** | **A** | **G** | **G** | **A** | **G** | **T** | **A** | **G** | **A** | **T** | **G** | **T** | **T** | **A** | **A** | **A** | **A** | **T** | **T** | **A** | **T** | **G** | **A** | **T** | **T** | **C** | **C** | **A** | **G** | **A** | **T** | **A** | **A** | **G** | **C** | **C** | **C** | **G** | **A** | **C** | **C** | **A** | **T** | **C** | **C** | **G** | **T** | **T** | **T** | **A** | **T** | **T** | **T** | **A** | **T** | **C** | **G** | **A** | **G** | **C** | **A** | **A** | **C** | **A** | **C** | **A** | **G** | **T** | **A** | **T** | **T** | **A** | **C** | **G** | **G** | **G** | **C** | **G** | **C** | **T** | **T** | **G** | **T** | **T** | **G** | **A** | **T** | **G** | **A** | **A** | **A** | **G** | **A** | **A** | **A** | **A** | **T** | **A** | **T** | **T** | **G** | **A** | **A** | **A** | **T** | **T** | **T** | **T** | **A** | **A** | **T** | **T** | **T** | **A** | **T** | **A** | **A** | **C** | **G** | **G** | **T** | **G** | **G** | **T** | **T** | **T** | **C** | **T** | **T** | **A** | **C** | **A** | **T** | **G** | **C** | **G** | **A** | **A** | **A** | **A** | **C** | **A** | **A** | **T** | **G** | **A** | **T** | **T** | **A** | **T** | **G** | **G** | **A** | **T** | **G** | **A** | **T** | **G** | **A** | **A** | **G** | **T** | **C** | **T** | **G** | **C** | **A** | **C** | **A** | **G** | **T** | **T** | **G** | **G** | **T** | **T** | **C** | **A** | **G** | **C** | **C** | **A** | **A** | **T** | **C** | **A** | **A** | **G** | **A** | **T** | **A** | **T** | **C** | **C** | **G** | **A** | **A** | **G** | **T** | **T** | **A** | **C** | **A** | **A** | **A** | **T** | **T** | **A** | **A** | **A** | **C** | **T** | **T** | **T** | **G** | **A** | **A** | **G** | **C** | **A** | **A** | **A** | **T** | **G** | **C** | **T** | **G** | **T** | **G** | **T** | **T** | **A** | **T** | **A** | **T** | **G** | **A** | **T** | **A** | **A** | **A** | **A** | **A** | **A** | **A** | **T** | **C** | **A** | **T** | **T** | **G** | **A** | **T** | **C** | **A** | **A** | **T** | **T** | **A** | **G** | **A** | **A** | **G** | **C** | **A** | **A** | **T** | **T** | **T** | **T** | **C** | **T** | **T** | **A** | **G** | **A** | **A** | **G** | **A** | **T** | **C** | **G** | **A** | **A** | **A** | **A** | **A** | **A** | **A** | **T** | **G** | **T** | **A** | **C** | **A** | **A** | **C** | **A** | **A** | **T** | **G** | **A** | **C** | **T** | **C** | **C** | **A** | **G** | **A** | **A** | **G** | **T** | **T** | **G** | **T** | **T** | **C** | **G** | **C** | **G** | **A** | **C** | **A** | **T** | **G** | **T** | **C** | **A** | **A** | **A** | **A** | **T** | **G** | **G** | **T** | **T** | **G** | **A** | **T** | **T** | **T** | **T** | **T** | **A** | **A** | **A** | **C** | **A** | **A** | **C** | **A** | **A** | **A** | **T** | **T** | **T** | **C** | **A** | **C** | **G** | **A** | **T** | **T** | **A** | **T** | **T** | **C** | **T** | **C** | **A** | **C** | **C** | **A** | **A** | **T** | **T** | **C** | **T** | **T** | **-** | **-** | **-** |
| **N55338** | **T** | **T** | **G** | **A** | **T** | **T** | **C** | **T** | **T** | **A** | **G** | **C** | **G** | **T** | **C** | **T** | **T** | **A** | **A** | **C** | **A** | **G** | **T** | **T** | **A** | **T** | **T** | **T** | **A** | **T** | **T** | **T** | **T** | **A** | **T** | **T** | **A** | **A** | **T** | **G** | **C** | **A** | **A** | **T** | **T** | **A** | **T** | **T** | **G** | **C** | **G** | **G** | **G** | **A** | **A** | **T** | **T** | **A** | **C** | **C** | **A** | **T** | **T** | **T** | **T** | **G** | **T** | **T** | **A** | **A** | **A** | **A** | **C** | **C** | **T** | **C** | **G** | **A** | **G** | **A** | **T** | **G** | **T** | **A** | **G** | **C** | **A** | **G** | **C** | **A** | **A** | **T** | **T** | **T** | **G** | **G** | **G** | **C** | **A** | **T** | **G** | **G** | **C** | **T** | **A** | **C** | **T** | **C** | **G** | **T** | **A** | **C** | **T** | **G** | **A** | **T** | **T** | **G** | **C** | **T** | **C** | **T** | **A** | **C** | **C** | **C** | **G** | **T** | **T** | **T** | **T** | **T** | **G** | **G** | **T** | **T** | **T** | **T** | **T** | **T** | **C** | **T** | **T** | **A** | **T** | **A** | **T** | **T** | **T** | **A** | **T** | **T** | **C** | **T** | **T** | **T** | **G** | **G** | **G** | **C** | **G** | **C** | **G** | **G** | **T** | **T** | **T** | **A** | **A** | **C** | **C** | **G** | **A** | **C** | **A** | **A** | **A** | **A** | **A** | **G** | **A** | **A** | **A** | **T** | **T** | **T** | **T** | **A** | **T** | **T** | **T** | **G** | **C** | **A** | **A** | **C** | **A** | **A** | **A** | **G** | **T** | **G** | **A** | **T** | **T** | **T** | **A** | **C** | **G** | **G** | **G** | **A** | **A** | **T** | **T** | **A** | **G** | **A** | **G** | **A** | **A** | **T** | **T** | **T** | **C** | **C** | **A** | **G** | **A** | **A** | **T** | **T** | **T** | **C** | **C** | **A** | **A** | **G** | **A** | **A** | **G** | **A** | **A** | **A** | **G** | **T** | **T** | **T** | **T** | **G** | **A** | **A** | **C** | **T** | **T** | **T** | **A** | **T** | **A** | **G** | **T** | **C** | **A** | **A** | **A** | **A** | **G** | **A** | **T** | **G** | **C** | **C** | **G** | **A** | **C** | **G** | **G** | **A** | **G** | **G** | **A** | **G** | **C** | **A** | **A** | **C** | **A** | **A** | **C** | **A** | **G** | **T** | **T** | **T** | **A** | **C** | **A** | **G** | **A** | **C** | **T** | **T** | **T** | **T** | **T** | **T** | **T** | **C** | **C** | **T** | **C** | **G** | **T** | **T** | **A** | **A** | **A** | **T** | **A** | **G** | **G** | **A** | **T** | **G** | **C** | **C** | **T** | **C** | **T** | **G** | **A** | **C** | **T** | **A** | **A** | **G** | **A** | **A** | **A** | **A** | **A** | **T** | **G** | **A** | **T** | **G** | **T** | **T** | **G** | **A** | **A** | **A** | **T** | **T** | **T** | **T** | **T** | **A** | **C** | **G** | **G** | **A** | **T** | **G** | **G** | **T** | **A** | **C** | **G** | **G** | **A** | **A** | **A** | **A** | **A** | **T** | **T** | **C** | **A** | **A** | **T** | **G** | **C** | **G** | **T** | **T** | **G** | **A** | **T** | **G** | **G** | **C** | **G** | **G** | **A** | **T** | **A** | **T** | **A** | **A** | **A** | **A** | **A** | **A** | **A** | **G** | **C** | **T** | **C** | **A** | **G** | **C** | **A** | **C** | **T** | **C** | **C** | **A** | **T** | **T** | **C** | **A** | **T** | **A** | **T** | **T** | **G** | **A** | **A** | **T** | **A** | **T** | **T** | **A** | **T** | **G** | **C** | **C** | **T** | **T** | **T** | **G** | **T** | **A** | **A** | **C** | **G** | **G** | **A** | **T** | **C** | **A** | **T** | **A** | **T** | **C** | **G** | **G** | **C** | **A** | **C** | **C** | **A** | **A** | **A** | **A** | **T** | **T** | **T** | **T** | **G** | **A** | **A** | **C** | **T** | **T** | **A** | **T** | **T** | **A** | **G** | **A** | **A** | **G** | **A** | **A** | **A** | **A** | **G** | **G** | **C** | **G** | **G** | **C** | **T** | **G** | **A** | **A** | **G** | **G** | **G** | **G** | **T** | **T** | **G** | **A** | **A** | **G** | **T** | **G** | **C** | **G** | **G** | **T** | **T** | **G** | **C** | **T** | **T** | **T** | **A** | **C** | **G** | **A** | **T** | **G** | **C** | **C** | **T** | **T** | **T** | **G** | **G** | **C** | **T** | **C** | **A** | **A** | **A** | **G** | **G** | **G** | **C** | **A** | **C** | **C** | **A** | **A** | **G** | **G** | **T** | **T** | **C** | **A** | **T** | **C** | **A** | **T** | **T** | **T** | **G** | **A** | **A** | **T** | **G** | **A** | **A** | **T** | **T** | **G** | **A** | **A** | **A** | **A** | **A** | **A** | **A** | **A** | **C** | **G** | **G** | **C** | **G** | **G** | **T** | **T** | **T** | **T** | **G** | **T** | **C** | **C** | **A** | **A** | **A** | **C** | **G** | **T** | **T** | **T** | **A** | **T** | **T** | **A** | **C** | **T** | **T** | **C** | **T** | **C** | **A** | **A** | **A** | **A** | **A** | **G** | **C** | **A** | **C** | **T** | **T** | **T** | **T** | **G** | **A** | **A** | **G** | **T** | **T** | **T** | **C** | **G** | **T** | **T** | **T** | **G** | **A** | **A** | **T** | **T** | **A** | **T** | **C** | **A** | **T** | **G** | **A** | **T** | **C** | **A** | **C** | **C** | **G** | **G** | **A** | **A** | **A** | **A** | **T** | **T** | **G** | **T** | **T** | **G** | **T** | **T** | **A** | **T** | **T** | **G** | **A** | **C** | **G** | **G** | **A** | **A** | **A** | **A** | **G** | **T** | **A** | **G** | **G** | **C** | **T** | **A** | **C** | **A** | **T** | **T** | **G** | **G** | **C** | **G** | **G** | **C** | **T** | **T** | **T** | **A** | **A** | **T** | **G** | **T** | **T** | **G** | **C** | **C** | **G** | **A** | **T** | **C** | **A** | **A** | **T** | **A** | **T** | **G** | **C** | **T** | **G** | **G** | **A** | **A** | **C** | **G** | **A** | **C** | **T** | **A** | **A** | **A** | **A** | **A** | **G** | **T** | **T** | **T** | **G** | **G** | **C** | **T** | **A** | **T** | **T** | **G** | **G** | **C** | **G** | **G** | **G** | **A** | **T** | **A** | **C** | **G** | **C** | **A** | **T** | **T** | **T** | **A** | **C** | **G** | **G** | **A** | **T** | **T** | **C** | **A** | **A** | **G** | **G** | **G** | **C** | **C** | **A** | **G** | **C** | **A** | **G** | **C** | **C** | **T** | **C** | **A** | **T** | **T** | **A** | **C** | **T** | **G** | **C** | **A** | **A** | **A** | **T** | **G** | **C** | **G** | **T** | **T** | **T** | **T** | **T** | **T** | **A** | **A** | **T** | **G** | **G** | **A** | **T** | **T** | **G** | **G** | **A** | **A** | **C** | **G** | **T** | **C** | **T** | **C** | **T** | **T** | **C** | **C** | **C** | **C** | **C** | **G** | **A** | **G** | **A** | **A** | **A** | **A** | **A** | **T** | **C** | **G** | **T** | **G** | **T** | **G** | **G** | **C** | **G** | **T** | **A** | **T** | **C** | **A** | **A** | **T** | **T** | **G** | **G** | **A** | **T** | **T** | **A** | **T** | **T** | **T** | **C** | **T** | **T** | **T** | **A** | **A** | **A** | **C** | **T** | **T** | **G** | **A** | **A** | **G** | **C** | **A** | **T** | **T** | **G** | **G** | **T** | **G** | **C** | **C** | **A** | **G** | **A** | **G** | **G** | **C** | **A** | **A** | **A** | **T** | **A** | **C** | **A** | **T** | **C** | **C** | **A** | **T** | **T** | **C** | **A** | **G** | **A** | **T** | **G** | **A** | **T** | **T** | **G** | **C** | **C** | **A** | **G** | **T** | **G** | **G** | **T** | **C** | **C** | **T** | **A** | **A** | **C** | **A** | **G** | **T** | **G** | **A** | **C** | **C** | **G** | **T** | **G** | **A** | **A** | **C** | **A** | **A** | **A** | **T** | **T** | **A** | **A** | **A** | **T** | **T** | **G** | **G** | **C** | **C** | **T** | **T** | **T** | **A** | **T** | **T** | **A** | **A** | **A** | **T** | **T** | **G** | **A** | **T** | **T** | **A** | **C** | **T** | **T** | **C** | **T** | **G** | **C** | **C** | **A** | **A** | **G** | **A** | **A** | **A** | **A** | **G** | **A** | **G** | **T** | **C** | **T** | **G** | **G** | **A** | **T** | **T** | **C** | **A** | **A** | **A** | **C** | **G** | **C** | **C** | **G** | **T** | **A** | **T** | **T** | **T** | **A** | **G** | **T** | **T** | **C** | **C** | **T** | **G** | **A** | **T** | **G** | **A** | **T** | **A** | **G** | **T** | **G** | **T** | **C** | **T** | **T** | **G** | **G** | **C** | **T** | **G** | **C** | **T** | **T** | **T** | **A** | **A** | **A** | **G** | **G** | **T** | **T** | **G** | **C** | **T** | **G** | **C** | **G** | **G** | **C** | **T** | **T** | **C** | **A** | **G** | **G** | **A** | **G** | **T** | **A** | **G** | **A** | **T** | **G** | **T** | **T** | **A** | **A** | **A** | **A** | **T** | **T** | **A** | **T** | **G** | **A** | **T** | **T** | **C** | **C** | **A** | **G** | **A** | **T** | **A** | **A** | **G** | **C** | **C** | **C** | **G** | **A** | **C** | **C** | **A** | **T** | **C** | **C** | **G** | **T** | **T** | **T** | **A** | **T** | **T** | **T** | **A** | **T** | **C** | **G** | **A** | **G** | **C** | **A** | **A** | **C** | **A** | **C** | **A** | **G** | **T** | **A** | **T** | **T** | **A** | **C** | **G** | **G** | **C** | **C** | **G** | **C** | **T** | **T** | **A** | **T** | **T** | **G** | **A** | **T** | **G** | **A** | **A** | **A** | **G** | **A** | **A** | **A** | **A** | **T** | **A** | **T** | **T** | **G** | **A** | **A** | **A** | **T** | **T** | **T** | **T** | **A** | **A** | **T** | **T** | **T** | **A** | **T** | **A** | **A** | **C** | **G** | **G** | **T** | **G** | **G** | **T** | **T** | **T** | **C** | **T** | **T** | **A** | **C** | **A** | **T** | **G** | **C** | **G** | **A** | **A** | **A** | **A** | **C** | **A** | **A** | **T** | **G** | **A** | **T** | **T** | **A** | **T** | **G** | **G** | **A** | **T** | **G** | **A** | **T** | **G** | **A** | **A** | **G** | **T** | **C** | **T** | **G** | **T** | **A** | **C** | **A** | **G** | **T** | **T** | **G** | **G** | **T** | **T** | **C** | **A** | **G** | **C** | **C** | **A** | **A** | **T** | **C** | **A** | **A** | **G** | **A** | **T** | **A** | **T** | **C** | **C** | **G** | **A** | **A** | **G** | **T** | **T** | **A** | **C** | **A** | **A** | **A** | **T** | **T** | **A** | **A** | **A** | **C** | **T** | **T** | **T** | **G** | **A** | **A** | **G** | **C** | **A** | **A** | **A** | **T** | **G** | **C** | **T** | **G** | **T** | **G** | **T** | **T** | **A** | **T** | **A** | **T** | **G** | **A** | **T** | **A** | **A** | **A** | **A** | **A** | **A** | **A** | **T** | **C** | **A** | **T** | **T** | **G** | **A** | **T** | **C** | **A** | **A** | **T** | **T** | **A** | **G** | **A** | **A** | **G** | **C** | **A** | **A** | **T** | **T** | **T** | **T** | **C** | **T** | **T** | **A** | **G** | **A** | **A** | **G** | **A** | **T** | **C** | **G** | **A** | **A** | **A** | **A** | **A** | **A** | **A** | **T** | **G** | **T** | **A** | **C** | **A** | **A** | **C** | **A** | **A** | **T** | **G** | **A** | **C** | **T** | **C** | **C** | **A** | **G** | **A** | **A** | **G** | **T** | **T** | **G** | **T** | **T** | **C** | **G** | **C** | **G** | **A** | **C** | **A** | **T** | **G** | **T** | **C** | **A** | **A** | **A** | **A** | **T** | **G** | **G** | **T** | **T** | **G** | **A** | **T** | **T** | **T** | **T** | **T** | **A** | **A** | **A** | **C** | **A** | **A** | **C** | **A** | **A** | **A** | **T** | **T** | **T** | **C** | **A** | **C** | **G** | **A** | **T** | **T** | **A** | **T** | **T** | **T** | **T** | **C** | **A** | **C** | **C** | **A** | **A** | **T** | **T** | **C** | **T** | **T** | **-** | **-** | **-** |
| **N52587** | **T** | **T** | **G** | **A** | **T** | **T** | **C** | **T** | **T** | **A** | **G** | **T** | **G** | **T** | **C** | **T** | **T** | **A** | **A** | **C** | **A** | **G** | **T** | **T** | **A** | **T** | **T** | **T** | **A** | **T** | **T** | **T** | **T** | **A** | **T** | **T** | **A** | **A** | **T** | **G** | **C** | **A** | **A** | **T** | **T** | **A** | **T** | **T** | **G** | **C** | **G** | **G** | **G** | **A** | **A** | **T** | **T** | **A** | **C** | **C** | **A** | **T** | **C** | **T** | **T** | **G** | **T** | **T** | **A** | **A** | **A** | **A** | **C** | **C** | **T** | **C** | **G** | **A** | **G** | **A** | **T** | **G** | **T** | **A** | **G** | **C** | **G** | **G** | **C** | **A** | **A** | **T** | **T** | **T** | **G** | **G** | **G** | **C** | **A** | **T** | **G** | **G** | **T** | **T** | **A** | **C** | **T** | **C** | **G** | **T** | **A** | **C** | **T** | **G** | **A** | **T** | **T** | **G** | **C** | **T** | **C** | **T** | **A** | **C** | **C** | **C** | **G** | **T** | **T** | **T** | **T** | **T** | **G** | **G** | **T** | **T** | **T** | **T** | **T** | **T** | **C** | **T** | **T** | **A** | **T** | **A** | **T** | **T** | **T** | **A** | **T** | **T** | **C** | **T** | **T** | **T** | **G** | **G** | **T** | **C** | **G** | **A** | **G** | **G** | **C** | **T** | **T** | **G** | **A** | **C** | **C** | **G** | **A** | **C** | **A** | **A** | **A** | **A** | **A** | **G** | **A** | **A** | **A** | **T** | **T** | **T** | **T** | **A** | **T** | **T** | **T** | **G** | **C** | **A** | **A** | **C** | **A** | **A** | **A** | **G** | **T** | **G** | **A** | **T** | **T** | **T** | **A** | **C** | **G** | **G** | **G** | **A** | **A** | **T** | **T** | **A** | **G** | **A** | **G** | **A** | **A** | **T** | **T** | **T** | **T** | **C** | **A** | **G** | **A** | **A** | **T** | **T** | **T** | **C** | **C** | **A** | **A** | **G** | **A** | **A** | **G** | **A** | **A** | **A** | **G** | **T** | **T** | **T** | **T** | **G** | **A** | **A** | **C** | **T** | **T** | **T** | **A** | **T** | **A** | **G** | **T** | **C** | **A** | **A** | **A** | **A** | **G** | **A** | **T** | **G** | **C** | **C** | **G** | **A** | **C** | **G** | **G** | **A** | **G** | **G** | **A** | **G** | **C** | **A** | **A** | **C** | **A** | **G** | **C** | **A** | **G** | **T** | **T** | **T** | **A** | **C** | **A** | **G** | **A** | **C** | **T** | **T** | **T** | **T** | **T** | **C** | **T** | **C** | **C** | **T** | **C** | **G** | **T** | **T** | **A** | **A** | **A** | **T** | **A** | **G** | **G** | **A** | **T** | **G** | **C** | **C** | **T** | **C** | **T** | **G** | **A** | **C** | **T** | **A** | **A** | **G** | **A** | **A** | **A** | **A** | **A** | **T** | **G** | **A** | **C** | **G** | **T** | **T** | **G** | **A** | **G** | **A** | **T** | **T** | **T** | **T** | **T** | **A** | **C** | **G** | **G** | **A** | **T** | **G** | **G** | **T** | **A** | **T** | **G** | **G** | **A** | **A** | **A** | **A** | **A** | **T** | **T** | **C** | **A** | **A** | **T** | **G** | **C** | **G** | **T** | **T** | **G** | **A** | **T** | **G** | **G** | **C** | **G** | **G** | **A** | **C** | **A** | **T** | **A** | **A** | **A** | **A** | **A** | **A** | **A** | **G** | **C** | **T** | **C** | **A** | **G** | **C** | **A** | **T** | **T** | **C** | **C** | **A** | **T** | **T** | **C** | **A** | **T** | **A** | **T** | **T** | **G** | **A** | **A** | **T** | **A** | **T** | **T** | **A** | **T** | **G** | **C** | **C** | **T** | **T** | **T** | **G** | **T** | **A** | **A** | **C** | **G** | **G** | **A** | **T** | **C** | **A** | **T** | **A** | **T** | **C** | **G** | **G** | **C** | **A** | **C** | **C** | **A** | **A** | **A** | **A** | **T** | **T** | **T** | **T** | **G** | **A** | **A** | **C** | **T** | **T** | **A** | **T** | **T** | **A** | **G** | **A** | **A** | **G** | **A** | **A** | **A** | **A** | **G** | **G** | **C** | **G** | **G** | **C** | **T** | **G** | **A** | **A** | **G** | **G** | **G** | **G** | **T** | **T** | **G** | **A** | **A** | **G** | **T** | **G** | **C** | **G** | **G** | **T** | **T** | **G** | **C** | **T** | **T** | **T** | **A** | **C** | **G** | **A** | **T** | **G** | **C** | **C** | **T** | **T** | **T** | **G** | **G** | **C** | **T** | **C** | **A** | **A** | **A** | **G** | **G** | **G** | **C** | **A** | **C** | **C** | **A** | **A** | **G** | **G** | **T** | **T** | **C** | **A** | **T** | **C** | **A** | **T** | **T** | **T** | **G** | **A** | **A** | **T** | **G** | **A** | **A** | **T** | **T** | **G** | **A** | **A** | **A** | **A** | **A** | **A** | **A** | **A** | **C** | **G** | **G** | **C** | **G** | **G** | **T** | **T** | **T** | **T** | **G** | **T** | **C** | **C** | **A** | **A** | **A** | **C** | **G** | **T** | **T** | **T** | **A** | **T** | **T** | **A** | **C** | **T** | **T** | **C** | **T** | **C** | **A** | **A** | **A** | **A** | **A** | **G** | **C** | **A** | **C** | **T** | **C** | **T** | **T** | **G** | **A** | **A** | **G** | **T** | **T** | **T** | **C** | **G** | **T** | **T** | **T** | **G** | **A** | **A** | **T** | **T** | **A** | **T** | **C** | **A** | **T** | **G** | **A** | **T** | **C** | **A** | **C** | **C** | **G** | **G** | **A** | **A** | **A** | **A** | **T** | **T** | **G** | **T** | **C** | **G** | **T** | **T** | **A** | **T** | **T** | **G** | **A** | **C** | **G** | **G** | **A** | **A** | **A** | **A** | **G** | **T** | **A** | **G** | **G** | **C** | **T** | **A** | **C** | **A** | **T** | **T** | **G** | **G** | **T** | **G** | **G** | **T** | **T** | **T** | **T** | **A** | **A** | **T** | **G** | **T** | **T** | **G** | **C** | **C** | **G** | **A** | **T** | **C** | **A** | **A** | **T** | **A** | **T** | **G** | **C** | **T** | **G** | **G** | **A** | **A** | **C** | **G** | **A** | **C** | **T** | **A** | **A** | **A** | **A** | **A** | **G** | **T** | **T** | **T** | **G** | **G** | **C** | **T** | **A** | **T** | **T** | **G** | **G** | **C** | **G** | **G** | **G** | **A** | **T** | **A** | **C** | **G** | **C** | **A** | **T** | **T** | **T** | **A** | **C** | **G** | **G** | **A** | **T** | **T** | **C** | **A** | **A** | **G** | **G** | **G** | **C** | **C** | **A** | **G** | **C** | **A** | **G** | **C** | **C** | **T** | **C** | **A** | **T** | **T** | **A** | **C** | **T** | **G** | **C** | **A** | **A** | **A** | **T** | **G** | **C** | **G** | **T** | **T** | **T** | **T** | **T** | **T** | **A** | **A** | **T** | **G** | **G** | **A** | **T** | **T** | **G** | **G** | **A** | **A** | **C** | **G** | **T** | **C** | **T** | **C** | **T** | **T** | **C** | **C** | **C** | **C** | **T** | **G** | **A** | **G** | **A** | **A** | **A** | **A** | **A** | **T** | **C** | **G** | **T** | **G** | **T** | **G** | **G** | **C** | **G** | **T** | **A** | **T** | **C** | **A** | **A** | **T** | **T** | **G** | **G** | **A** | **T** | **T** | **A** | **T** | **T** | **T** | **C** | **T** | **T** | **T** | **A** | **A** | **A** | **C** | **T** | **T** | **G** | **A** | **A** | **G** | **C** | **G** | **T** | **T** | **G** | **G** | **T** | **G** | **C** | **C** | **A** | **G** | **A** | **G** | **G** | **C** | **A** | **A** | **A** | **T** | **A** | **C** | **A** | **T** | **C** | **C** | **A** | **T** | **T** | **C** | **A** | **G** | **A** | **T** | **G** | **A** | **T** | **T** | **G** | **C** | **A** | **A** | **G** | **T** | **G** | **G** | **A** | **C** | **C** | **T** | **A** | **A** | **C** | **A** | **G** | **T** | **G** | **A** | **C** | **C** | **G** | **T** | **G** | **A** | **A** | **C** | **A** | **A** | **A** | **T** | **T** | **A** | **A** | **A** | **C** | **T** | **G** | **G** | **C** | **C** | **T** | **T** | **T** | **A** | **T** | **T** | **A** | **A** | **A** | **T** | **T** | **G** | **A** | **T** | **T** | **A** | **C** | **T** | **T** | **C** | **T** | **G** | **C** | **C** | **A** | **A** | **G** | **A** | **A** | **A** | **A** | **G** | **A** | **G** | **T** | **C** | **T** | **G** | **G** | **A** | **T** | **T** | **C** | **A** | **A** | **A** | **C** | **G** | **C** | **C** | **G** | **T** | **A** | **T** | **T** | **T** | **A** | **G** | **T** | **T** | **C** | **C** | **T** | **G** | **A** | **T** | **G** | **A** | **T** | **A** | **G** | **T** | **G** | **T** | **C** | **T** | **T** | **G** | **G** | **C** | **T** | **G** | **C** | **T** | **T** | **T** | **A** | **A** | **A** | **G** | **G** | **T** | **T** | **G** | **C** | **T** | **G** | **C** | **G** | **G** | **C** | **T** | **T** | **C** | **A** | **G** | **G** | **A** | **G** | **T** | **A** | **G** | **A** | **T** | **G** | **T** | **T** | **A** | **A** | **A** | **A** | **T** | **T** | **A** | **T** | **G** | **A** | **T** | **T** | **C** | **C** | **A** | **G** | **A** | **T** | **A** | **A** | **G** | **C** | **C** | **C** | **G** | **A** | **C** | **C** | **A** | **T** | **C** | **C** | **G** | **T** | **T** | **T** | **A** | **T** | **T** | **T** | **A** | **T** | **C** | **G** | **A** | **G** | **C** | **A** | **A** | **C** | **A** | **C** | **A** | **G** | **T** | **A** | **T** | **T** | **A** | **C** | **G** | **G** | **C** | **C** | **G** | **C** | **T** | **T** | **G** | **T** | **T** | **A** | **A** | **T** | **G** | **A** | **A** | **A** | **G** | **A** | **A** | **A** | **A** | **T** | **A** | **T** | **T** | **G** | **A** | **A** | **A** | **T** | **T** | **T** | **T** | **A** | **A** | **T** | **T** | **T** | **A** | **T** | **A** | **A** | **C** | **G** | **G** | **T** | **G** | **G** | **T** | **T** | **T** | **C** | **T** | **T** | **A** | **C** | **A** | **T** | **G** | **C** | **G** | **A** | **A** | **A** | **A** | **C** | **A** | **A** | **T** | **G** | **A** | **T** | **T** | **A** | **T** | **G** | **G** | **A** | **T** | **G** | **A** | **T** | **G** | **A** | **A** | **G** | **T** | **C** | **T** | **G** | **C** | **A** | **C** | **A** | **G** | **T** | **T** | **G** | **G** | **T** | **T** | **C** | **A** | **G** | **C** | **T** | **A** | **A** | **T** | **C** | **A** | **A** | **G** | **A** | **T** | **A** | **T** | **C** | **C** | **G** | **A** | **A** | **G** | **T** | **T** | **A** | **C** | **A** | **A** | **A** | **T** | **T** | **A** | **A** | **A** | **C** | **T** | **T** | **T** | **G** | **A** | **A** | **G** | **C** | **A** | **A** | **A** | **T** | **G** | **C** | **T** | **G** | **T** | **G** | **T** | **T** | **A** | **T** | **A** | **T** | **G** | **A** | **T** | **A** | **A** | **A** | **A** | **A** | **A** | **A** | **T** | **C** | **A** | **T** | **T** | **G** | **A** | **T** | **C** | **A** | **A** | **T** | **T** | **A** | **G** | **A** | **A** | **G** | **C** | **A** | **A** | **T** | **T** | **T** | **T** | **C** | **T** | **T** | **A** | **G** | **A** | **A** | **G** | **A** | **T** | **C** | **G** | **A** | **A** | **A** | **A** | **A** | **A** | **A** | **T** | **G** | **T** | **A** | **C** | **A** | **A** | **C** | **A** | **A** | **T** | **G** | **A** | **C** | **T** | **C** | **C** | **A** | **G** | **A** | **A** | **G** | **T** | **T** | **G** | **T** | **T** | **C** | **G** | **C** | **G** | **A** | **C** | **A** | **T** | **G** | **T** | **C** | **A** | **A** | **A** | **A** | **T** | **G** | **G** | **T** | **T** | **G** | **A** | **T** | **T** | **T** | **T** | **T** | **A** | **A** | **A** | **C** | **A** | **A** | **C** | **A** | **A** | **A** | **T** | **T** | **T** | **C** | **A** | **C** | **G** | **A** | **T** | **T** | **A** | **T** | **T** | **T** | **T** | **C** | **A** | **C** | **C** | **A** | **A** | **T** | **T** | **C** | **T** | **T** | **-** | **-** | **-** |
| **N60271F** | **T** | **T** | **G** | **A** | **T** | **T** | **C** | **T** | **T** | **A** | **G** | **C** | **G** | **T** | **C** | **T** | **T** | **A** | **A** | **C** | **A** | **G** | **T** | **T** | **A** | **T** | **T** | **T** | **A** | **T** | **T** | **T** | **T** | **A** | **T** | **T** | **A** | **A** | **T** | **G** | **C** | **A** | **A** | **T** | **T** | **A** | **T** | **T** | **G** | **C** | **G** | **G** | **G** | **A** | **A** | **T** | **T** | **A** | **C** | **C** | **A** | **T** | **T** | **T** | **T** | **G** | **T** | **T** | **A** | **A** | **A** | **A** | **C** | **C** | **T** | **C** | **G** | **A** | **G** | **A** | **T** | **G** | **T** | **A** | **G** | **C** | **A** | **G** | **C** | **A** | **A** | **T** | **T** | **T** | **G** | **G** | **G** | **C** | **A** | **T** | **G** | **G** | **C** | **T** | **A** | **C** | **T** | **C** | **G** | **T** | **A** | **C** | **T** | **G** | **A** | **T** | **T** | **G** | **C** | **T** | **C** | **T** | **A** | **C** | **C** | **C** | **G** | **T** | **T** | **T** | **T** | **T** | **G** | **G** | **T** | **T** | **T** | **T** | **T** | **T** | **C** | **T** | **T** | **A** | **T** | **A** | **T** | **T** | **T** | **A** | **T** | **T** | **C** | **T** | **T** | **T** | **G** | **G** | **T** | **C** | **G** | **C** | **G** | **G** | **T** | **T** | **T** | **A** | **A** | **C** | **C** | **G** | **A** | **C** | **A** | **A** | **A** | **A** | **A** | **G** | **A** | **A** | **A** | **T** | **T** | **T** | **T** | **A** | **T** | **T** | **T** | **G** | **C** | **A** | **A** | **C** | **A** | **A** | **A** | **G** | **T** | **G** | **A** | **T** | **T** | **T** | **G** | **C** | **G** | **G** | **G** | **A** | **A** | **T** | **T** | **A** | **G** | **A** | **G** | **A** | **A** | **T** | **T** | **T** | **T** | **C** | **A** | **G** | **A** | **A** | **T** | **T** | **T** | **C** | **C** | **A** | **A** | **G** | **A** | **A** | **G** | **A** | **A** | **A** | **G** | **T** | **T** | **T** | **T** | **G** | **A** | **A** | **C** | **T** | **T** | **T** | **A** | **T** | **A** | **G** | **T** | **C** | **A** | **A** | **A** | **A** | **G** | **A** | **T** | **G** | **C** | **C** | **G** | **A** | **C** | **G** | **G** | **A** | **G** | **G** | **A** | **G** | **C** | **A** | **A** | **C** | **A** | **A** | **C** | **A** | **G** | **T** | **T** | **T** | **A** | **C** | **A** | **G** | **A** | **C** | **T** | **T** | **T** | **T** | **T** | **T** | **T** | **C** | **C** | **T** | **C** | **G** | **T** | **T** | **A** | **A** | **A** | **T** | **A** | **G** | **G** | **A** | **T** | **G** | **C** | **C** | **T** | **C** | **T** | **G** | **A** | **C** | **T** | **A** | **A** | **G** | **A** | **A** | **A** | **A** | **A** | **T** | **G** | **A** | **T** | **G** | **T** | **T** | **G** | **A** | **A** | **A** | **T** | **T** | **T** | **T** | **T** | **A** | **C** | **G** | **G** | **A** | **T** | **G** | **G** | **T** | **A** | **C** | **G** | **G** | **A** | **A** | **A** | **A** | **A** | **T** | **T** | **C** | **A** | **A** | **T** | **G** | **C** | **G** | **T** | **T** | **G** | **A** | **T** | **G** | **G** | **C** | **G** | **G** | **A** | **C** | **A** | **T** | **A** | **A** | **A** | **A** | **A** | **A** | **A** | **G** | **C** | **T** | **C** | **A** | **G** | **C** | **A** | **C** | **T** | **C** | **C** | **A** | **T** | **T** | **C** | **A** | **T** | **A** | **T** | **T** | **G** | **A** | **A** | **T** | **A** | **T** | **T** | **A** | **T** | **G** | **C** | **C** | **T** | **T** | **T** | **G** | **T** | **A** | **A** | **C** | **G** | **G** | **A** | **T** | **C** | **A** | **T** | **A** | **T** | **C** | **G** | **G** | **C** | **A** | **C** | **C** | **A** | **A** | **A** | **A** | **T** | **T** | **T** | **T** | **G** | **A** | **A** | **C** | **T** | **T** | **A** | **T** | **T** | **A** | **G** | **A** | **A** | **G** | **A** | **A** | **A** | **A** | **G** | **G** | **C** | **G** | **G** | **C** | **T** | **G** | **A** | **A** | **G** | **G** | **G** | **G** | **T** | **T** | **G** | **A** | **A** | **G** | **T** | **G** | **C** | **G** | **G** | **T** | **T** | **G** | **C** | **T** | **T** | **T** | **A** | **C** | **G** | **A** | **T** | **G** | **C** | **C** | **T** | **T** | **T** | **G** | **G** | **C** | **T** | **C** | **A** | **A** | **A** | **G** | **G** | **G** | **C** | **A** | **C** | **C** | **A** | **A** | **G** | **G** | **T** | **T** | **C** | **A** | **T** | **C** | **A** | **T** | **T** | **T** | **G** | **A** | **A** | **T** | **G** | **A** | **A** | **T** | **T** | **G** | **A** | **A** | **A** | **A** | **A** | **A** | **A** | **A** | **C** | **G** | **G** | **T** | **G** | **G** | **T** | **T** | **T** | **T** | **G** | **T** | **C** | **C** | **A** | **A** | **A** | **C** | **G** | **T** | **T** | **T** | **A** | **T** | **T** | **A** | **C** | **T** | **T** | **C** | **T** | **C** | **A** | **A** | **A** | **A** | **A** | **G** | **C** | **A** | **C** | **T** | **T** | **T** | **T** | **G** | **A** | **A** | **G** | **T** | **T** | **T** | **C** | **G** | **T** | **T** | **T** | **G** | **A** | **A** | **T** | **T** | **A** | **T** | **C** | **A** | **T** | **G** | **A** | **T** | **C** | **A** | **C** | **C** | **G** | **G** | **A** | **A** | **A** | **A** | **T** | **T** | **G** | **T** | **T** | **G** | **T** | **T** | **A** | **T** | **T** | **G** | **A** | **C** | **G** | **G** | **A** | **A** | **A** | **A** | **G** | **T** | **A** | **G** | **G** | **C** | **T** | **A** | **C** | **A** | **T** | **T** | **G** | **G** | **C** | **G** | **G** | **C** | **T** | **T** | **T** | **A** | **A** | **T** | **G** | **T** | **T** | **G** | **C** | **C** | **G** | **A** | **T** | **C** | **A** | **A** | **T** | **A** | **T** | **G** | **C** | **T** | **G** | **G** | **A** | **A** | **C** | **G** | **A** | **C** | **T** | **A** | **A** | **A** | **A** | **A** | **G** | **T** | **T** | **T** | **G** | **G** | **C** | **T** | **A** | **T** | **T** | **G** | **G** | **C** | **G** | **G** | **G** | **A** | **T** | **A** | **C** | **G** | **C** | **A** | **T** | **T** | **T** | **A** | **C** | **G** | **G** | **A** | **T** | **T** | **C** | **A** | **A** | **G** | **G** | **G** | **C** | **C** | **A** | **G** | **C** | **A** | **G** | **C** | **C** | **T** | **C** | **A** | **T** | **T** | **A** | **C** | **T** | **G** | **C** | **A** | **A** | **A** | **T** | **G** | **C** | **G** | **T** | **T** | **T** | **T** | **T** | **T** | **A** | **A** | **T** | **G** | **G** | **A** | **T** | **T** | **G** | **G** | **A** | **A** | **C** | **G** | **T** | **C** | **T** | **C** | **T** | **T** | **C** | **C** | **C** | **C** | **C** | **G** | **A** | **G** | **A** | **A** | **A** | **A** | **A** | **T** | **C** | **G** | **T** | **G** | **T** | **G** | **G** | **C** | **G** | **T** | **A** | **T** | **C** | **A** | **A** | **T** | **T** | **G** | **G** | **A** | **T** | **T** | **A** | **T** | **T** | **T** | **C** | **T** | **T** | **T** | **A** | **A** | **A** | **C** | **T** | **T** | **G** | **A** | **A** | **G** | **C** | **A** | **T** | **T** | **G** | **G** | **T** | **G** | **C** | **C** | **A** | **G** | **A** | **G** | **G** | **C** | **A** | **A** | **A** | **T** | **A** | **C** | **A** | **T** | **C** | **C** | **A** | **T** | **T** | **C** | **A** | **G** | **A** | **T** | **G** | **A** | **T** | **T** | **G** | **C** | **C** | **A** | **G** | **T** | **G** | **G** | **T** | **C** | **C** | **T** | **A** | **A** | **C** | **A** | **G** | **T** | **G** | **A** | **C** | **C** | **G** | **T** | **G** | **A** | **A** | **C** | **A** | **A** | **A** | **T** | **T** | **A** | **A** | **A** | **T** | **T** | **G** | **G** | **C** | **C** | **T** | **T** | **T** | **A** | **T** | **T** | **A** | **A** | **A** | **T** | **T** | **G** | **A** | **T** | **T** | **A** | **C** | **T** | **T** | **C** | **T** | **G** | **C** | **C** | **A** | **A** | **G** | **A** | **A** | **A** | **A** | **G** | **A** | **G** | **T** | **C** | **T** | **G** | **G** | **A** | **T** | **T** | **C** | **A** | **A** | **A** | **C** | **G** | **C** | **C** | **G** | **T** | **A** | **T** | **T** | **T** | **A** | **G** | **T** | **T** | **C** | **C** | **T** | **G** | **A** | **T** | **G** | **A** | **T** | **A** | **G** | **T** | **G** | **T** | **C** | **T** | **T** | **G** | **G** | **C** | **T** | **G** | **C** | **T** | **T** | **T** | **A** | **A** | **A** | **G** | **G** | **T** | **T** | **G** | **C** | **T** | **G** | **C** | **G** | **G** | **C** | **T** | **T** | **C** | **A** | **G** | **G** | **A** | **G** | **T** | **A** | **G** | **A** | **T** | **G** | **T** | **T** | **A** | **A** | **A** | **A** | **T** | **T** | **A** | **T** | **G** | **A** | **T** | **T** | **C** | **C** | **A** | **G** | **A** | **T** | **A** | **A** | **G** | **C** | **C** | **C** | **G** | **A** | **C** | **C** | **A** | **T** | **C** | **C** | **G** | **T** | **T** | **T** | **A** | **T** | **T** | **T** | **A** | **T** | **C** | **G** | **A** | **G** | **C** | **A** | **A** | **C** | **A** | **C** | **A** | **G** | **T** | **A** | **T** | **T** | **A** | **C** | **G** | **G** | **C** | **C** | **G** | **C** | **T** | **T** | **A** | **T** | **T** | **G** | **A** | **T** | **G** | **A** | **A** | **A** | **G** | **A** | **A** | **A** | **A** | **T** | **A** | **T** | **T** | **G** | **A** | **A** | **A** | **T** | **T** | **T** | **T** | **A** | **A** | **T** | **T** | **T** | **A** | **T** | **A** | **A** | **C** | **G** | **G** | **T** | **G** | **G** | **T** | **T** | **T** | **C** | **T** | **T** | **A** | **C** | **A** | **T** | **G** | **C** | **G** | **A** | **A** | **A** | **A** | **C** | **A** | **A** | **T** | **G** | **A** | **T** | **T** | **A** | **T** | **G** | **G** | **A** | **T** | **G** | **A** | **T** | **G** | **A** | **A** | **G** | **T** | **C** | **T** | **G** | **C** | **A** | **C** | **A** | **G** | **T** | **T** | **G** | **G** | **T** | **T** | **C** | **A** | **G** | **C** | **C** | **A** | **A** | **C** | **C** | **A** | **A** | **G** | **A** | **T** | **A** | **T** | **C** | **C** | **G** | **A** | **A** | **G** | **T** | **T** | **A** | **C** | **A** | **A** | **A** | **T** | **T** | **A** | **A** | **A** | **C** | **T** | **T** | **T** | **G** | **A** | **A** | **G** | **C** | **A** | **A** | **A** | **T** | **G** | **C** | **T** | **G** | **T** | **G** | **T** | **T** | **A** | **T** | **A** | **T** | **G** | **A** | **T** | **A** | **A** | **A** | **A** | **A** | **A** | **A** | **T** | **C** | **A** | **T** | **T** | **G** | **A** | **T** | **C** | **A** | **A** | **T** | **T** | **A** | **G** | **A** | **A** | **G** | **C** | **A** | **A** | **T** | **T** | **T** | **T** | **C** | **T** | **T** | **A** | **G** | **A** | **A** | **G** | **A** | **T** | **C** | **G** | **A** | **A** | **A** | **A** | **A** | **A** | **A** | **T** | **G** | **T** | **A** | **C** | **A** | **A** | **C** | **A** | **A** | **T** | **G** | **A** | **C** | **T** | **C** | **C** | **A** | **G** | **A** | **A** | **G** | **T** | **T** | **G** | **T** | **T** | **C** | **G** | **C** | **G** | **A** | **C** | **A** | **T** | **G** | **T** | **C** | **A** | **A** | **A** | **A** | **T** | **G** | **G** | **T** | **T** | **G** | **A** | **T** | **T** | **T** | **T** | **T** | **A** | **A** | **A** | **C** | **A** | **A** | **C** | **A** | **A** | **A** | **T** | **T** | **T** | **C** | **A** | **C** | **G** | **A** | **T** | **T** | **A** | **T** | **T** | **T** | **T** | **C** | **A** | **C** | **C** | **A** | **A** | **T** | **T** | **C** | **T** | **T** | **-** | **-** | **-** |
| **N59841F** | **T** | **T** | **G** | **A** | **T** | **T** | **C** | **T** | **T** | **A** | **G** | **C** | **G** | **T** | **C** | **T** | **T** | **A** | **A** | **C** | **A** | **G** | **T** | **T** | **A** | **T** | **T** | **T** | **A** | **T** | **T** | **T** | **T** | **A** | **T** | **T** | **A** | **A** | **T** | **G** | **C** | **A** | **A** | **T** | **T** | **A** | **T** | **T** | **G** | **C** | **G** | **G** | **G** | **A** | **A** | **T** | **T** | **A** | **C** | **C** | **A** | **T** | **T** | **T** | **T** | **G** | **T** | **T** | **A** | **A** | **A** | **A** | **C** | **C** | **T** | **C** | **G** | **A** | **G** | **A** | **T** | **G** | **T** | **A** | **G** | **C** | **A** | **G** | **C** | **A** | **A** | **T** | **T** | **T** | **G** | **G** | **G** | **C** | **A** | **T** | **G** | **G** | **C** | **T** | **A** | **C** | **T** | **C** | **G** | **T** | **A** | **C** | **T** | **G** | **A** | **T** | **T** | **G** | **C** | **T** | **C** | **T** | **A** | **C** | **C** | **C** | **G** | **T** | **T** | **T** | **T** | **T** | **G** | **G** | **T** | **T** | **T** | **T** | **T** | **T** | **C** | **T** | **T** | **A** | **T** | **A** | **T** | **T** | **T** | **A** | **T** | **T** | **C** | **T** | **T** | **T** | **G** | **G** | **G** | **C** | **G** | **C** | **G** | **G** | **T** | **T** | **T** | **A** | **A** | **C** | **C** | **G** | **A** | **C** | **A** | **A** | **A** | **A** | **A** | **G** | **A** | **A** | **A** | **T** | **T** | **T** | **T** | **A** | **T** | **T** | **T** | **G** | **C** | **A** | **A** | **C** | **A** | **A** | **A** | **G** | **T** | **G** | **A** | **T** | **T** | **T** | **A** | **C** | **G** | **G** | **G** | **A** | **A** | **T** | **T** | **A** | **G** | **A** | **G** | **A** | **A** | **T** | **T** | **T** | **T** | **C** | **A** | **G** | **A** | **A** | **C** | **T** | **T** | **C** | **C** | **A** | **A** | **G** | **A** | **A** | **G** | **A** | **A** | **A** | **G** | **T** | **T** | **T** | **T** | **G** | **A** | **A** | **C** | **T** | **T** | **T** | **A** | **T** | **A** | **G** | **T** | **C** | **A** | **A** | **A** | **A** | **G** | **A** | **T** | **G** | **C** | **C** | **G** | **A** | **C** | **G** | **G** | **A** | **G** | **G** | **A** | **G** | **C** | **A** | **A** | **C** | **A** | **A** | **C** | **A** | **G** | **T** | **T** | **T** | **A** | **C** | **A** | **G** | **A** | **C** | **T** | **T** | **T** | **T** | **T** | **T** | **T** | **C** | **C** | **T** | **C** | **G** | **T** | **T** | **A** | **A** | **A** | **T** | **A** | **G** | **G** | **A** | **T** | **G** | **C** | **C** | **C** | **C** | **T** | **G** | **A** | **C** | **T** | **A** | **A** | **G** | **A** | **A** | **A** | **A** | **A** | **T** | **G** | **A** | **T** | **G** | **T** | **T** | **G** | **A** | **A** | **A** | **T** | **T** | **T** | **T** | **T** | **A** | **C** | **G** | **G** | **A** | **T** | **G** | **G** | **T** | **A** | **C** | **G** | **G** | **A** | **A** | **A** | **A** | **A** | **T** | **T** | **C** | **A** | **A** | **T** | **G** | **C** | **G** | **T** | **T** | **G** | **A** | **T** | **G** | **G** | **C** | **G** | **G** | **A** | **C** | **A** | **T** | **A** | **A** | **A** | **A** | **A** | **A** | **A** | **G** | **C** | **T** | **C** | **A** | **G** | **C** | **A** | **C** | **T** | **C** | **C** | **A** | **T** | **T** | **C** | **A** | **T** | **A** | **T** | **T** | **G** | **A** | **A** | **T** | **A** | **T** | **T** | **A** | **T** | **G** | **C** | **C** | **T** | **T** | **T** | **G** | **T** | **A** | **A** | **C** | **G** | **G** | **A** | **T** | **C** | **A** | **T** | **A** | **T** | **C** | **G** | **G** | **T** | **A** | **C** | **C** | **A** | **A** | **A** | **A** | **T** | **T** | **T** | **T** | **G** | **A** | **A** | **C** | **T** | **T** | **A** | **T** | **T** | **A** | **G** | **A** | **A** | **G** | **A** | **A** | **A** | **A** | **G** | **G** | **C** | **G** | **G** | **C** | **T** | **G** | **A** | **A** | **G** | **G** | **G** | **G** | **T** | **T** | **G** | **A** | **A** | **G** | **T** | **G** | **C** | **G** | **G** | **T** | **T** | **G** | **C** | **T** | **T** | **T** | **A** | **C** | **G** | **A** | **T** | **G** | **C** | **C** | **T** | **T** | **T** | **G** | **G** | **C** | **T** | **C** | **A** | **A** | **A** | **G** | **G** | **G** | **C** | **A** | **C** | **C** | **A** | **A** | **G** | **G** | **T** | **T** | **C** | **A** | **T** | **C** | **A** | **T** | **T** | **T** | **G** | **A** | **A** | **T** | **G** | **A** | **A** | **T** | **T** | **G** | **A** | **A** | **A** | **A** | **A** | **A** | **A** | **A** | **C** | **G** | **G** | **C** | **G** | **G** | **T** | **T** | **T** | **T** | **G** | **T** | **C** | **C** | **A** | **A** | **A** | **C** | **G** | **T** | **T** | **T** | **A** | **T** | **T** | **A** | **C** | **T** | **T** | **C** | **T** | **C** | **A** | **A** | **A** | **A** | **A** | **G** | **C** | **A** | **C** | **T** | **T** | **T** | **T** | **G** | **A** | **A** | **G** | **T** | **T** | **T** | **C** | **G** | **T** | **T** | **T** | **G** | **A** | **A** | **T** | **T** | **A** | **T** | **C** | **A** | **T** | **G** | **A** | **T** | **C** | **A** | **C** | **C** | **G** | **G** | **A** | **A** | **A** | **A** | **T** | **T** | **G** | **T** | **T** | **G** | **T** | **T** | **A** | **T** | **T** | **G** | **A** | **T** | **G** | **G** | **G** | **A** | **A** | **A** | **G** | **T** | **A** | **G** | **G** | **C** | **T** | **A** | **C** | **A** | **T** | **T** | **G** | **G** | **C** | **G** | **G** | **C** | **T** | **T** | **T** | **A** | **A** | **T** | **G** | **T** | **T** | **G** | **C** | **C** | **G** | **A** | **T** | **C** | **A** | **A** | **T** | **A** | **T** | **G** | **C** | **T** | **G** | **G** | **A** | **A** | **C** | **G** | **A** | **C** | **T** | **A** | **A** | **A** | **A** | **A** | **G** | **T** | **T** | **T** | **G** | **G** | **C** | **T** | **A** | **T** | **T** | **G** | **G** | **C** | **G** | **G** | **G** | **A** | **T** | **A** | **C** | **A** | **C** | **A** | **T** | **T** | **T** | **A** | **C** | **G** | **G** | **A** | **T** | **T** | **C** | **A** | **A** | **G** | **G** | **G** | **C** | **C** | **A** | **G** | **C** | **A** | **G** | **C** | **C** | **T** | **C** | **A** | **T** | **T** | **A** | **C** | **T** | **G** | **C** | **A** | **A** | **A** | **T** | **G** | **C** | **G** | **T** | **T** | **T** | **T** | **T** | **T** | **A** | **A** | **T** | **G** | **G** | **A** | **T** | **T** | **G** | **G** | **A** | **A** | **C** | **G** | **T** | **C** | **T** | **C** | **T** | **T** | **C** | **C** | **C** | **C** | **T** | **G** | **A** | **G** | **A** | **A** | **A** | **A** | **A** | **T** | **C** | **G** | **T** | **G** | **T** | **G** | **G** | **C** | **G** | **T** | **A** | **T** | **C** | **A** | **A** | **T** | **T** | **G** | **G** | **A** | **T** | **T** | **A** | **T** | **T** | **T** | **C** | **T** | **T** | **T** | **A** | **A** | **A** | **C** | **T** | **T** | **G** | **A** | **A** | **G** | **C** | **G** | **T** | **T** | **G** | **G** | **T** | **G** | **C** | **C** | **A** | **G** | **A** | **G** | **G** | **C** | **A** | **A** | **A** | **T** | **A** | **C** | **A** | **T** | **C** | **C** | **A** | **T** | **T** | **C** | **A** | **G** | **A** | **T** | **G** | **A** | **T** | **T** | **G** | **C** | **C** | **A** | **G** | **T** | **G** | **G** | **T** | **C** | **C** | **T** | **A** | **A** | **T** | **A** | **G** | **T** | **G** | **A** | **C** | **C** | **G** | **T** | **G** | **A** | **A** | **C** | **A** | **A** | **A** | **T** | **T** | **A** | **A** | **A** | **T** | **T** | **G** | **G** | **C** | **C** | **T** | **T** | **T** | **A** | **T** | **T** | **A** | **A** | **A** | **T** | **T** | **G** | **A** | **T** | **T** | **A** | **C** | **T** | **T** | **C** | **T** | **G** | **C** | **C** | **A** | **A** | **G** | **A** | **A** | **A** | **A** | **G** | **A** | **G** | **T** | **C** | **T** | **G** | **G** | **A** | **T** | **T** | **C** | **A** | **A** | **A** | **C** | **G** | **C** | **C** | **G** | **T** | **A** | **T** | **T** | **T** | **A** | **G** | **T** | **T** | **C** | **C** | **T** | **G** | **A** | **T** | **G** | **A** | **T** | **A** | **G** | **T** | **G** | **T** | **C** | **T** | **T** | **G** | **G** | **C** | **G** | **G** | **C** | **T** | **T** | **T** | **A** | **A** | **A** | **G** | **G** | **T** | **T** | **G** | **C** | **T** | **G** | **C** | **G** | **G** | **C** | **T** | **T** | **C** | **A** | **G** | **G** | **A** | **G** | **T** | **A** | **G** | **A** | **T** | **G** | **T** | **T** | **A** | **A** | **A** | **A** | **T** | **T** | **A** | **T** | **G** | **A** | **T** | **T** | **C** | **C** | **A** | **G** | **A** | **T** | **A** | **A** | **G** | **C** | **C** | **C** | **G** | **A** | **C** | **C** | **A** | **T** | **C** | **C** | **G** | **T** | **T** | **T** | **A** | **T** | **T** | **T** | **A** | **T** | **C** | **G** | **A** | **G** | **C** | **A** | **A** | **C** | **A** | **C** | **A** | **G** | **T** | **A** | **T** | **T** | **A** | **C** | **G** | **G** | **G** | **C** | **G** | **C** | **T** | **T** | **G** | **T** | **T** | **G** | **A** | **T** | **G** | **A** | **A** | **A** | **G** | **A** | **A** | **A** | **A** | **T** | **A** | **T** | **T** | **G** | **A** | **A** | **A** | **T** | **T** | **T** | **T** | **A** | **A** | **T** | **T** | **T** | **A** | **T** | **A** | **A** | **C** | **G** | **G** | **T** | **G** | **G** | **T** | **T** | **T** | **C** | **T** | **T** | **A** | **C** | **A** | **T** | **G** | **C** | **G** | **A** | **A** | **A** | **A** | **C** | **A** | **A** | **T** | **G** | **A** | **T** | **T** | **A** | **T** | **G** | **G** | **A** | **T** | **G** | **A** | **T** | **G** | **A** | **A** | **G** | **T** | **C** | **T** | **G** | **C** | **A** | **C** | **A** | **G** | **T** | **A** | **G** | **G** | **T** | **T** | **C** | **A** | **G** | **C** | **C** | **A** | **A** | **T** | **C** | **A** | **A** | **G** | **A** | **T** | **A** | **T** | **C** | **C** | **G** | **A** | **A** | **G** | **T** | **T** | **A** | **C** | **A** | **A** | **A** | **T** | **T** | **A** | **A** | **A** | **C** | **T** | **T** | **T** | **G** | **A** | **A** | **G** | **C** | **A** | **A** | **A** | **T** | **G** | **C** | **T** | **G** | **T** | **G** | **T** | **T** | **A** | **T** | **A** | **T** | **G** | **A** | **T** | **A** | **A** | **A** | **A** | **A** | **A** | **A** | **T** | **C** | **A** | **T** | **T** | **G** | **A** | **T** | **C** | **A** | **A** | **T** | **T** | **A** | **G** | **A** | **A** | **G** | **C** | **A** | **A** | **T** | **T** | **T** | **T** | **C** | **T** | **T** | **A** | **G** | **A** | **A** | **G** | **A** | **T** | **C** | **G** | **A** | **A** | **A** | **A** | **A** | **A** | **A** | **T** | **G** | **T** | **A** | **C** | **A** | **A** | **C** | **A** | **A** | **T** | **G** | **A** | **C** | **T** | **C** | **C** | **A** | **G** | **A** | **A** | **G** | **T** | **T** | **G** | **T** | **T** | **C** | **G** | **C** | **G** | **A** | **C** | **A** | **T** | **G** | **T** | **C** | **A** | **A** | **A** | **A** | **T** | **G** | **G** | **T** | **T** | **G** | **A** | **T** | **T** | **T** | **T** | **T** | **A** | **A** | **A** | **C** | **A** | **A** | **C** | **A** | **A** | **A** | **T** | **T** | **T** | **C** | **A** | **C** | **G** | **A** | **T** | **T** | **A** | **T** | **T** | **C** | **T** | **C** | **A** | **C** | **C** | **A** | **A** | **T** | **T** | **C** | **T** | **T** | **-** | **-** | **-** |
| **N59509F** | **T** | **T** | **G** | **A** | **T** | **T** | **C** | **T** | **T** | **A** | **G** | **C** | **G** | **T** | **C** | **T** | **T** | **A** | **A** | **C** | **A** | **G** | **T** | **T** | **A** | **T** | **T** | **T** | **A** | **T** | **T** | **T** | **T** | **A** | **T** | **T** | **A** | **A** | **T** | **G** | **C** | **A** | **A** | **T** | **T** | **A** | **T** | **T** | **G** | **C** | **G** | **G** | **G** | **A** | **A** | **T** | **T** | **A** | **C** | **C** | **A** | **T** | **T** | **T** | **T** | **G** | **T** | **T** | **A** | **A** | **A** | **A** | **C** | **C** | **T** | **C** | **G** | **A** | **G** | **A** | **T** | **G** | **T** | **A** | **G** | **C** | **A** | **G** | **C** | **A** | **A** | **T** | **T** | **T** | **G** | **G** | **G** | **C** | **A** | **T** | **G** | **G** | **C** | **T** | **A** | **C** | **T** | **C** | **G** | **T** | **A** | **C** | **T** | **G** | **A** | **T** | **T** | **G** | **C** | **T** | **C** | **T** | **A** | **C** | **C** | **C** | **G** | **T** | **T** | **T** | **T** | **T** | **G** | **G** | **T** | **T** | **T** | **T** | **T** | **T** | **C** | **T** | **T** | **A** | **T** | **A** | **T** | **T** | **T** | **A** | **T** | **T** | **C** | **T** | **T** | **T** | **G** | **G** | **T** | **C** | **G** | **A** | **G** | **G** | **G** | **T** | **T** | **A** | **A** | **C** | **C** | **G** | **A** | **C** | **A** | **A** | **A** | **A** | **A** | **G** | **A** | **A** | **A** | **T** | **T** | **T** | **T** | **A** | **T** | **T** | **T** | **G** | **C** | **A** | **A** | **C** | **A** | **A** | **A** | **G** | **T** | **G** | **A** | **T** | **T** | **T** | **A** | **C** | **G** | **G** | **G** | **A** | **A** | **T** | **T** | **A** | **G** | **A** | **G** | **A** | **A** | **T** | **T** | **T** | **T** | **C** | **A** | **G** | **A** | **A** | **T** | **T** | **T** | **C** | **C** | **A** | **A** | **G** | **A** | **A** | **G** | **A** | **A** | **A** | **G** | **T** | **T** | **T** | **T** | **G** | **A** | **A** | **C** | **T** | **T** | **T** | **A** | **T** | **A** | **G** | **T** | **C** | **A** | **A** | **A** | **A** | **G** | **A** | **T** | **G** | **C** | **C** | **G** | **A** | **C** | **G** | **G** | **A** | **G** | **G** | **A** | **G** | **C** | **A** | **A** | **C** | **A** | **A** | **C** | **A** | **G** | **T** | **T** | **T** | **A** | **C** | **A** | **G** | **A** | **C** | **T** | **T** | **T** | **T** | **T** | **T** | **T** | **C** | **C** | **T** | **C** | **G** | **T** | **T** | **A** | **A** | **A** | **T** | **A** | **G** | **G** | **A** | **T** | **G** | **C** | **C** | **T** | **C** | **T** | **G** | **A** | **C** | **T** | **A** | **A** | **G** | **A** | **A** | **A** | **A** | **A** | **T** | **G** | **A** | **T** | **G** | **T** | **T** | **G** | **A** | **A** | **A** | **T** | **T** | **T** | **T** | **T** | **A** | **C** | **G** | **G** | **A** | **T** | **G** | **G** | **T** | **A** | **C** | **G** | **G** | **A** | **A** | **A** | **A** | **A** | **T** | **T** | **C** | **A** | **A** | **T** | **G** | **C** | **G** | **T** | **T** | **G** | **A** | **T** | **G** | **G** | **C** | **G** | **G** | **A** | **C** | **A** | **T** | **A** | **A** | **A** | **A** | **A** | **A** | **A** | **G** | **C** | **T** | **C** | **A** | **G** | **C** | **A** | **C** | **T** | **C** | **T** | **A** | **T** | **T** | **C** | **A** | **T** | **A** | **T** | **T** | **G** | **A** | **A** | **T** | **A** | **T** | **T** | **A** | **T** | **G** | **C** | **C** | **T** | **T** | **T** | **G** | **T** | **A** | **A** | **C** | **G** | **G** | **A** | **T** | **C** | **A** | **T** | **A** | **T** | **C** | **G** | **G** | **C** | **A** | **C** | **C** | **A** | **A** | **A** | **A** | **T** | **T** | **T** | **T** | **G** | **A** | **A** | **C** | **T** | **T** | **A** | **T** | **T** | **A** | **G** | **A** | **A** | **G** | **A** | **A** | **A** | **A** | **G** | **G** | **C** | **G** | **G** | **C** | **T** | **G** | **A** | **A** | **G** | **G** | **G** | **G** | **T** | **T** | **G** | **A** | **A** | **G** | **T** | **G** | **C** | **G** | **G** | **T** | **T** | **G** | **C** | **T** | **T** | **T** | **A** | **C** | **G** | **A** | **T** | **G** | **C** | **C** | **T** | **T** | **T** | **G** | **G** | **C** | **T** | **C** | **A** | **A** | **A** | **G** | **G** | **G** | **C** | **A** | **C** | **C** | **A** | **A** | **G** | **G** | **T** | **T** | **C** | **A** | **T** | **C** | **A** | **T** | **T** | **T** | **G** | **A** | **A** | **T** | **G** | **A** | **A** | **T** | **T** | **G** | **A** | **A** | **A** | **A** | **A** | **A** | **A** | **A** | **C** | **G** | **G** | **C** | **G** | **G** | **T** | **T** | **T** | **T** | **G** | **T** | **C** | **C** | **A** | **A** | **A** | **C** | **G** | **T** | **T** | **T** | **A** | **T** | **T** | **A** | **C** | **T** | **T** | **C** | **T** | **C** | **A** | **A** | **A** | **A** | **A** | **G** | **C** | **A** | **C** | **T** | **T** | **T** | **T** | **G** | **A** | **A** | **G** | **T** | **T** | **T** | **C** | **G** | **T** | **T** | **T** | **G** | **A** | **A** | **T** | **T** | **A** | **T** | **C** | **A** | **T** | **G** | **A** | **T** | **C** | **A** | **C** | **C** | **G** | **G** | **A** | **A** | **A** | **A** | **T** | **T** | **G** | **T** | **T** | **G** | **T** | **T** | **A** | **T** | **T** | **G** | **A** | **C** | **G** | **G** | **A** | **A** | **A** | **A** | **G** | **T** | **A** | **G** | **G** | **C** | **T** | **A** | **C** | **A** | **T** | **T** | **G** | **G** | **C** | **G** | **G** | **C** | **T** | **T** | **T** | **A** | **A** | **T** | **G** | **T** | **T** | **G** | **C** | **C** | **G** | **A** | **T** | **C** | **A** | **A** | **T** | **A** | **T** | **G** | **C** | **C** | **G** | **G** | **A** | **A** | **C** | **G** | **A** | **C** | **T** | **A** | **A** | **A** | **A** | **A** | **G** | **T** | **T** | **T** | **G** | **G** | **C** | **T** | **A** | **T** | **T** | **G** | **G** | **C** | **G** | **G** | **G** | **A** | **T** | **A** | **C** | **A** | **C** | **A** | **T** | **T** | **T** | **A** | **C** | **G** | **G** | **A** | **T** | **T** | **C** | **A** | **A** | **G** | **G** | **G** | **C** | **C** | **A** | **G** | **C** | **A** | **G** | **C** | **C** | **T** | **C** | **A** | **T** | **T** | **A** | **C** | **T** | **G** | **C** | **A** | **A** | **A** | **T** | **G** | **C** | **G** | **T** | **T** | **T** | **T** | **T** | **T** | **A** | **A** | **T** | **G** | **G** | **A** | **T** | **T** | **G** | **G** | **A** | **A** | **C** | **G** | **T** | **C** | **T** | **C** | **T** | **T** | **C** | **C** | **C** | **C** | **C** | **G** | **A** | **G** | **A** | **A** | **A** | **A** | **A** | **T** | **C** | **G** | **T** | **G** | **T** | **G** | **G** | **C** | **G** | **T** | **A** | **T** | **C** | **A** | **A** | **T** | **T** | **G** | **G** | **A** | **T** | **T** | **A** | **T** | **T** | **T** | **C** | **T** | **T** | **T** | **A** | **A** | **A** | **C** | **T** | **T** | **G** | **A** | **A** | **G** | **C** | **A** | **T** | **T** | **G** | **G** | **T** | **G** | **C** | **C** | **A** | **G** | **A** | **G** | **G** | **C** | **A** | **A** | **A** | **T** | **A** | **C** | **A** | **T** | **C** | **C** | **A** | **T** | **T** | **C** | **A** | **G** | **A** | **T** | **G** | **A** | **T** | **T** | **G** | **C** | **C** | **A** | **G** | **T** | **G** | **G** | **T** | **C** | **C** | **T** | **A** | **A** | **C** | **A** | **G** | **T** | **G** | **A** | **C** | **C** | **G** | **T** | **G** | **A** | **A** | **C** | **A** | **A** | **A** | **T** | **T** | **A** | **A** | **A** | **T** | **T** | **G** | **G** | **C** | **C** | **T** | **T** | **T** | **A** | **T** | **T** | **A** | **A** | **A** | **T** | **T** | **G** | **A** | **T** | **T** | **A** | **C** | **T** | **T** | **C** | **T** | **G** | **C** | **C** | **A** | **A** | **G** | **A** | **A** | **A** | **A** | **G** | **A** | **G** | **T** | **C** | **T** | **G** | **G** | **A** | **T** | **T** | **C** | **A** | **A** | **A** | **C** | **G** | **C** | **C** | **G** | **T** | **A** | **T** | **T** | **T** | **A** | **G** | **T** | **T** | **C** | **C** | **T** | **G** | **A** | **T** | **G** | **A** | **T** | **A** | **G** | **T** | **G** | **T** | **C** | **T** | **T** | **G** | **G** | **C** | **T** | **G** | **C** | **T** | **T** | **T** | **A** | **A** | **A** | **G** | **G** | **T** | **T** | **G** | **C** | **T** | **G** | **C** | **G** | **G** | **C** | **T** | **T** | **C** | **A** | **G** | **G** | **A** | **G** | **T** | **A** | **G** | **A** | **T** | **G** | **T** | **T** | **A** | **A** | **A** | **A** | **T** | **T** | **A** | **T** | **G** | **A** | **T** | **T** | **C** | **C** | **A** | **G** | **A** | **T** | **A** | **A** | **G** | **C** | **C** | **C** | **G** | **A** | **C** | **C** | **A** | **T** | **C** | **C** | **G** | **T** | **T** | **T** | **A** | **T** | **T** | **T** | **A** | **T** | **C** | **G** | **A** | **G** | **C** | **A** | **A** | **C** | **A** | **C** | **A** | **G** | **T** | **A** | **T** | **T** | **A** | **C** | **G** | **G** | **C** | **C** | **G** | **C** | **T** | **T** | **A** | **T** | **T** | **G** | **A** | **T** | **G** | **A** | **A** | **A** | **G** | **A** | **A** | **A** | **A** | **T** | **A** | **T** | **T** | **G** | **A** | **A** | **A** | **T** | **T** | **T** | **T** | **A** | **A** | **T** | **T** | **T** | **A** | **T** | **A** | **A** | **C** | **G** | **G** | **T** | **G** | **G** | **T** | **T** | **T** | **C** | **T** | **T** | **A** | **C** | **A** | **T** | **G** | **C** | **G** | **A** | **A** | **A** | **A** | **C** | **A** | **A** | **T** | **G** | **A** | **T** | **T** | **A** | **T** | **G** | **G** | **A** | **T** | **G** | **A** | **T** | **G** | **A** | **A** | **G** | **T** | **C** | **T** | **G** | **C** | **A** | **C** | **A** | **G** | **T** | **T** | **G** | **G** | **T** | **T** | **C** | **A** | **G** | **C** | **C** | **A** | **A** | **T** | **C** | **A** | **A** | **G** | **A** | **T** | **A** | **T** | **C** | **C** | **G** | **A** | **A** | **G** | **T** | **T** | **A** | **C** | **A** | **A** | **A** | **T** | **T** | **A** | **A** | **A** | **C** | **T** | **T** | **T** | **G** | **A** | **A** | **G** | **C** | **A** | **A** | **A** | **T** | **G** | **C** | **T** | **G** | **T** | **G** | **T** | **T** | **A** | **T** | **A** | **T** | **G** | **A** | **T** | **A** | **A** | **A** | **A** | **A** | **A** | **A** | **T** | **C** | **A** | **T** | **T** | **G** | **A** | **T** | **C** | **A** | **A** | **T** | **T** | **A** | **G** | **A** | **A** | **G** | **C** | **A** | **A** | **T** | **T** | **T** | **T** | **C** | **T** | **T** | **A** | **G** | **A** | **A** | **G** | **A** | **T** | **C** | **G** | **A** | **A** | **A** | **A** | **A** | **A** | **A** | **T** | **G** | **T** | **A** | **C** | **A** | **A** | **C** | **A** | **A** | **T** | **G** | **A** | **C** | **T** | **C** | **C** | **A** | **G** | **A** | **A** | **G** | **T** | **T** | **G** | **T** | **T** | **C** | **G** | **C** | **G** | **A** | **C** | **A** | **T** | **G** | **T** | **C** | **A** | **A** | **A** | **A** | **T** | **G** | **G** | **T** | **T** | **G** | **A** | **T** | **T** | **T** | **T** | **T** | **A** | **A** | **A** | **C** | **A** | **A** | **C** | **A** | **A** | **A** | **T** | **T** | **T** | **C** | **A** | **C** | **G** | **A** | **T** | **T** | **A** | **T** | **T** | **T** | **T** | **C** | **A** | **C** | **C** | **A** | **A** | **T** | **T** | **C** | **T** | **T** | **-** | **-** | **-** |
| **N59646F** | **T** | **T** | **G** | **A** | **T** | **T** | **C** | **T** | **T** | **A** | **G** | **C** | **G** | **T** | **C** | **T** | **T** | **A** | **A** | **C** | **A** | **G** | **T** | **T** | **A** | **T** | **T** | **T** | **A** | **T** | **T** | **T** | **T** | **A** | **T** | **T** | **A** | **A** | **T** | **G** | **C** | **A** | **A** | **T** | **T** | **A** | **T** | **T** | **G** | **C** | **G** | **G** | **G** | **A** | **A** | **T** | **T** | **A** | **C** | **C** | **A** | **T** | **T** | **T** | **T** | **G** | **T** | **T** | **A** | **A** | **A** | **A** | **C** | **C** | **T** | **C** | **G** | **A** | **G** | **A** | **T** | **G** | **T** | **A** | **G** | **C** | **A** | **G** | **C** | **A** | **A** | **T** | **T** | **T** | **G** | **G** | **G** | **C** | **A** | **T** | **G** | **G** | **C** | **T** | **A** | **C** | **T** | **A** | **G** | **T** | **A** | **C** | **T** | **G** | **A** | **T** | **T** | **G** | **C** | **T** | **C** | **T** | **A** | **C** | **C** | **C** | **G** | **T** | **T** | **T** | **T** | **T** | **G** | **G** | **T** | **T** | **T** | **T** | **T** | **T** | **C** | **T** | **T** | **A** | **T** | **A** | **T** | **T** | **T** | **A** | **T** | **T** | **C** | **T** | **T** | **T** | **G** | **G** | **G** | **C** | **G** | **C** | **G** | **G** | **T** | **T** | **T** | **A** | **A** | **C** | **C** | **G** | **A** | **C** | **A** | **A** | **A** | **A** | **A** | **G** | **A** | **A** | **A** | **T** | **T** | **T** | **T** | **A** | **T** | **T** | **T** | **G** | **C** | **A** | **A** | **C** | **A** | **A** | **A** | **G** | **T** | **G** | **A** | **T** | **T** | **T** | **A** | **C** | **G** | **G** | **G** | **A** | **A** | **T** | **T** | **A** | **G** | **A** | **G** | **A** | **A** | **T** | **T** | **T** | **C** | **C** | **A** | **G** | **A** | **A** | **T** | **T** | **T** | **C** | **C** | **A** | **A** | **G** | **A** | **A** | **G** | **A** | **A** | **A** | **G** | **T** | **T** | **T** | **T** | **G** | **A** | **A** | **C** | **T** | **T** | **T** | **A** | **T** | **A** | **G** | **T** | **C** | **A** | **A** | **A** | **A** | **G** | **A** | **T** | **G** | **C** | **C** | **G** | **A** | **C** | **G** | **G** | **A** | **G** | **G** | **A** | **G** | **C** | **A** | **A** | **C** | **A** | **A** | **C** | **A** | **G** | **T** | **T** | **T** | **A** | **C** | **A** | **G** | **A** | **C** | **T** | **T** | **T** | **T** | **T** | **T** | **T** | **C** | **C** | **T** | **C** | **G** | **T** | **T** | **A** | **A** | **A** | **T** | **A** | **G** | **G** | **A** | **T** | **G** | **C** | **C** | **T** | **C** | **T** | **G** | **A** | **C** | **T** | **A** | **A** | **G** | **A** | **A** | **A** | **A** | **A** | **T** | **G** | **A** | **T** | **G** | **T** | **T** | **G** | **A** | **A** | **A** | **T** | **T** | **T** | **T** | **T** | **A** | **C** | **G** | **G** | **A** | **T** | **G** | **G** | **T** | **A** | **C** | **G** | **G** | **A** | **A** | **A** | **A** | **A** | **T** | **T** | **C** | **A** | **A** | **T** | **G** | **C** | **G** | **T** | **T** | **G** | **A** | **T** | **G** | **G** | **C** | **G** | **G** | **A** | **C** | **A** | **T** | **A** | **A** | **A** | **A** | **A** | **A** | **A** | **G** | **C** | **T** | **C** | **A** | **G** | **C** | **A** | **C** | **T** | **C** | **C** | **A** | **T** | **T** | **C** | **A** | **T** | **A** | **T** | **T** | **G** | **A** | **A** | **T** | **A** | **T** | **T** | **A** | **T** | **G** | **C** | **C** | **T** | **T** | **T** | **G** | **T** | **A** | **A** | **C** | **G** | **G** | **A** | **T** | **C** | **A** | **T** | **A** | **T** | **C** | **G** | **G** | **C** | **A** | **C** | **C** | **A** | **A** | **A** | **A** | **T** | **T** | **T** | **T** | **G** | **A** | **A** | **C** | **T** | **T** | **A** | **T** | **T** | **A** | **G** | **A** | **A** | **G** | **A** | **A** | **A** | **A** | **G** | **G** | **C** | **G** | **G** | **C** | **T** | **G** | **A** | **A** | **G** | **G** | **G** | **G** | **T** | **T** | **G** | **A** | **A** | **G** | **T** | **G** | **C** | **G** | **G** | **T** | **T** | **G** | **C** | **T** | **T** | **T** | **A** | **C** | **G** | **A** | **T** | **G** | **C** | **C** | **T** | **T** | **T** | **G** | **G** | **C** | **T** | **C** | **A** | **A** | **A** | **G** | **G** | **G** | **C** | **A** | **C** | **C** | **A** | **A** | **G** | **G** | **T** | **T** | **C** | **A** | **T** | **C** | **A** | **T** | **T** | **T** | **G** | **A** | **A** | **T** | **G** | **A** | **A** | **T** | **T** | **G** | **A** | **A** | **A** | **A** | **A** | **A** | **A** | **A** | **C** | **G** | **G** | **C** | **G** | **G** | **T** | **T** | **T** | **T** | **G** | **T** | **C** | **C** | **A** | **A** | **A** | **C** | **A** | **T** | **T** | **T** | **A** | **T** | **T** | **A** | **C** | **T** | **T** | **C** | **T** | **C** | **A** | **A** | **A** | **A** | **A** | **G** | **C** | **A** | **C** | **T** | **T** | **T** | **T** | **G** | **A** | **A** | **G** | **T** | **T** | **T** | **C** | **G** | **T** | **T** | **T** | **G** | **A** | **A** | **T** | **T** | **A** | **T** | **C** | **A** | **T** | **G** | **A** | **T** | **C** | **A** | **C** | **C** | **G** | **G** | **A** | **A** | **A** | **A** | **T** | **T** | **G** | **T** | **T** | **G** | **T** | **T** | **A** | **T** | **T** | **G** | **A** | **C** | **G** | **G** | **A** | **A** | **A** | **A** | **G** | **T** | **A** | **G** | **G** | **C** | **T** | **A** | **C** | **A** | **T** | **T** | **G** | **G** | **C** | **G** | **G** | **C** | **T** | **T** | **T** | **A** | **A** | **T** | **G** | **T** | **T** | **G** | **C** | **C** | **G** | **A** | **T** | **C** | **A** | **A** | **T** | **A** | **T** | **G** | **C** | **C** | **G** | **G** | **A** | **A** | **C** | **G** | **A** | **C** | **T** | **A** | **A** | **A** | **A** | **A** | **G** | **T** | **T** | **T** | **G** | **G** | **C** | **T** | **A** | **T** | **T** | **G** | **G** | **C** | **G** | **G** | **G** | **A** | **T** | **A** | **C** | **G** | **C** | **A** | **T** | **T** | **T** | **A** | **C** | **G** | **G** | **A** | **T** | **T** | **C** | **A** | **A** | **G** | **G** | **G** | **C** | **C** | **A** | **G** | **C** | **A** | **G** | **C** | **C** | **T** | **C** | **A** | **T** | **T** | **A** | **C** | **T** | **G** | **C** | **A** | **A** | **A** | **T** | **G** | **C** | **G** | **T** | **T** | **T** | **T** | **T** | **T** | **A** | **A** | **T** | **G** | **G** | **A** | **T** | **T** | **G** | **G** | **A** | **A** | **C** | **G** | **T** | **C** | **T** | **C** | **T** | **T** | **C** | **C** | **C** | **C** | **C** | **G** | **A** | **G** | **A** | **A** | **A** | **A** | **A** | **T** | **C** | **G** | **T** | **G** | **T** | **G** | **G** | **C** | **G** | **T** | **A** | **T** | **C** | **A** | **A** | **T** | **T** | **G** | **G** | **A** | **T** | **T** | **A** | **T** | **T** | **T** | **C** | **T** | **T** | **T** | **A** | **A** | **A** | **C** | **T** | **T** | **G** | **A** | **A** | **G** | **C** | **A** | **T** | **T** | **G** | **G** | **T** | **G** | **C** | **C** | **A** | **G** | **A** | **G** | **G** | **C** | **A** | **A** | **A** | **T** | **A** | **C** | **A** | **T** | **C** | **C** | **A** | **T** | **T** | **C** | **A** | **G** | **A** | **T** | **G** | **A** | **T** | **T** | **G** | **C** | **C** | **A** | **G** | **T** | **G** | **G** | **T** | **C** | **C** | **T** | **A** | **A** | **C** | **A** | **G** | **T** | **G** | **A** | **C** | **C** | **G** | **T** | **G** | **A** | **A** | **C** | **A** | **A** | **A** | **T** | **T** | **A** | **A** | **A** | **T** | **T** | **G** | **G** | **C** | **C** | **T** | **T** | **T** | **A** | **T** | **T** | **A** | **A** | **A** | **T** | **T** | **G** | **A** | **T** | **T** | **A** | **C** | **T** | **T** | **C** | **T** | **G** | **C** | **C** | **A** | **A** | **G** | **A** | **A** | **A** | **A** | **G** | **A** | **G** | **T** | **C** | **T** | **G** | **G** | **A** | **T** | **T** | **C** | **A** | **A** | **A** | **C** | **G** | **C** | **C** | **G** | **T** | **A** | **T** | **T** | **T** | **A** | **G** | **T** | **T** | **C** | **C** | **T** | **G** | **A** | **T** | **G** | **A** | **T** | **A** | **G** | **T** | **G** | **T** | **C** | **T** | **T** | **G** | **G** | **C** | **T** | **G** | **C** | **T** | **T** | **T** | **A** | **A** | **A** | **G** | **G** | **T** | **T** | **G** | **C** | **T** | **G** | **C** | **G** | **G** | **C** | **T** | **T** | **C** | **A** | **G** | **G** | **A** | **G** | **T** | **A** | **G** | **A** | **T** | **G** | **T** | **T** | **A** | **A** | **A** | **A** | **T** | **T** | **A** | **T** | **G** | **A** | **T** | **T** | **C** | **C** | **A** | **G** | **A** | **T** | **A** | **A** | **G** | **C** | **C** | **C** | **G** | **A** | **C** | **C** | **A** | **T** | **C** | **C** | **G** | **T** | **T** | **T** | **A** | **T** | **T** | **T** | **A** | **T** | **C** | **G** | **A** | **G** | **C** | **A** | **A** | **C** | **A** | **C** | **A** | **G** | **T** | **A** | **T** | **T** | **A** | **C** | **G** | **G** | **C** | **C** | **G** | **C** | **T** | **T** | **A** | **T** | **T** | **G** | **A** | **T** | **G** | **A** | **A** | **A** | **G** | **A** | **A** | **A** | **A** | **T** | **A** | **T** | **T** | **G** | **A** | **A** | **A** | **T** | **T** | **T** | **T** | **A** | **A** | **T** | **T** | **T** | **A** | **T** | **A** | **A** | **C** | **G** | **G** | **T** | **G** | **G** | **T** | **T** | **T** | **C** | **T** | **T** | **A** | **C** | **A** | **T** | **G** | **C** | **G** | **A** | **A** | **A** | **A** | **C** | **A** | **A** | **T** | **G** | **A** | **T** | **T** | **A** | **T** | **G** | **G** | **A** | **T** | **G** | **A** | **T** | **G** | **A** | **A** | **G** | **T** | **C** | **T** | **G** | **C** | **A** | **C** | **A** | **G** | **T** | **T** | **G** | **G** | **T** | **T** | **C** | **A** | **G** | **C** | **C** | **A** | **A** | **T** | **C** | **A** | **A** | **G** | **A** | **T** | **A** | **T** | **C** | **C** | **G** | **A** | **A** | **G** | **T** | **T** | **A** | **C** | **A** | **A** | **A** | **T** | **T** | **A** | **A** | **A** | **C** | **T** | **T** | **T** | **G** | **A** | **A** | **G** | **C** | **A** | **A** | **A** | **T** | **G** | **C** | **T** | **G** | **T** | **G** | **T** | **T** | **A** | **T** | **A** | **T** | **G** | **A** | **T** | **A** | **A** | **A** | **A** | **A** | **A** | **A** | **T** | **C** | **A** | **T** | **T** | **G** | **A** | **T** | **C** | **A** | **A** | **T** | **T** | **A** | **G** | **A** | **A** | **G** | **C** | **A** | **A** | **T** | **T** | **T** | **T** | **C** | **T** | **T** | **A** | **G** | **A** | **A** | **G** | **A** | **T** | **C** | **G** | **A** | **A** | **A** | **A** | **A** | **A** | **A** | **T** | **G** | **T** | **A** | **C** | **A** | **A** | **C** | **A** | **A** | **T** | **G** | **A** | **C** | **T** | **C** | **C** | **A** | **G** | **A** | **A** | **G** | **T** | **T** | **G** | **T** | **T** | **C** | **G** | **C** | **G** | **A** | **T** | **A** | **T** | **G** | **T** | **C** | **A** | **A** | **A** | **A** | **T** | **G** | **G** | **T** | **T** | **G** | **A** | **T** | **T** | **T** | **T** | **T** | **A** | **A** | **A** | **C** | **A** | **A** | **C** | **A** | **A** | **A** | **T** | **T** | **T** | **C** | **A** | **C** | **G** | **A** | **T** | **T** | **A** | **T** | **T** | **T** | **T** | **C** | **A** | **C** | **C** | **A** | **A** | **T** | **T** | **C** | **T** | **T** | **-** | **-** | **-** |
| **N59466F** | **T** | **T** | **G** | **A** | **T** | **T** | **C** | **T** | **T** | **A** | **G** | **T** | **G** | **T** | **C** | **T** | **T** | **A** | **A** | **C** | **A** | **G** | **T** | **T** | **A** | **T** | **T** | **T** | **A** | **T** | **T** | **T** | **T** | **A** | **T** | **T** | **A** | **A** | **T** | **G** | **C** | **A** | **A** | **T** | **T** | **A** | **T** | **T** | **G** | **C** | **G** | **G** | **G** | **A** | **A** | **T** | **T** | **A** | **C** | **C** | **A** | **T** | **T** | **T** | **T** | **G** | **T** | **T** | **A** | **A** | **A** | **A** | **C** | **C** | **T** | **C** | **G** | **A** | **G** | **A** | **T** | **G** | **T** | **A** | **G** | **C** | **A** | **G** | **C** | **A** | **A** | **T** | **T** | **T** | **G** | **G** | **G** | **C** | **A** | **T** | **G** | **G** | **C** | **T** | **A** | **C** | **T** | **C** | **G** | **T** | **A** | **C** | **T** | **G** | **A** | **T** | **T** | **G** | **C** | **T** | **C** | **T** | **A** | **C** | **C** | **C** | **G** | **T** | **T** | **T** | **T** | **T** | **G** | **G** | **T** | **T** | **T** | **T** | **T** | **T** | **C** | **T** | **T** | **A** | **T** | **A** | **T** | **T** | **T** | **A** | **T** | **T** | **C** | **T** | **T** | **T** | **G** | **G** | **G** | **C** | **G** | **C** | **G** | **G** | **T** | **T** | **T** | **A** | **A** | **C** | **C** | **G** | **A** | **C** | **A** | **A** | **A** | **A** | **A** | **G** | **A** | **A** | **A** | **T** | **T** | **T** | **T** | **A** | **T** | **T** | **T** | **G** | **C** | **A** | **A** | **C** | **A** | **A** | **A** | **G** | **T** | **G** | **A** | **T** | **T** | **T** | **G** | **C** | **G** | **G** | **G** | **A** | **A** | **T** | **T** | **A** | **G** | **A** | **G** | **A** | **A** | **T** | **T** | **T** | **T** | **C** | **A** | **G** | **A** | **A** | **T** | **T** | **T** | **C** | **C** | **A** | **A** | **G** | **A** | **A** | **G** | **A** | **A** | **A** | **G** | **T** | **T** | **T** | **T** | **G** | **A** | **A** | **C** | **T** | **T** | **T** | **A** | **T** | **A** | **G** | **T** | **C** | **A** | **A** | **A** | **A** | **G** | **A** | **T** | **G** | **C** | **C** | **G** | **A** | **C** | **G** | **G** | **A** | **G** | **G** | **A** | **G** | **C** | **A** | **A** | **C** | **A** | **A** | **C** | **A** | **G** | **T** | **T** | **T** | **A** | **C** | **A** | **G** | **A** | **C** | **T** | **T** | **T** | **T** | **T** | **T** | **T** | **C** | **C** | **T** | **C** | **G** | **T** | **T** | **A** | **A** | **A** | **T** | **A** | **G** | **G** | **A** | **T** | **G** | **C** | **C** | **T** | **C** | **T** | **G** | **A** | **C** | **T** | **A** | **A** | **G** | **A** | **A** | **A** | **A** | **A** | **T** | **G** | **A** | **T** | **G** | **T** | **T** | **G** | **A** | **A** | **A** | **T** | **T** | **T** | **T** | **T** | **A** | **C** | **G** | **G** | **A** | **T** | **G** | **G** | **T** | **A** | **C** | **G** | **G** | **A** | **A** | **A** | **A** | **A** | **T** | **T** | **C** | **A** | **A** | **T** | **G** | **C** | **G** | **T** | **T** | **G** | **A** | **T** | **G** | **G** | **C** | **G** | **G** | **A** | **C** | **A** | **T** | **A** | **A** | **A** | **A** | **A** | **A** | **A** | **G** | **C** | **T** | **C** | **A** | **G** | **C** | **A** | **C** | **T** | **C** | **C** | **A** | **T** | **T** | **C** | **A** | **T** | **A** | **T** | **T** | **G** | **A** | **A** | **T** | **A** | **T** | **T** | **A** | **T** | **G** | **C** | **C** | **T** | **T** | **T** | **G** | **T** | **A** | **A** | **C** | **G** | **G** | **A** | **T** | **C** | **A** | **T** | **A** | **T** | **C** | **G** | **G** | **C** | **A** | **C** | **C** | **A** | **A** | **A** | **A** | **T** | **T** | **T** | **T** | **G** | **A** | **A** | **C** | **T** | **T** | **A** | **T** | **T** | **A** | **G** | **A** | **A** | **G** | **A** | **A** | **A** | **A** | **G** | **G** | **C** | **G** | **G** | **C** | **T** | **G** | **A** | **A** | **G** | **G** | **G** | **G** | **T** | **T** | **G** | **A** | **A** | **G** | **T** | **G** | **C** | **G** | **G** | **T** | **T** | **G** | **C** | **T** | **T** | **T** | **A** | **C** | **G** | **A** | **T** | **G** | **C** | **C** | **T** | **T** | **T** | **G** | **G** | **C** | **T** | **C** | **A** | **A** | **A** | **G** | **G** | **G** | **C** | **A** | **C** | **C** | **A** | **A** | **G** | **G** | **T** | **T** | **C** | **A** | **T** | **C** | **A** | **T** | **T** | **T** | **G** | **A** | **A** | **T** | **G** | **A** | **A** | **T** | **T** | **G** | **A** | **A** | **A** | **A** | **A** | **A** | **A** | **A** | **C** | **G** | **G** | **C** | **G** | **G** | **T** | **T** | **T** | **T** | **G** | **T** | **C** | **C** | **A** | **A** | **A** | **C** | **A** | **T** | **T** | **T** | **A** | **T** | **T** | **A** | **C** | **T** | **T** | **C** | **T** | **C** | **A** | **A** | **A** | **A** | **A** | **G** | **C** | **A** | **C** | **T** | **T** | **T** | **T** | **G** | **A** | **A** | **G** | **T** | **T** | **T** | **C** | **G** | **C** | **T** | **T** | **G** | **A** | **A** | **T** | **T** | **A** | **T** | **C** | **A** | **T** | **G** | **A** | **T** | **C** | **A** | **C** | **C** | **G** | **G** | **A** | **A** | **A** | **A** | **T** | **T** | **G** | **T** | **T** | **G** | **T** | **T** | **A** | **T** | **T** | **G** | **A** | **C** | **G** | **G** | **A** | **A** | **A** | **A** | **G** | **T** | **A** | **G** | **G** | **C** | **T** | **A** | **C** | **A** | **T** | **T** | **G** | **G** | **C** | **G** | **G** | **C** | **T** | **T** | **T** | **A** | **A** | **T** | **G** | **T** | **T** | **G** | **C** | **C** | **G** | **A** | **T** | **C** | **A** | **A** | **T** | **A** | **T** | **G** | **C** | **C** | **G** | **G** | **A** | **A** | **C** | **G** | **A** | **C** | **T** | **A** | **A** | **A** | **A** | **A** | **G** | **T** | **T** | **T** | **G** | **G** | **C** | **T** | **A** | **T** | **T** | **G** | **G** | **C** | **G** | **G** | **G** | **A** | **T** | **A** | **C** | **G** | **C** | **A** | **T** | **T** | **T** | **A** | **C** | **G** | **G** | **A** | **T** | **T** | **C** | **A** | **A** | **G** | **G** | **G** | **C** | **C** | **A** | **G** | **C** | **A** | **G** | **C** | **C** | **T** | **C** | **A** | **T** | **T** | **A** | **C** | **T** | **G** | **C** | **A** | **A** | **A** | **T** | **G** | **C** | **G** | **T** | **T** | **T** | **T** | **T** | **T** | **A** | **A** | **T** | **G** | **G** | **A** | **T** | **T** | **G** | **G** | **A** | **A** | **C** | **G** | **T** | **C** | **T** | **C** | **T** | **T** | **C** | **C** | **C** | **C** | **C** | **G** | **A** | **G** | **A** | **A** | **A** | **A** | **A** | **T** | **C** | **G** | **T** | **G** | **T** | **G** | **G** | **C** | **G** | **T** | **A** | **T** | **C** | **A** | **A** | **T** | **T** | **G** | **G** | **A** | **T** | **T** | **A** | **T** | **T** | **T** | **C** | **T** | **T** | **T** | **A** | **A** | **A** | **C** | **T** | **T** | **G** | **A** | **A** | **G** | **C** | **A** | **T** | **T** | **G** | **G** | **T** | **G** | **C** | **C** | **A** | **G** | **A** | **G** | **G** | **C** | **A** | **A** | **A** | **T** | **A** | **C** | **A** | **T** | **C** | **C** | **A** | **T** | **T** | **C** | **A** | **G** | **A** | **T** | **G** | **A** | **T** | **T** | **G** | **C** | **C** | **A** | **G** | **T** | **G** | **G** | **T** | **C** | **C** | **T** | **A** | **A** | **C** | **A** | **G** | **T** | **G** | **A** | **C** | **C** | **G** | **T** | **G** | **A** | **A** | **C** | **A** | **A** | **A** | **T** | **T** | **A** | **A** | **A** | **T** | **T** | **G** | **G** | **C** | **C** | **T** | **T** | **T** | **A** | **T** | **T** | **A** | **A** | **A** | **T** | **T** | **G** | **A** | **T** | **T** | **A** | **C** | **T** | **T** | **C** | **T** | **G** | **C** | **C** | **A** | **A** | **G** | **A** | **A** | **A** | **A** | **G** | **A** | **G** | **T** | **C** | **T** | **G** | **G** | **A** | **T** | **T** | **C** | **A** | **A** | **A** | **C** | **G** | **C** | **C** | **G** | **T** | **A** | **T** | **T** | **T** | **A** | **G** | **T** | **T** | **C** | **C** | **T** | **G** | **A** | **T** | **G** | **A** | **T** | **A** | **G** | **T** | **G** | **T** | **C** | **T** | **T** | **G** | **G** | **C** | **T** | **G** | **C** | **T** | **T** | **T** | **A** | **A** | **A** | **G** | **G** | **T** | **T** | **G** | **C** | **T** | **G** | **C** | **G** | **G** | **C** | **T** | **T** | **C** | **A** | **G** | **G** | **A** | **G** | **T** | **A** | **G** | **A** | **T** | **G** | **T** | **T** | **A** | **A** | **A** | **A** | **T** | **T** | **A** | **T** | **G** | **A** | **T** | **T** | **C** | **C** | **A** | **G** | **A** | **T** | **A** | **A** | **G** | **C** | **C** | **C** | **G** | **A** | **C** | **C** | **A** | **T** | **C** | **C** | **G** | **T** | **T** | **T** | **A** | **T** | **T** | **T** | **A** | **T** | **C** | **G** | **A** | **G** | **C** | **A** | **A** | **C** | **A** | **C** | **A** | **G** | **T** | **A** | **T** | **T** | **A** | **C** | **G** | **G** | **C** | **C** | **G** | **C** | **T** | **T** | **A** | **T** | **T** | **G** | **A** | **T** | **G** | **A** | **A** | **A** | **G** | **A** | **A** | **A** | **A** | **T** | **A** | **T** | **T** | **G** | **A** | **A** | **A** | **T** | **T** | **T** | **T** | **A** | **A** | **T** | **T** | **T** | **A** | **T** | **A** | **A** | **C** | **G** | **G** | **T** | **G** | **G** | **T** | **T** | **T** | **C** | **T** | **T** | **A** | **C** | **A** | **T** | **G** | **C** | **G** | **A** | **A** | **A** | **A** | **C** | **A** | **A** | **T** | **G** | **A** | **T** | **T** | **A** | **T** | **G** | **G** | **A** | **T** | **G** | **A** | **T** | **G** | **A** | **A** | **G** | **T** | **C** | **T** | **G** | **C** | **A** | **C** | **A** | **G** | **T** | **T** | **G** | **G** | **T** | **T** | **C** | **A** | **G** | **C** | **C** | **A** | **A** | **T** | **C** | **A** | **A** | **G** | **A** | **T** | **A** | **T** | **C** | **C** | **G** | **A** | **A** | **G** | **T** | **T** | **A** | **C** | **A** | **A** | **A** | **T** | **T** | **A** | **A** | **A** | **C** | **T** | **T** | **T** | **G** | **A** | **A** | **G** | **C** | **A** | **A** | **A** | **T** | **G** | **C** | **T** | **G** | **T** | **G** | **T** | **T** | **A** | **T** | **A** | **T** | **G** | **A** | **T** | **A** | **A** | **A** | **A** | **A** | **A** | **A** | **T** | **C** | **A** | **T** | **T** | **G** | **A** | **T** | **C** | **A** | **A** | **T** | **T** | **A** | **G** | **A** | **A** | **G** | **C** | **A** | **A** | **T** | **T** | **T** | **T** | **C** | **T** | **T** | **A** | **G** | **A** | **A** | **G** | **A** | **T** | **C** | **G** | **A** | **A** | **A** | **A** | **A** | **A** | **A** | **T** | **G** | **T** | **A** | **C** | **A** | **A** | **C** | **A** | **A** | **T** | **G** | **A** | **C** | **T** | **C** | **C** | **A** | **G** | **A** | **A** | **G** | **T** | **T** | **G** | **T** | **T** | **C** | **G** | **C** | **G** | **A** | **C** | **A** | **T** | **G** | **T** | **C** | **A** | **A** | **A** | **A** | **T** | **G** | **G** | **T** | **T** | **G** | **A** | **T** | **T** | **T** | **T** | **T** | **A** | **A** | **A** | **C** | **A** | **A** | **C** | **A** | **A** | **A** | **T** | **T** | **T** | **C** | **A** | **C** | **G** | **A** | **T** | **T** | **A** | **T** | **T** | **T** | **T** | **C** | **A** | **C** | **C** | **A** | **A** | **T** | **T** | **C** | **T** | **T** | **-** | **-** | **-** |
| **N54564** | **T** | **T** | **G** | **A** | **T** | **T** | **C** | **T** | **T** | **A** | **G** | **C** | **G** | **T** | **C** | **T** | **T** | **A** | **A** | **C** | **A** | **G** | **T** | **T** | **A** | **T** | **T** | **T** | **A** | **T** | **T** | **T** | **T** | **A** | **T** | **T** | **A** | **A** | **T** | **G** | **C** | **A** | **A** | **T** | **T** | **A** | **T** | **T** | **G** | **C** | **G** | **G** | **G** | **A** | **A** | **T** | **T** | **A** | **C** | **C** | **A** | **T** | **T** | **T** | **T** | **G** | **T** | **T** | **A** | **A** | **A** | **A** | **C** | **C** | **T** | **C** | **G** | **A** | **G** | **A** | **T** | **G** | **T** | **A** | **G** | **C** | **A** | **G** | **C** | **A** | **A** | **T** | **T** | **T** | **G** | **G** | **G** | **C** | **A** | **T** | **G** | **G** | **C** | **T** | **A** | **C** | **T** | **C** | **G** | **T** | **A** | **C** | **T** | **G** | **A** | **T** | **T** | **G** | **C** | **T** | **C** | **T** | **A** | **C** | **C** | **C** | **G** | **T** | **T** | **T** | **T** | **T** | **G** | **G** | **T** | **T** | **T** | **T** | **T** | **T** | **C** | **T** | **T** | **A** | **T** | **A** | **T** | **T** | **T** | **A** | **T** | **T** | **C** | **T** | **T** | **T** | **G** | **G** | **G** | **C** | **G** | **C** | **G** | **G** | **T** | **T** | **T** | **A** | **A** | **C** | **C** | **G** | **A** | **C** | **A** | **A** | **A** | **A** | **A** | **G** | **A** | **A** | **A** | **T** | **T** | **T** | **T** | **A** | **T** | **T** | **T** | **G** | **C** | **A** | **A** | **C** | **A** | **A** | **A** | **G** | **T** | **G** | **A** | **T** | **T** | **T** | **G** | **C** | **G** | **G** | **G** | **A** | **A** | **T** | **T** | **A** | **G** | **A** | **G** | **A** | **A** | **T** | **T** | **T** | **T** | **C** | **A** | **G** | **A** | **A** | **T** | **T** | **T** | **C** | **C** | **A** | **A** | **G** | **A** | **A** | **G** | **A** | **A** | **A** | **G** | **T** | **T** | **T** | **T** | **G** | **A** | **A** | **C** | **T** | **T** | **T** | **A** | **T** | **A** | **G** | **T** | **C** | **A** | **A** | **A** | **A** | **G** | **A** | **T** | **G** | **C** | **C** | **G** | **A** | **C** | **G** | **G** | **A** | **G** | **G** | **A** | **G** | **C** | **A** | **A** | **C** | **A** | **A** | **C** | **A** | **G** | **T** | **T** | **T** | **A** | **C** | **A** | **G** | **A** | **C** | **T** | **T** | **T** | **T** | **T** | **T** | **T** | **C** | **C** | **T** | **C** | **G** | **T** | **T** | **A** | **A** | **A** | **T** | **A** | **G** | **G** | **A** | **T** | **G** | **C** | **C** | **T** | **C** | **T** | **G** | **A** | **C** | **T** | **A** | **A** | **G** | **A** | **A** | **A** | **A** | **A** | **T** | **G** | **A** | **T** | **G** | **T** | **T** | **G** | **A** | **A** | **A** | **T** | **T** | **T** | **T** | **T** | **A** | **C** | **G** | **G** | **A** | **T** | **G** | **G** | **T** | **A** | **C** | **G** | **G** | **A** | **A** | **A** | **A** | **A** | **T** | **T** | **C** | **A** | **A** | **T** | **G** | **C** | **G** | **T** | **T** | **G** | **A** | **T** | **G** | **G** | **C** | **G** | **G** | **A** | **C** | **A** | **T** | **A** | **A** | **A** | **A** | **A** | **A** | **A** | **G** | **C** | **T** | **C** | **A** | **G** | **C** | **A** | **C** | **T** | **C** | **C** | **A** | **T** | **T** | **C** | **A** | **T** | **A** | **T** | **T** | **G** | **A** | **A** | **T** | **A** | **T** | **T** | **A** | **T** | **G** | **C** | **C** | **T** | **T** | **T** | **G** | **T** | **A** | **A** | **C** | **G** | **G** | **A** | **T** | **C** | **A** | **T** | **A** | **T** | **C** | **G** | **G** | **C** | **A** | **C** | **C** | **A** | **A** | **A** | **A** | **T** | **T** | **T** | **T** | **G** | **A** | **A** | **C** | **T** | **T** | **A** | **T** | **T** | **A** | **G** | **A** | **A** | **G** | **A** | **A** | **A** | **A** | **G** | **G** | **C** | **G** | **G** | **C** | **T** | **G** | **A** | **A** | **G** | **G** | **G** | **G** | **T** | **T** | **G** | **A** | **A** | **G** | **T** | **G** | **C** | **G** | **G** | **T** | **T** | **G** | **C** | **T** | **T** | **T** | **A** | **C** | **G** | **A** | **T** | **G** | **C** | **C** | **T** | **T** | **T** | **G** | **G** | **C** | **T** | **C** | **A** | **A** | **A** | **G** | **G** | **G** | **C** | **A** | **C** | **C** | **A** | **A** | **G** | **G** | **T** | **T** | **C** | **A** | **T** | **C** | **A** | **T** | **T** | **T** | **G** | **A** | **A** | **T** | **G** | **A** | **A** | **T** | **T** | **G** | **A** | **A** | **A** | **A** | **A** | **A** | **A** | **A** | **C** | **G** | **G** | **T** | **G** | **G** | **T** | **T** | **T** | **T** | **G** | **T** | **C** | **C** | **A** | **A** | **A** | **C** | **G** | **T** | **T** | **T** | **A** | **T** | **T** | **A** | **C** | **T** | **T** | **C** | **T** | **C** | **A** | **A** | **A** | **A** | **A** | **G** | **C** | **A** | **C** | **T** | **T** | **T** | **T** | **G** | **A** | **A** | **G** | **T** | **T** | **T** | **C** | **G** | **T** | **T** | **T** | **G** | **A** | **A** | **T** | **T** | **A** | **T** | **C** | **A** | **T** | **G** | **A** | **T** | **C** | **A** | **C** | **C** | **G** | **G** | **A** | **A** | **A** | **A** | **T** | **T** | **G** | **T** | **T** | **G** | **T** | **T** | **A** | **T** | **T** | **G** | **A** | **C** | **G** | **G** | **A** | **A** | **A** | **A** | **G** | **T** | **A** | **G** | **G** | **C** | **T** | **A** | **C** | **A** | **T** | **T** | **G** | **G** | **C** | **G** | **G** | **C** | **T** | **T** | **T** | **A** | **A** | **T** | **G** | **T** | **T** | **G** | **C** | **C** | **G** | **A** | **T** | **C** | **A** | **A** | **T** | **A** | **T** | **G** | **C** | **T** | **G** | **G** | **A** | **A** | **C** | **G** | **A** | **C** | **T** | **A** | **A** | **A** | **A** | **A** | **G** | **T** | **T** | **T** | **G** | **G** | **C** | **T** | **A** | **T** | **T** | **G** | **G** | **C** | **G** | **G** | **G** | **A** | **T** | **A** | **C** | **G** | **C** | **A** | **T** | **T** | **T** | **A** | **C** | **G** | **G** | **A** | **T** | **T** | **C** | **A** | **A** | **G** | **G** | **G** | **C** | **C** | **A** | **G** | **C** | **A** | **G** | **C** | **C** | **T** | **C** | **A** | **T** | **T** | **A** | **C** | **T** | **G** | **C** | **A** | **A** | **A** | **T** | **G** | **C** | **G** | **T** | **T** | **T** | **T** | **T** | **T** | **A** | **A** | **T** | **G** | **G** | **A** | **T** | **T** | **G** | **G** | **A** | **A** | **C** | **G** | **T** | **C** | **T** | **C** | **T** | **T** | **C** | **C** | **C** | **C** | **C** | **G** | **A** | **G** | **A** | **A** | **A** | **A** | **A** | **T** | **C** | **G** | **T** | **G** | **T** | **G** | **G** | **C** | **G** | **T** | **A** | **T** | **C** | **A** | **A** | **T** | **T** | **G** | **G** | **A** | **T** | **T** | **A** | **T** | **T** | **T** | **C** | **T** | **T** | **T** | **A** | **A** | **A** | **C** | **T** | **T** | **G** | **A** | **A** | **G** | **C** | **A** | **T** | **T** | **G** | **G** | **T** | **G** | **C** | **C** | **A** | **G** | **A** | **G** | **G** | **C** | **A** | **A** | **A** | **T** | **A** | **C** | **A** | **T** | **C** | **C** | **A** | **T** | **T** | **C** | **A** | **G** | **A** | **T** | **G** | **A** | **T** | **T** | **G** | **C** | **C** | **A** | **G** | **T** | **G** | **G** | **T** | **C** | **C** | **T** | **A** | **A** | **C** | **A** | **G** | **T** | **G** | **A** | **C** | **C** | **G** | **T** | **G** | **A** | **A** | **C** | **A** | **A** | **A** | **T** | **T** | **A** | **A** | **A** | **T** | **T** | **G** | **G** | **C** | **C** | **T** | **T** | **T** | **A** | **T** | **T** | **A** | **A** | **A** | **T** | **T** | **G** | **A** | **T** | **T** | **A** | **C** | **T** | **T** | **C** | **T** | **G** | **C** | **C** | **A** | **A** | **G** | **A** | **A** | **A** | **A** | **G** | **A** | **G** | **T** | **C** | **T** | **G** | **G** | **A** | **T** | **T** | **C** | **A** | **A** | **A** | **C** | **G** | **C** | **C** | **G** | **T** | **A** | **T** | **T** | **T** | **A** | **G** | **T** | **T** | **C** | **C** | **T** | **G** | **A** | **T** | **G** | **A** | **T** | **A** | **G** | **T** | **G** | **T** | **C** | **T** | **T** | **G** | **G** | **C** | **T** | **G** | **C** | **T** | **T** | **T** | **A** | **A** | **A** | **G** | **G** | **T** | **T** | **G** | **C** | **T** | **G** | **C** | **G** | **G** | **C** | **T** | **T** | **C** | **A** | **G** | **G** | **A** | **G** | **T** | **A** | **G** | **A** | **T** | **G** | **T** | **T** | **A** | **A** | **A** | **A** | **T** | **T** | **A** | **T** | **G** | **A** | **T** | **T** | **C** | **C** | **A** | **G** | **A** | **T** | **A** | **A** | **G** | **C** | **C** | **C** | **G** | **A** | **C** | **C** | **A** | **T** | **C** | **C** | **G** | **T** | **T** | **T** | **A** | **T** | **T** | **T** | **A** | **T** | **C** | **G** | **A** | **G** | **C** | **A** | **A** | **C** | **A** | **C** | **A** | **G** | **T** | **A** | **T** | **T** | **A** | **C** | **G** | **G** | **C** | **C** | **G** | **C** | **T** | **T** | **A** | **T** | **T** | **G** | **A** | **T** | **G** | **A** | **A** | **A** | **G** | **A** | **A** | **A** | **A** | **T** | **A** | **T** | **T** | **G** | **A** | **A** | **A** | **T** | **T** | **T** | **T** | **A** | **A** | **T** | **T** | **T** | **A** | **T** | **A** | **A** | **C** | **G** | **G** | **T** | **G** | **G** | **T** | **T** | **T** | **C** | **T** | **T** | **A** | **C** | **A** | **T** | **G** | **C** | **G** | **A** | **A** | **A** | **A** | **C** | **A** | **A** | **T** | **G** | **A** | **T** | **T** | **A** | **T** | **G** | **G** | **A** | **T** | **G** | **A** | **T** | **G** | **A** | **A** | **G** | **T** | **C** | **T** | **G** | **C** | **A** | **C** | **A** | **G** | **T** | **T** | **G** | **G** | **T** | **T** | **C** | **A** | **G** | **C** | **C** | **A** | **A** | **T** | **C** | **A** | **A** | **G** | **A** | **T** | **A** | **T** | **C** | **C** | **G** | **A** | **A** | **G** | **T** | **T** | **A** | **C** | **A** | **A** | **A** | **T** | **T** | **A** | **A** | **A** | **C** | **T** | **T** | **T** | **G** | **A** | **A** | **G** | **C** | **A** | **A** | **A** | **T** | **G** | **C** | **T** | **G** | **T** | **G** | **T** | **T** | **A** | **T** | **A** | **T** | **G** | **A** | **T** | **A** | **A** | **A** | **A** | **A** | **A** | **A** | **T** | **C** | **A** | **T** | **T** | **G** | **A** | **T** | **C** | **A** | **A** | **T** | **T** | **A** | **G** | **A** | **A** | **G** | **C** | **A** | **A** | **T** | **T** | **T** | **T** | **C** | **T** | **T** | **A** | **G** | **A** | **A** | **G** | **A** | **T** | **C** | **G** | **A** | **A** | **A** | **A** | **A** | **A** | **A** | **T** | **G** | **T** | **A** | **C** | **A** | **A** | **C** | **A** | **A** | **T** | **G** | **A** | **C** | **T** | **C** | **C** | **A** | **G** | **A** | **A** | **G** | **T** | **T** | **G** | **T** | **T** | **C** | **G** | **C** | **G** | **A** | **C** | **A** | **T** | **G** | **T** | **C** | **A** | **A** | **A** | **A** | **T** | **G** | **G** | **T** | **T** | **G** | **A** | **T** | **T** | **T** | **T** | **T** | **A** | **A** | **A** | **C** | **A** | **A** | **C** | **A** | **A** | **A** | **T** | **T** | **T** | **C** | **A** | **C** | **G** | **A** | **T** | **T** | **A** | **T** | **T** | **T** | **T** | **C** | **A** | **C** | **C** | **A** | **A** | **T** | **T** | **C** | **T** | **T** | **-** | **-** | **-** |
| **N55106** | **T** | **T** | **G** | **A** | **T** | **T** | **C** | **T** | **T** | **A** | **G** | **C** | **G** | **T** | **C** | **T** | **T** | **A** | **A** | **C** | **A** | **G** | **T** | **T** | **A** | **T** | **T** | **T** | **A** | **T** | **T** | **T** | **T** | **A** | **T** | **T** | **A** | **A** | **T** | **G** | **C** | **A** | **A** | **T** | **T** | **A** | **T** | **T** | **G** | **C** | **G** | **G** | **G** | **A** | **A** | **T** | **T** | **A** | **C** | **C** | **A** | **T** | **T** | **T** | **T** | **G** | **T** | **T** | **A** | **A** | **A** | **A** | **C** | **C** | **T** | **C** | **G** | **A** | **G** | **A** | **T** | **G** | **T** | **A** | **G** | **C** | **A** | **G** | **C** | **A** | **A** | **T** | **T** | **T** | **G** | **G** | **G** | **C** | **A** | **T** | **G** | **G** | **C** | **T** | **A** | **C** | **T** | **C** | **G** | **T** | **A** | **C** | **T** | **G** | **A** | **T** | **T** | **G** | **C** | **T** | **C** | **T** | **A** | **C** | **C** | **C** | **G** | **T** | **T** | **T** | **T** | **T** | **G** | **G** | **T** | **T** | **T** | **T** | **T** | **T** | **C** | **T** | **T** | **A** | **T** | **A** | **T** | **T** | **T** | **A** | **T** | **T** | **C** | **T** | **T** | **T** | **G** | **G** | **G** | **C** | **G** | **C** | **G** | **G** | **T** | **T** | **T** | **A** | **A** | **C** | **C** | **G** | **A** | **C** | **A** | **A** | **A** | **A** | **A** | **G** | **A** | **A** | **A** | **T** | **T** | **T** | **T** | **A** | **T** | **T** | **T** | **G** | **C** | **A** | **A** | **C** | **A** | **A** | **A** | **G** | **T** | **G** | **A** | **T** | **T** | **T** | **A** | **C** | **G** | **G** | **G** | **A** | **A** | **T** | **T** | **A** | **G** | **A** | **G** | **A** | **A** | **T** | **T** | **T** | **C** | **C** | **A** | **G** | **A** | **A** | **T** | **T** | **T** | **C** | **C** | **A** | **A** | **G** | **A** | **A** | **G** | **A** | **A** | **A** | **G** | **T** | **T** | **T** | **T** | **G** | **A** | **A** | **C** | **T** | **T** | **T** | **A** | **T** | **A** | **G** | **T** | **C** | **A** | **A** | **A** | **A** | **G** | **A** | **T** | **G** | **C** | **C** | **G** | **A** | **C** | **G** | **G** | **A** | **G** | **G** | **A** | **G** | **C** | **A** | **A** | **C** | **A** | **A** | **C** | **A** | **G** | **T** | **T** | **T** | **A** | **C** | **A** | **G** | **A** | **C** | **T** | **T** | **T** | **T** | **T** | **T** | **T** | **C** | **C** | **T** | **C** | **G** | **T** | **T** | **A** | **A** | **A** | **T** | **A** | **G** | **G** | **A** | **T** | **G** | **C** | **C** | **T** | **C** | **T** | **G** | **A** | **C** | **T** | **A** | **A** | **G** | **A** | **A** | **A** | **A** | **A** | **T** | **G** | **A** | **T** | **G** | **T** | **T** | **G** | **A** | **A** | **A** | **T** | **T** | **T** | **T** | **T** | **A** | **C** | **G** | **G** | **A** | **T** | **G** | **G** | **T** | **A** | **C** | **G** | **G** | **A** | **A** | **A** | **A** | **A** | **T** | **T** | **C** | **A** | **A** | **T** | **G** | **C** | **G** | **T** | **T** | **G** | **A** | **T** | **G** | **G** | **C** | **G** | **G** | **A** | **C** | **A** | **T** | **A** | **A** | **A** | **A** | **A** | **A** | **A** | **G** | **C** | **T** | **C** | **A** | **G** | **C** | **A** | **C** | **T** | **C** | **C** | **A** | **T** | **T** | **C** | **A** | **T** | **A** | **T** | **T** | **G** | **A** | **A** | **T** | **A** | **T** | **T** | **A** | **T** | **G** | **C** | **C** | **T** | **T** | **T** | **G** | **T** | **A** | **A** | **C** | **G** | **G** | **A** | **T** | **C** | **A** | **T** | **A** | **T** | **C** | **G** | **G** | **C** | **A** | **C** | **C** | **A** | **A** | **A** | **A** | **T** | **T** | **T** | **T** | **G** | **A** | **A** | **C** | **T** | **T** | **A** | **T** | **T** | **A** | **G** | **A** | **A** | **G** | **A** | **A** | **A** | **A** | **G** | **G** | **C** | **G** | **G** | **C** | **T** | **G** | **A** | **A** | **G** | **G** | **G** | **G** | **T** | **T** | **G** | **A** | **A** | **G** | **T** | **G** | **C** | **G** | **G** | **T** | **T** | **G** | **C** | **T** | **T** | **T** | **A** | **C** | **G** | **A** | **T** | **G** | **C** | **C** | **T** | **T** | **T** | **G** | **G** | **C** | **T** | **C** | **A** | **A** | **A** | **G** | **G** | **G** | **C** | **A** | **C** | **C** | **A** | **A** | **G** | **G** | **T** | **T** | **C** | **A** | **T** | **C** | **A** | **T** | **T** | **T** | **G** | **A** | **A** | **T** | **G** | **A** | **A** | **T** | **T** | **G** | **A** | **A** | **A** | **A** | **A** | **A** | **A** | **A** | **C** | **G** | **G** | **C** | **G** | **G** | **T** | **T** | **T** | **T** | **G** | **T** | **C** | **C** | **A** | **A** | **A** | **C** | **A** | **T** | **T** | **T** | **A** | **T** | **T** | **A** | **C** | **T** | **T** | **C** | **T** | **C** | **A** | **A** | **A** | **A** | **A** | **G** | **C** | **A** | **C** | **T** | **T** | **T** | **T** | **G** | **A** | **A** | **G** | **T** | **T** | **T** | **C** | **G** | **T** | **T** | **T** | **G** | **A** | **A** | **T** | **T** | **A** | **T** | **C** | **A** | **T** | **G** | **A** | **T** | **C** | **A** | **C** | **C** | **G** | **G** | **A** | **A** | **A** | **A** | **T** | **T** | **G** | **T** | **T** | **G** | **T** | **T** | **A** | **T** | **T** | **G** | **A** | **C** | **G** | **G** | **A** | **A** | **A** | **A** | **G** | **T** | **A** | **G** | **G** | **C** | **T** | **A** | **C** | **A** | **T** | **T** | **G** | **G** | **C** | **G** | **G** | **C** | **T** | **T** | **T** | **A** | **A** | **T** | **G** | **T** | **T** | **G** | **C** | **C** | **G** | **A** | **T** | **C** | **A** | **A** | **T** | **A** | **T** | **G** | **C** | **C** | **G** | **G** | **A** | **A** | **C** | **G** | **A** | **C** | **T** | **A** | **A** | **A** | **A** | **A** | **G** | **T** | **T** | **T** | **G** | **G** | **C** | **T** | **A** | **T** | **T** | **G** | **G** | **C** | **G** | **G** | **G** | **A** | **T** | **A** | **C** | **G** | **C** | **A** | **T** | **T** | **T** | **A** | **C** | **G** | **G** | **A** | **T** | **T** | **C** | **A** | **A** | **G** | **G** | **G** | **C** | **C** | **A** | **G** | **C** | **A** | **G** | **C** | **C** | **T** | **C** | **A** | **T** | **T** | **A** | **C** | **T** | **G** | **C** | **A** | **A** | **A** | **T** | **G** | **C** | **G** | **T** | **T** | **T** | **T** | **T** | **T** | **A** | **A** | **T** | **G** | **G** | **A** | **T** | **T** | **G** | **G** | **A** | **A** | **C** | **G** | **T** | **C** | **T** | **C** | **T** | **T** | **C** | **C** | **C** | **C** | **C** | **G** | **A** | **G** | **A** | **A** | **A** | **A** | **A** | **T** | **C** | **G** | **T** | **G** | **T** | **G** | **G** | **C** | **G** | **T** | **A** | **T** | **C** | **A** | **A** | **T** | **T** | **G** | **G** | **A** | **T** | **T** | **A** | **T** | **T** | **T** | **C** | **T** | **T** | **T** | **A** | **A** | **A** | **C** | **T** | **T** | **G** | **A** | **A** | **G** | **C** | **A** | **T** | **T** | **G** | **G** | **T** | **G** | **C** | **C** | **A** | **G** | **A** | **G** | **G** | **C** | **A** | **A** | **A** | **T** | **A** | **C** | **A** | **T** | **C** | **C** | **A** | **T** | **T** | **C** | **A** | **G** | **A** | **T** | **G** | **A** | **T** | **T** | **G** | **C** | **C** | **A** | **G** | **T** | **G** | **G** | **T** | **C** | **C** | **T** | **A** | **A** | **C** | **A** | **G** | **T** | **G** | **A** | **C** | **C** | **G** | **T** | **G** | **A** | **A** | **C** | **A** | **A** | **A** | **T** | **T** | **A** | **A** | **A** | **T** | **T** | **G** | **G** | **C** | **C** | **T** | **T** | **T** | **A** | **T** | **T** | **A** | **A** | **A** | **T** | **T** | **G** | **A** | **T** | **T** | **A** | **C** | **T** | **T** | **C** | **T** | **G** | **C** | **C** | **A** | **A** | **G** | **A** | **A** | **A** | **A** | **G** | **A** | **G** | **T** | **C** | **T** | **G** | **G** | **A** | **T** | **T** | **C** | **A** | **A** | **A** | **C** | **G** | **C** | **C** | **G** | **T** | **A** | **T** | **T** | **T** | **A** | **G** | **T** | **T** | **C** | **C** | **T** | **G** | **A** | **T** | **G** | **A** | **T** | **A** | **G** | **T** | **G** | **T** | **C** | **T** | **T** | **G** | **G** | **C** | **T** | **G** | **C** | **T** | **T** | **T** | **A** | **A** | **A** | **G** | **G** | **T** | **T** | **G** | **C** | **T** | **G** | **C** | **G** | **G** | **C** | **T** | **T** | **C** | **A** | **G** | **G** | **A** | **G** | **T** | **A** | **G** | **A** | **T** | **G** | **T** | **T** | **A** | **A** | **A** | **A** | **T** | **T** | **A** | **T** | **G** | **A** | **T** | **T** | **C** | **C** | **A** | **G** | **A** | **T** | **A** | **A** | **G** | **C** | **C** | **C** | **G** | **A** | **C** | **C** | **A** | **T** | **C** | **C** | **G** | **T** | **T** | **T** | **A** | **T** | **T** | **T** | **A** | **T** | **C** | **G** | **A** | **G** | **C** | **A** | **A** | **C** | **A** | **C** | **A** | **G** | **T** | **A** | **T** | **T** | **A** | **C** | **G** | **G** | **C** | **C** | **G** | **C** | **T** | **T** | **A** | **T** | **T** | **G** | **A** | **T** | **G** | **A** | **A** | **A** | **G** | **A** | **A** | **A** | **A** | **T** | **A** | **T** | **T** | **G** | **A** | **A** | **A** | **T** | **T** | **T** | **T** | **A** | **A** | **T** | **T** | **T** | **A** | **T** | **A** | **A** | **C** | **G** | **G** | **T** | **G** | **G** | **T** | **T** | **T** | **C** | **T** | **T** | **A** | **C** | **A** | **T** | **G** | **C** | **G** | **A** | **A** | **A** | **A** | **C** | **A** | **A** | **T** | **G** | **A** | **T** | **T** | **A** | **T** | **G** | **G** | **A** | **T** | **G** | **A** | **T** | **G** | **A** | **A** | **G** | **T** | **C** | **T** | **G** | **C** | **A** | **C** | **A** | **G** | **T** | **T** | **G** | **G** | **T** | **T** | **C** | **A** | **G** | **C** | **C** | **A** | **A** | **T** | **C** | **A** | **A** | **G** | **A** | **T** | **A** | **T** | **C** | **C** | **G** | **A** | **A** | **G** | **T** | **T** | **A** | **C** | **A** | **A** | **A** | **T** | **T** | **A** | **A** | **A** | **C** | **T** | **T** | **T** | **G** | **A** | **A** | **G** | **C** | **A** | **A** | **A** | **T** | **G** | **C** | **T** | **G** | **T** | **G** | **T** | **T** | **A** | **T** | **A** | **T** | **G** | **A** | **T** | **A** | **A** | **A** | **A** | **A** | **A** | **A** | **T** | **C** | **A** | **T** | **T** | **G** | **A** | **T** | **C** | **A** | **A** | **T** | **T** | **A** | **G** | **A** | **A** | **G** | **C** | **A** | **A** | **T** | **T** | **T** | **T** | **C** | **T** | **T** | **A** | **G** | **A** | **A** | **G** | **A** | **T** | **C** | **G** | **A** | **A** | **A** | **A** | **A** | **A** | **A** | **T** | **G** | **T** | **A** | **C** | **A** | **A** | **C** | **A** | **A** | **T** | **G** | **A** | **C** | **T** | **C** | **C** | **A** | **G** | **A** | **A** | **G** | **T** | **T** | **G** | **T** | **T** | **C** | **G** | **C** | **G** | **A** | **C** | **A** | **T** | **G** | **T** | **C** | **A** | **A** | **A** | **A** | **T** | **G** | **G** | **T** | **T** | **G** | **A** | **T** | **T** | **T** | **T** | **T** | **A** | **A** | **A** | **C** | **A** | **A** | **C** | **A** | **A** | **A** | **T** | **T** | **T** | **C** | **A** | **C** | **G** | **A** | **T** | **T** | **A** | **T** | **T** | **T** | **T** | **C** | **A** | **C** | **C** | **A** | **A** | **T** | **T** | **C** | **T** | **T** | **-** | **-** | **-** |
| **N55240** | **T** | **T** | **G** | **A** | **T** | **T** | **C** | **T** | **T** | **A** | **G** | **C** | **G** | **T** | **C** | **T** | **T** | **A** | **A** | **C** | **A** | **G** | **T** | **T** | **A** | **T** | **T** | **T** | **A** | **T** | **T** | **T** | **T** | **A** | **T** | **T** | **A** | **A** | **T** | **G** | **C** | **A** | **A** | **T** | **T** | **A** | **T** | **T** | **G** | **C** | **G** | **G** | **G** | **A** | **A** | **T** | **T** | **A** | **C** | **C** | **A** | **T** | **T** | **T** | **T** | **G** | **T** | **T** | **A** | **A** | **A** | **A** | **C** | **C** | **T** | **C** | **G** | **A** | **G** | **A** | **T** | **G** | **T** | **A** | **G** | **C** | **A** | **G** | **C** | **A** | **A** | **T** | **T** | **T** | **G** | **G** | **G** | **C** | **A** | **T** | **G** | **G** | **C** | **T** | **A** | **C** | **T** | **C** | **G** | **T** | **A** | **C** | **T** | **G** | **A** | **T** | **T** | **G** | **C** | **T** | **C** | **T** | **A** | **C** | **C** | **C** | **G** | **T** | **T** | **T** | **T** | **T** | **G** | **G** | **T** | **T** | **T** | **T** | **T** | **T** | **C** | **T** | **T** | **A** | **T** | **A** | **T** | **T** | **T** | **A** | **T** | **T** | **C** | **T** | **T** | **T** | **G** | **G** | **G** | **C** | **G** | **C** | **G** | **G** | **T** | **T** | **T** | **A** | **A** | **C** | **C** | **G** | **A** | **C** | **A** | **A** | **A** | **A** | **A** | **G** | **A** | **A** | **A** | **T** | **T** | **T** | **T** | **A** | **T** | **T** | **T** | **G** | **C** | **A** | **A** | **C** | **A** | **A** | **A** | **G** | **T** | **G** | **A** | **T** | **T** | **T** | **A** | **C** | **G** | **G** | **G** | **A** | **A** | **T** | **T** | **A** | **G** | **A** | **G** | **A** | **A** | **T** | **T** | **T** | **C** | **C** | **A** | **G** | **A** | **A** | **T** | **T** | **T** | **C** | **C** | **A** | **A** | **G** | **A** | **A** | **G** | **A** | **A** | **A** | **G** | **T** | **T** | **T** | **T** | **G** | **A** | **A** | **C** | **T** | **T** | **T** | **A** | **T** | **A** | **G** | **T** | **C** | **A** | **A** | **A** | **A** | **G** | **A** | **T** | **G** | **C** | **C** | **G** | **A** | **C** | **G** | **G** | **A** | **G** | **G** | **A** | **G** | **C** | **A** | **A** | **C** | **A** | **A** | **C** | **A** | **G** | **T** | **T** | **T** | **A** | **C** | **A** | **G** | **A** | **C** | **T** | **T** | **T** | **T** | **T** | **T** | **T** | **C** | **C** | **T** | **C** | **G** | **T** | **T** | **A** | **A** | **A** | **T** | **A** | **G** | **G** | **A** | **T** | **G** | **C** | **C** | **T** | **C** | **T** | **G** | **A** | **C** | **T** | **A** | **A** | **G** | **A** | **A** | **A** | **A** | **A** | **T** | **G** | **A** | **T** | **G** | **T** | **T** | **G** | **A** | **A** | **A** | **T** | **T** | **T** | **T** | **T** | **A** | **C** | **G** | **G** | **A** | **T** | **G** | **G** | **T** | **A** | **C** | **G** | **G** | **A** | **A** | **A** | **A** | **A** | **T** | **T** | **C** | **A** | **A** | **T** | **G** | **C** | **G** | **T** | **T** | **G** | **A** | **T** | **G** | **G** | **C** | **G** | **G** | **A** | **T** | **A** | **T** | **A** | **A** | **A** | **A** | **A** | **A** | **A** | **G** | **C** | **T** | **C** | **A** | **G** | **C** | **A** | **C** | **T** | **C** | **T** | **A** | **T** | **T** | **C** | **A** | **T** | **A** | **T** | **T** | **G** | **A** | **A** | **T** | **A** | **T** | **T** | **A** | **T** | **G** | **C** | **C** | **T** | **T** | **T** | **G** | **T** | **A** | **A** | **C** | **G** | **G** | **A** | **T** | **C** | **A** | **T** | **A** | **T** | **C** | **G** | **G** | **T** | **A** | **C** | **C** | **A** | **A** | **A** | **A** | **T** | **T** | **T** | **T** | **G** | **A** | **A** | **C** | **T** | **T** | **A** | **T** | **T** | **A** | **G** | **A** | **A** | **G** | **A** | **A** | **A** | **A** | **G** | **G** | **C** | **G** | **G** | **C** | **T** | **G** | **A** | **A** | **G** | **G** | **G** | **G** | **T** | **T** | **G** | **A** | **A** | **G** | **T** | **G** | **C** | **G** | **G** | **T** | **T** | **G** | **C** | **T** | **T** | **T** | **A** | **C** | **G** | **A** | **T** | **G** | **C** | **C** | **T** | **T** | **T** | **G** | **G** | **C** | **T** | **C** | **A** | **A** | **A** | **G** | **G** | **G** | **C** | **A** | **C** | **C** | **A** | **A** | **G** | **G** | **T** | **T** | **C** | **A** | **T** | **C** | **A** | **T** | **T** | **T** | **G** | **A** | **A** | **T** | **G** | **A** | **A** | **T** | **T** | **G** | **A** | **A** | **A** | **A** | **A** | **A** | **A** | **A** | **C** | **G** | **G** | **C** | **G** | **G** | **T** | **T** | **T** | **T** | **G** | **T** | **C** | **C** | **A** | **A** | **A** | **C** | **G** | **T** | **T** | **T** | **A** | **T** | **T** | **A** | **C** | **T** | **T** | **C** | **T** | **C** | **A** | **A** | **A** | **A** | **A** | **G** | **C** | **A** | **C** | **T** | **T** | **T** | **T** | **G** | **A** | **A** | **G** | **T** | **T** | **T** | **C** | **G** | **T** | **T** | **T** | **G** | **A** | **A** | **T** | **T** | **A** | **T** | **C** | **A** | **T** | **G** | **A** | **T** | **C** | **A** | **C** | **C** | **G** | **G** | **A** | **A** | **A** | **A** | **T** | **T** | **G** | **T** | **T** | **G** | **T** | **T** | **A** | **T** | **T** | **G** | **A** | **C** | **G** | **G** | **A** | **A** | **A** | **A** | **G** | **T** | **A** | **G** | **G** | **C** | **T** | **A** | **C** | **A** | **T** | **T** | **G** | **G** | **C** | **G** | **G** | **C** | **T** | **T** | **T** | **A** | **A** | **T** | **G** | **T** | **T** | **G** | **C** | **C** | **G** | **A** | **T** | **C** | **A** | **A** | **T** | **A** | **T** | **G** | **C** | **T** | **G** | **G** | **A** | **A** | **C** | **G** | **A** | **C** | **T** | **A** | **A** | **A** | **A** | **A** | **G** | **T** | **T** | **T** | **G** | **G** | **C** | **T** | **A** | **T** | **T** | **G** | **G** | **C** | **G** | **G** | **G** | **A** | **T** | **A** | **C** | **A** | **C** | **A** | **T** | **T** | **T** | **A** | **C** | **G** | **G** | **A** | **T** | **T** | **C** | **A** | **A** | **G** | **G** | **G** | **C** | **C** | **A** | **G** | **C** | **A** | **G** | **C** | **C** | **T** | **C** | **A** | **T** | **T** | **A** | **C** | **T** | **G** | **C** | **A** | **A** | **A** | **T** | **G** | **C** | **G** | **T** | **T** | **T** | **T** | **T** | **T** | **A** | **A** | **T** | **G** | **G** | **A** | **T** | **T** | **G** | **G** | **A** | **A** | **C** | **G** | **T** | **C** | **T** | **C** | **T** | **T** | **C** | **C** | **C** | **C** | **C** | **G** | **A** | **G** | **A** | **A** | **A** | **A** | **A** | **T** | **C** | **G** | **T** | **G** | **T** | **G** | **G** | **C** | **G** | **T** | **A** | **T** | **C** | **A** | **A** | **T** | **T** | **G** | **G** | **A** | **T** | **T** | **A** | **T** | **T** | **T** | **C** | **T** | **T** | **T** | **A** | **A** | **A** | **C** | **T** | **T** | **G** | **A** | **A** | **G** | **C** | **A** | **T** | **T** | **G** | **G** | **T** | **G** | **C** | **C** | **A** | **G** | **A** | **G** | **G** | **C** | **A** | **A** | **A** | **T** | **A** | **C** | **A** | **T** | **C** | **C** | **A** | **T** | **T** | **C** | **A** | **G** | **A** | **T** | **G** | **A** | **T** | **T** | **G** | **C** | **C** | **A** | **G** | **T** | **G** | **G** | **T** | **C** | **C** | **T** | **A** | **A** | **C** | **A** | **G** | **T** | **G** | **A** | **C** | **C** | **G** | **T** | **G** | **A** | **A** | **C** | **A** | **A** | **A** | **T** | **T** | **A** | **A** | **A** | **T** | **T** | **G** | **G** | **C** | **C** | **T** | **T** | **T** | **A** | **T** | **T** | **A** | **A** | **A** | **T** | **T** | **G** | **A** | **T** | **T** | **A** | **C** | **T** | **T** | **C** | **T** | **G** | **C** | **C** | **A** | **A** | **G** | **A** | **A** | **A** | **A** | **G** | **A** | **G** | **T** | **C** | **T** | **G** | **G** | **A** | **T** | **T** | **C** | **A** | **A** | **A** | **C** | **G** | **C** | **C** | **G** | **T** | **A** | **T** | **T** | **T** | **A** | **G** | **T** | **T** | **C** | **C** | **T** | **G** | **A** | **T** | **G** | **A** | **T** | **A** | **G** | **T** | **G** | **T** | **C** | **T** | **T** | **G** | **G** | **C** | **T** | **G** | **C** | **T** | **T** | **T** | **A** | **A** | **A** | **G** | **G** | **T** | **T** | **G** | **C** | **T** | **G** | **C** | **G** | **G** | **C** | **T** | **T** | **C** | **A** | **G** | **G** | **A** | **G** | **T** | **A** | **G** | **A** | **T** | **G** | **T** | **T** | **A** | **A** | **A** | **A** | **T** | **T** | **A** | **T** | **G** | **A** | **T** | **T** | **C** | **C** | **A** | **G** | **A** | **T** | **A** | **A** | **G** | **C** | **C** | **C** | **G** | **A** | **C** | **C** | **A** | **T** | **C** | **C** | **G** | **T** | **T** | **T** | **A** | **T** | **T** | **T** | **A** | **T** | **C** | **G** | **A** | **G** | **C** | **A** | **A** | **C** | **A** | **C** | **A** | **G** | **T** | **A** | **T** | **T** | **A** | **C** | **G** | **G** | **C** | **C** | **G** | **C** | **T** | **T** | **A** | **T** | **T** | **G** | **A** | **T** | **G** | **A** | **A** | **A** | **G** | **A** | **A** | **A** | **A** | **T** | **A** | **T** | **T** | **G** | **A** | **A** | **A** | **T** | **T** | **T** | **T** | **A** | **A** | **T** | **T** | **T** | **A** | **T** | **A** | **A** | **C** | **G** | **G** | **T** | **G** | **G** | **T** | **T** | **T** | **C** | **T** | **T** | **A** | **C** | **A** | **T** | **G** | **C** | **G** | **A** | **A** | **A** | **A** | **C** | **A** | **A** | **T** | **G** | **A** | **T** | **T** | **A** | **T** | **G** | **G** | **A** | **T** | **G** | **A** | **T** | **G** | **A** | **A** | **G** | **T** | **C** | **T** | **G** | **C** | **A** | **C** | **A** | **G** | **T** | **T** | **G** | **G** | **T** | **T** | **C** | **A** | **G** | **C** | **C** | **A** | **A** | **T** | **C** | **A** | **A** | **G** | **A** | **T** | **A** | **T** | **C** | **C** | **G** | **A** | **A** | **G** | **T** | **T** | **A** | **C** | **A** | **A** | **A** | **T** | **T** | **A** | **A** | **A** | **C** | **T** | **T** | **T** | **G** | **A** | **A** | **G** | **C** | **A** | **A** | **A** | **T** | **G** | **C** | **T** | **G** | **T** | **G** | **T** | **T** | **A** | **T** | **A** | **T** | **G** | **A** | **T** | **A** | **A** | **A** | **A** | **A** | **A** | **A** | **T** | **C** | **A** | **T** | **T** | **G** | **A** | **T** | **C** | **A** | **A** | **T** | **T** | **A** | **G** | **A** | **A** | **G** | **C** | **A** | **A** | **T** | **T** | **T** | **T** | **C** | **T** | **T** | **A** | **G** | **A** | **A** | **G** | **A** | **T** | **C** | **G** | **A** | **A** | **A** | **A** | **A** | **A** | **A** | **T** | **G** | **T** | **A** | **C** | **A** | **A** | **C** | **A** | **A** | **T** | **G** | **A** | **C** | **T** | **C** | **C** | **A** | **G** | **A** | **A** | **G** | **T** | **T** | **G** | **T** | **T** | **C** | **G** | **T** | **G** | **A** | **C** | **A** | **T** | **G** | **T** | **C** | **A** | **A** | **A** | **A** | **T** | **G** | **G** | **T** | **T** | **G** | **A** | **T** | **T** | **T** | **T** | **T** | **A** | **A** | **A** | **C** | **A** | **A** | **C** | **A** | **A** | **A** | **T** | **T** | **T** | **C** | **A** | **C** | **G** | **A** | **T** | **T** | **A** | **T** | **T** | **T** | **T** | **C** | **A** | **C** | **C** | **A** | **A** | **T** | **T** | **C** | **T** | **T** | **-** | **-** | **-** |
| **N55706** | **T** | **T** | **G** | **A** | **T** | **T** | **C** | **T** | **T** | **A** | **G** | **T** | **G** | **T** | **C** | **T** | **T** | **A** | **A** | **C** | **A** | **G** | **T** | **T** | **A** | **T** | **T** | **T** | **A** | **T** | **T** | **T** | **T** | **A** | **T** | **T** | **A** | **A** | **T** | **G** | **C** | **A** | **A** | **T** | **T** | **A** | **T** | **T** | **G** | **C** | **G** | **G** | **G** | **A** | **A** | **T** | **T** | **A** | **C** | **C** | **A** | **T** | **T** | **T** | **T** | **G** | **T** | **T** | **A** | **A** | **A** | **A** | **C** | **C** | **T** | **C** | **G** | **A** | **G** | **A** | **T** | **G** | **T** | **A** | **G** | **C** | **A** | **G** | **C** | **A** | **A** | **T** | **T** | **T** | **G** | **G** | **G** | **C** | **A** | **T** | **G** | **G** | **C** | **T** | **A** | **C** | **T** | **C** | **G** | **T** | **A** | **C** | **T** | **G** | **A** | **T** | **T** | **G** | **C** | **T** | **C** | **T** | **A** | **C** | **C** | **C** | **G** | **T** | **T** | **T** | **T** | **T** | **G** | **G** | **T** | **T** | **T** | **T** | **T** | **T** | **C** | **T** | **T** | **A** | **T** | **A** | **T** | **T** | **T** | **A** | **T** | **T** | **C** | **T** | **T** | **T** | **G** | **G** | **G** | **C** | **G** | **C** | **G** | **G** | **T** | **T** | **T** | **A** | **A** | **C** | **C** | **G** | **A** | **C** | **A** | **A** | **A** | **A** | **A** | **G** | **A** | **A** | **A** | **T** | **T** | **T** | **T** | **A** | **T** | **T** | **T** | **G** | **C** | **A** | **A** | **C** | **A** | **A** | **A** | **G** | **T** | **G** | **A** | **T** | **T** | **T** | **G** | **C** | **G** | **G** | **G** | **A** | **A** | **T** | **T** | **A** | **G** | **A** | **G** | **A** | **A** | **T** | **T** | **T** | **T** | **C** | **A** | **G** | **A** | **A** | **T** | **T** | **T** | **C** | **C** | **A** | **A** | **G** | **A** | **A** | **G** | **A** | **A** | **A** | **G** | **T** | **T** | **T** | **T** | **G** | **A** | **A** | **C** | **T** | **T** | **T** | **A** | **T** | **A** | **G** | **T** | **C** | **A** | **A** | **A** | **A** | **G** | **A** | **T** | **G** | **C** | **C** | **G** | **A** | **C** | **G** | **G** | **A** | **G** | **G** | **A** | **G** | **C** | **A** | **A** | **C** | **A** | **A** | **C** | **A** | **G** | **T** | **T** | **T** | **A** | **C** | **A** | **G** | **A** | **C** | **T** | **T** | **T** | **T** | **T** | **T** | **T** | **C** | **C** | **T** | **C** | **G** | **T** | **T** | **A** | **A** | **A** | **T** | **A** | **G** | **G** | **A** | **T** | **G** | **C** | **C** | **T** | **C** | **T** | **G** | **A** | **C** | **T** | **A** | **A** | **G** | **A** | **A** | **A** | **A** | **A** | **T** | **G** | **A** | **T** | **G** | **T** | **T** | **G** | **A** | **A** | **A** | **T** | **T** | **T** | **T** | **T** | **A** | **C** | **G** | **G** | **A** | **T** | **G** | **G** | **T** | **A** | **C** | **G** | **G** | **A** | **A** | **A** | **A** | **A** | **T** | **T** | **C** | **A** | **A** | **T** | **G** | **C** | **G** | **T** | **T** | **G** | **A** | **T** | **G** | **G** | **C** | **G** | **G** | **A** | **C** | **A** | **T** | **A** | **A** | **A** | **A** | **A** | **A** | **A** | **G** | **C** | **T** | **C** | **A** | **G** | **C** | **A** | **C** | **T** | **C** | **C** | **A** | **T** | **T** | **C** | **A** | **T** | **A** | **T** | **T** | **G** | **A** | **A** | **T** | **A** | **T** | **T** | **A** | **T** | **G** | **C** | **C** | **T** | **T** | **T** | **G** | **T** | **A** | **A** | **C** | **G** | **G** | **A** | **T** | **C** | **A** | **T** | **A** | **T** | **C** | **G** | **G** | **C** | **A** | **C** | **C** | **A** | **A** | **A** | **A** | **T** | **T** | **T** | **T** | **G** | **A** | **A** | **C** | **T** | **T** | **A** | **T** | **T** | **A** | **G** | **A** | **A** | **G** | **A** | **A** | **A** | **A** | **G** | **G** | **C** | **G** | **G** | **C** | **T** | **G** | **A** | **A** | **G** | **G** | **G** | **G** | **T** | **T** | **G** | **A** | **A** | **G** | **T** | **G** | **C** | **G** | **G** | **T** | **T** | **G** | **C** | **T** | **T** | **T** | **A** | **C** | **G** | **A** | **T** | **G** | **C** | **C** | **T** | **T** | **T** | **G** | **G** | **C** | **T** | **C** | **A** | **A** | **A** | **G** | **G** | **G** | **C** | **A** | **C** | **C** | **A** | **A** | **G** | **G** | **T** | **T** | **C** | **A** | **T** | **C** | **A** | **T** | **T** | **T** | **G** | **A** | **A** | **T** | **G** | **A** | **A** | **T** | **T** | **G** | **A** | **A** | **A** | **A** | **A** | **A** | **A** | **A** | **C** | **G** | **G** | **T** | **G** | **G** | **T** | **T** | **T** | **T** | **G** | **T** | **C** | **C** | **A** | **A** | **A** | **C** | **G** | **T** | **T** | **T** | **A** | **T** | **T** | **A** | **C** | **T** | **T** | **C** | **T** | **C** | **A** | **A** | **A** | **A** | **A** | **G** | **C** | **A** | **C** | **T** | **T** | **T** | **T** | **G** | **A** | **A** | **G** | **T** | **T** | **T** | **C** | **G** | **T** | **T** | **T** | **G** | **A** | **A** | **T** | **T** | **A** | **T** | **C** | **A** | **T** | **G** | **A** | **T** | **C** | **A** | **C** | **C** | **G** | **G** | **A** | **A** | **A** | **A** | **T** | **T** | **G** | **T** | **T** | **G** | **T** | **T** | **A** | **T** | **T** | **G** | **A** | **C** | **G** | **G** | **A** | **A** | **A** | **A** | **G** | **T** | **A** | **G** | **G** | **C** | **T** | **A** | **C** | **A** | **T** | **T** | **G** | **G** | **C** | **G** | **G** | **C** | **T** | **T** | **T** | **A** | **A** | **T** | **G** | **T** | **T** | **G** | **C** | **C** | **G** | **A** | **T** | **C** | **A** | **A** | **T** | **A** | **T** | **G** | **C** | **C** | **G** | **G** | **A** | **A** | **C** | **G** | **A** | **C** | **T** | **A** | **A** | **A** | **A** | **A** | **G** | **T** | **T** | **T** | **G** | **G** | **C** | **T** | **A** | **T** | **T** | **G** | **G** | **C** | **G** | **G** | **G** | **A** | **T** | **A** | **C** | **A** | **C** | **A** | **T** | **T** | **T** | **A** | **C** | **G** | **G** | **A** | **T** | **T** | **C** | **A** | **A** | **G** | **G** | **G** | **C** | **C** | **A** | **G** | **C** | **A** | **G** | **C** | **C** | **T** | **C** | **A** | **T** | **T** | **A** | **C** | **T** | **G** | **C** | **A** | **A** | **A** | **T** | **G** | **C** | **G** | **T** | **T** | **T** | **T** | **T** | **T** | **A** | **A** | **T** | **G** | **G** | **A** | **T** | **T** | **G** | **G** | **A** | **A** | **C** | **G** | **T** | **C** | **T** | **C** | **T** | **T** | **C** | **C** | **C** | **C** | **C** | **G** | **A** | **G** | **A** | **A** | **A** | **A** | **A** | **T** | **C** | **G** | **T** | **G** | **T** | **G** | **G** | **C** | **G** | **T** | **A** | **T** | **C** | **A** | **A** | **T** | **T** | **G** | **G** | **A** | **T** | **T** | **A** | **T** | **T** | **T** | **C** | **T** | **T** | **T** | **A** | **A** | **A** | **C** | **T** | **T** | **G** | **A** | **A** | **G** | **C** | **A** | **T** | **T** | **G** | **G** | **T** | **G** | **C** | **C** | **A** | **G** | **A** | **G** | **G** | **C** | **A** | **A** | **A** | **T** | **A** | **C** | **A** | **T** | **C** | **C** | **A** | **T** | **T** | **C** | **A** | **G** | **A** | **T** | **G** | **A** | **T** | **T** | **G** | **C** | **C** | **A** | **G** | **T** | **G** | **G** | **T** | **C** | **C** | **T** | **A** | **A** | **C** | **A** | **G** | **T** | **G** | **A** | **C** | **C** | **G** | **T** | **G** | **A** | **A** | **C** | **A** | **A** | **A** | **T** | **T** | **A** | **A** | **A** | **T** | **T** | **G** | **G** | **C** | **C** | **T** | **T** | **T** | **A** | **T** | **T** | **A** | **A** | **A** | **T** | **T** | **G** | **A** | **T** | **T** | **A** | **C** | **T** | **T** | **C** | **T** | **G** | **C** | **C** | **A** | **A** | **G** | **A** | **A** | **A** | **A** | **G** | **A** | **G** | **T** | **C** | **T** | **G** | **G** | **A** | **T** | **T** | **C** | **A** | **A** | **A** | **C** | **G** | **C** | **C** | **G** | **T** | **A** | **T** | **T** | **T** | **A** | **G** | **T** | **T** | **C** | **C** | **T** | **G** | **A** | **T** | **G** | **A** | **C** | **A** | **G** | **T** | **G** | **T** | **C** | **T** | **T** | **G** | **G** | **C** | **T** | **G** | **C** | **T** | **T** | **T** | **A** | **A** | **A** | **G** | **G** | **T** | **T** | **G** | **C** | **T** | **G** | **C** | **G** | **G** | **C** | **T** | **T** | **C** | **A** | **G** | **G** | **A** | **G** | **T** | **A** | **G** | **A** | **T** | **G** | **T** | **T** | **A** | **A** | **A** | **A** | **T** | **T** | **A** | **T** | **G** | **A** | **T** | **T** | **C** | **C** | **A** | **G** | **A** | **T** | **A** | **A** | **G** | **C** | **C** | **C** | **G** | **A** | **C** | **C** | **A** | **T** | **C** | **C** | **G** | **T** | **T** | **T** | **A** | **T** | **T** | **T** | **A** | **T** | **C** | **G** | **A** | **G** | **C** | **A** | **A** | **C** | **A** | **C** | **A** | **G** | **T** | **A** | **T** | **T** | **A** | **C** | **G** | **G** | **C** | **C** | **G** | **C** | **T** | **T** | **A** | **T** | **T** | **G** | **A** | **T** | **G** | **A** | **A** | **A** | **G** | **A** | **A** | **A** | **A** | **T** | **A** | **T** | **T** | **G** | **A** | **A** | **A** | **T** | **T** | **T** | **T** | **A** | **A** | **T** | **T** | **T** | **A** | **T** | **A** | **A** | **C** | **G** | **G** | **T** | **G** | **G** | **T** | **T** | **T** | **C** | **T** | **T** | **A** | **C** | **A** | **T** | **G** | **C** | **G** | **A** | **A** | **A** | **A** | **C** | **A** | **A** | **T** | **G** | **A** | **T** | **T** | **A** | **T** | **G** | **G** | **A** | **T** | **G** | **A** | **T** | **G** | **A** | **A** | **G** | **T** | **C** | **T** | **G** | **C** | **A** | **C** | **A** | **G** | **T** | **T** | **G** | **G** | **T** | **T** | **C** | **A** | **G** | **C** | **C** | **A** | **A** | **T** | **C** | **A** | **A** | **G** | **A** | **T** | **A** | **T** | **C** | **C** | **G** | **A** | **A** | **G** | **T** | **T** | **A** | **C** | **A** | **A** | **A** | **T** | **T** | **A** | **A** | **A** | **C** | **T** | **T** | **T** | **G** | **A** | **A** | **G** | **C** | **A** | **A** | **A** | **T** | **G** | **C** | **T** | **G** | **T** | **G** | **T** | **T** | **A** | **T** | **A** | **T** | **G** | **A** | **T** | **A** | **A** | **A** | **A** | **A** | **A** | **A** | **T** | **C** | **A** | **T** | **T** | **G** | **A** | **T** | **C** | **A** | **A** | **T** | **T** | **A** | **G** | **A** | **A** | **G** | **C** | **A** | **A** | **T** | **T** | **T** | **T** | **C** | **T** | **T** | **A** | **G** | **A** | **A** | **G** | **A** | **T** | **C** | **G** | **A** | **A** | **A** | **A** | **A** | **A** | **A** | **T** | **G** | **T** | **A** | **C** | **A** | **A** | **C** | **A** | **A** | **T** | **G** | **A** | **C** | **T** | **C** | **C** | **A** | **G** | **A** | **A** | **G** | **T** | **T** | **G** | **T** | **T** | **C** | **G** | **C** | **G** | **A** | **C** | **A** | **T** | **G** | **T** | **C** | **A** | **A** | **A** | **A** | **T** | **G** | **G** | **T** | **T** | **G** | **A** | **T** | **T** | **T** | **T** | **T** | **A** | **A** | **A** | **C** | **A** | **A** | **C** | **A** | **A** | **A** | **T** | **T** | **T** | **C** | **A** | **C** | **G** | **A** | **T** | **T** | **A** | **T** | **T** | **T** | **T** | **C** | **A** | **C** | **C** | **A** | **A** | **T** | **T** | **C** | **T** | **T** | **-** | **-** | **-** |
| **N54118** | **T** | **T** | **G** | **A** | **T** | **T** | **C** | **T** | **T** | **A** | **G** | **C** | **G** | **T** | **C** | **T** | **T** | **A** | **A** | **C** | **A** | **G** | **T** | **T** | **A** | **T** | **T** | **T** | **A** | **T** | **T** | **T** | **T** | **A** | **T** | **T** | **A** | **A** | **T** | **G** | **C** | **A** | **A** | **T** | **T** | **A** | **T** | **T** | **G** | **C** | **G** | **G** | **G** | **A** | **A** | **T** | **T** | **A** | **C** | **C** | **A** | **T** | **T** | **T** | **T** | **G** | **T** | **T** | **A** | **A** | **A** | **A** | **C** | **C** | **T** | **C** | **G** | **A** | **G** | **A** | **T** | **G** | **T** | **A** | **G** | **C** | **A** | **G** | **C** | **A** | **A** | **T** | **T** | **T** | **G** | **G** | **G** | **C** | **A** | **T** | **G** | **G** | **C** | **T** | **A** | **C** | **T** | **C** | **G** | **T** | **A** | **C** | **T** | **G** | **A** | **T** | **T** | **G** | **C** | **T** | **C** | **T** | **A** | **C** | **C** | **C** | **G** | **T** | **T** | **T** | **T** | **T** | **G** | **G** | **T** | **T** | **T** | **T** | **T** | **T** | **C** | **T** | **T** | **A** | **T** | **A** | **T** | **T** | **T** | **A** | **T** | **T** | **C** | **T** | **T** | **T** | **G** | **G** | **G** | **C** | **G** | **C** | **G** | **G** | **T** | **T** | **T** | **A** | **A** | **C** | **C** | **G** | **A** | **C** | **A** | **A** | **A** | **A** | **A** | **G** | **A** | **A** | **A** | **T** | **T** | **T** | **T** | **A** | **T** | **T** | **T** | **G** | **C** | **A** | **A** | **C** | **A** | **A** | **A** | **G** | **T** | **G** | **A** | **T** | **T** | **T** | **G** | **C** | **G** | **G** | **G** | **A** | **A** | **T** | **T** | **A** | **G** | **A** | **G** | **A** | **A** | **T** | **T** | **T** | **T** | **C** | **A** | **G** | **A** | **A** | **T** | **T** | **T** | **C** | **C** | **A** | **A** | **G** | **A** | **A** | **G** | **A** | **A** | **A** | **G** | **T** | **T** | **T** | **T** | **G** | **A** | **A** | **C** | **T** | **T** | **T** | **A** | **T** | **A** | **G** | **T** | **C** | **A** | **A** | **A** | **A** | **G** | **A** | **T** | **G** | **C** | **C** | **G** | **A** | **C** | **G** | **G** | **A** | **G** | **G** | **A** | **G** | **C** | **A** | **A** | **C** | **A** | **A** | **C** | **A** | **G** | **T** | **T** | **T** | **A** | **C** | **A** | **G** | **A** | **C** | **T** | **T** | **T** | **T** | **T** | **T** | **T** | **C** | **C** | **T** | **C** | **G** | **T** | **T** | **G** | **A** | **A** | **T** | **A** | **G** | **G** | **A** | **T** | **G** | **C** | **C** | **T** | **C** | **T** | **G** | **A** | **C** | **T** | **A** | **A** | **G** | **A** | **A** | **A** | **A** | **A** | **T** | **G** | **A** | **T** | **G** | **T** | **T** | **C** | **A** | **A** | **A** | **T** | **T** | **T** | **T** | **T** | **A** | **C** | **G** | **G** | **A** | **T** | **G** | **G** | **T** | **A** | **C** | **G** | **G** | **A** | **A** | **A** | **A** | **A** | **T** | **T** | **C** | **A** | **A** | **T** | **G** | **C** | **G** | **T** | **T** | **G** | **A** | **T** | **G** | **G** | **C** | **G** | **G** | **A** | **C** | **A** | **T** | **A** | **A** | **A** | **A** | **A** | **A** | **A** | **G** | **C** | **T** | **C** | **A** | **G** | **C** | **A** | **C** | **T** | **C** | **C** | **A** | **T** | **T** | **C** | **A** | **T** | **A** | **T** | **T** | **G** | **A** | **A** | **T** | **A** | **T** | **T** | **A** | **T** | **G** | **C** | **C** | **T** | **T** | **T** | **G** | **T** | **A** | **A** | **C** | **G** | **G** | **A** | **T** | **C** | **A** | **T** | **A** | **T** | **C** | **G** | **G** | **C** | **A** | **C** | **C** | **A** | **A** | **A** | **A** | **T** | **T** | **T** | **T** | **G** | **A** | **A** | **C** | **T** | **T** | **A** | **T** | **T** | **A** | **G** | **A** | **A** | **G** | **A** | **A** | **A** | **A** | **G** | **G** | **C** | **G** | **G** | **C** | **T** | **G** | **A** | **A** | **G** | **G** | **G** | **G** | **T** | **T** | **G** | **A** | **A** | **G** | **T** | **G** | **C** | **G** | **G** | **T** | **T** | **G** | **C** | **T** | **T** | **T** | **A** | **C** | **G** | **A** | **T** | **G** | **C** | **C** | **T** | **T** | **T** | **G** | **G** | **C** | **T** | **C** | **A** | **A** | **A** | **G** | **G** | **G** | **C** | **A** | **C** | **C** | **A** | **A** | **G** | **G** | **T** | **T** | **C** | **A** | **T** | **C** | **A** | **T** | **T** | **T** | **G** | **A** | **A** | **T** | **G** | **A** | **A** | **T** | **T** | **G** | **A** | **A** | **A** | **A** | **A** | **A** | **A** | **A** | **C** | **G** | **G** | **T** | **G** | **G** | **T** | **T** | **T** | **T** | **G** | **T** | **C** | **C** | **A** | **A** | **A** | **C** | **G** | **T** | **T** | **T** | **A** | **T** | **T** | **A** | **C** | **T** | **T** | **C** | **T** | **C** | **A** | **A** | **A** | **A** | **A** | **G** | **C** | **A** | **C** | **T** | **T** | **T** | **T** | **G** | **A** | **A** | **G** | **T** | **T** | **T** | **C** | **G** | **T** | **T** | **T** | **G** | **A** | **A** | **T** | **T** | **A** | **T** | **C** | **A** | **T** | **G** | **A** | **T** | **C** | **A** | **C** | **C** | **G** | **G** | **A** | **A** | **A** | **A** | **T** | **T** | **G** | **T** | **T** | **G** | **T** | **T** | **A** | **T** | **T** | **G** | **A** | **C** | **G** | **G** | **A** | **A** | **A** | **A** | **G** | **T** | **A** | **G** | **G** | **C** | **T** | **A** | **C** | **A** | **T** | **T** | **G** | **G** | **C** | **G** | **G** | **C** | **T** | **T** | **T** | **A** | **A** | **T** | **G** | **T** | **T** | **G** | **C** | **C** | **G** | **A** | **T** | **C** | **A** | **A** | **T** | **A** | **T** | **G** | **C** | **T** | **G** | **G** | **A** | **A** | **C** | **G** | **A** | **C** | **T** | **A** | **A** | **A** | **A** | **A** | **G** | **T** | **T** | **T** | **G** | **G** | **C** | **T** | **A** | **T** | **T** | **G** | **G** | **C** | **G** | **G** | **G** | **A** | **T** | **A** | **C** | **G** | **C** | **A** | **T** | **T** | **T** | **A** | **C** | **G** | **G** | **A** | **T** | **T** | **C** | **A** | **A** | **G** | **G** | **G** | **C** | **C** | **A** | **G** | **C** | **A** | **G** | **C** | **C** | **T** | **C** | **A** | **T** | **T** | **A** | **C** | **T** | **G** | **C** | **A** | **A** | **A** | **T** | **G** | **C** | **G** | **T** | **T** | **T** | **T** | **T** | **T** | **A** | **A** | **T** | **G** | **G** | **A** | **T** | **T** | **G** | **G** | **A** | **A** | **C** | **G** | **T** | **C** | **T** | **C** | **T** | **T** | **C** | **C** | **C** | **C** | **C** | **G** | **A** | **G** | **A** | **A** | **A** | **A** | **A** | **T** | **C** | **G** | **T** | **G** | **T** | **G** | **G** | **C** | **G** | **T** | **A** | **T** | **C** | **A** | **A** | **T** | **T** | **G** | **G** | **A** | **T** | **T** | **A** | **T** | **T** | **T** | **C** | **T** | **T** | **T** | **A** | **A** | **A** | **C** | **T** | **T** | **G** | **A** | **A** | **G** | **C** | **A** | **T** | **T** | **G** | **G** | **T** | **G** | **C** | **C** | **A** | **G** | **A** | **G** | **G** | **C** | **A** | **A** | **A** | **T** | **A** | **C** | **A** | **T** | **C** | **C** | **A** | **T** | **T** | **C** | **A** | **G** | **A** | **T** | **G** | **A** | **T** | **T** | **G** | **C** | **C** | **A** | **G** | **T** | **G** | **G** | **T** | **C** | **C** | **T** | **A** | **A** | **C** | **A** | **G** | **T** | **G** | **A** | **C** | **C** | **G** | **T** | **G** | **A** | **A** | **C** | **A** | **A** | **A** | **T** | **T** | **A** | **A** | **A** | **T** | **T** | **G** | **G** | **C** | **C** | **T** | **T** | **T** | **A** | **T** | **T** | **A** | **A** | **A** | **T** | **T** | **G** | **A** | **T** | **T** | **A** | **C** | **T** | **T** | **C** | **T** | **G** | **C** | **C** | **A** | **A** | **G** | **A** | **A** | **A** | **A** | **G** | **A** | **G** | **T** | **C** | **T** | **G** | **G** | **A** | **T** | **T** | **C** | **A** | **A** | **A** | **C** | **G** | **C** | **C** | **G** | **T** | **A** | **T** | **T** | **T** | **A** | **G** | **T** | **T** | **C** | **C** | **T** | **G** | **A** | **T** | **G** | **A** | **T** | **A** | **G** | **T** | **G** | **T** | **C** | **T** | **T** | **G** | **G** | **C** | **T** | **G** | **C** | **T** | **T** | **T** | **A** | **A** | **A** | **G** | **G** | **T** | **T** | **G** | **C** | **T** | **G** | **C** | **G** | **G** | **C** | **T** | **T** | **C** | **A** | **G** | **G** | **A** | **G** | **T** | **A** | **G** | **A** | **T** | **G** | **T** | **T** | **A** | **A** | **A** | **A** | **T** | **T** | **A** | **T** | **G** | **A** | **T** | **T** | **C** | **C** | **A** | **G** | **A** | **T** | **A** | **A** | **G** | **C** | **C** | **C** | **G** | **A** | **C** | **C** | **A** | **T** | **C** | **C** | **G** | **T** | **T** | **T** | **A** | **T** | **T** | **T** | **A** | **T** | **C** | **G** | **A** | **G** | **C** | **A** | **A** | **C** | **A** | **C** | **A** | **G** | **T** | **A** | **T** | **T** | **A** | **C** | **G** | **G** | **C** | **C** | **G** | **C** | **T** | **T** | **A** | **T** | **T** | **G** | **A** | **T** | **G** | **A** | **A** | **A** | **G** | **A** | **A** | **A** | **A** | **T** | **A** | **T** | **T** | **G** | **A** | **A** | **A** | **T** | **T** | **T** | **T** | **A** | **A** | **T** | **T** | **T** | **A** | **T** | **A** | **A** | **C** | **G** | **G** | **T** | **G** | **G** | **T** | **T** | **T** | **C** | **T** | **T** | **A** | **C** | **A** | **T** | **G** | **C** | **G** | **A** | **A** | **A** | **A** | **C** | **A** | **A** | **T** | **G** | **A** | **T** | **T** | **A** | **T** | **G** | **G** | **A** | **T** | **G** | **A** | **T** | **G** | **A** | **A** | **G** | **T** | **C** | **T** | **G** | **C** | **A** | **C** | **A** | **G** | **T** | **T** | **G** | **G** | **T** | **T** | **C** | **A** | **G** | **C** | **C** | **A** | **A** | **T** | **C** | **A** | **A** | **G** | **A** | **T** | **A** | **T** | **C** | **C** | **G** | **A** | **A** | **G** | **T** | **T** | **A** | **C** | **A** | **A** | **A** | **T** | **T** | **A** | **A** | **A** | **C** | **T** | **T** | **T** | **G** | **A** | **A** | **G** | **C** | **A** | **A** | **A** | **T** | **G** | **C** | **T** | **G** | **T** | **G** | **T** | **T** | **A** | **T** | **A** | **T** | **G** | **A** | **T** | **A** | **A** | **A** | **A** | **A** | **A** | **A** | **T** | **C** | **A** | **T** | **T** | **G** | **A** | **T** | **C** | **A** | **A** | **T** | **T** | **A** | **G** | **A** | **A** | **G** | **C** | **A** | **A** | **T** | **T** | **T** | **T** | **C** | **T** | **T** | **A** | **G** | **A** | **A** | **G** | **A** | **T** | **C** | **G** | **A** | **A** | **A** | **A** | **A** | **A** | **A** | **T** | **G** | **T** | **A** | **C** | **A** | **A** | **C** | **A** | **A** | **T** | **G** | **A** | **C** | **T** | **C** | **C** | **A** | **G** | **A** | **A** | **G** | **T** | **T** | **G** | **T** | **T** | **C** | **G** | **C** | **G** | **A** | **C** | **A** | **T** | **G** | **T** | **C** | **A** | **A** | **A** | **A** | **T** | **G** | **G** | **T** | **T** | **G** | **A** | **T** | **T** | **T** | **T** | **T** | **A** | **A** | **A** | **C** | **A** | **A** | **C** | **A** | **A** | **A** | **T** | **T** | **T** | **C** | **A** | **C** | **G** | **A** | **T** | **T** | **A** | **T** | **T** | **T** | **T** | **C** | **A** | **C** | **C** | **A** | **A** | **T** | **T** | **C** | **T** | **T** | **-** | **-** | **-** |
| **N55265** | **T** | **T** | **G** | **A** | **T** | **T** | **C** | **T** | **T** | **A** | **G** | **T** | **G** | **T** | **C** | **T** | **T** | **A** | **A** | **C** | **A** | **G** | **T** | **T** | **A** | **T** | **T** | **T** | **A** | **T** | **T** | **T** | **T** | **A** | **T** | **T** | **A** | **A** | **T** | **G** | **C** | **A** | **A** | **T** | **T** | **A** | **T** | **T** | **G** | **C** | **G** | **G** | **G** | **A** | **A** | **T** | **T** | **A** | **C** | **C** | **A** | **T** | **C** | **T** | **T** | **G** | **T** | **T** | **A** | **A** | **A** | **A** | **C** | **C** | **T** | **C** | **G** | **A** | **G** | **A** | **T** | **G** | **T** | **A** | **G** | **C** | **G** | **G** | **C** | **A** | **A** | **T** | **T** | **T** | **G** | **G** | **G** | **C** | **A** | **T** | **G** | **G** | **T** | **T** | **A** | **C** | **T** | **C** | **G** | **T** | **A** | **C** | **T** | **G** | **A** | **T** | **T** | **G** | **C** | **T** | **C** | **T** | **A** | **C** | **C** | **C** | **G** | **T** | **T** | **T** | **T** | **T** | **G** | **G** | **T** | **T** | **T** | **T** | **T** | **T** | **C** | **T** | **T** | **A** | **T** | **A** | **T** | **T** | **T** | **A** | **T** | **T** | **C** | **T** | **T** | **T** | **G** | **G** | **T** | **C** | **G** | **A** | **G** | **G** | **C** | **T** | **T** | **G** | **A** | **C** | **C** | **G** | **A** | **C** | **A** | **A** | **A** | **A** | **A** | **G** | **A** | **A** | **A** | **T** | **T** | **T** | **T** | **A** | **T** | **T** | **T** | **G** | **C** | **A** | **A** | **C** | **A** | **A** | **A** | **G** | **T** | **G** | **A** | **T** | **T** | **T** | **A** | **C** | **G** | **G** | **G** | **A** | **A** | **T** | **T** | **A** | **G** | **A** | **G** | **A** | **A** | **T** | **T** | **T** | **T** | **C** | **A** | **G** | **A** | **A** | **T** | **T** | **T** | **C** | **C** | **A** | **A** | **G** | **A** | **A** | **G** | **A** | **A** | **A** | **G** | **T** | **T** | **T** | **T** | **G** | **A** | **A** | **C** | **T** | **T** | **T** | **A** | **T** | **A** | **G** | **T** | **C** | **A** | **A** | **A** | **A** | **G** | **A** | **T** | **G** | **C** | **C** | **G** | **A** | **C** | **G** | **G** | **A** | **G** | **G** | **A** | **G** | **C** | **A** | **A** | **C** | **A** | **G** | **C** | **A** | **G** | **T** | **T** | **T** | **A** | **C** | **A** | **G** | **A** | **C** | **T** | **T** | **T** | **T** | **T** | **C** | **T** | **C** | **C** | **T** | **C** | **G** | **T** | **T** | **A** | **A** | **A** | **T** | **A** | **G** | **G** | **A** | **T** | **G** | **C** | **C** | **T** | **C** | **T** | **G** | **A** | **C** | **T** | **A** | **A** | **G** | **A** | **A** | **A** | **A** | **A** | **T** | **G** | **A** | **C** | **G** | **T** | **T** | **G** | **A** | **G** | **A** | **T** | **T** | **T** | **T** | **T** | **A** | **C** | **G** | **G** | **A** | **T** | **G** | **G** | **T** | **A** | **T** | **G** | **G** | **A** | **A** | **A** | **A** | **A** | **T** | **T** | **C** | **A** | **A** | **T** | **G** | **C** | **G** | **T** | **T** | **G** | **A** | **T** | **G** | **G** | **C** | **G** | **G** | **A** | **C** | **A** | **T** | **A** | **A** | **A** | **A** | **A** | **A** | **A** | **G** | **C** | **T** | **C** | **A** | **G** | **C** | **A** | **T** | **T** | **C** | **C** | **A** | **T** | **T** | **C** | **A** | **T** | **A** | **T** | **T** | **G** | **A** | **A** | **T** | **A** | **T** | **T** | **A** | **T** | **G** | **C** | **C** | **T** | **T** | **T** | **G** | **T** | **A** | **A** | **C** | **G** | **G** | **A** | **T** | **C** | **A** | **T** | **A** | **T** | **C** | **G** | **G** | **C** | **A** | **C** | **C** | **A** | **A** | **A** | **A** | **T** | **T** | **T** | **T** | **G** | **A** | **A** | **C** | **T** | **T** | **A** | **T** | **T** | **A** | **G** | **A** | **A** | **G** | **A** | **A** | **A** | **A** | **G** | **G** | **C** | **G** | **G** | **C** | **T** | **G** | **A** | **A** | **G** | **G** | **G** | **G** | **T** | **T** | **G** | **A** | **A** | **G** | **T** | **G** | **C** | **G** | **G** | **T** | **T** | **G** | **C** | **T** | **T** | **T** | **A** | **C** | **G** | **A** | **T** | **G** | **C** | **C** | **T** | **T** | **T** | **G** | **G** | **C** | **T** | **C** | **A** | **A** | **A** | **G** | **G** | **G** | **C** | **A** | **C** | **C** | **A** | **A** | **G** | **G** | **T** | **T** | **C** | **A** | **T** | **C** | **A** | **T** | **T** | **T** | **G** | **A** | **A** | **T** | **G** | **A** | **A** | **T** | **T** | **G** | **A** | **A** | **A** | **A** | **A** | **A** | **A** | **A** | **C** | **G** | **G** | **C** | **G** | **G** | **T** | **T** | **T** | **T** | **G** | **T** | **C** | **C** | **A** | **A** | **A** | **C** | **G** | **T** | **T** | **T** | **A** | **T** | **T** | **A** | **C** | **T** | **T** | **C** | **T** | **C** | **A** | **A** | **A** | **A** | **A** | **G** | **C** | **A** | **C** | **T** | **C** | **T** | **T** | **G** | **A** | **A** | **G** | **T** | **T** | **T** | **C** | **G** | **T** | **T** | **T** | **G** | **A** | **A** | **T** | **T** | **A** | **T** | **C** | **A** | **T** | **G** | **A** | **T** | **C** | **A** | **C** | **C** | **G** | **G** | **A** | **A** | **A** | **A** | **T** | **T** | **G** | **T** | **C** | **G** | **T** | **T** | **A** | **T** | **T** | **G** | **A** | **C** | **G** | **G** | **A** | **A** | **A** | **A** | **G** | **T** | **A** | **G** | **G** | **C** | **T** | **A** | **C** | **A** | **T** | **T** | **G** | **G** | **T** | **G** | **G** | **T** | **T** | **T** | **T** | **A** | **A** | **T** | **G** | **T** | **T** | **G** | **C** | **C** | **G** | **A** | **T** | **C** | **A** | **A** | **T** | **A** | **T** | **G** | **C** | **T** | **G** | **G** | **A** | **A** | **C** | **G** | **A** | **C** | **T** | **A** | **A** | **A** | **A** | **A** | **G** | **T** | **T** | **T** | **G** | **G** | **C** | **T** | **A** | **T** | **T** | **G** | **G** | **C** | **G** | **G** | **G** | **A** | **T** | **A** | **C** | **G** | **C** | **A** | **T** | **T** | **T** | **A** | **C** | **G** | **G** | **A** | **T** | **T** | **C** | **A** | **A** | **G** | **G** | **G** | **C** | **C** | **A** | **G** | **C** | **A** | **G** | **C** | **C** | **T** | **C** | **A** | **T** | **T** | **A** | **C** | **T** | **G** | **C** | **A** | **A** | **A** | **T** | **G** | **C** | **G** | **T** | **T** | **T** | **T** | **T** | **T** | **A** | **A** | **T** | **G** | **G** | **A** | **T** | **T** | **G** | **G** | **A** | **A** | **C** | **G** | **T** | **C** | **T** | **C** | **T** | **T** | **C** | **C** | **C** | **C** | **T** | **G** | **A** | **G** | **A** | **A** | **A** | **A** | **A** | **T** | **C** | **G** | **T** | **G** | **T** | **G** | **G** | **C** | **G** | **T** | **A** | **T** | **C** | **A** | **A** | **T** | **T** | **G** | **G** | **A** | **T** | **T** | **A** | **T** | **T** | **T** | **C** | **T** | **T** | **T** | **A** | **A** | **A** | **C** | **T** | **T** | **G** | **A** | **A** | **G** | **C** | **G** | **T** | **T** | **G** | **G** | **T** | **G** | **C** | **C** | **A** | **G** | **A** | **G** | **G** | **C** | **A** | **A** | **A** | **T** | **A** | **C** | **A** | **T** | **C** | **C** | **A** | **T** | **T** | **C** | **A** | **G** | **A** | **T** | **G** | **A** | **T** | **T** | **G** | **C** | **A** | **A** | **G** | **T** | **G** | **G** | **A** | **C** | **C** | **T** | **A** | **A** | **C** | **A** | **G** | **T** | **G** | **A** | **C** | **C** | **G** | **T** | **G** | **A** | **A** | **C** | **A** | **A** | **A** | **T** | **T** | **A** | **A** | **A** | **T** | **T** | **G** | **G** | **C** | **C** | **T** | **T** | **T** | **A** | **T** | **T** | **A** | **A** | **A** | **T** | **T** | **G** | **A** | **T** | **T** | **A** | **C** | **T** | **T** | **C** | **T** | **G** | **C** | **C** | **A** | **A** | **G** | **A** | **A** | **A** | **A** | **G** | **A** | **G** | **T** | **C** | **T** | **G** | **G** | **A** | **T** | **T** | **C** | **A** | **A** | **A** | **C** | **G** | **C** | **C** | **G** | **T** | **A** | **T** | **T** | **T** | **A** | **G** | **T** | **T** | **C** | **C** | **T** | **G** | **A** | **T** | **G** | **A** | **T** | **A** | **G** | **T** | **G** | **T** | **C** | **T** | **T** | **G** | **G** | **C** | **T** | **G** | **C** | **T** | **T** | **T** | **A** | **A** | **A** | **G** | **G** | **T** | **T** | **G** | **C** | **T** | **G** | **C** | **G** | **G** | **C** | **T** | **T** | **C** | **A** | **G** | **G** | **A** | **G** | **T** | **A** | **G** | **A** | **T** | **G** | **T** | **T** | **A** | **A** | **A** | **A** | **T** | **T** | **A** | **T** | **G** | **A** | **T** | **T** | **C** | **C** | **A** | **G** | **A** | **T** | **A** | **A** | **G** | **C** | **C** | **C** | **G** | **A** | **C** | **C** | **A** | **T** | **C** | **C** | **G** | **T** | **T** | **T** | **A** | **T** | **T** | **T** | **A** | **T** | **C** | **G** | **A** | **G** | **C** | **A** | **A** | **C** | **A** | **C** | **A** | **G** | **T** | **A** | **T** | **T** | **A** | **C** | **G** | **G** | **C** | **C** | **G** | **C** | **T** | **T** | **G** | **T** | **T** | **A** | **A** | **T** | **G** | **A** | **A** | **A** | **G** | **A** | **A** | **A** | **A** | **T** | **A** | **T** | **T** | **G** | **A** | **A** | **A** | **T** | **T** | **T** | **T** | **A** | **A** | **T** | **T** | **T** | **A** | **T** | **A** | **A** | **C** | **G** | **G** | **T** | **G** | **G** | **T** | **T** | **T** | **C** | **T** | **T** | **A** | **C** | **A** | **T** | **G** | **C** | **G** | **A** | **A** | **A** | **A** | **C** | **A** | **A** | **T** | **G** | **A** | **T** | **T** | **A** | **T** | **G** | **G** | **A** | **T** | **G** | **A** | **T** | **G** | **A** | **A** | **G** | **T** | **C** | **T** | **G** | **C** | **A** | **C** | **A** | **G** | **T** | **T** | **G** | **G** | **T** | **T** | **C** | **A** | **G** | **C** | **T** | **A** | **A** | **T** | **C** | **A** | **A** | **G** | **A** | **T** | **A** | **T** | **C** | **C** | **G** | **A** | **A** | **G** | **T** | **T** | **A** | **C** | **A** | **A** | **A** | **T** | **T** | **A** | **A** | **A** | **C** | **T** | **T** | **T** | **G** | **A** | **A** | **G** | **C** | **A** | **A** | **A** | **T** | **G** | **C** | **T** | **G** | **T** | **G** | **T** | **T** | **A** | **T** | **A** | **T** | **G** | **A** | **T** | **A** | **A** | **A** | **A** | **A** | **A** | **A** | **T** | **C** | **A** | **T** | **T** | **G** | **A** | **T** | **C** | **A** | **A** | **T** | **T** | **A** | **G** | **A** | **A** | **G** | **C** | **A** | **A** | **T** | **T** | **T** | **T** | **C** | **T** | **T** | **A** | **G** | **A** | **A** | **G** | **A** | **T** | **C** | **G** | **A** | **A** | **A** | **A** | **A** | **A** | **A** | **T** | **G** | **T** | **A** | **C** | **A** | **A** | **C** | **A** | **A** | **T** | **G** | **A** | **C** | **T** | **C** | **C** | **A** | **G** | **A** | **A** | **G** | **T** | **T** | **G** | **T** | **T** | **C** | **G** | **C** | **G** | **A** | **C** | **A** | **T** | **G** | **T** | **C** | **A** | **A** | **A** | **A** | **T** | **G** | **G** | **T** | **T** | **G** | **A** | **T** | **T** | **T** | **T** | **T** | **A** | **A** | **A** | **C** | **A** | **A** | **C** | **A** | **A** | **A** | **T** | **T** | **T** | **C** | **A** | **C** | **G** | **A** | **T** | **T** | **A** | **T** | **T** | **T** | **T** | **C** | **A** | **C** | **C** | **A** | **A** | **T** | **T** | **C** | **T** | **T** | **-** | **-** | **-** |
| **N54548** | **T** | **T** | **G** | **A** | **T** | **T** | **C** | **T** | **T** | **A** | **G** | **T** | **G** | **T** | **C** | **T** | **T** | **A** | **A** | **C** | **A** | **G** | **T** | **T** | **A** | **T** | **T** | **T** | **A** | **T** | **T** | **T** | **T** | **A** | **T** | **T** | **A** | **A** | **T** | **G** | **C** | **A** | **A** | **T** | **T** | **A** | **T** | **T** | **G** | **C** | **G** | **G** | **G** | **A** | **A** | **T** | **T** | **A** | **C** | **C** | **A** | **T** | **C** | **T** | **T** | **G** | **T** | **T** | **A** | **A** | **A** | **A** | **C** | **C** | **T** | **C** | **G** | **A** | **G** | **A** | **T** | **G** | **T** | **A** | **G** | **C** | **G** | **G** | **C** | **A** | **A** | **T** | **T** | **T** | **G** | **G** | **G** | **C** | **A** | **T** | **G** | **G** | **T** | **T** | **A** | **C** | **T** | **C** | **G** | **T** | **A** | **C** | **T** | **G** | **A** | **T** | **T** | **G** | **C** | **T** | **C** | **T** | **A** | **C** | **C** | **C** | **G** | **T** | **T** | **T** | **T** | **T** | **G** | **G** | **T** | **T** | **T** | **T** | **T** | **T** | **C** | **T** | **T** | **A** | **T** | **A** | **T** | **T** | **T** | **A** | **T** | **T** | **C** | **T** | **T** | **T** | **G** | **G** | **T** | **C** | **G** | **A** | **G** | **G** | **C** | **T** | **T** | **G** | **A** | **C** | **C** | **G** | **A** | **C** | **A** | **A** | **A** | **A** | **A** | **G** | **A** | **A** | **A** | **T** | **T** | **T** | **T** | **A** | **T** | **T** | **T** | **G** | **C** | **A** | **A** | **C** | **A** | **A** | **A** | **G** | **T** | **G** | **A** | **T** | **T** | **T** | **A** | **C** | **G** | **G** | **G** | **A** | **A** | **T** | **T** | **A** | **G** | **A** | **G** | **A** | **A** | **T** | **T** | **T** | **T** | **C** | **A** | **G** | **A** | **A** | **T** | **T** | **T** | **C** | **C** | **A** | **A** | **G** | **A** | **A** | **G** | **A** | **A** | **A** | **G** | **T** | **T** | **T** | **T** | **G** | **A** | **A** | **C** | **T** | **T** | **T** | **A** | **T** | **A** | **G** | **T** | **C** | **A** | **A** | **A** | **A** | **G** | **A** | **T** | **G** | **C** | **C** | **G** | **A** | **C** | **G** | **G** | **A** | **G** | **G** | **A** | **G** | **C** | **A** | **A** | **C** | **A** | **G** | **C** | **A** | **G** | **T** | **T** | **T** | **A** | **C** | **A** | **G** | **A** | **C** | **T** | **T** | **T** | **T** | **T** | **C** | **T** | **C** | **C** | **T** | **C** | **G** | **T** | **T** | **A** | **A** | **A** | **T** | **A** | **G** | **G** | **A** | **T** | **G** | **C** | **C** | **T** | **C** | **T** | **G** | **A** | **C** | **T** | **A** | **A** | **G** | **A** | **A** | **A** | **A** | **A** | **T** | **G** | **A** | **C** | **G** | **T** | **T** | **G** | **A** | **G** | **A** | **T** | **T** | **T** | **T** | **T** | **A** | **C** | **G** | **G** | **A** | **T** | **G** | **G** | **T** | **A** | **T** | **G** | **G** | **A** | **A** | **A** | **A** | **A** | **T** | **T** | **C** | **A** | **A** | **T** | **G** | **C** | **G** | **T** | **T** | **G** | **A** | **T** | **G** | **G** | **C** | **G** | **G** | **A** | **C** | **A** | **T** | **A** | **A** | **A** | **A** | **A** | **A** | **A** | **G** | **C** | **T** | **C** | **A** | **G** | **C** | **A** | **T** | **T** | **C** | **C** | **A** | **T** | **T** | **C** | **A** | **T** | **A** | **T** | **T** | **G** | **A** | **A** | **T** | **A** | **T** | **T** | **A** | **T** | **G** | **C** | **C** | **T** | **T** | **T** | **G** | **T** | **A** | **A** | **C** | **G** | **G** | **A** | **T** | **C** | **A** | **T** | **A** | **T** | **C** | **G** | **G** | **C** | **A** | **C** | **C** | **A** | **A** | **A** | **A** | **T** | **T** | **T** | **T** | **G** | **A** | **A** | **C** | **T** | **T** | **A** | **T** | **T** | **A** | **G** | **A** | **A** | **G** | **A** | **A** | **A** | **A** | **G** | **G** | **C** | **G** | **G** | **C** | **T** | **G** | **A** | **A** | **G** | **G** | **G** | **G** | **T** | **T** | **G** | **A** | **A** | **G** | **T** | **G** | **C** | **G** | **G** | **T** | **T** | **G** | **C** | **T** | **T** | **T** | **A** | **C** | **G** | **A** | **T** | **G** | **C** | **C** | **T** | **T** | **T** | **G** | **G** | **C** | **T** | **C** | **A** | **A** | **A** | **G** | **G** | **G** | **C** | **A** | **C** | **C** | **A** | **A** | **G** | **G** | **T** | **T** | **C** | **A** | **T** | **C** | **A** | **T** | **T** | **T** | **G** | **A** | **A** | **T** | **G** | **A** | **A** | **T** | **T** | **G** | **A** | **A** | **A** | **A** | **A** | **A** | **A** | **A** | **C** | **G** | **G** | **C** | **G** | **G** | **T** | **T** | **T** | **T** | **G** | **T** | **C** | **C** | **A** | **A** | **A** | **C** | **G** | **T** | **T** | **T** | **A** | **T** | **T** | **A** | **C** | **T** | **T** | **C** | **T** | **C** | **A** | **A** | **A** | **A** | **A** | **G** | **C** | **A** | **C** | **T** | **C** | **T** | **T** | **G** | **A** | **A** | **G** | **T** | **T** | **T** | **C** | **G** | **T** | **T** | **T** | **G** | **A** | **A** | **T** | **T** | **A** | **T** | **C** | **A** | **T** | **G** | **A** | **T** | **C** | **A** | **C** | **C** | **G** | **G** | **A** | **A** | **A** | **A** | **T** | **T** | **G** | **T** | **C** | **G** | **T** | **T** | **A** | **T** | **T** | **G** | **A** | **C** | **G** | **G** | **A** | **A** | **A** | **A** | **G** | **T** | **A** | **G** | **G** | **C** | **T** | **A** | **C** | **A** | **T** | **T** | **G** | **G** | **T** | **G** | **G** | **T** | **T** | **T** | **T** | **A** | **A** | **T** | **G** | **T** | **T** | **G** | **C** | **C** | **G** | **A** | **T** | **C** | **A** | **A** | **T** | **A** | **T** | **G** | **C** | **T** | **G** | **G** | **A** | **A** | **C** | **G** | **A** | **C** | **T** | **A** | **A** | **A** | **A** | **A** | **G** | **T** | **T** | **T** | **G** | **G** | **C** | **T** | **A** | **T** | **T** | **G** | **G** | **C** | **G** | **G** | **G** | **A** | **T** | **A** | **C** | **G** | **C** | **A** | **T** | **T** | **T** | **A** | **C** | **G** | **G** | **A** | **T** | **T** | **C** | **A** | **A** | **G** | **G** | **G** | **C** | **C** | **A** | **G** | **C** | **A** | **G** | **C** | **C** | **T** | **C** | **A** | **T** | **T** | **A** | **C** | **T** | **G** | **C** | **A** | **A** | **A** | **T** | **G** | **C** | **G** | **T** | **T** | **T** | **T** | **T** | **T** | **A** | **A** | **T** | **G** | **G** | **A** | **T** | **T** | **G** | **G** | **A** | **A** | **C** | **G** | **T** | **C** | **T** | **C** | **T** | **T** | **C** | **C** | **C** | **C** | **T** | **G** | **A** | **G** | **A** | **A** | **A** | **A** | **A** | **T** | **C** | **G** | **T** | **G** | **T** | **G** | **G** | **C** | **G** | **T** | **A** | **T** | **C** | **A** | **A** | **T** | **T** | **G** | **G** | **A** | **T** | **T** | **A** | **T** | **T** | **T** | **C** | **T** | **T** | **T** | **A** | **A** | **A** | **C** | **T** | **T** | **G** | **A** | **A** | **G** | **C** | **G** | **T** | **T** | **G** | **G** | **T** | **G** | **C** | **C** | **A** | **G** | **A** | **G** | **G** | **C** | **A** | **A** | **A** | **T** | **A** | **C** | **A** | **T** | **C** | **C** | **A** | **T** | **T** | **C** | **A** | **G** | **A** | **T** | **G** | **A** | **T** | **T** | **G** | **C** | **A** | **A** | **G** | **T** | **G** | **G** | **A** | **C** | **C** | **T** | **A** | **A** | **C** | **A** | **G** | **T** | **G** | **A** | **C** | **C** | **G** | **T** | **G** | **A** | **A** | **C** | **A** | **A** | **A** | **T** | **T** | **A** | **A** | **A** | **T** | **T** | **G** | **G** | **C** | **C** | **T** | **T** | **T** | **A** | **T** | **T** | **A** | **A** | **A** | **T** | **T** | **G** | **A** | **T** | **T** | **A** | **C** | **T** | **T** | **C** | **T** | **G** | **C** | **C** | **A** | **A** | **G** | **A** | **A** | **A** | **A** | **G** | **A** | **G** | **T** | **C** | **T** | **G** | **G** | **A** | **T** | **T** | **C** | **A** | **A** | **A** | **C** | **G** | **C** | **C** | **G** | **T** | **A** | **T** | **T** | **T** | **A** | **G** | **T** | **T** | **C** | **C** | **T** | **G** | **A** | **T** | **G** | **A** | **T** | **A** | **G** | **T** | **G** | **T** | **C** | **T** | **T** | **G** | **G** | **C** | **T** | **G** | **C** | **T** | **T** | **T** | **A** | **A** | **A** | **G** | **G** | **T** | **T** | **G** | **C** | **T** | **G** | **C** | **G** | **G** | **C** | **T** | **T** | **C** | **A** | **G** | **G** | **A** | **G** | **T** | **A** | **G** | **A** | **T** | **G** | **T** | **T** | **A** | **A** | **A** | **A** | **T** | **T** | **A** | **T** | **G** | **A** | **T** | **T** | **C** | **C** | **A** | **G** | **A** | **T** | **A** | **A** | **G** | **C** | **C** | **C** | **G** | **A** | **C** | **C** | **A** | **T** | **C** | **C** | **G** | **T** | **T** | **T** | **A** | **T** | **T** | **T** | **A** | **T** | **C** | **G** | **A** | **G** | **C** | **A** | **A** | **C** | **A** | **C** | **A** | **G** | **T** | **A** | **T** | **T** | **A** | **C** | **G** | **G** | **C** | **C** | **G** | **C** | **T** | **T** | **G** | **T** | **T** | **A** | **A** | **T** | **G** | **A** | **A** | **A** | **G** | **A** | **A** | **A** | **A** | **T** | **A** | **T** | **T** | **G** | **A** | **A** | **A** | **T** | **T** | **T** | **T** | **A** | **A** | **T** | **T** | **T** | **A** | **T** | **A** | **A** | **C** | **G** | **G** | **T** | **G** | **G** | **T** | **T** | **T** | **C** | **T** | **T** | **A** | **C** | **A** | **T** | **G** | **C** | **G** | **A** | **A** | **A** | **A** | **C** | **A** | **A** | **T** | **G** | **A** | **T** | **T** | **A** | **T** | **G** | **G** | **A** | **T** | **G** | **A** | **T** | **G** | **A** | **A** | **G** | **T** | **C** | **T** | **G** | **C** | **A** | **C** | **A** | **G** | **T** | **T** | **G** | **G** | **T** | **T** | **C** | **A** | **G** | **C** | **T** | **A** | **A** | **T** | **C** | **A** | **A** | **G** | **A** | **T** | **A** | **T** | **C** | **C** | **G** | **A** | **A** | **G** | **T** | **T** | **A** | **C** | **A** | **A** | **A** | **T** | **T** | **A** | **A** | **A** | **C** | **T** | **T** | **T** | **G** | **A** | **A** | **G** | **C** | **A** | **A** | **A** | **T** | **G** | **C** | **T** | **G** | **T** | **G** | **T** | **T** | **A** | **T** | **A** | **T** | **G** | **A** | **T** | **A** | **A** | **A** | **A** | **A** | **A** | **A** | **T** | **C** | **A** | **T** | **T** | **G** | **A** | **T** | **C** | **A** | **A** | **T** | **T** | **A** | **G** | **A** | **A** | **G** | **C** | **A** | **A** | **T** | **T** | **T** | **T** | **C** | **T** | **T** | **A** | **G** | **A** | **A** | **G** | **A** | **T** | **C** | **G** | **A** | **A** | **A** | **A** | **A** | **A** | **A** | **T** | **G** | **T** | **A** | **C** | **A** | **A** | **C** | **A** | **A** | **T** | **G** | **A** | **C** | **T** | **C** | **C** | **A** | **G** | **A** | **A** | **G** | **T** | **T** | **G** | **T** | **T** | **C** | **G** | **C** | **G** | **A** | **C** | **A** | **T** | **G** | **T** | **C** | **A** | **A** | **A** | **A** | **T** | **G** | **G** | **T** | **T** | **G** | **A** | **T** | **T** | **T** | **T** | **T** | **A** | **A** | **A** | **C** | **A** | **A** | **C** | **A** | **A** | **A** | **T** | **T** | **T** | **C** | **A** | **C** | **G** | **A** | **T** | **T** | **A** | **T** | **T** | **T** | **T** | **C** | **A** | **C** | **C** | **A** | **A** | **T** | **T** | **C** | **T** | **T** | **-** | **-** | **-** |
| **N59611F** | **T** | **T** | **G** | **A** | **T** | **T** | **C** | **T** | **T** | **A** | **G** | **C** | **G** | **T** | **C** | **T** | **T** | **A** | **A** | **C** | **A** | **G** | **T** | **T** | **A** | **T** | **T** | **T** | **A** | **T** | **T** | **T** | **T** | **A** | **T** | **T** | **A** | **A** | **T** | **G** | **C** | **A** | **A** | **T** | **T** | **A** | **T** | **T** | **G** | **C** | **G** | **G** | **G** | **A** | **A** | **T** | **T** | **A** | **C** | **C** | **A** | **T** | **T** | **T** | **T** | **G** | **T** | **T** | **A** | **A** | **A** | **A** | **C** | **C** | **T** | **C** | **G** | **A** | **G** | **A** | **T** | **G** | **T** | **A** | **G** | **C** | **A** | **G** | **C** | **A** | **A** | **T** | **T** | **T** | **G** | **G** | **G** | **C** | **A** | **T** | **G** | **G** | **C** | **T** | **A** | **C** | **T** | **C** | **G** | **T** | **A** | **C** | **T** | **G** | **A** | **T** | **T** | **G** | **C** | **T** | **C** | **T** | **A** | **C** | **C** | **C** | **G** | **T** | **T** | **T** | **T** | **T** | **G** | **G** | **T** | **T** | **T** | **T** | **T** | **T** | **C** | **T** | **T** | **A** | **T** | **A** | **T** | **T** | **T** | **A** | **T** | **T** | **C** | **T** | **T** | **T** | **G** | **G** | **G** | **C** | **G** | **C** | **G** | **G** | **T** | **T** | **T** | **A** | **A** | **C** | **C** | **G** | **A** | **C** | **A** | **A** | **A** | **A** | **A** | **G** | **A** | **A** | **A** | **T** | **T** | **T** | **T** | **A** | **T** | **T** | **T** | **G** | **C** | **A** | **A** | **C** | **A** | **A** | **A** | **G** | **T** | **G** | **A** | **T** | **T** | **T** | **A** | **C** | **G** | **G** | **G** | **A** | **A** | **T** | **T** | **A** | **G** | **A** | **G** | **A** | **A** | **T** | **T** | **T** | **C** | **C** | **A** | **G** | **A** | **A** | **T** | **T** | **T** | **C** | **C** | **A** | **A** | **G** | **A** | **A** | **G** | **A** | **A** | **A** | **G** | **T** | **T** | **T** | **T** | **G** | **A** | **A** | **C** | **T** | **T** | **T** | **A** | **T** | **A** | **G** | **T** | **C** | **A** | **A** | **A** | **A** | **G** | **A** | **T** | **G** | **C** | **C** | **G** | **A** | **C** | **G** | **G** | **A** | **G** | **G** | **A** | **G** | **C** | **A** | **A** | **C** | **A** | **A** | **C** | **A** | **G** | **T** | **T** | **T** | **A** | **C** | **A** | **G** | **A** | **C** | **T** | **T** | **T** | **T** | **T** | **T** | **T** | **C** | **C** | **T** | **C** | **G** | **T** | **T** | **A** | **A** | **A** | **T** | **A** | **G** | **G** | **A** | **T** | **G** | **C** | **C** | **T** | **C** | **T** | **G** | **A** | **C** | **T** | **A** | **A** | **G** | **A** | **A** | **A** | **A** | **A** | **T** | **G** | **A** | **T** | **G** | **T** | **T** | **G** | **A** | **A** | **A** | **T** | **T** | **T** | **T** | **T** | **A** | **C** | **G** | **G** | **A** | **T** | **G** | **G** | **T** | **A** | **C** | **G** | **G** | **A** | **A** | **A** | **A** | **A** | **T** | **T** | **C** | **A** | **A** | **T** | **G** | **C** | **G** | **T** | **T** | **G** | **A** | **T** | **G** | **G** | **C** | **G** | **G** | **A** | **C** | **A** | **T** | **A** | **A** | **A** | **A** | **A** | **A** | **A** | **G** | **C** | **T** | **C** | **A** | **G** | **C** | **A** | **C** | **T** | **C** | **C** | **A** | **T** | **T** | **C** | **A** | **T** | **A** | **T** | **T** | **G** | **A** | **A** | **T** | **A** | **T** | **T** | **A** | **T** | **G** | **C** | **C** | **T** | **T** | **T** | **G** | **T** | **A** | **A** | **C** | **G** | **G** | **A** | **T** | **C** | **A** | **T** | **A** | **T** | **C** | **G** | **G** | **T** | **A** | **C** | **C** | **A** | **A** | **A** | **A** | **T** | **T** | **T** | **T** | **G** | **A** | **A** | **C** | **T** | **T** | **A** | **T** | **T** | **A** | **G** | **A** | **A** | **G** | **A** | **A** | **A** | **A** | **G** | **G** | **C** | **G** | **G** | **C** | **T** | **G** | **A** | **A** | **G** | **G** | **G** | **G** | **T** | **T** | **G** | **A** | **A** | **G** | **T** | **G** | **C** | **G** | **G** | **T** | **T** | **G** | **C** | **T** | **T** | **T** | **A** | **C** | **G** | **A** | **T** | **G** | **C** | **C** | **T** | **T** | **T** | **G** | **G** | **C** | **T** | **C** | **A** | **A** | **A** | **G** | **G** | **G** | **C** | **A** | **C** | **C** | **A** | **A** | **G** | **G** | **T** | **T** | **C** | **A** | **T** | **C** | **A** | **T** | **T** | **T** | **G** | **A** | **A** | **T** | **G** | **A** | **A** | **T** | **T** | **G** | **A** | **A** | **A** | **A** | **A** | **A** | **A** | **A** | **C** | **G** | **G** | **T** | **G** | **G** | **T** | **T** | **T** | **T** | **G** | **T** | **C** | **C** | **A** | **A** | **A** | **C** | **G** | **T** | **T** | **T** | **A** | **T** | **T** | **A** | **C** | **T** | **T** | **C** | **T** | **C** | **A** | **A** | **A** | **A** | **A** | **G** | **C** | **A** | **C** | **T** | **T** | **T** | **T** | **G** | **A** | **A** | **G** | **T** | **T** | **T** | **C** | **G** | **T** | **T** | **T** | **G** | **A** | **A** | **T** | **T** | **A** | **T** | **C** | **A** | **T** | **G** | **A** | **T** | **C** | **A** | **C** | **C** | **G** | **G** | **A** | **A** | **A** | **A** | **T** | **T** | **G** | **T** | **T** | **G** | **T** | **T** | **A** | **T** | **T** | **G** | **A** | **C** | **G** | **G** | **A** | **A** | **A** | **A** | **G** | **T** | **A** | **G** | **G** | **C** | **T** | **A** | **C** | **A** | **T** | **T** | **G** | **G** | **C** | **G** | **G** | **C** | **T** | **T** | **T** | **A** | **A** | **T** | **G** | **T** | **T** | **G** | **C** | **C** | **G** | **A** | **T** | **C** | **A** | **A** | **T** | **A** | **T** | **G** | **C** | **T** | **G** | **G** | **A** | **A** | **C** | **G** | **A** | **C** | **T** | **A** | **A** | **A** | **A** | **A** | **G** | **T** | **T** | **T** | **G** | **G** | **C** | **T** | **A** | **T** | **T** | **G** | **G** | **C** | **G** | **G** | **G** | **A** | **T** | **A** | **C** | **G** | **C** | **A** | **T** | **T** | **T** | **A** | **C** | **G** | **G** | **A** | **T** | **T** | **C** | **A** | **A** | **G** | **G** | **G** | **C** | **C** | **A** | **G** | **C** | **A** | **G** | **C** | **C** | **T** | **C** | **A** | **T** | **T** | **A** | **C** | **T** | **G** | **C** | **A** | **A** | **A** | **T** | **G** | **C** | **G** | **T** | **T** | **T** | **T** | **T** | **T** | **A** | **A** | **T** | **G** | **G** | **A** | **T** | **T** | **G** | **G** | **A** | **A** | **C** | **G** | **T** | **C** | **T** | **C** | **T** | **T** | **C** | **C** | **C** | **C** | **C** | **G** | **A** | **G** | **A** | **A** | **A** | **A** | **A** | **T** | **C** | **A** | **T** | **G** | **T** | **G** | **G** | **C** | **G** | **T** | **A** | **T** | **C** | **A** | **A** | **T** | **T** | **G** | **G** | **A** | **T** | **T** | **A** | **T** | **T** | **T** | **C** | **T** | **T** | **T** | **A** | **A** | **A** | **C** | **T** | **T** | **G** | **A** | **A** | **G** | **C** | **A** | **T** | **T** | **G** | **G** | **T** | **G** | **C** | **C** | **A** | **G** | **A** | **G** | **G** | **C** | **A** | **A** | **A** | **T** | **A** | **C** | **A** | **T** | **C** | **C** | **A** | **T** | **T** | **C** | **A** | **G** | **A** | **T** | **G** | **A** | **T** | **T** | **G** | **C** | **C** | **A** | **G** | **T** | **G** | **G** | **T** | **C** | **C** | **T** | **A** | **A** | **C** | **A** | **G** | **T** | **G** | **A** | **C** | **C** | **G** | **T** | **G** | **A** | **A** | **C** | **A** | **A** | **A** | **T** | **T** | **A** | **A** | **A** | **T** | **T** | **G** | **G** | **C** | **C** | **T** | **T** | **T** | **A** | **T** | **T** | **A** | **A** | **A** | **T** | **T** | **G** | **A** | **T** | **T** | **A** | **C** | **T** | **T** | **C** | **T** | **G** | **C** | **C** | **A** | **A** | **G** | **A** | **A** | **A** | **A** | **G** | **A** | **G** | **T** | **C** | **T** | **G** | **G** | **A** | **T** | **T** | **C** | **A** | **A** | **A** | **C** | **G** | **C** | **C** | **G** | **T** | **A** | **T** | **T** | **T** | **A** | **G** | **T** | **T** | **C** | **C** | **T** | **G** | **A** | **T** | **G** | **A** | **T** | **A** | **G** | **T** | **G** | **T** | **C** | **T** | **T** | **G** | **G** | **C** | **T** | **G** | **C** | **T** | **T** | **T** | **A** | **A** | **A** | **G** | **G** | **T** | **T** | **G** | **C** | **T** | **G** | **C** | **G** | **G** | **C** | **T** | **T** | **C** | **A** | **G** | **G** | **A** | **G** | **T** | **A** | **G** | **A** | **T** | **G** | **T** | **T** | **A** | **A** | **A** | **A** | **T** | **T** | **A** | **T** | **G** | **A** | **T** | **T** | **C** | **C** | **A** | **G** | **A** | **T** | **A** | **A** | **G** | **C** | **C** | **C** | **G** | **A** | **C** | **C** | **A** | **T** | **C** | **C** | **G** | **T** | **T** | **T** | **A** | **T** | **T** | **T** | **A** | **T** | **C** | **G** | **A** | **G** | **C** | **A** | **A** | **C** | **A** | **C** | **A** | **G** | **T** | **A** | **T** | **T** | **A** | **C** | **G** | **G** | **C** | **C** | **G** | **C** | **T** | **T** | **A** | **T** | **T** | **G** | **A** | **T** | **G** | **A** | **A** | **A** | **G** | **A** | **A** | **A** | **A** | **T** | **A** | **T** | **T** | **G** | **A** | **A** | **A** | **T** | **T** | **T** | **T** | **A** | **A** | **T** | **T** | **T** | **A** | **T** | **A** | **A** | **C** | **G** | **G** | **T** | **G** | **G** | **T** | **T** | **T** | **C** | **T** | **T** | **A** | **C** | **A** | **T** | **G** | **C** | **G** | **A** | **A** | **A** | **A** | **C** | **A** | **A** | **T** | **G** | **A** | **T** | **T** | **A** | **T** | **G** | **G** | **A** | **T** | **G** | **A** | **T** | **G** | **A** | **A** | **G** | **T** | **C** | **T** | **G** | **T** | **A** | **C** | **A** | **G** | **T** | **T** | **G** | **G** | **T** | **T** | **C** | **A** | **G** | **C** | **C** | **A** | **A** | **T** | **C** | **A** | **A** | **G** | **A** | **T** | **A** | **T** | **C** | **C** | **G** | **A** | **A** | **G** | **T** | **T** | **A** | **C** | **A** | **A** | **A** | **T** | **T** | **A** | **A** | **A** | **C** | **T** | **T** | **T** | **G** | **A** | **A** | **G** | **C** | **A** | **A** | **A** | **T** | **G** | **C** | **T** | **G** | **T** | **G** | **T** | **T** | **A** | **T** | **A** | **T** | **G** | **A** | **T** | **A** | **A** | **A** | **A** | **A** | **A** | **A** | **T** | **C** | **A** | **T** | **T** | **G** | **A** | **T** | **C** | **A** | **A** | **T** | **T** | **A** | **G** | **A** | **A** | **G** | **C** | **A** | **A** | **T** | **T** | **T** | **T** | **C** | **T** | **T** | **A** | **G** | **A** | **A** | **G** | **A** | **T** | **C** | **G** | **A** | **A** | **A** | **A** | **A** | **A** | **A** | **T** | **G** | **T** | **A** | **C** | **A** | **A** | **C** | **A** | **A** | **T** | **G** | **A** | **C** | **A** | **C** | **C** | **A** | **G** | **A** | **A** | **G** | **T** | **T** | **G** | **T** | **T** | **C** | **G** | **A** | **G** | **A** | **C** | **A** | **T** | **G** | **T** | **C** | **C** | **A** | **A** | **A** | **T** | **G** | **G** | **T** | **T** | **G** | **A** | **T** | **T** | **T** | **T** | **T** | **A** | **A** | **A** | **C** | **A** | **A** | **C** | **A** | **A** | **A** | **T** | **T** | **T** | **C** | **A** | **C** | **G** | **A** | **T** | **T** | **A** | **T** | **T** | **T** | **T** | **C** | **C** | **C** | **C** | **A** | **A** | **T** | **T** | **C** | **T** | **T** | **-** | **-** | **-** |
| **N53476** | **T** | **T** | **G** | **A** | **T** | **T** | **C** | **T** | **T** | **A** | **G** | **C** | **G** | **T** | **C** | **T** | **T** | **A** | **A** | **C** | **A** | **G** | **T** | **T** | **A** | **T** | **T** | **T** | **A** | **T** | **T** | **T** | **T** | **A** | **T** | **T** | **A** | **A** | **T** | **G** | **C** | **A** | **A** | **T** | **T** | **A** | **T** | **T** | **G** | **C** | **G** | **G** | **G** | **A** | **A** | **T** | **T** | **A** | **C** | **C** | **A** | **T** | **T** | **T** | **T** | **G** | **T** | **T** | **A** | **A** | **A** | **A** | **C** | **C** | **T** | **C** | **G** | **A** | **G** | **A** | **T** | **G** | **T** | **A** | **G** | **C** | **A** | **G** | **C** | **A** | **A** | **T** | **T** | **T** | **G** | **G** | **G** | **C** | **A** | **T** | **G** | **G** | **C** | **T** | **A** | **C** | **T** | **C** | **G** | **T** | **A** | **C** | **T** | **G** | **A** | **T** | **T** | **G** | **C** | **T** | **C** | **T** | **A** | **C** | **C** | **C** | **G** | **T** | **T** | **T** | **T** | **T** | **G** | **G** | **T** | **T** | **T** | **T** | **T** | **T** | **C** | **T** | **T** | **A** | **T** | **A** | **T** | **T** | **T** | **A** | **T** | **T** | **C** | **T** | **T** | **T** | **G** | **G** | **G** | **C** | **G** | **C** | **G** | **G** | **T** | **T** | **T** | **A** | **A** | **C** | **C** | **G** | **A** | **C** | **A** | **A** | **A** | **A** | **A** | **G** | **A** | **A** | **A** | **T** | **T** | **T** | **T** | **A** | **T** | **T** | **T** | **G** | **C** | **A** | **A** | **C** | **A** | **A** | **A** | **G** | **T** | **G** | **A** | **T** | **T** | **T** | **A** | **C** | **G** | **G** | **G** | **A** | **A** | **T** | **T** | **A** | **G** | **A** | **G** | **A** | **A** | **T** | **T** | **T** | **C** | **C** | **A** | **G** | **A** | **A** | **T** | **T** | **T** | **C** | **C** | **A** | **A** | **G** | **A** | **A** | **G** | **A** | **A** | **A** | **G** | **T** | **T** | **T** | **T** | **G** | **A** | **A** | **C** | **T** | **T** | **T** | **A** | **T** | **A** | **G** | **T** | **C** | **A** | **A** | **A** | **A** | **G** | **A** | **T** | **G** | **C** | **C** | **G** | **A** | **C** | **G** | **G** | **A** | **G** | **G** | **A** | **G** | **C** | **A** | **A** | **C** | **A** | **A** | **C** | **A** | **G** | **T** | **T** | **T** | **A** | **C** | **A** | **G** | **A** | **C** | **T** | **T** | **T** | **T** | **T** | **T** | **T** | **C** | **C** | **T** | **C** | **G** | **T** | **T** | **A** | **A** | **A** | **T** | **A** | **G** | **G** | **A** | **T** | **G** | **C** | **C** | **T** | **C** | **T** | **G** | **A** | **C** | **T** | **A** | **A** | **G** | **A** | **A** | **A** | **A** | **A** | **T** | **G** | **A** | **T** | **G** | **T** | **T** | **G** | **A** | **A** | **A** | **T** | **T** | **T** | **T** | **T** | **A** | **C** | **G** | **G** | **A** | **T** | **G** | **G** | **T** | **A** | **C** | **G** | **G** | **A** | **A** | **A** | **A** | **A** | **T** | **T** | **C** | **A** | **A** | **T** | **G** | **C** | **G** | **T** | **T** | **G** | **A** | **T** | **G** | **G** | **C** | **G** | **G** | **A** | **T** | **A** | **T** | **A** | **A** | **A** | **A** | **A** | **A** | **A** | **G** | **C** | **T** | **C** | **A** | **G** | **C** | **A** | **C** | **T** | **C** | **C** | **A** | **T** | **T** | **C** | **A** | **T** | **A** | **T** | **T** | **G** | **A** | **A** | **T** | **A** | **T** | **T** | **A** | **T** | **G** | **C** | **C** | **T** | **T** | **T** | **G** | **T** | **A** | **A** | **C** | **G** | **G** | **A** | **T** | **C** | **A** | **T** | **A** | **T** | **C** | **G** | **G** | **C** | **A** | **C** | **C** | **A** | **A** | **A** | **A** | **T** | **T** | **T** | **T** | **G** | **A** | **A** | **C** | **T** | **T** | **A** | **T** | **T** | **A** | **G** | **A** | **A** | **G** | **A** | **A** | **A** | **A** | **G** | **G** | **C** | **G** | **G** | **C** | **T** | **G** | **A** | **A** | **G** | **G** | **G** | **G** | **T** | **T** | **G** | **A** | **A** | **G** | **T** | **G** | **C** | **G** | **G** | **T** | **T** | **G** | **C** | **T** | **T** | **T** | **A** | **C** | **G** | **A** | **T** | **G** | **C** | **C** | **T** | **T** | **T** | **G** | **G** | **C** | **T** | **C** | **A** | **A** | **A** | **G** | **G** | **G** | **C** | **A** | **C** | **C** | **A** | **A** | **G** | **G** | **T** | **T** | **C** | **A** | **T** | **C** | **A** | **T** | **T** | **T** | **G** | **A** | **A** | **T** | **G** | **A** | **A** | **T** | **T** | **G** | **A** | **A** | **A** | **A** | **A** | **A** | **A** | **A** | **C** | **G** | **G** | **C** | **G** | **G** | **T** | **T** | **T** | **T** | **G** | **T** | **C** | **C** | **A** | **A** | **A** | **C** | **G** | **T** | **T** | **T** | **A** | **T** | **T** | **A** | **C** | **T** | **T** | **C** | **T** | **C** | **A** | **A** | **A** | **A** | **A** | **G** | **C** | **A** | **C** | **T** | **T** | **T** | **T** | **G** | **A** | **A** | **G** | **T** | **T** | **T** | **C** | **G** | **T** | **T** | **T** | **G** | **A** | **A** | **T** | **T** | **A** | **T** | **C** | **A** | **T** | **G** | **A** | **T** | **C** | **A** | **C** | **C** | **G** | **G** | **A** | **A** | **A** | **A** | **T** | **T** | **G** | **T** | **T** | **G** | **T** | **T** | **A** | **T** | **T** | **G** | **A** | **C** | **G** | **G** | **A** | **A** | **A** | **A** | **G** | **T** | **A** | **G** | **G** | **C** | **T** | **A** | **C** | **A** | **T** | **T** | **G** | **G** | **C** | **G** | **G** | **C** | **T** | **T** | **T** | **A** | **A** | **T** | **G** | **T** | **T** | **G** | **C** | **C** | **G** | **A** | **T** | **C** | **A** | **A** | **T** | **A** | **T** | **G** | **C** | **T** | **G** | **G** | **A** | **A** | **C** | **G** | **A** | **C** | **T** | **A** | **A** | **A** | **A** | **A** | **G** | **T** | **T** | **T** | **G** | **G** | **C** | **T** | **A** | **T** | **T** | **G** | **G** | **C** | **G** | **G** | **G** | **A** | **T** | **A** | **C** | **G** | **C** | **A** | **T** | **T** | **T** | **A** | **C** | **G** | **G** | **A** | **T** | **T** | **C** | **A** | **A** | **G** | **G** | **G** | **C** | **C** | **A** | **G** | **C** | **A** | **G** | **C** | **C** | **T** | **C** | **A** | **T** | **T** | **A** | **C** | **T** | **G** | **C** | **A** | **A** | **A** | **T** | **G** | **C** | **G** | **T** | **T** | **T** | **T** | **T** | **T** | **A** | **A** | **T** | **G** | **G** | **A** | **T** | **T** | **G** | **G** | **A** | **A** | **C** | **G** | **T** | **C** | **T** | **C** | **T** | **T** | **C** | **C** | **C** | **C** | **C** | **G** | **A** | **G** | **A** | **A** | **A** | **A** | **A** | **T** | **C** | **G** | **T** | **G** | **T** | **G** | **G** | **C** | **G** | **T** | **A** | **T** | **C** | **A** | **A** | **T** | **T** | **G** | **G** | **A** | **T** | **T** | **A** | **T** | **T** | **T** | **C** | **T** | **T** | **T** | **A** | **A** | **A** | **C** | **T** | **T** | **G** | **A** | **A** | **G** | **C** | **A** | **T** | **T** | **G** | **G** | **T** | **G** | **C** | **C** | **A** | **G** | **A** | **G** | **G** | **C** | **A** | **A** | **A** | **T** | **A** | **C** | **A** | **T** | **C** | **C** | **A** | **T** | **T** | **C** | **A** | **G** | **A** | **T** | **G** | **A** | **T** | **T** | **G** | **C** | **C** | **A** | **G** | **T** | **G** | **G** | **T** | **C** | **C** | **T** | **A** | **A** | **C** | **A** | **G** | **T** | **G** | **A** | **C** | **C** | **G** | **T** | **G** | **A** | **A** | **C** | **A** | **A** | **A** | **T** | **T** | **A** | **A** | **A** | **T** | **T** | **G** | **G** | **C** | **C** | **T** | **T** | **T** | **A** | **T** | **T** | **A** | **A** | **A** | **T** | **T** | **G** | **A** | **T** | **T** | **A** | **C** | **T** | **T** | **C** | **T** | **G** | **C** | **C** | **A** | **A** | **G** | **A** | **A** | **A** | **A** | **G** | **A** | **G** | **T** | **C** | **T** | **G** | **G** | **A** | **T** | **T** | **C** | **A** | **A** | **A** | **C** | **G** | **C** | **C** | **G** | **T** | **A** | **T** | **T** | **T** | **A** | **G** | **T** | **T** | **C** | **C** | **T** | **G** | **A** | **T** | **G** | **A** | **T** | **A** | **G** | **T** | **G** | **T** | **C** | **T** | **T** | **G** | **G** | **C** | **T** | **G** | **C** | **T** | **T** | **T** | **A** | **A** | **A** | **G** | **G** | **T** | **T** | **G** | **C** | **T** | **G** | **C** | **G** | **G** | **C** | **T** | **T** | **C** | **A** | **G** | **G** | **A** | **G** | **T** | **A** | **G** | **A** | **T** | **G** | **T** | **T** | **A** | **A** | **A** | **A** | **T** | **T** | **A** | **T** | **G** | **A** | **T** | **T** | **C** | **C** | **A** | **G** | **A** | **T** | **A** | **A** | **G** | **C** | **C** | **C** | **G** | **A** | **C** | **C** | **A** | **T** | **C** | **C** | **G** | **T** | **T** | **T** | **A** | **T** | **T** | **T** | **A** | **T** | **C** | **G** | **A** | **G** | **C** | **A** | **A** | **C** | **A** | **C** | **A** | **G** | **T** | **A** | **T** | **T** | **A** | **C** | **G** | **G** | **C** | **C** | **G** | **C** | **T** | **T** | **A** | **T** | **T** | **G** | **A** | **T** | **G** | **A** | **A** | **A** | **G** | **A** | **A** | **A** | **A** | **T** | **A** | **T** | **T** | **G** | **A** | **A** | **A** | **T** | **T** | **T** | **T** | **A** | **A** | **T** | **T** | **T** | **A** | **T** | **A** | **A** | **C** | **G** | **G** | **T** | **G** | **G** | **T** | **T** | **T** | **C** | **T** | **T** | **A** | **C** | **A** | **T** | **G** | **C** | **G** | **A** | **A** | **A** | **A** | **C** | **A** | **A** | **T** | **G** | **A** | **T** | **T** | **A** | **T** | **G** | **G** | **A** | **T** | **G** | **A** | **T** | **G** | **A** | **A** | **G** | **T** | **C** | **T** | **G** | **T** | **A** | **C** | **A** | **G** | **T** | **T** | **G** | **G** | **T** | **T** | **C** | **A** | **G** | **C** | **C** | **A** | **A** | **T** | **C** | **A** | **A** | **G** | **A** | **T** | **A** | **T** | **C** | **C** | **G** | **A** | **A** | **G** | **T** | **T** | **A** | **C** | **A** | **A** | **A** | **T** | **T** | **A** | **A** | **A** | **C** | **T** | **T** | **T** | **G** | **A** | **A** | **G** | **C** | **A** | **A** | **A** | **T** | **G** | **C** | **T** | **G** | **T** | **G** | **T** | **T** | **A** | **T** | **A** | **T** | **G** | **A** | **T** | **A** | **A** | **A** | **A** | **A** | **A** | **A** | **T** | **C** | **A** | **T** | **T** | **G** | **A** | **T** | **C** | **A** | **A** | **T** | **T** | **A** | **G** | **A** | **A** | **G** | **C** | **A** | **A** | **T** | **T** | **T** | **T** | **C** | **T** | **T** | **A** | **G** | **A** | **A** | **G** | **A** | **T** | **C** | **G** | **A** | **A** | **A** | **A** | **A** | **A** | **A** | **T** | **G** | **T** | **A** | **C** | **A** | **A** | **C** | **A** | **A** | **T** | **G** | **A** | **C** | **T** | **C** | **C** | **A** | **G** | **A** | **A** | **G** | **T** | **T** | **G** | **T** | **T** | **C** | **G** | **C** | **G** | **A** | **C** | **A** | **T** | **G** | **T** | **C** | **A** | **A** | **A** | **A** | **T** | **G** | **G** | **T** | **T** | **G** | **A** | **T** | **T** | **T** | **T** | **T** | **A** | **A** | **A** | **C** | **A** | **A** | **C** | **A** | **A** | **A** | **T** | **T** | **T** | **C** | **A** | **C** | **G** | **A** | **T** | **T** | **A** | **T** | **T** | **T** | **T** | **C** | **A** | **C** | **C** | **A** | **A** | **T** | **T** | **C** | **T** | **T** | **-** | **-** | **-** |
| **N54156** | **T** | **T** | **G** | **A** | **T** | **T** | **C** | **T** | **T** | **A** | **G** | **C** | **G** | **T** | **C** | **T** | **T** | **A** | **A** | **C** | **A** | **G** | **T** | **T** | **A** | **T** | **T** | **T** | **A** | **T** | **T** | **T** | **T** | **A** | **T** | **T** | **A** | **A** | **T** | **G** | **C** | **A** | **A** | **T** | **T** | **A** | **T** | **T** | **G** | **C** | **G** | **G** | **G** | **A** | **A** | **T** | **T** | **A** | **C** | **C** | **A** | **T** | **T** | **T** | **T** | **G** | **T** | **T** | **A** | **A** | **A** | **A** | **C** | **C** | **T** | **C** | **G** | **A** | **G** | **A** | **T** | **G** | **T** | **A** | **G** | **C** | **A** | **G** | **C** | **A** | **A** | **T** | **T** | **T** | **G** | **G** | **G** | **C** | **A** | **T** | **G** | **G** | **C** | **T** | **A** | **C** | **T** | **C** | **G** | **T** | **A** | **C** | **T** | **G** | **A** | **T** | **T** | **G** | **C** | **T** | **C** | **T** | **A** | **C** | **C** | **C** | **G** | **T** | **T** | **T** | **T** | **T** | **G** | **G** | **T** | **T** | **T** | **T** | **T** | **T** | **C** | **T** | **T** | **A** | **T** | **A** | **T** | **T** | **T** | **A** | **T** | **T** | **C** | **T** | **T** | **T** | **G** | **G** | **G** | **C** | **G** | **C** | **G** | **G** | **T** | **T** | **T** | **A** | **A** | **C** | **C** | **G** | **A** | **C** | **A** | **A** | **A** | **A** | **A** | **G** | **A** | **A** | **A** | **T** | **T** | **T** | **T** | **A** | **T** | **T** | **T** | **G** | **C** | **A** | **A** | **C** | **A** | **A** | **A** | **G** | **T** | **G** | **A** | **T** | **T** | **T** | **A** | **C** | **G** | **G** | **G** | **A** | **A** | **T** | **T** | **A** | **G** | **A** | **G** | **A** | **A** | **T** | **T** | **T** | **C** | **C** | **A** | **G** | **A** | **A** | **T** | **T** | **T** | **C** | **C** | **A** | **A** | **G** | **A** | **A** | **G** | **A** | **A** | **A** | **G** | **T** | **T** | **T** | **T** | **G** | **A** | **A** | **C** | **T** | **T** | **T** | **A** | **T** | **A** | **G** | **T** | **C** | **A** | **A** | **A** | **A** | **G** | **A** | **T** | **G** | **C** | **C** | **G** | **A** | **C** | **G** | **G** | **A** | **G** | **G** | **A** | **G** | **C** | **A** | **A** | **C** | **A** | **A** | **C** | **A** | **G** | **T** | **T** | **T** | **A** | **C** | **A** | **G** | **A** | **C** | **T** | **T** | **T** | **T** | **T** | **T** | **T** | **C** | **C** | **T** | **C** | **G** | **T** | **T** | **A** | **A** | **A** | **T** | **A** | **G** | **G** | **A** | **T** | **G** | **C** | **C** | **T** | **C** | **T** | **G** | **A** | **C** | **T** | **A** | **A** | **G** | **A** | **A** | **A** | **A** | **A** | **T** | **G** | **A** | **T** | **G** | **T** | **T** | **G** | **A** | **A** | **A** | **T** | **T** | **T** | **T** | **T** | **A** | **C** | **G** | **G** | **A** | **T** | **G** | **G** | **T** | **A** | **C** | **G** | **G** | **A** | **A** | **A** | **A** | **A** | **T** | **T** | **C** | **A** | **A** | **T** | **G** | **C** | **G** | **T** | **T** | **G** | **A** | **T** | **G** | **G** | **C** | **G** | **G** | **A** | **T** | **A** | **T** | **A** | **A** | **A** | **A** | **A** | **A** | **A** | **G** | **C** | **T** | **C** | **A** | **G** | **C** | **A** | **C** | **T** | **C** | **T** | **A** | **T** | **T** | **C** | **A** | **T** | **A** | **T** | **T** | **G** | **A** | **A** | **T** | **A** | **T** | **T** | **A** | **T** | **G** | **C** | **C** | **T** | **T** | **T** | **G** | **T** | **A** | **A** | **C** | **G** | **G** | **A** | **T** | **C** | **A** | **T** | **A** | **T** | **C** | **G** | **G** | **T** | **A** | **C** | **C** | **A** | **A** | **A** | **A** | **T** | **T** | **T** | **T** | **G** | **A** | **A** | **C** | **T** | **T** | **A** | **T** | **T** | **A** | **G** | **A** | **A** | **G** | **A** | **A** | **A** | **A** | **G** | **G** | **C** | **G** | **G** | **C** | **T** | **G** | **A** | **A** | **G** | **G** | **G** | **G** | **T** | **T** | **G** | **A** | **A** | **G** | **T** | **G** | **C** | **G** | **G** | **T** | **T** | **G** | **C** | **T** | **T** | **T** | **A** | **C** | **G** | **A** | **T** | **G** | **C** | **C** | **T** | **T** | **T** | **G** | **G** | **C** | **T** | **C** | **A** | **A** | **A** | **G** | **G** | **G** | **C** | **A** | **C** | **C** | **A** | **A** | **G** | **G** | **T** | **T** | **C** | **A** | **T** | **C** | **A** | **T** | **T** | **T** | **G** | **A** | **A** | **T** | **G** | **A** | **A** | **T** | **T** | **G** | **A** | **A** | **A** | **A** | **A** | **A** | **A** | **A** | **C** | **G** | **G** | **C** | **G** | **G** | **T** | **T** | **T** | **T** | **G** | **T** | **C** | **C** | **A** | **A** | **A** | **C** | **G** | **T** | **T** | **T** | **A** | **T** | **T** | **A** | **C** | **T** | **T** | **C** | **T** | **C** | **A** | **A** | **A** | **A** | **A** | **G** | **C** | **A** | **C** | **T** | **T** | **T** | **T** | **G** | **A** | **A** | **G** | **T** | **T** | **T** | **C** | **G** | **T** | **T** | **T** | **G** | **A** | **A** | **T** | **T** | **A** | **T** | **C** | **A** | **T** | **G** | **A** | **T** | **C** | **A** | **C** | **C** | **G** | **G** | **A** | **A** | **A** | **A** | **T** | **T** | **G** | **T** | **T** | **G** | **T** | **T** | **A** | **T** | **T** | **G** | **A** | **C** | **G** | **G** | **A** | **A** | **A** | **A** | **G** | **T** | **A** | **G** | **G** | **C** | **T** | **A** | **C** | **A** | **T** | **T** | **G** | **G** | **C** | **G** | **G** | **C** | **T** | **T** | **T** | **A** | **A** | **T** | **G** | **T** | **T** | **G** | **C** | **C** | **G** | **A** | **T** | **C** | **A** | **A** | **T** | **A** | **T** | **G** | **C** | **T** | **G** | **G** | **A** | **A** | **C** | **G** | **A** | **C** | **T** | **A** | **A** | **A** | **A** | **A** | **G** | **T** | **T** | **T** | **G** | **G** | **C** | **T** | **A** | **T** | **T** | **G** | **G** | **C** | **G** | **G** | **G** | **A** | **T** | **A** | **C** | **A** | **C** | **A** | **T** | **T** | **T** | **A** | **C** | **G** | **G** | **A** | **T** | **T** | **C** | **A** | **A** | **G** | **G** | **G** | **C** | **C** | **A** | **G** | **C** | **A** | **G** | **C** | **C** | **T** | **C** | **A** | **T** | **T** | **A** | **C** | **T** | **G** | **C** | **A** | **A** | **A** | **T** | **G** | **C** | **G** | **T** | **T** | **T** | **T** | **T** | **T** | **A** | **A** | **T** | **G** | **G** | **A** | **T** | **T** | **G** | **G** | **A** | **A** | **C** | **G** | **T** | **C** | **T** | **C** | **T** | **T** | **C** | **C** | **C** | **C** | **C** | **G** | **A** | **G** | **A** | **A** | **A** | **A** | **A** | **T** | **C** | **G** | **T** | **G** | **T** | **G** | **G** | **C** | **G** | **T** | **A** | **T** | **C** | **A** | **A** | **T** | **T** | **G** | **G** | **A** | **T** | **T** | **A** | **T** | **T** | **T** | **C** | **T** | **T** | **T** | **A** | **A** | **A** | **C** | **T** | **T** | **G** | **A** | **A** | **G** | **C** | **A** | **T** | **T** | **G** | **G** | **T** | **G** | **C** | **C** | **A** | **G** | **A** | **G** | **G** | **C** | **A** | **A** | **A** | **T** | **A** | **C** | **A** | **T** | **C** | **C** | **A** | **T** | **T** | **C** | **A** | **G** | **A** | **T** | **G** | **A** | **T** | **T** | **G** | **C** | **C** | **A** | **G** | **T** | **G** | **G** | **T** | **C** | **C** | **T** | **A** | **A** | **C** | **A** | **G** | **T** | **G** | **A** | **C** | **C** | **G** | **T** | **G** | **A** | **A** | **C** | **A** | **A** | **A** | **T** | **T** | **A** | **A** | **A** | **T** | **T** | **G** | **G** | **C** | **C** | **T** | **T** | **T** | **A** | **T** | **T** | **A** | **A** | **A** | **T** | **T** | **G** | **A** | **T** | **T** | **A** | **C** | **T** | **T** | **C** | **T** | **G** | **C** | **C** | **A** | **A** | **G** | **A** | **A** | **A** | **A** | **G** | **A** | **G** | **T** | **C** | **T** | **G** | **G** | **A** | **T** | **T** | **C** | **A** | **A** | **A** | **C** | **G** | **C** | **C** | **G** | **T** | **A** | **T** | **T** | **T** | **A** | **G** | **T** | **T** | **C** | **C** | **T** | **G** | **A** | **T** | **G** | **A** | **T** | **A** | **G** | **T** | **G** | **T** | **C** | **T** | **T** | **G** | **G** | **C** | **T** | **G** | **C** | **T** | **T** | **T** | **A** | **A** | **A** | **G** | **G** | **T** | **T** | **G** | **C** | **T** | **G** | **C** | **G** | **G** | **C** | **T** | **T** | **C** | **A** | **G** | **G** | **A** | **G** | **T** | **A** | **G** | **A** | **T** | **G** | **T** | **T** | **A** | **A** | **A** | **A** | **T** | **T** | **A** | **T** | **G** | **A** | **T** | **T** | **C** | **C** | **A** | **G** | **A** | **T** | **A** | **A** | **G** | **C** | **C** | **C** | **G** | **A** | **C** | **C** | **A** | **T** | **C** | **C** | **G** | **T** | **T** | **T** | **A** | **T** | **T** | **T** | **A** | **T** | **C** | **G** | **A** | **G** | **C** | **A** | **A** | **C** | **A** | **C** | **A** | **G** | **T** | **A** | **T** | **T** | **A** | **C** | **G** | **G** | **C** | **C** | **G** | **C** | **T** | **T** | **A** | **T** | **T** | **G** | **A** | **T** | **G** | **A** | **A** | **A** | **G** | **A** | **A** | **A** | **A** | **T** | **A** | **T** | **T** | **G** | **A** | **A** | **A** | **T** | **T** | **T** | **T** | **A** | **A** | **T** | **T** | **T** | **A** | **T** | **A** | **A** | **C** | **G** | **G** | **T** | **G** | **G** | **T** | **T** | **T** | **C** | **T** | **T** | **A** | **C** | **A** | **T** | **G** | **C** | **G** | **A** | **A** | **A** | **A** | **C** | **A** | **A** | **T** | **G** | **A** | **T** | **T** | **A** | **T** | **G** | **G** | **A** | **T** | **G** | **A** | **T** | **G** | **A** | **A** | **G** | **T** | **C** | **T** | **G** | **C** | **A** | **C** | **A** | **G** | **T** | **T** | **G** | **G** | **T** | **T** | **C** | **A** | **G** | **C** | **C** | **A** | **A** | **T** | **C** | **A** | **A** | **G** | **A** | **T** | **A** | **T** | **C** | **C** | **G** | **A** | **A** | **G** | **T** | **T** | **A** | **C** | **A** | **A** | **A** | **T** | **T** | **A** | **A** | **A** | **C** | **T** | **T** | **T** | **G** | **A** | **A** | **G** | **C** | **A** | **A** | **A** | **T** | **G** | **C** | **T** | **G** | **T** | **G** | **T** | **T** | **A** | **T** | **A** | **T** | **G** | **A** | **T** | **A** | **A** | **A** | **A** | **A** | **A** | **A** | **T** | **C** | **A** | **T** | **T** | **G** | **A** | **T** | **C** | **A** | **A** | **T** | **T** | **A** | **G** | **A** | **A** | **G** | **C** | **A** | **A** | **T** | **T** | **T** | **T** | **C** | **T** | **T** | **A** | **G** | **A** | **A** | **G** | **A** | **T** | **C** | **G** | **A** | **A** | **A** | **A** | **A** | **A** | **A** | **T** | **G** | **T** | **A** | **C** | **A** | **A** | **C** | **A** | **A** | **T** | **G** | **A** | **C** | **T** | **C** | **C** | **A** | **G** | **A** | **A** | **G** | **T** | **T** | **G** | **T** | **T** | **C** | **G** | **T** | **G** | **A** | **C** | **A** | **T** | **G** | **T** | **C** | **A** | **A** | **A** | **A** | **T** | **G** | **G** | **T** | **T** | **G** | **A** | **T** | **T** | **T** | **T** | **T** | **A** | **A** | **A** | **C** | **A** | **A** | **C** | **A** | **A** | **A** | **T** | **T** | **T** | **C** | **A** | **C** | **G** | **A** | **T** | **T** | **A** | **T** | **T** | **T** | **T** | **C** | **A** | **C** | **C** | **A** | **A** | **T** | **T** | **C** | **T** | **T** | **-** | **-** | **-** |
| **N59554F** | **T** | **T** | **G** | **A** | **T** | **T** | **C** | **T** | **T** | **A** | **G** | **C** | **G** | **T** | **C** | **T** | **T** | **A** | **A** | **C** | **A** | **G** | **T** | **T** | **A** | **T** | **T** | **T** | **A** | **T** | **T** | **T** | **T** | **A** | **T** | **T** | **A** | **A** | **T** | **G** | **C** | **A** | **A** | **T** | **T** | **A** | **T** | **T** | **G** | **C** | **G** | **G** | **G** | **A** | **A** | **T** | **T** | **A** | **C** | **C** | **A** | **T** | **T** | **T** | **T** | **G** | **T** | **T** | **A** | **A** | **A** | **A** | **C** | **C** | **T** | **C** | **G** | **A** | **G** | **A** | **T** | **G** | **T** | **A** | **G** | **C** | **A** | **G** | **C** | **A** | **A** | **T** | **T** | **T** | **G** | **G** | **G** | **C** | **A** | **T** | **G** | **G** | **C** | **T** | **A** | **C** | **T** | **C** | **G** | **T** | **A** | **C** | **T** | **G** | **A** | **T** | **T** | **G** | **C** | **T** | **C** | **T** | **A** | **C** | **C** | **C** | **G** | **T** | **T** | **T** | **T** | **T** | **G** | **G** | **T** | **T** | **T** | **T** | **T** | **T** | **C** | **T** | **T** | **A** | **T** | **A** | **T** | **T** | **T** | **A** | **T** | **T** | **C** | **T** | **T** | **T** | **G** | **G** | **G** | **C** | **G** | **C** | **G** | **G** | **T** | **T** | **T** | **A** | **A** | **C** | **C** | **G** | **A** | **C** | **A** | **A** | **A** | **A** | **A** | **G** | **A** | **A** | **A** | **T** | **T** | **T** | **T** | **A** | **T** | **T** | **T** | **G** | **C** | **A** | **A** | **C** | **A** | **A** | **A** | **G** | **T** | **G** | **A** | **T** | **T** | **T** | **G** | **C** | **G** | **G** | **G** | **A** | **A** | **T** | **T** | **A** | **G** | **A** | **G** | **A** | **A** | **T** | **T** | **T** | **T** | **C** | **A** | **G** | **A** | **A** | **T** | **T** | **T** | **C** | **C** | **A** | **A** | **G** | **A** | **A** | **G** | **A** | **A** | **A** | **G** | **T** | **T** | **T** | **T** | **G** | **A** | **A** | **C** | **T** | **T** | **T** | **A** | **T** | **A** | **G** | **T** | **C** | **A** | **A** | **A** | **A** | **G** | **A** | **T** | **G** | **C** | **C** | **G** | **A** | **C** | **G** | **G** | **A** | **G** | **G** | **A** | **G** | **C** | **A** | **A** | **C** | **A** | **A** | **C** | **A** | **G** | **T** | **T** | **T** | **A** | **C** | **A** | **G** | **A** | **C** | **T** | **T** | **T** | **T** | **T** | **T** | **T** | **C** | **C** | **T** | **C** | **G** | **T** | **T** | **A** | **A** | **A** | **T** | **A** | **G** | **G** | **A** | **T** | **G** | **C** | **C** | **T** | **C** | **T** | **G** | **A** | **C** | **T** | **A** | **A** | **G** | **A** | **A** | **A** | **A** | **A** | **T** | **G** | **A** | **T** | **G** | **T** | **T** | **G** | **A** | **A** | **A** | **T** | **T** | **T** | **T** | **T** | **A** | **C** | **G** | **G** | **A** | **T** | **G** | **G** | **T** | **A** | **C** | **G** | **G** | **A** | **A** | **A** | **A** | **A** | **T** | **T** | **C** | **A** | **A** | **T** | **G** | **C** | **G** | **T** | **T** | **G** | **A** | **T** | **G** | **G** | **C** | **G** | **G** | **A** | **C** | **A** | **T** | **A** | **A** | **A** | **A** | **A** | **A** | **A** | **G** | **C** | **T** | **C** | **A** | **G** | **C** | **A** | **C** | **T** | **C** | **C** | **A** | **T** | **T** | **C** | **A** | **T** | **A** | **T** | **T** | **G** | **A** | **A** | **T** | **A** | **T** | **T** | **A** | **T** | **G** | **C** | **C** | **T** | **T** | **T** | **G** | **T** | **A** | **A** | **C** | **G** | **G** | **A** | **T** | **C** | **A** | **T** | **A** | **T** | **C** | **G** | **G** | **C** | **A** | **C** | **C** | **A** | **A** | **A** | **A** | **T** | **T** | **T** | **T** | **G** | **A** | **A** | **C** | **T** | **T** | **A** | **T** | **T** | **A** | **G** | **A** | **A** | **G** | **A** | **A** | **A** | **A** | **G** | **G** | **C** | **G** | **G** | **C** | **T** | **G** | **A** | **A** | **G** | **G** | **G** | **G** | **T** | **T** | **G** | **A** | **A** | **G** | **T** | **G** | **C** | **G** | **G** | **T** | **T** | **G** | **C** | **T** | **T** | **T** | **A** | **C** | **G** | **A** | **T** | **G** | **C** | **C** | **T** | **T** | **T** | **G** | **G** | **C** | **T** | **C** | **A** | **A** | **A** | **G** | **G** | **G** | **C** | **A** | **C** | **C** | **A** | **A** | **G** | **G** | **T** | **T** | **C** | **A** | **T** | **C** | **A** | **T** | **T** | **T** | **G** | **A** | **A** | **T** | **G** | **A** | **A** | **T** | **T** | **G** | **A** | **A** | **A** | **A** | **A** | **A** | **A** | **A** | **C** | **G** | **G** | **C** | **G** | **G** | **T** | **T** | **T** | **T** | **G** | **T** | **C** | **C** | **A** | **A** | **A** | **C** | **A** | **T** | **T** | **T** | **A** | **T** | **T** | **A** | **C** | **T** | **T** | **C** | **T** | **C** | **A** | **A** | **A** | **A** | **A** | **G** | **C** | **A** | **C** | **T** | **T** | **C** | **T** | **G** | **A** | **A** | **G** | **T** | **T** | **T** | **C** | **G** | **T** | **T** | **T** | **G** | **A** | **A** | **T** | **T** | **A** | **T** | **C** | **A** | **T** | **G** | **A** | **T** | **C** | **A** | **C** | **C** | **G** | **G** | **A** | **A** | **A** | **A** | **T** | **T** | **G** | **T** | **T** | **G** | **T** | **T** | **A** | **T** | **T** | **G** | **A** | **C** | **G** | **G** | **A** | **A** | **A** | **A** | **G** | **T** | **A** | **G** | **G** | **C** | **T** | **A** | **C** | **A** | **T** | **T** | **G** | **G** | **C** | **G** | **G** | **C** | **T** | **T** | **T** | **A** | **A** | **T** | **G** | **T** | **T** | **G** | **C** | **C** | **G** | **A** | **T** | **C** | **A** | **A** | **T** | **A** | **T** | **G** | **C** | **T** | **G** | **G** | **A** | **A** | **C** | **G** | **A** | **C** | **T** | **A** | **A** | **A** | **A** | **A** | **G** | **T** | **T** | **T** | **G** | **G** | **C** | **T** | **A** | **T** | **T** | **G** | **G** | **C** | **G** | **G** | **G** | **A** | **T** | **A** | **C** | **G** | **C** | **A** | **T** | **T** | **T** | **A** | **C** | **G** | **G** | **A** | **T** | **T** | **C** | **A** | **A** | **G** | **G** | **G** | **C** | **C** | **A** | **G** | **C** | **A** | **G** | **C** | **C** | **T** | **C** | **A** | **T** | **T** | **A** | **C** | **T** | **G** | **C** | **A** | **A** | **A** | **T** | **G** | **C** | **G** | **T** | **T** | **T** | **T** | **T** | **T** | **A** | **A** | **T** | **G** | **G** | **A** | **T** | **T** | **G** | **G** | **A** | **A** | **C** | **G** | **T** | **C** | **T** | **C** | **T** | **T** | **C** | **C** | **C** | **C** | **C** | **G** | **A** | **G** | **A** | **A** | **A** | **A** | **A** | **T** | **C** | **G** | **T** | **G** | **T** | **G** | **G** | **C** | **G** | **T** | **A** | **T** | **C** | **A** | **A** | **T** | **T** | **G** | **G** | **A** | **T** | **T** | **A** | **T** | **T** | **T** | **C** | **T** | **T** | **T** | **A** | **A** | **A** | **C** | **T** | **T** | **G** | **A** | **A** | **G** | **C** | **A** | **T** | **T** | **G** | **G** | **T** | **G** | **C** | **C** | **A** | **G** | **A** | **G** | **G** | **C** | **A** | **A** | **A** | **T** | **A** | **C** | **A** | **T** | **C** | **C** | **A** | **T** | **T** | **C** | **A** | **G** | **A** | **T** | **G** | **A** | **T** | **T** | **G** | **C** | **C** | **A** | **G** | **T** | **G** | **G** | **T** | **C** | **C** | **T** | **A** | **A** | **C** | **A** | **G** | **T** | **G** | **A** | **C** | **C** | **G** | **T** | **G** | **A** | **A** | **C** | **A** | **A** | **A** | **T** | **T** | **A** | **A** | **A** | **T** | **T** | **G** | **G** | **C** | **C** | **T** | **T** | **T** | **A** | **T** | **T** | **A** | **A** | **A** | **T** | **T** | **G** | **A** | **T** | **T** | **A** | **C** | **T** | **T** | **C** | **T** | **G** | **C** | **C** | **A** | **A** | **G** | **A** | **A** | **A** | **A** | **G** | **A** | **G** | **T** | **C** | **T** | **G** | **G** | **A** | **T** | **T** | **C** | **A** | **A** | **A** | **C** | **G** | **C** | **C** | **G** | **T** | **A** | **T** | **T** | **T** | **A** | **G** | **T** | **T** | **C** | **C** | **T** | **G** | **A** | **T** | **G** | **A** | **C** | **A** | **G** | **T** | **G** | **T** | **C** | **T** | **T** | **G** | **G** | **C** | **T** | **G** | **C** | **T** | **T** | **T** | **A** | **A** | **A** | **G** | **G** | **T** | **T** | **G** | **C** | **T** | **G** | **C** | **G** | **G** | **C** | **T** | **T** | **C** | **A** | **G** | **G** | **A** | **G** | **T** | **A** | **G** | **A** | **T** | **G** | **T** | **T** | **A** | **A** | **A** | **A** | **T** | **T** | **A** | **T** | **G** | **A** | **T** | **T** | **C** | **C** | **A** | **G** | **A** | **T** | **A** | **A** | **G** | **C** | **C** | **C** | **G** | **A** | **C** | **C** | **A** | **T** | **C** | **C** | **G** | **T** | **T** | **T** | **A** | **T** | **T** | **T** | **A** | **T** | **C** | **G** | **A** | **G** | **C** | **A** | **A** | **C** | **A** | **C** | **A** | **G** | **T** | **A** | **T** | **T** | **A** | **C** | **G** | **G** | **C** | **C** | **G** | **C** | **T** | **T** | **A** | **T** | **T** | **G** | **A** | **T** | **G** | **A** | **A** | **A** | **G** | **A** | **A** | **A** | **A** | **T** | **A** | **T** | **T** | **G** | **A** | **A** | **A** | **T** | **T** | **T** | **T** | **A** | **A** | **T** | **T** | **T** | **A** | **T** | **A** | **A** | **C** | **G** | **G** | **T** | **G** | **G** | **T** | **T** | **T** | **C** | **T** | **T** | **A** | **C** | **A** | **T** | **G** | **C** | **G** | **A** | **A** | **A** | **A** | **C** | **A** | **A** | **T** | **G** | **A** | **T** | **T** | **A** | **T** | **G** | **G** | **A** | **T** | **G** | **A** | **T** | **G** | **A** | **A** | **G** | **T** | **C** | **T** | **G** | **C** | **A** | **C** | **A** | **G** | **T** | **T** | **G** | **G** | **T** | **T** | **C** | **A** | **G** | **C** | **C** | **A** | **A** | **T** | **C** | **A** | **A** | **G** | **A** | **T** | **A** | **T** | **C** | **C** | **G** | **A** | **A** | **G** | **T** | **T** | **A** | **C** | **A** | **A** | **A** | **T** | **T** | **A** | **A** | **A** | **C** | **T** | **T** | **T** | **G** | **A** | **A** | **G** | **C** | **A** | **A** | **A** | **T** | **G** | **C** | **T** | **G** | **T** | **G** | **T** | **T** | **A** | **T** | **A** | **T** | **G** | **A** | **T** | **A** | **A** | **A** | **A** | **A** | **A** | **A** | **T** | **C** | **A** | **T** | **T** | **G** | **A** | **T** | **C** | **A** | **A** | **T** | **T** | **A** | **G** | **A** | **A** | **G** | **C** | **A** | **A** | **T** | **T** | **T** | **T** | **C** | **T** | **T** | **A** | **G** | **A** | **A** | **G** | **A** | **T** | **C** | **G** | **A** | **A** | **A** | **A** | **A** | **A** | **A** | **T** | **G** | **T** | **A** | **C** | **A** | **A** | **C** | **A** | **A** | **T** | **G** | **A** | **C** | **T** | **C** | **C** | **A** | **G** | **A** | **A** | **G** | **T** | **T** | **G** | **T** | **T** | **C** | **G** | **C** | **G** | **A** | **C** | **A** | **T** | **G** | **T** | **C** | **A** | **A** | **A** | **A** | **T** | **G** | **G** | **T** | **T** | **G** | **A** | **T** | **T** | **T** | **T** | **T** | **A** | **A** | **A** | **C** | **A** | **A** | **C** | **A** | **A** | **A** | **T** | **T** | **T** | **C** | **A** | **C** | **G** | **A** | **T** | **T** | **A** | **T** | **T** | **T** | **T** | **C** | **A** | **C** | **C** | **A** | **A** | **T** | **T** | **C** | **T** | **T** | **-** | **-** | **-** |
| **N60034F** | **T** | **T** | **G** | **A** | **T** | **T** | **C** | **T** | **T** | **A** | **G** | **C** | **G** | **T** | **C** | **T** | **T** | **A** | **A** | **C** | **A** | **G** | **T** | **T** | **A** | **T** | **T** | **T** | **A** | **T** | **T** | **T** | **T** | **A** | **T** | **T** | **A** | **A** | **T** | **G** | **C** | **A** | **A** | **T** | **T** | **A** | **T** | **T** | **G** | **C** | **G** | **G** | **G** | **A** | **A** | **T** | **T** | **A** | **C** | **C** | **A** | **T** | **T** | **T** | **T** | **G** | **T** | **T** | **A** | **A** | **A** | **A** | **C** | **C** | **T** | **C** | **G** | **A** | **G** | **A** | **T** | **G** | **T** | **A** | **G** | **C** | **A** | **G** | **C** | **A** | **A** | **T** | **T** | **T** | **G** | **G** | **G** | **C** | **A** | **T** | **G** | **G** | **C** | **T** | **A** | **C** | **T** | **C** | **G** | **T** | **A** | **C** | **T** | **G** | **A** | **T** | **T** | **G** | **C** | **T** | **C** | **T** | **A** | **C** | **C** | **C** | **G** | **T** | **T** | **T** | **T** | **T** | **G** | **G** | **T** | **T** | **T** | **T** | **T** | **T** | **C** | **T** | **T** | **A** | **T** | **A** | **T** | **T** | **T** | **A** | **T** | **T** | **C** | **T** | **T** | **T** | **G** | **G** | **G** | **C** | **G** | **C** | **G** | **G** | **T** | **T** | **T** | **A** | **A** | **C** | **C** | **G** | **A** | **C** | **A** | **A** | **A** | **A** | **A** | **G** | **A** | **A** | **A** | **T** | **T** | **T** | **T** | **A** | **T** | **T** | **T** | **G** | **C** | **A** | **A** | **C** | **A** | **A** | **A** | **G** | **T** | **G** | **A** | **T** | **T** | **T** | **G** | **C** | **G** | **G** | **G** | **A** | **A** | **T** | **T** | **A** | **G** | **A** | **G** | **A** | **A** | **T** | **T** | **T** | **T** | **C** | **A** | **G** | **A** | **A** | **T** | **T** | **T** | **C** | **C** | **A** | **A** | **G** | **A** | **A** | **G** | **A** | **A** | **A** | **G** | **T** | **T** | **T** | **T** | **G** | **A** | **A** | **C** | **T** | **T** | **T** | **A** | **T** | **A** | **G** | **T** | **C** | **A** | **A** | **A** | **A** | **G** | **A** | **T** | **G** | **C** | **C** | **G** | **A** | **C** | **G** | **G** | **A** | **G** | **G** | **A** | **G** | **C** | **A** | **A** | **C** | **A** | **A** | **C** | **A** | **G** | **T** | **T** | **T** | **A** | **C** | **A** | **G** | **A** | **C** | **T** | **T** | **T** | **T** | **T** | **T** | **T** | **C** | **C** | **T** | **C** | **G** | **T** | **T** | **A** | **A** | **A** | **T** | **A** | **G** | **G** | **A** | **T** | **G** | **C** | **C** | **T** | **C** | **T** | **G** | **A** | **C** | **T** | **A** | **A** | **G** | **A** | **A** | **A** | **A** | **A** | **T** | **G** | **A** | **T** | **G** | **T** | **T** | **G** | **A** | **A** | **A** | **T** | **T** | **T** | **T** | **T** | **A** | **C** | **G** | **G** | **A** | **T** | **G** | **G** | **T** | **A** | **C** | **G** | **G** | **A** | **A** | **A** | **A** | **A** | **T** | **T** | **C** | **A** | **A** | **T** | **G** | **C** | **G** | **T** | **T** | **G** | **A** | **T** | **G** | **G** | **C** | **G** | **G** | **A** | **C** | **A** | **T** | **A** | **A** | **A** | **A** | **A** | **A** | **A** | **G** | **C** | **T** | **C** | **A** | **G** | **C** | **A** | **C** | **T** | **C** | **C** | **A** | **T** | **T** | **C** | **A** | **T** | **A** | **T** | **T** | **G** | **A** | **A** | **T** | **A** | **T** | **T** | **A** | **T** | **G** | **C** | **C** | **T** | **T** | **T** | **G** | **T** | **A** | **A** | **C** | **G** | **G** | **A** | **T** | **C** | **A** | **T** | **A** | **T** | **C** | **G** | **G** | **C** | **A** | **C** | **C** | **A** | **A** | **A** | **A** | **T** | **T** | **T** | **T** | **G** | **A** | **A** | **C** | **T** | **T** | **A** | **T** | **T** | **A** | **G** | **A** | **A** | **G** | **A** | **A** | **A** | **A** | **G** | **G** | **C** | **G** | **G** | **C** | **T** | **G** | **A** | **A** | **G** | **G** | **G** | **G** | **T** | **T** | **G** | **A** | **A** | **G** | **T** | **G** | **C** | **G** | **G** | **T** | **T** | **G** | **C** | **T** | **T** | **T** | **A** | **C** | **G** | **A** | **T** | **G** | **C** | **C** | **T** | **T** | **T** | **G** | **G** | **C** | **T** | **C** | **A** | **A** | **A** | **G** | **G** | **G** | **C** | **A** | **C** | **C** | **A** | **A** | **G** | **G** | **T** | **T** | **C** | **A** | **T** | **C** | **A** | **T** | **T** | **T** | **G** | **A** | **A** | **T** | **G** | **A** | **A** | **T** | **T** | **G** | **A** | **A** | **A** | **A** | **A** | **A** | **A** | **A** | **C** | **G** | **G** | **C** | **G** | **G** | **T** | **T** | **T** | **T** | **G** | **T** | **C** | **C** | **A** | **A** | **A** | **C** | **G** | **T** | **T** | **T** | **A** | **T** | **T** | **A** | **C** | **T** | **T** | **C** | **T** | **C** | **A** | **A** | **A** | **A** | **A** | **G** | **C** | **A** | **C** | **T** | **T** | **T** | **T** | **G** | **A** | **A** | **G** | **T** | **T** | **T** | **C** | **G** | **T** | **T** | **T** | **G** | **A** | **A** | **T** | **T** | **A** | **T** | **C** | **A** | **T** | **G** | **A** | **T** | **C** | **A** | **C** | **C** | **G** | **G** | **A** | **A** | **A** | **A** | **T** | **T** | **G** | **T** | **T** | **G** | **T** | **T** | **A** | **T** | **T** | **G** | **A** | **C** | **G** | **G** | **A** | **A** | **A** | **A** | **G** | **T** | **A** | **G** | **G** | **C** | **T** | **A** | **C** | **A** | **T** | **T** | **G** | **G** | **C** | **G** | **G** | **C** | **T** | **T** | **T** | **A** | **A** | **T** | **G** | **T** | **T** | **G** | **C** | **C** | **G** | **A** | **T** | **C** | **A** | **A** | **T** | **A** | **T** | **G** | **C** | **C** | **G** | **G** | **A** | **A** | **C** | **G** | **A** | **C** | **T** | **A** | **A** | **A** | **A** | **A** | **G** | **T** | **T** | **T** | **G** | **G** | **C** | **T** | **A** | **T** | **T** | **G** | **G** | **C** | **G** | **G** | **G** | **A** | **T** | **A** | **C** | **A** | **C** | **A** | **T** | **T** | **T** | **A** | **C** | **G** | **G** | **A** | **T** | **T** | **C** | **A** | **A** | **G** | **G** | **G** | **C** | **C** | **A** | **G** | **C** | **A** | **G** | **C** | **C** | **T** | **C** | **A** | **T** | **T** | **A** | **C** | **T** | **G** | **C** | **A** | **A** | **A** | **T** | **G** | **C** | **G** | **T** | **T** | **T** | **T** | **T** | **T** | **A** | **A** | **T** | **G** | **G** | **A** | **T** | **T** | **G** | **G** | **A** | **A** | **C** | **G** | **T** | **C** | **T** | **C** | **T** | **T** | **C** | **C** | **C** | **C** | **C** | **G** | **A** | **G** | **A** | **A** | **A** | **A** | **A** | **T** | **C** | **G** | **T** | **G** | **T** | **G** | **G** | **C** | **G** | **T** | **A** | **T** | **C** | **A** | **A** | **T** | **T** | **G** | **G** | **A** | **T** | **T** | **A** | **T** | **T** | **T** | **C** | **T** | **T** | **T** | **A** | **A** | **A** | **C** | **T** | **T** | **G** | **A** | **A** | **G** | **C** | **A** | **T** | **T** | **G** | **G** | **T** | **G** | **C** | **C** | **A** | **G** | **A** | **G** | **G** | **C** | **A** | **A** | **A** | **T** | **A** | **C** | **A** | **T** | **C** | **C** | **A** | **T** | **T** | **C** | **A** | **G** | **A** | **T** | **G** | **A** | **T** | **T** | **G** | **C** | **C** | **A** | **G** | **T** | **G** | **G** | **T** | **C** | **C** | **T** | **A** | **A** | **C** | **A** | **G** | **T** | **G** | **A** | **C** | **C** | **G** | **T** | **G** | **A** | **A** | **C** | **A** | **A** | **A** | **T** | **T** | **A** | **A** | **A** | **T** | **T** | **G** | **G** | **C** | **C** | **T** | **T** | **T** | **A** | **T** | **T** | **A** | **A** | **A** | **T** | **T** | **G** | **A** | **T** | **T** | **A** | **C** | **T** | **T** | **C** | **T** | **G** | **C** | **C** | **A** | **A** | **G** | **A** | **A** | **A** | **A** | **G** | **A** | **G** | **T** | **C** | **T** | **G** | **G** | **A** | **T** | **T** | **C** | **A** | **A** | **A** | **C** | **G** | **C** | **C** | **G** | **T** | **A** | **T** | **T** | **T** | **A** | **G** | **T** | **T** | **C** | **C** | **T** | **G** | **A** | **T** | **G** | **A** | **T** | **A** | **G** | **T** | **G** | **T** | **C** | **T** | **T** | **G** | **G** | **C** | **T** | **G** | **C** | **T** | **T** | **T** | **A** | **A** | **A** | **G** | **G** | **T** | **T** | **G** | **C** | **T** | **G** | **C** | **G** | **G** | **C** | **T** | **T** | **C** | **A** | **G** | **G** | **A** | **G** | **T** | **A** | **G** | **A** | **T** | **G** | **T** | **T** | **A** | **A** | **A** | **A** | **T** | **T** | **A** | **T** | **G** | **A** | **T** | **T** | **C** | **C** | **A** | **G** | **A** | **T** | **A** | **A** | **G** | **C** | **C** | **C** | **G** | **A** | **C** | **C** | **A** | **T** | **C** | **C** | **G** | **T** | **T** | **T** | **A** | **T** | **T** | **T** | **A** | **T** | **C** | **G** | **A** | **G** | **C** | **A** | **A** | **C** | **A** | **C** | **A** | **G** | **T** | **A** | **T** | **T** | **A** | **C** | **G** | **G** | **C** | **C** | **G** | **C** | **T** | **T** | **A** | **T** | **T** | **G** | **A** | **T** | **G** | **A** | **A** | **A** | **G** | **A** | **A** | **A** | **A** | **T** | **A** | **T** | **T** | **G** | **A** | **A** | **A** | **T** | **T** | **T** | **T** | **A** | **A** | **T** | **T** | **T** | **A** | **T** | **A** | **A** | **C** | **G** | **G** | **T** | **G** | **G** | **T** | **T** | **T** | **C** | **T** | **T** | **A** | **C** | **A** | **T** | **G** | **C** | **G** | **A** | **A** | **A** | **A** | **C** | **A** | **A** | **T** | **G** | **A** | **T** | **T** | **A** | **T** | **G** | **G** | **A** | **T** | **G** | **A** | **T** | **G** | **A** | **A** | **G** | **T** | **C** | **T** | **G** | **C** | **A** | **C** | **A** | **G** | **T** | **T** | **G** | **G** | **T** | **T** | **C** | **A** | **G** | **C** | **C** | **A** | **A** | **T** | **C** | **A** | **A** | **G** | **A** | **T** | **A** | **T** | **C** | **C** | **G** | **A** | **A** | **G** | **T** | **T** | **A** | **C** | **A** | **A** | **A** | **T** | **T** | **A** | **A** | **A** | **C** | **T** | **T** | **T** | **G** | **A** | **A** | **G** | **C** | **A** | **A** | **A** | **T** | **G** | **C** | **T** | **G** | **T** | **G** | **T** | **T** | **A** | **T** | **A** | **T** | **G** | **A** | **T** | **A** | **A** | **A** | **A** | **A** | **A** | **A** | **T** | **C** | **A** | **T** | **T** | **G** | **A** | **T** | **C** | **A** | **A** | **T** | **T** | **A** | **G** | **A** | **A** | **G** | **C** | **A** | **A** | **T** | **T** | **T** | **T** | **C** | **T** | **T** | **A** | **G** | **A** | **A** | **G** | **A** | **T** | **C** | **G** | **A** | **A** | **A** | **A** | **A** | **A** | **A** | **T** | **G** | **T** | **A** | **C** | **A** | **A** | **C** | **A** | **A** | **T** | **G** | **A** | **C** | **T** | **C** | **C** | **A** | **G** | **A** | **A** | **G** | **T** | **T** | **G** | **T** | **T** | **C** | **G** | **C** | **G** | **A** | **C** | **A** | **T** | **G** | **T** | **C** | **A** | **A** | **A** | **A** | **T** | **G** | **G** | **T** | **T** | **G** | **A** | **T** | **T** | **T** | **T** | **T** | **A** | **A** | **A** | **C** | **A** | **A** | **C** | **A** | **A** | **A** | **T** | **T** | **T** | **C** | **A** | **C** | **G** | **A** | **T** | **T** | **A** | **T** | **T** | **T** | **T** | **C** | **A** | **C** | **C** | **A** | **A** | **T** | **T** | **C** | **T** | **T** | **-** | **-** | **-** |
| **N54595** | **T** | **T** | **G** | **A** | **T** | **T** | **C** | **T** | **T** | **A** | **G** | **C** | **G** | **T** | **C** | **T** | **T** | **A** | **A** | **C** | **A** | **G** | **T** | **T** | **A** | **T** | **T** | **T** | **A** | **T** | **T** | **T** | **T** | **A** | **T** | **T** | **A** | **A** | **T** | **G** | **C** | **A** | **A** | **T** | **T** | **A** | **T** | **T** | **G** | **C** | **G** | **G** | **G** | **A** | **A** | **T** | **T** | **A** | **C** | **C** | **A** | **T** | **T** | **T** | **T** | **G** | **T** | **T** | **A** | **A** | **A** | **A** | **C** | **C** | **T** | **C** | **G** | **A** | **G** | **A** | **T** | **G** | **T** | **A** | **G** | **C** | **A** | **G** | **C** | **A** | **A** | **T** | **T** | **T** | **G** | **G** | **G** | **C** | **A** | **T** | **G** | **G** | **C** | **T** | **A** | **C** | **T** | **C** | **G** | **T** | **A** | **C** | **T** | **G** | **A** | **T** | **T** | **G** | **C** | **T** | **C** | **T** | **A** | **C** | **C** | **C** | **G** | **T** | **T** | **T** | **T** | **T** | **G** | **G** | **T** | **T** | **T** | **T** | **T** | **T** | **C** | **T** | **T** | **A** | **T** | **A** | **T** | **T** | **T** | **A** | **T** | **T** | **C** | **T** | **T** | **T** | **G** | **G** | **G** | **C** | **G** | **C** | **G** | **G** | **T** | **T** | **T** | **A** | **A** | **C** | **C** | **G** | **A** | **C** | **A** | **A** | **A** | **A** | **A** | **G** | **A** | **A** | **A** | **T** | **T** | **T** | **T** | **A** | **T** | **T** | **T** | **G** | **C** | **A** | **A** | **C** | **A** | **A** | **A** | **G** | **T** | **G** | **A** | **T** | **T** | **T** | **A** | **C** | **G** | **G** | **G** | **A** | **A** | **T** | **T** | **A** | **G** | **A** | **G** | **A** | **A** | **T** | **T** | **T** | **C** | **C** | **A** | **G** | **A** | **A** | **T** | **T** | **T** | **C** | **C** | **A** | **A** | **G** | **A** | **A** | **G** | **A** | **A** | **A** | **G** | **T** | **T** | **T** | **T** | **G** | **A** | **A** | **C** | **T** | **T** | **T** | **A** | **T** | **A** | **G** | **T** | **C** | **T** | **A** | **A** | **A** | **G** | **A** | **T** | **G** | **C** | **C** | **G** | **A** | **C** | **G** | **G** | **A** | **G** | **G** | **A** | **G** | **C** | **A** | **A** | **C** | **A** | **A** | **C** | **A** | **G** | **T** | **T** | **T** | **A** | **C** | **A** | **G** | **A** | **C** | **T** | **T** | **T** | **T** | **T** | **T** | **T** | **C** | **C** | **T** | **C** | **G** | **T** | **T** | **A** | **A** | **A** | **T** | **A** | **G** | **G** | **A** | **T** | **G** | **C** | **C** | **C** | **C** | **T** | **G** | **A** | **C** | **T** | **A** | **A** | **G** | **A** | **A** | **A** | **A** | **A** | **T** | **G** | **A** | **T** | **G** | **T** | **T** | **G** | **A** | **A** | **A** | **T** | **T** | **T** | **T** | **T** | **A** | **C** | **G** | **G** | **A** | **T** | **G** | **G** | **T** | **A** | **C** | **G** | **G** | **A** | **A** | **A** | **A** | **A** | **T** | **T** | **C** | **A** | **A** | **T** | **G** | **C** | **G** | **T** | **T** | **G** | **A** | **T** | **G** | **G** | **C** | **G** | **G** | **A** | **C** | **A** | **T** | **A** | **A** | **A** | **A** | **A** | **A** | **A** | **G** | **C** | **T** | **C** | **A** | **G** | **C** | **A** | **C** | **T** | **C** | **C** | **A** | **T** | **T** | **C** | **A** | **T** | **A** | **T** | **T** | **G** | **A** | **A** | **T** | **A** | **T** | **T** | **A** | **T** | **G** | **C** | **C** | **T** | **T** | **T** | **G** | **T** | **A** | **A** | **C** | **G** | **G** | **A** | **T** | **C** | **A** | **T** | **A** | **T** | **C** | **G** | **G** | **T** | **A** | **C** | **C** | **A** | **A** | **A** | **A** | **T** | **T** | **T** | **T** | **G** | **A** | **A** | **C** | **T** | **T** | **A** | **T** | **T** | **A** | **G** | **A** | **A** | **G** | **A** | **A** | **A** | **A** | **G** | **G** | **C** | **G** | **G** | **C** | **T** | **G** | **A** | **A** | **G** | **G** | **G** | **G** | **T** | **T** | **G** | **A** | **A** | **G** | **T** | **G** | **C** | **G** | **G** | **T** | **T** | **G** | **C** | **T** | **T** | **T** | **A** | **C** | **G** | **A** | **T** | **G** | **C** | **C** | **T** | **T** | **T** | **G** | **G** | **C** | **T** | **C** | **A** | **A** | **A** | **G** | **G** | **G** | **C** | **A** | **C** | **C** | **A** | **A** | **G** | **G** | **T** | **T** | **C** | **A** | **T** | **C** | **A** | **T** | **T** | **T** | **G** | **A** | **A** | **T** | **G** | **A** | **A** | **T** | **T** | **G** | **A** | **A** | **A** | **A** | **A** | **A** | **A** | **A** | **C** | **G** | **G** | **C** | **G** | **G** | **T** | **T** | **T** | **T** | **G** | **T** | **C** | **C** | **A** | **A** | **A** | **C** | **G** | **T** | **T** | **T** | **A** | **T** | **T** | **A** | **C** | **T** | **T** | **C** | **T** | **C** | **A** | **A** | **A** | **A** | **A** | **G** | **C** | **A** | **C** | **T** | **T** | **T** | **T** | **G** | **A** | **A** | **G** | **T** | **T** | **T** | **C** | **G** | **T** | **T** | **T** | **G** | **A** | **A** | **T** | **T** | **A** | **T** | **C** | **A** | **T** | **G** | **A** | **T** | **C** | **A** | **C** | **C** | **G** | **G** | **A** | **A** | **A** | **A** | **T** | **T** | **G** | **T** | **T** | **G** | **T** | **T** | **A** | **T** | **T** | **G** | **A** | **C** | **G** | **G** | **A** | **A** | **A** | **A** | **G** | **T** | **A** | **G** | **G** | **C** | **T** | **A** | **C** | **A** | **T** | **T** | **G** | **G** | **C** | **G** | **G** | **C** | **T** | **T** | **T** | **A** | **A** | **T** | **G** | **T** | **T** | **G** | **C** | **C** | **G** | **A** | **T** | **C** | **A** | **A** | **T** | **A** | **T** | **G** | **C** | **C** | **G** | **G** | **A** | **A** | **C** | **G** | **A** | **C** | **T** | **A** | **A** | **A** | **A** | **A** | **G** | **T** | **T** | **T** | **G** | **G** | **C** | **T** | **A** | **T** | **T** | **G** | **G** | **C** | **G** | **G** | **G** | **A** | **T** | **A** | **C** | **A** | **C** | **A** | **T** | **T** | **T** | **A** | **C** | **G** | **G** | **A** | **T** | **T** | **C** | **A** | **A** | **G** | **G** | **G** | **C** | **C** | **A** | **G** | **C** | **A** | **G** | **C** | **C** | **T** | **C** | **A** | **T** | **T** | **A** | **C** | **T** | **G** | **C** | **A** | **A** | **A** | **T** | **G** | **C** | **G** | **T** | **T** | **T** | **T** | **T** | **T** | **A** | **A** | **T** | **G** | **G** | **A** | **T** | **T** | **G** | **G** | **A** | **A** | **C** | **G** | **T** | **C** | **T** | **C** | **T** | **T** | **C** | **C** | **C** | **C** | **C** | **G** | **A** | **G** | **A** | **A** | **A** | **A** | **A** | **T** | **C** | **G** | **T** | **G** | **T** | **G** | **G** | **C** | **G** | **T** | **A** | **T** | **C** | **A** | **A** | **T** | **T** | **G** | **G** | **A** | **T** | **T** | **A** | **T** | **T** | **T** | **C** | **T** | **T** | **T** | **A** | **A** | **A** | **C** | **T** | **T** | **G** | **A** | **A** | **G** | **C** | **A** | **T** | **T** | **G** | **G** | **T** | **G** | **C** | **C** | **A** | **G** | **A** | **G** | **G** | **C** | **A** | **A** | **A** | **T** | **A** | **C** | **A** | **T** | **C** | **C** | **A** | **T** | **T** | **C** | **A** | **G** | **A** | **T** | **G** | **A** | **T** | **T** | **G** | **C** | **C** | **A** | **G** | **T** | **G** | **G** | **T** | **C** | **C** | **T** | **A** | **A** | **C** | **A** | **G** | **T** | **G** | **A** | **C** | **C** | **G** | **T** | **G** | **A** | **A** | **C** | **A** | **A** | **A** | **T** | **T** | **A** | **A** | **A** | **T** | **T** | **G** | **G** | **C** | **C** | **T** | **T** | **T** | **A** | **T** | **T** | **A** | **A** | **A** | **T** | **T** | **G** | **A** | **T** | **T** | **A** | **C** | **T** | **T** | **C** | **T** | **G** | **C** | **C** | **A** | **A** | **G** | **A** | **A** | **A** | **A** | **G** | **A** | **G** | **T** | **C** | **T** | **G** | **G** | **A** | **T** | **T** | **C** | **A** | **A** | **A** | **C** | **G** | **C** | **C** | **G** | **T** | **A** | **T** | **T** | **T** | **A** | **G** | **T** | **T** | **C** | **C** | **T** | **G** | **A** | **T** | **G** | **A** | **T** | **A** | **G** | **T** | **G** | **T** | **C** | **T** | **T** | **G** | **G** | **C** | **T** | **G** | **C** | **T** | **T** | **T** | **A** | **A** | **A** | **G** | **G** | **T** | **T** | **G** | **C** | **T** | **G** | **C** | **G** | **G** | **C** | **T** | **T** | **C** | **A** | **G** | **G** | **A** | **G** | **T** | **A** | **G** | **A** | **T** | **G** | **T** | **T** | **A** | **A** | **A** | **A** | **T** | **T** | **A** | **T** | **G** | **A** | **T** | **T** | **C** | **C** | **A** | **G** | **A** | **T** | **A** | **A** | **G** | **C** | **C** | **C** | **G** | **A** | **C** | **C** | **A** | **T** | **C** | **C** | **G** | **T** | **T** | **T** | **A** | **T** | **T** | **T** | **A** | **T** | **C** | **G** | **A** | **G** | **C** | **A** | **A** | **C** | **A** | **C** | **A** | **G** | **T** | **A** | **T** | **T** | **A** | **C** | **G** | **G** | **C** | **C** | **G** | **C** | **T** | **T** | **A** | **T** | **T** | **G** | **A** | **T** | **G** | **A** | **A** | **A** | **G** | **A** | **A** | **A** | **A** | **T** | **A** | **T** | **T** | **G** | **A** | **A** | **A** | **T** | **T** | **T** | **T** | **A** | **A** | **T** | **T** | **T** | **A** | **T** | **A** | **A** | **C** | **G** | **G** | **T** | **G** | **G** | **T** | **T** | **T** | **C** | **T** | **T** | **A** | **C** | **A** | **T** | **G** | **C** | **G** | **A** | **A** | **A** | **A** | **C** | **A** | **A** | **T** | **G** | **A** | **T** | **T** | **A** | **T** | **G** | **G** | **A** | **T** | **G** | **A** | **T** | **G** | **A** | **A** | **G** | **T** | **C** | **T** | **G** | **C** | **A** | **C** | **A** | **G** | **T** | **T** | **G** | **G** | **T** | **T** | **C** | **A** | **G** | **C** | **C** | **A** | **A** | **T** | **C** | **A** | **A** | **G** | **A** | **T** | **A** | **T** | **C** | **C** | **G** | **A** | **A** | **G** | **T** | **T** | **A** | **C** | **A** | **A** | **A** | **T** | **T** | **A** | **A** | **A** | **C** | **T** | **T** | **T** | **G** | **A** | **A** | **G** | **C** | **A** | **A** | **A** | **T** | **G** | **C** | **T** | **G** | **T** | **G** | **T** | **T** | **A** | **T** | **A** | **T** | **G** | **A** | **T** | **A** | **A** | **A** | **A** | **A** | **A** | **A** | **T** | **C** | **A** | **T** | **T** | **G** | **A** | **T** | **C** | **A** | **A** | **T** | **T** | **A** | **G** | **A** | **A** | **G** | **C** | **A** | **A** | **T** | **T** | **T** | **T** | **C** | **T** | **T** | **A** | **G** | **A** | **A** | **G** | **A** | **T** | **C** | **G** | **A** | **A** | **A** | **A** | **A** | **A** | **A** | **T** | **G** | **T** | **A** | **C** | **A** | **A** | **C** | **A** | **A** | **T** | **G** | **-** | **-** | **-** | **-** | **-** | **-** | **-** | **-** | **-** | **-** | **-** | **-** | **-** | **-** | **-** | **-** | **-** | **-** | **-** | **-** | **-** | **-** | **-** | **-** | **-** | **-** | **-** | **-** | **-** | **-** | **-** | **-** | **-** | **-** | **-** | **-** | **-** | **-** | **-** | **-** | **-** | **-** | **-** | **-** | **-** | **-** | **-** | **-** | **-** | **-** | **-** | **-** | **-** | **-** | **-** | **-** | **-** | **-** | **-** | **-** | **-** | **-** | **-** | **-** | **-** | **-** | **-** | **-** | **-** | **-** | **-** | **-** | **-** | **-** | **-** | **-** | **-** | **-** | **-** | **-** | **-** |
| **N59409F** | **T** | **T** | **G** | **A** | **T** | **T** | **C** | **T** | **T** | **A** | **G** | **C** | **G** | **T** | **C** | **T** | **T** | **A** | **A** | **C** | **A** | **G** | **T** | **T** | **A** | **T** | **T** | **T** | **A** | **T** | **T** | **T** | **T** | **A** | **T** | **T** | **A** | **A** | **T** | **G** | **C** | **A** | **A** | **T** | **T** | **A** | **T** | **T** | **G** | **C** | **G** | **G** | **G** | **A** | **A** | **T** | **T** | **A** | **C** | **C** | **A** | **T** | **T** | **T** | **T** | **G** | **T** | **T** | **A** | **A** | **A** | **A** | **C** | **C** | **T** | **C** | **G** | **A** | **G** | **A** | **T** | **G** | **T** | **A** | **G** | **C** | **A** | **G** | **C** | **A** | **A** | **T** | **T** | **T** | **G** | **G** | **G** | **C** | **A** | **T** | **G** | **G** | **C** | **T** | **A** | **C** | **T** | **C** | **G** | **T** | **A** | **C** | **T** | **G** | **A** | **T** | **T** | **G** | **C** | **T** | **C** | **T** | **A** | **C** | **C** | **C** | **G** | **T** | **T** | **T** | **T** | **T** | **G** | **G** | **T** | **T** | **T** | **T** | **T** | **T** | **C** | **T** | **T** | **A** | **T** | **A** | **T** | **T** | **T** | **A** | **T** | **T** | **C** | **T** | **T** | **T** | **G** | **G** | **T** | **C** | **G** | **A** | **G** | **G** | **T** | **T** | **T** | **A** | **A** | **C** | **C** | **G** | **A** | **C** | **A** | **A** | **A** | **A** | **A** | **G** | **A** | **A** | **A** | **T** | **T** | **T** | **T** | **A** | **T** | **T** | **T** | **G** | **C** | **A** | **A** | **C** | **A** | **A** | **A** | **G** | **T** | **G** | **A** | **T** | **T** | **T** | **A** | **C** | **G** | **G** | **G** | **A** | **A** | **T** | **T** | **A** | **G** | **A** | **G** | **A** | **A** | **T** | **T** | **T** | **C** | **C** | **A** | **G** | **A** | **A** | **T** | **T** | **T** | **C** | **C** | **A** | **A** | **G** | **A** | **A** | **G** | **A** | **A** | **A** | **G** | **T** | **T** | **T** | **T** | **G** | **A** | **A** | **C** | **T** | **T** | **T** | **A** | **T** | **A** | **G** | **T** | **C** | **A** | **A** | **A** | **A** | **G** | **A** | **T** | **G** | **C** | **C** | **G** | **A** | **C** | **G** | **G** | **A** | **G** | **G** | **A** | **G** | **C** | **A** | **A** | **C** | **A** | **A** | **C** | **A** | **G** | **T** | **T** | **T** | **A** | **C** | **A** | **G** | **A** | **C** | **T** | **T** | **T** | **T** | **T** | **T** | **T** | **C** | **C** | **T** | **C** | **G** | **T** | **T** | **A** | **A** | **A** | **T** | **A** | **G** | **G** | **A** | **T** | **G** | **C** | **C** | **T** | **C** | **T** | **G** | **A** | **C** | **T** | **A** | **A** | **G** | **A** | **A** | **A** | **A** | **A** | **T** | **G** | **A** | **T** | **G** | **T** | **T** | **G** | **A** | **A** | **A** | **T** | **T** | **T** | **T** | **T** | **A** | **C** | **G** | **G** | **A** | **T** | **G** | **G** | **T** | **A** | **C** | **G** | **G** | **A** | **A** | **A** | **A** | **A** | **T** | **T** | **C** | **A** | **A** | **T** | **G** | **C** | **G** | **T** | **T** | **G** | **A** | **T** | **G** | **G** | **C** | **G** | **G** | **A** | **T** | **A** | **T** | **A** | **A** | **A** | **A** | **A** | **A** | **A** | **G** | **C** | **T** | **C** | **A** | **G** | **C** | **A** | **C** | **T** | **C** | **C** | **A** | **T** | **T** | **C** | **A** | **T** | **A** | **T** | **T** | **G** | **A** | **A** | **T** | **A** | **T** | **T** | **A** | **T** | **G** | **C** | **C** | **T** | **T** | **T** | **G** | **T** | **A** | **A** | **C** | **G** | **G** | **A** | **T** | **C** | **A** | **T** | **A** | **T** | **C** | **G** | **G** | **C** | **A** | **C** | **C** | **A** | **A** | **A** | **A** | **T** | **T** | **T** | **T** | **G** | **A** | **A** | **C** | **T** | **T** | **A** | **T** | **T** | **A** | **G** | **A** | **A** | **G** | **A** | **A** | **A** | **A** | **G** | **G** | **C** | **G** | **G** | **C** | **T** | **G** | **A** | **A** | **G** | **G** | **G** | **G** | **T** | **T** | **G** | **A** | **A** | **G** | **T** | **G** | **C** | **G** | **G** | **T** | **T** | **G** | **C** | **T** | **T** | **T** | **A** | **C** | **G** | **A** | **T** | **G** | **C** | **C** | **T** | **T** | **T** | **G** | **G** | **C** | **T** | **C** | **A** | **A** | **A** | **G** | **G** | **G** | **C** | **A** | **C** | **C** | **A** | **A** | **G** | **G** | **T** | **T** | **C** | **A** | **T** | **C** | **A** | **T** | **T** | **T** | **G** | **A** | **A** | **T** | **G** | **A** | **A** | **T** | **T** | **G** | **A** | **A** | **A** | **A** | **A** | **A** | **A** | **A** | **C** | **G** | **G** | **C** | **G** | **G** | **T** | **T** | **T** | **T** | **G** | **T** | **C** | **C** | **A** | **A** | **A** | **C** | **A** | **T** | **T** | **T** | **A** | **T** | **T** | **A** | **C** | **T** | **T** | **C** | **T** | **C** | **A** | **A** | **A** | **A** | **A** | **G** | **C** | **A** | **C** | **T** | **T** | **T** | **T** | **G** | **A** | **A** | **G** | **T** | **T** | **T** | **C** | **G** | **T** | **T** | **T** | **G** | **A** | **A** | **T** | **T** | **A** | **T** | **C** | **A** | **T** | **G** | **A** | **T** | **C** | **A** | **C** | **C** | **G** | **G** | **A** | **A** | **A** | **A** | **T** | **T** | **G** | **T** | **T** | **G** | **T** | **T** | **A** | **T** | **T** | **G** | **A** | **C** | **G** | **G** | **A** | **A** | **A** | **A** | **G** | **T** | **A** | **G** | **G** | **C** | **T** | **A** | **C** | **A** | **T** | **T** | **G** | **G** | **C** | **G** | **G** | **C** | **T** | **T** | **T** | **A** | **A** | **T** | **G** | **T** | **T** | **G** | **C** | **C** | **G** | **A** | **T** | **C** | **A** | **A** | **T** | **A** | **T** | **G** | **C** | **T** | **G** | **G** | **A** | **A** | **C** | **G** | **A** | **C** | **T** | **A** | **A** | **A** | **A** | **A** | **G** | **T** | **T** | **T** | **G** | **G** | **C** | **T** | **A** | **T** | **T** | **G** | **G** | **C** | **G** | **G** | **G** | **A** | **T** | **A** | **C** | **G** | **C** | **A** | **T** | **T** | **T** | **A** | **C** | **G** | **G** | **A** | **T** | **T** | **C** | **A** | **A** | **G** | **G** | **G** | **C** | **C** | **A** | **G** | **C** | **A** | **G** | **C** | **C** | **T** | **C** | **A** | **T** | **T** | **A** | **C** | **T** | **G** | **C** | **A** | **A** | **A** | **T** | **G** | **C** | **G** | **T** | **T** | **T** | **T** | **T** | **T** | **A** | **A** | **T** | **G** | **G** | **A** | **T** | **T** | **G** | **G** | **A** | **A** | **C** | **G** | **T** | **C** | **T** | **C** | **T** | **T** | **C** | **C** | **C** | **C** | **C** | **G** | **A** | **G** | **A** | **A** | **A** | **A** | **A** | **T** | **C** | **G** | **T** | **G** | **T** | **G** | **G** | **C** | **G** | **T** | **A** | **T** | **C** | **A** | **A** | **T** | **T** | **G** | **G** | **A** | **T** | **T** | **A** | **T** | **T** | **T** | **C** | **T** | **T** | **T** | **A** | **A** | **A** | **C** | **T** | **T** | **G** | **A** | **A** | **G** | **C** | **A** | **T** | **T** | **G** | **G** | **T** | **G** | **C** | **C** | **A** | **G** | **A** | **G** | **G** | **C** | **A** | **A** | **A** | **T** | **A** | **C** | **A** | **T** | **C** | **C** | **A** | **T** | **T** | **C** | **A** | **G** | **A** | **T** | **G** | **A** | **T** | **T** | **G** | **C** | **C** | **A** | **G** | **T** | **G** | **G** | **T** | **C** | **C** | **T** | **A** | **A** | **C** | **A** | **G** | **T** | **G** | **A** | **C** | **C** | **G** | **T** | **G** | **A** | **A** | **C** | **A** | **A** | **A** | **T** | **T** | **A** | **A** | **A** | **T** | **T** | **G** | **G** | **C** | **C** | **T** | **T** | **T** | **A** | **T** | **T** | **A** | **A** | **A** | **T** | **T** | **G** | **A** | **T** | **T** | **A** | **C** | **T** | **T** | **C** | **T** | **G** | **C** | **C** | **A** | **A** | **G** | **A** | **A** | **A** | **A** | **G** | **A** | **G** | **T** | **C** | **T** | **G** | **G** | **A** | **T** | **T** | **C** | **A** | **A** | **A** | **C** | **G** | **C** | **C** | **G** | **T** | **A** | **T** | **T** | **T** | **A** | **G** | **T** | **T** | **C** | **C** | **T** | **G** | **A** | **T** | **G** | **A** | **T** | **A** | **G** | **T** | **G** | **T** | **C** | **T** | **T** | **G** | **G** | **C** | **T** | **G** | **C** | **T** | **T** | **T** | **A** | **A** | **A** | **G** | **G** | **T** | **T** | **G** | **C** | **T** | **G** | **C** | **G** | **G** | **C** | **T** | **T** | **C** | **A** | **G** | **G** | **A** | **G** | **T** | **A** | **G** | **A** | **T** | **G** | **T** | **T** | **A** | **A** | **A** | **A** | **T** | **T** | **A** | **T** | **G** | **A** | **T** | **T** | **C** | **C** | **A** | **G** | **A** | **T** | **A** | **A** | **G** | **C** | **C** | **C** | **G** | **A** | **C** | **C** | **A** | **T** | **C** | **C** | **G** | **T** | **T** | **T** | **A** | **T** | **T** | **T** | **A** | **T** | **C** | **G** | **A** | **G** | **C** | **A** | **A** | **C** | **A** | **C** | **A** | **G** | **T** | **A** | **T** | **T** | **A** | **C** | **G** | **G** | **C** | **C** | **G** | **C** | **T** | **T** | **A** | **T** | **T** | **G** | **A** | **T** | **G** | **A** | **A** | **A** | **G** | **A** | **A** | **A** | **A** | **T** | **A** | **T** | **T** | **G** | **A** | **A** | **A** | **T** | **T** | **T** | **T** | **A** | **A** | **T** | **T** | **T** | **A** | **T** | **A** | **A** | **C** | **G** | **G** | **T** | **G** | **G** | **T** | **T** | **T** | **C** | **T** | **T** | **A** | **C** | **A** | **T** | **G** | **C** | **G** | **A** | **A** | **A** | **A** | **C** | **A** | **A** | **T** | **G** | **A** | **T** | **T** | **A** | **T** | **G** | **G** | **A** | **T** | **G** | **A** | **T** | **G** | **A** | **A** | **G** | **T** | **C** | **T** | **G** | **T** | **A** | **C** | **A** | **G** | **T** | **T** | **G** | **G** | **T** | **T** | **C** | **A** | **G** | **C** | **C** | **A** | **A** | **T** | **C** | **A** | **A** | **G** | **A** | **T** | **A** | **T** | **C** | **C** | **G** | **A** | **A** | **G** | **T** | **T** | **A** | **C** | **A** | **A** | **A** | **T** | **T** | **A** | **A** | **A** | **C** | **T** | **T** | **T** | **G** | **A** | **A** | **G** | **C** | **A** | **A** | **A** | **T** | **G** | **C** | **T** | **G** | **T** | **G** | **T** | **T** | **A** | **T** | **A** | **T** | **G** | **A** | **T** | **A** | **A** | **A** | **A** | **A** | **A** | **A** | **T** | **C** | **A** | **T** | **T** | **G** | **A** | **T** | **C** | **A** | **A** | **T** | **T** | **A** | **G** | **A** | **A** | **G** | **C** | **A** | **A** | **T** | **T** | **T** | **T** | **C** | **T** | **T** | **A** | **G** | **A** | **A** | **G** | **A** | **T** | **C** | **G** | **A** | **A** | **A** | **A** | **A** | **A** | **A** | **T** | **G** | **T** | **A** | **C** | **A** | **A** | **C** | **A** | **A** | **T** | **G** | **A** | **C** | **T** | **C** | **C** | **A** | **G** | **A** | **A** | **G** | **T** | **T** | **G** | **T** | **T** | **C** | **G** | **C** | **G** | **A** | **C** | **A** | **T** | **G** | **T** | **C** | **A** | **A** | **A** | **A** | **T** | **G** | **G** | **T** | **T** | **G** | **A** | **T** | **T** | **T** | **T** | **T** | **A** | **A** | **A** | **C** | **A** | **A** | **C** | **A** | **A** | **A** | **T** | **T** | **T** | **C** | **A** | **C** | **G** | **A** | **T** | **T** | **A** | **T** | **T** | **T** | **T** | **C** | **A** | **C** | **C** | **A** | **A** | **T** | **T** | **C** | **T** | **T** | **-** | **-** | **-** |
| **N54676** | **T** | **T** | **G** | **A** | **T** | **T** | **C** | **T** | **T** | **A** | **G** | **C** | **G** | **T** | **C** | **T** | **T** | **A** | **A** | **C** | **A** | **G** | **T** | **T** | **A** | **T** | **T** | **T** | **A** | **T** | **T** | **T** | **T** | **A** | **T** | **T** | **A** | **A** | **T** | **G** | **C** | **A** | **A** | **T** | **T** | **A** | **T** | **T** | **G** | **C** | **G** | **G** | **G** | **A** | **A** | **T** | **T** | **A** | **C** | **C** | **A** | **T** | **T** | **T** | **T** | **G** | **T** | **T** | **A** | **A** | **A** | **A** | **C** | **C** | **T** | **C** | **G** | **A** | **G** | **A** | **T** | **G** | **T** | **A** | **G** | **C** | **A** | **G** | **C** | **A** | **A** | **T** | **T** | **T** | **G** | **G** | **G** | **C** | **A** | **T** | **G** | **G** | **C** | **T** | **A** | **C** | **T** | **C** | **G** | **T** | **A** | **C** | **T** | **G** | **A** | **T** | **T** | **G** | **C** | **T** | **C** | **T** | **A** | **C** | **C** | **C** | **G** | **T** | **T** | **T** | **T** | **T** | **G** | **G** | **T** | **T** | **T** | **T** | **T** | **T** | **C** | **T** | **T** | **A** | **T** | **A** | **T** | **T** | **T** | **A** | **T** | **T** | **C** | **T** | **T** | **T** | **G** | **G** | **G** | **C** | **G** | **C** | **G** | **G** | **T** | **T** | **T** | **A** | **A** | **C** | **C** | **G** | **A** | **C** | **A** | **A** | **A** | **A** | **A** | **G** | **A** | **A** | **A** | **T** | **T** | **T** | **T** | **A** | **T** | **T** | **T** | **G** | **C** | **A** | **A** | **C** | **A** | **A** | **A** | **G** | **T** | **G** | **A** | **T** | **T** | **T** | **A** | **C** | **G** | **G** | **G** | **A** | **A** | **T** | **T** | **A** | **G** | **A** | **G** | **A** | **A** | **T** | **T** | **T** | **T** | **C** | **A** | **G** | **A** | **A** | **C** | **T** | **T** | **C** | **C** | **A** | **A** | **G** | **A** | **A** | **G** | **A** | **A** | **A** | **G** | **T** | **T** | **T** | **T** | **G** | **A** | **A** | **C** | **T** | **T** | **T** | **A** | **T** | **A** | **G** | **T** | **C** | **A** | **A** | **A** | **A** | **G** | **A** | **T** | **G** | **C** | **C** | **G** | **A** | **C** | **G** | **G** | **A** | **G** | **G** | **A** | **G** | **C** | **A** | **A** | **C** | **A** | **A** | **C** | **A** | **G** | **T** | **T** | **T** | **A** | **C** | **A** | **G** | **A** | **C** | **T** | **T** | **T** | **T** | **T** | **T** | **T** | **C** | **C** | **T** | **C** | **G** | **T** | **T** | **A** | **A** | **A** | **T** | **A** | **G** | **G** | **A** | **T** | **G** | **C** | **C** | **C** | **C** | **T** | **G** | **A** | **C** | **T** | **A** | **A** | **G** | **A** | **A** | **A** | **A** | **A** | **T** | **G** | **A** | **T** | **G** | **T** | **T** | **G** | **A** | **A** | **A** | **T** | **T** | **T** | **T** | **T** | **A** | **C** | **G** | **G** | **A** | **T** | **G** | **G** | **T** | **A** | **C** | **G** | **G** | **A** | **A** | **A** | **A** | **A** | **T** | **T** | **C** | **A** | **A** | **T** | **G** | **C** | **G** | **T** | **T** | **G** | **A** | **T** | **G** | **G** | **C** | **G** | **G** | **A** | **C** | **A** | **T** | **A** | **A** | **A** | **A** | **A** | **A** | **A** | **G** | **C** | **T** | **C** | **A** | **G** | **C** | **A** | **C** | **T** | **C** | **C** | **A** | **T** | **T** | **C** | **A** | **T** | **A** | **T** | **T** | **G** | **A** | **A** | **T** | **A** | **T** | **T** | **A** | **T** | **G** | **C** | **C** | **T** | **T** | **T** | **G** | **T** | **A** | **A** | **C** | **G** | **G** | **A** | **T** | **C** | **A** | **T** | **A** | **T** | **C** | **G** | **G** | **T** | **A** | **C** | **C** | **A** | **A** | **A** | **A** | **T** | **T** | **T** | **T** | **G** | **A** | **A** | **C** | **T** | **T** | **A** | **T** | **T** | **A** | **G** | **A** | **A** | **G** | **A** | **A** | **A** | **A** | **G** | **G** | **C** | **G** | **G** | **C** | **T** | **G** | **A** | **A** | **G** | **G** | **G** | **G** | **T** | **T** | **G** | **A** | **A** | **G** | **T** | **G** | **C** | **G** | **G** | **T** | **T** | **G** | **C** | **T** | **T** | **T** | **A** | **C** | **G** | **A** | **T** | **G** | **C** | **C** | **T** | **T** | **T** | **G** | **G** | **C** | **T** | **C** | **A** | **A** | **A** | **G** | **G** | **G** | **C** | **A** | **C** | **C** | **A** | **A** | **G** | **G** | **T** | **T** | **C** | **A** | **T** | **C** | **A** | **T** | **T** | **T** | **G** | **A** | **A** | **T** | **G** | **A** | **A** | **T** | **T** | **G** | **A** | **A** | **A** | **A** | **A** | **A** | **A** | **A** | **C** | **G** | **G** | **C** | **G** | **G** | **T** | **T** | **T** | **T** | **G** | **T** | **C** | **C** | **A** | **A** | **A** | **C** | **G** | **T** | **T** | **T** | **A** | **T** | **T** | **A** | **C** | **T** | **T** | **C** | **T** | **C** | **A** | **A** | **A** | **A** | **A** | **G** | **C** | **A** | **C** | **T** | **T** | **T** | **T** | **G** | **A** | **A** | **G** | **T** | **T** | **T** | **C** | **G** | **T** | **T** | **T** | **G** | **A** | **A** | **T** | **T** | **A** | **T** | **C** | **A** | **T** | **G** | **A** | **T** | **C** | **A** | **C** | **C** | **G** | **G** | **A** | **A** | **A** | **A** | **T** | **T** | **G** | **T** | **T** | **G** | **T** | **T** | **A** | **T** | **T** | **G** | **A** | **T** | **G** | **G** | **G** | **A** | **A** | **A** | **G** | **T** | **A** | **G** | **G** | **C** | **T** | **A** | **C** | **A** | **T** | **T** | **G** | **G** | **C** | **G** | **G** | **C** | **T** | **T** | **T** | **A** | **A** | **T** | **G** | **T** | **T** | **G** | **C** | **C** | **G** | **A** | **T** | **C** | **A** | **A** | **T** | **A** | **T** | **G** | **C** | **C** | **G** | **G** | **A** | **A** | **C** | **G** | **A** | **C** | **T** | **A** | **A** | **A** | **A** | **A** | **G** | **T** | **T** | **T** | **G** | **G** | **C** | **T** | **A** | **T** | **T** | **G** | **G** | **C** | **G** | **G** | **G** | **A** | **T** | **A** | **C** | **A** | **C** | **A** | **T** | **T** | **T** | **A** | **C** | **G** | **G** | **A** | **T** | **T** | **C** | **A** | **A** | **G** | **G** | **G** | **C** | **C** | **A** | **G** | **C** | **A** | **G** | **C** | **C** | **T** | **C** | **A** | **T** | **T** | **A** | **C** | **T** | **G** | **C** | **A** | **A** | **A** | **T** | **G** | **C** | **G** | **T** | **T** | **T** | **T** | **T** | **T** | **A** | **A** | **T** | **G** | **G** | **A** | **T** | **T** | **G** | **G** | **A** | **A** | **C** | **G** | **T** | **C** | **T** | **C** | **T** | **T** | **C** | **C** | **C** | **C** | **T** | **G** | **A** | **G** | **A** | **A** | **A** | **A** | **A** | **T** | **C** | **G** | **T** | **G** | **T** | **G** | **G** | **C** | **G** | **T** | **A** | **T** | **C** | **A** | **A** | **T** | **T** | **G** | **G** | **A** | **T** | **T** | **A** | **T** | **T** | **T** | **C** | **T** | **T** | **T** | **A** | **A** | **A** | **C** | **T** | **T** | **G** | **A** | **A** | **G** | **C** | **G** | **T** | **T** | **G** | **G** | **T** | **G** | **C** | **C** | **A** | **G** | **A** | **G** | **G** | **C** | **A** | **A** | **A** | **T** | **A** | **C** | **A** | **T** | **C** | **C** | **A** | **T** | **T** | **C** | **A** | **G** | **A** | **T** | **G** | **A** | **T** | **T** | **G** | **C** | **C** | **A** | **G** | **T** | **G** | **G** | **T** | **C** | **C** | **T** | **A** | **A** | **T** | **A** | **G** | **T** | **G** | **A** | **C** | **C** | **G** | **T** | **G** | **A** | **A** | **C** | **A** | **A** | **A** | **T** | **T** | **A** | **A** | **A** | **T** | **T** | **G** | **G** | **C** | **T** | **T** | **T** | **T** | **A** | **T** | **T** | **A** | **A** | **A** | **T** | **T** | **G** | **A** | **T** | **T** | **A** | **C** | **T** | **T** | **C** | **T** | **G** | **C** | **C** | **A** | **A** | **G** | **A** | **A** | **A** | **A** | **G** | **A** | **G** | **T** | **C** | **T** | **G** | **G** | **A** | **T** | **T** | **C** | **A** | **A** | **A** | **C** | **G** | **C** | **C** | **G** | **T** | **A** | **T** | **T** | **T** | **A** | **G** | **T** | **T** | **C** | **C** | **T** | **G** | **A** | **T** | **G** | **A** | **T** | **A** | **G** | **T** | **G** | **T** | **C** | **T** | **T** | **G** | **G** | **C** | **G** | **G** | **C** | **T** | **T** | **T** | **A** | **A** | **A** | **G** | **G** | **T** | **T** | **G** | **C** | **T** | **G** | **C** | **G** | **G** | **C** | **T** | **T** | **C** | **A** | **G** | **G** | **A** | **G** | **T** | **A** | **G** | **A** | **T** | **G** | **T** | **T** | **A** | **A** | **A** | **A** | **T** | **T** | **A** | **T** | **G** | **A** | **T** | **T** | **C** | **C** | **A** | **G** | **A** | **T** | **A** | **A** | **G** | **C** | **C** | **C** | **G** | **A** | **C** | **C** | **A** | **T** | **C** | **C** | **G** | **T** | **T** | **T** | **A** | **T** | **T** | **T** | **A** | **T** | **C** | **G** | **A** | **G** | **C** | **A** | **A** | **C** | **A** | **C** | **A** | **G** | **T** | **A** | **T** | **T** | **A** | **C** | **G** | **G** | **G** | **C** | **G** | **C** | **T** | **T** | **G** | **T** | **T** | **G** | **A** | **T** | **G** | **A** | **A** | **A** | **G** | **A** | **A** | **A** | **A** | **T** | **A** | **T** | **T** | **G** | **A** | **A** | **A** | **T** | **T** | **T** | **T** | **A** | **A** | **T** | **T** | **T** | **A** | **T** | **A** | **A** | **C** | **G** | **G** | **T** | **G** | **G** | **T** | **T** | **T** | **C** | **T** | **T** | **A** | **C** | **A** | **T** | **G** | **C** | **G** | **A** | **A** | **A** | **A** | **C** | **A** | **A** | **T** | **G** | **A** | **T** | **T** | **A** | **T** | **G** | **G** | **A** | **T** | **G** | **A** | **T** | **G** | **A** | **A** | **G** | **T** | **C** | **T** | **G** | **C** | **A** | **C** | **A** | **G** | **T** | **A** | **G** | **G** | **T** | **T** | **C** | **A** | **G** | **C** | **C** | **A** | **A** | **T** | **C** | **A** | **A** | **G** | **A** | **T** | **A** | **T** | **C** | **C** | **G** | **A** | **A** | **G** | **T** | **T** | **A** | **C** | **A** | **A** | **A** | **T** | **T** | **A** | **A** | **A** | **C** | **T** | **T** | **T** | **G** | **A** | **A** | **G** | **C** | **A** | **A** | **A** | **T** | **G** | **C** | **T** | **G** | **T** | **G** | **T** | **T** | **A** | **T** | **A** | **T** | **G** | **A** | **T** | **A** | **A** | **A** | **A** | **A** | **A** | **A** | **T** | **C** | **A** | **T** | **T** | **G** | **A** | **T** | **C** | **A** | **A** | **T** | **T** | **A** | **G** | **A** | **A** | **G** | **C** | **A** | **A** | **T** | **T** | **T** | **T** | **C** | **T** | **T** | **A** | **G** | **A** | **A** | **G** | **A** | **T** | **C** | **G** | **A** | **A** | **A** | **A** | **A** | **A** | **A** | **T** | **G** | **T** | **A** | **C** | **A** | **A** | **C** | **A** | **A** | **T** | **G** | **A** | **C** | **T** | **C** | **C** | **A** | **G** | **A** | **A** | **G** | **T** | **T** | **G** | **T** | **T** | **C** | **G** | **C** | **G** | **A** | **C** | **A** | **T** | **G** | **T** | **C** | **A** | **A** | **A** | **A** | **T** | **G** | **G** | **T** | **T** | **G** | **A** | **T** | **T** | **T** | **T** | **T** | **A** | **A** | **A** | **C** | **A** | **A** | **C** | **A** | **A** | **A** | **T** | **T** | **T** | **C** | **A** | **C** | **G** | **A** | **T** | **T** | **A** | **T** | **T** | **C** | **T** | **C** | **A** | **C** | **C** | **A** | **A** | **T** | **T** | **C** | **T** | **T** | **-** | **-** | **-** |
| **N60063F** | **T** | **T** | **G** | **A** | **T** | **T** | **C** | **T** | **T** | **A** | **G** | **C** | **G** | **T** | **C** | **T** | **T** | **A** | **A** | **C** | **A** | **G** | **T** | **T** | **A** | **T** | **T** | **T** | **A** | **T** | **T** | **T** | **T** | **A** | **T** | **T** | **A** | **A** | **T** | **G** | **C** | **A** | **A** | **T** | **T** | **A** | **T** | **T** | **G** | **C** | **G** | **G** | **G** | **A** | **A** | **T** | **T** | **A** | **C** | **C** | **A** | **T** | **T** | **T** | **T** | **G** | **T** | **T** | **A** | **A** | **A** | **A** | **C** | **C** | **T** | **C** | **G** | **A** | **G** | **A** | **T** | **G** | **T** | **A** | **G** | **C** | **A** | **G** | **C** | **A** | **A** | **T** | **T** | **T** | **G** | **G** | **G** | **C** | **A** | **T** | **G** | **G** | **C** | **T** | **A** | **C** | **T** | **C** | **G** | **T** | **A** | **C** | **T** | **G** | **A** | **T** | **T** | **G** | **C** | **T** | **C** | **T** | **A** | **C** | **C** | **C** | **G** | **T** | **T** | **T** | **T** | **T** | **G** | **G** | **T** | **T** | **T** | **T** | **T** | **T** | **C** | **T** | **T** | **A** | **T** | **A** | **T** | **T** | **T** | **A** | **T** | **T** | **C** | **T** | **T** | **T** | **G** | **G** | **G** | **C** | **G** | **C** | **G** | **G** | **T** | **T** | **T** | **A** | **A** | **C** | **C** | **G** | **A** | **C** | **A** | **A** | **A** | **A** | **A** | **G** | **A** | **A** | **A** | **T** | **T** | **T** | **T** | **A** | **T** | **T** | **T** | **G** | **C** | **A** | **A** | **C** | **A** | **A** | **A** | **G** | **T** | **G** | **A** | **T** | **T** | **T** | **A** | **C** | **G** | **G** | **G** | **A** | **A** | **T** | **T** | **A** | **G** | **A** | **G** | **A** | **A** | **T** | **T** | **T** | **C** | **C** | **A** | **G** | **A** | **A** | **T** | **T** | **T** | **C** | **C** | **A** | **A** | **G** | **A** | **A** | **G** | **A** | **A** | **A** | **G** | **T** | **T** | **T** | **T** | **G** | **A** | **A** | **C** | **T** | **T** | **T** | **A** | **T** | **A** | **G** | **T** | **C** | **A** | **A** | **A** | **A** | **G** | **A** | **T** | **G** | **C** | **C** | **G** | **A** | **C** | **G** | **G** | **A** | **G** | **G** | **A** | **G** | **C** | **A** | **A** | **C** | **A** | **A** | **C** | **A** | **G** | **T** | **T** | **T** | **A** | **C** | **A** | **G** | **A** | **C** | **T** | **T** | **T** | **T** | **T** | **T** | **T** | **C** | **C** | **T** | **C** | **G** | **T** | **T** | **A** | **A** | **A** | **T** | **A** | **G** | **G** | **A** | **T** | **G** | **C** | **C** | **T** | **C** | **T** | **G** | **A** | **C** | **T** | **A** | **A** | **G** | **A** | **A** | **A** | **A** | **A** | **T** | **G** | **A** | **T** | **G** | **T** | **T** | **G** | **A** | **A** | **A** | **T** | **T** | **T** | **T** | **T** | **A** | **C** | **G** | **G** | **A** | **T** | **G** | **G** | **T** | **A** | **C** | **G** | **G** | **A** | **A** | **A** | **A** | **A** | **T** | **T** | **C** | **A** | **A** | **T** | **G** | **C** | **G** | **T** | **T** | **G** | **A** | **T** | **G** | **G** | **C** | **G** | **G** | **A** | **C** | **A** | **T** | **A** | **A** | **A** | **A** | **A** | **A** | **A** | **G** | **C** | **T** | **C** | **A** | **G** | **C** | **A** | **C** | **T** | **C** | **C** | **A** | **T** | **T** | **C** | **A** | **T** | **A** | **T** | **T** | **G** | **A** | **A** | **T** | **A** | **T** | **T** | **A** | **T** | **G** | **C** | **C** | **T** | **T** | **T** | **G** | **T** | **A** | **A** | **C** | **G** | **G** | **A** | **T** | **C** | **A** | **T** | **A** | **T** | **C** | **G** | **G** | **C** | **A** | **C** | **C** | **A** | **A** | **A** | **A** | **T** | **T** | **T** | **T** | **G** | **A** | **A** | **C** | **T** | **T** | **A** | **T** | **T** | **A** | **G** | **A** | **A** | **G** | **A** | **A** | **A** | **A** | **G** | **G** | **C** | **G** | **G** | **C** | **T** | **G** | **A** | **A** | **G** | **G** | **G** | **G** | **T** | **T** | **G** | **A** | **A** | **G** | **T** | **G** | **C** | **G** | **G** | **T** | **T** | **G** | **C** | **T** | **T** | **T** | **A** | **C** | **G** | **A** | **T** | **G** | **C** | **C** | **T** | **T** | **T** | **G** | **G** | **C** | **T** | **C** | **A** | **A** | **A** | **G** | **G** | **G** | **C** | **A** | **C** | **C** | **A** | **A** | **G** | **G** | **T** | **T** | **C** | **A** | **T** | **C** | **A** | **T** | **T** | **T** | **G** | **A** | **A** | **T** | **G** | **A** | **A** | **T** | **T** | **G** | **A** | **A** | **A** | **A** | **A** | **A** | **A** | **A** | **C** | **G** | **G** | **C** | **G** | **G** | **T** | **T** | **T** | **T** | **G** | **T** | **C** | **C** | **A** | **A** | **A** | **C** | **A** | **T** | **T** | **T** | **A** | **T** | **T** | **A** | **C** | **T** | **T** | **C** | **T** | **C** | **A** | **A** | **A** | **A** | **A** | **G** | **C** | **A** | **C** | **T** | **T** | **T** | **T** | **G** | **A** | **A** | **G** | **T** | **T** | **T** | **C** | **G** | **T** | **T** | **T** | **G** | **A** | **A** | **T** | **T** | **A** | **T** | **C** | **A** | **T** | **G** | **A** | **T** | **C** | **A** | **C** | **C** | **G** | **G** | **A** | **A** | **A** | **A** | **T** | **T** | **G** | **T** | **T** | **G** | **T** | **T** | **A** | **T** | **T** | **G** | **A** | **C** | **G** | **G** | **A** | **A** | **A** | **A** | **G** | **T** | **A** | **G** | **G** | **C** | **T** | **A** | **C** | **A** | **T** | **T** | **G** | **G** | **C** | **G** | **G** | **C** | **T** | **T** | **T** | **A** | **A** | **T** | **G** | **T** | **T** | **G** | **C** | **C** | **G** | **A** | **T** | **C** | **A** | **A** | **T** | **A** | **T** | **G** | **C** | **C** | **G** | **G** | **A** | **A** | **C** | **G** | **A** | **C** | **T** | **A** | **A** | **A** | **A** | **A** | **G** | **T** | **T** | **T** | **G** | **G** | **C** | **T** | **A** | **T** | **T** | **G** | **G** | **C** | **G** | **G** | **G** | **A** | **T** | **A** | **C** | **G** | **C** | **A** | **T** | **T** | **T** | **A** | **C** | **G** | **G** | **A** | **T** | **T** | **C** | **A** | **A** | **G** | **G** | **G** | **C** | **C** | **A** | **G** | **C** | **A** | **G** | **C** | **C** | **T** | **C** | **A** | **T** | **T** | **A** | **C** | **T** | **G** | **C** | **A** | **A** | **A** | **T** | **G** | **C** | **G** | **T** | **T** | **T** | **T** | **T** | **T** | **A** | **A** | **T** | **G** | **G** | **A** | **T** | **T** | **G** | **G** | **A** | **A** | **C** | **G** | **T** | **C** | **T** | **C** | **T** | **T** | **C** | **C** | **C** | **C** | **C** | **G** | **A** | **G** | **A** | **A** | **A** | **A** | **A** | **T** | **C** | **G** | **T** | **G** | **T** | **G** | **G** | **C** | **G** | **T** | **A** | **T** | **C** | **A** | **A** | **T** | **T** | **G** | **G** | **A** | **T** | **T** | **A** | **T** | **T** | **T** | **C** | **T** | **T** | **T** | **A** | **A** | **A** | **C** | **T** | **T** | **G** | **A** | **A** | **G** | **C** | **A** | **T** | **T** | **G** | **G** | **T** | **G** | **C** | **C** | **A** | **G** | **A** | **G** | **G** | **C** | **A** | **A** | **A** | **T** | **A** | **C** | **A** | **T** | **C** | **C** | **A** | **T** | **T** | **C** | **A** | **G** | **A** | **T** | **G** | **A** | **T** | **T** | **G** | **C** | **C** | **A** | **G** | **T** | **G** | **G** | **T** | **C** | **C** | **T** | **A** | **A** | **C** | **A** | **G** | **T** | **G** | **A** | **C** | **C** | **G** | **T** | **G** | **A** | **A** | **C** | **A** | **A** | **A** | **T** | **T** | **A** | **A** | **A** | **T** | **T** | **G** | **G** | **C** | **C** | **T** | **T** | **T** | **A** | **T** | **T** | **A** | **A** | **A** | **T** | **T** | **G** | **A** | **T** | **T** | **A** | **C** | **T** | **T** | **C** | **T** | **G** | **C** | **C** | **A** | **A** | **G** | **A** | **A** | **A** | **A** | **G** | **A** | **G** | **T** | **C** | **T** | **G** | **G** | **A** | **T** | **T** | **C** | **A** | **A** | **A** | **C** | **G** | **C** | **C** | **G** | **T** | **A** | **T** | **T** | **T** | **A** | **G** | **T** | **T** | **C** | **C** | **T** | **G** | **A** | **T** | **G** | **A** | **T** | **A** | **G** | **T** | **G** | **T** | **C** | **T** | **T** | **G** | **G** | **C** | **T** | **G** | **C** | **T** | **T** | **T** | **A** | **A** | **A** | **G** | **G** | **T** | **T** | **G** | **C** | **T** | **G** | **C** | **G** | **G** | **C** | **T** | **T** | **C** | **A** | **G** | **G** | **A** | **G** | **T** | **A** | **G** | **A** | **T** | **G** | **T** | **T** | **A** | **A** | **A** | **A** | **T** | **T** | **A** | **T** | **G** | **A** | **T** | **T** | **C** | **C** | **A** | **G** | **A** | **T** | **A** | **A** | **G** | **C** | **C** | **C** | **G** | **A** | **C** | **C** | **A** | **T** | **C** | **C** | **G** | **T** | **T** | **T** | **A** | **T** | **T** | **T** | **A** | **T** | **C** | **G** | **A** | **G** | **C** | **A** | **A** | **C** | **A** | **C** | **A** | **G** | **T** | **A** | **T** | **T** | **A** | **T** | **G** | **G** | **C** | **C** | **G** | **C** | **T** | **T** | **A** | **T** | **T** | **G** | **A** | **T** | **G** | **A** | **A** | **A** | **G** | **A** | **A** | **A** | **A** | **T** | **A** | **T** | **T** | **G** | **A** | **A** | **A** | **T** | **T** | **T** | **T** | **A** | **A** | **T** | **T** | **T** | **A** | **T** | **A** | **A** | **C** | **G** | **G** | **T** | **G** | **G** | **T** | **T** | **T** | **C** | **T** | **T** | **A** | **C** | **A** | **T** | **G** | **C** | **G** | **A** | **A** | **A** | **A** | **C** | **A** | **A** | **T** | **G** | **A** | **T** | **T** | **A** | **T** | **G** | **G** | **A** | **T** | **G** | **A** | **T** | **G** | **A** | **A** | **G** | **T** | **C** | **T** | **G** | **T** | **A** | **C** | **A** | **G** | **T** | **T** | **G** | **G** | **T** | **T** | **C** | **A** | **G** | **C** | **C** | **A** | **A** | **T** | **C** | **A** | **A** | **G** | **A** | **T** | **A** | **T** | **C** | **C** | **G** | **A** | **A** | **G** | **T** | **T** | **A** | **C** | **A** | **A** | **A** | **T** | **T** | **A** | **A** | **A** | **C** | **T** | **T** | **T** | **G** | **A** | **A** | **G** | **C** | **A** | **A** | **A** | **T** | **G** | **C** | **T** | **G** | **T** | **G** | **T** | **T** | **A** | **T** | **A** | **T** | **G** | **A** | **T** | **A** | **A** | **A** | **A** | **A** | **A** | **A** | **T** | **C** | **A** | **T** | **T** | **G** | **A** | **T** | **C** | **A** | **A** | **T** | **T** | **A** | **G** | **A** | **A** | **G** | **C** | **A** | **A** | **T** | **T** | **T** | **T** | **C** | **T** | **T** | **A** | **G** | **A** | **A** | **G** | **A** | **T** | **C** | **G** | **A** | **A** | **A** | **A** | **A** | **A** | **A** | **T** | **G** | **T** | **A** | **C** | **A** | **A** | **C** | **A** | **A** | **T** | **G** | **A** | **C** | **A** | **C** | **C** | **A** | **G** | **A** | **A** | **G** | **T** | **T** | **G** | **T** | **T** | **C** | **G** | **A** | **G** | **A** | **C** | **A** | **T** | **G** | **T** | **C** | **C** | **A** | **A** | **A** | **T** | **G** | **G** | **T** | **T** | **G** | **A** | **T** | **T** | **T** | **T** | **T** | **A** | **A** | **A** | **C** | **A** | **A** | **C** | **A** | **A** | **A** | **T** | **T** | **T** | **C** | **A** | **C** | **G** | **A** | **T** | **T** | **A** | **T** | **T** | **T** | **T** | **C** | **C** | **C** | **C** | **A** | **A** | **T** | **T** | **C** | **T** | **T** | **-** | **-** | **-** |
| **N59936F** | **T** | **T** | **G** | **A** | **T** | **T** | **C** | **T** | **T** | **A** | **G** | **C** | **G** | **T** | **C** | **T** | **T** | **A** | **A** | **C** | **A** | **G** | **T** | **T** | **A** | **T** | **T** | **T** | **A** | **T** | **T** | **T** | **T** | **A** | **T** | **T** | **A** | **A** | **T** | **G** | **C** | **A** | **A** | **T** | **T** | **A** | **T** | **T** | **G** | **C** | **G** | **G** | **G** | **A** | **A** | **T** | **T** | **A** | **C** | **C** | **A** | **T** | **T** | **T** | **T** | **G** | **T** | **T** | **A** | **A** | **A** | **A** | **C** | **C** | **T** | **C** | **G** | **A** | **G** | **A** | **T** | **G** | **T** | **A** | **G** | **C** | **A** | **G** | **C** | **A** | **A** | **T** | **T** | **T** | **G** | **G** | **G** | **C** | **A** | **T** | **G** | **G** | **C** | **T** | **A** | **C** | **T** | **C** | **G** | **T** | **A** | **C** | **T** | **G** | **A** | **T** | **T** | **G** | **C** | **T** | **C** | **T** | **A** | **C** | **C** | **C** | **G** | **T** | **T** | **T** | **T** | **T** | **G** | **G** | **T** | **T** | **T** | **T** | **T** | **T** | **C** | **T** | **T** | **A** | **T** | **A** | **T** | **T** | **T** | **A** | **T** | **T** | **C** | **T** | **T** | **T** | **G** | **G** | **G** | **C** | **G** | **C** | **G** | **G** | **T** | **T** | **T** | **A** | **A** | **C** | **C** | **G** | **A** | **C** | **A** | **A** | **A** | **A** | **A** | **G** | **A** | **A** | **A** | **T** | **T** | **T** | **T** | **A** | **T** | **T** | **T** | **G** | **C** | **A** | **A** | **C** | **A** | **A** | **A** | **G** | **T** | **G** | **A** | **T** | **T** | **T** | **A** | **C** | **G** | **G** | **G** | **A** | **A** | **T** | **T** | **A** | **G** | **A** | **G** | **A** | **A** | **T** | **T** | **T** | **T** | **C** | **A** | **G** | **A** | **A** | **C** | **T** | **T** | **C** | **C** | **A** | **A** | **G** | **A** | **A** | **G** | **A** | **A** | **A** | **G** | **T** | **T** | **T** | **T** | **G** | **A** | **A** | **C** | **T** | **T** | **T** | **A** | **T** | **A** | **G** | **T** | **C** | **A** | **A** | **A** | **A** | **G** | **A** | **T** | **G** | **C** | **C** | **G** | **A** | **C** | **G** | **G** | **A** | **G** | **G** | **A** | **G** | **C** | **A** | **A** | **C** | **A** | **A** | **C** | **A** | **G** | **T** | **T** | **T** | **A** | **C** | **A** | **G** | **A** | **C** | **T** | **T** | **T** | **T** | **T** | **T** | **T** | **C** | **C** | **T** | **C** | **G** | **T** | **T** | **A** | **A** | **A** | **T** | **A** | **G** | **G** | **A** | **T** | **G** | **C** | **C** | **C** | **C** | **T** | **G** | **A** | **C** | **T** | **A** | **A** | **G** | **A** | **A** | **A** | **A** | **A** | **T** | **G** | **A** | **T** | **G** | **T** | **T** | **G** | **A** | **A** | **A** | **T** | **T** | **T** | **T** | **T** | **A** | **C** | **G** | **G** | **A** | **T** | **G** | **G** | **T** | **A** | **C** | **G** | **G** | **A** | **A** | **A** | **A** | **A** | **T** | **T** | **C** | **A** | **A** | **T** | **G** | **C** | **G** | **T** | **T** | **G** | **A** | **T** | **G** | **G** | **C** | **G** | **G** | **A** | **C** | **A** | **T** | **A** | **A** | **A** | **A** | **A** | **A** | **A** | **G** | **C** | **T** | **C** | **A** | **G** | **C** | **A** | **C** | **T** | **C** | **C** | **A** | **T** | **T** | **C** | **A** | **T** | **A** | **T** | **T** | **G** | **A** | **A** | **T** | **A** | **T** | **T** | **A** | **T** | **G** | **C** | **C** | **T** | **T** | **T** | **G** | **T** | **A** | **A** | **C** | **G** | **G** | **A** | **T** | **C** | **A** | **T** | **A** | **T** | **C** | **G** | **G** | **T** | **A** | **C** | **C** | **A** | **A** | **A** | **A** | **T** | **T** | **T** | **T** | **G** | **A** | **A** | **C** | **T** | **T** | **A** | **T** | **T** | **A** | **G** | **A** | **A** | **G** | **A** | **A** | **A** | **A** | **G** | **G** | **C** | **G** | **G** | **C** | **T** | **G** | **A** | **A** | **G** | **G** | **G** | **G** | **T** | **T** | **G** | **A** | **A** | **G** | **T** | **G** | **C** | **G** | **G** | **T** | **T** | **G** | **C** | **T** | **T** | **T** | **A** | **C** | **G** | **A** | **T** | **G** | **C** | **C** | **T** | **T** | **T** | **G** | **G** | **C** | **T** | **C** | **A** | **A** | **A** | **G** | **G** | **G** | **C** | **A** | **C** | **C** | **A** | **A** | **G** | **G** | **T** | **T** | **C** | **A** | **T** | **C** | **A** | **T** | **T** | **T** | **G** | **A** | **A** | **T** | **G** | **A** | **A** | **T** | **T** | **G** | **A** | **A** | **A** | **A** | **A** | **A** | **A** | **A** | **C** | **G** | **G** | **C** | **G** | **G** | **T** | **T** | **T** | **T** | **G** | **T** | **C** | **C** | **A** | **A** | **A** | **C** | **G** | **T** | **T** | **T** | **A** | **T** | **T** | **A** | **C** | **T** | **T** | **C** | **T** | **C** | **A** | **A** | **A** | **A** | **A** | **G** | **C** | **A** | **C** | **T** | **T** | **T** | **T** | **G** | **A** | **A** | **G** | **T** | **T** | **T** | **C** | **G** | **T** | **T** | **T** | **G** | **A** | **A** | **T** | **T** | **A** | **T** | **C** | **A** | **T** | **G** | **A** | **T** | **C** | **A** | **C** | **C** | **G** | **G** | **A** | **A** | **A** | **A** | **T** | **T** | **G** | **T** | **T** | **G** | **T** | **T** | **A** | **T** | **T** | **G** | **A** | **T** | **G** | **G** | **G** | **A** | **A** | **A** | **G** | **T** | **A** | **G** | **G** | **C** | **T** | **A** | **C** | **A** | **T** | **T** | **G** | **G** | **C** | **G** | **G** | **C** | **T** | **T** | **T** | **A** | **A** | **T** | **G** | **T** | **T** | **G** | **C** | **C** | **G** | **A** | **T** | **C** | **A** | **A** | **T** | **A** | **T** | **G** | **C** | **C** | **G** | **G** | **A** | **A** | **C** | **G** | **A** | **C** | **T** | **A** | **A** | **A** | **A** | **A** | **G** | **T** | **T** | **T** | **G** | **G** | **C** | **T** | **A** | **T** | **T** | **G** | **G** | **C** | **G** | **G** | **G** | **A** | **T** | **A** | **C** | **A** | **C** | **A** | **T** | **T** | **T** | **A** | **C** | **G** | **G** | **A** | **T** | **T** | **C** | **A** | **A** | **G** | **G** | **G** | **C** | **C** | **A** | **G** | **C** | **A** | **G** | **C** | **C** | **T** | **C** | **A** | **T** | **T** | **A** | **C** | **T** | **G** | **C** | **A** | **A** | **A** | **T** | **G** | **C** | **G** | **T** | **T** | **T** | **T** | **T** | **T** | **A** | **A** | **T** | **G** | **G** | **A** | **T** | **T** | **G** | **G** | **A** | **A** | **C** | **G** | **T** | **C** | **T** | **C** | **T** | **T** | **C** | **C** | **C** | **C** | **T** | **G** | **A** | **G** | **A** | **A** | **A** | **A** | **A** | **T** | **C** | **G** | **T** | **G** | **T** | **G** | **G** | **C** | **G** | **T** | **A** | **T** | **C** | **A** | **A** | **T** | **T** | **G** | **G** | **A** | **T** | **T** | **A** | **T** | **T** | **T** | **C** | **T** | **T** | **T** | **A** | **A** | **A** | **C** | **T** | **T** | **G** | **A** | **A** | **G** | **C** | **G** | **T** | **T** | **G** | **G** | **T** | **G** | **C** | **C** | **A** | **G** | **A** | **G** | **G** | **C** | **A** | **A** | **A** | **T** | **A** | **C** | **A** | **T** | **C** | **C** | **A** | **T** | **T** | **C** | **A** | **G** | **A** | **T** | **G** | **A** | **T** | **T** | **G** | **C** | **C** | **A** | **G** | **T** | **G** | **G** | **T** | **C** | **C** | **T** | **A** | **A** | **T** | **A** | **G** | **T** | **G** | **A** | **C** | **C** | **G** | **T** | **G** | **A** | **A** | **C** | **A** | **A** | **A** | **T** | **T** | **A** | **A** | **A** | **T** | **T** | **G** | **G** | **C** | **T** | **T** | **T** | **T** | **A** | **T** | **T** | **A** | **A** | **A** | **T** | **T** | **G** | **A** | **T** | **T** | **A** | **C** | **T** | **T** | **C** | **T** | **G** | **C** | **C** | **A** | **A** | **G** | **A** | **A** | **A** | **A** | **G** | **A** | **G** | **T** | **C** | **T** | **G** | **G** | **A** | **T** | **T** | **C** | **A** | **A** | **A** | **C** | **G** | **C** | **C** | **G** | **T** | **A** | **T** | **T** | **T** | **A** | **G** | **T** | **T** | **C** | **C** | **T** | **G** | **A** | **T** | **G** | **A** | **T** | **A** | **G** | **T** | **G** | **T** | **C** | **T** | **T** | **G** | **G** | **C** | **G** | **G** | **C** | **T** | **T** | **T** | **A** | **A** | **A** | **G** | **G** | **T** | **T** | **G** | **C** | **T** | **G** | **C** | **G** | **G** | **C** | **T** | **T** | **C** | **A** | **G** | **G** | **A** | **G** | **T** | **A** | **G** | **A** | **T** | **G** | **T** | **T** | **A** | **A** | **A** | **A** | **T** | **T** | **A** | **T** | **G** | **A** | **T** | **T** | **C** | **C** | **A** | **G** | **A** | **T** | **A** | **A** | **G** | **C** | **C** | **C** | **G** | **A** | **C** | **C** | **A** | **T** | **C** | **C** | **G** | **T** | **T** | **T** | **A** | **T** | **T** | **T** | **A** | **T** | **C** | **G** | **A** | **G** | **C** | **A** | **A** | **C** | **A** | **C** | **A** | **G** | **T** | **A** | **T** | **T** | **A** | **C** | **G** | **G** | **G** | **C** | **G** | **C** | **T** | **T** | **G** | **T** | **T** | **G** | **A** | **T** | **G** | **A** | **A** | **A** | **G** | **A** | **A** | **A** | **A** | **T** | **A** | **T** | **T** | **G** | **A** | **A** | **A** | **T** | **T** | **T** | **T** | **A** | **A** | **T** | **T** | **T** | **A** | **T** | **A** | **A** | **C** | **G** | **G** | **T** | **G** | **G** | **T** | **T** | **T** | **C** | **T** | **T** | **A** | **C** | **A** | **T** | **G** | **C** | **G** | **A** | **A** | **A** | **A** | **C** | **A** | **A** | **T** | **G** | **A** | **T** | **T** | **A** | **T** | **G** | **G** | **A** | **T** | **G** | **A** | **T** | **G** | **A** | **A** | **G** | **T** | **C** | **T** | **G** | **C** | **A** | **C** | **A** | **G** | **T** | **A** | **G** | **G** | **T** | **T** | **C** | **A** | **G** | **C** | **C** | **A** | **A** | **T** | **C** | **A** | **A** | **G** | **A** | **T** | **A** | **T** | **C** | **C** | **G** | **A** | **A** | **G** | **T** | **T** | **A** | **C** | **A** | **A** | **A** | **T** | **T** | **A** | **A** | **A** | **C** | **T** | **T** | **T** | **G** | **A** | **A** | **G** | **C** | **A** | **A** | **A** | **T** | **G** | **C** | **T** | **G** | **T** | **G** | **T** | **T** | **A** | **T** | **A** | **T** | **G** | **A** | **T** | **A** | **A** | **A** | **A** | **A** | **A** | **A** | **T** | **C** | **A** | **T** | **T** | **G** | **A** | **T** | **C** | **A** | **A** | **T** | **T** | **A** | **G** | **A** | **A** | **G** | **C** | **A** | **A** | **T** | **T** | **T** | **T** | **C** | **T** | **T** | **A** | **G** | **A** | **A** | **G** | **A** | **T** | **C** | **G** | **A** | **A** | **A** | **A** | **A** | **A** | **A** | **T** | **G** | **T** | **A** | **C** | **A** | **A** | **C** | **A** | **A** | **T** | **G** | **A** | **C** | **T** | **C** | **C** | **A** | **G** | **A** | **A** | **G** | **T** | **T** | **G** | **T** | **T** | **C** | **G** | **C** | **G** | **A** | **C** | **A** | **T** | **G** | **T** | **C** | **A** | **A** | **A** | **A** | **T** | **G** | **G** | **T** | **T** | **G** | **A** | **T** | **T** | **T** | **T** | **T** | **A** | **A** | **A** | **C** | **A** | **A** | **C** | **A** | **A** | **A** | **T** | **T** | **T** | **C** | **A** | **C** | **G** | **A** | **T** | **T** | **A** | **T** | **T** | **C** | **T** | **C** | **A** | **C** | **C** | **A** | **A** | **T** | **T** | **C** | **T** | **T** | **-** | **-** | **-** |
| **N53405** | **T** | **T** | **G** | **A** | **T** | **T** | **C** | **T** | **T** | **A** | **G** | **T** | **G** | **T** | **C** | **T** | **T** | **A** | **A** | **C** | **A** | **G** | **T** | **T** | **A** | **T** | **T** | **T** | **A** | **T** | **T** | **T** | **T** | **A** | **T** | **T** | **A** | **A** | **T** | **G** | **C** | **A** | **A** | **T** | **T** | **A** | **T** | **T** | **G** | **C** | **G** | **G** | **G** | **A** | **A** | **T** | **T** | **A** | **C** | **C** | **A** | **T** | **T** | **T** | **T** | **G** | **T** | **T** | **A** | **A** | **A** | **A** | **C** | **C** | **T** | **C** | **G** | **A** | **G** | **A** | **T** | **G** | **T** | **A** | **G** | **C** | **A** | **G** | **C** | **A** | **A** | **T** | **T** | **T** | **G** | **G** | **G** | **C** | **A** | **T** | **G** | **G** | **C** | **T** | **A** | **C** | **T** | **C** | **G** | **T** | **A** | **C** | **T** | **G** | **A** | **T** | **T** | **G** | **C** | **T** | **C** | **T** | **A** | **C** | **C** | **C** | **G** | **T** | **T** | **T** | **T** | **T** | **G** | **G** | **T** | **T** | **T** | **T** | **T** | **T** | **C** | **T** | **T** | **A** | **T** | **A** | **T** | **T** | **T** | **A** | **T** | **T** | **C** | **T** | **T** | **T** | **G** | **G** | **G** | **C** | **G** | **C** | **G** | **G** | **T** | **T** | **T** | **A** | **A** | **C** | **C** | **G** | **A** | **C** | **A** | **A** | **A** | **A** | **A** | **G** | **A** | **A** | **A** | **T** | **T** | **T** | **T** | **A** | **T** | **T** | **T** | **G** | **C** | **A** | **A** | **C** | **A** | **A** | **A** | **G** | **T** | **G** | **A** | **T** | **T** | **T** | **G** | **C** | **G** | **G** | **G** | **A** | **A** | **T** | **T** | **A** | **G** | **A** | **G** | **A** | **A** | **T** | **T** | **T** | **T** | **C** | **A** | **G** | **A** | **A** | **T** | **T** | **T** | **C** | **C** | **A** | **A** | **G** | **A** | **A** | **G** | **A** | **A** | **A** | **G** | **T** | **T** | **T** | **T** | **G** | **A** | **A** | **C** | **T** | **T** | **T** | **A** | **T** | **A** | **G** | **T** | **C** | **A** | **A** | **A** | **A** | **G** | **A** | **T** | **G** | **C** | **C** | **G** | **A** | **C** | **G** | **G** | **A** | **G** | **G** | **A** | **G** | **C** | **A** | **A** | **C** | **A** | **A** | **C** | **A** | **G** | **T** | **T** | **T** | **A** | **C** | **A** | **G** | **A** | **C** | **T** | **T** | **T** | **T** | **T** | **T** | **T** | **C** | **C** | **T** | **C** | **G** | **T** | **T** | **A** | **A** | **A** | **T** | **A** | **G** | **G** | **A** | **T** | **G** | **C** | **C** | **T** | **C** | **T** | **G** | **A** | **C** | **T** | **A** | **A** | **G** | **A** | **A** | **A** | **A** | **A** | **T** | **G** | **A** | **T** | **G** | **T** | **T** | **G** | **A** | **A** | **A** | **T** | **T** | **T** | **T** | **T** | **A** | **C** | **G** | **G** | **A** | **T** | **G** | **G** | **T** | **A** | **C** | **G** | **G** | **A** | **A** | **A** | **A** | **A** | **T** | **T** | **C** | **A** | **A** | **T** | **G** | **C** | **G** | **T** | **T** | **G** | **A** | **T** | **G** | **G** | **C** | **G** | **G** | **A** | **C** | **A** | **T** | **A** | **A** | **A** | **A** | **A** | **A** | **A** | **G** | **C** | **T** | **C** | **A** | **G** | **C** | **A** | **C** | **T** | **C** | **C** | **A** | **T** | **T** | **C** | **A** | **T** | **A** | **T** | **T** | **G** | **A** | **A** | **T** | **A** | **T** | **T** | **A** | **T** | **G** | **C** | **C** | **T** | **T** | **T** | **G** | **T** | **A** | **A** | **C** | **G** | **G** | **A** | **T** | **C** | **A** | **T** | **A** | **T** | **C** | **G** | **G** | **C** | **A** | **C** | **C** | **A** | **A** | **A** | **A** | **T** | **T** | **T** | **T** | **G** | **A** | **A** | **C** | **T** | **T** | **A** | **T** | **T** | **A** | **G** | **A** | **A** | **G** | **A** | **A** | **A** | **A** | **G** | **G** | **C** | **G** | **G** | **C** | **T** | **G** | **A** | **A** | **G** | **G** | **G** | **G** | **T** | **T** | **G** | **A** | **A** | **G** | **T** | **G** | **C** | **G** | **G** | **T** | **T** | **G** | **C** | **T** | **T** | **T** | **A** | **C** | **G** | **A** | **T** | **G** | **C** | **C** | **T** | **T** | **T** | **G** | **G** | **C** | **T** | **C** | **A** | **A** | **A** | **G** | **G** | **G** | **C** | **A** | **C** | **C** | **A** | **A** | **G** | **G** | **T** | **T** | **C** | **A** | **T** | **C** | **A** | **T** | **T** | **T** | **G** | **A** | **A** | **T** | **G** | **A** | **A** | **T** | **T** | **G** | **A** | **A** | **A** | **A** | **A** | **A** | **A** | **A** | **C** | **G** | **G** | **T** | **G** | **G** | **T** | **T** | **T** | **T** | **G** | **T** | **C** | **C** | **A** | **A** | **A** | **C** | **G** | **T** | **T** | **T** | **A** | **T** | **T** | **A** | **C** | **T** | **T** | **C** | **T** | **C** | **A** | **A** | **A** | **A** | **A** | **G** | **C** | **A** | **C** | **T** | **T** | **T** | **T** | **G** | **A** | **A** | **G** | **T** | **T** | **T** | **C** | **G** | **T** | **T** | **T** | **G** | **A** | **A** | **T** | **T** | **A** | **T** | **C** | **A** | **T** | **G** | **A** | **T** | **C** | **A** | **C** | **C** | **G** | **G** | **A** | **A** | **A** | **A** | **T** | **T** | **G** | **T** | **T** | **G** | **T** | **T** | **A** | **T** | **T** | **G** | **A** | **C** | **G** | **G** | **A** | **A** | **A** | **A** | **G** | **T** | **A** | **G** | **G** | **C** | **T** | **A** | **C** | **A** | **T** | **T** | **G** | **G** | **C** | **G** | **G** | **C** | **T** | **T** | **T** | **A** | **A** | **T** | **G** | **T** | **T** | **G** | **C** | **C** | **G** | **A** | **T** | **C** | **A** | **A** | **T** | **A** | **T** | **G** | **C** | **C** | **G** | **G** | **A** | **A** | **C** | **G** | **A** | **C** | **T** | **A** | **A** | **A** | **A** | **A** | **G** | **T** | **T** | **T** | **G** | **G** | **C** | **T** | **A** | **T** | **T** | **G** | **G** | **C** | **G** | **G** | **G** | **A** | **T** | **A** | **C** | **A** | **C** | **A** | **T** | **T** | **T** | **A** | **C** | **G** | **G** | **A** | **T** | **T** | **C** | **A** | **A** | **G** | **G** | **G** | **C** | **C** | **A** | **G** | **C** | **A** | **G** | **C** | **C** | **T** | **C** | **A** | **T** | **T** | **A** | **C** | **T** | **G** | **C** | **A** | **A** | **A** | **T** | **G** | **C** | **G** | **T** | **T** | **T** | **T** | **T** | **T** | **A** | **A** | **T** | **G** | **G** | **A** | **T** | **T** | **G** | **G** | **A** | **A** | **C** | **G** | **T** | **C** | **T** | **C** | **T** | **T** | **C** | **C** | **C** | **C** | **C** | **G** | **A** | **G** | **A** | **A** | **A** | **A** | **A** | **T** | **C** | **G** | **T** | **G** | **T** | **G** | **G** | **C** | **G** | **T** | **A** | **T** | **C** | **A** | **A** | **T** | **T** | **G** | **G** | **A** | **T** | **T** | **A** | **T** | **T** | **T** | **C** | **T** | **T** | **T** | **A** | **A** | **A** | **C** | **T** | **T** | **G** | **A** | **A** | **G** | **C** | **A** | **T** | **T** | **G** | **G** | **T** | **G** | **C** | **C** | **A** | **G** | **A** | **G** | **G** | **C** | **A** | **A** | **A** | **T** | **A** | **C** | **A** | **T** | **C** | **C** | **A** | **T** | **T** | **C** | **A** | **G** | **A** | **T** | **G** | **A** | **T** | **T** | **G** | **C** | **C** | **A** | **G** | **T** | **G** | **G** | **T** | **C** | **C** | **T** | **A** | **A** | **C** | **A** | **G** | **T** | **G** | **A** | **C** | **C** | **G** | **T** | **G** | **A** | **A** | **C** | **A** | **A** | **A** | **T** | **T** | **A** | **A** | **A** | **T** | **T** | **G** | **G** | **C** | **C** | **T** | **T** | **T** | **A** | **T** | **T** | **A** | **A** | **A** | **T** | **T** | **G** | **A** | **T** | **T** | **A** | **C** | **T** | **T** | **C** | **T** | **G** | **C** | **C** | **A** | **A** | **G** | **A** | **A** | **A** | **A** | **G** | **A** | **G** | **T** | **C** | **T** | **G** | **G** | **A** | **T** | **T** | **C** | **A** | **A** | **A** | **C** | **G** | **C** | **C** | **G** | **T** | **A** | **T** | **T** | **T** | **A** | **G** | **T** | **T** | **C** | **C** | **T** | **G** | **A** | **T** | **G** | **A** | **C** | **A** | **G** | **T** | **G** | **T** | **C** | **T** | **T** | **G** | **G** | **C** | **T** | **G** | **C** | **T** | **T** | **T** | **A** | **A** | **A** | **G** | **G** | **T** | **T** | **G** | **C** | **T** | **G** | **C** | **G** | **G** | **C** | **T** | **T** | **C** | **A** | **G** | **G** | **A** | **G** | **T** | **A** | **G** | **A** | **T** | **G** | **T** | **T** | **A** | **A** | **A** | **A** | **T** | **T** | **A** | **T** | **G** | **A** | **T** | **T** | **C** | **C** | **A** | **G** | **A** | **T** | **A** | **A** | **G** | **C** | **C** | **C** | **G** | **A** | **C** | **C** | **A** | **T** | **C** | **C** | **G** | **T** | **T** | **T** | **A** | **T** | **T** | **T** | **A** | **T** | **C** | **G** | **A** | **G** | **C** | **A** | **A** | **C** | **A** | **C** | **A** | **G** | **T** | **A** | **T** | **T** | **A** | **C** | **G** | **G** | **C** | **C** | **G** | **C** | **T** | **T** | **A** | **T** | **T** | **G** | **A** | **T** | **G** | **A** | **A** | **A** | **G** | **A** | **A** | **A** | **A** | **T** | **A** | **T** | **T** | **G** | **A** | **A** | **A** | **T** | **T** | **T** | **T** | **A** | **A** | **T** | **T** | **T** | **A** | **T** | **A** | **A** | **C** | **G** | **G** | **T** | **G** | **G** | **T** | **T** | **T** | **C** | **T** | **T** | **A** | **C** | **A** | **T** | **G** | **C** | **G** | **A** | **A** | **A** | **A** | **C** | **A** | **A** | **T** | **G** | **A** | **T** | **T** | **A** | **T** | **G** | **G** | **A** | **T** | **G** | **A** | **T** | **G** | **A** | **A** | **G** | **T** | **C** | **T** | **G** | **C** | **A** | **C** | **A** | **G** | **T** | **T** | **G** | **G** | **T** | **T** | **C** | **A** | **G** | **C** | **C** | **A** | **A** | **T** | **C** | **A** | **A** | **G** | **A** | **T** | **A** | **T** | **C** | **C** | **G** | **A** | **A** | **G** | **T** | **T** | **A** | **C** | **A** | **A** | **A** | **T** | **T** | **A** | **A** | **A** | **C** | **T** | **T** | **T** | **G** | **A** | **A** | **G** | **C** | **A** | **A** | **A** | **T** | **G** | **C** | **T** | **G** | **T** | **G** | **T** | **T** | **A** | **T** | **A** | **T** | **G** | **A** | **T** | **A** | **A** | **A** | **A** | **A** | **A** | **A** | **T** | **C** | **A** | **T** | **T** | **G** | **A** | **T** | **C** | **A** | **A** | **T** | **T** | **A** | **G** | **A** | **A** | **G** | **C** | **A** | **A** | **T** | **T** | **T** | **T** | **C** | **T** | **T** | **A** | **G** | **A** | **A** | **G** | **A** | **T** | **C** | **G** | **A** | **A** | **A** | **A** | **A** | **A** | **A** | **T** | **G** | **T** | **A** | **C** | **A** | **A** | **C** | **A** | **A** | **T** | **G** | **A** | **C** | **T** | **C** | **C** | **A** | **G** | **A** | **A** | **G** | **T** | **T** | **G** | **T** | **T** | **C** | **G** | **C** | **G** | **A** | **C** | **A** | **T** | **G** | **T** | **C** | **A** | **A** | **A** | **A** | **T** | **G** | **G** | **T** | **T** | **G** | **A** | **T** | **T** | **T** | **T** | **T** | **A** | **A** | **A** | **C** | **A** | **A** | **C** | **A** | **A** | **A** | **T** | **T** | **T** | **C** | **A** | **C** | **G** | **A** | **T** | **T** | **A** | **T** | **T** | **T** | **T** | **C** | **A** | **C** | **C** | **A** | **A** | **T** | **T** | **C** | **T** | **T** | **-** | **-** | **-** |
| **N59544F** | **T** | **T** | **G** | **A** | **T** | **T** | **C** | **T** | **T** | **A** | **G** | **C** | **G** | **T** | **C** | **T** | **T** | **A** | **A** | **C** | **A** | **G** | **T** | **T** | **A** | **T** | **T** | **T** | **A** | **T** | **T** | **T** | **T** | **A** | **T** | **T** | **A** | **A** | **T** | **G** | **C** | **A** | **A** | **T** | **T** | **A** | **T** | **T** | **G** | **C** | **G** | **G** | **G** | **A** | **A** | **T** | **T** | **A** | **C** | **C** | **A** | **T** | **T** | **T** | **T** | **G** | **T** | **T** | **A** | **A** | **A** | **A** | **C** | **C** | **T** | **C** | **G** | **A** | **G** | **A** | **T** | **G** | **T** | **A** | **G** | **C** | **A** | **G** | **C** | **A** | **A** | **T** | **T** | **T** | **G** | **G** | **G** | **C** | **A** | **T** | **G** | **G** | **C** | **T** | **A** | **C** | **T** | **C** | **G** | **T** | **A** | **C** | **T** | **G** | **A** | **T** | **T** | **G** | **C** | **T** | **C** | **T** | **A** | **C** | **C** | **C** | **G** | **T** | **T** | **T** | **T** | **T** | **G** | **G** | **T** | **T** | **T** | **T** | **T** | **T** | **C** | **T** | **T** | **A** | **T** | **A** | **T** | **T** | **T** | **A** | **T** | **T** | **C** | **T** | **T** | **T** | **G** | **G** | **T** | **C** | **G** | **A** | **G** | **G** | **T** | **T** | **T** | **A** | **A** | **C** | **C** | **G** | **A** | **C** | **A** | **A** | **A** | **A** | **A** | **G** | **A** | **A** | **A** | **T** | **T** | **T** | **T** | **A** | **T** | **T** | **T** | **G** | **C** | **A** | **A** | **C** | **A** | **A** | **A** | **G** | **T** | **G** | **A** | **T** | **T** | **T** | **A** | **C** | **G** | **G** | **G** | **A** | **A** | **T** | **T** | **A** | **G** | **A** | **G** | **A** | **A** | **T** | **T** | **T** | **C** | **C** | **A** | **G** | **A** | **A** | **T** | **T** | **T** | **C** | **C** | **A** | **A** | **G** | **A** | **A** | **G** | **A** | **A** | **A** | **G** | **T** | **T** | **T** | **T** | **G** | **A** | **A** | **C** | **T** | **T** | **T** | **A** | **T** | **A** | **G** | **T** | **C** | **A** | **A** | **A** | **A** | **G** | **A** | **T** | **G** | **C** | **C** | **G** | **A** | **C** | **G** | **G** | **A** | **G** | **G** | **A** | **G** | **C** | **A** | **A** | **C** | **A** | **A** | **C** | **A** | **G** | **T** | **T** | **T** | **A** | **C** | **A** | **G** | **A** | **C** | **T** | **T** | **T** | **T** | **T** | **T** | **T** | **C** | **C** | **T** | **C** | **G** | **T** | **T** | **A** | **A** | **A** | **T** | **A** | **G** | **G** | **A** | **T** | **G** | **C** | **C** | **T** | **C** | **T** | **G** | **A** | **C** | **T** | **A** | **A** | **G** | **A** | **A** | **A** | **A** | **A** | **T** | **G** | **A** | **T** | **G** | **T** | **T** | **G** | **A** | **A** | **A** | **T** | **T** | **T** | **T** | **T** | **A** | **C** | **G** | **G** | **A** | **T** | **G** | **G** | **T** | **A** | **C** | **G** | **G** | **A** | **A** | **A** | **A** | **A** | **T** | **T** | **C** | **A** | **A** | **T** | **G** | **C** | **G** | **T** | **T** | **G** | **A** | **T** | **G** | **G** | **C** | **G** | **G** | **A** | **T** | **A** | **T** | **A** | **A** | **A** | **A** | **A** | **A** | **A** | **G** | **C** | **T** | **C** | **A** | **G** | **C** | **A** | **C** | **T** | **C** | **C** | **A** | **T** | **T** | **C** | **A** | **T** | **A** | **T** | **T** | **G** | **A** | **A** | **T** | **A** | **T** | **T** | **A** | **T** | **G** | **C** | **C** | **T** | **T** | **T** | **G** | **T** | **A** | **A** | **C** | **G** | **G** | **A** | **T** | **C** | **A** | **T** | **A** | **T** | **C** | **G** | **G** | **C** | **A** | **C** | **C** | **A** | **A** | **A** | **A** | **T** | **T** | **T** | **T** | **G** | **A** | **A** | **C** | **T** | **T** | **A** | **T** | **T** | **A** | **G** | **A** | **A** | **G** | **A** | **A** | **A** | **A** | **G** | **G** | **C** | **G** | **G** | **C** | **T** | **G** | **A** | **A** | **G** | **G** | **G** | **G** | **T** | **T** | **G** | **A** | **A** | **G** | **T** | **G** | **C** | **G** | **G** | **T** | **T** | **G** | **C** | **T** | **T** | **T** | **A** | **C** | **G** | **A** | **T** | **G** | **C** | **C** | **T** | **T** | **T** | **G** | **G** | **C** | **T** | **C** | **A** | **A** | **A** | **G** | **G** | **G** | **C** | **A** | **C** | **C** | **A** | **A** | **G** | **G** | **T** | **T** | **C** | **A** | **T** | **C** | **A** | **T** | **T** | **T** | **G** | **A** | **A** | **T** | **G** | **A** | **A** | **T** | **T** | **G** | **A** | **A** | **A** | **A** | **A** | **A** | **A** | **A** | **C** | **G** | **G** | **T** | **G** | **G** | **T** | **T** | **T** | **T** | **G** | **T** | **C** | **C** | **A** | **A** | **A** | **C** | **G** | **T** | **T** | **T** | **A** | **T** | **T** | **A** | **C** | **T** | **T** | **C** | **T** | **C** | **A** | **A** | **A** | **A** | **A** | **G** | **C** | **A** | **C** | **T** | **T** | **T** | **T** | **G** | **A** | **A** | **G** | **T** | **T** | **T** | **C** | **G** | **T** | **T** | **T** | **G** | **A** | **A** | **T** | **T** | **A** | **T** | **C** | **A** | **T** | **G** | **A** | **T** | **C** | **A** | **C** | **C** | **G** | **G** | **A** | **A** | **A** | **A** | **T** | **T** | **G** | **T** | **T** | **G** | **T** | **T** | **A** | **T** | **T** | **G** | **A** | **C** | **G** | **G** | **A** | **A** | **A** | **A** | **G** | **T** | **A** | **G** | **G** | **C** | **T** | **A** | **C** | **A** | **T** | **T** | **G** | **G** | **C** | **G** | **G** | **C** | **T** | **T** | **T** | **A** | **A** | **T** | **G** | **T** | **T** | **G** | **C** | **C** | **G** | **A** | **T** | **C** | **A** | **A** | **T** | **A** | **T** | **G** | **C** | **C** | **G** | **G** | **A** | **A** | **C** | **G** | **A** | **C** | **T** | **A** | **A** | **A** | **A** | **A** | **G** | **T** | **T** | **T** | **G** | **G** | **C** | **T** | **A** | **T** | **T** | **G** | **G** | **C** | **G** | **G** | **G** | **A** | **T** | **A** | **C** | **A** | **C** | **A** | **T** | **T** | **T** | **A** | **C** | **G** | **G** | **A** | **T** | **T** | **C** | **A** | **A** | **G** | **G** | **G** | **C** | **C** | **A** | **G** | **C** | **A** | **G** | **C** | **C** | **T** | **C** | **A** | **T** | **T** | **A** | **C** | **T** | **G** | **C** | **A** | **A** | **A** | **T** | **G** | **C** | **G** | **T** | **T** | **T** | **T** | **T** | **T** | **A** | **A** | **T** | **G** | **G** | **A** | **T** | **T** | **G** | **G** | **A** | **A** | **C** | **G** | **T** | **C** | **T** | **C** | **T** | **T** | **C** | **C** | **C** | **C** | **C** | **G** | **A** | **G** | **A** | **A** | **A** | **A** | **A** | **T** | **C** | **G** | **T** | **G** | **T** | **G** | **G** | **C** | **G** | **T** | **A** | **T** | **C** | **A** | **A** | **T** | **T** | **G** | **G** | **A** | **T** | **T** | **A** | **T** | **T** | **T** | **C** | **T** | **T** | **T** | **A** | **A** | **A** | **C** | **T** | **T** | **G** | **A** | **A** | **G** | **C** | **A** | **T** | **T** | **G** | **G** | **T** | **G** | **C** | **C** | **A** | **G** | **A** | **G** | **G** | **C** | **A** | **A** | **A** | **T** | **A** | **C** | **A** | **T** | **C** | **C** | **A** | **T** | **T** | **C** | **A** | **G** | **A** | **T** | **G** | **A** | **T** | **T** | **G** | **C** | **C** | **A** | **G** | **T** | **G** | **G** | **T** | **C** | **C** | **T** | **A** | **A** | **C** | **A** | **G** | **T** | **G** | **A** | **C** | **C** | **G** | **T** | **G** | **A** | **A** | **C** | **A** | **A** | **A** | **T** | **T** | **A** | **A** | **A** | **T** | **T** | **G** | **G** | **C** | **C** | **T** | **T** | **T** | **A** | **T** | **T** | **A** | **A** | **A** | **T** | **T** | **G** | **A** | **T** | **T** | **A** | **C** | **T** | **T** | **C** | **T** | **G** | **C** | **C** | **A** | **A** | **G** | **A** | **A** | **A** | **A** | **G** | **A** | **G** | **T** | **C** | **T** | **G** | **G** | **A** | **T** | **T** | **C** | **A** | **A** | **A** | **C** | **G** | **C** | **C** | **G** | **T** | **A** | **T** | **T** | **T** | **A** | **G** | **T** | **T** | **C** | **C** | **T** | **G** | **A** | **T** | **G** | **A** | **T** | **A** | **G** | **T** | **G** | **T** | **C** | **T** | **T** | **G** | **G** | **C** | **T** | **G** | **C** | **T** | **T** | **T** | **A** | **A** | **A** | **G** | **G** | **T** | **T** | **G** | **C** | **T** | **G** | **C** | **G** | **G** | **C** | **T** | **T** | **C** | **A** | **G** | **G** | **A** | **G** | **T** | **A** | **G** | **A** | **T** | **G** | **T** | **T** | **A** | **A** | **A** | **A** | **T** | **T** | **A** | **T** | **G** | **A** | **T** | **T** | **C** | **C** | **A** | **G** | **A** | **T** | **A** | **A** | **G** | **C** | **C** | **C** | **G** | **A** | **C** | **C** | **A** | **T** | **C** | **C** | **G** | **T** | **T** | **T** | **A** | **T** | **T** | **T** | **A** | **T** | **C** | **G** | **A** | **G** | **C** | **A** | **A** | **C** | **A** | **C** | **A** | **G** | **T** | **A** | **T** | **T** | **A** | **C** | **G** | **G** | **C** | **C** | **G** | **C** | **T** | **T** | **A** | **T** | **T** | **G** | **A** | **T** | **G** | **A** | **A** | **A** | **G** | **A** | **A** | **A** | **A** | **T** | **A** | **T** | **T** | **G** | **A** | **A** | **A** | **T** | **T** | **T** | **T** | **A** | **A** | **T** | **T** | **T** | **A** | **T** | **A** | **A** | **C** | **G** | **G** | **T** | **G** | **G** | **T** | **T** | **T** | **C** | **T** | **T** | **A** | **C** | **A** | **T** | **G** | **C** | **G** | **A** | **A** | **A** | **A** | **C** | **A** | **A** | **T** | **G** | **A** | **T** | **T** | **A** | **T** | **G** | **G** | **A** | **T** | **G** | **A** | **T** | **G** | **A** | **A** | **G** | **T** | **C** | **T** | **G** | **C** | **A** | **C** | **A** | **G** | **T** | **T** | **G** | **G** | **T** | **T** | **C** | **A** | **G** | **C** | **C** | **A** | **A** | **T** | **C** | **A** | **A** | **G** | **A** | **T** | **A** | **T** | **C** | **C** | **G** | **A** | **A** | **G** | **T** | **T** | **A** | **C** | **A** | **A** | **A** | **T** | **T** | **A** | **A** | **A** | **C** | **T** | **T** | **T** | **G** | **A** | **A** | **G** | **C** | **A** | **A** | **A** | **T** | **G** | **C** | **T** | **G** | **T** | **G** | **T** | **T** | **A** | **T** | **A** | **T** | **G** | **A** | **T** | **A** | **A** | **A** | **A** | **A** | **A** | **A** | **T** | **C** | **A** | **T** | **T** | **G** | **A** | **T** | **C** | **A** | **A** | **T** | **T** | **A** | **G** | **A** | **A** | **G** | **C** | **A** | **A** | **T** | **T** | **T** | **T** | **C** | **T** | **T** | **A** | **G** | **A** | **A** | **G** | **A** | **T** | **C** | **G** | **A** | **A** | **A** | **A** | **A** | **A** | **A** | **T** | **G** | **T** | **A** | **C** | **A** | **A** | **C** | **A** | **A** | **T** | **G** | **A** | **C** | **T** | **C** | **C** | **A** | **G** | **A** | **A** | **G** | **T** | **T** | **G** | **T** | **T** | **C** | **G** | **C** | **G** | **A** | **C** | **A** | **T** | **G** | **T** | **C** | **A** | **A** | **A** | **A** | **T** | **G** | **G** | **T** | **T** | **G** | **A** | **T** | **T** | **T** | **T** | **T** | **A** | **A** | **A** | **C** | **A** | **A** | **C** | **A** | **A** | **A** | **T** | **T** | **T** | **C** | **A** | **C** | **G** | **A** | **T** | **T** | **A** | **T** | **T** | **T** | **T** | **C** | **A** | **C** | **C** | **A** | **A** | **T** | **T** | **C** | **T** | **T** | **-** | **-** | **-** |
| **N59689F** | **T** | **T** | **G** | **A** | **T** | **T** | **C** | **T** | **T** | **A** | **G** | **C** | **G** | **T** | **C** | **T** | **T** | **A** | **A** | **C** | **A** | **G** | **T** | **T** | **A** | **T** | **T** | **T** | **A** | **T** | **T** | **T** | **T** | **A** | **T** | **T** | **A** | **A** | **T** | **G** | **C** | **A** | **A** | **T** | **T** | **A** | **T** | **T** | **G** | **C** | **G** | **G** | **G** | **A** | **A** | **T** | **T** | **A** | **C** | **C** | **A** | **T** | **T** | **T** | **T** | **G** | **T** | **T** | **A** | **A** | **A** | **A** | **C** | **C** | **T** | **C** | **G** | **A** | **G** | **A** | **T** | **G** | **T** | **A** | **G** | **C** | **A** | **G** | **C** | **A** | **A** | **T** | **T** | **T** | **G** | **G** | **G** | **C** | **A** | **T** | **G** | **G** | **C** | **T** | **A** | **C** | **T** | **C** | **G** | **T** | **A** | **C** | **T** | **G** | **A** | **T** | **T** | **G** | **C** | **T** | **C** | **T** | **A** | **C** | **C** | **C** | **G** | **T** | **T** | **T** | **T** | **T** | **G** | **G** | **T** | **T** | **T** | **T** | **T** | **T** | **C** | **T** | **T** | **A** | **T** | **A** | **T** | **T** | **T** | **A** | **T** | **T** | **C** | **T** | **T** | **T** | **G** | **G** | **T** | **C** | **G** | **A** | **G** | **G** | **T** | **T** | **T** | **A** | **A** | **C** | **C** | **G** | **A** | **C** | **A** | **A** | **A** | **A** | **A** | **G** | **A** | **A** | **A** | **T** | **T** | **T** | **T** | **A** | **T** | **T** | **T** | **G** | **C** | **A** | **A** | **C** | **A** | **A** | **A** | **G** | **T** | **G** | **A** | **T** | **T** | **T** | **A** | **C** | **G** | **G** | **G** | **A** | **A** | **T** | **T** | **A** | **G** | **A** | **G** | **A** | **A** | **T** | **T** | **T** | **C** | **C** | **A** | **G** | **A** | **A** | **T** | **T** | **T** | **C** | **C** | **A** | **A** | **G** | **A** | **A** | **G** | **A** | **A** | **A** | **G** | **T** | **T** | **T** | **T** | **G** | **A** | **A** | **C** | **T** | **T** | **T** | **A** | **T** | **A** | **G** | **T** | **C** | **A** | **A** | **A** | **A** | **G** | **A** | **T** | **G** | **C** | **C** | **G** | **A** | **C** | **G** | **G** | **A** | **G** | **G** | **A** | **G** | **C** | **A** | **A** | **C** | **A** | **A** | **C** | **A** | **G** | **T** | **T** | **T** | **A** | **C** | **A** | **G** | **A** | **C** | **T** | **T** | **T** | **T** | **T** | **T** | **T** | **C** | **C** | **T** | **C** | **G** | **T** | **T** | **A** | **A** | **A** | **T** | **A** | **G** | **G** | **A** | **T** | **G** | **C** | **C** | **T** | **C** | **T** | **G** | **A** | **C** | **T** | **A** | **A** | **G** | **A** | **A** | **A** | **A** | **A** | **T** | **G** | **A** | **T** | **G** | **T** | **T** | **G** | **A** | **A** | **A** | **T** | **T** | **T** | **T** | **T** | **A** | **C** | **G** | **G** | **A** | **T** | **G** | **G** | **T** | **A** | **C** | **G** | **G** | **A** | **A** | **A** | **A** | **A** | **T** | **T** | **C** | **A** | **A** | **T** | **G** | **C** | **G** | **T** | **T** | **G** | **A** | **T** | **G** | **G** | **C** | **G** | **G** | **A** | **C** | **A** | **T** | **A** | **A** | **A** | **A** | **A** | **A** | **A** | **G** | **C** | **T** | **C** | **A** | **G** | **C** | **A** | **C** | **T** | **C** | **C** | **A** | **T** | **T** | **C** | **A** | **T** | **A** | **T** | **T** | **G** | **A** | **A** | **T** | **A** | **T** | **T** | **A** | **T** | **G** | **C** | **C** | **T** | **T** | **T** | **G** | **T** | **A** | **A** | **C** | **G** | **G** | **A** | **T** | **C** | **A** | **T** | **A** | **T** | **C** | **G** | **G** | **T** | **A** | **C** | **C** | **A** | **A** | **A** | **A** | **T** | **T** | **T** | **T** | **G** | **A** | **A** | **C** | **T** | **T** | **A** | **T** | **T** | **A** | **G** | **A** | **A** | **G** | **A** | **A** | **A** | **A** | **G** | **G** | **C** | **G** | **G** | **C** | **T** | **G** | **A** | **A** | **G** | **G** | **G** | **G** | **T** | **T** | **G** | **A** | **A** | **G** | **T** | **G** | **C** | **G** | **G** | **T** | **T** | **G** | **C** | **T** | **T** | **T** | **A** | **C** | **G** | **A** | **T** | **G** | **C** | **C** | **T** | **T** | **T** | **G** | **G** | **C** | **T** | **C** | **A** | **A** | **A** | **G** | **G** | **G** | **C** | **A** | **C** | **C** | **A** | **A** | **G** | **G** | **T** | **T** | **C** | **A** | **T** | **C** | **A** | **T** | **T** | **T** | **G** | **A** | **A** | **T** | **G** | **A** | **A** | **T** | **T** | **G** | **A** | **A** | **A** | **A** | **A** | **A** | **A** | **A** | **C** | **G** | **G** | **T** | **G** | **G** | **T** | **T** | **T** | **T** | **G** | **T** | **C** | **C** | **A** | **A** | **A** | **C** | **G** | **T** | **T** | **T** | **A** | **T** | **T** | **A** | **C** | **T** | **T** | **C** | **T** | **C** | **A** | **A** | **A** | **A** | **A** | **G** | **C** | **A** | **C** | **T** | **T** | **T** | **T** | **G** | **A** | **A** | **G** | **T** | **T** | **T** | **C** | **G** | **T** | **T** | **T** | **G** | **A** | **A** | **T** | **T** | **A** | **T** | **C** | **A** | **T** | **G** | **A** | **T** | **C** | **A** | **C** | **C** | **G** | **G** | **A** | **A** | **A** | **A** | **T** | **T** | **G** | **T** | **T** | **G** | **T** | **T** | **A** | **T** | **T** | **G** | **A** | **C** | **G** | **G** | **A** | **A** | **A** | **A** | **G** | **T** | **A** | **G** | **G** | **C** | **T** | **A** | **C** | **A** | **T** | **T** | **G** | **G** | **C** | **G** | **G** | **C** | **T** | **T** | **T** | **A** | **A** | **T** | **G** | **T** | **T** | **G** | **C** | **C** | **G** | **A** | **T** | **C** | **A** | **A** | **T** | **A** | **T** | **G** | **C** | **C** | **G** | **G** | **A** | **A** | **C** | **G** | **A** | **C** | **T** | **A** | **A** | **A** | **A** | **A** | **G** | **T** | **T** | **T** | **G** | **G** | **C** | **T** | **A** | **T** | **T** | **G** | **G** | **C** | **G** | **G** | **G** | **A** | **T** | **A** | **C** | **A** | **C** | **A** | **T** | **T** | **T** | **A** | **C** | **G** | **G** | **A** | **T** | **T** | **C** | **A** | **A** | **G** | **G** | **G** | **C** | **C** | **A** | **G** | **C** | **A** | **G** | **C** | **C** | **T** | **C** | **A** | **T** | **T** | **A** | **C** | **T** | **G** | **C** | **A** | **A** | **A** | **T** | **G** | **C** | **G** | **T** | **T** | **T** | **T** | **T** | **T** | **A** | **A** | **T** | **G** | **G** | **A** | **T** | **T** | **G** | **G** | **A** | **A** | **C** | **G** | **T** | **C** | **T** | **C** | **T** | **T** | **C** | **C** | **C** | **C** | **C** | **G** | **A** | **G** | **A** | **A** | **A** | **A** | **A** | **T** | **C** | **G** | **T** | **G** | **T** | **G** | **G** | **C** | **G** | **T** | **A** | **T** | **C** | **A** | **A** | **T** | **T** | **G** | **G** | **A** | **T** | **T** | **A** | **T** | **T** | **T** | **C** | **T** | **T** | **T** | **A** | **A** | **A** | **C** | **T** | **T** | **G** | **A** | **A** | **G** | **C** | **A** | **T** | **T** | **G** | **G** | **T** | **G** | **C** | **C** | **A** | **G** | **A** | **G** | **G** | **C** | **A** | **A** | **A** | **T** | **A** | **C** | **A** | **T** | **C** | **C** | **A** | **T** | **T** | **C** | **A** | **G** | **A** | **T** | **G** | **A** | **T** | **T** | **G** | **C** | **C** | **A** | **G** | **T** | **G** | **G** | **T** | **C** | **C** | **T** | **A** | **A** | **C** | **A** | **G** | **T** | **G** | **A** | **C** | **C** | **G** | **T** | **G** | **A** | **A** | **C** | **A** | **A** | **A** | **T** | **T** | **A** | **A** | **A** | **T** | **T** | **G** | **G** | **C** | **C** | **T** | **T** | **T** | **A** | **T** | **T** | **A** | **A** | **A** | **T** | **T** | **G** | **A** | **T** | **T** | **A** | **C** | **T** | **T** | **C** | **T** | **G** | **C** | **C** | **A** | **A** | **G** | **A** | **A** | **A** | **A** | **G** | **A** | **G** | **T** | **C** | **T** | **G** | **G** | **A** | **T** | **T** | **C** | **A** | **A** | **A** | **C** | **G** | **C** | **C** | **G** | **T** | **A** | **T** | **T** | **T** | **A** | **G** | **T** | **T** | **C** | **C** | **T** | **G** | **A** | **T** | **G** | **A** | **T** | **A** | **G** | **T** | **G** | **T** | **C** | **T** | **T** | **G** | **G** | **C** | **T** | **G** | **C** | **T** | **T** | **T** | **A** | **A** | **A** | **G** | **G** | **T** | **T** | **G** | **C** | **T** | **G** | **C** | **G** | **G** | **C** | **T** | **T** | **C** | **A** | **G** | **G** | **A** | **G** | **T** | **A** | **G** | **A** | **T** | **G** | **T** | **T** | **A** | **A** | **A** | **A** | **T** | **T** | **A** | **T** | **G** | **A** | **T** | **T** | **C** | **C** | **A** | **G** | **A** | **T** | **A** | **A** | **G** | **C** | **C** | **C** | **G** | **A** | **C** | **C** | **A** | **T** | **C** | **C** | **G** | **T** | **T** | **T** | **A** | **T** | **T** | **T** | **A** | **T** | **C** | **G** | **A** | **G** | **C** | **A** | **A** | **C** | **A** | **C** | **A** | **G** | **T** | **A** | **T** | **T** | **A** | **C** | **G** | **G** | **C** | **C** | **G** | **C** | **T** | **T** | **A** | **T** | **T** | **G** | **A** | **T** | **G** | **A** | **A** | **A** | **G** | **A** | **A** | **A** | **A** | **T** | **A** | **T** | **T** | **G** | **A** | **A** | **A** | **T** | **T** | **T** | **T** | **A** | **A** | **T** | **T** | **T** | **A** | **T** | **A** | **A** | **C** | **G** | **G** | **T** | **G** | **G** | **T** | **T** | **T** | **C** | **T** | **T** | **A** | **C** | **A** | **T** | **G** | **C** | **G** | **A** | **A** | **A** | **A** | **C** | **A** | **A** | **T** | **G** | **A** | **T** | **T** | **A** | **T** | **G** | **G** | **A** | **T** | **G** | **A** | **T** | **G** | **A** | **A** | **G** | **T** | **C** | **T** | **G** | **C** | **A** | **C** | **A** | **G** | **T** | **T** | **G** | **G** | **T** | **T** | **C** | **A** | **G** | **C** | **C** | **A** | **A** | **T** | **C** | **A** | **A** | **G** | **A** | **T** | **A** | **T** | **C** | **C** | **G** | **A** | **A** | **G** | **T** | **T** | **A** | **C** | **A** | **A** | **A** | **T** | **T** | **A** | **A** | **A** | **C** | **T** | **T** | **T** | **G** | **A** | **A** | **G** | **C** | **A** | **A** | **A** | **T** | **G** | **C** | **T** | **G** | **T** | **G** | **T** | **T** | **A** | **T** | **A** | **T** | **G** | **A** | **T** | **A** | **A** | **A** | **A** | **A** | **A** | **A** | **T** | **C** | **A** | **T** | **T** | **G** | **A** | **T** | **C** | **A** | **A** | **T** | **T** | **A** | **G** | **A** | **A** | **G** | **C** | **A** | **A** | **T** | **T** | **T** | **T** | **C** | **T** | **T** | **A** | **G** | **A** | **A** | **G** | **A** | **T** | **C** | **G** | **A** | **A** | **A** | **A** | **A** | **A** | **A** | **T** | **G** | **T** | **A** | **C** | **A** | **A** | **C** | **A** | **A** | **T** | **G** | **A** | **C** | **T** | **C** | **C** | **A** | **G** | **A** | **A** | **G** | **T** | **T** | **G** | **T** | **T** | **C** | **G** | **C** | **G** | **A** | **C** | **A** | **T** | **G** | **T** | **C** | **A** | **A** | **A** | **A** | **T** | **G** | **G** | **T** | **T** | **G** | **A** | **T** | **T** | **T** | **T** | **T** | **A** | **A** | **A** | **C** | **A** | **A** | **C** | **A** | **A** | **A** | **T** | **T** | **T** | **C** | **A** | **C** | **G** | **A** | **T** | **T** | **A** | **T** | **T** | **T** | **T** | **C** | **A** | **C** | **C** | **A** | **A** | **T** | **T** | **C** | **T** | **T** | **-** | **-** | **-** |
| **N52454** | **T** | **T** | **G** | **A** | **T** | **T** | **C** | **T** | **T** | **A** | **G** | **C** | **G** | **T** | **C** | **T** | **T** | **A** | **A** | **C** | **A** | **G** | **T** | **T** | **A** | **T** | **T** | **T** | **A** | **T** | **T** | **T** | **T** | **A** | **T** | **T** | **A** | **A** | **T** | **G** | **C** | **A** | **A** | **T** | **T** | **A** | **T** | **T** | **G** | **C** | **G** | **G** | **G** | **A** | **A** | **T** | **T** | **A** | **C** | **C** | **A** | **T** | **T** | **T** | **T** | **G** | **T** | **T** | **A** | **A** | **A** | **A** | **C** | **C** | **T** | **C** | **G** | **A** | **G** | **A** | **T** | **G** | **T** | **A** | **G** | **C** | **A** | **G** | **C** | **A** | **A** | **T** | **T** | **T** | **G** | **G** | **G** | **C** | **A** | **T** | **G** | **G** | **C** | **T** | **A** | **C** | **T** | **C** | **G** | **T** | **A** | **C** | **T** | **G** | **A** | **T** | **T** | **G** | **C** | **T** | **C** | **T** | **A** | **C** | **C** | **C** | **G** | **T** | **T** | **T** | **T** | **T** | **G** | **G** | **T** | **T** | **T** | **T** | **T** | **T** | **C** | **T** | **T** | **A** | **T** | **A** | **T** | **T** | **T** | **A** | **T** | **T** | **C** | **T** | **T** | **T** | **G** | **G** | **G** | **C** | **G** | **C** | **G** | **G** | **T** | **T** | **T** | **A** | **A** | **C** | **C** | **G** | **A** | **C** | **A** | **A** | **A** | **A** | **A** | **G** | **A** | **A** | **A** | **T** | **T** | **T** | **T** | **A** | **T** | **T** | **T** | **G** | **C** | **A** | **A** | **C** | **A** | **A** | **A** | **G** | **T** | **G** | **A** | **T** | **T** | **T** | **A** | **C** | **G** | **G** | **G** | **A** | **A** | **T** | **T** | **A** | **G** | **A** | **G** | **A** | **A** | **T** | **T** | **T** | **C** | **C** | **A** | **G** | **A** | **A** | **T** | **T** | **T** | **C** | **C** | **A** | **A** | **G** | **A** | **A** | **G** | **A** | **A** | **A** | **G** | **T** | **T** | **T** | **T** | **G** | **A** | **A** | **C** | **T** | **T** | **T** | **A** | **T** | **A** | **G** | **T** | **C** | **A** | **A** | **A** | **A** | **G** | **A** | **T** | **G** | **C** | **C** | **G** | **A** | **C** | **G** | **G** | **A** | **G** | **G** | **A** | **G** | **C** | **A** | **A** | **C** | **A** | **A** | **C** | **A** | **G** | **T** | **T** | **T** | **A** | **C** | **A** | **G** | **A** | **C** | **T** | **T** | **T** | **T** | **T** | **T** | **T** | **C** | **C** | **T** | **C** | **G** | **T** | **T** | **A** | **A** | **A** | **T** | **A** | **G** | **G** | **A** | **T** | **G** | **C** | **C** | **T** | **C** | **T** | **G** | **A** | **C** | **T** | **A** | **A** | **G** | **A** | **A** | **A** | **A** | **A** | **T** | **G** | **A** | **T** | **G** | **T** | **T** | **G** | **A** | **A** | **A** | **T** | **T** | **T** | **T** | **T** | **A** | **C** | **G** | **G** | **A** | **T** | **G** | **G** | **T** | **A** | **C** | **G** | **G** | **A** | **A** | **A** | **A** | **A** | **T** | **T** | **C** | **A** | **A** | **T** | **G** | **C** | **G** | **T** | **T** | **G** | **A** | **T** | **G** | **G** | **C** | **G** | **G** | **A** | **T** | **A** | **T** | **A** | **A** | **A** | **A** | **A** | **A** | **A** | **G** | **C** | **T** | **C** | **A** | **G** | **C** | **A** | **C** | **T** | **C** | **T** | **A** | **T** | **T** | **C** | **A** | **T** | **A** | **T** | **T** | **G** | **A** | **A** | **T** | **A** | **T** | **T** | **A** | **T** | **G** | **C** | **C** | **T** | **T** | **T** | **G** | **T** | **A** | **A** | **C** | **G** | **G** | **A** | **T** | **C** | **A** | **T** | **A** | **T** | **C** | **G** | **G** | **T** | **A** | **C** | **C** | **A** | **A** | **A** | **A** | **T** | **T** | **T** | **T** | **G** | **A** | **A** | **C** | **T** | **T** | **A** | **T** | **T** | **A** | **G** | **A** | **A** | **G** | **A** | **A** | **A** | **A** | **G** | **G** | **C** | **G** | **G** | **C** | **T** | **G** | **A** | **A** | **G** | **G** | **G** | **G** | **T** | **T** | **G** | **A** | **A** | **G** | **T** | **G** | **C** | **G** | **G** | **T** | **T** | **G** | **C** | **T** | **T** | **T** | **A** | **C** | **G** | **A** | **T** | **G** | **C** | **C** | **T** | **T** | **T** | **G** | **G** | **C** | **T** | **C** | **A** | **A** | **A** | **G** | **G** | **G** | **C** | **A** | **C** | **C** | **A** | **A** | **G** | **G** | **T** | **T** | **C** | **A** | **T** | **C** | **A** | **T** | **T** | **T** | **G** | **A** | **A** | **T** | **G** | **A** | **A** | **T** | **T** | **G** | **A** | **A** | **A** | **A** | **A** | **A** | **A** | **A** | **C** | **G** | **G** | **C** | **G** | **G** | **T** | **T** | **T** | **T** | **G** | **T** | **C** | **C** | **A** | **A** | **A** | **C** | **G** | **T** | **T** | **T** | **A** | **T** | **T** | **A** | **C** | **T** | **T** | **C** | **T** | **C** | **A** | **A** | **A** | **A** | **A** | **G** | **C** | **A** | **C** | **T** | **T** | **T** | **T** | **G** | **A** | **A** | **G** | **T** | **T** | **T** | **C** | **G** | **T** | **T** | **T** | **G** | **A** | **A** | **T** | **T** | **A** | **T** | **C** | **A** | **T** | **G** | **A** | **T** | **C** | **A** | **C** | **C** | **G** | **G** | **A** | **A** | **A** | **A** | **T** | **T** | **G** | **T** | **T** | **G** | **T** | **T** | **A** | **T** | **T** | **G** | **A** | **C** | **G** | **G** | **A** | **A** | **A** | **A** | **G** | **T** | **A** | **G** | **G** | **C** | **T** | **A** | **C** | **A** | **T** | **T** | **G** | **G** | **C** | **G** | **G** | **C** | **T** | **T** | **T** | **A** | **A** | **T** | **G** | **T** | **T** | **G** | **C** | **C** | **G** | **A** | **T** | **C** | **A** | **A** | **T** | **A** | **T** | **G** | **C** | **T** | **G** | **G** | **A** | **A** | **C** | **G** | **A** | **C** | **T** | **A** | **A** | **A** | **A** | **A** | **G** | **T** | **T** | **T** | **G** | **G** | **C** | **T** | **A** | **T** | **T** | **G** | **G** | **C** | **G** | **G** | **G** | **A** | **T** | **A** | **C** | **A** | **C** | **A** | **T** | **T** | **T** | **A** | **C** | **G** | **G** | **A** | **T** | **T** | **C** | **A** | **A** | **G** | **G** | **G** | **C** | **C** | **A** | **G** | **C** | **A** | **G** | **C** | **C** | **T** | **C** | **A** | **T** | **T** | **A** | **C** | **T** | **G** | **C** | **A** | **A** | **A** | **T** | **G** | **C** | **G** | **T** | **T** | **T** | **T** | **T** | **T** | **A** | **A** | **T** | **G** | **G** | **A** | **T** | **T** | **G** | **G** | **A** | **A** | **C** | **G** | **T** | **C** | **T** | **C** | **T** | **T** | **C** | **C** | **C** | **C** | **C** | **G** | **A** | **G** | **A** | **A** | **A** | **A** | **A** | **T** | **C** | **G** | **T** | **G** | **T** | **G** | **G** | **C** | **G** | **T** | **A** | **T** | **C** | **A** | **A** | **T** | **T** | **G** | **G** | **A** | **T** | **T** | **A** | **T** | **T** | **T** | **C** | **T** | **T** | **T** | **A** | **A** | **A** | **C** | **T** | **T** | **G** | **A** | **A** | **G** | **C** | **A** | **T** | **T** | **G** | **G** | **T** | **G** | **C** | **C** | **A** | **G** | **A** | **G** | **G** | **C** | **A** | **A** | **A** | **T** | **A** | **C** | **A** | **T** | **C** | **C** | **A** | **T** | **T** | **C** | **A** | **G** | **A** | **T** | **G** | **A** | **T** | **T** | **G** | **C** | **C** | **A** | **G** | **T** | **G** | **G** | **T** | **C** | **C** | **T** | **A** | **A** | **C** | **A** | **G** | **T** | **G** | **A** | **C** | **C** | **G** | **T** | **G** | **A** | **A** | **C** | **A** | **A** | **A** | **T** | **T** | **A** | **A** | **A** | **T** | **T** | **G** | **G** | **C** | **C** | **T** | **T** | **T** | **A** | **T** | **T** | **A** | **A** | **A** | **T** | **T** | **G** | **A** | **T** | **T** | **A** | **C** | **T** | **T** | **C** | **T** | **G** | **C** | **C** | **A** | **A** | **G** | **A** | **A** | **A** | **A** | **G** | **A** | **G** | **T** | **C** | **T** | **G** | **G** | **A** | **T** | **T** | **C** | **A** | **A** | **A** | **C** | **G** | **C** | **C** | **G** | **T** | **A** | **T** | **T** | **T** | **A** | **G** | **T** | **T** | **C** | **C** | **T** | **G** | **A** | **T** | **G** | **A** | **T** | **A** | **G** | **T** | **G** | **T** | **C** | **T** | **T** | **G** | **G** | **C** | **T** | **G** | **C** | **T** | **T** | **T** | **A** | **A** | **A** | **G** | **G** | **T** | **T** | **G** | **C** | **T** | **G** | **C** | **G** | **G** | **C** | **T** | **T** | **C** | **A** | **G** | **G** | **A** | **G** | **T** | **A** | **G** | **A** | **T** | **G** | **T** | **T** | **A** | **A** | **A** | **A** | **T** | **T** | **A** | **T** | **G** | **A** | **T** | **T** | **C** | **C** | **A** | **G** | **A** | **T** | **A** | **A** | **G** | **C** | **C** | **C** | **G** | **A** | **C** | **C** | **A** | **T** | **C** | **C** | **G** | **T** | **T** | **T** | **A** | **T** | **T** | **T** | **A** | **T** | **C** | **G** | **A** | **G** | **C** | **A** | **A** | **C** | **A** | **C** | **A** | **G** | **T** | **A** | **T** | **T** | **A** | **C** | **G** | **G** | **C** | **C** | **G** | **C** | **T** | **T** | **A** | **T** | **T** | **G** | **A** | **T** | **G** | **A** | **A** | **A** | **G** | **A** | **A** | **A** | **A** | **T** | **A** | **T** | **T** | **G** | **A** | **A** | **A** | **T** | **T** | **T** | **T** | **A** | **A** | **T** | **T** | **T** | **A** | **T** | **A** | **A** | **C** | **G** | **G** | **T** | **G** | **G** | **T** | **T** | **T** | **C** | **T** | **T** | **A** | **C** | **A** | **T** | **G** | **C** | **G** | **A** | **A** | **A** | **A** | **C** | **A** | **A** | **T** | **G** | **A** | **T** | **T** | **A** | **T** | **G** | **G** | **A** | **T** | **G** | **A** | **T** | **G** | **A** | **A** | **G** | **T** | **C** | **T** | **G** | **C** | **A** | **C** | **A** | **G** | **T** | **T** | **G** | **G** | **T** | **T** | **C** | **A** | **G** | **C** | **C** | **A** | **A** | **T** | **C** | **A** | **A** | **G** | **A** | **T** | **A** | **T** | **C** | **C** | **G** | **A** | **A** | **G** | **T** | **T** | **A** | **C** | **A** | **A** | **A** | **T** | **T** | **A** | **A** | **A** | **C** | **T** | **T** | **T** | **G** | **A** | **A** | **G** | **C** | **A** | **A** | **A** | **T** | **G** | **C** | **T** | **G** | **T** | **G** | **T** | **T** | **A** | **T** | **A** | **T** | **G** | **A** | **T** | **A** | **A** | **A** | **A** | **A** | **A** | **A** | **T** | **C** | **A** | **T** | **T** | **G** | **A** | **T** | **C** | **A** | **A** | **T** | **T** | **A** | **G** | **A** | **A** | **G** | **C** | **A** | **A** | **T** | **T** | **T** | **T** | **C** | **T** | **T** | **A** | **G** | **A** | **A** | **G** | **A** | **T** | **C** | **G** | **A** | **A** | **A** | **A** | **A** | **A** | **A** | **T** | **G** | **T** | **A** | **C** | **A** | **A** | **C** | **A** | **A** | **T** | **G** | **A** | **C** | **T** | **C** | **C** | **A** | **G** | **A** | **A** | **G** | **T** | **T** | **G** | **T** | **T** | **C** | **G** | **T** | **G** | **A** | **C** | **A** | **T** | **G** | **T** | **C** | **A** | **A** | **A** | **A** | **T** | **G** | **G** | **T** | **T** | **G** | **A** | **T** | **T** | **T** | **T** | **T** | **A** | **A** | **A** | **C** | **A** | **A** | **C** | **A** | **A** | **A** | **T** | **T** | **T** | **C** | **A** | **C** | **G** | **A** | **T** | **T** | **A** | **T** | **T** | **T** | **T** | **C** | **A** | **C** | **C** | **A** | **A** | **T** | **T** | **C** | **T** | **T** | **-** | **-** | **-** |
| **N59420F** | **T** | **T** | **G** | **A** | **T** | **T** | **C** | **T** | **T** | **A** | **G** | **C** | **G** | **T** | **C** | **T** | **T** | **A** | **A** | **C** | **A** | **G** | **T** | **T** | **A** | **T** | **T** | **T** | **A** | **T** | **T** | **T** | **T** | **A** | **T** | **T** | **A** | **A** | **T** | **G** | **C** | **A** | **A** | **T** | **T** | **A** | **T** | **T** | **G** | **C** | **G** | **G** | **G** | **A** | **A** | **T** | **T** | **A** | **C** | **C** | **A** | **T** | **T** | **T** | **T** | **G** | **T** | **T** | **A** | **A** | **A** | **A** | **C** | **C** | **T** | **C** | **G** | **A** | **G** | **A** | **T** | **G** | **T** | **A** | **G** | **C** | **A** | **G** | **C** | **A** | **A** | **T** | **T** | **T** | **G** | **G** | **G** | **C** | **A** | **T** | **G** | **G** | **C** | **T** | **A** | **C** | **T** | **C** | **G** | **T** | **A** | **C** | **T** | **G** | **A** | **T** | **T** | **G** | **C** | **T** | **C** | **T** | **A** | **C** | **C** | **C** | **G** | **T** | **T** | **T** | **T** | **T** | **G** | **G** | **T** | **T** | **T** | **T** | **T** | **T** | **C** | **T** | **T** | **A** | **T** | **A** | **T** | **T** | **T** | **A** | **T** | **T** | **C** | **T** | **T** | **T** | **G** | **G** | **G** | **C** | **G** | **C** | **G** | **G** | **T** | **T** | **T** | **A** | **A** | **C** | **C** | **G** | **A** | **C** | **A** | **A** | **A** | **A** | **A** | **G** | **A** | **A** | **A** | **T** | **T** | **T** | **T** | **A** | **T** | **T** | **T** | **G** | **C** | **A** | **A** | **C** | **A** | **A** | **A** | **G** | **T** | **G** | **A** | **T** | **T** | **T** | **A** | **C** | **G** | **G** | **G** | **A** | **A** | **T** | **T** | **A** | **G** | **A** | **G** | **A** | **A** | **T** | **T** | **T** | **C** | **C** | **A** | **G** | **A** | **A** | **T** | **T** | **T** | **C** | **C** | **A** | **A** | **G** | **A** | **A** | **G** | **A** | **A** | **A** | **G** | **T** | **T** | **T** | **T** | **G** | **A** | **A** | **C** | **T** | **T** | **T** | **A** | **T** | **A** | **G** | **T** | **C** | **A** | **A** | **A** | **A** | **G** | **A** | **T** | **G** | **C** | **C** | **G** | **A** | **C** | **G** | **G** | **A** | **G** | **G** | **A** | **G** | **C** | **A** | **A** | **C** | **A** | **A** | **C** | **A** | **G** | **T** | **T** | **T** | **A** | **C** | **A** | **G** | **A** | **C** | **T** | **T** | **T** | **T** | **T** | **T** | **T** | **C** | **C** | **T** | **C** | **G** | **T** | **T** | **A** | **A** | **A** | **T** | **A** | **G** | **G** | **A** | **T** | **G** | **C** | **C** | **C** | **C** | **T** | **G** | **A** | **C** | **T** | **A** | **A** | **G** | **A** | **A** | **A** | **A** | **A** | **T** | **G** | **A** | **T** | **G** | **T** | **T** | **G** | **A** | **A** | **A** | **T** | **T** | **T** | **T** | **T** | **A** | **C** | **G** | **G** | **A** | **T** | **G** | **G** | **T** | **A** | **C** | **G** | **G** | **A** | **A** | **A** | **A** | **A** | **T** | **T** | **C** | **A** | **A** | **T** | **G** | **C** | **G** | **T** | **T** | **G** | **A** | **T** | **G** | **G** | **C** | **A** | **G** | **A** | **C** | **A** | **T** | **A** | **A** | **A** | **A** | **A** | **A** | **A** | **G** | **C** | **T** | **C** | **A** | **G** | **C** | **A** | **C** | **T** | **C** | **T** | **A** | **T** | **T** | **C** | **A** | **T** | **A** | **T** | **T** | **G** | **A** | **A** | **T** | **A** | **T** | **T** | **A** | **T** | **G** | **C** | **C** | **T** | **T** | **T** | **G** | **T** | **A** | **A** | **C** | **G** | **G** | **A** | **T** | **C** | **A** | **T** | **A** | **T** | **C** | **G** | **G** | **T** | **A** | **C** | **C** | **A** | **A** | **A** | **A** | **T** | **T** | **T** | **T** | **G** | **A** | **A** | **C** | **T** | **T** | **A** | **T** | **T** | **A** | **G** | **A** | **A** | **G** | **A** | **A** | **A** | **A** | **G** | **G** | **C** | **G** | **G** | **C** | **T** | **G** | **A** | **A** | **G** | **G** | **G** | **G** | **T** | **T** | **G** | **A** | **A** | **G** | **T** | **G** | **C** | **G** | **G** | **T** | **T** | **A** | **C** | **T** | **T** | **T** | **A** | **C** | **G** | **A** | **T** | **G** | **C** | **C** | **T** | **T** | **T** | **G** | **G** | **C** | **T** | **C** | **A** | **A** | **A** | **G** | **G** | **G** | **C** | **A** | **C** | **C** | **A** | **A** | **G** | **G** | **T** | **T** | **C** | **A** | **T** | **C** | **A** | **T** | **T** | **T** | **G** | **A** | **A** | **T** | **G** | **A** | **A** | **T** | **T** | **G** | **A** | **A** | **A** | **A** | **A** | **A** | **A** | **A** | **C** | **G** | **G** | **C** | **G** | **G** | **T** | **T** | **T** | **T** | **G** | **T** | **C** | **C** | **A** | **A** | **A** | **C** | **G** | **T** | **T** | **T** | **A** | **T** | **T** | **A** | **C** | **T** | **T** | **C** | **T** | **C** | **A** | **A** | **A** | **A** | **A** | **G** | **C** | **A** | **C** | **T** | **T** | **T** | **T** | **G** | **A** | **A** | **G** | **T** | **T** | **T** | **C** | **G** | **T** | **T** | **T** | **G** | **A** | **A** | **T** | **T** | **A** | **T** | **C** | **A** | **T** | **G** | **A** | **T** | **C** | **A** | **C** | **C** | **G** | **G** | **A** | **A** | **A** | **A** | **T** | **T** | **G** | **T** | **T** | **G** | **T** | **T** | **A** | **T** | **T** | **G** | **A** | **C** | **G** | **G** | **A** | **A** | **A** | **A** | **G** | **T** | **A** | **G** | **G** | **C** | **T** | **A** | **C** | **A** | **T** | **T** | **G** | **G** | **C** | **G** | **G** | **C** | **T** | **T** | **T** | **A** | **A** | **T** | **G** | **T** | **T** | **G** | **C** | **C** | **G** | **A** | **T** | **C** | **A** | **A** | **T** | **A** | **T** | **G** | **C** | **C** | **G** | **G** | **A** | **A** | **C** | **G** | **A** | **C** | **T** | **A** | **A** | **A** | **A** | **A** | **G** | **T** | **T** | **T** | **G** | **G** | **C** | **T** | **A** | **T** | **T** | **G** | **G** | **C** | **G** | **G** | **G** | **A** | **T** | **A** | **C** | **A** | **C** | **A** | **T** | **T** | **T** | **A** | **C** | **G** | **G** | **A** | **T** | **T** | **C** | **A** | **A** | **G** | **G** | **G** | **C** | **C** | **A** | **G** | **C** | **A** | **G** | **C** | **C** | **T** | **C** | **A** | **T** | **T** | **A** | **C** | **T** | **G** | **C** | **A** | **A** | **A** | **T** | **G** | **C** | **G** | **T** | **T** | **T** | **T** | **T** | **T** | **A** | **A** | **T** | **G** | **G** | **A** | **T** | **T** | **G** | **G** | **A** | **A** | **C** | **G** | **T** | **C** | **T** | **C** | **T** | **T** | **C** | **C** | **C** | **C** | **C** | **G** | **A** | **G** | **A** | **A** | **A** | **A** | **A** | **T** | **C** | **G** | **T** | **G** | **T** | **G** | **G** | **C** | **G** | **T** | **A** | **T** | **C** | **A** | **A** | **T** | **T** | **G** | **G** | **A** | **T** | **T** | **A** | **T** | **T** | **T** | **C** | **T** | **T** | **T** | **A** | **A** | **A** | **C** | **T** | **T** | **G** | **A** | **A** | **G** | **C** | **A** | **T** | **T** | **G** | **G** | **T** | **G** | **C** | **C** | **A** | **G** | **A** | **G** | **G** | **C** | **A** | **A** | **A** | **T** | **A** | **C** | **A** | **T** | **C** | **C** | **A** | **T** | **T** | **C** | **A** | **G** | **A** | **T** | **G** | **A** | **T** | **T** | **G** | **C** | **C** | **A** | **G** | **T** | **G** | **G** | **T** | **C** | **C** | **T** | **A** | **A** | **C** | **A** | **G** | **T** | **G** | **A** | **C** | **C** | **G** | **T** | **G** | **A** | **A** | **C** | **A** | **A** | **A** | **T** | **T** | **A** | **A** | **A** | **T** | **T** | **G** | **G** | **C** | **C** | **T** | **T** | **T** | **A** | **T** | **T** | **A** | **A** | **A** | **T** | **T** | **G** | **A** | **T** | **T** | **A** | **C** | **T** | **T** | **C** | **T** | **G** | **C** | **C** | **A** | **A** | **G** | **A** | **A** | **A** | **A** | **G** | **A** | **G** | **T** | **C** | **T** | **G** | **G** | **A** | **T** | **T** | **C** | **A** | **A** | **A** | **C** | **G** | **C** | **C** | **G** | **T** | **A** | **T** | **T** | **T** | **A** | **G** | **T** | **T** | **C** | **C** | **T** | **G** | **A** | **T** | **G** | **A** | **C** | **A** | **G** | **T** | **G** | **T** | **C** | **T** | **T** | **G** | **G** | **C** | **T** | **G** | **C** | **T** | **T** | **T** | **A** | **A** | **A** | **G** | **G** | **T** | **T** | **G** | **C** | **T** | **G** | **C** | **G** | **G** | **C** | **T** | **T** | **C** | **A** | **G** | **G** | **A** | **G** | **T** | **A** | **G** | **A** | **T** | **G** | **T** | **T** | **A** | **A** | **A** | **A** | **T** | **T** | **A** | **T** | **G** | **A** | **T** | **T** | **C** | **C** | **A** | **G** | **A** | **T** | **A** | **A** | **G** | **C** | **C** | **C** | **G** | **A** | **C** | **C** | **A** | **T** | **C** | **C** | **G** | **T** | **T** | **T** | **A** | **T** | **T** | **T** | **A** | **T** | **C** | **G** | **A** | **G** | **C** | **A** | **A** | **C** | **A** | **C** | **A** | **G** | **T** | **A** | **T** | **T** | **A** | **C** | **G** | **G** | **C** | **C** | **G** | **C** | **T** | **T** | **A** | **T** | **T** | **G** | **A** | **T** | **G** | **A** | **A** | **A** | **G** | **A** | **A** | **A** | **A** | **T** | **A** | **T** | **T** | **G** | **A** | **A** | **A** | **T** | **T** | **T** | **T** | **A** | **A** | **T** | **T** | **T** | **A** | **T** | **A** | **A** | **C** | **G** | **G** | **T** | **G** | **G** | **T** | **T** | **T** | **C** | **T** | **T** | **A** | **C** | **A** | **T** | **G** | **C** | **G** | **A** | **A** | **A** | **A** | **C** | **A** | **A** | **T** | **G** | **A** | **T** | **T** | **A** | **T** | **G** | **G** | **A** | **T** | **G** | **A** | **T** | **G** | **A** | **A** | **G** | **T** | **C** | **T** | **G** | **C** | **A** | **C** | **A** | **G** | **T** | **T** | **G** | **G** | **T** | **T** | **C** | **A** | **G** | **C** | **C** | **A** | **A** | **T** | **C** | **A** | **A** | **G** | **A** | **T** | **A** | **T** | **C** | **C** | **G** | **A** | **A** | **G** | **T** | **T** | **A** | **C** | **A** | **A** | **A** | **T** | **T** | **A** | **A** | **A** | **C** | **T** | **T** | **T** | **G** | **A** | **A** | **G** | **C** | **A** | **A** | **A** | **T** | **G** | **C** | **T** | **G** | **T** | **G** | **T** | **T** | **A** | **T** | **A** | **T** | **G** | **A** | **T** | **A** | **A** | **A** | **A** | **A** | **A** | **A** | **T** | **C** | **A** | **T** | **T** | **G** | **A** | **T** | **C** | **A** | **A** | **T** | **T** | **A** | **G** | **A** | **A** | **G** | **C** | **A** | **A** | **T** | **T** | **T** | **T** | **C** | **T** | **T** | **A** | **G** | **A** | **A** | **G** | **A** | **T** | **C** | **G** | **A** | **A** | **A** | **A** | **A** | **A** | **A** | **T** | **G** | **T** | **A** | **C** | **A** | **A** | **C** | **A** | **A** | **T** | **G** | **A** | **C** | **T** | **C** | **C** | **A** | **G** | **A** | **A** | **G** | **T** | **T** | **G** | **T** | **T** | **C** | **G** | **T** | **G** | **A** | **C** | **A** | **T** | **G** | **T** | **C** | **A** | **A** | **A** | **A** | **T** | **G** | **G** | **T** | **T** | **G** | **A** | **T** | **T** | **T** | **T** | **T** | **A** | **A** | **A** | **C** | **A** | **A** | **C** | **A** | **A** | **A** | **T** | **T** | **T** | **C** | **A** | **C** | **G** | **A** | **T** | **T** | **A** | **T** | **T** | **T** | **T** | **C** | **A** | **C** | **C** | **A** | **A** | **T** | **T** | **C** | **T** | **T** | **-** | **-** | **-** |
| **N55268** | **T** | **T** | **G** | **A** | **T** | **T** | **C** | **T** | **T** | **A** | **G** | **C** | **G** | **T** | **C** | **T** | **T** | **A** | **A** | **C** | **A** | **G** | **T** | **T** | **A** | **T** | **T** | **T** | **A** | **T** | **T** | **T** | **T** | **A** | **T** | **T** | **A** | **A** | **T** | **G** | **C** | **A** | **A** | **T** | **T** | **A** | **T** | **T** | **G** | **C** | **G** | **G** | **G** | **A** | **A** | **T** | **T** | **A** | **C** | **C** | **A** | **T** | **T** | **T** | **T** | **G** | **T** | **T** | **A** | **A** | **A** | **A** | **C** | **C** | **T** | **C** | **G** | **A** | **G** | **A** | **T** | **G** | **T** | **A** | **G** | **C** | **A** | **G** | **C** | **A** | **A** | **T** | **T** | **T** | **G** | **G** | **G** | **C** | **A** | **T** | **G** | **G** | **C** | **T** | **A** | **C** | **T** | **C** | **G** | **T** | **A** | **C** | **T** | **G** | **A** | **T** | **T** | **G** | **C** | **T** | **C** | **T** | **A** | **C** | **C** | **C** | **G** | **T** | **T** | **T** | **T** | **T** | **G** | **G** | **T** | **T** | **T** | **T** | **T** | **T** | **C** | **T** | **T** | **A** | **T** | **A** | **T** | **T** | **T** | **A** | **T** | **T** | **C** | **T** | **T** | **T** | **G** | **G** | **T** | **C** | **G** | **C** | **G** | **G** | **T** | **T** | **T** | **A** | **A** | **C** | **C** | **G** | **A** | **C** | **A** | **A** | **A** | **A** | **A** | **G** | **A** | **A** | **A** | **T** | **T** | **T** | **T** | **A** | **T** | **T** | **T** | **G** | **C** | **A** | **A** | **C** | **A** | **A** | **A** | **G** | **T** | **G** | **A** | **T** | **T** | **T** | **G** | **C** | **G** | **G** | **G** | **A** | **A** | **T** | **T** | **A** | **G** | **A** | **G** | **A** | **A** | **T** | **T** | **T** | **T** | **C** | **A** | **G** | **A** | **A** | **T** | **T** | **T** | **C** | **C** | **A** | **A** | **G** | **A** | **A** | **G** | **A** | **A** | **A** | **G** | **T** | **T** | **T** | **T** | **G** | **A** | **A** | **C** | **T** | **T** | **T** | **A** | **T** | **A** | **G** | **T** | **C** | **A** | **A** | **A** | **A** | **G** | **A** | **T** | **G** | **C** | **C** | **G** | **A** | **C** | **G** | **G** | **A** | **G** | **G** | **A** | **G** | **C** | **A** | **A** | **C** | **A** | **A** | **C** | **A** | **G** | **T** | **T** | **T** | **A** | **C** | **A** | **G** | **A** | **C** | **T** | **T** | **T** | **T** | **T** | **T** | **T** | **C** | **C** | **T** | **C** | **G** | **T** | **T** | **A** | **A** | **A** | **T** | **A** | **G** | **G** | **A** | **T** | **G** | **C** | **C** | **T** | **C** | **T** | **G** | **A** | **C** | **T** | **A** | **A** | **G** | **A** | **A** | **A** | **A** | **A** | **T** | **G** | **A** | **T** | **G** | **T** | **T** | **G** | **A** | **A** | **A** | **T** | **T** | **T** | **T** | **T** | **A** | **C** | **G** | **G** | **A** | **T** | **G** | **G** | **T** | **A** | **C** | **G** | **G** | **A** | **A** | **A** | **A** | **A** | **T** | **T** | **C** | **A** | **A** | **T** | **G** | **C** | **G** | **T** | **T** | **G** | **A** | **T** | **G** | **G** | **C** | **G** | **G** | **A** | **T** | **A** | **T** | **A** | **A** | **A** | **A** | **A** | **A** | **A** | **G** | **C** | **T** | **C** | **A** | **G** | **C** | **A** | **C** | **T** | **C** | **T** | **A** | **T** | **T** | **C** | **A** | **T** | **A** | **T** | **T** | **G** | **A** | **A** | **T** | **A** | **T** | **T** | **A** | **T** | **G** | **C** | **C** | **T** | **T** | **T** | **G** | **T** | **A** | **A** | **C** | **G** | **G** | **A** | **T** | **C** | **A** | **T** | **A** | **T** | **C** | **G** | **G** | **T** | **A** | **C** | **C** | **A** | **A** | **A** | **A** | **T** | **T** | **T** | **T** | **G** | **A** | **A** | **C** | **T** | **T** | **A** | **T** | **T** | **A** | **G** | **A** | **A** | **G** | **A** | **A** | **A** | **A** | **G** | **G** | **C** | **G** | **G** | **C** | **T** | **G** | **A** | **A** | **G** | **G** | **G** | **G** | **T** | **T** | **G** | **A** | **A** | **G** | **T** | **G** | **C** | **G** | **G** | **T** | **T** | **G** | **C** | **T** | **T** | **T** | **A** | **C** | **G** | **A** | **T** | **G** | **C** | **C** | **T** | **T** | **T** | **G** | **G** | **C** | **T** | **C** | **A** | **A** | **A** | **G** | **G** | **G** | **C** | **A** | **C** | **C** | **A** | **A** | **G** | **G** | **T** | **T** | **C** | **A** | **T** | **C** | **A** | **T** | **T** | **T** | **G** | **A** | **A** | **T** | **G** | **A** | **A** | **T** | **T** | **G** | **A** | **A** | **A** | **A** | **A** | **A** | **A** | **A** | **C** | **G** | **G** | **T** | **G** | **G** | **T** | **T** | **T** | **T** | **G** | **T** | **C** | **C** | **A** | **A** | **A** | **C** | **G** | **T** | **T** | **T** | **A** | **T** | **T** | **A** | **C** | **T** | **T** | **C** | **T** | **C** | **A** | **A** | **A** | **A** | **A** | **G** | **C** | **A** | **C** | **T** | **T** | **T** | **T** | **G** | **A** | **A** | **G** | **T** | **T** | **T** | **C** | **G** | **T** | **T** | **T** | **G** | **A** | **A** | **T** | **T** | **A** | **T** | **C** | **A** | **T** | **G** | **A** | **T** | **C** | **A** | **C** | **C** | **G** | **G** | **A** | **A** | **A** | **A** | **T** | **T** | **G** | **T** | **T** | **G** | **T** | **T** | **A** | **T** | **T** | **G** | **A** | **C** | **G** | **G** | **A** | **A** | **A** | **A** | **G** | **T** | **A** | **G** | **G** | **C** | **T** | **A** | **C** | **A** | **T** | **T** | **G** | **G** | **C** | **G** | **G** | **C** | **T** | **T** | **T** | **A** | **A** | **T** | **G** | **T** | **T** | **G** | **C** | **C** | **G** | **A** | **T** | **C** | **A** | **A** | **T** | **A** | **T** | **G** | **C** | **T** | **G** | **G** | **A** | **A** | **C** | **G** | **A** | **C** | **T** | **A** | **A** | **A** | **A** | **A** | **G** | **T** | **T** | **T** | **G** | **G** | **C** | **T** | **A** | **T** | **T** | **G** | **G** | **C** | **G** | **G** | **G** | **A** | **T** | **A** | **C** | **G** | **C** | **A** | **T** | **T** | **T** | **A** | **C** | **G** | **G** | **A** | **T** | **T** | **C** | **A** | **A** | **G** | **G** | **G** | **C** | **C** | **A** | **G** | **C** | **A** | **G** | **C** | **C** | **T** | **C** | **A** | **T** | **T** | **A** | **C** | **T** | **G** | **C** | **A** | **A** | **A** | **T** | **G** | **C** | **G** | **T** | **T** | **T** | **T** | **T** | **T** | **A** | **A** | **T** | **G** | **G** | **A** | **T** | **T** | **G** | **G** | **A** | **A** | **C** | **G** | **T** | **C** | **T** | **C** | **T** | **T** | **C** | **C** | **C** | **C** | **C** | **G** | **A** | **G** | **A** | **A** | **A** | **A** | **A** | **T** | **C** | **G** | **T** | **G** | **T** | **G** | **G** | **C** | **G** | **T** | **A** | **T** | **C** | **A** | **A** | **T** | **T** | **G** | **G** | **A** | **T** | **T** | **A** | **T** | **T** | **T** | **C** | **T** | **T** | **T** | **A** | **A** | **A** | **C** | **T** | **T** | **G** | **A** | **A** | **G** | **C** | **A** | **T** | **T** | **G** | **G** | **T** | **G** | **C** | **C** | **A** | **G** | **A** | **G** | **G** | **C** | **A** | **A** | **A** | **T** | **A** | **C** | **A** | **T** | **C** | **C** | **A** | **T** | **T** | **C** | **A** | **G** | **A** | **T** | **G** | **A** | **T** | **T** | **G** | **C** | **C** | **A** | **G** | **T** | **G** | **G** | **T** | **C** | **C** | **T** | **A** | **A** | **C** | **A** | **G** | **T** | **G** | **A** | **C** | **C** | **G** | **T** | **G** | **A** | **A** | **C** | **A** | **A** | **A** | **T** | **T** | **A** | **A** | **A** | **T** | **T** | **G** | **G** | **C** | **C** | **T** | **T** | **T** | **A** | **T** | **T** | **A** | **A** | **A** | **T** | **T** | **G** | **A** | **T** | **T** | **A** | **C** | **T** | **T** | **C** | **T** | **G** | **C** | **C** | **A** | **A** | **G** | **A** | **A** | **A** | **A** | **G** | **A** | **G** | **T** | **C** | **T** | **G** | **G** | **A** | **T** | **T** | **C** | **A** | **A** | **A** | **C** | **G** | **C** | **C** | **G** | **T** | **A** | **T** | **T** | **T** | **A** | **G** | **T** | **T** | **C** | **C** | **T** | **G** | **A** | **T** | **G** | **A** | **T** | **A** | **G** | **T** | **G** | **T** | **C** | **T** | **T** | **G** | **G** | **C** | **T** | **G** | **C** | **T** | **T** | **T** | **A** | **A** | **A** | **G** | **G** | **T** | **T** | **G** | **C** | **T** | **G** | **C** | **G** | **G** | **C** | **T** | **T** | **C** | **A** | **G** | **G** | **A** | **G** | **T** | **A** | **G** | **A** | **T** | **G** | **T** | **T** | **A** | **A** | **A** | **A** | **T** | **T** | **A** | **T** | **G** | **A** | **T** | **T** | **C** | **C** | **A** | **G** | **A** | **T** | **A** | **A** | **G** | **C** | **C** | **C** | **G** | **A** | **C** | **C** | **A** | **T** | **C** | **C** | **G** | **T** | **T** | **T** | **A** | **T** | **T** | **T** | **A** | **T** | **C** | **G** | **A** | **G** | **C** | **A** | **A** | **C** | **A** | **C** | **A** | **G** | **T** | **A** | **T** | **T** | **A** | **C** | **G** | **G** | **C** | **C** | **G** | **C** | **T** | **T** | **A** | **T** | **T** | **G** | **A** | **T** | **G** | **A** | **A** | **A** | **G** | **A** | **A** | **A** | **A** | **T** | **A** | **T** | **T** | **G** | **A** | **A** | **A** | **T** | **T** | **T** | **T** | **A** | **A** | **T** | **T** | **T** | **A** | **T** | **A** | **A** | **C** | **G** | **G** | **T** | **G** | **G** | **T** | **T** | **T** | **C** | **T** | **T** | **A** | **C** | **A** | **T** | **G** | **C** | **G** | **A** | **A** | **A** | **A** | **C** | **A** | **A** | **T** | **G** | **A** | **T** | **T** | **A** | **T** | **G** | **G** | **A** | **T** | **G** | **A** | **T** | **G** | **A** | **A** | **G** | **T** | **C** | **T** | **G** | **C** | **A** | **C** | **A** | **G** | **T** | **T** | **G** | **G** | **T** | **T** | **C** | **A** | **G** | **C** | **C** | **A** | **A** | **T** | **C** | **A** | **A** | **G** | **A** | **T** | **A** | **T** | **C** | **C** | **G** | **A** | **A** | **G** | **T** | **T** | **A** | **C** | **A** | **A** | **A** | **T** | **T** | **A** | **A** | **A** | **C** | **T** | **T** | **T** | **G** | **A** | **A** | **G** | **C** | **A** | **A** | **A** | **T** | **G** | **C** | **T** | **G** | **T** | **G** | **T** | **T** | **A** | **T** | **A** | **T** | **G** | **A** | **T** | **A** | **A** | **A** | **A** | **A** | **A** | **A** | **T** | **C** | **A** | **T** | **T** | **G** | **A** | **T** | **C** | **A** | **A** | **T** | **T** | **A** | **G** | **A** | **A** | **G** | **C** | **A** | **A** | **T** | **T** | **T** | **T** | **C** | **T** | **T** | **A** | **G** | **A** | **A** | **G** | **A** | **T** | **C** | **G** | **A** | **A** | **A** | **A** | **A** | **A** | **A** | **T** | **G** | **T** | **A** | **C** | **A** | **A** | **C** | **A** | **A** | **T** | **G** | **A** | **C** | **T** | **C** | **C** | **A** | **G** | **A** | **A** | **G** | **T** | **T** | **G** | **T** | **T** | **C** | **G** | **C** | **G** | **A** | **C** | **A** | **T** | **G** | **T** | **C** | **A** | **A** | **A** | **A** | **T** | **G** | **G** | **T** | **T** | **G** | **A** | **T** | **T** | **T** | **T** | **T** | **A** | **A** | **A** | **C** | **A** | **G** | **C** | **A** | **A** | **A** | **T** | **T** | **T** | **C** | **A** | **C** | **G** | **A** | **T** | **T** | **A** | **T** | **T** | **T** | **T** | **C** | **A** | **C** | **C** | **A** | **A** | **T** | **T** | **C** | **T** | **T** | **-** | **-** | **-** |
| **N52564** | **T** | **T** | **G** | **A** | **T** | **T** | **C** | **T** | **T** | **A** | **G** | **C** | **G** | **T** | **C** | **T** | **T** | **A** | **A** | **C** | **A** | **G** | **T** | **T** | **A** | **T** | **T** | **T** | **A** | **T** | **T** | **T** | **T** | **A** | **T** | **T** | **A** | **A** | **T** | **G** | **C** | **A** | **A** | **T** | **T** | **A** | **T** | **T** | **G** | **C** | **G** | **G** | **G** | **A** | **A** | **T** | **T** | **A** | **C** | **C** | **A** | **T** | **T** | **T** | **T** | **G** | **T** | **T** | **A** | **A** | **A** | **A** | **C** | **C** | **T** | **C** | **G** | **A** | **G** | **A** | **T** | **G** | **T** | **A** | **G** | **C** | **A** | **G** | **C** | **A** | **A** | **T** | **T** | **T** | **G** | **G** | **G** | **C** | **A** | **T** | **G** | **G** | **C** | **T** | **A** | **C** | **T** | **C** | **G** | **T** | **A** | **C** | **T** | **G** | **A** | **T** | **T** | **G** | **C** | **T** | **C** | **T** | **A** | **C** | **C** | **C** | **G** | **T** | **T** | **T** | **T** | **T** | **G** | **G** | **T** | **T** | **T** | **T** | **T** | **T** | **C** | **T** | **T** | **A** | **T** | **A** | **T** | **T** | **T** | **A** | **T** | **T** | **C** | **T** | **T** | **T** | **G** | **G** | **G** | **C** | **G** | **C** | **G** | **G** | **T** | **T** | **T** | **A** | **A** | **C** | **C** | **G** | **A** | **C** | **A** | **A** | **A** | **A** | **A** | **G** | **A** | **A** | **A** | **T** | **T** | **T** | **T** | **A** | **T** | **T** | **T** | **G** | **C** | **A** | **A** | **C** | **A** | **A** | **A** | **G** | **T** | **G** | **A** | **T** | **T** | **T** | **A** | **C** | **G** | **G** | **G** | **A** | **A** | **T** | **T** | **A** | **G** | **A** | **G** | **A** | **A** | **T** | **T** | **T** | **C** | **C** | **A** | **G** | **A** | **A** | **T** | **T** | **T** | **C** | **C** | **A** | **A** | **G** | **A** | **A** | **G** | **A** | **A** | **A** | **G** | **T** | **T** | **T** | **T** | **G** | **A** | **A** | **C** | **T** | **T** | **T** | **A** | **T** | **A** | **G** | **T** | **C** | **A** | **A** | **A** | **A** | **G** | **A** | **T** | **G** | **C** | **C** | **G** | **A** | **C** | **G** | **G** | **A** | **G** | **G** | **A** | **G** | **C** | **A** | **A** | **C** | **A** | **A** | **C** | **A** | **G** | **T** | **T** | **T** | **A** | **C** | **A** | **G** | **A** | **C** | **T** | **T** | **T** | **T** | **T** | **T** | **T** | **C** | **C** | **T** | **C** | **G** | **T** | **T** | **A** | **A** | **A** | **T** | **A** | **G** | **G** | **A** | **T** | **G** | **C** | **C** | **T** | **C** | **T** | **G** | **A** | **C** | **T** | **A** | **A** | **G** | **A** | **A** | **A** | **A** | **A** | **T** | **G** | **A** | **T** | **G** | **T** | **T** | **G** | **A** | **A** | **A** | **T** | **T** | **T** | **T** | **T** | **A** | **C** | **G** | **G** | **A** | **T** | **G** | **G** | **T** | **A** | **C** | **G** | **G** | **A** | **A** | **A** | **A** | **A** | **T** | **T** | **C** | **A** | **A** | **T** | **G** | **C** | **G** | **T** | **T** | **G** | **A** | **T** | **G** | **G** | **C** | **G** | **G** | **A** | **T** | **A** | **T** | **A** | **A** | **A** | **A** | **A** | **A** | **A** | **G** | **C** | **T** | **C** | **A** | **G** | **C** | **A** | **C** | **T** | **C** | **T** | **A** | **T** | **T** | **C** | **A** | **T** | **A** | **T** | **T** | **G** | **A** | **A** | **T** | **A** | **T** | **T** | **A** | **T** | **G** | **C** | **C** | **T** | **T** | **T** | **G** | **T** | **A** | **A** | **C** | **G** | **G** | **A** | **T** | **C** | **A** | **T** | **A** | **T** | **C** | **G** | **G** | **T** | **A** | **C** | **C** | **A** | **A** | **A** | **A** | **T** | **T** | **T** | **T** | **G** | **A** | **A** | **C** | **T** | **T** | **A** | **T** | **T** | **A** | **G** | **A** | **A** | **G** | **A** | **A** | **A** | **A** | **G** | **G** | **C** | **G** | **G** | **C** | **T** | **G** | **A** | **A** | **G** | **G** | **G** | **G** | **T** | **T** | **G** | **A** | **A** | **G** | **T** | **G** | **C** | **G** | **G** | **T** | **T** | **G** | **C** | **T** | **T** | **T** | **A** | **C** | **G** | **A** | **T** | **G** | **C** | **C** | **T** | **T** | **T** | **G** | **G** | **C** | **T** | **C** | **A** | **A** | **A** | **G** | **G** | **G** | **C** | **A** | **C** | **C** | **A** | **A** | **G** | **G** | **T** | **T** | **C** | **A** | **T** | **C** | **A** | **T** | **T** | **T** | **G** | **A** | **A** | **T** | **G** | **A** | **A** | **T** | **T** | **G** | **A** | **A** | **A** | **A** | **A** | **A** | **A** | **A** | **C** | **G** | **G** | **C** | **G** | **G** | **T** | **T** | **T** | **T** | **G** | **T** | **C** | **C** | **A** | **A** | **A** | **C** | **G** | **T** | **T** | **T** | **A** | **T** | **T** | **A** | **C** | **T** | **T** | **C** | **T** | **C** | **A** | **A** | **A** | **A** | **A** | **G** | **C** | **A** | **C** | **T** | **T** | **T** | **T** | **G** | **A** | **A** | **G** | **T** | **T** | **T** | **C** | **G** | **T** | **T** | **T** | **G** | **A** | **A** | **T** | **T** | **A** | **T** | **C** | **A** | **T** | **G** | **A** | **T** | **C** | **A** | **C** | **C** | **G** | **G** | **A** | **A** | **A** | **A** | **T** | **T** | **G** | **T** | **T** | **G** | **T** | **T** | **A** | **T** | **T** | **G** | **A** | **C** | **G** | **G** | **A** | **A** | **A** | **A** | **G** | **T** | **A** | **G** | **G** | **C** | **T** | **A** | **C** | **A** | **T** | **T** | **G** | **G** | **C** | **G** | **G** | **C** | **T** | **T** | **T** | **A** | **A** | **T** | **G** | **T** | **T** | **G** | **C** | **C** | **G** | **A** | **T** | **C** | **A** | **A** | **T** | **A** | **T** | **G** | **C** | **T** | **G** | **G** | **A** | **A** | **C** | **G** | **A** | **C** | **T** | **A** | **A** | **A** | **A** | **A** | **G** | **T** | **T** | **T** | **G** | **G** | **C** | **T** | **A** | **T** | **T** | **G** | **G** | **C** | **G** | **G** | **G** | **A** | **T** | **A** | **C** | **A** | **C** | **A** | **T** | **T** | **T** | **A** | **C** | **G** | **G** | **A** | **T** | **T** | **C** | **A** | **A** | **G** | **G** | **G** | **C** | **C** | **A** | **G** | **C** | **A** | **G** | **C** | **C** | **T** | **C** | **A** | **T** | **T** | **A** | **C** | **T** | **G** | **C** | **A** | **A** | **A** | **T** | **G** | **C** | **G** | **T** | **T** | **T** | **T** | **T** | **T** | **A** | **A** | **T** | **G** | **G** | **A** | **T** | **T** | **G** | **G** | **A** | **A** | **C** | **G** | **T** | **C** | **T** | **C** | **T** | **T** | **C** | **C** | **C** | **C** | **C** | **G** | **A** | **G** | **A** | **A** | **A** | **A** | **A** | **T** | **C** | **G** | **T** | **G** | **T** | **G** | **G** | **C** | **G** | **T** | **A** | **T** | **C** | **A** | **A** | **T** | **T** | **G** | **G** | **A** | **T** | **T** | **A** | **T** | **T** | **T** | **C** | **T** | **T** | **T** | **A** | **A** | **A** | **C** | **T** | **T** | **G** | **A** | **A** | **G** | **C** | **A** | **T** | **T** | **G** | **G** | **T** | **G** | **C** | **C** | **A** | **G** | **A** | **G** | **G** | **C** | **A** | **A** | **A** | **T** | **A** | **C** | **A** | **T** | **C** | **C** | **A** | **T** | **T** | **C** | **A** | **G** | **A** | **T** | **G** | **A** | **T** | **T** | **G** | **C** | **C** | **A** | **G** | **T** | **G** | **G** | **T** | **C** | **C** | **T** | **A** | **A** | **C** | **A** | **G** | **T** | **G** | **A** | **C** | **C** | **G** | **T** | **G** | **A** | **A** | **C** | **A** | **A** | **A** | **T** | **T** | **A** | **A** | **A** | **T** | **T** | **G** | **G** | **C** | **C** | **T** | **T** | **T** | **A** | **T** | **T** | **A** | **A** | **A** | **T** | **T** | **G** | **A** | **T** | **T** | **A** | **C** | **T** | **T** | **C** | **T** | **G** | **C** | **C** | **A** | **A** | **G** | **A** | **A** | **A** | **A** | **G** | **A** | **G** | **T** | **C** | **T** | **G** | **G** | **A** | **T** | **T** | **C** | **A** | **A** | **A** | **C** | **G** | **C** | **C** | **G** | **T** | **A** | **T** | **T** | **T** | **A** | **G** | **T** | **T** | **C** | **C** | **T** | **G** | **A** | **T** | **G** | **A** | **T** | **A** | **G** | **T** | **G** | **T** | **C** | **T** | **T** | **G** | **G** | **C** | **T** | **G** | **C** | **T** | **T** | **T** | **A** | **A** | **A** | **G** | **G** | **T** | **T** | **G** | **C** | **T** | **G** | **C** | **G** | **G** | **C** | **T** | **T** | **C** | **A** | **G** | **G** | **A** | **G** | **T** | **A** | **G** | **A** | **T** | **G** | **T** | **T** | **A** | **A** | **A** | **A** | **T** | **T** | **A** | **T** | **G** | **A** | **T** | **T** | **C** | **C** | **A** | **G** | **A** | **T** | **A** | **A** | **G** | **C** | **C** | **C** | **G** | **A** | **C** | **C** | **A** | **T** | **C** | **C** | **G** | **T** | **T** | **T** | **A** | **T** | **T** | **T** | **A** | **T** | **C** | **G** | **A** | **G** | **C** | **A** | **A** | **C** | **A** | **C** | **A** | **G** | **T** | **A** | **T** | **T** | **A** | **C** | **G** | **G** | **C** | **C** | **G** | **C** | **T** | **T** | **A** | **T** | **T** | **G** | **A** | **T** | **G** | **A** | **A** | **A** | **G** | **A** | **A** | **A** | **A** | **T** | **A** | **T** | **T** | **G** | **A** | **A** | **A** | **T** | **T** | **T** | **T** | **A** | **A** | **T** | **T** | **T** | **A** | **T** | **A** | **A** | **C** | **G** | **G** | **T** | **G** | **G** | **T** | **T** | **T** | **C** | **T** | **T** | **A** | **C** | **A** | **T** | **G** | **C** | **G** | **A** | **A** | **A** | **A** | **C** | **A** | **A** | **T** | **G** | **A** | **T** | **T** | **A** | **T** | **G** | **G** | **A** | **T** | **G** | **A** | **T** | **G** | **A** | **A** | **G** | **T** | **C** | **T** | **G** | **C** | **A** | **C** | **A** | **G** | **T** | **T** | **G** | **G** | **T** | **T** | **C** | **A** | **G** | **C** | **C** | **A** | **A** | **T** | **C** | **A** | **A** | **G** | **A** | **T** | **A** | **T** | **C** | **C** | **G** | **A** | **A** | **G** | **T** | **T** | **A** | **C** | **A** | **A** | **A** | **T** | **T** | **A** | **A** | **A** | **C** | **T** | **T** | **T** | **G** | **A** | **A** | **G** | **C** | **A** | **A** | **A** | **T** | **G** | **C** | **T** | **G** | **T** | **G** | **T** | **T** | **A** | **T** | **A** | **T** | **G** | **A** | **T** | **A** | **A** | **A** | **A** | **A** | **A** | **A** | **T** | **C** | **A** | **T** | **T** | **G** | **A** | **T** | **C** | **A** | **A** | **T** | **T** | **A** | **G** | **A** | **A** | **G** | **C** | **A** | **A** | **T** | **T** | **T** | **T** | **C** | **T** | **T** | **A** | **G** | **A** | **A** | **G** | **A** | **T** | **C** | **G** | **A** | **A** | **A** | **A** | **A** | **A** | **A** | **T** | **G** | **T** | **A** | **C** | **A** | **A** | **C** | **A** | **A** | **T** | **G** | **A** | **C** | **T** | **C** | **C** | **A** | **G** | **A** | **A** | **G** | **T** | **T** | **G** | **T** | **T** | **C** | **G** | **T** | **G** | **A** | **C** | **A** | **T** | **G** | **T** | **C** | **A** | **A** | **A** | **A** | **T** | **G** | **G** | **T** | **T** | **G** | **A** | **T** | **T** | **T** | **T** | **T** | **A** | **A** | **A** | **C** | **A** | **A** | **C** | **A** | **A** | **A** | **T** | **T** | **T** | **C** | **A** | **C** | **G** | **A** | **T** | **T** | **A** | **T** | **T** | **T** | **T** | **C** | **A** | **C** | **C** | **A** | **A** | **T** | **T** | **C** | **T** | **T** | **-** | **-** | **-** |
| **N59860F** | **T** | **T** | **G** | **A** | **T** | **T** | **C** | **T** | **T** | **A** | **G** | **C** | **G** | **T** | **C** | **T** | **T** | **A** | **A** | **C** | **A** | **G** | **T** | **T** | **A** | **T** | **T** | **T** | **A** | **T** | **T** | **T** | **T** | **A** | **T** | **T** | **A** | **A** | **T** | **G** | **C** | **A** | **A** | **T** | **T** | **A** | **T** | **T** | **G** | **C** | **G** | **G** | **G** | **A** | **A** | **T** | **T** | **A** | **C** | **C** | **A** | **T** | **T** | **T** | **T** | **G** | **T** | **T** | **A** | **A** | **A** | **A** | **C** | **C** | **T** | **C** | **G** | **A** | **G** | **A** | **T** | **G** | **T** | **A** | **G** | **C** | **A** | **G** | **C** | **A** | **A** | **T** | **T** | **T** | **G** | **G** | **G** | **C** | **A** | **T** | **G** | **G** | **C** | **T** | **A** | **C** | **T** | **C** | **G** | **T** | **A** | **C** | **T** | **G** | **A** | **T** | **T** | **G** | **C** | **T** | **C** | **T** | **A** | **C** | **C** | **C** | **G** | **T** | **T** | **T** | **T** | **T** | **G** | **G** | **T** | **T** | **T** | **T** | **T** | **T** | **C** | **T** | **T** | **A** | **T** | **A** | **T** | **T** | **T** | **A** | **T** | **T** | **C** | **T** | **T** | **T** | **G** | **G** | **G** | **C** | **G** | **C** | **G** | **G** | **T** | **T** | **T** | **A** | **A** | **C** | **C** | **G** | **A** | **C** | **A** | **A** | **A** | **A** | **A** | **G** | **A** | **A** | **A** | **T** | **T** | **T** | **T** | **A** | **T** | **T** | **T** | **G** | **C** | **A** | **A** | **C** | **A** | **A** | **A** | **G** | **T** | **G** | **A** | **T** | **T** | **T** | **A** | **C** | **G** | **G** | **G** | **A** | **A** | **T** | **T** | **A** | **G** | **A** | **G** | **A** | **A** | **T** | **T** | **T** | **C** | **C** | **A** | **G** | **A** | **A** | **T** | **T** | **T** | **C** | **C** | **A** | **A** | **G** | **A** | **A** | **G** | **A** | **A** | **A** | **G** | **T** | **T** | **T** | **T** | **G** | **A** | **A** | **C** | **T** | **T** | **T** | **A** | **T** | **A** | **G** | **T** | **C** | **A** | **A** | **A** | **A** | **G** | **A** | **T** | **G** | **C** | **C** | **G** | **A** | **C** | **G** | **G** | **A** | **G** | **G** | **A** | **G** | **C** | **A** | **A** | **C** | **A** | **A** | **C** | **A** | **G** | **T** | **T** | **T** | **A** | **C** | **A** | **G** | **A** | **C** | **T** | **T** | **T** | **T** | **T** | **T** | **T** | **C** | **C** | **T** | **C** | **G** | **T** | **T** | **A** | **A** | **A** | **T** | **A** | **G** | **G** | **A** | **T** | **G** | **C** | **C** | **T** | **C** | **T** | **G** | **A** | **C** | **T** | **A** | **A** | **G** | **A** | **A** | **A** | **A** | **A** | **T** | **G** | **A** | **T** | **G** | **T** | **T** | **G** | **A** | **A** | **A** | **T** | **T** | **T** | **T** | **T** | **A** | **C** | **G** | **G** | **A** | **T** | **G** | **G** | **T** | **A** | **C** | **G** | **G** | **A** | **A** | **A** | **A** | **A** | **T** | **T** | **C** | **A** | **A** | **T** | **G** | **C** | **G** | **T** | **T** | **G** | **A** | **T** | **G** | **G** | **C** | **G** | **G** | **A** | **T** | **A** | **T** | **A** | **A** | **A** | **A** | **A** | **A** | **A** | **G** | **C** | **T** | **C** | **A** | **G** | **C** | **A** | **C** | **T** | **C** | **T** | **A** | **T** | **T** | **C** | **A** | **T** | **A** | **T** | **T** | **G** | **A** | **A** | **T** | **A** | **T** | **T** | **A** | **T** | **G** | **C** | **C** | **T** | **T** | **T** | **G** | **T** | **A** | **A** | **C** | **G** | **G** | **A** | **T** | **C** | **A** | **T** | **A** | **T** | **C** | **G** | **G** | **T** | **A** | **C** | **C** | **A** | **A** | **A** | **A** | **T** | **T** | **T** | **T** | **G** | **A** | **A** | **C** | **T** | **T** | **A** | **T** | **T** | **A** | **G** | **A** | **A** | **G** | **A** | **A** | **A** | **A** | **G** | **G** | **C** | **G** | **G** | **C** | **T** | **G** | **A** | **A** | **G** | **G** | **G** | **G** | **T** | **T** | **G** | **A** | **A** | **G** | **T** | **G** | **C** | **G** | **G** | **T** | **T** | **G** | **C** | **T** | **T** | **T** | **A** | **C** | **G** | **A** | **T** | **G** | **C** | **C** | **T** | **T** | **T** | **G** | **G** | **C** | **T** | **C** | **A** | **A** | **A** | **G** | **G** | **G** | **C** | **A** | **C** | **C** | **A** | **A** | **G** | **G** | **T** | **T** | **C** | **A** | **T** | **C** | **A** | **T** | **T** | **T** | **G** | **A** | **A** | **T** | **G** | **A** | **A** | **T** | **T** | **G** | **A** | **A** | **A** | **A** | **A** | **A** | **A** | **A** | **C** | **G** | **G** | **C** | **G** | **G** | **T** | **T** | **T** | **T** | **G** | **T** | **C** | **C** | **A** | **A** | **A** | **C** | **G** | **T** | **T** | **T** | **A** | **T** | **T** | **A** | **C** | **T** | **T** | **C** | **T** | **C** | **A** | **A** | **A** | **A** | **A** | **G** | **C** | **A** | **C** | **T** | **T** | **T** | **T** | **G** | **A** | **A** | **G** | **T** | **T** | **T** | **C** | **G** | **T** | **T** | **T** | **G** | **A** | **A** | **T** | **T** | **A** | **T** | **C** | **A** | **T** | **G** | **A** | **T** | **C** | **A** | **C** | **C** | **G** | **G** | **A** | **A** | **A** | **A** | **T** | **T** | **G** | **T** | **T** | **G** | **T** | **T** | **A** | **T** | **T** | **G** | **A** | **C** | **G** | **G** | **A** | **A** | **A** | **A** | **G** | **T** | **A** | **G** | **G** | **C** | **T** | **A** | **C** | **A** | **T** | **T** | **G** | **G** | **C** | **G** | **G** | **C** | **T** | **T** | **T** | **A** | **A** | **T** | **G** | **T** | **T** | **G** | **C** | **C** | **G** | **A** | **T** | **C** | **A** | **A** | **T** | **A** | **T** | **G** | **C** | **T** | **G** | **G** | **A** | **A** | **C** | **G** | **A** | **C** | **T** | **A** | **A** | **A** | **A** | **A** | **G** | **T** | **T** | **T** | **G** | **G** | **C** | **T** | **A** | **T** | **T** | **G** | **G** | **C** | **G** | **G** | **G** | **A** | **T** | **A** | **C** | **A** | **C** | **A** | **T** | **T** | **T** | **A** | **C** | **G** | **G** | **A** | **T** | **T** | **C** | **A** | **A** | **G** | **G** | **G** | **C** | **C** | **A** | **G** | **C** | **A** | **G** | **C** | **C** | **T** | **C** | **A** | **T** | **T** | **A** | **C** | **T** | **G** | **C** | **A** | **A** | **A** | **T** | **G** | **C** | **G** | **T** | **T** | **T** | **T** | **T** | **T** | **A** | **A** | **T** | **G** | **G** | **A** | **T** | **T** | **G** | **G** | **A** | **A** | **C** | **G** | **T** | **C** | **T** | **C** | **T** | **T** | **C** | **C** | **C** | **C** | **C** | **G** | **A** | **G** | **A** | **A** | **A** | **A** | **A** | **T** | **C** | **G** | **T** | **G** | **T** | **G** | **G** | **C** | **G** | **T** | **A** | **T** | **C** | **A** | **A** | **T** | **T** | **G** | **G** | **A** | **T** | **T** | **A** | **T** | **T** | **T** | **C** | **T** | **T** | **T** | **A** | **A** | **A** | **C** | **T** | **T** | **G** | **A** | **A** | **G** | **C** | **A** | **T** | **T** | **G** | **G** | **T** | **G** | **C** | **C** | **A** | **G** | **A** | **G** | **G** | **C** | **A** | **A** | **A** | **T** | **A** | **C** | **A** | **T** | **C** | **C** | **A** | **T** | **T** | **C** | **A** | **G** | **A** | **T** | **G** | **A** | **T** | **T** | **G** | **C** | **C** | **A** | **G** | **T** | **G** | **G** | **T** | **C** | **C** | **T** | **A** | **A** | **C** | **A** | **G** | **T** | **G** | **A** | **C** | **C** | **G** | **T** | **G** | **A** | **A** | **C** | **A** | **A** | **A** | **T** | **T** | **A** | **A** | **A** | **T** | **T** | **G** | **G** | **C** | **C** | **T** | **T** | **T** | **A** | **T** | **T** | **A** | **A** | **A** | **T** | **T** | **G** | **A** | **T** | **T** | **A** | **C** | **T** | **T** | **C** | **T** | **G** | **C** | **C** | **A** | **A** | **G** | **A** | **A** | **A** | **A** | **G** | **A** | **G** | **T** | **C** | **T** | **G** | **G** | **A** | **T** | **T** | **C** | **A** | **A** | **A** | **C** | **G** | **C** | **C** | **G** | **T** | **A** | **T** | **T** | **T** | **A** | **G** | **T** | **T** | **C** | **C** | **T** | **G** | **A** | **T** | **G** | **A** | **T** | **A** | **G** | **T** | **G** | **T** | **C** | **T** | **T** | **G** | **G** | **C** | **T** | **G** | **C** | **T** | **T** | **T** | **A** | **A** | **A** | **G** | **G** | **T** | **T** | **G** | **C** | **T** | **G** | **C** | **G** | **G** | **C** | **T** | **T** | **C** | **A** | **G** | **G** | **A** | **G** | **T** | **A** | **G** | **A** | **T** | **G** | **T** | **T** | **A** | **A** | **A** | **A** | **T** | **T** | **A** | **T** | **G** | **A** | **T** | **T** | **C** | **C** | **A** | **G** | **A** | **T** | **A** | **A** | **G** | **C** | **C** | **C** | **G** | **A** | **C** | **C** | **A** | **T** | **C** | **C** | **G** | **T** | **T** | **T** | **A** | **T** | **T** | **T** | **A** | **T** | **C** | **G** | **A** | **G** | **C** | **A** | **A** | **C** | **A** | **C** | **A** | **G** | **T** | **A** | **T** | **T** | **A** | **C** | **G** | **G** | **C** | **C** | **G** | **C** | **T** | **T** | **A** | **T** | **T** | **G** | **A** | **T** | **G** | **A** | **A** | **A** | **G** | **A** | **A** | **A** | **A** | **T** | **A** | **T** | **T** | **G** | **A** | **A** | **A** | **T** | **T** | **T** | **T** | **A** | **A** | **T** | **T** | **T** | **A** | **T** | **A** | **A** | **C** | **G** | **G** | **T** | **G** | **G** | **T** | **T** | **T** | **C** | **T** | **T** | **A** | **C** | **A** | **T** | **G** | **C** | **G** | **A** | **A** | **A** | **A** | **C** | **A** | **A** | **T** | **G** | **A** | **T** | **T** | **A** | **T** | **G** | **G** | **A** | **T** | **G** | **A** | **T** | **G** | **A** | **A** | **G** | **T** | **C** | **T** | **G** | **C** | **A** | **C** | **A** | **G** | **T** | **T** | **G** | **G** | **T** | **T** | **C** | **A** | **G** | **C** | **C** | **A** | **A** | **T** | **C** | **A** | **A** | **G** | **A** | **T** | **A** | **T** | **C** | **C** | **G** | **A** | **A** | **G** | **T** | **T** | **A** | **C** | **A** | **A** | **A** | **T** | **T** | **A** | **A** | **A** | **C** | **T** | **T** | **T** | **G** | **A** | **A** | **G** | **C** | **A** | **A** | **A** | **T** | **G** | **C** | **T** | **G** | **T** | **G** | **T** | **T** | **A** | **T** | **A** | **T** | **G** | **A** | **T** | **A** | **A** | **A** | **A** | **A** | **A** | **A** | **T** | **C** | **A** | **T** | **T** | **G** | **A** | **T** | **C** | **A** | **A** | **T** | **T** | **A** | **G** | **A** | **A** | **G** | **C** | **A** | **A** | **T** | **T** | **T** | **T** | **C** | **T** | **T** | **A** | **G** | **A** | **A** | **G** | **A** | **T** | **C** | **G** | **A** | **A** | **A** | **A** | **A** | **A** | **A** | **T** | **G** | **T** | **A** | **C** | **A** | **A** | **C** | **A** | **A** | **T** | **G** | **A** | **C** | **T** | **C** | **C** | **A** | **G** | **A** | **A** | **G** | **T** | **T** | **G** | **T** | **T** | **C** | **G** | **T** | **G** | **A** | **C** | **A** | **T** | **G** | **T** | **C** | **A** | **A** | **A** | **A** | **T** | **G** | **G** | **T** | **T** | **G** | **A** | **T** | **T** | **T** | **T** | **T** | **A** | **A** | **A** | **C** | **A** | **A** | **C** | **A** | **A** | **A** | **T** | **T** | **T** | **C** | **A** | **C** | **G** | **A** | **T** | **T** | **A** | **T** | **T** | **T** | **T** | **C** | **A** | **C** | **C** | **A** | **A** | **T** | **T** | **C** | **T** | **T** | **-** | **-** | **-** |
| **N52623** | **T** | **T** | **G** | **A** | **T** | **T** | **C** | **T** | **T** | **A** | **G** | **C** | **G** | **T** | **C** | **T** | **T** | **A** | **A** | **C** | **A** | **G** | **T** | **T** | **A** | **T** | **T** | **T** | **A** | **T** | **T** | **T** | **T** | **A** | **T** | **T** | **A** | **A** | **T** | **G** | **C** | **A** | **A** | **T** | **T** | **A** | **T** | **T** | **G** | **C** | **G** | **G** | **G** | **A** | **A** | **T** | **T** | **A** | **C** | **C** | **A** | **T** | **T** | **T** | **T** | **G** | **T** | **T** | **A** | **A** | **A** | **A** | **C** | **C** | **T** | **C** | **G** | **A** | **G** | **A** | **T** | **G** | **T** | **A** | **G** | **C** | **A** | **G** | **C** | **A** | **A** | **T** | **T** | **T** | **G** | **G** | **G** | **C** | **A** | **T** | **G** | **G** | **C** | **T** | **A** | **C** | **T** | **C** | **G** | **T** | **A** | **C** | **T** | **G** | **A** | **T** | **T** | **G** | **C** | **T** | **C** | **T** | **A** | **C** | **C** | **C** | **G** | **T** | **T** | **T** | **T** | **T** | **G** | **G** | **T** | **T** | **T** | **T** | **T** | **T** | **C** | **T** | **T** | **A** | **T** | **A** | **T** | **T** | **T** | **A** | **T** | **T** | **C** | **T** | **T** | **T** | **G** | **G** | **G** | **C** | **G** | **C** | **G** | **G** | **T** | **T** | **T** | **A** | **A** | **C** | **C** | **G** | **A** | **C** | **A** | **A** | **A** | **A** | **A** | **G** | **A** | **A** | **A** | **T** | **T** | **T** | **T** | **A** | **T** | **T** | **T** | **G** | **C** | **A** | **A** | **C** | **A** | **A** | **A** | **G** | **T** | **G** | **A** | **T** | **T** | **T** | **A** | **C** | **G** | **G** | **G** | **A** | **A** | **T** | **T** | **A** | **G** | **A** | **G** | **A** | **A** | **T** | **T** | **T** | **C** | **C** | **A** | **G** | **A** | **A** | **T** | **T** | **T** | **C** | **C** | **A** | **A** | **G** | **A** | **A** | **G** | **A** | **A** | **A** | **G** | **T** | **T** | **T** | **T** | **G** | **A** | **A** | **C** | **T** | **T** | **T** | **A** | **T** | **A** | **G** | **T** | **C** | **A** | **A** | **A** | **A** | **G** | **A** | **T** | **G** | **C** | **C** | **G** | **A** | **C** | **G** | **G** | **A** | **G** | **G** | **A** | **G** | **C** | **A** | **A** | **C** | **A** | **A** | **C** | **A** | **G** | **T** | **T** | **T** | **A** | **C** | **A** | **G** | **A** | **C** | **T** | **T** | **T** | **T** | **T** | **T** | **T** | **C** | **C** | **T** | **C** | **G** | **T** | **T** | **A** | **A** | **A** | **T** | **A** | **G** | **G** | **A** | **T** | **G** | **C** | **C** | **T** | **C** | **T** | **G** | **A** | **C** | **T** | **A** | **A** | **G** | **A** | **A** | **A** | **A** | **A** | **T** | **G** | **A** | **T** | **G** | **T** | **T** | **G** | **A** | **A** | **A** | **T** | **T** | **T** | **T** | **T** | **A** | **C** | **G** | **G** | **A** | **T** | **G** | **G** | **T** | **A** | **C** | **G** | **G** | **A** | **A** | **A** | **A** | **A** | **T** | **T** | **C** | **A** | **A** | **T** | **G** | **C** | **G** | **T** | **T** | **G** | **A** | **T** | **G** | **G** | **C** | **G** | **G** | **A** | **C** | **A** | **T** | **A** | **A** | **A** | **A** | **A** | **A** | **A** | **G** | **C** | **T** | **C** | **A** | **G** | **C** | **A** | **C** | **T** | **C** | **C** | **A** | **T** | **T** | **C** | **A** | **T** | **A** | **T** | **T** | **G** | **A** | **A** | **T** | **A** | **T** | **T** | **A** | **T** | **G** | **C** | **C** | **T** | **T** | **T** | **G** | **T** | **A** | **A** | **C** | **G** | **G** | **A** | **T** | **C** | **A** | **T** | **A** | **T** | **C** | **G** | **G** | **C** | **A** | **C** | **C** | **A** | **A** | **A** | **A** | **T** | **T** | **T** | **T** | **G** | **A** | **A** | **C** | **T** | **T** | **A** | **T** | **T** | **A** | **G** | **A** | **A** | **G** | **A** | **A** | **A** | **A** | **G** | **G** | **C** | **G** | **G** | **C** | **T** | **G** | **A** | **A** | **G** | **G** | **G** | **G** | **T** | **T** | **G** | **A** | **A** | **G** | **T** | **G** | **C** | **G** | **G** | **T** | **T** | **G** | **C** | **T** | **T** | **T** | **A** | **C** | **G** | **A** | **T** | **G** | **C** | **C** | **T** | **T** | **T** | **G** | **G** | **C** | **T** | **C** | **A** | **A** | **A** | **G** | **G** | **G** | **C** | **A** | **C** | **C** | **A** | **A** | **G** | **G** | **T** | **T** | **C** | **A** | **T** | **C** | **A** | **T** | **T** | **T** | **G** | **A** | **A** | **T** | **G** | **A** | **A** | **T** | **T** | **G** | **A** | **A** | **A** | **A** | **A** | **A** | **A** | **A** | **C** | **G** | **G** | **C** | **G** | **G** | **T** | **T** | **T** | **T** | **G** | **T** | **C** | **C** | **A** | **A** | **A** | **C** | **A** | **T** | **T** | **T** | **A** | **T** | **T** | **A** | **C** | **T** | **T** | **C** | **T** | **C** | **A** | **A** | **A** | **A** | **A** | **G** | **C** | **A** | **C** | **T** | **T** | **T** | **T** | **G** | **A** | **A** | **G** | **T** | **T** | **T** | **C** | **G** | **T** | **T** | **T** | **G** | **A** | **A** | **T** | **T** | **A** | **T** | **C** | **A** | **T** | **G** | **A** | **T** | **C** | **A** | **C** | **C** | **G** | **G** | **A** | **A** | **A** | **A** | **T** | **T** | **G** | **T** | **T** | **G** | **T** | **T** | **A** | **T** | **T** | **G** | **A** | **C** | **G** | **G** | **A** | **A** | **A** | **A** | **G** | **T** | **A** | **G** | **G** | **C** | **T** | **A** | **C** | **A** | **T** | **T** | **G** | **G** | **C** | **G** | **G** | **C** | **T** | **T** | **T** | **A** | **A** | **T** | **G** | **T** | **T** | **G** | **C** | **C** | **G** | **A** | **T** | **C** | **A** | **A** | **T** | **A** | **T** | **G** | **C** | **C** | **G** | **G** | **A** | **A** | **C** | **G** | **A** | **C** | **T** | **A** | **A** | **A** | **A** | **A** | **G** | **T** | **T** | **T** | **G** | **G** | **C** | **T** | **A** | **T** | **T** | **G** | **G** | **C** | **G** | **G** | **G** | **A** | **T** | **A** | **C** | **G** | **C** | **A** | **T** | **T** | **T** | **A** | **C** | **G** | **G** | **A** | **T** | **T** | **C** | **A** | **A** | **G** | **G** | **G** | **C** | **C** | **A** | **G** | **C** | **A** | **G** | **C** | **C** | **T** | **C** | **A** | **T** | **T** | **A** | **C** | **T** | **G** | **C** | **A** | **A** | **A** | **T** | **G** | **C** | **G** | **T** | **T** | **T** | **T** | **T** | **T** | **A** | **A** | **T** | **G** | **G** | **A** | **T** | **T** | **G** | **G** | **A** | **A** | **C** | **G** | **T** | **C** | **T** | **C** | **T** | **T** | **C** | **C** | **C** | **C** | **C** | **G** | **A** | **G** | **A** | **A** | **A** | **A** | **A** | **T** | **C** | **G** | **T** | **G** | **T** | **G** | **G** | **C** | **G** | **T** | **A** | **T** | **C** | **A** | **A** | **T** | **T** | **G** | **G** | **A** | **T** | **T** | **A** | **T** | **T** | **T** | **C** | **T** | **T** | **T** | **A** | **A** | **A** | **C** | **T** | **T** | **G** | **A** | **A** | **G** | **C** | **A** | **T** | **T** | **G** | **G** | **T** | **G** | **C** | **C** | **A** | **G** | **A** | **G** | **G** | **C** | **A** | **A** | **A** | **T** | **A** | **C** | **A** | **T** | **C** | **C** | **A** | **T** | **T** | **C** | **A** | **G** | **A** | **T** | **G** | **A** | **T** | **T** | **G** | **C** | **C** | **A** | **G** | **T** | **G** | **G** | **T** | **C** | **C** | **T** | **A** | **A** | **C** | **A** | **G** | **T** | **G** | **A** | **C** | **C** | **G** | **T** | **G** | **A** | **A** | **C** | **A** | **A** | **A** | **T** | **T** | **A** | **A** | **A** | **T** | **T** | **G** | **G** | **C** | **C** | **T** | **T** | **T** | **A** | **T** | **T** | **A** | **A** | **A** | **T** | **T** | **G** | **A** | **T** | **T** | **A** | **C** | **T** | **T** | **C** | **T** | **G** | **C** | **C** | **A** | **A** | **G** | **A** | **A** | **A** | **A** | **G** | **A** | **G** | **T** | **C** | **T** | **G** | **G** | **A** | **T** | **T** | **C** | **A** | **A** | **A** | **C** | **G** | **C** | **C** | **G** | **T** | **A** | **T** | **T** | **T** | **A** | **G** | **T** | **T** | **C** | **C** | **T** | **G** | **A** | **T** | **G** | **A** | **T** | **A** | **G** | **T** | **G** | **T** | **C** | **T** | **T** | **G** | **G** | **C** | **T** | **G** | **C** | **T** | **T** | **T** | **A** | **A** | **A** | **G** | **G** | **T** | **T** | **G** | **C** | **T** | **G** | **C** | **G** | **G** | **C** | **T** | **T** | **C** | **A** | **G** | **G** | **A** | **G** | **T** | **A** | **G** | **A** | **T** | **G** | **T** | **T** | **A** | **A** | **A** | **A** | **T** | **T** | **A** | **T** | **G** | **A** | **T** | **T** | **C** | **C** | **A** | **G** | **A** | **T** | **A** | **A** | **G** | **C** | **C** | **C** | **G** | **A** | **C** | **C** | **A** | **T** | **C** | **C** | **G** | **T** | **T** | **T** | **A** | **T** | **T** | **T** | **A** | **T** | **C** | **G** | **A** | **G** | **C** | **A** | **A** | **C** | **A** | **C** | **A** | **G** | **T** | **A** | **T** | **T** | **A** | **C** | **G** | **G** | **C** | **C** | **G** | **C** | **T** | **T** | **A** | **T** | **T** | **G** | **A** | **T** | **G** | **A** | **A** | **A** | **G** | **A** | **A** | **A** | **A** | **T** | **A** | **T** | **T** | **G** | **A** | **A** | **A** | **T** | **T** | **T** | **T** | **A** | **A** | **T** | **T** | **T** | **A** | **T** | **A** | **A** | **C** | **G** | **G** | **T** | **G** | **G** | **T** | **T** | **T** | **C** | **T** | **T** | **A** | **C** | **A** | **T** | **G** | **C** | **G** | **A** | **A** | **A** | **A** | **C** | **A** | **A** | **T** | **G** | **A** | **T** | **T** | **A** | **T** | **G** | **G** | **A** | **T** | **G** | **A** | **T** | **G** | **A** | **A** | **G** | **T** | **C** | **T** | **G** | **C** | **A** | **C** | **A** | **G** | **T** | **T** | **G** | **G** | **T** | **T** | **C** | **A** | **G** | **C** | **C** | **A** | **A** | **T** | **C** | **A** | **A** | **G** | **A** | **T** | **A** | **T** | **C** | **C** | **G** | **A** | **A** | **G** | **T** | **T** | **A** | **C** | **A** | **A** | **A** | **T** | **T** | **A** | **A** | **A** | **C** | **T** | **T** | **T** | **G** | **A** | **A** | **G** | **C** | **A** | **A** | **A** | **T** | **G** | **C** | **T** | **G** | **T** | **G** | **T** | **T** | **A** | **T** | **A** | **T** | **G** | **A** | **T** | **A** | **A** | **A** | **A** | **A** | **A** | **A** | **T** | **C** | **A** | **T** | **T** | **G** | **A** | **T** | **C** | **A** | **A** | **T** | **T** | **A** | **G** | **A** | **A** | **G** | **C** | **A** | **A** | **T** | **T** | **T** | **T** | **C** | **T** | **T** | **A** | **G** | **A** | **A** | **G** | **A** | **T** | **C** | **G** | **A** | **A** | **A** | **A** | **A** | **A** | **A** | **T** | **G** | **T** | **A** | **C** | **A** | **A** | **C** | **A** | **A** | **T** | **G** | **A** | **C** | **T** | **C** | **C** | **A** | **G** | **A** | **A** | **G** | **T** | **T** | **G** | **T** | **T** | **C** | **G** | **C** | **G** | **A** | **C** | **A** | **T** | **G** | **T** | **C** | **A** | **A** | **A** | **A** | **T** | **G** | **G** | **T** | **T** | **G** | **A** | **T** | **T** | **T** | **T** | **T** | **A** | **A** | **A** | **C** | **A** | **A** | **C** | **A** | **A** | **A** | **T** | **T** | **T** | **C** | **A** | **C** | **G** | **A** | **T** | **T** | **A** | **T** | **T** | **T** | **T** | **C** | **A** | **C** | **C** | **A** | **A** | **T** | **T** | **C** | **T** | **T** | **-** | **-** | **-** |
| **N55113** | **T** | **T** | **G** | **A** | **T** | **T** | **C** | **T** | **T** | **A** | **G** | **C** | **G** | **T** | **C** | **T** | **T** | **A** | **A** | **C** | **A** | **G** | **T** | **T** | **A** | **T** | **T** | **T** | **A** | **T** | **T** | **T** | **T** | **A** | **T** | **T** | **A** | **A** | **T** | **G** | **C** | **A** | **A** | **T** | **T** | **A** | **T** | **T** | **G** | **C** | **G** | **G** | **G** | **A** | **A** | **T** | **T** | **A** | **C** | **C** | **A** | **T** | **T** | **T** | **T** | **G** | **T** | **T** | **A** | **A** | **A** | **A** | **C** | **C** | **T** | **C** | **G** | **A** | **G** | **A** | **T** | **G** | **T** | **A** | **G** | **C** | **A** | **G** | **C** | **A** | **A** | **T** | **T** | **T** | **G** | **G** | **G** | **C** | **A** | **T** | **G** | **G** | **C** | **T** | **A** | **C** | **T** | **C** | **G** | **T** | **A** | **C** | **T** | **G** | **A** | **T** | **T** | **G** | **C** | **T** | **C** | **T** | **A** | **C** | **C** | **C** | **G** | **T** | **T** | **T** | **T** | **T** | **G** | **G** | **T** | **T** | **T** | **T** | **T** | **T** | **C** | **T** | **T** | **A** | **T** | **A** | **T** | **T** | **T** | **A** | **T** | **T** | **C** | **T** | **T** | **T** | **G** | **G** | **T** | **C** | **G** | **C** | **G** | **G** | **T** | **T** | **T** | **A** | **A** | **C** | **C** | **G** | **A** | **C** | **A** | **A** | **A** | **A** | **A** | **G** | **A** | **A** | **A** | **T** | **T** | **T** | **T** | **A** | **T** | **T** | **T** | **G** | **C** | **A** | **A** | **C** | **A** | **A** | **A** | **G** | **T** | **G** | **A** | **T** | **T** | **T** | **G** | **C** | **G** | **G** | **G** | **A** | **A** | **T** | **T** | **A** | **G** | **A** | **G** | **A** | **A** | **T** | **T** | **T** | **T** | **C** | **A** | **G** | **A** | **A** | **T** | **T** | **T** | **C** | **C** | **A** | **A** | **G** | **A** | **A** | **G** | **A** | **A** | **A** | **G** | **T** | **T** | **T** | **T** | **G** | **A** | **A** | **C** | **T** | **T** | **T** | **A** | **T** | **A** | **G** | **T** | **C** | **A** | **A** | **A** | **A** | **G** | **A** | **T** | **G** | **C** | **C** | **G** | **A** | **C** | **G** | **G** | **A** | **G** | **G** | **A** | **G** | **C** | **A** | **A** | **C** | **A** | **A** | **C** | **A** | **G** | **T** | **T** | **T** | **A** | **C** | **A** | **G** | **A** | **C** | **T** | **T** | **T** | **T** | **T** | **T** | **T** | **C** | **C** | **T** | **C** | **G** | **T** | **T** | **A** | **A** | **A** | **T** | **A** | **G** | **G** | **A** | **T** | **G** | **C** | **C** | **T** | **C** | **T** | **G** | **A** | **C** | **T** | **A** | **A** | **G** | **A** | **A** | **A** | **A** | **A** | **T** | **G** | **A** | **T** | **G** | **T** | **T** | **G** | **A** | **A** | **A** | **T** | **T** | **T** | **T** | **T** | **A** | **C** | **G** | **G** | **A** | **T** | **G** | **G** | **T** | **A** | **C** | **G** | **G** | **A** | **A** | **A** | **A** | **A** | **T** | **T** | **C** | **A** | **A** | **T** | **G** | **C** | **G** | **T** | **T** | **G** | **A** | **T** | **G** | **G** | **C** | **G** | **G** | **A** | **T** | **A** | **T** | **A** | **A** | **A** | **A** | **A** | **A** | **A** | **G** | **C** | **T** | **C** | **A** | **G** | **C** | **A** | **C** | **T** | **C** | **T** | **A** | **T** | **T** | **C** | **A** | **T** | **A** | **T** | **T** | **G** | **A** | **A** | **T** | **A** | **T** | **T** | **A** | **T** | **G** | **C** | **C** | **T** | **T** | **T** | **G** | **T** | **A** | **A** | **C** | **G** | **G** | **A** | **T** | **C** | **A** | **T** | **A** | **T** | **C** | **G** | **G** | **T** | **A** | **C** | **C** | **A** | **A** | **A** | **A** | **T** | **T** | **T** | **T** | **G** | **A** | **A** | **C** | **T** | **T** | **A** | **T** | **T** | **A** | **G** | **A** | **A** | **G** | **A** | **A** | **A** | **A** | **G** | **G** | **C** | **G** | **G** | **C** | **T** | **G** | **A** | **A** | **G** | **G** | **G** | **G** | **T** | **T** | **G** | **A** | **A** | **G** | **T** | **G** | **C** | **G** | **G** | **T** | **T** | **G** | **C** | **T** | **T** | **T** | **A** | **C** | **G** | **A** | **T** | **G** | **C** | **C** | **T** | **T** | **T** | **G** | **G** | **C** | **T** | **C** | **A** | **A** | **A** | **G** | **G** | **G** | **C** | **A** | **C** | **C** | **A** | **A** | **G** | **G** | **T** | **T** | **C** | **A** | **T** | **C** | **A** | **T** | **T** | **T** | **G** | **A** | **A** | **T** | **G** | **A** | **A** | **T** | **T** | **G** | **A** | **A** | **A** | **A** | **A** | **A** | **A** | **A** | **C** | **G** | **G** | **T** | **G** | **G** | **T** | **T** | **T** | **T** | **G** | **T** | **C** | **C** | **A** | **A** | **A** | **C** | **G** | **T** | **T** | **T** | **A** | **T** | **T** | **A** | **C** | **T** | **T** | **C** | **T** | **C** | **A** | **A** | **A** | **A** | **A** | **G** | **C** | **A** | **C** | **T** | **T** | **T** | **T** | **G** | **A** | **A** | **G** | **T** | **T** | **T** | **C** | **G** | **T** | **T** | **T** | **G** | **A** | **A** | **T** | **T** | **A** | **T** | **C** | **A** | **T** | **G** | **A** | **T** | **C** | **A** | **C** | **C** | **G** | **G** | **A** | **A** | **A** | **A** | **T** | **T** | **G** | **T** | **T** | **G** | **T** | **T** | **A** | **T** | **T** | **G** | **A** | **C** | **G** | **G** | **A** | **A** | **A** | **A** | **G** | **T** | **A** | **G** | **G** | **C** | **T** | **A** | **C** | **A** | **T** | **T** | **G** | **G** | **C** | **G** | **G** | **C** | **T** | **T** | **T** | **A** | **A** | **T** | **G** | **T** | **T** | **G** | **C** | **C** | **G** | **A** | **T** | **C** | **A** | **A** | **T** | **A** | **T** | **G** | **C** | **T** | **G** | **G** | **A** | **A** | **C** | **G** | **A** | **C** | **T** | **A** | **A** | **A** | **A** | **A** | **G** | **T** | **T** | **T** | **G** | **G** | **C** | **T** | **A** | **T** | **T** | **G** | **G** | **C** | **G** | **G** | **G** | **A** | **T** | **A** | **C** | **G** | **C** | **A** | **T** | **T** | **T** | **A** | **C** | **G** | **G** | **A** | **T** | **T** | **C** | **A** | **A** | **G** | **G** | **G** | **C** | **C** | **A** | **G** | **C** | **A** | **G** | **C** | **C** | **T** | **C** | **A** | **T** | **T** | **A** | **C** | **T** | **G** | **C** | **A** | **A** | **A** | **T** | **G** | **C** | **G** | **T** | **T** | **T** | **T** | **T** | **T** | **A** | **A** | **T** | **G** | **G** | **A** | **T** | **T** | **G** | **G** | **A** | **A** | **C** | **G** | **T** | **C** | **T** | **C** | **T** | **T** | **C** | **C** | **C** | **C** | **C** | **G** | **A** | **G** | **A** | **A** | **A** | **A** | **A** | **T** | **C** | **G** | **T** | **G** | **T** | **G** | **G** | **C** | **G** | **T** | **A** | **T** | **C** | **A** | **A** | **T** | **T** | **G** | **G** | **A** | **T** | **T** | **A** | **T** | **T** | **T** | **C** | **T** | **T** | **T** | **A** | **A** | **A** | **C** | **T** | **T** | **G** | **A** | **A** | **G** | **C** | **A** | **T** | **T** | **G** | **G** | **T** | **G** | **C** | **C** | **A** | **G** | **A** | **G** | **G** | **C** | **A** | **A** | **A** | **T** | **A** | **C** | **A** | **T** | **C** | **C** | **A** | **T** | **T** | **C** | **A** | **G** | **A** | **T** | **G** | **A** | **T** | **T** | **G** | **C** | **C** | **A** | **G** | **T** | **G** | **G** | **T** | **C** | **C** | **T** | **A** | **A** | **C** | **A** | **G** | **T** | **G** | **A** | **C** | **C** | **G** | **T** | **G** | **A** | **A** | **C** | **A** | **A** | **A** | **T** | **T** | **A** | **A** | **A** | **T** | **T** | **G** | **G** | **C** | **C** | **T** | **T** | **T** | **A** | **T** | **T** | **A** | **A** | **A** | **T** | **T** | **G** | **A** | **T** | **T** | **A** | **C** | **T** | **T** | **C** | **T** | **G** | **C** | **C** | **A** | **A** | **G** | **A** | **A** | **A** | **A** | **G** | **A** | **G** | **T** | **C** | **T** | **G** | **G** | **A** | **T** | **T** | **C** | **A** | **A** | **A** | **C** | **G** | **C** | **C** | **G** | **T** | **A** | **T** | **T** | **T** | **A** | **G** | **T** | **T** | **C** | **C** | **T** | **G** | **A** | **T** | **G** | **A** | **T** | **A** | **G** | **T** | **G** | **T** | **C** | **T** | **T** | **G** | **G** | **C** | **T** | **G** | **C** | **T** | **T** | **T** | **A** | **A** | **A** | **G** | **G** | **T** | **T** | **G** | **C** | **T** | **G** | **C** | **G** | **G** | **C** | **T** | **T** | **C** | **A** | **G** | **G** | **A** | **G** | **T** | **A** | **G** | **A** | **T** | **G** | **T** | **T** | **A** | **A** | **A** | **A** | **T** | **T** | **A** | **T** | **G** | **A** | **T** | **T** | **C** | **C** | **A** | **G** | **A** | **T** | **A** | **A** | **G** | **C** | **C** | **C** | **G** | **A** | **C** | **C** | **A** | **T** | **C** | **C** | **G** | **T** | **T** | **T** | **A** | **T** | **T** | **T** | **A** | **T** | **C** | **G** | **A** | **G** | **C** | **A** | **A** | **C** | **A** | **C** | **A** | **G** | **T** | **A** | **T** | **T** | **A** | **C** | **G** | **G** | **C** | **C** | **G** | **C** | **T** | **T** | **A** | **T** | **T** | **G** | **A** | **T** | **G** | **A** | **A** | **A** | **G** | **A** | **A** | **A** | **A** | **T** | **A** | **T** | **T** | **G** | **A** | **A** | **A** | **T** | **T** | **T** | **T** | **A** | **A** | **T** | **T** | **T** | **A** | **T** | **A** | **A** | **C** | **G** | **G** | **T** | **G** | **G** | **T** | **T** | **T** | **C** | **T** | **T** | **A** | **C** | **A** | **T** | **G** | **C** | **G** | **A** | **A** | **A** | **A** | **C** | **A** | **A** | **T** | **G** | **A** | **T** | **T** | **A** | **T** | **G** | **G** | **A** | **T** | **G** | **A** | **T** | **G** | **A** | **A** | **G** | **T** | **C** | **T** | **G** | **C** | **A** | **C** | **A** | **G** | **T** | **T** | **G** | **G** | **T** | **T** | **C** | **A** | **G** | **C** | **C** | **A** | **A** | **T** | **C** | **A** | **A** | **G** | **A** | **T** | **A** | **T** | **C** | **C** | **G** | **A** | **A** | **G** | **T** | **T** | **A** | **C** | **A** | **A** | **A** | **T** | **T** | **A** | **A** | **A** | **C** | **T** | **T** | **T** | **G** | **A** | **A** | **G** | **C** | **A** | **A** | **A** | **T** | **G** | **C** | **T** | **G** | **T** | **G** | **T** | **T** | **A** | **T** | **A** | **T** | **G** | **A** | **T** | **A** | **A** | **A** | **A** | **A** | **A** | **A** | **T** | **C** | **A** | **T** | **T** | **G** | **A** | **T** | **C** | **A** | **A** | **T** | **T** | **A** | **G** | **A** | **A** | **G** | **C** | **A** | **A** | **T** | **T** | **T** | **T** | **C** | **T** | **T** | **A** | **G** | **A** | **A** | **G** | **A** | **T** | **C** | **G** | **A** | **A** | **A** | **A** | **A** | **A** | **A** | **T** | **G** | **T** | **A** | **C** | **A** | **A** | **C** | **A** | **A** | **T** | **G** | **A** | **C** | **T** | **C** | **C** | **A** | **G** | **A** | **A** | **G** | **T** | **T** | **G** | **T** | **T** | **C** | **G** | **C** | **G** | **A** | **C** | **A** | **T** | **G** | **T** | **C** | **A** | **A** | **A** | **A** | **T** | **G** | **G** | **T** | **T** | **G** | **A** | **T** | **T** | **T** | **T** | **T** | **A** | **A** | **A** | **C** | **A** | **G** | **C** | **A** | **A** | **A** | **T** | **T** | **T** | **C** | **A** | **C** | **G** | **A** | **T** | **T** | **A** | **T** | **T** | **T** | **T** | **C** | **A** | **C** | **C** | **A** | **A** | **T** | **T** | **C** | **T** | **T** | **-** | **-** | **-** |
| **N59896F** | **T** | **T** | **G** | **A** | **T** | **T** | **C** | **T** | **T** | **A** | **G** | **T** | **G** | **T** | **C** | **T** | **T** | **A** | **A** | **C** | **A** | **G** | **T** | **T** | **A** | **T** | **T** | **T** | **A** | **T** | **T** | **T** | **T** | **A** | **T** | **T** | **A** | **A** | **T** | **G** | **C** | **A** | **A** | **T** | **T** | **A** | **T** | **T** | **G** | **C** | **G** | **G** | **G** | **A** | **A** | **T** | **T** | **A** | **C** | **C** | **A** | **T** | **T** | **T** | **T** | **G** | **T** | **T** | **A** | **A** | **A** | **A** | **C** | **C** | **T** | **C** | **G** | **A** | **G** | **A** | **T** | **G** | **T** | **A** | **G** | **C** | **A** | **G** | **C** | **A** | **A** | **T** | **T** | **T** | **G** | **G** | **G** | **C** | **A** | **T** | **G** | **G** | **C** | **T** | **A** | **C** | **T** | **C** | **G** | **T** | **A** | **C** | **T** | **G** | **A** | **T** | **T** | **G** | **C** | **T** | **C** | **T** | **A** | **C** | **C** | **C** | **G** | **T** | **T** | **T** | **T** | **T** | **G** | **G** | **T** | **T** | **T** | **T** | **T** | **T** | **C** | **T** | **T** | **A** | **T** | **A** | **T** | **T** | **T** | **A** | **T** | **T** | **C** | **T** | **T** | **T** | **G** | **G** | **G** | **C** | **G** | **C** | **G** | **G** | **T** | **T** | **T** | **A** | **A** | **C** | **C** | **G** | **A** | **C** | **A** | **A** | **A** | **A** | **A** | **G** | **A** | **A** | **A** | **T** | **T** | **T** | **T** | **A** | **T** | **T** | **T** | **G** | **C** | **A** | **A** | **C** | **A** | **A** | **A** | **G** | **T** | **G** | **A** | **T** | **T** | **T** | **G** | **C** | **G** | **G** | **G** | **A** | **A** | **T** | **T** | **A** | **G** | **A** | **G** | **A** | **A** | **T** | **T** | **T** | **T** | **C** | **A** | **G** | **A** | **A** | **T** | **T** | **T** | **C** | **C** | **A** | **A** | **G** | **A** | **A** | **G** | **A** | **A** | **A** | **G** | **T** | **T** | **T** | **T** | **G** | **A** | **A** | **C** | **T** | **T** | **T** | **A** | **T** | **A** | **G** | **T** | **C** | **A** | **A** | **A** | **A** | **G** | **A** | **T** | **G** | **C** | **C** | **G** | **A** | **C** | **G** | **G** | **A** | **G** | **G** | **A** | **G** | **C** | **A** | **A** | **C** | **A** | **A** | **C** | **A** | **G** | **T** | **T** | **T** | **A** | **C** | **A** | **G** | **A** | **C** | **T** | **T** | **T** | **T** | **T** | **T** | **T** | **C** | **C** | **T** | **C** | **G** | **T** | **T** | **A** | **A** | **A** | **T** | **A** | **G** | **G** | **A** | **T** | **G** | **C** | **C** | **T** | **C** | **T** | **G** | **A** | **C** | **T** | **A** | **A** | **G** | **A** | **A** | **A** | **A** | **A** | **T** | **G** | **A** | **T** | **G** | **T** | **T** | **G** | **A** | **A** | **A** | **T** | **T** | **T** | **T** | **T** | **A** | **C** | **G** | **G** | **A** | **T** | **G** | **G** | **T** | **A** | **C** | **G** | **G** | **A** | **A** | **A** | **A** | **A** | **T** | **T** | **C** | **A** | **A** | **T** | **G** | **C** | **G** | **T** | **T** | **G** | **A** | **T** | **G** | **G** | **C** | **G** | **G** | **A** | **C** | **A** | **T** | **A** | **A** | **A** | **A** | **A** | **A** | **A** | **G** | **C** | **T** | **C** | **A** | **G** | **C** | **A** | **C** | **T** | **C** | **C** | **A** | **T** | **T** | **C** | **A** | **T** | **A** | **T** | **T** | **G** | **A** | **A** | **T** | **A** | **T** | **T** | **A** | **T** | **G** | **C** | **C** | **T** | **T** | **T** | **G** | **T** | **A** | **A** | **C** | **G** | **G** | **A** | **T** | **C** | **A** | **T** | **A** | **T** | **C** | **G** | **G** | **C** | **A** | **C** | **C** | **A** | **A** | **A** | **A** | **T** | **T** | **T** | **T** | **G** | **A** | **A** | **C** | **T** | **T** | **A** | **T** | **T** | **A** | **G** | **A** | **A** | **G** | **A** | **A** | **A** | **A** | **G** | **G** | **C** | **G** | **G** | **C** | **T** | **G** | **A** | **A** | **G** | **G** | **G** | **G** | **T** | **T** | **G** | **A** | **A** | **G** | **T** | **G** | **C** | **G** | **G** | **T** | **T** | **G** | **C** | **T** | **T** | **T** | **A** | **C** | **G** | **A** | **T** | **G** | **C** | **C** | **T** | **T** | **T** | **G** | **G** | **C** | **T** | **C** | **A** | **A** | **A** | **G** | **G** | **G** | **C** | **A** | **C** | **C** | **A** | **A** | **G** | **G** | **T** | **T** | **C** | **A** | **T** | **C** | **A** | **T** | **T** | **T** | **G** | **A** | **A** | **T** | **G** | **A** | **A** | **T** | **T** | **G** | **A** | **A** | **A** | **A** | **A** | **A** | **A** | **A** | **C** | **G** | **G** | **T** | **G** | **G** | **T** | **T** | **T** | **T** | **G** | **T** | **C** | **C** | **A** | **A** | **A** | **C** | **G** | **T** | **T** | **T** | **A** | **T** | **T** | **A** | **C** | **T** | **T** | **C** | **T** | **C** | **A** | **A** | **A** | **A** | **A** | **G** | **C** | **A** | **C** | **T** | **T** | **T** | **T** | **G** | **A** | **A** | **G** | **T** | **T** | **T** | **C** | **G** | **T** | **T** | **T** | **G** | **A** | **A** | **T** | **T** | **A** | **T** | **C** | **A** | **T** | **G** | **A** | **T** | **C** | **A** | **C** | **C** | **G** | **G** | **A** | **A** | **A** | **A** | **T** | **T** | **G** | **T** | **T** | **G** | **T** | **T** | **A** | **T** | **T** | **G** | **A** | **C** | **G** | **G** | **A** | **A** | **A** | **A** | **G** | **T** | **A** | **G** | **G** | **C** | **T** | **A** | **C** | **A** | **T** | **T** | **G** | **G** | **C** | **G** | **G** | **C** | **T** | **T** | **T** | **A** | **A** | **T** | **G** | **T** | **T** | **G** | **C** | **C** | **G** | **A** | **T** | **C** | **A** | **A** | **T** | **A** | **T** | **G** | **C** | **C** | **G** | **G** | **A** | **A** | **C** | **G** | **A** | **C** | **T** | **A** | **A** | **A** | **A** | **A** | **G** | **T** | **T** | **T** | **G** | **G** | **C** | **T** | **A** | **T** | **T** | **G** | **G** | **C** | **G** | **G** | **G** | **A** | **T** | **A** | **C** | **A** | **C** | **A** | **T** | **T** | **T** | **A** | **C** | **G** | **G** | **A** | **T** | **T** | **C** | **A** | **A** | **G** | **G** | **G** | **C** | **C** | **A** | **G** | **C** | **A** | **G** | **C** | **C** | **T** | **C** | **A** | **T** | **T** | **A** | **C** | **T** | **G** | **C** | **A** | **A** | **A** | **T** | **G** | **C** | **G** | **T** | **T** | **T** | **T** | **T** | **T** | **A** | **A** | **T** | **G** | **G** | **A** | **T** | **T** | **G** | **G** | **A** | **A** | **C** | **G** | **T** | **C** | **T** | **C** | **T** | **T** | **C** | **C** | **C** | **C** | **C** | **G** | **A** | **G** | **A** | **A** | **A** | **A** | **A** | **T** | **C** | **G** | **T** | **G** | **T** | **G** | **G** | **C** | **G** | **T** | **A** | **T** | **C** | **A** | **A** | **T** | **T** | **G** | **G** | **A** | **T** | **T** | **A** | **T** | **T** | **T** | **C** | **T** | **T** | **T** | **A** | **A** | **A** | **C** | **T** | **T** | **G** | **A** | **A** | **G** | **C** | **A** | **T** | **T** | **G** | **G** | **T** | **G** | **C** | **C** | **A** | **G** | **A** | **G** | **G** | **C** | **A** | **A** | **A** | **T** | **A** | **C** | **A** | **T** | **C** | **C** | **A** | **T** | **T** | **C** | **A** | **G** | **A** | **T** | **G** | **A** | **T** | **T** | **G** | **C** | **C** | **A** | **G** | **T** | **G** | **G** | **T** | **C** | **C** | **T** | **A** | **A** | **C** | **A** | **G** | **T** | **G** | **A** | **C** | **C** | **G** | **T** | **G** | **A** | **A** | **C** | **A** | **A** | **A** | **T** | **T** | **A** | **A** | **A** | **T** | **T** | **G** | **G** | **C** | **C** | **T** | **T** | **T** | **A** | **T** | **T** | **A** | **A** | **A** | **T** | **T** | **G** | **A** | **T** | **T** | **A** | **C** | **T** | **T** | **C** | **T** | **G** | **C** | **C** | **A** | **A** | **G** | **A** | **A** | **A** | **A** | **G** | **A** | **G** | **T** | **C** | **T** | **G** | **G** | **A** | **T** | **T** | **C** | **A** | **A** | **A** | **C** | **G** | **C** | **C** | **G** | **T** | **A** | **T** | **T** | **T** | **A** | **G** | **T** | **T** | **C** | **C** | **T** | **G** | **A** | **T** | **G** | **A** | **C** | **A** | **G** | **T** | **G** | **T** | **C** | **T** | **T** | **G** | **G** | **C** | **T** | **G** | **C** | **T** | **T** | **T** | **A** | **A** | **A** | **G** | **G** | **T** | **T** | **G** | **C** | **T** | **G** | **C** | **G** | **G** | **C** | **T** | **T** | **C** | **A** | **G** | **G** | **A** | **G** | **T** | **A** | **G** | **A** | **T** | **G** | **T** | **T** | **A** | **A** | **A** | **A** | **T** | **T** | **A** | **T** | **G** | **A** | **T** | **T** | **C** | **C** | **A** | **G** | **A** | **T** | **A** | **A** | **G** | **C** | **C** | **C** | **G** | **A** | **C** | **C** | **A** | **T** | **C** | **C** | **G** | **T** | **T** | **T** | **A** | **T** | **T** | **T** | **A** | **T** | **C** | **G** | **A** | **G** | **C** | **A** | **A** | **C** | **A** | **C** | **A** | **G** | **T** | **A** | **T** | **T** | **A** | **C** | **G** | **G** | **C** | **C** | **G** | **C** | **T** | **T** | **A** | **T** | **T** | **G** | **A** | **T** | **G** | **A** | **A** | **A** | **G** | **A** | **A** | **A** | **A** | **T** | **A** | **T** | **T** | **G** | **A** | **A** | **A** | **T** | **T** | **T** | **T** | **A** | **A** | **T** | **T** | **T** | **A** | **T** | **A** | **A** | **C** | **G** | **G** | **T** | **G** | **G** | **T** | **T** | **T** | **C** | **T** | **T** | **A** | **C** | **A** | **T** | **G** | **C** | **G** | **A** | **A** | **A** | **A** | **C** | **A** | **A** | **T** | **G** | **A** | **T** | **T** | **A** | **T** | **G** | **G** | **A** | **T** | **G** | **A** | **T** | **G** | **A** | **A** | **G** | **T** | **C** | **T** | **G** | **C** | **A** | **C** | **A** | **G** | **T** | **T** | **G** | **G** | **T** | **T** | **C** | **A** | **G** | **C** | **C** | **A** | **A** | **T** | **C** | **A** | **A** | **G** | **A** | **T** | **A** | **T** | **C** | **C** | **G** | **A** | **A** | **G** | **T** | **T** | **A** | **C** | **A** | **A** | **A** | **T** | **T** | **A** | **A** | **A** | **C** | **T** | **T** | **T** | **G** | **A** | **A** | **G** | **C** | **A** | **A** | **A** | **T** | **G** | **C** | **T** | **G** | **T** | **G** | **T** | **T** | **A** | **T** | **A** | **T** | **G** | **A** | **T** | **A** | **A** | **A** | **A** | **A** | **A** | **A** | **T** | **C** | **A** | **T** | **T** | **G** | **A** | **T** | **C** | **A** | **A** | **T** | **T** | **A** | **G** | **A** | **A** | **G** | **C** | **A** | **A** | **T** | **T** | **T** | **T** | **C** | **T** | **T** | **A** | **G** | **A** | **A** | **G** | **A** | **T** | **C** | **G** | **A** | **A** | **A** | **A** | **A** | **A** | **A** | **T** | **G** | **T** | **A** | **C** | **A** | **A** | **C** | **A** | **A** | **T** | **G** | **A** | **C** | **T** | **C** | **C** | **A** | **G** | **A** | **A** | **G** | **T** | **T** | **G** | **T** | **T** | **C** | **G** | **C** | **G** | **A** | **C** | **A** | **T** | **G** | **T** | **C** | **A** | **A** | **A** | **A** | **T** | **G** | **G** | **T** | **T** | **G** | **A** | **T** | **T** | **T** | **T** | **T** | **A** | **A** | **A** | **C** | **A** | **A** | **C** | **A** | **A** | **A** | **T** | **T** | **T** | **C** | **A** | **C** | **G** | **A** | **T** | **T** | **A** | **T** | **T** | **T** | **T** | **C** | **A** | **C** | **C** | **A** | **A** | **T** | **T** | **C** | **T** | **T** | **-** | **-** | **-** |
| **N52729** | **T** | **T** | **G** | **A** | **T** | **T** | **C** | **T** | **T** | **A** | **G** | **C** | **G** | **T** | **C** | **T** | **T** | **A** | **A** | **C** | **A** | **G** | **T** | **T** | **A** | **T** | **T** | **T** | **A** | **T** | **T** | **T** | **T** | **A** | **T** | **T** | **A** | **A** | **T** | **G** | **C** | **A** | **A** | **T** | **T** | **A** | **T** | **T** | **G** | **C** | **G** | **G** | **G** | **A** | **A** | **T** | **T** | **A** | **C** | **C** | **A** | **T** | **T** | **T** | **T** | **G** | **T** | **T** | **A** | **A** | **A** | **A** | **C** | **C** | **T** | **C** | **G** | **A** | **G** | **A** | **T** | **G** | **T** | **A** | **G** | **C** | **A** | **G** | **C** | **A** | **A** | **T** | **T** | **T** | **G** | **G** | **G** | **C** | **A** | **T** | **G** | **G** | **C** | **T** | **A** | **C** | **T** | **C** | **G** | **T** | **A** | **C** | **T** | **G** | **A** | **T** | **T** | **G** | **C** | **T** | **C** | **T** | **A** | **C** | **C** | **C** | **G** | **T** | **T** | **T** | **T** | **T** | **G** | **G** | **T** | **T** | **T** | **T** | **T** | **T** | **C** | **T** | **T** | **A** | **T** | **A** | **T** | **T** | **T** | **A** | **T** | **T** | **C** | **T** | **T** | **T** | **G** | **G** | **G** | **C** | **G** | **C** | **G** | **G** | **T** | **T** | **T** | **A** | **A** | **C** | **C** | **G** | **A** | **C** | **A** | **A** | **A** | **A** | **A** | **G** | **A** | **A** | **A** | **T** | **T** | **T** | **T** | **A** | **T** | **T** | **T** | **G** | **C** | **A** | **A** | **C** | **A** | **A** | **A** | **G** | **T** | **G** | **A** | **T** | **T** | **T** | **G** | **C** | **G** | **G** | **G** | **A** | **A** | **T** | **T** | **A** | **G** | **A** | **G** | **A** | **A** | **T** | **T** | **T** | **T** | **C** | **A** | **G** | **A** | **A** | **T** | **T** | **T** | **C** | **C** | **A** | **A** | **G** | **A** | **A** | **G** | **A** | **A** | **A** | **G** | **T** | **T** | **T** | **T** | **G** | **A** | **A** | **C** | **T** | **T** | **T** | **A** | **T** | **A** | **G** | **T** | **C** | **A** | **A** | **A** | **A** | **G** | **A** | **T** | **G** | **C** | **C** | **G** | **A** | **C** | **G** | **G** | **A** | **G** | **G** | **A** | **G** | **C** | **A** | **A** | **C** | **A** | **A** | **C** | **A** | **G** | **T** | **T** | **T** | **A** | **C** | **A** | **G** | **A** | **C** | **T** | **T** | **T** | **T** | **T** | **T** | **T** | **C** | **C** | **T** | **C** | **G** | **T** | **T** | **G** | **A** | **A** | **T** | **A** | **G** | **G** | **A** | **T** | **G** | **C** | **C** | **T** | **C** | **T** | **G** | **A** | **C** | **T** | **A** | **A** | **G** | **A** | **A** | **A** | **A** | **A** | **T** | **G** | **A** | **T** | **G** | **T** | **T** | **C** | **A** | **A** | **A** | **T** | **T** | **T** | **T** | **T** | **A** | **C** | **G** | **G** | **A** | **T** | **G** | **G** | **T** | **A** | **C** | **G** | **G** | **A** | **A** | **A** | **A** | **A** | **T** | **T** | **C** | **A** | **A** | **T** | **G** | **C** | **G** | **T** | **T** | **G** | **A** | **T** | **G** | **G** | **C** | **G** | **G** | **A** | **C** | **A** | **T** | **A** | **A** | **A** | **A** | **A** | **A** | **A** | **G** | **C** | **T** | **C** | **A** | **G** | **C** | **A** | **C** | **T** | **C** | **C** | **A** | **T** | **T** | **C** | **A** | **T** | **A** | **T** | **T** | **G** | **A** | **A** | **T** | **A** | **T** | **T** | **A** | **T** | **G** | **C** | **C** | **T** | **T** | **T** | **G** | **T** | **A** | **A** | **C** | **G** | **G** | **A** | **T** | **C** | **A** | **T** | **A** | **T** | **C** | **G** | **G** | **C** | **A** | **C** | **C** | **A** | **A** | **A** | **A** | **T** | **T** | **T** | **T** | **G** | **A** | **A** | **C** | **T** | **T** | **A** | **T** | **T** | **A** | **G** | **A** | **A** | **G** | **A** | **A** | **A** | **A** | **G** | **G** | **C** | **G** | **G** | **C** | **T** | **G** | **A** | **A** | **G** | **G** | **G** | **G** | **T** | **T** | **G** | **A** | **A** | **G** | **T** | **G** | **C** | **G** | **G** | **T** | **T** | **G** | **C** | **T** | **T** | **T** | **A** | **C** | **G** | **A** | **T** | **G** | **C** | **C** | **T** | **T** | **T** | **G** | **G** | **C** | **T** | **C** | **A** | **A** | **A** | **G** | **G** | **G** | **C** | **A** | **C** | **C** | **A** | **A** | **G** | **G** | **T** | **T** | **C** | **A** | **T** | **C** | **A** | **T** | **T** | **T** | **G** | **A** | **A** | **T** | **G** | **A** | **A** | **T** | **T** | **G** | **A** | **A** | **A** | **A** | **A** | **A** | **A** | **A** | **C** | **G** | **G** | **T** | **G** | **G** | **T** | **T** | **T** | **T** | **G** | **T** | **C** | **C** | **A** | **A** | **A** | **C** | **G** | **T** | **T** | **T** | **A** | **T** | **T** | **A** | **C** | **T** | **T** | **C** | **T** | **C** | **A** | **A** | **A** | **A** | **A** | **G** | **C** | **A** | **C** | **T** | **T** | **T** | **T** | **G** | **A** | **A** | **G** | **T** | **T** | **T** | **C** | **G** | **T** | **T** | **T** | **G** | **A** | **A** | **T** | **T** | **A** | **T** | **C** | **A** | **T** | **G** | **A** | **T** | **C** | **A** | **C** | **C** | **G** | **G** | **A** | **A** | **A** | **A** | **T** | **T** | **G** | **T** | **T** | **G** | **T** | **T** | **A** | **T** | **T** | **G** | **A** | **C** | **G** | **G** | **A** | **A** | **A** | **A** | **G** | **T** | **A** | **G** | **G** | **C** | **T** | **A** | **C** | **A** | **T** | **T** | **G** | **G** | **C** | **G** | **G** | **C** | **T** | **T** | **T** | **A** | **A** | **T** | **G** | **T** | **T** | **G** | **C** | **C** | **G** | **A** | **T** | **C** | **A** | **A** | **T** | **A** | **T** | **G** | **C** | **C** | **G** | **G** | **A** | **A** | **C** | **G** | **A** | **C** | **T** | **A** | **A** | **A** | **A** | **A** | **G** | **T** | **T** | **T** | **G** | **G** | **C** | **T** | **A** | **T** | **T** | **G** | **G** | **C** | **G** | **G** | **G** | **A** | **T** | **A** | **C** | **A** | **C** | **A** | **T** | **T** | **T** | **A** | **C** | **G** | **G** | **A** | **T** | **T** | **C** | **A** | **A** | **G** | **G** | **G** | **C** | **C** | **A** | **G** | **C** | **A** | **G** | **C** | **C** | **T** | **C** | **A** | **T** | **T** | **A** | **C** | **T** | **G** | **C** | **A** | **A** | **A** | **T** | **G** | **C** | **G** | **T** | **T** | **T** | **T** | **T** | **T** | **A** | **A** | **T** | **G** | **G** | **A** | **T** | **T** | **G** | **G** | **A** | **A** | **C** | **G** | **T** | **C** | **T** | **C** | **T** | **T** | **C** | **C** | **C** | **C** | **C** | **G** | **A** | **G** | **A** | **A** | **A** | **A** | **A** | **T** | **C** | **G** | **T** | **G** | **T** | **G** | **G** | **C** | **G** | **T** | **A** | **T** | **C** | **A** | **A** | **T** | **T** | **G** | **G** | **A** | **T** | **T** | **A** | **T** | **T** | **T** | **C** | **T** | **T** | **T** | **A** | **A** | **A** | **C** | **T** | **T** | **G** | **A** | **A** | **G** | **C** | **A** | **T** | **T** | **G** | **G** | **T** | **G** | **C** | **C** | **A** | **G** | **A** | **G** | **G** | **C** | **A** | **A** | **A** | **T** | **A** | **C** | **A** | **T** | **C** | **C** | **A** | **T** | **T** | **C** | **A** | **G** | **A** | **T** | **G** | **A** | **T** | **T** | **G** | **C** | **C** | **A** | **G** | **T** | **G** | **G** | **T** | **C** | **C** | **T** | **A** | **A** | **C** | **A** | **G** | **T** | **G** | **A** | **C** | **C** | **G** | **T** | **G** | **A** | **A** | **C** | **A** | **A** | **A** | **T** | **T** | **A** | **A** | **A** | **T** | **T** | **G** | **G** | **C** | **C** | **T** | **T** | **T** | **A** | **T** | **T** | **A** | **A** | **A** | **T** | **T** | **G** | **A** | **T** | **T** | **A** | **C** | **T** | **T** | **C** | **T** | **G** | **C** | **C** | **A** | **A** | **G** | **A** | **A** | **A** | **A** | **G** | **A** | **G** | **T** | **C** | **T** | **G** | **G** | **A** | **T** | **T** | **C** | **A** | **A** | **A** | **C** | **G** | **C** | **C** | **G** | **T** | **A** | **T** | **T** | **T** | **A** | **G** | **T** | **T** | **C** | **C** | **T** | **G** | **A** | **T** | **G** | **A** | **T** | **A** | **G** | **T** | **G** | **T** | **C** | **T** | **T** | **G** | **G** | **C** | **T** | **G** | **C** | **T** | **T** | **T** | **A** | **A** | **A** | **G** | **G** | **T** | **T** | **G** | **C** | **T** | **G** | **C** | **G** | **G** | **C** | **T** | **T** | **C** | **A** | **G** | **G** | **A** | **G** | **T** | **A** | **G** | **A** | **T** | **G** | **T** | **T** | **A** | **A** | **A** | **A** | **T** | **T** | **A** | **T** | **G** | **A** | **T** | **T** | **C** | **C** | **A** | **G** | **A** | **T** | **A** | **A** | **G** | **C** | **C** | **C** | **G** | **A** | **C** | **C** | **A** | **T** | **C** | **C** | **G** | **T** | **T** | **T** | **A** | **T** | **T** | **T** | **A** | **T** | **C** | **G** | **A** | **G** | **C** | **A** | **A** | **C** | **A** | **C** | **A** | **G** | **T** | **A** | **T** | **T** | **A** | **C** | **G** | **G** | **C** | **C** | **G** | **C** | **T** | **T** | **A** | **T** | **T** | **G** | **A** | **T** | **G** | **A** | **A** | **A** | **G** | **A** | **A** | **A** | **A** | **T** | **A** | **T** | **T** | **G** | **A** | **A** | **A** | **T** | **T** | **T** | **T** | **A** | **A** | **T** | **T** | **T** | **A** | **T** | **A** | **A** | **C** | **G** | **G** | **T** | **G** | **G** | **T** | **T** | **T** | **C** | **T** | **T** | **A** | **C** | **A** | **T** | **G** | **C** | **G** | **A** | **A** | **A** | **A** | **C** | **A** | **A** | **T** | **G** | **A** | **T** | **T** | **A** | **T** | **G** | **G** | **A** | **T** | **G** | **A** | **T** | **G** | **A** | **A** | **G** | **T** | **C** | **T** | **G** | **C** | **A** | **C** | **A** | **G** | **T** | **T** | **G** | **G** | **T** | **T** | **C** | **A** | **G** | **C** | **C** | **A** | **A** | **T** | **C** | **A** | **A** | **G** | **A** | **T** | **A** | **T** | **C** | **C** | **G** | **A** | **A** | **G** | **T** | **T** | **A** | **C** | **A** | **A** | **A** | **T** | **T** | **A** | **A** | **A** | **C** | **T** | **T** | **T** | **G** | **A** | **A** | **G** | **C** | **A** | **A** | **A** | **T** | **G** | **C** | **T** | **G** | **T** | **G** | **T** | **T** | **A** | **T** | **A** | **T** | **G** | **A** | **T** | **A** | **A** | **A** | **A** | **A** | **A** | **A** | **T** | **C** | **A** | **T** | **T** | **G** | **A** | **T** | **C** | **A** | **A** | **T** | **T** | **A** | **G** | **A** | **A** | **G** | **C** | **A** | **A** | **T** | **T** | **T** | **T** | **C** | **T** | **T** | **A** | **G** | **A** | **A** | **G** | **A** | **T** | **C** | **G** | **A** | **A** | **A** | **A** | **A** | **A** | **A** | **T** | **G** | **T** | **A** | **C** | **A** | **A** | **C** | **A** | **A** | **T** | **G** | **A** | **C** | **T** | **C** | **C** | **A** | **G** | **A** | **A** | **G** | **T** | **T** | **G** | **T** | **T** | **C** | **G** | **C** | **G** | **A** | **C** | **A** | **T** | **G** | **T** | **C** | **A** | **A** | **A** | **A** | **T** | **G** | **G** | **T** | **T** | **G** | **A** | **T** | **T** | **T** | **T** | **T** | **A** | **A** | **A** | **C** | **A** | **A** | **C** | **A** | **A** | **A** | **T** | **T** | **T** | **C** | **A** | **C** | **G** | **A** | **T** | **T** | **A** | **T** | **T** | **T** | **T** | **C** | **A** | **C** | **C** | **A** | **A** | **T** | **T** | **C** | **T** | **T** | **-** | **-** | **-** |
| **N59959F** | **T** | **T** | **G** | **A** | **T** | **T** | **C** | **T** | **T** | **A** | **G** | **C** | **G** | **T** | **C** | **T** | **T** | **A** | **A** | **C** | **A** | **G** | **T** | **T** | **A** | **T** | **T** | **T** | **A** | **T** | **T** | **T** | **T** | **A** | **T** | **T** | **A** | **A** | **T** | **G** | **C** | **A** | **A** | **T** | **T** | **A** | **T** | **T** | **G** | **C** | **G** | **G** | **G** | **A** | **A** | **T** | **T** | **A** | **C** | **C** | **A** | **T** | **T** | **T** | **T** | **G** | **T** | **T** | **A** | **A** | **A** | **A** | **C** | **C** | **T** | **C** | **G** | **A** | **G** | **A** | **T** | **G** | **T** | **A** | **G** | **C** | **A** | **G** | **C** | **A** | **A** | **T** | **T** | **T** | **G** | **G** | **G** | **C** | **A** | **T** | **G** | **G** | **C** | **T** | **A** | **C** | **T** | **C** | **G** | **T** | **A** | **C** | **T** | **G** | **A** | **T** | **T** | **G** | **C** | **T** | **C** | **T** | **A** | **C** | **C** | **C** | **G** | **T** | **T** | **T** | **T** | **T** | **G** | **G** | **T** | **T** | **T** | **T** | **T** | **T** | **C** | **T** | **T** | **A** | **T** | **A** | **T** | **T** | **T** | **A** | **T** | **T** | **C** | **T** | **T** | **T** | **G** | **G** | **G** | **C** | **G** | **C** | **G** | **G** | **T** | **T** | **T** | **A** | **A** | **C** | **C** | **G** | **A** | **C** | **A** | **A** | **A** | **A** | **A** | **G** | **A** | **A** | **A** | **T** | **T** | **T** | **T** | **A** | **T** | **T** | **T** | **G** | **C** | **A** | **A** | **C** | **A** | **A** | **A** | **G** | **T** | **G** | **A** | **T** | **T** | **T** | **G** | **C** | **G** | **G** | **G** | **A** | **A** | **T** | **T** | **A** | **G** | **A** | **G** | **A** | **A** | **T** | **T** | **T** | **T** | **C** | **A** | **G** | **A** | **A** | **T** | **T** | **T** | **C** | **C** | **A** | **A** | **G** | **A** | **A** | **G** | **A** | **A** | **A** | **G** | **T** | **T** | **T** | **T** | **G** | **A** | **A** | **C** | **T** | **T** | **T** | **A** | **T** | **A** | **G** | **T** | **C** | **A** | **A** | **A** | **A** | **G** | **A** | **T** | **G** | **C** | **C** | **G** | **A** | **C** | **G** | **G** | **A** | **G** | **G** | **A** | **G** | **C** | **A** | **A** | **C** | **A** | **A** | **C** | **A** | **G** | **T** | **T** | **T** | **A** | **C** | **A** | **G** | **A** | **C** | **T** | **T** | **T** | **T** | **T** | **T** | **T** | **C** | **C** | **T** | **C** | **G** | **T** | **T** | **A** | **A** | **A** | **T** | **A** | **G** | **G** | **A** | **T** | **G** | **C** | **C** | **T** | **C** | **T** | **G** | **A** | **C** | **T** | **A** | **A** | **G** | **A** | **A** | **A** | **A** | **A** | **T** | **G** | **A** | **T** | **G** | **T** | **T** | **G** | **A** | **A** | **A** | **T** | **T** | **T** | **T** | **T** | **A** | **C** | **G** | **G** | **A** | **T** | **G** | **G** | **T** | **A** | **C** | **G** | **G** | **A** | **A** | **A** | **A** | **A** | **T** | **T** | **C** | **A** | **A** | **T** | **G** | **C** | **G** | **T** | **T** | **G** | **A** | **T** | **G** | **G** | **C** | **A** | **G** | **A** | **C** | **A** | **T** | **A** | **A** | **A** | **A** | **A** | **A** | **A** | **G** | **C** | **T** | **C** | **A** | **G** | **C** | **A** | **C** | **T** | **C** | **C** | **A** | **T** | **T** | **C** | **A** | **T** | **A** | **T** | **T** | **G** | **A** | **A** | **T** | **A** | **T** | **T** | **A** | **T** | **G** | **C** | **C** | **T** | **T** | **T** | **G** | **T** | **A** | **A** | **C** | **G** | **G** | **A** | **T** | **C** | **A** | **T** | **A** | **T** | **C** | **G** | **G** | **T** | **A** | **C** | **C** | **A** | **A** | **A** | **A** | **T** | **T** | **T** | **T** | **G** | **A** | **A** | **C** | **T** | **T** | **A** | **T** | **T** | **A** | **G** | **A** | **A** | **G** | **A** | **A** | **A** | **A** | **G** | **G** | **C** | **G** | **G** | **C** | **T** | **G** | **A** | **A** | **G** | **G** | **G** | **G** | **T** | **T** | **G** | **A** | **A** | **G** | **T** | **G** | **C** | **G** | **G** | **T** | **T** | **G** | **C** | **T** | **T** | **T** | **A** | **C** | **G** | **A** | **T** | **G** | **C** | **C** | **T** | **T** | **T** | **G** | **G** | **C** | **T** | **C** | **A** | **A** | **A** | **G** | **G** | **G** | **T** | **A** | **C** | **C** | **A** | **A** | **G** | **G** | **T** | **T** | **C** | **A** | **T** | **C** | **A** | **T** | **T** | **T** | **G** | **A** | **A** | **T** | **G** | **A** | **A** | **T** | **T** | **G** | **A** | **A** | **A** | **A** | **A** | **A** | **A** | **A** | **C** | **G** | **G** | **C** | **G** | **G** | **T** | **T** | **T** | **T** | **G** | **T** | **C** | **C** | **A** | **A** | **A** | **C** | **G** | **T** | **T** | **T** | **A** | **T** | **T** | **A** | **C** | **T** | **T** | **C** | **T** | **C** | **A** | **A** | **A** | **A** | **A** | **G** | **C** | **A** | **C** | **T** | **T** | **T** | **T** | **G** | **A** | **A** | **G** | **T** | **T** | **T** | **C** | **G** | **T** | **T** | **T** | **G** | **A** | **A** | **T** | **T** | **A** | **T** | **C** | **A** | **T** | **G** | **A** | **T** | **C** | **A** | **C** | **C** | **G** | **G** | **A** | **A** | **A** | **A** | **T** | **T** | **G** | **T** | **T** | **G** | **T** | **T** | **A** | **T** | **T** | **G** | **A** | **C** | **G** | **G** | **A** | **A** | **A** | **A** | **G** | **T** | **A** | **G** | **G** | **C** | **T** | **A** | **C** | **A** | **T** | **T** | **G** | **G** | **C** | **G** | **G** | **C** | **T** | **T** | **T** | **A** | **A** | **T** | **G** | **T** | **T** | **G** | **C** | **C** | **G** | **A** | **T** | **C** | **A** | **A** | **T** | **A** | **T** | **G** | **C** | **C** | **G** | **G** | **A** | **A** | **C** | **G** | **A** | **C** | **T** | **A** | **A** | **A** | **A** | **A** | **G** | **T** | **T** | **T** | **G** | **G** | **C** | **T** | **A** | **T** | **T** | **G** | **G** | **C** | **G** | **G** | **G** | **A** | **T** | **A** | **C** | **A** | **C** | **A** | **T** | **T** | **T** | **A** | **C** | **G** | **G** | **A** | **T** | **T** | **C** | **A** | **A** | **G** | **G** | **G** | **C** | **C** | **A** | **G** | **C** | **A** | **G** | **C** | **C** | **T** | **C** | **A** | **T** | **T** | **A** | **C** | **T** | **G** | **C** | **A** | **A** | **A** | **T** | **G** | **C** | **G** | **T** | **T** | **T** | **T** | **T** | **T** | **A** | **A** | **T** | **G** | **G** | **A** | **T** | **T** | **G** | **G** | **A** | **A** | **C** | **G** | **T** | **C** | **T** | **C** | **T** | **T** | **C** | **C** | **C** | **C** | **C** | **G** | **A** | **G** | **A** | **A** | **A** | **A** | **A** | **T** | **C** | **G** | **T** | **G** | **T** | **G** | **G** | **C** | **G** | **T** | **A** | **T** | **C** | **A** | **A** | **T** | **T** | **G** | **G** | **A** | **T** | **T** | **A** | **T** | **T** | **T** | **C** | **T** | **T** | **T** | **A** | **A** | **A** | **C** | **T** | **T** | **G** | **A** | **A** | **G** | **C** | **A** | **T** | **T** | **G** | **G** | **T** | **G** | **C** | **C** | **A** | **G** | **A** | **G** | **G** | **C** | **A** | **A** | **A** | **T** | **A** | **C** | **A** | **T** | **C** | **C** | **A** | **T** | **T** | **C** | **A** | **G** | **A** | **T** | **G** | **A** | **T** | **T** | **G** | **C** | **C** | **A** | **G** | **T** | **G** | **G** | **T** | **C** | **C** | **T** | **A** | **A** | **C** | **A** | **G** | **T** | **G** | **A** | **C** | **C** | **G** | **T** | **G** | **A** | **A** | **C** | **A** | **A** | **A** | **T** | **T** | **A** | **A** | **A** | **T** | **T** | **G** | **G** | **C** | **C** | **T** | **T** | **T** | **A** | **T** | **T** | **A** | **A** | **A** | **T** | **T** | **G** | **A** | **T** | **T** | **A** | **C** | **T** | **T** | **C** | **T** | **G** | **C** | **C** | **A** | **A** | **G** | **A** | **A** | **A** | **A** | **G** | **A** | **G** | **T** | **C** | **T** | **G** | **G** | **A** | **T** | **T** | **C** | **A** | **A** | **A** | **C** | **G** | **C** | **C** | **G** | **T** | **A** | **T** | **T** | **T** | **A** | **G** | **T** | **T** | **C** | **C** | **T** | **G** | **A** | **T** | **G** | **A** | **C** | **A** | **G** | **T** | **G** | **T** | **C** | **T** | **T** | **G** | **G** | **C** | **T** | **G** | **C** | **T** | **T** | **T** | **A** | **A** | **A** | **G** | **G** | **T** | **T** | **G** | **C** | **T** | **G** | **C** | **G** | **G** | **C** | **T** | **T** | **C** | **A** | **G** | **G** | **A** | **G** | **T** | **A** | **G** | **A** | **T** | **G** | **T** | **T** | **A** | **A** | **A** | **A** | **T** | **T** | **A** | **T** | **G** | **A** | **T** | **T** | **C** | **C** | **A** | **G** | **A** | **T** | **A** | **A** | **G** | **C** | **C** | **C** | **G** | **A** | **C** | **C** | **A** | **T** | **C** | **C** | **G** | **T** | **T** | **T** | **A** | **T** | **T** | **T** | **A** | **T** | **C** | **G** | **A** | **G** | **C** | **A** | **A** | **C** | **A** | **C** | **A** | **G** | **T** | **A** | **T** | **T** | **A** | **C** | **G** | **G** | **C** | **C** | **G** | **C** | **T** | **T** | **A** | **T** | **T** | **G** | **A** | **T** | **G** | **A** | **A** | **A** | **G** | **A** | **A** | **A** | **A** | **T** | **A** | **T** | **T** | **G** | **A** | **A** | **A** | **T** | **T** | **T** | **T** | **A** | **A** | **T** | **T** | **T** | **A** | **T** | **A** | **A** | **C** | **G** | **G** | **T** | **G** | **G** | **T** | **T** | **T** | **C** | **T** | **T** | **A** | **C** | **A** | **T** | **G** | **C** | **G** | **A** | **A** | **A** | **A** | **C** | **A** | **A** | **T** | **G** | **A** | **T** | **T** | **A** | **T** | **G** | **G** | **A** | **T** | **G** | **A** | **T** | **G** | **A** | **A** | **G** | **T** | **C** | **T** | **G** | **C** | **A** | **C** | **A** | **G** | **T** | **T** | **G** | **G** | **T** | **T** | **C** | **A** | **G** | **C** | **C** | **A** | **A** | **T** | **C** | **A** | **A** | **G** | **A** | **T** | **A** | **T** | **C** | **C** | **G** | **A** | **A** | **G** | **T** | **T** | **A** | **C** | **A** | **A** | **A** | **T** | **T** | **A** | **A** | **A** | **C** | **T** | **T** | **T** | **G** | **A** | **A** | **G** | **C** | **A** | **A** | **A** | **T** | **G** | **C** | **T** | **G** | **T** | **G** | **T** | **T** | **A** | **T** | **A** | **T** | **G** | **A** | **T** | **A** | **A** | **A** | **A** | **A** | **A** | **A** | **T** | **C** | **A** | **T** | **T** | **G** | **A** | **T** | **C** | **A** | **A** | **T** | **T** | **A** | **G** | **A** | **A** | **G** | **C** | **A** | **A** | **T** | **T** | **T** | **T** | **C** | **T** | **T** | **A** | **G** | **A** | **A** | **G** | **A** | **T** | **C** | **G** | **A** | **A** | **A** | **A** | **A** | **A** | **A** | **T** | **G** | **T** | **A** | **C** | **A** | **A** | **C** | **A** | **A** | **T** | **G** | **A** | **C** | **T** | **C** | **C** | **A** | **G** | **A** | **A** | **G** | **T** | **T** | **G** | **T** | **T** | **C** | **G** | **T** | **G** | **A** | **C** | **A** | **T** | **G** | **T** | **C** | **A** | **A** | **A** | **A** | **T** | **G** | **G** | **T** | **T** | **G** | **A** | **T** | **T** | **T** | **T** | **T** | **A** | **A** | **A** | **C** | **A** | **A** | **C** | **A** | **A** | **A** | **T** | **T** | **T** | **C** | **A** | **C** | **G** | **A** | **T** | **T** | **A** | **T** | **T** | **T** | **T** | **C** | **A** | **C** | **C** | **A** | **A** | **T** | **T** | **C** | **T** | **T** | **-** | **-** | **-** |
| **N55753** | **T** | **T** | **G** | **A** | **T** | **T** | **C** | **T** | **T** | **A** | **G** | **C** | **G** | **T** | **C** | **T** | **T** | **A** | **A** | **C** | **A** | **G** | **T** | **T** | **A** | **T** | **T** | **T** | **A** | **T** | **T** | **T** | **T** | **A** | **T** | **T** | **A** | **A** | **T** | **G** | **C** | **A** | **A** | **T** | **T** | **A** | **T** | **T** | **G** | **C** | **G** | **G** | **G** | **A** | **A** | **T** | **T** | **A** | **C** | **C** | **A** | **T** | **T** | **T** | **T** | **G** | **T** | **T** | **A** | **A** | **A** | **A** | **C** | **C** | **T** | **C** | **G** | **A** | **G** | **A** | **T** | **G** | **T** | **A** | **G** | **C** | **A** | **G** | **C** | **A** | **A** | **T** | **T** | **T** | **G** | **G** | **G** | **C** | **A** | **T** | **G** | **G** | **C** | **T** | **A** | **C** | **T** | **C** | **G** | **T** | **A** | **C** | **T** | **G** | **A** | **T** | **T** | **G** | **C** | **T** | **C** | **T** | **A** | **C** | **C** | **C** | **G** | **T** | **T** | **T** | **T** | **T** | **G** | **G** | **T** | **T** | **T** | **T** | **T** | **T** | **C** | **T** | **T** | **A** | **T** | **A** | **T** | **T** | **T** | **A** | **T** | **T** | **C** | **T** | **T** | **T** | **G** | **G** | **T** | **C** | **G** | **A** | **G** | **G** | **T** | **T** | **T** | **A** | **A** | **C** | **C** | **G** | **A** | **C** | **A** | **A** | **A** | **A** | **A** | **G** | **A** | **A** | **A** | **T** | **T** | **T** | **T** | **A** | **T** | **T** | **T** | **G** | **C** | **A** | **A** | **C** | **A** | **A** | **A** | **G** | **T** | **G** | **A** | **T** | **T** | **T** | **A** | **C** | **G** | **G** | **G** | **A** | **A** | **T** | **T** | **A** | **G** | **A** | **G** | **A** | **A** | **T** | **T** | **T** | **C** | **C** | **A** | **G** | **A** | **A** | **T** | **T** | **T** | **C** | **C** | **A** | **A** | **G** | **A** | **A** | **G** | **A** | **A** | **A** | **G** | **T** | **T** | **T** | **T** | **G** | **A** | **A** | **C** | **T** | **T** | **T** | **A** | **T** | **A** | **G** | **T** | **C** | **A** | **A** | **A** | **A** | **G** | **A** | **T** | **G** | **C** | **C** | **G** | **A** | **C** | **G** | **G** | **A** | **G** | **G** | **A** | **G** | **C** | **A** | **A** | **C** | **A** | **A** | **C** | **A** | **G** | **T** | **T** | **T** | **A** | **C** | **A** | **G** | **A** | **C** | **T** | **T** | **T** | **T** | **T** | **T** | **T** | **C** | **C** | **T** | **C** | **G** | **T** | **T** | **A** | **A** | **A** | **T** | **A** | **G** | **G** | **A** | **T** | **G** | **C** | **C** | **T** | **C** | **T** | **G** | **A** | **C** | **T** | **A** | **A** | **G** | **A** | **A** | **A** | **A** | **A** | **T** | **G** | **A** | **T** | **G** | **T** | **T** | **G** | **A** | **A** | **A** | **T** | **T** | **T** | **T** | **T** | **A** | **C** | **G** | **G** | **A** | **T** | **G** | **G** | **T** | **A** | **C** | **G** | **G** | **A** | **A** | **A** | **A** | **A** | **T** | **T** | **C** | **A** | **A** | **T** | **G** | **C** | **G** | **T** | **T** | **G** | **A** | **T** | **G** | **G** | **C** | **G** | **G** | **A** | **T** | **A** | **T** | **A** | **A** | **A** | **A** | **A** | **A** | **A** | **G** | **C** | **T** | **C** | **A** | **G** | **C** | **A** | **C** | **T** | **C** | **C** | **A** | **T** | **T** | **C** | **A** | **T** | **A** | **T** | **T** | **G** | **A** | **A** | **T** | **A** | **T** | **T** | **A** | **T** | **G** | **C** | **C** | **T** | **T** | **T** | **G** | **T** | **A** | **A** | **C** | **G** | **G** | **A** | **T** | **C** | **A** | **T** | **A** | **T** | **C** | **G** | **G** | **C** | **A** | **C** | **C** | **A** | **A** | **A** | **A** | **T** | **T** | **T** | **T** | **G** | **A** | **A** | **C** | **T** | **T** | **A** | **T** | **T** | **A** | **G** | **A** | **A** | **G** | **A** | **A** | **A** | **A** | **G** | **G** | **C** | **G** | **G** | **C** | **T** | **G** | **A** | **A** | **G** | **G** | **G** | **G** | **T** | **T** | **G** | **A** | **A** | **G** | **T** | **G** | **C** | **G** | **G** | **T** | **T** | **G** | **C** | **T** | **T** | **T** | **A** | **C** | **G** | **A** | **T** | **G** | **C** | **C** | **T** | **T** | **T** | **G** | **G** | **C** | **T** | **C** | **A** | **A** | **A** | **G** | **G** | **G** | **C** | **A** | **C** | **C** | **A** | **A** | **G** | **G** | **T** | **T** | **C** | **A** | **T** | **C** | **A** | **T** | **T** | **T** | **G** | **A** | **A** | **T** | **G** | **A** | **A** | **T** | **T** | **G** | **A** | **A** | **A** | **A** | **A** | **A** | **A** | **A** | **C** | **G** | **G** | **T** | **G** | **G** | **T** | **T** | **T** | **T** | **G** | **T** | **C** | **C** | **A** | **A** | **A** | **C** | **G** | **T** | **T** | **T** | **A** | **T** | **T** | **A** | **C** | **T** | **T** | **C** | **T** | **C** | **A** | **A** | **A** | **A** | **A** | **G** | **C** | **A** | **C** | **T** | **T** | **T** | **T** | **G** | **A** | **A** | **G** | **T** | **T** | **T** | **C** | **G** | **T** | **T** | **T** | **G** | **A** | **A** | **T** | **T** | **A** | **T** | **C** | **A** | **T** | **G** | **A** | **T** | **C** | **A** | **C** | **C** | **G** | **G** | **A** | **A** | **A** | **A** | **T** | **T** | **G** | **T** | **T** | **G** | **T** | **T** | **A** | **T** | **T** | **G** | **A** | **C** | **G** | **G** | **A** | **A** | **A** | **A** | **G** | **T** | **A** | **G** | **G** | **C** | **T** | **A** | **C** | **A** | **T** | **T** | **G** | **G** | **C** | **G** | **G** | **C** | **T** | **T** | **T** | **A** | **A** | **T** | **G** | **T** | **T** | **G** | **C** | **C** | **G** | **A** | **T** | **C** | **A** | **A** | **T** | **A** | **T** | **G** | **C** | **C** | **G** | **G** | **A** | **A** | **C** | **G** | **A** | **C** | **T** | **A** | **A** | **A** | **A** | **A** | **G** | **T** | **T** | **T** | **G** | **G** | **C** | **T** | **A** | **T** | **T** | **G** | **G** | **C** | **G** | **G** | **G** | **A** | **T** | **A** | **C** | **A** | **C** | **A** | **T** | **T** | **T** | **A** | **C** | **G** | **G** | **A** | **T** | **T** | **C** | **A** | **A** | **G** | **G** | **G** | **C** | **C** | **A** | **G** | **C** | **A** | **G** | **C** | **C** | **T** | **C** | **A** | **T** | **T** | **A** | **C** | **T** | **G** | **C** | **A** | **A** | **A** | **T** | **G** | **C** | **G** | **T** | **T** | **T** | **T** | **T** | **T** | **A** | **A** | **T** | **G** | **G** | **A** | **T** | **T** | **G** | **G** | **A** | **A** | **C** | **G** | **T** | **C** | **T** | **C** | **T** | **T** | **C** | **C** | **C** | **C** | **C** | **G** | **A** | **G** | **A** | **A** | **A** | **A** | **A** | **T** | **C** | **G** | **T** | **G** | **T** | **G** | **G** | **C** | **G** | **T** | **A** | **T** | **C** | **A** | **A** | **T** | **T** | **G** | **G** | **A** | **T** | **T** | **A** | **T** | **T** | **T** | **C** | **T** | **T** | **T** | **A** | **A** | **A** | **C** | **T** | **T** | **G** | **A** | **A** | **G** | **C** | **A** | **T** | **T** | **G** | **G** | **T** | **G** | **C** | **C** | **A** | **G** | **A** | **G** | **G** | **C** | **A** | **A** | **A** | **T** | **A** | **C** | **A** | **T** | **C** | **C** | **A** | **T** | **T** | **C** | **A** | **G** | **A** | **T** | **G** | **A** | **T** | **T** | **G** | **C** | **C** | **A** | **G** | **T** | **G** | **G** | **T** | **C** | **C** | **T** | **A** | **A** | **C** | **A** | **G** | **T** | **G** | **A** | **C** | **C** | **G** | **T** | **G** | **A** | **A** | **C** | **A** | **A** | **A** | **T** | **T** | **A** | **A** | **A** | **T** | **T** | **G** | **G** | **C** | **C** | **T** | **T** | **T** | **A** | **T** | **T** | **A** | **A** | **A** | **T** | **T** | **G** | **A** | **T** | **T** | **A** | **C** | **T** | **T** | **C** | **T** | **G** | **C** | **C** | **A** | **A** | **G** | **A** | **A** | **A** | **A** | **G** | **A** | **G** | **T** | **C** | **T** | **G** | **G** | **A** | **T** | **T** | **C** | **A** | **A** | **A** | **C** | **G** | **C** | **C** | **G** | **T** | **A** | **T** | **T** | **T** | **A** | **G** | **T** | **T** | **C** | **C** | **T** | **G** | **A** | **T** | **G** | **A** | **T** | **A** | **G** | **T** | **G** | **T** | **C** | **T** | **T** | **G** | **G** | **C** | **T** | **G** | **C** | **T** | **T** | **T** | **A** | **A** | **A** | **G** | **G** | **T** | **T** | **G** | **C** | **T** | **G** | **C** | **G** | **G** | **C** | **T** | **T** | **C** | **A** | **G** | **G** | **A** | **G** | **T** | **A** | **G** | **A** | **T** | **G** | **T** | **T** | **A** | **A** | **A** | **A** | **T** | **T** | **A** | **T** | **G** | **A** | **T** | **T** | **C** | **C** | **A** | **G** | **A** | **T** | **A** | **A** | **G** | **C** | **C** | **C** | **G** | **A** | **C** | **C** | **A** | **T** | **C** | **C** | **G** | **T** | **T** | **T** | **A** | **T** | **T** | **T** | **A** | **T** | **C** | **G** | **A** | **G** | **C** | **A** | **A** | **C** | **A** | **C** | **A** | **G** | **T** | **A** | **T** | **T** | **A** | **C** | **G** | **G** | **C** | **C** | **G** | **C** | **T** | **T** | **A** | **T** | **T** | **G** | **A** | **T** | **G** | **A** | **A** | **A** | **G** | **A** | **A** | **A** | **A** | **T** | **A** | **T** | **T** | **G** | **A** | **A** | **A** | **T** | **T** | **T** | **T** | **A** | **A** | **T** | **T** | **T** | **A** | **T** | **A** | **A** | **C** | **G** | **G** | **T** | **G** | **G** | **T** | **T** | **T** | **C** | **T** | **T** | **A** | **C** | **A** | **T** | **G** | **C** | **G** | **A** | **A** | **A** | **A** | **C** | **A** | **A** | **T** | **G** | **A** | **T** | **T** | **A** | **T** | **G** | **G** | **A** | **T** | **G** | **A** | **T** | **G** | **A** | **A** | **G** | **T** | **C** | **T** | **G** | **C** | **A** | **C** | **A** | **G** | **T** | **T** | **G** | **G** | **T** | **T** | **C** | **A** | **G** | **C** | **C** | **A** | **A** | **T** | **C** | **A** | **A** | **G** | **A** | **T** | **A** | **T** | **C** | **C** | **G** | **A** | **A** | **G** | **T** | **T** | **A** | **C** | **A** | **A** | **A** | **T** | **T** | **A** | **A** | **A** | **C** | **T** | **T** | **T** | **G** | **A** | **A** | **G** | **C** | **A** | **A** | **A** | **T** | **G** | **C** | **T** | **G** | **T** | **G** | **T** | **T** | **A** | **T** | **A** | **T** | **G** | **A** | **T** | **A** | **A** | **A** | **A** | **A** | **A** | **A** | **T** | **C** | **A** | **T** | **T** | **G** | **A** | **T** | **C** | **A** | **A** | **T** | **T** | **A** | **G** | **A** | **A** | **G** | **C** | **A** | **A** | **T** | **T** | **T** | **T** | **C** | **T** | **T** | **A** | **G** | **A** | **A** | **G** | **A** | **T** | **C** | **G** | **A** | **A** | **A** | **A** | **A** | **A** | **A** | **T** | **G** | **T** | **A** | **C** | **A** | **A** | **C** | **A** | **A** | **T** | **G** | **A** | **C** | **T** | **C** | **C** | **A** | **G** | **A** | **A** | **G** | **T** | **T** | **G** | **T** | **T** | **C** | **G** | **C** | **G** | **A** | **C** | **A** | **T** | **G** | **T** | **C** | **A** | **A** | **A** | **A** | **T** | **G** | **G** | **T** | **T** | **G** | **A** | **T** | **T** | **T** | **T** | **T** | **A** | **A** | **A** | **C** | **A** | **A** | **C** | **A** | **A** | **A** | **T** | **T** | **T** | **C** | **A** | **C** | **G** | **A** | **T** | **T** | **A** | **T** | **T** | **T** | **T** | **C** | **A** | **C** | **C** | **A** | **A** | **T** | **T** | **C** | **T** | **T** | **-** | **-** | **-** |
| **N59505F** | **T** | **T** | **G** | **A** | **T** | **T** | **C** | **T** | **T** | **A** | **G** | **C** | **G** | **T** | **C** | **T** | **T** | **A** | **A** | **C** | **A** | **G** | **T** | **T** | **A** | **T** | **T** | **T** | **A** | **T** | **T** | **T** | **T** | **A** | **T** | **T** | **A** | **A** | **T** | **G** | **C** | **A** | **A** | **T** | **T** | **A** | **T** | **T** | **G** | **C** | **G** | **G** | **G** | **A** | **A** | **T** | **T** | **A** | **C** | **C** | **A** | **T** | **T** | **T** | **T** | **G** | **T** | **T** | **A** | **A** | **A** | **A** | **C** | **C** | **T** | **C** | **G** | **A** | **G** | **A** | **T** | **G** | **T** | **A** | **G** | **C** | **A** | **G** | **C** | **A** | **A** | **T** | **T** | **T** | **G** | **G** | **G** | **C** | **A** | **T** | **G** | **G** | **C** | **T** | **A** | **C** | **T** | **C** | **G** | **T** | **A** | **C** | **T** | **G** | **A** | **T** | **T** | **G** | **C** | **T** | **C** | **T** | **A** | **C** | **C** | **C** | **G** | **T** | **T** | **T** | **T** | **T** | **G** | **G** | **T** | **T** | **T** | **T** | **T** | **T** | **C** | **T** | **T** | **A** | **T** | **A** | **T** | **T** | **T** | **A** | **T** | **T** | **C** | **T** | **T** | **T** | **G** | **G** | **G** | **C** | **G** | **C** | **G** | **G** | **T** | **T** | **T** | **A** | **A** | **C** | **C** | **G** | **A** | **C** | **A** | **A** | **A** | **A** | **A** | **G** | **A** | **A** | **A** | **T** | **T** | **T** | **T** | **A** | **T** | **T** | **T** | **G** | **C** | **A** | **A** | **C** | **A** | **A** | **A** | **G** | **T** | **G** | **A** | **T** | **T** | **T** | **A** | **C** | **G** | **G** | **G** | **A** | **A** | **T** | **T** | **A** | **G** | **A** | **G** | **A** | **A** | **T** | **T** | **T** | **T** | **C** | **A** | **G** | **A** | **A** | **C** | **T** | **T** | **C** | **C** | **A** | **A** | **G** | **A** | **A** | **G** | **A** | **A** | **A** | **G** | **T** | **T** | **T** | **T** | **G** | **A** | **A** | **C** | **T** | **T** | **T** | **A** | **T** | **A** | **G** | **T** | **C** | **A** | **A** | **A** | **A** | **G** | **A** | **T** | **G** | **C** | **C** | **G** | **A** | **C** | **G** | **G** | **A** | **G** | **G** | **A** | **G** | **C** | **A** | **A** | **C** | **A** | **A** | **C** | **A** | **G** | **T** | **T** | **T** | **A** | **C** | **A** | **G** | **A** | **C** | **T** | **T** | **T** | **T** | **T** | **T** | **T** | **C** | **C** | **T** | **C** | **G** | **T** | **T** | **A** | **A** | **A** | **T** | **A** | **G** | **G** | **A** | **T** | **G** | **C** | **C** | **C** | **C** | **T** | **G** | **A** | **C** | **T** | **A** | **A** | **G** | **A** | **A** | **A** | **A** | **A** | **T** | **G** | **A** | **T** | **G** | **T** | **T** | **G** | **A** | **A** | **A** | **T** | **T** | **T** | **T** | **T** | **A** | **C** | **G** | **G** | **A** | **T** | **G** | **G** | **T** | **A** | **C** | **G** | **G** | **A** | **A** | **A** | **A** | **A** | **T** | **T** | **C** | **A** | **A** | **T** | **G** | **C** | **G** | **T** | **T** | **G** | **A** | **T** | **G** | **G** | **C** | **G** | **G** | **A** | **C** | **A** | **T** | **A** | **A** | **A** | **A** | **A** | **A** | **A** | **G** | **C** | **T** | **C** | **A** | **G** | **C** | **A** | **C** | **T** | **C** | **C** | **A** | **T** | **T** | **C** | **A** | **T** | **A** | **T** | **T** | **G** | **A** | **A** | **T** | **A** | **T** | **T** | **A** | **T** | **G** | **C** | **C** | **T** | **T** | **T** | **G** | **T** | **A** | **A** | **C** | **G** | **G** | **A** | **T** | **C** | **A** | **T** | **A** | **T** | **C** | **G** | **G** | **T** | **A** | **C** | **C** | **A** | **A** | **A** | **A** | **T** | **T** | **T** | **T** | **G** | **A** | **A** | **C** | **T** | **T** | **A** | **T** | **T** | **A** | **G** | **A** | **A** | **G** | **A** | **A** | **A** | **A** | **G** | **G** | **C** | **G** | **G** | **C** | **T** | **G** | **A** | **A** | **G** | **G** | **G** | **G** | **T** | **T** | **G** | **A** | **A** | **G** | **T** | **G** | **C** | **G** | **G** | **T** | **T** | **G** | **C** | **T** | **T** | **T** | **A** | **C** | **G** | **A** | **T** | **G** | **C** | **C** | **T** | **T** | **T** | **G** | **G** | **C** | **T** | **C** | **A** | **A** | **A** | **G** | **G** | **G** | **C** | **A** | **C** | **C** | **A** | **A** | **G** | **G** | **T** | **T** | **C** | **A** | **T** | **C** | **A** | **T** | **T** | **T** | **G** | **A** | **A** | **T** | **G** | **A** | **A** | **T** | **T** | **G** | **A** | **A** | **A** | **A** | **A** | **A** | **A** | **A** | **C** | **G** | **G** | **C** | **G** | **G** | **T** | **T** | **T** | **T** | **G** | **T** | **C** | **C** | **A** | **A** | **A** | **C** | **G** | **T** | **T** | **T** | **A** | **T** | **T** | **A** | **C** | **T** | **T** | **C** | **T** | **C** | **A** | **A** | **A** | **A** | **A** | **G** | **C** | **A** | **C** | **T** | **T** | **T** | **T** | **G** | **A** | **A** | **G** | **T** | **T** | **T** | **C** | **G** | **T** | **T** | **T** | **G** | **A** | **A** | **T** | **T** | **A** | **T** | **C** | **A** | **T** | **G** | **A** | **T** | **C** | **A** | **C** | **C** | **G** | **G** | **A** | **A** | **A** | **A** | **T** | **T** | **G** | **T** | **T** | **G** | **T** | **T** | **A** | **T** | **T** | **G** | **A** | **T** | **G** | **G** | **G** | **A** | **A** | **A** | **G** | **T** | **A** | **G** | **G** | **C** | **T** | **A** | **C** | **A** | **T** | **T** | **G** | **G** | **C** | **G** | **G** | **C** | **T** | **T** | **T** | **A** | **A** | **T** | **G** | **T** | **T** | **G** | **C** | **C** | **G** | **A** | **T** | **C** | **A** | **A** | **T** | **A** | **T** | **G** | **C** | **T** | **G** | **G** | **A** | **A** | **C** | **G** | **A** | **C** | **T** | **A** | **A** | **A** | **A** | **A** | **G** | **T** | **T** | **T** | **G** | **G** | **C** | **T** | **A** | **T** | **T** | **G** | **G** | **C** | **G** | **G** | **G** | **A** | **T** | **A** | **C** | **A** | **C** | **A** | **T** | **T** | **T** | **A** | **C** | **G** | **G** | **A** | **T** | **T** | **C** | **A** | **A** | **G** | **G** | **G** | **C** | **C** | **A** | **G** | **C** | **A** | **G** | **C** | **C** | **T** | **C** | **A** | **T** | **T** | **A** | **C** | **T** | **G** | **C** | **A** | **A** | **A** | **T** | **G** | **C** | **G** | **T** | **T** | **T** | **T** | **T** | **T** | **A** | **A** | **T** | **G** | **G** | **A** | **T** | **T** | **G** | **G** | **A** | **A** | **C** | **G** | **T** | **C** | **T** | **C** | **T** | **T** | **C** | **C** | **C** | **C** | **T** | **G** | **A** | **G** | **A** | **A** | **A** | **A** | **A** | **T** | **C** | **G** | **T** | **G** | **T** | **G** | **G** | **C** | **G** | **T** | **A** | **T** | **C** | **A** | **A** | **T** | **T** | **G** | **G** | **A** | **T** | **T** | **A** | **T** | **T** | **T** | **C** | **T** | **T** | **T** | **A** | **A** | **A** | **C** | **T** | **T** | **G** | **A** | **A** | **G** | **C** | **G** | **T** | **T** | **G** | **G** | **T** | **G** | **C** | **C** | **A** | **G** | **A** | **G** | **G** | **C** | **A** | **A** | **A** | **T** | **A** | **C** | **A** | **T** | **C** | **C** | **A** | **T** | **T** | **C** | **A** | **G** | **A** | **T** | **G** | **A** | **T** | **T** | **G** | **C** | **C** | **A** | **G** | **T** | **G** | **G** | **T** | **C** | **C** | **T** | **A** | **A** | **T** | **A** | **G** | **T** | **G** | **A** | **C** | **C** | **G** | **T** | **G** | **A** | **A** | **C** | **A** | **A** | **A** | **T** | **T** | **A** | **A** | **A** | **T** | **T** | **G** | **G** | **C** | **C** | **T** | **T** | **T** | **A** | **T** | **T** | **A** | **A** | **A** | **T** | **T** | **G** | **A** | **T** | **T** | **A** | **C** | **T** | **T** | **C** | **T** | **G** | **C** | **C** | **A** | **A** | **G** | **A** | **A** | **A** | **A** | **G** | **A** | **G** | **T** | **C** | **T** | **G** | **G** | **A** | **T** | **T** | **C** | **A** | **A** | **A** | **C** | **G** | **C** | **C** | **G** | **T** | **A** | **T** | **T** | **T** | **A** | **G** | **T** | **T** | **C** | **C** | **T** | **G** | **A** | **T** | **G** | **A** | **T** | **A** | **G** | **T** | **G** | **T** | **C** | **T** | **T** | **G** | **G** | **C** | **G** | **G** | **C** | **T** | **T** | **T** | **A** | **A** | **A** | **G** | **G** | **T** | **T** | **G** | **C** | **T** | **G** | **C** | **G** | **G** | **C** | **T** | **T** | **C** | **A** | **G** | **G** | **A** | **G** | **T** | **A** | **G** | **A** | **T** | **G** | **T** | **T** | **A** | **A** | **A** | **A** | **T** | **T** | **A** | **T** | **G** | **A** | **T** | **T** | **C** | **C** | **A** | **G** | **A** | **T** | **A** | **A** | **G** | **C** | **C** | **C** | **G** | **A** | **C** | **C** | **A** | **T** | **C** | **C** | **G** | **T** | **T** | **T** | **A** | **T** | **T** | **T** | **A** | **T** | **C** | **G** | **A** | **G** | **C** | **A** | **A** | **C** | **A** | **C** | **A** | **G** | **T** | **A** | **T** | **T** | **A** | **C** | **G** | **G** | **G** | **C** | **G** | **C** | **T** | **T** | **G** | **T** | **T** | **G** | **A** | **T** | **G** | **A** | **A** | **A** | **G** | **A** | **A** | **A** | **A** | **T** | **A** | **T** | **T** | **G** | **A** | **A** | **A** | **T** | **T** | **T** | **T** | **A** | **A** | **T** | **T** | **T** | **A** | **T** | **A** | **A** | **C** | **G** | **G** | **T** | **G** | **G** | **T** | **T** | **T** | **C** | **T** | **T** | **A** | **C** | **A** | **T** | **G** | **C** | **G** | **A** | **A** | **A** | **A** | **C** | **A** | **A** | **T** | **G** | **A** | **T** | **T** | **A** | **T** | **G** | **G** | **A** | **T** | **G** | **A** | **T** | **G** | **A** | **A** | **G** | **T** | **C** | **T** | **G** | **C** | **A** | **C** | **A** | **G** | **T** | **A** | **G** | **G** | **T** | **T** | **C** | **A** | **G** | **C** | **C** | **A** | **A** | **T** | **C** | **A** | **A** | **G** | **A** | **T** | **A** | **T** | **C** | **C** | **G** | **A** | **A** | **G** | **T** | **T** | **A** | **C** | **A** | **A** | **A** | **T** | **T** | **A** | **A** | **A** | **C** | **T** | **T** | **T** | **G** | **A** | **A** | **G** | **C** | **A** | **A** | **A** | **T** | **G** | **C** | **T** | **G** | **T** | **G** | **T** | **T** | **A** | **T** | **A** | **T** | **G** | **A** | **T** | **A** | **A** | **A** | **A** | **A** | **A** | **A** | **T** | **C** | **A** | **T** | **T** | **G** | **A** | **T** | **C** | **A** | **A** | **T** | **T** | **A** | **G** | **A** | **A** | **G** | **C** | **A** | **A** | **T** | **T** | **T** | **T** | **C** | **T** | **T** | **A** | **G** | **A** | **A** | **G** | **A** | **T** | **C** | **G** | **A** | **A** | **A** | **A** | **A** | **A** | **A** | **T** | **G** | **T** | **A** | **C** | **A** | **A** | **C** | **A** | **A** | **T** | **G** | **A** | **C** | **T** | **C** | **C** | **A** | **G** | **A** | **A** | **G** | **T** | **T** | **G** | **T** | **T** | **C** | **G** | **C** | **G** | **A** | **C** | **A** | **T** | **G** | **T** | **C** | **A** | **A** | **A** | **A** | **T** | **G** | **G** | **T** | **T** | **G** | **A** | **T** | **T** | **T** | **T** | **T** | **A** | **A** | **A** | **C** | **A** | **A** | **C** | **A** | **A** | **A** | **T** | **T** | **T** | **C** | **A** | **C** | **G** | **A** | **T** | **T** | **A** | **T** | **T** | **C** | **T** | **C** | **A** | **C** | **C** | **A** | **A** | **T** | **T** | **C** | **T** | **T** | **-** | **-** | **-** |
| **N59845F** | **T** | **T** | **G** | **A** | **T** | **T** | **C** | **T** | **T** | **A** | **G** | **C** | **G** | **T** | **C** | **T** | **T** | **A** | **A** | **C** | **A** | **G** | **T** | **T** | **A** | **T** | **T** | **T** | **A** | **T** | **T** | **T** | **T** | **A** | **T** | **T** | **A** | **A** | **T** | **G** | **C** | **A** | **A** | **T** | **T** | **A** | **T** | **T** | **G** | **C** | **G** | **G** | **G** | **A** | **A** | **T** | **T** | **A** | **C** | **C** | **A** | **T** | **T** | **T** | **T** | **G** | **T** | **T** | **A** | **A** | **A** | **A** | **C** | **C** | **T** | **C** | **G** | **A** | **G** | **A** | **T** | **G** | **T** | **A** | **G** | **C** | **A** | **G** | **C** | **A** | **A** | **T** | **T** | **T** | **G** | **G** | **G** | **C** | **A** | **T** | **G** | **G** | **C** | **T** | **A** | **C** | **T** | **C** | **G** | **T** | **A** | **C** | **T** | **G** | **A** | **T** | **T** | **G** | **C** | **T** | **C** | **T** | **A** | **C** | **C** | **C** | **G** | **T** | **T** | **T** | **T** | **T** | **G** | **G** | **T** | **T** | **T** | **T** | **T** | **T** | **C** | **T** | **T** | **A** | **T** | **A** | **T** | **T** | **T** | **A** | **T** | **T** | **C** | **T** | **T** | **T** | **G** | **G** | **G** | **C** | **G** | **C** | **G** | **G** | **T** | **T** | **T** | **A** | **A** | **C** | **C** | **G** | **A** | **C** | **A** | **A** | **A** | **A** | **A** | **G** | **A** | **A** | **A** | **T** | **T** | **T** | **T** | **A** | **T** | **T** | **T** | **G** | **C** | **A** | **A** | **C** | **A** | **A** | **A** | **G** | **T** | **G** | **A** | **T** | **T** | **T** | **G** | **C** | **G** | **G** | **G** | **A** | **A** | **T** | **T** | **A** | **G** | **A** | **G** | **A** | **A** | **T** | **T** | **T** | **T** | **C** | **A** | **G** | **A** | **A** | **T** | **T** | **T** | **C** | **C** | **A** | **A** | **G** | **A** | **A** | **G** | **A** | **A** | **A** | **G** | **T** | **T** | **T** | **T** | **G** | **A** | **A** | **C** | **T** | **T** | **T** | **A** | **T** | **A** | **G** | **T** | **C** | **A** | **A** | **A** | **A** | **G** | **A** | **T** | **G** | **C** | **C** | **G** | **A** | **C** | **G** | **G** | **A** | **G** | **G** | **A** | **G** | **C** | **A** | **A** | **C** | **A** | **A** | **C** | **A** | **G** | **T** | **T** | **T** | **A** | **C** | **A** | **G** | **A** | **C** | **T** | **T** | **T** | **T** | **T** | **T** | **T** | **C** | **C** | **T** | **C** | **G** | **T** | **T** | **A** | **A** | **A** | **T** | **A** | **G** | **G** | **A** | **T** | **G** | **C** | **C** | **T** | **C** | **T** | **G** | **A** | **C** | **T** | **A** | **A** | **G** | **A** | **A** | **A** | **A** | **A** | **T** | **G** | **A** | **T** | **G** | **T** | **T** | **G** | **A** | **A** | **A** | **T** | **T** | **T** | **T** | **T** | **A** | **C** | **G** | **G** | **A** | **T** | **G** | **G** | **T** | **A** | **C** | **G** | **G** | **A** | **A** | **A** | **A** | **A** | **T** | **T** | **C** | **A** | **A** | **T** | **G** | **C** | **G** | **T** | **T** | **G** | **A** | **T** | **G** | **G** | **C** | **G** | **G** | **A** | **C** | **A** | **T** | **A** | **A** | **A** | **A** | **A** | **A** | **A** | **G** | **C** | **T** | **C** | **A** | **G** | **C** | **A** | **T** | **T** | **C** | **C** | **A** | **T** | **T** | **C** | **A** | **T** | **A** | **T** | **T** | **G** | **A** | **A** | **T** | **A** | **T** | **T** | **A** | **T** | **G** | **C** | **C** | **T** | **T** | **T** | **G** | **T** | **A** | **A** | **C** | **G** | **G** | **A** | **T** | **C** | **A** | **T** | **A** | **T** | **C** | **G** | **G** | **C** | **A** | **C** | **C** | **A** | **A** | **A** | **A** | **T** | **T** | **T** | **T** | **G** | **A** | **A** | **C** | **T** | **T** | **A** | **T** | **T** | **A** | **G** | **A** | **A** | **G** | **A** | **A** | **A** | **A** | **G** | **G** | **C** | **G** | **G** | **C** | **T** | **G** | **A** | **A** | **G** | **G** | **G** | **G** | **T** | **T** | **G** | **A** | **A** | **G** | **T** | **G** | **C** | **G** | **G** | **T** | **T** | **G** | **C** | **T** | **T** | **T** | **A** | **C** | **G** | **A** | **T** | **G** | **C** | **C** | **T** | **T** | **T** | **G** | **G** | **C** | **T** | **C** | **A** | **A** | **A** | **G** | **G** | **G** | **C** | **A** | **C** | **C** | **A** | **A** | **G** | **G** | **T** | **T** | **C** | **A** | **T** | **C** | **A** | **T** | **T** | **T** | **G** | **A** | **A** | **T** | **G** | **A** | **A** | **T** | **T** | **G** | **A** | **A** | **A** | **A** | **A** | **A** | **A** | **A** | **C** | **G** | **G** | **C** | **G** | **G** | **T** | **T** | **T** | **T** | **G** | **T** | **C** | **C** | **A** | **A** | **A** | **C** | **G** | **T** | **T** | **T** | **A** | **T** | **T** | **A** | **C** | **T** | **T** | **C** | **T** | **C** | **A** | **A** | **A** | **A** | **A** | **G** | **C** | **A** | **C** | **T** | **T** | **T** | **T** | **G** | **A** | **A** | **G** | **T** | **T** | **T** | **C** | **G** | **T** | **T** | **T** | **G** | **A** | **A** | **T** | **T** | **A** | **T** | **C** | **A** | **T** | **G** | **A** | **T** | **C** | **A** | **C** | **C** | **G** | **G** | **A** | **A** | **A** | **A** | **T** | **T** | **G** | **T** | **T** | **G** | **T** | **T** | **A** | **T** | **T** | **G** | **A** | **C** | **G** | **G** | **A** | **A** | **A** | **A** | **G** | **T** | **A** | **G** | **G** | **C** | **T** | **A** | **C** | **A** | **T** | **T** | **G** | **G** | **C** | **G** | **G** | **C** | **T** | **T** | **T** | **A** | **A** | **T** | **G** | **T** | **T** | **G** | **C** | **C** | **G** | **A** | **T** | **C** | **A** | **A** | **T** | **A** | **T** | **G** | **C** | **T** | **G** | **G** | **A** | **A** | **C** | **G** | **A** | **C** | **T** | **A** | **A** | **A** | **A** | **A** | **G** | **T** | **T** | **T** | **G** | **G** | **C** | **T** | **A** | **T** | **T** | **G** | **G** | **C** | **G** | **G** | **G** | **A** | **T** | **A** | **C** | **A** | **C** | **A** | **T** | **T** | **T** | **A** | **C** | **G** | **G** | **A** | **T** | **T** | **C** | **A** | **A** | **G** | **G** | **G** | **C** | **C** | **A** | **G** | **C** | **A** | **G** | **C** | **C** | **T** | **C** | **A** | **T** | **T** | **A** | **C** | **T** | **G** | **C** | **A** | **A** | **A** | **T** | **G** | **C** | **G** | **T** | **T** | **T** | **T** | **T** | **T** | **A** | **A** | **T** | **G** | **G** | **A** | **T** | **T** | **G** | **G** | **A** | **A** | **C** | **G** | **T** | **C** | **T** | **C** | **T** | **T** | **C** | **C** | **C** | **C** | **C** | **G** | **A** | **G** | **A** | **A** | **A** | **A** | **A** | **T** | **C** | **G** | **T** | **G** | **T** | **G** | **G** | **C** | **G** | **T** | **A** | **T** | **C** | **A** | **A** | **T** | **T** | **G** | **G** | **A** | **T** | **T** | **A** | **T** | **T** | **T** | **C** | **T** | **T** | **T** | **A** | **A** | **A** | **C** | **T** | **T** | **G** | **A** | **A** | **G** | **C** | **A** | **T** | **T** | **G** | **G** | **T** | **G** | **C** | **C** | **A** | **G** | **A** | **G** | **G** | **C** | **A** | **A** | **A** | **T** | **A** | **C** | **A** | **T** | **C** | **C** | **A** | **T** | **T** | **C** | **A** | **G** | **A** | **T** | **G** | **A** | **T** | **T** | **G** | **C** | **C** | **A** | **G** | **T** | **G** | **G** | **T** | **C** | **C** | **T** | **A** | **A** | **C** | **A** | **G** | **T** | **G** | **A** | **C** | **C** | **G** | **T** | **G** | **A** | **A** | **C** | **A** | **A** | **A** | **T** | **T** | **A** | **A** | **A** | **T** | **T** | **G** | **G** | **C** | **C** | **T** | **T** | **T** | **A** | **T** | **T** | **A** | **A** | **A** | **T** | **T** | **G** | **A** | **T** | **T** | **A** | **C** | **T** | **T** | **C** | **T** | **G** | **C** | **C** | **A** | **A** | **G** | **A** | **A** | **A** | **A** | **G** | **A** | **G** | **T** | **C** | **T** | **G** | **G** | **A** | **T** | **T** | **C** | **A** | **A** | **A** | **C** | **G** | **C** | **C** | **G** | **T** | **A** | **T** | **T** | **T** | **A** | **G** | **T** | **T** | **C** | **C** | **T** | **G** | **A** | **T** | **G** | **A** | **T** | **A** | **G** | **T** | **G** | **T** | **C** | **T** | **T** | **G** | **G** | **C** | **T** | **G** | **C** | **T** | **T** | **T** | **A** | **A** | **A** | **G** | **G** | **T** | **T** | **G** | **C** | **T** | **G** | **C** | **G** | **G** | **C** | **T** | **T** | **C** | **A** | **G** | **G** | **A** | **G** | **T** | **A** | **G** | **A** | **T** | **G** | **T** | **T** | **A** | **A** | **A** | **A** | **T** | **T** | **A** | **T** | **G** | **A** | **T** | **T** | **C** | **C** | **A** | **G** | **A** | **T** | **A** | **A** | **G** | **C** | **C** | **C** | **G** | **A** | **C** | **C** | **A** | **T** | **C** | **C** | **G** | **T** | **T** | **T** | **A** | **T** | **T** | **T** | **A** | **T** | **C** | **G** | **A** | **G** | **C** | **A** | **A** | **C** | **A** | **C** | **A** | **G** | **T** | **A** | **T** | **T** | **A** | **C** | **G** | **G** | **C** | **C** | **G** | **C** | **T** | **T** | **A** | **T** | **T** | **G** | **A** | **T** | **G** | **A** | **A** | **A** | **G** | **A** | **A** | **A** | **A** | **T** | **A** | **T** | **T** | **G** | **A** | **A** | **A** | **T** | **T** | **T** | **T** | **A** | **A** | **T** | **T** | **T** | **A** | **T** | **A** | **A** | **C** | **G** | **G** | **T** | **G** | **G** | **T** | **T** | **T** | **C** | **T** | **T** | **A** | **C** | **A** | **T** | **G** | **C** | **G** | **A** | **A** | **A** | **A** | **C** | **A** | **A** | **T** | **G** | **A** | **T** | **T** | **A** | **T** | **G** | **G** | **A** | **T** | **G** | **A** | **T** | **G** | **A** | **A** | **G** | **T** | **C** | **T** | **G** | **C** | **A** | **C** | **A** | **G** | **T** | **T** | **G** | **G** | **T** | **T** | **C** | **A** | **G** | **C** | **C** | **A** | **A** | **T** | **C** | **A** | **A** | **G** | **A** | **T** | **A** | **T** | **C** | **C** | **G** | **A** | **A** | **G** | **T** | **T** | **A** | **C** | **A** | **A** | **A** | **T** | **T** | **A** | **A** | **A** | **C** | **T** | **T** | **T** | **G** | **A** | **A** | **G** | **C** | **A** | **A** | **A** | **T** | **G** | **C** | **T** | **G** | **T** | **G** | **T** | **T** | **A** | **T** | **A** | **T** | **G** | **A** | **T** | **A** | **A** | **A** | **A** | **A** | **A** | **A** | **T** | **C** | **A** | **T** | **T** | **G** | **A** | **T** | **C** | **A** | **A** | **T** | **T** | **A** | **G** | **A** | **A** | **G** | **C** | **A** | **A** | **T** | **T** | **T** | **T** | **C** | **T** | **T** | **A** | **G** | **A** | **A** | **G** | **A** | **T** | **C** | **G** | **A** | **A** | **A** | **A** | **A** | **A** | **A** | **T** | **G** | **T** | **A** | **C** | **A** | **A** | **C** | **A** | **A** | **T** | **G** | **A** | **C** | **A** | **C** | **C** | **A** | **G** | **A** | **A** | **G** | **T** | **T** | **G** | **T** | **T** | **C** | **G** | **A** | **G** | **A** | **C** | **A** | **T** | **G** | **T** | **C** | **C** | **A** | **A** | **A** | **T** | **G** | **G** | **T** | **T** | **G** | **A** | **T** | **T** | **T** | **T** | **T** | **A** | **A** | **A** | **C** | **A** | **A** | **C** | **A** | **A** | **A** | **T** | **T** | **T** | **C** | **A** | **C** | **G** | **A** | **T** | **T** | **A** | **T** | **T** | **T** | **T** | **C** | **C** | **C** | **C** | **A** | **A** | **T** | **T** | **C** | **T** | **T** | **-** | **-** | **-** |
| **N55119** | **T** | **T** | **G** | **A** | **T** | **T** | **C** | **T** | **T** | **A** | **G** | **C** | **G** | **T** | **C** | **T** | **T** | **A** | **A** | **C** | **A** | **G** | **T** | **T** | **A** | **T** | **T** | **T** | **A** | **T** | **T** | **T** | **T** | **A** | **T** | **T** | **A** | **A** | **T** | **G** | **C** | **A** | **A** | **T** | **T** | **A** | **T** | **T** | **G** | **C** | **G** | **G** | **G** | **A** | **A** | **T** | **T** | **A** | **C** | **C** | **A** | **T** | **T** | **T** | **T** | **G** | **T** | **T** | **A** | **A** | **A** | **A** | **C** | **C** | **T** | **C** | **G** | **A** | **G** | **A** | **T** | **G** | **T** | **A** | **G** | **C** | **A** | **G** | **C** | **A** | **A** | **T** | **T** | **T** | **G** | **G** | **G** | **C** | **A** | **T** | **G** | **G** | **C** | **T** | **A** | **C** | **T** | **C** | **G** | **T** | **A** | **C** | **T** | **G** | **A** | **T** | **T** | **G** | **C** | **T** | **C** | **T** | **A** | **C** | **C** | **C** | **G** | **T** | **T** | **T** | **T** | **T** | **G** | **G** | **T** | **T** | **T** | **T** | **T** | **T** | **C** | **T** | **T** | **A** | **T** | **A** | **T** | **T** | **T** | **A** | **T** | **T** | **C** | **T** | **T** | **T** | **G** | **G** | **G** | **C** | **G** | **C** | **G** | **G** | **T** | **T** | **T** | **A** | **A** | **C** | **C** | **G** | **A** | **C** | **A** | **A** | **A** | **A** | **A** | **G** | **A** | **A** | **A** | **T** | **T** | **T** | **T** | **A** | **T** | **T** | **T** | **G** | **C** | **A** | **A** | **C** | **A** | **A** | **A** | **G** | **T** | **G** | **A** | **T** | **T** | **T** | **G** | **C** | **G** | **G** | **G** | **A** | **A** | **T** | **T** | **A** | **G** | **A** | **G** | **A** | **A** | **T** | **T** | **T** | **T** | **C** | **A** | **G** | **A** | **A** | **T** | **T** | **T** | **C** | **C** | **A** | **A** | **G** | **A** | **A** | **G** | **A** | **A** | **A** | **G** | **T** | **T** | **T** | **T** | **G** | **A** | **A** | **C** | **T** | **T** | **T** | **A** | **T** | **A** | **G** | **T** | **C** | **A** | **A** | **A** | **A** | **G** | **A** | **T** | **G** | **C** | **C** | **G** | **A** | **C** | **G** | **G** | **A** | **G** | **G** | **A** | **G** | **C** | **A** | **A** | **C** | **A** | **A** | **C** | **A** | **G** | **T** | **T** | **T** | **A** | **C** | **A** | **G** | **A** | **C** | **T** | **T** | **T** | **T** | **T** | **T** | **T** | **C** | **C** | **T** | **C** | **G** | **T** | **T** | **G** | **A** | **A** | **T** | **A** | **G** | **G** | **A** | **T** | **G** | **C** | **C** | **T** | **C** | **T** | **G** | **A** | **C** | **T** | **A** | **A** | **G** | **A** | **A** | **A** | **A** | **A** | **T** | **G** | **A** | **T** | **G** | **T** | **T** | **C** | **A** | **A** | **A** | **T** | **T** | **T** | **T** | **T** | **A** | **C** | **G** | **G** | **A** | **T** | **G** | **G** | **T** | **A** | **C** | **G** | **G** | **A** | **A** | **A** | **A** | **A** | **T** | **T** | **C** | **A** | **A** | **T** | **G** | **C** | **G** | **T** | **T** | **G** | **A** | **T** | **G** | **G** | **C** | **G** | **G** | **A** | **C** | **A** | **T** | **A** | **A** | **A** | **A** | **A** | **A** | **A** | **G** | **C** | **T** | **C** | **A** | **G** | **C** | **A** | **C** | **T** | **C** | **C** | **A** | **T** | **T** | **C** | **A** | **T** | **A** | **T** | **T** | **G** | **A** | **A** | **T** | **A** | **T** | **T** | **A** | **T** | **G** | **C** | **C** | **T** | **T** | **T** | **G** | **T** | **A** | **A** | **C** | **G** | **G** | **A** | **T** | **C** | **A** | **T** | **A** | **T** | **C** | **G** | **G** | **C** | **A** | **C** | **C** | **A** | **A** | **A** | **A** | **T** | **T** | **T** | **T** | **G** | **A** | **A** | **C** | **T** | **T** | **A** | **T** | **T** | **A** | **G** | **A** | **A** | **G** | **A** | **A** | **A** | **A** | **G** | **G** | **C** | **G** | **G** | **C** | **T** | **G** | **A** | **A** | **G** | **G** | **G** | **G** | **T** | **T** | **G** | **A** | **A** | **G** | **T** | **G** | **C** | **G** | **G** | **T** | **T** | **G** | **C** | **T** | **T** | **T** | **A** | **C** | **G** | **A** | **T** | **G** | **C** | **C** | **T** | **T** | **T** | **G** | **G** | **C** | **T** | **C** | **A** | **A** | **A** | **G** | **G** | **G** | **C** | **A** | **C** | **C** | **A** | **A** | **G** | **G** | **T** | **T** | **C** | **A** | **T** | **C** | **A** | **T** | **T** | **T** | **G** | **A** | **A** | **T** | **G** | **A** | **A** | **T** | **T** | **G** | **A** | **A** | **A** | **A** | **A** | **A** | **A** | **A** | **C** | **G** | **G** | **T** | **G** | **G** | **T** | **T** | **T** | **T** | **G** | **T** | **C** | **C** | **A** | **A** | **A** | **C** | **G** | **T** | **T** | **T** | **A** | **T** | **T** | **A** | **C** | **T** | **T** | **C** | **T** | **C** | **A** | **A** | **A** | **A** | **A** | **G** | **C** | **A** | **C** | **T** | **T** | **T** | **T** | **G** | **A** | **A** | **G** | **T** | **T** | **T** | **C** | **G** | **T** | **T** | **T** | **G** | **A** | **A** | **T** | **T** | **A** | **T** | **C** | **A** | **T** | **G** | **A** | **T** | **C** | **A** | **C** | **C** | **G** | **G** | **A** | **A** | **A** | **A** | **T** | **T** | **G** | **T** | **T** | **G** | **T** | **T** | **A** | **T** | **T** | **G** | **A** | **C** | **G** | **G** | **A** | **A** | **A** | **A** | **G** | **T** | **A** | **G** | **G** | **C** | **T** | **A** | **C** | **A** | **T** | **T** | **G** | **G** | **C** | **G** | **G** | **C** | **T** | **T** | **T** | **A** | **A** | **T** | **G** | **T** | **T** | **G** | **C** | **C** | **G** | **A** | **T** | **C** | **A** | **A** | **T** | **A** | **T** | **G** | **C** | **T** | **G** | **G** | **A** | **A** | **C** | **G** | **A** | **C** | **T** | **A** | **A** | **A** | **A** | **A** | **G** | **T** | **T** | **T** | **G** | **G** | **C** | **T** | **A** | **T** | **T** | **G** | **G** | **C** | **G** | **G** | **G** | **A** | **T** | **A** | **C** | **G** | **C** | **A** | **T** | **T** | **T** | **A** | **C** | **G** | **G** | **A** | **T** | **T** | **C** | **A** | **A** | **G** | **G** | **G** | **C** | **C** | **A** | **G** | **C** | **A** | **G** | **C** | **C** | **T** | **C** | **A** | **T** | **T** | **A** | **C** | **T** | **G** | **C** | **A** | **A** | **A** | **T** | **G** | **C** | **G** | **T** | **T** | **T** | **T** | **T** | **T** | **A** | **A** | **T** | **G** | **G** | **A** | **T** | **T** | **G** | **G** | **A** | **A** | **C** | **G** | **T** | **C** | **T** | **C** | **T** | **T** | **C** | **C** | **C** | **C** | **C** | **G** | **A** | **G** | **A** | **A** | **A** | **A** | **A** | **T** | **C** | **G** | **T** | **G** | **T** | **G** | **G** | **C** | **G** | **T** | **A** | **T** | **C** | **A** | **A** | **T** | **T** | **G** | **G** | **A** | **T** | **T** | **A** | **T** | **T** | **T** | **C** | **T** | **T** | **T** | **A** | **A** | **A** | **C** | **T** | **T** | **G** | **A** | **A** | **G** | **C** | **A** | **T** | **T** | **G** | **G** | **T** | **G** | **C** | **C** | **A** | **G** | **A** | **G** | **G** | **C** | **A** | **A** | **A** | **T** | **A** | **C** | **A** | **T** | **C** | **C** | **A** | **T** | **T** | **C** | **A** | **G** | **A** | **T** | **G** | **A** | **T** | **T** | **G** | **C** | **C** | **A** | **G** | **T** | **G** | **G** | **T** | **C** | **C** | **T** | **A** | **A** | **C** | **A** | **G** | **T** | **G** | **A** | **C** | **C** | **G** | **T** | **G** | **A** | **A** | **C** | **A** | **A** | **A** | **T** | **T** | **A** | **A** | **A** | **T** | **T** | **G** | **G** | **C** | **C** | **T** | **T** | **T** | **A** | **T** | **T** | **A** | **A** | **A** | **T** | **T** | **G** | **A** | **T** | **T** | **A** | **C** | **T** | **T** | **C** | **T** | **G** | **C** | **C** | **A** | **A** | **G** | **A** | **A** | **A** | **A** | **G** | **A** | **G** | **T** | **C** | **T** | **G** | **G** | **A** | **T** | **T** | **C** | **A** | **A** | **A** | **C** | **G** | **C** | **C** | **G** | **T** | **A** | **T** | **T** | **T** | **A** | **G** | **T** | **T** | **C** | **C** | **T** | **G** | **A** | **T** | **G** | **A** | **T** | **A** | **G** | **T** | **G** | **T** | **C** | **T** | **T** | **G** | **G** | **C** | **T** | **G** | **C** | **T** | **T** | **T** | **A** | **A** | **A** | **G** | **G** | **T** | **T** | **G** | **C** | **T** | **G** | **C** | **G** | **G** | **C** | **T** | **T** | **C** | **A** | **G** | **G** | **A** | **G** | **T** | **A** | **G** | **A** | **T** | **G** | **T** | **T** | **A** | **A** | **A** | **A** | **T** | **T** | **A** | **T** | **G** | **A** | **T** | **T** | **C** | **C** | **A** | **G** | **A** | **T** | **A** | **A** | **G** | **C** | **C** | **C** | **G** | **A** | **C** | **C** | **A** | **T** | **C** | **C** | **G** | **T** | **T** | **T** | **A** | **T** | **T** | **T** | **A** | **T** | **C** | **G** | **A** | **G** | **C** | **A** | **A** | **C** | **A** | **C** | **A** | **G** | **T** | **A** | **T** | **T** | **A** | **C** | **G** | **G** | **C** | **C** | **G** | **C** | **T** | **T** | **A** | **T** | **T** | **G** | **A** | **T** | **G** | **A** | **A** | **A** | **G** | **A** | **A** | **A** | **A** | **T** | **A** | **T** | **T** | **G** | **A** | **A** | **A** | **T** | **T** | **T** | **T** | **A** | **A** | **T** | **T** | **T** | **A** | **T** | **A** | **A** | **C** | **G** | **G** | **T** | **G** | **G** | **T** | **T** | **T** | **C** | **T** | **T** | **A** | **C** | **A** | **T** | **G** | **C** | **G** | **A** | **A** | **A** | **A** | **C** | **A** | **A** | **T** | **G** | **A** | **T** | **T** | **A** | **T** | **G** | **G** | **A** | **T** | **G** | **A** | **T** | **G** | **A** | **A** | **G** | **T** | **C** | **T** | **G** | **C** | **A** | **C** | **A** | **G** | **T** | **T** | **G** | **G** | **T** | **T** | **C** | **A** | **G** | **C** | **C** | **A** | **A** | **T** | **C** | **A** | **A** | **G** | **A** | **T** | **A** | **T** | **C** | **C** | **G** | **A** | **A** | **G** | **T** | **T** | **A** | **C** | **A** | **A** | **A** | **T** | **T** | **A** | **A** | **A** | **C** | **T** | **T** | **T** | **G** | **A** | **A** | **G** | **C** | **A** | **A** | **A** | **T** | **G** | **C** | **T** | **G** | **T** | **G** | **T** | **T** | **A** | **T** | **A** | **T** | **G** | **A** | **T** | **A** | **A** | **A** | **A** | **A** | **A** | **A** | **T** | **C** | **A** | **T** | **T** | **G** | **A** | **T** | **C** | **A** | **A** | **T** | **T** | **A** | **G** | **A** | **A** | **G** | **C** | **A** | **A** | **T** | **T** | **T** | **T** | **C** | **T** | **T** | **A** | **G** | **A** | **A** | **G** | **A** | **T** | **C** | **G** | **A** | **A** | **A** | **A** | **A** | **A** | **A** | **T** | **G** | **T** | **A** | **C** | **A** | **A** | **C** | **A** | **A** | **T** | **G** | **A** | **C** | **T** | **C** | **C** | **A** | **G** | **A** | **A** | **G** | **T** | **T** | **G** | **T** | **T** | **C** | **G** | **C** | **G** | **A** | **C** | **A** | **T** | **G** | **T** | **C** | **A** | **A** | **A** | **A** | **T** | **G** | **G** | **T** | **T** | **G** | **A** | **T** | **T** | **T** | **T** | **T** | **A** | **A** | **A** | **C** | **A** | **A** | **C** | **A** | **A** | **A** | **T** | **T** | **T** | **C** | **A** | **C** | **G** | **A** | **T** | **T** | **A** | **T** | **T** | **T** | **T** | **C** | **A** | **C** | **C** | **A** | **A** | **T** | **T** | **C** | **T** | **T** | **-** | **-** | **-** |
| **N59606F** | **T** | **T** | **G** | **A** | **T** | **T** | **C** | **T** | **T** | **A** | **G** | **C** | **G** | **T** | **C** | **T** | **T** | **A** | **A** | **C** | **A** | **G** | **T** | **T** | **A** | **T** | **T** | **T** | **A** | **T** | **T** | **T** | **T** | **A** | **T** | **T** | **A** | **A** | **T** | **G** | **C** | **A** | **A** | **T** | **T** | **A** | **T** | **T** | **G** | **C** | **G** | **G** | **G** | **A** | **A** | **T** | **T** | **A** | **C** | **C** | **A** | **T** | **T** | **T** | **T** | **G** | **T** | **T** | **A** | **A** | **A** | **A** | **C** | **C** | **T** | **C** | **G** | **A** | **G** | **A** | **T** | **G** | **T** | **A** | **G** | **C** | **A** | **G** | **C** | **A** | **A** | **T** | **T** | **T** | **G** | **G** | **G** | **C** | **A** | **T** | **G** | **G** | **C** | **T** | **A** | **C** | **T** | **C** | **G** | **T** | **A** | **C** | **T** | **G** | **A** | **T** | **T** | **G** | **C** | **T** | **C** | **T** | **A** | **C** | **C** | **C** | **G** | **T** | **T** | **T** | **T** | **T** | **G** | **G** | **T** | **T** | **T** | **T** | **T** | **T** | **C** | **T** | **T** | **A** | **T** | **A** | **T** | **T** | **T** | **A** | **T** | **T** | **C** | **T** | **T** | **T** | **G** | **G** | **G** | **C** | **G** | **C** | **G** | **G** | **T** | **T** | **T** | **A** | **A** | **C** | **C** | **G** | **A** | **C** | **A** | **A** | **A** | **A** | **A** | **G** | **A** | **A** | **A** | **T** | **T** | **T** | **T** | **A** | **T** | **T** | **T** | **G** | **C** | **A** | **A** | **C** | **A** | **A** | **A** | **G** | **T** | **G** | **A** | **T** | **T** | **T** | **G** | **C** | **G** | **G** | **G** | **A** | **A** | **T** | **T** | **A** | **G** | **A** | **G** | **A** | **A** | **T** | **T** | **T** | **T** | **C** | **A** | **G** | **A** | **A** | **T** | **T** | **T** | **C** | **C** | **A** | **A** | **G** | **A** | **A** | **G** | **A** | **A** | **A** | **G** | **T** | **T** | **T** | **T** | **G** | **A** | **A** | **C** | **T** | **T** | **T** | **A** | **T** | **A** | **G** | **T** | **C** | **A** | **A** | **A** | **A** | **G** | **A** | **T** | **G** | **C** | **C** | **G** | **A** | **C** | **G** | **G** | **A** | **G** | **G** | **A** | **G** | **C** | **A** | **A** | **C** | **A** | **A** | **C** | **A** | **G** | **T** | **T** | **T** | **A** | **C** | **A** | **G** | **A** | **C** | **T** | **T** | **T** | **T** | **T** | **T** | **T** | **C** | **C** | **T** | **C** | **G** | **T** | **T** | **G** | **A** | **A** | **T** | **A** | **G** | **G** | **A** | **T** | **G** | **C** | **C** | **T** | **C** | **T** | **G** | **A** | **C** | **T** | **A** | **A** | **G** | **A** | **A** | **A** | **A** | **A** | **T** | **G** | **A** | **T** | **G** | **T** | **T** | **C** | **A** | **A** | **A** | **T** | **T** | **T** | **T** | **T** | **A** | **C** | **G** | **G** | **A** | **T** | **G** | **G** | **T** | **A** | **C** | **G** | **G** | **A** | **A** | **A** | **A** | **A** | **T** | **T** | **C** | **A** | **A** | **T** | **G** | **C** | **G** | **T** | **T** | **G** | **A** | **T** | **G** | **G** | **C** | **G** | **G** | **A** | **C** | **A** | **T** | **A** | **A** | **A** | **A** | **A** | **A** | **A** | **G** | **C** | **T** | **C** | **A** | **G** | **C** | **A** | **C** | **T** | **C** | **C** | **A** | **T** | **T** | **C** | **A** | **T** | **A** | **T** | **T** | **G** | **A** | **A** | **T** | **A** | **T** | **T** | **A** | **T** | **G** | **C** | **C** | **T** | **T** | **T** | **G** | **T** | **A** | **A** | **C** | **G** | **G** | **A** | **T** | **C** | **A** | **T** | **A** | **T** | **C** | **G** | **G** | **C** | **A** | **C** | **C** | **A** | **A** | **A** | **A** | **T** | **T** | **T** | **T** | **G** | **A** | **A** | **C** | **T** | **T** | **A** | **T** | **T** | **A** | **G** | **A** | **A** | **G** | **A** | **A** | **A** | **A** | **G** | **G** | **C** | **G** | **G** | **C** | **T** | **G** | **A** | **A** | **G** | **G** | **G** | **G** | **T** | **T** | **G** | **A** | **A** | **G** | **T** | **G** | **C** | **G** | **G** | **T** | **T** | **G** | **C** | **T** | **T** | **T** | **A** | **C** | **G** | **A** | **T** | **G** | **C** | **C** | **T** | **T** | **T** | **G** | **G** | **C** | **T** | **C** | **A** | **A** | **A** | **G** | **G** | **G** | **C** | **A** | **C** | **C** | **A** | **A** | **G** | **G** | **T** | **T** | **C** | **A** | **T** | **C** | **A** | **T** | **T** | **T** | **G** | **A** | **A** | **T** | **G** | **A** | **A** | **T** | **T** | **G** | **A** | **A** | **A** | **A** | **A** | **A** | **A** | **A** | **C** | **G** | **G** | **T** | **G** | **G** | **T** | **T** | **T** | **T** | **G** | **T** | **C** | **C** | **A** | **A** | **A** | **C** | **G** | **T** | **T** | **T** | **A** | **T** | **T** | **A** | **C** | **T** | **T** | **C** | **T** | **C** | **A** | **A** | **A** | **A** | **A** | **G** | **C** | **A** | **C** | **T** | **T** | **T** | **T** | **G** | **A** | **A** | **G** | **T** | **T** | **T** | **C** | **G** | **T** | **T** | **T** | **G** | **A** | **A** | **T** | **T** | **A** | **T** | **C** | **A** | **T** | **G** | **A** | **T** | **C** | **A** | **C** | **C** | **G** | **G** | **A** | **A** | **A** | **A** | **T** | **T** | **G** | **T** | **T** | **G** | **T** | **T** | **A** | **T** | **T** | **G** | **A** | **C** | **G** | **G** | **A** | **A** | **A** | **A** | **G** | **T** | **A** | **G** | **G** | **C** | **T** | **A** | **C** | **A** | **T** | **T** | **G** | **G** | **C** | **G** | **G** | **C** | **T** | **T** | **T** | **A** | **A** | **T** | **G** | **T** | **T** | **G** | **C** | **C** | **G** | **A** | **T** | **C** | **A** | **A** | **T** | **A** | **T** | **G** | **C** | **C** | **G** | **G** | **A** | **A** | **C** | **G** | **A** | **C** | **T** | **A** | **A** | **A** | **A** | **A** | **G** | **T** | **T** | **T** | **G** | **G** | **C** | **T** | **A** | **T** | **T** | **G** | **G** | **C** | **G** | **G** | **G** | **A** | **T** | **A** | **C** | **A** | **C** | **A** | **T** | **T** | **T** | **A** | **C** | **G** | **G** | **A** | **T** | **T** | **C** | **A** | **A** | **G** | **G** | **G** | **C** | **C** | **A** | **G** | **C** | **A** | **G** | **C** | **C** | **T** | **C** | **A** | **T** | **T** | **A** | **C** | **T** | **G** | **C** | **A** | **A** | **A** | **T** | **G** | **C** | **G** | **T** | **T** | **T** | **T** | **T** | **T** | **A** | **A** | **T** | **G** | **G** | **A** | **T** | **T** | **G** | **G** | **A** | **A** | **C** | **G** | **T** | **C** | **T** | **C** | **T** | **T** | **C** | **C** | **C** | **C** | **C** | **G** | **A** | **G** | **A** | **A** | **A** | **A** | **A** | **T** | **C** | **G** | **T** | **G** | **T** | **G** | **G** | **C** | **G** | **T** | **A** | **T** | **C** | **A** | **A** | **T** | **T** | **G** | **G** | **A** | **T** | **T** | **A** | **T** | **T** | **T** | **C** | **T** | **T** | **T** | **A** | **A** | **A** | **C** | **T** | **T** | **G** | **A** | **A** | **G** | **C** | **A** | **T** | **T** | **G** | **G** | **T** | **G** | **C** | **C** | **A** | **G** | **A** | **G** | **G** | **C** | **A** | **A** | **A** | **T** | **A** | **C** | **A** | **T** | **C** | **C** | **A** | **T** | **T** | **C** | **A** | **G** | **A** | **T** | **G** | **A** | **T** | **T** | **G** | **C** | **C** | **A** | **G** | **T** | **G** | **G** | **T** | **C** | **C** | **T** | **A** | **A** | **C** | **A** | **G** | **T** | **G** | **A** | **C** | **C** | **G** | **T** | **G** | **A** | **A** | **C** | **A** | **A** | **A** | **T** | **T** | **A** | **A** | **A** | **T** | **T** | **G** | **G** | **C** | **C** | **T** | **T** | **T** | **A** | **T** | **T** | **A** | **A** | **A** | **T** | **T** | **G** | **A** | **T** | **T** | **A** | **C** | **T** | **T** | **C** | **T** | **G** | **C** | **C** | **A** | **A** | **G** | **A** | **A** | **A** | **A** | **G** | **A** | **G** | **T** | **C** | **T** | **G** | **G** | **A** | **T** | **T** | **C** | **A** | **A** | **A** | **C** | **G** | **C** | **C** | **G** | **T** | **A** | **T** | **T** | **T** | **A** | **G** | **T** | **T** | **C** | **C** | **T** | **G** | **A** | **T** | **G** | **A** | **T** | **A** | **G** | **T** | **G** | **T** | **C** | **T** | **T** | **G** | **G** | **C** | **T** | **G** | **C** | **T** | **T** | **T** | **A** | **A** | **A** | **G** | **G** | **T** | **T** | **G** | **C** | **T** | **G** | **C** | **G** | **G** | **C** | **T** | **T** | **C** | **A** | **G** | **G** | **A** | **G** | **T** | **A** | **G** | **A** | **T** | **G** | **T** | **T** | **A** | **A** | **A** | **A** | **T** | **T** | **A** | **T** | **G** | **A** | **T** | **T** | **C** | **C** | **A** | **G** | **A** | **T** | **A** | **A** | **G** | **C** | **C** | **C** | **G** | **A** | **C** | **C** | **A** | **T** | **C** | **C** | **G** | **T** | **T** | **T** | **A** | **T** | **T** | **T** | **A** | **T** | **C** | **G** | **A** | **G** | **C** | **A** | **A** | **C** | **A** | **C** | **A** | **G** | **T** | **A** | **T** | **T** | **A** | **C** | **G** | **G** | **C** | **C** | **G** | **C** | **T** | **T** | **G** | **T** | **T** | **G** | **A** | **T** | **G** | **A** | **A** | **A** | **G** | **A** | **A** | **A** | **A** | **T** | **A** | **T** | **T** | **G** | **A** | **A** | **A** | **T** | **T** | **T** | **T** | **A** | **A** | **T** | **T** | **T** | **A** | **T** | **A** | **A** | **C** | **G** | **G** | **T** | **G** | **G** | **T** | **T** | **T** | **C** | **T** | **T** | **A** | **C** | **A** | **T** | **G** | **C** | **G** | **A** | **A** | **A** | **A** | **C** | **A** | **A** | **T** | **G** | **A** | **T** | **T** | **A** | **T** | **G** | **G** | **A** | **T** | **G** | **A** | **T** | **G** | **A** | **A** | **G** | **T** | **C** | **T** | **G** | **C** | **A** | **C** | **A** | **G** | **T** | **A** | **G** | **G** | **T** | **T** | **C** | **A** | **G** | **C** | **C** | **A** | **A** | **T** | **C** | **A** | **A** | **G** | **A** | **T** | **A** | **T** | **C** | **C** | **G** | **A** | **A** | **G** | **T** | **T** | **A** | **C** | **A** | **A** | **A** | **T** | **T** | **A** | **A** | **A** | **C** | **T** | **T** | **T** | **G** | **A** | **A** | **G** | **C** | **A** | **A** | **A** | **T** | **G** | **C** | **T** | **G** | **T** | **G** | **T** | **T** | **A** | **T** | **A** | **T** | **G** | **A** | **T** | **A** | **A** | **A** | **A** | **A** | **A** | **A** | **T** | **C** | **A** | **T** | **T** | **G** | **A** | **T** | **C** | **A** | **A** | **T** | **T** | **A** | **G** | **A** | **A** | **G** | **C** | **A** | **A** | **T** | **T** | **T** | **T** | **C** | **T** | **T** | **A** | **G** | **A** | **A** | **G** | **A** | **T** | **C** | **G** | **A** | **A** | **A** | **A** | **A** | **A** | **A** | **T** | **G** | **T** | **A** | **C** | **A** | **A** | **C** | **A** | **A** | **T** | **G** | **A** | **C** | **T** | **C** | **C** | **A** | **G** | **A** | **A** | **G** | **T** | **T** | **G** | **T** | **T** | **C** | **G** | **C** | **G** | **A** | **C** | **A** | **T** | **G** | **T** | **C** | **C** | **A** | **A** | **A** | **T** | **G** | **G** | **T** | **T** | **G** | **A** | **T** | **T** | **T** | **T** | **T** | **A** | **A** | **A** | **C** | **A** | **A** | **C** | **A** | **A** | **A** | **T** | **T** | **T** | **C** | **A** | **C** | **G** | **A** | **T** | **T** | **A** | **T** | **T** | **T** | **T** | **C** | **A** | **C** | **C** | **A** | **A** | **T** | **T** | **C** | **T** | **T** | **-** | **-** | **-** |
 **SNP position** | . | . | . | . | . | . | . | . | . | . | . | 12 | . | . | . | . | . | . | . | . | . | . | . | . | . | . | . | . | . | . | . | . | . | . | . | . | . | . | . | . | . | . | . | . | . | . | . | . | . | . | . | . | . | . | . | . | . | . | . | . | . | . | 63 | . | . | . | . | . | . | . | . | . | . | . | . | . | . | . | . | . | . | . | . | . | . | . | 87 | . | . | . | . | . | . | . | . | . | . | . | . | . | . | . | 103 | . | . | . | . | 108 | . | . | . | . | . | . | . | . | . | . | . | . | . | . | . | . | . | . | . | . | . | . | . | . | . | . | . | . | . | . | . | . | . | . | . | . | . | . | . | . | . | . | . | . | . | . | . | . | . | . | 159 | . | . | 162 | . | . | 165 | . | . | 168 | . | . | . | . | . | . | . | . | . | . | . | . | . | . | . | . | . | . | . | . | . | . | . | . | . | . | . | . | . | . | . | . | . | . | . | . | . | . | 207 | . | . | . | . | . | . | . | . | . | . | . | . | . | . | . | . | . | 225 | . | . | . | . | . | 231 | . | . | . | . | . | . | . | . | . | . | . | . | . | . | . | . | . | . | . | . | . | . | . | . | . | . | . | . | . | . | . | 263 | . | . | . | . | . | . | . | . | . | . | . | . | . | . | . | . | . | . | . | . | . | 285 | . | . | 288 | . | . | . | . | . | . | . | . | . | . | . | . | . | . | . | . | . | 306 | . | . | . | . | . | . | . | . | 315 | . | . | . | . | . | . | . | . | 324 | . | . | 327 | . | . | . | . | . | . | . | . | . | . | . | . | . | . | . | . | . | 345 | . | . | . | 349 | . | 351 | . | . | . | . | . | . | . | . | . | . | . | . | . | . | . | . | 368 | . | . | . | . | . | . | . | . | . | . | . | . | . | . | . | . | . | . | . | . | . | . | . | 392 | 393 | . | . | 396 | . | . | . | . | . | . | . | . | . | . | . | . | . | . | . | . | . | 414 | . | . | 417 | . | . | . | . | . | . | . | . | . | . | . | . | . | . | . | . | . | . | . | . | . | . | . | . | . | . | . | . | . | . | . | . | . | . | . | . | . | . | . | . | . | 459 | . | . | . | . | . | . | . | . | . | . | . | . | . | . | . | . | . | . | . | . | . | . | . | . | . | . | . | . | . | . | . | . | . | . | . | . | . | . | . | . | . | . | . | . | . | . | . | . | . | . | . | . | . | . | . | . | 516 | . | . | . | . | . | . | . | . | 525 | . | . | . | . | . | . | . | . | . | . | . | . | . | . | . | . | . | 543 | . | . | . | . | . | . | . | . | . | . | . | . | . | . | . | . | . | . | . | . | . | . | . | . | . | . | . | . | . | . | . | . | . | . | . | . | . | . | 582 | . | . | . | . | . | . | . | . | . | . | . | . | . | . | 597 | . | . | . | . | . | . | . | . | . | . | . | . | . | . | . | . | . | . | . | . | . | . | . | 621 | 622 | . | . | . | . | . | . | . | . | . | . | 633 | . | . | . | . | . | . | . | . | . | . | . | . | . | . | . | . | . | . | . | . | . | . | . | . | . | . | . | . | . | 663 | . | . | . | . | . | . | . | . | 672 | . | . | 675 | . | . | . | . | . | . | . | . | . | . | . | . | . | . | . | . | . | 693 | . | . | 696 | . | . | . | . | . | . | . | . | . | . | . | . | . | . | . | . | . | . | . | . | . | . | . | 720 | . | . | . | . | . | . | . | . | . | . | . | . | . | . | . | . | . | . | . | . | . | . | . | . | . | . | . | . | . | . | . | . | . | . | . | 756 | . | . | . | . | . | . | . | . | . | . | . | . | . | . | . | . | . | . | . | . | . | . | . | . | 781 | . | . | . | . | . | . | . | . | . | . | . | . | . | . | . | . | . | . | . | . | . | . | . | . | . | . | . | . | . | . | . | . | . | . | . | . | . | . | . | . | . | . | . | . | . | . | . | . | . | 831 | . | . | . | . | . | . | . | . | . | . | 842 | . | . | . | . | . | . | . | . | . | . | . | . | . | . | . | . | . | . | . | . | . | . | . | . | . | . | . | . | . | . | . | . | . | . | . | . | . | . | . | 882 | . | . | . | . | . | 888 | . | . | . | . | . | . | . | . | . | . | . | . | . | . | . | . | . | . | . | . | . | . | . | . | . | . | . | . | . | . | . | . | 921 | . | . | . | . | . | 927 | . | . | . | . | . | 933 | . | . | . | . | . | . | . | . | . | . | . | . | . | . | . | . | . | . | . | . | . | 955 | . | . | . | . | 960 | . | . | . | . | . | . | . | . | . | . | . | . | . | . | . | . | . | . | . | . | . | . | . | . | . | . | . | . | . | . | . | . | . | . | . | . | . | . | . | . | . | . | . | . | . | . | . | 1008 | . | . | . | . | . | . | . | . | . | . | . | . | . | . | . | . | . | . | . | . | 1029 | . | . | . | . | . | . | . | . | . | . | . | 1041 | . | . | . | . | . | . | . | . | 1050 | . | . | . | . | . | . | . | . | . | . | . | . | . | . | . | . | . | . | . | . | . | . | . | . | . | . | . | . | . | . | . | . | . | . | . | . | . | . | . | . | . | . | . | . | . | . | . | . | . | . | . | . | . | . | . | . | . | . | . | . | . | . | . | . | . | . | . | . | . | . | . | . | . | . | . | . | . | . | . | . | . | . | . | . | . | . | 1137 | . | . | 1140 | . | . | . | . | . | 1146 | . | . | 1149 | . | . | . | . | . | . | . | . | . | . | . | . | . | . | . | . | . | . | . | . | . | . | . | . | . | . | . | . | . | . | . | . | . | . | . | . | . | . | . | . | . | . | . | . | . | . | . | . | . | . | 1200 | . | . | . | . | . | . | . | . | . | . | . | . | . | . | . | . | . | . | . | . | . | . | . | . | . | . | . | . | . | 1230 | . | . | . | . | . | 1236 | . | . | . | . | . | . | . | . | 1245 | . | . | 1248 | . | . | . | . | . | . | . | . | . | . | . | . | . | . | . | . | . | . | . | . | . | . | . | . | . | . | . | . | . | . | . | . | . | . | . | . | . | . | . | . | . | . | . | . | . | . | . | . | . | . | . | . | . | . | . | . | . | . | . | . | . | . | . | . | . | . | . | . | . | . | . | . | . | . | . | . | . | . | . | . | . | . | . | . | . | . | 1335 | . | . | . | . | . | . | . | . | . | . | . | . | . | . | . | . | . | . | . | . | . | . | . | . | . | . | . | . | . | . | . | . | . | . | . | . | . | . | . | . | . | . | . | . | . | . | . | . | . | . | . | . | . | . | . | . | . | . | . | . | . | . | . | . | . | . | . | . | . | . | . | . | . | . | . | . | . | . | . | . | . | . | . | . | . | . | . | . | . | . | . | . | . | . | . | . | . | . | . | . | . | . | . | . | . | . | . | . | . | . | . |

|  |
| --- |
|  |

|  |  |
| --- | --- |
|  |

|  |  |
| --- | --- |
| **Legend** | |
  | **Reference Sequence** |

  |
|  | **Detected SNPs** |
|  |  |
