# Supplementary material for: sraX: A Novel Comprehensive Resistome Analysis Tool
Source: Front Microbiol. 2020 Feb 5;11:52. doi: 10.3389/fmicb.2020.00052 (PMC7025521; doi:10.3389/fmicb.2020.00052)
Supplement: Supplementary file 4 [file Data_Sheet_3.ZIP › cls/cls_Efaecium_dna.html]

# cls

|  |
| --- |
|  |

|  |
| --- |
|  |

|  |  |
| --- | --- |
| **Definition:** | Cls or cardiolipin synthetase is an inner membrane protein that is involved in membrane synthesis. Specific mutations in Enterococcus can confer resistance to daptomycin. |

|  |
| --- |
|  |

|  |
| --- |
|  |

|  |  |  |
| --- | --- | --- |
| **Reference organism:** | *Enterococcus faecium* | [NCBI, CARD] |

|  |
| --- |
|  |

|  |
| --- |
|  |

|  |  |
| --- | --- |
| **Summary Data** | || **Parameter** | **Value_(n)_** || Total number of genomes | 101 |
| MSA length | 1452 |
| Total number of SNPs | 110 |
| SNP density (# SNPs/MSA length) | 0.076 |

|  |
| --- |
|  |

|  |
| --- |
|  |

|  |
| --- |
|  |

|  |  |  |  |  |  |  |  |  |  |  |  |  |  |  |  |  |  |  |  |  |  |  |  |  |  |  |  |  |  |  |  |  |  |  |  |  |  |  |  |  |  |  |  |  |  |  |  |  |  |  |  |  |  |  |  |  |  |  |  |  |  |  |  |  |  |  |  |  |  |  |  |  |  |  |  |  |  |  |  |  |  |  |  |  |  |  |  |  |  |  |  |  |  |  |  |  |  |  |  |  |  |  |  |  |  |  |  |  |  |  |  |  |  |  |  |  |  |  |  |  |  |  |  |  |  |  |  |  |  |  |  |  |  |  |  |  |  |  |  |  |  |  |  |  |  |  |  |  |  |  |  |  |  |  |  |  |  |  |  |  |  |  |  |  |  |  |  |  |  |  |  |  |  |  |  |  |  |  |  |  |  |  |  |  |  |  |  |  |  |  |  |  |  |  |  |  |  |  |  |  |  |  |  |  |  |  |  |  |  |  |  |  |  |  |  |  |  |  |  |  |  |  |  |  |  |  |  |  |  |  |  |  |  |  |  |  |  |  |  |  |  |  |  |  |  |  |  |  |  |  |  |  |  |  |  |  |  |  |  |  |  |  |  |  |  |  |  |  |  |  |  |  |  |  |  |  |  |  |  |  |  |  |  |  |  |  |  |  |  |  |  |  |  |  |  |  |  |  |  |  |  |  |  |  |  |  |  |  |  |  |  |  |  |  |  |  |  |  |  |  |  |  |  |  |  |  |  |  |  |  |  |  |  |  |  |  |  |  |  |  |  |  |  |  |  |  |  |  |  |  |  |  |  |  |  |  |  |  |  |  |  |  |  |  |  |  |  |  |  |  |  |  |  |  |  |  |  |  |  |  |  |  |  |  |  |  |  |  |  |  |  |  |  |  |  |  |  |  |  |  |  |  |  |  |  |  |  |  |  |  |  |  |  |  |  |  |  |  |  |  |  |  |  |  |  |  |  |  |  |  |  |  |  |  |  |  |  |  |  |  |  |  |  |  |  |  |  |  |  |  |  |  |  |  |  |  |  |  |  |  |  |  |  |  |  |  |  |  |  |  |  |  |  |  |  |  |  |  |  |  |  |  |  |  |  |  |  |  |  |  |  |  |  |  |  |  |  |  |  |  |  |  |  |  |  |  |  |  |  |  |  |  |  |  |  |  |  |  |  |  |  |  |  |  |  |  |  |  |  |  |  |  |  |  |  |  |  |  |  |  |  |  |  |  |  |  |  |  |  |  |  |  |  |  |  |  |  |  |  |  |  |  |  |  |  |  |  |  |  |  |  |  |  |  |  |  |  |  |  |  |  |  |  |  |  |  |  |  |  |  |  |  |  |  |  |  |  |  |  |  |  |  |  |  |  |  |  |  |  |  |  |  |  |  |  |  |  |  |  |  |  |  |  |  |  |  |  |  |  |  |  |  |  |  |  |  |  |  |  |  |  |  |  |  |  |  |  |  |  |  |  |  |  |  |  |  |  |  |  |  |  |  |  |  |  |  |  |  |  |  |  |  |  |  |  |  |  |  |  |  |  |  |  |  |  |  |  |  |  |  |  |  |  |  |  |  |  |  |  |  |  |  |  |  |  |  |  |  |  |  |  |  |  |  |  |  |  |  |  |  |  |  |  |  |  |  |  |  |  |  |  |  |  |  |  |  |  |  |  |  |  |  |  |  |  |  |  |  |  |  |  |  |  |  |  |  |  |  |  |  |  |  |  |  |  |  |  |  |  |  |  |  |  |  |  |  |  |  |  |  |  |  |  |  |  |  |  |  |  |  |  |  |  |  |  |  |  |  |  |  |  |  |  |  |  |  |  |  |  |  |  |  |  |  |  |  |  |  |  |  |  |  |  |  |  |  |  |  |  |  |  |  |  |  |  |  |  |  |  |  |  |  |  |  |  |  |  |  |  |  |  |  |  |  |  |  |  |  |  |  |  |  |  |  |  |  |  |  |  |  |  |  |  |  |  |  |  |  |  |  |  |  |  |  |  |  |  |  |  |  |  |  |  |  |  |  |  |  |  |  |  |  |  |  |  |  |  |  |  |  |  |  |  |  |  |  |  |  |  |  |  |  |  |  |  |  |  |  |  |  |  |  |  |  |  |  |  |  |  |  |  |  |  |  |  |  |  |  |  |  |  |  |  |  |  |  |  |  |  |  |  |  |  |  |  |  |  |  |  |  |  |  |  |  |  |  |  |  |  |  |  |  |  |  |  |  |  |  |  |  |  |  |  |  |  |  |  |  |  |  |  |  |  |  |  |  |  |  |  |  |  |  |  |  |  |  |  |  |  |  |  |  |  |  |  |  |  |  |  |  |  |  |  |  |  |  |  |  |  |  |  |  |  |  |  |  |  |  |  |  |  |  |  |  |  |  |  |  |  |  |  |  |  |  |  |  |  |  |  |  |  |  |  |  |  |  |  |  |  |  |  |  |  |  |  |  |  |  |  |  |  |  |  |  |  |  |  |  |  |  |  |  |  |  |  |  |  |  |  |  |  |  |  |  |  |  |  |  |  |  |  |  |  |  |  |  |  |  |  |  |  |  |  |  |  |  |  |  |  |  |  |  |  |  |  |  |  |  |  |  |  |  |  |  |  |  |  |  |  |  |  |  |  |  |  |  |  |  |  |  |  |  |  |  |  |  |  |  |  |  |  |  |  |  |  |  |  |  |  |  |  |  |  |  |  |  |  |  |  |  |  |  |  |  |  |  |  |  |  |  |  |  |  |  |  |  |  |  |  |  |  |  |  |  |  |  |  |  |  |  |  |  |  |  |  |  |  |  |  |  |  |  |  |  |  |  |  |  |  |  |  |  |  |  |  |  |  |  |  |  |  |  |  |  |  |  |  |  |  |  |  |  |  |  |  |  |  |  |  |  |  |  |  |  |  |  |  |  |  |  |  |  |  |  |  |  |  |  |  |  |  |  |  |  |  |  |  |  |  |  |  |  |  |  |  |  |  |  |  |  |  |  |  |  |  |  |  |  |  |  |  |  |  |  |  |  |  |  |  |  |  |  |  |  |  |  |  |  |  |  |  |  |  |  |  |  |  |  |  |  |  |  |  |  |  |  |  |  |  |  |  |  |  |  |  |  |  |  |  |  |  |  |  |  |  |  |  |  |  |  |  |  |  |  |  |  |  |  |  |  |  |  |  |  |  |  |  |  |  |  |  |  |  |  |  |  |  |  |  |  |  |  |  |  |  |  |  |  |  |  |  |  |  |  |  |  |  |  |  |  |  |  |  |  |  |  |  |  |  |  |  |  |  |
| --- | --- | --- | --- | --- | --- | --- | --- | --- | --- | --- | --- | --- | --- | --- | --- | --- | --- | --- | --- | --- | --- | --- | --- | --- | --- | --- | --- | --- | --- | --- | --- | --- | --- | --- | --- | --- | --- | --- | --- | --- | --- | --- | --- | --- | --- | --- | --- | --- | --- | --- | --- | --- | --- | --- | --- | --- | --- | --- | --- | --- | --- | --- | --- | --- | --- | --- | --- | --- | --- | --- | --- | --- | --- | --- | --- | --- | --- | --- | --- | --- | --- | --- | --- | --- | --- | --- | --- | --- | --- | --- | --- | --- | --- | --- | --- | --- | --- | --- | --- | --- | --- | --- | --- | --- | --- | --- | --- | --- | --- | --- | --- | --- | --- | --- | --- | --- | --- | --- | --- | --- | --- | --- | --- | --- | --- | --- | --- | --- | --- | --- | --- | --- | --- | --- | --- | --- | --- | --- | --- | --- | --- | --- | --- | --- | --- | --- | --- | --- | --- | --- | --- | --- | --- | --- | --- | --- | --- | --- | --- | --- | --- | --- | --- | --- | --- | --- | --- | --- | --- | --- | --- | --- | --- | --- | --- | --- | --- | --- | --- | --- | --- | --- | --- | --- | --- | --- | --- | --- | --- | --- | --- | --- | --- | --- | --- | --- | --- | --- | --- | --- | --- | --- | --- | --- | --- | --- | --- | --- | --- | --- | --- | --- | --- | --- | --- | --- | --- | --- | --- | --- | --- | --- | --- | --- | --- | --- | --- | --- | --- | --- | --- | --- | --- | --- | --- | --- | --- | --- | --- | --- | --- | --- | --- | --- | --- | --- | --- | --- | --- | --- | --- | --- | --- | --- | --- | --- | --- | --- | --- | --- | --- | --- | --- | --- | --- | --- | --- | --- | --- | --- | --- | --- | --- | --- | --- | --- | --- | --- | --- | --- | --- | --- | --- | --- | --- | --- | --- | --- | --- | --- | --- | --- | --- | --- | --- | --- | --- | --- | --- | --- | --- | --- | --- | --- | --- | --- | --- | --- | --- | --- | --- | --- | --- | --- | --- | --- | --- | --- | --- | --- | --- | --- | --- | --- | --- | --- | --- | --- | --- | --- | --- | --- | --- | --- | --- | --- | --- | --- | --- | --- | --- | --- | --- | --- | --- | --- | --- | --- | --- | --- | --- | --- | --- | --- | --- | --- | --- | --- | --- | --- | --- | --- | --- | --- | --- | --- | --- | --- | --- | --- | --- | --- | --- | --- | --- | --- | --- | --- | --- | --- | --- | --- | --- | --- | --- | --- | --- | --- | --- | --- | --- | --- | --- | --- | --- | --- | --- | --- | --- | --- | --- | --- | --- | --- | --- | --- | --- | --- | --- | --- | --- | --- | --- | --- | --- | --- | --- | --- | --- | --- | --- | --- | --- | --- | --- | --- | --- | --- | --- | --- | --- | --- | --- | --- | --- | --- | --- | --- | --- | --- | --- | --- | --- | --- | --- | --- | --- | --- | --- | --- | --- | --- | --- | --- | --- | --- | --- | --- | --- | --- | --- | --- | --- | --- | --- | --- | --- | --- | --- | --- | --- | --- | --- | --- | --- | --- | --- | --- | --- | --- | --- | --- | --- | --- | --- | --- | --- | --- | --- | --- | --- | --- | --- | --- | --- | --- | --- | --- | --- | --- | --- | --- | --- | --- | --- | --- | --- | --- | --- | --- | --- | --- | --- | --- | --- | --- | --- | --- | --- | --- | --- | --- | --- | --- | --- | --- | --- | --- | --- | --- | --- | --- | --- | --- | --- | --- | --- | --- | --- | --- | --- | --- | --- | --- | --- | --- | --- | --- | --- | --- | --- | --- | --- | --- | --- | --- | --- | --- | --- | --- | --- | --- | --- | --- | --- | --- | --- | --- | --- | --- | --- | --- | --- | --- | --- | --- | --- | --- | --- | --- | --- | --- | --- | --- | --- | --- | --- | --- | --- | --- | --- | --- | --- | --- | --- | --- | --- | --- | --- | --- | --- | --- | --- | --- | --- | --- | --- | --- | --- | --- | --- | --- | --- | --- | --- | --- | --- | --- | --- | --- | --- | --- | --- | --- | --- | --- | --- | --- | --- | --- | --- | --- | --- | --- | --- | --- | --- | --- | --- | --- | --- | --- | --- | --- | --- | --- | --- | --- | --- | --- | --- | --- | --- | --- | --- | --- | --- | --- | --- | --- | --- | --- | --- | --- | --- | --- | --- | --- | --- | --- | --- | --- | --- | --- | --- | --- | --- | --- | --- | --- | --- | --- | --- | --- | --- | --- | --- | --- | --- | --- | --- | --- | --- | --- | --- | --- | --- | --- | --- | --- | --- | --- | --- | --- | --- | --- | --- | --- | --- | --- | --- | --- | --- | --- | --- | --- | --- | --- | --- | --- | --- | --- | --- | --- | --- | --- | --- | --- | --- | --- | --- | --- | --- | --- | --- | --- | --- | --- | --- | --- | --- | --- | --- | --- | --- | --- | --- | --- | --- | --- | --- | --- | --- | --- | --- | --- | --- | --- | --- | --- | --- | --- | --- | --- | --- | --- | --- | --- | --- | --- | --- | --- | --- | --- | --- | --- | --- | --- | --- | --- | --- | --- | --- | --- | --- | --- | --- | --- | --- | --- | --- | --- | --- | --- | --- | --- | --- | --- | --- | --- | --- | --- | --- | --- | --- | --- | --- | --- | --- | --- | --- | --- | --- | --- | --- | --- | --- | --- | --- | --- | --- | --- | --- | --- | --- | --- | --- | --- | --- | --- | --- | --- | --- | --- | --- | --- | --- | --- | --- | --- | --- | --- | --- | --- | --- | --- | --- | --- | --- | --- | --- | --- | --- | --- | --- | --- | --- | --- | --- | --- | --- | --- | --- | --- | --- | --- | --- | --- | --- | --- | --- | --- | --- | --- | --- | --- | --- | --- | --- | --- | --- | --- | --- | --- | --- | --- | --- | --- | --- | --- | --- | --- | --- | --- | --- | --- | --- | --- | --- | --- | --- | --- | --- | --- | --- | --- | --- | --- | --- | --- | --- | --- | --- | --- | --- | --- | --- | --- | --- | --- | --- | --- | --- | --- | --- | --- | --- | --- | --- | --- | --- | --- | --- | --- | --- | --- | --- | --- | --- | --- | --- | --- | --- | --- | --- | --- | --- | --- | --- | --- | --- | --- | --- | --- | --- | --- | --- | --- | --- | --- | --- | --- | --- | --- | --- | --- | --- | --- | --- | --- | --- | --- | --- | --- | --- | --- | --- | --- | --- | --- | --- | --- | --- | --- | --- | --- | --- | --- | --- | --- | --- | --- | --- | --- | --- | --- | --- | --- | --- | --- | --- | --- | --- | --- | --- | --- | --- | --- | --- | --- | --- | --- | --- | --- | --- | --- | --- | --- | --- | --- | --- | --- | --- | --- | --- | --- | --- | --- | --- | --- | --- | --- | --- | --- | --- | --- | --- | --- | --- | --- | --- | --- | --- | --- | --- | --- | --- | --- | --- | --- | --- | --- | --- | --- | --- | --- | --- | --- | --- | --- | --- | --- | --- | --- | --- | --- | --- | --- | --- | --- | --- | --- | --- | --- | --- | --- | --- | --- | --- | --- | --- | --- | --- | --- | --- | --- | --- | --- | --- | --- | --- | --- | --- | --- | --- | --- | --- | --- | --- | --- | --- | --- | --- | --- | --- | --- | --- | --- | --- | --- | --- | --- | --- | --- | --- | --- | --- | --- | --- | --- | --- | --- | --- | --- | --- | --- | --- | --- | --- | --- | --- | --- | --- | --- | --- | --- | --- | --- | --- | --- | --- | --- | --- | --- | --- | --- | --- | --- | --- | --- | --- | --- | --- | --- | --- | --- | --- | --- | --- | --- | --- | --- | --- | --- | --- | --- | --- | --- | --- | --- | --- | --- | --- | --- | --- | --- | --- | --- | --- | --- | --- | --- | --- | --- | --- | --- | --- | --- | --- | --- | --- | --- | --- | --- | --- | --- | --- | --- | --- | --- | --- | --- | --- | --- | --- | --- | --- | --- | --- | --- | --- | --- | --- | --- | --- | --- | --- | --- | --- | --- | --- | --- | --- | --- | --- | --- | --- | --- | --- | --- | --- | --- | --- | --- | --- | --- | --- | --- | --- | --- | --- | --- | --- | --- | --- | --- | --- | --- | --- | --- | --- | --- | --- | --- | --- | --- | --- | --- | --- | --- | --- | --- | --- | --- | --- | --- | --- | --- | --- | --- | --- | --- | --- | --- | --- | --- | --- | --- | --- | --- | --- | --- | --- | --- | --- | --- | --- | --- | --- | --- | --- | --- | --- | --- | --- | --- | --- | --- | --- | --- | --- | --- | --- | --- | --- | --- | --- | --- | --- | --- | --- | --- | --- | --- | --- | --- | --- | --- | --- | --- | --- | --- | --- | --- | --- | --- | --- | --- | --- | --- | --- | --- | --- | --- | --- | --- | --- | --- | --- | --- | --- | --- | --- | --- | --- | --- | --- | --- | --- | --- | --- | --- | --- | --- | --- | --- | --- | --- | --- | --- | --- | --- | --- | --- | --- | --- | --- | --- | --- | --- | --- | --- | --- | --- | --- | --- | --- | --- | --- | --- | --- | --- | --- | --- | --- | --- | --- | --- | --- | --- | --- | --- | --- | --- | --- | --- | --- | --- | --- | --- | --- | --- | --- | --- | --- | --- | --- | --- | --- | --- | --- | --- | --- | --- | --- | --- | --- | --- | --- | --- | --- | --- | --- | --- | --- | --- | --- | --- | --- | --- | --- | --- | --- | --- | --- | --- | --- | --- | --- | --- | --- | --- | --- | --- | --- | --- | --- | --- | --- | --- | --- | --- | --- |
|  || **Enterococcus faecium (ALL09868.1)** | **G** | **T** | **G** | **G** | **T** | **A** | **T** | **C** | **T** | **A** | **G** | **T** | **A** | **T** | **T** | **A** | **T** | **A** | **A** | **C** | **C** | **G** | **C** | **C** | **C** | **T** | **T** | **T** | **A** | **T** | **C** | **T** | **A** | **T** | **T** | **A** | **A** | **A** | **T** | **G** | **C** | **A** | **C** | **T** | **T** | **A** | **T** | **T** | **G** | **C** | **T** | **T** | **T** | **G** | **G** | **T** | **G** | **G** | **C** | **T** | **A** | **T** | **T** | **T** | **T** | **G** | **A** | **T** | **C** | **A** | **A** | **A** | **C** | **C** | **C** | **C** | **G** | **A** | **G** | **A** | **T** | **G** | **T** | **A** | **G** | **C** | **A** | **G** | **C** | **C** | **A** | **T** | **T** | **T** | **G** | **G** | **G** | **C** | **A** | **T** | **G** | **G** | **C** | **T** | **C** | **T** | **T** | **A** | **G** | **T** | **A** | **T** | **T** | **A** | **T** | **T** | **T** | **G** | **C** | **C** | **C** | **T** | **T** | **C** | **C** | **T** | **G** | **G** | **G** | **G** | **T** | **G** | **G** | **G** | **C** | **T** | **T** | **T** | **G** | **T** | **A** | **T** | **T** | **A** | **T** | **A** | **T** | **T** | **T** | **A** | **T** | **T** | **T** | **T** | **T** | **C** | **G** | **G** | **A** | **C** | **G** | **T** | **G** | **G** | **A** | **T** | **T** | **A** | **A** | **C** | **G** | **G** | **A** | **T** | **A** | **A** | **G** | **A** | **A** | **A** | **A** | **A** | **A** | **T** | **T** | **C** | **T** | **A** | **T** | **C** | **T** | **C** | **C** | **G** | **A** | **C** | **A** | **A** | **A** | **G** | **T** | **G** | **A** | **C** | **T** | **T** | **G** | **A** | **A** | **A** | **G** | **A** | **A** | **T** | **T** | **A** | **G** | **A** | **A** | **A** | **A** | **C** | **T** | **T** | **T** | **C** | **A** | **G** | **T** | **C** | **T** | **T** | **T** | **T** | **A** | **G** | **A** | **G** | **G** | **A** | **G** | **A** | **T** | **A** | **C** | **C** | **A** | **T** | **T** | **G** | **A** | **A** | **C** | **A** | **T** | **T** | **A** | **C** | **G** | **A** | **T** | **C** | **C** | **T** | **G** | **A** | **C** | **A** | **T** | **G** | **G** | **G** | **C** | **G** | **A** | **T** | **A** | **A** | **A** | **G** | **A** | **C** | **A** | **A** | **A** | **C** | **A** | **G** | **C** | **A** | **A** | **T** | **T** | **T** | **G** | **T** | **T** | **G** | **A** | **C** | **T** | **T** | **C** | **T** | **T** | **C** | **T** | **C** | **T** | **T** | **C** | **A** | **T** | **T** | **A** | **A** | **A** | **T** | **C** | **G** | **T** | **A** | **T** | **G** | **C** | **C** | **G** | **C** | **T** | **G** | **A** | **C** | **A** | **A** | **G** | **A** | **A** | **T** | **G** | **A** | **A** | **T** | **T** | **C** | **T** | **G** | **T** | **C** | **A** | **C** | **T** | **C** | **T** | **T** | **C** | **T** | **C** | **A** | **C** | **A** | **G** | **A** | **C** | **G** | **G** | **A** | **C** | **A** | **A** | **G** | **A** | **G** | **A** | **A** | **A** | **T** | **T** | **G** | **G** | **A** | **T** | **T** | **C** | **A** | **C** | **T** | **G** | **C** | **T** | **T** | **C** | **A** | **A** | **G** | **A** | **T** | **C** | **T** | **A** | **A** | **A** | **A** | **A** | **A** | **A** | **G** | **C** | **C** | **A** | **A** | **A** | **C** | **A** | **T** | **T** | **C** | **G** | **A** | **T** | **C** | **C** | **A** | **T** | **A** | **T** | **C** | **G** | **A** | **A** | **T** | **A** | **T** | **T** | **A** | **C** | **G** | **C** | **A** | **T** | **T** | **T** | **G** | **T** | **G** | **A** | **C** | **A** | **G** | **A** | **T** | **A** | **A** | **T** | **A** | **T** | **C** | **G** | **G** | **C** | **C** | **A** | **G** | **C** | **A** | **A** | **G** | **T** | **C** | **T** | **T** | **A** | **C** | **A** | **T** | **G** | **T** | **T** | **T** | **T** | **A** | **G** | **A** | **A** | **G** | **A** | **A** | **A** | **A** | **A** | **G** | **C** | **C** | **G** | **C** | **A** | **G** | **A** | **A** | **G** | **G** | **C** | **G** | **T** | **G** | **G** | **A** | **A** | **G** | **T** | **T** | **C** | **G** | **A** | **A** | **T** | **A** | **T** | **T** | **A** | **T** | **A** | **T** | **G** | **A** | **T** | **G** | **C** | **A** | **T** | **T** | **T** | **G** | **G** | **C** | **T** | **C** | **T** | **C** | **A** | **T** | **G** | **G** | **C** | **A** | **C** | **A** | **A** | **A** | **A** | **G** | **C** | **A** | **A** | **A** | **A** | **G** | **A** | **T** | **T** | **T** | **C** | **A** | **A** | **T** | **C** | **G** | **T** | **C** | **T** | **A** | **A** | **T** | **C** | **A** | **A** | **A** | **A** | **A** | **T** | **G** | **G** | **T** | **G** | **G** | **A** | **C** | **A** | **T** | **G** | **T** | **C** | **C** | **A** | **T** | **A** | **C** | **A** | **T** | **T** | **T** | **G** | **T** | **T** | **A** | **C** | **C** | **T** | **C** | **A** | **C** | **A** | **A** | **A** | **G** | **G** | **G** | **C** | **A** | **T** | **T** | **A** | **C** | **T** | **T** | **C** | **G** | **T** | **T** | **T** | **C** | **C** | **G** | **A** | **T** | **T** | **G** | **A** | **A** | **T** | **T** | **A** | **C** | **C** | **A** | **T** | **G** | **A** | **T** | **C** | **A** | **C** | **C** | **G** | **A** | **A** | **A** | **A** | **A** | **T** | **C** | **G** | **T** | **T** | **G** | **T** | **G** | **A** | **T** | **C** | **G** | **A** | **T** | **G** | **G** | **A** | **A** | **A** | **G** | **A** | **T** | **T** | **A** | **G** | **T** | **T** | **A** | **T** | **A** | **C** | **C** | **G** | **G** | **T** | **G** | **G** | **T** | **T** | **T** | **C** | **A** | **A** | **T** | **A** | **T** | **T** | **G** | **C** | **C** | **A** | **A** | **T** | **C** | **A** | **A** | **T** | **A** | **T** | **G** | **T** | **A** | **A** | **A** | **T** | **A** | **C** | **A** | **A** | **C** | **A** | **A** | **A** | **A** | **A** | **A** | **A** | **T** | **T** | **C** | **G** | **G** | **C** | **T** | **A** | **T** | **T** | **G** | **G** | **C** | **G** | **C** | **G** | **A** | **T** | **A** | **C** | **G** | **C** | **A** | **T** | **A** | **T** | **A** | **C** | **G** | **G** | **A** | **T** | **T** | **T** | **T** | **C** | **G** | **G** | **T** | **G** | **C** | **C** | **G** | **C** | **T** | **T** | **C** | **T** | **T** | **C** | **T** | **T** | **T** | **G** | **C** | **T** | **C** | **C** | **A** | **G** | **C** | **T** | **T** | **C** | **G** | **C** | **T** | **T** | **C** | **T** | **T** | **A** | **A** | **C** | **A** | **G** | **A** | **C** | **T** | **G** | **G** | **A** | **A** | **C** | **G** | **T** | **C** | **T** | **C** | **G** | **G** | **T** | **A** | **C** | **C** | **T** | **G** | **A** | **A** | **G** | **A** | **A** | **A** | **A** | **A** | **A** | **A** | **G** | **G** | **T** | **C** | **G** | **G** | **C** | **T** | **A** | **T** | **C** | **A** | **T** | **T** | **T** | **G** | **A** | **A** | **T** | **T** | **A** | **T** | **T** | **T** | **C** | **T** | **T** | **T** | **A** | **A** | **A** | **A** | **A** | **A** | **G** | **C** | **A** | **G** | **A** | **T** | **C** | **G** | **A** | **G** | **A** | **T** | **G** | **A** | **A** | **T** | **C** | **T** | **A** | **A** | **G** | **C** | **T** | **T** | **G** | **C** | **T** | **G** | **A** | **T** | **A** | **C** | **A** | **T** | **C** | **C** | **A** | **T** | **C** | **C** | **A** | **G** | **C** | **T** | **T** | **G** | **T** | **T** | **T** | **C** | **A** | **A** | **G** | **C** | **G** | **G** | **A** | **C** | **C** | **G** | **A** | **A** | **T** | **A** | **A** | **C** | **G** | **A** | **A** | **A** | **G** | **G** | **G** | **A** | **A** | **C** | **A** | **A** | **A** | **T** | **C** | **A** | **A** | **G** | **C** | **T** | **T** | **T** | **C** | **A** | **T** | **T** | **T** | **A** | **T** | **C** | **A** | **A** | **A** | **T** | **T** | **G** | **A** | **T** | **T** | **A** | **C** | **T** | **T** | **C** | **T** | **G** | **C** | **T** | **A** | **A** | **A** | **A** | **A** | **A** | **C** | **G** | **T** | **G** | **T** | **T** | **T** | **G** | **G** | **A** | **T** | **A** | **C** | **A** | **G** | **A** | **C** | **A** | **C** | **C** | **T** | **T** | **A** | **C** | **C** | **T** | **T** | **G** | **T** | **T** | **C** | **C** | **T** | **G** | **A** | **T** | **G** | **A** | **A** | **A** | **G** | **T** | **G** | **T** | **C** | **A** | **T** | **T** | **G** | **C** | **T** | **G** | **C** | **T** | **T** | **T** | **A** | **A** | **A** | **A** | **A** | **T** | **C** | **G** | **C** | **A** | **A** | **C** | **T** | **G** | **C** | **C** | **T** | **C** | **T** | **G** | **G** | **T** | **G** | **T** | **A** | **G** | **A** | **T** | **G** | **T** | **G** | **A** | **A** | **A** | **A** | **T** | **C** | **A** | **T** | **G** | **A** | **T** | **T** | **C** | **C** | **C** | **A** | **A** | **C** | **A** | **A** | **A** | **C** | **C** | **G** | **G** | **A** | **T** | **C** | **A** | **T** | **C** | **C** | **T** | **T** | **T** | **T** | **A** | **T** | **T** | **T** | **A** | **T** | **C** | **G** | **A** | **G** | **C** | **A** | **A** | **C** | **A** | **C** | **A** | **A** | **T** | **A** | **T** | **T** | **A** | **T** | **G** | **C** | **T** | **C** | **G** | **G** | **C** | **A** | **G** | **C** | **T** | **G** | **A** | **T** | **C** | **A** | **A** | **G** | **G** | **A** | **A** | **A** | **A** | **T** | **G** | **T** | **A** | **C** | **A** | **A** | **A** | **T** | **C** | **C** | **T** | **T** | **G** | **T** | **C** | **T** | **A** | **T** | **G** | **A** | **G** | **A** | **A** | **C** | **G** | **G** | **C** | **T** | **T** | **C** | **C** | **T** | **C** | **C** | **A** | **T** | **G** | **C** | **A** | **A** | **A** | **A** | **A** | **C** | **A** | **T** | **T** | **G** | **A** | **T** | **A** | **A** | **T** | **G** | **G** | **A** | **T** | **G** | **A** | **T** | **G** | **A** | **A** | **A** | **T** | **C** | **T** | **G** | **C** | **A** | **T** | **G** | **G** | **T** | **A** | **G** | **G** | **T** | **T** | **C** | **A** | **G** | **C** | **A** | **A** | **A** | **T** | **C** | **A** | **A** | **G** | **A** | **T** | **A** | **T** | **T** | **C** | **G** | **T** | **A** | **G** | **C** | **T** | **A** | **C** | **C** | **G** | **A** | **T** | **T** | **G** | **A** | **A** | **T** | **T** | **T** | **T** | **G** | **A** | **A** | **A** | **C** | **A** | **A** | **G** | **T** | **G** | **C** | **T** | **G** | **T** | **C** | **A** | **T** | **T** | **T** | **A** | **C** | **G** | **A** | **T** | **C** | **C** | **T** | **G** | **A** | **G** | **T** | **T** | **T** | **T** | **T** | **A** | **G** | **A** | **A** | **G** | **A** | **A** | **C** | **T** | **T** | **G** | **C** | **T** | **A** | **C** | **T** | **C** | **A** | **G** | **T** | **T** | **C** | **A** | **A** | **A** | **G** | **A** | **A** | **G** | **A** | **T** | **G** | **A** | **G** | **A** | **C** | **A** | **C** | **A** | **T** | **T** | **G** | **T** | **T** | **C** | **A** | **T** | **C** | **C** | **A** | **T** | **G** | **A** | **C** | **A** | **A** | **C** | **T** | **G** | **A** | **A** | **A** | **C** | **A** | **G** | **T** | **C** | **A** | **A** | **G** | **G** | **A** | **A** | **A** | **T** | **G** | **T** | **C** | **T** | **A** | **A** | **C** | **T** | **G** | **G** | **C** | **T** | **A** | **T** | **T** | **A** | **T** | **T** | **C** | **A** | **A** | **G** | **C** | **A** | **A** | **C** | **A** | **A** | **A** | **T** | **T** | **T** | **C** | **T** | **C** | **G** | **A** | **T** | **T** | **A** | **T** | **T** | **T** | **T** | **C** | **T** | **C** | **C** | **A** | **A** | **T** | **C** | **C** | **T** | **A** | **T** | **A** | **A** |
| **N54567** | **-** | **-** | **-** | **-** | **-** | **-** | **-** | **-** | **-** | **-** | **-** | **-** | **-** | **-** | **-** | **-** | **-** | **-** | **-** | **-** | **-** | **-** | **-** | **-** | **-** | **-** | **-** | **-** | **-** | **-** | **-** | **-** | **-** | **-** | **-** | **-** | **-** | **-** | **-** | **-** | **-** | **-** | **-** | **-** | **-** | **-** | **-** | **-** | **-** | **-** | **-** | **-** | **-** | **-** | **-** | **-** | **-** | **-** | **-** | **-** | **-** | **-** | **-** | **-** | **-** | **-** | **-** | **-** | **-** | **A** | **A** | **A** | **C** | **C** | **C** | **C** | **G** | **A** | **G** | **A** | **T** | **G** | **T** | **A** | **G** | **C** | **A** | **G** | **C** | **C** | **A** | **T** | **T** | **T** | **G** | **G** | **G** | **C** | **A** | **T** | **G** | **G** | **C** | **T** | **C** | **T** | **T** | **A** | **G** | **T** | **A** | **T** | **T** | **G** | **T** | **T** | **T** | **G** | **C** | **C** | **C** | **T** | **T** | **C** | **C** | **T** | **G** | **G** | **G** | **G** | **T** | **G** | **G** | **G** | **C** | **T** | **T** | **T** | **G** | **T** | **A** | **T** | **T** | **A** | **T** | **A** | **T** | **T** | **T** | **A** | **T** | **T** | **T** | **T** | **T** | **C** | **G** | **G** | **A** | **C** | **G** | **T** | **G** | **G** | **A** | **T** | **T** | **A** | **A** | **C** | **G** | **G** | **A** | **T** | **A** | **A** | **G** | **A** | **A** | **A** | **A** | **A** | **A** | **T** | **T** | **C** | **T** | **A** | **T** | **C** | **T** | **C** | **C** | **G** | **A** | **C** | **A** | **A** | **A** | **G** | **T** | **G** | **A** | **C** | **T** | **T** | **G** | **A** | **A** | **A** | **G** | **A** | **A** | **T** | **T** | **A** | **G** | **A** | **A** | **A** | **A** | **C** | **T** | **T** | **T** | **C** | **A** | **G** | **T** | **C** | **T** | **T** | **T** | **T** | **A** | **G** | **A** | **G** | **G** | **A** | **G** | **A** | **T** | **A** | **A** | **C** | **A** | **T** | **T** | **G** | **A** | **A** | **C** | **A** | **T** | **T** | **A** | **C** | **G** | **A** | **T** | **C** | **C** | **T** | **G** | **A** | **C** | **A** | **T** | **G** | **G** | **G** | **C** | **G** | **A** | **T** | **A** | **A** | **A** | **G** | **A** | **C** | **A** | **A** | **A** | **C** | **A** | **G** | **C** | **A** | **A** | **T** | **T** | **T** | **G** | **T** | **T** | **G** | **A** | **C** | **T** | **T** | **C** | **T** | **T** | **C** | **T** | **C** | **T** | **T** | **C** | **A** | **T** | **T** | **A** | **A** | **A** | **T** | **C** | **G** | **T** | **A** | **T** | **G** | **C** | **C** | **G** | **C** | **T** | **G** | **A** | **C** | **A** | **A** | **G** | **A** | **A** | **T** | **G** | **A** | **A** | **T** | **T** | **C** | **T** | **G** | **T** | **C** | **A** | **C** | **T** | **C** | **T** | **T** | **C** | **T** | **C** | **A** | **C** | **A** | **G** | **A** | **C** | **G** | **G** | **A** | **C** | **A** | **A** | **G** | **A** | **G** | **A** | **A** | **A** | **T** | **T** | **G** | **G** | **A** | **T** | **T** | **C** | **A** | **C** | **T** | **G** | **C** | **T** | **T** | **C** | **A** | **A** | **G** | **A** | **T** | **C** | **T** | **A** | **A** | **A** | **A** | **A** | **A** | **A** | **G** | **C** | **C** | **A** | **A** | **A** | **C** | **A** | **T** | **T** | **C** | **G** | **A** | **T** | **C** | **C** | **A** | **T** | **A** | **T** | **C** | **G** | **A** | **A** | **T** | **A** | **T** | **T** | **A** | **C** | **G** | **C** | **A** | **T** | **T** | **T** | **G** | **T** | **G** | **A** | **C** | **A** | **G** | **A** | **T** | **A** | **A** | **T** | **A** | **T** | **C** | **G** | **G** | **C** | **C** | **A** | **G** | **C** | **A** | **A** | **G** | **T** | **C** | **T** | **T** | **A** | **C** | **A** | **T** | **G** | **T** | **T** | **T** | **T** | **A** | **G** | **A** | **A** | **G** | **A** | **A** | **A** | **A** | **A** | **G** | **C** | **C** | **G** | **C** | **A** | **G** | **A** | **A** | **G** | **G** | **C** | **G** | **T** | **G** | **G** | **A** | **A** | **G** | **T** | **T** | **C** | **G** | **A** | **A** | **T** | **A** | **T** | **T** | **A** | **T** | **A** | **T** | **G** | **A** | **T** | **G** | **C** | **A** | **T** | **T** | **T** | **G** | **G** | **C** | **T** | **C** | **T** | **C** | **A** | **T** | **G** | **G** | **C** | **A** | **C** | **A** | **A** | **A** | **A** | **G** | **C** | **A** | **A** | **A** | **A** | **G** | **A** | **T** | **T** | **T** | **C** | **A** | **A** | **T** | **C** | **G** | **T** | **C** | **T** | **A** | **A** | **T** | **C** | **A** | **A** | **A** | **A** | **A** | **T** | **G** | **G** | **T** | **G** | **G** | **A** | **C** | **A** | **T** | **G** | **T** | **C** | **C** | **A** | **T** | **A** | **C** | **A** | **T** | **T** | **T** | **G** | **T** | **T** | **A** | **C** | **C** | **T** | **C** | **A** | **C** | **A** | **A** | **A** | **A** | **G** | **G** | **C** | **A** | **T** | **T** | **A** | **C** | **T** | **T** | **C** | **G** | **T** | **T** | **T** | **C** | **C** | **G** | **A** | **T** | **T** | **G** | **A** | **A** | **T** | **T** | **A** | **C** | **C** | **A** | **T** | **G** | **A** | **T** | **C** | **A** | **C** | **C** | **G** | **A** | **A** | **A** | **A** | **A** | **T** | **C** | **G** | **T** | **T** | **G** | **T** | **G** | **A** | **T** | **C** | **G** | **A** | **T** | **G** | **G** | **A** | **A** | **A** | **G** | **A** | **T** | **T** | **A** | **G** | **T** | **T** | **A** | **T** | **A** | **C** | **C** | **G** | **G** | **T** | **G** | **G** | **T** | **T** | **T** | **C** | **A** | **A** | **T** | **A** | **T** | **T** | **G** | **C** | **C** | **A** | **A** | **T** | **C** | **A** | **A** | **T** | **A** | **T** | **G** | **T** | **A** | **A** | **A** | **T** | **A** | **C** | **A** | **A** | **C** | **A** | **A** | **A** | **A** | **A** | **A** | **A** | **T** | **T** | **C** | **G** | **G** | **C** | **T** | **A** | **T** | **T** | **G** | **G** | **C** | **G** | **C** | **G** | **A** | **T** | **A** | **C** | **G** | **C** | **A** | **T** | **A** | **T** | **A** | **C** | **G** | **G** | **A** | **T** | **T** | **T** | **T** | **C** | **G** | **G** | **T** | **G** | **C** | **C** | **G** | **C** | **T** | **T** | **C** | **T** | **T** | **C** | **T** | **T** | **T** | **G** | **C** | **T** | **C** | **C** | **A** | **G** | **C** | **T** | **T** | **C** | **G** | **C** | **T** | **T** | **C** | **T** | **T** | **A** | **A** | **C** | **A** | **G** | **A** | **C** | **T** | **G** | **G** | **A** | **A** | **C** | **G** | **T** | **C** | **T** | **C** | **G** | **G** | **T** | **A** | **C** | **C** | **T** | **G** | **A** | **A** | **G** | **A** | **A** | **A** | **A** | **A** | **A** | **A** | **G** | **G** | **T** | **C** | **G** | **G** | **C** | **T** | **A** | **T** | **C** | **A** | **T** | **T** | **T** | **G** | **A** | **A** | **T** | **T** | **A** | **T** | **T** | **T** | **C** | **T** | **T** | **T** | **A** | **A** | **A** | **A** | **A** | **A** | **G** | **C** | **A** | **G** | **A** | **T** | **C** | **G** | **A** | **G** | **A** | **T** | **G** | **A** | **A** | **T** | **C** | **T** | **A** | **A** | **G** | **C** | **T** | **T** | **G** | **C** | **T** | **G** | **A** | **T** | **A** | **C** | **A** | **T** | **C** | **C** | **A** | **T** | **C** | **C** | **A** | **G** | **C** | **T** | **T** | **G** | **T** | **T** | **T** | **C** | **A** | **A** | **G** | **C** | **G** | **G** | **A** | **C** | **C** | **G** | **A** | **A** | **T** | **A** | **A** | **C** | **G** | **A** | **A** | **A** | **G** | **G** | **G** | **A** | **A** | **C** | **A** | **A** | **A** | **T** | **C** | **A** | **A** | **G** | **C** | **T** | **T** | **T** | **C** | **A** | **T** | **T** | **T** | **A** | **T** | **C** | **A** | **A** | **A** | **T** | **T** | **G** | **A** | **T** | **T** | **A** | **C** | **T** | **T** | **C** | **T** | **G** | **C** | **T** | **A** | **A** | **A** | **A** | **A** | **A** | **C** | **G** | **T** | **G** | **T** | **T** | **T** | **G** | **G** | **A** | **T** | **A** | **C** | **A** | **G** | **A** | **C** | **A** | **C** | **C** | **T** | **T** | **A** | **C** | **C** | **T** | **T** | **G** | **T** | **T** | **C** | **C** | **T** | **G** | **A** | **T** | **G** | **A** | **A** | **A** | **G** | **T** | **G** | **T** | **C** | **A** | **T** | **T** | **G** | **C** | **T** | **G** | **C** | **T** | **T** | **T** | **A** | **A** | **A** | **A** | **A** | **T** | **C** | **G** | **C** | **A** | **A** | **C** | **T** | **G** | **C** | **C** | **T** | **C** | **T** | **G** | **G** | **T** | **G** | **T** | **A** | **G** | **A** | **T** | **G** | **T** | **G** | **A** | **A** | **A** | **A** | **T** | **C** | **A** | **T** | **G** | **A** | **T** | **T** | **C** | **C** | **C** | **A** | **A** | **C** | **A** | **A** | **A** | **C** | **C** | **G** | **G** | **A** | **T** | **C** | **A** | **T** | **C** | **C** | **T** | **T** | **T** | **T** | **A** | **T** | **T** | **T** | **A** | **T** | **C** | **G** | **A** | **G** | **C** | **A** | **A** | **C** | **A** | **C** | **A** | **A** | **T** | **A** | **T** | **T** | **A** | **T** | **G** | **C** | **T** | **C** | **G** | **G** | **C** | **A** | **G** | **C** | **T** | **G** | **A** | **T** | **C** | **A** | **A** | **G** | **G** | **A** | **A** | **A** | **A** | **T** | **G** | **T** | **A** | **C** | **A** | **A** | **A** | **T** | **C** | **C** | **T** | **T** | **G** | **T** | **C** | **T** | **A** | **T** | **G** | **A** | **G** | **A** | **A** | **C** | **G** | **G** | **C** | **T** | **T** | **C** | **C** | **T** | **C** | **C** | **A** | **T** | **G** | **C** | **A** | **A** | **A** | **A** | **A** | **C** | **A** | **T** | **T** | **G** | **A** | **T** | **A** | **A** | **T** | **G** | **G** | **A** | **T** | **G** | **A** | **T** | **G** | **A** | **A** | **A** | **T** | **C** | **T** | **G** | **C** | **A** | **T** | **G** | **G** | **T** | **A** | **G** | **G** | **T** | **T** | **C** | **A** | **G** | **C** | **A** | **A** | **A** | **T** | **C** | **A** | **A** | **G** | **A** | **T** | **A** | **T** | **T** | **C** | **G** | **T** | **A** | **G** | **C** | **T** | **A** | **C** | **C** | **G** | **A** | **T** | **T** | **G** | **A** | **A** | **T** | **T** | **T** | **T** | **G** | **A** | **A** | **A** | **C** | **A** | **A** | **G** | **T** | **G** | **C** | **T** | **G** | **T** | **C** | **A** | **T** | **T** | **T** | **A** | **C** | **G** | **A** | **T** | **C** | **C** | **T** | **G** | **A** | **G** | **T** | **T** | **T** | **T** | **T** | **A** | **G** | **A** | **A** | **G** | **A** | **A** | **C** | **T** | **T** | **G** | **C** | **T** | **A** | **C** | **T** | **C** | **A** | **G** | **T** | **T** | **C** | **A** | **A** | **A** | **G** | **A** | **A** | **G** | **A** | **T** | **G** | **A** | **G** | **A** | **C** | **A** | **C** | **A** | **T** | **T** | **G** | **T** | **T** | **C** | **A** | **T** | **C** | **C** | **A** | **T** | **G** | **A** | **C** | **A** | **A** | **C** | **T** | **G** | **A** | **A** | **A** | **C** | **A** | **G** | **T** | **C** | **A** | **A** | **G** | **G** | **A** | **A** | **A** | **T** | **G** | **T** | **C** | **T** | **A** | **A** | **C** | **T** | **G** | **G** | **C** | **T** | **A** | **T** | **T** | **A** | **T** | **T** | **C** | **A** | **A** | **G** | **C** | **A** | **A** | **C** | **A** | **A** | **A** | **T** | **T** | **T** | **C** | **T** | **C** | **G** | **A** | **T** | **T** | **A** | **T** | **T** | **T** | **T** | **C** | **T** | **C** | **C** | **A** | **A** | **T** | **C** | **C** | **T** | **A** | **-** | **-** | **-** |
| **N52777** | **-** | **-** | **-** | **-** | **-** | **-** | **-** | **-** | **-** | **-** | **-** | **-** | **-** | **-** | **-** | **-** | **-** | **-** | **-** | **-** | **-** | **-** | **-** | **-** | **-** | **-** | **-** | **-** | **-** | **-** | **-** | **-** | **-** | **-** | **-** | **-** | **-** | **-** | **-** | **-** | **-** | **-** | **-** | **-** | **-** | **-** | **-** | **-** | **-** | **-** | **-** | **-** | **-** | **-** | **-** | **-** | **-** | **-** | **-** | **-** | **-** | **-** | **-** | **-** | **-** | **-** | **-** | **-** | **-** | **A** | **A** | **A** | **C** | **C** | **C** | **C** | **G** | **A** | **G** | **A** | **T** | **G** | **T** | **A** | **G** | **C** | **A** | **G** | **C** | **C** | **A** | **T** | **T** | **T** | **G** | **G** | **G** | **C** | **A** | **T** | **G** | **G** | **C** | **T** | **C** | **T** | **T** | **A** | **G** | **T** | **A** | **T** | **T** | **G** | **T** | **T** | **T** | **G** | **C** | **C** | **C** | **T** | **T** | **C** | **C** | **T** | **G** | **G** | **G** | **A** | **T** | **G** | **G** | **G** | **T** | **T** | **T** | **T** | **G** | **T** | **A** | **T** | **T** | **A** | **T** | **A** | **T** | **T** | **T** | **A** | **T** | **T** | **T** | **T** | **T** | **C** | **G** | **G** | **A** | **C** | **G** | **T** | **G** | **G** | **A** | **T** | **T** | **A** | **A** | **C** | **G** | **G** | **A** | **T** | **A** | **A** | **G** | **A** | **A** | **A** | **A** | **A** | **A** | **T** | **T** | **C** | **T** | **A** | **T** | **C** | **T** | **C** | **C** | **G** | **A** | **C** | **A** | **A** | **A** | **G** | **T** | **G** | **A** | **C** | **T** | **T** | **G** | **A** | **A** | **A** | **G** | **A** | **A** | **T** | **T** | **A** | **G** | **A** | **A** | **A** | **A** | **C** | **T** | **T** | **T** | **C** | **A** | **G** | **T** | **C** | **T** | **T** | **T** | **T** | **A** | **G** | **A** | **G** | **G** | **A** | **G** | **A** | **T** | **A** | **C** | **C** | **A** | **T** | **T** | **G** | **A** | **A** | **C** | **A** | **T** | **T** | **A** | **C** | **G** | **A** | **T** | **C** | **C** | **T** | **G** | **A** | **C** | **A** | **T** | **G** | **G** | **G** | **C** | **G** | **A** | **T** | **A** | **A** | **A** | **G** | **A** | **C** | **A** | **A** | **A** | **C** | **A** | **G** | **C** | **A** | **A** | **T** | **T** | **T** | **G** | **T** | **C** | **G** | **A** | **C** | **T** | **T** | **C** | **T** | **T** | **C** | **T** | **C** | **T** | **T** | **C** | **A** | **T** | **T** | **A** | **A** | **A** | **T** | **C** | **G** | **T** | **A** | **T** | **G** | **C** | **C** | **G** | **C** | **T** | **G** | **A** | **C** | **A** | **A** | **G** | **A** | **A** | **T** | **G** | **A** | **A** | **T** | **T** | **C** | **T** | **G** | **T** | **C** | **A** | **C** | **T** | **C** | **T** | **T** | **C** | **T** | **C** | **A** | **C** | **A** | **G** | **A** | **C** | **G** | **G** | **A** | **C** | **A** | **A** | **G** | **A** | **G** | **A** | **A** | **A** | **T** | **T** | **G** | **G** | **A** | **T** | **T** | **C** | **A** | **C** | **T** | **G** | **C** | **T** | **T** | **C** | **A** | **A** | **G** | **A** | **T** | **C** | **T** | **A** | **A** | **A** | **A** | **A** | **A** | **A** | **G** | **C** | **C** | **A** | **A** | **A** | **C** | **A** | **T** | **T** | **C** | **G** | **A** | **T** | **C** | **C** | **A** | **T** | **A** | **T** | **C** | **G** | **A** | **A** | **T** | **A** | **T** | **T** | **A** | **C** | **G** | **C** | **A** | **T** | **T** | **T** | **G** | **T** | **G** | **A** | **C** | **A** | **G** | **A** | **T** | **A** | **A** | **T** | **A** | **T** | **C** | **G** | **G** | **C** | **C** | **A** | **G** | **C** | **A** | **A** | **G** | **T** | **C** | **T** | **T** | **A** | **C** | **A** | **T** | **G** | **T** | **T** | **T** | **T** | **A** | **G** | **A** | **A** | **G** | **A** | **A** | **A** | **A** | **A** | **G** | **C** | **C** | **G** | **C** | **A** | **G** | **A** | **A** | **G** | **G** | **C** | **G** | **T** | **G** | **G** | **A** | **A** | **G** | **T** | **T** | **C** | **G** | **A** | **A** | **T** | **A** | **T** | **T** | **A** | **T** | **A** | **T** | **G** | **A** | **T** | **G** | **C** | **A** | **T** | **T** | **T** | **G** | **G** | **C** | **T** | **C** | **T** | **C** | **A** | **T** | **G** | **G** | **C** | **A** | **C** | **A** | **A** | **A** | **A** | **G** | **C** | **A** | **A** | **A** | **A** | **G** | **A** | **T** | **T** | **T** | **C** | **A** | **A** | **T** | **C** | **G** | **T** | **C** | **T** | **A** | **A** | **T** | **C** | **A** | **A** | **A** | **A** | **A** | **T** | **G** | **G** | **T** | **G** | **G** | **A** | **C** | **A** | **T** | **G** | **T** | **C** | **C** | **A** | **T** | **A** | **C** | **A** | **T** | **T** | **T** | **G** | **T** | **T** | **A** | **C** | **C** | **T** | **C** | **A** | **C** | **A** | **A** | **A** | **A** | **G** | **G** | **C** | **A** | **T** | **T** | **A** | **C** | **T** | **T** | **C** | **G** | **T** | **T** | **T** | **C** | **C** | **G** | **A** | **T** | **T** | **G** | **A** | **A** | **T** | **T** | **A** | **C** | **C** | **A** | **T** | **G** | **A** | **T** | **C** | **A** | **C** | **C** | **G** | **A** | **A** | **A** | **A** | **A** | **T** | **C** | **G** | **T** | **T** | **G** | **T** | **G** | **A** | **T** | **C** | **G** | **A** | **T** | **G** | **G** | **A** | **A** | **A** | **G** | **A** | **T** | **T** | **A** | **G** | **T** | **T** | **A** | **T** | **A** | **C** | **C** | **G** | **G** | **T** | **G** | **G** | **T** | **T** | **T** | **C** | **A** | **A** | **T** | **A** | **T** | **T** | **G** | **C** | **C** | **A** | **A** | **T** | **C** | **A** | **A** | **T** | **A** | **T** | **G** | **T** | **A** | **A** | **A** | **T** | **A** | **C** | **A** | **A** | **C** | **A** | **A** | **A** | **A** | **A** | **A** | **A** | **T** | **T** | **C** | **G** | **G** | **C** | **T** | **A** | **T** | **T** | **G** | **G** | **C** | **G** | **C** | **G** | **A** | **T** | **A** | **C** | **G** | **C** | **A** | **T** | **A** | **T** | **A** | **C** | **G** | **G** | **A** | **T** | **T** | **T** | **T** | **C** | **G** | **G** | **T** | **G** | **C** | **C** | **G** | **C** | **T** | **T** | **C** | **T** | **T** | **C** | **T** | **T** | **T** | **G** | **C** | **T** | **C** | **C** | **A** | **G** | **C** | **T** | **T** | **C** | **G** | **C** | **T** | **T** | **C** | **T** | **T** | **A** | **A** | **C** | **A** | **G** | **A** | **C** | **T** | **G** | **G** | **A** | **A** | **C** | **G** | **T** | **C** | **T** | **C** | **G** | **G** | **T** | **A** | **C** | **C** | **T** | **G** | **A** | **A** | **G** | **A** | **A** | **A** | **A** | **A** | **A** | **A** | **G** | **G** | **T** | **C** | **G** | **G** | **C** | **T** | **A** | **T** | **C** | **A** | **T** | **T** | **T** | **G** | **A** | **A** | **T** | **T** | **A** | **T** | **T** | **T** | **C** | **T** | **T** | **T** | **A** | **A** | **A** | **A** | **A** | **A** | **G** | **C** | **A** | **G** | **A** | **T** | **C** | **G** | **A** | **G** | **A** | **T** | **G** | **A** | **A** | **T** | **C** | **T** | **A** | **A** | **G** | **C** | **T** | **T** | **G** | **C** | **T** | **G** | **A** | **T** | **A** | **C** | **A** | **T** | **C** | **C** | **A** | **T** | **C** | **C** | **A** | **G** | **C** | **T** | **T** | **G** | **T** | **T** | **T** | **C** | **A** | **A** | **G** | **C** | **G** | **G** | **A** | **C** | **C** | **G** | **A** | **A** | **T** | **A** | **A** | **C** | **G** | **A** | **A** | **A** | **G** | **G** | **G** | **A** | **A** | **C** | **A** | **A** | **A** | **T** | **C** | **A** | **A** | **G** | **C** | **T** | **T** | **T** | **C** | **A** | **T** | **T** | **T** | **A** | **T** | **C** | **A** | **A** | **A** | **T** | **T** | **G** | **A** | **T** | **T** | **A** | **C** | **T** | **T** | **C** | **T** | **G** | **C** | **T** | **A** | **A** | **A** | **A** | **A** | **A** | **C** | **G** | **T** | **G** | **T** | **T** | **T** | **G** | **G** | **A** | **T** | **A** | **C** | **A** | **G** | **A** | **C** | **A** | **C** | **C** | **T** | **T** | **A** | **C** | **C** | **T** | **T** | **G** | **T** | **T** | **C** | **C** | **T** | **G** | **A** | **T** | **G** | **A** | **A** | **A** | **G** | **T** | **G** | **T** | **C** | **A** | **T** | **T** | **G** | **C** | **T** | **G** | **C** | **T** | **T** | **T** | **A** | **A** | **A** | **A** | **A** | **T** | **C** | **G** | **C** | **A** | **A** | **C** | **T** | **G** | **C** | **C** | **T** | **C** | **T** | **G** | **G** | **T** | **G** | **T** | **A** | **G** | **A** | **T** | **G** | **T** | **G** | **A** | **A** | **A** | **A** | **T** | **C** | **A** | **T** | **G** | **A** | **T** | **T** | **C** | **C** | **C** | **A** | **A** | **C** | **A** | **A** | **A** | **C** | **C** | **G** | **G** | **A** | **T** | **C** | **A** | **T** | **C** | **C** | **T** | **T** | **T** | **T** | **A** | **T** | **T** | **T** | **A** | **T** | **C** | **G** | **A** | **G** | **C** | **A** | **A** | **C** | **A** | **C** | **A** | **A** | **T** | **A** | **T** | **T** | **A** | **T** | **G** | **C** | **T** | **C** | **G** | **G** | **C** | **A** | **G** | **C** | **T** | **G** | **A** | **T** | **C** | **A** | **A** | **G** | **G** | **A** | **A** | **A** | **A** | **T** | **G** | **T** | **A** | **C** | **A** | **A** | **A** | **T** | **C** | **C** | **T** | **T** | **G** | **T** | **C** | **T** | **A** | **T** | **G** | **A** | **G** | **A** | **A** | **C** | **G** | **G** | **C** | **T** | **T** | **C** | **C** | **T** | **C** | **C** | **A** | **T** | **G** | **C** | **A** | **A** | **A** | **A** | **A** | **C** | **A** | **T** | **T** | **G** | **A** | **T** | **A** | **A** | **T** | **G** | **G** | **A** | **T** | **G** | **A** | **T** | **G** | **A** | **A** | **A** | **T** | **C** | **T** | **G** | **C** | **A** | **T** | **G** | **G** | **T** | **A** | **G** | **G** | **T** | **T** | **C** | **A** | **G** | **C** | **A** | **A** | **A** | **T** | **C** | **A** | **A** | **G** | **A** | **T** | **A** | **T** | **T** | **C** | **G** | **T** | **A** | **G** | **C** | **T** | **A** | **C** | **C** | **G** | **A** | **T** | **T** | **G** | **A** | **A** | **T** | **T** | **T** | **T** | **G** | **A** | **A** | **A** | **C** | **A** | **A** | **G** | **T** | **G** | **C** | **T** | **G** | **T** | **C** | **A** | **T** | **T** | **T** | **A** | **C** | **G** | **A** | **T** | **C** | **C** | **T** | **G** | **A** | **G** | **T** | **T** | **T** | **T** | **T** | **A** | **G** | **A** | **A** | **G** | **A** | **A** | **C** | **T** | **T** | **G** | **C** | **T** | **A** | **C** | **T** | **C** | **A** | **G** | **T** | **T** | **C** | **A** | **A** | **A** | **G** | **A** | **A** | **G** | **A** | **T** | **G** | **A** | **G** | **A** | **C** | **A** | **C** | **A** | **T** | **T** | **G** | **T** | **T** | **C** | **A** | **T** | **C** | **C** | **A** | **T** | **G** | **A** | **C** | **A** | **A** | **C** | **T** | **G** | **A** | **A** | **A** | **C** | **A** | **G** | **T** | **C** | **A** | **A** | **A** | **G** | **A** | **A** | **A** | **T** | **G** | **T** | **C** | **T** | **A** | **A** | **C** | **T** | **G** | **G** | **C** | **T** | **A** | **T** | **T** | **A** | **T** | **T** | **C** | **A** | **A** | **G** | **C** | **A** | **A** | **C** | **A** | **A** | **A** | **T** | **T** | **T** | **C** | **T** | **C** | **G** | **A** | **T** | **T** | **A** | **T** | **T** | **T** | **T** | **C** | **T** | **C** | **C** | **A** | **A** | **T** | **C** | **C** | **T** | **A** | **-** | **-** | **-** |
| **N59531F** | **-** | **-** | **-** | **-** | **-** | **-** | **-** | **-** | **-** | **-** | **-** | **-** | **-** | **-** | **-** | **-** | **-** | **-** | **-** | **-** | **-** | **-** | **-** | **-** | **-** | **-** | **-** | **-** | **-** | **-** | **-** | **-** | **-** | **-** | **-** | **-** | **-** | **-** | **-** | **-** | **-** | **-** | **-** | **-** | **-** | **-** | **-** | **-** | **-** | **-** | **-** | **-** | **-** | **-** | **-** | **-** | **-** | **-** | **-** | **-** | **-** | **-** | **-** | **-** | **-** | **-** | **-** | **-** | **-** | **A** | **A** | **A** | **C** | **C** | **C** | **C** | **G** | **A** | **G** | **A** | **T** | **G** | **T** | **A** | **G** | **C** | **A** | **G** | **C** | **T** | **A** | **T** | **T** | **T** | **G** | **G** | **G** | **C** | **A** | **T** | **G** | **G** | **C** | **T** | **C** | **T** | **T** | **A** | **G** | **T** | **A** | **T** | **T** | **A** | **T** | **T** | **T** | **G** | **C** | **C** | **C** | **T** | **T** | **C** | **C** | **T** | **G** | **G** | **G** | **G** | **T** | **G** | **G** | **G** | **T** | **T** | **T** | **T** | **G** | **T** | **A** | **T** | **T** | **A** | **T** | **A** | **T** | **T** | **T** | **A** | **T** | **T** | **T** | **T** | **T** | **C** | **G** | **G** | **A** | **C** | **G** | **T** | **G** | **G** | **A** | **T** | **T** | **A** | **A** | **C** | **G** | **G** | **A** | **T** | **A** | **A** | **G** | **A** | **A** | **A** | **A** | **A** | **A** | **T** | **T** | **C** | **T** | **A** | **T** | **C** | **T** | **C** | **C** | **G** | **A** | **C** | **A** | **A** | **A** | **G** | **T** | **G** | **A** | **C** | **T** | **T** | **G** | **A** | **A** | **A** | **G** | **A** | **A** | **T** | **T** | **A** | **G** | **A** | **A** | **A** | **A** | **C** | **T** | **T** | **T** | **C** | **A** | **G** | **T** | **C** | **T** | **T** | **T** | **T** | **A** | **G** | **A** | **G** | **G** | **A** | **G** | **A** | **T** | **A** | **C** | **C** | **A** | **T** | **T** | **G** | **A** | **A** | **C** | **A** | **T** | **T** | **A** | **C** | **G** | **A** | **T** | **C** | **C** | **T** | **G** | **A** | **C** | **A** | **T** | **G** | **G** | **G** | **C** | **G** | **A** | **C** | **A** | **A** | **A** | **G** | **A** | **C** | **A** | **A** | **A** | **C** | **A** | **A** | **C** | **A** | **A** | **T** | **T** | **T** | **G** | **T** | **C** | **G** | **A** | **C** | **T** | **T** | **C** | **T** | **T** | **C** | **T** | **C** | **T** | **T** | **C** | **A** | **T** | **T** | **A** | **A** | **A** | **T** | **C** | **G** | **T** | **A** | **T** | **G** | **C** | **C** | **G** | **C** | **T** | **G** | **A** | **C** | **A** | **A** | **G** | **A** | **A** | **T** | **G** | **A** | **A** | **T** | **T** | **C** | **T** | **G** | **T** | **C** | **A** | **C** | **T** | **C** | **T** | **T** | **C** | **T** | **C** | **A** | **C** | **A** | **G** | **A** | **C** | **G** | **G** | **A** | **C** | **A** | **A** | **A** | **A** | **G** | **A** | **A** | **A** | **T** | **T** | **A** | **G** | **A** | **T** | **T** | **C** | **A** | **C** | **T** | **G** | **C** | **T** | **T** | **C** | **A** | **A** | **G** | **A** | **T** | **C** | **T** | **A** | **A** | **A** | **A** | **A** | **A** | **A** | **G** | **C** | **C** | **A** | **A** | **A** | **C** | **A** | **T** | **T** | **C** | **G** | **A** | **T** | **C** | **C** | **A** | **T** | **A** | **T** | **C** | **G** | **A** | **A** | **T** | **A** | **T** | **T** | **A** | **C** | **G** | **C** | **A** | **T** | **T** | **T** | **G** | **T** | **G** | **A** | **C** | **A** | **G** | **A** | **T** | **A** | **C** | **T** | **A** | **T** | **C** | **G** | **G** | **T** | **C** | **A** | **G** | **C** | **A** | **A** | **G** | **T** | **C** | **T** | **T** | **A** | **C** | **G** | **C** | **G** | **T** | **T** | **T** | **T** | **A** | **G** | **A** | **A** | **G** | **A** | **A** | **A** | **A** | **A** | **G** | **C** | **C** | **G** | **C** | **A** | **G** | **A** | **A** | **G** | **G** | **C** | **G** | **T** | **A** | **G** | **A** | **A** | **G** | **T** | **T** | **C** | **G** | **T** | **A** | **T** | **A** | **T** | **T** | **A** | **T** | **A** | **T** | **G** | **A** | **T** | **G** | **C** | **A** | **T** | **T** | **T** | **G** | **G** | **C** | **T** | **C** | **C** | **C** | **A** | **C** | **G** | **G** | **C** | **A** | **C** | **A** | **A** | **A** | **A** | **G** | **C** | **A** | **A** | **A** | **A** | **G** | **A** | **T** | **T** | **T** | **C** | **A** | **A** | **T** | **C** | **G** | **T** | **C** | **T** | **A** | **A** | **T** | **C** | **A** | **A** | **A** | **A** | **A** | **T** | **G** | **G** | **T** | **G** | **G** | **A** | **C** | **A** | **T** | **G** | **T** | **C** | **C** | **A** | **T** | **A** | **C** | **A** | **T** | **T** | **T** | **G** | **T** | **T** | **A** | **C** | **C** | **T** | **C** | **G** | **C** | **A** | **A** | **A** | **A** | **A** | **G** | **C** | **A** | **T** | **T** | **G** | **C** | **T** | **T** | **C** | **G** | **T** | **T** | **T** | **C** | **C** | **G** | **A** | **T** | **T** | **G** | **A** | **A** | **C** | **T** | **A** | **C** | **C** | **A** | **T** | **G** | **A** | **T** | **C** | **A** | **C** | **C** | **G** | **G** | **A** | **A** | **A** | **A** | **T** | **C** | **G** | **T** | **T** | **G** | **T** | **G** | **A** | **T** | **C** | **G** | **A** | **T** | **G** | **G** | **A** | **A** | **A** | **G** | **A** | **T** | **C** | **A** | **G** | **T** | **T** | **A** | **C** | **A** | **C** | **C** | **G** | **G** | **T** | **G** | **G** | **C** | **T** | **T** | **C** | **A** | **A** | **C** | **A** | **T** | **T** | **G** | **C** | **C** | **A** | **A** | **T** | **C** | **A** | **A** | **T** | **A** | **T** | **G** | **T** | **A** | **A** | **A** | **T** | **A** | **C** | **G** | **A** | **C** | **A** | **A** | **A** | **A** | **A** | **A** | **A** | **T** | **T** | **C** | **G** | **G** | **C** | **T** | **A** | **T** | **T** | **G** | **G** | **C** | **G** | **T** | **G** | **A** | **T** | **A** | **C** | **G** | **C** | **A** | **T** | **A** | **T** | **A** | **C** | **G** | **G** | **A** | **T** | **T** | **T** | **T** | **C** | **G** | **G** | **T** | **G** | **C** | **C** | **G** | **C** | **T** | **T** | **C** | **T** | **T** | **C** | **T** | **T** | **T** | **G** | **C** | **T** | **T** | **C** | **A** | **A** | **C** | **T** | **T** | **C** | **G** | **T** | **T** | **T** | **T** | **T** | **T** | **A** | **A** | **C** | **A** | **G** | **A** | **C** | **T** | **G** | **G** | **A** | **A** | **C** | **G** | **T** | **C** | **T** | **C** | **A** | **G** | **T** | **T** | **C** | **C** | **T** | **G** | **A** | **A** | **G** | **A** | **A** | **A** | **A** | **A** | **A** | **A** | **G** | **G** | **T** | **C** | **G** | **G** | **C** | **T** | **A** | **T** | **C** | **A** | **T** | **T** | **T** | **G** | **A** | **A** | **T** | **T** | **A** | **T** | **T** | **T** | **C** | **T** | **T** | **T** | **A** | **A** | **A** | **A** | **A** | **A** | **G** | **C** | **C** | **G** | **A** | **T** | **C** | **G** | **A** | **G** | **A** | **C** | **G** | **A** | **A** | **T** | **C** | **C** | **A** | **A** | **A** | **C** | **T** | **T** | **G** | **C** | **T** | **G** | **A** | **T** | **A** | **C** | **G** | **T** | **C** | **C** | **A** | **T** | **C** | **C** | **A** | **G** | **C** | **T** | **T** | **G** | **T** | **C** | **T** | **C** | **A** | **A** | **G** | **C** | **G** | **G** | **A** | **C** | **C** | **A** | **A** | **A** | **T** | **A** | **A** | **C** | **G** | **A** | **A** | **C** | **G** | **C** | **G** | **A** | **A** | **C** | **A** | **A** | **A** | **T** | **C** | **A** | **A** | **G** | **C** | **T** | **T** | **T** | **C** | **G** | **T** | **T** | **T** | **A** | **T** | **C** | **A** | **A** | **A** | **T** | **T** | **G** | **A** | **T** | **T** | **A** | **C** | **T** | **T** | **C** | **T** | **G** | **C** | **T** | **A** | **A** | **A** | **A** | **A** | **A** | **C** | **G** | **T** | **G** | **T** | **T** | **T** | **G** | **G** | **A** | **T** | **A** | **C** | **A** | **G** | **A** | **C** | **A** | **C** | **C** | **T** | **T** | **A** | **C** | **C** | **T** | **C** | **G** | **T** | **T** | **C** | **C** | **T** | **G** | **A** | **T** | **G** | **A** | **A** | **A** | **G** | **T** | **G** | **T** | **C** | **A** | **T** | **T** | **G** | **C** | **T** | **G** | **C** | **T** | **T** | **T** | **G** | **A** | **A** | **A** | **A** | **T** | **C** | **G** | **C** | **A** | **A** | **C** | **T** | **G** | **C** | **T** | **T** | **C** | **T** | **G** | **G** | **T** | **G** | **T** | **C** | **G** | **A** | **T** | **G** | **T** | **G** | **A** | **A** | **A** | **A** | **T** | **C** | **A** | **T** | **G** | **A** | **T** | **C** | **C** | **C** | **C** | **A** | **A** | **T** | **A** | **A** | **A** | **C** | **C** | **G** | **G** | **A** | **T** | **C** | **A** | **T** | **C** | **C** | **T** | **T** | **T** | **T** | **A** | **T** | **T** | **T** | **A** | **T** | **C** | **G** | **A** | **G** | **C** | **A** | **A** | **C** | **A** | **C** | **A** | **A** | **T** | **A** | **T** | **T** | **A** | **T** | **G** | **C** | **G** | **C** | **G** | **T** | **C** | **A** | **G** | **C** | **T** | **C** | **A** | **T** | **C** | **A** | **A** | **G** | **G** | **A** | **A** | **A** | **A** | **C** | **G** | **T** | **A** | **C** | **A** | **A** | **A** | **T** | **T** | **C** | **T** | **T** | **G** | **T** | **C** | **T** | **A** | **T** | **G** | **A** | **G** | **A** | **A** | **T** | **G** | **G** | **C** | **T** | **T** | **C** | **C** | **T** | **C** | **C** | **A** | **T** | **G** | **C** | **A** | **A** | **A** | **A** | **A** | **C** | **A** | **T** | **T** | **G** | **A** | **T** | **A** | **A** | **T** | **G** | **G** | **A** | **T** | **G** | **A** | **T** | **G** | **A** | **A** | **A** | **T** | **C** | **T** | **G** | **C** | **A** | **T** | **G** | **G** | **T** | **A** | **G** | **G** | **T** | **T** | **C** | **A** | **G** | **C** | **A** | **A** | **A** | **T** | **C** | **A** | **A** | **G** | **A** | **T** | **A** | **T** | **T** | **C** | **G** | **T** | **A** | **G** | **C** | **T** | **A** | **C** | **C** | **G** | **A** | **T** | **T** | **G** | **A** | **A** | **T** | **T** | **T** | **T** | **G** | **A** | **A** | **A** | **C** | **A** | **A** | **G** | **T** | **G** | **C** | **T** | **G** | **T** | **C** | **A** | **T** | **C** | **T** | **A** | **C** | **G** | **A** | **T** | **C** | **C** | **T** | **G** | **A** | **G** | **T** | **T** | **T** | **T** | **T** | **A** | **G** | **A** | **A** | **G** | **A** | **A** | **C** | **T** | **T** | **G** | **C** | **T** | **A** | **C** | **T** | **C** | **A** | **G** | **T** | **T** | **T** | **A** | **A** | **A** | **G** | **A** | **A** | **G** | **A** | **T** | **G** | **A** | **G** | **A** | **C** | **A** | **C** | **A** | **T** | **T** | **G** | **T** | **T** | **C** | **A** | **C** | **C** | **A** | **A** | **T** | **G** | **A** | **C** | **G** | **A** | **C** | **T** | **G** | **A** | **A** | **A** | **C** | **A** | **G** | **T** | **G** | **A** | **A** | **G** | **G** | **A** | **A** | **A** | **T** | **G** | **T** | **C** | **T** | **A** | **A** | **C** | **T** | **G** | **G** | **C** | **T** | **G** | **T** | **T** | **A** | **T** | **T** | **C** | **A** | **A** | **A** | **C** | **A** | **A** | **C** | **A** | **A** | **A** | **T** | **T** | **T** | **C** | **T** | **C** | **G** | **A** | **T** | **T** | **A** | **T** | **T** | **T** | **T** | **C** | **T** | **C** | **C** | **A** | **A** | **T** | **C** | **T** | **T** | **A** | **-** | **-** | **-** |
| **N52769** | **-** | **-** | **-** | **-** | **-** | **-** | **-** | **-** | **-** | **-** | **-** | **-** | **-** | **-** | **-** | **-** | **-** | **-** | **-** | **-** | **-** | **-** | **-** | **-** | **-** | **-** | **-** | **-** | **-** | **-** | **-** | **-** | **-** | **-** | **-** | **-** | **-** | **-** | **-** | **-** | **-** | **-** | **-** | **-** | **-** | **-** | **-** | **-** | **-** | **-** | **-** | **-** | **-** | **-** | **-** | **-** | **-** | **-** | **-** | **-** | **-** | **-** | **-** | **-** | **-** | **-** | **-** | **-** | **-** | **A** | **A** | **A** | **C** | **C** | **C** | **C** | **G** | **A** | **G** | **A** | **T** | **G** | **T** | **A** | **G** | **C** | **A** | **G** | **C** | **T** | **A** | **T** | **T** | **T** | **G** | **G** | **G** | **C** | **A** | **T** | **G** | **G** | **C** | **T** | **C** | **T** | **T** | **A** | **G** | **T** | **A** | **T** | **T** | **G** | **T** | **T** | **T** | **G** | **C** | **C** | **C** | **T** | **T** | **C** | **C** | **T** | **G** | **G** | **G** | **G** | **T** | **G** | **G** | **G** | **T** | **T** | **T** | **T** | **G** | **T** | **A** | **T** | **T** | **A** | **T** | **A** | **T** | **T** | **T** | **A** | **T** | **T** | **T** | **T** | **T** | **C** | **G** | **G** | **A** | **C** | **G** | **T** | **G** | **G** | **A** | **T** | **T** | **A** | **A** | **C** | **G** | **G** | **A** | **T** | **A** | **A** | **G** | **A** | **A** | **A** | **A** | **A** | **A** | **T** | **T** | **C** | **T** | **A** | **T** | **C** | **T** | **C** | **C** | **G** | **A** | **C** | **A** | **A** | **A** | **G** | **T** | **G** | **A** | **C** | **T** | **T** | **G** | **A** | **A** | **A** | **G** | **A** | **A** | **T** | **T** | **A** | **G** | **A** | **A** | **A** | **A** | **C** | **T** | **T** | **T** | **C** | **A** | **G** | **T** | **C** | **T** | **T** | **T** | **T** | **A** | **G** | **A** | **G** | **G** | **A** | **G** | **A** | **T** | **A** | **C** | **C** | **A** | **T** | **T** | **G** | **A** | **A** | **C** | **A** | **T** | **T** | **A** | **C** | **G** | **A** | **T** | **C** | **C** | **T** | **G** | **A** | **C** | **A** | **T** | **G** | **G** | **G** | **C** | **G** | **A** | **C** | **A** | **A** | **A** | **G** | **A** | **C** | **A** | **A** | **A** | **C** | **A** | **A** | **C** | **A** | **A** | **T** | **T** | **T** | **G** | **T** | **C** | **G** | **A** | **C** | **T** | **T** | **C** | **T** | **T** | **C** | **T** | **C** | **T** | **T** | **C** | **A** | **T** | **T** | **A** | **A** | **A** | **T** | **C** | **G** | **T** | **A** | **T** | **G** | **C** | **C** | **G** | **C** | **T** | **G** | **A** | **C** | **A** | **A** | **G** | **A** | **A** | **T** | **G** | **A** | **A** | **T** | **T** | **C** | **T** | **G** | **T** | **C** | **A** | **C** | **T** | **C** | **T** | **T** | **C** | **T** | **C** | **A** | **C** | **A** | **G** | **A** | **C** | **G** | **G** | **A** | **C** | **A** | **A** | **A** | **A** | **G** | **A** | **A** | **A** | **T** | **T** | **A** | **G** | **A** | **T** | **T** | **C** | **A** | **C** | **T** | **G** | **C** | **T** | **T** | **C** | **A** | **A** | **G** | **A** | **T** | **C** | **T** | **A** | **A** | **A** | **A** | **A** | **A** | **A** | **G** | **C** | **C** | **A** | **A** | **A** | **C** | **A** | **T** | **T** | **C** | **G** | **A** | **T** | **C** | **C** | **A** | **T** | **A** | **T** | **C** | **G** | **A** | **A** | **T** | **A** | **T** | **T** | **A** | **C** | **G** | **C** | **A** | **T** | **T** | **T** | **G** | **T** | **G** | **A** | **C** | **A** | **G** | **A** | **T** | **A** | **C** | **T** | **A** | **T** | **C** | **G** | **G** | **T** | **C** | **A** | **G** | **C** | **A** | **A** | **G** | **T** | **C** | **T** | **T** | **A** | **C** | **G** | **C** | **G** | **T** | **T** | **T** | **T** | **A** | **G** | **A** | **A** | **G** | **A** | **A** | **A** | **A** | **A** | **G** | **C** | **C** | **G** | **C** | **A** | **G** | **A** | **A** | **G** | **G** | **C** | **G** | **T** | **A** | **G** | **A** | **A** | **G** | **T** | **T** | **C** | **G** | **T** | **A** | **T** | **A** | **T** | **T** | **A** | **T** | **A** | **T** | **G** | **A** | **T** | **G** | **C** | **A** | **T** | **T** | **T** | **G** | **G** | **C** | **T** | **C** | **C** | **C** | **A** | **T** | **G** | **G** | **C** | **A** | **C** | **A** | **A** | **A** | **A** | **G** | **C** | **A** | **A** | **A** | **A** | **G** | **A** | **T** | **T** | **T** | **C** | **A** | **A** | **T** | **C** | **G** | **T** | **C** | **T** | **A** | **A** | **T** | **C** | **A** | **A** | **A** | **A** | **A** | **T** | **G** | **G** | **T** | **G** | **G** | **A** | **C** | **A** | **T** | **G** | **T** | **C** | **C** | **A** | **T** | **A** | **C** | **A** | **T** | **T** | **T** | **G** | **T** | **T** | **A** | **C** | **C** | **T** | **C** | **G** | **C** | **A** | **A** | **A** | **A** | **A** | **G** | **C** | **A** | **T** | **T** | **G** | **C** | **T** | **T** | **C** | **G** | **T** | **T** | **T** | **C** | **C** | **G** | **A** | **T** | **T** | **G** | **A** | **A** | **T** | **T** | **A** | **C** | **C** | **A** | **T** | **G** | **A** | **T** | **C** | **A** | **C** | **C** | **G** | **G** | **A** | **A** | **A** | **A** | **T** | **C** | **G** | **T** | **T** | **G** | **T** | **G** | **A** | **T** | **C** | **G** | **A** | **T** | **G** | **G** | **A** | **A** | **A** | **G** | **A** | **T** | **C** | **A** | **G** | **T** | **T** | **A** | **C** | **A** | **C** | **C** | **G** | **G** | **T** | **G** | **G** | **T** | **T** | **T** | **C** | **A** | **A** | **T** | **A** | **T** | **T** | **G** | **C** | **C** | **A** | **A** | **T** | **C** | **A** | **A** | **T** | **A** | **T** | **G** | **T** | **A** | **A** | **A** | **T** | **A** | **C** | **G** | **A** | **C** | **A** | **A** | **A** | **A** | **A** | **A** | **A** | **T** | **T** | **C** | **G** | **G** | **C** | **T** | **A** | **T** | **T** | **G** | **G** | **C** | **G** | **T** | **G** | **A** | **T** | **A** | **C** | **G** | **C** | **A** | **T** | **A** | **T** | **A** | **C** | **G** | **G** | **A** | **T** | **T** | **T** | **T** | **C** | **G** | **G** | **T** | **G** | **C** | **C** | **G** | **C** | **T** | **T** | **C** | **T** | **T** | **C** | **T** | **T** | **T** | **G** | **C** | **T** | **T** | **C** | **A** | **A** | **C** | **T** | **T** | **C** | **G** | **T** | **T** | **T** | **T** | **T** | **T** | **A** | **A** | **C** | **A** | **G** | **A** | **C** | **T** | **G** | **G** | **A** | **A** | **C** | **G** | **T** | **C** | **T** | **C** | **A** | **G** | **T** | **A** | **C** | **C** | **T** | **G** | **A** | **A** | **G** | **A** | **A** | **A** | **A** | **A** | **A** | **A** | **G** | **G** | **T** | **C** | **G** | **G** | **C** | **T** | **A** | **T** | **C** | **A** | **T** | **T** | **T** | **G** | **A** | **A** | **T** | **T** | **A** | **T** | **T** | **T** | **C** | **T** | **T** | **T** | **A** | **A** | **A** | **A** | **A** | **A** | **G** | **C** | **A** | **G** | **A** | **T** | **C** | **G** | **A** | **G** | **A** | **C** | **G** | **A** | **A** | **T** | **C** | **C** | **A** | **A** | **A** | **C** | **T** | **T** | **G** | **C** | **T** | **G** | **A** | **T** | **A** | **C** | **G** | **T** | **C** | **C** | **A** | **T** | **C** | **C** | **A** | **G** | **C** | **T** | **T** | **G** | **T** | **C** | **T** | **C** | **A** | **A** | **G** | **C** | **G** | **G** | **A** | **C** | **C** | **A** | **A** | **A** | **T** | **A** | **A** | **C** | **G** | **A** | **A** | **C** | **G** | **A** | **G** | **A** | **A** | **C** | **A** | **A** | **A** | **T** | **C** | **A** | **A** | **G** | **C** | **T** | **T** | **T** | **C** | **A** | **T** | **T** | **T** | **A** | **T** | **C** | **A** | **A** | **A** | **T** | **T** | **G** | **A** | **T** | **T** | **A** | **C** | **T** | **T** | **C** | **T** | **G** | **C** | **T** | **A** | **A** | **A** | **A** | **A** | **A** | **C** | **G** | **T** | **G** | **T** | **T** | **T** | **G** | **G** | **A** | **T** | **A** | **C** | **A** | **G** | **A** | **C** | **A** | **C** | **C** | **T** | **T** | **A** | **C** | **C** | **T** | **C** | **G** | **T** | **T** | **C** | **C** | **T** | **G** | **A** | **T** | **G** | **A** | **A** | **A** | **G** | **T** | **G** | **T** | **C** | **A** | **T** | **T** | **G** | **C** | **T** | **G** | **C** | **T** | **T** | **T** | **G** | **A** | **A** | **A** | **A** | **T** | **C** | **G** | **C** | **A** | **A** | **C** | **T** | **G** | **C** | **T** | **T** | **C** | **T** | **G** | **G** | **T** | **G** | **T** | **C** | **G** | **A** | **T** | **G** | **T** | **G** | **A** | **A** | **A** | **A** | **T** | **C** | **A** | **T** | **G** | **A** | **T** | **C** | **C** | **C** | **C** | **A** | **A** | **T** | **A** | **A** | **A** | **C** | **C** | **G** | **G** | **A** | **T** | **C** | **A** | **T** | **C** | **C** | **T** | **T** | **T** | **T** | **A** | **T** | **T** | **T** | **A** | **T** | **C** | **G** | **A** | **G** | **C** | **A** | **A** | **C** | **A** | **C** | **A** | **A** | **T** | **A** | **T** | **T** | **A** | **T** | **G** | **C** | **G** | **C** | **G** | **T** | **C** | **A** | **G** | **C** | **T** | **C** | **A** | **T** | **C** | **A** | **A** | **G** | **G** | **A** | **A** | **A** | **A** | **T** | **G** | **T** | **A** | **C** | **A** | **G** | **A** | **T** | **C** | **C** | **T** | **T** | **G** | **T** | **C** | **T** | **A** | **T** | **G** | **A** | **G** | **A** | **A** | **T** | **G** | **G** | **C** | **T** | **T** | **C** | **C** | **T** | **C** | **C** | **A** | **T** | **G** | **C** | **A** | **A** | **A** | **A** | **A** | **C** | **A** | **T** | **T** | **G** | **A** | **T** | **A** | **A** | **T** | **G** | **G** | **A** | **T** | **G** | **A** | **T** | **G** | **A** | **A** | **A** | **T** | **C** | **T** | **G** | **C** | **A** | **T** | **G** | **G** | **T** | **A** | **G** | **G** | **T** | **T** | **C** | **A** | **G** | **C** | **A** | **A** | **A** | **C** | **C** | **A** | **A** | **G** | **A** | **T** | **A** | **T** | **T** | **C** | **G** | **T** | **A** | **G** | **C** | **T** | **A** | **C** | **C** | **G** | **A** | **T** | **T** | **G** | **A** | **A** | **T** | **T** | **T** | **T** | **G** | **A** | **A** | **A** | **C** | **A** | **A** | **G** | **T** | **G** | **C** | **T** | **G** | **T** | **C** | **A** | **T** | **C** | **T** | **A** | **C** | **G** | **A** | **T** | **C** | **C** | **T** | **G** | **A** | **G** | **T** | **T** | **T** | **T** | **T** | **A** | **G** | **A** | **A** | **G** | **A** | **A** | **C** | **T** | **T** | **G** | **C** | **T** | **A** | **C** | **C** | **C** | **A** | **G** | **T** | **T** | **C** | **A** | **A** | **A** | **G** | **A** | **A** | **G** | **A** | **T** | **G** | **A** | **G** | **A** | **C** | **A** | **C** | **A** | **T** | **T** | **G** | **T** | **T** | **C** | **A** | **C** | **C** | **A** | **A** | **T** | **G** | **A** | **C** | **G** | **A** | **C** | **T** | **G** | **A** | **A** | **A** | **C** | **A** | **G** | **T** | **G** | **A** | **A** | **G** | **G** | **A** | **A** | **A** | **T** | **G** | **T** | **C** | **T** | **A** | **A** | **T** | **T** | **G** | **G** | **C** | **T** | **G** | **T** | **T** | **A** | **T** | **T** | **C** | **A** | **A** | **G** | **C** | **A** | **A** | **C** | **A** | **A** | **A** | **T** | **T** | **T** | **C** | **T** | **C** | **G** | **A** | **T** | **T** | **A** | **T** | **T** | **T** | **T** | **C** | **T** | **C** | **C** | **A** | **A** | **T** | **C** | **T** | **T** | **A** | **-** | **-** | **-** |
| **N54152** | **-** | **-** | **-** | **-** | **-** | **-** | **-** | **-** | **-** | **-** | **-** | **-** | **-** | **-** | **-** | **-** | **-** | **-** | **-** | **-** | **-** | **-** | **-** | **-** | **-** | **-** | **-** | **-** | **-** | **-** | **-** | **-** | **-** | **-** | **-** | **-** | **-** | **-** | **-** | **-** | **-** | **-** | **-** | **-** | **-** | **-** | **-** | **-** | **-** | **-** | **-** | **-** | **-** | **-** | **-** | **-** | **-** | **-** | **-** | **-** | **-** | **-** | **-** | **-** | **-** | **-** | **-** | **-** | **-** | **A** | **A** | **A** | **C** | **C** | **C** | **C** | **G** | **A** | **G** | **A** | **T** | **G** | **T** | **A** | **G** | **C** | **A** | **G** | **C** | **C** | **A** | **T** | **T** | **T** | **G** | **G** | **G** | **C** | **A** | **T** | **G** | **G** | **C** | **T** | **C** | **T** | **T** | **A** | **G** | **T** | **A** | **T** | **T** | **G** | **T** | **T** | **T** | **G** | **C** | **C** | **C** | **T** | **T** | **C** | **C** | **T** | **G** | **G** | **G** | **A** | **T** | **G** | **G** | **G** | **T** | **T** | **T** | **T** | **G** | **T** | **A** | **T** | **T** | **A** | **T** | **A** | **T** | **T** | **T** | **A** | **T** | **T** | **T** | **T** | **T** | **C** | **G** | **G** | **A** | **C** | **G** | **T** | **G** | **G** | **A** | **T** | **T** | **A** | **A** | **C** | **G** | **G** | **A** | **T** | **A** | **A** | **G** | **A** | **A** | **A** | **A** | **A** | **A** | **T** | **T** | **C** | **T** | **A** | **T** | **C** | **T** | **C** | **C** | **G** | **A** | **C** | **A** | **A** | **A** | **G** | **T** | **G** | **A** | **C** | **T** | **T** | **G** | **A** | **A** | **A** | **G** | **A** | **A** | **T** | **T** | **A** | **G** | **A** | **A** | **A** | **A** | **C** | **T** | **T** | **T** | **C** | **A** | **G** | **T** | **C** | **T** | **T** | **T** | **T** | **A** | **G** | **A** | **G** | **G** | **A** | **G** | **A** | **T** | **A** | **C** | **C** | **A** | **T** | **T** | **G** | **A** | **A** | **C** | **A** | **T** | **T** | **A** | **C** | **G** | **A** | **T** | **C** | **C** | **T** | **G** | **A** | **C** | **A** | **T** | **G** | **G** | **G** | **C** | **G** | **A** | **T** | **A** | **A** | **A** | **G** | **A** | **C** | **A** | **A** | **A** | **C** | **A** | **G** | **C** | **A** | **A** | **T** | **T** | **T** | **G** | **T** | **C** | **G** | **A** | **C** | **T** | **T** | **C** | **T** | **T** | **C** | **T** | **C** | **T** | **T** | **C** | **A** | **T** | **T** | **A** | **A** | **A** | **T** | **C** | **G** | **T** | **A** | **T** | **G** | **C** | **C** | **G** | **C** | **T** | **G** | **A** | **C** | **A** | **A** | **G** | **A** | **A** | **T** | **G** | **A** | **A** | **T** | **T** | **C** | **T** | **G** | **T** | **C** | **A** | **C** | **T** | **C** | **T** | **T** | **C** | **T** | **C** | **A** | **C** | **A** | **G** | **A** | **C** | **G** | **G** | **A** | **C** | **A** | **A** | **G** | **A** | **G** | **A** | **A** | **A** | **T** | **T** | **G** | **G** | **A** | **T** | **T** | **C** | **A** | **C** | **T** | **G** | **C** | **T** | **T** | **C** | **A** | **A** | **G** | **A** | **T** | **C** | **T** | **A** | **A** | **A** | **A** | **A** | **A** | **A** | **G** | **C** | **C** | **A** | **A** | **A** | **C** | **A** | **T** | **T** | **C** | **G** | **A** | **T** | **C** | **C** | **A** | **T** | **A** | **T** | **C** | **G** | **A** | **A** | **T** | **A** | **T** | **T** | **A** | **C** | **G** | **C** | **A** | **T** | **T** | **T** | **G** | **T** | **G** | **A** | **C** | **A** | **G** | **A** | **T** | **A** | **A** | **T** | **A** | **T** | **C** | **G** | **G** | **C** | **C** | **A** | **G** | **C** | **A** | **A** | **G** | **T** | **C** | **T** | **T** | **A** | **C** | **A** | **T** | **G** | **T** | **T** | **T** | **T** | **A** | **G** | **A** | **A** | **G** | **A** | **A** | **A** | **A** | **A** | **G** | **C** | **C** | **G** | **C** | **A** | **G** | **A** | **A** | **G** | **G** | **C** | **G** | **T** | **G** | **G** | **A** | **A** | **G** | **T** | **T** | **C** | **G** | **A** | **A** | **T** | **A** | **T** | **T** | **A** | **T** | **A** | **T** | **G** | **A** | **T** | **G** | **C** | **A** | **T** | **T** | **T** | **G** | **G** | **C** | **T** | **C** | **T** | **C** | **A** | **T** | **G** | **G** | **C** | **A** | **C** | **A** | **A** | **A** | **A** | **G** | **C** | **A** | **A** | **A** | **A** | **G** | **A** | **T** | **T** | **T** | **C** | **A** | **A** | **T** | **C** | **G** | **T** | **C** | **T** | **A** | **A** | **T** | **C** | **A** | **A** | **A** | **A** | **A** | **T** | **G** | **G** | **T** | **G** | **G** | **A** | **C** | **A** | **T** | **G** | **T** | **C** | **C** | **A** | **T** | **A** | **C** | **A** | **T** | **T** | **T** | **G** | **T** | **T** | **A** | **C** | **C** | **T** | **C** | **A** | **C** | **A** | **A** | **A** | **A** | **G** | **G** | **C** | **A** | **T** | **T** | **A** | **C** | **T** | **T** | **C** | **G** | **T** | **T** | **T** | **C** | **C** | **G** | **A** | **T** | **T** | **G** | **A** | **A** | **T** | **T** | **A** | **C** | **C** | **A** | **T** | **G** | **A** | **T** | **C** | **A** | **C** | **C** | **G** | **A** | **A** | **A** | **A** | **A** | **T** | **C** | **G** | **T** | **T** | **G** | **T** | **G** | **A** | **T** | **C** | **G** | **A** | **T** | **G** | **G** | **A** | **A** | **A** | **G** | **A** | **T** | **T** | **A** | **G** | **T** | **T** | **A** | **T** | **A** | **C** | **C** | **G** | **G** | **T** | **G** | **G** | **T** | **T** | **T** | **C** | **A** | **A** | **T** | **A** | **T** | **T** | **G** | **C** | **C** | **A** | **A** | **T** | **C** | **A** | **A** | **T** | **A** | **T** | **G** | **T** | **A** | **A** | **A** | **T** | **A** | **C** | **A** | **A** | **C** | **A** | **A** | **A** | **A** | **A** | **A** | **A** | **T** | **T** | **C** | **G** | **G** | **C** | **T** | **A** | **T** | **T** | **G** | **G** | **C** | **G** | **C** | **G** | **A** | **T** | **A** | **C** | **G** | **C** | **A** | **T** | **A** | **T** | **A** | **C** | **G** | **G** | **A** | **T** | **T** | **T** | **T** | **C** | **G** | **G** | **T** | **G** | **C** | **C** | **G** | **C** | **T** | **T** | **C** | **T** | **T** | **C** | **T** | **T** | **T** | **G** | **C** | **T** | **C** | **C** | **A** | **G** | **C** | **T** | **T** | **C** | **G** | **C** | **T** | **T** | **C** | **T** | **T** | **A** | **A** | **C** | **A** | **G** | **A** | **C** | **T** | **G** | **G** | **A** | **A** | **C** | **G** | **T** | **C** | **T** | **C** | **G** | **G** | **T** | **A** | **C** | **C** | **T** | **G** | **A** | **A** | **G** | **A** | **A** | **A** | **A** | **A** | **A** | **A** | **G** | **G** | **T** | **C** | **G** | **G** | **C** | **T** | **A** | **T** | **C** | **A** | **T** | **T** | **T** | **G** | **A** | **A** | **T** | **T** | **A** | **T** | **T** | **T** | **C** | **T** | **T** | **T** | **A** | **A** | **A** | **A** | **A** | **A** | **G** | **C** | **A** | **G** | **A** | **T** | **C** | **G** | **A** | **G** | **A** | **T** | **G** | **A** | **A** | **T** | **C** | **T** | **A** | **A** | **G** | **C** | **T** | **T** | **G** | **C** | **T** | **G** | **A** | **T** | **A** | **C** | **A** | **T** | **C** | **C** | **A** | **T** | **C** | **C** | **A** | **G** | **C** | **T** | **T** | **G** | **T** | **T** | **T** | **C** | **A** | **A** | **G** | **C** | **G** | **G** | **A** | **C** | **C** | **G** | **A** | **A** | **T** | **A** | **A** | **C** | **G** | **A** | **A** | **A** | **G** | **G** | **G** | **A** | **A** | **C** | **A** | **A** | **A** | **T** | **C** | **A** | **A** | **G** | **C** | **T** | **T** | **T** | **C** | **A** | **T** | **T** | **T** | **A** | **T** | **C** | **A** | **A** | **A** | **T** | **T** | **G** | **A** | **T** | **T** | **A** | **C** | **T** | **T** | **C** | **T** | **G** | **C** | **T** | **A** | **A** | **A** | **A** | **A** | **A** | **C** | **G** | **T** | **G** | **T** | **T** | **T** | **G** | **G** | **A** | **T** | **A** | **C** | **A** | **G** | **A** | **C** | **A** | **C** | **C** | **T** | **T** | **A** | **C** | **C** | **T** | **T** | **G** | **T** | **T** | **C** | **C** | **T** | **G** | **A** | **T** | **G** | **A** | **A** | **A** | **G** | **T** | **G** | **T** | **C** | **A** | **T** | **T** | **G** | **C** | **T** | **G** | **C** | **T** | **T** | **T** | **A** | **A** | **A** | **A** | **A** | **T** | **C** | **G** | **C** | **A** | **A** | **C** | **T** | **G** | **C** | **C** | **T** | **C** | **T** | **G** | **G** | **T** | **G** | **T** | **A** | **G** | **A** | **T** | **G** | **T** | **G** | **A** | **A** | **A** | **A** | **T** | **C** | **A** | **T** | **G** | **A** | **T** | **T** | **C** | **C** | **C** | **A** | **A** | **C** | **A** | **A** | **A** | **C** | **C** | **G** | **G** | **A** | **T** | **C** | **A** | **T** | **C** | **C** | **T** | **T** | **T** | **T** | **A** | **T** | **T** | **T** | **A** | **T** | **C** | **G** | **A** | **G** | **C** | **A** | **A** | **C** | **A** | **C** | **A** | **A** | **T** | **A** | **T** | **T** | **A** | **T** | **G** | **C** | **T** | **C** | **G** | **G** | **C** | **A** | **G** | **C** | **T** | **G** | **A** | **T** | **C** | **A** | **A** | **G** | **G** | **A** | **A** | **A** | **A** | **T** | **G** | **T** | **A** | **C** | **A** | **A** | **A** | **T** | **C** | **C** | **T** | **T** | **G** | **T** | **C** | **T** | **A** | **T** | **G** | **A** | **G** | **A** | **A** | **C** | **G** | **G** | **C** | **T** | **T** | **C** | **C** | **T** | **C** | **C** | **A** | **T** | **G** | **C** | **A** | **A** | **A** | **A** | **A** | **C** | **A** | **T** | **T** | **G** | **A** | **T** | **A** | **A** | **T** | **G** | **G** | **A** | **T** | **G** | **A** | **T** | **G** | **A** | **A** | **A** | **T** | **C** | **T** | **G** | **C** | **A** | **T** | **G** | **G** | **T** | **A** | **G** | **G** | **T** | **T** | **C** | **A** | **G** | **C** | **A** | **A** | **A** | **T** | **C** | **A** | **A** | **G** | **A** | **T** | **A** | **T** | **T** | **C** | **G** | **T** | **A** | **G** | **C** | **T** | **A** | **C** | **C** | **G** | **A** | **T** | **T** | **G** | **A** | **A** | **T** | **T** | **T** | **T** | **G** | **A** | **A** | **A** | **C** | **A** | **A** | **G** | **T** | **G** | **C** | **T** | **G** | **T** | **C** | **A** | **T** | **T** | **T** | **A** | **C** | **G** | **A** | **T** | **C** | **C** | **T** | **G** | **A** | **G** | **T** | **T** | **T** | **T** | **T** | **A** | **G** | **A** | **A** | **G** | **A** | **A** | **C** | **T** | **T** | **G** | **C** | **T** | **A** | **C** | **T** | **C** | **A** | **G** | **T** | **T** | **C** | **A** | **A** | **A** | **G** | **A** | **A** | **G** | **A** | **T** | **G** | **A** | **G** | **A** | **C** | **A** | **C** | **A** | **T** | **T** | **G** | **T** | **T** | **C** | **A** | **T** | **C** | **C** | **A** | **T** | **G** | **A** | **C** | **A** | **A** | **C** | **T** | **G** | **A** | **A** | **A** | **C** | **A** | **G** | **T** | **C** | **A** | **A** | **A** | **G** | **A** | **A** | **A** | **T** | **G** | **T** | **C** | **T** | **A** | **A** | **C** | **T** | **G** | **G** | **C** | **T** | **A** | **T** | **T** | **A** | **T** | **T** | **C** | **A** | **A** | **G** | **C** | **A** | **A** | **C** | **A** | **A** | **A** | **T** | **T** | **T** | **C** | **T** | **C** | **G** | **A** | **T** | **T** | **A** | **T** | **T** | **T** | **T** | **C** | **T** | **C** | **C** | **A** | **A** | **T** | **C** | **C** | **T** | **A** | **-** | **-** | **-** |
| **N54190** | **-** | **-** | **-** | **-** | **-** | **-** | **-** | **-** | **-** | **-** | **-** | **-** | **-** | **-** | **-** | **-** | **-** | **-** | **-** | **-** | **-** | **-** | **-** | **-** | **-** | **-** | **-** | **-** | **-** | **-** | **-** | **-** | **-** | **-** | **-** | **-** | **-** | **-** | **-** | **-** | **-** | **-** | **-** | **-** | **-** | **-** | **-** | **-** | **-** | **-** | **-** | **-** | **-** | **-** | **-** | **-** | **-** | **-** | **-** | **-** | **-** | **-** | **-** | **-** | **-** | **-** | **-** | **-** | **-** | **A** | **A** | **A** | **C** | **C** | **C** | **C** | **G** | **A** | **G** | **A** | **T** | **G** | **T** | **A** | **G** | **C** | **A** | **G** | **C** | **C** | **A** | **T** | **T** | **T** | **G** | **G** | **G** | **C** | **A** | **T** | **G** | **G** | **C** | **T** | **C** | **T** | **T** | **A** | **G** | **T** | **A** | **T** | **T** | **G** | **T** | **T** | **T** | **G** | **C** | **C** | **C** | **T** | **T** | **C** | **C** | **T** | **G** | **G** | **G** | **G** | **T** | **G** | **G** | **G** | **C** | **T** | **T** | **T** | **G** | **T** | **A** | **T** | **T** | **A** | **T** | **A** | **T** | **T** | **T** | **A** | **T** | **T** | **T** | **T** | **T** | **C** | **G** | **G** | **A** | **C** | **G** | **T** | **G** | **G** | **A** | **T** | **T** | **A** | **A** | **C** | **G** | **G** | **A** | **T** | **A** | **A** | **G** | **A** | **A** | **A** | **A** | **A** | **A** | **T** | **T** | **C** | **T** | **A** | **T** | **C** | **T** | **C** | **C** | **G** | **A** | **C** | **A** | **A** | **A** | **G** | **T** | **G** | **A** | **C** | **T** | **T** | **G** | **A** | **A** | **A** | **G** | **A** | **A** | **T** | **T** | **A** | **G** | **A** | **A** | **A** | **A** | **C** | **T** | **T** | **T** | **C** | **A** | **G** | **T** | **C** | **T** | **T** | **T** | **T** | **A** | **G** | **A** | **G** | **G** | **A** | **G** | **A** | **T** | **A** | **A** | **C** | **A** | **T** | **T** | **G** | **A** | **A** | **C** | **A** | **T** | **T** | **A** | **C** | **G** | **A** | **T** | **C** | **C** | **T** | **G** | **A** | **C** | **A** | **T** | **G** | **G** | **G** | **C** | **G** | **A** | **T** | **A** | **A** | **A** | **G** | **A** | **C** | **A** | **A** | **A** | **C** | **A** | **G** | **C** | **A** | **A** | **T** | **T** | **T** | **G** | **T** | **T** | **G** | **A** | **C** | **T** | **T** | **C** | **T** | **T** | **C** | **T** | **C** | **T** | **T** | **C** | **A** | **T** | **T** | **A** | **A** | **A** | **T** | **C** | **G** | **T** | **A** | **T** | **G** | **C** | **C** | **G** | **C** | **T** | **G** | **A** | **C** | **A** | **A** | **G** | **A** | **A** | **T** | **G** | **A** | **A** | **T** | **T** | **C** | **T** | **G** | **T** | **C** | **A** | **C** | **T** | **C** | **T** | **T** | **C** | **T** | **C** | **A** | **C** | **A** | **G** | **A** | **C** | **G** | **G** | **A** | **C** | **A** | **A** | **G** | **A** | **G** | **A** | **A** | **A** | **T** | **T** | **G** | **G** | **A** | **T** | **T** | **C** | **A** | **C** | **T** | **G** | **C** | **T** | **T** | **C** | **A** | **A** | **G** | **A** | **T** | **C** | **T** | **A** | **A** | **A** | **A** | **A** | **A** | **A** | **G** | **C** | **C** | **A** | **A** | **A** | **C** | **A** | **T** | **T** | **C** | **G** | **A** | **T** | **C** | **C** | **A** | **T** | **A** | **T** | **C** | **G** | **A** | **A** | **T** | **A** | **T** | **T** | **A** | **C** | **G** | **C** | **A** | **T** | **T** | **T** | **G** | **T** | **G** | **A** | **C** | **A** | **G** | **A** | **T** | **A** | **A** | **T** | **A** | **T** | **C** | **G** | **G** | **C** | **C** | **A** | **G** | **C** | **A** | **A** | **G** | **T** | **C** | **T** | **T** | **A** | **C** | **A** | **T** | **G** | **T** | **T** | **T** | **T** | **A** | **G** | **A** | **A** | **G** | **A** | **A** | **A** | **A** | **A** | **G** | **C** | **C** | **G** | **C** | **A** | **G** | **A** | **A** | **G** | **G** | **C** | **G** | **T** | **G** | **G** | **A** | **A** | **G** | **T** | **T** | **C** | **G** | **A** | **A** | **T** | **A** | **T** | **T** | **A** | **T** | **A** | **T** | **G** | **A** | **T** | **G** | **C** | **A** | **T** | **T** | **T** | **G** | **G** | **C** | **T** | **C** | **T** | **C** | **A** | **T** | **G** | **G** | **C** | **A** | **C** | **A** | **A** | **A** | **A** | **G** | **C** | **A** | **A** | **A** | **A** | **G** | **A** | **T** | **T** | **T** | **C** | **A** | **A** | **T** | **C** | **G** | **T** | **C** | **T** | **A** | **A** | **T** | **C** | **A** | **A** | **A** | **A** | **A** | **T** | **G** | **G** | **T** | **G** | **G** | **A** | **C** | **A** | **T** | **G** | **T** | **C** | **C** | **A** | **T** | **A** | **C** | **A** | **T** | **T** | **T** | **G** | **T** | **T** | **A** | **C** | **C** | **T** | **C** | **A** | **C** | **A** | **A** | **A** | **A** | **G** | **G** | **C** | **A** | **T** | **T** | **A** | **C** | **T** | **T** | **C** | **G** | **T** | **T** | **T** | **C** | **C** | **G** | **A** | **T** | **T** | **G** | **A** | **A** | **T** | **T** | **A** | **C** | **C** | **A** | **T** | **G** | **A** | **T** | **C** | **A** | **C** | **C** | **G** | **A** | **A** | **A** | **A** | **A** | **T** | **C** | **G** | **T** | **T** | **G** | **T** | **G** | **A** | **T** | **C** | **G** | **A** | **T** | **G** | **G** | **A** | **A** | **A** | **G** | **A** | **T** | **T** | **A** | **G** | **T** | **T** | **A** | **T** | **A** | **C** | **C** | **G** | **G** | **T** | **G** | **G** | **T** | **T** | **T** | **C** | **A** | **A** | **T** | **A** | **T** | **T** | **G** | **C** | **C** | **A** | **A** | **T** | **C** | **A** | **A** | **T** | **A** | **T** | **G** | **T** | **A** | **A** | **A** | **T** | **A** | **C** | **A** | **A** | **C** | **A** | **A** | **A** | **A** | **A** | **A** | **A** | **T** | **T** | **C** | **G** | **G** | **C** | **T** | **A** | **T** | **T** | **G** | **G** | **C** | **G** | **C** | **G** | **A** | **T** | **A** | **C** | **G** | **C** | **A** | **T** | **A** | **T** | **A** | **C** | **G** | **G** | **A** | **T** | **T** | **T** | **T** | **C** | **G** | **G** | **T** | **G** | **C** | **C** | **G** | **C** | **T** | **T** | **C** | **T** | **T** | **C** | **T** | **T** | **T** | **G** | **C** | **T** | **C** | **C** | **A** | **G** | **C** | **T** | **T** | **C** | **G** | **C** | **T** | **T** | **C** | **T** | **T** | **A** | **A** | **C** | **A** | **G** | **A** | **C** | **T** | **G** | **G** | **A** | **A** | **C** | **G** | **T** | **C** | **T** | **C** | **G** | **G** | **T** | **A** | **C** | **C** | **T** | **G** | **A** | **A** | **G** | **A** | **A** | **A** | **A** | **A** | **A** | **A** | **G** | **G** | **T** | **C** | **G** | **G** | **C** | **T** | **A** | **T** | **C** | **A** | **T** | **T** | **T** | **G** | **A** | **A** | **T** | **T** | **A** | **T** | **T** | **T** | **C** | **T** | **T** | **T** | **A** | **A** | **A** | **A** | **A** | **A** | **G** | **C** | **A** | **G** | **A** | **T** | **C** | **G** | **A** | **G** | **A** | **T** | **G** | **A** | **A** | **T** | **C** | **T** | **A** | **A** | **G** | **C** | **T** | **T** | **G** | **C** | **T** | **G** | **A** | **T** | **A** | **C** | **A** | **T** | **C** | **C** | **A** | **T** | **C** | **C** | **A** | **G** | **C** | **T** | **T** | **G** | **T** | **T** | **T** | **C** | **A** | **A** | **G** | **C** | **G** | **G** | **A** | **C** | **C** | **G** | **A** | **A** | **T** | **A** | **A** | **C** | **G** | **A** | **A** | **A** | **G** | **G** | **G** | **A** | **A** | **C** | **A** | **A** | **A** | **T** | **C** | **A** | **A** | **G** | **C** | **T** | **T** | **T** | **C** | **A** | **T** | **T** | **T** | **A** | **T** | **C** | **A** | **A** | **A** | **T** | **T** | **G** | **A** | **T** | **T** | **A** | **C** | **T** | **T** | **C** | **T** | **G** | **C** | **T** | **A** | **A** | **A** | **A** | **A** | **A** | **C** | **G** | **T** | **G** | **T** | **T** | **T** | **G** | **G** | **A** | **T** | **A** | **C** | **A** | **G** | **A** | **C** | **A** | **C** | **C** | **T** | **T** | **A** | **C** | **C** | **T** | **T** | **G** | **T** | **T** | **C** | **C** | **T** | **G** | **A** | **T** | **G** | **A** | **A** | **A** | **G** | **T** | **G** | **T** | **C** | **A** | **T** | **T** | **G** | **C** | **T** | **G** | **C** | **T** | **T** | **T** | **A** | **A** | **A** | **A** | **A** | **T** | **C** | **G** | **C** | **A** | **A** | **C** | **T** | **G** | **C** | **C** | **T** | **C** | **T** | **G** | **G** | **T** | **G** | **T** | **A** | **G** | **A** | **T** | **G** | **T** | **G** | **A** | **A** | **A** | **A** | **T** | **C** | **A** | **T** | **G** | **A** | **T** | **T** | **C** | **C** | **C** | **A** | **A** | **C** | **A** | **A** | **A** | **C** | **C** | **G** | **G** | **A** | **T** | **C** | **A** | **T** | **C** | **C** | **T** | **T** | **T** | **T** | **A** | **T** | **T** | **T** | **A** | **T** | **C** | **G** | **A** | **G** | **C** | **A** | **A** | **C** | **A** | **C** | **A** | **A** | **T** | **A** | **T** | **T** | **A** | **T** | **G** | **C** | **T** | **C** | **G** | **G** | **C** | **A** | **G** | **C** | **T** | **G** | **A** | **T** | **C** | **A** | **A** | **G** | **G** | **A** | **A** | **A** | **A** | **T** | **G** | **T** | **A** | **C** | **A** | **A** | **A** | **T** | **C** | **C** | **T** | **T** | **G** | **T** | **C** | **T** | **A** | **T** | **G** | **A** | **G** | **A** | **A** | **C** | **G** | **G** | **C** | **T** | **T** | **C** | **C** | **T** | **C** | **C** | **A** | **T** | **G** | **C** | **A** | **A** | **A** | **A** | **A** | **C** | **A** | **T** | **T** | **G** | **A** | **T** | **A** | **A** | **T** | **G** | **G** | **A** | **T** | **G** | **A** | **T** | **G** | **A** | **A** | **A** | **T** | **C** | **T** | **G** | **C** | **A** | **T** | **G** | **G** | **T** | **A** | **G** | **G** | **T** | **T** | **C** | **A** | **G** | **C** | **A** | **A** | **A** | **T** | **C** | **A** | **A** | **G** | **A** | **T** | **A** | **T** | **T** | **C** | **G** | **T** | **A** | **G** | **C** | **T** | **A** | **C** | **C** | **G** | **A** | **T** | **T** | **G** | **A** | **A** | **T** | **T** | **T** | **T** | **G** | **A** | **A** | **A** | **C** | **A** | **A** | **G** | **T** | **G** | **C** | **T** | **G** | **T** | **C** | **A** | **T** | **T** | **T** | **A** | **C** | **G** | **A** | **T** | **C** | **C** | **T** | **G** | **A** | **G** | **T** | **T** | **T** | **T** | **T** | **A** | **G** | **A** | **A** | **G** | **A** | **A** | **C** | **T** | **T** | **G** | **C** | **T** | **A** | **C** | **T** | **C** | **A** | **G** | **T** | **T** | **C** | **A** | **A** | **A** | **G** | **A** | **A** | **G** | **A** | **T** | **G** | **A** | **G** | **A** | **C** | **A** | **C** | **A** | **T** | **T** | **G** | **T** | **T** | **C** | **A** | **T** | **C** | **C** | **A** | **T** | **G** | **A** | **C** | **A** | **A** | **C** | **T** | **G** | **A** | **A** | **A** | **C** | **A** | **G** | **T** | **C** | **A** | **A** | **G** | **G** | **A** | **A** | **A** | **T** | **G** | **T** | **C** | **T** | **A** | **A** | **C** | **T** | **G** | **G** | **C** | **T** | **A** | **T** | **T** | **A** | **T** | **T** | **C** | **A** | **A** | **G** | **C** | **A** | **A** | **C** | **A** | **A** | **A** | **T** | **T** | **T** | **C** | **T** | **C** | **G** | **A** | **T** | **T** | **A** | **T** | **T** | **T** | **T** | **C** | **T** | **C** | **C** | **A** | **A** | **T** | **C** | **C** | **T** | **A** | **-** | **-** | **-** |
| **N60048F** | **-** | **-** | **-** | **-** | **-** | **-** | **-** | **-** | **-** | **-** | **-** | **-** | **-** | **-** | **-** | **-** | **-** | **-** | **-** | **-** | **-** | **-** | **-** | **-** | **-** | **-** | **-** | **-** | **-** | **-** | **-** | **-** | **-** | **-** | **-** | **-** | **-** | **-** | **-** | **-** | **-** | **-** | **-** | **-** | **-** | **-** | **-** | **-** | **-** | **-** | **-** | **-** | **-** | **-** | **-** | **-** | **-** | **-** | **-** | **-** | **-** | **-** | **-** | **-** | **-** | **-** | **-** | **-** | **-** | **A** | **A** | **A** | **C** | **C** | **C** | **C** | **G** | **A** | **G** | **A** | **T** | **G** | **T** | **A** | **G** | **C** | **A** | **G** | **C** | **C** | **A** | **T** | **T** | **T** | **G** | **G** | **G** | **C** | **A** | **T** | **G** | **G** | **C** | **T** | **C** | **T** | **T** | **A** | **G** | **T** | **A** | **T** | **T** | **G** | **T** | **T** | **T** | **G** | **C** | **C** | **C** | **T** | **T** | **C** | **C** | **T** | **G** | **G** | **G** | **G** | **T** | **G** | **G** | **G** | **T** | **T** | **T** | **T** | **G** | **T** | **A** | **T** | **T** | **A** | **T** | **A** | **T** | **T** | **T** | **A** | **T** | **T** | **T** | **T** | **T** | **C** | **G** | **G** | **A** | **C** | **G** | **T** | **G** | **G** | **A** | **T** | **T** | **A** | **A** | **C** | **G** | **G** | **A** | **T** | **A** | **A** | **G** | **A** | **A** | **A** | **A** | **A** | **A** | **T** | **T** | **C** | **T** | **A** | **T** | **C** | **T** | **C** | **C** | **G** | **A** | **C** | **A** | **A** | **A** | **G** | **T** | **G** | **A** | **C** | **T** | **T** | **G** | **A** | **A** | **A** | **G** | **A** | **A** | **T** | **T** | **A** | **G** | **A** | **A** | **A** | **A** | **C** | **T** | **T** | **T** | **C** | **A** | **G** | **T** | **C** | **T** | **T** | **T** | **T** | **A** | **G** | **A** | **G** | **G** | **A** | **G** | **A** | **T** | **A** | **C** | **C** | **A** | **T** | **T** | **G** | **A** | **A** | **C** | **A** | **T** | **T** | **A** | **C** | **G** | **A** | **T** | **C** | **C** | **T** | **G** | **A** | **C** | **A** | **T** | **G** | **G** | **G** | **C** | **G** | **A** | **T** | **A** | **A** | **A** | **G** | **A** | **C** | **A** | **A** | **A** | **C** | **A** | **G** | **C** | **A** | **A** | **T** | **T** | **T** | **G** | **T** | **T** | **G** | **A** | **C** | **T** | **T** | **C** | **T** | **T** | **C** | **T** | **C** | **T** | **T** | **C** | **A** | **T** | **T** | **A** | **A** | **A** | **T** | **C** | **G** | **T** | **A** | **T** | **G** | **C** | **C** | **G** | **C** | **T** | **G** | **A** | **C** | **A** | **A** | **G** | **A** | **A** | **T** | **G** | **A** | **A** | **T** | **T** | **C** | **T** | **G** | **T** | **C** | **A** | **C** | **T** | **C** | **T** | **T** | **C** | **T** | **C** | **A** | **C** | **A** | **G** | **A** | **C** | **G** | **G** | **A** | **C** | **A** | **A** | **G** | **A** | **G** | **A** | **A** | **A** | **T** | **T** | **G** | **G** | **A** | **T** | **T** | **C** | **A** | **C** | **T** | **G** | **C** | **T** | **T** | **C** | **A** | **A** | **G** | **A** | **T** | **C** | **T** | **A** | **A** | **A** | **A** | **A** | **A** | **A** | **G** | **C** | **C** | **A** | **A** | **A** | **C** | **A** | **T** | **T** | **C** | **G** | **A** | **T** | **C** | **C** | **A** | **T** | **A** | **T** | **C** | **G** | **A** | **A** | **T** | **A** | **T** | **T** | **A** | **C** | **G** | **C** | **A** | **T** | **T** | **T** | **G** | **T** | **G** | **A** | **C** | **A** | **G** | **A** | **T** | **A** | **A** | **T** | **A** | **T** | **C** | **G** | **G** | **C** | **C** | **A** | **G** | **C** | **A** | **A** | **G** | **T** | **C** | **T** | **T** | **A** | **C** | **A** | **T** | **G** | **T** | **T** | **T** | **T** | **A** | **G** | **A** | **A** | **G** | **A** | **A** | **A** | **A** | **A** | **G** | **C** | **C** | **G** | **C** | **A** | **G** | **A** | **A** | **G** | **G** | **C** | **G** | **T** | **G** | **G** | **A** | **A** | **G** | **T** | **T** | **C** | **G** | **A** | **A** | **T** | **A** | **T** | **T** | **A** | **T** | **A** | **T** | **G** | **A** | **T** | **G** | **C** | **A** | **T** | **T** | **T** | **G** | **G** | **C** | **T** | **C** | **T** | **C** | **A** | **T** | **G** | **G** | **C** | **A** | **C** | **A** | **A** | **A** | **A** | **G** | **C** | **A** | **A** | **A** | **A** | **G** | **A** | **T** | **T** | **T** | **C** | **A** | **A** | **T** | **C** | **G** | **T** | **C** | **T** | **A** | **A** | **T** | **C** | **A** | **A** | **A** | **A** | **A** | **T** | **G** | **G** | **T** | **G** | **G** | **A** | **C** | **A** | **T** | **G** | **T** | **C** | **C** | **A** | **T** | **A** | **C** | **A** | **T** | **T** | **T** | **G** | **T** | **T** | **A** | **C** | **C** | **T** | **C** | **A** | **C** | **A** | **A** | **A** | **A** | **G** | **G** | **C** | **A** | **T** | **T** | **A** | **C** | **T** | **T** | **C** | **G** | **T** | **T** | **T** | **C** | **C** | **G** | **A** | **T** | **T** | **G** | **A** | **A** | **T** | **T** | **A** | **C** | **C** | **A** | **T** | **G** | **A** | **T** | **C** | **A** | **C** | **C** | **G** | **A** | **A** | **A** | **A** | **A** | **T** | **C** | **G** | **T** | **T** | **G** | **T** | **G** | **A** | **T** | **C** | **G** | **A** | **T** | **G** | **G** | **A** | **A** | **A** | **G** | **A** | **T** | **T** | **A** | **G** | **T** | **T** | **A** | **T** | **A** | **C** | **C** | **G** | **G** | **T** | **G** | **G** | **T** | **T** | **T** | **C** | **A** | **A** | **T** | **A** | **T** | **T** | **G** | **C** | **C** | **A** | **A** | **T** | **C** | **A** | **A** | **T** | **A** | **T** | **G** | **T** | **A** | **A** | **A** | **T** | **A** | **C** | **A** | **A** | **C** | **A** | **A** | **A** | **A** | **A** | **A** | **A** | **T** | **T** | **C** | **G** | **G** | **C** | **T** | **A** | **T** | **T** | **G** | **G** | **C** | **G** | **C** | **G** | **A** | **T** | **A** | **C** | **G** | **C** | **A** | **T** | **A** | **T** | **A** | **C** | **G** | **G** | **A** | **T** | **T** | **T** | **T** | **C** | **G** | **G** | **T** | **G** | **C** | **C** | **G** | **C** | **T** | **T** | **C** | **T** | **T** | **C** | **T** | **T** | **T** | **G** | **C** | **T** | **C** | **C** | **A** | **G** | **C** | **T** | **T** | **C** | **G** | **C** | **T** | **T** | **C** | **T** | **T** | **A** | **A** | **C** | **A** | **G** | **A** | **C** | **T** | **G** | **G** | **A** | **A** | **C** | **G** | **T** | **C** | **T** | **C** | **G** | **G** | **T** | **A** | **C** | **C** | **T** | **G** | **A** | **A** | **G** | **A** | **A** | **A** | **A** | **A** | **A** | **A** | **G** | **G** | **T** | **C** | **G** | **G** | **C** | **T** | **A** | **T** | **C** | **A** | **T** | **T** | **T** | **G** | **A** | **A** | **T** | **T** | **A** | **T** | **T** | **T** | **C** | **T** | **T** | **T** | **A** | **A** | **A** | **A** | **A** | **A** | **G** | **C** | **A** | **G** | **A** | **T** | **C** | **G** | **A** | **G** | **A** | **T** | **G** | **A** | **A** | **T** | **C** | **T** | **A** | **A** | **G** | **C** | **T** | **T** | **G** | **C** | **T** | **G** | **A** | **A** | **A** | **C** | **A** | **T** | **C** | **C** | **A** | **T** | **C** | **C** | **A** | **G** | **C** | **T** | **T** | **G** | **T** | **T** | **T** | **C** | **A** | **A** | **G** | **C** | **G** | **G** | **A** | **C** | **C** | **G** | **A** | **A** | **T** | **A** | **A** | **C** | **G** | **A** | **A** | **A** | **G** | **G** | **G** | **A** | **A** | **C** | **A** | **A** | **A** | **T** | **C** | **A** | **A** | **G** | **C** | **T** | **T** | **T** | **C** | **A** | **T** | **T** | **T** | **A** | **T** | **C** | **A** | **A** | **A** | **T** | **T** | **G** | **A** | **T** | **T** | **A** | **C** | **T** | **T** | **C** | **T** | **G** | **C** | **T** | **A** | **A** | **A** | **A** | **A** | **G** | **C** | **G** | **T** | **G** | **T** | **T** | **T** | **G** | **G** | **A** | **T** | **A** | **C** | **A** | **G** | **A** | **C** | **A** | **C** | **C** | **T** | **T** | **A** | **C** | **C** | **T** | **T** | **G** | **T** | **T** | **C** | **C** | **T** | **G** | **A** | **T** | **G** | **A** | **A** | **A** | **G** | **T** | **G** | **T** | **C** | **A** | **T** | **T** | **G** | **C** | **T** | **G** | **C** | **T** | **T** | **T** | **A** | **A** | **A** | **A** | **A** | **T** | **C** | **G** | **C** | **A** | **A** | **C** | **T** | **G** | **C** | **C** | **T** | **C** | **T** | **G** | **G** | **T** | **G** | **T** | **A** | **G** | **A** | **T** | **G** | **T** | **G** | **A** | **A** | **A** | **A** | **T** | **C** | **A** | **T** | **G** | **A** | **T** | **T** | **C** | **C** | **C** | **A** | **A** | **C** | **A** | **A** | **A** | **C** | **C** | **G** | **G** | **A** | **T** | **C** | **A** | **T** | **C** | **C** | **T** | **T** | **T** | **T** | **A** | **T** | **T** | **T** | **A** | **T** | **C** | **G** | **A** | **G** | **C** | **A** | **A** | **C** | **A** | **C** | **A** | **A** | **T** | **A** | **T** | **T** | **A** | **T** | **G** | **C** | **T** | **C** | **G** | **G** | **C** | **A** | **G** | **C** | **T** | **G** | **A** | **T** | **C** | **A** | **A** | **G** | **G** | **A** | **A** | **A** | **A** | **T** | **G** | **T** | **A** | **C** | **A** | **A** | **A** | **T** | **C** | **C** | **T** | **T** | **G** | **T** | **C** | **T** | **A** | **T** | **G** | **A** | **G** | **A** | **A** | **C** | **G** | **G** | **C** | **T** | **T** | **C** | **C** | **T** | **C** | **C** | **A** | **T** | **G** | **C** | **A** | **A** | **A** | **A** | **A** | **C** | **A** | **T** | **T** | **G** | **A** | **T** | **A** | **A** | **T** | **G** | **G** | **A** | **T** | **G** | **A** | **T** | **G** | **A** | **A** | **A** | **T** | **C** | **T** | **G** | **C** | **A** | **T** | **G** | **G** | **T** | **A** | **G** | **G** | **T** | **T** | **C** | **A** | **G** | **C** | **A** | **A** | **A** | **T** | **C** | **A** | **A** | **G** | **A** | **T** | **A** | **T** | **T** | **C** | **G** | **T** | **A** | **G** | **C** | **T** | **A** | **C** | **A** | **G** | **A** | **T** | **T** | **G** | **A** | **A** | **T** | **T** | **T** | **T** | **G** | **A** | **A** | **A** | **C** | **A** | **A** | **G** | **T** | **G** | **C** | **T** | **G** | **T** | **C** | **A** | **T** | **T** | **T** | **A** | **C** | **G** | **A** | **T** | **C** | **C** | **T** | **G** | **A** | **G** | **T** | **T** | **T** | **T** | **T** | **A** | **G** | **A** | **A** | **G** | **A** | **A** | **C** | **T** | **T** | **G** | **C** | **T** | **A** | **C** | **T** | **C** | **A** | **G** | **T** | **T** | **C** | **A** | **A** | **A** | **G** | **A** | **A** | **G** | **A** | **T** | **G** | **A** | **G** | **A** | **C** | **A** | **C** | **A** | **T** | **T** | **G** | **T** | **T** | **C** | **A** | **T** | **C** | **C** | **A** | **T** | **G** | **A** | **C** | **A** | **A** | **C** | **T** | **G** | **A** | **A** | **A** | **C** | **A** | **G** | **T** | **T** | **A** | **A** | **A** | **G** | **A** | **A** | **A** | **T** | **G** | **T** | **C** | **T** | **A** | **A** | **C** | **T** | **G** | **G** | **C** | **T** | **A** | **T** | **T** | **A** | **T** | **T** | **C** | **A** | **A** | **G** | **C** | **A** | **A** | **C** | **A** | **A** | **A** | **T** | **T** | **T** | **C** | **T** | **C** | **G** | **A** | **T** | **T** | **A** | **T** | **T** | **T** | **T** | **C** | **T** | **C** | **C** | **A** | **A** | **T** | **C** | **C** | **T** | **A** | **-** | **-** | **-** |
| **N60001F** | **-** | **-** | **-** | **-** | **-** | **-** | **-** | **-** | **-** | **-** | **-** | **-** | **-** | **-** | **-** | **-** | **-** | **-** | **-** | **-** | **-** | **-** | **-** | **-** | **-** | **-** | **-** | **-** | **-** | **-** | **-** | **-** | **-** | **-** | **-** | **-** | **-** | **-** | **-** | **-** | **-** | **-** | **-** | **-** | **-** | **-** | **-** | **-** | **-** | **-** | **-** | **-** | **-** | **-** | **-** | **-** | **-** | **-** | **-** | **-** | **-** | **-** | **-** | **-** | **-** | **-** | **-** | **-** | **-** | **A** | **A** | **A** | **C** | **C** | **C** | **C** | **G** | **A** | **G** | **A** | **T** | **G** | **T** | **A** | **G** | **C** | **A** | **G** | **C** | **T** | **A** | **T** | **T** | **T** | **G** | **G** | **G** | **C** | **A** | **T** | **G** | **G** | **C** | **T** | **C** | **T** | **T** | **A** | **G** | **T** | **A** | **T** | **T** | **G** | **T** | **T** | **T** | **G** | **C** | **C** | **C** | **T** | **T** | **C** | **C** | **T** | **G** | **G** | **G** | **G** | **T** | **G** | **G** | **G** | **T** | **T** | **T** | **T** | **G** | **T** | **A** | **T** | **T** | **A** | **T** | **A** | **T** | **T** | **T** | **A** | **T** | **T** | **T** | **T** | **T** | **C** | **G** | **G** | **A** | **C** | **G** | **T** | **G** | **G** | **A** | **T** | **T** | **A** | **A** | **C** | **G** | **G** | **A** | **T** | **A** | **A** | **G** | **A** | **A** | **A** | **A** | **A** | **A** | **T** | **T** | **C** | **T** | **A** | **T** | **C** | **T** | **C** | **C** | **G** | **A** | **C** | **A** | **A** | **A** | **G** | **T** | **G** | **A** | **C** | **T** | **T** | **G** | **A** | **A** | **A** | **G** | **A** | **A** | **T** | **T** | **A** | **G** | **A** | **A** | **A** | **A** | **C** | **T** | **T** | **T** | **C** | **A** | **G** | **T** | **C** | **T** | **T** | **T** | **T** | **A** | **G** | **A** | **G** | **G** | **A** | **G** | **A** | **T** | **A** | **C** | **C** | **A** | **T** | **T** | **G** | **A** | **A** | **C** | **A** | **T** | **T** | **A** | **C** | **G** | **A** | **T** | **C** | **C** | **T** | **G** | **A** | **C** | **A** | **T** | **G** | **G** | **G** | **C** | **G** | **A** | **C** | **A** | **A** | **A** | **G** | **A** | **C** | **A** | **A** | **A** | **C** | **A** | **A** | **C** | **A** | **A** | **T** | **T** | **T** | **G** | **T** | **C** | **G** | **A** | **C** | **T** | **T** | **C** | **T** | **T** | **C** | **T** | **C** | **T** | **T** | **C** | **A** | **T** | **T** | **A** | **A** | **A** | **T** | **C** | **G** | **T** | **A** | **T** | **G** | **C** | **C** | **G** | **C** | **T** | **G** | **A** | **C** | **A** | **A** | **G** | **A** | **A** | **T** | **G** | **A** | **A** | **T** | **T** | **C** | **T** | **G** | **T** | **C** | **A** | **C** | **T** | **C** | **T** | **T** | **C** | **T** | **C** | **A** | **C** | **A** | **G** | **A** | **C** | **G** | **G** | **A** | **C** | **A** | **A** | **A** | **A** | **G** | **A** | **A** | **A** | **T** | **T** | **A** | **G** | **A** | **T** | **T** | **C** | **A** | **C** | **T** | **G** | **C** | **T** | **T** | **C** | **A** | **A** | **G** | **A** | **T** | **C** | **T** | **A** | **A** | **A** | **A** | **A** | **A** | **A** | **G** | **C** | **C** | **A** | **A** | **A** | **C** | **A** | **T** | **T** | **C** | **G** | **A** | **T** | **C** | **C** | **A** | **T** | **A** | **T** | **C** | **G** | **A** | **A** | **T** | **A** | **T** | **T** | **A** | **C** | **G** | **C** | **A** | **T** | **T** | **T** | **G** | **T** | **G** | **A** | **C** | **A** | **G** | **A** | **T** | **A** | **A** | **T** | **A** | **T** | **C** | **G** | **G** | **T** | **C** | **A** | **G** | **C** | **A** | **A** | **G** | **T** | **C** | **T** | **T** | **A** | **C** | **G** | **C** | **G** | **T** | **T** | **T** | **T** | **A** | **G** | **A** | **A** | **G** | **A** | **A** | **A** | **A** | **A** | **G** | **C** | **C** | **G** | **C** | **A** | **G** | **A** | **A** | **G** | **G** | **C** | **G** | **T** | **A** | **G** | **A** | **A** | **G** | **T** | **T** | **C** | **G** | **T** | **A** | **T** | **A** | **T** | **T** | **A** | **T** | **A** | **T** | **G** | **A** | **T** | **G** | **C** | **A** | **T** | **T** | **T** | **G** | **G** | **C** | **T** | **C** | **C** | **C** | **A** | **T** | **G** | **G** | **C** | **A** | **C** | **A** | **A** | **A** | **A** | **G** | **C** | **A** | **A** | **A** | **A** | **G** | **A** | **T** | **T** | **T** | **C** | **A** | **A** | **T** | **C** | **G** | **T** | **C** | **T** | **A** | **A** | **T** | **C** | **A** | **A** | **A** | **A** | **A** | **T** | **G** | **G** | **T** | **G** | **G** | **A** | **C** | **A** | **T** | **G** | **T** | **C** | **C** | **A** | **T** | **A** | **C** | **A** | **T** | **T** | **T** | **G** | **T** | **T** | **A** | **C** | **C** | **T** | **C** | **G** | **C** | **A** | **A** | **A** | **A** | **A** | **G** | **C** | **A** | **T** | **T** | **G** | **C** | **T** | **T** | **C** | **G** | **T** | **T** | **T** | **C** | **C** | **G** | **A** | **T** | **T** | **G** | **A** | **A** | **T** | **T** | **A** | **C** | **C** | **A** | **T** | **G** | **A** | **T** | **C** | **A** | **C** | **C** | **G** | **A** | **A** | **A** | **A** | **A** | **T** | **C** | **G** | **T** | **T** | **G** | **T** | **G** | **A** | **T** | **C** | **G** | **A** | **T** | **G** | **G** | **A** | **A** | **A** | **G** | **A** | **T** | **C** | **A** | **G** | **T** | **T** | **A** | **C** | **A** | **C** | **C** | **G** | **G** | **T** | **G** | **G** | **T** | **T** | **T** | **C** | **A** | **A** | **T** | **A** | **T** | **T** | **G** | **C** | **C** | **A** | **A** | **T** | **C** | **A** | **A** | **T** | **A** | **T** | **G** | **T** | **A** | **A** | **A** | **T** | **A** | **C** | **G** | **A** | **C** | **A** | **A** | **A** | **A** | **A** | **A** | **A** | **T** | **T** | **C** | **G** | **G** | **T** | **T** | **A** | **T** | **T** | **G** | **G** | **C** | **G** | **T** | **G** | **A** | **T** | **A** | **C** | **G** | **C** | **A** | **T** | **A** | **T** | **A** | **C** | **G** | **A** | **A** | **T** | **T** | **T** | **T** | **C** | **G** | **G** | **T** | **G** | **C** | **C** | **G** | **C** | **T** | **T** | **C** | **T** | **T** | **C** | **T** | **T** | **T** | **G** | **C** | **T** | **T** | **C** | **A** | **A** | **C** | **T** | **T** | **C** | **G** | **T** | **T** | **T** | **T** | **T** | **T** | **A** | **A** | **C** | **A** | **G** | **A** | **C** | **T** | **G** | **G** | **A** | **A** | **C** | **G** | **T** | **T** | **T** | **C** | **A** | **G** | **T** | **G** | **C** | **C** | **T** | **G** | **A** | **A** | **G** | **A** | **A** | **A** | **A** | **A** | **A** | **A** | **G** | **G** | **T** | **C** | **G** | **G** | **C** | **T** | **A** | **T** | **C** | **A** | **T** | **T** | **T** | **G** | **A** | **A** | **T** | **T** | **A** | **T** | **T** | **T** | **C** | **T** | **T** | **T** | **A** | **A** | **A** | **A** | **A** | **A** | **G** | **C** | **C** | **G** | **A** | **T** | **C** | **G** | **A** | **G** | **A** | **T** | **G** | **A** | **A** | **T** | **C** | **C** | **A** | **A** | **A** | **C** | **T** | **T** | **G** | **C** | **T** | **G** | **A** | **T** | **A** | **C** | **G** | **T** | **C** | **C** | **A** | **T** | **C** | **C** | **A** | **G** | **C** | **T** | **T** | **G** | **T** | **C** | **T** | **C** | **A** | **A** | **G** | **C** | **G** | **G** | **A** | **C** | **C** | **A** | **A** | **A** | **T** | **A** | **A** | **C** | **G** | **A** | **A** | **C** | **G** | **A** | **G** | **A** | **A** | **C** | **A** | **A** | **A** | **T** | **C** | **A** | **A** | **G** | **C** | **T** | **T** | **T** | **C** | **G** | **T** | **T** | **T** | **A** | **T** | **C** | **A** | **A** | **A** | **T** | **T** | **G** | **A** | **T** | **T** | **A** | **C** | **T** | **T** | **C** | **T** | **G** | **C** | **T** | **A** | **A** | **A** | **A** | **A** | **A** | **C** | **G** | **T** | **G** | **T** | **T** | **T** | **G** | **G** | **A** | **T** | **A** | **C** | **A** | **G** | **A** | **C** | **A** | **C** | **C** | **T** | **T** | **A** | **C** | **C** | **T** | **C** | **G** | **T** | **T** | **C** | **C** | **T** | **G** | **A** | **T** | **G** | **A** | **A** | **A** | **G** | **T** | **G** | **T** | **C** | **A** | **T** | **T** | **G** | **C** | **T** | **G** | **C** | **T** | **T** | **T** | **G** | **A** | **A** | **A** | **A** | **T** | **C** | **G** | **C** | **A** | **A** | **C** | **T** | **G** | **C** | **T** | **T** | **C** | **T** | **G** | **G** | **T** | **G** | **T** | **C** | **G** | **A** | **T** | **G** | **T** | **G** | **A** | **A** | **A** | **A** | **T** | **C** | **A** | **T** | **G** | **A** | **T** | **C** | **C** | **C** | **C** | **A** | **A** | **T** | **A** | **A** | **A** | **C** | **C** | **G** | **G** | **A** | **T** | **C** | **A** | **T** | **C** | **C** | **T** | **T** | **T** | **T** | **A** | **T** | **T** | **T** | **A** | **T** | **C** | **G** | **A** | **G** | **C** | **A** | **A** | **C** | **A** | **C** | **A** | **A** | **T** | **A** | **T** | **T** | **A** | **T** | **G** | **C** | **T** | **C** | **G** | **T** | **C** | **A** | **G** | **C** | **T** | **C** | **A** | **T** | **C** | **A** | **A** | **G** | **G** | **A** | **A** | **A** | **A** | **T** | **G** | **T** | **A** | **C** | **A** | **G** | **A** | **T** | **C** | **C** | **T** | **T** | **G** | **T** | **C** | **T** | **A** | **T** | **G** | **A** | **G** | **A** | **A** | **T** | **G** | **G** | **C** | **T** | **T** | **C** | **C** | **T** | **C** | **C** | **A** | **T** | **G** | **C** | **A** | **A** | **A** | **A** | **A** | **C** | **A** | **T** | **T** | **A** | **A** | **T** | **A** | **A** | **T** | **G** | **G** | **A** | **T** | **G** | **A** | **T** | **G** | **A** | **A** | **A** | **T** | **C** | **T** | **G** | **C** | **A** | **T** | **G** | **G** | **T** | **A** | **G** | **G** | **T** | **T** | **C** | **A** | **G** | **C** | **A** | **A** | **A** | **T** | **C** | **A** | **A** | **G** | **A** | **T** | **A** | **T** | **T** | **C** | **G** | **T** | **A** | **G** | **C** | **T** | **A** | **C** | **C** | **G** | **A** | **T** | **T** | **G** | **A** | **A** | **T** | **T** | **T** | **T** | **G** | **A** | **A** | **A** | **C** | **A** | **A** | **G** | **T** | **G** | **C** | **T** | **G** | **T** | **C** | **A** | **T** | **T** | **T** | **A** | **C** | **G** | **A** | **T** | **C** | **C** | **T** | **G** | **A** | **G** | **T** | **T** | **T** | **T** | **T** | **A** | **G** | **A** | **A** | **G** | **A** | **G** | **C** | **T** | **T** | **G** | **C** | **T** | **A** | **C** | **T** | **C** | **A** | **G** | **T** | **T** | **C** | **A** | **A** | **A** | **G** | **A** | **A** | **G** | **A** | **T** | **G** | **A** | **G** | **A** | **C** | **A** | **C** | **A** | **T** | **T** | **G** | **T** | **T** | **C** | **A** | **C** | **C** | **A** | **A** | **T** | **G** | **A** | **C** | **A** | **A** | **C** | **T** | **G** | **A** | **A** | **A** | **C** | **A** | **G** | **T** | **G** | **A** | **A** | **G** | **G** | **A** | **A** | **A** | **T** | **G** | **T** | **C** | **T** | **A** | **A** | **C** | **T** | **G** | **G** | **C** | **T** | **G** | **T** | **T** | **A** | **T** | **T** | **C** | **A** | **A** | **A** | **C** | **A** | **A** | **C** | **A** | **A** | **A** | **T** | **T** | **T** | **C** | **T** | **C** | **G** | **A** | **T** | **T** | **G** | **T** | **T** | **T** | **T** | **C** | **T** | **C** | **C** | **A** | **A** | **T** | **C** | **T** | **T** | **A** | **-** | **-** | **-** |
| **N60200F** | **-** | **-** | **-** | **-** | **-** | **-** | **-** | **-** | **-** | **-** | **-** | **-** | **-** | **-** | **-** | **-** | **-** | **-** | **-** | **-** | **-** | **-** | **-** | **-** | **-** | **-** | **-** | **-** | **-** | **-** | **-** | **-** | **-** | **-** | **-** | **-** | **-** | **-** | **-** | **-** | **-** | **-** | **-** | **-** | **-** | **-** | **-** | **-** | **-** | **-** | **-** | **-** | **-** | **-** | **-** | **-** | **-** | **-** | **-** | **-** | **-** | **-** | **-** | **-** | **-** | **-** | **-** | **-** | **-** | **A** | **A** | **A** | **C** | **C** | **C** | **C** | **G** | **A** | **G** | **A** | **T** | **G** | **T** | **A** | **G** | **C** | **A** | **G** | **C** | **C** | **A** | **T** | **T** | **T** | **G** | **G** | **G** | **C** | **A** | **T** | **G** | **G** | **C** | **T** | **C** | **T** | **T** | **A** | **G** | **T** | **A** | **T** | **T** | **G** | **T** | **T** | **T** | **G** | **C** | **C** | **C** | **T** | **T** | **C** | **C** | **T** | **G** | **G** | **G** | **G** | **T** | **G** | **G** | **G** | **C** | **T** | **T** | **T** | **G** | **T** | **A** | **T** | **T** | **A** | **T** | **A** | **T** | **T** | **T** | **A** | **T** | **T** | **T** | **T** | **T** | **C** | **G** | **G** | **A** | **C** | **G** | **T** | **G** | **G** | **A** | **T** | **T** | **A** | **A** | **C** | **G** | **G** | **A** | **T** | **A** | **A** | **G** | **A** | **A** | **A** | **A** | **A** | **A** | **T** | **T** | **C** | **T** | **A** | **T** | **C** | **T** | **C** | **C** | **G** | **A** | **C** | **A** | **A** | **A** | **G** | **T** | **G** | **A** | **C** | **T** | **T** | **G** | **A** | **A** | **A** | **G** | **A** | **A** | **T** | **T** | **A** | **G** | **A** | **A** | **A** | **A** | **C** | **T** | **T** | **T** | **C** | **A** | **G** | **T** | **C** | **T** | **T** | **T** | **T** | **A** | **G** | **A** | **G** | **G** | **A** | **G** | **A** | **T** | **A** | **A** | **C** | **A** | **T** | **T** | **G** | **A** | **A** | **C** | **A** | **T** | **T** | **A** | **C** | **G** | **A** | **T** | **C** | **C** | **T** | **G** | **A** | **C** | **A** | **T** | **G** | **G** | **G** | **C** | **G** | **A** | **T** | **A** | **A** | **A** | **G** | **A** | **C** | **A** | **A** | **A** | **C** | **A** | **G** | **C** | **A** | **A** | **T** | **T** | **T** | **G** | **T** | **T** | **G** | **A** | **C** | **T** | **T** | **C** | **T** | **T** | **C** | **T** | **C** | **T** | **T** | **C** | **A** | **T** | **T** | **A** | **A** | **A** | **T** | **C** | **G** | **T** | **A** | **T** | **G** | **C** | **C** | **G** | **C** | **T** | **G** | **A** | **C** | **A** | **A** | **G** | **A** | **A** | **T** | **G** | **A** | **A** | **T** | **T** | **C** | **T** | **G** | **T** | **C** | **A** | **C** | **T** | **C** | **T** | **T** | **C** | **T** | **C** | **A** | **C** | **A** | **G** | **A** | **C** | **G** | **G** | **A** | **C** | **A** | **A** | **G** | **A** | **G** | **A** | **A** | **A** | **T** | **T** | **G** | **G** | **A** | **T** | **T** | **C** | **A** | **C** | **T** | **G** | **C** | **T** | **T** | **C** | **A** | **A** | **G** | **A** | **T** | **C** | **T** | **A** | **A** | **A** | **A** | **A** | **A** | **A** | **G** | **C** | **C** | **A** | **A** | **A** | **C** | **A** | **T** | **T** | **C** | **G** | **A** | **T** | **C** | **C** | **A** | **T** | **A** | **T** | **C** | **G** | **A** | **A** | **T** | **A** | **T** | **T** | **A** | **C** | **G** | **C** | **A** | **T** | **T** | **T** | **G** | **T** | **G** | **A** | **C** | **A** | **G** | **A** | **T** | **A** | **A** | **T** | **A** | **T** | **C** | **G** | **G** | **C** | **C** | **A** | **G** | **C** | **A** | **A** | **G** | **T** | **C** | **T** | **T** | **A** | **C** | **A** | **T** | **G** | **T** | **T** | **T** | **T** | **A** | **G** | **A** | **A** | **G** | **A** | **A** | **A** | **A** | **A** | **G** | **C** | **C** | **G** | **C** | **A** | **G** | **A** | **A** | **G** | **G** | **C** | **G** | **T** | **G** | **G** | **A** | **A** | **G** | **T** | **T** | **C** | **G** | **A** | **A** | **T** | **A** | **T** | **T** | **A** | **T** | **A** | **T** | **G** | **A** | **T** | **G** | **C** | **A** | **T** | **T** | **T** | **G** | **G** | **C** | **T** | **C** | **T** | **C** | **A** | **T** | **G** | **G** | **C** | **A** | **C** | **A** | **A** | **A** | **A** | **G** | **C** | **A** | **A** | **A** | **A** | **G** | **A** | **T** | **T** | **T** | **C** | **A** | **A** | **T** | **C** | **G** | **T** | **C** | **T** | **A** | **A** | **T** | **C** | **A** | **A** | **A** | **A** | **A** | **T** | **G** | **G** | **T** | **G** | **G** | **A** | **C** | **A** | **T** | **G** | **T** | **C** | **C** | **A** | **T** | **A** | **C** | **A** | **T** | **T** | **T** | **G** | **T** | **T** | **A** | **C** | **C** | **T** | **C** | **A** | **C** | **A** | **A** | **A** | **A** | **G** | **G** | **C** | **A** | **T** | **T** | **A** | **C** | **T** | **T** | **C** | **G** | **T** | **T** | **T** | **C** | **C** | **G** | **A** | **T** | **T** | **G** | **A** | **A** | **T** | **T** | **A** | **C** | **C** | **A** | **T** | **G** | **A** | **T** | **C** | **A** | **C** | **C** | **G** | **A** | **A** | **A** | **A** | **A** | **T** | **C** | **G** | **T** | **T** | **G** | **T** | **G** | **A** | **T** | **C** | **G** | **A** | **T** | **G** | **G** | **A** | **A** | **A** | **G** | **A** | **T** | **T** | **A** | **G** | **T** | **T** | **A** | **T** | **A** | **C** | **C** | **G** | **G** | **T** | **G** | **G** | **T** | **T** | **T** | **C** | **A** | **A** | **T** | **A** | **T** | **T** | **G** | **C** | **C** | **A** | **A** | **T** | **C** | **A** | **A** | **T** | **A** | **T** | **G** | **T** | **A** | **A** | **A** | **T** | **A** | **C** | **A** | **A** | **C** | **A** | **A** | **A** | **A** | **A** | **A** | **A** | **T** | **T** | **C** | **G** | **G** | **C** | **T** | **A** | **T** | **T** | **G** | **G** | **C** | **G** | **C** | **G** | **A** | **T** | **A** | **C** | **G** | **C** | **A** | **T** | **A** | **T** | **A** | **C** | **G** | **G** | **A** | **T** | **T** | **T** | **T** | **C** | **G** | **G** | **T** | **G** | **C** | **C** | **G** | **C** | **T** | **T** | **C** | **T** | **T** | **C** | **T** | **T** | **T** | **G** | **C** | **T** | **C** | **C** | **A** | **G** | **C** | **T** | **T** | **C** | **G** | **C** | **T** | **T** | **C** | **T** | **T** | **A** | **A** | **C** | **A** | **G** | **A** | **C** | **T** | **G** | **G** | **A** | **A** | **C** | **G** | **T** | **C** | **T** | **C** | **G** | **G** | **T** | **A** | **C** | **C** | **T** | **G** | **A** | **A** | **G** | **A** | **A** | **A** | **A** | **A** | **A** | **A** | **G** | **G** | **T** | **C** | **G** | **G** | **C** | **T** | **A** | **T** | **C** | **A** | **T** | **T** | **T** | **G** | **A** | **A** | **T** | **T** | **A** | **T** | **T** | **T** | **C** | **T** | **T** | **T** | **A** | **A** | **A** | **A** | **A** | **A** | **G** | **C** | **A** | **G** | **A** | **T** | **C** | **G** | **A** | **G** | **A** | **T** | **G** | **A** | **A** | **T** | **C** | **T** | **A** | **A** | **G** | **C** | **T** | **T** | **G** | **C** | **T** | **G** | **A** | **T** | **A** | **C** | **A** | **T** | **C** | **C** | **A** | **T** | **C** | **C** | **A** | **G** | **C** | **T** | **T** | **G** | **T** | **T** | **T** | **C** | **A** | **A** | **G** | **C** | **G** | **G** | **A** | **C** | **C** | **G** | **A** | **A** | **T** | **A** | **A** | **C** | **G** | **A** | **A** | **A** | **G** | **G** | **G** | **A** | **A** | **C** | **A** | **A** | **A** | **T** | **C** | **A** | **A** | **G** | **C** | **T** | **T** | **T** | **C** | **A** | **T** | **T** | **T** | **A** | **T** | **C** | **A** | **A** | **A** | **T** | **T** | **G** | **A** | **T** | **T** | **A** | **C** | **T** | **T** | **C** | **T** | **G** | **C** | **T** | **A** | **A** | **A** | **A** | **A** | **A** | **C** | **G** | **T** | **G** | **T** | **T** | **T** | **G** | **G** | **A** | **T** | **A** | **C** | **A** | **G** | **A** | **C** | **A** | **C** | **C** | **T** | **T** | **A** | **C** | **C** | **T** | **T** | **G** | **T** | **T** | **C** | **C** | **T** | **G** | **A** | **T** | **G** | **A** | **A** | **A** | **G** | **T** | **G** | **T** | **C** | **A** | **T** | **T** | **G** | **C** | **T** | **G** | **C** | **T** | **T** | **T** | **A** | **A** | **A** | **A** | **A** | **T** | **C** | **G** | **C** | **A** | **A** | **C** | **T** | **G** | **C** | **C** | **T** | **C** | **T** | **G** | **G** | **T** | **G** | **T** | **A** | **G** | **A** | **T** | **G** | **T** | **G** | **A** | **A** | **A** | **A** | **T** | **C** | **A** | **T** | **G** | **A** | **T** | **T** | **C** | **C** | **C** | **A** | **A** | **C** | **A** | **A** | **A** | **C** | **C** | **G** | **G** | **A** | **T** | **C** | **A** | **T** | **C** | **C** | **T** | **T** | **T** | **T** | **A** | **T** | **T** | **T** | **A** | **T** | **C** | **G** | **A** | **G** | **C** | **A** | **A** | **C** | **A** | **C** | **A** | **A** | **T** | **A** | **T** | **T** | **A** | **T** | **G** | **C** | **T** | **C** | **G** | **G** | **C** | **A** | **G** | **C** | **T** | **G** | **A** | **T** | **C** | **A** | **A** | **G** | **G** | **A** | **A** | **A** | **A** | **T** | **G** | **T** | **A** | **C** | **A** | **A** | **A** | **T** | **C** | **C** | **T** | **T** | **G** | **T** | **C** | **T** | **A** | **T** | **G** | **A** | **G** | **A** | **A** | **C** | **G** | **G** | **C** | **T** | **T** | **C** | **C** | **T** | **C** | **C** | **A** | **T** | **G** | **C** | **A** | **A** | **A** | **A** | **A** | **C** | **A** | **T** | **T** | **G** | **A** | **T** | **A** | **A** | **T** | **G** | **G** | **A** | **T** | **G** | **A** | **T** | **G** | **A** | **A** | **A** | **T** | **C** | **T** | **G** | **C** | **A** | **T** | **G** | **G** | **T** | **A** | **G** | **G** | **T** | **T** | **C** | **A** | **G** | **C** | **A** | **A** | **A** | **T** | **C** | **A** | **A** | **G** | **A** | **T** | **A** | **T** | **T** | **C** | **G** | **T** | **A** | **G** | **C** | **T** | **A** | **C** | **C** | **G** | **A** | **T** | **T** | **G** | **A** | **A** | **T** | **T** | **T** | **T** | **G** | **A** | **A** | **A** | **C** | **A** | **A** | **G** | **T** | **G** | **C** | **T** | **G** | **T** | **C** | **A** | **T** | **T** | **T** | **A** | **C** | **G** | **A** | **T** | **C** | **C** | **T** | **G** | **A** | **G** | **T** | **T** | **T** | **T** | **T** | **A** | **G** | **A** | **A** | **G** | **A** | **A** | **C** | **T** | **T** | **G** | **C** | **T** | **A** | **C** | **T** | **C** | **A** | **G** | **T** | **T** | **C** | **A** | **A** | **A** | **G** | **A** | **A** | **G** | **A** | **T** | **G** | **A** | **G** | **A** | **C** | **A** | **C** | **A** | **T** | **T** | **G** | **T** | **T** | **C** | **A** | **T** | **C** | **C** | **A** | **T** | **G** | **A** | **C** | **A** | **A** | **C** | **T** | **G** | **A** | **A** | **A** | **C** | **A** | **G** | **T** | **C** | **A** | **A** | **G** | **G** | **A** | **A** | **A** | **T** | **G** | **T** | **C** | **T** | **A** | **A** | **C** | **T** | **G** | **G** | **C** | **T** | **A** | **T** | **T** | **A** | **T** | **T** | **C** | **A** | **A** | **G** | **C** | **A** | **A** | **C** | **A** | **A** | **A** | **T** | **T** | **T** | **C** | **T** | **C** | **G** | **A** | **T** | **T** | **A** | **T** | **T** | **T** | **T** | **C** | **T** | **C** | **C** | **A** | **A** | **T** | **C** | **C** | **T** | **A** | **-** | **-** | **-** |
| **N53559** | **-** | **-** | **-** | **-** | **-** | **-** | **-** | **-** | **-** | **-** | **-** | **-** | **-** | **-** | **-** | **-** | **-** | **-** | **-** | **-** | **-** | **-** | **-** | **-** | **-** | **-** | **-** | **-** | **-** | **-** | **-** | **-** | **-** | **-** | **-** | **-** | **-** | **-** | **-** | **-** | **-** | **-** | **-** | **-** | **-** | **-** | **-** | **-** | **-** | **-** | **-** | **-** | **-** | **-** | **-** | **-** | **-** | **-** | **-** | **-** | **-** | **-** | **-** | **-** | **-** | **-** | **-** | **-** | **-** | **A** | **A** | **A** | **C** | **C** | **C** | **C** | **G** | **A** | **G** | **A** | **T** | **G** | **T** | **A** | **G** | **C** | **A** | **G** | **C** | **C** | **A** | **T** | **T** | **T** | **G** | **G** | **G** | **C** | **A** | **T** | **G** | **G** | **C** | **T** | **C** | **T** | **T** | **A** | **G** | **T** | **A** | **T** | **T** | **G** | **T** | **T** | **T** | **G** | **T** | **C** | **C** | **T** | **T** | **C** | **C** | **T** | **G** | **G** | **G** | **G** | **T** | **G** | **G** | **G** | **C** | **T** | **T** | **T** | **G** | **T** | **A** | **T** | **T** | **A** | **T** | **A** | **T** | **T** | **T** | **A** | **T** | **T** | **T** | **T** | **T** | **C** | **G** | **G** | **A** | **C** | **G** | **T** | **G** | **G** | **A** | **T** | **T** | **A** | **A** | **C** | **G** | **G** | **A** | **T** | **A** | **A** | **G** | **A** | **A** | **A** | **A** | **A** | **A** | **T** | **T** | **C** | **T** | **A** | **T** | **C** | **T** | **C** | **C** | **G** | **A** | **C** | **A** | **A** | **A** | **G** | **T** | **G** | **A** | **C** | **T** | **T** | **G** | **A** | **A** | **A** | **G** | **A** | **A** | **T** | **T** | **A** | **G** | **A** | **A** | **A** | **A** | **C** | **T** | **T** | **T** | **C** | **A** | **G** | **T** | **C** | **T** | **T** | **T** | **T** | **A** | **G** | **A** | **G** | **G** | **A** | **G** | **A** | **T** | **A** | **C** | **C** | **A** | **T** | **T** | **G** | **A** | **A** | **C** | **A** | **T** | **T** | **A** | **C** | **G** | **A** | **T** | **C** | **C** | **T** | **G** | **A** | **C** | **A** | **T** | **G** | **G** | **G** | **C** | **G** | **A** | **T** | **A** | **A** | **A** | **G** | **A** | **C** | **A** | **A** | **A** | **C** | **A** | **G** | **C** | **A** | **A** | **T** | **T** | **T** | **G** | **T** | **T** | **G** | **A** | **C** | **T** | **T** | **C** | **T** | **T** | **C** | **T** | **C** | **T** | **T** | **C** | **A** | **T** | **T** | **A** | **A** | **A** | **T** | **C** | **G** | **T** | **A** | **T** | **G** | **C** | **C** | **G** | **C** | **T** | **G** | **A** | **C** | **A** | **A** | **G** | **A** | **A** | **T** | **G** | **A** | **A** | **T** | **T** | **C** | **T** | **G** | **T** | **C** | **A** | **C** | **T** | **C** | **T** | **T** | **C** | **T** | **C** | **A** | **C** | **A** | **G** | **A** | **C** | **G** | **G** | **A** | **C** | **A** | **A** | **G** | **A** | **G** | **A** | **A** | **A** | **T** | **T** | **G** | **G** | **A** | **T** | **T** | **C** | **A** | **C** | **T** | **G** | **C** | **T** | **T** | **C** | **A** | **A** | **G** | **A** | **T** | **C** | **T** | **A** | **A** | **A** | **A** | **A** | **A** | **A** | **G** | **C** | **C** | **A** | **A** | **A** | **C** | **A** | **T** | **T** | **C** | **G** | **A** | **T** | **C** | **C** | **A** | **T** | **A** | **T** | **C** | **G** | **A** | **A** | **T** | **A** | **T** | **T** | **A** | **C** | **G** | **C** | **A** | **T** | **T** | **T** | **G** | **T** | **G** | **A** | **C** | **A** | **G** | **A** | **T** | **A** | **A** | **T** | **A** | **T** | **C** | **G** | **G** | **C** | **C** | **A** | **G** | **C** | **A** | **A** | **G** | **T** | **C** | **T** | **T** | **A** | **C** | **A** | **T** | **G** | **T** | **T** | **T** | **T** | **A** | **G** | **A** | **A** | **G** | **A** | **A** | **A** | **A** | **A** | **G** | **C** | **C** | **G** | **C** | **A** | **G** | **A** | **A** | **G** | **G** | **C** | **G** | **T** | **G** | **G** | **A** | **A** | **G** | **T** | **T** | **C** | **G** | **A** | **A** | **T** | **A** | **T** | **T** | **A** | **T** | **A** | **T** | **G** | **A** | **T** | **G** | **C** | **A** | **T** | **T** | **T** | **G** | **G** | **C** | **T** | **C** | **T** | **C** | **A** | **T** | **G** | **G** | **C** | **A** | **C** | **A** | **A** | **A** | **A** | **G** | **C** | **A** | **A** | **A** | **A** | **G** | **A** | **T** | **T** | **T** | **C** | **A** | **A** | **T** | **C** | **G** | **T** | **C** | **T** | **A** | **A** | **T** | **C** | **A** | **A** | **A** | **A** | **A** | **T** | **G** | **G** | **T** | **G** | **G** | **A** | **C** | **A** | **T** | **G** | **T** | **C** | **C** | **A** | **T** | **A** | **C** | **A** | **T** | **T** | **T** | **G** | **T** | **T** | **A** | **C** | **C** | **T** | **C** | **A** | **C** | **A** | **A** | **A** | **A** | **G** | **G** | **C** | **A** | **T** | **T** | **A** | **C** | **T** | **T** | **C** | **G** | **T** | **T** | **T** | **C** | **C** | **G** | **A** | **T** | **T** | **G** | **A** | **A** | **T** | **T** | **A** | **C** | **C** | **A** | **T** | **G** | **A** | **T** | **C** | **A** | **C** | **C** | **G** | **A** | **A** | **A** | **A** | **A** | **T** | **C** | **G** | **T** | **T** | **G** | **T** | **G** | **A** | **T** | **C** | **G** | **A** | **T** | **G** | **G** | **A** | **A** | **A** | **G** | **A** | **T** | **T** | **A** | **G** | **T** | **T** | **A** | **T** | **A** | **C** | **C** | **G** | **G** | **T** | **G** | **G** | **T** | **T** | **T** | **C** | **A** | **A** | **T** | **A** | **T** | **T** | **G** | **C** | **C** | **A** | **A** | **T** | **C** | **A** | **A** | **T** | **A** | **T** | **G** | **T** | **A** | **A** | **A** | **T** | **A** | **C** | **A** | **A** | **C** | **A** | **A** | **A** | **A** | **A** | **A** | **A** | **T** | **T** | **C** | **G** | **G** | **C** | **T** | **A** | **T** | **T** | **G** | **G** | **C** | **G** | **C** | **G** | **A** | **T** | **A** | **C** | **G** | **C** | **A** | **T** | **A** | **T** | **A** | **C** | **G** | **G** | **A** | **T** | **T** | **T** | **T** | **C** | **G** | **G** | **T** | **G** | **C** | **C** | **G** | **C** | **T** | **T** | **C** | **T** | **T** | **C** | **T** | **T** | **T** | **G** | **C** | **T** | **C** | **C** | **A** | **G** | **C** | **T** | **T** | **C** | **G** | **C** | **T** | **T** | **C** | **T** | **T** | **A** | **A** | **C** | **A** | **G** | **A** | **C** | **T** | **G** | **G** | **A** | **A** | **C** | **G** | **T** | **C** | **T** | **C** | **G** | **G** | **T** | **A** | **C** | **C** | **T** | **G** | **A** | **A** | **G** | **A** | **A** | **A** | **A** | **A** | **A** | **A** | **G** | **G** | **T** | **C** | **G** | **G** | **C** | **T** | **A** | **T** | **C** | **A** | **T** | **T** | **T** | **G** | **A** | **A** | **T** | **T** | **A** | **T** | **T** | **T** | **C** | **T** | **T** | **T** | **A** | **A** | **A** | **A** | **A** | **A** | **G** | **C** | **A** | **G** | **A** | **T** | **C** | **G** | **A** | **G** | **A** | **T** | **G** | **A** | **A** | **T** | **C** | **T** | **A** | **A** | **G** | **C** | **T** | **T** | **G** | **C** | **T** | **G** | **A** | **T** | **A** | **C** | **A** | **T** | **C** | **C** | **A** | **T** | **C** | **C** | **A** | **G** | **C** | **T** | **T** | **G** | **T** | **T** | **T** | **C** | **A** | **A** | **G** | **C** | **G** | **G** | **A** | **C** | **C** | **G** | **A** | **A** | **T** | **A** | **A** | **C** | **G** | **A** | **A** | **A** | **G** | **G** | **G** | **A** | **A** | **C** | **A** | **A** | **A** | **T** | **C** | **A** | **A** | **G** | **C** | **T** | **T** | **T** | **C** | **A** | **T** | **T** | **T** | **A** | **T** | **C** | **A** | **A** | **A** | **T** | **T** | **G** | **A** | **T** | **T** | **A** | **C** | **T** | **T** | **C** | **T** | **G** | **C** | **T** | **A** | **A** | **A** | **A** | **A** | **A** | **C** | **G** | **T** | **G** | **T** | **T** | **T** | **G** | **G** | **A** | **T** | **A** | **C** | **A** | **G** | **A** | **C** | **A** | **C** | **C** | **T** | **T** | **A** | **C** | **C** | **T** | **T** | **G** | **T** | **T** | **C** | **C** | **T** | **G** | **A** | **T** | **G** | **A** | **A** | **A** | **G** | **T** | **G** | **T** | **C** | **A** | **T** | **T** | **G** | **C** | **T** | **G** | **C** | **T** | **T** | **T** | **A** | **A** | **A** | **A** | **A** | **T** | **C** | **G** | **C** | **A** | **A** | **C** | **T** | **G** | **C** | **C** | **T** | **C** | **T** | **G** | **G** | **T** | **G** | **T** | **A** | **G** | **A** | **T** | **G** | **T** | **G** | **A** | **A** | **A** | **A** | **T** | **C** | **A** | **T** | **G** | **A** | **T** | **T** | **C** | **C** | **C** | **A** | **A** | **C** | **A** | **A** | **A** | **C** | **C** | **G** | **G** | **A** | **T** | **C** | **A** | **T** | **C** | **C** | **T** | **T** | **T** | **T** | **A** | **T** | **T** | **T** | **A** | **T** | **C** | **G** | **A** | **G** | **C** | **A** | **A** | **C** | **A** | **C** | **A** | **A** | **T** | **A** | **T** | **T** | **A** | **T** | **G** | **C** | **T** | **C** | **G** | **G** | **C** | **A** | **G** | **C** | **T** | **G** | **A** | **T** | **C** | **A** | **A** | **G** | **G** | **A** | **A** | **A** | **A** | **T** | **G** | **T** | **A** | **C** | **A** | **A** | **A** | **T** | **C** | **C** | **T** | **T** | **G** | **T** | **C** | **T** | **A** | **T** | **G** | **A** | **G** | **A** | **A** | **C** | **G** | **G** | **C** | **T** | **T** | **C** | **C** | **T** | **C** | **C** | **A** | **T** | **G** | **C** | **A** | **A** | **A** | **A** | **A** | **C** | **A** | **T** | **T** | **G** | **A** | **T** | **A** | **A** | **T** | **G** | **G** | **A** | **T** | **G** | **A** | **T** | **G** | **A** | **A** | **A** | **T** | **C** | **T** | **G** | **C** | **A** | **T** | **G** | **G** | **T** | **A** | **G** | **G** | **T** | **T** | **C** | **A** | **G** | **C** | **A** | **A** | **A** | **T** | **C** | **A** | **A** | **G** | **A** | **T** | **A** | **T** | **T** | **C** | **G** | **T** | **A** | **G** | **C** | **T** | **A** | **C** | **C** | **G** | **A** | **T** | **T** | **G** | **A** | **A** | **T** | **T** | **T** | **T** | **G** | **A** | **A** | **A** | **C** | **A** | **A** | **G** | **T** | **G** | **C** | **T** | **G** | **T** | **C** | **A** | **T** | **T** | **T** | **A** | **C** | **G** | **A** | **T** | **C** | **C** | **T** | **G** | **A** | **G** | **T** | **T** | **T** | **T** | **T** | **A** | **G** | **A** | **A** | **G** | **A** | **A** | **C** | **T** | **T** | **G** | **C** | **T** | **A** | **C** | **T** | **C** | **A** | **G** | **T** | **T** | **C** | **A** | **A** | **A** | **G** | **A** | **A** | **G** | **A** | **T** | **G** | **A** | **G** | **A** | **C** | **A** | **C** | **A** | **T** | **T** | **G** | **T** | **T** | **C** | **A** | **T** | **C** | **C** | **A** | **T** | **G** | **A** | **C** | **A** | **A** | **C** | **T** | **G** | **A** | **A** | **A** | **C** | **A** | **G** | **T** | **C** | **A** | **A** | **G** | **G** | **A** | **A** | **A** | **T** | **G** | **T** | **C** | **T** | **A** | **A** | **C** | **T** | **G** | **G** | **C** | **T** | **A** | **T** | **T** | **A** | **T** | **T** | **C** | **A** | **A** | **G** | **C** | **A** | **A** | **C** | **A** | **A** | **A** | **T** | **T** | **T** | **C** | **T** | **C** | **G** | **A** | **T** | **T** | **A** | **T** | **T** | **T** | **T** | **C** | **T** | **C** | **C** | **A** | **A** | **T** | **C** | **C** | **T** | **A** | **-** | **-** | **-** |
| **N52643** | **-** | **-** | **-** | **-** | **-** | **-** | **-** | **-** | **-** | **-** | **-** | **-** | **-** | **-** | **-** | **-** | **-** | **-** | **-** | **-** | **-** | **-** | **-** | **-** | **-** | **-** | **-** | **-** | **-** | **-** | **-** | **-** | **-** | **-** | **-** | **-** | **-** | **-** | **-** | **-** | **-** | **-** | **-** | **-** | **-** | **-** | **-** | **-** | **-** | **-** | **-** | **-** | **-** | **-** | **-** | **-** | **-** | **-** | **-** | **-** | **-** | **-** | **-** | **-** | **-** | **-** | **-** | **-** | **-** | **A** | **A** | **A** | **C** | **C** | **C** | **C** | **G** | **A** | **G** | **A** | **T** | **G** | **T** | **A** | **G** | **C** | **A** | **G** | **C** | **C** | **A** | **T** | **T** | **T** | **G** | **G** | **G** | **C** | **A** | **T** | **G** | **G** | **C** | **T** | **C** | **T** | **T** | **A** | **G** | **T** | **A** | **T** | **T** | **G** | **T** | **T** | **T** | **G** | **C** | **C** | **C** | **T** | **T** | **C** | **C** | **T** | **G** | **G** | **G** | **G** | **T** | **G** | **G** | **G** | **T** | **T** | **T** | **T** | **G** | **T** | **A** | **T** | **T** | **A** | **T** | **A** | **T** | **T** | **T** | **A** | **T** | **T** | **T** | **T** | **T** | **C** | **G** | **G** | **A** | **C** | **G** | **T** | **G** | **G** | **A** | **T** | **T** | **A** | **A** | **C** | **G** | **G** | **A** | **T** | **A** | **A** | **G** | **A** | **A** | **A** | **A** | **A** | **A** | **T** | **T** | **C** | **T** | **A** | **T** | **C** | **T** | **C** | **C** | **G** | **A** | **C** | **A** | **A** | **A** | **G** | **T** | **G** | **A** | **C** | **T** | **T** | **G** | **A** | **A** | **A** | **G** | **A** | **A** | **T** | **T** | **A** | **G** | **A** | **A** | **A** | **A** | **C** | **T** | **T** | **T** | **C** | **A** | **G** | **T** | **C** | **T** | **T** | **T** | **T** | **A** | **G** | **A** | **G** | **G** | **A** | **G** | **A** | **T** | **A** | **C** | **C** | **A** | **T** | **T** | **G** | **A** | **A** | **C** | **A** | **T** | **T** | **A** | **C** | **G** | **A** | **T** | **C** | **C** | **T** | **G** | **A** | **C** | **A** | **T** | **G** | **G** | **G** | **C** | **G** | **A** | **T** | **A** | **A** | **A** | **G** | **A** | **C** | **A** | **A** | **A** | **C** | **A** | **G** | **C** | **A** | **A** | **T** | **T** | **T** | **G** | **T** | **C** | **G** | **A** | **C** | **T** | **T** | **C** | **T** | **T** | **C** | **T** | **C** | **T** | **T** | **C** | **A** | **T** | **T** | **A** | **A** | **A** | **T** | **C** | **G** | **T** | **A** | **T** | **G** | **C** | **C** | **G** | **C** | **T** | **G** | **A** | **C** | **A** | **A** | **G** | **A** | **A** | **T** | **G** | **A** | **A** | **T** | **T** | **C** | **T** | **G** | **T** | **C** | **A** | **C** | **T** | **C** | **T** | **T** | **C** | **T** | **C** | **A** | **C** | **A** | **G** | **A** | **C** | **G** | **G** | **A** | **C** | **A** | **A** | **G** | **A** | **G** | **A** | **A** | **A** | **T** | **T** | **G** | **G** | **A** | **T** | **T** | **C** | **A** | **C** | **T** | **G** | **C** | **T** | **T** | **C** | **A** | **A** | **G** | **A** | **T** | **C** | **T** | **A** | **A** | **A** | **A** | **A** | **A** | **A** | **G** | **C** | **C** | **A** | **A** | **A** | **C** | **A** | **T** | **T** | **C** | **G** | **A** | **T** | **C** | **C** | **A** | **T** | **A** | **T** | **C** | **G** | **A** | **A** | **T** | **A** | **T** | **T** | **A** | **C** | **G** | **C** | **A** | **T** | **T** | **T** | **G** | **T** | **G** | **A** | **C** | **A** | **G** | **A** | **T** | **A** | **A** | **T** | **A** | **T** | **C** | **G** | **G** | **C** | **C** | **A** | **G** | **C** | **A** | **A** | **G** | **T** | **C** | **T** | **T** | **A** | **C** | **A** | **T** | **G** | **T** | **T** | **T** | **T** | **A** | **G** | **A** | **A** | **G** | **A** | **A** | **A** | **A** | **A** | **G** | **C** | **C** | **G** | **C** | **A** | **G** | **A** | **A** | **G** | **G** | **C** | **G** | **T** | **G** | **G** | **A** | **A** | **G** | **T** | **T** | **C** | **G** | **A** | **A** | **T** | **A** | **T** | **T** | **A** | **T** | **A** | **T** | **G** | **A** | **T** | **G** | **C** | **A** | **T** | **T** | **T** | **G** | **G** | **C** | **T** | **C** | **T** | **C** | **A** | **T** | **G** | **G** | **C** | **A** | **C** | **A** | **A** | **A** | **A** | **G** | **C** | **A** | **A** | **A** | **A** | **G** | **A** | **T** | **T** | **T** | **C** | **A** | **A** | **T** | **C** | **G** | **T** | **C** | **T** | **A** | **A** | **T** | **C** | **A** | **A** | **A** | **A** | **A** | **T** | **G** | **G** | **T** | **G** | **G** | **A** | **C** | **A** | **T** | **G** | **T** | **C** | **C** | **A** | **T** | **A** | **C** | **A** | **T** | **T** | **T** | **G** | **T** | **T** | **A** | **C** | **C** | **T** | **C** | **A** | **C** | **A** | **A** | **A** | **A** | **G** | **G** | **C** | **A** | **T** | **T** | **A** | **C** | **T** | **T** | **C** | **G** | **T** | **T** | **T** | **C** | **C** | **G** | **A** | **T** | **T** | **G** | **A** | **A** | **T** | **T** | **A** | **C** | **C** | **A** | **T** | **G** | **A** | **T** | **C** | **A** | **C** | **C** | **G** | **A** | **A** | **A** | **A** | **A** | **T** | **C** | **G** | **T** | **T** | **G** | **T** | **G** | **A** | **T** | **C** | **G** | **A** | **T** | **G** | **G** | **A** | **A** | **A** | **G** | **A** | **T** | **T** | **A** | **G** | **T** | **T** | **A** | **T** | **A** | **C** | **C** | **G** | **G** | **T** | **G** | **G** | **T** | **T** | **T** | **C** | **A** | **A** | **T** | **A** | **T** | **T** | **G** | **C** | **C** | **A** | **A** | **T** | **C** | **A** | **A** | **T** | **A** | **T** | **G** | **T** | **A** | **A** | **A** | **T** | **A** | **C** | **A** | **A** | **C** | **A** | **A** | **A** | **A** | **A** | **A** | **A** | **T** | **T** | **C** | **G** | **G** | **C** | **T** | **A** | **T** | **T** | **G** | **G** | **C** | **G** | **C** | **G** | **A** | **T** | **A** | **C** | **G** | **C** | **A** | **T** | **A** | **T** | **A** | **C** | **G** | **G** | **A** | **T** | **T** | **T** | **T** | **C** | **G** | **G** | **T** | **G** | **C** | **C** | **G** | **C** | **T** | **T** | **C** | **T** | **T** | **C** | **T** | **T** | **T** | **G** | **C** | **T** | **C** | **C** | **A** | **G** | **C** | **T** | **T** | **C** | **G** | **C** | **T** | **T** | **C** | **T** | **T** | **A** | **A** | **C** | **A** | **G** | **A** | **C** | **T** | **G** | **G** | **A** | **A** | **C** | **G** | **T** | **C** | **T** | **C** | **G** | **G** | **T** | **A** | **C** | **C** | **T** | **G** | **A** | **A** | **G** | **A** | **A** | **A** | **A** | **A** | **A** | **A** | **G** | **G** | **T** | **C** | **G** | **G** | **C** | **T** | **A** | **T** | **C** | **A** | **T** | **T** | **T** | **G** | **A** | **A** | **T** | **T** | **A** | **T** | **T** | **T** | **C** | **T** | **T** | **T** | **A** | **A** | **A** | **A** | **A** | **A** | **G** | **C** | **A** | **G** | **A** | **T** | **C** | **G** | **A** | **G** | **A** | **T** | **G** | **A** | **A** | **T** | **C** | **T** | **A** | **A** | **G** | **C** | **T** | **T** | **G** | **C** | **T** | **G** | **A** | **T** | **A** | **C** | **A** | **T** | **C** | **C** | **A** | **T** | **C** | **C** | **A** | **G** | **C** | **T** | **T** | **G** | **T** | **T** | **T** | **C** | **A** | **A** | **G** | **C** | **G** | **G** | **A** | **C** | **C** | **G** | **A** | **A** | **T** | **A** | **A** | **C** | **G** | **A** | **A** | **A** | **G** | **G** | **G** | **A** | **A** | **C** | **A** | **A** | **A** | **T** | **C** | **A** | **A** | **G** | **C** | **T** | **T** | **T** | **C** | **A** | **T** | **T** | **T** | **A** | **T** | **C** | **A** | **A** | **A** | **T** | **T** | **G** | **A** | **T** | **T** | **A** | **C** | **T** | **T** | **C** | **T** | **G** | **C** | **T** | **A** | **A** | **A** | **A** | **A** | **A** | **C** | **G** | **T** | **G** | **T** | **T** | **T** | **G** | **G** | **A** | **T** | **A** | **C** | **A** | **G** | **A** | **C** | **A** | **C** | **C** | **T** | **T** | **A** | **C** | **C** | **T** | **T** | **G** | **T** | **T** | **C** | **C** | **T** | **G** | **A** | **T** | **G** | **A** | **A** | **A** | **G** | **T** | **G** | **T** | **C** | **A** | **T** | **T** | **G** | **C** | **T** | **G** | **C** | **T** | **T** | **T** | **A** | **A** | **A** | **A** | **A** | **T** | **C** | **G** | **C** | **A** | **A** | **C** | **T** | **G** | **C** | **C** | **T** | **C** | **T** | **G** | **G** | **T** | **G** | **T** | **A** | **G** | **A** | **T** | **G** | **T** | **G** | **A** | **A** | **A** | **A** | **T** | **C** | **A** | **T** | **G** | **A** | **T** | **T** | **C** | **C** | **C** | **A** | **A** | **C** | **A** | **A** | **A** | **C** | **C** | **G** | **G** | **A** | **T** | **C** | **A** | **T** | **C** | **C** | **T** | **T** | **T** | **T** | **A** | **T** | **T** | **T** | **A** | **T** | **C** | **G** | **A** | **G** | **C** | **A** | **A** | **C** | **A** | **C** | **A** | **A** | **T** | **A** | **T** | **T** | **A** | **T** | **G** | **C** | **T** | **C** | **G** | **G** | **C** | **A** | **G** | **C** | **T** | **G** | **A** | **T** | **C** | **A** | **A** | **G** | **G** | **A** | **A** | **A** | **A** | **T** | **G** | **T** | **A** | **C** | **A** | **A** | **A** | **T** | **C** | **C** | **T** | **T** | **G** | **T** | **C** | **T** | **A** | **T** | **G** | **A** | **G** | **A** | **A** | **C** | **G** | **G** | **C** | **T** | **T** | **C** | **C** | **T** | **C** | **C** | **A** | **T** | **G** | **C** | **A** | **A** | **A** | **A** | **A** | **C** | **A** | **T** | **T** | **G** | **A** | **T** | **A** | **A** | **T** | **G** | **G** | **A** | **T** | **G** | **A** | **T** | **G** | **A** | **A** | **A** | **T** | **C** | **T** | **G** | **C** | **A** | **T** | **G** | **G** | **T** | **A** | **G** | **G** | **T** | **T** | **C** | **A** | **G** | **C** | **A** | **A** | **A** | **T** | **C** | **A** | **A** | **G** | **A** | **T** | **A** | **T** | **T** | **C** | **G** | **T** | **A** | **G** | **C** | **T** | **A** | **C** | **C** | **G** | **A** | **T** | **T** | **G** | **A** | **A** | **T** | **T** | **T** | **T** | **G** | **A** | **A** | **A** | **C** | **A** | **A** | **G** | **T** | **G** | **C** | **T** | **G** | **T** | **C** | **A** | **T** | **T** | **T** | **A** | **C** | **G** | **A** | **T** | **C** | **C** | **T** | **G** | **A** | **G** | **T** | **T** | **T** | **T** | **T** | **A** | **G** | **A** | **A** | **G** | **A** | **A** | **C** | **T** | **T** | **G** | **C** | **T** | **A** | **C** | **T** | **C** | **A** | **G** | **T** | **T** | **C** | **A** | **A** | **A** | **G** | **A** | **A** | **G** | **A** | **T** | **G** | **A** | **G** | **A** | **C** | **A** | **C** | **A** | **T** | **T** | **G** | **T** | **T** | **C** | **A** | **T** | **C** | **C** | **A** | **T** | **G** | **A** | **C** | **A** | **A** | **C** | **T** | **G** | **A** | **A** | **A** | **C** | **A** | **G** | **T** | **C** | **A** | **A** | **G** | **G** | **A** | **A** | **A** | **T** | **G** | **T** | **C** | **T** | **A** | **A** | **C** | **T** | **G** | **G** | **C** | **T** | **A** | **T** | **T** | **A** | **T** | **T** | **C** | **A** | **A** | **G** | **C** | **A** | **A** | **C** | **A** | **A** | **A** | **T** | **T** | **T** | **C** | **T** | **C** | **G** | **A** | **T** | **T** | **A** | **T** | **T** | **T** | **T** | **C** | **T** | **C** | **C** | **A** | **A** | **T** | **C** | **C** | **T** | **A** | **-** | **-** | **-** |
| **N59951F** | **-** | **-** | **-** | **-** | **-** | **-** | **-** | **-** | **-** | **-** | **-** | **-** | **-** | **-** | **-** | **-** | **-** | **-** | **-** | **-** | **-** | **-** | **-** | **-** | **-** | **-** | **-** | **-** | **-** | **-** | **-** | **-** | **-** | **-** | **-** | **-** | **-** | **-** | **-** | **-** | **-** | **-** | **-** | **-** | **-** | **-** | **-** | **-** | **-** | **-** | **-** | **-** | **-** | **-** | **-** | **-** | **-** | **-** | **-** | **-** | **-** | **-** | **-** | **-** | **-** | **-** | **-** | **-** | **-** | **A** | **A** | **A** | **C** | **C** | **C** | **C** | **G** | **A** | **G** | **A** | **T** | **G** | **T** | **A** | **G** | **C** | **A** | **G** | **C** | **C** | **A** | **T** | **T** | **T** | **G** | **G** | **G** | **C** | **A** | **T** | **G** | **G** | **C** | **T** | **C** | **T** | **T** | **A** | **G** | **T** | **A** | **T** | **T** | **G** | **T** | **T** | **T** | **G** | **C** | **C** | **C** | **T** | **T** | **C** | **C** | **T** | **G** | **G** | **G** | **G** | **T** | **G** | **G** | **G** | **T** | **T** | **T** | **T** | **G** | **T** | **A** | **T** | **T** | **A** | **T** | **A** | **T** | **T** | **T** | **A** | **T** | **T** | **T** | **T** | **T** | **C** | **G** | **G** | **A** | **C** | **G** | **T** | **G** | **G** | **A** | **T** | **T** | **A** | **A** | **C** | **G** | **G** | **A** | **T** | **A** | **A** | **G** | **A** | **A** | **A** | **A** | **A** | **A** | **T** | **T** | **C** | **T** | **A** | **T** | **C** | **T** | **C** | **C** | **G** | **A** | **C** | **A** | **A** | **A** | **G** | **T** | **G** | **A** | **C** | **T** | **T** | **G** | **A** | **A** | **A** | **G** | **A** | **A** | **T** | **T** | **A** | **G** | **A** | **A** | **A** | **A** | **C** | **T** | **T** | **T** | **C** | **A** | **G** | **T** | **C** | **T** | **T** | **T** | **T** | **A** | **G** | **A** | **G** | **G** | **A** | **G** | **A** | **T** | **A** | **C** | **C** | **A** | **T** | **T** | **G** | **A** | **A** | **C** | **A** | **T** | **T** | **A** | **C** | **G** | **A** | **T** | **C** | **C** | **T** | **G** | **A** | **C** | **A** | **T** | **G** | **G** | **G** | **C** | **G** | **A** | **T** | **A** | **A** | **A** | **G** | **A** | **C** | **A** | **A** | **A** | **C** | **A** | **G** | **C** | **A** | **A** | **T** | **T** | **T** | **G** | **T** | **T** | **G** | **A** | **C** | **T** | **T** | **C** | **T** | **T** | **C** | **T** | **C** | **T** | **T** | **C** | **A** | **T** | **T** | **A** | **A** | **A** | **T** | **C** | **G** | **T** | **A** | **T** | **G** | **C** | **C** | **G** | **C** | **T** | **G** | **A** | **C** | **A** | **A** | **G** | **A** | **A** | **T** | **G** | **A** | **A** | **T** | **T** | **C** | **T** | **G** | **T** | **C** | **A** | **C** | **T** | **C** | **T** | **T** | **C** | **T** | **C** | **A** | **C** | **A** | **G** | **A** | **C** | **G** | **G** | **A** | **C** | **A** | **A** | **G** | **A** | **G** | **A** | **A** | **A** | **T** | **T** | **G** | **G** | **A** | **T** | **T** | **C** | **A** | **C** | **T** | **G** | **C** | **T** | **T** | **C** | **A** | **A** | **G** | **A** | **T** | **C** | **T** | **A** | **A** | **A** | **A** | **A** | **A** | **A** | **G** | **C** | **C** | **A** | **A** | **A** | **C** | **A** | **T** | **T** | **C** | **G** | **A** | **T** | **C** | **C** | **A** | **T** | **A** | **T** | **C** | **G** | **A** | **A** | **T** | **A** | **T** | **T** | **A** | **C** | **G** | **C** | **A** | **T** | **T** | **T** | **G** | **T** | **G** | **A** | **C** | **A** | **G** | **A** | **T** | **A** | **A** | **T** | **A** | **T** | **C** | **G** | **G** | **C** | **C** | **A** | **G** | **C** | **A** | **A** | **G** | **T** | **C** | **T** | **T** | **A** | **C** | **A** | **T** | **G** | **T** | **T** | **T** | **T** | **A** | **G** | **A** | **A** | **G** | **A** | **A** | **A** | **A** | **A** | **G** | **C** | **C** | **G** | **C** | **A** | **G** | **A** | **A** | **G** | **G** | **C** | **G** | **T** | **G** | **G** | **A** | **A** | **G** | **T** | **T** | **C** | **G** | **A** | **A** | **T** | **A** | **T** | **T** | **A** | **T** | **A** | **T** | **G** | **A** | **T** | **G** | **C** | **A** | **T** | **T** | **T** | **G** | **G** | **C** | **T** | **C** | **T** | **C** | **A** | **T** | **G** | **G** | **C** | **A** | **C** | **A** | **A** | **A** | **A** | **G** | **C** | **A** | **A** | **A** | **A** | **G** | **A** | **T** | **T** | **T** | **C** | **A** | **A** | **T** | **C** | **G** | **T** | **C** | **T** | **A** | **A** | **T** | **C** | **A** | **A** | **A** | **A** | **A** | **T** | **G** | **G** | **T** | **G** | **G** | **A** | **C** | **A** | **T** | **G** | **T** | **C** | **C** | **A** | **T** | **A** | **C** | **A** | **T** | **T** | **T** | **G** | **T** | **T** | **A** | **C** | **C** | **T** | **C** | **A** | **C** | **A** | **A** | **A** | **A** | **G** | **G** | **C** | **A** | **T** | **T** | **A** | **C** | **T** | **T** | **C** | **G** | **T** | **T** | **T** | **C** | **C** | **G** | **A** | **T** | **T** | **G** | **A** | **A** | **T** | **T** | **A** | **C** | **C** | **A** | **T** | **G** | **A** | **T** | **C** | **A** | **C** | **C** | **G** | **A** | **A** | **A** | **A** | **A** | **T** | **C** | **G** | **T** | **T** | **G** | **T** | **G** | **A** | **T** | **C** | **G** | **A** | **T** | **G** | **G** | **A** | **A** | **A** | **G** | **A** | **T** | **T** | **A** | **G** | **T** | **T** | **A** | **T** | **A** | **C** | **C** | **G** | **G** | **T** | **G** | **G** | **T** | **T** | **T** | **C** | **A** | **A** | **T** | **A** | **T** | **T** | **G** | **C** | **C** | **A** | **A** | **T** | **C** | **A** | **A** | **T** | **A** | **T** | **G** | **T** | **A** | **A** | **A** | **T** | **A** | **C** | **A** | **A** | **C** | **A** | **A** | **A** | **A** | **A** | **A** | **A** | **T** | **T** | **C** | **G** | **G** | **C** | **T** | **A** | **T** | **T** | **G** | **G** | **C** | **G** | **C** | **G** | **A** | **T** | **A** | **C** | **G** | **C** | **A** | **T** | **A** | **T** | **A** | **C** | **G** | **G** | **A** | **T** | **T** | **T** | **T** | **C** | **G** | **G** | **T** | **G** | **C** | **C** | **G** | **C** | **T** | **T** | **C** | **T** | **T** | **C** | **T** | **T** | **T** | **G** | **C** | **T** | **C** | **C** | **A** | **G** | **C** | **T** | **T** | **C** | **G** | **C** | **T** | **T** | **C** | **T** | **T** | **A** | **A** | **C** | **A** | **G** | **A** | **C** | **T** | **G** | **G** | **A** | **A** | **C** | **G** | **T** | **C** | **T** | **C** | **G** | **G** | **T** | **A** | **C** | **C** | **T** | **G** | **A** | **A** | **G** | **A** | **A** | **A** | **A** | **A** | **A** | **A** | **G** | **G** | **T** | **C** | **G** | **G** | **C** | **T** | **A** | **T** | **C** | **A** | **T** | **T** | **T** | **G** | **A** | **A** | **T** | **T** | **A** | **T** | **T** | **T** | **C** | **T** | **T** | **T** | **A** | **A** | **A** | **A** | **A** | **A** | **G** | **C** | **A** | **G** | **A** | **T** | **C** | **G** | **A** | **G** | **A** | **T** | **G** | **A** | **A** | **T** | **C** | **T** | **A** | **A** | **G** | **C** | **T** | **T** | **G** | **C** | **T** | **G** | **A** | **A** | **A** | **C** | **A** | **T** | **C** | **C** | **A** | **T** | **C** | **C** | **A** | **G** | **C** | **T** | **T** | **G** | **T** | **T** | **T** | **C** | **A** | **A** | **G** | **C** | **G** | **G** | **A** | **C** | **C** | **G** | **A** | **A** | **T** | **A** | **A** | **C** | **G** | **A** | **A** | **A** | **G** | **G** | **G** | **A** | **A** | **C** | **A** | **A** | **A** | **T** | **C** | **A** | **A** | **G** | **C** | **T** | **T** | **T** | **C** | **A** | **T** | **T** | **T** | **A** | **T** | **C** | **A** | **A** | **A** | **T** | **T** | **G** | **A** | **T** | **T** | **A** | **C** | **T** | **T** | **C** | **T** | **G** | **C** | **T** | **A** | **A** | **A** | **A** | **A** | **G** | **C** | **G** | **T** | **G** | **T** | **T** | **T** | **G** | **G** | **A** | **T** | **A** | **C** | **A** | **G** | **A** | **C** | **A** | **C** | **C** | **T** | **T** | **A** | **C** | **C** | **T** | **T** | **G** | **T** | **T** | **C** | **C** | **T** | **G** | **A** | **T** | **G** | **A** | **A** | **A** | **G** | **T** | **G** | **T** | **C** | **A** | **T** | **T** | **G** | **C** | **T** | **G** | **C** | **T** | **T** | **T** | **A** | **A** | **A** | **A** | **A** | **T** | **C** | **G** | **C** | **A** | **A** | **C** | **T** | **G** | **C** | **C** | **T** | **C** | **T** | **G** | **G** | **T** | **G** | **T** | **A** | **G** | **A** | **T** | **G** | **T** | **G** | **A** | **A** | **A** | **A** | **T** | **C** | **A** | **T** | **G** | **A** | **T** | **T** | **C** | **C** | **C** | **A** | **A** | **C** | **A** | **A** | **A** | **C** | **C** | **G** | **G** | **A** | **T** | **C** | **A** | **T** | **C** | **C** | **T** | **T** | **T** | **T** | **A** | **T** | **T** | **T** | **A** | **T** | **C** | **G** | **A** | **G** | **C** | **A** | **A** | **C** | **A** | **C** | **A** | **A** | **T** | **A** | **T** | **T** | **A** | **T** | **G** | **C** | **T** | **C** | **G** | **G** | **C** | **A** | **G** | **C** | **T** | **G** | **A** | **T** | **C** | **A** | **A** | **G** | **G** | **A** | **A** | **A** | **A** | **T** | **G** | **T** | **A** | **C** | **A** | **A** | **A** | **T** | **C** | **C** | **T** | **T** | **G** | **T** | **C** | **T** | **A** | **T** | **G** | **A** | **G** | **A** | **A** | **C** | **G** | **G** | **C** | **T** | **T** | **C** | **C** | **T** | **C** | **C** | **A** | **T** | **G** | **C** | **A** | **A** | **A** | **A** | **A** | **C** | **A** | **T** | **T** | **G** | **A** | **T** | **A** | **A** | **T** | **G** | **G** | **A** | **T** | **G** | **A** | **T** | **G** | **A** | **A** | **A** | **T** | **C** | **T** | **G** | **C** | **A** | **T** | **G** | **G** | **T** | **A** | **G** | **G** | **T** | **T** | **C** | **A** | **G** | **C** | **A** | **A** | **A** | **T** | **C** | **A** | **A** | **G** | **A** | **T** | **A** | **T** | **T** | **C** | **G** | **T** | **A** | **G** | **C** | **T** | **A** | **C** | **A** | **G** | **A** | **T** | **T** | **G** | **A** | **A** | **T** | **T** | **T** | **T** | **G** | **A** | **A** | **A** | **C** | **A** | **A** | **G** | **T** | **G** | **C** | **T** | **G** | **T** | **C** | **A** | **T** | **T** | **T** | **A** | **C** | **G** | **A** | **T** | **C** | **C** | **T** | **G** | **A** | **G** | **T** | **T** | **T** | **T** | **T** | **A** | **G** | **A** | **A** | **G** | **A** | **A** | **C** | **T** | **T** | **G** | **C** | **T** | **A** | **C** | **T** | **C** | **A** | **G** | **T** | **T** | **C** | **A** | **A** | **A** | **G** | **A** | **A** | **G** | **A** | **T** | **G** | **A** | **G** | **A** | **C** | **A** | **C** | **A** | **T** | **T** | **G** | **T** | **T** | **C** | **A** | **T** | **C** | **C** | **A** | **T** | **G** | **A** | **C** | **A** | **A** | **C** | **T** | **G** | **A** | **A** | **A** | **C** | **A** | **G** | **T** | **T** | **A** | **A** | **A** | **G** | **A** | **A** | **A** | **T** | **G** | **T** | **C** | **T** | **A** | **A** | **C** | **T** | **G** | **G** | **C** | **T** | **A** | **T** | **T** | **A** | **T** | **T** | **C** | **A** | **A** | **G** | **C** | **A** | **A** | **C** | **A** | **A** | **A** | **T** | **T** | **T** | **C** | **T** | **C** | **G** | **A** | **T** | **T** | **A** | **T** | **T** | **T** | **T** | **C** | **T** | **C** | **C** | **A** | **A** | **T** | **C** | **C** | **T** | **A** | **-** | **-** | **-** |
| **N60190F** | **-** | **-** | **-** | **-** | **-** | **-** | **-** | **-** | **-** | **-** | **-** | **-** | **-** | **-** | **-** | **-** | **-** | **-** | **-** | **-** | **-** | **-** | **-** | **-** | **-** | **-** | **-** | **-** | **-** | **-** | **-** | **-** | **-** | **-** | **-** | **-** | **-** | **-** | **-** | **-** | **-** | **-** | **-** | **-** | **-** | **-** | **-** | **-** | **-** | **-** | **-** | **-** | **-** | **-** | **-** | **-** | **-** | **-** | **-** | **-** | **-** | **-** | **-** | **-** | **-** | **-** | **-** | **-** | **-** | **A** | **A** | **A** | **C** | **C** | **C** | **C** | **G** | **A** | **G** | **A** | **T** | **G** | **T** | **A** | **G** | **C** | **A** | **G** | **C** | **T** | **A** | **T** | **T** | **T** | **G** | **G** | **G** | **C** | **A** | **T** | **G** | **G** | **C** | **T** | **C** | **T** | **T** | **A** | **G** | **T** | **A** | **T** | **T** | **G** | **T** | **T** | **T** | **G** | **C** | **C** | **C** | **T** | **T** | **C** | **C** | **T** | **G** | **G** | **G** | **G** | **T** | **G** | **G** | **G** | **T** | **T** | **T** | **T** | **G** | **T** | **A** | **T** | **T** | **A** | **T** | **A** | **T** | **T** | **T** | **A** | **T** | **T** | **T** | **T** | **T** | **C** | **G** | **G** | **A** | **C** | **G** | **T** | **G** | **G** | **A** | **T** | **T** | **A** | **A** | **C** | **G** | **G** | **A** | **T** | **A** | **A** | **G** | **A** | **A** | **A** | **A** | **A** | **A** | **T** | **T** | **C** | **T** | **A** | **T** | **C** | **T** | **C** | **C** | **G** | **A** | **C** | **A** | **A** | **A** | **G** | **T** | **G** | **A** | **C** | **T** | **T** | **G** | **A** | **A** | **A** | **G** | **A** | **A** | **T** | **T** | **A** | **G** | **A** | **A** | **A** | **A** | **C** | **T** | **T** | **T** | **C** | **A** | **G** | **T** | **C** | **T** | **T** | **T** | **T** | **A** | **G** | **A** | **G** | **G** | **A** | **G** | **A** | **T** | **A** | **C** | **C** | **A** | **T** | **T** | **G** | **A** | **A** | **C** | **A** | **T** | **T** | **A** | **C** | **G** | **A** | **T** | **C** | **C** | **T** | **G** | **A** | **C** | **A** | **T** | **G** | **G** | **G** | **C** | **G** | **A** | **C** | **A** | **A** | **A** | **G** | **A** | **C** | **A** | **A** | **A** | **C** | **A** | **A** | **C** | **A** | **A** | **T** | **T** | **T** | **G** | **T** | **C** | **G** | **A** | **C** | **T** | **T** | **C** | **T** | **T** | **C** | **T** | **C** | **T** | **T** | **C** | **A** | **T** | **T** | **A** | **A** | **A** | **T** | **C** | **G** | **T** | **A** | **T** | **G** | **C** | **C** | **G** | **C** | **T** | **G** | **A** | **C** | **A** | **A** | **G** | **A** | **A** | **T** | **G** | **A** | **A** | **T** | **T** | **C** | **T** | **G** | **T** | **C** | **A** | **C** | **T** | **C** | **T** | **T** | **C** | **T** | **C** | **A** | **C** | **A** | **G** | **A** | **C** | **G** | **G** | **A** | **C** | **A** | **A** | **A** | **A** | **G** | **A** | **A** | **A** | **T** | **T** | **A** | **G** | **A** | **T** | **T** | **C** | **A** | **C** | **T** | **G** | **C** | **T** | **T** | **C** | **A** | **A** | **G** | **A** | **T** | **C** | **T** | **A** | **A** | **A** | **A** | **A** | **A** | **A** | **G** | **C** | **C** | **A** | **A** | **A** | **C** | **A** | **T** | **T** | **C** | **G** | **A** | **T** | **C** | **C** | **A** | **T** | **A** | **T** | **C** | **G** | **A** | **A** | **T** | **A** | **T** | **T** | **A** | **C** | **G** | **C** | **A** | **T** | **T** | **T** | **G** | **T** | **G** | **A** | **C** | **A** | **G** | **A** | **T** | **A** | **A** | **T** | **A** | **T** | **C** | **G** | **G** | **T** | **C** | **A** | **G** | **C** | **A** | **A** | **G** | **T** | **C** | **T** | **T** | **A** | **C** | **G** | **C** | **G** | **T** | **T** | **T** | **T** | **A** | **G** | **A** | **A** | **G** | **A** | **A** | **A** | **A** | **A** | **G** | **C** | **C** | **G** | **C** | **A** | **G** | **A** | **A** | **G** | **G** | **C** | **G** | **T** | **A** | **G** | **A** | **A** | **G** | **T** | **T** | **C** | **G** | **T** | **A** | **T** | **A** | **T** | **T** | **A** | **T** | **A** | **T** | **G** | **A** | **T** | **G** | **C** | **A** | **T** | **T** | **T** | **G** | **G** | **C** | **T** | **C** | **C** | **C** | **A** | **T** | **G** | **G** | **C** | **A** | **C** | **A** | **A** | **A** | **A** | **G** | **C** | **A** | **A** | **A** | **A** | **G** | **A** | **T** | **T** | **T** | **C** | **A** | **A** | **T** | **C** | **G** | **T** | **C** | **T** | **A** | **A** | **T** | **C** | **A** | **A** | **A** | **A** | **A** | **T** | **G** | **G** | **T** | **G** | **G** | **A** | **C** | **A** | **T** | **G** | **T** | **C** | **C** | **A** | **T** | **A** | **C** | **A** | **T** | **T** | **T** | **G** | **T** | **T** | **A** | **C** | **C** | **T** | **C** | **G** | **C** | **A** | **A** | **A** | **A** | **A** | **G** | **C** | **A** | **T** | **T** | **G** | **C** | **T** | **T** | **C** | **G** | **T** | **T** | **T** | **C** | **C** | **G** | **A** | **T** | **T** | **G** | **A** | **A** | **T** | **T** | **A** | **C** | **C** | **A** | **T** | **G** | **A** | **T** | **C** | **A** | **C** | **C** | **G** | **A** | **A** | **A** | **A** | **A** | **T** | **C** | **G** | **T** | **T** | **G** | **T** | **G** | **A** | **T** | **C** | **G** | **A** | **T** | **G** | **G** | **A** | **A** | **A** | **G** | **A** | **T** | **C** | **A** | **G** | **T** | **T** | **A** | **C** | **A** | **C** | **C** | **G** | **G** | **T** | **G** | **G** | **T** | **T** | **T** | **C** | **A** | **A** | **T** | **A** | **T** | **T** | **G** | **C** | **C** | **A** | **A** | **T** | **C** | **A** | **A** | **T** | **A** | **T** | **G** | **T** | **A** | **A** | **A** | **T** | **A** | **C** | **G** | **A** | **C** | **A** | **A** | **A** | **A** | **A** | **A** | **A** | **T** | **T** | **C** | **G** | **G** | **T** | **T** | **A** | **T** | **T** | **G** | **G** | **C** | **G** | **T** | **G** | **A** | **T** | **A** | **C** | **G** | **C** | **A** | **T** | **A** | **T** | **A** | **C** | **G** | **A** | **A** | **T** | **T** | **T** | **T** | **C** | **G** | **G** | **T** | **G** | **C** | **T** | **G** | **C** | **T** | **T** | **C** | **T** | **T** | **C** | **T** | **T** | **T** | **G** | **C** | **T** | **T** | **C** | **A** | **A** | **C** | **T** | **T** | **C** | **G** | **T** | **T** | **T** | **T** | **T** | **T** | **A** | **A** | **C** | **A** | **G** | **A** | **C** | **T** | **G** | **G** | **A** | **A** | **C** | **G** | **T** | **T** | **T** | **C** | **A** | **G** | **T** | **G** | **T** | **C** | **T** | **G** | **A** | **A** | **G** | **A** | **A** | **A** | **A** | **A** | **A** | **A** | **G** | **G** | **T** | **C** | **G** | **G** | **C** | **T** | **A** | **T** | **C** | **A** | **T** | **T** | **T** | **G** | **A** | **A** | **T** | **T** | **A** | **T** | **T** | **T** | **C** | **T** | **T** | **T** | **A** | **A** | **A** | **A** | **A** | **A** | **G** | **C** | **C** | **G** | **A** | **T** | **C** | **G** | **A** | **G** | **A** | **T** | **G** | **A** | **A** | **T** | **C** | **C** | **A** | **A** | **A** | **C** | **T** | **T** | **G** | **C** | **T** | **G** | **A** | **T** | **A** | **C** | **G** | **T** | **C** | **C** | **A** | **T** | **C** | **C** | **A** | **G** | **C** | **T** | **T** | **G** | **T** | **C** | **T** | **C** | **A** | **A** | **G** | **C** | **G** | **G** | **A** | **C** | **C** | **A** | **A** | **A** | **T** | **A** | **A** | **C** | **G** | **A** | **A** | **C** | **G** | **A** | **G** | **A** | **A** | **C** | **A** | **A** | **A** | **T** | **C** | **A** | **A** | **G** | **C** | **T** | **T** | **T** | **C** | **G** | **T** | **T** | **T** | **A** | **T** | **C** | **A** | **A** | **A** | **T** | **T** | **G** | **A** | **T** | **T** | **A** | **C** | **T** | **T** | **C** | **T** | **G** | **C** | **T** | **A** | **A** | **A** | **A** | **A** | **A** | **C** | **G** | **T** | **G** | **T** | **T** | **T** | **G** | **G** | **A** | **T** | **A** | **C** | **A** | **G** | **A** | **C** | **A** | **C** | **C** | **T** | **T** | **A** | **C** | **C** | **T** | **C** | **G** | **T** | **T** | **C** | **C** | **T** | **G** | **A** | **T** | **G** | **A** | **A** | **A** | **G** | **T** | **G** | **T** | **C** | **A** | **T** | **T** | **G** | **C** | **T** | **G** | **C** | **T** | **T** | **T** | **G** | **A** | **A** | **A** | **A** | **T** | **C** | **G** | **C** | **A** | **A** | **C** | **T** | **G** | **C** | **T** | **T** | **C** | **T** | **G** | **G** | **T** | **G** | **T** | **C** | **G** | **A** | **T** | **G** | **T** | **G** | **A** | **A** | **A** | **A** | **T** | **C** | **A** | **T** | **G** | **A** | **T** | **C** | **C** | **C** | **C** | **A** | **A** | **T** | **A** | **A** | **A** | **C** | **C** | **G** | **G** | **A** | **T** | **C** | **A** | **T** | **C** | **C** | **T** | **T** | **T** | **T** | **A** | **T** | **T** | **T** | **A** | **T** | **C** | **G** | **A** | **G** | **C** | **A** | **A** | **C** | **A** | **C** | **A** | **A** | **T** | **A** | **T** | **T** | **A** | **T** | **G** | **C** | **T** | **C** | **G** | **T** | **C** | **A** | **G** | **C** | **T** | **C** | **A** | **T** | **C** | **A** | **A** | **G** | **G** | **A** | **A** | **A** | **A** | **T** | **G** | **T** | **A** | **C** | **A** | **G** | **A** | **T** | **C** | **C** | **T** | **T** | **G** | **T** | **C** | **T** | **A** | **T** | **G** | **A** | **G** | **A** | **A** | **T** | **G** | **G** | **C** | **T** | **T** | **C** | **C** | **T** | **C** | **C** | **A** | **T** | **G** | **C** | **A** | **A** | **A** | **A** | **A** | **C** | **A** | **T** | **T** | **A** | **A** | **T** | **A** | **A** | **T** | **G** | **G** | **A** | **T** | **G** | **A** | **T** | **G** | **A** | **A** | **A** | **T** | **C** | **T** | **G** | **C** | **A** | **T** | **G** | **G** | **T** | **A** | **G** | **G** | **T** | **T** | **C** | **A** | **G** | **C** | **A** | **A** | **A** | **T** | **C** | **A** | **A** | **G** | **A** | **T** | **A** | **T** | **T** | **C** | **G** | **T** | **A** | **G** | **C** | **T** | **A** | **C** | **C** | **G** | **A** | **T** | **T** | **G** | **A** | **A** | **T** | **T** | **T** | **T** | **G** | **A** | **A** | **A** | **C** | **A** | **A** | **G** | **T** | **G** | **C** | **T** | **G** | **T** | **C** | **A** | **T** | **T** | **T** | **A** | **C** | **G** | **A** | **T** | **C** | **C** | **T** | **G** | **A** | **G** | **T** | **T** | **T** | **T** | **T** | **A** | **G** | **A** | **A** | **G** | **A** | **G** | **C** | **T** | **T** | **G** | **C** | **T** | **A** | **C** | **T** | **C** | **A** | **G** | **T** | **T** | **C** | **A** | **A** | **A** | **G** | **A** | **A** | **G** | **A** | **T** | **G** | **A** | **G** | **A** | **C** | **A** | **C** | **A** | **T** | **T** | **G** | **T** | **T** | **C** | **A** | **C** | **C** | **A** | **A** | **T** | **G** | **A** | **C** | **A** | **A** | **C** | **T** | **G** | **A** | **A** | **A** | **C** | **A** | **G** | **T** | **G** | **A** | **A** | **G** | **G** | **A** | **A** | **A** | **T** | **G** | **T** | **C** | **T** | **A** | **A** | **C** | **T** | **G** | **G** | **C** | **T** | **G** | **T** | **T** | **A** | **T** | **T** | **C** | **A** | **A** | **A** | **C** | **A** | **A** | **C** | **A** | **A** | **A** | **T** | **T** | **T** | **C** | **T** | **C** | **G** | **A** | **T** | **T** | **G** | **T** | **T** | **T** | **T** | **C** | **T** | **C** | **C** | **A** | **A** | **T** | **C** | **T** | **T** | **A** | **-** | **-** | **-** |
| **N52649** | **-** | **-** | **-** | **-** | **-** | **-** | **-** | **-** | **-** | **-** | **-** | **-** | **-** | **-** | **-** | **-** | **-** | **-** | **-** | **-** | **-** | **-** | **-** | **-** | **-** | **-** | **-** | **-** | **-** | **-** | **-** | **-** | **-** | **-** | **-** | **-** | **-** | **-** | **-** | **-** | **-** | **-** | **-** | **-** | **-** | **-** | **-** | **-** | **-** | **-** | **-** | **-** | **-** | **-** | **-** | **-** | **-** | **-** | **-** | **-** | **-** | **-** | **-** | **-** | **-** | **-** | **-** | **-** | **-** | **A** | **A** | **A** | **C** | **C** | **C** | **C** | **G** | **A** | **G** | **A** | **T** | **G** | **T** | **A** | **G** | **C** | **A** | **G** | **C** | **C** | **A** | **T** | **T** | **T** | **G** | **G** | **G** | **C** | **A** | **T** | **G** | **G** | **C** | **T** | **C** | **T** | **T** | **A** | **G** | **T** | **A** | **T** | **T** | **G** | **T** | **T** | **T** | **G** | **C** | **C** | **C** | **T** | **T** | **C** | **C** | **T** | **G** | **G** | **G** | **G** | **T** | **G** | **G** | **G** | **T** | **T** | **T** | **T** | **G** | **T** | **A** | **T** | **T** | **A** | **T** | **A** | **T** | **T** | **T** | **A** | **T** | **T** | **T** | **T** | **T** | **C** | **G** | **G** | **A** | **C** | **G** | **T** | **G** | **G** | **A** | **T** | **T** | **A** | **A** | **C** | **G** | **G** | **A** | **T** | **A** | **A** | **G** | **A** | **A** | **A** | **A** | **A** | **A** | **T** | **T** | **C** | **T** | **A** | **T** | **C** | **T** | **C** | **C** | **G** | **A** | **C** | **A** | **A** | **A** | **G** | **T** | **G** | **A** | **C** | **T** | **T** | **G** | **A** | **A** | **A** | **G** | **A** | **A** | **T** | **T** | **A** | **G** | **A** | **A** | **A** | **A** | **C** | **T** | **T** | **T** | **C** | **A** | **G** | **T** | **C** | **T** | **T** | **T** | **T** | **A** | **G** | **A** | **G** | **G** | **A** | **G** | **A** | **T** | **A** | **C** | **C** | **A** | **T** | **T** | **G** | **A** | **A** | **C** | **A** | **T** | **T** | **A** | **C** | **G** | **A** | **T** | **C** | **C** | **T** | **G** | **A** | **C** | **A** | **T** | **G** | **G** | **G** | **C** | **G** | **A** | **T** | **A** | **A** | **A** | **G** | **A** | **C** | **A** | **A** | **A** | **C** | **A** | **G** | **C** | **A** | **A** | **T** | **T** | **T** | **G** | **T** | **C** | **G** | **A** | **C** | **T** | **T** | **C** | **T** | **T** | **C** | **T** | **C** | **T** | **T** | **C** | **A** | **T** | **T** | **A** | **A** | **A** | **T** | **C** | **G** | **T** | **A** | **T** | **G** | **C** | **C** | **G** | **C** | **T** | **G** | **A** | **C** | **A** | **A** | **G** | **A** | **A** | **T** | **G** | **A** | **A** | **T** | **T** | **C** | **T** | **G** | **T** | **C** | **A** | **C** | **T** | **C** | **T** | **T** | **C** | **T** | **C** | **A** | **C** | **A** | **G** | **A** | **C** | **G** | **G** | **A** | **C** | **A** | **A** | **G** | **A** | **G** | **A** | **A** | **A** | **T** | **T** | **G** | **G** | **A** | **T** | **T** | **C** | **A** | **C** | **T** | **G** | **C** | **T** | **T** | **C** | **A** | **A** | **G** | **A** | **T** | **C** | **T** | **A** | **A** | **A** | **A** | **A** | **A** | **A** | **G** | **C** | **C** | **A** | **A** | **A** | **C** | **A** | **T** | **T** | **C** | **G** | **A** | **T** | **C** | **C** | **A** | **T** | **A** | **T** | **C** | **G** | **A** | **A** | **T** | **A** | **T** | **T** | **A** | **C** | **G** | **C** | **A** | **T** | **T** | **T** | **G** | **T** | **G** | **A** | **C** | **A** | **G** | **A** | **T** | **A** | **A** | **T** | **A** | **T** | **C** | **G** | **G** | **C** | **C** | **A** | **G** | **C** | **A** | **A** | **G** | **T** | **C** | **T** | **T** | **A** | **C** | **A** | **T** | **G** | **T** | **T** | **T** | **T** | **A** | **G** | **A** | **A** | **G** | **A** | **A** | **A** | **A** | **A** | **G** | **C** | **C** | **G** | **C** | **A** | **G** | **A** | **A** | **G** | **G** | **C** | **G** | **T** | **G** | **G** | **A** | **A** | **G** | **T** | **T** | **C** | **G** | **A** | **A** | **T** | **A** | **T** | **T** | **A** | **T** | **A** | **T** | **G** | **A** | **T** | **G** | **C** | **A** | **T** | **T** | **T** | **G** | **G** | **C** | **T** | **C** | **T** | **C** | **A** | **T** | **G** | **G** | **C** | **A** | **C** | **A** | **A** | **A** | **A** | **G** | **C** | **A** | **A** | **A** | **A** | **G** | **A** | **T** | **T** | **T** | **C** | **A** | **A** | **T** | **C** | **G** | **T** | **C** | **T** | **A** | **A** | **T** | **C** | **A** | **A** | **A** | **A** | **A** | **T** | **G** | **G** | **T** | **G** | **G** | **A** | **C** | **A** | **T** | **G** | **T** | **C** | **C** | **A** | **T** | **A** | **C** | **A** | **T** | **T** | **T** | **G** | **T** | **T** | **A** | **C** | **C** | **T** | **C** | **A** | **C** | **A** | **A** | **A** | **A** | **G** | **G** | **C** | **A** | **T** | **T** | **A** | **C** | **T** | **T** | **C** | **G** | **T** | **T** | **T** | **C** | **C** | **G** | **A** | **T** | **T** | **G** | **A** | **A** | **T** | **T** | **A** | **C** | **C** | **A** | **T** | **G** | **A** | **T** | **C** | **A** | **C** | **C** | **G** | **A** | **A** | **A** | **A** | **A** | **T** | **C** | **G** | **T** | **T** | **G** | **T** | **G** | **A** | **T** | **C** | **G** | **A** | **T** | **G** | **G** | **A** | **A** | **A** | **G** | **A** | **T** | **T** | **A** | **G** | **T** | **T** | **A** | **T** | **A** | **C** | **C** | **G** | **G** | **T** | **G** | **G** | **T** | **T** | **T** | **C** | **A** | **A** | **T** | **A** | **T** | **T** | **G** | **C** | **C** | **A** | **A** | **T** | **C** | **A** | **A** | **T** | **A** | **T** | **G** | **T** | **A** | **A** | **A** | **T** | **A** | **C** | **A** | **A** | **C** | **A** | **A** | **A** | **A** | **A** | **A** | **A** | **T** | **T** | **C** | **G** | **G** | **C** | **T** | **A** | **T** | **T** | **G** | **G** | **C** | **G** | **C** | **G** | **A** | **T** | **A** | **C** | **G** | **C** | **A** | **T** | **A** | **T** | **A** | **C** | **G** | **G** | **A** | **T** | **T** | **T** | **T** | **C** | **G** | **G** | **T** | **G** | **C** | **C** | **G** | **C** | **T** | **T** | **C** | **T** | **T** | **C** | **T** | **T** | **T** | **G** | **C** | **T** | **C** | **C** | **A** | **G** | **C** | **T** | **T** | **C** | **G** | **C** | **T** | **T** | **C** | **T** | **T** | **A** | **A** | **C** | **A** | **G** | **A** | **C** | **T** | **G** | **G** | **A** | **A** | **C** | **G** | **T** | **C** | **T** | **C** | **G** | **G** | **T** | **A** | **C** | **C** | **T** | **G** | **A** | **A** | **G** | **A** | **A** | **A** | **A** | **A** | **A** | **A** | **G** | **G** | **T** | **C** | **G** | **G** | **C** | **T** | **A** | **T** | **C** | **A** | **T** | **T** | **T** | **G** | **A** | **A** | **T** | **T** | **A** | **T** | **T** | **T** | **C** | **T** | **T** | **T** | **A** | **A** | **A** | **A** | **A** | **A** | **G** | **C** | **A** | **G** | **A** | **T** | **C** | **G** | **A** | **G** | **A** | **T** | **G** | **A** | **A** | **T** | **C** | **T** | **A** | **A** | **G** | **C** | **T** | **T** | **G** | **C** | **T** | **G** | **A** | **T** | **A** | **C** | **A** | **T** | **C** | **C** | **A** | **T** | **C** | **C** | **A** | **G** | **C** | **T** | **T** | **G** | **T** | **T** | **T** | **C** | **A** | **A** | **G** | **C** | **G** | **G** | **A** | **C** | **C** | **G** | **A** | **A** | **T** | **A** | **A** | **C** | **G** | **A** | **A** | **A** | **G** | **G** | **G** | **A** | **A** | **C** | **A** | **A** | **A** | **T** | **C** | **A** | **A** | **G** | **C** | **T** | **T** | **T** | **C** | **A** | **T** | **T** | **T** | **A** | **T** | **C** | **A** | **A** | **A** | **T** | **T** | **G** | **A** | **T** | **T** | **A** | **C** | **T** | **T** | **C** | **T** | **G** | **C** | **T** | **A** | **A** | **A** | **A** | **A** | **A** | **C** | **G** | **T** | **G** | **T** | **T** | **T** | **G** | **G** | **A** | **T** | **A** | **C** | **A** | **G** | **A** | **C** | **A** | **C** | **C** | **T** | **T** | **A** | **C** | **C** | **T** | **T** | **G** | **T** | **T** | **C** | **C** | **T** | **G** | **A** | **T** | **G** | **A** | **A** | **A** | **G** | **T** | **G** | **T** | **C** | **A** | **T** | **T** | **G** | **C** | **T** | **G** | **C** | **T** | **T** | **T** | **A** | **A** | **A** | **A** | **A** | **T** | **C** | **G** | **C** | **A** | **A** | **C** | **T** | **G** | **C** | **C** | **T** | **C** | **T** | **G** | **G** | **T** | **G** | **T** | **A** | **G** | **A** | **T** | **G** | **T** | **G** | **A** | **A** | **A** | **A** | **T** | **C** | **A** | **T** | **G** | **A** | **T** | **T** | **C** | **C** | **C** | **A** | **A** | **C** | **A** | **A** | **A** | **C** | **C** | **G** | **G** | **A** | **T** | **C** | **A** | **T** | **C** | **C** | **T** | **T** | **T** | **T** | **A** | **T** | **T** | **T** | **A** | **T** | **C** | **G** | **A** | **G** | **C** | **A** | **A** | **C** | **A** | **C** | **A** | **A** | **T** | **A** | **T** | **T** | **A** | **T** | **G** | **C** | **T** | **C** | **G** | **G** | **C** | **A** | **G** | **C** | **T** | **G** | **A** | **T** | **C** | **A** | **A** | **G** | **G** | **A** | **A** | **A** | **A** | **T** | **G** | **T** | **A** | **C** | **A** | **A** | **A** | **T** | **C** | **C** | **T** | **T** | **G** | **T** | **C** | **T** | **A** | **T** | **G** | **A** | **G** | **A** | **A** | **C** | **G** | **G** | **C** | **T** | **T** | **C** | **C** | **T** | **C** | **C** | **A** | **T** | **G** | **C** | **A** | **A** | **A** | **A** | **A** | **C** | **A** | **T** | **T** | **G** | **A** | **T** | **A** | **A** | **T** | **G** | **G** | **A** | **T** | **G** | **A** | **T** | **G** | **A** | **A** | **A** | **T** | **C** | **T** | **G** | **C** | **A** | **T** | **G** | **G** | **T** | **A** | **G** | **G** | **T** | **T** | **C** | **A** | **G** | **C** | **A** | **A** | **A** | **T** | **C** | **A** | **A** | **G** | **A** | **T** | **A** | **T** | **T** | **C** | **G** | **T** | **A** | **G** | **C** | **T** | **A** | **C** | **C** | **G** | **A** | **T** | **T** | **G** | **A** | **A** | **T** | **T** | **T** | **T** | **G** | **A** | **A** | **A** | **C** | **A** | **A** | **G** | **T** | **G** | **C** | **T** | **G** | **T** | **C** | **A** | **T** | **T** | **T** | **A** | **C** | **G** | **A** | **T** | **C** | **C** | **T** | **G** | **A** | **G** | **T** | **T** | **T** | **T** | **T** | **A** | **G** | **A** | **A** | **G** | **A** | **A** | **C** | **T** | **T** | **G** | **C** | **T** | **A** | **C** | **T** | **C** | **A** | **G** | **T** | **T** | **C** | **A** | **A** | **A** | **G** | **A** | **A** | **G** | **A** | **T** | **G** | **A** | **G** | **A** | **C** | **A** | **C** | **A** | **T** | **T** | **G** | **T** | **T** | **C** | **A** | **T** | **C** | **C** | **A** | **T** | **G** | **A** | **C** | **A** | **A** | **C** | **T** | **G** | **A** | **A** | **A** | **C** | **A** | **G** | **T** | **C** | **A** | **A** | **A** | **G** | **A** | **A** | **A** | **T** | **G** | **T** | **C** | **T** | **A** | **A** | **C** | **T** | **G** | **G** | **C** | **T** | **A** | **T** | **T** | **A** | **T** | **T** | **C** | **A** | **A** | **G** | **C** | **A** | **A** | **C** | **A** | **A** | **A** | **T** | **T** | **T** | **C** | **T** | **C** | **G** | **A** | **T** | **T** | **A** | **T** | **T** | **T** | **T** | **C** | **T** | **C** | **C** | **A** | **A** | **T** | **C** | **C** | **T** | **A** | **-** | **-** | **-** |
| **N52702** | **-** | **-** | **-** | **-** | **-** | **-** | **-** | **-** | **-** | **-** | **-** | **-** | **-** | **-** | **-** | **-** | **-** | **-** | **-** | **-** | **-** | **-** | **-** | **-** | **-** | **-** | **-** | **-** | **-** | **-** | **-** | **-** | **-** | **-** | **-** | **-** | **-** | **-** | **-** | **-** | **-** | **-** | **-** | **-** | **-** | **-** | **-** | **-** | **-** | **-** | **-** | **-** | **-** | **-** | **-** | **-** | **-** | **-** | **-** | **-** | **-** | **-** | **-** | **-** | **-** | **-** | **-** | **-** | **-** | **A** | **A** | **A** | **C** | **C** | **C** | **C** | **G** | **A** | **G** | **A** | **T** | **G** | **T** | **A** | **G** | **C** | **A** | **G** | **C** | **C** | **A** | **T** | **T** | **T** | **G** | **G** | **G** | **C** | **A** | **T** | **G** | **G** | **C** | **T** | **C** | **T** | **T** | **A** | **G** | **T** | **A** | **T** | **T** | **G** | **T** | **T** | **T** | **G** | **C** | **C** | **C** | **T** | **T** | **C** | **C** | **T** | **G** | **G** | **G** | **G** | **T** | **G** | **G** | **G** | **C** | **T** | **T** | **T** | **G** | **T** | **A** | **T** | **T** | **A** | **T** | **A** | **T** | **T** | **T** | **A** | **T** | **T** | **T** | **T** | **T** | **C** | **G** | **G** | **A** | **C** | **G** | **T** | **G** | **G** | **A** | **T** | **T** | **A** | **A** | **C** | **G** | **G** | **A** | **T** | **A** | **A** | **G** | **A** | **A** | **A** | **A** | **A** | **A** | **T** | **T** | **C** | **T** | **A** | **T** | **C** | **T** | **C** | **C** | **G** | **A** | **C** | **A** | **A** | **A** | **G** | **T** | **G** | **A** | **C** | **T** | **T** | **G** | **A** | **A** | **A** | **G** | **A** | **A** | **T** | **T** | **A** | **G** | **A** | **A** | **A** | **A** | **C** | **T** | **T** | **T** | **C** | **A** | **G** | **T** | **C** | **T** | **T** | **T** | **T** | **A** | **G** | **A** | **G** | **G** | **A** | **G** | **A** | **T** | **A** | **A** | **C** | **A** | **T** | **T** | **G** | **A** | **A** | **C** | **A** | **T** | **T** | **A** | **C** | **G** | **A** | **T** | **C** | **C** | **T** | **G** | **A** | **C** | **A** | **T** | **G** | **G** | **G** | **C** | **G** | **A** | **T** | **A** | **A** | **A** | **G** | **A** | **C** | **A** | **A** | **A** | **C** | **A** | **G** | **C** | **A** | **A** | **T** | **T** | **T** | **G** | **T** | **T** | **G** | **A** | **C** | **T** | **T** | **C** | **T** | **T** | **C** | **T** | **C** | **T** | **T** | **C** | **A** | **T** | **T** | **A** | **A** | **A** | **T** | **C** | **G** | **T** | **A** | **T** | **G** | **C** | **C** | **G** | **C** | **T** | **G** | **A** | **C** | **A** | **A** | **G** | **A** | **A** | **T** | **G** | **A** | **A** | **T** | **T** | **C** | **T** | **G** | **T** | **C** | **A** | **C** | **T** | **C** | **T** | **T** | **C** | **T** | **C** | **A** | **C** | **A** | **G** | **A** | **C** | **G** | **G** | **A** | **C** | **A** | **A** | **G** | **A** | **G** | **A** | **A** | **A** | **T** | **T** | **G** | **G** | **A** | **T** | **T** | **C** | **A** | **C** | **T** | **G** | **C** | **T** | **T** | **C** | **A** | **A** | **G** | **A** | **T** | **C** | **T** | **A** | **A** | **A** | **A** | **A** | **A** | **A** | **G** | **C** | **C** | **A** | **A** | **A** | **C** | **A** | **T** | **T** | **C** | **G** | **A** | **T** | **C** | **C** | **A** | **T** | **A** | **T** | **C** | **G** | **A** | **A** | **T** | **A** | **T** | **T** | **A** | **C** | **G** | **C** | **A** | **T** | **T** | **T** | **G** | **T** | **G** | **A** | **C** | **A** | **G** | **A** | **T** | **A** | **A** | **T** | **A** | **T** | **C** | **G** | **G** | **C** | **C** | **A** | **G** | **C** | **A** | **A** | **G** | **T** | **C** | **T** | **T** | **A** | **C** | **A** | **T** | **G** | **T** | **T** | **T** | **T** | **A** | **G** | **A** | **A** | **G** | **A** | **A** | **A** | **A** | **A** | **G** | **C** | **C** | **G** | **C** | **A** | **G** | **A** | **A** | **G** | **G** | **C** | **G** | **T** | **G** | **G** | **A** | **A** | **G** | **T** | **T** | **C** | **G** | **A** | **A** | **T** | **A** | **T** | **T** | **A** | **T** | **A** | **T** | **G** | **A** | **T** | **G** | **C** | **A** | **T** | **T** | **T** | **G** | **G** | **C** | **T** | **C** | **T** | **C** | **A** | **T** | **G** | **G** | **C** | **A** | **C** | **A** | **A** | **A** | **A** | **G** | **C** | **A** | **A** | **A** | **A** | **G** | **A** | **T** | **T** | **T** | **C** | **A** | **A** | **T** | **C** | **G** | **T** | **C** | **T** | **A** | **A** | **T** | **C** | **A** | **A** | **A** | **A** | **A** | **T** | **G** | **G** | **T** | **G** | **G** | **A** | **C** | **A** | **T** | **G** | **T** | **C** | **C** | **A** | **T** | **A** | **C** | **A** | **T** | **T** | **T** | **G** | **T** | **T** | **A** | **C** | **C** | **T** | **C** | **A** | **C** | **A** | **A** | **A** | **A** | **G** | **G** | **C** | **A** | **T** | **T** | **A** | **C** | **T** | **T** | **C** | **G** | **T** | **T** | **T** | **C** | **C** | **G** | **A** | **T** | **T** | **G** | **A** | **A** | **T** | **T** | **A** | **C** | **C** | **A** | **T** | **G** | **A** | **T** | **C** | **A** | **C** | **C** | **G** | **A** | **A** | **A** | **A** | **A** | **T** | **C** | **G** | **T** | **T** | **G** | **T** | **G** | **A** | **T** | **C** | **G** | **A** | **T** | **G** | **G** | **A** | **A** | **A** | **G** | **A** | **T** | **T** | **A** | **G** | **T** | **T** | **A** | **T** | **A** | **C** | **C** | **G** | **G** | **T** | **G** | **G** | **T** | **T** | **T** | **C** | **A** | **A** | **T** | **A** | **T** | **T** | **G** | **C** | **C** | **A** | **A** | **T** | **C** | **A** | **A** | **T** | **A** | **T** | **G** | **T** | **A** | **A** | **A** | **T** | **A** | **C** | **A** | **A** | **C** | **A** | **A** | **A** | **A** | **A** | **A** | **A** | **T** | **T** | **C** | **G** | **G** | **C** | **T** | **A** | **T** | **T** | **G** | **G** | **C** | **G** | **C** | **G** | **A** | **T** | **A** | **C** | **G** | **C** | **A** | **T** | **A** | **T** | **A** | **C** | **G** | **G** | **A** | **T** | **T** | **T** | **T** | **C** | **G** | **G** | **T** | **G** | **C** | **C** | **G** | **C** | **T** | **T** | **C** | **T** | **T** | **C** | **T** | **T** | **T** | **G** | **C** | **T** | **C** | **C** | **A** | **G** | **C** | **T** | **T** | **C** | **G** | **C** | **T** | **T** | **C** | **T** | **T** | **A** | **A** | **C** | **A** | **G** | **A** | **C** | **T** | **G** | **G** | **A** | **A** | **C** | **G** | **T** | **C** | **T** | **C** | **G** | **G** | **T** | **A** | **C** | **C** | **T** | **G** | **A** | **A** | **G** | **A** | **A** | **A** | **A** | **A** | **A** | **A** | **G** | **G** | **T** | **C** | **G** | **G** | **C** | **T** | **A** | **T** | **C** | **A** | **T** | **T** | **T** | **G** | **A** | **A** | **T** | **T** | **A** | **T** | **T** | **T** | **C** | **T** | **T** | **T** | **A** | **A** | **A** | **A** | **A** | **A** | **G** | **C** | **A** | **G** | **A** | **T** | **C** | **G** | **A** | **G** | **A** | **T** | **G** | **A** | **A** | **T** | **C** | **T** | **A** | **A** | **G** | **C** | **T** | **T** | **G** | **C** | **T** | **G** | **A** | **T** | **A** | **C** | **A** | **T** | **C** | **C** | **A** | **T** | **C** | **C** | **A** | **G** | **C** | **T** | **T** | **G** | **T** | **T** | **T** | **C** | **A** | **A** | **G** | **C** | **G** | **G** | **A** | **C** | **C** | **G** | **A** | **A** | **T** | **A** | **A** | **C** | **G** | **A** | **A** | **A** | **G** | **G** | **G** | **A** | **A** | **C** | **A** | **A** | **A** | **T** | **C** | **A** | **A** | **G** | **C** | **T** | **T** | **T** | **C** | **A** | **T** | **T** | **T** | **A** | **T** | **C** | **A** | **A** | **A** | **T** | **T** | **G** | **A** | **T** | **T** | **A** | **C** | **T** | **T** | **C** | **T** | **G** | **C** | **T** | **A** | **A** | **A** | **A** | **A** | **A** | **C** | **G** | **T** | **G** | **T** | **T** | **T** | **G** | **G** | **A** | **T** | **A** | **C** | **A** | **G** | **A** | **C** | **A** | **C** | **C** | **T** | **T** | **A** | **C** | **C** | **T** | **T** | **G** | **T** | **T** | **C** | **C** | **T** | **G** | **A** | **T** | **G** | **A** | **A** | **A** | **G** | **T** | **G** | **T** | **C** | **A** | **T** | **T** | **G** | **C** | **T** | **G** | **C** | **T** | **T** | **T** | **A** | **A** | **A** | **A** | **A** | **T** | **C** | **G** | **C** | **A** | **A** | **C** | **T** | **G** | **C** | **C** | **T** | **C** | **T** | **G** | **G** | **T** | **G** | **T** | **A** | **G** | **A** | **T** | **G** | **T** | **G** | **A** | **A** | **A** | **A** | **T** | **C** | **A** | **T** | **G** | **A** | **T** | **T** | **C** | **C** | **C** | **A** | **A** | **C** | **A** | **A** | **A** | **C** | **C** | **G** | **G** | **A** | **T** | **C** | **A** | **T** | **C** | **C** | **T** | **T** | **T** | **T** | **A** | **T** | **T** | **T** | **A** | **T** | **C** | **G** | **A** | **G** | **C** | **A** | **A** | **C** | **A** | **C** | **A** | **A** | **T** | **A** | **T** | **T** | **A** | **T** | **G** | **C** | **T** | **C** | **G** | **G** | **C** | **A** | **G** | **C** | **T** | **G** | **A** | **T** | **C** | **A** | **A** | **G** | **G** | **A** | **A** | **A** | **A** | **T** | **G** | **T** | **A** | **C** | **A** | **A** | **A** | **T** | **C** | **C** | **T** | **T** | **G** | **T** | **C** | **T** | **A** | **T** | **G** | **A** | **G** | **A** | **A** | **C** | **G** | **G** | **C** | **T** | **T** | **C** | **C** | **T** | **C** | **C** | **A** | **T** | **G** | **C** | **A** | **A** | **A** | **A** | **A** | **C** | **A** | **T** | **T** | **G** | **A** | **T** | **A** | **A** | **T** | **G** | **G** | **A** | **T** | **G** | **A** | **T** | **G** | **A** | **A** | **A** | **T** | **C** | **T** | **G** | **C** | **A** | **T** | **G** | **G** | **T** | **A** | **G** | **G** | **T** | **T** | **C** | **A** | **G** | **C** | **A** | **A** | **A** | **T** | **C** | **A** | **A** | **G** | **A** | **T** | **A** | **T** | **T** | **C** | **G** | **T** | **A** | **G** | **C** | **T** | **A** | **C** | **C** | **G** | **A** | **T** | **T** | **G** | **A** | **A** | **T** | **T** | **T** | **T** | **G** | **A** | **A** | **A** | **C** | **A** | **A** | **G** | **T** | **G** | **C** | **T** | **G** | **T** | **C** | **A** | **T** | **T** | **T** | **A** | **C** | **G** | **A** | **T** | **C** | **C** | **T** | **G** | **A** | **G** | **T** | **T** | **T** | **T** | **T** | **A** | **G** | **A** | **A** | **G** | **A** | **A** | **C** | **T** | **T** | **G** | **C** | **T** | **A** | **C** | **T** | **C** | **A** | **G** | **T** | **T** | **C** | **A** | **A** | **A** | **G** | **A** | **A** | **G** | **A** | **T** | **G** | **A** | **G** | **A** | **C** | **A** | **C** | **A** | **T** | **T** | **G** | **T** | **T** | **C** | **A** | **T** | **C** | **C** | **A** | **T** | **G** | **A** | **C** | **A** | **A** | **C** | **T** | **G** | **A** | **A** | **A** | **C** | **A** | **G** | **T** | **C** | **A** | **A** | **G** | **G** | **A** | **A** | **A** | **T** | **G** | **T** | **C** | **T** | **A** | **A** | **C** | **T** | **G** | **G** | **C** | **T** | **A** | **T** | **T** | **A** | **T** | **T** | **C** | **A** | **A** | **G** | **C** | **A** | **A** | **C** | **A** | **A** | **A** | **T** | **T** | **T** | **C** | **T** | **C** | **G** | **A** | **T** | **T** | **A** | **T** | **T** | **T** | **T** | **C** | **T** | **C** | **C** | **A** | **A** | **T** | **C** | **C** | **T** | **A** | **-** | **-** | **-** |
| **N60299F** | **G** | **T** | **G** | **G** | **T** | **A** | **T** | **C** | **T** | **A** | **G** | **T** | **A** | **T** | **T** | **A** | **T** | **A** | **A** | **C** | **C** | **G** | **C** | **C** | **C** | **T** | **T** | **T** | **A** | **T** | **C** | **T** | **A** | **T** | **T** | **A** | **A** | **A** | **T** | **G** | **C** | **A** | **C** | **T** | **T** | **A** | **T** | **T** | **G** | **C** | **T** | **T** | **T** | **G** | **G** | **T** | **G** | **A** | **C** | **T** | **A** | **T** | **T** | **T** | **T** | **G** | **A** | **T** | **C** | **A** | **A** | **A** | **C** | **C** | **C** | **C** | **G** | **A** | **G** | **A** | **T** | **G** | **T** | **A** | **G** | **C** | **A** | **G** | **C** | **C** | **A** | **T** | **T** | **T** | **G** | **G** | **G** | **C** | **A** | **T** | **G** | **G** | **C** | **T** | **C** | **T** | **T** | **A** | **G** | **T** | **A** | **T** | **T** | **G** | **T** | **T** | **T** | **G** | **C** | **C** | **C** | **T** | **T** | **C** | **C** | **T** | **G** | **G** | **G** | **G** | **T** | **G** | **G** | **G** | **C** | **T** | **T** | **T** | **G** | **T** | **A** | **T** | **T** | **A** | **T** | **A** | **T** | **T** | **T** | **A** | **T** | **T** | **T** | **T** | **T** | **C** | **G** | **G** | **A** | **C** | **G** | **T** | **G** | **G** | **A** | **T** | **T** | **A** | **A** | **C** | **G** | **G** | **A** | **T** | **A** | **A** | **G** | **A** | **A** | **A** | **A** | **A** | **A** | **T** | **T** | **C** | **T** | **A** | **T** | **C** | **T** | **C** | **C** | **G** | **A** | **C** | **A** | **A** | **A** | **G** | **T** | **G** | **A** | **C** | **T** | **T** | **G** | **A** | **A** | **A** | **G** | **A** | **A** | **T** | **T** | **A** | **G** | **A** | **A** | **A** | **A** | **C** | **T** | **T** | **T** | **C** | **A** | **G** | **T** | **C** | **T** | **T** | **T** | **T** | **A** | **G** | **A** | **G** | **G** | **A** | **G** | **A** | **T** | **A** | **C** | **C** | **A** | **T** | **T** | **G** | **A** | **A** | **C** | **A** | **T** | **T** | **A** | **C** | **G** | **A** | **T** | **C** | **C** | **T** | **G** | **A** | **C** | **A** | **T** | **G** | **G** | **G** | **C** | **G** | **A** | **T** | **A** | **A** | **A** | **G** | **A** | **C** | **A** | **A** | **A** | **C** | **A** | **G** | **C** | **A** | **A** | **T** | **T** | **T** | **G** | **T** | **T** | **G** | **A** | **C** | **T** | **T** | **C** | **T** | **T** | **C** | **T** | **C** | **T** | **T** | **C** | **A** | **T** | **T** | **A** | **A** | **A** | **T** | **C** | **G** | **T** | **A** | **T** | **G** | **C** | **C** | **G** | **C** | **T** | **G** | **G** | **C** | **A** | **A** | **G** | **A** | **A** | **T** | **G** | **A** | **A** | **T** | **T** | **C** | **T** | **G** | **T** | **C** | **A** | **C** | **T** | **C** | **T** | **T** | **C** | **T** | **C** | **A** | **C** | **A** | **G** | **A** | **C** | **G** | **G** | **A** | **C** | **A** | **A** | **G** | **A** | **G** | **A** | **A** | **A** | **T** | **T** | **G** | **G** | **A** | **T** | **T** | **C** | **A** | **C** | **T** | **G** | **C** | **T** | **T** | **C** | **A** | **A** | **G** | **A** | **T** | **C** | **T** | **A** | **A** | **A** | **A** | **A** | **A** | **A** | **G** | **C** | **C** | **A** | **A** | **A** | **C** | **A** | **T** | **T** | **C** | **G** | **A** | **T** | **C** | **C** | **A** | **T** | **A** | **T** | **C** | **G** | **A** | **A** | **T** | **A** | **T** | **T** | **A** | **C** | **G** | **C** | **A** | **T** | **T** | **T** | **G** | **T** | **G** | **A** | **C** | **A** | **G** | **A** | **T** | **A** | **A** | **T** | **A** | **T** | **C** | **G** | **G** | **C** | **C** | **A** | **G** | **C** | **A** | **A** | **G** | **T** | **C** | **T** | **T** | **A** | **C** | **A** | **T** | **G** | **T** | **T** | **T** | **T** | **A** | **G** | **A** | **A** | **G** | **A** | **A** | **A** | **A** | **A** | **G** | **C** | **C** | **G** | **C** | **A** | **G** | **A** | **A** | **G** | **G** | **C** | **G** | **T** | **G** | **G** | **A** | **A** | **G** | **T** | **T** | **C** | **G** | **A** | **A** | **T** | **A** | **T** | **T** | **A** | **T** | **A** | **T** | **G** | **A** | **T** | **G** | **C** | **A** | **T** | **T** | **T** | **G** | **G** | **C** | **T** | **C** | **T** | **C** | **A** | **T** | **G** | **G** | **C** | **A** | **C** | **A** | **A** | **A** | **A** | **G** | **C** | **A** | **A** | **A** | **A** | **G** | **A** | **T** | **T** | **T** | **C** | **A** | **A** | **T** | **C** | **G** | **T** | **C** | **T** | **A** | **A** | **T** | **C** | **A** | **A** | **A** | **A** | **A** | **T** | **G** | **G** | **T** | **G** | **G** | **A** | **C** | **A** | **T** | **G** | **T** | **C** | **C** | **A** | **T** | **A** | **C** | **A** | **T** | **T** | **T** | **G** | **T** | **T** | **A** | **C** | **C** | **T** | **C** | **A** | **C** | **A** | **A** | **A** | **A** | **G** | **G** | **C** | **A** | **T** | **T** | **A** | **C** | **T** | **T** | **C** | **G** | **T** | **T** | **T** | **C** | **C** | **G** | **A** | **T** | **T** | **G** | **A** | **A** | **T** | **T** | **A** | **C** | **C** | **A** | **T** | **G** | **A** | **T** | **C** | **A** | **C** | **C** | **G** | **A** | **A** | **A** | **A** | **A** | **T** | **C** | **G** | **T** | **T** | **G** | **T** | **G** | **A** | **T** | **C** | **G** | **A** | **T** | **G** | **G** | **A** | **A** | **A** | **G** | **A** | **T** | **T** | **A** | **G** | **T** | **T** | **A** | **T** | **A** | **C** | **C** | **G** | **G** | **T** | **G** | **G** | **T** | **T** | **T** | **C** | **A** | **A** | **T** | **A** | **T** | **T** | **G** | **C** | **C** | **A** | **A** | **T** | **C** | **A** | **A** | **T** | **A** | **T** | **G** | **T** | **A** | **A** | **A** | **T** | **A** | **C** | **A** | **A** | **C** | **A** | **A** | **A** | **A** | **A** | **A** | **A** | **T** | **T** | **C** | **G** | **G** | **C** | **T** | **A** | **T** | **T** | **G** | **G** | **C** | **G** | **C** | **G** | **A** | **T** | **A** | **C** | **G** | **C** | **A** | **T** | **A** | **T** | **A** | **C** | **G** | **G** | **A** | **T** | **T** | **T** | **T** | **C** | **G** | **G** | **T** | **G** | **C** | **C** | **G** | **C** | **T** | **T** | **C** | **T** | **T** | **C** | **T** | **T** | **T** | **G** | **C** | **T** | **C** | **C** | **A** | **G** | **C** | **T** | **T** | **C** | **G** | **C** | **T** | **T** | **C** | **T** | **T** | **A** | **A** | **C** | **A** | **G** | **A** | **C** | **T** | **G** | **G** | **A** | **A** | **C** | **G** | **T** | **C** | **T** | **C** | **G** | **G** | **T** | **A** | **C** | **C** | **T** | **G** | **A** | **A** | **G** | **A** | **A** | **A** | **A** | **A** | **A** | **A** | **G** | **G** | **T** | **C** | **G** | **G** | **C** | **T** | **A** | **T** | **C** | **A** | **T** | **T** | **T** | **G** | **A** | **A** | **T** | **T** | **A** | **T** | **T** | **T** | **C** | **T** | **T** | **T** | **A** | **A** | **A** | **A** | **A** | **A** | **G** | **C** | **A** | **G** | **A** | **T** | **C** | **G** | **A** | **G** | **A** | **T** | **G** | **A** | **A** | **T** | **C** | **T** | **A** | **A** | **G** | **C** | **T** | **T** | **G** | **C** | **T** | **G** | **A** | **T** | **A** | **C** | **A** | **T** | **C** | **C** | **A** | **T** | **C** | **C** | **A** | **G** | **C** | **T** | **T** | **G** | **T** | **T** | **T** | **C** | **A** | **A** | **G** | **C** | **G** | **G** | **A** | **C** | **C** | **G** | **A** | **A** | **T** | **A** | **A** | **C** | **G** | **A** | **A** | **A** | **G** | **G** | **G** | **A** | **A** | **C** | **A** | **A** | **A** | **T** | **C** | **A** | **A** | **G** | **C** | **T** | **T** | **T** | **C** | **A** | **T** | **T** | **T** | **A** | **T** | **C** | **A** | **A** | **A** | **T** | **T** | **G** | **A** | **T** | **T** | **A** | **C** | **T** | **T** | **C** | **T** | **G** | **C** | **T** | **A** | **A** | **A** | **A** | **A** | **A** | **C** | **G** | **T** | **G** | **T** | **T** | **T** | **G** | **G** | **A** | **T** | **A** | **C** | **A** | **G** | **A** | **C** | **A** | **C** | **C** | **T** | **T** | **A** | **C** | **C** | **T** | **T** | **G** | **T** | **T** | **C** | **C** | **T** | **G** | **A** | **T** | **G** | **A** | **A** | **A** | **G** | **T** | **G** | **T** | **C** | **A** | **T** | **T** | **G** | **C** | **T** | **G** | **C** | **T** | **T** | **T** | **A** | **A** | **A** | **A** | **A** | **T** | **C** | **G** | **C** | **A** | **A** | **C** | **T** | **G** | **C** | **C** | **T** | **C** | **T** | **G** | **G** | **T** | **G** | **T** | **A** | **G** | **A** | **T** | **G** | **T** | **G** | **A** | **A** | **A** | **A** | **T** | **C** | **A** | **T** | **G** | **A** | **T** | **T** | **C** | **C** | **C** | **A** | **A** | **C** | **A** | **A** | **A** | **C** | **C** | **G** | **G** | **A** | **T** | **C** | **A** | **T** | **C** | **C** | **T** | **T** | **T** | **T** | **A** | **T** | **T** | **T** | **A** | **T** | **C** | **G** | **A** | **G** | **C** | **A** | **A** | **C** | **A** | **C** | **A** | **A** | **T** | **A** | **T** | **T** | **A** | **T** | **G** | **C** | **T** | **C** | **G** | **G** | **C** | **A** | **G** | **C** | **T** | **G** | **A** | **T** | **C** | **A** | **A** | **G** | **G** | **A** | **A** | **A** | **A** | **T** | **G** | **T** | **A** | **C** | **A** | **A** | **A** | **T** | **C** | **C** | **T** | **T** | **G** | **T** | **C** | **T** | **A** | **T** | **G** | **A** | **G** | **A** | **A** | **C** | **G** | **G** | **C** | **T** | **T** | **C** | **C** | **T** | **C** | **C** | **A** | **T** | **G** | **C** | **A** | **A** | **A** | **A** | **A** | **C** | **A** | **T** | **T** | **G** | **A** | **T** | **A** | **A** | **T** | **G** | **G** | **A** | **T** | **G** | **A** | **T** | **G** | **A** | **A** | **A** | **T** | **C** | **T** | **G** | **C** | **A** | **T** | **G** | **G** | **T** | **A** | **G** | **G** | **T** | **T** | **C** | **A** | **G** | **C** | **A** | **A** | **A** | **T** | **C** | **A** | **A** | **G** | **A** | **T** | **A** | **T** | **T** | **C** | **G** | **T** | **A** | **G** | **C** | **T** | **A** | **C** | **C** | **G** | **A** | **T** | **T** | **G** | **A** | **A** | **T** | **T** | **T** | **T** | **G** | **A** | **A** | **A** | **C** | **A** | **A** | **G** | **T** | **G** | **C** | **T** | **G** | **T** | **C** | **A** | **T** | **T** | **T** | **A** | **C** | **G** | **A** | **T** | **C** | **C** | **T** | **G** | **A** | **G** | **T** | **T** | **T** | **T** | **T** | **A** | **G** | **A** | **A** | **G** | **A** | **A** | **C** | **T** | **T** | **G** | **C** | **T** | **A** | **C** | **T** | **C** | **A** | **G** | **T** | **T** | **C** | **A** | **A** | **A** | **G** | **A** | **A** | **G** | **A** | **T** | **G** | **A** | **G** | **A** | **C** | **A** | **C** | **A** | **T** | **T** | **G** | **T** | **T** | **C** | **A** | **T** | **C** | **C** | **A** | **T** | **G** | **A** | **C** | **A** | **A** | **C** | **T** | **G** | **A** | **A** | **A** | **C** | **A** | **G** | **T** | **C** | **A** | **A** | **G** | **G** | **A** | **A** | **A** | **T** | **G** | **T** | **C** | **T** | **A** | **A** | **C** | **T** | **G** | **G** | **C** | **T** | **A** | **T** | **T** | **A** | **T** | **T** | **C** | **A** | **A** | **G** | **C** | **A** | **A** | **C** | **A** | **A** | **A** | **T** | **T** | **T** | **C** | **T** | **C** | **G** | **A** | **T** | **T** | **A** | **T** | **T** | **T** | **T** | **C** | **T** | **C** | **C** | **A** | **A** | **T** | **C** | **C** | **T** | **A** | **-** | **-** | **-** |
| **N60417F** | **-** | **-** | **-** | **-** | **-** | **-** | **-** | **-** | **-** | **-** | **-** | **-** | **-** | **-** | **-** | **-** | **-** | **-** | **-** | **-** | **-** | **-** | **-** | **-** | **-** | **-** | **-** | **-** | **-** | **-** | **-** | **-** | **-** | **-** | **-** | **-** | **-** | **-** | **-** | **-** | **-** | **-** | **-** | **-** | **-** | **-** | **-** | **-** | **-** | **-** | **-** | **-** | **-** | **-** | **-** | **-** | **-** | **-** | **-** | **-** | **-** | **-** | **-** | **-** | **-** | **-** | **-** | **-** | **-** | **A** | **A** | **A** | **C** | **C** | **C** | **C** | **G** | **A** | **G** | **A** | **T** | **G** | **T** | **A** | **G** | **C** | **A** | **G** | **C** | **T** | **A** | **T** | **T** | **T** | **G** | **G** | **G** | **C** | **A** | **T** | **G** | **G** | **C** | **T** | **C** | **T** | **T** | **A** | **G** | **T** | **A** | **T** | **T** | **A** | **T** | **T** | **T** | **G** | **C** | **C** | **C** | **T** | **T** | **C** | **C** | **T** | **G** | **G** | **G** | **G** | **T** | **G** | **G** | **G** | **T** | **T** | **T** | **T** | **G** | **T** | **A** | **T** | **T** | **A** | **T** | **A** | **T** | **T** | **T** | **A** | **T** | **T** | **T** | **T** | **T** | **C** | **G** | **G** | **A** | **C** | **G** | **T** | **G** | **G** | **A** | **T** | **T** | **A** | **A** | **C** | **G** | **G** | **A** | **T** | **A** | **A** | **G** | **A** | **A** | **A** | **A** | **A** | **A** | **T** | **T** | **C** | **T** | **A** | **T** | **C** | **T** | **C** | **C** | **G** | **A** | **C** | **A** | **A** | **A** | **G** | **T** | **G** | **A** | **C** | **T** | **T** | **G** | **A** | **A** | **A** | **G** | **A** | **A** | **T** | **T** | **A** | **G** | **A** | **A** | **A** | **A** | **C** | **T** | **T** | **T** | **C** | **A** | **G** | **T** | **C** | **T** | **T** | **T** | **T** | **A** | **G** | **A** | **G** | **G** | **A** | **G** | **A** | **T** | **A** | **C** | **C** | **A** | **T** | **T** | **G** | **A** | **A** | **C** | **A** | **T** | **T** | **A** | **C** | **G** | **A** | **T** | **C** | **C** | **T** | **G** | **A** | **C** | **A** | **T** | **G** | **G** | **G** | **C** | **G** | **A** | **C** | **A** | **A** | **A** | **G** | **A** | **C** | **A** | **A** | **A** | **C** | **A** | **A** | **C** | **A** | **A** | **T** | **T** | **T** | **G** | **T** | **C** | **G** | **A** | **C** | **T** | **T** | **C** | **T** | **T** | **C** | **T** | **C** | **T** | **T** | **C** | **A** | **T** | **T** | **A** | **A** | **A** | **T** | **C** | **G** | **T** | **A** | **T** | **G** | **C** | **C** | **G** | **C** | **T** | **G** | **A** | **C** | **A** | **A** | **G** | **A** | **A** | **T** | **G** | **A** | **A** | **T** | **T** | **C** | **T** | **G** | **T** | **C** | **A** | **C** | **T** | **C** | **T** | **T** | **C** | **T** | **C** | **A** | **C** | **A** | **G** | **A** | **C** | **G** | **G** | **A** | **C** | **A** | **A** | **A** | **A** | **G** | **A** | **A** | **A** | **T** | **T** | **A** | **G** | **A** | **T** | **T** | **C** | **A** | **C** | **T** | **G** | **C** | **T** | **T** | **C** | **A** | **A** | **G** | **A** | **T** | **C** | **T** | **A** | **A** | **A** | **A** | **A** | **A** | **A** | **G** | **C** | **C** | **A** | **A** | **A** | **C** | **A** | **T** | **T** | **C** | **G** | **A** | **T** | **C** | **C** | **A** | **T** | **A** | **T** | **C** | **G** | **A** | **A** | **T** | **A** | **T** | **T** | **A** | **C** | **G** | **C** | **A** | **T** | **T** | **T** | **G** | **T** | **G** | **A** | **C** | **A** | **G** | **A** | **T** | **A** | **C** | **T** | **A** | **T** | **C** | **G** | **G** | **T** | **C** | **A** | **G** | **C** | **A** | **A** | **G** | **T** | **C** | **T** | **T** | **A** | **C** | **G** | **C** | **G** | **T** | **T** | **T** | **T** | **A** | **G** | **A** | **A** | **G** | **A** | **A** | **A** | **A** | **A** | **G** | **C** | **C** | **G** | **C** | **A** | **G** | **A** | **A** | **G** | **G** | **C** | **G** | **T** | **A** | **G** | **A** | **A** | **G** | **T** | **T** | **C** | **G** | **T** | **A** | **T** | **A** | **T** | **T** | **A** | **T** | **A** | **T** | **G** | **A** | **T** | **G** | **C** | **A** | **T** | **T** | **T** | **G** | **G** | **C** | **T** | **C** | **C** | **C** | **A** | **C** | **G** | **G** | **C** | **A** | **C** | **A** | **A** | **A** | **A** | **G** | **C** | **A** | **A** | **A** | **A** | **G** | **A** | **T** | **T** | **T** | **C** | **A** | **A** | **T** | **C** | **G** | **T** | **C** | **T** | **A** | **A** | **T** | **C** | **A** | **A** | **A** | **A** | **A** | **T** | **G** | **G** | **T** | **G** | **G** | **A** | **C** | **A** | **T** | **G** | **T** | **C** | **C** | **A** | **T** | **A** | **C** | **A** | **T** | **T** | **T** | **G** | **T** | **T** | **A** | **C** | **C** | **T** | **C** | **G** | **C** | **A** | **A** | **A** | **A** | **A** | **G** | **C** | **A** | **T** | **T** | **G** | **C** | **T** | **T** | **C** | **G** | **T** | **T** | **T** | **C** | **C** | **G** | **A** | **T** | **T** | **G** | **A** | **A** | **C** | **T** | **A** | **C** | **C** | **A** | **T** | **G** | **A** | **T** | **C** | **A** | **C** | **C** | **G** | **G** | **A** | **A** | **A** | **A** | **T** | **C** | **G** | **T** | **T** | **G** | **T** | **G** | **A** | **T** | **C** | **G** | **A** | **T** | **G** | **G** | **A** | **A** | **A** | **G** | **A** | **T** | **C** | **A** | **G** | **T** | **T** | **A** | **C** | **A** | **C** | **C** | **G** | **G** | **T** | **G** | **G** | **C** | **T** | **T** | **C** | **A** | **A** | **C** | **A** | **T** | **T** | **G** | **C** | **C** | **A** | **A** | **T** | **C** | **A** | **A** | **T** | **A** | **T** | **G** | **T** | **A** | **A** | **A** | **T** | **A** | **C** | **G** | **A** | **C** | **A** | **A** | **A** | **A** | **A** | **A** | **A** | **T** | **T** | **C** | **G** | **G** | **C** | **T** | **A** | **T** | **T** | **G** | **G** | **C** | **G** | **T** | **G** | **A** | **T** | **A** | **C** | **G** | **C** | **A** | **T** | **A** | **T** | **A** | **C** | **G** | **G** | **A** | **T** | **T** | **T** | **T** | **C** | **G** | **G** | **T** | **G** | **C** | **C** | **G** | **C** | **T** | **T** | **C** | **T** | **T** | **C** | **T** | **T** | **T** | **G** | **C** | **T** | **T** | **C** | **A** | **A** | **C** | **T** | **T** | **C** | **G** | **T** | **T** | **T** | **T** | **T** | **T** | **A** | **A** | **C** | **A** | **G** | **A** | **C** | **T** | **G** | **G** | **A** | **A** | **C** | **G** | **T** | **C** | **T** | **C** | **A** | **G** | **T** | **T** | **C** | **C** | **T** | **G** | **A** | **A** | **G** | **A** | **A** | **A** | **A** | **A** | **A** | **A** | **G** | **G** | **T** | **C** | **G** | **G** | **C** | **T** | **A** | **T** | **C** | **A** | **T** | **T** | **T** | **G** | **A** | **A** | **T** | **T** | **A** | **T** | **T** | **T** | **C** | **T** | **T** | **T** | **A** | **A** | **A** | **A** | **A** | **A** | **G** | **C** | **C** | **G** | **A** | **T** | **C** | **G** | **A** | **G** | **A** | **C** | **G** | **A** | **A** | **T** | **C** | **C** | **A** | **A** | **A** | **C** | **T** | **T** | **G** | **C** | **T** | **G** | **A** | **T** | **A** | **C** | **A** | **T** | **C** | **C** | **A** | **T** | **C** | **C** | **A** | **G** | **C** | **T** | **T** | **G** | **T** | **C** | **T** | **C** | **A** | **A** | **G** | **C** | **G** | **G** | **A** | **C** | **C** | **A** | **A** | **A** | **T** | **A** | **A** | **C** | **G** | **A** | **A** | **C** | **G** | **C** | **G** | **A** | **A** | **C** | **A** | **A** | **A** | **T** | **C** | **A** | **A** | **G** | **C** | **T** | **T** | **T** | **C** | **G** | **T** | **T** | **T** | **A** | **T** | **C** | **A** | **A** | **A** | **T** | **T** | **G** | **A** | **T** | **T** | **A** | **C** | **T** | **T** | **C** | **T** | **G** | **C** | **T** | **A** | **A** | **A** | **A** | **A** | **A** | **C** | **G** | **T** | **G** | **T** | **T** | **T** | **G** | **G** | **A** | **T** | **A** | **C** | **A** | **G** | **A** | **C** | **G** | **C** | **C** | **T** | **T** | **A** | **C** | **C** | **T** | **C** | **G** | **T** | **T** | **C** | **C** | **T** | **G** | **A** | **T** | **G** | **A** | **A** | **A** | **G** | **T** | **G** | **T** | **C** | **A** | **T** | **T** | **G** | **C** | **T** | **G** | **C** | **T** | **T** | **T** | **G** | **A** | **A** | **A** | **A** | **T** | **C** | **G** | **C** | **A** | **A** | **C** | **T** | **G** | **C** | **T** | **T** | **C** | **T** | **G** | **G** | **T** | **G** | **T** | **C** | **G** | **A** | **T** | **G** | **T** | **G** | **A** | **A** | **A** | **A** | **T** | **C** | **A** | **T** | **G** | **A** | **T** | **C** | **C** | **C** | **C** | **A** | **A** | **T** | **A** | **A** | **A** | **C** | **C** | **G** | **G** | **A** | **T** | **C** | **A** | **T** | **C** | **C** | **T** | **T** | **T** | **T** | **A** | **T** | **T** | **T** | **A** | **T** | **C** | **G** | **A** | **G** | **C** | **A** | **A** | **C** | **A** | **C** | **A** | **A** | **T** | **A** | **T** | **T** | **A** | **T** | **G** | **C** | **G** | **C** | **G** | **T** | **C** | **A** | **G** | **C** | **T** | **C** | **A** | **T** | **C** | **A** | **A** | **G** | **G** | **A** | **A** | **A** | **A** | **C** | **G** | **T** | **A** | **C** | **A** | **A** | **A** | **T** | **T** | **C** | **T** | **T** | **G** | **T** | **C** | **T** | **A** | **T** | **G** | **A** | **G** | **A** | **A** | **T** | **G** | **G** | **C** | **T** | **T** | **C** | **C** | **T** | **C** | **C** | **A** | **T** | **G** | **C** | **A** | **A** | **A** | **A** | **A** | **C** | **A** | **T** | **T** | **G** | **A** | **T** | **A** | **A** | **T** | **G** | **G** | **A** | **T** | **G** | **A** | **T** | **G** | **A** | **A** | **A** | **T** | **C** | **T** | **G** | **C** | **A** | **T** | **G** | **G** | **T** | **A** | **G** | **G** | **T** | **T** | **C** | **A** | **G** | **C** | **A** | **A** | **A** | **T** | **C** | **A** | **A** | **G** | **A** | **T** | **A** | **T** | **T** | **C** | **G** | **T** | **A** | **G** | **C** | **T** | **A** | **C** | **C** | **G** | **A** | **T** | **T** | **G** | **A** | **A** | **T** | **T** | **T** | **T** | **G** | **A** | **A** | **A** | **C** | **A** | **A** | **G** | **T** | **G** | **C** | **T** | **G** | **T** | **C** | **A** | **T** | **C** | **T** | **A** | **C** | **G** | **A** | **T** | **C** | **C** | **T** | **G** | **A** | **G** | **T** | **T** | **T** | **T** | **T** | **A** | **G** | **A** | **A** | **G** | **A** | **A** | **C** | **T** | **T** | **G** | **C** | **T** | **A** | **C** | **T** | **C** | **A** | **G** | **T** | **T** | **T** | **A** | **A** | **A** | **G** | **A** | **A** | **G** | **A** | **T** | **G** | **A** | **G** | **A** | **C** | **A** | **C** | **A** | **T** | **T** | **G** | **T** | **T** | **C** | **A** | **C** | **C** | **A** | **A** | **T** | **G** | **A** | **C** | **G** | **A** | **C** | **T** | **G** | **A** | **A** | **A** | **C** | **A** | **G** | **T** | **G** | **A** | **A** | **G** | **G** | **A** | **A** | **A** | **T** | **G** | **T** | **C** | **T** | **A** | **A** | **T** | **T** | **G** | **G** | **C** | **T** | **G** | **T** | **T** | **A** | **T** | **T** | **C** | **A** | **A** | **G** | **C** | **A** | **A** | **C** | **A** | **A** | **A** | **T** | **T** | **T** | **C** | **T** | **C** | **G** | **A** | **T** | **T** | **A** | **T** | **T** | **T** | **T** | **C** | **T** | **C** | **C** | **A** | **A** | **T** | **C** | **T** | **T** | **A** | **-** | **-** | **-** |
| **N54551** | **-** | **-** | **-** | **-** | **-** | **-** | **-** | **-** | **-** | **-** | **-** | **-** | **-** | **-** | **-** | **-** | **-** | **-** | **-** | **-** | **-** | **-** | **-** | **-** | **-** | **-** | **-** | **-** | **-** | **-** | **-** | **-** | **-** | **-** | **-** | **-** | **-** | **-** | **-** | **-** | **-** | **-** | **-** | **-** | **-** | **-** | **-** | **-** | **-** | **-** | **-** | **-** | **-** | **-** | **-** | **-** | **-** | **-** | **-** | **-** | **-** | **-** | **-** | **-** | **-** | **-** | **-** | **-** | **-** | **A** | **A** | **A** | **C** | **C** | **C** | **C** | **G** | **A** | **G** | **A** | **T** | **G** | **T** | **A** | **G** | **C** | **A** | **G** | **C** | **C** | **A** | **T** | **T** | **T** | **G** | **G** | **G** | **C** | **A** | **T** | **G** | **G** | **C** | **T** | **C** | **T** | **T** | **A** | **G** | **T** | **A** | **T** | **T** | **G** | **T** | **T** | **T** | **G** | **C** | **C** | **C** | **T** | **T** | **C** | **C** | **T** | **G** | **G** | **G** | **G** | **T** | **G** | **G** | **G** | **T** | **T** | **T** | **T** | **G** | **T** | **A** | **T** | **T** | **A** | **T** | **A** | **T** | **T** | **T** | **A** | **T** | **T** | **T** | **T** | **T** | **C** | **G** | **G** | **A** | **C** | **G** | **T** | **G** | **G** | **A** | **T** | **T** | **A** | **A** | **C** | **G** | **G** | **A** | **T** | **A** | **A** | **G** | **A** | **A** | **A** | **A** | **A** | **A** | **T** | **T** | **C** | **T** | **A** | **T** | **C** | **T** | **C** | **C** | **G** | **A** | **C** | **A** | **A** | **A** | **G** | **T** | **G** | **A** | **C** | **T** | **T** | **G** | **A** | **A** | **A** | **G** | **A** | **A** | **T** | **T** | **A** | **G** | **A** | **A** | **A** | **A** | **C** | **T** | **T** | **T** | **C** | **A** | **G** | **T** | **C** | **T** | **T** | **T** | **T** | **A** | **G** | **A** | **G** | **G** | **A** | **G** | **A** | **T** | **A** | **C** | **C** | **A** | **T** | **T** | **G** | **A** | **A** | **C** | **A** | **T** | **T** | **A** | **C** | **G** | **A** | **T** | **C** | **C** | **T** | **G** | **A** | **C** | **A** | **T** | **G** | **G** | **G** | **C** | **G** | **A** | **T** | **A** | **A** | **A** | **G** | **A** | **C** | **A** | **A** | **A** | **C** | **A** | **G** | **C** | **A** | **A** | **T** | **T** | **T** | **G** | **T** | **C** | **G** | **A** | **C** | **T** | **T** | **C** | **T** | **T** | **C** | **T** | **C** | **T** | **T** | **C** | **A** | **T** | **T** | **A** | **A** | **A** | **T** | **C** | **G** | **T** | **A** | **T** | **G** | **C** | **C** | **G** | **C** | **T** | **G** | **A** | **C** | **A** | **A** | **G** | **A** | **A** | **T** | **G** | **A** | **A** | **T** | **T** | **C** | **T** | **G** | **T** | **C** | **A** | **C** | **T** | **C** | **T** | **T** | **C** | **T** | **C** | **A** | **C** | **A** | **G** | **A** | **C** | **G** | **G** | **A** | **C** | **A** | **A** | **G** | **A** | **G** | **A** | **A** | **A** | **T** | **T** | **G** | **G** | **A** | **T** | **T** | **C** | **A** | **C** | **T** | **G** | **C** | **T** | **T** | **C** | **A** | **A** | **G** | **A** | **T** | **C** | **T** | **A** | **A** | **A** | **A** | **A** | **A** | **A** | **G** | **C** | **C** | **A** | **A** | **A** | **C** | **A** | **T** | **T** | **C** | **G** | **A** | **T** | **C** | **C** | **A** | **T** | **A** | **T** | **C** | **G** | **A** | **A** | **T** | **A** | **T** | **T** | **A** | **C** | **G** | **C** | **A** | **T** | **T** | **T** | **G** | **T** | **G** | **A** | **C** | **A** | **G** | **A** | **T** | **A** | **A** | **T** | **A** | **T** | **C** | **G** | **G** | **C** | **C** | **A** | **G** | **C** | **A** | **A** | **G** | **T** | **C** | **T** | **T** | **A** | **C** | **A** | **T** | **G** | **T** | **T** | **T** | **T** | **A** | **G** | **A** | **A** | **G** | **A** | **A** | **A** | **A** | **A** | **G** | **C** | **C** | **G** | **C** | **A** | **G** | **A** | **A** | **G** | **G** | **C** | **G** | **T** | **G** | **G** | **A** | **A** | **G** | **T** | **T** | **C** | **G** | **A** | **A** | **T** | **A** | **T** | **T** | **A** | **T** | **A** | **T** | **G** | **A** | **T** | **G** | **C** | **A** | **T** | **T** | **T** | **G** | **G** | **C** | **T** | **C** | **T** | **C** | **A** | **T** | **G** | **G** | **C** | **A** | **C** | **A** | **A** | **A** | **A** | **G** | **C** | **A** | **A** | **A** | **A** | **G** | **A** | **T** | **T** | **T** | **C** | **A** | **A** | **T** | **C** | **G** | **T** | **C** | **T** | **A** | **A** | **T** | **C** | **A** | **A** | **A** | **A** | **A** | **T** | **G** | **G** | **T** | **G** | **G** | **A** | **C** | **A** | **T** | **G** | **T** | **C** | **C** | **A** | **T** | **A** | **C** | **A** | **T** | **T** | **T** | **G** | **T** | **T** | **A** | **C** | **C** | **T** | **C** | **A** | **C** | **A** | **A** | **A** | **A** | **G** | **G** | **C** | **A** | **T** | **T** | **A** | **C** | **T** | **T** | **C** | **G** | **T** | **T** | **T** | **C** | **C** | **G** | **A** | **T** | **T** | **G** | **A** | **A** | **T** | **T** | **A** | **C** | **C** | **A** | **T** | **G** | **A** | **T** | **C** | **A** | **C** | **C** | **G** | **A** | **A** | **A** | **A** | **A** | **T** | **C** | **G** | **T** | **T** | **G** | **T** | **G** | **A** | **T** | **C** | **G** | **A** | **T** | **G** | **G** | **A** | **A** | **A** | **G** | **A** | **T** | **T** | **A** | **G** | **T** | **T** | **A** | **T** | **A** | **C** | **C** | **G** | **G** | **T** | **G** | **G** | **T** | **T** | **T** | **C** | **A** | **A** | **T** | **A** | **T** | **T** | **G** | **C** | **C** | **A** | **A** | **T** | **C** | **A** | **A** | **T** | **A** | **T** | **G** | **T** | **A** | **A** | **A** | **T** | **A** | **C** | **A** | **A** | **C** | **A** | **A** | **A** | **A** | **A** | **A** | **A** | **T** | **T** | **C** | **G** | **G** | **C** | **T** | **A** | **T** | **T** | **G** | **G** | **C** | **G** | **C** | **G** | **A** | **T** | **A** | **C** | **G** | **C** | **A** | **T** | **A** | **T** | **A** | **C** | **G** | **G** | **A** | **T** | **T** | **T** | **T** | **C** | **G** | **G** | **T** | **G** | **C** | **C** | **G** | **C** | **T** | **T** | **C** | **T** | **T** | **C** | **T** | **T** | **T** | **G** | **C** | **T** | **C** | **C** | **A** | **G** | **C** | **T** | **T** | **C** | **G** | **C** | **T** | **T** | **C** | **T** | **T** | **A** | **A** | **C** | **A** | **G** | **A** | **C** | **T** | **G** | **G** | **A** | **A** | **C** | **G** | **T** | **C** | **T** | **C** | **G** | **G** | **T** | **A** | **C** | **C** | **T** | **G** | **A** | **A** | **G** | **A** | **A** | **A** | **A** | **A** | **A** | **A** | **G** | **G** | **T** | **C** | **G** | **G** | **C** | **T** | **A** | **T** | **C** | **A** | **T** | **T** | **T** | **G** | **A** | **A** | **T** | **T** | **A** | **T** | **T** | **T** | **C** | **T** | **T** | **T** | **A** | **A** | **A** | **A** | **A** | **A** | **G** | **C** | **A** | **G** | **A** | **T** | **C** | **G** | **A** | **G** | **A** | **T** | **G** | **A** | **A** | **T** | **C** | **T** | **A** | **A** | **G** | **C** | **T** | **T** | **G** | **C** | **T** | **G** | **A** | **T** | **A** | **C** | **A** | **T** | **C** | **C** | **A** | **T** | **C** | **C** | **A** | **G** | **C** | **T** | **T** | **G** | **T** | **T** | **T** | **C** | **A** | **A** | **G** | **C** | **G** | **G** | **A** | **C** | **C** | **G** | **A** | **A** | **T** | **A** | **A** | **C** | **G** | **A** | **A** | **A** | **G** | **G** | **G** | **A** | **A** | **C** | **A** | **A** | **A** | **T** | **C** | **A** | **A** | **G** | **C** | **T** | **T** | **T** | **C** | **A** | **T** | **T** | **T** | **A** | **T** | **C** | **A** | **A** | **A** | **T** | **T** | **G** | **A** | **T** | **T** | **A** | **C** | **T** | **T** | **C** | **T** | **G** | **C** | **T** | **A** | **A** | **A** | **A** | **A** | **A** | **C** | **G** | **T** | **G** | **T** | **T** | **T** | **G** | **G** | **A** | **T** | **A** | **C** | **A** | **G** | **A** | **C** | **A** | **C** | **C** | **T** | **T** | **A** | **C** | **C** | **T** | **T** | **G** | **T** | **T** | **C** | **C** | **T** | **G** | **A** | **T** | **G** | **A** | **A** | **A** | **G** | **T** | **G** | **T** | **C** | **A** | **T** | **T** | **G** | **C** | **T** | **G** | **C** | **T** | **T** | **T** | **A** | **A** | **A** | **A** | **A** | **T** | **C** | **G** | **C** | **A** | **A** | **C** | **T** | **G** | **C** | **C** | **T** | **C** | **T** | **G** | **G** | **T** | **G** | **T** | **A** | **G** | **A** | **T** | **G** | **T** | **G** | **A** | **A** | **A** | **A** | **T** | **C** | **A** | **T** | **G** | **A** | **T** | **T** | **C** | **C** | **C** | **A** | **A** | **C** | **A** | **A** | **A** | **C** | **C** | **G** | **G** | **A** | **T** | **C** | **A** | **T** | **C** | **C** | **T** | **T** | **T** | **T** | **A** | **T** | **T** | **T** | **A** | **T** | **C** | **G** | **A** | **G** | **C** | **A** | **A** | **C** | **A** | **C** | **A** | **A** | **T** | **A** | **T** | **T** | **A** | **T** | **G** | **C** | **T** | **C** | **G** | **G** | **C** | **A** | **G** | **C** | **T** | **G** | **A** | **T** | **C** | **A** | **A** | **G** | **G** | **A** | **A** | **A** | **A** | **T** | **G** | **T** | **A** | **C** | **A** | **A** | **A** | **T** | **C** | **C** | **T** | **T** | **G** | **T** | **C** | **T** | **A** | **T** | **G** | **A** | **G** | **A** | **A** | **C** | **G** | **G** | **C** | **T** | **T** | **C** | **C** | **T** | **C** | **C** | **A** | **T** | **G** | **C** | **A** | **A** | **A** | **A** | **A** | **C** | **A** | **T** | **T** | **G** | **A** | **T** | **A** | **A** | **T** | **G** | **G** | **A** | **T** | **G** | **A** | **T** | **G** | **A** | **A** | **A** | **T** | **C** | **T** | **G** | **C** | **A** | **T** | **G** | **G** | **T** | **A** | **G** | **G** | **T** | **T** | **C** | **A** | **G** | **C** | **A** | **A** | **A** | **T** | **C** | **A** | **A** | **G** | **A** | **T** | **A** | **T** | **T** | **C** | **G** | **T** | **A** | **G** | **C** | **T** | **A** | **C** | **C** | **G** | **A** | **T** | **T** | **G** | **A** | **A** | **T** | **T** | **T** | **T** | **G** | **A** | **A** | **A** | **C** | **A** | **A** | **G** | **T** | **G** | **C** | **T** | **G** | **T** | **C** | **A** | **T** | **T** | **T** | **A** | **C** | **G** | **A** | **T** | **C** | **C** | **T** | **G** | **A** | **G** | **T** | **T** | **T** | **T** | **T** | **A** | **G** | **A** | **A** | **G** | **A** | **A** | **C** | **T** | **T** | **G** | **C** | **T** | **A** | **C** | **T** | **C** | **A** | **G** | **T** | **T** | **C** | **A** | **A** | **A** | **G** | **A** | **A** | **G** | **A** | **T** | **G** | **A** | **G** | **A** | **C** | **A** | **C** | **A** | **T** | **T** | **G** | **T** | **T** | **C** | **A** | **T** | **C** | **C** | **A** | **T** | **G** | **A** | **C** | **A** | **A** | **C** | **T** | **G** | **A** | **A** | **A** | **C** | **A** | **G** | **T** | **C** | **A** | **A** | **G** | **G** | **A** | **A** | **A** | **T** | **G** | **T** | **C** | **T** | **A** | **A** | **C** | **T** | **G** | **G** | **C** | **T** | **A** | **T** | **T** | **A** | **T** | **T** | **C** | **A** | **A** | **G** | **C** | **A** | **A** | **C** | **A** | **A** | **A** | **T** | **T** | **T** | **C** | **T** | **C** | **G** | **A** | **T** | **T** | **A** | **T** | **T** | **T** | **T** | **C** | **T** | **C** | **C** | **A** | **A** | **T** | **C** | **C** | **T** | **A** | **-** | **-** | **-** |
| **N60260F** | **-** | **-** | **-** | **-** | **-** | **-** | **-** | **-** | **-** | **-** | **-** | **-** | **-** | **-** | **-** | **-** | **-** | **-** | **-** | **-** | **-** | **-** | **-** | **-** | **-** | **-** | **-** | **-** | **-** | **-** | **-** | **-** | **-** | **-** | **-** | **-** | **-** | **-** | **-** | **-** | **-** | **-** | **-** | **-** | **-** | **-** | **-** | **-** | **-** | **-** | **-** | **-** | **-** | **-** | **-** | **-** | **-** | **-** | **-** | **-** | **-** | **-** | **-** | **-** | **-** | **-** | **-** | **-** | **-** | **A** | **A** | **A** | **C** | **C** | **C** | **C** | **G** | **A** | **G** | **A** | **T** | **G** | **T** | **A** | **G** | **C** | **A** | **G** | **C** | **C** | **A** | **T** | **T** | **T** | **G** | **G** | **G** | **C** | **A** | **T** | **G** | **G** | **C** | **T** | **C** | **T** | **T** | **A** | **G** | **T** | **A** | **T** | **T** | **G** | **T** | **T** | **T** | **G** | **C** | **C** | **C** | **T** | **T** | **C** | **C** | **T** | **G** | **G** | **G** | **G** | **T** | **G** | **G** | **G** | **T** | **T** | **T** | **T** | **G** | **T** | **A** | **T** | **T** | **A** | **T** | **A** | **T** | **T** | **T** | **A** | **T** | **T** | **T** | **T** | **T** | **C** | **G** | **G** | **A** | **C** | **G** | **T** | **G** | **G** | **A** | **T** | **T** | **A** | **A** | **C** | **G** | **G** | **A** | **T** | **A** | **A** | **G** | **A** | **A** | **A** | **A** | **A** | **A** | **T** | **T** | **C** | **T** | **A** | **T** | **C** | **T** | **C** | **C** | **G** | **A** | **C** | **A** | **A** | **A** | **G** | **T** | **G** | **A** | **C** | **T** | **T** | **G** | **A** | **A** | **A** | **G** | **A** | **A** | **T** | **T** | **A** | **G** | **A** | **A** | **A** | **A** | **C** | **T** | **T** | **T** | **C** | **A** | **G** | **T** | **C** | **T** | **T** | **T** | **T** | **A** | **G** | **A** | **G** | **G** | **A** | **G** | **A** | **T** | **A** | **C** | **C** | **A** | **T** | **T** | **G** | **A** | **A** | **C** | **A** | **T** | **T** | **A** | **C** | **G** | **A** | **T** | **C** | **C** | **T** | **G** | **A** | **C** | **A** | **T** | **G** | **G** | **G** | **C** | **G** | **A** | **T** | **A** | **A** | **A** | **G** | **A** | **C** | **A** | **A** | **A** | **C** | **A** | **G** | **C** | **A** | **A** | **T** | **T** | **T** | **G** | **T** | **C** | **G** | **A** | **C** | **T** | **T** | **C** | **T** | **T** | **C** | **T** | **C** | **T** | **T** | **C** | **A** | **T** | **T** | **A** | **A** | **A** | **T** | **C** | **G** | **T** | **A** | **T** | **G** | **C** | **C** | **G** | **C** | **T** | **G** | **A** | **C** | **A** | **A** | **G** | **A** | **A** | **T** | **G** | **A** | **A** | **T** | **T** | **C** | **T** | **G** | **T** | **C** | **A** | **C** | **T** | **C** | **T** | **T** | **C** | **T** | **C** | **A** | **C** | **A** | **G** | **A** | **C** | **G** | **G** | **A** | **C** | **A** | **A** | **G** | **A** | **G** | **A** | **A** | **A** | **T** | **T** | **G** | **G** | **A** | **T** | **T** | **C** | **A** | **C** | **T** | **G** | **C** | **T** | **T** | **C** | **A** | **A** | **G** | **A** | **T** | **C** | **T** | **A** | **A** | **A** | **A** | **A** | **A** | **A** | **G** | **C** | **C** | **A** | **A** | **A** | **C** | **A** | **T** | **T** | **C** | **G** | **A** | **T** | **C** | **C** | **A** | **T** | **A** | **T** | **C** | **G** | **A** | **A** | **T** | **A** | **T** | **T** | **A** | **C** | **G** | **C** | **A** | **T** | **T** | **T** | **G** | **T** | **G** | **A** | **C** | **A** | **G** | **A** | **T** | **A** | **A** | **T** | **A** | **T** | **C** | **G** | **G** | **C** | **C** | **A** | **G** | **C** | **A** | **A** | **G** | **T** | **C** | **T** | **T** | **A** | **C** | **A** | **T** | **G** | **T** | **T** | **T** | **T** | **A** | **G** | **A** | **A** | **G** | **A** | **A** | **A** | **A** | **A** | **G** | **C** | **C** | **G** | **C** | **A** | **G** | **A** | **A** | **G** | **G** | **C** | **G** | **T** | **G** | **G** | **A** | **A** | **G** | **T** | **T** | **C** | **G** | **A** | **A** | **T** | **A** | **T** | **T** | **A** | **T** | **A** | **T** | **G** | **A** | **T** | **G** | **C** | **A** | **T** | **T** | **T** | **G** | **G** | **C** | **T** | **C** | **T** | **C** | **A** | **T** | **G** | **G** | **C** | **A** | **C** | **A** | **A** | **A** | **A** | **G** | **C** | **A** | **A** | **A** | **A** | **G** | **A** | **T** | **T** | **T** | **C** | **A** | **A** | **T** | **C** | **G** | **T** | **C** | **T** | **A** | **A** | **T** | **C** | **A** | **A** | **A** | **A** | **A** | **T** | **G** | **G** | **T** | **G** | **G** | **A** | **C** | **A** | **T** | **G** | **T** | **C** | **C** | **A** | **T** | **A** | **C** | **A** | **T** | **T** | **T** | **G** | **T** | **T** | **A** | **C** | **C** | **T** | **C** | **A** | **C** | **A** | **A** | **A** | **A** | **G** | **G** | **C** | **A** | **T** | **T** | **A** | **C** | **T** | **T** | **C** | **G** | **T** | **T** | **T** | **C** | **C** | **G** | **A** | **T** | **T** | **G** | **A** | **A** | **T** | **T** | **A** | **C** | **C** | **A** | **T** | **G** | **A** | **T** | **C** | **A** | **C** | **C** | **G** | **A** | **A** | **A** | **A** | **A** | **T** | **C** | **G** | **T** | **T** | **G** | **T** | **G** | **A** | **T** | **C** | **G** | **A** | **T** | **G** | **G** | **A** | **A** | **A** | **G** | **A** | **T** | **T** | **A** | **G** | **T** | **T** | **A** | **T** | **A** | **C** | **C** | **G** | **G** | **T** | **G** | **G** | **T** | **T** | **T** | **C** | **A** | **A** | **T** | **A** | **T** | **T** | **G** | **C** | **C** | **A** | **A** | **T** | **C** | **A** | **A** | **T** | **A** | **T** | **G** | **T** | **A** | **A** | **A** | **T** | **A** | **C** | **A** | **A** | **C** | **A** | **A** | **A** | **A** | **A** | **A** | **A** | **T** | **T** | **C** | **G** | **G** | **C** | **T** | **A** | **T** | **T** | **G** | **G** | **C** | **G** | **C** | **G** | **A** | **T** | **A** | **C** | **G** | **C** | **A** | **T** | **A** | **T** | **A** | **C** | **G** | **G** | **A** | **T** | **T** | **T** | **T** | **C** | **G** | **G** | **T** | **G** | **C** | **C** | **G** | **C** | **T** | **T** | **C** | **T** | **T** | **C** | **T** | **T** | **T** | **G** | **C** | **T** | **C** | **C** | **A** | **G** | **C** | **T** | **T** | **C** | **G** | **C** | **T** | **T** | **C** | **T** | **T** | **A** | **A** | **C** | **A** | **G** | **A** | **C** | **T** | **G** | **G** | **A** | **A** | **C** | **G** | **T** | **C** | **T** | **C** | **G** | **G** | **T** | **A** | **C** | **C** | **T** | **G** | **A** | **A** | **G** | **A** | **A** | **A** | **A** | **A** | **A** | **A** | **G** | **G** | **T** | **C** | **G** | **G** | **C** | **T** | **A** | **T** | **C** | **A** | **T** | **T** | **T** | **G** | **A** | **A** | **T** | **T** | **A** | **T** | **T** | **T** | **C** | **T** | **T** | **T** | **A** | **A** | **A** | **A** | **A** | **A** | **G** | **C** | **A** | **G** | **A** | **T** | **C** | **G** | **A** | **G** | **A** | **T** | **G** | **A** | **A** | **T** | **C** | **T** | **A** | **A** | **G** | **C** | **T** | **T** | **G** | **C** | **T** | **G** | **A** | **T** | **A** | **C** | **A** | **T** | **C** | **T** | **A** | **T** | **C** | **C** | **A** | **G** | **C** | **T** | **T** | **G** | **T** | **T** | **T** | **C** | **A** | **A** | **G** | **C** | **G** | **G** | **A** | **C** | **C** | **G** | **A** | **A** | **T** | **A** | **A** | **C** | **G** | **A** | **A** | **A** | **G** | **G** | **G** | **A** | **A** | **C** | **A** | **A** | **A** | **T** | **C** | **A** | **A** | **G** | **C** | **T** | **T** | **T** | **C** | **A** | **T** | **T** | **T** | **A** | **T** | **C** | **A** | **A** | **A** | **T** | **T** | **G** | **A** | **T** | **T** | **A** | **C** | **T** | **T** | **C** | **T** | **G** | **C** | **T** | **A** | **A** | **A** | **A** | **A** | **A** | **C** | **G** | **T** | **G** | **T** | **T** | **T** | **G** | **G** | **A** | **T** | **A** | **C** | **A** | **G** | **A** | **C** | **A** | **C** | **C** | **T** | **T** | **A** | **C** | **C** | **T** | **T** | **G** | **T** | **T** | **C** | **C** | **T** | **G** | **A** | **T** | **G** | **A** | **A** | **A** | **G** | **T** | **G** | **T** | **C** | **A** | **T** | **T** | **G** | **C** | **T** | **G** | **C** | **T** | **T** | **T** | **A** | **A** | **A** | **A** | **A** | **T** | **C** | **G** | **C** | **A** | **A** | **C** | **T** | **G** | **C** | **C** | **T** | **C** | **T** | **G** | **G** | **T** | **G** | **T** | **A** | **G** | **A** | **T** | **G** | **T** | **G** | **A** | **A** | **A** | **A** | **T** | **C** | **A** | **T** | **G** | **A** | **T** | **T** | **C** | **C** | **C** | **A** | **A** | **C** | **A** | **A** | **A** | **C** | **C** | **G** | **G** | **A** | **T** | **C** | **A** | **T** | **C** | **C** | **T** | **T** | **T** | **T** | **A** | **T** | **T** | **T** | **A** | **T** | **C** | **G** | **A** | **G** | **C** | **A** | **A** | **C** | **A** | **C** | **A** | **A** | **T** | **A** | **T** | **T** | **A** | **T** | **G** | **C** | **T** | **C** | **G** | **G** | **C** | **A** | **G** | **C** | **T** | **G** | **A** | **T** | **C** | **A** | **A** | **G** | **G** | **A** | **A** | **A** | **A** | **T** | **G** | **T** | **A** | **C** | **A** | **A** | **A** | **T** | **C** | **C** | **T** | **T** | **G** | **T** | **C** | **T** | **A** | **T** | **G** | **A** | **G** | **A** | **A** | **C** | **G** | **G** | **C** | **T** | **T** | **C** | **C** | **T** | **C** | **C** | **A** | **T** | **G** | **C** | **A** | **A** | **A** | **A** | **A** | **C** | **A** | **T** | **T** | **G** | **A** | **T** | **A** | **A** | **T** | **G** | **G** | **A** | **T** | **G** | **A** | **T** | **G** | **A** | **A** | **A** | **T** | **C** | **T** | **G** | **C** | **A** | **T** | **G** | **G** | **T** | **A** | **G** | **G** | **T** | **T** | **C** | **A** | **G** | **C** | **A** | **A** | **A** | **T** | **C** | **A** | **A** | **G** | **A** | **T** | **A** | **T** | **T** | **C** | **G** | **T** | **A** | **G** | **C** | **T** | **A** | **C** | **C** | **G** | **A** | **T** | **T** | **G** | **A** | **A** | **T** | **T** | **T** | **T** | **G** | **A** | **A** | **A** | **C** | **A** | **A** | **G** | **T** | **G** | **C** | **T** | **G** | **T** | **C** | **A** | **T** | **T** | **T** | **A** | **C** | **G** | **A** | **T** | **C** | **C** | **T** | **G** | **A** | **G** | **T** | **T** | **T** | **T** | **T** | **A** | **G** | **A** | **A** | **G** | **A** | **A** | **C** | **T** | **T** | **G** | **C** | **T** | **A** | **C** | **T** | **C** | **A** | **G** | **T** | **T** | **C** | **A** | **A** | **A** | **G** | **A** | **A** | **G** | **A** | **T** | **G** | **A** | **G** | **A** | **C** | **A** | **C** | **A** | **T** | **T** | **G** | **T** | **T** | **C** | **A** | **T** | **C** | **C** | **A** | **T** | **G** | **A** | **C** | **A** | **A** | **C** | **T** | **G** | **A** | **A** | **A** | **C** | **A** | **G** | **T** | **C** | **A** | **A** | **G** | **G** | **A** | **A** | **A** | **T** | **G** | **T** | **C** | **T** | **A** | **A** | **C** | **T** | **G** | **G** | **C** | **T** | **A** | **T** | **T** | **A** | **T** | **T** | **C** | **A** | **A** | **G** | **C** | **A** | **A** | **C** | **A** | **A** | **A** | **T** | **T** | **T** | **C** | **T** | **C** | **G** | **A** | **T** | **T** | **A** | **T** | **T** | **T** | **T** | **C** | **T** | **C** | **C** | **A** | **A** | **T** | **C** | **C** | **T** | **A** | **-** | **-** | **-** |
| **N53439** | **-** | **-** | **-** | **-** | **-** | **-** | **-** | **-** | **-** | **-** | **-** | **-** | **-** | **-** | **-** | **-** | **-** | **-** | **-** | **-** | **-** | **-** | **-** | **-** | **-** | **-** | **-** | **-** | **-** | **-** | **-** | **-** | **-** | **-** | **-** | **-** | **-** | **-** | **-** | **-** | **-** | **-** | **-** | **-** | **-** | **-** | **-** | **-** | **-** | **-** | **-** | **-** | **-** | **-** | **-** | **-** | **-** | **-** | **-** | **-** | **-** | **-** | **-** | **-** | **-** | **-** | **-** | **-** | **-** | **A** | **A** | **A** | **C** | **C** | **C** | **C** | **G** | **A** | **G** | **A** | **T** | **G** | **T** | **A** | **G** | **C** | **A** | **G** | **C** | **C** | **A** | **T** | **T** | **T** | **G** | **G** | **G** | **C** | **A** | **T** | **G** | **G** | **C** | **T** | **C** | **T** | **T** | **A** | **G** | **T** | **A** | **T** | **T** | **G** | **T** | **T** | **T** | **G** | **C** | **C** | **C** | **T** | **T** | **C** | **C** | **T** | **G** | **G** | **G** | **G** | **T** | **G** | **G** | **G** | **C** | **T** | **T** | **T** | **G** | **T** | **A** | **T** | **T** | **A** | **T** | **A** | **T** | **T** | **T** | **A** | **T** | **T** | **T** | **T** | **T** | **C** | **G** | **G** | **A** | **C** | **G** | **T** | **G** | **G** | **A** | **T** | **T** | **A** | **A** | **C** | **G** | **G** | **A** | **T** | **A** | **A** | **G** | **A** | **A** | **A** | **A** | **A** | **A** | **T** | **T** | **C** | **T** | **A** | **T** | **C** | **T** | **C** | **C** | **G** | **A** | **C** | **A** | **A** | **A** | **G** | **T** | **G** | **A** | **C** | **T** | **T** | **G** | **A** | **A** | **A** | **G** | **A** | **A** | **T** | **T** | **A** | **G** | **A** | **A** | **A** | **A** | **C** | **T** | **T** | **T** | **C** | **A** | **G** | **T** | **C** | **T** | **T** | **T** | **T** | **A** | **G** | **A** | **G** | **G** | **A** | **G** | **A** | **T** | **A** | **C** | **C** | **A** | **T** | **T** | **G** | **A** | **A** | **C** | **A** | **T** | **T** | **A** | **C** | **G** | **A** | **T** | **C** | **C** | **T** | **G** | **A** | **C** | **A** | **T** | **G** | **G** | **G** | **C** | **G** | **A** | **T** | **A** | **A** | **A** | **G** | **A** | **C** | **A** | **A** | **A** | **C** | **A** | **G** | **C** | **A** | **A** | **T** | **T** | **T** | **G** | **T** | **T** | **G** | **A** | **C** | **T** | **T** | **C** | **T** | **T** | **C** | **T** | **C** | **T** | **T** | **C** | **A** | **T** | **T** | **A** | **A** | **A** | **T** | **C** | **G** | **T** | **A** | **T** | **G** | **C** | **C** | **G** | **C** | **T** | **G** | **A** | **C** | **A** | **A** | **T** | **A** | **A** | **T** | **G** | **A** | **A** | **T** | **T** | **C** | **T** | **G** | **T** | **C** | **A** | **C** | **T** | **C** | **T** | **T** | **C** | **T** | **C** | **A** | **C** | **A** | **G** | **A** | **C** | **G** | **G** | **A** | **C** | **A** | **A** | **G** | **A** | **G** | **A** | **A** | **A** | **T** | **T** | **G** | **G** | **A** | **T** | **T** | **C** | **A** | **C** | **T** | **G** | **C** | **T** | **T** | **C** | **A** | **A** | **G** | **A** | **T** | **C** | **T** | **A** | **A** | **A** | **A** | **A** | **A** | **A** | **G** | **C** | **C** | **A** | **A** | **A** | **C** | **A** | **T** | **T** | **C** | **G** | **A** | **T** | **C** | **C** | **A** | **T** | **A** | **T** | **C** | **G** | **A** | **A** | **T** | **A** | **T** | **T** | **A** | **C** | **G** | **C** | **A** | **T** | **T** | **T** | **G** | **T** | **G** | **A** | **C** | **A** | **G** | **A** | **T** | **A** | **A** | **T** | **A** | **T** | **C** | **G** | **G** | **C** | **C** | **A** | **G** | **C** | **A** | **A** | **G** | **T** | **C** | **T** | **T** | **A** | **C** | **A** | **T** | **G** | **T** | **T** | **T** | **T** | **A** | **G** | **A** | **A** | **G** | **A** | **A** | **A** | **A** | **A** | **G** | **C** | **C** | **G** | **C** | **A** | **G** | **A** | **A** | **G** | **G** | **C** | **G** | **T** | **G** | **G** | **A** | **A** | **G** | **T** | **T** | **C** | **G** | **A** | **A** | **T** | **A** | **T** | **T** | **A** | **T** | **A** | **T** | **G** | **A** | **T** | **G** | **C** | **A** | **T** | **T** | **T** | **G** | **G** | **C** | **T** | **C** | **T** | **C** | **A** | **T** | **G** | **G** | **C** | **A** | **C** | **A** | **A** | **A** | **A** | **G** | **C** | **A** | **A** | **A** | **A** | **G** | **A** | **T** | **T** | **T** | **C** | **A** | **A** | **T** | **C** | **G** | **T** | **C** | **T** | **A** | **A** | **T** | **C** | **A** | **A** | **A** | **A** | **A** | **T** | **G** | **G** | **T** | **G** | **G** | **A** | **C** | **A** | **T** | **G** | **T** | **C** | **C** | **A** | **T** | **A** | **C** | **A** | **T** | **T** | **T** | **G** | **T** | **T** | **A** | **C** | **C** | **T** | **C** | **A** | **C** | **A** | **A** | **A** | **A** | **G** | **G** | **C** | **A** | **T** | **T** | **A** | **C** | **T** | **T** | **C** | **G** | **T** | **T** | **T** | **C** | **C** | **G** | **A** | **T** | **T** | **G** | **A** | **A** | **T** | **T** | **A** | **C** | **C** | **A** | **T** | **G** | **A** | **T** | **C** | **A** | **C** | **C** | **G** | **A** | **A** | **A** | **A** | **A** | **T** | **C** | **G** | **T** | **T** | **G** | **T** | **G** | **A** | **T** | **C** | **G** | **A** | **T** | **G** | **G** | **A** | **A** | **A** | **G** | **A** | **T** | **T** | **A** | **G** | **T** | **T** | **A** | **T** | **A** | **C** | **C** | **G** | **G** | **T** | **G** | **G** | **T** | **T** | **T** | **C** | **A** | **A** | **T** | **A** | **T** | **T** | **G** | **C** | **C** | **A** | **A** | **T** | **C** | **A** | **A** | **T** | **A** | **T** | **G** | **T** | **A** | **A** | **A** | **T** | **A** | **C** | **A** | **A** | **C** | **A** | **A** | **A** | **A** | **A** | **A** | **A** | **T** | **T** | **C** | **G** | **G** | **C** | **T** | **A** | **T** | **T** | **G** | **G** | **C** | **G** | **C** | **G** | **A** | **T** | **A** | **C** | **G** | **C** | **A** | **T** | **A** | **T** | **A** | **C** | **G** | **G** | **A** | **T** | **T** | **T** | **T** | **C** | **G** | **G** | **T** | **G** | **C** | **C** | **G** | **C** | **T** | **T** | **C** | **T** | **T** | **C** | **T** | **T** | **T** | **G** | **C** | **T** | **C** | **C** | **A** | **G** | **C** | **T** | **T** | **C** | **G** | **C** | **T** | **T** | **C** | **T** | **T** | **A** | **A** | **C** | **A** | **G** | **A** | **C** | **T** | **G** | **G** | **A** | **A** | **C** | **G** | **T** | **C** | **T** | **C** | **G** | **G** | **T** | **A** | **C** | **C** | **T** | **G** | **A** | **A** | **G** | **A** | **A** | **A** | **A** | **A** | **A** | **A** | **G** | **G** | **T** | **C** | **G** | **G** | **C** | **T** | **A** | **T** | **C** | **A** | **T** | **T** | **T** | **G** | **A** | **A** | **T** | **T** | **A** | **T** | **T** | **T** | **T** | **T** | **T** | **T** | **A** | **A** | **A** | **A** | **A** | **A** | **G** | **C** | **A** | **G** | **A** | **T** | **C** | **A** | **A** | **G** | **A** | **T** | **G** | **A** | **A** | **T** | **C** | **T** | **A** | **A** | **G** | **C** | **T** | **T** | **G** | **C** | **T** | **G** | **A** | **T** | **A** | **C** | **A** | **T** | **C** | **C** | **A** | **T** | **C** | **C** | **A** | **G** | **C** | **T** | **T** | **G** | **T** | **T** | **T** | **C** | **A** | **A** | **G** | **C** | **G** | **G** | **A** | **C** | **C** | **G** | **A** | **A** | **T** | **A** | **A** | **C** | **G** | **A** | **A** | **A** | **G** | **G** | **G** | **A** | **A** | **C** | **A** | **A** | **A** | **T** | **C** | **A** | **A** | **G** | **C** | **T** | **T** | **T** | **C** | **A** | **T** | **T** | **T** | **A** | **T** | **C** | **A** | **A** | **A** | **T** | **T** | **G** | **A** | **T** | **T** | **A** | **C** | **T** | **T** | **C** | **T** | **G** | **C** | **T** | **A** | **A** | **A** | **A** | **A** | **A** | **C** | **G** | **T** | **G** | **T** | **T** | **T** | **G** | **G** | **A** | **T** | **A** | **C** | **A** | **G** | **A** | **C** | **A** | **C** | **C** | **T** | **T** | **A** | **C** | **C** | **T** | **T** | **G** | **T** | **T** | **C** | **C** | **T** | **G** | **A** | **T** | **G** | **A** | **A** | **A** | **G** | **T** | **G** | **T** | **C** | **A** | **T** | **T** | **G** | **C** | **T** | **G** | **C** | **T** | **T** | **T** | **A** | **A** | **A** | **A** | **A** | **T** | **C** | **G** | **C** | **A** | **A** | **C** | **T** | **G** | **C** | **C** | **T** | **C** | **T** | **G** | **G** | **T** | **G** | **T** | **A** | **G** | **A** | **T** | **G** | **T** | **G** | **A** | **A** | **A** | **A** | **T** | **C** | **A** | **T** | **G** | **A** | **T** | **T** | **C** | **C** | **C** | **A** | **A** | **C** | **A** | **A** | **A** | **C** | **C** | **G** | **G** | **A** | **T** | **C** | **A** | **T** | **C** | **C** | **T** | **T** | **T** | **T** | **A** | **T** | **T** | **T** | **A** | **T** | **C** | **G** | **A** | **G** | **C** | **A** | **A** | **C** | **A** | **C** | **A** | **A** | **T** | **A** | **T** | **T** | **A** | **T** | **G** | **C** | **T** | **C** | **G** | **G** | **C** | **A** | **G** | **C** | **T** | **G** | **A** | **T** | **C** | **A** | **A** | **G** | **G** | **A** | **A** | **A** | **A** | **T** | **G** | **T** | **A** | **C** | **A** | **A** | **A** | **T** | **C** | **C** | **T** | **T** | **G** | **T** | **C** | **T** | **A** | **T** | **G** | **A** | **G** | **A** | **A** | **C** | **G** | **G** | **C** | **T** | **T** | **C** | **C** | **T** | **C** | **C** | **A** | **T** | **G** | **C** | **A** | **A** | **A** | **A** | **A** | **C** | **A** | **T** | **T** | **G** | **A** | **T** | **A** | **A** | **T** | **G** | **G** | **A** | **T** | **G** | **A** | **T** | **G** | **A** | **A** | **A** | **T** | **C** | **T** | **G** | **C** | **A** | **T** | **G** | **G** | **T** | **A** | **G** | **G** | **T** | **T** | **C** | **A** | **G** | **C** | **A** | **A** | **A** | **T** | **C** | **A** | **A** | **G** | **A** | **T** | **A** | **T** | **T** | **C** | **G** | **T** | **A** | **G** | **C** | **T** | **A** | **C** | **C** | **G** | **A** | **T** | **T** | **G** | **A** | **A** | **T** | **T** | **T** | **T** | **G** | **A** | **A** | **A** | **C** | **A** | **A** | **G** | **T** | **G** | **C** | **T** | **G** | **T** | **C** | **A** | **T** | **T** | **T** | **A** | **C** | **G** | **A** | **T** | **C** | **C** | **T** | **G** | **A** | **G** | **T** | **T** | **T** | **T** | **T** | **A** | **G** | **A** | **A** | **G** | **A** | **A** | **C** | **T** | **T** | **G** | **C** | **T** | **A** | **C** | **T** | **C** | **A** | **G** | **T** | **T** | **C** | **A** | **A** | **A** | **G** | **A** | **A** | **G** | **A** | **T** | **G** | **A** | **G** | **A** | **C** | **A** | **C** | **A** | **T** | **T** | **G** | **T** | **T** | **C** | **A** | **T** | **C** | **C** | **A** | **T** | **G** | **A** | **C** | **A** | **A** | **C** | **T** | **G** | **A** | **A** | **A** | **C** | **A** | **G** | **T** | **C** | **A** | **A** | **G** | **G** | **A** | **A** | **A** | **T** | **G** | **T** | **C** | **T** | **A** | **A** | **C** | **T** | **G** | **G** | **C** | **T** | **A** | **T** | **T** | **A** | **T** | **T** | **C** | **A** | **A** | **G** | **C** | **A** | **A** | **C** | **A** | **A** | **A** | **T** | **T** | **T** | **C** | **T** | **C** | **G** | **A** | **T** | **T** | **A** | **T** | **T** | **T** | **T** | **C** | **T** | **C** | **C** | **A** | **A** | **T** | **C** | **C** | **T** | **A** | **-** | **-** | **-** |
| **N55077** | **-** | **-** | **-** | **-** | **-** | **-** | **-** | **-** | **-** | **-** | **-** | **-** | **-** | **-** | **-** | **-** | **-** | **-** | **-** | **-** | **-** | **-** | **-** | **-** | **-** | **-** | **-** | **-** | **-** | **-** | **-** | **-** | **-** | **-** | **-** | **-** | **-** | **-** | **-** | **-** | **-** | **-** | **-** | **-** | **-** | **-** | **-** | **-** | **-** | **-** | **-** | **-** | **-** | **-** | **-** | **-** | **-** | **-** | **-** | **-** | **-** | **-** | **-** | **-** | **-** | **-** | **-** | **-** | **-** | **A** | **A** | **A** | **C** | **C** | **C** | **C** | **G** | **A** | **G** | **A** | **T** | **G** | **T** | **A** | **G** | **C** | **A** | **G** | **C** | **C** | **A** | **T** | **T** | **T** | **G** | **G** | **G** | **C** | **A** | **T** | **G** | **G** | **C** | **T** | **C** | **T** | **T** | **A** | **G** | **T** | **A** | **T** | **T** | **G** | **T** | **T** | **T** | **G** | **C** | **C** | **C** | **T** | **T** | **C** | **C** | **T** | **G** | **G** | **G** | **G** | **T** | **G** | **G** | **G** | **C** | **T** | **T** | **T** | **G** | **T** | **A** | **T** | **T** | **A** | **T** | **A** | **T** | **T** | **T** | **A** | **T** | **T** | **T** | **T** | **T** | **C** | **G** | **G** | **A** | **C** | **G** | **T** | **G** | **G** | **A** | **T** | **T** | **A** | **A** | **C** | **G** | **G** | **A** | **T** | **A** | **A** | **G** | **A** | **A** | **A** | **A** | **A** | **A** | **T** | **T** | **C** | **T** | **A** | **T** | **C** | **T** | **C** | **C** | **G** | **A** | **C** | **A** | **A** | **A** | **G** | **T** | **G** | **A** | **C** | **T** | **T** | **G** | **A** | **A** | **A** | **G** | **A** | **A** | **T** | **T** | **A** | **G** | **A** | **A** | **A** | **A** | **C** | **T** | **T** | **T** | **C** | **A** | **G** | **T** | **C** | **T** | **T** | **T** | **T** | **A** | **G** | **A** | **G** | **G** | **A** | **G** | **A** | **T** | **A** | **C** | **C** | **A** | **T** | **T** | **G** | **A** | **A** | **C** | **A** | **T** | **T** | **A** | **C** | **G** | **A** | **T** | **C** | **C** | **T** | **G** | **A** | **C** | **A** | **T** | **G** | **G** | **G** | **C** | **G** | **A** | **T** | **A** | **A** | **A** | **G** | **A** | **C** | **A** | **A** | **A** | **C** | **A** | **G** | **C** | **A** | **A** | **T** | **T** | **T** | **G** | **T** | **T** | **G** | **A** | **C** | **T** | **T** | **C** | **T** | **T** | **C** | **T** | **C** | **T** | **T** | **C** | **A** | **T** | **T** | **A** | **A** | **A** | **T** | **C** | **G** | **T** | **A** | **T** | **G** | **C** | **C** | **G** | **C** | **T** | **G** | **A** | **C** | **A** | **A** | **G** | **A** | **A** | **T** | **G** | **A** | **A** | **T** | **T** | **C** | **T** | **G** | **T** | **C** | **A** | **C** | **T** | **C** | **T** | **T** | **C** | **T** | **C** | **A** | **C** | **A** | **G** | **A** | **C** | **G** | **G** | **A** | **C** | **A** | **A** | **G** | **A** | **G** | **A** | **A** | **A** | **T** | **T** | **G** | **G** | **A** | **T** | **T** | **C** | **A** | **C** | **T** | **G** | **C** | **T** | **T** | **C** | **A** | **A** | **G** | **A** | **T** | **C** | **T** | **A** | **A** | **A** | **A** | **A** | **A** | **A** | **G** | **C** | **C** | **A** | **A** | **A** | **C** | **A** | **T** | **T** | **C** | **G** | **A** | **T** | **C** | **C** | **A** | **T** | **A** | **T** | **C** | **G** | **A** | **A** | **T** | **A** | **T** | **T** | **A** | **C** | **G** | **C** | **A** | **T** | **T** | **T** | **G** | **T** | **G** | **A** | **C** | **A** | **G** | **A** | **T** | **A** | **A** | **T** | **A** | **T** | **C** | **G** | **G** | **C** | **C** | **A** | **G** | **C** | **A** | **A** | **G** | **T** | **C** | **T** | **T** | **A** | **C** | **A** | **T** | **G** | **T** | **T** | **T** | **T** | **A** | **G** | **A** | **A** | **G** | **A** | **A** | **A** | **A** | **A** | **G** | **C** | **C** | **G** | **C** | **A** | **G** | **A** | **A** | **G** | **G** | **C** | **G** | **T** | **G** | **G** | **A** | **A** | **G** | **T** | **T** | **C** | **G** | **A** | **A** | **T** | **A** | **T** | **T** | **A** | **T** | **A** | **T** | **G** | **A** | **T** | **G** | **C** | **A** | **T** | **T** | **T** | **G** | **G** | **C** | **T** | **C** | **T** | **C** | **A** | **T** | **G** | **G** | **C** | **A** | **C** | **A** | **A** | **A** | **A** | **G** | **C** | **A** | **A** | **A** | **A** | **G** | **A** | **T** | **T** | **T** | **C** | **A** | **A** | **T** | **C** | **G** | **T** | **C** | **T** | **A** | **A** | **T** | **C** | **A** | **A** | **A** | **A** | **A** | **T** | **G** | **G** | **T** | **G** | **G** | **A** | **C** | **A** | **T** | **G** | **T** | **C** | **C** | **A** | **T** | **A** | **C** | **A** | **T** | **T** | **T** | **G** | **T** | **T** | **A** | **C** | **C** | **T** | **C** | **A** | **C** | **A** | **A** | **A** | **G** | **G** | **G** | **C** | **A** | **T** | **T** | **A** | **C** | **T** | **T** | **C** | **G** | **T** | **T** | **T** | **C** | **C** | **G** | **A** | **T** | **T** | **G** | **A** | **A** | **T** | **T** | **A** | **C** | **C** | **A** | **T** | **G** | **A** | **T** | **C** | **A** | **C** | **C** | **G** | **A** | **A** | **A** | **A** | **A** | **T** | **C** | **G** | **T** | **T** | **G** | **T** | **G** | **A** | **T** | **C** | **G** | **A** | **T** | **G** | **G** | **A** | **A** | **A** | **G** | **A** | **T** | **T** | **A** | **G** | **T** | **T** | **A** | **T** | **A** | **C** | **C** | **G** | **G** | **T** | **G** | **G** | **T** | **T** | **T** | **C** | **A** | **A** | **T** | **A** | **T** | **T** | **G** | **C** | **C** | **A** | **A** | **T** | **C** | **A** | **A** | **T** | **A** | **T** | **G** | **T** | **A** | **A** | **A** | **T** | **A** | **C** | **A** | **A** | **C** | **A** | **A** | **A** | **A** | **A** | **A** | **A** | **T** | **T** | **C** | **G** | **G** | **C** | **T** | **A** | **T** | **T** | **G** | **G** | **C** | **G** | **C** | **G** | **A** | **T** | **A** | **C** | **G** | **C** | **A** | **T** | **A** | **T** | **A** | **C** | **G** | **G** | **A** | **T** | **T** | **T** | **T** | **C** | **G** | **G** | **T** | **G** | **C** | **C** | **G** | **C** | **T** | **T** | **C** | **T** | **T** | **C** | **T** | **T** | **T** | **G** | **C** | **T** | **C** | **C** | **A** | **G** | **C** | **T** | **T** | **C** | **G** | **C** | **T** | **T** | **C** | **T** | **T** | **A** | **A** | **C** | **A** | **G** | **A** | **C** | **T** | **G** | **G** | **A** | **A** | **C** | **G** | **T** | **C** | **T** | **C** | **G** | **G** | **T** | **A** | **C** | **C** | **T** | **G** | **A** | **A** | **G** | **A** | **A** | **A** | **A** | **A** | **A** | **A** | **G** | **G** | **T** | **C** | **G** | **G** | **C** | **T** | **A** | **T** | **C** | **A** | **T** | **T** | **T** | **G** | **A** | **A** | **T** | **T** | **A** | **T** | **T** | **T** | **C** | **T** | **T** | **T** | **A** | **A** | **A** | **A** | **A** | **A** | **G** | **C** | **A** | **G** | **A** | **T** | **C** | **G** | **A** | **G** | **A** | **T** | **G** | **A** | **A** | **T** | **C** | **T** | **A** | **A** | **G** | **C** | **T** | **T** | **G** | **C** | **T** | **G** | **A** | **T** | **A** | **C** | **A** | **T** | **C** | **C** | **A** | **T** | **C** | **C** | **A** | **G** | **C** | **T** | **T** | **G** | **T** | **T** | **T** | **C** | **A** | **A** | **G** | **C** | **G** | **G** | **A** | **C** | **C** | **G** | **A** | **A** | **T** | **A** | **A** | **C** | **G** | **A** | **A** | **A** | **G** | **G** | **G** | **A** | **A** | **C** | **A** | **A** | **A** | **T** | **C** | **A** | **A** | **G** | **C** | **T** | **T** | **T** | **C** | **A** | **T** | **T** | **T** | **A** | **T** | **C** | **A** | **A** | **A** | **T** | **T** | **G** | **A** | **T** | **T** | **A** | **C** | **T** | **T** | **C** | **T** | **G** | **C** | **T** | **A** | **A** | **A** | **A** | **A** | **A** | **C** | **G** | **T** | **G** | **T** | **T** | **T** | **G** | **G** | **A** | **T** | **A** | **C** | **A** | **G** | **A** | **C** | **A** | **C** | **C** | **T** | **T** | **A** | **C** | **C** | **T** | **T** | **G** | **T** | **T** | **C** | **C** | **T** | **G** | **A** | **T** | **G** | **A** | **A** | **A** | **G** | **T** | **G** | **T** | **C** | **A** | **T** | **T** | **G** | **C** | **T** | **G** | **C** | **T** | **T** | **T** | **A** | **A** | **A** | **A** | **A** | **T** | **C** | **G** | **C** | **A** | **A** | **C** | **T** | **G** | **C** | **C** | **T** | **C** | **T** | **G** | **G** | **T** | **G** | **T** | **A** | **G** | **A** | **T** | **G** | **T** | **G** | **A** | **A** | **A** | **A** | **T** | **C** | **A** | **T** | **G** | **A** | **T** | **T** | **C** | **C** | **C** | **A** | **A** | **C** | **A** | **A** | **A** | **C** | **C** | **G** | **G** | **A** | **T** | **C** | **A** | **T** | **C** | **C** | **T** | **T** | **T** | **T** | **A** | **T** | **T** | **T** | **A** | **T** | **C** | **G** | **A** | **G** | **C** | **A** | **A** | **C** | **A** | **C** | **A** | **A** | **T** | **A** | **T** | **T** | **A** | **T** | **G** | **C** | **T** | **C** | **G** | **G** | **C** | **A** | **G** | **C** | **T** | **G** | **A** | **T** | **C** | **A** | **A** | **G** | **G** | **A** | **A** | **A** | **A** | **T** | **G** | **T** | **A** | **C** | **A** | **A** | **A** | **T** | **C** | **C** | **T** | **T** | **G** | **T** | **C** | **T** | **A** | **T** | **G** | **A** | **G** | **A** | **A** | **C** | **G** | **G** | **C** | **T** | **T** | **C** | **C** | **T** | **C** | **C** | **A** | **T** | **G** | **C** | **A** | **A** | **A** | **A** | **A** | **C** | **A** | **T** | **T** | **G** | **A** | **T** | **A** | **A** | **T** | **G** | **G** | **A** | **T** | **G** | **A** | **T** | **G** | **A** | **A** | **A** | **T** | **C** | **T** | **G** | **C** | **A** | **T** | **G** | **G** | **T** | **A** | **G** | **G** | **T** | **T** | **C** | **A** | **G** | **C** | **A** | **A** | **A** | **T** | **C** | **A** | **A** | **G** | **A** | **T** | **A** | **T** | **T** | **C** | **G** | **T** | **A** | **G** | **C** | **T** | **A** | **C** | **C** | **G** | **A** | **T** | **T** | **G** | **A** | **A** | **T** | **T** | **T** | **T** | **G** | **A** | **A** | **A** | **C** | **A** | **A** | **G** | **T** | **G** | **C** | **T** | **G** | **T** | **C** | **A** | **T** | **T** | **T** | **A** | **C** | **G** | **A** | **T** | **C** | **C** | **T** | **G** | **A** | **G** | **T** | **T** | **T** | **T** | **T** | **A** | **G** | **A** | **A** | **G** | **A** | **A** | **C** | **T** | **T** | **G** | **C** | **T** | **A** | **C** | **T** | **C** | **A** | **G** | **T** | **T** | **C** | **A** | **A** | **A** | **G** | **A** | **A** | **G** | **A** | **T** | **G** | **A** | **G** | **A** | **C** | **A** | **C** | **A** | **T** | **T** | **G** | **T** | **T** | **C** | **A** | **T** | **C** | **C** | **A** | **T** | **G** | **A** | **C** | **A** | **A** | **C** | **T** | **G** | **A** | **A** | **A** | **C** | **A** | **G** | **T** | **C** | **A** | **A** | **G** | **G** | **A** | **A** | **A** | **T** | **G** | **T** | **C** | **T** | **A** | **A** | **C** | **T** | **G** | **G** | **C** | **T** | **A** | **T** | **T** | **A** | **T** | **T** | **C** | **A** | **A** | **G** | **C** | **A** | **A** | **C** | **A** | **A** | **A** | **T** | **T** | **T** | **C** | **T** | **C** | **G** | **A** | **T** | **T** | **A** | **T** | **T** | **T** | **T** | **C** | **T** | **C** | **C** | **A** | **A** | **T** | **C** | **C** | **T** | **A** | **-** | **-** | **-** |
| **N54107** | **-** | **-** | **-** | **-** | **-** | **-** | **-** | **-** | **-** | **-** | **-** | **-** | **-** | **-** | **-** | **-** | **-** | **-** | **-** | **-** | **-** | **-** | **-** | **-** | **-** | **-** | **-** | **-** | **-** | **-** | **-** | **-** | **-** | **-** | **-** | **-** | **-** | **-** | **-** | **-** | **-** | **-** | **-** | **-** | **-** | **-** | **-** | **-** | **-** | **-** | **-** | **-** | **-** | **-** | **-** | **-** | **-** | **-** | **-** | **-** | **-** | **-** | **-** | **-** | **-** | **-** | **-** | **-** | **-** | **A** | **A** | **A** | **C** | **C** | **C** | **C** | **G** | **A** | **G** | **A** | **T** | **G** | **T** | **A** | **G** | **C** | **A** | **G** | **C** | **C** | **A** | **T** | **T** | **T** | **G** | **G** | **G** | **C** | **A** | **T** | **G** | **G** | **C** | **T** | **C** | **T** | **T** | **A** | **G** | **T** | **A** | **T** | **T** | **G** | **T** | **T** | **T** | **G** | **C** | **C** | **C** | **T** | **T** | **C** | **C** | **T** | **G** | **G** | **G** | **G** | **T** | **G** | **G** | **G** | **C** | **T** | **T** | **T** | **G** | **T** | **A** | **T** | **T** | **A** | **T** | **A** | **T** | **T** | **T** | **A** | **T** | **T** | **T** | **T** | **T** | **C** | **G** | **G** | **A** | **C** | **G** | **T** | **G** | **G** | **A** | **T** | **T** | **A** | **A** | **C** | **G** | **G** | **A** | **T** | **A** | **A** | **G** | **A** | **A** | **A** | **A** | **A** | **A** | **T** | **T** | **C** | **T** | **A** | **T** | **C** | **T** | **C** | **C** | **G** | **A** | **C** | **A** | **A** | **A** | **G** | **T** | **G** | **A** | **C** | **T** | **T** | **G** | **A** | **A** | **A** | **G** | **A** | **A** | **T** | **T** | **A** | **G** | **A** | **A** | **A** | **A** | **C** | **T** | **T** | **T** | **C** | **A** | **G** | **T** | **C** | **T** | **T** | **T** | **T** | **A** | **G** | **A** | **G** | **G** | **A** | **G** | **A** | **T** | **A** | **A** | **C** | **A** | **T** | **T** | **G** | **A** | **A** | **C** | **A** | **T** | **T** | **A** | **C** | **G** | **A** | **T** | **C** | **C** | **T** | **G** | **A** | **C** | **A** | **T** | **G** | **G** | **G** | **C** | **G** | **A** | **T** | **A** | **A** | **A** | **G** | **A** | **C** | **A** | **A** | **A** | **C** | **A** | **G** | **C** | **A** | **A** | **T** | **T** | **T** | **G** | **T** | **T** | **G** | **A** | **C** | **T** | **T** | **C** | **T** | **T** | **C** | **T** | **C** | **T** | **T** | **C** | **A** | **T** | **T** | **A** | **A** | **A** | **T** | **C** | **G** | **T** | **A** | **T** | **G** | **C** | **C** | **G** | **C** | **T** | **G** | **A** | **C** | **A** | **A** | **G** | **A** | **A** | **T** | **G** | **A** | **A** | **T** | **T** | **C** | **T** | **G** | **T** | **C** | **A** | **C** | **T** | **C** | **T** | **T** | **C** | **T** | **C** | **A** | **C** | **A** | **G** | **A** | **C** | **G** | **G** | **A** | **C** | **A** | **A** | **G** | **A** | **G** | **A** | **A** | **A** | **T** | **T** | **G** | **G** | **A** | **T** | **T** | **C** | **A** | **C** | **T** | **G** | **C** | **T** | **T** | **C** | **A** | **A** | **G** | **A** | **T** | **C** | **T** | **A** | **A** | **A** | **A** | **A** | **A** | **A** | **G** | **C** | **C** | **A** | **A** | **A** | **C** | **A** | **T** | **T** | **C** | **G** | **A** | **T** | **C** | **C** | **A** | **T** | **A** | **T** | **C** | **G** | **A** | **A** | **T** | **A** | **T** | **T** | **A** | **C** | **G** | **C** | **A** | **T** | **T** | **T** | **G** | **T** | **G** | **A** | **C** | **A** | **G** | **A** | **T** | **A** | **A** | **T** | **A** | **T** | **C** | **G** | **G** | **C** | **C** | **A** | **G** | **C** | **A** | **A** | **G** | **T** | **C** | **T** | **T** | **A** | **C** | **A** | **T** | **G** | **T** | **T** | **T** | **T** | **A** | **G** | **A** | **A** | **G** | **A** | **A** | **A** | **A** | **A** | **G** | **C** | **C** | **G** | **C** | **A** | **G** | **A** | **A** | **G** | **G** | **C** | **G** | **T** | **G** | **G** | **A** | **A** | **G** | **T** | **T** | **C** | **G** | **A** | **A** | **T** | **A** | **T** | **T** | **A** | **T** | **A** | **T** | **G** | **A** | **T** | **G** | **C** | **A** | **T** | **T** | **T** | **G** | **G** | **C** | **T** | **C** | **T** | **C** | **A** | **T** | **G** | **G** | **C** | **A** | **C** | **A** | **A** | **A** | **A** | **G** | **C** | **A** | **A** | **A** | **A** | **G** | **A** | **T** | **T** | **T** | **C** | **A** | **A** | **T** | **C** | **G** | **T** | **C** | **T** | **A** | **A** | **T** | **C** | **A** | **A** | **A** | **A** | **A** | **T** | **G** | **G** | **T** | **G** | **G** | **A** | **C** | **A** | **T** | **G** | **T** | **C** | **C** | **A** | **T** | **A** | **C** | **A** | **T** | **T** | **T** | **G** | **T** | **T** | **A** | **C** | **C** | **T** | **C** | **A** | **C** | **A** | **A** | **A** | **A** | **G** | **G** | **C** | **A** | **T** | **T** | **A** | **C** | **T** | **T** | **C** | **G** | **T** | **T** | **T** | **C** | **C** | **G** | **A** | **T** | **T** | **G** | **A** | **A** | **T** | **T** | **A** | **C** | **C** | **A** | **T** | **G** | **A** | **T** | **C** | **A** | **C** | **C** | **G** | **A** | **A** | **A** | **A** | **A** | **T** | **C** | **G** | **T** | **T** | **G** | **T** | **G** | **A** | **T** | **C** | **G** | **A** | **T** | **G** | **G** | **A** | **A** | **A** | **G** | **A** | **T** | **T** | **A** | **G** | **T** | **T** | **A** | **T** | **A** | **C** | **C** | **G** | **G** | **T** | **G** | **G** | **T** | **T** | **T** | **C** | **A** | **A** | **T** | **A** | **T** | **T** | **G** | **C** | **C** | **A** | **A** | **T** | **C** | **A** | **A** | **T** | **A** | **T** | **G** | **T** | **A** | **A** | **A** | **T** | **A** | **C** | **A** | **A** | **C** | **A** | **A** | **A** | **A** | **A** | **A** | **A** | **T** | **T** | **C** | **G** | **G** | **C** | **T** | **A** | **T** | **T** | **G** | **G** | **C** | **G** | **C** | **G** | **A** | **T** | **A** | **C** | **G** | **C** | **A** | **T** | **A** | **T** | **A** | **C** | **G** | **G** | **A** | **T** | **T** | **T** | **T** | **C** | **G** | **G** | **T** | **G** | **C** | **C** | **G** | **C** | **T** | **T** | **C** | **T** | **T** | **C** | **T** | **T** | **T** | **G** | **C** | **T** | **C** | **C** | **A** | **G** | **C** | **T** | **T** | **C** | **G** | **C** | **T** | **T** | **C** | **T** | **T** | **A** | **A** | **C** | **A** | **G** | **A** | **C** | **T** | **G** | **G** | **A** | **A** | **C** | **G** | **T** | **C** | **T** | **C** | **G** | **G** | **T** | **A** | **C** | **C** | **T** | **G** | **A** | **A** | **G** | **A** | **A** | **A** | **A** | **A** | **A** | **A** | **G** | **G** | **T** | **C** | **G** | **G** | **C** | **T** | **A** | **T** | **C** | **A** | **T** | **T** | **T** | **G** | **A** | **A** | **T** | **T** | **A** | **T** | **T** | **T** | **C** | **T** | **T** | **T** | **A** | **A** | **A** | **A** | **A** | **A** | **G** | **C** | **A** | **G** | **A** | **T** | **C** | **G** | **A** | **G** | **A** | **T** | **G** | **A** | **A** | **T** | **C** | **T** | **A** | **A** | **G** | **C** | **T** | **T** | **G** | **C** | **T** | **G** | **A** | **T** | **A** | **C** | **A** | **T** | **C** | **C** | **A** | **T** | **C** | **C** | **A** | **G** | **C** | **T** | **T** | **G** | **T** | **T** | **T** | **C** | **A** | **A** | **G** | **C** | **G** | **G** | **A** | **C** | **C** | **G** | **A** | **A** | **T** | **A** | **A** | **C** | **G** | **A** | **A** | **A** | **G** | **G** | **G** | **A** | **A** | **C** | **A** | **A** | **A** | **T** | **C** | **A** | **A** | **G** | **C** | **T** | **T** | **T** | **C** | **A** | **T** | **T** | **T** | **A** | **T** | **C** | **A** | **A** | **A** | **T** | **T** | **G** | **A** | **T** | **T** | **A** | **C** | **T** | **T** | **C** | **T** | **G** | **C** | **T** | **A** | **A** | **A** | **A** | **A** | **A** | **C** | **G** | **T** | **G** | **T** | **T** | **T** | **G** | **G** | **A** | **T** | **A** | **C** | **A** | **G** | **A** | **C** | **A** | **C** | **C** | **T** | **T** | **A** | **C** | **C** | **T** | **T** | **G** | **T** | **T** | **C** | **C** | **T** | **G** | **A** | **T** | **G** | **A** | **A** | **A** | **G** | **T** | **G** | **T** | **C** | **A** | **T** | **T** | **G** | **C** | **T** | **G** | **C** | **T** | **T** | **T** | **A** | **A** | **A** | **A** | **A** | **T** | **C** | **G** | **C** | **A** | **A** | **C** | **T** | **G** | **C** | **C** | **T** | **C** | **T** | **G** | **G** | **T** | **G** | **T** | **A** | **G** | **A** | **T** | **G** | **T** | **G** | **A** | **A** | **A** | **A** | **T** | **C** | **A** | **T** | **G** | **A** | **T** | **T** | **C** | **C** | **C** | **A** | **A** | **C** | **A** | **A** | **A** | **C** | **C** | **G** | **G** | **A** | **T** | **C** | **A** | **T** | **C** | **C** | **T** | **T** | **T** | **T** | **A** | **T** | **T** | **T** | **A** | **T** | **C** | **G** | **A** | **G** | **C** | **A** | **A** | **C** | **A** | **C** | **A** | **A** | **T** | **A** | **T** | **T** | **A** | **T** | **G** | **C** | **T** | **C** | **G** | **G** | **C** | **A** | **G** | **C** | **T** | **G** | **A** | **T** | **C** | **A** | **A** | **G** | **G** | **A** | **A** | **A** | **A** | **T** | **G** | **T** | **A** | **C** | **A** | **A** | **A** | **T** | **C** | **C** | **T** | **T** | **G** | **T** | **C** | **T** | **A** | **T** | **G** | **A** | **G** | **A** | **A** | **C** | **G** | **G** | **C** | **T** | **T** | **C** | **C** | **T** | **C** | **C** | **A** | **T** | **G** | **C** | **A** | **A** | **A** | **A** | **A** | **C** | **A** | **T** | **T** | **G** | **A** | **T** | **A** | **A** | **T** | **G** | **G** | **A** | **T** | **G** | **A** | **T** | **G** | **A** | **A** | **A** | **T** | **C** | **T** | **G** | **C** | **A** | **T** | **G** | **G** | **T** | **A** | **G** | **G** | **T** | **T** | **C** | **A** | **G** | **C** | **A** | **A** | **A** | **T** | **C** | **A** | **A** | **G** | **A** | **T** | **A** | **T** | **T** | **C** | **G** | **T** | **A** | **G** | **C** | **T** | **A** | **C** | **C** | **G** | **A** | **T** | **T** | **G** | **A** | **A** | **T** | **T** | **T** | **T** | **G** | **A** | **A** | **A** | **C** | **A** | **A** | **G** | **T** | **G** | **C** | **T** | **G** | **T** | **C** | **A** | **T** | **T** | **T** | **A** | **C** | **G** | **A** | **T** | **C** | **C** | **T** | **G** | **A** | **G** | **T** | **T** | **T** | **T** | **T** | **A** | **G** | **A** | **A** | **G** | **A** | **A** | **C** | **T** | **T** | **G** | **C** | **T** | **A** | **C** | **T** | **C** | **A** | **G** | **T** | **T** | **C** | **A** | **A** | **A** | **G** | **A** | **A** | **G** | **A** | **T** | **G** | **A** | **G** | **A** | **C** | **A** | **C** | **A** | **T** | **T** | **G** | **T** | **T** | **C** | **A** | **T** | **C** | **C** | **A** | **T** | **G** | **A** | **C** | **A** | **A** | **C** | **T** | **G** | **A** | **A** | **A** | **C** | **A** | **G** | **T** | **C** | **A** | **A** | **G** | **G** | **A** | **A** | **A** | **T** | **G** | **T** | **C** | **T** | **A** | **A** | **C** | **T** | **G** | **G** | **C** | **T** | **A** | **T** | **T** | **A** | **T** | **T** | **C** | **A** | **A** | **G** | **C** | **A** | **A** | **C** | **A** | **A** | **A** | **T** | **T** | **T** | **C** | **T** | **C** | **G** | **A** | **T** | **T** | **A** | **T** | **T** | **T** | **T** | **C** | **T** | **C** | **C** | **A** | **A** | **T** | **C** | **C** | **T** | **A** | **-** | **-** | **-** |
| **N54155** | **-** | **-** | **-** | **-** | **-** | **-** | **-** | **-** | **-** | **-** | **-** | **-** | **-** | **-** | **-** | **-** | **-** | **-** | **-** | **-** | **-** | **-** | **-** | **-** | **-** | **-** | **-** | **-** | **-** | **-** | **-** | **-** | **-** | **-** | **-** | **-** | **-** | **-** | **-** | **-** | **-** | **-** | **-** | **-** | **-** | **-** | **-** | **-** | **-** | **-** | **-** | **-** | **-** | **-** | **-** | **-** | **-** | **-** | **-** | **-** | **-** | **-** | **-** | **-** | **-** | **-** | **-** | **-** | **-** | **A** | **A** | **A** | **C** | **C** | **C** | **C** | **G** | **A** | **G** | **A** | **T** | **G** | **T** | **A** | **G** | **C** | **A** | **G** | **C** | **C** | **A** | **T** | **T** | **T** | **G** | **G** | **G** | **C** | **A** | **T** | **G** | **G** | **C** | **T** | **C** | **T** | **T** | **A** | **G** | **T** | **A** | **T** | **T** | **G** | **T** | **T** | **T** | **G** | **C** | **C** | **C** | **T** | **T** | **C** | **C** | **T** | **G** | **G** | **G** | **G** | **T** | **G** | **G** | **G** | **C** | **T** | **T** | **T** | **G** | **T** | **A** | **T** | **T** | **A** | **T** | **A** | **T** | **T** | **T** | **A** | **T** | **T** | **T** | **T** | **T** | **C** | **G** | **G** | **A** | **C** | **G** | **T** | **G** | **G** | **A** | **T** | **T** | **A** | **A** | **C** | **G** | **G** | **A** | **T** | **A** | **A** | **G** | **A** | **A** | **A** | **A** | **A** | **A** | **T** | **T** | **C** | **T** | **A** | **T** | **C** | **T** | **C** | **C** | **G** | **A** | **C** | **A** | **A** | **A** | **G** | **T** | **G** | **A** | **C** | **T** | **T** | **G** | **A** | **A** | **A** | **G** | **A** | **A** | **T** | **T** | **A** | **G** | **A** | **A** | **A** | **A** | **C** | **T** | **T** | **T** | **C** | **A** | **G** | **T** | **C** | **T** | **T** | **T** | **T** | **A** | **G** | **A** | **G** | **G** | **A** | **G** | **A** | **T** | **A** | **A** | **C** | **A** | **T** | **T** | **G** | **A** | **A** | **C** | **A** | **T** | **T** | **A** | **C** | **G** | **A** | **T** | **C** | **C** | **T** | **G** | **A** | **C** | **A** | **T** | **G** | **G** | **G** | **C** | **G** | **A** | **T** | **A** | **A** | **A** | **G** | **A** | **C** | **A** | **A** | **A** | **C** | **A** | **G** | **C** | **A** | **A** | **T** | **T** | **T** | **G** | **T** | **T** | **G** | **A** | **C** | **T** | **T** | **C** | **T** | **T** | **C** | **T** | **C** | **T** | **T** | **C** | **A** | **T** | **T** | **A** | **A** | **A** | **T** | **C** | **G** | **T** | **A** | **T** | **G** | **C** | **C** | **G** | **C** | **T** | **G** | **A** | **C** | **A** | **A** | **G** | **A** | **A** | **T** | **G** | **A** | **A** | **T** | **T** | **C** | **T** | **G** | **T** | **C** | **A** | **C** | **T** | **C** | **T** | **T** | **C** | **T** | **C** | **A** | **C** | **A** | **G** | **A** | **C** | **G** | **G** | **A** | **C** | **A** | **A** | **G** | **A** | **G** | **A** | **A** | **A** | **T** | **T** | **G** | **G** | **A** | **T** | **T** | **C** | **A** | **C** | **T** | **G** | **C** | **T** | **T** | **C** | **A** | **A** | **G** | **A** | **T** | **C** | **T** | **A** | **A** | **A** | **A** | **A** | **A** | **A** | **G** | **C** | **C** | **A** | **A** | **A** | **C** | **A** | **T** | **T** | **C** | **G** | **A** | **T** | **C** | **C** | **A** | **T** | **A** | **T** | **C** | **G** | **A** | **A** | **T** | **A** | **T** | **T** | **A** | **C** | **G** | **C** | **A** | **T** | **T** | **T** | **G** | **T** | **G** | **A** | **C** | **A** | **G** | **A** | **T** | **A** | **A** | **T** | **A** | **T** | **C** | **G** | **G** | **C** | **C** | **A** | **G** | **C** | **A** | **A** | **G** | **T** | **C** | **T** | **T** | **A** | **C** | **A** | **T** | **G** | **T** | **T** | **T** | **T** | **A** | **G** | **A** | **A** | **G** | **A** | **A** | **A** | **A** | **A** | **G** | **C** | **C** | **G** | **C** | **A** | **G** | **A** | **A** | **G** | **G** | **C** | **G** | **T** | **G** | **G** | **A** | **A** | **G** | **T** | **T** | **C** | **G** | **A** | **A** | **T** | **A** | **T** | **T** | **A** | **T** | **A** | **T** | **G** | **A** | **T** | **G** | **C** | **A** | **T** | **T** | **T** | **G** | **G** | **C** | **T** | **C** | **T** | **C** | **A** | **T** | **G** | **G** | **C** | **A** | **C** | **A** | **A** | **A** | **A** | **G** | **C** | **A** | **A** | **A** | **A** | **G** | **A** | **T** | **T** | **T** | **C** | **A** | **A** | **T** | **C** | **G** | **T** | **C** | **T** | **A** | **A** | **T** | **C** | **A** | **A** | **A** | **A** | **A** | **T** | **G** | **G** | **T** | **G** | **G** | **A** | **C** | **A** | **T** | **G** | **T** | **C** | **C** | **A** | **T** | **A** | **C** | **A** | **T** | **T** | **T** | **G** | **T** | **T** | **A** | **C** | **C** | **T** | **C** | **A** | **C** | **A** | **A** | **A** | **A** | **G** | **G** | **C** | **A** | **T** | **T** | **A** | **C** | **T** | **T** | **C** | **G** | **T** | **T** | **T** | **C** | **C** | **G** | **A** | **T** | **T** | **G** | **A** | **A** | **T** | **T** | **A** | **C** | **C** | **A** | **T** | **G** | **A** | **T** | **C** | **A** | **C** | **C** | **G** | **A** | **A** | **A** | **A** | **A** | **T** | **C** | **G** | **T** | **T** | **G** | **T** | **G** | **A** | **T** | **C** | **G** | **A** | **T** | **G** | **G** | **A** | **A** | **A** | **G** | **A** | **T** | **T** | **A** | **G** | **T** | **T** | **A** | **T** | **A** | **C** | **C** | **G** | **G** | **T** | **G** | **G** | **T** | **T** | **T** | **C** | **A** | **A** | **T** | **A** | **T** | **T** | **G** | **C** | **C** | **A** | **A** | **T** | **C** | **A** | **A** | **T** | **A** | **T** | **G** | **T** | **A** | **A** | **A** | **T** | **A** | **C** | **A** | **A** | **C** | **A** | **A** | **A** | **A** | **A** | **A** | **A** | **T** | **T** | **C** | **G** | **G** | **C** | **T** | **A** | **T** | **T** | **G** | **G** | **C** | **G** | **C** | **G** | **A** | **T** | **A** | **C** | **G** | **C** | **A** | **T** | **A** | **T** | **A** | **C** | **G** | **G** | **A** | **T** | **T** | **T** | **T** | **C** | **G** | **G** | **T** | **G** | **C** | **C** | **G** | **C** | **T** | **T** | **C** | **T** | **T** | **C** | **T** | **T** | **T** | **G** | **C** | **T** | **C** | **C** | **A** | **G** | **C** | **T** | **T** | **C** | **G** | **C** | **T** | **T** | **C** | **T** | **T** | **A** | **A** | **C** | **A** | **G** | **A** | **C** | **T** | **G** | **G** | **A** | **A** | **C** | **G** | **T** | **C** | **T** | **C** | **G** | **G** | **T** | **A** | **C** | **C** | **T** | **G** | **A** | **A** | **G** | **A** | **A** | **A** | **A** | **A** | **A** | **A** | **G** | **G** | **T** | **C** | **G** | **G** | **C** | **T** | **A** | **T** | **C** | **A** | **T** | **T** | **T** | **G** | **A** | **A** | **T** | **T** | **A** | **T** | **T** | **T** | **C** | **T** | **T** | **T** | **A** | **A** | **A** | **A** | **A** | **A** | **G** | **C** | **A** | **G** | **A** | **T** | **C** | **G** | **A** | **G** | **A** | **T** | **G** | **A** | **A** | **T** | **C** | **T** | **A** | **A** | **G** | **C** | **T** | **T** | **G** | **C** | **T** | **G** | **A** | **T** | **A** | **C** | **A** | **T** | **C** | **C** | **A** | **T** | **C** | **C** | **A** | **G** | **C** | **T** | **T** | **G** | **T** | **T** | **T** | **C** | **A** | **A** | **G** | **C** | **G** | **G** | **A** | **C** | **C** | **G** | **A** | **A** | **T** | **A** | **A** | **C** | **G** | **A** | **A** | **A** | **G** | **G** | **G** | **A** | **A** | **C** | **A** | **A** | **A** | **T** | **C** | **A** | **A** | **G** | **C** | **T** | **T** | **T** | **C** | **A** | **T** | **T** | **T** | **A** | **T** | **C** | **A** | **A** | **A** | **T** | **T** | **G** | **A** | **T** | **T** | **A** | **C** | **T** | **T** | **C** | **T** | **G** | **C** | **T** | **A** | **A** | **A** | **A** | **A** | **A** | **C** | **G** | **T** | **G** | **T** | **T** | **T** | **G** | **G** | **A** | **T** | **A** | **C** | **A** | **G** | **A** | **C** | **A** | **C** | **C** | **T** | **T** | **A** | **C** | **C** | **T** | **T** | **G** | **T** | **T** | **C** | **C** | **T** | **G** | **A** | **T** | **G** | **A** | **A** | **A** | **G** | **T** | **G** | **T** | **C** | **A** | **T** | **T** | **G** | **C** | **T** | **G** | **C** | **T** | **T** | **T** | **A** | **A** | **A** | **A** | **A** | **T** | **C** | **G** | **C** | **A** | **A** | **C** | **T** | **G** | **C** | **C** | **T** | **C** | **T** | **G** | **G** | **T** | **G** | **T** | **A** | **G** | **A** | **T** | **G** | **T** | **G** | **A** | **A** | **A** | **A** | **T** | **C** | **A** | **T** | **G** | **A** | **T** | **T** | **C** | **C** | **C** | **A** | **A** | **C** | **A** | **A** | **A** | **C** | **C** | **G** | **G** | **A** | **T** | **C** | **A** | **T** | **C** | **C** | **T** | **T** | **T** | **T** | **A** | **T** | **T** | **T** | **A** | **T** | **C** | **G** | **A** | **G** | **C** | **A** | **A** | **C** | **A** | **C** | **A** | **A** | **T** | **A** | **T** | **T** | **A** | **T** | **G** | **C** | **T** | **C** | **G** | **G** | **C** | **A** | **G** | **C** | **T** | **G** | **A** | **T** | **C** | **A** | **A** | **G** | **G** | **A** | **A** | **A** | **A** | **T** | **G** | **T** | **A** | **C** | **A** | **A** | **A** | **T** | **C** | **C** | **T** | **T** | **G** | **T** | **C** | **T** | **A** | **T** | **G** | **A** | **G** | **A** | **A** | **C** | **G** | **G** | **C** | **T** | **T** | **C** | **C** | **T** | **C** | **C** | **A** | **T** | **G** | **C** | **A** | **A** | **A** | **A** | **A** | **C** | **A** | **T** | **T** | **G** | **A** | **T** | **A** | **A** | **T** | **G** | **G** | **A** | **T** | **G** | **A** | **T** | **G** | **A** | **A** | **A** | **T** | **C** | **T** | **G** | **C** | **A** | **T** | **G** | **G** | **T** | **A** | **G** | **G** | **T** | **T** | **C** | **A** | **G** | **C** | **A** | **A** | **A** | **T** | **C** | **A** | **A** | **G** | **A** | **T** | **A** | **T** | **T** | **C** | **G** | **T** | **A** | **G** | **C** | **T** | **A** | **C** | **C** | **G** | **A** | **T** | **T** | **G** | **A** | **A** | **T** | **T** | **T** | **T** | **G** | **A** | **A** | **A** | **C** | **A** | **A** | **G** | **T** | **G** | **C** | **T** | **G** | **T** | **C** | **A** | **T** | **T** | **T** | **A** | **C** | **G** | **A** | **T** | **C** | **C** | **T** | **G** | **A** | **G** | **T** | **T** | **T** | **T** | **T** | **A** | **G** | **A** | **A** | **G** | **A** | **A** | **C** | **T** | **T** | **G** | **C** | **T** | **A** | **C** | **T** | **C** | **A** | **G** | **T** | **T** | **C** | **A** | **A** | **A** | **G** | **A** | **A** | **G** | **A** | **T** | **G** | **A** | **G** | **A** | **C** | **A** | **C** | **A** | **T** | **T** | **G** | **T** | **T** | **C** | **A** | **T** | **C** | **C** | **A** | **T** | **G** | **A** | **C** | **A** | **A** | **C** | **T** | **G** | **A** | **A** | **A** | **C** | **A** | **G** | **T** | **C** | **A** | **A** | **G** | **G** | **A** | **A** | **A** | **T** | **G** | **T** | **C** | **T** | **A** | **A** | **C** | **T** | **G** | **G** | **C** | **T** | **A** | **T** | **T** | **A** | **T** | **T** | **C** | **A** | **A** | **G** | **C** | **A** | **A** | **C** | **A** | **A** | **A** | **T** | **T** | **T** | **C** | **T** | **C** | **G** | **A** | **T** | **T** | **A** | **T** | **T** | **T** | **T** | **C** | **T** | **C** | **C** | **A** | **A** | **T** | **C** | **C** | **T** | **A** | **-** | **-** | **-** |
| **N55317** | **-** | **-** | **-** | **-** | **-** | **-** | **-** | **-** | **-** | **-** | **-** | **-** | **-** | **-** | **-** | **-** | **-** | **-** | **-** | **-** | **-** | **-** | **-** | **-** | **-** | **-** | **-** | **-** | **-** | **-** | **-** | **-** | **-** | **-** | **-** | **-** | **-** | **-** | **-** | **-** | **-** | **-** | **-** | **-** | **-** | **-** | **-** | **-** | **-** | **-** | **-** | **-** | **-** | **-** | **-** | **-** | **-** | **-** | **-** | **-** | **-** | **-** | **-** | **-** | **-** | **-** | **-** | **-** | **-** | **A** | **A** | **A** | **C** | **C** | **C** | **C** | **G** | **A** | **G** | **A** | **T** | **G** | **T** | **A** | **G** | **C** | **A** | **G** | **C** | **T** | **A** | **T** | **T** | **T** | **G** | **G** | **G** | **C** | **A** | **T** | **G** | **G** | **C** | **T** | **C** | **T** | **T** | **A** | **G** | **T** | **A** | **T** | **T** | **G** | **T** | **T** | **T** | **G** | **C** | **C** | **C** | **T** | **T** | **C** | **C** | **T** | **G** | **G** | **G** | **G** | **T** | **G** | **G** | **G** | **T** | **T** | **T** | **T** | **G** | **T** | **A** | **T** | **T** | **A** | **T** | **A** | **T** | **T** | **T** | **A** | **T** | **T** | **T** | **T** | **T** | **C** | **G** | **G** | **A** | **C** | **G** | **T** | **G** | **G** | **A** | **T** | **T** | **A** | **A** | **C** | **G** | **G** | **A** | **T** | **A** | **A** | **G** | **A** | **A** | **A** | **A** | **A** | **A** | **T** | **T** | **C** | **T** | **A** | **T** | **C** | **T** | **C** | **C** | **G** | **A** | **C** | **A** | **A** | **A** | **G** | **T** | **G** | **A** | **C** | **T** | **T** | **G** | **A** | **A** | **A** | **G** | **A** | **A** | **T** | **T** | **A** | **G** | **A** | **A** | **A** | **A** | **C** | **T** | **T** | **T** | **C** | **A** | **G** | **T** | **C** | **T** | **T** | **T** | **T** | **A** | **G** | **A** | **G** | **G** | **A** | **G** | **A** | **T** | **A** | **C** | **C** | **A** | **T** | **T** | **G** | **A** | **A** | **C** | **A** | **T** | **T** | **A** | **C** | **G** | **A** | **T** | **C** | **C** | **T** | **G** | **A** | **C** | **A** | **T** | **G** | **G** | **G** | **C** | **G** | **A** | **C** | **A** | **A** | **A** | **G** | **A** | **C** | **A** | **A** | **A** | **C** | **A** | **A** | **C** | **A** | **A** | **T** | **T** | **T** | **G** | **T** | **C** | **G** | **A** | **C** | **T** | **T** | **C** | **T** | **T** | **C** | **T** | **C** | **T** | **T** | **C** | **A** | **T** | **T** | **A** | **A** | **A** | **T** | **C** | **G** | **T** | **A** | **T** | **G** | **C** | **C** | **G** | **C** | **T** | **G** | **A** | **C** | **A** | **A** | **G** | **A** | **A** | **T** | **G** | **A** | **A** | **T** | **T** | **C** | **T** | **G** | **T** | **C** | **A** | **C** | **T** | **C** | **T** | **T** | **C** | **T** | **C** | **A** | **C** | **A** | **G** | **A** | **C** | **G** | **G** | **A** | **C** | **A** | **A** | **A** | **A** | **G** | **A** | **A** | **A** | **T** | **T** | **A** | **G** | **A** | **T** | **T** | **C** | **A** | **C** | **T** | **G** | **C** | **T** | **T** | **C** | **A** | **A** | **G** | **A** | **T** | **C** | **T** | **A** | **A** | **A** | **A** | **A** | **A** | **A** | **G** | **C** | **C** | **A** | **A** | **A** | **C** | **A** | **T** | **T** | **C** | **G** | **A** | **T** | **C** | **C** | **A** | **T** | **A** | **T** | **C** | **G** | **A** | **A** | **T** | **A** | **T** | **T** | **A** | **C** | **G** | **C** | **A** | **T** | **T** | **T** | **G** | **T** | **G** | **A** | **C** | **A** | **G** | **A** | **T** | **A** | **C** | **T** | **A** | **T** | **C** | **G** | **G** | **T** | **C** | **A** | **G** | **C** | **A** | **A** | **G** | **T** | **C** | **T** | **T** | **A** | **C** | **G** | **C** | **G** | **T** | **T** | **T** | **T** | **A** | **G** | **A** | **A** | **G** | **A** | **A** | **A** | **A** | **A** | **G** | **C** | **C** | **G** | **C** | **A** | **G** | **A** | **A** | **G** | **G** | **C** | **G** | **T** | **A** | **G** | **A** | **A** | **G** | **T** | **T** | **C** | **G** | **T** | **A** | **T** | **A** | **T** | **T** | **A** | **T** | **A** | **T** | **G** | **A** | **T** | **G** | **C** | **A** | **T** | **T** | **T** | **G** | **G** | **C** | **T** | **C** | **C** | **C** | **A** | **T** | **G** | **G** | **C** | **A** | **C** | **A** | **A** | **A** | **A** | **G** | **C** | **A** | **A** | **A** | **A** | **G** | **A** | **T** | **T** | **T** | **C** | **A** | **A** | **T** | **C** | **G** | **T** | **C** | **T** | **A** | **A** | **T** | **C** | **A** | **A** | **A** | **A** | **A** | **T** | **G** | **G** | **T** | **G** | **G** | **A** | **C** | **A** | **T** | **G** | **T** | **C** | **C** | **A** | **T** | **A** | **C** | **A** | **T** | **T** | **T** | **G** | **T** | **T** | **A** | **C** | **C** | **T** | **C** | **G** | **C** | **A** | **A** | **A** | **A** | **A** | **G** | **C** | **A** | **T** | **T** | **G** | **C** | **T** | **T** | **C** | **G** | **T** | **T** | **T** | **C** | **C** | **G** | **A** | **T** | **T** | **G** | **A** | **A** | **T** | **T** | **A** | **C** | **C** | **A** | **T** | **G** | **A** | **T** | **C** | **A** | **C** | **C** | **G** | **G** | **A** | **A** | **A** | **A** | **T** | **C** | **G** | **T** | **T** | **G** | **T** | **G** | **A** | **T** | **C** | **G** | **A** | **T** | **G** | **G** | **A** | **A** | **A** | **G** | **A** | **T** | **C** | **A** | **G** | **T** | **T** | **A** | **C** | **A** | **C** | **C** | **G** | **G** | **T** | **G** | **G** | **T** | **T** | **T** | **C** | **A** | **A** | **T** | **A** | **T** | **T** | **G** | **C** | **C** | **A** | **A** | **T** | **C** | **A** | **A** | **T** | **A** | **T** | **G** | **T** | **A** | **A** | **A** | **T** | **A** | **C** | **G** | **A** | **C** | **A** | **A** | **A** | **A** | **A** | **A** | **A** | **T** | **T** | **C** | **G** | **G** | **C** | **T** | **A** | **T** | **T** | **G** | **G** | **C** | **G** | **T** | **G** | **A** | **T** | **A** | **C** | **G** | **C** | **A** | **T** | **A** | **T** | **A** | **C** | **G** | **G** | **A** | **T** | **T** | **T** | **T** | **C** | **G** | **G** | **T** | **G** | **C** | **C** | **G** | **C** | **T** | **T** | **C** | **T** | **T** | **C** | **T** | **T** | **T** | **G** | **C** | **T** | **T** | **C** | **A** | **A** | **C** | **T** | **T** | **C** | **G** | **T** | **T** | **T** | **T** | **T** | **T** | **A** | **A** | **C** | **A** | **G** | **A** | **C** | **T** | **G** | **G** | **A** | **A** | **C** | **G** | **T** | **C** | **T** | **C** | **A** | **G** | **T** | **A** | **C** | **C** | **T** | **G** | **A** | **A** | **G** | **A** | **A** | **A** | **A** | **A** | **A** | **A** | **G** | **G** | **T** | **C** | **G** | **G** | **C** | **T** | **A** | **T** | **C** | **A** | **T** | **T** | **T** | **G** | **A** | **A** | **T** | **T** | **A** | **T** | **T** | **T** | **C** | **T** | **T** | **T** | **A** | **A** | **A** | **A** | **A** | **A** | **G** | **C** | **A** | **G** | **A** | **T** | **C** | **G** | **A** | **G** | **A** | **C** | **G** | **A** | **A** | **T** | **C** | **C** | **A** | **A** | **A** | **C** | **T** | **T** | **G** | **C** | **T** | **G** | **A** | **T** | **A** | **C** | **G** | **T** | **C** | **C** | **A** | **T** | **C** | **C** | **A** | **G** | **C** | **T** | **T** | **G** | **T** | **C** | **T** | **C** | **A** | **A** | **G** | **C** | **G** | **G** | **A** | **C** | **C** | **A** | **A** | **A** | **T** | **A** | **A** | **C** | **G** | **A** | **A** | **C** | **G** | **A** | **G** | **A** | **A** | **C** | **A** | **A** | **A** | **T** | **C** | **A** | **A** | **G** | **C** | **T** | **T** | **T** | **C** | **A** | **T** | **T** | **T** | **A** | **T** | **C** | **A** | **A** | **A** | **T** | **T** | **G** | **A** | **T** | **T** | **A** | **C** | **T** | **T** | **C** | **T** | **G** | **C** | **T** | **A** | **A** | **A** | **A** | **A** | **A** | **C** | **G** | **T** | **G** | **T** | **T** | **T** | **G** | **G** | **A** | **T** | **A** | **C** | **A** | **G** | **A** | **C** | **A** | **C** | **C** | **T** | **T** | **A** | **C** | **C** | **T** | **C** | **G** | **T** | **T** | **C** | **C** | **T** | **G** | **A** | **T** | **G** | **A** | **A** | **A** | **G** | **T** | **G** | **T** | **C** | **A** | **T** | **T** | **G** | **C** | **T** | **G** | **C** | **T** | **T** | **T** | **G** | **A** | **A** | **A** | **A** | **T** | **C** | **G** | **C** | **A** | **A** | **C** | **T** | **G** | **C** | **T** | **T** | **C** | **T** | **G** | **G** | **T** | **G** | **T** | **C** | **G** | **A** | **T** | **G** | **T** | **G** | **A** | **A** | **A** | **A** | **T** | **C** | **A** | **T** | **G** | **A** | **T** | **C** | **C** | **C** | **C** | **A** | **A** | **T** | **A** | **A** | **A** | **C** | **C** | **G** | **G** | **A** | **T** | **C** | **A** | **T** | **C** | **C** | **T** | **T** | **T** | **T** | **A** | **T** | **T** | **T** | **A** | **T** | **C** | **G** | **A** | **G** | **C** | **A** | **A** | **C** | **A** | **C** | **A** | **A** | **T** | **A** | **T** | **T** | **A** | **T** | **G** | **C** | **G** | **C** | **G** | **T** | **C** | **A** | **G** | **C** | **T** | **C** | **A** | **T** | **C** | **A** | **A** | **G** | **G** | **A** | **A** | **A** | **A** | **T** | **G** | **T** | **A** | **C** | **A** | **G** | **A** | **T** | **C** | **C** | **T** | **T** | **G** | **T** | **C** | **T** | **A** | **T** | **G** | **A** | **G** | **A** | **A** | **T** | **G** | **G** | **C** | **T** | **T** | **C** | **C** | **T** | **C** | **C** | **A** | **T** | **G** | **C** | **A** | **A** | **A** | **A** | **A** | **C** | **A** | **T** | **T** | **G** | **A** | **T** | **A** | **A** | **T** | **G** | **G** | **A** | **T** | **G** | **A** | **T** | **G** | **A** | **A** | **A** | **T** | **C** | **T** | **G** | **C** | **A** | **T** | **G** | **G** | **T** | **A** | **G** | **G** | **T** | **T** | **C** | **A** | **G** | **C** | **A** | **A** | **A** | **C** | **C** | **A** | **A** | **G** | **A** | **T** | **A** | **T** | **T** | **C** | **G** | **T** | **A** | **G** | **C** | **T** | **A** | **C** | **C** | **G** | **A** | **T** | **T** | **G** | **A** | **A** | **T** | **T** | **T** | **T** | **G** | **A** | **A** | **A** | **C** | **A** | **A** | **G** | **T** | **G** | **C** | **T** | **G** | **T** | **C** | **A** | **T** | **C** | **T** | **A** | **C** | **G** | **A** | **T** | **C** | **C** | **T** | **G** | **A** | **G** | **T** | **T** | **T** | **T** | **T** | **A** | **G** | **A** | **A** | **G** | **A** | **A** | **C** | **T** | **T** | **G** | **C** | **T** | **A** | **C** | **C** | **C** | **A** | **G** | **T** | **T** | **C** | **A** | **A** | **A** | **G** | **A** | **A** | **G** | **A** | **T** | **G** | **A** | **G** | **A** | **C** | **A** | **C** | **A** | **T** | **T** | **G** | **T** | **T** | **C** | **A** | **C** | **C** | **A** | **A** | **T** | **G** | **A** | **C** | **G** | **A** | **C** | **T** | **G** | **A** | **A** | **A** | **C** | **A** | **G** | **T** | **G** | **A** | **A** | **G** | **G** | **A** | **A** | **A** | **T** | **G** | **T** | **C** | **T** | **A** | **A** | **T** | **T** | **G** | **G** | **C** | **T** | **G** | **T** | **T** | **A** | **T** | **T** | **C** | **A** | **A** | **G** | **C** | **A** | **A** | **C** | **A** | **A** | **A** | **T** | **T** | **T** | **C** | **T** | **C** | **G** | **A** | **T** | **T** | **A** | **T** | **T** | **T** | **T** | **C** | **T** | **C** | **C** | **A** | **A** | **T** | **C** | **T** | **T** | **A** | **-** | **-** | **-** |
| **N59589F** | **-** | **-** | **-** | **-** | **-** | **-** | **-** | **-** | **-** | **-** | **-** | **-** | **-** | **-** | **-** | **-** | **-** | **-** | **-** | **-** | **-** | **-** | **-** | **-** | **-** | **-** | **-** | **-** | **-** | **-** | **-** | **-** | **-** | **-** | **-** | **-** | **-** | **-** | **-** | **-** | **-** | **-** | **-** | **-** | **-** | **-** | **-** | **-** | **-** | **-** | **-** | **-** | **-** | **-** | **-** | **-** | **-** | **-** | **-** | **-** | **-** | **-** | **-** | **-** | **-** | **-** | **-** | **-** | **-** | **A** | **A** | **A** | **C** | **C** | **C** | **C** | **G** | **A** | **G** | **A** | **T** | **G** | **T** | **A** | **G** | **C** | **A** | **G** | **C** | **T** | **A** | **T** | **T** | **T** | **G** | **G** | **G** | **C** | **A** | **T** | **G** | **G** | **C** | **T** | **C** | **T** | **T** | **A** | **G** | **T** | **A** | **T** | **T** | **G** | **T** | **T** | **T** | **G** | **C** | **C** | **C** | **T** | **T** | **C** | **C** | **T** | **G** | **G** | **G** | **G** | **T** | **G** | **G** | **G** | **T** | **T** | **T** | **T** | **G** | **T** | **A** | **T** | **T** | **A** | **T** | **A** | **T** | **T** | **T** | **A** | **T** | **T** | **T** | **T** | **T** | **C** | **G** | **G** | **A** | **C** | **G** | **T** | **G** | **G** | **A** | **T** | **T** | **A** | **A** | **C** | **G** | **G** | **A** | **T** | **A** | **A** | **G** | **A** | **A** | **A** | **A** | **A** | **A** | **T** | **T** | **C** | **T** | **A** | **T** | **C** | **T** | **C** | **C** | **G** | **A** | **C** | **A** | **A** | **A** | **G** | **T** | **G** | **A** | **C** | **T** | **T** | **G** | **A** | **A** | **A** | **G** | **A** | **A** | **T** | **T** | **A** | **G** | **A** | **A** | **A** | **A** | **C** | **T** | **T** | **T** | **C** | **A** | **G** | **T** | **C** | **T** | **T** | **T** | **T** | **A** | **G** | **A** | **G** | **G** | **A** | **G** | **A** | **T** | **A** | **C** | **C** | **A** | **T** | **T** | **G** | **A** | **A** | **C** | **A** | **T** | **T** | **A** | **C** | **G** | **A** | **T** | **C** | **C** | **T** | **G** | **A** | **C** | **A** | **T** | **G** | **G** | **G** | **C** | **G** | **A** | **C** | **A** | **A** | **A** | **G** | **A** | **C** | **A** | **A** | **A** | **C** | **A** | **A** | **C** | **A** | **A** | **T** | **T** | **T** | **G** | **T** | **C** | **G** | **A** | **C** | **T** | **T** | **C** | **T** | **T** | **C** | **T** | **C** | **T** | **T** | **C** | **A** | **T** | **T** | **A** | **A** | **A** | **T** | **C** | **G** | **T** | **A** | **T** | **G** | **C** | **C** | **G** | **C** | **T** | **G** | **A** | **C** | **A** | **A** | **G** | **A** | **A** | **T** | **G** | **A** | **A** | **T** | **T** | **C** | **T** | **G** | **T** | **C** | **A** | **C** | **T** | **C** | **T** | **T** | **C** | **T** | **C** | **A** | **C** | **A** | **G** | **A** | **C** | **G** | **G** | **A** | **C** | **A** | **A** | **A** | **A** | **G** | **A** | **A** | **A** | **T** | **T** | **A** | **G** | **A** | **T** | **T** | **C** | **A** | **C** | **T** | **G** | **C** | **T** | **T** | **C** | **A** | **A** | **G** | **A** | **T** | **C** | **T** | **A** | **A** | **A** | **A** | **A** | **A** | **A** | **G** | **C** | **C** | **A** | **A** | **A** | **C** | **A** | **T** | **T** | **C** | **G** | **A** | **T** | **C** | **C** | **A** | **T** | **A** | **T** | **C** | **G** | **A** | **A** | **T** | **A** | **T** | **T** | **A** | **C** | **G** | **C** | **A** | **T** | **T** | **T** | **G** | **T** | **G** | **A** | **C** | **A** | **G** | **A** | **T** | **A** | **A** | **T** | **A** | **T** | **C** | **G** | **G** | **T** | **C** | **A** | **G** | **C** | **A** | **A** | **G** | **T** | **C** | **T** | **T** | **A** | **C** | **G** | **C** | **G** | **T** | **T** | **T** | **T** | **A** | **G** | **A** | **A** | **G** | **A** | **A** | **A** | **A** | **A** | **G** | **C** | **C** | **G** | **C** | **A** | **G** | **A** | **A** | **G** | **G** | **C** | **G** | **T** | **A** | **G** | **A** | **A** | **G** | **T** | **T** | **C** | **G** | **T** | **A** | **T** | **A** | **T** | **T** | **A** | **T** | **A** | **T** | **G** | **A** | **T** | **G** | **C** | **A** | **T** | **T** | **T** | **G** | **G** | **C** | **T** | **C** | **C** | **C** | **A** | **T** | **G** | **G** | **C** | **A** | **C** | **A** | **A** | **A** | **A** | **G** | **C** | **A** | **A** | **A** | **A** | **G** | **A** | **T** | **T** | **T** | **C** | **A** | **A** | **T** | **C** | **G** | **T** | **C** | **T** | **A** | **A** | **T** | **C** | **A** | **A** | **A** | **A** | **A** | **T** | **G** | **G** | **T** | **G** | **G** | **A** | **C** | **A** | **T** | **G** | **T** | **C** | **C** | **A** | **T** | **A** | **C** | **A** | **T** | **T** | **T** | **G** | **T** | **T** | **A** | **C** | **C** | **T** | **C** | **G** | **C** | **A** | **A** | **A** | **A** | **A** | **G** | **C** | **A** | **T** | **T** | **G** | **C** | **T** | **T** | **C** | **G** | **T** | **T** | **T** | **C** | **C** | **G** | **A** | **T** | **T** | **G** | **A** | **A** | **T** | **T** | **A** | **C** | **C** | **A** | **T** | **G** | **A** | **T** | **C** | **A** | **C** | **C** | **G** | **A** | **A** | **A** | **A** | **A** | **T** | **C** | **G** | **T** | **T** | **G** | **T** | **G** | **A** | **T** | **C** | **G** | **A** | **T** | **G** | **G** | **A** | **A** | **A** | **G** | **A** | **T** | **C** | **A** | **G** | **T** | **T** | **A** | **C** | **A** | **C** | **C** | **G** | **G** | **T** | **G** | **G** | **T** | **T** | **T** | **C** | **A** | **A** | **T** | **A** | **T** | **T** | **G** | **C** | **C** | **A** | **A** | **T** | **C** | **A** | **A** | **T** | **A** | **T** | **G** | **T** | **A** | **A** | **A** | **T** | **A** | **C** | **G** | **A** | **C** | **A** | **A** | **A** | **A** | **A** | **A** | **A** | **T** | **T** | **C** | **G** | **G** | **T** | **T** | **A** | **T** | **T** | **G** | **G** | **C** | **G** | **T** | **G** | **A** | **T** | **A** | **C** | **G** | **C** | **A** | **T** | **A** | **T** | **A** | **C** | **G** | **A** | **A** | **T** | **T** | **T** | **T** | **C** | **G** | **G** | **T** | **G** | **C** | **T** | **G** | **C** | **T** | **T** | **C** | **T** | **T** | **C** | **T** | **T** | **T** | **G** | **C** | **T** | **T** | **C** | **A** | **A** | **C** | **T** | **T** | **C** | **G** | **T** | **T** | **T** | **T** | **T** | **T** | **A** | **A** | **C** | **A** | **G** | **A** | **C** | **T** | **G** | **G** | **A** | **A** | **C** | **G** | **T** | **T** | **T** | **C** | **A** | **G** | **T** | **G** | **T** | **C** | **T** | **G** | **A** | **A** | **G** | **A** | **A** | **A** | **A** | **A** | **A** | **A** | **G** | **G** | **T** | **C** | **G** | **G** | **C** | **T** | **A** | **T** | **C** | **A** | **T** | **T** | **T** | **G** | **A** | **A** | **T** | **T** | **A** | **T** | **T** | **T** | **C** | **T** | **T** | **T** | **A** | **A** | **A** | **A** | **A** | **A** | **G** | **C** | **C** | **G** | **A** | **T** | **C** | **G** | **A** | **G** | **A** | **T** | **G** | **A** | **A** | **T** | **C** | **C** | **A** | **A** | **A** | **C** | **T** | **T** | **G** | **C** | **T** | **G** | **A** | **T** | **A** | **C** | **G** | **T** | **C** | **C** | **A** | **T** | **C** | **C** | **A** | **G** | **C** | **T** | **T** | **G** | **T** | **C** | **T** | **C** | **A** | **A** | **G** | **C** | **G** | **G** | **A** | **C** | **C** | **A** | **A** | **A** | **T** | **A** | **A** | **C** | **G** | **A** | **A** | **C** | **G** | **A** | **G** | **A** | **A** | **C** | **A** | **A** | **A** | **T** | **C** | **A** | **A** | **G** | **C** | **T** | **T** | **T** | **C** | **G** | **T** | **T** | **T** | **A** | **T** | **C** | **A** | **A** | **A** | **T** | **T** | **G** | **A** | **T** | **T** | **A** | **C** | **T** | **T** | **C** | **T** | **G** | **C** | **T** | **A** | **A** | **A** | **A** | **A** | **A** | **C** | **G** | **T** | **G** | **T** | **T** | **T** | **G** | **G** | **A** | **T** | **A** | **C** | **A** | **G** | **A** | **C** | **A** | **C** | **C** | **T** | **T** | **A** | **C** | **C** | **T** | **C** | **G** | **T** | **T** | **C** | **C** | **T** | **G** | **A** | **T** | **G** | **A** | **A** | **A** | **G** | **T** | **G** | **T** | **C** | **A** | **T** | **T** | **G** | **C** | **T** | **G** | **C** | **T** | **T** | **T** | **G** | **A** | **A** | **A** | **A** | **T** | **C** | **G** | **C** | **A** | **A** | **C** | **T** | **G** | **C** | **T** | **T** | **C** | **T** | **G** | **G** | **T** | **G** | **T** | **C** | **G** | **A** | **T** | **G** | **T** | **G** | **A** | **A** | **A** | **A** | **T** | **C** | **A** | **T** | **G** | **A** | **T** | **C** | **C** | **C** | **C** | **A** | **A** | **T** | **A** | **A** | **A** | **C** | **C** | **G** | **G** | **A** | **T** | **C** | **A** | **T** | **C** | **C** | **T** | **T** | **T** | **T** | **A** | **T** | **T** | **T** | **A** | **T** | **C** | **G** | **A** | **G** | **C** | **A** | **A** | **C** | **A** | **C** | **A** | **A** | **T** | **A** | **T** | **T** | **A** | **T** | **G** | **C** | **T** | **C** | **G** | **T** | **C** | **A** | **G** | **C** | **T** | **C** | **A** | **T** | **C** | **A** | **A** | **G** | **G** | **A** | **A** | **A** | **A** | **T** | **G** | **T** | **A** | **C** | **A** | **G** | **A** | **T** | **C** | **C** | **T** | **T** | **G** | **T** | **C** | **T** | **A** | **T** | **G** | **A** | **G** | **A** | **A** | **T** | **G** | **G** | **C** | **T** | **T** | **C** | **C** | **T** | **C** | **C** | **A** | **T** | **G** | **C** | **A** | **A** | **A** | **A** | **A** | **C** | **A** | **T** | **T** | **A** | **A** | **T** | **A** | **A** | **T** | **G** | **G** | **A** | **T** | **G** | **A** | **T** | **G** | **A** | **A** | **A** | **T** | **C** | **T** | **G** | **C** | **A** | **T** | **G** | **G** | **T** | **A** | **G** | **G** | **T** | **T** | **C** | **A** | **G** | **C** | **A** | **A** | **A** | **T** | **C** | **A** | **A** | **G** | **A** | **T** | **A** | **T** | **T** | **C** | **G** | **T** | **A** | **G** | **C** | **T** | **A** | **C** | **C** | **G** | **A** | **T** | **T** | **G** | **A** | **A** | **T** | **T** | **T** | **T** | **G** | **A** | **A** | **A** | **C** | **A** | **A** | **G** | **T** | **G** | **C** | **T** | **G** | **T** | **C** | **A** | **T** | **T** | **T** | **A** | **C** | **G** | **A** | **T** | **C** | **C** | **T** | **G** | **A** | **G** | **T** | **T** | **T** | **T** | **T** | **A** | **G** | **A** | **A** | **G** | **A** | **G** | **C** | **T** | **T** | **G** | **C** | **T** | **A** | **C** | **T** | **C** | **A** | **G** | **T** | **T** | **C** | **A** | **A** | **A** | **G** | **A** | **A** | **G** | **A** | **T** | **G** | **A** | **G** | **A** | **C** | **A** | **C** | **A** | **T** | **T** | **G** | **T** | **T** | **C** | **A** | **C** | **C** | **A** | **A** | **T** | **G** | **A** | **C** | **A** | **A** | **C** | **T** | **G** | **A** | **A** | **A** | **C** | **A** | **G** | **T** | **G** | **A** | **A** | **G** | **G** | **A** | **A** | **A** | **T** | **G** | **T** | **C** | **T** | **A** | **A** | **C** | **T** | **G** | **G** | **C** | **T** | **G** | **T** | **T** | **A** | **T** | **T** | **C** | **A** | **A** | **A** | **C** | **A** | **A** | **C** | **A** | **A** | **A** | **T** | **T** | **T** | **C** | **T** | **C** | **G** | **A** | **T** | **T** | **G** | **T** | **T** | **T** | **T** | **C** | **T** | **C** | **C** | **A** | **A** | **T** | **C** | **T** | **T** | **A** | **-** | **-** | **-** |
| **N54599** | **-** | **-** | **-** | **-** | **-** | **-** | **-** | **-** | **-** | **-** | **-** | **-** | **-** | **-** | **-** | **-** | **-** | **-** | **-** | **-** | **-** | **-** | **-** | **-** | **-** | **-** | **-** | **-** | **-** | **-** | **-** | **-** | **-** | **-** | **-** | **-** | **-** | **-** | **-** | **-** | **-** | **-** | **-** | **-** | **-** | **-** | **-** | **-** | **-** | **-** | **-** | **-** | **-** | **-** | **-** | **-** | **-** | **-** | **-** | **-** | **-** | **-** | **-** | **-** | **-** | **-** | **-** | **-** | **-** | **A** | **A** | **A** | **C** | **C** | **C** | **C** | **G** | **A** | **G** | **A** | **T** | **G** | **T** | **A** | **G** | **C** | **A** | **G** | **C** | **T** | **A** | **T** | **T** | **T** | **G** | **G** | **G** | **C** | **A** | **T** | **G** | **G** | **C** | **T** | **C** | **T** | **T** | **A** | **G** | **T** | **A** | **T** | **T** | **A** | **T** | **T** | **T** | **G** | **C** | **C** | **C** | **T** | **T** | **C** | **C** | **T** | **G** | **G** | **G** | **G** | **T** | **G** | **G** | **G** | **T** | **T** | **T** | **T** | **G** | **T** | **A** | **T** | **T** | **A** | **T** | **A** | **T** | **T** | **T** | **A** | **T** | **T** | **T** | **T** | **T** | **C** | **G** | **G** | **A** | **C** | **G** | **T** | **G** | **G** | **A** | **T** | **T** | **A** | **A** | **C** | **G** | **G** | **A** | **T** | **A** | **A** | **G** | **A** | **A** | **A** | **A** | **A** | **A** | **T** | **T** | **C** | **T** | **A** | **T** | **C** | **T** | **C** | **C** | **G** | **A** | **C** | **A** | **A** | **A** | **G** | **T** | **G** | **A** | **C** | **T** | **T** | **G** | **A** | **A** | **A** | **G** | **A** | **A** | **T** | **T** | **A** | **G** | **A** | **A** | **A** | **A** | **C** | **T** | **T** | **T** | **C** | **A** | **G** | **T** | **C** | **T** | **T** | **T** | **T** | **A** | **G** | **A** | **G** | **G** | **A** | **G** | **A** | **T** | **A** | **C** | **C** | **A** | **T** | **T** | **G** | **A** | **A** | **C** | **A** | **T** | **T** | **A** | **C** | **G** | **A** | **T** | **C** | **C** | **T** | **G** | **A** | **C** | **A** | **T** | **G** | **G** | **G** | **C** | **G** | **A** | **C** | **A** | **A** | **A** | **G** | **A** | **C** | **A** | **A** | **A** | **C** | **A** | **A** | **C** | **A** | **A** | **T** | **T** | **T** | **G** | **T** | **C** | **G** | **A** | **C** | **T** | **T** | **C** | **T** | **T** | **C** | **T** | **C** | **T** | **T** | **C** | **A** | **T** | **T** | **A** | **A** | **A** | **T** | **C** | **G** | **T** | **A** | **T** | **G** | **C** | **C** | **G** | **C** | **T** | **G** | **A** | **C** | **A** | **A** | **G** | **A** | **A** | **T** | **G** | **A** | **A** | **T** | **T** | **C** | **T** | **G** | **T** | **C** | **A** | **C** | **T** | **C** | **T** | **T** | **C** | **T** | **C** | **A** | **C** | **A** | **G** | **A** | **C** | **G** | **G** | **A** | **C** | **A** | **A** | **A** | **A** | **G** | **A** | **A** | **A** | **T** | **T** | **A** | **G** | **A** | **T** | **T** | **C** | **A** | **C** | **T** | **G** | **C** | **T** | **T** | **C** | **A** | **A** | **G** | **A** | **T** | **C** | **T** | **A** | **A** | **A** | **A** | **A** | **A** | **A** | **G** | **C** | **C** | **A** | **A** | **A** | **C** | **A** | **T** | **T** | **C** | **G** | **A** | **T** | **C** | **C** | **A** | **T** | **A** | **T** | **C** | **G** | **A** | **A** | **T** | **A** | **T** | **T** | **A** | **C** | **G** | **C** | **A** | **T** | **T** | **T** | **G** | **T** | **G** | **A** | **C** | **A** | **G** | **A** | **T** | **A** | **C** | **T** | **A** | **T** | **C** | **G** | **G** | **T** | **C** | **A** | **G** | **C** | **A** | **A** | **G** | **T** | **C** | **T** | **T** | **A** | **C** | **G** | **C** | **G** | **T** | **T** | **T** | **T** | **A** | **G** | **A** | **A** | **G** | **A** | **A** | **A** | **A** | **A** | **G** | **T** | **C** | **G** | **C** | **A** | **G** | **A** | **A** | **G** | **G** | **C** | **G** | **T** | **A** | **G** | **A** | **A** | **G** | **T** | **T** | **C** | **G** | **T** | **A** | **T** | **A** | **T** | **T** | **A** | **T** | **A** | **T** | **G** | **A** | **T** | **G** | **C** | **A** | **T** | **T** | **T** | **G** | **G** | **C** | **T** | **C** | **C** | **C** | **A** | **C** | **G** | **G** | **C** | **A** | **C** | **A** | **A** | **A** | **A** | **G** | **C** | **A** | **A** | **A** | **A** | **G** | **A** | **T** | **T** | **T** | **C** | **A** | **A** | **T** | **C** | **G** | **T** | **C** | **T** | **A** | **A** | **T** | **C** | **A** | **A** | **A** | **A** | **A** | **T** | **G** | **G** | **T** | **G** | **G** | **A** | **C** | **A** | **T** | **G** | **T** | **C** | **C** | **A** | **T** | **A** | **C** | **A** | **T** | **T** | **T** | **G** | **T** | **T** | **A** | **C** | **C** | **T** | **C** | **G** | **C** | **A** | **A** | **A** | **A** | **A** | **G** | **C** | **A** | **T** | **T** | **G** | **C** | **T** | **T** | **C** | **G** | **T** | **T** | **T** | **C** | **C** | **G** | **A** | **T** | **T** | **G** | **A** | **A** | **C** | **T** | **A** | **C** | **C** | **A** | **T** | **G** | **A** | **T** | **C** | **A** | **C** | **C** | **G** | **G** | **A** | **A** | **A** | **A** | **T** | **C** | **G** | **T** | **T** | **G** | **T** | **G** | **A** | **T** | **C** | **G** | **A** | **T** | **G** | **G** | **A** | **A** | **A** | **G** | **A** | **T** | **C** | **A** | **G** | **T** | **T** | **A** | **C** | **A** | **C** | **C** | **G** | **G** | **T** | **G** | **G** | **C** | **T** | **T** | **C** | **A** | **A** | **C** | **A** | **T** | **T** | **G** | **C** | **C** | **A** | **A** | **T** | **C** | **A** | **A** | **T** | **A** | **T** | **G** | **T** | **A** | **A** | **A** | **T** | **A** | **C** | **G** | **A** | **C** | **A** | **A** | **A** | **A** | **A** | **A** | **A** | **T** | **T** | **C** | **G** | **G** | **C** | **T** | **A** | **T** | **T** | **G** | **G** | **C** | **G** | **T** | **G** | **A** | **T** | **A** | **C** | **G** | **C** | **A** | **T** | **A** | **T** | **A** | **C** | **G** | **G** | **A** | **T** | **T** | **T** | **T** | **C** | **G** | **G** | **T** | **G** | **C** | **C** | **G** | **C** | **T** | **T** | **C** | **T** | **T** | **C** | **T** | **T** | **T** | **G** | **C** | **T** | **T** | **C** | **A** | **A** | **C** | **T** | **T** | **C** | **G** | **T** | **T** | **T** | **T** | **T** | **T** | **A** | **A** | **C** | **A** | **G** | **A** | **C** | **T** | **G** | **G** | **A** | **A** | **C** | **G** | **T** | **C** | **T** | **C** | **A** | **G** | **T** | **T** | **C** | **C** | **T** | **G** | **A** | **A** | **G** | **A** | **A** | **A** | **A** | **A** | **A** | **A** | **G** | **G** | **T** | **C** | **G** | **G** | **C** | **T** | **A** | **T** | **C** | **A** | **T** | **T** | **T** | **G** | **A** | **A** | **T** | **T** | **A** | **T** | **T** | **T** | **C** | **T** | **T** | **T** | **A** | **A** | **A** | **A** | **A** | **A** | **G** | **C** | **C** | **G** | **A** | **T** | **C** | **G** | **A** | **G** | **A** | **C** | **G** | **A** | **A** | **T** | **C** | **C** | **A** | **A** | **A** | **C** | **T** | **T** | **G** | **C** | **T** | **G** | **A** | **T** | **A** | **C** | **G** | **T** | **C** | **C** | **A** | **T** | **C** | **C** | **A** | **G** | **C** | **T** | **T** | **G** | **T** | **C** | **T** | **C** | **A** | **A** | **G** | **C** | **G** | **G** | **A** | **C** | **C** | **A** | **A** | **A** | **T** | **A** | **A** | **C** | **G** | **A** | **A** | **C** | **G** | **C** | **G** | **A** | **A** | **C** | **A** | **A** | **A** | **T** | **C** | **A** | **A** | **G** | **C** | **T** | **T** | **T** | **C** | **G** | **T** | **T** | **T** | **A** | **T** | **C** | **A** | **A** | **A** | **T** | **T** | **G** | **A** | **T** | **T** | **A** | **C** | **T** | **T** | **C** | **T** | **G** | **C** | **T** | **A** | **A** | **A** | **A** | **A** | **A** | **C** | **G** | **T** | **G** | **T** | **T** | **T** | **G** | **G** | **A** | **T** | **A** | **C** | **A** | **G** | **A** | **C** | **A** | **C** | **C** | **T** | **T** | **A** | **C** | **C** | **T** | **C** | **G** | **T** | **T** | **C** | **C** | **T** | **G** | **A** | **T** | **G** | **A** | **A** | **A** | **G** | **T** | **G** | **T** | **C** | **A** | **T** | **T** | **G** | **C** | **T** | **G** | **C** | **T** | **T** | **T** | **G** | **A** | **A** | **A** | **A** | **T** | **C** | **G** | **C** | **A** | **A** | **C** | **T** | **G** | **C** | **T** | **T** | **C** | **T** | **G** | **G** | **T** | **G** | **T** | **C** | **G** | **A** | **T** | **G** | **T** | **G** | **A** | **A** | **A** | **A** | **T** | **C** | **A** | **T** | **G** | **A** | **T** | **C** | **C** | **C** | **C** | **A** | **A** | **T** | **A** | **A** | **A** | **C** | **C** | **G** | **G** | **A** | **T** | **C** | **A** | **T** | **C** | **C** | **T** | **T** | **T** | **T** | **A** | **T** | **T** | **T** | **A** | **T** | **C** | **G** | **A** | **G** | **C** | **A** | **A** | **C** | **A** | **C** | **A** | **A** | **T** | **A** | **T** | **T** | **A** | **T** | **G** | **C** | **G** | **C** | **G** | **T** | **C** | **A** | **G** | **C** | **T** | **C** | **A** | **T** | **C** | **A** | **A** | **G** | **G** | **A** | **A** | **A** | **A** | **C** | **G** | **T** | **A** | **C** | **A** | **A** | **A** | **T** | **T** | **C** | **T** | **T** | **G** | **T** | **C** | **T** | **A** | **T** | **G** | **A** | **G** | **A** | **A** | **T** | **G** | **G** | **C** | **T** | **T** | **C** | **C** | **T** | **C** | **C** | **A** | **T** | **G** | **C** | **A** | **A** | **A** | **A** | **A** | **C** | **A** | **T** | **T** | **G** | **A** | **T** | **A** | **A** | **T** | **G** | **G** | **A** | **T** | **G** | **A** | **T** | **G** | **A** | **A** | **A** | **T** | **C** | **T** | **G** | **C** | **A** | **T** | **G** | **G** | **T** | **A** | **G** | **G** | **T** | **T** | **C** | **A** | **G** | **C** | **A** | **A** | **A** | **T** | **C** | **A** | **A** | **G** | **A** | **T** | **A** | **T** | **T** | **C** | **G** | **T** | **A** | **G** | **C** | **T** | **A** | **C** | **C** | **G** | **A** | **T** | **T** | **G** | **A** | **A** | **T** | **T** | **T** | **T** | **G** | **A** | **A** | **A** | **C** | **A** | **A** | **G** | **T** | **G** | **C** | **T** | **G** | **T** | **C** | **A** | **T** | **C** | **T** | **A** | **C** | **G** | **A** | **T** | **C** | **C** | **T** | **G** | **A** | **G** | **T** | **T** | **T** | **T** | **T** | **A** | **G** | **A** | **A** | **G** | **A** | **A** | **C** | **T** | **T** | **G** | **C** | **T** | **A** | **C** | **T** | **C** | **A** | **G** | **T** | **T** | **T** | **A** | **A** | **A** | **G** | **A** | **A** | **G** | **A** | **T** | **G** | **A** | **G** | **A** | **C** | **A** | **C** | **A** | **T** | **T** | **G** | **T** | **T** | **C** | **A** | **C** | **C** | **A** | **A** | **T** | **G** | **A** | **C** | **G** | **A** | **C** | **T** | **G** | **A** | **A** | **A** | **C** | **A** | **G** | **T** | **G** | **A** | **A** | **G** | **G** | **A** | **A** | **A** | **T** | **G** | **T** | **C** | **T** | **A** | **A** | **C** | **T** | **G** | **G** | **C** | **T** | **G** | **T** | **T** | **A** | **T** | **T** | **C** | **A** | **A** | **A** | **C** | **A** | **A** | **C** | **A** | **A** | **A** | **T** | **T** | **T** | **C** | **T** | **C** | **G** | **A** | **T** | **T** | **A** | **T** | **T** | **T** | **T** | **C** | **T** | **C** | **C** | **A** | **A** | **T** | **C** | **T** | **T** | **A** | **-** | **-** | **-** |
| **N60252F** | **-** | **-** | **-** | **-** | **-** | **-** | **-** | **-** | **-** | **-** | **-** | **-** | **-** | **-** | **-** | **-** | **-** | **-** | **-** | **-** | **-** | **-** | **-** | **-** | **-** | **-** | **-** | **-** | **-** | **-** | **-** | **-** | **-** | **-** | **-** | **-** | **-** | **-** | **-** | **-** | **-** | **-** | **-** | **-** | **-** | **-** | **-** | **-** | **-** | **-** | **-** | **-** | **-** | **-** | **-** | **-** | **-** | **-** | **-** | **-** | **-** | **-** | **-** | **-** | **-** | **-** | **-** | **-** | **-** | **A** | **A** | **A** | **C** | **C** | **C** | **C** | **G** | **A** | **G** | **A** | **T** | **G** | **T** | **A** | **G** | **C** | **A** | **G** | **C** | **C** | **A** | **T** | **T** | **T** | **G** | **G** | **G** | **C** | **A** | **T** | **G** | **G** | **C** | **T** | **C** | **T** | **T** | **A** | **G** | **T** | **A** | **T** | **T** | **A** | **T** | **T** | **T** | **G** | **C** | **C** | **C** | **T** | **T** | **C** | **C** | **T** | **G** | **G** | **G** | **G** | **T** | **G** | **G** | **G** | **C** | **T** | **T** | **T** | **G** | **T** | **A** | **T** | **T** | **A** | **T** | **A** | **T** | **T** | **T** | **A** | **T** | **T** | **T** | **T** | **T** | **C** | **G** | **G** | **A** | **C** | **G** | **T** | **G** | **G** | **A** | **T** | **T** | **A** | **A** | **C** | **G** | **G** | **A** | **T** | **A** | **A** | **G** | **A** | **A** | **A** | **A** | **A** | **A** | **T** | **T** | **C** | **T** | **A** | **T** | **C** | **T** | **C** | **C** | **G** | **A** | **C** | **A** | **A** | **A** | **G** | **T** | **G** | **A** | **C** | **T** | **T** | **G** | **A** | **A** | **A** | **G** | **A** | **A** | **T** | **T** | **A** | **G** | **A** | **A** | **A** | **A** | **C** | **T** | **T** | **T** | **C** | **A** | **G** | **T** | **C** | **T** | **T** | **T** | **T** | **A** | **G** | **A** | **G** | **G** | **A** | **G** | **A** | **T** | **A** | **C** | **C** | **A** | **T** | **T** | **G** | **A** | **A** | **C** | **A** | **T** | **T** | **A** | **C** | **G** | **A** | **T** | **C** | **C** | **T** | **G** | **A** | **C** | **A** | **T** | **G** | **G** | **G** | **C** | **G** | **A** | **T** | **A** | **A** | **A** | **G** | **A** | **C** | **A** | **A** | **A** | **C** | **A** | **G** | **C** | **A** | **A** | **T** | **T** | **T** | **G** | **T** | **T** | **G** | **A** | **C** | **T** | **T** | **C** | **T** | **T** | **C** | **T** | **C** | **T** | **T** | **C** | **A** | **T** | **T** | **A** | **A** | **A** | **T** | **C** | **G** | **T** | **A** | **T** | **G** | **C** | **C** | **G** | **C** | **T** | **G** | **A** | **C** | **A** | **A** | **G** | **A** | **A** | **T** | **G** | **A** | **A** | **T** | **T** | **C** | **T** | **G** | **T** | **C** | **A** | **C** | **T** | **C** | **T** | **T** | **C** | **T** | **C** | **A** | **C** | **A** | **G** | **A** | **C** | **G** | **G** | **A** | **C** | **A** | **A** | **G** | **A** | **G** | **A** | **A** | **A** | **T** | **T** | **G** | **G** | **A** | **T** | **T** | **C** | **A** | **C** | **T** | **G** | **C** | **T** | **T** | **C** | **A** | **A** | **G** | **A** | **T** | **C** | **T** | **A** | **A** | **A** | **A** | **A** | **A** | **A** | **G** | **C** | **C** | **A** | **A** | **A** | **C** | **A** | **T** | **T** | **C** | **G** | **A** | **T** | **C** | **C** | **A** | **T** | **A** | **T** | **C** | **G** | **A** | **A** | **T** | **A** | **T** | **T** | **A** | **C** | **G** | **C** | **A** | **T** | **T** | **T** | **G** | **T** | **G** | **A** | **C** | **A** | **G** | **A** | **T** | **A** | **A** | **T** | **A** | **T** | **C** | **G** | **G** | **C** | **C** | **A** | **G** | **C** | **A** | **A** | **G** | **T** | **C** | **T** | **T** | **A** | **C** | **A** | **T** | **G** | **T** | **T** | **T** | **T** | **A** | **G** | **A** | **A** | **G** | **A** | **A** | **A** | **A** | **A** | **G** | **C** | **C** | **G** | **C** | **A** | **G** | **A** | **A** | **G** | **G** | **C** | **G** | **T** | **G** | **G** | **A** | **A** | **G** | **T** | **T** | **C** | **G** | **A** | **A** | **T** | **A** | **T** | **T** | **A** | **T** | **A** | **T** | **G** | **A** | **T** | **G** | **C** | **A** | **T** | **T** | **T** | **G** | **G** | **C** | **T** | **C** | **T** | **C** | **A** | **T** | **G** | **G** | **C** | **A** | **C** | **A** | **A** | **A** | **A** | **G** | **C** | **A** | **A** | **A** | **A** | **G** | **A** | **T** | **T** | **T** | **C** | **A** | **A** | **T** | **C** | **G** | **T** | **C** | **T** | **A** | **A** | **T** | **C** | **A** | **A** | **A** | **A** | **A** | **T** | **G** | **G** | **T** | **G** | **G** | **A** | **C** | **A** | **T** | **G** | **T** | **C** | **C** | **A** | **T** | **A** | **C** | **A** | **T** | **T** | **T** | **G** | **T** | **T** | **A** | **C** | **C** | **T** | **C** | **A** | **C** | **A** | **A** | **A** | **G** | **G** | **G** | **C** | **A** | **T** | **T** | **A** | **C** | **T** | **T** | **C** | **G** | **T** | **T** | **T** | **C** | **C** | **G** | **A** | **T** | **T** | **G** | **A** | **A** | **T** | **T** | **A** | **C** | **C** | **A** | **T** | **G** | **A** | **T** | **C** | **A** | **C** | **C** | **G** | **A** | **A** | **A** | **A** | **A** | **T** | **C** | **G** | **T** | **T** | **G** | **T** | **G** | **A** | **T** | **C** | **G** | **A** | **T** | **G** | **G** | **A** | **A** | **A** | **G** | **A** | **T** | **T** | **A** | **G** | **T** | **T** | **A** | **T** | **A** | **C** | **C** | **G** | **G** | **T** | **G** | **G** | **T** | **T** | **T** | **C** | **A** | **A** | **T** | **A** | **T** | **T** | **G** | **C** | **C** | **A** | **A** | **T** | **C** | **A** | **A** | **T** | **A** | **T** | **G** | **T** | **A** | **A** | **A** | **T** | **A** | **C** | **A** | **A** | **C** | **A** | **A** | **A** | **A** | **A** | **A** | **A** | **T** | **T** | **C** | **G** | **G** | **C** | **T** | **A** | **T** | **T** | **G** | **G** | **C** | **G** | **C** | **G** | **A** | **T** | **A** | **C** | **G** | **C** | **A** | **T** | **A** | **T** | **A** | **C** | **G** | **G** | **A** | **T** | **T** | **T** | **T** | **C** | **G** | **G** | **T** | **G** | **C** | **C** | **G** | **C** | **T** | **T** | **C** | **T** | **T** | **C** | **T** | **T** | **T** | **G** | **C** | **T** | **C** | **C** | **A** | **G** | **C** | **T** | **T** | **C** | **G** | **C** | **T** | **T** | **C** | **T** | **T** | **A** | **A** | **C** | **A** | **G** | **A** | **C** | **T** | **G** | **G** | **A** | **A** | **C** | **G** | **T** | **C** | **T** | **C** | **G** | **G** | **T** | **A** | **C** | **C** | **T** | **G** | **A** | **A** | **G** | **A** | **A** | **A** | **A** | **A** | **A** | **A** | **G** | **G** | **T** | **C** | **G** | **G** | **C** | **T** | **A** | **T** | **C** | **A** | **T** | **T** | **T** | **G** | **A** | **A** | **T** | **T** | **A** | **T** | **T** | **T** | **C** | **T** | **T** | **T** | **A** | **A** | **A** | **A** | **A** | **A** | **G** | **C** | **A** | **G** | **A** | **T** | **C** | **G** | **A** | **G** | **A** | **T** | **G** | **A** | **A** | **T** | **C** | **T** | **A** | **A** | **G** | **C** | **T** | **T** | **G** | **C** | **T** | **G** | **A** | **T** | **A** | **C** | **A** | **T** | **C** | **C** | **A** | **T** | **C** | **C** | **A** | **G** | **C** | **T** | **T** | **G** | **T** | **T** | **T** | **C** | **A** | **A** | **G** | **C** | **G** | **G** | **A** | **C** | **C** | **G** | **A** | **A** | **T** | **A** | **A** | **C** | **G** | **A** | **A** | **A** | **G** | **G** | **G** | **A** | **A** | **C** | **A** | **A** | **A** | **T** | **C** | **A** | **A** | **G** | **C** | **T** | **T** | **T** | **C** | **A** | **T** | **T** | **T** | **A** | **T** | **C** | **A** | **A** | **A** | **T** | **T** | **G** | **A** | **T** | **T** | **A** | **C** | **T** | **T** | **C** | **T** | **G** | **C** | **T** | **A** | **A** | **A** | **A** | **A** | **A** | **C** | **G** | **T** | **G** | **T** | **T** | **T** | **G** | **G** | **A** | **T** | **A** | **C** | **A** | **G** | **A** | **C** | **A** | **C** | **C** | **T** | **T** | **A** | **C** | **C** | **T** | **T** | **G** | **T** | **T** | **C** | **C** | **T** | **G** | **A** | **T** | **G** | **A** | **A** | **A** | **G** | **T** | **G** | **T** | **C** | **A** | **T** | **T** | **G** | **C** | **T** | **G** | **C** | **T** | **T** | **T** | **A** | **A** | **A** | **A** | **A** | **T** | **C** | **G** | **C** | **A** | **A** | **C** | **T** | **G** | **C** | **C** | **T** | **C** | **T** | **G** | **G** | **T** | **G** | **T** | **A** | **G** | **A** | **T** | **G** | **T** | **G** | **A** | **A** | **A** | **A** | **T** | **C** | **A** | **T** | **G** | **A** | **T** | **T** | **C** | **C** | **C** | **A** | **A** | **C** | **A** | **A** | **A** | **C** | **C** | **G** | **G** | **A** | **T** | **C** | **A** | **T** | **C** | **C** | **T** | **T** | **T** | **T** | **A** | **T** | **T** | **T** | **A** | **T** | **C** | **G** | **A** | **G** | **C** | **A** | **A** | **C** | **A** | **C** | **A** | **A** | **T** | **A** | **T** | **T** | **A** | **T** | **G** | **C** | **T** | **C** | **G** | **G** | **C** | **A** | **G** | **C** | **T** | **G** | **A** | **T** | **C** | **A** | **A** | **G** | **G** | **A** | **A** | **A** | **A** | **T** | **G** | **T** | **A** | **C** | **A** | **A** | **A** | **T** | **C** | **C** | **T** | **T** | **G** | **T** | **C** | **T** | **A** | **T** | **G** | **A** | **G** | **A** | **A** | **C** | **G** | **G** | **C** | **T** | **T** | **C** | **C** | **T** | **C** | **C** | **A** | **T** | **G** | **C** | **A** | **A** | **A** | **A** | **A** | **C** | **A** | **T** | **T** | **G** | **A** | **T** | **A** | **A** | **T** | **G** | **G** | **A** | **T** | **G** | **A** | **T** | **G** | **A** | **A** | **A** | **T** | **C** | **T** | **G** | **C** | **A** | **T** | **G** | **G** | **T** | **A** | **G** | **G** | **T** | **T** | **C** | **A** | **G** | **C** | **A** | **A** | **A** | **T** | **C** | **A** | **A** | **G** | **A** | **T** | **A** | **T** | **T** | **C** | **G** | **T** | **A** | **G** | **C** | **T** | **A** | **C** | **C** | **G** | **A** | **T** | **T** | **G** | **A** | **A** | **T** | **T** | **T** | **T** | **G** | **A** | **A** | **A** | **C** | **A** | **A** | **G** | **T** | **G** | **C** | **T** | **G** | **T** | **C** | **A** | **T** | **T** | **T** | **A** | **C** | **G** | **A** | **T** | **C** | **C** | **T** | **G** | **A** | **G** | **T** | **T** | **T** | **T** | **T** | **A** | **G** | **A** | **A** | **G** | **A** | **A** | **C** | **T** | **T** | **G** | **C** | **T** | **A** | **C** | **T** | **C** | **A** | **G** | **T** | **T** | **C** | **A** | **A** | **A** | **G** | **A** | **A** | **G** | **A** | **T** | **G** | **A** | **G** | **A** | **C** | **A** | **C** | **A** | **T** | **T** | **G** | **T** | **T** | **C** | **A** | **T** | **C** | **C** | **A** | **T** | **G** | **A** | **C** | **A** | **A** | **C** | **T** | **G** | **A** | **A** | **A** | **C** | **A** | **G** | **T** | **C** | **A** | **A** | **G** | **G** | **A** | **A** | **A** | **T** | **G** | **T** | **C** | **T** | **A** | **A** | **C** | **T** | **G** | **G** | **C** | **T** | **A** | **T** | **T** | **A** | **T** | **T** | **C** | **A** | **A** | **G** | **C** | **A** | **A** | **C** | **A** | **A** | **A** | **T** | **T** | **T** | **C** | **T** | **C** | **G** | **A** | **T** | **T** | **A** | **T** | **T** | **T** | **T** | **C** | **T** | **C** | **C** | **A** | **A** | **T** | **C** | **C** | **T** | **A** | **-** | **-** | **-** |
| **N59653F** | **-** | **-** | **-** | **-** | **-** | **-** | **-** | **-** | **-** | **-** | **-** | **-** | **-** | **-** | **-** | **-** | **-** | **-** | **-** | **-** | **-** | **-** | **-** | **-** | **-** | **-** | **-** | **-** | **-** | **-** | **-** | **-** | **-** | **-** | **-** | **-** | **-** | **-** | **-** | **-** | **-** | **-** | **-** | **-** | **-** | **-** | **-** | **-** | **-** | **-** | **-** | **-** | **-** | **-** | **-** | **-** | **-** | **-** | **-** | **-** | **-** | **-** | **-** | **-** | **-** | **-** | **-** | **-** | **-** | **A** | **A** | **A** | **C** | **C** | **C** | **C** | **G** | **A** | **G** | **A** | **T** | **G** | **T** | **A** | **G** | **C** | **A** | **G** | **C** | **T** | **A** | **T** | **T** | **T** | **G** | **G** | **G** | **C** | **A** | **T** | **G** | **G** | **C** | **T** | **C** | **T** | **T** | **A** | **G** | **T** | **A** | **T** | **T** | **G** | **T** | **T** | **T** | **G** | **C** | **C** | **C** | **T** | **T** | **C** | **C** | **T** | **G** | **G** | **G** | **G** | **T** | **G** | **G** | **G** | **T** | **T** | **T** | **T** | **G** | **T** | **A** | **T** | **T** | **A** | **T** | **A** | **T** | **T** | **T** | **A** | **T** | **T** | **T** | **T** | **T** | **C** | **G** | **G** | **A** | **C** | **G** | **T** | **G** | **G** | **A** | **T** | **T** | **A** | **A** | **C** | **G** | **G** | **A** | **T** | **A** | **A** | **G** | **A** | **A** | **A** | **A** | **A** | **A** | **T** | **T** | **C** | **T** | **A** | **T** | **C** | **T** | **C** | **C** | **G** | **A** | **C** | **A** | **A** | **A** | **G** | **T** | **G** | **A** | **C** | **T** | **T** | **G** | **A** | **A** | **A** | **G** | **A** | **A** | **T** | **T** | **A** | **G** | **A** | **A** | **A** | **A** | **C** | **T** | **T** | **T** | **C** | **A** | **G** | **T** | **C** | **T** | **T** | **T** | **T** | **A** | **G** | **A** | **G** | **G** | **A** | **G** | **A** | **T** | **A** | **C** | **C** | **A** | **T** | **T** | **G** | **A** | **A** | **C** | **A** | **T** | **T** | **A** | **C** | **G** | **A** | **T** | **C** | **C** | **T** | **G** | **A** | **C** | **A** | **T** | **G** | **G** | **G** | **C** | **G** | **A** | **C** | **A** | **A** | **A** | **G** | **A** | **C** | **A** | **A** | **A** | **C** | **A** | **A** | **C** | **A** | **A** | **T** | **T** | **T** | **G** | **T** | **C** | **G** | **A** | **C** | **T** | **T** | **C** | **T** | **T** | **C** | **T** | **C** | **T** | **T** | **C** | **A** | **T** | **T** | **A** | **A** | **A** | **T** | **C** | **G** | **T** | **A** | **T** | **G** | **C** | **C** | **G** | **C** | **T** | **G** | **A** | **C** | **A** | **A** | **G** | **A** | **A** | **T** | **G** | **A** | **A** | **T** | **T** | **C** | **T** | **G** | **T** | **C** | **A** | **C** | **T** | **C** | **T** | **T** | **C** | **T** | **C** | **A** | **C** | **A** | **G** | **A** | **C** | **G** | **G** | **A** | **C** | **A** | **A** | **A** | **A** | **G** | **A** | **A** | **A** | **T** | **T** | **A** | **G** | **A** | **T** | **T** | **C** | **A** | **C** | **T** | **G** | **C** | **T** | **T** | **C** | **A** | **A** | **G** | **A** | **T** | **C** | **T** | **A** | **A** | **A** | **A** | **A** | **A** | **A** | **G** | **C** | **C** | **A** | **A** | **A** | **C** | **A** | **T** | **T** | **C** | **G** | **A** | **T** | **C** | **C** | **A** | **T** | **A** | **T** | **C** | **G** | **A** | **A** | **T** | **A** | **T** | **T** | **A** | **C** | **G** | **C** | **A** | **T** | **T** | **T** | **G** | **T** | **G** | **A** | **C** | **A** | **G** | **A** | **T** | **A** | **T** | **T** | **A** | **T** | **C** | **G** | **G** | **T** | **C** | **A** | **G** | **C** | **A** | **A** | **G** | **T** | **C** | **T** | **T** | **A** | **C** | **G** | **C** | **G** | **T** | **T** | **T** | **T** | **A** | **G** | **A** | **A** | **G** | **A** | **A** | **A** | **A** | **A** | **G** | **C** | **C** | **G** | **C** | **A** | **G** | **A** | **A** | **G** | **G** | **C** | **G** | **T** | **A** | **G** | **A** | **A** | **G** | **T** | **T** | **C** | **G** | **T** | **A** | **T** | **A** | **T** | **T** | **A** | **T** | **A** | **T** | **G** | **A** | **T** | **G** | **C** | **A** | **T** | **T** | **T** | **G** | **G** | **C** | **T** | **C** | **C** | **C** | **A** | **T** | **G** | **G** | **C** | **A** | **C** | **A** | **A** | **A** | **A** | **G** | **C** | **A** | **A** | **A** | **A** | **G** | **A** | **T** | **T** | **T** | **C** | **A** | **A** | **T** | **C** | **G** | **T** | **C** | **T** | **A** | **A** | **T** | **C** | **A** | **A** | **A** | **A** | **A** | **T** | **G** | **G** | **T** | **G** | **G** | **A** | **C** | **A** | **T** | **G** | **T** | **C** | **C** | **A** | **T** | **A** | **C** | **A** | **T** | **T** | **T** | **G** | **T** | **T** | **A** | **C** | **C** | **T** | **C** | **G** | **C** | **A** | **A** | **A** | **A** | **A** | **G** | **C** | **A** | **T** | **T** | **G** | **C** | **T** | **T** | **C** | **G** | **T** | **T** | **T** | **C** | **C** | **G** | **A** | **T** | **T** | **G** | **A** | **A** | **T** | **T** | **A** | **C** | **C** | **A** | **T** | **G** | **A** | **T** | **C** | **A** | **C** | **C** | **G** | **A** | **A** | **A** | **A** | **A** | **T** | **C** | **G** | **T** | **T** | **G** | **T** | **G** | **A** | **T** | **C** | **G** | **A** | **T** | **G** | **G** | **A** | **A** | **A** | **G** | **A** | **T** | **C** | **A** | **G** | **T** | **T** | **A** | **C** | **A** | **C** | **C** | **G** | **G** | **T** | **G** | **G** | **T** | **T** | **T** | **C** | **A** | **A** | **T** | **A** | **T** | **T** | **G** | **C** | **C** | **A** | **A** | **T** | **C** | **A** | **A** | **T** | **A** | **T** | **G** | **T** | **A** | **A** | **A** | **T** | **A** | **C** | **G** | **A** | **C** | **A** | **A** | **A** | **A** | **A** | **A** | **A** | **T** | **T** | **C** | **G** | **G** | **T** | **T** | **A** | **T** | **T** | **G** | **G** | **C** | **G** | **T** | **G** | **A** | **T** | **A** | **C** | **G** | **C** | **A** | **T** | **A** | **T** | **A** | **C** | **G** | **A** | **A** | **T** | **T** | **T** | **T** | **C** | **G** | **G** | **T** | **G** | **C** | **C** | **G** | **C** | **T** | **T** | **C** | **T** | **T** | **C** | **T** | **T** | **T** | **G** | **C** | **T** | **T** | **C** | **A** | **A** | **C** | **T** | **T** | **C** | **G** | **T** | **T** | **T** | **T** | **T** | **T** | **A** | **A** | **C** | **A** | **G** | **A** | **C** | **T** | **G** | **G** | **A** | **A** | **C** | **G** | **T** | **T** | **T** | **C** | **A** | **G** | **T** | **G** | **C** | **C** | **T** | **G** | **A** | **A** | **G** | **A** | **A** | **A** | **A** | **A** | **A** | **A** | **G** | **G** | **T** | **C** | **G** | **G** | **C** | **T** | **A** | **T** | **C** | **A** | **T** | **T** | **T** | **G** | **A** | **A** | **T** | **T** | **A** | **T** | **T** | **T** | **C** | **T** | **T** | **T** | **A** | **A** | **A** | **A** | **A** | **A** | **G** | **C** | **C** | **G** | **A** | **T** | **C** | **G** | **A** | **G** | **A** | **T** | **G** | **A** | **A** | **T** | **C** | **C** | **A** | **A** | **A** | **C** | **T** | **T** | **G** | **C** | **T** | **G** | **A** | **T** | **A** | **T** | **G** | **T** | **C** | **C** | **A** | **T** | **C** | **C** | **A** | **G** | **C** | **T** | **T** | **G** | **T** | **C** | **T** | **C** | **A** | **A** | **G** | **C** | **G** | **G** | **A** | **C** | **C** | **A** | **A** | **A** | **T** | **A** | **A** | **C** | **G** | **A** | **A** | **C** | **G** | **A** | **G** | **A** | **A** | **C** | **A** | **A** | **A** | **T** | **C** | **A** | **A** | **G** | **C** | **T** | **T** | **T** | **C** | **G** | **T** | **T** | **T** | **A** | **T** | **C** | **A** | **A** | **A** | **T** | **T** | **G** | **A** | **T** | **T** | **A** | **C** | **T** | **T** | **C** | **T** | **G** | **C** | **T** | **A** | **A** | **A** | **A** | **A** | **A** | **C** | **G** | **T** | **G** | **T** | **T** | **T** | **G** | **G** | **A** | **T** | **A** | **C** | **A** | **G** | **A** | **C** | **A** | **C** | **C** | **T** | **T** | **A** | **C** | **C** | **T** | **C** | **G** | **T** | **T** | **C** | **C** | **T** | **G** | **A** | **T** | **G** | **A** | **A** | **A** | **G** | **T** | **G** | **T** | **C** | **A** | **T** | **T** | **G** | **C** | **T** | **G** | **C** | **T** | **T** | **T** | **G** | **A** | **A** | **A** | **A** | **T** | **C** | **G** | **C** | **A** | **A** | **C** | **T** | **G** | **C** | **T** | **T** | **C** | **T** | **G** | **G** | **T** | **G** | **T** | **C** | **G** | **A** | **T** | **G** | **T** | **G** | **A** | **A** | **A** | **A** | **T** | **C** | **A** | **T** | **G** | **A** | **T** | **C** | **C** | **C** | **C** | **A** | **A** | **T** | **A** | **A** | **A** | **C** | **C** | **G** | **G** | **A** | **T** | **C** | **A** | **T** | **C** | **C** | **T** | **T** | **T** | **T** | **A** | **T** | **T** | **T** | **A** | **T** | **C** | **G** | **A** | **G** | **C** | **A** | **A** | **C** | **A** | **C** | **A** | **A** | **T** | **A** | **T** | **T** | **A** | **T** | **G** | **C** | **T** | **C** | **G** | **T** | **C** | **A** | **G** | **C** | **T** | **C** | **A** | **T** | **C** | **A** | **A** | **G** | **G** | **A** | **A** | **A** | **A** | **T** | **G** | **T** | **A** | **C** | **A** | **G** | **A** | **T** | **C** | **C** | **T** | **T** | **G** | **T** | **C** | **T** | **A** | **T** | **G** | **A** | **G** | **A** | **A** | **T** | **G** | **G** | **C** | **T** | **T** | **C** | **C** | **T** | **C** | **C** | **A** | **T** | **G** | **C** | **A** | **A** | **A** | **A** | **A** | **C** | **A** | **T** | **T** | **A** | **A** | **T** | **A** | **A** | **T** | **G** | **G** | **A** | **T** | **G** | **A** | **T** | **G** | **A** | **A** | **A** | **T** | **C** | **T** | **G** | **C** | **A** | **T** | **G** | **G** | **T** | **A** | **G** | **G** | **T** | **T** | **C** | **A** | **G** | **C** | **A** | **A** | **A** | **T** | **C** | **A** | **A** | **G** | **A** | **T** | **A** | **T** | **T** | **C** | **G** | **T** | **A** | **G** | **C** | **T** | **A** | **C** | **C** | **G** | **A** | **T** | **T** | **G** | **A** | **A** | **T** | **T** | **T** | **T** | **G** | **A** | **A** | **A** | **C** | **A** | **A** | **G** | **T** | **G** | **C** | **T** | **G** | **T** | **C** | **A** | **T** | **T** | **T** | **A** | **C** | **G** | **A** | **T** | **C** | **C** | **T** | **G** | **A** | **G** | **T** | **T** | **T** | **T** | **T** | **A** | **G** | **A** | **A** | **G** | **A** | **G** | **C** | **T** | **T** | **G** | **C** | **T** | **A** | **C** | **T** | **C** | **A** | **G** | **T** | **T** | **C** | **A** | **A** | **A** | **G** | **A** | **A** | **G** | **A** | **T** | **G** | **A** | **G** | **A** | **C** | **A** | **C** | **A** | **T** | **T** | **G** | **T** | **T** | **C** | **A** | **C** | **C** | **A** | **A** | **T** | **G** | **A** | **C** | **A** | **A** | **C** | **T** | **G** | **A** | **A** | **A** | **C** | **A** | **G** | **T** | **G** | **A** | **A** | **G** | **G** | **A** | **A** | **A** | **T** | **G** | **T** | **C** | **T** | **A** | **A** | **C** | **T** | **G** | **G** | **C** | **T** | **G** | **T** | **T** | **A** | **T** | **T** | **C** | **A** | **A** | **A** | **C** | **A** | **A** | **C** | **A** | **A** | **A** | **T** | **T** | **T** | **C** | **T** | **C** | **G** | **A** | **T** | **T** | **G** | **T** | **T** | **T** | **T** | **C** | **T** | **C** | **C** | **A** | **A** | **T** | **C** | **T** | **T** | **A** | **-** | **-** | **-** |
| **N59779F** | **G** | **T** | **G** | **G** | **T** | **A** | **T** | **C** | **T** | **A** | **G** | **T** | **A** | **T** | **T** | **A** | **T** | **A** | **A** | **C** | **C** | **G** | **G** | **C** | **C** | **T** | **T** | **T** | **A** | **T** | **C** | **T** | **A** | **T** | **T** | **A** | **A** | **A** | **T** | **G** | **C** | **A** | **C** | **T** | **T** | **A** | **T** | **T** | **G** | **C** | **T** | **T** | **T** | **G** | **G** | **T** | **G** | **G** | **C** | **T** | **A** | **T** | **T** | **T** | **T** | **G** | **A** | **T** | **C** | **A** | **A** | **A** | **C** | **C** | **C** | **C** | **G** | **A** | **G** | **A** | **T** | **G** | **T** | **A** | **G** | **C** | **A** | **G** | **C** | **C** | **A** | **T** | **T** | **T** | **G** | **G** | **G** | **C** | **A** | **T** | **G** | **G** | **C** | **T** | **C** | **T** | **T** | **A** | **G** | **T** | **A** | **T** | **T** | **G** | **T** | **T** | **T** | **G** | **C** | **C** | **C** | **T** | **T** | **C** | **C** | **T** | **G** | **G** | **G** | **G** | **T** | **G** | **G** | **G** | **T** | **T** | **T** | **T** | **G** | **T** | **A** | **T** | **T** | **A** | **T** | **A** | **T** | **T** | **T** | **A** | **T** | **T** | **T** | **T** | **T** | **C** | **G** | **G** | **A** | **C** | **G** | **T** | **G** | **G** | **A** | **T** | **T** | **A** | **A** | **C** | **G** | **G** | **A** | **T** | **A** | **A** | **G** | **A** | **A** | **A** | **A** | **A** | **A** | **T** | **T** | **C** | **T** | **A** | **T** | **C** | **T** | **C** | **C** | **G** | **A** | **C** | **A** | **A** | **A** | **G** | **T** | **G** | **A** | **C** | **T** | **T** | **G** | **A** | **A** | **A** | **G** | **A** | **A** | **T** | **T** | **A** | **G** | **A** | **A** | **A** | **A** | **C** | **T** | **T** | **T** | **C** | **A** | **G** | **T** | **C** | **T** | **T** | **T** | **T** | **A** | **G** | **A** | **G** | **G** | **A** | **G** | **A** | **T** | **A** | **C** | **C** | **A** | **T** | **T** | **G** | **A** | **A** | **C** | **A** | **T** | **T** | **A** | **C** | **G** | **A** | **T** | **C** | **C** | **T** | **G** | **A** | **C** | **A** | **T** | **G** | **G** | **G** | **C** | **G** | **A** | **T** | **A** | **A** | **A** | **G** | **A** | **C** | **A** | **A** | **A** | **C** | **A** | **G** | **C** | **A** | **A** | **T** | **T** | **T** | **G** | **T** | **C** | **G** | **A** | **C** | **T** | **T** | **C** | **T** | **T** | **C** | **T** | **C** | **T** | **T** | **C** | **A** | **T** | **T** | **A** | **A** | **A** | **T** | **C** | **G** | **T** | **A** | **T** | **G** | **C** | **C** | **G** | **C** | **T** | **G** | **A** | **C** | **A** | **A** | **G** | **A** | **A** | **T** | **G** | **A** | **A** | **T** | **T** | **C** | **T** | **G** | **T** | **C** | **A** | **C** | **T** | **C** | **T** | **T** | **C** | **T** | **C** | **A** | **C** | **A** | **G** | **A** | **C** | **G** | **G** | **A** | **C** | **A** | **A** | **G** | **A** | **G** | **A** | **A** | **A** | **T** | **T** | **G** | **G** | **A** | **T** | **T** | **C** | **A** | **C** | **T** | **G** | **C** | **T** | **T** | **C** | **A** | **A** | **G** | **A** | **T** | **C** | **T** | **A** | **A** | **A** | **A** | **A** | **A** | **A** | **G** | **C** | **C** | **A** | **A** | **A** | **C** | **A** | **T** | **T** | **C** | **G** | **A** | **T** | **C** | **C** | **A** | **T** | **A** | **T** | **C** | **G** | **A** | **A** | **T** | **A** | **T** | **T** | **A** | **C** | **G** | **C** | **A** | **T** | **T** | **T** | **G** | **T** | **G** | **A** | **C** | **A** | **G** | **A** | **T** | **A** | **A** | **T** | **A** | **T** | **C** | **G** | **G** | **C** | **C** | **A** | **G** | **C** | **A** | **A** | **G** | **T** | **C** | **T** | **T** | **A** | **C** | **A** | **T** | **G** | **T** | **T** | **T** | **T** | **A** | **G** | **A** | **A** | **G** | **A** | **A** | **A** | **A** | **A** | **G** | **C** | **C** | **G** | **C** | **A** | **G** | **A** | **A** | **G** | **G** | **C** | **G** | **T** | **G** | **G** | **A** | **A** | **G** | **T** | **T** | **C** | **G** | **A** | **A** | **T** | **A** | **T** | **T** | **A** | **T** | **A** | **T** | **G** | **A** | **T** | **G** | **C** | **A** | **T** | **T** | **T** | **G** | **G** | **C** | **T** | **C** | **T** | **C** | **A** | **T** | **G** | **G** | **C** | **A** | **C** | **A** | **A** | **A** | **A** | **G** | **C** | **A** | **A** | **A** | **A** | **G** | **A** | **T** | **T** | **T** | **C** | **A** | **A** | **T** | **C** | **G** | **T** | **C** | **T** | **A** | **A** | **T** | **C** | **A** | **A** | **A** | **A** | **A** | **T** | **G** | **G** | **T** | **G** | **G** | **A** | **C** | **A** | **T** | **G** | **T** | **C** | **C** | **A** | **T** | **A** | **C** | **A** | **T** | **T** | **T** | **G** | **T** | **T** | **A** | **C** | **C** | **T** | **C** | **A** | **C** | **A** | **A** | **A** | **A** | **G** | **G** | **C** | **A** | **T** | **T** | **A** | **C** | **T** | **T** | **C** | **G** | **T** | **T** | **T** | **C** | **C** | **G** | **A** | **T** | **T** | **G** | **A** | **A** | **T** | **T** | **A** | **C** | **C** | **A** | **T** | **G** | **A** | **T** | **C** | **A** | **C** | **C** | **G** | **A** | **A** | **A** | **A** | **A** | **T** | **C** | **G** | **T** | **T** | **G** | **T** | **G** | **A** | **T** | **C** | **G** | **A** | **T** | **G** | **G** | **A** | **A** | **A** | **G** | **A** | **T** | **T** | **A** | **G** | **T** | **T** | **A** | **T** | **A** | **C** | **C** | **G** | **G** | **T** | **G** | **G** | **T** | **T** | **T** | **C** | **A** | **A** | **T** | **A** | **T** | **T** | **G** | **C** | **C** | **A** | **A** | **T** | **C** | **A** | **A** | **T** | **A** | **T** | **G** | **T** | **A** | **A** | **A** | **T** | **A** | **C** | **A** | **A** | **C** | **A** | **A** | **A** | **A** | **A** | **A** | **A** | **T** | **T** | **C** | **G** | **G** | **C** | **T** | **A** | **T** | **T** | **G** | **G** | **C** | **G** | **C** | **G** | **A** | **T** | **A** | **C** | **G** | **C** | **A** | **T** | **A** | **T** | **A** | **C** | **G** | **G** | **A** | **T** | **T** | **T** | **T** | **C** | **G** | **G** | **T** | **G** | **C** | **C** | **G** | **C** | **T** | **T** | **C** | **T** | **T** | **C** | **T** | **T** | **T** | **G** | **C** | **T** | **C** | **C** | **A** | **G** | **C** | **T** | **T** | **C** | **G** | **C** | **T** | **T** | **C** | **T** | **T** | **A** | **A** | **C** | **A** | **G** | **A** | **C** | **T** | **G** | **G** | **A** | **A** | **C** | **G** | **T** | **C** | **T** | **C** | **G** | **G** | **T** | **A** | **C** | **C** | **T** | **G** | **A** | **A** | **G** | **A** | **A** | **A** | **A** | **A** | **A** | **A** | **G** | **G** | **T** | **C** | **G** | **G** | **C** | **T** | **A** | **T** | **C** | **A** | **T** | **T** | **T** | **G** | **A** | **A** | **T** | **T** | **A** | **T** | **T** | **T** | **C** | **T** | **T** | **T** | **A** | **A** | **A** | **A** | **A** | **A** | **G** | **C** | **A** | **G** | **A** | **T** | **C** | **G** | **A** | **G** | **A** | **T** | **G** | **A** | **A** | **T** | **C** | **T** | **A** | **A** | **G** | **C** | **T** | **T** | **G** | **C** | **T** | **G** | **A** | **T** | **A** | **C** | **A** | **T** | **C** | **C** | **A** | **T** | **C** | **C** | **A** | **G** | **C** | **T** | **T** | **G** | **T** | **T** | **T** | **C** | **A** | **A** | **G** | **C** | **G** | **G** | **A** | **C** | **C** | **G** | **A** | **A** | **T** | **A** | **A** | **C** | **G** | **A** | **A** | **A** | **G** | **G** | **G** | **A** | **A** | **C** | **A** | **A** | **A** | **T** | **C** | **A** | **A** | **G** | **C** | **T** | **T** | **T** | **C** | **A** | **T** | **T** | **T** | **A** | **T** | **C** | **A** | **A** | **A** | **T** | **T** | **G** | **A** | **T** | **T** | **A** | **C** | **T** | **T** | **C** | **T** | **G** | **C** | **T** | **A** | **A** | **A** | **A** | **A** | **A** | **C** | **G** | **T** | **G** | **T** | **T** | **T** | **G** | **G** | **A** | **T** | **A** | **C** | **A** | **G** | **A** | **C** | **A** | **C** | **C** | **T** | **T** | **A** | **C** | **C** | **T** | **T** | **G** | **T** | **T** | **C** | **C** | **T** | **G** | **A** | **T** | **G** | **A** | **A** | **A** | **G** | **T** | **G** | **T** | **C** | **A** | **T** | **T** | **G** | **C** | **T** | **G** | **C** | **T** | **T** | **T** | **A** | **A** | **A** | **A** | **A** | **T** | **C** | **G** | **C** | **A** | **A** | **C** | **T** | **G** | **C** | **C** | **T** | **C** | **T** | **G** | **G** | **T** | **G** | **T** | **A** | **G** | **A** | **T** | **G** | **T** | **G** | **A** | **A** | **A** | **A** | **T** | **C** | **A** | **T** | **G** | **A** | **T** | **T** | **C** | **C** | **C** | **A** | **A** | **C** | **A** | **A** | **A** | **C** | **C** | **G** | **G** | **A** | **T** | **C** | **A** | **T** | **C** | **C** | **T** | **T** | **T** | **T** | **A** | **T** | **T** | **T** | **A** | **T** | **C** | **G** | **A** | **G** | **C** | **A** | **A** | **C** | **A** | **C** | **A** | **A** | **T** | **A** | **T** | **T** | **A** | **T** | **G** | **C** | **T** | **C** | **G** | **G** | **C** | **A** | **G** | **C** | **T** | **G** | **A** | **T** | **C** | **A** | **A** | **G** | **G** | **A** | **A** | **A** | **A** | **T** | **G** | **T** | **A** | **C** | **A** | **A** | **A** | **T** | **C** | **C** | **T** | **T** | **G** | **T** | **C** | **T** | **A** | **T** | **G** | **A** | **G** | **A** | **A** | **C** | **G** | **G** | **C** | **T** | **T** | **C** | **C** | **T** | **C** | **C** | **A** | **T** | **G** | **C** | **A** | **A** | **A** | **A** | **A** | **C** | **A** | **T** | **T** | **G** | **A** | **T** | **A** | **A** | **T** | **G** | **G** | **A** | **T** | **G** | **A** | **T** | **G** | **A** | **A** | **A** | **T** | **C** | **T** | **G** | **C** | **A** | **T** | **G** | **G** | **T** | **A** | **G** | **G** | **T** | **T** | **C** | **A** | **G** | **C** | **A** | **A** | **A** | **T** | **C** | **A** | **A** | **G** | **A** | **T** | **A** | **T** | **T** | **C** | **G** | **T** | **A** | **G** | **C** | **T** | **A** | **C** | **C** | **G** | **A** | **T** | **T** | **G** | **A** | **A** | **T** | **T** | **T** | **T** | **G** | **A** | **A** | **A** | **C** | **A** | **A** | **G** | **T** | **G** | **C** | **T** | **G** | **T** | **C** | **A** | **T** | **T** | **T** | **A** | **C** | **G** | **A** | **T** | **C** | **C** | **T** | **G** | **A** | **G** | **T** | **T** | **T** | **T** | **T** | **A** | **G** | **A** | **A** | **G** | **A** | **A** | **C** | **T** | **T** | **G** | **C** | **T** | **A** | **C** | **T** | **C** | **A** | **G** | **T** | **T** | **C** | **A** | **A** | **A** | **G** | **A** | **A** | **G** | **A** | **T** | **G** | **A** | **G** | **A** | **C** | **A** | **C** | **A** | **T** | **T** | **G** | **T** | **T** | **C** | **A** | **T** | **C** | **C** | **A** | **T** | **G** | **A** | **C** | **A** | **A** | **C** | **T** | **G** | **A** | **A** | **A** | **C** | **A** | **G** | **T** | **C** | **A** | **A** | **G** | **G** | **A** | **A** | **A** | **T** | **G** | **T** | **C** | **T** | **A** | **A** | **C** | **T** | **G** | **G** | **C** | **T** | **A** | **T** | **T** | **A** | **T** | **T** | **C** | **A** | **A** | **G** | **C** | **A** | **A** | **C** | **A** | **A** | **A** | **T** | **T** | **T** | **C** | **T** | **C** | **G** | **A** | **T** | **T** | **A** | **T** | **T** | **T** | **T** | **C** | **T** | **C** | **C** | **A** | **A** | **T** | **C** | **C** | **T** | **A** | **-** | **-** | **-** |
| **N59788F** | **-** | **-** | **-** | **-** | **-** | **-** | **-** | **-** | **-** | **-** | **-** | **-** | **-** | **-** | **-** | **-** | **-** | **-** | **-** | **-** | **-** | **-** | **-** | **-** | **-** | **-** | **-** | **-** | **-** | **-** | **-** | **-** | **-** | **-** | **-** | **-** | **-** | **-** | **-** | **-** | **-** | **-** | **-** | **-** | **-** | **-** | **-** | **-** | **-** | **-** | **-** | **-** | **-** | **-** | **-** | **-** | **-** | **-** | **-** | **-** | **-** | **-** | **-** | **-** | **-** | **-** | **-** | **-** | **-** | **A** | **A** | **A** | **C** | **C** | **C** | **C** | **G** | **A** | **G** | **A** | **T** | **G** | **T** | **A** | **G** | **C** | **A** | **G** | **C** | **C** | **A** | **T** | **T** | **T** | **G** | **G** | **G** | **C** | **A** | **T** | **G** | **G** | **C** | **T** | **C** | **T** | **T** | **A** | **G** | **T** | **A** | **T** | **T** | **G** | **T** | **T** | **T** | **G** | **C** | **C** | **C** | **T** | **T** | **C** | **C** | **T** | **G** | **G** | **G** | **G** | **T** | **G** | **G** | **G** | **T** | **T** | **T** | **T** | **G** | **T** | **A** | **T** | **T** | **A** | **T** | **A** | **T** | **T** | **T** | **A** | **T** | **T** | **T** | **T** | **T** | **C** | **G** | **G** | **A** | **C** | **G** | **T** | **G** | **G** | **A** | **T** | **T** | **A** | **A** | **C** | **G** | **G** | **A** | **T** | **A** | **A** | **G** | **A** | **A** | **A** | **A** | **A** | **A** | **T** | **T** | **C** | **T** | **A** | **T** | **C** | **T** | **C** | **C** | **G** | **A** | **C** | **A** | **A** | **A** | **G** | **T** | **G** | **A** | **C** | **T** | **T** | **G** | **A** | **A** | **A** | **G** | **A** | **A** | **T** | **T** | **A** | **G** | **A** | **A** | **A** | **A** | **C** | **T** | **T** | **T** | **C** | **A** | **G** | **T** | **C** | **T** | **T** | **T** | **T** | **A** | **G** | **A** | **G** | **G** | **G** | **G** | **A** | **T** | **A** | **C** | **C** | **A** | **T** | **T** | **G** | **A** | **A** | **C** | **A** | **T** | **T** | **A** | **C** | **G** | **A** | **T** | **C** | **C** | **T** | **G** | **A** | **C** | **A** | **T** | **G** | **G** | **G** | **C** | **G** | **A** | **T** | **A** | **A** | **A** | **G** | **A** | **C** | **A** | **A** | **A** | **C** | **A** | **G** | **C** | **A** | **A** | **T** | **T** | **T** | **G** | **T** | **T** | **G** | **A** | **C** | **T** | **T** | **C** | **T** | **T** | **C** | **T** | **C** | **T** | **T** | **C** | **A** | **T** | **T** | **A** | **A** | **A** | **T** | **C** | **G** | **T** | **A** | **T** | **G** | **C** | **C** | **G** | **C** | **T** | **G** | **A** | **C** | **A** | **A** | **G** | **A** | **A** | **T** | **G** | **A** | **A** | **T** | **T** | **C** | **T** | **G** | **T** | **C** | **A** | **C** | **T** | **C** | **T** | **T** | **C** | **T** | **C** | **A** | **C** | **A** | **G** | **A** | **C** | **G** | **G** | **A** | **C** | **A** | **A** | **G** | **A** | **G** | **A** | **A** | **A** | **T** | **T** | **G** | **G** | **A** | **T** | **T** | **C** | **A** | **C** | **T** | **G** | **C** | **T** | **T** | **C** | **A** | **A** | **G** | **A** | **T** | **C** | **T** | **A** | **A** | **A** | **A** | **A** | **A** | **A** | **G** | **C** | **C** | **A** | **A** | **A** | **C** | **A** | **T** | **T** | **C** | **G** | **A** | **T** | **C** | **C** | **A** | **T** | **A** | **T** | **C** | **G** | **A** | **A** | **T** | **A** | **T** | **T** | **A** | **C** | **G** | **C** | **A** | **T** | **T** | **T** | **G** | **T** | **G** | **A** | **C** | **A** | **G** | **A** | **T** | **A** | **A** | **T** | **A** | **T** | **C** | **G** | **G** | **C** | **C** | **A** | **G** | **C** | **A** | **A** | **G** | **T** | **C** | **T** | **T** | **A** | **C** | **A** | **T** | **G** | **T** | **T** | **T** | **T** | **A** | **G** | **A** | **A** | **G** | **A** | **A** | **A** | **A** | **A** | **G** | **C** | **C** | **G** | **C** | **A** | **G** | **A** | **A** | **G** | **G** | **C** | **G** | **T** | **G** | **G** | **A** | **A** | **G** | **T** | **T** | **C** | **G** | **A** | **A** | **T** | **A** | **T** | **T** | **A** | **T** | **A** | **T** | **G** | **A** | **T** | **G** | **C** | **A** | **T** | **T** | **T** | **G** | **G** | **C** | **T** | **C** | **T** | **C** | **A** | **T** | **G** | **G** | **C** | **A** | **C** | **A** | **A** | **A** | **A** | **G** | **C** | **A** | **A** | **A** | **A** | **G** | **A** | **T** | **T** | **T** | **C** | **A** | **A** | **T** | **C** | **G** | **T** | **C** | **T** | **A** | **A** | **T** | **C** | **A** | **A** | **A** | **A** | **A** | **T** | **G** | **G** | **T** | **G** | **G** | **A** | **C** | **A** | **T** | **G** | **T** | **C** | **C** | **A** | **T** | **A** | **C** | **A** | **T** | **T** | **T** | **G** | **T** | **T** | **A** | **C** | **C** | **T** | **C** | **A** | **C** | **A** | **A** | **A** | **A** | **G** | **G** | **C** | **A** | **T** | **T** | **A** | **C** | **T** | **T** | **C** | **G** | **T** | **T** | **T** | **C** | **C** | **G** | **A** | **T** | **T** | **G** | **A** | **A** | **T** | **T** | **A** | **C** | **C** | **A** | **T** | **G** | **A** | **T** | **C** | **A** | **C** | **C** | **G** | **A** | **A** | **A** | **A** | **A** | **T** | **C** | **G** | **T** | **T** | **G** | **T** | **G** | **A** | **T** | **C** | **G** | **A** | **T** | **G** | **G** | **A** | **A** | **A** | **G** | **A** | **T** | **T** | **A** | **G** | **T** | **T** | **A** | **T** | **A** | **C** | **C** | **G** | **G** | **T** | **G** | **G** | **T** | **T** | **T** | **C** | **A** | **A** | **T** | **A** | **T** | **T** | **G** | **C** | **C** | **A** | **A** | **T** | **C** | **A** | **A** | **T** | **A** | **T** | **G** | **T** | **A** | **A** | **A** | **T** | **A** | **C** | **A** | **A** | **C** | **A** | **A** | **A** | **A** | **A** | **A** | **A** | **T** | **T** | **C** | **G** | **G** | **C** | **T** | **A** | **T** | **T** | **G** | **G** | **C** | **G** | **C** | **G** | **A** | **T** | **A** | **C** | **G** | **C** | **A** | **T** | **A** | **T** | **A** | **C** | **G** | **G** | **A** | **T** | **T** | **T** | **T** | **C** | **G** | **G** | **T** | **G** | **C** | **C** | **G** | **C** | **T** | **T** | **C** | **T** | **T** | **C** | **T** | **T** | **T** | **G** | **C** | **T** | **C** | **C** | **A** | **G** | **C** | **T** | **T** | **C** | **G** | **C** | **T** | **T** | **C** | **T** | **T** | **A** | **A** | **C** | **A** | **G** | **A** | **C** | **T** | **G** | **G** | **A** | **A** | **C** | **G** | **T** | **C** | **T** | **C** | **G** | **G** | **T** | **A** | **C** | **C** | **T** | **G** | **A** | **A** | **G** | **A** | **A** | **A** | **A** | **A** | **A** | **A** | **G** | **G** | **T** | **C** | **G** | **G** | **C** | **T** | **A** | **T** | **C** | **A** | **T** | **T** | **T** | **G** | **A** | **A** | **T** | **T** | **A** | **T** | **T** | **T** | **C** | **T** | **T** | **T** | **A** | **A** | **A** | **A** | **A** | **A** | **G** | **C** | **A** | **G** | **A** | **T** | **C** | **G** | **A** | **G** | **A** | **T** | **G** | **A** | **A** | **T** | **C** | **T** | **A** | **A** | **G** | **C** | **T** | **T** | **G** | **C** | **T** | **G** | **A** | **T** | **A** | **C** | **A** | **T** | **C** | **C** | **A** | **T** | **C** | **C** | **A** | **G** | **C** | **T** | **T** | **G** | **T** | **T** | **T** | **C** | **A** | **A** | **G** | **C** | **G** | **G** | **A** | **C** | **C** | **G** | **A** | **A** | **T** | **A** | **A** | **C** | **G** | **A** | **A** | **A** | **G** | **G** | **G** | **A** | **A** | **C** | **A** | **A** | **A** | **T** | **C** | **A** | **A** | **G** | **C** | **T** | **T** | **T** | **C** | **A** | **T** | **T** | **T** | **A** | **T** | **C** | **A** | **A** | **A** | **T** | **T** | **G** | **A** | **T** | **T** | **A** | **C** | **T** | **T** | **C** | **T** | **G** | **C** | **T** | **A** | **A** | **A** | **A** | **A** | **A** | **C** | **G** | **T** | **G** | **T** | **T** | **T** | **G** | **G** | **A** | **T** | **A** | **C** | **A** | **G** | **A** | **C** | **A** | **C** | **C** | **T** | **T** | **A** | **C** | **C** | **T** | **T** | **G** | **T** | **T** | **C** | **C** | **T** | **G** | **A** | **T** | **G** | **A** | **A** | **A** | **G** | **T** | **G** | **T** | **C** | **A** | **T** | **T** | **G** | **C** | **T** | **G** | **C** | **T** | **T** | **T** | **A** | **A** | **A** | **A** | **A** | **T** | **C** | **G** | **C** | **A** | **A** | **C** | **T** | **G** | **C** | **C** | **T** | **C** | **T** | **G** | **G** | **T** | **G** | **T** | **A** | **G** | **A** | **T** | **G** | **T** | **G** | **A** | **A** | **A** | **A** | **T** | **C** | **A** | **T** | **G** | **A** | **T** | **T** | **C** | **C** | **C** | **A** | **A** | **C** | **A** | **A** | **A** | **C** | **C** | **G** | **G** | **A** | **T** | **C** | **A** | **T** | **C** | **C** | **T** | **T** | **T** | **T** | **A** | **T** | **T** | **T** | **A** | **T** | **C** | **G** | **A** | **G** | **C** | **A** | **A** | **C** | **A** | **C** | **A** | **A** | **T** | **A** | **T** | **T** | **A** | **T** | **G** | **C** | **T** | **C** | **G** | **G** | **C** | **A** | **G** | **C** | **T** | **G** | **A** | **T** | **C** | **A** | **A** | **G** | **G** | **A** | **A** | **A** | **A** | **T** | **G** | **T** | **A** | **C** | **A** | **A** | **A** | **T** | **C** | **C** | **T** | **T** | **G** | **T** | **C** | **T** | **A** | **T** | **G** | **A** | **G** | **A** | **A** | **C** | **G** | **G** | **C** | **T** | **T** | **C** | **C** | **T** | **C** | **C** | **A** | **T** | **G** | **C** | **A** | **A** | **A** | **A** | **A** | **C** | **A** | **T** | **T** | **G** | **A** | **T** | **A** | **A** | **T** | **G** | **G** | **A** | **T** | **G** | **A** | **T** | **G** | **A** | **A** | **A** | **T** | **C** | **T** | **G** | **C** | **A** | **T** | **G** | **G** | **T** | **A** | **G** | **G** | **T** | **T** | **C** | **A** | **G** | **C** | **A** | **A** | **A** | **T** | **C** | **A** | **A** | **G** | **A** | **T** | **A** | **T** | **T** | **C** | **G** | **T** | **A** | **G** | **C** | **T** | **A** | **C** | **C** | **G** | **A** | **T** | **T** | **G** | **A** | **A** | **T** | **T** | **T** | **T** | **G** | **A** | **A** | **A** | **C** | **A** | **A** | **G** | **T** | **G** | **C** | **T** | **G** | **T** | **C** | **A** | **T** | **T** | **T** | **A** | **C** | **G** | **A** | **T** | **C** | **C** | **T** | **G** | **A** | **G** | **T** | **T** | **T** | **T** | **T** | **A** | **G** | **A** | **A** | **G** | **A** | **A** | **C** | **T** | **T** | **G** | **C** | **T** | **A** | **C** | **T** | **C** | **A** | **G** | **T** | **T** | **C** | **A** | **A** | **A** | **G** | **A** | **A** | **G** | **A** | **T** | **G** | **A** | **G** | **A** | **C** | **A** | **C** | **A** | **T** | **T** | **G** | **T** | **T** | **C** | **A** | **T** | **C** | **C** | **A** | **T** | **G** | **A** | **C** | **A** | **A** | **C** | **T** | **G** | **A** | **A** | **A** | **C** | **A** | **G** | **T** | **C** | **A** | **A** | **G** | **G** | **A** | **A** | **A** | **T** | **G** | **T** | **C** | **T** | **A** | **A** | **C** | **T** | **G** | **G** | **C** | **T** | **A** | **T** | **T** | **A** | **T** | **T** | **C** | **A** | **A** | **G** | **C** | **A** | **A** | **C** | **A** | **A** | **A** | **T** | **T** | **T** | **C** | **T** | **C** | **G** | **A** | **T** | **T** | **A** | **T** | **T** | **T** | **T** | **C** | **T** | **C** | **C** | **A** | **A** | **T** | **C** | **C** | **T** | **A** | **-** | **-** | **-** |
| **N54671** | **-** | **-** | **-** | **-** | **-** | **-** | **-** | **-** | **-** | **-** | **-** | **-** | **-** | **-** | **-** | **-** | **-** | **-** | **-** | **-** | **-** | **-** | **-** | **-** | **-** | **-** | **-** | **-** | **-** | **-** | **-** | **-** | **-** | **-** | **-** | **-** | **-** | **-** | **-** | **-** | **-** | **-** | **-** | **-** | **-** | **-** | **-** | **-** | **-** | **-** | **-** | **-** | **-** | **-** | **-** | **-** | **-** | **-** | **-** | **-** | **-** | **-** | **-** | **-** | **-** | **-** | **-** | **-** | **-** | **A** | **A** | **A** | **C** | **C** | **C** | **C** | **G** | **A** | **G** | **A** | **T** | **G** | **T** | **A** | **G** | **C** | **A** | **G** | **C** | **C** | **A** | **T** | **T** | **T** | **G** | **G** | **G** | **C** | **A** | **T** | **G** | **G** | **C** | **T** | **C** | **T** | **T** | **A** | **G** | **T** | **A** | **T** | **T** | **G** | **T** | **T** | **T** | **G** | **C** | **C** | **C** | **T** | **T** | **C** | **C** | **T** | **G** | **G** | **G** | **G** | **T** | **G** | **G** | **G** | **C** | **T** | **T** | **T** | **G** | **T** | **A** | **T** | **T** | **A** | **T** | **A** | **T** | **T** | **T** | **A** | **T** | **T** | **T** | **T** | **T** | **C** | **G** | **G** | **A** | **C** | **G** | **T** | **G** | **G** | **A** | **T** | **T** | **A** | **A** | **C** | **G** | **G** | **A** | **T** | **A** | **A** | **G** | **A** | **A** | **A** | **A** | **A** | **A** | **T** | **T** | **C** | **T** | **A** | **T** | **C** | **T** | **C** | **C** | **G** | **A** | **C** | **A** | **A** | **A** | **G** | **T** | **G** | **A** | **C** | **T** | **T** | **G** | **A** | **A** | **A** | **G** | **A** | **A** | **T** | **T** | **A** | **G** | **A** | **A** | **A** | **A** | **C** | **T** | **T** | **T** | **C** | **A** | **G** | **T** | **C** | **T** | **T** | **T** | **T** | **A** | **G** | **A** | **G** | **G** | **A** | **G** | **A** | **T** | **A** | **A** | **C** | **A** | **T** | **T** | **G** | **A** | **A** | **C** | **A** | **T** | **T** | **A** | **C** | **G** | **A** | **T** | **C** | **C** | **T** | **G** | **A** | **C** | **A** | **T** | **G** | **G** | **G** | **C** | **G** | **A** | **T** | **A** | **A** | **A** | **G** | **A** | **C** | **A** | **A** | **A** | **C** | **A** | **G** | **C** | **A** | **A** | **T** | **T** | **T** | **G** | **T** | **T** | **G** | **A** | **C** | **T** | **T** | **C** | **T** | **T** | **C** | **T** | **C** | **T** | **T** | **C** | **A** | **T** | **T** | **A** | **A** | **A** | **T** | **C** | **G** | **T** | **A** | **T** | **G** | **C** | **C** | **G** | **C** | **T** | **G** | **A** | **C** | **A** | **A** | **G** | **A** | **A** | **T** | **G** | **A** | **A** | **T** | **T** | **C** | **T** | **G** | **T** | **C** | **A** | **C** | **T** | **C** | **T** | **T** | **C** | **T** | **C** | **A** | **C** | **A** | **G** | **A** | **C** | **G** | **G** | **A** | **C** | **A** | **A** | **G** | **A** | **G** | **A** | **A** | **A** | **T** | **T** | **G** | **G** | **A** | **T** | **T** | **C** | **A** | **C** | **T** | **G** | **C** | **T** | **T** | **C** | **A** | **A** | **G** | **A** | **T** | **C** | **T** | **A** | **A** | **A** | **A** | **A** | **A** | **A** | **G** | **C** | **C** | **A** | **A** | **A** | **C** | **A** | **T** | **T** | **C** | **G** | **A** | **T** | **C** | **C** | **A** | **T** | **A** | **T** | **C** | **G** | **A** | **A** | **T** | **A** | **T** | **T** | **A** | **C** | **G** | **C** | **A** | **T** | **T** | **T** | **G** | **T** | **G** | **A** | **C** | **A** | **G** | **A** | **T** | **A** | **A** | **T** | **A** | **T** | **C** | **G** | **G** | **C** | **C** | **A** | **G** | **C** | **A** | **A** | **G** | **T** | **C** | **T** | **T** | **A** | **C** | **A** | **T** | **G** | **T** | **T** | **T** | **T** | **A** | **G** | **A** | **A** | **G** | **A** | **A** | **A** | **A** | **A** | **G** | **C** | **C** | **G** | **C** | **A** | **G** | **A** | **A** | **G** | **G** | **C** | **G** | **T** | **G** | **G** | **A** | **A** | **G** | **T** | **T** | **C** | **G** | **A** | **A** | **T** | **A** | **T** | **T** | **A** | **T** | **A** | **T** | **G** | **A** | **T** | **G** | **C** | **A** | **T** | **T** | **T** | **G** | **G** | **C** | **T** | **C** | **T** | **C** | **A** | **T** | **G** | **G** | **C** | **A** | **C** | **A** | **A** | **A** | **A** | **G** | **C** | **A** | **A** | **A** | **A** | **G** | **A** | **T** | **T** | **T** | **C** | **A** | **A** | **T** | **C** | **G** | **T** | **C** | **T** | **A** | **A** | **T** | **C** | **A** | **A** | **A** | **A** | **A** | **T** | **G** | **G** | **T** | **G** | **G** | **A** | **C** | **A** | **T** | **G** | **T** | **C** | **C** | **A** | **T** | **A** | **C** | **A** | **T** | **T** | **T** | **G** | **T** | **T** | **A** | **C** | **C** | **T** | **C** | **A** | **C** | **A** | **A** | **A** | **A** | **G** | **G** | **C** | **A** | **T** | **T** | **A** | **C** | **T** | **T** | **C** | **G** | **T** | **T** | **T** | **C** | **C** | **G** | **A** | **T** | **T** | **G** | **A** | **A** | **T** | **T** | **A** | **C** | **C** | **A** | **T** | **G** | **A** | **T** | **C** | **A** | **C** | **C** | **G** | **A** | **A** | **A** | **A** | **A** | **T** | **C** | **G** | **T** | **T** | **G** | **T** | **G** | **A** | **T** | **C** | **G** | **A** | **T** | **G** | **G** | **A** | **A** | **A** | **G** | **A** | **T** | **T** | **A** | **G** | **T** | **T** | **A** | **T** | **A** | **C** | **C** | **G** | **G** | **T** | **G** | **G** | **T** | **T** | **T** | **C** | **A** | **A** | **T** | **A** | **T** | **T** | **G** | **C** | **C** | **A** | **A** | **T** | **C** | **A** | **A** | **T** | **A** | **T** | **G** | **T** | **A** | **A** | **A** | **T** | **A** | **C** | **A** | **A** | **C** | **A** | **A** | **A** | **A** | **A** | **A** | **A** | **T** | **T** | **C** | **G** | **G** | **C** | **T** | **A** | **T** | **T** | **G** | **G** | **C** | **G** | **C** | **G** | **A** | **T** | **A** | **C** | **G** | **C** | **A** | **T** | **A** | **T** | **A** | **C** | **G** | **G** | **A** | **T** | **T** | **T** | **T** | **C** | **G** | **G** | **T** | **G** | **C** | **C** | **G** | **C** | **T** | **T** | **C** | **T** | **T** | **C** | **T** | **T** | **T** | **G** | **C** | **T** | **C** | **C** | **A** | **G** | **C** | **T** | **T** | **C** | **G** | **C** | **T** | **T** | **C** | **T** | **T** | **A** | **A** | **C** | **A** | **G** | **A** | **C** | **T** | **G** | **G** | **A** | **A** | **C** | **G** | **T** | **C** | **T** | **C** | **G** | **G** | **T** | **A** | **C** | **C** | **T** | **G** | **A** | **A** | **G** | **A** | **A** | **A** | **A** | **A** | **A** | **A** | **G** | **G** | **T** | **C** | **G** | **G** | **C** | **T** | **A** | **T** | **C** | **A** | **T** | **T** | **T** | **G** | **A** | **A** | **T** | **T** | **A** | **T** | **T** | **T** | **C** | **T** | **T** | **T** | **A** | **A** | **A** | **A** | **A** | **A** | **G** | **C** | **A** | **G** | **A** | **T** | **C** | **G** | **A** | **G** | **A** | **T** | **G** | **A** | **A** | **T** | **C** | **T** | **A** | **A** | **G** | **C** | **T** | **T** | **G** | **C** | **T** | **G** | **A** | **T** | **A** | **C** | **A** | **T** | **C** | **C** | **A** | **T** | **C** | **C** | **A** | **G** | **C** | **T** | **T** | **G** | **T** | **T** | **T** | **C** | **A** | **A** | **G** | **C** | **G** | **G** | **A** | **C** | **C** | **G** | **A** | **A** | **T** | **A** | **A** | **C** | **G** | **A** | **A** | **A** | **G** | **G** | **G** | **A** | **A** | **C** | **A** | **A** | **A** | **T** | **C** | **A** | **A** | **G** | **C** | **T** | **T** | **T** | **C** | **A** | **T** | **T** | **T** | **A** | **T** | **C** | **A** | **A** | **A** | **T** | **T** | **G** | **A** | **T** | **T** | **A** | **C** | **T** | **T** | **C** | **T** | **G** | **C** | **T** | **A** | **A** | **A** | **A** | **A** | **A** | **C** | **G** | **T** | **G** | **T** | **T** | **T** | **G** | **G** | **A** | **T** | **A** | **C** | **A** | **G** | **A** | **C** | **A** | **C** | **C** | **T** | **T** | **A** | **C** | **C** | **T** | **T** | **G** | **T** | **T** | **C** | **C** | **T** | **G** | **A** | **T** | **G** | **A** | **A** | **A** | **G** | **T** | **G** | **T** | **C** | **A** | **T** | **T** | **G** | **C** | **T** | **G** | **C** | **T** | **T** | **T** | **A** | **A** | **A** | **A** | **A** | **T** | **C** | **G** | **C** | **A** | **A** | **C** | **T** | **G** | **C** | **C** | **T** | **C** | **T** | **G** | **G** | **T** | **G** | **T** | **A** | **G** | **A** | **T** | **G** | **T** | **G** | **A** | **A** | **A** | **A** | **T** | **C** | **A** | **T** | **G** | **A** | **T** | **T** | **C** | **C** | **C** | **A** | **A** | **C** | **A** | **A** | **A** | **C** | **C** | **G** | **G** | **A** | **T** | **C** | **A** | **T** | **C** | **C** | **T** | **T** | **T** | **T** | **A** | **T** | **T** | **T** | **A** | **T** | **C** | **G** | **A** | **G** | **C** | **A** | **A** | **C** | **A** | **C** | **A** | **A** | **T** | **A** | **T** | **T** | **A** | **T** | **G** | **C** | **T** | **C** | **G** | **G** | **C** | **A** | **G** | **C** | **T** | **G** | **A** | **T** | **C** | **A** | **A** | **G** | **G** | **A** | **A** | **A** | **A** | **T** | **G** | **T** | **A** | **C** | **A** | **A** | **A** | **T** | **C** | **C** | **T** | **T** | **G** | **T** | **C** | **T** | **A** | **T** | **G** | **A** | **G** | **A** | **A** | **C** | **G** | **G** | **C** | **T** | **T** | **C** | **C** | **T** | **C** | **C** | **A** | **T** | **G** | **C** | **A** | **A** | **A** | **A** | **A** | **C** | **A** | **T** | **T** | **G** | **A** | **T** | **A** | **A** | **T** | **G** | **G** | **A** | **T** | **G** | **A** | **T** | **G** | **A** | **A** | **A** | **T** | **C** | **T** | **G** | **C** | **A** | **T** | **G** | **G** | **T** | **A** | **G** | **G** | **T** | **T** | **C** | **A** | **G** | **C** | **A** | **A** | **A** | **T** | **C** | **A** | **A** | **G** | **A** | **T** | **A** | **T** | **T** | **C** | **G** | **T** | **A** | **G** | **C** | **T** | **A** | **C** | **C** | **G** | **A** | **T** | **T** | **G** | **A** | **A** | **T** | **T** | **T** | **T** | **G** | **A** | **A** | **A** | **C** | **A** | **A** | **G** | **T** | **G** | **C** | **T** | **G** | **T** | **C** | **A** | **T** | **T** | **T** | **A** | **C** | **G** | **A** | **T** | **C** | **C** | **T** | **G** | **A** | **G** | **T** | **T** | **T** | **T** | **T** | **A** | **G** | **A** | **A** | **G** | **A** | **A** | **C** | **T** | **T** | **G** | **C** | **T** | **A** | **C** | **T** | **C** | **A** | **G** | **T** | **T** | **C** | **A** | **A** | **A** | **G** | **A** | **A** | **G** | **A** | **T** | **G** | **A** | **G** | **A** | **C** | **A** | **C** | **A** | **T** | **T** | **G** | **T** | **T** | **C** | **A** | **T** | **C** | **C** | **A** | **T** | **G** | **A** | **C** | **A** | **A** | **C** | **T** | **G** | **A** | **A** | **A** | **C** | **A** | **G** | **T** | **C** | **A** | **A** | **G** | **G** | **A** | **A** | **A** | **T** | **G** | **T** | **C** | **T** | **A** | **A** | **C** | **T** | **G** | **G** | **C** | **T** | **A** | **T** | **T** | **A** | **T** | **T** | **C** | **A** | **A** | **G** | **C** | **A** | **A** | **C** | **A** | **A** | **A** | **T** | **T** | **T** | **C** | **T** | **C** | **G** | **A** | **T** | **T** | **A** | **T** | **T** | **T** | **T** | **C** | **T** | **C** | **C** | **A** | **A** | **T** | **C** | **C** | **T** | **A** | **-** | **-** | **-** |
| **N55337** | **-** | **-** | **-** | **-** | **-** | **-** | **-** | **-** | **-** | **-** | **-** | **-** | **-** | **-** | **-** | **-** | **-** | **-** | **-** | **-** | **-** | **-** | **-** | **-** | **-** | **-** | **-** | **-** | **-** | **-** | **-** | **-** | **-** | **-** | **-** | **-** | **-** | **-** | **-** | **-** | **-** | **-** | **-** | **-** | **-** | **-** | **-** | **-** | **-** | **-** | **-** | **-** | **-** | **-** | **-** | **-** | **-** | **-** | **-** | **-** | **-** | **-** | **-** | **-** | **-** | **-** | **-** | **-** | **-** | **A** | **A** | **A** | **C** | **C** | **C** | **C** | **G** | **A** | **G** | **A** | **T** | **G** | **T** | **A** | **G** | **C** | **A** | **G** | **C** | **C** | **A** | **T** | **T** | **T** | **G** | **G** | **G** | **C** | **A** | **T** | **G** | **G** | **C** | **T** | **C** | **T** | **T** | **A** | **G** | **T** | **A** | **T** | **T** | **G** | **T** | **T** | **T** | **G** | **T** | **C** | **C** | **T** | **T** | **C** | **C** | **T** | **G** | **G** | **G** | **G** | **T** | **G** | **G** | **G** | **C** | **T** | **T** | **T** | **G** | **T** | **A** | **T** | **T** | **A** | **T** | **A** | **T** | **T** | **T** | **A** | **T** | **T** | **T** | **T** | **T** | **C** | **G** | **G** | **A** | **C** | **G** | **T** | **G** | **G** | **A** | **T** | **T** | **A** | **A** | **C** | **G** | **G** | **A** | **T** | **A** | **A** | **G** | **A** | **A** | **A** | **A** | **A** | **A** | **T** | **T** | **C** | **T** | **A** | **T** | **C** | **T** | **C** | **C** | **G** | **A** | **C** | **A** | **A** | **A** | **G** | **T** | **G** | **A** | **C** | **T** | **T** | **G** | **A** | **A** | **A** | **G** | **A** | **A** | **T** | **T** | **A** | **G** | **A** | **A** | **A** | **A** | **C** | **T** | **T** | **T** | **C** | **A** | **G** | **T** | **C** | **T** | **T** | **T** | **T** | **A** | **G** | **A** | **G** | **G** | **A** | **G** | **A** | **T** | **A** | **C** | **C** | **A** | **T** | **T** | **G** | **A** | **A** | **C** | **A** | **T** | **T** | **A** | **C** | **G** | **A** | **T** | **C** | **C** | **T** | **G** | **A** | **C** | **A** | **T** | **G** | **G** | **G** | **C** | **G** | **A** | **T** | **A** | **A** | **A** | **G** | **A** | **C** | **A** | **A** | **A** | **C** | **A** | **G** | **C** | **A** | **A** | **T** | **T** | **T** | **G** | **T** | **T** | **G** | **A** | **C** | **T** | **T** | **C** | **T** | **T** | **C** | **T** | **C** | **T** | **T** | **C** | **A** | **T** | **T** | **A** | **A** | **A** | **T** | **C** | **G** | **T** | **A** | **T** | **G** | **C** | **C** | **G** | **C** | **T** | **G** | **A** | **C** | **A** | **A** | **G** | **A** | **A** | **T** | **G** | **A** | **A** | **T** | **T** | **C** | **T** | **G** | **T** | **C** | **A** | **C** | **T** | **C** | **T** | **T** | **C** | **T** | **C** | **A** | **C** | **A** | **G** | **A** | **C** | **G** | **G** | **A** | **C** | **A** | **A** | **G** | **A** | **G** | **A** | **A** | **A** | **T** | **T** | **G** | **G** | **A** | **T** | **T** | **C** | **A** | **C** | **T** | **G** | **C** | **T** | **T** | **C** | **A** | **A** | **G** | **A** | **T** | **C** | **T** | **A** | **A** | **A** | **A** | **A** | **A** | **A** | **G** | **C** | **C** | **A** | **A** | **A** | **C** | **A** | **T** | **T** | **C** | **G** | **A** | **T** | **C** | **C** | **A** | **T** | **A** | **T** | **C** | **G** | **A** | **A** | **T** | **A** | **T** | **T** | **A** | **C** | **G** | **C** | **A** | **T** | **T** | **T** | **G** | **T** | **G** | **A** | **C** | **A** | **G** | **A** | **T** | **A** | **A** | **T** | **A** | **T** | **C** | **G** | **G** | **C** | **C** | **A** | **G** | **C** | **A** | **A** | **G** | **T** | **C** | **T** | **T** | **A** | **C** | **A** | **T** | **G** | **T** | **T** | **T** | **T** | **A** | **G** | **A** | **A** | **G** | **A** | **A** | **A** | **A** | **A** | **G** | **C** | **C** | **G** | **C** | **A** | **G** | **A** | **A** | **G** | **G** | **C** | **G** | **T** | **G** | **G** | **A** | **A** | **G** | **T** | **T** | **C** | **G** | **A** | **A** | **T** | **A** | **T** | **T** | **A** | **T** | **A** | **T** | **G** | **A** | **T** | **G** | **C** | **A** | **T** | **T** | **T** | **G** | **G** | **C** | **T** | **C** | **T** | **C** | **A** | **T** | **G** | **G** | **C** | **A** | **C** | **A** | **A** | **A** | **A** | **G** | **C** | **A** | **A** | **A** | **A** | **G** | **A** | **T** | **T** | **T** | **C** | **A** | **A** | **T** | **C** | **G** | **T** | **C** | **T** | **A** | **A** | **T** | **C** | **A** | **A** | **A** | **A** | **A** | **T** | **G** | **G** | **T** | **G** | **G** | **A** | **C** | **A** | **T** | **G** | **T** | **C** | **C** | **A** | **T** | **A** | **C** | **A** | **T** | **T** | **T** | **G** | **T** | **T** | **A** | **C** | **C** | **T** | **C** | **A** | **C** | **A** | **A** | **A** | **A** | **G** | **G** | **C** | **A** | **T** | **T** | **A** | **C** | **T** | **T** | **C** | **G** | **T** | **T** | **T** | **C** | **C** | **G** | **A** | **T** | **T** | **G** | **A** | **A** | **T** | **T** | **A** | **C** | **C** | **A** | **T** | **G** | **A** | **T** | **C** | **A** | **C** | **C** | **G** | **A** | **A** | **A** | **A** | **A** | **T** | **C** | **G** | **T** | **T** | **G** | **T** | **G** | **A** | **T** | **C** | **G** | **A** | **T** | **G** | **G** | **A** | **A** | **A** | **G** | **A** | **T** | **T** | **A** | **G** | **T** | **T** | **A** | **T** | **A** | **C** | **C** | **G** | **G** | **T** | **G** | **G** | **T** | **T** | **T** | **C** | **A** | **A** | **T** | **A** | **T** | **T** | **G** | **C** | **C** | **A** | **A** | **T** | **C** | **A** | **A** | **T** | **A** | **T** | **G** | **T** | **A** | **A** | **A** | **T** | **A** | **C** | **A** | **A** | **C** | **A** | **A** | **A** | **A** | **A** | **A** | **A** | **T** | **T** | **C** | **G** | **G** | **C** | **T** | **A** | **T** | **T** | **G** | **G** | **C** | **G** | **C** | **G** | **A** | **T** | **A** | **C** | **G** | **C** | **A** | **T** | **A** | **T** | **A** | **C** | **G** | **G** | **A** | **T** | **T** | **T** | **T** | **C** | **G** | **G** | **T** | **G** | **C** | **C** | **G** | **C** | **T** | **T** | **C** | **T** | **T** | **C** | **T** | **T** | **T** | **G** | **C** | **T** | **C** | **C** | **A** | **G** | **C** | **T** | **T** | **C** | **G** | **C** | **T** | **T** | **C** | **T** | **T** | **A** | **A** | **C** | **A** | **G** | **A** | **C** | **T** | **G** | **G** | **A** | **A** | **C** | **G** | **T** | **C** | **T** | **C** | **G** | **G** | **T** | **A** | **C** | **C** | **T** | **G** | **A** | **A** | **G** | **A** | **A** | **A** | **A** | **A** | **A** | **A** | **G** | **G** | **T** | **C** | **G** | **G** | **C** | **T** | **A** | **T** | **C** | **A** | **T** | **T** | **T** | **G** | **A** | **A** | **T** | **T** | **A** | **T** | **T** | **T** | **C** | **T** | **T** | **T** | **A** | **A** | **A** | **A** | **A** | **A** | **G** | **C** | **A** | **G** | **A** | **T** | **C** | **G** | **A** | **G** | **A** | **T** | **G** | **A** | **A** | **T** | **C** | **T** | **A** | **A** | **G** | **C** | **T** | **T** | **G** | **C** | **T** | **G** | **A** | **T** | **A** | **C** | **A** | **T** | **C** | **C** | **A** | **T** | **C** | **C** | **A** | **G** | **C** | **T** | **T** | **G** | **T** | **T** | **T** | **C** | **A** | **A** | **G** | **C** | **G** | **G** | **A** | **C** | **C** | **G** | **A** | **A** | **T** | **A** | **A** | **C** | **G** | **A** | **A** | **A** | **G** | **G** | **G** | **A** | **A** | **C** | **A** | **A** | **A** | **T** | **C** | **A** | **A** | **G** | **C** | **T** | **T** | **T** | **C** | **A** | **T** | **T** | **T** | **A** | **T** | **C** | **A** | **A** | **A** | **T** | **T** | **G** | **A** | **T** | **T** | **A** | **C** | **T** | **T** | **C** | **T** | **G** | **C** | **T** | **A** | **A** | **A** | **A** | **A** | **A** | **C** | **G** | **T** | **G** | **T** | **T** | **T** | **G** | **G** | **A** | **T** | **A** | **C** | **A** | **G** | **A** | **C** | **A** | **C** | **C** | **T** | **T** | **A** | **C** | **C** | **T** | **T** | **G** | **T** | **T** | **C** | **C** | **T** | **G** | **A** | **T** | **G** | **A** | **A** | **A** | **G** | **T** | **G** | **T** | **C** | **A** | **T** | **T** | **G** | **C** | **T** | **G** | **C** | **T** | **T** | **T** | **A** | **A** | **A** | **A** | **A** | **T** | **C** | **G** | **C** | **A** | **A** | **C** | **T** | **G** | **C** | **C** | **T** | **C** | **T** | **G** | **G** | **T** | **G** | **T** | **A** | **G** | **A** | **T** | **G** | **T** | **G** | **A** | **A** | **A** | **A** | **T** | **C** | **A** | **T** | **G** | **A** | **T** | **T** | **C** | **C** | **C** | **A** | **A** | **C** | **A** | **A** | **A** | **C** | **C** | **G** | **G** | **A** | **T** | **C** | **A** | **T** | **C** | **C** | **T** | **T** | **T** | **T** | **A** | **T** | **T** | **T** | **A** | **T** | **C** | **G** | **A** | **G** | **C** | **A** | **A** | **C** | **A** | **C** | **A** | **A** | **T** | **A** | **T** | **T** | **A** | **T** | **G** | **C** | **T** | **C** | **G** | **G** | **C** | **A** | **G** | **C** | **T** | **G** | **A** | **T** | **C** | **A** | **A** | **G** | **G** | **A** | **A** | **A** | **A** | **T** | **G** | **T** | **A** | **C** | **A** | **A** | **A** | **T** | **C** | **C** | **T** | **T** | **G** | **T** | **C** | **T** | **A** | **T** | **G** | **A** | **G** | **A** | **A** | **C** | **G** | **G** | **C** | **T** | **T** | **C** | **C** | **T** | **C** | **C** | **A** | **T** | **G** | **C** | **A** | **A** | **A** | **A** | **A** | **C** | **A** | **T** | **T** | **G** | **A** | **T** | **A** | **A** | **T** | **G** | **G** | **A** | **T** | **G** | **A** | **T** | **G** | **A** | **A** | **A** | **T** | **C** | **T** | **G** | **C** | **A** | **T** | **G** | **G** | **T** | **A** | **G** | **G** | **T** | **T** | **C** | **A** | **G** | **C** | **A** | **A** | **A** | **T** | **C** | **A** | **A** | **G** | **A** | **T** | **A** | **T** | **T** | **C** | **G** | **T** | **A** | **G** | **C** | **T** | **A** | **C** | **C** | **G** | **A** | **T** | **T** | **G** | **A** | **A** | **T** | **T** | **T** | **T** | **G** | **A** | **A** | **A** | **C** | **A** | **A** | **G** | **T** | **G** | **C** | **T** | **G** | **T** | **C** | **A** | **T** | **T** | **T** | **A** | **C** | **G** | **A** | **T** | **C** | **C** | **T** | **G** | **A** | **G** | **T** | **T** | **T** | **T** | **T** | **A** | **G** | **A** | **A** | **G** | **A** | **A** | **C** | **T** | **T** | **G** | **C** | **T** | **A** | **C** | **T** | **C** | **A** | **G** | **T** | **T** | **C** | **A** | **A** | **A** | **G** | **A** | **A** | **G** | **A** | **T** | **G** | **A** | **G** | **A** | **C** | **A** | **C** | **A** | **T** | **T** | **G** | **T** | **T** | **C** | **A** | **T** | **C** | **C** | **A** | **T** | **G** | **A** | **C** | **A** | **A** | **C** | **T** | **G** | **A** | **A** | **A** | **C** | **A** | **G** | **T** | **C** | **A** | **A** | **G** | **G** | **A** | **A** | **A** | **T** | **G** | **T** | **C** | **T** | **A** | **A** | **C** | **T** | **G** | **G** | **C** | **T** | **A** | **T** | **T** | **A** | **T** | **T** | **C** | **A** | **A** | **G** | **C** | **A** | **A** | **C** | **A** | **A** | **A** | **T** | **T** | **T** | **C** | **T** | **C** | **G** | **A** | **T** | **T** | **A** | **T** | **T** | **T** | **T** | **C** | **T** | **C** | **C** | **A** | **A** | **T** | **C** | **C** | **T** | **A** | **-** | **-** | **-** |
| **N53702** | **-** | **-** | **-** | **-** | **-** | **-** | **-** | **-** | **-** | **-** | **-** | **-** | **-** | **-** | **-** | **-** | **-** | **-** | **-** | **-** | **-** | **-** | **-** | **-** | **-** | **-** | **-** | **-** | **-** | **-** | **-** | **-** | **-** | **-** | **-** | **-** | **-** | **-** | **-** | **-** | **-** | **-** | **-** | **-** | **-** | **-** | **-** | **-** | **-** | **-** | **-** | **-** | **-** | **-** | **-** | **-** | **-** | **-** | **-** | **-** | **-** | **-** | **-** | **-** | **-** | **-** | **-** | **-** | **-** | **A** | **A** | **A** | **C** | **C** | **C** | **C** | **G** | **A** | **G** | **A** | **T** | **G** | **T** | **A** | **G** | **C** | **A** | **G** | **C** | **C** | **A** | **T** | **T** | **T** | **G** | **G** | **G** | **C** | **A** | **T** | **G** | **G** | **C** | **T** | **C** | **T** | **T** | **A** | **G** | **T** | **A** | **T** | **T** | **G** | **T** | **T** | **T** | **G** | **C** | **C** | **C** | **T** | **T** | **C** | **C** | **T** | **G** | **G** | **G** | **G** | **T** | **G** | **G** | **G** | **T** | **T** | **T** | **T** | **G** | **T** | **A** | **T** | **T** | **A** | **T** | **A** | **T** | **T** | **T** | **A** | **T** | **T** | **T** | **T** | **T** | **C** | **G** | **G** | **A** | **C** | **G** | **T** | **G** | **G** | **A** | **T** | **T** | **A** | **A** | **C** | **G** | **G** | **A** | **T** | **A** | **A** | **G** | **A** | **A** | **A** | **A** | **A** | **A** | **T** | **T** | **C** | **T** | **A** | **T** | **C** | **T** | **C** | **C** | **G** | **A** | **C** | **A** | **A** | **A** | **G** | **T** | **G** | **A** | **C** | **T** | **T** | **G** | **A** | **A** | **A** | **G** | **A** | **A** | **T** | **T** | **A** | **G** | **A** | **A** | **A** | **A** | **C** | **T** | **T** | **T** | **C** | **A** | **G** | **T** | **C** | **T** | **T** | **T** | **T** | **A** | **G** | **A** | **G** | **G** | **A** | **G** | **A** | **T** | **A** | **C** | **C** | **A** | **T** | **T** | **G** | **A** | **A** | **C** | **A** | **T** | **T** | **A** | **C** | **G** | **A** | **T** | **C** | **C** | **T** | **G** | **A** | **C** | **A** | **T** | **G** | **G** | **G** | **C** | **G** | **A** | **T** | **A** | **A** | **A** | **G** | **A** | **C** | **A** | **A** | **A** | **C** | **A** | **G** | **C** | **A** | **A** | **T** | **T** | **T** | **G** | **T** | **C** | **G** | **A** | **C** | **T** | **T** | **C** | **T** | **T** | **C** | **T** | **C** | **T** | **T** | **C** | **A** | **T** | **T** | **A** | **A** | **A** | **T** | **C** | **G** | **T** | **A** | **T** | **G** | **C** | **C** | **G** | **C** | **T** | **G** | **A** | **C** | **A** | **A** | **G** | **A** | **A** | **T** | **G** | **A** | **A** | **T** | **T** | **C** | **T** | **G** | **T** | **C** | **A** | **C** | **T** | **C** | **T** | **T** | **C** | **T** | **C** | **A** | **C** | **A** | **G** | **A** | **C** | **G** | **G** | **A** | **C** | **A** | **A** | **G** | **A** | **G** | **A** | **A** | **A** | **T** | **T** | **G** | **G** | **A** | **T** | **T** | **C** | **A** | **C** | **T** | **G** | **C** | **T** | **T** | **C** | **A** | **A** | **G** | **A** | **T** | **C** | **T** | **A** | **A** | **A** | **A** | **A** | **A** | **A** | **G** | **C** | **C** | **A** | **A** | **A** | **C** | **A** | **T** | **T** | **C** | **G** | **A** | **T** | **C** | **C** | **A** | **T** | **A** | **T** | **C** | **G** | **A** | **A** | **T** | **A** | **T** | **T** | **A** | **C** | **G** | **C** | **A** | **T** | **T** | **T** | **G** | **T** | **G** | **A** | **C** | **A** | **G** | **A** | **T** | **A** | **A** | **T** | **A** | **T** | **C** | **G** | **G** | **C** | **C** | **A** | **G** | **C** | **A** | **A** | **G** | **T** | **C** | **T** | **T** | **A** | **C** | **A** | **T** | **G** | **T** | **T** | **T** | **T** | **A** | **G** | **A** | **A** | **G** | **A** | **A** | **A** | **A** | **A** | **G** | **C** | **C** | **G** | **C** | **A** | **G** | **A** | **A** | **G** | **G** | **C** | **G** | **T** | **G** | **G** | **A** | **A** | **G** | **T** | **T** | **C** | **G** | **A** | **A** | **T** | **A** | **T** | **T** | **A** | **T** | **A** | **T** | **G** | **A** | **T** | **G** | **C** | **A** | **T** | **T** | **T** | **G** | **G** | **C** | **T** | **C** | **T** | **C** | **A** | **T** | **G** | **G** | **C** | **A** | **C** | **A** | **A** | **A** | **A** | **G** | **C** | **A** | **A** | **A** | **A** | **G** | **A** | **T** | **T** | **T** | **C** | **A** | **A** | **T** | **C** | **G** | **T** | **C** | **T** | **A** | **A** | **T** | **C** | **A** | **A** | **A** | **A** | **A** | **T** | **G** | **G** | **T** | **G** | **G** | **A** | **C** | **A** | **T** | **G** | **T** | **C** | **C** | **A** | **T** | **A** | **C** | **A** | **T** | **T** | **T** | **G** | **T** | **T** | **A** | **C** | **C** | **T** | **C** | **A** | **C** | **A** | **A** | **A** | **A** | **G** | **G** | **C** | **A** | **T** | **T** | **A** | **C** | **T** | **T** | **C** | **G** | **T** | **T** | **T** | **C** | **C** | **G** | **A** | **T** | **T** | **G** | **A** | **A** | **T** | **T** | **A** | **C** | **C** | **A** | **T** | **G** | **A** | **T** | **C** | **A** | **C** | **C** | **G** | **A** | **A** | **A** | **A** | **A** | **T** | **C** | **G** | **T** | **T** | **G** | **T** | **G** | **A** | **T** | **C** | **G** | **A** | **T** | **G** | **G** | **A** | **A** | **A** | **G** | **A** | **T** | **T** | **A** | **G** | **T** | **T** | **A** | **T** | **A** | **C** | **C** | **G** | **G** | **T** | **G** | **G** | **T** | **T** | **T** | **C** | **A** | **A** | **T** | **A** | **T** | **T** | **G** | **C** | **C** | **A** | **A** | **T** | **C** | **A** | **A** | **T** | **A** | **T** | **G** | **T** | **A** | **A** | **A** | **T** | **A** | **C** | **A** | **A** | **C** | **A** | **A** | **A** | **A** | **A** | **A** | **A** | **T** | **T** | **C** | **G** | **G** | **C** | **T** | **A** | **T** | **T** | **G** | **G** | **C** | **G** | **C** | **G** | **A** | **T** | **A** | **C** | **G** | **C** | **A** | **T** | **A** | **T** | **A** | **C** | **G** | **G** | **A** | **T** | **T** | **T** | **T** | **C** | **G** | **G** | **T** | **G** | **C** | **C** | **G** | **C** | **T** | **T** | **C** | **T** | **T** | **C** | **T** | **T** | **T** | **G** | **C** | **T** | **C** | **C** | **A** | **G** | **C** | **T** | **T** | **C** | **G** | **C** | **T** | **T** | **C** | **T** | **T** | **A** | **A** | **C** | **A** | **G** | **A** | **C** | **T** | **G** | **G** | **A** | **A** | **C** | **G** | **T** | **C** | **T** | **C** | **G** | **G** | **T** | **A** | **C** | **C** | **T** | **G** | **A** | **A** | **G** | **A** | **A** | **A** | **A** | **A** | **A** | **A** | **G** | **G** | **T** | **C** | **G** | **G** | **C** | **T** | **A** | **T** | **C** | **A** | **T** | **T** | **T** | **G** | **A** | **A** | **T** | **T** | **A** | **T** | **T** | **T** | **C** | **T** | **T** | **T** | **A** | **A** | **A** | **A** | **A** | **A** | **G** | **C** | **A** | **G** | **A** | **T** | **C** | **G** | **A** | **G** | **A** | **T** | **G** | **A** | **A** | **T** | **C** | **T** | **A** | **A** | **G** | **C** | **T** | **T** | **G** | **C** | **T** | **G** | **A** | **T** | **A** | **C** | **A** | **T** | **C** | **C** | **A** | **T** | **C** | **C** | **A** | **G** | **C** | **T** | **T** | **G** | **T** | **T** | **T** | **C** | **A** | **A** | **G** | **C** | **G** | **G** | **A** | **C** | **C** | **G** | **A** | **A** | **T** | **A** | **A** | **C** | **G** | **A** | **A** | **A** | **G** | **G** | **G** | **A** | **A** | **C** | **A** | **A** | **A** | **T** | **C** | **A** | **A** | **G** | **C** | **T** | **T** | **T** | **C** | **A** | **T** | **T** | **T** | **A** | **T** | **C** | **A** | **A** | **A** | **T** | **T** | **G** | **A** | **T** | **T** | **A** | **C** | **T** | **T** | **C** | **T** | **G** | **C** | **T** | **A** | **A** | **A** | **A** | **A** | **A** | **C** | **G** | **T** | **G** | **T** | **T** | **T** | **G** | **G** | **A** | **T** | **A** | **C** | **A** | **G** | **A** | **C** | **A** | **C** | **C** | **T** | **T** | **A** | **C** | **C** | **T** | **T** | **G** | **T** | **T** | **C** | **C** | **T** | **G** | **A** | **T** | **G** | **A** | **A** | **A** | **G** | **T** | **G** | **T** | **C** | **A** | **T** | **T** | **G** | **C** | **T** | **G** | **C** | **T** | **T** | **T** | **A** | **A** | **A** | **A** | **A** | **T** | **C** | **G** | **C** | **A** | **A** | **C** | **T** | **G** | **C** | **C** | **T** | **C** | **T** | **G** | **G** | **T** | **G** | **T** | **A** | **G** | **A** | **T** | **G** | **T** | **G** | **A** | **A** | **A** | **A** | **T** | **C** | **A** | **T** | **G** | **A** | **T** | **T** | **C** | **C** | **C** | **A** | **A** | **C** | **A** | **A** | **A** | **C** | **C** | **G** | **G** | **A** | **T** | **C** | **A** | **T** | **C** | **C** | **T** | **T** | **T** | **T** | **A** | **T** | **T** | **T** | **A** | **T** | **C** | **G** | **A** | **G** | **C** | **A** | **A** | **C** | **A** | **C** | **A** | **A** | **T** | **A** | **T** | **T** | **A** | **T** | **G** | **C** | **T** | **C** | **G** | **G** | **C** | **A** | **G** | **C** | **T** | **G** | **A** | **T** | **C** | **A** | **A** | **G** | **G** | **A** | **A** | **A** | **A** | **T** | **G** | **T** | **A** | **C** | **A** | **A** | **A** | **T** | **C** | **C** | **T** | **T** | **G** | **T** | **C** | **T** | **A** | **T** | **G** | **A** | **G** | **A** | **A** | **C** | **G** | **G** | **C** | **T** | **T** | **C** | **C** | **T** | **C** | **C** | **A** | **T** | **G** | **C** | **A** | **A** | **A** | **A** | **A** | **C** | **A** | **T** | **T** | **G** | **A** | **T** | **A** | **A** | **T** | **G** | **G** | **A** | **T** | **G** | **A** | **T** | **G** | **A** | **A** | **A** | **T** | **C** | **T** | **G** | **C** | **A** | **T** | **G** | **G** | **T** | **A** | **G** | **G** | **T** | **T** | **C** | **A** | **G** | **C** | **A** | **A** | **A** | **T** | **C** | **A** | **A** | **G** | **A** | **T** | **A** | **T** | **T** | **C** | **G** | **T** | **A** | **G** | **C** | **T** | **A** | **C** | **C** | **G** | **A** | **T** | **T** | **G** | **A** | **A** | **T** | **T** | **T** | **T** | **G** | **A** | **A** | **A** | **C** | **A** | **A** | **G** | **T** | **G** | **C** | **T** | **G** | **T** | **C** | **A** | **T** | **T** | **T** | **A** | **C** | **G** | **A** | **T** | **C** | **C** | **T** | **G** | **A** | **G** | **T** | **T** | **T** | **T** | **T** | **A** | **G** | **A** | **A** | **G** | **A** | **A** | **C** | **T** | **T** | **G** | **C** | **T** | **A** | **C** | **T** | **C** | **A** | **G** | **T** | **T** | **C** | **A** | **A** | **A** | **G** | **A** | **A** | **G** | **A** | **T** | **G** | **A** | **G** | **A** | **C** | **A** | **C** | **A** | **T** | **T** | **G** | **T** | **T** | **C** | **A** | **T** | **C** | **C** | **A** | **T** | **G** | **A** | **C** | **A** | **A** | **C** | **T** | **G** | **A** | **A** | **A** | **C** | **A** | **G** | **T** | **C** | **A** | **A** | **G** | **G** | **A** | **A** | **A** | **T** | **G** | **T** | **C** | **T** | **A** | **A** | **C** | **T** | **G** | **G** | **C** | **T** | **A** | **T** | **T** | **A** | **T** | **T** | **C** | **A** | **A** | **G** | **C** | **A** | **A** | **C** | **A** | **A** | **A** | **T** | **T** | **T** | **C** | **T** | **C** | **G** | **A** | **T** | **T** | **A** | **T** | **T** | **T** | **T** | **C** | **T** | **C** | **C** | **A** | **A** | **T** | **C** | **C** | **T** | **A** | **-** | **-** | **-** |
| **N55655** | **-** | **-** | **-** | **-** | **-** | **-** | **-** | **-** | **-** | **-** | **-** | **-** | **-** | **-** | **-** | **-** | **-** | **-** | **-** | **-** | **-** | **-** | **-** | **-** | **-** | **-** | **-** | **-** | **-** | **-** | **-** | **-** | **-** | **-** | **-** | **-** | **-** | **-** | **-** | **-** | **-** | **-** | **-** | **-** | **-** | **-** | **-** | **-** | **-** | **-** | **-** | **-** | **-** | **-** | **-** | **-** | **-** | **-** | **-** | **-** | **-** | **-** | **-** | **-** | **-** | **-** | **-** | **-** | **-** | **A** | **A** | **A** | **C** | **C** | **C** | **C** | **G** | **A** | **G** | **A** | **T** | **G** | **T** | **A** | **G** | **C** | **A** | **G** | **C** | **C** | **A** | **T** | **T** | **T** | **G** | **G** | **G** | **C** | **A** | **T** | **G** | **G** | **C** | **T** | **C** | **T** | **T** | **A** | **G** | **T** | **A** | **T** | **T** | **G** | **T** | **T** | **T** | **G** | **T** | **C** | **C** | **T** | **T** | **C** | **C** | **T** | **G** | **G** | **G** | **G** | **T** | **G** | **G** | **G** | **C** | **T** | **T** | **T** | **G** | **T** | **A** | **T** | **T** | **A** | **T** | **A** | **T** | **T** | **T** | **A** | **T** | **T** | **T** | **T** | **T** | **C** | **G** | **G** | **A** | **C** | **G** | **T** | **G** | **G** | **A** | **T** | **T** | **A** | **A** | **C** | **G** | **G** | **A** | **T** | **A** | **A** | **G** | **A** | **A** | **A** | **A** | **A** | **A** | **T** | **T** | **C** | **T** | **A** | **T** | **C** | **T** | **C** | **C** | **G** | **A** | **C** | **A** | **A** | **A** | **G** | **T** | **G** | **A** | **C** | **T** | **T** | **G** | **A** | **A** | **A** | **G** | **A** | **A** | **T** | **T** | **A** | **G** | **A** | **A** | **A** | **A** | **C** | **T** | **T** | **T** | **C** | **A** | **G** | **T** | **C** | **T** | **T** | **T** | **T** | **A** | **G** | **A** | **G** | **G** | **A** | **G** | **A** | **T** | **A** | **C** | **C** | **A** | **T** | **T** | **G** | **A** | **A** | **C** | **A** | **T** | **T** | **A** | **C** | **G** | **A** | **T** | **C** | **C** | **T** | **G** | **A** | **C** | **A** | **T** | **G** | **G** | **G** | **C** | **G** | **A** | **T** | **A** | **A** | **A** | **G** | **A** | **C** | **A** | **A** | **A** | **C** | **A** | **G** | **C** | **A** | **A** | **T** | **T** | **T** | **G** | **T** | **T** | **G** | **A** | **C** | **T** | **T** | **C** | **T** | **T** | **C** | **T** | **C** | **T** | **T** | **C** | **A** | **T** | **T** | **A** | **A** | **A** | **T** | **C** | **G** | **T** | **A** | **T** | **G** | **C** | **C** | **G** | **C** | **T** | **G** | **A** | **C** | **A** | **A** | **G** | **A** | **A** | **T** | **G** | **A** | **A** | **T** | **T** | **C** | **T** | **G** | **T** | **C** | **A** | **C** | **T** | **C** | **T** | **T** | **C** | **T** | **C** | **A** | **C** | **A** | **G** | **A** | **C** | **G** | **G** | **A** | **C** | **A** | **A** | **G** | **A** | **G** | **A** | **A** | **A** | **T** | **T** | **G** | **G** | **A** | **T** | **T** | **C** | **A** | **C** | **T** | **G** | **C** | **T** | **T** | **C** | **A** | **A** | **G** | **A** | **T** | **C** | **T** | **A** | **A** | **A** | **A** | **A** | **A** | **A** | **G** | **C** | **C** | **A** | **A** | **A** | **C** | **A** | **T** | **T** | **C** | **G** | **A** | **T** | **C** | **C** | **A** | **T** | **A** | **T** | **C** | **G** | **A** | **A** | **T** | **A** | **T** | **T** | **A** | **C** | **G** | **C** | **A** | **T** | **T** | **T** | **G** | **T** | **G** | **A** | **C** | **A** | **G** | **A** | **T** | **A** | **A** | **T** | **A** | **T** | **C** | **G** | **G** | **C** | **C** | **A** | **G** | **C** | **A** | **A** | **G** | **T** | **C** | **T** | **T** | **A** | **C** | **A** | **T** | **G** | **T** | **T** | **T** | **T** | **A** | **G** | **A** | **A** | **G** | **A** | **A** | **A** | **A** | **A** | **G** | **C** | **C** | **G** | **C** | **A** | **G** | **A** | **A** | **G** | **G** | **C** | **G** | **T** | **G** | **G** | **A** | **A** | **G** | **T** | **T** | **C** | **G** | **A** | **A** | **T** | **A** | **T** | **T** | **A** | **T** | **A** | **T** | **G** | **A** | **T** | **G** | **C** | **A** | **T** | **T** | **T** | **G** | **G** | **C** | **T** | **C** | **T** | **C** | **A** | **T** | **G** | **G** | **C** | **A** | **C** | **A** | **A** | **A** | **A** | **G** | **C** | **A** | **A** | **A** | **A** | **G** | **A** | **T** | **T** | **T** | **C** | **A** | **A** | **T** | **C** | **G** | **T** | **C** | **T** | **A** | **A** | **T** | **C** | **A** | **A** | **A** | **A** | **A** | **T** | **G** | **G** | **T** | **G** | **G** | **A** | **C** | **A** | **T** | **G** | **T** | **C** | **C** | **A** | **T** | **A** | **C** | **A** | **T** | **T** | **T** | **G** | **T** | **T** | **A** | **C** | **C** | **T** | **C** | **A** | **C** | **A** | **A** | **A** | **A** | **G** | **G** | **C** | **A** | **T** | **T** | **A** | **C** | **T** | **T** | **C** | **G** | **T** | **T** | **T** | **C** | **C** | **G** | **A** | **T** | **T** | **G** | **A** | **A** | **T** | **T** | **A** | **C** | **C** | **A** | **T** | **G** | **A** | **T** | **C** | **A** | **C** | **C** | **G** | **A** | **A** | **A** | **A** | **A** | **T** | **C** | **G** | **T** | **T** | **G** | **T** | **G** | **A** | **T** | **C** | **G** | **A** | **T** | **G** | **G** | **A** | **A** | **A** | **G** | **A** | **T** | **T** | **A** | **G** | **T** | **T** | **A** | **T** | **A** | **C** | **C** | **G** | **G** | **T** | **G** | **G** | **T** | **T** | **T** | **C** | **A** | **A** | **T** | **A** | **T** | **T** | **G** | **C** | **C** | **A** | **A** | **T** | **C** | **A** | **A** | **T** | **A** | **T** | **G** | **T** | **A** | **A** | **A** | **T** | **A** | **C** | **A** | **A** | **C** | **A** | **A** | **A** | **A** | **A** | **A** | **A** | **T** | **T** | **C** | **G** | **G** | **C** | **T** | **A** | **T** | **T** | **G** | **G** | **C** | **G** | **C** | **G** | **A** | **T** | **A** | **C** | **G** | **C** | **A** | **T** | **A** | **T** | **A** | **C** | **G** | **G** | **A** | **T** | **T** | **T** | **T** | **C** | **G** | **G** | **T** | **G** | **C** | **C** | **G** | **C** | **T** | **T** | **C** | **T** | **T** | **C** | **T** | **T** | **T** | **G** | **C** | **T** | **C** | **C** | **A** | **G** | **C** | **T** | **T** | **C** | **G** | **C** | **T** | **T** | **C** | **T** | **T** | **A** | **A** | **C** | **A** | **G** | **A** | **C** | **T** | **G** | **G** | **A** | **A** | **C** | **G** | **T** | **C** | **T** | **C** | **G** | **G** | **T** | **A** | **C** | **C** | **T** | **G** | **A** | **A** | **G** | **A** | **A** | **A** | **A** | **A** | **A** | **A** | **G** | **G** | **T** | **C** | **G** | **G** | **C** | **T** | **A** | **T** | **C** | **A** | **T** | **T** | **T** | **G** | **A** | **A** | **T** | **T** | **A** | **T** | **T** | **T** | **C** | **T** | **T** | **T** | **A** | **A** | **A** | **A** | **A** | **A** | **G** | **C** | **A** | **G** | **A** | **T** | **C** | **G** | **A** | **G** | **A** | **T** | **G** | **A** | **A** | **T** | **C** | **T** | **A** | **A** | **G** | **C** | **T** | **T** | **G** | **C** | **T** | **G** | **A** | **T** | **A** | **C** | **A** | **T** | **C** | **C** | **A** | **T** | **C** | **C** | **A** | **G** | **C** | **T** | **T** | **G** | **T** | **T** | **T** | **C** | **A** | **A** | **G** | **C** | **G** | **G** | **A** | **C** | **C** | **G** | **A** | **A** | **T** | **A** | **A** | **C** | **G** | **A** | **A** | **A** | **G** | **G** | **G** | **A** | **A** | **C** | **A** | **A** | **A** | **T** | **C** | **A** | **A** | **G** | **C** | **T** | **T** | **T** | **C** | **A** | **T** | **T** | **T** | **A** | **T** | **C** | **A** | **A** | **A** | **T** | **T** | **G** | **A** | **T** | **T** | **A** | **C** | **T** | **T** | **C** | **T** | **G** | **C** | **T** | **A** | **A** | **A** | **A** | **A** | **A** | **C** | **G** | **T** | **G** | **T** | **T** | **T** | **G** | **G** | **A** | **T** | **A** | **C** | **A** | **G** | **A** | **C** | **A** | **C** | **C** | **T** | **T** | **A** | **C** | **C** | **T** | **T** | **G** | **T** | **T** | **C** | **C** | **T** | **G** | **A** | **T** | **G** | **A** | **A** | **A** | **G** | **T** | **G** | **T** | **C** | **A** | **T** | **T** | **G** | **C** | **T** | **G** | **C** | **T** | **T** | **T** | **A** | **A** | **A** | **A** | **A** | **T** | **C** | **G** | **C** | **A** | **A** | **C** | **T** | **G** | **C** | **C** | **T** | **C** | **T** | **G** | **G** | **T** | **G** | **T** | **A** | **G** | **A** | **T** | **G** | **T** | **G** | **A** | **A** | **A** | **A** | **T** | **C** | **A** | **T** | **G** | **A** | **T** | **T** | **C** | **C** | **C** | **A** | **A** | **C** | **A** | **A** | **A** | **C** | **C** | **G** | **G** | **A** | **T** | **C** | **A** | **T** | **C** | **C** | **T** | **T** | **T** | **T** | **A** | **T** | **T** | **T** | **A** | **T** | **C** | **G** | **A** | **G** | **C** | **A** | **A** | **C** | **A** | **C** | **A** | **A** | **T** | **A** | **T** | **T** | **A** | **T** | **G** | **C** | **T** | **C** | **G** | **G** | **C** | **A** | **G** | **C** | **T** | **G** | **A** | **T** | **C** | **A** | **A** | **G** | **G** | **A** | **A** | **A** | **A** | **T** | **G** | **T** | **A** | **C** | **A** | **A** | **A** | **T** | **C** | **C** | **T** | **T** | **G** | **T** | **C** | **T** | **A** | **T** | **G** | **A** | **G** | **A** | **A** | **C** | **G** | **G** | **C** | **T** | **T** | **C** | **C** | **T** | **C** | **C** | **A** | **T** | **G** | **C** | **A** | **A** | **A** | **A** | **A** | **C** | **A** | **T** | **T** | **G** | **A** | **T** | **A** | **A** | **T** | **G** | **G** | **A** | **T** | **G** | **A** | **T** | **G** | **A** | **A** | **A** | **T** | **C** | **T** | **G** | **C** | **A** | **T** | **G** | **G** | **T** | **A** | **G** | **G** | **T** | **T** | **C** | **A** | **G** | **C** | **A** | **A** | **A** | **T** | **C** | **A** | **A** | **G** | **A** | **T** | **A** | **T** | **T** | **C** | **G** | **T** | **A** | **G** | **C** | **T** | **A** | **C** | **C** | **G** | **A** | **T** | **T** | **G** | **A** | **A** | **T** | **T** | **T** | **T** | **G** | **A** | **A** | **A** | **C** | **A** | **A** | **G** | **T** | **G** | **C** | **T** | **G** | **T** | **C** | **A** | **T** | **T** | **T** | **A** | **C** | **G** | **A** | **T** | **C** | **C** | **T** | **G** | **A** | **G** | **T** | **T** | **T** | **T** | **T** | **A** | **G** | **A** | **A** | **G** | **A** | **A** | **C** | **T** | **T** | **G** | **C** | **T** | **A** | **C** | **T** | **C** | **A** | **G** | **T** | **T** | **C** | **A** | **A** | **A** | **G** | **A** | **A** | **G** | **A** | **T** | **G** | **A** | **G** | **A** | **C** | **A** | **C** | **A** | **T** | **T** | **G** | **T** | **T** | **C** | **A** | **T** | **C** | **C** | **A** | **T** | **G** | **A** | **C** | **A** | **A** | **C** | **T** | **G** | **A** | **A** | **A** | **C** | **A** | **G** | **T** | **C** | **A** | **A** | **G** | **G** | **A** | **A** | **A** | **T** | **G** | **T** | **C** | **T** | **A** | **A** | **C** | **T** | **G** | **G** | **C** | **T** | **A** | **T** | **T** | **A** | **T** | **T** | **C** | **A** | **A** | **G** | **C** | **A** | **A** | **C** | **A** | **A** | **A** | **T** | **T** | **T** | **C** | **T** | **C** | **G** | **A** | **T** | **T** | **A** | **T** | **T** | **T** | **T** | **C** | **T** | **C** | **C** | **A** | **A** | **T** | **C** | **C** | **T** | **A** | **-** | **-** | **-** |
| **N59559F** | **-** | **-** | **-** | **-** | **-** | **-** | **-** | **-** | **-** | **-** | **-** | **-** | **-** | **-** | **-** | **-** | **-** | **-** | **-** | **-** | **-** | **-** | **-** | **-** | **-** | **-** | **-** | **-** | **-** | **-** | **-** | **-** | **-** | **-** | **-** | **-** | **-** | **-** | **-** | **-** | **-** | **-** | **-** | **-** | **-** | **-** | **-** | **-** | **-** | **-** | **-** | **-** | **-** | **-** | **-** | **-** | **-** | **-** | **-** | **-** | **-** | **-** | **-** | **-** | **-** | **-** | **-** | **-** | **-** | **A** | **A** | **A** | **C** | **C** | **C** | **C** | **G** | **A** | **G** | **A** | **T** | **G** | **T** | **A** | **G** | **C** | **A** | **G** | **C** | **T** | **A** | **T** | **T** | **T** | **G** | **G** | **G** | **C** | **A** | **T** | **G** | **G** | **C** | **T** | **C** | **T** | **T** | **A** | **G** | **T** | **A** | **T** | **T** | **A** | **T** | **T** | **T** | **G** | **C** | **C** | **C** | **T** | **T** | **C** | **C** | **T** | **G** | **G** | **G** | **G** | **T** | **G** | **G** | **G** | **T** | **T** | **T** | **T** | **G** | **T** | **A** | **T** | **T** | **A** | **T** | **A** | **T** | **T** | **T** | **A** | **T** | **T** | **T** | **T** | **T** | **C** | **G** | **G** | **A** | **C** | **G** | **T** | **G** | **G** | **A** | **T** | **T** | **A** | **A** | **C** | **G** | **G** | **A** | **T** | **A** | **A** | **G** | **A** | **A** | **A** | **A** | **A** | **A** | **T** | **T** | **C** | **T** | **A** | **T** | **C** | **T** | **C** | **C** | **G** | **A** | **C** | **A** | **A** | **A** | **G** | **T** | **G** | **A** | **C** | **T** | **T** | **G** | **A** | **A** | **A** | **G** | **A** | **A** | **T** | **T** | **A** | **G** | **A** | **A** | **A** | **A** | **C** | **T** | **T** | **T** | **C** | **A** | **G** | **T** | **C** | **T** | **T** | **T** | **T** | **A** | **G** | **A** | **G** | **G** | **A** | **G** | **A** | **T** | **A** | **C** | **C** | **A** | **T** | **T** | **G** | **A** | **A** | **C** | **A** | **T** | **T** | **A** | **C** | **G** | **A** | **T** | **C** | **C** | **T** | **G** | **A** | **C** | **A** | **T** | **G** | **G** | **G** | **C** | **G** | **A** | **C** | **A** | **A** | **A** | **G** | **A** | **C** | **A** | **A** | **A** | **C** | **A** | **A** | **C** | **A** | **A** | **T** | **T** | **T** | **G** | **T** | **C** | **G** | **A** | **C** | **T** | **T** | **C** | **T** | **T** | **C** | **T** | **C** | **T** | **T** | **C** | **A** | **T** | **T** | **A** | **A** | **A** | **T** | **C** | **G** | **T** | **A** | **T** | **G** | **C** | **C** | **G** | **C** | **T** | **G** | **A** | **C** | **A** | **A** | **G** | **A** | **A** | **T** | **G** | **A** | **A** | **T** | **T** | **C** | **T** | **G** | **T** | **C** | **A** | **C** | **T** | **C** | **T** | **T** | **C** | **T** | **C** | **A** | **C** | **A** | **G** | **A** | **C** | **G** | **G** | **A** | **C** | **A** | **A** | **A** | **A** | **G** | **A** | **A** | **A** | **T** | **T** | **A** | **G** | **A** | **T** | **T** | **C** | **A** | **C** | **T** | **G** | **C** | **T** | **T** | **C** | **A** | **A** | **G** | **A** | **T** | **C** | **T** | **A** | **A** | **A** | **A** | **A** | **A** | **A** | **G** | **C** | **C** | **A** | **A** | **A** | **C** | **A** | **T** | **T** | **C** | **G** | **A** | **T** | **C** | **C** | **A** | **T** | **A** | **T** | **C** | **G** | **A** | **A** | **T** | **A** | **T** | **T** | **A** | **C** | **G** | **C** | **A** | **T** | **T** | **T** | **G** | **T** | **G** | **A** | **C** | **A** | **G** | **A** | **T** | **A** | **C** | **T** | **A** | **T** | **C** | **G** | **G** | **T** | **C** | **A** | **G** | **C** | **A** | **A** | **G** | **T** | **C** | **T** | **T** | **A** | **C** | **G** | **C** | **G** | **T** | **T** | **T** | **T** | **A** | **G** | **A** | **A** | **G** | **A** | **A** | **A** | **A** | **A** | **G** | **C** | **C** | **G** | **C** | **A** | **G** | **A** | **A** | **G** | **G** | **C** | **G** | **T** | **A** | **G** | **A** | **A** | **G** | **T** | **T** | **C** | **G** | **T** | **A** | **T** | **A** | **T** | **T** | **A** | **T** | **A** | **T** | **G** | **A** | **T** | **G** | **C** | **A** | **T** | **T** | **T** | **G** | **G** | **C** | **T** | **C** | **C** | **C** | **A** | **C** | **G** | **G** | **C** | **A** | **C** | **A** | **A** | **A** | **A** | **G** | **C** | **A** | **A** | **A** | **A** | **G** | **A** | **T** | **T** | **T** | **C** | **A** | **A** | **T** | **C** | **G** | **T** | **C** | **T** | **A** | **A** | **T** | **C** | **A** | **A** | **A** | **A** | **A** | **T** | **G** | **G** | **T** | **G** | **G** | **A** | **C** | **A** | **T** | **G** | **T** | **C** | **C** | **A** | **T** | **A** | **C** | **A** | **T** | **T** | **T** | **G** | **T** | **T** | **A** | **C** | **C** | **T** | **C** | **G** | **C** | **A** | **A** | **A** | **A** | **A** | **G** | **C** | **A** | **T** | **T** | **G** | **C** | **T** | **T** | **C** | **G** | **T** | **T** | **T** | **C** | **C** | **G** | **A** | **T** | **T** | **G** | **A** | **A** | **C** | **T** | **A** | **C** | **C** | **A** | **T** | **G** | **A** | **T** | **C** | **A** | **C** | **C** | **G** | **G** | **A** | **A** | **A** | **A** | **T** | **C** | **G** | **T** | **T** | **G** | **T** | **G** | **A** | **T** | **C** | **G** | **A** | **T** | **G** | **G** | **A** | **A** | **A** | **G** | **A** | **T** | **C** | **A** | **G** | **T** | **T** | **A** | **C** | **A** | **C** | **C** | **G** | **G** | **T** | **G** | **G** | **C** | **T** | **T** | **C** | **A** | **A** | **C** | **A** | **T** | **T** | **G** | **C** | **C** | **A** | **A** | **T** | **C** | **A** | **A** | **T** | **A** | **T** | **G** | **T** | **A** | **A** | **A** | **T** | **A** | **C** | **G** | **A** | **C** | **A** | **A** | **A** | **A** | **A** | **A** | **A** | **T** | **T** | **C** | **G** | **G** | **C** | **T** | **A** | **T** | **T** | **G** | **G** | **C** | **G** | **T** | **G** | **A** | **T** | **A** | **C** | **G** | **C** | **A** | **T** | **A** | **T** | **A** | **C** | **G** | **G** | **A** | **T** | **T** | **T** | **T** | **C** | **G** | **G** | **T** | **G** | **C** | **C** | **G** | **C** | **T** | **T** | **C** | **T** | **T** | **C** | **T** | **T** | **T** | **G** | **C** | **T** | **T** | **C** | **A** | **A** | **C** | **T** | **T** | **C** | **G** | **T** | **T** | **T** | **T** | **T** | **T** | **A** | **A** | **C** | **A** | **G** | **A** | **C** | **T** | **G** | **G** | **A** | **A** | **C** | **G** | **T** | **C** | **T** | **C** | **A** | **G** | **T** | **T** | **C** | **C** | **T** | **G** | **A** | **A** | **G** | **A** | **A** | **A** | **A** | **A** | **A** | **A** | **G** | **G** | **T** | **C** | **G** | **G** | **C** | **T** | **A** | **T** | **C** | **A** | **T** | **T** | **T** | **G** | **A** | **A** | **T** | **T** | **A** | **T** | **T** | **T** | **C** | **T** | **T** | **T** | **A** | **A** | **A** | **A** | **A** | **A** | **G** | **C** | **C** | **G** | **A** | **T** | **C** | **G** | **A** | **G** | **A** | **C** | **G** | **A** | **A** | **T** | **C** | **C** | **A** | **A** | **A** | **C** | **T** | **T** | **G** | **C** | **T** | **G** | **A** | **T** | **A** | **C** | **A** | **T** | **C** | **C** | **A** | **T** | **C** | **C** | **A** | **G** | **C** | **T** | **T** | **G** | **T** | **C** | **T** | **C** | **A** | **A** | **G** | **C** | **G** | **G** | **A** | **C** | **C** | **A** | **A** | **A** | **T** | **A** | **A** | **C** | **G** | **A** | **A** | **C** | **G** | **C** | **G** | **A** | **A** | **C** | **A** | **A** | **A** | **T** | **C** | **A** | **A** | **G** | **C** | **T** | **T** | **T** | **C** | **G** | **T** | **T** | **T** | **A** | **T** | **C** | **A** | **A** | **A** | **T** | **T** | **G** | **A** | **T** | **T** | **A** | **C** | **T** | **T** | **C** | **T** | **G** | **C** | **T** | **A** | **A** | **A** | **A** | **A** | **A** | **C** | **G** | **T** | **G** | **T** | **T** | **T** | **G** | **G** | **A** | **T** | **A** | **C** | **A** | **G** | **A** | **C** | **G** | **C** | **C** | **T** | **T** | **A** | **C** | **C** | **T** | **C** | **G** | **T** | **T** | **C** | **C** | **T** | **G** | **A** | **T** | **G** | **A** | **A** | **A** | **G** | **T** | **G** | **T** | **C** | **A** | **T** | **T** | **G** | **C** | **T** | **G** | **C** | **T** | **T** | **T** | **G** | **A** | **A** | **A** | **A** | **T** | **C** | **G** | **C** | **A** | **A** | **C** | **T** | **G** | **C** | **T** | **T** | **C** | **T** | **G** | **G** | **T** | **G** | **T** | **C** | **G** | **A** | **T** | **G** | **T** | **G** | **A** | **A** | **A** | **A** | **T** | **C** | **A** | **T** | **G** | **A** | **T** | **C** | **C** | **C** | **C** | **A** | **A** | **T** | **A** | **A** | **A** | **C** | **C** | **G** | **G** | **A** | **T** | **C** | **A** | **T** | **C** | **C** | **T** | **T** | **T** | **T** | **A** | **T** | **T** | **T** | **A** | **T** | **C** | **G** | **A** | **G** | **C** | **A** | **A** | **C** | **A** | **C** | **A** | **A** | **T** | **A** | **T** | **T** | **A** | **T** | **G** | **C** | **G** | **C** | **G** | **T** | **C** | **A** | **G** | **C** | **T** | **C** | **A** | **T** | **C** | **A** | **A** | **G** | **G** | **A** | **A** | **A** | **A** | **C** | **G** | **T** | **A** | **C** | **A** | **A** | **A** | **T** | **T** | **C** | **T** | **T** | **G** | **T** | **C** | **T** | **A** | **T** | **G** | **A** | **G** | **A** | **A** | **T** | **G** | **G** | **C** | **T** | **T** | **C** | **C** | **T** | **C** | **C** | **A** | **T** | **G** | **C** | **A** | **A** | **A** | **A** | **A** | **C** | **A** | **T** | **T** | **G** | **A** | **T** | **A** | **A** | **T** | **G** | **G** | **A** | **T** | **G** | **A** | **T** | **G** | **A** | **A** | **A** | **T** | **C** | **T** | **G** | **C** | **A** | **T** | **G** | **G** | **T** | **A** | **G** | **G** | **T** | **T** | **C** | **A** | **G** | **C** | **A** | **A** | **A** | **T** | **C** | **A** | **A** | **G** | **A** | **T** | **A** | **T** | **T** | **C** | **G** | **T** | **A** | **G** | **C** | **T** | **A** | **C** | **C** | **G** | **A** | **T** | **T** | **G** | **A** | **A** | **T** | **T** | **T** | **T** | **G** | **A** | **A** | **A** | **C** | **A** | **A** | **G** | **T** | **G** | **C** | **T** | **G** | **T** | **C** | **A** | **T** | **C** | **T** | **A** | **C** | **G** | **A** | **T** | **C** | **C** | **T** | **G** | **A** | **G** | **T** | **T** | **T** | **T** | **T** | **A** | **G** | **A** | **A** | **G** | **A** | **A** | **C** | **T** | **T** | **G** | **C** | **T** | **A** | **C** | **T** | **C** | **A** | **G** | **T** | **T** | **T** | **A** | **A** | **A** | **G** | **A** | **A** | **G** | **A** | **T** | **G** | **A** | **G** | **A** | **C** | **A** | **C** | **A** | **T** | **T** | **G** | **T** | **T** | **C** | **A** | **C** | **C** | **A** | **A** | **T** | **G** | **A** | **C** | **G** | **A** | **C** | **T** | **G** | **A** | **A** | **A** | **C** | **A** | **G** | **T** | **G** | **A** | **A** | **G** | **G** | **A** | **A** | **A** | **T** | **G** | **T** | **C** | **T** | **A** | **A** | **T** | **T** | **G** | **G** | **C** | **T** | **G** | **T** | **T** | **A** | **T** | **T** | **C** | **A** | **A** | **G** | **C** | **A** | **A** | **C** | **A** | **A** | **A** | **T** | **T** | **T** | **C** | **T** | **C** | **G** | **A** | **T** | **T** | **A** | **T** | **T** | **T** | **T** | **C** | **T** | **C** | **C** | **A** | **A** | **T** | **C** | **T** | **T** | **A** | **-** | **-** | **-** |
| **N52751** | **-** | **-** | **-** | **-** | **-** | **-** | **-** | **-** | **-** | **-** | **-** | **-** | **-** | **-** | **-** | **-** | **-** | **-** | **-** | **-** | **-** | **-** | **-** | **-** | **-** | **-** | **-** | **-** | **-** | **-** | **-** | **-** | **-** | **-** | **-** | **-** | **-** | **-** | **-** | **-** | **-** | **-** | **-** | **-** | **-** | **-** | **-** | **-** | **-** | **-** | **-** | **-** | **-** | **-** | **-** | **-** | **-** | **-** | **-** | **-** | **-** | **-** | **-** | **-** | **-** | **-** | **-** | **-** | **-** | **A** | **A** | **A** | **C** | **C** | **C** | **C** | **G** | **A** | **G** | **A** | **T** | **G** | **T** | **A** | **G** | **C** | **A** | **G** | **C** | **C** | **A** | **T** | **T** | **T** | **G** | **G** | **G** | **C** | **A** | **T** | **G** | **G** | **C** | **T** | **C** | **T** | **T** | **A** | **G** | **T** | **A** | **T** | **T** | **G** | **T** | **T** | **T** | **G** | **C** | **C** | **C** | **T** | **T** | **C** | **C** | **T** | **G** | **G** | **G** | **G** | **T** | **G** | **G** | **G** | **C** | **T** | **T** | **T** | **G** | **T** | **A** | **T** | **T** | **A** | **T** | **A** | **T** | **T** | **T** | **A** | **T** | **T** | **T** | **T** | **T** | **C** | **G** | **G** | **A** | **C** | **G** | **T** | **G** | **G** | **A** | **T** | **T** | **A** | **A** | **C** | **G** | **G** | **A** | **T** | **A** | **A** | **G** | **A** | **A** | **A** | **A** | **A** | **A** | **T** | **T** | **C** | **T** | **A** | **T** | **C** | **T** | **C** | **C** | **G** | **A** | **C** | **A** | **A** | **A** | **G** | **T** | **G** | **A** | **C** | **T** | **T** | **G** | **A** | **A** | **A** | **G** | **A** | **A** | **T** | **T** | **A** | **G** | **A** | **A** | **A** | **A** | **C** | **T** | **T** | **T** | **C** | **A** | **G** | **T** | **C** | **T** | **T** | **T** | **T** | **A** | **G** | **A** | **G** | **G** | **A** | **G** | **A** | **T** | **A** | **A** | **C** | **A** | **T** | **T** | **G** | **A** | **A** | **C** | **A** | **T** | **T** | **A** | **C** | **G** | **A** | **T** | **C** | **C** | **T** | **G** | **A** | **C** | **A** | **T** | **G** | **G** | **G** | **C** | **G** | **A** | **T** | **A** | **A** | **A** | **G** | **A** | **C** | **A** | **A** | **A** | **C** | **A** | **G** | **C** | **A** | **A** | **T** | **T** | **T** | **G** | **T** | **T** | **G** | **A** | **C** | **T** | **T** | **C** | **T** | **T** | **C** | **T** | **C** | **T** | **T** | **C** | **A** | **T** | **T** | **A** | **A** | **A** | **T** | **C** | **G** | **T** | **A** | **T** | **G** | **C** | **C** | **G** | **C** | **T** | **G** | **A** | **C** | **A** | **A** | **G** | **A** | **A** | **T** | **G** | **A** | **A** | **T** | **T** | **C** | **T** | **G** | **T** | **C** | **A** | **C** | **T** | **C** | **T** | **T** | **C** | **T** | **C** | **A** | **C** | **A** | **G** | **A** | **C** | **G** | **G** | **A** | **C** | **A** | **A** | **G** | **A** | **G** | **A** | **A** | **A** | **T** | **T** | **G** | **G** | **A** | **T** | **T** | **C** | **A** | **C** | **T** | **G** | **C** | **T** | **T** | **C** | **A** | **A** | **G** | **A** | **T** | **C** | **T** | **A** | **A** | **A** | **A** | **A** | **A** | **A** | **G** | **C** | **C** | **A** | **A** | **A** | **C** | **A** | **T** | **T** | **C** | **G** | **A** | **T** | **C** | **C** | **A** | **T** | **A** | **T** | **C** | **G** | **A** | **A** | **T** | **A** | **T** | **T** | **A** | **C** | **G** | **C** | **A** | **T** | **T** | **T** | **G** | **T** | **G** | **A** | **C** | **A** | **G** | **A** | **T** | **A** | **A** | **T** | **A** | **T** | **C** | **G** | **G** | **C** | **C** | **A** | **G** | **C** | **A** | **A** | **G** | **T** | **C** | **T** | **T** | **A** | **C** | **A** | **T** | **G** | **T** | **T** | **T** | **T** | **A** | **G** | **A** | **A** | **G** | **A** | **A** | **A** | **A** | **A** | **G** | **C** | **C** | **G** | **C** | **A** | **G** | **A** | **A** | **G** | **G** | **C** | **G** | **T** | **G** | **G** | **A** | **A** | **G** | **T** | **T** | **C** | **G** | **A** | **A** | **T** | **A** | **T** | **T** | **A** | **T** | **A** | **T** | **G** | **A** | **T** | **G** | **C** | **A** | **T** | **T** | **T** | **G** | **G** | **C** | **T** | **C** | **T** | **C** | **A** | **T** | **G** | **G** | **C** | **A** | **C** | **A** | **A** | **A** | **A** | **G** | **C** | **A** | **A** | **A** | **A** | **G** | **A** | **T** | **T** | **T** | **C** | **A** | **A** | **T** | **C** | **G** | **T** | **C** | **T** | **A** | **A** | **T** | **C** | **A** | **A** | **A** | **A** | **A** | **T** | **G** | **G** | **T** | **G** | **G** | **A** | **C** | **A** | **T** | **G** | **T** | **C** | **C** | **A** | **T** | **A** | **C** | **A** | **T** | **T** | **T** | **G** | **T** | **T** | **A** | **C** | **C** | **T** | **C** | **A** | **C** | **A** | **A** | **A** | **A** | **G** | **G** | **C** | **A** | **T** | **T** | **A** | **C** | **T** | **T** | **C** | **G** | **T** | **T** | **T** | **C** | **C** | **G** | **A** | **T** | **T** | **G** | **A** | **A** | **T** | **T** | **A** | **C** | **C** | **A** | **T** | **G** | **A** | **T** | **C** | **A** | **C** | **C** | **G** | **A** | **A** | **A** | **A** | **A** | **T** | **C** | **G** | **T** | **T** | **G** | **T** | **G** | **A** | **T** | **C** | **G** | **A** | **T** | **G** | **G** | **A** | **A** | **A** | **G** | **A** | **T** | **T** | **A** | **G** | **T** | **T** | **A** | **T** | **A** | **C** | **C** | **G** | **G** | **T** | **G** | **G** | **T** | **T** | **T** | **C** | **A** | **A** | **T** | **A** | **T** | **T** | **G** | **C** | **C** | **A** | **A** | **T** | **C** | **A** | **A** | **T** | **A** | **T** | **G** | **T** | **A** | **A** | **A** | **T** | **A** | **C** | **A** | **A** | **C** | **A** | **A** | **A** | **A** | **A** | **A** | **A** | **T** | **T** | **C** | **G** | **G** | **C** | **T** | **A** | **T** | **T** | **G** | **G** | **C** | **G** | **C** | **G** | **A** | **T** | **A** | **C** | **G** | **C** | **A** | **T** | **A** | **T** | **A** | **C** | **G** | **G** | **A** | **T** | **T** | **T** | **T** | **C** | **G** | **G** | **T** | **G** | **C** | **C** | **G** | **C** | **T** | **T** | **C** | **T** | **T** | **C** | **T** | **T** | **T** | **G** | **C** | **T** | **C** | **C** | **A** | **G** | **C** | **T** | **T** | **C** | **G** | **C** | **T** | **T** | **C** | **T** | **T** | **A** | **A** | **C** | **A** | **G** | **A** | **C** | **T** | **G** | **G** | **A** | **A** | **C** | **G** | **T** | **C** | **T** | **C** | **G** | **G** | **T** | **A** | **C** | **C** | **T** | **G** | **A** | **A** | **G** | **A** | **A** | **A** | **A** | **A** | **A** | **A** | **G** | **G** | **T** | **C** | **G** | **G** | **C** | **T** | **A** | **T** | **C** | **A** | **T** | **T** | **T** | **G** | **A** | **A** | **T** | **T** | **A** | **T** | **T** | **T** | **C** | **T** | **T** | **T** | **A** | **A** | **A** | **A** | **A** | **A** | **G** | **C** | **A** | **G** | **A** | **T** | **C** | **G** | **A** | **G** | **A** | **T** | **G** | **A** | **A** | **T** | **C** | **T** | **A** | **A** | **G** | **C** | **T** | **T** | **G** | **C** | **T** | **G** | **A** | **T** | **A** | **C** | **A** | **T** | **C** | **C** | **A** | **T** | **C** | **C** | **A** | **G** | **C** | **T** | **T** | **G** | **T** | **T** | **T** | **C** | **A** | **A** | **G** | **C** | **G** | **G** | **A** | **C** | **C** | **G** | **A** | **A** | **T** | **A** | **A** | **C** | **G** | **A** | **A** | **A** | **G** | **G** | **G** | **A** | **A** | **C** | **A** | **A** | **A** | **T** | **C** | **A** | **A** | **G** | **C** | **T** | **T** | **T** | **C** | **A** | **T** | **T** | **T** | **A** | **T** | **C** | **A** | **A** | **A** | **T** | **T** | **G** | **A** | **T** | **T** | **A** | **C** | **T** | **T** | **C** | **T** | **G** | **C** | **T** | **A** | **A** | **A** | **A** | **A** | **A** | **C** | **G** | **T** | **G** | **T** | **T** | **T** | **G** | **G** | **A** | **T** | **A** | **C** | **A** | **G** | **A** | **C** | **A** | **C** | **C** | **T** | **T** | **A** | **C** | **C** | **T** | **T** | **G** | **T** | **T** | **C** | **C** | **T** | **G** | **A** | **T** | **G** | **A** | **A** | **A** | **G** | **T** | **G** | **T** | **C** | **A** | **T** | **T** | **G** | **C** | **T** | **G** | **C** | **T** | **T** | **T** | **A** | **A** | **A** | **A** | **A** | **T** | **C** | **G** | **C** | **A** | **A** | **C** | **T** | **G** | **C** | **C** | **T** | **C** | **T** | **G** | **G** | **T** | **G** | **T** | **A** | **G** | **A** | **T** | **G** | **T** | **G** | **A** | **A** | **A** | **A** | **T** | **C** | **A** | **T** | **G** | **A** | **T** | **T** | **C** | **C** | **C** | **A** | **A** | **C** | **A** | **A** | **A** | **C** | **C** | **G** | **G** | **A** | **T** | **C** | **A** | **T** | **C** | **C** | **T** | **T** | **T** | **T** | **A** | **T** | **T** | **T** | **A** | **T** | **C** | **G** | **A** | **G** | **C** | **A** | **A** | **C** | **A** | **C** | **A** | **A** | **T** | **A** | **T** | **T** | **A** | **T** | **G** | **C** | **T** | **C** | **G** | **G** | **C** | **A** | **G** | **C** | **T** | **G** | **A** | **T** | **C** | **A** | **A** | **G** | **G** | **A** | **A** | **A** | **A** | **T** | **G** | **T** | **A** | **C** | **A** | **A** | **A** | **T** | **C** | **C** | **T** | **T** | **G** | **T** | **C** | **T** | **A** | **T** | **G** | **A** | **G** | **A** | **A** | **C** | **G** | **G** | **C** | **T** | **T** | **C** | **C** | **T** | **C** | **C** | **A** | **T** | **G** | **C** | **A** | **A** | **A** | **A** | **A** | **C** | **A** | **T** | **T** | **G** | **A** | **T** | **A** | **A** | **T** | **G** | **G** | **A** | **T** | **G** | **A** | **T** | **G** | **A** | **A** | **A** | **T** | **C** | **T** | **G** | **C** | **A** | **T** | **G** | **G** | **T** | **A** | **G** | **G** | **T** | **T** | **C** | **A** | **G** | **C** | **A** | **A** | **A** | **T** | **C** | **A** | **A** | **G** | **A** | **T** | **A** | **T** | **T** | **C** | **G** | **T** | **A** | **G** | **C** | **T** | **A** | **C** | **C** | **G** | **A** | **T** | **T** | **G** | **A** | **A** | **T** | **T** | **T** | **T** | **G** | **A** | **A** | **A** | **C** | **A** | **A** | **G** | **T** | **G** | **C** | **T** | **G** | **T** | **C** | **A** | **T** | **T** | **T** | **A** | **C** | **G** | **A** | **T** | **C** | **C** | **T** | **G** | **A** | **G** | **T** | **T** | **T** | **T** | **T** | **A** | **G** | **A** | **A** | **G** | **A** | **A** | **C** | **T** | **T** | **G** | **C** | **T** | **A** | **C** | **T** | **C** | **A** | **G** | **T** | **T** | **C** | **A** | **A** | **A** | **G** | **A** | **A** | **G** | **A** | **T** | **G** | **A** | **G** | **A** | **C** | **A** | **C** | **A** | **T** | **T** | **G** | **T** | **T** | **C** | **A** | **T** | **C** | **C** | **A** | **T** | **G** | **A** | **C** | **A** | **A** | **C** | **T** | **G** | **A** | **A** | **A** | **C** | **A** | **G** | **T** | **C** | **A** | **A** | **G** | **G** | **A** | **A** | **A** | **T** | **G** | **T** | **C** | **T** | **A** | **A** | **C** | **T** | **G** | **G** | **C** | **T** | **A** | **T** | **T** | **A** | **T** | **T** | **C** | **A** | **A** | **G** | **C** | **A** | **A** | **C** | **A** | **A** | **A** | **T** | **T** | **T** | **C** | **T** | **C** | **G** | **A** | **T** | **T** | **A** | **T** | **T** | **T** | **T** | **C** | **T** | **C** | **C** | **A** | **A** | **T** | **C** | **C** | **T** | **A** | **-** | **-** | **-** |
| **N53804** | **-** | **-** | **-** | **-** | **-** | **-** | **-** | **-** | **-** | **-** | **-** | **-** | **-** | **-** | **-** | **-** | **-** | **-** | **-** | **-** | **-** | **-** | **-** | **-** | **-** | **-** | **-** | **-** | **-** | **-** | **-** | **-** | **-** | **-** | **-** | **-** | **-** | **-** | **-** | **-** | **-** | **-** | **-** | **-** | **-** | **-** | **-** | **-** | **-** | **-** | **-** | **-** | **-** | **-** | **-** | **-** | **-** | **-** | **-** | **-** | **-** | **-** | **-** | **-** | **-** | **-** | **-** | **-** | **-** | **A** | **A** | **A** | **C** | **C** | **C** | **C** | **G** | **A** | **G** | **A** | **T** | **G** | **T** | **A** | **G** | **C** | **A** | **G** | **C** | **C** | **A** | **T** | **T** | **T** | **G** | **G** | **G** | **C** | **A** | **T** | **G** | **G** | **C** | **T** | **C** | **T** | **T** | **A** | **G** | **T** | **A** | **T** | **T** | **A** | **T** | **T** | **T** | **G** | **C** | **C** | **C** | **T** | **T** | **C** | **C** | **T** | **G** | **G** | **G** | **G** | **T** | **G** | **G** | **G** | **C** | **T** | **T** | **T** | **G** | **T** | **A** | **T** | **T** | **A** | **T** | **A** | **T** | **T** | **T** | **A** | **T** | **T** | **T** | **T** | **T** | **C** | **G** | **G** | **A** | **C** | **G** | **T** | **G** | **G** | **A** | **T** | **T** | **A** | **A** | **C** | **G** | **G** | **A** | **T** | **A** | **A** | **G** | **A** | **A** | **A** | **A** | **A** | **A** | **T** | **T** | **C** | **T** | **A** | **T** | **C** | **T** | **C** | **C** | **G** | **A** | **C** | **A** | **A** | **A** | **G** | **T** | **G** | **A** | **C** | **T** | **T** | **G** | **A** | **A** | **A** | **G** | **A** | **A** | **T** | **T** | **A** | **G** | **A** | **A** | **A** | **A** | **C** | **T** | **T** | **T** | **C** | **A** | **G** | **T** | **C** | **T** | **T** | **T** | **T** | **A** | **G** | **A** | **G** | **G** | **A** | **G** | **A** | **T** | **A** | **C** | **C** | **A** | **T** | **T** | **G** | **A** | **A** | **C** | **A** | **T** | **T** | **A** | **C** | **G** | **A** | **T** | **C** | **C** | **T** | **G** | **A** | **C** | **A** | **T** | **G** | **G** | **G** | **C** | **G** | **A** | **T** | **A** | **A** | **A** | **G** | **A** | **C** | **A** | **A** | **A** | **C** | **A** | **G** | **C** | **A** | **A** | **T** | **T** | **T** | **G** | **T** | **T** | **G** | **A** | **C** | **T** | **T** | **C** | **T** | **T** | **C** | **T** | **C** | **T** | **T** | **C** | **A** | **T** | **T** | **A** | **A** | **A** | **T** | **C** | **G** | **T** | **A** | **T** | **G** | **C** | **C** | **G** | **C** | **T** | **G** | **A** | **C** | **A** | **A** | **G** | **A** | **A** | **T** | **G** | **A** | **A** | **T** | **T** | **C** | **T** | **G** | **T** | **C** | **A** | **C** | **T** | **C** | **T** | **T** | **C** | **T** | **C** | **A** | **C** | **A** | **G** | **A** | **C** | **G** | **G** | **A** | **C** | **A** | **A** | **G** | **A** | **G** | **A** | **A** | **A** | **T** | **T** | **G** | **G** | **A** | **T** | **T** | **C** | **A** | **C** | **T** | **G** | **C** | **T** | **T** | **C** | **A** | **A** | **G** | **A** | **T** | **C** | **T** | **A** | **A** | **A** | **A** | **A** | **A** | **A** | **G** | **C** | **C** | **A** | **A** | **A** | **C** | **A** | **T** | **T** | **C** | **G** | **A** | **T** | **C** | **C** | **A** | **T** | **A** | **T** | **C** | **G** | **A** | **A** | **T** | **A** | **T** | **T** | **A** | **C** | **G** | **C** | **A** | **T** | **T** | **T** | **G** | **T** | **G** | **A** | **C** | **A** | **G** | **A** | **T** | **A** | **A** | **T** | **A** | **T** | **C** | **G** | **G** | **C** | **C** | **A** | **G** | **C** | **A** | **A** | **G** | **T** | **C** | **T** | **T** | **A** | **C** | **A** | **T** | **G** | **T** | **T** | **T** | **T** | **A** | **G** | **A** | **A** | **G** | **A** | **A** | **A** | **A** | **A** | **G** | **C** | **C** | **G** | **C** | **A** | **G** | **A** | **A** | **G** | **G** | **C** | **G** | **T** | **G** | **G** | **A** | **A** | **G** | **T** | **T** | **C** | **G** | **A** | **A** | **T** | **A** | **T** | **T** | **A** | **T** | **A** | **T** | **G** | **A** | **T** | **G** | **C** | **A** | **T** | **T** | **T** | **G** | **G** | **C** | **T** | **C** | **T** | **C** | **A** | **T** | **G** | **G** | **C** | **A** | **C** | **A** | **A** | **A** | **A** | **G** | **C** | **A** | **A** | **A** | **A** | **G** | **A** | **T** | **T** | **T** | **C** | **A** | **A** | **T** | **C** | **G** | **T** | **C** | **T** | **A** | **A** | **T** | **C** | **A** | **A** | **A** | **A** | **A** | **T** | **G** | **G** | **T** | **G** | **G** | **A** | **C** | **A** | **T** | **G** | **T** | **C** | **C** | **A** | **T** | **A** | **C** | **A** | **T** | **T** | **T** | **G** | **T** | **T** | **A** | **C** | **C** | **T** | **C** | **A** | **C** | **A** | **A** | **A** | **G** | **G** | **G** | **C** | **A** | **T** | **T** | **A** | **C** | **T** | **T** | **C** | **G** | **T** | **T** | **T** | **C** | **C** | **G** | **A** | **T** | **T** | **G** | **A** | **A** | **T** | **T** | **A** | **C** | **C** | **A** | **T** | **G** | **A** | **T** | **C** | **A** | **C** | **C** | **G** | **A** | **A** | **A** | **A** | **A** | **T** | **C** | **G** | **T** | **T** | **G** | **T** | **G** | **A** | **T** | **C** | **G** | **A** | **T** | **G** | **G** | **A** | **A** | **A** | **G** | **A** | **T** | **T** | **A** | **G** | **T** | **T** | **A** | **T** | **A** | **C** | **C** | **G** | **G** | **T** | **G** | **G** | **T** | **T** | **T** | **C** | **A** | **A** | **T** | **A** | **T** | **T** | **G** | **C** | **C** | **A** | **A** | **T** | **C** | **A** | **A** | **T** | **A** | **T** | **G** | **T** | **A** | **A** | **A** | **T** | **A** | **C** | **A** | **A** | **C** | **A** | **A** | **A** | **A** | **A** | **A** | **A** | **T** | **T** | **C** | **G** | **G** | **C** | **T** | **A** | **T** | **T** | **G** | **G** | **C** | **G** | **C** | **G** | **A** | **T** | **A** | **C** | **G** | **C** | **A** | **T** | **A** | **T** | **A** | **C** | **G** | **G** | **A** | **T** | **T** | **T** | **T** | **C** | **G** | **G** | **T** | **G** | **C** | **C** | **G** | **C** | **T** | **T** | **C** | **T** | **T** | **C** | **T** | **T** | **T** | **G** | **C** | **T** | **C** | **C** | **A** | **G** | **C** | **T** | **T** | **C** | **G** | **C** | **T** | **T** | **C** | **T** | **T** | **A** | **A** | **C** | **A** | **G** | **A** | **C** | **T** | **G** | **G** | **A** | **A** | **C** | **G** | **T** | **C** | **T** | **C** | **G** | **G** | **T** | **A** | **C** | **C** | **T** | **G** | **A** | **A** | **G** | **A** | **A** | **A** | **A** | **A** | **A** | **A** | **G** | **G** | **T** | **C** | **G** | **G** | **C** | **T** | **A** | **T** | **C** | **A** | **T** | **T** | **T** | **G** | **A** | **A** | **T** | **T** | **A** | **T** | **T** | **T** | **C** | **T** | **T** | **T** | **A** | **A** | **A** | **A** | **A** | **A** | **G** | **C** | **A** | **G** | **A** | **T** | **C** | **G** | **A** | **G** | **A** | **T** | **G** | **A** | **A** | **T** | **C** | **T** | **A** | **A** | **G** | **C** | **T** | **T** | **G** | **C** | **T** | **G** | **A** | **T** | **A** | **C** | **A** | **T** | **C** | **C** | **A** | **T** | **C** | **C** | **A** | **G** | **C** | **T** | **T** | **G** | **T** | **T** | **T** | **C** | **A** | **A** | **G** | **C** | **G** | **G** | **A** | **C** | **C** | **G** | **A** | **A** | **T** | **A** | **A** | **C** | **G** | **A** | **A** | **A** | **G** | **G** | **G** | **A** | **A** | **C** | **A** | **A** | **A** | **T** | **C** | **A** | **A** | **G** | **C** | **T** | **T** | **T** | **C** | **A** | **T** | **T** | **T** | **A** | **T** | **C** | **A** | **A** | **A** | **T** | **T** | **G** | **A** | **T** | **T** | **A** | **C** | **T** | **T** | **C** | **T** | **G** | **C** | **T** | **A** | **A** | **A** | **A** | **A** | **A** | **C** | **G** | **T** | **G** | **T** | **T** | **T** | **G** | **G** | **A** | **T** | **A** | **C** | **A** | **G** | **A** | **C** | **A** | **C** | **C** | **T** | **T** | **A** | **C** | **C** | **T** | **T** | **G** | **T** | **T** | **C** | **C** | **T** | **G** | **A** | **T** | **G** | **A** | **A** | **A** | **G** | **T** | **G** | **T** | **C** | **A** | **T** | **T** | **G** | **C** | **T** | **G** | **C** | **T** | **T** | **T** | **A** | **A** | **A** | **A** | **A** | **T** | **C** | **G** | **C** | **A** | **A** | **C** | **T** | **G** | **C** | **C** | **T** | **C** | **T** | **G** | **G** | **T** | **G** | **T** | **A** | **G** | **A** | **T** | **G** | **T** | **G** | **A** | **A** | **A** | **A** | **T** | **C** | **A** | **T** | **G** | **A** | **T** | **T** | **C** | **C** | **C** | **A** | **A** | **C** | **A** | **A** | **A** | **C** | **C** | **G** | **G** | **A** | **T** | **C** | **A** | **T** | **C** | **C** | **T** | **T** | **T** | **T** | **A** | **T** | **T** | **T** | **A** | **T** | **C** | **G** | **A** | **G** | **C** | **A** | **A** | **C** | **A** | **C** | **A** | **A** | **T** | **A** | **T** | **T** | **A** | **T** | **G** | **C** | **T** | **C** | **G** | **G** | **C** | **A** | **G** | **C** | **T** | **G** | **A** | **T** | **C** | **A** | **A** | **G** | **G** | **A** | **A** | **A** | **A** | **T** | **G** | **T** | **A** | **C** | **A** | **A** | **A** | **T** | **C** | **C** | **T** | **T** | **G** | **T** | **C** | **T** | **A** | **T** | **G** | **A** | **G** | **A** | **A** | **C** | **G** | **G** | **C** | **T** | **T** | **C** | **C** | **T** | **C** | **C** | **A** | **T** | **G** | **C** | **A** | **A** | **A** | **A** | **A** | **C** | **A** | **T** | **T** | **G** | **A** | **T** | **A** | **A** | **T** | **G** | **G** | **A** | **T** | **G** | **A** | **T** | **G** | **A** | **A** | **A** | **T** | **C** | **T** | **G** | **C** | **A** | **T** | **G** | **G** | **T** | **A** | **G** | **G** | **T** | **T** | **C** | **A** | **G** | **C** | **A** | **A** | **A** | **T** | **C** | **A** | **A** | **G** | **A** | **T** | **A** | **T** | **T** | **C** | **G** | **T** | **A** | **G** | **C** | **T** | **A** | **C** | **C** | **G** | **A** | **T** | **T** | **G** | **A** | **A** | **T** | **T** | **T** | **T** | **G** | **A** | **A** | **A** | **C** | **A** | **A** | **G** | **T** | **G** | **C** | **T** | **G** | **T** | **C** | **A** | **T** | **T** | **T** | **A** | **C** | **G** | **A** | **T** | **C** | **C** | **T** | **G** | **A** | **G** | **T** | **T** | **T** | **T** | **T** | **A** | **G** | **A** | **A** | **G** | **A** | **A** | **C** | **T** | **T** | **G** | **C** | **T** | **A** | **C** | **T** | **C** | **A** | **G** | **T** | **T** | **C** | **A** | **A** | **A** | **G** | **A** | **A** | **G** | **A** | **T** | **G** | **A** | **G** | **A** | **C** | **A** | **C** | **A** | **T** | **T** | **G** | **T** | **T** | **C** | **A** | **T** | **C** | **C** | **A** | **T** | **G** | **A** | **C** | **A** | **A** | **C** | **T** | **G** | **A** | **A** | **A** | **C** | **A** | **G** | **T** | **C** | **A** | **A** | **G** | **G** | **A** | **A** | **A** | **T** | **G** | **T** | **C** | **T** | **A** | **A** | **C** | **T** | **G** | **G** | **C** | **T** | **A** | **T** | **T** | **A** | **T** | **T** | **C** | **A** | **A** | **G** | **C** | **A** | **A** | **C** | **A** | **A** | **A** | **T** | **T** | **T** | **C** | **T** | **C** | **G** | **A** | **T** | **T** | **A** | **T** | **T** | **T** | **T** | **C** | **T** | **C** | **C** | **A** | **A** | **T** | **C** | **C** | **T** | **A** | **-** | **-** | **-** |
| **N55728** | **-** | **-** | **-** | **-** | **-** | **-** | **-** | **-** | **-** | **-** | **-** | **-** | **-** | **-** | **-** | **-** | **-** | **-** | **-** | **-** | **-** | **-** | **-** | **-** | **-** | **-** | **-** | **-** | **-** | **-** | **-** | **-** | **-** | **-** | **-** | **-** | **-** | **-** | **-** | **-** | **-** | **-** | **-** | **-** | **-** | **-** | **-** | **-** | **-** | **-** | **-** | **-** | **-** | **-** | **-** | **-** | **-** | **-** | **-** | **-** | **-** | **-** | **-** | **-** | **-** | **-** | **-** | **-** | **-** | **A** | **A** | **A** | **C** | **C** | **C** | **C** | **G** | **A** | **G** | **A** | **T** | **G** | **T** | **A** | **G** | **C** | **A** | **G** | **C** | **C** | **A** | **T** | **T** | **T** | **G** | **G** | **G** | **C** | **A** | **T** | **G** | **G** | **C** | **T** | **C** | **T** | **T** | **A** | **G** | **T** | **A** | **T** | **T** | **G** | **T** | **T** | **T** | **G** | **C** | **C** | **C** | **T** | **T** | **C** | **C** | **T** | **G** | **G** | **G** | **G** | **T** | **G** | **G** | **G** | **C** | **T** | **T** | **T** | **G** | **T** | **A** | **T** | **T** | **A** | **T** | **A** | **T** | **T** | **T** | **A** | **T** | **T** | **T** | **T** | **T** | **C** | **G** | **G** | **A** | **C** | **G** | **T** | **G** | **G** | **A** | **T** | **T** | **A** | **A** | **C** | **G** | **G** | **A** | **T** | **A** | **A** | **G** | **A** | **A** | **A** | **A** | **A** | **A** | **T** | **T** | **C** | **T** | **A** | **T** | **C** | **T** | **C** | **C** | **G** | **A** | **C** | **A** | **A** | **A** | **G** | **T** | **G** | **A** | **C** | **T** | **T** | **G** | **A** | **A** | **A** | **G** | **A** | **A** | **T** | **T** | **A** | **G** | **A** | **A** | **A** | **A** | **C** | **T** | **T** | **T** | **C** | **A** | **G** | **T** | **C** | **T** | **T** | **T** | **T** | **A** | **G** | **A** | **G** | **G** | **A** | **G** | **A** | **T** | **A** | **A** | **C** | **A** | **T** | **T** | **G** | **A** | **A** | **C** | **A** | **T** | **T** | **A** | **C** | **G** | **A** | **T** | **C** | **C** | **T** | **G** | **A** | **C** | **A** | **T** | **G** | **G** | **G** | **C** | **G** | **A** | **T** | **A** | **A** | **A** | **G** | **A** | **C** | **A** | **A** | **A** | **C** | **A** | **G** | **C** | **A** | **A** | **T** | **T** | **T** | **G** | **T** | **T** | **G** | **A** | **C** | **T** | **T** | **C** | **T** | **T** | **C** | **T** | **C** | **T** | **T** | **C** | **A** | **T** | **T** | **A** | **A** | **A** | **T** | **C** | **G** | **T** | **A** | **T** | **G** | **C** | **C** | **G** | **C** | **T** | **G** | **A** | **C** | **A** | **A** | **G** | **A** | **A** | **T** | **G** | **A** | **A** | **T** | **T** | **C** | **T** | **G** | **T** | **C** | **A** | **C** | **T** | **C** | **T** | **T** | **C** | **T** | **C** | **A** | **C** | **A** | **G** | **A** | **C** | **G** | **G** | **A** | **C** | **A** | **A** | **G** | **A** | **G** | **A** | **A** | **A** | **T** | **T** | **G** | **G** | **A** | **T** | **T** | **C** | **A** | **C** | **T** | **G** | **C** | **T** | **T** | **C** | **A** | **A** | **G** | **A** | **T** | **C** | **T** | **A** | **A** | **A** | **A** | **A** | **A** | **A** | **G** | **C** | **C** | **A** | **A** | **A** | **C** | **A** | **T** | **T** | **C** | **G** | **A** | **T** | **C** | **C** | **A** | **T** | **A** | **T** | **C** | **G** | **A** | **A** | **T** | **A** | **T** | **T** | **A** | **C** | **G** | **C** | **A** | **T** | **T** | **T** | **G** | **T** | **G** | **A** | **C** | **A** | **G** | **A** | **T** | **A** | **A** | **T** | **A** | **T** | **C** | **G** | **G** | **C** | **C** | **A** | **G** | **C** | **A** | **A** | **G** | **T** | **C** | **T** | **T** | **A** | **C** | **A** | **T** | **G** | **T** | **T** | **T** | **T** | **A** | **G** | **A** | **A** | **G** | **A** | **A** | **A** | **A** | **A** | **G** | **C** | **C** | **G** | **C** | **A** | **G** | **A** | **A** | **G** | **G** | **C** | **G** | **T** | **G** | **G** | **A** | **A** | **G** | **T** | **T** | **C** | **G** | **A** | **A** | **T** | **A** | **T** | **T** | **A** | **T** | **A** | **T** | **G** | **A** | **T** | **G** | **C** | **A** | **T** | **T** | **T** | **G** | **G** | **C** | **T** | **C** | **T** | **C** | **A** | **T** | **G** | **G** | **C** | **A** | **C** | **A** | **A** | **A** | **A** | **G** | **C** | **A** | **A** | **A** | **A** | **G** | **A** | **T** | **T** | **T** | **C** | **A** | **A** | **T** | **C** | **G** | **T** | **C** | **T** | **A** | **A** | **T** | **C** | **A** | **A** | **A** | **A** | **A** | **T** | **G** | **G** | **T** | **G** | **G** | **A** | **C** | **A** | **T** | **G** | **T** | **C** | **C** | **A** | **T** | **A** | **C** | **A** | **T** | **T** | **T** | **G** | **T** | **T** | **A** | **C** | **C** | **T** | **C** | **A** | **C** | **A** | **A** | **A** | **A** | **G** | **G** | **C** | **A** | **T** | **T** | **A** | **C** | **T** | **T** | **C** | **G** | **T** | **T** | **T** | **C** | **C** | **G** | **A** | **T** | **T** | **G** | **A** | **A** | **T** | **T** | **A** | **C** | **C** | **A** | **T** | **G** | **A** | **T** | **C** | **A** | **C** | **C** | **G** | **A** | **A** | **A** | **A** | **A** | **T** | **C** | **G** | **T** | **T** | **G** | **T** | **G** | **A** | **T** | **C** | **G** | **A** | **T** | **G** | **G** | **A** | **A** | **A** | **G** | **A** | **T** | **T** | **A** | **G** | **T** | **T** | **A** | **T** | **A** | **C** | **C** | **G** | **G** | **T** | **G** | **G** | **T** | **T** | **T** | **C** | **A** | **A** | **T** | **A** | **T** | **T** | **G** | **C** | **C** | **A** | **A** | **T** | **C** | **A** | **A** | **T** | **A** | **T** | **G** | **T** | **A** | **A** | **A** | **T** | **A** | **C** | **A** | **A** | **C** | **A** | **A** | **A** | **A** | **A** | **A** | **A** | **T** | **T** | **C** | **G** | **G** | **C** | **T** | **A** | **T** | **T** | **G** | **G** | **C** | **G** | **C** | **G** | **A** | **T** | **A** | **C** | **G** | **C** | **A** | **T** | **A** | **T** | **A** | **C** | **G** | **G** | **A** | **T** | **T** | **T** | **T** | **C** | **G** | **G** | **T** | **G** | **C** | **C** | **G** | **C** | **T** | **T** | **C** | **T** | **T** | **C** | **T** | **T** | **T** | **G** | **C** | **T** | **C** | **C** | **A** | **G** | **C** | **T** | **T** | **C** | **G** | **C** | **T** | **T** | **C** | **T** | **T** | **A** | **A** | **C** | **A** | **G** | **A** | **C** | **T** | **G** | **G** | **A** | **A** | **C** | **G** | **T** | **C** | **T** | **C** | **G** | **G** | **T** | **A** | **C** | **C** | **T** | **G** | **A** | **A** | **G** | **A** | **A** | **A** | **A** | **A** | **A** | **A** | **G** | **G** | **T** | **C** | **G** | **G** | **C** | **T** | **A** | **T** | **C** | **A** | **T** | **T** | **T** | **G** | **A** | **A** | **T** | **T** | **A** | **T** | **T** | **T** | **C** | **T** | **T** | **T** | **A** | **A** | **A** | **A** | **A** | **A** | **G** | **C** | **A** | **G** | **A** | **T** | **C** | **G** | **A** | **G** | **A** | **T** | **G** | **A** | **A** | **T** | **C** | **T** | **A** | **A** | **G** | **C** | **T** | **T** | **G** | **C** | **T** | **G** | **A** | **T** | **A** | **C** | **A** | **T** | **C** | **C** | **A** | **T** | **C** | **C** | **A** | **G** | **C** | **T** | **T** | **G** | **T** | **T** | **T** | **C** | **A** | **A** | **G** | **C** | **G** | **G** | **A** | **C** | **C** | **G** | **A** | **A** | **T** | **A** | **A** | **C** | **G** | **A** | **A** | **A** | **G** | **G** | **G** | **A** | **A** | **C** | **A** | **A** | **A** | **T** | **C** | **A** | **A** | **G** | **C** | **T** | **T** | **T** | **C** | **A** | **T** | **T** | **T** | **A** | **T** | **C** | **A** | **A** | **A** | **T** | **T** | **G** | **A** | **T** | **T** | **A** | **C** | **T** | **T** | **C** | **T** | **G** | **C** | **T** | **A** | **A** | **A** | **A** | **A** | **A** | **C** | **G** | **T** | **G** | **T** | **T** | **T** | **G** | **G** | **A** | **T** | **A** | **C** | **A** | **G** | **A** | **C** | **A** | **C** | **C** | **T** | **T** | **A** | **C** | **C** | **T** | **T** | **G** | **T** | **T** | **C** | **C** | **T** | **G** | **A** | **T** | **G** | **A** | **A** | **A** | **G** | **T** | **G** | **T** | **C** | **A** | **T** | **T** | **G** | **C** | **T** | **G** | **C** | **T** | **T** | **T** | **A** | **A** | **A** | **A** | **A** | **T** | **C** | **G** | **C** | **A** | **A** | **C** | **T** | **G** | **C** | **C** | **T** | **C** | **T** | **G** | **G** | **T** | **G** | **T** | **A** | **G** | **A** | **T** | **G** | **T** | **G** | **A** | **A** | **A** | **A** | **T** | **C** | **A** | **T** | **G** | **A** | **T** | **T** | **C** | **C** | **C** | **A** | **A** | **C** | **A** | **A** | **A** | **C** | **C** | **G** | **G** | **A** | **T** | **C** | **A** | **T** | **C** | **C** | **T** | **T** | **T** | **T** | **A** | **T** | **T** | **T** | **A** | **T** | **C** | **G** | **A** | **G** | **C** | **A** | **A** | **C** | **A** | **C** | **A** | **A** | **T** | **A** | **T** | **T** | **A** | **T** | **G** | **C** | **T** | **C** | **G** | **G** | **C** | **A** | **G** | **C** | **T** | **G** | **A** | **T** | **C** | **A** | **A** | **G** | **G** | **A** | **A** | **A** | **A** | **T** | **G** | **T** | **A** | **C** | **A** | **A** | **A** | **T** | **C** | **C** | **T** | **T** | **G** | **T** | **C** | **T** | **A** | **T** | **G** | **A** | **G** | **A** | **A** | **C** | **G** | **G** | **C** | **T** | **T** | **C** | **C** | **T** | **C** | **C** | **A** | **T** | **G** | **C** | **A** | **A** | **A** | **A** | **A** | **C** | **A** | **T** | **T** | **G** | **A** | **T** | **A** | **A** | **T** | **G** | **G** | **A** | **T** | **G** | **A** | **T** | **G** | **A** | **A** | **A** | **T** | **C** | **T** | **G** | **C** | **A** | **T** | **G** | **G** | **T** | **A** | **G** | **G** | **T** | **T** | **C** | **A** | **G** | **C** | **A** | **A** | **A** | **T** | **C** | **A** | **A** | **G** | **A** | **T** | **A** | **T** | **T** | **C** | **G** | **T** | **A** | **G** | **C** | **T** | **A** | **C** | **C** | **G** | **A** | **T** | **T** | **G** | **A** | **A** | **T** | **T** | **T** | **T** | **G** | **A** | **A** | **A** | **C** | **A** | **A** | **G** | **T** | **G** | **C** | **T** | **G** | **T** | **C** | **A** | **T** | **T** | **T** | **A** | **C** | **G** | **A** | **T** | **C** | **C** | **T** | **G** | **A** | **G** | **T** | **T** | **T** | **T** | **T** | **A** | **G** | **A** | **A** | **G** | **A** | **A** | **C** | **T** | **T** | **G** | **C** | **T** | **A** | **C** | **T** | **C** | **A** | **G** | **T** | **T** | **C** | **A** | **A** | **A** | **G** | **A** | **A** | **G** | **A** | **T** | **G** | **A** | **G** | **A** | **C** | **A** | **C** | **A** | **T** | **T** | **G** | **T** | **T** | **C** | **A** | **T** | **C** | **C** | **A** | **T** | **G** | **A** | **C** | **A** | **A** | **C** | **T** | **G** | **A** | **A** | **A** | **C** | **A** | **G** | **T** | **C** | **A** | **A** | **G** | **G** | **A** | **A** | **A** | **T** | **G** | **T** | **C** | **T** | **A** | **A** | **C** | **T** | **G** | **G** | **C** | **T** | **A** | **T** | **T** | **A** | **T** | **T** | **C** | **A** | **A** | **G** | **C** | **A** | **A** | **C** | **A** | **A** | **A** | **T** | **T** | **T** | **C** | **T** | **C** | **G** | **A** | **T** | **T** | **A** | **T** | **T** | **T** | **T** | **C** | **T** | **C** | **C** | **A** | **A** | **T** | **C** | **C** | **T** | **A** | **-** | **-** | **-** |
| **N54035** | **-** | **-** | **-** | **-** | **-** | **-** | **-** | **-** | **-** | **-** | **-** | **-** | **-** | **-** | **-** | **-** | **-** | **-** | **-** | **-** | **-** | **-** | **-** | **-** | **-** | **-** | **-** | **-** | **-** | **-** | **-** | **-** | **-** | **-** | **-** | **-** | **-** | **-** | **-** | **-** | **-** | **-** | **-** | **-** | **-** | **-** | **-** | **-** | **-** | **-** | **-** | **-** | **-** | **-** | **-** | **-** | **-** | **-** | **-** | **-** | **-** | **-** | **-** | **-** | **-** | **-** | **-** | **-** | **-** | **A** | **A** | **A** | **C** | **C** | **C** | **C** | **G** | **A** | **G** | **A** | **T** | **G** | **T** | **A** | **G** | **C** | **A** | **G** | **C** | **C** | **A** | **T** | **T** | **T** | **G** | **G** | **G** | **C** | **A** | **T** | **G** | **G** | **C** | **T** | **C** | **T** | **T** | **A** | **G** | **T** | **A** | **T** | **T** | **G** | **T** | **T** | **T** | **G** | **C** | **C** | **C** | **T** | **T** | **C** | **C** | **T** | **G** | **G** | **G** | **G** | **T** | **G** | **G** | **G** | **T** | **T** | **T** | **T** | **G** | **T** | **A** | **T** | **T** | **A** | **T** | **A** | **T** | **T** | **T** | **A** | **T** | **T** | **T** | **T** | **T** | **C** | **G** | **G** | **A** | **C** | **G** | **T** | **G** | **G** | **A** | **T** | **T** | **A** | **A** | **C** | **G** | **G** | **A** | **T** | **A** | **A** | **G** | **A** | **A** | **A** | **A** | **A** | **A** | **T** | **T** | **C** | **T** | **A** | **T** | **C** | **T** | **C** | **C** | **G** | **A** | **C** | **A** | **A** | **A** | **G** | **T** | **G** | **A** | **C** | **T** | **T** | **G** | **A** | **A** | **A** | **G** | **A** | **A** | **T** | **T** | **A** | **G** | **A** | **A** | **A** | **A** | **C** | **T** | **T** | **T** | **C** | **A** | **G** | **T** | **C** | **T** | **T** | **T** | **T** | **A** | **G** | **A** | **G** | **G** | **A** | **G** | **A** | **T** | **A** | **C** | **C** | **A** | **T** | **T** | **G** | **A** | **A** | **C** | **A** | **T** | **T** | **A** | **C** | **G** | **A** | **T** | **C** | **C** | **T** | **G** | **A** | **C** | **A** | **T** | **G** | **G** | **G** | **C** | **G** | **A** | **T** | **A** | **A** | **A** | **G** | **A** | **C** | **A** | **A** | **A** | **C** | **A** | **G** | **C** | **A** | **A** | **T** | **T** | **T** | **G** | **T** | **T** | **G** | **A** | **C** | **T** | **T** | **C** | **T** | **T** | **C** | **T** | **C** | **T** | **T** | **C** | **A** | **T** | **T** | **A** | **A** | **A** | **T** | **C** | **G** | **T** | **A** | **T** | **G** | **C** | **C** | **G** | **C** | **T** | **G** | **A** | **C** | **A** | **A** | **G** | **A** | **A** | **T** | **G** | **A** | **A** | **T** | **T** | **C** | **T** | **G** | **T** | **C** | **A** | **C** | **T** | **C** | **T** | **T** | **C** | **T** | **C** | **A** | **C** | **A** | **G** | **A** | **C** | **G** | **G** | **A** | **C** | **A** | **A** | **G** | **A** | **G** | **A** | **A** | **A** | **T** | **T** | **G** | **G** | **A** | **T** | **T** | **C** | **A** | **C** | **T** | **G** | **C** | **T** | **T** | **C** | **A** | **A** | **G** | **A** | **T** | **C** | **T** | **A** | **A** | **A** | **A** | **A** | **A** | **A** | **G** | **C** | **C** | **A** | **A** | **A** | **C** | **A** | **T** | **T** | **C** | **G** | **A** | **T** | **C** | **C** | **A** | **T** | **A** | **T** | **C** | **G** | **A** | **A** | **T** | **A** | **T** | **T** | **A** | **C** | **G** | **C** | **A** | **T** | **T** | **T** | **G** | **T** | **G** | **A** | **C** | **A** | **G** | **A** | **T** | **A** | **A** | **T** | **A** | **T** | **C** | **G** | **G** | **C** | **C** | **A** | **G** | **C** | **A** | **A** | **G** | **T** | **C** | **T** | **T** | **A** | **C** | **A** | **T** | **G** | **T** | **T** | **T** | **T** | **A** | **G** | **A** | **A** | **G** | **A** | **A** | **A** | **A** | **A** | **G** | **C** | **C** | **G** | **C** | **A** | **G** | **A** | **A** | **G** | **G** | **C** | **G** | **T** | **G** | **G** | **A** | **A** | **G** | **T** | **T** | **C** | **G** | **A** | **A** | **T** | **A** | **T** | **T** | **A** | **T** | **A** | **T** | **G** | **A** | **T** | **G** | **C** | **A** | **T** | **T** | **T** | **G** | **G** | **C** | **T** | **C** | **T** | **C** | **A** | **T** | **G** | **G** | **C** | **A** | **C** | **A** | **A** | **A** | **A** | **G** | **C** | **A** | **A** | **A** | **A** | **G** | **A** | **T** | **T** | **T** | **C** | **A** | **A** | **T** | **C** | **G** | **T** | **C** | **T** | **A** | **A** | **T** | **C** | **A** | **A** | **A** | **A** | **A** | **T** | **G** | **G** | **T** | **G** | **G** | **A** | **C** | **A** | **T** | **G** | **T** | **C** | **C** | **A** | **T** | **A** | **C** | **A** | **T** | **T** | **T** | **G** | **T** | **T** | **A** | **C** | **C** | **T** | **C** | **A** | **C** | **A** | **A** | **A** | **A** | **G** | **G** | **C** | **A** | **T** | **T** | **A** | **C** | **T** | **T** | **C** | **G** | **T** | **T** | **T** | **C** | **C** | **G** | **A** | **T** | **T** | **G** | **A** | **A** | **T** | **T** | **A** | **C** | **C** | **A** | **T** | **G** | **A** | **T** | **C** | **A** | **C** | **C** | **G** | **A** | **A** | **A** | **A** | **A** | **T** | **C** | **G** | **T** | **T** | **G** | **T** | **G** | **A** | **T** | **C** | **G** | **A** | **T** | **G** | **G** | **A** | **A** | **A** | **G** | **A** | **T** | **T** | **A** | **G** | **T** | **T** | **A** | **T** | **A** | **C** | **C** | **G** | **G** | **T** | **G** | **G** | **T** | **T** | **T** | **C** | **A** | **A** | **T** | **A** | **T** | **T** | **G** | **C** | **C** | **A** | **A** | **T** | **C** | **A** | **A** | **T** | **A** | **T** | **G** | **T** | **A** | **A** | **A** | **T** | **A** | **C** | **A** | **A** | **C** | **A** | **A** | **A** | **A** | **A** | **A** | **A** | **T** | **T** | **C** | **G** | **G** | **C** | **T** | **A** | **T** | **T** | **G** | **G** | **C** | **G** | **C** | **G** | **A** | **T** | **A** | **C** | **G** | **C** | **A** | **T** | **A** | **T** | **A** | **C** | **G** | **G** | **A** | **T** | **T** | **T** | **T** | **C** | **G** | **G** | **T** | **G** | **C** | **C** | **G** | **C** | **T** | **T** | **C** | **T** | **T** | **C** | **T** | **T** | **T** | **G** | **C** | **T** | **C** | **C** | **A** | **G** | **C** | **T** | **T** | **C** | **G** | **C** | **T** | **T** | **C** | **T** | **T** | **A** | **A** | **C** | **A** | **G** | **A** | **C** | **T** | **G** | **G** | **A** | **A** | **C** | **G** | **T** | **C** | **T** | **C** | **G** | **G** | **T** | **A** | **C** | **C** | **T** | **G** | **A** | **A** | **G** | **A** | **A** | **A** | **A** | **A** | **A** | **A** | **G** | **G** | **T** | **C** | **G** | **G** | **C** | **T** | **A** | **T** | **C** | **A** | **T** | **T** | **T** | **G** | **A** | **A** | **T** | **T** | **A** | **T** | **T** | **T** | **C** | **T** | **T** | **T** | **A** | **A** | **A** | **A** | **A** | **A** | **G** | **C** | **A** | **G** | **A** | **T** | **C** | **G** | **A** | **G** | **A** | **T** | **G** | **A** | **A** | **T** | **C** | **T** | **A** | **A** | **G** | **C** | **T** | **T** | **G** | **C** | **T** | **G** | **A** | **A** | **A** | **C** | **A** | **T** | **C** | **C** | **A** | **T** | **C** | **C** | **A** | **G** | **C** | **T** | **T** | **G** | **T** | **T** | **T** | **C** | **A** | **A** | **G** | **C** | **G** | **G** | **A** | **C** | **C** | **G** | **A** | **A** | **T** | **A** | **A** | **C** | **G** | **A** | **A** | **A** | **G** | **G** | **G** | **A** | **A** | **C** | **A** | **A** | **A** | **T** | **C** | **A** | **A** | **G** | **C** | **T** | **T** | **T** | **C** | **A** | **T** | **T** | **T** | **A** | **T** | **C** | **A** | **A** | **A** | **T** | **T** | **G** | **A** | **T** | **T** | **A** | **C** | **T** | **T** | **C** | **T** | **G** | **C** | **T** | **A** | **A** | **A** | **A** | **A** | **G** | **C** | **G** | **T** | **G** | **T** | **T** | **T** | **G** | **G** | **A** | **T** | **A** | **C** | **A** | **G** | **A** | **C** | **A** | **C** | **C** | **T** | **T** | **A** | **C** | **C** | **T** | **T** | **G** | **T** | **T** | **C** | **C** | **T** | **G** | **A** | **T** | **G** | **A** | **A** | **A** | **G** | **T** | **G** | **T** | **C** | **A** | **T** | **T** | **G** | **C** | **T** | **G** | **C** | **T** | **T** | **T** | **A** | **A** | **A** | **A** | **A** | **T** | **C** | **G** | **C** | **A** | **A** | **C** | **T** | **G** | **C** | **C** | **T** | **C** | **T** | **G** | **G** | **T** | **G** | **T** | **A** | **G** | **A** | **T** | **G** | **T** | **G** | **A** | **A** | **A** | **A** | **T** | **C** | **A** | **T** | **G** | **A** | **T** | **T** | **C** | **C** | **C** | **A** | **A** | **C** | **A** | **A** | **A** | **C** | **C** | **G** | **G** | **A** | **T** | **C** | **A** | **T** | **C** | **C** | **T** | **T** | **T** | **T** | **A** | **T** | **T** | **T** | **A** | **T** | **C** | **G** | **A** | **G** | **C** | **A** | **A** | **C** | **A** | **C** | **A** | **A** | **T** | **A** | **T** | **T** | **A** | **T** | **G** | **C** | **T** | **C** | **G** | **G** | **C** | **A** | **G** | **C** | **T** | **G** | **A** | **T** | **C** | **A** | **A** | **G** | **G** | **A** | **A** | **A** | **A** | **T** | **G** | **T** | **A** | **C** | **A** | **A** | **A** | **T** | **C** | **C** | **T** | **T** | **G** | **T** | **C** | **T** | **A** | **T** | **G** | **A** | **G** | **A** | **A** | **C** | **G** | **G** | **C** | **T** | **T** | **C** | **C** | **T** | **C** | **C** | **A** | **T** | **G** | **C** | **A** | **A** | **A** | **A** | **A** | **C** | **A** | **T** | **T** | **G** | **A** | **T** | **A** | **A** | **T** | **G** | **G** | **A** | **T** | **G** | **A** | **T** | **G** | **A** | **A** | **A** | **T** | **C** | **T** | **G** | **C** | **A** | **T** | **G** | **G** | **T** | **A** | **G** | **G** | **T** | **T** | **C** | **A** | **G** | **C** | **A** | **A** | **A** | **T** | **C** | **A** | **A** | **G** | **A** | **T** | **A** | **T** | **T** | **C** | **G** | **T** | **A** | **G** | **C** | **T** | **A** | **C** | **A** | **G** | **A** | **T** | **T** | **G** | **A** | **A** | **T** | **T** | **T** | **T** | **G** | **A** | **A** | **A** | **C** | **A** | **A** | **G** | **T** | **G** | **C** | **T** | **G** | **T** | **C** | **A** | **T** | **T** | **T** | **A** | **C** | **G** | **A** | **T** | **C** | **C** | **T** | **G** | **A** | **G** | **T** | **T** | **T** | **T** | **T** | **A** | **G** | **A** | **A** | **G** | **A** | **A** | **C** | **T** | **T** | **G** | **C** | **T** | **A** | **C** | **T** | **C** | **A** | **G** | **T** | **T** | **C** | **A** | **A** | **A** | **G** | **A** | **A** | **G** | **A** | **T** | **G** | **A** | **G** | **A** | **C** | **A** | **C** | **A** | **T** | **T** | **G** | **T** | **T** | **C** | **A** | **T** | **C** | **C** | **A** | **T** | **G** | **A** | **C** | **A** | **A** | **C** | **T** | **G** | **A** | **A** | **A** | **C** | **A** | **G** | **T** | **T** | **A** | **A** | **A** | **G** | **A** | **A** | **A** | **T** | **G** | **T** | **C** | **T** | **A** | **A** | **C** | **T** | **G** | **G** | **C** | **T** | **A** | **T** | **T** | **A** | **T** | **T** | **C** | **A** | **A** | **G** | **C** | **A** | **A** | **C** | **A** | **A** | **A** | **T** | **T** | **T** | **C** | **T** | **C** | **G** | **A** | **T** | **T** | **A** | **T** | **T** | **T** | **T** | **C** | **T** | **C** | **C** | **A** | **A** | **T** | **C** | **C** | **T** | **A** | **-** | **-** | **-** |
| **N60357F** | **-** | **-** | **-** | **-** | **-** | **-** | **-** | **-** | **-** | **-** | **-** | **-** | **-** | **-** | **-** | **-** | **-** | **-** | **-** | **-** | **-** | **-** | **-** | **-** | **-** | **-** | **-** | **-** | **-** | **-** | **-** | **-** | **-** | **-** | **-** | **-** | **-** | **-** | **-** | **-** | **-** | **-** | **-** | **-** | **-** | **-** | **-** | **-** | **-** | **-** | **-** | **-** | **-** | **-** | **-** | **-** | **-** | **-** | **-** | **-** | **-** | **-** | **-** | **-** | **-** | **-** | **-** | **-** | **-** | **A** | **A** | **A** | **C** | **C** | **C** | **C** | **G** | **A** | **G** | **A** | **T** | **G** | **T** | **A** | **G** | **C** | **A** | **G** | **C** | **C** | **A** | **T** | **T** | **T** | **G** | **G** | **G** | **C** | **A** | **T** | **G** | **G** | **C** | **T** | **C** | **T** | **T** | **A** | **G** | **T** | **A** | **T** | **T** | **G** | **T** | **T** | **T** | **G** | **C** | **C** | **C** | **T** | **T** | **C** | **C** | **T** | **G** | **G** | **G** | **G** | **T** | **G** | **G** | **G** | **C** | **T** | **T** | **T** | **G** | **T** | **A** | **T** | **T** | **A** | **T** | **A** | **T** | **T** | **T** | **A** | **T** | **T** | **T** | **T** | **T** | **C** | **G** | **G** | **A** | **C** | **G** | **T** | **G** | **G** | **A** | **T** | **T** | **A** | **A** | **C** | **G** | **G** | **A** | **T** | **A** | **A** | **G** | **A** | **A** | **A** | **A** | **A** | **A** | **T** | **T** | **C** | **T** | **A** | **T** | **C** | **T** | **C** | **C** | **G** | **A** | **C** | **A** | **A** | **A** | **G** | **T** | **G** | **A** | **C** | **T** | **T** | **G** | **A** | **A** | **A** | **G** | **A** | **A** | **T** | **T** | **A** | **G** | **A** | **A** | **A** | **A** | **C** | **T** | **T** | **T** | **C** | **A** | **G** | **T** | **C** | **T** | **T** | **T** | **T** | **A** | **G** | **A** | **G** | **G** | **A** | **G** | **A** | **T** | **A** | **C** | **C** | **A** | **T** | **T** | **G** | **A** | **A** | **C** | **A** | **T** | **T** | **A** | **C** | **G** | **A** | **T** | **C** | **C** | **T** | **G** | **A** | **C** | **A** | **T** | **G** | **G** | **G** | **C** | **G** | **A** | **T** | **A** | **A** | **A** | **G** | **A** | **C** | **A** | **A** | **A** | **C** | **A** | **G** | **C** | **A** | **A** | **T** | **T** | **T** | **G** | **T** | **T** | **G** | **A** | **C** | **T** | **T** | **C** | **T** | **T** | **C** | **T** | **C** | **T** | **T** | **C** | **A** | **T** | **T** | **A** | **A** | **A** | **T** | **C** | **G** | **T** | **A** | **T** | **G** | **C** | **C** | **G** | **C** | **T** | **G** | **A** | **C** | **A** | **A** | **T** | **A** | **A** | **T** | **G** | **A** | **A** | **T** | **T** | **C** | **T** | **G** | **T** | **C** | **A** | **C** | **T** | **C** | **T** | **T** | **C** | **T** | **C** | **A** | **C** | **A** | **G** | **A** | **C** | **G** | **G** | **A** | **C** | **A** | **A** | **G** | **A** | **G** | **A** | **A** | **A** | **T** | **T** | **G** | **G** | **A** | **T** | **T** | **C** | **A** | **C** | **T** | **G** | **C** | **T** | **T** | **C** | **A** | **A** | **G** | **A** | **T** | **C** | **T** | **A** | **A** | **A** | **A** | **A** | **A** | **A** | **G** | **C** | **C** | **A** | **A** | **A** | **C** | **A** | **T** | **T** | **C** | **G** | **A** | **T** | **C** | **C** | **A** | **T** | **A** | **T** | **C** | **G** | **A** | **A** | **T** | **A** | **T** | **T** | **A** | **C** | **G** | **C** | **A** | **T** | **T** | **T** | **G** | **T** | **G** | **A** | **C** | **A** | **G** | **A** | **T** | **A** | **A** | **T** | **A** | **T** | **C** | **G** | **G** | **C** | **C** | **A** | **G** | **C** | **A** | **A** | **G** | **T** | **C** | **T** | **T** | **A** | **C** | **A** | **T** | **G** | **T** | **T** | **T** | **T** | **A** | **G** | **A** | **A** | **G** | **A** | **A** | **A** | **A** | **A** | **G** | **C** | **C** | **G** | **C** | **A** | **G** | **A** | **A** | **G** | **G** | **C** | **G** | **T** | **G** | **G** | **A** | **A** | **G** | **T** | **T** | **C** | **G** | **A** | **A** | **T** | **A** | **T** | **T** | **A** | **T** | **A** | **T** | **G** | **A** | **T** | **G** | **C** | **A** | **T** | **T** | **T** | **G** | **G** | **C** | **T** | **C** | **T** | **C** | **A** | **T** | **G** | **G** | **C** | **A** | **C** | **A** | **A** | **A** | **A** | **G** | **C** | **A** | **A** | **A** | **A** | **G** | **A** | **T** | **T** | **T** | **C** | **A** | **A** | **T** | **C** | **G** | **T** | **C** | **T** | **A** | **A** | **T** | **C** | **A** | **A** | **A** | **A** | **A** | **T** | **G** | **G** | **T** | **G** | **G** | **A** | **C** | **A** | **T** | **G** | **T** | **C** | **C** | **A** | **T** | **A** | **C** | **A** | **T** | **T** | **T** | **G** | **T** | **T** | **A** | **C** | **C** | **T** | **C** | **A** | **C** | **A** | **A** | **A** | **A** | **G** | **G** | **C** | **A** | **T** | **T** | **A** | **C** | **T** | **T** | **C** | **G** | **T** | **T** | **T** | **C** | **C** | **G** | **A** | **T** | **T** | **G** | **A** | **A** | **T** | **T** | **A** | **C** | **C** | **A** | **T** | **G** | **A** | **T** | **C** | **A** | **C** | **C** | **G** | **A** | **A** | **A** | **A** | **A** | **T** | **C** | **G** | **T** | **T** | **G** | **T** | **G** | **A** | **T** | **C** | **G** | **A** | **T** | **G** | **G** | **A** | **A** | **A** | **G** | **A** | **T** | **T** | **A** | **G** | **T** | **T** | **A** | **T** | **A** | **C** | **C** | **G** | **G** | **T** | **G** | **G** | **T** | **T** | **T** | **C** | **A** | **A** | **T** | **A** | **T** | **T** | **G** | **C** | **C** | **A** | **A** | **T** | **C** | **A** | **A** | **T** | **A** | **T** | **G** | **T** | **A** | **A** | **A** | **T** | **A** | **C** | **A** | **A** | **C** | **A** | **A** | **A** | **A** | **A** | **A** | **A** | **T** | **T** | **C** | **G** | **G** | **C** | **T** | **A** | **T** | **T** | **G** | **G** | **C** | **G** | **C** | **G** | **A** | **T** | **A** | **C** | **G** | **C** | **A** | **T** | **A** | **T** | **A** | **C** | **G** | **G** | **A** | **T** | **T** | **T** | **T** | **C** | **G** | **G** | **T** | **G** | **C** | **C** | **G** | **C** | **T** | **T** | **C** | **T** | **T** | **C** | **T** | **T** | **T** | **G** | **C** | **T** | **C** | **C** | **A** | **G** | **C** | **T** | **T** | **C** | **G** | **C** | **T** | **T** | **C** | **T** | **T** | **A** | **A** | **C** | **A** | **G** | **A** | **C** | **T** | **G** | **G** | **A** | **A** | **C** | **G** | **T** | **C** | **T** | **C** | **G** | **G** | **T** | **A** | **C** | **C** | **T** | **G** | **A** | **A** | **G** | **A** | **A** | **A** | **A** | **A** | **A** | **A** | **G** | **G** | **T** | **C** | **G** | **G** | **C** | **T** | **A** | **T** | **C** | **A** | **T** | **T** | **T** | **G** | **A** | **A** | **T** | **T** | **A** | **T** | **T** | **T** | **C** | **T** | **T** | **T** | **A** | **A** | **A** | **A** | **A** | **A** | **G** | **C** | **A** | **G** | **A** | **T** | **C** | **G** | **A** | **G** | **A** | **T** | **G** | **A** | **A** | **T** | **C** | **T** | **A** | **A** | **G** | **C** | **T** | **T** | **G** | **C** | **T** | **G** | **A** | **T** | **A** | **C** | **A** | **T** | **C** | **C** | **A** | **T** | **C** | **C** | **A** | **G** | **C** | **T** | **T** | **G** | **T** | **T** | **T** | **C** | **A** | **A** | **G** | **C** | **G** | **G** | **A** | **C** | **C** | **G** | **A** | **A** | **T** | **A** | **A** | **C** | **G** | **A** | **A** | **A** | **G** | **G** | **G** | **A** | **A** | **C** | **A** | **A** | **A** | **T** | **C** | **A** | **A** | **G** | **C** | **T** | **T** | **T** | **C** | **A** | **T** | **T** | **T** | **A** | **T** | **C** | **A** | **A** | **A** | **T** | **T** | **G** | **A** | **T** | **T** | **A** | **C** | **T** | **T** | **C** | **T** | **G** | **C** | **T** | **A** | **A** | **A** | **A** | **A** | **A** | **C** | **G** | **T** | **G** | **T** | **T** | **T** | **G** | **G** | **A** | **T** | **A** | **C** | **A** | **G** | **A** | **C** | **A** | **C** | **C** | **T** | **T** | **A** | **C** | **C** | **T** | **T** | **G** | **T** | **T** | **C** | **C** | **T** | **G** | **A** | **T** | **G** | **A** | **A** | **A** | **G** | **T** | **G** | **T** | **C** | **A** | **T** | **T** | **G** | **C** | **T** | **G** | **C** | **T** | **T** | **T** | **A** | **A** | **A** | **A** | **A** | **T** | **C** | **G** | **C** | **A** | **A** | **C** | **T** | **G** | **C** | **C** | **T** | **C** | **T** | **G** | **G** | **T** | **G** | **T** | **A** | **G** | **A** | **T** | **G** | **T** | **G** | **A** | **A** | **A** | **A** | **T** | **C** | **A** | **T** | **G** | **A** | **T** | **T** | **C** | **C** | **C** | **A** | **A** | **C** | **A** | **A** | **A** | **C** | **C** | **G** | **G** | **A** | **T** | **C** | **A** | **T** | **C** | **C** | **T** | **T** | **T** | **T** | **A** | **T** | **T** | **T** | **A** | **T** | **C** | **G** | **A** | **G** | **C** | **A** | **A** | **C** | **A** | **C** | **A** | **A** | **T** | **A** | **T** | **T** | **A** | **T** | **G** | **C** | **T** | **C** | **G** | **G** | **C** | **A** | **G** | **C** | **T** | **G** | **A** | **T** | **C** | **A** | **A** | **G** | **G** | **A** | **A** | **A** | **A** | **T** | **G** | **T** | **A** | **C** | **A** | **A** | **A** | **T** | **C** | **C** | **T** | **T** | **G** | **T** | **C** | **T** | **A** | **T** | **G** | **A** | **G** | **A** | **A** | **C** | **G** | **G** | **C** | **T** | **T** | **C** | **C** | **T** | **C** | **C** | **A** | **T** | **G** | **C** | **A** | **A** | **A** | **A** | **A** | **C** | **A** | **T** | **T** | **G** | **A** | **T** | **A** | **A** | **T** | **G** | **G** | **A** | **T** | **G** | **A** | **T** | **G** | **A** | **A** | **A** | **T** | **C** | **T** | **G** | **C** | **A** | **T** | **G** | **G** | **T** | **A** | **G** | **G** | **T** | **T** | **C** | **A** | **G** | **C** | **A** | **A** | **A** | **T** | **C** | **A** | **A** | **G** | **A** | **T** | **A** | **T** | **T** | **C** | **G** | **T** | **A** | **G** | **C** | **T** | **A** | **C** | **C** | **G** | **A** | **T** | **T** | **G** | **A** | **A** | **T** | **T** | **T** | **T** | **G** | **A** | **A** | **A** | **C** | **A** | **A** | **G** | **T** | **G** | **C** | **T** | **G** | **T** | **C** | **A** | **T** | **T** | **T** | **A** | **C** | **G** | **A** | **T** | **C** | **C** | **T** | **G** | **A** | **G** | **T** | **T** | **T** | **T** | **T** | **A** | **G** | **A** | **A** | **G** | **A** | **A** | **C** | **T** | **T** | **G** | **C** | **T** | **A** | **C** | **T** | **C** | **A** | **G** | **T** | **T** | **C** | **A** | **A** | **A** | **G** | **A** | **A** | **G** | **A** | **T** | **G** | **A** | **G** | **A** | **C** | **A** | **T** | **A** | **T** | **T** | **G** | **T** | **T** | **C** | **A** | **T** | **C** | **C** | **A** | **T** | **G** | **A** | **C** | **A** | **A** | **C** | **T** | **G** | **A** | **A** | **A** | **C** | **A** | **G** | **T** | **C** | **A** | **A** | **G** | **G** | **A** | **A** | **A** | **T** | **G** | **T** | **C** | **T** | **A** | **A** | **C** | **T** | **G** | **G** | **C** | **T** | **A** | **T** | **T** | **A** | **T** | **T** | **C** | **A** | **A** | **G** | **C** | **A** | **A** | **C** | **A** | **A** | **A** | **T** | **T** | **T** | **C** | **T** | **C** | **G** | **A** | **T** | **T** | **A** | **T** | **T** | **T** | **T** | **C** | **T** | **C** | **C** | **A** | **A** | **T** | **C** | **C** | **T** | **A** | **-** | **-** | **-** |
| **N60363F** | **-** | **-** | **-** | **-** | **-** | **-** | **-** | **-** | **-** | **-** | **-** | **-** | **-** | **-** | **-** | **-** | **-** | **-** | **-** | **-** | **-** | **-** | **-** | **-** | **-** | **-** | **-** | **-** | **-** | **-** | **-** | **-** | **-** | **-** | **-** | **-** | **-** | **-** | **-** | **-** | **-** | **-** | **-** | **-** | **-** | **-** | **-** | **-** | **-** | **-** | **-** | **-** | **-** | **-** | **-** | **-** | **-** | **-** | **-** | **-** | **-** | **-** | **-** | **-** | **-** | **-** | **-** | **-** | **-** | **A** | **A** | **A** | **C** | **C** | **C** | **C** | **G** | **A** | **G** | **A** | **T** | **G** | **T** | **A** | **G** | **C** | **A** | **G** | **C** | **C** | **A** | **T** | **T** | **T** | **G** | **G** | **G** | **C** | **A** | **T** | **G** | **G** | **C** | **T** | **C** | **T** | **T** | **A** | **G** | **T** | **A** | **T** | **T** | **G** | **T** | **T** | **T** | **G** | **C** | **C** | **C** | **T** | **T** | **C** | **C** | **T** | **G** | **G** | **G** | **G** | **T** | **G** | **G** | **G** | **T** | **T** | **T** | **T** | **G** | **T** | **A** | **T** | **T** | **A** | **T** | **A** | **T** | **T** | **T** | **A** | **T** | **T** | **T** | **T** | **T** | **C** | **G** | **G** | **A** | **C** | **G** | **T** | **G** | **G** | **A** | **T** | **T** | **A** | **A** | **C** | **G** | **G** | **A** | **T** | **A** | **A** | **G** | **A** | **A** | **A** | **A** | **A** | **A** | **T** | **T** | **C** | **T** | **A** | **T** | **C** | **T** | **C** | **C** | **G** | **A** | **C** | **A** | **A** | **A** | **G** | **T** | **G** | **A** | **C** | **T** | **T** | **G** | **A** | **A** | **A** | **G** | **A** | **A** | **T** | **T** | **A** | **G** | **A** | **A** | **A** | **A** | **C** | **T** | **T** | **T** | **C** | **A** | **G** | **T** | **C** | **T** | **T** | **T** | **T** | **A** | **G** | **A** | **G** | **G** | **A** | **G** | **A** | **T** | **A** | **C** | **C** | **A** | **T** | **T** | **G** | **A** | **A** | **C** | **A** | **T** | **T** | **A** | **C** | **G** | **A** | **T** | **C** | **C** | **T** | **G** | **A** | **C** | **A** | **T** | **G** | **G** | **G** | **C** | **G** | **A** | **T** | **A** | **A** | **A** | **G** | **A** | **C** | **A** | **A** | **A** | **C** | **A** | **G** | **C** | **A** | **A** | **T** | **T** | **T** | **G** | **T** | **C** | **G** | **A** | **C** | **T** | **T** | **C** | **T** | **T** | **C** | **T** | **C** | **T** | **T** | **C** | **A** | **T** | **T** | **A** | **A** | **A** | **T** | **C** | **G** | **T** | **A** | **T** | **G** | **C** | **C** | **G** | **C** | **T** | **G** | **A** | **C** | **A** | **A** | **G** | **A** | **A** | **T** | **G** | **A** | **A** | **T** | **T** | **C** | **T** | **G** | **T** | **C** | **A** | **C** | **T** | **C** | **T** | **T** | **C** | **T** | **C** | **A** | **C** | **A** | **G** | **A** | **C** | **G** | **G** | **A** | **C** | **A** | **A** | **G** | **A** | **G** | **A** | **A** | **A** | **T** | **T** | **G** | **G** | **A** | **T** | **T** | **C** | **A** | **C** | **T** | **G** | **C** | **T** | **T** | **C** | **A** | **A** | **G** | **A** | **T** | **C** | **T** | **A** | **A** | **A** | **A** | **A** | **A** | **A** | **G** | **C** | **C** | **A** | **A** | **A** | **C** | **A** | **T** | **T** | **C** | **G** | **A** | **T** | **C** | **C** | **A** | **T** | **A** | **T** | **C** | **G** | **A** | **A** | **T** | **A** | **T** | **T** | **A** | **C** | **G** | **C** | **A** | **T** | **T** | **T** | **G** | **T** | **G** | **A** | **C** | **A** | **G** | **A** | **T** | **A** | **A** | **T** | **A** | **T** | **C** | **G** | **G** | **C** | **C** | **A** | **G** | **C** | **A** | **A** | **G** | **T** | **C** | **T** | **T** | **A** | **C** | **A** | **T** | **G** | **T** | **T** | **T** | **T** | **A** | **G** | **A** | **A** | **G** | **A** | **A** | **A** | **A** | **A** | **G** | **C** | **C** | **G** | **C** | **A** | **G** | **A** | **A** | **G** | **G** | **C** | **G** | **T** | **G** | **G** | **A** | **A** | **G** | **T** | **T** | **C** | **G** | **A** | **A** | **T** | **A** | **T** | **T** | **A** | **T** | **A** | **T** | **G** | **A** | **T** | **G** | **C** | **A** | **T** | **T** | **T** | **G** | **G** | **C** | **T** | **C** | **T** | **C** | **A** | **T** | **G** | **G** | **C** | **A** | **C** | **A** | **A** | **A** | **A** | **G** | **C** | **A** | **A** | **A** | **A** | **G** | **A** | **T** | **T** | **T** | **C** | **A** | **A** | **T** | **C** | **G** | **T** | **C** | **T** | **A** | **A** | **T** | **C** | **A** | **A** | **A** | **A** | **A** | **T** | **G** | **G** | **T** | **G** | **G** | **A** | **C** | **A** | **T** | **G** | **T** | **C** | **C** | **A** | **T** | **A** | **C** | **A** | **T** | **T** | **T** | **G** | **T** | **T** | **A** | **C** | **C** | **T** | **C** | **A** | **C** | **A** | **A** | **A** | **A** | **G** | **G** | **C** | **A** | **T** | **T** | **A** | **C** | **T** | **T** | **C** | **G** | **T** | **T** | **T** | **C** | **C** | **G** | **A** | **T** | **T** | **G** | **A** | **A** | **T** | **T** | **A** | **C** | **C** | **A** | **T** | **G** | **A** | **T** | **C** | **A** | **C** | **C** | **G** | **A** | **A** | **A** | **A** | **A** | **T** | **C** | **G** | **T** | **T** | **G** | **T** | **G** | **A** | **T** | **C** | **G** | **A** | **T** | **G** | **G** | **A** | **A** | **A** | **G** | **A** | **T** | **T** | **A** | **G** | **T** | **T** | **A** | **T** | **A** | **C** | **C** | **G** | **G** | **T** | **G** | **G** | **T** | **T** | **T** | **C** | **A** | **A** | **T** | **A** | **T** | **T** | **G** | **C** | **C** | **A** | **A** | **T** | **C** | **A** | **A** | **T** | **A** | **T** | **G** | **T** | **A** | **A** | **A** | **T** | **A** | **C** | **A** | **A** | **C** | **A** | **A** | **A** | **A** | **A** | **A** | **A** | **T** | **T** | **C** | **G** | **G** | **C** | **T** | **A** | **T** | **T** | **G** | **G** | **C** | **G** | **C** | **G** | **A** | **T** | **A** | **C** | **G** | **C** | **A** | **T** | **A** | **T** | **A** | **C** | **G** | **G** | **A** | **T** | **T** | **T** | **T** | **C** | **G** | **G** | **T** | **G** | **C** | **C** | **G** | **C** | **T** | **T** | **C** | **T** | **T** | **C** | **T** | **T** | **T** | **G** | **C** | **T** | **C** | **C** | **A** | **G** | **C** | **T** | **T** | **C** | **G** | **C** | **T** | **T** | **C** | **T** | **T** | **A** | **A** | **C** | **A** | **G** | **A** | **C** | **T** | **G** | **G** | **A** | **A** | **C** | **G** | **T** | **C** | **T** | **C** | **G** | **G** | **T** | **A** | **C** | **C** | **T** | **G** | **A** | **A** | **G** | **A** | **A** | **A** | **A** | **A** | **A** | **A** | **G** | **G** | **T** | **C** | **G** | **G** | **C** | **T** | **A** | **T** | **C** | **A** | **T** | **T** | **T** | **G** | **A** | **A** | **T** | **T** | **A** | **T** | **T** | **T** | **C** | **T** | **T** | **T** | **A** | **A** | **A** | **A** | **A** | **A** | **G** | **C** | **A** | **G** | **A** | **T** | **C** | **G** | **A** | **G** | **A** | **T** | **G** | **A** | **A** | **T** | **C** | **T** | **A** | **A** | **G** | **C** | **T** | **T** | **G** | **C** | **T** | **G** | **A** | **T** | **A** | **C** | **A** | **T** | **C** | **C** | **A** | **T** | **C** | **C** | **A** | **G** | **C** | **T** | **T** | **G** | **T** | **T** | **T** | **C** | **A** | **A** | **G** | **C** | **G** | **G** | **A** | **C** | **C** | **G** | **A** | **A** | **T** | **A** | **A** | **C** | **G** | **A** | **A** | **A** | **G** | **G** | **G** | **A** | **A** | **C** | **A** | **A** | **A** | **T** | **C** | **A** | **A** | **G** | **C** | **T** | **T** | **T** | **C** | **A** | **T** | **T** | **T** | **A** | **T** | **C** | **A** | **A** | **A** | **T** | **T** | **G** | **A** | **T** | **T** | **A** | **C** | **T** | **T** | **C** | **T** | **G** | **C** | **T** | **A** | **A** | **A** | **A** | **A** | **A** | **C** | **G** | **T** | **G** | **T** | **T** | **T** | **G** | **G** | **A** | **T** | **A** | **C** | **A** | **G** | **A** | **C** | **A** | **C** | **C** | **T** | **T** | **A** | **C** | **C** | **T** | **T** | **G** | **T** | **T** | **C** | **C** | **T** | **G** | **A** | **T** | **G** | **A** | **A** | **A** | **G** | **T** | **G** | **T** | **C** | **A** | **T** | **T** | **G** | **C** | **T** | **G** | **C** | **T** | **T** | **T** | **A** | **A** | **A** | **A** | **A** | **T** | **C** | **G** | **C** | **A** | **A** | **C** | **T** | **G** | **C** | **C** | **T** | **C** | **T** | **G** | **G** | **T** | **G** | **T** | **A** | **G** | **A** | **T** | **G** | **T** | **G** | **A** | **A** | **A** | **A** | **T** | **C** | **A** | **T** | **G** | **A** | **T** | **T** | **C** | **C** | **C** | **A** | **A** | **C** | **A** | **A** | **A** | **C** | **C** | **G** | **G** | **A** | **T** | **C** | **A** | **T** | **C** | **C** | **T** | **T** | **T** | **T** | **A** | **T** | **T** | **T** | **A** | **T** | **C** | **G** | **A** | **G** | **C** | **A** | **A** | **C** | **A** | **C** | **A** | **A** | **T** | **A** | **T** | **T** | **A** | **T** | **G** | **C** | **T** | **C** | **G** | **G** | **C** | **A** | **G** | **C** | **T** | **G** | **A** | **T** | **C** | **A** | **A** | **G** | **G** | **A** | **A** | **A** | **A** | **T** | **G** | **T** | **A** | **C** | **A** | **A** | **A** | **T** | **C** | **C** | **T** | **T** | **G** | **T** | **C** | **T** | **A** | **T** | **G** | **A** | **G** | **A** | **A** | **C** | **G** | **G** | **C** | **T** | **T** | **C** | **C** | **T** | **C** | **C** | **A** | **T** | **G** | **C** | **A** | **A** | **A** | **A** | **A** | **C** | **A** | **T** | **T** | **G** | **A** | **T** | **A** | **A** | **T** | **G** | **G** | **A** | **T** | **G** | **A** | **T** | **G** | **A** | **A** | **A** | **T** | **C** | **T** | **G** | **C** | **A** | **T** | **G** | **G** | **T** | **A** | **G** | **G** | **T** | **T** | **C** | **A** | **G** | **C** | **A** | **A** | **A** | **T** | **C** | **A** | **A** | **G** | **A** | **T** | **A** | **T** | **T** | **C** | **G** | **T** | **A** | **G** | **C** | **T** | **A** | **C** | **C** | **G** | **A** | **T** | **T** | **G** | **A** | **A** | **T** | **T** | **T** | **T** | **G** | **A** | **A** | **A** | **C** | **A** | **A** | **G** | **T** | **G** | **C** | **T** | **G** | **T** | **C** | **A** | **T** | **T** | **T** | **A** | **C** | **G** | **A** | **T** | **C** | **C** | **T** | **G** | **A** | **G** | **T** | **T** | **T** | **T** | **T** | **A** | **G** | **A** | **A** | **G** | **A** | **A** | **C** | **T** | **T** | **G** | **C** | **T** | **A** | **C** | **T** | **C** | **A** | **G** | **T** | **T** | **C** | **A** | **A** | **A** | **G** | **A** | **A** | **G** | **A** | **T** | **G** | **A** | **G** | **A** | **C** | **A** | **C** | **A** | **T** | **T** | **G** | **T** | **T** | **C** | **A** | **T** | **C** | **C** | **A** | **T** | **G** | **A** | **C** | **A** | **A** | **C** | **T** | **G** | **A** | **A** | **A** | **C** | **A** | **G** | **T** | **C** | **A** | **A** | **G** | **G** | **A** | **A** | **A** | **T** | **G** | **T** | **C** | **T** | **A** | **A** | **C** | **T** | **G** | **G** | **C** | **T** | **A** | **T** | **T** | **A** | **T** | **T** | **C** | **A** | **A** | **G** | **C** | **A** | **A** | **C** | **A** | **A** | **A** | **T** | **T** | **T** | **C** | **T** | **C** | **G** | **A** | **T** | **T** | **A** | **T** | **T** | **T** | **T** | **C** | **T** | **C** | **C** | **A** | **A** | **T** | **C** | **C** | **T** | **A** | **-** | **-** | **-** |
| **N59517F** | **-** | **-** | **-** | **-** | **-** | **-** | **-** | **-** | **-** | **-** | **-** | **-** | **-** | **-** | **-** | **-** | **-** | **-** | **-** | **-** | **-** | **-** | **-** | **-** | **-** | **-** | **-** | **-** | **-** | **-** | **-** | **-** | **-** | **-** | **-** | **-** | **-** | **-** | **-** | **-** | **-** | **-** | **-** | **-** | **-** | **-** | **-** | **-** | **-** | **-** | **-** | **-** | **-** | **-** | **-** | **-** | **-** | **-** | **-** | **-** | **-** | **-** | **-** | **-** | **-** | **-** | **-** | **-** | **-** | **A** | **A** | **A** | **C** | **C** | **C** | **C** | **G** | **A** | **G** | **A** | **T** | **G** | **T** | **A** | **G** | **C** | **A** | **G** | **C** | **C** | **A** | **T** | **T** | **T** | **G** | **G** | **G** | **C** | **A** | **T** | **G** | **G** | **C** | **T** | **C** | **T** | **T** | **A** | **G** | **T** | **A** | **T** | **T** | **A** | **T** | **T** | **T** | **G** | **C** | **C** | **C** | **T** | **T** | **C** | **C** | **T** | **G** | **G** | **G** | **G** | **T** | **G** | **G** | **G** | **C** | **T** | **T** | **T** | **G** | **T** | **A** | **T** | **T** | **A** | **T** | **A** | **T** | **T** | **T** | **A** | **T** | **T** | **T** | **T** | **T** | **C** | **G** | **G** | **A** | **C** | **G** | **T** | **G** | **G** | **A** | **T** | **T** | **A** | **A** | **C** | **G** | **G** | **A** | **T** | **A** | **A** | **G** | **A** | **A** | **A** | **A** | **A** | **A** | **T** | **T** | **C** | **T** | **A** | **T** | **C** | **T** | **C** | **C** | **G** | **A** | **C** | **A** | **A** | **A** | **G** | **T** | **G** | **A** | **C** | **T** | **T** | **G** | **A** | **A** | **A** | **G** | **A** | **A** | **T** | **T** | **A** | **G** | **A** | **A** | **A** | **A** | **C** | **T** | **T** | **T** | **C** | **A** | **G** | **T** | **C** | **T** | **T** | **T** | **T** | **A** | **G** | **A** | **G** | **G** | **A** | **G** | **A** | **T** | **A** | **C** | **C** | **A** | **T** | **T** | **G** | **A** | **A** | **C** | **A** | **T** | **T** | **A** | **C** | **G** | **A** | **T** | **C** | **C** | **T** | **G** | **A** | **C** | **A** | **T** | **G** | **G** | **G** | **C** | **G** | **A** | **T** | **A** | **A** | **A** | **G** | **A** | **C** | **A** | **A** | **A** | **C** | **A** | **G** | **C** | **A** | **A** | **T** | **T** | **T** | **G** | **T** | **T** | **G** | **A** | **C** | **T** | **T** | **C** | **T** | **T** | **C** | **T** | **C** | **T** | **T** | **C** | **A** | **T** | **T** | **A** | **A** | **A** | **T** | **C** | **G** | **T** | **A** | **T** | **G** | **C** | **C** | **G** | **C** | **T** | **G** | **A** | **C** | **A** | **A** | **G** | **A** | **A** | **T** | **G** | **A** | **A** | **T** | **T** | **C** | **T** | **G** | **T** | **C** | **A** | **C** | **T** | **C** | **T** | **T** | **C** | **T** | **C** | **A** | **C** | **A** | **G** | **A** | **C** | **G** | **G** | **A** | **C** | **A** | **A** | **G** | **A** | **G** | **A** | **A** | **A** | **T** | **T** | **G** | **G** | **A** | **T** | **T** | **C** | **A** | **C** | **T** | **G** | **C** | **T** | **T** | **C** | **A** | **A** | **G** | **A** | **T** | **C** | **T** | **A** | **A** | **A** | **A** | **A** | **A** | **A** | **G** | **C** | **C** | **A** | **A** | **A** | **C** | **A** | **T** | **T** | **C** | **G** | **A** | **T** | **C** | **C** | **A** | **T** | **A** | **T** | **C** | **G** | **A** | **A** | **T** | **A** | **T** | **T** | **A** | **C** | **G** | **C** | **A** | **T** | **T** | **T** | **G** | **T** | **G** | **A** | **C** | **A** | **G** | **A** | **T** | **A** | **A** | **T** | **A** | **T** | **C** | **G** | **G** | **C** | **C** | **A** | **G** | **C** | **A** | **A** | **G** | **T** | **C** | **T** | **T** | **A** | **C** | **A** | **T** | **G** | **T** | **T** | **T** | **T** | **A** | **G** | **A** | **A** | **G** | **A** | **A** | **A** | **A** | **A** | **G** | **C** | **C** | **G** | **C** | **A** | **G** | **A** | **A** | **G** | **G** | **C** | **G** | **T** | **G** | **G** | **A** | **A** | **G** | **T** | **T** | **C** | **G** | **A** | **A** | **T** | **A** | **T** | **T** | **A** | **T** | **A** | **T** | **G** | **A** | **T** | **G** | **C** | **A** | **T** | **T** | **T** | **G** | **G** | **C** | **T** | **C** | **T** | **C** | **A** | **T** | **G** | **G** | **C** | **A** | **C** | **A** | **A** | **A** | **A** | **G** | **C** | **A** | **A** | **A** | **A** | **G** | **A** | **T** | **T** | **T** | **C** | **A** | **A** | **T** | **C** | **G** | **T** | **C** | **T** | **A** | **A** | **T** | **C** | **A** | **A** | **A** | **A** | **A** | **T** | **G** | **G** | **T** | **G** | **G** | **A** | **C** | **A** | **T** | **G** | **T** | **C** | **C** | **A** | **T** | **A** | **C** | **A** | **T** | **T** | **T** | **G** | **T** | **T** | **A** | **C** | **C** | **T** | **C** | **A** | **C** | **A** | **A** | **A** | **G** | **G** | **G** | **C** | **A** | **T** | **T** | **A** | **C** | **T** | **T** | **C** | **G** | **T** | **T** | **T** | **C** | **C** | **G** | **A** | **T** | **T** | **G** | **A** | **A** | **T** | **T** | **A** | **C** | **C** | **A** | **T** | **G** | **A** | **T** | **C** | **A** | **C** | **C** | **G** | **A** | **A** | **A** | **A** | **A** | **T** | **C** | **G** | **T** | **T** | **G** | **T** | **G** | **A** | **T** | **C** | **G** | **A** | **T** | **G** | **G** | **A** | **A** | **A** | **G** | **A** | **T** | **T** | **A** | **G** | **T** | **T** | **A** | **T** | **A** | **C** | **C** | **G** | **G** | **T** | **G** | **G** | **T** | **T** | **T** | **C** | **A** | **A** | **T** | **A** | **T** | **T** | **G** | **C** | **C** | **A** | **A** | **T** | **C** | **A** | **A** | **T** | **A** | **T** | **G** | **T** | **A** | **A** | **A** | **T** | **A** | **C** | **A** | **A** | **C** | **A** | **A** | **A** | **A** | **A** | **A** | **A** | **T** | **T** | **C** | **G** | **G** | **C** | **T** | **A** | **T** | **T** | **G** | **G** | **C** | **G** | **C** | **G** | **A** | **T** | **A** | **C** | **G** | **C** | **A** | **T** | **A** | **T** | **A** | **C** | **G** | **G** | **A** | **T** | **T** | **T** | **T** | **C** | **G** | **G** | **T** | **G** | **C** | **C** | **G** | **C** | **T** | **T** | **C** | **T** | **T** | **C** | **T** | **T** | **T** | **G** | **C** | **T** | **C** | **C** | **A** | **G** | **C** | **T** | **T** | **C** | **G** | **C** | **T** | **T** | **C** | **T** | **T** | **A** | **A** | **C** | **A** | **G** | **A** | **C** | **T** | **G** | **G** | **A** | **A** | **C** | **G** | **T** | **C** | **T** | **C** | **G** | **G** | **T** | **A** | **C** | **C** | **T** | **G** | **A** | **A** | **G** | **A** | **A** | **A** | **A** | **A** | **A** | **A** | **G** | **G** | **T** | **C** | **G** | **G** | **C** | **T** | **A** | **T** | **C** | **A** | **T** | **T** | **T** | **G** | **A** | **A** | **T** | **T** | **A** | **T** | **T** | **T** | **C** | **T** | **T** | **T** | **A** | **A** | **A** | **A** | **A** | **A** | **G** | **C** | **A** | **G** | **A** | **T** | **C** | **G** | **A** | **G** | **A** | **T** | **G** | **A** | **A** | **T** | **C** | **T** | **A** | **A** | **G** | **C** | **T** | **T** | **G** | **C** | **T** | **G** | **A** | **T** | **A** | **C** | **A** | **T** | **C** | **C** | **A** | **T** | **C** | **C** | **A** | **G** | **C** | **T** | **T** | **G** | **T** | **T** | **T** | **C** | **A** | **A** | **G** | **C** | **G** | **G** | **A** | **C** | **C** | **G** | **A** | **A** | **T** | **A** | **A** | **C** | **G** | **A** | **A** | **A** | **G** | **G** | **G** | **A** | **A** | **C** | **A** | **A** | **A** | **T** | **C** | **A** | **A** | **G** | **C** | **T** | **T** | **T** | **C** | **A** | **T** | **T** | **T** | **A** | **T** | **C** | **A** | **A** | **A** | **T** | **T** | **G** | **A** | **T** | **T** | **A** | **C** | **T** | **T** | **C** | **T** | **G** | **C** | **T** | **A** | **A** | **A** | **A** | **A** | **A** | **C** | **G** | **T** | **G** | **T** | **T** | **T** | **G** | **G** | **A** | **T** | **A** | **C** | **A** | **G** | **A** | **C** | **A** | **C** | **C** | **T** | **T** | **A** | **C** | **C** | **T** | **T** | **G** | **T** | **T** | **C** | **C** | **T** | **G** | **A** | **T** | **G** | **A** | **A** | **A** | **G** | **T** | **G** | **T** | **C** | **A** | **T** | **T** | **G** | **C** | **T** | **G** | **C** | **T** | **T** | **T** | **A** | **A** | **A** | **A** | **A** | **T** | **C** | **G** | **C** | **A** | **A** | **C** | **T** | **G** | **C** | **C** | **T** | **C** | **T** | **G** | **G** | **T** | **G** | **T** | **A** | **G** | **A** | **T** | **G** | **T** | **G** | **A** | **A** | **A** | **A** | **T** | **C** | **A** | **T** | **G** | **A** | **T** | **T** | **C** | **C** | **C** | **A** | **A** | **C** | **A** | **A** | **A** | **C** | **C** | **G** | **G** | **A** | **T** | **C** | **A** | **T** | **C** | **C** | **T** | **T** | **T** | **T** | **A** | **T** | **T** | **T** | **A** | **T** | **C** | **G** | **A** | **G** | **C** | **A** | **A** | **C** | **A** | **C** | **A** | **A** | **T** | **A** | **T** | **T** | **A** | **T** | **G** | **C** | **T** | **C** | **G** | **G** | **C** | **A** | **G** | **C** | **T** | **G** | **A** | **T** | **C** | **A** | **A** | **G** | **G** | **A** | **A** | **A** | **A** | **T** | **G** | **T** | **A** | **C** | **A** | **A** | **A** | **T** | **C** | **C** | **T** | **T** | **G** | **T** | **C** | **T** | **A** | **T** | **G** | **A** | **G** | **A** | **A** | **C** | **G** | **G** | **C** | **T** | **T** | **C** | **C** | **T** | **C** | **C** | **A** | **T** | **G** | **C** | **A** | **A** | **A** | **A** | **A** | **C** | **A** | **T** | **T** | **G** | **A** | **T** | **A** | **A** | **T** | **G** | **G** | **A** | **T** | **G** | **A** | **T** | **G** | **A** | **A** | **A** | **T** | **C** | **T** | **G** | **C** | **A** | **T** | **G** | **G** | **T** | **A** | **G** | **G** | **T** | **T** | **C** | **A** | **G** | **C** | **A** | **A** | **A** | **T** | **C** | **A** | **A** | **G** | **A** | **T** | **A** | **T** | **T** | **C** | **G** | **T** | **A** | **G** | **C** | **T** | **A** | **C** | **C** | **G** | **A** | **T** | **T** | **G** | **A** | **A** | **T** | **T** | **T** | **T** | **G** | **A** | **A** | **A** | **C** | **A** | **A** | **G** | **T** | **G** | **C** | **T** | **G** | **T** | **C** | **A** | **T** | **T** | **T** | **A** | **C** | **G** | **A** | **T** | **C** | **C** | **T** | **G** | **A** | **G** | **T** | **T** | **T** | **T** | **T** | **A** | **G** | **A** | **A** | **G** | **A** | **A** | **C** | **T** | **T** | **G** | **C** | **T** | **A** | **C** | **T** | **C** | **A** | **G** | **T** | **T** | **C** | **A** | **A** | **A** | **G** | **A** | **A** | **G** | **A** | **T** | **G** | **A** | **G** | **A** | **C** | **A** | **C** | **A** | **T** | **T** | **G** | **T** | **T** | **C** | **A** | **T** | **C** | **C** | **A** | **T** | **G** | **A** | **C** | **A** | **A** | **C** | **T** | **G** | **A** | **A** | **A** | **C** | **A** | **G** | **T** | **C** | **A** | **A** | **G** | **G** | **A** | **A** | **A** | **T** | **G** | **T** | **C** | **T** | **A** | **A** | **C** | **T** | **G** | **G** | **C** | **T** | **A** | **T** | **T** | **A** | **T** | **T** | **C** | **A** | **A** | **G** | **C** | **A** | **A** | **C** | **A** | **A** | **A** | **T** | **T** | **T** | **C** | **T** | **C** | **G** | **A** | **T** | **T** | **A** | **T** | **T** | **T** | **T** | **C** | **T** | **C** | **C** | **A** | **A** | **T** | **C** | **C** | **T** | **A** | **-** | **-** | **-** |
| **N54546** | **-** | **-** | **-** | **-** | **-** | **-** | **-** | **-** | **-** | **-** | **-** | **-** | **-** | **-** | **-** | **-** | **-** | **-** | **-** | **-** | **-** | **-** | **-** | **-** | **-** | **-** | **-** | **-** | **-** | **-** | **-** | **-** | **-** | **-** | **-** | **-** | **-** | **-** | **-** | **-** | **-** | **-** | **-** | **-** | **-** | **-** | **-** | **-** | **-** | **-** | **-** | **-** | **-** | **-** | **-** | **-** | **-** | **-** | **-** | **-** | **-** | **-** | **-** | **-** | **-** | **-** | **-** | **-** | **-** | **A** | **A** | **A** | **C** | **C** | **C** | **C** | **G** | **A** | **G** | **A** | **T** | **G** | **T** | **A** | **G** | **C** | **A** | **G** | **C** | **C** | **A** | **T** | **T** | **T** | **G** | **G** | **G** | **C** | **A** | **T** | **G** | **G** | **C** | **T** | **C** | **T** | **T** | **A** | **G** | **T** | **A** | **T** | **T** | **G** | **T** | **T** | **T** | **G** | **T** | **C** | **C** | **T** | **T** | **C** | **C** | **T** | **G** | **G** | **G** | **G** | **T** | **G** | **G** | **G** | **C** | **T** | **T** | **T** | **G** | **T** | **A** | **T** | **T** | **A** | **T** | **A** | **T** | **T** | **T** | **A** | **T** | **T** | **T** | **T** | **T** | **C** | **G** | **G** | **A** | **C** | **G** | **T** | **G** | **G** | **A** | **T** | **T** | **A** | **A** | **C** | **G** | **G** | **A** | **T** | **A** | **A** | **G** | **A** | **A** | **A** | **A** | **A** | **A** | **T** | **T** | **C** | **T** | **A** | **T** | **C** | **T** | **C** | **C** | **G** | **A** | **C** | **A** | **A** | **A** | **G** | **T** | **G** | **A** | **C** | **T** | **T** | **G** | **A** | **A** | **A** | **G** | **A** | **A** | **T** | **T** | **A** | **G** | **A** | **A** | **A** | **A** | **C** | **T** | **T** | **T** | **C** | **A** | **G** | **T** | **C** | **T** | **T** | **T** | **T** | **A** | **G** | **A** | **G** | **G** | **A** | **G** | **A** | **T** | **A** | **C** | **C** | **A** | **T** | **T** | **G** | **A** | **A** | **C** | **A** | **T** | **T** | **A** | **C** | **G** | **A** | **T** | **C** | **C** | **T** | **G** | **A** | **C** | **A** | **T** | **G** | **G** | **G** | **C** | **G** | **A** | **T** | **A** | **A** | **A** | **G** | **A** | **C** | **A** | **A** | **A** | **C** | **A** | **G** | **C** | **A** | **A** | **T** | **T** | **T** | **G** | **T** | **T** | **G** | **A** | **C** | **T** | **T** | **C** | **T** | **T** | **C** | **T** | **C** | **T** | **T** | **C** | **A** | **T** | **T** | **A** | **A** | **A** | **T** | **C** | **G** | **T** | **A** | **T** | **G** | **C** | **C** | **G** | **C** | **T** | **G** | **A** | **C** | **A** | **A** | **G** | **A** | **A** | **T** | **G** | **A** | **A** | **T** | **T** | **C** | **T** | **G** | **T** | **C** | **A** | **C** | **T** | **C** | **T** | **T** | **C** | **T** | **C** | **A** | **C** | **A** | **G** | **A** | **C** | **G** | **G** | **A** | **C** | **A** | **A** | **G** | **A** | **G** | **A** | **A** | **A** | **T** | **T** | **G** | **G** | **A** | **T** | **T** | **C** | **A** | **C** | **T** | **G** | **C** | **T** | **T** | **C** | **A** | **A** | **G** | **A** | **T** | **C** | **T** | **A** | **A** | **A** | **A** | **A** | **A** | **A** | **G** | **C** | **C** | **A** | **A** | **A** | **C** | **A** | **T** | **T** | **C** | **G** | **A** | **T** | **C** | **C** | **A** | **T** | **A** | **T** | **C** | **G** | **A** | **A** | **T** | **A** | **T** | **T** | **A** | **C** | **G** | **C** | **A** | **T** | **T** | **T** | **G** | **T** | **G** | **A** | **C** | **A** | **G** | **A** | **T** | **A** | **A** | **T** | **A** | **T** | **C** | **G** | **G** | **C** | **C** | **A** | **G** | **C** | **A** | **A** | **G** | **T** | **C** | **T** | **T** | **A** | **C** | **A** | **T** | **G** | **T** | **T** | **T** | **T** | **A** | **G** | **A** | **A** | **G** | **A** | **A** | **A** | **A** | **A** | **G** | **C** | **C** | **G** | **C** | **A** | **G** | **A** | **A** | **G** | **G** | **C** | **G** | **T** | **G** | **G** | **A** | **A** | **G** | **T** | **T** | **C** | **G** | **A** | **A** | **T** | **A** | **T** | **T** | **A** | **T** | **A** | **T** | **G** | **A** | **T** | **G** | **C** | **A** | **T** | **T** | **T** | **G** | **G** | **C** | **T** | **C** | **T** | **C** | **A** | **T** | **G** | **G** | **C** | **A** | **C** | **A** | **A** | **A** | **A** | **G** | **C** | **A** | **A** | **A** | **A** | **G** | **A** | **T** | **T** | **T** | **C** | **A** | **A** | **T** | **C** | **G** | **T** | **C** | **T** | **A** | **A** | **T** | **C** | **A** | **A** | **A** | **A** | **A** | **T** | **G** | **G** | **T** | **G** | **G** | **A** | **C** | **A** | **T** | **G** | **T** | **C** | **C** | **A** | **T** | **A** | **C** | **A** | **T** | **T** | **T** | **G** | **T** | **T** | **A** | **C** | **C** | **T** | **C** | **A** | **C** | **A** | **A** | **A** | **A** | **G** | **G** | **C** | **A** | **T** | **T** | **A** | **C** | **T** | **T** | **C** | **G** | **T** | **T** | **T** | **C** | **C** | **G** | **A** | **T** | **T** | **G** | **A** | **A** | **T** | **T** | **A** | **C** | **C** | **A** | **T** | **G** | **A** | **T** | **C** | **A** | **C** | **C** | **G** | **A** | **A** | **A** | **A** | **A** | **T** | **C** | **G** | **T** | **T** | **G** | **T** | **G** | **A** | **T** | **C** | **G** | **A** | **T** | **G** | **G** | **A** | **A** | **A** | **G** | **A** | **T** | **T** | **A** | **G** | **T** | **T** | **A** | **T** | **A** | **C** | **C** | **G** | **G** | **T** | **G** | **G** | **T** | **T** | **T** | **C** | **A** | **A** | **T** | **A** | **T** | **T** | **G** | **C** | **C** | **A** | **A** | **T** | **C** | **A** | **A** | **T** | **A** | **T** | **G** | **T** | **A** | **A** | **A** | **T** | **A** | **C** | **A** | **A** | **C** | **A** | **A** | **A** | **A** | **A** | **A** | **A** | **T** | **T** | **C** | **G** | **G** | **C** | **T** | **A** | **T** | **T** | **G** | **G** | **C** | **G** | **C** | **G** | **A** | **T** | **A** | **C** | **G** | **C** | **A** | **T** | **A** | **T** | **A** | **C** | **G** | **G** | **A** | **T** | **T** | **T** | **T** | **C** | **G** | **G** | **T** | **G** | **C** | **C** | **G** | **C** | **T** | **T** | **C** | **T** | **T** | **C** | **T** | **T** | **T** | **G** | **C** | **T** | **C** | **C** | **A** | **G** | **C** | **T** | **T** | **C** | **G** | **C** | **T** | **T** | **C** | **T** | **T** | **A** | **A** | **C** | **A** | **G** | **A** | **C** | **T** | **G** | **G** | **A** | **A** | **C** | **G** | **T** | **C** | **T** | **C** | **G** | **G** | **T** | **A** | **C** | **C** | **T** | **G** | **A** | **A** | **G** | **A** | **A** | **A** | **A** | **A** | **A** | **A** | **G** | **G** | **T** | **C** | **G** | **G** | **C** | **T** | **A** | **T** | **C** | **A** | **T** | **T** | **T** | **G** | **A** | **A** | **T** | **T** | **A** | **T** | **T** | **T** | **C** | **T** | **T** | **T** | **A** | **A** | **A** | **A** | **A** | **A** | **G** | **C** | **A** | **G** | **A** | **T** | **C** | **G** | **A** | **G** | **A** | **T** | **G** | **A** | **A** | **T** | **C** | **T** | **A** | **A** | **G** | **C** | **T** | **T** | **G** | **C** | **T** | **G** | **A** | **T** | **A** | **C** | **A** | **T** | **C** | **C** | **A** | **T** | **C** | **C** | **A** | **G** | **C** | **T** | **T** | **G** | **T** | **T** | **T** | **C** | **A** | **A** | **G** | **C** | **G** | **G** | **A** | **C** | **C** | **G** | **A** | **A** | **T** | **A** | **A** | **C** | **G** | **A** | **A** | **A** | **G** | **G** | **G** | **A** | **A** | **C** | **A** | **A** | **A** | **T** | **C** | **A** | **A** | **G** | **C** | **T** | **T** | **T** | **C** | **A** | **T** | **T** | **T** | **A** | **T** | **C** | **A** | **A** | **A** | **T** | **T** | **G** | **A** | **T** | **T** | **A** | **C** | **T** | **T** | **C** | **T** | **G** | **C** | **T** | **A** | **A** | **A** | **A** | **A** | **A** | **C** | **G** | **T** | **G** | **T** | **T** | **T** | **G** | **G** | **A** | **T** | **A** | **C** | **A** | **G** | **A** | **C** | **A** | **C** | **C** | **T** | **T** | **A** | **C** | **C** | **T** | **T** | **G** | **T** | **T** | **C** | **C** | **T** | **G** | **A** | **T** | **G** | **A** | **A** | **A** | **G** | **T** | **G** | **T** | **C** | **A** | **T** | **T** | **G** | **C** | **T** | **G** | **C** | **T** | **T** | **T** | **A** | **A** | **A** | **A** | **A** | **T** | **C** | **G** | **C** | **A** | **A** | **C** | **T** | **G** | **C** | **C** | **T** | **C** | **T** | **G** | **G** | **T** | **G** | **T** | **A** | **G** | **A** | **T** | **G** | **T** | **G** | **A** | **A** | **A** | **A** | **T** | **C** | **A** | **T** | **G** | **A** | **T** | **T** | **C** | **C** | **C** | **A** | **A** | **C** | **A** | **A** | **A** | **C** | **C** | **G** | **G** | **A** | **T** | **C** | **A** | **T** | **C** | **C** | **T** | **T** | **T** | **T** | **A** | **T** | **T** | **T** | **A** | **T** | **C** | **G** | **A** | **G** | **C** | **A** | **A** | **C** | **A** | **C** | **A** | **A** | **T** | **A** | **T** | **T** | **A** | **T** | **G** | **C** | **T** | **C** | **G** | **G** | **C** | **A** | **G** | **C** | **T** | **G** | **A** | **T** | **C** | **A** | **A** | **G** | **G** | **A** | **A** | **A** | **A** | **T** | **G** | **T** | **A** | **C** | **A** | **A** | **A** | **T** | **C** | **C** | **T** | **T** | **G** | **T** | **C** | **T** | **A** | **T** | **G** | **A** | **G** | **A** | **A** | **C** | **G** | **G** | **C** | **T** | **T** | **C** | **C** | **T** | **C** | **C** | **A** | **T** | **G** | **C** | **A** | **A** | **A** | **A** | **A** | **C** | **A** | **T** | **T** | **G** | **A** | **T** | **A** | **A** | **T** | **G** | **G** | **A** | **T** | **G** | **A** | **T** | **G** | **A** | **A** | **A** | **T** | **C** | **T** | **G** | **C** | **A** | **T** | **G** | **G** | **T** | **A** | **G** | **G** | **T** | **T** | **C** | **A** | **G** | **C** | **A** | **A** | **A** | **T** | **C** | **A** | **A** | **G** | **A** | **T** | **A** | **T** | **T** | **C** | **G** | **T** | **A** | **G** | **C** | **T** | **A** | **C** | **C** | **G** | **A** | **T** | **T** | **G** | **A** | **A** | **T** | **T** | **T** | **T** | **G** | **A** | **A** | **A** | **C** | **A** | **A** | **G** | **T** | **G** | **C** | **T** | **G** | **T** | **C** | **A** | **T** | **T** | **T** | **A** | **C** | **G** | **A** | **T** | **C** | **C** | **T** | **G** | **A** | **G** | **T** | **T** | **T** | **T** | **T** | **A** | **G** | **A** | **A** | **G** | **A** | **A** | **C** | **T** | **T** | **G** | **C** | **T** | **A** | **C** | **T** | **C** | **A** | **G** | **T** | **T** | **C** | **A** | **A** | **A** | **G** | **A** | **A** | **G** | **A** | **T** | **G** | **A** | **G** | **A** | **C** | **A** | **C** | **A** | **T** | **T** | **G** | **T** | **T** | **C** | **A** | **T** | **C** | **C** | **A** | **T** | **G** | **A** | **C** | **A** | **A** | **C** | **T** | **G** | **A** | **A** | **A** | **C** | **A** | **G** | **T** | **C** | **A** | **A** | **G** | **G** | **A** | **A** | **A** | **T** | **G** | **T** | **C** | **T** | **A** | **A** | **C** | **T** | **G** | **G** | **C** | **T** | **A** | **T** | **T** | **A** | **T** | **T** | **C** | **A** | **A** | **G** | **C** | **A** | **A** | **C** | **A** | **A** | **A** | **T** | **T** | **T** | **C** | **T** | **C** | **G** | **A** | **T** | **T** | **A** | **T** | **T** | **T** | **T** | **C** | **T** | **C** | **C** | **A** | **A** | **T** | **C** | **C** | **T** | **A** | **-** | **-** | **-** |
| **N59567F** | **-** | **-** | **-** | **-** | **-** | **-** | **-** | **-** | **-** | **-** | **-** | **-** | **-** | **-** | **-** | **-** | **-** | **-** | **-** | **-** | **-** | **-** | **-** | **-** | **-** | **-** | **-** | **-** | **-** | **-** | **-** | **-** | **-** | **-** | **-** | **-** | **-** | **-** | **-** | **-** | **-** | **-** | **-** | **-** | **-** | **-** | **-** | **-** | **-** | **-** | **-** | **-** | **-** | **-** | **-** | **-** | **-** | **-** | **-** | **-** | **-** | **-** | **-** | **-** | **-** | **-** | **-** | **-** | **-** | **A** | **A** | **A** | **C** | **C** | **C** | **C** | **G** | **A** | **G** | **A** | **T** | **G** | **T** | **A** | **G** | **C** | **A** | **G** | **C** | **C** | **A** | **T** | **T** | **T** | **G** | **G** | **G** | **C** | **A** | **T** | **G** | **G** | **C** | **T** | **C** | **T** | **T** | **A** | **G** | **T** | **A** | **T** | **T** | **G** | **T** | **T** | **T** | **G** | **C** | **C** | **C** | **T** | **T** | **C** | **C** | **T** | **G** | **G** | **G** | **G** | **T** | **G** | **G** | **G** | **T** | **T** | **T** | **T** | **G** | **T** | **A** | **T** | **T** | **A** | **T** | **A** | **T** | **T** | **T** | **A** | **T** | **T** | **T** | **T** | **T** | **C** | **G** | **G** | **A** | **C** | **G** | **T** | **G** | **G** | **A** | **T** | **T** | **A** | **A** | **C** | **G** | **G** | **A** | **T** | **A** | **A** | **G** | **A** | **A** | **A** | **A** | **A** | **A** | **T** | **T** | **C** | **T** | **A** | **T** | **C** | **T** | **C** | **C** | **G** | **A** | **C** | **A** | **A** | **A** | **G** | **T** | **G** | **A** | **C** | **T** | **T** | **G** | **A** | **A** | **A** | **G** | **A** | **A** | **T** | **T** | **A** | **G** | **A** | **A** | **A** | **A** | **C** | **T** | **T** | **T** | **C** | **A** | **G** | **T** | **C** | **T** | **T** | **T** | **T** | **A** | **G** | **A** | **G** | **G** | **A** | **G** | **A** | **T** | **A** | **C** | **C** | **A** | **T** | **T** | **G** | **A** | **A** | **C** | **A** | **T** | **T** | **A** | **C** | **G** | **A** | **T** | **C** | **C** | **T** | **G** | **A** | **C** | **A** | **T** | **G** | **G** | **G** | **C** | **G** | **A** | **T** | **A** | **A** | **A** | **G** | **A** | **C** | **A** | **A** | **A** | **C** | **A** | **G** | **C** | **A** | **A** | **T** | **T** | **T** | **G** | **T** | **C** | **G** | **A** | **C** | **T** | **T** | **C** | **T** | **T** | **C** | **T** | **C** | **T** | **T** | **C** | **A** | **T** | **T** | **A** | **A** | **A** | **T** | **C** | **G** | **T** | **A** | **T** | **G** | **C** | **C** | **G** | **C** | **T** | **G** | **A** | **C** | **A** | **A** | **G** | **A** | **A** | **T** | **G** | **A** | **A** | **T** | **T** | **C** | **T** | **G** | **T** | **C** | **A** | **C** | **T** | **C** | **T** | **T** | **C** | **T** | **C** | **A** | **C** | **A** | **G** | **A** | **C** | **G** | **G** | **A** | **C** | **A** | **A** | **G** | **A** | **G** | **A** | **A** | **A** | **T** | **T** | **G** | **G** | **A** | **T** | **T** | **C** | **A** | **C** | **T** | **G** | **C** | **T** | **T** | **C** | **A** | **A** | **G** | **A** | **T** | **C** | **T** | **A** | **A** | **A** | **A** | **A** | **A** | **A** | **G** | **C** | **C** | **A** | **A** | **A** | **C** | **A** | **T** | **T** | **C** | **G** | **A** | **T** | **C** | **C** | **A** | **T** | **A** | **T** | **C** | **G** | **A** | **A** | **T** | **A** | **T** | **T** | **A** | **C** | **G** | **C** | **A** | **T** | **T** | **T** | **G** | **T** | **G** | **A** | **C** | **A** | **G** | **A** | **T** | **A** | **A** | **T** | **A** | **T** | **C** | **G** | **G** | **C** | **C** | **A** | **G** | **C** | **A** | **A** | **G** | **T** | **C** | **T** | **T** | **A** | **C** | **A** | **T** | **G** | **T** | **T** | **T** | **T** | **A** | **G** | **A** | **A** | **G** | **A** | **A** | **A** | **A** | **A** | **G** | **C** | **C** | **G** | **C** | **A** | **G** | **A** | **A** | **G** | **G** | **C** | **G** | **T** | **G** | **G** | **A** | **A** | **G** | **T** | **T** | **C** | **G** | **A** | **A** | **T** | **A** | **T** | **T** | **A** | **T** | **A** | **T** | **G** | **A** | **T** | **G** | **C** | **A** | **T** | **T** | **T** | **G** | **G** | **C** | **T** | **C** | **T** | **C** | **A** | **T** | **G** | **G** | **C** | **A** | **C** | **A** | **A** | **A** | **A** | **G** | **C** | **A** | **A** | **A** | **A** | **G** | **A** | **T** | **T** | **T** | **C** | **A** | **A** | **T** | **C** | **G** | **T** | **C** | **T** | **A** | **A** | **T** | **C** | **A** | **A** | **A** | **A** | **A** | **T** | **G** | **G** | **T** | **G** | **G** | **A** | **C** | **A** | **T** | **G** | **T** | **C** | **C** | **A** | **T** | **A** | **C** | **A** | **T** | **T** | **T** | **G** | **T** | **T** | **A** | **C** | **C** | **T** | **C** | **A** | **C** | **A** | **A** | **A** | **A** | **G** | **G** | **C** | **A** | **T** | **T** | **A** | **C** | **T** | **T** | **C** | **G** | **T** | **T** | **T** | **C** | **C** | **G** | **A** | **T** | **T** | **G** | **A** | **A** | **T** | **T** | **A** | **C** | **C** | **A** | **T** | **G** | **A** | **T** | **C** | **A** | **C** | **C** | **G** | **A** | **A** | **A** | **A** | **A** | **T** | **C** | **G** | **T** | **T** | **G** | **T** | **G** | **A** | **T** | **C** | **G** | **A** | **T** | **G** | **G** | **A** | **A** | **A** | **G** | **A** | **T** | **T** | **A** | **G** | **T** | **T** | **A** | **T** | **A** | **C** | **C** | **G** | **G** | **T** | **G** | **G** | **T** | **T** | **T** | **C** | **A** | **A** | **T** | **A** | **T** | **T** | **G** | **C** | **C** | **A** | **A** | **T** | **C** | **A** | **A** | **T** | **A** | **T** | **G** | **T** | **A** | **A** | **A** | **T** | **A** | **C** | **A** | **A** | **C** | **A** | **A** | **A** | **A** | **A** | **A** | **A** | **T** | **T** | **C** | **G** | **G** | **C** | **T** | **A** | **T** | **T** | **G** | **G** | **C** | **G** | **C** | **G** | **A** | **T** | **A** | **C** | **G** | **C** | **A** | **T** | **A** | **T** | **A** | **C** | **G** | **G** | **A** | **T** | **T** | **T** | **T** | **C** | **G** | **G** | **T** | **G** | **C** | **C** | **G** | **C** | **T** | **T** | **C** | **T** | **T** | **C** | **T** | **T** | **T** | **G** | **C** | **T** | **C** | **C** | **A** | **G** | **C** | **T** | **T** | **C** | **G** | **C** | **T** | **T** | **C** | **T** | **T** | **A** | **A** | **C** | **A** | **G** | **A** | **C** | **T** | **G** | **G** | **A** | **A** | **C** | **G** | **T** | **C** | **T** | **C** | **G** | **G** | **T** | **A** | **C** | **C** | **T** | **G** | **A** | **A** | **G** | **A** | **A** | **A** | **A** | **A** | **A** | **A** | **G** | **G** | **T** | **C** | **G** | **G** | **C** | **T** | **A** | **T** | **C** | **A** | **T** | **T** | **T** | **G** | **A** | **A** | **T** | **T** | **A** | **T** | **T** | **T** | **C** | **T** | **T** | **T** | **A** | **A** | **A** | **A** | **A** | **A** | **G** | **C** | **A** | **G** | **A** | **T** | **C** | **G** | **A** | **G** | **A** | **T** | **G** | **A** | **A** | **T** | **C** | **T** | **A** | **A** | **G** | **C** | **T** | **T** | **G** | **C** | **T** | **G** | **A** | **T** | **A** | **C** | **A** | **T** | **C** | **C** | **A** | **T** | **C** | **C** | **A** | **G** | **C** | **T** | **T** | **G** | **T** | **T** | **T** | **C** | **A** | **A** | **G** | **C** | **G** | **G** | **A** | **C** | **C** | **G** | **A** | **A** | **T** | **A** | **A** | **C** | **G** | **A** | **A** | **A** | **G** | **G** | **G** | **A** | **A** | **C** | **A** | **A** | **A** | **T** | **C** | **A** | **A** | **G** | **C** | **T** | **T** | **T** | **C** | **A** | **T** | **T** | **T** | **A** | **T** | **C** | **A** | **A** | **A** | **T** | **T** | **G** | **A** | **T** | **T** | **A** | **C** | **T** | **T** | **C** | **T** | **G** | **C** | **T** | **A** | **A** | **A** | **A** | **A** | **A** | **C** | **G** | **T** | **G** | **T** | **T** | **T** | **G** | **G** | **A** | **T** | **A** | **C** | **A** | **G** | **A** | **C** | **A** | **C** | **C** | **T** | **T** | **A** | **C** | **C** | **T** | **T** | **G** | **T** | **T** | **C** | **C** | **T** | **G** | **A** | **T** | **G** | **A** | **A** | **A** | **G** | **T** | **G** | **T** | **C** | **A** | **T** | **T** | **G** | **C** | **T** | **G** | **C** | **T** | **T** | **T** | **A** | **A** | **A** | **A** | **A** | **T** | **C** | **G** | **C** | **A** | **A** | **C** | **T** | **G** | **C** | **C** | **T** | **C** | **T** | **G** | **G** | **T** | **G** | **T** | **A** | **G** | **A** | **T** | **G** | **T** | **G** | **A** | **A** | **A** | **A** | **T** | **C** | **A** | **T** | **G** | **A** | **T** | **T** | **C** | **C** | **C** | **A** | **A** | **C** | **A** | **A** | **A** | **C** | **C** | **G** | **G** | **A** | **T** | **C** | **A** | **T** | **C** | **C** | **T** | **T** | **T** | **T** | **A** | **T** | **T** | **T** | **A** | **T** | **C** | **G** | **A** | **G** | **C** | **A** | **A** | **C** | **A** | **C** | **A** | **A** | **T** | **A** | **T** | **T** | **A** | **T** | **G** | **C** | **T** | **C** | **G** | **G** | **C** | **A** | **G** | **C** | **T** | **G** | **A** | **T** | **C** | **A** | **A** | **G** | **G** | **A** | **A** | **A** | **A** | **T** | **G** | **T** | **A** | **C** | **A** | **A** | **A** | **T** | **C** | **C** | **T** | **T** | **G** | **T** | **C** | **T** | **A** | **T** | **G** | **A** | **G** | **A** | **A** | **C** | **G** | **G** | **C** | **T** | **T** | **C** | **C** | **T** | **C** | **C** | **A** | **T** | **G** | **C** | **A** | **A** | **A** | **A** | **A** | **C** | **A** | **T** | **T** | **G** | **A** | **T** | **A** | **A** | **T** | **G** | **G** | **A** | **T** | **G** | **A** | **T** | **G** | **A** | **A** | **A** | **T** | **C** | **T** | **G** | **C** | **A** | **T** | **G** | **G** | **T** | **A** | **G** | **G** | **T** | **T** | **C** | **A** | **G** | **C** | **A** | **A** | **A** | **T** | **C** | **A** | **A** | **G** | **A** | **T** | **A** | **T** | **T** | **C** | **G** | **T** | **A** | **G** | **C** | **T** | **A** | **C** | **C** | **G** | **A** | **T** | **T** | **G** | **A** | **A** | **T** | **T** | **T** | **T** | **G** | **A** | **A** | **A** | **C** | **A** | **A** | **G** | **T** | **G** | **C** | **T** | **G** | **T** | **C** | **A** | **T** | **T** | **T** | **A** | **C** | **G** | **A** | **T** | **C** | **C** | **T** | **G** | **A** | **G** | **T** | **T** | **T** | **T** | **T** | **A** | **G** | **A** | **A** | **G** | **A** | **A** | **C** | **T** | **T** | **G** | **C** | **T** | **A** | **C** | **T** | **C** | **A** | **G** | **T** | **T** | **C** | **A** | **A** | **A** | **G** | **A** | **A** | **G** | **A** | **T** | **G** | **A** | **G** | **A** | **C** | **A** | **C** | **A** | **T** | **T** | **G** | **T** | **T** | **C** | **A** | **T** | **C** | **C** | **A** | **T** | **G** | **A** | **C** | **A** | **A** | **C** | **T** | **G** | **A** | **A** | **A** | **C** | **A** | **G** | **T** | **C** | **A** | **A** | **G** | **G** | **A** | **A** | **A** | **T** | **G** | **T** | **C** | **T** | **A** | **A** | **C** | **T** | **G** | **G** | **C** | **T** | **A** | **T** | **T** | **A** | **T** | **T** | **C** | **A** | **A** | **G** | **C** | **A** | **A** | **C** | **A** | **A** | **A** | **T** | **T** | **T** | **C** | **T** | **C** | **G** | **A** | **T** | **T** | **A** | **T** | **T** | **T** | **T** | **C** | **T** | **C** | **C** | **A** | **A** | **T** | **C** | **C** | **T** | **A** | **-** | **-** | **-** |
| **N59947F** | **-** | **-** | **-** | **-** | **-** | **-** | **-** | **-** | **-** | **-** | **-** | **-** | **-** | **-** | **-** | **-** | **-** | **-** | **-** | **-** | **-** | **-** | **-** | **-** | **-** | **-** | **-** | **-** | **-** | **-** | **-** | **-** | **-** | **-** | **-** | **-** | **-** | **-** | **-** | **-** | **-** | **-** | **-** | **-** | **-** | **-** | **-** | **-** | **-** | **-** | **-** | **-** | **-** | **-** | **-** | **-** | **-** | **-** | **-** | **-** | **-** | **-** | **-** | **-** | **-** | **-** | **-** | **-** | **-** | **A** | **A** | **A** | **C** | **C** | **C** | **C** | **G** | **A** | **G** | **A** | **T** | **G** | **T** | **A** | **G** | **C** | **A** | **G** | **C** | **T** | **A** | **T** | **T** | **T** | **G** | **G** | **G** | **C** | **A** | **T** | **G** | **G** | **C** | **T** | **C** | **T** | **T** | **A** | **G** | **T** | **A** | **T** | **T** | **G** | **T** | **T** | **T** | **G** | **C** | **C** | **C** | **T** | **T** | **C** | **C** | **T** | **G** | **G** | **G** | **G** | **T** | **G** | **G** | **G** | **T** | **T** | **T** | **T** | **G** | **T** | **A** | **T** | **T** | **A** | **T** | **A** | **T** | **T** | **T** | **A** | **T** | **T** | **T** | **T** | **T** | **C** | **G** | **G** | **A** | **C** | **G** | **T** | **G** | **G** | **A** | **T** | **T** | **A** | **A** | **C** | **G** | **G** | **A** | **T** | **A** | **A** | **G** | **A** | **A** | **A** | **A** | **A** | **A** | **T** | **T** | **C** | **T** | **A** | **T** | **C** | **T** | **C** | **C** | **G** | **A** | **C** | **A** | **A** | **A** | **G** | **T** | **G** | **A** | **C** | **T** | **T** | **G** | **A** | **A** | **A** | **G** | **A** | **A** | **T** | **T** | **A** | **G** | **A** | **A** | **A** | **A** | **C** | **T** | **T** | **T** | **C** | **A** | **G** | **T** | **C** | **T** | **T** | **T** | **T** | **A** | **G** | **A** | **G** | **G** | **A** | **G** | **A** | **T** | **A** | **C** | **C** | **A** | **T** | **T** | **G** | **A** | **A** | **C** | **A** | **T** | **T** | **A** | **C** | **G** | **A** | **T** | **C** | **C** | **T** | **G** | **A** | **C** | **A** | **T** | **G** | **G** | **G** | **C** | **G** | **A** | **C** | **A** | **A** | **A** | **G** | **A** | **C** | **A** | **A** | **A** | **C** | **A** | **A** | **C** | **A** | **A** | **T** | **T** | **T** | **G** | **T** | **C** | **G** | **A** | **C** | **T** | **T** | **C** | **T** | **T** | **C** | **T** | **C** | **T** | **T** | **C** | **A** | **T** | **T** | **A** | **A** | **A** | **T** | **C** | **G** | **T** | **A** | **T** | **G** | **C** | **C** | **G** | **C** | **T** | **G** | **A** | **C** | **A** | **A** | **G** | **A** | **A** | **T** | **G** | **A** | **A** | **T** | **T** | **C** | **T** | **G** | **T** | **C** | **A** | **C** | **T** | **C** | **T** | **T** | **C** | **T** | **C** | **A** | **C** | **A** | **G** | **A** | **C** | **G** | **G** | **A** | **C** | **A** | **A** | **A** | **A** | **G** | **A** | **A** | **A** | **T** | **T** | **A** | **G** | **A** | **T** | **T** | **C** | **A** | **C** | **T** | **G** | **C** | **T** | **T** | **C** | **A** | **A** | **G** | **A** | **T** | **C** | **T** | **A** | **A** | **A** | **A** | **A** | **A** | **A** | **G** | **C** | **C** | **A** | **A** | **A** | **C** | **A** | **T** | **T** | **C** | **G** | **A** | **T** | **C** | **C** | **A** | **T** | **A** | **T** | **C** | **G** | **A** | **A** | **T** | **A** | **T** | **T** | **A** | **C** | **G** | **C** | **A** | **T** | **T** | **T** | **G** | **T** | **G** | **A** | **C** | **A** | **G** | **A** | **T** | **A** | **A** | **T** | **A** | **T** | **C** | **G** | **G** | **T** | **C** | **A** | **G** | **C** | **A** | **A** | **G** | **T** | **C** | **T** | **T** | **A** | **C** | **G** | **C** | **G** | **T** | **T** | **T** | **T** | **A** | **G** | **A** | **A** | **G** | **A** | **A** | **A** | **A** | **A** | **G** | **C** | **C** | **G** | **C** | **A** | **G** | **A** | **A** | **G** | **G** | **C** | **G** | **T** | **A** | **G** | **A** | **A** | **G** | **T** | **T** | **C** | **G** | **T** | **A** | **T** | **A** | **T** | **T** | **A** | **T** | **A** | **T** | **G** | **A** | **T** | **G** | **C** | **A** | **T** | **T** | **T** | **G** | **G** | **C** | **T** | **C** | **C** | **C** | **A** | **T** | **G** | **G** | **C** | **A** | **C** | **A** | **A** | **A** | **A** | **G** | **C** | **A** | **A** | **A** | **A** | **G** | **A** | **T** | **T** | **T** | **C** | **A** | **A** | **T** | **C** | **G** | **T** | **C** | **T** | **A** | **A** | **T** | **C** | **A** | **A** | **A** | **A** | **A** | **T** | **G** | **G** | **T** | **G** | **G** | **A** | **C** | **A** | **T** | **G** | **T** | **C** | **C** | **A** | **T** | **A** | **C** | **A** | **T** | **T** | **T** | **G** | **T** | **T** | **A** | **C** | **C** | **T** | **C** | **G** | **C** | **A** | **A** | **A** | **A** | **A** | **G** | **C** | **A** | **T** | **T** | **G** | **C** | **T** | **T** | **C** | **G** | **T** | **T** | **T** | **C** | **C** | **G** | **A** | **T** | **T** | **G** | **A** | **A** | **T** | **T** | **A** | **C** | **C** | **A** | **T** | **G** | **A** | **T** | **C** | **A** | **C** | **C** | **G** | **A** | **A** | **A** | **A** | **A** | **T** | **C** | **G** | **T** | **T** | **G** | **T** | **G** | **A** | **T** | **C** | **G** | **A** | **T** | **G** | **G** | **A** | **A** | **A** | **G** | **A** | **T** | **C** | **A** | **G** | **T** | **T** | **A** | **C** | **A** | **C** | **C** | **G** | **G** | **T** | **G** | **G** | **T** | **T** | **T** | **C** | **A** | **A** | **T** | **A** | **T** | **T** | **G** | **C** | **C** | **A** | **A** | **T** | **C** | **A** | **A** | **T** | **A** | **T** | **G** | **T** | **A** | **A** | **A** | **T** | **A** | **C** | **G** | **A** | **C** | **A** | **A** | **A** | **A** | **A** | **A** | **A** | **T** | **T** | **C** | **G** | **G** | **T** | **T** | **A** | **T** | **T** | **G** | **G** | **C** | **G** | **T** | **G** | **A** | **T** | **A** | **C** | **G** | **C** | **A** | **T** | **A** | **T** | **A** | **C** | **G** | **A** | **A** | **T** | **T** | **T** | **T** | **C** | **G** | **G** | **T** | **G** | **C** | **T** | **G** | **C** | **T** | **T** | **C** | **T** | **T** | **C** | **T** | **T** | **T** | **G** | **C** | **T** | **T** | **C** | **A** | **A** | **C** | **T** | **T** | **C** | **G** | **T** | **T** | **T** | **T** | **T** | **T** | **A** | **A** | **C** | **A** | **G** | **A** | **C** | **T** | **G** | **G** | **A** | **A** | **C** | **G** | **T** | **T** | **T** | **C** | **A** | **G** | **T** | **G** | **T** | **C** | **T** | **G** | **A** | **A** | **G** | **A** | **A** | **A** | **A** | **A** | **A** | **A** | **G** | **G** | **T** | **C** | **G** | **G** | **C** | **T** | **A** | **T** | **C** | **A** | **T** | **T** | **T** | **G** | **A** | **A** | **T** | **T** | **A** | **T** | **T** | **T** | **C** | **T** | **T** | **T** | **A** | **A** | **A** | **A** | **A** | **A** | **G** | **C** | **C** | **G** | **A** | **T** | **C** | **G** | **A** | **G** | **A** | **T** | **G** | **A** | **A** | **T** | **C** | **C** | **A** | **A** | **A** | **C** | **T** | **T** | **G** | **C** | **T** | **G** | **A** | **T** | **A** | **C** | **G** | **T** | **C** | **C** | **A** | **T** | **C** | **C** | **A** | **G** | **C** | **T** | **T** | **G** | **T** | **C** | **T** | **C** | **A** | **A** | **G** | **C** | **G** | **G** | **A** | **C** | **C** | **A** | **A** | **A** | **T** | **A** | **A** | **C** | **G** | **A** | **A** | **C** | **G** | **A** | **G** | **A** | **A** | **C** | **A** | **A** | **A** | **T** | **C** | **A** | **A** | **G** | **C** | **T** | **T** | **T** | **C** | **G** | **T** | **T** | **T** | **A** | **T** | **C** | **A** | **A** | **A** | **T** | **T** | **G** | **A** | **T** | **T** | **A** | **C** | **T** | **T** | **C** | **T** | **G** | **C** | **T** | **A** | **A** | **A** | **A** | **A** | **A** | **C** | **G** | **T** | **G** | **T** | **T** | **T** | **G** | **G** | **A** | **T** | **A** | **C** | **A** | **G** | **A** | **C** | **A** | **C** | **C** | **T** | **T** | **A** | **C** | **C** | **T** | **C** | **G** | **T** | **T** | **C** | **C** | **T** | **G** | **A** | **T** | **G** | **A** | **A** | **A** | **G** | **T** | **G** | **T** | **C** | **A** | **T** | **T** | **G** | **C** | **T** | **G** | **C** | **T** | **T** | **T** | **G** | **A** | **A** | **A** | **A** | **T** | **C** | **G** | **C** | **A** | **A** | **C** | **T** | **G** | **C** | **T** | **T** | **C** | **T** | **G** | **G** | **T** | **G** | **T** | **C** | **G** | **A** | **T** | **G** | **T** | **G** | **A** | **A** | **A** | **A** | **T** | **C** | **A** | **T** | **G** | **A** | **T** | **C** | **C** | **C** | **C** | **A** | **A** | **T** | **A** | **A** | **A** | **C** | **C** | **G** | **G** | **A** | **T** | **C** | **A** | **T** | **C** | **C** | **T** | **T** | **T** | **T** | **A** | **T** | **T** | **T** | **A** | **T** | **C** | **G** | **A** | **G** | **C** | **A** | **A** | **C** | **A** | **C** | **A** | **A** | **T** | **A** | **T** | **T** | **A** | **T** | **G** | **C** | **T** | **C** | **G** | **T** | **C** | **A** | **G** | **C** | **T** | **C** | **A** | **T** | **C** | **A** | **A** | **G** | **G** | **A** | **A** | **A** | **A** | **T** | **G** | **T** | **A** | **C** | **A** | **G** | **A** | **T** | **C** | **C** | **T** | **T** | **G** | **T** | **C** | **T** | **A** | **T** | **G** | **A** | **G** | **A** | **A** | **T** | **G** | **G** | **C** | **T** | **T** | **C** | **C** | **T** | **C** | **C** | **A** | **T** | **G** | **C** | **A** | **A** | **A** | **A** | **A** | **C** | **A** | **T** | **T** | **A** | **A** | **T** | **A** | **A** | **T** | **G** | **G** | **A** | **T** | **G** | **A** | **T** | **G** | **A** | **A** | **A** | **T** | **C** | **T** | **G** | **C** | **A** | **T** | **G** | **G** | **T** | **A** | **G** | **G** | **T** | **T** | **C** | **A** | **G** | **C** | **A** | **A** | **A** | **T** | **C** | **A** | **A** | **G** | **A** | **T** | **A** | **T** | **T** | **C** | **G** | **T** | **A** | **G** | **C** | **T** | **A** | **C** | **C** | **G** | **A** | **T** | **T** | **G** | **A** | **A** | **T** | **T** | **T** | **T** | **G** | **A** | **A** | **A** | **C** | **A** | **A** | **G** | **T** | **G** | **C** | **T** | **G** | **T** | **C** | **A** | **T** | **T** | **T** | **A** | **C** | **G** | **A** | **T** | **C** | **C** | **T** | **G** | **A** | **G** | **T** | **T** | **T** | **T** | **T** | **A** | **G** | **A** | **A** | **G** | **A** | **G** | **C** | **T** | **T** | **G** | **C** | **T** | **A** | **C** | **T** | **C** | **A** | **G** | **T** | **T** | **C** | **A** | **A** | **A** | **G** | **A** | **A** | **G** | **A** | **T** | **G** | **A** | **G** | **A** | **C** | **A** | **C** | **A** | **T** | **T** | **G** | **T** | **T** | **C** | **A** | **C** | **C** | **A** | **A** | **T** | **G** | **A** | **C** | **A** | **A** | **C** | **T** | **G** | **A** | **A** | **A** | **C** | **A** | **G** | **T** | **G** | **A** | **A** | **G** | **G** | **A** | **A** | **A** | **T** | **G** | **T** | **C** | **T** | **A** | **A** | **C** | **T** | **G** | **G** | **C** | **T** | **G** | **T** | **T** | **A** | **T** | **T** | **C** | **A** | **A** | **A** | **C** | **A** | **A** | **C** | **A** | **A** | **A** | **T** | **T** | **T** | **C** | **T** | **C** | **G** | **A** | **T** | **T** | **G** | **T** | **T** | **T** | **T** | **C** | **T** | **C** | **C** | **A** | **A** | **T** | **C** | **T** | **T** | **A** | **-** | **-** | **-** |
| **N53655** | **-** | **-** | **-** | **-** | **-** | **-** | **-** | **-** | **-** | **-** | **-** | **-** | **-** | **-** | **-** | **-** | **-** | **-** | **-** | **-** | **-** | **-** | **-** | **-** | **-** | **-** | **-** | **-** | **-** | **-** | **-** | **-** | **-** | **-** | **-** | **-** | **-** | **-** | **-** | **-** | **-** | **-** | **-** | **-** | **-** | **-** | **-** | **-** | **-** | **-** | **-** | **-** | **-** | **-** | **-** | **-** | **-** | **-** | **-** | **-** | **-** | **-** | **-** | **-** | **-** | **-** | **-** | **-** | **-** | **A** | **A** | **A** | **C** | **C** | **C** | **C** | **G** | **A** | **G** | **A** | **T** | **G** | **T** | **A** | **G** | **C** | **A** | **G** | **C** | **C** | **A** | **T** | **T** | **T** | **G** | **G** | **G** | **C** | **A** | **T** | **G** | **G** | **C** | **T** | **C** | **T** | **T** | **A** | **G** | **T** | **A** | **T** | **T** | **G** | **T** | **T** | **T** | **G** | **C** | **C** | **C** | **T** | **T** | **C** | **C** | **T** | **G** | **G** | **G** | **G** | **T** | **G** | **G** | **G** | **C** | **T** | **T** | **T** | **G** | **T** | **A** | **T** | **T** | **A** | **T** | **A** | **T** | **T** | **T** | **A** | **T** | **T** | **T** | **T** | **T** | **C** | **G** | **G** | **A** | **C** | **G** | **T** | **G** | **G** | **A** | **T** | **T** | **A** | **A** | **C** | **G** | **G** | **A** | **T** | **A** | **A** | **G** | **A** | **A** | **A** | **A** | **A** | **A** | **T** | **T** | **C** | **T** | **A** | **T** | **C** | **T** | **C** | **C** | **G** | **A** | **C** | **A** | **A** | **A** | **G** | **T** | **G** | **A** | **C** | **T** | **T** | **G** | **A** | **A** | **A** | **G** | **A** | **A** | **T** | **T** | **A** | **G** | **A** | **A** | **A** | **A** | **C** | **T** | **T** | **T** | **C** | **A** | **G** | **T** | **C** | **T** | **T** | **T** | **T** | **A** | **G** | **A** | **G** | **G** | **A** | **G** | **A** | **T** | **A** | **A** | **C** | **A** | **T** | **T** | **G** | **A** | **A** | **C** | **A** | **T** | **T** | **A** | **C** | **G** | **A** | **T** | **C** | **C** | **T** | **G** | **A** | **C** | **A** | **T** | **G** | **G** | **G** | **C** | **G** | **A** | **T** | **A** | **A** | **A** | **G** | **A** | **C** | **A** | **A** | **A** | **C** | **A** | **G** | **C** | **A** | **A** | **T** | **T** | **T** | **G** | **T** | **T** | **G** | **A** | **C** | **T** | **T** | **C** | **T** | **T** | **C** | **T** | **C** | **T** | **T** | **C** | **A** | **T** | **T** | **A** | **A** | **A** | **T** | **C** | **G** | **T** | **A** | **T** | **G** | **C** | **C** | **G** | **C** | **T** | **G** | **A** | **C** | **A** | **A** | **G** | **A** | **A** | **T** | **G** | **A** | **A** | **T** | **T** | **C** | **T** | **G** | **T** | **C** | **A** | **C** | **T** | **C** | **T** | **T** | **C** | **T** | **C** | **A** | **C** | **A** | **G** | **A** | **C** | **G** | **G** | **A** | **C** | **A** | **A** | **G** | **A** | **G** | **A** | **A** | **A** | **T** | **T** | **G** | **G** | **A** | **T** | **T** | **C** | **A** | **C** | **T** | **G** | **C** | **T** | **T** | **C** | **A** | **A** | **G** | **A** | **T** | **C** | **T** | **A** | **A** | **A** | **A** | **A** | **A** | **A** | **G** | **C** | **C** | **A** | **A** | **A** | **C** | **A** | **T** | **T** | **C** | **G** | **A** | **T** | **C** | **C** | **A** | **T** | **A** | **T** | **C** | **G** | **A** | **A** | **T** | **A** | **T** | **T** | **A** | **C** | **G** | **C** | **A** | **T** | **T** | **T** | **G** | **T** | **G** | **A** | **C** | **A** | **G** | **A** | **T** | **A** | **A** | **T** | **A** | **T** | **C** | **G** | **G** | **C** | **C** | **A** | **G** | **C** | **A** | **A** | **G** | **T** | **C** | **T** | **T** | **A** | **C** | **A** | **T** | **G** | **T** | **T** | **T** | **T** | **A** | **G** | **A** | **A** | **G** | **A** | **A** | **A** | **A** | **A** | **G** | **C** | **C** | **G** | **C** | **A** | **G** | **A** | **A** | **G** | **G** | **C** | **G** | **T** | **G** | **G** | **A** | **A** | **G** | **T** | **T** | **C** | **G** | **A** | **A** | **T** | **A** | **T** | **T** | **A** | **T** | **A** | **T** | **G** | **A** | **T** | **G** | **C** | **A** | **T** | **T** | **T** | **G** | **G** | **C** | **T** | **C** | **T** | **C** | **A** | **T** | **G** | **G** | **C** | **A** | **C** | **A** | **A** | **A** | **A** | **G** | **C** | **A** | **A** | **A** | **A** | **G** | **A** | **T** | **T** | **T** | **C** | **A** | **A** | **T** | **C** | **G** | **T** | **C** | **T** | **A** | **A** | **T** | **C** | **A** | **A** | **A** | **A** | **A** | **T** | **G** | **G** | **T** | **G** | **G** | **A** | **C** | **A** | **T** | **G** | **T** | **C** | **C** | **A** | **T** | **A** | **C** | **A** | **T** | **T** | **T** | **G** | **T** | **T** | **A** | **C** | **C** | **T** | **C** | **A** | **C** | **A** | **A** | **A** | **A** | **G** | **G** | **C** | **A** | **T** | **T** | **A** | **C** | **T** | **T** | **C** | **G** | **T** | **T** | **T** | **C** | **C** | **G** | **A** | **T** | **T** | **G** | **A** | **A** | **T** | **T** | **A** | **C** | **C** | **A** | **T** | **G** | **A** | **T** | **C** | **A** | **C** | **C** | **G** | **A** | **A** | **A** | **A** | **A** | **T** | **C** | **G** | **T** | **T** | **G** | **T** | **G** | **A** | **T** | **C** | **G** | **A** | **T** | **G** | **G** | **A** | **A** | **A** | **G** | **A** | **T** | **T** | **A** | **G** | **T** | **T** | **A** | **T** | **A** | **C** | **C** | **G** | **G** | **T** | **G** | **G** | **T** | **T** | **T** | **C** | **A** | **A** | **T** | **A** | **T** | **T** | **G** | **C** | **C** | **A** | **A** | **T** | **C** | **A** | **A** | **T** | **A** | **T** | **G** | **T** | **A** | **A** | **A** | **T** | **A** | **C** | **A** | **A** | **C** | **A** | **A** | **A** | **A** | **A** | **A** | **A** | **T** | **T** | **C** | **G** | **G** | **C** | **T** | **A** | **T** | **T** | **G** | **G** | **C** | **G** | **C** | **G** | **A** | **T** | **A** | **C** | **G** | **C** | **A** | **T** | **A** | **T** | **A** | **C** | **G** | **G** | **A** | **T** | **T** | **T** | **T** | **C** | **G** | **G** | **T** | **G** | **C** | **C** | **G** | **C** | **T** | **T** | **C** | **T** | **T** | **C** | **T** | **T** | **T** | **G** | **C** | **T** | **C** | **C** | **A** | **G** | **C** | **T** | **T** | **C** | **G** | **C** | **T** | **T** | **C** | **T** | **T** | **A** | **A** | **C** | **A** | **G** | **A** | **C** | **T** | **G** | **G** | **A** | **A** | **C** | **G** | **T** | **C** | **T** | **C** | **G** | **G** | **T** | **A** | **C** | **C** | **T** | **G** | **A** | **A** | **G** | **A** | **A** | **A** | **A** | **A** | **A** | **A** | **G** | **G** | **T** | **C** | **G** | **G** | **C** | **T** | **A** | **T** | **C** | **A** | **T** | **T** | **T** | **G** | **A** | **A** | **T** | **T** | **A** | **T** | **T** | **T** | **C** | **T** | **T** | **T** | **A** | **A** | **A** | **A** | **A** | **A** | **G** | **C** | **A** | **G** | **A** | **T** | **C** | **G** | **A** | **G** | **A** | **T** | **G** | **A** | **A** | **T** | **C** | **T** | **A** | **A** | **G** | **C** | **T** | **T** | **G** | **C** | **T** | **G** | **A** | **T** | **A** | **C** | **A** | **T** | **C** | **C** | **A** | **T** | **C** | **C** | **A** | **G** | **C** | **T** | **T** | **G** | **T** | **T** | **T** | **C** | **A** | **A** | **G** | **C** | **G** | **G** | **A** | **C** | **C** | **G** | **A** | **A** | **T** | **A** | **A** | **C** | **G** | **A** | **A** | **A** | **G** | **G** | **G** | **A** | **A** | **C** | **A** | **A** | **A** | **T** | **C** | **A** | **A** | **G** | **C** | **T** | **T** | **T** | **C** | **A** | **T** | **T** | **T** | **A** | **T** | **C** | **A** | **A** | **A** | **T** | **T** | **G** | **A** | **T** | **T** | **A** | **C** | **T** | **T** | **C** | **T** | **G** | **C** | **T** | **A** | **A** | **A** | **A** | **A** | **A** | **C** | **G** | **T** | **G** | **T** | **T** | **T** | **G** | **G** | **A** | **T** | **A** | **C** | **A** | **G** | **A** | **C** | **A** | **C** | **C** | **T** | **T** | **A** | **C** | **C** | **T** | **T** | **G** | **T** | **T** | **C** | **C** | **T** | **G** | **A** | **T** | **G** | **A** | **A** | **A** | **G** | **T** | **G** | **T** | **C** | **A** | **T** | **T** | **G** | **C** | **T** | **G** | **C** | **T** | **T** | **T** | **A** | **A** | **A** | **A** | **A** | **T** | **C** | **G** | **C** | **A** | **A** | **C** | **T** | **G** | **C** | **C** | **T** | **C** | **T** | **G** | **G** | **T** | **G** | **T** | **A** | **G** | **A** | **T** | **G** | **T** | **G** | **A** | **A** | **A** | **A** | **T** | **C** | **A** | **T** | **G** | **A** | **T** | **T** | **C** | **C** | **C** | **A** | **A** | **C** | **A** | **A** | **A** | **C** | **C** | **G** | **G** | **A** | **T** | **C** | **A** | **T** | **C** | **C** | **T** | **T** | **T** | **T** | **A** | **T** | **T** | **T** | **A** | **T** | **C** | **G** | **A** | **G** | **C** | **A** | **A** | **C** | **A** | **C** | **A** | **A** | **T** | **A** | **T** | **T** | **A** | **T** | **G** | **C** | **T** | **C** | **G** | **G** | **C** | **A** | **G** | **C** | **T** | **G** | **A** | **T** | **C** | **A** | **A** | **G** | **G** | **A** | **A** | **A** | **A** | **T** | **G** | **T** | **A** | **C** | **A** | **A** | **A** | **T** | **C** | **C** | **T** | **T** | **G** | **T** | **C** | **T** | **A** | **T** | **G** | **A** | **G** | **A** | **A** | **C** | **G** | **G** | **C** | **T** | **T** | **C** | **C** | **T** | **C** | **C** | **A** | **T** | **G** | **C** | **A** | **A** | **A** | **A** | **A** | **C** | **A** | **T** | **T** | **G** | **A** | **T** | **A** | **A** | **T** | **G** | **G** | **A** | **T** | **G** | **A** | **T** | **G** | **A** | **A** | **A** | **T** | **C** | **T** | **G** | **C** | **A** | **T** | **G** | **G** | **T** | **A** | **G** | **G** | **T** | **T** | **C** | **A** | **G** | **C** | **A** | **A** | **A** | **T** | **C** | **A** | **A** | **G** | **A** | **T** | **A** | **T** | **T** | **C** | **G** | **T** | **A** | **G** | **C** | **T** | **A** | **C** | **C** | **G** | **A** | **T** | **T** | **G** | **A** | **A** | **T** | **T** | **T** | **T** | **G** | **A** | **A** | **A** | **C** | **A** | **A** | **G** | **T** | **G** | **C** | **T** | **G** | **T** | **C** | **A** | **T** | **T** | **T** | **A** | **C** | **G** | **A** | **T** | **C** | **C** | **T** | **G** | **A** | **G** | **T** | **T** | **T** | **T** | **T** | **A** | **G** | **A** | **A** | **G** | **A** | **A** | **C** | **T** | **T** | **G** | **C** | **T** | **A** | **C** | **T** | **C** | **A** | **G** | **T** | **T** | **C** | **A** | **A** | **A** | **G** | **A** | **A** | **G** | **A** | **T** | **G** | **A** | **G** | **A** | **C** | **A** | **C** | **A** | **T** | **T** | **G** | **T** | **T** | **C** | **A** | **T** | **C** | **C** | **A** | **T** | **G** | **A** | **C** | **A** | **A** | **C** | **T** | **G** | **A** | **A** | **A** | **C** | **A** | **G** | **T** | **C** | **A** | **A** | **G** | **G** | **A** | **A** | **A** | **T** | **G** | **T** | **C** | **T** | **A** | **A** | **C** | **T** | **G** | **G** | **C** | **T** | **A** | **T** | **T** | **A** | **T** | **T** | **C** | **A** | **A** | **G** | **C** | **A** | **A** | **C** | **A** | **A** | **A** | **T** | **T** | **T** | **C** | **T** | **C** | **G** | **A** | **T** | **T** | **A** | **T** | **T** | **T** | **T** | **C** | **T** | **C** | **C** | **A** | **A** | **T** | **C** | **C** | **T** | **A** | **-** | **-** | **-** |
| **N60463F** | **-** | **-** | **-** | **-** | **-** | **-** | **-** | **-** | **-** | **-** | **-** | **-** | **-** | **-** | **-** | **-** | **-** | **-** | **-** | **-** | **-** | **-** | **-** | **-** | **-** | **-** | **-** | **-** | **-** | **-** | **-** | **-** | **-** | **-** | **-** | **-** | **-** | **-** | **-** | **-** | **-** | **-** | **-** | **-** | **-** | **-** | **-** | **-** | **-** | **-** | **-** | **-** | **-** | **-** | **-** | **-** | **-** | **-** | **-** | **-** | **-** | **-** | **-** | **-** | **-** | **-** | **-** | **-** | **-** | **A** | **A** | **A** | **C** | **C** | **C** | **C** | **G** | **A** | **G** | **A** | **T** | **G** | **T** | **A** | **G** | **C** | **A** | **G** | **C** | **C** | **A** | **T** | **T** | **T** | **G** | **G** | **G** | **C** | **A** | **T** | **G** | **G** | **C** | **T** | **C** | **T** | **T** | **A** | **G** | **T** | **A** | **T** | **T** | **G** | **T** | **T** | **T** | **G** | **C** | **C** | **C** | **T** | **T** | **C** | **C** | **T** | **G** | **G** | **G** | **G** | **T** | **G** | **G** | **G** | **T** | **T** | **T** | **T** | **G** | **T** | **A** | **T** | **T** | **A** | **T** | **A** | **T** | **T** | **T** | **A** | **T** | **T** | **T** | **T** | **T** | **C** | **G** | **G** | **A** | **C** | **G** | **T** | **G** | **G** | **A** | **T** | **T** | **A** | **A** | **C** | **G** | **G** | **A** | **T** | **A** | **A** | **G** | **A** | **A** | **A** | **A** | **A** | **A** | **T** | **T** | **C** | **T** | **A** | **T** | **C** | **T** | **C** | **C** | **G** | **A** | **C** | **A** | **A** | **A** | **G** | **T** | **G** | **A** | **C** | **T** | **T** | **G** | **A** | **A** | **A** | **G** | **A** | **A** | **T** | **T** | **A** | **G** | **A** | **A** | **A** | **A** | **C** | **T** | **T** | **T** | **C** | **A** | **G** | **T** | **C** | **T** | **T** | **T** | **T** | **A** | **G** | **A** | **G** | **G** | **A** | **G** | **A** | **T** | **A** | **C** | **C** | **A** | **T** | **T** | **G** | **A** | **A** | **C** | **A** | **T** | **T** | **A** | **C** | **G** | **A** | **T** | **C** | **C** | **T** | **G** | **A** | **C** | **A** | **T** | **G** | **G** | **G** | **C** | **G** | **A** | **T** | **A** | **A** | **A** | **G** | **A** | **C** | **A** | **A** | **A** | **C** | **A** | **G** | **C** | **A** | **A** | **T** | **T** | **T** | **G** | **T** | **C** | **G** | **A** | **C** | **T** | **T** | **C** | **T** | **T** | **C** | **T** | **C** | **T** | **T** | **C** | **A** | **T** | **T** | **A** | **A** | **A** | **T** | **C** | **G** | **T** | **A** | **T** | **G** | **C** | **C** | **G** | **C** | **T** | **G** | **A** | **C** | **A** | **A** | **G** | **A** | **A** | **T** | **G** | **A** | **A** | **T** | **T** | **C** | **T** | **G** | **T** | **C** | **A** | **C** | **T** | **C** | **T** | **T** | **C** | **T** | **C** | **A** | **C** | **A** | **G** | **A** | **C** | **G** | **G** | **A** | **C** | **A** | **A** | **G** | **A** | **G** | **A** | **A** | **A** | **T** | **T** | **G** | **G** | **A** | **T** | **T** | **C** | **A** | **C** | **T** | **G** | **C** | **T** | **T** | **C** | **A** | **A** | **G** | **A** | **T** | **C** | **T** | **A** | **A** | **A** | **A** | **A** | **A** | **A** | **G** | **C** | **C** | **A** | **A** | **A** | **C** | **A** | **T** | **T** | **C** | **G** | **A** | **T** | **C** | **C** | **A** | **T** | **A** | **T** | **C** | **G** | **A** | **A** | **T** | **A** | **T** | **T** | **A** | **C** | **G** | **C** | **A** | **T** | **T** | **T** | **G** | **T** | **G** | **A** | **C** | **A** | **G** | **A** | **T** | **A** | **A** | **T** | **A** | **T** | **C** | **G** | **G** | **C** | **C** | **A** | **G** | **C** | **A** | **A** | **G** | **T** | **C** | **T** | **T** | **A** | **C** | **A** | **T** | **G** | **T** | **T** | **T** | **T** | **A** | **G** | **A** | **A** | **G** | **A** | **A** | **A** | **A** | **A** | **G** | **C** | **C** | **G** | **C** | **A** | **G** | **A** | **A** | **G** | **G** | **C** | **G** | **T** | **G** | **G** | **A** | **A** | **G** | **T** | **T** | **C** | **G** | **A** | **A** | **T** | **A** | **T** | **T** | **A** | **T** | **A** | **T** | **G** | **A** | **T** | **G** | **C** | **A** | **T** | **T** | **T** | **G** | **G** | **C** | **T** | **C** | **T** | **C** | **A** | **T** | **G** | **G** | **C** | **A** | **C** | **A** | **A** | **A** | **A** | **G** | **C** | **A** | **A** | **A** | **A** | **G** | **A** | **T** | **T** | **T** | **C** | **A** | **A** | **T** | **C** | **G** | **T** | **C** | **T** | **A** | **A** | **T** | **C** | **A** | **A** | **A** | **A** | **A** | **T** | **G** | **G** | **T** | **G** | **G** | **A** | **C** | **A** | **T** | **G** | **T** | **C** | **C** | **A** | **T** | **A** | **C** | **A** | **T** | **T** | **T** | **G** | **T** | **T** | **A** | **C** | **C** | **T** | **C** | **A** | **C** | **A** | **A** | **A** | **A** | **G** | **G** | **C** | **A** | **T** | **T** | **A** | **C** | **T** | **T** | **C** | **G** | **T** | **T** | **T** | **C** | **C** | **G** | **A** | **T** | **T** | **G** | **A** | **A** | **T** | **T** | **A** | **C** | **C** | **A** | **T** | **G** | **A** | **T** | **C** | **A** | **C** | **C** | **G** | **A** | **A** | **A** | **A** | **A** | **T** | **C** | **G** | **T** | **T** | **G** | **T** | **G** | **A** | **T** | **C** | **G** | **A** | **T** | **G** | **G** | **A** | **A** | **A** | **G** | **A** | **T** | **T** | **A** | **G** | **T** | **T** | **A** | **T** | **A** | **C** | **C** | **G** | **G** | **T** | **G** | **G** | **T** | **T** | **T** | **C** | **A** | **A** | **T** | **A** | **T** | **T** | **G** | **C** | **C** | **A** | **A** | **T** | **C** | **A** | **A** | **T** | **A** | **T** | **G** | **T** | **A** | **A** | **A** | **T** | **A** | **C** | **A** | **A** | **C** | **A** | **A** | **A** | **A** | **A** | **A** | **A** | **T** | **T** | **C** | **G** | **G** | **C** | **T** | **A** | **T** | **T** | **G** | **G** | **C** | **G** | **C** | **G** | **A** | **T** | **A** | **C** | **G** | **C** | **A** | **T** | **A** | **T** | **A** | **C** | **G** | **G** | **A** | **T** | **T** | **T** | **T** | **C** | **G** | **G** | **T** | **G** | **C** | **C** | **G** | **C** | **T** | **T** | **C** | **T** | **T** | **C** | **T** | **T** | **T** | **G** | **C** | **T** | **C** | **C** | **A** | **G** | **C** | **T** | **T** | **C** | **G** | **C** | **T** | **T** | **C** | **T** | **T** | **A** | **A** | **C** | **A** | **G** | **A** | **C** | **T** | **G** | **G** | **A** | **A** | **C** | **G** | **T** | **C** | **T** | **C** | **G** | **G** | **T** | **A** | **C** | **C** | **T** | **G** | **A** | **A** | **G** | **A** | **A** | **A** | **A** | **A** | **A** | **A** | **G** | **G** | **T** | **C** | **G** | **G** | **C** | **T** | **A** | **T** | **C** | **A** | **T** | **T** | **T** | **G** | **A** | **A** | **T** | **T** | **A** | **T** | **T** | **T** | **C** | **T** | **T** | **T** | **A** | **A** | **A** | **A** | **A** | **A** | **G** | **C** | **A** | **G** | **A** | **T** | **C** | **G** | **A** | **G** | **A** | **T** | **G** | **A** | **A** | **T** | **C** | **T** | **A** | **A** | **G** | **C** | **T** | **T** | **G** | **C** | **T** | **G** | **A** | **T** | **A** | **C** | **A** | **T** | **C** | **C** | **A** | **T** | **C** | **C** | **A** | **G** | **C** | **T** | **T** | **G** | **T** | **T** | **T** | **C** | **A** | **A** | **G** | **C** | **G** | **G** | **A** | **C** | **C** | **G** | **A** | **A** | **T** | **A** | **A** | **C** | **G** | **A** | **A** | **A** | **G** | **G** | **G** | **A** | **A** | **C** | **A** | **A** | **A** | **T** | **C** | **A** | **A** | **G** | **C** | **T** | **T** | **T** | **C** | **A** | **T** | **T** | **T** | **A** | **T** | **C** | **A** | **A** | **A** | **T** | **T** | **G** | **A** | **T** | **T** | **A** | **C** | **T** | **T** | **C** | **T** | **G** | **C** | **T** | **A** | **A** | **A** | **A** | **A** | **A** | **C** | **G** | **T** | **G** | **T** | **T** | **T** | **G** | **G** | **A** | **T** | **A** | **C** | **A** | **G** | **A** | **C** | **A** | **C** | **C** | **T** | **T** | **A** | **C** | **C** | **T** | **T** | **G** | **T** | **T** | **C** | **C** | **T** | **G** | **A** | **T** | **G** | **A** | **A** | **A** | **G** | **T** | **G** | **T** | **C** | **A** | **T** | **T** | **G** | **C** | **T** | **G** | **C** | **T** | **T** | **T** | **A** | **A** | **A** | **A** | **A** | **T** | **C** | **G** | **C** | **A** | **A** | **C** | **T** | **G** | **C** | **C** | **T** | **C** | **T** | **G** | **G** | **T** | **G** | **T** | **A** | **G** | **A** | **T** | **G** | **T** | **G** | **A** | **A** | **A** | **A** | **T** | **C** | **A** | **T** | **G** | **A** | **T** | **T** | **C** | **C** | **C** | **A** | **A** | **C** | **A** | **A** | **A** | **C** | **C** | **G** | **G** | **A** | **T** | **C** | **A** | **T** | **C** | **C** | **T** | **T** | **T** | **T** | **A** | **T** | **T** | **T** | **A** | **T** | **C** | **G** | **A** | **G** | **C** | **A** | **A** | **C** | **A** | **C** | **A** | **A** | **T** | **A** | **T** | **T** | **A** | **T** | **G** | **C** | **T** | **C** | **G** | **G** | **C** | **A** | **G** | **C** | **T** | **G** | **A** | **T** | **C** | **A** | **A** | **G** | **G** | **A** | **A** | **A** | **A** | **T** | **G** | **T** | **A** | **C** | **A** | **A** | **A** | **T** | **C** | **C** | **T** | **T** | **G** | **T** | **C** | **T** | **A** | **T** | **G** | **A** | **G** | **A** | **A** | **C** | **G** | **G** | **C** | **T** | **T** | **C** | **C** | **T** | **C** | **C** | **A** | **T** | **G** | **C** | **A** | **A** | **A** | **A** | **A** | **C** | **A** | **T** | **T** | **G** | **A** | **T** | **A** | **A** | **T** | **G** | **G** | **A** | **T** | **G** | **A** | **T** | **G** | **A** | **A** | **A** | **T** | **C** | **T** | **G** | **C** | **A** | **T** | **G** | **G** | **T** | **A** | **G** | **G** | **T** | **T** | **C** | **A** | **G** | **C** | **A** | **A** | **A** | **T** | **C** | **A** | **A** | **G** | **A** | **T** | **A** | **T** | **T** | **C** | **G** | **T** | **A** | **G** | **C** | **T** | **A** | **C** | **C** | **G** | **A** | **T** | **T** | **G** | **A** | **A** | **T** | **T** | **T** | **T** | **G** | **A** | **A** | **A** | **C** | **A** | **A** | **G** | **T** | **G** | **C** | **T** | **G** | **T** | **C** | **A** | **T** | **T** | **T** | **A** | **C** | **G** | **A** | **T** | **C** | **C** | **T** | **G** | **A** | **G** | **T** | **T** | **T** | **T** | **T** | **A** | **G** | **A** | **A** | **G** | **A** | **A** | **C** | **T** | **T** | **G** | **C** | **T** | **A** | **C** | **T** | **C** | **A** | **G** | **T** | **T** | **C** | **A** | **A** | **A** | **G** | **A** | **A** | **G** | **A** | **T** | **G** | **A** | **G** | **A** | **C** | **A** | **C** | **A** | **T** | **T** | **G** | **T** | **T** | **C** | **A** | **T** | **C** | **C** | **A** | **T** | **G** | **A** | **C** | **A** | **A** | **C** | **T** | **G** | **A** | **A** | **A** | **C** | **A** | **G** | **T** | **C** | **A** | **A** | **G** | **G** | **A** | **A** | **A** | **T** | **G** | **T** | **C** | **T** | **A** | **A** | **C** | **T** | **G** | **G** | **C** | **T** | **A** | **T** | **T** | **A** | **T** | **T** | **C** | **A** | **A** | **G** | **C** | **A** | **A** | **C** | **A** | **A** | **A** | **T** | **T** | **T** | **C** | **T** | **C** | **G** | **A** | **T** | **T** | **A** | **T** | **T** | **T** | **T** | **C** | **T** | **C** | **C** | **A** | **A** | **T** | **C** | **C** | **T** | **A** | **-** | **-** | **-** |
| **N60123F** | **-** | **-** | **-** | **-** | **-** | **-** | **-** | **-** | **-** | **-** | **-** | **-** | **-** | **-** | **-** | **-** | **-** | **-** | **-** | **-** | **-** | **-** | **-** | **-** | **-** | **-** | **-** | **-** | **-** | **-** | **-** | **-** | **-** | **-** | **-** | **-** | **-** | **-** | **-** | **-** | **-** | **-** | **-** | **-** | **-** | **-** | **-** | **-** | **-** | **-** | **-** | **-** | **-** | **-** | **-** | **-** | **-** | **-** | **-** | **-** | **-** | **-** | **-** | **-** | **-** | **-** | **-** | **-** | **-** | **A** | **A** | **A** | **C** | **C** | **C** | **C** | **G** | **A** | **G** | **A** | **T** | **G** | **T** | **A** | **G** | **C** | **A** | **G** | **C** | **C** | **A** | **T** | **T** | **T** | **G** | **G** | **G** | **C** | **A** | **T** | **G** | **G** | **C** | **T** | **C** | **T** | **T** | **A** | **G** | **T** | **A** | **T** | **T** | **G** | **T** | **T** | **T** | **G** | **C** | **C** | **C** | **T** | **T** | **C** | **C** | **T** | **G** | **G** | **G** | **G** | **T** | **G** | **G** | **G** | **T** | **T** | **T** | **T** | **G** | **T** | **A** | **T** | **T** | **A** | **T** | **A** | **T** | **T** | **T** | **A** | **T** | **T** | **T** | **T** | **T** | **C** | **G** | **G** | **A** | **C** | **G** | **T** | **G** | **G** | **A** | **T** | **T** | **A** | **A** | **C** | **G** | **G** | **A** | **T** | **A** | **A** | **G** | **A** | **A** | **A** | **A** | **A** | **A** | **T** | **T** | **C** | **T** | **A** | **T** | **C** | **T** | **C** | **C** | **G** | **A** | **C** | **A** | **A** | **A** | **G** | **T** | **G** | **A** | **C** | **T** | **T** | **G** | **A** | **A** | **A** | **G** | **A** | **A** | **T** | **T** | **A** | **G** | **A** | **A** | **A** | **A** | **C** | **T** | **T** | **T** | **C** | **A** | **G** | **T** | **C** | **T** | **T** | **T** | **T** | **A** | **G** | **A** | **G** | **G** | **A** | **G** | **A** | **T** | **A** | **C** | **C** | **A** | **T** | **T** | **G** | **A** | **A** | **C** | **A** | **T** | **T** | **A** | **C** | **G** | **A** | **T** | **C** | **C** | **T** | **G** | **A** | **C** | **A** | **T** | **G** | **G** | **G** | **C** | **G** | **A** | **T** | **A** | **A** | **A** | **G** | **A** | **C** | **A** | **A** | **A** | **C** | **A** | **G** | **C** | **A** | **A** | **T** | **T** | **T** | **G** | **T** | **C** | **G** | **A** | **C** | **T** | **T** | **C** | **T** | **T** | **C** | **T** | **C** | **T** | **T** | **C** | **A** | **T** | **T** | **A** | **A** | **A** | **T** | **C** | **G** | **T** | **A** | **T** | **G** | **C** | **C** | **G** | **C** | **T** | **G** | **A** | **C** | **A** | **A** | **G** | **A** | **A** | **T** | **G** | **A** | **A** | **T** | **T** | **C** | **T** | **G** | **T** | **C** | **A** | **C** | **T** | **C** | **T** | **T** | **C** | **T** | **C** | **A** | **C** | **A** | **G** | **A** | **C** | **G** | **G** | **A** | **C** | **A** | **A** | **G** | **A** | **G** | **A** | **A** | **A** | **T** | **T** | **G** | **G** | **A** | **T** | **T** | **C** | **A** | **C** | **T** | **G** | **C** | **T** | **T** | **C** | **A** | **A** | **G** | **A** | **T** | **C** | **T** | **A** | **A** | **A** | **A** | **A** | **A** | **A** | **G** | **C** | **C** | **A** | **A** | **A** | **C** | **A** | **T** | **T** | **C** | **G** | **A** | **T** | **C** | **C** | **A** | **T** | **A** | **T** | **C** | **G** | **A** | **A** | **T** | **A** | **T** | **T** | **A** | **C** | **G** | **C** | **A** | **T** | **T** | **T** | **G** | **T** | **G** | **A** | **C** | **A** | **G** | **A** | **T** | **A** | **A** | **T** | **A** | **T** | **C** | **G** | **G** | **C** | **C** | **A** | **G** | **C** | **A** | **A** | **G** | **T** | **C** | **T** | **T** | **A** | **C** | **A** | **T** | **G** | **T** | **T** | **T** | **T** | **A** | **G** | **A** | **A** | **G** | **A** | **A** | **A** | **A** | **A** | **G** | **C** | **C** | **G** | **C** | **A** | **G** | **A** | **A** | **G** | **G** | **C** | **G** | **T** | **G** | **G** | **A** | **A** | **G** | **T** | **T** | **C** | **G** | **A** | **A** | **T** | **A** | **T** | **T** | **A** | **T** | **A** | **T** | **G** | **A** | **T** | **G** | **C** | **A** | **T** | **T** | **T** | **G** | **G** | **C** | **T** | **C** | **T** | **C** | **A** | **T** | **G** | **G** | **C** | **A** | **C** | **A** | **A** | **A** | **A** | **G** | **C** | **A** | **A** | **A** | **A** | **G** | **A** | **T** | **T** | **T** | **C** | **A** | **A** | **T** | **C** | **G** | **T** | **C** | **T** | **A** | **A** | **T** | **C** | **A** | **A** | **A** | **A** | **A** | **T** | **G** | **G** | **T** | **G** | **G** | **A** | **C** | **A** | **T** | **G** | **T** | **C** | **C** | **A** | **T** | **A** | **C** | **A** | **T** | **T** | **T** | **G** | **T** | **T** | **A** | **C** | **C** | **T** | **C** | **A** | **C** | **A** | **A** | **A** | **A** | **G** | **G** | **C** | **A** | **T** | **T** | **A** | **C** | **T** | **T** | **C** | **G** | **T** | **T** | **T** | **C** | **C** | **G** | **A** | **T** | **T** | **G** | **A** | **A** | **T** | **T** | **A** | **C** | **C** | **A** | **T** | **G** | **A** | **T** | **C** | **A** | **C** | **C** | **G** | **A** | **A** | **A** | **A** | **A** | **T** | **C** | **G** | **T** | **T** | **G** | **T** | **G** | **A** | **T** | **C** | **G** | **A** | **T** | **G** | **G** | **A** | **A** | **A** | **G** | **A** | **T** | **T** | **A** | **G** | **T** | **T** | **A** | **T** | **A** | **C** | **C** | **G** | **G** | **T** | **G** | **G** | **T** | **T** | **T** | **C** | **A** | **A** | **T** | **A** | **T** | **T** | **G** | **C** | **C** | **A** | **A** | **T** | **C** | **A** | **A** | **T** | **A** | **T** | **G** | **T** | **A** | **A** | **A** | **T** | **A** | **C** | **A** | **A** | **C** | **A** | **A** | **A** | **A** | **A** | **A** | **A** | **T** | **T** | **C** | **G** | **G** | **C** | **T** | **A** | **T** | **T** | **G** | **G** | **C** | **G** | **C** | **G** | **A** | **T** | **A** | **C** | **G** | **C** | **A** | **T** | **A** | **T** | **A** | **C** | **G** | **G** | **A** | **T** | **T** | **T** | **T** | **C** | **G** | **G** | **T** | **G** | **C** | **C** | **G** | **C** | **T** | **T** | **C** | **T** | **T** | **C** | **T** | **T** | **T** | **G** | **C** | **T** | **C** | **C** | **A** | **G** | **C** | **T** | **T** | **C** | **G** | **C** | **T** | **T** | **C** | **T** | **T** | **A** | **A** | **C** | **A** | **G** | **A** | **C** | **T** | **G** | **G** | **A** | **A** | **C** | **G** | **T** | **C** | **T** | **C** | **G** | **G** | **T** | **A** | **C** | **C** | **T** | **G** | **A** | **A** | **G** | **A** | **A** | **A** | **A** | **A** | **A** | **A** | **G** | **G** | **T** | **C** | **G** | **G** | **C** | **T** | **A** | **T** | **C** | **A** | **T** | **T** | **T** | **G** | **A** | **A** | **T** | **T** | **A** | **T** | **T** | **T** | **C** | **T** | **T** | **T** | **A** | **A** | **A** | **A** | **A** | **A** | **G** | **C** | **A** | **G** | **A** | **T** | **C** | **G** | **A** | **G** | **A** | **T** | **G** | **A** | **A** | **T** | **C** | **T** | **A** | **A** | **G** | **C** | **T** | **T** | **G** | **C** | **T** | **G** | **A** | **T** | **A** | **C** | **A** | **T** | **C** | **C** | **A** | **T** | **C** | **C** | **A** | **G** | **C** | **T** | **T** | **G** | **T** | **T** | **T** | **C** | **A** | **A** | **G** | **C** | **G** | **G** | **A** | **C** | **C** | **G** | **A** | **A** | **T** | **A** | **A** | **C** | **G** | **A** | **A** | **A** | **G** | **G** | **G** | **A** | **A** | **C** | **A** | **A** | **A** | **T** | **C** | **A** | **A** | **G** | **C** | **T** | **T** | **T** | **C** | **A** | **T** | **T** | **T** | **A** | **T** | **C** | **A** | **A** | **A** | **T** | **T** | **G** | **A** | **T** | **T** | **A** | **C** | **T** | **T** | **C** | **T** | **G** | **C** | **T** | **A** | **A** | **A** | **A** | **A** | **A** | **C** | **G** | **T** | **G** | **T** | **T** | **T** | **G** | **G** | **A** | **T** | **A** | **C** | **A** | **G** | **A** | **C** | **A** | **C** | **C** | **T** | **T** | **A** | **C** | **C** | **T** | **T** | **G** | **T** | **T** | **C** | **C** | **T** | **G** | **A** | **T** | **G** | **A** | **A** | **A** | **G** | **T** | **G** | **T** | **C** | **A** | **T** | **T** | **G** | **C** | **T** | **G** | **C** | **T** | **T** | **T** | **A** | **A** | **A** | **A** | **A** | **T** | **C** | **G** | **C** | **A** | **A** | **C** | **T** | **G** | **C** | **C** | **T** | **C** | **T** | **G** | **G** | **T** | **G** | **T** | **A** | **G** | **A** | **T** | **G** | **T** | **G** | **A** | **A** | **A** | **A** | **T** | **C** | **A** | **T** | **G** | **A** | **T** | **T** | **C** | **C** | **C** | **A** | **A** | **C** | **A** | **A** | **A** | **C** | **C** | **G** | **G** | **A** | **T** | **C** | **A** | **T** | **C** | **C** | **T** | **T** | **T** | **T** | **A** | **T** | **T** | **T** | **A** | **T** | **C** | **G** | **A** | **G** | **C** | **A** | **A** | **C** | **A** | **C** | **A** | **A** | **T** | **A** | **T** | **T** | **A** | **T** | **G** | **C** | **T** | **C** | **G** | **G** | **C** | **A** | **G** | **C** | **T** | **G** | **A** | **T** | **C** | **A** | **A** | **G** | **G** | **A** | **A** | **A** | **A** | **T** | **G** | **T** | **A** | **C** | **A** | **A** | **A** | **T** | **C** | **C** | **T** | **T** | **G** | **T** | **C** | **T** | **A** | **T** | **G** | **A** | **G** | **A** | **A** | **C** | **G** | **G** | **C** | **T** | **T** | **C** | **C** | **T** | **C** | **C** | **A** | **T** | **G** | **C** | **A** | **A** | **A** | **A** | **A** | **C** | **A** | **T** | **T** | **G** | **A** | **T** | **A** | **A** | **T** | **G** | **G** | **A** | **T** | **G** | **A** | **T** | **G** | **A** | **A** | **A** | **T** | **C** | **T** | **G** | **C** | **A** | **T** | **G** | **G** | **T** | **A** | **G** | **G** | **T** | **T** | **C** | **A** | **G** | **C** | **A** | **A** | **A** | **T** | **C** | **A** | **A** | **G** | **A** | **T** | **A** | **T** | **T** | **C** | **G** | **T** | **A** | **G** | **C** | **T** | **A** | **C** | **C** | **G** | **A** | **T** | **T** | **G** | **A** | **A** | **T** | **T** | **T** | **T** | **G** | **A** | **A** | **A** | **C** | **A** | **A** | **G** | **T** | **G** | **C** | **T** | **G** | **T** | **C** | **A** | **T** | **T** | **T** | **A** | **C** | **G** | **A** | **T** | **C** | **C** | **T** | **G** | **A** | **G** | **T** | **T** | **T** | **T** | **T** | **A** | **G** | **A** | **A** | **G** | **A** | **A** | **C** | **T** | **T** | **G** | **C** | **T** | **A** | **C** | **T** | **C** | **A** | **G** | **T** | **T** | **C** | **A** | **A** | **A** | **G** | **A** | **A** | **G** | **A** | **T** | **G** | **A** | **G** | **A** | **C** | **A** | **C** | **A** | **T** | **T** | **G** | **T** | **T** | **C** | **A** | **T** | **C** | **C** | **A** | **T** | **G** | **A** | **C** | **A** | **A** | **C** | **T** | **G** | **A** | **A** | **A** | **C** | **A** | **G** | **T** | **C** | **A** | **A** | **G** | **G** | **A** | **A** | **A** | **T** | **G** | **T** | **C** | **T** | **A** | **A** | **C** | **T** | **G** | **G** | **C** | **T** | **A** | **T** | **T** | **A** | **T** | **T** | **C** | **A** | **A** | **G** | **C** | **A** | **A** | **C** | **A** | **A** | **A** | **T** | **T** | **T** | **C** | **T** | **C** | **G** | **A** | **T** | **T** | **A** | **T** | **T** | **T** | **T** | **C** | **T** | **C** | **C** | **A** | **A** | **T** | **C** | **C** | **T** | **A** | **-** | **-** | **-** |
| **N53595** | **-** | **-** | **-** | **-** | **-** | **-** | **-** | **-** | **-** | **-** | **-** | **-** | **-** | **-** | **-** | **-** | **-** | **-** | **-** | **-** | **-** | **-** | **-** | **-** | **-** | **-** | **-** | **-** | **-** | **-** | **-** | **-** | **-** | **-** | **-** | **-** | **-** | **-** | **-** | **-** | **-** | **-** | **-** | **-** | **-** | **-** | **-** | **-** | **-** | **-** | **-** | **-** | **-** | **-** | **-** | **-** | **-** | **-** | **-** | **-** | **-** | **-** | **-** | **-** | **-** | **-** | **-** | **-** | **-** | **A** | **A** | **A** | **C** | **C** | **C** | **C** | **G** | **A** | **G** | **A** | **T** | **G** | **T** | **A** | **G** | **C** | **A** | **G** | **C** | **C** | **A** | **T** | **T** | **T** | **G** | **G** | **G** | **C** | **A** | **T** | **G** | **G** | **C** | **T** | **C** | **T** | **T** | **A** | **G** | **T** | **A** | **T** | **T** | **G** | **T** | **T** | **T** | **G** | **C** | **C** | **C** | **T** | **T** | **C** | **C** | **T** | **G** | **G** | **G** | **G** | **T** | **G** | **G** | **G** | **C** | **T** | **T** | **T** | **G** | **T** | **A** | **T** | **T** | **A** | **T** | **A** | **T** | **T** | **T** | **A** | **T** | **T** | **T** | **T** | **T** | **C** | **G** | **G** | **A** | **C** | **G** | **T** | **G** | **G** | **A** | **T** | **T** | **A** | **A** | **C** | **G** | **G** | **A** | **T** | **A** | **A** | **G** | **A** | **A** | **A** | **A** | **A** | **A** | **T** | **T** | **C** | **T** | **A** | **T** | **C** | **T** | **C** | **C** | **G** | **A** | **C** | **A** | **A** | **A** | **G** | **T** | **G** | **A** | **C** | **T** | **T** | **G** | **A** | **A** | **A** | **G** | **A** | **A** | **T** | **T** | **A** | **G** | **A** | **A** | **A** | **A** | **C** | **T** | **T** | **T** | **C** | **A** | **G** | **T** | **C** | **T** | **T** | **T** | **T** | **A** | **G** | **A** | **G** | **G** | **A** | **G** | **A** | **T** | **A** | **A** | **C** | **A** | **T** | **T** | **G** | **A** | **A** | **C** | **A** | **T** | **T** | **A** | **C** | **G** | **A** | **T** | **C** | **C** | **T** | **G** | **A** | **C** | **A** | **T** | **G** | **G** | **G** | **C** | **G** | **A** | **T** | **A** | **A** | **A** | **G** | **A** | **C** | **A** | **A** | **A** | **C** | **A** | **G** | **C** | **A** | **A** | **T** | **T** | **T** | **G** | **T** | **T** | **G** | **A** | **C** | **T** | **T** | **C** | **T** | **T** | **C** | **T** | **C** | **T** | **T** | **C** | **A** | **T** | **T** | **A** | **A** | **A** | **T** | **C** | **G** | **T** | **A** | **T** | **G** | **C** | **C** | **G** | **C** | **T** | **G** | **A** | **C** | **A** | **A** | **G** | **A** | **A** | **T** | **G** | **A** | **A** | **T** | **T** | **C** | **T** | **G** | **T** | **C** | **A** | **C** | **T** | **C** | **T** | **T** | **C** | **T** | **C** | **A** | **C** | **A** | **G** | **A** | **C** | **G** | **G** | **A** | **C** | **A** | **A** | **G** | **A** | **G** | **A** | **A** | **A** | **T** | **T** | **G** | **G** | **A** | **T** | **T** | **C** | **A** | **C** | **T** | **G** | **C** | **T** | **T** | **C** | **A** | **A** | **G** | **A** | **T** | **C** | **T** | **A** | **A** | **A** | **A** | **A** | **A** | **A** | **G** | **C** | **C** | **A** | **A** | **A** | **C** | **A** | **T** | **T** | **C** | **G** | **A** | **T** | **C** | **C** | **A** | **T** | **A** | **T** | **C** | **G** | **A** | **A** | **T** | **A** | **T** | **T** | **A** | **C** | **G** | **C** | **A** | **T** | **T** | **T** | **G** | **T** | **G** | **A** | **C** | **A** | **G** | **A** | **T** | **A** | **A** | **T** | **A** | **T** | **C** | **G** | **G** | **C** | **C** | **A** | **G** | **C** | **A** | **A** | **G** | **T** | **C** | **T** | **T** | **A** | **C** | **A** | **T** | **G** | **T** | **T** | **T** | **T** | **A** | **G** | **A** | **A** | **G** | **A** | **A** | **A** | **A** | **A** | **G** | **C** | **C** | **G** | **C** | **A** | **G** | **A** | **A** | **G** | **G** | **C** | **G** | **T** | **G** | **G** | **A** | **A** | **G** | **T** | **T** | **C** | **G** | **A** | **A** | **T** | **A** | **T** | **T** | **A** | **T** | **A** | **T** | **G** | **A** | **T** | **G** | **C** | **A** | **T** | **T** | **T** | **G** | **G** | **C** | **T** | **C** | **T** | **C** | **A** | **T** | **G** | **G** | **C** | **A** | **C** | **A** | **A** | **A** | **A** | **G** | **C** | **A** | **A** | **A** | **A** | **G** | **A** | **T** | **T** | **T** | **C** | **A** | **A** | **T** | **C** | **G** | **T** | **C** | **T** | **A** | **A** | **T** | **C** | **A** | **A** | **A** | **A** | **A** | **T** | **G** | **G** | **T** | **G** | **G** | **A** | **C** | **A** | **T** | **G** | **T** | **C** | **C** | **A** | **T** | **A** | **C** | **A** | **T** | **T** | **T** | **G** | **T** | **T** | **A** | **C** | **C** | **T** | **C** | **A** | **C** | **A** | **A** | **A** | **A** | **G** | **G** | **C** | **A** | **T** | **T** | **A** | **C** | **T** | **T** | **C** | **G** | **T** | **T** | **T** | **C** | **C** | **G** | **A** | **T** | **T** | **G** | **A** | **A** | **T** | **T** | **A** | **C** | **C** | **A** | **T** | **G** | **A** | **T** | **C** | **A** | **C** | **C** | **G** | **A** | **A** | **A** | **A** | **A** | **T** | **C** | **G** | **T** | **T** | **G** | **T** | **G** | **A** | **T** | **C** | **G** | **A** | **T** | **G** | **G** | **A** | **A** | **A** | **G** | **A** | **T** | **T** | **A** | **G** | **T** | **T** | **A** | **T** | **A** | **C** | **C** | **G** | **G** | **T** | **G** | **G** | **T** | **T** | **T** | **C** | **A** | **A** | **T** | **A** | **T** | **T** | **G** | **C** | **C** | **A** | **A** | **T** | **C** | **A** | **A** | **T** | **A** | **T** | **G** | **T** | **A** | **A** | **A** | **T** | **A** | **C** | **A** | **A** | **C** | **A** | **A** | **A** | **A** | **A** | **A** | **A** | **T** | **T** | **C** | **G** | **G** | **C** | **T** | **A** | **T** | **T** | **G** | **G** | **C** | **G** | **C** | **G** | **A** | **T** | **A** | **C** | **G** | **C** | **A** | **T** | **A** | **T** | **A** | **C** | **G** | **G** | **A** | **T** | **T** | **T** | **T** | **C** | **G** | **G** | **T** | **G** | **C** | **C** | **G** | **C** | **T** | **T** | **C** | **T** | **T** | **C** | **T** | **T** | **T** | **G** | **C** | **T** | **C** | **C** | **A** | **G** | **C** | **T** | **T** | **C** | **G** | **C** | **T** | **T** | **C** | **T** | **T** | **A** | **A** | **C** | **A** | **G** | **A** | **C** | **T** | **G** | **G** | **A** | **A** | **C** | **G** | **T** | **C** | **T** | **C** | **G** | **G** | **T** | **A** | **C** | **C** | **T** | **G** | **A** | **A** | **G** | **A** | **A** | **A** | **A** | **A** | **A** | **A** | **G** | **G** | **T** | **C** | **G** | **G** | **C** | **T** | **A** | **T** | **C** | **A** | **T** | **T** | **T** | **G** | **A** | **A** | **T** | **T** | **A** | **T** | **T** | **T** | **C** | **T** | **T** | **T** | **A** | **A** | **A** | **A** | **A** | **A** | **G** | **C** | **A** | **G** | **A** | **T** | **C** | **G** | **A** | **G** | **A** | **T** | **G** | **A** | **A** | **T** | **C** | **T** | **A** | **A** | **G** | **C** | **T** | **T** | **G** | **C** | **T** | **G** | **A** | **T** | **A** | **C** | **A** | **T** | **C** | **C** | **A** | **T** | **C** | **C** | **A** | **G** | **C** | **T** | **T** | **G** | **T** | **T** | **T** | **C** | **A** | **A** | **G** | **C** | **G** | **G** | **A** | **C** | **C** | **G** | **A** | **A** | **T** | **A** | **A** | **C** | **G** | **A** | **A** | **A** | **G** | **G** | **G** | **A** | **A** | **C** | **A** | **A** | **A** | **T** | **C** | **A** | **A** | **G** | **C** | **T** | **T** | **T** | **C** | **A** | **T** | **T** | **T** | **A** | **T** | **C** | **A** | **A** | **A** | **T** | **T** | **G** | **A** | **T** | **T** | **A** | **C** | **T** | **T** | **C** | **T** | **G** | **C** | **T** | **A** | **A** | **A** | **A** | **A** | **A** | **C** | **G** | **T** | **G** | **T** | **T** | **T** | **G** | **G** | **A** | **T** | **A** | **C** | **A** | **G** | **A** | **C** | **A** | **C** | **C** | **T** | **T** | **A** | **C** | **C** | **T** | **T** | **G** | **T** | **T** | **C** | **C** | **T** | **G** | **A** | **T** | **G** | **A** | **A** | **A** | **G** | **T** | **G** | **T** | **C** | **A** | **T** | **T** | **G** | **C** | **T** | **G** | **C** | **T** | **T** | **T** | **A** | **A** | **A** | **A** | **A** | **T** | **C** | **G** | **C** | **A** | **A** | **C** | **T** | **G** | **C** | **C** | **T** | **C** | **T** | **G** | **G** | **T** | **G** | **T** | **A** | **G** | **A** | **T** | **G** | **T** | **G** | **A** | **A** | **A** | **A** | **T** | **C** | **A** | **T** | **G** | **A** | **T** | **T** | **C** | **C** | **C** | **A** | **A** | **C** | **A** | **A** | **A** | **C** | **C** | **G** | **G** | **A** | **T** | **C** | **A** | **T** | **C** | **C** | **T** | **T** | **T** | **T** | **A** | **T** | **T** | **T** | **A** | **T** | **C** | **G** | **A** | **G** | **C** | **A** | **A** | **C** | **A** | **C** | **A** | **A** | **T** | **A** | **T** | **T** | **A** | **T** | **G** | **C** | **T** | **C** | **G** | **G** | **C** | **A** | **G** | **C** | **T** | **G** | **A** | **T** | **C** | **A** | **A** | **G** | **G** | **A** | **A** | **A** | **A** | **T** | **G** | **T** | **A** | **C** | **A** | **A** | **A** | **T** | **C** | **C** | **T** | **T** | **G** | **T** | **C** | **T** | **A** | **T** | **G** | **A** | **G** | **A** | **A** | **C** | **G** | **G** | **C** | **T** | **T** | **C** | **C** | **T** | **C** | **C** | **A** | **T** | **G** | **C** | **A** | **A** | **A** | **A** | **A** | **C** | **A** | **T** | **T** | **G** | **A** | **T** | **A** | **A** | **T** | **G** | **G** | **A** | **T** | **G** | **A** | **T** | **G** | **A** | **A** | **A** | **T** | **C** | **T** | **G** | **C** | **A** | **T** | **G** | **G** | **T** | **A** | **G** | **G** | **T** | **T** | **C** | **A** | **G** | **C** | **A** | **A** | **A** | **T** | **C** | **A** | **A** | **G** | **A** | **T** | **A** | **T** | **T** | **C** | **G** | **T** | **A** | **G** | **C** | **T** | **A** | **C** | **C** | **G** | **A** | **T** | **T** | **G** | **A** | **A** | **T** | **T** | **T** | **T** | **G** | **A** | **A** | **A** | **C** | **A** | **A** | **G** | **T** | **G** | **C** | **T** | **G** | **T** | **C** | **A** | **T** | **T** | **T** | **A** | **C** | **G** | **A** | **T** | **C** | **C** | **T** | **G** | **A** | **G** | **T** | **T** | **T** | **T** | **T** | **A** | **G** | **A** | **A** | **G** | **A** | **A** | **C** | **T** | **T** | **G** | **C** | **T** | **A** | **C** | **T** | **C** | **A** | **G** | **T** | **T** | **C** | **A** | **A** | **A** | **G** | **A** | **A** | **G** | **A** | **T** | **G** | **A** | **G** | **A** | **C** | **A** | **C** | **A** | **T** | **T** | **G** | **T** | **T** | **C** | **A** | **T** | **C** | **C** | **A** | **T** | **G** | **A** | **C** | **A** | **A** | **C** | **T** | **G** | **A** | **A** | **A** | **C** | **A** | **G** | **T** | **C** | **A** | **A** | **G** | **G** | **A** | **A** | **A** | **T** | **G** | **T** | **C** | **T** | **A** | **A** | **C** | **T** | **G** | **G** | **C** | **T** | **A** | **T** | **T** | **A** | **T** | **T** | **C** | **A** | **A** | **G** | **C** | **A** | **A** | **C** | **A** | **A** | **A** | **T** | **T** | **T** | **C** | **T** | **C** | **G** | **A** | **T** | **T** | **A** | **T** | **T** | **T** | **T** | **C** | **T** | **C** | **C** | **A** | **A** | **T** | **C** | **C** | **T** | **A** | **-** | **-** | **-** |
| **N59439F** | **-** | **-** | **-** | **-** | **-** | **-** | **-** | **-** | **-** | **-** | **-** | **-** | **-** | **-** | **-** | **-** | **-** | **-** | **-** | **-** | **-** | **-** | **-** | **-** | **-** | **-** | **-** | **-** | **-** | **-** | **-** | **-** | **-** | **-** | **-** | **-** | **-** | **-** | **-** | **-** | **-** | **-** | **-** | **-** | **-** | **-** | **-** | **-** | **-** | **-** | **-** | **-** | **-** | **-** | **-** | **-** | **-** | **-** | **-** | **-** | **-** | **-** | **-** | **-** | **-** | **-** | **-** | **-** | **-** | **A** | **A** | **A** | **C** | **C** | **C** | **C** | **G** | **A** | **G** | **A** | **T** | **G** | **T** | **A** | **G** | **C** | **A** | **G** | **C** | **C** | **A** | **T** | **T** | **T** | **G** | **G** | **G** | **C** | **A** | **T** | **G** | **G** | **C** | **T** | **C** | **T** | **T** | **A** | **G** | **T** | **A** | **T** | **T** | **G** | **T** | **T** | **T** | **G** | **C** | **C** | **C** | **T** | **T** | **C** | **C** | **T** | **G** | **G** | **G** | **G** | **T** | **G** | **G** | **G** | **T** | **T** | **T** | **T** | **G** | **T** | **A** | **T** | **T** | **A** | **T** | **A** | **T** | **T** | **T** | **A** | **T** | **T** | **T** | **T** | **T** | **C** | **G** | **G** | **A** | **C** | **G** | **T** | **G** | **G** | **A** | **T** | **T** | **A** | **A** | **C** | **G** | **G** | **A** | **T** | **A** | **A** | **G** | **A** | **A** | **A** | **A** | **A** | **A** | **T** | **T** | **C** | **T** | **A** | **T** | **C** | **T** | **C** | **C** | **G** | **A** | **C** | **A** | **A** | **A** | **G** | **T** | **G** | **A** | **C** | **T** | **T** | **G** | **A** | **A** | **A** | **G** | **A** | **A** | **T** | **T** | **A** | **G** | **A** | **A** | **A** | **A** | **C** | **T** | **T** | **T** | **C** | **A** | **G** | **T** | **C** | **T** | **T** | **T** | **T** | **A** | **G** | **A** | **G** | **G** | **A** | **G** | **A** | **T** | **A** | **C** | **C** | **A** | **T** | **T** | **G** | **A** | **A** | **C** | **A** | **T** | **T** | **A** | **C** | **G** | **A** | **T** | **C** | **C** | **T** | **G** | **A** | **C** | **A** | **T** | **G** | **G** | **G** | **C** | **G** | **A** | **T** | **A** | **A** | **A** | **G** | **A** | **C** | **A** | **A** | **A** | **C** | **A** | **G** | **C** | **A** | **A** | **T** | **T** | **T** | **G** | **T** | **C** | **G** | **A** | **C** | **T** | **T** | **C** | **T** | **T** | **C** | **T** | **C** | **T** | **T** | **C** | **A** | **T** | **T** | **A** | **A** | **A** | **T** | **C** | **G** | **T** | **A** | **T** | **G** | **C** | **C** | **G** | **C** | **T** | **G** | **A** | **C** | **A** | **A** | **G** | **A** | **A** | **T** | **G** | **A** | **A** | **T** | **T** | **C** | **T** | **G** | **T** | **C** | **A** | **C** | **T** | **C** | **T** | **T** | **C** | **T** | **C** | **A** | **C** | **A** | **G** | **A** | **C** | **G** | **G** | **A** | **C** | **A** | **A** | **G** | **A** | **G** | **A** | **A** | **A** | **T** | **T** | **G** | **G** | **A** | **T** | **T** | **C** | **A** | **C** | **T** | **G** | **C** | **T** | **T** | **C** | **A** | **A** | **G** | **A** | **T** | **C** | **T** | **A** | **A** | **A** | **A** | **A** | **A** | **A** | **G** | **C** | **C** | **A** | **A** | **A** | **C** | **A** | **T** | **T** | **C** | **G** | **A** | **T** | **C** | **C** | **A** | **T** | **A** | **T** | **C** | **G** | **A** | **A** | **T** | **A** | **T** | **T** | **A** | **C** | **G** | **C** | **A** | **T** | **T** | **T** | **G** | **T** | **G** | **A** | **C** | **A** | **G** | **A** | **T** | **A** | **A** | **T** | **A** | **T** | **C** | **G** | **G** | **C** | **C** | **A** | **G** | **C** | **A** | **A** | **G** | **T** | **C** | **T** | **T** | **A** | **C** | **A** | **T** | **G** | **T** | **T** | **T** | **T** | **A** | **G** | **A** | **A** | **G** | **A** | **A** | **A** | **A** | **A** | **G** | **C** | **C** | **G** | **C** | **A** | **G** | **A** | **A** | **G** | **G** | **C** | **G** | **T** | **G** | **G** | **A** | **A** | **G** | **T** | **T** | **C** | **G** | **A** | **A** | **T** | **A** | **T** | **T** | **A** | **T** | **A** | **T** | **G** | **A** | **T** | **G** | **C** | **A** | **T** | **T** | **T** | **G** | **G** | **C** | **T** | **C** | **T** | **C** | **A** | **T** | **G** | **G** | **C** | **A** | **C** | **A** | **A** | **A** | **A** | **G** | **C** | **A** | **A** | **A** | **A** | **G** | **A** | **T** | **T** | **T** | **C** | **A** | **A** | **T** | **C** | **G** | **T** | **C** | **T** | **A** | **A** | **T** | **C** | **A** | **A** | **A** | **A** | **A** | **T** | **G** | **G** | **T** | **G** | **G** | **A** | **C** | **A** | **T** | **G** | **T** | **C** | **C** | **A** | **T** | **A** | **C** | **A** | **T** | **T** | **T** | **G** | **T** | **T** | **A** | **C** | **C** | **T** | **C** | **A** | **C** | **A** | **A** | **A** | **A** | **G** | **G** | **C** | **A** | **T** | **T** | **A** | **C** | **T** | **T** | **C** | **G** | **T** | **T** | **T** | **C** | **C** | **G** | **A** | **T** | **T** | **G** | **A** | **A** | **T** | **T** | **A** | **C** | **C** | **A** | **T** | **G** | **A** | **T** | **C** | **A** | **C** | **C** | **G** | **A** | **A** | **A** | **A** | **A** | **T** | **C** | **G** | **T** | **T** | **G** | **T** | **G** | **A** | **T** | **C** | **G** | **A** | **T** | **G** | **G** | **A** | **A** | **A** | **G** | **A** | **T** | **T** | **A** | **G** | **T** | **T** | **A** | **T** | **A** | **C** | **C** | **G** | **G** | **T** | **G** | **G** | **T** | **T** | **T** | **C** | **A** | **A** | **T** | **A** | **T** | **T** | **G** | **C** | **C** | **A** | **A** | **T** | **C** | **A** | **A** | **T** | **A** | **T** | **G** | **T** | **A** | **A** | **A** | **T** | **A** | **C** | **A** | **A** | **C** | **A** | **A** | **A** | **A** | **A** | **A** | **A** | **T** | **T** | **C** | **G** | **G** | **C** | **T** | **A** | **T** | **T** | **G** | **G** | **C** | **G** | **C** | **G** | **A** | **T** | **A** | **C** | **G** | **C** | **A** | **T** | **A** | **T** | **A** | **C** | **G** | **G** | **A** | **T** | **T** | **T** | **T** | **C** | **G** | **G** | **T** | **G** | **C** | **C** | **G** | **C** | **T** | **T** | **C** | **T** | **T** | **C** | **T** | **T** | **T** | **G** | **C** | **T** | **C** | **C** | **A** | **G** | **C** | **T** | **T** | **C** | **G** | **C** | **T** | **T** | **C** | **T** | **T** | **A** | **A** | **C** | **A** | **G** | **A** | **C** | **T** | **G** | **G** | **A** | **A** | **C** | **G** | **T** | **C** | **T** | **C** | **G** | **G** | **T** | **A** | **C** | **C** | **T** | **G** | **A** | **A** | **G** | **A** | **A** | **A** | **A** | **A** | **A** | **A** | **G** | **G** | **T** | **C** | **G** | **G** | **C** | **T** | **A** | **T** | **C** | **A** | **T** | **T** | **T** | **G** | **A** | **A** | **T** | **T** | **A** | **T** | **T** | **T** | **C** | **T** | **T** | **T** | **A** | **A** | **A** | **A** | **A** | **A** | **G** | **C** | **A** | **G** | **A** | **T** | **C** | **G** | **A** | **G** | **A** | **T** | **G** | **A** | **A** | **T** | **C** | **T** | **A** | **A** | **G** | **C** | **T** | **T** | **G** | **C** | **T** | **G** | **A** | **T** | **A** | **C** | **A** | **T** | **C** | **C** | **A** | **T** | **C** | **C** | **A** | **G** | **C** | **T** | **T** | **G** | **T** | **T** | **T** | **C** | **A** | **A** | **G** | **C** | **G** | **G** | **A** | **C** | **C** | **G** | **A** | **A** | **T** | **A** | **A** | **C** | **G** | **A** | **A** | **A** | **G** | **G** | **G** | **A** | **A** | **C** | **A** | **A** | **A** | **T** | **C** | **A** | **A** | **G** | **C** | **T** | **T** | **T** | **C** | **A** | **T** | **T** | **T** | **A** | **T** | **C** | **A** | **A** | **A** | **T** | **T** | **G** | **A** | **T** | **T** | **A** | **C** | **T** | **T** | **C** | **T** | **G** | **C** | **T** | **A** | **A** | **A** | **A** | **A** | **A** | **C** | **G** | **T** | **G** | **T** | **T** | **T** | **G** | **G** | **A** | **T** | **A** | **C** | **A** | **G** | **A** | **C** | **A** | **C** | **C** | **T** | **T** | **A** | **C** | **C** | **T** | **T** | **G** | **T** | **T** | **C** | **C** | **T** | **G** | **A** | **T** | **G** | **A** | **A** | **A** | **G** | **T** | **G** | **T** | **C** | **A** | **T** | **T** | **G** | **C** | **T** | **G** | **C** | **T** | **T** | **T** | **A** | **A** | **A** | **A** | **A** | **T** | **C** | **G** | **C** | **A** | **A** | **C** | **T** | **G** | **C** | **C** | **T** | **C** | **T** | **G** | **G** | **T** | **G** | **T** | **A** | **G** | **A** | **T** | **G** | **T** | **G** | **A** | **A** | **A** | **A** | **T** | **C** | **A** | **T** | **G** | **A** | **T** | **T** | **C** | **C** | **C** | **A** | **A** | **C** | **A** | **A** | **A** | **C** | **C** | **G** | **G** | **A** | **T** | **C** | **A** | **T** | **C** | **C** | **T** | **T** | **T** | **T** | **A** | **T** | **T** | **T** | **A** | **T** | **C** | **G** | **A** | **G** | **C** | **A** | **A** | **C** | **A** | **C** | **A** | **A** | **T** | **A** | **T** | **T** | **A** | **T** | **G** | **C** | **T** | **C** | **G** | **G** | **C** | **A** | **G** | **C** | **T** | **G** | **A** | **T** | **C** | **A** | **A** | **G** | **G** | **A** | **A** | **A** | **A** | **T** | **G** | **T** | **A** | **C** | **A** | **A** | **A** | **T** | **C** | **C** | **T** | **T** | **G** | **T** | **C** | **T** | **A** | **T** | **G** | **A** | **G** | **A** | **A** | **C** | **G** | **G** | **C** | **T** | **T** | **C** | **C** | **T** | **C** | **C** | **A** | **T** | **G** | **C** | **A** | **A** | **A** | **A** | **A** | **C** | **A** | **T** | **T** | **G** | **A** | **T** | **A** | **A** | **T** | **G** | **G** | **A** | **T** | **G** | **A** | **T** | **G** | **A** | **A** | **A** | **T** | **C** | **T** | **G** | **C** | **A** | **T** | **G** | **G** | **T** | **A** | **G** | **G** | **T** | **T** | **C** | **A** | **G** | **C** | **A** | **A** | **A** | **T** | **C** | **A** | **A** | **G** | **A** | **T** | **A** | **T** | **T** | **C** | **G** | **T** | **A** | **G** | **C** | **T** | **A** | **C** | **C** | **G** | **A** | **T** | **T** | **G** | **A** | **A** | **T** | **T** | **T** | **T** | **G** | **A** | **A** | **A** | **C** | **A** | **A** | **G** | **T** | **G** | **C** | **T** | **G** | **T** | **C** | **A** | **T** | **T** | **T** | **A** | **C** | **G** | **A** | **T** | **C** | **C** | **T** | **G** | **A** | **G** | **T** | **T** | **T** | **T** | **T** | **A** | **G** | **A** | **A** | **G** | **A** | **A** | **C** | **T** | **T** | **G** | **C** | **T** | **A** | **C** | **T** | **C** | **A** | **G** | **T** | **T** | **C** | **A** | **A** | **A** | **G** | **A** | **A** | **G** | **A** | **T** | **G** | **A** | **G** | **A** | **C** | **A** | **C** | **A** | **T** | **T** | **G** | **T** | **T** | **C** | **A** | **T** | **C** | **C** | **A** | **T** | **G** | **A** | **C** | **A** | **A** | **C** | **T** | **G** | **A** | **A** | **A** | **C** | **A** | **G** | **T** | **C** | **A** | **A** | **G** | **G** | **A** | **A** | **A** | **T** | **G** | **T** | **C** | **T** | **A** | **A** | **C** | **T** | **G** | **G** | **C** | **T** | **A** | **T** | **T** | **A** | **T** | **T** | **C** | **A** | **A** | **G** | **C** | **A** | **A** | **C** | **A** | **A** | **A** | **T** | **T** | **T** | **C** | **T** | **C** | **G** | **A** | **T** | **T** | **A** | **T** | **T** | **T** | **T** | **C** | **T** | **C** | **C** | **A** | **A** | **T** | **C** | **C** | **T** | **A** | **-** | **-** | **-** |
| **N53805** | **-** | **-** | **-** | **-** | **-** | **-** | **-** | **-** | **-** | **-** | **-** | **-** | **-** | **-** | **-** | **-** | **-** | **-** | **-** | **-** | **-** | **-** | **-** | **-** | **-** | **-** | **-** | **-** | **-** | **-** | **-** | **-** | **-** | **-** | **-** | **-** | **-** | **-** | **-** | **-** | **-** | **-** | **-** | **-** | **-** | **-** | **-** | **-** | **-** | **-** | **-** | **-** | **-** | **-** | **-** | **-** | **-** | **-** | **-** | **-** | **-** | **-** | **-** | **-** | **-** | **-** | **-** | **-** | **-** | **A** | **A** | **A** | **C** | **C** | **C** | **C** | **G** | **A** | **G** | **A** | **T** | **G** | **T** | **A** | **G** | **C** | **A** | **G** | **C** | **C** | **A** | **T** | **T** | **T** | **G** | **G** | **G** | **C** | **A** | **T** | **G** | **G** | **C** | **T** | **C** | **T** | **T** | **A** | **G** | **T** | **A** | **T** | **T** | **G** | **T** | **T** | **T** | **G** | **T** | **C** | **C** | **T** | **T** | **C** | **C** | **T** | **G** | **G** | **G** | **G** | **T** | **G** | **G** | **G** | **C** | **T** | **T** | **T** | **G** | **T** | **A** | **T** | **T** | **A** | **T** | **A** | **T** | **T** | **T** | **A** | **T** | **T** | **T** | **T** | **T** | **C** | **G** | **G** | **A** | **C** | **G** | **T** | **G** | **G** | **A** | **T** | **T** | **A** | **A** | **C** | **G** | **G** | **A** | **T** | **A** | **A** | **G** | **A** | **A** | **A** | **A** | **A** | **A** | **T** | **T** | **C** | **T** | **A** | **T** | **C** | **T** | **C** | **C** | **G** | **A** | **C** | **A** | **A** | **A** | **G** | **T** | **G** | **A** | **C** | **T** | **T** | **G** | **A** | **A** | **A** | **G** | **A** | **A** | **T** | **T** | **A** | **G** | **A** | **A** | **A** | **A** | **C** | **T** | **T** | **T** | **C** | **A** | **G** | **T** | **C** | **T** | **T** | **T** | **T** | **A** | **G** | **A** | **G** | **G** | **A** | **G** | **A** | **T** | **A** | **C** | **C** | **A** | **T** | **T** | **G** | **A** | **A** | **C** | **A** | **T** | **T** | **A** | **C** | **G** | **A** | **T** | **C** | **C** | **T** | **G** | **A** | **C** | **A** | **T** | **G** | **G** | **G** | **C** | **G** | **A** | **T** | **A** | **A** | **A** | **G** | **A** | **C** | **A** | **A** | **A** | **C** | **A** | **G** | **C** | **A** | **A** | **T** | **T** | **T** | **G** | **T** | **T** | **G** | **A** | **C** | **T** | **T** | **C** | **T** | **T** | **C** | **T** | **C** | **T** | **T** | **C** | **A** | **T** | **T** | **A** | **A** | **A** | **T** | **C** | **G** | **T** | **A** | **T** | **G** | **C** | **C** | **G** | **C** | **T** | **G** | **A** | **C** | **A** | **A** | **G** | **A** | **A** | **T** | **G** | **A** | **A** | **T** | **T** | **C** | **T** | **G** | **T** | **C** | **A** | **C** | **T** | **C** | **T** | **T** | **C** | **T** | **C** | **A** | **C** | **A** | **G** | **A** | **C** | **G** | **G** | **A** | **C** | **A** | **A** | **G** | **A** | **G** | **A** | **A** | **A** | **T** | **T** | **G** | **G** | **A** | **T** | **T** | **C** | **A** | **C** | **T** | **G** | **C** | **T** | **T** | **C** | **A** | **A** | **G** | **A** | **T** | **C** | **T** | **A** | **A** | **A** | **A** | **A** | **A** | **A** | **G** | **C** | **C** | **A** | **A** | **A** | **C** | **A** | **T** | **T** | **C** | **G** | **A** | **T** | **C** | **C** | **A** | **T** | **A** | **T** | **C** | **G** | **A** | **A** | **T** | **A** | **T** | **T** | **A** | **C** | **G** | **C** | **A** | **T** | **T** | **T** | **G** | **T** | **G** | **A** | **C** | **A** | **G** | **A** | **T** | **A** | **A** | **T** | **A** | **T** | **C** | **G** | **G** | **C** | **C** | **A** | **G** | **C** | **A** | **A** | **G** | **T** | **C** | **T** | **T** | **A** | **C** | **A** | **T** | **G** | **T** | **T** | **T** | **T** | **A** | **G** | **A** | **A** | **G** | **A** | **A** | **A** | **A** | **A** | **G** | **C** | **C** | **G** | **C** | **A** | **G** | **A** | **A** | **G** | **G** | **C** | **G** | **T** | **G** | **G** | **A** | **A** | **G** | **T** | **T** | **C** | **G** | **A** | **A** | **T** | **A** | **T** | **T** | **A** | **T** | **A** | **T** | **G** | **A** | **T** | **G** | **C** | **A** | **T** | **T** | **T** | **G** | **G** | **C** | **T** | **C** | **T** | **C** | **A** | **T** | **G** | **G** | **C** | **A** | **C** | **A** | **A** | **A** | **A** | **G** | **C** | **A** | **A** | **A** | **A** | **G** | **A** | **T** | **T** | **T** | **C** | **A** | **A** | **T** | **C** | **G** | **T** | **C** | **T** | **A** | **A** | **T** | **C** | **A** | **A** | **A** | **A** | **A** | **T** | **G** | **G** | **T** | **G** | **G** | **A** | **C** | **A** | **T** | **G** | **T** | **C** | **C** | **A** | **T** | **A** | **C** | **A** | **T** | **T** | **T** | **G** | **T** | **T** | **A** | **C** | **C** | **T** | **C** | **A** | **C** | **A** | **A** | **A** | **A** | **G** | **G** | **C** | **A** | **T** | **T** | **A** | **C** | **T** | **T** | **C** | **G** | **T** | **T** | **T** | **C** | **C** | **G** | **A** | **T** | **T** | **G** | **A** | **A** | **T** | **T** | **A** | **C** | **C** | **A** | **T** | **G** | **A** | **T** | **C** | **A** | **C** | **C** | **G** | **A** | **A** | **A** | **A** | **A** | **T** | **C** | **G** | **T** | **T** | **G** | **T** | **G** | **A** | **T** | **C** | **G** | **A** | **T** | **G** | **G** | **A** | **A** | **A** | **G** | **A** | **T** | **T** | **A** | **G** | **T** | **T** | **A** | **T** | **A** | **C** | **C** | **G** | **G** | **T** | **G** | **G** | **T** | **T** | **T** | **C** | **A** | **A** | **T** | **A** | **T** | **T** | **G** | **C** | **C** | **A** | **A** | **T** | **C** | **A** | **A** | **T** | **A** | **T** | **G** | **T** | **A** | **A** | **A** | **T** | **A** | **C** | **A** | **A** | **C** | **A** | **A** | **A** | **A** | **A** | **A** | **A** | **T** | **T** | **C** | **G** | **G** | **C** | **T** | **A** | **T** | **T** | **G** | **G** | **C** | **G** | **C** | **G** | **A** | **T** | **A** | **C** | **G** | **C** | **A** | **T** | **A** | **T** | **A** | **C** | **G** | **G** | **A** | **T** | **T** | **T** | **T** | **C** | **G** | **G** | **T** | **G** | **C** | **C** | **G** | **C** | **T** | **T** | **C** | **T** | **T** | **C** | **T** | **T** | **T** | **G** | **C** | **T** | **C** | **C** | **A** | **G** | **C** | **T** | **T** | **C** | **G** | **C** | **T** | **T** | **C** | **T** | **T** | **A** | **A** | **C** | **A** | **G** | **A** | **C** | **T** | **G** | **G** | **A** | **A** | **C** | **G** | **T** | **C** | **T** | **C** | **G** | **G** | **T** | **A** | **C** | **C** | **T** | **G** | **A** | **A** | **G** | **A** | **A** | **A** | **A** | **A** | **A** | **A** | **G** | **G** | **T** | **C** | **G** | **G** | **C** | **T** | **A** | **T** | **C** | **A** | **T** | **T** | **T** | **G** | **A** | **A** | **T** | **T** | **A** | **T** | **T** | **T** | **C** | **T** | **T** | **T** | **A** | **A** | **A** | **A** | **A** | **A** | **G** | **C** | **A** | **G** | **A** | **T** | **C** | **G** | **A** | **G** | **A** | **T** | **G** | **A** | **A** | **T** | **C** | **T** | **A** | **A** | **G** | **C** | **T** | **T** | **G** | **C** | **T** | **G** | **A** | **T** | **A** | **C** | **A** | **T** | **C** | **C** | **A** | **T** | **C** | **C** | **A** | **G** | **C** | **T** | **T** | **G** | **T** | **T** | **T** | **C** | **A** | **A** | **G** | **C** | **G** | **G** | **A** | **C** | **C** | **G** | **A** | **A** | **T** | **A** | **A** | **C** | **G** | **A** | **A** | **A** | **G** | **G** | **G** | **A** | **A** | **C** | **A** | **A** | **A** | **T** | **C** | **A** | **A** | **G** | **C** | **T** | **T** | **T** | **C** | **A** | **T** | **T** | **T** | **A** | **T** | **C** | **A** | **A** | **A** | **T** | **T** | **G** | **A** | **T** | **T** | **A** | **C** | **T** | **T** | **C** | **T** | **G** | **C** | **T** | **A** | **A** | **A** | **A** | **A** | **A** | **C** | **G** | **T** | **G** | **T** | **T** | **T** | **G** | **G** | **A** | **T** | **A** | **C** | **A** | **G** | **A** | **C** | **A** | **C** | **C** | **T** | **T** | **A** | **C** | **C** | **T** | **T** | **G** | **T** | **T** | **C** | **C** | **T** | **G** | **A** | **T** | **G** | **A** | **A** | **A** | **G** | **T** | **G** | **T** | **C** | **A** | **T** | **T** | **G** | **C** | **T** | **G** | **C** | **T** | **T** | **T** | **A** | **A** | **A** | **A** | **A** | **T** | **C** | **G** | **C** | **A** | **A** | **C** | **T** | **G** | **C** | **C** | **T** | **C** | **T** | **G** | **G** | **T** | **G** | **T** | **A** | **G** | **A** | **T** | **G** | **T** | **G** | **A** | **A** | **A** | **A** | **T** | **C** | **A** | **T** | **G** | **A** | **T** | **T** | **C** | **C** | **C** | **A** | **A** | **C** | **A** | **A** | **A** | **C** | **C** | **G** | **G** | **A** | **T** | **C** | **A** | **T** | **C** | **C** | **T** | **T** | **T** | **T** | **A** | **T** | **T** | **T** | **A** | **T** | **C** | **G** | **A** | **G** | **C** | **A** | **A** | **C** | **A** | **C** | **A** | **A** | **T** | **A** | **T** | **T** | **A** | **T** | **G** | **C** | **T** | **C** | **G** | **G** | **C** | **A** | **G** | **C** | **T** | **G** | **A** | **T** | **C** | **A** | **A** | **G** | **G** | **A** | **A** | **A** | **A** | **T** | **G** | **T** | **A** | **C** | **A** | **A** | **A** | **T** | **C** | **C** | **T** | **T** | **G** | **T** | **C** | **T** | **A** | **T** | **G** | **A** | **G** | **A** | **A** | **C** | **G** | **G** | **C** | **T** | **T** | **C** | **C** | **T** | **C** | **C** | **A** | **T** | **G** | **C** | **A** | **A** | **A** | **A** | **A** | **C** | **A** | **T** | **T** | **G** | **A** | **T** | **A** | **A** | **T** | **G** | **G** | **A** | **T** | **G** | **A** | **T** | **G** | **A** | **A** | **A** | **T** | **C** | **T** | **G** | **C** | **A** | **T** | **G** | **G** | **T** | **A** | **G** | **G** | **T** | **T** | **C** | **A** | **G** | **C** | **A** | **A** | **A** | **T** | **C** | **A** | **A** | **G** | **A** | **T** | **A** | **T** | **T** | **C** | **G** | **T** | **A** | **G** | **C** | **T** | **A** | **C** | **C** | **G** | **A** | **T** | **T** | **G** | **A** | **A** | **T** | **T** | **T** | **T** | **G** | **A** | **A** | **A** | **C** | **A** | **A** | **G** | **T** | **G** | **C** | **T** | **G** | **T** | **C** | **A** | **T** | **T** | **T** | **A** | **C** | **G** | **A** | **T** | **C** | **C** | **T** | **G** | **A** | **G** | **T** | **T** | **T** | **T** | **T** | **A** | **G** | **A** | **A** | **G** | **A** | **A** | **C** | **T** | **T** | **G** | **C** | **T** | **A** | **C** | **T** | **C** | **A** | **G** | **T** | **T** | **C** | **A** | **A** | **A** | **G** | **A** | **A** | **G** | **A** | **T** | **G** | **A** | **G** | **A** | **C** | **A** | **C** | **A** | **T** | **T** | **G** | **T** | **T** | **C** | **A** | **T** | **C** | **C** | **A** | **T** | **G** | **A** | **C** | **A** | **A** | **C** | **T** | **G** | **A** | **A** | **A** | **C** | **A** | **G** | **T** | **C** | **A** | **A** | **G** | **G** | **A** | **A** | **A** | **T** | **G** | **T** | **C** | **T** | **A** | **A** | **C** | **T** | **G** | **G** | **C** | **T** | **A** | **T** | **T** | **A** | **T** | **T** | **C** | **A** | **A** | **G** | **C** | **A** | **A** | **C** | **A** | **A** | **A** | **T** | **T** | **T** | **C** | **T** | **C** | **G** | **A** | **T** | **T** | **A** | **T** | **T** | **T** | **T** | **C** | **T** | **C** | **C** | **A** | **A** | **T** | **C** | **C** | **T** | **A** | **-** | **-** | **-** |
| **N52690** | **-** | **-** | **-** | **-** | **-** | **-** | **-** | **-** | **-** | **-** | **-** | **-** | **-** | **-** | **-** | **-** | **-** | **-** | **-** | **-** | **-** | **-** | **-** | **-** | **-** | **-** | **-** | **-** | **-** | **-** | **-** | **-** | **-** | **-** | **-** | **-** | **-** | **-** | **-** | **-** | **-** | **-** | **-** | **-** | **-** | **-** | **-** | **-** | **-** | **-** | **-** | **-** | **-** | **-** | **-** | **-** | **-** | **-** | **-** | **-** | **-** | **-** | **-** | **-** | **-** | **-** | **-** | **-** | **-** | **A** | **A** | **A** | **C** | **C** | **C** | **C** | **G** | **A** | **G** | **A** | **T** | **G** | **T** | **A** | **G** | **C** | **A** | **G** | **C** | **C** | **A** | **T** | **T** | **T** | **G** | **G** | **G** | **C** | **A** | **T** | **G** | **G** | **C** | **T** | **C** | **T** | **T** | **A** | **G** | **T** | **A** | **T** | **T** | **G** | **T** | **T** | **T** | **G** | **C** | **C** | **C** | **T** | **T** | **C** | **C** | **T** | **G** | **G** | **G** | **G** | **T** | **G** | **G** | **G** | **T** | **T** | **T** | **T** | **G** | **T** | **A** | **T** | **T** | **A** | **T** | **A** | **T** | **T** | **T** | **A** | **T** | **T** | **T** | **T** | **T** | **C** | **G** | **G** | **A** | **C** | **G** | **T** | **G** | **G** | **A** | **T** | **T** | **A** | **A** | **C** | **G** | **G** | **A** | **T** | **A** | **A** | **G** | **A** | **A** | **A** | **A** | **A** | **A** | **T** | **T** | **C** | **T** | **A** | **T** | **C** | **T** | **C** | **C** | **G** | **A** | **C** | **A** | **A** | **A** | **G** | **T** | **G** | **A** | **C** | **T** | **T** | **G** | **A** | **A** | **A** | **G** | **A** | **A** | **T** | **T** | **A** | **G** | **A** | **A** | **A** | **A** | **C** | **T** | **T** | **T** | **C** | **A** | **G** | **T** | **C** | **T** | **T** | **T** | **T** | **A** | **G** | **A** | **G** | **G** | **A** | **G** | **A** | **T** | **A** | **C** | **C** | **A** | **T** | **T** | **G** | **A** | **A** | **C** | **A** | **T** | **T** | **A** | **C** | **G** | **A** | **T** | **C** | **C** | **T** | **G** | **A** | **C** | **A** | **T** | **G** | **G** | **G** | **C** | **G** | **A** | **T** | **A** | **A** | **A** | **G** | **A** | **C** | **A** | **A** | **A** | **C** | **A** | **G** | **C** | **A** | **A** | **T** | **T** | **T** | **G** | **T** | **C** | **G** | **A** | **C** | **T** | **T** | **C** | **T** | **T** | **C** | **T** | **C** | **T** | **T** | **C** | **A** | **T** | **T** | **A** | **A** | **A** | **T** | **C** | **G** | **T** | **A** | **T** | **G** | **C** | **C** | **G** | **C** | **T** | **G** | **A** | **C** | **A** | **A** | **G** | **A** | **A** | **T** | **G** | **A** | **A** | **T** | **T** | **C** | **T** | **G** | **T** | **C** | **A** | **C** | **T** | **C** | **T** | **T** | **C** | **T** | **C** | **A** | **C** | **A** | **G** | **A** | **C** | **G** | **G** | **A** | **C** | **A** | **A** | **G** | **A** | **G** | **A** | **A** | **A** | **T** | **T** | **G** | **G** | **A** | **T** | **T** | **C** | **A** | **C** | **T** | **G** | **C** | **T** | **T** | **C** | **A** | **A** | **G** | **A** | **T** | **C** | **T** | **A** | **A** | **A** | **A** | **A** | **A** | **A** | **G** | **C** | **C** | **A** | **A** | **A** | **C** | **A** | **T** | **T** | **C** | **G** | **A** | **T** | **C** | **C** | **A** | **T** | **A** | **T** | **C** | **G** | **A** | **A** | **T** | **A** | **T** | **T** | **A** | **C** | **G** | **C** | **A** | **T** | **T** | **T** | **G** | **T** | **G** | **A** | **C** | **A** | **G** | **A** | **T** | **A** | **A** | **T** | **A** | **T** | **C** | **G** | **G** | **C** | **C** | **A** | **G** | **C** | **A** | **A** | **G** | **T** | **C** | **T** | **T** | **A** | **C** | **A** | **T** | **G** | **T** | **T** | **T** | **T** | **A** | **G** | **A** | **A** | **G** | **A** | **A** | **A** | **A** | **A** | **G** | **C** | **C** | **G** | **C** | **A** | **G** | **A** | **A** | **G** | **G** | **C** | **G** | **T** | **G** | **G** | **A** | **A** | **G** | **T** | **T** | **C** | **G** | **A** | **A** | **T** | **A** | **T** | **T** | **A** | **T** | **A** | **T** | **G** | **A** | **T** | **G** | **C** | **A** | **T** | **T** | **T** | **G** | **G** | **C** | **T** | **C** | **T** | **C** | **A** | **T** | **G** | **G** | **C** | **A** | **C** | **A** | **A** | **A** | **A** | **G** | **C** | **A** | **A** | **A** | **A** | **G** | **A** | **T** | **T** | **T** | **C** | **A** | **A** | **T** | **C** | **G** | **T** | **C** | **T** | **A** | **A** | **T** | **C** | **A** | **A** | **A** | **A** | **A** | **T** | **G** | **G** | **T** | **G** | **G** | **A** | **C** | **A** | **T** | **G** | **T** | **C** | **C** | **A** | **T** | **A** | **C** | **A** | **T** | **T** | **T** | **G** | **T** | **T** | **A** | **C** | **C** | **T** | **C** | **A** | **C** | **A** | **A** | **A** | **A** | **G** | **G** | **C** | **A** | **T** | **T** | **A** | **C** | **T** | **T** | **C** | **G** | **T** | **T** | **T** | **C** | **C** | **G** | **A** | **T** | **T** | **G** | **A** | **A** | **T** | **T** | **A** | **C** | **C** | **A** | **T** | **G** | **A** | **T** | **C** | **A** | **C** | **C** | **G** | **A** | **A** | **A** | **A** | **A** | **T** | **C** | **G** | **T** | **T** | **G** | **T** | **G** | **A** | **T** | **C** | **G** | **A** | **T** | **G** | **G** | **A** | **A** | **A** | **G** | **A** | **T** | **T** | **A** | **G** | **T** | **T** | **A** | **T** | **A** | **T** | **C** | **G** | **G** | **T** | **G** | **G** | **T** | **T** | **T** | **C** | **A** | **A** | **T** | **A** | **T** | **T** | **G** | **C** | **C** | **A** | **A** | **T** | **C** | **A** | **A** | **T** | **A** | **T** | **G** | **T** | **A** | **A** | **A** | **T** | **A** | **C** | **A** | **A** | **C** | **A** | **A** | **A** | **A** | **A** | **A** | **A** | **T** | **T** | **C** | **G** | **G** | **C** | **T** | **A** | **T** | **T** | **G** | **G** | **C** | **G** | **C** | **G** | **A** | **T** | **A** | **C** | **G** | **C** | **A** | **T** | **A** | **T** | **A** | **C** | **G** | **G** | **A** | **T** | **T** | **T** | **T** | **C** | **G** | **G** | **T** | **G** | **C** | **C** | **G** | **C** | **T** | **T** | **C** | **T** | **T** | **C** | **T** | **T** | **T** | **G** | **C** | **T** | **C** | **C** | **A** | **G** | **C** | **T** | **T** | **C** | **G** | **C** | **T** | **T** | **C** | **T** | **T** | **A** | **A** | **C** | **A** | **G** | **A** | **C** | **T** | **G** | **G** | **A** | **A** | **C** | **G** | **T** | **C** | **T** | **C** | **G** | **G** | **T** | **A** | **C** | **C** | **T** | **G** | **A** | **A** | **G** | **A** | **A** | **A** | **A** | **A** | **A** | **A** | **G** | **G** | **T** | **C** | **G** | **G** | **C** | **T** | **A** | **T** | **C** | **A** | **T** | **T** | **T** | **G** | **A** | **A** | **T** | **T** | **A** | **T** | **T** | **T** | **C** | **T** | **T** | **T** | **A** | **A** | **A** | **A** | **A** | **A** | **G** | **C** | **A** | **G** | **A** | **T** | **C** | **G** | **A** | **G** | **A** | **T** | **G** | **A** | **A** | **T** | **C** | **T** | **A** | **A** | **G** | **C** | **T** | **T** | **G** | **C** | **T** | **G** | **A** | **T** | **A** | **C** | **A** | **T** | **C** | **C** | **A** | **T** | **C** | **C** | **A** | **G** | **C** | **T** | **T** | **G** | **T** | **T** | **T** | **C** | **A** | **A** | **G** | **C** | **G** | **G** | **A** | **C** | **C** | **G** | **A** | **A** | **T** | **A** | **A** | **C** | **G** | **A** | **A** | **A** | **G** | **G** | **G** | **A** | **A** | **C** | **A** | **A** | **A** | **T** | **C** | **A** | **A** | **G** | **C** | **T** | **T** | **T** | **C** | **A** | **T** | **T** | **T** | **A** | **T** | **C** | **A** | **A** | **A** | **T** | **T** | **G** | **A** | **T** | **T** | **A** | **C** | **T** | **T** | **C** | **T** | **G** | **C** | **T** | **A** | **A** | **A** | **A** | **A** | **A** | **C** | **G** | **T** | **G** | **T** | **T** | **T** | **G** | **G** | **A** | **T** | **A** | **C** | **A** | **G** | **A** | **C** | **A** | **C** | **C** | **T** | **T** | **A** | **C** | **C** | **T** | **T** | **G** | **T** | **T** | **C** | **C** | **T** | **G** | **A** | **T** | **G** | **A** | **A** | **A** | **G** | **T** | **G** | **T** | **C** | **A** | **T** | **T** | **G** | **C** | **T** | **G** | **C** | **T** | **T** | **T** | **A** | **A** | **A** | **A** | **A** | **T** | **C** | **G** | **C** | **A** | **A** | **C** | **T** | **G** | **C** | **C** | **T** | **C** | **T** | **G** | **G** | **T** | **G** | **T** | **A** | **G** | **A** | **T** | **G** | **T** | **G** | **A** | **A** | **A** | **A** | **T** | **C** | **A** | **T** | **G** | **A** | **T** | **T** | **C** | **C** | **C** | **A** | **A** | **C** | **A** | **A** | **A** | **C** | **C** | **G** | **G** | **A** | **T** | **C** | **A** | **T** | **C** | **C** | **T** | **T** | **T** | **T** | **A** | **T** | **T** | **T** | **A** | **T** | **C** | **G** | **A** | **G** | **C** | **A** | **A** | **C** | **A** | **C** | **A** | **A** | **T** | **A** | **T** | **T** | **A** | **T** | **G** | **C** | **T** | **C** | **G** | **G** | **C** | **A** | **G** | **C** | **T** | **G** | **A** | **T** | **C** | **A** | **A** | **G** | **G** | **A** | **A** | **A** | **A** | **T** | **G** | **T** | **A** | **C** | **A** | **A** | **A** | **T** | **C** | **C** | **T** | **T** | **G** | **T** | **C** | **T** | **A** | **T** | **G** | **A** | **G** | **A** | **A** | **C** | **G** | **G** | **C** | **T** | **T** | **C** | **C** | **T** | **C** | **C** | **A** | **T** | **G** | **C** | **A** | **A** | **A** | **A** | **A** | **C** | **A** | **T** | **T** | **G** | **A** | **T** | **A** | **A** | **T** | **G** | **G** | **A** | **T** | **G** | **A** | **T** | **G** | **A** | **A** | **A** | **T** | **C** | **T** | **G** | **C** | **A** | **T** | **G** | **G** | **T** | **A** | **G** | **G** | **T** | **T** | **C** | **A** | **G** | **C** | **A** | **A** | **A** | **T** | **C** | **A** | **A** | **G** | **A** | **T** | **A** | **T** | **T** | **C** | **G** | **T** | **A** | **G** | **C** | **T** | **A** | **C** | **C** | **G** | **A** | **T** | **T** | **G** | **A** | **A** | **T** | **T** | **T** | **T** | **G** | **A** | **A** | **A** | **C** | **A** | **A** | **G** | **T** | **G** | **C** | **T** | **G** | **T** | **T** | **A** | **T** | **T** | **T** | **A** | **C** | **G** | **A** | **T** | **C** | **C** | **T** | **G** | **A** | **G** | **T** | **T** | **T** | **T** | **T** | **A** | **G** | **A** | **A** | **G** | **A** | **A** | **C** | **T** | **T** | **G** | **C** | **T** | **A** | **C** | **T** | **C** | **A** | **G** | **T** | **T** | **C** | **A** | **A** | **A** | **G** | **A** | **A** | **G** | **A** | **T** | **G** | **A** | **G** | **A** | **C** | **A** | **C** | **A** | **T** | **T** | **G** | **T** | **T** | **C** | **A** | **T** | **C** | **C** | **A** | **T** | **G** | **A** | **C** | **A** | **A** | **C** | **T** | **G** | **A** | **A** | **A** | **C** | **A** | **G** | **T** | **C** | **A** | **A** | **A** | **G** | **A** | **A** | **A** | **T** | **G** | **T** | **C** | **T** | **A** | **A** | **C** | **T** | **G** | **G** | **C** | **T** | **A** | **T** | **T** | **A** | **T** | **T** | **C** | **A** | **A** | **G** | **C** | **A** | **A** | **C** | **A** | **A** | **A** | **T** | **T** | **T** | **C** | **T** | **C** | **G** | **A** | **T** | **T** | **A** | **T** | **T** | **T** | **T** | **C** | **T** | **C** | **C** | **A** | **A** | **T** | **C** | **C** | **T** | **A** | **-** | **-** | **-** |
| **N59613F** | **-** | **-** | **-** | **-** | **-** | **-** | **-** | **-** | **-** | **-** | **-** | **-** | **-** | **-** | **-** | **-** | **-** | **-** | **-** | **-** | **-** | **-** | **-** | **-** | **-** | **-** | **-** | **-** | **-** | **-** | **-** | **-** | **-** | **-** | **-** | **-** | **-** | **-** | **-** | **-** | **-** | **-** | **-** | **-** | **-** | **-** | **-** | **-** | **-** | **-** | **-** | **-** | **-** | **-** | **-** | **-** | **-** | **-** | **-** | **-** | **-** | **-** | **-** | **-** | **-** | **-** | **-** | **-** | **-** | **A** | **A** | **A** | **C** | **C** | **C** | **C** | **G** | **A** | **G** | **A** | **T** | **G** | **T** | **A** | **G** | **C** | **A** | **G** | **C** | **T** | **A** | **T** | **T** | **T** | **G** | **G** | **G** | **C** | **A** | **T** | **G** | **G** | **C** | **T** | **C** | **T** | **T** | **A** | **G** | **T** | **A** | **T** | **T** | **G** | **T** | **T** | **T** | **G** | **C** | **C** | **C** | **T** | **T** | **C** | **C** | **T** | **G** | **G** | **G** | **G** | **T** | **G** | **G** | **G** | **T** | **T** | **T** | **T** | **G** | **T** | **A** | **T** | **T** | **A** | **T** | **A** | **T** | **T** | **T** | **A** | **T** | **T** | **T** | **T** | **T** | **C** | **G** | **G** | **A** | **C** | **G** | **T** | **G** | **G** | **A** | **T** | **T** | **A** | **A** | **C** | **G** | **G** | **A** | **T** | **A** | **A** | **G** | **A** | **A** | **A** | **A** | **A** | **A** | **T** | **T** | **C** | **T** | **A** | **T** | **C** | **T** | **C** | **C** | **G** | **A** | **C** | **A** | **A** | **A** | **G** | **T** | **G** | **A** | **C** | **T** | **T** | **G** | **A** | **A** | **A** | **G** | **A** | **A** | **T** | **T** | **A** | **G** | **A** | **A** | **A** | **A** | **C** | **T** | **T** | **T** | **C** | **A** | **G** | **T** | **C** | **T** | **T** | **T** | **T** | **A** | **G** | **A** | **G** | **G** | **A** | **G** | **A** | **T** | **A** | **C** | **C** | **A** | **T** | **T** | **G** | **A** | **A** | **C** | **A** | **T** | **T** | **A** | **C** | **G** | **A** | **T** | **C** | **C** | **T** | **G** | **A** | **C** | **A** | **T** | **G** | **G** | **G** | **C** | **G** | **A** | **C** | **A** | **A** | **A** | **G** | **A** | **C** | **A** | **A** | **A** | **C** | **A** | **A** | **C** | **A** | **A** | **T** | **T** | **T** | **G** | **T** | **C** | **G** | **A** | **C** | **T** | **T** | **C** | **T** | **T** | **C** | **T** | **C** | **T** | **T** | **C** | **A** | **T** | **T** | **A** | **A** | **A** | **T** | **C** | **G** | **T** | **A** | **T** | **G** | **C** | **C** | **G** | **C** | **T** | **G** | **A** | **C** | **A** | **A** | **G** | **A** | **A** | **T** | **G** | **A** | **A** | **T** | **T** | **C** | **T** | **G** | **T** | **C** | **A** | **C** | **T** | **C** | **T** | **T** | **C** | **T** | **C** | **A** | **C** | **A** | **G** | **A** | **C** | **G** | **G** | **A** | **C** | **A** | **A** | **A** | **A** | **G** | **A** | **A** | **A** | **T** | **T** | **A** | **G** | **A** | **T** | **T** | **C** | **A** | **C** | **T** | **G** | **C** | **T** | **T** | **C** | **A** | **A** | **G** | **A** | **T** | **C** | **T** | **A** | **A** | **A** | **A** | **A** | **A** | **A** | **G** | **C** | **C** | **A** | **A** | **A** | **C** | **A** | **T** | **T** | **C** | **G** | **A** | **T** | **C** | **C** | **A** | **T** | **A** | **T** | **C** | **G** | **A** | **A** | **T** | **A** | **T** | **T** | **A** | **C** | **G** | **C** | **A** | **T** | **T** | **T** | **G** | **T** | **G** | **A** | **C** | **A** | **G** | **A** | **T** | **A** | **A** | **T** | **A** | **T** | **C** | **G** | **G** | **T** | **C** | **A** | **G** | **C** | **A** | **A** | **G** | **T** | **C** | **T** | **T** | **A** | **C** | **G** | **C** | **G** | **T** | **T** | **T** | **T** | **A** | **G** | **A** | **A** | **G** | **A** | **A** | **A** | **A** | **A** | **G** | **C** | **C** | **G** | **C** | **A** | **G** | **A** | **A** | **G** | **G** | **C** | **G** | **T** | **A** | **G** | **A** | **A** | **G** | **T** | **T** | **C** | **G** | **T** | **A** | **T** | **A** | **T** | **T** | **A** | **T** | **A** | **T** | **G** | **A** | **T** | **G** | **C** | **A** | **T** | **T** | **T** | **G** | **G** | **C** | **T** | **C** | **C** | **C** | **A** | **T** | **G** | **G** | **C** | **A** | **C** | **A** | **A** | **A** | **A** | **G** | **C** | **A** | **A** | **A** | **A** | **G** | **A** | **T** | **T** | **T** | **C** | **A** | **A** | **T** | **C** | **G** | **T** | **C** | **T** | **A** | **A** | **T** | **C** | **A** | **A** | **A** | **A** | **A** | **T** | **G** | **G** | **T** | **G** | **G** | **A** | **C** | **A** | **T** | **G** | **T** | **C** | **C** | **A** | **T** | **A** | **C** | **A** | **T** | **T** | **T** | **G** | **T** | **T** | **A** | **C** | **C** | **T** | **C** | **G** | **C** | **A** | **A** | **A** | **A** | **A** | **G** | **C** | **A** | **T** | **T** | **G** | **C** | **T** | **T** | **C** | **G** | **T** | **T** | **T** | **C** | **C** | **G** | **A** | **T** | **T** | **G** | **A** | **A** | **T** | **T** | **A** | **C** | **C** | **A** | **T** | **G** | **A** | **T** | **C** | **A** | **C** | **C** | **G** | **A** | **A** | **A** | **A** | **A** | **T** | **C** | **G** | **T** | **T** | **G** | **T** | **G** | **A** | **T** | **C** | **G** | **A** | **T** | **G** | **G** | **A** | **A** | **A** | **G** | **A** | **T** | **C** | **A** | **G** | **T** | **T** | **A** | **C** | **A** | **C** | **C** | **G** | **G** | **T** | **G** | **G** | **T** | **T** | **T** | **C** | **A** | **A** | **T** | **A** | **T** | **T** | **G** | **C** | **C** | **A** | **A** | **T** | **C** | **A** | **A** | **T** | **A** | **T** | **G** | **T** | **A** | **A** | **A** | **T** | **A** | **C** | **G** | **A** | **C** | **A** | **A** | **A** | **A** | **A** | **A** | **A** | **T** | **T** | **C** | **G** | **G** | **T** | **T** | **A** | **T** | **T** | **G** | **G** | **C** | **G** | **T** | **G** | **A** | **T** | **A** | **C** | **G** | **C** | **A** | **T** | **A** | **T** | **A** | **C** | **G** | **A** | **A** | **T** | **T** | **T** | **T** | **C** | **G** | **G** | **T** | **G** | **C** | **T** | **G** | **C** | **T** | **T** | **C** | **T** | **T** | **C** | **T** | **T** | **T** | **G** | **C** | **T** | **T** | **C** | **A** | **A** | **C** | **T** | **T** | **C** | **G** | **T** | **T** | **T** | **T** | **T** | **T** | **A** | **A** | **C** | **A** | **G** | **A** | **C** | **T** | **G** | **G** | **A** | **A** | **C** | **G** | **T** | **T** | **T** | **C** | **A** | **G** | **T** | **G** | **T** | **C** | **T** | **G** | **A** | **A** | **G** | **A** | **A** | **A** | **A** | **A** | **A** | **A** | **G** | **G** | **T** | **C** | **G** | **G** | **C** | **T** | **A** | **T** | **C** | **A** | **T** | **T** | **T** | **G** | **A** | **A** | **T** | **T** | **A** | **T** | **T** | **T** | **C** | **T** | **T** | **T** | **A** | **A** | **A** | **A** | **A** | **A** | **G** | **C** | **C** | **G** | **A** | **T** | **C** | **G** | **A** | **G** | **A** | **T** | **G** | **A** | **A** | **T** | **C** | **C** | **A** | **A** | **A** | **C** | **T** | **T** | **G** | **C** | **T** | **G** | **A** | **T** | **A** | **C** | **G** | **T** | **C** | **C** | **A** | **T** | **C** | **C** | **A** | **G** | **C** | **T** | **T** | **G** | **T** | **C** | **T** | **C** | **A** | **A** | **G** | **C** | **G** | **G** | **A** | **C** | **C** | **A** | **A** | **A** | **T** | **A** | **A** | **C** | **G** | **A** | **A** | **C** | **G** | **A** | **G** | **A** | **A** | **C** | **A** | **A** | **A** | **T** | **C** | **A** | **A** | **G** | **C** | **T** | **T** | **T** | **C** | **G** | **T** | **T** | **T** | **A** | **T** | **C** | **A** | **A** | **A** | **T** | **T** | **G** | **A** | **T** | **T** | **A** | **C** | **T** | **T** | **C** | **T** | **G** | **C** | **T** | **A** | **A** | **A** | **A** | **A** | **A** | **C** | **G** | **T** | **G** | **T** | **T** | **T** | **G** | **G** | **A** | **T** | **A** | **C** | **A** | **G** | **A** | **C** | **A** | **C** | **C** | **T** | **T** | **A** | **C** | **C** | **T** | **C** | **G** | **T** | **T** | **C** | **C** | **T** | **G** | **A** | **T** | **G** | **A** | **A** | **A** | **G** | **T** | **G** | **T** | **C** | **A** | **T** | **T** | **G** | **C** | **T** | **G** | **C** | **T** | **T** | **T** | **G** | **A** | **A** | **A** | **A** | **T** | **C** | **G** | **C** | **A** | **A** | **C** | **T** | **G** | **C** | **T** | **T** | **C** | **T** | **G** | **G** | **T** | **G** | **T** | **C** | **G** | **A** | **T** | **G** | **T** | **G** | **A** | **A** | **A** | **A** | **T** | **C** | **A** | **T** | **G** | **A** | **T** | **C** | **C** | **C** | **C** | **A** | **A** | **T** | **A** | **A** | **A** | **C** | **C** | **G** | **G** | **A** | **T** | **C** | **A** | **T** | **C** | **C** | **T** | **T** | **T** | **T** | **A** | **T** | **T** | **T** | **A** | **T** | **C** | **G** | **A** | **G** | **C** | **A** | **A** | **C** | **A** | **C** | **A** | **A** | **T** | **A** | **T** | **T** | **A** | **T** | **G** | **C** | **T** | **C** | **G** | **T** | **C** | **A** | **G** | **C** | **T** | **C** | **A** | **T** | **C** | **A** | **A** | **G** | **G** | **A** | **A** | **A** | **A** | **T** | **G** | **T** | **A** | **C** | **A** | **G** | **A** | **T** | **C** | **C** | **T** | **T** | **G** | **T** | **C** | **T** | **A** | **T** | **G** | **A** | **G** | **A** | **A** | **T** | **G** | **G** | **C** | **T** | **T** | **C** | **C** | **T** | **C** | **C** | **A** | **T** | **G** | **C** | **A** | **A** | **A** | **A** | **A** | **C** | **A** | **T** | **T** | **A** | **A** | **T** | **A** | **A** | **T** | **G** | **G** | **A** | **T** | **G** | **A** | **T** | **G** | **A** | **A** | **A** | **T** | **C** | **T** | **G** | **C** | **A** | **T** | **G** | **G** | **T** | **A** | **G** | **G** | **T** | **T** | **C** | **A** | **G** | **C** | **A** | **A** | **A** | **T** | **C** | **A** | **A** | **G** | **A** | **T** | **A** | **T** | **T** | **C** | **G** | **T** | **A** | **G** | **C** | **T** | **A** | **C** | **C** | **G** | **A** | **T** | **T** | **G** | **A** | **A** | **T** | **T** | **T** | **T** | **G** | **A** | **A** | **A** | **C** | **A** | **A** | **G** | **T** | **G** | **C** | **T** | **G** | **T** | **C** | **A** | **T** | **T** | **T** | **A** | **C** | **G** | **A** | **T** | **C** | **C** | **T** | **G** | **A** | **G** | **T** | **T** | **T** | **T** | **T** | **A** | **G** | **A** | **A** | **G** | **A** | **G** | **C** | **T** | **T** | **G** | **C** | **T** | **A** | **C** | **T** | **C** | **A** | **G** | **T** | **T** | **C** | **A** | **A** | **A** | **G** | **A** | **A** | **G** | **A** | **T** | **G** | **A** | **G** | **A** | **C** | **A** | **C** | **A** | **T** | **T** | **G** | **T** | **T** | **C** | **A** | **C** | **C** | **A** | **A** | **T** | **G** | **A** | **C** | **A** | **A** | **C** | **T** | **G** | **A** | **A** | **A** | **C** | **A** | **G** | **T** | **G** | **A** | **A** | **G** | **G** | **A** | **A** | **A** | **T** | **G** | **T** | **C** | **T** | **A** | **A** | **C** | **T** | **G** | **G** | **C** | **T** | **G** | **T** | **T** | **A** | **T** | **T** | **C** | **A** | **A** | **A** | **C** | **A** | **A** | **C** | **A** | **A** | **A** | **T** | **T** | **T** | **C** | **T** | **C** | **G** | **A** | **T** | **T** | **G** | **T** | **T** | **T** | **T** | **C** | **T** | **C** | **C** | **A** | **A** | **T** | **C** | **T** | **T** | **A** | **-** | **-** | **-** |
| **N59469F** | **-** | **-** | **-** | **-** | **-** | **-** | **-** | **-** | **-** | **-** | **-** | **-** | **-** | **-** | **-** | **-** | **-** | **-** | **-** | **-** | **-** | **-** | **-** | **-** | **-** | **-** | **-** | **-** | **-** | **-** | **-** | **-** | **-** | **-** | **-** | **-** | **-** | **-** | **-** | **-** | **-** | **-** | **-** | **-** | **-** | **-** | **-** | **-** | **-** | **-** | **-** | **-** | **-** | **-** | **-** | **-** | **-** | **-** | **-** | **-** | **-** | **-** | **-** | **-** | **-** | **-** | **-** | **-** | **-** | **A** | **A** | **A** | **C** | **C** | **C** | **C** | **G** | **A** | **G** | **A** | **T** | **G** | **T** | **A** | **G** | **C** | **A** | **G** | **C** | **C** | **A** | **T** | **T** | **T** | **G** | **G** | **G** | **C** | **A** | **T** | **G** | **G** | **C** | **T** | **C** | **T** | **T** | **A** | **G** | **T** | **A** | **T** | **T** | **G** | **T** | **T** | **T** | **G** | **C** | **C** | **C** | **T** | **T** | **C** | **C** | **T** | **G** | **G** | **G** | **G** | **T** | **G** | **G** | **G** | **T** | **T** | **T** | **T** | **G** | **T** | **A** | **T** | **T** | **A** | **T** | **A** | **T** | **T** | **T** | **A** | **T** | **T** | **T** | **T** | **T** | **C** | **G** | **G** | **A** | **C** | **G** | **T** | **G** | **G** | **A** | **T** | **T** | **A** | **A** | **C** | **G** | **G** | **A** | **T** | **A** | **A** | **G** | **A** | **A** | **A** | **A** | **A** | **A** | **T** | **T** | **C** | **T** | **A** | **T** | **C** | **T** | **C** | **C** | **G** | **A** | **C** | **A** | **A** | **A** | **G** | **T** | **G** | **A** | **C** | **T** | **T** | **G** | **A** | **A** | **A** | **G** | **A** | **A** | **T** | **T** | **A** | **G** | **A** | **A** | **A** | **A** | **C** | **T** | **T** | **T** | **C** | **A** | **G** | **T** | **C** | **T** | **T** | **T** | **T** | **A** | **G** | **A** | **G** | **G** | **A** | **G** | **A** | **T** | **A** | **C** | **C** | **A** | **T** | **T** | **G** | **A** | **A** | **C** | **A** | **T** | **T** | **A** | **C** | **G** | **A** | **T** | **C** | **C** | **T** | **G** | **A** | **C** | **A** | **T** | **G** | **G** | **G** | **C** | **G** | **A** | **T** | **A** | **A** | **A** | **G** | **A** | **C** | **A** | **A** | **A** | **C** | **A** | **G** | **C** | **A** | **A** | **T** | **T** | **T** | **G** | **T** | **T** | **G** | **A** | **C** | **T** | **T** | **C** | **T** | **T** | **C** | **T** | **C** | **T** | **T** | **C** | **A** | **T** | **T** | **A** | **A** | **A** | **T** | **C** | **G** | **T** | **A** | **T** | **G** | **C** | **C** | **G** | **C** | **T** | **G** | **A** | **C** | **A** | **A** | **G** | **A** | **A** | **T** | **G** | **A** | **A** | **T** | **T** | **C** | **T** | **G** | **T** | **C** | **A** | **C** | **T** | **C** | **T** | **T** | **C** | **T** | **C** | **A** | **C** | **A** | **G** | **A** | **C** | **G** | **G** | **A** | **C** | **A** | **A** | **G** | **A** | **G** | **A** | **A** | **A** | **T** | **T** | **G** | **G** | **A** | **T** | **T** | **C** | **A** | **C** | **T** | **G** | **C** | **T** | **T** | **C** | **A** | **A** | **G** | **A** | **T** | **C** | **T** | **A** | **A** | **A** | **A** | **A** | **A** | **A** | **G** | **C** | **C** | **A** | **A** | **A** | **C** | **A** | **T** | **T** | **C** | **G** | **A** | **T** | **C** | **C** | **A** | **T** | **A** | **T** | **C** | **G** | **A** | **A** | **T** | **A** | **T** | **T** | **A** | **C** | **G** | **C** | **A** | **T** | **T** | **T** | **G** | **T** | **G** | **A** | **C** | **A** | **G** | **A** | **T** | **A** | **A** | **T** | **A** | **T** | **C** | **G** | **G** | **C** | **C** | **A** | **G** | **C** | **A** | **A** | **G** | **T** | **C** | **T** | **T** | **A** | **C** | **A** | **T** | **G** | **T** | **T** | **T** | **T** | **A** | **G** | **A** | **A** | **G** | **A** | **A** | **A** | **A** | **A** | **G** | **C** | **C** | **G** | **C** | **A** | **G** | **A** | **A** | **G** | **G** | **C** | **G** | **T** | **G** | **G** | **A** | **A** | **G** | **T** | **T** | **C** | **G** | **A** | **A** | **T** | **A** | **T** | **T** | **A** | **T** | **A** | **T** | **G** | **A** | **T** | **G** | **C** | **A** | **T** | **T** | **T** | **G** | **G** | **C** | **T** | **C** | **T** | **C** | **A** | **T** | **G** | **G** | **C** | **A** | **C** | **A** | **A** | **A** | **A** | **G** | **C** | **A** | **A** | **A** | **A** | **G** | **A** | **T** | **T** | **T** | **C** | **A** | **A** | **T** | **C** | **G** | **T** | **C** | **T** | **A** | **A** | **T** | **C** | **A** | **A** | **A** | **A** | **A** | **T** | **G** | **G** | **T** | **G** | **G** | **A** | **C** | **A** | **T** | **G** | **T** | **C** | **C** | **A** | **T** | **A** | **C** | **A** | **T** | **T** | **T** | **G** | **T** | **T** | **A** | **C** | **C** | **T** | **C** | **A** | **C** | **A** | **A** | **A** | **A** | **G** | **G** | **C** | **A** | **T** | **T** | **A** | **C** | **T** | **T** | **C** | **G** | **T** | **T** | **T** | **C** | **C** | **G** | **A** | **T** | **T** | **G** | **A** | **A** | **T** | **T** | **A** | **C** | **C** | **A** | **T** | **G** | **A** | **T** | **C** | **A** | **C** | **C** | **G** | **A** | **A** | **A** | **A** | **A** | **T** | **C** | **G** | **T** | **T** | **G** | **T** | **G** | **A** | **T** | **C** | **G** | **A** | **T** | **G** | **G** | **A** | **A** | **A** | **G** | **A** | **T** | **T** | **A** | **G** | **T** | **T** | **A** | **T** | **A** | **C** | **C** | **G** | **G** | **T** | **G** | **G** | **T** | **T** | **T** | **C** | **A** | **A** | **T** | **A** | **T** | **T** | **G** | **C** | **C** | **A** | **A** | **T** | **C** | **A** | **A** | **T** | **A** | **T** | **G** | **T** | **A** | **A** | **A** | **T** | **A** | **C** | **A** | **A** | **C** | **A** | **A** | **A** | **A** | **A** | **A** | **A** | **T** | **T** | **C** | **G** | **G** | **C** | **T** | **A** | **T** | **T** | **G** | **G** | **C** | **G** | **C** | **G** | **A** | **T** | **A** | **C** | **G** | **C** | **A** | **T** | **A** | **T** | **A** | **C** | **G** | **G** | **A** | **T** | **T** | **T** | **T** | **C** | **G** | **G** | **T** | **G** | **C** | **C** | **G** | **C** | **T** | **T** | **C** | **T** | **T** | **C** | **T** | **T** | **T** | **G** | **C** | **T** | **C** | **C** | **A** | **G** | **C** | **T** | **T** | **C** | **G** | **C** | **T** | **T** | **C** | **T** | **T** | **A** | **A** | **C** | **A** | **G** | **A** | **C** | **T** | **G** | **G** | **A** | **A** | **C** | **G** | **T** | **C** | **T** | **C** | **G** | **G** | **T** | **A** | **C** | **C** | **T** | **G** | **A** | **A** | **G** | **A** | **A** | **A** | **A** | **A** | **A** | **A** | **G** | **G** | **T** | **C** | **G** | **G** | **C** | **T** | **A** | **T** | **C** | **A** | **T** | **T** | **T** | **G** | **A** | **A** | **T** | **T** | **A** | **T** | **T** | **T** | **C** | **T** | **T** | **T** | **A** | **A** | **A** | **A** | **A** | **A** | **G** | **C** | **A** | **G** | **A** | **T** | **C** | **G** | **A** | **G** | **A** | **T** | **G** | **A** | **A** | **T** | **C** | **T** | **A** | **A** | **G** | **C** | **T** | **T** | **G** | **C** | **T** | **G** | **A** | **A** | **A** | **C** | **A** | **T** | **C** | **C** | **A** | **T** | **C** | **C** | **A** | **G** | **C** | **T** | **T** | **G** | **T** | **T** | **T** | **C** | **A** | **A** | **G** | **C** | **G** | **G** | **A** | **C** | **C** | **G** | **A** | **A** | **T** | **A** | **A** | **C** | **G** | **A** | **A** | **A** | **G** | **G** | **G** | **A** | **A** | **C** | **A** | **A** | **A** | **T** | **C** | **A** | **A** | **G** | **C** | **T** | **T** | **T** | **C** | **A** | **T** | **T** | **T** | **A** | **T** | **C** | **A** | **A** | **A** | **T** | **T** | **G** | **A** | **T** | **T** | **A** | **C** | **T** | **T** | **C** | **T** | **G** | **C** | **T** | **A** | **A** | **A** | **A** | **A** | **G** | **C** | **G** | **T** | **G** | **T** | **T** | **T** | **G** | **G** | **A** | **T** | **A** | **C** | **A** | **G** | **A** | **C** | **A** | **C** | **C** | **T** | **T** | **A** | **C** | **C** | **T** | **T** | **G** | **T** | **T** | **C** | **C** | **T** | **G** | **A** | **T** | **G** | **A** | **A** | **A** | **G** | **T** | **G** | **T** | **C** | **A** | **T** | **T** | **G** | **C** | **T** | **G** | **C** | **T** | **T** | **T** | **A** | **A** | **A** | **A** | **A** | **T** | **C** | **G** | **C** | **A** | **A** | **C** | **T** | **G** | **C** | **C** | **T** | **C** | **T** | **G** | **G** | **T** | **G** | **T** | **A** | **G** | **A** | **T** | **G** | **T** | **G** | **A** | **A** | **A** | **A** | **T** | **C** | **A** | **T** | **G** | **A** | **T** | **T** | **C** | **C** | **C** | **A** | **A** | **C** | **A** | **A** | **A** | **C** | **C** | **G** | **G** | **A** | **T** | **C** | **A** | **T** | **C** | **C** | **T** | **T** | **T** | **T** | **A** | **T** | **T** | **T** | **A** | **T** | **C** | **G** | **A** | **G** | **C** | **A** | **A** | **C** | **A** | **C** | **A** | **A** | **T** | **A** | **T** | **T** | **A** | **T** | **G** | **C** | **T** | **C** | **G** | **G** | **C** | **A** | **G** | **C** | **T** | **G** | **A** | **T** | **C** | **A** | **A** | **G** | **G** | **A** | **A** | **A** | **A** | **T** | **G** | **T** | **A** | **C** | **A** | **A** | **A** | **T** | **C** | **C** | **T** | **T** | **G** | **T** | **C** | **T** | **A** | **T** | **G** | **A** | **G** | **A** | **A** | **C** | **G** | **G** | **C** | **T** | **T** | **C** | **C** | **T** | **C** | **C** | **A** | **T** | **G** | **C** | **A** | **A** | **A** | **A** | **A** | **C** | **A** | **T** | **T** | **G** | **A** | **T** | **A** | **A** | **T** | **G** | **G** | **A** | **T** | **G** | **A** | **T** | **G** | **A** | **A** | **A** | **T** | **C** | **T** | **G** | **C** | **A** | **T** | **G** | **G** | **T** | **A** | **G** | **G** | **T** | **T** | **C** | **A** | **G** | **C** | **A** | **A** | **A** | **T** | **C** | **A** | **A** | **G** | **A** | **T** | **A** | **T** | **T** | **C** | **G** | **T** | **A** | **G** | **C** | **T** | **A** | **C** | **A** | **G** | **A** | **T** | **T** | **G** | **A** | **A** | **T** | **T** | **T** | **T** | **G** | **A** | **A** | **A** | **C** | **A** | **A** | **G** | **T** | **G** | **C** | **T** | **G** | **T** | **C** | **A** | **T** | **T** | **T** | **A** | **C** | **G** | **A** | **T** | **C** | **C** | **T** | **G** | **A** | **G** | **T** | **T** | **T** | **T** | **T** | **A** | **G** | **A** | **A** | **G** | **A** | **A** | **C** | **T** | **T** | **G** | **C** | **T** | **A** | **C** | **T** | **C** | **A** | **G** | **T** | **T** | **C** | **A** | **A** | **A** | **G** | **A** | **A** | **G** | **A** | **T** | **G** | **A** | **G** | **A** | **C** | **A** | **C** | **A** | **T** | **T** | **G** | **T** | **T** | **C** | **A** | **T** | **C** | **C** | **A** | **T** | **G** | **A** | **C** | **A** | **A** | **C** | **T** | **G** | **A** | **A** | **A** | **C** | **A** | **G** | **T** | **T** | **A** | **A** | **A** | **G** | **A** | **A** | **A** | **T** | **G** | **T** | **C** | **T** | **A** | **A** | **C** | **T** | **G** | **G** | **C** | **T** | **A** | **T** | **T** | **A** | **T** | **T** | **C** | **A** | **A** | **G** | **C** | **A** | **A** | **C** | **A** | **A** | **A** | **T** | **T** | **T** | **C** | **T** | **C** | **G** | **A** | **T** | **T** | **A** | **T** | **T** | **T** | **T** | **C** | **T** | **C** | **C** | **A** | **A** | **T** | **C** | **C** | **T** | **A** | **-** | **-** | **-** |
| **N59754F** | **-** | **-** | **-** | **-** | **-** | **-** | **-** | **-** | **-** | **-** | **-** | **-** | **-** | **-** | **-** | **-** | **-** | **-** | **-** | **-** | **-** | **-** | **-** | **-** | **-** | **-** | **-** | **-** | **-** | **-** | **-** | **-** | **-** | **-** | **-** | **-** | **-** | **-** | **-** | **-** | **-** | **-** | **-** | **-** | **-** | **-** | **-** | **-** | **-** | **-** | **-** | **-** | **-** | **-** | **-** | **-** | **-** | **-** | **-** | **-** | **-** | **-** | **-** | **-** | **-** | **-** | **-** | **-** | **-** | **A** | **A** | **A** | **C** | **C** | **C** | **C** | **G** | **A** | **G** | **A** | **T** | **G** | **T** | **A** | **G** | **C** | **A** | **G** | **C** | **C** | **A** | **T** | **T** | **T** | **G** | **G** | **G** | **C** | **A** | **T** | **G** | **G** | **C** | **T** | **C** | **T** | **T** | **A** | **G** | **T** | **A** | **T** | **T** | **G** | **T** | **T** | **T** | **G** | **C** | **C** | **C** | **T** | **T** | **C** | **C** | **T** | **G** | **G** | **G** | **G** | **T** | **G** | **G** | **G** | **C** | **T** | **T** | **T** | **G** | **T** | **A** | **T** | **T** | **A** | **T** | **A** | **T** | **T** | **T** | **A** | **T** | **T** | **T** | **T** | **T** | **C** | **G** | **G** | **A** | **C** | **G** | **T** | **G** | **G** | **A** | **T** | **T** | **A** | **A** | **C** | **G** | **G** | **A** | **T** | **A** | **A** | **G** | **A** | **A** | **A** | **A** | **A** | **A** | **T** | **T** | **C** | **T** | **A** | **T** | **C** | **T** | **C** | **C** | **A** | **A** | **C** | **A** | **A** | **A** | **G** | **T** | **G** | **A** | **C** | **T** | **T** | **G** | **A** | **A** | **A** | **G** | **A** | **A** | **T** | **T** | **A** | **G** | **A** | **A** | **A** | **A** | **C** | **T** | **T** | **T** | **C** | **A** | **G** | **T** | **C** | **T** | **T** | **T** | **T** | **A** | **G** | **A** | **G** | **G** | **A** | **G** | **A** | **T** | **A** | **C** | **C** | **A** | **T** | **T** | **G** | **A** | **A** | **C** | **A** | **T** | **T** | **A** | **C** | **G** | **A** | **T** | **C** | **C** | **T** | **G** | **A** | **C** | **A** | **T** | **G** | **G** | **G** | **C** | **G** | **A** | **T** | **A** | **A** | **A** | **G** | **A** | **C** | **A** | **A** | **A** | **C** | **A** | **G** | **C** | **A** | **A** | **T** | **T** | **T** | **G** | **T** | **T** | **G** | **A** | **C** | **T** | **T** | **C** | **T** | **T** | **C** | **T** | **C** | **T** | **T** | **C** | **A** | **T** | **T** | **A** | **A** | **A** | **T** | **C** | **G** | **T** | **A** | **T** | **G** | **C** | **C** | **G** | **C** | **T** | **G** | **A** | **C** | **A** | **A** | **G** | **A** | **A** | **T** | **G** | **A** | **A** | **T** | **T** | **C** | **T** | **G** | **T** | **C** | **A** | **C** | **T** | **C** | **T** | **T** | **C** | **T** | **C** | **A** | **C** | **A** | **G** | **A** | **C** | **G** | **G** | **A** | **C** | **A** | **A** | **G** | **A** | **G** | **A** | **A** | **A** | **T** | **T** | **G** | **G** | **A** | **T** | **T** | **C** | **A** | **C** | **T** | **G** | **C** | **T** | **T** | **C** | **A** | **A** | **G** | **A** | **T** | **C** | **T** | **A** | **A** | **A** | **A** | **A** | **A** | **A** | **G** | **C** | **C** | **A** | **A** | **A** | **C** | **A** | **T** | **T** | **C** | **G** | **A** | **T** | **C** | **C** | **A** | **T** | **A** | **T** | **C** | **G** | **A** | **A** | **T** | **A** | **T** | **T** | **A** | **C** | **G** | **C** | **A** | **T** | **T** | **T** | **G** | **T** | **G** | **A** | **C** | **A** | **G** | **A** | **T** | **A** | **A** | **T** | **A** | **T** | **C** | **G** | **G** | **C** | **C** | **A** | **G** | **C** | **A** | **A** | **G** | **T** | **C** | **T** | **T** | **A** | **C** | **A** | **T** | **G** | **T** | **T** | **T** | **T** | **A** | **G** | **A** | **A** | **G** | **A** | **A** | **A** | **A** | **A** | **G** | **C** | **C** | **G** | **C** | **A** | **G** | **A** | **A** | **G** | **G** | **C** | **G** | **T** | **G** | **G** | **A** | **A** | **G** | **T** | **T** | **C** | **G** | **A** | **A** | **T** | **A** | **T** | **T** | **A** | **T** | **A** | **T** | **G** | **A** | **T** | **G** | **C** | **A** | **T** | **T** | **T** | **G** | **G** | **C** | **T** | **C** | **T** | **C** | **A** | **T** | **G** | **G** | **C** | **A** | **C** | **A** | **A** | **A** | **A** | **G** | **C** | **A** | **A** | **A** | **A** | **G** | **A** | **T** | **T** | **T** | **C** | **A** | **A** | **T** | **C** | **G** | **T** | **C** | **T** | **A** | **A** | **T** | **C** | **A** | **A** | **A** | **A** | **A** | **T** | **G** | **G** | **T** | **G** | **G** | **A** | **C** | **A** | **T** | **G** | **T** | **C** | **C** | **A** | **T** | **A** | **C** | **A** | **T** | **T** | **T** | **G** | **T** | **T** | **A** | **C** | **C** | **T** | **C** | **A** | **C** | **A** | **A** | **A** | **A** | **G** | **G** | **C** | **A** | **T** | **T** | **A** | **C** | **T** | **T** | **C** | **G** | **T** | **T** | **T** | **C** | **C** | **G** | **A** | **T** | **T** | **G** | **A** | **A** | **T** | **T** | **A** | **C** | **C** | **A** | **T** | **G** | **A** | **T** | **C** | **A** | **C** | **C** | **G** | **A** | **A** | **A** | **A** | **A** | **T** | **C** | **G** | **T** | **T** | **G** | **T** | **G** | **A** | **T** | **C** | **G** | **A** | **T** | **G** | **G** | **A** | **A** | **A** | **G** | **A** | **T** | **T** | **A** | **G** | **T** | **T** | **A** | **T** | **A** | **C** | **C** | **G** | **G** | **T** | **G** | **G** | **T** | **T** | **T** | **C** | **A** | **A** | **T** | **A** | **T** | **T** | **G** | **C** | **C** | **A** | **A** | **T** | **C** | **A** | **A** | **T** | **A** | **T** | **G** | **T** | **A** | **A** | **A** | **T** | **A** | **C** | **A** | **A** | **C** | **A** | **A** | **A** | **A** | **A** | **A** | **A** | **T** | **T** | **C** | **G** | **G** | **C** | **T** | **A** | **T** | **T** | **G** | **G** | **C** | **G** | **C** | **G** | **A** | **T** | **A** | **C** | **G** | **C** | **A** | **T** | **A** | **T** | **A** | **C** | **G** | **G** | **A** | **T** | **T** | **T** | **T** | **C** | **G** | **G** | **T** | **G** | **C** | **C** | **G** | **C** | **T** | **T** | **C** | **T** | **T** | **C** | **T** | **T** | **T** | **G** | **C** | **T** | **C** | **C** | **A** | **G** | **C** | **T** | **T** | **C** | **G** | **C** | **T** | **T** | **C** | **T** | **T** | **A** | **A** | **C** | **A** | **G** | **A** | **C** | **T** | **G** | **G** | **A** | **A** | **C** | **G** | **T** | **C** | **T** | **C** | **G** | **G** | **T** | **A** | **C** | **C** | **T** | **G** | **A** | **A** | **G** | **A** | **A** | **A** | **A** | **A** | **A** | **A** | **G** | **G** | **T** | **C** | **G** | **G** | **C** | **T** | **A** | **T** | **C** | **A** | **T** | **T** | **T** | **G** | **A** | **A** | **T** | **T** | **A** | **T** | **T** | **T** | **C** | **T** | **T** | **T** | **A** | **A** | **A** | **A** | **A** | **A** | **G** | **C** | **A** | **G** | **A** | **T** | **C** | **G** | **A** | **G** | **A** | **T** | **G** | **A** | **A** | **T** | **C** | **T** | **A** | **A** | **G** | **C** | **T** | **T** | **G** | **C** | **T** | **G** | **A** | **T** | **A** | **C** | **A** | **T** | **C** | **C** | **A** | **T** | **C** | **C** | **A** | **G** | **C** | **T** | **T** | **G** | **T** | **T** | **T** | **C** | **A** | **A** | **G** | **C** | **G** | **G** | **A** | **C** | **C** | **G** | **A** | **A** | **T** | **A** | **A** | **C** | **G** | **A** | **A** | **A** | **G** | **G** | **G** | **A** | **A** | **C** | **A** | **A** | **A** | **T** | **C** | **A** | **A** | **G** | **C** | **T** | **T** | **T** | **C** | **A** | **T** | **T** | **T** | **A** | **T** | **C** | **A** | **A** | **A** | **T** | **T** | **G** | **A** | **T** | **T** | **A** | **C** | **T** | **T** | **C** | **T** | **G** | **C** | **T** | **A** | **A** | **A** | **A** | **A** | **A** | **C** | **G** | **T** | **G** | **T** | **T** | **T** | **G** | **G** | **A** | **T** | **A** | **C** | **A** | **G** | **A** | **C** | **A** | **C** | **C** | **T** | **T** | **A** | **C** | **C** | **T** | **T** | **G** | **T** | **T** | **C** | **C** | **T** | **G** | **A** | **T** | **G** | **A** | **A** | **A** | **G** | **T** | **G** | **T** | **C** | **A** | **T** | **T** | **G** | **C** | **T** | **G** | **C** | **T** | **T** | **T** | **A** | **A** | **A** | **A** | **A** | **T** | **C** | **G** | **C** | **A** | **A** | **C** | **T** | **G** | **C** | **C** | **T** | **C** | **T** | **G** | **G** | **T** | **G** | **T** | **A** | **G** | **A** | **T** | **G** | **T** | **G** | **A** | **A** | **A** | **A** | **T** | **C** | **A** | **T** | **G** | **A** | **T** | **T** | **C** | **C** | **C** | **A** | **A** | **C** | **A** | **A** | **A** | **C** | **C** | **G** | **G** | **A** | **T** | **C** | **A** | **T** | **C** | **C** | **T** | **T** | **T** | **T** | **A** | **T** | **T** | **T** | **A** | **T** | **C** | **G** | **A** | **G** | **C** | **A** | **A** | **C** | **A** | **C** | **A** | **A** | **T** | **A** | **T** | **T** | **A** | **T** | **G** | **C** | **T** | **C** | **G** | **G** | **C** | **A** | **G** | **C** | **T** | **G** | **A** | **T** | **C** | **A** | **A** | **G** | **G** | **A** | **A** | **A** | **A** | **T** | **G** | **T** | **A** | **C** | **A** | **A** | **A** | **T** | **C** | **C** | **T** | **T** | **G** | **T** | **C** | **T** | **A** | **T** | **G** | **A** | **G** | **A** | **A** | **C** | **G** | **G** | **C** | **T** | **T** | **C** | **C** | **T** | **C** | **C** | **A** | **T** | **G** | **C** | **A** | **A** | **A** | **A** | **A** | **C** | **A** | **T** | **T** | **G** | **A** | **T** | **A** | **A** | **T** | **G** | **G** | **A** | **T** | **G** | **A** | **T** | **G** | **A** | **A** | **A** | **T** | **C** | **T** | **G** | **C** | **A** | **T** | **G** | **G** | **T** | **A** | **G** | **G** | **T** | **T** | **C** | **A** | **G** | **C** | **A** | **A** | **A** | **T** | **C** | **A** | **A** | **G** | **A** | **T** | **A** | **T** | **T** | **C** | **G** | **T** | **A** | **G** | **C** | **T** | **A** | **C** | **C** | **G** | **A** | **T** | **T** | **G** | **A** | **A** | **T** | **T** | **T** | **T** | **G** | **A** | **A** | **A** | **C** | **A** | **A** | **G** | **T** | **G** | **C** | **T** | **G** | **T** | **C** | **A** | **T** | **T** | **T** | **A** | **C** | **G** | **A** | **T** | **C** | **C** | **T** | **G** | **A** | **G** | **T** | **T** | **T** | **T** | **T** | **A** | **G** | **A** | **A** | **G** | **A** | **A** | **C** | **T** | **T** | **G** | **C** | **T** | **A** | **C** | **T** | **C** | **A** | **G** | **T** | **T** | **C** | **A** | **A** | **A** | **G** | **A** | **A** | **G** | **A** | **T** | **G** | **A** | **G** | **A** | **C** | **A** | **C** | **A** | **T** | **T** | **G** | **T** | **T** | **C** | **A** | **T** | **C** | **C** | **A** | **T** | **G** | **A** | **C** | **A** | **A** | **C** | **T** | **G** | **A** | **A** | **A** | **C** | **A** | **G** | **T** | **C** | **A** | **A** | **G** | **G** | **A** | **A** | **A** | **T** | **G** | **T** | **C** | **T** | **A** | **A** | **C** | **T** | **G** | **G** | **C** | **T** | **A** | **T** | **T** | **A** | **T** | **T** | **C** | **A** | **A** | **G** | **C** | **A** | **A** | **C** | **A** | **A** | **A** | **T** | **T** | **T** | **C** | **T** | **C** | **G** | **A** | **T** | **T** | **A** | **T** | **T** | **T** | **T** | **C** | **T** | **C** | **C** | **A** | **A** | **T** | **C** | **C** | **T** | **A** | **-** | **-** | **-** |
| **N55279** | **-** | **-** | **-** | **-** | **-** | **-** | **-** | **-** | **-** | **-** | **-** | **-** | **-** | **-** | **-** | **-** | **-** | **-** | **-** | **-** | **-** | **-** | **-** | **-** | **-** | **-** | **-** | **-** | **-** | **-** | **-** | **-** | **-** | **-** | **-** | **-** | **-** | **-** | **-** | **-** | **-** | **-** | **-** | **-** | **-** | **-** | **-** | **-** | **-** | **-** | **-** | **-** | **-** | **-** | **-** | **-** | **-** | **-** | **-** | **-** | **-** | **-** | **-** | **-** | **-** | **-** | **-** | **-** | **-** | **A** | **A** | **A** | **C** | **C** | **C** | **C** | **G** | **A** | **G** | **A** | **T** | **G** | **T** | **A** | **G** | **C** | **A** | **G** | **C** | **T** | **A** | **T** | **T** | **T** | **G** | **G** | **G** | **C** | **A** | **T** | **G** | **G** | **C** | **T** | **C** | **T** | **T** | **A** | **G** | **T** | **A** | **T** | **T** | **G** | **T** | **T** | **T** | **G** | **C** | **C** | **C** | **T** | **T** | **C** | **C** | **T** | **G** | **G** | **G** | **G** | **T** | **G** | **G** | **G** | **T** | **T** | **T** | **T** | **G** | **T** | **A** | **T** | **T** | **A** | **T** | **A** | **T** | **T** | **T** | **A** | **T** | **T** | **T** | **T** | **T** | **C** | **G** | **G** | **A** | **C** | **G** | **T** | **G** | **G** | **A** | **T** | **T** | **A** | **A** | **C** | **G** | **G** | **A** | **T** | **A** | **A** | **G** | **A** | **A** | **A** | **A** | **A** | **A** | **T** | **T** | **C** | **T** | **A** | **T** | **C** | **T** | **C** | **C** | **G** | **A** | **C** | **A** | **A** | **A** | **G** | **T** | **G** | **A** | **C** | **T** | **T** | **G** | **A** | **A** | **A** | **G** | **A** | **A** | **T** | **T** | **A** | **G** | **A** | **A** | **A** | **A** | **C** | **T** | **T** | **T** | **C** | **A** | **G** | **T** | **C** | **T** | **T** | **T** | **T** | **A** | **G** | **A** | **G** | **G** | **A** | **G** | **A** | **T** | **A** | **C** | **C** | **A** | **T** | **T** | **G** | **A** | **A** | **C** | **A** | **T** | **T** | **A** | **C** | **G** | **A** | **T** | **C** | **C** | **T** | **G** | **A** | **C** | **A** | **T** | **G** | **G** | **G** | **C** | **G** | **A** | **C** | **A** | **A** | **A** | **G** | **A** | **C** | **A** | **A** | **A** | **C** | **A** | **A** | **C** | **A** | **A** | **T** | **T** | **T** | **G** | **T** | **C** | **G** | **A** | **C** | **T** | **T** | **C** | **T** | **T** | **C** | **T** | **C** | **T** | **T** | **C** | **A** | **T** | **T** | **A** | **A** | **A** | **T** | **C** | **G** | **T** | **A** | **T** | **G** | **C** | **C** | **G** | **C** | **T** | **G** | **A** | **C** | **A** | **A** | **G** | **A** | **A** | **T** | **G** | **A** | **A** | **T** | **T** | **C** | **T** | **G** | **T** | **C** | **A** | **C** | **T** | **C** | **T** | **T** | **C** | **T** | **C** | **A** | **C** | **A** | **G** | **A** | **C** | **G** | **G** | **A** | **C** | **A** | **A** | **A** | **A** | **G** | **A** | **A** | **A** | **T** | **T** | **A** | **G** | **A** | **T** | **T** | **C** | **A** | **C** | **T** | **G** | **C** | **T** | **T** | **C** | **A** | **A** | **G** | **A** | **T** | **C** | **T** | **A** | **A** | **A** | **A** | **A** | **A** | **A** | **G** | **C** | **C** | **A** | **A** | **A** | **C** | **A** | **T** | **T** | **C** | **G** | **A** | **T** | **C** | **C** | **A** | **T** | **A** | **T** | **C** | **G** | **A** | **A** | **T** | **A** | **T** | **T** | **A** | **C** | **G** | **C** | **A** | **T** | **T** | **T** | **G** | **T** | **G** | **A** | **C** | **A** | **G** | **A** | **T** | **A** | **A** | **T** | **A** | **T** | **C** | **G** | **G** | **T** | **C** | **A** | **G** | **C** | **A** | **A** | **G** | **T** | **C** | **T** | **T** | **A** | **C** | **G** | **C** | **G** | **T** | **T** | **T** | **T** | **A** | **G** | **A** | **A** | **G** | **A** | **A** | **A** | **A** | **A** | **G** | **C** | **C** | **G** | **C** | **A** | **G** | **A** | **A** | **G** | **G** | **C** | **G** | **T** | **A** | **G** | **A** | **A** | **G** | **T** | **T** | **C** | **G** | **T** | **A** | **T** | **A** | **T** | **T** | **A** | **T** | **A** | **T** | **G** | **A** | **T** | **G** | **C** | **A** | **T** | **T** | **T** | **G** | **G** | **C** | **T** | **C** | **C** | **C** | **A** | **T** | **G** | **G** | **C** | **A** | **C** | **A** | **A** | **A** | **A** | **G** | **C** | **A** | **A** | **A** | **A** | **G** | **A** | **T** | **T** | **T** | **C** | **A** | **A** | **T** | **C** | **G** | **T** | **C** | **T** | **A** | **A** | **T** | **C** | **A** | **A** | **A** | **A** | **A** | **T** | **G** | **G** | **T** | **G** | **G** | **A** | **C** | **A** | **T** | **G** | **T** | **C** | **C** | **A** | **T** | **A** | **C** | **A** | **T** | **T** | **T** | **G** | **T** | **T** | **A** | **C** | **C** | **T** | **C** | **G** | **C** | **A** | **A** | **A** | **A** | **A** | **G** | **C** | **A** | **T** | **T** | **G** | **C** | **T** | **T** | **C** | **G** | **T** | **T** | **T** | **C** | **C** | **G** | **A** | **T** | **T** | **G** | **A** | **A** | **T** | **T** | **A** | **C** | **C** | **A** | **T** | **G** | **A** | **T** | **C** | **A** | **C** | **C** | **G** | **A** | **A** | **A** | **A** | **A** | **T** | **C** | **G** | **T** | **T** | **G** | **T** | **G** | **A** | **T** | **C** | **G** | **A** | **T** | **G** | **G** | **A** | **A** | **A** | **G** | **A** | **T** | **C** | **A** | **G** | **T** | **T** | **A** | **C** | **A** | **C** | **C** | **G** | **G** | **T** | **G** | **G** | **T** | **T** | **T** | **C** | **A** | **A** | **T** | **A** | **T** | **T** | **G** | **C** | **C** | **A** | **A** | **T** | **C** | **A** | **A** | **T** | **A** | **T** | **G** | **T** | **A** | **A** | **A** | **T** | **A** | **C** | **G** | **A** | **C** | **A** | **A** | **A** | **A** | **A** | **A** | **A** | **T** | **T** | **C** | **G** | **G** | **T** | **T** | **A** | **T** | **T** | **G** | **G** | **C** | **G** | **T** | **G** | **A** | **T** | **A** | **C** | **G** | **C** | **A** | **T** | **A** | **T** | **A** | **C** | **G** | **A** | **A** | **T** | **T** | **T** | **T** | **C** | **G** | **G** | **T** | **G** | **C** | **T** | **G** | **C** | **T** | **T** | **C** | **T** | **T** | **C** | **T** | **T** | **T** | **G** | **C** | **T** | **T** | **C** | **A** | **A** | **C** | **T** | **T** | **C** | **G** | **T** | **T** | **T** | **T** | **T** | **T** | **A** | **A** | **C** | **A** | **G** | **A** | **C** | **T** | **G** | **G** | **A** | **A** | **C** | **G** | **T** | **T** | **T** | **C** | **A** | **G** | **T** | **G** | **T** | **C** | **T** | **G** | **A** | **A** | **G** | **A** | **A** | **A** | **A** | **A** | **A** | **A** | **G** | **G** | **T** | **C** | **G** | **G** | **C** | **T** | **A** | **T** | **C** | **A** | **T** | **T** | **T** | **G** | **A** | **A** | **T** | **T** | **A** | **T** | **T** | **T** | **C** | **T** | **T** | **T** | **A** | **A** | **A** | **A** | **A** | **A** | **G** | **C** | **C** | **G** | **A** | **T** | **C** | **G** | **A** | **G** | **A** | **T** | **G** | **A** | **A** | **T** | **C** | **C** | **A** | **A** | **A** | **C** | **T** | **T** | **G** | **C** | **T** | **G** | **A** | **T** | **A** | **C** | **G** | **T** | **C** | **C** | **A** | **T** | **C** | **C** | **A** | **G** | **C** | **T** | **T** | **G** | **T** | **C** | **T** | **C** | **A** | **A** | **G** | **C** | **G** | **G** | **A** | **C** | **C** | **A** | **A** | **A** | **T** | **A** | **A** | **C** | **G** | **A** | **A** | **C** | **G** | **A** | **G** | **A** | **A** | **C** | **A** | **A** | **A** | **T** | **C** | **A** | **A** | **G** | **C** | **T** | **T** | **T** | **C** | **G** | **T** | **T** | **T** | **A** | **T** | **C** | **A** | **A** | **A** | **T** | **T** | **G** | **A** | **T** | **T** | **A** | **C** | **T** | **T** | **C** | **T** | **G** | **C** | **T** | **A** | **A** | **A** | **A** | **A** | **A** | **C** | **G** | **T** | **G** | **T** | **T** | **T** | **G** | **G** | **A** | **T** | **A** | **C** | **A** | **G** | **A** | **C** | **A** | **C** | **C** | **T** | **T** | **A** | **C** | **C** | **T** | **C** | **G** | **T** | **T** | **C** | **C** | **T** | **G** | **A** | **T** | **G** | **A** | **A** | **A** | **G** | **T** | **G** | **T** | **C** | **A** | **T** | **T** | **G** | **C** | **T** | **G** | **C** | **T** | **T** | **T** | **G** | **A** | **A** | **A** | **A** | **T** | **C** | **G** | **C** | **A** | **A** | **C** | **T** | **G** | **C** | **T** | **T** | **C** | **T** | **G** | **G** | **T** | **G** | **T** | **C** | **G** | **A** | **T** | **G** | **T** | **G** | **A** | **A** | **A** | **A** | **T** | **C** | **A** | **T** | **G** | **A** | **T** | **C** | **C** | **C** | **C** | **A** | **A** | **T** | **A** | **A** | **A** | **C** | **C** | **G** | **G** | **A** | **T** | **C** | **A** | **T** | **C** | **C** | **T** | **T** | **T** | **T** | **A** | **T** | **T** | **T** | **A** | **T** | **C** | **G** | **A** | **G** | **C** | **A** | **A** | **C** | **A** | **C** | **A** | **A** | **T** | **A** | **T** | **T** | **A** | **T** | **G** | **C** | **T** | **C** | **G** | **T** | **C** | **A** | **G** | **C** | **T** | **C** | **A** | **T** | **C** | **A** | **A** | **G** | **G** | **A** | **A** | **A** | **A** | **T** | **G** | **T** | **A** | **C** | **A** | **G** | **A** | **T** | **C** | **C** | **T** | **T** | **G** | **T** | **C** | **T** | **A** | **T** | **G** | **A** | **G** | **A** | **A** | **T** | **G** | **G** | **C** | **T** | **T** | **C** | **C** | **T** | **C** | **C** | **A** | **T** | **G** | **C** | **A** | **A** | **A** | **A** | **A** | **C** | **A** | **T** | **T** | **A** | **A** | **T** | **A** | **A** | **T** | **G** | **G** | **A** | **T** | **G** | **A** | **T** | **G** | **A** | **A** | **A** | **T** | **C** | **T** | **G** | **C** | **A** | **T** | **G** | **G** | **T** | **A** | **G** | **G** | **T** | **T** | **C** | **A** | **G** | **C** | **A** | **A** | **A** | **T** | **C** | **A** | **A** | **G** | **A** | **T** | **A** | **T** | **T** | **C** | **G** | **T** | **A** | **G** | **C** | **T** | **A** | **C** | **C** | **G** | **A** | **T** | **T** | **G** | **A** | **A** | **T** | **T** | **T** | **T** | **G** | **A** | **A** | **A** | **C** | **A** | **A** | **G** | **T** | **G** | **C** | **T** | **G** | **T** | **C** | **A** | **T** | **T** | **T** | **A** | **C** | **G** | **A** | **T** | **C** | **C** | **T** | **G** | **A** | **G** | **T** | **T** | **T** | **T** | **T** | **A** | **G** | **A** | **A** | **G** | **A** | **G** | **C** | **T** | **T** | **G** | **C** | **T** | **A** | **C** | **T** | **C** | **A** | **G** | **T** | **T** | **C** | **A** | **A** | **A** | **G** | **A** | **A** | **G** | **A** | **T** | **G** | **A** | **G** | **A** | **C** | **A** | **C** | **A** | **T** | **T** | **G** | **T** | **T** | **C** | **A** | **C** | **C** | **A** | **A** | **T** | **G** | **A** | **C** | **A** | **A** | **C** | **T** | **G** | **A** | **A** | **A** | **C** | **A** | **G** | **T** | **G** | **A** | **A** | **G** | **G** | **A** | **A** | **A** | **T** | **G** | **T** | **C** | **T** | **A** | **A** | **C** | **T** | **G** | **G** | **C** | **T** | **G** | **T** | **T** | **A** | **T** | **T** | **C** | **A** | **A** | **A** | **C** | **A** | **A** | **C** | **A** | **A** | **A** | **T** | **T** | **T** | **C** | **T** | **C** | **G** | **A** | **T** | **T** | **G** | **T** | **T** | **T** | **T** | **C** | **T** | **C** | **C** | **A** | **A** | **T** | **C** | **T** | **T** | **A** | **-** | **-** | **-** |
| **N59943F** | **-** | **-** | **-** | **-** | **-** | **-** | **-** | **-** | **-** | **-** | **-** | **-** | **-** | **-** | **-** | **-** | **-** | **-** | **-** | **-** | **-** | **-** | **-** | **-** | **-** | **-** | **-** | **-** | **-** | **-** | **-** | **-** | **-** | **-** | **-** | **-** | **-** | **-** | **-** | **-** | **-** | **-** | **-** | **-** | **-** | **-** | **-** | **-** | **-** | **-** | **-** | **-** | **-** | **-** | **-** | **-** | **-** | **-** | **-** | **-** | **-** | **-** | **-** | **-** | **-** | **-** | **-** | **-** | **-** | **A** | **A** | **A** | **C** | **C** | **C** | **C** | **G** | **A** | **G** | **A** | **T** | **G** | **T** | **A** | **G** | **C** | **A** | **G** | **C** | **T** | **A** | **T** | **T** | **T** | **G** | **G** | **G** | **C** | **A** | **T** | **G** | **G** | **C** | **T** | **C** | **T** | **T** | **A** | **G** | **T** | **A** | **T** | **T** | **A** | **T** | **T** | **T** | **G** | **C** | **C** | **C** | **T** | **T** | **C** | **C** | **T** | **G** | **G** | **G** | **G** | **T** | **G** | **G** | **G** | **T** | **T** | **T** | **T** | **G** | **T** | **A** | **T** | **T** | **A** | **T** | **A** | **T** | **T** | **T** | **A** | **T** | **T** | **T** | **T** | **T** | **C** | **G** | **G** | **A** | **C** | **G** | **T** | **G** | **G** | **A** | **T** | **T** | **A** | **A** | **C** | **G** | **G** | **A** | **T** | **A** | **A** | **G** | **A** | **A** | **A** | **A** | **A** | **A** | **T** | **T** | **C** | **T** | **A** | **T** | **C** | **T** | **C** | **C** | **G** | **A** | **C** | **A** | **A** | **A** | **G** | **T** | **G** | **A** | **C** | **T** | **T** | **G** | **A** | **A** | **A** | **G** | **A** | **A** | **T** | **T** | **A** | **G** | **A** | **A** | **A** | **A** | **C** | **T** | **T** | **T** | **C** | **A** | **G** | **T** | **C** | **T** | **T** | **T** | **T** | **A** | **G** | **A** | **G** | **G** | **A** | **G** | **A** | **T** | **A** | **C** | **C** | **A** | **T** | **T** | **G** | **A** | **A** | **C** | **A** | **T** | **T** | **A** | **C** | **G** | **A** | **T** | **C** | **C** | **T** | **G** | **A** | **C** | **A** | **T** | **G** | **G** | **G** | **C** | **G** | **A** | **C** | **A** | **A** | **A** | **G** | **A** | **C** | **A** | **A** | **A** | **C** | **A** | **A** | **C** | **A** | **A** | **T** | **T** | **T** | **G** | **T** | **C** | **G** | **A** | **C** | **T** | **T** | **C** | **T** | **T** | **C** | **T** | **C** | **T** | **T** | **C** | **A** | **T** | **T** | **A** | **A** | **A** | **T** | **C** | **G** | **T** | **A** | **T** | **G** | **C** | **C** | **G** | **C** | **T** | **G** | **A** | **C** | **A** | **A** | **G** | **A** | **A** | **T** | **G** | **A** | **A** | **T** | **T** | **C** | **T** | **G** | **T** | **C** | **A** | **C** | **T** | **C** | **T** | **T** | **C** | **T** | **C** | **A** | **C** | **A** | **G** | **A** | **C** | **G** | **G** | **A** | **C** | **A** | **A** | **A** | **A** | **G** | **A** | **A** | **A** | **T** | **T** | **A** | **G** | **A** | **T** | **T** | **C** | **A** | **C** | **T** | **G** | **C** | **T** | **T** | **C** | **A** | **A** | **G** | **A** | **T** | **C** | **T** | **A** | **A** | **A** | **A** | **A** | **A** | **A** | **G** | **C** | **C** | **A** | **A** | **A** | **C** | **A** | **T** | **T** | **C** | **G** | **A** | **T** | **C** | **C** | **A** | **T** | **A** | **T** | **C** | **G** | **A** | **A** | **T** | **A** | **T** | **T** | **A** | **C** | **G** | **C** | **A** | **T** | **T** | **T** | **G** | **T** | **G** | **A** | **C** | **A** | **G** | **A** | **T** | **A** | **C** | **T** | **A** | **T** | **C** | **G** | **G** | **T** | **C** | **A** | **G** | **C** | **A** | **A** | **G** | **T** | **C** | **T** | **T** | **A** | **C** | **G** | **C** | **G** | **T** | **T** | **T** | **T** | **A** | **G** | **A** | **A** | **G** | **A** | **A** | **A** | **A** | **A** | **G** | **C** | **C** | **G** | **C** | **A** | **G** | **A** | **A** | **G** | **G** | **C** | **G** | **T** | **A** | **G** | **A** | **A** | **G** | **T** | **T** | **C** | **G** | **T** | **A** | **T** | **A** | **T** | **T** | **A** | **T** | **A** | **T** | **G** | **A** | **T** | **G** | **C** | **A** | **T** | **T** | **T** | **G** | **G** | **C** | **T** | **C** | **C** | **C** | **A** | **C** | **G** | **G** | **C** | **A** | **C** | **A** | **A** | **A** | **A** | **G** | **C** | **A** | **A** | **A** | **A** | **G** | **A** | **T** | **T** | **T** | **C** | **A** | **A** | **T** | **C** | **G** | **T** | **C** | **T** | **A** | **A** | **T** | **C** | **A** | **A** | **A** | **A** | **A** | **T** | **G** | **G** | **T** | **G** | **G** | **A** | **C** | **A** | **T** | **G** | **T** | **C** | **C** | **A** | **T** | **A** | **C** | **A** | **T** | **T** | **T** | **G** | **T** | **T** | **A** | **C** | **C** | **T** | **C** | **G** | **C** | **A** | **A** | **A** | **A** | **A** | **G** | **C** | **A** | **T** | **T** | **G** | **C** | **T** | **T** | **C** | **G** | **T** | **T** | **T** | **C** | **C** | **G** | **A** | **T** | **T** | **G** | **A** | **A** | **C** | **T** | **A** | **C** | **C** | **A** | **T** | **G** | **A** | **T** | **C** | **A** | **C** | **C** | **G** | **G** | **A** | **A** | **A** | **A** | **T** | **C** | **G** | **T** | **T** | **G** | **T** | **G** | **A** | **T** | **C** | **G** | **A** | **T** | **G** | **G** | **A** | **A** | **A** | **G** | **A** | **T** | **C** | **A** | **G** | **T** | **T** | **A** | **C** | **A** | **C** | **C** | **G** | **G** | **T** | **G** | **G** | **C** | **T** | **T** | **C** | **A** | **A** | **C** | **A** | **T** | **T** | **G** | **C** | **C** | **A** | **A** | **T** | **C** | **A** | **A** | **T** | **A** | **T** | **G** | **T** | **A** | **A** | **A** | **T** | **A** | **C** | **G** | **A** | **C** | **A** | **A** | **A** | **A** | **A** | **A** | **A** | **T** | **T** | **C** | **G** | **G** | **C** | **T** | **A** | **T** | **T** | **G** | **G** | **C** | **G** | **T** | **G** | **A** | **T** | **A** | **C** | **G** | **C** | **A** | **T** | **A** | **T** | **A** | **C** | **G** | **G** | **A** | **T** | **T** | **T** | **T** | **C** | **G** | **G** | **T** | **G** | **C** | **C** | **G** | **C** | **T** | **T** | **C** | **T** | **T** | **C** | **T** | **T** | **T** | **G** | **C** | **T** | **T** | **C** | **A** | **A** | **C** | **T** | **T** | **C** | **G** | **T** | **T** | **T** | **T** | **T** | **T** | **A** | **A** | **C** | **A** | **G** | **A** | **C** | **T** | **G** | **G** | **A** | **A** | **C** | **G** | **T** | **C** | **T** | **C** | **A** | **G** | **T** | **T** | **C** | **C** | **T** | **G** | **A** | **A** | **G** | **A** | **A** | **A** | **A** | **A** | **A** | **A** | **G** | **G** | **T** | **C** | **G** | **G** | **C** | **T** | **A** | **T** | **C** | **A** | **T** | **T** | **T** | **G** | **A** | **A** | **T** | **T** | **A** | **T** | **T** | **T** | **C** | **T** | **T** | **T** | **A** | **A** | **A** | **A** | **A** | **A** | **G** | **C** | **C** | **G** | **A** | **T** | **C** | **G** | **A** | **G** | **A** | **C** | **G** | **A** | **A** | **T** | **C** | **C** | **A** | **A** | **A** | **C** | **T** | **T** | **G** | **C** | **T** | **G** | **A** | **T** | **A** | **C** | **A** | **T** | **C** | **C** | **A** | **T** | **C** | **C** | **A** | **G** | **C** | **T** | **T** | **G** | **T** | **C** | **T** | **C** | **A** | **A** | **G** | **C** | **G** | **G** | **A** | **C** | **C** | **A** | **A** | **A** | **T** | **A** | **A** | **C** | **G** | **A** | **A** | **C** | **G** | **C** | **G** | **A** | **A** | **C** | **A** | **A** | **A** | **T** | **C** | **A** | **A** | **G** | **C** | **T** | **T** | **T** | **C** | **G** | **T** | **T** | **T** | **A** | **T** | **C** | **A** | **A** | **A** | **T** | **T** | **G** | **A** | **T** | **T** | **A** | **C** | **T** | **T** | **C** | **T** | **G** | **C** | **T** | **A** | **A** | **A** | **A** | **A** | **A** | **C** | **G** | **T** | **G** | **T** | **T** | **T** | **G** | **G** | **A** | **T** | **A** | **C** | **A** | **G** | **A** | **C** | **G** | **C** | **C** | **T** | **T** | **A** | **C** | **C** | **T** | **C** | **G** | **T** | **T** | **C** | **C** | **T** | **G** | **A** | **T** | **G** | **A** | **A** | **A** | **G** | **T** | **G** | **T** | **C** | **A** | **T** | **T** | **G** | **C** | **T** | **G** | **C** | **T** | **T** | **T** | **G** | **A** | **A** | **A** | **A** | **T** | **C** | **G** | **C** | **A** | **A** | **C** | **T** | **G** | **C** | **T** | **T** | **C** | **T** | **G** | **G** | **T** | **G** | **T** | **C** | **G** | **A** | **T** | **G** | **T** | **G** | **A** | **A** | **A** | **A** | **T** | **C** | **A** | **T** | **G** | **A** | **T** | **C** | **C** | **C** | **C** | **A** | **A** | **T** | **A** | **A** | **A** | **C** | **C** | **G** | **G** | **A** | **T** | **C** | **A** | **T** | **C** | **C** | **T** | **T** | **T** | **T** | **A** | **T** | **T** | **T** | **A** | **T** | **C** | **G** | **A** | **G** | **C** | **A** | **A** | **C** | **A** | **C** | **A** | **A** | **T** | **A** | **T** | **T** | **A** | **T** | **G** | **C** | **G** | **C** | **G** | **T** | **C** | **A** | **G** | **C** | **T** | **C** | **A** | **T** | **C** | **A** | **A** | **G** | **G** | **A** | **A** | **A** | **A** | **C** | **G** | **T** | **A** | **C** | **A** | **A** | **A** | **T** | **T** | **C** | **T** | **T** | **G** | **T** | **C** | **T** | **A** | **T** | **G** | **A** | **G** | **A** | **A** | **T** | **G** | **G** | **C** | **T** | **T** | **C** | **C** | **T** | **C** | **C** | **A** | **T** | **G** | **C** | **A** | **A** | **A** | **A** | **A** | **C** | **A** | **T** | **T** | **G** | **A** | **T** | **A** | **A** | **T** | **G** | **G** | **A** | **T** | **G** | **A** | **T** | **G** | **A** | **A** | **A** | **T** | **C** | **T** | **G** | **C** | **A** | **T** | **G** | **G** | **T** | **A** | **G** | **G** | **T** | **T** | **C** | **A** | **G** | **C** | **A** | **A** | **A** | **T** | **C** | **A** | **A** | **G** | **A** | **T** | **A** | **T** | **T** | **C** | **G** | **T** | **A** | **G** | **C** | **T** | **A** | **C** | **C** | **G** | **A** | **T** | **T** | **G** | **A** | **A** | **T** | **T** | **T** | **T** | **G** | **A** | **A** | **A** | **C** | **A** | **A** | **G** | **T** | **G** | **C** | **T** | **G** | **T** | **C** | **A** | **T** | **C** | **T** | **A** | **C** | **G** | **A** | **T** | **C** | **C** | **T** | **G** | **A** | **G** | **T** | **T** | **T** | **T** | **T** | **A** | **G** | **A** | **A** | **G** | **A** | **A** | **C** | **T** | **T** | **G** | **C** | **T** | **A** | **C** | **T** | **C** | **A** | **G** | **T** | **T** | **T** | **A** | **A** | **A** | **G** | **A** | **A** | **G** | **A** | **T** | **G** | **A** | **G** | **A** | **C** | **A** | **C** | **A** | **T** | **T** | **G** | **T** | **T** | **C** | **A** | **C** | **C** | **A** | **A** | **T** | **G** | **A** | **C** | **G** | **A** | **C** | **T** | **G** | **A** | **A** | **A** | **C** | **A** | **G** | **T** | **G** | **A** | **A** | **G** | **G** | **A** | **A** | **A** | **T** | **G** | **T** | **C** | **T** | **A** | **A** | **T** | **T** | **G** | **G** | **C** | **T** | **G** | **T** | **T** | **A** | **T** | **T** | **C** | **A** | **A** | **G** | **C** | **A** | **A** | **C** | **A** | **A** | **A** | **T** | **T** | **T** | **C** | **T** | **C** | **G** | **A** | **T** | **T** | **A** | **T** | **T** | **T** | **T** | **C** | **T** | **C** | **C** | **A** | **A** | **T** | **C** | **T** | **T** | **A** | **-** | **-** | **-** |
| **N59711F** | **-** | **-** | **-** | **-** | **-** | **-** | **-** | **-** | **-** | **-** | **-** | **-** | **-** | **-** | **-** | **-** | **-** | **-** | **-** | **-** | **-** | **-** | **-** | **-** | **-** | **-** | **-** | **-** | **-** | **-** | **-** | **-** | **-** | **-** | **-** | **-** | **-** | **-** | **-** | **-** | **-** | **-** | **-** | **-** | **-** | **-** | **-** | **-** | **-** | **-** | **-** | **-** | **-** | **-** | **-** | **-** | **-** | **-** | **-** | **-** | **-** | **-** | **-** | **-** | **-** | **-** | **-** | **-** | **-** | **A** | **A** | **A** | **C** | **C** | **C** | **C** | **G** | **A** | **G** | **A** | **T** | **G** | **T** | **A** | **G** | **C** | **A** | **G** | **C** | **T** | **A** | **T** | **T** | **T** | **G** | **G** | **G** | **C** | **A** | **T** | **G** | **G** | **C** | **T** | **C** | **T** | **T** | **A** | **G** | **T** | **A** | **T** | **T** | **G** | **T** | **T** | **T** | **G** | **C** | **C** | **C** | **T** | **T** | **C** | **C** | **T** | **G** | **G** | **G** | **G** | **T** | **G** | **G** | **G** | **T** | **T** | **T** | **T** | **G** | **T** | **A** | **T** | **T** | **A** | **T** | **A** | **T** | **T** | **T** | **A** | **T** | **T** | **T** | **T** | **T** | **C** | **G** | **G** | **A** | **C** | **G** | **T** | **G** | **G** | **A** | **T** | **T** | **A** | **A** | **C** | **G** | **G** | **A** | **T** | **A** | **A** | **G** | **A** | **A** | **A** | **A** | **A** | **A** | **T** | **T** | **C** | **T** | **A** | **T** | **C** | **T** | **C** | **C** | **G** | **A** | **C** | **A** | **A** | **A** | **G** | **T** | **G** | **A** | **C** | **T** | **T** | **G** | **A** | **A** | **A** | **G** | **A** | **A** | **T** | **T** | **A** | **G** | **A** | **A** | **A** | **A** | **C** | **T** | **T** | **T** | **C** | **A** | **G** | **T** | **C** | **T** | **T** | **T** | **T** | **A** | **G** | **A** | **G** | **G** | **A** | **G** | **A** | **T** | **A** | **C** | **C** | **A** | **T** | **T** | **G** | **A** | **A** | **C** | **A** | **T** | **T** | **A** | **C** | **G** | **A** | **T** | **C** | **C** | **T** | **G** | **A** | **C** | **A** | **T** | **G** | **G** | **G** | **C** | **G** | **A** | **C** | **A** | **A** | **A** | **G** | **A** | **C** | **A** | **A** | **A** | **C** | **A** | **A** | **C** | **A** | **A** | **T** | **T** | **T** | **G** | **T** | **C** | **G** | **A** | **C** | **T** | **T** | **C** | **T** | **T** | **C** | **T** | **C** | **T** | **T** | **C** | **A** | **T** | **T** | **A** | **A** | **A** | **T** | **C** | **G** | **T** | **A** | **T** | **G** | **C** | **C** | **G** | **C** | **T** | **G** | **A** | **C** | **A** | **A** | **G** | **A** | **A** | **T** | **G** | **A** | **A** | **T** | **T** | **C** | **T** | **G** | **T** | **C** | **A** | **C** | **T** | **C** | **T** | **T** | **C** | **T** | **C** | **A** | **C** | **A** | **G** | **A** | **C** | **G** | **G** | **A** | **C** | **A** | **A** | **A** | **A** | **G** | **A** | **A** | **A** | **T** | **T** | **A** | **G** | **A** | **T** | **T** | **C** | **A** | **C** | **T** | **G** | **C** | **T** | **T** | **C** | **A** | **A** | **G** | **A** | **T** | **C** | **T** | **A** | **A** | **A** | **A** | **A** | **A** | **A** | **G** | **C** | **C** | **A** | **A** | **A** | **C** | **A** | **T** | **T** | **C** | **G** | **A** | **T** | **C** | **C** | **A** | **T** | **A** | **T** | **C** | **G** | **A** | **A** | **T** | **A** | **T** | **T** | **A** | **C** | **G** | **C** | **A** | **T** | **T** | **T** | **G** | **T** | **G** | **A** | **C** | **A** | **G** | **A** | **T** | **A** | **A** | **T** | **A** | **T** | **C** | **G** | **G** | **T** | **C** | **A** | **G** | **C** | **A** | **A** | **G** | **T** | **C** | **T** | **T** | **A** | **C** | **G** | **C** | **G** | **T** | **T** | **T** | **T** | **A** | **G** | **A** | **A** | **G** | **A** | **A** | **A** | **A** | **A** | **G** | **C** | **C** | **G** | **C** | **A** | **G** | **A** | **A** | **G** | **G** | **C** | **G** | **T** | **A** | **G** | **A** | **A** | **G** | **T** | **T** | **C** | **G** | **T** | **A** | **T** | **A** | **T** | **T** | **A** | **T** | **A** | **T** | **G** | **A** | **T** | **G** | **C** | **A** | **T** | **T** | **T** | **G** | **G** | **C** | **T** | **C** | **C** | **C** | **A** | **T** | **G** | **G** | **C** | **A** | **C** | **A** | **A** | **A** | **A** | **G** | **C** | **A** | **A** | **A** | **A** | **G** | **A** | **T** | **T** | **T** | **C** | **A** | **A** | **T** | **C** | **G** | **T** | **C** | **T** | **A** | **A** | **T** | **C** | **A** | **A** | **A** | **A** | **A** | **T** | **G** | **G** | **T** | **G** | **G** | **A** | **C** | **A** | **T** | **G** | **T** | **C** | **C** | **A** | **T** | **A** | **C** | **A** | **T** | **T** | **T** | **G** | **T** | **T** | **A** | **C** | **C** | **T** | **C** | **G** | **C** | **A** | **A** | **A** | **A** | **A** | **G** | **C** | **A** | **T** | **T** | **G** | **C** | **T** | **T** | **C** | **G** | **T** | **T** | **T** | **C** | **C** | **G** | **A** | **T** | **T** | **G** | **A** | **A** | **T** | **T** | **A** | **C** | **C** | **A** | **T** | **G** | **A** | **T** | **C** | **A** | **C** | **C** | **G** | **A** | **A** | **A** | **A** | **A** | **T** | **C** | **G** | **T** | **T** | **G** | **T** | **G** | **A** | **T** | **C** | **G** | **A** | **T** | **G** | **G** | **A** | **A** | **A** | **G** | **A** | **T** | **C** | **A** | **G** | **T** | **T** | **A** | **C** | **A** | **C** | **C** | **G** | **G** | **T** | **G** | **G** | **T** | **T** | **T** | **C** | **A** | **A** | **T** | **A** | **T** | **T** | **G** | **C** | **C** | **A** | **A** | **T** | **C** | **A** | **A** | **T** | **A** | **T** | **G** | **T** | **A** | **A** | **A** | **T** | **A** | **C** | **G** | **A** | **C** | **A** | **A** | **A** | **A** | **A** | **A** | **A** | **T** | **T** | **C** | **G** | **G** | **T** | **T** | **A** | **T** | **T** | **G** | **G** | **C** | **G** | **T** | **G** | **A** | **T** | **A** | **C** | **G** | **C** | **A** | **T** | **A** | **T** | **A** | **C** | **G** | **A** | **A** | **T** | **T** | **T** | **T** | **C** | **G** | **G** | **T** | **G** | **C** | **T** | **G** | **C** | **T** | **T** | **C** | **T** | **T** | **C** | **T** | **T** | **T** | **G** | **C** | **T** | **T** | **C** | **A** | **A** | **C** | **T** | **T** | **C** | **G** | **T** | **T** | **T** | **T** | **T** | **T** | **A** | **A** | **C** | **A** | **G** | **A** | **C** | **T** | **G** | **G** | **A** | **A** | **C** | **G** | **T** | **T** | **T** | **C** | **A** | **G** | **T** | **G** | **T** | **C** | **T** | **G** | **A** | **A** | **G** | **A** | **A** | **A** | **A** | **A** | **A** | **A** | **G** | **G** | **T** | **C** | **G** | **G** | **C** | **T** | **A** | **T** | **C** | **A** | **T** | **T** | **T** | **G** | **A** | **A** | **T** | **T** | **A** | **T** | **T** | **T** | **C** | **T** | **T** | **T** | **A** | **A** | **A** | **A** | **A** | **A** | **G** | **C** | **C** | **G** | **A** | **T** | **C** | **G** | **A** | **G** | **A** | **T** | **G** | **A** | **A** | **T** | **C** | **C** | **A** | **A** | **A** | **C** | **T** | **T** | **G** | **C** | **T** | **G** | **A** | **T** | **A** | **C** | **G** | **T** | **C** | **C** | **A** | **T** | **C** | **C** | **A** | **G** | **C** | **T** | **T** | **G** | **T** | **C** | **T** | **C** | **A** | **A** | **G** | **C** | **G** | **G** | **A** | **C** | **C** | **A** | **A** | **A** | **T** | **A** | **A** | **C** | **G** | **A** | **A** | **C** | **G** | **A** | **G** | **A** | **A** | **C** | **A** | **A** | **A** | **T** | **C** | **A** | **A** | **G** | **C** | **T** | **T** | **T** | **C** | **G** | **T** | **T** | **T** | **A** | **T** | **C** | **A** | **A** | **A** | **T** | **T** | **G** | **A** | **T** | **T** | **A** | **C** | **T** | **T** | **C** | **T** | **G** | **C** | **T** | **A** | **A** | **A** | **A** | **A** | **A** | **C** | **G** | **T** | **G** | **T** | **T** | **T** | **G** | **G** | **A** | **T** | **A** | **C** | **A** | **G** | **A** | **C** | **A** | **C** | **C** | **T** | **T** | **A** | **C** | **C** | **T** | **C** | **G** | **T** | **T** | **C** | **C** | **T** | **G** | **A** | **T** | **G** | **A** | **A** | **A** | **G** | **T** | **G** | **T** | **C** | **A** | **T** | **T** | **G** | **C** | **T** | **G** | **C** | **T** | **T** | **T** | **G** | **A** | **A** | **A** | **A** | **T** | **C** | **G** | **C** | **A** | **A** | **C** | **T** | **G** | **C** | **T** | **T** | **C** | **T** | **G** | **G** | **T** | **G** | **T** | **C** | **G** | **A** | **T** | **G** | **T** | **G** | **A** | **A** | **A** | **A** | **T** | **C** | **A** | **T** | **G** | **A** | **T** | **C** | **C** | **C** | **C** | **A** | **A** | **T** | **A** | **A** | **A** | **C** | **C** | **G** | **G** | **A** | **T** | **C** | **A** | **T** | **C** | **C** | **T** | **T** | **T** | **T** | **A** | **T** | **T** | **T** | **A** | **T** | **C** | **G** | **A** | **G** | **C** | **A** | **A** | **C** | **A** | **C** | **A** | **A** | **T** | **A** | **T** | **T** | **A** | **T** | **G** | **C** | **T** | **C** | **G** | **T** | **C** | **A** | **G** | **C** | **T** | **C** | **A** | **T** | **C** | **A** | **A** | **G** | **G** | **A** | **A** | **A** | **A** | **T** | **G** | **T** | **A** | **C** | **A** | **G** | **A** | **T** | **C** | **C** | **T** | **T** | **G** | **T** | **C** | **T** | **A** | **T** | **G** | **A** | **G** | **A** | **A** | **T** | **G** | **G** | **C** | **T** | **T** | **C** | **C** | **T** | **C** | **C** | **A** | **T** | **G** | **C** | **A** | **A** | **A** | **A** | **A** | **C** | **A** | **T** | **T** | **A** | **A** | **T** | **A** | **A** | **T** | **G** | **G** | **A** | **T** | **G** | **A** | **T** | **G** | **A** | **A** | **A** | **T** | **C** | **T** | **G** | **C** | **A** | **T** | **G** | **G** | **T** | **A** | **G** | **G** | **T** | **T** | **C** | **A** | **G** | **C** | **A** | **A** | **A** | **T** | **C** | **A** | **A** | **G** | **A** | **T** | **A** | **T** | **T** | **C** | **G** | **T** | **A** | **G** | **C** | **T** | **A** | **C** | **C** | **G** | **A** | **T** | **T** | **G** | **A** | **A** | **T** | **T** | **T** | **T** | **G** | **A** | **A** | **A** | **C** | **A** | **A** | **G** | **T** | **G** | **C** | **T** | **G** | **T** | **C** | **A** | **T** | **T** | **T** | **A** | **C** | **G** | **A** | **T** | **C** | **C** | **T** | **G** | **A** | **G** | **T** | **T** | **T** | **T** | **T** | **A** | **G** | **A** | **A** | **G** | **A** | **G** | **C** | **T** | **T** | **G** | **C** | **T** | **A** | **C** | **T** | **C** | **A** | **G** | **T** | **T** | **C** | **A** | **A** | **A** | **G** | **A** | **A** | **G** | **A** | **T** | **G** | **A** | **G** | **A** | **C** | **A** | **C** | **A** | **T** | **T** | **G** | **T** | **T** | **C** | **A** | **C** | **C** | **A** | **A** | **T** | **G** | **A** | **C** | **A** | **A** | **C** | **T** | **G** | **A** | **A** | **A** | **C** | **A** | **G** | **T** | **G** | **A** | **A** | **G** | **G** | **A** | **A** | **A** | **T** | **G** | **T** | **C** | **T** | **A** | **A** | **C** | **T** | **G** | **G** | **C** | **T** | **G** | **T** | **T** | **A** | **T** | **T** | **C** | **A** | **A** | **A** | **C** | **A** | **A** | **C** | **A** | **A** | **A** | **T** | **T** | **T** | **C** | **T** | **C** | **G** | **A** | **T** | **T** | **G** | **T** | **T** | **T** | **T** | **C** | **T** | **C** | **C** | **A** | **A** | **T** | **C** | **T** | **T** | **A** | **-** | **-** | **-** |
| **N59891F** | **-** | **-** | **-** | **-** | **-** | **-** | **-** | **-** | **-** | **-** | **-** | **-** | **-** | **-** | **-** | **-** | **-** | **-** | **-** | **-** | **-** | **-** | **-** | **-** | **-** | **-** | **-** | **-** | **-** | **-** | **-** | **-** | **-** | **-** | **-** | **-** | **-** | **-** | **-** | **-** | **-** | **-** | **-** | **-** | **-** | **-** | **-** | **-** | **-** | **-** | **-** | **-** | **-** | **-** | **-** | **-** | **-** | **-** | **-** | **-** | **-** | **-** | **-** | **-** | **-** | **-** | **-** | **-** | **-** | **A** | **A** | **A** | **C** | **C** | **C** | **C** | **G** | **A** | **G** | **A** | **T** | **G** | **T** | **A** | **G** | **C** | **A** | **G** | **C** | **C** | **A** | **T** | **T** | **T** | **G** | **G** | **G** | **C** | **A** | **T** | **G** | **G** | **C** | **T** | **C** | **T** | **T** | **A** | **G** | **T** | **A** | **T** | **T** | **G** | **T** | **T** | **T** | **G** | **C** | **C** | **C** | **T** | **T** | **C** | **C** | **T** | **G** | **G** | **G** | **G** | **T** | **G** | **G** | **G** | **C** | **T** | **T** | **T** | **G** | **T** | **A** | **T** | **T** | **A** | **T** | **A** | **T** | **T** | **T** | **A** | **T** | **T** | **T** | **T** | **T** | **C** | **G** | **G** | **A** | **C** | **G** | **T** | **G** | **G** | **A** | **T** | **T** | **A** | **A** | **C** | **G** | **G** | **A** | **T** | **A** | **A** | **G** | **A** | **A** | **A** | **A** | **A** | **A** | **T** | **T** | **C** | **T** | **A** | **T** | **C** | **T** | **C** | **C** | **G** | **A** | **C** | **A** | **A** | **A** | **G** | **T** | **G** | **A** | **C** | **T** | **T** | **G** | **A** | **A** | **A** | **G** | **A** | **A** | **T** | **T** | **A** | **G** | **A** | **A** | **A** | **A** | **C** | **T** | **T** | **T** | **C** | **A** | **G** | **T** | **C** | **T** | **T** | **T** | **T** | **A** | **G** | **A** | **G** | **G** | **A** | **G** | **A** | **T** | **A** | **C** | **C** | **A** | **T** | **T** | **G** | **A** | **A** | **C** | **A** | **T** | **T** | **A** | **C** | **G** | **A** | **T** | **C** | **C** | **T** | **G** | **A** | **C** | **A** | **T** | **G** | **G** | **G** | **C** | **G** | **A** | **T** | **A** | **A** | **A** | **G** | **A** | **C** | **A** | **A** | **A** | **C** | **A** | **G** | **C** | **A** | **A** | **T** | **T** | **T** | **G** | **T** | **T** | **G** | **A** | **C** | **T** | **T** | **C** | **T** | **T** | **C** | **T** | **C** | **T** | **T** | **C** | **A** | **T** | **T** | **A** | **A** | **A** | **T** | **C** | **G** | **T** | **A** | **T** | **G** | **C** | **C** | **G** | **C** | **T** | **G** | **A** | **C** | **A** | **A** | **G** | **A** | **A** | **T** | **G** | **A** | **A** | **T** | **T** | **C** | **T** | **G** | **T** | **C** | **A** | **C** | **T** | **C** | **T** | **T** | **C** | **T** | **C** | **A** | **C** | **A** | **G** | **A** | **C** | **G** | **G** | **A** | **C** | **A** | **A** | **G** | **A** | **G** | **A** | **A** | **A** | **T** | **T** | **G** | **G** | **A** | **T** | **T** | **C** | **A** | **C** | **T** | **G** | **C** | **T** | **T** | **C** | **A** | **A** | **G** | **A** | **T** | **C** | **T** | **A** | **A** | **A** | **A** | **A** | **A** | **A** | **G** | **C** | **C** | **A** | **A** | **A** | **C** | **A** | **T** | **T** | **C** | **G** | **A** | **T** | **C** | **C** | **A** | **T** | **A** | **T** | **C** | **G** | **A** | **A** | **T** | **A** | **T** | **T** | **A** | **C** | **G** | **C** | **A** | **T** | **T** | **T** | **G** | **T** | **G** | **A** | **C** | **A** | **G** | **A** | **T** | **A** | **A** | **T** | **A** | **T** | **C** | **G** | **G** | **C** | **C** | **A** | **G** | **C** | **A** | **A** | **G** | **T** | **C** | **T** | **T** | **A** | **C** | **A** | **T** | **G** | **T** | **T** | **T** | **T** | **A** | **G** | **A** | **A** | **G** | **A** | **A** | **A** | **A** | **A** | **G** | **C** | **C** | **G** | **C** | **A** | **G** | **A** | **A** | **G** | **G** | **C** | **G** | **T** | **G** | **G** | **A** | **A** | **G** | **T** | **T** | **C** | **G** | **A** | **A** | **T** | **A** | **T** | **T** | **A** | **T** | **A** | **T** | **G** | **A** | **T** | **G** | **C** | **A** | **T** | **T** | **T** | **G** | **G** | **C** | **T** | **C** | **T** | **C** | **A** | **T** | **G** | **G** | **C** | **A** | **C** | **A** | **A** | **A** | **A** | **G** | **C** | **A** | **A** | **A** | **A** | **G** | **A** | **T** | **T** | **T** | **C** | **A** | **A** | **T** | **C** | **G** | **T** | **C** | **T** | **A** | **A** | **T** | **C** | **A** | **A** | **A** | **A** | **A** | **T** | **G** | **G** | **T** | **G** | **G** | **A** | **C** | **A** | **T** | **G** | **T** | **C** | **C** | **A** | **T** | **A** | **C** | **A** | **T** | **T** | **T** | **G** | **T** | **T** | **A** | **C** | **C** | **T** | **C** | **A** | **C** | **A** | **A** | **A** | **G** | **G** | **G** | **C** | **A** | **T** | **T** | **A** | **C** | **T** | **T** | **C** | **G** | **T** | **T** | **T** | **C** | **C** | **G** | **A** | **T** | **T** | **G** | **A** | **A** | **T** | **T** | **A** | **C** | **C** | **A** | **T** | **G** | **A** | **T** | **C** | **A** | **C** | **C** | **G** | **A** | **A** | **A** | **A** | **A** | **T** | **C** | **G** | **T** | **T** | **G** | **T** | **G** | **A** | **T** | **C** | **G** | **A** | **T** | **G** | **G** | **A** | **A** | **A** | **G** | **A** | **T** | **T** | **A** | **G** | **T** | **T** | **A** | **T** | **A** | **C** | **C** | **G** | **G** | **T** | **G** | **G** | **T** | **T** | **T** | **C** | **A** | **A** | **T** | **A** | **T** | **T** | **G** | **C** | **C** | **A** | **A** | **T** | **C** | **A** | **A** | **T** | **A** | **T** | **G** | **T** | **A** | **A** | **A** | **T** | **A** | **C** | **A** | **A** | **C** | **A** | **A** | **A** | **A** | **A** | **A** | **A** | **T** | **T** | **C** | **G** | **G** | **C** | **T** | **A** | **T** | **T** | **G** | **G** | **C** | **G** | **C** | **G** | **A** | **T** | **A** | **C** | **G** | **C** | **A** | **T** | **A** | **T** | **A** | **C** | **G** | **G** | **A** | **T** | **T** | **T** | **T** | **C** | **G** | **G** | **T** | **G** | **C** | **C** | **G** | **C** | **T** | **T** | **C** | **T** | **T** | **C** | **T** | **T** | **T** | **G** | **C** | **T** | **C** | **C** | **A** | **G** | **C** | **T** | **T** | **C** | **G** | **C** | **T** | **T** | **C** | **T** | **T** | **A** | **A** | **C** | **A** | **G** | **A** | **C** | **T** | **G** | **G** | **A** | **A** | **C** | **G** | **T** | **C** | **T** | **C** | **G** | **G** | **T** | **A** | **C** | **C** | **T** | **G** | **A** | **A** | **G** | **A** | **A** | **A** | **A** | **A** | **A** | **A** | **G** | **G** | **T** | **C** | **G** | **G** | **C** | **T** | **A** | **T** | **C** | **A** | **T** | **T** | **T** | **G** | **A** | **A** | **T** | **T** | **A** | **T** | **T** | **T** | **C** | **T** | **T** | **T** | **A** | **A** | **A** | **A** | **A** | **A** | **G** | **C** | **A** | **G** | **A** | **T** | **C** | **G** | **A** | **G** | **A** | **T** | **G** | **A** | **A** | **T** | **C** | **T** | **A** | **A** | **G** | **C** | **T** | **T** | **G** | **C** | **T** | **G** | **A** | **T** | **A** | **C** | **A** | **T** | **C** | **C** | **A** | **T** | **C** | **C** | **A** | **G** | **C** | **T** | **T** | **G** | **T** | **T** | **T** | **C** | **A** | **A** | **G** | **C** | **G** | **G** | **A** | **C** | **C** | **G** | **A** | **A** | **T** | **A** | **A** | **C** | **G** | **A** | **A** | **A** | **G** | **G** | **G** | **A** | **A** | **C** | **A** | **A** | **A** | **T** | **C** | **A** | **A** | **G** | **C** | **T** | **T** | **T** | **C** | **A** | **T** | **T** | **T** | **A** | **T** | **C** | **A** | **A** | **A** | **T** | **T** | **G** | **A** | **T** | **T** | **A** | **C** | **T** | **T** | **C** | **T** | **G** | **C** | **T** | **A** | **A** | **A** | **A** | **A** | **A** | **C** | **G** | **T** | **G** | **T** | **T** | **T** | **G** | **G** | **A** | **T** | **A** | **C** | **A** | **G** | **A** | **C** | **A** | **C** | **C** | **T** | **T** | **A** | **C** | **C** | **T** | **T** | **G** | **T** | **T** | **C** | **C** | **T** | **G** | **A** | **T** | **G** | **A** | **A** | **A** | **G** | **T** | **G** | **T** | **C** | **A** | **T** | **T** | **G** | **C** | **T** | **G** | **C** | **T** | **T** | **T** | **A** | **A** | **A** | **A** | **A** | **T** | **C** | **G** | **C** | **A** | **A** | **C** | **T** | **G** | **C** | **C** | **T** | **C** | **T** | **G** | **G** | **T** | **G** | **T** | **A** | **G** | **A** | **T** | **G** | **T** | **G** | **A** | **A** | **A** | **A** | **T** | **C** | **A** | **T** | **G** | **A** | **T** | **T** | **C** | **C** | **C** | **A** | **A** | **C** | **A** | **A** | **A** | **C** | **C** | **G** | **G** | **A** | **T** | **C** | **A** | **T** | **C** | **C** | **T** | **T** | **T** | **T** | **A** | **T** | **T** | **T** | **A** | **T** | **C** | **G** | **A** | **G** | **C** | **A** | **A** | **C** | **A** | **C** | **A** | **A** | **T** | **A** | **T** | **T** | **A** | **T** | **G** | **C** | **T** | **C** | **G** | **G** | **C** | **A** | **G** | **C** | **T** | **G** | **A** | **T** | **C** | **A** | **A** | **G** | **G** | **A** | **A** | **A** | **A** | **T** | **G** | **T** | **A** | **C** | **A** | **A** | **A** | **T** | **C** | **C** | **T** | **T** | **G** | **T** | **C** | **T** | **A** | **T** | **G** | **A** | **G** | **A** | **A** | **C** | **G** | **G** | **C** | **T** | **T** | **C** | **C** | **T** | **C** | **C** | **A** | **T** | **G** | **C** | **A** | **A** | **A** | **A** | **A** | **C** | **A** | **T** | **T** | **G** | **A** | **T** | **A** | **A** | **T** | **G** | **G** | **A** | **T** | **G** | **A** | **T** | **G** | **A** | **A** | **A** | **T** | **C** | **T** | **G** | **C** | **A** | **T** | **G** | **G** | **T** | **A** | **G** | **G** | **T** | **T** | **C** | **A** | **G** | **C** | **A** | **A** | **A** | **T** | **C** | **A** | **A** | **G** | **A** | **T** | **A** | **T** | **T** | **C** | **G** | **T** | **A** | **G** | **C** | **T** | **A** | **C** | **C** | **G** | **A** | **T** | **T** | **G** | **A** | **A** | **T** | **T** | **T** | **T** | **G** | **A** | **A** | **A** | **C** | **A** | **A** | **G** | **T** | **G** | **C** | **T** | **G** | **T** | **C** | **A** | **T** | **T** | **T** | **A** | **C** | **G** | **A** | **T** | **C** | **C** | **T** | **G** | **A** | **G** | **T** | **T** | **T** | **T** | **T** | **A** | **G** | **A** | **A** | **G** | **A** | **A** | **C** | **T** | **T** | **G** | **C** | **T** | **A** | **C** | **T** | **C** | **A** | **G** | **T** | **T** | **C** | **A** | **A** | **A** | **G** | **A** | **A** | **G** | **A** | **T** | **G** | **A** | **G** | **A** | **C** | **A** | **C** | **A** | **T** | **T** | **G** | **T** | **T** | **C** | **A** | **T** | **C** | **C** | **A** | **T** | **G** | **A** | **C** | **A** | **A** | **C** | **T** | **G** | **A** | **A** | **A** | **C** | **A** | **G** | **T** | **C** | **A** | **A** | **G** | **G** | **A** | **A** | **A** | **T** | **G** | **T** | **C** | **T** | **A** | **A** | **C** | **T** | **G** | **G** | **C** | **T** | **A** | **T** | **T** | **A** | **T** | **T** | **C** | **A** | **A** | **G** | **C** | **A** | **A** | **C** | **A** | **A** | **A** | **T** | **T** | **T** | **C** | **T** | **C** | **G** | **A** | **T** | **T** | **A** | **T** | **T** | **T** | **T** | **C** | **T** | **C** | **C** | **A** | **A** | **T** | **C** | **C** | **T** | **A** | **-** | **-** | **-** |
| **N55229** | **-** | **-** | **-** | **-** | **-** | **-** | **-** | **-** | **-** | **-** | **-** | **-** | **-** | **-** | **-** | **-** | **-** | **-** | **-** | **-** | **-** | **-** | **-** | **-** | **-** | **-** | **-** | **-** | **-** | **-** | **-** | **-** | **-** | **-** | **-** | **-** | **-** | **-** | **-** | **-** | **-** | **-** | **-** | **-** | **-** | **-** | **-** | **-** | **-** | **-** | **-** | **-** | **-** | **-** | **-** | **-** | **-** | **-** | **-** | **-** | **-** | **-** | **-** | **-** | **-** | **-** | **-** | **-** | **-** | **A** | **A** | **A** | **C** | **C** | **C** | **C** | **G** | **A** | **G** | **A** | **T** | **G** | **T** | **A** | **G** | **C** | **A** | **G** | **C** | **C** | **A** | **T** | **T** | **T** | **G** | **G** | **G** | **C** | **A** | **T** | **G** | **G** | **C** | **T** | **C** | **T** | **T** | **A** | **G** | **T** | **A** | **T** | **T** | **G** | **T** | **T** | **T** | **G** | **C** | **C** | **C** | **T** | **T** | **C** | **C** | **T** | **G** | **G** | **G** | **G** | **T** | **G** | **G** | **G** | **T** | **T** | **T** | **T** | **G** | **T** | **A** | **T** | **T** | **A** | **T** | **A** | **T** | **T** | **T** | **A** | **T** | **T** | **T** | **T** | **T** | **C** | **G** | **G** | **A** | **C** | **G** | **T** | **G** | **G** | **A** | **T** | **T** | **A** | **A** | **C** | **G** | **G** | **A** | **T** | **A** | **A** | **G** | **A** | **A** | **A** | **A** | **A** | **A** | **T** | **T** | **C** | **T** | **A** | **T** | **C** | **T** | **C** | **C** | **G** | **A** | **C** | **A** | **A** | **A** | **G** | **T** | **G** | **A** | **C** | **T** | **T** | **G** | **A** | **A** | **A** | **G** | **A** | **A** | **T** | **T** | **A** | **G** | **A** | **A** | **A** | **A** | **C** | **T** | **T** | **T** | **C** | **A** | **G** | **T** | **C** | **T** | **T** | **T** | **T** | **A** | **G** | **A** | **G** | **G** | **A** | **G** | **A** | **T** | **A** | **C** | **C** | **A** | **T** | **T** | **G** | **A** | **A** | **C** | **A** | **T** | **T** | **A** | **C** | **G** | **A** | **T** | **C** | **C** | **T** | **G** | **A** | **C** | **A** | **T** | **G** | **G** | **G** | **C** | **G** | **A** | **T** | **A** | **A** | **A** | **G** | **A** | **C** | **A** | **A** | **A** | **C** | **A** | **G** | **C** | **A** | **A** | **T** | **T** | **T** | **G** | **T** | **C** | **G** | **A** | **C** | **T** | **T** | **C** | **T** | **T** | **C** | **T** | **C** | **T** | **T** | **C** | **A** | **T** | **T** | **A** | **A** | **A** | **T** | **C** | **G** | **T** | **A** | **T** | **G** | **C** | **C** | **G** | **C** | **T** | **G** | **A** | **C** | **A** | **A** | **G** | **A** | **A** | **T** | **G** | **A** | **A** | **T** | **T** | **C** | **T** | **G** | **T** | **C** | **A** | **C** | **T** | **C** | **T** | **T** | **C** | **T** | **C** | **A** | **C** | **A** | **G** | **A** | **C** | **G** | **G** | **A** | **C** | **A** | **A** | **G** | **A** | **G** | **A** | **A** | **A** | **T** | **T** | **G** | **G** | **A** | **T** | **T** | **C** | **A** | **C** | **T** | **G** | **C** | **T** | **T** | **C** | **A** | **A** | **G** | **A** | **T** | **C** | **T** | **A** | **A** | **A** | **A** | **A** | **A** | **A** | **G** | **C** | **C** | **A** | **A** | **A** | **C** | **A** | **T** | **T** | **C** | **G** | **A** | **T** | **C** | **C** | **A** | **T** | **A** | **T** | **C** | **G** | **A** | **A** | **T** | **A** | **T** | **T** | **A** | **C** | **G** | **C** | **A** | **T** | **T** | **T** | **G** | **T** | **G** | **A** | **C** | **A** | **G** | **A** | **T** | **A** | **A** | **T** | **A** | **T** | **C** | **G** | **G** | **C** | **C** | **A** | **G** | **C** | **A** | **A** | **G** | **T** | **C** | **T** | **T** | **A** | **C** | **A** | **T** | **G** | **T** | **T** | **T** | **T** | **A** | **G** | **A** | **A** | **G** | **A** | **A** | **A** | **A** | **A** | **G** | **C** | **C** | **G** | **C** | **A** | **G** | **A** | **A** | **G** | **G** | **C** | **G** | **T** | **G** | **G** | **A** | **A** | **G** | **T** | **T** | **C** | **G** | **A** | **A** | **T** | **A** | **T** | **T** | **A** | **T** | **A** | **T** | **G** | **A** | **T** | **G** | **C** | **A** | **T** | **T** | **T** | **G** | **G** | **C** | **T** | **C** | **T** | **C** | **A** | **T** | **G** | **G** | **C** | **A** | **C** | **A** | **A** | **A** | **A** | **G** | **C** | **A** | **A** | **A** | **A** | **G** | **A** | **T** | **T** | **T** | **C** | **A** | **A** | **T** | **C** | **G** | **T** | **C** | **T** | **A** | **A** | **T** | **C** | **A** | **A** | **A** | **A** | **A** | **T** | **G** | **G** | **T** | **G** | **G** | **A** | **C** | **A** | **T** | **G** | **T** | **C** | **C** | **A** | **T** | **A** | **C** | **A** | **T** | **T** | **T** | **G** | **T** | **T** | **A** | **C** | **C** | **T** | **C** | **A** | **C** | **A** | **A** | **A** | **A** | **G** | **G** | **C** | **A** | **T** | **T** | **A** | **C** | **T** | **T** | **C** | **G** | **T** | **T** | **T** | **C** | **C** | **G** | **A** | **T** | **T** | **G** | **A** | **A** | **T** | **T** | **A** | **C** | **C** | **A** | **T** | **G** | **A** | **T** | **C** | **A** | **C** | **C** | **G** | **A** | **A** | **A** | **A** | **A** | **T** | **C** | **G** | **T** | **T** | **G** | **T** | **G** | **A** | **T** | **C** | **G** | **A** | **T** | **G** | **G** | **A** | **A** | **A** | **G** | **A** | **T** | **T** | **A** | **G** | **T** | **T** | **A** | **T** | **A** | **C** | **C** | **G** | **G** | **T** | **G** | **G** | **T** | **T** | **T** | **C** | **A** | **A** | **T** | **A** | **T** | **T** | **G** | **C** | **C** | **A** | **A** | **T** | **C** | **A** | **A** | **T** | **A** | **T** | **G** | **T** | **A** | **A** | **A** | **T** | **A** | **C** | **A** | **A** | **C** | **A** | **A** | **A** | **A** | **A** | **A** | **A** | **T** | **T** | **C** | **G** | **G** | **C** | **T** | **A** | **T** | **T** | **G** | **G** | **C** | **G** | **C** | **G** | **A** | **T** | **A** | **C** | **G** | **C** | **A** | **T** | **A** | **T** | **A** | **C** | **G** | **G** | **A** | **T** | **T** | **T** | **T** | **C** | **G** | **G** | **T** | **G** | **C** | **C** | **G** | **C** | **T** | **T** | **C** | **T** | **T** | **C** | **T** | **T** | **T** | **G** | **C** | **T** | **C** | **C** | **A** | **G** | **C** | **T** | **T** | **C** | **G** | **C** | **T** | **T** | **C** | **T** | **T** | **A** | **A** | **C** | **A** | **G** | **A** | **C** | **T** | **G** | **G** | **A** | **A** | **C** | **G** | **T** | **C** | **T** | **C** | **G** | **G** | **T** | **A** | **C** | **C** | **T** | **G** | **A** | **A** | **G** | **A** | **A** | **A** | **A** | **A** | **A** | **A** | **G** | **G** | **T** | **C** | **G** | **G** | **C** | **T** | **A** | **T** | **C** | **A** | **T** | **T** | **T** | **G** | **A** | **A** | **T** | **T** | **A** | **T** | **T** | **T** | **C** | **T** | **T** | **T** | **A** | **A** | **A** | **A** | **A** | **A** | **G** | **C** | **A** | **G** | **A** | **T** | **C** | **G** | **A** | **G** | **A** | **T** | **G** | **A** | **A** | **T** | **C** | **T** | **A** | **A** | **G** | **C** | **T** | **T** | **G** | **C** | **T** | **G** | **A** | **T** | **A** | **C** | **A** | **T** | **C** | **C** | **A** | **T** | **C** | **C** | **A** | **G** | **C** | **T** | **T** | **G** | **T** | **T** | **T** | **C** | **A** | **A** | **G** | **C** | **G** | **G** | **A** | **C** | **C** | **G** | **A** | **A** | **T** | **A** | **A** | **C** | **G** | **A** | **A** | **A** | **G** | **G** | **G** | **A** | **A** | **C** | **A** | **A** | **A** | **T** | **C** | **A** | **A** | **G** | **C** | **T** | **T** | **T** | **C** | **A** | **T** | **T** | **T** | **A** | **T** | **C** | **A** | **A** | **A** | **T** | **T** | **G** | **A** | **T** | **T** | **A** | **C** | **T** | **T** | **C** | **T** | **G** | **C** | **T** | **A** | **A** | **A** | **A** | **A** | **A** | **C** | **G** | **T** | **G** | **T** | **T** | **T** | **G** | **G** | **A** | **T** | **A** | **C** | **A** | **G** | **A** | **C** | **A** | **C** | **C** | **T** | **T** | **A** | **C** | **C** | **T** | **T** | **G** | **T** | **T** | **C** | **C** | **T** | **G** | **A** | **T** | **G** | **A** | **A** | **A** | **G** | **T** | **G** | **T** | **C** | **A** | **T** | **T** | **G** | **C** | **T** | **G** | **C** | **T** | **T** | **T** | **A** | **A** | **A** | **A** | **A** | **T** | **C** | **G** | **C** | **A** | **A** | **C** | **T** | **G** | **C** | **C** | **T** | **C** | **T** | **G** | **G** | **T** | **G** | **T** | **A** | **G** | **A** | **T** | **G** | **T** | **G** | **A** | **A** | **A** | **A** | **T** | **C** | **A** | **T** | **G** | **A** | **T** | **T** | **C** | **C** | **C** | **A** | **A** | **C** | **A** | **A** | **A** | **C** | **C** | **G** | **G** | **A** | **T** | **C** | **A** | **T** | **C** | **C** | **T** | **T** | **T** | **T** | **A** | **T** | **T** | **T** | **A** | **T** | **C** | **G** | **A** | **G** | **C** | **A** | **A** | **C** | **A** | **C** | **A** | **A** | **T** | **A** | **T** | **T** | **A** | **T** | **G** | **C** | **T** | **C** | **G** | **G** | **C** | **A** | **G** | **C** | **T** | **G** | **A** | **T** | **C** | **A** | **A** | **G** | **G** | **A** | **A** | **A** | **A** | **T** | **G** | **T** | **A** | **C** | **A** | **A** | **A** | **T** | **C** | **C** | **T** | **T** | **G** | **T** | **C** | **T** | **A** | **T** | **G** | **A** | **G** | **A** | **A** | **C** | **G** | **G** | **C** | **T** | **T** | **C** | **C** | **T** | **C** | **C** | **A** | **T** | **G** | **C** | **A** | **A** | **A** | **A** | **A** | **C** | **A** | **T** | **T** | **G** | **A** | **T** | **A** | **A** | **T** | **G** | **G** | **A** | **T** | **G** | **A** | **T** | **G** | **A** | **A** | **A** | **T** | **C** | **T** | **G** | **C** | **A** | **T** | **G** | **G** | **T** | **A** | **G** | **G** | **T** | **T** | **C** | **A** | **G** | **C** | **A** | **A** | **A** | **T** | **C** | **A** | **A** | **G** | **A** | **T** | **A** | **T** | **T** | **C** | **G** | **T** | **A** | **G** | **C** | **T** | **A** | **C** | **C** | **G** | **A** | **T** | **T** | **G** | **A** | **A** | **T** | **T** | **T** | **T** | **G** | **A** | **A** | **A** | **C** | **A** | **A** | **G** | **T** | **G** | **C** | **T** | **G** | **T** | **C** | **A** | **T** | **T** | **T** | **A** | **C** | **G** | **A** | **T** | **C** | **C** | **T** | **G** | **A** | **G** | **T** | **T** | **T** | **T** | **T** | **A** | **G** | **A** | **A** | **G** | **A** | **A** | **C** | **T** | **T** | **G** | **C** | **T** | **A** | **C** | **T** | **C** | **A** | **G** | **T** | **T** | **C** | **A** | **A** | **A** | **G** | **A** | **A** | **G** | **A** | **T** | **G** | **A** | **G** | **A** | **C** | **A** | **C** | **A** | **T** | **T** | **G** | **T** | **T** | **C** | **A** | **T** | **C** | **C** | **A** | **T** | **G** | **A** | **C** | **A** | **A** | **C** | **T** | **G** | **A** | **A** | **A** | **C** | **A** | **G** | **T** | **C** | **A** | **A** | **A** | **G** | **A** | **A** | **A** | **T** | **G** | **T** | **C** | **T** | **A** | **A** | **C** | **T** | **G** | **G** | **C** | **T** | **A** | **T** | **T** | **A** | **T** | **T** | **C** | **A** | **A** | **G** | **C** | **A** | **A** | **C** | **A** | **A** | **A** | **T** | **T** | **T** | **C** | **T** | **C** | **G** | **A** | **T** | **T** | **A** | **T** | **T** | **T** | **T** | **C** | **T** | **C** | **C** | **A** | **A** | **T** | **C** | **C** | **T** | **A** | **-** | **-** | **-** |
| **N54150** | **-** | **-** | **-** | **-** | **-** | **-** | **-** | **-** | **-** | **-** | **-** | **-** | **-** | **-** | **-** | **-** | **-** | **-** | **-** | **-** | **-** | **-** | **-** | **-** | **-** | **-** | **-** | **-** | **-** | **-** | **-** | **-** | **-** | **-** | **-** | **-** | **-** | **-** | **-** | **-** | **-** | **-** | **-** | **-** | **-** | **-** | **-** | **-** | **-** | **-** | **-** | **-** | **-** | **-** | **-** | **-** | **-** | **-** | **-** | **-** | **-** | **-** | **-** | **-** | **-** | **-** | **-** | **-** | **-** | **A** | **A** | **A** | **C** | **C** | **C** | **C** | **G** | **A** | **G** | **A** | **T** | **G** | **T** | **A** | **G** | **C** | **A** | **G** | **C** | **C** | **A** | **T** | **T** | **T** | **G** | **G** | **G** | **C** | **A** | **T** | **G** | **G** | **C** | **T** | **C** | **T** | **T** | **A** | **G** | **T** | **A** | **T** | **T** | **G** | **T** | **T** | **T** | **G** | **C** | **C** | **C** | **T** | **T** | **C** | **C** | **T** | **G** | **G** | **G** | **G** | **T** | **G** | **G** | **G** | **C** | **T** | **T** | **T** | **G** | **T** | **A** | **T** | **T** | **A** | **T** | **A** | **T** | **T** | **T** | **A** | **T** | **T** | **T** | **T** | **T** | **C** | **G** | **G** | **A** | **C** | **G** | **T** | **G** | **G** | **A** | **T** | **T** | **A** | **A** | **C** | **G** | **G** | **A** | **T** | **A** | **A** | **G** | **A** | **A** | **A** | **A** | **A** | **A** | **T** | **T** | **C** | **T** | **A** | **T** | **C** | **T** | **C** | **C** | **G** | **A** | **C** | **A** | **A** | **A** | **G** | **T** | **G** | **A** | **C** | **T** | **T** | **G** | **A** | **A** | **A** | **G** | **A** | **A** | **T** | **T** | **A** | **G** | **A** | **A** | **A** | **A** | **C** | **T** | **T** | **T** | **C** | **A** | **G** | **T** | **C** | **T** | **T** | **T** | **T** | **A** | **G** | **A** | **G** | **G** | **A** | **G** | **A** | **T** | **A** | **C** | **C** | **A** | **T** | **T** | **G** | **A** | **A** | **C** | **A** | **T** | **T** | **A** | **C** | **G** | **A** | **T** | **C** | **C** | **T** | **G** | **A** | **C** | **A** | **T** | **G** | **G** | **G** | **C** | **G** | **A** | **T** | **A** | **A** | **A** | **G** | **A** | **C** | **A** | **A** | **A** | **C** | **A** | **G** | **C** | **A** | **A** | **T** | **T** | **T** | **G** | **T** | **T** | **G** | **A** | **C** | **T** | **T** | **C** | **T** | **T** | **C** | **T** | **C** | **T** | **T** | **C** | **A** | **T** | **T** | **A** | **A** | **A** | **T** | **C** | **G** | **T** | **A** | **T** | **G** | **C** | **C** | **G** | **C** | **T** | **G** | **A** | **C** | **A** | **A** | **G** | **A** | **A** | **T** | **G** | **A** | **A** | **T** | **T** | **C** | **T** | **G** | **T** | **C** | **A** | **C** | **T** | **C** | **T** | **T** | **C** | **T** | **C** | **A** | **C** | **A** | **G** | **A** | **C** | **G** | **G** | **A** | **C** | **A** | **A** | **G** | **A** | **G** | **A** | **A** | **A** | **T** | **T** | **G** | **G** | **A** | **T** | **T** | **C** | **A** | **C** | **T** | **G** | **C** | **T** | **T** | **C** | **A** | **A** | **G** | **A** | **T** | **C** | **T** | **A** | **A** | **A** | **A** | **A** | **A** | **A** | **G** | **C** | **C** | **A** | **A** | **A** | **C** | **A** | **T** | **T** | **C** | **G** | **A** | **T** | **C** | **C** | **A** | **T** | **A** | **T** | **C** | **G** | **A** | **A** | **T** | **A** | **T** | **T** | **A** | **C** | **G** | **C** | **A** | **T** | **T** | **T** | **G** | **T** | **G** | **A** | **C** | **A** | **G** | **A** | **T** | **A** | **A** | **T** | **A** | **T** | **C** | **G** | **G** | **C** | **C** | **A** | **G** | **C** | **A** | **A** | **G** | **T** | **C** | **T** | **T** | **A** | **C** | **A** | **T** | **G** | **T** | **T** | **T** | **T** | **A** | **G** | **A** | **A** | **G** | **A** | **A** | **A** | **A** | **A** | **G** | **C** | **C** | **G** | **C** | **A** | **G** | **A** | **A** | **G** | **G** | **C** | **G** | **T** | **G** | **G** | **A** | **A** | **G** | **T** | **T** | **C** | **G** | **A** | **A** | **T** | **A** | **T** | **T** | **A** | **T** | **A** | **T** | **G** | **A** | **T** | **G** | **C** | **A** | **T** | **T** | **T** | **G** | **G** | **C** | **T** | **C** | **T** | **C** | **A** | **T** | **G** | **G** | **C** | **A** | **C** | **A** | **A** | **A** | **A** | **G** | **C** | **A** | **A** | **A** | **A** | **G** | **A** | **T** | **T** | **T** | **C** | **A** | **A** | **T** | **C** | **G** | **T** | **C** | **T** | **A** | **A** | **T** | **C** | **A** | **A** | **A** | **A** | **A** | **T** | **G** | **G** | **T** | **G** | **G** | **A** | **C** | **A** | **T** | **G** | **T** | **C** | **C** | **A** | **T** | **A** | **C** | **A** | **T** | **T** | **T** | **G** | **T** | **T** | **A** | **C** | **C** | **T** | **C** | **A** | **C** | **A** | **A** | **A** | **A** | **G** | **G** | **C** | **A** | **T** | **T** | **A** | **C** | **T** | **T** | **C** | **G** | **T** | **T** | **T** | **C** | **C** | **G** | **A** | **T** | **T** | **G** | **A** | **A** | **T** | **T** | **A** | **C** | **C** | **A** | **T** | **G** | **A** | **T** | **C** | **A** | **C** | **C** | **G** | **A** | **A** | **A** | **A** | **A** | **T** | **C** | **G** | **T** | **T** | **G** | **T** | **G** | **A** | **T** | **C** | **G** | **A** | **T** | **G** | **G** | **A** | **A** | **A** | **G** | **A** | **T** | **T** | **A** | **G** | **T** | **T** | **A** | **T** | **A** | **C** | **C** | **G** | **G** | **T** | **G** | **G** | **T** | **T** | **T** | **C** | **A** | **A** | **T** | **A** | **T** | **T** | **G** | **C** | **C** | **A** | **A** | **T** | **C** | **A** | **A** | **T** | **A** | **T** | **G** | **T** | **A** | **A** | **A** | **T** | **A** | **T** | **A** | **A** | **C** | **A** | **A** | **A** | **A** | **A** | **A** | **A** | **T** | **T** | **C** | **G** | **G** | **C** | **T** | **A** | **T** | **T** | **G** | **G** | **C** | **G** | **C** | **G** | **A** | **T** | **A** | **C** | **G** | **C** | **A** | **T** | **A** | **T** | **A** | **C** | **G** | **G** | **A** | **T** | **T** | **T** | **T** | **C** | **G** | **G** | **T** | **G** | **C** | **C** | **G** | **C** | **T** | **T** | **C** | **T** | **T** | **C** | **T** | **T** | **T** | **G** | **C** | **T** | **C** | **C** | **A** | **G** | **C** | **T** | **T** | **C** | **G** | **C** | **T** | **T** | **C** | **T** | **T** | **A** | **A** | **C** | **A** | **G** | **A** | **C** | **T** | **G** | **G** | **A** | **A** | **C** | **G** | **T** | **C** | **T** | **C** | **G** | **G** | **T** | **A** | **C** | **C** | **T** | **G** | **A** | **A** | **G** | **A** | **A** | **A** | **A** | **A** | **A** | **A** | **G** | **G** | **T** | **C** | **G** | **G** | **C** | **T** | **A** | **T** | **C** | **A** | **T** | **T** | **T** | **G** | **A** | **A** | **T** | **T** | **A** | **T** | **T** | **T** | **C** | **T** | **T** | **T** | **A** | **A** | **A** | **A** | **A** | **A** | **G** | **C** | **A** | **G** | **A** | **T** | **C** | **G** | **A** | **G** | **A** | **T** | **G** | **A** | **A** | **T** | **C** | **T** | **A** | **A** | **G** | **C** | **T** | **T** | **G** | **C** | **T** | **G** | **A** | **T** | **A** | **C** | **A** | **T** | **C** | **C** | **A** | **T** | **C** | **C** | **A** | **G** | **C** | **T** | **T** | **G** | **T** | **T** | **T** | **C** | **A** | **A** | **G** | **C** | **G** | **G** | **A** | **C** | **C** | **G** | **A** | **A** | **T** | **A** | **A** | **C** | **G** | **A** | **A** | **A** | **G** | **G** | **G** | **A** | **A** | **C** | **A** | **A** | **A** | **T** | **C** | **A** | **A** | **G** | **C** | **T** | **T** | **T** | **C** | **A** | **T** | **T** | **T** | **A** | **T** | **C** | **A** | **A** | **A** | **T** | **T** | **G** | **A** | **T** | **T** | **A** | **C** | **T** | **T** | **C** | **T** | **G** | **C** | **T** | **A** | **A** | **A** | **A** | **A** | **A** | **C** | **G** | **T** | **G** | **T** | **T** | **T** | **G** | **G** | **A** | **T** | **A** | **C** | **A** | **G** | **A** | **C** | **A** | **C** | **C** | **T** | **T** | **A** | **C** | **C** | **T** | **T** | **G** | **T** | **T** | **C** | **C** | **T** | **G** | **A** | **T** | **G** | **A** | **A** | **A** | **G** | **T** | **G** | **T** | **C** | **A** | **T** | **T** | **G** | **C** | **T** | **G** | **C** | **T** | **T** | **T** | **A** | **A** | **A** | **A** | **A** | **T** | **C** | **G** | **C** | **A** | **A** | **C** | **T** | **G** | **C** | **C** | **T** | **C** | **T** | **G** | **G** | **T** | **G** | **T** | **A** | **G** | **A** | **T** | **G** | **T** | **G** | **A** | **A** | **A** | **A** | **T** | **C** | **A** | **T** | **G** | **A** | **T** | **T** | **C** | **C** | **C** | **A** | **A** | **C** | **A** | **A** | **A** | **C** | **C** | **G** | **G** | **A** | **T** | **C** | **A** | **T** | **C** | **C** | **T** | **T** | **T** | **T** | **A** | **T** | **T** | **T** | **A** | **T** | **C** | **G** | **A** | **G** | **C** | **A** | **A** | **C** | **A** | **C** | **A** | **A** | **T** | **A** | **T** | **T** | **A** | **T** | **G** | **C** | **T** | **C** | **G** | **G** | **C** | **A** | **G** | **C** | **T** | **G** | **A** | **T** | **C** | **A** | **A** | **G** | **G** | **A** | **A** | **A** | **A** | **T** | **G** | **T** | **A** | **C** | **A** | **A** | **A** | **T** | **C** | **C** | **T** | **T** | **G** | **T** | **C** | **T** | **A** | **T** | **G** | **A** | **G** | **A** | **A** | **C** | **G** | **G** | **C** | **T** | **T** | **C** | **C** | **T** | **C** | **C** | **A** | **T** | **G** | **C** | **A** | **A** | **A** | **A** | **A** | **C** | **A** | **T** | **T** | **G** | **A** | **T** | **A** | **A** | **T** | **G** | **G** | **A** | **T** | **G** | **A** | **T** | **G** | **A** | **A** | **A** | **T** | **C** | **T** | **G** | **C** | **A** | **T** | **G** | **G** | **T** | **A** | **G** | **G** | **T** | **T** | **C** | **A** | **G** | **C** | **A** | **A** | **A** | **T** | **C** | **A** | **A** | **G** | **A** | **T** | **A** | **T** | **T** | **C** | **G** | **T** | **A** | **G** | **C** | **T** | **A** | **C** | **C** | **G** | **A** | **T** | **T** | **G** | **A** | **A** | **T** | **T** | **T** | **T** | **G** | **A** | **A** | **A** | **C** | **A** | **A** | **G** | **T** | **G** | **C** | **T** | **G** | **T** | **C** | **A** | **T** | **T** | **T** | **A** | **C** | **G** | **A** | **T** | **C** | **C** | **T** | **G** | **A** | **G** | **T** | **T** | **T** | **T** | **T** | **A** | **G** | **A** | **A** | **G** | **A** | **A** | **C** | **T** | **T** | **G** | **C** | **T** | **A** | **C** | **T** | **C** | **A** | **G** | **T** | **T** | **C** | **A** | **A** | **A** | **G** | **A** | **A** | **G** | **A** | **T** | **G** | **A** | **G** | **A** | **C** | **A** | **C** | **A** | **T** | **T** | **G** | **T** | **T** | **C** | **A** | **T** | **C** | **C** | **A** | **T** | **G** | **A** | **C** | **A** | **A** | **C** | **T** | **G** | **A** | **A** | **A** | **C** | **A** | **G** | **T** | **C** | **A** | **A** | **G** | **G** | **A** | **A** | **A** | **T** | **G** | **T** | **C** | **T** | **A** | **A** | **C** | **T** | **G** | **G** | **C** | **T** | **A** | **T** | **T** | **A** | **T** | **T** | **C** | **A** | **A** | **G** | **C** | **A** | **A** | **C** | **A** | **A** | **A** | **T** | **T** | **T** | **C** | **T** | **C** | **G** | **A** | **T** | **T** | **A** | **T** | **T** | **T** | **T** | **C** | **T** | **C** | **C** | **A** | **A** | **T** | **C** | **C** | **T** | **A** | **-** | **-** | **-** |
| **N52656** | **-** | **-** | **-** | **-** | **-** | **-** | **-** | **-** | **-** | **-** | **-** | **-** | **-** | **-** | **-** | **-** | **-** | **-** | **-** | **-** | **-** | **-** | **-** | **-** | **-** | **-** | **-** | **-** | **-** | **-** | **-** | **-** | **-** | **-** | **-** | **-** | **-** | **-** | **-** | **-** | **-** | **-** | **-** | **-** | **-** | **-** | **-** | **-** | **-** | **-** | **-** | **-** | **-** | **-** | **-** | **-** | **-** | **-** | **-** | **-** | **-** | **-** | **-** | **-** | **-** | **-** | **-** | **-** | **-** | **A** | **A** | **A** | **C** | **C** | **C** | **C** | **G** | **A** | **G** | **A** | **T** | **G** | **T** | **A** | **G** | **C** | **A** | **G** | **C** | **T** | **A** | **T** | **T** | **T** | **G** | **G** | **G** | **C** | **A** | **T** | **G** | **G** | **C** | **T** | **C** | **T** | **T** | **A** | **G** | **T** | **A** | **T** | **T** | **A** | **T** | **T** | **T** | **G** | **C** | **C** | **C** | **T** | **T** | **C** | **C** | **T** | **G** | **G** | **G** | **G** | **T** | **G** | **G** | **G** | **T** | **T** | **T** | **T** | **G** | **T** | **A** | **T** | **T** | **A** | **T** | **A** | **T** | **T** | **T** | **A** | **T** | **T** | **T** | **T** | **T** | **C** | **G** | **G** | **A** | **C** | **G** | **T** | **G** | **G** | **A** | **T** | **T** | **A** | **A** | **C** | **G** | **G** | **A** | **T** | **A** | **A** | **G** | **A** | **A** | **A** | **A** | **A** | **A** | **T** | **T** | **C** | **T** | **A** | **T** | **C** | **T** | **C** | **C** | **G** | **A** | **C** | **A** | **A** | **A** | **G** | **T** | **G** | **A** | **C** | **T** | **T** | **G** | **A** | **A** | **A** | **G** | **A** | **A** | **T** | **T** | **A** | **G** | **A** | **A** | **A** | **A** | **C** | **T** | **T** | **T** | **C** | **A** | **G** | **T** | **C** | **T** | **T** | **T** | **T** | **A** | **G** | **A** | **G** | **G** | **A** | **G** | **A** | **T** | **A** | **C** | **C** | **A** | **T** | **T** | **G** | **A** | **A** | **C** | **A** | **T** | **T** | **A** | **C** | **G** | **A** | **T** | **C** | **C** | **T** | **G** | **A** | **C** | **A** | **T** | **G** | **G** | **G** | **C** | **G** | **A** | **C** | **A** | **A** | **A** | **G** | **A** | **C** | **A** | **A** | **A** | **C** | **A** | **A** | **C** | **A** | **A** | **T** | **T** | **T** | **G** | **T** | **C** | **G** | **A** | **C** | **T** | **T** | **C** | **T** | **T** | **C** | **T** | **C** | **T** | **T** | **C** | **A** | **T** | **T** | **A** | **A** | **A** | **T** | **C** | **G** | **T** | **A** | **T** | **G** | **C** | **C** | **G** | **C** | **T** | **G** | **A** | **C** | **A** | **A** | **G** | **A** | **A** | **T** | **G** | **A** | **A** | **T** | **T** | **C** | **T** | **G** | **T** | **C** | **A** | **C** | **T** | **C** | **T** | **T** | **C** | **T** | **C** | **A** | **C** | **A** | **G** | **A** | **C** | **G** | **G** | **A** | **C** | **A** | **A** | **A** | **A** | **G** | **A** | **A** | **A** | **T** | **T** | **A** | **G** | **A** | **T** | **T** | **C** | **A** | **C** | **T** | **G** | **C** | **T** | **T** | **C** | **A** | **A** | **G** | **A** | **T** | **C** | **T** | **A** | **A** | **A** | **A** | **A** | **A** | **A** | **G** | **C** | **C** | **A** | **A** | **A** | **C** | **A** | **T** | **T** | **C** | **G** | **A** | **T** | **C** | **C** | **A** | **T** | **A** | **T** | **C** | **G** | **A** | **A** | **T** | **A** | **T** | **T** | **A** | **C** | **G** | **C** | **A** | **T** | **T** | **T** | **G** | **T** | **G** | **A** | **C** | **A** | **G** | **A** | **T** | **A** | **C** | **T** | **A** | **T** | **C** | **G** | **G** | **T** | **C** | **A** | **G** | **C** | **A** | **A** | **G** | **T** | **C** | **T** | **T** | **A** | **C** | **G** | **C** | **G** | **T** | **T** | **T** | **T** | **A** | **G** | **A** | **A** | **G** | **A** | **A** | **A** | **A** | **A** | **G** | **C** | **C** | **G** | **C** | **A** | **G** | **A** | **A** | **G** | **G** | **C** | **G** | **T** | **A** | **G** | **A** | **A** | **G** | **T** | **T** | **C** | **G** | **T** | **A** | **T** | **A** | **T** | **T** | **A** | **T** | **A** | **T** | **G** | **A** | **T** | **G** | **C** | **A** | **T** | **T** | **T** | **G** | **G** | **C** | **T** | **C** | **C** | **C** | **A** | **C** | **G** | **G** | **C** | **A** | **C** | **A** | **A** | **A** | **A** | **G** | **C** | **A** | **A** | **A** | **A** | **G** | **A** | **T** | **T** | **T** | **C** | **A** | **A** | **T** | **C** | **G** | **T** | **C** | **T** | **A** | **A** | **T** | **C** | **A** | **A** | **A** | **A** | **A** | **T** | **G** | **G** | **T** | **G** | **G** | **A** | **C** | **A** | **T** | **G** | **T** | **C** | **C** | **A** | **T** | **A** | **C** | **A** | **T** | **T** | **T** | **G** | **T** | **T** | **A** | **C** | **C** | **T** | **C** | **G** | **C** | **A** | **A** | **A** | **A** | **A** | **G** | **C** | **A** | **T** | **T** | **G** | **C** | **T** | **T** | **C** | **G** | **T** | **T** | **T** | **C** | **C** | **G** | **A** | **T** | **T** | **G** | **A** | **A** | **C** | **T** | **A** | **C** | **C** | **A** | **T** | **G** | **A** | **T** | **C** | **A** | **C** | **C** | **G** | **G** | **A** | **A** | **A** | **A** | **T** | **C** | **G** | **T** | **T** | **G** | **T** | **G** | **A** | **T** | **C** | **G** | **A** | **T** | **G** | **G** | **A** | **A** | **A** | **G** | **A** | **T** | **C** | **A** | **G** | **T** | **T** | **A** | **C** | **A** | **C** | **C** | **G** | **G** | **T** | **G** | **G** | **C** | **T** | **T** | **C** | **A** | **A** | **C** | **A** | **T** | **T** | **G** | **C** | **C** | **A** | **A** | **T** | **C** | **A** | **A** | **T** | **A** | **T** | **G** | **T** | **A** | **A** | **A** | **T** | **A** | **C** | **G** | **A** | **C** | **A** | **A** | **A** | **A** | **A** | **A** | **A** | **T** | **T** | **C** | **G** | **G** | **C** | **T** | **A** | **T** | **T** | **G** | **G** | **C** | **G** | **T** | **G** | **A** | **T** | **A** | **C** | **G** | **C** | **A** | **T** | **A** | **T** | **A** | **C** | **G** | **G** | **A** | **T** | **T** | **T** | **T** | **C** | **G** | **G** | **T** | **G** | **C** | **C** | **G** | **C** | **T** | **T** | **C** | **T** | **T** | **C** | **T** | **T** | **T** | **G** | **C** | **T** | **T** | **C** | **A** | **A** | **C** | **T** | **T** | **C** | **G** | **T** | **T** | **T** | **T** | **T** | **T** | **A** | **A** | **C** | **A** | **G** | **A** | **C** | **T** | **G** | **G** | **A** | **A** | **C** | **G** | **T** | **C** | **T** | **C** | **A** | **G** | **T** | **T** | **C** | **C** | **T** | **G** | **A** | **A** | **G** | **A** | **A** | **A** | **A** | **A** | **A** | **A** | **G** | **G** | **T** | **C** | **G** | **G** | **C** | **T** | **A** | **T** | **C** | **A** | **T** | **T** | **T** | **G** | **A** | **A** | **T** | **T** | **A** | **T** | **T** | **T** | **C** | **T** | **T** | **T** | **A** | **A** | **A** | **A** | **A** | **A** | **G** | **C** | **C** | **G** | **A** | **T** | **C** | **G** | **A** | **G** | **A** | **C** | **G** | **A** | **A** | **T** | **C** | **C** | **A** | **A** | **A** | **C** | **T** | **T** | **G** | **C** | **T** | **G** | **A** | **T** | **A** | **C** | **A** | **T** | **C** | **C** | **A** | **T** | **C** | **C** | **A** | **G** | **C** | **T** | **T** | **G** | **T** | **C** | **T** | **C** | **A** | **A** | **G** | **C** | **G** | **G** | **A** | **C** | **C** | **A** | **A** | **A** | **T** | **A** | **A** | **C** | **G** | **A** | **A** | **C** | **G** | **C** | **G** | **A** | **A** | **C** | **A** | **A** | **A** | **T** | **C** | **A** | **A** | **G** | **C** | **T** | **T** | **T** | **C** | **G** | **T** | **T** | **T** | **A** | **T** | **C** | **A** | **A** | **A** | **T** | **T** | **G** | **A** | **T** | **T** | **A** | **C** | **T** | **T** | **C** | **T** | **G** | **C** | **T** | **A** | **A** | **A** | **A** | **A** | **A** | **C** | **G** | **T** | **G** | **T** | **T** | **T** | **G** | **G** | **A** | **T** | **A** | **C** | **A** | **G** | **A** | **C** | **G** | **C** | **C** | **T** | **T** | **A** | **C** | **C** | **T** | **C** | **G** | **T** | **T** | **C** | **C** | **T** | **G** | **A** | **T** | **G** | **A** | **A** | **A** | **G** | **T** | **G** | **T** | **C** | **A** | **T** | **T** | **G** | **C** | **T** | **G** | **C** | **T** | **T** | **T** | **G** | **A** | **A** | **A** | **A** | **T** | **C** | **G** | **C** | **A** | **A** | **C** | **T** | **G** | **C** | **T** | **T** | **C** | **T** | **G** | **G** | **T** | **G** | **T** | **C** | **G** | **A** | **T** | **G** | **T** | **G** | **A** | **A** | **A** | **A** | **T** | **C** | **A** | **T** | **G** | **A** | **T** | **C** | **C** | **C** | **C** | **A** | **A** | **T** | **A** | **A** | **A** | **C** | **C** | **G** | **G** | **A** | **T** | **C** | **A** | **T** | **C** | **C** | **T** | **T** | **T** | **T** | **A** | **T** | **T** | **T** | **A** | **T** | **C** | **G** | **A** | **G** | **C** | **A** | **A** | **C** | **A** | **C** | **A** | **A** | **T** | **A** | **T** | **T** | **A** | **T** | **G** | **C** | **G** | **C** | **G** | **T** | **C** | **A** | **G** | **C** | **T** | **C** | **A** | **T** | **C** | **A** | **A** | **G** | **G** | **A** | **A** | **A** | **A** | **C** | **G** | **T** | **A** | **C** | **A** | **A** | **A** | **T** | **T** | **C** | **T** | **T** | **G** | **T** | **C** | **T** | **A** | **T** | **G** | **A** | **G** | **A** | **A** | **T** | **G** | **G** | **C** | **T** | **T** | **C** | **C** | **T** | **C** | **C** | **A** | **T** | **G** | **C** | **A** | **A** | **A** | **A** | **A** | **C** | **A** | **T** | **T** | **G** | **A** | **T** | **A** | **A** | **T** | **G** | **G** | **A** | **T** | **G** | **A** | **T** | **G** | **A** | **A** | **A** | **T** | **C** | **T** | **G** | **C** | **A** | **T** | **G** | **G** | **T** | **A** | **G** | **G** | **T** | **T** | **C** | **A** | **G** | **C** | **A** | **A** | **A** | **T** | **C** | **A** | **A** | **G** | **A** | **T** | **A** | **T** | **T** | **C** | **G** | **T** | **A** | **G** | **C** | **T** | **A** | **C** | **C** | **G** | **A** | **T** | **T** | **G** | **A** | **A** | **T** | **T** | **T** | **T** | **G** | **A** | **A** | **A** | **C** | **A** | **A** | **G** | **T** | **G** | **C** | **T** | **G** | **T** | **C** | **A** | **T** | **C** | **T** | **A** | **C** | **G** | **A** | **T** | **C** | **C** | **T** | **G** | **A** | **G** | **T** | **T** | **T** | **T** | **T** | **A** | **G** | **A** | **A** | **G** | **A** | **A** | **C** | **T** | **T** | **G** | **C** | **T** | **A** | **C** | **T** | **C** | **A** | **G** | **T** | **T** | **T** | **A** | **A** | **A** | **G** | **A** | **A** | **G** | **A** | **T** | **G** | **A** | **G** | **A** | **C** | **A** | **C** | **A** | **T** | **T** | **G** | **T** | **T** | **C** | **A** | **C** | **C** | **A** | **A** | **T** | **G** | **A** | **C** | **G** | **A** | **C** | **T** | **G** | **A** | **A** | **A** | **C** | **A** | **G** | **T** | **G** | **A** | **A** | **G** | **G** | **A** | **A** | **A** | **T** | **G** | **T** | **C** | **T** | **A** | **A** | **T** | **T** | **G** | **G** | **C** | **T** | **G** | **T** | **T** | **A** | **T** | **T** | **C** | **A** | **A** | **G** | **C** | **A** | **A** | **C** | **A** | **A** | **A** | **T** | **T** | **T** | **C** | **T** | **C** | **G** | **A** | **T** | **T** | **A** | **T** | **T** | **T** | **T** | **C** | **T** | **C** | **C** | **A** | **A** | **T** | **C** | **T** | **T** | **A** | **-** | **-** | **-** |
| **N55299** | **-** | **-** | **-** | **-** | **-** | **-** | **-** | **-** | **-** | **-** | **-** | **-** | **-** | **-** | **-** | **-** | **-** | **-** | **-** | **-** | **-** | **-** | **-** | **-** | **-** | **-** | **-** | **-** | **-** | **-** | **-** | **-** | **-** | **-** | **-** | **-** | **-** | **-** | **-** | **-** | **-** | **-** | **-** | **-** | **-** | **-** | **-** | **-** | **-** | **-** | **-** | **-** | **-** | **-** | **-** | **-** | **-** | **-** | **-** | **-** | **-** | **-** | **-** | **-** | **-** | **-** | **-** | **-** | **-** | **A** | **A** | **A** | **C** | **C** | **C** | **C** | **G** | **A** | **G** | **A** | **T** | **G** | **T** | **A** | **G** | **C** | **A** | **G** | **C** | **C** | **A** | **T** | **T** | **T** | **G** | **G** | **G** | **C** | **A** | **T** | **G** | **G** | **C** | **T** | **C** | **T** | **T** | **A** | **G** | **T** | **A** | **T** | **T** | **G** | **T** | **T** | **T** | **G** | **C** | **C** | **C** | **T** | **T** | **C** | **C** | **T** | **G** | **G** | **G** | **G** | **T** | **G** | **G** | **G** | **T** | **T** | **T** | **T** | **G** | **T** | **A** | **T** | **T** | **A** | **T** | **A** | **T** | **T** | **T** | **A** | **T** | **T** | **T** | **T** | **T** | **C** | **G** | **G** | **A** | **C** | **G** | **T** | **G** | **G** | **A** | **T** | **T** | **A** | **A** | **C** | **G** | **G** | **A** | **T** | **A** | **A** | **G** | **A** | **A** | **A** | **A** | **A** | **A** | **T** | **T** | **C** | **T** | **A** | **T** | **C** | **T** | **C** | **C** | **G** | **A** | **C** | **A** | **A** | **A** | **G** | **T** | **G** | **A** | **C** | **T** | **T** | **G** | **A** | **A** | **A** | **G** | **A** | **A** | **T** | **T** | **A** | **G** | **A** | **A** | **A** | **A** | **C** | **T** | **T** | **T** | **C** | **A** | **G** | **T** | **C** | **T** | **T** | **T** | **T** | **A** | **G** | **A** | **G** | **G** | **A** | **G** | **A** | **T** | **A** | **C** | **C** | **A** | **T** | **T** | **G** | **A** | **A** | **C** | **A** | **T** | **T** | **A** | **C** | **G** | **A** | **T** | **C** | **C** | **T** | **G** | **A** | **C** | **A** | **T** | **G** | **G** | **G** | **C** | **G** | **A** | **T** | **A** | **A** | **A** | **G** | **A** | **C** | **A** | **A** | **A** | **C** | **A** | **G** | **C** | **A** | **A** | **T** | **T** | **T** | **G** | **T** | **C** | **G** | **A** | **C** | **T** | **T** | **C** | **T** | **T** | **C** | **T** | **C** | **T** | **T** | **C** | **A** | **T** | **T** | **A** | **A** | **A** | **T** | **C** | **G** | **T** | **A** | **T** | **G** | **C** | **C** | **G** | **C** | **T** | **G** | **A** | **C** | **A** | **A** | **G** | **A** | **A** | **T** | **G** | **A** | **A** | **T** | **T** | **C** | **T** | **G** | **T** | **C** | **A** | **C** | **T** | **C** | **T** | **T** | **C** | **T** | **C** | **A** | **C** | **A** | **G** | **A** | **C** | **G** | **G** | **A** | **C** | **A** | **A** | **G** | **A** | **G** | **A** | **A** | **A** | **T** | **T** | **G** | **G** | **A** | **T** | **T** | **C** | **A** | **C** | **T** | **G** | **C** | **T** | **T** | **C** | **A** | **A** | **G** | **A** | **T** | **C** | **T** | **A** | **A** | **A** | **A** | **A** | **A** | **A** | **G** | **C** | **C** | **A** | **A** | **A** | **C** | **A** | **T** | **T** | **C** | **G** | **A** | **T** | **C** | **C** | **A** | **T** | **A** | **T** | **C** | **G** | **A** | **A** | **T** | **A** | **T** | **T** | **A** | **C** | **G** | **C** | **A** | **T** | **T** | **T** | **G** | **T** | **G** | **A** | **C** | **A** | **G** | **A** | **T** | **A** | **A** | **T** | **A** | **T** | **C** | **G** | **G** | **C** | **C** | **A** | **G** | **C** | **A** | **A** | **G** | **T** | **C** | **T** | **T** | **A** | **C** | **A** | **T** | **G** | **T** | **T** | **T** | **T** | **A** | **G** | **A** | **A** | **G** | **A** | **A** | **A** | **A** | **A** | **G** | **C** | **C** | **G** | **C** | **A** | **G** | **A** | **A** | **G** | **G** | **C** | **G** | **T** | **G** | **G** | **A** | **A** | **G** | **T** | **T** | **C** | **G** | **A** | **A** | **T** | **A** | **T** | **T** | **A** | **T** | **A** | **T** | **G** | **A** | **T** | **G** | **C** | **A** | **T** | **T** | **T** | **G** | **G** | **C** | **T** | **C** | **T** | **C** | **A** | **T** | **G** | **G** | **C** | **A** | **C** | **A** | **A** | **A** | **A** | **G** | **C** | **A** | **A** | **A** | **A** | **G** | **A** | **T** | **T** | **T** | **C** | **A** | **A** | **T** | **C** | **G** | **T** | **C** | **T** | **A** | **A** | **T** | **C** | **A** | **A** | **A** | **A** | **A** | **T** | **G** | **G** | **T** | **G** | **G** | **A** | **C** | **A** | **T** | **G** | **T** | **C** | **C** | **A** | **T** | **A** | **C** | **A** | **T** | **T** | **T** | **G** | **T** | **T** | **A** | **C** | **C** | **T** | **C** | **A** | **C** | **A** | **A** | **A** | **A** | **G** | **G** | **C** | **A** | **T** | **T** | **A** | **C** | **T** | **T** | **C** | **G** | **T** | **T** | **T** | **C** | **C** | **G** | **A** | **T** | **T** | **G** | **A** | **A** | **T** | **T** | **A** | **C** | **C** | **A** | **T** | **G** | **A** | **T** | **C** | **A** | **C** | **C** | **G** | **A** | **A** | **A** | **A** | **A** | **T** | **C** | **G** | **T** | **T** | **G** | **T** | **G** | **A** | **T** | **C** | **G** | **A** | **T** | **G** | **G** | **A** | **A** | **A** | **G** | **A** | **T** | **T** | **A** | **G** | **T** | **T** | **A** | **T** | **A** | **C** | **C** | **G** | **G** | **T** | **G** | **G** | **T** | **T** | **T** | **C** | **A** | **A** | **T** | **A** | **T** | **T** | **G** | **C** | **C** | **A** | **A** | **T** | **C** | **A** | **A** | **T** | **A** | **T** | **G** | **T** | **A** | **A** | **A** | **T** | **A** | **C** | **A** | **A** | **C** | **A** | **A** | **A** | **A** | **A** | **A** | **A** | **T** | **T** | **C** | **G** | **G** | **C** | **T** | **A** | **T** | **T** | **G** | **G** | **C** | **G** | **C** | **G** | **A** | **T** | **A** | **C** | **G** | **C** | **A** | **T** | **A** | **T** | **A** | **C** | **G** | **G** | **A** | **T** | **T** | **T** | **T** | **C** | **G** | **G** | **T** | **G** | **C** | **C** | **G** | **C** | **T** | **T** | **C** | **T** | **T** | **C** | **T** | **T** | **T** | **G** | **C** | **T** | **C** | **C** | **A** | **G** | **C** | **T** | **T** | **C** | **G** | **C** | **T** | **T** | **C** | **T** | **T** | **A** | **A** | **C** | **A** | **G** | **A** | **C** | **T** | **G** | **G** | **A** | **A** | **C** | **G** | **T** | **C** | **T** | **C** | **G** | **G** | **T** | **A** | **C** | **C** | **T** | **G** | **A** | **A** | **G** | **A** | **A** | **A** | **A** | **A** | **A** | **A** | **G** | **G** | **T** | **C** | **G** | **G** | **C** | **T** | **A** | **T** | **C** | **A** | **T** | **T** | **T** | **G** | **A** | **A** | **T** | **T** | **A** | **T** | **T** | **T** | **C** | **T** | **T** | **T** | **A** | **A** | **A** | **A** | **A** | **A** | **G** | **C** | **A** | **G** | **A** | **T** | **C** | **G** | **A** | **G** | **A** | **T** | **G** | **A** | **A** | **T** | **C** | **T** | **A** | **A** | **G** | **C** | **T** | **T** | **G** | **C** | **T** | **G** | **A** | **T** | **A** | **C** | **A** | **T** | **C** | **C** | **A** | **T** | **C** | **C** | **A** | **G** | **C** | **T** | **T** | **G** | **T** | **T** | **T** | **C** | **A** | **A** | **G** | **C** | **G** | **G** | **A** | **C** | **C** | **G** | **A** | **A** | **T** | **A** | **A** | **C** | **G** | **A** | **A** | **A** | **G** | **G** | **G** | **A** | **A** | **C** | **A** | **A** | **A** | **T** | **C** | **A** | **A** | **G** | **C** | **T** | **T** | **T** | **C** | **A** | **T** | **T** | **T** | **A** | **T** | **C** | **A** | **A** | **A** | **T** | **T** | **G** | **A** | **T** | **T** | **A** | **C** | **T** | **T** | **C** | **T** | **G** | **C** | **T** | **A** | **A** | **A** | **A** | **A** | **A** | **C** | **G** | **T** | **G** | **T** | **T** | **T** | **G** | **G** | **A** | **T** | **A** | **C** | **A** | **G** | **A** | **C** | **A** | **C** | **C** | **T** | **T** | **A** | **C** | **C** | **T** | **T** | **G** | **T** | **T** | **C** | **C** | **T** | **G** | **A** | **T** | **G** | **A** | **A** | **A** | **G** | **T** | **G** | **T** | **C** | **A** | **T** | **T** | **G** | **C** | **T** | **G** | **C** | **T** | **T** | **T** | **A** | **A** | **A** | **A** | **A** | **T** | **C** | **G** | **C** | **A** | **A** | **C** | **T** | **G** | **C** | **C** | **T** | **C** | **T** | **G** | **G** | **T** | **G** | **T** | **A** | **G** | **A** | **T** | **G** | **T** | **G** | **A** | **A** | **A** | **A** | **T** | **C** | **A** | **T** | **G** | **A** | **T** | **T** | **C** | **C** | **C** | **A** | **A** | **C** | **A** | **A** | **A** | **C** | **C** | **G** | **G** | **A** | **T** | **C** | **A** | **T** | **C** | **C** | **T** | **T** | **T** | **T** | **A** | **T** | **T** | **T** | **A** | **T** | **C** | **G** | **A** | **G** | **C** | **A** | **A** | **C** | **A** | **C** | **A** | **A** | **T** | **A** | **T** | **T** | **A** | **T** | **G** | **C** | **T** | **C** | **G** | **G** | **C** | **A** | **G** | **C** | **T** | **G** | **A** | **T** | **C** | **A** | **A** | **G** | **G** | **A** | **A** | **A** | **A** | **T** | **G** | **T** | **A** | **C** | **A** | **A** | **A** | **T** | **C** | **C** | **T** | **T** | **G** | **T** | **C** | **T** | **A** | **T** | **G** | **A** | **G** | **A** | **A** | **C** | **G** | **G** | **C** | **T** | **T** | **C** | **C** | **T** | **C** | **C** | **A** | **T** | **G** | **C** | **A** | **A** | **A** | **A** | **A** | **C** | **A** | **T** | **T** | **G** | **A** | **T** | **A** | **A** | **T** | **G** | **G** | **A** | **T** | **G** | **A** | **T** | **G** | **A** | **A** | **A** | **T** | **C** | **T** | **G** | **C** | **A** | **T** | **G** | **G** | **T** | **A** | **G** | **G** | **T** | **T** | **C** | **A** | **G** | **C** | **A** | **A** | **A** | **T** | **C** | **A** | **A** | **G** | **A** | **T** | **A** | **T** | **T** | **C** | **G** | **T** | **A** | **G** | **C** | **T** | **A** | **C** | **C** | **G** | **A** | **T** | **T** | **G** | **A** | **A** | **T** | **T** | **T** | **T** | **G** | **A** | **A** | **A** | **C** | **A** | **A** | **G** | **T** | **G** | **C** | **T** | **G** | **T** | **C** | **A** | **T** | **T** | **T** | **A** | **C** | **G** | **A** | **T** | **C** | **C** | **T** | **G** | **A** | **G** | **T** | **T** | **T** | **T** | **T** | **A** | **G** | **A** | **A** | **G** | **A** | **A** | **C** | **T** | **T** | **G** | **C** | **T** | **A** | **C** | **T** | **C** | **A** | **G** | **T** | **T** | **C** | **A** | **A** | **A** | **G** | **A** | **A** | **G** | **A** | **T** | **G** | **A** | **G** | **A** | **C** | **A** | **C** | **A** | **T** | **T** | **G** | **T** | **T** | **C** | **A** | **T** | **C** | **C** | **A** | **T** | **G** | **A** | **C** | **A** | **A** | **C** | **T** | **G** | **A** | **A** | **A** | **C** | **A** | **G** | **T** | **C** | **A** | **A** | **G** | **G** | **A** | **A** | **A** | **T** | **G** | **T** | **C** | **T** | **A** | **A** | **C** | **T** | **G** | **G** | **C** | **T** | **A** | **T** | **T** | **A** | **T** | **T** | **C** | **A** | **A** | **G** | **C** | **A** | **A** | **C** | **A** | **A** | **A** | **T** | **T** | **T** | **C** | **T** | **C** | **G** | **A** | **T** | **T** | **A** | **T** | **T** | **T** | **T** | **C** | **T** | **C** | **C** | **A** | **A** | **T** | **C** | **C** | **T** | **A** | **-** | **-** | **-** |
| **N59504F** | **-** | **-** | **-** | **-** | **-** | **-** | **-** | **-** | **-** | **-** | **-** | **-** | **-** | **-** | **-** | **-** | **-** | **-** | **-** | **-** | **-** | **-** | **-** | **-** | **-** | **-** | **-** | **-** | **-** | **-** | **-** | **-** | **-** | **-** | **-** | **-** | **-** | **-** | **-** | **-** | **-** | **-** | **-** | **-** | **-** | **-** | **-** | **-** | **-** | **-** | **-** | **-** | **-** | **-** | **-** | **-** | **-** | **-** | **-** | **-** | **-** | **-** | **-** | **-** | **-** | **-** | **-** | **-** | **-** | **A** | **A** | **A** | **C** | **C** | **C** | **C** | **G** | **A** | **G** | **A** | **T** | **G** | **T** | **A** | **G** | **C** | **A** | **G** | **C** | **C** | **A** | **T** | **T** | **T** | **G** | **G** | **G** | **C** | **A** | **T** | **G** | **G** | **C** | **T** | **C** | **T** | **T** | **A** | **G** | **T** | **A** | **T** | **T** | **G** | **T** | **T** | **T** | **G** | **C** | **C** | **C** | **T** | **T** | **C** | **C** | **T** | **G** | **G** | **G** | **G** | **T** | **G** | **G** | **G** | **T** | **T** | **T** | **T** | **G** | **T** | **A** | **T** | **T** | **A** | **T** | **A** | **T** | **T** | **T** | **A** | **T** | **T** | **T** | **T** | **T** | **C** | **G** | **G** | **A** | **C** | **G** | **T** | **G** | **G** | **A** | **T** | **T** | **A** | **A** | **C** | **G** | **G** | **A** | **T** | **A** | **A** | **G** | **A** | **A** | **A** | **A** | **A** | **A** | **T** | **T** | **C** | **T** | **A** | **T** | **C** | **T** | **C** | **C** | **G** | **A** | **C** | **A** | **A** | **A** | **G** | **T** | **G** | **A** | **C** | **T** | **T** | **G** | **A** | **A** | **A** | **G** | **A** | **A** | **T** | **T** | **A** | **G** | **A** | **A** | **A** | **A** | **C** | **T** | **T** | **T** | **C** | **A** | **G** | **T** | **C** | **T** | **T** | **T** | **T** | **A** | **G** | **A** | **G** | **G** | **A** | **G** | **A** | **T** | **A** | **C** | **C** | **A** | **T** | **T** | **G** | **A** | **A** | **C** | **A** | **T** | **T** | **A** | **C** | **G** | **A** | **T** | **C** | **C** | **T** | **G** | **A** | **C** | **A** | **T** | **G** | **G** | **G** | **C** | **G** | **A** | **T** | **A** | **A** | **A** | **G** | **A** | **C** | **A** | **A** | **A** | **C** | **A** | **G** | **C** | **A** | **A** | **T** | **T** | **T** | **G** | **T** | **C** | **G** | **A** | **C** | **T** | **T** | **C** | **T** | **T** | **C** | **T** | **C** | **T** | **T** | **C** | **A** | **T** | **T** | **A** | **A** | **A** | **T** | **C** | **G** | **T** | **A** | **T** | **G** | **C** | **C** | **G** | **C** | **T** | **G** | **A** | **C** | **A** | **A** | **G** | **A** | **A** | **T** | **G** | **A** | **A** | **T** | **T** | **C** | **T** | **G** | **T** | **C** | **A** | **C** | **T** | **C** | **T** | **T** | **C** | **T** | **C** | **A** | **C** | **A** | **G** | **A** | **C** | **G** | **G** | **A** | **C** | **A** | **A** | **G** | **A** | **G** | **A** | **A** | **A** | **T** | **T** | **G** | **G** | **A** | **T** | **T** | **C** | **A** | **C** | **T** | **G** | **C** | **T** | **T** | **C** | **A** | **A** | **G** | **A** | **T** | **C** | **T** | **A** | **A** | **A** | **A** | **A** | **A** | **A** | **G** | **C** | **C** | **A** | **A** | **A** | **C** | **A** | **T** | **T** | **C** | **G** | **A** | **T** | **C** | **C** | **A** | **T** | **A** | **T** | **C** | **G** | **A** | **A** | **T** | **A** | **T** | **T** | **A** | **C** | **G** | **C** | **A** | **T** | **T** | **T** | **G** | **T** | **G** | **A** | **C** | **A** | **G** | **A** | **T** | **A** | **A** | **T** | **A** | **T** | **C** | **G** | **G** | **C** | **C** | **A** | **G** | **C** | **A** | **A** | **G** | **T** | **C** | **T** | **T** | **A** | **C** | **A** | **T** | **G** | **T** | **T** | **T** | **T** | **A** | **G** | **A** | **A** | **G** | **A** | **A** | **A** | **A** | **A** | **G** | **C** | **C** | **G** | **C** | **A** | **G** | **A** | **A** | **G** | **G** | **C** | **G** | **T** | **G** | **G** | **A** | **A** | **G** | **T** | **T** | **C** | **G** | **A** | **A** | **T** | **A** | **T** | **T** | **A** | **T** | **A** | **T** | **G** | **A** | **T** | **G** | **C** | **A** | **T** | **T** | **T** | **G** | **G** | **C** | **T** | **C** | **T** | **C** | **A** | **T** | **G** | **G** | **C** | **A** | **C** | **A** | **A** | **A** | **A** | **G** | **C** | **A** | **A** | **A** | **A** | **G** | **A** | **T** | **T** | **T** | **C** | **A** | **A** | **T** | **C** | **G** | **T** | **C** | **T** | **A** | **A** | **T** | **C** | **A** | **A** | **A** | **A** | **A** | **T** | **G** | **G** | **T** | **G** | **G** | **A** | **C** | **A** | **T** | **G** | **T** | **C** | **C** | **A** | **T** | **A** | **C** | **A** | **T** | **T** | **T** | **G** | **T** | **T** | **A** | **C** | **C** | **T** | **C** | **A** | **C** | **A** | **A** | **A** | **A** | **G** | **G** | **C** | **A** | **T** | **T** | **A** | **C** | **T** | **T** | **C** | **G** | **T** | **T** | **T** | **C** | **C** | **G** | **A** | **T** | **T** | **G** | **A** | **A** | **T** | **T** | **A** | **C** | **C** | **A** | **T** | **G** | **A** | **T** | **C** | **A** | **C** | **C** | **G** | **A** | **A** | **A** | **A** | **A** | **T** | **C** | **G** | **T** | **T** | **G** | **T** | **G** | **A** | **T** | **C** | **G** | **A** | **T** | **G** | **G** | **A** | **A** | **A** | **G** | **A** | **T** | **T** | **A** | **G** | **T** | **T** | **A** | **T** | **A** | **C** | **C** | **G** | **G** | **T** | **G** | **G** | **T** | **T** | **T** | **C** | **A** | **A** | **T** | **A** | **T** | **T** | **G** | **C** | **C** | **A** | **A** | **T** | **C** | **A** | **A** | **T** | **A** | **T** | **G** | **T** | **A** | **A** | **A** | **T** | **A** | **C** | **A** | **A** | **C** | **A** | **A** | **A** | **A** | **A** | **A** | **A** | **T** | **T** | **C** | **G** | **G** | **C** | **T** | **A** | **T** | **T** | **G** | **G** | **C** | **G** | **C** | **G** | **A** | **T** | **A** | **C** | **G** | **C** | **A** | **T** | **A** | **T** | **A** | **C** | **G** | **G** | **A** | **T** | **T** | **T** | **T** | **C** | **G** | **G** | **T** | **G** | **C** | **C** | **G** | **C** | **T** | **T** | **C** | **T** | **T** | **C** | **T** | **T** | **T** | **G** | **C** | **T** | **C** | **C** | **A** | **G** | **C** | **T** | **T** | **C** | **G** | **C** | **T** | **T** | **C** | **T** | **T** | **A** | **A** | **C** | **A** | **G** | **A** | **C** | **T** | **G** | **G** | **A** | **A** | **C** | **G** | **T** | **C** | **T** | **C** | **G** | **G** | **T** | **A** | **C** | **C** | **T** | **G** | **A** | **A** | **G** | **A** | **A** | **A** | **A** | **A** | **A** | **A** | **G** | **G** | **T** | **C** | **G** | **G** | **C** | **T** | **A** | **T** | **C** | **A** | **T** | **T** | **T** | **G** | **A** | **A** | **T** | **T** | **A** | **T** | **T** | **T** | **C** | **T** | **T** | **T** | **A** | **A** | **A** | **A** | **A** | **A** | **G** | **C** | **A** | **G** | **A** | **T** | **C** | **G** | **A** | **G** | **A** | **T** | **G** | **A** | **A** | **T** | **C** | **T** | **A** | **A** | **G** | **C** | **T** | **T** | **G** | **C** | **T** | **G** | **A** | **T** | **A** | **C** | **A** | **T** | **C** | **C** | **A** | **T** | **C** | **C** | **A** | **G** | **C** | **T** | **T** | **G** | **T** | **T** | **T** | **C** | **A** | **A** | **G** | **C** | **G** | **G** | **A** | **C** | **C** | **G** | **A** | **A** | **T** | **A** | **A** | **C** | **G** | **A** | **A** | **A** | **G** | **G** | **G** | **A** | **A** | **C** | **A** | **A** | **A** | **T** | **A** | **A** | **A** | **G** | **C** | **T** | **T** | **T** | **C** | **A** | **T** | **T** | **T** | **A** | **T** | **C** | **A** | **A** | **A** | **T** | **T** | **G** | **A** | **T** | **T** | **A** | **C** | **T** | **T** | **C** | **T** | **G** | **C** | **T** | **A** | **A** | **A** | **A** | **A** | **A** | **C** | **G** | **T** | **G** | **T** | **T** | **T** | **G** | **G** | **A** | **T** | **A** | **C** | **A** | **G** | **A** | **C** | **A** | **C** | **C** | **T** | **T** | **A** | **C** | **C** | **T** | **T** | **G** | **T** | **T** | **C** | **C** | **T** | **G** | **A** | **T** | **G** | **A** | **A** | **A** | **G** | **T** | **G** | **T** | **C** | **A** | **T** | **T** | **G** | **C** | **T** | **G** | **C** | **T** | **T** | **T** | **A** | **A** | **A** | **A** | **A** | **T** | **C** | **G** | **C** | **A** | **A** | **C** | **T** | **G** | **C** | **C** | **T** | **C** | **T** | **G** | **G** | **T** | **G** | **T** | **A** | **G** | **A** | **T** | **G** | **T** | **G** | **A** | **A** | **A** | **A** | **T** | **C** | **A** | **T** | **G** | **A** | **T** | **T** | **C** | **C** | **C** | **A** | **A** | **C** | **A** | **A** | **A** | **C** | **C** | **G** | **G** | **A** | **T** | **C** | **A** | **T** | **C** | **C** | **T** | **T** | **T** | **T** | **A** | **T** | **T** | **T** | **A** | **T** | **C** | **G** | **A** | **G** | **C** | **A** | **A** | **C** | **A** | **C** | **A** | **A** | **T** | **A** | **T** | **T** | **A** | **T** | **G** | **C** | **T** | **C** | **G** | **G** | **C** | **A** | **G** | **C** | **T** | **G** | **A** | **T** | **C** | **A** | **A** | **G** | **G** | **A** | **A** | **A** | **A** | **T** | **G** | **T** | **A** | **C** | **A** | **A** | **A** | **T** | **C** | **C** | **T** | **T** | **G** | **T** | **C** | **T** | **A** | **T** | **G** | **A** | **G** | **A** | **A** | **C** | **G** | **G** | **C** | **T** | **T** | **C** | **C** | **T** | **C** | **C** | **A** | **T** | **G** | **C** | **A** | **A** | **A** | **A** | **A** | **C** | **A** | **T** | **T** | **G** | **A** | **T** | **A** | **A** | **T** | **G** | **G** | **A** | **T** | **G** | **A** | **T** | **G** | **A** | **A** | **A** | **T** | **C** | **T** | **G** | **C** | **A** | **T** | **G** | **G** | **T** | **A** | **G** | **G** | **T** | **T** | **C** | **A** | **G** | **C** | **A** | **A** | **A** | **T** | **C** | **A** | **A** | **G** | **A** | **T** | **A** | **T** | **T** | **C** | **G** | **T** | **A** | **G** | **C** | **T** | **A** | **C** | **C** | **G** | **A** | **T** | **T** | **G** | **A** | **A** | **T** | **T** | **T** | **T** | **G** | **A** | **A** | **A** | **C** | **A** | **A** | **G** | **T** | **G** | **C** | **T** | **G** | **T** | **C** | **A** | **T** | **T** | **T** | **A** | **C** | **G** | **A** | **T** | **C** | **C** | **T** | **G** | **A** | **G** | **T** | **T** | **T** | **T** | **T** | **A** | **G** | **A** | **A** | **G** | **A** | **A** | **C** | **T** | **T** | **G** | **C** | **T** | **A** | **C** | **T** | **C** | **A** | **G** | **T** | **T** | **C** | **A** | **A** | **A** | **G** | **A** | **A** | **G** | **A** | **T** | **G** | **A** | **G** | **A** | **C** | **A** | **C** | **A** | **T** | **T** | **G** | **T** | **T** | **C** | **A** | **T** | **C** | **C** | **A** | **T** | **G** | **A** | **C** | **A** | **A** | **C** | **T** | **G** | **A** | **A** | **A** | **C** | **A** | **G** | **T** | **C** | **A** | **A** | **G** | **G** | **A** | **A** | **A** | **T** | **G** | **T** | **C** | **T** | **A** | **A** | **C** | **T** | **G** | **G** | **C** | **T** | **A** | **T** | **T** | **A** | **T** | **T** | **C** | **A** | **A** | **G** | **C** | **A** | **A** | **C** | **A** | **A** | **A** | **T** | **T** | **T** | **C** | **T** | **C** | **G** | **A** | **T** | **T** | **A** | **T** | **T** | **T** | **T** | **C** | **T** | **C** | **C** | **A** | **A** | **T** | **C** | **C** | **T** | **A** | **-** | **-** | **-** |
| **N60425F** | **-** | **-** | **-** | **-** | **-** | **-** | **-** | **-** | **-** | **-** | **-** | **-** | **-** | **-** | **-** | **-** | **-** | **-** | **-** | **-** | **-** | **-** | **-** | **-** | **-** | **-** | **-** | **-** | **-** | **-** | **-** | **-** | **-** | **-** | **-** | **-** | **-** | **-** | **-** | **-** | **-** | **-** | **-** | **-** | **-** | **-** | **-** | **-** | **-** | **-** | **-** | **-** | **-** | **-** | **-** | **-** | **-** | **-** | **-** | **-** | **-** | **-** | **-** | **-** | **-** | **-** | **-** | **-** | **-** | **A** | **A** | **A** | **C** | **C** | **C** | **C** | **G** | **A** | **G** | **A** | **T** | **G** | **T** | **A** | **G** | **C** | **A** | **G** | **C** | **C** | **A** | **T** | **T** | **T** | **G** | **G** | **G** | **C** | **A** | **T** | **G** | **G** | **C** | **T** | **C** | **T** | **T** | **A** | **G** | **T** | **A** | **T** | **T** | **G** | **T** | **T** | **T** | **G** | **C** | **C** | **C** | **T** | **T** | **C** | **C** | **T** | **G** | **G** | **G** | **G** | **T** | **G** | **G** | **G** | **T** | **T** | **T** | **T** | **G** | **T** | **A** | **T** | **T** | **A** | **T** | **A** | **T** | **T** | **T** | **A** | **T** | **T** | **T** | **T** | **T** | **C** | **G** | **G** | **A** | **C** | **G** | **T** | **G** | **G** | **A** | **T** | **T** | **A** | **A** | **C** | **G** | **G** | **A** | **T** | **A** | **A** | **G** | **A** | **A** | **A** | **A** | **A** | **A** | **T** | **T** | **C** | **T** | **A** | **T** | **C** | **T** | **C** | **C** | **G** | **A** | **C** | **A** | **A** | **A** | **G** | **T** | **G** | **A** | **C** | **T** | **T** | **G** | **A** | **A** | **A** | **G** | **A** | **A** | **T** | **T** | **A** | **G** | **A** | **A** | **A** | **A** | **C** | **T** | **T** | **T** | **C** | **A** | **G** | **T** | **C** | **T** | **T** | **T** | **T** | **A** | **G** | **A** | **G** | **G** | **A** | **G** | **A** | **T** | **A** | **C** | **C** | **A** | **T** | **T** | **G** | **A** | **A** | **C** | **A** | **T** | **T** | **A** | **C** | **G** | **A** | **T** | **C** | **C** | **T** | **G** | **A** | **C** | **A** | **T** | **G** | **G** | **G** | **C** | **G** | **A** | **T** | **A** | **A** | **A** | **G** | **A** | **C** | **A** | **A** | **A** | **C** | **A** | **G** | **C** | **A** | **A** | **T** | **T** | **T** | **G** | **T** | **T** | **G** | **A** | **C** | **T** | **T** | **C** | **T** | **T** | **C** | **T** | **C** | **T** | **T** | **C** | **A** | **T** | **T** | **A** | **A** | **A** | **T** | **C** | **G** | **T** | **A** | **T** | **G** | **C** | **C** | **G** | **C** | **T** | **G** | **A** | **C** | **A** | **A** | **G** | **A** | **A** | **T** | **G** | **A** | **A** | **T** | **T** | **C** | **T** | **G** | **T** | **C** | **A** | **C** | **T** | **C** | **T** | **T** | **C** | **T** | **C** | **A** | **C** | **A** | **G** | **A** | **C** | **G** | **G** | **A** | **C** | **A** | **A** | **G** | **A** | **G** | **A** | **A** | **A** | **T** | **T** | **G** | **G** | **A** | **T** | **T** | **C** | **A** | **C** | **T** | **G** | **C** | **T** | **T** | **C** | **A** | **A** | **G** | **A** | **T** | **C** | **T** | **A** | **A** | **A** | **A** | **A** | **A** | **A** | **G** | **C** | **C** | **A** | **A** | **A** | **C** | **A** | **T** | **T** | **C** | **G** | **A** | **T** | **C** | **C** | **A** | **T** | **A** | **T** | **C** | **G** | **A** | **A** | **T** | **A** | **T** | **T** | **A** | **C** | **G** | **C** | **A** | **T** | **T** | **T** | **G** | **T** | **G** | **A** | **C** | **A** | **G** | **A** | **T** | **A** | **A** | **T** | **A** | **T** | **C** | **G** | **G** | **C** | **C** | **A** | **G** | **C** | **A** | **A** | **G** | **T** | **C** | **T** | **T** | **A** | **C** | **A** | **T** | **G** | **T** | **T** | **T** | **T** | **A** | **G** | **A** | **A** | **G** | **A** | **A** | **A** | **A** | **A** | **G** | **C** | **C** | **G** | **C** | **A** | **G** | **A** | **A** | **G** | **G** | **C** | **G** | **T** | **G** | **G** | **A** | **A** | **G** | **T** | **T** | **C** | **G** | **A** | **A** | **T** | **A** | **T** | **T** | **A** | **T** | **A** | **T** | **G** | **A** | **T** | **G** | **C** | **A** | **T** | **T** | **T** | **G** | **G** | **C** | **T** | **C** | **T** | **C** | **A** | **T** | **G** | **G** | **C** | **A** | **C** | **A** | **A** | **A** | **A** | **G** | **C** | **A** | **A** | **A** | **A** | **G** | **A** | **T** | **T** | **T** | **C** | **A** | **A** | **T** | **C** | **G** | **T** | **C** | **T** | **A** | **A** | **T** | **C** | **A** | **A** | **A** | **A** | **A** | **T** | **G** | **G** | **T** | **G** | **G** | **A** | **C** | **A** | **T** | **G** | **T** | **C** | **C** | **A** | **T** | **A** | **C** | **A** | **T** | **T** | **T** | **G** | **T** | **T** | **A** | **C** | **C** | **T** | **C** | **A** | **C** | **A** | **A** | **A** | **A** | **G** | **G** | **C** | **A** | **T** | **T** | **A** | **C** | **T** | **T** | **C** | **G** | **T** | **T** | **T** | **C** | **C** | **G** | **A** | **T** | **T** | **G** | **A** | **A** | **T** | **T** | **A** | **C** | **C** | **A** | **T** | **G** | **A** | **T** | **C** | **A** | **C** | **C** | **G** | **A** | **A** | **A** | **A** | **A** | **T** | **C** | **G** | **T** | **T** | **G** | **T** | **G** | **A** | **T** | **C** | **G** | **A** | **T** | **G** | **G** | **A** | **A** | **A** | **G** | **A** | **T** | **T** | **A** | **G** | **T** | **T** | **A** | **T** | **A** | **C** | **C** | **G** | **G** | **T** | **G** | **G** | **T** | **T** | **T** | **C** | **A** | **A** | **T** | **A** | **T** | **T** | **G** | **C** | **C** | **A** | **A** | **T** | **C** | **A** | **A** | **T** | **A** | **T** | **G** | **T** | **A** | **A** | **A** | **T** | **A** | **C** | **A** | **A** | **C** | **A** | **A** | **A** | **A** | **A** | **A** | **A** | **T** | **T** | **C** | **G** | **G** | **C** | **T** | **A** | **T** | **T** | **G** | **G** | **C** | **G** | **C** | **G** | **A** | **T** | **A** | **C** | **G** | **C** | **A** | **T** | **A** | **T** | **A** | **C** | **G** | **G** | **A** | **T** | **T** | **T** | **T** | **C** | **G** | **G** | **T** | **G** | **C** | **C** | **G** | **C** | **T** | **T** | **C** | **T** | **T** | **C** | **T** | **T** | **T** | **G** | **C** | **T** | **C** | **C** | **A** | **G** | **C** | **T** | **T** | **C** | **G** | **C** | **T** | **T** | **C** | **T** | **T** | **A** | **A** | **C** | **A** | **G** | **A** | **C** | **T** | **G** | **G** | **A** | **A** | **C** | **G** | **T** | **C** | **T** | **C** | **G** | **G** | **T** | **A** | **C** | **C** | **T** | **G** | **A** | **A** | **G** | **A** | **A** | **A** | **A** | **A** | **A** | **A** | **G** | **G** | **T** | **C** | **G** | **G** | **C** | **T** | **A** | **T** | **C** | **A** | **T** | **T** | **T** | **G** | **A** | **A** | **T** | **T** | **A** | **T** | **T** | **T** | **C** | **T** | **T** | **T** | **A** | **A** | **A** | **A** | **A** | **A** | **G** | **C** | **A** | **G** | **A** | **T** | **C** | **G** | **A** | **G** | **A** | **T** | **G** | **A** | **A** | **T** | **C** | **T** | **A** | **A** | **G** | **C** | **T** | **T** | **G** | **C** | **T** | **G** | **A** | **A** | **A** | **C** | **A** | **T** | **C** | **C** | **A** | **T** | **C** | **C** | **A** | **G** | **C** | **T** | **T** | **G** | **T** | **T** | **T** | **C** | **A** | **A** | **G** | **C** | **G** | **G** | **A** | **C** | **C** | **G** | **A** | **A** | **T** | **A** | **A** | **C** | **G** | **A** | **A** | **A** | **G** | **G** | **G** | **A** | **A** | **C** | **A** | **A** | **A** | **T** | **C** | **A** | **A** | **G** | **C** | **T** | **T** | **T** | **C** | **A** | **T** | **T** | **T** | **A** | **T** | **C** | **A** | **A** | **A** | **T** | **T** | **G** | **A** | **T** | **T** | **A** | **C** | **T** | **T** | **C** | **T** | **G** | **C** | **T** | **A** | **A** | **A** | **A** | **A** | **G** | **C** | **G** | **T** | **G** | **T** | **T** | **T** | **G** | **G** | **A** | **T** | **A** | **C** | **A** | **G** | **A** | **C** | **A** | **C** | **C** | **T** | **T** | **A** | **C** | **C** | **T** | **T** | **G** | **T** | **T** | **C** | **C** | **T** | **G** | **A** | **T** | **G** | **A** | **A** | **A** | **G** | **T** | **G** | **T** | **C** | **A** | **T** | **T** | **G** | **C** | **T** | **G** | **C** | **T** | **T** | **T** | **A** | **A** | **A** | **A** | **A** | **T** | **C** | **G** | **C** | **A** | **A** | **C** | **T** | **G** | **C** | **C** | **T** | **C** | **T** | **G** | **G** | **T** | **G** | **T** | **A** | **G** | **A** | **T** | **G** | **T** | **G** | **A** | **A** | **A** | **A** | **T** | **C** | **A** | **T** | **G** | **A** | **T** | **T** | **C** | **C** | **C** | **A** | **A** | **C** | **A** | **A** | **A** | **C** | **C** | **G** | **G** | **A** | **T** | **C** | **A** | **T** | **C** | **C** | **T** | **T** | **T** | **T** | **A** | **T** | **T** | **T** | **A** | **T** | **C** | **G** | **A** | **G** | **C** | **A** | **A** | **C** | **A** | **C** | **A** | **A** | **T** | **A** | **T** | **T** | **A** | **T** | **G** | **C** | **T** | **C** | **G** | **G** | **C** | **A** | **G** | **C** | **T** | **G** | **A** | **T** | **C** | **A** | **A** | **G** | **G** | **A** | **A** | **A** | **A** | **T** | **G** | **T** | **A** | **C** | **A** | **A** | **A** | **T** | **C** | **C** | **T** | **T** | **G** | **T** | **C** | **T** | **A** | **T** | **G** | **A** | **G** | **A** | **A** | **C** | **G** | **G** | **C** | **T** | **T** | **C** | **C** | **T** | **C** | **C** | **A** | **T** | **G** | **C** | **A** | **A** | **A** | **A** | **A** | **C** | **A** | **T** | **T** | **G** | **A** | **T** | **A** | **A** | **T** | **G** | **G** | **A** | **T** | **G** | **A** | **T** | **G** | **A** | **A** | **A** | **T** | **C** | **T** | **G** | **C** | **A** | **T** | **G** | **G** | **T** | **A** | **G** | **G** | **T** | **T** | **C** | **A** | **G** | **C** | **A** | **A** | **A** | **T** | **C** | **A** | **A** | **G** | **A** | **T** | **A** | **T** | **T** | **C** | **G** | **T** | **A** | **G** | **C** | **T** | **A** | **C** | **A** | **G** | **A** | **T** | **T** | **G** | **A** | **A** | **T** | **T** | **T** | **T** | **G** | **A** | **A** | **A** | **C** | **A** | **A** | **G** | **T** | **G** | **C** | **T** | **G** | **T** | **C** | **A** | **T** | **T** | **T** | **A** | **C** | **G** | **A** | **T** | **C** | **C** | **T** | **G** | **A** | **G** | **T** | **T** | **T** | **T** | **T** | **A** | **G** | **A** | **A** | **G** | **A** | **A** | **C** | **T** | **T** | **G** | **C** | **T** | **A** | **C** | **T** | **C** | **A** | **G** | **T** | **T** | **C** | **A** | **A** | **A** | **G** | **A** | **A** | **G** | **A** | **T** | **G** | **A** | **G** | **A** | **C** | **A** | **C** | **A** | **T** | **T** | **G** | **T** | **T** | **C** | **A** | **T** | **C** | **C** | **A** | **T** | **G** | **A** | **C** | **A** | **A** | **C** | **T** | **G** | **A** | **A** | **A** | **C** | **A** | **G** | **T** | **T** | **A** | **A** | **A** | **G** | **A** | **A** | **A** | **T** | **G** | **T** | **C** | **T** | **A** | **A** | **C** | **T** | **G** | **G** | **C** | **T** | **A** | **T** | **T** | **A** | **T** | **T** | **C** | **A** | **A** | **G** | **C** | **A** | **A** | **C** | **A** | **A** | **A** | **T** | **T** | **T** | **C** | **T** | **C** | **G** | **A** | **T** | **T** | **A** | **T** | **T** | **T** | **T** | **C** | **T** | **C** | **C** | **A** | **A** | **T** | **C** | **C** | **T** | **A** | **-** | **-** | **-** |
| **N60449F** | **-** | **-** | **-** | **-** | **-** | **-** | **-** | **-** | **-** | **-** | **-** | **-** | **-** | **-** | **-** | **-** | **-** | **-** | **-** | **-** | **-** | **-** | **-** | **-** | **-** | **-** | **-** | **-** | **-** | **-** | **-** | **-** | **-** | **-** | **-** | **-** | **-** | **-** | **-** | **-** | **-** | **-** | **-** | **-** | **-** | **-** | **-** | **-** | **-** | **-** | **-** | **-** | **-** | **-** | **-** | **-** | **-** | **-** | **-** | **-** | **-** | **-** | **-** | **-** | **-** | **-** | **-** | **-** | **-** | **A** | **A** | **A** | **C** | **C** | **C** | **C** | **G** | **A** | **G** | **A** | **T** | **G** | **T** | **A** | **G** | **C** | **A** | **G** | **C** | **C** | **A** | **T** | **T** | **T** | **G** | **G** | **G** | **C** | **A** | **T** | **G** | **G** | **C** | **T** | **C** | **T** | **T** | **A** | **G** | **T** | **A** | **T** | **T** | **G** | **T** | **T** | **T** | **G** | **C** | **C** | **C** | **T** | **T** | **C** | **C** | **T** | **G** | **G** | **G** | **G** | **T** | **G** | **G** | **G** | **C** | **T** | **T** | **T** | **G** | **T** | **A** | **T** | **T** | **A** | **T** | **A** | **T** | **T** | **T** | **A** | **T** | **T** | **T** | **T** | **T** | **C** | **G** | **G** | **A** | **C** | **G** | **T** | **G** | **G** | **A** | **T** | **T** | **A** | **A** | **C** | **G** | **G** | **A** | **T** | **A** | **A** | **G** | **A** | **A** | **A** | **A** | **A** | **A** | **T** | **T** | **C** | **T** | **A** | **T** | **C** | **T** | **C** | **C** | **G** | **A** | **C** | **A** | **A** | **A** | **G** | **T** | **G** | **A** | **C** | **T** | **T** | **G** | **A** | **A** | **A** | **G** | **A** | **A** | **T** | **T** | **A** | **G** | **A** | **A** | **A** | **A** | **C** | **T** | **T** | **T** | **C** | **A** | **G** | **T** | **C** | **T** | **T** | **T** | **T** | **A** | **G** | **A** | **G** | **G** | **A** | **G** | **A** | **T** | **A** | **C** | **C** | **A** | **T** | **T** | **G** | **A** | **A** | **C** | **A** | **T** | **T** | **A** | **C** | **G** | **A** | **T** | **C** | **C** | **T** | **G** | **A** | **C** | **A** | **T** | **G** | **G** | **G** | **C** | **G** | **A** | **T** | **A** | **A** | **A** | **G** | **A** | **C** | **A** | **A** | **A** | **C** | **A** | **G** | **C** | **A** | **A** | **T** | **T** | **T** | **G** | **T** | **T** | **G** | **A** | **C** | **T** | **T** | **C** | **T** | **T** | **C** | **T** | **C** | **T** | **T** | **C** | **A** | **T** | **T** | **A** | **A** | **A** | **T** | **C** | **G** | **T** | **A** | **T** | **G** | **C** | **C** | **G** | **C** | **T** | **G** | **A** | **C** | **A** | **A** | **G** | **A** | **A** | **T** | **G** | **A** | **A** | **T** | **T** | **C** | **T** | **G** | **T** | **C** | **A** | **C** | **T** | **C** | **T** | **T** | **C** | **T** | **C** | **A** | **C** | **A** | **G** | **A** | **C** | **G** | **G** | **A** | **C** | **A** | **A** | **G** | **A** | **G** | **A** | **A** | **A** | **T** | **T** | **G** | **G** | **A** | **T** | **T** | **C** | **A** | **C** | **T** | **G** | **C** | **T** | **T** | **C** | **A** | **A** | **G** | **A** | **T** | **C** | **T** | **A** | **A** | **A** | **A** | **A** | **A** | **A** | **G** | **C** | **C** | **A** | **A** | **A** | **C** | **A** | **T** | **T** | **C** | **G** | **A** | **T** | **C** | **C** | **A** | **T** | **A** | **T** | **C** | **G** | **A** | **A** | **T** | **A** | **T** | **T** | **A** | **C** | **G** | **C** | **A** | **T** | **T** | **T** | **G** | **T** | **G** | **A** | **C** | **A** | **G** | **A** | **T** | **A** | **A** | **T** | **A** | **T** | **C** | **G** | **G** | **C** | **C** | **A** | **G** | **C** | **A** | **A** | **G** | **T** | **C** | **T** | **T** | **A** | **C** | **A** | **T** | **G** | **T** | **T** | **T** | **T** | **A** | **G** | **A** | **A** | **G** | **A** | **A** | **A** | **A** | **A** | **G** | **C** | **C** | **G** | **C** | **A** | **G** | **A** | **A** | **G** | **G** | **C** | **G** | **T** | **G** | **G** | **A** | **A** | **G** | **T** | **T** | **C** | **G** | **A** | **A** | **T** | **A** | **T** | **T** | **A** | **T** | **A** | **T** | **G** | **A** | **T** | **G** | **C** | **A** | **T** | **T** | **T** | **G** | **G** | **C** | **T** | **C** | **T** | **C** | **A** | **T** | **G** | **G** | **C** | **A** | **C** | **A** | **A** | **A** | **A** | **G** | **C** | **A** | **A** | **A** | **A** | **G** | **A** | **T** | **T** | **T** | **C** | **A** | **A** | **T** | **C** | **G** | **T** | **C** | **T** | **A** | **A** | **T** | **C** | **A** | **A** | **A** | **A** | **A** | **T** | **G** | **G** | **T** | **G** | **G** | **A** | **C** | **A** | **T** | **G** | **T** | **C** | **C** | **A** | **T** | **A** | **C** | **A** | **T** | **T** | **T** | **G** | **T** | **T** | **A** | **C** | **C** | **T** | **C** | **A** | **C** | **A** | **A** | **A** | **G** | **G** | **G** | **C** | **A** | **T** | **T** | **A** | **C** | **T** | **T** | **C** | **G** | **T** | **T** | **T** | **C** | **C** | **G** | **A** | **T** | **T** | **G** | **A** | **A** | **T** | **T** | **A** | **C** | **C** | **A** | **T** | **G** | **A** | **T** | **C** | **A** | **C** | **C** | **G** | **A** | **A** | **A** | **A** | **A** | **T** | **C** | **G** | **T** | **T** | **G** | **T** | **G** | **A** | **T** | **C** | **G** | **A** | **T** | **G** | **G** | **A** | **A** | **A** | **G** | **A** | **T** | **T** | **A** | **G** | **T** | **T** | **A** | **T** | **A** | **C** | **C** | **G** | **G** | **T** | **G** | **G** | **T** | **T** | **T** | **C** | **A** | **A** | **T** | **A** | **T** | **T** | **G** | **C** | **C** | **A** | **A** | **T** | **C** | **A** | **A** | **T** | **A** | **T** | **G** | **T** | **A** | **A** | **A** | **T** | **A** | **C** | **A** | **A** | **C** | **A** | **A** | **A** | **A** | **A** | **A** | **A** | **T** | **T** | **C** | **G** | **G** | **C** | **T** | **A** | **T** | **T** | **G** | **G** | **C** | **G** | **C** | **G** | **A** | **T** | **A** | **C** | **G** | **C** | **A** | **T** | **A** | **T** | **A** | **C** | **G** | **G** | **A** | **T** | **T** | **T** | **T** | **C** | **G** | **G** | **T** | **G** | **C** | **C** | **G** | **C** | **T** | **T** | **C** | **T** | **T** | **C** | **T** | **T** | **T** | **G** | **C** | **T** | **C** | **C** | **A** | **G** | **C** | **T** | **T** | **C** | **G** | **C** | **T** | **T** | **C** | **T** | **T** | **A** | **A** | **C** | **A** | **G** | **A** | **C** | **T** | **G** | **G** | **A** | **A** | **C** | **G** | **T** | **C** | **T** | **C** | **G** | **G** | **T** | **A** | **C** | **C** | **T** | **G** | **A** | **A** | **G** | **A** | **A** | **A** | **A** | **A** | **A** | **A** | **G** | **G** | **T** | **C** | **G** | **G** | **C** | **T** | **A** | **T** | **C** | **A** | **T** | **T** | **T** | **G** | **A** | **A** | **T** | **T** | **A** | **T** | **T** | **T** | **C** | **T** | **T** | **T** | **A** | **A** | **A** | **A** | **A** | **A** | **G** | **C** | **A** | **G** | **A** | **T** | **C** | **G** | **A** | **G** | **A** | **T** | **G** | **A** | **A** | **T** | **C** | **T** | **A** | **A** | **G** | **C** | **T** | **T** | **G** | **C** | **T** | **G** | **A** | **T** | **A** | **C** | **A** | **T** | **C** | **C** | **A** | **T** | **C** | **C** | **A** | **G** | **C** | **T** | **T** | **G** | **T** | **T** | **T** | **C** | **A** | **A** | **G** | **C** | **G** | **G** | **A** | **C** | **C** | **G** | **A** | **A** | **T** | **A** | **A** | **C** | **G** | **A** | **A** | **A** | **G** | **G** | **G** | **A** | **A** | **C** | **A** | **A** | **A** | **T** | **C** | **A** | **A** | **G** | **C** | **T** | **T** | **T** | **C** | **A** | **T** | **T** | **T** | **A** | **T** | **C** | **A** | **A** | **A** | **T** | **T** | **G** | **A** | **T** | **T** | **A** | **C** | **T** | **T** | **C** | **T** | **G** | **C** | **T** | **A** | **A** | **A** | **A** | **A** | **A** | **C** | **G** | **T** | **G** | **T** | **T** | **T** | **G** | **G** | **A** | **T** | **A** | **C** | **A** | **G** | **A** | **C** | **A** | **C** | **C** | **T** | **T** | **A** | **C** | **C** | **T** | **T** | **G** | **T** | **T** | **C** | **C** | **T** | **G** | **A** | **T** | **G** | **A** | **A** | **A** | **G** | **T** | **G** | **T** | **C** | **A** | **T** | **T** | **G** | **C** | **T** | **G** | **C** | **T** | **T** | **T** | **A** | **A** | **A** | **A** | **A** | **T** | **C** | **G** | **C** | **A** | **A** | **C** | **T** | **G** | **C** | **C** | **T** | **C** | **T** | **G** | **G** | **T** | **G** | **T** | **A** | **G** | **A** | **T** | **G** | **T** | **G** | **A** | **A** | **A** | **A** | **T** | **C** | **A** | **T** | **G** | **A** | **T** | **T** | **C** | **C** | **C** | **A** | **A** | **C** | **A** | **A** | **A** | **C** | **C** | **G** | **G** | **A** | **T** | **C** | **A** | **T** | **C** | **C** | **T** | **T** | **T** | **T** | **A** | **T** | **T** | **T** | **A** | **T** | **C** | **G** | **A** | **G** | **C** | **A** | **A** | **C** | **A** | **C** | **A** | **A** | **T** | **A** | **T** | **T** | **A** | **T** | **G** | **C** | **T** | **C** | **G** | **G** | **C** | **A** | **G** | **C** | **T** | **G** | **A** | **T** | **C** | **A** | **A** | **G** | **G** | **A** | **A** | **A** | **A** | **T** | **G** | **T** | **A** | **C** | **A** | **A** | **A** | **T** | **C** | **C** | **T** | **T** | **G** | **T** | **C** | **T** | **A** | **T** | **G** | **A** | **G** | **A** | **A** | **C** | **G** | **G** | **C** | **T** | **T** | **C** | **C** | **T** | **C** | **C** | **A** | **T** | **G** | **C** | **A** | **A** | **A** | **A** | **A** | **C** | **A** | **T** | **T** | **G** | **A** | **T** | **A** | **A** | **T** | **G** | **G** | **A** | **T** | **G** | **A** | **T** | **G** | **A** | **A** | **A** | **T** | **C** | **T** | **G** | **C** | **A** | **T** | **G** | **G** | **T** | **A** | **G** | **G** | **T** | **T** | **C** | **A** | **G** | **C** | **A** | **A** | **A** | **T** | **C** | **A** | **A** | **G** | **A** | **T** | **A** | **T** | **T** | **C** | **G** | **T** | **A** | **G** | **C** | **T** | **A** | **C** | **C** | **G** | **A** | **T** | **T** | **G** | **A** | **A** | **T** | **T** | **T** | **T** | **G** | **A** | **A** | **A** | **C** | **A** | **A** | **G** | **T** | **G** | **C** | **T** | **G** | **T** | **C** | **A** | **T** | **T** | **T** | **A** | **C** | **G** | **A** | **T** | **C** | **C** | **T** | **G** | **A** | **G** | **T** | **T** | **T** | **T** | **T** | **A** | **G** | **A** | **A** | **G** | **A** | **A** | **C** | **T** | **T** | **G** | **C** | **T** | **A** | **C** | **T** | **C** | **A** | **G** | **T** | **T** | **C** | **A** | **A** | **A** | **G** | **A** | **A** | **G** | **A** | **T** | **G** | **A** | **G** | **A** | **C** | **A** | **C** | **A** | **T** | **T** | **G** | **T** | **T** | **C** | **A** | **T** | **C** | **C** | **A** | **T** | **G** | **A** | **C** | **A** | **A** | **C** | **T** | **G** | **A** | **A** | **A** | **C** | **A** | **G** | **T** | **C** | **A** | **A** | **G** | **G** | **A** | **A** | **A** | **T** | **G** | **T** | **C** | **T** | **A** | **A** | **C** | **T** | **G** | **G** | **C** | **T** | **A** | **T** | **T** | **A** | **T** | **T** | **C** | **A** | **A** | **G** | **C** | **A** | **A** | **C** | **A** | **A** | **A** | **T** | **T** | **T** | **C** | **T** | **C** | **G** | **A** | **T** | **T** | **A** | **T** | **T** | **T** | **T** | **C** | **T** | **C** | **C** | **A** | **A** | **T** | **C** | **C** | **T** | **A** | **-** | **-** | **-** |
| **N54070** | **-** | **-** | **-** | **-** | **-** | **-** | **-** | **-** | **-** | **-** | **-** | **-** | **-** | **-** | **-** | **-** | **-** | **-** | **-** | **-** | **-** | **-** | **-** | **-** | **-** | **-** | **-** | **-** | **-** | **-** | **-** | **-** | **-** | **-** | **-** | **-** | **-** | **-** | **-** | **-** | **-** | **-** | **-** | **-** | **-** | **-** | **-** | **-** | **-** | **-** | **-** | **-** | **-** | **-** | **-** | **-** | **-** | **-** | **-** | **-** | **-** | **-** | **-** | **-** | **-** | **-** | **-** | **-** | **-** | **A** | **A** | **A** | **C** | **C** | **C** | **C** | **G** | **A** | **G** | **A** | **T** | **G** | **T** | **A** | **G** | **C** | **A** | **G** | **C** | **C** | **A** | **T** | **T** | **T** | **G** | **G** | **G** | **C** | **A** | **T** | **G** | **G** | **C** | **T** | **C** | **T** | **T** | **A** | **G** | **T** | **A** | **T** | **T** | **G** | **T** | **T** | **T** | **G** | **C** | **C** | **C** | **T** | **T** | **C** | **C** | **T** | **G** | **G** | **G** | **G** | **T** | **G** | **G** | **G** | **T** | **T** | **T** | **T** | **G** | **T** | **A** | **T** | **T** | **A** | **T** | **A** | **T** | **T** | **T** | **A** | **T** | **T** | **T** | **T** | **T** | **C** | **G** | **G** | **A** | **C** | **G** | **T** | **G** | **G** | **A** | **T** | **T** | **A** | **A** | **C** | **G** | **G** | **A** | **T** | **A** | **A** | **G** | **A** | **A** | **A** | **A** | **A** | **A** | **T** | **T** | **C** | **T** | **A** | **T** | **C** | **T** | **C** | **C** | **G** | **A** | **C** | **A** | **A** | **A** | **G** | **T** | **G** | **A** | **C** | **T** | **T** | **G** | **A** | **A** | **A** | **G** | **A** | **A** | **T** | **T** | **A** | **G** | **A** | **A** | **A** | **A** | **C** | **T** | **T** | **T** | **C** | **A** | **G** | **T** | **C** | **T** | **T** | **T** | **T** | **A** | **G** | **A** | **G** | **G** | **A** | **G** | **A** | **T** | **A** | **C** | **C** | **A** | **T** | **T** | **G** | **A** | **A** | **C** | **A** | **T** | **T** | **A** | **C** | **G** | **A** | **T** | **C** | **C** | **T** | **G** | **A** | **C** | **A** | **T** | **G** | **G** | **G** | **C** | **G** | **A** | **T** | **A** | **A** | **A** | **G** | **A** | **C** | **A** | **A** | **A** | **C** | **A** | **G** | **C** | **A** | **A** | **T** | **T** | **T** | **G** | **T** | **C** | **G** | **A** | **C** | **T** | **T** | **C** | **T** | **T** | **C** | **T** | **C** | **T** | **T** | **C** | **A** | **T** | **T** | **A** | **A** | **A** | **T** | **C** | **G** | **T** | **A** | **T** | **G** | **C** | **C** | **G** | **C** | **T** | **G** | **A** | **C** | **A** | **A** | **G** | **A** | **A** | **T** | **G** | **A** | **A** | **T** | **T** | **C** | **T** | **G** | **T** | **C** | **A** | **C** | **T** | **C** | **T** | **T** | **C** | **T** | **C** | **A** | **C** | **A** | **G** | **A** | **C** | **G** | **G** | **A** | **C** | **A** | **A** | **G** | **A** | **G** | **A** | **A** | **A** | **T** | **T** | **G** | **G** | **A** | **T** | **T** | **C** | **A** | **C** | **T** | **G** | **C** | **T** | **T** | **C** | **A** | **A** | **G** | **A** | **T** | **C** | **T** | **A** | **A** | **A** | **A** | **A** | **A** | **A** | **G** | **C** | **C** | **A** | **A** | **A** | **C** | **A** | **T** | **T** | **C** | **G** | **A** | **T** | **C** | **C** | **A** | **T** | **A** | **T** | **C** | **G** | **A** | **A** | **T** | **A** | **T** | **T** | **A** | **C** | **G** | **C** | **A** | **T** | **T** | **T** | **G** | **T** | **G** | **A** | **C** | **A** | **G** | **A** | **T** | **A** | **A** | **T** | **A** | **T** | **C** | **G** | **G** | **C** | **C** | **A** | **G** | **C** | **A** | **A** | **G** | **T** | **C** | **T** | **T** | **A** | **C** | **A** | **T** | **G** | **T** | **T** | **T** | **T** | **A** | **G** | **A** | **A** | **G** | **A** | **A** | **A** | **A** | **A** | **G** | **C** | **C** | **G** | **C** | **A** | **G** | **A** | **A** | **G** | **G** | **C** | **G** | **T** | **G** | **G** | **A** | **A** | **G** | **T** | **T** | **C** | **G** | **A** | **A** | **T** | **A** | **T** | **T** | **A** | **T** | **A** | **T** | **G** | **A** | **T** | **G** | **C** | **A** | **T** | **T** | **T** | **G** | **G** | **C** | **T** | **C** | **T** | **C** | **A** | **T** | **G** | **G** | **C** | **A** | **C** | **A** | **A** | **A** | **A** | **G** | **C** | **A** | **A** | **A** | **A** | **G** | **A** | **T** | **T** | **T** | **C** | **A** | **A** | **T** | **C** | **G** | **T** | **C** | **T** | **A** | **A** | **T** | **C** | **A** | **A** | **A** | **A** | **A** | **T** | **G** | **G** | **T** | **G** | **G** | **A** | **C** | **A** | **T** | **G** | **T** | **C** | **C** | **A** | **T** | **A** | **C** | **A** | **T** | **T** | **T** | **G** | **T** | **T** | **A** | **C** | **C** | **T** | **C** | **A** | **C** | **A** | **A** | **A** | **A** | **G** | **G** | **C** | **A** | **T** | **T** | **A** | **C** | **T** | **T** | **C** | **G** | **T** | **T** | **T** | **C** | **C** | **G** | **A** | **T** | **T** | **G** | **A** | **A** | **T** | **T** | **A** | **C** | **C** | **A** | **T** | **G** | **A** | **T** | **C** | **A** | **C** | **C** | **G** | **A** | **A** | **A** | **A** | **A** | **T** | **C** | **G** | **T** | **T** | **G** | **T** | **G** | **A** | **T** | **C** | **G** | **A** | **T** | **G** | **G** | **A** | **A** | **A** | **G** | **A** | **T** | **T** | **A** | **G** | **T** | **T** | **A** | **T** | **A** | **C** | **C** | **G** | **G** | **T** | **G** | **G** | **T** | **T** | **T** | **C** | **A** | **A** | **T** | **A** | **T** | **T** | **G** | **C** | **C** | **A** | **A** | **T** | **C** | **A** | **A** | **T** | **A** | **T** | **G** | **T** | **A** | **A** | **A** | **T** | **A** | **C** | **A** | **A** | **C** | **A** | **A** | **A** | **A** | **A** | **A** | **A** | **T** | **T** | **C** | **G** | **G** | **C** | **T** | **A** | **T** | **T** | **G** | **G** | **C** | **G** | **C** | **G** | **A** | **T** | **A** | **C** | **G** | **C** | **A** | **T** | **A** | **T** | **A** | **C** | **G** | **G** | **A** | **T** | **T** | **T** | **T** | **C** | **G** | **G** | **T** | **G** | **C** | **C** | **G** | **C** | **T** | **T** | **C** | **T** | **T** | **C** | **T** | **T** | **T** | **G** | **C** | **T** | **C** | **C** | **A** | **G** | **C** | **T** | **T** | **C** | **G** | **C** | **T** | **T** | **C** | **T** | **T** | **A** | **A** | **C** | **A** | **G** | **A** | **C** | **T** | **G** | **G** | **A** | **A** | **C** | **G** | **T** | **C** | **T** | **C** | **G** | **G** | **T** | **A** | **C** | **C** | **T** | **G** | **A** | **A** | **G** | **A** | **A** | **A** | **A** | **A** | **A** | **A** | **G** | **G** | **T** | **C** | **G** | **G** | **C** | **T** | **A** | **T** | **C** | **A** | **T** | **T** | **T** | **G** | **A** | **A** | **T** | **T** | **A** | **T** | **T** | **T** | **C** | **T** | **T** | **T** | **A** | **A** | **A** | **A** | **A** | **A** | **G** | **C** | **A** | **G** | **A** | **T** | **C** | **G** | **A** | **G** | **A** | **T** | **G** | **A** | **A** | **T** | **C** | **T** | **A** | **A** | **G** | **C** | **T** | **T** | **G** | **C** | **T** | **G** | **A** | **T** | **A** | **C** | **A** | **T** | **C** | **C** | **A** | **T** | **C** | **C** | **A** | **G** | **C** | **T** | **T** | **G** | **T** | **T** | **T** | **C** | **A** | **A** | **G** | **C** | **G** | **G** | **A** | **C** | **C** | **G** | **A** | **A** | **T** | **A** | **A** | **C** | **G** | **A** | **A** | **A** | **G** | **G** | **G** | **A** | **A** | **C** | **A** | **A** | **A** | **T** | **C** | **A** | **A** | **G** | **C** | **T** | **T** | **T** | **C** | **A** | **T** | **T** | **T** | **A** | **T** | **C** | **A** | **A** | **A** | **T** | **T** | **G** | **A** | **T** | **T** | **A** | **C** | **T** | **T** | **C** | **T** | **G** | **C** | **T** | **A** | **A** | **A** | **A** | **A** | **A** | **C** | **G** | **T** | **G** | **T** | **T** | **T** | **G** | **G** | **A** | **T** | **A** | **C** | **A** | **G** | **A** | **C** | **A** | **C** | **C** | **T** | **T** | **A** | **C** | **C** | **T** | **T** | **G** | **T** | **T** | **C** | **C** | **T** | **G** | **A** | **T** | **G** | **A** | **A** | **A** | **G** | **T** | **G** | **T** | **C** | **A** | **T** | **T** | **G** | **C** | **T** | **G** | **C** | **T** | **T** | **T** | **A** | **A** | **A** | **A** | **A** | **T** | **C** | **G** | **C** | **A** | **A** | **C** | **T** | **G** | **C** | **C** | **T** | **C** | **T** | **G** | **G** | **T** | **G** | **T** | **A** | **G** | **A** | **T** | **G** | **T** | **G** | **A** | **A** | **A** | **A** | **T** | **C** | **A** | **T** | **G** | **A** | **T** | **T** | **C** | **C** | **C** | **A** | **A** | **C** | **A** | **A** | **A** | **C** | **C** | **G** | **G** | **A** | **T** | **C** | **A** | **T** | **C** | **C** | **T** | **T** | **T** | **T** | **A** | **T** | **T** | **T** | **A** | **T** | **C** | **G** | **A** | **G** | **C** | **A** | **A** | **C** | **A** | **C** | **A** | **A** | **T** | **A** | **T** | **T** | **A** | **T** | **G** | **C** | **T** | **C** | **G** | **G** | **C** | **A** | **G** | **C** | **T** | **G** | **A** | **T** | **C** | **A** | **A** | **G** | **G** | **A** | **A** | **A** | **A** | **T** | **G** | **T** | **A** | **C** | **A** | **A** | **A** | **T** | **C** | **C** | **T** | **T** | **G** | **T** | **C** | **T** | **A** | **T** | **G** | **A** | **G** | **A** | **A** | **C** | **G** | **G** | **C** | **T** | **T** | **C** | **C** | **T** | **C** | **C** | **A** | **T** | **G** | **C** | **A** | **A** | **A** | **A** | **A** | **C** | **A** | **T** | **T** | **G** | **A** | **T** | **A** | **A** | **T** | **G** | **G** | **A** | **T** | **G** | **A** | **T** | **G** | **A** | **A** | **A** | **T** | **C** | **T** | **G** | **C** | **A** | **T** | **G** | **G** | **T** | **A** | **G** | **G** | **T** | **T** | **C** | **A** | **G** | **C** | **A** | **A** | **A** | **T** | **C** | **A** | **A** | **G** | **A** | **T** | **A** | **T** | **T** | **C** | **G** | **T** | **A** | **G** | **C** | **T** | **A** | **C** | **C** | **G** | **A** | **T** | **T** | **G** | **A** | **A** | **T** | **T** | **T** | **T** | **G** | **A** | **A** | **A** | **C** | **A** | **A** | **G** | **T** | **G** | **C** | **T** | **G** | **T** | **C** | **A** | **T** | **T** | **T** | **A** | **C** | **G** | **A** | **T** | **C** | **C** | **T** | **G** | **A** | **G** | **T** | **T** | **T** | **T** | **T** | **A** | **G** | **A** | **A** | **G** | **A** | **A** | **C** | **T** | **T** | **G** | **C** | **T** | **A** | **C** | **T** | **C** | **A** | **G** | **T** | **T** | **C** | **A** | **A** | **A** | **G** | **A** | **A** | **G** | **A** | **T** | **G** | **A** | **G** | **A** | **C** | **A** | **C** | **A** | **T** | **T** | **G** | **T** | **T** | **C** | **A** | **T** | **C** | **C** | **A** | **T** | **G** | **A** | **C** | **A** | **A** | **C** | **T** | **G** | **A** | **A** | **A** | **C** | **A** | **G** | **T** | **C** | **A** | **A** | **G** | **G** | **A** | **A** | **A** | **T** | **G** | **T** | **C** | **T** | **A** | **A** | **C** | **T** | **G** | **G** | **C** | **T** | **A** | **T** | **T** | **A** | **T** | **T** | **C** | **A** | **A** | **G** | **C** | **A** | **A** | **C** | **A** | **A** | **A** | **T** | **T** | **T** | **C** | **T** | **C** | **G** | **A** | **T** | **T** | **A** | **T** | **T** | **T** | **T** | **C** | **T** | **C** | **C** | **A** | **A** | **T** | **C** | **C** | **T** | **A** | **-** | **-** | **-** |
| **N59624F** | **-** | **-** | **-** | **-** | **-** | **-** | **-** | **-** | **-** | **-** | **-** | **-** | **-** | **-** | **-** | **-** | **-** | **-** | **-** | **-** | **-** | **-** | **-** | **-** | **-** | **-** | **-** | **-** | **-** | **-** | **-** | **-** | **-** | **-** | **-** | **-** | **-** | **-** | **-** | **-** | **-** | **-** | **-** | **-** | **-** | **-** | **-** | **-** | **-** | **-** | **-** | **-** | **-** | **-** | **-** | **-** | **-** | **-** | **-** | **-** | **-** | **-** | **-** | **-** | **-** | **-** | **-** | **-** | **-** | **A** | **A** | **A** | **C** | **C** | **C** | **C** | **G** | **A** | **G** | **A** | **T** | **G** | **T** | **A** | **G** | **C** | **A** | **G** | **C** | **T** | **A** | **T** | **T** | **T** | **G** | **G** | **G** | **C** | **A** | **T** | **G** | **G** | **C** | **T** | **C** | **T** | **T** | **A** | **G** | **T** | **A** | **T** | **T** | **G** | **T** | **T** | **T** | **G** | **C** | **C** | **C** | **T** | **T** | **C** | **C** | **T** | **G** | **G** | **G** | **G** | **T** | **G** | **G** | **G** | **T** | **T** | **T** | **T** | **G** | **T** | **A** | **T** | **T** | **A** | **T** | **A** | **T** | **T** | **T** | **A** | **T** | **T** | **T** | **T** | **T** | **C** | **G** | **G** | **A** | **C** | **G** | **T** | **G** | **G** | **A** | **T** | **T** | **A** | **A** | **C** | **G** | **G** | **A** | **T** | **A** | **A** | **G** | **A** | **A** | **A** | **A** | **A** | **A** | **T** | **T** | **C** | **T** | **A** | **T** | **C** | **T** | **C** | **C** | **G** | **A** | **C** | **A** | **A** | **A** | **G** | **T** | **G** | **A** | **C** | **T** | **T** | **G** | **A** | **A** | **A** | **G** | **A** | **A** | **T** | **T** | **A** | **G** | **A** | **A** | **A** | **A** | **C** | **T** | **T** | **T** | **C** | **A** | **G** | **T** | **C** | **T** | **T** | **T** | **T** | **A** | **G** | **A** | **G** | **G** | **A** | **G** | **A** | **T** | **A** | **C** | **C** | **A** | **T** | **T** | **G** | **A** | **A** | **C** | **A** | **T** | **T** | **A** | **C** | **G** | **A** | **T** | **C** | **C** | **T** | **G** | **A** | **C** | **A** | **T** | **G** | **G** | **G** | **C** | **G** | **A** | **C** | **A** | **A** | **A** | **G** | **A** | **C** | **A** | **A** | **A** | **C** | **A** | **A** | **C** | **A** | **A** | **T** | **T** | **T** | **G** | **T** | **C** | **G** | **A** | **C** | **T** | **T** | **C** | **T** | **T** | **C** | **T** | **C** | **T** | **T** | **C** | **A** | **T** | **T** | **A** | **A** | **A** | **T** | **C** | **G** | **T** | **A** | **T** | **G** | **C** | **C** | **G** | **C** | **T** | **G** | **A** | **C** | **A** | **A** | **G** | **A** | **A** | **T** | **G** | **A** | **A** | **T** | **T** | **C** | **T** | **G** | **T** | **C** | **A** | **C** | **T** | **C** | **T** | **T** | **C** | **T** | **C** | **A** | **C** | **A** | **G** | **A** | **C** | **G** | **G** | **A** | **C** | **A** | **A** | **A** | **A** | **G** | **A** | **A** | **A** | **T** | **T** | **A** | **G** | **A** | **T** | **T** | **C** | **A** | **C** | **T** | **G** | **C** | **T** | **T** | **C** | **A** | **A** | **G** | **A** | **T** | **C** | **T** | **A** | **A** | **A** | **A** | **A** | **A** | **A** | **G** | **C** | **C** | **A** | **A** | **A** | **C** | **A** | **T** | **T** | **C** | **G** | **A** | **T** | **C** | **C** | **A** | **T** | **A** | **T** | **C** | **G** | **A** | **A** | **T** | **A** | **T** | **T** | **A** | **C** | **G** | **C** | **A** | **T** | **T** | **T** | **G** | **T** | **G** | **A** | **C** | **A** | **G** | **A** | **T** | **A** | **A** | **T** | **A** | **T** | **C** | **G** | **G** | **T** | **C** | **A** | **G** | **C** | **A** | **A** | **G** | **T** | **C** | **T** | **T** | **A** | **C** | **G** | **C** | **G** | **T** | **T** | **T** | **T** | **A** | **G** | **A** | **A** | **G** | **A** | **A** | **A** | **A** | **A** | **G** | **C** | **C** | **G** | **C** | **A** | **G** | **A** | **A** | **G** | **G** | **C** | **G** | **T** | **A** | **G** | **A** | **A** | **G** | **T** | **T** | **C** | **G** | **T** | **A** | **T** | **A** | **T** | **T** | **A** | **T** | **A** | **T** | **G** | **A** | **T** | **G** | **C** | **A** | **T** | **T** | **T** | **G** | **G** | **C** | **T** | **C** | **C** | **C** | **A** | **T** | **G** | **G** | **C** | **A** | **C** | **A** | **A** | **A** | **A** | **G** | **C** | **A** | **A** | **A** | **A** | **G** | **A** | **T** | **T** | **T** | **C** | **A** | **A** | **T** | **C** | **G** | **T** | **C** | **T** | **A** | **A** | **T** | **C** | **A** | **A** | **A** | **A** | **A** | **T** | **G** | **G** | **T** | **G** | **G** | **A** | **C** | **A** | **T** | **G** | **T** | **C** | **C** | **A** | **T** | **A** | **C** | **A** | **T** | **T** | **T** | **G** | **T** | **T** | **A** | **C** | **C** | **T** | **C** | **G** | **C** | **A** | **A** | **A** | **A** | **A** | **G** | **C** | **A** | **T** | **T** | **G** | **C** | **T** | **T** | **C** | **G** | **T** | **T** | **T** | **C** | **C** | **G** | **A** | **T** | **T** | **G** | **A** | **A** | **T** | **T** | **A** | **C** | **C** | **A** | **T** | **G** | **A** | **T** | **C** | **A** | **C** | **C** | **G** | **A** | **A** | **A** | **A** | **A** | **T** | **C** | **G** | **T** | **T** | **G** | **T** | **G** | **A** | **T** | **C** | **G** | **A** | **T** | **G** | **G** | **A** | **A** | **A** | **G** | **A** | **T** | **C** | **A** | **G** | **T** | **T** | **A** | **C** | **A** | **C** | **C** | **G** | **G** | **T** | **G** | **G** | **T** | **T** | **T** | **C** | **A** | **A** | **T** | **A** | **T** | **T** | **G** | **C** | **C** | **A** | **A** | **T** | **C** | **A** | **A** | **T** | **A** | **T** | **G** | **T** | **A** | **A** | **A** | **T** | **A** | **C** | **G** | **A** | **C** | **A** | **A** | **A** | **A** | **A** | **A** | **A** | **T** | **T** | **C** | **G** | **G** | **T** | **T** | **A** | **T** | **T** | **G** | **G** | **C** | **G** | **T** | **G** | **A** | **T** | **A** | **C** | **G** | **C** | **A** | **T** | **A** | **T** | **A** | **C** | **G** | **A** | **A** | **T** | **T** | **T** | **T** | **C** | **G** | **G** | **T** | **G** | **C** | **T** | **G** | **C** | **T** | **T** | **C** | **T** | **T** | **C** | **T** | **T** | **T** | **G** | **C** | **T** | **T** | **C** | **A** | **A** | **C** | **T** | **T** | **C** | **G** | **T** | **T** | **T** | **T** | **T** | **T** | **A** | **A** | **C** | **A** | **G** | **A** | **C** | **T** | **G** | **G** | **A** | **A** | **C** | **G** | **T** | **T** | **T** | **C** | **A** | **G** | **T** | **G** | **T** | **C** | **T** | **G** | **A** | **A** | **G** | **A** | **A** | **A** | **A** | **A** | **A** | **A** | **G** | **G** | **T** | **C** | **G** | **G** | **C** | **T** | **A** | **T** | **C** | **A** | **T** | **T** | **T** | **G** | **A** | **A** | **T** | **T** | **A** | **T** | **T** | **T** | **C** | **T** | **T** | **T** | **A** | **A** | **A** | **A** | **A** | **A** | **G** | **C** | **C** | **G** | **A** | **T** | **C** | **G** | **A** | **G** | **A** | **T** | **G** | **A** | **A** | **T** | **C** | **C** | **A** | **A** | **A** | **C** | **T** | **T** | **G** | **C** | **T** | **G** | **A** | **T** | **A** | **C** | **G** | **T** | **C** | **C** | **A** | **T** | **C** | **C** | **A** | **G** | **C** | **T** | **T** | **G** | **T** | **C** | **T** | **C** | **A** | **A** | **G** | **C** | **G** | **G** | **A** | **C** | **C** | **A** | **A** | **A** | **T** | **A** | **A** | **C** | **G** | **A** | **A** | **C** | **G** | **A** | **G** | **A** | **A** | **C** | **A** | **A** | **A** | **T** | **C** | **A** | **A** | **G** | **C** | **T** | **T** | **T** | **C** | **G** | **T** | **T** | **T** | **A** | **T** | **C** | **A** | **A** | **A** | **T** | **T** | **G** | **A** | **T** | **T** | **A** | **C** | **T** | **T** | **C** | **T** | **G** | **C** | **T** | **A** | **A** | **A** | **A** | **A** | **A** | **C** | **G** | **T** | **G** | **T** | **T** | **T** | **G** | **G** | **A** | **T** | **A** | **C** | **A** | **G** | **A** | **C** | **A** | **C** | **C** | **T** | **T** | **A** | **C** | **C** | **T** | **C** | **G** | **T** | **T** | **C** | **C** | **T** | **G** | **A** | **T** | **G** | **A** | **A** | **A** | **G** | **T** | **G** | **T** | **C** | **A** | **T** | **T** | **G** | **C** | **T** | **G** | **C** | **T** | **T** | **T** | **G** | **A** | **A** | **A** | **A** | **T** | **C** | **G** | **C** | **A** | **A** | **C** | **T** | **G** | **C** | **T** | **T** | **C** | **T** | **G** | **G** | **T** | **G** | **T** | **C** | **G** | **A** | **T** | **G** | **T** | **G** | **A** | **A** | **A** | **A** | **T** | **C** | **A** | **T** | **G** | **A** | **T** | **C** | **C** | **C** | **C** | **A** | **A** | **T** | **A** | **A** | **A** | **C** | **C** | **G** | **G** | **A** | **T** | **C** | **A** | **T** | **C** | **C** | **T** | **T** | **T** | **T** | **A** | **T** | **T** | **T** | **A** | **T** | **C** | **G** | **A** | **G** | **C** | **A** | **A** | **C** | **A** | **C** | **A** | **A** | **T** | **A** | **T** | **T** | **A** | **T** | **G** | **C** | **T** | **C** | **G** | **T** | **C** | **A** | **G** | **C** | **T** | **C** | **A** | **T** | **C** | **A** | **A** | **G** | **G** | **A** | **A** | **A** | **A** | **T** | **G** | **T** | **A** | **C** | **A** | **G** | **A** | **T** | **C** | **C** | **T** | **T** | **G** | **T** | **C** | **T** | **A** | **T** | **G** | **A** | **G** | **A** | **A** | **T** | **G** | **G** | **C** | **T** | **T** | **C** | **C** | **T** | **C** | **C** | **A** | **T** | **G** | **C** | **A** | **A** | **A** | **A** | **A** | **C** | **A** | **T** | **T** | **A** | **A** | **T** | **A** | **A** | **T** | **G** | **G** | **A** | **T** | **G** | **A** | **T** | **G** | **A** | **A** | **A** | **T** | **C** | **T** | **G** | **C** | **A** | **T** | **G** | **G** | **T** | **A** | **G** | **G** | **T** | **T** | **C** | **A** | **G** | **C** | **A** | **A** | **A** | **T** | **C** | **A** | **A** | **G** | **A** | **T** | **A** | **T** | **T** | **C** | **G** | **T** | **A** | **G** | **C** | **T** | **A** | **C** | **C** | **G** | **A** | **T** | **T** | **G** | **A** | **A** | **T** | **T** | **T** | **T** | **G** | **A** | **A** | **A** | **C** | **A** | **A** | **G** | **T** | **G** | **C** | **T** | **G** | **T** | **C** | **A** | **T** | **T** | **T** | **A** | **C** | **G** | **A** | **T** | **C** | **C** | **T** | **G** | **A** | **G** | **T** | **T** | **T** | **T** | **T** | **A** | **G** | **A** | **A** | **G** | **A** | **G** | **C** | **T** | **T** | **G** | **C** | **T** | **A** | **C** | **T** | **C** | **A** | **G** | **T** | **T** | **C** | **A** | **A** | **A** | **G** | **A** | **A** | **G** | **A** | **T** | **G** | **A** | **G** | **A** | **C** | **A** | **C** | **A** | **T** | **T** | **G** | **T** | **T** | **C** | **A** | **C** | **C** | **A** | **A** | **T** | **G** | **A** | **C** | **A** | **A** | **C** | **T** | **G** | **A** | **A** | **A** | **C** | **A** | **G** | **T** | **G** | **A** | **A** | **G** | **G** | **A** | **A** | **A** | **T** | **G** | **T** | **C** | **T** | **A** | **A** | **C** | **T** | **G** | **G** | **C** | **T** | **G** | **T** | **T** | **A** | **T** | **T** | **C** | **A** | **A** | **A** | **C** | **A** | **A** | **C** | **A** | **A** | **A** | **T** | **T** | **T** | **C** | **T** | **C** | **G** | **A** | **T** | **T** | **G** | **T** | **T** | **T** | **T** | **C** | **T** | **C** | **C** | **A** | **A** | **T** | **C** | **T** | **T** | **A** | **-** | **-** | **-** |
| **N60284F** | **G** | **T** | **G** | **G** | **T** | **A** | **T** | **C** | **T** | **A** | **G** | **T** | **A** | **T** | **T** | **A** | **T** | **A** | **A** | **C** | **C** | **G** | **G** | **C** | **C** | **T** | **T** | **T** | **A** | **T** | **C** | **T** | **A** | **T** | **T** | **A** | **A** | **A** | **T** | **G** | **C** | **A** | **C** | **T** | **T** | **A** | **T** | **T** | **G** | **C** | **T** | **T** | **T** | **G** | **G** | **T** | **G** | **G** | **C** | **T** | **A** | **T** | **T** | **T** | **T** | **G** | **A** | **T** | **C** | **A** | **A** | **A** | **C** | **C** | **C** | **C** | **G** | **A** | **G** | **A** | **T** | **G** | **T** | **A** | **G** | **C** | **A** | **G** | **C** | **C** | **A** | **T** | **T** | **T** | **G** | **G** | **G** | **C** | **A** | **T** | **G** | **G** | **C** | **T** | **C** | **T** | **T** | **A** | **G** | **T** | **A** | **T** | **T** | **G** | **T** | **T** | **T** | **G** | **C** | **C** | **C** | **T** | **T** | **C** | **C** | **T** | **G** | **G** | **G** | **G** | **T** | **G** | **G** | **G** | **T** | **T** | **T** | **T** | **G** | **T** | **A** | **T** | **T** | **A** | **T** | **A** | **T** | **T** | **T** | **A** | **T** | **T** | **T** | **T** | **T** | **C** | **G** | **G** | **A** | **C** | **G** | **T** | **G** | **G** | **A** | **T** | **T** | **A** | **A** | **C** | **G** | **G** | **A** | **T** | **A** | **A** | **G** | **A** | **A** | **A** | **A** | **A** | **A** | **T** | **T** | **C** | **T** | **A** | **T** | **C** | **T** | **C** | **C** | **G** | **A** | **C** | **A** | **A** | **A** | **G** | **T** | **G** | **A** | **C** | **T** | **T** | **G** | **A** | **A** | **A** | **G** | **A** | **A** | **T** | **T** | **A** | **G** | **A** | **A** | **A** | **A** | **C** | **T** | **T** | **T** | **C** | **A** | **G** | **T** | **C** | **T** | **T** | **T** | **T** | **A** | **G** | **A** | **G** | **G** | **A** | **G** | **A** | **T** | **A** | **C** | **C** | **A** | **T** | **T** | **G** | **A** | **A** | **C** | **A** | **T** | **T** | **A** | **C** | **G** | **A** | **T** | **C** | **C** | **T** | **G** | **A** | **C** | **A** | **T** | **G** | **G** | **G** | **C** | **G** | **A** | **T** | **A** | **A** | **A** | **G** | **A** | **C** | **A** | **A** | **A** | **C** | **A** | **G** | **C** | **A** | **A** | **T** | **T** | **T** | **G** | **T** | **C** | **G** | **A** | **C** | **T** | **T** | **C** | **T** | **T** | **C** | **T** | **C** | **T** | **T** | **C** | **A** | **T** | **T** | **A** | **A** | **A** | **T** | **C** | **G** | **T** | **A** | **T** | **G** | **C** | **C** | **G** | **C** | **T** | **G** | **A** | **C** | **A** | **A** | **G** | **A** | **A** | **T** | **G** | **A** | **A** | **T** | **T** | **C** | **T** | **G** | **T** | **C** | **A** | **C** | **T** | **C** | **T** | **T** | **C** | **T** | **C** | **A** | **C** | **A** | **G** | **A** | **C** | **G** | **G** | **A** | **C** | **A** | **A** | **G** | **A** | **G** | **A** | **A** | **A** | **T** | **T** | **G** | **G** | **A** | **T** | **T** | **C** | **A** | **C** | **T** | **G** | **C** | **T** | **T** | **C** | **A** | **A** | **G** | **A** | **T** | **C** | **T** | **A** | **A** | **A** | **A** | **A** | **A** | **A** | **G** | **C** | **C** | **A** | **A** | **A** | **C** | **A** | **T** | **T** | **C** | **G** | **A** | **T** | **C** | **C** | **A** | **T** | **A** | **T** | **C** | **G** | **A** | **A** | **T** | **A** | **T** | **T** | **A** | **C** | **G** | **C** | **A** | **T** | **T** | **T** | **G** | **T** | **G** | **A** | **C** | **A** | **G** | **A** | **T** | **A** | **A** | **T** | **A** | **T** | **C** | **G** | **G** | **C** | **C** | **A** | **G** | **C** | **A** | **A** | **G** | **T** | **C** | **T** | **T** | **A** | **C** | **A** | **T** | **G** | **T** | **T** | **T** | **T** | **A** | **G** | **A** | **A** | **G** | **A** | **A** | **A** | **A** | **A** | **G** | **C** | **C** | **G** | **C** | **A** | **G** | **A** | **A** | **G** | **G** | **C** | **G** | **T** | **G** | **G** | **A** | **A** | **G** | **T** | **T** | **C** | **G** | **A** | **A** | **T** | **A** | **T** | **T** | **A** | **T** | **A** | **T** | **G** | **A** | **T** | **G** | **C** | **A** | **T** | **T** | **T** | **G** | **G** | **C** | **T** | **C** | **T** | **C** | **A** | **T** | **G** | **G** | **C** | **A** | **C** | **A** | **A** | **A** | **A** | **G** | **C** | **A** | **A** | **A** | **A** | **G** | **A** | **T** | **T** | **T** | **C** | **A** | **A** | **T** | **C** | **G** | **T** | **C** | **T** | **A** | **A** | **T** | **C** | **A** | **A** | **A** | **A** | **A** | **T** | **G** | **G** | **T** | **G** | **G** | **A** | **C** | **A** | **T** | **G** | **T** | **C** | **C** | **A** | **T** | **A** | **C** | **A** | **T** | **T** | **T** | **G** | **T** | **T** | **A** | **C** | **C** | **T** | **C** | **A** | **C** | **A** | **A** | **A** | **A** | **G** | **G** | **C** | **A** | **T** | **T** | **A** | **C** | **T** | **T** | **C** | **G** | **T** | **T** | **T** | **C** | **C** | **G** | **A** | **T** | **T** | **G** | **A** | **A** | **T** | **T** | **A** | **C** | **C** | **A** | **T** | **G** | **A** | **T** | **C** | **A** | **C** | **C** | **G** | **A** | **A** | **A** | **A** | **A** | **T** | **C** | **G** | **T** | **T** | **G** | **T** | **G** | **A** | **T** | **C** | **G** | **A** | **T** | **G** | **G** | **A** | **A** | **A** | **G** | **A** | **T** | **T** | **A** | **G** | **T** | **T** | **A** | **T** | **A** | **C** | **C** | **G** | **G** | **T** | **G** | **G** | **T** | **T** | **T** | **C** | **A** | **A** | **T** | **A** | **T** | **T** | **G** | **C** | **C** | **A** | **A** | **T** | **C** | **A** | **A** | **T** | **A** | **T** | **G** | **T** | **A** | **A** | **A** | **T** | **A** | **C** | **A** | **A** | **C** | **A** | **A** | **A** | **A** | **A** | **A** | **A** | **T** | **T** | **C** | **G** | **G** | **C** | **T** | **A** | **T** | **T** | **G** | **G** | **C** | **G** | **C** | **G** | **A** | **T** | **A** | **C** | **G** | **C** | **A** | **T** | **A** | **T** | **A** | **C** | **G** | **G** | **A** | **T** | **T** | **T** | **T** | **C** | **G** | **G** | **T** | **G** | **C** | **C** | **G** | **C** | **T** | **T** | **C** | **T** | **T** | **C** | **T** | **T** | **T** | **G** | **C** | **T** | **C** | **C** | **A** | **G** | **C** | **T** | **T** | **C** | **G** | **C** | **T** | **T** | **C** | **T** | **T** | **A** | **A** | **C** | **A** | **G** | **A** | **C** | **T** | **G** | **G** | **A** | **A** | **C** | **G** | **T** | **C** | **T** | **C** | **G** | **G** | **T** | **A** | **C** | **C** | **T** | **G** | **A** | **A** | **G** | **A** | **A** | **A** | **A** | **A** | **A** | **A** | **G** | **G** | **T** | **C** | **G** | **G** | **C** | **T** | **A** | **T** | **C** | **A** | **T** | **T** | **T** | **G** | **A** | **A** | **T** | **T** | **A** | **T** | **T** | **T** | **C** | **T** | **T** | **T** | **A** | **A** | **A** | **A** | **A** | **A** | **G** | **C** | **A** | **G** | **A** | **T** | **C** | **G** | **A** | **G** | **A** | **T** | **G** | **A** | **A** | **T** | **C** | **T** | **A** | **A** | **G** | **C** | **T** | **T** | **G** | **C** | **T** | **G** | **A** | **T** | **A** | **C** | **A** | **T** | **C** | **C** | **A** | **T** | **C** | **C** | **A** | **G** | **C** | **T** | **T** | **G** | **T** | **T** | **T** | **C** | **A** | **A** | **G** | **C** | **G** | **G** | **A** | **C** | **C** | **G** | **A** | **A** | **T** | **A** | **A** | **C** | **G** | **A** | **A** | **A** | **G** | **G** | **G** | **A** | **A** | **C** | **A** | **A** | **A** | **T** | **C** | **A** | **A** | **G** | **C** | **T** | **T** | **T** | **C** | **A** | **T** | **T** | **T** | **A** | **T** | **C** | **A** | **A** | **A** | **T** | **T** | **G** | **A** | **T** | **T** | **A** | **C** | **T** | **T** | **C** | **T** | **G** | **C** | **T** | **A** | **A** | **A** | **A** | **A** | **A** | **C** | **G** | **T** | **G** | **T** | **T** | **T** | **G** | **G** | **A** | **T** | **A** | **C** | **A** | **G** | **A** | **C** | **A** | **C** | **C** | **T** | **T** | **A** | **C** | **C** | **T** | **T** | **G** | **T** | **T** | **C** | **C** | **T** | **G** | **A** | **T** | **G** | **A** | **A** | **A** | **G** | **T** | **G** | **T** | **C** | **A** | **T** | **T** | **G** | **C** | **T** | **G** | **C** | **T** | **T** | **T** | **A** | **A** | **A** | **A** | **A** | **T** | **C** | **G** | **C** | **A** | **A** | **C** | **T** | **G** | **C** | **C** | **T** | **C** | **T** | **G** | **G** | **T** | **G** | **T** | **A** | **G** | **A** | **T** | **G** | **T** | **G** | **A** | **A** | **A** | **A** | **T** | **C** | **A** | **T** | **G** | **A** | **T** | **T** | **C** | **C** | **C** | **A** | **A** | **C** | **A** | **A** | **A** | **C** | **C** | **G** | **G** | **A** | **T** | **C** | **A** | **T** | **C** | **C** | **T** | **T** | **T** | **T** | **A** | **T** | **T** | **T** | **A** | **T** | **C** | **G** | **A** | **G** | **C** | **A** | **A** | **C** | **A** | **C** | **A** | **A** | **T** | **A** | **T** | **T** | **A** | **T** | **G** | **C** | **T** | **C** | **G** | **G** | **C** | **A** | **G** | **C** | **T** | **G** | **A** | **T** | **C** | **A** | **A** | **G** | **G** | **A** | **A** | **A** | **A** | **T** | **G** | **T** | **A** | **C** | **A** | **A** | **A** | **T** | **C** | **C** | **T** | **T** | **G** | **T** | **C** | **T** | **A** | **T** | **G** | **A** | **G** | **A** | **A** | **C** | **G** | **G** | **C** | **T** | **T** | **C** | **C** | **T** | **C** | **C** | **A** | **T** | **G** | **C** | **A** | **A** | **A** | **A** | **A** | **C** | **A** | **T** | **T** | **G** | **A** | **T** | **A** | **A** | **T** | **G** | **G** | **A** | **T** | **G** | **A** | **T** | **G** | **A** | **A** | **A** | **T** | **C** | **T** | **G** | **C** | **A** | **T** | **G** | **G** | **T** | **A** | **G** | **G** | **T** | **T** | **C** | **A** | **G** | **C** | **A** | **A** | **A** | **T** | **C** | **A** | **A** | **G** | **A** | **T** | **A** | **T** | **T** | **C** | **G** | **T** | **A** | **G** | **C** | **T** | **A** | **C** | **C** | **G** | **A** | **T** | **T** | **G** | **A** | **A** | **T** | **T** | **T** | **T** | **G** | **A** | **A** | **A** | **C** | **A** | **A** | **G** | **T** | **G** | **C** | **T** | **G** | **T** | **C** | **A** | **T** | **T** | **T** | **A** | **C** | **G** | **A** | **T** | **C** | **C** | **T** | **G** | **A** | **G** | **T** | **T** | **T** | **T** | **T** | **A** | **G** | **A** | **A** | **G** | **A** | **A** | **C** | **T** | **T** | **G** | **C** | **T** | **A** | **C** | **T** | **C** | **A** | **G** | **T** | **T** | **C** | **A** | **A** | **A** | **G** | **A** | **A** | **G** | **A** | **T** | **G** | **A** | **G** | **A** | **C** | **A** | **C** | **A** | **T** | **T** | **G** | **T** | **T** | **C** | **A** | **T** | **C** | **C** | **A** | **T** | **G** | **A** | **C** | **A** | **A** | **C** | **T** | **G** | **A** | **A** | **A** | **C** | **A** | **G** | **T** | **C** | **A** | **A** | **G** | **G** | **A** | **A** | **A** | **T** | **G** | **T** | **C** | **T** | **A** | **A** | **C** | **T** | **G** | **G** | **C** | **T** | **A** | **T** | **T** | **A** | **T** | **T** | **C** | **A** | **A** | **G** | **C** | **A** | **A** | **C** | **A** | **A** | **A** | **T** | **T** | **T** | **C** | **T** | **C** | **G** | **A** | **T** | **T** | **A** | **T** | **T** | **T** | **T** | **C** | **T** | **C** | **C** | **A** | **A** | **T** | **C** | **C** | **T** | **A** | **-** | **-** | **-** |
| **N52732** | **-** | **-** | **-** | **-** | **-** | **-** | **-** | **-** | **-** | **-** | **-** | **-** | **-** | **-** | **-** | **-** | **-** | **-** | **-** | **-** | **-** | **-** | **-** | **-** | **-** | **-** | **-** | **-** | **-** | **-** | **-** | **-** | **-** | **-** | **-** | **-** | **-** | **-** | **-** | **-** | **-** | **-** | **-** | **-** | **-** | **-** | **-** | **-** | **-** | **-** | **-** | **-** | **-** | **-** | **-** | **-** | **-** | **-** | **-** | **-** | **-** | **-** | **-** | **-** | **-** | **-** | **-** | **-** | **-** | **A** | **A** | **A** | **C** | **C** | **C** | **C** | **G** | **A** | **G** | **A** | **T** | **G** | **T** | **A** | **G** | **C** | **A** | **G** | **C** | **T** | **A** | **T** | **T** | **T** | **G** | **G** | **G** | **C** | **A** | **T** | **G** | **G** | **C** | **T** | **C** | **T** | **T** | **A** | **G** | **T** | **A** | **T** | **T** | **A** | **T** | **T** | **T** | **G** | **C** | **C** | **C** | **T** | **T** | **C** | **C** | **T** | **G** | **G** | **G** | **G** | **T** | **G** | **G** | **G** | **T** | **T** | **T** | **T** | **G** | **T** | **A** | **T** | **T** | **A** | **T** | **A** | **T** | **T** | **T** | **A** | **T** | **T** | **T** | **T** | **T** | **C** | **G** | **G** | **A** | **C** | **G** | **T** | **G** | **G** | **A** | **T** | **T** | **A** | **A** | **C** | **G** | **G** | **A** | **T** | **A** | **A** | **G** | **A** | **A** | **A** | **A** | **A** | **A** | **T** | **T** | **C** | **T** | **A** | **T** | **C** | **T** | **C** | **C** | **G** | **A** | **C** | **A** | **A** | **A** | **G** | **T** | **G** | **A** | **C** | **T** | **T** | **G** | **A** | **A** | **A** | **G** | **A** | **A** | **T** | **T** | **A** | **G** | **A** | **A** | **A** | **A** | **C** | **T** | **T** | **T** | **C** | **A** | **G** | **T** | **C** | **T** | **T** | **T** | **T** | **A** | **G** | **A** | **G** | **G** | **A** | **G** | **A** | **T** | **A** | **C** | **C** | **A** | **T** | **T** | **G** | **A** | **A** | **C** | **A** | **T** | **T** | **A** | **C** | **G** | **A** | **T** | **C** | **C** | **T** | **G** | **A** | **C** | **A** | **T** | **G** | **G** | **G** | **C** | **G** | **A** | **C** | **A** | **A** | **A** | **G** | **A** | **C** | **A** | **A** | **A** | **C** | **A** | **A** | **C** | **A** | **A** | **T** | **T** | **T** | **G** | **T** | **C** | **G** | **A** | **C** | **T** | **T** | **C** | **T** | **T** | **C** | **T** | **C** | **T** | **T** | **C** | **A** | **T** | **T** | **A** | **A** | **A** | **T** | **C** | **G** | **T** | **A** | **T** | **G** | **C** | **C** | **G** | **C** | **T** | **G** | **A** | **C** | **A** | **A** | **G** | **A** | **A** | **T** | **G** | **A** | **A** | **T** | **T** | **C** | **T** | **G** | **T** | **C** | **A** | **C** | **T** | **C** | **T** | **T** | **C** | **T** | **C** | **A** | **C** | **A** | **G** | **A** | **C** | **G** | **G** | **A** | **C** | **A** | **A** | **A** | **A** | **G** | **A** | **A** | **A** | **T** | **T** | **A** | **G** | **A** | **T** | **T** | **C** | **A** | **C** | **T** | **G** | **C** | **T** | **T** | **C** | **A** | **A** | **G** | **A** | **T** | **C** | **T** | **A** | **A** | **A** | **A** | **A** | **A** | **A** | **G** | **C** | **C** | **A** | **A** | **A** | **C** | **A** | **T** | **T** | **C** | **G** | **A** | **T** | **C** | **C** | **A** | **T** | **A** | **T** | **C** | **G** | **A** | **A** | **T** | **A** | **T** | **T** | **A** | **C** | **G** | **C** | **A** | **T** | **T** | **T** | **G** | **T** | **G** | **A** | **C** | **A** | **G** | **A** | **T** | **A** | **C** | **T** | **A** | **T** | **C** | **G** | **G** | **T** | **C** | **A** | **G** | **C** | **A** | **A** | **G** | **T** | **C** | **T** | **T** | **A** | **C** | **G** | **C** | **G** | **T** | **T** | **T** | **T** | **A** | **G** | **A** | **A** | **G** | **A** | **A** | **A** | **A** | **A** | **G** | **C** | **C** | **G** | **C** | **A** | **G** | **A** | **A** | **G** | **G** | **C** | **G** | **T** | **A** | **G** | **A** | **A** | **G** | **T** | **T** | **C** | **G** | **T** | **A** | **T** | **A** | **T** | **T** | **A** | **T** | **A** | **T** | **G** | **A** | **T** | **G** | **C** | **A** | **T** | **T** | **T** | **G** | **G** | **C** | **T** | **C** | **C** | **C** | **A** | **C** | **G** | **G** | **C** | **A** | **C** | **A** | **A** | **A** | **A** | **G** | **C** | **A** | **A** | **A** | **A** | **G** | **A** | **T** | **T** | **T** | **C** | **A** | **A** | **T** | **C** | **G** | **T** | **C** | **T** | **A** | **A** | **T** | **C** | **A** | **A** | **A** | **A** | **A** | **T** | **G** | **G** | **T** | **G** | **G** | **A** | **C** | **A** | **T** | **G** | **T** | **C** | **C** | **A** | **T** | **A** | **C** | **A** | **T** | **T** | **T** | **G** | **T** | **T** | **A** | **C** | **C** | **T** | **C** | **G** | **C** | **A** | **A** | **A** | **A** | **A** | **G** | **C** | **A** | **T** | **T** | **G** | **C** | **T** | **T** | **C** | **G** | **T** | **T** | **T** | **C** | **C** | **G** | **A** | **T** | **T** | **G** | **A** | **A** | **C** | **T** | **A** | **C** | **C** | **A** | **T** | **G** | **A** | **T** | **C** | **A** | **C** | **C** | **G** | **G** | **A** | **A** | **A** | **A** | **T** | **C** | **G** | **T** | **T** | **G** | **T** | **G** | **A** | **T** | **C** | **G** | **A** | **T** | **G** | **G** | **A** | **A** | **A** | **G** | **A** | **T** | **C** | **A** | **G** | **T** | **T** | **A** | **C** | **A** | **C** | **C** | **G** | **G** | **T** | **G** | **G** | **C** | **T** | **T** | **C** | **A** | **A** | **C** | **A** | **T** | **T** | **G** | **C** | **C** | **A** | **A** | **T** | **C** | **A** | **A** | **T** | **A** | **T** | **G** | **T** | **A** | **A** | **A** | **T** | **A** | **C** | **G** | **A** | **C** | **A** | **A** | **A** | **A** | **A** | **A** | **A** | **T** | **T** | **C** | **G** | **G** | **C** | **T** | **A** | **T** | **T** | **G** | **G** | **C** | **G** | **T** | **G** | **A** | **T** | **A** | **C** | **G** | **C** | **A** | **T** | **A** | **T** | **A** | **C** | **G** | **G** | **A** | **T** | **T** | **T** | **T** | **C** | **G** | **G** | **T** | **G** | **C** | **C** | **G** | **C** | **T** | **T** | **C** | **T** | **T** | **C** | **T** | **T** | **T** | **G** | **C** | **T** | **T** | **C** | **A** | **A** | **C** | **T** | **T** | **C** | **G** | **T** | **T** | **T** | **T** | **T** | **T** | **A** | **A** | **C** | **A** | **G** | **A** | **C** | **T** | **G** | **G** | **A** | **A** | **C** | **G** | **T** | **C** | **T** | **C** | **A** | **G** | **T** | **T** | **C** | **C** | **T** | **G** | **A** | **A** | **G** | **A** | **A** | **A** | **A** | **A** | **A** | **A** | **G** | **G** | **T** | **C** | **G** | **G** | **C** | **T** | **A** | **T** | **C** | **A** | **T** | **T** | **T** | **G** | **A** | **A** | **T** | **T** | **A** | **T** | **T** | **T** | **C** | **T** | **T** | **T** | **A** | **A** | **A** | **A** | **A** | **A** | **G** | **C** | **C** | **G** | **A** | **T** | **C** | **G** | **A** | **G** | **A** | **C** | **G** | **A** | **A** | **T** | **C** | **C** | **A** | **A** | **A** | **C** | **T** | **T** | **G** | **C** | **T** | **G** | **A** | **T** | **A** | **C** | **A** | **T** | **C** | **C** | **A** | **T** | **C** | **C** | **A** | **G** | **C** | **T** | **T** | **G** | **T** | **C** | **T** | **C** | **A** | **A** | **G** | **C** | **G** | **G** | **A** | **C** | **C** | **A** | **A** | **A** | **T** | **A** | **A** | **C** | **G** | **A** | **A** | **C** | **G** | **C** | **G** | **A** | **A** | **C** | **A** | **A** | **A** | **T** | **C** | **A** | **A** | **G** | **C** | **T** | **T** | **T** | **C** | **G** | **T** | **T** | **T** | **A** | **T** | **C** | **A** | **A** | **A** | **T** | **T** | **G** | **A** | **T** | **T** | **A** | **C** | **T** | **T** | **C** | **T** | **G** | **C** | **T** | **A** | **A** | **A** | **A** | **A** | **A** | **C** | **G** | **T** | **G** | **T** | **T** | **T** | **G** | **G** | **A** | **T** | **A** | **C** | **A** | **G** | **A** | **C** | **G** | **C** | **C** | **T** | **T** | **A** | **C** | **C** | **T** | **C** | **G** | **T** | **T** | **C** | **C** | **T** | **G** | **A** | **T** | **G** | **A** | **A** | **A** | **G** | **T** | **G** | **T** | **C** | **A** | **T** | **T** | **G** | **C** | **T** | **G** | **C** | **T** | **T** | **T** | **G** | **A** | **A** | **A** | **A** | **T** | **C** | **G** | **C** | **A** | **A** | **C** | **T** | **G** | **C** | **T** | **T** | **C** | **T** | **G** | **G** | **T** | **G** | **T** | **C** | **G** | **A** | **T** | **G** | **T** | **G** | **A** | **A** | **A** | **A** | **T** | **C** | **A** | **T** | **G** | **A** | **T** | **C** | **C** | **C** | **C** | **A** | **A** | **T** | **A** | **A** | **A** | **C** | **C** | **G** | **G** | **A** | **T** | **C** | **A** | **T** | **C** | **C** | **T** | **T** | **T** | **T** | **A** | **T** | **T** | **T** | **A** | **T** | **C** | **G** | **A** | **G** | **C** | **A** | **A** | **C** | **A** | **C** | **A** | **A** | **T** | **A** | **T** | **T** | **A** | **T** | **G** | **C** | **G** | **C** | **G** | **T** | **C** | **A** | **G** | **C** | **T** | **C** | **A** | **T** | **C** | **A** | **A** | **G** | **G** | **A** | **A** | **A** | **A** | **C** | **G** | **T** | **A** | **C** | **A** | **A** | **A** | **T** | **T** | **C** | **T** | **T** | **G** | **T** | **C** | **T** | **A** | **T** | **G** | **A** | **G** | **A** | **A** | **T** | **G** | **G** | **C** | **T** | **T** | **C** | **C** | **T** | **C** | **C** | **A** | **T** | **G** | **C** | **A** | **A** | **A** | **A** | **A** | **C** | **A** | **T** | **T** | **G** | **A** | **T** | **A** | **A** | **T** | **G** | **G** | **A** | **T** | **G** | **A** | **T** | **G** | **A** | **A** | **A** | **T** | **C** | **T** | **G** | **C** | **A** | **T** | **G** | **G** | **T** | **A** | **G** | **G** | **T** | **T** | **C** | **A** | **G** | **C** | **A** | **A** | **A** | **T** | **C** | **A** | **A** | **G** | **A** | **T** | **A** | **T** | **T** | **C** | **G** | **T** | **A** | **G** | **C** | **T** | **A** | **C** | **C** | **G** | **A** | **T** | **T** | **G** | **A** | **A** | **T** | **T** | **T** | **T** | **G** | **A** | **A** | **A** | **C** | **A** | **A** | **G** | **T** | **G** | **C** | **T** | **G** | **T** | **C** | **A** | **T** | **C** | **T** | **A** | **C** | **G** | **A** | **T** | **C** | **C** | **T** | **G** | **A** | **G** | **T** | **T** | **T** | **T** | **T** | **A** | **G** | **A** | **A** | **G** | **A** | **A** | **C** | **T** | **T** | **G** | **C** | **T** | **A** | **C** | **T** | **C** | **A** | **G** | **T** | **T** | **T** | **A** | **A** | **A** | **G** | **A** | **A** | **G** | **A** | **T** | **G** | **A** | **G** | **A** | **C** | **A** | **C** | **A** | **T** | **T** | **G** | **T** | **T** | **C** | **A** | **C** | **C** | **A** | **A** | **T** | **G** | **A** | **C** | **G** | **A** | **C** | **T** | **G** | **A** | **A** | **A** | **C** | **A** | **G** | **T** | **G** | **A** | **A** | **G** | **G** | **A** | **A** | **A** | **T** | **G** | **T** | **C** | **T** | **A** | **A** | **T** | **T** | **G** | **G** | **C** | **T** | **G** | **T** | **T** | **A** | **T** | **T** | **C** | **A** | **A** | **G** | **C** | **A** | **A** | **C** | **A** | **A** | **A** | **T** | **T** | **T** | **C** | **T** | **C** | **G** | **A** | **T** | **T** | **A** | **T** | **T** | **T** | **T** | **C** | **T** | **C** | **C** | **A** | **A** | **T** | **C** | **T** | **T** | **A** | **-** | **-** | **-** |
| **N54560** | **-** | **-** | **-** | **-** | **-** | **-** | **-** | **-** | **-** | **-** | **-** | **-** | **-** | **-** | **-** | **-** | **-** | **-** | **-** | **-** | **-** | **-** | **-** | **-** | **-** | **-** | **-** | **-** | **-** | **-** | **-** | **-** | **-** | **-** | **-** | **-** | **-** | **-** | **-** | **-** | **-** | **-** | **-** | **-** | **-** | **-** | **-** | **-** | **-** | **-** | **-** | **-** | **-** | **-** | **-** | **-** | **-** | **-** | **-** | **-** | **-** | **-** | **-** | **-** | **-** | **-** | **-** | **-** | **-** | **A** | **A** | **A** | **C** | **C** | **C** | **C** | **G** | **A** | **G** | **A** | **T** | **G** | **T** | **A** | **G** | **C** | **A** | **G** | **C** | **C** | **A** | **T** | **T** | **T** | **G** | **G** | **G** | **C** | **A** | **T** | **G** | **G** | **C** | **T** | **C** | **T** | **T** | **A** | **G** | **T** | **A** | **T** | **T** | **G** | **T** | **T** | **T** | **G** | **C** | **C** | **C** | **T** | **T** | **C** | **C** | **T** | **G** | **G** | **G** | **G** | **T** | **G** | **G** | **G** | **T** | **T** | **T** | **T** | **G** | **T** | **A** | **T** | **T** | **A** | **T** | **A** | **T** | **T** | **T** | **A** | **T** | **T** | **T** | **T** | **T** | **C** | **G** | **G** | **A** | **C** | **G** | **T** | **G** | **G** | **A** | **T** | **T** | **A** | **A** | **C** | **G** | **G** | **A** | **T** | **A** | **A** | **G** | **A** | **A** | **A** | **A** | **A** | **A** | **T** | **T** | **C** | **T** | **A** | **T** | **C** | **T** | **C** | **C** | **G** | **A** | **C** | **A** | **A** | **A** | **G** | **T** | **G** | **A** | **C** | **T** | **T** | **G** | **A** | **A** | **A** | **G** | **A** | **A** | **T** | **T** | **A** | **G** | **A** | **A** | **A** | **A** | **C** | **T** | **T** | **T** | **C** | **A** | **G** | **T** | **C** | **T** | **T** | **T** | **T** | **A** | **G** | **A** | **G** | **G** | **A** | **G** | **A** | **T** | **A** | **C** | **C** | **A** | **T** | **T** | **G** | **A** | **A** | **C** | **A** | **T** | **T** | **A** | **C** | **G** | **A** | **T** | **C** | **C** | **T** | **G** | **A** | **C** | **A** | **T** | **G** | **G** | **G** | **C** | **G** | **A** | **T** | **A** | **A** | **A** | **G** | **A** | **C** | **A** | **A** | **A** | **C** | **A** | **G** | **C** | **A** | **A** | **T** | **T** | **T** | **G** | **T** | **T** | **G** | **A** | **C** | **T** | **T** | **C** | **T** | **T** | **C** | **T** | **C** | **T** | **T** | **C** | **A** | **T** | **T** | **A** | **A** | **A** | **T** | **C** | **G** | **T** | **A** | **T** | **G** | **C** | **C** | **G** | **C** | **T** | **G** | **A** | **C** | **A** | **A** | **G** | **A** | **A** | **T** | **G** | **A** | **A** | **T** | **T** | **C** | **T** | **G** | **T** | **C** | **A** | **C** | **T** | **C** | **T** | **T** | **C** | **T** | **C** | **A** | **C** | **A** | **G** | **A** | **C** | **G** | **G** | **A** | **C** | **A** | **A** | **G** | **A** | **G** | **A** | **A** | **A** | **T** | **T** | **G** | **G** | **A** | **T** | **T** | **C** | **A** | **C** | **T** | **G** | **C** | **T** | **T** | **C** | **A** | **A** | **G** | **A** | **T** | **C** | **T** | **A** | **A** | **A** | **A** | **A** | **A** | **A** | **G** | **C** | **C** | **A** | **A** | **A** | **C** | **A** | **T** | **T** | **C** | **G** | **A** | **T** | **C** | **C** | **A** | **T** | **A** | **T** | **C** | **G** | **A** | **A** | **T** | **A** | **T** | **T** | **A** | **C** | **G** | **C** | **A** | **T** | **T** | **T** | **G** | **T** | **G** | **A** | **C** | **A** | **G** | **A** | **T** | **A** | **A** | **T** | **A** | **T** | **C** | **G** | **G** | **C** | **C** | **A** | **G** | **C** | **A** | **A** | **G** | **T** | **C** | **T** | **T** | **A** | **C** | **A** | **T** | **G** | **T** | **T** | **T** | **T** | **A** | **G** | **A** | **A** | **G** | **A** | **A** | **A** | **A** | **A** | **G** | **C** | **C** | **G** | **C** | **A** | **G** | **A** | **A** | **G** | **G** | **C** | **G** | **T** | **G** | **G** | **A** | **A** | **G** | **T** | **T** | **C** | **G** | **A** | **A** | **T** | **A** | **T** | **T** | **A** | **T** | **A** | **T** | **G** | **A** | **T** | **G** | **C** | **A** | **T** | **T** | **T** | **G** | **G** | **C** | **T** | **C** | **T** | **C** | **A** | **T** | **G** | **G** | **C** | **A** | **C** | **A** | **A** | **A** | **A** | **G** | **C** | **A** | **A** | **A** | **A** | **G** | **A** | **T** | **T** | **T** | **C** | **A** | **A** | **T** | **C** | **G** | **T** | **C** | **T** | **A** | **A** | **T** | **C** | **A** | **A** | **A** | **A** | **A** | **T** | **G** | **G** | **T** | **G** | **G** | **A** | **C** | **A** | **T** | **G** | **T** | **C** | **C** | **A** | **T** | **A** | **C** | **A** | **T** | **T** | **T** | **G** | **T** | **T** | **A** | **C** | **C** | **T** | **C** | **A** | **C** | **A** | **A** | **A** | **A** | **G** | **G** | **C** | **A** | **T** | **T** | **A** | **C** | **T** | **T** | **C** | **G** | **T** | **T** | **T** | **C** | **C** | **G** | **A** | **T** | **T** | **G** | **A** | **A** | **T** | **T** | **A** | **C** | **C** | **A** | **T** | **G** | **A** | **T** | **C** | **A** | **C** | **C** | **G** | **A** | **A** | **A** | **A** | **A** | **T** | **C** | **G** | **T** | **T** | **G** | **T** | **G** | **A** | **T** | **C** | **G** | **A** | **T** | **G** | **G** | **A** | **A** | **A** | **G** | **A** | **T** | **T** | **A** | **G** | **T** | **T** | **A** | **T** | **A** | **C** | **C** | **G** | **G** | **T** | **G** | **G** | **T** | **T** | **T** | **C** | **A** | **A** | **T** | **A** | **T** | **T** | **G** | **C** | **C** | **A** | **A** | **T** | **C** | **A** | **A** | **T** | **A** | **T** | **G** | **T** | **A** | **A** | **A** | **T** | **A** | **C** | **A** | **A** | **C** | **A** | **A** | **A** | **A** | **A** | **A** | **A** | **T** | **T** | **C** | **G** | **G** | **C** | **T** | **A** | **T** | **T** | **G** | **G** | **C** | **G** | **C** | **G** | **A** | **T** | **A** | **C** | **G** | **C** | **A** | **T** | **A** | **T** | **A** | **C** | **G** | **G** | **A** | **T** | **T** | **T** | **T** | **C** | **G** | **G** | **T** | **G** | **C** | **C** | **G** | **C** | **T** | **T** | **C** | **T** | **T** | **C** | **T** | **T** | **T** | **G** | **C** | **T** | **C** | **C** | **A** | **G** | **C** | **T** | **T** | **C** | **G** | **C** | **T** | **T** | **C** | **T** | **T** | **A** | **A** | **C** | **A** | **G** | **A** | **C** | **T** | **G** | **G** | **A** | **A** | **C** | **G** | **T** | **C** | **T** | **C** | **G** | **G** | **T** | **A** | **C** | **C** | **T** | **G** | **A** | **A** | **G** | **A** | **A** | **A** | **A** | **A** | **A** | **A** | **G** | **G** | **T** | **C** | **G** | **G** | **C** | **T** | **A** | **T** | **C** | **A** | **T** | **T** | **T** | **G** | **A** | **A** | **T** | **T** | **A** | **T** | **T** | **T** | **C** | **T** | **T** | **T** | **A** | **A** | **A** | **A** | **A** | **A** | **G** | **C** | **A** | **G** | **A** | **T** | **C** | **G** | **A** | **G** | **A** | **T** | **G** | **A** | **A** | **T** | **C** | **T** | **A** | **A** | **G** | **C** | **T** | **T** | **G** | **C** | **T** | **G** | **A** | **A** | **A** | **C** | **A** | **T** | **C** | **C** | **A** | **T** | **C** | **C** | **A** | **G** | **C** | **T** | **T** | **G** | **T** | **T** | **T** | **C** | **A** | **A** | **G** | **C** | **G** | **G** | **A** | **C** | **C** | **G** | **A** | **A** | **T** | **A** | **A** | **C** | **G** | **A** | **A** | **A** | **G** | **G** | **G** | **A** | **A** | **C** | **A** | **A** | **A** | **T** | **C** | **A** | **A** | **G** | **C** | **T** | **T** | **T** | **C** | **A** | **T** | **T** | **T** | **A** | **T** | **C** | **A** | **A** | **A** | **T** | **T** | **G** | **A** | **T** | **T** | **A** | **C** | **T** | **T** | **C** | **T** | **G** | **C** | **T** | **A** | **A** | **A** | **A** | **A** | **G** | **C** | **G** | **T** | **G** | **T** | **T** | **T** | **G** | **G** | **A** | **T** | **A** | **C** | **A** | **G** | **A** | **C** | **A** | **C** | **C** | **T** | **T** | **A** | **C** | **C** | **T** | **T** | **G** | **T** | **T** | **C** | **C** | **T** | **G** | **A** | **T** | **G** | **A** | **A** | **A** | **G** | **T** | **G** | **T** | **C** | **A** | **T** | **T** | **G** | **C** | **T** | **G** | **C** | **T** | **T** | **T** | **A** | **A** | **A** | **A** | **A** | **T** | **C** | **G** | **C** | **A** | **A** | **C** | **T** | **G** | **C** | **C** | **T** | **C** | **T** | **G** | **G** | **T** | **G** | **T** | **A** | **G** | **A** | **T** | **G** | **T** | **G** | **A** | **A** | **A** | **A** | **T** | **C** | **A** | **T** | **G** | **A** | **T** | **T** | **C** | **C** | **C** | **A** | **A** | **C** | **A** | **A** | **A** | **C** | **C** | **G** | **G** | **A** | **T** | **C** | **A** | **T** | **C** | **C** | **T** | **T** | **T** | **T** | **A** | **T** | **T** | **T** | **A** | **T** | **C** | **G** | **A** | **G** | **C** | **A** | **A** | **C** | **A** | **C** | **A** | **A** | **T** | **A** | **T** | **T** | **A** | **T** | **G** | **C** | **T** | **C** | **G** | **G** | **C** | **A** | **G** | **C** | **T** | **G** | **A** | **T** | **C** | **A** | **A** | **G** | **G** | **A** | **A** | **A** | **A** | **T** | **G** | **T** | **A** | **C** | **A** | **A** | **A** | **T** | **C** | **C** | **T** | **T** | **G** | **T** | **C** | **T** | **A** | **T** | **G** | **A** | **G** | **A** | **A** | **C** | **G** | **G** | **C** | **T** | **T** | **C** | **C** | **T** | **C** | **C** | **A** | **T** | **G** | **C** | **A** | **A** | **A** | **A** | **A** | **C** | **A** | **T** | **T** | **G** | **A** | **T** | **A** | **A** | **T** | **G** | **G** | **A** | **T** | **G** | **A** | **T** | **G** | **A** | **A** | **A** | **T** | **C** | **T** | **G** | **C** | **A** | **T** | **G** | **G** | **T** | **A** | **G** | **G** | **T** | **T** | **C** | **A** | **G** | **C** | **A** | **A** | **A** | **T** | **C** | **A** | **A** | **G** | **A** | **T** | **A** | **T** | **T** | **C** | **G** | **T** | **A** | **G** | **C** | **T** | **A** | **C** | **A** | **G** | **A** | **T** | **T** | **G** | **A** | **A** | **T** | **T** | **T** | **T** | **G** | **A** | **A** | **A** | **C** | **A** | **A** | **G** | **T** | **G** | **C** | **T** | **G** | **T** | **C** | **A** | **T** | **T** | **T** | **A** | **C** | **G** | **A** | **T** | **C** | **C** | **T** | **G** | **A** | **G** | **T** | **T** | **T** | **T** | **T** | **A** | **G** | **A** | **A** | **G** | **A** | **A** | **C** | **T** | **T** | **G** | **C** | **T** | **A** | **C** | **T** | **C** | **A** | **G** | **T** | **T** | **C** | **A** | **A** | **A** | **G** | **A** | **A** | **G** | **A** | **T** | **G** | **A** | **G** | **A** | **C** | **A** | **C** | **A** | **T** | **T** | **G** | **T** | **T** | **C** | **A** | **T** | **C** | **C** | **A** | **T** | **G** | **A** | **C** | **A** | **A** | **C** | **T** | **G** | **A** | **A** | **A** | **C** | **A** | **G** | **T** | **T** | **A** | **A** | **A** | **G** | **A** | **A** | **A** | **T** | **G** | **T** | **C** | **T** | **A** | **A** | **C** | **T** | **G** | **G** | **C** | **T** | **A** | **T** | **T** | **A** | **T** | **T** | **C** | **A** | **A** | **G** | **C** | **A** | **A** | **C** | **A** | **A** | **A** | **T** | **T** | **T** | **C** | **T** | **C** | **G** | **A** | **T** | **T** | **A** | **T** | **T** | **T** | **T** | **C** | **T** | **C** | **C** | **A** | **A** | **T** | **C** | **C** | **T** | **A** | **-** | **-** | **-** |
| **N52829** | **-** | **-** | **-** | **-** | **-** | **-** | **-** | **-** | **-** | **-** | **-** | **-** | **-** | **-** | **-** | **-** | **-** | **-** | **-** | **-** | **-** | **-** | **-** | **-** | **-** | **-** | **-** | **-** | **-** | **-** | **-** | **-** | **-** | **-** | **-** | **-** | **-** | **-** | **-** | **-** | **-** | **-** | **-** | **-** | **-** | **-** | **-** | **-** | **-** | **-** | **-** | **-** | **-** | **-** | **-** | **-** | **-** | **-** | **-** | **-** | **-** | **-** | **-** | **-** | **-** | **-** | **-** | **-** | **-** | **A** | **A** | **A** | **C** | **C** | **C** | **C** | **G** | **A** | **G** | **A** | **T** | **G** | **T** | **A** | **G** | **C** | **A** | **G** | **C** | **C** | **A** | **T** | **T** | **T** | **G** | **G** | **G** | **C** | **A** | **T** | **G** | **G** | **C** | **T** | **C** | **T** | **T** | **A** | **G** | **T** | **A** | **T** | **T** | **G** | **T** | **T** | **T** | **G** | **C** | **C** | **C** | **T** | **T** | **C** | **C** | **T** | **G** | **G** | **G** | **G** | **T** | **G** | **G** | **G** | **C** | **T** | **T** | **T** | **G** | **T** | **A** | **T** | **T** | **A** | **T** | **A** | **T** | **T** | **T** | **A** | **T** | **T** | **T** | **T** | **T** | **C** | **G** | **G** | **A** | **C** | **G** | **T** | **G** | **G** | **A** | **T** | **T** | **A** | **A** | **C** | **G** | **G** | **A** | **T** | **A** | **A** | **G** | **A** | **A** | **A** | **A** | **A** | **A** | **T** | **T** | **C** | **T** | **A** | **T** | **C** | **T** | **C** | **C** | **G** | **A** | **C** | **A** | **A** | **A** | **G** | **T** | **G** | **A** | **C** | **T** | **T** | **G** | **A** | **A** | **A** | **G** | **A** | **A** | **T** | **T** | **A** | **G** | **A** | **A** | **A** | **A** | **C** | **T** | **T** | **T** | **C** | **A** | **G** | **T** | **C** | **T** | **T** | **T** | **T** | **A** | **G** | **A** | **G** | **G** | **A** | **G** | **A** | **T** | **A** | **C** | **C** | **A** | **T** | **T** | **G** | **A** | **A** | **C** | **A** | **T** | **T** | **A** | **C** | **G** | **A** | **T** | **C** | **C** | **T** | **G** | **A** | **C** | **A** | **T** | **G** | **G** | **G** | **C** | **G** | **A** | **T** | **A** | **A** | **A** | **G** | **A** | **C** | **A** | **A** | **A** | **C** | **A** | **G** | **C** | **A** | **A** | **T** | **T** | **T** | **G** | **T** | **T** | **G** | **A** | **C** | **T** | **T** | **C** | **T** | **T** | **C** | **T** | **C** | **T** | **T** | **C** | **A** | **T** | **T** | **A** | **A** | **A** | **T** | **C** | **G** | **T** | **A** | **T** | **G** | **C** | **C** | **G** | **C** | **T** | **G** | **G** | **C** | **A** | **A** | **G** | **A** | **A** | **T** | **G** | **A** | **A** | **T** | **T** | **C** | **T** | **G** | **T** | **C** | **A** | **C** | **T** | **C** | **T** | **T** | **C** | **T** | **C** | **A** | **C** | **A** | **G** | **A** | **C** | **G** | **G** | **A** | **C** | **A** | **A** | **G** | **A** | **G** | **A** | **A** | **A** | **T** | **T** | **G** | **G** | **A** | **T** | **T** | **C** | **A** | **C** | **T** | **G** | **C** | **T** | **T** | **C** | **A** | **A** | **G** | **A** | **T** | **C** | **T** | **A** | **A** | **A** | **A** | **A** | **A** | **A** | **G** | **C** | **C** | **A** | **A** | **A** | **C** | **A** | **T** | **T** | **C** | **G** | **A** | **T** | **C** | **C** | **A** | **T** | **A** | **T** | **C** | **G** | **A** | **A** | **T** | **A** | **T** | **T** | **A** | **C** | **G** | **C** | **A** | **T** | **T** | **T** | **G** | **T** | **G** | **A** | **C** | **A** | **G** | **A** | **T** | **A** | **A** | **T** | **A** | **T** | **C** | **G** | **G** | **C** | **C** | **A** | **G** | **C** | **A** | **A** | **G** | **T** | **C** | **T** | **T** | **A** | **C** | **A** | **T** | **G** | **T** | **T** | **T** | **T** | **A** | **G** | **A** | **A** | **G** | **A** | **A** | **A** | **A** | **A** | **G** | **C** | **C** | **G** | **C** | **A** | **G** | **A** | **A** | **G** | **G** | **C** | **G** | **T** | **G** | **G** | **A** | **A** | **G** | **T** | **T** | **C** | **G** | **A** | **A** | **T** | **A** | **T** | **T** | **A** | **T** | **A** | **T** | **G** | **A** | **T** | **G** | **C** | **A** | **T** | **T** | **T** | **G** | **G** | **C** | **T** | **C** | **T** | **C** | **A** | **T** | **G** | **G** | **C** | **A** | **C** | **A** | **A** | **A** | **A** | **G** | **C** | **A** | **A** | **A** | **A** | **G** | **A** | **T** | **T** | **T** | **C** | **A** | **A** | **T** | **C** | **G** | **T** | **C** | **T** | **A** | **A** | **T** | **C** | **A** | **A** | **A** | **A** | **A** | **T** | **G** | **G** | **T** | **G** | **G** | **A** | **C** | **A** | **T** | **G** | **T** | **C** | **C** | **A** | **T** | **A** | **C** | **A** | **T** | **T** | **T** | **G** | **T** | **T** | **A** | **C** | **C** | **T** | **C** | **A** | **C** | **A** | **A** | **A** | **A** | **G** | **G** | **C** | **A** | **T** | **T** | **A** | **C** | **T** | **T** | **C** | **G** | **T** | **T** | **T** | **C** | **C** | **G** | **A** | **T** | **T** | **G** | **A** | **A** | **T** | **T** | **A** | **C** | **C** | **A** | **T** | **G** | **A** | **T** | **C** | **A** | **C** | **C** | **G** | **A** | **A** | **A** | **A** | **A** | **T** | **C** | **G** | **T** | **T** | **G** | **T** | **G** | **A** | **T** | **C** | **G** | **A** | **T** | **G** | **G** | **A** | **A** | **A** | **G** | **A** | **T** | **T** | **A** | **G** | **T** | **T** | **A** | **T** | **A** | **C** | **C** | **G** | **G** | **T** | **G** | **G** | **T** | **T** | **T** | **C** | **A** | **A** | **T** | **A** | **T** | **T** | **G** | **C** | **C** | **A** | **A** | **T** | **C** | **A** | **A** | **T** | **A** | **T** | **G** | **T** | **A** | **A** | **A** | **T** | **A** | **C** | **A** | **A** | **C** | **A** | **A** | **A** | **A** | **A** | **A** | **A** | **T** | **T** | **C** | **G** | **G** | **C** | **T** | **A** | **T** | **T** | **G** | **G** | **C** | **G** | **C** | **G** | **A** | **T** | **A** | **C** | **G** | **C** | **A** | **T** | **A** | **T** | **A** | **C** | **G** | **G** | **A** | **T** | **T** | **T** | **T** | **C** | **G** | **G** | **T** | **G** | **C** | **C** | **G** | **C** | **T** | **T** | **C** | **T** | **T** | **C** | **T** | **T** | **T** | **G** | **C** | **T** | **C** | **C** | **A** | **G** | **C** | **T** | **T** | **C** | **G** | **C** | **T** | **T** | **C** | **T** | **T** | **A** | **A** | **C** | **A** | **G** | **A** | **C** | **T** | **G** | **G** | **A** | **A** | **C** | **G** | **T** | **C** | **T** | **C** | **G** | **G** | **T** | **A** | **C** | **C** | **T** | **G** | **A** | **A** | **G** | **A** | **A** | **A** | **A** | **A** | **A** | **A** | **G** | **G** | **T** | **C** | **G** | **G** | **C** | **T** | **A** | **T** | **C** | **A** | **T** | **T** | **T** | **G** | **A** | **A** | **T** | **T** | **A** | **T** | **T** | **T** | **C** | **T** | **T** | **T** | **A** | **A** | **A** | **A** | **A** | **A** | **G** | **C** | **A** | **G** | **A** | **T** | **C** | **G** | **A** | **G** | **A** | **T** | **G** | **A** | **A** | **T** | **C** | **T** | **A** | **A** | **G** | **C** | **T** | **T** | **G** | **C** | **T** | **G** | **A** | **T** | **A** | **C** | **A** | **T** | **C** | **C** | **A** | **T** | **C** | **C** | **A** | **G** | **C** | **T** | **T** | **G** | **T** | **T** | **T** | **C** | **A** | **A** | **G** | **C** | **G** | **G** | **A** | **C** | **C** | **G** | **A** | **A** | **T** | **A** | **A** | **C** | **G** | **A** | **A** | **A** | **G** | **G** | **G** | **A** | **A** | **C** | **A** | **A** | **A** | **T** | **C** | **A** | **A** | **G** | **C** | **T** | **T** | **T** | **C** | **A** | **T** | **T** | **T** | **A** | **T** | **C** | **A** | **A** | **A** | **T** | **T** | **G** | **A** | **T** | **T** | **A** | **C** | **T** | **T** | **C** | **T** | **G** | **C** | **T** | **A** | **A** | **A** | **A** | **A** | **A** | **C** | **G** | **T** | **G** | **T** | **T** | **T** | **G** | **G** | **A** | **T** | **A** | **C** | **A** | **G** | **A** | **C** | **A** | **C** | **C** | **T** | **T** | **A** | **C** | **C** | **T** | **T** | **G** | **T** | **T** | **C** | **C** | **T** | **G** | **A** | **T** | **G** | **A** | **A** | **A** | **G** | **T** | **G** | **T** | **C** | **A** | **T** | **T** | **G** | **C** | **T** | **G** | **C** | **T** | **T** | **T** | **A** | **A** | **A** | **A** | **A** | **T** | **C** | **G** | **C** | **A** | **A** | **C** | **T** | **G** | **C** | **C** | **T** | **C** | **T** | **G** | **G** | **T** | **G** | **T** | **A** | **G** | **A** | **T** | **G** | **T** | **G** | **A** | **A** | **A** | **A** | **T** | **C** | **A** | **T** | **G** | **A** | **T** | **T** | **C** | **C** | **C** | **A** | **A** | **C** | **A** | **A** | **A** | **C** | **C** | **G** | **G** | **A** | **T** | **C** | **A** | **T** | **C** | **C** | **T** | **T** | **T** | **T** | **A** | **T** | **T** | **T** | **A** | **T** | **C** | **G** | **A** | **G** | **C** | **A** | **A** | **C** | **A** | **C** | **A** | **A** | **T** | **A** | **T** | **T** | **A** | **T** | **G** | **C** | **T** | **C** | **G** | **G** | **C** | **A** | **G** | **C** | **T** | **G** | **A** | **T** | **C** | **A** | **A** | **G** | **G** | **A** | **A** | **A** | **A** | **T** | **G** | **T** | **A** | **C** | **A** | **A** | **A** | **T** | **C** | **C** | **T** | **T** | **G** | **T** | **C** | **T** | **A** | **T** | **G** | **A** | **G** | **A** | **A** | **C** | **G** | **G** | **C** | **T** | **T** | **C** | **C** | **T** | **C** | **C** | **A** | **T** | **G** | **C** | **A** | **A** | **A** | **A** | **A** | **C** | **A** | **T** | **T** | **G** | **A** | **T** | **A** | **A** | **T** | **G** | **G** | **A** | **T** | **G** | **A** | **T** | **G** | **A** | **A** | **A** | **T** | **C** | **T** | **G** | **C** | **A** | **T** | **G** | **G** | **T** | **A** | **G** | **G** | **T** | **T** | **C** | **A** | **G** | **C** | **A** | **A** | **A** | **T** | **C** | **A** | **A** | **G** | **A** | **T** | **A** | **T** | **T** | **C** | **G** | **T** | **A** | **G** | **C** | **T** | **A** | **C** | **C** | **G** | **A** | **T** | **T** | **G** | **A** | **A** | **T** | **T** | **T** | **T** | **G** | **A** | **A** | **A** | **C** | **A** | **A** | **G** | **T** | **G** | **C** | **T** | **G** | **T** | **C** | **A** | **T** | **T** | **T** | **A** | **C** | **G** | **A** | **T** | **C** | **C** | **T** | **G** | **A** | **G** | **T** | **T** | **T** | **T** | **T** | **A** | **G** | **A** | **A** | **G** | **A** | **A** | **C** | **T** | **T** | **G** | **C** | **T** | **A** | **C** | **T** | **C** | **A** | **G** | **T** | **T** | **C** | **A** | **A** | **A** | **G** | **A** | **A** | **G** | **A** | **T** | **G** | **A** | **G** | **A** | **C** | **A** | **C** | **A** | **T** | **T** | **G** | **T** | **T** | **C** | **A** | **T** | **C** | **C** | **A** | **T** | **G** | **A** | **C** | **A** | **A** | **C** | **T** | **G** | **A** | **A** | **A** | **C** | **A** | **G** | **T** | **C** | **A** | **A** | **G** | **G** | **A** | **A** | **A** | **T** | **G** | **T** | **C** | **T** | **A** | **A** | **C** | **T** | **G** | **G** | **C** | **T** | **A** | **T** | **T** | **A** | **T** | **T** | **C** | **A** | **A** | **G** | **C** | **A** | **A** | **C** | **A** | **A** | **A** | **T** | **T** | **T** | **C** | **T** | **C** | **G** | **A** | **T** | **T** | **A** | **T** | **T** | **T** | **T** | **C** | **T** | **C** | **C** | **A** | **A** | **T** | **C** | **C** | **T** | **A** | **-** | **-** | **-** |
| **N59745F** | **-** | **-** | **-** | **-** | **-** | **-** | **-** | **-** | **-** | **-** | **-** | **-** | **-** | **-** | **-** | **-** | **-** | **-** | **-** | **-** | **-** | **-** | **-** | **-** | **-** | **-** | **-** | **-** | **-** | **-** | **-** | **-** | **-** | **-** | **-** | **-** | **-** | **-** | **-** | **-** | **-** | **-** | **-** | **-** | **-** | **-** | **-** | **-** | **-** | **-** | **-** | **-** | **-** | **-** | **-** | **-** | **-** | **-** | **-** | **-** | **-** | **-** | **-** | **-** | **-** | **-** | **-** | **-** | **-** | **A** | **A** | **A** | **C** | **C** | **C** | **C** | **G** | **A** | **G** | **A** | **T** | **G** | **T** | **A** | **G** | **C** | **A** | **G** | **C** | **T** | **A** | **T** | **T** | **T** | **G** | **G** | **G** | **C** | **A** | **T** | **G** | **G** | **C** | **T** | **C** | **T** | **T** | **A** | **G** | **T** | **A** | **T** | **T** | **G** | **T** | **T** | **T** | **G** | **C** | **C** | **C** | **T** | **T** | **C** | **C** | **T** | **G** | **G** | **G** | **G** | **T** | **G** | **G** | **G** | **T** | **T** | **T** | **T** | **G** | **T** | **A** | **T** | **T** | **A** | **T** | **A** | **T** | **T** | **T** | **A** | **T** | **T** | **T** | **T** | **T** | **C** | **G** | **G** | **A** | **C** | **G** | **T** | **G** | **G** | **A** | **T** | **T** | **A** | **A** | **C** | **G** | **G** | **A** | **T** | **A** | **A** | **G** | **A** | **A** | **A** | **A** | **A** | **A** | **T** | **T** | **C** | **T** | **A** | **T** | **C** | **T** | **C** | **C** | **G** | **A** | **C** | **A** | **A** | **A** | **G** | **T** | **G** | **A** | **C** | **T** | **T** | **G** | **A** | **A** | **A** | **G** | **A** | **A** | **T** | **T** | **A** | **G** | **A** | **A** | **A** | **A** | **C** | **T** | **T** | **T** | **C** | **A** | **G** | **T** | **C** | **T** | **T** | **T** | **T** | **A** | **G** | **A** | **G** | **G** | **A** | **G** | **A** | **T** | **A** | **C** | **C** | **A** | **T** | **T** | **G** | **A** | **A** | **C** | **A** | **T** | **T** | **A** | **C** | **G** | **A** | **T** | **C** | **C** | **T** | **G** | **A** | **C** | **A** | **T** | **G** | **G** | **G** | **C** | **G** | **A** | **C** | **A** | **A** | **A** | **G** | **A** | **C** | **A** | **A** | **A** | **C** | **A** | **A** | **C** | **A** | **A** | **T** | **T** | **T** | **G** | **T** | **C** | **G** | **A** | **C** | **T** | **T** | **C** | **T** | **T** | **C** | **T** | **C** | **T** | **T** | **C** | **A** | **T** | **T** | **A** | **A** | **A** | **T** | **C** | **G** | **T** | **A** | **T** | **G** | **C** | **C** | **G** | **C** | **T** | **G** | **A** | **C** | **A** | **A** | **G** | **A** | **A** | **T** | **G** | **A** | **A** | **T** | **T** | **C** | **T** | **G** | **T** | **C** | **A** | **C** | **T** | **C** | **T** | **T** | **C** | **T** | **C** | **A** | **C** | **A** | **G** | **A** | **C** | **G** | **G** | **A** | **C** | **A** | **A** | **A** | **A** | **G** | **A** | **A** | **A** | **T** | **T** | **A** | **G** | **A** | **T** | **T** | **C** | **A** | **C** | **T** | **G** | **C** | **T** | **T** | **C** | **A** | **A** | **G** | **A** | **T** | **C** | **T** | **A** | **A** | **A** | **A** | **A** | **A** | **A** | **G** | **C** | **C** | **A** | **A** | **A** | **C** | **A** | **T** | **T** | **C** | **G** | **A** | **T** | **C** | **C** | **A** | **T** | **A** | **T** | **C** | **G** | **A** | **A** | **T** | **A** | **T** | **T** | **A** | **C** | **G** | **C** | **A** | **T** | **T** | **T** | **G** | **T** | **G** | **A** | **C** | **A** | **G** | **A** | **T** | **A** | **A** | **T** | **A** | **T** | **C** | **G** | **G** | **T** | **C** | **A** | **G** | **C** | **A** | **A** | **G** | **T** | **C** | **T** | **T** | **A** | **C** | **G** | **C** | **G** | **T** | **T** | **T** | **T** | **A** | **G** | **A** | **A** | **G** | **A** | **A** | **A** | **A** | **A** | **G** | **C** | **C** | **G** | **C** | **A** | **G** | **A** | **A** | **G** | **G** | **C** | **G** | **T** | **A** | **G** | **A** | **A** | **G** | **T** | **T** | **C** | **G** | **T** | **A** | **T** | **A** | **T** | **T** | **A** | **T** | **A** | **T** | **G** | **A** | **T** | **G** | **C** | **A** | **T** | **T** | **T** | **G** | **G** | **C** | **T** | **C** | **C** | **C** | **A** | **T** | **G** | **G** | **C** | **A** | **C** | **A** | **A** | **A** | **A** | **G** | **C** | **A** | **A** | **A** | **A** | **G** | **A** | **T** | **T** | **T** | **C** | **A** | **A** | **T** | **C** | **G** | **T** | **C** | **T** | **A** | **A** | **T** | **C** | **A** | **A** | **A** | **A** | **A** | **T** | **G** | **G** | **T** | **G** | **G** | **A** | **C** | **A** | **T** | **G** | **T** | **C** | **C** | **A** | **T** | **A** | **C** | **A** | **T** | **T** | **T** | **G** | **T** | **T** | **A** | **C** | **C** | **T** | **C** | **G** | **C** | **A** | **A** | **A** | **A** | **A** | **G** | **C** | **A** | **T** | **T** | **G** | **C** | **T** | **T** | **C** | **G** | **T** | **T** | **T** | **C** | **C** | **G** | **A** | **T** | **T** | **G** | **A** | **A** | **T** | **T** | **A** | **C** | **C** | **A** | **T** | **G** | **A** | **T** | **C** | **A** | **C** | **C** | **G** | **A** | **A** | **A** | **A** | **A** | **T** | **C** | **G** | **T** | **T** | **G** | **T** | **G** | **A** | **T** | **C** | **G** | **A** | **T** | **G** | **G** | **A** | **A** | **A** | **G** | **A** | **T** | **C** | **A** | **G** | **T** | **T** | **A** | **C** | **A** | **C** | **C** | **G** | **G** | **T** | **G** | **G** | **T** | **T** | **T** | **C** | **A** | **A** | **T** | **A** | **T** | **T** | **G** | **C** | **C** | **A** | **A** | **T** | **C** | **A** | **A** | **T** | **A** | **T** | **G** | **T** | **A** | **A** | **A** | **T** | **A** | **C** | **G** | **A** | **C** | **A** | **A** | **A** | **A** | **A** | **A** | **A** | **T** | **T** | **C** | **G** | **G** | **T** | **T** | **A** | **T** | **T** | **G** | **G** | **C** | **G** | **T** | **G** | **A** | **T** | **A** | **C** | **G** | **C** | **A** | **T** | **A** | **T** | **A** | **C** | **G** | **A** | **A** | **T** | **T** | **T** | **T** | **C** | **G** | **G** | **T** | **G** | **C** | **T** | **G** | **C** | **T** | **T** | **C** | **T** | **T** | **C** | **T** | **T** | **T** | **G** | **C** | **T** | **T** | **C** | **A** | **A** | **C** | **T** | **T** | **C** | **G** | **T** | **T** | **T** | **T** | **T** | **T** | **A** | **A** | **C** | **A** | **G** | **A** | **C** | **T** | **G** | **G** | **A** | **A** | **C** | **G** | **T** | **T** | **T** | **C** | **A** | **G** | **T** | **G** | **T** | **C** | **T** | **G** | **A** | **A** | **G** | **A** | **A** | **A** | **A** | **A** | **A** | **A** | **G** | **G** | **T** | **C** | **G** | **G** | **C** | **T** | **A** | **T** | **C** | **A** | **T** | **T** | **T** | **G** | **A** | **A** | **T** | **T** | **A** | **T** | **T** | **T** | **C** | **T** | **T** | **T** | **A** | **A** | **A** | **A** | **A** | **A** | **G** | **C** | **C** | **G** | **A** | **T** | **C** | **G** | **A** | **G** | **A** | **T** | **G** | **A** | **A** | **T** | **C** | **C** | **A** | **A** | **A** | **C** | **T** | **T** | **G** | **C** | **T** | **G** | **A** | **T** | **A** | **C** | **G** | **T** | **C** | **C** | **A** | **T** | **C** | **C** | **A** | **G** | **C** | **T** | **T** | **G** | **T** | **C** | **T** | **C** | **A** | **A** | **G** | **C** | **G** | **G** | **A** | **C** | **C** | **A** | **A** | **A** | **T** | **A** | **A** | **C** | **G** | **A** | **A** | **C** | **G** | **A** | **G** | **A** | **A** | **C** | **A** | **A** | **A** | **T** | **C** | **A** | **A** | **G** | **C** | **T** | **T** | **T** | **C** | **G** | **T** | **T** | **T** | **A** | **T** | **C** | **A** | **A** | **A** | **T** | **T** | **G** | **A** | **T** | **T** | **A** | **C** | **T** | **T** | **C** | **T** | **G** | **C** | **T** | **A** | **A** | **A** | **A** | **A** | **A** | **C** | **G** | **T** | **G** | **T** | **T** | **T** | **G** | **G** | **A** | **T** | **A** | **C** | **A** | **G** | **A** | **C** | **A** | **C** | **C** | **T** | **T** | **A** | **C** | **C** | **T** | **C** | **G** | **T** | **T** | **C** | **C** | **T** | **G** | **A** | **T** | **G** | **A** | **A** | **A** | **G** | **T** | **G** | **T** | **C** | **A** | **T** | **T** | **G** | **C** | **T** | **G** | **C** | **T** | **T** | **T** | **G** | **A** | **A** | **A** | **A** | **T** | **C** | **G** | **C** | **A** | **A** | **C** | **T** | **G** | **C** | **T** | **T** | **C** | **T** | **G** | **G** | **T** | **G** | **T** | **C** | **G** | **A** | **T** | **G** | **T** | **G** | **A** | **A** | **A** | **A** | **T** | **C** | **A** | **T** | **G** | **A** | **T** | **C** | **C** | **C** | **C** | **A** | **A** | **T** | **A** | **A** | **A** | **C** | **C** | **G** | **G** | **A** | **T** | **C** | **A** | **T** | **C** | **C** | **T** | **T** | **T** | **T** | **A** | **T** | **T** | **T** | **A** | **T** | **C** | **G** | **A** | **G** | **C** | **A** | **A** | **C** | **A** | **C** | **A** | **A** | **T** | **A** | **T** | **T** | **A** | **T** | **G** | **C** | **T** | **C** | **G** | **T** | **C** | **A** | **G** | **C** | **T** | **C** | **A** | **T** | **C** | **A** | **A** | **G** | **G** | **A** | **A** | **A** | **A** | **T** | **G** | **T** | **A** | **C** | **A** | **G** | **A** | **T** | **C** | **C** | **T** | **T** | **G** | **T** | **C** | **T** | **A** | **T** | **G** | **A** | **G** | **A** | **A** | **T** | **G** | **G** | **C** | **T** | **T** | **C** | **C** | **T** | **C** | **C** | **A** | **T** | **G** | **C** | **A** | **A** | **A** | **A** | **A** | **C** | **A** | **T** | **T** | **A** | **A** | **T** | **A** | **A** | **T** | **G** | **G** | **A** | **T** | **G** | **A** | **T** | **G** | **A** | **A** | **A** | **T** | **C** | **T** | **G** | **C** | **A** | **T** | **G** | **G** | **T** | **A** | **G** | **G** | **T** | **T** | **C** | **A** | **G** | **C** | **A** | **A** | **A** | **T** | **C** | **A** | **A** | **G** | **A** | **T** | **A** | **T** | **T** | **C** | **G** | **T** | **A** | **G** | **C** | **T** | **A** | **C** | **C** | **G** | **A** | **T** | **T** | **G** | **A** | **A** | **T** | **T** | **T** | **T** | **G** | **A** | **A** | **A** | **C** | **A** | **A** | **G** | **T** | **G** | **C** | **T** | **G** | **T** | **C** | **A** | **T** | **T** | **T** | **A** | **C** | **G** | **A** | **T** | **C** | **C** | **T** | **G** | **A** | **G** | **T** | **T** | **T** | **T** | **T** | **A** | **G** | **A** | **A** | **G** | **A** | **G** | **C** | **T** | **T** | **G** | **C** | **T** | **A** | **C** | **T** | **C** | **A** | **G** | **T** | **T** | **C** | **A** | **A** | **A** | **G** | **A** | **A** | **G** | **A** | **T** | **G** | **A** | **G** | **A** | **C** | **A** | **C** | **A** | **T** | **T** | **G** | **T** | **T** | **C** | **A** | **C** | **C** | **A** | **A** | **T** | **G** | **A** | **C** | **A** | **A** | **C** | **T** | **G** | **A** | **A** | **A** | **C** | **A** | **G** | **T** | **G** | **A** | **A** | **G** | **G** | **A** | **A** | **A** | **T** | **G** | **T** | **C** | **T** | **A** | **A** | **C** | **T** | **G** | **G** | **C** | **T** | **G** | **T** | **T** | **A** | **T** | **T** | **C** | **A** | **A** | **A** | **C** | **A** | **A** | **C** | **A** | **A** | **A** | **T** | **T** | **T** | **C** | **T** | **C** | **G** | **A** | **T** | **T** | **G** | **T** | **T** | **T** | **T** | **C** | **T** | **C** | **C** | **A** | **A** | **T** | **C** | **T** | **T** | **A** | **-** | **-** | **-** |
| **N59484F** | **-** | **-** | **-** | **-** | **-** | **-** | **-** | **-** | **-** | **-** | **-** | **-** | **-** | **-** | **-** | **-** | **-** | **-** | **-** | **-** | **-** | **-** | **-** | **-** | **-** | **-** | **-** | **-** | **-** | **-** | **-** | **-** | **-** | **-** | **-** | **-** | **-** | **-** | **-** | **-** | **-** | **-** | **-** | **-** | **-** | **-** | **-** | **-** | **-** | **-** | **-** | **-** | **-** | **-** | **-** | **-** | **-** | **-** | **-** | **-** | **-** | **-** | **-** | **-** | **-** | **-** | **-** | **-** | **-** | **A** | **A** | **A** | **C** | **C** | **C** | **C** | **G** | **A** | **G** | **A** | **T** | **G** | **T** | **A** | **G** | **C** | **A** | **G** | **C** | **C** | **A** | **T** | **T** | **T** | **G** | **G** | **G** | **C** | **A** | **T** | **G** | **G** | **C** | **T** | **C** | **T** | **T** | **A** | **G** | **T** | **A** | **T** | **T** | **G** | **T** | **T** | **T** | **G** | **C** | **C** | **C** | **T** | **T** | **C** | **C** | **T** | **G** | **G** | **G** | **G** | **T** | **G** | **G** | **G** | **T** | **T** | **T** | **T** | **G** | **T** | **A** | **T** | **T** | **A** | **T** | **A** | **T** | **T** | **T** | **A** | **T** | **T** | **T** | **T** | **T** | **C** | **G** | **G** | **A** | **C** | **G** | **T** | **G** | **G** | **A** | **T** | **T** | **A** | **A** | **C** | **G** | **G** | **A** | **T** | **A** | **A** | **G** | **A** | **A** | **A** | **A** | **A** | **A** | **T** | **T** | **C** | **T** | **A** | **T** | **C** | **T** | **C** | **C** | **G** | **A** | **C** | **A** | **A** | **A** | **G** | **T** | **G** | **A** | **C** | **T** | **T** | **G** | **A** | **A** | **A** | **G** | **A** | **A** | **T** | **T** | **A** | **G** | **A** | **A** | **A** | **A** | **C** | **T** | **T** | **T** | **C** | **A** | **G** | **T** | **C** | **T** | **T** | **T** | **T** | **A** | **G** | **A** | **G** | **G** | **A** | **G** | **A** | **T** | **A** | **C** | **C** | **A** | **T** | **T** | **G** | **A** | **A** | **C** | **A** | **T** | **T** | **A** | **C** | **G** | **A** | **T** | **C** | **C** | **T** | **G** | **A** | **C** | **A** | **T** | **G** | **G** | **G** | **C** | **G** | **A** | **T** | **A** | **A** | **A** | **G** | **A** | **C** | **A** | **A** | **A** | **C** | **A** | **G** | **C** | **A** | **A** | **T** | **T** | **T** | **G** | **T** | **C** | **G** | **A** | **C** | **T** | **T** | **C** | **T** | **T** | **C** | **T** | **C** | **T** | **T** | **C** | **A** | **T** | **T** | **A** | **A** | **A** | **T** | **C** | **G** | **T** | **A** | **T** | **G** | **C** | **C** | **G** | **C** | **T** | **G** | **A** | **C** | **A** | **A** | **G** | **A** | **A** | **T** | **G** | **A** | **A** | **T** | **T** | **C** | **T** | **G** | **T** | **C** | **A** | **C** | **T** | **C** | **T** | **T** | **C** | **T** | **C** | **A** | **C** | **A** | **G** | **A** | **C** | **G** | **G** | **A** | **C** | **A** | **A** | **G** | **A** | **G** | **A** | **A** | **A** | **T** | **T** | **G** | **G** | **A** | **T** | **T** | **C** | **A** | **C** | **T** | **G** | **C** | **T** | **T** | **C** | **A** | **A** | **G** | **A** | **T** | **C** | **T** | **A** | **A** | **A** | **A** | **A** | **A** | **A** | **G** | **C** | **C** | **A** | **A** | **A** | **C** | **A** | **T** | **T** | **C** | **G** | **A** | **T** | **C** | **C** | **A** | **T** | **A** | **T** | **C** | **G** | **A** | **A** | **T** | **A** | **T** | **T** | **A** | **C** | **G** | **C** | **A** | **T** | **T** | **T** | **G** | **T** | **G** | **A** | **C** | **A** | **G** | **A** | **T** | **A** | **A** | **T** | **A** | **T** | **C** | **G** | **G** | **C** | **C** | **A** | **G** | **C** | **A** | **A** | **G** | **T** | **C** | **T** | **T** | **A** | **C** | **A** | **T** | **G** | **T** | **T** | **T** | **T** | **A** | **G** | **A** | **A** | **G** | **A** | **A** | **A** | **A** | **A** | **G** | **C** | **C** | **G** | **C** | **A** | **G** | **A** | **A** | **G** | **G** | **C** | **G** | **T** | **G** | **G** | **A** | **A** | **G** | **T** | **T** | **C** | **G** | **A** | **A** | **T** | **A** | **T** | **T** | **A** | **T** | **A** | **T** | **G** | **A** | **T** | **G** | **C** | **A** | **T** | **T** | **T** | **G** | **G** | **C** | **T** | **C** | **T** | **C** | **A** | **T** | **G** | **G** | **C** | **A** | **C** | **A** | **A** | **A** | **A** | **G** | **C** | **A** | **A** | **A** | **A** | **G** | **A** | **T** | **T** | **T** | **C** | **A** | **A** | **T** | **C** | **G** | **T** | **C** | **T** | **A** | **A** | **T** | **C** | **A** | **A** | **A** | **A** | **A** | **T** | **G** | **G** | **T** | **G** | **G** | **A** | **C** | **A** | **T** | **G** | **T** | **C** | **C** | **A** | **T** | **A** | **C** | **A** | **T** | **T** | **T** | **G** | **T** | **T** | **A** | **C** | **C** | **T** | **C** | **A** | **C** | **A** | **A** | **A** | **A** | **G** | **G** | **C** | **A** | **T** | **T** | **A** | **C** | **T** | **T** | **C** | **G** | **T** | **T** | **T** | **C** | **C** | **G** | **A** | **T** | **T** | **G** | **A** | **A** | **T** | **T** | **A** | **C** | **C** | **A** | **T** | **G** | **A** | **T** | **C** | **A** | **C** | **C** | **G** | **A** | **A** | **A** | **A** | **A** | **T** | **C** | **G** | **T** | **T** | **G** | **T** | **G** | **A** | **T** | **C** | **G** | **A** | **T** | **G** | **G** | **A** | **A** | **A** | **G** | **A** | **T** | **T** | **A** | **G** | **T** | **T** | **A** | **T** | **A** | **C** | **C** | **G** | **G** | **T** | **G** | **G** | **T** | **T** | **T** | **C** | **A** | **A** | **T** | **A** | **T** | **T** | **G** | **C** | **C** | **A** | **A** | **T** | **C** | **A** | **A** | **T** | **A** | **T** | **G** | **T** | **A** | **A** | **A** | **T** | **A** | **C** | **A** | **A** | **C** | **A** | **A** | **A** | **A** | **A** | **A** | **A** | **T** | **T** | **C** | **G** | **G** | **C** | **T** | **A** | **T** | **T** | **G** | **G** | **C** | **G** | **C** | **G** | **A** | **T** | **A** | **C** | **G** | **C** | **A** | **T** | **A** | **T** | **A** | **C** | **G** | **G** | **A** | **T** | **T** | **T** | **T** | **C** | **G** | **G** | **T** | **G** | **C** | **C** | **G** | **C** | **T** | **T** | **C** | **T** | **T** | **C** | **T** | **T** | **T** | **G** | **C** | **T** | **C** | **C** | **A** | **G** | **C** | **T** | **T** | **C** | **G** | **C** | **T** | **T** | **C** | **T** | **T** | **A** | **A** | **C** | **A** | **G** | **A** | **C** | **T** | **G** | **G** | **A** | **A** | **C** | **G** | **T** | **C** | **T** | **C** | **G** | **G** | **T** | **A** | **C** | **C** | **T** | **G** | **A** | **A** | **G** | **A** | **A** | **A** | **A** | **A** | **A** | **A** | **G** | **G** | **T** | **C** | **G** | **G** | **C** | **T** | **A** | **T** | **C** | **A** | **T** | **T** | **T** | **G** | **A** | **A** | **T** | **T** | **A** | **T** | **T** | **T** | **C** | **T** | **T** | **T** | **A** | **A** | **A** | **A** | **A** | **A** | **G** | **C** | **A** | **G** | **A** | **T** | **C** | **G** | **A** | **G** | **A** | **T** | **G** | **A** | **A** | **T** | **C** | **T** | **A** | **A** | **G** | **C** | **T** | **T** | **G** | **C** | **T** | **G** | **A** | **T** | **A** | **C** | **A** | **T** | **C** | **C** | **A** | **T** | **C** | **C** | **A** | **G** | **C** | **T** | **T** | **G** | **T** | **T** | **T** | **C** | **A** | **A** | **G** | **C** | **G** | **G** | **A** | **C** | **C** | **G** | **A** | **A** | **T** | **A** | **A** | **C** | **G** | **A** | **A** | **A** | **G** | **G** | **G** | **A** | **A** | **C** | **A** | **A** | **A** | **T** | **C** | **A** | **A** | **G** | **C** | **T** | **T** | **T** | **C** | **A** | **T** | **T** | **T** | **A** | **T** | **C** | **A** | **A** | **A** | **T** | **T** | **G** | **A** | **T** | **T** | **A** | **C** | **T** | **T** | **C** | **T** | **G** | **C** | **T** | **A** | **A** | **A** | **A** | **A** | **A** | **C** | **G** | **T** | **G** | **T** | **T** | **T** | **G** | **G** | **A** | **T** | **A** | **C** | **A** | **G** | **A** | **C** | **A** | **C** | **C** | **T** | **T** | **A** | **C** | **C** | **T** | **T** | **G** | **T** | **T** | **C** | **C** | **T** | **G** | **A** | **T** | **G** | **A** | **A** | **A** | **G** | **T** | **G** | **T** | **C** | **A** | **T** | **T** | **G** | **C** | **T** | **G** | **C** | **T** | **T** | **T** | **A** | **A** | **A** | **A** | **A** | **T** | **C** | **G** | **C** | **A** | **A** | **C** | **T** | **G** | **C** | **C** | **T** | **C** | **T** | **G** | **G** | **T** | **G** | **T** | **A** | **G** | **A** | **T** | **G** | **T** | **G** | **A** | **A** | **A** | **A** | **T** | **C** | **A** | **T** | **G** | **A** | **T** | **T** | **C** | **C** | **C** | **A** | **A** | **C** | **A** | **A** | **A** | **C** | **C** | **G** | **G** | **A** | **T** | **C** | **A** | **T** | **C** | **C** | **T** | **T** | **T** | **T** | **A** | **T** | **T** | **T** | **A** | **T** | **C** | **G** | **A** | **G** | **C** | **A** | **A** | **C** | **A** | **C** | **A** | **A** | **T** | **A** | **T** | **T** | **A** | **T** | **G** | **C** | **T** | **C** | **G** | **G** | **C** | **A** | **G** | **C** | **T** | **G** | **A** | **T** | **C** | **A** | **A** | **G** | **G** | **A** | **A** | **A** | **A** | **T** | **G** | **T** | **A** | **C** | **A** | **A** | **A** | **T** | **C** | **C** | **T** | **T** | **G** | **T** | **C** | **T** | **A** | **T** | **G** | **A** | **G** | **A** | **A** | **C** | **G** | **G** | **C** | **T** | **T** | **C** | **C** | **T** | **C** | **C** | **A** | **T** | **G** | **C** | **A** | **A** | **A** | **A** | **A** | **C** | **A** | **T** | **T** | **G** | **A** | **T** | **A** | **A** | **T** | **G** | **G** | **A** | **T** | **G** | **A** | **T** | **G** | **A** | **A** | **A** | **T** | **C** | **T** | **G** | **C** | **A** | **T** | **G** | **G** | **T** | **A** | **G** | **G** | **T** | **T** | **C** | **A** | **G** | **C** | **A** | **A** | **A** | **T** | **C** | **A** | **A** | **G** | **A** | **T** | **A** | **T** | **T** | **C** | **G** | **T** | **A** | **G** | **C** | **T** | **A** | **C** | **C** | **G** | **A** | **T** | **T** | **G** | **A** | **A** | **T** | **T** | **T** | **T** | **G** | **A** | **A** | **A** | **C** | **A** | **A** | **G** | **T** | **G** | **C** | **T** | **G** | **T** | **C** | **A** | **T** | **T** | **T** | **A** | **C** | **G** | **A** | **T** | **C** | **C** | **T** | **G** | **A** | **G** | **T** | **T** | **T** | **T** | **T** | **A** | **G** | **A** | **A** | **G** | **A** | **A** | **C** | **T** | **T** | **G** | **C** | **T** | **A** | **C** | **T** | **C** | **A** | **G** | **T** | **T** | **C** | **A** | **A** | **A** | **G** | **A** | **A** | **G** | **A** | **T** | **G** | **A** | **G** | **A** | **C** | **A** | **C** | **A** | **T** | **T** | **G** | **T** | **T** | **C** | **A** | **T** | **C** | **C** | **A** | **T** | **G** | **A** | **C** | **A** | **A** | **C** | **T** | **G** | **A** | **A** | **A** | **C** | **A** | **G** | **T** | **C** | **A** | **A** | **G** | **G** | **A** | **A** | **A** | **T** | **G** | **T** | **C** | **T** | **A** | **A** | **C** | **T** | **G** | **G** | **C** | **T** | **A** | **T** | **T** | **A** | **T** | **T** | **C** | **A** | **A** | **G** | **C** | **A** | **A** | **C** | **A** | **A** | **A** | **T** | **T** | **T** | **C** | **T** | **C** | **G** | **A** | **T** | **T** | **A** | **T** | **T** | **T** | **T** | **C** | **T** | **C** | **C** | **A** | **A** | **T** | **C** | **C** | **T** | **A** | **-** | **-** | **-** |
| **N60490F** | **-** | **-** | **-** | **-** | **-** | **-** | **-** | **-** | **-** | **-** | **-** | **-** | **-** | **-** | **-** | **-** | **-** | **-** | **-** | **-** | **-** | **-** | **-** | **-** | **-** | **-** | **-** | **-** | **-** | **-** | **-** | **-** | **-** | **-** | **-** | **-** | **-** | **-** | **-** | **-** | **-** | **-** | **-** | **-** | **-** | **-** | **-** | **-** | **-** | **-** | **-** | **-** | **-** | **-** | **-** | **-** | **-** | **-** | **-** | **-** | **-** | **-** | **-** | **-** | **-** | **-** | **-** | **-** | **-** | **A** | **A** | **A** | **C** | **C** | **C** | **C** | **G** | **A** | **G** | **A** | **T** | **G** | **T** | **A** | **G** | **C** | **A** | **G** | **C** | **C** | **A** | **T** | **T** | **T** | **G** | **G** | **G** | **C** | **A** | **T** | **G** | **G** | **C** | **T** | **C** | **T** | **T** | **A** | **G** | **T** | **A** | **T** | **T** | **G** | **T** | **T** | **T** | **G** | **C** | **C** | **C** | **T** | **T** | **C** | **C** | **T** | **G** | **G** | **G** | **G** | **T** | **G** | **G** | **G** | **T** | **T** | **T** | **T** | **G** | **T** | **A** | **T** | **T** | **A** | **T** | **A** | **T** | **T** | **T** | **A** | **T** | **T** | **T** | **T** | **T** | **C** | **G** | **G** | **A** | **C** | **G** | **T** | **G** | **G** | **A** | **T** | **T** | **A** | **A** | **C** | **G** | **G** | **A** | **T** | **A** | **A** | **G** | **A** | **A** | **A** | **A** | **A** | **A** | **T** | **T** | **C** | **T** | **A** | **T** | **C** | **T** | **C** | **C** | **G** | **A** | **C** | **A** | **A** | **A** | **G** | **T** | **G** | **A** | **C** | **T** | **T** | **G** | **A** | **A** | **A** | **G** | **A** | **A** | **T** | **T** | **A** | **G** | **A** | **A** | **A** | **A** | **C** | **T** | **T** | **T** | **C** | **A** | **G** | **T** | **C** | **T** | **T** | **T** | **T** | **A** | **G** | **A** | **G** | **G** | **A** | **G** | **A** | **T** | **A** | **C** | **C** | **A** | **T** | **T** | **G** | **A** | **A** | **C** | **A** | **T** | **T** | **A** | **C** | **G** | **A** | **T** | **C** | **C** | **T** | **G** | **A** | **C** | **A** | **T** | **G** | **G** | **G** | **C** | **G** | **A** | **T** | **A** | **A** | **A** | **G** | **A** | **C** | **A** | **A** | **A** | **C** | **A** | **G** | **C** | **A** | **A** | **T** | **T** | **T** | **G** | **T** | **C** | **G** | **A** | **C** | **T** | **T** | **C** | **T** | **T** | **C** | **T** | **C** | **T** | **T** | **C** | **A** | **T** | **T** | **A** | **A** | **A** | **T** | **C** | **G** | **T** | **A** | **T** | **G** | **C** | **C** | **G** | **C** | **T** | **G** | **A** | **C** | **A** | **A** | **G** | **A** | **A** | **T** | **G** | **A** | **A** | **T** | **T** | **C** | **T** | **G** | **T** | **C** | **A** | **C** | **T** | **C** | **T** | **T** | **C** | **T** | **C** | **A** | **C** | **A** | **G** | **A** | **C** | **G** | **G** | **A** | **C** | **A** | **A** | **G** | **A** | **G** | **A** | **A** | **A** | **T** | **T** | **G** | **G** | **A** | **T** | **T** | **C** | **A** | **C** | **T** | **G** | **C** | **T** | **T** | **C** | **A** | **A** | **G** | **A** | **T** | **C** | **T** | **A** | **A** | **A** | **A** | **A** | **A** | **A** | **G** | **C** | **C** | **A** | **A** | **A** | **C** | **A** | **T** | **T** | **C** | **G** | **A** | **T** | **C** | **C** | **A** | **T** | **A** | **T** | **C** | **G** | **A** | **A** | **T** | **A** | **T** | **T** | **A** | **C** | **G** | **C** | **A** | **T** | **T** | **T** | **G** | **T** | **G** | **A** | **C** | **A** | **G** | **A** | **T** | **A** | **A** | **T** | **A** | **T** | **C** | **G** | **G** | **C** | **C** | **A** | **G** | **C** | **A** | **A** | **G** | **T** | **C** | **T** | **T** | **A** | **C** | **A** | **T** | **G** | **T** | **T** | **T** | **T** | **A** | **G** | **A** | **A** | **G** | **A** | **A** | **A** | **A** | **A** | **G** | **C** | **C** | **G** | **C** | **A** | **G** | **A** | **A** | **G** | **G** | **C** | **G** | **T** | **G** | **G** | **A** | **A** | **G** | **T** | **T** | **C** | **G** | **A** | **A** | **T** | **A** | **T** | **T** | **A** | **T** | **A** | **T** | **G** | **A** | **T** | **G** | **C** | **A** | **T** | **T** | **T** | **G** | **G** | **C** | **T** | **C** | **T** | **C** | **A** | **T** | **G** | **G** | **C** | **A** | **C** | **A** | **A** | **A** | **A** | **G** | **C** | **A** | **A** | **A** | **A** | **G** | **A** | **T** | **T** | **T** | **C** | **A** | **A** | **T** | **C** | **G** | **T** | **C** | **T** | **A** | **A** | **T** | **C** | **A** | **A** | **A** | **A** | **A** | **T** | **G** | **G** | **T** | **G** | **G** | **A** | **C** | **A** | **T** | **G** | **T** | **C** | **C** | **A** | **T** | **A** | **C** | **A** | **T** | **T** | **T** | **G** | **T** | **T** | **A** | **C** | **C** | **T** | **C** | **A** | **C** | **A** | **A** | **A** | **A** | **G** | **G** | **C** | **A** | **T** | **T** | **A** | **C** | **T** | **T** | **C** | **G** | **T** | **T** | **T** | **C** | **C** | **G** | **A** | **T** | **T** | **G** | **A** | **A** | **T** | **T** | **A** | **C** | **C** | **A** | **T** | **G** | **A** | **T** | **C** | **A** | **C** | **C** | **G** | **A** | **A** | **A** | **A** | **A** | **T** | **C** | **G** | **T** | **T** | **G** | **T** | **G** | **A** | **T** | **C** | **G** | **A** | **T** | **G** | **G** | **A** | **A** | **A** | **G** | **A** | **T** | **T** | **A** | **G** | **T** | **T** | **A** | **T** | **A** | **C** | **C** | **G** | **G** | **T** | **G** | **G** | **T** | **T** | **T** | **C** | **A** | **A** | **T** | **A** | **T** | **T** | **G** | **C** | **C** | **A** | **A** | **T** | **C** | **A** | **A** | **T** | **A** | **T** | **G** | **T** | **A** | **A** | **A** | **T** | **A** | **C** | **A** | **A** | **C** | **A** | **A** | **A** | **A** | **A** | **A** | **A** | **T** | **T** | **C** | **G** | **G** | **C** | **T** | **A** | **T** | **T** | **G** | **G** | **C** | **G** | **C** | **G** | **A** | **T** | **A** | **C** | **G** | **C** | **A** | **T** | **A** | **T** | **A** | **C** | **G** | **G** | **A** | **T** | **T** | **T** | **T** | **C** | **G** | **G** | **T** | **G** | **C** | **C** | **G** | **C** | **T** | **T** | **C** | **T** | **T** | **C** | **T** | **T** | **T** | **G** | **C** | **T** | **C** | **C** | **A** | **G** | **C** | **T** | **T** | **C** | **G** | **C** | **T** | **T** | **C** | **T** | **T** | **A** | **A** | **C** | **A** | **G** | **A** | **C** | **T** | **G** | **G** | **A** | **A** | **C** | **G** | **T** | **C** | **T** | **C** | **G** | **G** | **T** | **A** | **C** | **C** | **T** | **G** | **A** | **A** | **G** | **A** | **A** | **A** | **A** | **A** | **A** | **A** | **G** | **G** | **T** | **C** | **G** | **G** | **C** | **T** | **A** | **T** | **C** | **A** | **T** | **T** | **T** | **G** | **A** | **A** | **T** | **T** | **A** | **T** | **T** | **T** | **C** | **T** | **T** | **T** | **A** | **A** | **A** | **A** | **A** | **A** | **G** | **C** | **A** | **G** | **A** | **T** | **C** | **G** | **A** | **G** | **A** | **T** | **G** | **A** | **A** | **T** | **C** | **T** | **A** | **A** | **G** | **C** | **T** | **T** | **G** | **C** | **T** | **G** | **A** | **T** | **A** | **C** | **A** | **T** | **C** | **C** | **A** | **T** | **C** | **C** | **A** | **G** | **C** | **T** | **T** | **G** | **T** | **T** | **T** | **C** | **A** | **A** | **G** | **C** | **G** | **G** | **A** | **C** | **C** | **G** | **A** | **A** | **T** | **A** | **A** | **C** | **G** | **A** | **A** | **A** | **G** | **G** | **G** | **A** | **A** | **C** | **A** | **A** | **A** | **T** | **C** | **A** | **A** | **G** | **C** | **T** | **T** | **T** | **C** | **A** | **T** | **T** | **T** | **A** | **T** | **C** | **A** | **A** | **A** | **T** | **T** | **G** | **A** | **T** | **T** | **A** | **C** | **T** | **T** | **C** | **T** | **G** | **C** | **T** | **A** | **A** | **A** | **A** | **A** | **A** | **C** | **G** | **T** | **G** | **T** | **T** | **T** | **G** | **G** | **A** | **T** | **A** | **C** | **A** | **G** | **A** | **C** | **A** | **C** | **C** | **T** | **T** | **A** | **C** | **C** | **T** | **T** | **G** | **T** | **T** | **C** | **C** | **T** | **G** | **A** | **T** | **G** | **A** | **A** | **A** | **G** | **T** | **G** | **T** | **C** | **A** | **T** | **T** | **G** | **C** | **T** | **G** | **C** | **T** | **T** | **T** | **A** | **A** | **A** | **A** | **A** | **T** | **C** | **G** | **C** | **A** | **A** | **C** | **T** | **G** | **C** | **C** | **T** | **C** | **T** | **G** | **G** | **T** | **G** | **T** | **A** | **G** | **A** | **T** | **G** | **T** | **G** | **A** | **A** | **A** | **A** | **T** | **C** | **A** | **T** | **G** | **A** | **T** | **T** | **C** | **C** | **C** | **A** | **A** | **C** | **A** | **A** | **A** | **C** | **C** | **G** | **G** | **A** | **T** | **C** | **A** | **T** | **C** | **C** | **T** | **T** | **T** | **T** | **A** | **T** | **T** | **T** | **A** | **T** | **C** | **G** | **A** | **G** | **C** | **A** | **A** | **C** | **A** | **C** | **A** | **A** | **T** | **A** | **T** | **T** | **A** | **T** | **G** | **C** | **T** | **C** | **G** | **G** | **C** | **A** | **G** | **C** | **T** | **G** | **A** | **T** | **C** | **A** | **A** | **G** | **G** | **A** | **A** | **A** | **A** | **T** | **G** | **T** | **A** | **C** | **A** | **A** | **A** | **T** | **C** | **C** | **T** | **T** | **G** | **T** | **C** | **T** | **A** | **T** | **G** | **A** | **G** | **A** | **A** | **C** | **G** | **G** | **C** | **T** | **T** | **C** | **C** | **T** | **C** | **C** | **A** | **T** | **G** | **C** | **A** | **A** | **A** | **A** | **A** | **C** | **A** | **T** | **T** | **G** | **A** | **T** | **A** | **A** | **T** | **G** | **G** | **A** | **T** | **G** | **A** | **T** | **G** | **A** | **A** | **A** | **T** | **C** | **T** | **G** | **C** | **A** | **T** | **G** | **G** | **T** | **A** | **G** | **G** | **T** | **T** | **C** | **A** | **G** | **C** | **A** | **A** | **A** | **T** | **C** | **A** | **A** | **G** | **A** | **T** | **A** | **T** | **T** | **C** | **G** | **T** | **A** | **G** | **C** | **T** | **A** | **C** | **C** | **G** | **A** | **T** | **T** | **G** | **A** | **A** | **T** | **T** | **T** | **T** | **G** | **A** | **A** | **A** | **C** | **A** | **A** | **G** | **T** | **G** | **C** | **T** | **G** | **T** | **C** | **A** | **T** | **T** | **T** | **A** | **C** | **G** | **A** | **T** | **C** | **C** | **T** | **G** | **A** | **G** | **T** | **T** | **T** | **T** | **T** | **A** | **G** | **A** | **A** | **G** | **A** | **A** | **C** | **T** | **T** | **G** | **C** | **T** | **A** | **C** | **T** | **C** | **A** | **G** | **T** | **T** | **C** | **A** | **A** | **A** | **G** | **A** | **A** | **G** | **A** | **T** | **G** | **A** | **G** | **A** | **C** | **A** | **C** | **A** | **T** | **T** | **G** | **T** | **T** | **C** | **A** | **T** | **C** | **C** | **A** | **T** | **G** | **A** | **C** | **A** | **A** | **C** | **T** | **G** | **A** | **A** | **A** | **C** | **A** | **G** | **T** | **C** | **A** | **A** | **G** | **G** | **A** | **A** | **A** | **T** | **G** | **T** | **C** | **T** | **A** | **A** | **C** | **T** | **G** | **G** | **C** | **T** | **A** | **T** | **T** | **A** | **T** | **T** | **C** | **A** | **A** | **G** | **C** | **A** | **A** | **C** | **A** | **A** | **A** | **T** | **T** | **T** | **C** | **T** | **C** | **G** | **A** | **T** | **T** | **A** | **T** | **T** | **T** | **T** | **C** | **T** | **C** | **C** | **A** | **A** | **T** | **C** | **C** | **T** | **A** | **-** | **-** | **-** |
| **N59542F** | **-** | **-** | **-** | **-** | **-** | **-** | **-** | **-** | **-** | **-** | **-** | **-** | **-** | **-** | **-** | **-** | **-** | **-** | **-** | **-** | **-** | **-** | **-** | **-** | **-** | **-** | **-** | **-** | **-** | **-** | **-** | **-** | **-** | **-** | **-** | **-** | **-** | **-** | **-** | **-** | **-** | **-** | **-** | **-** | **-** | **-** | **-** | **-** | **-** | **-** | **-** | **-** | **-** | **-** | **-** | **-** | **-** | **-** | **-** | **-** | **-** | **-** | **-** | **-** | **-** | **-** | **-** | **-** | **-** | **A** | **A** | **A** | **C** | **C** | **C** | **C** | **G** | **A** | **G** | **A** | **T** | **G** | **T** | **A** | **G** | **C** | **A** | **G** | **C** | **C** | **A** | **T** | **T** | **T** | **G** | **G** | **G** | **C** | **A** | **T** | **G** | **G** | **C** | **T** | **C** | **T** | **T** | **A** | **G** | **T** | **A** | **T** | **T** | **G** | **T** | **T** | **T** | **G** | **C** | **C** | **C** | **T** | **T** | **C** | **C** | **T** | **G** | **G** | **G** | **G** | **T** | **G** | **G** | **G** | **T** | **T** | **T** | **T** | **G** | **T** | **A** | **T** | **T** | **A** | **T** | **A** | **T** | **T** | **T** | **A** | **T** | **T** | **T** | **T** | **T** | **C** | **G** | **G** | **A** | **C** | **G** | **T** | **G** | **G** | **A** | **T** | **T** | **A** | **A** | **C** | **G** | **G** | **A** | **T** | **A** | **A** | **G** | **A** | **A** | **A** | **A** | **A** | **A** | **T** | **T** | **C** | **T** | **A** | **T** | **C** | **T** | **C** | **C** | **G** | **A** | **C** | **A** | **A** | **A** | **G** | **T** | **G** | **A** | **C** | **T** | **T** | **G** | **A** | **A** | **A** | **G** | **A** | **A** | **T** | **T** | **A** | **G** | **A** | **A** | **A** | **A** | **C** | **T** | **T** | **T** | **C** | **A** | **G** | **T** | **C** | **T** | **T** | **T** | **T** | **A** | **G** | **A** | **G** | **G** | **A** | **G** | **A** | **T** | **A** | **C** | **C** | **A** | **T** | **T** | **G** | **A** | **A** | **C** | **A** | **T** | **T** | **A** | **C** | **G** | **A** | **T** | **C** | **C** | **T** | **G** | **A** | **C** | **A** | **T** | **G** | **G** | **G** | **C** | **G** | **A** | **T** | **A** | **A** | **A** | **G** | **A** | **C** | **A** | **A** | **A** | **C** | **A** | **G** | **C** | **A** | **A** | **T** | **T** | **T** | **G** | **T** | **C** | **G** | **A** | **C** | **T** | **T** | **C** | **T** | **T** | **C** | **T** | **C** | **T** | **T** | **C** | **A** | **T** | **T** | **A** | **A** | **A** | **T** | **C** | **G** | **T** | **A** | **T** | **G** | **C** | **C** | **G** | **C** | **T** | **G** | **A** | **C** | **A** | **A** | **G** | **A** | **A** | **T** | **G** | **A** | **A** | **T** | **T** | **C** | **T** | **G** | **T** | **C** | **A** | **C** | **T** | **C** | **T** | **T** | **C** | **T** | **C** | **A** | **C** | **A** | **G** | **A** | **C** | **G** | **G** | **A** | **C** | **A** | **A** | **G** | **A** | **G** | **A** | **A** | **A** | **T** | **T** | **G** | **G** | **A** | **T** | **T** | **C** | **A** | **C** | **T** | **G** | **C** | **T** | **T** | **C** | **A** | **A** | **G** | **A** | **T** | **C** | **T** | **A** | **A** | **A** | **A** | **A** | **A** | **A** | **G** | **C** | **C** | **A** | **A** | **A** | **C** | **A** | **T** | **T** | **C** | **G** | **A** | **T** | **C** | **C** | **A** | **T** | **A** | **T** | **C** | **G** | **A** | **A** | **T** | **A** | **T** | **T** | **A** | **C** | **G** | **C** | **A** | **T** | **T** | **T** | **G** | **T** | **G** | **A** | **C** | **A** | **G** | **A** | **T** | **A** | **A** | **T** | **A** | **T** | **C** | **G** | **G** | **C** | **C** | **A** | **G** | **C** | **A** | **A** | **G** | **T** | **C** | **T** | **T** | **A** | **C** | **A** | **T** | **G** | **T** | **T** | **T** | **T** | **A** | **G** | **A** | **A** | **G** | **A** | **A** | **A** | **A** | **A** | **G** | **C** | **C** | **G** | **C** | **A** | **G** | **A** | **A** | **G** | **G** | **C** | **G** | **T** | **G** | **G** | **A** | **A** | **G** | **T** | **T** | **C** | **G** | **A** | **A** | **T** | **A** | **T** | **T** | **A** | **T** | **A** | **T** | **G** | **A** | **T** | **G** | **C** | **A** | **T** | **T** | **T** | **G** | **G** | **C** | **T** | **C** | **T** | **C** | **A** | **T** | **G** | **G** | **C** | **A** | **C** | **A** | **A** | **A** | **A** | **G** | **C** | **A** | **A** | **A** | **A** | **G** | **A** | **T** | **T** | **T** | **C** | **A** | **A** | **T** | **C** | **G** | **T** | **C** | **T** | **A** | **A** | **T** | **C** | **A** | **A** | **A** | **A** | **A** | **T** | **G** | **G** | **T** | **G** | **G** | **A** | **C** | **A** | **T** | **G** | **T** | **C** | **C** | **A** | **T** | **A** | **C** | **A** | **T** | **T** | **T** | **G** | **T** | **T** | **A** | **C** | **C** | **T** | **C** | **A** | **C** | **A** | **A** | **A** | **A** | **G** | **G** | **C** | **A** | **T** | **T** | **A** | **C** | **T** | **T** | **C** | **G** | **T** | **T** | **T** | **C** | **C** | **G** | **A** | **T** | **T** | **G** | **A** | **A** | **T** | **T** | **A** | **C** | **C** | **A** | **T** | **G** | **A** | **T** | **C** | **A** | **C** | **C** | **G** | **A** | **A** | **A** | **A** | **A** | **T** | **C** | **G** | **T** | **T** | **G** | **T** | **G** | **A** | **T** | **C** | **G** | **A** | **T** | **G** | **G** | **A** | **A** | **A** | **G** | **A** | **T** | **T** | **A** | **G** | **T** | **T** | **A** | **T** | **A** | **C** | **C** | **G** | **G** | **T** | **G** | **G** | **T** | **T** | **T** | **C** | **A** | **A** | **T** | **A** | **T** | **T** | **G** | **C** | **C** | **A** | **A** | **T** | **C** | **A** | **A** | **T** | **A** | **T** | **G** | **T** | **A** | **A** | **A** | **T** | **A** | **C** | **A** | **A** | **C** | **A** | **A** | **A** | **A** | **A** | **A** | **A** | **T** | **T** | **C** | **G** | **G** | **C** | **T** | **A** | **T** | **T** | **G** | **G** | **C** | **G** | **C** | **G** | **A** | **T** | **A** | **C** | **G** | **C** | **A** | **T** | **A** | **T** | **A** | **C** | **G** | **G** | **A** | **T** | **T** | **T** | **T** | **C** | **G** | **G** | **T** | **G** | **C** | **C** | **G** | **C** | **T** | **T** | **C** | **T** | **T** | **C** | **T** | **T** | **T** | **G** | **C** | **T** | **C** | **C** | **A** | **G** | **C** | **T** | **T** | **C** | **G** | **C** | **T** | **T** | **C** | **T** | **T** | **A** | **A** | **C** | **A** | **G** | **A** | **C** | **T** | **G** | **G** | **A** | **A** | **C** | **G** | **T** | **C** | **T** | **C** | **G** | **G** | **T** | **A** | **C** | **C** | **T** | **G** | **A** | **A** | **G** | **A** | **A** | **A** | **A** | **A** | **A** | **A** | **G** | **G** | **T** | **C** | **G** | **G** | **C** | **T** | **A** | **T** | **C** | **A** | **T** | **T** | **T** | **G** | **A** | **A** | **T** | **T** | **A** | **T** | **T** | **T** | **C** | **T** | **T** | **T** | **A** | **A** | **A** | **A** | **A** | **A** | **G** | **C** | **A** | **G** | **A** | **T** | **C** | **G** | **A** | **G** | **A** | **T** | **G** | **A** | **A** | **T** | **C** | **T** | **A** | **A** | **G** | **C** | **T** | **T** | **G** | **C** | **T** | **G** | **A** | **T** | **A** | **C** | **A** | **T** | **C** | **C** | **A** | **T** | **C** | **C** | **A** | **G** | **C** | **T** | **T** | **G** | **T** | **T** | **T** | **C** | **A** | **A** | **G** | **C** | **G** | **G** | **A** | **C** | **C** | **G** | **A** | **A** | **T** | **A** | **A** | **C** | **G** | **A** | **A** | **A** | **G** | **G** | **G** | **A** | **A** | **C** | **A** | **A** | **A** | **T** | **C** | **A** | **A** | **G** | **C** | **T** | **T** | **T** | **C** | **A** | **T** | **T** | **T** | **A** | **T** | **C** | **A** | **A** | **A** | **T** | **T** | **G** | **A** | **T** | **T** | **A** | **C** | **T** | **T** | **C** | **T** | **G** | **C** | **T** | **A** | **A** | **A** | **A** | **A** | **A** | **C** | **G** | **T** | **G** | **T** | **T** | **T** | **G** | **G** | **A** | **T** | **A** | **C** | **A** | **G** | **A** | **C** | **A** | **C** | **C** | **T** | **T** | **A** | **C** | **C** | **T** | **T** | **G** | **T** | **T** | **C** | **C** | **T** | **G** | **A** | **T** | **G** | **A** | **A** | **A** | **G** | **T** | **G** | **T** | **C** | **A** | **T** | **T** | **G** | **C** | **T** | **G** | **C** | **T** | **T** | **T** | **A** | **A** | **A** | **A** | **A** | **T** | **C** | **G** | **C** | **A** | **A** | **C** | **T** | **G** | **C** | **C** | **T** | **C** | **T** | **G** | **G** | **T** | **G** | **T** | **A** | **G** | **A** | **T** | **G** | **T** | **G** | **A** | **A** | **A** | **A** | **T** | **C** | **A** | **T** | **G** | **A** | **T** | **T** | **C** | **C** | **C** | **A** | **A** | **C** | **A** | **A** | **A** | **C** | **C** | **G** | **G** | **A** | **T** | **C** | **A** | **T** | **C** | **C** | **T** | **T** | **T** | **T** | **A** | **T** | **T** | **T** | **A** | **T** | **C** | **G** | **A** | **G** | **C** | **A** | **A** | **C** | **A** | **C** | **A** | **A** | **T** | **A** | **T** | **T** | **A** | **T** | **G** | **C** | **T** | **C** | **G** | **G** | **C** | **A** | **G** | **C** | **T** | **G** | **A** | **T** | **C** | **A** | **A** | **G** | **G** | **A** | **A** | **A** | **A** | **T** | **G** | **T** | **A** | **C** | **A** | **A** | **A** | **T** | **C** | **C** | **T** | **T** | **G** | **T** | **C** | **T** | **A** | **T** | **G** | **A** | **G** | **A** | **A** | **C** | **G** | **G** | **C** | **T** | **T** | **C** | **C** | **T** | **C** | **C** | **A** | **T** | **G** | **C** | **A** | **A** | **A** | **A** | **A** | **C** | **A** | **T** | **T** | **G** | **A** | **T** | **A** | **A** | **T** | **G** | **G** | **A** | **T** | **G** | **A** | **T** | **G** | **A** | **A** | **A** | **T** | **C** | **T** | **G** | **C** | **A** | **T** | **G** | **G** | **T** | **A** | **G** | **G** | **T** | **T** | **C** | **A** | **G** | **C** | **A** | **A** | **A** | **T** | **C** | **A** | **A** | **G** | **A** | **T** | **A** | **T** | **T** | **C** | **G** | **T** | **A** | **G** | **C** | **T** | **A** | **C** | **C** | **G** | **A** | **T** | **T** | **G** | **A** | **A** | **T** | **T** | **T** | **T** | **G** | **A** | **A** | **A** | **C** | **A** | **A** | **G** | **T** | **G** | **C** | **T** | **G** | **T** | **C** | **A** | **T** | **T** | **T** | **A** | **C** | **G** | **A** | **T** | **C** | **C** | **T** | **G** | **A** | **G** | **T** | **T** | **T** | **T** | **T** | **A** | **G** | **A** | **A** | **G** | **A** | **A** | **C** | **T** | **T** | **G** | **C** | **T** | **A** | **C** | **T** | **C** | **A** | **G** | **T** | **T** | **C** | **A** | **A** | **A** | **G** | **A** | **A** | **G** | **A** | **T** | **G** | **A** | **G** | **A** | **C** | **A** | **C** | **A** | **T** | **T** | **G** | **T** | **T** | **C** | **A** | **T** | **C** | **C** | **A** | **T** | **G** | **A** | **C** | **A** | **A** | **C** | **T** | **G** | **A** | **A** | **A** | **C** | **A** | **G** | **T** | **C** | **A** | **A** | **G** | **G** | **A** | **A** | **A** | **T** | **G** | **T** | **C** | **T** | **A** | **A** | **C** | **T** | **G** | **G** | **C** | **T** | **A** | **T** | **T** | **A** | **T** | **T** | **C** | **A** | **A** | **G** | **C** | **A** | **A** | **C** | **A** | **A** | **A** | **T** | **T** | **T** | **C** | **T** | **C** | **G** | **A** | **T** | **T** | **A** | **T** | **T** | **T** | **T** | **C** | **T** | **C** | **C** | **A** | **A** | **T** | **C** | **C** | **T** | **A** | **-** | **-** | **-** |
| **N54519** | **-** | **-** | **-** | **-** | **-** | **-** | **-** | **-** | **-** | **-** | **-** | **-** | **-** | **-** | **-** | **-** | **-** | **-** | **-** | **-** | **-** | **-** | **-** | **-** | **-** | **-** | **-** | **-** | **-** | **-** | **-** | **-** | **-** | **-** | **-** | **-** | **-** | **-** | **-** | **-** | **-** | **-** | **-** | **-** | **-** | **-** | **-** | **-** | **-** | **-** | **-** | **-** | **-** | **-** | **-** | **-** | **-** | **-** | **-** | **-** | **-** | **-** | **-** | **-** | **-** | **-** | **-** | **-** | **-** | **A** | **A** | **A** | **C** | **C** | **C** | **C** | **G** | **A** | **G** | **A** | **T** | **G** | **T** | **A** | **G** | **C** | **A** | **G** | **C** | **T** | **A** | **T** | **T** | **T** | **G** | **G** | **G** | **C** | **A** | **T** | **G** | **G** | **C** | **T** | **C** | **T** | **T** | **A** | **G** | **T** | **A** | **T** | **T** | **A** | **T** | **T** | **T** | **G** | **C** | **C** | **C** | **T** | **T** | **C** | **C** | **T** | **G** | **G** | **G** | **G** | **T** | **G** | **G** | **G** | **T** | **T** | **T** | **T** | **G** | **T** | **A** | **T** | **T** | **A** | **T** | **A** | **T** | **T** | **T** | **A** | **T** | **T** | **T** | **T** | **T** | **C** | **G** | **G** | **A** | **C** | **G** | **T** | **G** | **G** | **A** | **T** | **T** | **A** | **A** | **C** | **G** | **G** | **A** | **T** | **A** | **A** | **G** | **A** | **A** | **A** | **A** | **A** | **A** | **T** | **T** | **C** | **T** | **A** | **T** | **C** | **T** | **C** | **C** | **G** | **A** | **C** | **A** | **A** | **A** | **G** | **T** | **G** | **A** | **C** | **T** | **T** | **G** | **A** | **A** | **A** | **G** | **A** | **A** | **T** | **T** | **A** | **G** | **A** | **A** | **A** | **A** | **C** | **T** | **T** | **T** | **C** | **A** | **G** | **T** | **C** | **T** | **T** | **T** | **T** | **A** | **G** | **A** | **G** | **G** | **A** | **G** | **A** | **T** | **A** | **C** | **C** | **A** | **T** | **T** | **G** | **A** | **A** | **C** | **A** | **T** | **T** | **A** | **C** | **G** | **A** | **T** | **C** | **C** | **T** | **G** | **A** | **C** | **A** | **T** | **G** | **G** | **G** | **C** | **G** | **A** | **C** | **A** | **A** | **A** | **G** | **A** | **C** | **A** | **A** | **A** | **C** | **A** | **A** | **C** | **A** | **A** | **T** | **T** | **T** | **G** | **T** | **C** | **G** | **A** | **C** | **T** | **T** | **C** | **T** | **T** | **C** | **T** | **C** | **T** | **T** | **C** | **A** | **T** | **T** | **A** | **A** | **A** | **T** | **C** | **G** | **T** | **A** | **T** | **G** | **C** | **C** | **G** | **C** | **T** | **G** | **A** | **C** | **A** | **A** | **G** | **A** | **A** | **T** | **G** | **A** | **A** | **T** | **T** | **C** | **T** | **G** | **T** | **C** | **A** | **C** | **T** | **C** | **T** | **T** | **C** | **T** | **C** | **A** | **C** | **A** | **G** | **A** | **C** | **G** | **G** | **A** | **C** | **A** | **A** | **A** | **A** | **G** | **A** | **A** | **A** | **T** | **T** | **A** | **G** | **A** | **T** | **T** | **C** | **A** | **C** | **T** | **G** | **C** | **T** | **T** | **C** | **A** | **A** | **G** | **A** | **T** | **C** | **T** | **A** | **A** | **A** | **A** | **A** | **A** | **A** | **G** | **C** | **C** | **A** | **A** | **A** | **C** | **A** | **T** | **T** | **C** | **G** | **A** | **T** | **C** | **C** | **A** | **T** | **A** | **T** | **C** | **G** | **A** | **A** | **T** | **A** | **T** | **T** | **A** | **C** | **G** | **C** | **A** | **T** | **T** | **T** | **G** | **T** | **G** | **A** | **C** | **A** | **G** | **A** | **T** | **A** | **C** | **T** | **A** | **T** | **C** | **G** | **G** | **T** | **C** | **A** | **G** | **C** | **A** | **A** | **G** | **T** | **C** | **T** | **T** | **A** | **C** | **G** | **C** | **G** | **T** | **T** | **T** | **T** | **A** | **G** | **A** | **A** | **G** | **A** | **A** | **A** | **A** | **A** | **G** | **C** | **C** | **G** | **C** | **A** | **G** | **A** | **A** | **G** | **G** | **C** | **G** | **T** | **A** | **G** | **A** | **A** | **G** | **T** | **T** | **C** | **G** | **T** | **A** | **T** | **A** | **T** | **T** | **A** | **T** | **A** | **T** | **G** | **A** | **T** | **G** | **C** | **A** | **T** | **T** | **T** | **G** | **G** | **C** | **T** | **C** | **C** | **C** | **A** | **C** | **G** | **G** | **C** | **A** | **C** | **A** | **A** | **A** | **A** | **G** | **C** | **A** | **A** | **A** | **A** | **G** | **A** | **T** | **T** | **T** | **C** | **A** | **A** | **T** | **C** | **G** | **T** | **C** | **T** | **A** | **A** | **T** | **C** | **A** | **A** | **A** | **A** | **A** | **T** | **G** | **G** | **T** | **G** | **G** | **A** | **C** | **A** | **T** | **G** | **T** | **C** | **C** | **A** | **T** | **A** | **C** | **A** | **T** | **T** | **T** | **G** | **T** | **T** | **A** | **C** | **C** | **T** | **C** | **G** | **C** | **A** | **A** | **A** | **A** | **A** | **G** | **C** | **A** | **T** | **T** | **G** | **C** | **T** | **T** | **C** | **G** | **T** | **T** | **T** | **C** | **C** | **G** | **A** | **T** | **T** | **G** | **A** | **A** | **C** | **T** | **A** | **C** | **C** | **A** | **T** | **G** | **A** | **T** | **C** | **A** | **C** | **C** | **G** | **G** | **A** | **A** | **A** | **A** | **T** | **C** | **G** | **T** | **T** | **G** | **T** | **G** | **A** | **T** | **C** | **G** | **A** | **T** | **G** | **G** | **A** | **A** | **A** | **G** | **A** | **T** | **C** | **A** | **G** | **T** | **T** | **A** | **C** | **A** | **C** | **C** | **G** | **G** | **T** | **G** | **G** | **C** | **T** | **T** | **C** | **A** | **A** | **C** | **A** | **T** | **T** | **G** | **C** | **C** | **A** | **A** | **T** | **C** | **A** | **A** | **T** | **A** | **T** | **G** | **T** | **A** | **A** | **A** | **T** | **A** | **C** | **G** | **A** | **C** | **A** | **A** | **A** | **A** | **A** | **A** | **A** | **T** | **T** | **C** | **G** | **G** | **C** | **T** | **A** | **T** | **T** | **G** | **G** | **C** | **G** | **T** | **G** | **A** | **T** | **A** | **C** | **G** | **C** | **A** | **T** | **A** | **T** | **A** | **C** | **G** | **G** | **A** | **T** | **T** | **T** | **T** | **C** | **G** | **G** | **T** | **G** | **C** | **C** | **G** | **C** | **T** | **T** | **C** | **T** | **T** | **C** | **T** | **T** | **T** | **G** | **C** | **T** | **T** | **C** | **A** | **A** | **C** | **T** | **T** | **C** | **G** | **T** | **T** | **T** | **T** | **T** | **T** | **A** | **A** | **C** | **A** | **G** | **A** | **C** | **T** | **G** | **G** | **A** | **A** | **C** | **G** | **T** | **C** | **T** | **C** | **A** | **G** | **T** | **T** | **C** | **C** | **T** | **G** | **A** | **A** | **G** | **A** | **A** | **A** | **A** | **A** | **A** | **A** | **G** | **G** | **T** | **C** | **G** | **G** | **C** | **T** | **A** | **T** | **C** | **A** | **T** | **T** | **T** | **G** | **A** | **A** | **T** | **T** | **A** | **T** | **T** | **T** | **C** | **T** | **T** | **T** | **A** | **A** | **A** | **A** | **A** | **A** | **G** | **C** | **C** | **G** | **A** | **T** | **C** | **G** | **A** | **G** | **A** | **C** | **G** | **A** | **A** | **T** | **C** | **C** | **A** | **A** | **A** | **C** | **T** | **T** | **G** | **C** | **T** | **G** | **A** | **T** | **A** | **C** | **A** | **T** | **C** | **C** | **A** | **T** | **C** | **C** | **A** | **G** | **C** | **T** | **T** | **G** | **T** | **C** | **T** | **C** | **A** | **A** | **G** | **C** | **G** | **G** | **A** | **C** | **C** | **A** | **A** | **A** | **T** | **A** | **A** | **C** | **G** | **A** | **A** | **C** | **G** | **C** | **G** | **A** | **A** | **C** | **A** | **A** | **A** | **T** | **C** | **A** | **A** | **G** | **C** | **T** | **T** | **T** | **C** | **G** | **T** | **T** | **T** | **A** | **T** | **C** | **A** | **A** | **A** | **T** | **T** | **G** | **A** | **T** | **T** | **A** | **C** | **T** | **T** | **C** | **T** | **G** | **C** | **T** | **A** | **A** | **A** | **A** | **A** | **A** | **C** | **G** | **T** | **G** | **T** | **T** | **T** | **G** | **G** | **A** | **T** | **A** | **C** | **A** | **G** | **A** | **C** | **G** | **C** | **C** | **T** | **T** | **A** | **C** | **C** | **T** | **C** | **G** | **T** | **T** | **C** | **C** | **T** | **G** | **A** | **T** | **G** | **A** | **A** | **A** | **G** | **T** | **G** | **T** | **C** | **A** | **T** | **T** | **G** | **C** | **T** | **G** | **C** | **T** | **T** | **T** | **G** | **A** | **A** | **A** | **A** | **T** | **C** | **G** | **C** | **A** | **A** | **C** | **T** | **G** | **C** | **T** | **T** | **C** | **T** | **G** | **G** | **T** | **G** | **T** | **C** | **G** | **A** | **T** | **G** | **T** | **G** | **A** | **A** | **A** | **A** | **T** | **C** | **A** | **T** | **G** | **A** | **T** | **C** | **C** | **C** | **C** | **A** | **A** | **T** | **A** | **A** | **A** | **C** | **C** | **G** | **G** | **A** | **T** | **C** | **A** | **T** | **C** | **C** | **T** | **T** | **T** | **T** | **A** | **T** | **T** | **T** | **A** | **T** | **C** | **G** | **A** | **G** | **C** | **A** | **A** | **C** | **A** | **C** | **A** | **A** | **T** | **A** | **T** | **T** | **A** | **T** | **G** | **C** | **G** | **C** | **G** | **T** | **C** | **A** | **G** | **C** | **T** | **C** | **A** | **T** | **C** | **A** | **A** | **G** | **G** | **A** | **A** | **A** | **A** | **C** | **G** | **T** | **A** | **C** | **A** | **A** | **A** | **T** | **T** | **C** | **T** | **T** | **G** | **T** | **C** | **T** | **A** | **T** | **G** | **A** | **G** | **A** | **A** | **T** | **G** | **G** | **C** | **T** | **T** | **C** | **C** | **T** | **C** | **C** | **A** | **T** | **G** | **C** | **A** | **A** | **A** | **A** | **A** | **C** | **A** | **T** | **T** | **G** | **A** | **T** | **A** | **A** | **T** | **G** | **G** | **A** | **T** | **G** | **A** | **T** | **G** | **A** | **A** | **A** | **T** | **C** | **T** | **G** | **C** | **A** | **T** | **G** | **G** | **T** | **A** | **G** | **G** | **T** | **T** | **C** | **A** | **G** | **C** | **A** | **A** | **A** | **T** | **C** | **A** | **A** | **G** | **A** | **T** | **A** | **T** | **T** | **C** | **G** | **T** | **A** | **G** | **C** | **T** | **A** | **C** | **C** | **G** | **A** | **T** | **T** | **G** | **A** | **A** | **T** | **T** | **T** | **T** | **G** | **A** | **A** | **A** | **C** | **A** | **A** | **G** | **T** | **G** | **C** | **T** | **G** | **T** | **C** | **A** | **T** | **C** | **T** | **A** | **C** | **G** | **A** | **T** | **C** | **C** | **T** | **G** | **A** | **G** | **T** | **T** | **T** | **T** | **T** | **A** | **G** | **A** | **A** | **G** | **A** | **A** | **C** | **T** | **T** | **G** | **C** | **T** | **A** | **C** | **T** | **C** | **A** | **G** | **T** | **T** | **T** | **A** | **A** | **A** | **G** | **A** | **A** | **G** | **A** | **T** | **G** | **A** | **G** | **A** | **C** | **A** | **C** | **A** | **T** | **T** | **G** | **T** | **T** | **C** | **A** | **C** | **C** | **A** | **A** | **T** | **G** | **A** | **C** | **G** | **A** | **C** | **T** | **G** | **A** | **A** | **A** | **C** | **A** | **G** | **T** | **G** | **A** | **A** | **G** | **G** | **A** | **A** | **A** | **T** | **G** | **T** | **C** | **T** | **A** | **A** | **T** | **T** | **G** | **G** | **C** | **T** | **G** | **T** | **T** | **A** | **T** | **T** | **C** | **A** | **A** | **G** | **C** | **A** | **A** | **C** | **A** | **A** | **A** | **T** | **T** | **T** | **C** | **T** | **C** | **G** | **A** | **T** | **T** | **A** | **T** | **T** | **T** | **T** | **C** | **T** | **C** | **C** | **A** | **A** | **T** | **C** | **T** | **T** | **A** | **-** | **-** | **-** |
| **N53533** | **-** | **-** | **-** | **-** | **-** | **-** | **-** | **-** | **-** | **-** | **-** | **-** | **-** | **-** | **-** | **-** | **-** | **-** | **-** | **-** | **-** | **-** | **-** | **-** | **-** | **-** | **-** | **-** | **-** | **-** | **-** | **-** | **-** | **-** | **-** | **-** | **-** | **-** | **-** | **-** | **-** | **-** | **-** | **-** | **-** | **-** | **-** | **-** | **-** | **-** | **-** | **-** | **-** | **-** | **-** | **-** | **-** | **-** | **-** | **-** | **-** | **-** | **-** | **-** | **-** | **-** | **-** | **-** | **-** | **A** | **A** | **A** | **C** | **C** | **C** | **C** | **G** | **A** | **G** | **A** | **T** | **G** | **T** | **A** | **G** | **C** | **A** | **G** | **C** | **C** | **A** | **T** | **T** | **T** | **G** | **G** | **G** | **C** | **A** | **T** | **G** | **G** | **C** | **T** | **C** | **T** | **T** | **A** | **G** | **T** | **A** | **T** | **T** | **G** | **T** | **T** | **T** | **G** | **C** | **C** | **C** | **T** | **T** | **C** | **C** | **T** | **G** | **G** | **G** | **G** | **T** | **G** | **G** | **G** | **T** | **T** | **T** | **T** | **G** | **T** | **A** | **T** | **T** | **A** | **T** | **A** | **T** | **T** | **T** | **A** | **T** | **T** | **T** | **T** | **T** | **C** | **G** | **G** | **A** | **C** | **G** | **T** | **G** | **G** | **A** | **T** | **T** | **A** | **A** | **C** | **G** | **G** | **A** | **T** | **A** | **A** | **G** | **A** | **A** | **A** | **A** | **A** | **A** | **T** | **T** | **C** | **T** | **A** | **T** | **C** | **T** | **C** | **C** | **G** | **A** | **C** | **A** | **A** | **A** | **G** | **T** | **G** | **A** | **C** | **T** | **T** | **G** | **A** | **A** | **A** | **G** | **A** | **A** | **T** | **T** | **A** | **G** | **A** | **A** | **A** | **A** | **C** | **T** | **T** | **T** | **C** | **A** | **G** | **T** | **C** | **T** | **T** | **T** | **T** | **A** | **G** | **A** | **G** | **G** | **A** | **G** | **A** | **T** | **A** | **C** | **C** | **A** | **T** | **T** | **G** | **A** | **A** | **C** | **A** | **T** | **T** | **A** | **C** | **G** | **A** | **T** | **C** | **C** | **T** | **G** | **A** | **C** | **A** | **T** | **G** | **G** | **G** | **C** | **G** | **A** | **T** | **A** | **A** | **A** | **G** | **A** | **C** | **A** | **A** | **A** | **C** | **A** | **G** | **C** | **A** | **A** | **T** | **T** | **T** | **G** | **T** | **C** | **G** | **A** | **C** | **T** | **T** | **C** | **T** | **T** | **C** | **T** | **C** | **T** | **T** | **C** | **A** | **T** | **T** | **A** | **A** | **A** | **T** | **C** | **G** | **T** | **A** | **T** | **G** | **C** | **C** | **G** | **C** | **T** | **G** | **A** | **C** | **A** | **A** | **G** | **A** | **A** | **T** | **G** | **A** | **A** | **T** | **T** | **C** | **T** | **G** | **T** | **C** | **A** | **C** | **T** | **C** | **T** | **T** | **C** | **T** | **C** | **A** | **C** | **A** | **G** | **A** | **C** | **G** | **G** | **A** | **C** | **A** | **A** | **G** | **A** | **G** | **A** | **A** | **A** | **T** | **T** | **G** | **G** | **A** | **T** | **T** | **C** | **A** | **C** | **T** | **G** | **C** | **T** | **T** | **C** | **A** | **A** | **G** | **A** | **T** | **C** | **T** | **A** | **A** | **A** | **A** | **A** | **A** | **A** | **G** | **C** | **C** | **A** | **A** | **A** | **C** | **A** | **T** | **T** | **C** | **G** | **A** | **T** | **C** | **C** | **A** | **T** | **A** | **T** | **C** | **G** | **A** | **A** | **T** | **A** | **T** | **T** | **A** | **C** | **G** | **C** | **A** | **T** | **T** | **T** | **G** | **T** | **G** | **A** | **C** | **A** | **G** | **A** | **T** | **A** | **A** | **T** | **A** | **T** | **C** | **G** | **G** | **C** | **C** | **A** | **G** | **C** | **A** | **A** | **G** | **T** | **C** | **T** | **T** | **A** | **C** | **A** | **T** | **G** | **T** | **T** | **T** | **T** | **A** | **G** | **A** | **A** | **G** | **A** | **A** | **A** | **A** | **A** | **G** | **C** | **C** | **G** | **C** | **A** | **G** | **A** | **A** | **G** | **G** | **C** | **G** | **T** | **G** | **G** | **A** | **A** | **G** | **T** | **T** | **C** | **G** | **A** | **A** | **T** | **A** | **T** | **T** | **A** | **T** | **A** | **T** | **G** | **A** | **T** | **G** | **C** | **A** | **T** | **T** | **T** | **G** | **G** | **C** | **T** | **C** | **T** | **C** | **A** | **T** | **G** | **G** | **C** | **A** | **C** | **A** | **A** | **A** | **A** | **G** | **C** | **A** | **A** | **A** | **A** | **G** | **A** | **T** | **T** | **T** | **C** | **A** | **A** | **T** | **C** | **G** | **T** | **C** | **T** | **A** | **A** | **T** | **C** | **A** | **A** | **A** | **A** | **A** | **T** | **G** | **G** | **T** | **G** | **G** | **A** | **C** | **A** | **T** | **G** | **T** | **C** | **C** | **A** | **T** | **A** | **C** | **A** | **T** | **T** | **T** | **G** | **T** | **T** | **A** | **C** | **C** | **T** | **C** | **A** | **C** | **A** | **A** | **A** | **A** | **G** | **G** | **C** | **A** | **T** | **T** | **A** | **C** | **T** | **T** | **C** | **G** | **T** | **T** | **T** | **C** | **C** | **G** | **A** | **T** | **T** | **G** | **A** | **A** | **T** | **T** | **A** | **C** | **C** | **A** | **T** | **G** | **A** | **T** | **C** | **A** | **C** | **C** | **G** | **A** | **A** | **A** | **A** | **A** | **T** | **C** | **G** | **T** | **T** | **G** | **T** | **G** | **A** | **T** | **C** | **G** | **A** | **T** | **G** | **G** | **A** | **A** | **A** | **G** | **A** | **T** | **T** | **A** | **G** | **T** | **T** | **A** | **T** | **A** | **C** | **C** | **G** | **G** | **T** | **G** | **G** | **T** | **T** | **T** | **C** | **A** | **A** | **T** | **A** | **T** | **T** | **G** | **C** | **C** | **A** | **A** | **T** | **C** | **A** | **A** | **T** | **A** | **T** | **G** | **T** | **A** | **A** | **A** | **T** | **A** | **C** | **A** | **A** | **C** | **A** | **A** | **A** | **A** | **A** | **A** | **A** | **T** | **T** | **C** | **G** | **G** | **C** | **T** | **A** | **T** | **T** | **G** | **G** | **C** | **G** | **C** | **G** | **A** | **T** | **A** | **C** | **G** | **C** | **A** | **T** | **A** | **T** | **A** | **C** | **G** | **G** | **A** | **T** | **T** | **T** | **T** | **C** | **G** | **G** | **T** | **G** | **C** | **C** | **G** | **C** | **T** | **T** | **C** | **T** | **T** | **C** | **T** | **T** | **T** | **G** | **C** | **T** | **C** | **C** | **A** | **G** | **C** | **T** | **T** | **C** | **G** | **C** | **T** | **T** | **C** | **T** | **T** | **A** | **A** | **C** | **A** | **G** | **A** | **C** | **T** | **G** | **G** | **A** | **A** | **C** | **G** | **T** | **C** | **T** | **C** | **G** | **G** | **T** | **A** | **C** | **C** | **T** | **G** | **A** | **A** | **G** | **A** | **A** | **A** | **A** | **A** | **A** | **A** | **G** | **G** | **T** | **C** | **G** | **G** | **C** | **T** | **A** | **T** | **C** | **A** | **T** | **T** | **T** | **G** | **A** | **A** | **T** | **T** | **A** | **T** | **T** | **T** | **C** | **T** | **T** | **T** | **A** | **A** | **A** | **A** | **A** | **A** | **G** | **C** | **A** | **G** | **A** | **T** | **C** | **G** | **A** | **G** | **A** | **T** | **G** | **A** | **A** | **T** | **C** | **T** | **A** | **A** | **G** | **C** | **T** | **T** | **G** | **C** | **T** | **G** | **A** | **T** | **A** | **C** | **A** | **T** | **C** | **C** | **A** | **T** | **C** | **C** | **A** | **G** | **C** | **T** | **T** | **G** | **T** | **T** | **T** | **C** | **A** | **A** | **G** | **C** | **G** | **G** | **A** | **C** | **C** | **G** | **A** | **A** | **T** | **A** | **A** | **C** | **G** | **A** | **A** | **A** | **G** | **G** | **G** | **A** | **A** | **C** | **A** | **A** | **A** | **T** | **C** | **A** | **A** | **G** | **C** | **T** | **T** | **T** | **C** | **A** | **T** | **T** | **T** | **A** | **T** | **C** | **A** | **A** | **A** | **T** | **T** | **G** | **A** | **T** | **T** | **A** | **C** | **T** | **T** | **C** | **T** | **G** | **C** | **T** | **A** | **A** | **A** | **A** | **A** | **A** | **C** | **G** | **T** | **G** | **T** | **T** | **T** | **G** | **G** | **A** | **T** | **A** | **C** | **A** | **G** | **A** | **C** | **A** | **C** | **C** | **T** | **T** | **A** | **C** | **C** | **T** | **T** | **G** | **T** | **T** | **C** | **C** | **T** | **G** | **A** | **T** | **G** | **A** | **A** | **A** | **G** | **T** | **G** | **T** | **C** | **A** | **T** | **T** | **G** | **C** | **T** | **G** | **C** | **T** | **T** | **T** | **A** | **A** | **A** | **A** | **A** | **T** | **C** | **G** | **C** | **A** | **A** | **C** | **T** | **G** | **C** | **C** | **T** | **C** | **T** | **G** | **G** | **T** | **G** | **T** | **A** | **G** | **A** | **T** | **G** | **T** | **G** | **A** | **A** | **A** | **A** | **T** | **C** | **A** | **T** | **G** | **A** | **T** | **T** | **C** | **C** | **C** | **A** | **A** | **C** | **A** | **A** | **A** | **C** | **C** | **G** | **G** | **A** | **T** | **C** | **A** | **T** | **C** | **C** | **T** | **T** | **T** | **T** | **A** | **T** | **T** | **T** | **A** | **T** | **C** | **G** | **A** | **G** | **C** | **A** | **A** | **C** | **A** | **C** | **A** | **A** | **T** | **A** | **T** | **T** | **A** | **T** | **G** | **C** | **T** | **C** | **G** | **G** | **C** | **A** | **G** | **C** | **T** | **G** | **A** | **T** | **C** | **A** | **A** | **G** | **G** | **A** | **A** | **A** | **A** | **T** | **G** | **T** | **A** | **C** | **A** | **A** | **A** | **T** | **C** | **C** | **T** | **T** | **G** | **T** | **C** | **T** | **A** | **T** | **G** | **A** | **G** | **A** | **A** | **C** | **G** | **G** | **C** | **T** | **T** | **C** | **C** | **T** | **C** | **C** | **A** | **T** | **G** | **C** | **A** | **A** | **A** | **A** | **A** | **C** | **A** | **T** | **T** | **G** | **A** | **T** | **A** | **A** | **T** | **G** | **G** | **A** | **T** | **G** | **A** | **T** | **G** | **A** | **A** | **A** | **T** | **C** | **T** | **G** | **C** | **A** | **T** | **G** | **G** | **T** | **A** | **G** | **G** | **T** | **T** | **C** | **A** | **G** | **C** | **A** | **A** | **A** | **T** | **C** | **A** | **A** | **G** | **A** | **T** | **A** | **T** | **T** | **C** | **G** | **T** | **A** | **G** | **C** | **T** | **A** | **C** | **C** | **G** | **A** | **T** | **T** | **G** | **A** | **A** | **T** | **T** | **T** | **T** | **G** | **A** | **A** | **A** | **C** | **A** | **A** | **G** | **T** | **G** | **C** | **T** | **G** | **T** | **C** | **A** | **T** | **T** | **T** | **A** | **C** | **G** | **A** | **T** | **C** | **C** | **T** | **G** | **A** | **G** | **T** | **T** | **T** | **T** | **T** | **A** | **G** | **A** | **A** | **G** | **A** | **A** | **C** | **T** | **T** | **G** | **C** | **T** | **A** | **C** | **T** | **C** | **A** | **G** | **T** | **T** | **C** | **A** | **A** | **A** | **G** | **A** | **A** | **G** | **A** | **T** | **G** | **A** | **G** | **A** | **C** | **A** | **C** | **A** | **T** | **T** | **G** | **T** | **T** | **C** | **A** | **T** | **C** | **C** | **A** | **T** | **G** | **A** | **C** | **A** | **A** | **C** | **T** | **G** | **A** | **A** | **A** | **C** | **A** | **G** | **T** | **C** | **A** | **A** | **G** | **G** | **A** | **A** | **A** | **T** | **G** | **T** | **C** | **T** | **A** | **A** | **C** | **T** | **G** | **G** | **C** | **T** | **A** | **T** | **T** | **A** | **T** | **T** | **C** | **A** | **A** | **G** | **C** | **A** | **A** | **C** | **A** | **A** | **A** | **T** | **T** | **T** | **C** | **T** | **C** | **G** | **A** | **T** | **T** | **A** | **T** | **T** | **T** | **T** | **C** | **T** | **C** | **C** | **A** | **A** | **T** | **C** | **C** | **T** | **A** | **-** | **-** | **-** |
| **N59492F** | **-** | **-** | **-** | **-** | **-** | **-** | **-** | **-** | **-** | **-** | **-** | **-** | **-** | **-** | **-** | **-** | **-** | **-** | **-** | **-** | **-** | **-** | **-** | **-** | **-** | **-** | **-** | **-** | **-** | **-** | **-** | **-** | **-** | **-** | **-** | **-** | **-** | **-** | **-** | **-** | **-** | **-** | **-** | **-** | **-** | **-** | **-** | **-** | **-** | **-** | **-** | **-** | **-** | **-** | **-** | **-** | **-** | **-** | **-** | **-** | **-** | **-** | **-** | **-** | **-** | **-** | **-** | **-** | **-** | **A** | **A** | **A** | **C** | **C** | **C** | **C** | **G** | **A** | **G** | **A** | **T** | **G** | **T** | **A** | **G** | **C** | **A** | **G** | **C** | **C** | **A** | **T** | **T** | **T** | **G** | **G** | **G** | **C** | **A** | **T** | **G** | **G** | **C** | **T** | **C** | **T** | **T** | **A** | **G** | **T** | **A** | **T** | **T** | **G** | **T** | **T** | **T** | **G** | **C** | **C** | **C** | **T** | **T** | **C** | **C** | **T** | **G** | **G** | **G** | **G** | **T** | **G** | **G** | **G** | **T** | **T** | **T** | **T** | **G** | **T** | **A** | **T** | **T** | **A** | **T** | **A** | **T** | **T** | **T** | **A** | **T** | **T** | **T** | **T** | **T** | **C** | **G** | **G** | **A** | **C** | **G** | **T** | **G** | **G** | **A** | **T** | **T** | **A** | **A** | **C** | **G** | **G** | **A** | **T** | **A** | **A** | **G** | **A** | **A** | **A** | **A** | **A** | **A** | **T** | **T** | **C** | **T** | **A** | **T** | **C** | **T** | **C** | **C** | **G** | **A** | **C** | **A** | **A** | **A** | **G** | **T** | **G** | **A** | **C** | **T** | **T** | **G** | **A** | **A** | **A** | **G** | **A** | **A** | **T** | **T** | **A** | **G** | **A** | **A** | **A** | **A** | **C** | **T** | **T** | **T** | **C** | **A** | **G** | **T** | **C** | **T** | **T** | **T** | **T** | **A** | **G** | **A** | **G** | **G** | **A** | **G** | **A** | **T** | **A** | **C** | **C** | **A** | **T** | **T** | **G** | **A** | **A** | **C** | **A** | **T** | **T** | **A** | **C** | **G** | **A** | **T** | **C** | **C** | **T** | **G** | **A** | **C** | **A** | **T** | **G** | **G** | **G** | **C** | **G** | **A** | **T** | **A** | **A** | **A** | **G** | **A** | **C** | **A** | **A** | **A** | **C** | **A** | **G** | **C** | **A** | **A** | **T** | **T** | **T** | **G** | **T** | **C** | **G** | **A** | **C** | **T** | **T** | **C** | **T** | **T** | **C** | **T** | **C** | **T** | **T** | **C** | **A** | **T** | **T** | **A** | **A** | **A** | **T** | **C** | **G** | **T** | **A** | **T** | **G** | **C** | **C** | **G** | **C** | **T** | **G** | **A** | **C** | **A** | **A** | **G** | **A** | **A** | **T** | **G** | **A** | **A** | **T** | **T** | **C** | **T** | **G** | **T** | **C** | **A** | **C** | **T** | **C** | **T** | **T** | **C** | **T** | **C** | **A** | **C** | **A** | **G** | **A** | **C** | **G** | **G** | **A** | **C** | **A** | **A** | **G** | **A** | **G** | **A** | **A** | **A** | **T** | **T** | **G** | **G** | **A** | **T** | **T** | **C** | **A** | **C** | **T** | **G** | **C** | **T** | **T** | **C** | **A** | **A** | **G** | **A** | **T** | **C** | **T** | **A** | **A** | **A** | **A** | **A** | **A** | **A** | **G** | **C** | **C** | **A** | **A** | **A** | **C** | **A** | **T** | **T** | **C** | **G** | **A** | **T** | **C** | **C** | **A** | **T** | **A** | **T** | **C** | **G** | **A** | **A** | **T** | **A** | **T** | **T** | **A** | **C** | **G** | **C** | **A** | **T** | **T** | **T** | **G** | **T** | **G** | **A** | **C** | **A** | **G** | **A** | **T** | **A** | **A** | **T** | **A** | **T** | **C** | **G** | **G** | **C** | **C** | **A** | **G** | **C** | **A** | **A** | **G** | **T** | **C** | **T** | **T** | **A** | **C** | **A** | **T** | **G** | **T** | **T** | **T** | **T** | **A** | **G** | **A** | **A** | **G** | **A** | **A** | **A** | **A** | **A** | **G** | **C** | **C** | **G** | **C** | **A** | **G** | **A** | **A** | **G** | **G** | **C** | **G** | **T** | **G** | **G** | **A** | **A** | **G** | **T** | **T** | **C** | **G** | **A** | **A** | **T** | **A** | **T** | **T** | **A** | **T** | **A** | **T** | **G** | **A** | **T** | **G** | **C** | **A** | **T** | **T** | **T** | **G** | **G** | **C** | **T** | **C** | **T** | **C** | **A** | **T** | **G** | **G** | **C** | **A** | **C** | **A** | **A** | **A** | **A** | **G** | **C** | **A** | **A** | **A** | **A** | **G** | **A** | **T** | **T** | **T** | **C** | **A** | **A** | **T** | **C** | **G** | **T** | **C** | **T** | **A** | **A** | **T** | **C** | **A** | **A** | **A** | **A** | **A** | **T** | **G** | **G** | **T** | **G** | **G** | **A** | **C** | **A** | **T** | **G** | **T** | **C** | **C** | **A** | **T** | **A** | **C** | **A** | **T** | **T** | **T** | **G** | **T** | **T** | **A** | **C** | **C** | **T** | **C** | **A** | **C** | **A** | **A** | **A** | **A** | **G** | **G** | **C** | **A** | **T** | **T** | **A** | **C** | **T** | **T** | **C** | **G** | **T** | **T** | **T** | **C** | **C** | **G** | **A** | **T** | **T** | **G** | **A** | **A** | **T** | **T** | **A** | **C** | **C** | **A** | **T** | **G** | **A** | **T** | **C** | **A** | **C** | **C** | **G** | **A** | **A** | **A** | **A** | **A** | **T** | **C** | **G** | **T** | **T** | **G** | **T** | **G** | **A** | **T** | **C** | **G** | **A** | **T** | **G** | **G** | **A** | **A** | **A** | **G** | **A** | **T** | **T** | **A** | **G** | **T** | **T** | **A** | **T** | **A** | **C** | **C** | **G** | **G** | **T** | **G** | **G** | **T** | **T** | **T** | **C** | **A** | **A** | **T** | **A** | **T** | **T** | **G** | **C** | **C** | **A** | **A** | **T** | **C** | **A** | **A** | **T** | **A** | **T** | **G** | **T** | **A** | **A** | **A** | **T** | **A** | **C** | **A** | **A** | **C** | **A** | **A** | **A** | **A** | **A** | **A** | **A** | **T** | **T** | **C** | **G** | **G** | **C** | **T** | **A** | **T** | **T** | **G** | **G** | **C** | **G** | **C** | **G** | **A** | **T** | **A** | **C** | **G** | **C** | **A** | **T** | **A** | **T** | **A** | **C** | **G** | **G** | **A** | **T** | **T** | **T** | **T** | **C** | **G** | **G** | **T** | **G** | **C** | **C** | **G** | **C** | **T** | **T** | **C** | **T** | **T** | **C** | **T** | **T** | **T** | **G** | **C** | **T** | **C** | **C** | **A** | **G** | **C** | **T** | **T** | **C** | **G** | **C** | **T** | **T** | **C** | **T** | **T** | **A** | **A** | **C** | **A** | **G** | **A** | **C** | **T** | **G** | **G** | **A** | **A** | **C** | **G** | **T** | **C** | **T** | **C** | **G** | **G** | **T** | **A** | **C** | **C** | **T** | **G** | **A** | **A** | **G** | **A** | **A** | **A** | **A** | **A** | **A** | **A** | **G** | **G** | **T** | **C** | **G** | **G** | **C** | **T** | **A** | **T** | **C** | **A** | **T** | **T** | **T** | **G** | **A** | **A** | **T** | **T** | **A** | **T** | **T** | **T** | **C** | **T** | **T** | **T** | **A** | **A** | **A** | **A** | **A** | **A** | **G** | **C** | **A** | **G** | **A** | **T** | **C** | **G** | **A** | **G** | **A** | **T** | **G** | **A** | **A** | **T** | **C** | **T** | **A** | **A** | **G** | **C** | **T** | **T** | **G** | **C** | **T** | **G** | **A** | **T** | **A** | **C** | **A** | **T** | **C** | **C** | **A** | **T** | **C** | **C** | **A** | **G** | **C** | **T** | **T** | **G** | **T** | **T** | **T** | **C** | **A** | **A** | **G** | **C** | **G** | **G** | **A** | **C** | **C** | **G** | **A** | **A** | **T** | **A** | **A** | **C** | **G** | **A** | **A** | **A** | **G** | **G** | **G** | **A** | **A** | **C** | **A** | **A** | **A** | **T** | **C** | **A** | **A** | **G** | **C** | **T** | **T** | **T** | **C** | **A** | **T** | **T** | **T** | **A** | **T** | **C** | **A** | **A** | **A** | **T** | **T** | **G** | **A** | **T** | **T** | **A** | **C** | **T** | **T** | **C** | **T** | **G** | **C** | **T** | **A** | **A** | **A** | **A** | **A** | **A** | **C** | **G** | **T** | **G** | **T** | **T** | **T** | **G** | **G** | **A** | **T** | **A** | **C** | **A** | **G** | **A** | **C** | **A** | **C** | **C** | **T** | **T** | **A** | **C** | **C** | **T** | **T** | **G** | **T** | **T** | **C** | **C** | **T** | **G** | **A** | **T** | **G** | **A** | **A** | **A** | **G** | **T** | **G** | **T** | **C** | **A** | **T** | **T** | **G** | **C** | **T** | **G** | **C** | **T** | **T** | **T** | **A** | **A** | **A** | **A** | **A** | **T** | **C** | **G** | **C** | **A** | **A** | **C** | **T** | **G** | **C** | **C** | **T** | **C** | **T** | **G** | **G** | **T** | **G** | **T** | **A** | **G** | **A** | **T** | **G** | **T** | **G** | **A** | **A** | **A** | **A** | **T** | **C** | **A** | **T** | **G** | **A** | **T** | **T** | **C** | **C** | **C** | **A** | **A** | **C** | **A** | **A** | **A** | **C** | **C** | **G** | **G** | **A** | **T** | **C** | **A** | **T** | **C** | **C** | **T** | **T** | **T** | **T** | **A** | **T** | **T** | **T** | **A** | **T** | **C** | **G** | **A** | **G** | **C** | **A** | **A** | **C** | **A** | **C** | **A** | **A** | **T** | **A** | **T** | **T** | **A** | **T** | **G** | **C** | **T** | **C** | **G** | **G** | **C** | **A** | **G** | **C** | **T** | **G** | **A** | **T** | **C** | **A** | **A** | **G** | **G** | **A** | **A** | **A** | **A** | **T** | **G** | **T** | **A** | **C** | **A** | **A** | **A** | **T** | **C** | **C** | **T** | **T** | **G** | **T** | **C** | **T** | **A** | **T** | **G** | **A** | **G** | **A** | **A** | **C** | **G** | **G** | **C** | **T** | **T** | **C** | **C** | **T** | **C** | **C** | **A** | **T** | **G** | **C** | **A** | **A** | **A** | **A** | **A** | **C** | **A** | **T** | **T** | **G** | **A** | **T** | **A** | **A** | **T** | **G** | **G** | **A** | **T** | **G** | **A** | **T** | **G** | **A** | **A** | **A** | **T** | **C** | **T** | **G** | **C** | **A** | **T** | **G** | **G** | **T** | **A** | **G** | **G** | **T** | **T** | **C** | **A** | **G** | **C** | **A** | **A** | **A** | **T** | **C** | **A** | **A** | **G** | **A** | **T** | **A** | **T** | **T** | **C** | **G** | **T** | **A** | **G** | **C** | **T** | **A** | **C** | **C** | **G** | **A** | **T** | **T** | **G** | **A** | **A** | **T** | **T** | **T** | **T** | **G** | **A** | **A** | **A** | **C** | **A** | **A** | **G** | **T** | **G** | **C** | **T** | **G** | **T** | **C** | **A** | **T** | **T** | **T** | **A** | **C** | **G** | **A** | **T** | **C** | **C** | **T** | **G** | **A** | **G** | **T** | **T** | **T** | **T** | **T** | **A** | **G** | **A** | **A** | **G** | **A** | **A** | **C** | **T** | **T** | **G** | **C** | **T** | **A** | **C** | **T** | **C** | **A** | **G** | **T** | **T** | **C** | **A** | **A** | **A** | **G** | **A** | **A** | **G** | **A** | **T** | **G** | **A** | **G** | **A** | **C** | **A** | **C** | **A** | **T** | **T** | **G** | **T** | **T** | **C** | **A** | **T** | **C** | **C** | **A** | **T** | **G** | **A** | **C** | **A** | **A** | **C** | **T** | **G** | **A** | **A** | **A** | **C** | **A** | **G** | **T** | **C** | **A** | **A** | **G** | **G** | **A** | **A** | **A** | **T** | **G** | **T** | **C** | **T** | **A** | **A** | **C** | **T** | **G** | **G** | **C** | **T** | **A** | **T** | **T** | **A** | **T** | **T** | **C** | **A** | **A** | **G** | **C** | **A** | **A** | **C** | **A** | **A** | **A** | **T** | **T** | **T** | **C** | **T** | **C** | **G** | **A** | **T** | **T** | **A** | **T** | **T** | **T** | **T** | **C** | **T** | **C** | **C** | **A** | **A** | **T** | **C** | **C** | **T** | **A** | **-** | **-** | **-** |
| **N59907F** | **-** | **-** | **-** | **-** | **-** | **-** | **-** | **-** | **-** | **-** | **-** | **-** | **-** | **-** | **-** | **-** | **-** | **-** | **-** | **-** | **-** | **-** | **-** | **-** | **-** | **-** | **-** | **-** | **-** | **-** | **-** | **-** | **-** | **-** | **-** | **-** | **-** | **-** | **-** | **-** | **-** | **-** | **-** | **-** | **-** | **-** | **-** | **-** | **-** | **-** | **-** | **-** | **-** | **-** | **-** | **-** | **-** | **-** | **-** | **-** | **-** | **-** | **-** | **-** | **-** | **-** | **-** | **-** | **-** | **A** | **A** | **A** | **C** | **C** | **C** | **C** | **G** | **A** | **G** | **A** | **T** | **G** | **T** | **A** | **G** | **C** | **A** | **G** | **C** | **C** | **A** | **T** | **T** | **T** | **G** | **G** | **G** | **C** | **A** | **T** | **G** | **G** | **C** | **T** | **C** | **T** | **T** | **A** | **G** | **T** | **A** | **T** | **T** | **G** | **T** | **T** | **T** | **G** | **C** | **C** | **C** | **T** | **T** | **C** | **C** | **T** | **G** | **G** | **G** | **G** | **T** | **G** | **G** | **G** | **T** | **T** | **T** | **T** | **G** | **T** | **A** | **T** | **T** | **A** | **T** | **A** | **T** | **T** | **T** | **A** | **T** | **T** | **T** | **T** | **T** | **C** | **G** | **G** | **A** | **C** | **G** | **T** | **G** | **G** | **A** | **T** | **T** | **A** | **A** | **C** | **G** | **G** | **A** | **T** | **A** | **A** | **G** | **A** | **A** | **A** | **A** | **A** | **A** | **T** | **T** | **C** | **T** | **A** | **T** | **C** | **T** | **C** | **C** | **G** | **A** | **C** | **A** | **A** | **A** | **G** | **T** | **G** | **A** | **C** | **T** | **T** | **G** | **A** | **A** | **A** | **G** | **A** | **A** | **T** | **T** | **A** | **G** | **A** | **A** | **A** | **A** | **C** | **T** | **T** | **T** | **C** | **A** | **G** | **T** | **C** | **T** | **T** | **T** | **T** | **A** | **G** | **A** | **G** | **G** | **A** | **G** | **A** | **T** | **A** | **C** | **C** | **A** | **T** | **T** | **G** | **A** | **A** | **C** | **A** | **T** | **T** | **A** | **C** | **G** | **A** | **T** | **C** | **C** | **T** | **G** | **A** | **C** | **A** | **T** | **G** | **G** | **G** | **C** | **G** | **A** | **T** | **A** | **A** | **A** | **G** | **A** | **C** | **A** | **A** | **A** | **C** | **A** | **G** | **C** | **A** | **A** | **T** | **T** | **T** | **G** | **T** | **T** | **G** | **A** | **C** | **T** | **T** | **C** | **T** | **T** | **C** | **T** | **C** | **T** | **T** | **C** | **A** | **T** | **T** | **A** | **A** | **A** | **T** | **C** | **G** | **T** | **A** | **T** | **G** | **C** | **C** | **G** | **C** | **T** | **G** | **A** | **C** | **A** | **A** | **G** | **A** | **A** | **T** | **G** | **A** | **A** | **T** | **T** | **C** | **T** | **G** | **T** | **C** | **A** | **C** | **T** | **C** | **T** | **T** | **C** | **T** | **C** | **A** | **C** | **A** | **G** | **A** | **C** | **G** | **G** | **A** | **C** | **A** | **A** | **G** | **A** | **G** | **A** | **A** | **A** | **T** | **T** | **G** | **G** | **A** | **T** | **T** | **C** | **A** | **C** | **T** | **G** | **C** | **T** | **T** | **C** | **A** | **A** | **G** | **A** | **T** | **C** | **T** | **A** | **A** | **A** | **A** | **A** | **A** | **A** | **G** | **C** | **C** | **A** | **A** | **A** | **C** | **A** | **T** | **T** | **C** | **G** | **A** | **T** | **C** | **C** | **A** | **T** | **A** | **T** | **C** | **G** | **A** | **A** | **T** | **A** | **T** | **T** | **A** | **C** | **G** | **C** | **A** | **T** | **T** | **T** | **G** | **T** | **G** | **A** | **C** | **A** | **G** | **A** | **T** | **A** | **A** | **T** | **A** | **T** | **C** | **G** | **G** | **C** | **C** | **A** | **G** | **C** | **A** | **A** | **G** | **T** | **C** | **T** | **T** | **A** | **C** | **A** | **T** | **G** | **T** | **T** | **T** | **T** | **A** | **G** | **A** | **A** | **G** | **A** | **A** | **A** | **A** | **A** | **G** | **C** | **C** | **G** | **C** | **A** | **G** | **A** | **A** | **G** | **G** | **C** | **G** | **T** | **G** | **G** | **A** | **A** | **G** | **T** | **T** | **C** | **G** | **A** | **A** | **T** | **A** | **T** | **T** | **A** | **T** | **A** | **T** | **G** | **A** | **T** | **G** | **C** | **A** | **T** | **T** | **T** | **G** | **G** | **C** | **T** | **C** | **T** | **C** | **A** | **T** | **G** | **G** | **C** | **A** | **C** | **A** | **A** | **A** | **A** | **G** | **C** | **A** | **A** | **A** | **A** | **G** | **A** | **T** | **T** | **T** | **C** | **A** | **A** | **T** | **C** | **G** | **T** | **C** | **T** | **A** | **A** | **T** | **C** | **A** | **A** | **A** | **A** | **A** | **T** | **G** | **G** | **T** | **G** | **G** | **A** | **C** | **A** | **T** | **G** | **T** | **C** | **C** | **A** | **T** | **A** | **C** | **A** | **T** | **T** | **T** | **G** | **T** | **T** | **A** | **C** | **C** | **T** | **C** | **A** | **C** | **A** | **A** | **A** | **A** | **G** | **G** | **C** | **A** | **T** | **T** | **A** | **C** | **T** | **T** | **C** | **G** | **T** | **T** | **T** | **C** | **C** | **G** | **A** | **T** | **T** | **G** | **A** | **A** | **T** | **T** | **A** | **C** | **C** | **A** | **T** | **G** | **A** | **T** | **C** | **A** | **C** | **C** | **G** | **A** | **A** | **A** | **A** | **A** | **T** | **C** | **G** | **T** | **T** | **G** | **T** | **G** | **A** | **T** | **C** | **G** | **A** | **T** | **G** | **G** | **A** | **A** | **A** | **G** | **A** | **T** | **T** | **A** | **G** | **T** | **T** | **A** | **T** | **A** | **C** | **C** | **G** | **G** | **T** | **G** | **G** | **T** | **T** | **T** | **C** | **A** | **A** | **T** | **A** | **T** | **T** | **G** | **C** | **C** | **A** | **A** | **T** | **C** | **A** | **A** | **T** | **A** | **T** | **G** | **T** | **A** | **A** | **A** | **T** | **A** | **C** | **A** | **A** | **C** | **A** | **A** | **A** | **A** | **A** | **A** | **A** | **T** | **T** | **C** | **G** | **G** | **C** | **T** | **A** | **T** | **T** | **G** | **G** | **C** | **G** | **C** | **G** | **A** | **T** | **A** | **C** | **G** | **C** | **A** | **T** | **A** | **T** | **A** | **C** | **G** | **G** | **A** | **T** | **T** | **T** | **T** | **C** | **G** | **G** | **T** | **G** | **C** | **C** | **G** | **C** | **T** | **T** | **C** | **T** | **T** | **C** | **T** | **T** | **T** | **G** | **C** | **T** | **C** | **C** | **A** | **G** | **C** | **T** | **T** | **C** | **G** | **C** | **T** | **T** | **C** | **T** | **T** | **A** | **A** | **C** | **A** | **G** | **A** | **C** | **T** | **G** | **G** | **A** | **A** | **C** | **G** | **T** | **C** | **T** | **C** | **G** | **G** | **T** | **A** | **C** | **C** | **T** | **G** | **A** | **A** | **G** | **A** | **A** | **A** | **A** | **A** | **A** | **A** | **G** | **G** | **T** | **C** | **G** | **G** | **C** | **T** | **A** | **T** | **C** | **A** | **T** | **T** | **T** | **G** | **A** | **A** | **T** | **T** | **A** | **T** | **T** | **T** | **C** | **T** | **T** | **T** | **A** | **A** | **A** | **A** | **A** | **A** | **G** | **C** | **A** | **G** | **A** | **T** | **C** | **G** | **A** | **G** | **A** | **T** | **G** | **A** | **A** | **T** | **C** | **T** | **A** | **A** | **G** | **C** | **T** | **T** | **G** | **C** | **T** | **G** | **A** | **A** | **A** | **C** | **A** | **T** | **C** | **C** | **A** | **T** | **C** | **C** | **A** | **G** | **C** | **T** | **T** | **G** | **T** | **T** | **T** | **C** | **A** | **A** | **G** | **C** | **G** | **G** | **A** | **C** | **C** | **G** | **A** | **A** | **T** | **A** | **A** | **C** | **G** | **A** | **A** | **A** | **G** | **G** | **G** | **A** | **A** | **C** | **A** | **A** | **A** | **T** | **C** | **A** | **A** | **G** | **C** | **T** | **T** | **T** | **C** | **A** | **T** | **T** | **T** | **A** | **T** | **C** | **A** | **A** | **A** | **T** | **T** | **G** | **A** | **T** | **T** | **A** | **C** | **T** | **T** | **C** | **T** | **G** | **C** | **T** | **A** | **A** | **A** | **A** | **A** | **G** | **C** | **G** | **T** | **G** | **T** | **T** | **T** | **G** | **G** | **A** | **T** | **A** | **C** | **A** | **G** | **A** | **C** | **A** | **C** | **C** | **T** | **T** | **A** | **C** | **C** | **T** | **T** | **G** | **T** | **T** | **C** | **C** | **T** | **G** | **A** | **T** | **G** | **A** | **A** | **A** | **G** | **T** | **G** | **T** | **C** | **A** | **T** | **T** | **G** | **C** | **T** | **G** | **C** | **T** | **T** | **T** | **A** | **A** | **A** | **A** | **A** | **T** | **C** | **G** | **C** | **A** | **A** | **C** | **T** | **G** | **C** | **C** | **T** | **C** | **T** | **G** | **G** | **T** | **G** | **T** | **A** | **G** | **A** | **T** | **G** | **T** | **G** | **A** | **A** | **A** | **A** | **T** | **C** | **A** | **T** | **G** | **A** | **T** | **T** | **C** | **C** | **C** | **A** | **A** | **C** | **A** | **A** | **A** | **C** | **C** | **G** | **G** | **A** | **T** | **C** | **A** | **T** | **C** | **C** | **T** | **T** | **T** | **T** | **A** | **T** | **T** | **T** | **A** | **T** | **C** | **G** | **A** | **G** | **C** | **A** | **A** | **C** | **A** | **C** | **A** | **A** | **T** | **A** | **T** | **T** | **A** | **T** | **G** | **C** | **T** | **C** | **G** | **G** | **C** | **A** | **G** | **C** | **T** | **G** | **A** | **T** | **C** | **A** | **A** | **G** | **G** | **A** | **A** | **A** | **A** | **T** | **G** | **T** | **A** | **C** | **A** | **A** | **A** | **T** | **C** | **C** | **T** | **T** | **G** | **T** | **C** | **T** | **A** | **T** | **G** | **A** | **G** | **A** | **A** | **C** | **G** | **G** | **C** | **T** | **T** | **C** | **C** | **T** | **C** | **C** | **A** | **T** | **G** | **C** | **A** | **A** | **A** | **A** | **A** | **C** | **A** | **T** | **T** | **G** | **A** | **T** | **A** | **A** | **T** | **G** | **G** | **A** | **T** | **G** | **A** | **T** | **G** | **A** | **A** | **A** | **T** | **C** | **T** | **G** | **C** | **A** | **T** | **G** | **G** | **T** | **A** | **G** | **G** | **T** | **T** | **C** | **A** | **G** | **C** | **A** | **A** | **A** | **T** | **C** | **A** | **A** | **G** | **A** | **T** | **A** | **T** | **T** | **C** | **G** | **T** | **A** | **G** | **C** | **T** | **A** | **C** | **A** | **G** | **A** | **T** | **T** | **G** | **A** | **A** | **T** | **T** | **T** | **T** | **G** | **A** | **A** | **A** | **C** | **A** | **A** | **G** | **T** | **G** | **C** | **T** | **G** | **T** | **C** | **A** | **T** | **T** | **T** | **A** | **C** | **G** | **A** | **T** | **C** | **C** | **T** | **G** | **A** | **G** | **T** | **T** | **T** | **T** | **T** | **A** | **G** | **A** | **A** | **G** | **A** | **A** | **C** | **T** | **T** | **G** | **C** | **T** | **A** | **C** | **T** | **C** | **A** | **G** | **T** | **T** | **C** | **A** | **A** | **A** | **G** | **A** | **A** | **G** | **A** | **T** | **G** | **A** | **G** | **A** | **C** | **A** | **C** | **A** | **T** | **T** | **G** | **T** | **T** | **C** | **A** | **T** | **C** | **C** | **A** | **T** | **G** | **A** | **C** | **A** | **A** | **C** | **T** | **G** | **A** | **A** | **A** | **C** | **A** | **G** | **T** | **T** | **A** | **A** | **A** | **G** | **A** | **A** | **A** | **T** | **G** | **T** | **C** | **T** | **A** | **A** | **C** | **T** | **G** | **G** | **C** | **T** | **A** | **T** | **T** | **A** | **T** | **T** | **C** | **A** | **A** | **G** | **C** | **A** | **A** | **C** | **A** | **A** | **A** | **T** | **T** | **T** | **C** | **T** | **C** | **G** | **A** | **T** | **T** | **A** | **T** | **T** | **T** | **T** | **C** | **T** | **C** | **C** | **A** | **A** | **T** | **C** | **C** | **T** | **A** | **-** | **-** | **-** |
| **N59513F** | **-** | **-** | **-** | **-** | **-** | **-** | **-** | **-** | **-** | **-** | **-** | **-** | **-** | **-** | **-** | **-** | **-** | **-** | **-** | **-** | **-** | **-** | **-** | **-** | **-** | **-** | **-** | **-** | **-** | **-** | **-** | **-** | **-** | **-** | **-** | **-** | **-** | **-** | **-** | **-** | **-** | **-** | **-** | **-** | **-** | **-** | **-** | **-** | **-** | **-** | **-** | **-** | **-** | **-** | **-** | **-** | **-** | **-** | **-** | **-** | **-** | **-** | **-** | **-** | **-** | **-** | **-** | **-** | **-** | **A** | **A** | **A** | **C** | **C** | **C** | **C** | **G** | **A** | **G** | **A** | **T** | **G** | **T** | **A** | **G** | **C** | **A** | **G** | **C** | **T** | **A** | **T** | **T** | **T** | **G** | **G** | **G** | **C** | **A** | **T** | **G** | **G** | **C** | **T** | **C** | **T** | **T** | **A** | **G** | **T** | **A** | **T** | **T** | **G** | **T** | **T** | **T** | **G** | **C** | **C** | **C** | **T** | **T** | **C** | **C** | **T** | **G** | **G** | **G** | **G** | **T** | **G** | **G** | **G** | **T** | **T** | **T** | **T** | **G** | **T** | **A** | **T** | **T** | **A** | **T** | **A** | **T** | **T** | **T** | **A** | **T** | **T** | **T** | **T** | **T** | **C** | **G** | **G** | **A** | **C** | **G** | **T** | **G** | **G** | **A** | **T** | **T** | **A** | **A** | **C** | **G** | **G** | **A** | **T** | **A** | **A** | **G** | **A** | **A** | **A** | **A** | **A** | **A** | **T** | **T** | **C** | **T** | **A** | **T** | **C** | **T** | **C** | **C** | **G** | **A** | **C** | **A** | **A** | **A** | **G** | **T** | **G** | **A** | **C** | **T** | **T** | **G** | **A** | **A** | **A** | **G** | **A** | **A** | **T** | **T** | **A** | **G** | **A** | **A** | **A** | **A** | **C** | **T** | **T** | **T** | **C** | **A** | **G** | **T** | **C** | **T** | **T** | **T** | **T** | **A** | **G** | **A** | **G** | **G** | **A** | **G** | **A** | **T** | **A** | **C** | **C** | **A** | **T** | **T** | **G** | **A** | **A** | **C** | **A** | **T** | **T** | **A** | **C** | **G** | **A** | **T** | **C** | **C** | **T** | **G** | **A** | **C** | **A** | **T** | **G** | **G** | **G** | **C** | **G** | **A** | **C** | **A** | **A** | **A** | **G** | **A** | **C** | **A** | **A** | **A** | **C** | **A** | **A** | **C** | **A** | **A** | **T** | **T** | **T** | **G** | **T** | **C** | **G** | **A** | **C** | **T** | **T** | **C** | **T** | **T** | **C** | **T** | **C** | **T** | **T** | **C** | **A** | **T** | **T** | **A** | **A** | **A** | **T** | **C** | **G** | **T** | **A** | **T** | **G** | **C** | **C** | **G** | **C** | **T** | **G** | **A** | **C** | **A** | **A** | **G** | **A** | **A** | **T** | **G** | **A** | **A** | **T** | **T** | **C** | **T** | **G** | **T** | **C** | **A** | **C** | **T** | **C** | **T** | **T** | **C** | **T** | **C** | **A** | **C** | **A** | **G** | **A** | **C** | **G** | **G** | **A** | **C** | **A** | **A** | **A** | **A** | **G** | **A** | **A** | **A** | **T** | **T** | **A** | **G** | **A** | **T** | **T** | **C** | **A** | **C** | **T** | **G** | **C** | **T** | **T** | **C** | **A** | **A** | **G** | **A** | **T** | **C** | **T** | **A** | **A** | **A** | **A** | **A** | **A** | **A** | **G** | **C** | **C** | **A** | **A** | **A** | **C** | **A** | **T** | **T** | **C** | **G** | **A** | **T** | **C** | **C** | **A** | **T** | **A** | **T** | **C** | **G** | **A** | **A** | **T** | **A** | **T** | **T** | **A** | **C** | **G** | **C** | **A** | **T** | **T** | **T** | **G** | **T** | **G** | **A** | **C** | **A** | **G** | **A** | **T** | **A** | **A** | **T** | **A** | **T** | **C** | **G** | **G** | **T** | **C** | **A** | **G** | **C** | **A** | **A** | **G** | **T** | **C** | **T** | **T** | **A** | **C** | **G** | **C** | **G** | **T** | **T** | **T** | **T** | **A** | **G** | **A** | **A** | **G** | **A** | **A** | **A** | **A** | **A** | **G** | **C** | **C** | **G** | **C** | **A** | **G** | **A** | **A** | **G** | **G** | **C** | **G** | **T** | **A** | **G** | **A** | **A** | **G** | **T** | **T** | **C** | **G** | **T** | **A** | **T** | **A** | **T** | **T** | **A** | **T** | **A** | **T** | **G** | **A** | **T** | **G** | **C** | **A** | **T** | **T** | **T** | **G** | **G** | **C** | **T** | **C** | **C** | **C** | **A** | **T** | **G** | **G** | **C** | **A** | **C** | **A** | **A** | **A** | **A** | **G** | **C** | **A** | **A** | **A** | **A** | **G** | **A** | **T** | **T** | **T** | **C** | **A** | **A** | **T** | **C** | **G** | **T** | **C** | **T** | **A** | **A** | **T** | **C** | **A** | **A** | **A** | **A** | **A** | **T** | **G** | **G** | **T** | **G** | **G** | **A** | **C** | **A** | **T** | **G** | **T** | **C** | **C** | **A** | **T** | **A** | **C** | **A** | **T** | **T** | **T** | **G** | **T** | **T** | **A** | **C** | **C** | **T** | **C** | **G** | **C** | **A** | **A** | **A** | **A** | **A** | **G** | **C** | **A** | **T** | **T** | **G** | **C** | **T** | **T** | **C** | **G** | **T** | **T** | **T** | **C** | **C** | **G** | **A** | **T** | **T** | **G** | **A** | **A** | **T** | **T** | **A** | **C** | **C** | **A** | **T** | **G** | **A** | **T** | **C** | **A** | **C** | **C** | **G** | **A** | **A** | **A** | **A** | **A** | **T** | **C** | **G** | **T** | **T** | **G** | **T** | **G** | **A** | **T** | **C** | **G** | **A** | **T** | **G** | **G** | **A** | **A** | **A** | **G** | **A** | **T** | **C** | **A** | **G** | **T** | **T** | **A** | **C** | **A** | **C** | **C** | **G** | **G** | **T** | **G** | **G** | **T** | **T** | **T** | **C** | **A** | **A** | **T** | **A** | **T** | **T** | **G** | **C** | **C** | **A** | **A** | **T** | **C** | **A** | **A** | **T** | **A** | **T** | **G** | **T** | **A** | **A** | **A** | **T** | **A** | **C** | **G** | **A** | **C** | **A** | **A** | **A** | **A** | **A** | **A** | **A** | **T** | **T** | **C** | **G** | **G** | **T** | **T** | **A** | **T** | **T** | **G** | **G** | **C** | **G** | **T** | **G** | **A** | **T** | **A** | **C** | **G** | **C** | **A** | **T** | **A** | **T** | **A** | **C** | **G** | **A** | **A** | **T** | **T** | **T** | **T** | **C** | **G** | **G** | **T** | **G** | **C** | **C** | **G** | **C** | **T** | **T** | **C** | **T** | **T** | **C** | **T** | **T** | **T** | **G** | **C** | **T** | **T** | **C** | **A** | **A** | **C** | **T** | **T** | **C** | **G** | **T** | **T** | **T** | **T** | **T** | **T** | **A** | **A** | **C** | **A** | **G** | **A** | **C** | **T** | **G** | **G** | **A** | **A** | **C** | **G** | **T** | **T** | **T** | **C** | **A** | **G** | **T** | **G** | **C** | **C** | **T** | **G** | **A** | **A** | **G** | **A** | **A** | **A** | **A** | **A** | **A** | **A** | **G** | **G** | **T** | **C** | **G** | **G** | **C** | **T** | **A** | **T** | **C** | **A** | **T** | **T** | **T** | **G** | **A** | **A** | **T** | **T** | **A** | **T** | **T** | **T** | **C** | **T** | **T** | **T** | **A** | **A** | **A** | **A** | **A** | **A** | **G** | **C** | **C** | **G** | **A** | **T** | **C** | **G** | **A** | **G** | **A** | **T** | **G** | **A** | **A** | **T** | **C** | **C** | **A** | **A** | **A** | **C** | **T** | **T** | **G** | **C** | **T** | **G** | **A** | **T** | **A** | **C** | **G** | **T** | **C** | **C** | **A** | **T** | **C** | **C** | **A** | **G** | **C** | **T** | **T** | **G** | **T** | **C** | **T** | **C** | **A** | **A** | **G** | **C** | **G** | **G** | **A** | **C** | **C** | **A** | **A** | **A** | **T** | **A** | **A** | **C** | **G** | **A** | **A** | **C** | **G** | **A** | **G** | **A** | **A** | **C** | **A** | **A** | **A** | **T** | **C** | **A** | **A** | **G** | **C** | **T** | **T** | **T** | **C** | **G** | **T** | **T** | **T** | **A** | **T** | **C** | **A** | **A** | **A** | **T** | **T** | **G** | **A** | **T** | **T** | **A** | **C** | **T** | **T** | **C** | **T** | **G** | **C** | **T** | **A** | **A** | **A** | **A** | **A** | **A** | **C** | **G** | **T** | **G** | **T** | **T** | **T** | **G** | **G** | **A** | **T** | **A** | **C** | **A** | **G** | **A** | **C** | **A** | **C** | **C** | **T** | **T** | **A** | **C** | **C** | **T** | **C** | **G** | **T** | **T** | **C** | **C** | **T** | **G** | **A** | **T** | **G** | **A** | **A** | **A** | **G** | **T** | **G** | **T** | **C** | **A** | **T** | **T** | **G** | **C** | **T** | **G** | **C** | **T** | **T** | **T** | **G** | **A** | **A** | **A** | **A** | **T** | **C** | **G** | **C** | **A** | **A** | **C** | **T** | **G** | **C** | **T** | **T** | **C** | **T** | **G** | **G** | **T** | **G** | **T** | **C** | **G** | **A** | **T** | **G** | **T** | **G** | **A** | **A** | **A** | **A** | **T** | **C** | **A** | **T** | **G** | **A** | **T** | **C** | **C** | **C** | **C** | **A** | **A** | **T** | **A** | **A** | **A** | **C** | **C** | **G** | **G** | **A** | **T** | **C** | **A** | **T** | **C** | **C** | **T** | **T** | **T** | **T** | **A** | **T** | **T** | **T** | **A** | **T** | **C** | **G** | **A** | **G** | **C** | **A** | **A** | **C** | **A** | **C** | **A** | **A** | **T** | **A** | **T** | **T** | **A** | **T** | **G** | **C** | **T** | **C** | **G** | **T** | **C** | **A** | **G** | **C** | **T** | **C** | **A** | **T** | **C** | **A** | **A** | **G** | **G** | **A** | **A** | **A** | **A** | **T** | **G** | **T** | **A** | **C** | **A** | **G** | **A** | **T** | **C** | **C** | **T** | **T** | **G** | **T** | **C** | **T** | **A** | **T** | **G** | **A** | **G** | **A** | **A** | **T** | **G** | **G** | **C** | **T** | **T** | **C** | **C** | **T** | **C** | **C** | **A** | **T** | **G** | **C** | **A** | **A** | **A** | **A** | **A** | **C** | **A** | **T** | **T** | **A** | **A** | **T** | **A** | **A** | **T** | **G** | **G** | **A** | **T** | **G** | **A** | **T** | **G** | **A** | **A** | **A** | **T** | **C** | **T** | **G** | **C** | **A** | **T** | **G** | **G** | **T** | **A** | **G** | **G** | **T** | **T** | **C** | **A** | **G** | **C** | **A** | **A** | **A** | **T** | **C** | **A** | **A** | **G** | **A** | **T** | **A** | **T** | **T** | **C** | **G** | **T** | **A** | **G** | **C** | **T** | **A** | **C** | **C** | **G** | **A** | **T** | **T** | **G** | **A** | **A** | **T** | **T** | **T** | **T** | **G** | **A** | **A** | **A** | **C** | **A** | **A** | **G** | **T** | **G** | **C** | **T** | **G** | **T** | **C** | **A** | **T** | **T** | **T** | **A** | **C** | **G** | **A** | **T** | **C** | **C** | **T** | **G** | **A** | **G** | **T** | **T** | **T** | **T** | **T** | **A** | **G** | **A** | **A** | **G** | **A** | **G** | **C** | **T** | **T** | **G** | **C** | **T** | **A** | **C** | **T** | **C** | **A** | **G** | **T** | **T** | **C** | **A** | **A** | **A** | **G** | **A** | **A** | **G** | **A** | **T** | **G** | **A** | **G** | **A** | **C** | **A** | **C** | **A** | **T** | **T** | **G** | **T** | **T** | **C** | **A** | **C** | **C** | **A** | **A** | **T** | **G** | **A** | **C** | **A** | **A** | **C** | **T** | **G** | **A** | **A** | **A** | **C** | **A** | **G** | **T** | **G** | **A** | **A** | **G** | **G** | **A** | **A** | **A** | **T** | **G** | **T** | **C** | **T** | **A** | **A** | **C** | **T** | **G** | **G** | **C** | **T** | **G** | **T** | **T** | **A** | **T** | **T** | **C** | **A** | **A** | **A** | **C** | **A** | **A** | **C** | **A** | **A** | **A** | **T** | **T** | **T** | **C** | **T** | **C** | **G** | **A** | **T** | **T** | **G** | **T** | **T** | **T** | **T** | **C** | **T** | **C** | **C** | **A** | **A** | **T** | **C** | **T** | **T** | **A** | **-** | **-** | **-** |
| **N59998F** | **-** | **-** | **-** | **-** | **-** | **-** | **-** | **-** | **-** | **-** | **-** | **-** | **-** | **-** | **-** | **-** | **-** | **-** | **-** | **-** | **-** | **-** | **-** | **-** | **-** | **-** | **-** | **-** | **-** | **-** | **-** | **-** | **-** | **-** | **-** | **-** | **-** | **-** | **-** | **-** | **-** | **-** | **-** | **-** | **-** | **-** | **-** | **-** | **-** | **-** | **-** | **-** | **-** | **-** | **-** | **-** | **-** | **-** | **-** | **-** | **-** | **-** | **-** | **-** | **-** | **-** | **-** | **-** | **-** | **A** | **A** | **A** | **C** | **C** | **C** | **C** | **G** | **A** | **G** | **A** | **T** | **G** | **T** | **A** | **G** | **C** | **A** | **G** | **C** | **C** | **A** | **T** | **T** | **T** | **G** | **G** | **G** | **C** | **A** | **T** | **G** | **G** | **C** | **T** | **C** | **T** | **T** | **A** | **G** | **T** | **A** | **T** | **T** | **G** | **T** | **T** | **T** | **G** | **C** | **C** | **C** | **T** | **T** | **C** | **C** | **T** | **G** | **G** | **G** | **G** | **T** | **G** | **G** | **G** | **T** | **T** | **T** | **T** | **G** | **T** | **A** | **T** | **T** | **A** | **T** | **A** | **T** | **T** | **T** | **A** | **T** | **T** | **T** | **T** | **T** | **C** | **G** | **G** | **A** | **C** | **G** | **T** | **G** | **G** | **A** | **T** | **T** | **A** | **A** | **C** | **G** | **G** | **A** | **T** | **A** | **A** | **G** | **A** | **A** | **A** | **A** | **A** | **A** | **T** | **T** | **C** | **T** | **A** | **T** | **C** | **T** | **C** | **C** | **G** | **A** | **C** | **A** | **A** | **A** | **G** | **T** | **G** | **A** | **C** | **T** | **T** | **G** | **A** | **A** | **A** | **G** | **A** | **A** | **T** | **T** | **A** | **G** | **A** | **A** | **A** | **A** | **C** | **T** | **T** | **T** | **C** | **A** | **G** | **T** | **C** | **T** | **T** | **T** | **T** | **A** | **G** | **A** | **G** | **G** | **A** | **G** | **A** | **T** | **A** | **C** | **C** | **A** | **T** | **T** | **G** | **A** | **A** | **C** | **A** | **T** | **T** | **A** | **C** | **G** | **A** | **T** | **C** | **C** | **T** | **G** | **A** | **C** | **A** | **T** | **G** | **G** | **G** | **C** | **G** | **A** | **T** | **A** | **A** | **A** | **G** | **A** | **C** | **A** | **A** | **A** | **C** | **A** | **G** | **C** | **A** | **A** | **T** | **T** | **T** | **G** | **T** | **C** | **G** | **A** | **C** | **T** | **T** | **C** | **T** | **T** | **C** | **T** | **C** | **T** | **T** | **C** | **A** | **T** | **T** | **A** | **A** | **A** | **T** | **C** | **G** | **T** | **A** | **T** | **G** | **C** | **C** | **G** | **C** | **T** | **G** | **A** | **C** | **A** | **A** | **G** | **A** | **A** | **T** | **G** | **A** | **A** | **T** | **T** | **C** | **T** | **G** | **T** | **C** | **A** | **C** | **T** | **C** | **T** | **T** | **C** | **T** | **C** | **A** | **C** | **A** | **G** | **A** | **C** | **G** | **G** | **A** | **C** | **A** | **A** | **G** | **A** | **G** | **A** | **A** | **A** | **T** | **T** | **G** | **G** | **A** | **T** | **T** | **C** | **A** | **C** | **T** | **G** | **C** | **T** | **T** | **C** | **A** | **A** | **G** | **A** | **T** | **C** | **T** | **A** | **A** | **A** | **A** | **A** | **A** | **A** | **G** | **C** | **C** | **A** | **A** | **A** | **C** | **A** | **T** | **T** | **C** | **G** | **A** | **T** | **C** | **C** | **A** | **T** | **A** | **T** | **C** | **G** | **A** | **A** | **T** | **A** | **T** | **T** | **A** | **C** | **G** | **C** | **A** | **T** | **T** | **T** | **G** | **T** | **G** | **A** | **C** | **A** | **G** | **A** | **T** | **A** | **A** | **T** | **A** | **T** | **C** | **G** | **G** | **C** | **C** | **A** | **G** | **C** | **A** | **A** | **G** | **T** | **C** | **T** | **T** | **A** | **C** | **A** | **T** | **G** | **T** | **T** | **T** | **T** | **A** | **G** | **A** | **A** | **G** | **A** | **A** | **A** | **A** | **A** | **G** | **C** | **C** | **G** | **C** | **A** | **G** | **A** | **A** | **G** | **G** | **C** | **G** | **T** | **G** | **G** | **A** | **A** | **G** | **T** | **T** | **C** | **G** | **A** | **A** | **T** | **A** | **T** | **T** | **A** | **T** | **A** | **T** | **G** | **A** | **T** | **G** | **C** | **A** | **T** | **T** | **T** | **G** | **G** | **C** | **T** | **C** | **T** | **C** | **A** | **T** | **G** | **G** | **C** | **A** | **C** | **A** | **A** | **A** | **A** | **G** | **C** | **A** | **A** | **A** | **A** | **G** | **A** | **T** | **T** | **T** | **C** | **A** | **A** | **T** | **C** | **G** | **T** | **C** | **T** | **A** | **A** | **T** | **C** | **A** | **A** | **A** | **A** | **A** | **T** | **G** | **G** | **T** | **G** | **G** | **A** | **C** | **A** | **T** | **G** | **T** | **C** | **C** | **A** | **T** | **A** | **C** | **A** | **T** | **T** | **T** | **G** | **T** | **T** | **A** | **C** | **C** | **T** | **C** | **A** | **C** | **A** | **A** | **A** | **A** | **G** | **G** | **C** | **A** | **T** | **T** | **A** | **C** | **T** | **T** | **C** | **G** | **T** | **T** | **T** | **C** | **C** | **G** | **A** | **T** | **T** | **G** | **A** | **A** | **T** | **T** | **A** | **C** | **C** | **A** | **T** | **G** | **A** | **T** | **C** | **A** | **C** | **C** | **G** | **A** | **A** | **A** | **A** | **A** | **T** | **C** | **G** | **T** | **T** | **G** | **T** | **G** | **A** | **T** | **C** | **G** | **A** | **T** | **G** | **G** | **A** | **A** | **A** | **G** | **A** | **T** | **T** | **A** | **G** | **T** | **T** | **A** | **T** | **A** | **C** | **C** | **G** | **G** | **T** | **G** | **G** | **T** | **T** | **T** | **C** | **A** | **A** | **T** | **A** | **T** | **T** | **G** | **C** | **C** | **A** | **A** | **T** | **C** | **A** | **A** | **T** | **A** | **T** | **G** | **T** | **A** | **A** | **A** | **T** | **A** | **C** | **A** | **A** | **C** | **A** | **A** | **A** | **A** | **A** | **A** | **A** | **T** | **T** | **C** | **G** | **G** | **C** | **T** | **A** | **T** | **T** | **G** | **G** | **C** | **G** | **C** | **G** | **A** | **T** | **A** | **C** | **G** | **C** | **A** | **T** | **A** | **T** | **A** | **C** | **G** | **G** | **A** | **T** | **T** | **T** | **T** | **C** | **G** | **G** | **T** | **G** | **C** | **C** | **G** | **C** | **T** | **T** | **C** | **T** | **T** | **C** | **T** | **T** | **T** | **G** | **C** | **T** | **C** | **C** | **A** | **G** | **C** | **T** | **T** | **C** | **G** | **C** | **T** | **T** | **C** | **T** | **T** | **A** | **A** | **C** | **A** | **G** | **A** | **C** | **T** | **G** | **G** | **A** | **A** | **C** | **G** | **T** | **C** | **T** | **C** | **G** | **G** | **T** | **A** | **C** | **C** | **T** | **G** | **A** | **A** | **G** | **A** | **A** | **A** | **A** | **A** | **A** | **A** | **G** | **G** | **T** | **C** | **G** | **G** | **C** | **T** | **A** | **T** | **C** | **A** | **T** | **T** | **T** | **G** | **A** | **A** | **T** | **T** | **A** | **T** | **T** | **T** | **C** | **T** | **T** | **T** | **A** | **A** | **A** | **A** | **A** | **A** | **G** | **C** | **A** | **G** | **A** | **T** | **C** | **G** | **A** | **G** | **A** | **T** | **G** | **A** | **A** | **T** | **C** | **T** | **A** | **A** | **G** | **C** | **T** | **T** | **G** | **C** | **T** | **G** | **A** | **T** | **A** | **C** | **A** | **T** | **C** | **C** | **A** | **T** | **C** | **C** | **A** | **G** | **C** | **T** | **T** | **G** | **T** | **T** | **T** | **C** | **A** | **A** | **G** | **C** | **G** | **G** | **A** | **C** | **C** | **G** | **A** | **A** | **T** | **A** | **A** | **C** | **G** | **A** | **A** | **A** | **G** | **G** | **G** | **A** | **A** | **C** | **A** | **A** | **A** | **T** | **C** | **A** | **A** | **G** | **C** | **T** | **T** | **T** | **C** | **A** | **T** | **T** | **T** | **A** | **T** | **C** | **A** | **A** | **A** | **T** | **T** | **G** | **A** | **T** | **T** | **A** | **C** | **T** | **T** | **C** | **T** | **G** | **C** | **T** | **A** | **A** | **A** | **A** | **A** | **A** | **C** | **G** | **T** | **G** | **T** | **T** | **T** | **G** | **G** | **A** | **T** | **A** | **C** | **A** | **G** | **A** | **C** | **A** | **C** | **C** | **T** | **T** | **A** | **C** | **C** | **T** | **T** | **G** | **T** | **T** | **C** | **C** | **T** | **G** | **A** | **T** | **G** | **A** | **A** | **A** | **G** | **T** | **G** | **T** | **C** | **A** | **T** | **T** | **G** | **C** | **T** | **G** | **C** | **T** | **T** | **T** | **A** | **A** | **A** | **A** | **A** | **T** | **C** | **G** | **C** | **A** | **A** | **C** | **T** | **G** | **C** | **C** | **T** | **C** | **T** | **G** | **G** | **T** | **G** | **T** | **A** | **G** | **A** | **T** | **G** | **T** | **G** | **A** | **A** | **A** | **A** | **T** | **C** | **A** | **T** | **G** | **A** | **T** | **T** | **C** | **C** | **C** | **A** | **A** | **C** | **A** | **A** | **A** | **C** | **C** | **G** | **G** | **A** | **T** | **C** | **A** | **T** | **C** | **C** | **T** | **T** | **T** | **T** | **A** | **T** | **T** | **T** | **A** | **T** | **C** | **G** | **A** | **G** | **C** | **A** | **A** | **C** | **A** | **C** | **A** | **A** | **T** | **A** | **T** | **T** | **A** | **T** | **G** | **C** | **T** | **C** | **G** | **G** | **C** | **A** | **G** | **C** | **T** | **G** | **A** | **T** | **C** | **A** | **A** | **G** | **G** | **A** | **A** | **A** | **A** | **T** | **G** | **T** | **A** | **C** | **A** | **A** | **A** | **T** | **C** | **C** | **T** | **T** | **G** | **T** | **C** | **T** | **A** | **T** | **G** | **A** | **G** | **A** | **A** | **C** | **G** | **G** | **C** | **T** | **T** | **C** | **C** | **T** | **C** | **C** | **A** | **T** | **G** | **C** | **A** | **A** | **A** | **A** | **A** | **C** | **A** | **T** | **T** | **G** | **A** | **T** | **A** | **A** | **T** | **G** | **G** | **A** | **T** | **G** | **A** | **T** | **G** | **A** | **A** | **A** | **T** | **C** | **T** | **G** | **C** | **A** | **T** | **G** | **G** | **T** | **A** | **G** | **G** | **T** | **T** | **C** | **A** | **G** | **C** | **A** | **A** | **A** | **T** | **C** | **A** | **A** | **G** | **A** | **T** | **A** | **T** | **T** | **C** | **G** | **T** | **A** | **G** | **C** | **T** | **A** | **C** | **C** | **G** | **A** | **T** | **T** | **G** | **A** | **A** | **T** | **T** | **T** | **T** | **G** | **A** | **A** | **A** | **C** | **A** | **A** | **G** | **T** | **G** | **C** | **T** | **G** | **T** | **C** | **A** | **T** | **T** | **T** | **A** | **C** | **G** | **A** | **T** | **C** | **C** | **T** | **G** | **A** | **G** | **T** | **T** | **T** | **T** | **T** | **A** | **G** | **A** | **A** | **G** | **A** | **A** | **C** | **T** | **T** | **G** | **C** | **T** | **A** | **C** | **T** | **C** | **A** | **G** | **T** | **T** | **C** | **A** | **A** | **A** | **G** | **A** | **A** | **G** | **A** | **T** | **G** | **A** | **G** | **A** | **C** | **A** | **C** | **A** | **T** | **T** | **G** | **T** | **T** | **C** | **A** | **T** | **C** | **C** | **A** | **T** | **G** | **A** | **C** | **A** | **A** | **C** | **T** | **G** | **A** | **A** | **A** | **C** | **A** | **G** | **T** | **C** | **A** | **A** | **G** | **G** | **A** | **A** | **A** | **T** | **G** | **T** | **C** | **T** | **A** | **A** | **C** | **T** | **G** | **G** | **C** | **T** | **A** | **T** | **T** | **A** | **T** | **T** | **C** | **A** | **A** | **G** | **C** | **A** | **A** | **C** | **A** | **A** | **A** | **T** | **T** | **T** | **C** | **T** | **C** | **G** | **A** | **T** | **T** | **A** | **T** | **T** | **T** | **T** | **C** | **T** | **C** | **C** | **A** | **A** | **T** | **C** | **C** | **T** | **A** | **-** | **-** | **-** |
| **N59503F** | **-** | **-** | **-** | **-** | **-** | **-** | **-** | **-** | **-** | **-** | **-** | **-** | **-** | **-** | **-** | **-** | **-** | **-** | **-** | **-** | **-** | **-** | **-** | **-** | **-** | **-** | **-** | **-** | **-** | **-** | **-** | **-** | **-** | **-** | **-** | **-** | **-** | **-** | **-** | **-** | **-** | **-** | **-** | **-** | **-** | **-** | **-** | **-** | **-** | **-** | **-** | **-** | **-** | **-** | **-** | **-** | **-** | **-** | **-** | **-** | **-** | **-** | **-** | **-** | **-** | **-** | **-** | **-** | **-** | **A** | **A** | **A** | **C** | **C** | **C** | **C** | **G** | **A** | **G** | **A** | **T** | **G** | **T** | **A** | **G** | **C** | **A** | **G** | **C** | **C** | **A** | **T** | **T** | **T** | **G** | **G** | **G** | **C** | **A** | **T** | **G** | **G** | **C** | **T** | **C** | **T** | **T** | **A** | **G** | **T** | **A** | **T** | **T** | **G** | **T** | **T** | **T** | **G** | **C** | **C** | **C** | **T** | **T** | **C** | **C** | **T** | **G** | **G** | **G** | **G** | **T** | **G** | **G** | **G** | **T** | **T** | **T** | **T** | **G** | **T** | **A** | **T** | **T** | **A** | **T** | **A** | **T** | **T** | **T** | **A** | **T** | **T** | **T** | **T** | **T** | **C** | **G** | **G** | **A** | **C** | **G** | **T** | **G** | **G** | **A** | **T** | **T** | **A** | **A** | **C** | **G** | **G** | **A** | **T** | **A** | **A** | **G** | **A** | **A** | **A** | **A** | **A** | **A** | **T** | **T** | **C** | **T** | **A** | **T** | **C** | **T** | **C** | **C** | **G** | **A** | **C** | **A** | **A** | **A** | **G** | **T** | **G** | **A** | **C** | **T** | **T** | **G** | **A** | **A** | **A** | **G** | **A** | **A** | **T** | **T** | **A** | **G** | **A** | **A** | **A** | **A** | **C** | **T** | **T** | **T** | **C** | **A** | **G** | **T** | **C** | **T** | **T** | **T** | **T** | **A** | **G** | **A** | **G** | **G** | **A** | **G** | **A** | **T** | **A** | **C** | **C** | **A** | **T** | **T** | **G** | **A** | **A** | **C** | **A** | **T** | **T** | **A** | **C** | **G** | **A** | **T** | **C** | **C** | **T** | **G** | **A** | **C** | **A** | **T** | **G** | **G** | **G** | **C** | **G** | **A** | **T** | **A** | **A** | **A** | **G** | **A** | **C** | **A** | **A** | **A** | **C** | **A** | **G** | **C** | **A** | **A** | **T** | **T** | **T** | **G** | **T** | **C** | **G** | **A** | **C** | **T** | **T** | **C** | **T** | **T** | **C** | **T** | **C** | **T** | **T** | **C** | **A** | **T** | **T** | **A** | **A** | **A** | **T** | **C** | **G** | **T** | **A** | **T** | **G** | **C** | **C** | **G** | **C** | **T** | **G** | **A** | **C** | **A** | **A** | **G** | **A** | **A** | **T** | **G** | **A** | **A** | **T** | **T** | **C** | **T** | **G** | **T** | **C** | **A** | **C** | **T** | **C** | **T** | **T** | **C** | **T** | **C** | **A** | **C** | **A** | **G** | **A** | **C** | **G** | **G** | **A** | **C** | **A** | **A** | **G** | **A** | **G** | **A** | **A** | **A** | **T** | **T** | **G** | **G** | **A** | **T** | **T** | **C** | **A** | **C** | **T** | **G** | **C** | **T** | **T** | **C** | **A** | **A** | **G** | **A** | **T** | **C** | **T** | **A** | **A** | **A** | **A** | **A** | **A** | **A** | **G** | **C** | **C** | **A** | **A** | **A** | **C** | **A** | **T** | **T** | **C** | **G** | **A** | **T** | **C** | **C** | **A** | **T** | **A** | **T** | **C** | **G** | **A** | **A** | **T** | **A** | **T** | **T** | **A** | **C** | **G** | **C** | **A** | **T** | **T** | **T** | **G** | **T** | **G** | **A** | **C** | **A** | **G** | **A** | **T** | **A** | **A** | **T** | **A** | **T** | **C** | **G** | **G** | **C** | **C** | **A** | **G** | **C** | **A** | **A** | **G** | **T** | **C** | **T** | **T** | **A** | **C** | **A** | **T** | **G** | **T** | **T** | **T** | **T** | **A** | **G** | **A** | **A** | **G** | **A** | **A** | **A** | **A** | **A** | **G** | **C** | **C** | **G** | **C** | **A** | **G** | **A** | **A** | **G** | **G** | **C** | **G** | **T** | **G** | **G** | **A** | **A** | **G** | **T** | **T** | **C** | **G** | **A** | **A** | **T** | **A** | **T** | **T** | **A** | **T** | **A** | **T** | **G** | **A** | **T** | **G** | **C** | **A** | **T** | **T** | **T** | **G** | **G** | **C** | **T** | **C** | **T** | **C** | **A** | **T** | **G** | **G** | **C** | **A** | **C** | **A** | **A** | **A** | **A** | **G** | **C** | **A** | **A** | **A** | **A** | **G** | **A** | **T** | **T** | **T** | **C** | **A** | **A** | **T** | **C** | **G** | **T** | **C** | **T** | **A** | **A** | **T** | **C** | **A** | **A** | **A** | **A** | **A** | **T** | **G** | **G** | **T** | **G** | **G** | **A** | **C** | **A** | **T** | **G** | **T** | **C** | **C** | **A** | **T** | **A** | **C** | **A** | **T** | **T** | **T** | **G** | **T** | **T** | **A** | **C** | **C** | **T** | **C** | **A** | **C** | **A** | **A** | **A** | **A** | **G** | **G** | **C** | **A** | **T** | **T** | **A** | **C** | **T** | **T** | **C** | **G** | **T** | **T** | **T** | **C** | **C** | **G** | **A** | **T** | **T** | **G** | **A** | **A** | **T** | **T** | **A** | **C** | **C** | **A** | **T** | **G** | **A** | **T** | **C** | **A** | **C** | **C** | **G** | **A** | **A** | **A** | **A** | **A** | **T** | **C** | **G** | **T** | **T** | **G** | **T** | **G** | **A** | **T** | **C** | **G** | **A** | **T** | **G** | **G** | **A** | **A** | **A** | **G** | **A** | **T** | **T** | **A** | **G** | **T** | **T** | **A** | **T** | **A** | **C** | **C** | **G** | **G** | **T** | **G** | **G** | **T** | **T** | **T** | **C** | **A** | **A** | **T** | **A** | **T** | **T** | **G** | **C** | **C** | **A** | **A** | **T** | **C** | **A** | **A** | **T** | **A** | **T** | **G** | **T** | **A** | **A** | **A** | **T** | **A** | **C** | **A** | **A** | **C** | **A** | **A** | **A** | **A** | **A** | **A** | **A** | **T** | **T** | **C** | **G** | **G** | **C** | **T** | **A** | **T** | **T** | **G** | **G** | **C** | **G** | **C** | **G** | **A** | **T** | **A** | **C** | **G** | **C** | **A** | **T** | **A** | **T** | **A** | **C** | **G** | **G** | **A** | **T** | **T** | **T** | **T** | **C** | **G** | **G** | **T** | **G** | **C** | **C** | **G** | **C** | **T** | **T** | **C** | **T** | **T** | **C** | **T** | **T** | **T** | **G** | **C** | **T** | **C** | **C** | **A** | **G** | **C** | **T** | **T** | **C** | **G** | **C** | **T** | **T** | **C** | **T** | **T** | **A** | **A** | **C** | **A** | **G** | **A** | **C** | **T** | **G** | **G** | **A** | **A** | **C** | **G** | **T** | **C** | **T** | **C** | **G** | **G** | **T** | **A** | **C** | **C** | **T** | **G** | **A** | **A** | **G** | **A** | **A** | **A** | **A** | **A** | **A** | **A** | **G** | **G** | **T** | **C** | **G** | **G** | **C** | **T** | **A** | **T** | **C** | **A** | **T** | **T** | **T** | **G** | **A** | **A** | **T** | **T** | **A** | **T** | **T** | **T** | **C** | **T** | **T** | **T** | **A** | **A** | **A** | **A** | **A** | **A** | **G** | **C** | **A** | **G** | **A** | **T** | **C** | **G** | **A** | **G** | **A** | **T** | **G** | **A** | **A** | **T** | **C** | **T** | **A** | **A** | **G** | **C** | **T** | **T** | **G** | **C** | **T** | **G** | **A** | **T** | **A** | **C** | **A** | **T** | **C** | **C** | **A** | **T** | **C** | **C** | **A** | **G** | **C** | **T** | **T** | **G** | **T** | **T** | **T** | **C** | **A** | **A** | **G** | **C** | **G** | **G** | **A** | **C** | **C** | **G** | **A** | **A** | **T** | **A** | **A** | **C** | **G** | **A** | **A** | **A** | **G** | **G** | **G** | **A** | **A** | **C** | **A** | **A** | **A** | **T** | **C** | **A** | **A** | **G** | **C** | **T** | **T** | **T** | **C** | **A** | **T** | **T** | **T** | **A** | **T** | **C** | **A** | **A** | **A** | **T** | **T** | **G** | **A** | **T** | **T** | **A** | **C** | **T** | **T** | **C** | **T** | **G** | **C** | **T** | **A** | **A** | **A** | **A** | **A** | **A** | **C** | **G** | **T** | **G** | **T** | **T** | **T** | **G** | **G** | **A** | **T** | **A** | **C** | **A** | **G** | **A** | **C** | **A** | **C** | **C** | **T** | **T** | **A** | **C** | **C** | **T** | **T** | **G** | **T** | **T** | **C** | **C** | **T** | **G** | **A** | **T** | **G** | **A** | **A** | **A** | **G** | **T** | **G** | **T** | **C** | **A** | **T** | **T** | **G** | **C** | **T** | **G** | **C** | **T** | **T** | **T** | **A** | **A** | **A** | **A** | **A** | **T** | **C** | **G** | **C** | **A** | **A** | **C** | **T** | **G** | **C** | **C** | **T** | **C** | **T** | **G** | **G** | **T** | **G** | **T** | **A** | **G** | **A** | **T** | **G** | **T** | **G** | **A** | **A** | **A** | **A** | **T** | **C** | **A** | **T** | **G** | **A** | **T** | **T** | **C** | **C** | **C** | **A** | **A** | **C** | **A** | **A** | **A** | **C** | **C** | **G** | **G** | **A** | **T** | **C** | **A** | **T** | **C** | **C** | **T** | **T** | **T** | **T** | **A** | **T** | **T** | **T** | **A** | **T** | **C** | **G** | **A** | **G** | **C** | **A** | **A** | **C** | **A** | **C** | **A** | **A** | **T** | **A** | **T** | **T** | **A** | **T** | **G** | **C** | **T** | **C** | **G** | **G** | **C** | **A** | **G** | **C** | **T** | **G** | **A** | **T** | **C** | **A** | **A** | **G** | **G** | **A** | **A** | **A** | **A** | **T** | **G** | **T** | **A** | **C** | **A** | **A** | **A** | **T** | **C** | **C** | **T** | **T** | **G** | **T** | **C** | **T** | **A** | **T** | **G** | **A** | **G** | **A** | **A** | **C** | **G** | **G** | **C** | **T** | **T** | **C** | **C** | **T** | **C** | **C** | **A** | **T** | **G** | **C** | **A** | **A** | **A** | **A** | **A** | **C** | **A** | **T** | **T** | **G** | **A** | **T** | **A** | **A** | **T** | **G** | **G** | **A** | **T** | **G** | **A** | **T** | **G** | **A** | **A** | **A** | **T** | **C** | **T** | **G** | **C** | **A** | **T** | **G** | **G** | **T** | **A** | **G** | **G** | **T** | **T** | **C** | **A** | **G** | **C** | **A** | **A** | **A** | **T** | **C** | **A** | **A** | **G** | **A** | **T** | **A** | **T** | **T** | **C** | **G** | **T** | **A** | **G** | **C** | **T** | **A** | **C** | **C** | **G** | **A** | **T** | **T** | **G** | **A** | **A** | **T** | **T** | **T** | **T** | **G** | **A** | **A** | **A** | **C** | **A** | **A** | **G** | **T** | **G** | **C** | **T** | **G** | **T** | **C** | **A** | **T** | **T** | **T** | **A** | **C** | **G** | **A** | **T** | **C** | **C** | **T** | **G** | **A** | **G** | **T** | **T** | **T** | **T** | **T** | **A** | **G** | **A** | **A** | **G** | **A** | **A** | **C** | **T** | **T** | **G** | **C** | **T** | **A** | **C** | **T** | **C** | **A** | **G** | **T** | **T** | **C** | **A** | **A** | **A** | **G** | **A** | **A** | **G** | **A** | **T** | **G** | **A** | **G** | **A** | **C** | **A** | **C** | **A** | **T** | **T** | **G** | **T** | **T** | **C** | **A** | **T** | **C** | **C** | **A** | **T** | **G** | **A** | **C** | **A** | **A** | **C** | **T** | **G** | **A** | **A** | **A** | **C** | **A** | **G** | **T** | **C** | **A** | **A** | **G** | **G** | **A** | **A** | **A** | **T** | **G** | **T** | **C** | **T** | **A** | **A** | **C** | **T** | **G** | **G** | **C** | **T** | **A** | **T** | **T** | **A** | **T** | **T** | **C** | **A** | **A** | **G** | **C** | **A** | **A** | **C** | **A** | **A** | **A** | **T** | **T** | **T** | **C** | **T** | **C** | **G** | **A** | **T** | **T** | **A** | **T** | **T** | **T** | **T** | **C** | **T** | **C** | **C** | **A** | **A** | **T** | **C** | **C** | **T** | **A** | **-** | **-** | **-** |
| **N54550** | **-** | **-** | **-** | **-** | **-** | **-** | **-** | **-** | **-** | **-** | **-** | **-** | **-** | **-** | **-** | **-** | **-** | **-** | **-** | **-** | **-** | **-** | **-** | **-** | **-** | **-** | **-** | **-** | **-** | **-** | **-** | **-** | **-** | **-** | **-** | **-** | **-** | **-** | **-** | **-** | **-** | **-** | **-** | **-** | **-** | **-** | **-** | **-** | **-** | **-** | **-** | **-** | **-** | **-** | **-** | **-** | **-** | **-** | **-** | **-** | **-** | **-** | **-** | **-** | **-** | **-** | **-** | **-** | **-** | **A** | **A** | **A** | **C** | **C** | **C** | **C** | **G** | **A** | **G** | **A** | **T** | **G** | **T** | **A** | **G** | **C** | **A** | **G** | **C** | **C** | **A** | **T** | **T** | **T** | **G** | **G** | **G** | **C** | **A** | **T** | **G** | **G** | **C** | **T** | **C** | **T** | **T** | **A** | **G** | **T** | **A** | **T** | **T** | **G** | **T** | **T** | **T** | **G** | **C** | **C** | **C** | **T** | **T** | **C** | **C** | **T** | **G** | **G** | **G** | **G** | **T** | **G** | **G** | **G** | **T** | **T** | **T** | **T** | **G** | **T** | **A** | **T** | **T** | **A** | **T** | **A** | **T** | **T** | **T** | **A** | **T** | **T** | **T** | **T** | **T** | **C** | **G** | **G** | **A** | **C** | **G** | **T** | **G** | **G** | **A** | **T** | **T** | **A** | **A** | **C** | **G** | **G** | **A** | **T** | **A** | **A** | **G** | **A** | **A** | **A** | **A** | **A** | **A** | **T** | **T** | **C** | **T** | **A** | **T** | **C** | **T** | **C** | **C** | **G** | **A** | **C** | **A** | **A** | **A** | **G** | **T** | **G** | **A** | **C** | **T** | **T** | **G** | **A** | **A** | **A** | **G** | **A** | **A** | **T** | **T** | **A** | **G** | **A** | **A** | **A** | **A** | **C** | **T** | **T** | **T** | **C** | **A** | **G** | **T** | **C** | **T** | **T** | **T** | **T** | **A** | **G** | **A** | **G** | **G** | **A** | **G** | **A** | **T** | **A** | **C** | **C** | **A** | **T** | **T** | **G** | **A** | **A** | **C** | **A** | **T** | **T** | **A** | **C** | **G** | **A** | **T** | **C** | **C** | **T** | **G** | **A** | **C** | **A** | **T** | **G** | **G** | **G** | **C** | **G** | **A** | **T** | **A** | **A** | **A** | **G** | **A** | **C** | **A** | **A** | **A** | **C** | **A** | **G** | **C** | **A** | **A** | **T** | **T** | **T** | **G** | **T** | **C** | **G** | **A** | **C** | **T** | **T** | **C** | **T** | **T** | **C** | **T** | **C** | **T** | **T** | **C** | **A** | **T** | **T** | **A** | **A** | **A** | **T** | **C** | **G** | **T** | **A** | **T** | **G** | **C** | **C** | **G** | **C** | **T** | **G** | **A** | **C** | **A** | **A** | **G** | **A** | **A** | **T** | **G** | **A** | **A** | **T** | **T** | **C** | **T** | **G** | **T** | **C** | **A** | **C** | **T** | **C** | **T** | **T** | **C** | **T** | **C** | **A** | **C** | **A** | **G** | **A** | **C** | **G** | **G** | **A** | **C** | **A** | **A** | **G** | **A** | **G** | **A** | **A** | **A** | **T** | **T** | **G** | **G** | **A** | **T** | **T** | **C** | **A** | **C** | **T** | **G** | **C** | **T** | **T** | **C** | **A** | **A** | **G** | **A** | **T** | **C** | **T** | **A** | **A** | **A** | **A** | **A** | **A** | **A** | **G** | **C** | **C** | **A** | **A** | **A** | **C** | **A** | **T** | **T** | **C** | **G** | **A** | **T** | **C** | **C** | **A** | **T** | **A** | **T** | **C** | **G** | **A** | **A** | **T** | **A** | **T** | **T** | **A** | **C** | **G** | **C** | **A** | **T** | **T** | **T** | **G** | **T** | **G** | **A** | **C** | **A** | **G** | **A** | **T** | **A** | **A** | **T** | **A** | **T** | **C** | **G** | **G** | **C** | **C** | **A** | **G** | **C** | **A** | **A** | **G** | **T** | **C** | **T** | **T** | **A** | **C** | **A** | **T** | **G** | **T** | **T** | **T** | **T** | **A** | **G** | **A** | **A** | **G** | **A** | **A** | **A** | **A** | **A** | **G** | **C** | **C** | **G** | **C** | **A** | **G** | **A** | **A** | **G** | **G** | **C** | **G** | **T** | **G** | **G** | **A** | **A** | **G** | **T** | **T** | **C** | **G** | **A** | **A** | **T** | **A** | **T** | **T** | **A** | **T** | **A** | **T** | **G** | **A** | **T** | **G** | **C** | **A** | **T** | **T** | **T** | **G** | **G** | **C** | **T** | **C** | **T** | **C** | **A** | **T** | **G** | **G** | **C** | **A** | **C** | **A** | **A** | **A** | **A** | **G** | **C** | **A** | **A** | **A** | **A** | **G** | **A** | **T** | **T** | **T** | **C** | **A** | **A** | **T** | **C** | **G** | **T** | **C** | **T** | **A** | **A** | **T** | **C** | **A** | **A** | **A** | **A** | **A** | **T** | **G** | **G** | **T** | **G** | **G** | **A** | **C** | **A** | **T** | **G** | **T** | **C** | **C** | **A** | **T** | **A** | **C** | **A** | **T** | **T** | **T** | **G** | **T** | **T** | **A** | **C** | **C** | **T** | **C** | **A** | **C** | **A** | **A** | **A** | **A** | **G** | **G** | **C** | **A** | **T** | **T** | **A** | **C** | **T** | **T** | **C** | **G** | **T** | **T** | **T** | **C** | **C** | **G** | **A** | **T** | **T** | **G** | **A** | **A** | **T** | **T** | **A** | **C** | **C** | **A** | **T** | **G** | **A** | **T** | **C** | **A** | **C** | **C** | **G** | **A** | **A** | **A** | **A** | **A** | **T** | **C** | **G** | **T** | **T** | **G** | **T** | **G** | **A** | **T** | **C** | **G** | **A** | **T** | **G** | **G** | **A** | **A** | **A** | **G** | **A** | **T** | **T** | **A** | **G** | **T** | **T** | **A** | **T** | **A** | **C** | **C** | **G** | **G** | **T** | **G** | **G** | **T** | **T** | **T** | **C** | **A** | **A** | **T** | **A** | **T** | **T** | **G** | **C** | **C** | **A** | **A** | **T** | **C** | **A** | **A** | **T** | **A** | **T** | **G** | **T** | **A** | **A** | **A** | **T** | **A** | **C** | **A** | **A** | **C** | **A** | **A** | **A** | **A** | **A** | **A** | **A** | **T** | **T** | **C** | **G** | **G** | **C** | **T** | **A** | **T** | **T** | **G** | **G** | **C** | **G** | **C** | **G** | **A** | **T** | **A** | **C** | **G** | **C** | **A** | **T** | **A** | **T** | **A** | **C** | **G** | **G** | **A** | **T** | **T** | **T** | **T** | **C** | **G** | **G** | **T** | **G** | **C** | **C** | **G** | **C** | **T** | **T** | **C** | **T** | **T** | **C** | **T** | **T** | **T** | **G** | **C** | **T** | **C** | **C** | **A** | **G** | **C** | **T** | **T** | **C** | **G** | **C** | **T** | **T** | **C** | **T** | **T** | **A** | **A** | **C** | **A** | **G** | **A** | **C** | **T** | **G** | **G** | **A** | **A** | **C** | **G** | **T** | **C** | **T** | **C** | **G** | **G** | **T** | **A** | **C** | **C** | **T** | **G** | **A** | **A** | **G** | **A** | **A** | **A** | **A** | **A** | **A** | **A** | **G** | **G** | **T** | **C** | **G** | **G** | **C** | **T** | **A** | **T** | **C** | **A** | **T** | **T** | **T** | **G** | **A** | **A** | **T** | **T** | **A** | **T** | **T** | **T** | **C** | **T** | **T** | **T** | **A** | **A** | **A** | **A** | **A** | **A** | **G** | **C** | **A** | **G** | **A** | **T** | **C** | **G** | **A** | **G** | **A** | **T** | **G** | **A** | **A** | **T** | **C** | **T** | **A** | **A** | **G** | **C** | **T** | **T** | **G** | **C** | **T** | **G** | **A** | **T** | **A** | **C** | **A** | **T** | **C** | **C** | **A** | **T** | **C** | **C** | **A** | **G** | **C** | **T** | **T** | **G** | **T** | **T** | **T** | **C** | **A** | **A** | **G** | **C** | **G** | **G** | **A** | **C** | **C** | **G** | **A** | **A** | **T** | **A** | **A** | **C** | **G** | **A** | **A** | **A** | **G** | **G** | **G** | **A** | **A** | **C** | **A** | **A** | **A** | **T** | **C** | **A** | **A** | **G** | **C** | **T** | **T** | **T** | **C** | **A** | **T** | **T** | **T** | **A** | **T** | **C** | **A** | **A** | **A** | **T** | **T** | **G** | **A** | **T** | **T** | **A** | **C** | **T** | **T** | **C** | **T** | **G** | **C** | **T** | **A** | **A** | **A** | **A** | **A** | **A** | **C** | **G** | **T** | **G** | **T** | **T** | **T** | **G** | **G** | **A** | **T** | **A** | **C** | **A** | **G** | **A** | **C** | **A** | **C** | **C** | **T** | **T** | **A** | **C** | **C** | **T** | **T** | **G** | **T** | **T** | **C** | **C** | **T** | **G** | **A** | **T** | **G** | **A** | **A** | **A** | **G** | **T** | **G** | **T** | **C** | **A** | **T** | **T** | **G** | **C** | **T** | **G** | **C** | **T** | **T** | **T** | **A** | **A** | **A** | **A** | **A** | **T** | **C** | **G** | **C** | **A** | **A** | **C** | **T** | **G** | **C** | **C** | **T** | **C** | **T** | **G** | **G** | **T** | **G** | **T** | **A** | **G** | **A** | **T** | **G** | **T** | **G** | **A** | **A** | **A** | **A** | **T** | **C** | **A** | **T** | **G** | **A** | **T** | **T** | **C** | **C** | **C** | **A** | **A** | **C** | **A** | **A** | **A** | **C** | **C** | **G** | **G** | **A** | **T** | **C** | **A** | **T** | **C** | **C** | **T** | **T** | **T** | **T** | **A** | **T** | **T** | **T** | **A** | **T** | **C** | **G** | **A** | **G** | **C** | **A** | **A** | **C** | **A** | **C** | **A** | **A** | **T** | **A** | **T** | **T** | **A** | **T** | **G** | **C** | **T** | **C** | **G** | **G** | **C** | **A** | **G** | **C** | **T** | **G** | **A** | **T** | **C** | **A** | **A** | **G** | **G** | **A** | **A** | **A** | **A** | **T** | **G** | **T** | **A** | **C** | **A** | **A** | **A** | **T** | **C** | **C** | **T** | **T** | **G** | **T** | **C** | **T** | **A** | **T** | **G** | **A** | **G** | **A** | **A** | **C** | **G** | **G** | **C** | **T** | **T** | **C** | **C** | **T** | **C** | **C** | **A** | **T** | **G** | **C** | **A** | **A** | **A** | **A** | **A** | **C** | **A** | **T** | **T** | **G** | **A** | **T** | **A** | **A** | **T** | **G** | **G** | **A** | **T** | **G** | **A** | **T** | **G** | **A** | **A** | **A** | **T** | **C** | **T** | **G** | **C** | **A** | **T** | **G** | **G** | **T** | **A** | **G** | **G** | **T** | **T** | **C** | **A** | **G** | **C** | **A** | **A** | **A** | **T** | **C** | **A** | **A** | **G** | **A** | **T** | **A** | **T** | **T** | **C** | **G** | **T** | **A** | **G** | **C** | **T** | **A** | **C** | **C** | **G** | **A** | **T** | **T** | **G** | **A** | **A** | **T** | **T** | **T** | **T** | **G** | **A** | **A** | **A** | **C** | **A** | **A** | **G** | **T** | **G** | **C** | **T** | **G** | **T** | **C** | **A** | **T** | **T** | **T** | **A** | **C** | **G** | **A** | **T** | **C** | **C** | **T** | **G** | **A** | **G** | **T** | **T** | **T** | **T** | **T** | **A** | **G** | **A** | **A** | **G** | **A** | **A** | **C** | **T** | **T** | **G** | **C** | **T** | **A** | **C** | **T** | **C** | **A** | **G** | **T** | **T** | **C** | **A** | **A** | **A** | **G** | **A** | **A** | **G** | **A** | **T** | **G** | **A** | **G** | **A** | **C** | **A** | **C** | **A** | **T** | **T** | **G** | **T** | **T** | **C** | **A** | **T** | **C** | **C** | **A** | **T** | **G** | **A** | **C** | **A** | **A** | **C** | **T** | **G** | **A** | **A** | **A** | **C** | **A** | **G** | **T** | **C** | **A** | **A** | **G** | **G** | **A** | **A** | **A** | **T** | **G** | **T** | **C** | **T** | **A** | **A** | **C** | **T** | **G** | **G** | **C** | **T** | **A** | **T** | **T** | **A** | **T** | **T** | **C** | **A** | **A** | **G** | **C** | **A** | **A** | **C** | **A** | **A** | **A** | **T** | **T** | **T** | **C** | **T** | **C** | **G** | **A** | **T** | **T** | **A** | **T** | **T** | **T** | **T** | **C** | **T** | **C** | **C** | **A** | **A** | **T** | **C** | **C** | **T** | **A** | **-** | **-** | **-** |
| **N60045F** | **-** | **-** | **-** | **-** | **-** | **-** | **-** | **-** | **-** | **-** | **-** | **-** | **-** | **-** | **-** | **-** | **-** | **-** | **-** | **-** | **-** | **-** | **-** | **-** | **-** | **-** | **-** | **-** | **-** | **-** | **-** | **-** | **-** | **-** | **-** | **-** | **-** | **-** | **-** | **-** | **-** | **-** | **-** | **-** | **-** | **-** | **-** | **-** | **-** | **-** | **-** | **-** | **-** | **-** | **-** | **-** | **-** | **-** | **-** | **-** | **-** | **-** | **-** | **-** | **-** | **-** | **-** | **-** | **-** | **A** | **A** | **A** | **C** | **C** | **C** | **C** | **G** | **A** | **G** | **A** | **T** | **G** | **T** | **A** | **G** | **C** | **A** | **G** | **C** | **C** | **A** | **T** | **T** | **T** | **G** | **G** | **G** | **C** | **A** | **T** | **G** | **G** | **C** | **T** | **C** | **T** | **T** | **A** | **G** | **T** | **A** | **T** | **T** | **G** | **T** | **T** | **T** | **G** | **C** | **C** | **C** | **T** | **T** | **C** | **C** | **T** | **G** | **G** | **G** | **G** | **T** | **G** | **G** | **G** | **T** | **T** | **T** | **T** | **G** | **T** | **A** | **T** | **T** | **A** | **T** | **A** | **T** | **T** | **T** | **A** | **T** | **T** | **T** | **T** | **T** | **C** | **G** | **G** | **A** | **C** | **G** | **T** | **G** | **G** | **A** | **T** | **T** | **A** | **A** | **C** | **G** | **G** | **A** | **T** | **A** | **A** | **G** | **A** | **A** | **A** | **A** | **A** | **A** | **T** | **T** | **C** | **T** | **A** | **T** | **C** | **T** | **C** | **C** | **G** | **A** | **C** | **A** | **A** | **A** | **G** | **T** | **G** | **A** | **C** | **T** | **T** | **G** | **A** | **A** | **A** | **G** | **A** | **A** | **T** | **T** | **A** | **G** | **A** | **A** | **A** | **A** | **C** | **T** | **T** | **T** | **C** | **A** | **G** | **T** | **C** | **T** | **T** | **T** | **T** | **A** | **G** | **A** | **G** | **G** | **A** | **G** | **A** | **T** | **A** | **C** | **C** | **A** | **T** | **T** | **G** | **A** | **A** | **C** | **A** | **T** | **T** | **A** | **C** | **G** | **A** | **T** | **C** | **C** | **T** | **G** | **A** | **C** | **A** | **T** | **G** | **G** | **G** | **C** | **G** | **A** | **T** | **A** | **A** | **A** | **G** | **A** | **C** | **A** | **A** | **A** | **C** | **A** | **G** | **C** | **A** | **A** | **T** | **T** | **T** | **G** | **T** | **T** | **G** | **A** | **C** | **T** | **T** | **C** | **T** | **T** | **C** | **T** | **C** | **T** | **T** | **C** | **A** | **T** | **T** | **A** | **A** | **A** | **T** | **C** | **G** | **T** | **A** | **T** | **G** | **C** | **C** | **G** | **C** | **T** | **G** | **A** | **C** | **A** | **A** | **G** | **A** | **A** | **T** | **G** | **A** | **A** | **T** | **T** | **C** | **T** | **G** | **T** | **C** | **A** | **C** | **T** | **C** | **T** | **T** | **C** | **T** | **C** | **A** | **C** | **A** | **G** | **A** | **C** | **G** | **G** | **A** | **C** | **A** | **A** | **G** | **A** | **G** | **A** | **A** | **A** | **T** | **T** | **G** | **G** | **A** | **T** | **T** | **C** | **A** | **C** | **T** | **G** | **C** | **T** | **T** | **C** | **A** | **A** | **G** | **A** | **T** | **C** | **T** | **A** | **A** | **A** | **A** | **A** | **A** | **A** | **G** | **C** | **C** | **A** | **A** | **A** | **C** | **A** | **T** | **T** | **C** | **G** | **A** | **T** | **C** | **C** | **A** | **T** | **A** | **T** | **C** | **G** | **A** | **A** | **T** | **A** | **T** | **T** | **A** | **C** | **G** | **C** | **A** | **T** | **T** | **T** | **G** | **T** | **G** | **A** | **C** | **A** | **G** | **A** | **T** | **A** | **A** | **T** | **A** | **T** | **C** | **G** | **G** | **C** | **C** | **A** | **G** | **C** | **A** | **A** | **G** | **T** | **C** | **T** | **T** | **A** | **C** | **A** | **T** | **G** | **T** | **T** | **T** | **T** | **A** | **G** | **A** | **A** | **G** | **A** | **A** | **A** | **A** | **A** | **G** | **C** | **C** | **G** | **C** | **A** | **G** | **A** | **A** | **G** | **G** | **C** | **G** | **T** | **G** | **G** | **A** | **A** | **G** | **T** | **T** | **C** | **G** | **A** | **A** | **T** | **A** | **T** | **T** | **A** | **T** | **A** | **T** | **G** | **A** | **T** | **G** | **C** | **A** | **T** | **T** | **T** | **G** | **G** | **C** | **T** | **C** | **T** | **C** | **A** | **T** | **G** | **G** | **C** | **A** | **C** | **A** | **A** | **A** | **A** | **G** | **C** | **A** | **A** | **A** | **A** | **G** | **A** | **T** | **T** | **T** | **C** | **A** | **A** | **T** | **C** | **G** | **T** | **C** | **T** | **A** | **A** | **T** | **C** | **A** | **A** | **A** | **A** | **A** | **T** | **G** | **G** | **T** | **G** | **G** | **A** | **C** | **A** | **T** | **G** | **T** | **C** | **C** | **A** | **T** | **A** | **C** | **A** | **T** | **T** | **T** | **G** | **T** | **T** | **A** | **C** | **C** | **T** | **C** | **A** | **C** | **A** | **A** | **A** | **A** | **G** | **G** | **C** | **A** | **T** | **T** | **A** | **C** | **T** | **T** | **C** | **G** | **T** | **T** | **T** | **C** | **C** | **G** | **A** | **T** | **T** | **G** | **A** | **A** | **T** | **T** | **A** | **C** | **C** | **A** | **T** | **G** | **A** | **T** | **C** | **A** | **C** | **C** | **G** | **A** | **A** | **A** | **A** | **A** | **T** | **C** | **G** | **T** | **T** | **G** | **T** | **G** | **A** | **T** | **C** | **G** | **A** | **T** | **G** | **G** | **A** | **A** | **A** | **G** | **A** | **T** | **T** | **A** | **G** | **T** | **T** | **A** | **T** | **A** | **C** | **C** | **G** | **G** | **T** | **G** | **G** | **T** | **T** | **T** | **C** | **A** | **A** | **T** | **A** | **T** | **T** | **G** | **C** | **C** | **A** | **A** | **T** | **C** | **A** | **A** | **T** | **A** | **T** | **G** | **T** | **A** | **A** | **A** | **T** | **A** | **C** | **A** | **A** | **C** | **A** | **A** | **A** | **A** | **A** | **A** | **A** | **T** | **T** | **C** | **G** | **G** | **C** | **T** | **A** | **T** | **T** | **G** | **G** | **C** | **G** | **C** | **G** | **A** | **T** | **A** | **C** | **G** | **C** | **A** | **T** | **A** | **T** | **A** | **C** | **G** | **G** | **A** | **T** | **T** | **T** | **T** | **C** | **G** | **G** | **T** | **G** | **C** | **C** | **G** | **C** | **T** | **T** | **C** | **T** | **T** | **C** | **T** | **T** | **T** | **G** | **C** | **T** | **C** | **C** | **A** | **G** | **C** | **T** | **T** | **C** | **G** | **C** | **T** | **T** | **C** | **T** | **T** | **A** | **A** | **C** | **A** | **G** | **A** | **C** | **T** | **G** | **G** | **A** | **A** | **C** | **G** | **T** | **C** | **T** | **C** | **G** | **G** | **T** | **A** | **C** | **C** | **T** | **G** | **A** | **A** | **G** | **A** | **A** | **A** | **A** | **A** | **A** | **A** | **G** | **G** | **T** | **C** | **G** | **G** | **C** | **T** | **A** | **T** | **C** | **A** | **T** | **T** | **T** | **G** | **A** | **A** | **T** | **T** | **A** | **T** | **T** | **T** | **C** | **T** | **T** | **T** | **A** | **A** | **A** | **A** | **A** | **A** | **G** | **C** | **A** | **G** | **A** | **T** | **C** | **G** | **A** | **G** | **A** | **T** | **G** | **A** | **A** | **T** | **C** | **T** | **A** | **A** | **G** | **C** | **T** | **T** | **G** | **C** | **T** | **G** | **A** | **A** | **A** | **C** | **A** | **T** | **C** | **C** | **A** | **T** | **C** | **C** | **A** | **G** | **C** | **T** | **T** | **G** | **T** | **T** | **T** | **C** | **A** | **A** | **G** | **C** | **G** | **G** | **A** | **C** | **C** | **G** | **A** | **A** | **T** | **A** | **A** | **C** | **G** | **A** | **A** | **A** | **G** | **G** | **G** | **A** | **A** | **C** | **A** | **A** | **A** | **T** | **C** | **A** | **A** | **G** | **C** | **T** | **T** | **T** | **C** | **A** | **T** | **T** | **T** | **A** | **T** | **C** | **A** | **A** | **A** | **T** | **T** | **G** | **A** | **T** | **T** | **A** | **C** | **T** | **T** | **C** | **T** | **G** | **C** | **T** | **A** | **A** | **A** | **A** | **A** | **G** | **C** | **G** | **T** | **G** | **T** | **T** | **T** | **G** | **G** | **A** | **T** | **A** | **C** | **A** | **G** | **A** | **C** | **A** | **C** | **C** | **T** | **T** | **A** | **C** | **C** | **T** | **T** | **G** | **T** | **T** | **C** | **C** | **T** | **G** | **A** | **T** | **G** | **A** | **A** | **A** | **G** | **T** | **G** | **T** | **C** | **A** | **T** | **T** | **G** | **C** | **T** | **G** | **C** | **T** | **T** | **T** | **A** | **A** | **A** | **A** | **A** | **T** | **C** | **G** | **C** | **A** | **A** | **C** | **T** | **G** | **C** | **C** | **T** | **C** | **T** | **G** | **G** | **T** | **G** | **T** | **A** | **G** | **A** | **T** | **G** | **T** | **G** | **A** | **A** | **A** | **A** | **T** | **C** | **A** | **T** | **G** | **A** | **T** | **T** | **C** | **C** | **C** | **A** | **A** | **C** | **A** | **A** | **A** | **C** | **C** | **G** | **G** | **A** | **T** | **C** | **A** | **T** | **C** | **C** | **T** | **T** | **T** | **T** | **A** | **T** | **T** | **T** | **A** | **T** | **C** | **G** | **A** | **G** | **C** | **A** | **A** | **C** | **A** | **C** | **A** | **A** | **T** | **A** | **T** | **T** | **A** | **T** | **G** | **C** | **T** | **C** | **G** | **G** | **C** | **A** | **G** | **C** | **T** | **G** | **A** | **T** | **C** | **A** | **A** | **G** | **G** | **A** | **A** | **A** | **A** | **T** | **G** | **T** | **A** | **C** | **A** | **A** | **A** | **T** | **C** | **C** | **T** | **T** | **G** | **T** | **C** | **T** | **A** | **T** | **G** | **A** | **G** | **A** | **A** | **C** | **G** | **G** | **C** | **T** | **T** | **C** | **C** | **T** | **C** | **C** | **A** | **T** | **G** | **C** | **A** | **A** | **A** | **A** | **A** | **C** | **A** | **T** | **T** | **G** | **A** | **T** | **A** | **A** | **T** | **G** | **G** | **A** | **T** | **G** | **A** | **T** | **G** | **A** | **A** | **A** | **T** | **C** | **T** | **G** | **C** | **A** | **T** | **G** | **G** | **T** | **A** | **G** | **G** | **T** | **T** | **C** | **A** | **G** | **C** | **A** | **A** | **A** | **T** | **C** | **A** | **A** | **G** | **A** | **T** | **A** | **T** | **T** | **C** | **G** | **T** | **A** | **G** | **C** | **T** | **A** | **C** | **A** | **G** | **A** | **T** | **T** | **G** | **A** | **A** | **T** | **T** | **T** | **T** | **G** | **A** | **A** | **A** | **C** | **A** | **A** | **G** | **T** | **G** | **C** | **T** | **G** | **T** | **C** | **A** | **T** | **T** | **T** | **A** | **C** | **G** | **A** | **T** | **C** | **C** | **T** | **G** | **A** | **G** | **T** | **T** | **T** | **T** | **T** | **A** | **G** | **A** | **A** | **G** | **A** | **A** | **C** | **T** | **T** | **G** | **C** | **T** | **A** | **C** | **T** | **C** | **A** | **G** | **T** | **T** | **C** | **A** | **A** | **A** | **G** | **A** | **A** | **G** | **A** | **T** | **G** | **A** | **G** | **A** | **C** | **A** | **C** | **A** | **T** | **T** | **G** | **T** | **T** | **C** | **A** | **T** | **C** | **C** | **A** | **T** | **G** | **A** | **C** | **A** | **A** | **C** | **T** | **G** | **A** | **A** | **A** | **C** | **A** | **G** | **T** | **T** | **A** | **A** | **A** | **G** | **A** | **A** | **A** | **T** | **G** | **T** | **C** | **T** | **A** | **A** | **C** | **T** | **G** | **G** | **C** | **T** | **A** | **T** | **T** | **A** | **T** | **T** | **C** | **A** | **A** | **G** | **C** | **A** | **A** | **C** | **A** | **A** | **A** | **T** | **T** | **T** | **C** | **T** | **C** | **G** | **A** | **T** | **T** | **A** | **T** | **T** | **T** | **T** | **C** | **T** | **C** | **C** | **A** | **A** | **T** | **C** | **C** | **T** | **A** | **-** | **-** | **-** |
| **N55290** | **-** | **-** | **-** | **-** | **-** | **-** | **-** | **-** | **-** | **-** | **-** | **-** | **-** | **-** | **-** | **-** | **-** | **-** | **-** | **-** | **-** | **-** | **-** | **-** | **-** | **-** | **-** | **-** | **-** | **-** | **-** | **-** | **-** | **-** | **-** | **-** | **-** | **-** | **-** | **-** | **-** | **-** | **-** | **-** | **-** | **-** | **-** | **-** | **-** | **-** | **-** | **-** | **-** | **-** | **-** | **-** | **-** | **-** | **-** | **-** | **-** | **-** | **-** | **-** | **-** | **-** | **-** | **-** | **-** | **A** | **A** | **A** | **C** | **C** | **C** | **C** | **G** | **A** | **G** | **A** | **T** | **G** | **T** | **A** | **G** | **C** | **A** | **G** | **C** | **T** | **A** | **T** | **T** | **T** | **G** | **G** | **G** | **C** | **A** | **T** | **G** | **G** | **C** | **T** | **C** | **T** | **T** | **A** | **G** | **T** | **A** | **T** | **T** | **G** | **T** | **T** | **T** | **G** | **C** | **C** | **C** | **T** | **T** | **C** | **C** | **T** | **G** | **G** | **G** | **G** | **T** | **G** | **G** | **G** | **T** | **T** | **T** | **T** | **G** | **T** | **A** | **T** | **T** | **A** | **T** | **A** | **T** | **T** | **T** | **A** | **T** | **T** | **T** | **T** | **T** | **C** | **G** | **G** | **A** | **C** | **G** | **T** | **G** | **G** | **A** | **T** | **T** | **A** | **A** | **C** | **G** | **G** | **A** | **T** | **A** | **A** | **G** | **A** | **A** | **A** | **A** | **A** | **A** | **T** | **T** | **C** | **T** | **A** | **T** | **C** | **T** | **C** | **C** | **G** | **A** | **C** | **A** | **A** | **A** | **G** | **T** | **G** | **A** | **C** | **T** | **T** | **G** | **A** | **A** | **A** | **G** | **A** | **A** | **T** | **T** | **A** | **G** | **A** | **A** | **A** | **A** | **C** | **T** | **T** | **T** | **C** | **A** | **G** | **T** | **C** | **T** | **T** | **T** | **T** | **A** | **G** | **A** | **G** | **G** | **A** | **G** | **A** | **T** | **A** | **C** | **C** | **A** | **T** | **T** | **G** | **A** | **A** | **C** | **A** | **T** | **T** | **A** | **C** | **G** | **A** | **T** | **C** | **C** | **T** | **G** | **A** | **C** | **A** | **T** | **G** | **G** | **G** | **C** | **G** | **A** | **C** | **A** | **A** | **A** | **G** | **A** | **C** | **A** | **A** | **A** | **C** | **A** | **A** | **C** | **A** | **A** | **T** | **T** | **T** | **G** | **T** | **C** | **G** | **A** | **C** | **T** | **T** | **C** | **T** | **T** | **C** | **T** | **C** | **T** | **T** | **C** | **A** | **T** | **T** | **A** | **A** | **A** | **T** | **C** | **G** | **T** | **A** | **T** | **G** | **C** | **C** | **G** | **C** | **T** | **G** | **A** | **C** | **A** | **A** | **G** | **A** | **A** | **T** | **G** | **A** | **A** | **T** | **T** | **C** | **T** | **G** | **T** | **C** | **A** | **C** | **T** | **C** | **T** | **T** | **C** | **T** | **C** | **A** | **C** | **A** | **G** | **A** | **C** | **G** | **G** | **A** | **C** | **A** | **A** | **A** | **A** | **G** | **A** | **A** | **A** | **T** | **T** | **A** | **G** | **A** | **T** | **T** | **C** | **A** | **C** | **T** | **G** | **C** | **T** | **T** | **C** | **A** | **A** | **G** | **A** | **T** | **C** | **T** | **A** | **A** | **A** | **A** | **A** | **A** | **A** | **G** | **C** | **C** | **A** | **A** | **A** | **C** | **A** | **T** | **T** | **C** | **G** | **A** | **T** | **C** | **C** | **A** | **T** | **A** | **T** | **C** | **G** | **A** | **A** | **T** | **A** | **T** | **T** | **A** | **C** | **G** | **C** | **A** | **T** | **T** | **T** | **G** | **T** | **G** | **A** | **C** | **A** | **G** | **A** | **T** | **A** | **C** | **T** | **A** | **T** | **C** | **G** | **G** | **T** | **C** | **A** | **G** | **C** | **A** | **A** | **G** | **T** | **C** | **T** | **T** | **A** | **C** | **G** | **C** | **G** | **T** | **T** | **T** | **T** | **A** | **G** | **A** | **A** | **G** | **A** | **A** | **A** | **A** | **A** | **G** | **C** | **C** | **G** | **C** | **A** | **G** | **A** | **A** | **G** | **G** | **C** | **G** | **T** | **A** | **G** | **A** | **A** | **G** | **T** | **T** | **C** | **G** | **T** | **A** | **T** | **A** | **T** | **T** | **A** | **T** | **A** | **T** | **G** | **A** | **T** | **G** | **C** | **A** | **T** | **T** | **T** | **G** | **G** | **C** | **T** | **C** | **C** | **C** | **A** | **T** | **G** | **G** | **C** | **A** | **C** | **A** | **A** | **A** | **A** | **G** | **C** | **A** | **A** | **A** | **A** | **G** | **A** | **T** | **T** | **T** | **C** | **A** | **A** | **T** | **C** | **G** | **T** | **C** | **T** | **A** | **A** | **T** | **C** | **A** | **A** | **A** | **A** | **A** | **T** | **G** | **G** | **T** | **G** | **G** | **A** | **C** | **A** | **T** | **G** | **T** | **C** | **C** | **A** | **T** | **A** | **C** | **A** | **T** | **T** | **T** | **G** | **T** | **T** | **A** | **C** | **C** | **T** | **C** | **G** | **C** | **A** | **A** | **A** | **A** | **A** | **G** | **C** | **A** | **T** | **T** | **G** | **C** | **T** | **T** | **C** | **G** | **T** | **T** | **T** | **C** | **C** | **G** | **A** | **T** | **T** | **G** | **A** | **A** | **T** | **T** | **A** | **C** | **C** | **A** | **T** | **G** | **A** | **T** | **C** | **A** | **C** | **C** | **G** | **G** | **A** | **A** | **A** | **A** | **T** | **C** | **G** | **T** | **T** | **G** | **T** | **G** | **A** | **T** | **C** | **G** | **A** | **T** | **G** | **G** | **A** | **A** | **A** | **G** | **A** | **T** | **C** | **A** | **G** | **T** | **T** | **A** | **C** | **A** | **C** | **C** | **G** | **G** | **T** | **G** | **G** | **T** | **T** | **T** | **C** | **A** | **A** | **T** | **A** | **T** | **T** | **G** | **C** | **C** | **A** | **A** | **T** | **C** | **A** | **A** | **T** | **A** | **T** | **G** | **T** | **A** | **A** | **A** | **T** | **A** | **C** | **G** | **A** | **C** | **A** | **A** | **A** | **A** | **A** | **A** | **A** | **T** | **T** | **C** | **G** | **G** | **C** | **T** | **A** | **T** | **T** | **G** | **G** | **C** | **G** | **T** | **G** | **A** | **T** | **A** | **C** | **G** | **C** | **A** | **T** | **A** | **T** | **A** | **C** | **G** | **G** | **A** | **T** | **T** | **T** | **T** | **C** | **G** | **G** | **T** | **G** | **C** | **C** | **G** | **C** | **T** | **T** | **C** | **T** | **T** | **C** | **T** | **T** | **T** | **G** | **C** | **T** | **T** | **C** | **A** | **A** | **C** | **T** | **T** | **C** | **G** | **T** | **T** | **T** | **T** | **T** | **T** | **A** | **A** | **C** | **A** | **G** | **A** | **C** | **T** | **G** | **G** | **A** | **A** | **C** | **G** | **T** | **C** | **T** | **C** | **A** | **G** | **T** | **A** | **C** | **C** | **T** | **G** | **A** | **A** | **G** | **A** | **A** | **A** | **A** | **A** | **A** | **A** | **G** | **G** | **T** | **C** | **G** | **G** | **C** | **T** | **A** | **T** | **C** | **A** | **T** | **T** | **T** | **G** | **A** | **A** | **T** | **T** | **A** | **T** | **T** | **T** | **C** | **T** | **T** | **T** | **A** | **A** | **A** | **A** | **A** | **A** | **G** | **C** | **A** | **G** | **A** | **T** | **C** | **G** | **A** | **G** | **A** | **C** | **G** | **A** | **A** | **T** | **C** | **C** | **A** | **A** | **A** | **C** | **T** | **T** | **G** | **C** | **T** | **G** | **A** | **T** | **A** | **C** | **G** | **T** | **C** | **C** | **A** | **T** | **C** | **C** | **A** | **G** | **C** | **T** | **T** | **G** | **T** | **C** | **T** | **C** | **A** | **A** | **G** | **C** | **G** | **G** | **A** | **C** | **C** | **A** | **A** | **A** | **T** | **A** | **A** | **C** | **G** | **A** | **A** | **C** | **G** | **A** | **G** | **A** | **A** | **C** | **A** | **A** | **A** | **T** | **C** | **A** | **A** | **G** | **C** | **T** | **T** | **T** | **C** | **A** | **T** | **T** | **T** | **A** | **T** | **C** | **A** | **A** | **A** | **T** | **T** | **G** | **A** | **T** | **T** | **A** | **C** | **T** | **T** | **C** | **T** | **G** | **C** | **T** | **A** | **A** | **A** | **A** | **A** | **A** | **C** | **G** | **T** | **G** | **T** | **T** | **T** | **G** | **G** | **A** | **T** | **A** | **C** | **A** | **G** | **A** | **C** | **A** | **C** | **C** | **T** | **T** | **A** | **C** | **C** | **T** | **C** | **G** | **T** | **T** | **C** | **C** | **T** | **G** | **A** | **T** | **G** | **A** | **A** | **A** | **G** | **T** | **G** | **T** | **C** | **A** | **T** | **T** | **G** | **C** | **T** | **G** | **C** | **T** | **T** | **T** | **G** | **A** | **A** | **A** | **A** | **T** | **C** | **G** | **C** | **A** | **A** | **C** | **T** | **G** | **C** | **T** | **T** | **C** | **T** | **G** | **G** | **T** | **G** | **T** | **C** | **G** | **A** | **T** | **G** | **T** | **G** | **A** | **A** | **A** | **A** | **T** | **C** | **A** | **T** | **G** | **A** | **T** | **C** | **C** | **C** | **C** | **A** | **A** | **T** | **A** | **A** | **A** | **C** | **C** | **G** | **G** | **A** | **T** | **C** | **A** | **T** | **C** | **C** | **T** | **T** | **T** | **T** | **A** | **T** | **T** | **T** | **A** | **T** | **C** | **G** | **A** | **G** | **C** | **A** | **A** | **C** | **A** | **C** | **A** | **A** | **T** | **A** | **T** | **T** | **A** | **T** | **G** | **C** | **G** | **C** | **G** | **T** | **C** | **A** | **G** | **C** | **T** | **C** | **A** | **T** | **C** | **A** | **A** | **G** | **G** | **A** | **A** | **A** | **A** | **T** | **G** | **T** | **A** | **C** | **A** | **G** | **A** | **T** | **C** | **C** | **T** | **T** | **G** | **T** | **C** | **T** | **A** | **T** | **G** | **A** | **G** | **A** | **A** | **T** | **G** | **G** | **C** | **T** | **T** | **C** | **C** | **T** | **C** | **C** | **A** | **T** | **G** | **C** | **A** | **A** | **A** | **A** | **A** | **C** | **A** | **T** | **T** | **G** | **A** | **T** | **A** | **A** | **T** | **G** | **G** | **A** | **T** | **G** | **A** | **T** | **G** | **A** | **A** | **A** | **T** | **C** | **T** | **G** | **C** | **A** | **T** | **G** | **G** | **T** | **A** | **G** | **G** | **T** | **T** | **C** | **A** | **G** | **C** | **A** | **A** | **A** | **C** | **C** | **A** | **A** | **G** | **A** | **T** | **A** | **T** | **T** | **C** | **G** | **T** | **A** | **G** | **C** | **T** | **A** | **C** | **C** | **G** | **A** | **T** | **T** | **G** | **A** | **A** | **T** | **T** | **T** | **T** | **G** | **A** | **A** | **A** | **C** | **A** | **A** | **G** | **T** | **G** | **C** | **T** | **G** | **T** | **C** | **A** | **T** | **C** | **T** | **A** | **C** | **G** | **A** | **T** | **C** | **C** | **T** | **G** | **A** | **G** | **T** | **T** | **T** | **T** | **T** | **A** | **G** | **A** | **A** | **G** | **A** | **A** | **C** | **T** | **T** | **G** | **C** | **T** | **A** | **C** | **C** | **C** | **A** | **G** | **T** | **T** | **C** | **A** | **A** | **A** | **G** | **A** | **A** | **G** | **A** | **T** | **G** | **A** | **G** | **A** | **C** | **A** | **C** | **A** | **T** | **T** | **G** | **T** | **T** | **C** | **A** | **C** | **C** | **A** | **A** | **T** | **G** | **A** | **C** | **G** | **A** | **C** | **T** | **G** | **A** | **A** | **A** | **C** | **A** | **G** | **T** | **G** | **A** | **A** | **G** | **G** | **A** | **A** | **A** | **T** | **G** | **T** | **C** | **T** | **A** | **A** | **T** | **T** | **G** | **G** | **C** | **T** | **G** | **T** | **T** | **A** | **T** | **T** | **C** | **A** | **A** | **G** | **C** | **A** | **A** | **C** | **A** | **A** | **A** | **T** | **T** | **T** | **C** | **T** | **C** | **G** | **A** | **T** | **T** | **A** | **T** | **T** | **T** | **T** | **C** | **T** | **C** | **C** | **A** | **A** | **T** | **C** | **T** | **T** | **A** | **-** | **-** | **-** |
| **N53548** | **-** | **-** | **-** | **-** | **-** | **-** | **-** | **-** | **-** | **-** | **-** | **-** | **-** | **-** | **-** | **-** | **-** | **-** | **-** | **-** | **-** | **-** | **-** | **-** | **-** | **-** | **-** | **-** | **-** | **-** | **-** | **-** | **-** | **-** | **-** | **-** | **-** | **-** | **-** | **-** | **-** | **-** | **-** | **-** | **-** | **-** | **-** | **-** | **-** | **-** | **-** | **-** | **-** | **-** | **-** | **-** | **-** | **-** | **-** | **-** | **-** | **-** | **-** | **-** | **-** | **-** | **-** | **-** | **-** | **A** | **A** | **A** | **C** | **C** | **C** | **C** | **G** | **A** | **G** | **A** | **T** | **G** | **T** | **A** | **G** | **C** | **A** | **G** | **C** | **C** | **A** | **T** | **T** | **T** | **G** | **G** | **G** | **C** | **A** | **T** | **G** | **G** | **C** | **T** | **C** | **T** | **T** | **A** | **G** | **T** | **A** | **T** | **T** | **G** | **T** | **T** | **T** | **G** | **C** | **C** | **C** | **T** | **T** | **C** | **C** | **T** | **G** | **G** | **G** | **G** | **T** | **G** | **G** | **G** | **C** | **T** | **T** | **T** | **G** | **T** | **A** | **T** | **T** | **A** | **T** | **A** | **T** | **T** | **T** | **A** | **T** | **T** | **T** | **T** | **T** | **C** | **G** | **G** | **A** | **C** | **G** | **T** | **G** | **G** | **A** | **T** | **T** | **A** | **A** | **C** | **G** | **G** | **A** | **T** | **A** | **A** | **G** | **A** | **A** | **A** | **A** | **A** | **A** | **T** | **T** | **C** | **T** | **A** | **T** | **C** | **T** | **C** | **C** | **G** | **A** | **C** | **A** | **A** | **A** | **G** | **T** | **G** | **A** | **C** | **T** | **T** | **G** | **A** | **A** | **A** | **G** | **A** | **A** | **T** | **T** | **A** | **G** | **A** | **A** | **A** | **A** | **C** | **T** | **T** | **T** | **C** | **A** | **G** | **T** | **C** | **T** | **T** | **T** | **T** | **A** | **G** | **A** | **G** | **G** | **A** | **G** | **A** | **T** | **A** | **C** | **C** | **A** | **T** | **T** | **G** | **A** | **A** | **C** | **A** | **T** | **T** | **A** | **C** | **G** | **A** | **T** | **C** | **C** | **T** | **G** | **A** | **C** | **A** | **T** | **G** | **G** | **G** | **C** | **G** | **A** | **T** | **A** | **A** | **A** | **G** | **A** | **C** | **A** | **A** | **A** | **C** | **A** | **G** | **C** | **A** | **A** | **T** | **T** | **T** | **G** | **T** | **T** | **G** | **A** | **C** | **T** | **T** | **C** | **T** | **T** | **C** | **T** | **C** | **T** | **T** | **C** | **A** | **T** | **T** | **A** | **A** | **A** | **T** | **C** | **G** | **T** | **A** | **T** | **G** | **C** | **C** | **G** | **C** | **T** | **G** | **A** | **C** | **A** | **A** | **G** | **A** | **A** | **T** | **G** | **A** | **A** | **T** | **T** | **C** | **T** | **G** | **T** | **C** | **A** | **C** | **T** | **C** | **T** | **T** | **C** | **T** | **C** | **A** | **C** | **A** | **G** | **A** | **C** | **G** | **G** | **A** | **C** | **A** | **A** | **G** | **A** | **G** | **A** | **A** | **A** | **T** | **T** | **G** | **G** | **A** | **T** | **T** | **C** | **A** | **C** | **T** | **G** | **C** | **T** | **T** | **C** | **A** | **A** | **G** | **A** | **T** | **C** | **T** | **A** | **A** | **A** | **A** | **A** | **A** | **A** | **G** | **C** | **C** | **A** | **A** | **A** | **C** | **A** | **T** | **T** | **C** | **G** | **A** | **T** | **C** | **C** | **A** | **T** | **A** | **T** | **C** | **G** | **A** | **A** | **T** | **A** | **T** | **T** | **A** | **C** | **G** | **C** | **A** | **T** | **T** | **T** | **G** | **T** | **G** | **A** | **C** | **A** | **G** | **A** | **T** | **A** | **A** | **T** | **A** | **T** | **C** | **G** | **G** | **C** | **C** | **A** | **G** | **C** | **A** | **A** | **G** | **T** | **C** | **T** | **T** | **A** | **C** | **A** | **T** | **G** | **T** | **T** | **T** | **T** | **A** | **G** | **A** | **A** | **G** | **A** | **A** | **A** | **A** | **A** | **G** | **C** | **C** | **G** | **C** | **A** | **G** | **A** | **A** | **G** | **G** | **C** | **G** | **T** | **G** | **G** | **A** | **A** | **G** | **T** | **T** | **C** | **G** | **A** | **A** | **T** | **A** | **T** | **T** | **A** | **T** | **A** | **T** | **G** | **A** | **T** | **G** | **C** | **A** | **T** | **T** | **T** | **G** | **G** | **C** | **T** | **C** | **T** | **C** | **A** | **T** | **G** | **G** | **C** | **A** | **C** | **A** | **A** | **A** | **A** | **G** | **C** | **A** | **A** | **A** | **A** | **G** | **A** | **T** | **T** | **T** | **C** | **A** | **A** | **T** | **C** | **G** | **T** | **C** | **T** | **A** | **A** | **T** | **C** | **A** | **A** | **A** | **A** | **A** | **T** | **G** | **G** | **T** | **G** | **G** | **A** | **C** | **A** | **T** | **G** | **T** | **C** | **C** | **A** | **T** | **A** | **C** | **A** | **T** | **T** | **T** | **G** | **T** | **T** | **A** | **C** | **C** | **T** | **C** | **A** | **C** | **A** | **A** | **A** | **G** | **G** | **G** | **C** | **A** | **T** | **T** | **A** | **C** | **T** | **T** | **C** | **G** | **T** | **T** | **T** | **C** | **C** | **G** | **A** | **T** | **T** | **G** | **A** | **A** | **T** | **T** | **A** | **C** | **C** | **A** | **T** | **G** | **A** | **T** | **C** | **A** | **C** | **C** | **G** | **A** | **A** | **A** | **A** | **A** | **T** | **C** | **G** | **T** | **T** | **G** | **T** | **G** | **A** | **T** | **C** | **G** | **A** | **T** | **G** | **G** | **A** | **A** | **A** | **G** | **A** | **T** | **T** | **A** | **G** | **T** | **T** | **A** | **T** | **A** | **C** | **C** | **G** | **G** | **T** | **G** | **G** | **T** | **T** | **T** | **C** | **A** | **A** | **T** | **A** | **T** | **T** | **G** | **C** | **C** | **A** | **A** | **T** | **C** | **A** | **A** | **T** | **A** | **T** | **G** | **T** | **A** | **A** | **A** | **T** | **A** | **C** | **A** | **A** | **C** | **A** | **A** | **A** | **A** | **A** | **A** | **A** | **T** | **T** | **C** | **G** | **G** | **C** | **T** | **A** | **T** | **T** | **G** | **G** | **C** | **G** | **C** | **G** | **A** | **T** | **A** | **C** | **G** | **C** | **A** | **T** | **A** | **T** | **A** | **C** | **G** | **G** | **A** | **T** | **T** | **T** | **T** | **C** | **G** | **G** | **T** | **G** | **C** | **C** | **G** | **C** | **T** | **T** | **C** | **T** | **T** | **C** | **T** | **T** | **T** | **G** | **C** | **T** | **C** | **C** | **A** | **G** | **C** | **T** | **T** | **C** | **G** | **C** | **T** | **T** | **C** | **T** | **T** | **A** | **A** | **C** | **A** | **G** | **A** | **C** | **T** | **G** | **G** | **A** | **A** | **C** | **G** | **T** | **C** | **T** | **C** | **G** | **G** | **T** | **A** | **C** | **C** | **T** | **G** | **A** | **A** | **G** | **A** | **A** | **A** | **A** | **A** | **A** | **A** | **G** | **G** | **T** | **C** | **G** | **G** | **C** | **T** | **A** | **T** | **C** | **A** | **T** | **T** | **T** | **G** | **A** | **A** | **T** | **T** | **A** | **T** | **T** | **T** | **C** | **T** | **T** | **T** | **A** | **A** | **A** | **A** | **A** | **A** | **G** | **C** | **A** | **G** | **A** | **T** | **C** | **G** | **A** | **G** | **A** | **T** | **G** | **A** | **A** | **T** | **C** | **T** | **A** | **A** | **G** | **C** | **T** | **T** | **G** | **C** | **T** | **G** | **A** | **T** | **A** | **C** | **A** | **T** | **C** | **C** | **A** | **T** | **C** | **C** | **A** | **G** | **C** | **T** | **T** | **G** | **T** | **T** | **T** | **C** | **A** | **A** | **G** | **C** | **G** | **G** | **A** | **C** | **C** | **G** | **A** | **A** | **T** | **A** | **A** | **C** | **G** | **A** | **A** | **A** | **G** | **G** | **G** | **A** | **A** | **C** | **A** | **A** | **A** | **T** | **C** | **A** | **A** | **G** | **C** | **T** | **T** | **T** | **C** | **A** | **T** | **T** | **T** | **A** | **T** | **C** | **A** | **A** | **A** | **T** | **T** | **G** | **A** | **T** | **T** | **A** | **C** | **T** | **T** | **C** | **T** | **G** | **C** | **T** | **A** | **A** | **A** | **A** | **A** | **A** | **C** | **G** | **T** | **G** | **T** | **T** | **T** | **G** | **G** | **A** | **T** | **A** | **C** | **A** | **G** | **A** | **C** | **A** | **C** | **C** | **T** | **T** | **A** | **C** | **C** | **T** | **T** | **G** | **T** | **T** | **C** | **C** | **T** | **G** | **A** | **T** | **G** | **A** | **A** | **A** | **G** | **T** | **G** | **T** | **C** | **A** | **T** | **T** | **G** | **C** | **T** | **G** | **C** | **T** | **T** | **T** | **A** | **A** | **A** | **A** | **A** | **T** | **C** | **G** | **C** | **A** | **A** | **C** | **T** | **G** | **C** | **C** | **T** | **C** | **T** | **G** | **G** | **T** | **G** | **T** | **A** | **G** | **A** | **T** | **G** | **T** | **G** | **A** | **A** | **A** | **A** | **T** | **C** | **A** | **T** | **G** | **A** | **T** | **T** | **C** | **C** | **C** | **A** | **A** | **C** | **A** | **A** | **A** | **C** | **C** | **G** | **G** | **A** | **T** | **C** | **A** | **T** | **C** | **C** | **T** | **T** | **T** | **T** | **A** | **T** | **T** | **T** | **A** | **T** | **C** | **G** | **A** | **G** | **C** | **A** | **A** | **C** | **A** | **C** | **A** | **A** | **T** | **A** | **T** | **T** | **A** | **T** | **G** | **C** | **T** | **C** | **G** | **G** | **C** | **A** | **G** | **C** | **T** | **G** | **A** | **T** | **C** | **A** | **A** | **G** | **G** | **A** | **A** | **A** | **A** | **T** | **G** | **T** | **A** | **C** | **A** | **A** | **A** | **T** | **C** | **C** | **T** | **T** | **G** | **T** | **C** | **T** | **A** | **T** | **G** | **A** | **G** | **A** | **A** | **C** | **G** | **G** | **C** | **T** | **T** | **C** | **C** | **T** | **C** | **C** | **A** | **T** | **G** | **C** | **A** | **A** | **A** | **A** | **A** | **C** | **A** | **T** | **T** | **G** | **A** | **T** | **A** | **A** | **T** | **G** | **G** | **A** | **T** | **G** | **A** | **T** | **G** | **A** | **A** | **A** | **T** | **C** | **T** | **G** | **C** | **A** | **T** | **G** | **G** | **T** | **A** | **G** | **G** | **T** | **T** | **C** | **A** | **G** | **C** | **A** | **A** | **A** | **T** | **C** | **A** | **A** | **G** | **A** | **T** | **A** | **T** | **T** | **C** | **G** | **T** | **A** | **G** | **C** | **T** | **A** | **C** | **C** | **G** | **A** | **T** | **T** | **G** | **A** | **A** | **T** | **T** | **T** | **T** | **G** | **A** | **A** | **A** | **C** | **A** | **A** | **G** | **T** | **G** | **C** | **T** | **G** | **T** | **C** | **A** | **T** | **T** | **T** | **A** | **C** | **G** | **A** | **T** | **C** | **C** | **T** | **G** | **A** | **G** | **T** | **T** | **T** | **T** | **T** | **A** | **G** | **A** | **A** | **G** | **A** | **A** | **C** | **T** | **T** | **G** | **C** | **T** | **A** | **C** | **T** | **C** | **A** | **G** | **T** | **T** | **C** | **A** | **A** | **A** | **G** | **A** | **A** | **G** | **A** | **T** | **G** | **A** | **G** | **A** | **C** | **A** | **C** | **A** | **T** | **T** | **G** | **T** | **T** | **C** | **A** | **T** | **C** | **C** | **A** | **T** | **G** | **A** | **C** | **A** | **A** | **C** | **T** | **G** | **A** | **A** | **A** | **C** | **A** | **G** | **T** | **C** | **A** | **A** | **G** | **G** | **A** | **A** | **A** | **T** | **G** | **T** | **C** | **T** | **A** | **A** | **C** | **T** | **G** | **G** | **C** | **T** | **A** | **T** | **T** | **A** | **T** | **T** | **C** | **A** | **A** | **G** | **C** | **A** | **A** | **C** | **A** | **A** | **A** | **T** | **T** | **T** | **C** | **T** | **C** | **G** | **A** | **T** | **T** | **A** | **T** | **T** | **T** | **T** | **C** | **T** | **C** | **C** | **A** | **A** | **T** | **C** | **C** | **T** | **A** | **-** | **-** | **-** |
| **N53479** | **-** | **-** | **-** | **-** | **-** | **-** | **-** | **-** | **-** | **-** | **-** | **-** | **-** | **-** | **-** | **-** | **-** | **-** | **-** | **-** | **-** | **-** | **-** | **-** | **-** | **-** | **-** | **-** | **-** | **-** | **-** | **-** | **-** | **-** | **-** | **-** | **-** | **-** | **-** | **-** | **-** | **-** | **-** | **-** | **-** | **-** | **-** | **-** | **-** | **-** | **-** | **-** | **-** | **-** | **-** | **-** | **-** | **-** | **-** | **-** | **-** | **-** | **-** | **-** | **-** | **-** | **-** | **-** | **-** | **A** | **A** | **A** | **C** | **C** | **C** | **C** | **G** | **A** | **G** | **A** | **T** | **G** | **T** | **A** | **G** | **C** | **A** | **G** | **C** | **C** | **A** | **T** | **T** | **T** | **G** | **G** | **G** | **C** | **A** | **T** | **G** | **G** | **C** | **T** | **C** | **T** | **T** | **A** | **G** | **T** | **A** | **T** | **T** | **G** | **T** | **T** | **T** | **G** | **C** | **C** | **C** | **T** | **T** | **C** | **C** | **T** | **G** | **G** | **G** | **G** | **T** | **G** | **G** | **G** | **C** | **T** | **T** | **T** | **G** | **T** | **A** | **T** | **T** | **A** | **T** | **A** | **T** | **T** | **T** | **A** | **T** | **T** | **T** | **T** | **T** | **C** | **G** | **G** | **A** | **C** | **G** | **T** | **G** | **G** | **A** | **T** | **T** | **A** | **A** | **C** | **G** | **G** | **A** | **T** | **A** | **A** | **G** | **A** | **A** | **A** | **A** | **A** | **A** | **T** | **T** | **C** | **T** | **A** | **T** | **C** | **T** | **C** | **C** | **G** | **A** | **C** | **A** | **A** | **A** | **G** | **T** | **G** | **A** | **C** | **T** | **T** | **G** | **A** | **A** | **A** | **G** | **A** | **A** | **T** | **T** | **A** | **G** | **A** | **A** | **A** | **A** | **C** | **T** | **T** | **T** | **C** | **A** | **G** | **T** | **C** | **T** | **T** | **T** | **T** | **A** | **G** | **A** | **G** | **G** | **A** | **G** | **A** | **T** | **A** | **C** | **C** | **A** | **T** | **T** | **G** | **A** | **A** | **C** | **A** | **T** | **T** | **A** | **C** | **G** | **A** | **T** | **C** | **C** | **T** | **G** | **A** | **C** | **A** | **T** | **G** | **G** | **G** | **C** | **G** | **A** | **T** | **A** | **A** | **A** | **G** | **A** | **C** | **A** | **A** | **A** | **C** | **A** | **G** | **C** | **A** | **A** | **T** | **T** | **T** | **G** | **T** | **T** | **G** | **A** | **C** | **T** | **T** | **C** | **T** | **T** | **C** | **T** | **C** | **T** | **T** | **C** | **A** | **T** | **T** | **A** | **A** | **A** | **T** | **C** | **G** | **T** | **A** | **T** | **G** | **C** | **C** | **G** | **C** | **T** | **G** | **A** | **C** | **A** | **A** | **G** | **A** | **A** | **T** | **G** | **A** | **A** | **T** | **T** | **C** | **T** | **G** | **T** | **C** | **A** | **C** | **T** | **C** | **T** | **T** | **C** | **T** | **C** | **A** | **C** | **A** | **G** | **A** | **C** | **G** | **G** | **A** | **C** | **A** | **A** | **G** | **A** | **G** | **A** | **A** | **A** | **T** | **T** | **G** | **G** | **A** | **T** | **T** | **C** | **A** | **C** | **T** | **G** | **C** | **T** | **T** | **C** | **A** | **A** | **G** | **A** | **T** | **C** | **T** | **A** | **A** | **A** | **A** | **A** | **A** | **A** | **G** | **C** | **C** | **A** | **A** | **A** | **C** | **A** | **T** | **T** | **C** | **G** | **A** | **T** | **C** | **C** | **A** | **T** | **A** | **T** | **C** | **G** | **A** | **A** | **T** | **A** | **T** | **T** | **A** | **C** | **G** | **C** | **A** | **T** | **T** | **T** | **G** | **T** | **G** | **A** | **C** | **A** | **G** | **A** | **T** | **A** | **A** | **T** | **A** | **T** | **C** | **G** | **G** | **C** | **C** | **A** | **G** | **C** | **A** | **A** | **G** | **T** | **C** | **T** | **T** | **A** | **C** | **A** | **T** | **G** | **T** | **T** | **T** | **T** | **A** | **G** | **A** | **A** | **G** | **A** | **A** | **A** | **A** | **A** | **G** | **C** | **C** | **G** | **C** | **A** | **G** | **A** | **A** | **G** | **G** | **C** | **G** | **T** | **G** | **G** | **A** | **A** | **G** | **T** | **T** | **C** | **G** | **A** | **A** | **T** | **A** | **T** | **T** | **A** | **T** | **A** | **T** | **G** | **A** | **T** | **G** | **C** | **A** | **T** | **T** | **T** | **G** | **G** | **C** | **T** | **C** | **T** | **C** | **A** | **T** | **G** | **G** | **C** | **A** | **C** | **A** | **A** | **A** | **A** | **G** | **C** | **A** | **A** | **A** | **A** | **G** | **A** | **T** | **T** | **T** | **C** | **A** | **A** | **T** | **C** | **G** | **T** | **C** | **T** | **A** | **A** | **T** | **C** | **A** | **A** | **A** | **A** | **A** | **T** | **G** | **G** | **T** | **G** | **G** | **A** | **C** | **A** | **T** | **G** | **T** | **C** | **C** | **A** | **T** | **A** | **C** | **A** | **T** | **T** | **T** | **G** | **T** | **T** | **A** | **C** | **C** | **T** | **C** | **A** | **C** | **A** | **A** | **A** | **G** | **G** | **G** | **C** | **A** | **T** | **T** | **A** | **C** | **T** | **T** | **C** | **G** | **T** | **T** | **T** | **C** | **C** | **G** | **A** | **T** | **T** | **G** | **A** | **A** | **T** | **T** | **A** | **C** | **C** | **A** | **T** | **G** | **A** | **T** | **C** | **A** | **C** | **C** | **G** | **A** | **A** | **A** | **A** | **A** | **T** | **C** | **G** | **T** | **T** | **G** | **T** | **G** | **A** | **T** | **C** | **G** | **A** | **T** | **G** | **G** | **A** | **A** | **A** | **G** | **A** | **T** | **T** | **A** | **G** | **T** | **T** | **A** | **T** | **A** | **C** | **C** | **G** | **G** | **T** | **G** | **G** | **T** | **T** | **T** | **C** | **A** | **A** | **T** | **A** | **T** | **T** | **G** | **C** | **C** | **A** | **A** | **T** | **C** | **A** | **A** | **T** | **A** | **T** | **A** | **T** | **A** | **A** | **A** | **T** | **A** | **C** | **A** | **A** | **C** | **A** | **A** | **A** | **A** | **A** | **A** | **A** | **T** | **T** | **C** | **G** | **G** | **C** | **T** | **A** | **T** | **T** | **G** | **G** | **C** | **G** | **C** | **G** | **A** | **T** | **A** | **C** | **G** | **C** | **A** | **T** | **A** | **T** | **A** | **C** | **G** | **G** | **A** | **T** | **T** | **T** | **T** | **C** | **G** | **G** | **T** | **G** | **C** | **C** | **G** | **C** | **T** | **T** | **C** | **T** | **T** | **C** | **T** | **T** | **T** | **G** | **C** | **T** | **C** | **C** | **A** | **G** | **C** | **T** | **T** | **C** | **G** | **C** | **T** | **T** | **C** | **T** | **T** | **A** | **A** | **C** | **A** | **G** | **A** | **C** | **T** | **G** | **G** | **A** | **A** | **C** | **G** | **T** | **C** | **T** | **C** | **G** | **G** | **T** | **A** | **C** | **C** | **T** | **G** | **A** | **A** | **G** | **A** | **A** | **A** | **A** | **A** | **A** | **A** | **G** | **G** | **T** | **C** | **G** | **G** | **C** | **T** | **A** | **T** | **C** | **A** | **T** | **T** | **T** | **G** | **A** | **A** | **T** | **T** | **A** | **T** | **T** | **T** | **C** | **T** | **T** | **T** | **A** | **A** | **A** | **A** | **A** | **A** | **G** | **C** | **A** | **G** | **A** | **T** | **C** | **G** | **A** | **G** | **A** | **T** | **G** | **A** | **A** | **T** | **C** | **T** | **A** | **A** | **G** | **C** | **T** | **T** | **G** | **C** | **T** | **G** | **A** | **T** | **A** | **C** | **A** | **T** | **C** | **C** | **A** | **T** | **C** | **C** | **A** | **G** | **C** | **T** | **T** | **G** | **T** | **T** | **T** | **C** | **A** | **A** | **G** | **C** | **G** | **G** | **A** | **C** | **C** | **G** | **A** | **A** | **T** | **A** | **A** | **C** | **G** | **A** | **A** | **A** | **G** | **G** | **G** | **A** | **A** | **C** | **A** | **A** | **A** | **T** | **C** | **A** | **A** | **G** | **C** | **T** | **T** | **T** | **C** | **A** | **T** | **T** | **T** | **A** | **T** | **C** | **A** | **A** | **A** | **T** | **T** | **G** | **A** | **T** | **T** | **A** | **C** | **T** | **T** | **C** | **T** | **G** | **C** | **T** | **A** | **A** | **A** | **A** | **A** | **A** | **C** | **G** | **T** | **G** | **T** | **T** | **T** | **G** | **G** | **A** | **T** | **A** | **C** | **A** | **G** | **A** | **C** | **A** | **C** | **C** | **T** | **T** | **A** | **C** | **C** | **T** | **T** | **G** | **T** | **T** | **C** | **C** | **T** | **G** | **A** | **T** | **G** | **A** | **A** | **A** | **G** | **T** | **G** | **T** | **C** | **A** | **T** | **T** | **G** | **C** | **T** | **G** | **C** | **T** | **T** | **T** | **A** | **A** | **A** | **A** | **A** | **T** | **C** | **G** | **C** | **A** | **A** | **C** | **T** | **G** | **C** | **C** | **T** | **C** | **T** | **G** | **G** | **T** | **G** | **T** | **A** | **G** | **A** | **T** | **G** | **T** | **G** | **A** | **A** | **A** | **A** | **T** | **C** | **A** | **T** | **G** | **A** | **T** | **T** | **C** | **C** | **C** | **A** | **A** | **C** | **A** | **A** | **A** | **C** | **C** | **G** | **G** | **A** | **T** | **C** | **A** | **T** | **C** | **C** | **T** | **T** | **T** | **T** | **A** | **T** | **T** | **T** | **A** | **T** | **C** | **G** | **A** | **G** | **C** | **A** | **A** | **C** | **A** | **C** | **A** | **A** | **T** | **A** | **T** | **T** | **A** | **T** | **G** | **C** | **T** | **C** | **G** | **G** | **C** | **A** | **G** | **C** | **T** | **G** | **A** | **T** | **C** | **A** | **A** | **G** | **G** | **A** | **A** | **A** | **A** | **T** | **G** | **T** | **A** | **C** | **A** | **A** | **A** | **T** | **C** | **C** | **T** | **T** | **G** | **T** | **C** | **T** | **A** | **T** | **G** | **A** | **G** | **A** | **A** | **C** | **G** | **G** | **C** | **T** | **T** | **C** | **C** | **T** | **C** | **C** | **A** | **T** | **G** | **C** | **A** | **A** | **A** | **A** | **A** | **C** | **A** | **T** | **T** | **G** | **A** | **T** | **A** | **A** | **T** | **G** | **G** | **A** | **T** | **G** | **A** | **T** | **G** | **A** | **A** | **A** | **T** | **C** | **T** | **G** | **C** | **A** | **T** | **G** | **G** | **T** | **A** | **G** | **G** | **T** | **T** | **C** | **A** | **G** | **C** | **A** | **A** | **A** | **T** | **C** | **A** | **A** | **G** | **A** | **T** | **A** | **T** | **T** | **C** | **G** | **T** | **A** | **G** | **C** | **T** | **A** | **C** | **C** | **G** | **A** | **T** | **T** | **G** | **A** | **A** | **T** | **T** | **T** | **T** | **G** | **A** | **A** | **A** | **C** | **A** | **A** | **G** | **T** | **G** | **C** | **T** | **G** | **T** | **C** | **A** | **T** | **T** | **T** | **A** | **C** | **G** | **A** | **T** | **C** | **C** | **T** | **G** | **A** | **G** | **T** | **T** | **T** | **T** | **T** | **A** | **G** | **A** | **A** | **G** | **A** | **A** | **C** | **T** | **T** | **G** | **C** | **T** | **A** | **C** | **T** | **C** | **A** | **G** | **T** | **T** | **C** | **A** | **A** | **A** | **G** | **A** | **A** | **G** | **A** | **T** | **G** | **A** | **G** | **A** | **C** | **A** | **C** | **A** | **T** | **T** | **G** | **T** | **T** | **C** | **A** | **T** | **C** | **C** | **A** | **T** | **G** | **A** | **C** | **A** | **A** | **C** | **T** | **G** | **A** | **A** | **A** | **C** | **A** | **G** | **T** | **C** | **A** | **A** | **G** | **G** | **A** | **A** | **A** | **T** | **G** | **T** | **C** | **T** | **A** | **A** | **C** | **T** | **G** | **G** | **C** | **T** | **A** | **T** | **T** | **A** | **T** | **T** | **C** | **A** | **A** | **G** | **C** | **A** | **A** | **C** | **A** | **A** | **A** | **T** | **T** | **T** | **C** | **T** | **C** | **G** | **A** | **T** | **T** | **A** | **T** | **T** | **T** | **T** | **C** | **T** | **C** | **C** | **A** | **A** | **T** | **C** | **C** | **T** | **A** | **-** | **-** | **-** |
| **N54538** | **G** | **T** | **G** | **G** | **T** | **A** | **T** | **C** | **T** | **A** | **G** | **T** | **A** | **T** | **T** | **A** | **T** | **A** | **A** | **C** | **C** | **G** | **C** | **C** | **C** | **T** | **T** | **T** | **A** | **T** | **C** | **T** | **A** | **T** | **T** | **A** | **A** | **A** | **T** | **G** | **C** | **A** | **C** | **T** | **T** | **A** | **T** | **T** | **G** | **C** | **T** | **T** | **T** | **G** | **G** | **T** | **G** | **A** | **C** | **T** | **A** | **T** | **T** | **T** | **T** | **G** | **A** | **T** | **C** | **A** | **A** | **A** | **C** | **C** | **C** | **C** | **G** | **A** | **G** | **A** | **T** | **G** | **T** | **A** | **G** | **C** | **A** | **G** | **C** | **C** | **A** | **T** | **T** | **T** | **G** | **G** | **G** | **C** | **A** | **T** | **G** | **G** | **C** | **T** | **C** | **T** | **T** | **A** | **G** | **T** | **A** | **T** | **T** | **G** | **T** | **T** | **T** | **G** | **C** | **C** | **C** | **T** | **T** | **C** | **C** | **T** | **G** | **G** | **G** | **G** | **T** | **G** | **G** | **G** | **C** | **T** | **T** | **T** | **G** | **T** | **A** | **T** | **T** | **A** | **T** | **A** | **T** | **T** | **T** | **A** | **T** | **T** | **T** | **T** | **T** | **C** | **G** | **G** | **A** | **C** | **G** | **T** | **G** | **G** | **A** | **T** | **T** | **A** | **A** | **C** | **G** | **G** | **A** | **T** | **A** | **A** | **G** | **A** | **A** | **A** | **A** | **A** | **A** | **T** | **T** | **C** | **T** | **A** | **T** | **C** | **T** | **C** | **C** | **G** | **A** | **C** | **A** | **A** | **A** | **G** | **T** | **G** | **A** | **C** | **T** | **T** | **G** | **A** | **A** | **A** | **G** | **A** | **A** | **T** | **T** | **A** | **G** | **A** | **A** | **A** | **A** | **C** | **T** | **T** | **T** | **C** | **A** | **G** | **T** | **C** | **T** | **T** | **T** | **T** | **A** | **G** | **A** | **G** | **G** | **A** | **G** | **A** | **T** | **A** | **C** | **C** | **A** | **T** | **T** | **G** | **A** | **A** | **C** | **A** | **T** | **T** | **A** | **C** | **G** | **A** | **T** | **C** | **C** | **T** | **G** | **A** | **C** | **A** | **T** | **G** | **G** | **G** | **C** | **G** | **A** | **T** | **A** | **A** | **A** | **G** | **A** | **C** | **A** | **A** | **A** | **C** | **A** | **G** | **C** | **A** | **A** | **T** | **T** | **T** | **G** | **T** | **T** | **G** | **A** | **C** | **T** | **T** | **C** | **T** | **T** | **C** | **T** | **C** | **T** | **T** | **C** | **A** | **T** | **T** | **A** | **A** | **A** | **T** | **C** | **G** | **T** | **A** | **T** | **G** | **C** | **C** | **G** | **C** | **T** | **G** | **G** | **C** | **A** | **A** | **G** | **A** | **A** | **T** | **G** | **A** | **A** | **T** | **T** | **C** | **T** | **G** | **T** | **C** | **A** | **C** | **T** | **C** | **T** | **T** | **C** | **T** | **C** | **A** | **C** | **A** | **G** | **A** | **C** | **G** | **G** | **A** | **C** | **A** | **A** | **G** | **A** | **G** | **A** | **A** | **A** | **T** | **T** | **G** | **G** | **A** | **T** | **T** | **C** | **A** | **C** | **T** | **G** | **C** | **T** | **T** | **C** | **A** | **A** | **G** | **A** | **T** | **C** | **T** | **A** | **A** | **A** | **A** | **A** | **A** | **A** | **G** | **C** | **C** | **A** | **A** | **A** | **C** | **A** | **T** | **T** | **C** | **G** | **A** | **T** | **C** | **C** | **A** | **T** | **A** | **T** | **C** | **G** | **A** | **A** | **T** | **A** | **T** | **T** | **A** | **C** | **G** | **C** | **A** | **T** | **T** | **T** | **G** | **T** | **G** | **A** | **C** | **A** | **G** | **A** | **T** | **A** | **A** | **T** | **A** | **T** | **C** | **G** | **G** | **C** | **C** | **A** | **G** | **C** | **A** | **A** | **G** | **T** | **C** | **T** | **T** | **A** | **C** | **A** | **T** | **G** | **T** | **T** | **T** | **T** | **A** | **G** | **A** | **A** | **G** | **A** | **A** | **A** | **A** | **A** | **G** | **C** | **C** | **G** | **C** | **A** | **G** | **A** | **A** | **G** | **G** | **C** | **G** | **T** | **G** | **G** | **A** | **A** | **G** | **T** | **T** | **C** | **G** | **A** | **A** | **T** | **A** | **T** | **T** | **A** | **T** | **A** | **T** | **G** | **A** | **T** | **G** | **C** | **A** | **T** | **T** | **T** | **G** | **G** | **C** | **T** | **C** | **T** | **C** | **A** | **T** | **G** | **G** | **C** | **A** | **C** | **A** | **A** | **A** | **A** | **G** | **C** | **A** | **A** | **A** | **A** | **G** | **A** | **T** | **T** | **T** | **C** | **A** | **A** | **T** | **C** | **G** | **T** | **C** | **T** | **A** | **A** | **T** | **C** | **A** | **A** | **A** | **A** | **A** | **T** | **G** | **G** | **T** | **G** | **G** | **A** | **C** | **A** | **T** | **G** | **T** | **C** | **C** | **A** | **T** | **A** | **C** | **A** | **T** | **T** | **T** | **G** | **T** | **T** | **A** | **C** | **C** | **T** | **C** | **A** | **C** | **A** | **A** | **A** | **A** | **G** | **G** | **C** | **A** | **T** | **T** | **A** | **C** | **T** | **T** | **C** | **G** | **T** | **T** | **T** | **C** | **C** | **G** | **A** | **T** | **T** | **G** | **A** | **A** | **T** | **T** | **A** | **C** | **C** | **A** | **T** | **G** | **A** | **T** | **C** | **A** | **C** | **C** | **G** | **A** | **A** | **A** | **A** | **A** | **T** | **C** | **G** | **T** | **T** | **G** | **T** | **G** | **A** | **T** | **C** | **G** | **A** | **T** | **G** | **G** | **A** | **A** | **A** | **G** | **A** | **T** | **T** | **A** | **G** | **T** | **T** | **A** | **T** | **A** | **C** | **C** | **G** | **G** | **T** | **G** | **G** | **T** | **T** | **T** | **C** | **A** | **A** | **T** | **A** | **T** | **T** | **G** | **C** | **C** | **A** | **A** | **T** | **C** | **A** | **A** | **T** | **A** | **T** | **G** | **T** | **A** | **A** | **A** | **T** | **A** | **C** | **A** | **A** | **C** | **A** | **A** | **A** | **A** | **A** | **A** | **A** | **T** | **T** | **C** | **G** | **G** | **C** | **T** | **A** | **T** | **T** | **G** | **G** | **C** | **G** | **C** | **G** | **A** | **T** | **A** | **C** | **G** | **C** | **A** | **T** | **A** | **T** | **A** | **C** | **G** | **G** | **A** | **T** | **T** | **T** | **T** | **C** | **G** | **G** | **T** | **G** | **C** | **C** | **G** | **C** | **T** | **T** | **C** | **T** | **T** | **C** | **T** | **T** | **T** | **G** | **C** | **T** | **C** | **C** | **A** | **G** | **C** | **T** | **T** | **C** | **G** | **C** | **T** | **T** | **C** | **T** | **T** | **A** | **A** | **C** | **A** | **G** | **A** | **C** | **T** | **G** | **G** | **A** | **A** | **C** | **G** | **T** | **C** | **T** | **C** | **G** | **G** | **T** | **A** | **C** | **C** | **T** | **G** | **A** | **A** | **G** | **A** | **A** | **A** | **A** | **A** | **A** | **A** | **G** | **G** | **T** | **C** | **G** | **G** | **C** | **T** | **A** | **T** | **C** | **A** | **T** | **T** | **T** | **G** | **A** | **A** | **T** | **T** | **A** | **T** | **T** | **T** | **C** | **T** | **T** | **T** | **A** | **A** | **A** | **A** | **A** | **A** | **G** | **C** | **A** | **G** | **A** | **T** | **C** | **G** | **A** | **G** | **A** | **T** | **G** | **A** | **A** | **T** | **C** | **T** | **A** | **A** | **G** | **C** | **T** | **T** | **G** | **C** | **T** | **G** | **A** | **T** | **A** | **C** | **A** | **T** | **C** | **C** | **A** | **T** | **C** | **C** | **A** | **G** | **C** | **T** | **T** | **G** | **T** | **T** | **T** | **C** | **A** | **A** | **G** | **C** | **G** | **G** | **A** | **C** | **C** | **G** | **A** | **A** | **T** | **A** | **A** | **C** | **G** | **A** | **A** | **A** | **G** | **G** | **G** | **A** | **A** | **C** | **A** | **A** | **A** | **T** | **C** | **A** | **A** | **G** | **C** | **T** | **T** | **T** | **C** | **A** | **T** | **T** | **T** | **A** | **T** | **C** | **A** | **A** | **A** | **T** | **T** | **G** | **A** | **T** | **T** | **A** | **C** | **T** | **T** | **C** | **T** | **G** | **C** | **T** | **A** | **A** | **A** | **A** | **A** | **A** | **C** | **G** | **T** | **G** | **T** | **T** | **T** | **G** | **G** | **A** | **T** | **A** | **C** | **A** | **G** | **A** | **C** | **A** | **C** | **C** | **T** | **T** | **A** | **C** | **C** | **T** | **T** | **G** | **T** | **T** | **C** | **C** | **T** | **G** | **A** | **T** | **G** | **A** | **A** | **A** | **G** | **T** | **G** | **T** | **C** | **A** | **T** | **T** | **G** | **C** | **T** | **G** | **C** | **T** | **T** | **T** | **A** | **A** | **A** | **A** | **A** | **T** | **C** | **G** | **C** | **A** | **A** | **C** | **T** | **G** | **C** | **C** | **T** | **C** | **T** | **G** | **G** | **T** | **G** | **T** | **A** | **G** | **A** | **T** | **G** | **T** | **G** | **A** | **A** | **A** | **A** | **T** | **C** | **A** | **T** | **G** | **A** | **T** | **T** | **C** | **C** | **C** | **A** | **A** | **C** | **A** | **A** | **A** | **C** | **C** | **G** | **G** | **A** | **T** | **C** | **A** | **T** | **C** | **C** | **T** | **T** | **T** | **T** | **A** | **T** | **T** | **T** | **A** | **T** | **C** | **G** | **A** | **G** | **C** | **A** | **A** | **C** | **A** | **C** | **A** | **A** | **T** | **A** | **T** | **T** | **A** | **T** | **G** | **C** | **T** | **C** | **G** | **G** | **C** | **A** | **G** | **C** | **T** | **G** | **A** | **T** | **C** | **A** | **A** | **G** | **G** | **A** | **A** | **A** | **A** | **T** | **G** | **T** | **A** | **C** | **A** | **A** | **A** | **T** | **C** | **C** | **T** | **T** | **G** | **T** | **C** | **T** | **A** | **T** | **G** | **A** | **G** | **A** | **A** | **C** | **G** | **G** | **C** | **T** | **T** | **C** | **C** | **T** | **C** | **C** | **A** | **T** | **G** | **C** | **A** | **A** | **A** | **A** | **A** | **C** | **A** | **T** | **T** | **G** | **A** | **T** | **A** | **A** | **T** | **G** | **G** | **A** | **T** | **G** | **A** | **T** | **G** | **A** | **A** | **A** | **T** | **C** | **T** | **G** | **C** | **A** | **T** | **G** | **G** | **T** | **A** | **G** | **G** | **T** | **T** | **C** | **A** | **G** | **C** | **A** | **A** | **A** | **T** | **C** | **A** | **A** | **G** | **A** | **T** | **A** | **T** | **T** | **C** | **G** | **T** | **A** | **G** | **C** | **T** | **A** | **C** | **C** | **G** | **A** | **T** | **T** | **G** | **A** | **A** | **T** | **T** | **T** | **T** | **G** | **A** | **A** | **A** | **C** | **A** | **A** | **G** | **T** | **G** | **C** | **T** | **G** | **T** | **C** | **A** | **T** | **T** | **T** | **A** | **C** | **G** | **A** | **T** | **C** | **C** | **T** | **G** | **A** | **G** | **T** | **T** | **T** | **T** | **T** | **A** | **G** | **A** | **A** | **G** | **A** | **A** | **C** | **T** | **T** | **G** | **C** | **T** | **A** | **C** | **T** | **C** | **A** | **G** | **T** | **T** | **C** | **A** | **A** | **A** | **G** | **A** | **A** | **G** | **A** | **T** | **G** | **A** | **G** | **A** | **C** | **A** | **C** | **A** | **T** | **T** | **G** | **T** | **T** | **C** | **A** | **T** | **C** | **C** | **A** | **T** | **G** | **A** | **C** | **A** | **A** | **C** | **T** | **G** | **A** | **A** | **A** | **C** | **A** | **G** | **T** | **C** | **A** | **A** | **G** | **G** | **A** | **A** | **A** | **T** | **G** | **T** | **C** | **T** | **A** | **A** | **C** | **T** | **G** | **G** | **C** | **T** | **A** | **T** | **T** | **A** | **T** | **T** | **C** | **A** | **A** | **G** | **C** | **A** | **A** | **C** | **A** | **A** | **A** | **T** | **T** | **T** | **C** | **T** | **C** | **G** | **A** | **T** | **T** | **A** | **T** | **T** | **T** | **T** | **C** | **T** | **C** | **C** | **A** | **A** | **T** | **C** | **C** | **T** | **A** | **-** | **-** | **-** |
| **N60336F** | **-** | **-** | **-** | **-** | **-** | **-** | **-** | **-** | **-** | **-** | **-** | **-** | **-** | **-** | **-** | **-** | **-** | **-** | **-** | **-** | **-** | **-** | **-** | **-** | **-** | **-** | **-** | **-** | **-** | **-** | **-** | **-** | **-** | **-** | **-** | **-** | **-** | **-** | **-** | **-** | **-** | **-** | **-** | **-** | **-** | **-** | **-** | **-** | **-** | **-** | **-** | **-** | **-** | **-** | **-** | **-** | **-** | **-** | **-** | **-** | **-** | **-** | **-** | **-** | **-** | **-** | **-** | **-** | **-** | **A** | **A** | **A** | **C** | **C** | **C** | **C** | **G** | **A** | **G** | **A** | **T** | **G** | **T** | **A** | **G** | **C** | **A** | **G** | **C** | **C** | **A** | **T** | **T** | **T** | **G** | **G** | **G** | **C** | **A** | **T** | **G** | **G** | **C** | **T** | **C** | **T** | **T** | **A** | **G** | **T** | **A** | **T** | **T** | **G** | **T** | **T** | **T** | **G** | **C** | **C** | **C** | **T** | **T** | **C** | **C** | **T** | **G** | **G** | **G** | **G** | **T** | **G** | **G** | **G** | **T** | **T** | **T** | **T** | **G** | **T** | **A** | **T** | **T** | **A** | **T** | **A** | **T** | **T** | **T** | **A** | **T** | **T** | **T** | **T** | **T** | **C** | **G** | **G** | **A** | **C** | **G** | **T** | **G** | **G** | **A** | **T** | **T** | **A** | **A** | **C** | **G** | **G** | **A** | **T** | **A** | **A** | **G** | **A** | **A** | **A** | **A** | **A** | **A** | **T** | **T** | **C** | **T** | **A** | **T** | **C** | **T** | **C** | **C** | **G** | **A** | **C** | **A** | **A** | **A** | **G** | **T** | **G** | **A** | **C** | **T** | **T** | **G** | **A** | **A** | **A** | **G** | **A** | **A** | **T** | **T** | **A** | **G** | **A** | **A** | **A** | **A** | **C** | **T** | **T** | **T** | **C** | **A** | **G** | **T** | **C** | **T** | **T** | **T** | **T** | **A** | **G** | **A** | **G** | **G** | **A** | **G** | **A** | **T** | **A** | **C** | **C** | **A** | **T** | **T** | **G** | **A** | **A** | **C** | **A** | **T** | **T** | **A** | **C** | **G** | **A** | **T** | **C** | **C** | **T** | **G** | **A** | **C** | **A** | **T** | **G** | **G** | **G** | **C** | **G** | **A** | **T** | **A** | **A** | **A** | **G** | **A** | **C** | **A** | **A** | **A** | **C** | **A** | **G** | **C** | **A** | **A** | **T** | **T** | **T** | **G** | **T** | **C** | **G** | **A** | **C** | **T** | **T** | **C** | **T** | **T** | **C** | **T** | **C** | **T** | **T** | **C** | **A** | **T** | **T** | **A** | **A** | **A** | **T** | **C** | **G** | **T** | **A** | **T** | **G** | **C** | **C** | **G** | **C** | **T** | **G** | **A** | **C** | **A** | **A** | **G** | **A** | **A** | **T** | **G** | **A** | **A** | **T** | **T** | **C** | **T** | **G** | **T** | **C** | **A** | **C** | **T** | **C** | **T** | **T** | **C** | **T** | **C** | **A** | **C** | **A** | **G** | **A** | **C** | **G** | **G** | **A** | **C** | **A** | **A** | **G** | **A** | **G** | **A** | **A** | **A** | **T** | **T** | **G** | **G** | **A** | **T** | **T** | **C** | **A** | **C** | **T** | **G** | **C** | **T** | **T** | **C** | **A** | **A** | **G** | **A** | **T** | **C** | **T** | **A** | **A** | **A** | **A** | **A** | **A** | **A** | **G** | **C** | **C** | **A** | **A** | **A** | **C** | **A** | **T** | **T** | **C** | **G** | **A** | **T** | **C** | **C** | **A** | **T** | **A** | **T** | **C** | **G** | **A** | **A** | **T** | **A** | **T** | **T** | **A** | **C** | **G** | **C** | **A** | **T** | **T** | **T** | **G** | **T** | **G** | **A** | **C** | **A** | **G** | **A** | **T** | **A** | **A** | **T** | **A** | **T** | **C** | **G** | **G** | **C** | **C** | **A** | **G** | **C** | **A** | **A** | **G** | **T** | **C** | **T** | **T** | **A** | **C** | **A** | **T** | **G** | **T** | **T** | **T** | **T** | **A** | **G** | **A** | **A** | **G** | **A** | **A** | **A** | **A** | **A** | **G** | **C** | **C** | **G** | **C** | **A** | **G** | **A** | **A** | **G** | **G** | **C** | **G** | **T** | **G** | **G** | **A** | **A** | **G** | **T** | **T** | **C** | **G** | **A** | **A** | **T** | **A** | **T** | **T** | **A** | **T** | **A** | **T** | **G** | **A** | **T** | **G** | **C** | **A** | **T** | **T** | **T** | **G** | **G** | **C** | **T** | **C** | **T** | **C** | **A** | **T** | **G** | **G** | **C** | **A** | **C** | **A** | **A** | **A** | **A** | **G** | **C** | **A** | **A** | **A** | **A** | **G** | **A** | **T** | **T** | **T** | **C** | **A** | **A** | **T** | **C** | **G** | **T** | **C** | **T** | **A** | **A** | **T** | **C** | **A** | **A** | **A** | **A** | **A** | **T** | **G** | **G** | **T** | **G** | **G** | **A** | **C** | **A** | **T** | **G** | **T** | **C** | **C** | **A** | **T** | **A** | **C** | **A** | **T** | **T** | **T** | **G** | **T** | **T** | **A** | **C** | **C** | **T** | **C** | **A** | **C** | **A** | **A** | **A** | **A** | **G** | **G** | **C** | **A** | **T** | **T** | **A** | **C** | **T** | **T** | **C** | **G** | **T** | **T** | **T** | **C** | **C** | **G** | **A** | **T** | **T** | **G** | **A** | **A** | **T** | **T** | **A** | **C** | **C** | **A** | **T** | **G** | **A** | **T** | **C** | **A** | **C** | **C** | **G** | **A** | **A** | **A** | **A** | **A** | **T** | **C** | **G** | **T** | **T** | **G** | **T** | **G** | **A** | **T** | **C** | **G** | **A** | **T** | **G** | **G** | **A** | **A** | **A** | **G** | **A** | **T** | **T** | **A** | **G** | **T** | **T** | **A** | **T** | **A** | **C** | **C** | **G** | **G** | **T** | **G** | **G** | **T** | **T** | **T** | **C** | **A** | **A** | **T** | **A** | **T** | **T** | **G** | **C** | **C** | **A** | **A** | **T** | **C** | **A** | **A** | **T** | **A** | **T** | **G** | **T** | **A** | **A** | **A** | **T** | **A** | **C** | **A** | **A** | **C** | **A** | **A** | **A** | **A** | **A** | **A** | **A** | **T** | **T** | **C** | **G** | **G** | **C** | **T** | **A** | **T** | **T** | **G** | **G** | **C** | **G** | **C** | **G** | **A** | **T** | **A** | **C** | **G** | **C** | **A** | **T** | **A** | **T** | **A** | **C** | **G** | **G** | **A** | **T** | **T** | **T** | **T** | **C** | **G** | **G** | **T** | **G** | **C** | **C** | **G** | **C** | **T** | **T** | **C** | **T** | **T** | **C** | **T** | **T** | **T** | **G** | **C** | **T** | **C** | **C** | **A** | **G** | **C** | **T** | **T** | **C** | **G** | **C** | **T** | **T** | **C** | **T** | **T** | **A** | **A** | **C** | **A** | **G** | **A** | **C** | **T** | **G** | **G** | **A** | **A** | **C** | **G** | **T** | **C** | **T** | **C** | **G** | **G** | **T** | **A** | **C** | **C** | **T** | **G** | **A** | **A** | **G** | **A** | **A** | **A** | **A** | **A** | **A** | **A** | **G** | **G** | **T** | **C** | **G** | **G** | **C** | **T** | **A** | **T** | **C** | **A** | **T** | **T** | **T** | **G** | **A** | **A** | **T** | **T** | **A** | **T** | **T** | **T** | **C** | **T** | **T** | **T** | **A** | **A** | **A** | **A** | **A** | **A** | **G** | **C** | **A** | **G** | **A** | **T** | **C** | **G** | **A** | **G** | **A** | **T** | **G** | **A** | **A** | **T** | **C** | **T** | **A** | **A** | **G** | **C** | **T** | **T** | **G** | **C** | **T** | **G** | **A** | **T** | **A** | **C** | **A** | **T** | **C** | **C** | **A** | **T** | **C** | **C** | **A** | **G** | **C** | **T** | **T** | **G** | **T** | **T** | **T** | **C** | **A** | **A** | **G** | **C** | **G** | **G** | **A** | **C** | **C** | **G** | **A** | **A** | **T** | **A** | **A** | **C** | **G** | **A** | **A** | **A** | **G** | **G** | **G** | **A** | **A** | **C** | **A** | **A** | **A** | **T** | **C** | **A** | **A** | **G** | **C** | **T** | **T** | **T** | **C** | **A** | **T** | **T** | **T** | **A** | **T** | **C** | **A** | **A** | **A** | **T** | **T** | **G** | **A** | **T** | **T** | **A** | **C** | **T** | **T** | **C** | **T** | **G** | **C** | **T** | **A** | **A** | **A** | **A** | **A** | **A** | **C** | **G** | **T** | **G** | **T** | **T** | **T** | **G** | **G** | **A** | **T** | **A** | **C** | **A** | **G** | **A** | **C** | **A** | **C** | **C** | **T** | **T** | **A** | **C** | **C** | **T** | **T** | **G** | **T** | **T** | **C** | **C** | **T** | **G** | **A** | **T** | **G** | **A** | **A** | **A** | **G** | **T** | **G** | **T** | **C** | **A** | **T** | **T** | **G** | **C** | **T** | **G** | **C** | **T** | **T** | **T** | **A** | **A** | **A** | **A** | **A** | **T** | **C** | **G** | **C** | **A** | **A** | **C** | **T** | **G** | **C** | **C** | **T** | **C** | **T** | **G** | **G** | **T** | **G** | **T** | **A** | **G** | **A** | **T** | **G** | **T** | **G** | **A** | **A** | **A** | **A** | **T** | **C** | **A** | **T** | **G** | **A** | **T** | **T** | **C** | **C** | **C** | **A** | **A** | **C** | **A** | **A** | **A** | **C** | **C** | **G** | **G** | **A** | **T** | **C** | **A** | **T** | **C** | **C** | **T** | **T** | **T** | **T** | **A** | **T** | **T** | **T** | **A** | **T** | **C** | **G** | **A** | **G** | **C** | **A** | **A** | **C** | **A** | **C** | **A** | **A** | **T** | **A** | **T** | **T** | **A** | **T** | **G** | **C** | **T** | **C** | **G** | **G** | **C** | **A** | **G** | **C** | **T** | **G** | **A** | **T** | **C** | **A** | **A** | **G** | **G** | **A** | **A** | **A** | **A** | **T** | **G** | **T** | **A** | **C** | **A** | **A** | **A** | **T** | **C** | **C** | **T** | **T** | **G** | **T** | **C** | **T** | **A** | **T** | **G** | **A** | **G** | **A** | **A** | **C** | **G** | **G** | **C** | **T** | **T** | **C** | **C** | **T** | **C** | **C** | **A** | **T** | **G** | **C** | **A** | **A** | **A** | **A** | **A** | **C** | **A** | **T** | **T** | **G** | **A** | **T** | **A** | **A** | **T** | **G** | **G** | **A** | **T** | **G** | **A** | **T** | **G** | **A** | **A** | **A** | **T** | **C** | **T** | **G** | **C** | **A** | **T** | **G** | **G** | **T** | **A** | **G** | **G** | **T** | **T** | **C** | **A** | **G** | **C** | **A** | **A** | **A** | **T** | **C** | **A** | **A** | **G** | **A** | **T** | **A** | **T** | **T** | **C** | **G** | **T** | **A** | **G** | **C** | **T** | **A** | **C** | **C** | **G** | **A** | **T** | **T** | **G** | **A** | **A** | **T** | **T** | **T** | **T** | **G** | **A** | **A** | **A** | **C** | **A** | **A** | **G** | **T** | **G** | **C** | **T** | **G** | **T** | **C** | **A** | **T** | **T** | **T** | **A** | **C** | **G** | **A** | **T** | **C** | **C** | **T** | **G** | **A** | **G** | **T** | **T** | **T** | **T** | **T** | **A** | **G** | **A** | **A** | **G** | **A** | **A** | **C** | **T** | **T** | **G** | **C** | **T** | **A** | **C** | **T** | **C** | **A** | **G** | **T** | **T** | **C** | **A** | **A** | **A** | **G** | **A** | **A** | **G** | **A** | **T** | **G** | **A** | **G** | **A** | **C** | **A** | **C** | **A** | **T** | **T** | **G** | **T** | **T** | **C** | **A** | **T** | **C** | **C** | **A** | **T** | **G** | **A** | **C** | **A** | **A** | **C** | **T** | **G** | **A** | **A** | **A** | **C** | **A** | **G** | **T** | **C** | **A** | **A** | **A** | **G** | **A** | **A** | **A** | **T** | **G** | **T** | **C** | **T** | **A** | **A** | **C** | **T** | **G** | **G** | **C** | **T** | **A** | **T** | **T** | **A** | **T** | **T** | **C** | **A** | **A** | **G** | **C** | **A** | **A** | **C** | **A** | **A** | **A** | **T** | **T** | **T** | **C** | **T** | **C** | **G** | **A** | **T** | **T** | **A** | **T** | **T** | **T** | **T** | **C** | **T** | **C** | **C** | **A** | **A** | **T** | **C** | **C** | **T** | **A** | **-** | **-** | **-** |
| **N52459** | **-** | **-** | **-** | **-** | **-** | **-** | **-** | **-** | **-** | **-** | **-** | **-** | **-** | **-** | **-** | **-** | **-** | **-** | **-** | **-** | **-** | **-** | **-** | **-** | **-** | **-** | **-** | **-** | **-** | **-** | **-** | **-** | **-** | **-** | **-** | **-** | **-** | **-** | **-** | **-** | **-** | **-** | **-** | **-** | **-** | **-** | **-** | **-** | **-** | **-** | **-** | **-** | **-** | **-** | **-** | **-** | **-** | **-** | **-** | **-** | **-** | **-** | **-** | **-** | **-** | **-** | **-** | **-** | **-** | **A** | **A** | **A** | **C** | **C** | **C** | **C** | **G** | **A** | **G** | **A** | **T** | **G** | **T** | **A** | **G** | **C** | **A** | **G** | **C** | **C** | **A** | **T** | **T** | **T** | **G** | **G** | **G** | **C** | **A** | **T** | **G** | **G** | **C** | **T** | **C** | **T** | **T** | **A** | **G** | **T** | **A** | **T** | **T** | **G** | **T** | **T** | **T** | **G** | **C** | **C** | **C** | **T** | **T** | **C** | **C** | **T** | **G** | **G** | **G** | **G** | **T** | **G** | **G** | **G** | **T** | **T** | **T** | **T** | **G** | **T** | **A** | **T** | **T** | **A** | **T** | **A** | **T** | **T** | **T** | **A** | **T** | **T** | **T** | **T** | **T** | **C** | **G** | **G** | **A** | **C** | **G** | **T** | **G** | **G** | **A** | **T** | **T** | **A** | **A** | **C** | **G** | **G** | **A** | **T** | **A** | **A** | **G** | **A** | **A** | **A** | **A** | **A** | **A** | **T** | **T** | **C** | **T** | **A** | **T** | **C** | **T** | **C** | **C** | **G** | **A** | **C** | **A** | **A** | **A** | **G** | **T** | **G** | **A** | **C** | **T** | **T** | **G** | **A** | **A** | **A** | **G** | **A** | **A** | **T** | **T** | **A** | **G** | **A** | **A** | **A** | **A** | **C** | **T** | **T** | **T** | **C** | **A** | **G** | **T** | **C** | **T** | **T** | **T** | **T** | **A** | **G** | **A** | **G** | **G** | **A** | **G** | **A** | **T** | **A** | **C** | **C** | **A** | **T** | **T** | **G** | **A** | **A** | **C** | **A** | **T** | **T** | **A** | **C** | **G** | **A** | **T** | **C** | **C** | **T** | **G** | **A** | **C** | **A** | **T** | **G** | **G** | **G** | **C** | **G** | **A** | **T** | **A** | **A** | **A** | **G** | **A** | **C** | **A** | **A** | **A** | **C** | **A** | **G** | **C** | **A** | **A** | **T** | **T** | **T** | **G** | **T** | **C** | **G** | **A** | **C** | **T** | **T** | **C** | **T** | **T** | **C** | **T** | **C** | **T** | **T** | **C** | **A** | **T** | **T** | **A** | **A** | **A** | **T** | **C** | **G** | **T** | **A** | **T** | **G** | **C** | **C** | **G** | **C** | **T** | **G** | **A** | **C** | **A** | **A** | **G** | **A** | **A** | **T** | **G** | **A** | **A** | **T** | **T** | **C** | **T** | **G** | **T** | **C** | **A** | **C** | **T** | **C** | **T** | **T** | **C** | **T** | **C** | **A** | **C** | **A** | **G** | **A** | **C** | **G** | **G** | **A** | **C** | **A** | **A** | **G** | **A** | **G** | **A** | **A** | **A** | **T** | **T** | **G** | **G** | **A** | **T** | **T** | **C** | **A** | **C** | **T** | **G** | **C** | **T** | **T** | **C** | **A** | **A** | **G** | **A** | **T** | **C** | **T** | **A** | **A** | **A** | **A** | **A** | **A** | **A** | **G** | **C** | **C** | **A** | **A** | **A** | **C** | **A** | **T** | **T** | **C** | **G** | **A** | **T** | **C** | **C** | **A** | **T** | **A** | **T** | **C** | **G** | **A** | **A** | **T** | **A** | **T** | **T** | **A** | **C** | **G** | **C** | **A** | **T** | **T** | **T** | **G** | **T** | **G** | **A** | **C** | **A** | **G** | **A** | **T** | **A** | **A** | **T** | **A** | **T** | **C** | **G** | **G** | **C** | **C** | **A** | **G** | **C** | **A** | **A** | **G** | **T** | **C** | **T** | **T** | **A** | **C** | **A** | **T** | **G** | **T** | **T** | **T** | **T** | **A** | **G** | **A** | **A** | **G** | **A** | **A** | **A** | **A** | **A** | **G** | **C** | **C** | **G** | **C** | **A** | **G** | **A** | **A** | **G** | **G** | **C** | **G** | **T** | **G** | **G** | **A** | **A** | **G** | **T** | **T** | **C** | **G** | **A** | **A** | **T** | **A** | **T** | **T** | **A** | **T** | **A** | **T** | **G** | **A** | **T** | **G** | **C** | **A** | **T** | **T** | **T** | **G** | **G** | **C** | **T** | **C** | **T** | **C** | **A** | **T** | **G** | **G** | **C** | **A** | **C** | **A** | **A** | **A** | **A** | **G** | **C** | **A** | **A** | **A** | **A** | **G** | **A** | **T** | **T** | **T** | **C** | **A** | **A** | **T** | **C** | **G** | **T** | **C** | **T** | **A** | **A** | **T** | **C** | **A** | **A** | **A** | **A** | **A** | **T** | **G** | **G** | **T** | **G** | **G** | **A** | **C** | **A** | **T** | **G** | **T** | **C** | **C** | **A** | **T** | **A** | **C** | **A** | **T** | **T** | **T** | **G** | **T** | **T** | **A** | **C** | **C** | **T** | **C** | **A** | **C** | **A** | **A** | **A** | **A** | **G** | **G** | **C** | **A** | **T** | **T** | **A** | **C** | **T** | **T** | **C** | **G** | **T** | **T** | **T** | **C** | **C** | **G** | **A** | **T** | **T** | **G** | **A** | **A** | **T** | **T** | **A** | **C** | **C** | **A** | **T** | **G** | **A** | **T** | **C** | **A** | **C** | **C** | **G** | **A** | **A** | **A** | **A** | **A** | **T** | **C** | **G** | **T** | **T** | **G** | **T** | **G** | **A** | **T** | **C** | **G** | **A** | **T** | **G** | **G** | **A** | **A** | **A** | **G** | **A** | **T** | **T** | **A** | **G** | **T** | **T** | **A** | **T** | **A** | **C** | **C** | **G** | **G** | **T** | **G** | **G** | **T** | **T** | **T** | **C** | **A** | **A** | **T** | **A** | **T** | **T** | **G** | **C** | **C** | **A** | **A** | **T** | **C** | **A** | **A** | **T** | **A** | **T** | **G** | **T** | **A** | **A** | **A** | **T** | **A** | **C** | **A** | **A** | **C** | **A** | **A** | **A** | **A** | **A** | **A** | **A** | **T** | **T** | **C** | **G** | **G** | **C** | **T** | **A** | **T** | **T** | **G** | **G** | **C** | **G** | **C** | **G** | **A** | **T** | **A** | **C** | **G** | **C** | **A** | **T** | **A** | **T** | **A** | **C** | **G** | **G** | **A** | **T** | **T** | **T** | **T** | **C** | **G** | **G** | **T** | **G** | **C** | **C** | **G** | **C** | **T** | **T** | **C** | **T** | **T** | **C** | **T** | **T** | **T** | **G** | **C** | **T** | **C** | **C** | **A** | **G** | **C** | **T** | **T** | **C** | **G** | **C** | **T** | **T** | **C** | **T** | **T** | **A** | **A** | **C** | **A** | **G** | **A** | **C** | **T** | **G** | **G** | **A** | **A** | **C** | **G** | **T** | **C** | **T** | **C** | **G** | **G** | **T** | **A** | **C** | **C** | **T** | **G** | **A** | **A** | **G** | **A** | **A** | **A** | **A** | **A** | **A** | **A** | **G** | **G** | **T** | **C** | **G** | **G** | **C** | **T** | **A** | **T** | **C** | **A** | **T** | **T** | **T** | **G** | **A** | **A** | **T** | **T** | **A** | **T** | **T** | **T** | **C** | **T** | **T** | **T** | **A** | **A** | **A** | **A** | **A** | **A** | **G** | **C** | **A** | **G** | **A** | **T** | **C** | **G** | **A** | **G** | **A** | **T** | **G** | **A** | **A** | **T** | **C** | **T** | **A** | **A** | **G** | **C** | **T** | **T** | **G** | **C** | **T** | **G** | **A** | **T** | **A** | **C** | **A** | **T** | **C** | **C** | **A** | **T** | **C** | **C** | **A** | **G** | **C** | **T** | **T** | **G** | **T** | **T** | **T** | **C** | **A** | **A** | **G** | **C** | **G** | **G** | **A** | **C** | **C** | **G** | **A** | **A** | **T** | **A** | **A** | **C** | **G** | **A** | **A** | **A** | **G** | **G** | **G** | **A** | **A** | **C** | **A** | **A** | **A** | **T** | **C** | **A** | **A** | **G** | **C** | **T** | **T** | **T** | **C** | **A** | **T** | **T** | **T** | **A** | **T** | **C** | **A** | **A** | **A** | **T** | **T** | **G** | **A** | **T** | **T** | **A** | **C** | **T** | **T** | **C** | **T** | **G** | **C** | **T** | **A** | **A** | **A** | **A** | **A** | **A** | **C** | **G** | **T** | **G** | **T** | **T** | **T** | **G** | **G** | **A** | **T** | **A** | **C** | **A** | **G** | **A** | **C** | **A** | **C** | **C** | **T** | **T** | **A** | **C** | **C** | **T** | **T** | **G** | **T** | **T** | **C** | **C** | **T** | **G** | **A** | **T** | **G** | **A** | **A** | **A** | **G** | **T** | **G** | **T** | **C** | **A** | **T** | **T** | **G** | **C** | **T** | **G** | **C** | **T** | **T** | **T** | **A** | **A** | **A** | **A** | **A** | **T** | **C** | **G** | **C** | **A** | **A** | **C** | **T** | **G** | **C** | **C** | **T** | **C** | **T** | **G** | **G** | **T** | **G** | **T** | **A** | **G** | **A** | **T** | **G** | **T** | **G** | **A** | **A** | **A** | **A** | **T** | **C** | **A** | **T** | **G** | **A** | **T** | **T** | **C** | **C** | **C** | **A** | **A** | **C** | **A** | **A** | **A** | **C** | **C** | **G** | **G** | **A** | **T** | **C** | **A** | **T** | **C** | **C** | **T** | **T** | **T** | **T** | **A** | **T** | **T** | **T** | **A** | **T** | **C** | **G** | **A** | **G** | **C** | **A** | **A** | **C** | **A** | **C** | **A** | **A** | **T** | **A** | **T** | **T** | **A** | **T** | **G** | **C** | **T** | **C** | **G** | **G** | **C** | **A** | **G** | **C** | **T** | **G** | **A** | **T** | **C** | **A** | **A** | **G** | **G** | **A** | **A** | **A** | **A** | **T** | **G** | **T** | **A** | **C** | **A** | **A** | **A** | **T** | **C** | **C** | **T** | **T** | **G** | **T** | **C** | **T** | **A** | **T** | **G** | **A** | **G** | **A** | **A** | **C** | **G** | **G** | **C** | **T** | **T** | **C** | **C** | **T** | **C** | **C** | **A** | **T** | **G** | **C** | **A** | **A** | **A** | **A** | **A** | **C** | **A** | **T** | **T** | **G** | **A** | **T** | **A** | **A** | **T** | **G** | **G** | **A** | **T** | **G** | **A** | **T** | **G** | **A** | **A** | **A** | **T** | **C** | **T** | **G** | **C** | **A** | **T** | **G** | **G** | **T** | **A** | **G** | **G** | **T** | **T** | **C** | **A** | **G** | **C** | **A** | **A** | **A** | **T** | **C** | **A** | **A** | **G** | **A** | **T** | **A** | **T** | **T** | **C** | **G** | **T** | **A** | **G** | **C** | **T** | **A** | **C** | **C** | **G** | **A** | **T** | **T** | **G** | **A** | **A** | **T** | **T** | **T** | **T** | **G** | **A** | **A** | **A** | **C** | **A** | **A** | **G** | **T** | **G** | **C** | **T** | **G** | **T** | **C** | **A** | **T** | **T** | **T** | **A** | **C** | **G** | **A** | **T** | **C** | **C** | **T** | **G** | **A** | **G** | **T** | **T** | **T** | **T** | **T** | **A** | **G** | **A** | **A** | **G** | **A** | **A** | **C** | **T** | **T** | **G** | **C** | **T** | **A** | **C** | **T** | **C** | **A** | **G** | **T** | **T** | **C** | **A** | **A** | **A** | **G** | **A** | **A** | **G** | **A** | **T** | **G** | **A** | **G** | **A** | **C** | **A** | **C** | **A** | **T** | **T** | **G** | **T** | **T** | **C** | **A** | **T** | **C** | **C** | **A** | **T** | **G** | **A** | **C** | **A** | **A** | **C** | **T** | **G** | **A** | **A** | **A** | **C** | **A** | **G** | **T** | **C** | **A** | **A** | **G** | **G** | **A** | **A** | **A** | **T** | **G** | **T** | **C** | **T** | **A** | **A** | **C** | **T** | **G** | **G** | **C** | **T** | **A** | **T** | **T** | **A** | **T** | **T** | **C** | **A** | **A** | **G** | **C** | **A** | **A** | **C** | **A** | **A** | **A** | **T** | **T** | **T** | **C** | **T** | **C** | **G** | **A** | **T** | **T** | **A** | **T** | **T** | **T** | **T** | **C** | **T** | **C** | **C** | **A** | **A** | **T** | **C** | **C** | **T** | **A** | **-** | **-** | **-** |
| **N60061F** | **-** | **-** | **-** | **-** | **-** | **-** | **-** | **-** | **-** | **-** | **-** | **-** | **-** | **-** | **-** | **-** | **-** | **-** | **-** | **-** | **-** | **-** | **-** | **-** | **-** | **-** | **-** | **-** | **-** | **-** | **-** | **-** | **-** | **-** | **-** | **-** | **-** | **-** | **-** | **-** | **-** | **-** | **-** | **-** | **-** | **-** | **-** | **-** | **-** | **-** | **-** | **-** | **-** | **-** | **-** | **-** | **-** | **-** | **-** | **-** | **-** | **-** | **-** | **-** | **-** | **-** | **-** | **-** | **-** | **A** | **A** | **A** | **C** | **C** | **C** | **C** | **G** | **A** | **G** | **A** | **T** | **G** | **T** | **A** | **G** | **C** | **A** | **G** | **C** | **C** | **A** | **T** | **T** | **T** | **G** | **G** | **G** | **C** | **A** | **T** | **G** | **G** | **C** | **T** | **C** | **T** | **T** | **A** | **G** | **T** | **A** | **T** | **T** | **G** | **T** | **T** | **T** | **G** | **C** | **C** | **C** | **T** | **T** | **C** | **C** | **T** | **G** | **G** | **G** | **G** | **T** | **G** | **G** | **G** | **T** | **T** | **T** | **T** | **G** | **T** | **A** | **T** | **T** | **A** | **T** | **A** | **T** | **T** | **T** | **A** | **T** | **T** | **T** | **T** | **T** | **C** | **G** | **G** | **A** | **C** | **G** | **T** | **G** | **G** | **A** | **T** | **T** | **A** | **A** | **C** | **G** | **G** | **A** | **T** | **A** | **A** | **G** | **A** | **A** | **A** | **A** | **A** | **A** | **T** | **T** | **C** | **T** | **A** | **T** | **C** | **T** | **C** | **C** | **G** | **A** | **C** | **A** | **A** | **A** | **G** | **T** | **G** | **A** | **C** | **T** | **T** | **G** | **A** | **A** | **A** | **G** | **A** | **A** | **T** | **T** | **A** | **G** | **A** | **A** | **A** | **A** | **C** | **T** | **T** | **T** | **C** | **A** | **G** | **T** | **C** | **T** | **T** | **T** | **T** | **A** | **G** | **A** | **G** | **G** | **A** | **G** | **A** | **T** | **A** | **C** | **C** | **A** | **T** | **T** | **G** | **A** | **A** | **C** | **A** | **T** | **T** | **A** | **C** | **G** | **A** | **T** | **C** | **C** | **T** | **G** | **A** | **C** | **A** | **T** | **G** | **G** | **G** | **C** | **G** | **A** | **T** | **A** | **A** | **A** | **G** | **A** | **C** | **A** | **A** | **A** | **C** | **A** | **G** | **C** | **A** | **A** | **T** | **T** | **T** | **G** | **T** | **T** | **G** | **A** | **C** | **T** | **T** | **C** | **T** | **T** | **C** | **T** | **C** | **T** | **T** | **C** | **A** | **T** | **T** | **A** | **A** | **A** | **T** | **C** | **G** | **T** | **A** | **T** | **G** | **C** | **C** | **G** | **C** | **T** | **G** | **A** | **C** | **A** | **A** | **G** | **A** | **A** | **T** | **G** | **A** | **A** | **T** | **T** | **C** | **T** | **G** | **T** | **C** | **A** | **C** | **T** | **C** | **T** | **T** | **C** | **T** | **C** | **A** | **C** | **A** | **G** | **A** | **C** | **G** | **G** | **A** | **C** | **A** | **A** | **G** | **A** | **G** | **A** | **A** | **A** | **T** | **T** | **G** | **G** | **A** | **T** | **T** | **C** | **A** | **C** | **T** | **G** | **C** | **T** | **T** | **C** | **A** | **A** | **G** | **A** | **T** | **C** | **T** | **A** | **A** | **A** | **A** | **A** | **A** | **A** | **G** | **C** | **C** | **A** | **A** | **A** | **C** | **A** | **T** | **T** | **C** | **G** | **A** | **T** | **C** | **C** | **A** | **T** | **A** | **T** | **C** | **G** | **A** | **A** | **T** | **A** | **T** | **T** | **A** | **C** | **G** | **C** | **A** | **T** | **T** | **T** | **G** | **T** | **G** | **A** | **C** | **A** | **G** | **A** | **T** | **A** | **A** | **T** | **A** | **T** | **C** | **G** | **G** | **C** | **C** | **A** | **G** | **C** | **A** | **A** | **G** | **T** | **C** | **T** | **T** | **A** | **C** | **A** | **T** | **G** | **T** | **T** | **T** | **T** | **A** | **G** | **A** | **A** | **G** | **A** | **A** | **A** | **A** | **A** | **G** | **C** | **C** | **G** | **C** | **A** | **G** | **A** | **A** | **G** | **G** | **C** | **G** | **T** | **G** | **G** | **A** | **A** | **G** | **T** | **T** | **C** | **G** | **A** | **A** | **T** | **A** | **T** | **T** | **A** | **T** | **A** | **T** | **G** | **A** | **T** | **G** | **C** | **A** | **T** | **T** | **T** | **G** | **G** | **C** | **T** | **C** | **T** | **C** | **A** | **T** | **G** | **G** | **C** | **A** | **C** | **A** | **A** | **A** | **A** | **G** | **C** | **A** | **A** | **A** | **A** | **G** | **A** | **T** | **T** | **T** | **C** | **A** | **A** | **T** | **C** | **G** | **T** | **C** | **T** | **A** | **A** | **T** | **C** | **A** | **A** | **A** | **A** | **A** | **T** | **G** | **G** | **T** | **G** | **G** | **A** | **C** | **A** | **T** | **G** | **T** | **C** | **C** | **A** | **T** | **A** | **C** | **A** | **T** | **T** | **T** | **G** | **T** | **T** | **A** | **C** | **C** | **T** | **C** | **A** | **C** | **A** | **A** | **A** | **A** | **G** | **G** | **C** | **A** | **T** | **T** | **A** | **C** | **T** | **T** | **C** | **G** | **T** | **T** | **T** | **C** | **C** | **G** | **A** | **T** | **T** | **G** | **A** | **A** | **T** | **T** | **A** | **C** | **C** | **A** | **T** | **G** | **A** | **T** | **C** | **A** | **C** | **C** | **G** | **A** | **A** | **A** | **A** | **A** | **T** | **C** | **G** | **T** | **T** | **G** | **T** | **G** | **A** | **T** | **C** | **G** | **A** | **T** | **G** | **G** | **A** | **A** | **A** | **G** | **A** | **T** | **T** | **A** | **G** | **T** | **T** | **A** | **T** | **A** | **C** | **C** | **G** | **G** | **T** | **G** | **G** | **T** | **T** | **T** | **C** | **A** | **A** | **T** | **A** | **T** | **T** | **G** | **C** | **C** | **A** | **A** | **T** | **C** | **A** | **A** | **T** | **A** | **T** | **G** | **T** | **A** | **A** | **A** | **T** | **A** | **C** | **A** | **A** | **C** | **A** | **A** | **A** | **A** | **A** | **A** | **A** | **T** | **T** | **C** | **G** | **G** | **C** | **T** | **A** | **T** | **T** | **G** | **G** | **C** | **G** | **C** | **G** | **A** | **T** | **A** | **C** | **G** | **C** | **A** | **T** | **A** | **T** | **A** | **C** | **G** | **G** | **A** | **T** | **T** | **T** | **T** | **C** | **G** | **G** | **T** | **G** | **C** | **C** | **G** | **C** | **T** | **T** | **C** | **T** | **T** | **C** | **T** | **T** | **T** | **G** | **C** | **T** | **C** | **C** | **A** | **G** | **C** | **T** | **T** | **C** | **G** | **C** | **T** | **T** | **C** | **T** | **T** | **A** | **A** | **C** | **A** | **G** | **A** | **C** | **T** | **G** | **G** | **A** | **A** | **C** | **G** | **T** | **C** | **T** | **C** | **G** | **G** | **T** | **A** | **C** | **C** | **T** | **G** | **A** | **A** | **G** | **A** | **A** | **A** | **A** | **A** | **A** | **A** | **G** | **G** | **T** | **C** | **G** | **G** | **C** | **T** | **A** | **T** | **C** | **A** | **T** | **T** | **T** | **G** | **A** | **A** | **T** | **T** | **A** | **T** | **T** | **T** | **C** | **T** | **T** | **T** | **A** | **A** | **A** | **A** | **A** | **A** | **G** | **C** | **A** | **G** | **A** | **T** | **C** | **G** | **A** | **G** | **A** | **T** | **G** | **A** | **A** | **T** | **C** | **T** | **A** | **A** | **G** | **C** | **T** | **T** | **G** | **C** | **T** | **G** | **A** | **A** | **A** | **C** | **A** | **T** | **C** | **C** | **A** | **T** | **C** | **C** | **A** | **G** | **C** | **T** | **T** | **G** | **T** | **T** | **T** | **C** | **A** | **A** | **G** | **C** | **G** | **G** | **A** | **C** | **C** | **G** | **A** | **A** | **T** | **A** | **A** | **C** | **G** | **A** | **A** | **A** | **G** | **G** | **G** | **A** | **A** | **C** | **A** | **A** | **A** | **T** | **C** | **A** | **A** | **G** | **C** | **T** | **T** | **T** | **C** | **A** | **T** | **T** | **T** | **A** | **T** | **C** | **A** | **A** | **A** | **T** | **T** | **G** | **A** | **T** | **T** | **A** | **C** | **T** | **T** | **C** | **T** | **G** | **C** | **T** | **A** | **A** | **A** | **A** | **A** | **G** | **C** | **G** | **T** | **G** | **T** | **T** | **T** | **G** | **G** | **A** | **T** | **A** | **C** | **A** | **G** | **A** | **C** | **A** | **C** | **C** | **T** | **T** | **A** | **C** | **C** | **T** | **T** | **G** | **T** | **T** | **C** | **C** | **T** | **G** | **A** | **T** | **G** | **A** | **A** | **A** | **G** | **T** | **G** | **T** | **C** | **A** | **T** | **T** | **G** | **C** | **T** | **G** | **C** | **T** | **T** | **T** | **A** | **A** | **A** | **A** | **A** | **T** | **C** | **G** | **C** | **A** | **A** | **C** | **T** | **G** | **C** | **C** | **T** | **C** | **T** | **G** | **G** | **T** | **G** | **T** | **A** | **G** | **A** | **T** | **G** | **T** | **G** | **A** | **A** | **A** | **A** | **T** | **C** | **A** | **T** | **G** | **A** | **T** | **T** | **C** | **C** | **C** | **A** | **A** | **C** | **A** | **A** | **A** | **C** | **C** | **G** | **G** | **A** | **T** | **C** | **A** | **T** | **C** | **C** | **T** | **T** | **T** | **T** | **A** | **T** | **T** | **T** | **A** | **T** | **C** | **G** | **A** | **G** | **C** | **A** | **A** | **C** | **A** | **C** | **A** | **A** | **T** | **A** | **T** | **T** | **A** | **T** | **G** | **C** | **T** | **C** | **G** | **G** | **C** | **A** | **G** | **C** | **T** | **G** | **A** | **T** | **C** | **A** | **A** | **G** | **G** | **A** | **A** | **A** | **A** | **T** | **G** | **T** | **A** | **C** | **A** | **A** | **A** | **T** | **C** | **C** | **T** | **T** | **G** | **T** | **C** | **T** | **A** | **T** | **G** | **A** | **G** | **A** | **A** | **C** | **G** | **G** | **C** | **T** | **T** | **C** | **C** | **T** | **C** | **C** | **A** | **T** | **G** | **C** | **A** | **A** | **A** | **A** | **A** | **C** | **A** | **T** | **T** | **G** | **A** | **T** | **A** | **A** | **T** | **G** | **G** | **A** | **T** | **G** | **A** | **T** | **G** | **A** | **A** | **A** | **T** | **C** | **T** | **G** | **C** | **A** | **T** | **G** | **G** | **T** | **A** | **G** | **G** | **T** | **T** | **C** | **A** | **G** | **C** | **A** | **A** | **A** | **T** | **C** | **A** | **A** | **G** | **A** | **T** | **A** | **T** | **T** | **C** | **G** | **T** | **A** | **G** | **C** | **T** | **A** | **C** | **A** | **G** | **A** | **T** | **T** | **G** | **A** | **A** | **T** | **T** | **T** | **T** | **G** | **A** | **A** | **A** | **C** | **A** | **A** | **G** | **T** | **G** | **C** | **T** | **G** | **T** | **C** | **A** | **T** | **T** | **T** | **A** | **C** | **G** | **A** | **T** | **C** | **C** | **T** | **G** | **A** | **G** | **T** | **T** | **T** | **T** | **T** | **A** | **G** | **A** | **A** | **G** | **A** | **A** | **C** | **T** | **T** | **G** | **C** | **T** | **A** | **C** | **T** | **C** | **A** | **G** | **T** | **T** | **C** | **A** | **A** | **A** | **G** | **A** | **A** | **G** | **A** | **T** | **G** | **A** | **G** | **A** | **C** | **A** | **C** | **A** | **T** | **T** | **G** | **T** | **T** | **C** | **A** | **T** | **C** | **C** | **A** | **T** | **G** | **A** | **C** | **A** | **A** | **C** | **T** | **G** | **A** | **A** | **A** | **C** | **A** | **G** | **T** | **T** | **A** | **A** | **A** | **G** | **A** | **A** | **A** | **T** | **G** | **T** | **C** | **T** | **A** | **A** | **C** | **T** | **G** | **G** | **C** | **T** | **A** | **T** | **T** | **A** | **T** | **T** | **C** | **A** | **A** | **G** | **C** | **A** | **A** | **C** | **A** | **A** | **A** | **T** | **T** | **T** | **C** | **T** | **C** | **G** | **A** | **T** | **T** | **A** | **T** | **T** | **T** | **T** | **C** | **T** | **C** | **C** | **A** | **A** | **T** | **C** | **C** | **T** | **A** | **-** | **-** | **-** |
| **N59435F** | **-** | **-** | **-** | **-** | **-** | **-** | **-** | **-** | **-** | **-** | **-** | **-** | **-** | **-** | **-** | **-** | **-** | **-** | **-** | **-** | **-** | **-** | **-** | **-** | **-** | **-** | **-** | **-** | **-** | **-** | **-** | **-** | **-** | **-** | **-** | **-** | **-** | **-** | **-** | **-** | **-** | **-** | **-** | **-** | **-** | **-** | **-** | **-** | **-** | **-** | **-** | **-** | **-** | **-** | **-** | **-** | **-** | **-** | **-** | **-** | **-** | **-** | **-** | **-** | **-** | **-** | **-** | **-** | **-** | **A** | **A** | **A** | **C** | **C** | **C** | **C** | **G** | **A** | **G** | **A** | **T** | **G** | **T** | **A** | **G** | **C** | **A** | **G** | **C** | **C** | **A** | **T** | **T** | **T** | **G** | **G** | **G** | **C** | **A** | **T** | **G** | **G** | **C** | **T** | **C** | **T** | **T** | **A** | **G** | **T** | **A** | **T** | **T** | **G** | **T** | **T** | **T** | **G** | **C** | **C** | **C** | **T** | **T** | **C** | **C** | **T** | **G** | **G** | **G** | **G** | **T** | **G** | **G** | **G** | **T** | **T** | **T** | **T** | **G** | **T** | **A** | **T** | **T** | **A** | **T** | **A** | **T** | **T** | **T** | **A** | **T** | **T** | **T** | **T** | **T** | **C** | **G** | **G** | **A** | **C** | **G** | **T** | **G** | **G** | **A** | **T** | **T** | **A** | **A** | **C** | **G** | **G** | **A** | **T** | **A** | **A** | **G** | **A** | **A** | **A** | **A** | **A** | **A** | **T** | **T** | **C** | **T** | **A** | **T** | **C** | **T** | **C** | **C** | **G** | **A** | **C** | **A** | **A** | **A** | **G** | **T** | **G** | **A** | **C** | **T** | **T** | **G** | **A** | **A** | **A** | **G** | **A** | **A** | **T** | **T** | **A** | **G** | **A** | **A** | **A** | **A** | **C** | **T** | **T** | **T** | **C** | **A** | **G** | **T** | **C** | **T** | **T** | **T** | **T** | **A** | **G** | **A** | **G** | **G** | **A** | **G** | **A** | **T** | **A** | **C** | **C** | **A** | **T** | **T** | **G** | **A** | **A** | **C** | **A** | **T** | **T** | **A** | **C** | **G** | **A** | **T** | **C** | **C** | **T** | **G** | **A** | **C** | **A** | **T** | **G** | **G** | **G** | **C** | **G** | **A** | **T** | **A** | **A** | **A** | **G** | **A** | **C** | **A** | **A** | **A** | **C** | **A** | **G** | **C** | **A** | **A** | **T** | **T** | **T** | **G** | **T** | **C** | **G** | **A** | **C** | **T** | **T** | **C** | **T** | **T** | **C** | **T** | **C** | **T** | **T** | **C** | **A** | **T** | **T** | **A** | **A** | **A** | **T** | **C** | **G** | **T** | **A** | **T** | **G** | **C** | **C** | **G** | **C** | **T** | **G** | **A** | **C** | **A** | **A** | **G** | **A** | **A** | **T** | **G** | **A** | **A** | **T** | **T** | **C** | **T** | **G** | **T** | **C** | **A** | **C** | **T** | **C** | **T** | **T** | **C** | **T** | **C** | **A** | **C** | **A** | **G** | **A** | **C** | **G** | **G** | **A** | **C** | **A** | **A** | **G** | **A** | **G** | **A** | **A** | **A** | **T** | **T** | **G** | **G** | **A** | **T** | **T** | **C** | **A** | **C** | **T** | **G** | **C** | **T** | **T** | **C** | **A** | **A** | **G** | **A** | **T** | **C** | **T** | **A** | **A** | **A** | **A** | **A** | **A** | **A** | **G** | **C** | **C** | **A** | **A** | **A** | **C** | **A** | **T** | **T** | **C** | **G** | **A** | **T** | **C** | **C** | **A** | **T** | **A** | **T** | **C** | **G** | **A** | **A** | **T** | **A** | **T** | **T** | **A** | **C** | **G** | **C** | **A** | **T** | **T** | **T** | **G** | **T** | **G** | **A** | **C** | **A** | **G** | **A** | **T** | **A** | **A** | **T** | **A** | **T** | **C** | **G** | **G** | **C** | **C** | **A** | **G** | **C** | **A** | **A** | **G** | **T** | **C** | **T** | **T** | **A** | **C** | **A** | **T** | **G** | **T** | **T** | **T** | **T** | **A** | **G** | **A** | **A** | **G** | **A** | **A** | **A** | **A** | **A** | **G** | **C** | **C** | **G** | **C** | **A** | **G** | **A** | **A** | **G** | **G** | **C** | **G** | **T** | **G** | **G** | **A** | **A** | **G** | **T** | **T** | **C** | **G** | **A** | **A** | **T** | **A** | **T** | **T** | **A** | **T** | **A** | **T** | **G** | **A** | **T** | **G** | **C** | **A** | **T** | **T** | **T** | **G** | **G** | **C** | **T** | **C** | **T** | **C** | **A** | **T** | **G** | **G** | **C** | **A** | **C** | **A** | **A** | **A** | **A** | **G** | **C** | **A** | **A** | **A** | **A** | **G** | **A** | **T** | **T** | **T** | **C** | **A** | **A** | **T** | **C** | **G** | **T** | **C** | **T** | **A** | **A** | **T** | **C** | **A** | **A** | **A** | **A** | **A** | **T** | **G** | **G** | **T** | **G** | **G** | **A** | **C** | **A** | **T** | **G** | **T** | **C** | **C** | **A** | **T** | **A** | **C** | **A** | **T** | **T** | **T** | **G** | **T** | **T** | **A** | **C** | **C** | **T** | **C** | **A** | **C** | **A** | **A** | **A** | **A** | **G** | **G** | **C** | **A** | **T** | **T** | **A** | **C** | **T** | **T** | **C** | **G** | **T** | **T** | **T** | **C** | **C** | **G** | **A** | **T** | **T** | **G** | **A** | **A** | **T** | **T** | **A** | **C** | **C** | **A** | **T** | **G** | **A** | **T** | **C** | **A** | **C** | **C** | **G** | **A** | **A** | **A** | **A** | **A** | **T** | **C** | **G** | **T** | **T** | **G** | **T** | **G** | **A** | **T** | **C** | **G** | **A** | **T** | **G** | **G** | **A** | **A** | **A** | **G** | **A** | **T** | **T** | **A** | **G** | **T** | **T** | **A** | **T** | **A** | **C** | **C** | **G** | **G** | **T** | **G** | **G** | **T** | **T** | **T** | **C** | **A** | **A** | **T** | **A** | **T** | **T** | **G** | **C** | **C** | **A** | **A** | **T** | **C** | **A** | **A** | **T** | **A** | **T** | **G** | **T** | **A** | **A** | **A** | **T** | **A** | **C** | **A** | **A** | **C** | **A** | **A** | **A** | **A** | **A** | **A** | **A** | **T** | **T** | **C** | **G** | **G** | **C** | **T** | **A** | **T** | **T** | **G** | **G** | **C** | **G** | **C** | **G** | **A** | **T** | **A** | **C** | **G** | **C** | **A** | **T** | **A** | **T** | **A** | **C** | **G** | **G** | **A** | **T** | **T** | **T** | **T** | **C** | **G** | **G** | **T** | **G** | **C** | **C** | **G** | **C** | **T** | **T** | **C** | **T** | **T** | **C** | **T** | **T** | **T** | **G** | **C** | **T** | **C** | **C** | **A** | **G** | **C** | **T** | **T** | **C** | **G** | **C** | **T** | **T** | **C** | **T** | **T** | **A** | **A** | **C** | **A** | **G** | **A** | **C** | **T** | **G** | **G** | **A** | **A** | **C** | **G** | **T** | **C** | **T** | **C** | **G** | **G** | **T** | **A** | **C** | **C** | **T** | **G** | **A** | **A** | **G** | **A** | **A** | **A** | **A** | **A** | **A** | **A** | **G** | **G** | **T** | **C** | **G** | **G** | **C** | **T** | **A** | **T** | **C** | **A** | **T** | **T** | **T** | **G** | **A** | **A** | **T** | **T** | **A** | **T** | **T** | **T** | **C** | **T** | **T** | **T** | **A** | **A** | **A** | **A** | **A** | **A** | **G** | **C** | **A** | **G** | **A** | **T** | **C** | **G** | **A** | **G** | **A** | **T** | **G** | **A** | **A** | **T** | **C** | **T** | **A** | **A** | **G** | **C** | **T** | **T** | **G** | **C** | **T** | **G** | **A** | **T** | **A** | **C** | **A** | **T** | **C** | **C** | **A** | **T** | **C** | **C** | **A** | **G** | **C** | **T** | **T** | **G** | **T** | **T** | **T** | **C** | **A** | **A** | **G** | **C** | **G** | **G** | **A** | **C** | **C** | **G** | **A** | **A** | **T** | **A** | **A** | **C** | **G** | **A** | **A** | **A** | **G** | **G** | **G** | **A** | **A** | **C** | **A** | **A** | **A** | **T** | **C** | **A** | **A** | **G** | **C** | **T** | **T** | **T** | **C** | **A** | **T** | **T** | **T** | **A** | **T** | **C** | **A** | **A** | **A** | **T** | **T** | **G** | **A** | **T** | **T** | **A** | **C** | **T** | **T** | **C** | **T** | **G** | **C** | **T** | **A** | **A** | **A** | **A** | **A** | **A** | **C** | **G** | **T** | **G** | **T** | **T** | **T** | **G** | **G** | **A** | **T** | **A** | **C** | **A** | **G** | **A** | **C** | **A** | **C** | **C** | **T** | **T** | **A** | **C** | **C** | **T** | **T** | **G** | **T** | **T** | **C** | **C** | **T** | **G** | **A** | **T** | **G** | **A** | **A** | **A** | **G** | **T** | **G** | **T** | **C** | **A** | **T** | **T** | **G** | **C** | **T** | **G** | **C** | **T** | **T** | **T** | **A** | **A** | **A** | **A** | **A** | **T** | **C** | **G** | **C** | **A** | **A** | **C** | **T** | **G** | **C** | **C** | **T** | **C** | **T** | **G** | **G** | **T** | **G** | **T** | **A** | **G** | **A** | **T** | **G** | **T** | **G** | **A** | **A** | **A** | **A** | **T** | **C** | **A** | **T** | **G** | **A** | **T** | **T** | **C** | **C** | **C** | **A** | **A** | **C** | **A** | **A** | **A** | **C** | **C** | **G** | **G** | **A** | **T** | **C** | **A** | **T** | **C** | **C** | **T** | **T** | **T** | **T** | **A** | **T** | **T** | **T** | **A** | **T** | **C** | **G** | **A** | **G** | **C** | **A** | **A** | **C** | **A** | **C** | **A** | **A** | **T** | **A** | **T** | **T** | **A** | **T** | **G** | **C** | **T** | **C** | **G** | **G** | **C** | **A** | **G** | **C** | **T** | **G** | **A** | **T** | **C** | **A** | **A** | **G** | **G** | **A** | **A** | **A** | **A** | **T** | **G** | **T** | **A** | **C** | **A** | **A** | **A** | **T** | **C** | **C** | **T** | **T** | **G** | **T** | **C** | **T** | **A** | **T** | **G** | **A** | **G** | **A** | **A** | **C** | **G** | **G** | **C** | **T** | **T** | **C** | **C** | **T** | **C** | **C** | **A** | **T** | **G** | **C** | **A** | **A** | **A** | **A** | **A** | **C** | **A** | **T** | **T** | **G** | **A** | **T** | **A** | **A** | **T** | **G** | **G** | **A** | **T** | **G** | **A** | **T** | **G** | **A** | **A** | **A** | **T** | **C** | **T** | **G** | **C** | **A** | **T** | **G** | **G** | **T** | **A** | **G** | **G** | **T** | **T** | **C** | **A** | **G** | **C** | **A** | **A** | **A** | **T** | **C** | **A** | **A** | **G** | **A** | **T** | **A** | **T** | **T** | **C** | **G** | **T** | **A** | **G** | **C** | **T** | **A** | **C** | **C** | **G** | **A** | **T** | **T** | **G** | **A** | **A** | **T** | **T** | **T** | **T** | **G** | **A** | **A** | **A** | **C** | **A** | **A** | **G** | **T** | **G** | **C** | **T** | **G** | **T** | **C** | **A** | **T** | **T** | **T** | **A** | **C** | **G** | **A** | **T** | **C** | **C** | **T** | **G** | **A** | **G** | **T** | **T** | **T** | **T** | **T** | **A** | **G** | **A** | **A** | **G** | **A** | **A** | **C** | **T** | **T** | **G** | **C** | **T** | **A** | **C** | **T** | **C** | **A** | **G** | **T** | **T** | **C** | **A** | **A** | **A** | **G** | **A** | **A** | **G** | **A** | **T** | **G** | **A** | **G** | **A** | **C** | **A** | **C** | **A** | **T** | **T** | **G** | **T** | **T** | **C** | **A** | **T** | **C** | **C** | **A** | **T** | **G** | **A** | **C** | **A** | **A** | **C** | **T** | **G** | **A** | **A** | **A** | **C** | **A** | **G** | **T** | **C** | **A** | **A** | **G** | **G** | **A** | **A** | **A** | **T** | **G** | **T** | **C** | **T** | **A** | **A** | **C** | **T** | **G** | **G** | **C** | **T** | **A** | **T** | **T** | **A** | **T** | **T** | **C** | **A** | **A** | **G** | **C** | **A** | **A** | **C** | **A** | **A** | **A** | **T** | **T** | **T** | **C** | **T** | **C** | **G** | **A** | **T** | **T** | **A** | **T** | **T** | **T** | **T** | **C** | **T** | **C** | **C** | **A** | **A** | **T** | **C** | **C** | **T** | **A** | **-** | **-** | **-** |
| **N52636** | **-** | **-** | **-** | **-** | **-** | **-** | **-** | **-** | **-** | **-** | **-** | **-** | **-** | **-** | **-** | **-** | **-** | **-** | **-** | **-** | **-** | **-** | **-** | **-** | **-** | **-** | **-** | **-** | **-** | **-** | **-** | **-** | **-** | **-** | **-** | **-** | **-** | **-** | **-** | **-** | **-** | **-** | **-** | **-** | **-** | **-** | **-** | **-** | **-** | **-** | **-** | **-** | **-** | **-** | **-** | **-** | **-** | **-** | **-** | **-** | **-** | **-** | **-** | **-** | **-** | **-** | **-** | **-** | **-** | **A** | **A** | **A** | **C** | **C** | **C** | **C** | **G** | **A** | **G** | **A** | **T** | **G** | **T** | **A** | **G** | **C** | **A** | **G** | **C** | **C** | **A** | **T** | **T** | **T** | **G** | **G** | **G** | **C** | **A** | **T** | **G** | **G** | **C** | **T** | **C** | **T** | **T** | **A** | **G** | **T** | **A** | **T** | **T** | **G** | **T** | **T** | **T** | **G** | **C** | **C** | **C** | **T** | **T** | **C** | **C** | **T** | **G** | **G** | **G** | **G** | **T** | **G** | **G** | **G** | **C** | **T** | **T** | **T** | **G** | **T** | **A** | **T** | **T** | **A** | **T** | **A** | **T** | **T** | **T** | **A** | **T** | **T** | **T** | **T** | **T** | **C** | **G** | **G** | **A** | **C** | **G** | **T** | **G** | **G** | **A** | **T** | **T** | **A** | **A** | **C** | **G** | **G** | **A** | **T** | **A** | **A** | **G** | **A** | **A** | **A** | **A** | **A** | **A** | **T** | **T** | **C** | **T** | **A** | **T** | **C** | **T** | **C** | **C** | **G** | **A** | **C** | **A** | **A** | **A** | **G** | **T** | **G** | **A** | **C** | **T** | **T** | **G** | **A** | **A** | **A** | **G** | **A** | **A** | **T** | **T** | **A** | **G** | **A** | **A** | **A** | **A** | **C** | **T** | **T** | **T** | **C** | **A** | **G** | **T** | **C** | **T** | **T** | **T** | **T** | **A** | **G** | **A** | **G** | **G** | **A** | **G** | **A** | **T** | **A** | **C** | **C** | **A** | **T** | **T** | **G** | **A** | **A** | **C** | **A** | **T** | **T** | **A** | **C** | **G** | **A** | **T** | **C** | **C** | **T** | **G** | **A** | **C** | **A** | **T** | **G** | **G** | **G** | **C** | **G** | **A** | **T** | **A** | **A** | **A** | **G** | **A** | **C** | **A** | **A** | **A** | **C** | **A** | **G** | **C** | **A** | **A** | **T** | **T** | **T** | **G** | **T** | **T** | **G** | **A** | **C** | **T** | **T** | **C** | **T** | **T** | **C** | **T** | **C** | **T** | **T** | **C** | **A** | **T** | **T** | **A** | **A** | **A** | **T** | **C** | **G** | **T** | **A** | **T** | **G** | **C** | **C** | **G** | **C** | **T** | **G** | **A** | **C** | **A** | **A** | **G** | **A** | **A** | **T** | **G** | **A** | **A** | **T** | **T** | **C** | **T** | **G** | **T** | **C** | **A** | **C** | **T** | **C** | **T** | **T** | **C** | **T** | **C** | **A** | **C** | **A** | **G** | **A** | **C** | **G** | **G** | **A** | **C** | **A** | **A** | **G** | **A** | **G** | **A** | **A** | **A** | **T** | **T** | **G** | **G** | **A** | **T** | **T** | **C** | **A** | **C** | **T** | **G** | **C** | **T** | **T** | **C** | **A** | **A** | **G** | **A** | **T** | **C** | **T** | **A** | **A** | **A** | **A** | **A** | **A** | **A** | **G** | **C** | **C** | **A** | **A** | **A** | **C** | **A** | **T** | **T** | **C** | **G** | **A** | **T** | **C** | **C** | **A** | **T** | **A** | **T** | **C** | **G** | **A** | **A** | **T** | **A** | **T** | **T** | **A** | **C** | **G** | **C** | **A** | **T** | **T** | **T** | **G** | **T** | **G** | **A** | **C** | **A** | **G** | **A** | **T** | **A** | **A** | **T** | **A** | **T** | **C** | **G** | **G** | **C** | **C** | **A** | **G** | **C** | **A** | **A** | **G** | **T** | **C** | **T** | **T** | **A** | **C** | **A** | **T** | **G** | **T** | **T** | **T** | **T** | **A** | **G** | **A** | **A** | **G** | **A** | **A** | **A** | **A** | **A** | **G** | **C** | **C** | **G** | **C** | **A** | **G** | **A** | **A** | **G** | **G** | **C** | **G** | **T** | **G** | **G** | **A** | **A** | **G** | **T** | **T** | **C** | **G** | **A** | **A** | **T** | **A** | **T** | **T** | **A** | **T** | **A** | **T** | **G** | **A** | **T** | **G** | **C** | **A** | **T** | **T** | **T** | **G** | **G** | **C** | **T** | **C** | **T** | **C** | **A** | **T** | **G** | **G** | **C** | **A** | **C** | **A** | **A** | **A** | **A** | **G** | **C** | **A** | **A** | **A** | **A** | **G** | **A** | **T** | **T** | **T** | **C** | **A** | **A** | **T** | **C** | **G** | **T** | **C** | **T** | **A** | **A** | **T** | **C** | **A** | **A** | **A** | **A** | **A** | **T** | **G** | **G** | **T** | **G** | **G** | **A** | **C** | **A** | **T** | **G** | **T** | **C** | **C** | **A** | **T** | **A** | **C** | **A** | **T** | **T** | **T** | **G** | **T** | **T** | **A** | **C** | **C** | **T** | **C** | **A** | **C** | **A** | **A** | **A** | **A** | **G** | **G** | **C** | **A** | **T** | **T** | **A** | **C** | **T** | **T** | **C** | **G** | **T** | **T** | **T** | **C** | **C** | **G** | **A** | **T** | **T** | **G** | **A** | **A** | **T** | **T** | **A** | **C** | **C** | **A** | **T** | **G** | **A** | **T** | **C** | **A** | **C** | **C** | **G** | **A** | **A** | **A** | **A** | **A** | **T** | **C** | **G** | **T** | **T** | **G** | **T** | **G** | **A** | **T** | **C** | **G** | **A** | **T** | **G** | **G** | **A** | **A** | **A** | **G** | **A** | **T** | **T** | **A** | **G** | **T** | **T** | **A** | **T** | **A** | **C** | **C** | **G** | **G** | **T** | **G** | **G** | **T** | **T** | **T** | **C** | **A** | **A** | **T** | **A** | **T** | **T** | **G** | **C** | **C** | **A** | **A** | **T** | **C** | **A** | **A** | **T** | **A** | **T** | **G** | **T** | **A** | **A** | **A** | **T** | **A** | **T** | **A** | **A** | **C** | **A** | **A** | **A** | **A** | **A** | **A** | **A** | **T** | **T** | **C** | **G** | **G** | **C** | **T** | **A** | **T** | **T** | **G** | **G** | **C** | **G** | **C** | **G** | **A** | **T** | **A** | **C** | **G** | **C** | **A** | **T** | **A** | **T** | **A** | **C** | **G** | **G** | **A** | **T** | **T** | **T** | **T** | **C** | **G** | **G** | **T** | **G** | **C** | **C** | **G** | **C** | **T** | **T** | **C** | **T** | **T** | **C** | **T** | **T** | **T** | **G** | **C** | **T** | **C** | **C** | **A** | **G** | **C** | **T** | **T** | **C** | **G** | **C** | **T** | **T** | **C** | **T** | **T** | **A** | **A** | **C** | **A** | **G** | **A** | **C** | **T** | **G** | **G** | **A** | **A** | **C** | **G** | **T** | **C** | **T** | **C** | **G** | **G** | **T** | **A** | **C** | **C** | **T** | **G** | **A** | **A** | **G** | **A** | **A** | **A** | **A** | **A** | **A** | **A** | **G** | **G** | **T** | **C** | **G** | **G** | **C** | **T** | **A** | **T** | **C** | **A** | **T** | **T** | **T** | **G** | **A** | **A** | **T** | **T** | **A** | **T** | **T** | **T** | **C** | **T** | **T** | **T** | **A** | **A** | **A** | **A** | **A** | **A** | **G** | **C** | **A** | **G** | **A** | **T** | **C** | **G** | **A** | **G** | **A** | **T** | **G** | **A** | **A** | **T** | **C** | **T** | **A** | **A** | **G** | **C** | **T** | **T** | **G** | **C** | **T** | **G** | **A** | **T** | **A** | **C** | **A** | **T** | **C** | **C** | **A** | **T** | **C** | **C** | **A** | **G** | **C** | **T** | **T** | **G** | **T** | **T** | **T** | **C** | **A** | **A** | **G** | **C** | **G** | **G** | **A** | **C** | **C** | **G** | **A** | **A** | **T** | **A** | **A** | **C** | **G** | **A** | **A** | **A** | **G** | **G** | **G** | **A** | **A** | **C** | **A** | **A** | **A** | **T** | **C** | **A** | **A** | **G** | **C** | **T** | **T** | **T** | **C** | **A** | **T** | **T** | **T** | **A** | **T** | **C** | **A** | **A** | **A** | **T** | **T** | **G** | **A** | **T** | **T** | **A** | **C** | **T** | **T** | **C** | **T** | **G** | **C** | **T** | **A** | **A** | **A** | **A** | **A** | **A** | **C** | **G** | **T** | **G** | **T** | **T** | **T** | **G** | **G** | **A** | **T** | **A** | **C** | **A** | **G** | **A** | **C** | **A** | **C** | **C** | **T** | **T** | **A** | **C** | **C** | **T** | **T** | **G** | **T** | **T** | **C** | **C** | **T** | **G** | **A** | **T** | **G** | **A** | **A** | **A** | **G** | **T** | **G** | **T** | **C** | **A** | **T** | **T** | **G** | **C** | **T** | **G** | **C** | **T** | **T** | **T** | **A** | **A** | **A** | **A** | **A** | **T** | **C** | **G** | **C** | **A** | **A** | **C** | **T** | **G** | **C** | **C** | **T** | **C** | **T** | **G** | **G** | **T** | **G** | **T** | **A** | **G** | **A** | **T** | **G** | **T** | **G** | **A** | **A** | **A** | **A** | **T** | **C** | **A** | **T** | **G** | **A** | **T** | **T** | **C** | **C** | **C** | **A** | **A** | **C** | **A** | **A** | **A** | **C** | **C** | **G** | **G** | **A** | **T** | **C** | **A** | **T** | **C** | **C** | **T** | **T** | **T** | **T** | **A** | **T** | **T** | **T** | **A** | **T** | **C** | **G** | **A** | **G** | **C** | **A** | **A** | **C** | **A** | **C** | **A** | **A** | **T** | **A** | **T** | **T** | **A** | **T** | **G** | **C** | **T** | **C** | **G** | **G** | **C** | **A** | **G** | **C** | **T** | **G** | **A** | **T** | **C** | **A** | **A** | **G** | **G** | **A** | **A** | **A** | **A** | **T** | **G** | **T** | **A** | **C** | **A** | **A** | **A** | **T** | **C** | **C** | **T** | **T** | **G** | **T** | **C** | **T** | **A** | **T** | **G** | **A** | **G** | **A** | **A** | **C** | **G** | **G** | **C** | **T** | **T** | **C** | **C** | **T** | **C** | **C** | **A** | **T** | **G** | **C** | **A** | **A** | **A** | **A** | **A** | **C** | **A** | **T** | **T** | **G** | **A** | **T** | **A** | **A** | **T** | **G** | **G** | **A** | **T** | **G** | **A** | **T** | **G** | **A** | **A** | **A** | **T** | **C** | **T** | **G** | **C** | **A** | **T** | **G** | **G** | **T** | **A** | **G** | **G** | **T** | **T** | **C** | **A** | **G** | **C** | **A** | **A** | **A** | **T** | **C** | **A** | **A** | **G** | **A** | **T** | **A** | **T** | **T** | **C** | **G** | **T** | **A** | **G** | **C** | **T** | **A** | **C** | **C** | **G** | **A** | **T** | **T** | **G** | **A** | **A** | **T** | **T** | **T** | **T** | **G** | **A** | **A** | **A** | **C** | **A** | **A** | **G** | **T** | **G** | **C** | **T** | **G** | **T** | **C** | **A** | **T** | **T** | **T** | **A** | **C** | **G** | **A** | **T** | **C** | **C** | **T** | **G** | **A** | **G** | **T** | **T** | **T** | **T** | **T** | **A** | **G** | **A** | **A** | **G** | **A** | **A** | **C** | **T** | **T** | **G** | **C** | **T** | **A** | **C** | **T** | **C** | **A** | **G** | **T** | **T** | **C** | **A** | **A** | **A** | **G** | **A** | **A** | **G** | **A** | **T** | **G** | **A** | **G** | **A** | **C** | **A** | **C** | **A** | **T** | **T** | **G** | **T** | **T** | **C** | **A** | **T** | **C** | **C** | **A** | **T** | **G** | **A** | **C** | **A** | **A** | **C** | **T** | **G** | **A** | **A** | **A** | **C** | **A** | **G** | **T** | **C** | **A** | **A** | **G** | **G** | **A** | **A** | **A** | **T** | **G** | **T** | **C** | **T** | **A** | **A** | **C** | **T** | **G** | **G** | **C** | **T** | **A** | **T** | **T** | **A** | **T** | **T** | **C** | **A** | **A** | **G** | **C** | **A** | **A** | **C** | **A** | **A** | **A** | **T** | **T** | **T** | **C** | **T** | **C** | **G** | **A** | **T** | **T** | **A** | **T** | **T** | **T** | **T** | **C** | **T** | **C** | **C** | **A** | **A** | **T** | **C** | **C** | **T** | **A** | **-** | **-** | **-** |
| **N60519F** | **-** | **-** | **-** | **-** | **-** | **-** | **-** | **-** | **-** | **-** | **-** | **-** | **-** | **-** | **-** | **-** | **-** | **-** | **-** | **-** | **-** | **-** | **-** | **-** | **-** | **-** | **-** | **-** | **-** | **-** | **-** | **-** | **-** | **-** | **-** | **-** | **-** | **-** | **-** | **-** | **-** | **-** | **-** | **-** | **-** | **-** | **-** | **-** | **-** | **-** | **-** | **-** | **-** | **-** | **-** | **-** | **-** | **-** | **-** | **-** | **-** | **-** | **-** | **-** | **-** | **-** | **-** | **-** | **-** | **A** | **A** | **A** | **C** | **C** | **C** | **C** | **G** | **A** | **G** | **A** | **T** | **G** | **T** | **A** | **G** | **C** | **A** | **G** | **C** | **C** | **A** | **T** | **T** | **T** | **G** | **G** | **G** | **C** | **A** | **T** | **G** | **G** | **C** | **T** | **C** | **T** | **T** | **A** | **G** | **T** | **A** | **T** | **T** | **G** | **T** | **T** | **T** | **G** | **C** | **C** | **C** | **T** | **T** | **C** | **C** | **T** | **G** | **G** | **G** | **G** | **T** | **G** | **G** | **G** | **T** | **T** | **T** | **T** | **G** | **T** | **A** | **T** | **T** | **A** | **T** | **A** | **T** | **T** | **T** | **A** | **T** | **T** | **T** | **T** | **T** | **C** | **G** | **G** | **A** | **C** | **G** | **T** | **G** | **G** | **A** | **T** | **T** | **A** | **A** | **C** | **G** | **G** | **A** | **T** | **A** | **A** | **G** | **A** | **A** | **A** | **A** | **A** | **A** | **T** | **T** | **C** | **T** | **A** | **T** | **C** | **T** | **C** | **C** | **G** | **A** | **C** | **A** | **A** | **A** | **G** | **T** | **G** | **A** | **C** | **T** | **T** | **G** | **A** | **A** | **A** | **G** | **A** | **A** | **T** | **T** | **A** | **G** | **A** | **A** | **A** | **A** | **C** | **T** | **T** | **T** | **C** | **A** | **G** | **T** | **C** | **T** | **T** | **T** | **T** | **A** | **G** | **A** | **G** | **G** | **G** | **G** | **A** | **T** | **A** | **C** | **C** | **A** | **T** | **T** | **G** | **A** | **A** | **C** | **A** | **T** | **T** | **A** | **C** | **G** | **A** | **T** | **C** | **C** | **T** | **G** | **A** | **C** | **A** | **T** | **G** | **G** | **G** | **C** | **G** | **A** | **T** | **A** | **A** | **A** | **G** | **A** | **C** | **A** | **A** | **A** | **C** | **A** | **G** | **C** | **A** | **A** | **T** | **T** | **T** | **G** | **T** | **T** | **G** | **A** | **C** | **T** | **T** | **C** | **T** | **T** | **C** | **T** | **C** | **T** | **T** | **C** | **A** | **T** | **T** | **A** | **A** | **A** | **T** | **C** | **G** | **T** | **A** | **T** | **G** | **C** | **C** | **G** | **C** | **T** | **G** | **A** | **C** | **A** | **A** | **G** | **A** | **A** | **T** | **G** | **A** | **A** | **T** | **T** | **C** | **T** | **G** | **T** | **C** | **A** | **C** | **T** | **C** | **T** | **T** | **C** | **T** | **C** | **A** | **C** | **A** | **G** | **A** | **C** | **G** | **G** | **A** | **C** | **A** | **A** | **G** | **A** | **G** | **A** | **A** | **A** | **T** | **T** | **G** | **G** | **A** | **T** | **T** | **C** | **A** | **C** | **T** | **G** | **C** | **T** | **T** | **C** | **A** | **A** | **G** | **A** | **T** | **C** | **T** | **A** | **A** | **A** | **A** | **A** | **A** | **A** | **G** | **C** | **C** | **A** | **A** | **A** | **C** | **A** | **T** | **T** | **C** | **G** | **A** | **T** | **C** | **C** | **A** | **T** | **A** | **T** | **C** | **G** | **A** | **A** | **T** | **A** | **T** | **T** | **A** | **C** | **G** | **C** | **A** | **T** | **T** | **T** | **G** | **T** | **G** | **A** | **C** | **A** | **G** | **A** | **T** | **A** | **A** | **T** | **A** | **T** | **C** | **G** | **G** | **C** | **C** | **A** | **G** | **C** | **A** | **A** | **G** | **T** | **C** | **T** | **T** | **A** | **C** | **A** | **T** | **G** | **T** | **T** | **T** | **T** | **A** | **G** | **A** | **A** | **G** | **A** | **A** | **A** | **A** | **A** | **G** | **C** | **C** | **G** | **C** | **A** | **G** | **A** | **A** | **G** | **G** | **C** | **G** | **T** | **G** | **G** | **A** | **A** | **G** | **T** | **T** | **C** | **G** | **A** | **A** | **T** | **A** | **T** | **T** | **A** | **T** | **A** | **T** | **G** | **A** | **T** | **G** | **C** | **A** | **T** | **T** | **T** | **G** | **G** | **C** | **T** | **C** | **T** | **C** | **A** | **T** | **G** | **G** | **C** | **A** | **C** | **A** | **A** | **A** | **A** | **G** | **C** | **A** | **A** | **A** | **A** | **G** | **A** | **T** | **T** | **T** | **C** | **A** | **A** | **T** | **C** | **G** | **T** | **C** | **T** | **A** | **A** | **T** | **C** | **A** | **A** | **A** | **A** | **A** | **T** | **G** | **G** | **T** | **G** | **G** | **A** | **C** | **A** | **T** | **G** | **T** | **C** | **C** | **A** | **T** | **A** | **C** | **A** | **T** | **T** | **T** | **G** | **T** | **T** | **A** | **C** | **C** | **T** | **C** | **A** | **C** | **A** | **A** | **A** | **A** | **G** | **G** | **C** | **A** | **T** | **T** | **A** | **C** | **T** | **T** | **C** | **G** | **T** | **T** | **T** | **C** | **C** | **G** | **A** | **T** | **T** | **G** | **A** | **A** | **T** | **T** | **A** | **C** | **C** | **A** | **T** | **G** | **A** | **T** | **C** | **A** | **C** | **C** | **G** | **A** | **A** | **A** | **A** | **A** | **T** | **C** | **G** | **T** | **T** | **G** | **T** | **G** | **A** | **T** | **C** | **G** | **A** | **T** | **G** | **G** | **A** | **A** | **A** | **G** | **A** | **T** | **T** | **A** | **G** | **T** | **T** | **A** | **T** | **A** | **C** | **C** | **G** | **G** | **T** | **G** | **G** | **T** | **T** | **T** | **C** | **A** | **A** | **T** | **A** | **T** | **T** | **G** | **C** | **C** | **A** | **A** | **T** | **C** | **A** | **A** | **T** | **A** | **T** | **G** | **T** | **A** | **A** | **A** | **T** | **A** | **C** | **A** | **A** | **C** | **A** | **A** | **A** | **A** | **A** | **A** | **A** | **T** | **T** | **C** | **G** | **G** | **C** | **T** | **A** | **T** | **T** | **G** | **G** | **C** | **G** | **C** | **G** | **A** | **T** | **A** | **C** | **G** | **C** | **A** | **T** | **A** | **T** | **A** | **C** | **G** | **G** | **A** | **T** | **T** | **T** | **T** | **C** | **G** | **G** | **T** | **G** | **C** | **C** | **G** | **C** | **T** | **T** | **C** | **T** | **T** | **C** | **T** | **T** | **T** | **G** | **C** | **T** | **C** | **C** | **A** | **G** | **C** | **T** | **T** | **C** | **G** | **C** | **T** | **T** | **C** | **T** | **T** | **A** | **A** | **C** | **A** | **G** | **A** | **C** | **T** | **G** | **G** | **A** | **A** | **C** | **G** | **T** | **C** | **T** | **C** | **G** | **G** | **T** | **A** | **C** | **C** | **T** | **G** | **A** | **A** | **G** | **A** | **A** | **A** | **A** | **A** | **A** | **A** | **G** | **G** | **T** | **C** | **G** | **G** | **C** | **T** | **A** | **T** | **C** | **A** | **T** | **T** | **T** | **G** | **A** | **A** | **T** | **T** | **A** | **T** | **T** | **T** | **C** | **T** | **T** | **T** | **A** | **A** | **A** | **A** | **A** | **A** | **G** | **C** | **A** | **G** | **A** | **T** | **C** | **G** | **A** | **G** | **A** | **T** | **G** | **A** | **A** | **T** | **C** | **T** | **A** | **A** | **G** | **C** | **T** | **T** | **G** | **C** | **T** | **G** | **A** | **T** | **A** | **C** | **A** | **T** | **C** | **C** | **A** | **T** | **C** | **C** | **A** | **G** | **C** | **T** | **T** | **G** | **T** | **T** | **T** | **C** | **A** | **A** | **G** | **C** | **G** | **G** | **A** | **C** | **C** | **G** | **A** | **A** | **T** | **A** | **A** | **C** | **G** | **A** | **A** | **A** | **G** | **G** | **G** | **A** | **A** | **C** | **A** | **A** | **A** | **T** | **C** | **A** | **A** | **G** | **C** | **T** | **T** | **T** | **C** | **A** | **T** | **T** | **T** | **A** | **T** | **C** | **A** | **A** | **A** | **T** | **T** | **G** | **A** | **T** | **T** | **A** | **C** | **T** | **T** | **C** | **T** | **G** | **C** | **T** | **A** | **A** | **A** | **A** | **A** | **A** | **C** | **G** | **T** | **G** | **T** | **T** | **T** | **G** | **G** | **A** | **T** | **A** | **C** | **A** | **G** | **A** | **C** | **A** | **C** | **C** | **T** | **T** | **A** | **C** | **C** | **T** | **T** | **G** | **T** | **T** | **C** | **C** | **T** | **G** | **A** | **T** | **G** | **A** | **A** | **A** | **G** | **T** | **G** | **T** | **C** | **A** | **T** | **T** | **G** | **C** | **T** | **G** | **C** | **T** | **T** | **T** | **A** | **A** | **A** | **A** | **A** | **T** | **C** | **G** | **C** | **A** | **A** | **C** | **T** | **G** | **C** | **C** | **T** | **C** | **T** | **G** | **G** | **T** | **G** | **T** | **A** | **G** | **A** | **T** | **G** | **T** | **G** | **A** | **A** | **A** | **A** | **T** | **C** | **A** | **T** | **G** | **A** | **T** | **T** | **C** | **C** | **C** | **A** | **A** | **C** | **A** | **A** | **A** | **C** | **C** | **G** | **G** | **A** | **T** | **C** | **A** | **T** | **C** | **C** | **T** | **T** | **T** | **T** | **A** | **T** | **T** | **T** | **A** | **T** | **C** | **G** | **A** | **G** | **C** | **A** | **A** | **C** | **A** | **C** | **A** | **A** | **T** | **A** | **T** | **T** | **A** | **T** | **G** | **C** | **T** | **C** | **G** | **G** | **C** | **A** | **G** | **C** | **T** | **G** | **A** | **T** | **C** | **A** | **A** | **G** | **G** | **A** | **A** | **A** | **A** | **T** | **G** | **T** | **A** | **C** | **A** | **A** | **A** | **T** | **C** | **C** | **T** | **T** | **G** | **T** | **C** | **T** | **A** | **T** | **G** | **A** | **G** | **A** | **A** | **C** | **G** | **G** | **C** | **T** | **T** | **C** | **C** | **T** | **C** | **C** | **A** | **T** | **G** | **C** | **A** | **A** | **A** | **A** | **A** | **C** | **A** | **T** | **T** | **G** | **A** | **T** | **A** | **A** | **T** | **G** | **G** | **A** | **T** | **G** | **A** | **T** | **G** | **A** | **A** | **A** | **T** | **C** | **T** | **G** | **C** | **A** | **T** | **G** | **G** | **T** | **A** | **G** | **G** | **T** | **T** | **C** | **A** | **G** | **C** | **A** | **A** | **A** | **T** | **C** | **A** | **A** | **G** | **A** | **T** | **A** | **T** | **T** | **C** | **G** | **T** | **A** | **G** | **C** | **T** | **A** | **C** | **C** | **G** | **A** | **T** | **T** | **G** | **A** | **A** | **T** | **T** | **T** | **T** | **G** | **A** | **A** | **A** | **C** | **A** | **A** | **G** | **T** | **G** | **C** | **T** | **G** | **T** | **C** | **A** | **T** | **T** | **T** | **A** | **C** | **G** | **A** | **T** | **C** | **C** | **T** | **G** | **A** | **G** | **T** | **T** | **T** | **T** | **T** | **A** | **G** | **A** | **A** | **G** | **A** | **A** | **C** | **T** | **T** | **G** | **C** | **T** | **A** | **C** | **T** | **C** | **A** | **G** | **T** | **T** | **C** | **A** | **A** | **A** | **G** | **A** | **A** | **G** | **A** | **T** | **G** | **A** | **G** | **A** | **C** | **A** | **C** | **A** | **T** | **T** | **G** | **T** | **T** | **C** | **A** | **T** | **C** | **C** | **A** | **T** | **G** | **A** | **C** | **A** | **A** | **C** | **T** | **G** | **A** | **A** | **A** | **C** | **A** | **G** | **T** | **C** | **A** | **A** | **G** | **G** | **A** | **A** | **A** | **T** | **G** | **T** | **C** | **T** | **A** | **A** | **C** | **T** | **G** | **G** | **C** | **T** | **A** | **T** | **T** | **A** | **T** | **T** | **C** | **A** | **A** | **G** | **C** | **A** | **A** | **C** | **A** | **A** | **A** | **T** | **T** | **T** | **C** | **T** | **C** | **G** | **A** | **T** | **T** | **A** | **T** | **T** | **T** | **T** | **C** | **T** | **C** | **C** | **A** | **A** | **T** | **C** | **C** | **T** | **A** | **-** | **-** | **-** |
| **N52824** | **-** | **-** | **-** | **-** | **-** | **-** | **-** | **-** | **-** | **-** | **-** | **-** | **-** | **-** | **-** | **-** | **-** | **-** | **-** | **-** | **-** | **-** | **-** | **-** | **-** | **-** | **-** | **-** | **-** | **-** | **-** | **-** | **-** | **-** | **-** | **-** | **-** | **-** | **-** | **-** | **-** | **-** | **-** | **-** | **-** | **-** | **-** | **-** | **-** | **-** | **-** | **-** | **-** | **-** | **-** | **-** | **-** | **-** | **-** | **-** | **-** | **-** | **-** | **-** | **-** | **-** | **-** | **-** | **-** | **A** | **A** | **A** | **C** | **C** | **C** | **C** | **G** | **A** | **G** | **A** | **T** | **G** | **T** | **A** | **G** | **C** | **A** | **G** | **C** | **C** | **A** | **T** | **T** | **T** | **G** | **G** | **G** | **C** | **A** | **T** | **G** | **G** | **C** | **T** | **C** | **T** | **T** | **A** | **G** | **T** | **A** | **T** | **T** | **G** | **T** | **T** | **T** | **G** | **C** | **C** | **C** | **T** | **T** | **C** | **C** | **T** | **G** | **G** | **G** | **G** | **T** | **G** | **G** | **G** | **C** | **T** | **T** | **T** | **G** | **T** | **A** | **T** | **T** | **A** | **T** | **A** | **T** | **T** | **T** | **A** | **T** | **T** | **T** | **T** | **T** | **C** | **G** | **G** | **A** | **C** | **G** | **T** | **G** | **G** | **A** | **T** | **T** | **A** | **A** | **C** | **G** | **G** | **A** | **T** | **A** | **A** | **G** | **A** | **A** | **A** | **A** | **A** | **A** | **T** | **T** | **C** | **T** | **A** | **T** | **C** | **T** | **C** | **C** | **G** | **A** | **C** | **A** | **A** | **A** | **G** | **T** | **G** | **A** | **C** | **T** | **T** | **G** | **A** | **A** | **A** | **G** | **A** | **A** | **T** | **T** | **A** | **G** | **A** | **A** | **A** | **A** | **C** | **T** | **T** | **T** | **C** | **A** | **G** | **T** | **C** | **T** | **T** | **T** | **T** | **A** | **G** | **A** | **G** | **G** | **A** | **G** | **A** | **T** | **A** | **A** | **C** | **A** | **T** | **T** | **G** | **A** | **A** | **C** | **A** | **T** | **T** | **A** | **C** | **G** | **A** | **T** | **C** | **C** | **T** | **G** | **A** | **C** | **A** | **T** | **G** | **G** | **G** | **C** | **G** | **A** | **T** | **A** | **A** | **A** | **G** | **A** | **C** | **A** | **A** | **A** | **C** | **A** | **G** | **C** | **A** | **A** | **T** | **T** | **T** | **G** | **T** | **T** | **G** | **A** | **C** | **T** | **T** | **C** | **T** | **T** | **C** | **T** | **C** | **T** | **T** | **C** | **A** | **T** | **T** | **A** | **A** | **A** | **T** | **C** | **G** | **T** | **A** | **T** | **G** | **C** | **C** | **G** | **C** | **T** | **G** | **A** | **C** | **A** | **A** | **G** | **A** | **A** | **T** | **G** | **A** | **A** | **T** | **T** | **C** | **T** | **G** | **T** | **C** | **A** | **C** | **T** | **C** | **T** | **T** | **C** | **T** | **C** | **A** | **C** | **A** | **G** | **A** | **C** | **G** | **G** | **A** | **C** | **A** | **A** | **G** | **A** | **G** | **A** | **A** | **A** | **T** | **T** | **G** | **G** | **A** | **T** | **T** | **C** | **A** | **C** | **T** | **G** | **C** | **T** | **T** | **C** | **A** | **A** | **G** | **A** | **T** | **C** | **T** | **A** | **A** | **A** | **A** | **A** | **A** | **A** | **G** | **C** | **C** | **A** | **A** | **A** | **C** | **A** | **T** | **T** | **C** | **G** | **A** | **T** | **C** | **C** | **A** | **T** | **A** | **T** | **C** | **G** | **A** | **A** | **T** | **A** | **T** | **T** | **A** | **C** | **G** | **C** | **A** | **T** | **T** | **T** | **G** | **T** | **G** | **A** | **C** | **A** | **G** | **A** | **T** | **A** | **A** | **T** | **A** | **T** | **C** | **G** | **G** | **C** | **C** | **A** | **G** | **C** | **A** | **A** | **G** | **T** | **C** | **T** | **T** | **A** | **C** | **A** | **T** | **G** | **T** | **T** | **T** | **T** | **A** | **G** | **A** | **A** | **G** | **A** | **A** | **A** | **A** | **A** | **G** | **C** | **C** | **G** | **C** | **A** | **G** | **A** | **A** | **G** | **G** | **C** | **G** | **T** | **G** | **G** | **A** | **A** | **G** | **T** | **T** | **C** | **G** | **A** | **A** | **T** | **A** | **T** | **T** | **A** | **T** | **A** | **T** | **G** | **A** | **T** | **G** | **C** | **A** | **T** | **T** | **T** | **G** | **G** | **C** | **T** | **C** | **T** | **C** | **A** | **T** | **G** | **G** | **C** | **A** | **C** | **A** | **A** | **A** | **A** | **G** | **C** | **A** | **A** | **A** | **A** | **G** | **A** | **T** | **T** | **T** | **C** | **A** | **A** | **T** | **C** | **G** | **T** | **C** | **T** | **A** | **A** | **T** | **C** | **A** | **A** | **A** | **A** | **A** | **T** | **G** | **G** | **T** | **G** | **G** | **A** | **C** | **A** | **T** | **G** | **T** | **C** | **C** | **A** | **T** | **A** | **C** | **A** | **T** | **T** | **T** | **G** | **T** | **T** | **A** | **C** | **C** | **T** | **C** | **A** | **C** | **A** | **A** | **A** | **A** | **G** | **G** | **C** | **A** | **T** | **T** | **A** | **C** | **T** | **T** | **C** | **G** | **T** | **T** | **T** | **C** | **C** | **G** | **A** | **T** | **T** | **G** | **A** | **A** | **T** | **T** | **A** | **C** | **C** | **A** | **T** | **G** | **A** | **T** | **C** | **A** | **C** | **C** | **G** | **A** | **A** | **A** | **A** | **A** | **T** | **C** | **G** | **T** | **T** | **G** | **T** | **G** | **A** | **T** | **C** | **G** | **A** | **T** | **G** | **G** | **A** | **A** | **A** | **G** | **A** | **T** | **T** | **A** | **G** | **T** | **T** | **A** | **T** | **A** | **C** | **C** | **G** | **G** | **T** | **G** | **G** | **T** | **T** | **T** | **C** | **A** | **A** | **T** | **A** | **T** | **T** | **G** | **C** | **C** | **A** | **A** | **T** | **C** | **A** | **A** | **T** | **A** | **T** | **G** | **T** | **A** | **A** | **A** | **T** | **A** | **C** | **A** | **A** | **C** | **A** | **A** | **A** | **A** | **A** | **A** | **A** | **T** | **T** | **C** | **G** | **G** | **C** | **T** | **A** | **T** | **T** | **G** | **G** | **C** | **G** | **C** | **G** | **A** | **T** | **A** | **C** | **G** | **C** | **A** | **T** | **A** | **T** | **A** | **C** | **G** | **G** | **A** | **T** | **T** | **T** | **T** | **C** | **G** | **G** | **T** | **G** | **C** | **C** | **G** | **C** | **T** | **T** | **C** | **T** | **T** | **C** | **T** | **T** | **T** | **G** | **C** | **T** | **C** | **C** | **A** | **G** | **C** | **T** | **T** | **C** | **G** | **C** | **T** | **T** | **C** | **T** | **T** | **A** | **A** | **C** | **A** | **G** | **A** | **C** | **T** | **G** | **G** | **A** | **A** | **C** | **G** | **T** | **C** | **T** | **C** | **G** | **G** | **T** | **A** | **C** | **C** | **T** | **G** | **A** | **A** | **G** | **A** | **A** | **A** | **A** | **A** | **A** | **A** | **G** | **G** | **T** | **C** | **G** | **G** | **C** | **T** | **A** | **T** | **C** | **A** | **T** | **T** | **T** | **G** | **A** | **A** | **T** | **T** | **A** | **T** | **T** | **T** | **C** | **T** | **T** | **T** | **A** | **A** | **A** | **A** | **A** | **A** | **G** | **C** | **A** | **G** | **A** | **T** | **C** | **G** | **A** | **G** | **A** | **T** | **G** | **A** | **A** | **T** | **C** | **T** | **A** | **A** | **G** | **C** | **T** | **T** | **G** | **C** | **T** | **G** | **A** | **T** | **A** | **C** | **A** | **T** | **C** | **C** | **A** | **T** | **C** | **C** | **A** | **G** | **C** | **T** | **T** | **G** | **T** | **T** | **T** | **C** | **A** | **A** | **G** | **C** | **G** | **G** | **A** | **C** | **C** | **G** | **A** | **A** | **T** | **A** | **A** | **C** | **G** | **A** | **A** | **A** | **G** | **G** | **G** | **A** | **A** | **C** | **A** | **A** | **A** | **T** | **C** | **A** | **A** | **G** | **C** | **T** | **T** | **T** | **C** | **A** | **T** | **T** | **T** | **A** | **T** | **C** | **A** | **A** | **A** | **T** | **T** | **G** | **A** | **T** | **T** | **A** | **C** | **T** | **T** | **C** | **T** | **G** | **C** | **T** | **A** | **A** | **A** | **A** | **A** | **A** | **C** | **G** | **T** | **G** | **T** | **T** | **T** | **G** | **G** | **A** | **T** | **A** | **C** | **A** | **G** | **A** | **C** | **A** | **C** | **C** | **T** | **T** | **A** | **C** | **C** | **T** | **T** | **G** | **T** | **T** | **C** | **C** | **T** | **G** | **A** | **T** | **G** | **A** | **A** | **A** | **G** | **T** | **G** | **T** | **C** | **A** | **T** | **T** | **G** | **C** | **T** | **G** | **C** | **T** | **T** | **T** | **A** | **A** | **A** | **A** | **A** | **T** | **C** | **G** | **C** | **A** | **A** | **C** | **T** | **G** | **C** | **C** | **T** | **C** | **T** | **G** | **G** | **T** | **G** | **T** | **A** | **G** | **A** | **T** | **G** | **T** | **G** | **A** | **A** | **A** | **A** | **T** | **C** | **A** | **T** | **G** | **A** | **T** | **T** | **C** | **C** | **C** | **A** | **A** | **C** | **A** | **A** | **A** | **C** | **C** | **G** | **G** | **A** | **T** | **C** | **A** | **T** | **C** | **C** | **T** | **T** | **T** | **T** | **A** | **T** | **T** | **T** | **A** | **T** | **C** | **G** | **A** | **G** | **C** | **A** | **A** | **C** | **A** | **C** | **A** | **A** | **T** | **A** | **T** | **T** | **A** | **T** | **G** | **C** | **T** | **C** | **G** | **G** | **C** | **A** | **G** | **C** | **T** | **G** | **A** | **T** | **C** | **A** | **A** | **G** | **G** | **A** | **A** | **A** | **A** | **T** | **G** | **T** | **A** | **C** | **A** | **A** | **A** | **T** | **C** | **C** | **T** | **T** | **G** | **T** | **C** | **T** | **A** | **T** | **G** | **A** | **G** | **A** | **A** | **C** | **G** | **G** | **C** | **T** | **T** | **C** | **C** | **T** | **C** | **C** | **A** | **T** | **G** | **C** | **A** | **A** | **A** | **A** | **A** | **C** | **A** | **T** | **T** | **G** | **A** | **T** | **A** | **A** | **T** | **G** | **G** | **A** | **T** | **G** | **A** | **T** | **G** | **A** | **A** | **A** | **T** | **C** | **T** | **G** | **C** | **A** | **T** | **G** | **G** | **T** | **A** | **G** | **G** | **T** | **T** | **C** | **A** | **G** | **C** | **A** | **A** | **A** | **T** | **C** | **A** | **A** | **G** | **A** | **T** | **A** | **T** | **T** | **C** | **G** | **T** | **A** | **G** | **C** | **T** | **A** | **C** | **C** | **G** | **A** | **T** | **T** | **G** | **A** | **A** | **T** | **T** | **T** | **T** | **G** | **A** | **A** | **A** | **C** | **A** | **A** | **G** | **T** | **G** | **C** | **T** | **G** | **T** | **C** | **A** | **T** | **T** | **T** | **A** | **C** | **G** | **A** | **T** | **C** | **C** | **T** | **G** | **A** | **G** | **T** | **T** | **T** | **T** | **T** | **A** | **G** | **A** | **A** | **G** | **A** | **A** | **C** | **T** | **T** | **G** | **C** | **T** | **A** | **C** | **T** | **C** | **A** | **G** | **T** | **T** | **C** | **A** | **A** | **A** | **G** | **A** | **A** | **G** | **A** | **T** | **G** | **A** | **G** | **A** | **C** | **A** | **C** | **A** | **T** | **T** | **G** | **T** | **T** | **C** | **A** | **T** | **C** | **C** | **A** | **T** | **G** | **A** | **C** | **A** | **A** | **C** | **T** | **G** | **A** | **A** | **A** | **C** | **A** | **G** | **T** | **C** | **A** | **A** | **G** | **G** | **A** | **A** | **A** | **T** | **G** | **T** | **C** | **T** | **A** | **A** | **C** | **T** | **G** | **G** | **C** | **T** | **A** | **T** | **T** | **A** | **T** | **T** | **C** | **A** | **A** | **G** | **C** | **A** | **A** | **C** | **A** | **A** | **A** | **T** | **T** | **T** | **C** | **T** | **C** | **G** | **A** | **T** | **T** | **A** | **T** | **T** | **T** | **T** | **C** | **T** | **C** | **C** | **A** | **A** | **T** | **C** | **C** | **T** | **A** | **-** | **-** | **-** |
| **N60365F** | **-** | **-** | **-** | **-** | **-** | **-** | **-** | **-** | **-** | **-** | **-** | **-** | **-** | **-** | **-** | **-** | **-** | **-** | **-** | **-** | **-** | **-** | **-** | **-** | **-** | **-** | **-** | **-** | **-** | **-** | **-** | **-** | **-** | **-** | **-** | **-** | **-** | **-** | **-** | **-** | **-** | **-** | **-** | **-** | **-** | **-** | **-** | **-** | **-** | **-** | **-** | **-** | **-** | **-** | **-** | **-** | **-** | **-** | **-** | **-** | **-** | **-** | **-** | **-** | **-** | **-** | **-** | **-** | **-** | **A** | **A** | **A** | **C** | **C** | **C** | **C** | **G** | **A** | **G** | **A** | **T** | **G** | **T** | **A** | **G** | **C** | **A** | **G** | **C** | **C** | **A** | **T** | **T** | **T** | **G** | **G** | **G** | **C** | **A** | **T** | **G** | **G** | **C** | **T** | **C** | **T** | **T** | **A** | **G** | **T** | **A** | **T** | **T** | **G** | **T** | **T** | **T** | **G** | **C** | **C** | **C** | **T** | **T** | **C** | **C** | **T** | **G** | **G** | **G** | **G** | **T** | **G** | **G** | **G** | **T** | **T** | **T** | **T** | **G** | **T** | **A** | **T** | **T** | **A** | **T** | **A** | **T** | **T** | **T** | **A** | **T** | **T** | **T** | **T** | **T** | **C** | **G** | **G** | **A** | **C** | **G** | **T** | **G** | **G** | **A** | **T** | **T** | **A** | **A** | **C** | **G** | **G** | **A** | **T** | **A** | **A** | **G** | **A** | **A** | **A** | **A** | **A** | **A** | **T** | **T** | **C** | **T** | **A** | **T** | **C** | **T** | **C** | **C** | **G** | **A** | **C** | **A** | **A** | **A** | **G** | **T** | **G** | **A** | **C** | **T** | **T** | **G** | **A** | **A** | **A** | **G** | **A** | **A** | **T** | **T** | **A** | **G** | **A** | **A** | **A** | **A** | **C** | **T** | **T** | **T** | **C** | **A** | **G** | **T** | **C** | **T** | **T** | **T** | **T** | **A** | **G** | **A** | **G** | **G** | **A** | **G** | **A** | **T** | **A** | **C** | **C** | **A** | **T** | **T** | **G** | **A** | **A** | **C** | **A** | **T** | **T** | **A** | **C** | **G** | **A** | **T** | **C** | **C** | **T** | **G** | **A** | **C** | **A** | **T** | **G** | **G** | **G** | **C** | **G** | **A** | **T** | **A** | **A** | **A** | **G** | **A** | **C** | **A** | **A** | **A** | **C** | **A** | **G** | **C** | **A** | **A** | **T** | **T** | **T** | **G** | **T** | **C** | **G** | **A** | **C** | **T** | **T** | **C** | **T** | **T** | **C** | **T** | **C** | **T** | **T** | **C** | **A** | **T** | **T** | **A** | **A** | **A** | **T** | **C** | **G** | **T** | **A** | **T** | **G** | **C** | **C** | **G** | **C** | **T** | **G** | **A** | **C** | **A** | **A** | **G** | **A** | **A** | **T** | **G** | **A** | **A** | **T** | **T** | **C** | **T** | **G** | **T** | **C** | **A** | **C** | **T** | **C** | **T** | **T** | **C** | **T** | **C** | **A** | **C** | **A** | **G** | **A** | **C** | **G** | **G** | **A** | **C** | **A** | **A** | **G** | **A** | **G** | **A** | **A** | **A** | **T** | **T** | **G** | **G** | **A** | **T** | **T** | **C** | **A** | **C** | **T** | **G** | **C** | **T** | **T** | **C** | **A** | **A** | **G** | **A** | **T** | **C** | **T** | **A** | **A** | **A** | **A** | **A** | **A** | **A** | **G** | **C** | **C** | **A** | **A** | **A** | **C** | **A** | **T** | **T** | **C** | **G** | **A** | **T** | **C** | **C** | **A** | **T** | **A** | **T** | **C** | **G** | **A** | **A** | **T** | **A** | **T** | **T** | **A** | **C** | **G** | **C** | **A** | **T** | **T** | **T** | **G** | **T** | **G** | **A** | **C** | **A** | **G** | **A** | **T** | **A** | **A** | **T** | **A** | **T** | **C** | **G** | **G** | **C** | **C** | **A** | **G** | **C** | **A** | **A** | **G** | **T** | **C** | **T** | **T** | **A** | **C** | **A** | **T** | **G** | **T** | **T** | **T** | **T** | **A** | **G** | **A** | **A** | **G** | **A** | **A** | **A** | **A** | **A** | **G** | **C** | **C** | **G** | **C** | **A** | **G** | **A** | **A** | **G** | **G** | **C** | **G** | **T** | **G** | **G** | **A** | **A** | **G** | **T** | **T** | **C** | **G** | **A** | **A** | **T** | **A** | **T** | **T** | **A** | **T** | **A** | **T** | **G** | **A** | **T** | **G** | **C** | **A** | **T** | **T** | **T** | **G** | **G** | **C** | **T** | **C** | **T** | **C** | **A** | **T** | **G** | **G** | **C** | **A** | **C** | **A** | **A** | **A** | **A** | **G** | **C** | **A** | **A** | **A** | **A** | **G** | **A** | **T** | **T** | **T** | **C** | **A** | **A** | **T** | **C** | **G** | **T** | **C** | **T** | **A** | **A** | **T** | **C** | **A** | **A** | **A** | **A** | **A** | **T** | **G** | **G** | **T** | **G** | **G** | **A** | **C** | **A** | **T** | **G** | **T** | **C** | **C** | **A** | **T** | **A** | **C** | **A** | **T** | **T** | **T** | **G** | **T** | **T** | **A** | **C** | **C** | **T** | **C** | **A** | **C** | **A** | **A** | **A** | **A** | **G** | **G** | **C** | **A** | **T** | **T** | **A** | **C** | **T** | **T** | **C** | **G** | **T** | **T** | **T** | **C** | **C** | **G** | **A** | **T** | **T** | **G** | **A** | **A** | **T** | **T** | **A** | **C** | **C** | **A** | **T** | **G** | **A** | **T** | **C** | **A** | **C** | **C** | **G** | **A** | **A** | **A** | **A** | **A** | **T** | **C** | **G** | **T** | **T** | **G** | **T** | **G** | **A** | **T** | **C** | **G** | **A** | **T** | **G** | **G** | **A** | **A** | **A** | **G** | **A** | **T** | **T** | **A** | **G** | **T** | **T** | **A** | **T** | **A** | **C** | **C** | **G** | **G** | **T** | **G** | **G** | **T** | **T** | **T** | **C** | **A** | **A** | **T** | **A** | **T** | **T** | **G** | **C** | **C** | **A** | **A** | **T** | **C** | **A** | **A** | **T** | **A** | **T** | **G** | **T** | **A** | **A** | **A** | **T** | **A** | **C** | **A** | **A** | **C** | **A** | **A** | **A** | **A** | **A** | **A** | **A** | **T** | **T** | **C** | **G** | **G** | **C** | **T** | **A** | **T** | **T** | **G** | **G** | **C** | **G** | **C** | **G** | **A** | **T** | **A** | **C** | **G** | **C** | **A** | **T** | **A** | **T** | **A** | **C** | **G** | **G** | **A** | **T** | **T** | **T** | **T** | **C** | **G** | **G** | **T** | **G** | **C** | **C** | **G** | **C** | **T** | **T** | **C** | **T** | **T** | **C** | **T** | **T** | **T** | **G** | **C** | **T** | **C** | **C** | **A** | **G** | **C** | **T** | **T** | **C** | **G** | **C** | **T** | **T** | **C** | **T** | **T** | **A** | **A** | **C** | **A** | **G** | **A** | **C** | **T** | **G** | **G** | **A** | **A** | **C** | **G** | **T** | **C** | **T** | **C** | **G** | **G** | **T** | **A** | **C** | **C** | **T** | **G** | **A** | **A** | **G** | **A** | **A** | **A** | **A** | **A** | **A** | **A** | **G** | **G** | **T** | **C** | **G** | **G** | **C** | **T** | **A** | **T** | **C** | **A** | **T** | **T** | **T** | **G** | **A** | **A** | **T** | **T** | **A** | **T** | **T** | **T** | **C** | **T** | **T** | **T** | **A** | **A** | **A** | **A** | **A** | **A** | **G** | **C** | **A** | **G** | **A** | **T** | **C** | **G** | **A** | **G** | **A** | **T** | **G** | **A** | **A** | **T** | **C** | **T** | **A** | **A** | **G** | **C** | **T** | **T** | **G** | **C** | **T** | **G** | **A** | **T** | **A** | **C** | **A** | **T** | **C** | **C** | **A** | **T** | **C** | **C** | **A** | **G** | **C** | **T** | **T** | **G** | **T** | **T** | **T** | **C** | **A** | **A** | **G** | **C** | **G** | **G** | **A** | **C** | **C** | **G** | **A** | **A** | **T** | **A** | **A** | **C** | **G** | **A** | **A** | **A** | **G** | **G** | **G** | **A** | **A** | **C** | **A** | **A** | **A** | **T** | **C** | **A** | **A** | **G** | **C** | **T** | **T** | **T** | **C** | **A** | **T** | **T** | **T** | **A** | **T** | **C** | **A** | **A** | **A** | **T** | **T** | **G** | **A** | **T** | **T** | **A** | **C** | **T** | **T** | **C** | **T** | **G** | **C** | **T** | **A** | **A** | **A** | **A** | **A** | **A** | **C** | **G** | **T** | **G** | **T** | **T** | **T** | **G** | **G** | **A** | **T** | **A** | **C** | **A** | **G** | **A** | **C** | **A** | **C** | **C** | **T** | **T** | **A** | **C** | **C** | **T** | **T** | **G** | **T** | **T** | **C** | **C** | **T** | **G** | **A** | **T** | **G** | **A** | **A** | **A** | **G** | **T** | **G** | **T** | **C** | **A** | **T** | **T** | **G** | **C** | **T** | **G** | **C** | **T** | **T** | **T** | **A** | **A** | **A** | **A** | **A** | **T** | **C** | **G** | **C** | **A** | **A** | **C** | **T** | **G** | **C** | **C** | **T** | **C** | **T** | **G** | **G** | **T** | **G** | **T** | **A** | **G** | **A** | **T** | **G** | **T** | **G** | **A** | **A** | **A** | **A** | **T** | **C** | **A** | **T** | **G** | **A** | **T** | **T** | **C** | **C** | **C** | **A** | **A** | **C** | **A** | **A** | **A** | **C** | **C** | **G** | **G** | **A** | **T** | **C** | **A** | **T** | **C** | **C** | **T** | **T** | **T** | **T** | **A** | **T** | **T** | **T** | **A** | **T** | **C** | **G** | **A** | **G** | **C** | **A** | **A** | **C** | **A** | **C** | **A** | **A** | **T** | **A** | **T** | **T** | **A** | **T** | **G** | **C** | **T** | **C** | **G** | **G** | **C** | **A** | **G** | **C** | **T** | **G** | **A** | **T** | **C** | **A** | **A** | **G** | **G** | **A** | **A** | **A** | **A** | **T** | **G** | **T** | **A** | **C** | **A** | **A** | **A** | **T** | **C** | **C** | **T** | **T** | **G** | **T** | **C** | **T** | **A** | **T** | **G** | **A** | **G** | **A** | **A** | **C** | **G** | **G** | **C** | **T** | **T** | **C** | **C** | **T** | **C** | **C** | **A** | **T** | **G** | **C** | **A** | **A** | **A** | **A** | **A** | **C** | **A** | **T** | **T** | **G** | **A** | **T** | **A** | **A** | **T** | **G** | **G** | **A** | **T** | **G** | **A** | **T** | **G** | **A** | **A** | **A** | **T** | **C** | **T** | **G** | **C** | **A** | **T** | **G** | **G** | **T** | **A** | **G** | **G** | **T** | **T** | **C** | **A** | **G** | **C** | **A** | **A** | **A** | **T** | **C** | **A** | **A** | **G** | **A** | **T** | **A** | **T** | **T** | **C** | **G** | **T** | **A** | **G** | **C** | **T** | **A** | **C** | **C** | **G** | **A** | **T** | **T** | **G** | **A** | **A** | **T** | **T** | **T** | **T** | **G** | **A** | **A** | **A** | **C** | **A** | **A** | **G** | **T** | **G** | **C** | **T** | **G** | **T** | **C** | **A** | **T** | **T** | **T** | **A** | **C** | **G** | **A** | **T** | **C** | **C** | **T** | **G** | **A** | **G** | **T** | **T** | **T** | **T** | **T** | **A** | **G** | **A** | **A** | **G** | **A** | **A** | **C** | **T** | **T** | **G** | **C** | **T** | **A** | **C** | **T** | **C** | **A** | **G** | **T** | **T** | **C** | **A** | **A** | **A** | **G** | **A** | **A** | **G** | **A** | **T** | **G** | **A** | **G** | **A** | **C** | **A** | **C** | **A** | **T** | **T** | **G** | **T** | **T** | **C** | **A** | **T** | **C** | **C** | **A** | **T** | **G** | **A** | **C** | **A** | **A** | **C** | **T** | **G** | **A** | **A** | **A** | **C** | **A** | **G** | **T** | **C** | **A** | **A** | **G** | **G** | **A** | **A** | **A** | **T** | **G** | **T** | **C** | **T** | **A** | **A** | **C** | **T** | **G** | **G** | **C** | **T** | **A** | **T** | **T** | **A** | **T** | **T** | **C** | **A** | **A** | **G** | **C** | **A** | **A** | **C** | **A** | **A** | **A** | **T** | **T** | **T** | **C** | **T** | **C** | **G** | **A** | **T** | **T** | **A** | **T** | **T** | **T** | **T** | **C** | **T** | **C** | **C** | **A** | **A** | **T** | **C** | **C** | **T** | **A** | **-** | **-** | **-** |
| **N52629** | **-** | **-** | **-** | **-** | **-** | **-** | **-** | **-** | **-** | **-** | **-** | **-** | **-** | **-** | **-** | **-** | **-** | **-** | **-** | **-** | **-** | **-** | **-** | **-** | **-** | **-** | **-** | **-** | **-** | **-** | **-** | **-** | **-** | **-** | **-** | **-** | **-** | **-** | **-** | **-** | **-** | **-** | **-** | **-** | **-** | **-** | **-** | **-** | **-** | **-** | **-** | **-** | **-** | **-** | **-** | **-** | **-** | **-** | **-** | **-** | **-** | **-** | **-** | **-** | **-** | **-** | **-** | **-** | **-** | **A** | **A** | **A** | **C** | **C** | **C** | **C** | **G** | **A** | **G** | **A** | **T** | **G** | **T** | **A** | **G** | **C** | **A** | **G** | **C** | **C** | **A** | **T** | **T** | **T** | **G** | **G** | **G** | **C** | **A** | **T** | **G** | **G** | **C** | **T** | **C** | **T** | **T** | **A** | **G** | **T** | **A** | **T** | **T** | **G** | **T** | **T** | **T** | **G** | **C** | **C** | **C** | **T** | **T** | **C** | **C** | **T** | **G** | **G** | **G** | **G** | **T** | **G** | **G** | **G** | **C** | **T** | **T** | **T** | **G** | **T** | **A** | **T** | **T** | **A** | **T** | **A** | **T** | **T** | **T** | **A** | **T** | **T** | **T** | **T** | **T** | **C** | **G** | **G** | **A** | **C** | **G** | **T** | **G** | **G** | **A** | **T** | **T** | **A** | **A** | **C** | **G** | **G** | **A** | **T** | **A** | **A** | **G** | **A** | **A** | **A** | **A** | **A** | **A** | **T** | **T** | **C** | **T** | **A** | **T** | **C** | **T** | **C** | **C** | **G** | **A** | **C** | **A** | **A** | **A** | **G** | **T** | **G** | **A** | **C** | **T** | **T** | **G** | **A** | **A** | **A** | **G** | **A** | **A** | **T** | **T** | **A** | **G** | **A** | **A** | **A** | **A** | **C** | **T** | **T** | **T** | **C** | **A** | **G** | **T** | **C** | **T** | **T** | **T** | **T** | **A** | **G** | **A** | **G** | **G** | **A** | **G** | **A** | **T** | **A** | **C** | **C** | **A** | **T** | **T** | **G** | **A** | **A** | **C** | **A** | **T** | **T** | **A** | **C** | **G** | **A** | **T** | **C** | **C** | **T** | **G** | **A** | **C** | **A** | **T** | **G** | **G** | **G** | **C** | **G** | **A** | **T** | **A** | **A** | **A** | **G** | **A** | **C** | **A** | **A** | **A** | **C** | **A** | **G** | **C** | **A** | **A** | **T** | **T** | **T** | **G** | **T** | **T** | **G** | **A** | **C** | **T** | **T** | **C** | **T** | **T** | **C** | **T** | **C** | **T** | **T** | **C** | **A** | **T** | **T** | **A** | **A** | **A** | **T** | **C** | **G** | **T** | **A** | **T** | **G** | **C** | **C** | **G** | **C** | **T** | **G** | **A** | **C** | **A** | **A** | **G** | **A** | **A** | **T** | **G** | **A** | **A** | **T** | **T** | **C** | **T** | **G** | **T** | **C** | **A** | **C** | **T** | **C** | **T** | **T** | **C** | **T** | **C** | **A** | **C** | **A** | **G** | **A** | **C** | **G** | **G** | **A** | **C** | **A** | **A** | **G** | **A** | **G** | **A** | **A** | **A** | **T** | **T** | **G** | **G** | **A** | **T** | **T** | **C** | **A** | **C** | **T** | **G** | **C** | **T** | **T** | **C** | **A** | **A** | **G** | **A** | **T** | **C** | **T** | **A** | **A** | **A** | **A** | **A** | **A** | **A** | **G** | **C** | **C** | **A** | **A** | **A** | **C** | **A** | **T** | **T** | **C** | **G** | **A** | **T** | **C** | **C** | **A** | **T** | **A** | **T** | **C** | **G** | **A** | **A** | **T** | **A** | **T** | **T** | **A** | **C** | **G** | **C** | **A** | **T** | **T** | **T** | **G** | **T** | **G** | **A** | **C** | **A** | **G** | **A** | **T** | **A** | **A** | **T** | **A** | **T** | **C** | **G** | **G** | **C** | **C** | **A** | **G** | **C** | **A** | **A** | **G** | **T** | **C** | **T** | **T** | **A** | **C** | **A** | **T** | **G** | **T** | **T** | **T** | **T** | **A** | **G** | **A** | **A** | **G** | **A** | **A** | **A** | **A** | **A** | **G** | **C** | **C** | **G** | **C** | **A** | **G** | **A** | **A** | **G** | **G** | **C** | **G** | **T** | **G** | **G** | **A** | **A** | **G** | **T** | **T** | **C** | **G** | **A** | **A** | **T** | **A** | **T** | **T** | **A** | **T** | **A** | **T** | **G** | **A** | **T** | **G** | **C** | **A** | **T** | **T** | **T** | **G** | **G** | **C** | **T** | **C** | **T** | **C** | **A** | **T** | **G** | **G** | **C** | **A** | **C** | **A** | **A** | **A** | **A** | **G** | **C** | **A** | **A** | **A** | **A** | **G** | **A** | **T** | **T** | **T** | **C** | **A** | **A** | **T** | **C** | **G** | **T** | **C** | **T** | **A** | **A** | **T** | **C** | **A** | **A** | **A** | **A** | **A** | **T** | **G** | **G** | **T** | **G** | **G** | **A** | **C** | **A** | **T** | **G** | **T** | **C** | **C** | **A** | **T** | **A** | **C** | **A** | **T** | **T** | **T** | **G** | **T** | **T** | **A** | **C** | **C** | **T** | **C** | **A** | **C** | **A** | **A** | **A** | **A** | **G** | **G** | **C** | **A** | **T** | **T** | **A** | **C** | **T** | **T** | **C** | **G** | **T** | **T** | **T** | **C** | **C** | **G** | **A** | **T** | **T** | **G** | **A** | **A** | **T** | **T** | **A** | **C** | **C** | **A** | **T** | **G** | **A** | **T** | **C** | **A** | **C** | **C** | **G** | **A** | **A** | **A** | **A** | **A** | **T** | **C** | **G** | **T** | **T** | **G** | **T** | **G** | **A** | **T** | **C** | **G** | **A** | **T** | **G** | **G** | **A** | **A** | **A** | **G** | **A** | **T** | **T** | **A** | **G** | **T** | **T** | **A** | **T** | **A** | **C** | **C** | **G** | **G** | **T** | **G** | **G** | **T** | **T** | **T** | **C** | **A** | **A** | **T** | **A** | **T** | **T** | **G** | **C** | **C** | **A** | **A** | **T** | **C** | **A** | **A** | **T** | **A** | **T** | **G** | **T** | **A** | **A** | **A** | **T** | **A** | **C** | **A** | **A** | **C** | **A** | **A** | **A** | **A** | **A** | **A** | **A** | **T** | **T** | **C** | **G** | **G** | **C** | **T** | **A** | **T** | **T** | **G** | **G** | **C** | **G** | **C** | **G** | **A** | **T** | **A** | **C** | **G** | **C** | **A** | **T** | **A** | **T** | **A** | **C** | **G** | **G** | **A** | **T** | **T** | **T** | **T** | **C** | **G** | **G** | **T** | **G** | **C** | **C** | **G** | **C** | **T** | **T** | **C** | **T** | **T** | **C** | **T** | **T** | **T** | **G** | **C** | **T** | **C** | **C** | **A** | **G** | **C** | **T** | **T** | **C** | **G** | **C** | **T** | **T** | **C** | **T** | **T** | **A** | **A** | **C** | **A** | **G** | **A** | **C** | **T** | **G** | **G** | **A** | **A** | **C** | **G** | **T** | **C** | **T** | **C** | **G** | **G** | **T** | **A** | **C** | **C** | **T** | **G** | **A** | **A** | **G** | **A** | **A** | **A** | **A** | **A** | **A** | **A** | **G** | **G** | **T** | **C** | **G** | **G** | **C** | **T** | **A** | **T** | **C** | **A** | **T** | **T** | **T** | **G** | **A** | **A** | **T** | **T** | **A** | **T** | **T** | **T** | **C** | **T** | **T** | **T** | **A** | **A** | **A** | **A** | **A** | **A** | **G** | **C** | **A** | **G** | **A** | **T** | **C** | **G** | **A** | **G** | **A** | **T** | **G** | **A** | **A** | **T** | **C** | **T** | **A** | **A** | **G** | **C** | **T** | **T** | **G** | **C** | **T** | **G** | **A** | **T** | **A** | **C** | **A** | **T** | **C** | **C** | **A** | **T** | **C** | **C** | **A** | **G** | **C** | **T** | **T** | **G** | **T** | **T** | **T** | **C** | **A** | **A** | **G** | **C** | **G** | **G** | **A** | **C** | **C** | **G** | **A** | **A** | **T** | **A** | **A** | **C** | **G** | **A** | **A** | **A** | **G** | **G** | **G** | **A** | **A** | **C** | **A** | **A** | **A** | **T** | **C** | **A** | **A** | **G** | **C** | **T** | **T** | **T** | **C** | **A** | **T** | **T** | **T** | **A** | **T** | **C** | **A** | **A** | **A** | **T** | **T** | **G** | **A** | **T** | **T** | **A** | **C** | **T** | **T** | **C** | **T** | **G** | **C** | **T** | **A** | **A** | **A** | **A** | **A** | **A** | **C** | **G** | **T** | **G** | **T** | **T** | **T** | **G** | **G** | **A** | **T** | **A** | **C** | **A** | **G** | **A** | **C** | **A** | **C** | **C** | **T** | **T** | **A** | **C** | **C** | **T** | **T** | **G** | **T** | **T** | **C** | **C** | **T** | **G** | **A** | **T** | **G** | **A** | **A** | **A** | **G** | **T** | **G** | **T** | **C** | **A** | **T** | **T** | **G** | **C** | **T** | **G** | **C** | **T** | **T** | **T** | **A** | **A** | **A** | **A** | **A** | **T** | **C** | **G** | **C** | **A** | **A** | **C** | **T** | **G** | **C** | **C** | **T** | **C** | **T** | **G** | **G** | **T** | **G** | **T** | **A** | **G** | **A** | **T** | **G** | **T** | **G** | **A** | **A** | **A** | **A** | **T** | **C** | **A** | **T** | **G** | **A** | **T** | **T** | **C** | **C** | **C** | **A** | **A** | **C** | **A** | **A** | **A** | **C** | **C** | **G** | **G** | **A** | **T** | **C** | **A** | **T** | **C** | **C** | **T** | **T** | **T** | **T** | **A** | **T** | **T** | **T** | **A** | **T** | **C** | **G** | **A** | **G** | **C** | **A** | **A** | **C** | **A** | **C** | **A** | **A** | **T** | **A** | **T** | **T** | **A** | **T** | **G** | **C** | **T** | **C** | **G** | **G** | **C** | **A** | **G** | **C** | **T** | **G** | **A** | **T** | **C** | **A** | **A** | **G** | **G** | **A** | **A** | **A** | **A** | **T** | **G** | **T** | **A** | **A** | **A** | **A** | **A** | **T** | **C** | **C** | **T** | **T** | **G** | **T** | **C** | **T** | **A** | **T** | **G** | **A** | **G** | **A** | **A** | **C** | **G** | **G** | **C** | **T** | **T** | **C** | **C** | **T** | **C** | **C** | **A** | **T** | **G** | **C** | **A** | **A** | **A** | **A** | **A** | **C** | **A** | **T** | **T** | **G** | **A** | **T** | **A** | **A** | **T** | **G** | **G** | **A** | **T** | **G** | **A** | **T** | **G** | **A** | **A** | **A** | **T** | **C** | **T** | **G** | **C** | **A** | **T** | **G** | **G** | **T** | **A** | **G** | **G** | **T** | **T** | **C** | **A** | **G** | **C** | **A** | **A** | **A** | **T** | **C** | **A** | **A** | **G** | **A** | **T** | **A** | **T** | **T** | **C** | **G** | **T** | **A** | **G** | **C** | **T** | **A** | **C** | **C** | **G** | **A** | **T** | **T** | **G** | **A** | **A** | **T** | **T** | **T** | **T** | **G** | **A** | **A** | **A** | **C** | **A** | **A** | **G** | **T** | **G** | **C** | **T** | **G** | **T** | **C** | **A** | **T** | **T** | **T** | **A** | **C** | **G** | **A** | **T** | **C** | **C** | **T** | **G** | **A** | **G** | **T** | **T** | **T** | **T** | **T** | **A** | **G** | **A** | **A** | **G** | **A** | **A** | **C** | **T** | **T** | **G** | **C** | **T** | **A** | **C** | **T** | **C** | **A** | **G** | **T** | **T** | **C** | **A** | **A** | **A** | **G** | **A** | **A** | **G** | **A** | **T** | **G** | **A** | **G** | **A** | **C** | **A** | **C** | **A** | **T** | **T** | **G** | **T** | **T** | **C** | **A** | **T** | **C** | **C** | **A** | **T** | **G** | **A** | **C** | **A** | **A** | **C** | **T** | **G** | **A** | **A** | **A** | **C** | **A** | **G** | **T** | **C** | **A** | **A** | **G** | **G** | **A** | **A** | **A** | **T** | **G** | **T** | **C** | **T** | **A** | **A** | **C** | **T** | **G** | **G** | **C** | **T** | **A** | **T** | **T** | **A** | **T** | **T** | **C** | **A** | **A** | **G** | **C** | **A** | **A** | **C** | **A** | **A** | **A** | **T** | **T** | **T** | **C** | **T** | **C** | **G** | **A** | **T** | **T** | **A** | **T** | **T** | **T** | **T** | **C** | **T** | **C** | **C** | **A** | **A** | **T** | **C** | **C** | **T** | **A** | **-** | **-** | **-** |
| **N55072** | **-** | **-** | **-** | **-** | **-** | **-** | **-** | **-** | **-** | **-** | **-** | **-** | **-** | **-** | **-** | **-** | **-** | **-** | **-** | **-** | **-** | **-** | **-** | **-** | **-** | **-** | **-** | **-** | **-** | **-** | **-** | **-** | **-** | **-** | **-** | **-** | **-** | **-** | **-** | **-** | **-** | **-** | **-** | **-** | **-** | **-** | **-** | **-** | **-** | **-** | **-** | **-** | **-** | **-** | **-** | **-** | **-** | **-** | **-** | **-** | **-** | **-** | **-** | **-** | **-** | **-** | **-** | **-** | **-** | **A** | **A** | **A** | **C** | **C** | **C** | **C** | **G** | **A** | **G** | **A** | **T** | **G** | **T** | **A** | **G** | **C** | **A** | **G** | **C** | **C** | **A** | **T** | **T** | **T** | **G** | **G** | **G** | **C** | **A** | **T** | **G** | **G** | **C** | **T** | **C** | **T** | **T** | **A** | **G** | **T** | **A** | **T** | **T** | **G** | **T** | **T** | **T** | **G** | **C** | **C** | **C** | **T** | **T** | **C** | **C** | **T** | **G** | **G** | **G** | **G** | **T** | **G** | **G** | **G** | **T** | **T** | **T** | **T** | **G** | **T** | **A** | **T** | **T** | **A** | **T** | **A** | **T** | **T** | **T** | **A** | **T** | **T** | **T** | **T** | **T** | **C** | **G** | **G** | **A** | **C** | **G** | **T** | **G** | **G** | **A** | **T** | **T** | **A** | **A** | **C** | **G** | **G** | **A** | **T** | **A** | **A** | **G** | **A** | **A** | **A** | **A** | **A** | **A** | **T** | **T** | **C** | **T** | **A** | **T** | **C** | **T** | **C** | **C** | **G** | **A** | **C** | **A** | **A** | **A** | **G** | **T** | **G** | **A** | **C** | **T** | **T** | **G** | **A** | **A** | **A** | **G** | **A** | **A** | **T** | **T** | **A** | **G** | **A** | **A** | **A** | **A** | **C** | **T** | **T** | **T** | **C** | **A** | **G** | **T** | **C** | **T** | **T** | **T** | **T** | **A** | **G** | **A** | **G** | **G** | **A** | **G** | **A** | **T** | **A** | **C** | **C** | **A** | **T** | **T** | **G** | **A** | **A** | **C** | **A** | **T** | **T** | **A** | **C** | **G** | **A** | **T** | **C** | **C** | **T** | **G** | **A** | **C** | **A** | **T** | **G** | **G** | **G** | **C** | **G** | **A** | **T** | **A** | **A** | **A** | **G** | **A** | **C** | **A** | **A** | **A** | **C** | **A** | **G** | **C** | **A** | **A** | **T** | **T** | **T** | **G** | **T** | **C** | **G** | **A** | **C** | **T** | **T** | **C** | **T** | **T** | **C** | **T** | **C** | **T** | **T** | **C** | **A** | **T** | **T** | **A** | **A** | **A** | **T** | **C** | **G** | **T** | **A** | **T** | **G** | **C** | **C** | **G** | **C** | **T** | **G** | **A** | **C** | **A** | **A** | **G** | **A** | **A** | **T** | **G** | **A** | **A** | **T** | **T** | **C** | **T** | **G** | **T** | **C** | **A** | **C** | **T** | **C** | **T** | **T** | **C** | **T** | **C** | **A** | **C** | **A** | **G** | **A** | **C** | **G** | **G** | **A** | **C** | **A** | **A** | **G** | **A** | **G** | **A** | **A** | **A** | **T** | **T** | **G** | **G** | **A** | **T** | **T** | **C** | **A** | **C** | **T** | **G** | **C** | **T** | **T** | **C** | **A** | **A** | **G** | **A** | **T** | **C** | **T** | **A** | **A** | **A** | **A** | **A** | **A** | **A** | **G** | **C** | **C** | **A** | **A** | **A** | **C** | **A** | **T** | **T** | **C** | **G** | **A** | **T** | **C** | **C** | **A** | **T** | **A** | **T** | **C** | **G** | **A** | **A** | **T** | **A** | **T** | **T** | **A** | **C** | **G** | **C** | **A** | **T** | **T** | **T** | **G** | **T** | **G** | **A** | **C** | **A** | **G** | **A** | **T** | **A** | **A** | **T** | **A** | **T** | **C** | **G** | **G** | **C** | **C** | **A** | **G** | **C** | **A** | **A** | **G** | **T** | **C** | **T** | **T** | **A** | **C** | **A** | **T** | **G** | **T** | **T** | **T** | **T** | **A** | **G** | **A** | **A** | **G** | **A** | **A** | **A** | **A** | **A** | **G** | **C** | **C** | **G** | **C** | **A** | **G** | **A** | **A** | **G** | **G** | **C** | **G** | **T** | **G** | **G** | **A** | **A** | **G** | **T** | **T** | **C** | **G** | **A** | **A** | **T** | **A** | **T** | **T** | **A** | **T** | **A** | **T** | **G** | **A** | **T** | **G** | **C** | **A** | **T** | **T** | **T** | **G** | **G** | **C** | **T** | **C** | **T** | **C** | **A** | **T** | **G** | **G** | **C** | **A** | **C** | **A** | **A** | **A** | **A** | **G** | **C** | **A** | **A** | **A** | **A** | **G** | **A** | **T** | **T** | **T** | **C** | **A** | **A** | **T** | **C** | **G** | **T** | **C** | **T** | **A** | **A** | **T** | **C** | **A** | **A** | **A** | **A** | **A** | **T** | **G** | **G** | **T** | **G** | **G** | **A** | **C** | **A** | **T** | **G** | **T** | **C** | **C** | **A** | **T** | **A** | **C** | **A** | **T** | **T** | **T** | **G** | **T** | **T** | **A** | **C** | **C** | **T** | **C** | **A** | **C** | **A** | **A** | **A** | **A** | **G** | **G** | **C** | **A** | **T** | **T** | **A** | **C** | **T** | **T** | **C** | **G** | **T** | **T** | **T** | **C** | **C** | **G** | **A** | **T** | **T** | **G** | **A** | **A** | **T** | **T** | **A** | **C** | **C** | **A** | **T** | **G** | **A** | **T** | **C** | **A** | **C** | **C** | **G** | **A** | **A** | **A** | **A** | **A** | **T** | **C** | **G** | **T** | **T** | **G** | **T** | **G** | **A** | **T** | **C** | **G** | **A** | **T** | **A** | **G** | **A** | **A** | **A** | **G** | **A** | **T** | **T** | **A** | **G** | **T** | **T** | **A** | **T** | **A** | **C** | **C** | **G** | **G** | **T** | **G** | **G** | **T** | **T** | **T** | **C** | **A** | **A** | **T** | **A** | **T** | **T** | **G** | **C** | **C** | **A** | **A** | **T** | **C** | **A** | **A** | **T** | **A** | **T** | **G** | **T** | **A** | **A** | **A** | **T** | **A** | **C** | **A** | **A** | **C** | **A** | **A** | **A** | **A** | **A** | **A** | **A** | **T** | **T** | **C** | **G** | **G** | **C** | **T** | **A** | **T** | **T** | **G** | **G** | **C** | **G** | **C** | **G** | **A** | **T** | **A** | **C** | **G** | **C** | **A** | **T** | **A** | **T** | **A** | **C** | **G** | **G** | **A** | **T** | **T** | **T** | **T** | **C** | **G** | **G** | **T** | **G** | **C** | **C** | **G** | **C** | **T** | **T** | **C** | **T** | **T** | **C** | **T** | **T** | **T** | **G** | **C** | **T** | **C** | **C** | **A** | **G** | **C** | **T** | **T** | **C** | **G** | **C** | **T** | **T** | **C** | **T** | **T** | **A** | **A** | **C** | **A** | **G** | **A** | **C** | **T** | **G** | **G** | **A** | **A** | **C** | **G** | **T** | **C** | **T** | **C** | **G** | **G** | **T** | **A** | **C** | **C** | **T** | **G** | **A** | **A** | **G** | **A** | **A** | **A** | **A** | **A** | **A** | **A** | **G** | **G** | **T** | **C** | **G** | **G** | **C** | **T** | **A** | **T** | **C** | **A** | **T** | **T** | **T** | **G** | **A** | **A** | **T** | **T** | **A** | **T** | **T** | **T** | **C** | **T** | **T** | **T** | **A** | **A** | **A** | **A** | **A** | **A** | **G** | **C** | **A** | **G** | **A** | **T** | **C** | **G** | **A** | **G** | **A** | **T** | **G** | **A** | **A** | **T** | **C** | **T** | **A** | **A** | **G** | **C** | **T** | **T** | **G** | **C** | **T** | **G** | **A** | **T** | **A** | **C** | **A** | **T** | **C** | **C** | **A** | **T** | **C** | **C** | **A** | **G** | **C** | **T** | **T** | **G** | **T** | **T** | **T** | **C** | **A** | **A** | **G** | **C** | **G** | **G** | **A** | **C** | **C** | **G** | **A** | **A** | **T** | **A** | **A** | **C** | **G** | **A** | **A** | **A** | **G** | **G** | **G** | **A** | **A** | **C** | **A** | **A** | **A** | **T** | **C** | **A** | **A** | **G** | **C** | **T** | **T** | **T** | **C** | **A** | **T** | **T** | **T** | **A** | **T** | **C** | **A** | **A** | **A** | **T** | **T** | **G** | **A** | **T** | **T** | **A** | **C** | **T** | **T** | **C** | **T** | **G** | **C** | **T** | **A** | **A** | **A** | **A** | **A** | **A** | **C** | **G** | **T** | **G** | **T** | **T** | **T** | **G** | **G** | **A** | **T** | **A** | **C** | **A** | **G** | **A** | **C** | **A** | **C** | **C** | **T** | **T** | **A** | **C** | **C** | **T** | **T** | **G** | **T** | **T** | **C** | **C** | **T** | **G** | **A** | **T** | **G** | **A** | **A** | **A** | **G** | **T** | **G** | **T** | **C** | **A** | **T** | **T** | **G** | **C** | **T** | **G** | **C** | **T** | **T** | **T** | **A** | **A** | **A** | **A** | **A** | **T** | **C** | **G** | **C** | **A** | **A** | **C** | **T** | **G** | **C** | **C** | **T** | **C** | **T** | **G** | **G** | **T** | **G** | **T** | **A** | **G** | **A** | **T** | **G** | **T** | **G** | **A** | **A** | **A** | **A** | **T** | **C** | **A** | **T** | **G** | **A** | **T** | **T** | **C** | **C** | **C** | **A** | **A** | **C** | **A** | **A** | **A** | **C** | **C** | **G** | **G** | **A** | **T** | **C** | **A** | **T** | **C** | **C** | **T** | **T** | **T** | **T** | **A** | **T** | **T** | **T** | **A** | **T** | **C** | **G** | **A** | **G** | **C** | **A** | **A** | **C** | **A** | **C** | **A** | **A** | **T** | **A** | **T** | **T** | **A** | **T** | **G** | **C** | **T** | **C** | **G** | **G** | **C** | **A** | **G** | **C** | **T** | **G** | **A** | **T** | **C** | **A** | **A** | **G** | **G** | **A** | **A** | **A** | **A** | **T** | **G** | **T** | **A** | **C** | **A** | **A** | **A** | **T** | **C** | **C** | **T** | **T** | **G** | **T** | **C** | **T** | **A** | **T** | **G** | **A** | **G** | **A** | **A** | **C** | **G** | **G** | **C** | **T** | **T** | **C** | **C** | **T** | **C** | **C** | **A** | **T** | **G** | **C** | **A** | **A** | **A** | **A** | **A** | **C** | **A** | **T** | **T** | **G** | **A** | **T** | **A** | **A** | **T** | **G** | **G** | **A** | **T** | **G** | **A** | **T** | **G** | **A** | **A** | **A** | **T** | **C** | **T** | **G** | **C** | **A** | **T** | **G** | **G** | **T** | **A** | **G** | **G** | **T** | **T** | **C** | **A** | **G** | **C** | **A** | **A** | **A** | **T** | **C** | **A** | **A** | **G** | **A** | **T** | **A** | **T** | **T** | **C** | **G** | **T** | **A** | **G** | **C** | **T** | **A** | **C** | **C** | **G** | **A** | **T** | **T** | **G** | **A** | **A** | **T** | **T** | **T** | **T** | **G** | **A** | **A** | **A** | **C** | **A** | **A** | **G** | **T** | **G** | **C** | **T** | **G** | **T** | **C** | **A** | **T** | **T** | **T** | **A** | **C** | **G** | **A** | **T** | **C** | **C** | **T** | **G** | **A** | **G** | **T** | **T** | **T** | **T** | **T** | **A** | **G** | **A** | **A** | **G** | **A** | **A** | **C** | **T** | **T** | **G** | **C** | **T** | **A** | **C** | **T** | **C** | **A** | **G** | **T** | **T** | **C** | **A** | **A** | **A** | **G** | **A** | **A** | **G** | **A** | **T** | **G** | **A** | **G** | **A** | **C** | **A** | **C** | **A** | **T** | **T** | **G** | **T** | **T** | **C** | **A** | **T** | **C** | **C** | **A** | **T** | **G** | **A** | **C** | **A** | **A** | **C** | **T** | **G** | **A** | **A** | **A** | **C** | **A** | **G** | **T** | **C** | **A** | **A** | **G** | **G** | **A** | **A** | **A** | **T** | **G** | **T** | **C** | **T** | **A** | **A** | **C** | **T** | **G** | **G** | **C** | **T** | **A** | **T** | **T** | **A** | **T** | **T** | **C** | **A** | **A** | **G** | **C** | **A** | **A** | **C** | **A** | **A** | **A** | **T** | **T** | **T** | **C** | **T** | **C** | **G** | **A** | **T** | **T** | **A** | **T** | **T** | **T** | **T** | **C** | **T** | **C** | **C** | **A** | **A** | **T** | **C** | **C** | **T** | **A** | **-** | **-** | **-** |
| **N60324F** | **G** | **T** | **G** | **G** | **T** | **A** | **T** | **C** | **T** | **A** | **G** | **T** | **A** | **T** | **T** | **A** | **T** | **A** | **A** | **C** | **C** | **G** | **C** | **C** | **C** | **T** | **T** | **T** | **A** | **T** | **C** | **T** | **A** | **T** | **T** | **A** | **A** | **A** | **T** | **G** | **C** | **A** | **C** | **T** | **T** | **A** | **T** | **T** | **G** | **C** | **T** | **T** | **T** | **G** | **G** | **T** | **G** | **A** | **C** | **T** | **A** | **T** | **T** | **T** | **T** | **G** | **A** | **T** | **C** | **A** | **A** | **A** | **C** | **C** | **C** | **C** | **G** | **A** | **G** | **A** | **T** | **G** | **T** | **A** | **G** | **C** | **A** | **G** | **C** | **C** | **A** | **T** | **T** | **T** | **G** | **G** | **G** | **C** | **A** | **T** | **G** | **G** | **C** | **T** | **C** | **T** | **T** | **A** | **G** | **T** | **A** | **T** | **T** | **G** | **T** | **T** | **T** | **G** | **C** | **C** | **C** | **T** | **T** | **C** | **C** | **T** | **G** | **G** | **G** | **G** | **T** | **G** | **G** | **G** | **C** | **T** | **T** | **T** | **G** | **T** | **A** | **T** | **T** | **A** | **T** | **A** | **T** | **T** | **T** | **A** | **T** | **T** | **T** | **T** | **T** | **C** | **G** | **G** | **A** | **C** | **G** | **T** | **G** | **G** | **A** | **T** | **T** | **A** | **A** | **C** | **G** | **G** | **A** | **T** | **A** | **A** | **G** | **A** | **A** | **A** | **A** | **A** | **A** | **T** | **T** | **C** | **T** | **A** | **T** | **C** | **T** | **C** | **C** | **G** | **A** | **C** | **A** | **A** | **A** | **G** | **T** | **G** | **A** | **C** | **T** | **T** | **G** | **A** | **A** | **A** | **G** | **A** | **A** | **T** | **T** | **A** | **G** | **A** | **A** | **A** | **A** | **C** | **T** | **T** | **T** | **C** | **A** | **G** | **T** | **C** | **T** | **T** | **T** | **T** | **A** | **G** | **A** | **G** | **G** | **A** | **G** | **A** | **T** | **A** | **C** | **C** | **A** | **T** | **T** | **G** | **A** | **A** | **C** | **A** | **T** | **T** | **A** | **C** | **G** | **A** | **T** | **C** | **C** | **T** | **G** | **A** | **C** | **A** | **T** | **G** | **G** | **G** | **C** | **G** | **A** | **T** | **A** | **A** | **A** | **G** | **A** | **C** | **A** | **A** | **A** | **C** | **A** | **G** | **C** | **A** | **A** | **T** | **T** | **T** | **G** | **T** | **T** | **G** | **A** | **C** | **T** | **T** | **C** | **T** | **T** | **C** | **T** | **C** | **T** | **T** | **C** | **A** | **T** | **T** | **A** | **A** | **A** | **T** | **C** | **G** | **T** | **A** | **T** | **G** | **C** | **C** | **G** | **C** | **T** | **G** | **G** | **C** | **A** | **A** | **G** | **A** | **A** | **T** | **G** | **A** | **A** | **T** | **T** | **C** | **T** | **G** | **T** | **C** | **A** | **C** | **T** | **C** | **T** | **T** | **C** | **T** | **C** | **A** | **C** | **A** | **G** | **A** | **C** | **G** | **G** | **A** | **C** | **A** | **A** | **G** | **A** | **G** | **A** | **A** | **A** | **T** | **T** | **G** | **G** | **A** | **T** | **T** | **C** | **A** | **C** | **T** | **G** | **C** | **T** | **T** | **C** | **A** | **A** | **G** | **A** | **T** | **C** | **T** | **A** | **A** | **A** | **A** | **A** | **A** | **A** | **G** | **C** | **C** | **A** | **A** | **A** | **C** | **A** | **T** | **T** | **C** | **G** | **A** | **T** | **C** | **C** | **A** | **T** | **A** | **T** | **C** | **G** | **A** | **A** | **T** | **A** | **T** | **T** | **A** | **C** | **G** | **C** | **A** | **T** | **T** | **T** | **G** | **T** | **G** | **A** | **C** | **A** | **G** | **A** | **T** | **A** | **A** | **T** | **A** | **T** | **C** | **G** | **G** | **C** | **C** | **A** | **G** | **C** | **A** | **A** | **G** | **T** | **C** | **T** | **T** | **A** | **C** | **A** | **T** | **G** | **T** | **T** | **T** | **T** | **A** | **G** | **A** | **A** | **G** | **A** | **A** | **A** | **A** | **A** | **G** | **C** | **C** | **G** | **C** | **A** | **G** | **A** | **A** | **G** | **G** | **C** | **G** | **T** | **G** | **G** | **A** | **A** | **G** | **T** | **T** | **C** | **G** | **A** | **A** | **T** | **A** | **T** | **T** | **A** | **T** | **A** | **T** | **G** | **A** | **T** | **G** | **C** | **A** | **T** | **T** | **T** | **G** | **G** | **C** | **T** | **C** | **T** | **C** | **A** | **T** | **G** | **G** | **C** | **A** | **C** | **A** | **A** | **A** | **A** | **G** | **C** | **A** | **A** | **A** | **A** | **G** | **A** | **T** | **T** | **T** | **C** | **A** | **A** | **T** | **C** | **G** | **T** | **C** | **T** | **A** | **A** | **T** | **C** | **A** | **A** | **A** | **A** | **A** | **T** | **G** | **G** | **T** | **G** | **G** | **A** | **C** | **A** | **T** | **G** | **T** | **C** | **C** | **A** | **T** | **A** | **C** | **A** | **T** | **T** | **T** | **G** | **T** | **T** | **A** | **C** | **C** | **T** | **C** | **A** | **C** | **A** | **A** | **A** | **A** | **G** | **G** | **C** | **A** | **T** | **T** | **A** | **C** | **T** | **T** | **C** | **G** | **T** | **T** | **T** | **C** | **C** | **G** | **A** | **T** | **T** | **G** | **A** | **A** | **T** | **T** | **A** | **C** | **C** | **A** | **T** | **G** | **A** | **T** | **C** | **A** | **C** | **C** | **G** | **A** | **A** | **A** | **A** | **A** | **T** | **C** | **G** | **T** | **T** | **G** | **T** | **G** | **A** | **T** | **C** | **G** | **A** | **T** | **G** | **G** | **A** | **A** | **A** | **G** | **A** | **T** | **T** | **A** | **G** | **T** | **T** | **A** | **T** | **A** | **C** | **C** | **G** | **G** | **T** | **G** | **G** | **T** | **T** | **T** | **C** | **A** | **A** | **T** | **A** | **T** | **T** | **G** | **C** | **C** | **A** | **A** | **T** | **C** | **A** | **A** | **T** | **A** | **T** | **G** | **T** | **A** | **A** | **A** | **T** | **A** | **C** | **A** | **A** | **C** | **A** | **A** | **A** | **A** | **A** | **A** | **A** | **T** | **T** | **C** | **G** | **G** | **C** | **T** | **A** | **T** | **T** | **G** | **G** | **C** | **G** | **C** | **G** | **A** | **T** | **A** | **C** | **G** | **C** | **A** | **T** | **A** | **T** | **A** | **C** | **G** | **G** | **A** | **T** | **T** | **T** | **T** | **C** | **G** | **G** | **T** | **G** | **C** | **C** | **G** | **C** | **T** | **T** | **C** | **T** | **T** | **C** | **T** | **T** | **T** | **G** | **C** | **T** | **C** | **C** | **A** | **G** | **C** | **T** | **T** | **C** | **G** | **C** | **T** | **T** | **C** | **T** | **T** | **A** | **A** | **C** | **A** | **G** | **A** | **C** | **T** | **G** | **G** | **A** | **A** | **C** | **G** | **T** | **C** | **T** | **C** | **G** | **G** | **T** | **A** | **C** | **C** | **T** | **G** | **A** | **A** | **G** | **A** | **A** | **A** | **A** | **A** | **A** | **A** | **G** | **G** | **T** | **C** | **G** | **G** | **C** | **T** | **A** | **T** | **C** | **A** | **T** | **T** | **T** | **G** | **A** | **A** | **T** | **T** | **A** | **T** | **T** | **T** | **C** | **T** | **T** | **T** | **A** | **A** | **A** | **A** | **A** | **A** | **G** | **C** | **A** | **G** | **A** | **T** | **C** | **G** | **A** | **G** | **A** | **T** | **G** | **A** | **A** | **T** | **C** | **T** | **A** | **A** | **G** | **C** | **T** | **T** | **G** | **C** | **T** | **G** | **A** | **T** | **A** | **C** | **A** | **T** | **C** | **C** | **A** | **T** | **C** | **C** | **A** | **G** | **C** | **T** | **T** | **G** | **T** | **T** | **T** | **C** | **A** | **A** | **G** | **C** | **G** | **G** | **A** | **C** | **C** | **G** | **A** | **A** | **T** | **A** | **A** | **C** | **G** | **A** | **A** | **A** | **G** | **G** | **G** | **A** | **A** | **C** | **A** | **A** | **A** | **T** | **C** | **A** | **A** | **G** | **C** | **T** | **T** | **T** | **C** | **A** | **T** | **T** | **T** | **A** | **T** | **C** | **A** | **A** | **A** | **T** | **T** | **G** | **A** | **T** | **T** | **A** | **C** | **T** | **T** | **C** | **T** | **G** | **C** | **T** | **A** | **A** | **A** | **A** | **A** | **A** | **C** | **G** | **T** | **G** | **T** | **T** | **T** | **G** | **G** | **A** | **T** | **A** | **C** | **A** | **G** | **A** | **C** | **A** | **C** | **C** | **T** | **T** | **A** | **C** | **C** | **T** | **T** | **G** | **T** | **T** | **C** | **C** | **T** | **G** | **A** | **T** | **G** | **A** | **A** | **A** | **G** | **T** | **G** | **T** | **C** | **A** | **T** | **T** | **G** | **C** | **T** | **G** | **C** | **T** | **T** | **T** | **A** | **A** | **A** | **A** | **A** | **T** | **C** | **G** | **C** | **A** | **A** | **C** | **T** | **G** | **C** | **C** | **T** | **C** | **T** | **G** | **G** | **T** | **G** | **T** | **A** | **G** | **A** | **T** | **G** | **T** | **G** | **A** | **A** | **A** | **A** | **T** | **C** | **A** | **T** | **G** | **A** | **T** | **T** | **C** | **C** | **C** | **A** | **A** | **C** | **A** | **A** | **A** | **C** | **C** | **G** | **G** | **A** | **T** | **C** | **A** | **T** | **C** | **C** | **T** | **T** | **T** | **T** | **A** | **T** | **T** | **T** | **A** | **T** | **C** | **G** | **A** | **G** | **C** | **A** | **A** | **C** | **A** | **C** | **A** | **A** | **T** | **A** | **T** | **T** | **A** | **T** | **G** | **C** | **T** | **C** | **G** | **G** | **C** | **A** | **G** | **C** | **T** | **G** | **A** | **T** | **C** | **A** | **A** | **G** | **G** | **A** | **A** | **A** | **A** | **T** | **G** | **T** | **A** | **C** | **A** | **A** | **A** | **T** | **C** | **C** | **T** | **T** | **G** | **T** | **C** | **T** | **A** | **T** | **G** | **A** | **G** | **A** | **A** | **C** | **G** | **G** | **C** | **T** | **T** | **C** | **C** | **T** | **C** | **C** | **A** | **T** | **G** | **C** | **A** | **A** | **A** | **A** | **A** | **C** | **A** | **T** | **T** | **G** | **A** | **T** | **A** | **A** | **T** | **G** | **G** | **A** | **T** | **G** | **A** | **T** | **G** | **A** | **A** | **A** | **T** | **C** | **T** | **G** | **C** | **A** | **T** | **G** | **G** | **T** | **A** | **G** | **G** | **T** | **T** | **C** | **A** | **G** | **C** | **A** | **A** | **A** | **T** | **C** | **A** | **A** | **G** | **A** | **T** | **A** | **T** | **T** | **C** | **G** | **T** | **A** | **G** | **C** | **T** | **A** | **C** | **C** | **G** | **A** | **T** | **T** | **G** | **A** | **A** | **T** | **T** | **T** | **T** | **G** | **A** | **A** | **A** | **C** | **A** | **A** | **G** | **T** | **G** | **C** | **T** | **G** | **T** | **C** | **A** | **T** | **T** | **T** | **A** | **C** | **G** | **A** | **T** | **C** | **C** | **T** | **G** | **A** | **G** | **T** | **T** | **T** | **T** | **T** | **A** | **G** | **A** | **A** | **G** | **A** | **A** | **C** | **T** | **T** | **G** | **C** | **T** | **A** | **C** | **T** | **C** | **A** | **G** | **T** | **T** | **C** | **A** | **A** | **A** | **G** | **A** | **A** | **G** | **A** | **T** | **G** | **A** | **G** | **A** | **C** | **A** | **C** | **A** | **T** | **T** | **G** | **T** | **T** | **C** | **A** | **T** | **C** | **C** | **A** | **T** | **G** | **A** | **C** | **A** | **A** | **C** | **T** | **G** | **A** | **A** | **A** | **C** | **A** | **G** | **T** | **C** | **A** | **A** | **G** | **G** | **A** | **A** | **A** | **T** | **G** | **T** | **C** | **T** | **A** | **A** | **C** | **T** | **G** | **G** | **C** | **T** | **A** | **T** | **T** | **A** | **T** | **T** | **C** | **A** | **A** | **G** | **C** | **A** | **A** | **C** | **A** | **A** | **A** | **T** | **T** | **T** | **C** | **T** | **C** | **G** | **A** | **T** | **T** | **A** | **T** | **T** | **T** | **T** | **C** | **T** | **C** | **C** | **A** | **A** | **T** | **C** | **C** | **T** | **A** | **-** | **-** | **-** |
 **SNP position** | . | . | . | . | . | . | . | . | . | . | . | . | . | . | . | . | . | . | . | . | . | . | . | . | . | . | . | . | . | . | . | . | . | . | . | . | . | . | . | . | . | . | . | . | . | . | . | . | . | . | . | . | . | . | . | . | . | . | . | . | . | . | . | . | . | . | . | . | . | . | . | . | . | . | . | . | . | . | . | . | . | . | . | . | . | . | . | . | . | 90 | . | . | . | . | . | . | . | . | . | . | . | . | . | . | . | . | . | . | . | . | . | . | . | 114 | . | . | . | . | 119 | . | . | . | . | . | . | . | . | . | . | 130 | . | . | . | . | 135 | . | . | . | . | . | . | . | . | . | . | . | . | . | . | . | . | . | . | . | . | . | . | . | . | . | . | . | . | . | . | . | . | . | . | . | . | . | . | . | . | . | . | . | . | . | . | . | . | . | . | . | . | . | . | . | . | . | . | 194 | . | . | . | . | . | . | . | . | . | . | . | . | . | . | . | . | . | . | . | . | . | . | . | . | . | . | . | . | . | . | . | . | . | . | . | . | . | . | . | . | . | . | . | . | . | 240 | . | . | . | . | 245 | . | . | . | . | . | . | . | . | . | . | . | . | . | . | . | . | . | . | . | . | . | . | . | . | . | . | . | . | . | . | 276 | . | . | . | . | . | . | . | . | . | . | . | 288 | . | . | . | . | . | . | . | . | 297 | . | . | . | . | . | . | . | . | . | . | . | . | . | . | . | . | . | . | . | . | . | . | . | . | . | . | . | . | . | . | . | . | . | 331 | . | . | . | 335 | . | . | . | . | . | . | . | . | . | . | . | . | . | . | . | . | . | . | . | . | . | . | . | . | . | . | . | . | . | . | . | . | . | . | 370 | . | . | . | . | . | . | . | 378 | . | . | . | . | . | . | . | . | . | . | . | . | . | . | . | . | . | . | . | . | . | . | . | . | . | . | . | . | . | . | . | . | . | . | . | . | . | . | . | . | . | . | . | . | . | . | . | . | . | . | . | . | . | . | . | . | . | . | . | . | . | . | . | . | . | . | . | . | . | . | . | . | . | 452 | . | . | . | . | . | . | 459 | . | . | . | . | . | . | . | . | . | . | . | . | . | 473 | 474 | . | . | . | . | . | . | . | . | . | . | . | . | . | . | . | . | 491 | . | . | . | . | . | . | . | . | . | . | . | . | 504 | . | . | . | . | . | . | . | . | 513 | . | . | . | . | . | . | . | . | . | . | . | . | . | . | . | . | . | . | . | . | . | . | . | 537 | . | . | 540 | . | . | . | . | . | . | . | . | . | . | . | . | . | . | . | . | . | . | . | . | . | . | . | . | . | . | . | . | . | . | . | . | . | . | . | . | . | . | . | . | . | . | . | . | . | . | . | . | . | . | . | . | . | . | . | . | . | . | . | . | . | . | . | . | . | . | . | . | 609 | . | . | . | . | 614 | 615 | . | . | . | . | . | 621 | . | . | . | . | . | . | . | . | . | . | . | . | . | . | . | . | . | 639 | . | . | . | . | . | . | . | . | . | . | . | . | . | . | 654 | . | . | . | . | . | . | . | . | . | . | . | . | . | . | . | . | . | . | 673 | . | . | . | . | . | . | . | 681 | . | . | . | . | . | 687 | . | 689 | . | . | . | . | . | . | 696 | . | . | . | . | . | 702 | . | . | . | . | . | . | . | . | . | . | . | . | . | . | . | 718 | . | . | . | . | . | . | 725 | 726 | . | . | . | . | . | . | . | . | . | . | . | . | . | . | 741 | . | . | . | . | . | . | . | . | 750 | . | . | . | . | . | . | . | . | . | . | . | . | . | . | 765 | . | . | . | . | . | . | . | . | . | . | . | 777 | . | . | . | . | . | . | . | . | . | . | . | . | . | . | 792 | . | . | 795 | . | . | . | . | . | 801 | . | . | 804 | . | . | . | . | . | . | . | . | . | . | . | . | . | . | . | . | . | 822 | . | . | 825 | . | . | 828 | 829 | . | . | . | . | . | . | . | . | . | . | . | . | . | . | . | . | . | . | . | . | . | . | . | . | . | . | . | . | . | . | . | . | . | . | . | . | . | 867 | . | . | . | . | . | . | . | . | . | . | . | 879 | . | . | . | . | 884 | . | . | . | 888 | . | . | . | . | . | 894 | . | . | 897 | . | . | . | . | . | . | . | . | 906 | . | 908 | 909 | . | . | 912 | . | . | . | . | . | . | . | . | . | . | . | 924 | . | . | . | . | . | . | . | . | . | . | . | 936 | . | . | . | . | . | . | . | . | . | 946 | . | 948 | . | . | . | . | . | . | . | . | 957 | . | . | . | . | . | . | . | . | 966 | . | . | . | . | . | . | . | . | . | . | . | . | . | . | . | . | . | . | . | . | . | . | . | . | . | . | . | . | . | 996 | . | . | . | . | . | . | . | . | . | . | . | . | . | . | . | . | . | 1014 | . | . | . | . | . | . | . | . | 1023 | . | . | . | . | . | . | . | . | . | . | . | . | . | . | . | . | . | . | . | . | . | . | . | . | . | . | . | . | . | 1053 | . | . | . | . | . | . | . | . | . | . | . | . | . | . | 1068 | . | . | . | . | . | . | . | . | 1077 | . | . | . | . | . | . | . | . | . | . | . | . | . | . | . | . | . | 1095 | . | . | . | . | . | 1101 | . | . | . | . | . | . | . | . | . | . | . | . | . | . | . | . | . | . | . | . | . | . | . | . | . | . | . | . | . | . | . | . | . | . | . | . | . | . | . | . | . | . | . | . | 1146 | . | . | 1149 | . | . | . | . | . | 1155 | . | . | . | . | . | . | . | . | . | . | . | 1167 | . | . | . | 1171 | . | 1173 | . | . | 1176 | . | . | . | . | . | . | . | . | . | . | . | . | . | . | 1191 | . | . | . | . | . | . | . | . | . | . | . | . | . | . | . | . | . | . | . | . | . | . | . | 1215 | . | . | . | . | . | . | . | . | . | . | . | . | . | . | . | . | . | . | . | . | . | . | . | . | . | . | . | . | . | . | . | . | . | . | . | . | . | . | 1254 | . | . | . | . | . | . | . | . | . | . | . | . | . | . | . | . | . | . | 1273 | . | . | . | . | . | . | . | . | . | . | . | . | . | . | . | . | . | . | . | . | . | . | . | . | . | 1299 | . | . | 1302 | . | . | . | . | . | . | . | . | . | . | . | . | . | . | . | . | . | . | . | . | . | . | . | 1326 | . | . | . | . | . | . | . | . | 1335 | . | . | . | . | . | 1341 | . | . | . | . | . | . | . | . | . | . | . | . | . | . | . | 1357 | . | . | . | . | . | . | . | . | 1366 | . | 1368 | . | . | . | . | . | 1374 | . | . | . | . | . | . | . | . | . | . | . | 1386 | . | . | 1389 | . | . | . | . | . | . | . | . | . | . | . | 1401 | . | . | . | . | . | 1407 | . | . | . | . | . | . | . | . | 1416 | . | . | . | . | . | . | . | . | . | . | . | . | . | . | . | . | . | 1434 | . | . | . | . | . | . | . | . | . | . | . | . | 1447 | . | . | . | . | . |

|  |
| --- |
|  |

|  |  |
| --- | --- |
|  |

|  |  |
| --- | --- |
| **Legend** | |
  | **Reference Sequence** |

  |
|  | **Detected SNPs** |
|  |  |
