# Supplementary material for: sraX: A Novel Comprehensive Resistome Analysis Tool
Source: Front Microbiol. 2020 Feb 5;11:52. doi: 10.3389/fmicb.2020.00052 (PMC7025521; doi:10.3389/fmicb.2020.00052)
Supplement: Supplementary file 4 [file Data_Sheet_3.ZIP › cls/cls_Efaecium_aa.html]

# cls

|  |
| --- |
|  |

|  |
| --- |
|  |

|  |  |
| --- | --- |
| **Definition:** | Cls or cardiolipin synthetase is an inner membrane protein that is involved in membrane synthesis. Specific mutations in Enterococcus can confer resistance to daptomycin. |

|  |
| --- |
|  |

|  |
| --- |
|  |

|  |  |  |
| --- | --- | --- |
| **Reference organism:** | *Enterococcus faecium* | [NCBI, CARD] |

|  |
| --- |
|  |

|  |
| --- |
|  |

|  |  |
| --- | --- |
| **Summary Data** | || **Parameter** | **Value_(n)_** || Total number of genomes | 101 |
| MSA length | 483 |
| Total number of SNPs | 29 |
| SNP density (# SNPs/MSA length) | 0.060 |

|  |
| --- |
|  |

|  |
| --- |
|  |

|  |
| --- |
|  |

|  |
| --- |
|  |

|  |  |  |  |  |
| --- | --- | --- | --- | --- |
| **SNPs conferring antibiotic resistance** | | | | || **Reference gene** | **Seq. pos.** | **Ref\_base** | **Alt\_base** | **Confirmation SNP?** |||  |  |  |  |  |
| --- | --- | --- | --- | --- |
| cls 13 N I **No** | | | | |
||  |  |  |  |  |
| --- | --- | --- | --- | --- |
| cls 13 N T **No** | | | | |
||  |  |  |  |  |
| --- | --- | --- | --- | --- |
| cls 20 A D **SNP variant (A20T)** | | | | |
||  |  |  |  |  |
| --- | --- | --- | --- | --- |
| cls 27 D N **No** | | | | |
||  |  |  |  |  |
| --- | --- | --- | --- | --- |
| cls 59 K T **No** | | | | |
||  |  |  |  |  |
| --- | --- | --- | --- | --- |
| cls 178 G T **No** | | | | |
||  |  |  |  |  |
| --- | --- | --- | --- | --- |
| cls 215 H R **No** | | | | |
||  |  |  |  |  |
| --- | --- | --- | --- | --- |
| cls 218 R Q **No** | | | | |
||  |  |  |  |  |
| --- | --- | --- | --- | --- |
| cls 267 R H **No** | | | | |

|  |
| --- |
|  |

|  |
| --- |
|  |

|  |
| --- |
|  |

|  |  |  |  |  |  |  |  |  |  |  |  |  |  |  |  |  |  |  |  |  |  |  |  |  |  |  |  |  |  |  |  |  |  |  |  |  |  |  |  |  |  |  |  |  |  |  |  |  |  |  |  |  |  |  |  |  |  |  |  |  |  |  |  |  |  |  |  |  |  |  |  |  |  |  |  |  |  |  |  |  |  |  |  |  |  |  |  |  |  |  |  |  |  |  |  |  |  |  |  |  |  |  |  |  |  |  |  |  |  |  |  |  |  |  |  |  |  |  |  |  |  |  |  |  |  |  |  |  |  |  |  |  |  |  |  |  |  |  |  |  |  |  |  |  |  |  |  |  |  |  |  |  |  |  |  |  |  |  |  |  |  |  |  |  |  |  |  |  |  |  |  |  |  |  |  |  |  |  |  |  |  |  |  |  |  |  |  |  |  |  |  |  |  |  |  |  |  |  |  |  |  |  |  |  |  |  |  |  |  |  |  |  |  |  |  |  |  |  |  |  |  |  |  |  |  |  |  |  |  |  |  |  |  |  |  |  |  |  |  |  |  |  |  |  |  |  |  |  |  |  |  |  |  |  |  |  |  |  |  |  |  |  |  |  |  |  |  |  |  |  |  |  |  |  |  |  |  |  |  |  |  |  |  |  |  |  |  |  |  |  |  |  |  |  |  |  |  |  |  |  |  |  |  |  |  |  |  |  |  |  |  |  |  |  |  |  |  |  |  |  |  |  |  |  |  |  |  |  |  |  |  |  |  |  |  |  |  |  |  |  |  |  |  |  |  |  |  |  |  |  |  |  |  |  |  |  |  |  |  |  |  |  |  |  |  |  |  |  |  |  |  |  |  |  |  |  |  |  |  |  |  |  |  |  |  |  |  |  |  |  |  |  |  |  |  |  |  |  |  |  |  |  |  |  |  |  |  |  |  |  |  |  |  |  |  |  |  |  |  |  |  |  |  |  |  |  |  |  |  |  |  |  |  |  |  |  |  |  |  |  |  |  |  |  |  |  |  |  |  |  |  |  |  |  |  |  |  |  |  |  |  |  |  |  |  |  |  |  |  |  |  |  |  |  |  |  |  |  |  |  |  |  |  |  |
| --- | --- | --- | --- | --- | --- | --- | --- | --- | --- | --- | --- | --- | --- | --- | --- | --- | --- | --- | --- | --- | --- | --- | --- | --- | --- | --- | --- | --- | --- | --- | --- | --- | --- | --- | --- | --- | --- | --- | --- | --- | --- | --- | --- | --- | --- | --- | --- | --- | --- | --- | --- | --- | --- | --- | --- | --- | --- | --- | --- | --- | --- | --- | --- | --- | --- | --- | --- | --- | --- | --- | --- | --- | --- | --- | --- | --- | --- | --- | --- | --- | --- | --- | --- | --- | --- | --- | --- | --- | --- | --- | --- | --- | --- | --- | --- | --- | --- | --- | --- | --- | --- | --- | --- | --- | --- | --- | --- | --- | --- | --- | --- | --- | --- | --- | --- | --- | --- | --- | --- | --- | --- | --- | --- | --- | --- | --- | --- | --- | --- | --- | --- | --- | --- | --- | --- | --- | --- | --- | --- | --- | --- | --- | --- | --- | --- | --- | --- | --- | --- | --- | --- | --- | --- | --- | --- | --- | --- | --- | --- | --- | --- | --- | --- | --- | --- | --- | --- | --- | --- | --- | --- | --- | --- | --- | --- | --- | --- | --- | --- | --- | --- | --- | --- | --- | --- | --- | --- | --- | --- | --- | --- | --- | --- | --- | --- | --- | --- | --- | --- | --- | --- | --- | --- | --- | --- | --- | --- | --- | --- | --- | --- | --- | --- | --- | --- | --- | --- | --- | --- | --- | --- | --- | --- | --- | --- | --- | --- | --- | --- | --- | --- | --- | --- | --- | --- | --- | --- | --- | --- | --- | --- | --- | --- | --- | --- | --- | --- | --- | --- | --- | --- | --- | --- | --- | --- | --- | --- | --- | --- | --- | --- | --- | --- | --- | --- | --- | --- | --- | --- | --- | --- | --- | --- | --- | --- | --- | --- | --- | --- | --- | --- | --- | --- | --- | --- | --- | --- | --- | --- | --- | --- | --- | --- | --- | --- | --- | --- | --- | --- | --- | --- | --- | --- | --- | --- | --- | --- | --- | --- | --- | --- | --- | --- | --- | --- | --- | --- | --- | --- | --- | --- | --- | --- | --- | --- | --- | --- | --- | --- | --- | --- | --- | --- | --- | --- | --- | --- | --- | --- | --- | --- | --- | --- | --- | --- | --- | --- | --- | --- | --- | --- | --- | --- | --- | --- | --- | --- | --- | --- | --- | --- | --- | --- | --- | --- | --- | --- | --- | --- | --- | --- | --- | --- | --- | --- | --- | --- | --- | --- | --- | --- | --- | --- | --- | --- | --- | --- | --- | --- | --- | --- | --- | --- | --- | --- | --- | --- | --- | --- | --- | --- | --- | --- | --- | --- | --- | --- | --- | --- | --- | --- | --- | --- | --- | --- | --- | --- | --- | --- | --- | --- | --- | --- | --- | --- | --- | --- | --- | --- | --- | --- | --- | --- | --- | --- | --- | --- | --- | --- | --- | --- | --- | --- | --- | --- | --- | --- | --- | --- | --- | --- | --- | --- | --- | --- | --- | --- | --- | --- | --- | --- | --- | --- | --- | --- | --- | --- | --- | --- | --- | --- | --- | --- | --- | --- | --- | --- | --- | --- | --- | --- | --- | --- | --- |
|  || **Enterococcus faecium (ALL09868.1)** | **M** | **V** | **S** | **S** | **I** | **I** | **T** | **A** | **L** | **Y** | **L** | **L** | **N** | **A** | **L** | **I** | **A** | **L** | **V** | **A** | **I** | **L** | **I** | **K** | **P** | **R** | **D** | **V** | **A** | **A** | **I** | **W** | **A** | **W** | **L** | **L** | **V** | **L** | **F** | **A** | **L** | **P** | **G** | **V** | **G** | **F** | **V** | **L** | **Y** | **L** | **F** | **F** | **G** | **R** | **G** | **L** | **T** | **D** | **K** | **K** | **K** | **F** | **Y** | **L** | **R** | **Q** | **S** | **D** | **L** | **K** | **E** | **L** | **E** | **N** | **F** | **Q** | **S** | **F** | **R** | **G** | **D** | **T** | **I** | **E** | **H** | **Y** | **D** | **P** | **D** | **M** | **G** | **D** | **K** | **D** | **K** | **Q** | **Q** | **F** | **V** | **D** | **F** | **F** | **S** | **S** | **L** | **N** | **R** | **M** | **P** | **L** | **T** | **R** | **M** | **N** | **S** | **V** | **T** | **L** | **L** | **T** | **D** | **G** | **Q** | **E** | **K** | **L** | **D** | **S** | **L** | **L** | **Q** | **D** | **L** | **K** | **K** | **A** | **K** | **H** | **S** | **I** | **H** | **I** | **E** | **Y** | **Y** | **A** | **F** | **V** | **T** | **D** | **N** | **I** | **G** | **Q** | **Q** | **V** | **L** | **H** | **V** | **L** | **E** | **E** | **K** | **A** | **A** | **E** | **G** | **V** | **E** | **V** | **R** | **I** | **L** | **Y** | **D** | **A** | **F** | **G** | **S** | **H** | **G** | **T** | **K** | **A** | **K** | **D** | **F** | **N** | **R** | **L** | **I** | **K** | **N** | **G** | **G** | **H** | **V** | **H** | **T** | **F** | **V** | **T** | **S** | **Q** | **R** | **A** | **L** | **L** | **R** | **F** | **R** | **L** | **N** | **Y** | **H** | **D** | **H** | **R** | **K** | **I** | **V** | **V** | **I** | **D** | **G** | **K** | **I** | **S** | **Y** | **T** | **G** | **G** | **F** | **N** | **I** | **A** | **N** | **Q** | **Y** | **V** | **N** | **T** | **T** | **K** | **K** | **F** | **G** | **Y** | **W** | **R** | **D** | **T** | **H** | **I** | **R** | **I** | **F** | **G** | **A** | **A** | **S** | **S** | **L** | **L** | **Q** | **L** | **R** | **F** | **L** | **T** | **D** | **W** | **N** | **V** | **S** | **V** | **P** | **E** | **E** | **K** | **K** | **V** | **G** | **Y** | **H** | **L** | **N** | **Y** | **F** | **F** | **K** | **K** | **A** | **D** | **R** | **D** | **E** | **S** | **K** | **L** | **A** | **D** | **T** | **S** | **I** | **Q** | **L** | **V** | **S** | **S** | **G** | **P** | **N** | **N** | **E** | **R** | **E** | **Q** | **I** | **K** | **L** | **S** | **F** | **I** | **K** | **L** | **I** | **T** | **S** | **A** | **K** | **K** | **R** | **V** | **W** | **I** | **Q** | **T** | **P** | **Y** | **L** | **V** | **P** | **D** | **E** | **S** | **V** | **I** | **A** | **A** | **L** | **K** | **I** | **A** | **T** | **A** | **S** | **G** | **V** | **D** | **V** | **K** | **I** | **M** | **I** | **P** | **N** | **K** | **P** | **D** | **H** | **P** | **F** | **I** | **Y** | **R** | **A** | **T** | **Q** | **Y** | **Y** | **A** | **R** | **Q** | **L** | **I** | **K** | **E** | **N** | **V** | **Q** | **I** | **L** | **V** | **Y** | **E** | **N** | **G** | **F** | **L** | **H** | **A** | **K** | **T** | **L** | **I** | **M** | **D** | **D** | **E** | **I** | **C** | **M** | **V** | **G** | **S** | **A** | **N** | **Q** | **D** | **I** | **R** | **S** | **Y** | **R** | **L** | **N** | **F** | **E** | **T** | **S** | **A** | **V** | **I** | **Y** | **D** | **P** | **E** | **F** | **L** | **E** | **E** | **L** | **A** | **T** | **Q** | **F** | **K** | **E** | **D** | **E** | **T** | **H** | **C** | **S** | **S** | **M** | **T** | **T** | **E** | **T** | **V** | **K** | **E** | **M** | **S** | **N** | **W** | **L** | **L** | **F** | **K** | **Q** | **Q** | **I** | **S** | **R** | **L** | **F** | **S** | **P** | **I** | **L** |
| **N52751** | **-** | **-** | **-** | **-** | **-** | **-** | **-** | **-** | **-** | **-** | **-** | **-** | **-** | **-** | **-** | **-** | **-** | **-** | **-** | **-** | **-** | **-** | **-** | **K** | **P** | **R** | **D** | **V** | **A** | **A** | **I** | **W** | **A** | **W** | **L** | **L** | **V** | **L** | **F** | **A** | **L** | **P** | **G** | **V** | **G** | **F** | **V** | **L** | **Y** | **L** | **F** | **F** | **G** | **R** | **G** | **L** | **T** | **D** | **K** | **K** | **K** | **F** | **Y** | **L** | **R** | **Q** | **S** | **D** | **L** | **K** | **E** | **L** | **E** | **N** | **F** | **Q** | **S** | **F** | **R** | **G** | **D** | **N** | **I** | **E** | **H** | **Y** | **D** | **P** | **D** | **M** | **G** | **D** | **K** | **D** | **K** | **Q** | **Q** | **F** | **V** | **D** | **F** | **F** | **S** | **S** | **L** | **N** | **R** | **M** | **P** | **L** | **T** | **R** | **M** | **N** | **S** | **V** | **T** | **L** | **L** | **T** | **D** | **G** | **Q** | **E** | **K** | **L** | **D** | **S** | **L** | **L** | **Q** | **D** | **L** | **K** | **K** | **A** | **K** | **H** | **S** | **I** | **H** | **I** | **E** | **Y** | **Y** | **A** | **F** | **V** | **T** | **D** | **N** | **I** | **G** | **Q** | **Q** | **V** | **L** | **H** | **V** | **L** | **E** | **E** | **K** | **A** | **A** | **E** | **G** | **V** | **E** | **V** | **R** | **I** | **L** | **Y** | **D** | **A** | **F** | **G** | **S** | **H** | **G** | **T** | **K** | **A** | **K** | **D** | **F** | **N** | **R** | **L** | **I** | **K** | **N** | **G** | **G** | **H** | **V** | **H** | **T** | **F** | **V** | **T** | **S** | **Q** | **K** | **A** | **L** | **L** | **R** | **F** | **R** | **L** | **N** | **Y** | **H** | **D** | **H** | **R** | **K** | **I** | **V** | **V** | **I** | **D** | **G** | **K** | **I** | **S** | **Y** | **T** | **G** | **G** | **F** | **N** | **I** | **A** | **N** | **Q** | **Y** | **V** | **N** | **T** | **T** | **K** | **K** | **F** | **G** | **Y** | **W** | **R** | **D** | **T** | **H** | **I** | **R** | **I** | **F** | **G** | **A** | **A** | **S** | **S** | **L** | **L** | **Q** | **L** | **R** | **F** | **L** | **T** | **D** | **W** | **N** | **V** | **S** | **V** | **P** | **E** | **E** | **K** | **K** | **V** | **G** | **Y** | **H** | **L** | **N** | **Y** | **F** | **F** | **K** | **K** | **A** | **D** | **R** | **D** | **E** | **S** | **K** | **L** | **A** | **D** | **T** | **S** | **I** | **Q** | **L** | **V** | **S** | **S** | **G** | **P** | **N** | **N** | **E** | **R** | **E** | **Q** | **I** | **K** | **L** | **S** | **F** | **I** | **K** | **L** | **I** | **T** | **S** | **A** | **K** | **K** | **R** | **V** | **W** | **I** | **Q** | **T** | **P** | **Y** | **L** | **V** | **P** | **D** | **E** | **S** | **V** | **I** | **A** | **A** | **L** | **K** | **I** | **A** | **T** | **A** | **S** | **G** | **V** | **D** | **V** | **K** | **I** | **M** | **I** | **P** | **N** | **K** | **P** | **D** | **H** | **P** | **F** | **I** | **Y** | **R** | **A** | **T** | **Q** | **Y** | **Y** | **A** | **R** | **Q** | **L** | **I** | **K** | **E** | **N** | **V** | **Q** | **I** | **L** | **V** | **Y** | **E** | **N** | **G** | **F** | **L** | **H** | **A** | **K** | **T** | **L** | **I** | **M** | **D** | **D** | **E** | **I** | **C** | **M** | **V** | **G** | **S** | **A** | **N** | **Q** | **D** | **I** | **R** | **S** | **Y** | **R** | **L** | **N** | **F** | **E** | **T** | **S** | **A** | **V** | **I** | **Y** | **D** | **P** | **E** | **F** | **L** | **E** | **E** | **L** | **A** | **T** | **Q** | **F** | **K** | **E** | **D** | **E** | **T** | **H** | **C** | **S** | **S** | **M** | **T** | **T** | **E** | **T** | **V** | **K** | **E** | **M** | **S** | **N** | **W** | **L** | **L** | **F** | **K** | **Q** | **Q** | **I** | **S** | **R** | **L** | **F** | **S** | **P** | **I** | **L** |
| **N55279** | **-** | **-** | **-** | **-** | **-** | **-** | **-** | **-** | **-** | **-** | **-** | **-** | **-** | **-** | **-** | **-** | **-** | **-** | **-** | **-** | **-** | **-** | **-** | **K** | **P** | **R** | **D** | **V** | **A** | **A** | **I** | **W** | **A** | **W** | **L** | **L** | **V** | **L** | **F** | **A** | **L** | **P** | **G** | **V** | **G** | **F** | **V** | **L** | **Y** | **L** | **F** | **F** | **G** | **R** | **G** | **L** | **T** | **D** | **K** | **K** | **K** | **F** | **Y** | **L** | **R** | **Q** | **S** | **D** | **L** | **K** | **E** | **L** | **E** | **N** | **F** | **Q** | **S** | **F** | **R** | **G** | **D** | **T** | **I** | **E** | **H** | **Y** | **D** | **P** | **D** | **M** | **G** | **D** | **K** | **D** | **K** | **Q** | **Q** | **F** | **V** | **D** | **F** | **F** | **S** | **S** | **L** | **N** | **R** | **M** | **P** | **L** | **T** | **R** | **M** | **N** | **S** | **V** | **T** | **L** | **L** | **T** | **D** | **G** | **Q** | **K** | **K** | **L** | **D** | **S** | **L** | **L** | **Q** | **D** | **L** | **K** | **K** | **A** | **K** | **H** | **S** | **I** | **H** | **I** | **E** | **Y** | **Y** | **A** | **F** | **V** | **T** | **D** | **N** | **I** | **G** | **Q** | **Q** | **V** | **L** | **R** | **V** | **L** | **E** | **E** | **K** | **A** | **A** | **E** | **G** | **V** | **E** | **V** | **R** | **I** | **L** | **Y** | **D** | **A** | **F** | **G** | **S** | **H** | **G** | **T** | **K** | **A** | **K** | **D** | **F** | **N** | **R** | **L** | **I** | **K** | **N** | **G** | **G** | **H** | **V** | **H** | **T** | **F** | **V** | **T** | **S** | **Q** | **K** | **A** | **L** | **L** | **R** | **F** | **R** | **L** | **N** | **Y** | **H** | **D** | **H** | **R** | **K** | **I** | **V** | **V** | **I** | **D** | **G** | **K** | **I** | **S** | **Y** | **T** | **G** | **G** | **F** | **N** | **I** | **A** | **N** | **Q** | **Y** | **V** | **N** | **T** | **T** | **K** | **K** | **F** | **G** | **Y** | **W** | **R** | **D** | **T** | **H** | **I** | **R** | **I** | **F** | **G** | **A** | **A** | **S** | **S** | **L** | **L** | **Q** | **L** | **R** | **F** | **L** | **T** | **D** | **W** | **N** | **V** | **S** | **V** | **S** | **E** | **E** | **K** | **K** | **V** | **G** | **Y** | **H** | **L** | **N** | **Y** | **F** | **F** | **K** | **K** | **A** | **D** | **R** | **D** | **E** | **S** | **K** | **L** | **A** | **D** | **T** | **S** | **I** | **Q** | **L** | **V** | **S** | **S** | **G** | **P** | **N** | **N** | **E** | **R** | **E** | **Q** | **I** | **K** | **L** | **S** | **F** | **I** | **K** | **L** | **I** | **T** | **S** | **A** | **K** | **K** | **R** | **V** | **W** | **I** | **Q** | **T** | **P** | **Y** | **L** | **V** | **P** | **D** | **E** | **S** | **V** | **I** | **A** | **A** | **L** | **K** | **I** | **A** | **T** | **A** | **S** | **G** | **V** | **D** | **V** | **K** | **I** | **M** | **I** | **P** | **N** | **K** | **P** | **D** | **H** | **P** | **F** | **I** | **Y** | **R** | **A** | **T** | **Q** | **Y** | **Y** | **A** | **R** | **Q** | **L** | **I** | **K** | **E** | **N** | **V** | **Q** | **I** | **L** | **V** | **Y** | **E** | **N** | **G** | **F** | **L** | **H** | **A** | **K** | **T** | **L** | **I** | **M** | **D** | **D** | **E** | **I** | **C** | **M** | **V** | **G** | **S** | **A** | **N** | **Q** | **D** | **I** | **R** | **S** | **Y** | **R** | **L** | **N** | **F** | **E** | **T** | **S** | **A** | **V** | **I** | **Y** | **D** | **P** | **E** | **F** | **L** | **E** | **E** | **L** | **A** | **T** | **Q** | **F** | **K** | **E** | **D** | **E** | **T** | **H** | **C** | **S** | **P** | **M** | **T** | **T** | **E** | **T** | **V** | **K** | **E** | **M** | **S** | **N** | **W** | **L** | **L** | **F** | **K** | **Q** | **Q** | **I** | **S** | **R** | **L** | **F** | **S** | **P** | **I** | **L** |
| **N54190** | **-** | **-** | **-** | **-** | **-** | **-** | **-** | **-** | **-** | **-** | **-** | **-** | **-** | **-** | **-** | **-** | **-** | **-** | **-** | **-** | **-** | **-** | **-** | **K** | **P** | **R** | **D** | **V** | **A** | **A** | **I** | **W** | **A** | **W** | **L** | **L** | **V** | **L** | **F** | **A** | **L** | **P** | **G** | **V** | **G** | **F** | **V** | **L** | **Y** | **L** | **F** | **F** | **G** | **R** | **G** | **L** | **T** | **D** | **K** | **K** | **K** | **F** | **Y** | **L** | **R** | **Q** | **S** | **D** | **L** | **K** | **E** | **L** | **E** | **N** | **F** | **Q** | **S** | **F** | **R** | **G** | **D** | **N** | **I** | **E** | **H** | **Y** | **D** | **P** | **D** | **M** | **G** | **D** | **K** | **D** | **K** | **Q** | **Q** | **F** | **V** | **D** | **F** | **F** | **S** | **S** | **L** | **N** | **R** | **M** | **P** | **L** | **T** | **R** | **M** | **N** | **S** | **V** | **T** | **L** | **L** | **T** | **D** | **G** | **Q** | **E** | **K** | **L** | **D** | **S** | **L** | **L** | **Q** | **D** | **L** | **K** | **K** | **A** | **K** | **H** | **S** | **I** | **H** | **I** | **E** | **Y** | **Y** | **A** | **F** | **V** | **T** | **D** | **N** | **I** | **G** | **Q** | **Q** | **V** | **L** | **H** | **V** | **L** | **E** | **E** | **K** | **A** | **A** | **E** | **G** | **V** | **E** | **V** | **R** | **I** | **L** | **Y** | **D** | **A** | **F** | **G** | **S** | **H** | **G** | **T** | **K** | **A** | **K** | **D** | **F** | **N** | **R** | **L** | **I** | **K** | **N** | **G** | **G** | **H** | **V** | **H** | **T** | **F** | **V** | **T** | **S** | **Q** | **K** | **A** | **L** | **L** | **R** | **F** | **R** | **L** | **N** | **Y** | **H** | **D** | **H** | **R** | **K** | **I** | **V** | **V** | **I** | **D** | **G** | **K** | **I** | **S** | **Y** | **T** | **G** | **G** | **F** | **N** | **I** | **A** | **N** | **Q** | **Y** | **V** | **N** | **T** | **T** | **K** | **K** | **F** | **G** | **Y** | **W** | **R** | **D** | **T** | **H** | **I** | **R** | **I** | **F** | **G** | **A** | **A** | **S** | **S** | **L** | **L** | **Q** | **L** | **R** | **F** | **L** | **T** | **D** | **W** | **N** | **V** | **S** | **V** | **P** | **E** | **E** | **K** | **K** | **V** | **G** | **Y** | **H** | **L** | **N** | **Y** | **F** | **F** | **K** | **K** | **A** | **D** | **R** | **D** | **E** | **S** | **K** | **L** | **A** | **D** | **T** | **S** | **I** | **Q** | **L** | **V** | **S** | **S** | **G** | **P** | **N** | **N** | **E** | **R** | **E** | **Q** | **I** | **K** | **L** | **S** | **F** | **I** | **K** | **L** | **I** | **T** | **S** | **A** | **K** | **K** | **R** | **V** | **W** | **I** | **Q** | **T** | **P** | **Y** | **L** | **V** | **P** | **D** | **E** | **S** | **V** | **I** | **A** | **A** | **L** | **K** | **I** | **A** | **T** | **A** | **S** | **G** | **V** | **D** | **V** | **K** | **I** | **M** | **I** | **P** | **N** | **K** | **P** | **D** | **H** | **P** | **F** | **I** | **Y** | **R** | **A** | **T** | **Q** | **Y** | **Y** | **A** | **R** | **Q** | **L** | **I** | **K** | **E** | **N** | **V** | **Q** | **I** | **L** | **V** | **Y** | **E** | **N** | **G** | **F** | **L** | **H** | **A** | **K** | **T** | **L** | **I** | **M** | **D** | **D** | **E** | **I** | **C** | **M** | **V** | **G** | **S** | **A** | **N** | **Q** | **D** | **I** | **R** | **S** | **Y** | **R** | **L** | **N** | **F** | **E** | **T** | **S** | **A** | **V** | **I** | **Y** | **D** | **P** | **E** | **F** | **L** | **E** | **E** | **L** | **A** | **T** | **Q** | **F** | **K** | **E** | **D** | **E** | **T** | **H** | **C** | **S** | **S** | **M** | **T** | **T** | **E** | **T** | **V** | **K** | **E** | **M** | **S** | **N** | **W** | **L** | **L** | **F** | **K** | **Q** | **Q** | **I** | **S** | **R** | **L** | **F** | **S** | **P** | **I** | **L** |
| **N59435F** | **-** | **-** | **-** | **-** | **-** | **-** | **-** | **-** | **-** | **-** | **-** | **-** | **-** | **-** | **-** | **-** | **-** | **-** | **-** | **-** | **-** | **-** | **-** | **K** | **P** | **R** | **D** | **V** | **A** | **A** | **I** | **W** | **A** | **W** | **L** | **L** | **V** | **L** | **F** | **A** | **L** | **P** | **G** | **V** | **G** | **F** | **V** | **L** | **Y** | **L** | **F** | **F** | **G** | **R** | **G** | **L** | **T** | **D** | **K** | **K** | **K** | **F** | **Y** | **L** | **R** | **Q** | **S** | **D** | **L** | **K** | **E** | **L** | **E** | **N** | **F** | **Q** | **S** | **F** | **R** | **G** | **D** | **T** | **I** | **E** | **H** | **Y** | **D** | **P** | **D** | **M** | **G** | **D** | **K** | **D** | **K** | **Q** | **Q** | **F** | **V** | **D** | **F** | **F** | **S** | **S** | **L** | **N** | **R** | **M** | **P** | **L** | **T** | **R** | **M** | **N** | **S** | **V** | **T** | **L** | **L** | **T** | **D** | **G** | **Q** | **E** | **K** | **L** | **D** | **S** | **L** | **L** | **Q** | **D** | **L** | **K** | **K** | **A** | **K** | **H** | **S** | **I** | **H** | **I** | **E** | **Y** | **Y** | **A** | **F** | **V** | **T** | **D** | **N** | **I** | **G** | **Q** | **Q** | **V** | **L** | **H** | **V** | **L** | **E** | **E** | **K** | **A** | **A** | **E** | **G** | **V** | **E** | **V** | **R** | **I** | **L** | **Y** | **D** | **A** | **F** | **G** | **S** | **H** | **G** | **T** | **K** | **A** | **K** | **D** | **F** | **N** | **R** | **L** | **I** | **K** | **N** | **G** | **G** | **H** | **V** | **H** | **T** | **F** | **V** | **T** | **S** | **Q** | **K** | **A** | **L** | **L** | **R** | **F** | **R** | **L** | **N** | **Y** | **H** | **D** | **H** | **R** | **K** | **I** | **V** | **V** | **I** | **D** | **G** | **K** | **I** | **S** | **Y** | **T** | **G** | **G** | **F** | **N** | **I** | **A** | **N** | **Q** | **Y** | **V** | **N** | **T** | **T** | **K** | **K** | **F** | **G** | **Y** | **W** | **R** | **D** | **T** | **H** | **I** | **R** | **I** | **F** | **G** | **A** | **A** | **S** | **S** | **L** | **L** | **Q** | **L** | **R** | **F** | **L** | **T** | **D** | **W** | **N** | **V** | **S** | **V** | **P** | **E** | **E** | **K** | **K** | **V** | **G** | **Y** | **H** | **L** | **N** | **Y** | **F** | **F** | **K** | **K** | **A** | **D** | **R** | **D** | **E** | **S** | **K** | **L** | **A** | **D** | **T** | **S** | **I** | **Q** | **L** | **V** | **S** | **S** | **G** | **P** | **N** | **N** | **E** | **R** | **E** | **Q** | **I** | **K** | **L** | **S** | **F** | **I** | **K** | **L** | **I** | **T** | **S** | **A** | **K** | **K** | **R** | **V** | **W** | **I** | **Q** | **T** | **P** | **Y** | **L** | **V** | **P** | **D** | **E** | **S** | **V** | **I** | **A** | **A** | **L** | **K** | **I** | **A** | **T** | **A** | **S** | **G** | **V** | **D** | **V** | **K** | **I** | **M** | **I** | **P** | **N** | **K** | **P** | **D** | **H** | **P** | **F** | **I** | **Y** | **R** | **A** | **T** | **Q** | **Y** | **Y** | **A** | **R** | **Q** | **L** | **I** | **K** | **E** | **N** | **V** | **Q** | **I** | **L** | **V** | **Y** | **E** | **N** | **G** | **F** | **L** | **H** | **A** | **K** | **T** | **L** | **I** | **M** | **D** | **D** | **E** | **I** | **C** | **M** | **V** | **G** | **S** | **A** | **N** | **Q** | **D** | **I** | **R** | **S** | **Y** | **R** | **L** | **N** | **F** | **E** | **T** | **S** | **A** | **V** | **I** | **Y** | **D** | **P** | **E** | **F** | **L** | **E** | **E** | **L** | **A** | **T** | **Q** | **F** | **K** | **E** | **D** | **E** | **T** | **H** | **C** | **S** | **S** | **M** | **T** | **T** | **E** | **T** | **V** | **K** | **E** | **M** | **S** | **N** | **W** | **L** | **L** | **F** | **K** | **Q** | **Q** | **I** | **S** | **R** | **L** | **F** | **S** | **P** | **I** | **L** |
| **N59653F** | **-** | **-** | **-** | **-** | **-** | **-** | **-** | **-** | **-** | **-** | **-** | **-** | **-** | **-** | **-** | **-** | **-** | **-** | **-** | **-** | **-** | **-** | **-** | **K** | **P** | **R** | **D** | **V** | **A** | **A** | **I** | **W** | **A** | **W** | **L** | **L** | **V** | **L** | **F** | **A** | **L** | **P** | **G** | **V** | **G** | **F** | **V** | **L** | **Y** | **L** | **F** | **F** | **G** | **R** | **G** | **L** | **T** | **D** | **K** | **K** | **K** | **F** | **Y** | **L** | **R** | **Q** | **S** | **D** | **L** | **K** | **E** | **L** | **E** | **N** | **F** | **Q** | **S** | **F** | **R** | **G** | **D** | **T** | **I** | **E** | **H** | **Y** | **D** | **P** | **D** | **M** | **G** | **D** | **K** | **D** | **K** | **Q** | **Q** | **F** | **V** | **D** | **F** | **F** | **S** | **S** | **L** | **N** | **R** | **M** | **P** | **L** | **T** | **R** | **M** | **N** | **S** | **V** | **T** | **L** | **L** | **T** | **D** | **G** | **Q** | **K** | **K** | **L** | **D** | **S** | **L** | **L** | **Q** | **D** | **L** | **K** | **K** | **A** | **K** | **H** | **S** | **I** | **H** | **I** | **E** | **Y** | **Y** | **A** | **F** | **V** | **T** | **D** | **I** | **I** | **G** | **Q** | **Q** | **V** | **L** | **R** | **V** | **L** | **E** | **E** | **K** | **A** | **A** | **E** | **G** | **V** | **E** | **V** | **R** | **I** | **L** | **Y** | **D** | **A** | **F** | **G** | **S** | **H** | **G** | **T** | **K** | **A** | **K** | **D** | **F** | **N** | **R** | **L** | **I** | **K** | **N** | **G** | **G** | **H** | **V** | **H** | **T** | **F** | **V** | **T** | **S** | **Q** | **K** | **A** | **L** | **L** | **R** | **F** | **R** | **L** | **N** | **Y** | **H** | **D** | **H** | **R** | **K** | **I** | **V** | **V** | **I** | **D** | **G** | **K** | **I** | **S** | **Y** | **T** | **G** | **G** | **F** | **N** | **I** | **A** | **N** | **Q** | **Y** | **V** | **N** | **T** | **T** | **K** | **K** | **F** | **G** | **Y** | **W** | **R** | **D** | **T** | **H** | **I** | **R** | **I** | **F** | **G** | **A** | **A** | **S** | **S** | **L** | **L** | **Q** | **L** | **R** | **F** | **L** | **T** | **D** | **W** | **N** | **V** | **S** | **V** | **P** | **E** | **E** | **K** | **K** | **V** | **G** | **Y** | **H** | **L** | **N** | **Y** | **F** | **F** | **K** | **K** | **A** | **D** | **R** | **D** | **E** | **S** | **K** | **L** | **A** | **D** | **M** | **S** | **I** | **Q** | **L** | **V** | **S** | **S** | **G** | **P** | **N** | **N** | **E** | **R** | **E** | **Q** | **I** | **K** | **L** | **S** | **F** | **I** | **K** | **L** | **I** | **T** | **S** | **A** | **K** | **K** | **R** | **V** | **W** | **I** | **Q** | **T** | **P** | **Y** | **L** | **V** | **P** | **D** | **E** | **S** | **V** | **I** | **A** | **A** | **L** | **K** | **I** | **A** | **T** | **A** | **S** | **G** | **V** | **D** | **V** | **K** | **I** | **M** | **I** | **P** | **N** | **K** | **P** | **D** | **H** | **P** | **F** | **I** | **Y** | **R** | **A** | **T** | **Q** | **Y** | **Y** | **A** | **R** | **Q** | **L** | **I** | **K** | **E** | **N** | **V** | **Q** | **I** | **L** | **V** | **Y** | **E** | **N** | **G** | **F** | **L** | **H** | **A** | **K** | **T** | **L** | **I** | **M** | **D** | **D** | **E** | **I** | **C** | **M** | **V** | **G** | **S** | **A** | **N** | **Q** | **D** | **I** | **R** | **S** | **Y** | **R** | **L** | **N** | **F** | **E** | **T** | **S** | **A** | **V** | **I** | **Y** | **D** | **P** | **E** | **F** | **L** | **E** | **E** | **L** | **A** | **T** | **Q** | **F** | **K** | **E** | **D** | **E** | **T** | **H** | **C** | **S** | **P** | **M** | **T** | **T** | **E** | **T** | **V** | **K** | **E** | **M** | **S** | **N** | **W** | **L** | **L** | **F** | **K** | **Q** | **Q** | **I** | **S** | **R** | **L** | **F** | **S** | **P** | **I** | **L** |
| **N55317** | **-** | **-** | **-** | **-** | **-** | **-** | **-** | **-** | **-** | **-** | **-** | **-** | **-** | **-** | **-** | **-** | **-** | **-** | **-** | **-** | **-** | **-** | **-** | **K** | **P** | **R** | **D** | **V** | **A** | **A** | **I** | **W** | **A** | **W** | **L** | **L** | **V** | **L** | **F** | **A** | **L** | **P** | **G** | **V** | **G** | **F** | **V** | **L** | **Y** | **L** | **F** | **F** | **G** | **R** | **G** | **L** | **T** | **D** | **K** | **K** | **K** | **F** | **Y** | **L** | **R** | **Q** | **S** | **D** | **L** | **K** | **E** | **L** | **E** | **N** | **F** | **Q** | **S** | **F** | **R** | **G** | **D** | **T** | **I** | **E** | **H** | **Y** | **D** | **P** | **D** | **M** | **G** | **D** | **K** | **D** | **K** | **Q** | **Q** | **F** | **V** | **D** | **F** | **F** | **S** | **S** | **L** | **N** | **R** | **M** | **P** | **L** | **T** | **R** | **M** | **N** | **S** | **V** | **T** | **L** | **L** | **T** | **D** | **G** | **Q** | **K** | **K** | **L** | **D** | **S** | **L** | **L** | **Q** | **D** | **L** | **K** | **K** | **A** | **K** | **H** | **S** | **I** | **H** | **I** | **E** | **Y** | **Y** | **A** | **F** | **V** | **T** | **D** | **T** | **I** | **G** | **Q** | **Q** | **V** | **L** | **R** | **V** | **L** | **E** | **E** | **K** | **A** | **A** | **E** | **G** | **V** | **E** | **V** | **R** | **I** | **L** | **Y** | **D** | **A** | **F** | **G** | **S** | **H** | **G** | **T** | **K** | **A** | **K** | **D** | **F** | **N** | **R** | **L** | **I** | **K** | **N** | **G** | **G** | **H** | **V** | **H** | **T** | **F** | **V** | **T** | **S** | **Q** | **K** | **A** | **L** | **L** | **R** | **F** | **R** | **L** | **N** | **Y** | **H** | **D** | **H** | **R** | **K** | **I** | **V** | **V** | **I** | **D** | **G** | **K** | **I** | **S** | **Y** | **T** | **G** | **G** | **F** | **N** | **I** | **A** | **N** | **Q** | **Y** | **V** | **N** | **T** | **T** | **K** | **K** | **F** | **G** | **Y** | **W** | **R** | **D** | **T** | **H** | **I** | **R** | **I** | **F** | **G** | **A** | **A** | **S** | **S** | **L** | **L** | **Q** | **L** | **R** | **F** | **L** | **T** | **D** | **W** | **N** | **V** | **S** | **V** | **P** | **E** | **E** | **K** | **K** | **V** | **G** | **Y** | **H** | **L** | **N** | **Y** | **F** | **F** | **K** | **K** | **A** | **D** | **R** | **D** | **E** | **S** | **K** | **L** | **A** | **D** | **T** | **S** | **I** | **Q** | **L** | **V** | **S** | **S** | **G** | **P** | **N** | **N** | **E** | **R** | **E** | **Q** | **I** | **K** | **L** | **S** | **F** | **I** | **K** | **L** | **I** | **T** | **S** | **A** | **K** | **K** | **R** | **V** | **W** | **I** | **Q** | **T** | **P** | **Y** | **L** | **V** | **P** | **D** | **E** | **S** | **V** | **I** | **A** | **A** | **L** | **K** | **I** | **A** | **T** | **A** | **S** | **G** | **V** | **D** | **V** | **K** | **I** | **M** | **I** | **P** | **N** | **K** | **P** | **D** | **H** | **P** | **F** | **I** | **Y** | **R** | **A** | **T** | **Q** | **Y** | **Y** | **A** | **R** | **Q** | **L** | **I** | **K** | **E** | **N** | **V** | **Q** | **I** | **L** | **V** | **Y** | **E** | **N** | **G** | **F** | **L** | **H** | **A** | **K** | **T** | **L** | **I** | **M** | **D** | **D** | **E** | **I** | **C** | **M** | **V** | **G** | **S** | **A** | **N** | **Q** | **D** | **I** | **R** | **S** | **Y** | **R** | **L** | **N** | **F** | **E** | **T** | **S** | **A** | **V** | **I** | **Y** | **D** | **P** | **E** | **F** | **L** | **E** | **E** | **L** | **A** | **T** | **Q** | **F** | **K** | **E** | **D** | **E** | **T** | **H** | **C** | **S** | **P** | **M** | **T** | **T** | **E** | **T** | **V** | **K** | **E** | **M** | **S** | **N** | **W** | **L** | **L** | **F** | **K** | **Q** | **Q** | **I** | **S** | **R** | **L** | **F** | **S** | **P** | **I** | **L** |
| **N60299F** | **V** | **V** | **S** | **S** | **I** | **I** | **T** | **A** | **L** | **Y** | **L** | **L** | **N** | **A** | **L** | **I** | **A** | **L** | **V** | **T** | **I** | **L** | **I** | **K** | **P** | **R** | **D** | **V** | **A** | **A** | **I** | **W** | **A** | **W** | **L** | **L** | **V** | **L** | **F** | **A** | **L** | **P** | **G** | **V** | **G** | **F** | **V** | **L** | **Y** | **L** | **F** | **F** | **G** | **R** | **G** | **L** | **T** | **D** | **K** | **K** | **K** | **F** | **Y** | **L** | **R** | **Q** | **S** | **D** | **L** | **K** | **E** | **L** | **E** | **N** | **F** | **Q** | **S** | **F** | **R** | **G** | **D** | **T** | **I** | **E** | **H** | **Y** | **D** | **P** | **D** | **M** | **G** | **D** | **K** | **D** | **K** | **Q** | **Q** | **F** | **V** | **D** | **F** | **F** | **S** | **S** | **L** | **N** | **R** | **M** | **P** | **L** | **A** | **R** | **M** | **N** | **S** | **V** | **T** | **L** | **L** | **T** | **D** | **G** | **Q** | **E** | **K** | **L** | **D** | **S** | **L** | **L** | **Q** | **D** | **L** | **K** | **K** | **A** | **K** | **H** | **S** | **I** | **H** | **I** | **E** | **Y** | **Y** | **A** | **F** | **V** | **T** | **D** | **N** | **I** | **G** | **Q** | **Q** | **V** | **L** | **H** | **V** | **L** | **E** | **E** | **K** | **A** | **A** | **E** | **G** | **V** | **E** | **V** | **R** | **I** | **L** | **Y** | **D** | **A** | **F** | **G** | **S** | **H** | **G** | **T** | **K** | **A** | **K** | **D** | **F** | **N** | **R** | **L** | **I** | **K** | **N** | **G** | **G** | **H** | **V** | **H** | **T** | **F** | **V** | **T** | **S** | **Q** | **K** | **A** | **L** | **L** | **R** | **F** | **R** | **L** | **N** | **Y** | **H** | **D** | **H** | **R** | **K** | **I** | **V** | **V** | **I** | **D** | **G** | **K** | **I** | **S** | **Y** | **T** | **G** | **G** | **F** | **N** | **I** | **A** | **N** | **Q** | **Y** | **V** | **N** | **T** | **T** | **K** | **K** | **F** | **G** | **Y** | **W** | **R** | **D** | **T** | **H** | **I** | **R** | **I** | **F** | **G** | **A** | **A** | **S** | **S** | **L** | **L** | **Q** | **L** | **R** | **F** | **L** | **T** | **D** | **W** | **N** | **V** | **S** | **V** | **P** | **E** | **E** | **K** | **K** | **V** | **G** | **Y** | **H** | **L** | **N** | **Y** | **F** | **F** | **K** | **K** | **A** | **D** | **R** | **D** | **E** | **S** | **K** | **L** | **A** | **D** | **T** | **S** | **I** | **Q** | **L** | **V** | **S** | **S** | **G** | **P** | **N** | **N** | **E** | **R** | **E** | **Q** | **I** | **K** | **L** | **S** | **F** | **I** | **K** | **L** | **I** | **T** | **S** | **A** | **K** | **K** | **R** | **V** | **W** | **I** | **Q** | **T** | **P** | **Y** | **L** | **V** | **P** | **D** | **E** | **S** | **V** | **I** | **A** | **A** | **L** | **K** | **I** | **A** | **T** | **A** | **S** | **G** | **V** | **D** | **V** | **K** | **I** | **M** | **I** | **P** | **N** | **K** | **P** | **D** | **H** | **P** | **F** | **I** | **Y** | **R** | **A** | **T** | **Q** | **Y** | **Y** | **A** | **R** | **Q** | **L** | **I** | **K** | **E** | **N** | **V** | **Q** | **I** | **L** | **V** | **Y** | **E** | **N** | **G** | **F** | **L** | **H** | **A** | **K** | **T** | **L** | **I** | **M** | **D** | **D** | **E** | **I** | **C** | **M** | **V** | **G** | **S** | **A** | **N** | **Q** | **D** | **I** | **R** | **S** | **Y** | **R** | **L** | **N** | **F** | **E** | **T** | **S** | **A** | **V** | **I** | **Y** | **D** | **P** | **E** | **F** | **L** | **E** | **E** | **L** | **A** | **T** | **Q** | **F** | **K** | **E** | **D** | **E** | **T** | **H** | **C** | **S** | **S** | **M** | **T** | **T** | **E** | **T** | **V** | **K** | **E** | **M** | **S** | **N** | **W** | **L** | **L** | **F** | **K** | **Q** | **Q** | **I** | **S** | **R** | **L** | **F** | **S** | **P** | **I** | **L** |
| **N60363F** | **-** | **-** | **-** | **-** | **-** | **-** | **-** | **-** | **-** | **-** | **-** | **-** | **-** | **-** | **-** | **-** | **-** | **-** | **-** | **-** | **-** | **-** | **-** | **K** | **P** | **R** | **D** | **V** | **A** | **A** | **I** | **W** | **A** | **W** | **L** | **L** | **V** | **L** | **F** | **A** | **L** | **P** | **G** | **V** | **G** | **F** | **V** | **L** | **Y** | **L** | **F** | **F** | **G** | **R** | **G** | **L** | **T** | **D** | **K** | **K** | **K** | **F** | **Y** | **L** | **R** | **Q** | **S** | **D** | **L** | **K** | **E** | **L** | **E** | **N** | **F** | **Q** | **S** | **F** | **R** | **G** | **D** | **T** | **I** | **E** | **H** | **Y** | **D** | **P** | **D** | **M** | **G** | **D** | **K** | **D** | **K** | **Q** | **Q** | **F** | **V** | **D** | **F** | **F** | **S** | **S** | **L** | **N** | **R** | **M** | **P** | **L** | **T** | **R** | **M** | **N** | **S** | **V** | **T** | **L** | **L** | **T** | **D** | **G** | **Q** | **E** | **K** | **L** | **D** | **S** | **L** | **L** | **Q** | **D** | **L** | **K** | **K** | **A** | **K** | **H** | **S** | **I** | **H** | **I** | **E** | **Y** | **Y** | **A** | **F** | **V** | **T** | **D** | **N** | **I** | **G** | **Q** | **Q** | **V** | **L** | **H** | **V** | **L** | **E** | **E** | **K** | **A** | **A** | **E** | **G** | **V** | **E** | **V** | **R** | **I** | **L** | **Y** | **D** | **A** | **F** | **G** | **S** | **H** | **G** | **T** | **K** | **A** | **K** | **D** | **F** | **N** | **R** | **L** | **I** | **K** | **N** | **G** | **G** | **H** | **V** | **H** | **T** | **F** | **V** | **T** | **S** | **Q** | **K** | **A** | **L** | **L** | **R** | **F** | **R** | **L** | **N** | **Y** | **H** | **D** | **H** | **R** | **K** | **I** | **V** | **V** | **I** | **D** | **G** | **K** | **I** | **S** | **Y** | **T** | **G** | **G** | **F** | **N** | **I** | **A** | **N** | **Q** | **Y** | **V** | **N** | **T** | **T** | **K** | **K** | **F** | **G** | **Y** | **W** | **R** | **D** | **T** | **H** | **I** | **R** | **I** | **F** | **G** | **A** | **A** | **S** | **S** | **L** | **L** | **Q** | **L** | **R** | **F** | **L** | **T** | **D** | **W** | **N** | **V** | **S** | **V** | **P** | **E** | **E** | **K** | **K** | **V** | **G** | **Y** | **H** | **L** | **N** | **Y** | **F** | **F** | **K** | **K** | **A** | **D** | **R** | **D** | **E** | **S** | **K** | **L** | **A** | **D** | **T** | **S** | **I** | **Q** | **L** | **V** | **S** | **S** | **G** | **P** | **N** | **N** | **E** | **R** | **E** | **Q** | **I** | **K** | **L** | **S** | **F** | **I** | **K** | **L** | **I** | **T** | **S** | **A** | **K** | **K** | **R** | **V** | **W** | **I** | **Q** | **T** | **P** | **Y** | **L** | **V** | **P** | **D** | **E** | **S** | **V** | **I** | **A** | **A** | **L** | **K** | **I** | **A** | **T** | **A** | **S** | **G** | **V** | **D** | **V** | **K** | **I** | **M** | **I** | **P** | **N** | **K** | **P** | **D** | **H** | **P** | **F** | **I** | **Y** | **R** | **A** | **T** | **Q** | **Y** | **Y** | **A** | **R** | **Q** | **L** | **I** | **K** | **E** | **N** | **V** | **Q** | **I** | **L** | **V** | **Y** | **E** | **N** | **G** | **F** | **L** | **H** | **A** | **K** | **T** | **L** | **I** | **M** | **D** | **D** | **E** | **I** | **C** | **M** | **V** | **G** | **S** | **A** | **N** | **Q** | **D** | **I** | **R** | **S** | **Y** | **R** | **L** | **N** | **F** | **E** | **T** | **S** | **A** | **V** | **I** | **Y** | **D** | **P** | **E** | **F** | **L** | **E** | **E** | **L** | **A** | **T** | **Q** | **F** | **K** | **E** | **D** | **E** | **T** | **H** | **C** | **S** | **S** | **M** | **T** | **T** | **E** | **T** | **V** | **K** | **E** | **M** | **S** | **N** | **W** | **L** | **L** | **F** | **K** | **Q** | **Q** | **I** | **S** | **R** | **L** | **F** | **S** | **P** | **I** | **L** |
| **N59779F** | **V** | **V** | **S** | **S** | **I** | **I** | **T** | **G** | **L** | **Y** | **L** | **L** | **N** | **A** | **L** | **I** | **A** | **L** | **V** | **A** | **I** | **L** | **I** | **K** | **P** | **R** | **D** | **V** | **A** | **A** | **I** | **W** | **A** | **W** | **L** | **L** | **V** | **L** | **F** | **A** | **L** | **P** | **G** | **V** | **G** | **F** | **V** | **L** | **Y** | **L** | **F** | **F** | **G** | **R** | **G** | **L** | **T** | **D** | **K** | **K** | **K** | **F** | **Y** | **L** | **R** | **Q** | **S** | **D** | **L** | **K** | **E** | **L** | **E** | **N** | **F** | **Q** | **S** | **F** | **R** | **G** | **D** | **T** | **I** | **E** | **H** | **Y** | **D** | **P** | **D** | **M** | **G** | **D** | **K** | **D** | **K** | **Q** | **Q** | **F** | **V** | **D** | **F** | **F** | **S** | **S** | **L** | **N** | **R** | **M** | **P** | **L** | **T** | **R** | **M** | **N** | **S** | **V** | **T** | **L** | **L** | **T** | **D** | **G** | **Q** | **E** | **K** | **L** | **D** | **S** | **L** | **L** | **Q** | **D** | **L** | **K** | **K** | **A** | **K** | **H** | **S** | **I** | **H** | **I** | **E** | **Y** | **Y** | **A** | **F** | **V** | **T** | **D** | **N** | **I** | **G** | **Q** | **Q** | **V** | **L** | **H** | **V** | **L** | **E** | **E** | **K** | **A** | **A** | **E** | **G** | **V** | **E** | **V** | **R** | **I** | **L** | **Y** | **D** | **A** | **F** | **G** | **S** | **H** | **G** | **T** | **K** | **A** | **K** | **D** | **F** | **N** | **R** | **L** | **I** | **K** | **N** | **G** | **G** | **H** | **V** | **H** | **T** | **F** | **V** | **T** | **S** | **Q** | **K** | **A** | **L** | **L** | **R** | **F** | **R** | **L** | **N** | **Y** | **H** | **D** | **H** | **R** | **K** | **I** | **V** | **V** | **I** | **D** | **G** | **K** | **I** | **S** | **Y** | **T** | **G** | **G** | **F** | **N** | **I** | **A** | **N** | **Q** | **Y** | **V** | **N** | **T** | **T** | **K** | **K** | **F** | **G** | **Y** | **W** | **R** | **D** | **T** | **H** | **I** | **R** | **I** | **F** | **G** | **A** | **A** | **S** | **S** | **L** | **L** | **Q** | **L** | **R** | **F** | **L** | **T** | **D** | **W** | **N** | **V** | **S** | **V** | **P** | **E** | **E** | **K** | **K** | **V** | **G** | **Y** | **H** | **L** | **N** | **Y** | **F** | **F** | **K** | **K** | **A** | **D** | **R** | **D** | **E** | **S** | **K** | **L** | **A** | **D** | **T** | **S** | **I** | **Q** | **L** | **V** | **S** | **S** | **G** | **P** | **N** | **N** | **E** | **R** | **E** | **Q** | **I** | **K** | **L** | **S** | **F** | **I** | **K** | **L** | **I** | **T** | **S** | **A** | **K** | **K** | **R** | **V** | **W** | **I** | **Q** | **T** | **P** | **Y** | **L** | **V** | **P** | **D** | **E** | **S** | **V** | **I** | **A** | **A** | **L** | **K** | **I** | **A** | **T** | **A** | **S** | **G** | **V** | **D** | **V** | **K** | **I** | **M** | **I** | **P** | **N** | **K** | **P** | **D** | **H** | **P** | **F** | **I** | **Y** | **R** | **A** | **T** | **Q** | **Y** | **Y** | **A** | **R** | **Q** | **L** | **I** | **K** | **E** | **N** | **V** | **Q** | **I** | **L** | **V** | **Y** | **E** | **N** | **G** | **F** | **L** | **H** | **A** | **K** | **T** | **L** | **I** | **M** | **D** | **D** | **E** | **I** | **C** | **M** | **V** | **G** | **S** | **A** | **N** | **Q** | **D** | **I** | **R** | **S** | **Y** | **R** | **L** | **N** | **F** | **E** | **T** | **S** | **A** | **V** | **I** | **Y** | **D** | **P** | **E** | **F** | **L** | **E** | **E** | **L** | **A** | **T** | **Q** | **F** | **K** | **E** | **D** | **E** | **T** | **H** | **C** | **S** | **S** | **M** | **T** | **T** | **E** | **T** | **V** | **K** | **E** | **M** | **S** | **N** | **W** | **L** | **L** | **F** | **K** | **Q** | **Q** | **I** | **S** | **R** | **L** | **F** | **S** | **P** | **I** | **L** |
| **N59484F** | **-** | **-** | **-** | **-** | **-** | **-** | **-** | **-** | **-** | **-** | **-** | **-** | **-** | **-** | **-** | **-** | **-** | **-** | **-** | **-** | **-** | **-** | **-** | **K** | **P** | **R** | **D** | **V** | **A** | **A** | **I** | **W** | **A** | **W** | **L** | **L** | **V** | **L** | **F** | **A** | **L** | **P** | **G** | **V** | **G** | **F** | **V** | **L** | **Y** | **L** | **F** | **F** | **G** | **R** | **G** | **L** | **T** | **D** | **K** | **K** | **K** | **F** | **Y** | **L** | **R** | **Q** | **S** | **D** | **L** | **K** | **E** | **L** | **E** | **N** | **F** | **Q** | **S** | **F** | **R** | **G** | **D** | **T** | **I** | **E** | **H** | **Y** | **D** | **P** | **D** | **M** | **G** | **D** | **K** | **D** | **K** | **Q** | **Q** | **F** | **V** | **D** | **F** | **F** | **S** | **S** | **L** | **N** | **R** | **M** | **P** | **L** | **T** | **R** | **M** | **N** | **S** | **V** | **T** | **L** | **L** | **T** | **D** | **G** | **Q** | **E** | **K** | **L** | **D** | **S** | **L** | **L** | **Q** | **D** | **L** | **K** | **K** | **A** | **K** | **H** | **S** | **I** | **H** | **I** | **E** | **Y** | **Y** | **A** | **F** | **V** | **T** | **D** | **N** | **I** | **G** | **Q** | **Q** | **V** | **L** | **H** | **V** | **L** | **E** | **E** | **K** | **A** | **A** | **E** | **G** | **V** | **E** | **V** | **R** | **I** | **L** | **Y** | **D** | **A** | **F** | **G** | **S** | **H** | **G** | **T** | **K** | **A** | **K** | **D** | **F** | **N** | **R** | **L** | **I** | **K** | **N** | **G** | **G** | **H** | **V** | **H** | **T** | **F** | **V** | **T** | **S** | **Q** | **K** | **A** | **L** | **L** | **R** | **F** | **R** | **L** | **N** | **Y** | **H** | **D** | **H** | **R** | **K** | **I** | **V** | **V** | **I** | **D** | **G** | **K** | **I** | **S** | **Y** | **T** | **G** | **G** | **F** | **N** | **I** | **A** | **N** | **Q** | **Y** | **V** | **N** | **T** | **T** | **K** | **K** | **F** | **G** | **Y** | **W** | **R** | **D** | **T** | **H** | **I** | **R** | **I** | **F** | **G** | **A** | **A** | **S** | **S** | **L** | **L** | **Q** | **L** | **R** | **F** | **L** | **T** | **D** | **W** | **N** | **V** | **S** | **V** | **P** | **E** | **E** | **K** | **K** | **V** | **G** | **Y** | **H** | **L** | **N** | **Y** | **F** | **F** | **K** | **K** | **A** | **D** | **R** | **D** | **E** | **S** | **K** | **L** | **A** | **D** | **T** | **S** | **I** | **Q** | **L** | **V** | **S** | **S** | **G** | **P** | **N** | **N** | **E** | **R** | **E** | **Q** | **I** | **K** | **L** | **S** | **F** | **I** | **K** | **L** | **I** | **T** | **S** | **A** | **K** | **K** | **R** | **V** | **W** | **I** | **Q** | **T** | **P** | **Y** | **L** | **V** | **P** | **D** | **E** | **S** | **V** | **I** | **A** | **A** | **L** | **K** | **I** | **A** | **T** | **A** | **S** | **G** | **V** | **D** | **V** | **K** | **I** | **M** | **I** | **P** | **N** | **K** | **P** | **D** | **H** | **P** | **F** | **I** | **Y** | **R** | **A** | **T** | **Q** | **Y** | **Y** | **A** | **R** | **Q** | **L** | **I** | **K** | **E** | **N** | **V** | **Q** | **I** | **L** | **V** | **Y** | **E** | **N** | **G** | **F** | **L** | **H** | **A** | **K** | **T** | **L** | **I** | **M** | **D** | **D** | **E** | **I** | **C** | **M** | **V** | **G** | **S** | **A** | **N** | **Q** | **D** | **I** | **R** | **S** | **Y** | **R** | **L** | **N** | **F** | **E** | **T** | **S** | **A** | **V** | **I** | **Y** | **D** | **P** | **E** | **F** | **L** | **E** | **E** | **L** | **A** | **T** | **Q** | **F** | **K** | **E** | **D** | **E** | **T** | **H** | **C** | **S** | **S** | **M** | **T** | **T** | **E** | **T** | **V** | **K** | **E** | **M** | **S** | **N** | **W** | **L** | **L** | **F** | **K** | **Q** | **Q** | **I** | **S** | **R** | **L** | **F** | **S** | **P** | **I** | **L** |
| **N53805** | **-** | **-** | **-** | **-** | **-** | **-** | **-** | **-** | **-** | **-** | **-** | **-** | **-** | **-** | **-** | **-** | **-** | **-** | **-** | **-** | **-** | **-** | **-** | **K** | **P** | **R** | **D** | **V** | **A** | **A** | **I** | **W** | **A** | **W** | **L** | **L** | **V** | **L** | **F** | **V** | **L** | **P** | **G** | **V** | **G** | **F** | **V** | **L** | **Y** | **L** | **F** | **F** | **G** | **R** | **G** | **L** | **T** | **D** | **K** | **K** | **K** | **F** | **Y** | **L** | **R** | **Q** | **S** | **D** | **L** | **K** | **E** | **L** | **E** | **N** | **F** | **Q** | **S** | **F** | **R** | **G** | **D** | **T** | **I** | **E** | **H** | **Y** | **D** | **P** | **D** | **M** | **G** | **D** | **K** | **D** | **K** | **Q** | **Q** | **F** | **V** | **D** | **F** | **F** | **S** | **S** | **L** | **N** | **R** | **M** | **P** | **L** | **T** | **R** | **M** | **N** | **S** | **V** | **T** | **L** | **L** | **T** | **D** | **G** | **Q** | **E** | **K** | **L** | **D** | **S** | **L** | **L** | **Q** | **D** | **L** | **K** | **K** | **A** | **K** | **H** | **S** | **I** | **H** | **I** | **E** | **Y** | **Y** | **A** | **F** | **V** | **T** | **D** | **N** | **I** | **G** | **Q** | **Q** | **V** | **L** | **H** | **V** | **L** | **E** | **E** | **K** | **A** | **A** | **E** | **G** | **V** | **E** | **V** | **R** | **I** | **L** | **Y** | **D** | **A** | **F** | **G** | **S** | **H** | **G** | **T** | **K** | **A** | **K** | **D** | **F** | **N** | **R** | **L** | **I** | **K** | **N** | **G** | **G** | **H** | **V** | **H** | **T** | **F** | **V** | **T** | **S** | **Q** | **K** | **A** | **L** | **L** | **R** | **F** | **R** | **L** | **N** | **Y** | **H** | **D** | **H** | **R** | **K** | **I** | **V** | **V** | **I** | **D** | **G** | **K** | **I** | **S** | **Y** | **T** | **G** | **G** | **F** | **N** | **I** | **A** | **N** | **Q** | **Y** | **V** | **N** | **T** | **T** | **K** | **K** | **F** | **G** | **Y** | **W** | **R** | **D** | **T** | **H** | **I** | **R** | **I** | **F** | **G** | **A** | **A** | **S** | **S** | **L** | **L** | **Q** | **L** | **R** | **F** | **L** | **T** | **D** | **W** | **N** | **V** | **S** | **V** | **P** | **E** | **E** | **K** | **K** | **V** | **G** | **Y** | **H** | **L** | **N** | **Y** | **F** | **F** | **K** | **K** | **A** | **D** | **R** | **D** | **E** | **S** | **K** | **L** | **A** | **D** | **T** | **S** | **I** | **Q** | **L** | **V** | **S** | **S** | **G** | **P** | **N** | **N** | **E** | **R** | **E** | **Q** | **I** | **K** | **L** | **S** | **F** | **I** | **K** | **L** | **I** | **T** | **S** | **A** | **K** | **K** | **R** | **V** | **W** | **I** | **Q** | **T** | **P** | **Y** | **L** | **V** | **P** | **D** | **E** | **S** | **V** | **I** | **A** | **A** | **L** | **K** | **I** | **A** | **T** | **A** | **S** | **G** | **V** | **D** | **V** | **K** | **I** | **M** | **I** | **P** | **N** | **K** | **P** | **D** | **H** | **P** | **F** | **I** | **Y** | **R** | **A** | **T** | **Q** | **Y** | **Y** | **A** | **R** | **Q** | **L** | **I** | **K** | **E** | **N** | **V** | **Q** | **I** | **L** | **V** | **Y** | **E** | **N** | **G** | **F** | **L** | **H** | **A** | **K** | **T** | **L** | **I** | **M** | **D** | **D** | **E** | **I** | **C** | **M** | **V** | **G** | **S** | **A** | **N** | **Q** | **D** | **I** | **R** | **S** | **Y** | **R** | **L** | **N** | **F** | **E** | **T** | **S** | **A** | **V** | **I** | **Y** | **D** | **P** | **E** | **F** | **L** | **E** | **E** | **L** | **A** | **T** | **Q** | **F** | **K** | **E** | **D** | **E** | **T** | **H** | **C** | **S** | **S** | **M** | **T** | **T** | **E** | **T** | **V** | **K** | **E** | **M** | **S** | **N** | **W** | **L** | **L** | **F** | **K** | **Q** | **Q** | **I** | **S** | **R** | **L** | **F** | **S** | **P** | **I** | **L** |
| **N53595** | **-** | **-** | **-** | **-** | **-** | **-** | **-** | **-** | **-** | **-** | **-** | **-** | **-** | **-** | **-** | **-** | **-** | **-** | **-** | **-** | **-** | **-** | **-** | **K** | **P** | **R** | **D** | **V** | **A** | **A** | **I** | **W** | **A** | **W** | **L** | **L** | **V** | **L** | **F** | **A** | **L** | **P** | **G** | **V** | **G** | **F** | **V** | **L** | **Y** | **L** | **F** | **F** | **G** | **R** | **G** | **L** | **T** | **D** | **K** | **K** | **K** | **F** | **Y** | **L** | **R** | **Q** | **S** | **D** | **L** | **K** | **E** | **L** | **E** | **N** | **F** | **Q** | **S** | **F** | **R** | **G** | **D** | **N** | **I** | **E** | **H** | **Y** | **D** | **P** | **D** | **M** | **G** | **D** | **K** | **D** | **K** | **Q** | **Q** | **F** | **V** | **D** | **F** | **F** | **S** | **S** | **L** | **N** | **R** | **M** | **P** | **L** | **T** | **R** | **M** | **N** | **S** | **V** | **T** | **L** | **L** | **T** | **D** | **G** | **Q** | **E** | **K** | **L** | **D** | **S** | **L** | **L** | **Q** | **D** | **L** | **K** | **K** | **A** | **K** | **H** | **S** | **I** | **H** | **I** | **E** | **Y** | **Y** | **A** | **F** | **V** | **T** | **D** | **N** | **I** | **G** | **Q** | **Q** | **V** | **L** | **H** | **V** | **L** | **E** | **E** | **K** | **A** | **A** | **E** | **G** | **V** | **E** | **V** | **R** | **I** | **L** | **Y** | **D** | **A** | **F** | **G** | **S** | **H** | **G** | **T** | **K** | **A** | **K** | **D** | **F** | **N** | **R** | **L** | **I** | **K** | **N** | **G** | **G** | **H** | **V** | **H** | **T** | **F** | **V** | **T** | **S** | **Q** | **K** | **A** | **L** | **L** | **R** | **F** | **R** | **L** | **N** | **Y** | **H** | **D** | **H** | **R** | **K** | **I** | **V** | **V** | **I** | **D** | **G** | **K** | **I** | **S** | **Y** | **T** | **G** | **G** | **F** | **N** | **I** | **A** | **N** | **Q** | **Y** | **V** | **N** | **T** | **T** | **K** | **K** | **F** | **G** | **Y** | **W** | **R** | **D** | **T** | **H** | **I** | **R** | **I** | **F** | **G** | **A** | **A** | **S** | **S** | **L** | **L** | **Q** | **L** | **R** | **F** | **L** | **T** | **D** | **W** | **N** | **V** | **S** | **V** | **P** | **E** | **E** | **K** | **K** | **V** | **G** | **Y** | **H** | **L** | **N** | **Y** | **F** | **F** | **K** | **K** | **A** | **D** | **R** | **D** | **E** | **S** | **K** | **L** | **A** | **D** | **T** | **S** | **I** | **Q** | **L** | **V** | **S** | **S** | **G** | **P** | **N** | **N** | **E** | **R** | **E** | **Q** | **I** | **K** | **L** | **S** | **F** | **I** | **K** | **L** | **I** | **T** | **S** | **A** | **K** | **K** | **R** | **V** | **W** | **I** | **Q** | **T** | **P** | **Y** | **L** | **V** | **P** | **D** | **E** | **S** | **V** | **I** | **A** | **A** | **L** | **K** | **I** | **A** | **T** | **A** | **S** | **G** | **V** | **D** | **V** | **K** | **I** | **M** | **I** | **P** | **N** | **K** | **P** | **D** | **H** | **P** | **F** | **I** | **Y** | **R** | **A** | **T** | **Q** | **Y** | **Y** | **A** | **R** | **Q** | **L** | **I** | **K** | **E** | **N** | **V** | **Q** | **I** | **L** | **V** | **Y** | **E** | **N** | **G** | **F** | **L** | **H** | **A** | **K** | **T** | **L** | **I** | **M** | **D** | **D** | **E** | **I** | **C** | **M** | **V** | **G** | **S** | **A** | **N** | **Q** | **D** | **I** | **R** | **S** | **Y** | **R** | **L** | **N** | **F** | **E** | **T** | **S** | **A** | **V** | **I** | **Y** | **D** | **P** | **E** | **F** | **L** | **E** | **E** | **L** | **A** | **T** | **Q** | **F** | **K** | **E** | **D** | **E** | **T** | **H** | **C** | **S** | **S** | **M** | **T** | **T** | **E** | **T** | **V** | **K** | **E** | **M** | **S** | **N** | **W** | **L** | **L** | **F** | **K** | **Q** | **Q** | **I** | **S** | **R** | **L** | **F** | **S** | **P** | **I** | **L** |
| **N60425F** | **-** | **-** | **-** | **-** | **-** | **-** | **-** | **-** | **-** | **-** | **-** | **-** | **-** | **-** | **-** | **-** | **-** | **-** | **-** | **-** | **-** | **-** | **-** | **K** | **P** | **R** | **D** | **V** | **A** | **A** | **I** | **W** | **A** | **W** | **L** | **L** | **V** | **L** | **F** | **A** | **L** | **P** | **G** | **V** | **G** | **F** | **V** | **L** | **Y** | **L** | **F** | **F** | **G** | **R** | **G** | **L** | **T** | **D** | **K** | **K** | **K** | **F** | **Y** | **L** | **R** | **Q** | **S** | **D** | **L** | **K** | **E** | **L** | **E** | **N** | **F** | **Q** | **S** | **F** | **R** | **G** | **D** | **T** | **I** | **E** | **H** | **Y** | **D** | **P** | **D** | **M** | **G** | **D** | **K** | **D** | **K** | **Q** | **Q** | **F** | **V** | **D** | **F** | **F** | **S** | **S** | **L** | **N** | **R** | **M** | **P** | **L** | **T** | **R** | **M** | **N** | **S** | **V** | **T** | **L** | **L** | **T** | **D** | **G** | **Q** | **E** | **K** | **L** | **D** | **S** | **L** | **L** | **Q** | **D** | **L** | **K** | **K** | **A** | **K** | **H** | **S** | **I** | **H** | **I** | **E** | **Y** | **Y** | **A** | **F** | **V** | **T** | **D** | **N** | **I** | **G** | **Q** | **Q** | **V** | **L** | **H** | **V** | **L** | **E** | **E** | **K** | **A** | **A** | **E** | **G** | **V** | **E** | **V** | **R** | **I** | **L** | **Y** | **D** | **A** | **F** | **G** | **S** | **H** | **G** | **T** | **K** | **A** | **K** | **D** | **F** | **N** | **R** | **L** | **I** | **K** | **N** | **G** | **G** | **H** | **V** | **H** | **T** | **F** | **V** | **T** | **S** | **Q** | **K** | **A** | **L** | **L** | **R** | **F** | **R** | **L** | **N** | **Y** | **H** | **D** | **H** | **R** | **K** | **I** | **V** | **V** | **I** | **D** | **G** | **K** | **I** | **S** | **Y** | **T** | **G** | **G** | **F** | **N** | **I** | **A** | **N** | **Q** | **Y** | **V** | **N** | **T** | **T** | **K** | **K** | **F** | **G** | **Y** | **W** | **R** | **D** | **T** | **H** | **I** | **R** | **I** | **F** | **G** | **A** | **A** | **S** | **S** | **L** | **L** | **Q** | **L** | **R** | **F** | **L** | **T** | **D** | **W** | **N** | **V** | **S** | **V** | **P** | **E** | **E** | **K** | **K** | **V** | **G** | **Y** | **H** | **L** | **N** | **Y** | **F** | **F** | **K** | **K** | **A** | **D** | **R** | **D** | **E** | **S** | **K** | **L** | **A** | **E** | **T** | **S** | **I** | **Q** | **L** | **V** | **S** | **S** | **G** | **P** | **N** | **N** | **E** | **R** | **E** | **Q** | **I** | **K** | **L** | **S** | **F** | **I** | **K** | **L** | **I** | **T** | **S** | **A** | **K** | **K** | **R** | **V** | **W** | **I** | **Q** | **T** | **P** | **Y** | **L** | **V** | **P** | **D** | **E** | **S** | **V** | **I** | **A** | **A** | **L** | **K** | **I** | **A** | **T** | **A** | **S** | **G** | **V** | **D** | **V** | **K** | **I** | **M** | **I** | **P** | **N** | **K** | **P** | **D** | **H** | **P** | **F** | **I** | **Y** | **R** | **A** | **T** | **Q** | **Y** | **Y** | **A** | **R** | **Q** | **L** | **I** | **K** | **E** | **N** | **V** | **Q** | **I** | **L** | **V** | **Y** | **E** | **N** | **G** | **F** | **L** | **H** | **A** | **K** | **T** | **L** | **I** | **M** | **D** | **D** | **E** | **I** | **C** | **M** | **V** | **G** | **S** | **A** | **N** | **Q** | **D** | **I** | **R** | **S** | **Y** | **R** | **L** | **N** | **F** | **E** | **T** | **S** | **A** | **V** | **I** | **Y** | **D** | **P** | **E** | **F** | **L** | **E** | **E** | **L** | **A** | **T** | **Q** | **F** | **K** | **E** | **D** | **E** | **T** | **H** | **C** | **S** | **S** | **M** | **T** | **T** | **E** | **T** | **V** | **K** | **E** | **M** | **S** | **N** | **W** | **L** | **L** | **F** | **K** | **Q** | **Q** | **I** | **S** | **R** | **L** | **F** | **S** | **P** | **I** | **L** |
| **N59504F** | **-** | **-** | **-** | **-** | **-** | **-** | **-** | **-** | **-** | **-** | **-** | **-** | **-** | **-** | **-** | **-** | **-** | **-** | **-** | **-** | **-** | **-** | **-** | **K** | **P** | **R** | **D** | **V** | **A** | **A** | **I** | **W** | **A** | **W** | **L** | **L** | **V** | **L** | **F** | **A** | **L** | **P** | **G** | **V** | **G** | **F** | **V** | **L** | **Y** | **L** | **F** | **F** | **G** | **R** | **G** | **L** | **T** | **D** | **K** | **K** | **K** | **F** | **Y** | **L** | **R** | **Q** | **S** | **D** | **L** | **K** | **E** | **L** | **E** | **N** | **F** | **Q** | **S** | **F** | **R** | **G** | **D** | **T** | **I** | **E** | **H** | **Y** | **D** | **P** | **D** | **M** | **G** | **D** | **K** | **D** | **K** | **Q** | **Q** | **F** | **V** | **D** | **F** | **F** | **S** | **S** | **L** | **N** | **R** | **M** | **P** | **L** | **T** | **R** | **M** | **N** | **S** | **V** | **T** | **L** | **L** | **T** | **D** | **G** | **Q** | **E** | **K** | **L** | **D** | **S** | **L** | **L** | **Q** | **D** | **L** | **K** | **K** | **A** | **K** | **H** | **S** | **I** | **H** | **I** | **E** | **Y** | **Y** | **A** | **F** | **V** | **T** | **D** | **N** | **I** | **G** | **Q** | **Q** | **V** | **L** | **H** | **V** | **L** | **E** | **E** | **K** | **A** | **A** | **E** | **G** | **V** | **E** | **V** | **R** | **I** | **L** | **Y** | **D** | **A** | **F** | **G** | **S** | **H** | **G** | **T** | **K** | **A** | **K** | **D** | **F** | **N** | **R** | **L** | **I** | **K** | **N** | **G** | **G** | **H** | **V** | **H** | **T** | **F** | **V** | **T** | **S** | **Q** | **K** | **A** | **L** | **L** | **R** | **F** | **R** | **L** | **N** | **Y** | **H** | **D** | **H** | **R** | **K** | **I** | **V** | **V** | **I** | **D** | **G** | **K** | **I** | **S** | **Y** | **T** | **G** | **G** | **F** | **N** | **I** | **A** | **N** | **Q** | **Y** | **V** | **N** | **T** | **T** | **K** | **K** | **F** | **G** | **Y** | **W** | **R** | **D** | **T** | **H** | **I** | **R** | **I** | **F** | **G** | **A** | **A** | **S** | **S** | **L** | **L** | **Q** | **L** | **R** | **F** | **L** | **T** | **D** | **W** | **N** | **V** | **S** | **V** | **P** | **E** | **E** | **K** | **K** | **V** | **G** | **Y** | **H** | **L** | **N** | **Y** | **F** | **F** | **K** | **K** | **A** | **D** | **R** | **D** | **E** | **S** | **K** | **L** | **A** | **D** | **T** | **S** | **I** | **Q** | **L** | **V** | **S** | **S** | **G** | **P** | **N** | **N** | **E** | **R** | **E** | **Q** | **I** | **K** | **L** | **S** | **F** | **I** | **K** | **L** | **I** | **T** | **S** | **A** | **K** | **K** | **R** | **V** | **W** | **I** | **Q** | **T** | **P** | **Y** | **L** | **V** | **P** | **D** | **E** | **S** | **V** | **I** | **A** | **A** | **L** | **K** | **I** | **A** | **T** | **A** | **S** | **G** | **V** | **D** | **V** | **K** | **I** | **M** | **I** | **P** | **N** | **K** | **P** | **D** | **H** | **P** | **F** | **I** | **Y** | **R** | **A** | **T** | **Q** | **Y** | **Y** | **A** | **R** | **Q** | **L** | **I** | **K** | **E** | **N** | **V** | **Q** | **I** | **L** | **V** | **Y** | **E** | **N** | **G** | **F** | **L** | **H** | **A** | **K** | **T** | **L** | **I** | **M** | **D** | **D** | **E** | **I** | **C** | **M** | **V** | **G** | **S** | **A** | **N** | **Q** | **D** | **I** | **R** | **S** | **Y** | **R** | **L** | **N** | **F** | **E** | **T** | **S** | **A** | **V** | **I** | **Y** | **D** | **P** | **E** | **F** | **L** | **E** | **E** | **L** | **A** | **T** | **Q** | **F** | **K** | **E** | **D** | **E** | **T** | **H** | **C** | **S** | **S** | **M** | **T** | **T** | **E** | **T** | **V** | **K** | **E** | **M** | **S** | **N** | **W** | **L** | **L** | **F** | **K** | **Q** | **Q** | **I** | **S** | **R** | **L** | **F** | **S** | **P** | **I** | **L** |
| **N60357F** | **-** | **-** | **-** | **-** | **-** | **-** | **-** | **-** | **-** | **-** | **-** | **-** | **-** | **-** | **-** | **-** | **-** | **-** | **-** | **-** | **-** | **-** | **-** | **K** | **P** | **R** | **D** | **V** | **A** | **A** | **I** | **W** | **A** | **W** | **L** | **L** | **V** | **L** | **F** | **A** | **L** | **P** | **G** | **V** | **G** | **F** | **V** | **L** | **Y** | **L** | **F** | **F** | **G** | **R** | **G** | **L** | **T** | **D** | **K** | **K** | **K** | **F** | **Y** | **L** | **R** | **Q** | **S** | **D** | **L** | **K** | **E** | **L** | **E** | **N** | **F** | **Q** | **S** | **F** | **R** | **G** | **D** | **T** | **I** | **E** | **H** | **Y** | **D** | **P** | **D** | **M** | **G** | **D** | **K** | **D** | **K** | **Q** | **Q** | **F** | **V** | **D** | **F** | **F** | **S** | **S** | **L** | **N** | **R** | **M** | **P** | **L** | **T** | **I** | **M** | **N** | **S** | **V** | **T** | **L** | **L** | **T** | **D** | **G** | **Q** | **E** | **K** | **L** | **D** | **S** | **L** | **L** | **Q** | **D** | **L** | **K** | **K** | **A** | **K** | **H** | **S** | **I** | **H** | **I** | **E** | **Y** | **Y** | **A** | **F** | **V** | **T** | **D** | **N** | **I** | **G** | **Q** | **Q** | **V** | **L** | **H** | **V** | **L** | **E** | **E** | **K** | **A** | **A** | **E** | **G** | **V** | **E** | **V** | **R** | **I** | **L** | **Y** | **D** | **A** | **F** | **G** | **S** | **H** | **G** | **T** | **K** | **A** | **K** | **D** | **F** | **N** | **R** | **L** | **I** | **K** | **N** | **G** | **G** | **H** | **V** | **H** | **T** | **F** | **V** | **T** | **S** | **Q** | **K** | **A** | **L** | **L** | **R** | **F** | **R** | **L** | **N** | **Y** | **H** | **D** | **H** | **R** | **K** | **I** | **V** | **V** | **I** | **D** | **G** | **K** | **I** | **S** | **Y** | **T** | **G** | **G** | **F** | **N** | **I** | **A** | **N** | **Q** | **Y** | **V** | **N** | **T** | **T** | **K** | **K** | **F** | **G** | **Y** | **W** | **R** | **D** | **T** | **H** | **I** | **R** | **I** | **F** | **G** | **A** | **A** | **S** | **S** | **L** | **L** | **Q** | **L** | **R** | **F** | **L** | **T** | **D** | **W** | **N** | **V** | **S** | **V** | **P** | **E** | **E** | **K** | **K** | **V** | **G** | **Y** | **H** | **L** | **N** | **Y** | **F** | **F** | **K** | **K** | **A** | **D** | **R** | **D** | **E** | **S** | **K** | **L** | **A** | **D** | **T** | **S** | **I** | **Q** | **L** | **V** | **S** | **S** | **G** | **P** | **N** | **N** | **E** | **R** | **E** | **Q** | **I** | **K** | **L** | **S** | **F** | **I** | **K** | **L** | **I** | **T** | **S** | **A** | **K** | **K** | **R** | **V** | **W** | **I** | **Q** | **T** | **P** | **Y** | **L** | **V** | **P** | **D** | **E** | **S** | **V** | **I** | **A** | **A** | **L** | **K** | **I** | **A** | **T** | **A** | **S** | **G** | **V** | **D** | **V** | **K** | **I** | **M** | **I** | **P** | **N** | **K** | **P** | **D** | **H** | **P** | **F** | **I** | **Y** | **R** | **A** | **T** | **Q** | **Y** | **Y** | **A** | **R** | **Q** | **L** | **I** | **K** | **E** | **N** | **V** | **Q** | **I** | **L** | **V** | **Y** | **E** | **N** | **G** | **F** | **L** | **H** | **A** | **K** | **T** | **L** | **I** | **M** | **D** | **D** | **E** | **I** | **C** | **M** | **V** | **G** | **S** | **A** | **N** | **Q** | **D** | **I** | **R** | **S** | **Y** | **R** | **L** | **N** | **F** | **E** | **T** | **S** | **A** | **V** | **I** | **Y** | **D** | **P** | **E** | **F** | **L** | **E** | **E** | **L** | **A** | **T** | **Q** | **F** | **K** | **E** | **D** | **E** | **T** | **Y** | **C** | **S** | **S** | **M** | **T** | **T** | **E** | **T** | **V** | **K** | **E** | **M** | **S** | **N** | **W** | **L** | **L** | **F** | **K** | **Q** | **Q** | **I** | **S** | **R** | **L** | **F** | **S** | **P** | **I** | **L** |
| **N60190F** | **-** | **-** | **-** | **-** | **-** | **-** | **-** | **-** | **-** | **-** | **-** | **-** | **-** | **-** | **-** | **-** | **-** | **-** | **-** | **-** | **-** | **-** | **-** | **K** | **P** | **R** | **D** | **V** | **A** | **A** | **I** | **W** | **A** | **W** | **L** | **L** | **V** | **L** | **F** | **A** | **L** | **P** | **G** | **V** | **G** | **F** | **V** | **L** | **Y** | **L** | **F** | **F** | **G** | **R** | **G** | **L** | **T** | **D** | **K** | **K** | **K** | **F** | **Y** | **L** | **R** | **Q** | **S** | **D** | **L** | **K** | **E** | **L** | **E** | **N** | **F** | **Q** | **S** | **F** | **R** | **G** | **D** | **T** | **I** | **E** | **H** | **Y** | **D** | **P** | **D** | **M** | **G** | **D** | **K** | **D** | **K** | **Q** | **Q** | **F** | **V** | **D** | **F** | **F** | **S** | **S** | **L** | **N** | **R** | **M** | **P** | **L** | **T** | **R** | **M** | **N** | **S** | **V** | **T** | **L** | **L** | **T** | **D** | **G** | **Q** | **K** | **K** | **L** | **D** | **S** | **L** | **L** | **Q** | **D** | **L** | **K** | **K** | **A** | **K** | **H** | **S** | **I** | **H** | **I** | **E** | **Y** | **Y** | **A** | **F** | **V** | **T** | **D** | **N** | **I** | **G** | **Q** | **Q** | **V** | **L** | **R** | **V** | **L** | **E** | **E** | **K** | **A** | **A** | **E** | **G** | **V** | **E** | **V** | **R** | **I** | **L** | **Y** | **D** | **A** | **F** | **G** | **S** | **H** | **G** | **T** | **K** | **A** | **K** | **D** | **F** | **N** | **R** | **L** | **I** | **K** | **N** | **G** | **G** | **H** | **V** | **H** | **T** | **F** | **V** | **T** | **S** | **Q** | **K** | **A** | **L** | **L** | **R** | **F** | **R** | **L** | **N** | **Y** | **H** | **D** | **H** | **R** | **K** | **I** | **V** | **V** | **I** | **D** | **G** | **K** | **I** | **S** | **Y** | **T** | **G** | **G** | **F** | **N** | **I** | **A** | **N** | **Q** | **Y** | **V** | **N** | **T** | **T** | **K** | **K** | **F** | **G** | **Y** | **W** | **R** | **D** | **T** | **H** | **I** | **R** | **I** | **F** | **G** | **A** | **A** | **S** | **S** | **L** | **L** | **Q** | **L** | **R** | **F** | **L** | **T** | **D** | **W** | **N** | **V** | **S** | **V** | **S** | **E** | **E** | **K** | **K** | **V** | **G** | **Y** | **H** | **L** | **N** | **Y** | **F** | **F** | **K** | **K** | **A** | **D** | **R** | **D** | **E** | **S** | **K** | **L** | **A** | **D** | **T** | **S** | **I** | **Q** | **L** | **V** | **S** | **S** | **G** | **P** | **N** | **N** | **E** | **R** | **E** | **Q** | **I** | **K** | **L** | **S** | **F** | **I** | **K** | **L** | **I** | **T** | **S** | **A** | **K** | **K** | **R** | **V** | **W** | **I** | **Q** | **T** | **P** | **Y** | **L** | **V** | **P** | **D** | **E** | **S** | **V** | **I** | **A** | **A** | **L** | **K** | **I** | **A** | **T** | **A** | **S** | **G** | **V** | **D** | **V** | **K** | **I** | **M** | **I** | **P** | **N** | **K** | **P** | **D** | **H** | **P** | **F** | **I** | **Y** | **R** | **A** | **T** | **Q** | **Y** | **Y** | **A** | **R** | **Q** | **L** | **I** | **K** | **E** | **N** | **V** | **Q** | **I** | **L** | **V** | **Y** | **E** | **N** | **G** | **F** | **L** | **H** | **A** | **K** | **T** | **L** | **I** | **M** | **D** | **D** | **E** | **I** | **C** | **M** | **V** | **G** | **S** | **A** | **N** | **Q** | **D** | **I** | **R** | **S** | **Y** | **R** | **L** | **N** | **F** | **E** | **T** | **S** | **A** | **V** | **I** | **Y** | **D** | **P** | **E** | **F** | **L** | **E** | **E** | **L** | **A** | **T** | **Q** | **F** | **K** | **E** | **D** | **E** | **T** | **H** | **C** | **S** | **P** | **M** | **T** | **T** | **E** | **T** | **V** | **K** | **E** | **M** | **S** | **N** | **W** | **L** | **L** | **F** | **K** | **Q** | **Q** | **I** | **S** | **R** | **L** | **F** | **S** | **P** | **I** | **L** |
| **N54155** | **-** | **-** | **-** | **-** | **-** | **-** | **-** | **-** | **-** | **-** | **-** | **-** | **-** | **-** | **-** | **-** | **-** | **-** | **-** | **-** | **-** | **-** | **-** | **K** | **P** | **R** | **D** | **V** | **A** | **A** | **I** | **W** | **A** | **W** | **L** | **L** | **V** | **L** | **F** | **A** | **L** | **P** | **G** | **V** | **G** | **F** | **V** | **L** | **Y** | **L** | **F** | **F** | **G** | **R** | **G** | **L** | **T** | **D** | **K** | **K** | **K** | **F** | **Y** | **L** | **R** | **Q** | **S** | **D** | **L** | **K** | **E** | **L** | **E** | **N** | **F** | **Q** | **S** | **F** | **R** | **G** | **D** | **N** | **I** | **E** | **H** | **Y** | **D** | **P** | **D** | **M** | **G** | **D** | **K** | **D** | **K** | **Q** | **Q** | **F** | **V** | **D** | **F** | **F** | **S** | **S** | **L** | **N** | **R** | **M** | **P** | **L** | **T** | **R** | **M** | **N** | **S** | **V** | **T** | **L** | **L** | **T** | **D** | **G** | **Q** | **E** | **K** | **L** | **D** | **S** | **L** | **L** | **Q** | **D** | **L** | **K** | **K** | **A** | **K** | **H** | **S** | **I** | **H** | **I** | **E** | **Y** | **Y** | **A** | **F** | **V** | **T** | **D** | **N** | **I** | **G** | **Q** | **Q** | **V** | **L** | **H** | **V** | **L** | **E** | **E** | **K** | **A** | **A** | **E** | **G** | **V** | **E** | **V** | **R** | **I** | **L** | **Y** | **D** | **A** | **F** | **G** | **S** | **H** | **G** | **T** | **K** | **A** | **K** | **D** | **F** | **N** | **R** | **L** | **I** | **K** | **N** | **G** | **G** | **H** | **V** | **H** | **T** | **F** | **V** | **T** | **S** | **Q** | **K** | **A** | **L** | **L** | **R** | **F** | **R** | **L** | **N** | **Y** | **H** | **D** | **H** | **R** | **K** | **I** | **V** | **V** | **I** | **D** | **G** | **K** | **I** | **S** | **Y** | **T** | **G** | **G** | **F** | **N** | **I** | **A** | **N** | **Q** | **Y** | **V** | **N** | **T** | **T** | **K** | **K** | **F** | **G** | **Y** | **W** | **R** | **D** | **T** | **H** | **I** | **R** | **I** | **F** | **G** | **A** | **A** | **S** | **S** | **L** | **L** | **Q** | **L** | **R** | **F** | **L** | **T** | **D** | **W** | **N** | **V** | **S** | **V** | **P** | **E** | **E** | **K** | **K** | **V** | **G** | **Y** | **H** | **L** | **N** | **Y** | **F** | **F** | **K** | **K** | **A** | **D** | **R** | **D** | **E** | **S** | **K** | **L** | **A** | **D** | **T** | **S** | **I** | **Q** | **L** | **V** | **S** | **S** | **G** | **P** | **N** | **N** | **E** | **R** | **E** | **Q** | **I** | **K** | **L** | **S** | **F** | **I** | **K** | **L** | **I** | **T** | **S** | **A** | **K** | **K** | **R** | **V** | **W** | **I** | **Q** | **T** | **P** | **Y** | **L** | **V** | **P** | **D** | **E** | **S** | **V** | **I** | **A** | **A** | **L** | **K** | **I** | **A** | **T** | **A** | **S** | **G** | **V** | **D** | **V** | **K** | **I** | **M** | **I** | **P** | **N** | **K** | **P** | **D** | **H** | **P** | **F** | **I** | **Y** | **R** | **A** | **T** | **Q** | **Y** | **Y** | **A** | **R** | **Q** | **L** | **I** | **K** | **E** | **N** | **V** | **Q** | **I** | **L** | **V** | **Y** | **E** | **N** | **G** | **F** | **L** | **H** | **A** | **K** | **T** | **L** | **I** | **M** | **D** | **D** | **E** | **I** | **C** | **M** | **V** | **G** | **S** | **A** | **N** | **Q** | **D** | **I** | **R** | **S** | **Y** | **R** | **L** | **N** | **F** | **E** | **T** | **S** | **A** | **V** | **I** | **Y** | **D** | **P** | **E** | **F** | **L** | **E** | **E** | **L** | **A** | **T** | **Q** | **F** | **K** | **E** | **D** | **E** | **T** | **H** | **C** | **S** | **S** | **M** | **T** | **T** | **E** | **T** | **V** | **K** | **E** | **M** | **S** | **N** | **W** | **L** | **L** | **F** | **K** | **Q** | **Q** | **I** | **S** | **R** | **L** | **F** | **S** | **P** | **I** | **L** |
| **N55229** | **-** | **-** | **-** | **-** | **-** | **-** | **-** | **-** | **-** | **-** | **-** | **-** | **-** | **-** | **-** | **-** | **-** | **-** | **-** | **-** | **-** | **-** | **-** | **K** | **P** | **R** | **D** | **V** | **A** | **A** | **I** | **W** | **A** | **W** | **L** | **L** | **V** | **L** | **F** | **A** | **L** | **P** | **G** | **V** | **G** | **F** | **V** | **L** | **Y** | **L** | **F** | **F** | **G** | **R** | **G** | **L** | **T** | **D** | **K** | **K** | **K** | **F** | **Y** | **L** | **R** | **Q** | **S** | **D** | **L** | **K** | **E** | **L** | **E** | **N** | **F** | **Q** | **S** | **F** | **R** | **G** | **D** | **T** | **I** | **E** | **H** | **Y** | **D** | **P** | **D** | **M** | **G** | **D** | **K** | **D** | **K** | **Q** | **Q** | **F** | **V** | **D** | **F** | **F** | **S** | **S** | **L** | **N** | **R** | **M** | **P** | **L** | **T** | **R** | **M** | **N** | **S** | **V** | **T** | **L** | **L** | **T** | **D** | **G** | **Q** | **E** | **K** | **L** | **D** | **S** | **L** | **L** | **Q** | **D** | **L** | **K** | **K** | **A** | **K** | **H** | **S** | **I** | **H** | **I** | **E** | **Y** | **Y** | **A** | **F** | **V** | **T** | **D** | **N** | **I** | **G** | **Q** | **Q** | **V** | **L** | **H** | **V** | **L** | **E** | **E** | **K** | **A** | **A** | **E** | **G** | **V** | **E** | **V** | **R** | **I** | **L** | **Y** | **D** | **A** | **F** | **G** | **S** | **H** | **G** | **T** | **K** | **A** | **K** | **D** | **F** | **N** | **R** | **L** | **I** | **K** | **N** | **G** | **G** | **H** | **V** | **H** | **T** | **F** | **V** | **T** | **S** | **Q** | **K** | **A** | **L** | **L** | **R** | **F** | **R** | **L** | **N** | **Y** | **H** | **D** | **H** | **R** | **K** | **I** | **V** | **V** | **I** | **D** | **G** | **K** | **I** | **S** | **Y** | **T** | **G** | **G** | **F** | **N** | **I** | **A** | **N** | **Q** | **Y** | **V** | **N** | **T** | **T** | **K** | **K** | **F** | **G** | **Y** | **W** | **R** | **D** | **T** | **H** | **I** | **R** | **I** | **F** | **G** | **A** | **A** | **S** | **S** | **L** | **L** | **Q** | **L** | **R** | **F** | **L** | **T** | **D** | **W** | **N** | **V** | **S** | **V** | **P** | **E** | **E** | **K** | **K** | **V** | **G** | **Y** | **H** | **L** | **N** | **Y** | **F** | **F** | **K** | **K** | **A** | **D** | **R** | **D** | **E** | **S** | **K** | **L** | **A** | **D** | **T** | **S** | **I** | **Q** | **L** | **V** | **S** | **S** | **G** | **P** | **N** | **N** | **E** | **R** | **E** | **Q** | **I** | **K** | **L** | **S** | **F** | **I** | **K** | **L** | **I** | **T** | **S** | **A** | **K** | **K** | **R** | **V** | **W** | **I** | **Q** | **T** | **P** | **Y** | **L** | **V** | **P** | **D** | **E** | **S** | **V** | **I** | **A** | **A** | **L** | **K** | **I** | **A** | **T** | **A** | **S** | **G** | **V** | **D** | **V** | **K** | **I** | **M** | **I** | **P** | **N** | **K** | **P** | **D** | **H** | **P** | **F** | **I** | **Y** | **R** | **A** | **T** | **Q** | **Y** | **Y** | **A** | **R** | **Q** | **L** | **I** | **K** | **E** | **N** | **V** | **Q** | **I** | **L** | **V** | **Y** | **E** | **N** | **G** | **F** | **L** | **H** | **A** | **K** | **T** | **L** | **I** | **M** | **D** | **D** | **E** | **I** | **C** | **M** | **V** | **G** | **S** | **A** | **N** | **Q** | **D** | **I** | **R** | **S** | **Y** | **R** | **L** | **N** | **F** | **E** | **T** | **S** | **A** | **V** | **I** | **Y** | **D** | **P** | **E** | **F** | **L** | **E** | **E** | **L** | **A** | **T** | **Q** | **F** | **K** | **E** | **D** | **E** | **T** | **H** | **C** | **S** | **S** | **M** | **T** | **T** | **E** | **T** | **V** | **K** | **E** | **M** | **S** | **N** | **W** | **L** | **L** | **F** | **K** | **Q** | **Q** | **I** | **S** | **R** | **L** | **F** | **S** | **P** | **I** | **L** |
| **N60284F** | **V** | **V** | **S** | **S** | **I** | **I** | **T** | **G** | **L** | **Y** | **L** | **L** | **N** | **A** | **L** | **I** | **A** | **L** | **V** | **A** | **I** | **L** | **I** | **K** | **P** | **R** | **D** | **V** | **A** | **A** | **I** | **W** | **A** | **W** | **L** | **L** | **V** | **L** | **F** | **A** | **L** | **P** | **G** | **V** | **G** | **F** | **V** | **L** | **Y** | **L** | **F** | **F** | **G** | **R** | **G** | **L** | **T** | **D** | **K** | **K** | **K** | **F** | **Y** | **L** | **R** | **Q** | **S** | **D** | **L** | **K** | **E** | **L** | **E** | **N** | **F** | **Q** | **S** | **F** | **R** | **G** | **D** | **T** | **I** | **E** | **H** | **Y** | **D** | **P** | **D** | **M** | **G** | **D** | **K** | **D** | **K** | **Q** | **Q** | **F** | **V** | **D** | **F** | **F** | **S** | **S** | **L** | **N** | **R** | **M** | **P** | **L** | **T** | **R** | **M** | **N** | **S** | **V** | **T** | **L** | **L** | **T** | **D** | **G** | **Q** | **E** | **K** | **L** | **D** | **S** | **L** | **L** | **Q** | **D** | **L** | **K** | **K** | **A** | **K** | **H** | **S** | **I** | **H** | **I** | **E** | **Y** | **Y** | **A** | **F** | **V** | **T** | **D** | **N** | **I** | **G** | **Q** | **Q** | **V** | **L** | **H** | **V** | **L** | **E** | **E** | **K** | **A** | **A** | **E** | **G** | **V** | **E** | **V** | **R** | **I** | **L** | **Y** | **D** | **A** | **F** | **G** | **S** | **H** | **G** | **T** | **K** | **A** | **K** | **D** | **F** | **N** | **R** | **L** | **I** | **K** | **N** | **G** | **G** | **H** | **V** | **H** | **T** | **F** | **V** | **T** | **S** | **Q** | **K** | **A** | **L** | **L** | **R** | **F** | **R** | **L** | **N** | **Y** | **H** | **D** | **H** | **R** | **K** | **I** | **V** | **V** | **I** | **D** | **G** | **K** | **I** | **S** | **Y** | **T** | **G** | **G** | **F** | **N** | **I** | **A** | **N** | **Q** | **Y** | **V** | **N** | **T** | **T** | **K** | **K** | **F** | **G** | **Y** | **W** | **R** | **D** | **T** | **H** | **I** | **R** | **I** | **F** | **G** | **A** | **A** | **S** | **S** | **L** | **L** | **Q** | **L** | **R** | **F** | **L** | **T** | **D** | **W** | **N** | **V** | **S** | **V** | **P** | **E** | **E** | **K** | **K** | **V** | **G** | **Y** | **H** | **L** | **N** | **Y** | **F** | **F** | **K** | **K** | **A** | **D** | **R** | **D** | **E** | **S** | **K** | **L** | **A** | **D** | **T** | **S** | **I** | **Q** | **L** | **V** | **S** | **S** | **G** | **P** | **N** | **N** | **E** | **R** | **E** | **Q** | **I** | **K** | **L** | **S** | **F** | **I** | **K** | **L** | **I** | **T** | **S** | **A** | **K** | **K** | **R** | **V** | **W** | **I** | **Q** | **T** | **P** | **Y** | **L** | **V** | **P** | **D** | **E** | **S** | **V** | **I** | **A** | **A** | **L** | **K** | **I** | **A** | **T** | **A** | **S** | **G** | **V** | **D** | **V** | **K** | **I** | **M** | **I** | **P** | **N** | **K** | **P** | **D** | **H** | **P** | **F** | **I** | **Y** | **R** | **A** | **T** | **Q** | **Y** | **Y** | **A** | **R** | **Q** | **L** | **I** | **K** | **E** | **N** | **V** | **Q** | **I** | **L** | **V** | **Y** | **E** | **N** | **G** | **F** | **L** | **H** | **A** | **K** | **T** | **L** | **I** | **M** | **D** | **D** | **E** | **I** | **C** | **M** | **V** | **G** | **S** | **A** | **N** | **Q** | **D** | **I** | **R** | **S** | **Y** | **R** | **L** | **N** | **F** | **E** | **T** | **S** | **A** | **V** | **I** | **Y** | **D** | **P** | **E** | **F** | **L** | **E** | **E** | **L** | **A** | **T** | **Q** | **F** | **K** | **E** | **D** | **E** | **T** | **H** | **C** | **S** | **S** | **M** | **T** | **T** | **E** | **T** | **V** | **K** | **E** | **M** | **S** | **N** | **W** | **L** | **L** | **F** | **K** | **Q** | **Q** | **I** | **S** | **R** | **L** | **F** | **S** | **P** | **I** | **L** |
| **N60061F** | **-** | **-** | **-** | **-** | **-** | **-** | **-** | **-** | **-** | **-** | **-** | **-** | **-** | **-** | **-** | **-** | **-** | **-** | **-** | **-** | **-** | **-** | **-** | **K** | **P** | **R** | **D** | **V** | **A** | **A** | **I** | **W** | **A** | **W** | **L** | **L** | **V** | **L** | **F** | **A** | **L** | **P** | **G** | **V** | **G** | **F** | **V** | **L** | **Y** | **L** | **F** | **F** | **G** | **R** | **G** | **L** | **T** | **D** | **K** | **K** | **K** | **F** | **Y** | **L** | **R** | **Q** | **S** | **D** | **L** | **K** | **E** | **L** | **E** | **N** | **F** | **Q** | **S** | **F** | **R** | **G** | **D** | **T** | **I** | **E** | **H** | **Y** | **D** | **P** | **D** | **M** | **G** | **D** | **K** | **D** | **K** | **Q** | **Q** | **F** | **V** | **D** | **F** | **F** | **S** | **S** | **L** | **N** | **R** | **M** | **P** | **L** | **T** | **R** | **M** | **N** | **S** | **V** | **T** | **L** | **L** | **T** | **D** | **G** | **Q** | **E** | **K** | **L** | **D** | **S** | **L** | **L** | **Q** | **D** | **L** | **K** | **K** | **A** | **K** | **H** | **S** | **I** | **H** | **I** | **E** | **Y** | **Y** | **A** | **F** | **V** | **T** | **D** | **N** | **I** | **G** | **Q** | **Q** | **V** | **L** | **H** | **V** | **L** | **E** | **E** | **K** | **A** | **A** | **E** | **G** | **V** | **E** | **V** | **R** | **I** | **L** | **Y** | **D** | **A** | **F** | **G** | **S** | **H** | **G** | **T** | **K** | **A** | **K** | **D** | **F** | **N** | **R** | **L** | **I** | **K** | **N** | **G** | **G** | **H** | **V** | **H** | **T** | **F** | **V** | **T** | **S** | **Q** | **K** | **A** | **L** | **L** | **R** | **F** | **R** | **L** | **N** | **Y** | **H** | **D** | **H** | **R** | **K** | **I** | **V** | **V** | **I** | **D** | **G** | **K** | **I** | **S** | **Y** | **T** | **G** | **G** | **F** | **N** | **I** | **A** | **N** | **Q** | **Y** | **V** | **N** | **T** | **T** | **K** | **K** | **F** | **G** | **Y** | **W** | **R** | **D** | **T** | **H** | **I** | **R** | **I** | **F** | **G** | **A** | **A** | **S** | **S** | **L** | **L** | **Q** | **L** | **R** | **F** | **L** | **T** | **D** | **W** | **N** | **V** | **S** | **V** | **P** | **E** | **E** | **K** | **K** | **V** | **G** | **Y** | **H** | **L** | **N** | **Y** | **F** | **F** | **K** | **K** | **A** | **D** | **R** | **D** | **E** | **S** | **K** | **L** | **A** | **E** | **T** | **S** | **I** | **Q** | **L** | **V** | **S** | **S** | **G** | **P** | **N** | **N** | **E** | **R** | **E** | **Q** | **I** | **K** | **L** | **S** | **F** | **I** | **K** | **L** | **I** | **T** | **S** | **A** | **K** | **K** | **R** | **V** | **W** | **I** | **Q** | **T** | **P** | **Y** | **L** | **V** | **P** | **D** | **E** | **S** | **V** | **I** | **A** | **A** | **L** | **K** | **I** | **A** | **T** | **A** | **S** | **G** | **V** | **D** | **V** | **K** | **I** | **M** | **I** | **P** | **N** | **K** | **P** | **D** | **H** | **P** | **F** | **I** | **Y** | **R** | **A** | **T** | **Q** | **Y** | **Y** | **A** | **R** | **Q** | **L** | **I** | **K** | **E** | **N** | **V** | **Q** | **I** | **L** | **V** | **Y** | **E** | **N** | **G** | **F** | **L** | **H** | **A** | **K** | **T** | **L** | **I** | **M** | **D** | **D** | **E** | **I** | **C** | **M** | **V** | **G** | **S** | **A** | **N** | **Q** | **D** | **I** | **R** | **S** | **Y** | **R** | **L** | **N** | **F** | **E** | **T** | **S** | **A** | **V** | **I** | **Y** | **D** | **P** | **E** | **F** | **L** | **E** | **E** | **L** | **A** | **T** | **Q** | **F** | **K** | **E** | **D** | **E** | **T** | **H** | **C** | **S** | **S** | **M** | **T** | **T** | **E** | **T** | **V** | **K** | **E** | **M** | **S** | **N** | **W** | **L** | **L** | **F** | **K** | **Q** | **Q** | **I** | **S** | **R** | **L** | **F** | **S** | **P** | **I** | **L** |
| **N59943F** | **-** | **-** | **-** | **-** | **-** | **-** | **-** | **-** | **-** | **-** | **-** | **-** | **-** | **-** | **-** | **-** | **-** | **-** | **-** | **-** | **-** | **-** | **-** | **K** | **P** | **R** | **D** | **V** | **A** | **A** | **I** | **W** | **A** | **W** | **L** | **L** | **V** | **L** | **F** | **A** | **L** | **P** | **G** | **V** | **G** | **F** | **V** | **L** | **Y** | **L** | **F** | **F** | **G** | **R** | **G** | **L** | **T** | **D** | **K** | **K** | **K** | **F** | **Y** | **L** | **R** | **Q** | **S** | **D** | **L** | **K** | **E** | **L** | **E** | **N** | **F** | **Q** | **S** | **F** | **R** | **G** | **D** | **T** | **I** | **E** | **H** | **Y** | **D** | **P** | **D** | **M** | **G** | **D** | **K** | **D** | **K** | **Q** | **Q** | **F** | **V** | **D** | **F** | **F** | **S** | **S** | **L** | **N** | **R** | **M** | **P** | **L** | **T** | **R** | **M** | **N** | **S** | **V** | **T** | **L** | **L** | **T** | **D** | **G** | **Q** | **K** | **K** | **L** | **D** | **S** | **L** | **L** | **Q** | **D** | **L** | **K** | **K** | **A** | **K** | **H** | **S** | **I** | **H** | **I** | **E** | **Y** | **Y** | **A** | **F** | **V** | **T** | **D** | **T** | **I** | **G** | **Q** | **Q** | **V** | **L** | **R** | **V** | **L** | **E** | **E** | **K** | **A** | **A** | **E** | **G** | **V** | **E** | **V** | **R** | **I** | **L** | **Y** | **D** | **A** | **F** | **G** | **S** | **H** | **G** | **T** | **K** | **A** | **K** | **D** | **F** | **N** | **R** | **L** | **I** | **K** | **N** | **G** | **G** | **H** | **V** | **H** | **T** | **F** | **V** | **T** | **S** | **Q** | **K** | **A** | **L** | **L** | **R** | **F** | **R** | **L** | **N** | **Y** | **H** | **D** | **H** | **R** | **K** | **I** | **V** | **V** | **I** | **D** | **G** | **K** | **I** | **S** | **Y** | **T** | **G** | **G** | **F** | **N** | **I** | **A** | **N** | **Q** | **Y** | **V** | **N** | **T** | **T** | **K** | **K** | **F** | **G** | **Y** | **W** | **R** | **D** | **T** | **H** | **I** | **R** | **I** | **F** | **G** | **A** | **A** | **S** | **S** | **L** | **L** | **Q** | **L** | **R** | **F** | **L** | **T** | **D** | **W** | **N** | **V** | **S** | **V** | **P** | **E** | **E** | **K** | **K** | **V** | **G** | **Y** | **H** | **L** | **N** | **Y** | **F** | **F** | **K** | **K** | **A** | **D** | **R** | **D** | **E** | **S** | **K** | **L** | **A** | **D** | **T** | **S** | **I** | **Q** | **L** | **V** | **S** | **S** | **G** | **P** | **N** | **N** | **E** | **R** | **E** | **Q** | **I** | **K** | **L** | **S** | **F** | **I** | **K** | **L** | **I** | **T** | **S** | **A** | **K** | **K** | **R** | **V** | **W** | **I** | **Q** | **T** | **P** | **Y** | **L** | **V** | **P** | **D** | **E** | **S** | **V** | **I** | **A** | **A** | **L** | **K** | **I** | **A** | **T** | **A** | **S** | **G** | **V** | **D** | **V** | **K** | **I** | **M** | **I** | **P** | **N** | **K** | **P** | **D** | **H** | **P** | **F** | **I** | **Y** | **R** | **A** | **T** | **Q** | **Y** | **Y** | **A** | **R** | **Q** | **L** | **I** | **K** | **E** | **N** | **V** | **Q** | **I** | **L** | **V** | **Y** | **E** | **N** | **G** | **F** | **L** | **H** | **A** | **K** | **T** | **L** | **I** | **M** | **D** | **D** | **E** | **I** | **C** | **M** | **V** | **G** | **S** | **A** | **N** | **Q** | **D** | **I** | **R** | **S** | **Y** | **R** | **L** | **N** | **F** | **E** | **T** | **S** | **A** | **V** | **I** | **Y** | **D** | **P** | **E** | **F** | **L** | **E** | **E** | **L** | **A** | **T** | **Q** | **F** | **K** | **E** | **D** | **E** | **T** | **H** | **C** | **S** | **P** | **M** | **T** | **T** | **E** | **T** | **V** | **K** | **E** | **M** | **S** | **N** | **W** | **L** | **L** | **F** | **K** | **Q** | **Q** | **I** | **S** | **R** | **L** | **F** | **S** | **P** | **I** | **L** |
| **N59531F** | **-** | **-** | **-** | **-** | **-** | **-** | **-** | **-** | **-** | **-** | **-** | **-** | **-** | **-** | **-** | **-** | **-** | **-** | **-** | **-** | **-** | **-** | **-** | **K** | **P** | **R** | **D** | **V** | **A** | **A** | **I** | **W** | **A** | **W** | **L** | **L** | **V** | **L** | **F** | **A** | **L** | **P** | **G** | **V** | **G** | **F** | **V** | **L** | **Y** | **L** | **F** | **F** | **G** | **R** | **G** | **L** | **T** | **D** | **K** | **K** | **K** | **F** | **Y** | **L** | **R** | **Q** | **S** | **D** | **L** | **K** | **E** | **L** | **E** | **N** | **F** | **Q** | **S** | **F** | **R** | **G** | **D** | **T** | **I** | **E** | **H** | **Y** | **D** | **P** | **D** | **M** | **G** | **D** | **K** | **D** | **K** | **Q** | **Q** | **F** | **V** | **D** | **F** | **F** | **S** | **S** | **L** | **N** | **R** | **M** | **P** | **L** | **T** | **R** | **M** | **N** | **S** | **V** | **T** | **L** | **L** | **T** | **D** | **G** | **Q** | **K** | **K** | **L** | **D** | **S** | **L** | **L** | **Q** | **D** | **L** | **K** | **K** | **A** | **K** | **H** | **S** | **I** | **H** | **I** | **E** | **Y** | **Y** | **A** | **F** | **V** | **T** | **D** | **T** | **I** | **G** | **Q** | **Q** | **V** | **L** | **R** | **V** | **L** | **E** | **E** | **K** | **A** | **A** | **E** | **G** | **V** | **E** | **V** | **R** | **I** | **L** | **Y** | **D** | **A** | **F** | **G** | **S** | **H** | **G** | **T** | **K** | **A** | **K** | **D** | **F** | **N** | **R** | **L** | **I** | **K** | **N** | **G** | **G** | **H** | **V** | **H** | **T** | **F** | **V** | **T** | **S** | **Q** | **K** | **A** | **L** | **L** | **R** | **F** | **R** | **L** | **N** | **Y** | **H** | **D** | **H** | **R** | **K** | **I** | **V** | **V** | **I** | **D** | **G** | **K** | **I** | **S** | **Y** | **T** | **G** | **G** | **F** | **N** | **I** | **A** | **N** | **Q** | **Y** | **V** | **N** | **T** | **T** | **K** | **K** | **F** | **G** | **Y** | **W** | **R** | **D** | **T** | **H** | **I** | **R** | **I** | **F** | **G** | **A** | **A** | **S** | **S** | **L** | **L** | **Q** | **L** | **R** | **F** | **L** | **T** | **D** | **W** | **N** | **V** | **S** | **V** | **P** | **E** | **E** | **K** | **K** | **V** | **G** | **Y** | **H** | **L** | **N** | **Y** | **F** | **F** | **K** | **K** | **A** | **D** | **R** | **D** | **E** | **S** | **K** | **L** | **A** | **D** | **T** | **S** | **I** | **Q** | **L** | **V** | **S** | **S** | **G** | **P** | **N** | **N** | **E** | **R** | **E** | **Q** | **I** | **K** | **L** | **S** | **F** | **I** | **K** | **L** | **I** | **T** | **S** | **A** | **K** | **K** | **R** | **V** | **W** | **I** | **Q** | **T** | **P** | **Y** | **L** | **V** | **P** | **D** | **E** | **S** | **V** | **I** | **A** | **A** | **L** | **K** | **I** | **A** | **T** | **A** | **S** | **G** | **V** | **D** | **V** | **K** | **I** | **M** | **I** | **P** | **N** | **K** | **P** | **D** | **H** | **P** | **F** | **I** | **Y** | **R** | **A** | **T** | **Q** | **Y** | **Y** | **A** | **R** | **Q** | **L** | **I** | **K** | **E** | **N** | **V** | **Q** | **I** | **L** | **V** | **Y** | **E** | **N** | **G** | **F** | **L** | **H** | **A** | **K** | **T** | **L** | **I** | **M** | **D** | **D** | **E** | **I** | **C** | **M** | **V** | **G** | **S** | **A** | **N** | **Q** | **D** | **I** | **R** | **S** | **Y** | **R** | **L** | **N** | **F** | **E** | **T** | **S** | **A** | **V** | **I** | **Y** | **D** | **P** | **E** | **F** | **L** | **E** | **E** | **L** | **A** | **T** | **Q** | **F** | **K** | **E** | **D** | **E** | **T** | **H** | **C** | **S** | **P** | **M** | **T** | **T** | **E** | **T** | **V** | **K** | **E** | **M** | **S** | **N** | **W** | **L** | **L** | **F** | **K** | **Q** | **Q** | **I** | **S** | **R** | **L** | **F** | **S** | **P** | **I** | **L** |
| **N52459** | **-** | **-** | **-** | **-** | **-** | **-** | **-** | **-** | **-** | **-** | **-** | **-** | **-** | **-** | **-** | **-** | **-** | **-** | **-** | **-** | **-** | **-** | **-** | **K** | **P** | **R** | **D** | **V** | **A** | **A** | **I** | **W** | **A** | **W** | **L** | **L** | **V** | **L** | **F** | **A** | **L** | **P** | **G** | **V** | **G** | **F** | **V** | **L** | **Y** | **L** | **F** | **F** | **G** | **R** | **G** | **L** | **T** | **D** | **K** | **K** | **K** | **F** | **Y** | **L** | **R** | **Q** | **S** | **D** | **L** | **K** | **E** | **L** | **E** | **N** | **F** | **Q** | **S** | **F** | **R** | **G** | **D** | **T** | **I** | **E** | **H** | **Y** | **D** | **P** | **D** | **M** | **G** | **D** | **K** | **D** | **K** | **Q** | **Q** | **F** | **V** | **D** | **F** | **F** | **S** | **S** | **L** | **N** | **R** | **M** | **P** | **L** | **T** | **R** | **M** | **N** | **S** | **V** | **T** | **L** | **L** | **T** | **D** | **G** | **Q** | **E** | **K** | **L** | **D** | **S** | **L** | **L** | **Q** | **D** | **L** | **K** | **K** | **A** | **K** | **H** | **S** | **I** | **H** | **I** | **E** | **Y** | **Y** | **A** | **F** | **V** | **T** | **D** | **N** | **I** | **G** | **Q** | **Q** | **V** | **L** | **H** | **V** | **L** | **E** | **E** | **K** | **A** | **A** | **E** | **G** | **V** | **E** | **V** | **R** | **I** | **L** | **Y** | **D** | **A** | **F** | **G** | **S** | **H** | **G** | **T** | **K** | **A** | **K** | **D** | **F** | **N** | **R** | **L** | **I** | **K** | **N** | **G** | **G** | **H** | **V** | **H** | **T** | **F** | **V** | **T** | **S** | **Q** | **K** | **A** | **L** | **L** | **R** | **F** | **R** | **L** | **N** | **Y** | **H** | **D** | **H** | **R** | **K** | **I** | **V** | **V** | **I** | **D** | **G** | **K** | **I** | **S** | **Y** | **T** | **G** | **G** | **F** | **N** | **I** | **A** | **N** | **Q** | **Y** | **V** | **N** | **T** | **T** | **K** | **K** | **F** | **G** | **Y** | **W** | **R** | **D** | **T** | **H** | **I** | **R** | **I** | **F** | **G** | **A** | **A** | **S** | **S** | **L** | **L** | **Q** | **L** | **R** | **F** | **L** | **T** | **D** | **W** | **N** | **V** | **S** | **V** | **P** | **E** | **E** | **K** | **K** | **V** | **G** | **Y** | **H** | **L** | **N** | **Y** | **F** | **F** | **K** | **K** | **A** | **D** | **R** | **D** | **E** | **S** | **K** | **L** | **A** | **D** | **T** | **S** | **I** | **Q** | **L** | **V** | **S** | **S** | **G** | **P** | **N** | **N** | **E** | **R** | **E** | **Q** | **I** | **K** | **L** | **S** | **F** | **I** | **K** | **L** | **I** | **T** | **S** | **A** | **K** | **K** | **R** | **V** | **W** | **I** | **Q** | **T** | **P** | **Y** | **L** | **V** | **P** | **D** | **E** | **S** | **V** | **I** | **A** | **A** | **L** | **K** | **I** | **A** | **T** | **A** | **S** | **G** | **V** | **D** | **V** | **K** | **I** | **M** | **I** | **P** | **N** | **K** | **P** | **D** | **H** | **P** | **F** | **I** | **Y** | **R** | **A** | **T** | **Q** | **Y** | **Y** | **A** | **R** | **Q** | **L** | **I** | **K** | **E** | **N** | **V** | **Q** | **I** | **L** | **V** | **Y** | **E** | **N** | **G** | **F** | **L** | **H** | **A** | **K** | **T** | **L** | **I** | **M** | **D** | **D** | **E** | **I** | **C** | **M** | **V** | **G** | **S** | **A** | **N** | **Q** | **D** | **I** | **R** | **S** | **Y** | **R** | **L** | **N** | **F** | **E** | **T** | **S** | **A** | **V** | **I** | **Y** | **D** | **P** | **E** | **F** | **L** | **E** | **E** | **L** | **A** | **T** | **Q** | **F** | **K** | **E** | **D** | **E** | **T** | **H** | **C** | **S** | **S** | **M** | **T** | **T** | **E** | **T** | **V** | **K** | **E** | **M** | **S** | **N** | **W** | **L** | **L** | **F** | **K** | **Q** | **Q** | **I** | **S** | **R** | **L** | **F** | **S** | **P** | **I** | **L** |
| **N59613F** | **-** | **-** | **-** | **-** | **-** | **-** | **-** | **-** | **-** | **-** | **-** | **-** | **-** | **-** | **-** | **-** | **-** | **-** | **-** | **-** | **-** | **-** | **-** | **K** | **P** | **R** | **D** | **V** | **A** | **A** | **I** | **W** | **A** | **W** | **L** | **L** | **V** | **L** | **F** | **A** | **L** | **P** | **G** | **V** | **G** | **F** | **V** | **L** | **Y** | **L** | **F** | **F** | **G** | **R** | **G** | **L** | **T** | **D** | **K** | **K** | **K** | **F** | **Y** | **L** | **R** | **Q** | **S** | **D** | **L** | **K** | **E** | **L** | **E** | **N** | **F** | **Q** | **S** | **F** | **R** | **G** | **D** | **T** | **I** | **E** | **H** | **Y** | **D** | **P** | **D** | **M** | **G** | **D** | **K** | **D** | **K** | **Q** | **Q** | **F** | **V** | **D** | **F** | **F** | **S** | **S** | **L** | **N** | **R** | **M** | **P** | **L** | **T** | **R** | **M** | **N** | **S** | **V** | **T** | **L** | **L** | **T** | **D** | **G** | **Q** | **K** | **K** | **L** | **D** | **S** | **L** | **L** | **Q** | **D** | **L** | **K** | **K** | **A** | **K** | **H** | **S** | **I** | **H** | **I** | **E** | **Y** | **Y** | **A** | **F** | **V** | **T** | **D** | **N** | **I** | **G** | **Q** | **Q** | **V** | **L** | **R** | **V** | **L** | **E** | **E** | **K** | **A** | **A** | **E** | **G** | **V** | **E** | **V** | **R** | **I** | **L** | **Y** | **D** | **A** | **F** | **G** | **S** | **H** | **G** | **T** | **K** | **A** | **K** | **D** | **F** | **N** | **R** | **L** | **I** | **K** | **N** | **G** | **G** | **H** | **V** | **H** | **T** | **F** | **V** | **T** | **S** | **Q** | **K** | **A** | **L** | **L** | **R** | **F** | **R** | **L** | **N** | **Y** | **H** | **D** | **H** | **R** | **K** | **I** | **V** | **V** | **I** | **D** | **G** | **K** | **I** | **S** | **Y** | **T** | **G** | **G** | **F** | **N** | **I** | **A** | **N** | **Q** | **Y** | **V** | **N** | **T** | **T** | **K** | **K** | **F** | **G** | **Y** | **W** | **R** | **D** | **T** | **H** | **I** | **R** | **I** | **F** | **G** | **A** | **A** | **S** | **S** | **L** | **L** | **Q** | **L** | **R** | **F** | **L** | **T** | **D** | **W** | **N** | **V** | **S** | **V** | **S** | **E** | **E** | **K** | **K** | **V** | **G** | **Y** | **H** | **L** | **N** | **Y** | **F** | **F** | **K** | **K** | **A** | **D** | **R** | **D** | **E** | **S** | **K** | **L** | **A** | **D** | **T** | **S** | **I** | **Q** | **L** | **V** | **S** | **S** | **G** | **P** | **N** | **N** | **E** | **R** | **E** | **Q** | **I** | **K** | **L** | **S** | **F** | **I** | **K** | **L** | **I** | **T** | **S** | **A** | **K** | **K** | **R** | **V** | **W** | **I** | **Q** | **T** | **P** | **Y** | **L** | **V** | **P** | **D** | **E** | **S** | **V** | **I** | **A** | **A** | **L** | **K** | **I** | **A** | **T** | **A** | **S** | **G** | **V** | **D** | **V** | **K** | **I** | **M** | **I** | **P** | **N** | **K** | **P** | **D** | **H** | **P** | **F** | **I** | **Y** | **R** | **A** | **T** | **Q** | **Y** | **Y** | **A** | **R** | **Q** | **L** | **I** | **K** | **E** | **N** | **V** | **Q** | **I** | **L** | **V** | **Y** | **E** | **N** | **G** | **F** | **L** | **H** | **A** | **K** | **T** | **L** | **I** | **M** | **D** | **D** | **E** | **I** | **C** | **M** | **V** | **G** | **S** | **A** | **N** | **Q** | **D** | **I** | **R** | **S** | **Y** | **R** | **L** | **N** | **F** | **E** | **T** | **S** | **A** | **V** | **I** | **Y** | **D** | **P** | **E** | **F** | **L** | **E** | **E** | **L** | **A** | **T** | **Q** | **F** | **K** | **E** | **D** | **E** | **T** | **H** | **C** | **S** | **P** | **M** | **T** | **T** | **E** | **T** | **V** | **K** | **E** | **M** | **S** | **N** | **W** | **L** | **L** | **F** | **K** | **Q** | **Q** | **I** | **S** | **R** | **L** | **F** | **S** | **P** | **I** | **L** |
| **N54599** | **-** | **-** | **-** | **-** | **-** | **-** | **-** | **-** | **-** | **-** | **-** | **-** | **-** | **-** | **-** | **-** | **-** | **-** | **-** | **-** | **-** | **-** | **-** | **K** | **P** | **R** | **D** | **V** | **A** | **A** | **I** | **W** | **A** | **W** | **L** | **L** | **V** | **L** | **F** | **A** | **L** | **P** | **G** | **V** | **G** | **F** | **V** | **L** | **Y** | **L** | **F** | **F** | **G** | **R** | **G** | **L** | **T** | **D** | **K** | **K** | **K** | **F** | **Y** | **L** | **R** | **Q** | **S** | **D** | **L** | **K** | **E** | **L** | **E** | **N** | **F** | **Q** | **S** | **F** | **R** | **G** | **D** | **T** | **I** | **E** | **H** | **Y** | **D** | **P** | **D** | **M** | **G** | **D** | **K** | **D** | **K** | **Q** | **Q** | **F** | **V** | **D** | **F** | **F** | **S** | **S** | **L** | **N** | **R** | **M** | **P** | **L** | **T** | **R** | **M** | **N** | **S** | **V** | **T** | **L** | **L** | **T** | **D** | **G** | **Q** | **K** | **K** | **L** | **D** | **S** | **L** | **L** | **Q** | **D** | **L** | **K** | **K** | **A** | **K** | **H** | **S** | **I** | **H** | **I** | **E** | **Y** | **Y** | **A** | **F** | **V** | **T** | **D** | **T** | **I** | **G** | **Q** | **Q** | **V** | **L** | **R** | **V** | **L** | **E** | **E** | **K** | **V** | **A** | **E** | **G** | **V** | **E** | **V** | **R** | **I** | **L** | **Y** | **D** | **A** | **F** | **G** | **S** | **H** | **G** | **T** | **K** | **A** | **K** | **D** | **F** | **N** | **R** | **L** | **I** | **K** | **N** | **G** | **G** | **H** | **V** | **H** | **T** | **F** | **V** | **T** | **S** | **Q** | **K** | **A** | **L** | **L** | **R** | **F** | **R** | **L** | **N** | **Y** | **H** | **D** | **H** | **R** | **K** | **I** | **V** | **V** | **I** | **D** | **G** | **K** | **I** | **S** | **Y** | **T** | **G** | **G** | **F** | **N** | **I** | **A** | **N** | **Q** | **Y** | **V** | **N** | **T** | **T** | **K** | **K** | **F** | **G** | **Y** | **W** | **R** | **D** | **T** | **H** | **I** | **R** | **I** | **F** | **G** | **A** | **A** | **S** | **S** | **L** | **L** | **Q** | **L** | **R** | **F** | **L** | **T** | **D** | **W** | **N** | **V** | **S** | **V** | **P** | **E** | **E** | **K** | **K** | **V** | **G** | **Y** | **H** | **L** | **N** | **Y** | **F** | **F** | **K** | **K** | **A** | **D** | **R** | **D** | **E** | **S** | **K** | **L** | **A** | **D** | **T** | **S** | **I** | **Q** | **L** | **V** | **S** | **S** | **G** | **P** | **N** | **N** | **E** | **R** | **E** | **Q** | **I** | **K** | **L** | **S** | **F** | **I** | **K** | **L** | **I** | **T** | **S** | **A** | **K** | **K** | **R** | **V** | **W** | **I** | **Q** | **T** | **P** | **Y** | **L** | **V** | **P** | **D** | **E** | **S** | **V** | **I** | **A** | **A** | **L** | **K** | **I** | **A** | **T** | **A** | **S** | **G** | **V** | **D** | **V** | **K** | **I** | **M** | **I** | **P** | **N** | **K** | **P** | **D** | **H** | **P** | **F** | **I** | **Y** | **R** | **A** | **T** | **Q** | **Y** | **Y** | **A** | **R** | **Q** | **L** | **I** | **K** | **E** | **N** | **V** | **Q** | **I** | **L** | **V** | **Y** | **E** | **N** | **G** | **F** | **L** | **H** | **A** | **K** | **T** | **L** | **I** | **M** | **D** | **D** | **E** | **I** | **C** | **M** | **V** | **G** | **S** | **A** | **N** | **Q** | **D** | **I** | **R** | **S** | **Y** | **R** | **L** | **N** | **F** | **E** | **T** | **S** | **A** | **V** | **I** | **Y** | **D** | **P** | **E** | **F** | **L** | **E** | **E** | **L** | **A** | **T** | **Q** | **F** | **K** | **E** | **D** | **E** | **T** | **H** | **C** | **S** | **P** | **M** | **T** | **T** | **E** | **T** | **V** | **K** | **E** | **M** | **S** | **N** | **W** | **L** | **L** | **F** | **K** | **Q** | **Q** | **I** | **S** | **R** | **L** | **F** | **S** | **P** | **I** | **L** |
| **N52777** | **-** | **-** | **-** | **-** | **-** | **-** | **-** | **-** | **-** | **-** | **-** | **-** | **-** | **-** | **-** | **-** | **-** | **-** | **-** | **-** | **-** | **-** | **-** | **K** | **P** | **R** | **D** | **V** | **A** | **A** | **I** | **W** | **A** | **W** | **L** | **L** | **V** | **L** | **F** | **A** | **L** | **P** | **G** | **M** | **G** | **F** | **V** | **L** | **Y** | **L** | **F** | **F** | **G** | **R** | **G** | **L** | **T** | **D** | **K** | **K** | **K** | **F** | **Y** | **L** | **R** | **Q** | **S** | **D** | **L** | **K** | **E** | **L** | **E** | **N** | **F** | **Q** | **S** | **F** | **R** | **G** | **D** | **T** | **I** | **E** | **H** | **Y** | **D** | **P** | **D** | **M** | **G** | **D** | **K** | **D** | **K** | **Q** | **Q** | **F** | **V** | **D** | **F** | **F** | **S** | **S** | **L** | **N** | **R** | **M** | **P** | **L** | **T** | **R** | **M** | **N** | **S** | **V** | **T** | **L** | **L** | **T** | **D** | **G** | **Q** | **E** | **K** | **L** | **D** | **S** | **L** | **L** | **Q** | **D** | **L** | **K** | **K** | **A** | **K** | **H** | **S** | **I** | **H** | **I** | **E** | **Y** | **Y** | **A** | **F** | **V** | **T** | **D** | **N** | **I** | **G** | **Q** | **Q** | **V** | **L** | **H** | **V** | **L** | **E** | **E** | **K** | **A** | **A** | **E** | **G** | **V** | **E** | **V** | **R** | **I** | **L** | **Y** | **D** | **A** | **F** | **G** | **S** | **H** | **G** | **T** | **K** | **A** | **K** | **D** | **F** | **N** | **R** | **L** | **I** | **K** | **N** | **G** | **G** | **H** | **V** | **H** | **T** | **F** | **V** | **T** | **S** | **Q** | **K** | **A** | **L** | **L** | **R** | **F** | **R** | **L** | **N** | **Y** | **H** | **D** | **H** | **R** | **K** | **I** | **V** | **V** | **I** | **D** | **G** | **K** | **I** | **S** | **Y** | **T** | **G** | **G** | **F** | **N** | **I** | **A** | **N** | **Q** | **Y** | **V** | **N** | **T** | **T** | **K** | **K** | **F** | **G** | **Y** | **W** | **R** | **D** | **T** | **H** | **I** | **R** | **I** | **F** | **G** | **A** | **A** | **S** | **S** | **L** | **L** | **Q** | **L** | **R** | **F** | **L** | **T** | **D** | **W** | **N** | **V** | **S** | **V** | **P** | **E** | **E** | **K** | **K** | **V** | **G** | **Y** | **H** | **L** | **N** | **Y** | **F** | **F** | **K** | **K** | **A** | **D** | **R** | **D** | **E** | **S** | **K** | **L** | **A** | **D** | **T** | **S** | **I** | **Q** | **L** | **V** | **S** | **S** | **G** | **P** | **N** | **N** | **E** | **R** | **E** | **Q** | **I** | **K** | **L** | **S** | **F** | **I** | **K** | **L** | **I** | **T** | **S** | **A** | **K** | **K** | **R** | **V** | **W** | **I** | **Q** | **T** | **P** | **Y** | **L** | **V** | **P** | **D** | **E** | **S** | **V** | **I** | **A** | **A** | **L** | **K** | **I** | **A** | **T** | **A** | **S** | **G** | **V** | **D** | **V** | **K** | **I** | **M** | **I** | **P** | **N** | **K** | **P** | **D** | **H** | **P** | **F** | **I** | **Y** | **R** | **A** | **T** | **Q** | **Y** | **Y** | **A** | **R** | **Q** | **L** | **I** | **K** | **E** | **N** | **V** | **Q** | **I** | **L** | **V** | **Y** | **E** | **N** | **G** | **F** | **L** | **H** | **A** | **K** | **T** | **L** | **I** | **M** | **D** | **D** | **E** | **I** | **C** | **M** | **V** | **G** | **S** | **A** | **N** | **Q** | **D** | **I** | **R** | **S** | **Y** | **R** | **L** | **N** | **F** | **E** | **T** | **S** | **A** | **V** | **I** | **Y** | **D** | **P** | **E** | **F** | **L** | **E** | **E** | **L** | **A** | **T** | **Q** | **F** | **K** | **E** | **D** | **E** | **T** | **H** | **C** | **S** | **S** | **M** | **T** | **T** | **E** | **T** | **V** | **K** | **E** | **M** | **S** | **N** | **W** | **L** | **L** | **F** | **K** | **Q** | **Q** | **I** | **S** | **R** | **L** | **F** | **S** | **P** | **I** | **L** |
| **N54550** | **-** | **-** | **-** | **-** | **-** | **-** | **-** | **-** | **-** | **-** | **-** | **-** | **-** | **-** | **-** | **-** | **-** | **-** | **-** | **-** | **-** | **-** | **-** | **K** | **P** | **R** | **D** | **V** | **A** | **A** | **I** | **W** | **A** | **W** | **L** | **L** | **V** | **L** | **F** | **A** | **L** | **P** | **G** | **V** | **G** | **F** | **V** | **L** | **Y** | **L** | **F** | **F** | **G** | **R** | **G** | **L** | **T** | **D** | **K** | **K** | **K** | **F** | **Y** | **L** | **R** | **Q** | **S** | **D** | **L** | **K** | **E** | **L** | **E** | **N** | **F** | **Q** | **S** | **F** | **R** | **G** | **D** | **T** | **I** | **E** | **H** | **Y** | **D** | **P** | **D** | **M** | **G** | **D** | **K** | **D** | **K** | **Q** | **Q** | **F** | **V** | **D** | **F** | **F** | **S** | **S** | **L** | **N** | **R** | **M** | **P** | **L** | **T** | **R** | **M** | **N** | **S** | **V** | **T** | **L** | **L** | **T** | **D** | **G** | **Q** | **E** | **K** | **L** | **D** | **S** | **L** | **L** | **Q** | **D** | **L** | **K** | **K** | **A** | **K** | **H** | **S** | **I** | **H** | **I** | **E** | **Y** | **Y** | **A** | **F** | **V** | **T** | **D** | **N** | **I** | **G** | **Q** | **Q** | **V** | **L** | **H** | **V** | **L** | **E** | **E** | **K** | **A** | **A** | **E** | **G** | **V** | **E** | **V** | **R** | **I** | **L** | **Y** | **D** | **A** | **F** | **G** | **S** | **H** | **G** | **T** | **K** | **A** | **K** | **D** | **F** | **N** | **R** | **L** | **I** | **K** | **N** | **G** | **G** | **H** | **V** | **H** | **T** | **F** | **V** | **T** | **S** | **Q** | **K** | **A** | **L** | **L** | **R** | **F** | **R** | **L** | **N** | **Y** | **H** | **D** | **H** | **R** | **K** | **I** | **V** | **V** | **I** | **D** | **G** | **K** | **I** | **S** | **Y** | **T** | **G** | **G** | **F** | **N** | **I** | **A** | **N** | **Q** | **Y** | **V** | **N** | **T** | **T** | **K** | **K** | **F** | **G** | **Y** | **W** | **R** | **D** | **T** | **H** | **I** | **R** | **I** | **F** | **G** | **A** | **A** | **S** | **S** | **L** | **L** | **Q** | **L** | **R** | **F** | **L** | **T** | **D** | **W** | **N** | **V** | **S** | **V** | **P** | **E** | **E** | **K** | **K** | **V** | **G** | **Y** | **H** | **L** | **N** | **Y** | **F** | **F** | **K** | **K** | **A** | **D** | **R** | **D** | **E** | **S** | **K** | **L** | **A** | **D** | **T** | **S** | **I** | **Q** | **L** | **V** | **S** | **S** | **G** | **P** | **N** | **N** | **E** | **R** | **E** | **Q** | **I** | **K** | **L** | **S** | **F** | **I** | **K** | **L** | **I** | **T** | **S** | **A** | **K** | **K** | **R** | **V** | **W** | **I** | **Q** | **T** | **P** | **Y** | **L** | **V** | **P** | **D** | **E** | **S** | **V** | **I** | **A** | **A** | **L** | **K** | **I** | **A** | **T** | **A** | **S** | **G** | **V** | **D** | **V** | **K** | **I** | **M** | **I** | **P** | **N** | **K** | **P** | **D** | **H** | **P** | **F** | **I** | **Y** | **R** | **A** | **T** | **Q** | **Y** | **Y** | **A** | **R** | **Q** | **L** | **I** | **K** | **E** | **N** | **V** | **Q** | **I** | **L** | **V** | **Y** | **E** | **N** | **G** | **F** | **L** | **H** | **A** | **K** | **T** | **L** | **I** | **M** | **D** | **D** | **E** | **I** | **C** | **M** | **V** | **G** | **S** | **A** | **N** | **Q** | **D** | **I** | **R** | **S** | **Y** | **R** | **L** | **N** | **F** | **E** | **T** | **S** | **A** | **V** | **I** | **Y** | **D** | **P** | **E** | **F** | **L** | **E** | **E** | **L** | **A** | **T** | **Q** | **F** | **K** | **E** | **D** | **E** | **T** | **H** | **C** | **S** | **S** | **M** | **T** | **T** | **E** | **T** | **V** | **K** | **E** | **M** | **S** | **N** | **W** | **L** | **L** | **F** | **K** | **Q** | **Q** | **I** | **S** | **R** | **L** | **F** | **S** | **P** | **I** | **L** |
| **N59517F** | **-** | **-** | **-** | **-** | **-** | **-** | **-** | **-** | **-** | **-** | **-** | **-** | **-** | **-** | **-** | **-** | **-** | **-** | **-** | **-** | **-** | **-** | **-** | **K** | **P** | **R** | **D** | **V** | **A** | **A** | **I** | **W** | **A** | **W** | **L** | **L** | **V** | **L** | **F** | **A** | **L** | **P** | **G** | **V** | **G** | **F** | **V** | **L** | **Y** | **L** | **F** | **F** | **G** | **R** | **G** | **L** | **T** | **D** | **K** | **K** | **K** | **F** | **Y** | **L** | **R** | **Q** | **S** | **D** | **L** | **K** | **E** | **L** | **E** | **N** | **F** | **Q** | **S** | **F** | **R** | **G** | **D** | **T** | **I** | **E** | **H** | **Y** | **D** | **P** | **D** | **M** | **G** | **D** | **K** | **D** | **K** | **Q** | **Q** | **F** | **V** | **D** | **F** | **F** | **S** | **S** | **L** | **N** | **R** | **M** | **P** | **L** | **T** | **R** | **M** | **N** | **S** | **V** | **T** | **L** | **L** | **T** | **D** | **G** | **Q** | **E** | **K** | **L** | **D** | **S** | **L** | **L** | **Q** | **D** | **L** | **K** | **K** | **A** | **K** | **H** | **S** | **I** | **H** | **I** | **E** | **Y** | **Y** | **A** | **F** | **V** | **T** | **D** | **N** | **I** | **G** | **Q** | **Q** | **V** | **L** | **H** | **V** | **L** | **E** | **E** | **K** | **A** | **A** | **E** | **G** | **V** | **E** | **V** | **R** | **I** | **L** | **Y** | **D** | **A** | **F** | **G** | **S** | **H** | **G** | **T** | **K** | **A** | **K** | **D** | **F** | **N** | **R** | **L** | **I** | **K** | **N** | **G** | **G** | **H** | **V** | **H** | **T** | **F** | **V** | **T** | **S** | **Q** | **R** | **A** | **L** | **L** | **R** | **F** | **R** | **L** | **N** | **Y** | **H** | **D** | **H** | **R** | **K** | **I** | **V** | **V** | **I** | **D** | **G** | **K** | **I** | **S** | **Y** | **T** | **G** | **G** | **F** | **N** | **I** | **A** | **N** | **Q** | **Y** | **V** | **N** | **T** | **T** | **K** | **K** | **F** | **G** | **Y** | **W** | **R** | **D** | **T** | **H** | **I** | **R** | **I** | **F** | **G** | **A** | **A** | **S** | **S** | **L** | **L** | **Q** | **L** | **R** | **F** | **L** | **T** | **D** | **W** | **N** | **V** | **S** | **V** | **P** | **E** | **E** | **K** | **K** | **V** | **G** | **Y** | **H** | **L** | **N** | **Y** | **F** | **F** | **K** | **K** | **A** | **D** | **R** | **D** | **E** | **S** | **K** | **L** | **A** | **D** | **T** | **S** | **I** | **Q** | **L** | **V** | **S** | **S** | **G** | **P** | **N** | **N** | **E** | **R** | **E** | **Q** | **I** | **K** | **L** | **S** | **F** | **I** | **K** | **L** | **I** | **T** | **S** | **A** | **K** | **K** | **R** | **V** | **W** | **I** | **Q** | **T** | **P** | **Y** | **L** | **V** | **P** | **D** | **E** | **S** | **V** | **I** | **A** | **A** | **L** | **K** | **I** | **A** | **T** | **A** | **S** | **G** | **V** | **D** | **V** | **K** | **I** | **M** | **I** | **P** | **N** | **K** | **P** | **D** | **H** | **P** | **F** | **I** | **Y** | **R** | **A** | **T** | **Q** | **Y** | **Y** | **A** | **R** | **Q** | **L** | **I** | **K** | **E** | **N** | **V** | **Q** | **I** | **L** | **V** | **Y** | **E** | **N** | **G** | **F** | **L** | **H** | **A** | **K** | **T** | **L** | **I** | **M** | **D** | **D** | **E** | **I** | **C** | **M** | **V** | **G** | **S** | **A** | **N** | **Q** | **D** | **I** | **R** | **S** | **Y** | **R** | **L** | **N** | **F** | **E** | **T** | **S** | **A** | **V** | **I** | **Y** | **D** | **P** | **E** | **F** | **L** | **E** | **E** | **L** | **A** | **T** | **Q** | **F** | **K** | **E** | **D** | **E** | **T** | **H** | **C** | **S** | **S** | **M** | **T** | **T** | **E** | **T** | **V** | **K** | **E** | **M** | **S** | **N** | **W** | **L** | **L** | **F** | **K** | **Q** | **Q** | **I** | **S** | **R** | **L** | **F** | **S** | **P** | **I** | **L** |
| **N53533** | **-** | **-** | **-** | **-** | **-** | **-** | **-** | **-** | **-** | **-** | **-** | **-** | **-** | **-** | **-** | **-** | **-** | **-** | **-** | **-** | **-** | **-** | **-** | **K** | **P** | **R** | **D** | **V** | **A** | **A** | **I** | **W** | **A** | **W** | **L** | **L** | **V** | **L** | **F** | **A** | **L** | **P** | **G** | **V** | **G** | **F** | **V** | **L** | **Y** | **L** | **F** | **F** | **G** | **R** | **G** | **L** | **T** | **D** | **K** | **K** | **K** | **F** | **Y** | **L** | **R** | **Q** | **S** | **D** | **L** | **K** | **E** | **L** | **E** | **N** | **F** | **Q** | **S** | **F** | **R** | **G** | **D** | **T** | **I** | **E** | **H** | **Y** | **D** | **P** | **D** | **M** | **G** | **D** | **K** | **D** | **K** | **Q** | **Q** | **F** | **V** | **D** | **F** | **F** | **S** | **S** | **L** | **N** | **R** | **M** | **P** | **L** | **T** | **R** | **M** | **N** | **S** | **V** | **T** | **L** | **L** | **T** | **D** | **G** | **Q** | **E** | **K** | **L** | **D** | **S** | **L** | **L** | **Q** | **D** | **L** | **K** | **K** | **A** | **K** | **H** | **S** | **I** | **H** | **I** | **E** | **Y** | **Y** | **A** | **F** | **V** | **T** | **D** | **N** | **I** | **G** | **Q** | **Q** | **V** | **L** | **H** | **V** | **L** | **E** | **E** | **K** | **A** | **A** | **E** | **G** | **V** | **E** | **V** | **R** | **I** | **L** | **Y** | **D** | **A** | **F** | **G** | **S** | **H** | **G** | **T** | **K** | **A** | **K** | **D** | **F** | **N** | **R** | **L** | **I** | **K** | **N** | **G** | **G** | **H** | **V** | **H** | **T** | **F** | **V** | **T** | **S** | **Q** | **K** | **A** | **L** | **L** | **R** | **F** | **R** | **L** | **N** | **Y** | **H** | **D** | **H** | **R** | **K** | **I** | **V** | **V** | **I** | **D** | **G** | **K** | **I** | **S** | **Y** | **T** | **G** | **G** | **F** | **N** | **I** | **A** | **N** | **Q** | **Y** | **V** | **N** | **T** | **T** | **K** | **K** | **F** | **G** | **Y** | **W** | **R** | **D** | **T** | **H** | **I** | **R** | **I** | **F** | **G** | **A** | **A** | **S** | **S** | **L** | **L** | **Q** | **L** | **R** | **F** | **L** | **T** | **D** | **W** | **N** | **V** | **S** | **V** | **P** | **E** | **E** | **K** | **K** | **V** | **G** | **Y** | **H** | **L** | **N** | **Y** | **F** | **F** | **K** | **K** | **A** | **D** | **R** | **D** | **E** | **S** | **K** | **L** | **A** | **D** | **T** | **S** | **I** | **Q** | **L** | **V** | **S** | **S** | **G** | **P** | **N** | **N** | **E** | **R** | **E** | **Q** | **I** | **K** | **L** | **S** | **F** | **I** | **K** | **L** | **I** | **T** | **S** | **A** | **K** | **K** | **R** | **V** | **W** | **I** | **Q** | **T** | **P** | **Y** | **L** | **V** | **P** | **D** | **E** | **S** | **V** | **I** | **A** | **A** | **L** | **K** | **I** | **A** | **T** | **A** | **S** | **G** | **V** | **D** | **V** | **K** | **I** | **M** | **I** | **P** | **N** | **K** | **P** | **D** | **H** | **P** | **F** | **I** | **Y** | **R** | **A** | **T** | **Q** | **Y** | **Y** | **A** | **R** | **Q** | **L** | **I** | **K** | **E** | **N** | **V** | **Q** | **I** | **L** | **V** | **Y** | **E** | **N** | **G** | **F** | **L** | **H** | **A** | **K** | **T** | **L** | **I** | **M** | **D** | **D** | **E** | **I** | **C** | **M** | **V** | **G** | **S** | **A** | **N** | **Q** | **D** | **I** | **R** | **S** | **Y** | **R** | **L** | **N** | **F** | **E** | **T** | **S** | **A** | **V** | **I** | **Y** | **D** | **P** | **E** | **F** | **L** | **E** | **E** | **L** | **A** | **T** | **Q** | **F** | **K** | **E** | **D** | **E** | **T** | **H** | **C** | **S** | **S** | **M** | **T** | **T** | **E** | **T** | **V** | **K** | **E** | **M** | **S** | **N** | **W** | **L** | **L** | **F** | **K** | **Q** | **Q** | **I** | **S** | **R** | **L** | **F** | **S** | **P** | **I** | **L** |
| **N59907F** | **-** | **-** | **-** | **-** | **-** | **-** | **-** | **-** | **-** | **-** | **-** | **-** | **-** | **-** | **-** | **-** | **-** | **-** | **-** | **-** | **-** | **-** | **-** | **K** | **P** | **R** | **D** | **V** | **A** | **A** | **I** | **W** | **A** | **W** | **L** | **L** | **V** | **L** | **F** | **A** | **L** | **P** | **G** | **V** | **G** | **F** | **V** | **L** | **Y** | **L** | **F** | **F** | **G** | **R** | **G** | **L** | **T** | **D** | **K** | **K** | **K** | **F** | **Y** | **L** | **R** | **Q** | **S** | **D** | **L** | **K** | **E** | **L** | **E** | **N** | **F** | **Q** | **S** | **F** | **R** | **G** | **D** | **T** | **I** | **E** | **H** | **Y** | **D** | **P** | **D** | **M** | **G** | **D** | **K** | **D** | **K** | **Q** | **Q** | **F** | **V** | **D** | **F** | **F** | **S** | **S** | **L** | **N** | **R** | **M** | **P** | **L** | **T** | **R** | **M** | **N** | **S** | **V** | **T** | **L** | **L** | **T** | **D** | **G** | **Q** | **E** | **K** | **L** | **D** | **S** | **L** | **L** | **Q** | **D** | **L** | **K** | **K** | **A** | **K** | **H** | **S** | **I** | **H** | **I** | **E** | **Y** | **Y** | **A** | **F** | **V** | **T** | **D** | **N** | **I** | **G** | **Q** | **Q** | **V** | **L** | **H** | **V** | **L** | **E** | **E** | **K** | **A** | **A** | **E** | **G** | **V** | **E** | **V** | **R** | **I** | **L** | **Y** | **D** | **A** | **F** | **G** | **S** | **H** | **G** | **T** | **K** | **A** | **K** | **D** | **F** | **N** | **R** | **L** | **I** | **K** | **N** | **G** | **G** | **H** | **V** | **H** | **T** | **F** | **V** | **T** | **S** | **Q** | **K** | **A** | **L** | **L** | **R** | **F** | **R** | **L** | **N** | **Y** | **H** | **D** | **H** | **R** | **K** | **I** | **V** | **V** | **I** | **D** | **G** | **K** | **I** | **S** | **Y** | **T** | **G** | **G** | **F** | **N** | **I** | **A** | **N** | **Q** | **Y** | **V** | **N** | **T** | **T** | **K** | **K** | **F** | **G** | **Y** | **W** | **R** | **D** | **T** | **H** | **I** | **R** | **I** | **F** | **G** | **A** | **A** | **S** | **S** | **L** | **L** | **Q** | **L** | **R** | **F** | **L** | **T** | **D** | **W** | **N** | **V** | **S** | **V** | **P** | **E** | **E** | **K** | **K** | **V** | **G** | **Y** | **H** | **L** | **N** | **Y** | **F** | **F** | **K** | **K** | **A** | **D** | **R** | **D** | **E** | **S** | **K** | **L** | **A** | **E** | **T** | **S** | **I** | **Q** | **L** | **V** | **S** | **S** | **G** | **P** | **N** | **N** | **E** | **R** | **E** | **Q** | **I** | **K** | **L** | **S** | **F** | **I** | **K** | **L** | **I** | **T** | **S** | **A** | **K** | **K** | **R** | **V** | **W** | **I** | **Q** | **T** | **P** | **Y** | **L** | **V** | **P** | **D** | **E** | **S** | **V** | **I** | **A** | **A** | **L** | **K** | **I** | **A** | **T** | **A** | **S** | **G** | **V** | **D** | **V** | **K** | **I** | **M** | **I** | **P** | **N** | **K** | **P** | **D** | **H** | **P** | **F** | **I** | **Y** | **R** | **A** | **T** | **Q** | **Y** | **Y** | **A** | **R** | **Q** | **L** | **I** | **K** | **E** | **N** | **V** | **Q** | **I** | **L** | **V** | **Y** | **E** | **N** | **G** | **F** | **L** | **H** | **A** | **K** | **T** | **L** | **I** | **M** | **D** | **D** | **E** | **I** | **C** | **M** | **V** | **G** | **S** | **A** | **N** | **Q** | **D** | **I** | **R** | **S** | **Y** | **R** | **L** | **N** | **F** | **E** | **T** | **S** | **A** | **V** | **I** | **Y** | **D** | **P** | **E** | **F** | **L** | **E** | **E** | **L** | **A** | **T** | **Q** | **F** | **K** | **E** | **D** | **E** | **T** | **H** | **C** | **S** | **S** | **M** | **T** | **T** | **E** | **T** | **V** | **K** | **E** | **M** | **S** | **N** | **W** | **L** | **L** | **F** | **K** | **Q** | **Q** | **I** | **S** | **R** | **L** | **F** | **S** | **P** | **I** | **L** |
| **N52690** | **-** | **-** | **-** | **-** | **-** | **-** | **-** | **-** | **-** | **-** | **-** | **-** | **-** | **-** | **-** | **-** | **-** | **-** | **-** | **-** | **-** | **-** | **-** | **K** | **P** | **R** | **D** | **V** | **A** | **A** | **I** | **W** | **A** | **W** | **L** | **L** | **V** | **L** | **F** | **A** | **L** | **P** | **G** | **V** | **G** | **F** | **V** | **L** | **Y** | **L** | **F** | **F** | **G** | **R** | **G** | **L** | **T** | **D** | **K** | **K** | **K** | **F** | **Y** | **L** | **R** | **Q** | **S** | **D** | **L** | **K** | **E** | **L** | **E** | **N** | **F** | **Q** | **S** | **F** | **R** | **G** | **D** | **T** | **I** | **E** | **H** | **Y** | **D** | **P** | **D** | **M** | **G** | **D** | **K** | **D** | **K** | **Q** | **Q** | **F** | **V** | **D** | **F** | **F** | **S** | **S** | **L** | **N** | **R** | **M** | **P** | **L** | **T** | **R** | **M** | **N** | **S** | **V** | **T** | **L** | **L** | **T** | **D** | **G** | **Q** | **E** | **K** | **L** | **D** | **S** | **L** | **L** | **Q** | **D** | **L** | **K** | **K** | **A** | **K** | **H** | **S** | **I** | **H** | **I** | **E** | **Y** | **Y** | **A** | **F** | **V** | **T** | **D** | **N** | **I** | **G** | **Q** | **Q** | **V** | **L** | **H** | **V** | **L** | **E** | **E** | **K** | **A** | **A** | **E** | **G** | **V** | **E** | **V** | **R** | **I** | **L** | **Y** | **D** | **A** | **F** | **G** | **S** | **H** | **G** | **T** | **K** | **A** | **K** | **D** | **F** | **N** | **R** | **L** | **I** | **K** | **N** | **G** | **G** | **H** | **V** | **H** | **T** | **F** | **V** | **T** | **S** | **Q** | **K** | **A** | **L** | **L** | **R** | **F** | **R** | **L** | **N** | **Y** | **H** | **D** | **H** | **R** | **K** | **I** | **V** | **V** | **I** | **D** | **G** | **K** | **I** | **S** | **Y** | **I** | **G** | **G** | **F** | **N** | **I** | **A** | **N** | **Q** | **Y** | **V** | **N** | **T** | **T** | **K** | **K** | **F** | **G** | **Y** | **W** | **R** | **D** | **T** | **H** | **I** | **R** | **I** | **F** | **G** | **A** | **A** | **S** | **S** | **L** | **L** | **Q** | **L** | **R** | **F** | **L** | **T** | **D** | **W** | **N** | **V** | **S** | **V** | **P** | **E** | **E** | **K** | **K** | **V** | **G** | **Y** | **H** | **L** | **N** | **Y** | **F** | **F** | **K** | **K** | **A** | **D** | **R** | **D** | **E** | **S** | **K** | **L** | **A** | **D** | **T** | **S** | **I** | **Q** | **L** | **V** | **S** | **S** | **G** | **P** | **N** | **N** | **E** | **R** | **E** | **Q** | **I** | **K** | **L** | **S** | **F** | **I** | **K** | **L** | **I** | **T** | **S** | **A** | **K** | **K** | **R** | **V** | **W** | **I** | **Q** | **T** | **P** | **Y** | **L** | **V** | **P** | **D** | **E** | **S** | **V** | **I** | **A** | **A** | **L** | **K** | **I** | **A** | **T** | **A** | **S** | **G** | **V** | **D** | **V** | **K** | **I** | **M** | **I** | **P** | **N** | **K** | **P** | **D** | **H** | **P** | **F** | **I** | **Y** | **R** | **A** | **T** | **Q** | **Y** | **Y** | **A** | **R** | **Q** | **L** | **I** | **K** | **E** | **N** | **V** | **Q** | **I** | **L** | **V** | **Y** | **E** | **N** | **G** | **F** | **L** | **H** | **A** | **K** | **T** | **L** | **I** | **M** | **D** | **D** | **E** | **I** | **C** | **M** | **V** | **G** | **S** | **A** | **N** | **Q** | **D** | **I** | **R** | **S** | **Y** | **R** | **L** | **N** | **F** | **E** | **T** | **S** | **A** | **V** | **I** | **Y** | **D** | **P** | **E** | **F** | **L** | **E** | **E** | **L** | **A** | **T** | **Q** | **F** | **K** | **E** | **D** | **E** | **T** | **H** | **C** | **S** | **S** | **M** | **T** | **T** | **E** | **T** | **V** | **K** | **E** | **M** | **S** | **N** | **W** | **L** | **L** | **F** | **K** | **Q** | **Q** | **I** | **S** | **R** | **L** | **F** | **S** | **P** | **I** | **L** |
| **N59542F** | **-** | **-** | **-** | **-** | **-** | **-** | **-** | **-** | **-** | **-** | **-** | **-** | **-** | **-** | **-** | **-** | **-** | **-** | **-** | **-** | **-** | **-** | **-** | **K** | **P** | **R** | **D** | **V** | **A** | **A** | **I** | **W** | **A** | **W** | **L** | **L** | **V** | **L** | **F** | **A** | **L** | **P** | **G** | **V** | **G** | **F** | **V** | **L** | **Y** | **L** | **F** | **F** | **G** | **R** | **G** | **L** | **T** | **D** | **K** | **K** | **K** | **F** | **Y** | **L** | **R** | **Q** | **S** | **D** | **L** | **K** | **E** | **L** | **E** | **N** | **F** | **Q** | **S** | **F** | **R** | **G** | **D** | **T** | **I** | **E** | **H** | **Y** | **D** | **P** | **D** | **M** | **G** | **D** | **K** | **D** | **K** | **Q** | **Q** | **F** | **V** | **D** | **F** | **F** | **S** | **S** | **L** | **N** | **R** | **M** | **P** | **L** | **T** | **R** | **M** | **N** | **S** | **V** | **T** | **L** | **L** | **T** | **D** | **G** | **Q** | **E** | **K** | **L** | **D** | **S** | **L** | **L** | **Q** | **D** | **L** | **K** | **K** | **A** | **K** | **H** | **S** | **I** | **H** | **I** | **E** | **Y** | **Y** | **A** | **F** | **V** | **T** | **D** | **N** | **I** | **G** | **Q** | **Q** | **V** | **L** | **H** | **V** | **L** | **E** | **E** | **K** | **A** | **A** | **E** | **G** | **V** | **E** | **V** | **R** | **I** | **L** | **Y** | **D** | **A** | **F** | **G** | **S** | **H** | **G** | **T** | **K** | **A** | **K** | **D** | **F** | **N** | **R** | **L** | **I** | **K** | **N** | **G** | **G** | **H** | **V** | **H** | **T** | **F** | **V** | **T** | **S** | **Q** | **K** | **A** | **L** | **L** | **R** | **F** | **R** | **L** | **N** | **Y** | **H** | **D** | **H** | **R** | **K** | **I** | **V** | **V** | **I** | **D** | **G** | **K** | **I** | **S** | **Y** | **T** | **G** | **G** | **F** | **N** | **I** | **A** | **N** | **Q** | **Y** | **V** | **N** | **T** | **T** | **K** | **K** | **F** | **G** | **Y** | **W** | **R** | **D** | **T** | **H** | **I** | **R** | **I** | **F** | **G** | **A** | **A** | **S** | **S** | **L** | **L** | **Q** | **L** | **R** | **F** | **L** | **T** | **D** | **W** | **N** | **V** | **S** | **V** | **P** | **E** | **E** | **K** | **K** | **V** | **G** | **Y** | **H** | **L** | **N** | **Y** | **F** | **F** | **K** | **K** | **A** | **D** | **R** | **D** | **E** | **S** | **K** | **L** | **A** | **D** | **T** | **S** | **I** | **Q** | **L** | **V** | **S** | **S** | **G** | **P** | **N** | **N** | **E** | **R** | **E** | **Q** | **I** | **K** | **L** | **S** | **F** | **I** | **K** | **L** | **I** | **T** | **S** | **A** | **K** | **K** | **R** | **V** | **W** | **I** | **Q** | **T** | **P** | **Y** | **L** | **V** | **P** | **D** | **E** | **S** | **V** | **I** | **A** | **A** | **L** | **K** | **I** | **A** | **T** | **A** | **S** | **G** | **V** | **D** | **V** | **K** | **I** | **M** | **I** | **P** | **N** | **K** | **P** | **D** | **H** | **P** | **F** | **I** | **Y** | **R** | **A** | **T** | **Q** | **Y** | **Y** | **A** | **R** | **Q** | **L** | **I** | **K** | **E** | **N** | **V** | **Q** | **I** | **L** | **V** | **Y** | **E** | **N** | **G** | **F** | **L** | **H** | **A** | **K** | **T** | **L** | **I** | **M** | **D** | **D** | **E** | **I** | **C** | **M** | **V** | **G** | **S** | **A** | **N** | **Q** | **D** | **I** | **R** | **S** | **Y** | **R** | **L** | **N** | **F** | **E** | **T** | **S** | **A** | **V** | **I** | **Y** | **D** | **P** | **E** | **F** | **L** | **E** | **E** | **L** | **A** | **T** | **Q** | **F** | **K** | **E** | **D** | **E** | **T** | **H** | **C** | **S** | **S** | **M** | **T** | **T** | **E** | **T** | **V** | **K** | **E** | **M** | **S** | **N** | **W** | **L** | **L** | **F** | **K** | **Q** | **Q** | **I** | **S** | **R** | **L** | **F** | **S** | **P** | **I** | **L** |
| **N59998F** | **-** | **-** | **-** | **-** | **-** | **-** | **-** | **-** | **-** | **-** | **-** | **-** | **-** | **-** | **-** | **-** | **-** | **-** | **-** | **-** | **-** | **-** | **-** | **K** | **P** | **R** | **D** | **V** | **A** | **A** | **I** | **W** | **A** | **W** | **L** | **L** | **V** | **L** | **F** | **A** | **L** | **P** | **G** | **V** | **G** | **F** | **V** | **L** | **Y** | **L** | **F** | **F** | **G** | **R** | **G** | **L** | **T** | **D** | **K** | **K** | **K** | **F** | **Y** | **L** | **R** | **Q** | **S** | **D** | **L** | **K** | **E** | **L** | **E** | **N** | **F** | **Q** | **S** | **F** | **R** | **G** | **D** | **T** | **I** | **E** | **H** | **Y** | **D** | **P** | **D** | **M** | **G** | **D** | **K** | **D** | **K** | **Q** | **Q** | **F** | **V** | **D** | **F** | **F** | **S** | **S** | **L** | **N** | **R** | **M** | **P** | **L** | **T** | **R** | **M** | **N** | **S** | **V** | **T** | **L** | **L** | **T** | **D** | **G** | **Q** | **E** | **K** | **L** | **D** | **S** | **L** | **L** | **Q** | **D** | **L** | **K** | **K** | **A** | **K** | **H** | **S** | **I** | **H** | **I** | **E** | **Y** | **Y** | **A** | **F** | **V** | **T** | **D** | **N** | **I** | **G** | **Q** | **Q** | **V** | **L** | **H** | **V** | **L** | **E** | **E** | **K** | **A** | **A** | **E** | **G** | **V** | **E** | **V** | **R** | **I** | **L** | **Y** | **D** | **A** | **F** | **G** | **S** | **H** | **G** | **T** | **K** | **A** | **K** | **D** | **F** | **N** | **R** | **L** | **I** | **K** | **N** | **G** | **G** | **H** | **V** | **H** | **T** | **F** | **V** | **T** | **S** | **Q** | **K** | **A** | **L** | **L** | **R** | **F** | **R** | **L** | **N** | **Y** | **H** | **D** | **H** | **R** | **K** | **I** | **V** | **V** | **I** | **D** | **G** | **K** | **I** | **S** | **Y** | **T** | **G** | **G** | **F** | **N** | **I** | **A** | **N** | **Q** | **Y** | **V** | **N** | **T** | **T** | **K** | **K** | **F** | **G** | **Y** | **W** | **R** | **D** | **T** | **H** | **I** | **R** | **I** | **F** | **G** | **A** | **A** | **S** | **S** | **L** | **L** | **Q** | **L** | **R** | **F** | **L** | **T** | **D** | **W** | **N** | **V** | **S** | **V** | **P** | **E** | **E** | **K** | **K** | **V** | **G** | **Y** | **H** | **L** | **N** | **Y** | **F** | **F** | **K** | **K** | **A** | **D** | **R** | **D** | **E** | **S** | **K** | **L** | **A** | **D** | **T** | **S** | **I** | **Q** | **L** | **V** | **S** | **S** | **G** | **P** | **N** | **N** | **E** | **R** | **E** | **Q** | **I** | **K** | **L** | **S** | **F** | **I** | **K** | **L** | **I** | **T** | **S** | **A** | **K** | **K** | **R** | **V** | **W** | **I** | **Q** | **T** | **P** | **Y** | **L** | **V** | **P** | **D** | **E** | **S** | **V** | **I** | **A** | **A** | **L** | **K** | **I** | **A** | **T** | **A** | **S** | **G** | **V** | **D** | **V** | **K** | **I** | **M** | **I** | **P** | **N** | **K** | **P** | **D** | **H** | **P** | **F** | **I** | **Y** | **R** | **A** | **T** | **Q** | **Y** | **Y** | **A** | **R** | **Q** | **L** | **I** | **K** | **E** | **N** | **V** | **Q** | **I** | **L** | **V** | **Y** | **E** | **N** | **G** | **F** | **L** | **H** | **A** | **K** | **T** | **L** | **I** | **M** | **D** | **D** | **E** | **I** | **C** | **M** | **V** | **G** | **S** | **A** | **N** | **Q** | **D** | **I** | **R** | **S** | **Y** | **R** | **L** | **N** | **F** | **E** | **T** | **S** | **A** | **V** | **I** | **Y** | **D** | **P** | **E** | **F** | **L** | **E** | **E** | **L** | **A** | **T** | **Q** | **F** | **K** | **E** | **D** | **E** | **T** | **H** | **C** | **S** | **S** | **M** | **T** | **T** | **E** | **T** | **V** | **K** | **E** | **M** | **S** | **N** | **W** | **L** | **L** | **F** | **K** | **Q** | **Q** | **I** | **S** | **R** | **L** | **F** | **S** | **P** | **I** | **L** |
| **N60045F** | **-** | **-** | **-** | **-** | **-** | **-** | **-** | **-** | **-** | **-** | **-** | **-** | **-** | **-** | **-** | **-** | **-** | **-** | **-** | **-** | **-** | **-** | **-** | **K** | **P** | **R** | **D** | **V** | **A** | **A** | **I** | **W** | **A** | **W** | **L** | **L** | **V** | **L** | **F** | **A** | **L** | **P** | **G** | **V** | **G** | **F** | **V** | **L** | **Y** | **L** | **F** | **F** | **G** | **R** | **G** | **L** | **T** | **D** | **K** | **K** | **K** | **F** | **Y** | **L** | **R** | **Q** | **S** | **D** | **L** | **K** | **E** | **L** | **E** | **N** | **F** | **Q** | **S** | **F** | **R** | **G** | **D** | **T** | **I** | **E** | **H** | **Y** | **D** | **P** | **D** | **M** | **G** | **D** | **K** | **D** | **K** | **Q** | **Q** | **F** | **V** | **D** | **F** | **F** | **S** | **S** | **L** | **N** | **R** | **M** | **P** | **L** | **T** | **R** | **M** | **N** | **S** | **V** | **T** | **L** | **L** | **T** | **D** | **G** | **Q** | **E** | **K** | **L** | **D** | **S** | **L** | **L** | **Q** | **D** | **L** | **K** | **K** | **A** | **K** | **H** | **S** | **I** | **H** | **I** | **E** | **Y** | **Y** | **A** | **F** | **V** | **T** | **D** | **N** | **I** | **G** | **Q** | **Q** | **V** | **L** | **H** | **V** | **L** | **E** | **E** | **K** | **A** | **A** | **E** | **G** | **V** | **E** | **V** | **R** | **I** | **L** | **Y** | **D** | **A** | **F** | **G** | **S** | **H** | **G** | **T** | **K** | **A** | **K** | **D** | **F** | **N** | **R** | **L** | **I** | **K** | **N** | **G** | **G** | **H** | **V** | **H** | **T** | **F** | **V** | **T** | **S** | **Q** | **K** | **A** | **L** | **L** | **R** | **F** | **R** | **L** | **N** | **Y** | **H** | **D** | **H** | **R** | **K** | **I** | **V** | **V** | **I** | **D** | **G** | **K** | **I** | **S** | **Y** | **T** | **G** | **G** | **F** | **N** | **I** | **A** | **N** | **Q** | **Y** | **V** | **N** | **T** | **T** | **K** | **K** | **F** | **G** | **Y** | **W** | **R** | **D** | **T** | **H** | **I** | **R** | **I** | **F** | **G** | **A** | **A** | **S** | **S** | **L** | **L** | **Q** | **L** | **R** | **F** | **L** | **T** | **D** | **W** | **N** | **V** | **S** | **V** | **P** | **E** | **E** | **K** | **K** | **V** | **G** | **Y** | **H** | **L** | **N** | **Y** | **F** | **F** | **K** | **K** | **A** | **D** | **R** | **D** | **E** | **S** | **K** | **L** | **A** | **E** | **T** | **S** | **I** | **Q** | **L** | **V** | **S** | **S** | **G** | **P** | **N** | **N** | **E** | **R** | **E** | **Q** | **I** | **K** | **L** | **S** | **F** | **I** | **K** | **L** | **I** | **T** | **S** | **A** | **K** | **K** | **R** | **V** | **W** | **I** | **Q** | **T** | **P** | **Y** | **L** | **V** | **P** | **D** | **E** | **S** | **V** | **I** | **A** | **A** | **L** | **K** | **I** | **A** | **T** | **A** | **S** | **G** | **V** | **D** | **V** | **K** | **I** | **M** | **I** | **P** | **N** | **K** | **P** | **D** | **H** | **P** | **F** | **I** | **Y** | **R** | **A** | **T** | **Q** | **Y** | **Y** | **A** | **R** | **Q** | **L** | **I** | **K** | **E** | **N** | **V** | **Q** | **I** | **L** | **V** | **Y** | **E** | **N** | **G** | **F** | **L** | **H** | **A** | **K** | **T** | **L** | **I** | **M** | **D** | **D** | **E** | **I** | **C** | **M** | **V** | **G** | **S** | **A** | **N** | **Q** | **D** | **I** | **R** | **S** | **Y** | **R** | **L** | **N** | **F** | **E** | **T** | **S** | **A** | **V** | **I** | **Y** | **D** | **P** | **E** | **F** | **L** | **E** | **E** | **L** | **A** | **T** | **Q** | **F** | **K** | **E** | **D** | **E** | **T** | **H** | **C** | **S** | **S** | **M** | **T** | **T** | **E** | **T** | **V** | **K** | **E** | **M** | **S** | **N** | **W** | **L** | **L** | **F** | **K** | **Q** | **Q** | **I** | **S** | **R** | **L** | **F** | **S** | **P** | **I** | **L** |
| **N55077** | **-** | **-** | **-** | **-** | **-** | **-** | **-** | **-** | **-** | **-** | **-** | **-** | **-** | **-** | **-** | **-** | **-** | **-** | **-** | **-** | **-** | **-** | **-** | **K** | **P** | **R** | **D** | **V** | **A** | **A** | **I** | **W** | **A** | **W** | **L** | **L** | **V** | **L** | **F** | **A** | **L** | **P** | **G** | **V** | **G** | **F** | **V** | **L** | **Y** | **L** | **F** | **F** | **G** | **R** | **G** | **L** | **T** | **D** | **K** | **K** | **K** | **F** | **Y** | **L** | **R** | **Q** | **S** | **D** | **L** | **K** | **E** | **L** | **E** | **N** | **F** | **Q** | **S** | **F** | **R** | **G** | **D** | **T** | **I** | **E** | **H** | **Y** | **D** | **P** | **D** | **M** | **G** | **D** | **K** | **D** | **K** | **Q** | **Q** | **F** | **V** | **D** | **F** | **F** | **S** | **S** | **L** | **N** | **R** | **M** | **P** | **L** | **T** | **R** | **M** | **N** | **S** | **V** | **T** | **L** | **L** | **T** | **D** | **G** | **Q** | **E** | **K** | **L** | **D** | **S** | **L** | **L** | **Q** | **D** | **L** | **K** | **K** | **A** | **K** | **H** | **S** | **I** | **H** | **I** | **E** | **Y** | **Y** | **A** | **F** | **V** | **T** | **D** | **N** | **I** | **G** | **Q** | **Q** | **V** | **L** | **H** | **V** | **L** | **E** | **E** | **K** | **A** | **A** | **E** | **G** | **V** | **E** | **V** | **R** | **I** | **L** | **Y** | **D** | **A** | **F** | **G** | **S** | **H** | **G** | **T** | **K** | **A** | **K** | **D** | **F** | **N** | **R** | **L** | **I** | **K** | **N** | **G** | **G** | **H** | **V** | **H** | **T** | **F** | **V** | **T** | **S** | **Q** | **R** | **A** | **L** | **L** | **R** | **F** | **R** | **L** | **N** | **Y** | **H** | **D** | **H** | **R** | **K** | **I** | **V** | **V** | **I** | **D** | **G** | **K** | **I** | **S** | **Y** | **T** | **G** | **G** | **F** | **N** | **I** | **A** | **N** | **Q** | **Y** | **V** | **N** | **T** | **T** | **K** | **K** | **F** | **G** | **Y** | **W** | **R** | **D** | **T** | **H** | **I** | **R** | **I** | **F** | **G** | **A** | **A** | **S** | **S** | **L** | **L** | **Q** | **L** | **R** | **F** | **L** | **T** | **D** | **W** | **N** | **V** | **S** | **V** | **P** | **E** | **E** | **K** | **K** | **V** | **G** | **Y** | **H** | **L** | **N** | **Y** | **F** | **F** | **K** | **K** | **A** | **D** | **R** | **D** | **E** | **S** | **K** | **L** | **A** | **D** | **T** | **S** | **I** | **Q** | **L** | **V** | **S** | **S** | **G** | **P** | **N** | **N** | **E** | **R** | **E** | **Q** | **I** | **K** | **L** | **S** | **F** | **I** | **K** | **L** | **I** | **T** | **S** | **A** | **K** | **K** | **R** | **V** | **W** | **I** | **Q** | **T** | **P** | **Y** | **L** | **V** | **P** | **D** | **E** | **S** | **V** | **I** | **A** | **A** | **L** | **K** | **I** | **A** | **T** | **A** | **S** | **G** | **V** | **D** | **V** | **K** | **I** | **M** | **I** | **P** | **N** | **K** | **P** | **D** | **H** | **P** | **F** | **I** | **Y** | **R** | **A** | **T** | **Q** | **Y** | **Y** | **A** | **R** | **Q** | **L** | **I** | **K** | **E** | **N** | **V** | **Q** | **I** | **L** | **V** | **Y** | **E** | **N** | **G** | **F** | **L** | **H** | **A** | **K** | **T** | **L** | **I** | **M** | **D** | **D** | **E** | **I** | **C** | **M** | **V** | **G** | **S** | **A** | **N** | **Q** | **D** | **I** | **R** | **S** | **Y** | **R** | **L** | **N** | **F** | **E** | **T** | **S** | **A** | **V** | **I** | **Y** | **D** | **P** | **E** | **F** | **L** | **E** | **E** | **L** | **A** | **T** | **Q** | **F** | **K** | **E** | **D** | **E** | **T** | **H** | **C** | **S** | **S** | **M** | **T** | **T** | **E** | **T** | **V** | **K** | **E** | **M** | **S** | **N** | **W** | **L** | **L** | **F** | **K** | **Q** | **Q** | **I** | **S** | **R** | **L** | **F** | **S** | **P** | **I** | **L** |
| **N59745F** | **-** | **-** | **-** | **-** | **-** | **-** | **-** | **-** | **-** | **-** | **-** | **-** | **-** | **-** | **-** | **-** | **-** | **-** | **-** | **-** | **-** | **-** | **-** | **K** | **P** | **R** | **D** | **V** | **A** | **A** | **I** | **W** | **A** | **W** | **L** | **L** | **V** | **L** | **F** | **A** | **L** | **P** | **G** | **V** | **G** | **F** | **V** | **L** | **Y** | **L** | **F** | **F** | **G** | **R** | **G** | **L** | **T** | **D** | **K** | **K** | **K** | **F** | **Y** | **L** | **R** | **Q** | **S** | **D** | **L** | **K** | **E** | **L** | **E** | **N** | **F** | **Q** | **S** | **F** | **R** | **G** | **D** | **T** | **I** | **E** | **H** | **Y** | **D** | **P** | **D** | **M** | **G** | **D** | **K** | **D** | **K** | **Q** | **Q** | **F** | **V** | **D** | **F** | **F** | **S** | **S** | **L** | **N** | **R** | **M** | **P** | **L** | **T** | **R** | **M** | **N** | **S** | **V** | **T** | **L** | **L** | **T** | **D** | **G** | **Q** | **K** | **K** | **L** | **D** | **S** | **L** | **L** | **Q** | **D** | **L** | **K** | **K** | **A** | **K** | **H** | **S** | **I** | **H** | **I** | **E** | **Y** | **Y** | **A** | **F** | **V** | **T** | **D** | **N** | **I** | **G** | **Q** | **Q** | **V** | **L** | **R** | **V** | **L** | **E** | **E** | **K** | **A** | **A** | **E** | **G** | **V** | **E** | **V** | **R** | **I** | **L** | **Y** | **D** | **A** | **F** | **G** | **S** | **H** | **G** | **T** | **K** | **A** | **K** | **D** | **F** | **N** | **R** | **L** | **I** | **K** | **N** | **G** | **G** | **H** | **V** | **H** | **T** | **F** | **V** | **T** | **S** | **Q** | **K** | **A** | **L** | **L** | **R** | **F** | **R** | **L** | **N** | **Y** | **H** | **D** | **H** | **R** | **K** | **I** | **V** | **V** | **I** | **D** | **G** | **K** | **I** | **S** | **Y** | **T** | **G** | **G** | **F** | **N** | **I** | **A** | **N** | **Q** | **Y** | **V** | **N** | **T** | **T** | **K** | **K** | **F** | **G** | **Y** | **W** | **R** | **D** | **T** | **H** | **I** | **R** | **I** | **F** | **G** | **A** | **A** | **S** | **S** | **L** | **L** | **Q** | **L** | **R** | **F** | **L** | **T** | **D** | **W** | **N** | **V** | **S** | **V** | **S** | **E** | **E** | **K** | **K** | **V** | **G** | **Y** | **H** | **L** | **N** | **Y** | **F** | **F** | **K** | **K** | **A** | **D** | **R** | **D** | **E** | **S** | **K** | **L** | **A** | **D** | **T** | **S** | **I** | **Q** | **L** | **V** | **S** | **S** | **G** | **P** | **N** | **N** | **E** | **R** | **E** | **Q** | **I** | **K** | **L** | **S** | **F** | **I** | **K** | **L** | **I** | **T** | **S** | **A** | **K** | **K** | **R** | **V** | **W** | **I** | **Q** | **T** | **P** | **Y** | **L** | **V** | **P** | **D** | **E** | **S** | **V** | **I** | **A** | **A** | **L** | **K** | **I** | **A** | **T** | **A** | **S** | **G** | **V** | **D** | **V** | **K** | **I** | **M** | **I** | **P** | **N** | **K** | **P** | **D** | **H** | **P** | **F** | **I** | **Y** | **R** | **A** | **T** | **Q** | **Y** | **Y** | **A** | **R** | **Q** | **L** | **I** | **K** | **E** | **N** | **V** | **Q** | **I** | **L** | **V** | **Y** | **E** | **N** | **G** | **F** | **L** | **H** | **A** | **K** | **T** | **L** | **I** | **M** | **D** | **D** | **E** | **I** | **C** | **M** | **V** | **G** | **S** | **A** | **N** | **Q** | **D** | **I** | **R** | **S** | **Y** | **R** | **L** | **N** | **F** | **E** | **T** | **S** | **A** | **V** | **I** | **Y** | **D** | **P** | **E** | **F** | **L** | **E** | **E** | **L** | **A** | **T** | **Q** | **F** | **K** | **E** | **D** | **E** | **T** | **H** | **C** | **S** | **P** | **M** | **T** | **T** | **E** | **T** | **V** | **K** | **E** | **M** | **S** | **N** | **W** | **L** | **L** | **F** | **K** | **Q** | **Q** | **I** | **S** | **R** | **L** | **F** | **S** | **P** | **I** | **L** |
| **N53559** | **-** | **-** | **-** | **-** | **-** | **-** | **-** | **-** | **-** | **-** | **-** | **-** | **-** | **-** | **-** | **-** | **-** | **-** | **-** | **-** | **-** | **-** | **-** | **K** | **P** | **R** | **D** | **V** | **A** | **A** | **I** | **W** | **A** | **W** | **L** | **L** | **V** | **L** | **F** | **V** | **L** | **P** | **G** | **V** | **G** | **F** | **V** | **L** | **Y** | **L** | **F** | **F** | **G** | **R** | **G** | **L** | **T** | **D** | **K** | **K** | **K** | **F** | **Y** | **L** | **R** | **Q** | **S** | **D** | **L** | **K** | **E** | **L** | **E** | **N** | **F** | **Q** | **S** | **F** | **R** | **G** | **D** | **T** | **I** | **E** | **H** | **Y** | **D** | **P** | **D** | **M** | **G** | **D** | **K** | **D** | **K** | **Q** | **Q** | **F** | **V** | **D** | **F** | **F** | **S** | **S** | **L** | **N** | **R** | **M** | **P** | **L** | **T** | **R** | **M** | **N** | **S** | **V** | **T** | **L** | **L** | **T** | **D** | **G** | **Q** | **E** | **K** | **L** | **D** | **S** | **L** | **L** | **Q** | **D** | **L** | **K** | **K** | **A** | **K** | **H** | **S** | **I** | **H** | **I** | **E** | **Y** | **Y** | **A** | **F** | **V** | **T** | **D** | **N** | **I** | **G** | **Q** | **Q** | **V** | **L** | **H** | **V** | **L** | **E** | **E** | **K** | **A** | **A** | **E** | **G** | **V** | **E** | **V** | **R** | **I** | **L** | **Y** | **D** | **A** | **F** | **G** | **S** | **H** | **G** | **T** | **K** | **A** | **K** | **D** | **F** | **N** | **R** | **L** | **I** | **K** | **N** | **G** | **G** | **H** | **V** | **H** | **T** | **F** | **V** | **T** | **S** | **Q** | **K** | **A** | **L** | **L** | **R** | **F** | **R** | **L** | **N** | **Y** | **H** | **D** | **H** | **R** | **K** | **I** | **V** | **V** | **I** | **D** | **G** | **K** | **I** | **S** | **Y** | **T** | **G** | **G** | **F** | **N** | **I** | **A** | **N** | **Q** | **Y** | **V** | **N** | **T** | **T** | **K** | **K** | **F** | **G** | **Y** | **W** | **R** | **D** | **T** | **H** | **I** | **R** | **I** | **F** | **G** | **A** | **A** | **S** | **S** | **L** | **L** | **Q** | **L** | **R** | **F** | **L** | **T** | **D** | **W** | **N** | **V** | **S** | **V** | **P** | **E** | **E** | **K** | **K** | **V** | **G** | **Y** | **H** | **L** | **N** | **Y** | **F** | **F** | **K** | **K** | **A** | **D** | **R** | **D** | **E** | **S** | **K** | **L** | **A** | **D** | **T** | **S** | **I** | **Q** | **L** | **V** | **S** | **S** | **G** | **P** | **N** | **N** | **E** | **R** | **E** | **Q** | **I** | **K** | **L** | **S** | **F** | **I** | **K** | **L** | **I** | **T** | **S** | **A** | **K** | **K** | **R** | **V** | **W** | **I** | **Q** | **T** | **P** | **Y** | **L** | **V** | **P** | **D** | **E** | **S** | **V** | **I** | **A** | **A** | **L** | **K** | **I** | **A** | **T** | **A** | **S** | **G** | **V** | **D** | **V** | **K** | **I** | **M** | **I** | **P** | **N** | **K** | **P** | **D** | **H** | **P** | **F** | **I** | **Y** | **R** | **A** | **T** | **Q** | **Y** | **Y** | **A** | **R** | **Q** | **L** | **I** | **K** | **E** | **N** | **V** | **Q** | **I** | **L** | **V** | **Y** | **E** | **N** | **G** | **F** | **L** | **H** | **A** | **K** | **T** | **L** | **I** | **M** | **D** | **D** | **E** | **I** | **C** | **M** | **V** | **G** | **S** | **A** | **N** | **Q** | **D** | **I** | **R** | **S** | **Y** | **R** | **L** | **N** | **F** | **E** | **T** | **S** | **A** | **V** | **I** | **Y** | **D** | **P** | **E** | **F** | **L** | **E** | **E** | **L** | **A** | **T** | **Q** | **F** | **K** | **E** | **D** | **E** | **T** | **H** | **C** | **S** | **S** | **M** | **T** | **T** | **E** | **T** | **V** | **K** | **E** | **M** | **S** | **N** | **W** | **L** | **L** | **F** | **K** | **Q** | **Q** | **I** | **S** | **R** | **L** | **F** | **S** | **P** | **I** | **L** |
| **N60200F** | **-** | **-** | **-** | **-** | **-** | **-** | **-** | **-** | **-** | **-** | **-** | **-** | **-** | **-** | **-** | **-** | **-** | **-** | **-** | **-** | **-** | **-** | **-** | **K** | **P** | **R** | **D** | **V** | **A** | **A** | **I** | **W** | **A** | **W** | **L** | **L** | **V** | **L** | **F** | **A** | **L** | **P** | **G** | **V** | **G** | **F** | **V** | **L** | **Y** | **L** | **F** | **F** | **G** | **R** | **G** | **L** | **T** | **D** | **K** | **K** | **K** | **F** | **Y** | **L** | **R** | **Q** | **S** | **D** | **L** | **K** | **E** | **L** | **E** | **N** | **F** | **Q** | **S** | **F** | **R** | **G** | **D** | **N** | **I** | **E** | **H** | **Y** | **D** | **P** | **D** | **M** | **G** | **D** | **K** | **D** | **K** | **Q** | **Q** | **F** | **V** | **D** | **F** | **F** | **S** | **S** | **L** | **N** | **R** | **M** | **P** | **L** | **T** | **R** | **M** | **N** | **S** | **V** | **T** | **L** | **L** | **T** | **D** | **G** | **Q** | **E** | **K** | **L** | **D** | **S** | **L** | **L** | **Q** | **D** | **L** | **K** | **K** | **A** | **K** | **H** | **S** | **I** | **H** | **I** | **E** | **Y** | **Y** | **A** | **F** | **V** | **T** | **D** | **N** | **I** | **G** | **Q** | **Q** | **V** | **L** | **H** | **V** | **L** | **E** | **E** | **K** | **A** | **A** | **E** | **G** | **V** | **E** | **V** | **R** | **I** | **L** | **Y** | **D** | **A** | **F** | **G** | **S** | **H** | **G** | **T** | **K** | **A** | **K** | **D** | **F** | **N** | **R** | **L** | **I** | **K** | **N** | **G** | **G** | **H** | **V** | **H** | **T** | **F** | **V** | **T** | **S** | **Q** | **K** | **A** | **L** | **L** | **R** | **F** | **R** | **L** | **N** | **Y** | **H** | **D** | **H** | **R** | **K** | **I** | **V** | **V** | **I** | **D** | **G** | **K** | **I** | **S** | **Y** | **T** | **G** | **G** | **F** | **N** | **I** | **A** | **N** | **Q** | **Y** | **V** | **N** | **T** | **T** | **K** | **K** | **F** | **G** | **Y** | **W** | **R** | **D** | **T** | **H** | **I** | **R** | **I** | **F** | **G** | **A** | **A** | **S** | **S** | **L** | **L** | **Q** | **L** | **R** | **F** | **L** | **T** | **D** | **W** | **N** | **V** | **S** | **V** | **P** | **E** | **E** | **K** | **K** | **V** | **G** | **Y** | **H** | **L** | **N** | **Y** | **F** | **F** | **K** | **K** | **A** | **D** | **R** | **D** | **E** | **S** | **K** | **L** | **A** | **D** | **T** | **S** | **I** | **Q** | **L** | **V** | **S** | **S** | **G** | **P** | **N** | **N** | **E** | **R** | **E** | **Q** | **I** | **K** | **L** | **S** | **F** | **I** | **K** | **L** | **I** | **T** | **S** | **A** | **K** | **K** | **R** | **V** | **W** | **I** | **Q** | **T** | **P** | **Y** | **L** | **V** | **P** | **D** | **E** | **S** | **V** | **I** | **A** | **A** | **L** | **K** | **I** | **A** | **T** | **A** | **S** | **G** | **V** | **D** | **V** | **K** | **I** | **M** | **I** | **P** | **N** | **K** | **P** | **D** | **H** | **P** | **F** | **I** | **Y** | **R** | **A** | **T** | **Q** | **Y** | **Y** | **A** | **R** | **Q** | **L** | **I** | **K** | **E** | **N** | **V** | **Q** | **I** | **L** | **V** | **Y** | **E** | **N** | **G** | **F** | **L** | **H** | **A** | **K** | **T** | **L** | **I** | **M** | **D** | **D** | **E** | **I** | **C** | **M** | **V** | **G** | **S** | **A** | **N** | **Q** | **D** | **I** | **R** | **S** | **Y** | **R** | **L** | **N** | **F** | **E** | **T** | **S** | **A** | **V** | **I** | **Y** | **D** | **P** | **E** | **F** | **L** | **E** | **E** | **L** | **A** | **T** | **Q** | **F** | **K** | **E** | **D** | **E** | **T** | **H** | **C** | **S** | **S** | **M** | **T** | **T** | **E** | **T** | **V** | **K** | **E** | **M** | **S** | **N** | **W** | **L** | **L** | **F** | **K** | **Q** | **Q** | **I** | **S** | **R** | **L** | **F** | **S** | **P** | **I** | **L** |
| **N52732** | **-** | **-** | **-** | **-** | **-** | **-** | **-** | **-** | **-** | **-** | **-** | **-** | **-** | **-** | **-** | **-** | **-** | **-** | **-** | **-** | **-** | **-** | **-** | **K** | **P** | **R** | **D** | **V** | **A** | **A** | **I** | **W** | **A** | **W** | **L** | **L** | **V** | **L** | **F** | **A** | **L** | **P** | **G** | **V** | **G** | **F** | **V** | **L** | **Y** | **L** | **F** | **F** | **G** | **R** | **G** | **L** | **T** | **D** | **K** | **K** | **K** | **F** | **Y** | **L** | **R** | **Q** | **S** | **D** | **L** | **K** | **E** | **L** | **E** | **N** | **F** | **Q** | **S** | **F** | **R** | **G** | **D** | **T** | **I** | **E** | **H** | **Y** | **D** | **P** | **D** | **M** | **G** | **D** | **K** | **D** | **K** | **Q** | **Q** | **F** | **V** | **D** | **F** | **F** | **S** | **S** | **L** | **N** | **R** | **M** | **P** | **L** | **T** | **R** | **M** | **N** | **S** | **V** | **T** | **L** | **L** | **T** | **D** | **G** | **Q** | **K** | **K** | **L** | **D** | **S** | **L** | **L** | **Q** | **D** | **L** | **K** | **K** | **A** | **K** | **H** | **S** | **I** | **H** | **I** | **E** | **Y** | **Y** | **A** | **F** | **V** | **T** | **D** | **T** | **I** | **G** | **Q** | **Q** | **V** | **L** | **R** | **V** | **L** | **E** | **E** | **K** | **A** | **A** | **E** | **G** | **V** | **E** | **V** | **R** | **I** | **L** | **Y** | **D** | **A** | **F** | **G** | **S** | **H** | **G** | **T** | **K** | **A** | **K** | **D** | **F** | **N** | **R** | **L** | **I** | **K** | **N** | **G** | **G** | **H** | **V** | **H** | **T** | **F** | **V** | **T** | **S** | **Q** | **K** | **A** | **L** | **L** | **R** | **F** | **R** | **L** | **N** | **Y** | **H** | **D** | **H** | **R** | **K** | **I** | **V** | **V** | **I** | **D** | **G** | **K** | **I** | **S** | **Y** | **T** | **G** | **G** | **F** | **N** | **I** | **A** | **N** | **Q** | **Y** | **V** | **N** | **T** | **T** | **K** | **K** | **F** | **G** | **Y** | **W** | **R** | **D** | **T** | **H** | **I** | **R** | **I** | **F** | **G** | **A** | **A** | **S** | **S** | **L** | **L** | **Q** | **L** | **R** | **F** | **L** | **T** | **D** | **W** | **N** | **V** | **S** | **V** | **P** | **E** | **E** | **K** | **K** | **V** | **G** | **Y** | **H** | **L** | **N** | **Y** | **F** | **F** | **K** | **K** | **A** | **D** | **R** | **D** | **E** | **S** | **K** | **L** | **A** | **D** | **T** | **S** | **I** | **Q** | **L** | **V** | **S** | **S** | **G** | **P** | **N** | **N** | **E** | **R** | **E** | **Q** | **I** | **K** | **L** | **S** | **F** | **I** | **K** | **L** | **I** | **T** | **S** | **A** | **K** | **K** | **R** | **V** | **W** | **I** | **Q** | **T** | **P** | **Y** | **L** | **V** | **P** | **D** | **E** | **S** | **V** | **I** | **A** | **A** | **L** | **K** | **I** | **A** | **T** | **A** | **S** | **G** | **V** | **D** | **V** | **K** | **I** | **M** | **I** | **P** | **N** | **K** | **P** | **D** | **H** | **P** | **F** | **I** | **Y** | **R** | **A** | **T** | **Q** | **Y** | **Y** | **A** | **R** | **Q** | **L** | **I** | **K** | **E** | **N** | **V** | **Q** | **I** | **L** | **V** | **Y** | **E** | **N** | **G** | **F** | **L** | **H** | **A** | **K** | **T** | **L** | **I** | **M** | **D** | **D** | **E** | **I** | **C** | **M** | **V** | **G** | **S** | **A** | **N** | **Q** | **D** | **I** | **R** | **S** | **Y** | **R** | **L** | **N** | **F** | **E** | **T** | **S** | **A** | **V** | **I** | **Y** | **D** | **P** | **E** | **F** | **L** | **E** | **E** | **L** | **A** | **T** | **Q** | **F** | **K** | **E** | **D** | **E** | **T** | **H** | **C** | **S** | **P** | **M** | **T** | **T** | **E** | **T** | **V** | **K** | **E** | **M** | **S** | **N** | **W** | **L** | **L** | **F** | **K** | **Q** | **Q** | **I** | **S** | **R** | **L** | **F** | **S** | **P** | **I** | **L** |
| **N54519** | **-** | **-** | **-** | **-** | **-** | **-** | **-** | **-** | **-** | **-** | **-** | **-** | **-** | **-** | **-** | **-** | **-** | **-** | **-** | **-** | **-** | **-** | **-** | **K** | **P** | **R** | **D** | **V** | **A** | **A** | **I** | **W** | **A** | **W** | **L** | **L** | **V** | **L** | **F** | **A** | **L** | **P** | **G** | **V** | **G** | **F** | **V** | **L** | **Y** | **L** | **F** | **F** | **G** | **R** | **G** | **L** | **T** | **D** | **K** | **K** | **K** | **F** | **Y** | **L** | **R** | **Q** | **S** | **D** | **L** | **K** | **E** | **L** | **E** | **N** | **F** | **Q** | **S** | **F** | **R** | **G** | **D** | **T** | **I** | **E** | **H** | **Y** | **D** | **P** | **D** | **M** | **G** | **D** | **K** | **D** | **K** | **Q** | **Q** | **F** | **V** | **D** | **F** | **F** | **S** | **S** | **L** | **N** | **R** | **M** | **P** | **L** | **T** | **R** | **M** | **N** | **S** | **V** | **T** | **L** | **L** | **T** | **D** | **G** | **Q** | **K** | **K** | **L** | **D** | **S** | **L** | **L** | **Q** | **D** | **L** | **K** | **K** | **A** | **K** | **H** | **S** | **I** | **H** | **I** | **E** | **Y** | **Y** | **A** | **F** | **V** | **T** | **D** | **T** | **I** | **G** | **Q** | **Q** | **V** | **L** | **R** | **V** | **L** | **E** | **E** | **K** | **A** | **A** | **E** | **G** | **V** | **E** | **V** | **R** | **I** | **L** | **Y** | **D** | **A** | **F** | **G** | **S** | **H** | **G** | **T** | **K** | **A** | **K** | **D** | **F** | **N** | **R** | **L** | **I** | **K** | **N** | **G** | **G** | **H** | **V** | **H** | **T** | **F** | **V** | **T** | **S** | **Q** | **K** | **A** | **L** | **L** | **R** | **F** | **R** | **L** | **N** | **Y** | **H** | **D** | **H** | **R** | **K** | **I** | **V** | **V** | **I** | **D** | **G** | **K** | **I** | **S** | **Y** | **T** | **G** | **G** | **F** | **N** | **I** | **A** | **N** | **Q** | **Y** | **V** | **N** | **T** | **T** | **K** | **K** | **F** | **G** | **Y** | **W** | **R** | **D** | **T** | **H** | **I** | **R** | **I** | **F** | **G** | **A** | **A** | **S** | **S** | **L** | **L** | **Q** | **L** | **R** | **F** | **L** | **T** | **D** | **W** | **N** | **V** | **S** | **V** | **P** | **E** | **E** | **K** | **K** | **V** | **G** | **Y** | **H** | **L** | **N** | **Y** | **F** | **F** | **K** | **K** | **A** | **D** | **R** | **D** | **E** | **S** | **K** | **L** | **A** | **D** | **T** | **S** | **I** | **Q** | **L** | **V** | **S** | **S** | **G** | **P** | **N** | **N** | **E** | **R** | **E** | **Q** | **I** | **K** | **L** | **S** | **F** | **I** | **K** | **L** | **I** | **T** | **S** | **A** | **K** | **K** | **R** | **V** | **W** | **I** | **Q** | **T** | **P** | **Y** | **L** | **V** | **P** | **D** | **E** | **S** | **V** | **I** | **A** | **A** | **L** | **K** | **I** | **A** | **T** | **A** | **S** | **G** | **V** | **D** | **V** | **K** | **I** | **M** | **I** | **P** | **N** | **K** | **P** | **D** | **H** | **P** | **F** | **I** | **Y** | **R** | **A** | **T** | **Q** | **Y** | **Y** | **A** | **R** | **Q** | **L** | **I** | **K** | **E** | **N** | **V** | **Q** | **I** | **L** | **V** | **Y** | **E** | **N** | **G** | **F** | **L** | **H** | **A** | **K** | **T** | **L** | **I** | **M** | **D** | **D** | **E** | **I** | **C** | **M** | **V** | **G** | **S** | **A** | **N** | **Q** | **D** | **I** | **R** | **S** | **Y** | **R** | **L** | **N** | **F** | **E** | **T** | **S** | **A** | **V** | **I** | **Y** | **D** | **P** | **E** | **F** | **L** | **E** | **E** | **L** | **A** | **T** | **Q** | **F** | **K** | **E** | **D** | **E** | **T** | **H** | **C** | **S** | **P** | **M** | **T** | **T** | **E** | **T** | **V** | **K** | **E** | **M** | **S** | **N** | **W** | **L** | **L** | **F** | **K** | **Q** | **Q** | **I** | **S** | **R** | **L** | **F** | **S** | **P** | **I** | **L** |
| **N53548** | **-** | **-** | **-** | **-** | **-** | **-** | **-** | **-** | **-** | **-** | **-** | **-** | **-** | **-** | **-** | **-** | **-** | **-** | **-** | **-** | **-** | **-** | **-** | **K** | **P** | **R** | **D** | **V** | **A** | **A** | **I** | **W** | **A** | **W** | **L** | **L** | **V** | **L** | **F** | **A** | **L** | **P** | **G** | **V** | **G** | **F** | **V** | **L** | **Y** | **L** | **F** | **F** | **G** | **R** | **G** | **L** | **T** | **D** | **K** | **K** | **K** | **F** | **Y** | **L** | **R** | **Q** | **S** | **D** | **L** | **K** | **E** | **L** | **E** | **N** | **F** | **Q** | **S** | **F** | **R** | **G** | **D** | **T** | **I** | **E** | **H** | **Y** | **D** | **P** | **D** | **M** | **G** | **D** | **K** | **D** | **K** | **Q** | **Q** | **F** | **V** | **D** | **F** | **F** | **S** | **S** | **L** | **N** | **R** | **M** | **P** | **L** | **T** | **R** | **M** | **N** | **S** | **V** | **T** | **L** | **L** | **T** | **D** | **G** | **Q** | **E** | **K** | **L** | **D** | **S** | **L** | **L** | **Q** | **D** | **L** | **K** | **K** | **A** | **K** | **H** | **S** | **I** | **H** | **I** | **E** | **Y** | **Y** | **A** | **F** | **V** | **T** | **D** | **N** | **I** | **G** | **Q** | **Q** | **V** | **L** | **H** | **V** | **L** | **E** | **E** | **K** | **A** | **A** | **E** | **G** | **V** | **E** | **V** | **R** | **I** | **L** | **Y** | **D** | **A** | **F** | **G** | **S** | **H** | **G** | **T** | **K** | **A** | **K** | **D** | **F** | **N** | **R** | **L** | **I** | **K** | **N** | **G** | **G** | **H** | **V** | **H** | **T** | **F** | **V** | **T** | **S** | **Q** | **R** | **A** | **L** | **L** | **R** | **F** | **R** | **L** | **N** | **Y** | **H** | **D** | **H** | **R** | **K** | **I** | **V** | **V** | **I** | **D** | **G** | **K** | **I** | **S** | **Y** | **T** | **G** | **G** | **F** | **N** | **I** | **A** | **N** | **Q** | **Y** | **V** | **N** | **T** | **T** | **K** | **K** | **F** | **G** | **Y** | **W** | **R** | **D** | **T** | **H** | **I** | **R** | **I** | **F** | **G** | **A** | **A** | **S** | **S** | **L** | **L** | **Q** | **L** | **R** | **F** | **L** | **T** | **D** | **W** | **N** | **V** | **S** | **V** | **P** | **E** | **E** | **K** | **K** | **V** | **G** | **Y** | **H** | **L** | **N** | **Y** | **F** | **F** | **K** | **K** | **A** | **D** | **R** | **D** | **E** | **S** | **K** | **L** | **A** | **D** | **T** | **S** | **I** | **Q** | **L** | **V** | **S** | **S** | **G** | **P** | **N** | **N** | **E** | **R** | **E** | **Q** | **I** | **K** | **L** | **S** | **F** | **I** | **K** | **L** | **I** | **T** | **S** | **A** | **K** | **K** | **R** | **V** | **W** | **I** | **Q** | **T** | **P** | **Y** | **L** | **V** | **P** | **D** | **E** | **S** | **V** | **I** | **A** | **A** | **L** | **K** | **I** | **A** | **T** | **A** | **S** | **G** | **V** | **D** | **V** | **K** | **I** | **M** | **I** | **P** | **N** | **K** | **P** | **D** | **H** | **P** | **F** | **I** | **Y** | **R** | **A** | **T** | **Q** | **Y** | **Y** | **A** | **R** | **Q** | **L** | **I** | **K** | **E** | **N** | **V** | **Q** | **I** | **L** | **V** | **Y** | **E** | **N** | **G** | **F** | **L** | **H** | **A** | **K** | **T** | **L** | **I** | **M** | **D** | **D** | **E** | **I** | **C** | **M** | **V** | **G** | **S** | **A** | **N** | **Q** | **D** | **I** | **R** | **S** | **Y** | **R** | **L** | **N** | **F** | **E** | **T** | **S** | **A** | **V** | **I** | **Y** | **D** | **P** | **E** | **F** | **L** | **E** | **E** | **L** | **A** | **T** | **Q** | **F** | **K** | **E** | **D** | **E** | **T** | **H** | **C** | **S** | **S** | **M** | **T** | **T** | **E** | **T** | **V** | **K** | **E** | **M** | **S** | **N** | **W** | **L** | **L** | **F** | **K** | **Q** | **Q** | **I** | **S** | **R** | **L** | **F** | **S** | **P** | **I** | **L** |
| **N53655** | **-** | **-** | **-** | **-** | **-** | **-** | **-** | **-** | **-** | **-** | **-** | **-** | **-** | **-** | **-** | **-** | **-** | **-** | **-** | **-** | **-** | **-** | **-** | **K** | **P** | **R** | **D** | **V** | **A** | **A** | **I** | **W** | **A** | **W** | **L** | **L** | **V** | **L** | **F** | **A** | **L** | **P** | **G** | **V** | **G** | **F** | **V** | **L** | **Y** | **L** | **F** | **F** | **G** | **R** | **G** | **L** | **T** | **D** | **K** | **K** | **K** | **F** | **Y** | **L** | **R** | **Q** | **S** | **D** | **L** | **K** | **E** | **L** | **E** | **N** | **F** | **Q** | **S** | **F** | **R** | **G** | **D** | **N** | **I** | **E** | **H** | **Y** | **D** | **P** | **D** | **M** | **G** | **D** | **K** | **D** | **K** | **Q** | **Q** | **F** | **V** | **D** | **F** | **F** | **S** | **S** | **L** | **N** | **R** | **M** | **P** | **L** | **T** | **R** | **M** | **N** | **S** | **V** | **T** | **L** | **L** | **T** | **D** | **G** | **Q** | **E** | **K** | **L** | **D** | **S** | **L** | **L** | **Q** | **D** | **L** | **K** | **K** | **A** | **K** | **H** | **S** | **I** | **H** | **I** | **E** | **Y** | **Y** | **A** | **F** | **V** | **T** | **D** | **N** | **I** | **G** | **Q** | **Q** | **V** | **L** | **H** | **V** | **L** | **E** | **E** | **K** | **A** | **A** | **E** | **G** | **V** | **E** | **V** | **R** | **I** | **L** | **Y** | **D** | **A** | **F** | **G** | **S** | **H** | **G** | **T** | **K** | **A** | **K** | **D** | **F** | **N** | **R** | **L** | **I** | **K** | **N** | **G** | **G** | **H** | **V** | **H** | **T** | **F** | **V** | **T** | **S** | **Q** | **K** | **A** | **L** | **L** | **R** | **F** | **R** | **L** | **N** | **Y** | **H** | **D** | **H** | **R** | **K** | **I** | **V** | **V** | **I** | **D** | **G** | **K** | **I** | **S** | **Y** | **T** | **G** | **G** | **F** | **N** | **I** | **A** | **N** | **Q** | **Y** | **V** | **N** | **T** | **T** | **K** | **K** | **F** | **G** | **Y** | **W** | **R** | **D** | **T** | **H** | **I** | **R** | **I** | **F** | **G** | **A** | **A** | **S** | **S** | **L** | **L** | **Q** | **L** | **R** | **F** | **L** | **T** | **D** | **W** | **N** | **V** | **S** | **V** | **P** | **E** | **E** | **K** | **K** | **V** | **G** | **Y** | **H** | **L** | **N** | **Y** | **F** | **F** | **K** | **K** | **A** | **D** | **R** | **D** | **E** | **S** | **K** | **L** | **A** | **D** | **T** | **S** | **I** | **Q** | **L** | **V** | **S** | **S** | **G** | **P** | **N** | **N** | **E** | **R** | **E** | **Q** | **I** | **K** | **L** | **S** | **F** | **I** | **K** | **L** | **I** | **T** | **S** | **A** | **K** | **K** | **R** | **V** | **W** | **I** | **Q** | **T** | **P** | **Y** | **L** | **V** | **P** | **D** | **E** | **S** | **V** | **I** | **A** | **A** | **L** | **K** | **I** | **A** | **T** | **A** | **S** | **G** | **V** | **D** | **V** | **K** | **I** | **M** | **I** | **P** | **N** | **K** | **P** | **D** | **H** | **P** | **F** | **I** | **Y** | **R** | **A** | **T** | **Q** | **Y** | **Y** | **A** | **R** | **Q** | **L** | **I** | **K** | **E** | **N** | **V** | **Q** | **I** | **L** | **V** | **Y** | **E** | **N** | **G** | **F** | **L** | **H** | **A** | **K** | **T** | **L** | **I** | **M** | **D** | **D** | **E** | **I** | **C** | **M** | **V** | **G** | **S** | **A** | **N** | **Q** | **D** | **I** | **R** | **S** | **Y** | **R** | **L** | **N** | **F** | **E** | **T** | **S** | **A** | **V** | **I** | **Y** | **D** | **P** | **E** | **F** | **L** | **E** | **E** | **L** | **A** | **T** | **Q** | **F** | **K** | **E** | **D** | **E** | **T** | **H** | **C** | **S** | **S** | **M** | **T** | **T** | **E** | **T** | **V** | **K** | **E** | **M** | **S** | **N** | **W** | **L** | **L** | **F** | **K** | **Q** | **Q** | **I** | **S** | **R** | **L** | **F** | **S** | **P** | **I** | **L** |
| **N60324F** | **V** | **V** | **S** | **S** | **I** | **I** | **T** | **A** | **L** | **Y** | **L** | **L** | **N** | **A** | **L** | **I** | **A** | **L** | **V** | **T** | **I** | **L** | **I** | **K** | **P** | **R** | **D** | **V** | **A** | **A** | **I** | **W** | **A** | **W** | **L** | **L** | **V** | **L** | **F** | **A** | **L** | **P** | **G** | **V** | **G** | **F** | **V** | **L** | **Y** | **L** | **F** | **F** | **G** | **R** | **G** | **L** | **T** | **D** | **K** | **K** | **K** | **F** | **Y** | **L** | **R** | **Q** | **S** | **D** | **L** | **K** | **E** | **L** | **E** | **N** | **F** | **Q** | **S** | **F** | **R** | **G** | **D** | **T** | **I** | **E** | **H** | **Y** | **D** | **P** | **D** | **M** | **G** | **D** | **K** | **D** | **K** | **Q** | **Q** | **F** | **V** | **D** | **F** | **F** | **S** | **S** | **L** | **N** | **R** | **M** | **P** | **L** | **A** | **R** | **M** | **N** | **S** | **V** | **T** | **L** | **L** | **T** | **D** | **G** | **Q** | **E** | **K** | **L** | **D** | **S** | **L** | **L** | **Q** | **D** | **L** | **K** | **K** | **A** | **K** | **H** | **S** | **I** | **H** | **I** | **E** | **Y** | **Y** | **A** | **F** | **V** | **T** | **D** | **N** | **I** | **G** | **Q** | **Q** | **V** | **L** | **H** | **V** | **L** | **E** | **E** | **K** | **A** | **A** | **E** | **G** | **V** | **E** | **V** | **R** | **I** | **L** | **Y** | **D** | **A** | **F** | **G** | **S** | **H** | **G** | **T** | **K** | **A** | **K** | **D** | **F** | **N** | **R** | **L** | **I** | **K** | **N** | **G** | **G** | **H** | **V** | **H** | **T** | **F** | **V** | **T** | **S** | **Q** | **K** | **A** | **L** | **L** | **R** | **F** | **R** | **L** | **N** | **Y** | **H** | **D** | **H** | **R** | **K** | **I** | **V** | **V** | **I** | **D** | **G** | **K** | **I** | **S** | **Y** | **T** | **G** | **G** | **F** | **N** | **I** | **A** | **N** | **Q** | **Y** | **V** | **N** | **T** | **T** | **K** | **K** | **F** | **G** | **Y** | **W** | **R** | **D** | **T** | **H** | **I** | **R** | **I** | **F** | **G** | **A** | **A** | **S** | **S** | **L** | **L** | **Q** | **L** | **R** | **F** | **L** | **T** | **D** | **W** | **N** | **V** | **S** | **V** | **P** | **E** | **E** | **K** | **K** | **V** | **G** | **Y** | **H** | **L** | **N** | **Y** | **F** | **F** | **K** | **K** | **A** | **D** | **R** | **D** | **E** | **S** | **K** | **L** | **A** | **D** | **T** | **S** | **I** | **Q** | **L** | **V** | **S** | **S** | **G** | **P** | **N** | **N** | **E** | **R** | **E** | **Q** | **I** | **K** | **L** | **S** | **F** | **I** | **K** | **L** | **I** | **T** | **S** | **A** | **K** | **K** | **R** | **V** | **W** | **I** | **Q** | **T** | **P** | **Y** | **L** | **V** | **P** | **D** | **E** | **S** | **V** | **I** | **A** | **A** | **L** | **K** | **I** | **A** | **T** | **A** | **S** | **G** | **V** | **D** | **V** | **K** | **I** | **M** | **I** | **P** | **N** | **K** | **P** | **D** | **H** | **P** | **F** | **I** | **Y** | **R** | **A** | **T** | **Q** | **Y** | **Y** | **A** | **R** | **Q** | **L** | **I** | **K** | **E** | **N** | **V** | **Q** | **I** | **L** | **V** | **Y** | **E** | **N** | **G** | **F** | **L** | **H** | **A** | **K** | **T** | **L** | **I** | **M** | **D** | **D** | **E** | **I** | **C** | **M** | **V** | **G** | **S** | **A** | **N** | **Q** | **D** | **I** | **R** | **S** | **Y** | **R** | **L** | **N** | **F** | **E** | **T** | **S** | **A** | **V** | **I** | **Y** | **D** | **P** | **E** | **F** | **L** | **E** | **E** | **L** | **A** | **T** | **Q** | **F** | **K** | **E** | **D** | **E** | **T** | **H** | **C** | **S** | **S** | **M** | **T** | **T** | **E** | **T** | **V** | **K** | **E** | **M** | **S** | **N** | **W** | **L** | **L** | **F** | **K** | **Q** | **Q** | **I** | **S** | **R** | **L** | **F** | **S** | **P** | **I** | **L** |
| **N55290** | **-** | **-** | **-** | **-** | **-** | **-** | **-** | **-** | **-** | **-** | **-** | **-** | **-** | **-** | **-** | **-** | **-** | **-** | **-** | **-** | **-** | **-** | **-** | **K** | **P** | **R** | **D** | **V** | **A** | **A** | **I** | **W** | **A** | **W** | **L** | **L** | **V** | **L** | **F** | **A** | **L** | **P** | **G** | **V** | **G** | **F** | **V** | **L** | **Y** | **L** | **F** | **F** | **G** | **R** | **G** | **L** | **T** | **D** | **K** | **K** | **K** | **F** | **Y** | **L** | **R** | **Q** | **S** | **D** | **L** | **K** | **E** | **L** | **E** | **N** | **F** | **Q** | **S** | **F** | **R** | **G** | **D** | **T** | **I** | **E** | **H** | **Y** | **D** | **P** | **D** | **M** | **G** | **D** | **K** | **D** | **K** | **Q** | **Q** | **F** | **V** | **D** | **F** | **F** | **S** | **S** | **L** | **N** | **R** | **M** | **P** | **L** | **T** | **R** | **M** | **N** | **S** | **V** | **T** | **L** | **L** | **T** | **D** | **G** | **Q** | **K** | **K** | **L** | **D** | **S** | **L** | **L** | **Q** | **D** | **L** | **K** | **K** | **A** | **K** | **H** | **S** | **I** | **H** | **I** | **E** | **Y** | **Y** | **A** | **F** | **V** | **T** | **D** | **T** | **I** | **G** | **Q** | **Q** | **V** | **L** | **R** | **V** | **L** | **E** | **E** | **K** | **A** | **A** | **E** | **G** | **V** | **E** | **V** | **R** | **I** | **L** | **Y** | **D** | **A** | **F** | **G** | **S** | **H** | **G** | **T** | **K** | **A** | **K** | **D** | **F** | **N** | **R** | **L** | **I** | **K** | **N** | **G** | **G** | **H** | **V** | **H** | **T** | **F** | **V** | **T** | **S** | **Q** | **K** | **A** | **L** | **L** | **R** | **F** | **R** | **L** | **N** | **Y** | **H** | **D** | **H** | **R** | **K** | **I** | **V** | **V** | **I** | **D** | **G** | **K** | **I** | **S** | **Y** | **T** | **G** | **G** | **F** | **N** | **I** | **A** | **N** | **Q** | **Y** | **V** | **N** | **T** | **T** | **K** | **K** | **F** | **G** | **Y** | **W** | **R** | **D** | **T** | **H** | **I** | **R** | **I** | **F** | **G** | **A** | **A** | **S** | **S** | **L** | **L** | **Q** | **L** | **R** | **F** | **L** | **T** | **D** | **W** | **N** | **V** | **S** | **V** | **P** | **E** | **E** | **K** | **K** | **V** | **G** | **Y** | **H** | **L** | **N** | **Y** | **F** | **F** | **K** | **K** | **A** | **D** | **R** | **D** | **E** | **S** | **K** | **L** | **A** | **D** | **T** | **S** | **I** | **Q** | **L** | **V** | **S** | **S** | **G** | **P** | **N** | **N** | **E** | **R** | **E** | **Q** | **I** | **K** | **L** | **S** | **F** | **I** | **K** | **L** | **I** | **T** | **S** | **A** | **K** | **K** | **R** | **V** | **W** | **I** | **Q** | **T** | **P** | **Y** | **L** | **V** | **P** | **D** | **E** | **S** | **V** | **I** | **A** | **A** | **L** | **K** | **I** | **A** | **T** | **A** | **S** | **G** | **V** | **D** | **V** | **K** | **I** | **M** | **I** | **P** | **N** | **K** | **P** | **D** | **H** | **P** | **F** | **I** | **Y** | **R** | **A** | **T** | **Q** | **Y** | **Y** | **A** | **R** | **Q** | **L** | **I** | **K** | **E** | **N** | **V** | **Q** | **I** | **L** | **V** | **Y** | **E** | **N** | **G** | **F** | **L** | **H** | **A** | **K** | **T** | **L** | **I** | **M** | **D** | **D** | **E** | **I** | **C** | **M** | **V** | **G** | **S** | **A** | **N** | **Q** | **D** | **I** | **R** | **S** | **Y** | **R** | **L** | **N** | **F** | **E** | **T** | **S** | **A** | **V** | **I** | **Y** | **D** | **P** | **E** | **F** | **L** | **E** | **E** | **L** | **A** | **T** | **Q** | **F** | **K** | **E** | **D** | **E** | **T** | **H** | **C** | **S** | **P** | **M** | **T** | **T** | **E** | **T** | **V** | **K** | **E** | **M** | **S** | **N** | **W** | **L** | **L** | **F** | **K** | **Q** | **Q** | **I** | **S** | **R** | **L** | **F** | **S** | **P** | **I** | **L** |
| **N54152** | **-** | **-** | **-** | **-** | **-** | **-** | **-** | **-** | **-** | **-** | **-** | **-** | **-** | **-** | **-** | **-** | **-** | **-** | **-** | **-** | **-** | **-** | **-** | **K** | **P** | **R** | **D** | **V** | **A** | **A** | **I** | **W** | **A** | **W** | **L** | **L** | **V** | **L** | **F** | **A** | **L** | **P** | **G** | **M** | **G** | **F** | **V** | **L** | **Y** | **L** | **F** | **F** | **G** | **R** | **G** | **L** | **T** | **D** | **K** | **K** | **K** | **F** | **Y** | **L** | **R** | **Q** | **S** | **D** | **L** | **K** | **E** | **L** | **E** | **N** | **F** | **Q** | **S** | **F** | **R** | **G** | **D** | **T** | **I** | **E** | **H** | **Y** | **D** | **P** | **D** | **M** | **G** | **D** | **K** | **D** | **K** | **Q** | **Q** | **F** | **V** | **D** | **F** | **F** | **S** | **S** | **L** | **N** | **R** | **M** | **P** | **L** | **T** | **R** | **M** | **N** | **S** | **V** | **T** | **L** | **L** | **T** | **D** | **G** | **Q** | **E** | **K** | **L** | **D** | **S** | **L** | **L** | **Q** | **D** | **L** | **K** | **K** | **A** | **K** | **H** | **S** | **I** | **H** | **I** | **E** | **Y** | **Y** | **A** | **F** | **V** | **T** | **D** | **N** | **I** | **G** | **Q** | **Q** | **V** | **L** | **H** | **V** | **L** | **E** | **E** | **K** | **A** | **A** | **E** | **G** | **V** | **E** | **V** | **R** | **I** | **L** | **Y** | **D** | **A** | **F** | **G** | **S** | **H** | **G** | **T** | **K** | **A** | **K** | **D** | **F** | **N** | **R** | **L** | **I** | **K** | **N** | **G** | **G** | **H** | **V** | **H** | **T** | **F** | **V** | **T** | **S** | **Q** | **K** | **A** | **L** | **L** | **R** | **F** | **R** | **L** | **N** | **Y** | **H** | **D** | **H** | **R** | **K** | **I** | **V** | **V** | **I** | **D** | **G** | **K** | **I** | **S** | **Y** | **T** | **G** | **G** | **F** | **N** | **I** | **A** | **N** | **Q** | **Y** | **V** | **N** | **T** | **T** | **K** | **K** | **F** | **G** | **Y** | **W** | **R** | **D** | **T** | **H** | **I** | **R** | **I** | **F** | **G** | **A** | **A** | **S** | **S** | **L** | **L** | **Q** | **L** | **R** | **F** | **L** | **T** | **D** | **W** | **N** | **V** | **S** | **V** | **P** | **E** | **E** | **K** | **K** | **V** | **G** | **Y** | **H** | **L** | **N** | **Y** | **F** | **F** | **K** | **K** | **A** | **D** | **R** | **D** | **E** | **S** | **K** | **L** | **A** | **D** | **T** | **S** | **I** | **Q** | **L** | **V** | **S** | **S** | **G** | **P** | **N** | **N** | **E** | **R** | **E** | **Q** | **I** | **K** | **L** | **S** | **F** | **I** | **K** | **L** | **I** | **T** | **S** | **A** | **K** | **K** | **R** | **V** | **W** | **I** | **Q** | **T** | **P** | **Y** | **L** | **V** | **P** | **D** | **E** | **S** | **V** | **I** | **A** | **A** | **L** | **K** | **I** | **A** | **T** | **A** | **S** | **G** | **V** | **D** | **V** | **K** | **I** | **M** | **I** | **P** | **N** | **K** | **P** | **D** | **H** | **P** | **F** | **I** | **Y** | **R** | **A** | **T** | **Q** | **Y** | **Y** | **A** | **R** | **Q** | **L** | **I** | **K** | **E** | **N** | **V** | **Q** | **I** | **L** | **V** | **Y** | **E** | **N** | **G** | **F** | **L** | **H** | **A** | **K** | **T** | **L** | **I** | **M** | **D** | **D** | **E** | **I** | **C** | **M** | **V** | **G** | **S** | **A** | **N** | **Q** | **D** | **I** | **R** | **S** | **Y** | **R** | **L** | **N** | **F** | **E** | **T** | **S** | **A** | **V** | **I** | **Y** | **D** | **P** | **E** | **F** | **L** | **E** | **E** | **L** | **A** | **T** | **Q** | **F** | **K** | **E** | **D** | **E** | **T** | **H** | **C** | **S** | **S** | **M** | **T** | **T** | **E** | **T** | **V** | **K** | **E** | **M** | **S** | **N** | **W** | **L** | **L** | **F** | **K** | **Q** | **Q** | **I** | **S** | **R** | **L** | **F** | **S** | **P** | **I** | **L** |
| **N54546** | **-** | **-** | **-** | **-** | **-** | **-** | **-** | **-** | **-** | **-** | **-** | **-** | **-** | **-** | **-** | **-** | **-** | **-** | **-** | **-** | **-** | **-** | **-** | **K** | **P** | **R** | **D** | **V** | **A** | **A** | **I** | **W** | **A** | **W** | **L** | **L** | **V** | **L** | **F** | **V** | **L** | **P** | **G** | **V** | **G** | **F** | **V** | **L** | **Y** | **L** | **F** | **F** | **G** | **R** | **G** | **L** | **T** | **D** | **K** | **K** | **K** | **F** | **Y** | **L** | **R** | **Q** | **S** | **D** | **L** | **K** | **E** | **L** | **E** | **N** | **F** | **Q** | **S** | **F** | **R** | **G** | **D** | **T** | **I** | **E** | **H** | **Y** | **D** | **P** | **D** | **M** | **G** | **D** | **K** | **D** | **K** | **Q** | **Q** | **F** | **V** | **D** | **F** | **F** | **S** | **S** | **L** | **N** | **R** | **M** | **P** | **L** | **T** | **R** | **M** | **N** | **S** | **V** | **T** | **L** | **L** | **T** | **D** | **G** | **Q** | **E** | **K** | **L** | **D** | **S** | **L** | **L** | **Q** | **D** | **L** | **K** | **K** | **A** | **K** | **H** | **S** | **I** | **H** | **I** | **E** | **Y** | **Y** | **A** | **F** | **V** | **T** | **D** | **N** | **I** | **G** | **Q** | **Q** | **V** | **L** | **H** | **V** | **L** | **E** | **E** | **K** | **A** | **A** | **E** | **G** | **V** | **E** | **V** | **R** | **I** | **L** | **Y** | **D** | **A** | **F** | **G** | **S** | **H** | **G** | **T** | **K** | **A** | **K** | **D** | **F** | **N** | **R** | **L** | **I** | **K** | **N** | **G** | **G** | **H** | **V** | **H** | **T** | **F** | **V** | **T** | **S** | **Q** | **K** | **A** | **L** | **L** | **R** | **F** | **R** | **L** | **N** | **Y** | **H** | **D** | **H** | **R** | **K** | **I** | **V** | **V** | **I** | **D** | **G** | **K** | **I** | **S** | **Y** | **T** | **G** | **G** | **F** | **N** | **I** | **A** | **N** | **Q** | **Y** | **V** | **N** | **T** | **T** | **K** | **K** | **F** | **G** | **Y** | **W** | **R** | **D** | **T** | **H** | **I** | **R** | **I** | **F** | **G** | **A** | **A** | **S** | **S** | **L** | **L** | **Q** | **L** | **R** | **F** | **L** | **T** | **D** | **W** | **N** | **V** | **S** | **V** | **P** | **E** | **E** | **K** | **K** | **V** | **G** | **Y** | **H** | **L** | **N** | **Y** | **F** | **F** | **K** | **K** | **A** | **D** | **R** | **D** | **E** | **S** | **K** | **L** | **A** | **D** | **T** | **S** | **I** | **Q** | **L** | **V** | **S** | **S** | **G** | **P** | **N** | **N** | **E** | **R** | **E** | **Q** | **I** | **K** | **L** | **S** | **F** | **I** | **K** | **L** | **I** | **T** | **S** | **A** | **K** | **K** | **R** | **V** | **W** | **I** | **Q** | **T** | **P** | **Y** | **L** | **V** | **P** | **D** | **E** | **S** | **V** | **I** | **A** | **A** | **L** | **K** | **I** | **A** | **T** | **A** | **S** | **G** | **V** | **D** | **V** | **K** | **I** | **M** | **I** | **P** | **N** | **K** | **P** | **D** | **H** | **P** | **F** | **I** | **Y** | **R** | **A** | **T** | **Q** | **Y** | **Y** | **A** | **R** | **Q** | **L** | **I** | **K** | **E** | **N** | **V** | **Q** | **I** | **L** | **V** | **Y** | **E** | **N** | **G** | **F** | **L** | **H** | **A** | **K** | **T** | **L** | **I** | **M** | **D** | **D** | **E** | **I** | **C** | **M** | **V** | **G** | **S** | **A** | **N** | **Q** | **D** | **I** | **R** | **S** | **Y** | **R** | **L** | **N** | **F** | **E** | **T** | **S** | **A** | **V** | **I** | **Y** | **D** | **P** | **E** | **F** | **L** | **E** | **E** | **L** | **A** | **T** | **Q** | **F** | **K** | **E** | **D** | **E** | **T** | **H** | **C** | **S** | **S** | **M** | **T** | **T** | **E** | **T** | **V** | **K** | **E** | **M** | **S** | **N** | **W** | **L** | **L** | **F** | **K** | **Q** | **Q** | **I** | **S** | **R** | **L** | **F** | **S** | **P** | **I** | **L** |
| **N60463F** | **-** | **-** | **-** | **-** | **-** | **-** | **-** | **-** | **-** | **-** | **-** | **-** | **-** | **-** | **-** | **-** | **-** | **-** | **-** | **-** | **-** | **-** | **-** | **K** | **P** | **R** | **D** | **V** | **A** | **A** | **I** | **W** | **A** | **W** | **L** | **L** | **V** | **L** | **F** | **A** | **L** | **P** | **G** | **V** | **G** | **F** | **V** | **L** | **Y** | **L** | **F** | **F** | **G** | **R** | **G** | **L** | **T** | **D** | **K** | **K** | **K** | **F** | **Y** | **L** | **R** | **Q** | **S** | **D** | **L** | **K** | **E** | **L** | **E** | **N** | **F** | **Q** | **S** | **F** | **R** | **G** | **D** | **T** | **I** | **E** | **H** | **Y** | **D** | **P** | **D** | **M** | **G** | **D** | **K** | **D** | **K** | **Q** | **Q** | **F** | **V** | **D** | **F** | **F** | **S** | **S** | **L** | **N** | **R** | **M** | **P** | **L** | **T** | **R** | **M** | **N** | **S** | **V** | **T** | **L** | **L** | **T** | **D** | **G** | **Q** | **E** | **K** | **L** | **D** | **S** | **L** | **L** | **Q** | **D** | **L** | **K** | **K** | **A** | **K** | **H** | **S** | **I** | **H** | **I** | **E** | **Y** | **Y** | **A** | **F** | **V** | **T** | **D** | **N** | **I** | **G** | **Q** | **Q** | **V** | **L** | **H** | **V** | **L** | **E** | **E** | **K** | **A** | **A** | **E** | **G** | **V** | **E** | **V** | **R** | **I** | **L** | **Y** | **D** | **A** | **F** | **G** | **S** | **H** | **G** | **T** | **K** | **A** | **K** | **D** | **F** | **N** | **R** | **L** | **I** | **K** | **N** | **G** | **G** | **H** | **V** | **H** | **T** | **F** | **V** | **T** | **S** | **Q** | **K** | **A** | **L** | **L** | **R** | **F** | **R** | **L** | **N** | **Y** | **H** | **D** | **H** | **R** | **K** | **I** | **V** | **V** | **I** | **D** | **G** | **K** | **I** | **S** | **Y** | **T** | **G** | **G** | **F** | **N** | **I** | **A** | **N** | **Q** | **Y** | **V** | **N** | **T** | **T** | **K** | **K** | **F** | **G** | **Y** | **W** | **R** | **D** | **T** | **H** | **I** | **R** | **I** | **F** | **G** | **A** | **A** | **S** | **S** | **L** | **L** | **Q** | **L** | **R** | **F** | **L** | **T** | **D** | **W** | **N** | **V** | **S** | **V** | **P** | **E** | **E** | **K** | **K** | **V** | **G** | **Y** | **H** | **L** | **N** | **Y** | **F** | **F** | **K** | **K** | **A** | **D** | **R** | **D** | **E** | **S** | **K** | **L** | **A** | **D** | **T** | **S** | **I** | **Q** | **L** | **V** | **S** | **S** | **G** | **P** | **N** | **N** | **E** | **R** | **E** | **Q** | **I** | **K** | **L** | **S** | **F** | **I** | **K** | **L** | **I** | **T** | **S** | **A** | **K** | **K** | **R** | **V** | **W** | **I** | **Q** | **T** | **P** | **Y** | **L** | **V** | **P** | **D** | **E** | **S** | **V** | **I** | **A** | **A** | **L** | **K** | **I** | **A** | **T** | **A** | **S** | **G** | **V** | **D** | **V** | **K** | **I** | **M** | **I** | **P** | **N** | **K** | **P** | **D** | **H** | **P** | **F** | **I** | **Y** | **R** | **A** | **T** | **Q** | **Y** | **Y** | **A** | **R** | **Q** | **L** | **I** | **K** | **E** | **N** | **V** | **Q** | **I** | **L** | **V** | **Y** | **E** | **N** | **G** | **F** | **L** | **H** | **A** | **K** | **T** | **L** | **I** | **M** | **D** | **D** | **E** | **I** | **C** | **M** | **V** | **G** | **S** | **A** | **N** | **Q** | **D** | **I** | **R** | **S** | **Y** | **R** | **L** | **N** | **F** | **E** | **T** | **S** | **A** | **V** | **I** | **Y** | **D** | **P** | **E** | **F** | **L** | **E** | **E** | **L** | **A** | **T** | **Q** | **F** | **K** | **E** | **D** | **E** | **T** | **H** | **C** | **S** | **S** | **M** | **T** | **T** | **E** | **T** | **V** | **K** | **E** | **M** | **S** | **N** | **W** | **L** | **L** | **F** | **K** | **Q** | **Q** | **I** | **S** | **R** | **L** | **F** | **S** | **P** | **I** | **L** |
| **N52643** | **-** | **-** | **-** | **-** | **-** | **-** | **-** | **-** | **-** | **-** | **-** | **-** | **-** | **-** | **-** | **-** | **-** | **-** | **-** | **-** | **-** | **-** | **-** | **K** | **P** | **R** | **D** | **V** | **A** | **A** | **I** | **W** | **A** | **W** | **L** | **L** | **V** | **L** | **F** | **A** | **L** | **P** | **G** | **V** | **G** | **F** | **V** | **L** | **Y** | **L** | **F** | **F** | **G** | **R** | **G** | **L** | **T** | **D** | **K** | **K** | **K** | **F** | **Y** | **L** | **R** | **Q** | **S** | **D** | **L** | **K** | **E** | **L** | **E** | **N** | **F** | **Q** | **S** | **F** | **R** | **G** | **D** | **T** | **I** | **E** | **H** | **Y** | **D** | **P** | **D** | **M** | **G** | **D** | **K** | **D** | **K** | **Q** | **Q** | **F** | **V** | **D** | **F** | **F** | **S** | **S** | **L** | **N** | **R** | **M** | **P** | **L** | **T** | **R** | **M** | **N** | **S** | **V** | **T** | **L** | **L** | **T** | **D** | **G** | **Q** | **E** | **K** | **L** | **D** | **S** | **L** | **L** | **Q** | **D** | **L** | **K** | **K** | **A** | **K** | **H** | **S** | **I** | **H** | **I** | **E** | **Y** | **Y** | **A** | **F** | **V** | **T** | **D** | **N** | **I** | **G** | **Q** | **Q** | **V** | **L** | **H** | **V** | **L** | **E** | **E** | **K** | **A** | **A** | **E** | **G** | **V** | **E** | **V** | **R** | **I** | **L** | **Y** | **D** | **A** | **F** | **G** | **S** | **H** | **G** | **T** | **K** | **A** | **K** | **D** | **F** | **N** | **R** | **L** | **I** | **K** | **N** | **G** | **G** | **H** | **V** | **H** | **T** | **F** | **V** | **T** | **S** | **Q** | **K** | **A** | **L** | **L** | **R** | **F** | **R** | **L** | **N** | **Y** | **H** | **D** | **H** | **R** | **K** | **I** | **V** | **V** | **I** | **D** | **G** | **K** | **I** | **S** | **Y** | **T** | **G** | **G** | **F** | **N** | **I** | **A** | **N** | **Q** | **Y** | **V** | **N** | **T** | **T** | **K** | **K** | **F** | **G** | **Y** | **W** | **R** | **D** | **T** | **H** | **I** | **R** | **I** | **F** | **G** | **A** | **A** | **S** | **S** | **L** | **L** | **Q** | **L** | **R** | **F** | **L** | **T** | **D** | **W** | **N** | **V** | **S** | **V** | **P** | **E** | **E** | **K** | **K** | **V** | **G** | **Y** | **H** | **L** | **N** | **Y** | **F** | **F** | **K** | **K** | **A** | **D** | **R** | **D** | **E** | **S** | **K** | **L** | **A** | **D** | **T** | **S** | **I** | **Q** | **L** | **V** | **S** | **S** | **G** | **P** | **N** | **N** | **E** | **R** | **E** | **Q** | **I** | **K** | **L** | **S** | **F** | **I** | **K** | **L** | **I** | **T** | **S** | **A** | **K** | **K** | **R** | **V** | **W** | **I** | **Q** | **T** | **P** | **Y** | **L** | **V** | **P** | **D** | **E** | **S** | **V** | **I** | **A** | **A** | **L** | **K** | **I** | **A** | **T** | **A** | **S** | **G** | **V** | **D** | **V** | **K** | **I** | **M** | **I** | **P** | **N** | **K** | **P** | **D** | **H** | **P** | **F** | **I** | **Y** | **R** | **A** | **T** | **Q** | **Y** | **Y** | **A** | **R** | **Q** | **L** | **I** | **K** | **E** | **N** | **V** | **Q** | **I** | **L** | **V** | **Y** | **E** | **N** | **G** | **F** | **L** | **H** | **A** | **K** | **T** | **L** | **I** | **M** | **D** | **D** | **E** | **I** | **C** | **M** | **V** | **G** | **S** | **A** | **N** | **Q** | **D** | **I** | **R** | **S** | **Y** | **R** | **L** | **N** | **F** | **E** | **T** | **S** | **A** | **V** | **I** | **Y** | **D** | **P** | **E** | **F** | **L** | **E** | **E** | **L** | **A** | **T** | **Q** | **F** | **K** | **E** | **D** | **E** | **T** | **H** | **C** | **S** | **S** | **M** | **T** | **T** | **E** | **T** | **V** | **K** | **E** | **M** | **S** | **N** | **W** | **L** | **L** | **F** | **K** | **Q** | **Q** | **I** | **S** | **R** | **L** | **F** | **S** | **P** | **I** | **L** |
| **N52702** | **-** | **-** | **-** | **-** | **-** | **-** | **-** | **-** | **-** | **-** | **-** | **-** | **-** | **-** | **-** | **-** | **-** | **-** | **-** | **-** | **-** | **-** | **-** | **K** | **P** | **R** | **D** | **V** | **A** | **A** | **I** | **W** | **A** | **W** | **L** | **L** | **V** | **L** | **F** | **A** | **L** | **P** | **G** | **V** | **G** | **F** | **V** | **L** | **Y** | **L** | **F** | **F** | **G** | **R** | **G** | **L** | **T** | **D** | **K** | **K** | **K** | **F** | **Y** | **L** | **R** | **Q** | **S** | **D** | **L** | **K** | **E** | **L** | **E** | **N** | **F** | **Q** | **S** | **F** | **R** | **G** | **D** | **N** | **I** | **E** | **H** | **Y** | **D** | **P** | **D** | **M** | **G** | **D** | **K** | **D** | **K** | **Q** | **Q** | **F** | **V** | **D** | **F** | **F** | **S** | **S** | **L** | **N** | **R** | **M** | **P** | **L** | **T** | **R** | **M** | **N** | **S** | **V** | **T** | **L** | **L** | **T** | **D** | **G** | **Q** | **E** | **K** | **L** | **D** | **S** | **L** | **L** | **Q** | **D** | **L** | **K** | **K** | **A** | **K** | **H** | **S** | **I** | **H** | **I** | **E** | **Y** | **Y** | **A** | **F** | **V** | **T** | **D** | **N** | **I** | **G** | **Q** | **Q** | **V** | **L** | **H** | **V** | **L** | **E** | **E** | **K** | **A** | **A** | **E** | **G** | **V** | **E** | **V** | **R** | **I** | **L** | **Y** | **D** | **A** | **F** | **G** | **S** | **H** | **G** | **T** | **K** | **A** | **K** | **D** | **F** | **N** | **R** | **L** | **I** | **K** | **N** | **G** | **G** | **H** | **V** | **H** | **T** | **F** | **V** | **T** | **S** | **Q** | **K** | **A** | **L** | **L** | **R** | **F** | **R** | **L** | **N** | **Y** | **H** | **D** | **H** | **R** | **K** | **I** | **V** | **V** | **I** | **D** | **G** | **K** | **I** | **S** | **Y** | **T** | **G** | **G** | **F** | **N** | **I** | **A** | **N** | **Q** | **Y** | **V** | **N** | **T** | **T** | **K** | **K** | **F** | **G** | **Y** | **W** | **R** | **D** | **T** | **H** | **I** | **R** | **I** | **F** | **G** | **A** | **A** | **S** | **S** | **L** | **L** | **Q** | **L** | **R** | **F** | **L** | **T** | **D** | **W** | **N** | **V** | **S** | **V** | **P** | **E** | **E** | **K** | **K** | **V** | **G** | **Y** | **H** | **L** | **N** | **Y** | **F** | **F** | **K** | **K** | **A** | **D** | **R** | **D** | **E** | **S** | **K** | **L** | **A** | **D** | **T** | **S** | **I** | **Q** | **L** | **V** | **S** | **S** | **G** | **P** | **N** | **N** | **E** | **R** | **E** | **Q** | **I** | **K** | **L** | **S** | **F** | **I** | **K** | **L** | **I** | **T** | **S** | **A** | **K** | **K** | **R** | **V** | **W** | **I** | **Q** | **T** | **P** | **Y** | **L** | **V** | **P** | **D** | **E** | **S** | **V** | **I** | **A** | **A** | **L** | **K** | **I** | **A** | **T** | **A** | **S** | **G** | **V** | **D** | **V** | **K** | **I** | **M** | **I** | **P** | **N** | **K** | **P** | **D** | **H** | **P** | **F** | **I** | **Y** | **R** | **A** | **T** | **Q** | **Y** | **Y** | **A** | **R** | **Q** | **L** | **I** | **K** | **E** | **N** | **V** | **Q** | **I** | **L** | **V** | **Y** | **E** | **N** | **G** | **F** | **L** | **H** | **A** | **K** | **T** | **L** | **I** | **M** | **D** | **D** | **E** | **I** | **C** | **M** | **V** | **G** | **S** | **A** | **N** | **Q** | **D** | **I** | **R** | **S** | **Y** | **R** | **L** | **N** | **F** | **E** | **T** | **S** | **A** | **V** | **I** | **Y** | **D** | **P** | **E** | **F** | **L** | **E** | **E** | **L** | **A** | **T** | **Q** | **F** | **K** | **E** | **D** | **E** | **T** | **H** | **C** | **S** | **S** | **M** | **T** | **T** | **E** | **T** | **V** | **K** | **E** | **M** | **S** | **N** | **W** | **L** | **L** | **F** | **K** | **Q** | **Q** | **I** | **S** | **R** | **L** | **F** | **S** | **P** | **I** | **L** |
| **N54567** | **-** | **-** | **-** | **-** | **-** | **-** | **-** | **-** | **-** | **-** | **-** | **-** | **-** | **-** | **-** | **-** | **-** | **-** | **-** | **-** | **-** | **-** | **-** | **K** | **P** | **R** | **D** | **V** | **A** | **A** | **I** | **W** | **A** | **W** | **L** | **L** | **V** | **L** | **F** | **A** | **L** | **P** | **G** | **V** | **G** | **F** | **V** | **L** | **Y** | **L** | **F** | **F** | **G** | **R** | **G** | **L** | **T** | **D** | **K** | **K** | **K** | **F** | **Y** | **L** | **R** | **Q** | **S** | **D** | **L** | **K** | **E** | **L** | **E** | **N** | **F** | **Q** | **S** | **F** | **R** | **G** | **D** | **N** | **I** | **E** | **H** | **Y** | **D** | **P** | **D** | **M** | **G** | **D** | **K** | **D** | **K** | **Q** | **Q** | **F** | **V** | **D** | **F** | **F** | **S** | **S** | **L** | **N** | **R** | **M** | **P** | **L** | **T** | **R** | **M** | **N** | **S** | **V** | **T** | **L** | **L** | **T** | **D** | **G** | **Q** | **E** | **K** | **L** | **D** | **S** | **L** | **L** | **Q** | **D** | **L** | **K** | **K** | **A** | **K** | **H** | **S** | **I** | **H** | **I** | **E** | **Y** | **Y** | **A** | **F** | **V** | **T** | **D** | **N** | **I** | **G** | **Q** | **Q** | **V** | **L** | **H** | **V** | **L** | **E** | **E** | **K** | **A** | **A** | **E** | **G** | **V** | **E** | **V** | **R** | **I** | **L** | **Y** | **D** | **A** | **F** | **G** | **S** | **H** | **G** | **T** | **K** | **A** | **K** | **D** | **F** | **N** | **R** | **L** | **I** | **K** | **N** | **G** | **G** | **H** | **V** | **H** | **T** | **F** | **V** | **T** | **S** | **Q** | **K** | **A** | **L** | **L** | **R** | **F** | **R** | **L** | **N** | **Y** | **H** | **D** | **H** | **R** | **K** | **I** | **V** | **V** | **I** | **D** | **G** | **K** | **I** | **S** | **Y** | **T** | **G** | **G** | **F** | **N** | **I** | **A** | **N** | **Q** | **Y** | **V** | **N** | **T** | **T** | **K** | **K** | **F** | **G** | **Y** | **W** | **R** | **D** | **T** | **H** | **I** | **R** | **I** | **F** | **G** | **A** | **A** | **S** | **S** | **L** | **L** | **Q** | **L** | **R** | **F** | **L** | **T** | **D** | **W** | **N** | **V** | **S** | **V** | **P** | **E** | **E** | **K** | **K** | **V** | **G** | **Y** | **H** | **L** | **N** | **Y** | **F** | **F** | **K** | **K** | **A** | **D** | **R** | **D** | **E** | **S** | **K** | **L** | **A** | **D** | **T** | **S** | **I** | **Q** | **L** | **V** | **S** | **S** | **G** | **P** | **N** | **N** | **E** | **R** | **E** | **Q** | **I** | **K** | **L** | **S** | **F** | **I** | **K** | **L** | **I** | **T** | **S** | **A** | **K** | **K** | **R** | **V** | **W** | **I** | **Q** | **T** | **P** | **Y** | **L** | **V** | **P** | **D** | **E** | **S** | **V** | **I** | **A** | **A** | **L** | **K** | **I** | **A** | **T** | **A** | **S** | **G** | **V** | **D** | **V** | **K** | **I** | **M** | **I** | **P** | **N** | **K** | **P** | **D** | **H** | **P** | **F** | **I** | **Y** | **R** | **A** | **T** | **Q** | **Y** | **Y** | **A** | **R** | **Q** | **L** | **I** | **K** | **E** | **N** | **V** | **Q** | **I** | **L** | **V** | **Y** | **E** | **N** | **G** | **F** | **L** | **H** | **A** | **K** | **T** | **L** | **I** | **M** | **D** | **D** | **E** | **I** | **C** | **M** | **V** | **G** | **S** | **A** | **N** | **Q** | **D** | **I** | **R** | **S** | **Y** | **R** | **L** | **N** | **F** | **E** | **T** | **S** | **A** | **V** | **I** | **Y** | **D** | **P** | **E** | **F** | **L** | **E** | **E** | **L** | **A** | **T** | **Q** | **F** | **K** | **E** | **D** | **E** | **T** | **H** | **C** | **S** | **S** | **M** | **T** | **T** | **E** | **T** | **V** | **K** | **E** | **M** | **S** | **N** | **W** | **L** | **L** | **F** | **K** | **Q** | **Q** | **I** | **S** | **R** | **L** | **F** | **S** | **P** | **I** | **L** |
| **N59567F** | **-** | **-** | **-** | **-** | **-** | **-** | **-** | **-** | **-** | **-** | **-** | **-** | **-** | **-** | **-** | **-** | **-** | **-** | **-** | **-** | **-** | **-** | **-** | **K** | **P** | **R** | **D** | **V** | **A** | **A** | **I** | **W** | **A** | **W** | **L** | **L** | **V** | **L** | **F** | **A** | **L** | **P** | **G** | **V** | **G** | **F** | **V** | **L** | **Y** | **L** | **F** | **F** | **G** | **R** | **G** | **L** | **T** | **D** | **K** | **K** | **K** | **F** | **Y** | **L** | **R** | **Q** | **S** | **D** | **L** | **K** | **E** | **L** | **E** | **N** | **F** | **Q** | **S** | **F** | **R** | **G** | **D** | **T** | **I** | **E** | **H** | **Y** | **D** | **P** | **D** | **M** | **G** | **D** | **K** | **D** | **K** | **Q** | **Q** | **F** | **V** | **D** | **F** | **F** | **S** | **S** | **L** | **N** | **R** | **M** | **P** | **L** | **T** | **R** | **M** | **N** | **S** | **V** | **T** | **L** | **L** | **T** | **D** | **G** | **Q** | **E** | **K** | **L** | **D** | **S** | **L** | **L** | **Q** | **D** | **L** | **K** | **K** | **A** | **K** | **H** | **S** | **I** | **H** | **I** | **E** | **Y** | **Y** | **A** | **F** | **V** | **T** | **D** | **N** | **I** | **G** | **Q** | **Q** | **V** | **L** | **H** | **V** | **L** | **E** | **E** | **K** | **A** | **A** | **E** | **G** | **V** | **E** | **V** | **R** | **I** | **L** | **Y** | **D** | **A** | **F** | **G** | **S** | **H** | **G** | **T** | **K** | **A** | **K** | **D** | **F** | **N** | **R** | **L** | **I** | **K** | **N** | **G** | **G** | **H** | **V** | **H** | **T** | **F** | **V** | **T** | **S** | **Q** | **K** | **A** | **L** | **L** | **R** | **F** | **R** | **L** | **N** | **Y** | **H** | **D** | **H** | **R** | **K** | **I** | **V** | **V** | **I** | **D** | **G** | **K** | **I** | **S** | **Y** | **T** | **G** | **G** | **F** | **N** | **I** | **A** | **N** | **Q** | **Y** | **V** | **N** | **T** | **T** | **K** | **K** | **F** | **G** | **Y** | **W** | **R** | **D** | **T** | **H** | **I** | **R** | **I** | **F** | **G** | **A** | **A** | **S** | **S** | **L** | **L** | **Q** | **L** | **R** | **F** | **L** | **T** | **D** | **W** | **N** | **V** | **S** | **V** | **P** | **E** | **E** | **K** | **K** | **V** | **G** | **Y** | **H** | **L** | **N** | **Y** | **F** | **F** | **K** | **K** | **A** | **D** | **R** | **D** | **E** | **S** | **K** | **L** | **A** | **D** | **T** | **S** | **I** | **Q** | **L** | **V** | **S** | **S** | **G** | **P** | **N** | **N** | **E** | **R** | **E** | **Q** | **I** | **K** | **L** | **S** | **F** | **I** | **K** | **L** | **I** | **T** | **S** | **A** | **K** | **K** | **R** | **V** | **W** | **I** | **Q** | **T** | **P** | **Y** | **L** | **V** | **P** | **D** | **E** | **S** | **V** | **I** | **A** | **A** | **L** | **K** | **I** | **A** | **T** | **A** | **S** | **G** | **V** | **D** | **V** | **K** | **I** | **M** | **I** | **P** | **N** | **K** | **P** | **D** | **H** | **P** | **F** | **I** | **Y** | **R** | **A** | **T** | **Q** | **Y** | **Y** | **A** | **R** | **Q** | **L** | **I** | **K** | **E** | **N** | **V** | **Q** | **I** | **L** | **V** | **Y** | **E** | **N** | **G** | **F** | **L** | **H** | **A** | **K** | **T** | **L** | **I** | **M** | **D** | **D** | **E** | **I** | **C** | **M** | **V** | **G** | **S** | **A** | **N** | **Q** | **D** | **I** | **R** | **S** | **Y** | **R** | **L** | **N** | **F** | **E** | **T** | **S** | **A** | **V** | **I** | **Y** | **D** | **P** | **E** | **F** | **L** | **E** | **E** | **L** | **A** | **T** | **Q** | **F** | **K** | **E** | **D** | **E** | **T** | **H** | **C** | **S** | **S** | **M** | **T** | **T** | **E** | **T** | **V** | **K** | **E** | **M** | **S** | **N** | **W** | **L** | **L** | **F** | **K** | **Q** | **Q** | **I** | **S** | **R** | **L** | **F** | **S** | **P** | **I** | **L** |
| **N60490F** | **-** | **-** | **-** | **-** | **-** | **-** | **-** | **-** | **-** | **-** | **-** | **-** | **-** | **-** | **-** | **-** | **-** | **-** | **-** | **-** | **-** | **-** | **-** | **K** | **P** | **R** | **D** | **V** | **A** | **A** | **I** | **W** | **A** | **W** | **L** | **L** | **V** | **L** | **F** | **A** | **L** | **P** | **G** | **V** | **G** | **F** | **V** | **L** | **Y** | **L** | **F** | **F** | **G** | **R** | **G** | **L** | **T** | **D** | **K** | **K** | **K** | **F** | **Y** | **L** | **R** | **Q** | **S** | **D** | **L** | **K** | **E** | **L** | **E** | **N** | **F** | **Q** | **S** | **F** | **R** | **G** | **D** | **T** | **I** | **E** | **H** | **Y** | **D** | **P** | **D** | **M** | **G** | **D** | **K** | **D** | **K** | **Q** | **Q** | **F** | **V** | **D** | **F** | **F** | **S** | **S** | **L** | **N** | **R** | **M** | **P** | **L** | **T** | **R** | **M** | **N** | **S** | **V** | **T** | **L** | **L** | **T** | **D** | **G** | **Q** | **E** | **K** | **L** | **D** | **S** | **L** | **L** | **Q** | **D** | **L** | **K** | **K** | **A** | **K** | **H** | **S** | **I** | **H** | **I** | **E** | **Y** | **Y** | **A** | **F** | **V** | **T** | **D** | **N** | **I** | **G** | **Q** | **Q** | **V** | **L** | **H** | **V** | **L** | **E** | **E** | **K** | **A** | **A** | **E** | **G** | **V** | **E** | **V** | **R** | **I** | **L** | **Y** | **D** | **A** | **F** | **G** | **S** | **H** | **G** | **T** | **K** | **A** | **K** | **D** | **F** | **N** | **R** | **L** | **I** | **K** | **N** | **G** | **G** | **H** | **V** | **H** | **T** | **F** | **V** | **T** | **S** | **Q** | **K** | **A** | **L** | **L** | **R** | **F** | **R** | **L** | **N** | **Y** | **H** | **D** | **H** | **R** | **K** | **I** | **V** | **V** | **I** | **D** | **G** | **K** | **I** | **S** | **Y** | **T** | **G** | **G** | **F** | **N** | **I** | **A** | **N** | **Q** | **Y** | **V** | **N** | **T** | **T** | **K** | **K** | **F** | **G** | **Y** | **W** | **R** | **D** | **T** | **H** | **I** | **R** | **I** | **F** | **G** | **A** | **A** | **S** | **S** | **L** | **L** | **Q** | **L** | **R** | **F** | **L** | **T** | **D** | **W** | **N** | **V** | **S** | **V** | **P** | **E** | **E** | **K** | **K** | **V** | **G** | **Y** | **H** | **L** | **N** | **Y** | **F** | **F** | **K** | **K** | **A** | **D** | **R** | **D** | **E** | **S** | **K** | **L** | **A** | **D** | **T** | **S** | **I** | **Q** | **L** | **V** | **S** | **S** | **G** | **P** | **N** | **N** | **E** | **R** | **E** | **Q** | **I** | **K** | **L** | **S** | **F** | **I** | **K** | **L** | **I** | **T** | **S** | **A** | **K** | **K** | **R** | **V** | **W** | **I** | **Q** | **T** | **P** | **Y** | **L** | **V** | **P** | **D** | **E** | **S** | **V** | **I** | **A** | **A** | **L** | **K** | **I** | **A** | **T** | **A** | **S** | **G** | **V** | **D** | **V** | **K** | **I** | **M** | **I** | **P** | **N** | **K** | **P** | **D** | **H** | **P** | **F** | **I** | **Y** | **R** | **A** | **T** | **Q** | **Y** | **Y** | **A** | **R** | **Q** | **L** | **I** | **K** | **E** | **N** | **V** | **Q** | **I** | **L** | **V** | **Y** | **E** | **N** | **G** | **F** | **L** | **H** | **A** | **K** | **T** | **L** | **I** | **M** | **D** | **D** | **E** | **I** | **C** | **M** | **V** | **G** | **S** | **A** | **N** | **Q** | **D** | **I** | **R** | **S** | **Y** | **R** | **L** | **N** | **F** | **E** | **T** | **S** | **A** | **V** | **I** | **Y** | **D** | **P** | **E** | **F** | **L** | **E** | **E** | **L** | **A** | **T** | **Q** | **F** | **K** | **E** | **D** | **E** | **T** | **H** | **C** | **S** | **S** | **M** | **T** | **T** | **E** | **T** | **V** | **K** | **E** | **M** | **S** | **N** | **W** | **L** | **L** | **F** | **K** | **Q** | **Q** | **I** | **S** | **R** | **L** | **F** | **S** | **P** | **I** | **L** |
| **N55728** | **-** | **-** | **-** | **-** | **-** | **-** | **-** | **-** | **-** | **-** | **-** | **-** | **-** | **-** | **-** | **-** | **-** | **-** | **-** | **-** | **-** | **-** | **-** | **K** | **P** | **R** | **D** | **V** | **A** | **A** | **I** | **W** | **A** | **W** | **L** | **L** | **V** | **L** | **F** | **A** | **L** | **P** | **G** | **V** | **G** | **F** | **V** | **L** | **Y** | **L** | **F** | **F** | **G** | **R** | **G** | **L** | **T** | **D** | **K** | **K** | **K** | **F** | **Y** | **L** | **R** | **Q** | **S** | **D** | **L** | **K** | **E** | **L** | **E** | **N** | **F** | **Q** | **S** | **F** | **R** | **G** | **D** | **N** | **I** | **E** | **H** | **Y** | **D** | **P** | **D** | **M** | **G** | **D** | **K** | **D** | **K** | **Q** | **Q** | **F** | **V** | **D** | **F** | **F** | **S** | **S** | **L** | **N** | **R** | **M** | **P** | **L** | **T** | **R** | **M** | **N** | **S** | **V** | **T** | **L** | **L** | **T** | **D** | **G** | **Q** | **E** | **K** | **L** | **D** | **S** | **L** | **L** | **Q** | **D** | **L** | **K** | **K** | **A** | **K** | **H** | **S** | **I** | **H** | **I** | **E** | **Y** | **Y** | **A** | **F** | **V** | **T** | **D** | **N** | **I** | **G** | **Q** | **Q** | **V** | **L** | **H** | **V** | **L** | **E** | **E** | **K** | **A** | **A** | **E** | **G** | **V** | **E** | **V** | **R** | **I** | **L** | **Y** | **D** | **A** | **F** | **G** | **S** | **H** | **G** | **T** | **K** | **A** | **K** | **D** | **F** | **N** | **R** | **L** | **I** | **K** | **N** | **G** | **G** | **H** | **V** | **H** | **T** | **F** | **V** | **T** | **S** | **Q** | **K** | **A** | **L** | **L** | **R** | **F** | **R** | **L** | **N** | **Y** | **H** | **D** | **H** | **R** | **K** | **I** | **V** | **V** | **I** | **D** | **G** | **K** | **I** | **S** | **Y** | **T** | **G** | **G** | **F** | **N** | **I** | **A** | **N** | **Q** | **Y** | **V** | **N** | **T** | **T** | **K** | **K** | **F** | **G** | **Y** | **W** | **R** | **D** | **T** | **H** | **I** | **R** | **I** | **F** | **G** | **A** | **A** | **S** | **S** | **L** | **L** | **Q** | **L** | **R** | **F** | **L** | **T** | **D** | **W** | **N** | **V** | **S** | **V** | **P** | **E** | **E** | **K** | **K** | **V** | **G** | **Y** | **H** | **L** | **N** | **Y** | **F** | **F** | **K** | **K** | **A** | **D** | **R** | **D** | **E** | **S** | **K** | **L** | **A** | **D** | **T** | **S** | **I** | **Q** | **L** | **V** | **S** | **S** | **G** | **P** | **N** | **N** | **E** | **R** | **E** | **Q** | **I** | **K** | **L** | **S** | **F** | **I** | **K** | **L** | **I** | **T** | **S** | **A** | **K** | **K** | **R** | **V** | **W** | **I** | **Q** | **T** | **P** | **Y** | **L** | **V** | **P** | **D** | **E** | **S** | **V** | **I** | **A** | **A** | **L** | **K** | **I** | **A** | **T** | **A** | **S** | **G** | **V** | **D** | **V** | **K** | **I** | **M** | **I** | **P** | **N** | **K** | **P** | **D** | **H** | **P** | **F** | **I** | **Y** | **R** | **A** | **T** | **Q** | **Y** | **Y** | **A** | **R** | **Q** | **L** | **I** | **K** | **E** | **N** | **V** | **Q** | **I** | **L** | **V** | **Y** | **E** | **N** | **G** | **F** | **L** | **H** | **A** | **K** | **T** | **L** | **I** | **M** | **D** | **D** | **E** | **I** | **C** | **M** | **V** | **G** | **S** | **A** | **N** | **Q** | **D** | **I** | **R** | **S** | **Y** | **R** | **L** | **N** | **F** | **E** | **T** | **S** | **A** | **V** | **I** | **Y** | **D** | **P** | **E** | **F** | **L** | **E** | **E** | **L** | **A** | **T** | **Q** | **F** | **K** | **E** | **D** | **E** | **T** | **H** | **C** | **S** | **S** | **M** | **T** | **T** | **E** | **T** | **V** | **K** | **E** | **M** | **S** | **N** | **W** | **L** | **L** | **F** | **K** | **Q** | **Q** | **I** | **S** | **R** | **L** | **F** | **S** | **P** | **I** | **L** |
| **N59513F** | **-** | **-** | **-** | **-** | **-** | **-** | **-** | **-** | **-** | **-** | **-** | **-** | **-** | **-** | **-** | **-** | **-** | **-** | **-** | **-** | **-** | **-** | **-** | **K** | **P** | **R** | **D** | **V** | **A** | **A** | **I** | **W** | **A** | **W** | **L** | **L** | **V** | **L** | **F** | **A** | **L** | **P** | **G** | **V** | **G** | **F** | **V** | **L** | **Y** | **L** | **F** | **F** | **G** | **R** | **G** | **L** | **T** | **D** | **K** | **K** | **K** | **F** | **Y** | **L** | **R** | **Q** | **S** | **D** | **L** | **K** | **E** | **L** | **E** | **N** | **F** | **Q** | **S** | **F** | **R** | **G** | **D** | **T** | **I** | **E** | **H** | **Y** | **D** | **P** | **D** | **M** | **G** | **D** | **K** | **D** | **K** | **Q** | **Q** | **F** | **V** | **D** | **F** | **F** | **S** | **S** | **L** | **N** | **R** | **M** | **P** | **L** | **T** | **R** | **M** | **N** | **S** | **V** | **T** | **L** | **L** | **T** | **D** | **G** | **Q** | **K** | **K** | **L** | **D** | **S** | **L** | **L** | **Q** | **D** | **L** | **K** | **K** | **A** | **K** | **H** | **S** | **I** | **H** | **I** | **E** | **Y** | **Y** | **A** | **F** | **V** | **T** | **D** | **N** | **I** | **G** | **Q** | **Q** | **V** | **L** | **R** | **V** | **L** | **E** | **E** | **K** | **A** | **A** | **E** | **G** | **V** | **E** | **V** | **R** | **I** | **L** | **Y** | **D** | **A** | **F** | **G** | **S** | **H** | **G** | **T** | **K** | **A** | **K** | **D** | **F** | **N** | **R** | **L** | **I** | **K** | **N** | **G** | **G** | **H** | **V** | **H** | **T** | **F** | **V** | **T** | **S** | **Q** | **K** | **A** | **L** | **L** | **R** | **F** | **R** | **L** | **N** | **Y** | **H** | **D** | **H** | **R** | **K** | **I** | **V** | **V** | **I** | **D** | **G** | **K** | **I** | **S** | **Y** | **T** | **G** | **G** | **F** | **N** | **I** | **A** | **N** | **Q** | **Y** | **V** | **N** | **T** | **T** | **K** | **K** | **F** | **G** | **Y** | **W** | **R** | **D** | **T** | **H** | **I** | **R** | **I** | **F** | **G** | **A** | **A** | **S** | **S** | **L** | **L** | **Q** | **L** | **R** | **F** | **L** | **T** | **D** | **W** | **N** | **V** | **S** | **V** | **P** | **E** | **E** | **K** | **K** | **V** | **G** | **Y** | **H** | **L** | **N** | **Y** | **F** | **F** | **K** | **K** | **A** | **D** | **R** | **D** | **E** | **S** | **K** | **L** | **A** | **D** | **T** | **S** | **I** | **Q** | **L** | **V** | **S** | **S** | **G** | **P** | **N** | **N** | **E** | **R** | **E** | **Q** | **I** | **K** | **L** | **S** | **F** | **I** | **K** | **L** | **I** | **T** | **S** | **A** | **K** | **K** | **R** | **V** | **W** | **I** | **Q** | **T** | **P** | **Y** | **L** | **V** | **P** | **D** | **E** | **S** | **V** | **I** | **A** | **A** | **L** | **K** | **I** | **A** | **T** | **A** | **S** | **G** | **V** | **D** | **V** | **K** | **I** | **M** | **I** | **P** | **N** | **K** | **P** | **D** | **H** | **P** | **F** | **I** | **Y** | **R** | **A** | **T** | **Q** | **Y** | **Y** | **A** | **R** | **Q** | **L** | **I** | **K** | **E** | **N** | **V** | **Q** | **I** | **L** | **V** | **Y** | **E** | **N** | **G** | **F** | **L** | **H** | **A** | **K** | **T** | **L** | **I** | **M** | **D** | **D** | **E** | **I** | **C** | **M** | **V** | **G** | **S** | **A** | **N** | **Q** | **D** | **I** | **R** | **S** | **Y** | **R** | **L** | **N** | **F** | **E** | **T** | **S** | **A** | **V** | **I** | **Y** | **D** | **P** | **E** | **F** | **L** | **E** | **E** | **L** | **A** | **T** | **Q** | **F** | **K** | **E** | **D** | **E** | **T** | **H** | **C** | **S** | **P** | **M** | **T** | **T** | **E** | **T** | **V** | **K** | **E** | **M** | **S** | **N** | **W** | **L** | **L** | **F** | **K** | **Q** | **Q** | **I** | **S** | **R** | **L** | **F** | **S** | **P** | **I** | **L** |
| **N54150** | **-** | **-** | **-** | **-** | **-** | **-** | **-** | **-** | **-** | **-** | **-** | **-** | **-** | **-** | **-** | **-** | **-** | **-** | **-** | **-** | **-** | **-** | **-** | **K** | **P** | **R** | **D** | **V** | **A** | **A** | **I** | **W** | **A** | **W** | **L** | **L** | **V** | **L** | **F** | **A** | **L** | **P** | **G** | **V** | **G** | **F** | **V** | **L** | **Y** | **L** | **F** | **F** | **G** | **R** | **G** | **L** | **T** | **D** | **K** | **K** | **K** | **F** | **Y** | **L** | **R** | **Q** | **S** | **D** | **L** | **K** | **E** | **L** | **E** | **N** | **F** | **Q** | **S** | **F** | **R** | **G** | **D** | **T** | **I** | **E** | **H** | **Y** | **D** | **P** | **D** | **M** | **G** | **D** | **K** | **D** | **K** | **Q** | **Q** | **F** | **V** | **D** | **F** | **F** | **S** | **S** | **L** | **N** | **R** | **M** | **P** | **L** | **T** | **R** | **M** | **N** | **S** | **V** | **T** | **L** | **L** | **T** | **D** | **G** | **Q** | **E** | **K** | **L** | **D** | **S** | **L** | **L** | **Q** | **D** | **L** | **K** | **K** | **A** | **K** | **H** | **S** | **I** | **H** | **I** | **E** | **Y** | **Y** | **A** | **F** | **V** | **T** | **D** | **N** | **I** | **G** | **Q** | **Q** | **V** | **L** | **H** | **V** | **L** | **E** | **E** | **K** | **A** | **A** | **E** | **G** | **V** | **E** | **V** | **R** | **I** | **L** | **Y** | **D** | **A** | **F** | **G** | **S** | **H** | **G** | **T** | **K** | **A** | **K** | **D** | **F** | **N** | **R** | **L** | **I** | **K** | **N** | **G** | **G** | **H** | **V** | **H** | **T** | **F** | **V** | **T** | **S** | **Q** | **K** | **A** | **L** | **L** | **R** | **F** | **R** | **L** | **N** | **Y** | **H** | **D** | **H** | **R** | **K** | **I** | **V** | **V** | **I** | **D** | **G** | **K** | **I** | **S** | **Y** | **T** | **G** | **G** | **F** | **N** | **I** | **A** | **N** | **Q** | **Y** | **V** | **N** | **I** | **T** | **K** | **K** | **F** | **G** | **Y** | **W** | **R** | **D** | **T** | **H** | **I** | **R** | **I** | **F** | **G** | **A** | **A** | **S** | **S** | **L** | **L** | **Q** | **L** | **R** | **F** | **L** | **T** | **D** | **W** | **N** | **V** | **S** | **V** | **P** | **E** | **E** | **K** | **K** | **V** | **G** | **Y** | **H** | **L** | **N** | **Y** | **F** | **F** | **K** | **K** | **A** | **D** | **R** | **D** | **E** | **S** | **K** | **L** | **A** | **D** | **T** | **S** | **I** | **Q** | **L** | **V** | **S** | **S** | **G** | **P** | **N** | **N** | **E** | **R** | **E** | **Q** | **I** | **K** | **L** | **S** | **F** | **I** | **K** | **L** | **I** | **T** | **S** | **A** | **K** | **K** | **R** | **V** | **W** | **I** | **Q** | **T** | **P** | **Y** | **L** | **V** | **P** | **D** | **E** | **S** | **V** | **I** | **A** | **A** | **L** | **K** | **I** | **A** | **T** | **A** | **S** | **G** | **V** | **D** | **V** | **K** | **I** | **M** | **I** | **P** | **N** | **K** | **P** | **D** | **H** | **P** | **F** | **I** | **Y** | **R** | **A** | **T** | **Q** | **Y** | **Y** | **A** | **R** | **Q** | **L** | **I** | **K** | **E** | **N** | **V** | **Q** | **I** | **L** | **V** | **Y** | **E** | **N** | **G** | **F** | **L** | **H** | **A** | **K** | **T** | **L** | **I** | **M** | **D** | **D** | **E** | **I** | **C** | **M** | **V** | **G** | **S** | **A** | **N** | **Q** | **D** | **I** | **R** | **S** | **Y** | **R** | **L** | **N** | **F** | **E** | **T** | **S** | **A** | **V** | **I** | **Y** | **D** | **P** | **E** | **F** | **L** | **E** | **E** | **L** | **A** | **T** | **Q** | **F** | **K** | **E** | **D** | **E** | **T** | **H** | **C** | **S** | **S** | **M** | **T** | **T** | **E** | **T** | **V** | **K** | **E** | **M** | **S** | **N** | **W** | **L** | **L** | **F** | **K** | **Q** | **Q** | **I** | **S** | **R** | **L** | **F** | **S** | **P** | **I** | **L** |
| **N59951F** | **-** | **-** | **-** | **-** | **-** | **-** | **-** | **-** | **-** | **-** | **-** | **-** | **-** | **-** | **-** | **-** | **-** | **-** | **-** | **-** | **-** | **-** | **-** | **K** | **P** | **R** | **D** | **V** | **A** | **A** | **I** | **W** | **A** | **W** | **L** | **L** | **V** | **L** | **F** | **A** | **L** | **P** | **G** | **V** | **G** | **F** | **V** | **L** | **Y** | **L** | **F** | **F** | **G** | **R** | **G** | **L** | **T** | **D** | **K** | **K** | **K** | **F** | **Y** | **L** | **R** | **Q** | **S** | **D** | **L** | **K** | **E** | **L** | **E** | **N** | **F** | **Q** | **S** | **F** | **R** | **G** | **D** | **T** | **I** | **E** | **H** | **Y** | **D** | **P** | **D** | **M** | **G** | **D** | **K** | **D** | **K** | **Q** | **Q** | **F** | **V** | **D** | **F** | **F** | **S** | **S** | **L** | **N** | **R** | **M** | **P** | **L** | **T** | **R** | **M** | **N** | **S** | **V** | **T** | **L** | **L** | **T** | **D** | **G** | **Q** | **E** | **K** | **L** | **D** | **S** | **L** | **L** | **Q** | **D** | **L** | **K** | **K** | **A** | **K** | **H** | **S** | **I** | **H** | **I** | **E** | **Y** | **Y** | **A** | **F** | **V** | **T** | **D** | **N** | **I** | **G** | **Q** | **Q** | **V** | **L** | **H** | **V** | **L** | **E** | **E** | **K** | **A** | **A** | **E** | **G** | **V** | **E** | **V** | **R** | **I** | **L** | **Y** | **D** | **A** | **F** | **G** | **S** | **H** | **G** | **T** | **K** | **A** | **K** | **D** | **F** | **N** | **R** | **L** | **I** | **K** | **N** | **G** | **G** | **H** | **V** | **H** | **T** | **F** | **V** | **T** | **S** | **Q** | **K** | **A** | **L** | **L** | **R** | **F** | **R** | **L** | **N** | **Y** | **H** | **D** | **H** | **R** | **K** | **I** | **V** | **V** | **I** | **D** | **G** | **K** | **I** | **S** | **Y** | **T** | **G** | **G** | **F** | **N** | **I** | **A** | **N** | **Q** | **Y** | **V** | **N** | **T** | **T** | **K** | **K** | **F** | **G** | **Y** | **W** | **R** | **D** | **T** | **H** | **I** | **R** | **I** | **F** | **G** | **A** | **A** | **S** | **S** | **L** | **L** | **Q** | **L** | **R** | **F** | **L** | **T** | **D** | **W** | **N** | **V** | **S** | **V** | **P** | **E** | **E** | **K** | **K** | **V** | **G** | **Y** | **H** | **L** | **N** | **Y** | **F** | **F** | **K** | **K** | **A** | **D** | **R** | **D** | **E** | **S** | **K** | **L** | **A** | **E** | **T** | **S** | **I** | **Q** | **L** | **V** | **S** | **S** | **G** | **P** | **N** | **N** | **E** | **R** | **E** | **Q** | **I** | **K** | **L** | **S** | **F** | **I** | **K** | **L** | **I** | **T** | **S** | **A** | **K** | **K** | **R** | **V** | **W** | **I** | **Q** | **T** | **P** | **Y** | **L** | **V** | **P** | **D** | **E** | **S** | **V** | **I** | **A** | **A** | **L** | **K** | **I** | **A** | **T** | **A** | **S** | **G** | **V** | **D** | **V** | **K** | **I** | **M** | **I** | **P** | **N** | **K** | **P** | **D** | **H** | **P** | **F** | **I** | **Y** | **R** | **A** | **T** | **Q** | **Y** | **Y** | **A** | **R** | **Q** | **L** | **I** | **K** | **E** | **N** | **V** | **Q** | **I** | **L** | **V** | **Y** | **E** | **N** | **G** | **F** | **L** | **H** | **A** | **K** | **T** | **L** | **I** | **M** | **D** | **D** | **E** | **I** | **C** | **M** | **V** | **G** | **S** | **A** | **N** | **Q** | **D** | **I** | **R** | **S** | **Y** | **R** | **L** | **N** | **F** | **E** | **T** | **S** | **A** | **V** | **I** | **Y** | **D** | **P** | **E** | **F** | **L** | **E** | **E** | **L** | **A** | **T** | **Q** | **F** | **K** | **E** | **D** | **E** | **T** | **H** | **C** | **S** | **S** | **M** | **T** | **T** | **E** | **T** | **V** | **K** | **E** | **M** | **S** | **N** | **W** | **L** | **L** | **F** | **K** | **Q** | **Q** | **I** | **S** | **R** | **L** | **F** | **S** | **P** | **I** | **L** |
| **N60001F** | **-** | **-** | **-** | **-** | **-** | **-** | **-** | **-** | **-** | **-** | **-** | **-** | **-** | **-** | **-** | **-** | **-** | **-** | **-** | **-** | **-** | **-** | **-** | **K** | **P** | **R** | **D** | **V** | **A** | **A** | **I** | **W** | **A** | **W** | **L** | **L** | **V** | **L** | **F** | **A** | **L** | **P** | **G** | **V** | **G** | **F** | **V** | **L** | **Y** | **L** | **F** | **F** | **G** | **R** | **G** | **L** | **T** | **D** | **K** | **K** | **K** | **F** | **Y** | **L** | **R** | **Q** | **S** | **D** | **L** | **K** | **E** | **L** | **E** | **N** | **F** | **Q** | **S** | **F** | **R** | **G** | **D** | **T** | **I** | **E** | **H** | **Y** | **D** | **P** | **D** | **M** | **G** | **D** | **K** | **D** | **K** | **Q** | **Q** | **F** | **V** | **D** | **F** | **F** | **S** | **S** | **L** | **N** | **R** | **M** | **P** | **L** | **T** | **R** | **M** | **N** | **S** | **V** | **T** | **L** | **L** | **T** | **D** | **G** | **Q** | **K** | **K** | **L** | **D** | **S** | **L** | **L** | **Q** | **D** | **L** | **K** | **K** | **A** | **K** | **H** | **S** | **I** | **H** | **I** | **E** | **Y** | **Y** | **A** | **F** | **V** | **T** | **D** | **N** | **I** | **G** | **Q** | **Q** | **V** | **L** | **R** | **V** | **L** | **E** | **E** | **K** | **A** | **A** | **E** | **G** | **V** | **E** | **V** | **R** | **I** | **L** | **Y** | **D** | **A** | **F** | **G** | **S** | **H** | **G** | **T** | **K** | **A** | **K** | **D** | **F** | **N** | **R** | **L** | **I** | **K** | **N** | **G** | **G** | **H** | **V** | **H** | **T** | **F** | **V** | **T** | **S** | **Q** | **K** | **A** | **L** | **L** | **R** | **F** | **R** | **L** | **N** | **Y** | **H** | **D** | **H** | **R** | **K** | **I** | **V** | **V** | **I** | **D** | **G** | **K** | **I** | **S** | **Y** | **T** | **G** | **G** | **F** | **N** | **I** | **A** | **N** | **Q** | **Y** | **V** | **N** | **T** | **T** | **K** | **K** | **F** | **G** | **Y** | **W** | **R** | **D** | **T** | **H** | **I** | **R** | **I** | **F** | **G** | **A** | **A** | **S** | **S** | **L** | **L** | **Q** | **L** | **R** | **F** | **L** | **T** | **D** | **W** | **N** | **V** | **S** | **V** | **P** | **E** | **E** | **K** | **K** | **V** | **G** | **Y** | **H** | **L** | **N** | **Y** | **F** | **F** | **K** | **K** | **A** | **D** | **R** | **D** | **E** | **S** | **K** | **L** | **A** | **D** | **T** | **S** | **I** | **Q** | **L** | **V** | **S** | **S** | **G** | **P** | **N** | **N** | **E** | **R** | **E** | **Q** | **I** | **K** | **L** | **S** | **F** | **I** | **K** | **L** | **I** | **T** | **S** | **A** | **K** | **K** | **R** | **V** | **W** | **I** | **Q** | **T** | **P** | **Y** | **L** | **V** | **P** | **D** | **E** | **S** | **V** | **I** | **A** | **A** | **L** | **K** | **I** | **A** | **T** | **A** | **S** | **G** | **V** | **D** | **V** | **K** | **I** | **M** | **I** | **P** | **N** | **K** | **P** | **D** | **H** | **P** | **F** | **I** | **Y** | **R** | **A** | **T** | **Q** | **Y** | **Y** | **A** | **R** | **Q** | **L** | **I** | **K** | **E** | **N** | **V** | **Q** | **I** | **L** | **V** | **Y** | **E** | **N** | **G** | **F** | **L** | **H** | **A** | **K** | **T** | **L** | **I** | **M** | **D** | **D** | **E** | **I** | **C** | **M** | **V** | **G** | **S** | **A** | **N** | **Q** | **D** | **I** | **R** | **S** | **Y** | **R** | **L** | **N** | **F** | **E** | **T** | **S** | **A** | **V** | **I** | **Y** | **D** | **P** | **E** | **F** | **L** | **E** | **E** | **L** | **A** | **T** | **Q** | **F** | **K** | **E** | **D** | **E** | **T** | **H** | **C** | **S** | **P** | **M** | **T** | **T** | **E** | **T** | **V** | **K** | **E** | **M** | **S** | **N** | **W** | **L** | **L** | **F** | **K** | **Q** | **Q** | **I** | **S** | **R** | **L** | **F** | **S** | **P** | **I** | **L** |
| **N52656** | **-** | **-** | **-** | **-** | **-** | **-** | **-** | **-** | **-** | **-** | **-** | **-** | **-** | **-** | **-** | **-** | **-** | **-** | **-** | **-** | **-** | **-** | **-** | **K** | **P** | **R** | **D** | **V** | **A** | **A** | **I** | **W** | **A** | **W** | **L** | **L** | **V** | **L** | **F** | **A** | **L** | **P** | **G** | **V** | **G** | **F** | **V** | **L** | **Y** | **L** | **F** | **F** | **G** | **R** | **G** | **L** | **T** | **D** | **K** | **K** | **K** | **F** | **Y** | **L** | **R** | **Q** | **S** | **D** | **L** | **K** | **E** | **L** | **E** | **N** | **F** | **Q** | **S** | **F** | **R** | **G** | **D** | **T** | **I** | **E** | **H** | **Y** | **D** | **P** | **D** | **M** | **G** | **D** | **K** | **D** | **K** | **Q** | **Q** | **F** | **V** | **D** | **F** | **F** | **S** | **S** | **L** | **N** | **R** | **M** | **P** | **L** | **T** | **R** | **M** | **N** | **S** | **V** | **T** | **L** | **L** | **T** | **D** | **G** | **Q** | **K** | **K** | **L** | **D** | **S** | **L** | **L** | **Q** | **D** | **L** | **K** | **K** | **A** | **K** | **H** | **S** | **I** | **H** | **I** | **E** | **Y** | **Y** | **A** | **F** | **V** | **T** | **D** | **T** | **I** | **G** | **Q** | **Q** | **V** | **L** | **R** | **V** | **L** | **E** | **E** | **K** | **A** | **A** | **E** | **G** | **V** | **E** | **V** | **R** | **I** | **L** | **Y** | **D** | **A** | **F** | **G** | **S** | **H** | **G** | **T** | **K** | **A** | **K** | **D** | **F** | **N** | **R** | **L** | **I** | **K** | **N** | **G** | **G** | **H** | **V** | **H** | **T** | **F** | **V** | **T** | **S** | **Q** | **K** | **A** | **L** | **L** | **R** | **F** | **R** | **L** | **N** | **Y** | **H** | **D** | **H** | **R** | **K** | **I** | **V** | **V** | **I** | **D** | **G** | **K** | **I** | **S** | **Y** | **T** | **G** | **G** | **F** | **N** | **I** | **A** | **N** | **Q** | **Y** | **V** | **N** | **T** | **T** | **K** | **K** | **F** | **G** | **Y** | **W** | **R** | **D** | **T** | **H** | **I** | **R** | **I** | **F** | **G** | **A** | **A** | **S** | **S** | **L** | **L** | **Q** | **L** | **R** | **F** | **L** | **T** | **D** | **W** | **N** | **V** | **S** | **V** | **P** | **E** | **E** | **K** | **K** | **V** | **G** | **Y** | **H** | **L** | **N** | **Y** | **F** | **F** | **K** | **K** | **A** | **D** | **R** | **D** | **E** | **S** | **K** | **L** | **A** | **D** | **T** | **S** | **I** | **Q** | **L** | **V** | **S** | **S** | **G** | **P** | **N** | **N** | **E** | **R** | **E** | **Q** | **I** | **K** | **L** | **S** | **F** | **I** | **K** | **L** | **I** | **T** | **S** | **A** | **K** | **K** | **R** | **V** | **W** | **I** | **Q** | **T** | **P** | **Y** | **L** | **V** | **P** | **D** | **E** | **S** | **V** | **I** | **A** | **A** | **L** | **K** | **I** | **A** | **T** | **A** | **S** | **G** | **V** | **D** | **V** | **K** | **I** | **M** | **I** | **P** | **N** | **K** | **P** | **D** | **H** | **P** | **F** | **I** | **Y** | **R** | **A** | **T** | **Q** | **Y** | **Y** | **A** | **R** | **Q** | **L** | **I** | **K** | **E** | **N** | **V** | **Q** | **I** | **L** | **V** | **Y** | **E** | **N** | **G** | **F** | **L** | **H** | **A** | **K** | **T** | **L** | **I** | **M** | **D** | **D** | **E** | **I** | **C** | **M** | **V** | **G** | **S** | **A** | **N** | **Q** | **D** | **I** | **R** | **S** | **Y** | **R** | **L** | **N** | **F** | **E** | **T** | **S** | **A** | **V** | **I** | **Y** | **D** | **P** | **E** | **F** | **L** | **E** | **E** | **L** | **A** | **T** | **Q** | **F** | **K** | **E** | **D** | **E** | **T** | **H** | **C** | **S** | **P** | **M** | **T** | **T** | **E** | **T** | **V** | **K** | **E** | **M** | **S** | **N** | **W** | **L** | **L** | **F** | **K** | **Q** | **Q** | **I** | **S** | **R** | **L** | **F** | **S** | **P** | **I** | **L** |
| **N59891F** | **-** | **-** | **-** | **-** | **-** | **-** | **-** | **-** | **-** | **-** | **-** | **-** | **-** | **-** | **-** | **-** | **-** | **-** | **-** | **-** | **-** | **-** | **-** | **K** | **P** | **R** | **D** | **V** | **A** | **A** | **I** | **W** | **A** | **W** | **L** | **L** | **V** | **L** | **F** | **A** | **L** | **P** | **G** | **V** | **G** | **F** | **V** | **L** | **Y** | **L** | **F** | **F** | **G** | **R** | **G** | **L** | **T** | **D** | **K** | **K** | **K** | **F** | **Y** | **L** | **R** | **Q** | **S** | **D** | **L** | **K** | **E** | **L** | **E** | **N** | **F** | **Q** | **S** | **F** | **R** | **G** | **D** | **T** | **I** | **E** | **H** | **Y** | **D** | **P** | **D** | **M** | **G** | **D** | **K** | **D** | **K** | **Q** | **Q** | **F** | **V** | **D** | **F** | **F** | **S** | **S** | **L** | **N** | **R** | **M** | **P** | **L** | **T** | **R** | **M** | **N** | **S** | **V** | **T** | **L** | **L** | **T** | **D** | **G** | **Q** | **E** | **K** | **L** | **D** | **S** | **L** | **L** | **Q** | **D** | **L** | **K** | **K** | **A** | **K** | **H** | **S** | **I** | **H** | **I** | **E** | **Y** | **Y** | **A** | **F** | **V** | **T** | **D** | **N** | **I** | **G** | **Q** | **Q** | **V** | **L** | **H** | **V** | **L** | **E** | **E** | **K** | **A** | **A** | **E** | **G** | **V** | **E** | **V** | **R** | **I** | **L** | **Y** | **D** | **A** | **F** | **G** | **S** | **H** | **G** | **T** | **K** | **A** | **K** | **D** | **F** | **N** | **R** | **L** | **I** | **K** | **N** | **G** | **G** | **H** | **V** | **H** | **T** | **F** | **V** | **T** | **S** | **Q** | **R** | **A** | **L** | **L** | **R** | **F** | **R** | **L** | **N** | **Y** | **H** | **D** | **H** | **R** | **K** | **I** | **V** | **V** | **I** | **D** | **G** | **K** | **I** | **S** | **Y** | **T** | **G** | **G** | **F** | **N** | **I** | **A** | **N** | **Q** | **Y** | **V** | **N** | **T** | **T** | **K** | **K** | **F** | **G** | **Y** | **W** | **R** | **D** | **T** | **H** | **I** | **R** | **I** | **F** | **G** | **A** | **A** | **S** | **S** | **L** | **L** | **Q** | **L** | **R** | **F** | **L** | **T** | **D** | **W** | **N** | **V** | **S** | **V** | **P** | **E** | **E** | **K** | **K** | **V** | **G** | **Y** | **H** | **L** | **N** | **Y** | **F** | **F** | **K** | **K** | **A** | **D** | **R** | **D** | **E** | **S** | **K** | **L** | **A** | **D** | **T** | **S** | **I** | **Q** | **L** | **V** | **S** | **S** | **G** | **P** | **N** | **N** | **E** | **R** | **E** | **Q** | **I** | **K** | **L** | **S** | **F** | **I** | **K** | **L** | **I** | **T** | **S** | **A** | **K** | **K** | **R** | **V** | **W** | **I** | **Q** | **T** | **P** | **Y** | **L** | **V** | **P** | **D** | **E** | **S** | **V** | **I** | **A** | **A** | **L** | **K** | **I** | **A** | **T** | **A** | **S** | **G** | **V** | **D** | **V** | **K** | **I** | **M** | **I** | **P** | **N** | **K** | **P** | **D** | **H** | **P** | **F** | **I** | **Y** | **R** | **A** | **T** | **Q** | **Y** | **Y** | **A** | **R** | **Q** | **L** | **I** | **K** | **E** | **N** | **V** | **Q** | **I** | **L** | **V** | **Y** | **E** | **N** | **G** | **F** | **L** | **H** | **A** | **K** | **T** | **L** | **I** | **M** | **D** | **D** | **E** | **I** | **C** | **M** | **V** | **G** | **S** | **A** | **N** | **Q** | **D** | **I** | **R** | **S** | **Y** | **R** | **L** | **N** | **F** | **E** | **T** | **S** | **A** | **V** | **I** | **Y** | **D** | **P** | **E** | **F** | **L** | **E** | **E** | **L** | **A** | **T** | **Q** | **F** | **K** | **E** | **D** | **E** | **T** | **H** | **C** | **S** | **S** | **M** | **T** | **T** | **E** | **T** | **V** | **K** | **E** | **M** | **S** | **N** | **W** | **L** | **L** | **F** | **K** | **Q** | **Q** | **I** | **S** | **R** | **L** | **F** | **S** | **P** | **I** | **L** |
| **N54671** | **-** | **-** | **-** | **-** | **-** | **-** | **-** | **-** | **-** | **-** | **-** | **-** | **-** | **-** | **-** | **-** | **-** | **-** | **-** | **-** | **-** | **-** | **-** | **K** | **P** | **R** | **D** | **V** | **A** | **A** | **I** | **W** | **A** | **W** | **L** | **L** | **V** | **L** | **F** | **A** | **L** | **P** | **G** | **V** | **G** | **F** | **V** | **L** | **Y** | **L** | **F** | **F** | **G** | **R** | **G** | **L** | **T** | **D** | **K** | **K** | **K** | **F** | **Y** | **L** | **R** | **Q** | **S** | **D** | **L** | **K** | **E** | **L** | **E** | **N** | **F** | **Q** | **S** | **F** | **R** | **G** | **D** | **N** | **I** | **E** | **H** | **Y** | **D** | **P** | **D** | **M** | **G** | **D** | **K** | **D** | **K** | **Q** | **Q** | **F** | **V** | **D** | **F** | **F** | **S** | **S** | **L** | **N** | **R** | **M** | **P** | **L** | **T** | **R** | **M** | **N** | **S** | **V** | **T** | **L** | **L** | **T** | **D** | **G** | **Q** | **E** | **K** | **L** | **D** | **S** | **L** | **L** | **Q** | **D** | **L** | **K** | **K** | **A** | **K** | **H** | **S** | **I** | **H** | **I** | **E** | **Y** | **Y** | **A** | **F** | **V** | **T** | **D** | **N** | **I** | **G** | **Q** | **Q** | **V** | **L** | **H** | **V** | **L** | **E** | **E** | **K** | **A** | **A** | **E** | **G** | **V** | **E** | **V** | **R** | **I** | **L** | **Y** | **D** | **A** | **F** | **G** | **S** | **H** | **G** | **T** | **K** | **A** | **K** | **D** | **F** | **N** | **R** | **L** | **I** | **K** | **N** | **G** | **G** | **H** | **V** | **H** | **T** | **F** | **V** | **T** | **S** | **Q** | **K** | **A** | **L** | **L** | **R** | **F** | **R** | **L** | **N** | **Y** | **H** | **D** | **H** | **R** | **K** | **I** | **V** | **V** | **I** | **D** | **G** | **K** | **I** | **S** | **Y** | **T** | **G** | **G** | **F** | **N** | **I** | **A** | **N** | **Q** | **Y** | **V** | **N** | **T** | **T** | **K** | **K** | **F** | **G** | **Y** | **W** | **R** | **D** | **T** | **H** | **I** | **R** | **I** | **F** | **G** | **A** | **A** | **S** | **S** | **L** | **L** | **Q** | **L** | **R** | **F** | **L** | **T** | **D** | **W** | **N** | **V** | **S** | **V** | **P** | **E** | **E** | **K** | **K** | **V** | **G** | **Y** | **H** | **L** | **N** | **Y** | **F** | **F** | **K** | **K** | **A** | **D** | **R** | **D** | **E** | **S** | **K** | **L** | **A** | **D** | **T** | **S** | **I** | **Q** | **L** | **V** | **S** | **S** | **G** | **P** | **N** | **N** | **E** | **R** | **E** | **Q** | **I** | **K** | **L** | **S** | **F** | **I** | **K** | **L** | **I** | **T** | **S** | **A** | **K** | **K** | **R** | **V** | **W** | **I** | **Q** | **T** | **P** | **Y** | **L** | **V** | **P** | **D** | **E** | **S** | **V** | **I** | **A** | **A** | **L** | **K** | **I** | **A** | **T** | **A** | **S** | **G** | **V** | **D** | **V** | **K** | **I** | **M** | **I** | **P** | **N** | **K** | **P** | **D** | **H** | **P** | **F** | **I** | **Y** | **R** | **A** | **T** | **Q** | **Y** | **Y** | **A** | **R** | **Q** | **L** | **I** | **K** | **E** | **N** | **V** | **Q** | **I** | **L** | **V** | **Y** | **E** | **N** | **G** | **F** | **L** | **H** | **A** | **K** | **T** | **L** | **I** | **M** | **D** | **D** | **E** | **I** | **C** | **M** | **V** | **G** | **S** | **A** | **N** | **Q** | **D** | **I** | **R** | **S** | **Y** | **R** | **L** | **N** | **F** | **E** | **T** | **S** | **A** | **V** | **I** | **Y** | **D** | **P** | **E** | **F** | **L** | **E** | **E** | **L** | **A** | **T** | **Q** | **F** | **K** | **E** | **D** | **E** | **T** | **H** | **C** | **S** | **S** | **M** | **T** | **T** | **E** | **T** | **V** | **K** | **E** | **M** | **S** | **N** | **W** | **L** | **L** | **F** | **K** | **Q** | **Q** | **I** | **S** | **R** | **L** | **F** | **S** | **P** | **I** | **L** |
| **N60260F** | **-** | **-** | **-** | **-** | **-** | **-** | **-** | **-** | **-** | **-** | **-** | **-** | **-** | **-** | **-** | **-** | **-** | **-** | **-** | **-** | **-** | **-** | **-** | **K** | **P** | **R** | **D** | **V** | **A** | **A** | **I** | **W** | **A** | **W** | **L** | **L** | **V** | **L** | **F** | **A** | **L** | **P** | **G** | **V** | **G** | **F** | **V** | **L** | **Y** | **L** | **F** | **F** | **G** | **R** | **G** | **L** | **T** | **D** | **K** | **K** | **K** | **F** | **Y** | **L** | **R** | **Q** | **S** | **D** | **L** | **K** | **E** | **L** | **E** | **N** | **F** | **Q** | **S** | **F** | **R** | **G** | **D** | **T** | **I** | **E** | **H** | **Y** | **D** | **P** | **D** | **M** | **G** | **D** | **K** | **D** | **K** | **Q** | **Q** | **F** | **V** | **D** | **F** | **F** | **S** | **S** | **L** | **N** | **R** | **M** | **P** | **L** | **T** | **R** | **M** | **N** | **S** | **V** | **T** | **L** | **L** | **T** | **D** | **G** | **Q** | **E** | **K** | **L** | **D** | **S** | **L** | **L** | **Q** | **D** | **L** | **K** | **K** | **A** | **K** | **H** | **S** | **I** | **H** | **I** | **E** | **Y** | **Y** | **A** | **F** | **V** | **T** | **D** | **N** | **I** | **G** | **Q** | **Q** | **V** | **L** | **H** | **V** | **L** | **E** | **E** | **K** | **A** | **A** | **E** | **G** | **V** | **E** | **V** | **R** | **I** | **L** | **Y** | **D** | **A** | **F** | **G** | **S** | **H** | **G** | **T** | **K** | **A** | **K** | **D** | **F** | **N** | **R** | **L** | **I** | **K** | **N** | **G** | **G** | **H** | **V** | **H** | **T** | **F** | **V** | **T** | **S** | **Q** | **K** | **A** | **L** | **L** | **R** | **F** | **R** | **L** | **N** | **Y** | **H** | **D** | **H** | **R** | **K** | **I** | **V** | **V** | **I** | **D** | **G** | **K** | **I** | **S** | **Y** | **T** | **G** | **G** | **F** | **N** | **I** | **A** | **N** | **Q** | **Y** | **V** | **N** | **T** | **T** | **K** | **K** | **F** | **G** | **Y** | **W** | **R** | **D** | **T** | **H** | **I** | **R** | **I** | **F** | **G** | **A** | **A** | **S** | **S** | **L** | **L** | **Q** | **L** | **R** | **F** | **L** | **T** | **D** | **W** | **N** | **V** | **S** | **V** | **P** | **E** | **E** | **K** | **K** | **V** | **G** | **Y** | **H** | **L** | **N** | **Y** | **F** | **F** | **K** | **K** | **A** | **D** | **R** | **D** | **E** | **S** | **K** | **L** | **A** | **D** | **T** | **S** | **I** | **Q** | **L** | **V** | **S** | **S** | **G** | **P** | **N** | **N** | **E** | **R** | **E** | **Q** | **I** | **K** | **L** | **S** | **F** | **I** | **K** | **L** | **I** | **T** | **S** | **A** | **K** | **K** | **R** | **V** | **W** | **I** | **Q** | **T** | **P** | **Y** | **L** | **V** | **P** | **D** | **E** | **S** | **V** | **I** | **A** | **A** | **L** | **K** | **I** | **A** | **T** | **A** | **S** | **G** | **V** | **D** | **V** | **K** | **I** | **M** | **I** | **P** | **N** | **K** | **P** | **D** | **H** | **P** | **F** | **I** | **Y** | **R** | **A** | **T** | **Q** | **Y** | **Y** | **A** | **R** | **Q** | **L** | **I** | **K** | **E** | **N** | **V** | **Q** | **I** | **L** | **V** | **Y** | **E** | **N** | **G** | **F** | **L** | **H** | **A** | **K** | **T** | **L** | **I** | **M** | **D** | **D** | **E** | **I** | **C** | **M** | **V** | **G** | **S** | **A** | **N** | **Q** | **D** | **I** | **R** | **S** | **Y** | **R** | **L** | **N** | **F** | **E** | **T** | **S** | **A** | **V** | **I** | **Y** | **D** | **P** | **E** | **F** | **L** | **E** | **E** | **L** | **A** | **T** | **Q** | **F** | **K** | **E** | **D** | **E** | **T** | **H** | **C** | **S** | **S** | **M** | **T** | **T** | **E** | **T** | **V** | **K** | **E** | **M** | **S** | **N** | **W** | **L** | **L** | **F** | **K** | **Q** | **Q** | **I** | **S** | **R** | **L** | **F** | **S** | **P** | **I** | **L** |
| **N59589F** | **-** | **-** | **-** | **-** | **-** | **-** | **-** | **-** | **-** | **-** | **-** | **-** | **-** | **-** | **-** | **-** | **-** | **-** | **-** | **-** | **-** | **-** | **-** | **K** | **P** | **R** | **D** | **V** | **A** | **A** | **I** | **W** | **A** | **W** | **L** | **L** | **V** | **L** | **F** | **A** | **L** | **P** | **G** | **V** | **G** | **F** | **V** | **L** | **Y** | **L** | **F** | **F** | **G** | **R** | **G** | **L** | **T** | **D** | **K** | **K** | **K** | **F** | **Y** | **L** | **R** | **Q** | **S** | **D** | **L** | **K** | **E** | **L** | **E** | **N** | **F** | **Q** | **S** | **F** | **R** | **G** | **D** | **T** | **I** | **E** | **H** | **Y** | **D** | **P** | **D** | **M** | **G** | **D** | **K** | **D** | **K** | **Q** | **Q** | **F** | **V** | **D** | **F** | **F** | **S** | **S** | **L** | **N** | **R** | **M** | **P** | **L** | **T** | **R** | **M** | **N** | **S** | **V** | **T** | **L** | **L** | **T** | **D** | **G** | **Q** | **K** | **K** | **L** | **D** | **S** | **L** | **L** | **Q** | **D** | **L** | **K** | **K** | **A** | **K** | **H** | **S** | **I** | **H** | **I** | **E** | **Y** | **Y** | **A** | **F** | **V** | **T** | **D** | **N** | **I** | **G** | **Q** | **Q** | **V** | **L** | **R** | **V** | **L** | **E** | **E** | **K** | **A** | **A** | **E** | **G** | **V** | **E** | **V** | **R** | **I** | **L** | **Y** | **D** | **A** | **F** | **G** | **S** | **H** | **G** | **T** | **K** | **A** | **K** | **D** | **F** | **N** | **R** | **L** | **I** | **K** | **N** | **G** | **G** | **H** | **V** | **H** | **T** | **F** | **V** | **T** | **S** | **Q** | **K** | **A** | **L** | **L** | **R** | **F** | **R** | **L** | **N** | **Y** | **H** | **D** | **H** | **R** | **K** | **I** | **V** | **V** | **I** | **D** | **G** | **K** | **I** | **S** | **Y** | **T** | **G** | **G** | **F** | **N** | **I** | **A** | **N** | **Q** | **Y** | **V** | **N** | **T** | **T** | **K** | **K** | **F** | **G** | **Y** | **W** | **R** | **D** | **T** | **H** | **I** | **R** | **I** | **F** | **G** | **A** | **A** | **S** | **S** | **L** | **L** | **Q** | **L** | **R** | **F** | **L** | **T** | **D** | **W** | **N** | **V** | **S** | **V** | **S** | **E** | **E** | **K** | **K** | **V** | **G** | **Y** | **H** | **L** | **N** | **Y** | **F** | **F** | **K** | **K** | **A** | **D** | **R** | **D** | **E** | **S** | **K** | **L** | **A** | **D** | **T** | **S** | **I** | **Q** | **L** | **V** | **S** | **S** | **G** | **P** | **N** | **N** | **E** | **R** | **E** | **Q** | **I** | **K** | **L** | **S** | **F** | **I** | **K** | **L** | **I** | **T** | **S** | **A** | **K** | **K** | **R** | **V** | **W** | **I** | **Q** | **T** | **P** | **Y** | **L** | **V** | **P** | **D** | **E** | **S** | **V** | **I** | **A** | **A** | **L** | **K** | **I** | **A** | **T** | **A** | **S** | **G** | **V** | **D** | **V** | **K** | **I** | **M** | **I** | **P** | **N** | **K** | **P** | **D** | **H** | **P** | **F** | **I** | **Y** | **R** | **A** | **T** | **Q** | **Y** | **Y** | **A** | **R** | **Q** | **L** | **I** | **K** | **E** | **N** | **V** | **Q** | **I** | **L** | **V** | **Y** | **E** | **N** | **G** | **F** | **L** | **H** | **A** | **K** | **T** | **L** | **I** | **M** | **D** | **D** | **E** | **I** | **C** | **M** | **V** | **G** | **S** | **A** | **N** | **Q** | **D** | **I** | **R** | **S** | **Y** | **R** | **L** | **N** | **F** | **E** | **T** | **S** | **A** | **V** | **I** | **Y** | **D** | **P** | **E** | **F** | **L** | **E** | **E** | **L** | **A** | **T** | **Q** | **F** | **K** | **E** | **D** | **E** | **T** | **H** | **C** | **S** | **P** | **M** | **T** | **T** | **E** | **T** | **V** | **K** | **E** | **M** | **S** | **N** | **W** | **L** | **L** | **F** | **K** | **Q** | **Q** | **I** | **S** | **R** | **L** | **F** | **S** | **P** | **I** | **L** |
| **N60123F** | **-** | **-** | **-** | **-** | **-** | **-** | **-** | **-** | **-** | **-** | **-** | **-** | **-** | **-** | **-** | **-** | **-** | **-** | **-** | **-** | **-** | **-** | **-** | **K** | **P** | **R** | **D** | **V** | **A** | **A** | **I** | **W** | **A** | **W** | **L** | **L** | **V** | **L** | **F** | **A** | **L** | **P** | **G** | **V** | **G** | **F** | **V** | **L** | **Y** | **L** | **F** | **F** | **G** | **R** | **G** | **L** | **T** | **D** | **K** | **K** | **K** | **F** | **Y** | **L** | **R** | **Q** | **S** | **D** | **L** | **K** | **E** | **L** | **E** | **N** | **F** | **Q** | **S** | **F** | **R** | **G** | **D** | **T** | **I** | **E** | **H** | **Y** | **D** | **P** | **D** | **M** | **G** | **D** | **K** | **D** | **K** | **Q** | **Q** | **F** | **V** | **D** | **F** | **F** | **S** | **S** | **L** | **N** | **R** | **M** | **P** | **L** | **T** | **R** | **M** | **N** | **S** | **V** | **T** | **L** | **L** | **T** | **D** | **G** | **Q** | **E** | **K** | **L** | **D** | **S** | **L** | **L** | **Q** | **D** | **L** | **K** | **K** | **A** | **K** | **H** | **S** | **I** | **H** | **I** | **E** | **Y** | **Y** | **A** | **F** | **V** | **T** | **D** | **N** | **I** | **G** | **Q** | **Q** | **V** | **L** | **H** | **V** | **L** | **E** | **E** | **K** | **A** | **A** | **E** | **G** | **V** | **E** | **V** | **R** | **I** | **L** | **Y** | **D** | **A** | **F** | **G** | **S** | **H** | **G** | **T** | **K** | **A** | **K** | **D** | **F** | **N** | **R** | **L** | **I** | **K** | **N** | **G** | **G** | **H** | **V** | **H** | **T** | **F** | **V** | **T** | **S** | **Q** | **K** | **A** | **L** | **L** | **R** | **F** | **R** | **L** | **N** | **Y** | **H** | **D** | **H** | **R** | **K** | **I** | **V** | **V** | **I** | **D** | **G** | **K** | **I** | **S** | **Y** | **T** | **G** | **G** | **F** | **N** | **I** | **A** | **N** | **Q** | **Y** | **V** | **N** | **T** | **T** | **K** | **K** | **F** | **G** | **Y** | **W** | **R** | **D** | **T** | **H** | **I** | **R** | **I** | **F** | **G** | **A** | **A** | **S** | **S** | **L** | **L** | **Q** | **L** | **R** | **F** | **L** | **T** | **D** | **W** | **N** | **V** | **S** | **V** | **P** | **E** | **E** | **K** | **K** | **V** | **G** | **Y** | **H** | **L** | **N** | **Y** | **F** | **F** | **K** | **K** | **A** | **D** | **R** | **D** | **E** | **S** | **K** | **L** | **A** | **D** | **T** | **S** | **I** | **Q** | **L** | **V** | **S** | **S** | **G** | **P** | **N** | **N** | **E** | **R** | **E** | **Q** | **I** | **K** | **L** | **S** | **F** | **I** | **K** | **L** | **I** | **T** | **S** | **A** | **K** | **K** | **R** | **V** | **W** | **I** | **Q** | **T** | **P** | **Y** | **L** | **V** | **P** | **D** | **E** | **S** | **V** | **I** | **A** | **A** | **L** | **K** | **I** | **A** | **T** | **A** | **S** | **G** | **V** | **D** | **V** | **K** | **I** | **M** | **I** | **P** | **N** | **K** | **P** | **D** | **H** | **P** | **F** | **I** | **Y** | **R** | **A** | **T** | **Q** | **Y** | **Y** | **A** | **R** | **Q** | **L** | **I** | **K** | **E** | **N** | **V** | **Q** | **I** | **L** | **V** | **Y** | **E** | **N** | **G** | **F** | **L** | **H** | **A** | **K** | **T** | **L** | **I** | **M** | **D** | **D** | **E** | **I** | **C** | **M** | **V** | **G** | **S** | **A** | **N** | **Q** | **D** | **I** | **R** | **S** | **Y** | **R** | **L** | **N** | **F** | **E** | **T** | **S** | **A** | **V** | **I** | **Y** | **D** | **P** | **E** | **F** | **L** | **E** | **E** | **L** | **A** | **T** | **Q** | **F** | **K** | **E** | **D** | **E** | **T** | **H** | **C** | **S** | **S** | **M** | **T** | **T** | **E** | **T** | **V** | **K** | **E** | **M** | **S** | **N** | **W** | **L** | **L** | **F** | **K** | **Q** | **Q** | **I** | **S** | **R** | **L** | **F** | **S** | **P** | **I** | **L** |
| **N53804** | **-** | **-** | **-** | **-** | **-** | **-** | **-** | **-** | **-** | **-** | **-** | **-** | **-** | **-** | **-** | **-** | **-** | **-** | **-** | **-** | **-** | **-** | **-** | **K** | **P** | **R** | **D** | **V** | **A** | **A** | **I** | **W** | **A** | **W** | **L** | **L** | **V** | **L** | **F** | **A** | **L** | **P** | **G** | **V** | **G** | **F** | **V** | **L** | **Y** | **L** | **F** | **F** | **G** | **R** | **G** | **L** | **T** | **D** | **K** | **K** | **K** | **F** | **Y** | **L** | **R** | **Q** | **S** | **D** | **L** | **K** | **E** | **L** | **E** | **N** | **F** | **Q** | **S** | **F** | **R** | **G** | **D** | **T** | **I** | **E** | **H** | **Y** | **D** | **P** | **D** | **M** | **G** | **D** | **K** | **D** | **K** | **Q** | **Q** | **F** | **V** | **D** | **F** | **F** | **S** | **S** | **L** | **N** | **R** | **M** | **P** | **L** | **T** | **R** | **M** | **N** | **S** | **V** | **T** | **L** | **L** | **T** | **D** | **G** | **Q** | **E** | **K** | **L** | **D** | **S** | **L** | **L** | **Q** | **D** | **L** | **K** | **K** | **A** | **K** | **H** | **S** | **I** | **H** | **I** | **E** | **Y** | **Y** | **A** | **F** | **V** | **T** | **D** | **N** | **I** | **G** | **Q** | **Q** | **V** | **L** | **H** | **V** | **L** | **E** | **E** | **K** | **A** | **A** | **E** | **G** | **V** | **E** | **V** | **R** | **I** | **L** | **Y** | **D** | **A** | **F** | **G** | **S** | **H** | **G** | **T** | **K** | **A** | **K** | **D** | **F** | **N** | **R** | **L** | **I** | **K** | **N** | **G** | **G** | **H** | **V** | **H** | **T** | **F** | **V** | **T** | **S** | **Q** | **R** | **A** | **L** | **L** | **R** | **F** | **R** | **L** | **N** | **Y** | **H** | **D** | **H** | **R** | **K** | **I** | **V** | **V** | **I** | **D** | **G** | **K** | **I** | **S** | **Y** | **T** | **G** | **G** | **F** | **N** | **I** | **A** | **N** | **Q** | **Y** | **V** | **N** | **T** | **T** | **K** | **K** | **F** | **G** | **Y** | **W** | **R** | **D** | **T** | **H** | **I** | **R** | **I** | **F** | **G** | **A** | **A** | **S** | **S** | **L** | **L** | **Q** | **L** | **R** | **F** | **L** | **T** | **D** | **W** | **N** | **V** | **S** | **V** | **P** | **E** | **E** | **K** | **K** | **V** | **G** | **Y** | **H** | **L** | **N** | **Y** | **F** | **F** | **K** | **K** | **A** | **D** | **R** | **D** | **E** | **S** | **K** | **L** | **A** | **D** | **T** | **S** | **I** | **Q** | **L** | **V** | **S** | **S** | **G** | **P** | **N** | **N** | **E** | **R** | **E** | **Q** | **I** | **K** | **L** | **S** | **F** | **I** | **K** | **L** | **I** | **T** | **S** | **A** | **K** | **K** | **R** | **V** | **W** | **I** | **Q** | **T** | **P** | **Y** | **L** | **V** | **P** | **D** | **E** | **S** | **V** | **I** | **A** | **A** | **L** | **K** | **I** | **A** | **T** | **A** | **S** | **G** | **V** | **D** | **V** | **K** | **I** | **M** | **I** | **P** | **N** | **K** | **P** | **D** | **H** | **P** | **F** | **I** | **Y** | **R** | **A** | **T** | **Q** | **Y** | **Y** | **A** | **R** | **Q** | **L** | **I** | **K** | **E** | **N** | **V** | **Q** | **I** | **L** | **V** | **Y** | **E** | **N** | **G** | **F** | **L** | **H** | **A** | **K** | **T** | **L** | **I** | **M** | **D** | **D** | **E** | **I** | **C** | **M** | **V** | **G** | **S** | **A** | **N** | **Q** | **D** | **I** | **R** | **S** | **Y** | **R** | **L** | **N** | **F** | **E** | **T** | **S** | **A** | **V** | **I** | **Y** | **D** | **P** | **E** | **F** | **L** | **E** | **E** | **L** | **A** | **T** | **Q** | **F** | **K** | **E** | **D** | **E** | **T** | **H** | **C** | **S** | **S** | **M** | **T** | **T** | **E** | **T** | **V** | **K** | **E** | **M** | **S** | **N** | **W** | **L** | **L** | **F** | **K** | **Q** | **Q** | **I** | **S** | **R** | **L** | **F** | **S** | **P** | **I** | **L** |
| **N60048F** | **-** | **-** | **-** | **-** | **-** | **-** | **-** | **-** | **-** | **-** | **-** | **-** | **-** | **-** | **-** | **-** | **-** | **-** | **-** | **-** | **-** | **-** | **-** | **K** | **P** | **R** | **D** | **V** | **A** | **A** | **I** | **W** | **A** | **W** | **L** | **L** | **V** | **L** | **F** | **A** | **L** | **P** | **G** | **V** | **G** | **F** | **V** | **L** | **Y** | **L** | **F** | **F** | **G** | **R** | **G** | **L** | **T** | **D** | **K** | **K** | **K** | **F** | **Y** | **L** | **R** | **Q** | **S** | **D** | **L** | **K** | **E** | **L** | **E** | **N** | **F** | **Q** | **S** | **F** | **R** | **G** | **D** | **T** | **I** | **E** | **H** | **Y** | **D** | **P** | **D** | **M** | **G** | **D** | **K** | **D** | **K** | **Q** | **Q** | **F** | **V** | **D** | **F** | **F** | **S** | **S** | **L** | **N** | **R** | **M** | **P** | **L** | **T** | **R** | **M** | **N** | **S** | **V** | **T** | **L** | **L** | **T** | **D** | **G** | **Q** | **E** | **K** | **L** | **D** | **S** | **L** | **L** | **Q** | **D** | **L** | **K** | **K** | **A** | **K** | **H** | **S** | **I** | **H** | **I** | **E** | **Y** | **Y** | **A** | **F** | **V** | **T** | **D** | **N** | **I** | **G** | **Q** | **Q** | **V** | **L** | **H** | **V** | **L** | **E** | **E** | **K** | **A** | **A** | **E** | **G** | **V** | **E** | **V** | **R** | **I** | **L** | **Y** | **D** | **A** | **F** | **G** | **S** | **H** | **G** | **T** | **K** | **A** | **K** | **D** | **F** | **N** | **R** | **L** | **I** | **K** | **N** | **G** | **G** | **H** | **V** | **H** | **T** | **F** | **V** | **T** | **S** | **Q** | **K** | **A** | **L** | **L** | **R** | **F** | **R** | **L** | **N** | **Y** | **H** | **D** | **H** | **R** | **K** | **I** | **V** | **V** | **I** | **D** | **G** | **K** | **I** | **S** | **Y** | **T** | **G** | **G** | **F** | **N** | **I** | **A** | **N** | **Q** | **Y** | **V** | **N** | **T** | **T** | **K** | **K** | **F** | **G** | **Y** | **W** | **R** | **D** | **T** | **H** | **I** | **R** | **I** | **F** | **G** | **A** | **A** | **S** | **S** | **L** | **L** | **Q** | **L** | **R** | **F** | **L** | **T** | **D** | **W** | **N** | **V** | **S** | **V** | **P** | **E** | **E** | **K** | **K** | **V** | **G** | **Y** | **H** | **L** | **N** | **Y** | **F** | **F** | **K** | **K** | **A** | **D** | **R** | **D** | **E** | **S** | **K** | **L** | **A** | **E** | **T** | **S** | **I** | **Q** | **L** | **V** | **S** | **S** | **G** | **P** | **N** | **N** | **E** | **R** | **E** | **Q** | **I** | **K** | **L** | **S** | **F** | **I** | **K** | **L** | **I** | **T** | **S** | **A** | **K** | **K** | **R** | **V** | **W** | **I** | **Q** | **T** | **P** | **Y** | **L** | **V** | **P** | **D** | **E** | **S** | **V** | **I** | **A** | **A** | **L** | **K** | **I** | **A** | **T** | **A** | **S** | **G** | **V** | **D** | **V** | **K** | **I** | **M** | **I** | **P** | **N** | **K** | **P** | **D** | **H** | **P** | **F** | **I** | **Y** | **R** | **A** | **T** | **Q** | **Y** | **Y** | **A** | **R** | **Q** | **L** | **I** | **K** | **E** | **N** | **V** | **Q** | **I** | **L** | **V** | **Y** | **E** | **N** | **G** | **F** | **L** | **H** | **A** | **K** | **T** | **L** | **I** | **M** | **D** | **D** | **E** | **I** | **C** | **M** | **V** | **G** | **S** | **A** | **N** | **Q** | **D** | **I** | **R** | **S** | **Y** | **R** | **L** | **N** | **F** | **E** | **T** | **S** | **A** | **V** | **I** | **Y** | **D** | **P** | **E** | **F** | **L** | **E** | **E** | **L** | **A** | **T** | **Q** | **F** | **K** | **E** | **D** | **E** | **T** | **H** | **C** | **S** | **S** | **M** | **T** | **T** | **E** | **T** | **V** | **K** | **E** | **M** | **S** | **N** | **W** | **L** | **L** | **F** | **K** | **Q** | **Q** | **I** | **S** | **R** | **L** | **F** | **S** | **P** | **I** | **L** |
| **N53479** | **-** | **-** | **-** | **-** | **-** | **-** | **-** | **-** | **-** | **-** | **-** | **-** | **-** | **-** | **-** | **-** | **-** | **-** | **-** | **-** | **-** | **-** | **-** | **K** | **P** | **R** | **D** | **V** | **A** | **A** | **I** | **W** | **A** | **W** | **L** | **L** | **V** | **L** | **F** | **A** | **L** | **P** | **G** | **V** | **G** | **F** | **V** | **L** | **Y** | **L** | **F** | **F** | **G** | **R** | **G** | **L** | **T** | **D** | **K** | **K** | **K** | **F** | **Y** | **L** | **R** | **Q** | **S** | **D** | **L** | **K** | **E** | **L** | **E** | **N** | **F** | **Q** | **S** | **F** | **R** | **G** | **D** | **T** | **I** | **E** | **H** | **Y** | **D** | **P** | **D** | **M** | **G** | **D** | **K** | **D** | **K** | **Q** | **Q** | **F** | **V** | **D** | **F** | **F** | **S** | **S** | **L** | **N** | **R** | **M** | **P** | **L** | **T** | **R** | **M** | **N** | **S** | **V** | **T** | **L** | **L** | **T** | **D** | **G** | **Q** | **E** | **K** | **L** | **D** | **S** | **L** | **L** | **Q** | **D** | **L** | **K** | **K** | **A** | **K** | **H** | **S** | **I** | **H** | **I** | **E** | **Y** | **Y** | **A** | **F** | **V** | **T** | **D** | **N** | **I** | **G** | **Q** | **Q** | **V** | **L** | **H** | **V** | **L** | **E** | **E** | **K** | **A** | **A** | **E** | **G** | **V** | **E** | **V** | **R** | **I** | **L** | **Y** | **D** | **A** | **F** | **G** | **S** | **H** | **G** | **T** | **K** | **A** | **K** | **D** | **F** | **N** | **R** | **L** | **I** | **K** | **N** | **G** | **G** | **H** | **V** | **H** | **T** | **F** | **V** | **T** | **S** | **Q** | **R** | **A** | **L** | **L** | **R** | **F** | **R** | **L** | **N** | **Y** | **H** | **D** | **H** | **R** | **K** | **I** | **V** | **V** | **I** | **D** | **G** | **K** | **I** | **S** | **Y** | **T** | **G** | **G** | **F** | **N** | **I** | **A** | **N** | **Q** | **Y** | **I** | **N** | **T** | **T** | **K** | **K** | **F** | **G** | **Y** | **W** | **R** | **D** | **T** | **H** | **I** | **R** | **I** | **F** | **G** | **A** | **A** | **S** | **S** | **L** | **L** | **Q** | **L** | **R** | **F** | **L** | **T** | **D** | **W** | **N** | **V** | **S** | **V** | **P** | **E** | **E** | **K** | **K** | **V** | **G** | **Y** | **H** | **L** | **N** | **Y** | **F** | **F** | **K** | **K** | **A** | **D** | **R** | **D** | **E** | **S** | **K** | **L** | **A** | **D** | **T** | **S** | **I** | **Q** | **L** | **V** | **S** | **S** | **G** | **P** | **N** | **N** | **E** | **R** | **E** | **Q** | **I** | **K** | **L** | **S** | **F** | **I** | **K** | **L** | **I** | **T** | **S** | **A** | **K** | **K** | **R** | **V** | **W** | **I** | **Q** | **T** | **P** | **Y** | **L** | **V** | **P** | **D** | **E** | **S** | **V** | **I** | **A** | **A** | **L** | **K** | **I** | **A** | **T** | **A** | **S** | **G** | **V** | **D** | **V** | **K** | **I** | **M** | **I** | **P** | **N** | **K** | **P** | **D** | **H** | **P** | **F** | **I** | **Y** | **R** | **A** | **T** | **Q** | **Y** | **Y** | **A** | **R** | **Q** | **L** | **I** | **K** | **E** | **N** | **V** | **Q** | **I** | **L** | **V** | **Y** | **E** | **N** | **G** | **F** | **L** | **H** | **A** | **K** | **T** | **L** | **I** | **M** | **D** | **D** | **E** | **I** | **C** | **M** | **V** | **G** | **S** | **A** | **N** | **Q** | **D** | **I** | **R** | **S** | **Y** | **R** | **L** | **N** | **F** | **E** | **T** | **S** | **A** | **V** | **I** | **Y** | **D** | **P** | **E** | **F** | **L** | **E** | **E** | **L** | **A** | **T** | **Q** | **F** | **K** | **E** | **D** | **E** | **T** | **H** | **C** | **S** | **S** | **M** | **T** | **T** | **E** | **T** | **V** | **K** | **E** | **M** | **S** | **N** | **W** | **L** | **L** | **F** | **K** | **Q** | **Q** | **I** | **S** | **R** | **L** | **F** | **S** | **P** | **I** | **L** |
| **N55655** | **-** | **-** | **-** | **-** | **-** | **-** | **-** | **-** | **-** | **-** | **-** | **-** | **-** | **-** | **-** | **-** | **-** | **-** | **-** | **-** | **-** | **-** | **-** | **K** | **P** | **R** | **D** | **V** | **A** | **A** | **I** | **W** | **A** | **W** | **L** | **L** | **V** | **L** | **F** | **V** | **L** | **P** | **G** | **V** | **G** | **F** | **V** | **L** | **Y** | **L** | **F** | **F** | **G** | **R** | **G** | **L** | **T** | **D** | **K** | **K** | **K** | **F** | **Y** | **L** | **R** | **Q** | **S** | **D** | **L** | **K** | **E** | **L** | **E** | **N** | **F** | **Q** | **S** | **F** | **R** | **G** | **D** | **T** | **I** | **E** | **H** | **Y** | **D** | **P** | **D** | **M** | **G** | **D** | **K** | **D** | **K** | **Q** | **Q** | **F** | **V** | **D** | **F** | **F** | **S** | **S** | **L** | **N** | **R** | **M** | **P** | **L** | **T** | **R** | **M** | **N** | **S** | **V** | **T** | **L** | **L** | **T** | **D** | **G** | **Q** | **E** | **K** | **L** | **D** | **S** | **L** | **L** | **Q** | **D** | **L** | **K** | **K** | **A** | **K** | **H** | **S** | **I** | **H** | **I** | **E** | **Y** | **Y** | **A** | **F** | **V** | **T** | **D** | **N** | **I** | **G** | **Q** | **Q** | **V** | **L** | **H** | **V** | **L** | **E** | **E** | **K** | **A** | **A** | **E** | **G** | **V** | **E** | **V** | **R** | **I** | **L** | **Y** | **D** | **A** | **F** | **G** | **S** | **H** | **G** | **T** | **K** | **A** | **K** | **D** | **F** | **N** | **R** | **L** | **I** | **K** | **N** | **G** | **G** | **H** | **V** | **H** | **T** | **F** | **V** | **T** | **S** | **Q** | **K** | **A** | **L** | **L** | **R** | **F** | **R** | **L** | **N** | **Y** | **H** | **D** | **H** | **R** | **K** | **I** | **V** | **V** | **I** | **D** | **G** | **K** | **I** | **S** | **Y** | **T** | **G** | **G** | **F** | **N** | **I** | **A** | **N** | **Q** | **Y** | **V** | **N** | **T** | **T** | **K** | **K** | **F** | **G** | **Y** | **W** | **R** | **D** | **T** | **H** | **I** | **R** | **I** | **F** | **G** | **A** | **A** | **S** | **S** | **L** | **L** | **Q** | **L** | **R** | **F** | **L** | **T** | **D** | **W** | **N** | **V** | **S** | **V** | **P** | **E** | **E** | **K** | **K** | **V** | **G** | **Y** | **H** | **L** | **N** | **Y** | **F** | **F** | **K** | **K** | **A** | **D** | **R** | **D** | **E** | **S** | **K** | **L** | **A** | **D** | **T** | **S** | **I** | **Q** | **L** | **V** | **S** | **S** | **G** | **P** | **N** | **N** | **E** | **R** | **E** | **Q** | **I** | **K** | **L** | **S** | **F** | **I** | **K** | **L** | **I** | **T** | **S** | **A** | **K** | **K** | **R** | **V** | **W** | **I** | **Q** | **T** | **P** | **Y** | **L** | **V** | **P** | **D** | **E** | **S** | **V** | **I** | **A** | **A** | **L** | **K** | **I** | **A** | **T** | **A** | **S** | **G** | **V** | **D** | **V** | **K** | **I** | **M** | **I** | **P** | **N** | **K** | **P** | **D** | **H** | **P** | **F** | **I** | **Y** | **R** | **A** | **T** | **Q** | **Y** | **Y** | **A** | **R** | **Q** | **L** | **I** | **K** | **E** | **N** | **V** | **Q** | **I** | **L** | **V** | **Y** | **E** | **N** | **G** | **F** | **L** | **H** | **A** | **K** | **T** | **L** | **I** | **M** | **D** | **D** | **E** | **I** | **C** | **M** | **V** | **G** | **S** | **A** | **N** | **Q** | **D** | **I** | **R** | **S** | **Y** | **R** | **L** | **N** | **F** | **E** | **T** | **S** | **A** | **V** | **I** | **Y** | **D** | **P** | **E** | **F** | **L** | **E** | **E** | **L** | **A** | **T** | **Q** | **F** | **K** | **E** | **D** | **E** | **T** | **H** | **C** | **S** | **S** | **M** | **T** | **T** | **E** | **T** | **V** | **K** | **E** | **M** | **S** | **N** | **W** | **L** | **L** | **F** | **K** | **Q** | **Q** | **I** | **S** | **R** | **L** | **F** | **S** | **P** | **I** | **L** |
| **N55299** | **-** | **-** | **-** | **-** | **-** | **-** | **-** | **-** | **-** | **-** | **-** | **-** | **-** | **-** | **-** | **-** | **-** | **-** | **-** | **-** | **-** | **-** | **-** | **K** | **P** | **R** | **D** | **V** | **A** | **A** | **I** | **W** | **A** | **W** | **L** | **L** | **V** | **L** | **F** | **A** | **L** | **P** | **G** | **V** | **G** | **F** | **V** | **L** | **Y** | **L** | **F** | **F** | **G** | **R** | **G** | **L** | **T** | **D** | **K** | **K** | **K** | **F** | **Y** | **L** | **R** | **Q** | **S** | **D** | **L** | **K** | **E** | **L** | **E** | **N** | **F** | **Q** | **S** | **F** | **R** | **G** | **D** | **T** | **I** | **E** | **H** | **Y** | **D** | **P** | **D** | **M** | **G** | **D** | **K** | **D** | **K** | **Q** | **Q** | **F** | **V** | **D** | **F** | **F** | **S** | **S** | **L** | **N** | **R** | **M** | **P** | **L** | **T** | **R** | **M** | **N** | **S** | **V** | **T** | **L** | **L** | **T** | **D** | **G** | **Q** | **E** | **K** | **L** | **D** | **S** | **L** | **L** | **Q** | **D** | **L** | **K** | **K** | **A** | **K** | **H** | **S** | **I** | **H** | **I** | **E** | **Y** | **Y** | **A** | **F** | **V** | **T** | **D** | **N** | **I** | **G** | **Q** | **Q** | **V** | **L** | **H** | **V** | **L** | **E** | **E** | **K** | **A** | **A** | **E** | **G** | **V** | **E** | **V** | **R** | **I** | **L** | **Y** | **D** | **A** | **F** | **G** | **S** | **H** | **G** | **T** | **K** | **A** | **K** | **D** | **F** | **N** | **R** | **L** | **I** | **K** | **N** | **G** | **G** | **H** | **V** | **H** | **T** | **F** | **V** | **T** | **S** | **Q** | **K** | **A** | **L** | **L** | **R** | **F** | **R** | **L** | **N** | **Y** | **H** | **D** | **H** | **R** | **K** | **I** | **V** | **V** | **I** | **D** | **G** | **K** | **I** | **S** | **Y** | **T** | **G** | **G** | **F** | **N** | **I** | **A** | **N** | **Q** | **Y** | **V** | **N** | **T** | **T** | **K** | **K** | **F** | **G** | **Y** | **W** | **R** | **D** | **T** | **H** | **I** | **R** | **I** | **F** | **G** | **A** | **A** | **S** | **S** | **L** | **L** | **Q** | **L** | **R** | **F** | **L** | **T** | **D** | **W** | **N** | **V** | **S** | **V** | **P** | **E** | **E** | **K** | **K** | **V** | **G** | **Y** | **H** | **L** | **N** | **Y** | **F** | **F** | **K** | **K** | **A** | **D** | **R** | **D** | **E** | **S** | **K** | **L** | **A** | **D** | **T** | **S** | **I** | **Q** | **L** | **V** | **S** | **S** | **G** | **P** | **N** | **N** | **E** | **R** | **E** | **Q** | **I** | **K** | **L** | **S** | **F** | **I** | **K** | **L** | **I** | **T** | **S** | **A** | **K** | **K** | **R** | **V** | **W** | **I** | **Q** | **T** | **P** | **Y** | **L** | **V** | **P** | **D** | **E** | **S** | **V** | **I** | **A** | **A** | **L** | **K** | **I** | **A** | **T** | **A** | **S** | **G** | **V** | **D** | **V** | **K** | **I** | **M** | **I** | **P** | **N** | **K** | **P** | **D** | **H** | **P** | **F** | **I** | **Y** | **R** | **A** | **T** | **Q** | **Y** | **Y** | **A** | **R** | **Q** | **L** | **I** | **K** | **E** | **N** | **V** | **Q** | **I** | **L** | **V** | **Y** | **E** | **N** | **G** | **F** | **L** | **H** | **A** | **K** | **T** | **L** | **I** | **M** | **D** | **D** | **E** | **I** | **C** | **M** | **V** | **G** | **S** | **A** | **N** | **Q** | **D** | **I** | **R** | **S** | **Y** | **R** | **L** | **N** | **F** | **E** | **T** | **S** | **A** | **V** | **I** | **Y** | **D** | **P** | **E** | **F** | **L** | **E** | **E** | **L** | **A** | **T** | **Q** | **F** | **K** | **E** | **D** | **E** | **T** | **H** | **C** | **S** | **S** | **M** | **T** | **T** | **E** | **T** | **V** | **K** | **E** | **M** | **S** | **N** | **W** | **L** | **L** | **F** | **K** | **Q** | **Q** | **I** | **S** | **R** | **L** | **F** | **S** | **P** | **I** | **L** |
| **N60252F** | **-** | **-** | **-** | **-** | **-** | **-** | **-** | **-** | **-** | **-** | **-** | **-** | **-** | **-** | **-** | **-** | **-** | **-** | **-** | **-** | **-** | **-** | **-** | **K** | **P** | **R** | **D** | **V** | **A** | **A** | **I** | **W** | **A** | **W** | **L** | **L** | **V** | **L** | **F** | **A** | **L** | **P** | **G** | **V** | **G** | **F** | **V** | **L** | **Y** | **L** | **F** | **F** | **G** | **R** | **G** | **L** | **T** | **D** | **K** | **K** | **K** | **F** | **Y** | **L** | **R** | **Q** | **S** | **D** | **L** | **K** | **E** | **L** | **E** | **N** | **F** | **Q** | **S** | **F** | **R** | **G** | **D** | **T** | **I** | **E** | **H** | **Y** | **D** | **P** | **D** | **M** | **G** | **D** | **K** | **D** | **K** | **Q** | **Q** | **F** | **V** | **D** | **F** | **F** | **S** | **S** | **L** | **N** | **R** | **M** | **P** | **L** | **T** | **R** | **M** | **N** | **S** | **V** | **T** | **L** | **L** | **T** | **D** | **G** | **Q** | **E** | **K** | **L** | **D** | **S** | **L** | **L** | **Q** | **D** | **L** | **K** | **K** | **A** | **K** | **H** | **S** | **I** | **H** | **I** | **E** | **Y** | **Y** | **A** | **F** | **V** | **T** | **D** | **N** | **I** | **G** | **Q** | **Q** | **V** | **L** | **H** | **V** | **L** | **E** | **E** | **K** | **A** | **A** | **E** | **G** | **V** | **E** | **V** | **R** | **I** | **L** | **Y** | **D** | **A** | **F** | **G** | **S** | **H** | **G** | **T** | **K** | **A** | **K** | **D** | **F** | **N** | **R** | **L** | **I** | **K** | **N** | **G** | **G** | **H** | **V** | **H** | **T** | **F** | **V** | **T** | **S** | **Q** | **R** | **A** | **L** | **L** | **R** | **F** | **R** | **L** | **N** | **Y** | **H** | **D** | **H** | **R** | **K** | **I** | **V** | **V** | **I** | **D** | **G** | **K** | **I** | **S** | **Y** | **T** | **G** | **G** | **F** | **N** | **I** | **A** | **N** | **Q** | **Y** | **V** | **N** | **T** | **T** | **K** | **K** | **F** | **G** | **Y** | **W** | **R** | **D** | **T** | **H** | **I** | **R** | **I** | **F** | **G** | **A** | **A** | **S** | **S** | **L** | **L** | **Q** | **L** | **R** | **F** | **L** | **T** | **D** | **W** | **N** | **V** | **S** | **V** | **P** | **E** | **E** | **K** | **K** | **V** | **G** | **Y** | **H** | **L** | **N** | **Y** | **F** | **F** | **K** | **K** | **A** | **D** | **R** | **D** | **E** | **S** | **K** | **L** | **A** | **D** | **T** | **S** | **I** | **Q** | **L** | **V** | **S** | **S** | **G** | **P** | **N** | **N** | **E** | **R** | **E** | **Q** | **I** | **K** | **L** | **S** | **F** | **I** | **K** | **L** | **I** | **T** | **S** | **A** | **K** | **K** | **R** | **V** | **W** | **I** | **Q** | **T** | **P** | **Y** | **L** | **V** | **P** | **D** | **E** | **S** | **V** | **I** | **A** | **A** | **L** | **K** | **I** | **A** | **T** | **A** | **S** | **G** | **V** | **D** | **V** | **K** | **I** | **M** | **I** | **P** | **N** | **K** | **P** | **D** | **H** | **P** | **F** | **I** | **Y** | **R** | **A** | **T** | **Q** | **Y** | **Y** | **A** | **R** | **Q** | **L** | **I** | **K** | **E** | **N** | **V** | **Q** | **I** | **L** | **V** | **Y** | **E** | **N** | **G** | **F** | **L** | **H** | **A** | **K** | **T** | **L** | **I** | **M** | **D** | **D** | **E** | **I** | **C** | **M** | **V** | **G** | **S** | **A** | **N** | **Q** | **D** | **I** | **R** | **S** | **Y** | **R** | **L** | **N** | **F** | **E** | **T** | **S** | **A** | **V** | **I** | **Y** | **D** | **P** | **E** | **F** | **L** | **E** | **E** | **L** | **A** | **T** | **Q** | **F** | **K** | **E** | **D** | **E** | **T** | **H** | **C** | **S** | **S** | **M** | **T** | **T** | **E** | **T** | **V** | **K** | **E** | **M** | **S** | **N** | **W** | **L** | **L** | **F** | **K** | **Q** | **Q** | **I** | **S** | **R** | **L** | **F** | **S** | **P** | **I** | **L** |
| **N59469F** | **-** | **-** | **-** | **-** | **-** | **-** | **-** | **-** | **-** | **-** | **-** | **-** | **-** | **-** | **-** | **-** | **-** | **-** | **-** | **-** | **-** | **-** | **-** | **K** | **P** | **R** | **D** | **V** | **A** | **A** | **I** | **W** | **A** | **W** | **L** | **L** | **V** | **L** | **F** | **A** | **L** | **P** | **G** | **V** | **G** | **F** | **V** | **L** | **Y** | **L** | **F** | **F** | **G** | **R** | **G** | **L** | **T** | **D** | **K** | **K** | **K** | **F** | **Y** | **L** | **R** | **Q** | **S** | **D** | **L** | **K** | **E** | **L** | **E** | **N** | **F** | **Q** | **S** | **F** | **R** | **G** | **D** | **T** | **I** | **E** | **H** | **Y** | **D** | **P** | **D** | **M** | **G** | **D** | **K** | **D** | **K** | **Q** | **Q** | **F** | **V** | **D** | **F** | **F** | **S** | **S** | **L** | **N** | **R** | **M** | **P** | **L** | **T** | **R** | **M** | **N** | **S** | **V** | **T** | **L** | **L** | **T** | **D** | **G** | **Q** | **E** | **K** | **L** | **D** | **S** | **L** | **L** | **Q** | **D** | **L** | **K** | **K** | **A** | **K** | **H** | **S** | **I** | **H** | **I** | **E** | **Y** | **Y** | **A** | **F** | **V** | **T** | **D** | **N** | **I** | **G** | **Q** | **Q** | **V** | **L** | **H** | **V** | **L** | **E** | **E** | **K** | **A** | **A** | **E** | **G** | **V** | **E** | **V** | **R** | **I** | **L** | **Y** | **D** | **A** | **F** | **G** | **S** | **H** | **G** | **T** | **K** | **A** | **K** | **D** | **F** | **N** | **R** | **L** | **I** | **K** | **N** | **G** | **G** | **H** | **V** | **H** | **T** | **F** | **V** | **T** | **S** | **Q** | **K** | **A** | **L** | **L** | **R** | **F** | **R** | **L** | **N** | **Y** | **H** | **D** | **H** | **R** | **K** | **I** | **V** | **V** | **I** | **D** | **G** | **K** | **I** | **S** | **Y** | **T** | **G** | **G** | **F** | **N** | **I** | **A** | **N** | **Q** | **Y** | **V** | **N** | **T** | **T** | **K** | **K** | **F** | **G** | **Y** | **W** | **R** | **D** | **T** | **H** | **I** | **R** | **I** | **F** | **G** | **A** | **A** | **S** | **S** | **L** | **L** | **Q** | **L** | **R** | **F** | **L** | **T** | **D** | **W** | **N** | **V** | **S** | **V** | **P** | **E** | **E** | **K** | **K** | **V** | **G** | **Y** | **H** | **L** | **N** | **Y** | **F** | **F** | **K** | **K** | **A** | **D** | **R** | **D** | **E** | **S** | **K** | **L** | **A** | **E** | **T** | **S** | **I** | **Q** | **L** | **V** | **S** | **S** | **G** | **P** | **N** | **N** | **E** | **R** | **E** | **Q** | **I** | **K** | **L** | **S** | **F** | **I** | **K** | **L** | **I** | **T** | **S** | **A** | **K** | **K** | **R** | **V** | **W** | **I** | **Q** | **T** | **P** | **Y** | **L** | **V** | **P** | **D** | **E** | **S** | **V** | **I** | **A** | **A** | **L** | **K** | **I** | **A** | **T** | **A** | **S** | **G** | **V** | **D** | **V** | **K** | **I** | **M** | **I** | **P** | **N** | **K** | **P** | **D** | **H** | **P** | **F** | **I** | **Y** | **R** | **A** | **T** | **Q** | **Y** | **Y** | **A** | **R** | **Q** | **L** | **I** | **K** | **E** | **N** | **V** | **Q** | **I** | **L** | **V** | **Y** | **E** | **N** | **G** | **F** | **L** | **H** | **A** | **K** | **T** | **L** | **I** | **M** | **D** | **D** | **E** | **I** | **C** | **M** | **V** | **G** | **S** | **A** | **N** | **Q** | **D** | **I** | **R** | **S** | **Y** | **R** | **L** | **N** | **F** | **E** | **T** | **S** | **A** | **V** | **I** | **Y** | **D** | **P** | **E** | **F** | **L** | **E** | **E** | **L** | **A** | **T** | **Q** | **F** | **K** | **E** | **D** | **E** | **T** | **H** | **C** | **S** | **S** | **M** | **T** | **T** | **E** | **T** | **V** | **K** | **E** | **M** | **S** | **N** | **W** | **L** | **L** | **F** | **K** | **Q** | **Q** | **I** | **S** | **R** | **L** | **F** | **S** | **P** | **I** | **L** |
| **N59947F** | **-** | **-** | **-** | **-** | **-** | **-** | **-** | **-** | **-** | **-** | **-** | **-** | **-** | **-** | **-** | **-** | **-** | **-** | **-** | **-** | **-** | **-** | **-** | **K** | **P** | **R** | **D** | **V** | **A** | **A** | **I** | **W** | **A** | **W** | **L** | **L** | **V** | **L** | **F** | **A** | **L** | **P** | **G** | **V** | **G** | **F** | **V** | **L** | **Y** | **L** | **F** | **F** | **G** | **R** | **G** | **L** | **T** | **D** | **K** | **K** | **K** | **F** | **Y** | **L** | **R** | **Q** | **S** | **D** | **L** | **K** | **E** | **L** | **E** | **N** | **F** | **Q** | **S** | **F** | **R** | **G** | **D** | **T** | **I** | **E** | **H** | **Y** | **D** | **P** | **D** | **M** | **G** | **D** | **K** | **D** | **K** | **Q** | **Q** | **F** | **V** | **D** | **F** | **F** | **S** | **S** | **L** | **N** | **R** | **M** | **P** | **L** | **T** | **R** | **M** | **N** | **S** | **V** | **T** | **L** | **L** | **T** | **D** | **G** | **Q** | **K** | **K** | **L** | **D** | **S** | **L** | **L** | **Q** | **D** | **L** | **K** | **K** | **A** | **K** | **H** | **S** | **I** | **H** | **I** | **E** | **Y** | **Y** | **A** | **F** | **V** | **T** | **D** | **N** | **I** | **G** | **Q** | **Q** | **V** | **L** | **R** | **V** | **L** | **E** | **E** | **K** | **A** | **A** | **E** | **G** | **V** | **E** | **V** | **R** | **I** | **L** | **Y** | **D** | **A** | **F** | **G** | **S** | **H** | **G** | **T** | **K** | **A** | **K** | **D** | **F** | **N** | **R** | **L** | **I** | **K** | **N** | **G** | **G** | **H** | **V** | **H** | **T** | **F** | **V** | **T** | **S** | **Q** | **K** | **A** | **L** | **L** | **R** | **F** | **R** | **L** | **N** | **Y** | **H** | **D** | **H** | **R** | **K** | **I** | **V** | **V** | **I** | **D** | **G** | **K** | **I** | **S** | **Y** | **T** | **G** | **G** | **F** | **N** | **I** | **A** | **N** | **Q** | **Y** | **V** | **N** | **T** | **T** | **K** | **K** | **F** | **G** | **Y** | **W** | **R** | **D** | **T** | **H** | **I** | **R** | **I** | **F** | **G** | **A** | **A** | **S** | **S** | **L** | **L** | **Q** | **L** | **R** | **F** | **L** | **T** | **D** | **W** | **N** | **V** | **S** | **V** | **S** | **E** | **E** | **K** | **K** | **V** | **G** | **Y** | **H** | **L** | **N** | **Y** | **F** | **F** | **K** | **K** | **A** | **D** | **R** | **D** | **E** | **S** | **K** | **L** | **A** | **D** | **T** | **S** | **I** | **Q** | **L** | **V** | **S** | **S** | **G** | **P** | **N** | **N** | **E** | **R** | **E** | **Q** | **I** | **K** | **L** | **S** | **F** | **I** | **K** | **L** | **I** | **T** | **S** | **A** | **K** | **K** | **R** | **V** | **W** | **I** | **Q** | **T** | **P** | **Y** | **L** | **V** | **P** | **D** | **E** | **S** | **V** | **I** | **A** | **A** | **L** | **K** | **I** | **A** | **T** | **A** | **S** | **G** | **V** | **D** | **V** | **K** | **I** | **M** | **I** | **P** | **N** | **K** | **P** | **D** | **H** | **P** | **F** | **I** | **Y** | **R** | **A** | **T** | **Q** | **Y** | **Y** | **A** | **R** | **Q** | **L** | **I** | **K** | **E** | **N** | **V** | **Q** | **I** | **L** | **V** | **Y** | **E** | **N** | **G** | **F** | **L** | **H** | **A** | **K** | **T** | **L** | **I** | **M** | **D** | **D** | **E** | **I** | **C** | **M** | **V** | **G** | **S** | **A** | **N** | **Q** | **D** | **I** | **R** | **S** | **Y** | **R** | **L** | **N** | **F** | **E** | **T** | **S** | **A** | **V** | **I** | **Y** | **D** | **P** | **E** | **F** | **L** | **E** | **E** | **L** | **A** | **T** | **Q** | **F** | **K** | **E** | **D** | **E** | **T** | **H** | **C** | **S** | **P** | **M** | **T** | **T** | **E** | **T** | **V** | **K** | **E** | **M** | **S** | **N** | **W** | **L** | **L** | **F** | **K** | **Q** | **Q** | **I** | **S** | **R** | **L** | **F** | **S** | **P** | **I** | **L** |
| **N60336F** | **-** | **-** | **-** | **-** | **-** | **-** | **-** | **-** | **-** | **-** | **-** | **-** | **-** | **-** | **-** | **-** | **-** | **-** | **-** | **-** | **-** | **-** | **-** | **K** | **P** | **R** | **D** | **V** | **A** | **A** | **I** | **W** | **A** | **W** | **L** | **L** | **V** | **L** | **F** | **A** | **L** | **P** | **G** | **V** | **G** | **F** | **V** | **L** | **Y** | **L** | **F** | **F** | **G** | **R** | **G** | **L** | **T** | **D** | **K** | **K** | **K** | **F** | **Y** | **L** | **R** | **Q** | **S** | **D** | **L** | **K** | **E** | **L** | **E** | **N** | **F** | **Q** | **S** | **F** | **R** | **G** | **D** | **T** | **I** | **E** | **H** | **Y** | **D** | **P** | **D** | **M** | **G** | **D** | **K** | **D** | **K** | **Q** | **Q** | **F** | **V** | **D** | **F** | **F** | **S** | **S** | **L** | **N** | **R** | **M** | **P** | **L** | **T** | **R** | **M** | **N** | **S** | **V** | **T** | **L** | **L** | **T** | **D** | **G** | **Q** | **E** | **K** | **L** | **D** | **S** | **L** | **L** | **Q** | **D** | **L** | **K** | **K** | **A** | **K** | **H** | **S** | **I** | **H** | **I** | **E** | **Y** | **Y** | **A** | **F** | **V** | **T** | **D** | **N** | **I** | **G** | **Q** | **Q** | **V** | **L** | **H** | **V** | **L** | **E** | **E** | **K** | **A** | **A** | **E** | **G** | **V** | **E** | **V** | **R** | **I** | **L** | **Y** | **D** | **A** | **F** | **G** | **S** | **H** | **G** | **T** | **K** | **A** | **K** | **D** | **F** | **N** | **R** | **L** | **I** | **K** | **N** | **G** | **G** | **H** | **V** | **H** | **T** | **F** | **V** | **T** | **S** | **Q** | **K** | **A** | **L** | **L** | **R** | **F** | **R** | **L** | **N** | **Y** | **H** | **D** | **H** | **R** | **K** | **I** | **V** | **V** | **I** | **D** | **G** | **K** | **I** | **S** | **Y** | **T** | **G** | **G** | **F** | **N** | **I** | **A** | **N** | **Q** | **Y** | **V** | **N** | **T** | **T** | **K** | **K** | **F** | **G** | **Y** | **W** | **R** | **D** | **T** | **H** | **I** | **R** | **I** | **F** | **G** | **A** | **A** | **S** | **S** | **L** | **L** | **Q** | **L** | **R** | **F** | **L** | **T** | **D** | **W** | **N** | **V** | **S** | **V** | **P** | **E** | **E** | **K** | **K** | **V** | **G** | **Y** | **H** | **L** | **N** | **Y** | **F** | **F** | **K** | **K** | **A** | **D** | **R** | **D** | **E** | **S** | **K** | **L** | **A** | **D** | **T** | **S** | **I** | **Q** | **L** | **V** | **S** | **S** | **G** | **P** | **N** | **N** | **E** | **R** | **E** | **Q** | **I** | **K** | **L** | **S** | **F** | **I** | **K** | **L** | **I** | **T** | **S** | **A** | **K** | **K** | **R** | **V** | **W** | **I** | **Q** | **T** | **P** | **Y** | **L** | **V** | **P** | **D** | **E** | **S** | **V** | **I** | **A** | **A** | **L** | **K** | **I** | **A** | **T** | **A** | **S** | **G** | **V** | **D** | **V** | **K** | **I** | **M** | **I** | **P** | **N** | **K** | **P** | **D** | **H** | **P** | **F** | **I** | **Y** | **R** | **A** | **T** | **Q** | **Y** | **Y** | **A** | **R** | **Q** | **L** | **I** | **K** | **E** | **N** | **V** | **Q** | **I** | **L** | **V** | **Y** | **E** | **N** | **G** | **F** | **L** | **H** | **A** | **K** | **T** | **L** | **I** | **M** | **D** | **D** | **E** | **I** | **C** | **M** | **V** | **G** | **S** | **A** | **N** | **Q** | **D** | **I** | **R** | **S** | **Y** | **R** | **L** | **N** | **F** | **E** | **T** | **S** | **A** | **V** | **I** | **Y** | **D** | **P** | **E** | **F** | **L** | **E** | **E** | **L** | **A** | **T** | **Q** | **F** | **K** | **E** | **D** | **E** | **T** | **H** | **C** | **S** | **S** | **M** | **T** | **T** | **E** | **T** | **V** | **K** | **E** | **M** | **S** | **N** | **W** | **L** | **L** | **F** | **K** | **Q** | **Q** | **I** | **S** | **R** | **L** | **F** | **S** | **P** | **I** | **L** |
| **N55337** | **-** | **-** | **-** | **-** | **-** | **-** | **-** | **-** | **-** | **-** | **-** | **-** | **-** | **-** | **-** | **-** | **-** | **-** | **-** | **-** | **-** | **-** | **-** | **K** | **P** | **R** | **D** | **V** | **A** | **A** | **I** | **W** | **A** | **W** | **L** | **L** | **V** | **L** | **F** | **V** | **L** | **P** | **G** | **V** | **G** | **F** | **V** | **L** | **Y** | **L** | **F** | **F** | **G** | **R** | **G** | **L** | **T** | **D** | **K** | **K** | **K** | **F** | **Y** | **L** | **R** | **Q** | **S** | **D** | **L** | **K** | **E** | **L** | **E** | **N** | **F** | **Q** | **S** | **F** | **R** | **G** | **D** | **T** | **I** | **E** | **H** | **Y** | **D** | **P** | **D** | **M** | **G** | **D** | **K** | **D** | **K** | **Q** | **Q** | **F** | **V** | **D** | **F** | **F** | **S** | **S** | **L** | **N** | **R** | **M** | **P** | **L** | **T** | **R** | **M** | **N** | **S** | **V** | **T** | **L** | **L** | **T** | **D** | **G** | **Q** | **E** | **K** | **L** | **D** | **S** | **L** | **L** | **Q** | **D** | **L** | **K** | **K** | **A** | **K** | **H** | **S** | **I** | **H** | **I** | **E** | **Y** | **Y** | **A** | **F** | **V** | **T** | **D** | **N** | **I** | **G** | **Q** | **Q** | **V** | **L** | **H** | **V** | **L** | **E** | **E** | **K** | **A** | **A** | **E** | **G** | **V** | **E** | **V** | **R** | **I** | **L** | **Y** | **D** | **A** | **F** | **G** | **S** | **H** | **G** | **T** | **K** | **A** | **K** | **D** | **F** | **N** | **R** | **L** | **I** | **K** | **N** | **G** | **G** | **H** | **V** | **H** | **T** | **F** | **V** | **T** | **S** | **Q** | **K** | **A** | **L** | **L** | **R** | **F** | **R** | **L** | **N** | **Y** | **H** | **D** | **H** | **R** | **K** | **I** | **V** | **V** | **I** | **D** | **G** | **K** | **I** | **S** | **Y** | **T** | **G** | **G** | **F** | **N** | **I** | **A** | **N** | **Q** | **Y** | **V** | **N** | **T** | **T** | **K** | **K** | **F** | **G** | **Y** | **W** | **R** | **D** | **T** | **H** | **I** | **R** | **I** | **F** | **G** | **A** | **A** | **S** | **S** | **L** | **L** | **Q** | **L** | **R** | **F** | **L** | **T** | **D** | **W** | **N** | **V** | **S** | **V** | **P** | **E** | **E** | **K** | **K** | **V** | **G** | **Y** | **H** | **L** | **N** | **Y** | **F** | **F** | **K** | **K** | **A** | **D** | **R** | **D** | **E** | **S** | **K** | **L** | **A** | **D** | **T** | **S** | **I** | **Q** | **L** | **V** | **S** | **S** | **G** | **P** | **N** | **N** | **E** | **R** | **E** | **Q** | **I** | **K** | **L** | **S** | **F** | **I** | **K** | **L** | **I** | **T** | **S** | **A** | **K** | **K** | **R** | **V** | **W** | **I** | **Q** | **T** | **P** | **Y** | **L** | **V** | **P** | **D** | **E** | **S** | **V** | **I** | **A** | **A** | **L** | **K** | **I** | **A** | **T** | **A** | **S** | **G** | **V** | **D** | **V** | **K** | **I** | **M** | **I** | **P** | **N** | **K** | **P** | **D** | **H** | **P** | **F** | **I** | **Y** | **R** | **A** | **T** | **Q** | **Y** | **Y** | **A** | **R** | **Q** | **L** | **I** | **K** | **E** | **N** | **V** | **Q** | **I** | **L** | **V** | **Y** | **E** | **N** | **G** | **F** | **L** | **H** | **A** | **K** | **T** | **L** | **I** | **M** | **D** | **D** | **E** | **I** | **C** | **M** | **V** | **G** | **S** | **A** | **N** | **Q** | **D** | **I** | **R** | **S** | **Y** | **R** | **L** | **N** | **F** | **E** | **T** | **S** | **A** | **V** | **I** | **Y** | **D** | **P** | **E** | **F** | **L** | **E** | **E** | **L** | **A** | **T** | **Q** | **F** | **K** | **E** | **D** | **E** | **T** | **H** | **C** | **S** | **S** | **M** | **T** | **T** | **E** | **T** | **V** | **K** | **E** | **M** | **S** | **N** | **W** | **L** | **L** | **F** | **K** | **Q** | **Q** | **I** | **S** | **R** | **L** | **F** | **S** | **P** | **I** | **L** |
| **N54107** | **-** | **-** | **-** | **-** | **-** | **-** | **-** | **-** | **-** | **-** | **-** | **-** | **-** | **-** | **-** | **-** | **-** | **-** | **-** | **-** | **-** | **-** | **-** | **K** | **P** | **R** | **D** | **V** | **A** | **A** | **I** | **W** | **A** | **W** | **L** | **L** | **V** | **L** | **F** | **A** | **L** | **P** | **G** | **V** | **G** | **F** | **V** | **L** | **Y** | **L** | **F** | **F** | **G** | **R** | **G** | **L** | **T** | **D** | **K** | **K** | **K** | **F** | **Y** | **L** | **R** | **Q** | **S** | **D** | **L** | **K** | **E** | **L** | **E** | **N** | **F** | **Q** | **S** | **F** | **R** | **G** | **D** | **N** | **I** | **E** | **H** | **Y** | **D** | **P** | **D** | **M** | **G** | **D** | **K** | **D** | **K** | **Q** | **Q** | **F** | **V** | **D** | **F** | **F** | **S** | **S** | **L** | **N** | **R** | **M** | **P** | **L** | **T** | **R** | **M** | **N** | **S** | **V** | **T** | **L** | **L** | **T** | **D** | **G** | **Q** | **E** | **K** | **L** | **D** | **S** | **L** | **L** | **Q** | **D** | **L** | **K** | **K** | **A** | **K** | **H** | **S** | **I** | **H** | **I** | **E** | **Y** | **Y** | **A** | **F** | **V** | **T** | **D** | **N** | **I** | **G** | **Q** | **Q** | **V** | **L** | **H** | **V** | **L** | **E** | **E** | **K** | **A** | **A** | **E** | **G** | **V** | **E** | **V** | **R** | **I** | **L** | **Y** | **D** | **A** | **F** | **G** | **S** | **H** | **G** | **T** | **K** | **A** | **K** | **D** | **F** | **N** | **R** | **L** | **I** | **K** | **N** | **G** | **G** | **H** | **V** | **H** | **T** | **F** | **V** | **T** | **S** | **Q** | **K** | **A** | **L** | **L** | **R** | **F** | **R** | **L** | **N** | **Y** | **H** | **D** | **H** | **R** | **K** | **I** | **V** | **V** | **I** | **D** | **G** | **K** | **I** | **S** | **Y** | **T** | **G** | **G** | **F** | **N** | **I** | **A** | **N** | **Q** | **Y** | **V** | **N** | **T** | **T** | **K** | **K** | **F** | **G** | **Y** | **W** | **R** | **D** | **T** | **H** | **I** | **R** | **I** | **F** | **G** | **A** | **A** | **S** | **S** | **L** | **L** | **Q** | **L** | **R** | **F** | **L** | **T** | **D** | **W** | **N** | **V** | **S** | **V** | **P** | **E** | **E** | **K** | **K** | **V** | **G** | **Y** | **H** | **L** | **N** | **Y** | **F** | **F** | **K** | **K** | **A** | **D** | **R** | **D** | **E** | **S** | **K** | **L** | **A** | **D** | **T** | **S** | **I** | **Q** | **L** | **V** | **S** | **S** | **G** | **P** | **N** | **N** | **E** | **R** | **E** | **Q** | **I** | **K** | **L** | **S** | **F** | **I** | **K** | **L** | **I** | **T** | **S** | **A** | **K** | **K** | **R** | **V** | **W** | **I** | **Q** | **T** | **P** | **Y** | **L** | **V** | **P** | **D** | **E** | **S** | **V** | **I** | **A** | **A** | **L** | **K** | **I** | **A** | **T** | **A** | **S** | **G** | **V** | **D** | **V** | **K** | **I** | **M** | **I** | **P** | **N** | **K** | **P** | **D** | **H** | **P** | **F** | **I** | **Y** | **R** | **A** | **T** | **Q** | **Y** | **Y** | **A** | **R** | **Q** | **L** | **I** | **K** | **E** | **N** | **V** | **Q** | **I** | **L** | **V** | **Y** | **E** | **N** | **G** | **F** | **L** | **H** | **A** | **K** | **T** | **L** | **I** | **M** | **D** | **D** | **E** | **I** | **C** | **M** | **V** | **G** | **S** | **A** | **N** | **Q** | **D** | **I** | **R** | **S** | **Y** | **R** | **L** | **N** | **F** | **E** | **T** | **S** | **A** | **V** | **I** | **Y** | **D** | **P** | **E** | **F** | **L** | **E** | **E** | **L** | **A** | **T** | **Q** | **F** | **K** | **E** | **D** | **E** | **T** | **H** | **C** | **S** | **S** | **M** | **T** | **T** | **E** | **T** | **V** | **K** | **E** | **M** | **S** | **N** | **W** | **L** | **L** | **F** | **K** | **Q** | **Q** | **I** | **S** | **R** | **L** | **F** | **S** | **P** | **I** | **L** |
| **N59492F** | **-** | **-** | **-** | **-** | **-** | **-** | **-** | **-** | **-** | **-** | **-** | **-** | **-** | **-** | **-** | **-** | **-** | **-** | **-** | **-** | **-** | **-** | **-** | **K** | **P** | **R** | **D** | **V** | **A** | **A** | **I** | **W** | **A** | **W** | **L** | **L** | **V** | **L** | **F** | **A** | **L** | **P** | **G** | **V** | **G** | **F** | **V** | **L** | **Y** | **L** | **F** | **F** | **G** | **R** | **G** | **L** | **T** | **D** | **K** | **K** | **K** | **F** | **Y** | **L** | **R** | **Q** | **S** | **D** | **L** | **K** | **E** | **L** | **E** | **N** | **F** | **Q** | **S** | **F** | **R** | **G** | **D** | **T** | **I** | **E** | **H** | **Y** | **D** | **P** | **D** | **M** | **G** | **D** | **K** | **D** | **K** | **Q** | **Q** | **F** | **V** | **D** | **F** | **F** | **S** | **S** | **L** | **N** | **R** | **M** | **P** | **L** | **T** | **R** | **M** | **N** | **S** | **V** | **T** | **L** | **L** | **T** | **D** | **G** | **Q** | **E** | **K** | **L** | **D** | **S** | **L** | **L** | **Q** | **D** | **L** | **K** | **K** | **A** | **K** | **H** | **S** | **I** | **H** | **I** | **E** | **Y** | **Y** | **A** | **F** | **V** | **T** | **D** | **N** | **I** | **G** | **Q** | **Q** | **V** | **L** | **H** | **V** | **L** | **E** | **E** | **K** | **A** | **A** | **E** | **G** | **V** | **E** | **V** | **R** | **I** | **L** | **Y** | **D** | **A** | **F** | **G** | **S** | **H** | **G** | **T** | **K** | **A** | **K** | **D** | **F** | **N** | **R** | **L** | **I** | **K** | **N** | **G** | **G** | **H** | **V** | **H** | **T** | **F** | **V** | **T** | **S** | **Q** | **K** | **A** | **L** | **L** | **R** | **F** | **R** | **L** | **N** | **Y** | **H** | **D** | **H** | **R** | **K** | **I** | **V** | **V** | **I** | **D** | **G** | **K** | **I** | **S** | **Y** | **T** | **G** | **G** | **F** | **N** | **I** | **A** | **N** | **Q** | **Y** | **V** | **N** | **T** | **T** | **K** | **K** | **F** | **G** | **Y** | **W** | **R** | **D** | **T** | **H** | **I** | **R** | **I** | **F** | **G** | **A** | **A** | **S** | **S** | **L** | **L** | **Q** | **L** | **R** | **F** | **L** | **T** | **D** | **W** | **N** | **V** | **S** | **V** | **P** | **E** | **E** | **K** | **K** | **V** | **G** | **Y** | **H** | **L** | **N** | **Y** | **F** | **F** | **K** | **K** | **A** | **D** | **R** | **D** | **E** | **S** | **K** | **L** | **A** | **D** | **T** | **S** | **I** | **Q** | **L** | **V** | **S** | **S** | **G** | **P** | **N** | **N** | **E** | **R** | **E** | **Q** | **I** | **K** | **L** | **S** | **F** | **I** | **K** | **L** | **I** | **T** | **S** | **A** | **K** | **K** | **R** | **V** | **W** | **I** | **Q** | **T** | **P** | **Y** | **L** | **V** | **P** | **D** | **E** | **S** | **V** | **I** | **A** | **A** | **L** | **K** | **I** | **A** | **T** | **A** | **S** | **G** | **V** | **D** | **V** | **K** | **I** | **M** | **I** | **P** | **N** | **K** | **P** | **D** | **H** | **P** | **F** | **I** | **Y** | **R** | **A** | **T** | **Q** | **Y** | **Y** | **A** | **R** | **Q** | **L** | **I** | **K** | **E** | **N** | **V** | **Q** | **I** | **L** | **V** | **Y** | **E** | **N** | **G** | **F** | **L** | **H** | **A** | **K** | **T** | **L** | **I** | **M** | **D** | **D** | **E** | **I** | **C** | **M** | **V** | **G** | **S** | **A** | **N** | **Q** | **D** | **I** | **R** | **S** | **Y** | **R** | **L** | **N** | **F** | **E** | **T** | **S** | **A** | **V** | **I** | **Y** | **D** | **P** | **E** | **F** | **L** | **E** | **E** | **L** | **A** | **T** | **Q** | **F** | **K** | **E** | **D** | **E** | **T** | **H** | **C** | **S** | **S** | **M** | **T** | **T** | **E** | **T** | **V** | **K** | **E** | **M** | **S** | **N** | **W** | **L** | **L** | **F** | **K** | **Q** | **Q** | **I** | **S** | **R** | **L** | **F** | **S** | **P** | **I** | **L** |
| **N59439F** | **-** | **-** | **-** | **-** | **-** | **-** | **-** | **-** | **-** | **-** | **-** | **-** | **-** | **-** | **-** | **-** | **-** | **-** | **-** | **-** | **-** | **-** | **-** | **K** | **P** | **R** | **D** | **V** | **A** | **A** | **I** | **W** | **A** | **W** | **L** | **L** | **V** | **L** | **F** | **A** | **L** | **P** | **G** | **V** | **G** | **F** | **V** | **L** | **Y** | **L** | **F** | **F** | **G** | **R** | **G** | **L** | **T** | **D** | **K** | **K** | **K** | **F** | **Y** | **L** | **R** | **Q** | **S** | **D** | **L** | **K** | **E** | **L** | **E** | **N** | **F** | **Q** | **S** | **F** | **R** | **G** | **D** | **T** | **I** | **E** | **H** | **Y** | **D** | **P** | **D** | **M** | **G** | **D** | **K** | **D** | **K** | **Q** | **Q** | **F** | **V** | **D** | **F** | **F** | **S** | **S** | **L** | **N** | **R** | **M** | **P** | **L** | **T** | **R** | **M** | **N** | **S** | **V** | **T** | **L** | **L** | **T** | **D** | **G** | **Q** | **E** | **K** | **L** | **D** | **S** | **L** | **L** | **Q** | **D** | **L** | **K** | **K** | **A** | **K** | **H** | **S** | **I** | **H** | **I** | **E** | **Y** | **Y** | **A** | **F** | **V** | **T** | **D** | **N** | **I** | **G** | **Q** | **Q** | **V** | **L** | **H** | **V** | **L** | **E** | **E** | **K** | **A** | **A** | **E** | **G** | **V** | **E** | **V** | **R** | **I** | **L** | **Y** | **D** | **A** | **F** | **G** | **S** | **H** | **G** | **T** | **K** | **A** | **K** | **D** | **F** | **N** | **R** | **L** | **I** | **K** | **N** | **G** | **G** | **H** | **V** | **H** | **T** | **F** | **V** | **T** | **S** | **Q** | **K** | **A** | **L** | **L** | **R** | **F** | **R** | **L** | **N** | **Y** | **H** | **D** | **H** | **R** | **K** | **I** | **V** | **V** | **I** | **D** | **G** | **K** | **I** | **S** | **Y** | **T** | **G** | **G** | **F** | **N** | **I** | **A** | **N** | **Q** | **Y** | **V** | **N** | **T** | **T** | **K** | **K** | **F** | **G** | **Y** | **W** | **R** | **D** | **T** | **H** | **I** | **R** | **I** | **F** | **G** | **A** | **A** | **S** | **S** | **L** | **L** | **Q** | **L** | **R** | **F** | **L** | **T** | **D** | **W** | **N** | **V** | **S** | **V** | **P** | **E** | **E** | **K** | **K** | **V** | **G** | **Y** | **H** | **L** | **N** | **Y** | **F** | **F** | **K** | **K** | **A** | **D** | **R** | **D** | **E** | **S** | **K** | **L** | **A** | **D** | **T** | **S** | **I** | **Q** | **L** | **V** | **S** | **S** | **G** | **P** | **N** | **N** | **E** | **R** | **E** | **Q** | **I** | **K** | **L** | **S** | **F** | **I** | **K** | **L** | **I** | **T** | **S** | **A** | **K** | **K** | **R** | **V** | **W** | **I** | **Q** | **T** | **P** | **Y** | **L** | **V** | **P** | **D** | **E** | **S** | **V** | **I** | **A** | **A** | **L** | **K** | **I** | **A** | **T** | **A** | **S** | **G** | **V** | **D** | **V** | **K** | **I** | **M** | **I** | **P** | **N** | **K** | **P** | **D** | **H** | **P** | **F** | **I** | **Y** | **R** | **A** | **T** | **Q** | **Y** | **Y** | **A** | **R** | **Q** | **L** | **I** | **K** | **E** | **N** | **V** | **Q** | **I** | **L** | **V** | **Y** | **E** | **N** | **G** | **F** | **L** | **H** | **A** | **K** | **T** | **L** | **I** | **M** | **D** | **D** | **E** | **I** | **C** | **M** | **V** | **G** | **S** | **A** | **N** | **Q** | **D** | **I** | **R** | **S** | **Y** | **R** | **L** | **N** | **F** | **E** | **T** | **S** | **A** | **V** | **I** | **Y** | **D** | **P** | **E** | **F** | **L** | **E** | **E** | **L** | **A** | **T** | **Q** | **F** | **K** | **E** | **D** | **E** | **T** | **H** | **C** | **S** | **S** | **M** | **T** | **T** | **E** | **T** | **V** | **K** | **E** | **M** | **S** | **N** | **W** | **L** | **L** | **F** | **K** | **Q** | **Q** | **I** | **S** | **R** | **L** | **F** | **S** | **P** | **I** | **L** |
| **N59788F** | **-** | **-** | **-** | **-** | **-** | **-** | **-** | **-** | **-** | **-** | **-** | **-** | **-** | **-** | **-** | **-** | **-** | **-** | **-** | **-** | **-** | **-** | **-** | **K** | **P** | **R** | **D** | **V** | **A** | **A** | **I** | **W** | **A** | **W** | **L** | **L** | **V** | **L** | **F** | **A** | **L** | **P** | **G** | **V** | **G** | **F** | **V** | **L** | **Y** | **L** | **F** | **F** | **G** | **R** | **G** | **L** | **T** | **D** | **K** | **K** | **K** | **F** | **Y** | **L** | **R** | **Q** | **S** | **D** | **L** | **K** | **E** | **L** | **E** | **N** | **F** | **Q** | **S** | **F** | **R** | **G** | **D** | **T** | **I** | **E** | **H** | **Y** | **D** | **P** | **D** | **M** | **G** | **D** | **K** | **D** | **K** | **Q** | **Q** | **F** | **V** | **D** | **F** | **F** | **S** | **S** | **L** | **N** | **R** | **M** | **P** | **L** | **T** | **R** | **M** | **N** | **S** | **V** | **T** | **L** | **L** | **T** | **D** | **G** | **Q** | **E** | **K** | **L** | **D** | **S** | **L** | **L** | **Q** | **D** | **L** | **K** | **K** | **A** | **K** | **H** | **S** | **I** | **H** | **I** | **E** | **Y** | **Y** | **A** | **F** | **V** | **T** | **D** | **N** | **I** | **G** | **Q** | **Q** | **V** | **L** | **H** | **V** | **L** | **E** | **E** | **K** | **A** | **A** | **E** | **G** | **V** | **E** | **V** | **R** | **I** | **L** | **Y** | **D** | **A** | **F** | **G** | **S** | **H** | **G** | **T** | **K** | **A** | **K** | **D** | **F** | **N** | **R** | **L** | **I** | **K** | **N** | **G** | **G** | **H** | **V** | **H** | **T** | **F** | **V** | **T** | **S** | **Q** | **K** | **A** | **L** | **L** | **R** | **F** | **R** | **L** | **N** | **Y** | **H** | **D** | **H** | **R** | **K** | **I** | **V** | **V** | **I** | **D** | **G** | **K** | **I** | **S** | **Y** | **T** | **G** | **G** | **F** | **N** | **I** | **A** | **N** | **Q** | **Y** | **V** | **N** | **T** | **T** | **K** | **K** | **F** | **G** | **Y** | **W** | **R** | **D** | **T** | **H** | **I** | **R** | **I** | **F** | **G** | **A** | **A** | **S** | **S** | **L** | **L** | **Q** | **L** | **R** | **F** | **L** | **T** | **D** | **W** | **N** | **V** | **S** | **V** | **P** | **E** | **E** | **K** | **K** | **V** | **G** | **Y** | **H** | **L** | **N** | **Y** | **F** | **F** | **K** | **K** | **A** | **D** | **R** | **D** | **E** | **S** | **K** | **L** | **A** | **D** | **T** | **S** | **I** | **Q** | **L** | **V** | **S** | **S** | **G** | **P** | **N** | **N** | **E** | **R** | **E** | **Q** | **I** | **K** | **L** | **S** | **F** | **I** | **K** | **L** | **I** | **T** | **S** | **A** | **K** | **K** | **R** | **V** | **W** | **I** | **Q** | **T** | **P** | **Y** | **L** | **V** | **P** | **D** | **E** | **S** | **V** | **I** | **A** | **A** | **L** | **K** | **I** | **A** | **T** | **A** | **S** | **G** | **V** | **D** | **V** | **K** | **I** | **M** | **I** | **P** | **N** | **K** | **P** | **D** | **H** | **P** | **F** | **I** | **Y** | **R** | **A** | **T** | **Q** | **Y** | **Y** | **A** | **R** | **Q** | **L** | **I** | **K** | **E** | **N** | **V** | **Q** | **I** | **L** | **V** | **Y** | **E** | **N** | **G** | **F** | **L** | **H** | **A** | **K** | **T** | **L** | **I** | **M** | **D** | **D** | **E** | **I** | **C** | **M** | **V** | **G** | **S** | **A** | **N** | **Q** | **D** | **I** | **R** | **S** | **Y** | **R** | **L** | **N** | **F** | **E** | **T** | **S** | **A** | **V** | **I** | **Y** | **D** | **P** | **E** | **F** | **L** | **E** | **E** | **L** | **A** | **T** | **Q** | **F** | **K** | **E** | **D** | **E** | **T** | **H** | **C** | **S** | **S** | **M** | **T** | **T** | **E** | **T** | **V** | **K** | **E** | **M** | **S** | **N** | **W** | **L** | **L** | **F** | **K** | **Q** | **Q** | **I** | **S** | **R** | **L** | **F** | **S** | **P** | **I** | **L** |
| **N52629** | **-** | **-** | **-** | **-** | **-** | **-** | **-** | **-** | **-** | **-** | **-** | **-** | **-** | **-** | **-** | **-** | **-** | **-** | **-** | **-** | **-** | **-** | **-** | **K** | **P** | **R** | **D** | **V** | **A** | **A** | **I** | **W** | **A** | **W** | **L** | **L** | **V** | **L** | **F** | **A** | **L** | **P** | **G** | **V** | **G** | **F** | **V** | **L** | **Y** | **L** | **F** | **F** | **G** | **R** | **G** | **L** | **T** | **D** | **K** | **K** | **K** | **F** | **Y** | **L** | **R** | **Q** | **S** | **D** | **L** | **K** | **E** | **L** | **E** | **N** | **F** | **Q** | **S** | **F** | **R** | **G** | **D** | **T** | **I** | **E** | **H** | **Y** | **D** | **P** | **D** | **M** | **G** | **D** | **K** | **D** | **K** | **Q** | **Q** | **F** | **V** | **D** | **F** | **F** | **S** | **S** | **L** | **N** | **R** | **M** | **P** | **L** | **T** | **R** | **M** | **N** | **S** | **V** | **T** | **L** | **L** | **T** | **D** | **G** | **Q** | **E** | **K** | **L** | **D** | **S** | **L** | **L** | **Q** | **D** | **L** | **K** | **K** | **A** | **K** | **H** | **S** | **I** | **H** | **I** | **E** | **Y** | **Y** | **A** | **F** | **V** | **T** | **D** | **N** | **I** | **G** | **Q** | **Q** | **V** | **L** | **H** | **V** | **L** | **E** | **E** | **K** | **A** | **A** | **E** | **G** | **V** | **E** | **V** | **R** | **I** | **L** | **Y** | **D** | **A** | **F** | **G** | **S** | **H** | **G** | **T** | **K** | **A** | **K** | **D** | **F** | **N** | **R** | **L** | **I** | **K** | **N** | **G** | **G** | **H** | **V** | **H** | **T** | **F** | **V** | **T** | **S** | **Q** | **K** | **A** | **L** | **L** | **R** | **F** | **R** | **L** | **N** | **Y** | **H** | **D** | **H** | **R** | **K** | **I** | **V** | **V** | **I** | **D** | **G** | **K** | **I** | **S** | **Y** | **T** | **G** | **G** | **F** | **N** | **I** | **A** | **N** | **Q** | **Y** | **V** | **N** | **T** | **T** | **K** | **K** | **F** | **G** | **Y** | **W** | **R** | **D** | **T** | **H** | **I** | **R** | **I** | **F** | **G** | **A** | **A** | **S** | **S** | **L** | **L** | **Q** | **L** | **R** | **F** | **L** | **T** | **D** | **W** | **N** | **V** | **S** | **V** | **P** | **E** | **E** | **K** | **K** | **V** | **G** | **Y** | **H** | **L** | **N** | **Y** | **F** | **F** | **K** | **K** | **A** | **D** | **R** | **D** | **E** | **S** | **K** | **L** | **A** | **D** | **T** | **S** | **I** | **Q** | **L** | **V** | **S** | **S** | **G** | **P** | **N** | **N** | **E** | **R** | **E** | **Q** | **I** | **K** | **L** | **S** | **F** | **I** | **K** | **L** | **I** | **T** | **S** | **A** | **K** | **K** | **R** | **V** | **W** | **I** | **Q** | **T** | **P** | **Y** | **L** | **V** | **P** | **D** | **E** | **S** | **V** | **I** | **A** | **A** | **L** | **K** | **I** | **A** | **T** | **A** | **S** | **G** | **V** | **D** | **V** | **K** | **I** | **M** | **I** | **P** | **N** | **K** | **P** | **D** | **H** | **P** | **F** | **I** | **Y** | **R** | **A** | **T** | **Q** | **Y** | **Y** | **A** | **R** | **Q** | **L** | **I** | **K** | **E** | **N** | **V** | **K** | **I** | **L** | **V** | **Y** | **E** | **N** | **G** | **F** | **L** | **H** | **A** | **K** | **T** | **L** | **I** | **M** | **D** | **D** | **E** | **I** | **C** | **M** | **V** | **G** | **S** | **A** | **N** | **Q** | **D** | **I** | **R** | **S** | **Y** | **R** | **L** | **N** | **F** | **E** | **T** | **S** | **A** | **V** | **I** | **Y** | **D** | **P** | **E** | **F** | **L** | **E** | **E** | **L** | **A** | **T** | **Q** | **F** | **K** | **E** | **D** | **E** | **T** | **H** | **C** | **S** | **S** | **M** | **T** | **T** | **E** | **T** | **V** | **K** | **E** | **M** | **S** | **N** | **W** | **L** | **L** | **F** | **K** | **Q** | **Q** | **I** | **S** | **R** | **L** | **F** | **S** | **P** | **I** | **L** |
| **N52829** | **-** | **-** | **-** | **-** | **-** | **-** | **-** | **-** | **-** | **-** | **-** | **-** | **-** | **-** | **-** | **-** | **-** | **-** | **-** | **-** | **-** | **-** | **-** | **K** | **P** | **R** | **D** | **V** | **A** | **A** | **I** | **W** | **A** | **W** | **L** | **L** | **V** | **L** | **F** | **A** | **L** | **P** | **G** | **V** | **G** | **F** | **V** | **L** | **Y** | **L** | **F** | **F** | **G** | **R** | **G** | **L** | **T** | **D** | **K** | **K** | **K** | **F** | **Y** | **L** | **R** | **Q** | **S** | **D** | **L** | **K** | **E** | **L** | **E** | **N** | **F** | **Q** | **S** | **F** | **R** | **G** | **D** | **T** | **I** | **E** | **H** | **Y** | **D** | **P** | **D** | **M** | **G** | **D** | **K** | **D** | **K** | **Q** | **Q** | **F** | **V** | **D** | **F** | **F** | **S** | **S** | **L** | **N** | **R** | **M** | **P** | **L** | **A** | **R** | **M** | **N** | **S** | **V** | **T** | **L** | **L** | **T** | **D** | **G** | **Q** | **E** | **K** | **L** | **D** | **S** | **L** | **L** | **Q** | **D** | **L** | **K** | **K** | **A** | **K** | **H** | **S** | **I** | **H** | **I** | **E** | **Y** | **Y** | **A** | **F** | **V** | **T** | **D** | **N** | **I** | **G** | **Q** | **Q** | **V** | **L** | **H** | **V** | **L** | **E** | **E** | **K** | **A** | **A** | **E** | **G** | **V** | **E** | **V** | **R** | **I** | **L** | **Y** | **D** | **A** | **F** | **G** | **S** | **H** | **G** | **T** | **K** | **A** | **K** | **D** | **F** | **N** | **R** | **L** | **I** | **K** | **N** | **G** | **G** | **H** | **V** | **H** | **T** | **F** | **V** | **T** | **S** | **Q** | **K** | **A** | **L** | **L** | **R** | **F** | **R** | **L** | **N** | **Y** | **H** | **D** | **H** | **R** | **K** | **I** | **V** | **V** | **I** | **D** | **G** | **K** | **I** | **S** | **Y** | **T** | **G** | **G** | **F** | **N** | **I** | **A** | **N** | **Q** | **Y** | **V** | **N** | **T** | **T** | **K** | **K** | **F** | **G** | **Y** | **W** | **R** | **D** | **T** | **H** | **I** | **R** | **I** | **F** | **G** | **A** | **A** | **S** | **S** | **L** | **L** | **Q** | **L** | **R** | **F** | **L** | **T** | **D** | **W** | **N** | **V** | **S** | **V** | **P** | **E** | **E** | **K** | **K** | **V** | **G** | **Y** | **H** | **L** | **N** | **Y** | **F** | **F** | **K** | **K** | **A** | **D** | **R** | **D** | **E** | **S** | **K** | **L** | **A** | **D** | **T** | **S** | **I** | **Q** | **L** | **V** | **S** | **S** | **G** | **P** | **N** | **N** | **E** | **R** | **E** | **Q** | **I** | **K** | **L** | **S** | **F** | **I** | **K** | **L** | **I** | **T** | **S** | **A** | **K** | **K** | **R** | **V** | **W** | **I** | **Q** | **T** | **P** | **Y** | **L** | **V** | **P** | **D** | **E** | **S** | **V** | **I** | **A** | **A** | **L** | **K** | **I** | **A** | **T** | **A** | **S** | **G** | **V** | **D** | **V** | **K** | **I** | **M** | **I** | **P** | **N** | **K** | **P** | **D** | **H** | **P** | **F** | **I** | **Y** | **R** | **A** | **T** | **Q** | **Y** | **Y** | **A** | **R** | **Q** | **L** | **I** | **K** | **E** | **N** | **V** | **Q** | **I** | **L** | **V** | **Y** | **E** | **N** | **G** | **F** | **L** | **H** | **A** | **K** | **T** | **L** | **I** | **M** | **D** | **D** | **E** | **I** | **C** | **M** | **V** | **G** | **S** | **A** | **N** | **Q** | **D** | **I** | **R** | **S** | **Y** | **R** | **L** | **N** | **F** | **E** | **T** | **S** | **A** | **V** | **I** | **Y** | **D** | **P** | **E** | **F** | **L** | **E** | **E** | **L** | **A** | **T** | **Q** | **F** | **K** | **E** | **D** | **E** | **T** | **H** | **C** | **S** | **S** | **M** | **T** | **T** | **E** | **T** | **V** | **K** | **E** | **M** | **S** | **N** | **W** | **L** | **L** | **F** | **K** | **Q** | **Q** | **I** | **S** | **R** | **L** | **F** | **S** | **P** | **I** | **L** |
| **N59754F** | **-** | **-** | **-** | **-** | **-** | **-** | **-** | **-** | **-** | **-** | **-** | **-** | **-** | **-** | **-** | **-** | **-** | **-** | **-** | **-** | **-** | **-** | **-** | **K** | **P** | **R** | **D** | **V** | **A** | **A** | **I** | **W** | **A** | **W** | **L** | **L** | **V** | **L** | **F** | **A** | **L** | **P** | **G** | **V** | **G** | **F** | **V** | **L** | **Y** | **L** | **F** | **F** | **G** | **R** | **G** | **L** | **T** | **D** | **K** | **K** | **K** | **F** | **Y** | **L** | **Q** | **Q** | **S** | **D** | **L** | **K** | **E** | **L** | **E** | **N** | **F** | **Q** | **S** | **F** | **R** | **G** | **D** | **T** | **I** | **E** | **H** | **Y** | **D** | **P** | **D** | **M** | **G** | **D** | **K** | **D** | **K** | **Q** | **Q** | **F** | **V** | **D** | **F** | **F** | **S** | **S** | **L** | **N** | **R** | **M** | **P** | **L** | **T** | **R** | **M** | **N** | **S** | **V** | **T** | **L** | **L** | **T** | **D** | **G** | **Q** | **E** | **K** | **L** | **D** | **S** | **L** | **L** | **Q** | **D** | **L** | **K** | **K** | **A** | **K** | **H** | **S** | **I** | **H** | **I** | **E** | **Y** | **Y** | **A** | **F** | **V** | **T** | **D** | **N** | **I** | **G** | **Q** | **Q** | **V** | **L** | **H** | **V** | **L** | **E** | **E** | **K** | **A** | **A** | **E** | **G** | **V** | **E** | **V** | **R** | **I** | **L** | **Y** | **D** | **A** | **F** | **G** | **S** | **H** | **G** | **T** | **K** | **A** | **K** | **D** | **F** | **N** | **R** | **L** | **I** | **K** | **N** | **G** | **G** | **H** | **V** | **H** | **T** | **F** | **V** | **T** | **S** | **Q** | **K** | **A** | **L** | **L** | **R** | **F** | **R** | **L** | **N** | **Y** | **H** | **D** | **H** | **R** | **K** | **I** | **V** | **V** | **I** | **D** | **G** | **K** | **I** | **S** | **Y** | **T** | **G** | **G** | **F** | **N** | **I** | **A** | **N** | **Q** | **Y** | **V** | **N** | **T** | **T** | **K** | **K** | **F** | **G** | **Y** | **W** | **R** | **D** | **T** | **H** | **I** | **R** | **I** | **F** | **G** | **A** | **A** | **S** | **S** | **L** | **L** | **Q** | **L** | **R** | **F** | **L** | **T** | **D** | **W** | **N** | **V** | **S** | **V** | **P** | **E** | **E** | **K** | **K** | **V** | **G** | **Y** | **H** | **L** | **N** | **Y** | **F** | **F** | **K** | **K** | **A** | **D** | **R** | **D** | **E** | **S** | **K** | **L** | **A** | **D** | **T** | **S** | **I** | **Q** | **L** | **V** | **S** | **S** | **G** | **P** | **N** | **N** | **E** | **R** | **E** | **Q** | **I** | **K** | **L** | **S** | **F** | **I** | **K** | **L** | **I** | **T** | **S** | **A** | **K** | **K** | **R** | **V** | **W** | **I** | **Q** | **T** | **P** | **Y** | **L** | **V** | **P** | **D** | **E** | **S** | **V** | **I** | **A** | **A** | **L** | **K** | **I** | **A** | **T** | **A** | **S** | **G** | **V** | **D** | **V** | **K** | **I** | **M** | **I** | **P** | **N** | **K** | **P** | **D** | **H** | **P** | **F** | **I** | **Y** | **R** | **A** | **T** | **Q** | **Y** | **Y** | **A** | **R** | **Q** | **L** | **I** | **K** | **E** | **N** | **V** | **Q** | **I** | **L** | **V** | **Y** | **E** | **N** | **G** | **F** | **L** | **H** | **A** | **K** | **T** | **L** | **I** | **M** | **D** | **D** | **E** | **I** | **C** | **M** | **V** | **G** | **S** | **A** | **N** | **Q** | **D** | **I** | **R** | **S** | **Y** | **R** | **L** | **N** | **F** | **E** | **T** | **S** | **A** | **V** | **I** | **Y** | **D** | **P** | **E** | **F** | **L** | **E** | **E** | **L** | **A** | **T** | **Q** | **F** | **K** | **E** | **D** | **E** | **T** | **H** | **C** | **S** | **S** | **M** | **T** | **T** | **E** | **T** | **V** | **K** | **E** | **M** | **S** | **N** | **W** | **L** | **L** | **F** | **K** | **Q** | **Q** | **I** | **S** | **R** | **L** | **F** | **S** | **P** | **I** | **L** |
| **N60449F** | **-** | **-** | **-** | **-** | **-** | **-** | **-** | **-** | **-** | **-** | **-** | **-** | **-** | **-** | **-** | **-** | **-** | **-** | **-** | **-** | **-** | **-** | **-** | **K** | **P** | **R** | **D** | **V** | **A** | **A** | **I** | **W** | **A** | **W** | **L** | **L** | **V** | **L** | **F** | **A** | **L** | **P** | **G** | **V** | **G** | **F** | **V** | **L** | **Y** | **L** | **F** | **F** | **G** | **R** | **G** | **L** | **T** | **D** | **K** | **K** | **K** | **F** | **Y** | **L** | **R** | **Q** | **S** | **D** | **L** | **K** | **E** | **L** | **E** | **N** | **F** | **Q** | **S** | **F** | **R** | **G** | **D** | **T** | **I** | **E** | **H** | **Y** | **D** | **P** | **D** | **M** | **G** | **D** | **K** | **D** | **K** | **Q** | **Q** | **F** | **V** | **D** | **F** | **F** | **S** | **S** | **L** | **N** | **R** | **M** | **P** | **L** | **T** | **R** | **M** | **N** | **S** | **V** | **T** | **L** | **L** | **T** | **D** | **G** | **Q** | **E** | **K** | **L** | **D** | **S** | **L** | **L** | **Q** | **D** | **L** | **K** | **K** | **A** | **K** | **H** | **S** | **I** | **H** | **I** | **E** | **Y** | **Y** | **A** | **F** | **V** | **T** | **D** | **N** | **I** | **G** | **Q** | **Q** | **V** | **L** | **H** | **V** | **L** | **E** | **E** | **K** | **A** | **A** | **E** | **G** | **V** | **E** | **V** | **R** | **I** | **L** | **Y** | **D** | **A** | **F** | **G** | **S** | **H** | **G** | **T** | **K** | **A** | **K** | **D** | **F** | **N** | **R** | **L** | **I** | **K** | **N** | **G** | **G** | **H** | **V** | **H** | **T** | **F** | **V** | **T** | **S** | **Q** | **R** | **A** | **L** | **L** | **R** | **F** | **R** | **L** | **N** | **Y** | **H** | **D** | **H** | **R** | **K** | **I** | **V** | **V** | **I** | **D** | **G** | **K** | **I** | **S** | **Y** | **T** | **G** | **G** | **F** | **N** | **I** | **A** | **N** | **Q** | **Y** | **V** | **N** | **T** | **T** | **K** | **K** | **F** | **G** | **Y** | **W** | **R** | **D** | **T** | **H** | **I** | **R** | **I** | **F** | **G** | **A** | **A** | **S** | **S** | **L** | **L** | **Q** | **L** | **R** | **F** | **L** | **T** | **D** | **W** | **N** | **V** | **S** | **V** | **P** | **E** | **E** | **K** | **K** | **V** | **G** | **Y** | **H** | **L** | **N** | **Y** | **F** | **F** | **K** | **K** | **A** | **D** | **R** | **D** | **E** | **S** | **K** | **L** | **A** | **D** | **T** | **S** | **I** | **Q** | **L** | **V** | **S** | **S** | **G** | **P** | **N** | **N** | **E** | **R** | **E** | **Q** | **I** | **K** | **L** | **S** | **F** | **I** | **K** | **L** | **I** | **T** | **S** | **A** | **K** | **K** | **R** | **V** | **W** | **I** | **Q** | **T** | **P** | **Y** | **L** | **V** | **P** | **D** | **E** | **S** | **V** | **I** | **A** | **A** | **L** | **K** | **I** | **A** | **T** | **A** | **S** | **G** | **V** | **D** | **V** | **K** | **I** | **M** | **I** | **P** | **N** | **K** | **P** | **D** | **H** | **P** | **F** | **I** | **Y** | **R** | **A** | **T** | **Q** | **Y** | **Y** | **A** | **R** | **Q** | **L** | **I** | **K** | **E** | **N** | **V** | **Q** | **I** | **L** | **V** | **Y** | **E** | **N** | **G** | **F** | **L** | **H** | **A** | **K** | **T** | **L** | **I** | **M** | **D** | **D** | **E** | **I** | **C** | **M** | **V** | **G** | **S** | **A** | **N** | **Q** | **D** | **I** | **R** | **S** | **Y** | **R** | **L** | **N** | **F** | **E** | **T** | **S** | **A** | **V** | **I** | **Y** | **D** | **P** | **E** | **F** | **L** | **E** | **E** | **L** | **A** | **T** | **Q** | **F** | **K** | **E** | **D** | **E** | **T** | **H** | **C** | **S** | **S** | **M** | **T** | **T** | **E** | **T** | **V** | **K** | **E** | **M** | **S** | **N** | **W** | **L** | **L** | **F** | **K** | **Q** | **Q** | **I** | **S** | **R** | **L** | **F** | **S** | **P** | **I** | **L** |
| **N60519F** | **-** | **-** | **-** | **-** | **-** | **-** | **-** | **-** | **-** | **-** | **-** | **-** | **-** | **-** | **-** | **-** | **-** | **-** | **-** | **-** | **-** | **-** | **-** | **K** | **P** | **R** | **D** | **V** | **A** | **A** | **I** | **W** | **A** | **W** | **L** | **L** | **V** | **L** | **F** | **A** | **L** | **P** | **G** | **V** | **G** | **F** | **V** | **L** | **Y** | **L** | **F** | **F** | **G** | **R** | **G** | **L** | **T** | **D** | **K** | **K** | **K** | **F** | **Y** | **L** | **R** | **Q** | **S** | **D** | **L** | **K** | **E** | **L** | **E** | **N** | **F** | **Q** | **S** | **F** | **R** | **G** | **D** | **T** | **I** | **E** | **H** | **Y** | **D** | **P** | **D** | **M** | **G** | **D** | **K** | **D** | **K** | **Q** | **Q** | **F** | **V** | **D** | **F** | **F** | **S** | **S** | **L** | **N** | **R** | **M** | **P** | **L** | **T** | **R** | **M** | **N** | **S** | **V** | **T** | **L** | **L** | **T** | **D** | **G** | **Q** | **E** | **K** | **L** | **D** | **S** | **L** | **L** | **Q** | **D** | **L** | **K** | **K** | **A** | **K** | **H** | **S** | **I** | **H** | **I** | **E** | **Y** | **Y** | **A** | **F** | **V** | **T** | **D** | **N** | **I** | **G** | **Q** | **Q** | **V** | **L** | **H** | **V** | **L** | **E** | **E** | **K** | **A** | **A** | **E** | **G** | **V** | **E** | **V** | **R** | **I** | **L** | **Y** | **D** | **A** | **F** | **G** | **S** | **H** | **G** | **T** | **K** | **A** | **K** | **D** | **F** | **N** | **R** | **L** | **I** | **K** | **N** | **G** | **G** | **H** | **V** | **H** | **T** | **F** | **V** | **T** | **S** | **Q** | **K** | **A** | **L** | **L** | **R** | **F** | **R** | **L** | **N** | **Y** | **H** | **D** | **H** | **R** | **K** | **I** | **V** | **V** | **I** | **D** | **G** | **K** | **I** | **S** | **Y** | **T** | **G** | **G** | **F** | **N** | **I** | **A** | **N** | **Q** | **Y** | **V** | **N** | **T** | **T** | **K** | **K** | **F** | **G** | **Y** | **W** | **R** | **D** | **T** | **H** | **I** | **R** | **I** | **F** | **G** | **A** | **A** | **S** | **S** | **L** | **L** | **Q** | **L** | **R** | **F** | **L** | **T** | **D** | **W** | **N** | **V** | **S** | **V** | **P** | **E** | **E** | **K** | **K** | **V** | **G** | **Y** | **H** | **L** | **N** | **Y** | **F** | **F** | **K** | **K** | **A** | **D** | **R** | **D** | **E** | **S** | **K** | **L** | **A** | **D** | **T** | **S** | **I** | **Q** | **L** | **V** | **S** | **S** | **G** | **P** | **N** | **N** | **E** | **R** | **E** | **Q** | **I** | **K** | **L** | **S** | **F** | **I** | **K** | **L** | **I** | **T** | **S** | **A** | **K** | **K** | **R** | **V** | **W** | **I** | **Q** | **T** | **P** | **Y** | **L** | **V** | **P** | **D** | **E** | **S** | **V** | **I** | **A** | **A** | **L** | **K** | **I** | **A** | **T** | **A** | **S** | **G** | **V** | **D** | **V** | **K** | **I** | **M** | **I** | **P** | **N** | **K** | **P** | **D** | **H** | **P** | **F** | **I** | **Y** | **R** | **A** | **T** | **Q** | **Y** | **Y** | **A** | **R** | **Q** | **L** | **I** | **K** | **E** | **N** | **V** | **Q** | **I** | **L** | **V** | **Y** | **E** | **N** | **G** | **F** | **L** | **H** | **A** | **K** | **T** | **L** | **I** | **M** | **D** | **D** | **E** | **I** | **C** | **M** | **V** | **G** | **S** | **A** | **N** | **Q** | **D** | **I** | **R** | **S** | **Y** | **R** | **L** | **N** | **F** | **E** | **T** | **S** | **A** | **V** | **I** | **Y** | **D** | **P** | **E** | **F** | **L** | **E** | **E** | **L** | **A** | **T** | **Q** | **F** | **K** | **E** | **D** | **E** | **T** | **H** | **C** | **S** | **S** | **M** | **T** | **T** | **E** | **T** | **V** | **K** | **E** | **M** | **S** | **N** | **W** | **L** | **L** | **F** | **K** | **Q** | **Q** | **I** | **S** | **R** | **L** | **F** | **S** | **P** | **I** | **L** |
| **N59624F** | **-** | **-** | **-** | **-** | **-** | **-** | **-** | **-** | **-** | **-** | **-** | **-** | **-** | **-** | **-** | **-** | **-** | **-** | **-** | **-** | **-** | **-** | **-** | **K** | **P** | **R** | **D** | **V** | **A** | **A** | **I** | **W** | **A** | **W** | **L** | **L** | **V** | **L** | **F** | **A** | **L** | **P** | **G** | **V** | **G** | **F** | **V** | **L** | **Y** | **L** | **F** | **F** | **G** | **R** | **G** | **L** | **T** | **D** | **K** | **K** | **K** | **F** | **Y** | **L** | **R** | **Q** | **S** | **D** | **L** | **K** | **E** | **L** | **E** | **N** | **F** | **Q** | **S** | **F** | **R** | **G** | **D** | **T** | **I** | **E** | **H** | **Y** | **D** | **P** | **D** | **M** | **G** | **D** | **K** | **D** | **K** | **Q** | **Q** | **F** | **V** | **D** | **F** | **F** | **S** | **S** | **L** | **N** | **R** | **M** | **P** | **L** | **T** | **R** | **M** | **N** | **S** | **V** | **T** | **L** | **L** | **T** | **D** | **G** | **Q** | **K** | **K** | **L** | **D** | **S** | **L** | **L** | **Q** | **D** | **L** | **K** | **K** | **A** | **K** | **H** | **S** | **I** | **H** | **I** | **E** | **Y** | **Y** | **A** | **F** | **V** | **T** | **D** | **N** | **I** | **G** | **Q** | **Q** | **V** | **L** | **R** | **V** | **L** | **E** | **E** | **K** | **A** | **A** | **E** | **G** | **V** | **E** | **V** | **R** | **I** | **L** | **Y** | **D** | **A** | **F** | **G** | **S** | **H** | **G** | **T** | **K** | **A** | **K** | **D** | **F** | **N** | **R** | **L** | **I** | **K** | **N** | **G** | **G** | **H** | **V** | **H** | **T** | **F** | **V** | **T** | **S** | **Q** | **K** | **A** | **L** | **L** | **R** | **F** | **R** | **L** | **N** | **Y** | **H** | **D** | **H** | **R** | **K** | **I** | **V** | **V** | **I** | **D** | **G** | **K** | **I** | **S** | **Y** | **T** | **G** | **G** | **F** | **N** | **I** | **A** | **N** | **Q** | **Y** | **V** | **N** | **T** | **T** | **K** | **K** | **F** | **G** | **Y** | **W** | **R** | **D** | **T** | **H** | **I** | **R** | **I** | **F** | **G** | **A** | **A** | **S** | **S** | **L** | **L** | **Q** | **L** | **R** | **F** | **L** | **T** | **D** | **W** | **N** | **V** | **S** | **V** | **S** | **E** | **E** | **K** | **K** | **V** | **G** | **Y** | **H** | **L** | **N** | **Y** | **F** | **F** | **K** | **K** | **A** | **D** | **R** | **D** | **E** | **S** | **K** | **L** | **A** | **D** | **T** | **S** | **I** | **Q** | **L** | **V** | **S** | **S** | **G** | **P** | **N** | **N** | **E** | **R** | **E** | **Q** | **I** | **K** | **L** | **S** | **F** | **I** | **K** | **L** | **I** | **T** | **S** | **A** | **K** | **K** | **R** | **V** | **W** | **I** | **Q** | **T** | **P** | **Y** | **L** | **V** | **P** | **D** | **E** | **S** | **V** | **I** | **A** | **A** | **L** | **K** | **I** | **A** | **T** | **A** | **S** | **G** | **V** | **D** | **V** | **K** | **I** | **M** | **I** | **P** | **N** | **K** | **P** | **D** | **H** | **P** | **F** | **I** | **Y** | **R** | **A** | **T** | **Q** | **Y** | **Y** | **A** | **R** | **Q** | **L** | **I** | **K** | **E** | **N** | **V** | **Q** | **I** | **L** | **V** | **Y** | **E** | **N** | **G** | **F** | **L** | **H** | **A** | **K** | **T** | **L** | **I** | **M** | **D** | **D** | **E** | **I** | **C** | **M** | **V** | **G** | **S** | **A** | **N** | **Q** | **D** | **I** | **R** | **S** | **Y** | **R** | **L** | **N** | **F** | **E** | **T** | **S** | **A** | **V** | **I** | **Y** | **D** | **P** | **E** | **F** | **L** | **E** | **E** | **L** | **A** | **T** | **Q** | **F** | **K** | **E** | **D** | **E** | **T** | **H** | **C** | **S** | **P** | **M** | **T** | **T** | **E** | **T** | **V** | **K** | **E** | **M** | **S** | **N** | **W** | **L** | **L** | **F** | **K** | **Q** | **Q** | **I** | **S** | **R** | **L** | **F** | **S** | **P** | **I** | **L** |
| **N54538** | **V** | **V** | **S** | **S** | **I** | **I** | **T** | **A** | **L** | **Y** | **L** | **L** | **N** | **A** | **L** | **I** | **A** | **L** | **V** | **T** | **I** | **L** | **I** | **K** | **P** | **R** | **D** | **V** | **A** | **A** | **I** | **W** | **A** | **W** | **L** | **L** | **V** | **L** | **F** | **A** | **L** | **P** | **G** | **V** | **G** | **F** | **V** | **L** | **Y** | **L** | **F** | **F** | **G** | **R** | **G** | **L** | **T** | **D** | **K** | **K** | **K** | **F** | **Y** | **L** | **R** | **Q** | **S** | **D** | **L** | **K** | **E** | **L** | **E** | **N** | **F** | **Q** | **S** | **F** | **R** | **G** | **D** | **T** | **I** | **E** | **H** | **Y** | **D** | **P** | **D** | **M** | **G** | **D** | **K** | **D** | **K** | **Q** | **Q** | **F** | **V** | **D** | **F** | **F** | **S** | **S** | **L** | **N** | **R** | **M** | **P** | **L** | **A** | **R** | **M** | **N** | **S** | **V** | **T** | **L** | **L** | **T** | **D** | **G** | **Q** | **E** | **K** | **L** | **D** | **S** | **L** | **L** | **Q** | **D** | **L** | **K** | **K** | **A** | **K** | **H** | **S** | **I** | **H** | **I** | **E** | **Y** | **Y** | **A** | **F** | **V** | **T** | **D** | **N** | **I** | **G** | **Q** | **Q** | **V** | **L** | **H** | **V** | **L** | **E** | **E** | **K** | **A** | **A** | **E** | **G** | **V** | **E** | **V** | **R** | **I** | **L** | **Y** | **D** | **A** | **F** | **G** | **S** | **H** | **G** | **T** | **K** | **A** | **K** | **D** | **F** | **N** | **R** | **L** | **I** | **K** | **N** | **G** | **G** | **H** | **V** | **H** | **T** | **F** | **V** | **T** | **S** | **Q** | **K** | **A** | **L** | **L** | **R** | **F** | **R** | **L** | **N** | **Y** | **H** | **D** | **H** | **R** | **K** | **I** | **V** | **V** | **I** | **D** | **G** | **K** | **I** | **S** | **Y** | **T** | **G** | **G** | **F** | **N** | **I** | **A** | **N** | **Q** | **Y** | **V** | **N** | **T** | **T** | **K** | **K** | **F** | **G** | **Y** | **W** | **R** | **D** | **T** | **H** | **I** | **R** | **I** | **F** | **G** | **A** | **A** | **S** | **S** | **L** | **L** | **Q** | **L** | **R** | **F** | **L** | **T** | **D** | **W** | **N** | **V** | **S** | **V** | **P** | **E** | **E** | **K** | **K** | **V** | **G** | **Y** | **H** | **L** | **N** | **Y** | **F** | **F** | **K** | **K** | **A** | **D** | **R** | **D** | **E** | **S** | **K** | **L** | **A** | **D** | **T** | **S** | **I** | **Q** | **L** | **V** | **S** | **S** | **G** | **P** | **N** | **N** | **E** | **R** | **E** | **Q** | **I** | **K** | **L** | **S** | **F** | **I** | **K** | **L** | **I** | **T** | **S** | **A** | **K** | **K** | **R** | **V** | **W** | **I** | **Q** | **T** | **P** | **Y** | **L** | **V** | **P** | **D** | **E** | **S** | **V** | **I** | **A** | **A** | **L** | **K** | **I** | **A** | **T** | **A** | **S** | **G** | **V** | **D** | **V** | **K** | **I** | **M** | **I** | **P** | **N** | **K** | **P** | **D** | **H** | **P** | **F** | **I** | **Y** | **R** | **A** | **T** | **Q** | **Y** | **Y** | **A** | **R** | **Q** | **L** | **I** | **K** | **E** | **N** | **V** | **Q** | **I** | **L** | **V** | **Y** | **E** | **N** | **G** | **F** | **L** | **H** | **A** | **K** | **T** | **L** | **I** | **M** | **D** | **D** | **E** | **I** | **C** | **M** | **V** | **G** | **S** | **A** | **N** | **Q** | **D** | **I** | **R** | **S** | **Y** | **R** | **L** | **N** | **F** | **E** | **T** | **S** | **A** | **V** | **I** | **Y** | **D** | **P** | **E** | **F** | **L** | **E** | **E** | **L** | **A** | **T** | **Q** | **F** | **K** | **E** | **D** | **E** | **T** | **H** | **C** | **S** | **S** | **M** | **T** | **T** | **E** | **T** | **V** | **K** | **E** | **M** | **S** | **N** | **W** | **L** | **L** | **F** | **K** | **Q** | **Q** | **I** | **S** | **R** | **L** | **F** | **S** | **P** | **I** | **L** |
| **N59711F** | **-** | **-** | **-** | **-** | **-** | **-** | **-** | **-** | **-** | **-** | **-** | **-** | **-** | **-** | **-** | **-** | **-** | **-** | **-** | **-** | **-** | **-** | **-** | **K** | **P** | **R** | **D** | **V** | **A** | **A** | **I** | **W** | **A** | **W** | **L** | **L** | **V** | **L** | **F** | **A** | **L** | **P** | **G** | **V** | **G** | **F** | **V** | **L** | **Y** | **L** | **F** | **F** | **G** | **R** | **G** | **L** | **T** | **D** | **K** | **K** | **K** | **F** | **Y** | **L** | **R** | **Q** | **S** | **D** | **L** | **K** | **E** | **L** | **E** | **N** | **F** | **Q** | **S** | **F** | **R** | **G** | **D** | **T** | **I** | **E** | **H** | **Y** | **D** | **P** | **D** | **M** | **G** | **D** | **K** | **D** | **K** | **Q** | **Q** | **F** | **V** | **D** | **F** | **F** | **S** | **S** | **L** | **N** | **R** | **M** | **P** | **L** | **T** | **R** | **M** | **N** | **S** | **V** | **T** | **L** | **L** | **T** | **D** | **G** | **Q** | **K** | **K** | **L** | **D** | **S** | **L** | **L** | **Q** | **D** | **L** | **K** | **K** | **A** | **K** | **H** | **S** | **I** | **H** | **I** | **E** | **Y** | **Y** | **A** | **F** | **V** | **T** | **D** | **N** | **I** | **G** | **Q** | **Q** | **V** | **L** | **R** | **V** | **L** | **E** | **E** | **K** | **A** | **A** | **E** | **G** | **V** | **E** | **V** | **R** | **I** | **L** | **Y** | **D** | **A** | **F** | **G** | **S** | **H** | **G** | **T** | **K** | **A** | **K** | **D** | **F** | **N** | **R** | **L** | **I** | **K** | **N** | **G** | **G** | **H** | **V** | **H** | **T** | **F** | **V** | **T** | **S** | **Q** | **K** | **A** | **L** | **L** | **R** | **F** | **R** | **L** | **N** | **Y** | **H** | **D** | **H** | **R** | **K** | **I** | **V** | **V** | **I** | **D** | **G** | **K** | **I** | **S** | **Y** | **T** | **G** | **G** | **F** | **N** | **I** | **A** | **N** | **Q** | **Y** | **V** | **N** | **T** | **T** | **K** | **K** | **F** | **G** | **Y** | **W** | **R** | **D** | **T** | **H** | **I** | **R** | **I** | **F** | **G** | **A** | **A** | **S** | **S** | **L** | **L** | **Q** | **L** | **R** | **F** | **L** | **T** | **D** | **W** | **N** | **V** | **S** | **V** | **S** | **E** | **E** | **K** | **K** | **V** | **G** | **Y** | **H** | **L** | **N** | **Y** | **F** | **F** | **K** | **K** | **A** | **D** | **R** | **D** | **E** | **S** | **K** | **L** | **A** | **D** | **T** | **S** | **I** | **Q** | **L** | **V** | **S** | **S** | **G** | **P** | **N** | **N** | **E** | **R** | **E** | **Q** | **I** | **K** | **L** | **S** | **F** | **I** | **K** | **L** | **I** | **T** | **S** | **A** | **K** | **K** | **R** | **V** | **W** | **I** | **Q** | **T** | **P** | **Y** | **L** | **V** | **P** | **D** | **E** | **S** | **V** | **I** | **A** | **A** | **L** | **K** | **I** | **A** | **T** | **A** | **S** | **G** | **V** | **D** | **V** | **K** | **I** | **M** | **I** | **P** | **N** | **K** | **P** | **D** | **H** | **P** | **F** | **I** | **Y** | **R** | **A** | **T** | **Q** | **Y** | **Y** | **A** | **R** | **Q** | **L** | **I** | **K** | **E** | **N** | **V** | **Q** | **I** | **L** | **V** | **Y** | **E** | **N** | **G** | **F** | **L** | **H** | **A** | **K** | **T** | **L** | **I** | **M** | **D** | **D** | **E** | **I** | **C** | **M** | **V** | **G** | **S** | **A** | **N** | **Q** | **D** | **I** | **R** | **S** | **Y** | **R** | **L** | **N** | **F** | **E** | **T** | **S** | **A** | **V** | **I** | **Y** | **D** | **P** | **E** | **F** | **L** | **E** | **E** | **L** | **A** | **T** | **Q** | **F** | **K** | **E** | **D** | **E** | **T** | **H** | **C** | **S** | **P** | **M** | **T** | **T** | **E** | **T** | **V** | **K** | **E** | **M** | **S** | **N** | **W** | **L** | **L** | **F** | **K** | **Q** | **Q** | **I** | **S** | **R** | **L** | **F** | **S** | **P** | **I** | **L** |
| **N59559F** | **-** | **-** | **-** | **-** | **-** | **-** | **-** | **-** | **-** | **-** | **-** | **-** | **-** | **-** | **-** | **-** | **-** | **-** | **-** | **-** | **-** | **-** | **-** | **K** | **P** | **R** | **D** | **V** | **A** | **A** | **I** | **W** | **A** | **W** | **L** | **L** | **V** | **L** | **F** | **A** | **L** | **P** | **G** | **V** | **G** | **F** | **V** | **L** | **Y** | **L** | **F** | **F** | **G** | **R** | **G** | **L** | **T** | **D** | **K** | **K** | **K** | **F** | **Y** | **L** | **R** | **Q** | **S** | **D** | **L** | **K** | **E** | **L** | **E** | **N** | **F** | **Q** | **S** | **F** | **R** | **G** | **D** | **T** | **I** | **E** | **H** | **Y** | **D** | **P** | **D** | **M** | **G** | **D** | **K** | **D** | **K** | **Q** | **Q** | **F** | **V** | **D** | **F** | **F** | **S** | **S** | **L** | **N** | **R** | **M** | **P** | **L** | **T** | **R** | **M** | **N** | **S** | **V** | **T** | **L** | **L** | **T** | **D** | **G** | **Q** | **K** | **K** | **L** | **D** | **S** | **L** | **L** | **Q** | **D** | **L** | **K** | **K** | **A** | **K** | **H** | **S** | **I** | **H** | **I** | **E** | **Y** | **Y** | **A** | **F** | **V** | **T** | **D** | **T** | **I** | **G** | **Q** | **Q** | **V** | **L** | **R** | **V** | **L** | **E** | **E** | **K** | **A** | **A** | **E** | **G** | **V** | **E** | **V** | **R** | **I** | **L** | **Y** | **D** | **A** | **F** | **G** | **S** | **H** | **G** | **T** | **K** | **A** | **K** | **D** | **F** | **N** | **R** | **L** | **I** | **K** | **N** | **G** | **G** | **H** | **V** | **H** | **T** | **F** | **V** | **T** | **S** | **Q** | **K** | **A** | **L** | **L** | **R** | **F** | **R** | **L** | **N** | **Y** | **H** | **D** | **H** | **R** | **K** | **I** | **V** | **V** | **I** | **D** | **G** | **K** | **I** | **S** | **Y** | **T** | **G** | **G** | **F** | **N** | **I** | **A** | **N** | **Q** | **Y** | **V** | **N** | **T** | **T** | **K** | **K** | **F** | **G** | **Y** | **W** | **R** | **D** | **T** | **H** | **I** | **R** | **I** | **F** | **G** | **A** | **A** | **S** | **S** | **L** | **L** | **Q** | **L** | **R** | **F** | **L** | **T** | **D** | **W** | **N** | **V** | **S** | **V** | **P** | **E** | **E** | **K** | **K** | **V** | **G** | **Y** | **H** | **L** | **N** | **Y** | **F** | **F** | **K** | **K** | **A** | **D** | **R** | **D** | **E** | **S** | **K** | **L** | **A** | **D** | **T** | **S** | **I** | **Q** | **L** | **V** | **S** | **S** | **G** | **P** | **N** | **N** | **E** | **R** | **E** | **Q** | **I** | **K** | **L** | **S** | **F** | **I** | **K** | **L** | **I** | **T** | **S** | **A** | **K** | **K** | **R** | **V** | **W** | **I** | **Q** | **T** | **P** | **Y** | **L** | **V** | **P** | **D** | **E** | **S** | **V** | **I** | **A** | **A** | **L** | **K** | **I** | **A** | **T** | **A** | **S** | **G** | **V** | **D** | **V** | **K** | **I** | **M** | **I** | **P** | **N** | **K** | **P** | **D** | **H** | **P** | **F** | **I** | **Y** | **R** | **A** | **T** | **Q** | **Y** | **Y** | **A** | **R** | **Q** | **L** | **I** | **K** | **E** | **N** | **V** | **Q** | **I** | **L** | **V** | **Y** | **E** | **N** | **G** | **F** | **L** | **H** | **A** | **K** | **T** | **L** | **I** | **M** | **D** | **D** | **E** | **I** | **C** | **M** | **V** | **G** | **S** | **A** | **N** | **Q** | **D** | **I** | **R** | **S** | **Y** | **R** | **L** | **N** | **F** | **E** | **T** | **S** | **A** | **V** | **I** | **Y** | **D** | **P** | **E** | **F** | **L** | **E** | **E** | **L** | **A** | **T** | **Q** | **F** | **K** | **E** | **D** | **E** | **T** | **H** | **C** | **S** | **P** | **M** | **T** | **T** | **E** | **T** | **V** | **K** | **E** | **M** | **S** | **N** | **W** | **L** | **L** | **F** | **K** | **Q** | **Q** | **I** | **S** | **R** | **L** | **F** | **S** | **P** | **I** | **L** |
| **N53439** | **-** | **-** | **-** | **-** | **-** | **-** | **-** | **-** | **-** | **-** | **-** | **-** | **-** | **-** | **-** | **-** | **-** | **-** | **-** | **-** | **-** | **-** | **-** | **K** | **P** | **R** | **D** | **V** | **A** | **A** | **I** | **W** | **A** | **W** | **L** | **L** | **V** | **L** | **F** | **A** | **L** | **P** | **G** | **V** | **G** | **F** | **V** | **L** | **Y** | **L** | **F** | **F** | **G** | **R** | **G** | **L** | **T** | **D** | **K** | **K** | **K** | **F** | **Y** | **L** | **R** | **Q** | **S** | **D** | **L** | **K** | **E** | **L** | **E** | **N** | **F** | **Q** | **S** | **F** | **R** | **G** | **D** | **T** | **I** | **E** | **H** | **Y** | **D** | **P** | **D** | **M** | **G** | **D** | **K** | **D** | **K** | **Q** | **Q** | **F** | **V** | **D** | **F** | **F** | **S** | **S** | **L** | **N** | **R** | **M** | **P** | **L** | **T** | **I** | **M** | **N** | **S** | **V** | **T** | **L** | **L** | **T** | **D** | **G** | **Q** | **E** | **K** | **L** | **D** | **S** | **L** | **L** | **Q** | **D** | **L** | **K** | **K** | **A** | **K** | **H** | **S** | **I** | **H** | **I** | **E** | **Y** | **Y** | **A** | **F** | **V** | **T** | **D** | **N** | **I** | **G** | **Q** | **Q** | **V** | **L** | **H** | **V** | **L** | **E** | **E** | **K** | **A** | **A** | **E** | **G** | **V** | **E** | **V** | **R** | **I** | **L** | **Y** | **D** | **A** | **F** | **G** | **S** | **H** | **G** | **T** | **K** | **A** | **K** | **D** | **F** | **N** | **R** | **L** | **I** | **K** | **N** | **G** | **G** | **H** | **V** | **H** | **T** | **F** | **V** | **T** | **S** | **Q** | **K** | **A** | **L** | **L** | **R** | **F** | **R** | **L** | **N** | **Y** | **H** | **D** | **H** | **R** | **K** | **I** | **V** | **V** | **I** | **D** | **G** | **K** | **I** | **S** | **Y** | **T** | **G** | **G** | **F** | **N** | **I** | **A** | **N** | **Q** | **Y** | **V** | **N** | **T** | **T** | **K** | **K** | **F** | **G** | **Y** | **W** | **R** | **D** | **T** | **H** | **I** | **R** | **I** | **F** | **G** | **A** | **A** | **S** | **S** | **L** | **L** | **Q** | **L** | **R** | **F** | **L** | **T** | **D** | **W** | **N** | **V** | **S** | **V** | **P** | **E** | **E** | **K** | **K** | **V** | **G** | **Y** | **H** | **L** | **N** | **Y** | **F** | **F** | **K** | **K** | **A** | **D** | **Q** | **D** | **E** | **S** | **K** | **L** | **A** | **D** | **T** | **S** | **I** | **Q** | **L** | **V** | **S** | **S** | **G** | **P** | **N** | **N** | **E** | **R** | **E** | **Q** | **I** | **K** | **L** | **S** | **F** | **I** | **K** | **L** | **I** | **T** | **S** | **A** | **K** | **K** | **R** | **V** | **W** | **I** | **Q** | **T** | **P** | **Y** | **L** | **V** | **P** | **D** | **E** | **S** | **V** | **I** | **A** | **A** | **L** | **K** | **I** | **A** | **T** | **A** | **S** | **G** | **V** | **D** | **V** | **K** | **I** | **M** | **I** | **P** | **N** | **K** | **P** | **D** | **H** | **P** | **F** | **I** | **Y** | **R** | **A** | **T** | **Q** | **Y** | **Y** | **A** | **R** | **Q** | **L** | **I** | **K** | **E** | **N** | **V** | **Q** | **I** | **L** | **V** | **Y** | **E** | **N** | **G** | **F** | **L** | **H** | **A** | **K** | **T** | **L** | **I** | **M** | **D** | **D** | **E** | **I** | **C** | **M** | **V** | **G** | **S** | **A** | **N** | **Q** | **D** | **I** | **R** | **S** | **Y** | **R** | **L** | **N** | **F** | **E** | **T** | **S** | **A** | **V** | **I** | **Y** | **D** | **P** | **E** | **F** | **L** | **E** | **E** | **L** | **A** | **T** | **Q** | **F** | **K** | **E** | **D** | **E** | **T** | **H** | **C** | **S** | **S** | **M** | **T** | **T** | **E** | **T** | **V** | **K** | **E** | **M** | **S** | **N** | **W** | **L** | **L** | **F** | **K** | **Q** | **Q** | **I** | **S** | **R** | **L** | **F** | **S** | **P** | **I** | **L** |
| **N52769** | **-** | **-** | **-** | **-** | **-** | **-** | **-** | **-** | **-** | **-** | **-** | **-** | **-** | **-** | **-** | **-** | **-** | **-** | **-** | **-** | **-** | **-** | **-** | **K** | **P** | **R** | **D** | **V** | **A** | **A** | **I** | **W** | **A** | **W** | **L** | **L** | **V** | **L** | **F** | **A** | **L** | **P** | **G** | **V** | **G** | **F** | **V** | **L** | **Y** | **L** | **F** | **F** | **G** | **R** | **G** | **L** | **T** | **D** | **K** | **K** | **K** | **F** | **Y** | **L** | **R** | **Q** | **S** | **D** | **L** | **K** | **E** | **L** | **E** | **N** | **F** | **Q** | **S** | **F** | **R** | **G** | **D** | **T** | **I** | **E** | **H** | **Y** | **D** | **P** | **D** | **M** | **G** | **D** | **K** | **D** | **K** | **Q** | **Q** | **F** | **V** | **D** | **F** | **F** | **S** | **S** | **L** | **N** | **R** | **M** | **P** | **L** | **T** | **R** | **M** | **N** | **S** | **V** | **T** | **L** | **L** | **T** | **D** | **G** | **Q** | **K** | **K** | **L** | **D** | **S** | **L** | **L** | **Q** | **D** | **L** | **K** | **K** | **A** | **K** | **H** | **S** | **I** | **H** | **I** | **E** | **Y** | **Y** | **A** | **F** | **V** | **T** | **D** | **T** | **I** | **G** | **Q** | **Q** | **V** | **L** | **R** | **V** | **L** | **E** | **E** | **K** | **A** | **A** | **E** | **G** | **V** | **E** | **V** | **R** | **I** | **L** | **Y** | **D** | **A** | **F** | **G** | **S** | **H** | **G** | **T** | **K** | **A** | **K** | **D** | **F** | **N** | **R** | **L** | **I** | **K** | **N** | **G** | **G** | **H** | **V** | **H** | **T** | **F** | **V** | **T** | **S** | **Q** | **K** | **A** | **L** | **L** | **R** | **F** | **R** | **L** | **N** | **Y** | **H** | **D** | **H** | **R** | **K** | **I** | **V** | **V** | **I** | **D** | **G** | **K** | **I** | **S** | **Y** | **T** | **G** | **G** | **F** | **N** | **I** | **A** | **N** | **Q** | **Y** | **V** | **N** | **T** | **T** | **K** | **K** | **F** | **G** | **Y** | **W** | **R** | **D** | **T** | **H** | **I** | **R** | **I** | **F** | **G** | **A** | **A** | **S** | **S** | **L** | **L** | **Q** | **L** | **R** | **F** | **L** | **T** | **D** | **W** | **N** | **V** | **S** | **V** | **P** | **E** | **E** | **K** | **K** | **V** | **G** | **Y** | **H** | **L** | **N** | **Y** | **F** | **F** | **K** | **K** | **A** | **D** | **R** | **D** | **E** | **S** | **K** | **L** | **A** | **D** | **T** | **S** | **I** | **Q** | **L** | **V** | **S** | **S** | **G** | **P** | **N** | **N** | **E** | **R** | **E** | **Q** | **I** | **K** | **L** | **S** | **F** | **I** | **K** | **L** | **I** | **T** | **S** | **A** | **K** | **K** | **R** | **V** | **W** | **I** | **Q** | **T** | **P** | **Y** | **L** | **V** | **P** | **D** | **E** | **S** | **V** | **I** | **A** | **A** | **L** | **K** | **I** | **A** | **T** | **A** | **S** | **G** | **V** | **D** | **V** | **K** | **I** | **M** | **I** | **P** | **N** | **K** | **P** | **D** | **H** | **P** | **F** | **I** | **Y** | **R** | **A** | **T** | **Q** | **Y** | **Y** | **A** | **R** | **Q** | **L** | **I** | **K** | **E** | **N** | **V** | **Q** | **I** | **L** | **V** | **Y** | **E** | **N** | **G** | **F** | **L** | **H** | **A** | **K** | **T** | **L** | **I** | **M** | **D** | **D** | **E** | **I** | **C** | **M** | **V** | **G** | **S** | **A** | **N** | **Q** | **D** | **I** | **R** | **S** | **Y** | **R** | **L** | **N** | **F** | **E** | **T** | **S** | **A** | **V** | **I** | **Y** | **D** | **P** | **E** | **F** | **L** | **E** | **E** | **L** | **A** | **T** | **Q** | **F** | **K** | **E** | **D** | **E** | **T** | **H** | **C** | **S** | **P** | **M** | **T** | **T** | **E** | **T** | **V** | **K** | **E** | **M** | **S** | **N** | **W** | **L** | **L** | **F** | **K** | **Q** | **Q** | **I** | **S** | **R** | **L** | **F** | **S** | **P** | **I** | **L** |
| **N54560** | **-** | **-** | **-** | **-** | **-** | **-** | **-** | **-** | **-** | **-** | **-** | **-** | **-** | **-** | **-** | **-** | **-** | **-** | **-** | **-** | **-** | **-** | **-** | **K** | **P** | **R** | **D** | **V** | **A** | **A** | **I** | **W** | **A** | **W** | **L** | **L** | **V** | **L** | **F** | **A** | **L** | **P** | **G** | **V** | **G** | **F** | **V** | **L** | **Y** | **L** | **F** | **F** | **G** | **R** | **G** | **L** | **T** | **D** | **K** | **K** | **K** | **F** | **Y** | **L** | **R** | **Q** | **S** | **D** | **L** | **K** | **E** | **L** | **E** | **N** | **F** | **Q** | **S** | **F** | **R** | **G** | **D** | **T** | **I** | **E** | **H** | **Y** | **D** | **P** | **D** | **M** | **G** | **D** | **K** | **D** | **K** | **Q** | **Q** | **F** | **V** | **D** | **F** | **F** | **S** | **S** | **L** | **N** | **R** | **M** | **P** | **L** | **T** | **R** | **M** | **N** | **S** | **V** | **T** | **L** | **L** | **T** | **D** | **G** | **Q** | **E** | **K** | **L** | **D** | **S** | **L** | **L** | **Q** | **D** | **L** | **K** | **K** | **A** | **K** | **H** | **S** | **I** | **H** | **I** | **E** | **Y** | **Y** | **A** | **F** | **V** | **T** | **D** | **N** | **I** | **G** | **Q** | **Q** | **V** | **L** | **H** | **V** | **L** | **E** | **E** | **K** | **A** | **A** | **E** | **G** | **V** | **E** | **V** | **R** | **I** | **L** | **Y** | **D** | **A** | **F** | **G** | **S** | **H** | **G** | **T** | **K** | **A** | **K** | **D** | **F** | **N** | **R** | **L** | **I** | **K** | **N** | **G** | **G** | **H** | **V** | **H** | **T** | **F** | **V** | **T** | **S** | **Q** | **K** | **A** | **L** | **L** | **R** | **F** | **R** | **L** | **N** | **Y** | **H** | **D** | **H** | **R** | **K** | **I** | **V** | **V** | **I** | **D** | **G** | **K** | **I** | **S** | **Y** | **T** | **G** | **G** | **F** | **N** | **I** | **A** | **N** | **Q** | **Y** | **V** | **N** | **T** | **T** | **K** | **K** | **F** | **G** | **Y** | **W** | **R** | **D** | **T** | **H** | **I** | **R** | **I** | **F** | **G** | **A** | **A** | **S** | **S** | **L** | **L** | **Q** | **L** | **R** | **F** | **L** | **T** | **D** | **W** | **N** | **V** | **S** | **V** | **P** | **E** | **E** | **K** | **K** | **V** | **G** | **Y** | **H** | **L** | **N** | **Y** | **F** | **F** | **K** | **K** | **A** | **D** | **R** | **D** | **E** | **S** | **K** | **L** | **A** | **E** | **T** | **S** | **I** | **Q** | **L** | **V** | **S** | **S** | **G** | **P** | **N** | **N** | **E** | **R** | **E** | **Q** | **I** | **K** | **L** | **S** | **F** | **I** | **K** | **L** | **I** | **T** | **S** | **A** | **K** | **K** | **R** | **V** | **W** | **I** | **Q** | **T** | **P** | **Y** | **L** | **V** | **P** | **D** | **E** | **S** | **V** | **I** | **A** | **A** | **L** | **K** | **I** | **A** | **T** | **A** | **S** | **G** | **V** | **D** | **V** | **K** | **I** | **M** | **I** | **P** | **N** | **K** | **P** | **D** | **H** | **P** | **F** | **I** | **Y** | **R** | **A** | **T** | **Q** | **Y** | **Y** | **A** | **R** | **Q** | **L** | **I** | **K** | **E** | **N** | **V** | **Q** | **I** | **L** | **V** | **Y** | **E** | **N** | **G** | **F** | **L** | **H** | **A** | **K** | **T** | **L** | **I** | **M** | **D** | **D** | **E** | **I** | **C** | **M** | **V** | **G** | **S** | **A** | **N** | **Q** | **D** | **I** | **R** | **S** | **Y** | **R** | **L** | **N** | **F** | **E** | **T** | **S** | **A** | **V** | **I** | **Y** | **D** | **P** | **E** | **F** | **L** | **E** | **E** | **L** | **A** | **T** | **Q** | **F** | **K** | **E** | **D** | **E** | **T** | **H** | **C** | **S** | **S** | **M** | **T** | **T** | **E** | **T** | **V** | **K** | **E** | **M** | **S** | **N** | **W** | **L** | **L** | **F** | **K** | **Q** | **Q** | **I** | **S** | **R** | **L** | **F** | **S** | **P** | **I** | **L** |
| **N54035** | **-** | **-** | **-** | **-** | **-** | **-** | **-** | **-** | **-** | **-** | **-** | **-** | **-** | **-** | **-** | **-** | **-** | **-** | **-** | **-** | **-** | **-** | **-** | **K** | **P** | **R** | **D** | **V** | **A** | **A** | **I** | **W** | **A** | **W** | **L** | **L** | **V** | **L** | **F** | **A** | **L** | **P** | **G** | **V** | **G** | **F** | **V** | **L** | **Y** | **L** | **F** | **F** | **G** | **R** | **G** | **L** | **T** | **D** | **K** | **K** | **K** | **F** | **Y** | **L** | **R** | **Q** | **S** | **D** | **L** | **K** | **E** | **L** | **E** | **N** | **F** | **Q** | **S** | **F** | **R** | **G** | **D** | **T** | **I** | **E** | **H** | **Y** | **D** | **P** | **D** | **M** | **G** | **D** | **K** | **D** | **K** | **Q** | **Q** | **F** | **V** | **D** | **F** | **F** | **S** | **S** | **L** | **N** | **R** | **M** | **P** | **L** | **T** | **R** | **M** | **N** | **S** | **V** | **T** | **L** | **L** | **T** | **D** | **G** | **Q** | **E** | **K** | **L** | **D** | **S** | **L** | **L** | **Q** | **D** | **L** | **K** | **K** | **A** | **K** | **H** | **S** | **I** | **H** | **I** | **E** | **Y** | **Y** | **A** | **F** | **V** | **T** | **D** | **N** | **I** | **G** | **Q** | **Q** | **V** | **L** | **H** | **V** | **L** | **E** | **E** | **K** | **A** | **A** | **E** | **G** | **V** | **E** | **V** | **R** | **I** | **L** | **Y** | **D** | **A** | **F** | **G** | **S** | **H** | **G** | **T** | **K** | **A** | **K** | **D** | **F** | **N** | **R** | **L** | **I** | **K** | **N** | **G** | **G** | **H** | **V** | **H** | **T** | **F** | **V** | **T** | **S** | **Q** | **K** | **A** | **L** | **L** | **R** | **F** | **R** | **L** | **N** | **Y** | **H** | **D** | **H** | **R** | **K** | **I** | **V** | **V** | **I** | **D** | **G** | **K** | **I** | **S** | **Y** | **T** | **G** | **G** | **F** | **N** | **I** | **A** | **N** | **Q** | **Y** | **V** | **N** | **T** | **T** | **K** | **K** | **F** | **G** | **Y** | **W** | **R** | **D** | **T** | **H** | **I** | **R** | **I** | **F** | **G** | **A** | **A** | **S** | **S** | **L** | **L** | **Q** | **L** | **R** | **F** | **L** | **T** | **D** | **W** | **N** | **V** | **S** | **V** | **P** | **E** | **E** | **K** | **K** | **V** | **G** | **Y** | **H** | **L** | **N** | **Y** | **F** | **F** | **K** | **K** | **A** | **D** | **R** | **D** | **E** | **S** | **K** | **L** | **A** | **E** | **T** | **S** | **I** | **Q** | **L** | **V** | **S** | **S** | **G** | **P** | **N** | **N** | **E** | **R** | **E** | **Q** | **I** | **K** | **L** | **S** | **F** | **I** | **K** | **L** | **I** | **T** | **S** | **A** | **K** | **K** | **R** | **V** | **W** | **I** | **Q** | **T** | **P** | **Y** | **L** | **V** | **P** | **D** | **E** | **S** | **V** | **I** | **A** | **A** | **L** | **K** | **I** | **A** | **T** | **A** | **S** | **G** | **V** | **D** | **V** | **K** | **I** | **M** | **I** | **P** | **N** | **K** | **P** | **D** | **H** | **P** | **F** | **I** | **Y** | **R** | **A** | **T** | **Q** | **Y** | **Y** | **A** | **R** | **Q** | **L** | **I** | **K** | **E** | **N** | **V** | **Q** | **I** | **L** | **V** | **Y** | **E** | **N** | **G** | **F** | **L** | **H** | **A** | **K** | **T** | **L** | **I** | **M** | **D** | **D** | **E** | **I** | **C** | **M** | **V** | **G** | **S** | **A** | **N** | **Q** | **D** | **I** | **R** | **S** | **Y** | **R** | **L** | **N** | **F** | **E** | **T** | **S** | **A** | **V** | **I** | **Y** | **D** | **P** | **E** | **F** | **L** | **E** | **E** | **L** | **A** | **T** | **Q** | **F** | **K** | **E** | **D** | **E** | **T** | **H** | **C** | **S** | **S** | **M** | **T** | **T** | **E** | **T** | **V** | **K** | **E** | **M** | **S** | **N** | **W** | **L** | **L** | **F** | **K** | **Q** | **Q** | **I** | **S** | **R** | **L** | **F** | **S** | **P** | **I** | **L** |
| **N60365F** | **-** | **-** | **-** | **-** | **-** | **-** | **-** | **-** | **-** | **-** | **-** | **-** | **-** | **-** | **-** | **-** | **-** | **-** | **-** | **-** | **-** | **-** | **-** | **K** | **P** | **R** | **D** | **V** | **A** | **A** | **I** | **W** | **A** | **W** | **L** | **L** | **V** | **L** | **F** | **A** | **L** | **P** | **G** | **V** | **G** | **F** | **V** | **L** | **Y** | **L** | **F** | **F** | **G** | **R** | **G** | **L** | **T** | **D** | **K** | **K** | **K** | **F** | **Y** | **L** | **R** | **Q** | **S** | **D** | **L** | **K** | **E** | **L** | **E** | **N** | **F** | **Q** | **S** | **F** | **R** | **G** | **D** | **T** | **I** | **E** | **H** | **Y** | **D** | **P** | **D** | **M** | **G** | **D** | **K** | **D** | **K** | **Q** | **Q** | **F** | **V** | **D** | **F** | **F** | **S** | **S** | **L** | **N** | **R** | **M** | **P** | **L** | **T** | **R** | **M** | **N** | **S** | **V** | **T** | **L** | **L** | **T** | **D** | **G** | **Q** | **E** | **K** | **L** | **D** | **S** | **L** | **L** | **Q** | **D** | **L** | **K** | **K** | **A** | **K** | **H** | **S** | **I** | **H** | **I** | **E** | **Y** | **Y** | **A** | **F** | **V** | **T** | **D** | **N** | **I** | **G** | **Q** | **Q** | **V** | **L** | **H** | **V** | **L** | **E** | **E** | **K** | **A** | **A** | **E** | **G** | **V** | **E** | **V** | **R** | **I** | **L** | **Y** | **D** | **A** | **F** | **G** | **S** | **H** | **G** | **T** | **K** | **A** | **K** | **D** | **F** | **N** | **R** | **L** | **I** | **K** | **N** | **G** | **G** | **H** | **V** | **H** | **T** | **F** | **V** | **T** | **S** | **Q** | **K** | **A** | **L** | **L** | **R** | **F** | **R** | **L** | **N** | **Y** | **H** | **D** | **H** | **R** | **K** | **I** | **V** | **V** | **I** | **D** | **G** | **K** | **I** | **S** | **Y** | **T** | **G** | **G** | **F** | **N** | **I** | **A** | **N** | **Q** | **Y** | **V** | **N** | **T** | **T** | **K** | **K** | **F** | **G** | **Y** | **W** | **R** | **D** | **T** | **H** | **I** | **R** | **I** | **F** | **G** | **A** | **A** | **S** | **S** | **L** | **L** | **Q** | **L** | **R** | **F** | **L** | **T** | **D** | **W** | **N** | **V** | **S** | **V** | **P** | **E** | **E** | **K** | **K** | **V** | **G** | **Y** | **H** | **L** | **N** | **Y** | **F** | **F** | **K** | **K** | **A** | **D** | **R** | **D** | **E** | **S** | **K** | **L** | **A** | **D** | **T** | **S** | **I** | **Q** | **L** | **V** | **S** | **S** | **G** | **P** | **N** | **N** | **E** | **R** | **E** | **Q** | **I** | **K** | **L** | **S** | **F** | **I** | **K** | **L** | **I** | **T** | **S** | **A** | **K** | **K** | **R** | **V** | **W** | **I** | **Q** | **T** | **P** | **Y** | **L** | **V** | **P** | **D** | **E** | **S** | **V** | **I** | **A** | **A** | **L** | **K** | **I** | **A** | **T** | **A** | **S** | **G** | **V** | **D** | **V** | **K** | **I** | **M** | **I** | **P** | **N** | **K** | **P** | **D** | **H** | **P** | **F** | **I** | **Y** | **R** | **A** | **T** | **Q** | **Y** | **Y** | **A** | **R** | **Q** | **L** | **I** | **K** | **E** | **N** | **V** | **Q** | **I** | **L** | **V** | **Y** | **E** | **N** | **G** | **F** | **L** | **H** | **A** | **K** | **T** | **L** | **I** | **M** | **D** | **D** | **E** | **I** | **C** | **M** | **V** | **G** | **S** | **A** | **N** | **Q** | **D** | **I** | **R** | **S** | **Y** | **R** | **L** | **N** | **F** | **E** | **T** | **S** | **A** | **V** | **I** | **Y** | **D** | **P** | **E** | **F** | **L** | **E** | **E** | **L** | **A** | **T** | **Q** | **F** | **K** | **E** | **D** | **E** | **T** | **H** | **C** | **S** | **S** | **M** | **T** | **T** | **E** | **T** | **V** | **K** | **E** | **M** | **S** | **N** | **W** | **L** | **L** | **F** | **K** | **Q** | **Q** | **I** | **S** | **R** | **L** | **F** | **S** | **P** | **I** | **L** |
| **N52649** | **-** | **-** | **-** | **-** | **-** | **-** | **-** | **-** | **-** | **-** | **-** | **-** | **-** | **-** | **-** | **-** | **-** | **-** | **-** | **-** | **-** | **-** | **-** | **K** | **P** | **R** | **D** | **V** | **A** | **A** | **I** | **W** | **A** | **W** | **L** | **L** | **V** | **L** | **F** | **A** | **L** | **P** | **G** | **V** | **G** | **F** | **V** | **L** | **Y** | **L** | **F** | **F** | **G** | **R** | **G** | **L** | **T** | **D** | **K** | **K** | **K** | **F** | **Y** | **L** | **R** | **Q** | **S** | **D** | **L** | **K** | **E** | **L** | **E** | **N** | **F** | **Q** | **S** | **F** | **R** | **G** | **D** | **T** | **I** | **E** | **H** | **Y** | **D** | **P** | **D** | **M** | **G** | **D** | **K** | **D** | **K** | **Q** | **Q** | **F** | **V** | **D** | **F** | **F** | **S** | **S** | **L** | **N** | **R** | **M** | **P** | **L** | **T** | **R** | **M** | **N** | **S** | **V** | **T** | **L** | **L** | **T** | **D** | **G** | **Q** | **E** | **K** | **L** | **D** | **S** | **L** | **L** | **Q** | **D** | **L** | **K** | **K** | **A** | **K** | **H** | **S** | **I** | **H** | **I** | **E** | **Y** | **Y** | **A** | **F** | **V** | **T** | **D** | **N** | **I** | **G** | **Q** | **Q** | **V** | **L** | **H** | **V** | **L** | **E** | **E** | **K** | **A** | **A** | **E** | **G** | **V** | **E** | **V** | **R** | **I** | **L** | **Y** | **D** | **A** | **F** | **G** | **S** | **H** | **G** | **T** | **K** | **A** | **K** | **D** | **F** | **N** | **R** | **L** | **I** | **K** | **N** | **G** | **G** | **H** | **V** | **H** | **T** | **F** | **V** | **T** | **S** | **Q** | **K** | **A** | **L** | **L** | **R** | **F** | **R** | **L** | **N** | **Y** | **H** | **D** | **H** | **R** | **K** | **I** | **V** | **V** | **I** | **D** | **G** | **K** | **I** | **S** | **Y** | **T** | **G** | **G** | **F** | **N** | **I** | **A** | **N** | **Q** | **Y** | **V** | **N** | **T** | **T** | **K** | **K** | **F** | **G** | **Y** | **W** | **R** | **D** | **T** | **H** | **I** | **R** | **I** | **F** | **G** | **A** | **A** | **S** | **S** | **L** | **L** | **Q** | **L** | **R** | **F** | **L** | **T** | **D** | **W** | **N** | **V** | **S** | **V** | **P** | **E** | **E** | **K** | **K** | **V** | **G** | **Y** | **H** | **L** | **N** | **Y** | **F** | **F** | **K** | **K** | **A** | **D** | **R** | **D** | **E** | **S** | **K** | **L** | **A** | **D** | **T** | **S** | **I** | **Q** | **L** | **V** | **S** | **S** | **G** | **P** | **N** | **N** | **E** | **R** | **E** | **Q** | **I** | **K** | **L** | **S** | **F** | **I** | **K** | **L** | **I** | **T** | **S** | **A** | **K** | **K** | **R** | **V** | **W** | **I** | **Q** | **T** | **P** | **Y** | **L** | **V** | **P** | **D** | **E** | **S** | **V** | **I** | **A** | **A** | **L** | **K** | **I** | **A** | **T** | **A** | **S** | **G** | **V** | **D** | **V** | **K** | **I** | **M** | **I** | **P** | **N** | **K** | **P** | **D** | **H** | **P** | **F** | **I** | **Y** | **R** | **A** | **T** | **Q** | **Y** | **Y** | **A** | **R** | **Q** | **L** | **I** | **K** | **E** | **N** | **V** | **Q** | **I** | **L** | **V** | **Y** | **E** | **N** | **G** | **F** | **L** | **H** | **A** | **K** | **T** | **L** | **I** | **M** | **D** | **D** | **E** | **I** | **C** | **M** | **V** | **G** | **S** | **A** | **N** | **Q** | **D** | **I** | **R** | **S** | **Y** | **R** | **L** | **N** | **F** | **E** | **T** | **S** | **A** | **V** | **I** | **Y** | **D** | **P** | **E** | **F** | **L** | **E** | **E** | **L** | **A** | **T** | **Q** | **F** | **K** | **E** | **D** | **E** | **T** | **H** | **C** | **S** | **S** | **M** | **T** | **T** | **E** | **T** | **V** | **K** | **E** | **M** | **S** | **N** | **W** | **L** | **L** | **F** | **K** | **Q** | **Q** | **I** | **S** | **R** | **L** | **F** | **S** | **P** | **I** | **L** |
| **N55072** | **-** | **-** | **-** | **-** | **-** | **-** | **-** | **-** | **-** | **-** | **-** | **-** | **-** | **-** | **-** | **-** | **-** | **-** | **-** | **-** | **-** | **-** | **-** | **K** | **P** | **R** | **D** | **V** | **A** | **A** | **I** | **W** | **A** | **W** | **L** | **L** | **V** | **L** | **F** | **A** | **L** | **P** | **G** | **V** | **G** | **F** | **V** | **L** | **Y** | **L** | **F** | **F** | **G** | **R** | **G** | **L** | **T** | **D** | **K** | **K** | **K** | **F** | **Y** | **L** | **R** | **Q** | **S** | **D** | **L** | **K** | **E** | **L** | **E** | **N** | **F** | **Q** | **S** | **F** | **R** | **G** | **D** | **T** | **I** | **E** | **H** | **Y** | **D** | **P** | **D** | **M** | **G** | **D** | **K** | **D** | **K** | **Q** | **Q** | **F** | **V** | **D** | **F** | **F** | **S** | **S** | **L** | **N** | **R** | **M** | **P** | **L** | **T** | **R** | **M** | **N** | **S** | **V** | **T** | **L** | **L** | **T** | **D** | **G** | **Q** | **E** | **K** | **L** | **D** | **S** | **L** | **L** | **Q** | **D** | **L** | **K** | **K** | **A** | **K** | **H** | **S** | **I** | **H** | **I** | **E** | **Y** | **Y** | **A** | **F** | **V** | **T** | **D** | **N** | **I** | **G** | **Q** | **Q** | **V** | **L** | **H** | **V** | **L** | **E** | **E** | **K** | **A** | **A** | **E** | **G** | **V** | **E** | **V** | **R** | **I** | **L** | **Y** | **D** | **A** | **F** | **G** | **S** | **H** | **G** | **T** | **K** | **A** | **K** | **D** | **F** | **N** | **R** | **L** | **I** | **K** | **N** | **G** | **G** | **H** | **V** | **H** | **T** | **F** | **V** | **T** | **S** | **Q** | **K** | **A** | **L** | **L** | **R** | **F** | **R** | **L** | **N** | **Y** | **H** | **D** | **H** | **R** | **K** | **I** | **V** | **V** | **I** | **D** | **R** | **K** | **I** | **S** | **Y** | **T** | **G** | **G** | **F** | **N** | **I** | **A** | **N** | **Q** | **Y** | **V** | **N** | **T** | **T** | **K** | **K** | **F** | **G** | **Y** | **W** | **R** | **D** | **T** | **H** | **I** | **R** | **I** | **F** | **G** | **A** | **A** | **S** | **S** | **L** | **L** | **Q** | **L** | **R** | **F** | **L** | **T** | **D** | **W** | **N** | **V** | **S** | **V** | **P** | **E** | **E** | **K** | **K** | **V** | **G** | **Y** | **H** | **L** | **N** | **Y** | **F** | **F** | **K** | **K** | **A** | **D** | **R** | **D** | **E** | **S** | **K** | **L** | **A** | **D** | **T** | **S** | **I** | **Q** | **L** | **V** | **S** | **S** | **G** | **P** | **N** | **N** | **E** | **R** | **E** | **Q** | **I** | **K** | **L** | **S** | **F** | **I** | **K** | **L** | **I** | **T** | **S** | **A** | **K** | **K** | **R** | **V** | **W** | **I** | **Q** | **T** | **P** | **Y** | **L** | **V** | **P** | **D** | **E** | **S** | **V** | **I** | **A** | **A** | **L** | **K** | **I** | **A** | **T** | **A** | **S** | **G** | **V** | **D** | **V** | **K** | **I** | **M** | **I** | **P** | **N** | **K** | **P** | **D** | **H** | **P** | **F** | **I** | **Y** | **R** | **A** | **T** | **Q** | **Y** | **Y** | **A** | **R** | **Q** | **L** | **I** | **K** | **E** | **N** | **V** | **Q** | **I** | **L** | **V** | **Y** | **E** | **N** | **G** | **F** | **L** | **H** | **A** | **K** | **T** | **L** | **I** | **M** | **D** | **D** | **E** | **I** | **C** | **M** | **V** | **G** | **S** | **A** | **N** | **Q** | **D** | **I** | **R** | **S** | **Y** | **R** | **L** | **N** | **F** | **E** | **T** | **S** | **A** | **V** | **I** | **Y** | **D** | **P** | **E** | **F** | **L** | **E** | **E** | **L** | **A** | **T** | **Q** | **F** | **K** | **E** | **D** | **E** | **T** | **H** | **C** | **S** | **S** | **M** | **T** | **T** | **E** | **T** | **V** | **K** | **E** | **M** | **S** | **N** | **W** | **L** | **L** | **F** | **K** | **Q** | **Q** | **I** | **S** | **R** | **L** | **F** | **S** | **P** | **I** | **L** |
| **N52824** | **-** | **-** | **-** | **-** | **-** | **-** | **-** | **-** | **-** | **-** | **-** | **-** | **-** | **-** | **-** | **-** | **-** | **-** | **-** | **-** | **-** | **-** | **-** | **K** | **P** | **R** | **D** | **V** | **A** | **A** | **I** | **W** | **A** | **W** | **L** | **L** | **V** | **L** | **F** | **A** | **L** | **P** | **G** | **V** | **G** | **F** | **V** | **L** | **Y** | **L** | **F** | **F** | **G** | **R** | **G** | **L** | **T** | **D** | **K** | **K** | **K** | **F** | **Y** | **L** | **R** | **Q** | **S** | **D** | **L** | **K** | **E** | **L** | **E** | **N** | **F** | **Q** | **S** | **F** | **R** | **G** | **D** | **N** | **I** | **E** | **H** | **Y** | **D** | **P** | **D** | **M** | **G** | **D** | **K** | **D** | **K** | **Q** | **Q** | **F** | **V** | **D** | **F** | **F** | **S** | **S** | **L** | **N** | **R** | **M** | **P** | **L** | **T** | **R** | **M** | **N** | **S** | **V** | **T** | **L** | **L** | **T** | **D** | **G** | **Q** | **E** | **K** | **L** | **D** | **S** | **L** | **L** | **Q** | **D** | **L** | **K** | **K** | **A** | **K** | **H** | **S** | **I** | **H** | **I** | **E** | **Y** | **Y** | **A** | **F** | **V** | **T** | **D** | **N** | **I** | **G** | **Q** | **Q** | **V** | **L** | **H** | **V** | **L** | **E** | **E** | **K** | **A** | **A** | **E** | **G** | **V** | **E** | **V** | **R** | **I** | **L** | **Y** | **D** | **A** | **F** | **G** | **S** | **H** | **G** | **T** | **K** | **A** | **K** | **D** | **F** | **N** | **R** | **L** | **I** | **K** | **N** | **G** | **G** | **H** | **V** | **H** | **T** | **F** | **V** | **T** | **S** | **Q** | **K** | **A** | **L** | **L** | **R** | **F** | **R** | **L** | **N** | **Y** | **H** | **D** | **H** | **R** | **K** | **I** | **V** | **V** | **I** | **D** | **G** | **K** | **I** | **S** | **Y** | **T** | **G** | **G** | **F** | **N** | **I** | **A** | **N** | **Q** | **Y** | **V** | **N** | **T** | **T** | **K** | **K** | **F** | **G** | **Y** | **W** | **R** | **D** | **T** | **H** | **I** | **R** | **I** | **F** | **G** | **A** | **A** | **S** | **S** | **L** | **L** | **Q** | **L** | **R** | **F** | **L** | **T** | **D** | **W** | **N** | **V** | **S** | **V** | **P** | **E** | **E** | **K** | **K** | **V** | **G** | **Y** | **H** | **L** | **N** | **Y** | **F** | **F** | **K** | **K** | **A** | **D** | **R** | **D** | **E** | **S** | **K** | **L** | **A** | **D** | **T** | **S** | **I** | **Q** | **L** | **V** | **S** | **S** | **G** | **P** | **N** | **N** | **E** | **R** | **E** | **Q** | **I** | **K** | **L** | **S** | **F** | **I** | **K** | **L** | **I** | **T** | **S** | **A** | **K** | **K** | **R** | **V** | **W** | **I** | **Q** | **T** | **P** | **Y** | **L** | **V** | **P** | **D** | **E** | **S** | **V** | **I** | **A** | **A** | **L** | **K** | **I** | **A** | **T** | **A** | **S** | **G** | **V** | **D** | **V** | **K** | **I** | **M** | **I** | **P** | **N** | **K** | **P** | **D** | **H** | **P** | **F** | **I** | **Y** | **R** | **A** | **T** | **Q** | **Y** | **Y** | **A** | **R** | **Q** | **L** | **I** | **K** | **E** | **N** | **V** | **Q** | **I** | **L** | **V** | **Y** | **E** | **N** | **G** | **F** | **L** | **H** | **A** | **K** | **T** | **L** | **I** | **M** | **D** | **D** | **E** | **I** | **C** | **M** | **V** | **G** | **S** | **A** | **N** | **Q** | **D** | **I** | **R** | **S** | **Y** | **R** | **L** | **N** | **F** | **E** | **T** | **S** | **A** | **V** | **I** | **Y** | **D** | **P** | **E** | **F** | **L** | **E** | **E** | **L** | **A** | **T** | **Q** | **F** | **K** | **E** | **D** | **E** | **T** | **H** | **C** | **S** | **S** | **M** | **T** | **T** | **E** | **T** | **V** | **K** | **E** | **M** | **S** | **N** | **W** | **L** | **L** | **F** | **K** | **Q** | **Q** | **I** | **S** | **R** | **L** | **F** | **S** | **P** | **I** | **L** |
| **N59503F** | **-** | **-** | **-** | **-** | **-** | **-** | **-** | **-** | **-** | **-** | **-** | **-** | **-** | **-** | **-** | **-** | **-** | **-** | **-** | **-** | **-** | **-** | **-** | **K** | **P** | **R** | **D** | **V** | **A** | **A** | **I** | **W** | **A** | **W** | **L** | **L** | **V** | **L** | **F** | **A** | **L** | **P** | **G** | **V** | **G** | **F** | **V** | **L** | **Y** | **L** | **F** | **F** | **G** | **R** | **G** | **L** | **T** | **D** | **K** | **K** | **K** | **F** | **Y** | **L** | **R** | **Q** | **S** | **D** | **L** | **K** | **E** | **L** | **E** | **N** | **F** | **Q** | **S** | **F** | **R** | **G** | **D** | **T** | **I** | **E** | **H** | **Y** | **D** | **P** | **D** | **M** | **G** | **D** | **K** | **D** | **K** | **Q** | **Q** | **F** | **V** | **D** | **F** | **F** | **S** | **S** | **L** | **N** | **R** | **M** | **P** | **L** | **T** | **R** | **M** | **N** | **S** | **V** | **T** | **L** | **L** | **T** | **D** | **G** | **Q** | **E** | **K** | **L** | **D** | **S** | **L** | **L** | **Q** | **D** | **L** | **K** | **K** | **A** | **K** | **H** | **S** | **I** | **H** | **I** | **E** | **Y** | **Y** | **A** | **F** | **V** | **T** | **D** | **N** | **I** | **G** | **Q** | **Q** | **V** | **L** | **H** | **V** | **L** | **E** | **E** | **K** | **A** | **A** | **E** | **G** | **V** | **E** | **V** | **R** | **I** | **L** | **Y** | **D** | **A** | **F** | **G** | **S** | **H** | **G** | **T** | **K** | **A** | **K** | **D** | **F** | **N** | **R** | **L** | **I** | **K** | **N** | **G** | **G** | **H** | **V** | **H** | **T** | **F** | **V** | **T** | **S** | **Q** | **K** | **A** | **L** | **L** | **R** | **F** | **R** | **L** | **N** | **Y** | **H** | **D** | **H** | **R** | **K** | **I** | **V** | **V** | **I** | **D** | **G** | **K** | **I** | **S** | **Y** | **T** | **G** | **G** | **F** | **N** | **I** | **A** | **N** | **Q** | **Y** | **V** | **N** | **T** | **T** | **K** | **K** | **F** | **G** | **Y** | **W** | **R** | **D** | **T** | **H** | **I** | **R** | **I** | **F** | **G** | **A** | **A** | **S** | **S** | **L** | **L** | **Q** | **L** | **R** | **F** | **L** | **T** | **D** | **W** | **N** | **V** | **S** | **V** | **P** | **E** | **E** | **K** | **K** | **V** | **G** | **Y** | **H** | **L** | **N** | **Y** | **F** | **F** | **K** | **K** | **A** | **D** | **R** | **D** | **E** | **S** | **K** | **L** | **A** | **D** | **T** | **S** | **I** | **Q** | **L** | **V** | **S** | **S** | **G** | **P** | **N** | **N** | **E** | **R** | **E** | **Q** | **I** | **K** | **L** | **S** | **F** | **I** | **K** | **L** | **I** | **T** | **S** | **A** | **K** | **K** | **R** | **V** | **W** | **I** | **Q** | **T** | **P** | **Y** | **L** | **V** | **P** | **D** | **E** | **S** | **V** | **I** | **A** | **A** | **L** | **K** | **I** | **A** | **T** | **A** | **S** | **G** | **V** | **D** | **V** | **K** | **I** | **M** | **I** | **P** | **N** | **K** | **P** | **D** | **H** | **P** | **F** | **I** | **Y** | **R** | **A** | **T** | **Q** | **Y** | **Y** | **A** | **R** | **Q** | **L** | **I** | **K** | **E** | **N** | **V** | **Q** | **I** | **L** | **V** | **Y** | **E** | **N** | **G** | **F** | **L** | **H** | **A** | **K** | **T** | **L** | **I** | **M** | **D** | **D** | **E** | **I** | **C** | **M** | **V** | **G** | **S** | **A** | **N** | **Q** | **D** | **I** | **R** | **S** | **Y** | **R** | **L** | **N** | **F** | **E** | **T** | **S** | **A** | **V** | **I** | **Y** | **D** | **P** | **E** | **F** | **L** | **E** | **E** | **L** | **A** | **T** | **Q** | **F** | **K** | **E** | **D** | **E** | **T** | **H** | **C** | **S** | **S** | **M** | **T** | **T** | **E** | **T** | **V** | **K** | **E** | **M** | **S** | **N** | **W** | **L** | **L** | **F** | **K** | **Q** | **Q** | **I** | **S** | **R** | **L** | **F** | **S** | **P** | **I** | **L** |
| **N54551** | **-** | **-** | **-** | **-** | **-** | **-** | **-** | **-** | **-** | **-** | **-** | **-** | **-** | **-** | **-** | **-** | **-** | **-** | **-** | **-** | **-** | **-** | **-** | **K** | **P** | **R** | **D** | **V** | **A** | **A** | **I** | **W** | **A** | **W** | **L** | **L** | **V** | **L** | **F** | **A** | **L** | **P** | **G** | **V** | **G** | **F** | **V** | **L** | **Y** | **L** | **F** | **F** | **G** | **R** | **G** | **L** | **T** | **D** | **K** | **K** | **K** | **F** | **Y** | **L** | **R** | **Q** | **S** | **D** | **L** | **K** | **E** | **L** | **E** | **N** | **F** | **Q** | **S** | **F** | **R** | **G** | **D** | **T** | **I** | **E** | **H** | **Y** | **D** | **P** | **D** | **M** | **G** | **D** | **K** | **D** | **K** | **Q** | **Q** | **F** | **V** | **D** | **F** | **F** | **S** | **S** | **L** | **N** | **R** | **M** | **P** | **L** | **T** | **R** | **M** | **N** | **S** | **V** | **T** | **L** | **L** | **T** | **D** | **G** | **Q** | **E** | **K** | **L** | **D** | **S** | **L** | **L** | **Q** | **D** | **L** | **K** | **K** | **A** | **K** | **H** | **S** | **I** | **H** | **I** | **E** | **Y** | **Y** | **A** | **F** | **V** | **T** | **D** | **N** | **I** | **G** | **Q** | **Q** | **V** | **L** | **H** | **V** | **L** | **E** | **E** | **K** | **A** | **A** | **E** | **G** | **V** | **E** | **V** | **R** | **I** | **L** | **Y** | **D** | **A** | **F** | **G** | **S** | **H** | **G** | **T** | **K** | **A** | **K** | **D** | **F** | **N** | **R** | **L** | **I** | **K** | **N** | **G** | **G** | **H** | **V** | **H** | **T** | **F** | **V** | **T** | **S** | **Q** | **K** | **A** | **L** | **L** | **R** | **F** | **R** | **L** | **N** | **Y** | **H** | **D** | **H** | **R** | **K** | **I** | **V** | **V** | **I** | **D** | **G** | **K** | **I** | **S** | **Y** | **T** | **G** | **G** | **F** | **N** | **I** | **A** | **N** | **Q** | **Y** | **V** | **N** | **T** | **T** | **K** | **K** | **F** | **G** | **Y** | **W** | **R** | **D** | **T** | **H** | **I** | **R** | **I** | **F** | **G** | **A** | **A** | **S** | **S** | **L** | **L** | **Q** | **L** | **R** | **F** | **L** | **T** | **D** | **W** | **N** | **V** | **S** | **V** | **P** | **E** | **E** | **K** | **K** | **V** | **G** | **Y** | **H** | **L** | **N** | **Y** | **F** | **F** | **K** | **K** | **A** | **D** | **R** | **D** | **E** | **S** | **K** | **L** | **A** | **D** | **T** | **S** | **I** | **Q** | **L** | **V** | **S** | **S** | **G** | **P** | **N** | **N** | **E** | **R** | **E** | **Q** | **I** | **K** | **L** | **S** | **F** | **I** | **K** | **L** | **I** | **T** | **S** | **A** | **K** | **K** | **R** | **V** | **W** | **I** | **Q** | **T** | **P** | **Y** | **L** | **V** | **P** | **D** | **E** | **S** | **V** | **I** | **A** | **A** | **L** | **K** | **I** | **A** | **T** | **A** | **S** | **G** | **V** | **D** | **V** | **K** | **I** | **M** | **I** | **P** | **N** | **K** | **P** | **D** | **H** | **P** | **F** | **I** | **Y** | **R** | **A** | **T** | **Q** | **Y** | **Y** | **A** | **R** | **Q** | **L** | **I** | **K** | **E** | **N** | **V** | **Q** | **I** | **L** | **V** | **Y** | **E** | **N** | **G** | **F** | **L** | **H** | **A** | **K** | **T** | **L** | **I** | **M** | **D** | **D** | **E** | **I** | **C** | **M** | **V** | **G** | **S** | **A** | **N** | **Q** | **D** | **I** | **R** | **S** | **Y** | **R** | **L** | **N** | **F** | **E** | **T** | **S** | **A** | **V** | **I** | **Y** | **D** | **P** | **E** | **F** | **L** | **E** | **E** | **L** | **A** | **T** | **Q** | **F** | **K** | **E** | **D** | **E** | **T** | **H** | **C** | **S** | **S** | **M** | **T** | **T** | **E** | **T** | **V** | **K** | **E** | **M** | **S** | **N** | **W** | **L** | **L** | **F** | **K** | **Q** | **Q** | **I** | **S** | **R** | **L** | **F** | **S** | **P** | **I** | **L** |
| **N54070** | **-** | **-** | **-** | **-** | **-** | **-** | **-** | **-** | **-** | **-** | **-** | **-** | **-** | **-** | **-** | **-** | **-** | **-** | **-** | **-** | **-** | **-** | **-** | **K** | **P** | **R** | **D** | **V** | **A** | **A** | **I** | **W** | **A** | **W** | **L** | **L** | **V** | **L** | **F** | **A** | **L** | **P** | **G** | **V** | **G** | **F** | **V** | **L** | **Y** | **L** | **F** | **F** | **G** | **R** | **G** | **L** | **T** | **D** | **K** | **K** | **K** | **F** | **Y** | **L** | **R** | **Q** | **S** | **D** | **L** | **K** | **E** | **L** | **E** | **N** | **F** | **Q** | **S** | **F** | **R** | **G** | **D** | **T** | **I** | **E** | **H** | **Y** | **D** | **P** | **D** | **M** | **G** | **D** | **K** | **D** | **K** | **Q** | **Q** | **F** | **V** | **D** | **F** | **F** | **S** | **S** | **L** | **N** | **R** | **M** | **P** | **L** | **T** | **R** | **M** | **N** | **S** | **V** | **T** | **L** | **L** | **T** | **D** | **G** | **Q** | **E** | **K** | **L** | **D** | **S** | **L** | **L** | **Q** | **D** | **L** | **K** | **K** | **A** | **K** | **H** | **S** | **I** | **H** | **I** | **E** | **Y** | **Y** | **A** | **F** | **V** | **T** | **D** | **N** | **I** | **G** | **Q** | **Q** | **V** | **L** | **H** | **V** | **L** | **E** | **E** | **K** | **A** | **A** | **E** | **G** | **V** | **E** | **V** | **R** | **I** | **L** | **Y** | **D** | **A** | **F** | **G** | **S** | **H** | **G** | **T** | **K** | **A** | **K** | **D** | **F** | **N** | **R** | **L** | **I** | **K** | **N** | **G** | **G** | **H** | **V** | **H** | **T** | **F** | **V** | **T** | **S** | **Q** | **K** | **A** | **L** | **L** | **R** | **F** | **R** | **L** | **N** | **Y** | **H** | **D** | **H** | **R** | **K** | **I** | **V** | **V** | **I** | **D** | **G** | **K** | **I** | **S** | **Y** | **T** | **G** | **G** | **F** | **N** | **I** | **A** | **N** | **Q** | **Y** | **V** | **N** | **T** | **T** | **K** | **K** | **F** | **G** | **Y** | **W** | **R** | **D** | **T** | **H** | **I** | **R** | **I** | **F** | **G** | **A** | **A** | **S** | **S** | **L** | **L** | **Q** | **L** | **R** | **F** | **L** | **T** | **D** | **W** | **N** | **V** | **S** | **V** | **P** | **E** | **E** | **K** | **K** | **V** | **G** | **Y** | **H** | **L** | **N** | **Y** | **F** | **F** | **K** | **K** | **A** | **D** | **R** | **D** | **E** | **S** | **K** | **L** | **A** | **D** | **T** | **S** | **I** | **Q** | **L** | **V** | **S** | **S** | **G** | **P** | **N** | **N** | **E** | **R** | **E** | **Q** | **I** | **K** | **L** | **S** | **F** | **I** | **K** | **L** | **I** | **T** | **S** | **A** | **K** | **K** | **R** | **V** | **W** | **I** | **Q** | **T** | **P** | **Y** | **L** | **V** | **P** | **D** | **E** | **S** | **V** | **I** | **A** | **A** | **L** | **K** | **I** | **A** | **T** | **A** | **S** | **G** | **V** | **D** | **V** | **K** | **I** | **M** | **I** | **P** | **N** | **K** | **P** | **D** | **H** | **P** | **F** | **I** | **Y** | **R** | **A** | **T** | **Q** | **Y** | **Y** | **A** | **R** | **Q** | **L** | **I** | **K** | **E** | **N** | **V** | **Q** | **I** | **L** | **V** | **Y** | **E** | **N** | **G** | **F** | **L** | **H** | **A** | **K** | **T** | **L** | **I** | **M** | **D** | **D** | **E** | **I** | **C** | **M** | **V** | **G** | **S** | **A** | **N** | **Q** | **D** | **I** | **R** | **S** | **Y** | **R** | **L** | **N** | **F** | **E** | **T** | **S** | **A** | **V** | **I** | **Y** | **D** | **P** | **E** | **F** | **L** | **E** | **E** | **L** | **A** | **T** | **Q** | **F** | **K** | **E** | **D** | **E** | **T** | **H** | **C** | **S** | **S** | **M** | **T** | **T** | **E** | **T** | **V** | **K** | **E** | **M** | **S** | **N** | **W** | **L** | **L** | **F** | **K** | **Q** | **Q** | **I** | **S** | **R** | **L** | **F** | **S** | **P** | **I** | **L** |
| **N52636** | **-** | **-** | **-** | **-** | **-** | **-** | **-** | **-** | **-** | **-** | **-** | **-** | **-** | **-** | **-** | **-** | **-** | **-** | **-** | **-** | **-** | **-** | **-** | **K** | **P** | **R** | **D** | **V** | **A** | **A** | **I** | **W** | **A** | **W** | **L** | **L** | **V** | **L** | **F** | **A** | **L** | **P** | **G** | **V** | **G** | **F** | **V** | **L** | **Y** | **L** | **F** | **F** | **G** | **R** | **G** | **L** | **T** | **D** | **K** | **K** | **K** | **F** | **Y** | **L** | **R** | **Q** | **S** | **D** | **L** | **K** | **E** | **L** | **E** | **N** | **F** | **Q** | **S** | **F** | **R** | **G** | **D** | **T** | **I** | **E** | **H** | **Y** | **D** | **P** | **D** | **M** | **G** | **D** | **K** | **D** | **K** | **Q** | **Q** | **F** | **V** | **D** | **F** | **F** | **S** | **S** | **L** | **N** | **R** | **M** | **P** | **L** | **T** | **R** | **M** | **N** | **S** | **V** | **T** | **L** | **L** | **T** | **D** | **G** | **Q** | **E** | **K** | **L** | **D** | **S** | **L** | **L** | **Q** | **D** | **L** | **K** | **K** | **A** | **K** | **H** | **S** | **I** | **H** | **I** | **E** | **Y** | **Y** | **A** | **F** | **V** | **T** | **D** | **N** | **I** | **G** | **Q** | **Q** | **V** | **L** | **H** | **V** | **L** | **E** | **E** | **K** | **A** | **A** | **E** | **G** | **V** | **E** | **V** | **R** | **I** | **L** | **Y** | **D** | **A** | **F** | **G** | **S** | **H** | **G** | **T** | **K** | **A** | **K** | **D** | **F** | **N** | **R** | **L** | **I** | **K** | **N** | **G** | **G** | **H** | **V** | **H** | **T** | **F** | **V** | **T** | **S** | **Q** | **K** | **A** | **L** | **L** | **R** | **F** | **R** | **L** | **N** | **Y** | **H** | **D** | **H** | **R** | **K** | **I** | **V** | **V** | **I** | **D** | **G** | **K** | **I** | **S** | **Y** | **T** | **G** | **G** | **F** | **N** | **I** | **A** | **N** | **Q** | **Y** | **V** | **N** | **I** | **T** | **K** | **K** | **F** | **G** | **Y** | **W** | **R** | **D** | **T** | **H** | **I** | **R** | **I** | **F** | **G** | **A** | **A** | **S** | **S** | **L** | **L** | **Q** | **L** | **R** | **F** | **L** | **T** | **D** | **W** | **N** | **V** | **S** | **V** | **P** | **E** | **E** | **K** | **K** | **V** | **G** | **Y** | **H** | **L** | **N** | **Y** | **F** | **F** | **K** | **K** | **A** | **D** | **R** | **D** | **E** | **S** | **K** | **L** | **A** | **D** | **T** | **S** | **I** | **Q** | **L** | **V** | **S** | **S** | **G** | **P** | **N** | **N** | **E** | **R** | **E** | **Q** | **I** | **K** | **L** | **S** | **F** | **I** | **K** | **L** | **I** | **T** | **S** | **A** | **K** | **K** | **R** | **V** | **W** | **I** | **Q** | **T** | **P** | **Y** | **L** | **V** | **P** | **D** | **E** | **S** | **V** | **I** | **A** | **A** | **L** | **K** | **I** | **A** | **T** | **A** | **S** | **G** | **V** | **D** | **V** | **K** | **I** | **M** | **I** | **P** | **N** | **K** | **P** | **D** | **H** | **P** | **F** | **I** | **Y** | **R** | **A** | **T** | **Q** | **Y** | **Y** | **A** | **R** | **Q** | **L** | **I** | **K** | **E** | **N** | **V** | **Q** | **I** | **L** | **V** | **Y** | **E** | **N** | **G** | **F** | **L** | **H** | **A** | **K** | **T** | **L** | **I** | **M** | **D** | **D** | **E** | **I** | **C** | **M** | **V** | **G** | **S** | **A** | **N** | **Q** | **D** | **I** | **R** | **S** | **Y** | **R** | **L** | **N** | **F** | **E** | **T** | **S** | **A** | **V** | **I** | **Y** | **D** | **P** | **E** | **F** | **L** | **E** | **E** | **L** | **A** | **T** | **Q** | **F** | **K** | **E** | **D** | **E** | **T** | **H** | **C** | **S** | **S** | **M** | **T** | **T** | **E** | **T** | **V** | **K** | **E** | **M** | **S** | **N** | **W** | **L** | **L** | **F** | **K** | **Q** | **Q** | **I** | **S** | **R** | **L** | **F** | **S** | **P** | **I** | **L** |
| **N53702** | **-** | **-** | **-** | **-** | **-** | **-** | **-** | **-** | **-** | **-** | **-** | **-** | **-** | **-** | **-** | **-** | **-** | **-** | **-** | **-** | **-** | **-** | **-** | **K** | **P** | **R** | **D** | **V** | **A** | **A** | **I** | **W** | **A** | **W** | **L** | **L** | **V** | **L** | **F** | **A** | **L** | **P** | **G** | **V** | **G** | **F** | **V** | **L** | **Y** | **L** | **F** | **F** | **G** | **R** | **G** | **L** | **T** | **D** | **K** | **K** | **K** | **F** | **Y** | **L** | **R** | **Q** | **S** | **D** | **L** | **K** | **E** | **L** | **E** | **N** | **F** | **Q** | **S** | **F** | **R** | **G** | **D** | **T** | **I** | **E** | **H** | **Y** | **D** | **P** | **D** | **M** | **G** | **D** | **K** | **D** | **K** | **Q** | **Q** | **F** | **V** | **D** | **F** | **F** | **S** | **S** | **L** | **N** | **R** | **M** | **P** | **L** | **T** | **R** | **M** | **N** | **S** | **V** | **T** | **L** | **L** | **T** | **D** | **G** | **Q** | **E** | **K** | **L** | **D** | **S** | **L** | **L** | **Q** | **D** | **L** | **K** | **K** | **A** | **K** | **H** | **S** | **I** | **H** | **I** | **E** | **Y** | **Y** | **A** | **F** | **V** | **T** | **D** | **N** | **I** | **G** | **Q** | **Q** | **V** | **L** | **H** | **V** | **L** | **E** | **E** | **K** | **A** | **A** | **E** | **G** | **V** | **E** | **V** | **R** | **I** | **L** | **Y** | **D** | **A** | **F** | **G** | **S** | **H** | **G** | **T** | **K** | **A** | **K** | **D** | **F** | **N** | **R** | **L** | **I** | **K** | **N** | **G** | **G** | **H** | **V** | **H** | **T** | **F** | **V** | **T** | **S** | **Q** | **K** | **A** | **L** | **L** | **R** | **F** | **R** | **L** | **N** | **Y** | **H** | **D** | **H** | **R** | **K** | **I** | **V** | **V** | **I** | **D** | **G** | **K** | **I** | **S** | **Y** | **T** | **G** | **G** | **F** | **N** | **I** | **A** | **N** | **Q** | **Y** | **V** | **N** | **T** | **T** | **K** | **K** | **F** | **G** | **Y** | **W** | **R** | **D** | **T** | **H** | **I** | **R** | **I** | **F** | **G** | **A** | **A** | **S** | **S** | **L** | **L** | **Q** | **L** | **R** | **F** | **L** | **T** | **D** | **W** | **N** | **V** | **S** | **V** | **P** | **E** | **E** | **K** | **K** | **V** | **G** | **Y** | **H** | **L** | **N** | **Y** | **F** | **F** | **K** | **K** | **A** | **D** | **R** | **D** | **E** | **S** | **K** | **L** | **A** | **D** | **T** | **S** | **I** | **Q** | **L** | **V** | **S** | **S** | **G** | **P** | **N** | **N** | **E** | **R** | **E** | **Q** | **I** | **K** | **L** | **S** | **F** | **I** | **K** | **L** | **I** | **T** | **S** | **A** | **K** | **K** | **R** | **V** | **W** | **I** | **Q** | **T** | **P** | **Y** | **L** | **V** | **P** | **D** | **E** | **S** | **V** | **I** | **A** | **A** | **L** | **K** | **I** | **A** | **T** | **A** | **S** | **G** | **V** | **D** | **V** | **K** | **I** | **M** | **I** | **P** | **N** | **K** | **P** | **D** | **H** | **P** | **F** | **I** | **Y** | **R** | **A** | **T** | **Q** | **Y** | **Y** | **A** | **R** | **Q** | **L** | **I** | **K** | **E** | **N** | **V** | **Q** | **I** | **L** | **V** | **Y** | **E** | **N** | **G** | **F** | **L** | **H** | **A** | **K** | **T** | **L** | **I** | **M** | **D** | **D** | **E** | **I** | **C** | **M** | **V** | **G** | **S** | **A** | **N** | **Q** | **D** | **I** | **R** | **S** | **Y** | **R** | **L** | **N** | **F** | **E** | **T** | **S** | **A** | **V** | **I** | **Y** | **D** | **P** | **E** | **F** | **L** | **E** | **E** | **L** | **A** | **T** | **Q** | **F** | **K** | **E** | **D** | **E** | **T** | **H** | **C** | **S** | **S** | **M** | **T** | **T** | **E** | **T** | **V** | **K** | **E** | **M** | **S** | **N** | **W** | **L** | **L** | **F** | **K** | **Q** | **Q** | **I** | **S** | **R** | **L** | **F** | **S** | **P** | **I** | **L** |
| **N60417F** | **-** | **-** | **-** | **-** | **-** | **-** | **-** | **-** | **-** | **-** | **-** | **-** | **-** | **-** | **-** | **-** | **-** | **-** | **-** | **-** | **-** | **-** | **-** | **K** | **P** | **R** | **D** | **V** | **A** | **A** | **I** | **W** | **A** | **W** | **L** | **L** | **V** | **L** | **F** | **A** | **L** | **P** | **G** | **V** | **G** | **F** | **V** | **L** | **Y** | **L** | **F** | **F** | **G** | **R** | **G** | **L** | **T** | **D** | **K** | **K** | **K** | **F** | **Y** | **L** | **R** | **Q** | **S** | **D** | **L** | **K** | **E** | **L** | **E** | **N** | **F** | **Q** | **S** | **F** | **R** | **G** | **D** | **T** | **I** | **E** | **H** | **Y** | **D** | **P** | **D** | **M** | **G** | **D** | **K** | **D** | **K** | **Q** | **Q** | **F** | **V** | **D** | **F** | **F** | **S** | **S** | **L** | **N** | **R** | **M** | **P** | **L** | **T** | **R** | **M** | **N** | **S** | **V** | **T** | **L** | **L** | **T** | **D** | **G** | **Q** | **K** | **K** | **L** | **D** | **S** | **L** | **L** | **Q** | **D** | **L** | **K** | **K** | **A** | **K** | **H** | **S** | **I** | **H** | **I** | **E** | **Y** | **Y** | **A** | **F** | **V** | **T** | **D** | **T** | **I** | **G** | **Q** | **Q** | **V** | **L** | **R** | **V** | **L** | **E** | **E** | **K** | **A** | **A** | **E** | **G** | **V** | **E** | **V** | **R** | **I** | **L** | **Y** | **D** | **A** | **F** | **G** | **S** | **H** | **G** | **T** | **K** | **A** | **K** | **D** | **F** | **N** | **R** | **L** | **I** | **K** | **N** | **G** | **G** | **H** | **V** | **H** | **T** | **F** | **V** | **T** | **S** | **Q** | **K** | **A** | **L** | **L** | **R** | **F** | **R** | **L** | **N** | **Y** | **H** | **D** | **H** | **R** | **K** | **I** | **V** | **V** | **I** | **D** | **G** | **K** | **I** | **S** | **Y** | **T** | **G** | **G** | **F** | **N** | **I** | **A** | **N** | **Q** | **Y** | **V** | **N** | **T** | **T** | **K** | **K** | **F** | **G** | **Y** | **W** | **R** | **D** | **T** | **H** | **I** | **R** | **I** | **F** | **G** | **A** | **A** | **S** | **S** | **L** | **L** | **Q** | **L** | **R** | **F** | **L** | **T** | **D** | **W** | **N** | **V** | **S** | **V** | **P** | **E** | **E** | **K** | **K** | **V** | **G** | **Y** | **H** | **L** | **N** | **Y** | **F** | **F** | **K** | **K** | **A** | **D** | **R** | **D** | **E** | **S** | **K** | **L** | **A** | **D** | **T** | **S** | **I** | **Q** | **L** | **V** | **S** | **S** | **G** | **P** | **N** | **N** | **E** | **R** | **E** | **Q** | **I** | **K** | **L** | **S** | **F** | **I** | **K** | **L** | **I** | **T** | **S** | **A** | **K** | **K** | **R** | **V** | **W** | **I** | **Q** | **T** | **P** | **Y** | **L** | **V** | **P** | **D** | **E** | **S** | **V** | **I** | **A** | **A** | **L** | **K** | **I** | **A** | **T** | **A** | **S** | **G** | **V** | **D** | **V** | **K** | **I** | **M** | **I** | **P** | **N** | **K** | **P** | **D** | **H** | **P** | **F** | **I** | **Y** | **R** | **A** | **T** | **Q** | **Y** | **Y** | **A** | **R** | **Q** | **L** | **I** | **K** | **E** | **N** | **V** | **Q** | **I** | **L** | **V** | **Y** | **E** | **N** | **G** | **F** | **L** | **H** | **A** | **K** | **T** | **L** | **I** | **M** | **D** | **D** | **E** | **I** | **C** | **M** | **V** | **G** | **S** | **A** | **N** | **Q** | **D** | **I** | **R** | **S** | **Y** | **R** | **L** | **N** | **F** | **E** | **T** | **S** | **A** | **V** | **I** | **Y** | **D** | **P** | **E** | **F** | **L** | **E** | **E** | **L** | **A** | **T** | **Q** | **F** | **K** | **E** | **D** | **E** | **T** | **H** | **C** | **S** | **P** | **M** | **T** | **T** | **E** | **T** | **V** | **K** | **E** | **M** | **S** | **N** | **W** | **L** | **L** | **F** | **K** | **Q** | **Q** | **I** | **S** | **R** | **L** | **F** | **S** | **P** | **I** | **L** |
 **SNP position** | . | . | . | . | . | . | . | . | . | . | . | . | 13 | . | . | . | . | . | . | 20 | . | . | . | . | . | . | 27 | . | . | . | . | . | . | . | . | . | . | . | . | 40 | . | . | . | 44 | . | . | . | . | . | . | . | . | . | . | . | . | . | . | 59 | . | . | . | . | . | 65 | . | . | . | . | . | . | . | . | . | . | . | . | . | . | . | . | 82 | . | . | . | . | . | . | . | . | . | . | . | . | . | . | . | . | . | . | . | . | . | . | . | . | . | . | . | . | 111 | 112 | . | . | . | . | . | . | . | . | . | . | . | 124 | . | . | . | . | . | . | . | . | . | . | . | . | . | . | . | . | . | . | . | . | . | . | . | . | . | . | 151 | . | . | . | . | . | . | 158 | . | . | . | . | . | 164 | . | . | . | . | . | . | . | . | . | . | . | . | . | 178 | . | . | . | . | . | . | . | . | . | . | . | . | . | . | . | . | . | . | . | . | . | . | . | . | . | . | 205 | . | . | . | . | . | . | . | . | . | 215 | . | . | 218 | . | . | . | . | . | . | 225 | . | . | . | . | 230 | . | . | . | . | . | . | . | . | . | 240 | . | 242 | . | . | . | . | . | . | . | . | . | . | . | . | . | . | . | . | . | . | . | . | . | . | . | . | 267 | . | . | . | . | . | . | . | . | . | 277 | . | . | . | . | . | . | . | . | . | . | . | . | . | . | . | . | . | 295 | . | . | . | . | . | . | 302 | 303 | . | . | . | . | . | . | . | . | . | . | . | . | . | . | . | . | . | . | . | . | . | . | . | . | . | . | . | . | . | . | . | . | . | . | . | . | . | . | . | . | . | . | . | . | . | . | . | . | . | . | . | . | . | . | . | . | . | . | . | . | . | . | . | . | . | . | . | . | . | . | . | . | . | . | . | . | . | . | . | . | . | . | . | . | . | . | . | 391 | . | . | . | . | . | . | . | . | . | . | . | . | . | . | . | . | . | . | . | . | . | . | . | . | . | . | . | . | . | . | . | . | . | . | . | . | . | . | . | . | . | . | . | . | . | . | . | . | . | . | . | . | . | . | . | . | . | . | . | . | . | 453 | . | . | 456 | . | . | . | . | . | . | . | . | . | . | . | . | . | . | . | . | . | . | . | . | . | . | . | . | . | . | . |

|  |  |
| --- | --- |
|  |

|  |  |
| --- | --- |
|  |

|  |  |
| --- | --- |
| **Legend** | |
  | **Reference Sequence** |

  |
|  | **Validated SNPs** |
|  | **Non-validated SNPs** |
|  | **New SNPs** |
|  | **GAPs** |
|  |  |

  
  |
|  |  |
